
JOURNAL INFORMATION
==============================
NLM Title Abbreviation: Hemasphere
Journal ID: Hemasphere
NLM Journal ID: 101740619
Journal ID: HEM3

HemaSphere

EISSN: 2572-9241
Publisher: Wiley

ARTICLE INFORMATION
==============================
PMCID: PMC13254489
DOI: 10.1002/hem3.70421
Article ID: HEM370421
Article version: 1
Subjects: Abstract Book, ABSTRACT BOOK, Publication Only

Publication Only

Electronic publication date: 2026 Jun 10
Publication date: 2026 Jun
Volume: 10
Issue: Suppl 1
Electronic Location ID: e70421
Copyright: © 2026 The Author(s). HemaSphere published by John Wiley & Sons Ltd. on behalf of European Hematology Association.
License: This is an open access article under the terms of the http://creativecommons.org/licenses/by/4.0/ License, which permits use, distribution and reproduction in any medium, provided the original work is properly cited.
License URL: https://creativecommons.org/licenses/by/4.0/

Figure count: 0
Table count: 0
Page count: 3765

source-schema-version-number: 2.0
cover-date: June 2026
details-of-publishers-convertor: Converter:WILEY_ML3GV2_TO_JATSPMC version:6.8.0 mode:remove_FC converted:11.06.2026

==============================

eha

HemaSphere

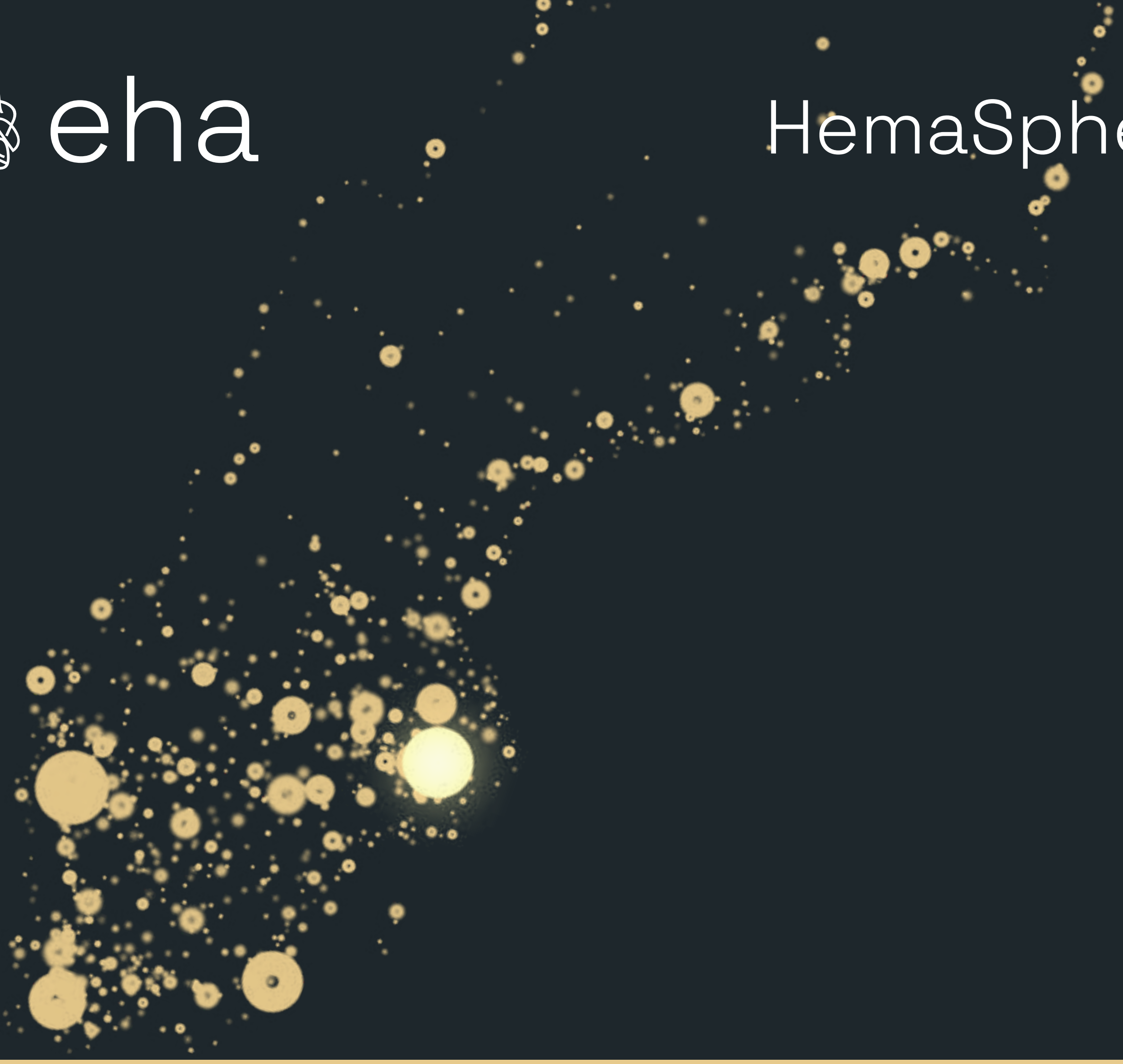

### Abstract Book

EHA2026
Congress

Volume 10
Issue S1

June 11-14
Stockholm, Sweden www.hemaspherejournal.com

Medical Education

### Attend MM & CLL symposia at EHA2026!

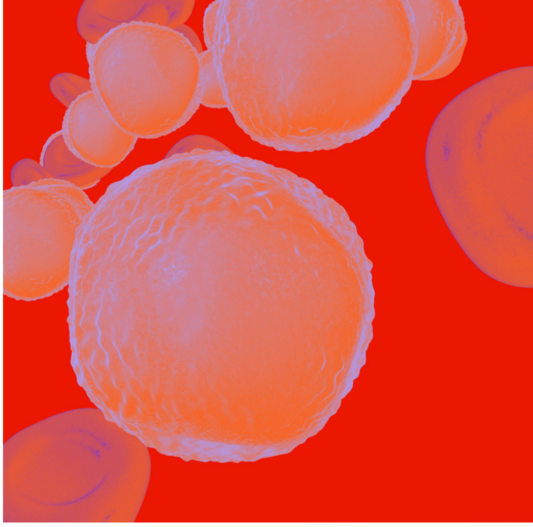

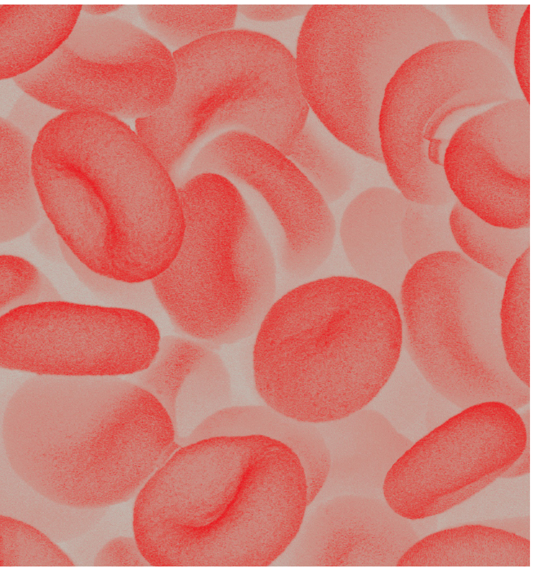

On **Thursday 11th, Friday 12th and Saturday 13th June**, follow the red thread with us throughout an exciting programme of satellite symposia supported by Johnson & Johnson and hosted by the esteemed Symposium Committee.

Prepare your burning questions for the expert panel and get ready to participate in stimulating Q&A sessions and interactive polling activities.

Thursday 11th, Friday 12th and Saturday 13th June 2026
10:00–11:30 CEST
Nobel Hall, Stockholmsmässan, Stockholm

Thursday 11th June
10:00–11:30 CEST

Weaving the red thread towards cure in newly diagnosed multiple myeloma (NDMM)

Prof. María-Victoria Mateos (Chair & Symposium Committee)
Prof. Philippe Moreau (Symposium Committee)
Prof. Paula Rodriguez-Otero
Dr Charlotte Pawlyn

Friday 12th June
10:00–11:30 CEST

Following the red thread in multiple myeloma (MM): aligning innovation with clinical practice

Prof. María-Victoria Mateos (Chair & Symposium Committee)
Dr Rakesh Popat (Symposium Committee)
Prof. Elena Zamagni
Dr Roberto Mina

Saturday 13th June
10:00-11:30 CEST

Following the red thread of evidence: guiding optimal decision-making in first-line (1L) chronic lymphocytic leukaemia (CLL)

Prof. Martin Trepel (Symposium Committee)
Dr Alessandra Tedeschi (Symposium Committee)
Dr Anna Panovská (Co-chair)
Prof. Paolo Sportoletti (Co-chair)
Dr José-Ángel Hernández-Rivas

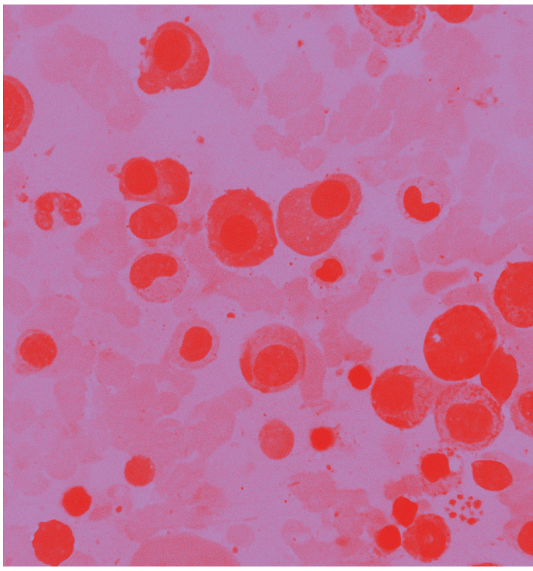

*This symposium is organised and sponsored by Johnson & Johnson and is not part of the official EHA scientific programme.*

Johnson&Johnson

Organised and supported by Janssen Pharmaceutica NV,
a Johnson & Johnson company
Janssen Pharmaceutica NV, Turnhoutseweg 30, 2340 Beerse Belgium
Copyright © 2026 Janssen Pharmaceutica NV

Job code: EM-199756 Date of prep: May 2026

31st Congress of the
European Hematology Association

EHA2026 Annual Congress Edition
June 2026

### ABSTRACT BOOK

### SECTION 03

HemaSphere eha

**Author Information**

***HemaSphere*** is an open access journal powered by the European Hematology Association and dedicated to supporting hematology patient care, research, and education worldwide.

This Abstract Book of the European Hematology Association's 31st Annual Congress - EHA2026 Congress - is published as a supplement of *HemaSphere* and available online at www.hemaspherejournal.com.

Please read our author guidelines for preparing and submitting articles. Manuscripts must be submitted online via the Research Exchange submission portal.

**Article Processing Charges (APC)**

| Article types | EHA members | Non-members |
|---|---|---|
| Tier 1: Original articles, review articles, guidelines | 10%* | €2500 |
| Tier 2: Letters, perspectives | 10%* | €1500 |
| Tier 3: Editorials, comments, HemaTopics, HemaPolicy | Free | Free |
| All invited manuscripts** | Free | Free |

*2026 Promotional Discounts on APCs: 10% discount for EHA members who are lead or co-authors on an accepted submission
**APCs for all contributions on *invitation* by the Editors will be waived

**Copyright Information**
(Online) ISSN: 2572-9241
© 2026 The Author(s). *HemaSphere* published by John Wiley & Sons Ltd on behalf of European Hematology Association. This is an open access Abstract Book distributed under the Attribution-NonCommercial-NoDerivs (CC BY-NC-ND), which allows third parties to download the articles and share them with others as long as they credit the author and the Abstract Book, but they cannot change the content in any way or use them commercially.

**Rights and Permissions**
For reprints and to order copies of the Abstract Book, please email commercialreprints@wiley.com. Questions regarding permissions should be addressed to hemasphere@ehaweb.org.

**Abstract Book Citations:** Authors, Title, *HemaSphere*, 2026;10:(S1):pages.

**DOIs:**
Oral section: 10.1002/hem3.70151
Poster section: 10.1002/hem3.70152
Publication only section: 10.1002/hem3.70153

**Disclaimer**
Articles published in the journal *HemaSphere* exclusively reflect the opinions of the authors. The authors are responsible for all content in their abstracts including accuracy of the facts, statements, citing resources, etc.

### Editorial Board

### Editor in Chief
**Professor Jan Cools**
*VIB-KU Leuven Center for Cancer Biology, Belgium*

### Deputy Editor in Chief
**Professor Claire Harrison**
*Guy's and St Thomas' NHS Foundation Trust, London, United Kingdom*

### Associate Editors
Simone Ferrero, *University of Torino, Italy*
Adele Fielding, *University of York, Hull York Medical School, York, United Kingdom*
Simon Hallam, *St. Bartholomew's Hospital, Barts Health NHS Trust, London, United Kingdom*
Steffen Koschmieder, *University Medical Center RWTH, Aachen, Germany*
Riitta Lassila, *University of Helsinki, Helsinki, Finland*
Marek Mraz, *CEITEC Masaryk University and University Hospital Brno, Czech Republic*
Paula Rodríguez Otero, *Clínica Universidad de Navarra, Spain*
Jürg Schwaller, *University Hospital Basel, Switzerland*
Francesca Vinchi, *Emory University School of Medicine, Atlanta, Georgia, United States*

### Scientific Editors
Charles de Bock, *Children's Cancer Institute, Sydney, Australia*
Stephen Hibbs, *Queen Mary University of London, United Kingdom*
Mandy Lauw, *Erasmus University MC, Rotterdam, The Netherlands*
Lauren Merz, *University of Michigan, Ann Arbor, Michigan, United States*
Edeghonghon Olayemi, *Vancouver Coastal Health (Richmond Hospital Site), Richmond, British Columbia, Canada*

### Statistics Editor
Robert Hills, *University of Oxford, United Kingdom*

### Editorial Manager, European Hematology Association (EHA)
Jessica Mastrodomenico, *EHA/HemaSphere, The Hague, The Netherlands*

### Editorial Board
Frederic Baron, *University of Liège, GIGA Institute, Liège, Belgium*
Grzegorz Basak, *Medical University of Warsaw, Poland*
Andrea Biondi, *University of Milano-Bicocca, Italy*
Jonathan Bond, *University College Dublin, Ireland*
Dominique Bonnet, *The Francis Crick Institute, London, United Kingdom*
Arndt Borkhardt, *University of Düsseldorf, Germany*
Monika Brüggemann, *University Hospital Schleswig-Holstein, Kiel, Germany*
David Bryder, *Lund University, Sweden*
Ryan Cassaday, *Fred Hutchinson Cancer Center, Seattle, Washington, United States*
Giancarlo Castaman, *Azienda Ospedaliera Universitaria Careggi, Florence, Italy*
Anna Castleton, *The Christie Hospital NHS Foundation Trust, Manchester, United Kingdom*
Nicholas Chiorazzi, *Northwell Health, Manhasset, New York, United States*
Emmanuelle Clappier, *Hôpital Saint-Louis, Paris, France*
Paolo Corradini, *Università degli Studi di Milano, Italy*
Lucia De Franceschi, *University of Verona, Italy*
Kim De Keersmaecker, *University of Leuven, Belgium*

Marianne De Montalembert, *Hôpital Universitaire Necker-Enfants Malades, Paris, France*
Michel Delforge, *Leuven Cancer Institute, Leuven, Belgium*
Catherine Diefenbach, *Perlmutter Cancer Center, New York, United States*
Carlos Fernández De Larrea, *Hospital Clínic Barcelona, Spain*
Francesca Gay, *University of Turin, Italy*
Paolo Ghia, *Università Vita Salute San Raffaele, Milan, Italy*
Domenico Girelli, *Universita degli Studi di Verona, Italy*
Florian Grebien, *University of Veterinary Medicine Vienna, Austria*
Hildegard Greinix, *Medical University of Graz, Austria*
Kirsten Grønbæk, *Rigshospitalet, Copenhagen, Denmark*
Iqbal Hamza, *University of Maryland School of Medicine, Baltimore, Maryland, United States*
Florian Heidel, *University Medical Center Greifswald, Germany*
Olaf Heidenreich, *Princess Máxima Center, Utrecht, The Netherlands*
Brian Huntly, *University of Cambridge, United Kingdom*
Elaine Jaffe, N*ational Cancer Institute, Bethesda, Maryland, United States*
Mats Jerkeman, *Lund University, Sweden*
Efstathios Kastritis, *National and Kapodistrian University of Athens, Greece*
Léon Kautz, *Inserm, Toulouse, France*
David Kent, *University of York, United Kingdom*
Ulf Klein, *University of Leeds, United Kingdom*
Joost Kluiver, *Universitair Medisch Centrum Groningen, The Netherlands*
Daniela Krause, *University of Frankfurt, Germany*
Nicolaus Kröger, *University Hospital Hamburg-Eppendorf, Germany*
Steven Lane, *QIMR Berghofer Medical Research Institute, Australia*
Frank Leebeek, *Erasmus MC Cancer Institute, Rotterdam, The Netherlands*
Florent Malard, *INSERM, Saint-Antoine Hospital, Paris, France*
Maria-Victoria Mateos, *University of Salamanca, Spain*
Francesco Maura, *Memorial Sloan Kettering Cancer Center, New York, United States*
Simon Mendez-Ferrer, *University of Cambridge, United Kingdom*
Thomas Mercher, *Gustave Roussy Institute, Villejuif, France*
Martina Muckenthaler, *Heidelberg University Hospital, Heidelberg, Germany*
Cesar Nombela Arrieta, *University Hospital Zurich, Switzerland*
James O'Donnell, *Royal College of Surgeons in Ireland, Dublin, Ireland*
Kostas Pantopoulos, *McGill University, Montreal, Quebec, Canada*
Jakob Passweg, *University of Basel, Switzerland*
Heike Pfeifer, *University Hospital Frankfurt, Germany*
Marc Raab, *Heidelberg University Hospital, Heidelberg, Germany*
Marc Raaijmakers, *Erasmus Medical Center Cancer Institute, Rotterdam, The Netherlands*
Alejo Rodriguez-Fratelli, *Institute for Research in Biomedicine, Barcelona, Spain*
Richard Rosenquist, *Karolinska Institutet, Stockholm, Sweden*
Deepa Sampath, *MD Anderson Cancer Center, Houston, Texas, United States*
Rebekka Schneider, *Erasmus MC, Rotterdam, The Netherlands*
Marie Scully, *University College London Hospitals, NHS Foundation Trust, London, United Kingdom*
Kostas Stamatopoulos, *CERTH, Centre for Research and Technology Hellas, Thessaloniki, Greece*
Anna Maria Sureda Balari, *Institut Català d'Oncologia, Barcelona, Spain*
Evangelos Terpos, *National and Kapodistrian University of Athens, Greece*
Alexandre Theocharides, *University Hospital Zürich, Switzerland*
Jan Trka, *University of Prague, Czech Republic*
Karen Vanhoorelbeke, *KU Leuven, Laboratory for Thrombosis Research, Kortrijk, Belgium*
Carlo Visco, *University of Verona, Italy*
Clive Zent, *University of Rochester Medical Center, Rochester, New York, United States*

eha

### Borderless Hematology

### Join us, and let's make a difference together.

Welcome to a passionate, global network that improves patient care, advances scientific discovery, and drives innovation in hematology.

Here, every voice is heard, and every contribution matters. It's a place where we learn, inspire, and support each other, facing challenges and celebrating victories as one.

Learn more about our community

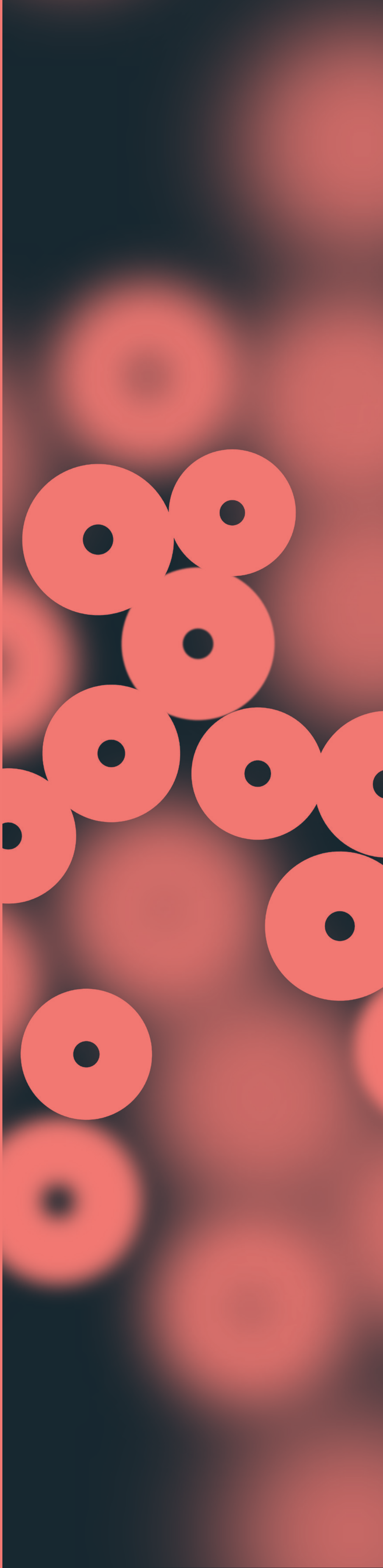

**EHA Board and Organization EHA2026 Congress**

**Executive Board**
K Döhner, *Germany* (President)
A Almeida, *Portugal* (Past President)
B Huntly, *United Kingdom* (President Elect)
F Leebeek, *The Netherlands* (Treasurer)
K Grønbæk, *Denmark* (Secretary)
M Alberich Jorda, *Czechia* (General member)

**Councillors**
D Bonnet, *United Kingdom*
J Delgado, *Spain*
L De Franceschi, *Italy*
M Guenova, *Bulgaria*
M Hudecek, *Germany*
S Leppä, *Finland*
S Mustjoki, *Finland*
E Terpos, *Greece*
J Trka, *Czechia*

**EHA2026 Congress Scientific Program Committee**
M Alberich Jorda, *Czechia* (Chair)
D Bonnet, *United Kingdom* (Chair Elect)
M Dreyling, *Germany* (Past Chair)
M Carlsten, *Sweden* (Local representative)
V Brousse, *France*
V Buxhofer-Ausch, *Austria*
A Cvejic, *Denmark*
M D'Agostino, *Italy*
K Giannopoulos, *Poland*
M Gomes da Silva, *Portugal*
J Hauer, *Germany*
D Hodson, *United Kingdom*
S Lehmann, *Sweden*
S Leppä, *Finland*
T Masszi, *Hungary*
K Meijer, *The Netherlands*
M Morado, *Spain*
C Papayannidis, *Italy*
D Rea, *France*
S Šaulytė Trakymienė, *Lithuania*
B Skopec, *Slovenia*
E Solomou, *Greece*
C So-Osman, *The Netherlands*
G Vassiliou, *United Kingdom*
MT Voso, *Italy*
G Weiss, *Austria*

**EHA2026 Congress SPC Advisory Board**
L Arranz, *Norway*
Y Aydinok, *Türkiye*
C Bödör, *Hungary*
M Buschbeck, *Spain*
J Caers, *Belgium*
M Della Porta, *Italy*
M Doubek, *Czechia*
F Fioredda, *Italy*
C Fox, *United Kingdom*
S Garciaz, *France*
M Gavriatopoulou, *Greece*
E Giné, *Spain*
A Glenthøj, *Denmark*
C Halsey, *United Kingdom*
V Helgason, *United Kingdom*
C João, *Portugal*
M Martello, *Italy*
J O'Sullivan, *Ireland*
F Pirenne, *France*
C Röllig, *Germany*
S Roulland, *France*
E Sala, *Germany*
M Sobas, *Poland*
A Stathis, *Switzerland*
J Thaler, *Austria*
M Themeli, *The Netherlands*
A Theocharides, *Switzerland*
C Touzeau, *France*
E van Beers, *The Netherlands*
J Werner Hansen, *Denmark*
P Woll, *Sweden*

**EHA2026 Congress Scientific Program Committee - Observers**
N Borges, *United Kingdom (Young EHA Committee)*
S Nier, *Switzerland (Patient Advocacy Committee)*

**EHA Executive Office**
Koninginnegracht 12b
2514 AA The Hague
The Netherlands
Tel: +31 (0)70 345 55 63
Fax: +31 (0)70 392 36 63
E-mail: info@ehaweb.org
Website: www.ehaweb.org

**Abstract Reviewers**

EHA would like to thank the following experts for their time and efforts reviewing abstracts for this Congress

R Advani, United States
A Agathangelidis, Greece
I Age Kos, Germany
J Aguadé-Gorgorió, Spain
S Akyol, Türkiye
A Al-Ani, Iraq
A Alanya, Belgium
J Alberge, France
M Alberich Jorda, Czechia
A Alhajahjeh, Jordan
A Almeida, Portugal
M Almowaled, Saudi Arabia
A Anastasiadi, Greece
A Anastasopoulou, Greece
A Aprile, Italy
S Aras Gürcan, Türkiye
A Armatys, Poland
L Arranz, Norway
F Auer, Germany
I Aurer, Croatia
F Awan, United States
Y Aydinok, Türkiye
E Azzoni, Italy
A Bachev, Bulgaria
E Bachy, France
A Bacquelaine Veloso, Belgium
D Baggio, United Kingdom
A Balduini, Italy
A Barrett, Ireland
A Baruchel, France
K Basso, United States
B Bátai, Hungary
J Batinic, Croatia
A Bazarbachi, Lebanon
S Bea, Spain
R Beekman, Spain
F Beier, Germany
C Bellodi, Denmark
S Bertoli, France
A Bigas, Spain
M Bishton, United Kingdom
N Bizymi, Greece
S Bjelosevic, United States
J Blatny, Czechia
I Bodo, Hungary
C Bödör, Hungary
D Bogdanic, Croatia
C Bonini, Italy
D Bonnet, United Kingdom
F Borzooee, Canada
F Bosch, Spain
C Brierley, United Kingdom
S Bringhen, Italy
J Broseus, France
V Brousse, France
P Bruch, Germany
T Brümmendorf, Germany
V Bücklein, Germany
B Burak, United States
M Buschbeck, Spain
C Buske, Germany
V Buxhofer-Ausch, Austria
J Caers, Belgium
M Callanan, France
V Camus, France
A Candoni, Italy
J Carda, Portugal
B Cardoso, Portugal
M Carlsten, Sweden
M Carpenedo, Italy
M Carter, United Kingdom
F Castagnetti, Italy
H Cavé, France
G Cengiz, Türkiye
C Chabannon, France
T Chatzikonstantinou, Greece
G Cheminet, France
X Chen, China
A Chiappella, Italy
P Christoforou, Greece
G Collins, United Kingdom
A Condoluci, Switzerland
E Crisà, Italy
J Croden, Canada
N Cross, United Kingdom
N Crump, United Kingdom
M Csanadi, Hungary
S Cuartero, Spain
A Cvejic, Denmark
M D'Agostino, Italy
F Damm, Germany
A Davies, United States
S de Botton, France
L de Franceschi, Italy
S de la Iglesia, Spain
C de Larrea, Spain
P de Nully Brown, Denmark
Z DeFilipp, United States
A Degulys, Lithuania
S Delicou, Greece
M Della Porta, Italy
O Demska, Poland
T Devos, Belgium
A Di Rocco, Italy
M Dickinson, Australia
A Dimitrijevic, Denmark
M Dimopoulos, Greece
A Ditadi, Italy
M Długosz-Danecka, Poland
M Doubek, Czechia
M Dreyling, Germany
Ľ Drgoňa, Slovakia
R Duléry, France
A Duminuco, Italy
B Eichhorst, Germany
T Eipe, India

A Eiring, United States
I El Serafi, United Arab Emirates
G Enblad, Sweden
T Eyre, United Kingdom
T Facon, France
B Faganel Kotnik, Slovenia
A Fagnan, France
S Faict, Belgium
L Falchi, United States
M Farina, Italy
B Fattizzo, Italy
G Faulhaber, New Zealand
E Fermo, Italy
H Feys, Belgium
W Fibbe, The Netherlands
F Fioredda, Italy
R Fiskesund, Sweden
S Fleming, Australia
F Forconi, United Kingdom
S Forté, Canada
D Fotiou, Greece
C Fox, United Kingdom
v Frank, The Netherlands
K Freson, Belgium
E Fronkova, Czechia
S Gandhi, United Kingdom
V Garcia, Spain
S Garciaz, France
M Gavriatopoulo, Greece
E Genesca, Spain
H Ghesquières, France
A Ghiaur, Romania
K Giannopoulos, Poland
A Gimbert, Spain
E Giné, Spain
V Gjylameti Cavolli, Kosovo
S Glavey, Ireland
H Gleitz, The Netherlands
A Glenthøj, Denmark
C Glytsou, United States
S Gohil, United Kingdom
A Gołos, Poland
M Gomes da Silva, Portugal
M Gomez Alvarez, Spain
F Gonzales, Switzerland
A Gozzetti, Italy
M Griffin, United Kingdom
E Grignano, Canada
A Gullà, Italy
L Haaksma, The Netherlands
S Haebe, Germany
C Halsey, United Kingdom
E Hatjiharissi, Greece
J Hauer, Germany
O Havranek, Czechia
J Heger, Germany
S Heibl, Austria
A Heine, Austria
J Helby Petersen, Denmark
G Held, Germany
V Helgason, United Kingdom
C Herbaux, France
D Hodson, United Kingdom
I Horvat, Croatia
K Hosokawa, Japan
S Hrabovsky, Czechia
M Hultcrantz, United States
A Hundebøll Torpe, Denmark
M Hutchings, Denmark
G Iacoboni, Spain
V Iacomi, Moldova
G Illerhaus, Germany
J Infante, Portugal
I Ionita, Romania
T Ionova, Russia
M Iqbal, United States
R Irimia, Romania
S Iyengar, United Kingdom
R Jaeger, Austria
F Jardin, France
C João, Portugal
A Jonasova, Czechia
A Jurak Begonja, Croatia
W Jurczak, Poland
B Kalaja, Albania
I Kalashnikov, Finland
T Kalina, Czechia
D Karapınar, Türkiye
A Karow, Austria
I Karsten, Germany
A Kater, The Netherlands
K Keeshan, United Kingdom
W Kern, Germany
C Khadanpour, Germany
A Khan, United Kingdom
A Kittai, United States
R Klaassen, Canada
M Klimiankou, Germany
S Klobuch, The Netherlands
C Koch, Switzerland
S Koschmieder, Germany
F Köseoğlu, Türkiye
A Kramer, The Netherlands
I Krečak, Croatia
V Krishnan, Singapore
M Ku, Australia
M Kulis, Spain
K Kuo, Canada
J Kuruvilla, Canada
H Kuusanmäki, Finland
L Lanikov, Czechia
M Lauw, The Netherlands
S Lehmann, Sweden
D Lekovic, Serbia
J Lengefeld, Sweden
S Leppä, Finland
K Lewandowski, Poland
K Liang, Belgium
H Lindqvist, Sweden
P Ljungman, Sweden
C Lo Celso, United Kingdom
L Lobo de Figueiredo Pontes, Brazil
O Lohi, Finland
C Loke, United Kingdom

C Lopez, United Kingdom
Y López García, Mexico
A Lopez Guillermo, Spain
M Lordkipanidze, Canada
S Loughran, United Kingdom
M Ludvigsen, Denmark
S Luminari, Italy
M Lupo-Stanghellini, Italy
F Lussana, Italy
E Magalhaes Rego, Brazil
M Mahevas, France
A Maiques, Spain
P Malandrakis, Greece
S Manier, France
A Marin Niebla, Spain
O Marques, Portugal
M Martello, Italy
A Martín García-Sancho, Spain
I Martin-Subero, Spain
M Massaia, Italy
T Masszi, Hungary
E McCormack, Norway
H Medyouf, Germany
K Meijer, The Netherlands
M Merz, Germany
M Metzler, Germany
M Miano, Italy
M Minnema, The Netherlands
R Moia, Italy
M Morado, Spain
A Mosquera Orgueira, Spain
D Mott, United Kingdom
I Motta, Italy
C Mueller-Tidow, Germany
A Müller, Austria
A Mussetti, Spain
M Myllymäki, Finland
N Narayan, United Kingdom
R Nelles, Australia
S Nier, Switzerland
S Nier, Switzerland
M Nijland, The Netherlands
O Nnodu, Nigeria
J Nordlund, Sweden
F Onida, Italy
R Origa, Italy
M Ormhoj, Denmark
J O'Sullivan, United Kingdom
C Owen, Canada
I Pagani, Australia
L Pagano, Italy
J Panse, Germany
H Papadaki, Greece
C Papayannidis, Italy
A Pavlovsky, Argentina
E Payne, United Kingdom
Z Peric, Croatia
F Peters, Austria
A Piekarska, Poland
F Piel, United Kingdom
L Pinczes, Hungary
F Pirenne, France
M Pirosa, Switzerland
A Plesa, France
K Plevova, Czechia
I Plo, France
C Pontikoglou, Greece
R Popat, United Kingdom
K Porkka, Finland
L Pour, Czechia
I Preloznik Zupan, Slovenia
A Puissant, France
P Quarello, Italy
A Queirós, Portugal
L Quek, United Kingdom
R Rääty, Finland
M Rab, The Netherlands
J Radocha, Czechia
S Ragaini, Italy
D Rea, France
A Reinisch, Austria
M Reiterová, Czechia
R Renella, Switzerland
S Richardson, United Kingdom
L Rigacci, Italy
I Roberts, United Kingdom
P Rodriguez, Argentina
E Roldan Galvan, United Kingdom
C Röllig, Germany
A Romano, Italy
A Roque, Portugal
S Roulland, France
A Rovó, Switzerland
E Ruggiero, Italy
N Sabljic, Serbia
E Sala, United States
I Salaverria, Spain
E Salido, Spain
J Salmanton-García, Germany
V Sampath, Singapore
M Sanchez, Spain
J Sanz, Spain
A Sarmento, Portugal
B Sarni Sanches, Brazil
J Sarry, France
T Sauer, Germany
S Šaulytė Trakymienė, Lithuania
K Savage, Canada
A Schavgoulidze, France
S Scheding, Sweden
M Schemionek, Germany
C Schinke, United States
L Schmalbrock, United States
C Schneider, United States
P Schneider, United States
A Schurich, United Kingdom
V Šeda, Czechia
L Sehn, Canada
D Serruggia, Austria
C Skedgel, United Kingdom
J Skokowa, Germany
B Skopec, Slovenia
A Slomka, Poland

L Smit, The Netherlands
M Sobas, Poland
E Solomou, Greece
C So-Osman, The Netherlands
T Standal, Norway
A Stathis, Switzerland
U Steidl, United States
F Stella, Italy
M Stelljes, Germany
Y Stepanishyna, Belgium
T Stoklosa, Poland
J Stuchly, Czechia
A Sureda, Spain
M Syed, United Kingdom
M Szopa, Poland
Ł Szukalski, Poland
A Tatic, Romania
E Tausch, Germany
P te Boekhorst, The Netherlands
J Thaler, Austria
M Themeli, The Netherlands
A Theocharides, Switzerland
M Tobiasson, Sweden
D Todorieva, Bulgaria
C Tomuleasa, Romania
T Toubai, Japan
C Touzeau, France
A Trinquand, Ireland
J Trka, Czechia
C Tsioutis, Cyprus
V Turati, Sweden
A Turki, Germany
T Tzenou, Greece
A Ukmeraj, Kosovo
E van Beers, The Netherlands
M Van Duin, The Netherlands
R van Wijk, The Netherlands
G Vassiliou, United Kingdom
L Velten, Spain
P Verhamme, Belgium
K Vettenranta, Finland
A Viardot, Germany
C Visco, Italy
M von Lilienfeld-Toal, Germany
B von Tresckow, Germany
A Voskanyan, Armenia
MT Voso, Italy
E Wagner-Drouet, Germany
Y Wang, Taiwan
U Wartiovaara-Kautto, Finland
V Wauters, Argentina
T Weber, Germany
G Weiss, Austria
J Werner Hansen, Denmark
A Wierzbowska, Poland
M Wilson, United Kingdom
R Winkler, Spain
A Woelfler, Austria
P Woll, Sweden
T Wrobel, Poland
N Yasin, Malaysia
U Yilmaz, Türkiye
S Yilmaz, Türkiye
L Zamora, Spain
S Zanwar, United States
J Zaugg, Switzerland
R Zeiser, Germany
X Zhong, Sweden
J Zubicaray, Spain
A Žučenka, Lithuania
J Zuna, Czechia
S Zver, Slovenia
M Zwaan, The Netherlands eha

### Borderless Careers

### Igniting progress in hematology by enriching your professional journey

Advance your career at every stage with the European Hematology Association. Whether you're a student, an early-career professional, or a seasoned specialist, EHA offers a vibrant community and invaluable resources to support your growth. Join us to connect with peers, access cutting-edge research, and enhance your skills through workshops and events.

Our network is not just a professional association; it is a community that nurtures growth, mentorship, and innovation in a manner that transcends geographical and intellectual borders.

To us, this is Borderless Hematology.

**Find out more about our career path here!**

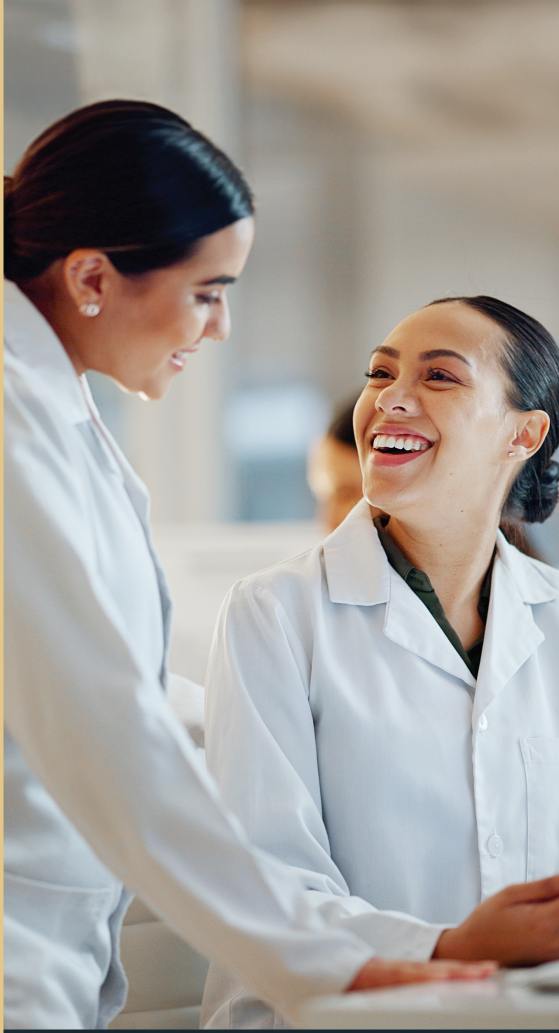

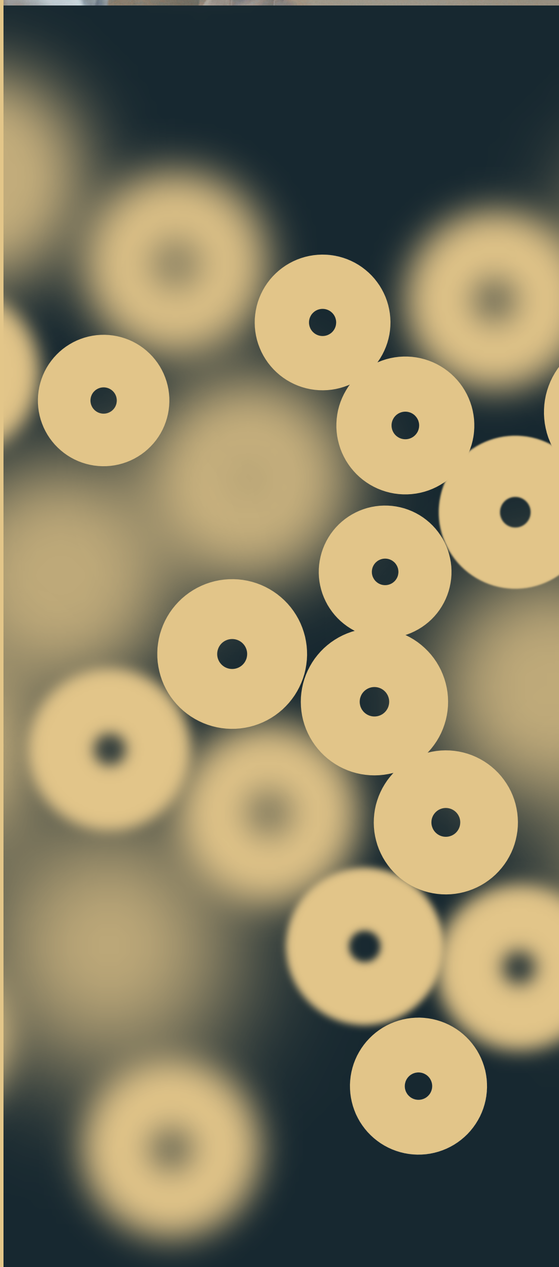

### Words of Welcome

On behalf of the EHA Board and the Scientific Program Committee, we are pleased to present to you this year's Abstract Program for the EHA2026 Congress.

We have received a record number of 4738 abstract submissions from across the globe. After a thorough and careful review process, the Scientific Program Committee has designed a comprehensive program comprising both oral and poster presentations. We are also happy to announce an increased number of Oral Sessions to accommodate the high-quality number of submissions representing all fields of hematology. The highest scoring abstracts will be presented during the Plenary Abstract Session on Saturday, June 13 from 12:00 - 13:30 CEST, one of the highlights of the Congress program.

This year we continue with the new Congress program structure  introduced last year with the scientific sessions spread out of 3,5 days (Thursday to Sunday), which also positively impacts the abstract program. This change provides you with ample opportunity to attend many of them and get updated on a broad range of topics in clinical and basic science areas.

We aim to give maximum exposure to scientific work and allow for direct interaction between the authors and the Congress delegates, so do not miss this unique opportunity to discuss and network during the Poster Sessions, which have been increased from one to two! Don't miss the opportunity to attend both on Friday and Saturday. In addition, all accepted posters can be viewed on (e)poster screens in Stockholm and on the Congress platform as of Thursday, June 11 at 09:00 CEST.

Supporting the next generation of hematologists is of the highest priority for EHA and this year we are awarding close to 300 travel grants to junior members who are presenting an abstract. In addition to the grants, EHA is again awarding Young EHA Best Abstract Awards in four categories to trainees with diverse scientific backgrounds and at different stages in their career. The names of all grant winners and awardees can be found on the next pages. EHA warmly congratulates them all.

The Late-Breaking Abstract Program is designed to provide an opportunity for investigators to submit abstracts with novel data that were not yet available at the time of the regular abstract submission deadline. Multiple abstracts of the highest scientific quality have been selected for presentation during the Late-Breaking Oral Session on Sunday, June 14 from 09:15 - 10:45 CEST, so please join us there.

All abstracts are available on the EHA Library and the Congress platform.

On behalf of the EHA Board, Scientific Program Committee & Advisory Board, all reviewers, and others involved in this year's Abstract Program, we trust you will have an interesting and inspiring congress of the European Hematology Association!

Looking forward to a lively conference in Stockholm!

Prof Meritxell Alberich Jorda
Chair Scientific Program Committee of the EHA2026 Congress

### Grant Winners

For this Congress, 300 travel grants have been awarded to junior members of EHA, based on the mean score of their abstracts as well as them being the first, submitting and presenting authors.

EHA congratulates the following grant awardees:

Abarca Rios E, Czechia
Age Kos I, Germany
Aguadé-Gorgorió J, Spain
Akhlaghi T, United States
Almabadi R, United Kingdom
Angotzi F, Italy
Appelman M, The Netherlands
Aquilina C, Italy
Argiro E, The Netherlands
Arnhardt I, Germany
Arrigo G, Italy
Bacarin F, Brazil
Baden D, Germany
Baech J, Denmark
Bähler L, Switzerland
Balantinakis A, Sweden
Barrena N, Spain
Bassani Lins De Miranda L, The Netherlands
Bataller A, United States
Becking AM, The Netherlands
Bertuglia G, Italy
Bhatnagar S, United States
Biosca C, Spain
Bock A, United States
Boldrin E, Sweden
Bonsdorff A, Finland
Booth C, United Kingdom
Boscaro E, Italy
Boudry A, France
Bouhlal J, Finland
Brieva Lopez A, Spain
Bushuieva M, Ukraine
Caduc M, Germany
Campoverde L, United States
Cantera Estefanía R, Spain
Capelletti MM, Italy
Carmody C, Ireland
Caruso M, Belgium
Cavalleri A, Italy
Ceran E, Germany
Chang YS, Taiwan
Chatzikalil E, Greece
Chatzikonstantinou T, Greece
Chen Z, United Kingdom
Cherng HJ, United States
Ciboddo M, Finland
Civettini I, Italy
Colmenero A, Spain
Consoli C, Italy
Corona L, Italy
Corsale AM, Italy
Craig R, United Kingdom
Cristóbal Vargas S, Spain
Croese K, The Netherlands
Croizier C, France
D'Addona M, Italy
Das Gupta D, Germany
de la Mata L, Spain
de Wit F, The Netherlands
Decombis S, Finland
Deyell J, United Kingdom
Doeven T, The Netherlands
Donato E, Germany
Dong M, China
Dreier T, Austria
Dulla D, Czechia
Duminuco A, Italy
Eipe T, India
Ellerington R, United Kingdom
Encinas Mayoral J, United States
Evangelidis P, Greece
Falodia R, India
Faria Zeni P, Czechia
Ferdinandus J, Germany
Fernandez-Luis S, Spain
Flight K, United Kingdom
Foo CY, United States
Foston A, United Kingdom
Galanopoulos A, Germany
Gariazzo C, Italy
Garrote-de-Barros A, Spain
Gil-Alós D, Spain
Giorgi M, United States
Globisch M, Sweden
Godard A, France
Gomez-Echarte N, Spain
Goulart H, United States
Gräßel L, Germany
Grau M, Spain
Grenier J, France
Grosu TI, Ireland
Gupta H, India
Gutierrez-Padilla C, Spain
Hamer L, United Kingdom
Hannuksela N, Finland
Herzberg F, Germany
Hoekx M, United Kingdom
Horváth R, Hungary
Htut TW, United States
Hu S, China
Hundebøll Torpe A, Denmark
Hunzeker Z, United States
Ibraheem A, United Kingdom
Idrizovic A, Spain
Jia Q, United Kingdom
Julia E, France
Kalafati E, Greece
Kamizela A, United Kingdom

Kamperman Z, The Netherlands
Kato J, Japan
Kato S, Japan
Kauer J, Germany
Kaulen LD, Germany
Ketharnathan S, Canada
Khan A, United Kingdom
Khan MA, United States
Kim R, France
Kiracı Şen B, Türkiye
Koca O, Türkiye
Koch C, Switzerland
Kolarik M, Czechia
Kolijn PM, The Netherlands
Kouroutzidou AZ, Greece
Koziel S, Germany
Kreuzbauer T, Australia
Krishnan V, United States
Laaouimir L, Belgium
Lam S, United Kingdom
Larios-Martinez KI, United Kingdom
Leonard E, Sweden
Li E, China
Li Q, China
Li S, United States
Liebaert J, Germany
Liem C, Germany
Lin F, China
Ling V, United Kingdom
Liu X, United Kingdom
Loap A, France
Löffler L, Denmark
Lui D, France
M Steiner T, Australia
MacAldaz M, Canada
MacPhee L, Canada
Maegaki M, Japan
Maeng C, Denmark
Maher N, Italy
Malik A, United Kingdom
MALJI A, India
Mandleywala K, United States
Manfreda L, Italy
Manzano-Muñoz A, Spain
Márquez-López I, Spain
Marugal MA, Spain
Marvin-Peek J, United States
Masnikov D, France
Massaar S, The Netherlands
Mato S, Spain
Mecklenbrauck R, United Kingdom
Medeiros Visentini L, Brazil
Merrion C, United Kingdom
Meskes N, The Netherlands
Mey F, Canada
Michaelou A, Czechia
Miedema SE, The Netherlands
Mikulski D, Poland
Milani M, Italy
Mönnig M, Germany
Montanaro A, Italy
Morcos MNF, Germany
Murali A, Canada
Myint PT, United States
Nasiri A, Saudi Arabia
Natani S, United Kingdom
Nikolaidou K, Greece
Nostroso A, Italy
Nyamondo K, United Kingdom
Ogłoza-Puchowska A, Poland
Ong XJ, Australia
Óskarsson JP, Iceland
Otasevic V, United States
Palmer M, United States
Palo S, Sweden
Palombella L, Switzerland
Pan M, China
Paschoudi K, Greece
Pasqualini G, Italy
Paterson J, United Kingdom
Pearce J, Germany
Pedone GL, Italy
Peek C, United States
Peng L, China
Pereira M, Portugal
Perpinello S, Italy
Perzolli A, The Netherlands
Portaluri M, Italy
Prummel K, Germany
Pula A, United States
Quero-Dotor C, Spain
Ramadani P, Canada
Ramanathan R, United States
Ramirez-Gamero A, United States
Ravattinen L, Finland
Ray G, United States
Renders S, Germany
Reuvekamp T, The Netherlands
Riccardi V, Italy
Richardson A, Iceland
Rigamonti C, Italy
RInkinen O, Finland
Rivero Equiza T, Spain
Rocha AL, Belgium
Rodriguez-Res C, Spain
Roehnert M, Germany
Rosato BE, Italy
Rosun T, United Kingdom
Roussos A, Greece
Ruiz Arenas C, Spain
Russo F, Italy
Salehi M, Switzerland
Sales L, Brazil
Sambruna E, Italy
Sánchez-Castillo A, Belgium
Scaramellini N, Italy
Schaefers C, United States
Schavgoulidze A, France
Schniederjohann C, Germany
Schönung M, Germany
Schovsbo J, Denmark
Schulze N, Germany
Seeling C, Germany
Sevim O, United Kingdom

Shah A, India
Shah P, United States
Shah R, United States
Sharif MI, Canada
Sharma s, India
Shimizu Y, Japan
Shokun N, Ukraine
Shuwaikan R, Spain
Simon F, Germany
Smits F, The Netherlands
Spadea M, Italy
Staehle H, Germany
Štefíková B, Czechia
Steinhäuser S, Germany
Stompór P, Poland
Sun R, Sweden
Swart K, The Netherlands
Szepesi G, Hungary
Tameni A, Italy
Tan M, United Kingdom
Tavakoli Shirazi P, Australia
Tavernari L, Italy
Tharmaseelan H, Germany
Toho M, Japan
Tseng TC, Taiwan
Turkulainen E, Finland
Ullahammer WM, Denmark
Urizar-Compains E, Spain
Vallvé i Oliva M, Spain
Van den Bergh S, Belgium
van der Velde J, The Netherlands
Van Trimpont M, Belgium
Vasileiou E, Germany
Vedenjuoksu J, Finland
Veltmaat N, The Netherlands
Vodicka P, Czechia
Voigt S, Germany
Wan H, Sweden
Wang S, China
Wang W, China
Wannberg F, Sweden
Wei Y, China
Weinhäuser I, The Netherlands
Wencel J, France
Wiechers L, Germany
Wikström F, Sweden
Yamamoto Y, Japan
Yami A, Italy
YANG M, Japan
Yuan Y, Australia
Zaratiegui A, Spain
Zeng A, Canada
Zhang F, China
Zhang X, China
Zhu J, Finland
Zhu J, United Kingdom
Zhu Y, United Kingdom
Žučenka A, Lithuania
Zugasti I, Spain
Zuleeg MR, Germany
Zwolsman M, The Netherlands

### Young EHA Best Abstract Awards

One of the primary missions of the European Hematology Association is to support young hematology clinicians and researchers. For the fourth year after launching in 2017, we are proud to announce the Young EHA Best Abstract Awards for 2024. These are awarded to the highest-ranking abstracts in the following four categories: Clinicians or medical students training for a PhD degree, PhD research students, postdoctoral fellows, and clinical hematology trainees. We are honored that these outstanding Young EHA trainees will be presenting during the EHA Congress - they are the future of hematology!

### CLINICIANS OR MEDICAL STUDENTS TRAINING FOR A PHD DEGREE
W Wang (China)

### POSTDOCTORAL FELLOWS
C Brieghel (Denmark)

### CLINICAL HEMATOLOGY TRAINEES
A Zhang (China)

### PHD RESEARCH STUDENTS
H Bergugnat (France)

abbvie
Join Us
as we share our commitment and passion in treating patients with hematologic malignancies
Visit the AbbVie booth B2.03 in Hall C
Don’t miss our AML Product Theater on
Friday, June 12, 09:35 – 09:55
EHA Theater in Hall C
abbvie.com
ALL-ONC-260008 V1.0 Approved 3/2026

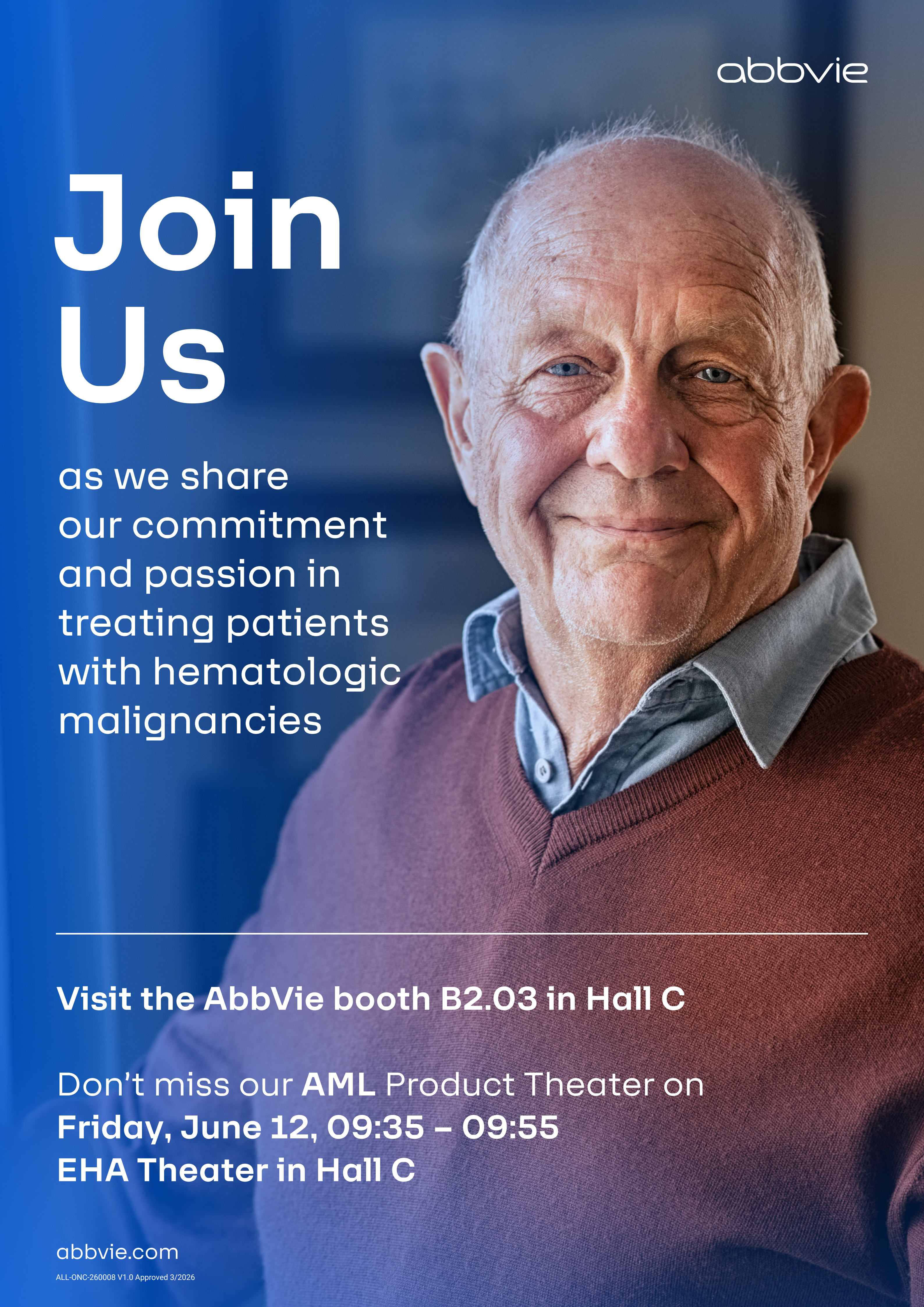

### COME SEE WHAT'S NEW IN MULTIPLE MYELOMA

Stop by the Sanofi booth to learn more sanofi

©2026 Sanofi-aventis Groupe. All rights reserved. Sanofi is a registered trademark of Sanofi or an affiliate.
MAT-GLB-2601772-v2.0-04/2026

### Have questions about multiple myeloma or myelofibrosis treatment options for your patients?

### Visit GSK Booth #E3/F3

Trademarks are owned by or licensed to the GSK group of companies.
©2026 GSK or licensor.
NP-GBL-MML-BNNR-260001 April 2026

BeOne
It's time to push the limits
of what BCL-2 inhibition can achieve
BeOne is unlocking the true power of BCL-2 inhibition with a more comprehensive development approach.
Speak with a representative at Booth G2.01
Redefine your expectations
Visit BCL2i.com
BCL-2=B-cell lymphoma-2.
BeOne Medicines is a trademark owned by BeOne Medicines I GmbH or its affiliates.
© BeOne Medicines I GmbH, 2026 All Rights Reserved. 0426-NP-PRC-077 6/2026

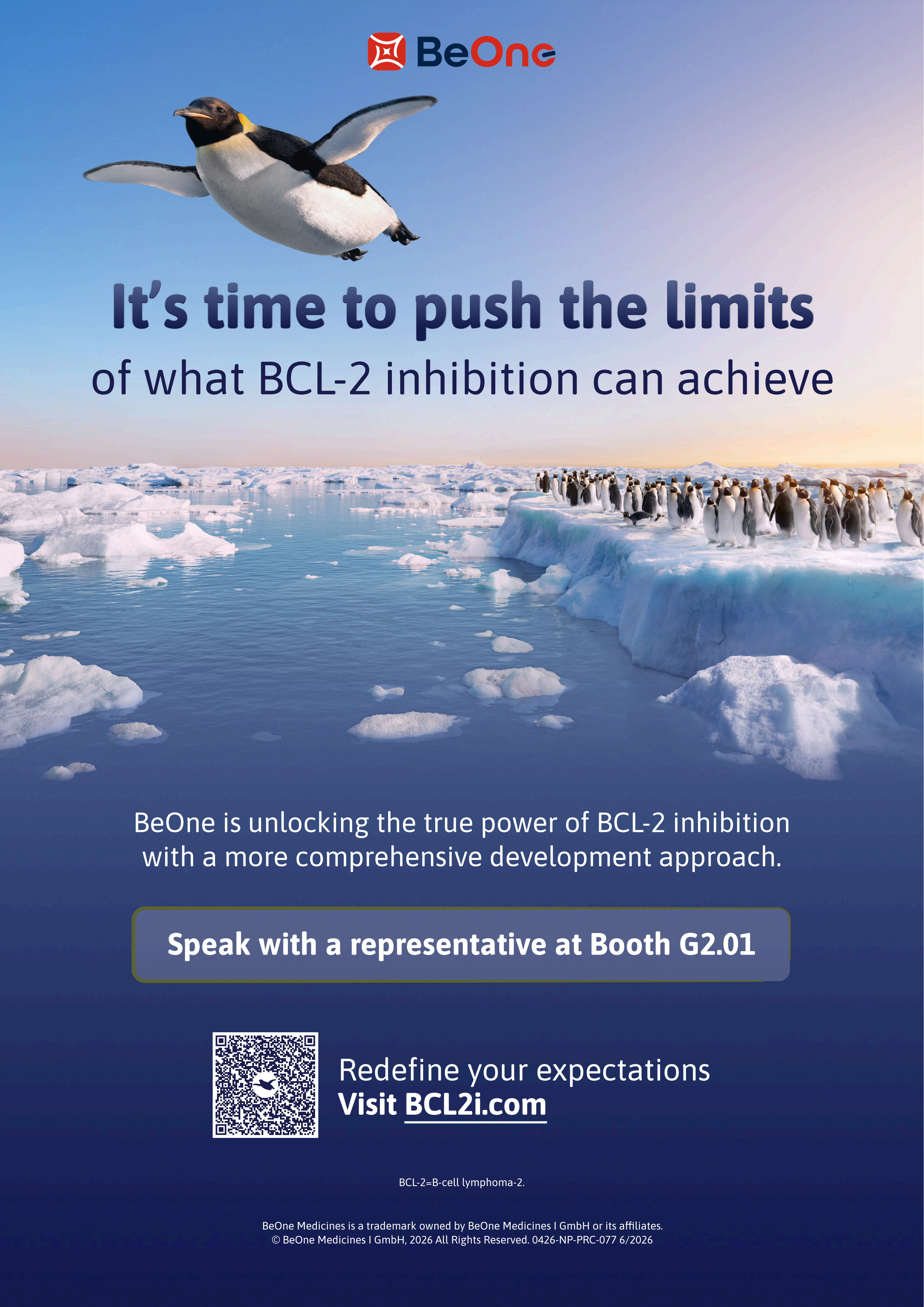

Syndax

### THE LEADER IN
### MENIN CLINICAL RESEARCH

REVEAL
ND NPM1

Revumenib Evaluation in Acute Leukemia (REVEAL-ND NPM1)

A global, actively recruiting phase 3 clinical trial investigating revumenib in combination with intensive chemotherapy for the treatment of patients aged 12 years and older with newly diagnosed AML with an *NPM1* mutation

This study is sponsored by Syndax Pharmaceuticals, Inc.

### Learn more about REVEAL-ND NPM1

**RevealTrialAML.com**

**revealsiteinquiries@syndax.com**
Please do not include any identifiable patient information in your email.
Note: Your message will be directed to a US-based email inbox.

---

### EVOLVE-2

A global, actively recruiting phase 3 clinical trial of revumenib in combination with azacitidine + venetoclax in adult patients with newly diagnosed *NPM1*-mutated or *KMT2A*-rearranged AML ineligible for intensive chemotherapy

This study is sponsored by HOVON, conducted by HOVON, AMLSG, UKAMLRN, ALFA, FILO, CETLAM, GIMEMA, ALLG, SAKK, Nordic AML Group, and the Beat AML Consortium, in collaboration with and funded by Syndax.

### Learn more about EVOLVE-2

**EUClinicalTrials.EU**
2024-512733-32-00

**HO177@erasmusmc.nl**
Please do not include any identifiable patient information in your email.
Note: Your message will be directed to a non-US-based email inbox.

VISIT US AT **BOOTH #A4.06**

Use of revumenib in this setting is under investigation and has not been approved by any regulatory authority worldwide for the uses under investigation.

This material is for scientific purposes only and is intended solely for healthcare practitioners.
Patient enrollment is dependent on approval by local ethics committees.

AML, acute myeloid leukemia; *KMT2A*, lysine methyltransferase 2A; *NPM1*, nucleophosmin 1.

© 2026 Syndax Pharmaceuticals, Inc. All rights reserved. The Syndax logo is a registered trademark of Syndax. All other referenced trademarks are the property of their respective owners.
MED-REV-260016 5/2026

Learn more at
**syndaxmedical.com**

### B.H.D.M

BHDM is a **global initiative confronting inequality in healthcare, research and treatment** access for Black communities affected by underfunded conditions like **Sickle Cell Disease**.
Through research, advocacy and storytelling, **we challenge a system where health innovation follows profit instead of need** — pushing to make equitable healthcare impossible to ignore.

DATA.
DIGNITY.
CHANGE.
NOT OPTIONAL.

**20+ MILLION**
people worldwide live with Sickle Cell Disease.

**<1%**
of NIH funding for SCD compared to diseases with lower mortality.

**MILLIONS**
lack access to adequate care, treatment and safe infrastructure.

### OUR CAMPAIGN

**REPURPOSING.
INNOVATING.
SAVING LIVES.**

We are advancing research on an affordable NMDA receptor antagonist to help reduce pain crises and hospitalizations in Sickle Cell Disease.

Our goal: advance a global Phase 3 trial and improve treatment accessibility worldwide.

### JOIN the movement

Where research meets action. Join the conversation on advancing toward a Global Phase 3 Trial

### READ the research

Find the evidence recently published in **HemaSphere**, 2026, Jan 11; 10 (1): e70278

### WATCH the documentary

Discover the human stories behind the data. A film about resilience, inequity and hope.

EQUITABLE HEALTHCARE SHOULDN'T BE RADICAL

Click for more!

**SCAN NOW**
**to discover what's missing from the conversation**

### Table of Contents

### Publication Only

| | |
|---|---|
| 01. Acute lymphoblastic leukemia - Biology & translational research | PB2538-PB2579 |
| 02. Acute lymphoblastic leukemia - Clinical | PB2581-PB2655 |
| 03. Acute myeloid leukemia - Biology & translational research | PB2656-PB2726 |
| 04. Acute myeloid leukemia - Clinical | PB2727-PB2861 |
| 05. Chronic lymphocytic leukemia and related disorders - Biology & translational research | PB2862-PB2886 |
| 06. Chronic lymphocytic leukemia and related disorders - Clinical | PB2887-PB2973 |
| 07. Chronic myeloid leukemia - Biology & translational research | PB2975-PB2981 |
| 08. Chronic myeloid leukemia - Clinical | PB2982-PB3050 |
| 09. Myelodysplastic syndromes - Biology & translational research | PB3051-PB3074 |
| 10. Myelodysplastic syndromes - Clinical | PB3075-PB3112 |
| 11. Bone marrow failure syndromes incl. PNH - Biology & translational research | PB3113-PB3127 |
| 12. Bone marrow failure syndromes incl. PNH - Clinical | PB2535-PB3172 |
| 13. Myeloma and other monoclonal gammopathies - Biology & translational research | PB3173-PB3222 |
| 14. Myeloma and other monoclonal gammopathies - Clinical | PB3223-PB3388 |
| 15. Myeloproliferative neoplasms - Biology & translational research | PB3389-PB3414 |
| 16. Myeloproliferative neoplasms - Clinical | PB3415-PB3554 |
| 17. Hodgkin lymphoma - Clinical | PB3556-PB3598 |
| 18. Indolent and mantle-cell non-Hodgkin lymphoma - Clinical | PB3599-PB3699 |
| 19. Large B cell lymphomas - Clinical | PB3700-PB3839 |
| 20. Other aggressive lymphomas | PB3840-PB3911 |
| 21. Lymphoma biology & translational research | PB3912-PB3986 |
| 22. Stem cell transplantation - Experimental | PB3987-PB3991 |
| 23. Stem cell transplantation - Clinical | PB3992-PB4078 |
| 24. Hematopoiesis, stem cells and microenvironment | PB4079-PB4096 |
| 25. Gene therapy, cellular immunotherapy and vaccination - Biology & translational research | PB2536-PB4104 |
| 26. Gene therapy, cellular immunotherapy and vaccination - Clinical | PB4106-PB4129 |
| 27. Sickle cell disease | PB4130-PB4159 |
| 28. Thalassemias | PB4160-PB4182 |
| 29. Enzymopathies, membranopathies and other red blood cell disorders | PB4183-PB4214 |
| 30. Iron metabolism, deficiency and overload | PB4215-PB4224 |
| 31. Infections in hematology (incl. supportive care/therapy) | PB4225-PB4266 |
| 32. Transfusion medicine | PB4267-PB4572 |
| 33. Platelet disorders | PB4282-PB4321 |
| 34. Bleeding disorders (congenital and acquired) | PB4322-PB4358 |
| 35. Thrombosis and vascular biology | PB4359-PB4402 |
| 36. Quality of life, ethics, supportive and palliative care | PB4403-PB4464 |
| 37. Health economics | PB4465-PB4475 |
| 38. Novel technologies, techniques and digital analytical tools in hematology | PB4476-PB4567 |

### (PB2538) A DOMINANT NATIONAL DIETARY PATTERN SHAPES GLOBAL VARIATION IN ACUTE MYELOID LEUKEMIA–RELATED DISABILITY BURDEN

**Topic:** 01. Acute lymphoblastic leukemia - Biology & translational research

Diguang Wen*[1]

*[1]The Second Affiliated Hospital of Chongqing Medical University, Chongqing, China;*

### Background

Acute myeloid leukemia (AML) exhibits marked international variation in disability burden. While intrinsic genetic heterogeneity has been extensively characterized, population-level metabolic environments that may influence leukemogenesis remain insufficiently explored.

### Aims

To identify dominant national dietary patterns associated with AML-related disability-adjusted life years (DALYs) and evaluate their age-dependent effects.

### Methods

Country-level dietary exposure data (1990–2018) from the Global Dietary Database were integrated with AML-related DALYs from the Global Burden of Disease study. After excluding variables with >15% missingness, 41 dietary factors were retained. Associations between individual dietary exposures and AML-related DALYs were assessed using generalized linear mixed models (GLMMs). Dietary variables demonstrating statistically robust and biologically plausible associations were incorporated into principal component analysis (PCA) to derive dominant dietary patterns. Associations between principal components and AML-related DALYs were evaluated using GLMMs with country and age group specified as random effects. Temporal trends and projections to 2030 were estimated using Bayesian hierarchical modeling.

### Results

Twenty-six dietary factors were associated with AML-related DALYs in single-exposure analyses. The first principal component (PC1) explained the largest variance and represented a dietary pattern characterized by higher total carbohydrate intake and lower intake of saturated fat, animal protein, and high-fat dairy products. Higher PC1 scores were associated with lower AML-related DALYs (RR per 100-point increase = 0.80; 95% CI 0.50–1.27). Notably, the protective association was substantially stronger among adults ≥60 years (RR = 0.30; 95% CI 0.16–0.56), suggesting cumulative metabolic exposure may influence AML burden in aging populations.

### Summary/Conclusion

A dominant dietary pattern is associated with global variation in AML-related disability burden, with age-dependent effects. These findings highlight the potential role of long-term metabolic environments in shaping population-level AML risk.

**Copyright Information:** (Online) ISSN: 2572-9241
©2026 The Author(s). HemaSphere is published by John Wiley & Sons Ltd on behalf of European Hematology Association. This is an open access Abstract Book distributed under the Attribution-NonCommercial-NoDerivs (CC BY-NC-ND), which allows third parties to download the articles and share them with others as long as they credit the author and the Abstract Book, but they cannot change the content in any way or use them commercially.
**Abstract Book Citations:** Authors, Title, HemaSphere, 2026;10:(S1):pages. The abstract book DOIs can be found on the “Author Information” page.
**Disclaimer:** Articles published in the journal HemaSphere exclusively reflect the opinions of the authors. The authors are responsible for all content in their abstracts including accuracy of the facts, statements, citing resources, etc.

### (PB2539) THERAPEUTIC EFFECT OF TARGETING CASEIN KINASE II / PLK1 ONCOGENIC SIGNALING IN T-CELL ACUTE LYMPHOBLASTIC LEUKEMIA

**Topic:** 01. Acute lymphoblastic leukemia - Biology & translational research

Qi Han[1], Tong Wu[1], Sinisa Dovat[2], Chunhua Song[3], Zheng Ge*[1]

*[1]Zhongda Hospital Southeast University, Institute of Hematology Southeast University, Department of Hematology, Nanjing, China; [2]Division of Hematology and Oncology, Department of Pediatrics, Hershey Medical Center, Pennsylvania State University Medical College, Hershey, United States of America; [3]Division of Hematology, The Ohio State University Wexner Medical Center, The James Cancer Hospital, Columbus, United States of America;*

### Background

T-cell acute lymphoblastic leukemia (T-ALL) is an aggressive hematological malignancy with high recurrence and poor prognosis. PLK1 is a crucial cell cycle regulator often overexpressed in cancer, which drives uncontrolled proliferation by promoting mitotic progression, making it a significant therapeutic target for drug development. We reported that targeting the Casein Kinase II (CK2)/Ikaros axis by CK2 inhibitor CX-4945 demonstrated the therapeutic efficacy in T-ALL. However, the synergy of the PLK1 inhibitor with CX-4945 in T-ALL remains unknown.

### Aims

To investigate the synergistic effect of the combination of PLK1 inhibitor Volasertib and CK2 inhibitor CX-4945 in T-ALL.

### Methods

RNA-seq was performed for CEM cells treated with CX-4945 and the vehicle control for 48 hours. CCK-8 assay was performed to analyze the synergistic effect of Volasertib combined with CX-4945 in T-ALL cell lines and the patient samples. Luciferase assay was conducted to verify the regulation of PLK1 by IKAROS. Patient-derived xenograft (PDX) mouse models were used to explore the synergistic effect of Volasertib and CX-4945 in T-ALL.

### Results

*PLK1* expression is significantly downregulated by the CK2 inhibitor CX-4945 in CEM cells (Fig. 1A). QPCR confirmed that the expression of *PLK1* is significantly reduced upon CX-4945 treatment in T-ALL patient samples ($P$<0.001) (Fig. 1B). IKAROS significantly reduced the promoter activity of PLK1 ($P$<0.001) (Fig. 1C). CX-4945 combined PLK1 inhibitor Volasertib exhibited more significant effect on cell proliferation arrest in T-ALL samples compared to either single drug controls ($P$<0.001) (Fig. 1D); and Calcusyn analysis indicated that the significant synergistic effect (Fig. 1E). Furthermore, the T-ALL PDX mouse model was established and treated with vehicle control, Volasertib alone, CX-4945 alone, or the combination. Results showed that the combination of Volasertib and CX-4945 significantly reduced the spleen sizes (Fig. 1F), spleen weights ($P$<0.001) (Fig. 1G), % of human $CD45^{+}CD7^{+}$ T-ALL cells in the mouse spleens ($P$<0.001) (Fig. 1H), bone marrow ($P$<0.001) (Fig. 1I) and significantly prolonged mouse survival ($P$<0.001) (Fig. 1J) compared with either single drug controls.

### Summary/Conclusion

Our results indicated that Volasertib and CX-4945 exhibit synergistic efficacy in T-ALL by inhibiting the oncogenic CK2-PLK1 signaling pathway. Dual targeting PLK1 with CK2 may be a potential therapeutic option for T-ALL.

**Copyright Information:** (Online) ISSN: 2572-9241
©2026 The Author(s). HemaSphere is published by John Wiley & Sons Ltd on behalf of European Hematology Association. This is an open access Abstract Book distributed under the Attribution-NonCommercial-NoDerivs (CC BY-NC-ND), which allows third parties to download the articles and share them with others as long as they credit the author and the Abstract Book, but they cannot change the content in any way or use them commercially.
**Abstract Book Citations:** Authors, Title, HemaSphere, 2026;10:(S1):pages. The abstract book DOIs can be found on the “Author Information” page.
**Disclaimer:** Articles published in the journal HemaSphere exclusively reflect the opinions of the authors. The authors are responsible for all content in their abstracts including accuracy of the facts, statements, citing resources, etc.

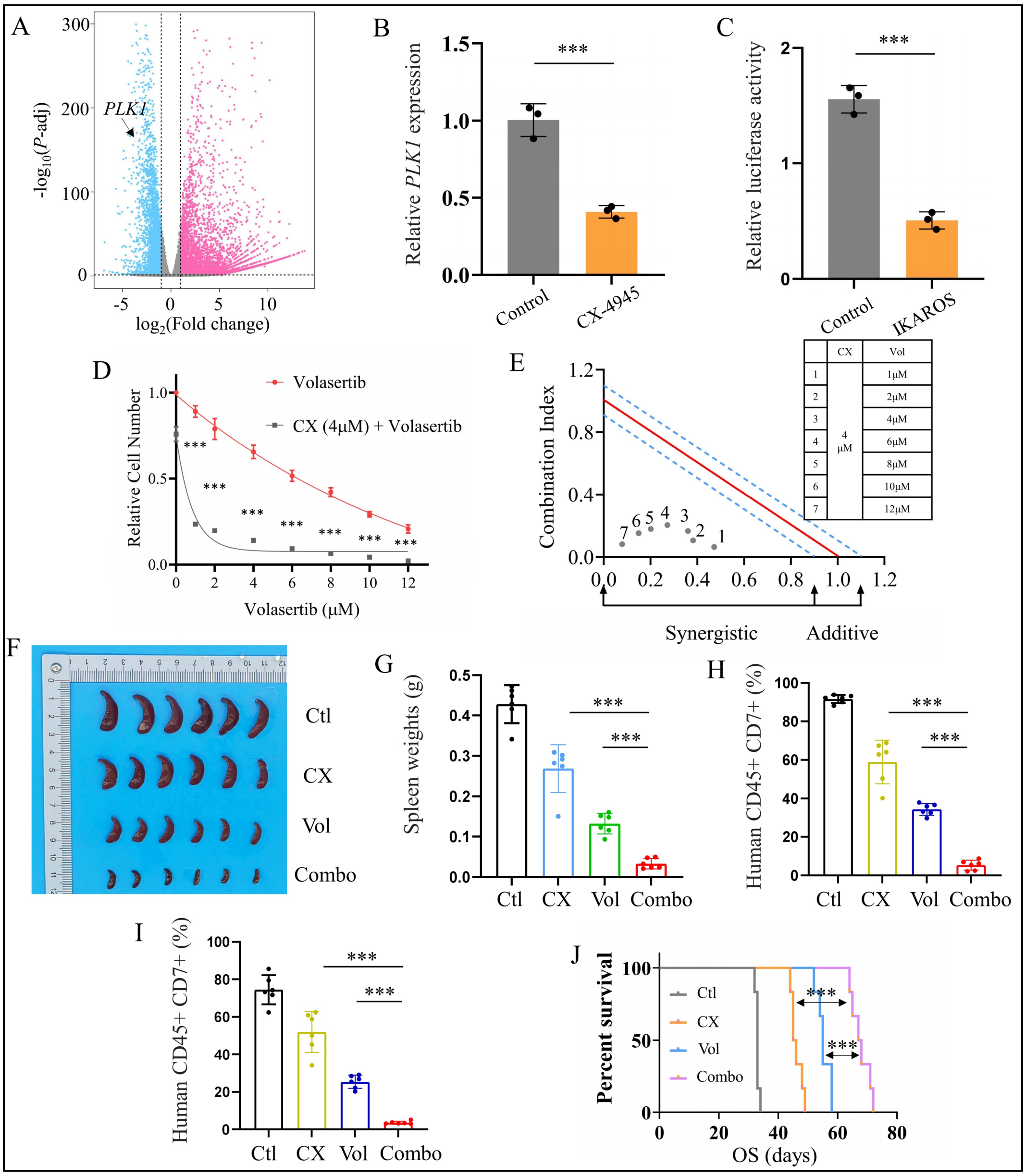

Fig 1 Therapeutic effect of targeting Casein Kinase II / PLK1 axis in T-cell Acute Lymphoblastic Leukemia. (A) Volcano plot of differential expressed genes after CEM cells treated with CX-4945 for 48 hours. (B) Expression of PLK1 in mRNA level after T-ALL patient primary cells treated with CX-4945. (C) The impact of IKAROS on PLK1 promotor activity by luciferase. (D-E) Effect (D) and synergistic analysis (E) of the combination of CX-4945 with various doses of Volasertib on cell proliferation in T-ALL patient primary cells. (F-J) Spleen image (F), Spleen weights (G), % of human CD45 and CD7 positive T-ALL cells in the spleens (H) and bone morrow (I) and the survival (J) after T-ALL patient derived mouse model treated with Volasertib, CX-4945 and the combination for 28 days. *** P<0.001.

**Copyright Information:** (Online) ISSN: 2572-9241
©2026 The Author(s). HemaSphere is published by John Wiley & Sons Ltd on behalf of European Hematology Association. This is an open access Abstract Book distributed under the Attribution-NonCommercial-NoDerivs (CC BY-NC-ND), which allows third parties to download the articles and share them with others as long as they credit the author and the Abstract Book, but they cannot change the content in any way or use them commercially.
**Abstract Book Citations:** Authors, Title, HemaSphere, 2026;10:(S1):pages. The abstract book DOIs can be found on the "Author Information" page.
**Disclaimer:** Articles published in the journal HemaSphere exclusively reflect the opinions of the authors. The authors are responsible for all content in their abstracts including accuracy of the facts, statements, citing resources, etc.

### (PB2540) SYNERGISTIC THERAPEUTIC EFFICACY OF CDK9 INHIBITOR WITH CX-4945 BY REPRESSING ASNS TRANSCRIPTION IN HIGH-RISK B-ALL

**Topic:** 01. Acute lymphoblastic leukemia - Biology & translational research

Huimin Xiang[1], Qi Han[1], Chunhua Song[2], Zheng Ge*[1]

*[1]Zhongda Hospital Southeast University, Institute of Hematology Southeast University, Department of Hematology, Nanjing, China; [2]Division of Hematology, The Ohio State University Wexner Medical Center, The James Cancer Hospital, Columbus, United States of America;*

### Background

Acute lymphoblastic leukemia (ALL) is caused by the clonal proliferation of lymphoid stem cells or progenitor cells, with more than 80% originating from B-cell progenitors (B-ALL). As a member of the cyclin-dependent kinase family, CDK9 plays an important role in the occurrence and development of leukemia. CDKI-73, as a new highly selective CDK9 inhibitor, has been shown to exhibit antitumor effects in hematologic malignancies. CX-4945, a selective CK2 inhibitor, has shown strong CK2 activity inhibition as well as anti-leukemia efficacy in both in vivo and in vitro experiments. However, it remains unclear whether the combination of the two drugs exerts a synergistic anti-tumor effect.

### Aims

To investigate the synergistic effect and the underlying mechanism of CX-4945 combined with CDKI-73 in B-ALL.

### Methods

CCK-8 cell proliferation assay, Annexin V apoptosis assay, and colony formation assay were performed in B-ALL cells in the presence of CX-4945 only, CDKI-73 only, CX-4945 + CDKI-73, and vehicle control for 48h. Gene knockdown was performed by lentiviral shRNA. Student *T-test* was employed for statistical analysis to determine the significant difference between groups. A synergistic effect was determined by the combination indices (CIs) with CalcuSyn software. The B-ALL patient-derived xenograft (PDX) mouse models were applied for in vivo assay.

### Results

Treatment of CDKI-73 significantly results in cell proliferation arrest of Nalm6 cells in a dose-dependent manner. The combination of various doses of CX-4945 with 1/2IC50 of CDKI-73 significantly decreases the cell viability of the cells *versus* single-drug control, and CalcuSyn analysis showed the significant synergistic effects of the two drugs (**Fig.1A-B**). CDKI-73 increased the % apoptotic cells in a dose-dependent manner, and the combination also significantly increased the % apoptotic cells compared to the single-drug controls in the Nalm6 cells (P<.001 or .01, **Fig.1C-D**). Moreover, the combination produced significantly less colony-forming *vs* either single drug in Nalm6 cells (**Fig.1E-F**). The combination significantly decreases the cell viability and apoptosis in the patient samples compared with single-drug controls; CalcuSyn analysis showed the synergistic effects of the two drugs on the proliferation arrest (**Fig.1G-H**). Moreover, the combination significantly reduced the spleen size, decreased % $hCD45^{+}hCD19^{+}$ cells in the spleen and bone marrow, and prolonged the survival of the B-ALL PDX mouse models (**Fig.1I-L**). To further understand the underlying mechanism of the synergy, RNA-seq analysis was performed in Nalm6 cells treated with either CX-4945 or CDKI-73 *vs* vehicle control, respectively. The overlapped altered DEGs were identified, and asparagine synthetase (ASNS) is one of the top DEGs; its mRNA level is dramatically down-regulated. The combination of the two drugs *vs* either single drug controls significantly suppressed the *ASNS* expression in Nalm6 cells (P<0.001,**Fig.1M-N**). Moreover, *ASNS* knockdown significantly suppresses the proliferation of Nalm6 cells (P<0.001, **Fig.1O**).

**Copyright Information:** (Online) ISSN: 2572-9241
©2026 The Author(s). HemaSphere is published by John Wiley & Sons Ltd on behalf of European Hematology Association. This is an open access Abstract Book distributed under the Attribution-NonCommercial-NoDerivs (CC BY-NC-ND), which allows third parties to download the articles and share them with others as long as they credit the author and the Abstract Book, but they cannot change the content in any way or use them commercially.
**Abstract Book Citations:** Authors, Title, HemaSphere, 2026;10:(S1):pages. The abstract book DOIs can be found on the “Author Information” page.
**Disclaimer:** Articles published in the journal HemaSphere exclusively reflect the opinions of the authors. The authors are responsible for all content in their abstracts including accuracy of the facts, statements, citing resources, etc.

**Summary/Conclusion**

Combination of CX-4945 with CDKI-73 has a synergistic efficacy in B-ALL cells by down-regulation of *ASNS* in B-ALL. Our data provide experimental evidence for the combination as a potential option for high-risk B-ALL therapy.

**Copyright Information:** (Online) ISSN: 2572-9241
©2026 The Author(s). HemaSphere is published by John Wiley & Sons Ltd on behalf of European Hematology Association. This is an open access Abstract Book distributed under the Attribution-NonCommercial-NoDerivs (CC BY-NC-ND), which allows third parties to download the articles and share them with others as long as they credit the author and the Abstract Book, but they cannot change the content in any way or use them commercially.
**Abstract Book Citations:** Authors, Title, HemaSphere, 2026;10:(S1):pages. The abstract book DOIs can be found on the "Author Information" page.
**Disclaimer:** Articles published in the journal HemaSphere exclusively reflect the opinions of the authors. The authors are responsible for all content in their abstracts including accuracy of the facts, statements, citing resources, etc.

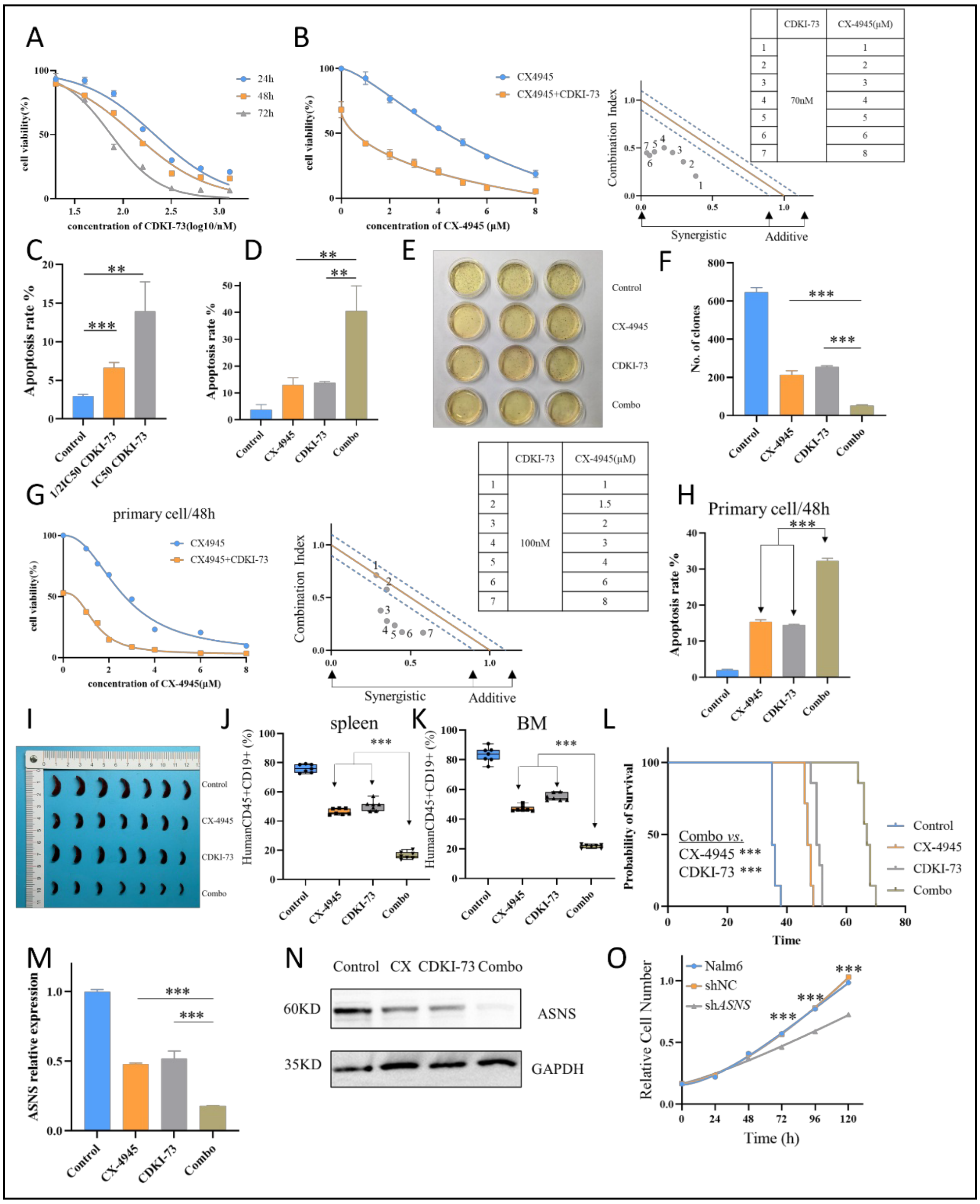

Fig. 1 Synergy of CX-4945 with CDKI-73 on therapeutic efficacy by repressing ASNS on transcription in high-risk B-ALL (A-B) The cell proliferation arrest effect of CDKI-73 alone with gradient concentration for 24, 48, and 72h in Nalm6 cells (A). Effect of the combination of CX-4945 and CDKI-73 vs single drug on cell proliferation and the synergistic analysis of the combination in Nalm6 cells(B). (C-D) The statistic histogram of apoptosis in Nalm6 cells treated by CDKI-73 alone with ½ IC50 and IC50 concentration for 48h. The statistic histogram of apoptosis in Nalm6 cells that were treated with CX-4945, CDKI-73 alone, and two drugs combination for 48h (D). (E-F) Effect of the combination of CX-4945 and CDKI-73 vs single drug on colony-forming and the statistic histogram of colony-forming in Nalm6 cells. (G) Effect and synergistic analysis of the combination of CX-4945 and CDKI-73 vs single drug on cell proliferation in B-ALL patient primary cells. (H) The statistic histogram of apoptosis in B-ALL patient primary cells that were treated with CX-4945, CDKI-73 alone, and two drugs combination for 48h. (I) Spleen image of 4 groups of mice post-treatment period. (J-K) The percentage of hCD45+ hCD19+ cells in the spleen (J) and bone marrow (K) from 4 groups of mice post-treatment period. (L) Comparison of Kaplan–Meier survival curves in the combination of CX-4945 with CDKI-73 compared with either single drug controls. (M-N) The expression of ASNS in Nalm6 cells treated with CX-4945 alone, CDKI-73 alone, and two drugs combination for 48 h detected by RT-qPCR (M) and western blot (N). (O) Effect of ASNS knockdown versus shNC on cell proliferation in Nalm6 cells.

**Copyright Information:** (Online) ISSN: 2572-9241
©2026 The Author(s). HemaSphere is published by John Wiley & Sons Ltd on behalf of European Hematology Association. This is an open access Abstract Book distributed under the Attribution-NonCommercial-NoDerivs (CC BY-NC-ND), which allows third parties to download the articles and share them with others as long as they credit the author and the Abstract Book, but they cannot change the content in any way or use them commercially.
**Abstract Book Citations:** Authors, Title, HemaSphere, 2026;10:(S1):pages. The abstract book DOIs can be found on the "Author Information" page.
**Disclaimer:** Articles published in the journal HemaSphere exclusively reflect the opinions of the authors. The authors are responsible for all content in their abstracts including accuracy of the facts, statements, citing resources, etc.

### (PB2541) ONCOGENIC ROLE AND DRUG SENSITIVITY OF A NEW IKZF1 MUTATION IN B-CELL ACUTE LYMPHOBLASTIC LEUKEMIA

**Topic:** 01. Acute lymphoblastic leukemia - Biology & translational research

Huimin Xiang[1], Qi Han[1], Chunhua Song[2], Zheng Ge*[1]

*[1]Zhongda Hospital Southeast University, Institute of Hematology Southeast University, Department of Hematology, Nanjing, China; [2]Division of Hematology, The Ohio State University Wexner Medical Center, The James Cancer Hospital, Columbus, United States of America;*

### Background

The *IKZF1* gene encoded IKAROS protein, a zinc finger transcription factor, plays a crucial role in the differentiation and development of lymphocytes. Deletion /mutation of the *IKZF1* gene, as a hallmark of high-risk B-ALL, leads to IKAROS loss of function and is associated with poor prognosis in high-risk B-cell acute lymphoblastic leukemia (B-ALL). We have identified a novel *IKZF1* mutation (c.643T>C: p.S215P) in a patient who exhibited treatment resistance and a poor prognosis. The underlying molecular mechanism of this mutation and drug sensitivity remains to be further elucidated.

### Aims

To investigate the oncogenic role of the new *IKZF1* mutation (c.643T>C: p.S215P) and the sensitivity of CK2 inhibitor CX-4945 and HDAC inhibitor Chidamide to B-ALL with the mutation.

### Methods

Nalm6 cells with vector only, $IKZF1^{WT}$ and $IKZF1^{S215P}$ were established by lentiviral transduction. CCK-8 cell proliferation assay, Annexin V apoptosis assay, and colony formation assay were performed in the Nalm6-vector only, $IKZF1^{WT}$ and $IKZF1^{S215P}$ cells for 48 hours. The synergistic effect was determined by the combination indices (CIs) with CalcuSyn software. Human leukemia cell-derived xenograft (CDX) mouse models were established by using Nalm6-vector only, $IKZF1^{WT}$ and $IKZF1^{S215P}$ cells.

### Results

Lentiviral IKZF1 wild type ($IKZF1^{WT}$), $IKZF1^{S215P}$ mutant were successfully transduced into Nalm6 cells with comparable expression levels (**Fig.1A**). The cell proliferation, apoptosis, and colony-forming assay showed that $IKZF1^{S215P}$ significantly promotes cell proliferation (P<0.05, **Fig.1B**), inhibits apoptosis (P<0.001, **Fig.1C**), and suppresses colony-forming (P<0.01, **Fig.1D, 1E**). In vivo CDX experiments demonstrated that $IKZF1^{WT}$ reduced the spleen size and weight, as well as % $mCD45^{-}hCD19^{+}$ cells in both the spleen and bone marrow; while the $IKZF1^{S215P}$ mutant increased spleen size (**Fig.1F**) and weight (P<0.001, **Fig.1G**), along with an elevated % $mCD45^{-}hCD19^{+}$ cells in the spleen (P<0.001, **Fig.1H**) and bone marrow (P<0.001, **Fig.1I**). We found that the combination of CX-4945 and Chidamide also has a synergistic effect on the proliferation (P<0.001, **Fig.1J**) and apoptosis (P<0.001, **Fig.1K**) in $IKZF1^{S215P}$ mutant cells. Moreover, the expression of TCL1A, a crucial coactivator of AKT signaling, is significantly up-regulated in $IKZF1^{S215P}$ mutant cells compared with vector-only control (P<0.001, **Fig.1L**). CX-4945 or Chidamide down-regulates *TCL1A* expression in $IKZF1^{S215P}$ mutant cells, and the reduction is more significant in the combination of CX-4945 and Chidamide compared with either single drug alone (P<0.001, **Fig.1M**).

**Copyright Information:** (Online) ISSN: 2572-9241
©2026 The Author(s). HemaSphere is published by John Wiley & Sons Ltd on behalf of European Hematology Association. This is an open access Abstract Book distributed under the Attribution-NonCommercial-NoDerivs (CC BY-NC-ND), which allows third parties to download the articles and share them with others as long as they credit the author and the Abstract Book, but they cannot change the content in any way or use them commercially.
**Abstract Book Citations:** Authors, Title, HemaSphere, 2026;10:(S1):pages. The abstract book DOIs can be found on the "Author Information" page.
**Disclaimer:** Articles published in the journal HemaSphere exclusively reflect the opinions of the authors. The authors are responsible for all content in their abstracts including accuracy of the facts, statements, citing resources, etc.

**Summary/Conclusion**

Our results indicate that the IKZF1$^{S215P}$ mutation promotes oncogenesis in B-ALL. The combination of CX-4945 with Chidamide demonstrates potent synergistic efficacy by suppressing TCL1A expression in IKZF1$^{S215P}$ mutant cells. This study supports the combination therapy as a promising strategy for high-risk IKZF1-mutated B-ALL.

**Copyright Information:** (Online) ISSN: 2572-9241
©2026 The Author(s). HemaSphere is published by John Wiley & Sons Ltd on behalf of European Hematology Association. This is an open access Abstract Book distributed under the Attribution-NonCommercial-NoDerivs (CC BY-NC-ND), which allows third parties to download the articles and share them with others as long as they credit the author and the Abstract Book, but they cannot change the content in any way or use them commercially.
**Abstract Book Citations:** Authors, Title, HemaSphere, 2026;10:(S1):pages. The abstract book DOIs can be found on the "Author Information" page.
**Disclaimer:** Articles published in the journal HemaSphere exclusively reflect the opinions of the authors. The authors are responsible for all content in their abstracts including accuracy of the facts, statements, citing resources, etc.

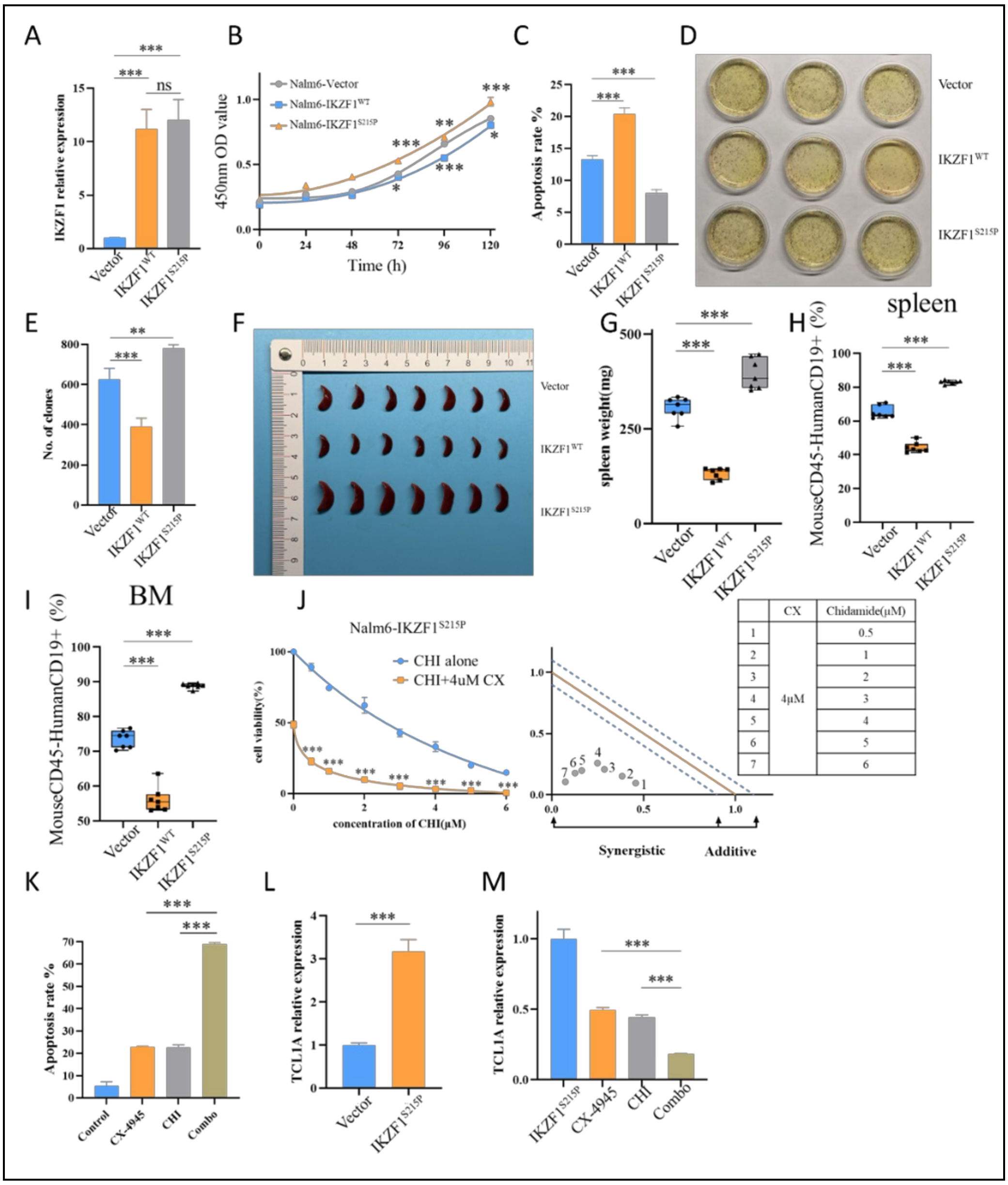

Fig. 1 Novel Oncogenic IKZF1S215P Mutation is Sensitive to CX-4945 and Chidamide in B-ALL (A) Expression level of IKZF1WT and IKZF1S215P in Nalm6 cells by qPCR. Cells were stably expressed with lentiviral IKZF1WT, IKZF1S215P, and vector only. (B-E) Effect of IKZF1WT and IKZF1S215P on cell proliferation (B), apoptosis (C) and colony-forming (D-E) in the Nalm6 cells compared with that of vector only. (F-G) Spleen image (F) and spleen weight (G) of the mice with IKZF1WT, IKZF1S215P, and vector only cells. (H-I) The percentage of mCD45-hCD19+ cells in the spleen (H) and bone marrow (I) from IKZF1WT, IKZF1S215P, and vector only in Nalm6 cells of mice. (J) Effect and synergistic analysis of the combination of CX-4945 and Chidamide vs single drug on cell proliferation in IKZF1S215P cells. (K) The statistic histogram of apoptosis in IKZF1S215P mutant cells that were treated with CX-4945, Chidamide alone, and two drugs combination for 48h. (L) The expression of TCL1A in IKZF1S215P and vector only in Nalm6 cells by qPCR. (M) The expression of TCL1A in IKZF1S215P cells treated with CX-4945 alone, Chidamide alone, and two drugs combination for 48 h detected by RT-qPCR.

**Copyright Information:** (Online) ISSN: 2572-9241
©2026 The Author(s). HemaSphere is published by John Wiley & Sons Ltd on behalf of European Hematology Association. This is an open access Abstract Book distributed under the Attribution-NonCommercial-NoDerivs (CC BY-NC-ND), which allows third parties to download the articles and share them with others as long as they credit the author and the Abstract Book, but they cannot change the content in any way or use them commercially.
**Abstract Book Citations:** Authors, Title, HemaSphere, 2026;10:(S1):pages. The abstract book DOIs can be found on the “Author Information” page.
**Disclaimer:** Articles published in the journal HemaSphere exclusively reflect the opinions of the authors. The authors are responsible for all content in their abstracts including accuracy of the facts, statements, citing resources, etc.

### (PB2542) SYNERGISTIC TARGETING OF JAK-STAT AND PI3K PATHWAYS BY GOLIDOCITINIB AND LINPERLISIB IN T-ALL/LBL: INVOLVEMENT OF RAG1/2 REGULATION

**Topic:** 01. Acute lymphoblastic leukemia - Biology & translational research

Jie Wang[1], Yue Zhang[1], Shiji Wang[1], Mengke Huang[1], Jionglin Chen[1], Chen Zhang[2], Xudong Zhang[1], Mingzhi Zhang[1], Hui Yu*[1]

*[1]The First Affiliated Hospital of Zhengzhou University, zhengzhou, China;[2]Beijing Friendship Hospital, Capital Medical University, Beijing, China;*

### Background

T-ALL/LBL is an aggressive precursor T-cell malignancy with rapid progression and high drug resistance, often leading to poor prognosis. The JAK-STAT and PI3K pathways are key drivers of T-cell tumorigenesis, and their crosstalk supports the rationale for combined targeting. However, the molecular mechanisms underlying the response to combination therapy remain incompletely understood. Therefore, this study investigates the therapeutic efficacy and underlying mechanisms of Golidocitinib (a next-generation oral JAK-1 inhibitor) in combination with Linperlisib (an oral PI3Kδ inhibitor) in T-ALL/LBL.

### Aims

To assess the antitumor efficacy of Golidocitinib and Linperlisib in T-ALL/LBL and elucidate underlying mechanisms.

### Methods

Human T-ALL/LBL cell lines (Supt-1, Jurkat, Molt-3) were treated with graded concentrations (5–80 μg/mL) of each drug alone or in combination for 24, 48, and 72 hours. Synergistic effects were assessed after 72 h using the Chou-Talalay method. Cell viability was measured by CCK-8 assay; apoptosis and cell cycle were analyzed by Annexin V/PI and PI staining followed by flow cytometry. Protein levels of p53, Bax, Bcl-2, Cyclin D1, and P21 were examined by Western blot. Transcriptomic profiling was performed in Supt-1 cells, and qPCR validated key gene expression, with a focus on RAG1/2 changes to explore underlying mechanisms.

### Results

Golidocitinib and Linperlisib inhibited proliferation in Supt-1, Jurkat, and Molt-3 cells in a dose- and time-dependent manner. After 72 h, the IC50 values of Golidocitinib were 9.61, 4.27, and 6.41 μg/mL, and those of Linperlisib were 5.50, 14.91, and 8.06 μg/mL, respectively. All combinations showed synergistic effects (CI < 1), with optimal concentrations of 12.5+6.25, 12.5+4.0, and 6.25+6.25 μg/mL. Apoptosis rates increased significantly with combination treatment: in Jurkat cells from 8.54% to 58.8%, in Molt-3 from 6.9% to 70.6%, and in Supt-1 from 8.84% to 49.8% (P<0.0001), accompanied by upregulation of p53, Bax, and cleaved caspase-3, and downregulation of Bcl-2, consistent with mitochondrial apoptosis. The combination also induced G0/G1 arrest, with the proportion of cells in G0/G1 phase increasing from 47.4% to 77.3% in Supt-1, from 41.8% to 50.7% in Jurkat, and from 42.4% to 65.7% in Molt-3 (P<0.05). Downregulation of Cyclin D1 and upregulation of P21 were also observed. Transcriptomic profiling identified 157 upregulated and 127 downregulated genes in drug-treated groups versus controls, including 93 genes commonly and concordantly regulated by both monotherapy and combination. Notably, RAG1 and RAG2 were among the most significantly upregulated. KEGG/GSEA enrichment highlighted p53 signaling, DNA damage response, TP53 axis activation, and PI3K-AKT inhibition, along with impaired eukaryotic translation elongation.

**Copyright Information:** (Online) ISSN: 2572-9241
©2026 The Author(s). HemaSphere is published by John Wiley & Sons Ltd on behalf of European Hematology Association. This is an open access Abstract Book distributed under the Attribution-NonCommercial-NoDerivs (CC BY-NC-ND), which allows third parties to download the articles and share them with others as long as they credit the author and the Abstract Book, but they cannot change the content in any way or use them commercially.
**Abstract Book Citations:** Authors, Title, HemaSphere, 2026;10:(S1):pages. The abstract book DOIs can be found on the "Author Information" page.
**Disclaimer:** Articles published in the journal HemaSphere exclusively reflect the opinions of the authors. The authors are responsible for all content in their abstracts including accuracy of the facts, statements, citing resources, etc.

### Summary/Conclusion

This study demonstrates that Golidocitinib and Linperlisib synergistically suppress T-ALL/LBL by activating the p53-mediated apoptotic pathway and inducing G0/G1 arrest. Given its role in DNA damage response, the combination-induced upregulation of RAG1/2 may enhance genomic instability to sensitize tumor cells and serve as a mechanistic marker or therapeutic target in T-ALL/LBL. Based on this dual modulation of the TP53 and PI3K-AKT signaling axes, a "pro-apoptotic and anti-survival" strategy is established, offering a promising therapeutic avenue for T-ALL/LBL. Ongoing validation in patient-derived xenograft (PDX) models will support its clinical translation.

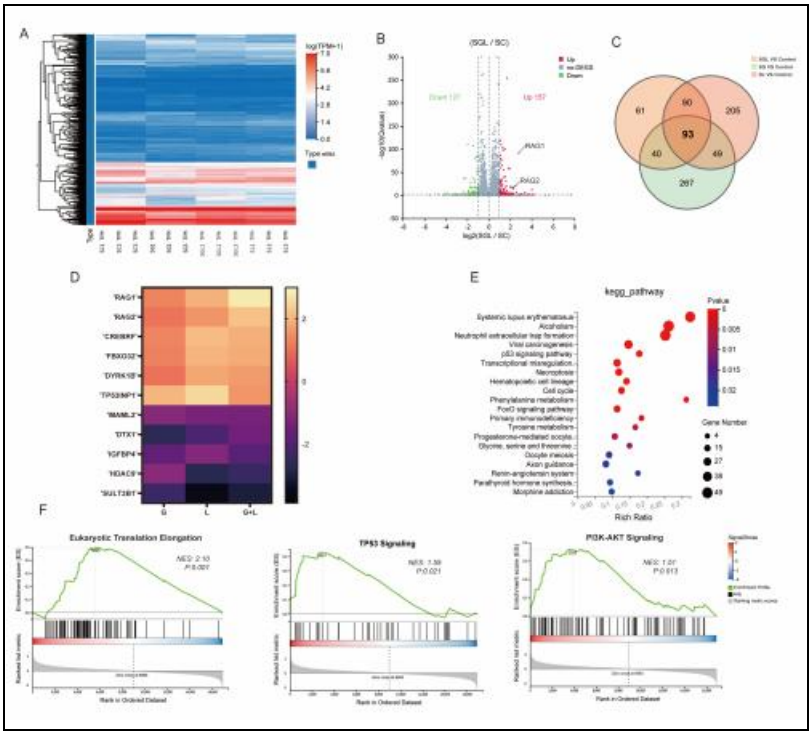

**Copyright Information:** (Online) ISSN: 2572-9241
©2026 The Author(s). HemaSphere is published by John Wiley & Sons Ltd on behalf of European Hematology Association. This is an open access Abstract Book distributed under the Attribution-NonCommercial-NoDerivs (CC BY-NC-ND), which allows third parties to download the articles and share them with others as long as they credit the author and the Abstract Book, but they cannot change the content in any way or use them commercially.
**Abstract Book Citations:** Authors, Title, HemaSphere, 2026;10:(S1):pages. The abstract book DOIs can be found on the "Author Information" page.
**Disclaimer:** Articles published in the journal HemaSphere exclusively reflect the opinions of the authors. The authors are responsible for all content in their abstracts including accuracy of the facts, statements, citing resources, etc.

### (PB2543) LIPID METABOLIC VULNERABILITIES IN ETV6::RUNX1-POSITIVE B-CELL ACUTE LYMPHOBLASTIC LEUKEMIA

**Topic:** 01. Acute lymphoblastic leukemia - Biology & translational research

Iiris Leino*[1], Laura Oksa[1], Merja Heinäniemi[2], Olli Lohi[1]

*[1]Center for Cancer Eradication Research, Faculty of Medicine and Health Technology, Tampere University and Tays Cancer Center, Tampere University Hospital, Tampere, Finland; [2]The Institute of Biomedicine, University of Eastern Finland, Kuopio, Finland;*

### Background

Acute lymphoblastic leukemia (ALL) is the most common pediatric malignancy, and *ETV6::RUNX1*-positive B-ALL accounts for approximately 25% of childhood cases. Although overall prognosis is favorable, patients with a slow early response to induction therapy remain at increased risk of relapse and inferior outcomes. Identifying subtype-specific dependencies that contribute to treatment resistance may reveal new therapeutic vulnerabilities.

### Aims

To investigate the functional role of lipid metabolism, specifically through *Acyl-CoA Synthetase Long-Chain Family Member 4 (ACSL4)* in *ETV6::RUNX1* B-ALL and to identify associated therapeutic vulnerabilities linked to ferroptosis.

### Methods

CRISPR-Cas9 knockout and inducible shRNA knockdown models of *ACSL4* were generated in the *ETV6::RUNX1*-positive B-ALL cell line REH. Functional assays assessed colony formation, cell viability, proliferation, apoptosis, and cell cycle progression. Lipidomic and transcriptomic profiling were performed to characterize molecular changes following target depletion. In parallel, RNA-sequencing data from primary *ETV6::RUNX1*-positive patient samples (n=51) were analyzed to evaluate gene expression patterns and their association with end-of-induction (d29) minimal residual disease.

### Results

A genome-wide perturbation screen (DepMap) identified *ACSL4* as a selective dependency in *ETV6::RUNX1* B-ALL. Functional validation demonstrated that genetic ablation of *ACSL4* significantly impaired clonogenic growth, while overall cell viability, proliferation, apoptosis, and cell cycle distribution were largely unaffected. Lipidomic profiling following *ACSL4* knockdown revealed accumulation of multiple membrane lipids and ceramides, consistent with altered membrane homeostasis. Transcriptomic analyses identified differential expression of genes involved in membrane raft organization and mitochondrial structure.
In primary patient samples, *ACSL4* expression did not significantly differ between fast and slow responders. However, genes implicated in the negative regulation of ferroptosis were significantly upregulated in patients with slow response, suggesting enhanced ferroptosis resistance in this subgroup of patients.

### Summary/Conclusion

These findings identify *ACSL4* as a regulator of clonogenic capacity and lipid homeostasis in *ETV6::RUNX1*-positive B-ALL. Although *ACSL4* expression itself does not stratify early treatment response, enrichment of ferroptosis-suppressing programs in slow responders suggests that resistance to lipid peroxidation-induced cell death may contribute to treatment failure. Targeting ferroptosis regulatory pathways may therefore represent a therapeutically exploitable vulnerability in this leukemia subtype.

**Copyright Information:** (Online) ISSN: 2572-9241
©2026 The Author(s). HemaSphere is published by John Wiley & Sons Ltd on behalf of European Hematology Association. This is an open access Abstract Book distributed under the Attribution-NonCommercial-NoDerivs (CC BY-NC-ND), which allows third parties to download the articles and share them with others as long as they credit the author and the Abstract Book, but they cannot change the content in any way or use them commercially.
**Abstract Book Citations:** Authors, Title, HemaSphere, 2026;10:(S1):pages. The abstract book DOIs can be found on the "Author Information" page.
**Disclaimer:** Articles published in the journal HemaSphere exclusively reflect the opinions of the authors. The authors are responsible for all content in their abstracts including accuracy of the facts, statements, citing resources, etc.

### (PB2545) NOVEL CD38-CD3 BISPECIFIC ANTIBODIES FOR IMMUNOTHERAPY OF ACUTE LEUKEMIA WITH T-CELL PHENOTYPE

**Topic:** 01. Acute lymphoblastic leukemia - Biology & translational research

Shisen Wang[1], Manling Chen[1], Tong Zhou[1], Leling Xie[1], Chengcai Guo[1], Zhifeng Yan[1], Yingxi Xu[1], Haiyan Xing[1], Kejing Tang[1], Zheng Tian[1], Qing Rao[1], Shaowei Qiu[1], Ying Wang[1], Runxia Gu[1], Min Wang[1], Jianxiang Wang*[1]

*[1]State Key Laboratory of Experimental Hematology, National Clinical Research Center for Blood Diseases, Tianjin Key Laboratory of Cell Therapy for Blood Diseases, Haihe Laboratory of Cell Ecosystem, Institute of Hematology and Blood Diseases Hospital, Tianjin Institutes of Health Science, Chinese Academy of Medical Sciences & Peking Union Medical College, Tianjin, China;*

### Background

Acute T-cell lymphoblastic leukemia (T-ALL) is a highly aggressive hematologic malignancy with limited immunotherapeutic options. T/myeloid mixed phenotype acute leukemia (T/M-MPAL), another acute leukemia bearing T-cell phenotype, poses even greater therapeutic challenges. Ideal immunotherapeutic targets for T-cell phenotype acute leukemia remain elusive, and no bispecific antibodies (BsAbs) have been approved for the treatment of these malignancies.

### Aims

To investigate the therapeutic potential of two novel in-house developed CD38-targeting BsAbs against acute leukemia with T-cell phenotype, including T-ALL and T/M-MPAL.

### Methods

We designed and produced two CD38-CD3 BsAbs featuring a unique loop structure: 38-3-loop-BsAb and 38-3-loopFc-BsAb, the latter fusing a human IgG1 Fc domain to yield an enhanced version. The cytotoxic activity of both BsAbs was evaluated in vitro by T cells co-culture killing assays with leukemia cell lines and primary patients blast cells. For in vivo assessment, both cell line-derived xenograft (CDX) and patient-derived xenograft (PDX) mouse models of T-ALL and T/M-MPAL were established to evaluate therapeutic efficacy.

### Results

Both 38-3-BsAbs exhibited potent anti-leukemic activity against T-ALL and T/M-MPAL cells in vitro at low BsAb concentrations. In vivo, both BsAbs significantly prolonged survival in CDX mouse models engrafted with either $CD3^-$ or $CD3^+$ T-ALL cells, as well as in T/M-MPAL CDX models. Furthermore, PDX mouse models derived from patients with T-ALL and T/M-MPAL were independently constructed to evaluate therapeutic efficacy, corroborating the in vivo findings.

### Summary/Conclusion

Our CD38-targeting BsAbs exerted significant therapeutic effects against acute leukemia with a T-cell phenotype, encompassing both T-ALL and T/M-MPAL. Our findings established a novel BsAb immunotherapy for these aggressive malignancies.

**Copyright Information:** (Online) ISSN: 2572-9241
©2026 The Author(s). HemaSphere is published by John Wiley & Sons Ltd on behalf of European Hematology Association. This is an open access Abstract Book distributed under the Attribution-NonCommercial-NoDerivs (CC BY-NC-ND), which allows third parties to download the articles and share them with others as long as they credit the author and the Abstract Book, but they cannot change the content in any way or use them commercially.
**Abstract Book Citations:** Authors, Title, HemaSphere, 2026;10:(S1):pages. The abstract book DOIs can be found on the "Author Information" page.
**Disclaimer:** Articles published in the journal HemaSphere exclusively reflect the opinions of the authors. The authors are responsible for all content in their abstracts including accuracy of the facts, statements, citing resources, etc.

### (PB2546) LEUKAEMOGENESIS IS INDUCED IN CYTOKINE DEPENDENT PARENTAL BA/F3 CELLS BY EXTRACELLULAR VESICLES FROM CYTOKINE INDEPENDENT CRLF2 P.F232C BA/F3 CELLS

**Topic:** 01. Acute lymphoblastic leukemia - Biology & translational research

Maxim Buckley*[1, 2], Elyse Page[1, 2], Cherie Blenkiron[3], Fiona Whelan[1], Deborah White[1, 2], Laura Eadie[1, 2]

*[1]Adelaide University, Faculty of Health and Medical Sciences - Adelaide Medical School, Adelaide Central Business District, Australia; [2]South Australian Health and Medical Research Institute, Precision Cancer Medicine - Leukaemia Research Group, Adelaide Central Business District, Australia; [3]University of Auckland Faculty of Medical & Health Sciences, Molecular Medicine & Pathology, Grafton, New Zealand;*

### Background

Extracellular vesicles (EVs) are lipid bodies produced by all cells. Medium EVs (mEVs) are 100-1000 nm in diameter and are formed by budding of cell membranes, potentially taking with them surface receptors. Small EVs (sEVs), historically referred to as exosomes, are 50-150 nm in diameter and formed intracellularly using endosomal machinery. Both populations of EV carry nucleic acids and proteins, and have been demonstrated in other cancers to transfer oncogenic properties. *CRLF2* p.F232C B-ALL results in a constitutively active TSLP membrane-bound receptor, activating JAK/STAT signalling pathways, resulting in leukaemogenesis.

### Aims

We investigated if either EV population could transfer the constitutively active *CRLF2* p.F232C receptor (TSLPR) and induce leukaemic transformation.

### Methods

SWATH proteomics and cryo-TEM were used to analyse vesicle populations. Cytokine-dependent (IL-3) parental Ba/F3 cells were treated daily for 20 days in the presence of IL-3 plus mEVs or sEVs derived from *CRLF2* p.F232C Ba/F3 cells. Vehicle-treated cells were used as control. Flow cytometry detected TSLPR expression on Ba/F3 cells and mEVs. Proteinase K was used was used to shear external TSLP from mEVs. mEV-treated Ba/F3 cells were FACS sorted based on TSLPR+ after 20 days. Phenol-chloroform was used to extract gDNA, and PCR to amplify genomic sequences. CellTiter-Glo determined growth in the absence of IL-3. Phosphoflow cytometry investigated changes in STAT5 and ERK phosphorylation.

### Results mEVs and sEVs differed in size and protein composition. mEV-treated Ba/F3 cells demonstrated significantly increased TSLPR expression compared to vehicle and sEV-treated cells after 20 days of treatment (51; vs 2204, $p$=0.0087 respectively). A purified population of TSLPR+ Ba/F3 cells contained the *CRLF2* gene with the p.F232C point mutation and demonstrated cytokine-independent growth that was significantly increased compared to parental control (day 4 fold change=2516 vs. 49, $p$=0.0104). TSLPR+ Ba/F3 cells also demonstrated significantly increased phosphorylated(p)-STAT5 and pERK compared to parental control ($p$=0.008; $p$=0.0052 respectively). mEVs from *CRLF2* p.F232C Ba/F3 cells expressed the TSLP receptor; expression was significantly reduced after proteinase K treatment ($p$ = 0.009). *CRLF2* gDNA was detected in both mEVS and sEVs.

**Copyright Information:** (Online) ISSN: 2572-9241
©2026 The Author(s). HemaSphere is published by John Wiley & Sons Ltd on behalf of European Hematology Association. This is an open access Abstract Book distributed under the Attribution-NonCommercial-NoDerivs (CC BY-NC-ND), which allows third parties to download the articles and share them with others as long as they credit the author and the Abstract Book, but they cannot change the content in any way or use them commercially.
**Abstract Book Citations:** Authors, Title, HemaSphere, 2026;10:(S1):pages. The abstract book DOIs can be found on the “Author Information” page.
**Disclaimer:** Articles published in the journal HemaSphere exclusively reflect the opinions of the authors. The authors are responsible for all content in their abstracts including accuracy of the facts, statements, citing resources, etc.

**Summary/Conclusion**

For the first time, we have demonstrated horizontal transfer of protein and genomic material via mEVs in an ALL setting. This horizontal transfer induced leukaemic transformation, altering growth and intracellular signalling profiles. These results indicate a broader role for EVs in leukaemia and have potential implications for leukaemia pathogenesis and relapse.

**Copyright Information:** (Online) ISSN: 2572-9241
©2026 The Author(s). HemaSphere is published by John Wiley & Sons Ltd on behalf of European Hematology Association. This is an open access Abstract Book distributed under the Attribution-NonCommercial-NoDerivs (CC BY-NC-ND), which allows third parties to download the articles and share them with others as long as they credit the author and the Abstract Book, but they cannot change the content in any way or use them commercially.
**Abstract Book Citations:** Authors, Title, HemaSphere, 2026;10:(S1):pages. The abstract book DOIs can be found on the “Author Information” page.
**Disclaimer:** Articles published in the journal HemaSphere exclusively reflect the opinions of the authors. The authors are responsible for all content in their abstracts including accuracy of the facts, statements, citing resources, etc.

### (PB2547) PRMT7 OVEREXPRESSION PREDICTS POOR SURVIVAL IN B-CELL PRECURSOR ALL AND PHARMACOLOGIC INHIBITION ENHANCES ANTI-LEUKEMIA EFFECTS IN VITRO AND VIVO

**Topic:** 01. Acute lymphoblastic leukemia - Biology & translational research

Hongbo He*[1], Shilai Bao[2], Huyong Zheng[1]

*[1]Beijing Children's Hospital, Hematology Center, Beijing, China;[2]Institute of Genetics and Developmental Biology, Chinese Academy of Sciences, Beijing, China;*

### Background

Protein arginine methyltransferase 7 (PRMT7) plays a critical role in tumorigenesis and cancer progression. To explore the role of PRMT7 in B-cell precursor acute lymphoblastic leukemia (BCP-ALL), we assessed PRMT7 expression in patient samples and its association with clinical outcomes, and we evaluated the impact of genetic and pharmacologic PRMT7 inhibition in vitro and in vivo.

### Aims

This study aims to investigate the expression and clinical significance of PRMT7 in pediatric B-cell precursor acute lymphoblastic leukemia (BCP-ALL), evaluate its functional role in leukemia cell proliferation and survival through genetic and pharmacological inhibition, explore the underlying mechanisms involving the SLC7A11-GPX4 axis and ferroptosis, and assess the therapeutic potential of the PRMT7 inhibitor SGC3027 in vitro and in vivo.

### Methods

PRMT7 mRNA levels were quantified by RT-PCR in 249 pediatric BCP-ALL cases. Patients were stratified into high vs low PRMT7 expression groups by the median value, and clinical characteristics and outcomes were compared. PRMT7 expression was also examined in paired bone marrow samples collected at new diagnosis (ND) and complete remission (CR). Functional studies were performed in Nalm-6 and Pre-B-697 cells following PRMT7 knockdown to assess cell proliferation, apoptosis, and colony formation. Mechanistic studies (Western blot analysis, qPCR, RNA sequencing and Metabolomics) were used to explore pathways regulated by PRMT7. Pharmacological inhibition with the PRMT7 inhibitor SGC3027 was tested in vitro and in a mouse model of leukemia to evaluate its therapeutic potential.

### Results

PRMT7 expression as higher in ND than in CR samples. Patients with high PRMT7 high expression had significantly shorter overall survival (OS) and event free survival (EFS) than those with low expression (OS, P=0.0063 and EFS, P = 0.01). PRMT7 knockdown reduced viability, increased apoptosis, and caused G2/M arrest in Nalm-6 and Pre-B-697 cells. Pharmacological inhibition of PRMT7 with SGC3027 similarly reduced cell growth and increased apoptosis, supporting PRMT7 as a potential therapeutic target for BCP-ALL. RNA sequencing and metabolomics suggested PRMT7 promote leukemia cell proliferation via the regulation of the SLC7A11-GPX4 axis and ferroptosis. Strikingly, the concentration of cystine and cysteine in bone marrow cells were significantly higher at CR than at ND. In vivo, SGC3027 attenuated the development of BCP-ALL and prolonged survival.

### Summary/Conclusion

High PRMT7 expression predicts inferior survival in pediatric BCP-ALL. Genetic deletion of PRMT7 or PRMT7 inhibitor SGC3027 could suppress leukemia cell growth and prolong survival in vivo, supporting PRMT7 as a prognostic biomarker and potential therapeutic target in pediatric BCP-ALL.

**Copyright Information:** (Online) ISSN: 2572-9241
©2026 The Author(s). HemaSphere is published by John Wiley & Sons Ltd on behalf of European Hematology Association. This is an open access Abstract Book distributed under the Attribution-NonCommercial-NoDerivs (CC BY-NC-ND), which allows third parties to download the articles and share them with others as long as they credit the author and the Abstract Book, but they cannot change the content in any way or use them commercially.
**Abstract Book Citations:** Authors, Title, HemaSphere, 2026;10:(S1):pages. The abstract book DOIs can be found on the "Author Information" page.
**Disclaimer:** Articles published in the journal HemaSphere exclusively reflect the opinions of the authors. The authors are responsible for all content in their abstracts including accuracy of the facts, statements, citing resources, etc.

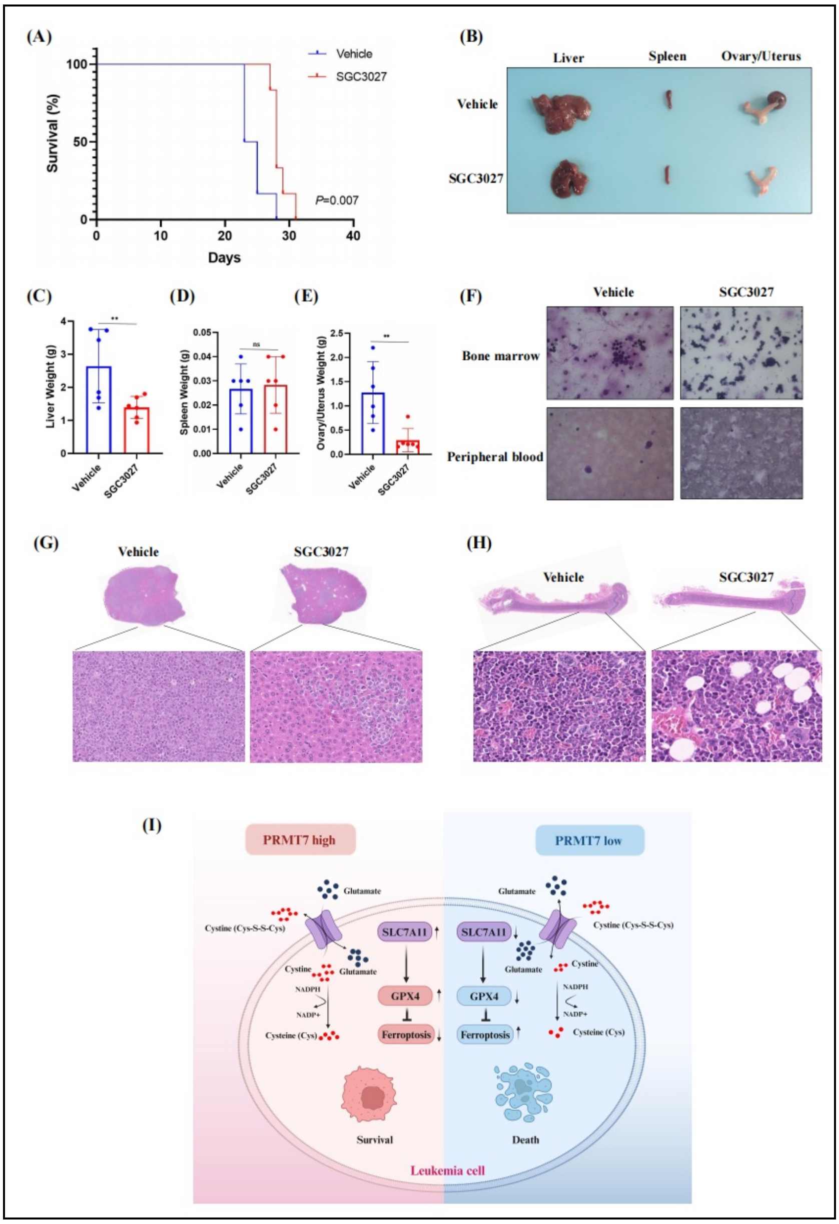

**Copyright Information:** (Online) ISSN: 2572-9241
©2026 The Author(s). HemaSphere is published by John Wiley & Sons Ltd on behalf of European Hematology Association. This is an open access Abstract Book distributed under the Attribution-NonCommercial-NoDerivs (CC BY-NC-ND), which allows third parties to download the articles and share them with others as long as they credit the author and the Abstract Book, but they cannot change the content in any way or use them commercially.
**Abstract Book Citations:** Authors, Title, HemaSphere, 2026;10:(S1):pages. The abstract book DOIs can be found on the "Author Information" page.
**Disclaimer:** Articles published in the journal HemaSphere exclusively reflect the opinions of the authors. The authors are responsible for all content in their abstracts including accuracy of the facts, statements, citing resources, etc.

### (PB2548) STIL-TAL1 DELETED T-ALLS TOLERATE STIL HAPLOSUFFICIENCY THROUGH A THERAPEUTICALLY EXPLOITABLE FBXW7 AXIS

**Topic:** 01. Acute lymphoblastic leukemia - Biology & translational research

Daisy Gresham*[1, 2], Jamie Deyell[2], Tanya Rapoz-D'silva[2], Marc Mansour[1, 2]

*[1]UCL Great Ormond Street Institute of Child Health, London, United Kingdom;[2]UCL Cancer Institute, London, United Kingdom;*

### Background

In 25% of T-ALL cases, the interstitial STIL-TAL1 deletion juxtaposes TAL1 to the STIL promoter, driving TAL1 overexpression while causing monoallelic loss of STIL, a critical regulator of centriole assembly and oncogenic driver in multiple malignancies. FBXW7, a tumour suppressor E3 ubiquitin ligase mutated in ~20% of T-ALL, is suppressed by TAL1 via microRNA-223, highlighting functional interplay between TAL1 activation and FBXW7 pathway disruption. Cyclin G-associated kinase (GAK) has recently been identified as a synthetic lethal vulnerability in FBXW7-deficient contexts; however, the mechanism remains unknown. Here, we define a previously unrecognized STIL regulatory axis in TAL1-driven T-ALL that is therapeutically exploitable through GAK inhibition.

### Aims

We investigated whether monoallelic loss of STIL provides a therapeutic vulnerability in STIL-TAL1-driven T-ALL.

### Methods

STIL mRNA expression in T-ALL cell lines was analysed by RNA-seq and qRT-PCR, and protein levels by Western blotting and dual STIL-propidium iodide flow cytometry. Protein stability was assessed by cycloheximide chase. TAL1 was silenced in S1C1 Jurkat cells using doxycycline-inducible shRNA, and isogenic FBXW7-knockout SUPT1 cells were generated by CRISPR–Cas9. GAK was inhibited using SGC-GAK, and cell viability measured by CellTitre-Glo. Public RNA-seq data were analysed from primary T-ALL samples (COGALL0434).

### Results

RNA-seq and qRT-PCR demonstrated ~50% reduction of STIL mRNA in STIL-TAL1$^+$ T-ALL compared to WT in both patient samples and cell lines; surprisingly, STIL protein levels were maintained or increased, indicating post-translational stabilisation. Using a doxycycline-inducible TAL1 knockdown model, TAL1 suppression increased FBXW7 protein and reduced canonical substrates (c-MYC, NOTCH1, cyclin E), while unexpectedly decreasing STIL, suggesting FBXW7-mediated regulation. STIL expression was significantly elevated in isogenic FBXW7-KO cells in G1 ($p<0.05$) and G2 ($p<0.001$), with cycloheximide chase assays showing ~50% slower STIL degradation versus WT. This phenotype was recapitulated in TAL1-high versus TAL1-low Jurkat cells (G1 $p<0.01$; G2 $p<0.0001$), supporting a TAL1-driven, FBXW7-dependent STIL stabilization axis. Targeting FBXW7 synthetic lethal partner GAK revealed increased sensitivity in TAL1-high ($IC_{50}$ 0.24 µM) vs TAL1-low Jurkats ($IC_{50}$ 2.55 µM), and FBXW7-KO ($IC_{50}$ 0.69 µM) vs WT ($IC_{50}$ 2.05 µM) isogenic SUPT1s. Furthermore, GAK inhibition significantly reduced STIL expression in FBXW7-KO versus WT cells (G1 $p<0.01$; G2 $p<0.05$). We hypothesize that STIL-TAL1-deleted T-ALL cases tolerate STIL haplosufficiency through FBXW7-dependent stabilisation of STIL that can be exploited through GAK inhibition. Analysis of primary T-ALL RNA-seq samples demonstrated significantly higher CA20 and CIN25 centrosome amplification signatures in TAL1-driven cases, consistent with functional consequences of STIL upregulation.

**Copyright Information:** (Online) ISSN: 2572-9241
©2026 The Author(s). HemaSphere is published by John Wiley & Sons Ltd on behalf of European Hematology Association. This is an open access Abstract Book distributed under the Attribution-NonCommercial-NoDerivs (CC BY-NC-ND), which allows third parties to download the articles and share them with others as long as they credit the author and the Abstract Book, but they cannot change the content in any way or use them commercially.
**Abstract Book Citations:** Authors, Title, HemaSphere, 2026;10:(S1):pages. The abstract book DOIs can be found on the "Author Information" page.
**Disclaimer:** Articles published in the journal HemaSphere exclusively reflect the opinions of the authors. The authors are responsible for all content in their abstracts including accuracy of the facts, statements, citing resources, etc.

**Summary/Conclusion**

These findings define a FBXW7-dependent mechanism of STIL stabilisation in TAL1-driven T-ALL. We also identify GAK as a regulator of STIL in FBXW7-deficient models, consistent with its role in mitosis and spindle assembly checkpoint control. As STIL functions as an oncogene in multiple cancers by driving centrosome amplification and tumour progression, our data suggest a novel therapeutic option of GAK inhibition in TAL1-driven FBXW7-mutant T-ALL. Given the high frequency of FBXW7 alterations across cancers, this mechanism may have broader relevance beyond T-ALL.

**Copyright Information:** (Online) ISSN: 2572-9241
©2026 The Author(s). HemaSphere is published by John Wiley & Sons Ltd on behalf of European Hematology Association. This is an open access Abstract Book distributed under the Attribution-NonCommercial-NoDerivs (CC BY-NC-ND), which allows third parties to download the articles and share them with others as long as they credit the author and the Abstract Book, but they cannot change the content in any way or use them commercially.
**Abstract Book Citations:** Authors, Title, HemaSphere, 2026;10:(S1):pages. The abstract book DOIs can be found on the "Author Information" page.
**Disclaimer:** Articles published in the journal HemaSphere exclusively reflect the opinions of the authors. The authors are responsible for all content in their abstracts including accuracy of the facts, statements, citing resources, etc.

### (PB2549) PH+ ALL CELLS INDUCE ENDOGENOUS PD-L1 UPREGULATION IN BONE MARROW MESENCHYMAL STROMAL CELLS VIA THE CX43-XBP1-STING AXIS TO PROMOTE MICROENVIRONMENTAL IMMUNOSUPPRESSION

**Topic:** 01. Acute lymphoblastic leukemia - Biology & translational research

Chengyan Chai*[1], Xi Zhang[2], Zheng Wang[3]

*[1]Xinqiao Hospital of Army Medical University, CHONGQING, China;[2]Xinqiao Hospital of Army Medical University, Medical Center of Hematology, Chongqing, China; [3]Xinqiao Hospital of Army Medical University, Chongqing, China;*

### Background

The immunosuppressive microenvironment is an important factor in immune escape of Philadelphia chromosome-positive (Ph+) acute lymphoblastic leukemia (ALL). In our previous study, we found that exosomes derived from Ph+ ALL cells downregulate the expression of connexin 43 (Cx43) in bone marrow mesenchymal stromal cells (BMSCs), induce the upregulation of the immune checkpoint molecule PD-L1, and promote T-cell exhaustion.However, the molecular mechanism by which Cx43 regulates PD-L1 remains unclear.

### Aims

To investigate the molecular mechanism underlying Cx43-mediated regulation of endogenous PD-L1 in BMSCs, and to explore whether blocking this signaling axis can reverse the inhibitory effect of BMSCs on T cells and enhance anti-tumor immunity.

### Methods

First, flow cytometry, immunofluorescence and other methods were used to preliminarily confirm that the downregulation of Cx43 in patient samples with Ph+ ALL promotes immunosuppression in the bone marrow microenvironment.Subsequently, transcriptome sequencing and validation were performed to demonstrate that Ph+ ALL cells induce the upregulation of endogenous PD-L1 in BMSCs via the Cx43-XBP1-STING axis.Finally, in vivo animal experiments were conducted to verify that Cx43 downregulation induces endogenous PD-L1 expression in BMSCs, thereby driving immunosuppression and tumor immune escape in the bone marrow microenvironment.

### Results

Ph+ ALL cells drived exosome downregulate the expression of the gap junction protein Cx43 in BMSCs[illegible]downregulation of Cx43 promotes the nuclear translocation of XBP1 to transcriptionally regulate STING , inducing intrinsic PD-L1 (PD-1-independent) driven immunosuppressive function in BMSCs by activating IRF3 signaling and inhibiting NF-κB activation.

### Summary/Conclusion

We conducted in-depth studies on the molecular mechanism of the Cx43-STING-PD-L1 axis by clinical specimens, animal experiments, and  the cellular level, aiming to provide novel strategies for identifying new therapeutic targets and developing immunotherapies for Ph+ ALL.

**Copyright Information:** (Online) ISSN: 2572-9241
©2026 The Author(s). HemaSphere is published by John Wiley & Sons Ltd on behalf of European Hematology Association. This is an open access Abstract Book distributed under the Attribution-NonCommercial-NoDerivs (CC BY-NC-ND), which allows third parties to download the articles and share them with others as long as they credit the author and the Abstract Book, but they cannot change the content in any way or use them commercially.
**Abstract Book Citations:** Authors, Title, HemaSphere, 2026;10:(S1):pages. The abstract book DOIs can be found on the "Author Information" page.
**Disclaimer:** Articles published in the journal HemaSphere exclusively reflect the opinions of the authors. The authors are responsible for all content in their abstracts including accuracy of the facts, statements, citing resources, etc.

### (PB2550) KSR2 IS A NOVEL ONCOGENE IN TAL1 SUBTYPE OF T-CELL ACUTE LYMPHOBLASTIC LEUKEMIA WITH POTENTIAL CLINICAL AND PROGNOSTIC IMPLICATIONS

**Topic:** 01. Acute lymphoblastic leukemia - Biology & translational research

Monika Drobna-Sledzinska*[1], Natalia Makowska-Maslak[1], Roman Jaksik[2], Igor Fijalkowski[3], Maria Kosmalska[1], Jakub Czarny[4], Monika Lejman[5], Łukasz Sędek[6], Tomasz Szczepański[7], Agata Pastorczak[8], Katarzyna Derwich[9], Michał Witt[1], Panagiotis Ntziachristos[3], Małgorzata Dawidowska[1]

*[1]Institute of Human Genetics, Polish Academy of Sciences, Poznan, Poland;[2]Department of Systems Biology and Engineering and Biotechnology Centre, Silesian University of Technology, Gliwice, Poland; [3]Leukemia Therapy Resistance laboratory, Department of Biomolecular Medicine, Ghent University, Ghent, Belgium; [4]Faculty of Medicine, Poznan University of Medical Sciences, Poznan, Poland; [5]Independent Laboratory of Genetic Diagnostics, Medical University of Lublin, Lublin, Poland; [6]Department of Microbiology and Immunology, Medical University of Silesia, Zabrze, Poland;[7]Department of Pediatric Hematology and Oncology, Medical University of Silesia, Zabrze, Poland; [8]Department of Pediatrics, Oncology and Hematology, Medical University of Lodz, Łódź, Poland; [9]Department of Pediatric Oncology, Hematology and Transplantology, Institute of Pediatrics, Poznań University of Medical Sciences, Poznan, Poland;*

### Background

T-cell acute lymphoblastic leukemia (T-ALL) is an aggressive hematologic malignancy arising from aberrant thymic T-cell precursors. Although cure rates reach 75–90% in pediatric patients, outcomes remain poor in relapsed or refractory disease (~20% of cases). Recent integrative genomic and transcriptomic analyses of 982 T-ALL cases identified 15 molecular subtypes with distinct prognoses. However, comprehensive genome- and transcriptome-wide profiling is not routinely feasible in most clinical settings. Therefore, clinically applicable biomarkers reflecting these molecular subtypes are urgently needed for improved risk stratification and therapeutic targeting.

### Aims

Our aim was to define the biological function and clinical relevance of *KSR2*, a gene ectopically expressed in a subgroup of pediatric T-ALL patients.

### Methods

T-ALL cell lines were cultured under standard conditions. *KSR2* repression was achieved using CRISPR interference (CRISPRi) via lentiviral transduction of dCas9-KRAB constructs. For functional studies, cells were treated with APS-2-79 (5–15 μM), a small molecule inhibitor of KSR2, for 72 h; 0.5% DMSO served as vehicle control. Cell viability was assessed using Cell Counting Kit-8 and GloMax Microplate Reader.
Transcriptomic profiling of diagnostic samples from 88 Polish pediatric T-ALL patients was performed using TruSeq Stranded mRNA libraries and sequenced on NovaSeq 6000 (2×150 bp; 150M reads/sample). DNA methylation was analyzed with Infinium MethylationEPIC arrays. RNA-seq of transduced JURKAT cells was performed using Watchmaker total mRNA library preparation followed by AVITI 2×150 sequencing. Publicly available TAL1 ChIP-seq datasets were integrated with methylation and transcriptomic data.

**Copyright Information:** (Online) ISSN: 2572-9241
©2026 The Author(s). HemaSphere is published by John Wiley & Sons Ltd on behalf of European Hematology Association. This is an open access Abstract Book distributed under the Attribution-NonCommercial-NoDerivs (CC BY-NC-ND), which allows third parties to download the articles and share them with others as long as they credit the author and the Abstract Book, but they cannot change the content in any way or use them commercially.
**Abstract Book Citations:** Authors, Title, HemaSphere, 2026;10:(S1):pages. The abstract book DOIs can be found on the "Author Information" page.
**Disclaimer:** Articles published in the journal HemaSphere exclusively reflect the opinions of the authors. The authors are responsible for all content in their abstracts including accuracy of the facts, statements, citing resources, etc.

### Results

Ectopic *KSR2* expression was strongly associated with the TAL1 molecular subtype. Integration of TAL1 ChIP-seq, DNA methylation, and transcriptomic data demonstrated direct TAL1 binding within an intronic regulatory region of *KSR2* that is aberrantly demethylated in a subset of patients, resulting in transcriptional activation.
CRISPRi-mediated repression of *KSR2* significantly reduced proliferation of T-ALL cells. Similarly, pharmacologic inhibition with APS-2-79 decreased cell viability in a dose-dependent manner, indicating therapeutic vulnerability. Mechanistically, KSR2 supported AMPK signaling, which is critical for T-ALL cell survival.
Clinically, patients with ectopic *KSR2* expression exhibited inferior overall survival and a higher incidence of relapse compared with *KSR2*-negative cases.

### Summary/Conclusion

*KSR2* represents a novel TAL1-driven oncogenic dependency in pediatric T-ALL. Its expression identifies a high-risk subgroup and reveals a therapeutically actionable vulnerability, supporting its potential utility as both a prognostic biomarker and a targeted treatment candidate.
This project was supported by funding from the National Science Centre, Poland: 2017/25/N/NZ2/01132; 2021/41/N/NZ2/02247; 2022/47/B/NZ5/00663.

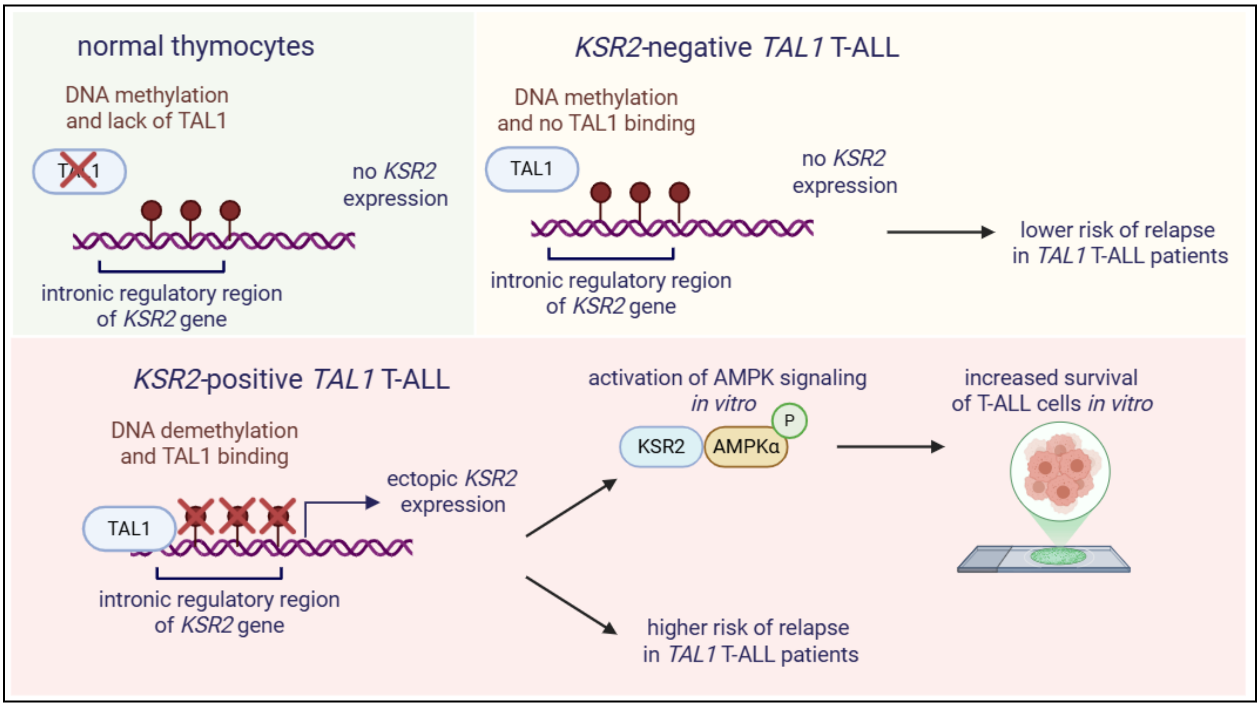

Proposed mechanism of aberrant KSR2 expression in T-ALL

**Copyright Information:** (Online) ISSN: 2572-9241
©2026 The Author(s). HemaSphere is published by John Wiley & Sons Ltd on behalf of European Hematology Association. This is an open access Abstract Book distributed under the Attribution-NonCommercial-NoDerivs (CC BY-NC-ND), which allows third parties to download the articles and share them with others as long as they credit the author and the Abstract Book, but they cannot change the content in any way or use them commercially.
**Abstract Book Citations:** Authors, Title, HemaSphere, 2026;10:(S1):pages. The abstract book DOIs can be found on the "Author Information" page.
**Disclaimer:** Articles published in the journal HemaSphere exclusively reflect the opinions of the authors. The authors are responsible for all content in their abstracts including accuracy of the facts, statements, citing resources, etc.

### (PB2551) POTENTIAL IMPACT OF CD8+ T-CELL IL-2 EXPRESSION AND LACK OF T-SENESCENT EXPANSION ON BLINATUMOMAB MRD RESPONSE: LONGITUDINAL IMMUNE MONITORING IN ALL-B PATIENTS

**Topic:** 01. Acute lymphoblastic leukemia - Biology & translational research

Uros Markovic[1], Andrea Duminuco*[1], Nardo Antonella[1], Sara Marino[1], Nunziatina Laura Parrinello[1], Luca Lo Nigro[1], Gaia Stanzione[1], Marina Parisi[1], Cinzia Maugeri[1], Giulio Antonio Milone[1], Benedetta Esposito[1], Paolo Fabio Fiumara[1], Elisa Mauro[1], Alessandra Cupri[1], Alessandra Romano[1], Francesco Di Raimondo[1], Salvatore Leotta[1]

*[1]Hematology and Bone Marrow Transplatation Unit, A.O.U. Policlinico "G. Rodolico - S.Marco", Catania, Italy;*

### Background

Blinatumomab (BLINA) is the first bispecific T-cell engager that binds cytotoxic CD3+ T- lymphocytes and CD19+ lymphoblastic cells in acute lymphoblastic B-cell leukemia (ALL-B) leading to targeted lysis. However, it is still not well understood which immune mediators specifically (immune-effector T-cells or cytokines) can be used as predictive response biomarkers in terms of minimal residual disease (MRD).

### Aims

Monitor T-cell and cytokine expression kinetics at baseline and in course of first BLINA cycle in order to identify potential biomarkers of MRD response.

### Methods

Eighteen ALL-B patients treated with BLINA, both adult and pediatric, were monitored using peripheral blood flow cytometry at baseline (pre-BLINA) and between day 14 and day 28 of the first cycle (post-BLINA), measuring percentages of CD4+ and CD8+ T-cell subsets and specific cytokine expression. The following T-cells subsets were evaluated: effector memory (TEM), regulatory (T-reg), central memory (TCM), effector memory re-expressing CD45RA (TEMRA) and senescent (T-sen). T-cell cytokine expression was assessed using intracellular staining followed by flow cytometry for IFN-g, IL-2, TNF-a and PD-1 by mean fluorescence intensity. Given the heterogeneity of the cohort the ratios between post- and pre-BLINA values were compared stratifying the patients by MRD response (MRD +/-) by using Mann-Whitney U-test. MRD was measured by RT-qPCR with a sensitivity of 10 -4  for clonal-IgH or for BCR-ABL respectively for Ph'- and Ph'+ patients.

**Copyright Information:** (Online) ISSN: 2572-9241
©2026 The Author(s). HemaSphere is published by John Wiley & Sons Ltd on behalf of European Hematology Association. This is an open access Abstract Book distributed under the Attribution-NonCommercial-NoDerivs (CC BY-NC-ND), which allows third parties to download the articles and share them with others as long as they credit the author and the Abstract Book, but they cannot change the content in any way or use them commercially.
**Abstract Book Citations:** Authors, Title, HemaSphere, 2026;10:(S1):pages. The abstract book DOIs can be found on the "Author Information" page.
**Disclaimer:** Articles published in the journal HemaSphere exclusively reflect the opinions of the authors. The authors are responsible for all content in their abstracts including accuracy of the facts, statements, citing resources, etc.

**Results**

Eighteen ALL-B patients received BLINA, with a median age of 29 years (6-76), and male prevalence (M/F: 13/5.). Seven subjects were Ph'+ (39%). Pre-BLINA treatment was "pediatric-inspired" chemotherapy and ponatinib and steroids, respectively in 13 (75 %) and in 5 (25%) of patients. Indications for nlinatumomab were respectively, MRD+ in ten and maintenance post-chemotherapy in eight MRD- patients not eligible for Hematopoietic Stem Cell Transplantation (HSCT). Median number of BLINA cycles was 2 (range 1-5). MRD negativity was reached in 6/10 (60%) and maintained in 8/8 of patient that received BLINA respectively for MRD+ and for maintenance.
Analysis of T-cell subsets and cytokine expression showed that post-BLINA/pre-BLINA IL-2+ CD8+ T-cells and post-BLINA /pre-BLINA CD8+ T-sen ratios were correlated with MRD-response (respectively, p=0.024, Fig. 1A, and p=0.045, Fig. 1B). ROC-analysis showed that the cut-off values for MRD-response were >0.81 (AUC 0.96 p<0.001) and <1.12 (AUC 0.84, p=0.001) respectively, for post- and pre-BLINA CD8+ IL-2+ and CD8+ T-sen ratio. Interestingly, there was also a difference between the median MRD- and MRD+ ratios in CD8+ TEMRA (respectively 1.67 vs 0.8) and CD4+ T-sen subsets (0.61 vs 2.73), that were not statistically significant. Given the limited number of patients, the exclusion of the MRD- patients treated for maintenance did not confirm the abovementioned results.

**Summary/Conclusion**

Longitudinal monitoring during the first BLINA cycle revealed CD8+ IL-2 expression and reduction of CD8+ T-senescence as potential biomarkers of MRD-response. However, the limited number of patients does not allow definitive conclusions.

**Copyright Information:** (Online) ISSN: 2572-9241
©2026 The Author(s). HemaSphere is published by John Wiley & Sons Ltd on behalf of European Hematology Association. This is an open access Abstract Book distributed under the Attribution-NonCommercial-NoDerivs (CC BY-NC-ND), which allows third parties to download the articles and share them with others as long as they credit the author and the Abstract Book, but they cannot change the content in any way or use them commercially.
**Abstract Book Citations:** Authors, Title, HemaSphere, 2026;10:(S1):pages. The abstract book DOIs can be found on the "Author Information" page.
**Disclaimer:** Articles published in the journal HemaSphere exclusively reflect the opinions of the authors. The authors are responsible for all content in their abstracts including accuracy of the facts, statements, citing resources, etc.

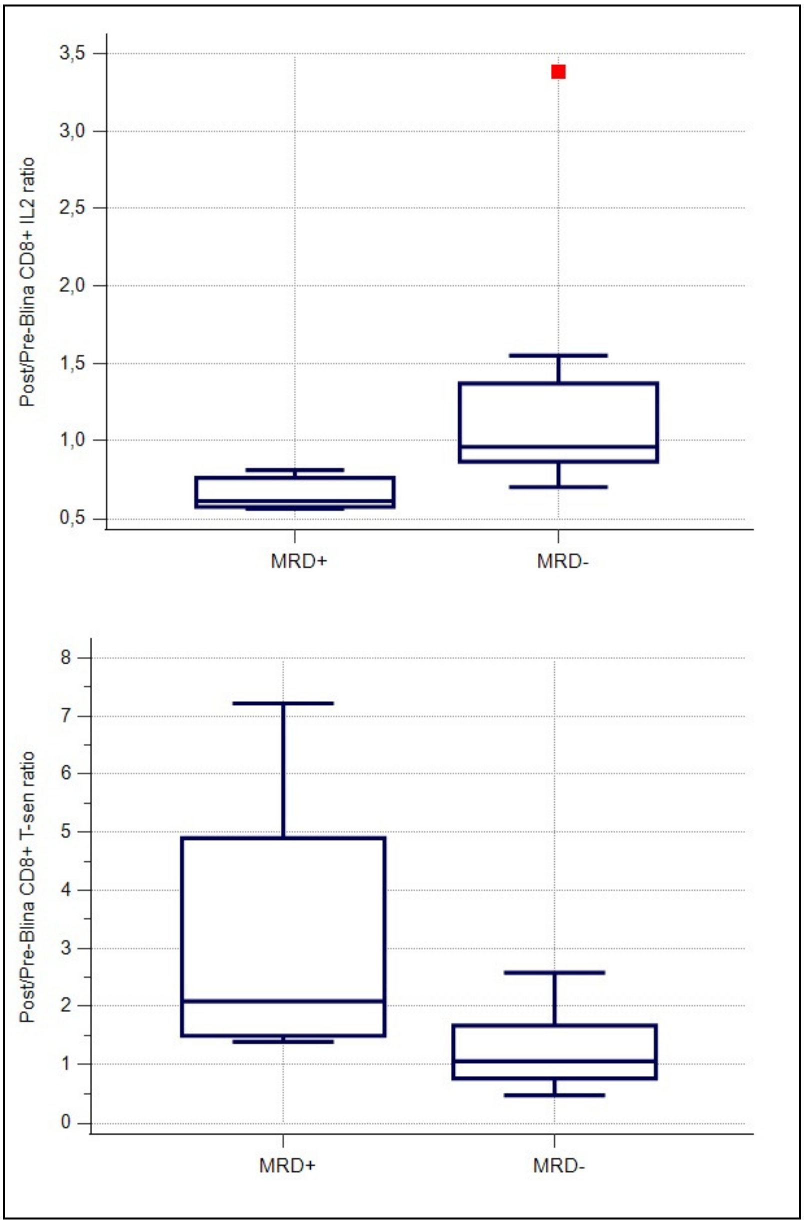

**Copyright Information:** (Online) ISSN: 2572-9241
©2026 The Author(s). HemaSphere is published by John Wiley & Sons Ltd on behalf of European Hematology Association. This is an open access Abstract Book distributed under the Attribution-NonCommercial-NoDerivs (CC BY-NC-ND), which allows third parties to download the articles and share them with others as long as they credit the author and the Abstract Book, but they cannot change the content in any way or use them commercially.
**Abstract Book Citations:** Authors, Title, HemaSphere, 2026;10:(S1):pages. The abstract book DOIs can be found on the "Author Information" page.
**Disclaimer:** Articles published in the journal HemaSphere exclusively reflect the opinions of the authors. The authors are responsible for all content in their abstracts including accuracy of the facts, statements, citing resources, etc.

### (PB2552) ENTROPY AND TRANSCRIPTIONAL PLASTICITY CHARACTERIZE CONVERGENT INFLAMMATORY–METABOLIC MACROSTATES IN ACUTE LYMPHOBLASTIC LEUKEMIA

**Topic:** 01. Acute lymphoblastic leukemia - Biology & translational research

Jairo Jattin-Balcázar*[1]

[1] *Universidad Nacional de Colombia, Facultad de Medicina, Bogotá, Colombia;*

### Background

B-cell acute lymphoblastic leukemia (B-ALL) is profoundly shaped by age and clinical status, with relapse representing a critical determinant of prognosis. While conventional differential expression analyses highlight gene-level changes, they often miss the broader structural reorganization of the transcriptome. Transcriptional entropy, grounded in statistical physics and information theory, provides a "state-centric" lens to capture plasticity and order, offering insight into how leukemia evolves across diagnosis, relapse, and therapeutic stress.

### Aims

The objective of this study was to determine whether entropy can distinguish newly diagnosed from relapsed B-ALL and pediatric from adult disease, and whether it adds an orthogonal layer of evidence beyond traditional gene-centric approaches.

### Methods

Bulk RNA-seq data from 272 patients were preprocessed by filtering low-expression genes, normalized with TMM factors and CPM + voom, and batch-corrected with ComBat. Shannon entropy (H) and normalized entropy (H_norm = H/logN) were calculated globally and across 50 MSigDB Hallmark gene sets, after mapping identifiers to ensure proper module definition. Group differences were assessed with Wilcoxon rank-sum tests, while linear models evaluated age–status interactions. Single-cell RNA-seq datasets (scCTDB) spanning pre-treatment, post-CAR-T, and relapse stages were used for validation, and entropy results were integrated with DEG profiles using Fisher's exact tests.

### Results

The analysis of 272 bulk RNA-seq profiles revealed age-dependent differences in transcriptomic entropy. Pediatric patients showed significantly higher global entropy than adults ($p = 0.01604$), a difference that was strongest at diagnosis ($p = 0.00048$) but disappeared in relapse ($p = 0.2455$), indicating convergence across age groups. Global entropy did not distinguish diagnosis from relapse overall ($p = 0.8807$), suggesting that relapse signals are concentrated in specific modules. Linear modeling of the relapse signature indicated reduced entropy linked to clinical status ($\beta = -0.10451$, $p = 0.0526$) and age ($\beta = -0.11533$, $p = 0.0265$), consistent with a shift toward rigidity.
Single-cell RNA-seq validation reinforced these findings. In one post-CAR-T patient, plasticity collapsed ($p < 2.2 \times 10^{-16}$) with activation of relapse programs ($p = 4.354 \times 10^{-8}$; $\beta = -0.00542$, $p = 5.13 \times 10^{-10}$). In a relapse case after CAR-T, both loss of plasticity and activation of inflammatory/metabolic pathways were extremely robust ($p < 2.2 \times 10^{-16}$; $\beta = -0.02165$ and $\beta = -0.03359$). Modular entropy confirmed consistent reductions in gene diversity, with $\Delta H$ values of −0.13 to −0.37 across IL6/JAK/STAT3, Glycolysis, and TNFα/NFκB signaling, defining rigid macrostates associated with resistance.

**Copyright Information:** (Online) ISSN: 2572-9241
©2026 The Author(s). HemaSphere is published by John Wiley & Sons Ltd on behalf of European Hematology Association. This is an open access Abstract Book distributed under the Attribution-NonCommercial-NoDerivs (CC BY-NC-ND), which allows third parties to download the articles and share them with others as long as they credit the author and the Abstract Book, but they cannot change the content in any way or use them commercially.
**Abstract Book Citations:** Authors, Title, HemaSphere, 2026;10:(S1):pages. The abstract book DOIs can be found on the "Author Information" page.
**Disclaimer:** Articles published in the journal HemaSphere exclusively reflect the opinions of the authors. The authors are responsible for all content in their abstracts including accuracy of the facts, statements, citing resources, etc.

**Summary/Conclusion**

The analysis suggests that relapse in B-ALL reshapes the transcriptome by reducing plasticity and erasing age-related distinctions, leading to a convergent resistant state characterized by rigidity and metabolic dominance. Entropy emerges as a complementary perspective to differential expression, highlighting structural reorganization rather than isolated gene changes and possibly providing a broader framework to understand how disease progression and therapeutic stress influence transcriptomic architecture, offering potential value for future strategies in risk assessment and treatment design.

**Copyright Information:** (Online) ISSN: 2572-9241
©2026 The Author(s). HemaSphere is published by John Wiley & Sons Ltd on behalf of European Hematology Association. This is an open access Abstract Book distributed under the Attribution-NonCommercial-NoDerivs (CC BY-NC-ND), which allows third parties to download the articles and share them with others as long as they credit the author and the Abstract Book, but they cannot change the content in any way or use them commercially.
**Abstract Book Citations:** Authors, Title, HemaSphere, 2026;10:(S1):pages. The abstract book DOIs can be found on the "Author Information" page.
**Disclaimer:** Articles published in the journal HemaSphere exclusively reflect the opinions of the authors. The authors are responsible for all content in their abstracts including accuracy of the facts, statements, citing resources, etc.

### (PB2553) TARGETING GPR183 REVERSES CHEMORESISTANCE AND SUPPRESSES LEUKEMIC PROGRESSION IN ACUTE MYELOID LEUKEMIA BY MODULATING GLYCOSPHINGOLIPID METABOLISM

**Topic:** 01. Acute lymphoblastic leukemia - Biology & translational research

Haihui Zhuang*[1], Fenglin Li[1], Xia Jiang[1], Wenjie Sha[1, 1], Ying Lu[1]

[1]*Department of Hematology, The Affiliated People's Hospital of Ningbo University, Ningbo, China;*

### Background

Treatment failure in acute myeloid leukemia (AML) is primarily attributed to chemoresistance and disease progression. Through constructing homoharringtonine (HHT)-resistant AML cell lines coupled with transcriptomic analysis, we previously identified GPR183 as significantly upregulated in resistant cells. GPR183 expression negatively correlated with HHT sensitivity in both cell lines and primary patient samples, and served as an independent prognostic factor for overall survival in AML patients. However, the underlying mechanisms and therapeutic targeting of GPR183 remain unexplored.

### Aims

To elucidate the mechanism by which GPR183 drives chemoresistance and malignant progression in AML, and to identify and characterize small-molecule inhibitors targeting GPR183 with anti-leukemic efficacy.

### Methods

GPR183 was knocked down in MV4-11 cells via lentiviral-mediated shRNA transfection. The effects on malignant phenotypes were assessed by MTS assay for proliferation, flow cytometry for apoptosis, and Transwell assay for migration. Gain-of-function studies were performed by overexpressing GPR183 in AML cells, and chemosensitivity to idarubicin (IDA) and daunorubicin (DNR) was evaluated. To explore downstream mechanisms, proteomic profiling was performed on GPR183-knockdown MV4-11 cells, followed by KEGG pathway enrichment analysis and correlation validation using TCGA database. For inhibitor screening, structure-based virtual screening of the ChemDiv database (~1.5 million compounds) was conducted to identify potential GPR183 binders. Compound N6 was selected based on molecular docking and drug sensitivity assays, and direct interaction with GPR183 was confirmed by drug affinity responsive target stability (DARTS) assay. Anti-leukemic activity was evaluated in 7 AML cell lines (including HHT-resistant sublines) and 13 primary patient samples using MTS and apoptosis assays. In vivo efficacy and safety were further assessed in an MV4-11-derived CDX mouse model.

### Results

GPR183 knockdown significantly inhibited proliferation, induced apoptosis, and impaired migration in MV4-11 cells. Conversely, GPR183 overexpression promoted cell proliferation and induced resistance to idarubicin and daunorubicin. In vivo, GPR183 knockdown reduced bone marrow infiltration and prolonged survival in CDX mice ($p<0.01$). Proteomic analysis revealed significant enrichment of the glycosphingolipid biosynthesis pathway upon GPR183 knockdown, with downregulation of key enzymes including ST3GAL5 and UGCG. TCGA data confirmed positive correlations between GPR183 and UGCG ($R=0.42$) as well as ST3GAL5 ($R=0.38$). Virtual screening identified compound N6, and DARTS assay confirmed its direct binding to GPR183. N6 exhibited potent cytotoxic effects across 7 AML cell lines and 13 primary AML samples, and induced substantial apoptosis in AML cells. In the CDX model, N6 administration (20mg/kg, i.p., thrice weekly) significantly reduced bone marrow leukemic burden and extended median survival compared to control ($p<0.01$), with no observable toxicity.

**Copyright Information:** (Online) ISSN: 2572-9241
©2026 The Author(s). HemaSphere is published by John Wiley & Sons Ltd on behalf of European Hematology Association. This is an open access Abstract Book distributed under the Attribution-NonCommercial-NoDerivs (CC BY-NC-ND), which allows third parties to download the articles and share them with others as long as they credit the author and the Abstract Book, but they cannot change the content in any way or use them commercially.
**Abstract Book Citations:** Authors, Title, HemaSphere, 2026;10:(S1):pages. The abstract book DOIs can be found on the “Author Information” page.
**Disclaimer:** Articles published in the journal HemaSphere exclusively reflect the opinions of the authors. The authors are responsible for all content in their abstracts including accuracy of the facts, statements, citing resources, etc.

Summary/Conclusion

**This study uncovers a novel mechanism by which GPR183 drives AML progression through modulating glycosphingolipid metabolism. We identify N6 as a GPR183-targeting inhibitor with potent and selective anti-leukemic activity in vitro and in vivo. Targeting GPR183 represents a promising therapeutic strategy to overcome chemoresistance and improve outcomes in AML.**

**Copyright Information:** (Online) ISSN: 2572-9241
©2026 The Author(s). HemaSphere is published by John Wiley & Sons Ltd on behalf of European Hematology Association. This is an open access Abstract Book distributed under the Attribution-NonCommercial-NoDerivs (CC BY-NC-ND), which allows third parties to download the articles and share them with others as long as they credit the author and the Abstract Book, but they cannot change the content in any way or use them commercially.
**Abstract Book Citations:** Authors, Title, HemaSphere, 2026;10:(S1):pages. The abstract book DOIs can be found on the "Author Information" page.
**Disclaimer:** Articles published in the journal HemaSphere exclusively reflect the opinions of the authors. The authors are responsible for all content in their abstracts including accuracy of the facts, statements, citing resources, etc.

### (PB2554) GERMLINE COHESIN GENES ALTERATIONS IN PEDIATRIC ACUTE LYMPHOBLASTIC LEUKEMIA

**Topic:** 01. Acute lymphoblastic leukemia - Biology & translational research

Silvia Rasetti*[1, 2], Stefano Rebellato[2], Domenico Gaspari[2], Claudia Saitta[2], Denise Acunzo[2], Chiara Palmi[2], Laura Bettini[1], Grazia Fazio[2], Giovanni Cazzaniga[1]

*[1]University of Milano Bicocca, Monza, Italy;[2]Fondazione Tettamanti, IRCCS San Gerardo Dei Tintori, Monza, Italy;*

### Background

Leukemia is a complex disease whose molecular mechanisms of malignant transformation are not fully understood. Cohesins are essential proteins forming a complex with key roles not only in canonical DNA binding and chromosome segregation but also in non-canonical regulation of gene expression in both proliferating and post-mitotic cells. Alterations or mutations in genes encoding cohesin components may impair chromosomal stability and contribute to leukemia pathogenesis. Germline mutations cause Cohesinopathies such as the Cornelia de Lange Spectrum (CdLSp), whereas Somatic cohesin mutations have been reported in myeloproliferative disorders, pediatric Acute Myeloblastic Leukemia (AML) and pediatric Acute Lymphoblastic Leukemia (ALL). We previously reported a DNA-targeted screening of 120 consecutive pediatric ALL cases which has identified 10 carriers of 11 germline cohesin gene variants in STAG1 and SMC3 (2 variants each), NIPBL and SMC1A (three variants) and STAG2 (one variant). Among those aberrations, 7 were exonic and 4 were located and the 5'-3' UTR regulatory regions (Rigotti, Rebellato et al. 2025). Investigations studies on the functional role of germline cohesin aberrations in ALL are needed to understand their contribution to predisposition and pathogenesis of ALL.

### Aims

This study aims to understand the molecular mechanisms affected by Cohesin genes alterations in B-ALL.

### Methods

Different tools were used to predict the pathogenicity of the 11 germline variants found in the DNA-targeted screening of120 consecutive pediatric ALL cases. Western blot analyses were performed on 2 of the 6 missense variants in patient-derived *STAG1*-mutated LCLs.
To develop an *in vitro* model for functional studies we generated two STAG1-mutated Lymphoblastoid Cell Lines (LCLs) through CRISPR-Cas9 strategy on which we performed RNA sequencing and western blot analyses.
Germline variants were validated in tumor and remission samples by using real-time qPCR.

### Results

The DNA-targeted screening of 120 consecutive pediatric ALL cases has shown that 9% (n=11) of patients carries a germline cohesin mutation: 6 missense, 4 in 3′ or 5′ UTRs, and a single splice-acceptor variant. Western blot analyses performed on 2 of the 6 missense variants in patient-derived *STAG1*-mutated LCLs showed a decreased and increased STAG1 protein level, respectively, compared to healthy controls.
Then, we also analyzed STAG1 protein levels in CRISPR-Cas9 mutated LCLs, and both lines showed a decreased level of STAG1 protein compared to healthy controls.
Moreover, lymphoblastoid cells lines were used to generate human induced pluripotent stem cells (hIPSCs) which will be differentiated into B cells and used as an *in vitro* model for functional studies.

**Copyright Information:** (Online) ISSN: 2572-9241
©2026 The Author(s). HemaSphere is published by John Wiley & Sons Ltd on behalf of European Hematology Association. This is an open access Abstract Book distributed under the Attribution-NonCommercial-NoDerivs (CC BY-NC-ND), which allows third parties to download the articles and share them with others as long as they credit the author and the Abstract Book, but they cannot change the content in any way or use them commercially.
**Abstract Book Citations:** Authors, Title, HemaSphere, 2026;10:(S1):pages. The abstract book DOIs can be found on the “Author Information” page.
**Disclaimer:** Articles published in the journal HemaSphere exclusively reflect the opinions of the authors. The authors are responsible for all content in their abstracts including accuracy of the facts, statements, citing resources, etc.

**Summary/Conclusion**

Collectively, these observations endeavor to elucidate the role of germline cohesin events in pediatric ALL. In particular, the investigation seeks to unveil the non-canonical functions of these proteins, thus paving the way for the identification and characterization of a novel and distinct subcategory of ALL within the pediatric population. Further studies will demonstrate whether germline variants in cohesin genes may represent new events predisposing to leukemia.

**Copyright Information:** (Online) ISSN: 2572-9241
©2026 The Author(s). HemaSphere is published by John Wiley & Sons Ltd on behalf of European Hematology Association. This is an open access Abstract Book distributed under the Attribution-NonCommercial-NoDerivs (CC BY-NC-ND), which allows third parties to download the articles and share them with others as long as they credit the author and the Abstract Book, but they cannot change the content in any way or use them commercially.
**Abstract Book Citations:** Authors, Title, HemaSphere, 2026;10:(S1):pages. The abstract book DOIs can be found on the "Author Information" page.
**Disclaimer:** Articles published in the journal HemaSphere exclusively reflect the opinions of the authors. The authors are responsible for all content in their abstracts including accuracy of the facts, statements, citing resources, etc.

### (PB2555) NOVEL CLONAL IMMUNOGLOBULIN GENE REARRANGEMENTS DETECTED BY NEXT-GENERATION SEQUENCING DURING MINIMAL RESIDUAL DISEASE MONITORING: DIFFERENTIATING CLONAL EVOLUTION FROM BENIGN REGENERATION

**Topic:** 01. Acute lymphoblastic leukemia - Biology & translational research

Jiao Chang*[1], Yujiao Jia[1], Bingqing Huang[1], Li Qin[1], Haoxu Wang[1], Ruixue Zhang[1], Jiawei Zhao[1], Lijuan Zhang[1], Qi Sun[1], Lihong Shi[1]

*[1]Institute of Hematology & Blood Diseases Hospital, Chinese Academy of Medical Sciences & Peking Union Medical College, tianjin, China;*

### Background

Measurable residual disease (MRD) is a crucial biomarker for risk stratification and individualized risk-directed therapeutic decision-making in hematologic malignancies. Compared with conventional flow cytometry or quantitative polymerase chain reaction, next-generation sequencing (NGS)-based detection of immunoglobulin (Ig) gene rearrangements offers higher sensitivity, reaching up to $10^{-6}$, enabling more accurate prognostic assessment during MRD monitoring. Our previous study identified that some novel clonal rearrangements detected by NGS-MRD during MRD surveillance in lymphoid malignancies were associated with CAR-T therapy. However, with the expansion of our case series, we have observed that the nature of these emerging novel clones is more diverse.

### Aims

This study seeks to characterize novel clonal rearrangements identified by NGS-Ig MRD testing in lymphoid malignancies, aiming to refine MRD assessment in this patient population.

### Methods

This study enrolled a total of 653 patients with newly diagnosed lymphoid malignancies, including 293 with B-cell acute lymphoblastic leukemia (B-ALL), 171 with chronic lymphocytic leukemia (CLL), and 189 with multiple myeloma (MM). Among them, 62 patients underwent at least two NGS-based Ig gene rearrangement MRD assessments. Clonal evolution was defined as the emergence of new malignant Ig clones or inconsistent changes in baseline clones before and after treatment. The nature of residual cells was comprehensively evaluated through dynamic changes in NGS-Ig rearrangement MRD, integrated with clinical treatment data, various laboratory molecular pathological indicators, and single-cell sequencing approaches.

### Results

Among the 62 patients who underwent at least two NGS-MRD assessments and had new clones detected during MRD monitoring, 24 patients exhibited new clones with consistent evolutionary trends with their initial clones, suggesting tumor-origin clonal evolution. In 38 patients, the new clones showed inconsistent evolutionary trends with the initial clones. Among these, 6 cases were associated with CAR-T therapy, 6 with allogeneic hematopoietic stem cell transplantation, and 1 with clinical infection; in 1 patient, the new clone might be related to another concurrent disease state; 6 patients presented with multiple new clones of varying nature; and 18 patients had new clones that persisted from baseline samples without significant changes following clinical treatment. Using single-cell sequencing, we further discovered that these persistent, relatively stable new clonal rearrangements might be associated with normal B cells.

**Copyright Information:** (Online) ISSN: 2572-9241
©2026 The Author(s). HemaSphere is published by John Wiley & Sons Ltd on behalf of European Hematology Association. This is an open access Abstract Book distributed under the Attribution-NonCommercial-NoDerivs (CC BY-NC-ND), which allows third parties to download the articles and share them with others as long as they credit the author and the Abstract Book, but they cannot change the content in any way or use them commercially.
**Abstract Book Citations:** Authors, Title, HemaSphere, 2026;10:(S1):pages. The abstract book DOIs can be found on the "Author Information" page.
**Disclaimer:** Articles published in the journal HemaSphere exclusively reflect the opinions of the authors. The authors are responsible for all content in their abstracts including accuracy of the facts, statements, citing resources, etc.

**Summary/Conclusion**

NGS-based MRD detection via Ig gene rearrangements not only enables highly sensitive assessment of MRD levels in patients with lymphoid malignancies but also allows for the detection of clonal evolution and facilitates the characterization of emerging novel clones. The identification of clonal evolution aids in the early recognition and intervention of patients at higher risk of relapse, while the determination of the nature of novel clonal rearrangements helps avoid misinterpretation caused by non-malignant emerging clones, serving as a powerful complement to conventional MRD assessment.

**Copyright Information:** (Online) ISSN: 2572-9241
©2026 The Author(s). HemaSphere is published by John Wiley & Sons Ltd on behalf of European Hematology Association. This is an open access Abstract Book distributed under the Attribution-NonCommercial-NoDerivs (CC BY-NC-ND), which allows third parties to download the articles and share them with others as long as they credit the author and the Abstract Book, but they cannot change the content in any way or use them commercially.
**Abstract Book Citations:** Authors, Title, HemaSphere, 2026;10:(S1):pages. The abstract book DOIs can be found on the "Author Information" page.
**Disclaimer:** Articles published in the journal HemaSphere exclusively reflect the opinions of the authors. The authors are responsible for all content in their abstracts including accuracy of the facts, statements, citing resources, etc.

### (PB2556) GL-V9, A NOVEL NATURAL FLAVONOID DERIVATIVE, DISPLAYING AN ANTI-TUMOR EFFECT IN T CELL ACUTE LYMPHOBLASTIC LEUKEMIA

**Topic:** 01. Acute lymphoblastic leukemia - Biology & translational research

Mengyue Li[1], Qi Han[1], Qinglong Guo[2], Hui Hui[2], Chunhua Song[3], Zheng Ge*[1]

*[1]Zhongda Hospital, Southeast University, Institute of Hematology Southeast University, Nanjing, China;[2]State Key Laboratory of Natural Medicines, Jiangsu Key Laboratory of Carcinogenesis and Intervention, School of Basic Medicine and Clinical Pharmacy, China Pharmaceutical University, Nanjing, China; [3]Division of Hematology, The Ohio State University Wexner Medical Center, the James Cancer Hospital, Columbus, State of Ohio, United States of America;*

### Background

T-cell acute lymphoblastic leukemia (T-ALL) is an aggressive hematological malignance with limited treatment choices and extremely poor prognosis. GL-V9, a nature compound derived from wogonin, has shown superior anti-tumor potential by inhibiting cancer cell growth and low toxicity, while the anti-leukemia effect and molecular mechanism against T-ALL remain elusive.

### Aims

In this study, we investigated the anti-leukemia effect of GL-V9 as a single drug in T-ALL cells both in vivo and in vitro levels, aiming to provide evidence for future clinical treatment.

### Methods

Treat all cells with GL-V9 from China Pharmaceutical University (Nanjing, China). We employed Cell Counting Kit-8 (CCK-8) to assess cell viability in T-ALL cells(CCRF-CEM and MOLT-4) and primary cells from patients with T-ALL from Zhongda Hospital (Nanjing, China), Annexin-V/PI staining followed by flow cytometry analysis to measure apoptosis. Preliminary establish mice model by injecting T-ALL primary cells into immunodeficient NCG mice intravenously and treat mice with GL-V9 at different concentrations or vehicle by gavage. Observe the survival difference between 4 groups of mice. Test T-ALL cell proliferation and infiltration condition in vivo by flow cytometry. Transcriptome was analyzed by RNA-seq in CCRF-CEM cells treated with 5μM GL-V9 for 48 hours,differentially expressed genes (DEGs) were identified using R-software and subjected to KEGG (Kyoto Encyclopedia of Genes andGenomes) analysis.

### Results

GL-V9 had a dose-/time-dependent effect on cell proliferation arrest with an IC50 at 2-5uM in T-ALL cell lines, even as a single drug (Fig.1A,E). Compared with the control group, GL-V9 monotherapy also significantly increased the percentage of apoptotic cells in CCRF-CEM, MOLT-4, and Jurkat cells ($p<0.0001$) (Fig.1B). In primary cells from several T-ALL patients, GL-V9 monotherapy also dose-dependently reduced cell viability with an IC50 at 3-5uM (Fig.1C) . Moreover, various doses of GL-V9 induced a substantial increase in cell apoptosis in primary cells compared to the control group (Fig.1D). In a mouse model, GL-V9 at 300mg/kg orally markedly suppressed T-ALL cell proliferation and infiltration(Fig.1F,G,H). Pathway analysis the DEGs showed that metabolic pathways were obviously altered after GL-V9 treated ($P<0.001$) (Fig 1I,K). In the GSE13159 database, the AHCYL2,NDST3,PHGDH and SHMT2 were upregulated in patients ($p < 0.001$). Sequencing results revealed that following GL-V9 treatment, these genes exhibited significant downregulation, suggesting they may play a role in the anti-tumour effects of GL-V9(Fig 1J).

**Copyright Information:** (Online) ISSN: 2572-9241
©2026 The Author(s). HemaSphere is published by John Wiley & Sons Ltd on behalf of European Hematology Association. This is an open access Abstract Book distributed under the Attribution-NonCommercial-NoDerivs (CC BY-NC-ND), which allows third parties to download the articles and share them with others as long as they credit the author and the Abstract Book, but they cannot change the content in any way or use them commercially.
**Abstract Book Citations:** Authors, Title, HemaSphere, 2026;10:(S1):pages. The abstract book DOIs can be found on the "Author Information" page.
**Disclaimer:** Articles published in the journal HemaSphere exclusively reflect the opinions of the authors. The authors are responsible for all content in their abstracts including accuracy of the facts, statements, citing resources, etc.

### Summary/Conclusion

Our results indicate novel natural flavonoid derivative GL-V9 monotherapy has high efficient anti-tumor effect on T-ALL whether in vivo or in vitro. With preliminary in vivo investigation, our results also provide a potential chemotherapy-free option for T-ALL patients.

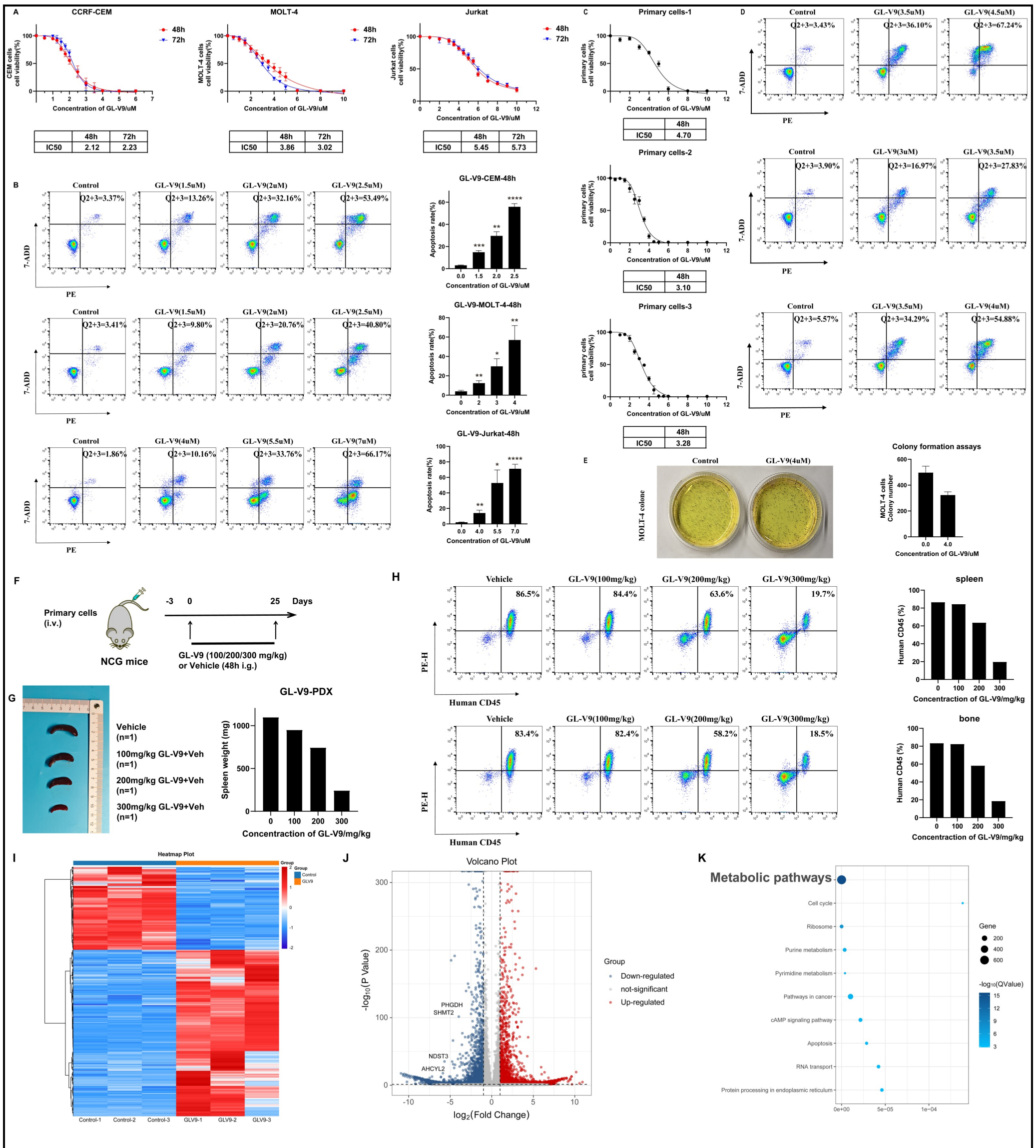

Fig 1: In vitro(A-E): effect of GL-V9 on cell proliferation arrest(A,E) and apoptosis(B) in T-ALL cell lines. Effect of GL-V9 on cell proliferation arrest(C) and apoptosis(D) in T-ALL primary cells. In vivo(F-H):GL-V9 remarkably suppressed T-ALL cell proliferation and infiltration in mice model. (F) Establish a patient-derived tumor xenograft (PDX) model. (G)Schematic diagram of spleen size and weight in mice treated with different concentrations of GL-V9 vs. control group. (H)GL-V9 reduces the proportion of huCD45+ cells of the spleen and bone marrow in a mouse model. Molecular Mechanism(I-K): Effects of GL-V9 on transcriptome Levels in T-ALL cells and signaling pathway analysis.Heatmp plot(I),volcano plot(J) and KEGG analysis results(K) of differentially expressed genes identified by RNA-seq analysis after 48 hours of 5uM GL-V9 treatment in CCRF-CEM cells.

**Copyright Information:** (Online) ISSN: 2572-9241
©2026 The Author(s). HemaSphere is published by John Wiley & Sons Ltd on behalf of European Hematology Association. This is an open access Abstract Book distributed under the Attribution-NonCommercial-NoDerivs (CC BY-NC-ND), which allows third parties to download the articles and share them with others as long as they credit the author and the Abstract Book, but they cannot change the content in any way or use them commercially.
**Abstract Book Citations:** Authors, Title, HemaSphere, 2026;10:(S1):pages. The abstract book DOIs can be found on the "Author Information" page.
**Disclaimer:** Articles published in the journal HemaSphere exclusively reflect the opinions of the authors. The authors are responsible for all content in their abstracts including accuracy of the facts, statements, citing resources, etc.

### (PB2557) HDAC6 INHIBITOR ACY-738 INDUCES FERRITINOPHAGY-MEDIATED FERROPTOSIS IN LEUKEMIA

**Topic:** 01. Acute lymphoblastic leukemia - Biology & translational research

Guancui Yang*[1, 2], Jiarun Li[1], Peijie Jiang[1], Xioalong Tian[1], Xueqing Yang[1, 3], Xi Zhang[1], Jinyi Liu[1]

*[1]Medical Center of Hematology, Second Affiliated Hospital, Army Medical University, Chongqing, China;[2]School of Medicine, Chongqing University, Chongqing, China; [3]Department of Hematology, Affiliated Hospital of North Sichuan Medical College, Nanchong, China;*

**Background**

Leukemia is one of the most challenging hematological system tumor with poor prognosis. Frequent recurrence and drug resistance seriously restrict the clinical efficacy. Ferroptosis is an iron-dependent form of regulated cell death and plays a vital role in tumor suppression. Ferroptosis inducing agents have been studied extensively as a novel promising way to fight against therapy resistant cancers. The aim of this study is to investigate the mechanism of ACY-738, a kind of HDAC6 inhibitor, as a novel ferroptosis inducer in tumor suppression.

**Methods**

The response of cells to ACY-738 was assessed by cell viability assay, EdU assay and clonogenic assay. Proteomic analysis and metabolomic analysis were used to identify downstream targets of GD3s. RNA sequencing analysis and metabolomic analysis was used to explore the possible biological processes, molecular functions and signaling pathways of ACY-738. The morphological changes of mitochondria were observed by transmission electron microscope. The level of ferroptosis was determined by the lipid peroxidation assay, cystine uptake assay, glutathione content detection assay and the level of free iron. The mechanism was verified by molecular approaches including Western blot, quantitative real-time PCR and immunofluorescence.

**Results**

ACY-738 inhibited the proliferation, DNA synthesis and colony formation of leukemia cells, and induced an oxidative cellular microenvironment resulting in ferroptosis of leukemia cells. ACY-738-induced ferroptosis was accompanied with the decreased of cystine uptaking, attenuation of glutathione levels and increased of lipid peroxidation. ACY-738 also increased the level of intracellular free iron and the level of labile iron pool, which also led to ferroptosis. Mechanistically, ACY-738 inhibited the binding of HDAC6 and NCOA4, reduced the ubiquitination degradation of NCOA4, increased the expression of autophagy-related protein LC3B and P62, promoted the autophagic degradation of NCOA4-mediated ferritin, led to the increase in the labile iron pool. ACY-738 also inhibited the expression of SLC7A11, resulting in decreased intracellular cystine levels and attenuation of glutathione levels, indirectly inhibiting GPX4 activity, which promoted lipid peroxidation and ultimately inducing ferroptosis in cells.

**Summary/Conclusion**

In summary, we provided the evidence that HDAC6 inhibitor ACY-738 inhibited the proliferation and induced ferroptosis in leukemia cells. Mechanistically, ACY-738 induces ferroptosis through inhibits the binding of HDAC6 and NCOA4 by targeting HDAC6, reduces the ubiquitination degradation of NCOA4, and promotes NCOA4-mediated ferritinophagy. ACY-738, identified as a novel ferroptosis inducer, may be effective chemosensitizer that can expand the efficacy and range of chemotherapeutic agents.

**Copyright Information:** (Online) ISSN: 2572-9241
©2026 The Author(s). HemaSphere is published by John Wiley & Sons Ltd on behalf of European Hematology Association. This is an open access Abstract Book distributed under the Attribution-NonCommercial-NoDerivs (CC BY-NC-ND), which allows third parties to download the articles and share them with others as long as they credit the author and the Abstract Book, but they cannot change the content in any way or use them commercially.
**Abstract Book Citations:** Authors, Title, HemaSphere, 2026;10:(S1):pages. The abstract book DOIs can be found on the "Author Information" page.
**Disclaimer:** Articles published in the journal HemaSphere exclusively reflect the opinions of the authors. The authors are responsible for all content in their abstracts including accuracy of the facts, statements, citing resources, etc.

### (PB2558) T-CELL KINETICS DURING FIRST-LINE BLINATUMOMAB THERAPY IN BCP-ALL PATIENTS

**Topic:** 01. Acute lymphoblastic leukemia - Biology & translational research

Rosan Olsman*[1], Eske van Baalen[2], Mattias Hofmans[3, 4], Patrycja Gradowska[2, 5], Anita Rijneveld[2], Vincent van der Velden[1]

*[1]Erasmus MC, Rotterdam, The Netherlands; [2]Erasmus MC Cancer Institute, Rotterdam, The Netherlands; [3]Ghent University Hospital, Ghent, Belgium; [4]Ghent University, Ghent, Belgium; [5]HOVON Foundation, Rotterdam, The Netherlands;*

### Background

Adult patients with B-cell precursor acute lymphoblastic leukemia (BCP-ALL), specifically those aged ≥40 years, have a discouraging prognosis, with only 25-57% achieving long-term survival following standard treatments. Blinatumomab, a bispecific T-cell engager targeting $CD19^+$ cells, improves patient outcomes in the relapsed/refractory setting through T-cell-mediated cytotoxicity.

### Aims

The contribution of T-cell subsets to treatment response in newly diagnosed adult BCP-ALL patients, both at baseline and during first-line blinatumomab therapy, is not fully understood. Flow cytometric immunophenotyping was performed to characterize baseline immune profiles, their dynamic changes during blinatumomab therapy, and its association with clinical response.

### Methods

In the HOVON146 (NCT03541083) single-arm phase II trial, newly diagnosed adult BCP-ALL patients received first-line blinatumomab. Peripheral blood leukocyte populations were analyzed at baseline (TP1), after 5 days of steroid pre-treatment (TP2), and after 7 (TP3) and 14 (TP4) days of blinatumomab. Leukocytes, with a specific focus on T-cell subsets, were characterized using four 8-color flow cytometry tubes. Immune populations were identified with FlowSOM and analyzed using generalized linear mixed models. Treatment response was assessed by cytological assessment of the bone marrow.

### Results

A total of 71 patients were included (median age, 53 years; range: 18-70), of whom 70 completed first-line blinatumomab (8 with toxicity-related interruption). Immune profiling was available for 66 patients, and 46/66 (70%) achieved complete remission (CR) at TP4. Non-responders (NR), defined as patients not achieving CR at TP4, showed no significant differences from responders in peripheral blood blast counts ($p = 0.628$) or bone marrow blast percentage ($p = 0.769$) at TP1 (*Fig. 1A*). Similarly, at TP1, no differences were observed in total $CD3^+$ T-cells, $CD4^+$ T-cells or $CD8^+$ T-cells, or their respective subsets, except for naïve T-regulatory cells, which were significantly higher in NR ($p = 0.017$; *Fig. 1C*). Moreover, the reduction in peripheral blood blasts during 5 days of steroid pre-treatment was markedly less pronounced in NR (TP1-TP2; $p = 0.02$), and this disparity persisted after the start of blinatumomab (*Fig. 1B*). The overall kinetics of most T-cell subsets did not differ significantly between responders and NR. However, at TP4, NR exhibited significantly higher frequencies of naïve T-regulatory cells ($p = 0.023$; *Fig. 1C*), naïve $CD8^+$ T-cells ($p = 0.003$; *Fig. D*), and naïve $CD4^+$ T-cells ($p = 0.03$; *Fig. 1E*).

**Copyright Information:** (Online) ISSN: 2572-9241
©2026 The Author(s). HemaSphere is published by John Wiley & Sons Ltd on behalf of European Hematology Association. This is an open access Abstract Book distributed under the Attribution-NonCommercial-NoDerivs (CC BY-NC-ND), which allows third parties to download the articles and share them with others as long as they credit the author and the Abstract Book, but they cannot change the content in any way or use them commercially.
**Abstract Book Citations:** Authors, Title, HemaSphere, 2026;10:(S1):pages. The abstract book DOIs can be found on the “Author Information” page.
**Disclaimer:** Articles published in the journal HemaSphere exclusively reflect the opinions of the authors. The authors are responsible for all content in their abstracts including accuracy of the facts, statements, citing resources, etc.

### Summary/Conclusion

Immunological dynamics in newly diagnosed adult BCP-ALL patients showed that baseline blast count did not distinguish responders from NR, whereas steroid pre-treatment sensitivity did. Additionally, NR displayed higher frequencies of naïve regulatory T-cells at TP1, which persisted after blinatumomab initiation, along with elevated naïve $CD8^{+}/CD4^{+}$ T-cells. This enrichment of naïve T-cells suggests a less differentiated T-cell compartment, which may result in less cytotoxic responses. Larger studies are needed to validate these findings and define immune biomarkers that can guide patient stratification.

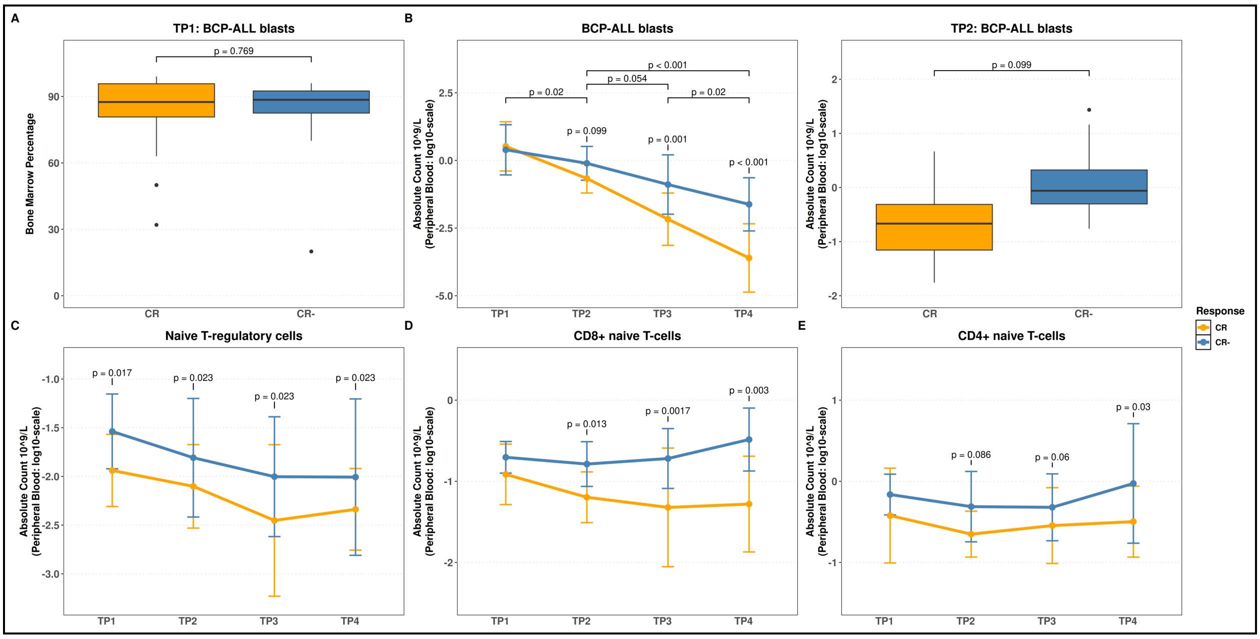

Figure 1: (A) Blast percentage in the bone marrow. (B) Absolute blast counts in peripheral blood. Absolute count kinetics of (C) naïve T-regulatory cells, (D) CD8+ naïve T-cells, and (E) CD4+ naïve T-cells. CR-: No Complete Remission. CR: Complete Remission.

**Copyright Information:** (Online) ISSN: 2572-9241
©2026 The Author(s). HemaSphere is published by John Wiley & Sons Ltd on behalf of European Hematology Association. This is an open access Abstract Book distributed under the Attribution-NonCommercial-NoDerivs (CC BY-NC-ND), which allows third parties to download the articles and share them with others as long as they credit the author and the Abstract Book, but they cannot change the content in any way or use them commercially.
**Abstract Book Citations:** Authors, Title, HemaSphere, 2026;10:(S1):pages. The abstract book DOIs can be found on the "Author Information" page.
**Disclaimer:** Articles published in the journal HemaSphere exclusively reflect the opinions of the authors. The authors are responsible for all content in their abstracts including accuracy of the facts, statements, citing resources, etc.

### (PB2559) CLINICAL AND GENOMIC LANDSCAPE OF STAG2/LMO2 SUBTYPE T-ALL IN YOUNG CHILDREN: A SINGLE-CENTER COHORT WITH SYSTEMATIC LITERATURE INTEGRATION

**Topic:** 01. Acute lymphoblastic leukemia - Biology & translational research

Senlin Zhang*[1], Peifang Xiao[1], Jun Lu[1], Yu Liu[2], Shaoyan Hu[1]

*[1]Children's Hospital of Soochow University, Department of Hematology and Oncology, Suzhou, China;[2]Shanghai Children's Medical Center, Shanghai, China;*

### Background

The STAG2/LMO2 subtype of pediatric T-cell acute lymphoblastic leukemia (T-ALL) is rare and not fully understood. To explore its clinical, genomic, and prognostic features, we conducted an international collaborative analysis. This study integrates cases from the Children's Hospital of Soochow University with published data from renowned cooperative groups, including COG (Children's Oncology Group) and GRAALL/FRALLE (French Society of Leukemia).

### Aims

We are aim to provide a comprehensive understanding of this subtype, offering insights into its molecular mechanisms, clinical presentation, and potential prognostic markers, which could guide more effective diagnosis and treatment strategies.

### Methods

Fifteen pediatric patients with STAG2/LMO2-associated T-ALL were retrospectively analyzed. Clinical characteristics, treatment response, and survival outcomes were collected. Genomic profiling included whole-genome sequencing, RNA sequencing, and targeted mutation analysis. Event-free survival (EFS) and overall survival (OS) were estimated and compared with other COG-defined pediatric T-ALL subtypes.

### Results

The cohort comprised 11 females and 4 males, with a median age of 1.4 years (range, 0.7–3.4) and a median presenting leukocyte count of 40.3×$10^9$/L (11.1–300), indicating a predominance in very young children. The principal driver event was LMO2::STAG2 fusion (11/15). Additional alterations included LMO2 rearrangements (e.g., LMO2::TCR, LMO2::CELF1), STAG2 truncating variants (including p.E177X; VAF 0.52), and LMO2 enhancer SNV/indels. NOTCH pathway alterations were highly prevalent, with NOTCH1 and/or FBXW7 mutations detected in 14/15 (93.3%) cases. Fourteen patients achieved morphologic complete remission (CR) after induction therapy. The 5-year EFS was 84.9% and the 5-year OS was 93.3%. Survival did not significantly differ from other COG T-ALL subtypes (5-year EFS 84.9% vs 85.7%, P>0.05).

### Summary/Conclusion

STAG2/LMO2 subtype pediatric T-ALL presents at very young age with frequent NOTCH pathway co-mutations and demonstrates survival outcomes comparable to other COG-defined T-ALL subtypes.

**Copyright Information:** (Online) ISSN: 2572-9241
©2026 The Author(s). HemaSphere is published by John Wiley & Sons Ltd on behalf of European Hematology Association. This is an open access Abstract Book distributed under the Attribution-NonCommercial-NoDerivs (CC BY-NC-ND), which allows third parties to download the articles and share them with others as long as they credit the author and the Abstract Book, but they cannot change the content in any way or use them commercially.
**Abstract Book Citations:** Authors, Title, HemaSphere, 2026;10:(S1):pages. The abstract book DOIs can be found on the "Author Information" page.
**Disclaimer:** Articles published in the journal HemaSphere exclusively reflect the opinions of the authors. The authors are responsible for all content in their abstracts including accuracy of the facts, statements, citing resources, etc.

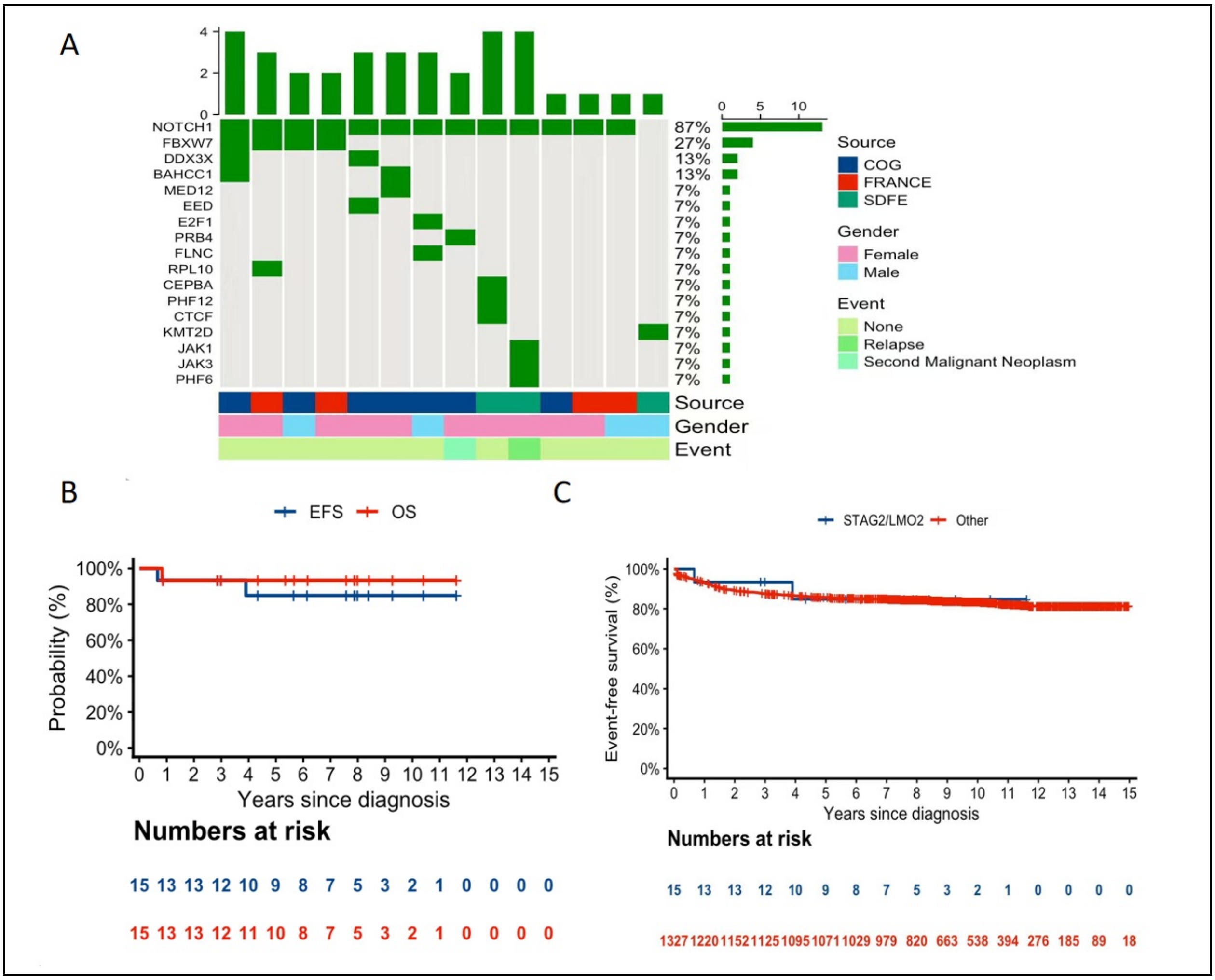

(A)The mutational distribution in the STAG2/LMO2 subtype of pediatric T-ALL. (B): The Event-Free Survival (EFS) and Overall Survival (OS) curves for the STAG2/LMO2 subtype. (C): EFS between the STAG2/LMO2 subtype and COG-other T-ALL subtypes.

Copyright Information: (Online) ISSN: 2572-9241
©2026 The Author(s). HemaSphere is published by John Wiley & Sons Ltd on behalf of European Hematology Association. This is an open access Abstract Book distributed under the Attribution-NonCommercial-NoDerivs (CC BY-NC-ND), which allows third parties to download the articles and share them with others as long as they credit the author and the Abstract Book, but they cannot change the content in any way or use them commercially.
Abstract Book Citations: Authors, Title, HemaSphere, 2026;10:(S1):pages. The abstract book DOIs can be found on the "Author Information" page.
Disclaimer: Articles published in the journal HemaSphere exclusively reflect the opinions of the authors. The authors are responsible for all content in their abstracts including accuracy of the facts, statements, citing resources, etc.

### (PB2560) ASSOCIATION OF VARIANTS IN CANDIDATE PHARMACOGENES WITH RESPONSE TO MERCAPTOPURINE AND METHOTREXATE IN PEDIATRIC ALL: A SINGLE-CENTRE EXPERIENCE FROM CROATIA

**Topic:** 01. Acute lymphoblastic leukemia - Biology & translational research

Isidora Ćurić*[1], Nikola Kotur[1], Bojan Ristivojević[1], Đorđe Pavlović[1], Ivana Grubiša[1], Mia Radosević[2, 2], Ana Đorđević[3], Sonja Pavlovic[1], Jelena Roganovic[4], Branka Zukić[1]

*[1]Institute of Molecular Genetics and Genetic Engineering, Human molecular genetics and genomics, Belgrade, Serbia;[2]Health Center of Primorsko-goranska County, Rijeka, Croatia; [3]Jadran-Galenski Laboratorij, Rijeka, Croatia; [4]Children's Hospital Zagreb, Department for Pediatric Oncology and Hematology, Zagreb, Croatia;*

### Background

Interindividual variability in the efficacy and toxicity of 6-mercaptopurine (6-MP) and methotrexate (MTX) drugs remains a major challenge in the treatment of pediatric acute lymphoblastic leukemia (ALL). Germline variation in pharmacogenes involved in drug metabolism, transport, and folate pathways may contribute to this variability.

### Aims

Variability in 6-MP and MTX tolerance remains a key limitation in pediatric ALL therapy and pharmacogenetic data beyond*TPMT* and *NUDT15* genes are limited in European populations.Hypotesis of this study was whether germline variation in thiopurine, folate and drug transport genes, alone or combined as a polygenic risk score (PRS), influences 6-MP dose tolerance and MTX pharmacokinetics and toxicity in pediatric ALL.

### Methods

In this retrospective cohort study, 43 pediatric ALL patients treated at a tertiary referral centre in Croatia according to ALL IC-BFM 2002 or 2009 protocols were genotyped for eleven variants in *TPMT, ITPA*, *NUDT15*, *PACSIN2*, *MTHFR*, *TYMS*, *SLC19A1* and *SLCO1B1* genes. Associations with average 6-MP dose during maintenance therapy, MTX pharmacokinetics during consolidation and MTX-related toxicity were analyzed. A PRS was constructed to assess cumulative genetic risk of developing toxicity when administering these two drugs.

### Results

Carriers of the *ITPA* rs1127354 variant required significantly lower average 6-MP doses compared with wild-type carriers. The *NUDT15* rs61973267 variant was associated with higher tolerated 6-MP doses. No significant associations were observed between individual variants and MTX clearance. A trend toward reduced risk of oral mucositis was observed in carriers of the *SLCO1B1* rs4149056 variant. PRS analyses that included carriers of the TPMT rs1142345 and ITPA rs1127354 variants showed a borderline association with the requirement for 6-MP dose reduction.

### Summary/Conclusion

Our findings support a role for *ITPA* and *NUDT15* variants in modulating 6-MP dose requirements, while single-variant associations with MTX pharmacokinetics and toxicity were limited and not supported. These results underscore the complexity of antimetabolite pharmacogenetics and suggest that integrative polygenic approaches may better capture clinically relevant pharmacogenetic risk.

**Copyright Information:** (Online) ISSN: 2572-9241
©2026 The Author(s). HemaSphere is published by John Wiley & Sons Ltd on behalf of European Hematology Association. This is an open access Abstract Book distributed under the Attribution-NonCommercial-NoDerivs (CC BY-NC-ND), which allows third parties to download the articles and share them with others as long as they credit the author and the Abstract Book, but they cannot change the content in any way or use them commercially.
**Abstract Book Citations:** Authors, Title, HemaSphere, 2026;10:(S1):pages. The abstract book DOIs can be found on the “Author Information” page.
**Disclaimer:** Articles published in the journal HemaSphere exclusively reflect the opinions of the authors. The authors are responsible for all content in their abstracts including accuracy of the facts, statements, citing resources, etc.

### (PB2562) THE TUMOR SUPPRESSOR WDR82 INHIBITED PROLIFERATION OF B-CELL ACUTE LYMPHOBLASTIC LEUKEMIA CELLS BY REGULATING RB1

**Topic:** 01. Acute lymphoblastic leukemia - Biology & translational research

Dai-Hong Xie*[1], Zhao-Yu Li[1], Zhao-Can Niu[1], Wen-Min Chen[1], Ya-Zhen Qin[1]

[1]*Peking University People's Hospital, Peking University Institute of Hematology, Beijing, China;*

### Background

Adult Philadelphia chromosome (Ph)-negative B-cell acute lymphoblastic leukemia (B-ALL) remains to be a hematologic malignancy with high relapse and poor long-term survival. Further elucidation of the molecular characteristics and prognostic stratification in these patients is crucial. We identified novel SFMBT1-related fusion genes derived from micro-deletion at chromosome 3p21 (Detailed in Abstract EHA-2863), a tumor suppressor-enriched region frequently altered in solid tumors. We focused on genes downstream of SFMBT1 on 3p21, analyzed the relationship between mRNA levels from RNA-sequencing and relapse-free survival (RFS), and identified WD repeat-containing protein 82 (WDR82) as one candidate gene. WDR82 is a component of the histone methyltransferase complex Set1A/Set1B and regulates transcription by recognizing phosphorylated RNA polymerase II. Although WDR82 functions as a tumor suppressor in solid tumors, its role in hematologic malignancies, including B-ALL, is not reported.

### Aims

This study aimed to analyze the expression and prognostic significance of WDR82 in adult Ph-negative B-ALL and explore its effects on B-ALL cell biology and molecular mechanisms.

### Methods

WDR82 mRNA levels in the diagnostic bone marrows of 140 consecutive Ph-negative B-ALL patients were measured by real-time quantitative PCR (RQ-PCR). WDR82-knockdown B-ALL cell lines were established in Nalm6 and BALL-1 via lentiviral transduction, and tested for cell proliferation, cell cycle, differentiation, apoptosis and drug sensitivity in vitro and subcutaneous tumorigenicity in BALB/c nude mice. Transcriptome data of cell lines and public datasets was used to screen potential downstream target gene, which was further validated by RQ-PCR and Western Blot in cell lines and primary samples. Chromatin Immunoprecipitation-quantitative PCR (ChIP-qPCR) was performed to determine direct transcriptional target of WDR82.

### Results

Patients with low WDR82 expression had significantly poorer RFS compared with those with high expression (p=0.034), and low WDR82 expression was an independent adverse prognostic factor of RFS (Hazard ratio (HR)(95%CI): 2.3 (1.0-5.0), p=0.040). In vitro, WDR82 knockdown in Nalm6 and BALL-1 cells significantly promoted cell proliferation, increased the S-phase proportion and reduced sensitivity to vincristine, without affecting differentiation and apoptosis. WDR82 knockdown promoted subcutaneous tumorigenicity in vivo (3/3 vs 1/3), accelerated tumor growth and significantly increased tumor weight (p=0.024) (Figure 1). KEGG enrichment of cell line transcriptome data revealed significant enrichment of cell cycle, including RB1, a key G1/S checkpoint regulator. RB1 mRNA levels was positively correlated with WDR82 in all 4 public transcriptome datasets of newly-diagnosed B-ALL patients. What's more, RB1 mRNA and protein levels were significantly downregulated after WDR82 knockdown in Nalm6 and BALL-1 cells. Both mRNA and protein levels were positively correlated between WDR82 and RB1 in B-ALL patients. Furthermore, ChIP-qPCR demonstrated that WDR82 protein directly bound to the transcription start sites (TSS) of RB1 gene.

**Copyright Information:** (Online) ISSN: 2572-9241
©2026 The Author(s). HemaSphere is published by John Wiley & Sons Ltd on behalf of European Hematology Association. This is an open access Abstract Book distributed under the Attribution-NonCommercial-NoDerivs (CC BY-NC-ND), which allows third parties to download the articles and share them with others as long as they credit the author and the Abstract Book, but they cannot change the content in any way or use them commercially.
**Abstract Book Citations:** Authors, Title, HemaSphere, 2026;10:(S1):pages. The abstract book DOIs can be found on the "Author Information" page.
**Disclaimer:** Articles published in the journal HemaSphere exclusively reflect the opinions of the authors. The authors are responsible for all content in their abstracts including accuracy of the facts, statements, citing resources, etc.

### Summary/Conclusion

Adult Ph-negative B-ALL patients with low WDR82 expression at diagnosis have high risk of relapse. WDR82 promotes RB1 transcription in B-ALL cells by directly binding to its TSS, thereby inhibiting the G1-to-S phase transition and suppressing proliferation. (Grant: The National Natural Science Foundation of China (82370160)).

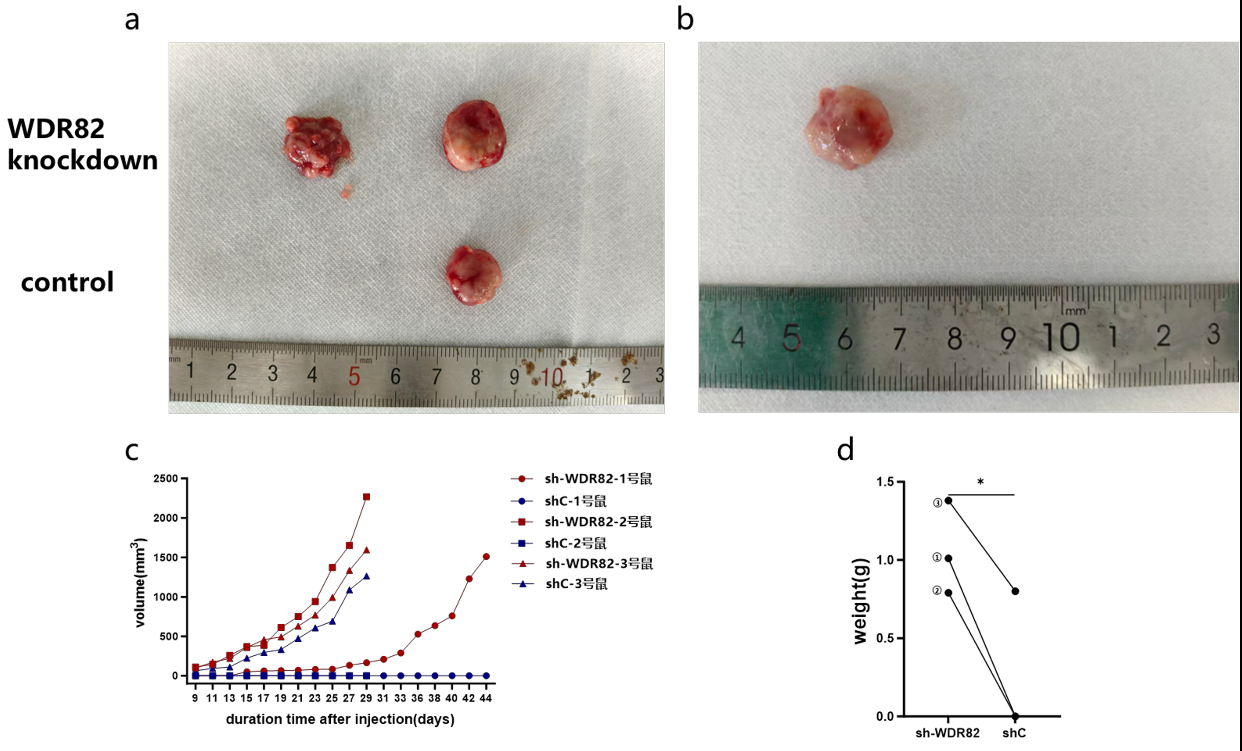

Figure 1. Effect of WDR82 knockdown on subcutaneous tumorigenicity of Nalm6 cells in BALB/c nude mice. (a, b) Representative images of tumors; (c) Tumor growth curves; (d) Tumor weights. * indicates $p < 0.05$.

Copyright Information: (Online) ISSN: 2572-9241
©2026 The Author(s). HemaSphere is published by John Wiley & Sons Ltd on behalf of European Hematology Association. This is an open access Abstract Book distributed under the Attribution-NonCommercial-NoDerivs (CC BY-NC-ND), which allows third parties to download the articles and share them with others as long as they credit the author and the Abstract Book, but they cannot change the content in any way or use them commercially.
Abstract Book Citations: Authors, Title, HemaSphere, 2026;10:(S1):pages. The abstract book DOIs can be found on the "Author Information" page.
Disclaimer: Articles published in the journal HemaSphere exclusively reflect the opinions of the authors. The authors are responsible for all content in their abstracts including accuracy of the facts, statements, citing resources, etc.

### (PB2563) TRANSCRIPTOME ANALYSIS IDENTIFIES A MYELOID-LIKE SIGNATURE IN RELAPSED OR REFRACTORY B-CELL ACUTE LYMPHOCYTIC LEUKEMIA PATIENTS WITH POOR RESPONSE TO CD19 CHIMERIC ANTIGEN RECEPTOR T CELL THERAPY

**Topic:** 01. Acute lymphoblastic leukemia - Biology & translational research

Shisen Wang[1], Wenbing Liu[1], Ying Wang[1], Yingxi Xu[1], Haiyan Xing[1], Kejing Tang[1], Zheng Tian[1], Qing Rao[1], Shaowei Qiu[1], Hui Wei[1], Runxia Gu[1], Min Wang[1], Jianxiang Wang*[1]

*[1]State Key Laboratory of Experimental Hematology, National Clinical Research Center for Blood Diseases, Tianjin Key Laboratory of Cell Therapy for Blood Diseases, Haihe Laboratory of Cell Ecosystem, Institute of Hematology and Blood Diseases Hospital, Tianjin Institutes of Health Science, Chinese Academy of Medical Sciences & Peking Union Medical College, Tianjin, China;*

### Background

Relapsed or refractory B-cell acute lymphoblastic leukemia (R/R B-ALL) patients exhibit heterogeneous responses to CD19 chimeric antigen receptor T-cell (CAR-T) therapy, with underlying molecular mechanisms poorly understood. Currently, CAR-T therapy research predominantly focus on the intrinsic functionality of CAR-T cells or strategies to address target antigen loss, thereby overlooking the influences exerted by the tumor and its microenvironment.

### Aims

This study aimed to identify the underlying molecular disparities in B-ALL patients with poor CD19 CAR-T efficacy.

### Methods

A cohort of 15 R/R B-ALL patients enrolled in a phase II, single-arm clinical trial (NCT04684147) was conducted and stratified into 8 good responders and 7 poor responders based on the occurrence of disease relapse. Differences were analyzed from clinical characteristics, genetic alterations to transcriptomics, and a CAR-T response predictive model was constructed.

### Results

A distinct myeloid-like signature and significant upregulation of myeloid-related genes were identified on poor CD19 CAR-T responders through differential expression analysis and enrichment analysis. The signature and genes expression were further validated in patients, cell lines and at the single-cell level of patients. A seven-gene LASSO model achieved decent predictive performance of CD19 CAR-T response (AUC of 1.00 in internal cohorts and AUC of 0.68 in external independent cohort).

### Summary/Conclusion

The transcriptomic profiling revealed a myeloid-like signature in R/R B-ALL patients with poor CD19 CAR-T response, suggesting a potential mechanism and clinical predictor. These findings provide biomarkers for patient stratification and inform strategies to enhance CD19 CAR-T efficacy.

**Copyright Information:** (Online) ISSN: 2572-9241
©2026 The Author(s). HemaSphere is published by John Wiley & Sons Ltd on behalf of European Hematology Association. This is an open access Abstract Book distributed under the Attribution-NonCommercial-NoDerivs (CC BY-NC-ND), which allows third parties to download the articles and share them with others as long as they credit the author and the Abstract Book, but they cannot change the content in any way or use them commercially.
**Abstract Book Citations:** Authors, Title, HemaSphere, 2026;10:(S1):pages. The abstract book DOIs can be found on the “Author Information” page.
**Disclaimer:** Articles published in the journal HemaSphere exclusively reflect the opinions of the authors. The authors are responsible for all content in their abstracts including accuracy of the facts, statements, citing resources, etc.

### (PB2564) THERAPEUTICAL POTENTIAL OF EPIGENETIC MODULATORS IN CLL AND ALL: IN VITRO AND EX VIVO STUDIES

**Topic:** 01. Acute lymphoblastic leukemia - Biology & translational research

Tânia Gaspar[1], Joana Jorge[1, 2, 3], Raquel Alves[1, 2, 3], Sara Duarte[1, 2, 3, 4], Ana Cristina Gonçalves[1, 2, 3], Ana Ribeiro*[1, 2, 3, 4]

*[1]University of Coimbra, Faculty of Medicine (FMUC), Coimbra, Portugal; [2]University of Coimbra, Coimbra Institute for Clinical and Biomedical Research (iCBR) – Environment, Genetics, and Oncobiology Group (CIMAGO) – FMUC, Center for Innovative Biomedicine and Biotechnology (CIBB), Coimbra, Portugal; [3]Academic Clinical Center, Coimbra, Portugal; [4]Coimbra Local Health Unit, Departament of Clinical Hematology, Coimbra, Portugal;*

### Background

Epigenetic modifications, including DNA methylation and histone modifications, play an important role in the development of acute lymphoblastic leukemia (ALL) and chronic lymphocytic leukemia (CLL). Although these alterations are emerging as therapeutic targets, their clinical relevance in ALL and CLL remains nuclear.

### Aims

This study evaluated the therapeutic potential of epigenetic modulators (epidrugs), namely DNA methyltransferase inhibitors (DNMTis) [azacytidine (AZA), decitabine (DAC)] and histone deacetylase inhibitors (HDACis) [vorinostat (SAHA), panobinostat (LBH-589)] – used alone or in combination in ALL and CLL cell lines (697 and HG3 cells, respectively), as well as in peripheral blood mononuclear cells (PBMCs) from CLL patients (*ex vivo*).

### Methods

HG3 and 697 cells and CLL PBMCs (n=22; 7F/15M; Binet A=13 and B/C=9) were treated with epigenetic drugs, alone or in combination, for 72h and 48h, respectively. Metabolic activity and survival index (SI) were assessed using resazurin and fluorometric microculture cytotoxicity assays. Apoptosis and cell cycle were analyzed by flow cytometry using Annexin V/7-AAD and PI/RNase staining, respectively, with morphological confirmation by May–Grünwald–Giemsa staining. Statistical analyses were performed at a 95% confidence level.

### Results

In both 697 and HG3 cell lines, epidrugs reduced the SI in a dose- and time-dependent manner, except for DAC in HG3 cells, which showed only a predominantly time-dependent effect (at 72h, the IC50 range: AZA 2-19 µM; DAC 3 µM; SAHA 0.5 µM; LBH-589 5-6 nM). In HG3 cells, all combinations significantly reduced metabolic activity compared with monotherapy, whereas in 697 cells no additional reduction was observed. All treatments induced apoptosis in both models (ALL and CLL), with DNMTis showing greater efficacy and DAC-HDACi combinations inducing higher apoptotic rates than AZA-based combinations. The cytostatic effect induced by the drugs differed between cell lines, occurring cell cycle arrest mainly at G2/M in 697 cells and at G0/G1 in HG3 cells, while panobinostat did not significantly affect cell cycle progression. In PBMCs obtained from CLL patients, the tested epidrugs reduced the SI after 48h (IC50: AZA 13 µM; DAC 131 µM; SAHA 0.5 µM; LBH-589 5 nM), and all combinations except AZA/SAHA significantly reduced the survival index compared with monotherapy. The stratification of patients by Binet stage revealed comparable sensitivity to the drugs between early- and advanced-stage disease patients, however with a trend toward increased sensitivity to AZA and panobinostat in cells from Binet B/C patients.

**Copyright Information:** (Online) ISSN: 2572-9241
©2026 The Author(s). HemaSphere is published by John Wiley & Sons Ltd on behalf of European Hematology Association. This is an open access Abstract Book distributed under the Attribution-NonCommercial-NoDerivs (CC BY-NC-ND), which allows third parties to download the articles and share them with others as long as they credit the author and the Abstract Book, but they cannot change the content in any way or use them commercially.
**Abstract Book Citations:** Authors, Title, HemaSphere, 2026;10:(S1):pages. The abstract book DOIs can be found on the "Author Information" page.
**Disclaimer:** Articles published in the journal HemaSphere exclusively reflect the opinions of the authors. The authors are responsible for all content in their abstracts including accuracy of the facts, statements, citing resources, etc.

**Summary/Conclusion**

Epigenetic modulators exhibited consistent antileukemic activity in ALL and CLL models, significantly reducing metabolic activity and survival in a dose- and time-dependent manner through both cytotoxic and cytostatic effects. DNMTis demonstrated stronger pro-apoptotic activity, and DAC-HDACis combinations enhanced apoptotic induction compared with monotherapy. Notably, primary CLL PBMCs were also responsive, with a trend toward increased sensitivity to AZA and panobinostat in advanced-stage (Binet B/C) patients. These findings reinforce the therapeutic relevance of epigenetic targeting in lymphoid leukemias and support further investigation into its clinical applicability.

**Copyright Information:** (Online) ISSN: 2572-9241
©2026 The Author(s). HemaSphere is published by John Wiley & Sons Ltd on behalf of European Hematology Association. This is an open access Abstract Book distributed under the Attribution-NonCommercial-NoDerivs (CC BY-NC-ND), which allows third parties to download the articles and share them with others as long as they credit the author and the Abstract Book, but they cannot change the content in any way or use them commercially.
**Abstract Book Citations:** Authors, Title, HemaSphere, 2026;10:(S1):pages. The abstract book DOIs can be found on the “Author Information” page.
**Disclaimer:** Articles published in the journal HemaSphere exclusively reflect the opinions of the authors. The authors are responsible for all content in their abstracts including accuracy of the facts, statements, citing resources, etc.

### (PB2565) A NOVEL GSPT1 DEGRADER 329 EXHIBITS IN VITRO EFFICACY IN T CELL ACUTE LYMPHOBLASTIC LEUKEMIA

**Topic:** 01. Acute lymphoblastic leukemia - Biology & translational research

Mengyue Li[1], Qi Han[1], Xi Xu[2], Chunhua Song[3], Zheng Ge*[1]

*[1]Zhongda Hospital, Southeast University, Institute of Hematology Southeast University, Nanjing, China;[2]School of Pharmacy, China Pharmaceutical University, Nanjing, China; [3]Division of Hematology, The Ohio State University Wexner Medical Center, the James Cancer Hospital, Columbus, State of Ohio, United States of America;*

### Background

Advancing cure rates for T cell acute lymphoblastic leukemia (T-ALL) has been limited by the lack of agents that effectively kill malignant cells. GSPT1 (G1 to S phase transition 1), or eRF3a, is a critical GTPase involved in translation termination, cell cycle progression, and mRNA decay. GSPT1 is overexpressed in various cancers, including AML. Molecular glues direct the ubiquitin ligase cellular machinery to target neosubstrates for protein degradation. We used a novel molecular glue degrader, 329, that exhibits potent and selective degradation of GSPT1 and cytotoxic activity against lymphoblastic leukemia cell lines.

### Aims

In this study, we investigated the anti-leukemia effect of 329 as a single drug in T-ALL cells, aiming to provide evidence for future clinical treatment.

### Methods

Treat all cells with 329 from China Pharmaceutical University (Nanjing, China). We employed Cell Counting Kit-8 (CCK-8) to assess cell viability in T-ALL cells(CCRF-CEM and MOLT-4) and primary cells from patients with T-ALL from Zhongda Hospital (Nanjing, China), Annexin-V/PI staining followed by flow cytometry analysis to measure apoptosis.

### Results

The GSPT1 molecular glue degraders 329 had a dose-dependent effect on cell proliferation arrest with an IC50 at 12-18nM in T-ALL cell lines, even as a single drug (Fig.1A,C). Compared with the control group, 329 monotherapy also significantly increased the percentage of apoptotic cells in CCRF-CEM and MOLT-4 cells ($p<0.05$) (Fig.1B). In primary cells from several T-ALL patients, 329 also dose-dependently reduced cell viability with an IC50 at 11-13nM (Fig.1C) . Moreover, various doses of 329 induced a substantial increase in cell apoptosis in primary cells compared to the control group (Fig.1D).

### Summary/Conclusion

Our results indicate novel GSPT1 molecular glue degrader 329 has high efficient anti-tumor effect on T-ALL. With further in vivo investigation, our results also provide a potential chemotherapy-free option for T-ALL patients.

**Copyright Information:** (Online) ISSN: 2572-9241
©2026 The Author(s). HemaSphere is published by John Wiley & Sons Ltd on behalf of European Hematology Association. This is an open access Abstract Book distributed under the Attribution-NonCommercial-NoDerivs (CC BY-NC-ND), which allows third parties to download the articles and share them with others as long as they credit the author and the Abstract Book, but they cannot change the content in any way or use them commercially.
**Abstract Book Citations:** Authors, Title, HemaSphere, 2026;10:(S1):pages. The abstract book DOIs can be found on the “Author Information” page.
**Disclaimer:** Articles published in the journal HemaSphere exclusively reflect the opinions of the authors. The authors are responsible for all content in their abstracts including accuracy of the facts, statements, citing resources, etc.

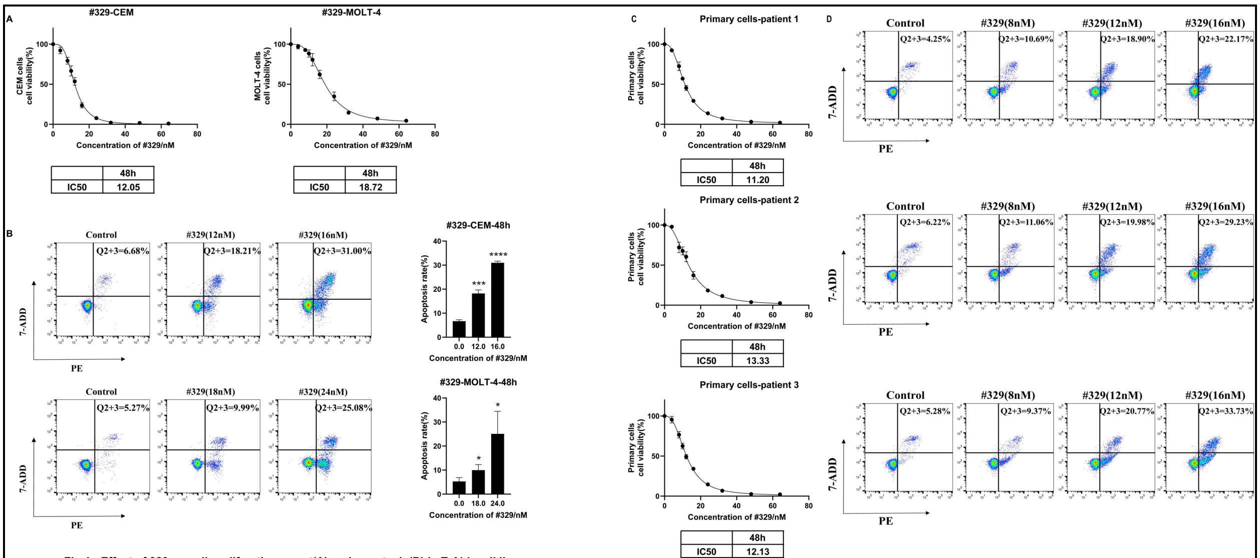

Fig 1 : Effect of 329 on cell proliferation arrest(A) and apoptosis(B) in T-ALL cell lines.
Effect of 329 on cell proliferation arrest(C) and apoptosis(D) in T-ALL primary cells.

**Copyright Information:** (Online) ISSN: 2572-9241
©2026 The Author(s). HemaSphere is published by John Wiley & Sons Ltd on behalf of European Hematology Association. This is an open access Abstract Book distributed under the Attribution-NonCommercial-NoDerivs (CC BY-NC-ND), which allows third parties to download the articles and share them with others as long as they credit the author and the Abstract Book, but they cannot change the content in any way or use them commercially.
**Abstract Book Citations:** Authors, Title, HemaSphere, 2026;10:(S1):pages. The abstract book DOIs can be found on the "Author Information" page.
**Disclaimer:** Articles published in the journal HemaSphere exclusively reflect the opinions of the authors. The authors are responsible for all content in their abstracts including accuracy of the facts, statements, citing resources, etc.

### (PB2566) SYNERGISTIC DRUG COMBINATIONS TARGETING NELARABINE RESISTANCE IN T-CELL ACUTE LYMPHOBLASTIC LEUKEMIA

**Topic:** 01. Acute lymphoblastic leukemia - Biology & translational research

Charlotte Rinne*[1], Pia Vähäkuopus[1], Laura Oksa[1], Jonne Nieminen[1], Merja Heinäniemi[2], Olli Lohi[1]

*[1]Center for Cancer Eradication Research, Faculty of Medicine and Health Technology, Tampere University and Tays Cancer Center, Tampere University Hospital,, Tampere, Finland; [2]Institute of Biomedicine, School of Medicine, Faculty of Health Sciences, University of Eastern Finland, Kuopio, Finland, Kuopio, Finland;*

### Background

Acute lymphoblastic leukemia (ALL) is the most common pediatric malignancy. T-lineage ALL (T-ALL) comprises approximately 15 % of ALL cases, and although survival rates for newly diagnosed T-ALL have improved considerably, the prognosis after relapse remains dismal with survival rates around 35%. Moreover, treatment options in the relapsed setting are limited. Nelarabine, a nucleoside analogue targeting T-cells, remains the only approved agent specifically for relapsed T-ALL. However, clinical response rates have been modest (~50 %), emphasizing the need for better understanding of the underlying mechanisms of resistance. Additionally, the identification of effective combination strategies with Nelarabine could help to improve clinical outcome.

### Aims

In this project we aimed to uncover molecular mechanisms that drive Nelarabine resistance and to identify drug combinations capable of enhancing therapeutic efficacy.

### Methods

RNA sequencing was performed on primary T-ALL patient samples (n = 54) to assess the expression of genes implicated in Nelarabine transportation and intracellular metabolism. Nelarabine sensitivity was evaluated across a panel of 15 T-ALL cell lines. In two resistant cell lines a high-throughput combination screen comprising 527 compounds was performed to identify potential synergistic partners. Promising combinations were further assessed using cell viability and apoptosis assays, as well as Western blot analyses to investigate treatment-induced modulation of key signalling pathways.

### Results

Patient samples did not reveal significant differences in the mRNA expression of known genes involved in Nelarabine transportation or metabolism. Drug sensitivity profiling demonstrated marked heterogeneity in Nelarabine responses across T-ALL cell lines. The combination screen identified synergistic interactions between Nelarabine and two drug classes, inhibitors of the PI3k/AKT/mTOR pathway and the BCL-2/BCL-xL family, characterized by lower cell viability and increased apoptosis.  Ongoing experiments, including extended signalling analyses by Western blot, aim to further characterize the molecular mechanisms underlying these effects and to validate the robustness of the observed synergy.

### Summary/Conclusion

Our data identify PI3K/AKT/mTOR inhibitors and anti-apoptotic BCL-2/BCL-xL inhibitors as promising combination partners for Nelarabine in resistant T-ALL models. These combinations enhance apoptosis and reduce cell viability, supporting further mechanistic validation to improve therapeutic strategies.

**Copyright Information:** (Online) ISSN: 2572-9241
©2026 The Author(s). HemaSphere is published by John Wiley & Sons Ltd on behalf of European Hematology Association. This is an open access Abstract Book distributed under the Attribution-NonCommercial-NoDerivs (CC BY-NC-ND), which allows third parties to download the articles and share them with others as long as they credit the author and the Abstract Book, but they cannot change the content in any way or use them commercially.
**Abstract Book Citations:** Authors, Title, HemaSphere, 2026;10:(S1):pages. The abstract book DOIs can be found on the “Author Information” page.
**Disclaimer:** Articles published in the journal HemaSphere exclusively reflect the opinions of the authors. The authors are responsible for all content in their abstracts including accuracy of the facts, statements, citing resources, etc.

### (PB2567) A NOVEL ANTI-T-ALL COMPOUND AP-3 INDUCES CENTROSOME DEFECTS AND CELL CYCLE ARREST BY TARGETING MRGBP AND DISRUPTING CEP78 STABILITY

**Topic:** 01. Acute lymphoblastic leukemia - Biology & translational research

Fenglin Li[1], Haihui Zhuang[2], Xia Jiang[2], Wenjie Sha[2], Ying Lu*[2]

*[1]The Affiliated People's Hospital of Ningbo University, Hematology, Ningbo, China;[2]The Affiliated People's Hospital of Ningbo University, Ningbo, China;*

### Background

Treatment options for adult T-cell acute lymphoblastic leukemia (T-ALL) are limited, and the prognosis remains poor. In our preliminary study, we identified a natural compound, AP-3, with potent anti-T-ALL activity through high-throughput screening, offering a new therapeutic candidate for T-ALL. However, its specific mechanism of action and molecular target remain unclear.

### Aims

This study aims to elucidate the anti-tumor mechanism of the novel compound AP-3 in T-ALL, particularly focusing on identifying its direct target and deciphering the downstream signaling pathways.

### Methods

Drug affinity responsive target stability coupled with mass spectrometry (DARTS-MS) was employed to identify the direct binding protein of AP-3. Comprehensive proteomic analysis and co-immunoprecipitation followed by mass spectrometry (IP-MS) were used to investigate the downstream interacting protein network. Western blotting and flow cytometry were performed in T-ALL cell lines to validate the mechanism. Functional rescue experiments were conducted using gene knockdown and overexpression approaches.

### Results

DARTS-MS, DARTS-WB, and thermal shift assays confirmed MRGBP as the direct molecular target of AP-3. Comprehensive proteomic analysis and IP-MS revealed that AP-3 treatment reduced MRGBP protein expression and disrupted its interaction with the centrosomal protein CEP78, leading to downregulation of CEP78. Depletion of CEP78 caused structural abnormalities in centrosomes (evidenced by degradation of proteins such as CEP152) and defective microtubule nucleation function. This ultimately activated the spindle assembly checkpoint, resulting in G2/M phase cell cycle arrest and induction of apoptosis. Knockdown of MRGBP in T-ALL cells phenocopied the effects of AP-3 treatment, whereas overexpression of MRGBP reversed its anti-tumor effects.

### Summary/Conclusion

AP-3 exerts its anti-T-ALL effect by directly targeting MRGBP, disrupting its function in stabilizing CEP78, thereby inducing centrosome dysfunction and cell cycle arrest. This study reveals the MRGBP-CEP78 axis as a novel regulatory pathway for centrosome stability and provides a potential new target for T-ALL therapy.

**Copyright Information:** (Online) ISSN: 2572-9241
©2026 The Author(s). HemaSphere is published by John Wiley & Sons Ltd on behalf of European Hematology Association. This is an open access Abstract Book distributed under the Attribution-NonCommercial-NoDerivs (CC BY-NC-ND), which allows third parties to download the articles and share them with others as long as they credit the author and the Abstract Book, but they cannot change the content in any way or use them commercially.
**Abstract Book Citations:** Authors, Title, HemaSphere, 2026;10:(S1):pages. The abstract book DOIs can be found on the "Author Information" page.
**Disclaimer:** Articles published in the journal HemaSphere exclusively reflect the opinions of the authors. The authors are responsible for all content in their abstracts including accuracy of the facts, statements, citing resources, etc.

### (PB2570) A COMPREHENSIVE EVALUATION OF NELARABINE (ARA-G) COMBINED WITH INDUCTION CHEMOTHERAPY AGENTS ACROSS T-CELL ACUTE LYMPHOBLASTIC LEUKEMIA CELL LINES

**Topic:** 01. Acute lymphoblastic leukemia - Biology & translational research

Femke M. Hormann*[1], Andreas T. Post[1], Aida Selimovic[2], Jelle Dylus[1], Ann-Kathrin Schlotterbeck[1], Rozbeh Jafari[1], Sean G. Rudd[1]

*[1]Science for Life Laboratory (SciLifeLab), Karolinska Institutet, Oncology-Pathology, Stockholm, Sweden;[2]Crown Princess Victoria Children's Hospital, Linköping University, Department of Biomedical and Clinical Sciences, Linköping, Sweden;*

### Background

Nelarabine is the only T-ALL–specific drug currently used in the clinic. Initially approved for relapsed and refractory disease, it is now incorporated into upfront treatment for high-risk T-ALL as part of multi-drug induction protocols. However, how nelarabine interacts with other ALL therapies remains poorly understood (Hormann & Rudd, *Leukemia*, 2025; 39: 531–42). Although administered sequentially rather than simultaneously, potential antagonistc or synergystic interactions remain important, as they may influence overall treatment efficacy and emergence of resistance.

### Aims

To characterize the interactions between the nelarabine active metabolite ara-G and five commonly used ALL induction agents across six T-ALL cell lines, and to explore transcriptional correlations of single-drug sensitivity and drug combination responses.

### Methods

Six T-ALL cell lines (CCRF-CEM, Jurkat, MOLT-4, HPB-ALL, MOLT-16, and Peer) were treated with ara-G and induction drugs (ara-C, vincristine, daunorubicin, 6-MP, and dexamethasone) in 7×7 matrix combinations. Cell viability was measured after 4-days using resazurin reduction and synergy was quantified using the Zero Interaction Potency (ZIP) model. Gene expression was evaluated in a publicly available dataset (GSE168386) using predetermined panels covering nucleoside metabolism, thiopurine pathways, glucocorticoid signalling, anthracycline response, microtubule/mitotic checkpoint biology, and DNA replication/G2-M cell-cycle regulators.

### Results

All six cell lines showed dose-dependent responses to most of the single drug treatments with variable sensitivity. HPB-ALL and Peer showed resistance to 6-MP and four lines (Peer, HPB-ALL, Jurkat, MOLT-4) lacked clear dexamethasone sensitivity. Ara-G sensitivity most closely resembled that of ara-C, with similar patterns also observed for 6-MP and daunorubicin. Across all five ara-G induction drug combinations, ZIP interaction maps revealed overall an additive effect without consistent synergy or antagonism. Instead, interaction patterns were highly cell-line specific and largely concentration-restricted. Several combinations exhibited discrete pockets of synergy or antagonism, with the highest level of antagonism in the combination of ara-G with ara-C or vincristine in HPB-ALL. This indicates that ara-G does not broadly synergize or antagonize with these drugs, supporting the beneficial addition of nelarabine to T-ALL treatment protocols. Gene expression analysis revealed that 6-MP, daunorubicin, and in part dexamethasone sensitivity correlated strongly with tubulin-related genes (*TUBA1A*, *TUBB*, *TUBB2A*) and nucleotide metabolism genes (*NT5C2*, *NT5C3A*), while vincristine sensitivity associated with *TSC22D3* and *IMPDH1*. Ara-G sensitivity showed a trend towards correlation with *ABCG2* expression. Expression of tubulin genes correlated with synergy scores for ara-G combinations with ara-C, daunorubicin, and vincristine, while *ABCG2* expression correlated with synergy for ara-G with 6-MP.

**Copyright Information:** (Online) ISSN: 2572-9241
©2026 The Author(s). HemaSphere is published by John Wiley & Sons Ltd on behalf of European Hematology Association. This is an open access Abstract Book distributed under the Attribution-NonCommercial-NoDerivs (CC BY-NC-ND), which allows third parties to download the articles and share them with others as long as they credit the author and the Abstract Book, but they cannot change the content in any way or use them commercially.
**Abstract Book Citations:** Authors, Title, HemaSphere, 2026;10:(S1):pages. The abstract book DOIs can be found on the "Author Information" page.
**Disclaimer:** Articles published in the journal HemaSphere exclusively reflect the opinions of the authors. The authors are responsible for all content in their abstracts including accuracy of the facts, statements, citing resources, etc.

### Summary/Conclusion

These findings suggest that ara-G does not exhibit broad synergistic or antagonistic interactions with commonly used induction agents, supporting the integration of nelarabine into multi-drug T-ALL regimens. Transcriptional correlates of sensitivity and interaction patterns highlight candidate pathways that may shape response heterogeneity across T-ALL subtypes. Further mechanistic validation is ongoing and will be presented at the meeting.

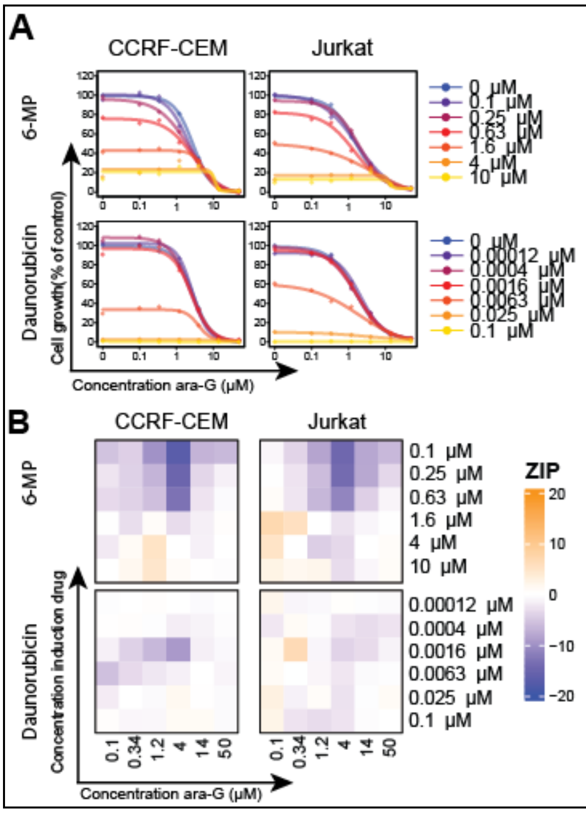

**Figure: Example data of 2 cell lines and 2 induction drugs.** Cells were exposed to ara-G and one of the synergy drugs in 7x7 matrices for 4 days and cell growth was evaluated with resazurin reduction assay. **A)** Dose-response curves evaluating synergy between ara-G and induction drugs. Each dot represents the average of 3 independent experiments. Dose-response curves were fitted using a four parameter log logistic model. **B)** Heatmaps showing the median zero-interaction potential (ZIP) score per concentration (n=3 independent experiments) using data from panel A.

**Copyright Information:** (Online) ISSN: 2572-9241
©2026 The Author(s). HemaSphere is published by John Wiley & Sons Ltd on behalf of European Hematology Association. This is an open access Abstract Book distributed under the Attribution-NonCommercial-NoDerivs (CC BY-NC-ND), which allows third parties to download the articles and share them with others as long as they credit the author and the Abstract Book, but they cannot change the content in any way or use them commercially.
**Abstract Book Citations:** Authors, Title, HemaSphere, 2026;10:(S1):pages. The abstract book DOIs can be found on the "Author Information" page.
**Disclaimer:** Articles published in the journal HemaSphere exclusively reflect the opinions of the authors. The authors are responsible for all content in their abstracts including accuracy of the facts, statements, citing resources, etc.

### (PB2571) TRANSCRIPTOMIC MATURATION IN B-ALL SHAPED BY AGE AND CLINICAL STATUS THROUGH INTEGRATIVE ANALYSIS OF PEDIATRIC AND ADULT DEG PROFILES

**Topic:** 01. Acute lymphoblastic leukemia - Biology & translational research

Jairo Jattin-Balcázar*[1]

[1] *Universidad Nacional de Colombia, Facultad de Medicina, Bogotá, Colombia;*

### Background

B-cell acute lymphoblastic leukemia (B-ALL) is the most common pediatric malignancy but also affects adults with poorer outcomes. Clinical status (diagnosis vs. relapse/refractory) critically shapes prognosis, yet integrated transcriptomic analyses across age groups remain scarce. Understanding how age influences relapse biology may reveal developmental constraints and prognostic biomarkers.

### Aims

To integrate pediatric and adult RNA-seq cohorts of B-ALL, identify transcriptomic signatures associated with clinical status, and evaluate how age modulates these patterns.

### Methods

Thirteen public RNA-seq studies (n=272 patients) were harmonized. Expression data were normalized with voom and batch-corrected using ComBat, preserving age group and clinical status. Differential expression analyses were performed globally, age-adjusted, and separately in pediatric and adult cohorts (FDR < 0.005; |logFC| ≥ 2). Gene modules were characterized through GO/KEGG enrichment. Pediatric-exclusive signatures were modeled against age using linear and interaction models. Prognostic relevance was assessed in TARGET via Cox regression, maximally selected rank statistics, and Kaplan–Meier survival.

### Results

In adult B-ALL, 617 protein-coding DEGs were identified, with relapse enriched for chromatin remodeling and immune inflammatory pathways, whereas pediatric B-ALL revealed 418 protein-coding DEGs dominated by proliferative and early lymphoid developmental signals. A strict intersection analysis yielded a 14-gene shared signature (FDR ≤ 0.005; |logFC| ≥ 2), which, although biologically coherent, failed to discriminate diagnosis from relapse in children (p = 0.4625). In contrast, a pediatric-exclusive set of 19 down-regulated genes, including *DNTT, RAG1,* and *ITK*, displayed a strong age-dependent decline (linear coefficient −0.0176, p < 0.001), with highest expression in young children and minimal levels in adults. Relapse shifted the baseline of this maturation trajectory toward more adult-like states without altering slope, as shown by intercept coefficients of −0.951 (p = 0.0034) in the full cohort and −1.05 (p = 0.0035) in patients younger than 21 years. External references confirmed minimal expression of these genes in healthy adults, with GTEx showing orders-of-magnitude differences for *DNTT* (~51,293 vs. 0 TPM) and *RAG1* (~45,107 vs. 0.0239 TPM). Survival analyses in TARGET highlighted *CDK3, GIMAP6, TMIGD2, ARPP21, DNTT,* and *PLXDC1* as prognostic, with Kaplan–Meier curves demonstrating improved survival for higher *CDK3* and *PLXDC1* expression (HR < 1, log-rank p < 0.05), and worse outcomes for elevated *GIMAP6, TMIGD2, ARPP21,* and *DNTT* (HR > 1, log-rank p < 0.05).

**Copyright Information:** (Online) ISSN: 2572-9241
©2026 The Author(s). HemaSphere is published by John Wiley & Sons Ltd on behalf of European Hematology Association. This is an open access Abstract Book distributed under the Attribution-NonCommercial-NoDerivs (CC BY-NC-ND), which allows third parties to download the articles and share them with others as long as they credit the author and the Abstract Book, but they cannot change the content in any way or use them commercially.
**Abstract Book Citations:** Authors, Title, HemaSphere, 2026;10:(S1):pages. The abstract book DOIs can be found on the "Author Information" page.
**Disclaimer:** Articles published in the journal HemaSphere exclusively reflect the opinions of the authors. The authors are responsible for all content in their abstracts including accuracy of the facts, statements, citing resources, etc.

### Summary/Conclusion

Transcriptomic maturation in B-ALL follows a continuous age-dependent trajectory (DOWN score coefficient −0.0176, $p < 0.001$). Pediatric relapse accelerates this process, producing adult-like profiles, while adult relapse emerges within immune-enriched landscapes. The pediatric-exclusive DOWN signature reflects developmental lymphoid programs with prognostic impact. These findings underscore transcriptomic maturation as a key axis modulating relapse biology and survival across age groups.

**Copyright Information:** (Online) ISSN: 2572-9241
©2026 The Author(s). HemaSphere is published by John Wiley & Sons Ltd on behalf of European Hematology Association. This is an open access Abstract Book distributed under the Attribution-NonCommercial-NoDerivs (CC BY-NC-ND), which allows third parties to download the articles and share them with others as long as they credit the author and the Abstract Book, but they cannot change the content in any way or use them commercially.
**Abstract Book Citations:** Authors, Title, HemaSphere, 2026;10:(S1):pages. The abstract book DOIs can be found on the "Author Information" page.
**Disclaimer:** Articles published in the journal HemaSphere exclusively reflect the opinions of the authors. The authors are responsible for all content in their abstracts including accuracy of the facts, statements, citing resources, etc.

### (PB2572) ASCIMINIB IN PHILADELPHIA CHROMOSOME-POSITIVE ACUTE LYMPHOBLASTIC LEUKEMIA: A CASE SERIES

**Topic:** 01. Acute lymphoblastic leukemia - Biology & translational research

Mostafa Mohammed Saleh[1], Abdulrahman Nasiri*[2], Ahmed Abdrabou[1], Hadeel Samarkandi[1], Ayman Saad[1], Aljurf Mahmoud[1], Amr Suleiman Hanbali[1], Ali Alahmari[1]

*[1]King Faisal Specialist Hospital & Research Centre, Department of Hematology, Riyadh, Saudi Arabia;[2]Imam Mohammad Ibn Saud Islamic University, College of Medicine, Riyadh, Saudi Arabia;*

### Background

Philadelphia chromosome-positive acute lymphoblastic leukemia (Ph+ ALL) remains associated with high relapse rates despite tyrosine kinase inhibitors, immunotherapy, and cellular therapies. Resistance, CNS sanctuary disease, and post-CAR-T relapse limit durable control. Asciminib, a first-in-class STAMP inhibitor targeting the ABL1 myristoyl pocket, has demonstrated efficacy in CML, but its clinical role in Ph+ ALL remains incompletely defined.

### Aims

To evaluate the clinical utility, safety, and therapeutic integration of asciminib in high-risk Ph+ ALL across diverse relapsed and refractory settings.

### Methods

We conducted a three-patient case series involving adults with multiply relapsed Ph+ ALL or lymphoid blast crisis treated with asciminib in combination or consolidation settings. Clinical scenarios included CNS involvement, T315I mutation, post-CAR-T relapse, and transplant bridging. Treatment response, molecular outcomes, CNS clearance, and safety were evaluated. Findings were contextualized within emerging clinical and mechanistic evidence.

### Results

Asciminib was administered at doses up to 200 mg twice daily as part of combination or consolidation strategies. Two patients achieved sustained molecular remission with undetectable BCR::ABL1 transcripts and successfully proceeded to or maintained remission following allogeneic transplantation or cellular therapy. Asciminib contributed to CNS disease clearance and durable molecular control, including in T315I-mutated and post-CAR-T relapse settings. The third patient achieved initial disease control but died from infectious complications unrelated to asciminib. Overall, asciminib demonstrated favorable tolerability without vascular toxicity or treatment-limiting adverse effects. Published evidence supports enhanced durability when asciminib is used in rational combinations and as maintenance or bridging therapy.

### Summary/Conclusion

Asciminib represents a versatile therapeutic option in Ph+ ALL, with greatest clinical benefit observed in combination regimens, maintenance strategies, and transplant bridging rather than monotherapy. Its activity in CNS disease and post-cellular therapy relapse highlights its potential role in high-risk settings. Prospective studies are needed to define optimal sequencing and integration.

**Copyright Information:** (Online) ISSN: 2572-9241
©2026 The Author(s). HemaSphere is published by John Wiley & Sons Ltd on behalf of European Hematology Association. This is an open access Abstract Book distributed under the Attribution-NonCommercial-NoDerivs (CC BY-NC-ND), which allows third parties to download the articles and share them with others as long as they credit the author and the Abstract Book, but they cannot change the content in any way or use them commercially.
**Abstract Book Citations:** Authors, Title, HemaSphere, 2026;10:(S1):pages. The abstract book DOIs can be found on the "Author Information" page.
**Disclaimer:** Articles published in the journal HemaSphere exclusively reflect the opinions of the authors. The authors are responsible for all content in their abstracts including accuracy of the facts, statements, citing resources, etc.

### (PB2573) MITOCHONDRIAL DNA CONTENT IS ELEVATED IN ADULT ACUTE LYMPHOBLASTIC LEUKEMIA

**Topic:** 01. Acute lymphoblastic leukemia - Biology & translational research

Juan Coelho-Silva*[1], Keli Lima[2], Frederico Lisboa Nogueira[2], Wellington Fernandes Da Silva Júnior[2], Israel Bendit[2], Eduardo Rego[2], João Agostinho MacHado Neto[1]

*[1]University of Sao Paulo, Department of Pharmacology, Institute of Biomedical Sciences, Sao Paulo, Brazil;[2]University of Sao Paulo, Hematology, Sao Paulo, Brazil;*

**Background**

Mitochondrial DNA content (mtDNAc) is a quantitative proxy of mitochondrial mass/biogenesis and cellular metabolic state. While mtDNAc has shown biological and clinical relevance in myeloid malignancies, its distribution and clinical associations in adult acute lymphoblastic leukemia (ALL) remain poorly defined.

**Aims**

To quantify mtDNAc in adult ALL, compare it with healthy donors (HD), and evaluate associations between mtDNAc strata and clinical, laboratory, and molecular features, and outcomes.

**Methods**

Bone marrow or peripheral blood from adult ALL patients treated at Hospital das Clínicas, FMUSP, were analyzed alongside HD controls (ALL, n=70; HD, n=142). mtDNAc was quantified by real-time PCR following a previously published protocol (PMID: 40653487). Group differences between ALL and HD were tested using a two-tailed Mann–Whitney test with exact p-values, and the effect size was summarized by median differences and the Hodges–Lehmann estimator. For within-ALL analyses, patients were categorized as mtDNAc-high if mtDNAc exceeded the 95th percentile of the HD distribution. Associations with continuous variables were evaluated by the Mann–Whitney test, and categorical biomarkers by the exact Fisher test or chi-square. Outcomes analyzed included overall survival (OS), disease-free survival (DFS), and time to complete remission (TTR).

**Results**

mtDNAc was markedly higher in adult ALL than in HD (median 364.5 vs 127.6; absolute median difference 236.9; Hodges–Lehmann difference 238.1; p<0.0001). Using the external threshold based on HD, 34/70 (48.6%) ALL cases were classified as mtDNAc-high. mtDNA category showed selective associations with laboratory parameters: mtDNAc-high differed from mtDNAc-low for white blood cell count (WBC; p=0.024), segmented neutrophils (Seg; p=0.007), and AST/TGO (p=0.045), with rank distributions consistent with lower values in the mtDNAc-high group. These results were consistent regardless of bone marrow or peripheral blood source (WBC p=0.032; Seg p=0.004; ASTp=0.023). Among categorical biomarkers, CRLF2 status was associated with mtDNA category (p=0.033), with CRLF2 positivity enriched in mtDNAc-high cases. In contrast, mtDNAc (as a continuous variable or by mtDNA category) was not associated with OS, DFS, or TTR in this adult ALL cohort.

**Copyright Information:** (Online) ISSN: 2572-9241
©2026 The Author(s). HemaSphere is published by John Wiley & Sons Ltd on behalf of European Hematology Association. This is an open access Abstract Book distributed under the Attribution-NonCommercial-NoDerivs (CC BY-NC-ND), which allows third parties to download the articles and share them with others as long as they credit the author and the Abstract Book, but they cannot change the content in any way or use them commercially.
**Abstract Book Citations:** Authors, Title, HemaSphere, 2026;10:(S1):pages. The abstract book DOIs can be found on the “Author Information” page.
**Disclaimer:** Articles published in the journal HemaSphere exclusively reflect the opinions of the authors. The authors are responsible for all content in their abstracts including accuracy of the facts, statements, citing resources, etc.

**Summary/Conclusion**

Adult ALL is characterized by substantially elevated mtDNA content relative to healthy donors, and nearly half of patients exceed an externally defined "abnormal" mtDNAc threshold. Although mtDNAc was not prognostic for OS, DFS, or time to remission, its association with specific laboratory features and CRLF2 status supports mtDNAc as a disease biology marker in adult ALL. These findings motivate future work to clarify the mechanistic basis of mtDNAc elevation and its relationship to molecular subtypes and treatment response.

**Copyright Information:** (Online) ISSN: 2572-9241
©2026 The Author(s). HemaSphere is published by John Wiley & Sons Ltd on behalf of European Hematology Association. This is an open access Abstract Book distributed under the Attribution-NonCommercial-NoDerivs (CC BY-NC-ND), which allows third parties to download the articles and share them with others as long as they credit the author and the Abstract Book, but they cannot change the content in any way or use them commercially.
**Abstract Book Citations:** Authors, Title, HemaSphere, 2026;10:(S1):pages. The abstract book DOIs can be found on the "Author Information" page.
**Disclaimer:** Articles published in the journal HemaSphere exclusively reflect the opinions of the authors. The authors are responsible for all content in their abstracts including accuracy of the facts, statements, citing resources, etc.

### (PB2574) METACHRONOUS KMT2A::BCL9L-DRIVEN T-ALL 11 YEARS AFTER CHILDHOOD B-ALL REMISSION: INTEGRATED GENOMICS AND IG/TCR CLONALITY RESOLVE RELAPSE VS SECOND PRIMARY LEUKEMIA

**Topic:** 01. Acute lymphoblastic leukemia - Biology & translational research

Xinni Bian*[1], Senlin Zhang[1], Qingwei Wang[1], Shaoyan Hu[1]

[1]*Children's Hospital of Soochow University, Suzhou, China;*

**Background**

Differentiating relapse from a metachronous second primary leukemia is challenging when acute lymphoblastic leukemia (ALL) reappears after prolonged remission with a discordant immunophenotype. Integrated genomics and immunoreceptor clonality tracking may provide decisive evidence.

**Aims**

To determine whether a late T-ALL emerging 11 years after childhood B-ALL represents lineage-switch relapse or a second primary leukemia, and to define the molecular drivers of the T-ALL.

**Methods**

Clinical course, flow cytometry, and serial minimal residual disease (MRD) assessments were reviewed. Whole-genome sequencing (WGS) and RNA sequencing were performed at recurrence. Immunoglobulin (IG) and T-cell receptor (TCR) clonality were assessed by next-generation sequencing. Key lesions at diagnosis and recurrence were compared.

**Results**

A boy was diagnosed at age 2 (2013) with precursor B-ALL presenting with hyperleukocytosis (WBC 164.11×10^9/L). Diagnostic immunophenotype supported B-lineage blasts (CD10^high, CD19^+, cytoplasmic CD79a^+, cytoplasmic μ^+; cytoplasmic CD3 negative) with TLX1/HOX11 overexpression. He achieved remission on CCCG-2008 (day 33) and remained in continuous remission for 11 years without radiotherapy or transplantation. In 2025, he re-presented with extreme hyperleukocytosis (WBC 524.97×10^9/L) and a T-ALL phenotype (CD7^bright, cytoplasmic CD3^+, CD5^+, TdT^+; B markers negative), again with TLX1/HOX11 overexpression. WGS/RNA-seq identified a KMT2A::BCL9L fusion (inv(11)(q23.3)) supported by fusion reads, accompanied by CDKN2A/B deletion and recurrent T-ALL signaling lesions including NOTCH1 p.R1598P and NRAS p.G13D, plus additional variants (e.g., WT1 and BCL11B frameshift). These lesions were not detected at diagnosis. Immunoreceptor profiling showed monoclonal IG rearrangements at diagnosis that were absent at recurrence, while TRD/TRG became monoclonal at recurrence, consistent with a distinct T-lineage clone. The patient received CCCG-ALL2020–based therapy, underwent allogeneic transplantation, relapsed early post-transplant with persistent KMT2A::BCL9L, and received salvage including a menin inhibitor (revumenib).

**Summary/Conclusion**

Non-overlapping driver landscapes and discordant IG versus TCR clonality support a metachronous second primary KMT2A::BCL9L-driven T-ALL rather than lineage-switch relapse. This case highlights the value of integrating WGS/RNA-seq with immunoreceptor tracking to resolve diagnostically ambiguous late “recurrences” and to guide mechanism-based therapy.

**Copyright Information:** (Online) ISSN: 2572-9241
©2026 The Author(s). HemaSphere is published by John Wiley & Sons Ltd on behalf of European Hematology Association. This is an open access Abstract Book distributed under the Attribution-NonCommercial-NoDerivs (CC BY-NC-ND), which allows third parties to download the articles and share them with others as long as they credit the author and the Abstract Book, but they cannot change the content in any way or use them commercially.
**Abstract Book Citations:** Authors, Title, HemaSphere, 2026;10:(S1):pages. The abstract book DOIs can be found on the “Author Information” page.
**Disclaimer:** Articles published in the journal HemaSphere exclusively reflect the opinions of the authors. The authors are responsible for all content in their abstracts including accuracy of the facts, statements, citing resources, etc.

| Category | Gene / Chromosomal Region | Type of Alteration | Initial Diagnosis (B-ALL) | Relapse (T-ALL) |
|---|---|---|---|---|
| Structural Variant | IKZF1 | Gene fusion / Structural rearrangement | Positive (IKZF1::CCL24) | Negative |
| Structural Variant | PAX5 | Gene fusion / Structural rearrangement | Positive (PAX5::ZCCHC7) | Negative |
| Structural Variant | MTAP / CDKN2B-AS1 | Structural rearrangement | Positive | Negative |
| Copy Number Variant | 22q11.22 | Deletion | Positive | Negative |
| Copy Number Variant | 9p21 (CDKN2A/B) | Deletion / CN-LOH | Deletion | CN-LOH with homozygous deletion |
| Fusion Gene | KMT2A | Fusion | Negative | Positive (KMT2A::BCL9L) |
| Somatic Mutation | NOTCH1 | Missense mutation | Negative | Positive |
| Somatic Mutation | BCL11B | Frameshift mutation | Negative | Positive |
| V(D)J Rearrangement | IGH | Clonal rearrangement | Positive | Negative |
| V(D)J Rearrangement | TCR | Clonal rearrangement | Positive (TRGV9/TRGJ2; TRGJ1) | Positive (TRDV1/TRDD3/TRDJ1, TRGV8/TRGJ2, TRGV5/TRGJ2) |

Genomic Landscape at Initial Diagnosis (B-ALL) and Relapse (T-ALL)

**Copyright Information:** (Online) ISSN: 2572-9241
©2026 The Author(s). HemaSphere is published by John Wiley & Sons Ltd on behalf of European Hematology Association. This is an open access Abstract Book distributed under the Attribution-NonCommercial-NoDerivs (CC BY-NC-ND), which allows third parties to download the articles and share them with others as long as they credit the author and the Abstract Book, but they cannot change the content in any way or use them commercially.
**Abstract Book Citations:** Authors, Title, HemaSphere, 2026;10:(S1):pages. The abstract book DOIs can be found on the "Author Information" page.
**Disclaimer:** Articles published in the journal HemaSphere exclusively reflect the opinions of the authors. The authors are responsible for all content in their abstracts including accuracy of the facts, statements, citing resources, etc.

### (PB2575) IMPLEMENTATION AND OPTIMIZATION OF AN NGS-BASED IG/TR CLONALITY ASSAY ON THE ILLUMINA NEXTSEQ 2000 PLATFORM IN PEDIATRIC B-CELL PRECURSOR ACUTE LYMPHOBLASTIC LEUKEMIA PATIENTS

**Topic:** 01. Acute lymphoblastic leukemia - Biology & translational research

Mercè Richarte-Franqués*[1, 2, 3], Clara Vicente Garcés[1, 2], Alba Crespo-Carrasco[1, 2, 4], Mireia Camos Guijosa[1, 5, 6], Sara Montesdeoca[5], Ignacio Isola[5], Anna Monter[5], Susana Rives[1, 2, 6, 7], Montserrat Torrebadell[1, 5, 6], Elena Esperanza Cebollada[1, 2], Nerea Vega-Garcia[1, 2, 5]

*[1]Institut de Recerca Sant Joan de Déu (IRSJD), Leukemia and Pediatric Hematology Disorders, Developmental Tumors Biology Group, Santa Rosa 39-57, 08950 Esplugues de Llobregat, Spain; [2]Institut de Recerca Sant Joan de Déu (IRSJD)-Josep Carreras Leukemia Research Institute (IJC), Leukemia and Pediatric Hematology Disorders, Pediatric Hematologic malignancies Research Program, Esplugues de Llobregat, Spain; [3]Facultat de Biologia, Universitat de Barcelona (UB), Av. Diagonal, 643, 08028, Barcelona, Spain; [4]Facultat de Medicina i Ciències de la Salut, Universitat de Barcelona (UB), c. Casanova, 143, 08036, Barcelona, Spain; [5]Hospital Sant Joan de Déu, Hematology Laboratory, Passeig Sant Joan de Déu 2 08950, Esplugues de Llobregat, Spain; [6]Instituto de Salud Carlos III, Centro de Investigación Biomédica en Red de Enfermedades Raras (CIBERER), Madrid, Spain; [7]Pediatric Cancer Center Barcelona (PCCB), Hospital Sant Joan de Déu, Leukemia and Lymphoma Department, Barcelona, Spain;*

**Background**

Immunoglobulin (IG) and T-cell receptor (TR) gene rearrangements are targets for monitoring measurable residual disease (MRD) in acute lymphoblastic leukemia (ALL). The EuroClonality-NGS Working Group has developed a Next-Generation Sequencing (NGS)-based protocol, validated on the Illumina MiSeq system, to assess all IG/TR repertoire. This protocol enables clonality assessment and MRD monitoring in ALL, offering high sensitivity and specificity, increasing the number of MRD markers identified, and is less time-consuming. Despite successful experience on MiSeq system, its sequencing output limits sample multiplexing capacity, leading to high cost per sample. The recently introduced Illumina NextSeq 2000 XLEAP flow cells provide substantially higher sequencing output, enabling greater sample multiplexing, shorter sequencing times, and improved cost-effectiveness.

**Aims**

To implement, optimize, and evaluate the analytical performance of the EuroClonality-NGS protocol on the Illumina NextSeq 2000 system for IG/TR clonality analysis in pediatric B-cell precursor (BCP)-ALL patients.

**Methods**

NGS libraries were prepared following the EuroClonality One-step IG/TR NGS-based MRD detection protocol and sequenced on a NextSeq 2000 P1 XLEAP flow cell. Data was analyzed using ARResT/Interrogate. We evaluated sequencing quality metrics including Q30 score, reads passing filter (PF), and flow cell occupancy. We studied DNA monoclonal controls from Invivoscribe ® (IVS-0019 and IVS-0021) and DNA samples from pediatric BCP-ALL patients previously characterized by Allele-Specific Oligonucleotide (ASO)-PCR at Hospital Sant Joan de Déu.

**Copyright Information:** (Online) ISSN: 2572-9241
©2026 The Author(s). HemaSphere is published by John Wiley & Sons Ltd on behalf of European Hematology Association. This is an open access Abstract Book distributed under the Attribution-NonCommercial-NoDerivs (CC BY-NC-ND), which allows third parties to download the articles and share them with others as long as they credit the author and the Abstract Book, but they cannot change the content in any way or use them commercially.
**Abstract Book Citations:** Authors, Title, HemaSphere, 2026;10:(S1):pages. The abstract book DOIs can be found on the "Author Information" page.
**Disclaimer:** Articles published in the journal HemaSphere exclusively reflect the opinions of the authors. The authors are responsible for all content in their abstracts including accuracy of the facts, statements, citing resources, etc.

### Results

Three independent runs were performed, each containing the same six samples (2 monoclonal controls, 3 pediatric BCP-ALL samples, and 1 polyclonal control of mononuclear cells). For the first run, the total reads PF and the flow cell occupancy achieved expected results (116M and 95%, respectively), but the Q30 score was below the accepted threshold of ≥85% (69%) (Figure 1). All rearrangements reached enough number of reads to be analyzed (>2000 reads). However, longer targets (IGH-VJ, mean length=500bp) obtained fewer reads than shorter amplicons (mean length=280-300bp) (117.778 reads vs 2.056.111 reads). This suggests lower binding efficiency of long fragments to the flow cell. To correct this imbalance and increase nucleotide diversity, pooling conditions and PhiX concentration were optimized for subsequent runs. After these improvements, a higher Q30 score (84% and 89%), higher reads PF (119M and 131M), and better read distribution across the different rearrangements were achieved, obtaining a great general performance of the runs (Figure 1). Regarding biological results, NGS identified clonal rearrangements (≥10% reads) in all the samples. All previously established clonal markers by ASO-PCR were confirmed (n=13/13, 100%). Importantly, 18 clonal markers (n=18/31, 58%) were identified only by NGS. Despite challenges in detecting TRB rearrangements in one patient across runs, all rearrangements showed a good performance.

### Summary/Conclusion

The results showed good reproducibility and confirmed previously characterized IG/TR markers while identifying additional gene rearrangements. Therefore, we confirm the feasibility of IG/TR clonality assessment in pediatric BCP-ALL patients using NextSeq 2000 system with the EuroClonality NGS-based protocol, which may also enable MRD monitoring at higher sensitivity, higher sample multiplexing capacity and cost efficiency.

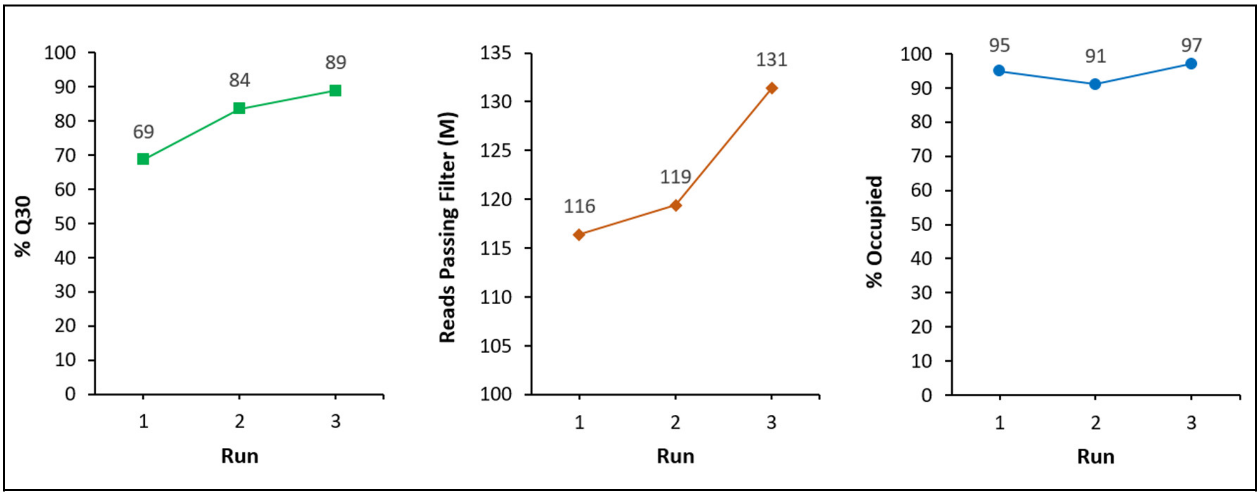

Figure 1. Representation of Q30 quality score, total reads passing filter, and occupancy of the flow cell obtained across the runs.

Copyright Information: (Online) ISSN: 2572-9241
©2026 The Author(s). HemaSphere is published by John Wiley & Sons Ltd on behalf of European Hematology Association. This is an open access Abstract Book distributed under the Attribution-NonCommercial-NoDerivs (CC BY-NC-ND), which allows third parties to download the articles and share them with others as long as they credit the author and the Abstract Book, but they cannot change the content in any way or use them commercially.
Abstract Book Citations: Authors, Title, HemaSphere, 2026;10:(S1):pages. The abstract book DOIs can be found on the "Author Information" page.
Disclaimer: Articles published in the journal HemaSphere exclusively reflect the opinions of the authors. The authors are responsible for all content in their abstracts including accuracy of the facts, statements, citing resources, etc.

### (PB2576) IN VITRO EVALUATION OF THE COMBINATION OF AZACITIDINE, VENETOCLAX AND VALPROIC ACID IN T-ACUTE LYMPHOBLASTIC LEUKEMIA

**Topic:** 01. Acute lymphoblastic leukemia - Biology & translational research

Iordanis Pelagiadis[1, 2, 3], Myrto Karkavitsa[1], Ioanna Lygerou*[1, 3], Ioannis Kyriakidis[2, 3], Maria Stratigaki[3], Nikolaos Katzilakis[3], Eftichia Stiakaki[1, 2, 3]

*[1]MSc Program "Hematology-Oncology of Childhood and Adolescence", School of Medicine, University of Crete, 71003, Heraklion, Greece; [2]Laboratory of Blood Diseases and Childhood Cancer Biology, School of Medicine, University of Crete, 71003, Heraklion, Greece; [3]Department of Pediatric Hematology-Oncology, University Hospital of Heraklion, 71500, Heraklion, Greece;*

### Background

T-cell acute lymphoblastic leukemia (T-ALL) continues to exhibit high relapse rates and poor prognosis despite intensive chemotherapy. BCL-2 overexpression and epigenetic dysregulation contribute to leukemic cell survival, providing a rationale for the combination of the BCL-2 inhibitor venetoclax with hypomethylating and histone deacetylase-inhibiting agents. Azacitidine and valproic acid may prime leukemic cells for apoptosis and cell cycle arrest, potentially enhancing venetoclax efficacy.

### Aims

To evaluate the in vitro antileukemic effects of azacitidine, venetoclax, and valproic acid, both alone and in combination, on a T-ALL Jurkat cell line, with a focus on growth inhibition, cell cycle distribution, and apoptosis.

### Methods

Jurkat cells were treated for 24 and 48 hours with azacitidine (500, 1000 µM), venetoclax (500, 1000 nM), and valproic acid (1, 2 mM) as monotherapies, as well as double and triple combinations. Growth inhibition was assessed using the MTT assay, and cell cycle and Sub-G1 fractions were analyzed by propidium iodide staining and flow cytometry. Data from at least six independent experiments were summarized as mean growth inhibition versus control and compared using Student's t-test ($p \leq 0.05$ significant).

### Results

Monotherapies induced modest, dose- and time-dependent inhibition. Azacitidine 1000 µM achieved maximal growth inhibition of 30% at 48h, while venetoclax and valproic acid alone achieved mild, comparable inhibition at 24h and showed reduced effects at 48h. Double combinations were more effective. At 48h, azacitidine 1000 µM + valproic acid 1–2 mM yielded up to 43% inhibition, and venetoclax 1000 nM + azacitidine 1000 µM achieved 35%. Triple regimens demonstrated the most pronounced effects, with the combination of venetoclax 500nM, azacitidine 500µM, and valproic acid 2 mM achieving 34% at a 24 hours and venetoclax 1000nM, azacitidine 1000µM, and valproic acid 1mM achieving a 53% growth inhibition at 48 hours.
Venetoclax and valproic acid-containing conditions induced S-phase accumulation, whereas azacitidine conditions caused $G_2$/M arrest. A marked increase in the Sub-$G_1$ population, particularly with high-dose azacitidine and its combinations, indicated activation of apoptotic pathways.

**Copyright Information:** (Online) ISSN: 2572-9241
©2026 The Author(s). HemaSphere is published by John Wiley & Sons Ltd on behalf of European Hematology Association. This is an open access Abstract Book distributed under the Attribution-NonCommercial-NoDerivs (CC BY-NC-ND), which allows third parties to download the articles and share them with others as long as they credit the author and the Abstract Book, but they cannot change the content in any way or use them commercially.
**Abstract Book Citations:** Authors, Title, HemaSphere, 2026;10:(S1):pages. The abstract book DOIs can be found on the "Author Information" page.
**Disclaimer:** Articles published in the journal HemaSphere exclusively reflect the opinions of the authors. The authors are responsible for all content in their abstracts including accuracy of the facts, statements, citing resources, etc.

**Summary/Conclusion**

The combination of azacitidine, venetoclax, and valproic acid exerts additive anti-leukemic activity in Jurkat T-ALL cells through enhanced growth inhibition, cell cycle arrest, and apoptosis. These findings support further preclinical evaluation of this epigenetic-BCL-2-targeting strategy, including studies in primary T-ALL samples and in vivo models, as a potential therapeutic option for relapsed/refractory T-ALL.

**Copyright Information:** (Online) ISSN: 2572-9241
©2026 The Author(s). HemaSphere is published by John Wiley & Sons Ltd on behalf of European Hematology Association. This is an open access Abstract Book distributed under the Attribution-NonCommercial-NoDerivs (CC BY-NC-ND), which allows third parties to download the articles and share them with others as long as they credit the author and the Abstract Book, but they cannot change the content in any way or use them commercially.
**Abstract Book Citations:** Authors, Title, HemaSphere, 2026;10:(S1):pages. The abstract book DOIs can be found on the "Author Information" page.
**Disclaimer:** Articles published in the journal HemaSphere exclusively reflect the opinions of the authors. The authors are responsible for all content in their abstracts including accuracy of the facts, statements, citing resources, etc.

### (PB2577) TRANSCRIPTOMIC MATURATION IN LEUKEMIA SHAPED BY AGE AND CLINICAL STATUS THROUGH INTEGRATIVE ANALYSIS OF PEDIATRIC AND ADULT DEG PROFILES

**Topic:** 01. Acute lymphoblastic leukemia - Biology & translational research

Jairo Jattin*[1]

[1]*Universidad Nacional de Colombia, Bogotá, Colombia;*

### Background

Age shapes the biology of leukemia, with pediatric and adult cases displaying distinct transcriptional programs linked to hematopoietic maturation and chromatin regulation. How these age-dependent expression patterns evolve during disease progression, including relapsed or refractory states, remains unclear. Integrative analyses comparing pediatric and adult cohorts offer an opportunity to identify conserved and age-specific molecular signatures and to determine whether relapse accelerates the transition toward differential transcriptomic profile.

### Aims

To identify conserved and age-specific transcriptomic signatures in pediatric and adult leukemia cohorts and the influence of age and clinical status on gene expression dynamics.

### Methods

Differential expression analysis was conducted on consolidated pediatric and adult leukemia cohorts using voom-normalized gene expression data. Within each age group, contrasts were defined between diagnosis and relapsed/refractory (RR) states. Strict thresholds (FDR ≤ 0.005, |LogFC| ≥ 2) identified robust DEGs, while relaxed criteria (FDR ≤ 0.05, |LogFC| ≥ 1.5) captured age-specific signals. Gene Ontology and KEGG enrichment analyses were performed on adult-specific, pediatric-specific, and shared gene modules. A transcriptomic score based on 19 pediatric-exclusive down-regulated genes was modeled across age using linear and polynomial regression. Expression values were contrasted against age-stratified reference profiles from voyAGEr to validate age-related trends, and against GTEx to assess general tissue expression.

### Results

Adult cohorts showed enrichment in chromatin-related processes and pathways such as nucleosome assembly and ATP-dependent chromatin remodeling. Pediatric cohorts were enriched for immune developmental processes, including hemopoiesis and T cell differentiation. A set of 404 protein-coding genes was differentially expressed exclusively in pediatric RR samples; 385 were up-regulated and 19 down-regulated. The 19 down-regulated genes showed a strong negative correlation with age ($p \approx 1.0e\text{-}07$), while the UP–DOWN balance index increased with age ($p \approx 7.04e\text{-}07$), suggesting a transcriptomic maturation gradient. RR status significantly shifted the intercepts of both scores ($p < 0.01$), suggesting an accelerated transition toward an *adult-like* profile. Comparisons with voyAGEr suggest that RR pediatric patients exhibit expression profiles closer to adult samples.

### Summary/Conclusion

Transcriptomic maturation in pediatric leukemia follows a robust age-dependent trajectory. In relapsed/refractory cases, this trajectory is significantly accelerated, resulting in an adult-like molecular profile. These findings highlight the importance of age-adjusted analyses and suggest that transcriptomic maturation markers may inform relapse risk and disease progression.

**Copyright Information:** (Online) ISSN: 2572-9241
©2026 The Author(s). HemaSphere is published by John Wiley & Sons Ltd on behalf of European Hematology Association. This is an open access Abstract Book distributed under the Attribution-NonCommercial-NoDerivs (CC BY-NC-ND), which allows third parties to download the articles and share them with others as long as they credit the author and the Abstract Book, but they cannot change the content in any way or use them commercially.
**Abstract Book Citations:** Authors, Title, HemaSphere, 2026;10:(S1):pages. The abstract book DOIs can be found on the “Author Information” page.
**Disclaimer:** Articles published in the journal HemaSphere exclusively reflect the opinions of the authors. The authors are responsible for all content in their abstracts including accuracy of the facts, statements, citing resources, etc.

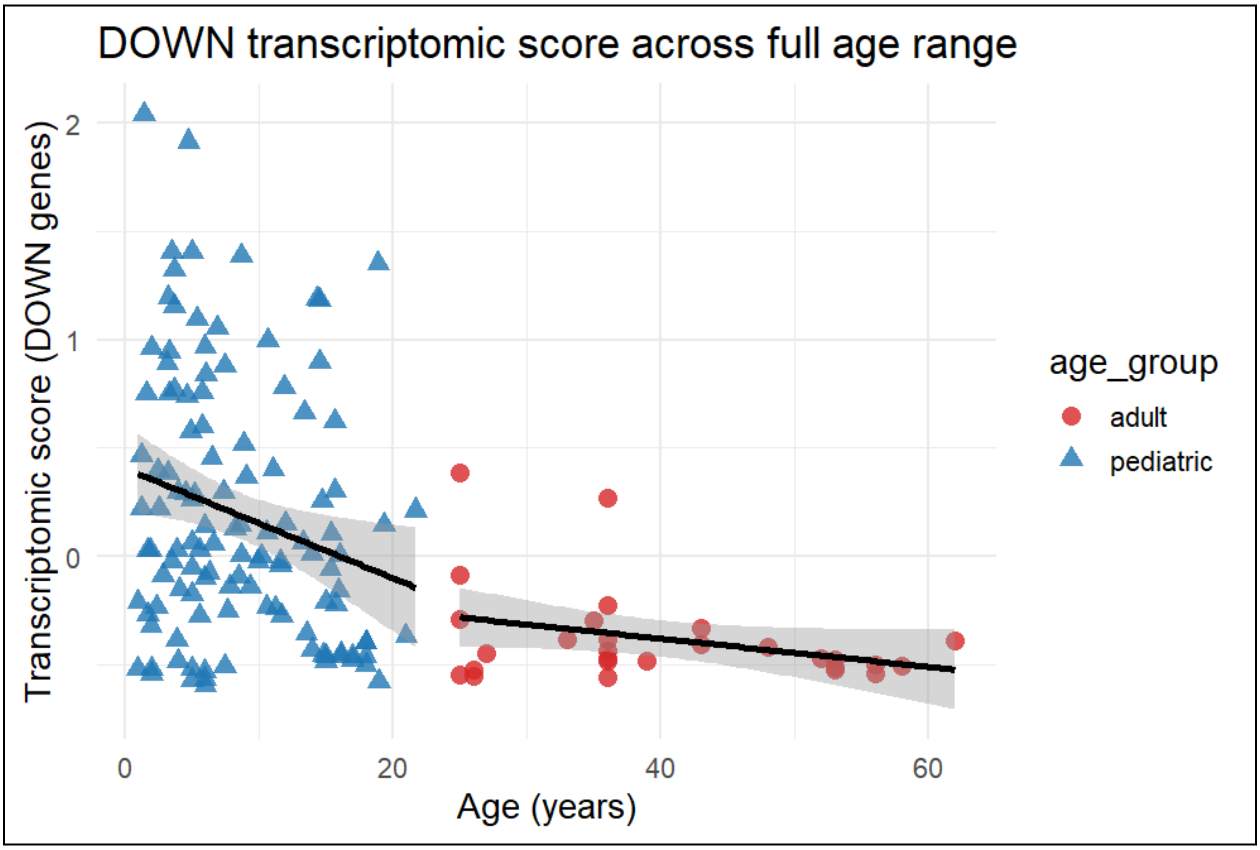

Copyright Information: (Online) ISSN: 2572-9241
©2026 The Author(s). HemaSphere is published by John Wiley & Sons Ltd on behalf of European Hematology Association. This is an open access Abstract Book distributed under the Attribution-NonCommercial-NoDerivs (CC BY-NC-ND), which allows third parties to download the articles and share them with others as long as they credit the author and the Abstract Book, but they cannot change the content in any way or use them commercially.
Abstract Book Citations: Authors, Title, HemaSphere, 2026;10:(S1):pages. The abstract book DOIs can be found on the "Author Information" page.
Disclaimer: Articles published in the journal HemaSphere exclusively reflect the opinions of the authors. The authors are responsible for all content in their abstracts including accuracy of the facts, statements, citing resources, etc.

### (PB2578) PROGNOSTIC VALUE OF ENDOGENIC CYTOKINES IN THE TUMOR MICROENVIRONMENT OF PERIPHERAL BLOOD IN ACUTE LEUKEMIA

**Topic:** 01. Acute lymphoblastic leukemia - Biology & translational research

Vira Barilka*[1], Volodymyr Matlan[2], Olha Shalay[1], Sofiya Prymak[1], Vasyl Novak[1]

*[1]State Institution "Institute of Blood Pathology and Transfusion Medicine of National Academy of Medical Science of Ukraine", Laboratory of Immunology and Genetics of Blood Neoplasms, Lviv, Ukraine; [2]«State Non-Profit Enterprise «Danylo Halytsky Lviv National Medical University», Faculty of Postgraduate Studies, Lviv, Ukraine;*

### Background

The state of neoplastic inflammation indicators, such as leukocytosis/neutropenia, lymphocytosis, blast count, cytotoxic activity (CA) of peripheral blood mononuclear cells (PBMC) under the influence of endogenous cytokines such as tumor necrosis factor (TNF), transforming growth factor beta 1 (TGFβ1) in peripheral blood (PB) of patients (pts), can influence the prognosis and course of acute leukemias (AL).

### Aims

To investigate the prognostic value of endogenic cytokines TNF, TGFβ1  in the tumor microenvironment of peripheral blood of AL pts

### Methods

The concentration of TNF and TGFβ1 in plasma and culture media (CM) of PBMC (a mixture of lymphocytes, monocytes with blasts), the cytotoxic activity (CA) of PBMC against allogeneic cells culture K562, in the ratio (effector:target; 1:20) were determined by biological methods. The study was conducted on 15 AL pts, who were divided into 2 groups (Gr.). Gr. A included pts with newly diagnosed AL, there were 5 pts with acute myeloid leukemia (AML) and 5 pts with acute lymphoblastic leukemia (ALL). The median survival of pts in Gr. A was 115 days. Gr. B included 5 pts with relapsed ALL before the start of treatment. The median survival in Gr. B was 32 days. The control group consisted of 15 blood donors.

### Results

The concentration of TNF in the plasma and CM of PBMC in the compared pts groups significantly exceeded the values in the control ($p < 0{,}01$), while the content of TGFβ1 was statistically different from the control only in the plasma of AL pts ($p < 0{,}05$). The CA of PBMC of AL pts was significantly higher than in control groups ($p < 0{,}05$). A direct correlation was established between CA PBMC and the level of TNF in the CM of PBMC in AL pts ($r = 0.52$); and an inverse correlation was established between the number of leukocytes ($r = -0.53$); lymphocytes ($r = -0.43$) in the general group of AL pts. In Gr. B, the number of leukocytes and lymphocytes was found to be lower than in Gr. A. The concentration of TGFβ1 in the plasma and the CM of PBMC of these pts significantly exceeded the indicators in Gr. A and in the control ($p < 0.05$, in both cases). At the same time, the CA of PBMC in Gr. B directly correlated with the number of blasts ($r = 0.72$); leukocytes ($r = 0.83$) and the concentration of TGFβ1 in CM of PBMC ($r = 0.8$).

**Copyright Information:** (Online) ISSN: 2572-9241
©2026 The Author(s). HemaSphere is published by John Wiley & Sons Ltd on behalf of European Hematology Association. This is an open access Abstract Book distributed under the Attribution-NonCommercial-NoDerivs (CC BY-NC-ND), which allows third parties to download the articles and share them with others as long as they credit the author and the Abstract Book, but they cannot change the content in any way or use them commercially.
**Abstract Book Citations:** Authors, Title, HemaSphere, 2026;10:(S1):pages. The abstract book DOIs can be found on the "Author Information" page.
**Disclaimer:** Articles published in the journal HemaSphere exclusively reflect the opinions of the authors. The authors are responsible for all content in their abstracts including accuracy of the facts, statements, citing resources, etc.

**Summary/Conclusion**

The concentration of cytokines, TNF, TGFβ1, in the plasma of AL pts significantly exceeds the indicators in healthy individuals and may be the result of the body's reactive response to the pathological process, and on the other hand, act as a paracrine source of growth stimuli for AL through modification of the number and activity of immune cells, indicators of neoplastic inflammation, cytokines. The accumulation of TGFβ1 in the plasma of AL pts, especially with relapsed ALL, may be an important cause of inhibition of the immune antitumor response and contribute to the progression of the disease. The obtained results may indicate a tumor-associated role of inflammation in the regulation of cytotoxic properties not only of PBMC in a mixture with blasts, as an alternative route of nutrition, or use the cytotoxicity of PBMC, in symbiosis with blasts, as a mechanism for the spread of ALL. On the other hand, we do not exclude that PBMC together with blasts may be promising targets for achieving remission provided that subpopulations of immune cells and concentrations of TGFβ1 and TNF are corrected at advanced stages of AL.

**Copyright Information:** (Online) ISSN: 2572-9241
©2026 The Author(s). HemaSphere is published by John Wiley & Sons Ltd on behalf of European Hematology Association. This is an open access Abstract Book distributed under the Attribution-NonCommercial-NoDerivs (CC BY-NC-ND), which allows third parties to download the articles and share them with others as long as they credit the author and the Abstract Book, but they cannot change the content in any way or use them commercially.
**Abstract Book Citations:** Authors, Title, HemaSphere, 2026;10:(S1):pages. The abstract book DOIs can be found on the "Author Information" page.
**Disclaimer:** Articles published in the journal HemaSphere exclusively reflect the opinions of the authors. The authors are responsible for all content in their abstracts including accuracy of the facts, statements, citing resources, etc.

### (PB2579) PATTERNS OF CHROMOSOMAL ALTERATIONS IN HIGH-HYPERDIPLOID CHILDHOOD ACUTE LYMPHOBLASTIC LEUKEMIAS IDENTIFIED BY WHOLE-GENOME SEQUENCING

**Topic:** 01. Acute lymphoblastic leukemia - Biology & translational research

Vladimir Kozeev*[1], Ruslan Abasov[1], Anna Kazakova[1]

*[1]Dmitry Rogachev National Medical Research Center of Pediatric Hematology, Oncology and Immunology, Moscow, Russia;*

### Background

High hyperdiploidy as a cytogenetic subtype of childhood B-cell precursor acute lymphoblastic leukemia (BCP-ALL) is defined by the presence of 51–67 chromosomes. This subtype accounts for 30–35% of all BCP-ALL cases and is the most common one. Conventional diagnostic methods do not always allow reliable determination of the modal chromosome number and aneuploidy of individual chromosomes. The use of whole-genome sequencing (WGS) enables precise detection of aneuploidies, providing new opportunities for clinical assessment of this genetic subtype of BCP-ALL.

### Aims

To characterize numerical and structural chromosomal abnormalities in pediatric patients with high-hyperdiploid BCP-ALL using WGS.

### Methods

We analyzed 124 bone marrow samples from patients with newly diagnosed BCP-ALL. Patient age at diagnosis ranged from 3 months to 17.6 years, with a median of 4.6 years. Inclusion criteria were the presence of ploidy changes detected by conventional karyotyping and FISH in the absence of recurrent chromosomal rearrangements. All samples underwent WGS per national genetic initiative "100000+Ya". Aneuploidies were identified using a read-depth–based approach relying on the correlation between sequencing coverage of a genomic region and its copy number.

### Results

In the studied cohort, the modal chromosome number ranged from 51 to 63, with the largest subgroup comprising cases with 55 chromosomes (23 cases, 19%).
The distribution of the modal number of chromosomes is shown in Figure 1A.
The most frequent aneuploidy was an additional chromosome 21, detected in 98% of cases: tetrasomy in 73%, trisomy in 19%, and pentasomy in 5%. Gain of chromosome 14 was observed in 89% of cases (tetrasomy in 71%, trisomy in 18%). Gain of the X chromosome was found in 88% of cases (86% of girls and 89% of boys). Other common aneuploidies included trisomy 6 (88%), 18 (80%), 4 (77%), 17 (73%), 10 (71%), 8 (39%), 5 (30%), 9 (16%), 11 (17%), 12 (16%), 22 (15%), Y (12%), and 16 (9%). Rarer aneuploidies included trisomies 7 (9%), 2 (4%), 20 (4%), 3 (3%), 13 (3%), 15 (3%), 19 (3%), and 1 (3%). Frequencies of chromosome copy-number gains in the cohort are shown in Figure 1B. In 81% of cases, additional subchromosomal copy-number alterations were detected. The most frequent lesions were dup(1q) (n=35), del(9p)(*CDKN2A/B*) (n=25), del(7p)(*IKZF1*) (n=16), del(17p)(*TP53*) (n=10), del(12p)(*ETV6*) (n=8), del(19p) (n=7), del(19q) (n=6), and dup(3q) (n=6).

Copyright Information: (Online) ISSN: 2572-9241
©2026 The Author(s). HemaSphere is published by John Wiley & Sons Ltd on behalf of European Hematology Association. This is an open access Abstract Book distributed under the Attribution-NonCommercial-NoDerivs (CC BY-NC-ND), which allows third parties to download the articles and share them with others as long as they credit the author and the Abstract Book, but they cannot change the content in any way or use them commercially.
Abstract Book Citations: Authors, Title, HemaSphere, 2026;10:(S1):pages. The abstract book DOIs can be found on the "Author Information" page.
Disclaimer: Articles published in the journal HemaSphere exclusively reflect the opinions of the authors. The authors are responsible for all content in their abstracts including accuracy of the facts, statements, citing resources, etc.

### Summary/Conclusion

In high-hyperdiploid BCP-ALL, WGS revealed that the most frequent subgroup had a modal chromosome number of 55. Gains of chromosome 21 were present in 98% of cases, while trisomies of chromosomes 14, X, 6, 18, 4, 17, and 10 occurred in 70–89% of cases. Additional subchromosomal copy-number alterations were identified in 81% of cases, with 1q duplication (n=35) being the most common lesion. WGS is an effective method for assessing both numerical and structural chromosomal abnormalities.

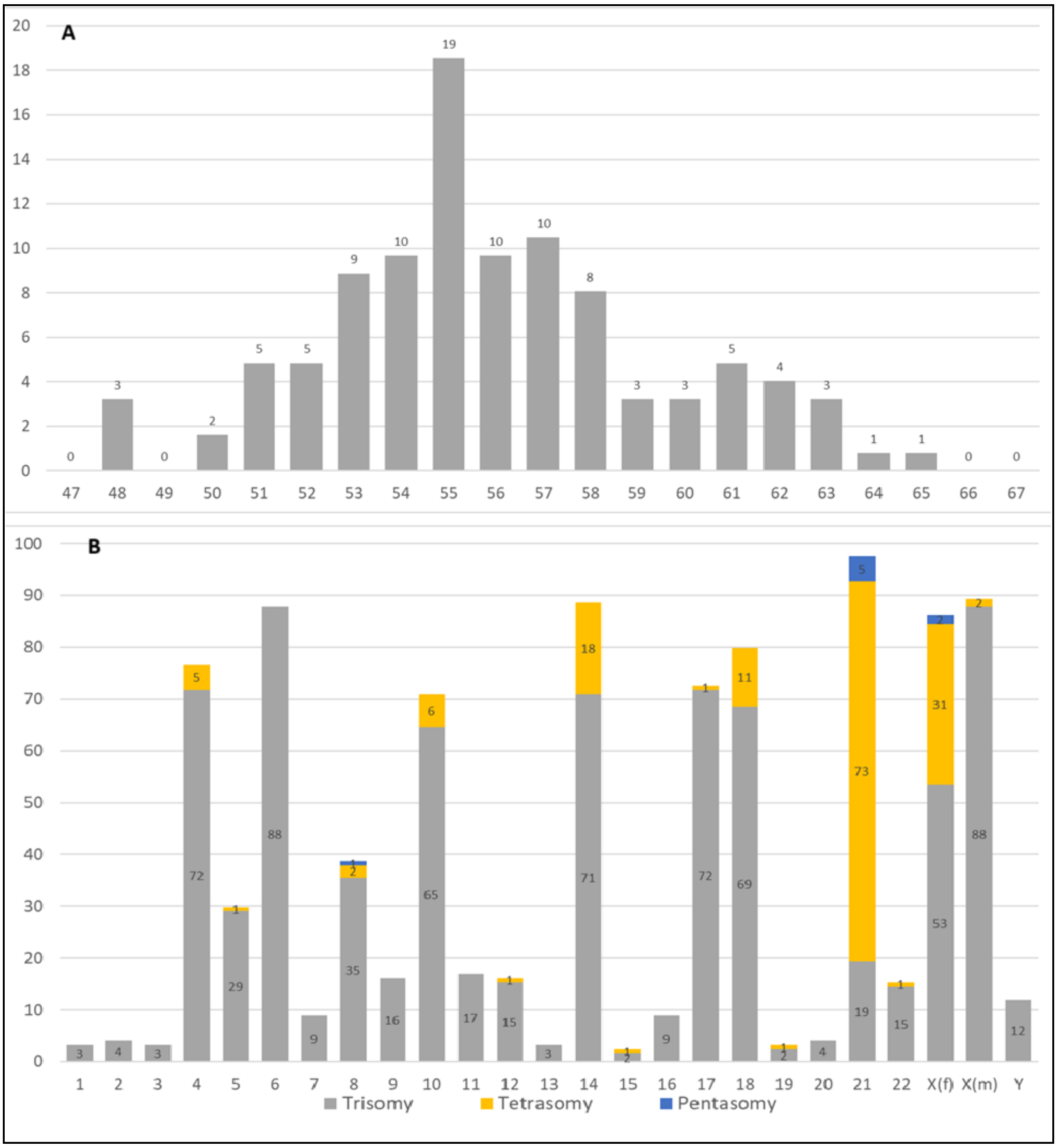

Figure 1. A. Modal number of chromosomes in high-hyperdiploid BCP-ALL in children. B. Frequency of chromosomal gains in high hyperdiploid BCP-ALL. X(f) indicates gain of the X chromosome in females; X(m) in males. “Trisomy” and “tetrasomy” for X(m) and Y correspond to one and two additional copies, respectively.

**Copyright Information:** (Online) ISSN: 2572-9241
©2026 The Author(s). HemaSphere is published by John Wiley & Sons Ltd on behalf of European Hematology Association. This is an open access Abstract Book distributed under the Attribution-NonCommercial-NoDerivs (CC BY-NC-ND), which allows third parties to download the articles and share them with others as long as they credit the author and the Abstract Book, but they cannot change the content in any way or use them commercially.
**Abstract Book Citations:** Authors, Title, HemaSphere, 2026;10:(S1):pages. The abstract book DOIs can be found on the “Author Information” page.
**Disclaimer:** Articles published in the journal HemaSphere exclusively reflect the opinions of the authors. The authors are responsible for all content in their abstracts including accuracy of the facts, statements, citing resources, etc.

### (PB2581) COMBINATION OF CD19-TARGETED CAR-T CELL THERAPY WITH ALLO-HSCT IN THE TREATMENT OF RELAPSED/REFRACTORY B-CELL ACUTE LYMPHOBLASTIC LEUKEMIA. A SINGLE CENTER, PROSPECTIVE, SINGLE-ARM STUDY

**Topic:** 02. Acute lymphoblastic leukemia - Clinical

Guopan Yu*[1], Zhi Liu[1], Qing Zhang[1], Yuanyuan Du[1], Jing Huang[1], Qi Zhong[1], Yangmin Zhu[1], Rong Hu[1], Liling Zheng[1], Xiaoting Zhang[1]

[1] *The Affiliated Guangdong Second Provincial General Hospital of Jinan University, Guangzhou, China;*

### Background

CD19-targeted Chimeric antigen receptor T (CD19 CAR-T) cell therapy plays a key role in the treatment of relapsed/refractory (RR) B-cell acute lymphoblastic leukemia (ALL) and has significantly improved the outcome. Whether allogeneic hematopoietic stem cell transplantation (allo-HSCT) is necessary after CAR-T cell therapy, and how to combine CAR-T cell therapy with allo-HSCT, in the treatment of RR B-cell ALL, remain controversy.

### Aims

Aim to work out how to combine allo-HSCT with CD19 CAR-T cell therap in the treatment of RR B-cell ALL.

### Methods

Here we conducted a single center, prospective, single-arm study (Ethics approval number2018YWPI-008) to savagely treat RR B-cell ALL with CD19 CAR-T cells, and analyzed those with combination treatment of CD19 CAR-T cell therapy and allo-HSCT.

### Results

Totally 26 patients met the criteria and were included for analysis, with male to female 15/11, median age of 28(18-57) years old. 15 patients received autologous CD19 CAR-T cell therapy, and after a median time of 78(42–345) days were followed by allo-HSCT (group A)The other 11 patients obtained allogeneic CD19 CAR-T cell therapy after relapse post allo-HSCT (group B), and only 1 patients referred to secondary transplantation. All the 15 (100%) patients acquired complete remission (CR) or CR with incomplete hematologic recovery (CRi) in the group A, while 8(72.7%) got CR/CRi in the group B (P=0.032). With a median follow-up of 17 (1-93) months, 4(26.7%) relapsed and 6(40%) died in the group A, while 3/8(37.5%) relapsed and 9(81.8%) died in the group B. There were not significantly different in the rate of 2-year disease-free survival (DFS) (64.5%±14.7% vs 66.7% ±19.2%, P=0.917) and overall survival (OS) (the whole cohort, 52.7%±14.1% vs 36.4%±14.5%, P=0.089; the CR/CRi cohort, 52.7%±14.1% vs 57.1%±18.7%, P=0.641) between the two groups. In the group A, 7 patients were bridged to haplo-HSCT, and 8 obtained HLA-matched-HSCT. Those with haplo-HSCT seemed to have a trend of lower cumulative relapse rate than those with HLA-matched-HSCT (1/8 versus 3/7, P=0.185). Side effects including cytokine release syndrome and immune effector cell-associated neurotoxicity syndrome in CAR-T cell therapy were well tolerated in both groups, and there was not CAR-T-related mortality.

### Summary/Conclusion

In conclusion, CD19 CAR-T cell therapy including autologous CAR-T cells before allo-HSCT and allogeneic CAR-T cell post allo-HSCT should be a promising strategy in RR B-cell ALL, with high response and well toleration. Haplo-HSCT might be a better choice than HLA-matched HSCT after CD19 CAR-T cell therapy. Large-scale clinical trials are needed to be guaranteed.

**Copyright Information:** (Online) ISSN: 2572-9241
©2026 The Author(s). HemaSphere is published by John Wiley & Sons Ltd on behalf of European Hematology Association. This is an open access Abstract Book distributed under the Attribution-NonCommercial-NoDerivs (CC BY-NC-ND), which allows third parties to download the articles and share them with others as long as they credit the author and the Abstract Book, but they cannot change the content in any way or use them commercially.
**Abstract Book Citations:** Authors, Title, HemaSphere, 2026;10:(S1):pages. The abstract book DOIs can be found on the "Author Information" page.
**Disclaimer:** Articles published in the journal HemaSphere exclusively reflect the opinions of the authors. The authors are responsible for all content in their abstracts including accuracy of the facts, statements, citing resources, etc.

### (PB2582) CLINICAL CHARACTERISTICS AND PROGNOSTIC ANALYSIS OF CENTRAL NERVOUS SYSTEM LEUKEMIA IN CHILDREN

**Topic:** 02. Acute lymphoblastic leukemia - Clinical

Henghui Chang[1], Huyong Zheng*[1]

[1]*Beijing Children's Hospital, Beijing, China;*

### Background

Central nervous system leukemia (CNSL) is the most common extramedullary leukemia. In pediatric leukemia, the rate of extramedullary relapse is considerably higher than in adults, and it significantly impacts the prognosis and quality of life of children with acute leukemia. In existing research, risk factors associated with CNSL are relatively well-defined, such as younger age at diagnosis, hyperleukocytosis, and the presence of high-risk cytogenetic abnormalities. However, conclusions regarding the survival rate of pediatric CNSL and its impact on prognosis remain unclear. Furthermore, the influence of patient clinical characteristics, particularly CNS status at initial diagnosis, on relapse and prognosis is still controversial.

### Aims

To investigate the clinical characteristics and prognostic risk factors of central nervous system leukemia (CNSL) in children, aiming to guide precise diagnosis and treatment and improve patient outcomes.

### Methods

A retrospective analysis was conducted on clinical data from 62 CNSL patients admitted to Beijing Children's Hospital between May 2016 and June 2022. The Kaplan-Meier method was used to plot survival curves and analyze prognostic factors. The Chi-square test and Fisher's exact test were employed to examine the relationships between immunophenotype, initial white blood cell count, and CNSL.

### Results

Among the 62 pediatric CNSL patients, there were 9 cases of acute myeloid leukemia (AML) and 53 cases of acute lymphoblastic leukemia (ALL), including 44 cases of B-ALL and 9 cases of T-ALL. Based on the timing of CNSL diagnosis, 36 cases were classified as initial CNSL at diagnosis, and 26 cases were CNS relapse after complete remission (including 13 cases of isolated CNS relapse and 13 cases of combined CNS and bone marrow relapse).

The median follow-up time was 38.9 (33.6–44.2) months. The 5-year event-free survival (EFS) rates were (83.3±6.2)% for initial CNSL patients and (47.6±13.3)% for relapse CNSL patients (*P*=0.019). The median survival time for the relapse group was 42.2 (0.2–90.1) months.

Among initial CNSL patients, those with CNS2 status and those diagnosed as CNS3($CSF^+$) (positive cerebrospinal fluid cytology) had poorer EFS compared to patients diagnosed as CNS3($CSF^-$) (negative cytology but positive by imaging and clinical manifestations) (*P*=0.018 and *P*=0.049, respectively). No significant difference in EFS was observed between CNS2 and CNS3($CSF^+$) patients (*P*=0.698).

Among relapse CNSL patients, very early, early, and late relapses accounted for 7, 10, and 9 cases, respectively. The 5-year EFS rates were (42.9±18.7)%, (48.0±16.4)%, and (65.6±20.9)%, respectively (*P*=0.093), with very early and early relapse patients having poorer outcomes and earlier subsequent events. No significant difference in 5-year EFS was found between patients with isolated CNS relapse and those with combined (bone marrow + CNS) relapse.

**Copyright Information:** (Online) ISSN: 2572-9241
©2026 The Author(s). HemaSphere is published by John Wiley & Sons Ltd on behalf of European Hematology Association. This is an open access Abstract Book distributed under the Attribution-NonCommercial-NoDerivs (CC BY-NC-ND), which allows third parties to download the articles and share them with others as long as they credit the author and the Abstract Book, but they cannot change the content in any way or use them commercially.
**Abstract Book Citations:** Authors, Title, HemaSphere, 2026;10:(S1):pages. The abstract book DOIs can be found on the "Author Information" page.
**Disclaimer:** Articles published in the journal HemaSphere exclusively reflect the opinions of the authors. The authors are responsible for all content in their abstracts including accuracy of the facts, statements, citing resources, etc.

**Summary/Conclusion**

Pediatric patients with relapsed CNSL, particularly those with very early or early relapse, have a poor prognosis. CNS2 status is associated with an unfavorable outcome. Among CNS3 patients, those diagnosed by positive CSF cytology (CNS3(CSF$^+$)) have a worse prognosis and a higher tendency for multiple relapses compared to those diagnosed by imaging and clinical manifestations (CNS3(CSF$^-$)). These findings suggest limitations in the current CNS status grading system, highlighting the need for further optimization of the diagnostic and grading criteria for CNSL.

**Copyright Information:** (Online) ISSN: 2572-9241
©2026 The Author(s). HemaSphere is published by John Wiley & Sons Ltd on behalf of European Hematology Association. This is an open access Abstract Book distributed under the Attribution-NonCommercial-NoDerivs (CC BY-NC-ND), which allows third parties to download the articles and share them with others as long as they credit the author and the Abstract Book, but they cannot change the content in any way or use them commercially.
**Abstract Book Citations:** Authors, Title, HemaSphere, 2026;10:(S1):pages. The abstract book DOIs can be found on the "Author Information" page.
**Disclaimer:** Articles published in the journal HemaSphere exclusively reflect the opinions of the authors. The authors are responsible for all content in their abstracts including accuracy of the facts, statements, citing resources, etc.

### (PB2583) LOW PLATELET COUNT AT DIAGNOSIS PREDICTS RELAPSE IN CHILDHOOD B-CELL ACUTE LYMPHOBLASTIC LEUKEMIA

**Topic:** 02. Acute lymphoblastic leukemia - Clinical

Zhiheng Li*[1], Lingzhen Wang[2], Jiaoyang Cai[3], Fenglin Xu[4], Fen Zhou[5], Ju Gao[6], Wenyu Yang[7], Lirong Sun[8], Ching-Hon Pui[9], Shaoyan Hu[10]

*[1]Children's hospital of Soochow university, Hematology and Oncology, Soochow, China;[2]the affiliated hospital of Qingdao University, Qingdao, China; [3]Shanghai Children's Medical Center, Shanghai Jiao Tong University, Shanghai, China;[4]Chongqing Medical University Affiliated Children's Hospital, Chongqing, China; [5]Union Hospital of Tongji Medical College, Huazhong University of Science and Technolog, Wuhan, China; [6]West China Second University Hospital, Sichuan University, Chengdu, China; [7]Institute of Hematology and Blood Diseases Hospital, Peking Union Medical College, Chinese Academy of Medical Sciences, Tianjin, China; [8]Jiangsu Pediatric Hematol&Oncol Center Affiliated Hospital of Qingdao University, Qingdao, China;[9]St. Jude Children's Research Hospital, Memphis, United States of America; [10]Children's Hospital of Soochow University, Soochow, China;*

### Background

Despite substantial improvements in survival of childhood acute lymphoblastic leukemia (ALL), relapse remains a major cause of treatment failure, highlighting the need for simple and broadly accessible prognostic markers to enhance risk stratification. Platelet counts, a key player in hemostasis, has had its prognostic significance comparatively underexplored.

### Aims

To investigate the prognostic significance of initial platelet count on outcomes in childhood ALL.

### Methods

Between Dec 31th, 2014 to Dec 30th, 2019, 7640 pediatric patients with newly diagnosed ALL were enrolled in the Chinese Children's Cancer Group ALL-2015 clinical trial (CCCG-ALL-2015). Platelet count at diagnosis of each patient was collected and patients were divided into two groups based on a cutoff of 50 x$10^9$/L.

### Results

Among 6,916 patients with B-cell ALL (B-ALL), a platelet count <50 × $10^9$/L at diagnosis was independently associated with inferior 5-year event-free survival (77.1% vs 84.7%, *P*<.001) and overall survival, whereas no such association was observed in T-ALL. Low platelet count independently predicted relapse (subdistribution hazard ratio [SHR], 1.41), including any central nervous system (CNS) relapse (SHR, 1.81) and isolated CNS relapse (SHR, 2.30). The cumulative incidence of any CNS relapse was significantly higher in patients with platelet counts <50 × $10^9$/L than in those with higher counts (3.12% vs 1.57%, *P*<.001). Notably, this association was most pronounced among patients with CNS1 status at diagnosis, in whom low platelet count predicted increased risks of overall relapse, any CNS relapse, and isolated CNS relapse. Platelet count was not correlated with early treatment response or measurable residual disease, indicating independent prognostic value.

### Summary/Conclusion

These findings identify pretreatment thrombocytopenia as a novel and readily available risk factor for CNS relapse in pediatric B-ALL, supporting consideration of platelet-guided strategies to optimize CNS-directed therapy.

**Copyright Information:** (Online) ISSN: 2572-9241
©2026 The Author(s). HemaSphere is published by John Wiley & Sons Ltd on behalf of European Hematology Association. This is an open access Abstract Book distributed under the Attribution-NonCommercial-NoDerivs (CC BY-NC-ND), which allows third parties to download the articles and share them with others as long as they credit the author and the Abstract Book, but they cannot change the content in any way or use them commercially.
**Abstract Book Citations:** Authors, Title, HemaSphere, 2026;10:(S1):pages. The abstract book DOIs can be found on the "Author Information" page.
**Disclaimer:** Articles published in the journal HemaSphere exclusively reflect the opinions of the authors. The authors are responsible for all content in their abstracts including accuracy of the facts, statements, citing resources, etc.

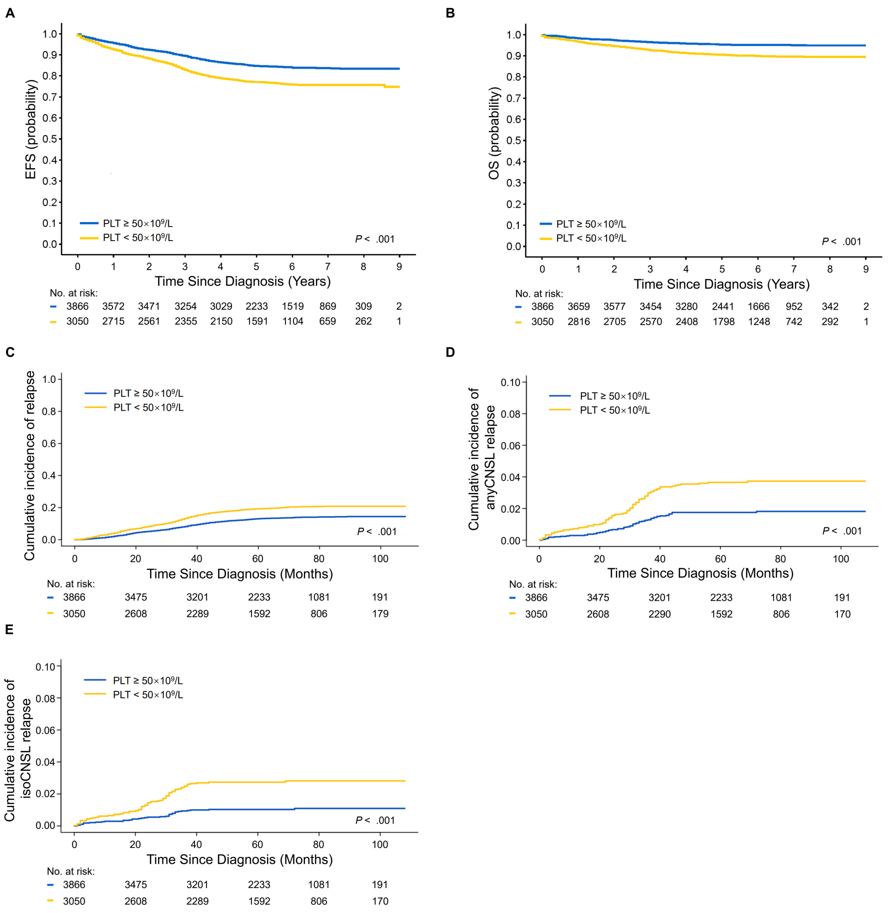

**Copyright Information:** (Online) ISSN: 2572-9241
©2026 The Author(s). HemaSphere is published by John Wiley & Sons Ltd on behalf of European Hematology Association. This is an open access Abstract Book distributed under the Attribution-NonCommercial-NoDerivs (CC BY-NC-ND), which allows third parties to download the articles and share them with others as long as they credit the author and the Abstract Book, but they cannot change the content in any way or use them commercially.
**Abstract Book Citations:** Authors, Title, HemaSphere, 2026;10:(S1):pages. The abstract book DOIs can be found on the "Author Information" page.
**Disclaimer:** Articles published in the journal HemaSphere exclusively reflect the opinions of the authors. The authors are responsible for all content in their abstracts including accuracy of the facts, statements, citing resources, etc.

### (PB2584) EFFICACY AND SAFETY OF THE FLUMATINIB, VENETOCLAX, VINCRISTINE, AND PREDNISONE (FVVP) REGIMEN IN NEWLY DIAGNOSED ADULT PHILADELPHIA CHROMOSOME-POSITIVE ACUTE LYMPHOBLASTIC LEUKEMIA

**Topic:** 02. Acute lymphoblastic leukemia - Clinical

Weimin Wang*[1], Jieke Cui[1], Shuyu Han[1], Yuxia Dai[1], Yanan Wang[1], Yan Yang[1], Hui Sun[1], Silin Gan[1]

[1]*the first affiliated hospital of Zhengzhou University, Zhengzhou, China;*

### Background

Tyrosine kinase inhibitors (TKIs) combined with chemotherapy and immunotherapy have evolved as the standard treatment for newly diagnosed (ND) Philadelphia chromosome-positive acute lymphoblastic leukemia (Ph+ ALL). However, many patients are intolerant to chemotherapy or lack access to immunotherapy, especially in developing country. Therefore, there is an urgent need to explore therapeutic regimens that are highly effective, low-toxicity, and economically viable. Venetoclax has demonstrated favorable efficacy in a variety of hematologic malignancies.Nonetheless, the efficacy and safety of combining Flumatinib with Venetoclax in ND Ph+ ALL have yet to be fully elucidated.

### Aims

We conducted a prospective clinical trial to evaluate the efficacy and safety of the flumatinib, venetoclax, vincristine, and prednisone (FVVP) regimen as frontline treatment in patients with ND Ph+ ALL.

### Methods

From May 2024 to January 2026, a total of 11 newly diagnosed adult patients with Ph+ ALL from the First Affiliated Hospital of Zhengzhou University were enrolled. Baseline characteristics included age, gender, leukocyte count, bone marrow blast percentage, fusion transcript type, cytogenetics, and mutational status. Treatment responses were evaluated, including the 3-month complete molecular response (CMR) rate, minimal residual disease (MRD) negativity, overall survival (OS), relapse-free survival (RFS), relapse incidence, adverse events, and central nervous system leukemia (CNSL) status.

### Results

Among 11 patients (median age 38 years [range 15–58]; 63.6% male), the median initial WBC was 28.7×10⁹/L (range 1.89–423.49×10⁹/L), with hyperleukocytosis (≥30×10⁹/L) in 45.5%. The mean percentage of bone marrow blasts was 90.8% (range 68.4%–98.8%). BCR-ABL1 isoforms: p190 in 54.5%, p210 in 36.4%, and rare E1A3 in 9.3%. Cytogenetics: normal karyotype 11.1%, isolated Ph 88.9%. Among 8 patients with next-generation sequencing, 37.5% harbored IKZF1 mutations, with no IKZF1plus genotype (IKZF1 deletion plus CDKN2A/B/PAX5/PAR1 loss without ERG deletion).
The FVVP regimen achieved CR rates of 100% at day 28, month 3, and month 6. MRD negativity by flow cytometry was 90.9%, 100%, and 100% at these timepoints. Major molecular response (MMR) rates were 72.7%, 100%, and 100%, while CMR rates reached 40%, 50%, and 100%, respectively.
The median follow-up duration was 10 months (range 0–21). As of February 1, 2026, all patients were alive. Only one patient relapsed and subsequently underwent allo-HSCT. The median RFS was not reached. The estimated 1-year RFS rate was 74.1%.
No deaths occurred during induction therapy. Hematologic adverse events were the main toxicities, with grade 4 neutropenia and thrombocytopenia occurring in 36.4% and 54.5% of patients, respectively—significantly lower than with traditional chemotherapy.

**Copyright Information:** (Online) ISSN: 2572-9241
©2026 The Author(s). HemaSphere is published by John Wiley & Sons Ltd on behalf of European Hematology Association. This is an open access Abstract Book distributed under the Attribution-NonCommercial-NoDerivs (CC BY-NC-ND), which allows third parties to download the articles and share them with others as long as they credit the author and the Abstract Book, but they cannot change the content in any way or use them commercially.
**Abstract Book Citations:** Authors, Title, HemaSphere, 2026;10:(S1):pages. The abstract book DOIs can be found on the "Author Information" page.
**Disclaimer:** Articles published in the journal HemaSphere exclusively reflect the opinions of the authors. The authors are responsible for all content in their abstracts including accuracy of the facts, statements, citing resources, etc.

**Summary/Conclusion**

The FVVP regimen represents an effective, safe and cost-effective therapeutic option for Ph+ ALL, warranting further multicenter prospective validation to optimize long-term efficacy.

**Copyright Information:** (Online) ISSN: 2572-9241
©2026 The Author(s). HemaSphere is published by John Wiley & Sons Ltd on behalf of European Hematology Association. This is an open access Abstract Book distributed under the Attribution-NonCommercial-NoDerivs (CC BY-NC-ND), which allows third parties to download the articles and share them with others as long as they credit the author and the Abstract Book, but they cannot change the content in any way or use them commercially.
**Abstract Book Citations:** Authors, Title, HemaSphere, 2026;10:(S1):pages. The abstract book DOIs can be found on the “Author Information” page.
**Disclaimer:** Articles published in the journal HemaSphere exclusively reflect the opinions of the authors. The authors are responsible for all content in their abstracts including accuracy of the facts, statements, citing resources, etc.

### (PB2585) PHILADELPHIA CHROMOSOME–NEGATIVE BUT BCR::ABL1-POSITIVE ACUTE LYMPHOBLASTIC LEUKEMIA: A REAL-WORLD MULTICENTER COHORT STUDY

**Topic:** 02. Acute lymphoblastic leukemia - Clinical

Shota Yokoyama*[1, 2], Masahiro Onozawa[1], Hiroyuki Kimura[1], Takahide Ara[1], Jun Nagai[1], Shota Yoshida[1], Naoki Miyashita[1], Toshihiro Matsukawa[1], Hideki Goto[1], Shinsuke Hirabayashi[3], Minoru Kanaya[4], Akio Mori[4], Daisuke Hidaka[5], Junichi Hashiguchi[6], Kentaro Wakasa[2], Makoto Ibata[7], Yukari Takeda[8], Takuto Miyagishima[9], Satoshi Yamamoto[10], Katsuya Fujimoto[11], Toma Suzuki[12], Tomoyuki Saga[13], Hajime Sakai[14], Yasutaka Kakinoki[15], Tatsuo Oyake[16], Takeshi Kondo[4], Takanori Teshima[1]

*[1]Hokkaido University Faculty of Medicine, Graduate School of Medicine, Department of Hematology, Sapporo, Japan;[2]Obihiro Kosei Hospital, Department of Hematology, Obihiro, Japan; [3]Hokkaido University Faculty of Medicine, Graduate School of Medicine, Department of Pediatrics, Sapporo, Japan; [4]Aiiku Hospital, Blood Disorders Center, Sapporo, Japan; [5]Sapporo Hokuyu Hospital, Department of Hematology, Sapporo, Japan; [6]Kitami Red Cross Hospital, Department of Internal Medicine, Kitami, Japan; [7]Sapporo-Kosei General Hospital, Sapporo, Japan; [8]Tonan Hospital, Department of Hematology, Sapporo, Japan; [9]Kushiro Rosai Hospital,, Department of Hematology, Kushiro, Japan; [10]Sapporo City General Hospital, Department of Hematology, Sapporo, Japan; [11]NHO Hokkaido Cancer Center, Department of Hematology, Sapporo, Japan; [12]Hakodate Municipal Hospital, Hakodate, Japan; [13]Kin-ikyo Chuo Hospital, Sapporo, Japan; [14]Teine Keijinkai Hospital, Department of Hematology, Sapporo, Japan; [15]Asahikawa City Hospital, Department of Hematology, Asahikawa, Japan; [16]Iwate Medical University, Department of Hematology and Oncology, Yahaba, Japan;*

### Background

The Philadelphia (Ph) chromosome is the product of the reciprocal translocation t(9;22)(q34;q11), and represents the hallmark of CML. About 5-10% of CML patients lack cytogenetic evidence of the Ph chromosome but demonstrate BCR::ABL1 fusion by FISH or RT-PCR. Patients with masked-Ph CML show comparable responses to tyrosine kinase inhibitors (TKIs) with cytogenetically detectable Ph chromosomes. In contrast, the prevalence and clinical characteristics of masked Ph in ALL remain poorly understood.To validate prevalence and clinical features of these Ph-/BCR::ABL1+ (Masked Ph) in the ALL.

### Aims

To validate prevalence and clinical features of these Masked Ph in the ALL.

### Methods

Hokkaido Leukemia Net (HLN) is a prospective cohort study collecting acute leukemia samples from affiliated hospitals in northern Japan. In this study, B-ALL patients registered in HLN between February 2017 and May 2024 were enrolled. Detection of major and minor BCR::ABL1 transcripts was centrally performed, using diagnostic bone marrow (BM) or peripheral blood samples. Conventional G-banding analysis was performed at certified commercial cytogenetic laboratories in accordance with internationally accepted methods, following recommendations of the International System for Human Cytogenomic Nomenclature (ISCN). FISH for BCR::ABL1 was performed at the commercial laboratories.

**Copyright Information:** (Online) ISSN: 2572-9241
©2026 The Author(s). HemaSphere is published by John Wiley & Sons Ltd on behalf of European Hematology Association. This is an open access Abstract Book distributed under the Attribution-NonCommercial-NoDerivs (CC BY-NC-ND), which allows third parties to download the articles and share them with others as long as they credit the author and the Abstract Book, but they cannot change the content in any way or use them commercially.
**Abstract Book Citations:** Authors, Title, HemaSphere, 2026;10:(S1):pages. The abstract book DOIs can be found on the "Author Information" page.
**Disclaimer:** Articles published in the journal HemaSphere exclusively reflect the opinions of the authors. The authors are responsible for all content in their abstracts including accuracy of the facts, statements, citing resources, etc.

### Results

Karyotype and BCR::ABL1 fusion gene status were identified in 160 B-ALL. Ph chromosome was identified in 73 cases and all cases were confirmed to have either major or minor BCR::ABL1 by RT-PCR (Ph+ ALL). Ph chromosome was not identified in 87 of 160 B-ALL, however, either major or minor BCR::ABL1 was identified in 19 (21.8%) (masked-Ph ALL). In 160 cases, 68 cases had neither the Ph chromosome nor the BCR::ABL1 fusion gene (non-Ph ALL). Most cases with Ph+ ALL (70/73, 95.9%) and all of the cases with masked-Ph ALL, received a TKI during induction therapy. The rate of achieving hematological complete remission (hCR) was comparable in Ph+ ALL and masked-Ph ALL (94.5% vs 94.7%, P=1). In contrast, all 68 cases with non-Ph ALL were treated with cytoreductive chemotherapy and steroids, and 59 of 68 (86.8%) achieved hCR. The 3-year overall survival rates of masked-Ph ALL cases, Ph+ ALL cases and non-Ph ALL cases were 83%, 79% and 56%, respectively (P=0.032). The prognosis of masked-Ph and Ph+ ALL were comparable. In addition, BCR::ABL1 positive ALL (masked-Ph and Ph+ ALL) showed better prognosis than non-Ph ALL (P=0.0087). The total analyzed cell count by G-banding of masked-Ph ALL was significantly lower than that of Ph+ ALL (P= 0.0061). This result suggests that a lower number of analyzed metaphases may contribute to underestimation of leukemic cells by conventional karyotyping. On the other hand, one masked-Ph ALL case had atypical FISH pattern (Figure 1). In this case, G-banding analysis in BM cells showed 46,XX,i(8)(q19) in all 24 analyzed cells. This result suggested that a microinsertion of the ABL gene into the BCR gene was formed, which is not visible in conventional G-banding.

### Summary/Conclusion

About 20% of BCR::ABL1-positive ALL cases lacked cytogenetic evidence of the Ph chromosome. These masked-Ph cases may result from cryptic translocations as well as a lower mitotic index of lymphoblasts. Importantly, patients with masked-Ph ALL who were treated with TKIs achieved CR remission and long-term outcomes comparable to those of patients with conventional Ph+ ALL.

**Figure 1. Micro insertion of ABL1 into BCR resulting in cryptic BCR::ABL1 detected by FISH**

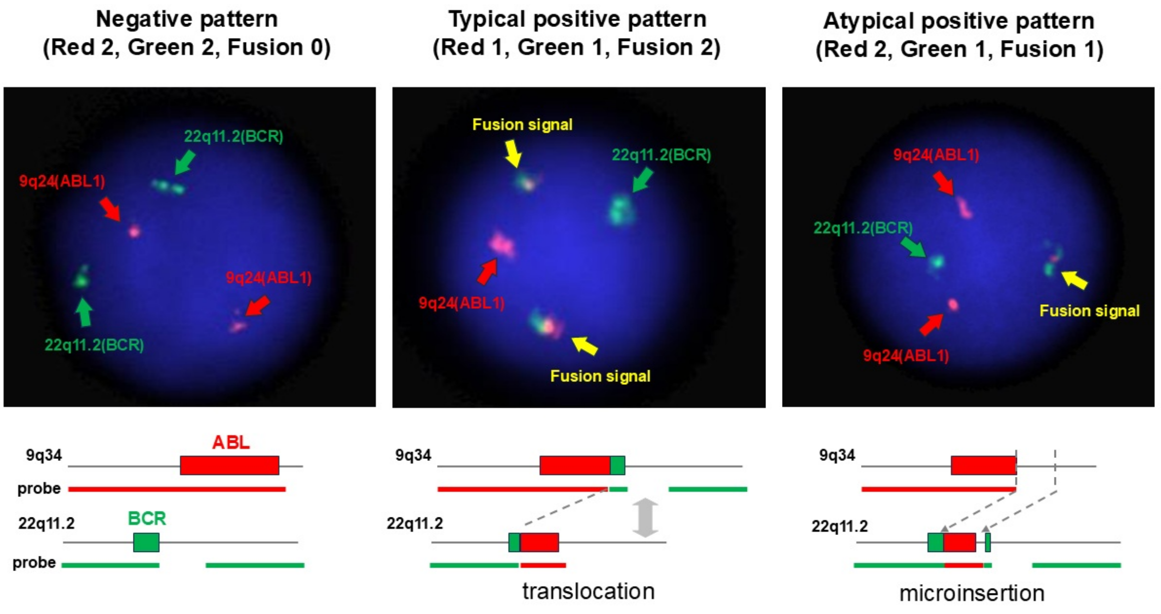

Copyright Information: (Online) ISSN: 2572-9241
©2026 The Author(s). HemaSphere is published by John Wiley & Sons Ltd on behalf of European Hematology Association. This is an open access Abstract Book distributed under the Attribution-NonCommercial-NoDerivs (CC BY-NC-ND), which allows third parties to download the articles and share them with others as long as they credit the author and the Abstract Book, but they cannot change the content in any way or use them commercially.
Abstract Book Citations: Authors, Title, HemaSphere, 2026;10:(S1):pages. The abstract book DOIs can be found on the "Author Information" page.
Disclaimer: Articles published in the journal HemaSphere exclusively reflect the opinions of the authors. The authors are responsible for all content in their abstracts including accuracy of the facts, statements, citing resources, etc.

### (PB2586) A NEW MODEL OF CHEMOTHERAPY-FREE INDUCTION FOR ADULT PH- ALL: VENETOCLAX AND BLINATUMOMAB DEMONSTRATE SUPERIOR EFFICACY AND T-CELL COMPATIBILITY

**Topic:** 02. Acute lymphoblastic leukemia - Clinical

Siyu Chen[1], Jing Li[1], Ying Chen*[1], Maihong Wang[1], Guo Chen[1], Xiannian Qin[1], Qin Wen[1], Hong Liu[1], Xi Zhang[1], Cheng Zhang[1]

[1]*Medical Center of Hematology, Xinqiao Hospital of Army Medical University, Chongqing, China;*

### Background

Standard induction for adult Philadelphia chromosome-negative acute lymphoblastic leukemia (Ph– ALL) relies on toxic chemotherapy with suboptimal MRD eradication. While Venetoclax (Bcl-2 inhibitor) and Blinatumomab (BiTE) show efficacy in refractory settings, their frontline combination has not been explored. Crucially, since Blinatumomab relies on host T-cells, determining whether Venetoclax impairs T-cell fitness is vital for therapeutic rationale.

### Aims

We evaluated the feasibility of a novel chemotherapy-free Venetoclax-Blinatumomab induction regimen in newly diagnosed Ph– ALL and mechanistically validated its compatibility by assessing *ex vivo* T-cell proliferation and immunophenotype under Bcl-2 inhibition.

### Methods

**Clinical:** In this pilot study, adults with newly diagnosed Ph– ALL received Venetoclax (ramp-up to 400 mg, days 1–7), followed sequentially by Blinatumomab (days 8–28). Endpoints included Complete Remission (CR), MRD (flow cytometry, sensitivity $10^{-4}$), and safety (CTCAE v4.0).

**Translational:** To verify T-cell preservation, healthy donor $CD3^+$ T-cells were isolated and physiologically activated using anti-CD3/CD28 magnetic beads. Cells were cultured with Venetoclax (0.24 nM) or vehicle. T-cell proliferation was quantified via CCK-8 assays, and subset distribution ($CD3^+/CD4^+/CD8^+$) was analyzed by flow cytometry to determine if Venetoclax alters the helper/cytotoxic ratio required for BiTE activity.

**Copyright Information:** (Online) ISSN: 2572-9241
©2026 The Author(s). HemaSphere is published by John Wiley & Sons Ltd on behalf of European Hematology Association. This is an open access Abstract Book distributed under the Attribution-NonCommercial-NoDerivs (CC BY-NC-ND), which allows third parties to download the articles and share them with others as long as they credit the author and the Abstract Book, but they cannot change the content in any way or use them commercially.
**Abstract Book Citations:** Authors, Title, HemaSphere, 2026;10:(S1):pages. The abstract book DOIs can be found on the "Author Information" page.
**Disclaimer:** Articles published in the journal HemaSphere exclusively reflect the opinions of the authors. The authors are responsible for all content in their abstracts including accuracy of the facts, statements, citing resources, etc.

### Results

Three patients (median age 51; including *MLL-AF4* fusion/complex karyotype) were enrolled. 100% completed induction without dose reductions.
**Clinical Efficacy:** All patients (3/3) achieved CR and, notably, 100% attained deep MRD negativity. Patient 1 achieved CR on day 8 with Venetoclax alone.
**Safety:** The regimen showed a superior safety profile compared to historical chemotherapy: no grade 3–4 myelosuppression, infections, bleeding events, CRS, or ICANS were observed. EORTC QLQ-C30 scores indicated improved quality of life during therapy (Figure D).
**Mechanistic Insights:** *In vitro* assays confirmed biological compatibility. Venetoclax did not inhibit the proliferation or viability of activated normal T-cells compared to controls (Figure A-B). Furthermore, flow cytometry demonstrated preserved frequencies of $CD3^{+}$, $CD4^{+}$, and $CD8^{+}$ subsets, confirming that Bcl-2 inhibition does not compromise the T-cell effector pool essential for Blinatumomab-mediated cytotoxicity (Figure C).

| | Patient 1 | Patient 2 | Patient 3 |
|---|---|---|---|
| Sex (Male/Female) | Female | Female | Female |
| Age (Year) | 37 | 51 | 55 |
| ECOG performance status | 0 | 0 | 0 |
| Hemogram at diagnosis | | | |
| WBC ($\times 10^9$/L) | 2.48 | 37.87 | 1.64 |
| ANC ($\times 10^9$/L) | 1.33 | 2.75 | 0.69 |
| HGB (g/L) | 87 | 58 | 94 |
| Platelet ($\times 10^9$/L) | 71 | 201 | 307 |
| FISH | +6,+14/14q+,+22/22q+,+21/21q+,+1/1q+, +19/19q+,+8/8q+,+14/14q+,+11/11q+,+8/8q+ | / | / |
| Chromosome karyotype | 46 xx [20] | 46 xx, t(4,11)(q21;q23)[7] | 46 xx [20] |
| Fusion gene | / | MLL-AF4 | / |
| Bone marrow blasts (%) | 39.77 | 94 | 34 |
| At the end of venetoclax | | | |
| Bone marrow blasts (%) | 0 | 17 | 0 |
| MRD (%) | NA | NA | P |
| At the end of induction treatment | | | |
| Bone marrow blasts (%) | 0 | 0 | 0 |
| MRD (%) | N | N | N |

### Summary/Conclusion

We report the world's first chemotherapy-free induction with Venetoclax and Blinatumomab for Ph– ALL, achieving 100% MRD-negative CR with minimal toxicity. Translational data provide the mechanistic underpinning for this strategy, demonstrating that Venetoclax spares normal T cells. This regimen represents a potential paradigm shift in frontline ALL management and warrants validation in larger trials.

**Copyright Information:** (Online) ISSN: 2572-9241
©2026 The Author(s). HemaSphere is published by John Wiley & Sons Ltd on behalf of European Hematology Association. This is an open access Abstract Book distributed under the Attribution-NonCommercial-NoDerivs (CC BY-NC-ND), which allows third parties to download the articles and share them with others as long as they credit the author and the Abstract Book, but they cannot change the content in any way or use them commercially.
**Abstract Book Citations:** Authors, Title, HemaSphere, 2026;10:(S1):pages. The abstract book DOIs can be found on the "Author Information" page.
**Disclaimer:** Articles published in the journal HemaSphere exclusively reflect the opinions of the authors. The authors are responsible for all content in their abstracts including accuracy of the facts, statements, citing resources, etc.

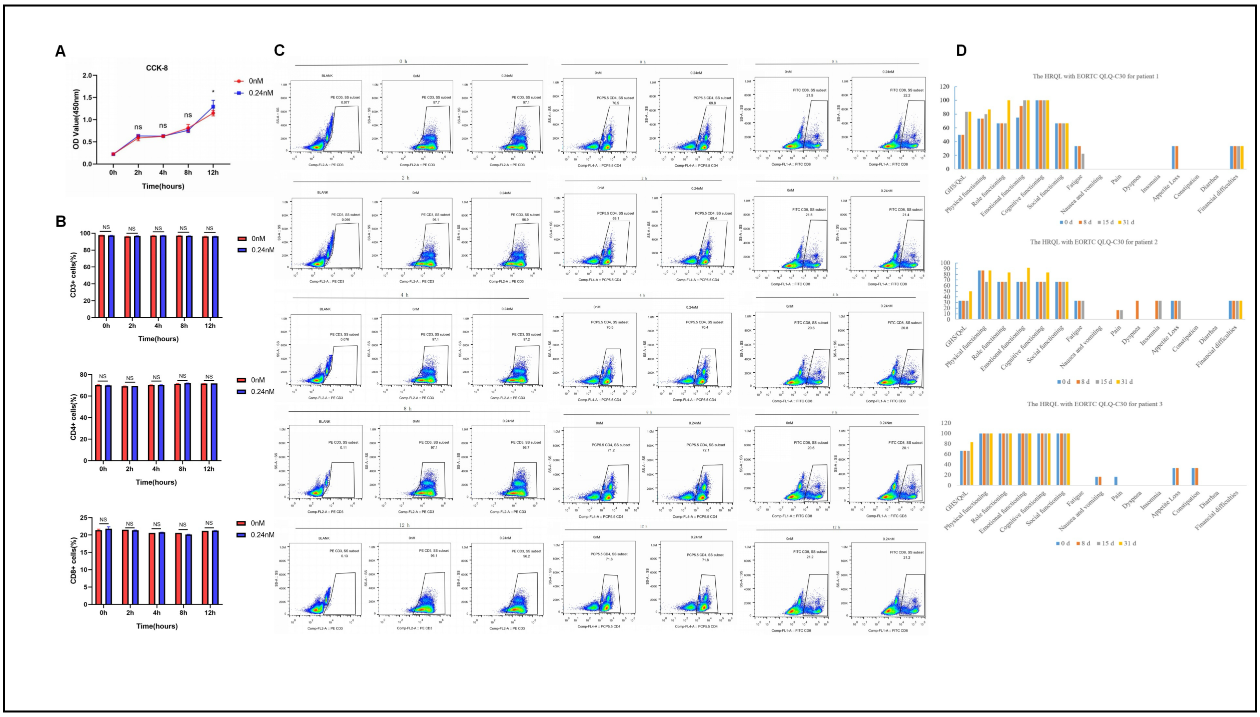

**Copyright Information:** (Online) ISSN: 2572-9241
©2026 The Author(s). HemaSphere is published by John Wiley & Sons Ltd on behalf of European Hematology Association. This is an open access Abstract Book distributed under the Attribution-NonCommercial-NoDerivs (CC BY-NC-ND), which allows third parties to download the articles and share them with others as long as they credit the author and the Abstract Book, but they cannot change the content in any way or use them commercially.
**Abstract Book Citations:** Authors, Title, HemaSphere, 2026;10:(S1):pages. The abstract book DOIs can be found on the "Author Information" page.
**Disclaimer:** Articles published in the journal HemaSphere exclusively reflect the opinions of the authors. The authors are responsible for all content in their abstracts including accuracy of the facts, statements, citing resources, etc.

### (PB2587) THE EFFICIENCY OF THERAPY IN PATIENTS WITH MPAL: SINGLE-CENTER STUDY RESULTS

**Topic:** 02. Acute lymphoblastic leukemia - Clinical

Anastasia Vasileva*[1], Mariam Abu-Khadir[2], Olga Aleshina (Gavrilina)[1], Ekaterina Kotova[1], Galina Isinova[1], Zalina Fidarova[1], Vera Troitskaya[1], Elena Parovichnikova[1]

*[1]National Medical Research Centre for Hematology, Moscow, Russia;[2]Lomonosov Moscow State University, Moscow, Russia;*

### Background

Mixed-phenotype acute leukemia (MPAL) is a clonal disease of the blood system. MPAL is characterized by proliferation of blasts, for which simultaneous expression of antigens of two and/or more differentiation lines is characteristic. MPAL constitutes about 1-3% of all acute leukemia (AL) in adults and is characterized by phenotypic and genetic diversity.

### Aims

To evaluate the long-term treatment results and risk factors of patients with MPAL who received therapy at the NMRC for hematology according to the RALL-2016, RALL-2009 or AML-2021

### Methods

The analysis included 32 patients with 2 and/or more populations of blast were identified or one population of blast expressing markers of 2 and/or more lines of differentiation according to the immunophenotypic analysis (IA). 18(56%) were male and 14 (44%) - female. The median age for men was 31 years (21-53), for women - 42 years (18-75). 21 (65%) matched the criteria of MPAL(consensus on MPAL 2023), 9 (28%) - AL with a second population of <5%, 2 (7%) - another hematological disease.

### Results

In the group of patients with MPAL (n=21), the ratio of men to women was 1:0.75. The median age among men with MPAL was 32 years (21-53), women - 34 years (18-75) (p>0.05). IA: 12 (57%) - T-ALL/AML, 8 (38%) – B-ALL/AML, 1 (5%) - T/B/AML. The median hemoglobin was 101 g/l (63-158), leukocytes - 8,01 x $10^9$/l (1.52 - 212.42), platelets - 81 x $10^9$/l (15 - 403), blast BM - 74.8% (20.4 - 94.8), LDH - 523 U/L(170 - 1666). 16 (80%) had lymphadenopathy, 13 (65%) - hepatosplenomegaly, neuroleukemia - 3 (15%) patients, and 1 (5%) - leukemids.
Karyotype: 7 (33%) – normal karyotype, 12 (57%) – abnormal karyotype (7 (33%) – complex karyotype) (CK)), 2 (10%) – no mitosis. A rearrangement of *BCR::ABL* gene was detected in 1 (5%), and the *KMT2A* gene - 2 (10%). These mutations were detected only in patients with B/AML phenotype (p=0.01). According to molecular studies: 3 (14%) had mutations in the *FLT3* gene, 3 (14%) in the *CEBPa* gene, and 1 (5%) in the *NPM1* gene.
The frequency of remission after the 1st line of therapy was (n=15) 75%, the proportion of primary refractory in the B/AML group was 28.6%, T/AML - 16%, T/B/AML - 100% (p>0.05). MRD- remission was achieved in (n=8) 53% among patients who achieved CR after the 1st line of therapy. The relapse rate in this group was 12.5% (in 1 out of 8) patients. MRD+ remission after line 1 was also achieved in 57% of patients, the relapse rate in this group was 57% (4 out of 7 patients) (p=0.07). 4-year OS in the MRD- group was 85%, in the MRD+ 42% group (p=0.044) (Fig.1). The 4-year RFS in the MRD- group was 86%, in the MRD+ group + 26% (p=0.08) (Fig.2).

**Copyright Information:** (Online) ISSN: 2572-9241
©2026 The Author(s). HemaSphere is published by John Wiley & Sons Ltd on behalf of European Hematology Association. This is an open access Abstract Book distributed under the Attribution-NonCommercial-NoDerivs (CC BY-NC-ND), which allows third parties to download the articles and share them with others as long as they credit the author and the Abstract Book, but they cannot change the content in any way or use them commercially.
**Abstract Book Citations:** Authors, Title, HemaSphere, 2026;10:(S1):pages. The abstract book DOIs can be found on the “Author Information” page.
**Disclaimer:** Articles published in the journal HemaSphere exclusively reflect the opinions of the authors. The authors are responsible for all content in their abstracts including accuracy of the facts, statements, citing resources, etc.

### Summary/Conclusion

The persistence of MRD after the end of induction therapy is an unfavorable prognostic factor for patients with MPAL. CK was detected in 33% of patients. CK is a risk factor for failure to achieve remission after subsequent lines of therapy in the group of refractory patients.

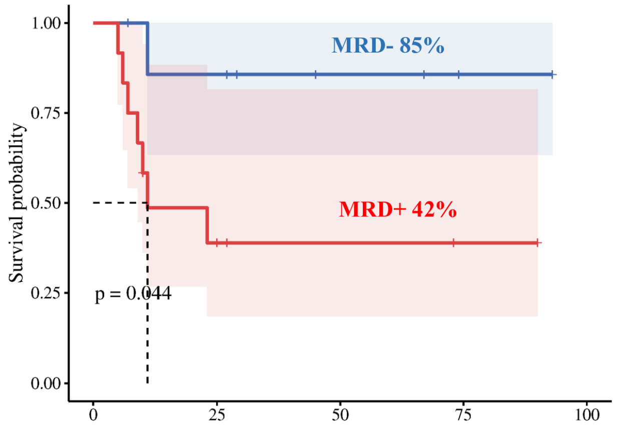

Fig. 1 Overall survival

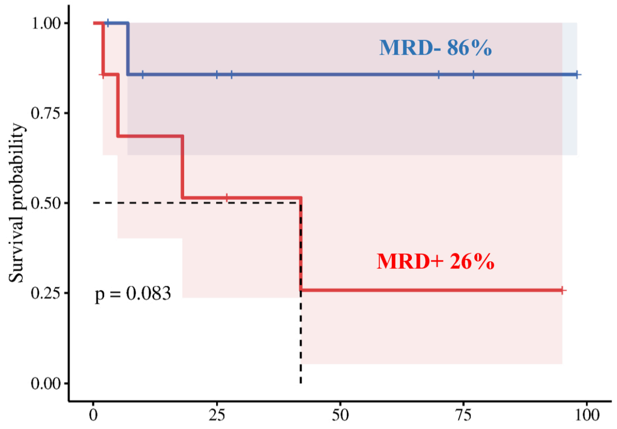

Fig. 2 Relapse free survival

**Copyright Information:** (Online) ISSN: 2572-9241
©2026 The Author(s). HemaSphere is published by John Wiley & Sons Ltd on behalf of European Hematology Association. This is an open access Abstract Book distributed under the Attribution-NonCommercial-NoDerivs (CC BY-NC-ND), which allows third parties to download the articles and share them with others as long as they credit the author and the Abstract Book, but they cannot change the content in any way or use them commercially.
**Abstract Book Citations:** Authors, Title, HemaSphere, 2026;10:(S1):pages. The abstract book DOIs can be found on the "Author Information" page.
**Disclaimer:** Articles published in the journal HemaSphere exclusively reflect the opinions of the authors. The authors are responsible for all content in their abstracts including accuracy of the facts, statements, citing resources, etc.

### (PB2588) IMPACT OF TP53 MUTATIONS IN ASIAN ADULTS WITH NEWLY DIAGNOSED B-CELL ACUTE LYMPHOBLASTIC LEUKEMIA

**Topic:** 02. Acute lymphoblastic leukemia - Clinical

Zihan Ding*[1], Ziyang Zhou[1], Yinjun Lou[1], Chenying Li[1], Jie Jin[1]

[1]*Department of Hematology, The First Affiliated Hospital, Zhejiang University School of Medicine, Hangzhou, China;*

### Background

TP53 mutations have been confirmed to be closely associated with poor survival outcomes in various hematologic malignancies and are recognized as an adverse prognostic molecular marker. However, in B-cell acute lymphoblastic leukemia (B-ALL), the specific impact of the mutations themselves and their variant allele frequency (VAF) on disease progression, treatment response, and survival prognosis remains poorly defined in current research.

### Aims

To investigate the molecular characteristics and clinical significance of TP53 mutations in B-ALL in the Asian population.

### Methods

We conducted a retrospective study of newly diagnosed B-ALL in Asian adults. A total of 635 patients who were treated at the First Affiliated Hospital, Zhejiang University School of Medicine from January 2020 to December 2025 were included. Next-generation sequencing was applied to identify the molecular characteristics of TP53 mutations. The clinical and follow-up data of TP53-mutated and TP53-wild-type patients were analyzed, and the VAF cutoff value of TP53 mutations related to poor survival was determined.

### Results

The overall incidence of TP53 mutations was 10.6% (67/635) in B-ALL and 16.2% (62/382) in Ph-negative B-ALL. Patients with TP53 mutations were predominantly male (70%), with lower WBC counts (median, 5.37 vs. $8.5\times10^9$/L, p=0.03) and higher CD20 expression (45% vs. 21%, p<0.001). TP53-mutated patients were more likely to exhibit high-risk cytogenetic features, including low-hypodiploid/near-triploid (4% vs. 0.2%, p<0.001) and KMT2A rearrangement (7% vs. 3%, p=0.03). The majority of TP53 mutations (50/71, 70%) were missense mutations and localized within the DNA-binding domain of the p53 protein. Common co-mutations included TET2 (13%) and NRAS (10%). In Ph-negative B-ALL, the optimal VAF cutoff was 64%: VAF<64% correlated with better 1-year EFS (77% vs. 59%, p=0.002) and OS (87% vs. 56%, p<0.001), and outcomes were comparable to those of TP53 wild-type patients with other high-risk factors.

### Summary/Conclusion

TP53 mutations occur in 10.6% of Asian adult B-ALL patients and are associated with a male predominance, low WBC counts, high CD20 expression, and high-risk cytogenetic features. TP53 VAF has important prognostic value in Ph-negative B-ALL, with 64% as the optimal cutoff value in the overall population and 63.7% in patients <60 years old. These findings provide real-world evidence for personalized risk stratification and treatment of TP53-mutated B-ALL in the Asian population.

**Copyright Information:** (Online) ISSN: 2572-9241
©2026 The Author(s). HemaSphere is published by John Wiley & Sons Ltd on behalf of European Hematology Association. This is an open access Abstract Book distributed under the Attribution-NonCommercial-NoDerivs (CC BY-NC-ND), which allows third parties to download the articles and share them with others as long as they credit the author and the Abstract Book, but they cannot change the content in any way or use them commercially.
**Abstract Book Citations:** Authors, Title, HemaSphere, 2026;10:(S1):pages. The abstract book DOIs can be found on the "Author Information" page.
**Disclaimer:** Articles published in the journal HemaSphere exclusively reflect the opinions of the authors. The authors are responsible for all content in their abstracts including accuracy of the facts, statements, citing resources, etc.

### (PB2589) PROPHYLACTIC AND EARLY INTERVENTION STRATEGIES REDUCE SEVERE CRS AND ICANS AFTER CAR-T CELL THERAPY: A SYSTEMATIC REVIEW AND META-ANALYSIS

**Topic:** 02. Acute lymphoblastic leukemia - Clinical

Yashasvi Srivastava*[1], Shankar Biswas[1], Kridhay Jindal[2]

*[1]Ivano-Frankivsk National Medical University, Ivano-Frankivsk, Ukraine; [2]GMC, Patiala, India;*

### Background

Cytokine release syndrome (CRS) and immune effector cell-associated neurotoxicity syndrome (ICANS) remain the principal toxicities of chimeric antigen receptor T-cell (CAR-T) therapy. Although reactive management with tocilizumab and corticosteroids is well established, prophylactic and early intervention strategies are increasingly adopted to prevent severe toxicities. However, evidence supporting these approaches derives from small, single-center, non-randomized studies, and no quantitative synthesis has been performed to date.

### Aims

To systematically evaluate the effect of prophylactic or early intervention strategies including corticosteroids, tocilizumab, and anakinra on grade ≥3 CRS, grade ≥3 ICANS, ICU admission, and treatment efficacy following CAR-T cell therapy.

### Methods

We searched PubMed, Embase, and Scopus through January 2026 for comparative studies evaluating prophylactic or early intervention versus standard reactive management of CRS/ICANS in patients receiving CAR-T therapy. Screening followed PRISMA 2020 guidelines (PROSPERO ID:CRD420261329674). Risk of bias was assessed using ROBINS-I. Pooled odds ratios (OR) were calculated using random-effects models (REML). Heterogeneity was assessed with $I^2$ and Cochran's Q. Subgroup analyses were performed by intervention agent, strategy type (prophylaxis vs early intervention), and disease. Sensitivity analyses excluded studies with serious risk of bias, ICD-code-defined outcomes, or conference-abstract-only data.

**Copyright Information:** (Online) ISSN: 2572-9241
©2026 The Author(s). HemaSphere is published by John Wiley & Sons Ltd on behalf of European Hematology Association. This is an open access Abstract Book distributed under the Attribution-NonCommercial-NoDerivs (CC BY-NC-ND), which allows third parties to download the articles and share them with others as long as they credit the author and the Abstract Book, but they cannot change the content in any way or use them commercially.
**Abstract Book Citations:** Authors, Title, HemaSphere, 2026;10:(S1):pages. The abstract book DOIs can be found on the “Author Information” page.
**Disclaimer:** Articles published in the journal HemaSphere exclusively reflect the opinions of the authors. The authors are responsible for all content in their abstracts including accuracy of the facts, statements, citing resources, etc.

### Results

Thirteen studies (2,252 patients) met inclusion criteria, encompassing corticosteroids (n=7), tocilizumab (n=3), anakinra (n=2), and combined approaches (n=1) across lymphoma (n=8), myeloma (n=3), and ALL (n=2). All were non-randomized; risk of bias was moderate in 7 studies and serious/critical in 6. Key findings (Table):

| Outcome | Studies | OR (95% CI) | P | I² |
|---|---|---|---|---|
| Grade ≥3 CRS | 5 | 0.18 (0.06–0.52) | 0.001 | 0% |
| Grade ≥3 ICANS | 7 | 0.59 (0.38–0.92) | 0.019 | 0% |
| Any CRS | 7 | 0.78 (0.37–1.61) | 0.50 | 64% |
| Any ICANS | 6 | 0.74 (0.49–1.13) | 0.16 | 0% |
| ICU admission | 4 | 0.27 (0.12–0.58) | <0.001 | 22% |
| ORR | 4 | 0.80 (0.40–1.62) | 0.54 | 23% |
| CR | 5 | 1.02 (0.42–2.45) | 0.97 | 55% |

Subgroup analyses showed consistent effects across corticosteroids, tocilizumab, and anakinra for grade ≥3 CRS (between-group P=0.99) and grade ≥3 ICANS (P=0.99), with no significant difference between prophylactic and early intervention strategies. Leave-one-out analysis confirmed robustness of primary estimates. Prediction intervals for grade ≥3 CRS (0.04–0.80) excluded 1.0, supporting generalizability.

### Summary/Conclusion

This first meta-analysis of prophylactic and early intervention strategies for CAR-T–associated toxicities demonstrates significant reductions in grade ≥3 CRS (82%), grade ≥3 ICANS (41%), and ICU admissions (73%) without compromising antitumor efficacy. These benefits were consistent across intervention agents and disease types. Although certainty of evidence is very low due to the observational nature of all included studies, the magnitude and consistency of effect support prospective randomized evaluation of prophylactic strategies. These findings may inform updates to current toxicity management guidelines.

**Copyright Information:** (Online) ISSN: 2572-9241
©2026 The Author(s). HemaSphere is published by John Wiley & Sons Ltd on behalf of European Hematology Association. This is an open access Abstract Book distributed under the Attribution-NonCommercial-NoDerivs (CC BY-NC-ND), which allows third parties to download the articles and share them with others as long as they credit the author and the Abstract Book, but they cannot change the content in any way or use them commercially.
**Abstract Book Citations:** Authors, Title, HemaSphere, 2026;10:(S1):pages. The abstract book DOIs can be found on the “Author Information” page.
**Disclaimer:** Articles published in the journal HemaSphere exclusively reflect the opinions of the authors. The authors are responsible for all content in their abstracts including accuracy of the facts, statements, citing resources, etc.

### (PB2590) EXPLORING THE PROGNOSTIC AND THERAPEUTIC GUIDANCE VALUE OF DYNAMICALLY MONITORED MINIMAL RESIDUAL DISEASE IN T-CELL ACUTE LYMPHOBLASTIC LEUKEMIA

**Topic:** 02. Acute lymphoblastic leukemia - Clinical

Yijie Wang[1], Xiangli Chen*[2], Yaru Wang[1], Tiantian Dai[3], Xiaoting Xu[1], Wenyi Lu[2], Pingfan Lu[2], Fei Ge[2], Xiaona Niu[2]

*[1]Henan Provincial People's Clinical Medical School of Zhengzhou University, Department of Hematology, Zhengzhou City, China; [2]Henan Provincial People's Hospital, Department of Hematology, Zhengzhou City, China;[3]People's Hospital of Henan University, Department of Hematology, Zhengzhou City, China;*

### Background

T-cell acute lymphoblastic leukemia (T-ALL) is an aggressive hematologic malignancy with inferior outcomes compared to B-ALL, particularly in adults, highlighting an unmet clinical need. Current risk stratification and treatment strategies for T-ALL are largely extrapolated from B-ALL protocols, despite its distinct biology. There is no internationally unified, T-ALL-specific risk classification system. Although minimal residual disease (MRD) is the most powerful prognostic factor in the ALL era, its application in T-ALL is hampered by lack of standardization regarding detection methods, timing, and thresholds, limiting its utility in guiding risk-adapted therapy.

### Aims

To evaluate the prognostic significance of MRD dynamics monitored by multiparameter flow cytometry (MFC) in T-ALL, and to identify an integrated set of risk factors combining MRD status with clinical features for improved risk stratification.

### Methods

We retrospectively analyzed 86 T-ALL patients diagnosed between Jan 2016 and Aug 2025. Bone marrow MRD was assessed by ≥8-color MFC (positivity threshold: ≥0.01%) at two timepoints: after induction therapy (TP1) and after two consolidation cycles (TP2). Survival analysis was performed using Kaplan-Meier method and Log-rank test. Cox proportional hazards models were used for univariate and multivariate analysis.

### Results

With a median age of 17 years (range 2-60), 16 patients experienced CNS relapse. Survival analysis revealed:
TP1 MRD as an early prognostic divider: TP1 MRD-positive patients (n=30) had significantly shorter median OS and RFS than MRD-negative patients (n=56) (OS: 24 mo vs NR, P=0.012; RFS: 10 mo vs 46 mo, P=0.010).
Dynamic change is crucial: Among TP1 MRD-positive patients, those who cleared MRD by TP2 (n=12) had superior OS and RFS vs. those with persistent positivity (n=18) (OS: NR vs 18 mo, P=0.019; RFS: 30 mo vs 9 mo, P=0.044). No survival difference was found between patients persistently negative (n=52) and those who converted to negative (n=12) at TP2.
Transplant benefit was MRD-dependent: HSCT significantly improved OS in the TP1 MRD-positive subgroup (P=0.029), but not in those MRD-negative at TP1 or TP2.
Independent risk factors: Multivariate analysis identified WBC >100×10⁹/L, MRD positivity at TP2, and CNS involvement at diagnosis as independent adverse prognostic factors for both OS and RFS.

**Copyright Information:** (Online) ISSN: 2572-9241
©2026 The Author(s). HemaSphere is published by John Wiley & Sons Ltd on behalf of European Hematology Association. This is an open access Abstract Book distributed under the Attribution-NonCommercial-NoDerivs (CC BY-NC-ND), which allows third parties to download the articles and share them with others as long as they credit the author and the Abstract Book, but they cannot change the content in any way or use them commercially.
**Abstract Book Citations:** Authors, Title, HemaSphere, 2026;10:(S1):pages. The abstract book DOIs can be found on the "Author Information" page.
**Disclaimer:** Articles published in the journal HemaSphere exclusively reflect the opinions of the authors. The authors are responsible for all content in their abstracts including accuracy of the facts, statements, citing resources, etc.

**Summary/Conclusion**

Dynamic MRD assessment by MFC effectively stratifies T-ALL prognosis. MRD positivity after induction is a strong poor prognostic marker, while MRD clearance after consolidation identifies a subset of high-risk patients with curable disease. Dynamic evaluation outperforms single-timepoint assessment. Combining the identified independent risk factors (high WBC, CNS involvement, TP2 MRD positivity) may form a practical risk model (Low: 0; Int: 1; High: ≥2 factors) to guide therapeutic decisions, such as intensification and transplant timing, towards personalized therapy for T-ALL.

**Copyright Information:** (Online) ISSN: 2572-9241
©2026 The Author(s). HemaSphere is published by John Wiley & Sons Ltd on behalf of European Hematology Association. This is an open access Abstract Book distributed under the Attribution-NonCommercial-NoDerivs (CC BY-NC-ND), which allows third parties to download the articles and share them with others as long as they credit the author and the Abstract Book, but they cannot change the content in any way or use them commercially.
**Abstract Book Citations:** Authors, Title, HemaSphere, 2026;10:(S1):pages. The abstract book DOIs can be found on the "Author Information" page.
**Disclaimer:** Articles published in the journal HemaSphere exclusively reflect the opinions of the authors. The authors are responsible for all content in their abstracts including accuracy of the facts, statements, citing resources, etc.

### (PB2591) THE IMPACT OF THE EWALL-PI INDEX COMPARED WTH CYTOGENETIC RISK STRATIFICATION ON CLINICAL OUTCOMES IN ACUTE LTMPHOBLASTIC LEUKEMIA

**Topic:** 02. Acute lymphoblastic leukemia - Clinical

Hina Tahir*[1]

[1]*INMOL Cancer Hospital, Lahore, Hemato-oncology, Lahore, Pakistan;*

**Background**

IN ALL patients, for successful treatment and improve prognostic outcome, appropriate risk stratification is essential and irreplaceable. This segregation can help curb the risk of relapse, which is the highest mortality contributor in the developing world after sepsis related deaths. Many of the available treatment trials apply mostly to children with minor moderations in regards to adults. Additionally, the allocation of patients to different treatment protocols solely due to age is the least acceptable. To serve this EWALL-Pi index was introduced. This study compares efficacy of EWALL-PI index to cytogenetic risk group.

**Aims**

**OBJECTIVE:** To evaluate the impact of the EWALL Prognostic Index (EWALL-PI) and cytogenetic risk stratification on clinical outcomes in patients with Acute Lymphoblastic Leukemia (ALL).

**Methods**

**STUDY DESIGN: Prospective Cohort Study**
**PLACE AND DURATION OF STUDY:** This study was done at the Department of Clinical Hematology, Inmol Cancer Hospital, Lahore since January 2022 till December 2025.
**METHODOLOGY:** Patients aged 13–55 years diagnosed with ALL were included. Risk stratification was performed using the EWALL Prognostic Index, incorporating age,
WBC count, cytogenetics, and end-of-induction MRD status. Patients were categorized into low- and high-risk groups. Clinical outcomes, including induction failure, relapse, composite outcomes, and mortality, were assessed.
Data were analyzed using SPSS version 26. Continuous variables were expressed as median (IQR) and categorical variables as frequencies and percentages. Associations were assessed using the Chi-square test. Univariate logistic regression was performed to evaluate predictors of poor outcomes. Variables with $p < 0.25$ were entered into multivariate logistic regression to identify independent predictors. A p-value $p < 0.05$ was considered statistically significant.

**Copyright Information:** (Online) ISSN: 2572-9241
©2026 The Author(s). HemaSphere is published by John Wiley & Sons Ltd on behalf of European Hematology Association. This is an open access Abstract Book distributed under the Attribution-NonCommercial-NoDerivs (CC BY-NC-ND), which allows third parties to download the articles and share them with others as long as they credit the author and the Abstract Book, but they cannot change the content in any way or use them commercially.
**Abstract Book Citations:** Authors, Title, HemaSphere, 2026;10:(S1):pages. The abstract book DOIs can be found on the “Author Information” page.
**Disclaimer:** Articles published in the journal HemaSphere exclusively reflect the opinions of the authors. The authors are responsible for all content in their abstracts including accuracy of the facts, statements, citing resources, etc.

**Results**

**RESULTS:**
A total of 57 patients were included, of whom 44 (77.2%) were male and 13 (22.8%) were female. Patients with low EWALL-PI scores had significantly better clinical outcomes compared to those with high EWALL-PI (p<0.001). MRD-negative patients achieved significantly more favorable outcomes than MRD-positive patients (p = 0.001). Patients with standard-risk cytogenetics demonstrated better outcomes compared to high-risk cytogenetics (p = 0.026).
In univariate analysis, high EWALL-PI was significantly associated with poor outcomes (p = 0.008). Older age was also significantly associated with adverse outcomes (p = 0.005). Although MRD positivity (OR = 62.37) and high cytogenetic risk (OR = 3.75) showed increased odds of poor outcomes, these did not reach statistical significance. WBC count at diagnosis was not significantly associated with outcome (p = 0.792).
Multivariate logistic regression demonstrated that high EWALL-PI (p = 0.003) and increasing age (p = 0.004) were independent predictors of poor clinical outcomes. MRD positivity did not retain statistical significance in multivariate analysis.

**Summary/Conclusion**

**CONCLUSIONS:**
These results indicate that low EWALL-PI, negative MRD and standard cytogenetic profile are associated with significantly higher rates of favorable outcomes in ALL patients. High EWALL-PI score and older age are significant independent predictors of poor clinical outcomes in patients with ALL. The EWALL Prognostic Index is a valuable tool for risk stratification and outcome prediction in clinical practice.

**Copyright Information:** (Online) ISSN: 2572-9241
©2026 The Author(s). HemaSphere is published by John Wiley & Sons Ltd on behalf of European Hematology Association. This is an open access Abstract Book distributed under the Attribution-NonCommercial-NoDerivs (CC BY-NC-ND), which allows third parties to download the articles and share them with others as long as they credit the author and the Abstract Book, but they cannot change the content in any way or use them commercially.
**Abstract Book Citations:** Authors, Title, HemaSphere, 2026;10:(S1):pages. The abstract book DOIs can be found on the "Author Information" page.
**Disclaimer:** Articles published in the journal HemaSphere exclusively reflect the opinions of the authors. The authors are responsible for all content in their abstracts including accuracy of the facts, statements, citing resources, etc.

### (PB2592) KINETICS OF MOLECULAR RESPONSE AND LONGITUDINAL TRAJECTORIES OF BLINATUMOMAB COMBINED WITH NOVEL TYROSINE KINASE INHIBITORS IN PH+ ALL

**Topic:** 02. Acute lymphoblastic leukemia - Clinical

Yanping Sun*[1], Zhaoxia Liu[2], Fei Lu[1], Jinlin Chen[1], Yixue Wang[1], Chunyan Ji[1], Xiaosheng Fang[3], Jingjing Ye[1]

[1]*Department of Hematology，Qilu Hospital, Cheeloo College of Medicine, Shandong University, Jinan, China;*[2]*Affiliated Hospital of Shandong University of Traditional Chinese Medicine, Department of Hematology, Jinan, China;* [3]*Department of Hematology, Shandong Provincial Hospital Affiliated to Shandong First Medical University, Jinan, China;*

### Background

Blinatumomab demonstrates broad efficacy in B-cell acute lymphoblastic leukemia (B-ALL). However, in Philadelphia chromosome-positive (Ph+) ALL, the precise molecular kinetics and long-term outcomes of combining it with different generations of tyrosine kinase inhibitors (TKIs) remain underexplored, especially in high-risk mutational subsets.

### Aims

This study aims to evaluate the depth and rapidity of BCR-ABL1 clearance when combining blinatumomab with varying generations of tyrosine kinase inhibitors (TKIs). Additionally, we explored the longitudinal survival trajectories of an ultra-high-risk subset (R/R Ph+ ALL and CML-LBC) to unveil the potential clinical impact of bridging to allogeneic hematopoietic stem cell transplantation (allo-HSCT).

### Methods

In this focused sub-analysis of a real-world cohort, 33 evaluable Ph+ ALL patients receiving blinatumomab plus TKIs for either induction or MRD clearance were analyzed for molecular dynamics. The magnitude of response was assessed via log10 reduction in BCR-ABL1 International Scale (IS). Early deep remission was evaluated at Day 14 of the first treatment cycle. Longitudinal survival trajectories were mapped for a predefined high-risk sub-cohort (n=8) receiving blinatumomab-based induction therapy. This subset comprised 7 patients with R/R Ph+ ALL and 1 with newly diagnosed CML-LBC, including 4 cases with resistant kinase domain mutations (three T315I, one E255M).

### Results

The magnitude of molecular clearance was comparable across TKI subgroups, with median log10 reductions of -3.19, -2.68, and -1.72 for the 1st-, 2nd-, and 3rd-generation TKI cohorts, respectively ($P = 0.85$, Kruskal-Wallis test). The blinatumomab regimen induced a rapid onset of deep remission. Among the 19 patients achieving complete molecular response (CMR), 11 (57.9%) reached CMR by Day 14. This early transcript clearance was driven by later-generation TKIs (7 patients with 2nd-generation, 4 with 3rd-generation), with no early CMR events observed in the 1st-generation group. Longitudinal tracking of the 8 ultra-high-risk patients indicated the aggressive disease biology. The blinatumomab/TKI combination induced initial CMR in several cases, including those harboring resistant mutations (e.g., T315I). However, maintaining durable remission remained challenging, with subsequent relapses occurring despite initial deep molecular clearance. Nevertheless, extended overall survival (2 to >3 years) was achieved in selected patients, including those who bridged to allo-HSCT, confirming the regimen's value in extending survival windows.

**Copyright Information:** (Online) ISSN: 2572-9241
©2026 The Author(s). HemaSphere is published by John Wiley & Sons Ltd on behalf of European Hematology Association. This is an open access Abstract Book distributed under the Attribution-NonCommercial-NoDerivs (CC BY-NC-ND), which allows third parties to download the articles and share them with others as long as they credit the author and the Abstract Book, but they cannot change the content in any way or use them commercially.
**Abstract Book Citations:** Authors, Title, HemaSphere, 2026;10:(S1):pages. The abstract book DOIs can be found on the "Author Information" page.
**Disclaimer:** Articles published in the journal HemaSphere exclusively reflect the opinions of the authors. The authors are responsible for all content in their abstracts including accuracy of the facts, statements, citing resources, etc.

### Summary/Conclusion

The combination of blinatumomab with 2nd- or 3rd-generation TKIs induces rapid and deep molecular responses in Ph+ ALL, equalizing the depth of clearance across TKI generations. For heavily pretreated patients or those harboring resistant mutations (T315I/E255M), this regimen may mainly serve as a crucial bridging strategy. Individualized consolidation, frequently involving allo-HSCT, therefore remains essential for sustaining long-term survival.

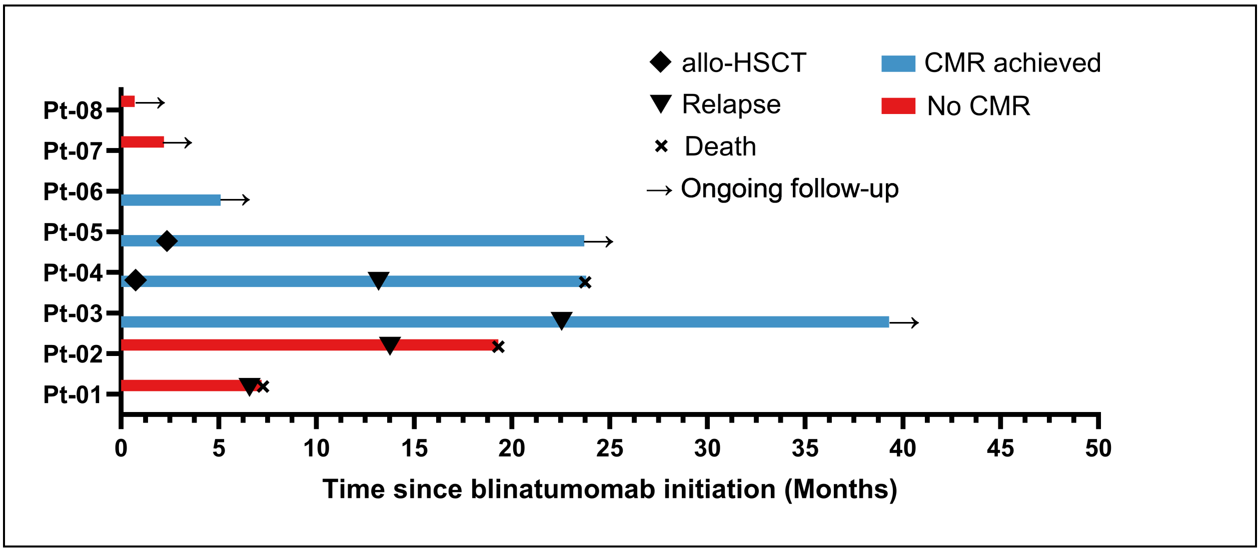

Longitudinal survival trajectories and clinical events of the ultra-high-risk sub-cohort (n=8). Each horizontal bar represents an individual patient, with the length indicating the overall time (in months) from blinatumomab initiation to the last follow-up or death. Bar colors define the molecular response status: blue bars denote patients who achieved complete molecular response (CMR), whereas red bars represent those who failed to achieve CMR. Key clinical events are annotated with symbols: black diamonds (◆) for allogeneic hematopoietic stem cell transplantation (allo-HSCT); inverted triangles (▼) for disease relapse; crosses (✖) for death; and arrows (→) for ongoing survival at the last follow-up. The plot illustrates diverse clinical outcomes in this pretreated, mutation-enriched (T315I/E255M) population. While the regimen effectively induces early CMR, patients often relapse. Still, this combination may act as a crucial bridge and extend the survival window.

**Copyright Information:** (Online) ISSN: 2572-9241
©2026 The Author(s). HemaSphere is published by John Wiley & Sons Ltd on behalf of European Hematology Association. This is an open access Abstract Book distributed under the Attribution-NonCommercial-NoDerivs (CC BY-NC-ND), which allows third parties to download the articles and share them with others as long as they credit the author and the Abstract Book, but they cannot change the content in any way or use them commercially.
**Abstract Book Citations:** Authors, Title, HemaSphere, 2026;10:(S1):pages. The abstract book DOIs can be found on the "Author Information" page.
**Disclaimer:** Articles published in the journal HemaSphere exclusively reflect the opinions of the authors. The authors are responsible for all content in their abstracts including accuracy of the facts, statements, citing resources, etc.

### (PB2593) CENTRAL NERVOUS SYSTEM EXPOSURE TO IMATINIB IN PEDIATRIC AND YOUNG ADULT ACUTE LYMPHOBLASTIC LEUKEMIA: PHARMACOKINETICS AND CLINICAL IMPLICATIONS

**Topic:** 02. Acute lymphoblastic leukemia - Clinical

Anna Sofie Buhl Rasmussen*[1], Cecilie Rank[2], Ib Christensen[3], Allan Weimann[1], Maria Thastrup[1], Tianwu Yang[4], Trine Lund[4], Hilde Wik[5], Hartmut Vogt[6, 7], Ulrika Norén-Nyström[8], Goda Vaitkevičienė[9], Birgitte Albertsen[10, 11], Peder Wehner[12], Bodil Als-Nielsen[1], Christen Bertel Lykkegaard Andersen[2, 13], Kim Dalhoff[14, 15], Kjeld Schmiegelow[1, 15]

*[1]Copenhagen University Hospital - Rigshospitalet, Dept. of Pediatrics and Adolescent Medicine, Copenhagen, Denmark; [2]Copenhagen University Hospital - Rigshospitalet, Dept. of Hematology, Copenhagen, Denmark; [3]Copenhagen University Hospital - Herlev, Dept. of Pathology, Herlev, Denmark; [4]University of Copenhagen, Department of Drug Design and Pharmacology, Copenhagen, Denmark; [5]Oslo University Hospital Rikshospitalet, Department of Hematology, Oslo, Norway; [6]Linköping University and University Hospital, Department of Biomedical and Clinical Science, Linköping, Sweden; [7]Crown Princess Victoria Children's Hospital, Linköping, Sweden; [8]Umeå University, Department of Clinical Sciences, Pediatrics, Umeå, Sweden; [9]Vilnius University, Clinic of Children's Diseases, Institute of Clinical Medicine, Vilnius, Lithuania; [10]Aarhus University Hospital, Department of Pediatrics and Adolescent Medicine,, Aarhus, Denmark; [11]Aarhus University, Department of Clinical Medicine, Aarhus, Denmark; [12]Odense University Hospital, Department of Pediatric Hematology and Oncology, H. C. Andersen Children's Hospital, Odense, Denmark; [13]University of Copenhagen, Research Unit for General Practice, Department of Public Health, Copenhagen, Denmark; [14]Copenhagen University Hospital - Bispebjerg, Department of Clinical Pharmacology, Copenhagen, Denmark; [15]University of Copenhagen, Department of Clinical Medicine, Faculty of Health and Medical Sciences, Copenhagen, Denmark;*

### Background

Tyrosine kinase inhibitors (TKIs) have improved outcomes in Philadelphia chromosome-positive (Ph+) acute lymphoblastic leukemia (ALL) and are increasingly incorporated into treatment protocols of Philadelphia chromosome-like (Ph-like, ABL-class) ALL. However, central nervous system (CNS) relapse remains a significant challenge despite intrathecal chemotherapy backbone. Imatinib, a first-generation TKI, demonstrates limited CNS penetration in adults, yet data in children are sparse.

### Aims

This study aimed to investigate the relationship between CSF and plasma levels of imatinib and its active metabolite in children and young adults with Ph+ or Ph-like ALL.

### Methods

This prospective, multicenter study investigated cerebrospinal fluid (CSF) and matched plasma concentrations of imatinib and its primary bioactive metabolite, N-desmethyl-imatinib, in children and young adults with Ph+ or Ph-like (ABL-class) ALL. Plasma and CSF samples were analyzed using liquid chromatography-tandem mass spectrometry (LC-MS/MS). Linear mixed-effects models were applied to assess concentrations across compartments and over time.

**Copyright Information:** (Online) ISSN: 2572-9241
©2026 The Author(s). HemaSphere is published by John Wiley & Sons Ltd on behalf of European Hematology Association. This is an open access Abstract Book distributed under the Attribution-NonCommercial-NoDerivs (CC BY-NC-ND), which allows third parties to download the articles and share them with others as long as they credit the author and the Abstract Book, but they cannot change the content in any way or use them commercially.
**Abstract Book Citations:** Authors, Title, HemaSphere, 2026;10:(S1):pages. The abstract book DOIs can be found on the "Author Information" page.
**Disclaimer:** Articles published in the journal HemaSphere exclusively reflect the opinions of the authors. The authors are responsible for all content in their abstracts including accuracy of the facts, statements, citing resources, etc.

**Results**

Between January 2023 and June 2025, 32 paired plasma and CSF samples were collected from ten patients (range 1–8 samples per patient; median 3). Overall, 78% of plasma imatinib concentrations exceeded 1,000 ng/mL, a therapeutic exposure threshold associated with improved molecular response in adult chronic myeloid leukemia. Plasma imatinib concentrations were on average 189-fold higher than in CSF (geometric mean ratio, 95% CI: 142–249, $p<0.001$) (Figure 1), which was even more profound for N-desmethyl-imatinib (geometric mean ratio 273, 95% CI: 218–339, $p<0.001$). The highest measured CSF imatinib concentration was 63 ng/mL. During therapy, both plasma and CSF imatinib concentrations declined by an average of 0.32% per day ($p<0.001$), resulting in stable CSF-to-plasma ratio over a median follow-up of 5.7 months (range: 0–14 months).

**Summary/Conclusion**

The very low CSF exposure to imatinib, despite systemic therapeutic plasma concentrations, supports the rationale for alternative TKIs with improved CNS distribution in ALL patients at risk of CNS relapse.

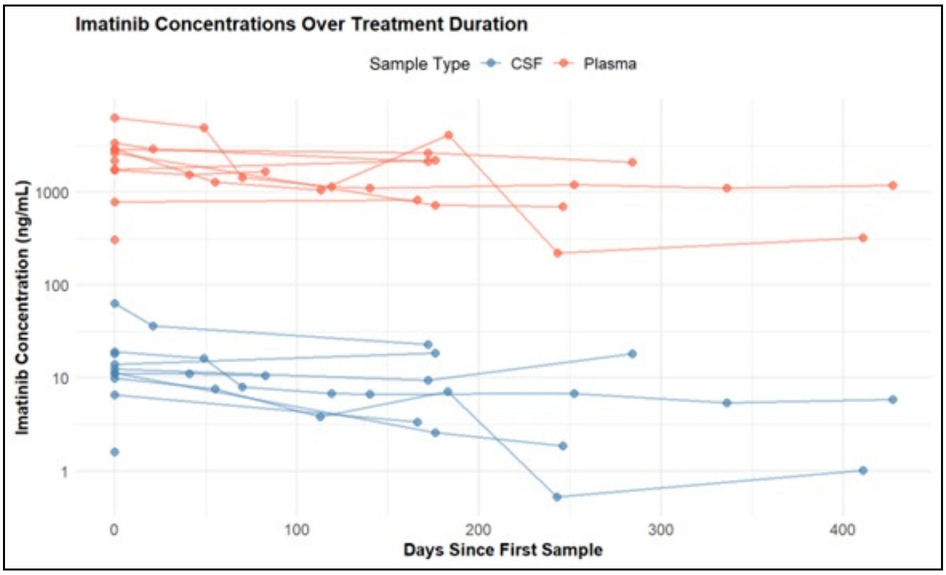

**Copyright Information:** (Online) ISSN: 2572-9241
©2026 The Author(s). HemaSphere is published by John Wiley & Sons Ltd on behalf of European Hematology Association. This is an open access Abstract Book distributed under the Attribution-NonCommercial-NoDerivs (CC BY-NC-ND), which allows third parties to download the articles and share them with others as long as they credit the author and the Abstract Book, but they cannot change the content in any way or use them commercially.
**Abstract Book Citations:** Authors, Title, HemaSphere, 2026;10:(S1):pages. The abstract book DOIs can be found on the "Author Information" page.
**Disclaimer:** Articles published in the journal HemaSphere exclusively reflect the opinions of the authors. The authors are responsible for all content in their abstracts including accuracy of the facts, statements, citing resources, etc.

### (PB2594) OLVEREMBATINIB AS FRONTLINE TREATMENT FOR ADULT NEWLY DIAGNOSED PHILADELPHIA CHROMOSOME-POSITIVE ACUTE LYMPHOBLASTIC LEUKEMIA

**Topic:** 02. Acute lymphoblastic leukemia - Clinical

Yonggong Yang*[1], Kai Shen[1], Peipei Xu[1], Jian Ou-Yang[1], Ting Xie[1], Yue-Yi Xu[1], Miao-Xin Peng[1]

*[1]Nanjing Drum Tower Hospital, Affiliated Hospital of Medical School, Nanjing University, Nanjing, China;*

### Background

In recent years, the therapeutic paradigm for Philadelphia chromosome-positive acute lymphoblastic leukemia ($Ph^+$ ALL) has been reshaped by potent BCR::ABL1 tyrosine kinase inhibitors (TKIs) and novel immunotherapies, shifting treatment from intensive chemotherapy toward reduced-intensity or chemotherapy-free regimens and reducing reliance on allogeneic hematopoietic stem cell transplantation (allo-HSCT). In contrast to earlier-generation TKIs, the third-generation TKI ponatinib demonstrates superior antileukemic activity in $Ph^+$ ALL by effectively addressing multiple ABL1-resistant mutations, notably the recalcitrant T315I mutation. Early attainment of complete molecular response (CMR) represents a key prognostic marker in Ph+ ALL, as it is strongly associated with excellent long-term survival, permitting the omission of allo-HSCT. Clinical trials demonstrate that ponatinib-based regimens—whether combined with H-CVAD chemotherapy or blinatumomab—are highly effective in newly diagnosed $Ph^+$ ALL, eliciting sustained complete molecular response (CMR) rates of 84% and 87%, respectively, and translating into impressive overall survival (OS) (6-year OS 75% and 3-year OS 91%). Despite the implementation of optimized dosing, which has mitigated overall ponatinib toxicity and eliminated treatment-related mortality, cardiovascular and peripheral vascular events continue to pose a significant challenge in combination regimens. As another effective third-generation TKI, olverembatinib (HQP1351) obtained approval in China in November 2021, indicated for adult patients with TKI-resistant chronic or accelerated phase chronic myeloid leukemia (CML) harboring the T315I mutation.

### Aims

This study aimed to evaluate the efficacy and safety of olverembatinib combined with immunotherapy or low-intensity chemotherapy as first-line treatment for adult patients with newly diagnosed Philadelphia chromosome-positive acute lymphoblastic leukemia ($Ph^+$ ALL)

### Methods

A retrospective analysis was conducted on 12 consecutive adult patients (median age 50 years) with newly diagnosed $Ph^+$ ALL who received first-line olverembatinib-based regimens at our institution between March 2022 and May 2025. The p190 BCR::ABL1 isoform was expressed in all patients. Induction regimens included olverembatinib combined with vindesine and prednisone (VP) (n=7), blinatumomab (n=4), or prednisone alone (n=1)

### Results

Patients achieved complete remission (CR) or CR with incomplete hematologic recovery (CRi) within 28 days. At the end of induction, 83.3% (10/12) of patients achieved flow cytometry-based measurable residual disease (MRD) negativity. The complete molecular response (CMR) rate was 83.3%, and the major molecular response (MMR) rate was 91.6%. The estimated 1-year overall survival (OS) and event-free survival (EFS) rates were 80.2% and 83.3%, respectively, at a median follow-up of 11.3 months. Grade 4 neutropenia and thrombocytopenia occurred in 66.7% and 50.0% of patients. Non-hematologic adverse events were mostly grade 1-2, with no treatment-related pancreatitis, hypertension, cardiac arrhythmias, or vascular thrombotic events observed

**Copyright Information:** (Online) ISSN: 2572-9241
©2026 The Author(s). HemaSphere is published by John Wiley & Sons Ltd on behalf of European Hematology Association. This is an open access Abstract Book distributed under the Attribution-NonCommercial-NoDerivs (CC BY-NC-ND), which allows third parties to download the articles and share them with others as long as they credit the author and the Abstract Book, but they cannot change the content in any way or use them commercially.
**Abstract Book Citations:** Authors, Title, HemaSphere, 2026;10:(S1):pages. The abstract book DOIs can be found on the "Author Information" page.
**Disclaimer:** Articles published in the journal HemaSphere exclusively reflect the opinions of the authors. The authors are responsible for all content in their abstracts including accuracy of the facts, statements, citing resources, etc.

**Summary/Conclusion**

The olverembatinib-based combination regimen demonstrates high molecular response rates and promising short-term survival with a manageable safety profile in newly diagnosed $Ph^{+}$ ALL, offering a potential new therapeutic option

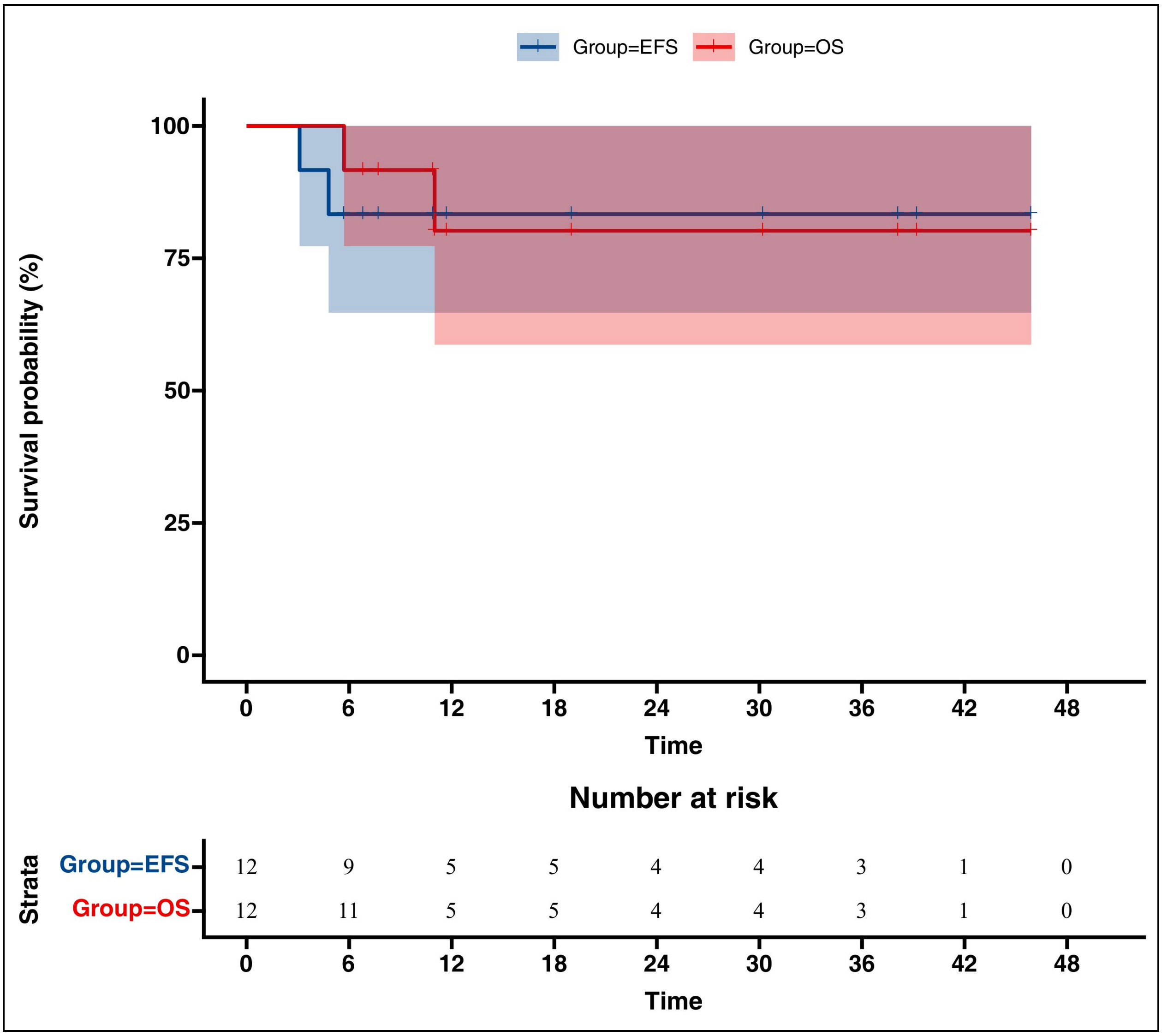

**Copyright Information:** (Online) ISSN: 2572-9241
©2026 The Author(s). HemaSphere is published by John Wiley & Sons Ltd on behalf of European Hematology Association. This is an open access Abstract Book distributed under the Attribution-NonCommercial-NoDerivs (CC BY-NC-ND), which allows third parties to download the articles and share them with others as long as they credit the author and the Abstract Book, but they cannot change the content in any way or use them commercially.
**Abstract Book Citations:** Authors, Title, HemaSphere, 2026;10:(S1):pages. The abstract book DOIs can be found on the "Author Information" page.
**Disclaimer:** Articles published in the journal HemaSphere exclusively reflect the opinions of the authors. The authors are responsible for all content in their abstracts including accuracy of the facts, statements, citing resources, etc.

### (PB2595) NUDT15 RS116855232 OUTPERFORMS TPMT RS1142345 FOR SEVERE HEPATIC AND HEMATOLOGIC TOXICITY IN CHILDHOOD ALL

**Topic:** 02. Acute lymphoblastic leukemia - Clinical

Ioannis Kyriakidis[1, 2], Iordanis Pelagiadis[1, 2], Ioanna Lygerou*[2], Maria Stratigaki[2], Nikolaos Katzilakis[2], Eftichia Stiakaki[1, 2]

[1]*Laboratory of Blood Diseases and Childhood Cancer Biology, School of Medicine, University of Crete, 71003, Heraklion, Greece;* [2]*Department of Pediatric Hematology-Oncology, University Hospital of Heraklion, 71500, Heraklion, Greece;*

### Background

Contemporary chemotherapy for acute lymphoblastic leukemia (ALL) achieves over 90% survival in children and adolescents. Pharmacogenetics plays a critical role, as toxicity accounts for up to one-third of mortality. The maintenance antimetabolite 6-mercaptopurine (6-MP) is predominantly metabolized by thiopurine S-methyltransferase (TPMT) and nudix hydrolase 15 (NUDT15).

### Aims

This study investigated the impact of TPMT rs1142345 T>C (*3A–*3C haplotypes) and NUDT15 rs116855232 C>T (*2–*3) variants, which are linked to impaired thiopurine metabolism, lower tolerated 6-mercaptopurine doses, reduced dose intensity, and, in some cases, treatment interruption due to Grade ≥3 hepatotoxicity and myelotoxicity.

### Methods

Peripheral blood samples from 90 children and adolescents with ALL in remission were analyzed. Genotyping was performed by RT-qPCR using TaqMan Genotyping Assays after genomic DNA extraction with the PureLink Genomic DNA Mini Kit. All experiments were performed in duplicate, with 100% concordance. No deviation from Hardy–Weinberg equilibrium was observed. Toxicity grades were coded according to CTCAE v6.0 criteria.

### Results

In the overall cohort, the median maximum hepatic and hematologic toxicity was grade 3 (range, 1–4; IQR, 1 for hepatotoxicity and 0 for hematologic toxicity), and all patients developed at least one type of toxicity. During maintenance therapy, grade ≥3 hepatotoxicity occurred in 73 % and grade ≥3 hematologic toxicity in 82 %. The median tolerated daily 6-MP dose was 46.2 mg/m²/day (range, 16.8–57.5; IQR, 6.8), the median relative dose intensity was 92.3% (range, 33.6–115; IQR, 13.7), and the median cumulative duration of zero-dose interruptions was 7 days/patient (range, 0–161; IQR, 21). Regarding TPMT rs1142345, 92% were normal metabolizers (NM; TT) and 8% were intermediate metabolizers (IM; CT). As regards NUDT15 rs116855232, 91% were NM, 8% were IM, and 1% were poor metabolizers. For both polymorphisms, carriers of the variant alleles had a lower tolerated daily 6-MP dose (38 vs. 46 mg/m2/day and p=0.02 for NUDT15*3, and 44 vs. 46 for TPMT*3C), lower relative dose intensity (92% vs. 96% and p=0.02 for NUDT15*3, and 88% vs. 92% for TPMT*3C), and more zero-dose days than major-allele carriers (statistically significant associations only for NUDT15*3). The combination of TPMT rs1142345 and NUDT15 rs116855232 genotypes showed lower tolerated 6-MP dosage for IM based on NUDT15 (Figure raincloud plots; p=0.05). No patient was determined as an intermediate metabolizer for both polymorphisms. Zero-dose days were not associated with relapse or death. Only rs116855232 was associated with hepatotoxicity grade (p=0.045) and concomitant hepatotoxicity/myelotoxicity (p=0.044). Binary logistic regression identified radiotherapy and high-risk protocols as significant predictors of severe 6-MP toxicity, primarily hematologic. Consistent with the existing literature, younger age correlated with higher tolerated 6-MP doses, regardless of the presence of *alleles.

**Copyright Information:** (Online) ISSN: 2572-9241
©2026 The Author(s). HemaSphere is published by John Wiley & Sons Ltd on behalf of European Hematology Association. This is an open access Abstract Book distributed under the Attribution-NonCommercial-NoDerivs (CC BY-NC-ND), which allows third parties to download the articles and share them with others as long as they credit the author and the Abstract Book, but they cannot change the content in any way or use them commercially.
**Abstract Book Citations:** Authors, Title, HemaSphere, 2026;10:(S1):pages. The abstract book DOIs can be found on the "Author Information" page.
**Disclaimer:** Articles published in the journal HemaSphere exclusively reflect the opinions of the authors. The authors are responsible for all content in their abstracts including accuracy of the facts, statements, citing resources, etc.

**Summary/Conclusion**

Thiopurine dose individualization based on pharmacogenetic studies (esp. NUDT15 rs116855232) underscores the importance of avoiding excess toxicity and prolonged zero-dose interruptions, both of which have been linked to ALL outcomes.

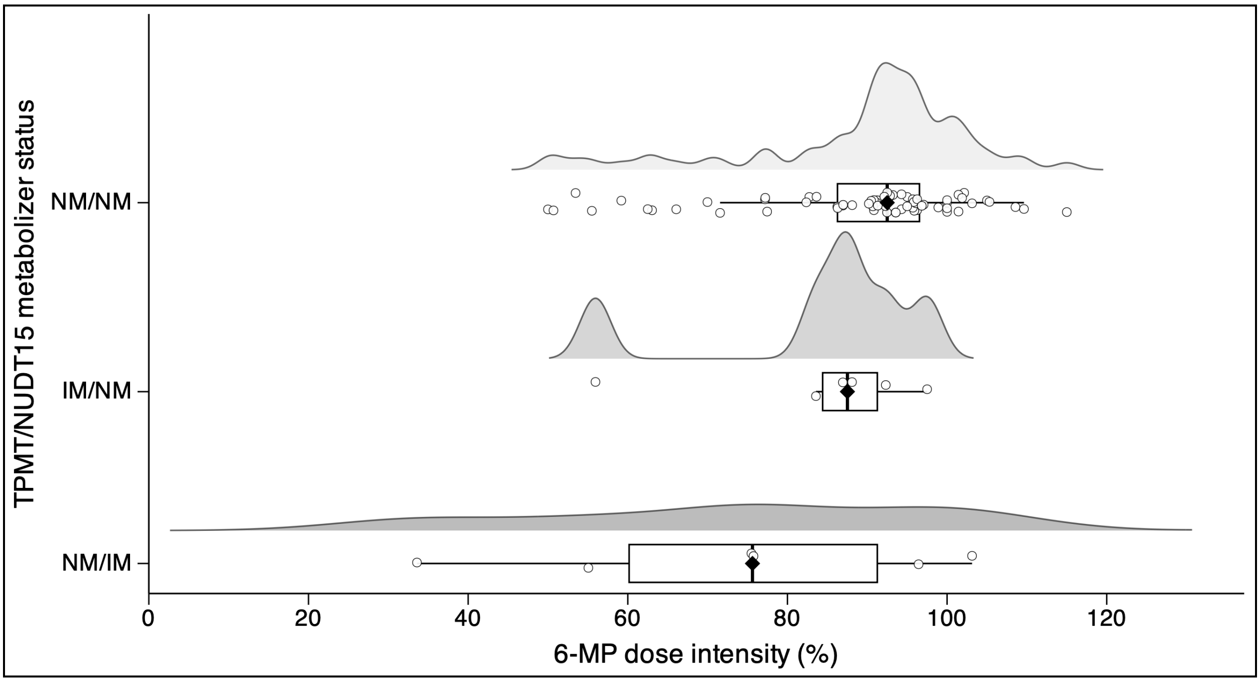

**Copyright Information:** (Online) ISSN: 2572-9241
©2026 The Author(s). HemaSphere is published by John Wiley & Sons Ltd on behalf of European Hematology Association. This is an open access Abstract Book distributed under the Attribution-NonCommercial-NoDerivs (CC BY-NC-ND), which allows third parties to download the articles and share them with others as long as they credit the author and the Abstract Book, but they cannot change the content in any way or use them commercially.
**Abstract Book Citations:** Authors, Title, HemaSphere, 2026;10:(S1):pages. The abstract book DOIs can be found on the "Author Information" page.
**Disclaimer:** Articles published in the journal HemaSphere exclusively reflect the opinions of the authors. The authors are responsible for all content in their abstracts including accuracy of the facts, statements, citing resources, etc.

### (PB2596) INTEGRATED MOLECULAR PROFILING AND MINIMAL RESIDUAL DISEASE-BASED RISK STRATIFICATION IN PAEDIATRIC B-LYMPHOBLASTIC LEUKAEMIA: RETROSPECTIVE COHORT ANALYSIS FROM A SOUTH ASIAN TERTIARY CARE CENTRE

**Topic:** 02. Acute lymphoblastic leukemia - Clinical

Neha Singh*[1, 2], Shabana Azad[2, 3], Raghwesh Ranjan[2, 4], Nilesh Dhole[1, 2], Ankita Pal[2, 5], Deepali Saxena[1, 2], Pradeep Arumugam[1, 2], Anil Yadav[2, 6], Rohit Kori[1, 2], Snehal Jaiswar[1, 2], Seema Biswas[1, 2], Parichay Singh[2, 4]

*[1]TATA MEMORIAL CENTRE (HBCH & MPMMCC), HEMATOPATHOLOGY, VARANASI, India;[2]HOMI BHABHA NATIONAL INSTITUTE, MUMBAI, Mumbai, India; [3]TATA MEMORIAL CENTRE (HBCH & MPMMCC), ONCOPATHOLOGY, VARANASI, India; [4]TATA MEMORIAL CENTRE (HBCH & MPMMCC), PEDIATRIC ONCOLOGY, VARANASI, India;[5]TATA MEMORIAL CENTRE (HBCH & MPMMCC), BIOSTATISTICS AND EPIDEMIOLOGY, VARANASI, India; [6]TATA MEMORIAL CENTRE (HBCH & MPMMCC), CANCER CYTOGENETICS, VARANASI, India;*

### Background

Paediatric B-cell acute lymphoblastic leukaemia (B-ALL) is the commonest childhood malignancy worldwide. While advances in molecular diagnostics and minimal residual disease (MRD) monitoring have substantially refined prognostication, data integrating both parameters from resource-constrained settings remain scarce. The revised 2022 WHO classification prioritises molecular subtyping; however, the relative prognostic hierarchy of cytogenetic alterations versus treatment-response parameters in such populations remains incompletely established.

### Aims

To delineate the molecular landscape of paediatric B-ALL at an Indian tertiary care centre and evaluate associations among molecular subtype, MRD kinetics, risk stratification, and long-term survival outcomes.

### Methods

This retrospective analysis enrolled 568 children with newly diagnosed B-ALL managed over five years on the ICiCLe protocol. Immunophenotyping was performed using 13 colour multiparametric flow cytometry. Cytogenetic characterisation included FISH and karyotyping, supplemented by RNA-sequencing for rare fusion transcripts. MRD was quantified at end-of-induction and post-consolidation timepoints (≥4,000,000 events). Pearson chi-square and Fisher exact tests, alongside Kaplan–Meier survival analysis with log-rank testing, were employed for statistical comparisons.

**Copyright Information:** (Online) ISSN: 2572-9241
©2026 The Author(s). HemaSphere is published by John Wiley & Sons Ltd on behalf of European Hematology Association. This is an open access Abstract Book distributed under the Attribution-NonCommercial-NoDerivs (CC BY-NC-ND), which allows third parties to download the articles and share them with others as long as they credit the author and the Abstract Book, but they cannot change the content in any way or use them commercially.
**Abstract Book Citations:** Authors, Title, HemaSphere, 2026;10:(S1):pages. The abstract book DOIs can be found on the "Author Information" page.
**Disclaimer:** Articles published in the journal HemaSphere exclusively reflect the opinions of the authors. The authors are responsible for all content in their abstracts including accuracy of the facts, statements, citing resources, etc.

**Results**

The cohort was predominantly male (66.5%) with a median age of 5 years (IQR 3–10). Risk stratification yielded Standard (12.5%), Intermediate (50.9%), and High Risk (36.6%) categories. Molecular subtypes comprised B-Others (35.4%), hyperdiploidy (21.7%), ETV6::RUNX1 (13.2%), BCR::ABL1 (10.2%), TCF3::PBX1 (9.3%), MLL-rearranged (3.3%), Ph-like (2.1%), PAX5-alteration (2.1%), ZNF384-rearranged (0.9%), and MEF2D-rearranged (0.7%). ETV6::RUNX1 and hyperdiploidy were significantly enriched in Standard Risk ($p<0.001$), whereas BCR::ABL1 and Ph-like subtypes predominated in the High Risk category ($p<0.001$).
End-of-induction MRD positivity was disproportionately elevated in Ph-like (4.5%; $p=0.011$) and TCF3::PBX1 (11.3%; $p=0.019$) subtypes. Persistent post-consolidation MRD was most prevalent in BCR::ABL1/Ph-like (22.5%; $p=0.049$) and MLL-rearranged (10.0%; $p=0.038$) cases. CNS-III involvement was significantly overrepresented in Ph-like (25.0%; $p=0.002$) and the combined BCR::ABL1/Ph-like group (41.7%; $p=0.009$).
Kaplan–Meier analysis demonstrated superior 2-year RFS for ETV6::RUNX1 (78.8%; median not reached) and hyperdiploidy (76.2%), contrasting with markedly inferior outcomes in Ph-like (50.0%; median 24 months), PAX5-alteration (48.6%; median 36 months), and MLL-rearranged (62.5%; median 36 months) groups. The BCR::ABL1/Ph-like combined category approached statistical significance for worse RFS ($p=0.058$). Ph-like exhibited the lowest 2-year OS (65.6%; $p=0.056$, borderline). Risk-based analysis confirmed the best 2-year RFS (82.1%) and OS (90.0%) in Standard Risk patients, while High Risk patients showed inferior RFS (66.1%) despite comparable 2-year OS to Intermediate Risk (81.0% vs. 81.4%).

**Summary/Conclusion**

In this large South Asian paediatric B-ALL cohort, Ph-like and MLL-rearranged subtypes were associated with the highest MRD burden and poorest survival, underscoring a critical unmet therapeutic need. Dual-timepoint MRD assessment substantively complemented molecular subtyping, demonstrating that integrated genomic-response frameworks are both feasible and clinically meaningful within resource-constrained healthcare settings.

**Copyright Information:** (Online) ISSN: 2572-9241
©2026 The Author(s). HemaSphere is published by John Wiley & Sons Ltd on behalf of European Hematology Association. This is an open access Abstract Book distributed under the Attribution-NonCommercial-NoDerivs (CC BY-NC-ND), which allows third parties to download the articles and share them with others as long as they credit the author and the Abstract Book, but they cannot change the content in any way or use them commercially.
**Abstract Book Citations:** Authors, Title, HemaSphere, 2026;10:(S1):pages. The abstract book DOIs can be found on the “Author Information” page.
**Disclaimer:** Articles published in the journal HemaSphere exclusively reflect the opinions of the authors. The authors are responsible for all content in their abstracts including accuracy of the facts, statements, citing resources, etc.

### (PB2597) PRE-TRANSPLANT NLR AND PLR IN RELATION TO OUTCOMES OF ALLO-HSCT IN ADULT LEUKEMIA: A RETROSPECTIVE COHORT STUDY

**Topic:** 02. Acute lymphoblastic leukemia - Clinical

Xiaoli Chen*[1], Yimei Feng[2, 2], Lei Gao[2], Xi Zhang[2]

*[1]hospital, chongqing, China; [2]Second Affiliated Hospital of Army Military Medical University, Hematology Center,, Chongqing, China;*

### Background

Allogeneic hematopoietic stem cell transplantation (allo-HSCT) is currently a crucial means that could potentially cure malignant hematological disorders, and an increasing number of patients with cured hematological malignancies achieve long-term survival. The condition of patients before transplantation plays an important role in the outcome of hematopoietic stem cell transplantation, and prognostic indicators help predict survival rates. Optimizing pre-transplant risk assessment is essential for the formulation of decisions regarding allogeneic stem cell transplantation.
In recent years, the use of inflammatory markers for cancer prognosis assessment has gained attention. Among these, inflammatory markers such as the Neutrophil to Lymphocyte Ratio (NLR) and Platelet to Lymphocyte Ratio (PLR) have shown promising prognostic value in various solid tumors and hematological malignancies. However, the prognostic significance of NLR and PLR in leukemias following allogeneic hematopoietic stem cell transplantation has not been widely reported so far. In this paper, we explore the relationship and the prognostic value between NLR⊠PLR and mortality in patients with allogeneic hematopoietic stem cell transplantation in Adult leukemia.

### Aims we explore the relationship and the prognostic value between NLR⊠PLR and mortality in patients with allogeneic hematopoietic stem cell transplantation in Adult leukemia.

### Methods

In this study, we evaluate pre-HSCT NLR and PLR as predictive markers in patients undergoing allogeneic HSCT.we examined a cohort of adults leukemia who underwent allogeneic HCT in first or second remission at our institution between June 2014 and June 2020 .We assessed the prognostic role of the Pre-transplant neutrophil to lymphocyte ratio and platelet-tolymphocyte ratio. Multivariable COX regression was used to explore the association between Pre-transplant NLR⊠PLR and All-cause mortality/outcome.Interaction and stratified analyses were conducted including age,HLA.match,HSCT type,CD34+ cells⊠Donor sex,sex.

### Results

This retrospective analysis included 753 adult leukemia patients who underwent their first allogeneic hematopoietic stem cell transplantation (allo-HSCT), with an overall all-cause mortality of 19.9% (150/753). Multivariate analysis demonstrated that each 100-unit increase in pre-transplant neutrophil-to-lymphocyte ratio (NLR) was associated with a significantly elevated mortality risk (adjusted HR 1.64, 95% CI 1.19–2.28, P=0.003), whereas platelet-to-lymphocyte ratio (PLR) showed a modest linear association with mortality (adjusted HR 1.04 per 100-unit increase, 95% CI 1.01–1.07, P=0.017). Notably, PLR exhibited a non-linear relationship with outcomes: when analyzed in quartiles, patients in Q3 (95.83 to <156.7) had the lowest mortality risk compared with Q1 (<57.6) (adjusted HR 0.47, 95% CI 0.29–0.77, P=0.003), and threshold analysis revealed divergent effects below and above 135 units (HR 0.992 vs. 1.0005), though the latter difference lacked statistical significance.

**Copyright Information:** (Online) ISSN: 2572-9241
©2026 The Author(s). HemaSphere is published by John Wiley & Sons Ltd on behalf of European Hematology Association. This is an open access Abstract Book distributed under the Attribution-NonCommercial-NoDerivs (CC BY-NC-ND), which allows third parties to download the articles and share them with others as long as they credit the author and the Abstract Book, but they cannot change the content in any way or use them commercially.
**Abstract Book Citations:** Authors, Title, HemaSphere, 2026;10:(S1):pages. The abstract book DOIs can be found on the "Author Information" page.
**Disclaimer:** Articles published in the journal HemaSphere exclusively reflect the opinions of the authors. The authors are responsible for all content in their abstracts including accuracy of the facts, statements, citing resources, etc.

**Summary/Conclusion**

This study indicate that Pre-transplant NLR、PLR is independently associated with post-HCT outcome in patients with Allo-HSCT in Adult leukemia.Pre-transplant NLR and PLR serve as significant biomarkers for predicting All-cause mortality in adult leukemia patients who have undergone Allo-HSCT. By monitoring these biomarkers, healthcare providers can better identify high-risk patient populations and develop tailored management strategies for post-transplant care.

**Copyright Information:** (Online) ISSN: 2572-9241
©2026 The Author(s). HemaSphere is published by John Wiley & Sons Ltd on behalf of European Hematology Association. This is an open access Abstract Book distributed under the Attribution-NonCommercial-NoDerivs (CC BY-NC-ND), which allows third parties to download the articles and share them with others as long as they credit the author and the Abstract Book, but they cannot change the content in any way or use them commercially.
**Abstract Book Citations:** Authors, Title, HemaSphere, 2026;10:(S1):pages. The abstract book DOIs can be found on the "Author Information" page.
**Disclaimer:** Articles published in the journal HemaSphere exclusively reflect the opinions of the authors. The authors are responsible for all content in their abstracts including accuracy of the facts, statements, citing resources, etc.

### (PB2598) EFFICACY OF ALLOGENEIC CAR-T IN ACUTE LEUKEMIA RELAPSED AFTER ALLOGENEIC HEMATOPOIETIC STEM CELL TRANSPLANTATION A RETROSPECTIVE CLINICAL STUDY

**Topic:** 02. Acute lymphoblastic leukemia - Clinical

Zhongqing Li[1], Lin Luo[1], Yang Yang[1], Na Sun[1], Bingyuan Chen[1], Jun Luo[1], Jing Li*[1]

*[1]The First Affiliated Hospital of Guangxi Medical University, Hematology, Nanning, China;*

### Background

Patients with acute lymphoblastic leukaemia(ALL) relapsed after allogeneic haematopoietic stem cell transplantation(allo-HSCT) represent an extremely high-risk subgroup with a dismal prognosis, exhibiting a long-term survival below 10%. Second allo-HSCT achieves limited efficacy and a high treatment-related mortality. Autologous CAR-T therapy is also hampered by a high relapse rate, with a 2-year cumulative relapse rate of 43.2%. These data confirm a considerable unmet clinical need in this patient group.

### Methods

We conducted a single-center retrospective study involving 6 patients with B-ALL and 1 patient with mixed-phenotype acute leukemia, B/myeloid (MPAL) evolving from chronic myeloid leukemia, who had relapsed after Allo-HSCT and received donor-derived anti-CD19 CAR-T cell therapy between January 2023 and November 2025. Comprehensive clinical data, including patient baseline characteristics, Allo-HSCT details, relapse features, CAR-T therapy procedures, treatment-related adverse events, and survival outcomes, were systematically collected and analyzed.

### Results

The median age of the enrolled patients was 33 years (range, 18–40). Five patients (71.4%) harbored high-risk genetic abnormalities. Donor types included HLA-matched sibling donors (n=5) and haploidentical donors (n=2). The median time from Allo-HSCT to relapse was 262 days (range, 59–888), and the median interval from relapse to CAR-T infusion was 46 days (range, 25–418). All patients received bridging therapy prior to CAR-T infusion. After BT, 5 patients (71.4%) achieved MRD-negative complete hematologic remission(CHR), 2(14.3%) patients achieved CHR with MRD-positive. And all achieved MRD-negative CHR after CAR-T cells infusion with in 4-weeks. Cytokine release syndrome occurred in 4 patients (57.1%), all of grade 1–2, which resolved completely with standard supportive management. Acute graft-versus-host disease developed in 1 patient (14.3%) (grade 1 skin GVHD) and responded well to methylprednisolone. No neurotoxicity was observed in any patient. With a median follow-up of 10 months (range,3–35), six patients (85.7%) remained in MRD-negative CHR. The patient who developed MRD positivity 5 months after the first CAR-T infusion received a second infusion from the same donor targeting the same antigen and maintained CHR with persistent MRD positivity at the 9-month follow-up. The duration of remission for the two-earliest treated patients was 34 months and 24 months, respectively. For the entire cohort, both the 10-month progression-free survival (PFS) and OS rates were 100%.
Table1: Efficacy Outcomes of Donor-derived Anti-CD19 CAR-T Cell Therapy

**Copyright Information:** (Online) ISSN: 2572-9241
©2026 The Author(s). HemaSphere is published by John Wiley & Sons Ltd on behalf of European Hematology Association. This is an open access Abstract Book distributed under the Attribution-NonCommercial-NoDerivs (CC BY-NC-ND), which allows third parties to download the articles and share them with others as long as they credit the author and the Abstract Book, but they cannot change the content in any way or use them commercially.
**Abstract Book Citations:** Authors, Title, HemaSphere, 2026;10:(S1):pages. The abstract book DOIs can be found on the "Author Information" page.
**Disclaimer:** Articles published in the journal HemaSphere exclusively reflect the opinions of the authors. The authors are responsible for all content in their abstracts including accuracy of the facts, statements, citing resources, etc.

| Outcome | Value (n=7) |
|---|---|
| MRD-negative CHR within 4 weeks, n (%) | 7 (100) |
| Median peak CAR-T copies (range), copies/μg gDNA | 178(0.16~402) |
| Median time to CAR-T peak expansion (range), days | 9 (7–33) |
| CAR-T persistence at 3 months, *n* (%) | 7(100) |
| Median follow-up (range), months | 10(3–35) |
| Sustained MRD negativity at last follow-up, *n* (%) | 6(85.7) |
| MRD positive at last follow-up, *n* (%) | 1 (14.3) |
| 10-month progression-free survival rate, % | 100 |
| 10-month overall survival rate, % | 100 |

### Summary/Conclusion

Allogeneic CAR-T therapy is a promising therapeutic option for patients with ALL relapsing after Allo-HSCT, showing high CR rates and manageable safety profile with potential for long-term remission. However, current evidence is constrained by small sample sizes and short follow-up, necessitating further validation through larger trials and extended observation to confirm its long-term efficacy and safety.

**Copyright Information:** (Online) ISSN: 2572-9241
©2026 The Author(s). HemaSphere is published by John Wiley & Sons Ltd on behalf of European Hematology Association. This is an open access Abstract Book distributed under the Attribution-NonCommercial-NoDerivs (CC BY-NC-ND), which allows third parties to download the articles and share them with others as long as they credit the author and the Abstract Book, but they cannot change the content in any way or use them commercially.
**Abstract Book Citations:** Authors, Title, HemaSphere, 2026;10:(S1):pages. The abstract book DOIs can be found on the "Author Information" page.
**Disclaimer:** Articles published in the journal HemaSphere exclusively reflect the opinions of the authors. The authors are responsible for all content in their abstracts including accuracy of the facts, statements, citing resources, etc.

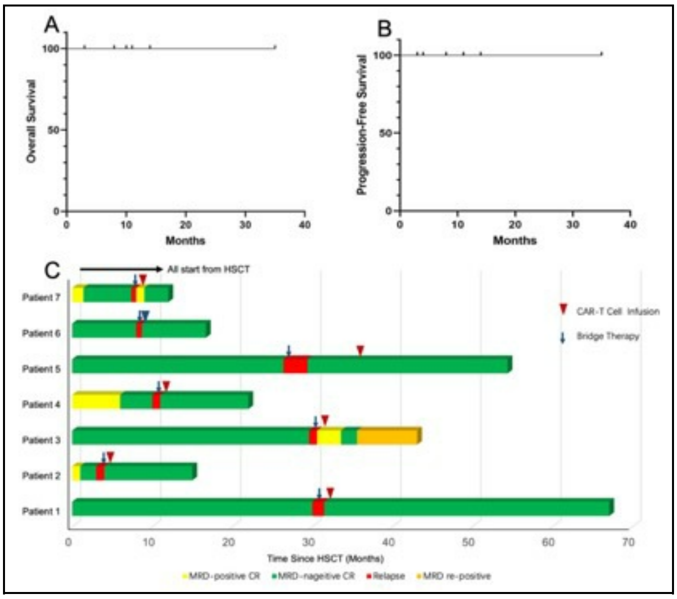

Figure1. Overall Survival, Progression-Free Survival and Longitudinal follow-up of the 7 patients. (A) Overall survival of the 7 patients. (B) Progression-Free Survival of the 7 patients. (C) Swim Plot: Longitudinal follow-up of the 7 patients starting from HSCT.

**Copyright Information:** (Online) ISSN: 2572-9241
©2026 The Author(s). HemaSphere is published by John Wiley & Sons Ltd on behalf of European Hematology Association. This is an open access Abstract Book distributed under the Attribution-NonCommercial-NoDerivs (CC BY-NC-ND), which allows third parties to download the articles and share them with others as long as they credit the author and the Abstract Book, but they cannot change the content in any way or use them commercially.
**Abstract Book Citations:** Authors, Title, HemaSphere, 2026;10:(S1):pages. The abstract book DOIs can be found on the "Author Information" page.
**Disclaimer:** Articles published in the journal HemaSphere exclusively reflect the opinions of the authors. The authors are responsible for all content in their abstracts including accuracy of the facts, statements, citing resources, etc.

### (PB2599) CLINICAL AND ADVERSE GENETIC FACTORS IN CHINESE PEDIATRIC B-ALL PATIENTS WITH ETV6::RUNX1 GENE FUSION

**Topic:** 02. Acute lymphoblastic leukemia - Clinical

Xiayu Xia[1], Keyan Yang[1], Qinlong Zheng*[1]

[1]*Beijing GoBroad Boren Hospital, Beijing, China;*

### Background

B-cell acute lymphoblastic leukemia (B-ALL) is a heterogeneous group of hematological malignancy generally occurs in children and adolescents. Recent omics studies have classified B-ALL into more than 20 subtypes with distinct oncogenic lesions or gene expression profiles, further promoted the risk-adapted treatment strategies. Among these subtypes, *ETV6::RUNX1* gene fusion is the most common in pediatric B-ALL patients, generally associated with a favorable prognosis, however, 10~20% of patients with *ETV6::RUNX1* gene fusion experience relapse, and the underlying mechanisms for this relapse are not yet clearly characterized.

### Aims

The current work aims to explore the adverse factors associated with Chinese pediatric B-ALL patients with *ETV6::RUNX1* gene fusion.

### Methods

Retrospective analyses were conducted for a total of 137 *ETV6::RUNX1* positive B-ALL cases, including 72 non-relapse patients with longitudinal follow-up, and 65 relapsed patients (including 19 with extramedullary relapse, 29.2%) who were admitted to Beijing GoBroad Boren hospital for further therapy. Genetic mutations were detected from their corresponding targeting sequencing data. The clinical and molecular features of non-relapsed and relapsed *ETV6::RUNX1* gene fusion cases were characterized, and adverse factors were explored.

### Results

The incidence of male patients was higher than that of female patients in both non-relapsed (61.1% vs. 38.9%) and relapsed (70.8% vs. 29.2%) *ETV6::RUNX1* positive B-ALL patients.
There was a median of 2 (IQR: 1~3) additional mutations for both of the 61 non-relapsed and 42 relapsed patients with available genetic mutation results. The most frequent mutated genes were *KRAS* (n=25, 24.3%), *NRAS* (n=13, 12.6%), *TP53* (n=8, 7.8%), *NSD2* (n=7, 6.8%), and *NR3C1* (n=7, 6.8%). Enrichment analyses indicated that *TP53* gene mutation was significantly more prevalent in the relapsed group (7/42 vs. 1/61, $p$<0.01), while *KRAS* gene mutations were less prevalent (6/42 vs. 19/61, $p$=0.05). Survival analyses were performed, with a median follow-up time of 41.7 (95% CI: 38.6-46.3) months. And the median time to first recurrence for patients with *TP53* gene mutation was 25.3 (95% CI: 14.5-NA) months, significantly shorter than 55.6 (95% CI: 45.0-71.9) months for *TP53* wild type patients ($p$<0.01), further confirming the poor prognosis.

### Summary/Conclusion

*ETV6::RUNX1* gene fusion B-ALL is generally associated with good prognosis, however, a relapse rate of 10~20% has been reported. *TP53* gene mutations were enriched in the relapsed group, and *TP53* mutation positive patients had a significantly shorter median time to first recurrence compared with *TP53* wild type patients. These findings suggest that *TP53* mutations are adverse genetic alterations in Chinese pediatric B-ALL patients with *ETV6::RUNX1* gene fusion.

**Copyright Information:** (Online) ISSN: 2572-9241
©2026 The Author(s). HemaSphere is published by John Wiley & Sons Ltd on behalf of European Hematology Association. This is an open access Abstract Book distributed under the Attribution-NonCommercial-NoDerivs (CC BY-NC-ND), which allows third parties to download the articles and share them with others as long as they credit the author and the Abstract Book, but they cannot change the content in any way or use them commercially.
**Abstract Book Citations:** Authors, Title, HemaSphere, 2026;10:(S1):pages. The abstract book DOIs can be found on the "Author Information" page.
**Disclaimer:** Articles published in the journal HemaSphere exclusively reflect the opinions of the authors. The authors are responsible for all content in their abstracts including accuracy of the facts, statements, citing resources, etc.

### (PB2600) INOTUZUMAB OZOGAMICIN INDUCES HIGH CR AND MRD CLEARANCE AND ENABLES SUBSEQUENT 2ND /3RD IN POST-ALLO-HSCT RELAPSED B-ALL

**Topic:** 02. Acute lymphoblastic leukemia - Clinical

Zhijie Wei*[1], Fei Pan[2], Jing Long[2], Peihua Lu[1, 2]

[1]*Hebei Yanda Lu Daopei Hospital, Langfang, China;*[2]*Beijing Lu Daopei Hospital, Beijing, China;*

### Background

Relapse after allogeneic hematopoietic stem cell transplantation (allo-HSCT) in relapsed/refractory B-cell acute lymphoblastic leukemia (R/R B-ALL) is associated with poor outcomes and limited salvage options. Achieving deep remission, particularly measurable residual disease (MRD) negativity, is critical for patients (pts) eligible for second or third ($2^{nd}$ /$3^{rd}$) transplantation .

### Aims

To assess the efficacy and safety of Inotuzumab Ozogamicin (INO) in post-transplant relapsed R/R B-ALL and its role as a potential bridge to $2^{nd}$ /$3^{rd}$ allo-HSCT.

### Methods

We enrolled 26 pts with R/R B-ALL treated at Yanda and Beijing Lu Daopei Hospitals between July 2022 and August 2025. All pts relapsed after prior allo-HSCT and expressed CD22. INO was administered using a fractionated dosing schedule (0.8 mg/m² on day 1 and 0.5 mg/m² on days 8 and 15). Pts achieving complete remission (CR) proceed to $2^{nd}$ /$3^{rd}$ allo-HSCT according to clinical evaluation and patient preference. Responses, survival outcomes, and adverse events (AEs) were assessed. Follow-up was censored on February 10, 2026.

### Results

Totally 25 pts were evaluable (one discontinued therapy due to infection). The median age was 22 years (range, 2–57), and 18 pts (72%) were male. Relapse occurred after first allo-HSCT in 22 pts (88%) and after second allo-HSCT in 3 pts; median time to relapse was 6 months (range, 1–36). Twelve pts (48%) had previously received CD19- or CD22-directed CAR-T therapy following transplant relapse.
Before INO treatment, 13 pts (52%) had morphologic relapse with a median bone marrow blast percentage of 46% (range, 7.5–94), while 12 pts (48%) had persistent disease detectable by flow cytometric MRD.
Response assessment within 7 days after completion of INO showed that 10 of 13 pts (77%) with morphologic relapse achieved CR, including MRD-negative CR in 8 and MRD-positive CR in 2, whereas 3 pts (23%) remained non-responsive. Among 12 MRD-positive pts, 11 (91.6%) converted to MRD negativity, and one remained MRD positive without response. No moderate or severe INO-related AEs, including hepatic sinusoidal obstruction syndrome (SOS/VOD), were observed.

Among 21 CR pts, 7 (33.3%) proceeded to $2^{nd}$ /$3^{rd}$ allo-HSCT, whereas 14 (66.7%) received non-transplant therapy. With a median follow-up of 348 days (range, 26–1219), bridged corhort demonstrated higher 2-year OS and LFS compared with non-transplanted corhort, although differences were not statistically significant (OS: 53.6% vs. 38.9%; LFS: 53.6% vs. 38.9%; both p>0.05; Fig. 1A–B). The cumulative incidence of relapse also favored the transplant corhort (14.2% vs. 45.2%, p=0.251).

Among four pts with persistent non-response, two underwent salvage $2^{nd}$ /$3^{rd}$ allo-HSCT; one achieved durable CR lasting 486 days, whereas the other relapsed and died at day 164. The remaining two non-transplanted pts relapsed and died at days 38 and 75, respectively. Notably, both MRD-positive CR pts eventually relapsed at 187 days (post- 2nd HSCT) and 26 days, respectively.

**Copyright Information:** (Online) ISSN: 2572-9241
©2026 The Author(s). HemaSphere is published by John Wiley & Sons Ltd on behalf of European Hematology Association. This is an open access Abstract Book distributed under the Attribution-NonCommercial-NoDerivs (CC BY-NC-ND), which allows third parties to download the articles and share them with others as long as they credit the author and the Abstract Book, but they cannot change the content in any way or use them commercially.
**Abstract Book Citations:** Authors, Title, HemaSphere, 2026;10:(S1):pages. The abstract book DOIs can be found on the "Author Information" page.
**Disclaimer:** Articles published in the journal HemaSphere exclusively reflect the opinions of the authors. The authors are responsible for all content in their abstracts including accuracy of the facts, statements, citing resources, etc.

**Summary/Conclusion**

INO induced high remission and MRD conversion rates with favorable safety in pts with post-allo-HSCT relapse and enabled successful bridging to 2nd /3rd allo-HSCT. These findings support INO as an effective salvage strategy to facilitate potentially curative transplantation in this high-risk population.

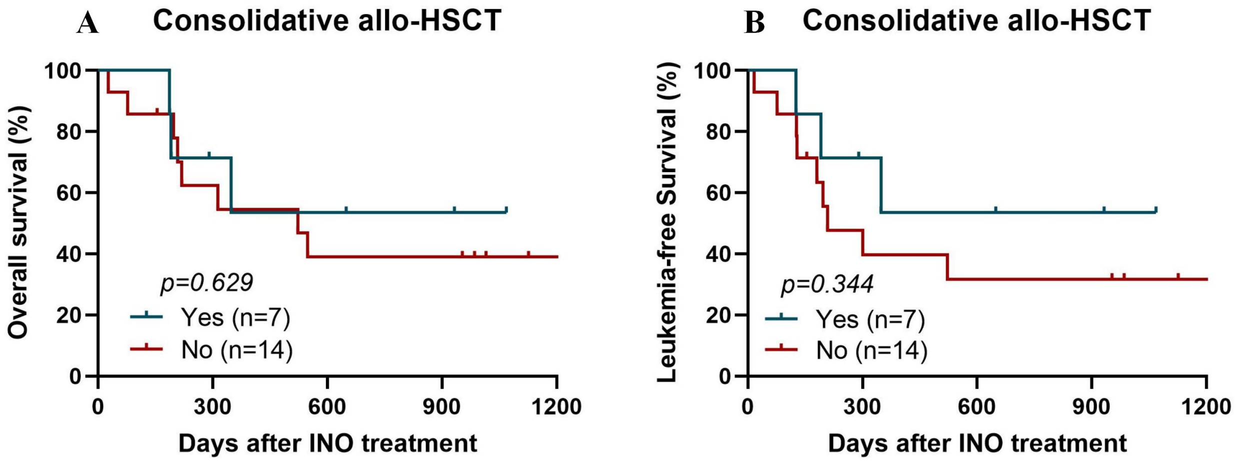

**Fig 1. Comparison of OS and LFS between CR patients bridged to2nd/3rd allo-HSCT and non-bridged patients (n=21).**

**Copyright Information:** (Online) ISSN: 2572-9241
©2026 The Author(s). HemaSphere is published by John Wiley & Sons Ltd on behalf of European Hematology Association. This is an open access Abstract Book distributed under the Attribution-NonCommercial-NoDerivs (CC BY-NC-ND), which allows third parties to download the articles and share them with others as long as they credit the author and the Abstract Book, but they cannot change the content in any way or use them commercially.
**Abstract Book Citations:** Authors, Title, HemaSphere, 2026;10:(S1):pages. The abstract book DOIs can be found on the "Author Information" page.
**Disclaimer:** Articles published in the journal HemaSphere exclusively reflect the opinions of the authors. The authors are responsible for all content in their abstracts including accuracy of the facts, statements, citing resources, etc.

### (PB2601) PHILADELPHIA POSITIVE T-ACUTE LYMPHOBLASTIC LEUKEMIA: A MAYO CLINIC SERIES

**Topic:** 02. Acute lymphoblastic leukemia - Clinical

Ashya Burgess*[1], Ali Husnain[1], Jabra Zarka[1], Dragan Jevremovic[1], Naseema Gangat[1], Nandita Khera[2], Aref Al-Kali[1], Brittany Kimball[1], Patricia Greipp[1], James M Foran[3], Mark Litzow[1], Hassan Alkhateeb[1], Antoine Saliba[1]

*[1]Mayo Clinic, Rochester, MN 55905, United States of America;[2]Mayo Clinic, Division of Hematology/Medical Oncology, Department of Medicine, Phoenix, AZ 85054, United States of America;[3]Mayo Clinic, Division of Hematology/Medical Oncology, Department of Medicine, Jacksonville, FL 32224, United States of America;*

### Background

The Philadelphia (Ph) chromosome is the defining cytogenetic abnormality in chronic myeloid leukemia (CML) and Ph-positive (Ph+) acute lymphoblastic leukemia (ALL), the latter predominantly of B-lineage. Ph+ T-lymphoblastic leukemia/lymphoma (T-ALL) and T-lymphoid blast phase (BP) of CML are exceedingly rare and poorly characterized. Prior reports suggest aggressive clinical behavior, frequent extramedullary disease, and poor outcomes. We describe the 3-site Mayo Clinic experience with Ph+ T-lineage ALL and CML-BP.

### Aims

To characterize clinical presentation, immunophenotype, treatment, and outcomes of patients with Ph+ T-lineage lymphoblastic neoplasms.

### Methods

We retrospectively identified patients diagnosed between 2014 and 2025 with T-lymphoblastic leukemia/lymphoma harboring *BCR::ABL1* rearrangement. Clinical, cytogenetic, molecular, treatment, and outcome data were abstracted from the medical record. Overall survival (OS) was calculated from diagnosis.

### Results

Five patients were identified (4 males, 1 female), with a median age of 73 years (range 49–74). Four (80%) had *de novo* Ph+ T-ALL and one (20%) developed T-lymphoid BP from antecedent CML (Table 1). All presented with lymphadenopathy and splenomegaly; none had CNS involvement at diagnosis. *BCR::ABL1* was detected by FISH in all cases. The p210 transcript was identified in 2 of 3 tested *de novo* cases and in the CML-BP case, suggesting predominance of p210 across both *de novo* and CML-BP presentations. Notably, none had prior TKI therapy at T-lineage diagnosis.
Immunophenotypes were consistent with immature T-lineage disease: cytoplasmic CD3 (100%) with CD2 (100%), CD5 (80%), CD7 (100%), and TdT (80%). No case demonstrated an early T-precursor phenotype. Aberrant myeloid antigen expression was limited (CD13 in 2, CD33 in 1). In patients with T-lymphoblastic leukemic involvement, marrow blasts ranged from 30% to 78%.
Treatment strategies varied and included hyperCVAD+dasatinib (n=1), mini-hyperCVD+dasatinib (n=1), vincristine+dexamethasone+dasatinib (n=2), and best supportive care (n=1). One patient underwent allogeneic stem cell transplantation in complete remission. Three patients achieved hematologic and/or molecular responses. The four patients treated achieved complete remission (CR). Two patients died within 3 months of diagnosis due to disease progression or treatment-related complications. The transplanted patient remained in complete molecular remission but died 5.3 years later from chronic GVHD-related complications. One patient remained alive at last follow-up in major molecular response on imatinib at 8.6 years. Median overall survival was 21 months (range 0.3–103).

**Copyright Information:** (Online) ISSN: 2572-9241
©2026 The Author(s). HemaSphere is published by John Wiley & Sons Ltd on behalf of European Hematology Association. This is an open access Abstract Book distributed under the Attribution-NonCommercial-NoDerivs (CC BY-NC-ND), which allows third parties to download the articles and share them with others as long as they credit the author and the Abstract Book, but they cannot change the content in any way or use them commercially.
**Abstract Book Citations:** Authors, Title, HemaSphere, 2026;10:(S1):pages. The abstract book DOIs can be found on the "Author Information" page.
**Disclaimer:** Articles published in the journal HemaSphere exclusively reflect the opinions of the authors. The authors are responsible for all content in their abstracts including accuracy of the facts, statements, citing resources, etc.

### Summary/Conclusion

Ph+ T-lineage lymphoblastic leukemias/lymphomas are rare and predominantly affect older adults and uniformly presented with nodal and splenic involvement in our series. None had prior TKI exposure, supporting *de novo* emergence rather than therapy-driven lineage shift. Durable remissions were achievable with chemotherapy+TKI-based strategies, including long-term survival beyond 8 years in one patient and successful transplantation in another. Outcomes were heterogeneous and influenced by comorbidity and treatment tolerability. Out of the four patients treated, the single p190 case had the shortest survival, suggesting possible transcript-specific biological differences. Multi-institutional collaboration is needed to better define classification and optimize management.

**Table 1. Clinical, Immunophenotypic, Molecular, Treatment, and Outcome Characteristics of Patients with Philadelphia Chromosome-Positive T-Cell Acute Lymphoblastic Leukemia**

| Case Number | Age at diagnosis, years | Sex (M/F) | Race/Ethnicity | CML-BP or *de novo* ALL | T-LL or T-ALL | *bcr::abl1* transcript type | ECOG PS | BMI (kg/m²) | Lymphadenopathy | Splenomegaly | Mediastinal mass | CNS involvement | LDH (U/L) | WBC (×10⁹/L) | Hemoglobin (g/dL) | Platelets (×10⁹/L) | PB blasts (%) | BM blasts (%) | TdT expression | Karyotype | Prior TKI therapy | Induction chemotherapy | TKI used | CR after induction | Allogeneic HSCT | Survival status | OS (months) | Disease status at last follow-up |
|---|---|---|---|---|---|---|---|---|---|---|---|---|---|---|---|---|---|---|---|---|---|---|---|---|---|---|---|---|
| 1 | 49 | M | Non-Hispanic White | CML-BP | T-LL | e14-a2 p210 | 0 | 23 | Y | Y | N | N | 485 | 185 | 9.2 | 501 | 0 | 2 | dim | 46,XY,t(9;22)(q34;q11.2)[13]/47,XY,idem,+8[6]/48,XY,idem,+8,-13,+19,+21[1] | N | Y | Dasatinib | Y | Y | Deceased | 63 | CMR |
| 2 | 49 | M | Non-Hispanic White | *de novo* ALL | T-ALL | Not done | 3 | 63 | Y | Y | N | N | 999 | 127 | 9.6 | 100 | 66 | 79 | - | 46,XY,t(9;22)(q34;q11.2)[8]/46,XY[12] | N | N | None | N/A | N | Deceased | 0.3 | PD |
| 3 | 74 | M | Non-Hispanic White | *de novo* ALL | T-ALL | e14-a2 p210 | 0 | 27 | Y | Y | N | N | 261 | 19 | 11.4 | 70 | 10 | 76 | + | 46,XY,t(9;22)(q34;q11.2)[2]/47,sl,+der(22)t(9;22) [7]/47,XY,der(9)t(9;22)(q34;q11.2),ider(22)(q11.2)t(9;22)[cp9]/46, XY[2] | N | Y | Dasatinib → imatinib | Y | N | Alive | 103 | MMR |
| 4 | 74 | M | Non-Hispanic White | *de novo* ALL | T-ALL | e1-a2 p190 | 0 | 26 | Y | Y | N | N | 203 | 29 | 11.4 | 80 | 50 | 30 | partial | 46,XY,t(9;22)(q34;q11.2)[1]/45,sl,del(11)(q13q21),-21[5]/46,XY[4] | N | Y | Dasatinib | Y | N | Deceased | 2.2 | CR with CCyR |
| 5 | 73 | F | African American | *de novo* ALL | T-ALL | e13/e14-a2 p210 | 3 | 65 | Y | N | N | N | 374 | 11 | 11.8 | 155 | 2 | 57 | + | 47,XX,t(9;22)(q34;q11.2),+19[4]/46,XX[16] | N | Y | Dasatinib | Y | N | Deceased | 21.4 | CR with CCyR |

**Abbreviations:** BM, bone marrow; BMI, body mass index; CCyR, complete cytogenetic response; CML-BP, chronic myeloid leukemia in blast phase; CMR, complete molecular response; CR, complete remission; CNS, central nervous system; ECOG PS, Eastern Cooperative Oncology Group performance status; F, female; M, male; LDH, lactate dehydrogenase; MMR, major molecular response; OS, overall survival; PD, progressive disease; T-ALL, T-cell acute lymphoblastic leukemia; T-LL, T-cell lymphoblastic lymphoma; TKI, tyrosine kinase inhibitor; WBC, white blood cells.

**Copyright Information:** (Online) ISSN: 2572-9241
©2026 The Author(s). HemaSphere is published by John Wiley & Sons Ltd on behalf of European Hematology Association. This is an open access Abstract Book distributed under the Attribution-NonCommercial-NoDerivs (CC BY-NC-ND), which allows third parties to download the articles and share them with others as long as they credit the author and the Abstract Book, but they cannot change the content in any way or use them commercially.
**Abstract Book Citations:** Authors, Title, HemaSphere, 2026;10:(S1):pages. The abstract book DOIs can be found on the "Author Information" page.
**Disclaimer:** Articles published in the journal HemaSphere exclusively reflect the opinions of the authors. The authors are responsible for all content in their abstracts including accuracy of the facts, statements, citing resources, etc.

### (PB2602) TRIAL IN PROGRESS: T-RREX - A PIVOTAL PHASE 2 STUDY OF WU-CART-007 (SOFI-CEL), CD7-DIRECTED ALLOGENEIC CAR-T CELL THERAPY, IN PATIENTS WITH R/R T-CELL ACUTE LYMPHOBLASTIC LEUKEMIA/LYMPHOMA

**Topic:** 02. Acute lymphoblastic leukemia - Clinical

Elias Jabbour*[1], Ibrahim Aldoss[2], Shannon Maude[3], Ashish Bajel[4], Seong L. Khaw[5], Rawan Faramand[6], Jeremy Rubinstein[7], Adam Lamble[8], Priti Tewari[1], Deepa Bhojwani[9], Jacqueline Blem[10], Alexander S. Hamil[10], Cherry T. Thomas[10], Armin Ghobadi[11]

*[1]University of Texas MD Anderson Cancer Center, Houston, United States of America;[2]City of Hope Hospital, Duarte, United States of America; [3]Children's Hospital of Philadelphia, Philadelphia, United States of America;[4]Peter McCallum Cancer Centre and The Royal Melbourne Hospital, Melbourne, Australia; [5]Royal Children's Hospital, Parkville, Australia;[6]Moffitt Cancer Center, Tampa, United States of America; [7]Cincinnati Children's Hospital Medical Center, Cincinnati, United States of America;[8]Seattle Children's Hospital, Seattle, United States of America; [9]Children's Hospital Los Angeles and Keck School of Medicine, Los Angeles, United States of America; [10]Wugen, Inc, St. Louis, United States of America; [11]Washington University, St. Louis, United States of America;*

### Background

T-cell acute lymphoblastic leukemia/lymphoblastic lymphoma (T-ALL/LBL) are aggressive hematologic cancers with high rates of mortality in both children and adults in the relapsed setting. Once relapsed, the disease is resistant to most therapies. CD7 is expressed on leukemic blasts in over 95% of patients with T-ALL/LBL. WU-CART-007 (soficabtagene geleucel "sofi-cel") is an allogeneic, CD7-targeted CAR-T cell therapy with a CD7 deletion to mitigate fratricide and a T-cell receptor alpha constant deletion to minimize graft-versus-host disease. In a global first-in-human, Phase 1 study, 26 adult and adolescent patients with relapsed/refractory (R/R) T-ALL/LBL were treated with sofi-cel (Ghobadi, *et al,* Blood 2025).

The recommended Phase 2 dose (RP2D) was 900x$10^6$ sofi-cel cells administered following lymphodepletion (LD). At the RP2D, sofi-cel demonstrated acceptable safety and anti-tumor activity. The overall response rate was 91%, with a composite complete remission (CRc) rate of 73%. The median duration of response was not reached (95% CI: 0.5 to not estimable; range, 0.3-10.7 months). CAR-T cell expansion peaked on Day 10 and was detectable through Day 90.

### Aims

To describe the design of T-RRex, a pivotal Phase 2 single-arm, open-label, multicenter study evaluating the efficacy of sofi-cel in patients with R/R T-ALL/LBL. The study also includes a planned exploratory cohort of patients with minimal residual disease (MRD)-positive T-ALL.

### Methods

Adult and pediatric patients ≥6 months old with R/R T-ALL/LBL, as defined by the World Health Organization classification, are eligible. R/R disease is defined as ≥5% bone marrow blasts or the presence of extramedullary disease after frontline induction and consolidation or in first or subsequent relapse. Patients receive sofi-cel at the target dose following LD.
The primary objective is to evaluate the CRc rate following administration of sofi-cel therapy. The CRc rate is defined as the proportion of patients achieving complete remission (CR), CR with partial hematologic recovery (CRh), or CR with incomplete hematologic recovery (CRi). Secondary endpoints include assessment of safety, duration of response, best overall response rate, and overall survival.
Response assessments are performed on Day 28, and at Months 3, 6, 9, 12, and 24. These evaluations include bone marrow aspirate and biopsy, with PET-CT imaging when clinically indicated.
An exploratory cohort is planned for pediatric and adult patients with MRD-positive T-ALL. MRD-positive status is defined as patients in CR with detectable MRD (0.1 to <5% bone marrow blasts).

**Copyright Information:** (Online) ISSN: 2572-9241
©2026 The Author(s). HemaSphere is published by John Wiley & Sons Ltd on behalf of European Hematology Association. This is an open access Abstract Book distributed under the Attribution-NonCommercial-NoDerivs (CC BY-NC-ND), which allows third parties to download the articles and share them with others as long as they credit the author and the Abstract Book, but they cannot change the content in any way or use them commercially.
**Abstract Book Citations:** Authors, Title, HemaSphere, 2026;10:(S1):pages. The abstract book DOIs can be found on the "Author Information" page.
**Disclaimer:** Articles published in the journal HemaSphere exclusively reflect the opinions of the authors. The authors are responsible for all content in their abstracts including accuracy of the facts, statements, citing resources, etc.

**Results**

The study is currently open and enrolling (NCT06514794) at 10 sites in the U.S. and Australia.

**Summary/Conclusion**

This pivotal study is designed to generate evidence supporting the potential of sofi-cel to address a significant unmet medical need in patients with R/R T-ALL/LBL.

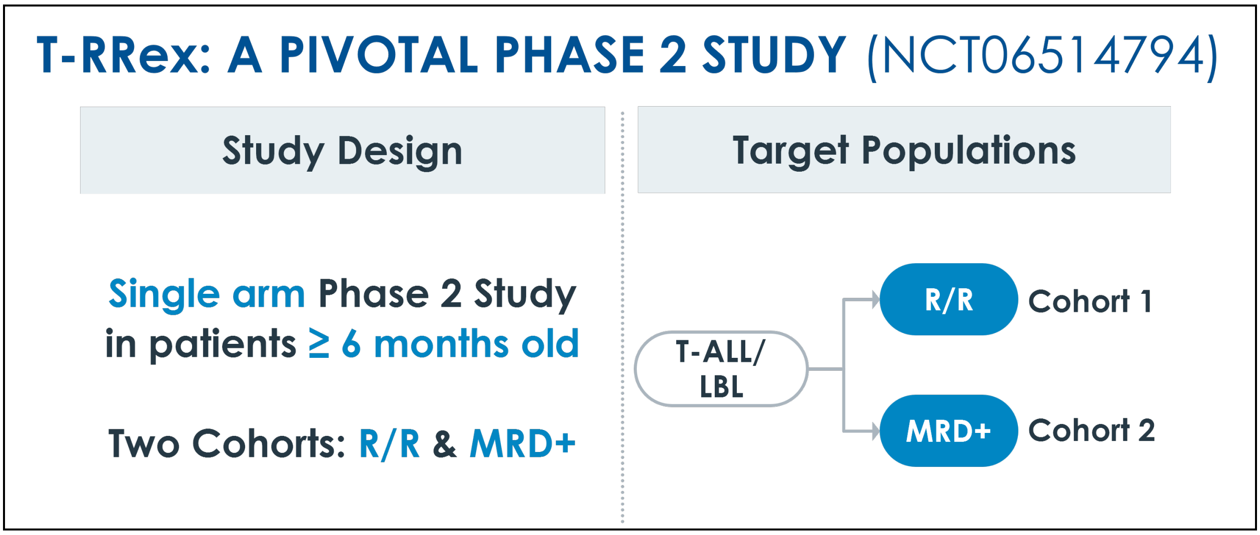

**Copyright Information:** (Online) ISSN: 2572-9241
©2026 The Author(s). HemaSphere is published by John Wiley & Sons Ltd on behalf of European Hematology Association. This is an open access Abstract Book distributed under the Attribution-NonCommercial-NoDerivs (CC BY-NC-ND), which allows third parties to download the articles and share them with others as long as they credit the author and the Abstract Book, but they cannot change the content in any way or use them commercially.
**Abstract Book Citations:** Authors, Title, HemaSphere, 2026;10:(S1):pages. The abstract book DOIs can be found on the "Author Information" page.
**Disclaimer:** Articles published in the journal HemaSphere exclusively reflect the opinions of the authors. The authors are responsible for all content in their abstracts including accuracy of the facts, statements, citing resources, etc.

### (PB2603) PATTERNS OF MULTIMODAL MRD RESPONSE AND TOXICITY WITH BLINATUMOMAB AND TYROSINE KINASE INHIBITORS IN PHILADELPHIA-POSITIVE ACUTE B-LYMPHOBLASTIC LEUKEMIA

**Topic:** 02. Acute lymphoblastic leukemia - Clinical

Sebastian Stolz[1], Jeremy Deuel[1], Wiebke Rösler[1], Rahel Schwotzer[1], Markus G. Manz[1], Stefan Balabanov[1], Max Jan Rieger*[1]

[1]*Universitätsspital Zürich, Medical Oncology and Hematology, Zürich, Switzerland;*

**Background**

Tyrosine kinase inhibitors (TKIs) and blinatumomab have gradually improved outcomes in Philadelphia positive B-lymphoblastic leukemia (Ph+ B-ALL) and have led to a fundamental change in patterns of response and toxicity.

**Methods**

We conducted a retrospective analysis of 24 adults with de novo or relapsed/refractory Ph+ B-ALL or chronic myeloid leukemia in lymphoid blast phase treated with blinatumomab plus TKI at our institution between 2018 and 2024.

**Results**

All patients achieved morphological complete remission (CR). Among those treated upfront (18/24), rapid and deep molecular responses were observed, with most achieving multiparameter minimal residual disease (MRD) negativity within two cycles of blinatumomab. In contrast, patients receiving therapy after inadequate BCR::ABL1 response to chemotherapy frequently demonstrated delayed or incomplete BCR::ABL1 clearance with blinatumomab, prior to transplantation. Several patients with persistent BCR::ABL1 transcripts and negative flow cytometry or Ig/TCR analysis remained in durable morphologic CR, potentially suggesting BCR::ABL persistence in non-lymphoblastic compartments of a ‘multilineage’ disease subtype. Whole-transcriptome sequencing enabled classification into lymphoid-only or multilineage subtypes in 11/24 patients, in whom MRD kinetics showed no obvious differences. Treatment was well tolerated, with no grade ≥3 cytokine release syndrome and low rates of neurotoxicity.

**Summary/Conclusion**

These findings support the safety and efficacy of TKI–blinatumomab combinations, highlight the importance of multiparametric MRD assessment, and challenge therapeutic strategies based solely on isolated BCR::ABL1 persistence.

**Copyright Information:** (Online) ISSN: 2572-9241
©2026 The Author(s). HemaSphere is published by John Wiley & Sons Ltd on behalf of European Hematology Association. This is an open access Abstract Book distributed under the Attribution-NonCommercial-NoDerivs (CC BY-NC-ND), which allows third parties to download the articles and share them with others as long as they credit the author and the Abstract Book, but they cannot change the content in any way or use them commercially.
**Abstract Book Citations:** Authors, Title, HemaSphere, 2026;10:(S1):pages. The abstract book DOIs can be found on the “Author Information” page.
**Disclaimer:** Articles published in the journal HemaSphere exclusively reflect the opinions of the authors. The authors are responsible for all content in their abstracts including accuracy of the facts, statements, citing resources, etc.

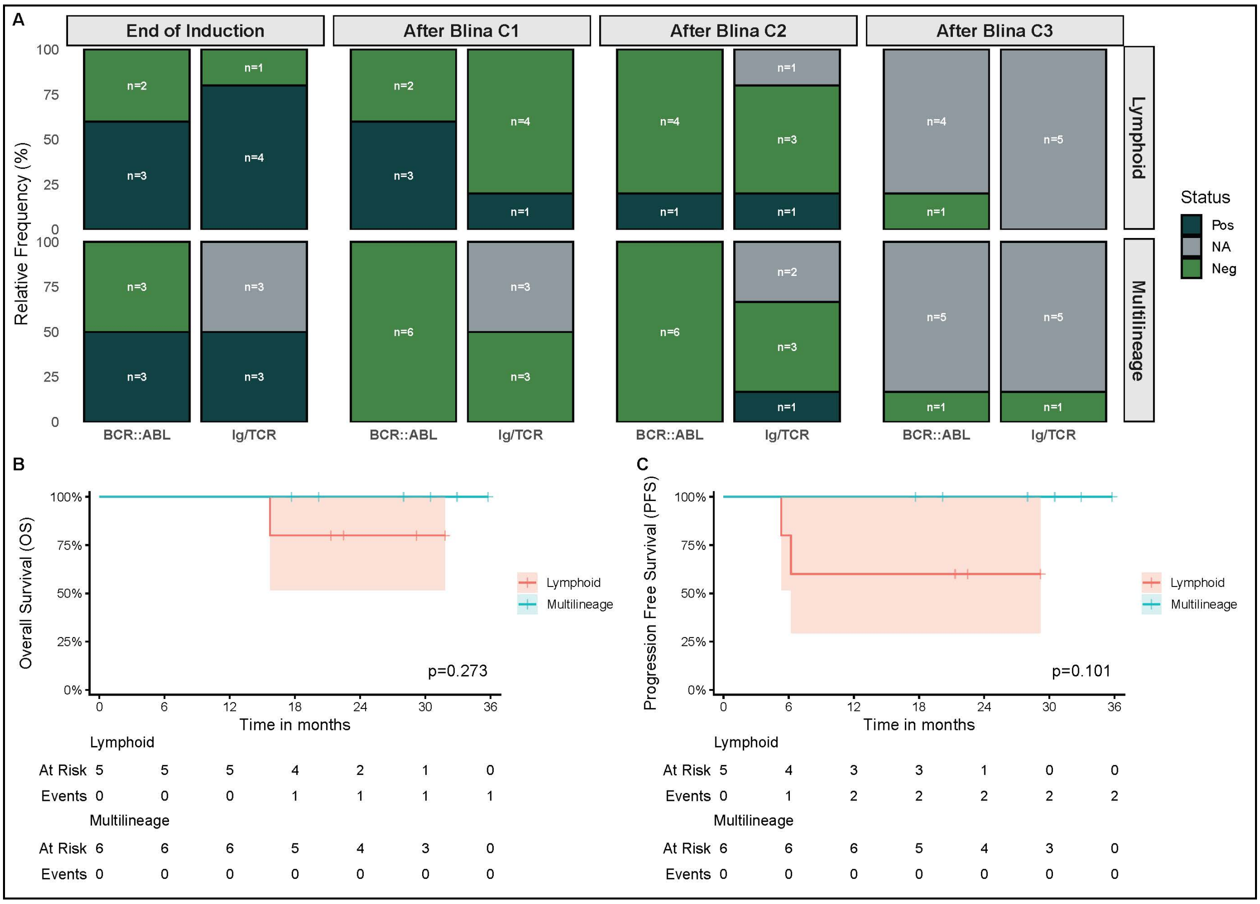

**Copyright Information:** (Online) ISSN: 2572-9241
©2026 The Author(s). HemaSphere is published by John Wiley & Sons Ltd on behalf of European Hematology Association. This is an open access Abstract Book distributed under the Attribution-NonCommercial-NoDerivs (CC BY-NC-ND), which allows third parties to download the articles and share them with others as long as they credit the author and the Abstract Book, but they cannot change the content in any way or use them commercially.
**Abstract Book Citations:** Authors, Title, HemaSphere, 2026;10:(S1):pages. The abstract book DOIs can be found on the "Author Information" page.
**Disclaimer:** Articles published in the journal HemaSphere exclusively reflect the opinions of the authors. The authors are responsible for all content in their abstracts including accuracy of the facts, statements, citing resources, etc.

### (PB2604) SAFETY AND EFFICACY OF CHIDAMIDE-BASED INTENSIFIED CONDITIONING REGIMEN IN ALLOGENEIC HEMATOPOIETIC STEM CELL TRANSPLANTATION IN PATIENTS WITH T-CELL ACUTE LYMPHOBLASTIC LEUKEMIA/LYMPHOMA

**Topic:** 02. Acute lymphoblastic leukemia - Clinical

□□ □*[1]

[1]*State Key Laboratory of Experimental Hematology, Senior Department of Hematology, the Fifth Medical Center of PLA General Hospital, beijing, China;*

### Aims

To analyze the safety and efficacy of chidamide-based intensified conditioning regimen in allogeneic hematopoietic stem cell transplantation(allo-HSCT) in patients with T-cell acute lymphoblastic leukemia/lymphoma (T-ALL/LBL).

### Methods

A retrospective analysis was conducted on 31 T-ALL/LBL patients who underwent allo-HSCT and received the chidamide combined with modified BuCy conditioning regimen in our Department from August 2020 to January 2025.Data of 54 T-ALL/LBL patients who underwent traditional modified BuCy conditioning regimen from March 2015 to December 2020 were collected as a control group. The adverse reactions and clinical outcomes of the two groups were observed and compared after transplantation.

### Results

The median time of neutrophil engraftment in chidamide and control groups was 14 (8 - 17) days and 12 (9 - 20) days, respectively (*P*=0.31). The median time of platelet engraftment was 15 (9 - 29) days in chidamide group and 13 (9 - 30) days in the control group (*P*=0.34). The incidence of grade II - IV acute GVHD (aGVHD) in chidamide and control groups was 35.5% and 40.7% (*P*=0.50), the incidence of grade III - IV aGVHD was 6.5% and 1.9% (*P*=0.28) and the incidence of chronic GVHD (cGVHD) at 2 years was 34.3% and 22.2% (*P*=0.39). The median follow-up time was 25.6 months and 87.9 months in chidamide and control groups. The 2-year cumulative incidence of relapse was 18.5% and 35.2%, respectively (*P*=0.065). the 2-year NRM was 11.2□ and 20.4%, respectively (*P*=0.22). The 2-year OS was 80.3□ and 50.0%, respectively □*P*=0.0029□. The 2-year DFS was 74.6% and 44.4%, respectively (*P* =0.005). Further subgroup analysis of high risk T-ALL/LBL patients, the 2-year cumulative incidence of relapse in chidamide and control groups was 21.4% and 45.2%(*P*=0.043), the 2-year NRM was 7.1% and 19.4%(*P*=0.10), the 2-year OS was 85.9% and 41.9%(*P*=0.00031), and the 2-year DFS was 77.9% and 35.5%,respectively (*P*=0.00081).

### Summary/Conclusion

The chidamide intensified regimen may be an effective and acceptable safety option for T-ALL/LBL undergoing allo-HSCT. Notably, for patients with high-risk T-ALL/LBL, this regimen could reduce relapse without increasing NRM and improve the prognosis.

**Copyright Information:** (Online) ISSN: 2572-9241
©2026 The Author(s). HemaSphere is published by John Wiley & Sons Ltd on behalf of European Hematology Association. This is an open access Abstract Book distributed under the Attribution-NonCommercial-NoDerivs (CC BY-NC-ND), which allows third parties to download the articles and share them with others as long as they credit the author and the Abstract Book, but they cannot change the content in any way or use them commercially.
**Abstract Book Citations:** Authors, Title, HemaSphere, 2026;10:(S1):pages. The abstract book DOIs can be found on the “Author Information” page.
**Disclaimer:** Articles published in the journal HemaSphere exclusively reflect the opinions of the authors. The authors are responsible for all content in their abstracts including accuracy of the facts, statements, citing resources, etc.

### (PB2605) SURVIVAL AND LOSS OF LIFETIME IN ACUTE MYELOID LEUKEMIA IN FIRST REMISSION FOLLOWING INTENSIVE CHEMOTHERAPY

**Topic:** 02. Acute lymphoblastic leukemia - Clinical

Silje Post Strøm*[1, 2], Mikkel Runason Simonsen[3, 4], Anne Stidsholt Roug[2, 5, 6], Claus Marcher[7], Andreas Ørskov[8], Gitte Thomsen[3], Claudia Schöllkopf[9], Henrik Sørensen[1, 2], Lars Pedersen[1, 2], Tarec El-Galaly[1, 2, 5, 6], Daniel Tuyet Kristensen[2, 5, 6]

*[1]Aarhus University and Aarhus University Hospital, Department of Clinical Epidemiology, Aarhus, Denmark;[2]Aarhus University, Department of Clinical Medicine, Aarhus, Denmark; [3]Aalborg University Hospital, Department of Hematology, Aalborg, Denmark; [4]Aalborg University, Department of Mathematical Sciences, Aalborg, Denmark;[5]Aarhus University Hospital, Department of Hematology, Aarhus, Denmark; [6]Aarhus University Hospital, Department of Molecular Medicine, Aarhus, Denmark; [7]Odense University Hospital, Department of Hematology, Odense, Denmark; [8]Zealand University Hospital, Department of Hematology, Roskilde, Denmark; [9]Copenhagen University Hospital, Department of Hematology, Copenhagen, Denmark;*

### Background

Most relapses of acute myeloid leukemia (AML) occur within the first 36 months after achieving complete remission (CR). Relapse-free survival (RFS) beyond this period may be critical for cure and long-term overall survival (OS).

### Aims

We examined how reaching specific RFS milestones in AML is associated with loss of lifetime (LOL) and normalization of survival.

### Methods

Patients diagnosed with AML between 2010-2024 and in CR after first-line intensive chemotherapy were identified from the Danish Acute Leukemia Registry, excluding acute promyelocytic leukemia patients. Their survival was compared with the expected survival of a standardized general population (GP) at pre-specified RFS landmarks (0, 12, 24, and 36 months) to compute 5-year LOL and standardized mortality ratios (SMRs). Subgroup analyses were conducted by sex, age-group (<60 and ≥60 years) and cytogenetic risk (favorable, intermediate, adverse), which was assigned based on the ELN 2022 risk stratification.

**Copyright Information:** (Online) ISSN: 2572-9241
©2026 The Author(s). HemaSphere is published by John Wiley & Sons Ltd on behalf of European Hematology Association. This is an open access Abstract Book distributed under the Attribution-NonCommercial-NoDerivs (CC BY-NC-ND), which allows third parties to download the articles and share them with others as long as they credit the author and the Abstract Book, but they cannot change the content in any way or use them commercially.
**Abstract Book Citations:** Authors, Title, HemaSphere, 2026;10:(S1):pages. The abstract book DOIs can be found on the "Author Information" page.
**Disclaimer:** Articles published in the journal HemaSphere exclusively reflect the opinions of the authors. The authors are responsible for all content in their abstracts including accuracy of the facts, statements, citing resources, etc.

### Results

A total of 1,207 patients fulfilled the inclusion criteria. The median age at diagnosis was 61 years (IQR, 51–68); 47% of patients were <60 years, and 52% were male. Performance status 0-1 was observed in 94% of patients, and 82% had a low comorbidity burden (Charlson Comorbidity Index (excluding leukemia) <2). Secondary AML was diagnosed in 18% of patients. The most frequently used induction regimen (81% of patients) was standard daunorubicin and cytarabine (“7+3”), with or without gemtuzumab, ozogamicin or midostaurin. Allogeneic hematopoietic stem cell transplantation in first CR was performed in 34% of patients. There were 659, 471, and 368 patients included for analysis at the 12, 24, and 36 months RFS landmarks, respectively.
The RFS landmarks showed progressively higher 5-year post-landmark OS: 48% (95% CI 45-51) for RFS0, 68% (95% CI 65-73) for RFS12, 79% (95% CI 75-83) for RFS24, and 86% (95% CI 82-90) for RFS36. However, the 5-year OS remained significantly lower than the corresponding 5-year survival in the standardized GP (Figure). Corresponding SMRs were 15 (95% CI 14-17) at RFS0, 6.8 (95% CI 5.9-7.9) at RFS12, 4.0 (95% CI 3.2–5.0) at RFS24, and 2.3 (95% CI 1.7-3.1) at RFS36.
The post-landmark 5-year LOL decreased with remission duration: 20 months (95% CI 18-21) for RFS0, 9.1 months (95% CI 7.5-11) for RFS12, 4.5 months (95% CI 3.2-5.9) for RFS24, and 1.2 months (95% CI 0.36-2.0) for RFS36.
In subgroup analyses, only patients with favorable risk disease approached normalized survival at RFS24, with 5-year post-landmark OS of 91% (95% CI 82-100) compared to 96% for the GP, resulting in an SMR of 2.1 (95%CI 0.79-5.6). Patients with intermediate risk disease did not achieve normalized survival at any timepoint, with SMRs of 4.5 (95% CI 3.5-5.7) at RFS24 and 2.7 (95% CI 1.9-3.8) at RFS36. Other subgroup analyses showed significantly elevated mortality after achieving RFS36 with SMRs in the range 1.8-3.3. The 5-year LOL decreased for all patients achieving RFS24, regardless of risk group, ranging from 0.5-7.1 months.

### Summary/Conclusion

Longer RFS in AML was associated with progressively improved outcomes and reduced LOL. However, even after RFS36, most patients continued to have higher mortality than the GP, with near-normal survival observed only in favorable-risk disease. This residual excess risk is likely multifactorial and may partly reflect late complications and transplant-related mortality and highlight the need for continued survivorship awareness beyond the RFS36 landmark.

**Copyright Information:** (Online) ISSN: 2572-9241
©2026 The Author(s). HemaSphere is published by John Wiley & Sons Ltd on behalf of European Hematology Association. This is an open access Abstract Book distributed under the Attribution-NonCommercial-NoDerivs (CC BY-NC-ND), which allows third parties to download the articles and share them with others as long as they credit the author and the Abstract Book, but they cannot change the content in any way or use them commercially.
**Abstract Book Citations:** Authors, Title, HemaSphere, 2026;10:(S1):pages. The abstract book DOIs can be found on the “Author Information” page.
**Disclaimer:** Articles published in the journal HemaSphere exclusively reflect the opinions of the authors. The authors are responsible for all content in their abstracts including accuracy of the facts, statements, citing resources, etc.

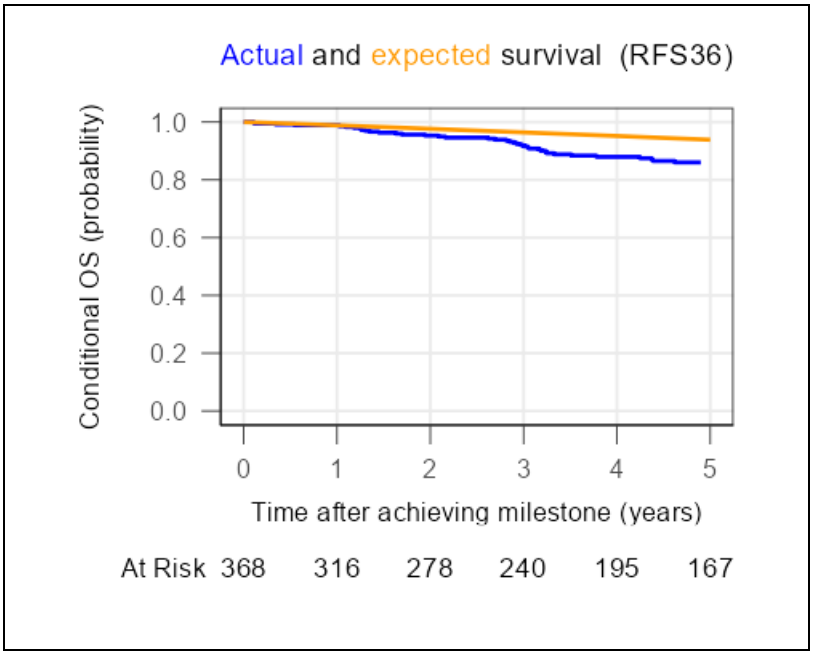

Figure: Overall survival (blue) conditional on achieving the RFS36 milestone and expected survival (yellow) for the standardized general population.

**Copyright Information:** (Online) ISSN: 2572-9241
©2026 The Author(s). HemaSphere is published by John Wiley & Sons Ltd on behalf of European Hematology Association. This is an open access Abstract Book distributed under the Attribution-NonCommercial-NoDerivs (CC BY-NC-ND), which allows third parties to download the articles and share them with others as long as they credit the author and the Abstract Book, but they cannot change the content in any way or use them commercially.
**Abstract Book Citations:** Authors, Title, HemaSphere, 2026;10:(S1):pages. The abstract book DOIs can be found on the “Author Information” page.
**Disclaimer:** Articles published in the journal HemaSphere exclusively reflect the opinions of the authors. The authors are responsible for all content in their abstracts including accuracy of the facts, statements, citing resources, etc.

### (PB2606) USE OF ASPARAGINASE IN PATIENTS WITH PHILADELPHIA CHROMOSOME-NEGATIVE ACUTE LYMPHOBLASTIC LEUKEMIA (ALL): A MULTICENTER ANALYSIS IN ARGENTINA AND CHILE

**Topic:** 02. Acute lymphoblastic leukemia - Clinical

Maria Victoria Lipera[1], Maria Moirano[2], Isolda Isabel Fernandez[3], Romina Montivero[4], Diego Wolhein[5], Erika Brulc[6], Liset Santoya[7], Claudia Vicente[8], Ines Paganini[9], Daniela Maymo[10], Roxana Ramirez[11], Silvina Andrea Palmer[12], Julian Matias Freue[13], Mariela Fernanda Gomez[14], Maria Manuela Clavijo[15], Miguel Lopez[16], Edgar Zapata[17], Ignacio Dominguez[17], Mariana Pereira[18], Nicolás Rodríguez[19], Gonzalo Guerrero[20], Alicia Enrico[21], Leticia Rapan[7], María Castro[4], Alberto Gimenez Conca[22], Maria Marta Rivas[23], Marcela Espinoza[9], Carolina Belli[24], Luciana Ferrari*[25]

*[1]GSK, Buenos Aires, Argentina; [2]Hospital Interzonal General de Agudos "General San Martín", La Plata, Argentina; [3]FUNDALEU, CABA, Argentina; [4]Hospital privado de Cordoba, Córdoba, Argentina; [5]Hospital escuela de agudos Dr. Ramon Madariaga, Posadas, Argentina; [6]Hospital Italiano de Buenos Aires, Buenos Aires, Argentina; [7]Sanatorio Sagrado Corazon, CABA, Argentina; [8]Hospital Interzonal General de Agudos "General San Martín", La plata, Argentina; [9]Hospital Austral, Pilar, Argentina; [10]Hospital Nacional Alejandro Posadas, Buenos Aires, Argentina; [11]Instituto conciencia, Neuquen, Argentina; [12]Hospital Britanico de buenos aires, Buenos Aires, Argentina; [13]Sanatorio Anchorena, Buenos Aires, Argentina; [14]Hospital de clínicas Jose de San Martín, Buenos aires, Argentina; [15]Hospital aleman, buenos aires, Argentina; [16]Fundacion arturo lopez perez, chile, Chile; [17]Hospital Dr sotero Del rio: Ignacio Dominguez, Santiago, Chile; [18]sanatorio de la mujer, Rosario, Argentina; [19]Hospital Barros Luco Trudeau, Santiago, Chile; [20]Clinica Red salud Vitacura, santiago, Chile; [21]Hospital italiano de La Plata, La Plata, Argentina; [22]Hospital Italiano de buenos aires, Buenos aires, Argentina; [23]Hospital universitario austral, Pilar, Argentina; [24]Conicet, buenos aires, Argentina; [25]FUNDALEU, Buenos Aires, Argentina;*

### Background

Pediatric-inspired protocols including asparaginase (ASP) improve outcomes in adolescents and young adults (AYA) with ALL. However, toxicity limits ASP use in older patients. Data on ASP safety in South America are scarce and needed to optimize real-world use.

### Aims

To assess ASP utilization, treatment-related adverse events, and their impact on outcomes.

### Methods

This retrospective study included patients (pts) ≥15 years treated with first-line protocols with or without ASP (2014-2025) at 28 institutions in Argentina (AR) and Chile (CH). Frequencies, contingency tables (Chi-square/Fisher), logistic regression, overall survival (OS) and event-free survival (EFS; events: refractory disease, relapse, death; Kaplan–Meier/Log-rank/Cox) were analyzed using SPSS v25.

**Copyright Information:** (Online) ISSN: 2572-9241
©2026 The Author(s). HemaSphere is published by John Wiley & Sons Ltd on behalf of European Hematology Association. This is an open access Abstract Book distributed under the Attribution-NonCommercial-NoDerivs (CC BY-NC-ND), which allows third parties to download the articles and share them with others as long as they credit the author and the Abstract Book, but they cannot change the content in any way or use them commercially.
**Abstract Book Citations:** Authors, Title, HemaSphere, 2026;10:(S1):pages. The abstract book DOIs can be found on the "Author Information" page.
**Disclaimer:** Articles published in the journal HemaSphere exclusively reflect the opinions of the authors. The authors are responsible for all content in their abstracts including accuracy of the facts, statements, citing resources, etc.

**Results**

A total of 409 patients were included (79%-AR, 21%-CH). Sex distribution was similar (53% male), with predominance of private institutions (61%) and B-cell phenotype (77%). Median (Mdn) age was 27 years (15–86): 74% AYA, 21% adults, 5% elderly. Overall, 89% (365) received ASP (278 AYA, 76 adults, 7 elderly; 4 age unknown). Formulations were L-ASP 41%, PEG-ASP 50%, L-ASP+PEG-ASP 5%, L-ASP+Erwinia 1%, PEG-ASP+Erwinia 2%, and all formulations 1%. Sixty-five percent received >60% of the planned dose. Pre-existing liver disease was present in 8%, and obesity (BMI ≥30) in 15%. Among ASP-treated patients, 92% received BFM-type protocols, 1% Hyper-CVAD, 7% unknown.
Among ASP-exposed patients, hypersensitivity (HS) occurred in 10%; hypofibrinogenemia in 54% (13% grade ≥3). Thrombosis occurred in 16% (12% CNS) and increased with cryoprecipitate administration (27% vs 11%, p<0.001), without impact of the thromboprophylaxis. Hyperbilirubinemia occurred in 41% (11% grade ≥3) and transaminitis in 50% (15% grade ≥3). Pre-existing liver disease increased grade ≥3 hyperbilirubinemia (44% vs 6%) and transaminitis (54% vs 13%) (both p<0.001). Obesity (BMI≥30) was not associated with grade ≥3 hepatotoxicity. Hypertriglyceridemia (25%), clinic pancreatitis (2%) and hypoglycemia (10%) showed no significant associations.
By ASP formulation (Table 1), thrombosis, hypofibrinogenemia, hyperbilirubinemia, transaminitis, hypertriglyceridemia, pancreatitis and hypoglycemia were more frequent with PEG-ASP. Multivariate analysis confirmed higher risk of hyperbilirubinemia (OR1.8, p=0.044) and hypoglycemia (OR6.4, p=0.014) with PEG-ASP. HS was more frequent with L-ASP.
Median follow-up in ASP-exposed patients was 18 months (1-201). Median OS/EFS were NR/55 months in AYA, 38/24 months in adults. Grade ≥3 hyperbilirubinemia was associated with worse OS (Mdn NR vs 18 months, HR 2.1, p=0.004) and EFS (Mdn 54 vs 18 months, HR 1.8, p=0.014). No differences were observed according to ASP formulation, obesity, pre-existing liver disease or other toxicities.

**Summary/Conclusion**

ASP was feasible in this large South American cohort. Cryoprecipitate use increased thrombosis risk, and pre-existing liver disease increased grade ≥3 hepatotoxicity. PEG-ASP was associated with higher rates of transaminitis, hepatotoxicity and hypoglycemia, whereas L-ASP with HS. Toxicities were manageable and only grade ≥3 hyperbilirubinemia adversely affected outcomes. These real-world data support optimization of ASP use in adult ALL.

**Copyright Information:** (Online) ISSN: 2572-9241
©2026 The Author(s). HemaSphere is published by John Wiley & Sons Ltd on behalf of European Hematology Association. This is an open access Abstract Book distributed under the Attribution-NonCommercial-NoDerivs (CC BY-NC-ND), which allows third parties to download the articles and share them with others as long as they credit the author and the Abstract Book, but they cannot change the content in any way or use them commercially.
**Abstract Book Citations:** Authors, Title, HemaSphere, 2026;10:(S1):pages. The abstract book DOIs can be found on the “Author Information” page.
**Disclaimer:** Articles published in the journal HemaSphere exclusively reflect the opinions of the authors. The authors are responsible for all content in their abstracts including accuracy of the facts, statements, citing resources, etc.

| | N exposed | PEG-ASP % (event/N) | NO PEG ASP % (event/N) | P |
|---|---|---|---|---|
| **Hypersensitivity** | 357 | 6 (11/184) | 14 (25/173) | **0,008** |
| **Hypofibrinogenemia** | 357 | 65 (134/207) | 40 (60/150) | **<0,001** |
| **Thrombosis** | 358 | 20 (41/208) | 11(16/150) | **0,027** |
| **Hyperbilirrubinemia** | 355 | 52 (107/206) | 24 (36/149) | **<0,001** |
| **Transaminitis** | 314 | 54 (114/204) | 37 (41/110) | **0,002** |
| **Hypertriglyceridemia** | 347 | 34 (69/200) | 14 (21/147) | **<0,001** |
| **Clinic Pancreatitis** | 357 | 3 (6/207) | - (-/150) | **0,042** |
| **Hypoglucemia** | 351 | 16 (32/206) | 2 (3/145) | **<0,001** |

**Copyright Information:** (Online) ISSN: 2572-9241
©2026 The Author(s). HemaSphere is published by John Wiley & Sons Ltd on behalf of European Hematology Association. This is an open access Abstract Book distributed under the Attribution-NonCommercial-NoDerivs (CC BY-NC-ND), which allows third parties to download the articles and share them with others as long as they credit the author and the Abstract Book, but they cannot change the content in any way or use them commercially.
**Abstract Book Citations:** Authors, Title, HemaSphere, 2026;10:(S1):pages. The abstract book DOIs can be found on the "Author Information" page.
**Disclaimer:** Articles published in the journal HemaSphere exclusively reflect the opinions of the authors. The authors are responsible for all content in their abstracts including accuracy of the facts, statements, citing resources, etc.

### (PB2607) BLINATUMOMAB USE IN PAEDIATRIC ACUTE LYMPHOBLASTIC LEUKAEMIA AND INCIDENCE OF SECONDARY HYPOGAMMAGLOBULINAEMIA REQUIRING IVIG REPLACEMENT: A SINGLE-CENTRE EXPERIENCE

**Topic:** 02. Acute lymphoblastic leukemia - Clinical

Sai Lon Wann*[1], Thomas McGrath[1], Arunthethy Mahendrayogam[1], Michael Gattens[1], Emmy Dickens[1]

[1]*Cambridge University Hospitals NHS Foundation Trust, Department of Haematology, Cambridge, United Kingdom;*

### Background

Blinatumomab is a bispecific T-cell engager (BiTE) targeting CD19-positive B cells and is increasingly used in paediatric B-cell acute lymphoblastic leukaemia (B-ALL), particularly in measurable residual disease (MRD)-positive and relapsed/refractory settings. Sustained B-cell aplasia following Blinatumomab may result in secondary hypogammaglobulinaemia and increased risk or severity of infections.

### Aims

To evaluate paediatric ALL patients treated with blinatumomab in our centre, assessing toxicity, disease response, post-treatment IgG levels, infection frequency, requirement for IVIG replacement, and associated complications.

### Methods

We conducted a retrospective review of 23 children with B-ALL diagnosed between 2015–2025 and treated with blinatumomab between 2020–2025 at Cambridge University Hospitals. Data collected included demographics, risk stratification, treatment protocol, MRD status, IgG levels, infectious complications, IVIG administration, and treatment-related toxicities. IgG levels were recorded at baseline and serially monitored following therapy. Neutrophil nadir, cytokine release syndrome (CRS), and immune effector cell-associated neurotoxicity syndrome (ICANS) were documented.

### Results

23 patients (9 male, 14 female) had a median age of 6.5 years (range 0–15). 13 were high-risk; 2 had Philadelphia chromosome positive ALL and 2 had Trisomy 21. Fourteen received blinatumomab during first-line therapy and nine in second-line treatment. Induction regimens included ALLTogether1 (n=11), UKALL 2011 (n=5), UKALL 2019 (n=4), Children's Cancer and Leukaemia Group protocol (n=3).

Twelve patients experienced no CRS while ten developed mild CRS (7 Grade 1, 3 Grade 2). One patient developed Grade 3 ICANS with seizure requiring treatment discontinuation and PICU admission. Eleven patients developed neutropenia ($<1.0 \times 10^9$/L), with a median nadir of $0.6 \times 10^9$/L occurring at a median of 5 days after initiation.

Twenty patients were MRD-positive prior to blinatumomab. After cycle 1, four remained MRD-positive. Sixteen received a second cycle, with only one remaining MRD-positive thereafter.

Median baseline IgG was 8.28 g/L (range 3.7–14.45). Median post-treatment IgG nadir was 2.43 g/L (1.4–11.53), occurring at a median of 230 days from first exposure to blinatumomab. Two patients (8.7%) required IVIG replacement for severe or recurrent infections: one following severe Klebsiella infection and one with multiple infections (Enterococcus UTI, Staphylococcus epidermidis with Abiotrophia defectiva bacteraemia, Moraxella osloensis bacteraemia, and Escherichia coli UTI). One mild IVIG-related reaction occurred.

Four patients underwent allogeneic transplantation, two received CAR-T therapy, and one received CAR-T followed by transplantation. Five additional patients received IVIG following CAR-T therapy or allogeneic. With a median follow-up of 35 months (range 7–126), all patients remain in remission.

**Copyright Information:** (Online) ISSN: 2572-9241
©2026 The Author(s). HemaSphere is published by John Wiley & Sons Ltd on behalf of European Hematology Association. This is an open access Abstract Book distributed under the Attribution-NonCommercial-NoDerivs (CC BY-NC-ND), which allows third parties to download the articles and share them with others as long as they credit the author and the Abstract Book, but they cannot change the content in any way or use them commercially.
**Abstract Book Citations:** Authors, Title, HemaSphere, 2026;10:(S1):pages. The abstract book DOIs can be found on the "Author Information" page.
**Disclaimer:** Articles published in the journal HemaSphere exclusively reflect the opinions of the authors. The authors are responsible for all content in their abstracts including accuracy of the facts, statements, citing resources, etc.

**Summary/Conclusion**

Blinatumomab was associated with significant reductions in IgG levels; however, clinically relevant secondary hypogammaglobulinaemia requiring IVIG was uncommon. Routine prophylactic IVIG does not appear necessary, but regular IgG monitoring and individualised replacement based on infection history and immune recovery are recommended.

**Copyright Information:** (Online) ISSN: 2572-9241
©2026 The Author(s). HemaSphere is published by John Wiley & Sons Ltd on behalf of European Hematology Association. This is an open access Abstract Book distributed under the Attribution-NonCommercial-NoDerivs (CC BY-NC-ND), which allows third parties to download the articles and share them with others as long as they credit the author and the Abstract Book, but they cannot change the content in any way or use them commercially.
**Abstract Book Citations:** Authors, Title, HemaSphere, 2026;10:(S1):pages. The abstract book DOIs can be found on the “Author Information” page.
**Disclaimer:** Articles published in the journal HemaSphere exclusively reflect the opinions of the authors. The authors are responsible for all content in their abstracts including accuracy of the facts, statements, citing resources, etc.

### (PB2608) EFFICACY AND SAFETY OF VENETOCLAX COMBINED WITH CHEMOTHERAPY IN THE TREATMENT OF NEWLY DIAGNOSED CORE-BINDING FACTOR-RELATED ACUTE MYELOID LEUKEMIA

**Topic:** 02. Acute lymphoblastic leukemia - Clinical

Congcong Zhang*[1], Suo Xiaohui[1], Xiaohui Zhang[1], Kaiqi Liu[2], Xiaohan Zhang[1]

*[1]Handan Central Hospital, Handan, Hebei, China;[2]Institute of Hematology & Blood Diseases Hospital, Chinese Academy of Medical Sciences & Peking Union Medical College, Tianjin, China;*

### Background

Core-binding factor acute myeloid leukemia (CBF-AML) is a subtype of AML defined by specific chromosomal translocations, including t(8;21)(q22;q22)/RUNX1::RUNX1T1 and inv(16)(p13q22) or t(16;16)(p13;q22)/CBFB::MYH11.CBF-AML is sensitive to conventional induction chemotherapy and classified into the favorable prognostic risk group, with a 3-year overall survival (OS) rate of 60%-70%. Despite its better overall prognosis compared to other AML subtypes, the 5-year relapse rate was still reaches 30%-40%. In recent years, with the application of several targeted agents, the efficacy of newly diagnosed AML has been significantly improved. Venetoclax (VEN), a small-molecule BCL-2 inhibitor, in combination with hypomethylating agents, has significantly improved the efficacy and survival rates in elderly or unfit patients newly diagnosed with AML. VEN combined with chemotherapy (DA, HAA, HAD regimens) has significantly increased the complete remission (CR) rate and measurable residual disease (MRD) negativity rate after one cycle of induction therapy. However, the efficacy of this combination in CBF-AML has not been reported. This retrospective study analyzed the clinical data of CBF-AML patients treated with VEN combined with chemotherapy, aiming to provide clinical evidence for optimizing the individualized treatment strategies for these patients.

### Aims

To evaluate the efficacy and safety of venetoclax (VEN) in combination with chemotherapy as induction therapy in patients with newly diagnosed core-binding factor acute myeloid leukemia (CBF-AML).

### Methods

We conducted a retrospective study of 60 consecutive patients with newly diagnosed CBF-AML who received VEN combined with chemotherapy as induction therapy. Baseline characteristics, genetic mutations, qPCR-measurable residual disease (MRD) levels, treatment response, survival outcomes, and adverse events were collected. Survival analysis was performed using the Kaplan-Meier method.

### Results

All patients were treated with VEN combined with DA, HAA, or HAD induction regimens. The overall response rate (ORR) was 95.00% with the complete remission (CR) rate was 93.33% after one cycle treatment. No significant differences were observed between the RUNX1::RUNX1T1 and CBFB::MYH11 subtypes, or among the three chemotherapy backbone regimens ($P_1$= 0.226; $P_2$= 0.604). As of November 2025, with a median follow-up of 22.5(4-43) months the estimated 2-year overall survival (OS), event-free survival (EFS), and relapse-free survival (RFS) rates were 86.67%, 80.00%, and 90.00%, respectively. The most frequent adverse events were grade Ⅳ myelosuppression (100%) and infection (85.00%), with no early treatment-related deaths. No patient deaths occurred during the induction therapy.

**Copyright Information:** (Online) ISSN: 2572-9241
©2026 The Author(s). HemaSphere is published by John Wiley & Sons Ltd on behalf of European Hematology Association. This is an open access Abstract Book distributed under the Attribution-NonCommercial-NoDerivs (CC BY-NC-ND), which allows third parties to download the articles and share them with others as long as they credit the author and the Abstract Book, but they cannot change the content in any way or use them commercially.
**Abstract Book Citations:** Authors, Title, HemaSphere, 2026;10:(S1):pages. The abstract book DOIs can be found on the "Author Information" page.
**Disclaimer:** Articles published in the journal HemaSphere exclusively reflect the opinions of the authors. The authors are responsible for all content in their abstracts including accuracy of the facts, statements, citing resources, etc.

**Summary/Conclusion**

Venetoclax combined with chemotherapy is a high efficacy and safty induction therapy in patients with newly diagnosed CBF-AML.

**Copyright Information:** (Online) ISSN: 2572-9241
©2026 The Author(s). HemaSphere is published by John Wiley & Sons Ltd on behalf of European Hematology Association. This is an open access Abstract Book distributed under the Attribution-NonCommercial-NoDerivs (CC BY-NC-ND), which allows third parties to download the articles and share them with others as long as they credit the author and the Abstract Book, but they cannot change the content in any way or use them commercially.
**Abstract Book Citations:** Authors, Title, HemaSphere, 2026;10:(S1):pages. The abstract book DOIs can be found on the "Author Information" page.
**Disclaimer:** Articles published in the journal HemaSphere exclusively reflect the opinions of the authors. The authors are responsible for all content in their abstracts including accuracy of the facts, statements, citing resources, etc.

### (PB2609) EFFICACY OF MINI-DOSED BLINATUMOMAB IN MINIMAL RESIDUAL DISEASE-POSITIVE ACUTE LYMPHOBLASTIC LEUKEMIA

**Topic:** 02. Acute lymphoblastic leukemia - Clinical

Xiao Wang*[1], Xu-Pai Zhang[1, 1], Guangcui He[1], Shan Zhang[1], Yan Deng[1], Lei MA[1], Hai Yi[1]

[1] *The General Hospital of Western Theater Command, Chengdu, China;*

### Background

Blinatumomab has emerged as a pivotal therapeutic modality in the management of relapsed/refractory B-cell acute lymphoblastic leukemia (R/R B-ALL). However, standard-dose regimens are frequently associated with substantial risks, including cytokine release syndrome (CRS), neurotoxicity, and infectious complications due to prolonged B-cell aplasia. For patients with R/R B-ALL and low tumor burden—defined as bone marrow blasts below 10% or minimal residual disease (MRD)-positive status—evidence-based guidance remains limited regarding how to optimize treatment intensity to reduce toxicity while preserving clinical efficacy. In addition, the high cost of standard protocols poses a significant barrier to broader implementation in certain patient populations.

### Aims

To investigate the safety and efficacy of mini-dosed blinatumomab in the treatment of minimal residual disease-positive R/R B-ALL.

### Methods

We performed a retrospective analysis of patients diagnosed with low-tumor-burden R/R B-ALL who received mini-dosed blinatumomab therapy at our institution between August 2021 and January 2025. The treatment protocol involved continuous intravenous infusion of blinatumomab at an initial dose of 8.75 μg/day for four days, followed by a maintenance dose of 28 μg/day thereafter. Therapy was limited to a maximum cumulative dose of 175 μg, equivalent to five vials.

### Results

A total of 15 patients were enrolled, comprising 8 females and 7 males, with ages ranging from 18 to 48 years. Among them, 10 patients had no prior history of hematopoietic stem cell transplantation (HSCT), while the remaining 5 had undergone previous transplantation. Baseline bone marrow tumor burden ranged from 0.1% to 10.0%. During treatment, 11 patients received 4 vials of blinatumomab, 3 patients received 5 vials, and 1 patient received 3 vials.
With regard to safety, treatment-emergent adverse events included pyrexia of varying severity in 12 patients, elevated transaminases in 4 patients, and altered mental status in 1 patient. All events resolved following appropriate supportive care. In terms of efficacy, post-treatment bone marrow assessment revealed minimal residual disease (MRD) negativity in 14 patients. One patient without prior transplantation exhibited primary treatment failure, followed by rapid disease progression and death.
Regarding subsequent therapy, all 9 transplant-naïve patients who achieved MRD negativity proceeded to allogeneic HSCT as consolidation. Among the 5 patients with a history of transplantation, 1 underwent a second allogeneic HSCT. The 1-year overall survival rate was 60.0%. Of the 9 patients who underwent bridging transplantation, 7 remained alive. Among the 5 previously transplanted patients, only the individual who received a second transplant survived, with one additional survivor among the remaining four. All surviving patients remained relapse-free at the last follow-up.

**Copyright Information:** (Online) ISSN: 2572-9241
©2026 The Author(s). HemaSphere is published by John Wiley & Sons Ltd on behalf of European Hematology Association. This is an open access Abstract Book distributed under the Attribution-NonCommercial-NoDerivs (CC BY-NC-ND), which allows third parties to download the articles and share them with others as long as they credit the author and the Abstract Book, but they cannot change the content in any way or use them commercially.
**Abstract Book Citations:** Authors, Title, HemaSphere, 2026;10:(S1):pages. The abstract book DOIs can be found on the "Author Information" page.
**Disclaimer:** Articles published in the journal HemaSphere exclusively reflect the opinions of the authors. The authors are responsible for all content in their abstracts including accuracy of the facts, statements, citing resources, etc.

**Summary/Conclusion**

Mini-dosed blinatumomab exhibits a favorable safety profile and demonstrates promising efficacy in patients with minimal residual disease-positive R/R B-ALL. However, the durability of these responses requires further validation in studies with larger cohorts.

**Copyright Information:** (Online) ISSN: 2572-9241
©2026 The Author(s). HemaSphere is published by John Wiley & Sons Ltd on behalf of European Hematology Association. This is an open access Abstract Book distributed under the Attribution-NonCommercial-NoDerivs (CC BY-NC-ND), which allows third parties to download the articles and share them with others as long as they credit the author and the Abstract Book, but they cannot change the content in any way or use them commercially.
**Abstract Book Citations:** Authors, Title, HemaSphere, 2026;10:(S1):pages. The abstract book DOIs can be found on the "Author Information" page.
**Disclaimer:** Articles published in the journal HemaSphere exclusively reflect the opinions of the authors. The authors are responsible for all content in their abstracts including accuracy of the facts, statements, citing resources, etc.

### (PB2610) REAL-WORLD TREATMENT PATTERNS AND OUTCOMES WITH OLUTASIDENIB AFTER VENETOCLAX IN IDH1-MUTATED AML USING EHR DATA

**Topic:** 02. Acute lymphoblastic leukemia - Clinical

Catherine Lai*[1], Pinkal Desai[2], Jorge Cortes[3], Tim Werwath[4], Amber Thomassen[5], Aaron Sheppard[5], Yasmin Abaza[6]

*[1]University of Pennsylvania, Division of Hematology & Oncology, Abramson Cancer Center, Philadelphia, United States of America; [2]Weill Cornell Medicine, Department of Medicine, Hematology/Oncology, New York, United States of America;[3]Georgia Cancer Center at Augusta University, Augusta, United States of America; [4]Putnam Associates, New York, United States of America; [5]Rigel Pharmaceuticals, Inc., South San Francisco, United States of America;[6]Northwestern University, Division of Hematology and Oncology, Robert H. Lurie Comprehensive Cancer Center, Feinberg School of Medicine, Chicago, United States of America;*

### Background

Venetoclax plus a hypomethylating agent (HMA) is a standard front-line therapy for patients with AML ineligible for intensive chemotherapy; however, most patients will ultimately relapse. Olutasidenib, a selective oral inhibitor of mutated *IDH1* (m*IDH1*), is approved for relapsed/refractory (R/R) m*IDH1* AML. In the pivotal Phase 2 trial, olutasidenib achieved CR/CRh in 35% of patients (median CR/CRh duration 25.3 months) with acceptable tolerability. Real-world data on olutasidenib utilization and outcomes in practice are limited.

### Aims

To describe real-world treatment sequencing and clinical outcomes (response, duration of response, and overall survival) among patients with m*IDH1* R/R AML treated with olutasidenib after venetoclax-based therapy using an EHR database.

### Methods

This retrospective cohort study used Loopback Analytics' EHR database (Oct 2016 to Oct 2025), incorporating structured and unstructured data. Eligible patients were ≥18 years old with confirmed m*IDH1* R/R AML, a prior venetoclax regimen, ≥25 days of olutasidenib, and a documented response assessment.
Treatment response was categorized as CR, CRh, CRi, or MLFS. Baseline demographics, treatment sequences, and clinical outcomes were summarized using descriptive statistics. Overall Survival (OS) and duration of response (DoR) were estimated using Kaplan-Meier methods.

**Copyright Information:** (Online) ISSN: 2572-9241
©2026 The Author(s). HemaSphere is published by John Wiley & Sons Ltd on behalf of European Hematology Association. This is an open access Abstract Book distributed under the Attribution-NonCommercial-NoDerivs (CC BY-NC-ND), which allows third parties to download the articles and share them with others as long as they credit the author and the Abstract Book, but they cannot change the content in any way or use them commercially.
**Abstract Book Citations:** Authors, Title, HemaSphere, 2026;10:(S1):pages. The abstract book DOIs can be found on the "Author Information" page.
**Disclaimer:** Articles published in the journal HemaSphere exclusively reflect the opinions of the authors. The authors are responsible for all content in their abstracts including accuracy of the facts, statements, citing resources, etc.

**Results**

Twenty-four olutasidenib-treated patients met the inclusion criteria. Median age at olutasidenib initiation was 67 years, and 63% of patients were male. Four patients (17%) received a hematopoietic stem cell transplant (HSCT) prior to olutasidenib. Most patients (75%) presented with at least one co-occurring mutation; most commonly FLT3 (33%), NPM1 (29%), and RUNX1 (21%). Median duration of olutasidenib treatment was 4.0 months (IQR 1.7-6.5 months) and olutasidenib was administered as monotherapy in 54% of patients. Over half (58%) of patients received venetoclax as front-line therapy, primarily with HMA. Olutasidenib was second-line therapy in 42% of cases and immediately followed venetoclax in 83% of cases. The median number of prior lines was 2. The most common reason for olutasidenib discontinuation was disease progression.
The ORR was 58% (4 CR, 5 CRi, 1 CRh, and 4 MLFS) with a CRc rate of 42%. Among patients who achieved any response, 10 (71%) received olutasidenib as the next line following venetoclax. Among patients with front-line venetoclax and second-line olutasidenib, the ORR was 60% and the CRc rate was 50%.
Median duration of CRc was 11.9 months (n=8 with evaluable DoR) with 2 having ongoing response at data cutoff and 2 having ongoing response at HSCT. In the overall population, four patients went to HSCT. Median OS from olutasidenib initiation was 11.6 months, with the proportion of patients surviving 6, 9, and 12 months estimated to be 80%, 72%, and 43%, respectively.

**Summary/Conclusion**

This study offers real-world insights into olutasidenib treatment and outcomes in post-venetoclax m*IDH1* R/R AML patients. Olutasidenib was used as monotherapy in half of patients, often directly following venetoclax. In this cohort, 58% of patients responded with 42% achieving CRc, consistent with efficacy observed in the subset of post-venetoclax patients from the pivotal Phase 2 trial. These findings support olutasidenib as a viable post-venetoclax option, although additional data are needed.

© 2026 American Society of Clinical Oncology, Inc. Reused with permission. This abstract was accepted and previously presented at the 2026 ASCO Annual Meeting. All rights reserved.

**Copyright Information:** (Online) ISSN: 2572-9241
©2026 The Author(s). HemaSphere is published by John Wiley & Sons Ltd on behalf of European Hematology Association. This is an open access Abstract Book distributed under the Attribution-NonCommercial-NoDerivs (CC BY-NC-ND), which allows third parties to download the articles and share them with others as long as they credit the author and the Abstract Book, but they cannot change the content in any way or use them commercially.
**Abstract Book Citations:** Authors, Title, HemaSphere, 2026;10:(S1):pages. The abstract book DOIs can be found on the “Author Information” page.
**Disclaimer:** Articles published in the journal HemaSphere exclusively reflect the opinions of the authors. The authors are responsible for all content in their abstracts including accuracy of the facts, statements, citing resources, etc.

### (PB2612) FACTORS IMPACTING PREFERRED FIRST-LINE TREATMENTS (TX) FOR ADOLESCENT AND YOUNG ADULT (AYA) PATIENTS (PTS) WITH ACUTE LYMPHOBLASTIC LEUKEMIA (ALL)/LYMPHOBLASTIC LYMPHOMA (LBL): AN ONLINE SURVEY

**Topic:** 02. Acute lymphoblastic leukemia - Clinical

Melisa Stricherz[1], Marlise Luskin[2], Amy Nguyen[1], Carl Mdibmun[1], Kristin Moore[1], Maryam Salehi[3], Rajkumar Manimaran[3], Andrew Hantel*[2]

*[1]Jazz Pharmaceuticals, Palo Alto, CA, United States of America; [2]Dana-Farber Cancer Institute, Harvard, Brookline, MA, United States of America; [3]Aptitude Health, Atlanta, GA, United States of America;*

### Background

Advances in pediatric ALL/LBL have led to a >90% 5-year (yr) overall survival (OS). Discordantly inferior OS for AYA pts aged ≥18 yrs suggests that Tx approaches may play a role in improved outcomes. NCCN/ASH guidelines advise asparaginase-containing pediatric-inspired regimens (PIRs) for AYA pts with ALL/LBL (Muffly; *JCO Oncol Pract;* 2023).

### Aims

We explored factors affecting US-based physicians' Tx choices for AYA pts with newly diagnosed Philadelphia negative (Ph-) ALL/LBL across care settings.

### Methods

A cross-sectional online survey (41 questions) was sent to 1805 randomly selected practicing oncologists in the US Axess Network, September–November 2024. Respondents had to be a medical/pediatric hematologist/oncologist treating pts with hematologic malignancies in the past yr. Respondents indicating they treated AYA pts were asked their preferred Tx for Ph- ALL/LBL AYA pts. Primary comparator groups were based on respondent's preference for using PIRs vs hyper-fractionated cyclophosphamide, vincristine, doxorubicin, and dexamethasone (hyper-CVAD) regimens. Unadjusted *P*-values <0.05 were considered statistically significant.

### Results

Of 105 respondents who completed the survey and were included in this analysis, 71 preferred a PIR (68%) and 34 (32%) preferred a hyper-CVAD regimen. Both groups were high-volume clinicians (~60% treated >200 pts/yr with varied solid and hematologic malignancies). A higher percentage of the PIR group than the hyper-CVAD group (30% vs 12%; *P*=0.04) reported that pts with hematologic malignancies comprised ≥75% of their overall pt population. A higher percentage of the hyper-CVAD group than the PIR group (21% vs 6%; *P*=0.04) practiced at a facility unable to administer PIRs. A larger percentage of the PIR group than the hyper-CVAD group (84% vs 65%; *P*=0.03) had access to a pediatric hematologist/oncologist. Avoiding hematopoietic stem cell transplant (HCT) until clinically necessary was rated higher in the PIR group (mean rating 6.86 out of 10 in a Likert-style format, with 10 being the most important) compared with the hyper-CVAD group (5.62/10; *P*=0.02); obtaining remission and referring pts to HCT was rated higher in the hyper-CVAD group (8.35/10) than the PIR group (7.17/10; *P*=0.005). Patient obesity did not differentially influence the choice of Tx regimen between groups (PIR, 5.45/10; hyper-CVAD, 5.08/10; *P*=0.53). The highest rated external factor influencing Tx choice was NCCN guidance for both groups (PIR, 8.03/10; hyper-CVAD, 8.26/10; *P*=0.49). In the hyper-CVAD group, toxicity concerns were the primary reason for not using a PIR. The hyper-CVAD group was more likely than the PIR group (38% vs 6%; *P*=0.03) to indicate that they would change their preferred therapy if toxicity was not a barrier.

**Copyright Information:** (Online) ISSN: 2572-9241
©2026 The Author(s). HemaSphere is published by John Wiley & Sons Ltd on behalf of European Hematology Association. This is an open access Abstract Book distributed under the Attribution-NonCommercial-NoDerivs (CC BY-NC-ND), which allows third parties to download the articles and share them with others as long as they credit the author and the Abstract Book, but they cannot change the content in any way or use them commercially.
**Abstract Book Citations:** Authors, Title, HemaSphere, 2026;10:(S1):pages. The abstract book DOIs can be found on the "Author Information" page.
**Disclaimer:** Articles published in the journal HemaSphere exclusively reflect the opinions of the authors. The authors are responsible for all content in their abstracts including accuracy of the facts, statements, citing resources, etc.

**Summary/Conclusion**

Despite NCCN/ASH guidance recommending the use of PIRs in AYAs with ALL/LBL, and physician respondents reporting that such guidance was the most influential factor during Tx decision-making, approximately one-third did not prefer PIRs. Opportunities to increase appropriate PIR use include improving access to pediatric consultation and to treaters experienced with hematologic malignancies, addressing toxicity concerns, and strengthening facility capabilities.

**Copyright Information:** (Online) ISSN: 2572-9241
©2026 The Author(s). HemaSphere is published by John Wiley & Sons Ltd on behalf of European Hematology Association. This is an open access Abstract Book distributed under the Attribution-NonCommercial-NoDerivs (CC BY-NC-ND), which allows third parties to download the articles and share them with others as long as they credit the author and the Abstract Book, but they cannot change the content in any way or use them commercially.
**Abstract Book Citations:** Authors, Title, HemaSphere, 2026;10:(S1):pages. The abstract book DOIs can be found on the “Author Information” page.
**Disclaimer:** Articles published in the journal HemaSphere exclusively reflect the opinions of the authors. The authors are responsible for all content in their abstracts including accuracy of the facts, statements, citing resources, etc.

### (PB2613) EFFICACY AND SAFETY OF INOTUZUMAB OZOGAMICIN IN ADULT CHINESE PATIENTS WITH RELAPSED OR REFRACTORY ACUTE LYMPHOBLASTIC LEUKEMIA: LONG-TERM RESULTS OF AN OPEN-LABEL, MULTI-CENTER, PHASE 4 TRIAL.

**Topic:** 02. Acute lymphoblastic leukemia - Clinical

Depei Wu*[1], Ying Wang[2], Yue Han[1], Shaoyuan Wang[3], Sujun Gao[4], Jie Jin[5], Xudong Wei[6], Hongsheng Zhou[7], Tiejun Gong[8], Mei Hong[9], Xiao Shuai[10], Shunqing Wang[11], Wei Huang[12], Hongmei Jing[13], Yang Liang[14], Erik Vandendries[15], Yang Dang[16], Yuankai Zhang[17], Guo Li[17], Zhijian Shen[18]

*[1]The First Affiliated Hospital of Soochow University, Suzhou, China;[2]Chinese Academy of Medical Sciences, Institute of Hematology and Blood Diseases Hospital, Tianjin, China; [3]Fujian Medical University Union Hospital, Fuzhou, China;[4]The First Hospital of Jilin University, Changchun, China; [5]The First Affiliated Hospital, Zhejiang University School of Medicine, Hangzhou, China; [6]Henan Cancer Hospital, Zhengzhou, China;[7]Nanfang Hospital of Southern Medical University, Guangzhou, China;[8]The First Hospital of Harbin, Harbin, China; [9]Union Hospital, Tongji Medical College of Huazhong University of Science and Technology, Wuhan, China; [10]West China Hospital of Sichuan University, Chengdu, China; [11]Guangzhou First People's Hospital, Guangzhou, China; [12]Tongji Hospital, Tongji Medical College of Huazhong University of Science and Technology, Wuhan, China; [13]Peking University Third Hospital, Beijing, China; [14]Sun Yat-Sen University Cancer Center, Guangzhou, China; [15]Pfizer Inc., Cambridge, United States of America; [16]Pfizer Inc., Beijing, China; [17]Pfizer Inc., Shanghai, China; [18]The First Affiliated Hospital of Wenzhou Medical University, Wenzhou, China;*

### Background

Inotuzumab ozogamicin (InO), a CD22-directed antibody-drug conjugate, is approved to treat adult patients (pts) with relapsed/refractory B-cell precursor acute lymphoblastic leukemia (R/R B-ALL). This postapproval commitment study of InO in pts with R/R B-ALL in China previously demonstrated a high rate of complete response/remission (CR) or CR with incomplete hematologic recovery (CRi) and durable responses with a generally manageable safety profile that was consistent with results observed in previous global studies.

### Aims

To confirm long-term efficacy and safety of InO in pts with R/R B-ALL in China.

### Methods

This phase 4, open-label, single-arm, multicenter study enrolled adults (aged ≥18 years) from mainland China who had CD22-positive, Philadelphia chromosome (Ph)–positive (Ph+) or Ph-negative R/R B-ALL and were due to receive salvage 1 or 2 therapy. The primary endpoint was CR/CRi rate. Secondary endpoints included duration of remission (DoR), progression-free survival (PFS), overall survival (OS), and safety; MRD negativity and pharmacokinetics were previously reported. Pts received InO monotherapy; the InO dosages were consistent with the approved label. Informed consent was obtained.

**Copyright Information:** (Online) ISSN: 2572-9241
©2026 The Author(s). HemaSphere is published by John Wiley & Sons Ltd on behalf of European Hematology Association. This is an open access Abstract Book distributed under the Attribution-NonCommercial-NoDerivs (CC BY-NC-ND), which allows third parties to download the articles and share them with others as long as they credit the author and the Abstract Book, but they cannot change the content in any way or use them commercially.
**Abstract Book Citations:** Authors, Title, HemaSphere, 2026;10:(S1):pages. The abstract book DOIs can be found on the "Author Information" page.
**Disclaimer:** Articles published in the journal HemaSphere exclusively reflect the opinions of the authors. The authors are responsible for all content in their abstracts including accuracy of the facts, statements, citing resources, etc.

**Results**

In total, 44 pts were enrolled and received InO treatment (data cutoff: Nov 25, 2025). The median age was 45 (range, 18-73) years, 50.0% were female, 25.0% had Ph+ disease, and the median bone marrow blasts was 61.0% (range, 5%-95%); 52.3% received salvage 1 therapy and 47.7% received salvage 2 therapy; and 5 pts (11.4%) had hematopoietic stem cell transplantation (HSCT) prior to InO treatment (3 were autologous HSCT and 2 were allogenic HSCT). A total of 36 pts (81.8%; 95% CI, 70.4%-93.2%) achieved CR/CRi (CR, n=21; CRi, n=15), median time from first dose to first documented CR/CRi was 0.72 (range, 0.53-3.02) months, and median DoR was 13.4 months (95% CI, 5.1-23.0). Median PFS was 10.4 months (95% CI, 4.0-18.8); the estimated 2-year event-free rate was 24.9%. With a median OS follow-up of 23.9 months (95% CI, 23.7-24.0), median OS was 23.1 months (95% CI, 9.6-NE); the estimated 2-year survival rate was 48.8%. Ten of 12 pts who had HSCT post InO treatment were alive (post-HSCT survival, 1.1-21.5 months). Efficacy results in Ph+ pts were consistent with the overall population.
The most frequently (≥50%) reported all-causality adverse events (AEs) of any grade were neutropenia (95.5%), thrombocytopenia (95.5%), leukopenia (88.6%), lymphopenia (79.5%), anemia (70.5%), hypokalemia (61.4%), AST increased (54.5%), blood lactate dehydrogenase increased (52.3%), and blood bilirubin increased (50.0%). All-causality serious AEs of any grade reported in >1 pt were thrombocytopenia (13.6%), pneumonia (9.1%), disease progression (4.5%), and pyrexia (4.5%). Two pts (4.5%) developed sinusoidal obstruction syndrome; 1 with prior HSCT and without post-InO HSCT and 1 without prior or post HSCT. The overall safety profile was acceptable, and no new safety signals were identified since the prior analysis.

**Summary/Conclusion**

InO maintained a high rate of CR/CRi and continued to show durable response with a generally manageable safety profile in Chinese pts with R/R B-ALL. The efficacy and safety profile of InO in this Chinese population remained broadly consistent with those observed in previous global studies.

**Copyright Information:** (Online) ISSN: 2572-9241
©2026 The Author(s). HemaSphere is published by John Wiley & Sons Ltd on behalf of European Hematology Association. This is an open access Abstract Book distributed under the Attribution-NonCommercial-NoDerivs (CC BY-NC-ND), which allows third parties to download the articles and share them with others as long as they credit the author and the Abstract Book, but they cannot change the content in any way or use them commercially.
**Abstract Book Citations:** Authors, Title, HemaSphere, 2026;10:(S1):pages. The abstract book DOIs can be found on the "Author Information" page.
**Disclaimer:** Articles published in the journal HemaSphere exclusively reflect the opinions of the authors. The authors are responsible for all content in their abstracts including accuracy of the facts, statements, citing resources, etc.

### (PB2614) BLINATUMOMAB TREATMENT OUTCOMES IN CHILDHOOD ACUTE LYMPHOBLASTIC LEUKEMIA PATIENTS: THE DATA FROM TÜRKİYE

**Topic:** 02. Acute lymphoblastic leukemia - Clinical

Adalet Meral Güneş*[1], Baris Malbora[2], Mehmet Ertem[3], Nese Yarali[4], Deniz Yilmaz Karapinar[5], Suar Caki Kilic[6], Hale Ören[7], Ali Fettah[8], Fatih Erbey[9], Nurşah Eker[10], Zühre Kaya[11], Zeynep Ozdemir[12], Rejin Kebudi[13], Sultan Aydin[14], Aysegul Unuvar[13], Sinan Akbayram[15], Arzu Akyay[16], Tuğba Belgemen Özer[17]

*[1]Bursa Uludag University, Pediatric Hematology, Bursa, Türkiye;[2]Yenıyuzyıl University, Pediatric Hematology, Istanbul, Türkiye; [3]Ankara University, Pediatric Hematology, Ankara, Türkiye;[4]Ankara Yıldırım Beyazıt University, Pediatric Hematology, Ankara, Türkiye; [5]Ege University, Pediatric Hematology, İzmir, Türkiye; [6]Umraniye Training and Research Hospital, Pediatric Hematology, İstanbul, Türkiye; [7]Dokuz Eylul University, Pediatric Hematology, İzmir, Türkiye;[8]Etlik Training and Research Hospital, Pediatric Hematology, Ankara, Türkiye; [9]Koc University, Pediatric Hematology, İstanbul, Türkiye; [10]Marmara University, Pediatric Hematology, İstanbul, Türkiye; [11]Gazi University, Pediatric Hematology, Ankara, Türkiye; [12]Eskisehir Osmangazi University, Pediatric Hematology, Eskisehir, Türkiye; [13]İstanbul University, Pediatric Hematology, İstanbul, Türkiye;[14]Antalya Training and Research Hospital, Pediatric Hematology, Antalya, Türkiye; [15]Gaziantep Universtiy, Pediatric Hematology, Gaziantep, Türkiye; [16]Inonu University, Pediatric Hematology, Malatya, Türkiye; [17]Goztepe City Hospital, Pediatric Hematology, İstanbul, Türkiye;*

### Background

Acute lymphoblastic leukemia (ALL) is the most common type of leukemia in children. Significant progress has been made in the treatment of pediatric ALL, with long-term survival exceeding 85%. Nevertheless, approximately 15-20% of children with ALL experience relapse after standard first-line chemotherapy. Blinatumomab is a targeted therapy successfully used to achieve remission in these children. It is a bispecific T cell-binding (BiTE) antibody that induces an antitumor response by pairing CD19-positive malignant B cells with cytotoxic T lymphocytes. In clinical practice, it offers an effective option, particularly in infant B-ALL patients who are KMT2A positive, in pediatric precursor B-ALL (pre-B ALL) patients resistant to chemotherapy and as bridging therapy to stem cell transplantation (SCT) in cases with MRD positivity.

### Aims

The aim of this study was to evaluate treatment responses and survival rates in childhood pre-B ALL patients treated with blinatumomab.

### Methods

This study included children diagnosed with CD-19-positive B-ALL in Türkiye between January 1, 2014, and December 30, 2024.

**Copyright Information:** (Online) ISSN: 2572-9241
©2026 The Author(s). HemaSphere is published by John Wiley & Sons Ltd on behalf of European Hematology Association. This is an open access Abstract Book distributed under the Attribution-NonCommercial-NoDerivs (CC BY-NC-ND), which allows third parties to download the articles and share them with others as long as they credit the author and the Abstract Book, but they cannot change the content in any way or use them commercially.
**Abstract Book Citations:** Authors, Title, HemaSphere, 2026;10:(S1):pages. The abstract book DOIs can be found on the "Author Information" page.
**Disclaimer:** Articles published in the journal HemaSphere exclusively reflect the opinions of the authors. The authors are responsible for all content in their abstracts including accuracy of the facts, statements, citing resources, etc.

### Results

147 patients diagnosed with CD-19-positive ALL were included in the study. Of the 10 children diagnosed with infant ALL, 2 received blinatumomab due to minimal residual disease (MRD) positivity and refractory disease, while 8 received it according to the protocol. Additionally, patients were divided into three groups based on the stage of treatment at which they received blinatumomab therapy:
Group A: Patients receiving Blinatumomab in complete remission 1 with positive MRD or overt blasts(CR1) (n=44)
Group B: Patients receiving Blinatumomab after relapse (n=67)
Group C: Patients receiving Blinatumomab due to relapse after HSCT (n=36).
The median (min-max) age of patients at initial diagnosis was 4.25 years, 4.91 years, and 6.75 years in groups A, B, and C, respectively. The majority of children (57%) were in the high-risk group (HRG) at initial diagnosis. Blinatumomab was administered in courses ranging from 1 to 3 cycles. HSCT was performed in 52%, 79%, and 39% of groups A, B, and C, respectively.
There were a total of 33 patients with M3 (blast ≥25%) bone marrow before Blinatumomab therapy. The distribution of these children was as follows: Group A 7% (n=3), Group B 25% (n=17), and Group C 36% (n=13).
In total, 54 out of 147 patients (37%) died. Mortality rates according to groups were found to be 9%, 43%, and 58% in groups A, B, and C, respectively ($p<0.05$). Children with M3 bone marrow in each group had significantly lower event-free survival (EFS) rates after induction compared to those with M1 and M2 bone marrow ($p<0.05$).
The 5-year overall survival (OS) for the entire cohort was 61%, and event-free survival (EFS) was 55%. Patients in group A had significantly higher OS(89%) and EFS (81%) compared to the others ($p<0.05$). In group B, the 5-year OS was 63%, and EFS 60%; in group C, OS was 43%, and EFS was 37%.

### Summary/Conclusion

The results showed that administration of blinatumomab in children group A who had positive MRD or blast percentage less than 25% had the highest OS and EFS rates ($p<0.05$). Survival rates were significantly reduced in cases of relapse after stem cell transplantation($p<0.05$). Therefore, early administration of blinatumomab in the treatment of ALL is crucial, especially in countries with limited resources.

**Copyright Information:** (Online) ISSN: 2572-9241
©2026 The Author(s). HemaSphere is published by John Wiley & Sons Ltd on behalf of European Hematology Association. This is an open access Abstract Book distributed under the Attribution-NonCommercial-NoDerivs (CC BY-NC-ND), which allows third parties to download the articles and share them with others as long as they credit the author and the Abstract Book, but they cannot change the content in any way or use them commercially.
**Abstract Book Citations:** Authors, Title, HemaSphere, 2026;10:(S1):pages. The abstract book DOIs can be found on the "Author Information" page.
**Disclaimer:** Articles published in the journal HemaSphere exclusively reflect the opinions of the authors. The authors are responsible for all content in their abstracts including accuracy of the facts, statements, citing resources, etc.

### (PB2615) THE ROLE OF MULTIPARAMETRIC FLOW CYTOMETRY IN DETECTING CENTRAL NERVOUS SYSTEM INVOLVEMENT AT DIAGNOSIS IN ADULT B-CELL ACUTE LYMPHOBLASTIC LEUKEMIA: EXPERIENCE FROM A SINGLE CENTER IN ARGENTINA.

**Topic:** 02. Acute lymphoblastic leukemia - Clinical

Tatiana Herlein[1], Marina Santas[1], Enrique Martins[1], Maira Masdea[1], Antonella Orsetti[1], Stella Loudet[1], Alicia Navickas[1], Fernanda Tosin*[1], Alejandra Martí[1], Diego Issouribehere[1]

*[1]Hospitel El Cruce, Alta Complejidad en Red, Florencio Varela, Buenos Aires, Argentina;*

### Background

Central nervous system involvement(CNSi) in B-cell Acute Lymphoblastic Leukemia (B-ALL) at diagnosis occurs in 5–11% of adult cases and the reported CNS relapse rate is approximately 15%, of which 75% occur with concomitant systemic involvement and the remaining 25% as isolated CNS relapse.

### Aims

This study aimed to evaluate the association between CNSi by B-lymphoid blasts and the presence of the BCR::ABL1 fusion transcript, hyperleukocytosis, and elevated LDH; to determine the proportion of patients with CNSi at diagnosis; to estimate the rates of isolated CNS relapse and relapse with concomitant systemic involvement; and to analyze post-induction overall survival.

### Methods

Eighty patients diagnosed with B-cell acute lymphoblastic leukemia (B-ALL) between February 2014 and January 2026 were included. All were treated according to the age-adapted GATLA, CNSi was assessed by MFC in 67 patients. The presence of the BCR::ABL1 rearrangement was evaluated by reverse transcription quantitative polymerase chain reaction (RT-qPCR) or fluorescence in situ hybridization (FISH). White blood cell (WBC) count (<30,000/µL vs. ≥30,000/µL) and lactate dehydrogenase (LDH) levels (>660 U/L) were recorded.
CSF samples collected at diagnosis in Transfix® (cell stabilizing preservative) were processed by MFC. CSF samples were concentrated by centrifugation and subsequently stained using an eight-color monoclonal antibody panel: CD15-FITC/CD64-PE/CD34-PerCP-Cy5.5/CD19-PE-Cy7/CD3+CD14-APC/CD38-APC-H7/CD20-V450/CD45-V500c. Data analysis was conducted using Infinicyt software according to EuroFlow standardization and optimization guidelines.
Associations were evaluated using the chi-square test, and the strength of association was estimated by calculating the odds ratio (OR).

### Results

A total of 80 patients diagnosed with B-cell ALL were studied. Of these, 52% (n=42) were male, with a median age of 33 years (range: 16-77 years). CNS status at diagnosis was evaluated in 67/80 patients. CNSi was detected in 54% (n=36) of cases. BCR::ABL1 was detectable in 31% (n=25) of patients, 39% (n=31) presented with hyperleukocytosis, and 34% (n=27) showed elevated LDH. An association was found between hyperleukocytosis and CNSi, with an OR of 5.600 (95% CI: 1.840–17.048; p = 0.002). No statistically significant association was found between CNSi and LDH levels or the presence of the BCR::ABL1 fusion transcript. The relapse rate for patients with CNSi at diagnosis was 19% (n=7/36) in bone marrow (BM) and 11% (n=4/36) in CSF. Regarding CSF relapses, 40% (n=2/5) were isolated, while 60% (n=3/5) presented with concomitant BM involvement. Post-induction survival was 39%.

**Copyright Information:** (Online) ISSN: 2572-9241
©2026 The Author(s). HemaSphere is published by John Wiley & Sons Ltd on behalf of European Hematology Association. This is an open access Abstract Book distributed under the Attribution-NonCommercial-NoDerivs (CC BY-NC-ND), which allows third parties to download the articles and share them with others as long as they credit the author and the Abstract Book, but they cannot change the content in any way or use them commercially.
**Abstract Book Citations:** Authors, Title, HemaSphere, 2026;10:(S1):pages. The abstract book DOIs can be found on the "Author Information" page.
**Disclaimer:** Articles published in the journal HemaSphere exclusively reflect the opinions of the authors. The authors are responsible for all content in their abstracts including accuracy of the facts, statements, citing resources, etc.

### Summary/Conclusion

Given the sensitivity of the method, FCM of the CSF is the technique of choice for identifying CNS status in B-cell ALL. Regarding the percentage of CNSi at diagnosis, we found a higher value than reported in the literature (54% in our cohort); this could be explained by the difference in sensitivity compared to other methods, such as cytospin. Consistent with literature reports, the CNS relapse rate was 11%. Relapses were more frequent with concomitant BM involvement than as isolated events. Regarding risk factors for CNSi in B-cell ALL, the results show a clear association with hyperleukocytosis.

**Copyright Information:** (Online) ISSN: 2572-9241
©2026 The Author(s). HemaSphere is published by John Wiley & Sons Ltd on behalf of European Hematology Association. This is an open access Abstract Book distributed under the Attribution-NonCommercial-NoDerivs (CC BY-NC-ND), which allows third parties to download the articles and share them with others as long as they credit the author and the Abstract Book, but they cannot change the content in any way or use them commercially.
**Abstract Book Citations:** Authors, Title, HemaSphere, 2026;10:(S1):pages. The abstract book DOIs can be found on the “Author Information” page.
**Disclaimer:** Articles published in the journal HemaSphere exclusively reflect the opinions of the authors. The authors are responsible for all content in their abstracts including accuracy of the facts, statements, citing resources, etc.

### (PB2616) A STUDY ON THE APPLICATION OF VIRTUAL REALITY DISTRACTION TECHNIQUES IN LUMBAR PUNCTURE CARE FOR PEDIATRIC LEUKEMIA PATIENTS

**Topic:** 02. Acute lymphoblastic leukemia - Clinical

Jun Tao*[1], [illegible][1]

[1]*Xinqiao Hospital of Army Medical University, CHONGQING, China;*

### Background

Virtual reality (VR) has demonstrated potential as a novel non-pharmacological intervention in medical pain management. This study focuses on lumbar puncture procedures for pediatric leukemia patients, investigating the efficacy of VR-based distraction interventions in alleviating procedure-related pain, anxiety, and behavioral resistance, with the aim of providing optimized analgesic support strategies for clinical practice.

### Aims

To investigate the efficacy of virtual reality (VR)-based distraction interventions during lumbar puncture procedures in pediatric leukemia patients, evaluating its effectiveness in alleviating procedural pain, anxiety, and behavioral resistance while enhancing treatment compliance and overall experience.

### Methods

A randomized controlled trial design was employed. Eighty-six pediatric leukemia patients requiring lumbar puncture were randomly assigned to an intervention group (n=43) and a control group (n=43). The control group received routine care and placebo interventions (e.g., conventional reassurance, conversation). The intervention group, in addition to routine care, wore VR headsets before and during the lumbar puncture to experience immersive, interactive games or scenarios (e.g., underwater exploration, space travel). Primary outcomes included children's self-reported pain levels during the procedure (using the Wong-Baker Facial Expression Pain Scale or Visual Analogue Scale) and observer-rated pain behaviors (e.g., FLACC scale). Secondary outcomes included pre- and postoperative anxiety levels (using the Modified Yale Preoperative Anxiety Scale or the Children's Anxiety Scale), behavioral cooperation, procedure duration, and patient/parent satisfaction.

### Results

Compared to the control group, children in the intervention group demonstrated significantly lower self-reported pain scores during lumbar puncture ($p<0.05$) and exhibited fewer observed pain behaviors ($p<0.05$). Additionally, the intervention group demonstrated better control of preoperative anxiety levels, higher behavioral compliance during the procedure (e.g., reduced crying and struggling), and shorter mean procedure duration ($p<0.05$). Both patients and parents reported high acceptance and satisfaction with the VR intervention, with most expressing willingness to continue its use in subsequent treatments.

### Summary/Conclusion

Virtual reality technology, as a novel non-pharmacological distraction method, effectively reduces pain perception and anxiety during lumbar puncture in pediatric leukemia patients, improves behavioral outcomes, and enhances procedural smoothness and comfort. This intervention is safe, feasible, and well-tolerated by children, offering an innovative adjunct strategy for invasive procedures in pediatric hematology/oncology care. It warrants further implementation in clinical practice.

**Copyright Information:** (Online) ISSN: 2572-9241
©2026 The Author(s). HemaSphere is published by John Wiley & Sons Ltd on behalf of European Hematology Association. This is an open access Abstract Book distributed under the Attribution-NonCommercial-NoDerivs (CC BY-NC-ND), which allows third parties to download the articles and share them with others as long as they credit the author and the Abstract Book, but they cannot change the content in any way or use them commercially.
**Abstract Book Citations:** Authors, Title, HemaSphere, 2026;10:(S1):pages. The abstract book DOIs can be found on the "Author Information" page.
**Disclaimer:** Articles published in the journal HemaSphere exclusively reflect the opinions of the authors. The authors are responsible for all content in their abstracts including accuracy of the facts, statements, citing resources, etc.

### (PB2617) CHILDHOOD ACUTE LYMPHOBLASTIC IN SOUTHERN TUNISIA: LONG TERM EXPERIENCE

**Topic:** 02. Acute lymphoblastic leukemia - Clinical

Ines Turki[1], Imen Frikha*[1], Khouloud Mezghani[1], Aicha Ben Kahla[1], Ikram Dammak[2], Nour Louati[3], Faten Kallel[1], Maha Charfi[1], Turki Fatma[4], Yousra Fakhfakh Derbel[1], Imen Ben Amor[1], Rahma Mallek[1], Rihab Werghi[5], Asma Koubaa[1], Insaf Ben Yaiche[5], Nour Siala[1], Hanen Charfi[1], Hend Elleuch[2], Ikram Ben Amor[3], Nour Ben Adejlil[5], Moez Medhaffar[1], Moez Elloumi[1]

*[1]Hedi Chaker University Hospital, Hematology Department, Sfax, Tunisia; [2]Hedi Chaker University Hospital, Hematology Laboratory, Sfax, Tunisia; [3]Regional blood Transfusion Center of Sfax, Sfax, Tunisia; [4]Hedi Chaker University Hospital, Genetic Department, Sfax, Tunisia; [5]National Bone Marrow Transplant Center, Tunis, Tunisia;*

### Background

The major challenge in the management of childhood Acute lymphoblastic leukemia (ALL) remains the high incidence of relapse. At our center, patients were treated with risk-adapted therapy according to the EORTC 58951 protocol.

### Aims

To describe the baseline futures of childhood ALL in Southern Tunisia and to evaluate long term treatment outcomes over a 24-years period.

### Methods

We conducted a retrospective cohort study of consecutive children (aged <16 years) diagnosed with ALL between 2000 and 2023. ALL were stratified into low-(LR), average-(AR), and high-risk (HR) groups and treated according to the EORTC 58951 protocol. Minimal residual desease (MRD) was assessed by flow cytometry with threshold positivity $\geq 10^{-2}$ for MRD1 and $\geq 10^{-3}$ for MRD. Clinical futures were analyzed. Outcomes analyzed included CR, relapse, overall survival (OS) and event free survival (EFS).

### Results

A total of 320 patients were included. The median age at diagnosis was 6 years and 26% were aged >10 years. Sex ratio was 1.46. There are 72,5% B-ALL and 27,5% T-ALL. Hyperleukocytic >100G/l was noted in 17% of patients. Central nervous system involvement was observed in 12 patients (3,7%). Bcr-Abl rearrangement was detected in 9 B-ALL patients (5%), and MLL-AF4 in 2. Fifteen percent of patients have favorable and 7% adverse cytogenetic risk. Corticosensitivity was observed in 78% of patients. ALL were stratified to LR (4%), AR1 (39%), AR2 (29%), and HR (28%). Induction mortality rate was 4,6%. CR was achieved in 92,5%. MRD1 and MRD2 negativity were observed in 80% and 71% of patients, respectively. Allogenic stem cell transplantation (ASCT) was indicated for 105 HR group patients. Only, 31 patients underwent ASCT. Relapse occurred in 98 patients (33%, mfollow-up of 146 months), including 6 after ASCT. Relapse rate was 21% in LR, 33% in AR1, 22% in AR2 and 39% in HR group (p=0,16). Relapse rate was lower in transplanted patients (14 vs 58%, p<0,001). Five-year OS and EFS were 62% and 57,2%, respectively.

### Summary/Conclusion

Our cohort showed an increased frequency of T-ALL (27,5 vs 10-15% in litterature) and a higher proportion of patients requiring HR tretment group. Relapse rate remains high despite the considerable proportion of ALL treated as high-risk group. Improved access to transplantation may help reducing relapses.

**Copyright Information:** (Online) ISSN: 2572-9241
©2026 The Author(s). HemaSphere is published by John Wiley & Sons Ltd on behalf of European Hematology Association. This is an open access Abstract Book distributed under the Attribution-NonCommercial-NoDerivs (CC BY-NC-ND), which allows third parties to download the articles and share them with others as long as they credit the author and the Abstract Book, but they cannot change the content in any way or use them commercially.
**Abstract Book Citations:** Authors, Title, HemaSphere, 2026;10:(S1):pages. The abstract book DOIs can be found on the "Author Information" page.
**Disclaimer:** Articles published in the journal HemaSphere exclusively reflect the opinions of the authors. The authors are responsible for all content in their abstracts including accuracy of the facts, statements, citing resources, etc.

### (PB2618) EVALUATION OF TESTICULAR RELAPSES IN PEDIATRIC PATIENTS WITH ACUTE LYMPHOBLASTIC LEUKEMIA

**Topic:** 02. Acute lymphoblastic leukemia - Clinical

Nihan Avcu Oral*[1], Gamze Gürsoy[1], Cem Kurtoğlu[1], Cagri Coskun[1], Tekin Aksu[1], Şule Ünal Cangül[1], Baris Kuskonmaz[1], Selin Aytaç[1]

[1]*Hacettepe University, Pediatric Hematology-Oncology, Ankara, Türkiye;*

**Background**

Testicular relapse is a rare but important extramedullary manifestation in pediatric acute lymphoblastic leukemia (ALL), associated with distinct biological characteristics and potential therapeutic challenges.

**Aims**

This study aimed to evaluate the clinical characteristics and outcomes of testicular relapses in pediatric patients diagnosed with ALL and followed at Hacettepe University Faculty of Medicine, Division of Pediatric Hematology.

**Methods**

The study included retrospectively ALL patients treated with the Modified St. Jude Total Therapy XV protocol between 2008–2016 and the ALL-IC-BFM 2009 protocol between 2016–2025.

**Results**

A total of 183 patients received the Modified St. Jude protocol, and relapse was detected in 16 patients (8.7%). The sites of relapse were bone marrow (BM) (n=9) and isolated central nervous system (CNS) (n=7). No testicular relapse was observed during this period. Among 115 patients treated with the ALL-IC-BFM 2009 protocol, relapse developed in 21 patients (18.2%). The distribution of relapse sites was as follows: BM (n=8), isolated CNS (n=5), BM+CNS (n=3), BM+testis (n=3), BM+CNS+testis (n=1), and isolated testis (n=1).
Of the 21 patients who relapsed after receiving the ALL-IC-BFM 2009 protocol, 15 were male (71.4%) and 6 were female (28.6%). Among male patients, relapse distribution was as follows: isolated CNS (n=4), BM (n=4), CNS+BM (n=2), BM+testis (n=3), BM+CNS+testis (n=1), and isolated testis (n=1).
The median age at diagnosis of patients with testicular relapse was 8.5 years (range: 3–11.5), while the median age at relapse was 11 years (range: 5.5–12.5). None of these patients had testicular involvement at initial diagnosis. The median white blood cell count at diagnosis was 183 $\times 10^3/\mu L$ (range: 7.4–446.4). Cytogenetic analysis revealed t(12;21) in three patients and t(4;11) and 11q23 positivity in one patient. Phenotypically, four patients had precursor B-cell ALL (Pre-B ALL) and one had early T-cell ALL. All patients demonstrated a good steroid response on day 8. End-of-induction complete remission was achieved in patients with Pre-B ALL. The patient with early T-cell ALL showed a partial response after induction and subsequently underwent allogeneic bone marrow transplantation; however, this patient developed bilateral testicular and bone marrow relapse after transplantation. Among patients with Pre-B ALL, relapse occurred after completion of maintenance therapy in three patients (median time from maintenance cessation to relapse: 7 months [range: 1–26 months]) and during ongoing primary therapy in one patient. In patients with Pre-B ALL, testicular relapse was unilateral in three cases and bilateral in one case. In the patient who developed unilateral testicular+BM+CNS relapse, t(9;22) positivity was detected at relapse. Of these patients, three died and two are alive.

**Copyright Information:** (Online) ISSN: 2572-9241
©2026 The Author(s). HemaSphere is published by John Wiley & Sons Ltd on behalf of European Hematology Association. This is an open access Abstract Book distributed under the Attribution-NonCommercial-NoDerivs (CC BY-NC-ND), which allows third parties to download the articles and share them with others as long as they credit the author and the Abstract Book, but they cannot change the content in any way or use them commercially.
**Abstract Book Citations:** Authors, Title, HemaSphere, 2026;10:(S1):pages. The abstract book DOIs can be found on the "Author Information" page.
**Disclaimer:** Articles published in the journal HemaSphere exclusively reflect the opinions of the authors. The authors are responsible for all content in their abstracts including accuracy of the facts, statements, citing resources, etc.

**Summary/Conclusion**

In our center, testicular relapse in pediatric ALL patients was observed particularly after 2016 in patients treated with the BFM protocol, predominantly in males older than 9 years. The impact of factors such as the COVID-19 pandemic period and drug resistance on testicular relapse should be investigated in further studies.

**Copyright Information:** (Online) ISSN: 2572-9241
©2026 The Author(s). HemaSphere is published by John Wiley & Sons Ltd on behalf of European Hematology Association. This is an open access Abstract Book distributed under the Attribution-NonCommercial-NoDerivs (CC BY-NC-ND), which allows third parties to download the articles and share them with others as long as they credit the author and the Abstract Book, but they cannot change the content in any way or use them commercially.
**Abstract Book Citations:** Authors, Title, HemaSphere, 2026;10:(S1):pages. The abstract book DOIs can be found on the "Author Information" page.
**Disclaimer:** Articles published in the journal HemaSphere exclusively reflect the opinions of the authors. The authors are responsible for all content in their abstracts including accuracy of the facts, statements, citing resources, etc.

### (PB2619) LEUKAPHERESIS FOR OPTIMAL CHIMERIC ANTIGEN RECEPTOR T-CELL MANUFACTURING – CAN WE DO BETTER?

**Topic:** 02. Acute lymphoblastic leukemia - Clinical

Filipa Fonseca-Dias*[1], Sara Lopes[2], Ana Filipa Bordalo[2], Catarina Pinho[2], Sérgio Lopes[2], Susana Roncon[2]

*[1]Santo António University Hospital, Immuno-haemotherapy Department, Porto, Portugal; [2]Portuguese Oncology Institute of Porto, Cellular Therapy Department, Porto, Portugal;*

### Background

Tisagenlecleucel is an autologous anti-CD19 chimeric antigen receptor (CAR) T-cell therapy approved for relapsed/refractory (R/R) B-cell acute lymphoblastic leukaemia (B-ALL). Although response rates are high, real-world effectiveness depends on successful cell collection and timely manufacturing.
Obtaining the initial material for CAR-T production requires leukapheresis, eventual cryopreservation, shipment to a central facility, where genetic modification, expansion, quality control, and return of the final product for infusion will be performed.
Patients are often heavily pretreated, with lymphotoxic exposure and aggressive disease, compromising T-cell yield and function. Understanding pathway-related delays is clinically relevant.

### Aims

To evaluate the feasibility, performance and timing of leukapheresis for CD19-directed CAR-T manufacturing in heavily pretreated paediatric and young adult patients with R/R B-ALL.

### Methods

We performed a retrospective single-centre analysis of consecutive patients with R/R B-ALL referred for CD19-directed CAR-T therapy between January 2019 and February 2026 at the Portuguese Oncology Institute of Porto. Mononuclear cells (MNC) were collected by leukapheresis, cryopreserved on the day of collection, and shipped for manufacturing. The final CAR-T product was stored at -150ºC until infusion. Peripheral blood $CD3^+$ counts, leukapheresis parameters, product specifications, need for a second collection day, and key time intervals across the CAR-T pathway were analysed.

### Results

Twelve patients (66.7% male), with a median age of 11.2 (4.4–45.0) years, all with relapsed/refractory B-ALL (41.7% with prior allogeneic haematopoietic transplantation), underwent leukapheresis (13 procedures, including 1 with a two-day collection); eight patients (66.7%) were infused. Patients were heavily pretreated (median 3 prior therapy lines) and median peripheral blood $CD3^+$ count prior to collection was 585 (128–2806)/µL, yet no collection failures occurred. Median $CD3^+$ content in the leukapheresis product was $2.48 \times 10^9$ (0.424–7.66), with a median proportion of 44%, demonstrating adequate cellular yield despite variable baseline lymphocyte counts. Notably, manufacturing success was achieved even in patients with low baseline $CD3^+$ counts ($<0.3 \times 10^9$/L), suggesting that pre-apheresis lymphopenia did not preclude adequate product generation. Median time from: referral to leukapheresis was 6 (1–11) days; leukapheresis to product receipt 39 (30–90) days; product receipt to infusion 25 (4–64) days. Among infused patients, the overall median time from referral to infusion was 71 (46–162) days. Manufacturing was successfully completed in all collected cases (100% manufacturing success rate), with no product out-of-specification. Cryopreservation occurred on the day of collection; for 3 patients on the day after.

**Copyright Information:** (Online) ISSN: 2572-9241
©2026 The Author(s). HemaSphere is published by John Wiley & Sons Ltd on behalf of European Hematology Association. This is an open access Abstract Book distributed under the Attribution-NonCommercial-NoDerivs (CC BY-NC-ND), which allows third parties to download the articles and share them with others as long as they credit the author and the Abstract Book, but they cannot change the content in any way or use them commercially.
**Abstract Book Citations:** Authors, Title, HemaSphere, 2026;10:(S1):pages. The abstract book DOIs can be found on the "Author Information" page.
**Disclaimer:** Articles published in the journal HemaSphere exclusively reflect the opinions of the authors. The authors are responsible for all content in their abstracts including accuracy of the facts, statements, citing resources, etc.

**Summary/Conclusion**

In this real-world cohort of paediatric and young adult patients with aggressive R/R B-ALL, leukapheresis was consistently feasible, not representing a limiting step. Streamlining manufacturing turnaround times may be critical to reducing time-to-infusion and maximising the clinical impact of CAR-T therapy in high-risk B-ALL.

**Copyright Information:** (Online) ISSN: 2572-9241
©2026 The Author(s). HemaSphere is published by John Wiley & Sons Ltd on behalf of European Hematology Association. This is an open access Abstract Book distributed under the Attribution-NonCommercial-NoDerivs (CC BY-NC-ND), which allows third parties to download the articles and share them with others as long as they credit the author and the Abstract Book, but they cannot change the content in any way or use them commercially.
**Abstract Book Citations:** Authors, Title, HemaSphere, 2026;10:(S1):pages. The abstract book DOIs can be found on the "Author Information" page.
**Disclaimer:** Articles published in the journal HemaSphere exclusively reflect the opinions of the authors. The authors are responsible for all content in their abstracts including accuracy of the facts, statements, citing resources, etc.

### (PB2620) ASC4ALL TRIAL-IN-PROGRESS: ASCIMINIB IN PEDIATRIC/YOUNG ADULT RELAPSED/REFRACTORY CD19+ PH+ B-CELL ALL

**Topic:** 02. Acute lymphoblastic leukemia - Clinical

C. Michel Zwaan*[1, 2], Sarah Tasian[3, 4], Maureen O'brien[5], Marlise Luskin[6], Veronica Leoni[7], Justin Wahlstrom[8], Vishal Dhamal[9], Haneen Shalabi[8], Thai Hoa Tran[10, 11]

*[1]Princess Maxima Center for Pediatric Oncology, Utrecht, The Netherlands;[2]Erasmus and MC-Sophia Children's Hospital, Rotterdam, The Netherlands; [3]Children's Hospital of Philadelphia, Philadelphia, PA, United States of America;[4]University of Pennsylvania School of Medicine, Philadelphia, PA, United States of America; [5]University of Colorado Anschutz Medical Campus, Aurora, OH, United States of America; [6]Department of Medical Oncology, Dana-Farber Cancer Institute, Boston, MA, United States of America; [7]Fondazione IRCCS San Gerardo dei Tintori, Monza, Italy; [8]Novartis Pharmaceuticals Corporation, East Hanover, NJ, United States of America; [9]Novartis Healthcare Private Limited, Mumbai, India; [10]CHU Sainte-Justine, University of Montreal, Montreal, QC, Canada; [11]CHU Sainte-Justine Research Center Azrieli, Montreal, QC, Canada;*

### Background

In pediatric patients, Philadelphia chromosome-positive (Ph+) acute lymphoblastic leukemia (ALL) is a rare, high-risk leukemia driven by the *BCR*::*ABL1* rearrangement resulting in constitutively active ABL1 kinase signaling. Tyrosine kinase inhibitors (TKIs) imatinib and dasatinib are approved for treatment of pediatric patients with newly diagnosed Ph+ ALL. However, approximately one-third of children and adolescents/young adults (AYAs) treated with TKI plus chemotherapy relapse. Chemotherapy followed by blinatumomab, is considered standard of care for children with relapsed/refractory (R/R) Ph- B-cell ALL, but there is no standard treatment approach for pediatric/AYA patients with R/R Ph+ ALL.
Asciminib, the first BCR::ABL1 inhibitor to Specifically Target the ABL Myristoyl Pocket, showed superior efficacy and favorable safety and tolerability compared with adenosine triphosphate-competitive TKIs in adults with chronic myeloid leukemia. A phase 1 trial of asciminib monotherapy in adults with R/R Ph+ ALL also demonstrated safety and preliminary antileukemic activity (NCT02081378).

### Aims

The ASC4ALL trial will assess the safety and efficacy of asciminib plus chemotherapy and immunotherapy to optimize treatment for children and AYAs with R/R CD19$^{+}$ Ph+ B-cell ALL. Patients with ABL1/ABL2-rearranged CD19+ 'Ph-like' ALL will be enrolled in a separate cohort.
Primary objectives are to identify the recommended phase 2 dose (RP2D) of asciminib with low-intensity debulking induction chemotherapy (i.e., dexamethasone and vincristine) and immunotherapy (blinatumomab) and to assess the rate of complete remission (CR) at the RP2D at the end of induction. Secondary objectives include assessing safety/tolerability and pharmacokinetics of asciminib.

**Copyright Information:** (Online) ISSN: 2572-9241
©2026 The Author(s). HemaSphere is published by John Wiley & Sons Ltd on behalf of European Hematology Association. This is an open access Abstract Book distributed under the Attribution-NonCommercial-NoDerivs (CC BY-NC-ND), which allows third parties to download the articles and share them with others as long as they credit the author and the Abstract Book, but they cannot change the content in any way or use them commercially.
**Abstract Book Citations:** Authors, Title, HemaSphere, 2026;10:(S1):pages. The abstract book DOIs can be found on the "Author Information" page.
**Disclaimer:** Articles published in the journal HemaSphere exclusively reflect the opinions of the authors. The authors are responsible for all content in their abstracts including accuracy of the facts, statements, citing resources, etc.

**Methods**

ASC4ALL (NCT07387926) is an international, phase 1/2, open-label, single-arm study of asciminib plus chemoimmunotherapy in children and AYAs (≥1 to ≤30 years old) with R/R CD19+ Ph+ or *ABL1/ABL2*-rearranged Ph-like ALL, including patients with the *BCR::ABL1* T315I mutation. Signed informed consent will be obtained prior to study participation.
The study will use a 2-part design with asciminib dose escalation to determine the RP2D (part 1) and dose expansion at RP2D (part 2). Both parts will consist of asciminib with low-intensity chemotherapy for 4 weeks, and asciminib with blinatumomab (consolidation) for 12 weeks. Intrathecal chemotherapy will be added for central nervous system prophylaxis or as treatment. These will be followed by a survival phase with allocation to definitive treatment (e.g., hematopoietic stem cell transplant or chimeric antigen receptor T-cell immunotherapy) and an optional asciminib monotherapy maintenance phase.

**Results**

ASC4ALL will begin recruitment in 2026 across 70 locations. Approximately 60 patients will be screened; at least 28 will be enrolled.

**Summary/Conclusion**

ASC4ALL results will evaluate the safety, efficacy, and optimal dosing of chemoimmunotherapy plus asciminib in children and AYAs with relapsed/refractory Ph+ or *ABL1*/*ABL2*-rearranged ‘Ph-like’ ALL.

**Copyright Information:** (Online) ISSN: 2572-9241
©2026 The Author(s). HemaSphere is published by John Wiley & Sons Ltd on behalf of European Hematology Association. This is an open access Abstract Book distributed under the Attribution-NonCommercial-NoDerivs (CC BY-NC-ND), which allows third parties to download the articles and share them with others as long as they credit the author and the Abstract Book, but they cannot change the content in any way or use them commercially.
**Abstract Book Citations:** Authors, Title, HemaSphere, 2026;10:(S1):pages. The abstract book DOIs can be found on the “Author Information” page.
**Disclaimer:** Articles published in the journal HemaSphere exclusively reflect the opinions of the authors. The authors are responsible for all content in their abstracts including accuracy of the facts, statements, citing resources, etc.

### (PB2621) THE EFFICACY AND SAFETY OF NELARABINE IN RELAPSED OR REFRACTORY T-CELL ACUTE LYMPHOBLASTIC LEUKEMIA: A SYSTEMATIC REVIEW AND META-ANALYSIS

**Topic:** 02. Acute lymphoblastic leukemia - Clinical

Aymar Akilimali*[1]

[1]*Medical Research Circle, Research, Goma, Congo (the Democratic Republic of the);*

### Background

T-cell Acute Lymphoblastic Leukemia (T-ALL) is a subtype of acute lymphoblastic leukemia characterized by the proliferation of abnormal T-cell precursors. Nelarabine, a purine analog, has been approved as a targeted therapy for patients with refractory or relapsed T-ALL.

### Aims

This study aims to evaluate the efficacy and safety of Nelarabine, either as monotherapy or in combination with other therapies, in treating T-ALL.

### Methods

A systematic review and meta-analysis were conducted following PRISMA guidelines. We searched Cochrane CENTRAL, PubMed, and Google Scholar up to August 2024 for studies evaluating Nelarabine's efficacy and safety in T-ALL patients. The primary outcome was complete response (CR), with secondary outcomes focusing on adverse events (AEs). Data were analyzed using a random effects model, with statistical significance set at $p \leq 0.05$.

### Results

Sixteen studies involving 1,865 patients were included, with 1,345 receiving Nelarabine. The pooled analysis revealed a CR rate of 37.9% (95% CI: 20.5-55.4%, $p < 0.001$) for Nelarabine monotherapy. Significant adverse events included neutropenia at 29.1% (95% CI: 9.1-49.1%, $p < 0.001$), thrombocytopenia at 32.4% (95% CI: 14.8-50.0%, $p < 0.001$), peripheral motor neuropathy at 17.1% (95% CI: 4.2-30.1%, $p = 0.001$), and peripheral sensory neuropathy at 15.3% (95% CI: 5.8-24.9%, $p = 0.003$). For combination therapy, infections occurred in 65.0% (95% CI: 27.1-103.0%, $p < 0.001$) of patients, febrile neutropenia in 48.7% (95% CI: -8.8-106.3%, $p < 0.001$), peripheral motor neuropathy in 10.5% (95% CI: 7.9-13.0%, $p < 0.001$), and peripheral sensory neuropathy in 23.1% (95% CI: 10.6-35.7%, $p < 0.001$).

### Summary/Conclusion

Nelarabine shows significant efficacy in treating refractory or relapsed T-ALL, with notable CR rates. However, its use, both as monotherapy and in combination therapy, is associated with considerable adverse events, particularly neurotoxicity and hematologic toxicities, necessitating careful monitoring. Further research is needed to optimize its application across diverse patient populations and to better manage its associated toxicities.

**Copyright Information:** (Online) ISSN: 2572-9241
©2026 The Author(s). HemaSphere is published by John Wiley & Sons Ltd on behalf of European Hematology Association. This is an open access Abstract Book distributed under the Attribution-NonCommercial-NoDerivs (CC BY-NC-ND), which allows third parties to download the articles and share them with others as long as they credit the author and the Abstract Book, but they cannot change the content in any way or use them commercially.
**Abstract Book Citations:** Authors, Title, HemaSphere, 2026;10:(S1):pages. The abstract book DOIs can be found on the “Author Information” page.
**Disclaimer:** Articles published in the journal HemaSphere exclusively reflect the opinions of the authors. The authors are responsible for all content in their abstracts including accuracy of the facts, statements, citing resources, etc.

### (PB2623) INTRAVENOUS VERSUS INTRAMUSCULAR ADMINISTRATION OF RECOMBINANT E. COLI ASPARAGINASE IN PEDIATRIC ACUTE LYMPHOBLASTIC LEUKEMIA: A RANDOMIZED COMPARATIVE STUDY

**Topic:** 02. Acute lymphoblastic leukemia - Clinical

Andrey Osipyants*[1], Julia Roumiantseva[1], Svetlana Lagoiko[1], Lyudmila Zharikova[1], Dmitry Blinov[1], Alexander Karachunskiy[1]

*[1]Dmitry Rogachev National Medical Research Center Of Pediatric Hematology, Oncology and Immunology, Moscow, Russia;*

### Background

Asparaginase is a key component of pediatric acute lymphoblastic leukemia (ALL) therapy. Both intravenous (IV) and intramuscular (IM) routes are used in clinical practice; however, prospective comparative data for recombinant E. coli asparaginase (Spectrila®) remain limited. Route of administration may influence pharmacodynamic exposure, tolerability, and risk of treatment inactivation.

### Aims

To compare pharmacodynamic parameters and toxicity of recombinant E. coli asparaginase administered intravenously versus intramuscularly in children with newly diagnosed ALL during consolidation therapy.

### Methods

This multicenter, randomized, open-label study was conducted within the ALL-MB 2015 treatment protocol. Pediatric patients (1–18 years) in remission after induction were randomized prior to consolidation part 1 to receive recombinant E. coli asparaginase either IV or IM once weekly for 6 weeks at doses of 5,000 IU/m² or 10,000 IU/m² depending on risk group. Serum asparaginase activity was assessed during therapy; toxicity and hypersensitivity reactions were recorded. Silent inactivation was defined by subtherapeutic enzyme activity in the absence of clinical hypersensitivity.

### Results

Twenty patients were included: 9 received 5,000 IU/m² (IM n=5; IV n=4) and 11 received 10,000 IU/m² (IM n=6; IV n=5). One patient in the IV 5,000 IU/m² group discontinued therapy before completion of third consolidation due to early relapse.
Known allergic reactions occurred in 4/9 patients treated IV and 3/11 treated IM. Silent inactivation was observed in 3/9 patients in the IV group and 3/11 in the IM group.
Mean serum asparaginase activity measured on day 4 after administration was comparable between routes. At the 5,000 IU/m² dose, mean activity was 185±31 IU/L for IV versus 202±81 IU/L for IM administration. At the 10,000 IU/m² dose, mean activity was 526±252 IU/L for IV and 522±188 IU/L for IM administration.

### Summary/Conclusion

In this randomized pilot cohort, intravenous and intramuscular administration of recombinant E. coli asparaginase demonstrated comparable pharmacodynamic exposure and similar rates of silent inactivation. Allergic reactions occurred in both groups without a clear advantage for either route. These data suggest that both IV and IM administration of recombinant E. coli asparaginase provide adequate systemic enzyme activity during consolidation therapy in pediatric ALL and may be used interchangeably based on clinical considerations and treatment logistics.
Larger, adequately powered prospective studies are required to confirm these results and to further evaluate potential differences in pharmacokinetics, immunogenicity, and clinical outcomes between administration routes.

**Copyright Information:** (Online) ISSN: 2572-9241
©2026 The Author(s). HemaSphere is published by John Wiley & Sons Ltd on behalf of European Hematology Association. This is an open access Abstract Book distributed under the Attribution-NonCommercial-NoDerivs (CC BY-NC-ND), which allows third parties to download the articles and share them with others as long as they credit the author and the Abstract Book, but they cannot change the content in any way or use them commercially.
**Abstract Book Citations:** Authors, Title, HemaSphere, 2026;10:(S1):pages. The abstract book DOIs can be found on the "Author Information" page.
**Disclaimer:** Articles published in the journal HemaSphere exclusively reflect the opinions of the authors. The authors are responsible for all content in their abstracts including accuracy of the facts, statements, citing resources, etc.

### (PB2625) EFFICACY OF ANTI-RELAPSE TREATMENT PROGRAMS IN ADULT PATIENTS WITH BCR::ABL1-NEGATIVE ACUTE LYMPHOBLASTIC LEUKEMIA.

**Topic:** 02. Acute lymphoblastic leukemia - Clinical

Ekaterina Kotova*[1], Olga Aleshina (Gavrilina)[1], Chabaeva Julia[1], Galina Isinova[1], Andrey Sokolov[1], Anastasia Vasileva[1], Vera Troitskaya[1], Elena Parovichnikova[1]

[1]*National Medical Research Center for Hematology,, moscow, Russia;*

### Background

Modifications to standard chemotherapy programs for adult patients with *de novo BCR::ABL1*-negative acute lymphoblastic leukemia (ALL) over the last decade have significantly improved overall survival (OS) from 24% to 67%. However, relapse occurs in 30–60% of patients. According to most research groups, the effectiveness of second-line and subsequent treatments in these patients does not exceed 20% over 3 years.

### Aims

To evaluate the effectiveness of anti-relapse treatment programs in adult patients with relapsed*BCR::ABL1*-negative ALL who received first-line treatment under the ALL-2016/ALL-2016m programs.

### Methods

The study included 185 patients with *de novo* ALL: 100 with *BCR::ABL1*-negative acute B-lymphoblastic leukemia (B-ALL) and 85 with acute T-lymphoblastic leukemia (T-ALL). The male to female ratio was 109 (59%):76 (41%). The median age was 30 (18–54) years. All patients were treated at the National Medical Research Center for Hematology from December 2016 to February 2022 according to the ALL-2016 protocol, and from March 2022 to December 2025 according to the ALL-2016m.

### Results

In 15 (15%) patients with B-ALL, disease relapse developed: in 10 - bone marrow, in 3 - mixed (in 2 - bone marrow and neuroleukemia, and in 1 -  involvement of bone marrow and extramedullary), in 2 -  extramedullary. Disease relapse was diagnosed in 15 (18%) patients with T-ALL: in 8 - bone marrow, in 3 - mixed (bone marrow and neuroleukemia), in 2 - extramedullary, in 2 - isolated neuroleukemia. As anti-relapse treatment programs, 15 patients with B-ALL received the following: blinatumomab -5, bortezomib-containing regimens - 3, inotuzumab ozogamicin - 1, anti-CD19 CAR-T - 2, no data for 4 patients; 15 patients with T-ALL: nelarabine-containing regimens  - 5, hypomethylating agents with venetoclax — 5, no data for 5 patients. Upon achieving second remission, allo-HSCT was performed in 7 patients with B-ALL and only 3 patients with T-ALL. The 3-year OS rate in patients with relapsed B-ALL and T-ALL  did not differ significantly: 34% and 29%, respectively (p=0.34) (Fig. 1).

### Summary/Conclusion

The effectiveness of anti-relapse treatment programs in this small group of patients remains unsatisfactory. Therefore, further modification of first-line therapy and the development of new anti-relapse treatment programs are relevant for patients with ALL.

**Copyright Information:** (Online) ISSN: 2572-9241
©2026 The Author(s). HemaSphere is published by John Wiley & Sons Ltd on behalf of European Hematology Association. This is an open access Abstract Book distributed under the Attribution-NonCommercial-NoDerivs (CC BY-NC-ND), which allows third parties to download the articles and share them with others as long as they credit the author and the Abstract Book, but they cannot change the content in any way or use them commercially.
**Abstract Book Citations:** Authors, Title, HemaSphere, 2026;10:(S1):pages. The abstract book DOIs can be found on the “Author Information” page.
**Disclaimer:** Articles published in the journal HemaSphere exclusively reflect the opinions of the authors. The authors are responsible for all content in their abstracts including accuracy of the facts, statements, citing resources, etc.

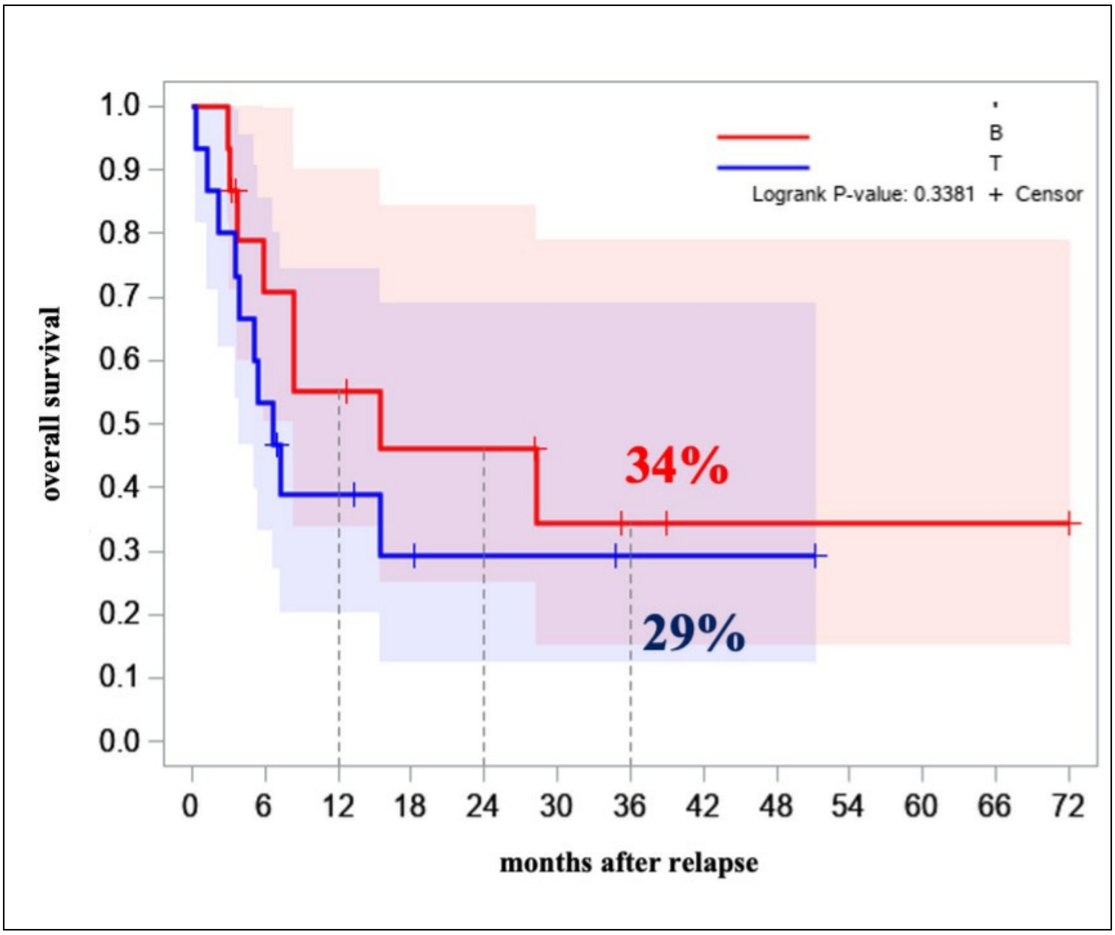

Fig 1. Three-year overall survival in adult patients with relapse BCR::ABL1-negative acute B-lymphoblastic leukemia and acute T-lymphoblastic leukemia

**Copyright Information:** (Online) ISSN: 2572-9241
©2026 The Author(s). HemaSphere is published by John Wiley & Sons Ltd on behalf of European Hematology Association. This is an open access Abstract Book distributed under the Attribution-NonCommercial-NoDerivs (CC BY-NC-ND), which allows third parties to download the articles and share them with others as long as they credit the author and the Abstract Book, but they cannot change the content in any way or use them commercially.
**Abstract Book Citations:** Authors, Title, HemaSphere, 2026;10:(S1):pages. The abstract book DOIs can be found on the "Author Information" page.
**Disclaimer:** Articles published in the journal HemaSphere exclusively reflect the opinions of the authors. The authors are responsible for all content in their abstracts including accuracy of the facts, statements, citing resources, etc.

### (PB2626) TIME TO CURE AND CURE FRACTIONS OF PRECURSOR LYMPHOID NEOPLASMS IN SPAIN, 1994-2018

**Topic:** 02. Acute lymphoblastic leukemia - Clinical

Jan Trallero*[1, 2, 3], Aina Romaguera[2, 4], Arantza Sanvisens[1, 2], Daniel Redondo-Sánchez[5, 6, 7], María Isabel Palacios[8], María Dolores Chirlaque[7, 9], Marcela Guevara[10], Marià Carulla[11], Xitama Álvarez[12], Montserrat Garrido[12], Ana Vizcaino-Batlles[13], Pilar Gutiérrez[14], Arantza Lopez-de-Munain[15], Silvia Sanclemente[15], Amaia Aizpurua[15], Maria Antònia Barceló[3, 7], Rafael Marcos-Gragera[1, 2, 3, 7, 16]

*[1]Epidemiology Unit and Girona Cancer Registry, Catalan Institute of Oncology, Girona Biomedical Research Institute Dr. Josep Trueta (IDIBGI-CERCA), Epidemiology Unit and Girona Cancer Registry, Girona, Spain; [2]Josep Carreras Leukaemia Research Institute, Badalona, Spain; [3]Research Group on Statistics, Econometrics and Health (GRECS), University of Girona, Girona, Spain; [4]Department of Computer Science, Applied Mathematics and Statistics, University of Girona, Girona, Spain;[5]Escuela Andaluza de Salud Pública (EASP), Granada, Spain; [6]Instituto de Investigación Biosanitaria ibs.GRANADA, Granada, Spain; [7]CIBER of Epidemiology and Public Health (CIBERESP), Madrid, Spain; [8]Epidemiology and Health Prevention Service. Directorate General of Public Health, Consumer Affairs and Care, Logroño, Spain; [9]Murcia Cancer Registry, Murcia, Spain; [10]Navarre Institute of Public and Occupational Health, Pamplona, Spain; [11]Cancer Epidemiology and Prevention Service, Hospital Universitari Sant Joan de Reus, Institut de Recerca Biomèdica Catalunya Sud, Reus, Spain; [12]Canarias Cancer Registry, Las Palmas de Gran Canaria, Spain; [13]Castellón Tumor Registry, Castellón, Spain; [14]Department of Health, Castilla y León Cancer Registry, Valladolid, Spain; [15]Basque Government, Basque Country Cancer Registry, Victoria-Gasteiz, Spain; [16]Department of Medical Sciences, University of Girona, Girona, Spain;*

### Background

In recent years, there has been a rise in the number of published studies on time to statistical cure (TTC) and cure fractions (CF) of cancer patients using population-based cancer data. In the framework of population-based cancer registries, TTC is considered as the time at which excess cancer mortality becomes negligible, reaching the same level as the general population. However, limited information is available on specific hematological neoplasms, such as precursor lymphoid neoplasms (PLN).

### Aims

To estimate the TTC and CF for PLN in the adult population in Spain.

### Methods

All primary malignant cases classified as PLN (International Classification for Oncology, 3rd Edition codes: 9727-9729, 9811, 9819, 9829, 9835-9837) in individuals aged 15 to 95 years from the Spanish Network of Cancer Registries database between 1994 and 2018 were included. These cases were followed up until December 31, 2021. TTC was defined as the time point at which the probability of dying from the disease falls below 5%, and the CF as the relative survival (RS) at TTC. To evaluate the cure assumption, graphical methods were employed to check for a flattening in the RS curve, and to determine if, at any point, the excess hazard of PLN cases reached the same level as that of the general population (Figure 1). To estimate TTC and CF, a flexible parametric non-mixture cure model, adjusted for age with a spline effect and year of diagnosis with a linear effect, was used to model the excess hazard.

**Copyright Information:** (Online) ISSN: 2572-9241
©2026 The Author(s). HemaSphere is published by John Wiley & Sons Ltd on behalf of European Hematology Association. This is an open access Abstract Book distributed under the Attribution-NonCommercial-NoDerivs (CC BY-NC-ND), which allows third parties to download the articles and share them with others as long as they credit the author and the Abstract Book, but they cannot change the content in any way or use them commercially.
**Abstract Book Citations:** Authors, Title, HemaSphere, 2026;10:(S1):pages. The abstract book DOIs can be found on the "Author Information" page.
**Disclaimer:** Articles published in the journal HemaSphere exclusively reflect the opinions of the authors. The authors are responsible for all content in their abstracts including accuracy of the facts, statements, citing resources, etc.

**Results**

The study included a total of 1,596 PLN cases, with a median age at diagnosis of 47 years [Interquartile range (IQR): 27-67]. A higher proportion of PLN cases were diagnosed in males (58.2%). The median follow-up time was 1.5 years [IQR: 0.3-6.8], with 71.7% of deaths at the end of the study. An overall TTC of 7.0 years [95% confidence intervals (CI): 6.9-7.1] and a CF of 32.3% [95%CI: 30.2-34.5] were observed. Significant differences were observed between age groups, with the youngest (15-39 years) showing a TTC of 5.8 years [95%CI: 5.7-5.8] and a CF of 49.8% [95%CI: 46.3-53.2], compared with the oldest (>69 years) who had a TTC of 8.8 years [95%CI: 8.7-8.9] and a CF of 12.7% [95%CI: 10.4-15.0]. A notable improvement in the prognosis of PLN was observed over time, as the CF improved from 24.6% [95%CI: 21.5-27.7] in the earliest period (1994-1998) to 38.9% [95%CI: 35.4-42.3] in the most recent period (2014-2018). Additionally, there was a reduction of 1.3 years in the TTC between these periods (1994-1998: 7.8 years [95%CI: 7.6-8.0] vs 2014-2018: 6.5 years [95%CI: 6.3-6.6]).

**Summary/Conclusion**

For the first time in Spain, TTC and CF for adult PLN cases have been reported, revealing a significant improvement in the prognosis of the disease over time. The reduction in the time required for patients to be considered statistically cured and the increase in percentage of cases considered cured may enhance understanding of this disease among clinicians, policymakers, and patients, which could contribute to improved resource allocation strategies.

**Copyright Information:** (Online) ISSN: 2572-9241
©2026 The Author(s). HemaSphere is published by John Wiley & Sons Ltd on behalf of European Hematology Association. This is an open access Abstract Book distributed under the Attribution-NonCommercial-NoDerivs (CC BY-NC-ND), which allows third parties to download the articles and share them with others as long as they credit the author and the Abstract Book, but they cannot change the content in any way or use them commercially.
**Abstract Book Citations:** Authors, Title, HemaSphere, 2026;10:(S1):pages. The abstract book DOIs can be found on the “Author Information” page.
**Disclaimer:** Articles published in the journal HemaSphere exclusively reflect the opinions of the authors. The authors are responsible for all content in their abstracts including accuracy of the facts, statements, citing resources, etc.

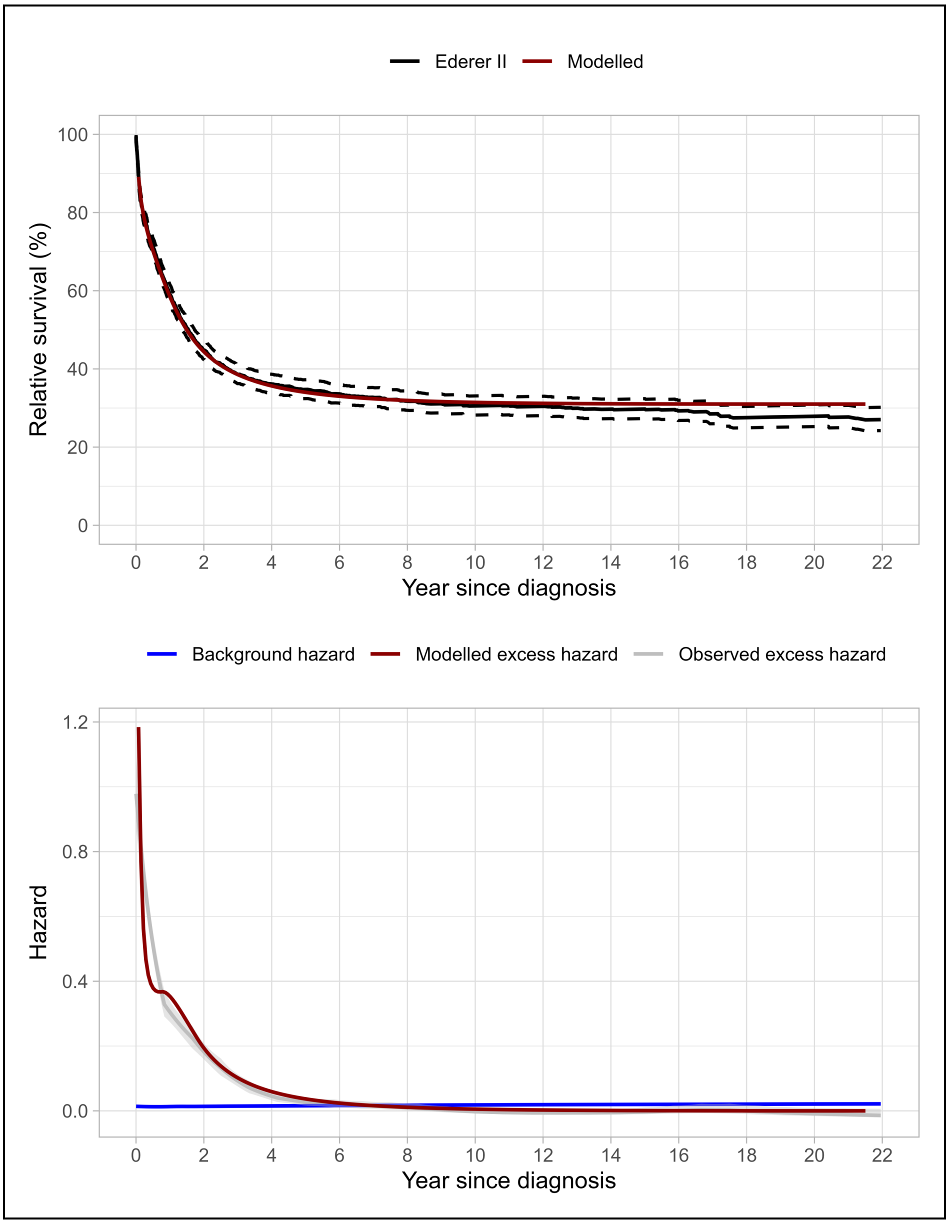

Figure 1: Relative survival and hazard of precursor lymphoid neoplasms in Spain, 1994-2018.

**Copyright Information:** (Online) ISSN: 2572-9241
©2026 The Author(s). HemaSphere is published by John Wiley & Sons Ltd on behalf of European Hematology Association. This is an open access Abstract Book distributed under the Attribution-NonCommercial-NoDerivs (CC BY-NC-ND), which allows third parties to download the articles and share them with others as long as they credit the author and the Abstract Book, but they cannot change the content in any way or use them commercially.
**Abstract Book Citations:** Authors, Title, HemaSphere, 2026;10:(S1):pages. The abstract book DOIs can be found on the "Author Information" page.
**Disclaimer:** Articles published in the journal HemaSphere exclusively reflect the opinions of the authors. The authors are responsible for all content in their abstracts including accuracy of the facts, statements, citing resources, etc.

### (PB2627) COMPLEMENTARITY OF DNA- AND RNA-BASED NEXT GENERATION SEQUENCING IN ACUTE LEUKEMIAS: IDENTIFICATION OF REARRANGEMENTS BY BOTH ANALYTES

**Topic:** 02. Acute lymphoblastic leukemia - Clinical

James S. Blachly*[1], Miriam Saffern[2], Dean Pavlick[2], Chelsea Marcus[2], Lincoln Pasquina[2]

[1]*The Ohio State University, Division of Hematology, Department of Internal Medicine, Columbus, United States of America;* [2]*Foundation Medicine, Inc., Boston, United States of America;*

### Background

Detection of many types of alterations is essential for diagnosis and treatment of patients with acute leukemia (AL). The optimal analyte for next generation sequencing (NGS) differs by alteration type to be assessed. For the detection of short variants, indels, and copy number alterations, DNA is preferred, while RNA is preferred for detection of rearrangements (REs) due to the range of potential breakpoints and RE partners.

### Aims

We describe the detection rate of disease-relevant variants of samples from patients with AL profiled by a single platform that analyzes both DNA and RNA. Further, we characterize REs that are potentially diagnostic identified by each analyte.

### Methods

We analyzed results from clinical NGS testing by FoundationOne Heme, a hybrid-capture based laboratory developed test that sequences DNA from 406 genes and RNA from 265 genes to profile hematologic malignancies as a laboratory developed test. Short variants (SNV), indels, and copy number alterations (CNA) are reported from DNA exclusively. REs are reported whether detected by DNA and/or RNA. Each sample is classified based on presumed diagnosis prior to sequencing.

**Copyright Information:** (Online) ISSN: 2572-9241
©2026 The Author(s). HemaSphere is published by John Wiley & Sons Ltd on behalf of European Hematology Association. This is an open access Abstract Book distributed under the Attribution-NonCommercial-NoDerivs (CC BY-NC-ND), which allows third parties to download the articles and share them with others as long as they credit the author and the Abstract Book, but they cannot change the content in any way or use them commercially.
**Abstract Book Citations:** Authors, Title, HemaSphere, 2026;10:(S1):pages. The abstract book DOIs can be found on the "Author Information" page.
**Disclaimer:** Articles published in the journal HemaSphere exclusively reflect the opinions of the authors. The authors are responsible for all content in their abstracts including accuracy of the facts, statements, citing resources, etc.

**Results**

Among AL, the most frequent REs found in 3753 samples submitted as acute lymphoblastic leukemia (ALL), including 3432 (91.4%) with results from both DNA and RNA, were *BCR-ABL1, ETV6-RUNX1*, and *IGH-CRLF2*. In 4820 samples submitted as acute myeloid leukemia (AML), including 4505 (93.5%) with results from both DNA and RNA, the most frequent REs were: *KMT2A-KMT2A* (including tandem duplications), *RUNX1-RUNX1T1*, and *MYH11-CBFB*. REs were detected by DNA, RNA, or both, each found across age ranges within B-ALL (0-14, 15-39, and 40+, **Table**).
Overall, of 3211 REs detected in ALL, 44.3% were detected by RNA only, and similarly for AML (2126 REs, 59.9% RNA only). 27.8% of ALL samples and 18.8% of AML samples had at least one RE reported only on RNA.
SNVs, indels, and CNAs were also detected. In ALL, CNAs (all losses) were detected in *CDKN2B* in 23.9% of samples, including 1.3% of samples co-occurring with a RE, and 1.5% with RE only (without CNA). SNVs and indels without a co-occurring RE in the same gene were also detected in *PAX5* (4.0% of samples), *ETV6* (2.9%), and *RUNX1* (2.5%). In AML, short variants in the same gene were detected in *NPM1* (14.8% of samples; RE in 0.3%), *RUNX1* (12.4%), and *ETV6* (1.4%).
We last assessed diagnostic REs. *PML-RARA* REs were found in 135 samples submitted as AML (n=118) or AL NOS (17). Of 137 distinct *PML-RARA* REs, 5 were detected by DNA only, 13 detected by RNA only, and 119 by both. A total of 1876 REs were found in 13 other diagnostic fusion pairs in the total AL cohort: 142 DNA only, 1082 detected by RNA only, and 652 both.

| | RE | DNA only | RNA only | Both |
|---|---|---|---|---|
| ALL | BCR-ABL1 | 13 | 328 | 93 |
| B-ALL age <15 | BCR-ABL1 | 0 | 5 | 3 |
| B-ALL age 15-39 | BCR-ABL1 | 0 | 43 | 6 |
| B-ALL age >=40 | BCR-ABL1 | 1 | 87 | 22 |
| ALL | ETV6-RUNX1 | 12 | 24 | 156 |
| ALL | IGH-CRLF2 | 36 | 3 | 166 |
| AML | KMT2A-KMT2A | 31 | 147 | 73 |
| AML | RUNX1-RUNX1T1 | 1 | 166 | 16 |
| AML | MYH11-CBFB | 0 | 133 | 8 |

**Summary/Conclusion**

This study provides evidence that DNA- and RNA-based NGS have complementary value. Treatment-informing alterations in AL are detected on DNA or RNA alone, or both, even within the same gene or fusion pair. Analysis of the same regions by different analytes may therefore be clinically warranted.
Factors affecting sensitivity of either analyte include assay design and sequenced regions, stability of the analyte (especially RNA), and success of sequencing. This can be especially true for samples where one analyte fails, typically RNA.

**Copyright Information:** (Online) ISSN: 2572-9241
©2026 The Author(s). HemaSphere is published by John Wiley & Sons Ltd on behalf of European Hematology Association. This is an open access Abstract Book distributed under the Attribution-NonCommercial-NoDerivs (CC BY-NC-ND), which allows third parties to download the articles and share them with others as long as they credit the author and the Abstract Book, but they cannot change the content in any way or use them commercially.
**Abstract Book Citations:** Authors, Title, HemaSphere, 2026;10:(S1):pages. The abstract book DOIs can be found on the “Author Information” page.
**Disclaimer:** Articles published in the journal HemaSphere exclusively reflect the opinions of the authors. The authors are responsible for all content in their abstracts including accuracy of the facts, statements, citing resources, etc.

### (PB2628) PREDICTIVE FACTORS OF RELAPSE IN ADULT T-CELL ACUTE LYMPHOBLASTIC LEUKEMIA

**Topic:** 02. Acute lymphoblastic leukemia - Clinical

Amel Meliane*[1], Hend Ben Neji[1]

[1]*hopital Aziza Othmana, Tunis, Tunisia;*

### Background

T-cell acute lymphoblastic leukemia (T-ALL) accounts for 25% of adult acute lymphoblastic leukemia  and it is characterized with a poor prognosis. Disease progression is marked by a high relapse rate, which further worsens the prognosis.

### Aims

The aim of our study was to investigate factors associated with relapse in adult T-ALL.

### Methods

This was a retrospective single-center study conducted in the adult clinical hematology department at Aziza Othmana Hospital, including patients aged 18 years or older followed for de novo T-ALL and treated according to the GRAALL05 protocol between January 2015 and December 2023.

### Results

Fifty patients were included. The median age was 37 years [24–62]. Eighteen patients (51%) relapsed, with a median time to relapse of 12 months [3–26]. Relapse sites were distributed as follows: isolated neuromeningeal relapse in 38% of cases, isolated medullary relapse in 33% of cases, combined medullary and neuromeningeal relapse in 11% of cases, medullary and cutaneous relapse in 5.5% of cases, and medullary, ocular, and testicular relapse in 5.5% of cases.
At diagnosis, hyperleukocytosis >100 G/L and mediastinal enlargement were observed in 66.6% and 50% of cases, respectively. Eight patients (44.4%) had a normal karyotype, and three patients  had a complex karyotype.
Two patients  had initial neuromeningeal involvement.
Among these patients, 13 received salvage therapy: high-dose cytarabine (HD Ara-C) : 5 patients, GRAALL05 reinduction : 3 patients, GRAALL14 reinduction : 2 patients, 2+5 regimen : 1 patient, hyper-CVAD : 1 patient, and high-dose methotrexate (HD MTX) : 1 patient.
In univariate analysis, the evaluation of age, sex, initial white blood cell count, presence of tumor syndrome, mediastinal enlargement, LDH level, cytogenetic risk, T-ALL stage, corticosteroid sensitivity, chemosensitivity, and post-induction MRD showed that only corticosteroid resistance had a statistically significant impact on relapse occurrence (P = 0.02).

### Summary/Conclusion

Salvage treatment options remain very limited in our country. The use of therapeutic agents such as nelarabine and venetoclax has led to improved therapeutic outcomes and thus to better prognosis.

**Copyright Information:** (Online) ISSN: 2572-9241
©2026 The Author(s). HemaSphere is published by John Wiley & Sons Ltd on behalf of European Hematology Association. This is an open access Abstract Book distributed under the Attribution-NonCommercial-NoDerivs (CC BY-NC-ND), which allows third parties to download the articles and share them with others as long as they credit the author and the Abstract Book, but they cannot change the content in any way or use them commercially.
**Abstract Book Citations:** Authors, Title, HemaSphere, 2026;10:(S1):pages. The abstract book DOIs can be found on the "Author Information" page.
**Disclaimer:** Articles published in the journal HemaSphere exclusively reflect the opinions of the authors. The authors are responsible for all content in their abstracts including accuracy of the facts, statements, citing resources, etc.

### (PB2629) EFFICACY OF BLINATUMOMAB FOR PLATELET TRANSFUSION REFRACTORINESS IN ACUTE LYMPHOBLASTIC LEUKEMIA

**Topic:** 02. Acute lymphoblastic leukemia - Clinical

Jieyu Ye[1], Baiwei Luo[1], Xuan Zhou[1], Wenhao Zhong[1], Zhixiang Wang[1], Bailin He[1], Guohuizhi Ye*[1], Hongsheng Zhou[1]

[1]*Department of Hematology, Nanfang Hospital, Southern Medical University, Guangzhou, China;*

### Background

Platelet transfusion refractoriness (PTR) is relatively common in patients with acute lymphoblastic leukemia (ALL). It increases bleeding risk, impairs therapeutic efficacy, and severely compromises patient prognosis, representing a critical issue in the management of ALL. Current therapeutic strategies include controlling infection, correcting DIC, transfusing HLA/HPA-matched platelets, using leukoreduced platelets, intravenous immunoglobulin (IVIG), or rituximab. Thrombopoietin (TPO) agonists may be added to stimulate endogenous platelet production and reduce bleeding risk. Nevertheless, many patients remain unresponsive to these approaches, highlighting the need for novel therapeutic interventions.Blinatumomab is the first bispecific T-cell engager (BiTE) immunotherapeutic agent worldwide and has become an important targeted therapy for ALL. This article presents the findings regarding the role of blinatumomab in the treatment of PTR.

### Aims

We herein reported 7 platelet transfusion refractoriness patients suffered by acute lymphoblastic leukemia who received blinatumomab.

### Methods

This study enrolled 7 patients with acute lymphoblastic leukemia and platelet transfusion refractoriness, who received blinatumomab treatment (days 1–14). The purpose of this study was to evaluate the efficacy of blinatumomab in the treatment of PTR.

### Results

Among the 7 ALL patients with platelet transfusion refractoriness, platelet-specific antibodies were detected in 3 cases (42.8%), and 6 cases (85.7%) no longer experienced PTR(Fig.A-C). Platelet transfusion refractoriness improved after treatment, with reduced platelet transfusion requirements and restored adequate platelet count increments(Fig.C). Blinatumomab was well-tolerated in all patients, with no transfusion-related reactions observed. It successfully solved the problem of PTR in some patients while treating ALL.

### Summary/Conclusion

In summary, we propose a method using blinatumomab for the management of platelet transfusion refractoriness in patients with acute lymphoblastic leukemia, which is partially effective and safe.

**Copyright Information:** (Online) ISSN: 2572-9241
©2026 The Author(s). HemaSphere is published by John Wiley & Sons Ltd on behalf of European Hematology Association. This is an open access Abstract Book distributed under the Attribution-NonCommercial-NoDerivs (CC BY-NC-ND), which allows third parties to download the articles and share them with others as long as they credit the author and the Abstract Book, but they cannot change the content in any way or use them commercially.
**Abstract Book Citations:** Authors, Title, HemaSphere, 2026;10:(S1):pages. The abstract book DOIs can be found on the “Author Information” page.
**Disclaimer:** Articles published in the journal HemaSphere exclusively reflect the opinions of the authors. The authors are responsible for all content in their abstracts including accuracy of the facts, statements, citing resources, etc.

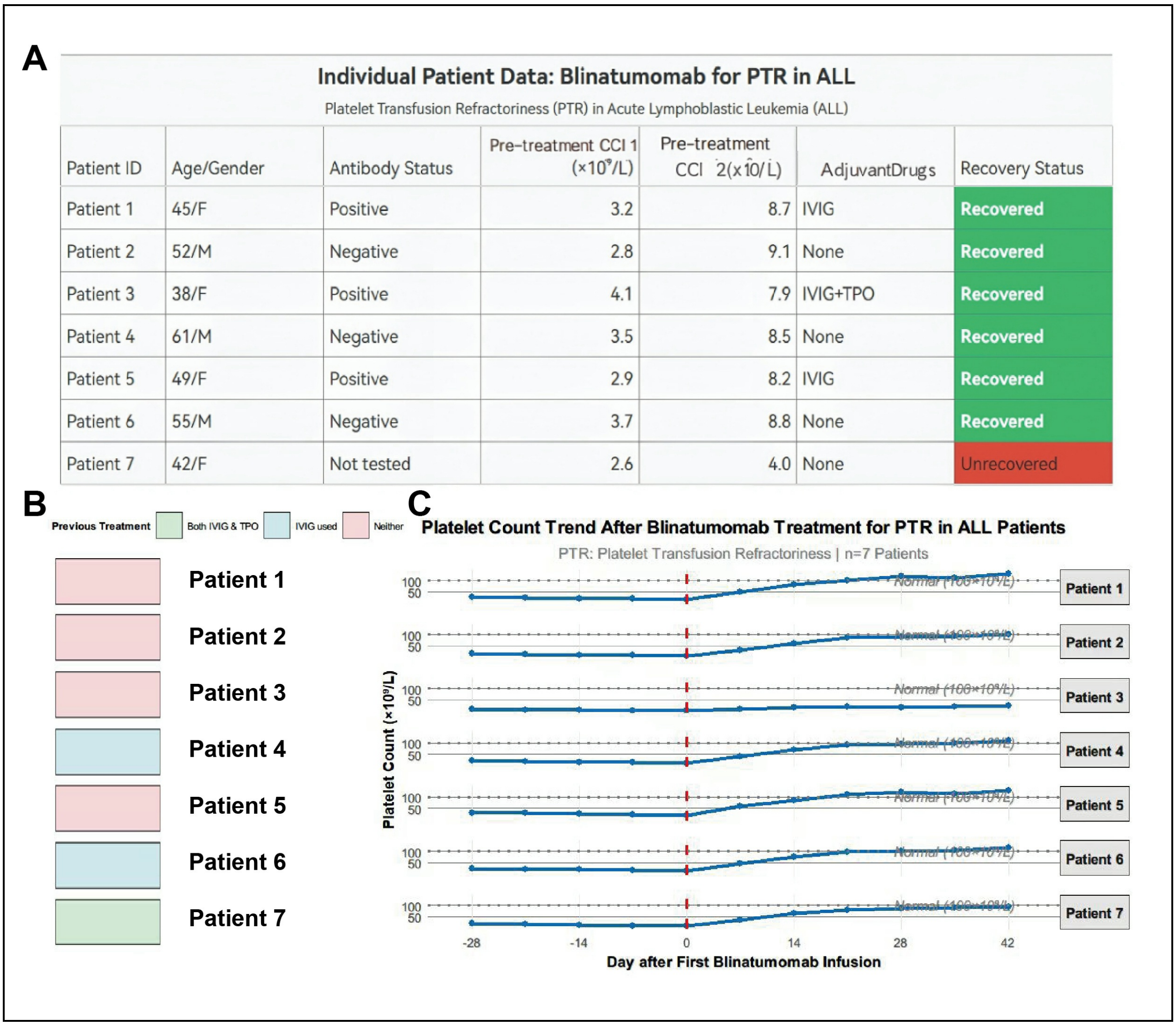

A

**Individual Patient Data: Blinatumomab for PTR in ALL**

Platelet Transfusion Refractoriness (PTR) in Acute Lymphoblastic Leukemia (ALL)

| Patient ID | Age/Gender | Antibody Status | Pre-treatment CCI 1 (×10⁹/L) | Pre-treatment CCI 2(x10/L) | AdjuvantDrugs | Recovery Status |
|---|---|---|---|---|---|---|
| Patient 1 | 45/F | Positive | 3.2 | 8.7 | IVIG | Recovered |
| Patient 2 | 52/M | Negative | 2.8 | 9.1 | None | Recovered |
| Patient 3 | 38/F | Positive | 4.1 | 7.9 | IVIG+TPO | Recovered |
| Patient 4 | 61/M | Negative | 3.5 | 8.5 | None | Recovered |
| Patient 5 | 49/F | Positive | 2.9 | 8.2 | IVIG | Recovered |
| Patient 6 | 55/M | Negative | 3.7 | 8.8 | None | Recovered |
| Patient 7 | 42/F | Not tested | 2.6 | 4.0 | None | Unrecovered |

Efficacy of blinatumomab for platelet transfusion refractoriness (PTR) in acute lymphoblastic leukemia (ALL). (A) Individual patient characteristics and treatment outcomes. (B) Previous treatment stratification showing IVIG and TPO usage. (C) Platelet count trends after blinatumomab treatment across 7 patients, with day 0 indicating the first blinatumomab infusion; red dashed line marks treatment initiation, and dotted lines indicate normal platelet count range (100-300 ×10⁹/L). CCI, corrected count increment; IVIG, intravenous immunoglobulin; TPO, thrombopoietin.

**Copyright Information:** (Online) ISSN: 2572-9241
©2026 The Author(s). HemaSphere is published by John Wiley & Sons Ltd on behalf of European Hematology Association. This is an open access Abstract Book distributed under the Attribution-NonCommercial-NoDerivs (CC BY-NC-ND), which allows third parties to download the articles and share them with others as long as they credit the author and the Abstract Book, but they cannot change the content in any way or use them commercially.
**Abstract Book Citations:** Authors, Title, HemaSphere, 2026;10:(S1):pages. The abstract book DOIs can be found on the “Author Information” page.
**Disclaimer:** Articles published in the journal HemaSphere exclusively reflect the opinions of the authors. The authors are responsible for all content in their abstracts including accuracy of the facts, statements, citing resources, etc.

### (PB2630) CONTEMPORARY REAL-WORLD TREATMENT PATTERNS, MRD TESTING PATTERNS AND OUTCOMES OF ADULT PATIENTS WITH B-CELL ACUTE LYMPHOBLASTIC LEUKEMIA (ALL) TREATED IN THE UNITED STATES IN 2018 OR LATER

**Topic:** 02. Acute lymphoblastic leukemia - Clinical

Olalekan Oluwole*[1], Enrico de Nigris[2], Jacob Ambrose[3], Jennifer Prescott[4], Razia Akhtar[4], Diana Chirovsky[4], Holger Keim[4], Christina Zettler[3], Laura Fernandes[3], Ming He[3]

*[1]Vanderbilt University Medical Center, Nashville, TN, United States of America;[2]MSD (UK) Limited, London, UK, London, United Kingdom; [3]COTA, Inc., New York, NY, United States of America;[4]Merck & Co., Inc., Rahway, NJ, USA, Rahway, NJ, United States of America;*

### Background

Historically, B-cell ALL in adults has been characterized by poor long-term outcomes, including high relapse rates even among frontline (1L)-treated patients (pts) who achieve complete response (CR). Targeted therapies, such as blinatumomab (blina), inotuzumab (ino) and chimeric antigen receptor T-cell (CAR-T) therapy, have revolutionized the treatment (tx) landscape. There is scant contemporaneous evidence of real-world tx patterns and outcomes among pts treated from the 1L through the relapsed/refractory (R/R) setting, including minimal residual disease (MRD) testing.

### Aims

To characterize contemporary real-world tx, MRD testing patterns, and outcomes among pts with 1L and R/R B-cell ALL.

### Methods

Eligible pts in COTA's real-world, electronic health records-based database met the following criteria: adults with diagnosis (dx) of B-cell ALL in 2018+ who initiated 1L tx on or before April 18, 2025. The index dates for 1L and R/R groups were tx initiation in each setting, and pts who received 1L tx and later had R/R disease were included in both groups. Time to event endpoints (real-world event-free survival (rwEFS; defined as time from index to tx for R/R disease or death) and overall survival (rwOS)) were analyzed using the Kaplan-Meier (KM) method, and CR rate (rwCRR) was calculated. Subgroup analyses by Ph status (derived with molecular marker, subtype, and tx data) and tx receipt (1L or R/R blina or ino) were conducted. MRD positivity was defined by a quantitative $1x10^{-4}$ threshold.

**Copyright Information:** (Online) ISSN: 2572-9241
©2026 The Author(s). HemaSphere is published by John Wiley & Sons Ltd on behalf of European Hematology Association. This is an open access Abstract Book distributed under the Attribution-NonCommercial-NoDerivs (CC BY-NC-ND), which allows third parties to download the articles and share them with others as long as they credit the author and the Abstract Book, but they cannot change the content in any way or use them commercially.
**Abstract Book Citations:** Authors, Title, HemaSphere, 2026;10:(S1):pages. The abstract book DOIs can be found on the "Author Information" page.
**Disclaimer:** Articles published in the journal HemaSphere exclusively reflect the opinions of the authors. The authors are responsible for all content in their abstracts including accuracy of the facts, statements, citing resources, etc.

### Results

N=598 1L-treated pts qualified. Median age at dx was 49 years, and pts were 50% male, 62% White, and 55% treated in the community. N=316 pts were Ph- (53%), 236 (39%) were Ph+, and 46 (8%) had unknown Ph status. Most pts received 1L chemotherapy without blina/ino (N=357; 60%), and 146 (24%) received a TKI. About 30% of pts received 1L blina (N=178), and 8% received 1L ino (N=46). Most (65%; N=116) 1L blina receipt occurred after 1L CR, while 67% (N=31) of 1L ino receipt occurred prior to/without 1L CR.

rwCRR in 1L was 83% overall and was similar across both tx and Ph status groups. Among pts with a 1L rwCR (N=499), 320 (64%) had at least one MRD test, and 227 (45%) had ≥ 2 MRD tests. Median time from 1L tx start to first MRD test was 69 days, and median time from 1L CR to first MRD test was 19 days. Among 115 pts with a quantitative MRD result, 49 (43%) were MRD+. Among 38 MRD+ pts with ≥ 2 MRD tests, 61% (N=23) converted to MRD- with 1L tx. Median rwEFS overall, among Ph+, and among Ph- groups was 18.4 (95% CI: 14.7, 22.1), 26.4 (95% CI: 18.0, 44.3), and 15.0 months (mos) (95% CI: 12.3, 21.0), respectively. (**Image**)

Over a median follow-up (reverse KM) of less than 3 years, 161 (27%) pts experienced R/R disease, and 2/3 (N=108) were Ph-. Chemotherapy was common in R/R tx (N=84; 52%), and 14 R/R pts (9%) received a TKI. N=62 (39%) received blina, 46 (29%) received ino, and 8 (5%) received CAR-T therapy. Median rwOS among the R/R population was 12.8 mos (95% CI: 10.4, 20.5).

### Summary/Conclusion

In this contemporaneous analysis of B-cell ALL, sequencing of targeted therapy with blina and ino is emerging. More than 83% of pts achieved 1L CR; however, in less than 3 years, relapse rates remain high, and survival outcomes meaningfully decline with disease progression. There remains substantial unmet need for more effective and durable novel therapies to prevent relapse and improve outcomes in both frontline and relapsed/refractory settings.

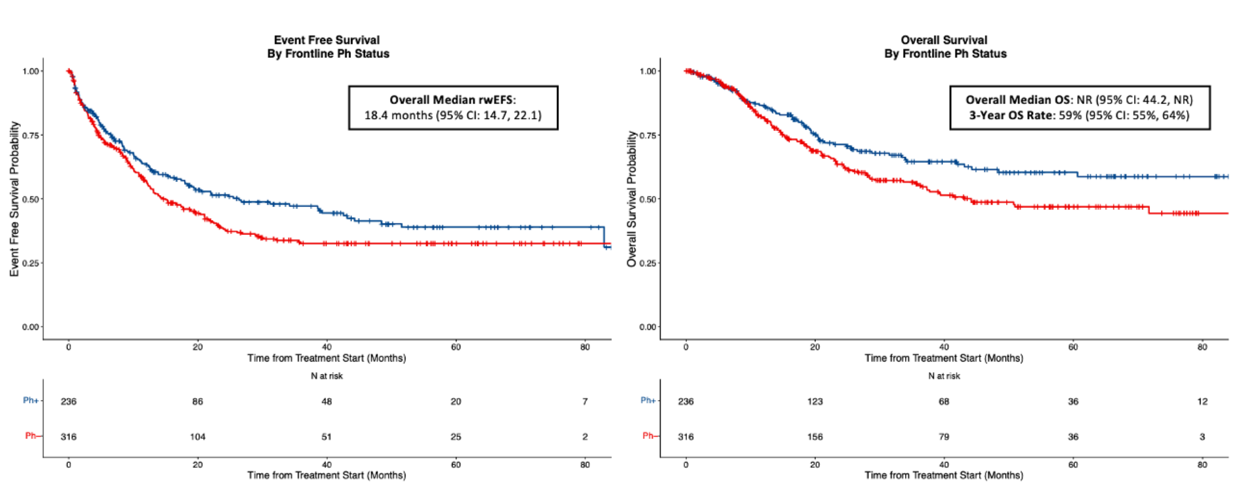

*CI: confidence interval; NR: not reached; OS: overall survival; rwEFS: real-world event-free survival*

**Copyright Information:** (Online) ISSN: 2572-9241
©2026 The Author(s). HemaSphere is published by John Wiley & Sons Ltd on behalf of European Hematology Association. This is an open access Abstract Book distributed under the Attribution-NonCommercial-NoDerivs (CC BY-NC-ND), which allows third parties to download the articles and share them with others as long as they credit the author and the Abstract Book, but they cannot change the content in any way or use them commercially.
**Abstract Book Citations:** Authors, Title, HemaSphere, 2026;10:(S1):pages. The abstract book DOIs can be found on the "Author Information" page.
**Disclaimer:** Articles published in the journal HemaSphere exclusively reflect the opinions of the authors. The authors are responsible for all content in their abstracts including accuracy of the facts, statements, citing resources, etc.

### (PB2631) MECHANISMS OF DEVELOPMENT OF ACUTE LEUKEMIA IN CHILDREN LIVING IN RADIOACTIVELY CONTAMINATED AREAS AFTER THE CHERNOBYL DISASTER

**Topic:** 02. Acute lymphoblastic leukemia - Clinical

Katerina Bruslova*[1], Lyidmila Lyashenko[1], Nataliia Tsvietkova[1], Tetiana Pushkaryova[1], Sergii Iatsemyrskyi[1], Larysa Gonchar[1]

[1] *"National Research Center for Radiation Medicine, Hematology and Oncology of The National Academy ofMedical Science of Ukraine", Kyiv, Ukraine;*

### Background

The condition of the stromal microenvironment (SM) of the bone marrow (BM) and bone structures plays a role in the mechanisms of leukogenesis.

### Aims

To evaluate osteogenesis processes during chemotherapy in children with acute lymphoblastic leukemia depending on iron metabolism and hormonal regulation for the formation of risk groups among children in the general population and residents of radioactively contaminated territories (RCT).

### Methods

We examined 136 children 6-18 years old with ALL (pro-B –10 patients, B-Common–106, pre-B –12, T-8). We studied BM cellularity and  different BM branches recovery(R) after chemotherapy (CT) protocol phase as  remission induction and consolidation. We assessed bone structures condition, total protein, amino acids involved in collagen methabolism, alkaline phosphatase(AF), vitamin D, calcium, iron(SI), ferritin(SF), transferrin, transferrin saturation coefficient, levels of cortisol, free thyroxine, thyroid-stimulating hormone (TSH) in blood serum, and bone mineral density (BMD). In age-and sex-matched group of 125 children from general population with osalgia and arthralgia the same data as for ALL patients were studied over the 2-3 year period. Mathematical statistics were used (Student, U-test, Pearson's $\chi 2$ criterion).

**Copyright Information:** (Online) ISSN: 2572-9241
©2026 The Author(s). HemaSphere is published by John Wiley & Sons Ltd on behalf of European Hematology Association. This is an open access Abstract Book distributed under the Attribution-NonCommercial-NoDerivs (CC BY-NC-ND), which allows third parties to download the articles and share them with others as long as they credit the author and the Abstract Book, but they cannot change the content in any way or use them commercially.
**Abstract Book Citations:** Authors, Title, HemaSphere, 2026;10:(S1):pages. The abstract book DOIs can be found on the "Author Information" page.
**Disclaimer:** Articles published in the journal HemaSphere exclusively reflect the opinions of the authors. The authors are responsible for all content in their abstracts including accuracy of the facts, statements, citing resources, etc.

**Results**

Hemopoiesis recovery in 63% of ALL children during CT was by the granulocytic type. Erythroid R was observed in 1/3 of children only during the remission induction CT. The monocytic type was characteristic of children at the start of the consolidation, indicating multivector mechanisms of hematopoiesis R regulation. During the stages of CT, the content of SI and SF in patients increased, and after CT, 24.6% had an excess of SF (($345.3 \pm 16.4$) ng/ml). A reverse correlation was established between SI and SF and the prognosis of ALL ($r = -1.0$ and $r = -0.44$, respectively) ($p < 0.001$). After CT, the highest SF level was in patients with pro-B-ALL ($485.0 \pm 22.7$) ng/ml. During the stages of CT, the BMD decreased, with deviations in 20%–22% due to changes in organic and mineral components and, accordingly, in SM. A direct relationship between AF and SI was established ($r = 0.25$, $p < 0.05$). In the initial period of ALL, 26% of children had manifestations of adrenal cortex hypofunction, and 6% had thyroid hypofunction. After CT, 51% of children had increased TSH levels, which amounted to 7.23±0.61 IU/l. To identify bone structure abnormalities in 136 children without oncohematological pathology, residents of the RCT, with complaints of osalgia, arthralgia, and a history of bone fractures, three groups were formed depending on the BMD. Group I included 63 children (46.3%) with normal BMD, group II included 47 children (34.6%) with 25-30% reduced BMD, and group III included 26 patients (19.1%) with BMD below 30%. Children in group I had no changes in bone structure. Children in group II had changes in the organic component of bone tissue: deficiency of glycine, lysine, increased AF, decreased calcium levels. Children in group II also had protein deficiency, changes in bone mineralization, thyroid disorders, and excess of iron. The BM radiation doses in ALL patients were (3.50±0.57) mSv, while in children without leukemia, the internal radiation doses were (1.45±0.17) mSv; these doses did not affect bone formation indicators.

**Summary/Conclusion**

Changes in bone structures and iron metabolism play a role in the mechanisms of ALL development, therefore children with such disorders are at risk of oncohematological pathology.

**Copyright Information:** (Online) ISSN: 2572-9241
©2026 The Author(s). HemaSphere is published by John Wiley & Sons Ltd on behalf of European Hematology Association. This is an open access Abstract Book distributed under the Attribution-NonCommercial-NoDerivs (CC BY-NC-ND), which allows third parties to download the articles and share them with others as long as they credit the author and the Abstract Book, but they cannot change the content in any way or use them commercially.
**Abstract Book Citations:** Authors, Title, HemaSphere, 2026;10:(S1):pages. The abstract book DOIs can be found on the "Author Information" page.
**Disclaimer:** Articles published in the journal HemaSphere exclusively reflect the opinions of the authors. The authors are responsible for all content in their abstracts including accuracy of the facts, statements, citing resources, etc.

### (PB2633) SFMBT1-RELATED GENE FUSIONS IDENTIFIED IN PATIENTS WITH B-CELL ACUTE LYMPHOBLASTIC LEUKEMIA

**Topic:** 02. Acute lymphoblastic leukemia - Clinical

Dai-Hong Xie*[1], Kai Sun[1], Zong-Yan Shi[1], Zhao-Yu Li[1], Wen-Min Chen[1], Ya-Zhen Qin[1]

*[1]Peking University People's Hospital, Peking University Institute of Hematology, Beijing, China;*

### Background

Gene fusions are major molecular abnormalities in B-cell acute lymphoblastic leukemia (B-ALL). They not only serve as markers for molecular subtyping and prognosis but also are valuable indicators for minimal residual disease (MRD) monitoring.

### Aims

This study aimed to identify novel fusion genes in B-ALL patients.

### Methods

Diagnostic bone marrow aspirates from 72 adult B-ALL patients were subjected to transcriptome sequencing. The expression levels of gene fusions mRNAs were evaluated using real-time quantitative PCR (RQ-PCR).

### Results

Novel SFMBT1-related gene fusion mRNAs were detected in 2 (2.8%) patients, namely SFMBT1::LTF and SFMBT1::STIMATE. SFMBT1, LTF and STIMATE are all located on the short arm of chromosome 3 at band 21(3p21). Moreover, both fusions had the same two breakpoints in SFMBT1 gene within the 5'UTR (chr3: 53046460 and 53045816), one in the promoter region and the other in exon 1. Both patients were classified as standard-risk. They achieved complete remission after one course of induction therapy but both subsequently developed hematologic relapse following the second course of standard consolidation therapy. Furthermore, the fusion mRNA levels during treatment were correlated with the proportion of blast cells (Figure 1) which indicated that fusion mRNA is specifically expressed in the patients' leukemic cells and may serve as a molecular marker for MRD monitoring.

### Summary/Conclusion

The recurrent SFMBT1-related gene fusions may be associated with poor prognosis in B-ALL patients, while their underlying mechanisms warrant further investigation. (Grant: The National Natural Science Foundation of China (82370160)).

**Copyright Information:** (Online) ISSN: 2572-9241
©2026 The Author(s). HemaSphere is published by John Wiley & Sons Ltd on behalf of European Hematology Association. This is an open access Abstract Book distributed under the Attribution-NonCommercial-NoDerivs (CC BY-NC-ND), which allows third parties to download the articles and share them with others as long as they credit the author and the Abstract Book, but they cannot change the content in any way or use them commercially.
**Abstract Book Citations:** Authors, Title, HemaSphere, 2026;10:(S1):pages. The abstract book DOIs can be found on the "Author Information" page.
**Disclaimer:** Articles published in the journal HemaSphere exclusively reflect the opinions of the authors. The authors are responsible for all content in their abstracts including accuracy of the facts, statements, citing resources, etc.

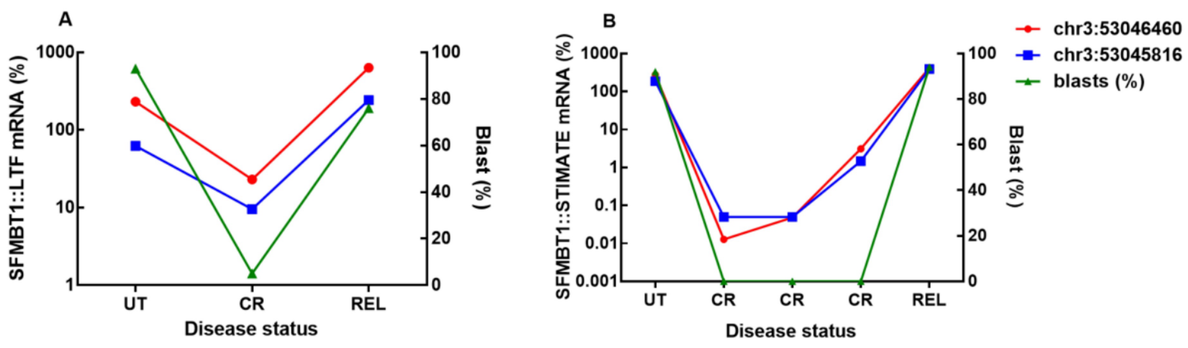

Figure 1. RQ-PCR analysis of SFMBT1 fusion mRNA levels at diagnosis and after treatment. (A) SFMBT1::LTF fusion and (B) SFMBT1::STIMATE fusion.

Copyright Information: (Online) ISSN: 2572-9241
©2026 The Author(s). HemaSphere is published by John Wiley & Sons Ltd on behalf of European Hematology Association. This is an open access Abstract Book distributed under the Attribution-NonCommercial-NoDerivs (CC BY-NC-ND), which allows third parties to download the articles and share them with others as long as they credit the author and the Abstract Book, but they cannot change the content in any way or use them commercially.
Abstract Book Citations: Authors, Title, HemaSphere, 2026;10:(S1):pages. The abstract book DOIs can be found on the "Author Information" page.
Disclaimer: Articles published in the journal HemaSphere exclusively reflect the opinions of the authors. The authors are responsible for all content in their abstracts including accuracy of the facts, statements, citing resources, etc.

### (PB2634) CLINICAL ANALYSIS AND TREATMENT OUTCOMES OF PEDIATRIC PATIENTS WITH FIRST RELAPSE OF B-CELL ACUTE LYMPHOBLASTIC LEUKEMIA FROM A SINGLE-CENTER

**Topic:** 02. Acute lymphoblastic leukemia - Clinical

Juan Xiao*[1], Zhouyang Liu[1], Yuan Sun[1]

*[1]Beijing Jingdu Children's Hospital, Department of Hematology, Beijing, China;*

### Background

Relapsed pediatric B-ALL carries a poor prognosis.This study analyzes the clinical characteristics, treatment regimens, and outcomes of 48 children with first-relapse B-cell acute lymphoblastic leukemia (B-ALL).

### Aims

It aims to investigate the clinical efficacy of VMLD regimen for re-induction of remission followed by allogeneic hematopoietic stem cell transplantation (allo-HSCT) in treating first-relapse B-ALL.

### Methods

A retrospective analysis was conducted on 43 children with first-relapse B-ALL who were admitted to Beijing Jingdu Children's Hospital from June 1, 2020, to May 31, 2025, following initial chemotherapy. Relapse risk stratification was performed according to the BFM criteria. Patients with relapse received re-induction chemotherapy with the VMLD regimen; patients with very early relapse additionally received ifosfamide (IFO). After achieving second complete remission (CR2), patients were assigned to either a transplantation group or a non-transplantation group. The transplantation group underwent 1-2 courses of consolidation therapy followed by allo-HSCT according to our hospital's high-risk ALL transplantation protocol. The non-transplantation group continued consolidation and maintenance chemotherapy according to the CCLG-ALL-2018 high-risk protocol. The primary endpoints were the re-induction remission rate and adverse effects of the VMLD±IFO regimen, as well as the overall survival (OS) and disease-free survival (DFS) rates of the patients.

### Results

The study included 48 children with first-relapse B-ALL. The median age was 4.8 years (range: 0.3-12.8 years), and the median time from diagnosis to relapse was 19.5 months (range: 2-94 months). According to BFM relapse risk stratification, 2 patients (4.2%) were classified as low-risk, 19 patients (39.6%) as intermediate-risk, and 27 patients (56.2%) as high-risk. 21 patients received the VMLD induction regimen, while 27 patients received the VMLD+IFO induction regimen. The CR2 rate was 93%, and the MRD negativity rate was 74.4%. No fatal severe infections occurred. 35 patients underwent allo-HSCT. The 3-year OS and DFS for this group were 84.7% and 77.3%, respectively. Specifically, the 3-year OS was 100% for the intermediate-risk group and 76.9% for the high-risk group. In contrast, the 3-year OS and DFS for non-transplanted patients were 35.7% and 25.8%, respectively.

### Summary/Conclusion

Allogeneic hematopoietic stem cell transplantation remains the standard treatment for relapsed B-ALL. Data from our center demonstrate that the VMLD regimen achieves a high re-induction remission rate with good tolerability, and subsequent bridging to transplantation significantly improves the clinical outcomes of relapsed ALL.

**Copyright Information:** (Online) ISSN: 2572-9241
©2026 The Author(s). HemaSphere is published by John Wiley & Sons Ltd on behalf of European Hematology Association. This is an open access Abstract Book distributed under the Attribution-NonCommercial-NoDerivs (CC BY-NC-ND), which allows third parties to download the articles and share them with others as long as they credit the author and the Abstract Book, but they cannot change the content in any way or use them commercially.
**Abstract Book Citations:** Authors, Title, HemaSphere, 2026;10:(S1):pages. The abstract book DOIs can be found on the "Author Information" page.
**Disclaimer:** Articles published in the journal HemaSphere exclusively reflect the opinions of the authors. The authors are responsible for all content in their abstracts including accuracy of the facts, statements, citing resources, etc.

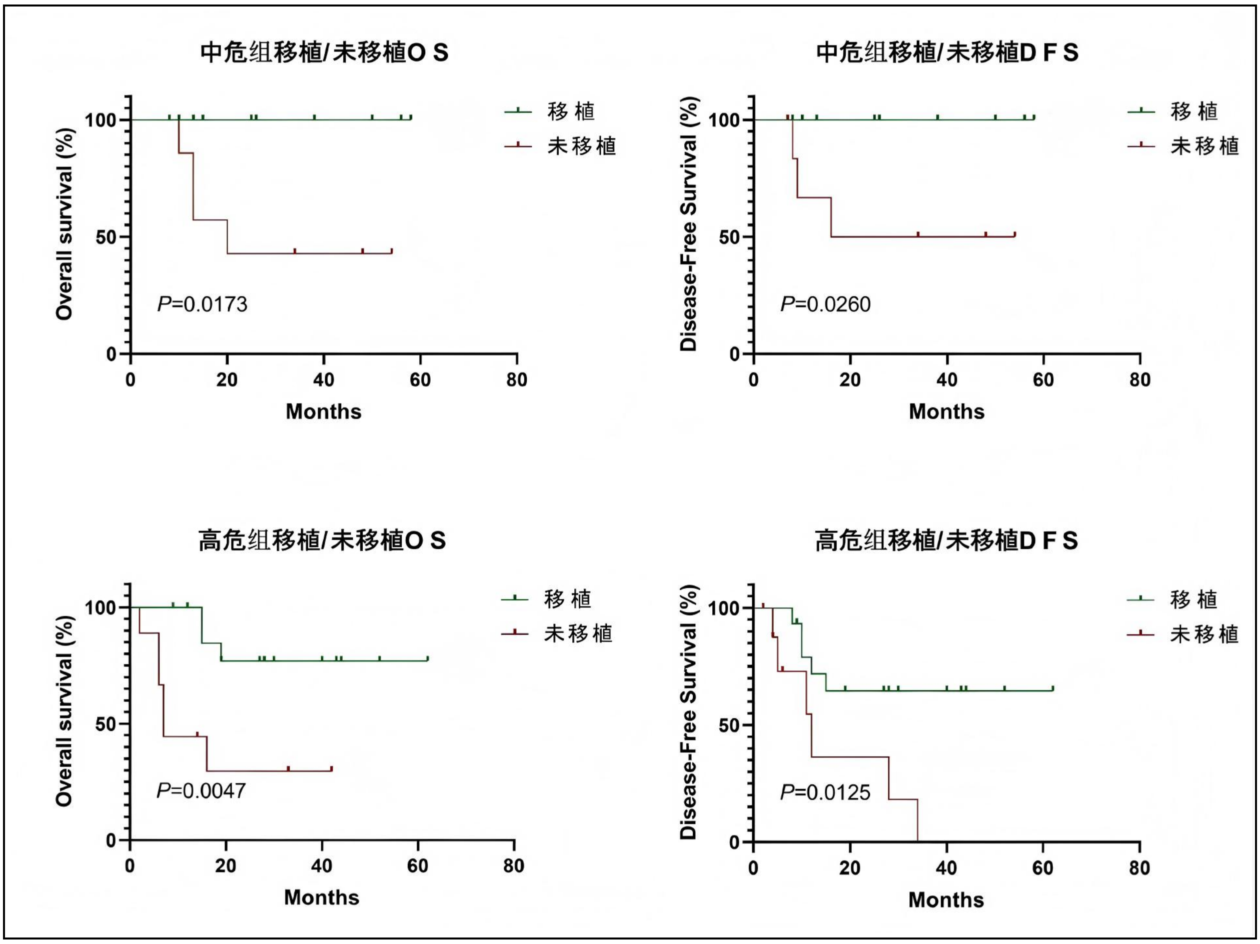

**Copyright Information:** (Online) ISSN: 2572-9241
©2026 The Author(s). HemaSphere is published by John Wiley & Sons Ltd on behalf of European Hematology Association. This is an open access Abstract Book distributed under the Attribution-NonCommercial-NoDerivs (CC BY-NC-ND), which allows third parties to download the articles and share them with others as long as they credit the author and the Abstract Book, but they cannot change the content in any way or use them commercially.
**Abstract Book Citations:** Authors, Title, HemaSphere, 2026;10:(S1):pages. The abstract book DOIs can be found on the "Author Information" page.
**Disclaimer:** Articles published in the journal HemaSphere exclusively reflect the opinions of the authors. The authors are responsible for all content in their abstracts including accuracy of the facts, statements, citing resources, etc.

### (PB2635) MEASURABLE RESIDUAL DISEASE AND SURVIVAL OUTCOMES IN ADULT B-CELL ACUTE LYMPHOBLASTIC LEUKEMIA: A SYSTEMATIC REVIEW OF THE LATIN AMERICAN EVIDENCE

**Topic:** 02. Acute lymphoblastic leukemia - Clinical

Jairo Jattin-Balcázar*[1], Karen Hurtado-Colorado[1]

[1]*Universidad Nacional de Colombia, Facultad de Medicina, Bogotá, Colombia;*

### Background

The prognostic significance of Measurable Residual Disease (MRD) in adult B-cell Acute Lymphoblastic Leukemia (B-ALL) is well-established in international guidelines; however, its specific impact within the Latin American clinical context remains poorly synthesized. Regional challenges, including genomic heterogeneity (high prevalence of Ph-like/CRLF2 alterations) and healthcare disparities, may influence both MRD clearance rates and the subsequent risk of relapse or death

### Aims

This systematic review aims to quantify the pooled rate of MRD negativity and evaluate its prognostic correlation with overall survival (OS) in adult and AYA B-ALL patients treated in Latin American centers

### Methods

A systematic search was conducted across PubMed, SciELO, and LILACS databases focusing on observational and clinical studies of adult B-ALL patients in Latin America reporting MRD data. Data extraction included induction protocols, transplant (HSCT) modalities, and survival outcomes stratified by MRD status. Quantitative synthesis was performed to estimate pooled MRD negativity rates at the end of induction (D28-35) and to analyze the magnitude of Hazard Ratios (HR) for OS across different treatment phases

### Results

Analysis of 113 evaluable patients from multiple regional cohorts (Mexico, Brazil, Chile, Colombia) revealed a pooled MRD negativity rate of 48.6% at the end of induction (D28-35). Patients achieving MRD negativity demonstrated a median OS gain of 30.2 months compared to MRD-positive counterparts, with the survival gap extending to 49 months in the post-HSCT setting. The prognostic weight of MRD persistence showed a marked escalation across treatment milestones: HR for OS was 5.75 (95% CI 1.82–18.18) at the end of induction, 8.8 (95% CI 1.44–53.73) at Day 30 post-HSCT, and reached 28.5 (95% CI 1.61–50.55) at Day 100 post-HSCT. Pediatric-inspired protocols showed higher MRD clearance rates compared to conventional adult regimens. Notably, a 14.1% loss of MRD negativity was observed between Day 30 and Day 100 post-HSCT, while non-relapse mortality (NRM) was significantly higher in MRD-positive patients (31.4% vs. 12.1%).

### Summary/Conclusion

MRD is a potent but heterogeneous biomarker in Latin American adult B-ALL. The pooled negativity rate of 48.6% suggests a lower initial molecular response compared to international benchmarks. The extreme magnitude of the hazard ratios, particularly in the post-transplant setting, identifies MRD persistence as a critical marker of therapeutic failure or high-risk biology in the region. These findings underscore the need for standardized MRD monitoring and early intervention strategies tailored to the specific risk profile and healthcare constraints of Latin American populations

**Copyright Information:** (Online) ISSN: 2572-9241
©2026 The Author(s). HemaSphere is published by John Wiley & Sons Ltd on behalf of European Hematology Association. This is an open access Abstract Book distributed under the Attribution-NonCommercial-NoDerivs (CC BY-NC-ND), which allows third parties to download the articles and share them with others as long as they credit the author and the Abstract Book, but they cannot change the content in any way or use them commercially.
**Abstract Book Citations:** Authors, Title, HemaSphere, 2026;10:(S1):pages. The abstract book DOIs can be found on the “Author Information” page.
**Disclaimer:** Articles published in the journal HemaSphere exclusively reflect the opinions of the authors. The authors are responsible for all content in their abstracts including accuracy of the facts, statements, citing resources, etc.

### (PB2636) INTRODUCTION OF TREATMENT FOR CHILDREN WITH ACUTE LYMPHOBLASTIC LEUKEMIA IN A RESOURCE LIMITED HOSPITAL IN CAMBODIA

**Topic:** 02. Acute lymphoblastic leukemia - Clinical

Sereyleak Bun*[1], Sam Lyvannak[1]

*[1]Angkor Hospital for Children, Oncology, Seim Reap, Cambodia;*

### Background

In developed country outcome ALL exceed 90%, however in resource-limited setting still poor, reasons for this included limitations in funding, equipment, laboratory testing , hospital facilities, drug accessibility ..etc.Prior to 2018, ALL was a uniformly fatal disease for children at Angkor Hospital for Children in Siem Reap Cambodia as they were able to receive palliative care only.In 2018, an ALL regimen was initiated that was comprised of a 3-drug induction, methotrexate (Capizzi dosing) intensification, and continuation (oral 6-mercaptopurine, oral methotrexate, vincristine and prednisone pulses); intrathecal central nervous system prophylaxis was included in all phases.

### Aims

- To evaluate the initial implement of low intensity regimen of ALL in resource setting-limited outcome at AHC over 5 years from 2018-2023.

### Methods

- Retrospective ,patient's chart( written consent form before started treatment) , who had diagnosed with ALL by flow cytometry from bone marrow aspiration or peripheral blood or sometime bone marrow core biopsy.
- Cytogenetics and molecular studies were not available.
- From March 2018 to December 2023 at Angkor Hospital for Children.Method for analysis Kaplan-Meier , log-rank test and Gray's test

### Results

Analysis of results after the first 5 years of implementation (2018-2023) with n=98 patients demonstrate a 2-year relapse free survival of 66.3% (95% CI 56.2-78.2) and relapse risk (RR) of 21% (95% CI 12.3-31.3). Treatment related mortality (TRM) was 7.2% (95% CI 3.2- 13.6) within the first month of therapy, and 2-year TRM of 12.7% (95% CI 6.6-21.0). Further improvements will likely require further improvement in dedicated cancer facilities, protocol intensification and continuing community and medical education.

**Copyright Information:** (Online) ISSN: 2572-9241
©2026 The Author(s). HemaSphere is published by John Wiley & Sons Ltd on behalf of European Hematology Association. This is an open access Abstract Book distributed under the Attribution-NonCommercial-NoDerivs (CC BY-NC-ND), which allows third parties to download the articles and share them with others as long as they credit the author and the Abstract Book, but they cannot change the content in any way or use them commercially.
**Abstract Book Citations:** Authors, Title, HemaSphere, 2026;10:(S1):pages. The abstract book DOIs can be found on the "Author Information" page.
**Disclaimer:** Articles published in the journal HemaSphere exclusively reflect the opinions of the authors. The authors are responsible for all content in their abstracts including accuracy of the facts, statements, citing resources, etc.

**Summary/Conclusion**

In conclusion, we describe the first results of an ALL protocol implemented by the ASHHVO-AHC collaboration in Siem Reap, Cambodia, which has dramatically improved the 2-year RFS from 0% to 66.3%. In efforts to continue to improve outcomes for these patients, further refinements in diagnostic techniques with a focus in utilizing more local resources, additional supportive care resources, ensuring drug accessibility, education of families and the referring community as well as protocol intensification will be needed.

**Copyright Information:** (Online) ISSN: 2572-9241
©2026 The Author(s). HemaSphere is published by John Wiley & Sons Ltd on behalf of European Hematology Association. This is an open access Abstract Book distributed under the Attribution-NonCommercial-NoDerivs (CC BY-NC-ND), which allows third parties to download the articles and share them with others as long as they credit the author and the Abstract Book, but they cannot change the content in any way or use them commercially.
**Abstract Book Citations:** Authors, Title, HemaSphere, 2026;10:(S1):pages. The abstract book DOIs can be found on the "Author Information" page.
**Disclaimer:** Articles published in the journal HemaSphere exclusively reflect the opinions of the authors. The authors are responsible for all content in their abstracts including accuracy of the facts, statements, citing resources, etc.

### (PB2637) FIRST-IN-WORLD SUBCUTANEOUS BLINATUMOMAB FOR CD19-POSITIVE MIXED PHENOTYPE ACUTE LEUKEMIA (MPAL): MULTICENTER PHASE II TRIAL AND FIRST PATIENT COMPLETE REMISSION WITH MINIMAL RESIDUAL DISEASE NEGATIVE

**Topic:** 02. Acute lymphoblastic leukemia - Clinical

Ashkan Emadi*[1], Abigail Swiger[1], Gary Monroe[1], Cara Randall[2], Austin Goncz[1], Jodi Aldinger[1], Christinea Marino[1], Morgan Bland[1], Angela Burrie[1], Anne Michels[1], Gabriel Kemp[1], Konstantinos Sdrimas[1], Lauren Veltri[1], Salah Ud Din Safi[1], Julia Deel[1], Faraz Zaman[3], Aaron Cumpston[1]

*[1]West Virginia University, Medical Oncology and WVU Cancer Institute, Morgantown, United States of America;[2]West Virginia University, Department of Pathology, Morgantown, United States of America; [3]Amgen, Inc., Thousand Oaks, United States of America;*

### Background

MPAL is an ultra-orphan acute leukemia (1-3% of cases) with no approved, disease-specific therapy and very poor outcomes in adults particularly with Philadelphia chromosome positive ($Ph^+$) (Matutes, *Blood* 2011). Outcomes remain dismal in older adults - median overall survival (OS) 7 months (mos.); 2-year (yr) OS 17%, declining to 5.5% in those >70 yrs. Post-relapse complete remission (CR) is ~10% with disease free survival <1 mo. (Murthy, *Clin. Lymph Myelo Leuk* 2017; Xu, *Haematologica* 2009).
In the absence of prospective trials, management of MPAL has relied on small case series, and expert consensus, underscoring the lack of an established standard of care. Blinatumomab (Blin), a CD19-directed BiTE approved for $CD19^+$ B-ALL, represents a rational therapeutic strategy for $CD19^+$ MPAL; however, conventional administration requires a 28-day continuous IV infusion, imposing logistical and patient (pt)-care burdens. A subcutaneous (SC) formulation, which delivers much higher dose, has the potential to enhance effective immunotargeted exposure while enabling an out-pt-centered approach with improved tolerability.

### Aims

To evaluate efficacy, safety, and feasibility of SC-blin in adults with $CD19^+$ MPAL, and to report the first pt treated on this first-in-world SC-blin MPAL trial, one of the very few prospective interventional trials open to accrual for adult $CD19^+$ MPAL.

### Methods

Multicenter, open-label phase II study (NCT07222579) with 3 parallel cohorts (planned n=75). Cohort A enrolls newly diagnosed $CD19^+$ MPAL pts who are ≥75 yrs or unfit for intensive chemotherapy (n=45, 2:1 randomization). Cohort B enrolls pts in CR/CRh/CRi with MRD ≥0.1% (n=10, single arm). Cohort C enrolls R/R $CD19^+$ MPAL (n=20, single arm). SC-blin cycles are 34 days (26-day treatment + 8-day break). Cycle 1: 250 mcg daily on days 1-7 (in-pt), then 500 mcg Mon/Wed/Fri (M/W/F) on days 8-26 (out-pt); subsequent cycles: 500 mcg M/W/F on days 1-26. Primary endpoints: OS (Cohort A), MRD-negative CR rate (Cohort B), and CR+CRh rate (Cohort C).

**Copyright Information:** (Online) ISSN: 2572-9241
©2026 The Author(s). HemaSphere is published by John Wiley & Sons Ltd on behalf of European Hematology Association. This is an open access Abstract Book distributed under the Attribution-NonCommercial-NoDerivs (CC BY-NC-ND), which allows third parties to download the articles and share them with others as long as they credit the author and the Abstract Book, but they cannot change the content in any way or use them commercially.
**Abstract Book Citations:** Authors, Title, HemaSphere, 2026;10:(S1):pages. The abstract book DOIs can be found on the "Author Information" page.
**Disclaimer:** Articles published in the journal HemaSphere exclusively reflect the opinions of the authors. The authors are responsible for all content in their abstracts including accuracy of the facts, statements, citing resources, etc.

**Results**

The first pt enrolled was a 77-yr old woman with newly diagnosed $Ph^+$ B/myeloid MPAL and significant comorbidities. Pathology revealed 97% bone marrow blasts by manual differential, diffusely positive for CD34, CD19, PAX5, and CD79a, with 60% of blasts positive for myeloperoxidase. Cytogenetics showed t(9;22) in 17/20 metaphases by karyotyping with 83% of nuclei positive for BCR::ABL1 fusion by FISH. Peripheral blood RT-PCR detected 63% p190 BCR-ABL1 transcripts. She received steroid cytoreduction then initiated SC-blin per protocol with concomitant dasatinib (100 mg orally daily). Early tumor lysis syndrome resolved with standard supportive care. Grade 1 cytokine release syndrome prompted brief admission and was managed with acetaminophen and empiric antibiotics per institutional guidelines; SC-blin was continued as scheduled. After a single treatment cycle, bone marrow evaluation demonstrated morphologic and immunophenotypic clearance of blasts with hematologic recovery (Figure 1), consistent with CR. No BCR::ABL1 fusion was detected by FISH. RT-PCR for the p190 transcript was undetectable (0.000), an unprecedented depth of MRD negativity after only one cycle.

**Summary/Conclusion**

This first patient achieved an unprecedented MRD-negative CR after a single cycle of SC-blin in $CD19^+$ MPAL, demonstrating potent activity with predominantly out-pt delivery. Accrual is ongoing across newly diagnosed elderly/unfit, MRD-positive, and R/R cohorts.

**Copyright Information:** (Online) ISSN: 2572-9241
©2026 The Author(s). HemaSphere is published by John Wiley & Sons Ltd on behalf of European Hematology Association. This is an open access Abstract Book distributed under the Attribution-NonCommercial-NoDerivs (CC BY-NC-ND), which allows third parties to download the articles and share them with others as long as they credit the author and the Abstract Book, but they cannot change the content in any way or use them commercially.
**Abstract Book Citations:** Authors, Title, HemaSphere, 2026;10:(S1):pages. The abstract book DOIs can be found on the "Author Information" page.
**Disclaimer:** Articles published in the journal HemaSphere exclusively reflect the opinions of the authors. The authors are responsible for all content in their abstracts including accuracy of the facts, statements, citing resources, etc.

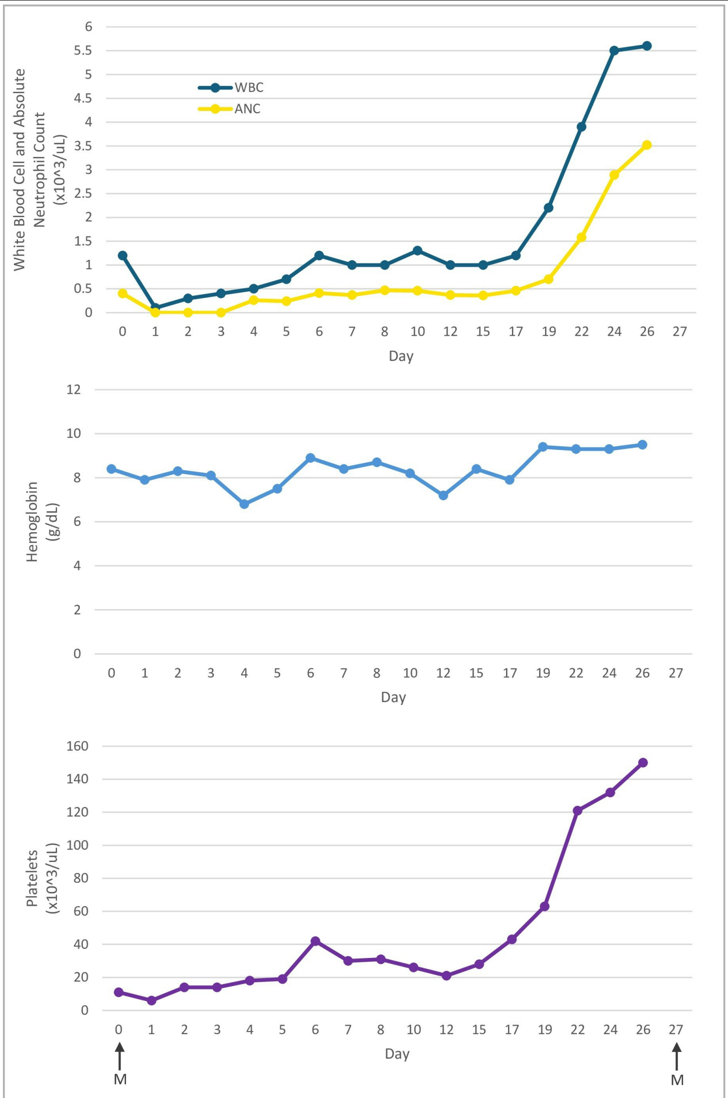

Figure 1. Timeline of days the patient received subcutaneous blinatumomab and associated blood counts. Day 1 was the start of cycle 1; day 26 was the last treatment day of a 34-day cycle. The patient underwent bone marrow evaluation on day 0 and 27 with biopsy and aspirate, denoted 'M' under arrows.

**Copyright Information:** (Online) ISSN: 2572-9241
©2026 The Author(s). HemaSphere is published by John Wiley & Sons Ltd on behalf of European Hematology Association. This is an open access Abstract Book distributed under the Attribution-NonCommercial-NoDerivs (CC BY-NC-ND), which allows third parties to download the articles and share them with others as long as they credit the author and the Abstract Book, but they cannot change the content in any way or use them commercially.
**Abstract Book Citations:** Authors, Title, HemaSphere, 2026;10:(S1):pages. The abstract book DOIs can be found on the "Author Information" page.
**Disclaimer:** Articles published in the journal HemaSphere exclusively reflect the opinions of the authors. The authors are responsible for all content in their abstracts including accuracy of the facts, statements, citing resources, etc.

### (PB2638) OLVEREMBATINIB AND HIGH-DOSE METHOTREXATE IN ACUTE LYMPHOBLASTIC LEUKEMIA: DELAYED METHOTREXATE CLEARANCE AND INCREASED NEPHROTOXICITY

**Topic:** 02. Acute lymphoblastic leukemia - Clinical

Jixin Tian[1], Wenjuan Miao*[1], Yingxi Liao[1], Ping Zhang[1]

*[1]State Key Laboratory of Experimental Hematology, National Clinical Research Center for Blood Diseases, Haihe Laboratory of Cell Ecosystem, Institute of Hematology & Blood Diseases Hospital, Chinese Academy of Medical Sciences & Peking Union Medical College, Tianjin, China;*

### Background

The novel tyrosine kinase inhibitors (TKIs), characterized by superior potency and selectivity against BCR-ABL1 kinase, are increasingly used in combination with high-dose methotrexate (HD-MTX) for BCR-ABL1-positive acute lymphoblastic leukemia (ALL). However, MTX pharmacokinetics are susceptible to drug-drug interactions, which may exacerbate the risk of HD-MTX-associated nephrotoxicity. A thorough understanding of the interaction profile between TKIs and MTX is therefore essential to mitigate unpredictable toxicities and optimize therapeutic safety.

### Aims

To date, no studies have investigated the interactions between HD-MTX and either flumatinib or olverembatinib. We conducted a retrospective analysis of patients with ALL who received TKIs concomitantly with HD-MTX to determine whether olverembatinib and flumatinib affect MTX clearance.

### Methods

This study retrospectively analyzed 49 adult patients treated with HD-MTX. Among them, 27 patients continued concurrent TKI therapy (with either olverembatinib or flumatinib) throughout the HD-MTX cycle. The remaining patients withheld TKI for at least 48 hours before HD-MTX infusion and resumed TKI treatment only after serum MTX levels decreased below 0.2 μmol/L. Blood samples were collected every 24 hours post-MTX infusion. Population pharmacokinetic analysis was performed to estimate MTX clearance for each patient.

**Copyright Information:** (Online) ISSN: 2572-9241
©2026 The Author(s). HemaSphere is published by John Wiley & Sons Ltd on behalf of European Hematology Association. This is an open access Abstract Book distributed under the Attribution-NonCommercial-NoDerivs (CC BY-NC-ND), which allows third parties to download the articles and share them with others as long as they credit the author and the Abstract Book, but they cannot change the content in any way or use them commercially.
**Abstract Book Citations:** Authors, Title, HemaSphere, 2026;10:(S1):pages. The abstract book DOIs can be found on the "Author Information" page.
**Disclaimer:** Articles published in the journal HemaSphere exclusively reflect the opinions of the authors. The authors are responsible for all content in their abstracts including accuracy of the facts, statements, citing resources, etc.

**Results**

Compared with HD-MTX monotherapy (n=22), patients receiving olverembatinib coadministration (n=10) exhibited a 35.1% reduction in MTX clearance ($p < 0.05$). Consequently, 80% of patients in the olverembatinib-combination group had MTX concentrations exceeding the 48-hour safety threshold (> 1 μmol/L), a proportion significantly higher than the 31.8% observed in the HD-MTX monotherapy group ($p = 0.02$). This pharmacokinetic interaction resulted in substantially increased clinical toxicity. The olverembatinib-combination group exhibited rates of 60% for ≥ grade 2 nephrotoxicity and 70% for acute kidney injury, significantly higher than the 4.6% and 27.3% observed in the MTX monotherapy group (both $p < 0.05$). In contrast, flumatinib coadministration (n=17) lowered MTX clearance by 20.4% and raised ≥ grade 2 nephrotoxicity to 29.4%, yet neither effect reached statistical significance.

Table. MTX concentration and nephrotoxicity incidence in different TKIs groups

| Group | No. of patients | MTX levels at 48 h > 1 μmol/L [n (%)] | MTX levels at 48 h > 5 μmol/L [n (%)] | MTX levels at 48 h [μmol/L, average (range)] | Clearance [L/h, average (SD)] | Nephrotoxicity [n (%)] | |
|---|---|---|---|---|---|---|---|
| | | | | | | AKI | grade 2-4 |
| Olverembatinib | 10 | 8 (80.00)* | 5 (50.00)* | 4.99 (0.33-10.32) | 5.21 (2.77) | 7 (70.00)* | 6 (60.00)* |
| Flumatinib | 17 | 10 (58.82) | 3 (17.65) | 3.44 (0.13-19.44) | 6.39 (2.74) | 9 (52.94) | 5 (29.41) |
| Without TKIs | 22 | 7 (31.82)* | 1 (4.55)* | 1.32 (0.16-7.74) | 8.03 (3.02) | 6 (27.27)* | 1 (4.55)* |
| *P*-value (Tri-group comparison) | | 0.030 | 0.008 | 0.018 | 0.029 | 0.056 | 0.002 |

*$p < 0.05$ by Fisher's exact test in pairwise comparison.

**Summary/Conclusion**

This study presents a preliminary investigation into the drug-drug interaction between HD-MTX and TKIs, providing valuable insights for treatment optimization. In light of the elevated risk of nephrotoxicity identified, temporarily withholding olverembatinib during the peri-infusion period of HD-MTX is recommended to avoid potentially severe adverse effects.

**Copyright Information:** (Online) ISSN: 2572-9241
©2026 The Author(s). HemaSphere is published by John Wiley & Sons Ltd on behalf of European Hematology Association. This is an open access Abstract Book distributed under the Attribution-NonCommercial-NoDerivs (CC BY-NC-ND), which allows third parties to download the articles and share them with others as long as they credit the author and the Abstract Book, but they cannot change the content in any way or use them commercially.
**Abstract Book Citations:** Authors, Title, HemaSphere, 2026;10:(S1):pages. The abstract book DOIs can be found on the "Author Information" page.
**Disclaimer:** Articles published in the journal HemaSphere exclusively reflect the opinions of the authors. The authors are responsible for all content in their abstracts including accuracy of the facts, statements, citing resources, etc.

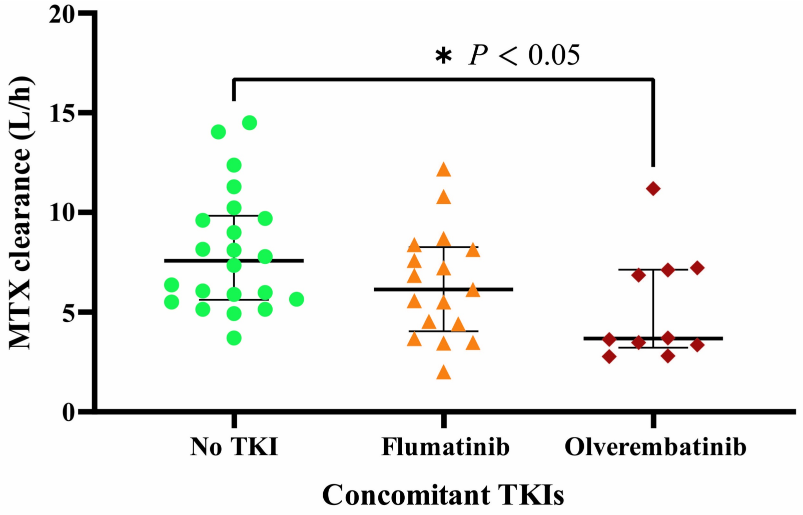

Figure legend:
Patients receiving olverembatinib exhibited slower clearance of MTX than those not receiving a TKI. The clearance of MTX was estimated for each patient with Phoenix NLME.

**Copyright Information:** (Online) ISSN: 2572-9241
©2026 The Author(s). HemaSphere is published by John Wiley & Sons Ltd on behalf of European Hematology Association. This is an open access Abstract Book distributed under the Attribution-NonCommercial-NoDerivs (CC BY-NC-ND), which allows third parties to download the articles and share them with others as long as they credit the author and the Abstract Book, but they cannot change the content in any way or use them commercially.
**Abstract Book Citations:** Authors, Title, HemaSphere, 2026;10:(S1):pages. The abstract book DOIs can be found on the "Author Information" page.
**Disclaimer:** Articles published in the journal HemaSphere exclusively reflect the opinions of the authors. The authors are responsible for all content in their abstracts including accuracy of the facts, statements, citing resources, etc.

### (PB2639) VENETOCLAX WITH INTENSIVE CHEMOTHERAPY IN HIGH RISK NEWLY DIAGNOSED PATIENTS WITH B-CELL ACUTE LYMPHOBLASTIC LEUKEMIA (B-ALL)

**Topic:** 02. Acute lymphoblastic leukemia - Clinical

Maher Salamoon*[1]

[1]*al bairouni university cancer center, hematology/oncology, Damascus, Syrian Arab Republic;*

### Background

Patients with high risk ALL have a relatively poor prognosis due to aggressive presentation and resistance to conventional treatment. The B-cell lymphoma 2 inhibitor venetoclax may enhance the efficacy of chemotherapy. This subgroup is treated with intensive chemotherapy such as HYPERCVAD before moving to allogeneic bone marrow transplant. Some patients are not eligible for transplants, however; they can do good with the addition of new agents to conventional chemotherapy

### Aims

In our study, we are evaluating the role of Venetoclax in newly diagnosed high risk patients with ALL. Protocol is adapted according to each case including dose dense and dose intense chemotherapy with Venetoclax during the induction and consolidation phase only.

### Methods

Patients are given HYPERCVAD with the standard dose for 2 cycles as induction followed by consolidation therapy for 6 cycles including high dose Cytarabine 3grams BID for 3 days and Methotrexate 3 grams/m² on day 4. 6 mercaptopurine 100mg/m² daily in continuous. Consolidation repeated for 6 cycles every 15 days or according to the white blood count. Maintenance included Cyclophosphamide 1gram/m², vincristine 2mg, 6 mercaptopurine 100mg/$m^2$ and oral Methotrexate 15mg every week for 2 years. Intrathecal Methotrexate every week in induction, every 2 weeks in consolidation and every 3 months in maintenance. Venetoclax was given as a single daily dose of 200 mg in continuous in both induction and consolidation setting

### Results

We have included 30 newly diagnosed patients with ALL with ages ranging between 18 and 55 (31 years in median). The male to female ratio was 1.5:1. The study was performed at al Bairouni university cancer center, Daffodil 88 cancer center and at the Italian hospital in Damascus, Syria. The complete response documented in 26 (86.6%) patients at the end of induction with a sensitivity of 0.0001. We have lost 1 patient in induction. 3 patients progressed in the consolidation phase. Toxicity included neutropenia in almost all patients in induction, tumor lysis syndrome in 3 patients and renal failure in 3 patients. 40% of patients could not tolerate the dose of Venetoclax and moved to intermittent dosing schedule according to their performance status. A follow-up of 36 months demonstrated an overall survival rate of 93.3% (95% CI: 87.8-95.2). change in Venetoclax schedule had no effect on response rate p value (0.04).

### Summary/Conclusion

Venetoclax shows to have a synergistic effect with chemotherapy through elimination of malignant cells expressing BCL-2. The safety profile and the tolerance make it a good partner of high dose chemotherapy in poor risk ALL patients. These findings need to be supported by comparative trials.

**Copyright Information:** (Online) ISSN: 2572-9241
©2026 The Author(s). HemaSphere is published by John Wiley & Sons Ltd on behalf of European Hematology Association. This is an open access Abstract Book distributed under the Attribution-NonCommercial-NoDerivs (CC BY-NC-ND), which allows third parties to download the articles and share them with others as long as they credit the author and the Abstract Book, but they cannot change the content in any way or use them commercially.
**Abstract Book Citations:** Authors, Title, HemaSphere, 2026;10:(S1):pages. The abstract book DOIs can be found on the "Author Information" page.
**Disclaimer:** Articles published in the journal HemaSphere exclusively reflect the opinions of the authors. The authors are responsible for all content in their abstracts including accuracy of the facts, statements, citing resources, etc.

### (PB2640) T-CELL ACUTE LYMPHOBLASTIC LEUKEMIA IN CHILDREN AND ADOLESCENTS/YOUNG ADULTS IN SOUTHERN TUNISIA: A 24-YEARS EXPERIENCE

**Topic:** 02. Acute lymphoblastic leukemia - Clinical

Khouloud Mezghani[1], Imen Frikha*[1], Ines Turki[2], Nour Louati[3], Ikram Ben Amor[3], Maha Charfi[1], Faten Kallel[1], Yousra Fakhfakh Derbel[1], Rahma Mallek[1], Asma Koubaa[1], Nour Siala[1], Hanen Charfi[1], Aicha Ben Kahla[1], Moez Medhaffar[1], Moez Elloumi[1]

*[1]Hedi Chaker Hospital, Hematology, Sfax, Tunisia;[2]CHU Hedi Chaker, Hematology, Sfax, Tunisia;[3]Sfax Regional Blood Transfusion Centre, University of Sfax, Sfax, Tunisia;*

### Background

T-cell acute lymphoblastic leukemia (T-ALL) accounts for 10–15% of pediatric ALL and is commonly associated with high leukocyte count and poorer early response.

### Aims

T-cell acute lymphoblastic leukemia (T-ALL) accounts for 10–15% of pediatric ALL and is commonly associated with high leukocyte count and poorer early response.

### Methods

This retrospective study included patients aged 1–30 years diagnosed with T-ALL between 2000 and 2023. Patients were treated according to the EORTC 58951 protocol. We analyzed the response to corticosteroid prephase, complete remission (CR), relapse rate, early mortality, overall survival (OS), and event-free survival (EFS).

### Results

A total of 136 patients were included: 88 children (65%) and 48 adolescents/young adults (AYA) (35%). The third quartile age (Q3) was 17 years. The male-to-female ratio was 1.95. Initial WBC was>100 G/L in 40% of cases. Conventional karyotype was normal in 53% and showed adverse cytogenetic risk in 12% including 11q23 rearrangements in 3 cases (2%) and complex karyotype in 10 cases (7%). A poor response to corticosteroid prephase was observed in 41% of patients. Among 127 evaluable patients, CR was achieved in 82.5% after the first induction course and 100% after the second. According to protocol stratification, 54% were classified as average risk (AR2) and 46% as high risk (HR) with no significant difference between children and AYA (p= 0.498). Mortality rate was 14% (11 in induction and 8 in consolidation).
Allogeneic stem cell transplantation (ASCT) was performed in 12 of the 45 indicated. Relapse occurred in 33 patients (24%) including 9% in AR2 and 15% in HR groups (p=0.01). Relapse was significantly associated with poor corticosteroid prophase response (p=0.018).
At 5 years, OS and EFS rates were 52% and 51%, respectively. Both prephase response (p=0.001) and therapeutic risk group (p=0.001) significantly influenced survival outcomes.

### Summary/Conclusion

Our study revealed a T-ALL cohort mainly composed of pediatric patients, with a higher rate of poor corticosteroid-response (41% vs 20–30% in major trials). However, the relapse rate (24%) remained within the range reported in the litterature (20–30%). Survival outcomes were lower than those reported and were likely driven by a higher mortality rate (14% vs 4-8% in literature), highlighting the need for more effective supportive care.

**Copyright Information:** (Online) ISSN: 2572-9241
©2026 The Author(s). HemaSphere is published by John Wiley & Sons Ltd on behalf of European Hematology Association. This is an open access Abstract Book distributed under the Attribution-NonCommercial-NoDerivs (CC BY-NC-ND), which allows third parties to download the articles and share them with others as long as they credit the author and the Abstract Book, but they cannot change the content in any way or use them commercially.
**Abstract Book Citations:** Authors, Title, HemaSphere, 2026;10:(S1):pages. The abstract book DOIs can be found on the “Author Information” page.
**Disclaimer:** Articles published in the journal HemaSphere exclusively reflect the opinions of the authors. The authors are responsible for all content in their abstracts including accuracy of the facts, statements, citing resources, etc.

### (PB2641) THERAPEUTIC RESULTS OF THE EORTC 58951 PROTOCOL IN HIGH-RISK CHILDHOOD AND AYA ACUTE LYMPHOBLASTIC LEUKEMIA

**Topic:** 02. Acute lymphoblastic leukemia - Clinical

Ines Turki[1], Imen Frikha*[1], Khouloud Mezghani[1], Aicha Ben Kahla[1], Nour Louati[2], Ikram Dammak[3], Maha Charfi[1], Faten Kallel[1], Yousra Fakhfakh Derbel[1], Imen Ben Amor[1], Rahma Mallek[1], Turki Fatma[4], Asma Koubaa[1], Nour Siala[1], Hanen Charfi[1], Insaf Ben Yaiche[5], Rihab Werghi[5], Hend Elleuch[3], Ikram Ben Amor[2], Nour Ben Adejlil[5], Moez Medhaffar[1], Moez Elloumi[1]

*[1]Hedi Chaker University Hospital, Hematology Department, Sfax, Tunisia; [2]Regional blood Transfusion Center of Sfax, Sfax, Tunisia; [3]Hedi Chaker University Hospital, Hematology Laboratory, Sfax, Tunisia; [4]Hedi Chaker University Hospital, Cytogenetic Laboratory, Sfax, Tunisia; [5]National Bone Marrow Transplant Center, Tunis, Tunisia;*

### Background

T-cell acute lymphoblastic leukemia (T-ALL) accounts for 10–15% of pediatric ALL and is commonly associated with high leukocyte count and poorer early response.

### Aims

Describe the clinical characteristics, treatment response, and survival outcomes of T-ALL patients treated in Southern Tunisia.

### Methods

This retrospective study included patients aged 1–30 years diagnosed with T-ALL between 2000 and 2023. Patients were treated according to the EORTC 58951 protocol. We analyzed the response to corticosteroid prephase, complete remission (CR), relapse rate, early mortality, overall survival (OS), and event-free survival (EFS).

### Results

A total of 136 patients were included: 88 children (65%) and 48 adolescents/young adults (AYA) (35%). The third quartile age (Q3) was 17 years. The male-to-female ratio was 1.95. Initial WBC was>100 G/L in 40% of cases. Conventional karyotype was normal in 53% and showed adverse cytogenetic risk in 12% including 11q23 rearrangements in 3 cases (2%) and complex karyotype in 10 cases (7%). A poor response to corticosteroid prephase was observed in 41% of patients. Among 127 evaluable patients, CR was achieved in 82.5% after the first induction course and 100% after the second. According to protocol stratification, 54% were classified as average risk (AR2) and 46% as high risk (HR) with no significant difference between children and AYA (p= 0.498). Mortality rate was 14% (11 in induction and 8 in consolidation).
Allogeneic stem cell transplantation (ASCT) was performed in 12 of the 45 indicated. Relapse occurred in 33 patients (24%) including 9% in AR2 and 15% in HR groups (p=0.01). Relapse was significantly associated with poor corticosteroid prophase response (p=0.018).
At 5 years, OS and EFS rates were 52% and 51%, respectively. Both prephase response (p=0.001) and therapeutic risk group (p=0.001) significantly influenced survival outcomes.

**Copyright Information:** (Online) ISSN: 2572-9241
©2026 The Author(s). HemaSphere is published by John Wiley & Sons Ltd on behalf of European Hematology Association. This is an open access Abstract Book distributed under the Attribution-NonCommercial-NoDerivs (CC BY-NC-ND), which allows third parties to download the articles and share them with others as long as they credit the author and the Abstract Book, but they cannot change the content in any way or use them commercially.
**Abstract Book Citations:** Authors, Title, HemaSphere, 2026;10:(S1):pages. The abstract book DOIs can be found on the “Author Information” page.
**Disclaimer:** Articles published in the journal HemaSphere exclusively reflect the opinions of the authors. The authors are responsible for all content in their abstracts including accuracy of the facts, statements, citing resources, etc.

**Summary/Conclusion**

Our study revealed a T-ALL cohort mainly composed of pediatric patients, with a higher rate of poor corticosteroid-response (41% vs 20–30% in major trials). However, the relapse rate (24%) remained within the range reported in the litterature (20–30%). Survival outcomes were lower than those reported and were likely driven by a higher mortality rate (14% vs 4-8% in literature), highlighting the need for more effective supportive care.

**Copyright Information:** (Online) ISSN: 2572-9241
©2026 The Author(s). HemaSphere is published by John Wiley & Sons Ltd on behalf of European Hematology Association. This is an open access Abstract Book distributed under the Attribution-NonCommercial-NoDerivs (CC BY-NC-ND), which allows third parties to download the articles and share them with others as long as they credit the author and the Abstract Book, but they cannot change the content in any way or use them commercially.
**Abstract Book Citations:** Authors, Title, HemaSphere, 2026;10:(S1):pages. The abstract book DOIs can be found on the "Author Information" page.
**Disclaimer:** Articles published in the journal HemaSphere exclusively reflect the opinions of the authors. The authors are responsible for all content in their abstracts including accuracy of the facts, statements, citing resources, etc.

### (PB2642) COMPLETE MOLECULAR REMISSION WITH SEQUENTIAL LOW-CELL-DOSE ASCT AND BISPECIFIC CD19/CD22 CAR-T THERAPY IN A HEMODIALYSIS-DEPENDENT PH-LIKE B-ALL PATIENT HABORING NUDT15 LOSS-OF-FUNCTION POLYMORPHISM

**Topic:** 02. Acute lymphoblastic leukemia - Clinical

Zhengyi Jia*[1], Liqiong Liu[1], Xiaoqing Jiang[1], Xiaofan Chen[1], Yuan Ye[1], Xiaoxuan Liu[1], Jizi Liu[1], Zhi Guo[1]

[1]*Affiliated Nanshan Hospital of Shenzhen University, Department of Hematology, Shenzhen, China;*

### Background

Philadelphia chromosome-like B acute lymphoblastic leukemia (Ph-like B ALL) with concurrent IKZF1 and JAK mutations exhibits profound resistance to conventional chemotherapy. This therapeutic challenge is markedly amplified in end-stage renal disease (ESRD) patients on maintenance hemodialysis. Coexisting NUDT15 loss-of-function polymorphisms preclude thiopurine-based therapy. Impaired metabolic clearance together with fluid balance restrictions further limit treatment options.

### Aims

To explore feasible and effective therapeutic strategies for  dialysis-dependent Ph-like B-ALL patients.

### Methods

We conducted a retrospective analysis of a hemodialysis-dependent patient with Ph-like B-ALL (JAK2 and IKZF1 mutations) bearing NUDT15 mutation who underwent sequential ASCT and CAR-T cell infusion, with evaluation of clinical features, treatment course, and efficacy.

### Results

Following induction of complete remission (CR) with vincristine plus prednisone and consolidation with an Ara-C/Dex/Peg regimen (cytarabine 1 g q12h on days 1–2, dexamethasone 15 mg q12h on days 1–2, pegaspargase 2500 IU/m² on day 3), sequential autologous stem cell transplantation (ASCT) and bispecific CD19/CD22 CAR-T therapy were planned. However, stem cell apheresis yielded only $0.61\times10^6$/kg $CD34^+$cells. After bridging therapy with selinexor 40 mg, a conditioning regimen comprising thiotepa 200 mg (day –6), fludarabine 15 mg/m² (days –5 to –3), and cyclophosphamide 250 mg/m² (days –5 to –3) was administered, with hemodialysis performed 12 hours after completion of fludarabine. Low-dose ASCT (day 0) followed by $3\times10^6$/Kg bispecific CD19/CD22 CAR-T cells (day +3) was subsequently delivered. Throughout the treatment course, the patient remained dialysis-dependent. Neutrophil engraftment occurred on day +12. Bone marrow evaluation on day +32 revealed confirming complete remission (CR) with demonstrated undetectable measurable residual disease (MRD-negative) by flow cytometry and next-generation sequencing confirmed complete molecular clearance with eradication of both IKZF1 and JAK2 mutations. This deep molecular response was maintained through day +82 with sustained MRD negativity. The patient experienced myelosuppression complicated by sepsis, Grade 1 CRS, and immune effector cell-associated hemophagocytic lymphohistiocytosis-like syndrome (IEC-HS), characterized by refractory cytopenias, hyperferritinemia (peak 7,627.24 ng/mL), progressive hypofibrinogenemia( nadir 1.44 g /L) and markedly elevated soluble CD25 (12,398 U/mL), together with renal function deterioration despite dialysis. Intensive management comprising intensified hemodialysis, broad-spectrum antimicrobials, dexamethasone, and ruxolitinib led to defervescence, ferritin reduction, stable hematopoietic reconstitution, and renal improvement. No immune effector cell-associated neurotoxicity syndrome (ICANS) occurred.

**Copyright Information:** (Online) ISSN: 2572-9241
©2026 The Author(s). HemaSphere is published by John Wiley & Sons Ltd on behalf of European Hematology Association. This is an open access Abstract Book distributed under the Attribution-NonCommercial-NoDerivs (CC BY-NC-ND), which allows third parties to download the articles and share them with others as long as they credit the author and the Abstract Book, but they cannot change the content in any way or use them commercially.
**Abstract Book Citations:** Authors, Title, HemaSphere, 2026;10:(S1):pages. The abstract book DOIs can be found on the "Author Information" page.
**Disclaimer:** Articles published in the journal HemaSphere exclusively reflect the opinions of the authors. The authors are responsible for all content in their abstracts including accuracy of the facts, statements, citing resources, etc.

### Summary/Conclusion

This proof-of-concept case illustrates that tandem ASCT-CAR-T therapy is both feasible and effective in high-risk Ph-like B-ALL with ESRD on maintenance hemodialysis. Selinexor bridging and thiotepa-containing conditioning contributed to adequate tumor burden reduction and lymphodepletion to enable CAR-T expansion, while low-dose stem cell apheresis proved adequate for hematopoietic rescue, underscoring that suboptimal stem cell yields need not preclude transplant consolidation. These findings offer a viable therapeutic paradigm for this historically high-risk population.

**Copyright Information:** (Online) ISSN: 2572-9241
©2026 The Author(s). HemaSphere is published by John Wiley & Sons Ltd on behalf of European Hematology Association. This is an open access Abstract Book distributed under the Attribution-NonCommercial-NoDerivs (CC BY-NC-ND), which allows third parties to download the articles and share them with others as long as they credit the author and the Abstract Book, but they cannot change the content in any way or use them commercially.
**Abstract Book Citations:** Authors, Title, HemaSphere, 2026;10:(S1):pages. The abstract book DOIs can be found on the “Author Information” page.
**Disclaimer:** Articles published in the journal HemaSphere exclusively reflect the opinions of the authors. The authors are responsible for all content in their abstracts including accuracy of the facts, statements, citing resources, etc.

### (PB2643) RITUXIMAB AS BRIDGE THERAPY FOR MANAGEMENT OF HEPATIC PRESENTATION OF B-CELL ACUTE LYMPHOBLASTIC LEUKEMIA IN CHILDREN

**Topic:** 02. Acute lymphoblastic leukemia - Clinical

Myrto Skafida*[1, 2], Waitman Aumann[1, 2], Tamara Miller[1, 2], Anna Bennett[1], Kelly MacDonald[1], Frank Keller[1, 2], Himalee Sabnis[1, 2]

*[1]Children's Healthcare of Atlanta, Aflac Cancer and Blood Disorders Center,, Atlanta, United States of America;[2]Emory University, Department of Pediatrics, Atlanta, United States of America;*

### Background

Pediatric patients with B-cell acute lymphoblastic leukemia (B-ALL) can present with hepatic dysfunction that is attributed to leukemic infiltration of the liver. Although this presentation is rare, it poses a significant therapeutic challenge. Abnormal liver function precludes use of standard of care (SOC) induction medications such as vincristine (VCR), asparaginase and daunorubicin. Specific guidelines for management are limited, and most groups favor a pre-phase with steroid monotherapy that can be effective but tedious. Approximately 50% of pediatric patients with B-ALL express CD20. Rituximab is a chimeric anti-CD20 monoclonal antibody that is used in the treatment of CD20+ B-cell malignancies including adult B-ALL. We describe 5 patients with B-ALL and significant liver dysfunction that were initially managed with steroids and Rituximab, allowing for subsequent treatment with SOC induction chemotherapy.

### Aims

To assess the efficacy and the toxicity of Rituximab as a pre-treatment for patients presenting with newly diagnosed B-ALL and significant liver dysfunction.

### Methods

Retrospective chart review including laboratory data, treatment approach and toxicities of five children with B-ALL with hepatic presentation, was manually completed from the electronic medical record.

**Copyright Information:** (Online) ISSN: 2572-9241
©2026 The Author(s). HemaSphere is published by John Wiley & Sons Ltd on behalf of European Hematology Association. This is an open access Abstract Book distributed under the Attribution-NonCommercial-NoDerivs (CC BY-NC-ND), which allows third parties to download the articles and share them with others as long as they credit the author and the Abstract Book, but they cannot change the content in any way or use them commercially.
**Abstract Book Citations:** Authors, Title, HemaSphere, 2026;10:(S1):pages. The abstract book DOIs can be found on the "Author Information" page.
**Disclaimer:** Articles published in the journal HemaSphere exclusively reflect the opinions of the authors. The authors are responsible for all content in their abstracts including accuracy of the facts, statements, citing resources, etc.

### Results

All patients presented with severe hepatocellular injury (LFT elevation) and/or direct hyperbilirubinemia (Table 1). Four were Hispanic and one African American; four were NCI high risk. Four received a steroid pre-phase with weekly Rituximab (1–3 doses) prior to SOC induction. One patient started dose-reduced VCR and daunorubicin before Rituximab, with subsequent worsening liver function. Treatment timeline for all patients is shown in Figure 1. All patients were MRD negative at end of induction (Day range: 36–48). Rituximab was well tolerated without severe infusion reactions. One patient developed HSV viremia and presumed fungal infection during late induction, and uncomplicated COVID during consolidation. No other viral infections occurred during induction and consolidation.

| Patient | Case 1 | Case 2 | Case 3 | Case 4 | Case 5 |
|---|---|---|---|---|---|
| **Patient's Characteristics** | | | | | |
| **Age at Dx (y)** | 14 | 15 | 7 | 12 | 16 |
| **Ethnicity** | Hispanic | African American | Hispanic | Hispanic | Hispanic |
| **BMI (kg/m$^2$)** | 14.03 | 24.9 | 18.5 | 22.6 | 24.6 |
| **Laboratory Evaluation at Diagnosis** | | | | | |
| **WBC(thou/ul)/Hb(g/dl)/PLT(thou/ul)** | 0.77/3.6/47 | 2.99/13.4/42 | 2.48/10.8/35 | 6.23/12.7/398 | 7.5/5.0/14 |
| **AST/ALT (U/L)** | 2,687/2,676 | 4,056/4,778 | 1548/3068 | 82/42 | 1,195/1251 |
| **Tbili/Dbili (mg/dl)** | 3.8/2.5 | 13.8/9.9 | 8.1/5.3 | 13.3/10.8 | 6.9/5.5 |
| **PT/PTT (sec)** | 18.4/29.0 | 17.1/32.0 | 17.8/31.8 | 14.6/27.7 | 13.9/32.3 |
| **Albumin (g/dl)** | 3.6 | 3.6 | 3.3 | 2.7 | 3.2/32.3 |
| **Disease Characteristics** | | | | | |
| **CNS Status** | CNS1 | CNS2 | CNS1 | CNS1 | CNS3 |
| **NCI Risk** | HR | HR | SR | HR | HR |
| **Complications and MRD Evaluation** | | | | | |
| **Complications during Induction** | None | F & N | None | Diabetes | Fungal Infection HSV stomatitis and viremia |
| **Complications during consolidation** | F & N | None | F & N (E.Coli bacteremia) | F & N | COVID (+) F & N |
| **Day of MRD evaluation** | 47 | 48 | 37 | 43 | 36 |
| **MRD** | Negative | Negative | Negative | Negative | Negative |

### Summary/Conclusion

Using Rituximab for induction therapy in de novo pediatric B-ALL is feasible. Rituximab can serve as an adjunctive pre-induction therapy in patients with hepatic presentation of ALL allowing the administration of full dose SOC chemotherapy.

**Copyright Information:** (Online) ISSN: 2572-9241
©2026 The Author(s). HemaSphere is published by John Wiley & Sons Ltd on behalf of European Hematology Association. This is an open access Abstract Book distributed under the Attribution-NonCommercial-NoDerivs (CC BY-NC-ND), which allows third parties to download the articles and share them with others as long as they credit the author and the Abstract Book, but they cannot change the content in any way or use them commercially.
**Abstract Book Citations:** Authors, Title, HemaSphere, 2026;10:(S1):pages. The abstract book DOIs can be found on the "Author Information" page.
**Disclaimer:** Articles published in the journal HemaSphere exclusively reflect the opinions of the authors. The authors are responsible for all content in their abstracts including accuracy of the facts, statements, citing resources, etc.

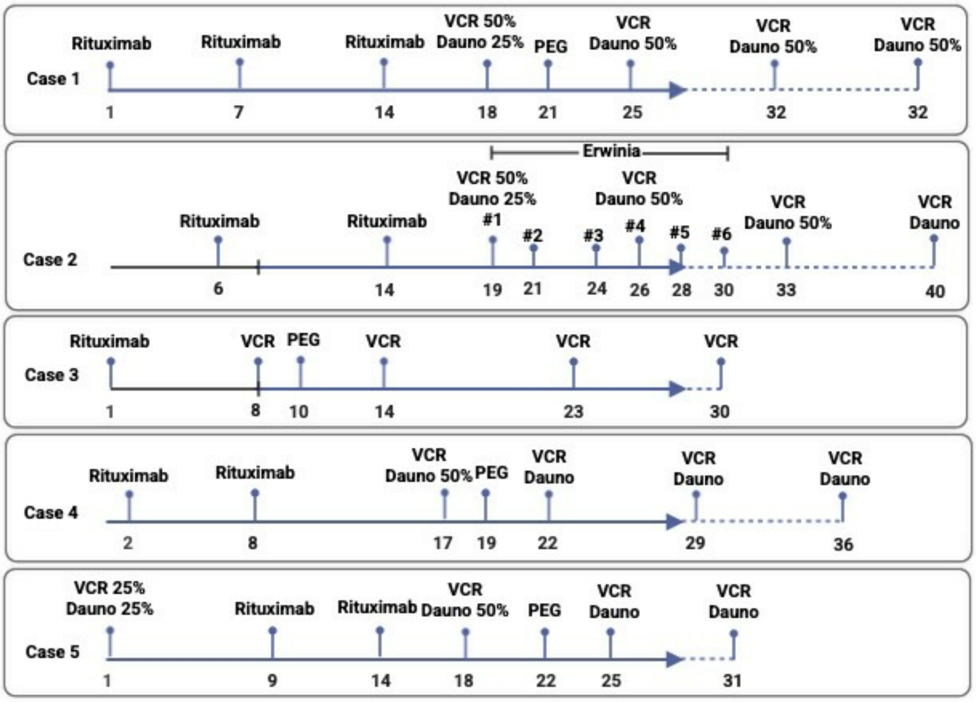

**Figure 1:** Treatment timeline of the presented cases. Solid arrows represent duration of steroid therapy; Doses are 100% unless otherwise specified. Days on the horizontal axis represent time from the first day of corticosteroid treatment. Created using biorender.

**Copyright Information:** (Online) ISSN: 2572-9241
©2026 The Author(s). HemaSphere is published by John Wiley & Sons Ltd on behalf of European Hematology Association. This is an open access Abstract Book distributed under the Attribution-NonCommercial-NoDerivs (CC BY-NC-ND), which allows third parties to download the articles and share them with others as long as they credit the author and the Abstract Book, but they cannot change the content in any way or use them commercially.
**Abstract Book Citations:** Authors, Title, HemaSphere, 2026;10:(S1):pages. The abstract book DOIs can be found on the "Author Information" page.
**Disclaimer:** Articles published in the journal HemaSphere exclusively reflect the opinions of the authors. The authors are responsible for all content in their abstracts including accuracy of the facts, statements, citing resources, etc.

### (PB2644) ALLOGENEIC STEM CELL TRANSPLANTATION FOR PHILADELPHIA CHROMOSOME POSITIVE ACUTE LYMPHOBLASTIC LEUKEMIA

**Topic:** 02. Acute lymphoblastic leukemia - Clinical

Hanane Bouarab[1], Farih Mehdid[1], Sabrina Akhrouf[1], Baazizi Mounira[1], Nadia Rahmoune[1], Ait Ouali Dina[1], Sarah Zerkout[1], Fouzia Louar[1], Rihab Benouattas[1], Farida Harieche[1], Rose Marie Hamladji[1], Redhouane Ahmed Nacer[1], Malek Benakli*[1]

[1]*Hematology and Bone Marrow Transplantation department, Pierre and Marie Curie Center, Algiers, Algeria;*

### Background

Philadelphia chromosome positive (Ph+) acute lymphoblastic leukemia (ALL) constitutes approximately 25% of adult and 3% of children B precursor ALL patients (pts). The prognosis was considered poor until the administration of tyrosine kinase inhibitors (TKIs) in association with intensive chemotherapy followed by allogenic hematopoietic stem cell transplantation (allo-HSCT). We report the results of this procedure over a long period of 13 years.

### Methods

From January 2012 to December 2023, 76 pts with Ph+ ALL underwent allo-HSCT from an HLA matched sibling donor (55 pts) and haplo-identical donor (20 pts). At the time of transplant, 64 pts were in first complete remission (CR1), 11 pts in second CR and one pt in active disease. Median age was 32 years (4-61) and sex-ratio (M/F): 1,37. The average time from diagnosis to transplant was 7 months (3-14). Three types of Busulfan-based conditioning regimen were used: Busulfan-Aracytine-Melphalan for 12 pts, Busulfan-cyclophosphamide-Etoposide for 15 pts, and Busulfan-Cyclophosphamide for 6 pts. For haplo-identical transplant, Busulfan-Aracytine-Cyclophosphamide and Thymoglobuline (Beijing Protocol) in 5 pts, Busulfan-Fludarabine-Thiotepa (TBF) in 15 pts. All pts received peripheral blood stem cells with an average CD34+ cell count: 8,18x10*6/Kg (3,89-41,08) with addition of bonre marrow G-CSF primed in Beijing Protocol. GVHD prophylaxis consisted on association of ciclosporine-methotrexate (HLA-identical sibling), ciclosporine-methotrexate-MMF (Beijing Protocol), and ciclosporine-MMF-PTCy (TBF). Relapse prevention with ITKs after transplantation was used in 44 pts (57,9%).

### Results

Aplasia was observed in all pts. Median day of neutrophils engraftment was 13 days (6-28). Fifty pts (64,1%) required red blood cells transfusions (2,5 unit/pt), and all pts needed platelets transfusion (2,1 unit/pt). One pt presented a moderate veino-occlusive disease. Acute GVHD occurred in 30 pts (40,5%) including 17 pts grade III-IV. Chronic GVHD was seen in 24 pts (42,85%) with extensive form in 09 pts. CMV reactivation is noted in 21 pts (27,6%); and hemorrhagic cystitis in 6 pts. Twenty-two pts (28,9%) relapsed within 16 pts received TKIs. At 30 June 2024, after a median follow-up of 50 months (7 128), 49 pts (64,4%) died of whom 32 pts (42%) from transplant-related complications (aGVHD 16 pts, cGVHD 3 pts, infection 09 pts, acute pancreatitis 1 pt, PRES 1 pt, liver toxicity 02 pts) and relapse (17 pts). The overall survival (0S) and disease-free survival (DFS) at 149 months are 27,7% et 22,5%.

### Summary/Conclusion

Allo-HSCT after a chemotherapy-alone conditioning regimen with TKIs have improved outcomes for pts with Ph+ ALL. Despite the use TKIs, we note à high rate of relapse. The advent of immunotherapy improves the results of transplantation due to better control of minimal residual disease

**Copyright Information:** (Online) ISSN: 2572-9241
©2026 The Author(s). HemaSphere is published by John Wiley & Sons Ltd on behalf of European Hematology Association. This is an open access Abstract Book distributed under the Attribution-NonCommercial-NoDerivs (CC BY-NC-ND), which allows third parties to download the articles and share them with others as long as they credit the author and the Abstract Book, but they cannot change the content in any way or use them commercially.
**Abstract Book Citations:** Authors, Title, HemaSphere, 2026;10:(S1):pages. The abstract book DOIs can be found on the "Author Information" page.
**Disclaimer:** Articles published in the journal HemaSphere exclusively reflect the opinions of the authors. The authors are responsible for all content in their abstracts including accuracy of the facts, statements, citing resources, etc.

### (PB2645) THERAPEUTIC OUTCOMES OF PEDIATRIC PHILADELPHIA CHROMOSOME-POSITIVE ACUTE LYMPHOBLASTIC LEUKEMIA

**Topic:** 02. Acute lymphoblastic leukemia - Clinical

Salma Sammoud[1], Emna Azza[1], Marwa Bahri[1], Ben Othmen Aya[1], Roua Hsasna[1], Raihane Ben Lakhal[1], Yosr Ben Abdennabi[1], Lamia Issaoui*[1]

[1]*Aziza Othmana Hospital, Tunis, Tunisia;*

### Background

The introduction of tyrosine kinase inhibitors (TKIs) has significantly transformed the management and prognosis of Philadelphia chromosome-positive acute lymphoblastic leukemia (Ph+ ALL).

### Aims

This study aimed to describe the therapeutic outcomes of pediatric patients with this high-risk leukemia.

### Methods

We conducted a descriptive, retrospective study at the clinical hematology department of Aziza Othmana Hospital in Tunisia covering the period from January 2005 to December 2023. All children diagnosed with Ph+ ALL were included and treated according to the EORTC 58951 protocol, VHR arm in combination with Imatinib.

### Results

Thirty three patients were included with a median age of 11 years (range: 3-18 years) and a sex ratio of 2.3. At diagnosis, 28 (85%) patients exhibited a tumor syndrome, 20 (60%) had hyperleukocytosis higher than 100G/L, and 3 (9%) presented with central nervous system involvement.
Twelve patients (36%) exhibited additional cytogenetic abnormalities. Imatinib was initiated after of median time of 9 days (rang: 2-36). Four patients (12%) died during induction. Seventeen patients (51%) were good prephase responders and 29 (87%) were good chemotherapy responders. A first complete remission (CR1) was achieved in 29 patients (87%). Molecular biology showed a minimal residual disease (MRD) $\leq 5 * 10^{-4}$ in 15 patients (45%). Nine patients underwent allogenic stem cell transplantation after CR1. One patient received an umbilical cord blood transplantation. Twelve patients (36%) relapsed within a median time of 31.7 months (range :4.8 – 115.2). At the study cutoff date, we recorded 11 (33%) disease-related deaths and 3 (9%) toxic deaths.
The 5-year overall survival (OS) was 55%. The prognostic factors affecting OS in univariate analysis were age (p=0.046), MRD post-induction (p=0.017) and relapse delay (p=0.013). In multivariate analysis, the significant factors were age (p=0.016) and the relapse delay (p< 0.0001).The 5-year event-free survival (EFS) was estimated at 39.7%. The prognostic factor affecting EFS in univariate analysis was MRD post-induction (p=0.046) but wasn't significant in multivariate analysis. The 5-year relapse-free (RFS) survival was estimated at 60.8%. No factor was found to be statistically significant for RFS in univariate analysis.

### Summary/Conclusion

Our results were inferior to those reported in the literature with a notably high rate of induction-related mortality and relapse. This could be improved through the use of a less intensive induction regimen, the introduction of immunotherapy, second-generation TKIs in high-risk cases, as well as the identification of molecular prognostic factors.

**Copyright Information:** (Online) ISSN: 2572-9241
©2026 The Author(s). HemaSphere is published by John Wiley & Sons Ltd on behalf of European Hematology Association. This is an open access Abstract Book distributed under the Attribution-NonCommercial-NoDerivs (CC BY-NC-ND), which allows third parties to download the articles and share them with others as long as they credit the author and the Abstract Book, but they cannot change the content in any way or use them commercially.
**Abstract Book Citations:** Authors, Title, HemaSphere, 2026;10:(S1):pages. The abstract book DOIs can be found on the "Author Information" page.
**Disclaimer:** Articles published in the journal HemaSphere exclusively reflect the opinions of the authors. The authors are responsible for all content in their abstracts including accuracy of the facts, statements, citing resources, etc.

### (PB2646) SECURED INPATIENT BLINATUMOMAB PATHWAY: A SINGLE-CENTER EXPERIENCE (PROPHYLAXIS, IMMUNOGLOBULIN SUPPLEMENTATION, DEDICATED VASCULAR ACCESS, CRS/ICANS ALGORITHMS)

**Topic:** 02. Acute lymphoblastic leukemia - Clinical

Sihem Kebaili*[1, 1]

[1]*chu constantine, hematology, constantine, Algeria;*

### Background

Blinatumomab is a standard therapy for B-cell precursor acute lymphoblastic leukemia (B-ALL). Its continuous infusion creates major logistical challenges and may increase infectious risk due to treatment-related immunosuppression. In Algeria, limited availability of home infusion services results in prolonged hospitalization, making a secured inpatient care pathway essential.

### Aims

To evaluate the safety and effectiveness of a structured inpatient pathway for blinatumomab administration in our center.

### Methods

Single-center prospective observational study (November 2023–December 2025) including 10 patients (9 relapsed/refractory; 1 consolidation). The pathway included: intrathecal CNS prophylaxis; immunoglobulin supplementation at day 0 guided by serum protein electrophoresis; dedicated central venous access (PICC line 90% [9/10], implanted port 10% [1/10]) with antiviral and anti-*Pneumocystis* prophylaxis; and protocolized grading/management of cytokine release syndrome (CRS) and immune effector cell-associated neurotoxicity syndrome (ICANS) per ASTCT 2019 criteria (corticosteroids/tocilizumab).

### Results

Median age 18 years; 90% Philadelphia-negative; one patient with trisomy 21. Complete response rate: 60%; 40% bridged to allogeneic transplant. Secondary hypogammaglobulinemia: 100%. CRS: 80% (8/10), including grade 3–4 in 30% (3/10) with one macrophage activation syndrome; grade 3 CRS controlled with tocilizumab, allowing re-initiation. ICANS: 60%, including one grade 3 event (seizures) in the patient with trisomy 21; resolved with rechallenge under levetiracetam. No documented infections (0%); one PICC-line thrombosis (10%). Three deaths (30%) from disease progression.

### Summary/Conclusion

A standardized secured inpatient pathway enabled blinatumomab administration with no documented infections and successful management of severe CRS/ICANS (60% complete responses, 40% bridged to transplant). This experience validates structured algorithms, prophylaxis, immunoglobulin supplementation, and dedicated vascular access for safe continuous-infusion blinatumomab in routine practice. As a next step, our center is developing a pilot multidisciplinary program supported by digital applications (patient/physician/nurse/pharmacist) to facilitate coordinated management and monitoring.

**Copyright Information:** (Online) ISSN: 2572-9241
©2026 The Author(s). HemaSphere is published by John Wiley & Sons Ltd on behalf of European Hematology Association. This is an open access Abstract Book distributed under the Attribution-NonCommercial-NoDerivs (CC BY-NC-ND), which allows third parties to download the articles and share them with others as long as they credit the author and the Abstract Book, but they cannot change the content in any way or use them commercially.
**Abstract Book Citations:** Authors, Title, HemaSphere, 2026;10:(S1):pages. The abstract book DOIs can be found on the "Author Information" page.
**Disclaimer:** Articles published in the journal HemaSphere exclusively reflect the opinions of the authors. The authors are responsible for all content in their abstracts including accuracy of the facts, statements, citing resources, etc.

### (PB2647) CLINICAL FEATURES AND PROGNOSIS OF DNMT3A AND CEBPA MUTATIONS IN PATIENTS WITH ACUTE MYELOID LEUKEMIA (NON-M3).

**Topic:** 02. Acute lymphoblastic leukemia - Clinical

Huihui An*[1], Tao Wu[1]

[1] *The 940th Hospital of Joint Logistics Support force of Chinese People's Liberation Army, hematology, Gansu, China;*

### Background

Acute myeloid leukemia (AML) is a hematologic malignancy characterized primarily by the malignant proliferation, differentiation impairment and apoptotic dysfunction of hematopoietic stem cells, accounting for 80% of adult acute leukemias and exhibiting a high degree of heterogeneity [1]. The cancer statistics report published by Bray et al. [2] indicated that there were 487,000 newly diagnosed leukemia cases and 305,000 leukemia-related deaths worldwide in 2022; the disease risk increases with age, with a median age at onset of 65 to 70 years [2]. DNA methyltransferase 3A (DNMT3A), a DNA methyltransferase, is associated with cell development, senescence and the early onset of cancer [3-4]. DNMT3A mutation represents a common genetic aberration in AML, occurring in 20% to 30% of adult AML patients and correlating with poor prognosis in these patients [5]. In this study, we conducted a retrospective analysis of prognosis-related clinical data from 162 newly diagnosed and untreated AML patients admitted to the 940th Hospital of the Joint Logistics Support Force, aiming to investigate the mutational profile of DNMT3A and its prognostic value in AML patients.

### Aims

To analyze the clinical characteristics, frequency and prognosis of gene mutations in patients with DNMT3A mutation acute myeloid leukemia (non-acute promyelocytic leukemia) (non-M3).

### Methods

A total of 162 patients with newly diagnosed AML (non-M3) who were admitted to the 940th Hospital of the Joint Logistics Support Force from January 2018 to December 2023 were selected as the research subjects. Among them, 82 were male patients and 80 were female; The median age of onset was 45 years (31,57 years). Retrospective research methods were used to collect clinical data such as age, gender, blood count (WBC) at initial diagnosis, bone marrow blast proportion, AML-related gene mutation frequency, European Leukemia Network (ELN) risk stratification, and allogeneic hematopoietic stem cell transplantation (allo-HSCT), and Cox proportional hazards regression model was used for univariate and multivariate analysis of patients' OS. The Kaplan-Meier method was used to draw the OS curves of the patients, and the log-rank method was used to compare the OS curves of patients with different clinical characteristics.

### Results

Among the 162 patients included in this study, the median OS of 40.0 months and 12.0 months for patients who received allo-HCCT and those who did not receive transplantation were 40.0 months and 12.0 months, respectively (P<0.05). The median OS of patients with low, intermediate, and high risk stratified by ELN was 18.0, 15.0, and 9.0 months (P>0.05). The median OS of patients with CEBPA mutation and without CEBPA mutation was 30.0 months and 13.0 months (P<0.05). The median OS of patients with DNMT3A mutation and no DNMT3A mutation was 8.5 months and 20.0 months (P<0.05), respectively. The median OS < 60-year-old patients and those aged ≥60-year-old were 23.0 months and 9.0 versus (P<0.05). Multivariate analysis showed that age of onset, CEBPA mutation, DNMT3A mutation, and hematopoietic stem cell transplantation were associated with OS.

**Copyright Information:** (Online) ISSN: 2572-9241
©2026 The Author(s). HemaSphere is published by John Wiley & Sons Ltd on behalf of European Hematology Association. This is an open access Abstract Book distributed under the Attribution-NonCommercial-NoDerivs (CC BY-NC-ND), which allows third parties to download the articles and share them with others as long as they credit the author and the Abstract Book, but they cannot change the content in any way or use them commercially.
**Abstract Book Citations:** Authors, Title, HemaSphere, 2026;10:(S1):pages. The abstract book DOIs can be found on the "Author Information" page.
**Disclaimer:** Articles published in the journal HemaSphere exclusively reflect the opinions of the authors. The authors are responsible for all content in their abstracts including accuracy of the facts, statements, citing resources, etc.

**Summary/Conclusion**

There are many factors affecting the prognosis of newly diagnosed AML (non-M3) patients, CEBPA mutation and hematopoietic stem cell transplantation are good prognosis indicators for AML (non-M3) patients, while DNMT3A mutation is a poor prognostic factor for AML.

**Copyright Information:** (Online) ISSN: 2572-9241
©2026 The Author(s). HemaSphere is published by John Wiley & Sons Ltd on behalf of European Hematology Association. This is an open access Abstract Book distributed under the Attribution-NonCommercial-NoDerivs (CC BY-NC-ND), which allows third parties to download the articles and share them with others as long as they credit the author and the Abstract Book, but they cannot change the content in any way or use them commercially.
**Abstract Book Citations:** Authors, Title, HemaSphere, 2026;10:(S1):pages. The abstract book DOIs can be found on the "Author Information" page.
**Disclaimer:** Articles published in the journal HemaSphere exclusively reflect the opinions of the authors. The authors are responsible for all content in their abstracts including accuracy of the facts, statements, citing resources, etc.

### (PB2648) FIRST RELAPSED ACUTE LYMPHOBLASTIC LEUKEMIA :THERAPEUTIC RESULTS OF COOPRALL REGIEMEN

**Topic:** 02. Acute lymphoblastic leukemia - Clinical

Aicha Ben Kahla*[1], Imen Frikha[1], Turki Ines[1], Khouloud Mezghani[1], Imen Ben Amor[1], Yousra Fakhfakh Derbel[1], Faten Kallel[1], Maha Charfi[1], Rahma Mallek[1], Asma Koubaa[1], Nour Siala[1], Hanen Charfi[1], Moez Medhaffar[1], Moez Elloumi[1]

[1]*hedi chaker hospital, hematology, sfax, Tunisia;*

### Background

Advances in the treatment of acute lymphoblasticleukemia (ALL) have led to dramatic improvements in outcome with survival rates more than 80%. However relapse remains one of the most important causes of treatment failure, occurring in 15 to 25 % of patients .

### Aims determine therapeutic results of COOPRALL regiemen in first relapsed ALL.

### Methods

In this study, we enrolled retrospectively all cases of patients under 30 years old, with first relapsed ALL, treated initially according to EORTC 58951 and diagnosed in the hematologydepartment of "Hedi Chaker" Hospital, Sfax, Tunisia, between January 2000 and December 2024.
Treatment is based on COOPRALL protocol that associate intensive chemotherapy, followed by radiotherapy to involved extramedullary sites (CNS or testis) and maintenance therapy to complete 2 years of relapse treatment. Allogenic stem cell transplantation is reserved for patients with HLA identical donor who achieved complete response. We studied therapeutic outcomes: complete remission, overall survival (OS) and event-free survival (EFS).

### Results

Between 2000 and 2024, we enrolled 52 cases of relapsed ALL treated with COOPRALL regiemen. The mean time of relapse was 2 years . The relapse was very early (≤18 months from diagnosis) in 31% of cases. Bone marrow relapse was noted in 88% .Relapse was isolated in 71% of cases. Nine patients died before evaluation. Among the evaluable patients, 22 cases (51%) were in complete response.Eight patients had HLA identical donor and have been allograft. Twelve patients are alive in complete remission and 10 patients died (second relapse in 9 cases and toxic death in 1 case). With a median follow up of 12 years, 5 years OS and EFS were 34% and 23% respectively.

### Summary/Conclusion

The rate of complete remission in our study (51%) is slightly inferior to the literature (55-60%). This can be explained by the presence of resistant clone and decreased tolerance to chemotherapy owing to previous treatment. Allogenic stem cell transplantation , which is performed in 8 cases only, is an important therapeutic arm that improve survival rates in patients who had minimal residual disease (MRD) less than 10-4 leukemia cells. Our survival rates (OS : 34%, EFS : 23%) are inferior to those reported in the literature (OS : 50 to 70%, EFS: 44% to 65%). Our results can be improved by using Mitoxantrone in reinduction course, stratification of patients according to the time of relapse and MRD, haploidentical transplantation if HLA identical donor is not available.

**Copyright Information:** (Online) ISSN: 2572-9241
©2026 The Author(s). HemaSphere is published by John Wiley & Sons Ltd on behalf of European Hematology Association. This is an open access Abstract Book distributed under the Attribution-NonCommercial-NoDerivs (CC BY-NC-ND), which allows third parties to download the articles and share them with others as long as they credit the author and the Abstract Book, but they cannot change the content in any way or use them commercially.
**Abstract Book Citations:** Authors, Title, HemaSphere, 2026;10:(S1):pages. The abstract book DOIs can be found on the "Author Information" page.
**Disclaimer:** Articles published in the journal HemaSphere exclusively reflect the opinions of the authors. The authors are responsible for all content in their abstracts including accuracy of the facts, statements, citing resources, etc.

### (PB2649) ZILOVERTAMAB VEDOTIN IN PEDIATRIC AND YOUNG ADULT HEMATOLOGIC MALIGNANCIES OR SOLID TUMORS: PHASE 1/2 LIGHTBEAM-U01A STUDY

**Topic:** 02. Acute lymphoblastic leukemia - Clinical

Hyoung Jin Kang[1], Pablo Berlanga[2], Quentin Campbell-Hewson[3], Steven G. Dubois[4], Kyung-Nam Koh[5], Christine Mauz-Koerholz*[6], Michael Sullivan[7], David Mangum[8], Rachael Windsor[9], David S. Dickens[10], Alexandre Duarte[11], Farzana Pashankar[12], Shifra Ash[13], Andrew Bellantoni[14], Scott J. Diede[15], Rohini Singh[15], Yulia Sidi[15], Michal Yalon[16]

*[1]Seoul National University College of Medicine, Seoul National University Cancer Research Institute, Seoul National University Children's Hospital, Seoul, Republic of Korea, Seoul, South Korea; [2]Gustave Roussy Cancer Campus, Villejuif, France, Villejuif, France; [3]Great North Children's Hospital, Newcastle Upon Tyne, United Kingdom, Newcastle Upon Tyne, United Kingdom; [4]Dana-Farber/Boston Children's Cancer and Blood Disorders Center, Harvard Medical School, Boston, MA, USA, Boston, United States of America; [5]Asan Medical Center Children's Hospital, University of Ulsan College of Medicine, Seoul, Republic of Korea, Seoul, South Korea; [6]Medical Faculty of the Martin-Luther-University of Halle-Wittenberg, Halle, Germany; Justus-Liebig University of Giessen, Giessen, Germany, Giessen, Germany; [7]Children's Cancer Centre, Royal Children's Hospital, Melbourne, Australia, Melbourne, Australia; [8]Intermountain - Primary Children's Hospital, Salt Lake City, UT, USA, Salt Lake City, United States of America; [9]University College London Hospital, London, UK, London, United Kingdom; [10]University of Iowa-Holden Comprehensive Cancer Center, Iowa City, IA, USA, Iowa City, United States of America; [11]Fundação Faculdade Regional de Medicina de São José do Rio Preto-Centro Integrado de Pesquisa, São José do Rio Preto, Brazil, São José do Rio Preto, Brazil; [12]Yale-New Haven Hospital, New Haven, CT, USA, New Haven, United States of America;[13]Rambam Health Care Campus-Pediatric Hemato-Oncology, Haifa, Israel, Haifa, Israel; [14]New York Medical College, Valhalla, NY, USA, Valhalla, United States of America; [15]Merck & Co., Inc., Rahway, NJ, USA, Rahway, United States of America;[16]Sheba Medical Center–Tel HaShomer, Ramat Gan, Israel, Ramat Gan, Israel;*

### Background

Zilovertamab vedotin (ZV) is an antibody-drug conjugate comprising an anti-ROR1 antibody, a proteolytically cleavable linker, and monomethyl auristatin E.

### Aims

Substudy 01A of LIGHTBEAM-U01 (NCT06395103) is a single-arm, open-label, phase 1/2 basket study designed to determine the preliminary recommended phase 2 dose (RP2D), safety, and antitumor activity of ZV in 4 disease cohorts: pediatric patients with relapsed/refractory (R/R) B-cell acute lymphoblastic leukemia (B-ALL), diffuse large B-cell lymphoma (DLBCL)/Burkitt lymphoma, or neuroblastoma, and pediatric or young adult patients with Ewing sarcoma.

**Copyright Information:** (Online) ISSN: 2572-9241
©2026 The Author(s). HemaSphere is published by John Wiley & Sons Ltd on behalf of European Hematology Association. This is an open access Abstract Book distributed under the Attribution-NonCommercial-NoDerivs (CC BY-NC-ND), which allows third parties to download the articles and share them with others as long as they credit the author and the Abstract Book, but they cannot change the content in any way or use them commercially.
**Abstract Book Citations:** Authors, Title, HemaSphere, 2026;10:(S1):pages. The abstract book DOIs can be found on the "Author Information" page.
**Disclaimer:** Articles published in the journal HemaSphere exclusively reflect the opinions of the authors. The authors are responsible for all content in their abstracts including accuracy of the facts, statements, citing resources, etc.

### Methods

Eligible patients are aged 0 months to ≤25 years. Patients with B-ALL or DLBCL/Burkitt lymphoma must have relapsed after ≥2 prior lines of therapy and exhausted all therapies of known clinical benefit. Patients with B-ALL must have ≥5% (M2 or M3) bone marrow blasts. Patients with DLBCL/Burkitt lymphoma must have radiographically measurable disease per International Pediatric Non-Hodgkin Lymphoma staging system response criteria. Patients with neuroblastoma or Ewing sarcoma must have measurable disease per RECIST v1.1 (or MIBG-avid evaluable neuroblastoma) and be refractory to front-line therapy and for whom further standard therapy is not available/appropriate. All patients must also have a Lansky play-performance scale ≥50 if aged ≤16 years, a Karnofsky performance status ≥50 if aged >16 to <18 years, or an ECOG PS of 0 or 1 if aged ≥18 years. Approximately 50 to 90 patients will be enrolled across the study, which will consist of 2 parts: dose escalation and confirmation (part 1) and efficacy expansion (part 2). In part 1, ≥3 patients will be enrolled for 2 age groups: 1 to <6 years and 6 to <18 years. Patients will receive ZV at a starting dose of 2 mg/kg IV Q3W, with escalation to 2.25 and 2.5 mg/kg or de-escalation to 1.75 mg/kg per a modified toxicity probability interval design-2. In part 2, the age of patients will be expanded to ≥6 months for all indications and ≤25 years for R/R Ewing sarcoma. Approximately 10 patients will be enrolled in each cohort and will receive ZV at the preliminary RP2D determined in part 1. For all patients except those with B-ALL, imaging/disease assessments will be performed Q8W for 6 months, then Q12W through 24 months, Q24W through 5 years, and annually thereafter. Disease assessments for B-ALL will be performed at the end of each cycle, at 6 months and 1 year, and then annually. Adverse events (AEs) will be monitored throughout the study and for 30 days after the last dose of study treatment (90 days for serious AEs or 30 days if new anticancer therapy is initiated). AEs will be graded per NCI CTCAE v5.0. Primary end points are safety and tolerability and objective response rate. Secondary end points include pharmacokinetics, immunogenicity, duration of response, and eligibility for transplant/CAR-T therapy in patients with B-ALL or DLBCL/Burkitt lymphoma. Recruitment is ongoing.

**Copyright Information:** (Online) ISSN: 2572-9241
©2026 The Author(s). HemaSphere is published by John Wiley & Sons Ltd on behalf of European Hematology Association. This is an open access Abstract Book distributed under the Attribution-NonCommercial-NoDerivs (CC BY-NC-ND), which allows third parties to download the articles and share them with others as long as they credit the author and the Abstract Book, but they cannot change the content in any way or use them commercially.
**Abstract Book Citations:** Authors, Title, HemaSphere, 2026;10:(S1):pages. The abstract book DOIs can be found on the “Author Information” page.
**Disclaimer:** Articles published in the journal HemaSphere exclusively reflect the opinions of the authors. The authors are responsible for all content in their abstracts including accuracy of the facts, statements, citing resources, etc.

### (PB2650) CLINICAL CHARACTERISTICS OF FIRST RELAPSED ACUTE LYMPHOBLASTIC LEUKEMIA IN CHILDREN, ADOLESCENT AND YOUNG ADULTS

**Topic:** 02. Acute lymphoblastic leukemia - Clinical

Aicha Ben Kahla*[1], Imen Frikha[1], Turki Ines[1], Khouloud Mezghani[1], Rahma Mallek[1], Imen Ben Amor[1], Yousra Fakhfakh Derbel[1], Faten Kallel[1], Maha Charfi[1], Asma Koubaa[1], Nour Siala[1], Hanen Charfi[1], Moez Medhaffar[1], Moez Elloumi[1]

[1]*hedi chaker hospital, hematology, sfax, Tunisia;*

### Background

Acute lymphoblastic leukemia (ALL) is the most frequent cancer in children. Outcomes of ALL have improved worldwide to more than 80%. Although, 15 to 20% of ALL patients relapsed. Prognosis of ALL after relapse remains poor.

### Aims

Determine the clinical characteristics of first relapsed ALL.

### Methods

In this study, we enrolled retrospectively all cases of ALL aged under 30 years old, treated initially according to EORTC 58951 regiemen, and who are relapsed. Our cases are diagnosed in the hematology department of "Hedi Chaker" Hospital, Sfax, Tunisia, between January 2000 and December 2025. Cases were stratified initially into low risk, medium 1 and 2 risk and high risk according to response to dexamethasone, cytogenetics abnormalities, complete remission after induction course, phenotype B or T, White blood cells rate at diagnosis, neuromeningeal and gonadic involvement. Time of relapse is defined as : very early if relapse occur 18 months or less from initial diagnosis ; early if relapse occur 18 months or more after initial diagnosis and less than 6 months after stopping first line treatment and late if relapse occur 6 months or more after stopping first line treatment. Bone marrow relapse is defined by reappearance of blastes (≥ 20%) in the blood and/or in bone marrow after a period of complete remission. Neuro meningeal relapse is the presence of a cytorachy ≥ 5 cells / mm with the presence of blasts and/or the existence of involvement of a cranial pair. Combined relapse is the association of bone marrow relapse with extramedullary, neuromeningeal or gonadal localization.
We studied the epidemiological, clinical and biological charachteristics.

### Results

We enrolled 128 cases of ALL (30%) of 423 cases in complete remission in the same study period. The mean time of relapse was 12 months. The relapse was very early in 58% of cases, early in 19% of cases and late in 23% of cases. Bone marrow relapse was noted in 93% and extramedullary relapse was noted in 7%. Relapse was isolated in 76%. The relapse rate was 80 cases for males and 48 cases for females .The relapse rate was 24% for patients aged more than 16 years and 76% for patients aged less than 16 years. The relapse rate was 24% for cases with white blood cells (WBC) more than 100 G/L compared to 36% and 40% for cases with WBC≤10 G/L and between 10-100 G/L. Relapse occured in 74% of cases with B ALL VS 26% of cases with T ALL. Patients with an abnormal Karyotype have a relapse rate of 51% compared to 48% in patients without cytogenetic abnormalities .The relapse rate was higher (38%) in the high risk group than the low risk , medium1 and 2 risk groups (2%, 36% and 24% in respectively.

**Copyright Information:** (Online) ISSN: 2572-9241
©2026 The Author(s). HemaSphere is published by John Wiley & Sons Ltd on behalf of European Hematology Association. This is an open access Abstract Book distributed under the Attribution-NonCommercial-NoDerivs (CC BY-NC-ND), which allows third parties to download the articles and share them with others as long as they credit the author and the Abstract Book, but they cannot change the content in any way or use them commercially.
**Abstract Book Citations:** Authors, Title, HemaSphere, 2026;10:(S1):pages. The abstract book DOIs can be found on the "Author Information" page.
**Disclaimer:** Articles published in the journal HemaSphere exclusively reflect the opinions of the authors. The authors are responsible for all content in their abstracts including accuracy of the facts, statements, citing resources, etc.

**Summary/Conclusion**

Between 2000 and 2025, relapse rate in our study is 30%, which is higher than in the literature (15 to 20%). This could be explained by the use of generic chemotherapy agents, chemotherapy resistance, the lack of early therapeutic intensification in all patients, the lack of genoidentical and phenoidentical stem cells transplantation and the lack of use of ASPARGINASE in some cases. The rate of early relapse (77%) is higher than in the literature (20 to 38%). Clinical biological characteristics of our patients are similar to those of other series.The relapse rate could be reduced by early intensification of therapy in all patients taking into account not only the blast rate but also the minimal residual disease.

**Copyright Information:** (Online) ISSN: 2572-9241
©2026 The Author(s). HemaSphere is published by John Wiley & Sons Ltd on behalf of European Hematology Association. This is an open access Abstract Book distributed under the Attribution-NonCommercial-NoDerivs (CC BY-NC-ND), which allows third parties to download the articles and share them with others as long as they credit the author and the Abstract Book, but they cannot change the content in any way or use them commercially.
**Abstract Book Citations:** Authors, Title, HemaSphere, 2026;10:(S1):pages. The abstract book DOIs can be found on the "Author Information" page.
**Disclaimer:** Articles published in the journal HemaSphere exclusively reflect the opinions of the authors. The authors are responsible for all content in their abstracts including accuracy of the facts, statements, citing resources, etc.

### (PB2651) COAGULATION ABNORMALITIES DEVELOPING DURING PRE-PHASE TREATMENT OF ADULT ACUTE LYMPHOBLASTIC LEUKEMIA PATIENTS

**Topic:** 02. Acute lymphoblastic leukemia - Clinical

Adi Karlin*[1], Miriam Neaman[1], Roy Vitkon[1], Tamir Shragai[1, 2], Yakir Moshe[1]

*[1]Tel Aviv Sourasky Medical Center, Department of Hematology, Tel Aviv, Israel;[2]Tel Aviv University, Gray Faculty of Medical and Health Sciences, Tel Aviv, Israel;*

### Background

Coagulopathy is a common issue in Patients (pts) with acute lymphoblastic leukemia (ALL) and can present at diagnosis or following asparaginase(asn)-based therapy. Coagulopathy may also develop during the cytoreductive steroid pre-phase, although its incidence and clinical impact remains unclear.

### Aims

To assess the prevalence of coagulation abnormalities during pre-phase therapy for ALL, and their association with patient- and disease-related characteristics and outcomes.

### Methods

We retrospectively reviewed all ALL pts treated at a tertiary medical center between Aug 2016 and Feb 2026. Eligible pts had coagulation studies at diagnosis and at least once during the first week of therapy. Treatment initiation was defined as the first day of steroid administration. We collected pt, disease and treatment-related characteristics, as well as lab results during the first week of treatment. We graded hypofibrinogenemia according to the CTCAE criteria. We collected data on pt outcomes, including complete remission (CR), overall survival (OS), relapse-free survival (RFS), and 30-day mortality.

### Results

Forty-four pts met inclusion criteria; 33 pts (75%) had B-ALL, 9 (20%) T-ALL, and 2 (5%) mixed phenotype acute leukemia. No pts received asn during the first week of treatment.
At diagnosis, 4 pts (9%) had grade ≥1 hypofibrinogenemia (hypoF), 5 (11%) had prolonged prothrombin time (PT), and none had prolonged partial thromboplastin time (PTT). Thirty-eight pts (86%) experienced a decline in fibrinogen, 27 (61%) had grade ≥1 hypoF and 16 (36%) developed grade ≥3 hypoF. HypoF of any grade was significantly associated with prolonged PT but not PTT or liver enzyme abnormalities. Philadelphia-negative (Ph-neg) B-ALL pts had a significantly higher incidence of HypoF compared with Philadelphia-positive (Ph-pos) B-ALL or T-ALL pts (88% vs. 56% vs. 22%, respectively; p=0.01). Pts with HypoF had significantly lower baseline platelet counts and higher bone marrow blast percentages at diagnosis. Cryoprecipitate was administered to 9 pts (20%) during the first week. Two pts developed clinically significant non-fatal bleeding, both intracranial hemorrhages; both had hypoF, including one with grade 3 hypoF. Only one pt died within 30 days. This pt had grade 2 hypoF. CR was achieved in 91% of pts. HypoF had no effect on CR rates (Fig 1a).
At a median follow-up of 60 months, median OS was not reached; estimated 60-month OS was 52.2%. Pts with HypoF had inferior 60-month OS compared with those with normal fibrinogen levels (36.2% vs. 74%, p=0.043). Of note, when stratified by disease subtype, Ph-neg B-ALL demonstrated a trend toward inferior OS compared with Ph-pos B-ALL and T-ALL (60-month OS 30.8% vs. 63.1% vs. 77.8%, p=0.051). Multivariate analysis was not feasible due to limited sample size. At 60 months, 55.3% of pts remained relapse-free. RFS did not significantly differ according to hypoF or disease subtype (Fig 1b).

**Copyright Information:** (Online) ISSN: 2572-9241
©2026 The Author(s). HemaSphere is published by John Wiley & Sons Ltd on behalf of European Hematology Association. This is an open access Abstract Book distributed under the Attribution-NonCommercial-NoDerivs (CC BY-NC-ND), which allows third parties to download the articles and share them with others as long as they credit the author and the Abstract Book, but they cannot change the content in any way or use them commercially.
**Abstract Book Citations:** Authors, Title, HemaSphere, 2026;10:(S1):pages. The abstract book DOIs can be found on the "Author Information" page.
**Disclaimer:** Articles published in the journal HemaSphere exclusively reflect the opinions of the authors. The authors are responsible for all content in their abstracts including accuracy of the facts, statements, citing resources, etc.

### Summary/Conclusion

Hypofibrinogenemia can occur during the steroid pre-phase of ALL treatment, prior to asn exposure. In our cohort, it was more common in Ph-neg B-ALL and was associated with markers of higher disease burden, but not with liver abnormalities. Low fibrinogen was present in the two major bleeding events and was associated with inferior OS in univariate analysis. Whether hypoF constitutes an independent risk factor for OS or is due to its higher prevalence in Ph-neg B-ALL is unclear. This study supports routine monitoring of fibrinogen levels during initial treatment of ALL pts.

1a

| Characteristic | All Patients (n=44) | Normal Fibrinogen ≥200 mg/dL (n=17) | Fibrinogen <200 mg/dL (n=27) | p value |
|---|---|---|---|---|
| Age at diagnosis, years | 51.5 (20–93)‡ | 48 (29.5–65.5)§ | 56 (34–70)§ | 0.39 |
| Male sex, n (%) | 23 (52.3) | 9 (52.9) | 14 (51.9) | 0.94 |
| Diagnosis, n (%) | | | | 0.01 |
| Ph-positive B-ALL | 16 (36.4) | 7 (41.2) | 9 (33.3) | |
| Ph-negative B-ALL | 17 (38.6) | 2 (11.8) | 15 (55.6) | |
| T-ALL | 9 (20.5) | 7 (41.2) | 2 (7.4) | |
| Mixed phenotype | 2 (4.5) | 1 (5.9) | 1 (3.7) | |
| Extramedullary disease, n (%) | 23 (52.3) | 7 (43.8) | 16 (59.3) | 0.32 |
| Bone marrow blasts, % | 82 (60–90.7)§ | 60 (42.5–86)§ | 88 (69–91)§ | 0.032 |
| Laboratory values at diagnosis | | | | |
| WBC, $\times 10^3/\mu L$ | 9 (2.8–35.5)§ | 11.3 (4.8–66.1)§ | 8.6 (2.4–22.6)§ | 0.347 |
| Hemoglobin, g/dL | 9.55 (8.27–11.9)§ | 11.6 (8.8–13.0)§ | 9.5 (7.7–10.8)§ | 0.067 |
| Platelets, $\times 10^3/\mu L$ | 65 (25–124)§ | 126 (31.5–176.0)§ | 44 (22–83)§ | 0.007 |
| Laboratory findings during days 1–7, n (%) | | | | |
| Prolonged PT | 16 (36.4) | 1 (5.8) | 15 (55.5) | 0.01 |
| Prolonged PTT | 4 (9.1) | 2 (11.7) | 2 (7.4) | 1.00 |
| Bilirubin >3× ULN | 2 (4.5) | 0 (0) | 2 (7.4) | 0.25 |
| ALT >3× ULN | 17 (38.6) | 5 (29.4) | 12 (44.4) | 0.32 |
| Cryoprecipitate use | 9 (20.5) | 1 (5.8) | 8 (29.6) | 0.11 |
| Bleeding | 2 (4.5) | 0 (0) | 2 (7.4) | 0.52 |
| Complete remission, n (%) | 40 (90.9) | 16 (94.1) | 24 (88.9) | 1 |
| 30-day mortality, n (%) | 1 (2.3) | 0 (0) | 1 (3.7) | 1.00 |

‡ - range, § - interquartile range,

1b

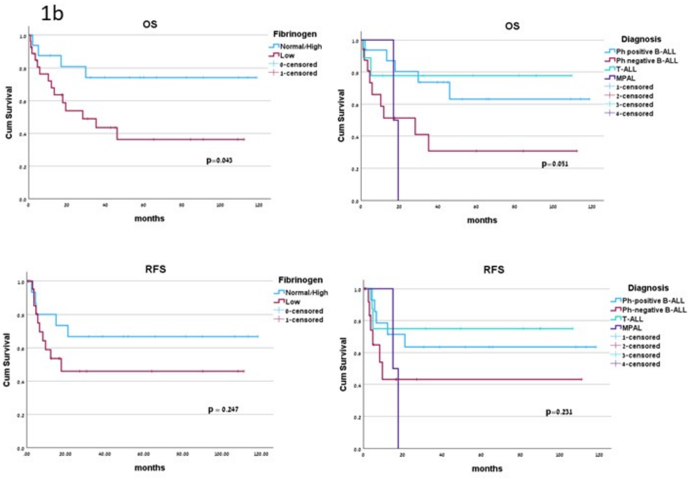

**Copyright Information:** (Online) ISSN: 2572-9241
©2026 The Author(s). HemaSphere is published by John Wiley & Sons Ltd on behalf of European Hematology Association. This is an open access Abstract Book distributed under the Attribution-NonCommercial-NoDerivs (CC BY-NC-ND), which allows third parties to download the articles and share them with others as long as they credit the author and the Abstract Book, but they cannot change the content in any way or use them commercially.
**Abstract Book Citations:** Authors, Title, HemaSphere, 2026;10:(S1):pages. The abstract book DOIs can be found on the "Author Information" page.
**Disclaimer:** Articles published in the journal HemaSphere exclusively reflect the opinions of the authors. The authors are responsible for all content in their abstracts including accuracy of the facts, statements, citing resources, etc.

### (PB2652) A REVIEW ON THE APPLICATION OF FLUORESCENCE IN SITU HYBRIDIZATION (FISH) IN PREDICTING POOR PROGNOSIS IN ACUTE LYMPHOBLASTIC LEUKEMIA (ALL)

**Topic:** 02. Acute lymphoblastic leukemia - Clinical

Xiaoting Xu[1], Xiangli Chen*[2], Yijie Wang[1], Yaru Wang[1], Tiantian Dai[2], Wenyi Lu[3], Pingfan Lu[2], Fei Ge[2], Xiaona Niu[2]

*[1]Henan Provincial People's Clinical Medical School of Zhengzhou University, Department of Hematology, Zhengzhou City, China; [2]People's Hospital of Henan University, Department of Hematology, Zhengzhou City, China;[3]Henan Provincial People's Hospital, Department of Hematology, Zhengzhou City, China;*

### Background

FISH, as a core method in molecular cytogenetics, employs fluorescently labeled nucleic acid probes to rapidly and sensitively detect chromosomal numerical abnormalities and structural rearrangements, while maintaining the integrity of cell morphology and nuclear structure. Acute lymphoblastic leukemia (ALL) is a highly heterogeneous hematological malignancy, with over 75% of ALL patients exhibiting acquired chromosomal abnormalities. Specific types of gene aberrations are key prognostic factors independent of traditional indicators such as age and white blood cell count. In recent years, with the continuous optimization and popularization of FISH technology, its detection rate for high-frequency prognostic-related gene abnormalities such as BCR-ABL1 and IKZF1 deletions has significantly increased, making it an important tool in the precision diagnosis system of ALL.

### Aims

This article aims to review the application progress of FISH in predicting poor prognosis in ALL, systematically elaborating on how it optimizes risk stratification and guides targeted drug selection by identifying high-risk cytogenetic abnormalities, and exploring its potential value in minimal residual disease monitoring.

### Methods

Additionally, by combining domestic and international diagnostic and treatment guidelines, this article analyzes the advantages and limitations of FISH compared to traditional karyotype analysis and high-throughput sequencing technologies.

### Results

With the goal of providing a more comprehensive laboratory basis for individualized treatment strategies for ALL patients.

**Copyright Information:** (Online) ISSN: 2572-9241
©2026 The Author(s). HemaSphere is published by John Wiley & Sons Ltd on behalf of European Hematology Association. This is an open access Abstract Book distributed under the Attribution-NonCommercial-NoDerivs (CC BY-NC-ND), which allows third parties to download the articles and share them with others as long as they credit the author and the Abstract Book, but they cannot change the content in any way or use them commercially.
**Abstract Book Citations:** Authors, Title, HemaSphere, 2026;10:(S1):pages. The abstract book DOIs can be found on the "Author Information" page.
**Disclaimer:** Articles published in the journal HemaSphere exclusively reflect the opinions of the authors. The authors are responsible for all content in their abstracts including accuracy of the facts, statements, citing resources, etc.

**Summary/Conclusion**

FISH, as a key molecular cytogenetic technique, enables sensitive and rapid detection of chromosomal numerical and structural abnormalities while preserving cell and nuclear integrity. Acute lymphoblastic leukemia (ALL) is a highly heterogeneous hematologic malignancy, and more than 75% of ALL patients carry acquired chromosomal abnormalities, among which specific gene aberrations serve as independent prognostic factors. With the optimization and application of FISH, the detection rate of high-frequency prognostic gene abnormalities such as BCR-ABL1 and IKZF1 deletions has been greatly improved, establishing FISH as an important tool in the precise diagnosis of ALL. This review summarizes the application of FISH in evaluating poor prognosis in ALL, highlighting its role in refining risk stratification and guiding targeted therapy by identifying high-risk cytogenetic abnormalities, as well as its potential value in minimal residual disease monitoring. By comparing FISH with conventional karyotyping and high-throughput sequencing based on clinical guidelines, its advantages and limitations are clarified. Taken together, FISH provides reliable laboratory evidence for individualized diagnosis and treatment strategies in ALL, and will continue to play an important role in promoting precise prognostic evaluation and personalized management of ALL.

**Copyright Information:** (Online) ISSN: 2572-9241
©2026 The Author(s). HemaSphere is published by John Wiley & Sons Ltd on behalf of European Hematology Association. This is an open access Abstract Book distributed under the Attribution-NonCommercial-NoDerivs (CC BY-NC-ND), which allows third parties to download the articles and share them with others as long as they credit the author and the Abstract Book, but they cannot change the content in any way or use them commercially.
**Abstract Book Citations:** Authors, Title, HemaSphere, 2026;10:(S1):pages. The abstract book DOIs can be found on the "Author Information" page.
**Disclaimer:** Articles published in the journal HemaSphere exclusively reflect the opinions of the authors. The authors are responsible for all content in their abstracts including accuracy of the facts, statements, citing resources, etc.

### (PB2653) CHARACTERISTICS OF RELAPSE IN PEDIATRIC PHILADELPHIA CHROMOSOME–POSITIVE ACUTE LYMPHOBLASTIC LEUKEMIA TREATED ACCORDING TO THE EORTC 58951 PROTOCOL

**Topic:** 02. Acute lymphoblastic leukemia - Clinical

Salma Sammoud[1, 1], Emna Azza[1], Marwa Bahri[1], Roua Hsasna[1], Ben Othmen Aya[1], Raihane Ben Lakhal[1], Yosr Ben Abdennabi[1], Lamia Issaoui*[1]

[1]*Aziza Othmana Hospital, Tunis, Tunisia;*

**Background**

Therapeutic outcomes of pediatric Philadelphia chromosome–positive B-cell acute lymphoblastic leukemia (Ph+ B-ALL) have significantly improved with the introduction of tyrosine kinase inhibitors (TKIs). However, the prognosis after relapse remains poor, and descriptive data specific to this population are still limited.

**Aims**

The aim of our study was to describe the clinical, biological, therapeutic, and outcome characteristics of relapsed patients.

**Methods**

We conducted a retrospective descriptive study at Aziza Othmana Hospital in Tunisia between January 2005 and December 2023. All patients diagnosed with Ph+ B-ALL, treated according to the EORTC 58951 high-risk arm in combination with Imatinib, and who subsequently experienced relapse were included.

**Results**

Among 33 treated patients, 12 (36%) experienced relapse.
The median age at diagnosis was 10 years (range: 3–18), with a male predominance (sex ratio: 2.3).
Tumor syndrome and hyperleukocytosis ≥100 G/L were observed in 11 (91%) and 7 patients (58%), respectively. No patient had central nervous system involvement at diagnosis. Good response to prephase at day 8 was noted in 6 patients (50%). All patients were good chemotherapy responders.
At day 35, 8 patients (67%) had minimal residual disease (MRD) $\geq 5 \times 10^{-4}$.
In our study, patients who were poor prephase responders and/or poor chemotherapy responders and/or had post-induction MRD $\geq 5 \times 10^{-4}$ were classified as high risk. Ten high-risk patients subsequently relapsed.
The median time of relapse was 31.7 months (range: 4.8–115.2). Six early relapses (50%) and six (50%) late ones were noted.
Side of relapse included: bone marrow in 9 cases (75%) and central nervous system in 3 cases (25%).
Regarding salvage therapy: 5 patients received reinduction chemotherapy according to the same protocol arm with a switch to Dasatinib, 6 patients received Vincristine–Dexamethasone combined with Dasatinib and one patient received the CAPIZZI regimen.
Six patients achieved a second complete remission, and two of them underwent haploidentical allogeneic stem cell transplantation.
Both transplanted patients subsequently relapsed and died. All non-transplanted patients also relapsed and died.

**Summary/Conclusion**

The prognosis of Ph+ B-ALL after relapse remains poor despite therapeutic advances. These descriptive data provide a rationale for further analysis of post-relapse prognostic factors in this population and highlight the need for innovative therapeutic strategies.

**Copyright Information:** (Online) ISSN: 2572-9241
©2026 The Author(s). HemaSphere is published by John Wiley & Sons Ltd on behalf of European Hematology Association. This is an open access Abstract Book distributed under the Attribution-NonCommercial-NoDerivs (CC BY-NC-ND), which allows third parties to download the articles and share them with others as long as they credit the author and the Abstract Book, but they cannot change the content in any way or use them commercially.
**Abstract Book Citations:** Authors, Title, HemaSphere, 2026;10:(S1):pages. The abstract book DOIs can be found on the "Author Information" page.
**Disclaimer:** Articles published in the journal HemaSphere exclusively reflect the opinions of the authors. The authors are responsible for all content in their abstracts including accuracy of the facts, statements, citing resources, etc.

### (PB2654) ADULT T-CELL ACUTE LYMPHOBLASTIC LEUKEMIA: EPIDEMIOLOGICAL, CLINICO-BIOLOGICAL, AND THERAPEUTIC PROFILE

**Topic:** 02. Acute lymphoblastic leukemia - Clinical

Amel Meliane*[1], Hend Ben Neji[1]

[1] *hopital Aziza Othmana, Tunis, Tunisia;*

### Background

T-cell acute lymphoblastic leukemia (T-ALL) is a malignant and aggressive hematologic disease. Despite the advent of new therapeutic agents, treatment outcomes remain unsatisfactory. In Tunisia, there are no available data on this pathology.

### Aims

The aim of our study was to describe the epidemiological, clinico-biological, and therapeutic profile of adult T-ALL.

### Methods

This was a retrospective and descriptive study conducted in the adult clinical hematology department at Aziza Othmana Hospital over a period of 9 years. We included patients aged 18 years or older who were followed for de novo T-ALL and treated according to the GRAALL 2005 protocol.

### Results

Fifty patients were collected . The median age was 37 years [24–62]. Central nervous system involvement was observed in 4% of cases. Mediastinal enlargement was noted in 38% of cases. Hyperleukocytosis ≥ 100 G/L was observed in 38% of cases. A pathological karyotype was present in 54.8% of cases. A complex karyotype was identified in 22% of patients. Corticosteroid resistance and chemoresistance were observed in 50% and 68% of cases, respectively. The complete remission (CR) rate post-induction was 87.5%. Mortality during induction was 20%. Post-induction minimal residual disease (MRD) was assessed in 31 patients (88.5%) and was $< 10^{-3}$ in 32% of cases. Post-first consolidation MRD was assessed in 71% of patients and was $> 10^{-4}$ in 48% of cases. Only three patients underwent allogeneic stem cell transplantation. During follow-up, 18 patients (51%) relapsed, with a median time of 12 months [3–26]. Central nervous system relapse was the most frequent (38%). Overall survival at 1 year and 5 years was 75.4% and 44.3%, respectively. Five-year relapse-free survival and event-free survival were 30.2% and 25%, respectively.

### Summary/Conclusion

Our results were lower than those reported in the literature. Further research in molecular biology, combined with strict MRD monitoring as well as the use if nelarabine, is essential to improve therapeutic outcomes.

**Copyright Information:** (Online) ISSN: 2572-9241
©2026 The Author(s). HemaSphere is published by John Wiley & Sons Ltd on behalf of European Hematology Association. This is an open access Abstract Book distributed under the Attribution-NonCommercial-NoDerivs (CC BY-NC-ND), which allows third parties to download the articles and share them with others as long as they credit the author and the Abstract Book, but they cannot change the content in any way or use them commercially.
**Abstract Book Citations:** Authors, Title, HemaSphere, 2026;10:(S1):pages. The abstract book DOIs can be found on the "Author Information" page.
**Disclaimer:** Articles published in the journal HemaSphere exclusively reflect the opinions of the authors. The authors are responsible for all content in their abstracts including accuracy of the facts, statements, citing resources, etc.

### (PB2655) ACUTE LEUKEMIA AND THROMBOSIS IN CHILDREN

**Topic:** 02. Acute lymphoblastic leukemia - Clinical

Selin Aytaç*[1], Cagri Coskun[1], Tekin Aksu[1], Sule Unal[1], Baris Kuskonmaz[1]

[1]*Hacettepe University Faculty of Medicine, Department of Pediatric Hematology, Ankara, Türkiye;*

### Background

Thrombosis is a serious complication in children with acute leukemia, and the incidence of venous thrombosis is reported to be similar between acute lymphoblastic leukemia (ALL) and acute myeloid leukemia (AML), ranging from 2% to 11%. In children with leukemia, thrombosis is often triggered by treatment (asparaginase, steroids), central venous catheters, and certain risk factors related to disease characteristics (high white blood cell count at the initial presentation, age at onset >10 years, T cell leukemia), and can lead to treatment delays, morbidity and mortality.

### Aims

Our aim is to present our experience with thrombosis in children with acute leukemia and to identify risk factors for thrombosis development and prognosis.

### Methods

Thrombosis observed in ALL and AML patients whose diagnosis and follow-up were completed at the Hacettepe University Faculty of Medicine , Department of Pediatric Hematology were retrospectively evaluated. Of the 41 children, 23 were boys and 18 were girls; 9 had been diagnosed with AML, and thrombosis was observed in 32 ALL patients. In 2 ALL patients, thrombosis developed during relapse, while in 39 patients it was observed shortly after diagnosis.The mean age was 8.1 years (1 month to 18 y) at the time of thrombosis and were the same both in ALL and AML groups. In the ALL group, the age range was 1.5 to 18 years, while in the AML group, the youngest patient diagnosed was 1 month old and the oldest was 14 years old.

**Copyright Information:** (Online) ISSN: 2572-9241
©2026 The Author(s). HemaSphere is published by John Wiley & Sons Ltd on behalf of European Hematology Association. This is an open access Abstract Book distributed under the Attribution-NonCommercial-NoDerivs (CC BY-NC-ND), which allows third parties to download the articles and share them with others as long as they credit the author and the Abstract Book, but they cannot change the content in any way or use them commercially.
**Abstract Book Citations:** Authors, Title, HemaSphere, 2026;10:(S1):pages. The abstract book DOIs can be found on the "Author Information" page.
**Disclaimer:** Articles published in the journal HemaSphere exclusively reflect the opinions of the authors. The authors are responsible for all content in their abstracts including accuracy of the facts, statements, citing resources, etc.

**Results**

When the distribution of observed thromboses was examined, deep vein thrombosis was the most common (n=18), and in half of these patients it was assessed as catheter-related (n=9)thrombosis .Cerebral venous sinus thrombosis was the second most common thrombosis (n=7), followed by cardiac thrombosis (n=6), and thrombophlebitis and superficial vein thrombosis were also observed in patients (n=7).One patient with AML and 1 patient with ALL were diagnosed as acute cerabral arterial ischemia due to thrombosis. Most of the ALL group was pre B cell ALL(n=26) and receiving modified St Jude Total XV protocol(N=24/26). In the entire group, thrombosis was observed in 29 patients  while receiving induction therapy and 12 patients while receiving maintenance therapy; thrombosis developed during induction therapy in all AML patients, and only 1 patient in the AML group was diagnosed with AML-M3. This patient with cardiac thrombosis underwent open heart surgery and recovered successfully. Thrombosis in superficial and deep veins was monitored with control imaging methods, and partial or near-complete resolution of thromboses was observed in 34 of 41 patients with LMWH treatment lasting an average of 4 months (1-6 months). Chronic thrombosis developed in 5 patients. None of them received thrombolytic therapy.

**Summary/Conclusion**

Thrombosis is one of the most common treatment-related complications in ALL, and it can be prevented or reduced by taking certain precautions.

**Copyright Information:** (Online) ISSN: 2572-9241
©2026 The Author(s). HemaSphere is published by John Wiley & Sons Ltd on behalf of European Hematology Association. This is an open access Abstract Book distributed under the Attribution-NonCommercial-NoDerivs (CC BY-NC-ND), which allows third parties to download the articles and share them with others as long as they credit the author and the Abstract Book, but they cannot change the content in any way or use them commercially.
**Abstract Book Citations:** Authors, Title, HemaSphere, 2026;10:(S1):pages. The abstract book DOIs can be found on the "Author Information" page.
**Disclaimer:** Articles published in the journal HemaSphere exclusively reflect the opinions of the authors. The authors are responsible for all content in their abstracts including accuracy of the facts, statements, citing resources, etc.

### (PB2656) BEYOND MORPHOLOGY: A FLOW CYTOMETRIC RISK SCORE FOR IMMEDIATE IDENTIFICATION OF BCR3-GENOTYPE AND FATAL OUTCOMES IN ACUTE PROMYELOCYTIC LEUKEMIA

**Topic:** 03. Acute myeloid leukemia - Biology & translational research

Manel Kasdallah*[1], Emna Sahli[1], Rym Cherni[2], Hassiba Ammouri[1], Raihane Ben Lakhal[2], Samia Menif[1]

*[1]Pasteur Institute of Tunis, Laboratory of cellular and molecular Hematology, Tunis, Tunisia;[2]Aziza Othmana Hospital, Hematology Clinic Department, Tunis, Tunisia;*

### Background

Acute Promyelocytic Leukemia (APL) is the most fulminant form of leukemia, where the "time-to-ATRA" and early risk-stratification are the primary determinants of survival. While the *PML-RARA* bcr3 (short) isoform is a major driver of aggressive biology, its identification via RT-PCR or FISH typically requires 24 to 48 hours—a window during which most early deaths (ED) occur. Multiparametric flow cytometry (MFC) provides results within 2 hours, but its potential to serve as a high-fidelity surrogate for molecular high-risk features and coagulopathy-related death remains under-exploited in clinical algorithms.

### Aims

To develop and validate an MFC-based biological score using Side-Scatter (SSC) intensity and aberrant marker expression (CD38/HLA-DR) as real-time proxies for the bcr3 genotype, high Sanz risk, and early mortality.

### Methods

We analyzed MFC data from 43 newly diagnosed APL patients (Tunis, 2018–2024). Beyond standard gating, we quantified SSC (Side-Scatter) intensity and the expression of CD34, HLA-DR, and CD38. These parameters were correlated with *PML-RARA* isoforms (RT-PCR), Sanz risk scores, clinical DIC (ISTH criteria), and overall survival (OS). Multivariate Cox regression and ROC curve analysis were utilized to determine the predictive power of immunophenotypic signatures.

### Results

**SSC as a Morpho-Molecular Link:** A low-SSC signal—characteristic of the microgranular (M3v) variant—was the strongest indicator of the **bcr3 genotype (p=0.004)** and extreme leukocytosis (WBC $>16.96\times10^9$/L; p<0.01). Patients with low-SSC signatures faced a nearly **20-fold increase in mortality risk (HR 19.8; 95% CI: 4.2-92.6; p=0.018)** compared to high-SSC cases.
**The "Fatal Triad" (HLA-DR/CD38/SSC):** * **HLA-DR:** Although rare (7.1%), aberrant HLA-DR expression was a specific harbinger of **early hemorrhagic death (HR 5.34; p=0.023)** and clustered with severe clinical DIC.**CD38:** CD38 positivity was significantly higher in Sanz high-risk patients (p=0.022) and correlated with resistance to standard induction.
**The MFC-Risk Score Performance:** We integrated these markers into a combined signature (**Low-SSC + CD38+**). This model achieved an **Area Under the Curve (AUC) of 0.84** for predicting unfavorable clinical evolution (ED or relapse), with a **sensitivity of 82% and specificity of 76%.** This signature successfully identified 71% of high-risk patients at the hour of diagnosis, effectively "pre-empting" molecular confirmation.

**Copyright Information:** (Online) ISSN: 2572-9241
©2026 The Author(s). HemaSphere is published by John Wiley & Sons Ltd on behalf of European Hematology Association. This is an open access Abstract Book distributed under the Attribution-NonCommercial-NoDerivs (CC BY-NC-ND), which allows third parties to download the articles and share them with others as long as they credit the author and the Abstract Book, but they cannot change the content in any way or use them commercially.
**Abstract Book Citations:** Authors, Title, HemaSphere, 2026;10:(S1):pages. The abstract book DOIs can be found on the "Author Information" page.
**Disclaimer:** Articles published in the journal HemaSphere exclusively reflect the opinions of the authors. The authors are responsible for all content in their abstracts including accuracy of the facts, statements, citing resources, etc.

**Summary/Conclusion**

Our study identifies a specific MFC "biological snapshot" (Low-SSC/CD38+/HLA-DR+) that functions as a robust, zero-cost, and real-time surrogate for the bcr3 isoform and fatal APL biology. This **"MFC-Risk Signature"** allows clinicians to bypass the molecular waiting period, justifying immediate therapeutic escalation—such as upfront ATRA (All-Trans Retinoic Acid) or intensified supportive care—in patients at highest risk of early death.

**Copyright Information:** (Online) ISSN: 2572-9241
©2026 The Author(s). HemaSphere is published by John Wiley & Sons Ltd on behalf of European Hematology Association. This is an open access Abstract Book distributed under the Attribution-NonCommercial-NoDerivs (CC BY-NC-ND), which allows third parties to download the articles and share them with others as long as they credit the author and the Abstract Book, but they cannot change the content in any way or use them commercially.
**Abstract Book Citations:** Authors, Title, HemaSphere, 2026;10:(S1):pages. The abstract book DOIs can be found on the "Author Information" page.
**Disclaimer:** Articles published in the journal HemaSphere exclusively reflect the opinions of the authors. The authors are responsible for all content in their abstracts including accuracy of the facts, statements, citing resources, etc.

### (PB2657) CLINICAL VALIDATION OF NEXT-GENERATION SEQUENCING-BASED LIQUID BIOPSY ASSAY FOR PROGNOSIS ASSESSMENT OF MEASURABLE RESIDUAL DISEASE IN ACUTE MYELOID LEUKEMIA

**Topic:** 03. Acute myeloid leukemia - Biology & translational research

Noemí Álvarez Sánchez-Redondo*[1], Alba Mora[1], Alejandro Martín Muñoz[2], Rafael Colmenares[3], Natalia Toledano[2], Irene Zamanillo[3], Patricia Losana[2], Gonzalo Carreño Gómez-Tarragona[3], Joaquín Martinez-Lopez[4], Rosa María Ayala Díaz[4]

*[1]Department of Translational Hematology, Research Institute Hospital 12 de Octubre (i+12), Hematological Malignancies Clinical Research Unit H120-CNIO, CIBERONC, MADRID, Spain; [2]Altum Sequencing Co., Madrid, Spain., MADRID, Spain;[3]Department of Translational Hematology, Research Institute Hospital 12 de Octubre (i+12), Hematological Malignancies Clinical Research Unit H120-CNIO, CIBERONC,, MADRID, Spain; [4]1.Department of Translational Hematology, Research Institute Hospital 12 de Octubre (i+12), Hematological Malignancies Clinical Research Unit H120-CNIO, CIBERONC, 3.Department of Medicine, Complutense University of Madrid, Madrid, Spain., MADRID, Spain;*

### Background

The quantification of measurable residual disease (MRD) has become a relevant marker, as it allows the detection of patients with AML at high risk of relapse. Although the reference method in AML is to quantify tumour cells present in the bone marrow (BM) or Peripheral Blood (PB), the study of cfDNA in PB is under investigation

### Aims

Quantification of MRD with an ultrasensitive test by NGS in DNA from leucocyte BM and PB (CTC), and cfDNA, analysing its concordance with MFC and validating the use of cfDNA as a minimally invasive tool for the early detection of relapses in AML

### Methods

This study included 48 patients with AML diagnosed at Hospital 12 de Octubre.The mutational profile was defined by NGS at diagnosis using a panel of 42 genes involved in myeloid pathologies.Somatic mutations were selected and used for monitoring in 61 BM samples, 76 CTCs samples, and 80 cfDNA samples.Quantification was performed in both whole blood cells and cfDNA fractions using the Altum TRACKseq® assay (Altum Sequencing,Madrid,Spain) test tumour informed for MRD follow-up with a sensitivity up to 10-6.The positivity threshold was set at 10-4.The outcome was defined by the biomarker with the highest VAF, excluding variants of uncertain significance, missense, non-frameshift variants, and variants classified as non-eligible by Robert P. Hasserjian(Hasserjian.Blood 2020) provided that the difference was less than one logarithm with respect to the diagnosis.The results were compared with MFC in BM

**Copyright Information:** (Online) ISSN: 2572-9241
©2026 The Author(s). HemaSphere is published by John Wiley & Sons Ltd on behalf of European Hematology Association. This is an open access Abstract Book distributed under the Attribution-NonCommercial-NoDerivs (CC BY-NC-ND), which allows third parties to download the articles and share them with others as long as they credit the author and the Abstract Book, but they cannot change the content in any way or use them commercially.
**Abstract Book Citations:** Authors, Title, HemaSphere, 2026;10:(S1):pages. The abstract book DOIs can be found on the “Author Information” page.
**Disclaimer:** Articles published in the journal HemaSphere exclusively reflect the opinions of the authors. The authors are responsible for all content in their abstracts including accuracy of the facts, statements, citing resources, etc.

**Results**

In this cohort, 17(35%) were males and 31(65%) were females.The median age at diagnosis was 55.5 years (range 18–84).Patients received intensive treatment, 3+7 or similar schemes (n=43) or treatments based on azacitidine, venetoclax or decitabine for non-eligible patients (n=5).Samples were collected at post-induction and post-consolidation follow-up points
MRD Biomarkers were detected in 45/48 patients (applicability 93.7%).Finally biomarkers were defined by variants in genes: *ASXL1,BCOR,BCORL1,CEBPA,DNMT3A,ETV6,FLT3,IDH1,IDH2,JAK2,KIT,NF1,NPM1,NRAS,PTPN11,RUNX1,SF3B1,SRSF2,TET2,TP53*, and *ZRSR2*
The correlations between different fractions (BM, CTCs and cfDNA) analysed by NGS showed a high correlation between CTCs/cfDNA(R2=0.792,p<0.001) and CTCs/BM(R2=0.920,p<0.0001)(Fig1A), and when MFC was evaluated: MFC/cfDNA(R2=0.131,p=0.003), MFC/BM(R2=0.354,p<0.001), MFC/CTCs(R2=0.256,p<0.001) y cfDNA/BM(R2=0.299,p<0.001)
The median OS was 21.6 months and RFS was 17 months. Samples MRD-positive were associated with shorter RFS (months): BM[49.2(43.1-55.2) vs 20.2(15.8-24.6),p<0.001][HR=8.2(CI 2.8-24),p<0.001]; CTCs[37.4(30.3-44.6) vs 17.1(13.8-20.3),p=0.003][HR=3.3(CI 1.8-6.3),p<0.001], cfDNA[40.5(32.7-48.3) vs 18.9(15.8-22),p=0.002] [HR=3.6(CI 1.8-7.2),p<0.001] and MFC[30.2(24.3-36.1) vs 17.2(13-21.4),p=0,017][HR=2,11(1,1-3,9),p=0,028]; and shorter OS (months):BM[51(45.9-56.1) vs 32(24.1-39.9),p<0.001][HR=7.9(CI 2.3-27.5),p<0.001]; CTCs[41.4(33.9-48.8) vs 35.3(27.2-43.3),p=0.036][HR=2.3(CI 1.02-5.03),p=0.040], cfDNA[47.1(40.8-53.3) vs 34.2(27.3-41),p=0.009][HR=3.1(CI 1.2-7.7),p=0.008] and MFC[44.1(37.9-50.3) vs 27.9(18.2-37.6),p<0,001][HR=3.1 (1.5-6.7),p=0,004](Fig1B)

**Summary/Conclusion**

The ultrasensitive method by NGS is used to predict prognosis in three different fractions (BM, cfDNA, and CTCs) in this cohort of AML patients.The quantification of MRD using NGS in liquid biopsy (cfDNA) shows a high correlation with BM and MFC, establishing itself as a minimally invasive tool with high prognostic value

**Copyright Information:** (Online) ISSN: 2572-9241
©2026 The Author(s). HemaSphere is published by John Wiley & Sons Ltd on behalf of European Hematology Association. This is an open access Abstract Book distributed under the Attribution-NonCommercial-NoDerivs (CC BY-NC-ND), which allows third parties to download the articles and share them with others as long as they credit the author and the Abstract Book, but they cannot change the content in any way or use them commercially.
**Abstract Book Citations:** Authors, Title, HemaSphere, 2026;10:(S1):pages. The abstract book DOIs can be found on the "Author Information" page.
**Disclaimer:** Articles published in the journal HemaSphere exclusively reflect the opinions of the authors. The authors are responsible for all content in their abstracts including accuracy of the facts, statements, citing resources, etc.

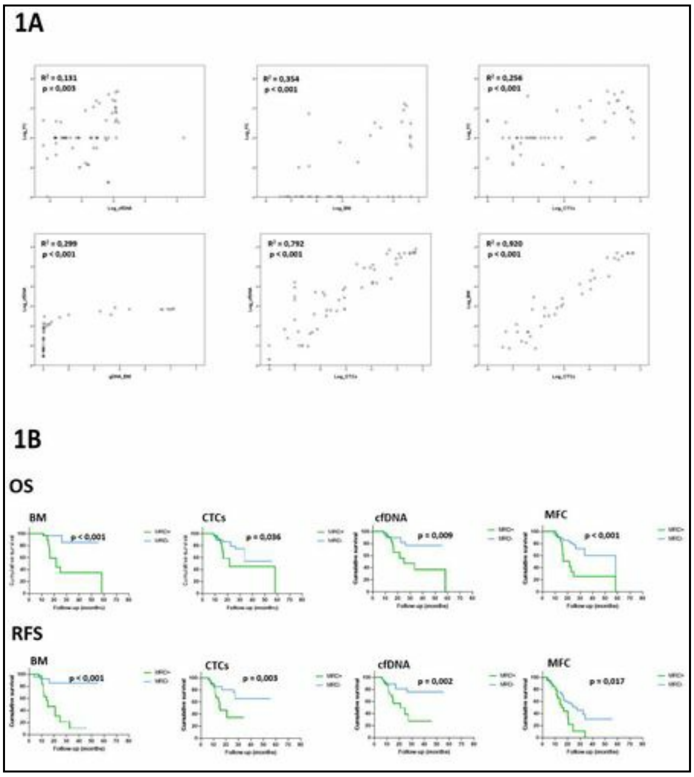

Figure 1A. Correlations in the study of measurable residual disease between circulating free DNA (cfDNA), circulating tumour cells (CTCs) and bone marrow (BM) samples analysed by NGS and multiparametric flow cytometry (MFC) samples. Figure 1B. Overall survival and relapse-free survival curves (Kaplan-Meier) for the study of measurable residual disease in cfDNA, CTCs and BM samples analysed by NGS and multiparametric flow cytometry (MFC) samples.

**Copyright Information:** (Online) ISSN: 2572-9241
©2026 The Author(s). HemaSphere is published by John Wiley & Sons Ltd on behalf of European Hematology Association. This is an open access Abstract Book distributed under the Attribution-NonCommercial-NoDerivs (CC BY-NC-ND), which allows third parties to download the articles and share them with others as long as they credit the author and the Abstract Book, but they cannot change the content in any way or use them commercially.
**Abstract Book Citations:** Authors, Title, HemaSphere, 2026;10:(S1):pages. The abstract book DOIs can be found on the "Author Information" page.
**Disclaimer:** Articles published in the journal HemaSphere exclusively reflect the opinions of the authors. The authors are responsible for all content in their abstracts including accuracy of the facts, statements, citing resources, etc.

### (PB2658) RAMAN SPECTROSCOPY COMBINED WITH MACHINE LEARNING PREDICTS INITIAL TREATMENT RESPONSE IN NEWLY DIAGNOSED ACUTE MYELOID LEUKEMIA

**Topic:** 03. Acute myeloid leukemia - Biology & translational research

Ming Zhang[1, 2], Long Su[1, 2], Wei Han[1, 2], Mingbo Chi[3], Fei Song[1, 2], Yu Fu[1, 2], Yihui Wu[3], Sujun Gao*[1, 2]

*[1]The First Hospital of Jilin University, Department of Hematology, Changchun, China;[2]The First Hospital of Jilin University, Key Laboratory of Hematology Precision Medicine of Jilin Province, Changchun, China; [3]Changchun Institute of Optics, Fine Mechanics and Physics, Chinese Academy of Sciences, Changchun, China;*

### Background

Acute myeloid leukemia (AML) displays marked heterogeneity in therapeutic response. Approximately 20%–40% of newly diagnosed patients fail to achieve complete remission (CR) following standard induction chemotherapy, which associated with poor prognosis. However, no reliable indicators are currently available to predict initial treatment response before induction chemotherapy. Raman spectroscopy (RS), a label-free optical analytical technique, generates single-cell profiles that reflect cellular biochemical composition and state. Studies have demonstrated its application in leukemia classification and diagnosis, and it may also serve as an emerging tool for drug resistance evaluation.

### Aims

This study aims to investigate the predictive value of RS for treatment response in patients with*de novo* AML.

### Methods

Bone marrow (BM) samples were prospectively collected from the newly diagnosed AML patients prior to induction therapy. Based on the treatment outcomes, these initial patients were classified as CR group and non-remission (NR) group. Single-cell Raman spectra were acquired from 30–50 leukemic blasts per patient over the 600–1800 $cm^{-1}$ fingerprint region using a Raman system (532 nm excitation, 40 mW power, 15 s integration time; three acquisitions per cell). The spectral data were analyzed using machine learning approaches: Principal Component Analysis-Linear Discriminant Analysis (PCA-LDA) was employed to establish a discriminant model, while Multivariate Curve Resolution-Alternating Least Squares (MCR-ALS) decomposed spectra into biochemically interpretable components.

### Results

Differential Raman analysis (CR:355 spectra; NR: 322 spectra) identified significant spectral distinctions (P<0.05) at 10 wavenumbers (662–1671 $cm^{-1}$) corresponding to nucleic acid, amino acid, and lipid vibrations, with 662 $cm^{-1}$ (AUC=0.823) and 1441 $cm^{-1}$ (AUC=0.800) showing high diagnostic efficacy. The training set analysis demonstrated that the PCA-LDA model robustly classified CR versus NR, revealing significant clustering differences. Employing 24 principal components (explaining 90.1% cumulative variance), the model achieved 89.3% ten-fold cross-validation accuracy. The discriminatory capability was confirmed on an independent cohort of four patients (two CR, two NR; 195 spectra total) with 94.8% overall accuracy (185/195 correct classifications), supported by key metrics: 92.0% specificity, 97.9% sensitivity, and ROC-AUC 0.949. To elucidate the biochemical basis of this discrimination, MCR-ALS analysis resolved four spectrally distinct components accounting for 96.9% total variance: protein (Component 1), lipid/carbohydrate (Component 2), nucleic acid (Component 3), and mixed signals (Component 4). NR patients showed significantly higher relative abundances of lipid/carbohydrate ($P$ = 0.012) and nucleic acid components ($P$ = 0.008) compared with CR patients, whereas protein content was higher in the CR group ($P$ = 0.023).

**Copyright Information:** (Online) ISSN: 2572-9241
©2026 The Author(s). HemaSphere is published by John Wiley & Sons Ltd on behalf of European Hematology Association. This is an open access Abstract Book distributed under the Attribution-NonCommercial-NoDerivs (CC BY-NC-ND), which allows third parties to download the articles and share them with others as long as they credit the author and the Abstract Book, but they cannot change the content in any way or use them commercially.
**Abstract Book Citations:** Authors, Title, HemaSphere, 2026;10:(S1):pages. The abstract book DOIs can be found on the "Author Information" page.
**Disclaimer:** Articles published in the journal HemaSphere exclusively reflect the opinions of the authors. The authors are responsible for all content in their abstracts including accuracy of the facts, statements, citing resources, etc.

**Summary/Conclusion**

RS enables rapid, label-free identification of distinct biomolecular signatures in BM cells from de novo AML patients, and when integrated with machine learning, these spectral profiles show significant potential for predicting initial therapeutic response prior to treatment initiation.

**Copyright Information:** (Online) ISSN: 2572-9241
©2026 The Author(s). HemaSphere is published by John Wiley & Sons Ltd on behalf of European Hematology Association. This is an open access Abstract Book distributed under the Attribution-NonCommercial-NoDerivs (CC BY-NC-ND), which allows third parties to download the articles and share them with others as long as they credit the author and the Abstract Book, but they cannot change the content in any way or use them commercially.
**Abstract Book Citations:** Authors, Title, HemaSphere, 2026;10:(S1):pages. The abstract book DOIs can be found on the "Author Information" page.
**Disclaimer:** Articles published in the journal HemaSphere exclusively reflect the opinions of the authors. The authors are responsible for all content in their abstracts including accuracy of the facts, statements, citing resources, etc.

### (PB2659) A CROSS-CANCER INTEGRATIVE FRAMEWORK COMBINING MULTI-OMICS AND MICROWELL IMAGING REVEALS THERAPEUTIC MITOTIC VULNERABILITIES IN AML

**Topic:** 03. Acute myeloid leukemia - Biology & translational research

Ryuta Niikura*[1], Tomohiro Yabushita[2, 3], Shohei Yamamoto[1], Hiroaki Suzuki[4], Susumu Goyama[5], Toshio Kitamura[3, 6], Takumi Chinen[1], Daiju Kitagawa[1]

*[1]The University of Tokyo, Laboratory of Physiological Chemistry, Graduate School of Pharmaceutical Sciences, Tokyo, Japan; [2]Kumamoto University, International Research Center for Medical Sciences, Kumamoto, Japan;[3]The University of Tokyo, Molecular Pharmacology of Malignant Diseases, Graduate School of Pharmaceutical Sciences, Tokyo, Japan; [4]Chuo University, Department of Precision Mechanics, Graduate School of Science and Engineering, Tokyo, Japan; [5]The University of Tokyo, Division of Molecular Oncology, Department of Computational Biology and Medical Sciences, Graduate School of Frontier Sciences, Tokyo, Japan; [6]Institute of Biomedical Research and Innovation, Foundation for Biomedical Research and Innovation at Kobe, Hyogo, Japan;*

### Background

Acute myeloid leukemia (AML) is a hematological malignancy with a poor prognosis. Despite recent advances in molecular targeted therapies, treatment outcomes for patients with complex genetic aberrations remain unsatisfactory. Exploring the phenotypic characteristics and unique vulnerabilities of AML cells beyond genetic profiling is essential for developing innovative therapeutic strategies.

### Aims

To identify novel therapeutic vulnerabilities in AML cells using large-scale drug sensitivity data and to evaluate the real-time cellular drug responses using a microwell-based long-term live cell imaging system (MW-imaging).

### Methods

We first used large-scale datasets from the Cancer Dependency Map (DepMap) to identify drugs with selective efficacy against AML cells and infer latent vulnerabilities. To observe these vulnerabilities at the cellular level, we established a MW-imaging system that allows continuous tracking of the drug responses of individual AML cells in suspension for more than 96 hours. These vulnerabilities were further characterized through comparative and mechanistic studies with other cell lines, identifying key vulnerability regulators whose suppression rescued cell death, as confirmed by real-time MW-imaging. We also performed proteomic analysis to identify changes in protein expression occurring at the onset of drug-induced cell death.

**Copyright Information:** (Online) ISSN: 2572-9241
©2026 The Author(s). HemaSphere is published by John Wiley & Sons Ltd on behalf of European Hematology Association. This is an open access Abstract Book distributed under the Attribution-NonCommercial-NoDerivs (CC BY-NC-ND), which allows third parties to download the articles and share them with others as long as they credit the author and the Abstract Book, but they cannot change the content in any way or use them commercially.
**Abstract Book Citations:** Authors, Title, HemaSphere, 2026;10:(S1):pages. The abstract book DOIs can be found on the "Author Information" page.
**Disclaimer:** Articles published in the journal HemaSphere exclusively reflect the opinions of the authors. The authors are responsible for all content in their abstracts including accuracy of the facts, statements, citing resources, etc.

**Results**

Bioinformatic analysis of DepMap drug sensitivity datasets identified 27 drugs exhibiting selective efficacy against AML cells, including 11 mitotic inhibitors, which suggests that AML cells are particularly vulnerable to mitotic perturbations. MW-imaging revealed that AML cells underwent mitotic cell death within 5 hours of mitotic arrest induced by vincristine, much earlier than other cell lines (e.g., ALL, CML, and adherent cell lines), indicating a heightened vulnerability to mitotic stress in AML. We explored factors correlated with mitotic vulnerability using multi-omics datasets and performing siRNA-based mini-screening. These analyses identified MYC and BCL2 as two genes highly expressed in AML cells that modulate mitotic cell fate in opposite directions. Specifically, MYC is an essential gene in AML and promoted rapid mitotic cell death, whereas BCL2 suppressed it. Proteomic analyses indicated that MCL1, a key anti-apoptotic factor in mitosis, decreased more rapidly in AML cells than in other cell lines. In contrast, the expression of BCL2, a suppressor of mitotic cell death, was maintained. Furthermore, targeted scavenging of mitochondrial ROS (mitoROS) reduced this cell death by 50%, indicating that mitoROS directly drive the mitotic cell death in AML. Given that BCL2 is known to buffer mitoROS, we hypothesized that it acts as a protective mechanism against mitoROS-induced mitotic cell death. Therefore, inhibiting BCL2 would eliminate this protection and fully exploit the inherent mitotic vulnerability of AML cells. Consistent with this, combining the BCL2 inhibitor venetoclax with vincristine synergistically enhanced AML cell death.

**Summary/Conclusion**

DepMap comparisons highlighted a mitosis-linked vulnerability in AML, and long-term single-cell MW-imaging enabled continuous tracking of AML-specific rapid mitotic cell death. Mechanistic analyses implicated opposing MYC and BCL2 functions, accelerated MCL1 degradation, and mitochondria-derived ROS in lowering the mitotic apoptotic threshold in AML cells. Guided by this framework, combined mitotic inhibition and venetoclax synergistically enhanced AML cell death.

**Copyright Information:** (Online) ISSN: 2572-9241
©2026 The Author(s). HemaSphere is published by John Wiley & Sons Ltd on behalf of European Hematology Association. This is an open access Abstract Book distributed under the Attribution-NonCommercial-NoDerivs (CC BY-NC-ND), which allows third parties to download the articles and share them with others as long as they credit the author and the Abstract Book, but they cannot change the content in any way or use them commercially.
**Abstract Book Citations:** Authors, Title, HemaSphere, 2026;10:(S1):pages. The abstract book DOIs can be found on the "Author Information" page.
**Disclaimer:** Articles published in the journal HemaSphere exclusively reflect the opinions of the authors. The authors are responsible for all content in their abstracts including accuracy of the facts, statements, citing resources, etc.

### (PB2660) OVEREXPRESSION OF MCPIP1 SUPPRESSES THE MTOR PATHWAY VIA TSC2 UPREGULATION AND SYNERGIZES WITH VENETOCLAX IN ACUTE MYELOID LEUKEMIA

**Topic:** 03. Acute myeloid leukemia - Biology & translational research

Liyuan Li[1], Weiguang Cui*[1], Xiaoyan Zhang[1], Zhenzhen Wang[1], Nan Zhou[1], Mengying Zheng[1], Fan Yang[1], Li Sun[1], Lin Yang[1, 2], Jianmin Luo[1, 2]

*[1]The Second Hospital of Hebei Medical University, Department of Hematology, Shijiazhuang, China;[2]Hebei Institute of Hematology, Hebei Key Laboratory of Hematology, Shijiazhuang, China;*

### Background

Venetoclax (VEN)-based therapies are standard for acute myeloid leukemia (AML), but their efficacy is limited by short responses and acquired resistance. The RNA-binding protein MCPIP1 acts as a tumor suppressor and induces apoptosis in AML. However, whether MCPIP1 overexpression sensitizes AML cells to VEN and overcomes resistance remains unexplored. This study investigates the synergistic anti-leukemic effects and underlying mechanisms of combining MCPIP1 overexpression with VEN in AML.

### Aims

This study specifically aims to establish the synergistic efficacy of MCPIP1 overexpression combined with VEN in AML and to elucidate the mechanistical interplays dictating this synergy.

### Methods

To investigate the biological impact of MCPIP1 modulation, we established stable MCPIP1-overexpressing and MCPIP1-knockout Kasumi-1, THP-1and MOLM13 AML cell lines via lentiviral transduction. For inducible models, cells were treated with doxycycline for 48 hours prior to transcriptomic profiling via RNA-sequencing (RNA-seq). Differential gene expression was subsequently validated using RT-qPCR and Western blotting. We extensively evaluated the regulatory cascade involving MCPIP1, the TSC2/mTOR signaling axis, and the anti-apoptotic protein MCL-1. Furthermore, the capacity of MCPIP1 overexpression to synergize with VEN in inducing cellular apoptosis was quantified using flow cytometry and comprehensive combination index (CI) tracking (using the Chou-Talalay method).

### Results

Clinical and in vitro profiling revealed that MCPIP1 expression is profoundly downregulated in AML patients and established cell lines compared to normal controls. RNA-seq of MCPIP1-overexpressing cells identified significant upregulation of TSC2, a finding stringently validated by RT-qPCR and Western blot analyses. Mechanistically, MCPIP1 overexpression suppressed the mTOR signaling pathway strictly through the upregulation of TSC2. This was evidenced by the markedly reduced phosphorylation of mTOR (p-mTOR) and its downstream effector 4EBP1 (p-4EBP1), concurrently leading to the downregulation of the anti-apoptotic protein MCL-1. Importantly, targeted knockout of TSC2 completely reversed these molecular events, confirming the TSC2/mTOR hub as the indispensable mediator of MCPIP1 function. Functionally, MCPIP1 overexpression not only autonomously induced apoptosis in leukemia cells but also profoundly sensitized them to VEN treatment. Interaction analyses yielded combination index (CI) values well below 1 (CI < 1), indicating a potent pharmacological synergy. Collectively, these findings demonstrate that MCPIP1 acts as a tumor suppressor by dampening the mTOR pathway via TSC2 upregulation, thereby abolishing MCL-1-mediated VEN resistance.

**Copyright Information:** (Online) ISSN: 2572-9241
©2026 The Author(s). HemaSphere is published by John Wiley & Sons Ltd on behalf of European Hematology Association. This is an open access Abstract Book distributed under the Attribution-NonCommercial-NoDerivs (CC BY-NC-ND), which allows third parties to download the articles and share them with others as long as they credit the author and the Abstract Book, but they cannot change the content in any way or use them commercially.
**Abstract Book Citations:** Authors, Title, HemaSphere, 2026;10:(S1):pages. The abstract book DOIs can be found on the "Author Information" page.
**Disclaimer:** Articles published in the journal HemaSphere exclusively reflect the opinions of the authors. The authors are responsible for all content in their abstracts including accuracy of the facts, statements, citing resources, etc.

### Summary/Conclusion

This study firmly establishes that MCPIP1 is downregulated in AML and functions as a critical tumor suppressor. By specifically upregulating TSC2, MCPIP1 expression impedes the mTOR signaling cascade—evidenced by depressed p-mTOR and p-4EBP1 levels—ultimately leading to the suppression of MCL-1. This targeted downregulation of MCL-1 seamlessly circumvents intrinsic resistance mechanisms, allowing MCPIP1 overexpression to forcefully synergize with VEN to maximize anti-leukemic efficacy (CI < 1). These discoveries identify the MCPIP1/TSC2/mTOR/MCL-1 axis as a vital vulnerability in AML cell survival and provide a compelling mechanistic rationale for combining MCPIP1 restoration strategies with BCL-2 inhibitors as a potent, novel therapeutic paradigm for AML.

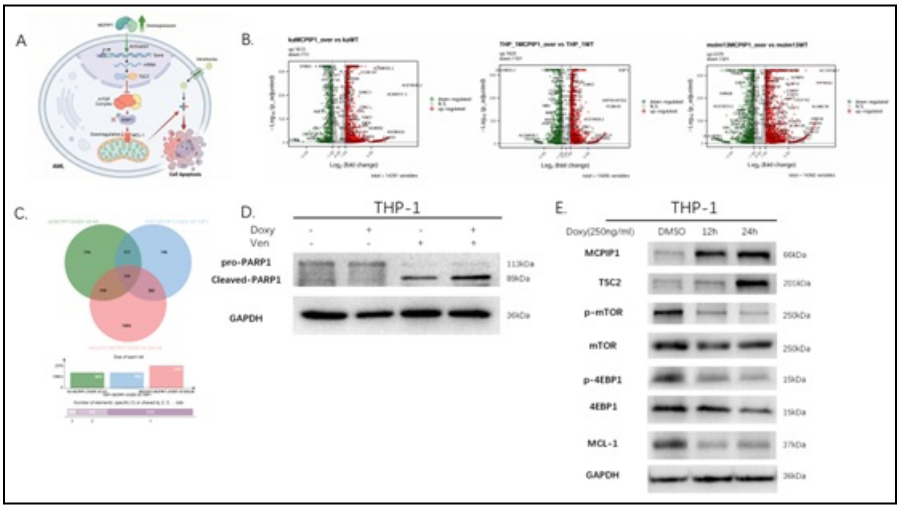

Figure 1. MCPIP1 synergizes with Venetoclax by suppressing the TSC2/mTOR/MCL-1 axis. (A) Schematic of the synergistic mechanism between MCPIP1 and Venetoclax (VEN). (B) Volcano plot showing differentially expressed genes (DEGs) in MCPIP1-overexpressing AML cells. (C) Venn diagram identifying TSC2/mTOR as a core regulatory target of MCPIP1. (D-E) Functional validation of synergistic apoptosis and targeted suppression of the mTOR/MCL-1 pathway.

**Copyright Information:** (Online) ISSN: 2572-9241
©2026 The Author(s). HemaSphere is published by John Wiley & Sons Ltd on behalf of European Hematology Association. This is an open access Abstract Book distributed under the Attribution-NonCommercial-NoDerivs (CC BY-NC-ND), which allows third parties to download the articles and share them with others as long as they credit the author and the Abstract Book, but they cannot change the content in any way or use them commercially.
**Abstract Book Citations:** Authors, Title, HemaSphere, 2026;10:(S1):pages. The abstract book DOIs can be found on the “Author Information” page.
**Disclaimer:** Articles published in the journal HemaSphere exclusively reflect the opinions of the authors. The authors are responsible for all content in their abstracts including accuracy of the facts, statements, citing resources, etc.

### (PB2661) AML WITH ATYPICAL MYELODYSPLASIA-RELATED CYTOGENETICS: CHALLENGING THE ROLE OF RARE ABERRATIONS IN AML-MR CLASSIFICATION

**Topic:** 03. Acute myeloid leukemia - Biology & translational research

Sandra Huber*[1], Anna Stengel[1], Gregor Hörmann[1], Christian Pohlkamp[1], Manja Meggendorfer[1], Torsten Haferlach[1], Isolde Summerer[1]

[1]*MLL Munich Leukemia Laboratory, Munich, Germany;*

### Background

The 5$^{th}$ edition of the WHO classification defines myelodysplasia-related AML (AML-MR) by showing either specific MR mutations (mutMR) or certain cytogenetic aberrations. Besides frequent MR-defining cytogenetic abnormalities like complex karyotype (CK), -5/del(5q), -7/del(7q) or del(17p) also the following abnormalities are considered MR-defining: del(11q), del(12p), -13/del(13q) and idic(X). The International Consensus Classification (ICC) further includes +8, del(20q), considers *RUNX1* as mutMR and recognizes AML with mutated *TP53* as separate entity.

### Aims

We aimed to analyze cases with non-frequent or WHO/ICC discrepant MR-defining cytogenetic abnormalities with respect to genetic and clinical characteristics.

### Methods

We retrospectively analyzed samples (received 2005-2025) of 460 patients (median age: 74 years; female: 38%) diagnosed as AML showing one of the following cytogenetic aberrations (atypical cytoMR): +8, del(20q), del(11q), del(12p), -13/del(13q) or idic(X) but no CK, -5/del(5q), -7/del(7q) or del(17p) (typical cytoMR), excluding non-MR genetically defined AML subtypes. All samples were analyzed by cytomorphology, cytogenetics and molecular genetics (targeted NGS panel for ≥24 myeloid genes). For comparison of survival we used a cohort of 204 WHO AML-MR (typical cytoMR and/or mutMR; median age: 74 years; female: 39%; WGS available).

### Results

Of the 460 atypical cytoMR cases 80% showed +8 (n=367), 10% del(20q) (n=46), 6% del(12p) (n=27), 4% del(11q) (n=19), 3% -13/del(13q) (n=14) and <1% had idic(X) (n=3). 36 cases (8%) fulfilled criteria of myeloid neoplasms associated with germline predisposition (*DDX41*; n=23) or AML with mutated *TP53* (n=13). Within the remaining 424 cases, WHO mutMR were detected in 365 cases (86%; median mutMR/case: 2 [1-5]; Fig.), most frequently mutations in *ASXL1* (59%) and *SRSF2* (49%). Of the cases with *RUNX1* mutations 95% (166/174) also showed WHO mutMR. Overall, 47% (215/460) of cases had therapeutically targetable mutations in *FLT3*-ITD/TKD and/or *IDH1/2.*
The 59 mutMR-negative cases with only atypical cytoMR showed superior overall survival compared to the WHO AML-MR comparison cohort (median: 10.7 vs. 0.5 years; p<0.001). This remained significant after exclusion of the prognostically very unfavorable AML with mutated *TP53* from the AML-MR comparison cohort (median: 10.7 vs. 1.0 years; p<0.001; Fig.). Even after excluding +8 cases (46/59; 78%), mutMR-negative atypical cytoMR cases showed superior survival compared to the comparison cohort (median: not reached vs. 1.0 years; p=0.004).

**Copyright Information:** (Online) ISSN: 2572-9241
©2026 The Author(s). HemaSphere is published by John Wiley & Sons Ltd on behalf of European Hematology Association. This is an open access Abstract Book distributed under the Attribution-NonCommercial-NoDerivs (CC BY-NC-ND), which allows third parties to download the articles and share them with others as long as they credit the author and the Abstract Book, but they cannot change the content in any way or use them commercially.
**Abstract Book Citations:** Authors, Title, HemaSphere, 2026;10:(S1):pages. The abstract book DOIs can be found on the “Author Information” page.
**Disclaimer:** Articles published in the journal HemaSphere exclusively reflect the opinions of the authors. The authors are responsible for all content in their abstracts including accuracy of the facts, statements, citing resources, etc.

**Summary/Conclusion**

Our analysis indicates, that cases with only atypical cytoMR abnormalities mainly (~80%) comprise +8, while all others are rare. As most cases (86%) also harbored WHO MR gene mutations (overlap to *RUNX1*: 95%) thereby qualifying for AML-MR irrespective of cytogenetic aberrations, few cases remain to be classified as AML-MR only due to atypical cytogenetic aberrations according to WHO and/or ICC. Together with the significantly longer survival of these cases compared to typical AML-MR, which is not only due to cases with +8, our data suggest that these aberrations should not per se qualify for AML-MR. In our study, cases with WHO MR mutations and frequent cytogenetic abnormalities (CK, -5/del(5q), -7/del(7q) or del(17p)) represent a distinct group with unfavorable prognosis suggesting only these aberrations as relevant for diagnosing AML-MR for a more precise and clinically relevant classification.

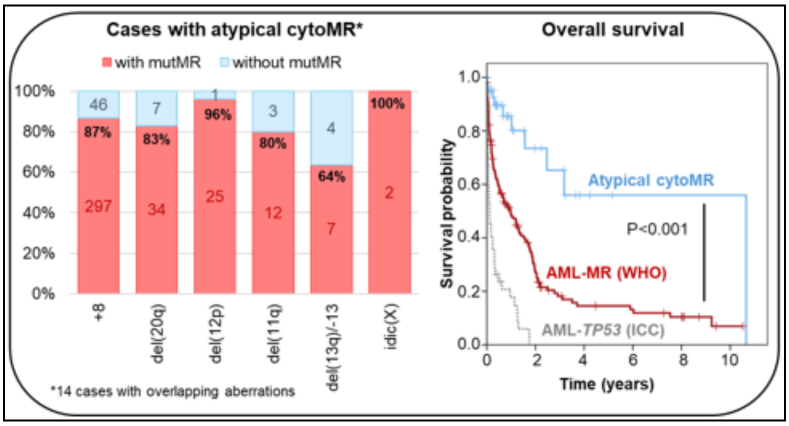

**Copyright Information:** (Online) ISSN: 2572-9241
©2026 The Author(s). HemaSphere is published by John Wiley & Sons Ltd on behalf of European Hematology Association. This is an open access Abstract Book distributed under the Attribution-NonCommercial-NoDerivs (CC BY-NC-ND), which allows third parties to download the articles and share them with others as long as they credit the author and the Abstract Book, but they cannot change the content in any way or use them commercially.
**Abstract Book Citations:** Authors, Title, HemaSphere, 2026;10:(S1):pages. The abstract book DOIs can be found on the "Author Information" page.
**Disclaimer:** Articles published in the journal HemaSphere exclusively reflect the opinions of the authors. The authors are responsible for all content in their abstracts including accuracy of the facts, statements, citing resources, etc.

### (PB2663) SERIALLY TRANSPLANTABLE PDX MODELS FOR AML SUBGROUPS FOR WHICH NO CELL LINE EXIST

**Topic:** 03. Acute myeloid leukemia - Biology & translational research

Binje Vick[1, 2], Tobias Herold[3], Klaus Metzeler[4], Martin Caroll[5], Christine Dierks[6], Martin Ebinger[7], Katharina Götze[8], Jan Henning Klusmann[9], Pablo Menendez[10], Christian Récher[11], Jean-Emmanuel Sarry[12], Christian Thiede[13], Sebastian Vosberg[3], Philipp Greif[3], Marion Subklewe[3], Wolfgang Hiddemann[3], Michael von Bergwelt[3], Karsten Spiekermann[3], Irmela Jeremias*[1, 2, 14]

*[1]Research Unit Apoptosis in Hematopoietic Stem Cells, Helmholtz Munich, Munich, Germany;[2]German Cancer Consortium (DKTK), partner site Munich, a partnership between DKFZ and University Hospital LMU Munich, Munich, Germany, Munich, Germany; [3]Ludwig Maximilians University Munich, Internal Medicine III, Munich, Germany; [4]University Leipzig, Leipzig, Germany; [5]University City Penn/penn, Philadelphia, United States of America; [6]Martin-Luther-Universität Löwengebäude, Halle (Saale), Germany; [7]Universitätsklinikum Tübingen, Tübingen, Germany;[8]Technical univeristy Munich, Munich, Germany;[9]University Frankfurt, Frankfurt, Germany; [10]Josep Carreras leukemia research Institute, Barcelona, Spain; [11]Service d'hématologie, Centre Hospitalier-universitaire de Toulouse, Institut Universitaire du Cancer de Toulouse-Oncopole, Université de Toulouse, Toulouse, France; [12]Institut Necker Enfants Malades, Toulouse, France; [13]Medizinische Klinik und Poliklinik, Universitätsklinikum Carl Gustav Carus an der Technischen Universität Dresden, Dresden, Germany; [14]Department of Pediatrics, Dr. von Hauner Children's Hospital, University Hospital, Ludwig-Maximilians-Universität München, Munich, Germany;*

### Background

Patients with acute myeloid leukemia (AML) suffer from poor prognosis and better treatment options are urgently needed. Preclinical research in the field of AML is hampered by a lack of tools and suitable models which represent the heterogeneity of the different AML subtypes.

### Aims

To complement the few existing AML cell lines and restricted primografts, we aimed at increasing the toolbox of AML models by establishing serially transplantable and robust patient-derived xenograft (*PDX) models.

### Methods

Primary tumor cells from patients with AML were transplanted into immunocompromised NSG mice and repetitively re-transplanted, if possible. Resulting PDX AML models were characterized in a multi-OMICs approach, genetically engineered using lentiviruses and subjected to preclinical in vivo treatment trials.

### Results

*PDX models represent individuals of all age groups and several WHO subgroups. They substantially outperform AML cell lines in preserving leukemia biology and include AML subgroups for which no cell lines exist, such as cytogenetically normal or *IDH1/2*-mutant AML or simultaneous *NPM1* mutation and *FLT3*-ITD. Their resilience to freeze-thaw cycles supports broad dissemination across research institutions through sharing via CancerModels.org. Using lentiviruses, we stably expressed luciferase for longitudinal, non-invasive and sensitive real-time monitoring of leukemia burden and therapeutic response *in vivo*. This approach enabled rigorously controlled preclinical studies, including phase II-like trials, which demonstrated highly variable treatment responses between different *PDX models, mimicking the heterogeneity in patient cohorts. Long-term treatment, including repeated cytarabine exposure over one year, showed a yet undescribed decrease in leukemia's proliferation rate. These insights underscore the platform's distinctive capacity to study treatment dynamics over clinically relevant timeframes.

**Copyright Information:** (Online) ISSN: 2572-9241
©2026 The Author(s). HemaSphere is published by John Wiley & Sons Ltd on behalf of European Hematology Association. This is an open access Abstract Book distributed under the Attribution-NonCommercial-NoDerivs (CC BY-NC-ND), which allows third parties to download the articles and share them with others as long as they credit the author and the Abstract Book, but they cannot change the content in any way or use them commercially.
**Abstract Book Citations:** Authors, Title, HemaSphere, 2026;10:(S1):pages. The abstract book DOIs can be found on the "Author Information" page.
**Disclaimer:** Articles published in the journal HemaSphere exclusively reflect the opinions of the authors. The authors are responsible for all content in their abstracts including accuracy of the facts, statements, citing resources, etc.

**Summary/Conclusion**

Collectively, our unique resource represents a powerful, robust, versatile and sharable platform with strong potential to accelerate translational AML research for the ultimate benefit of patients.

**Copyright Information:** (Online) ISSN: 2572-9241
©2026 The Author(s). HemaSphere is published by John Wiley & Sons Ltd on behalf of European Hematology Association. This is an open access Abstract Book distributed under the Attribution-NonCommercial-NoDerivs (CC BY-NC-ND), which allows third parties to download the articles and share them with others as long as they credit the author and the Abstract Book, but they cannot change the content in any way or use them commercially.
**Abstract Book Citations:** Authors, Title, HemaSphere, 2026;10:(S1):pages. The abstract book DOIs can be found on the "Author Information" page.
**Disclaimer:** Articles published in the journal HemaSphere exclusively reflect the opinions of the authors. The authors are responsible for all content in their abstracts including accuracy of the facts, statements, citing resources, etc.

### (PB2664) ALDH1A1 INHIBITION IN COMBINATION WITH HYPOMETHYLATING AGENTS AS A SALVAGE STRATEGY FOR RELAPSED/REFRACTORY AML

**Topic:** 03. Acute myeloid leukemia - Biology & translational research

Mehak Shaikh*[1, 2], Muhube Yazir[3], Celine Baier[3], Miroslava Kari Adamcova[1, 4], Rocio Rebollido-Rios[5, 6, 7], Andrada Constantinescu[3], Iris Manosalva[8], Lubomir Minarik[9], Guillaume Martin[3], Regis Costello[8], Anna Jonášová[9], Salvatore Spicuglia[8], Mileidys Perez[3], Merixtell Alberich Jorda[1, 4]

*[1]Institute of Molecular Genetics of the Czech Academy of Sciences, Laboratory of Hemato-Oncology, Prague, Czechia;[2]Faculty of Science, Charles University, Prague, Czechia; [3]Advanced BioDesign, Saint Priest, France; [4]Childhood Leukemia Investigation Prague, Department of Pediatric Hematology and Oncology, 2nd Faculty of Medicine, Charles University, Prague, Czechia; [5]Department I of Internal Medicine Center for Integrated Oncology Aachen Bonn Cologne Duesseldorf, Faculty of Medicine and University Hospital Cologne, University of Cologne, Cologne, Germany; [6]Center for Molecular Medicine Cologne, Cologne, Germany; [7]CECAD Center of Excellence on Cellular Stress Responses in Aging-Associated Diseases Cologne, Cologne, Germany; [8]Aix-Marseille University, INSERM, TAGC, UMR1090, Marseille, France; [9]General University Hospital and Charles University, Prague, Czechia;*

### Background

Aldehyde dehydrogenase 1A1 (ALDH1A1) contributes to leukemic cell survival by promoting proliferation, stemness, and chemoresistance through retinoic acid synthesis and aldehyde detoxification. Patients with relapsed or refractory acute myeloid leukemia (R/R AML) exhibit elevated ALDH1A1 levels and activity, which are associated with poor prognosis and reduced overall survival. Targeting ALDH1A has been reported to have a beneficial effect, however the mechanism of action remains largely elusive.

### Aims

1. To define the cellular and molecular mechanisms by which ALDH1A1 inhibition affects acute myeloid leukemia (AML) cells.
2. To evaluate the synergistic potential of ALDH1A1 inhibition in combination with hypomethylating agents in AML.

### Methods

Molecular and cellular techniques using protein expression analysis, capillary electrophoresis-based protein analysis, ALDH enzymatic tests, ELISAs, ROS measurements. drug interaction studies, annexin-PI staining, and colony formation assays were employed. Experiments were conducted using human AML cell lines, murine MLL-AF9 leukemia models, human AML mouse models, and R/R AML patient samples, analysis was performed by flow cytometry. In addition, the effect of ALDH1A1 inhibition in AML cell lines was analyzed by RNA sequencing.

**Copyright Information:** (Online) ISSN: 2572-9241
©2026 The Author(s). HemaSphere is published by John Wiley & Sons Ltd on behalf of European Hematology Association. This is an open access Abstract Book distributed under the Attribution-NonCommercial-NoDerivs (CC BY-NC-ND), which allows third parties to download the articles and share them with others as long as they credit the author and the Abstract Book, but they cannot change the content in any way or use them commercially.
**Abstract Book Citations:** Authors, Title, HemaSphere, 2026;10:(S1):pages. The abstract book DOIs can be found on the "Author Information" page.
**Disclaimer:** Articles published in the journal HemaSphere exclusively reflect the opinions of the authors. The authors are responsible for all content in their abstracts including accuracy of the facts, statements, citing resources, etc.

**Results**

To understand the effect of ALDH1A1 inhibition in AML, we carried out colony formation assays on AML cell lines, murine MLL-AF9 cells, and R/R AML patient samples. We observed that ALDH1A1 inhibition leads to impairment in colony forming ability of AML cells. Next, we assessed the effect of ALDH1A1 inhibition in a murine MLL-AF9 mouse model and observed that it leads to a reduction in the leukemic burden. Following this, to understand the mechanism of ALDH1A1 inhibition in AML cell lines, we carried out RNA sequencing, followed by capillary immuno-electrophoresis, reactive oxygen species test, Annexin-PI staining, and ELISAs. Together, our findings suggest that ALDH1A1 inhibition mediates leukemic cell death through extrinsic as well as intrinsic pathways of apoptosis, and by disruption of key cell proliferative pathways. We then screened for the combination of ALDH1A1 inhibition with standard of care chemotherapeutic drugs on AML cell lines. We observed that ALDH1A1 inhibition with hypomethylating agents ranked the highest in synergy scores. Accordingly, colony formation assays and Annexin-PI staining combining ALDH1A1 inhibition and hypomethylating agents (5-azacytidine and decitabine) exhibited reduced colony formation compared to the use of each agent alone. These effects were observed in AML cells lines, murine MLL-AF9 cells, as well as in R/R AML patient samples which were resistant to venetoclax. The combinatorial effect was further validated in vivo using the murine MLL-AF9 leukemic model. Ultimately, we determined that the beneficial effects were mediated by elevated levels of apoptosis. Altogether, our findings suggest that ALDH1A1 inhibition in combination with hypomethylating agents has a beneficial effect in preventing leukemic progression by inhibiting key cell survival pathways.

**Summary/Conclusion**

Combining ALDH1A1 inhibition with hypomethylating agents may serve as an effective salvage therapy for R/R AML. These findings support the clinical advancement of ALDH1A1 inhibitors for high-risk AML subtypes.

**Copyright Information:** (Online) ISSN: 2572-9241
©2026 The Author(s). HemaSphere is published by John Wiley & Sons Ltd on behalf of European Hematology Association. This is an open access Abstract Book distributed under the Attribution-NonCommercial-NoDerivs (CC BY-NC-ND), which allows third parties to download the articles and share them with others as long as they credit the author and the Abstract Book, but they cannot change the content in any way or use them commercially.
**Abstract Book Citations:** Authors, Title, HemaSphere, 2026;10:(S1):pages. The abstract book DOIs can be found on the “Author Information” page.
**Disclaimer:** Articles published in the journal HemaSphere exclusively reflect the opinions of the authors. The authors are responsible for all content in their abstracts including accuracy of the facts, statements, citing resources, etc.

### (PB2665) PARASYMPATHETIC CHOLINERGIC SIGNALING SUSTAINS STEMNESS IN ACUTE MYELOID LEUKEMIA VIA PI3K/MTOR SIGNALING

**Topic:** 03. Acute myeloid leukemia - Biology & translational research

Michelle Buri[1], Xiaochi MA[1], Benian Uzun[1], Chantal Bachmann[1], Ramin Radpour[1], Adrian Ochsenbein[1], Carsten Riether*[1]

[1]*Department of Medical Oncology, Inselspital, Bern University Hospital, University of Bern, Bern, Switzerland;*

### Background

Leukemia stem cells (LSCs) represent the cells of origin in acute myeloid leukemia (AML) and are thought to be implicated in disease relapse following an initial therapeutic response. Their maintenance and survival are not solely regulated cell-intrinsically but are critically influenced by extrinsic cues from the bone marrow microenvironment. Emerging evidence highlights dysregulated neural signaling as a component of the leukemic niche in hematologic malignancies (HemaSphere 2022;6:S3;S250; HemaSphere 2023;7:S3;P420). However, the functional contribution of aberrant neural regulation to LSC biology and AML progression remains insufficiently characterized.

### Aims

This study aimed to determine whether parasympathetic cholinergic signaling regulates LSC function in human and murine AML, and to elucidate the underlying molecular and cellular mechanisms by which this cholinergic neural input modulates LSC maintenance and disease progression.

### Methods

The role of acetylcholine (ACh) and vagal signaling in regulating LSCs was investigated using both syngeneic and patient-derived models of AML. To determine the contribution of parasympathetic cholinergic input in vivo, surgical vagotomy and pharmacologic inhibition of nicotinic ACh receptors were performed. The resulting effects on LSC frequency, functional capacity, and overall AML progression were systematically evaluated.
Complementary ex vivo experiments were conducted using ACh, the primary neurotransmitter of the cholinergic system, to characterize its functional and mechanistic effects on both human and murine LSCs. Additionally, RNA sequencing (RNA-seq) and assay for transposase-accessible chromatin sequencing (ATAC-seq) were employed to elucidate the transcriptional and epigenetic programs induced by cholinergic signaling in AML LSCs.

### Results

Here, we identify parasympathetic cholinergic signaling as a critical regulator of LSC function, while being dispensable for normal hematopoietic stem cell (HSC) maintenance. AML progression in mice was associated with increased systemic ACh levels, heightened vagal activity, and upregulation of CHRN subunits on LSCs. Surgical vagotomy or pharmacologic inhibition of nicotinic ACh receptors significantly reduced leukemic burden, impaired LSC self-renewal, and diminished leukemia-initiating capacity, while preserving normal hematopoiesis.
Mechanistically, ACh-mediated cholinergic signaling directly enhanced murine and human LSC stemness ex vivo through activation of the PI3K/mTOR pathway. In addition, cholinergic signaling induced epigenetic remodeling that increased chromatin accessibility at genes involved in neurotransmitter signaling and ion channel activity. Importantly, elevated serum ACh levels in patients with AML correlated with inferior overall survival and emerged as an independent adverse prognostic biomarker.

**Copyright Information:** (Online) ISSN: 2572-9241
©2026 The Author(s). HemaSphere is published by John Wiley & Sons Ltd on behalf of European Hematology Association. This is an open access Abstract Book distributed under the Attribution-NonCommercial-NoDerivs (CC BY-NC-ND), which allows third parties to download the articles and share them with others as long as they credit the author and the Abstract Book, but they cannot change the content in any way or use them commercially.
**Abstract Book Citations:** Authors, Title, HemaSphere, 2026;10:(S1):pages. The abstract book DOIs can be found on the “Author Information” page.
**Disclaimer:** Articles published in the journal HemaSphere exclusively reflect the opinions of the authors. The authors are responsible for all content in their abstracts including accuracy of the facts, statements, citing resources, etc.

**Summary/Conclusion**

Collectively, these findings establish a cholinergic–PI3K/mTOR axis as a driver of leukemic self-renewal and reveal parasympathetic signaling as a niche-derived vulnerability in AML.

**Copyright Information:** (Online) ISSN: 2572-9241
©2026 The Author(s). HemaSphere is published by John Wiley & Sons Ltd on behalf of European Hematology Association. This is an open access Abstract Book distributed under the Attribution-NonCommercial-NoDerivs (CC BY-NC-ND), which allows third parties to download the articles and share them with others as long as they credit the author and the Abstract Book, but they cannot change the content in any way or use them commercially.
**Abstract Book Citations:** Authors, Title, HemaSphere, 2026;10:(S1):pages. The abstract book DOIs can be found on the "Author Information" page.
**Disclaimer:** Articles published in the journal HemaSphere exclusively reflect the opinions of the authors. The authors are responsible for all content in their abstracts including accuracy of the facts, statements, citing resources, etc.

### (PB2666) IMPACT OF THE GERMLINE SH2B3 RS111340708 SPLICING VARIANT ON CLINICAL OUTCOMES AND SIGNALING DEREGULATION IN CORE BINDING FACTOR ACUTE MYELOID LEUKEMIA

**Topic:** 03. Acute myeloid leukemia - Biology & translational research

Alessandro Beghini*[1], Marta Rachele Stefanucci[2], Livia Leuzzi[3], Davide Bernasconi[4], Luca Cimaschi[2], Alessandra Trojani[2], Lorenzo Del Castello[4], Roberto Cairoli[2]

*[1]Università degli Studi di Milano, Deapartment of Health Sciences, Milan, Italy;[2]Grande Ospedale Metropolitano Niguarda, Department of Oncology, Milan, Italy; [3]ASST Fatebenefratelli Sacco, PO Fatebenefratelli, Oncology, Milan, Italy; [4]University of Milano-Bicocca, Bicocca Bioinformatics Biostatistics and Bioimaging Centre, Vedano al Lambro, Italy;*

### Background

*SH2B3* (LNK) encodes a key adaptor protein that negatively regulates hematopoietic signaling pathways (JAK-STAT, MAPK, and PI3K/AKT) by interacting with mediators such as JAK2, MPL, FLT3, and KIT. While loss-of-function mutations in *SH2B3* are known to predispose to myeloid malignancies, the role of recurrent germline variants in modulating signaling deregulation and clinical outcomes in Acute Myeloid Leukemia (AML) remains poorly defined.

### Aims

We aimed to characterize the distribution of the *SH2B3* rs111340708 polymorphism across AML molecular subtypes and evaluate its impact on splicing, protein expression, and survival, specifically focusing on Core Binding Factor Leukemias (CBFLs).

### Methods

Samples from 149 AML patients (CBFL, non-CBFL, and *CEBPA*-mutated) were analyzed using Next-Generation Sequencing (Oncomine Myeloid Research Assay). Functional consequences were assessed via *in vitro* assays and immunoblotting in primary samples and cell lines. Clinical impact in the CBFL cohort (n=32) was evaluated using Kaplan-Meier survival analysis and log-rank tests (significance at $p < 0.05$).

### Results

The intronic variant *SH2B3* rs111340708 (a deletion within IVS6) was significantly enriched in our AML cohort, appearing in 49% of patients with an allele frequency of 32.9% in CBFLs, markedly higher than the ~12% reported in European populations. In the CBFL subgroup, carriers of the variant had significantly shorter overall survival (OS) compared to non-carriers (median OS 866 days vs. not reached; p=0.011), while progression-free survival (PFS) showed a negative trend (median 285 vs. 998 days; p=0.15). Functional analyses demonstrated that this polymorphism promotes aberrant IVS6 retention, which predictably introduces premature stop codons. Consistently, immunoblotting revealed markedly reduced levels of functional SH2B3 and the expression of protein isoforms with altered molecular weights in carrier patients and cell lines.

### Summary/Conclusion

The *SH2B3* rs111340708 splicing polymorphism represents a novel germline mechanism of *SH2B3* inactivation in AML. By compromising hematopoietic homeostasis, this high-frequency variant contributes to leukemogenesis and may identifies a high-risk subgroup within CBFL. These findings highlight *SH2B3* as a significant prognostic biomarker and suggest that targeting downstream pathways, such as JAK signaling, may provide a tailored therapeutic strategy for *SH2B3*-deficient leukemias.

**Copyright Information:** (Online) ISSN: 2572-9241
©2026 The Author(s). HemaSphere is published by John Wiley & Sons Ltd on behalf of European Hematology Association. This is an open access Abstract Book distributed under the Attribution-NonCommercial-NoDerivs (CC BY-NC-ND), which allows third parties to download the articles and share them with others as long as they credit the author and the Abstract Book, but they cannot change the content in any way or use them commercially.
**Abstract Book Citations:** Authors, Title, HemaSphere, 2026;10:(S1):pages. The abstract book DOIs can be found on the "Author Information" page.
**Disclaimer:** Articles published in the journal HemaSphere exclusively reflect the opinions of the authors. The authors are responsible for all content in their abstracts including accuracy of the facts, statements, citing resources, etc.

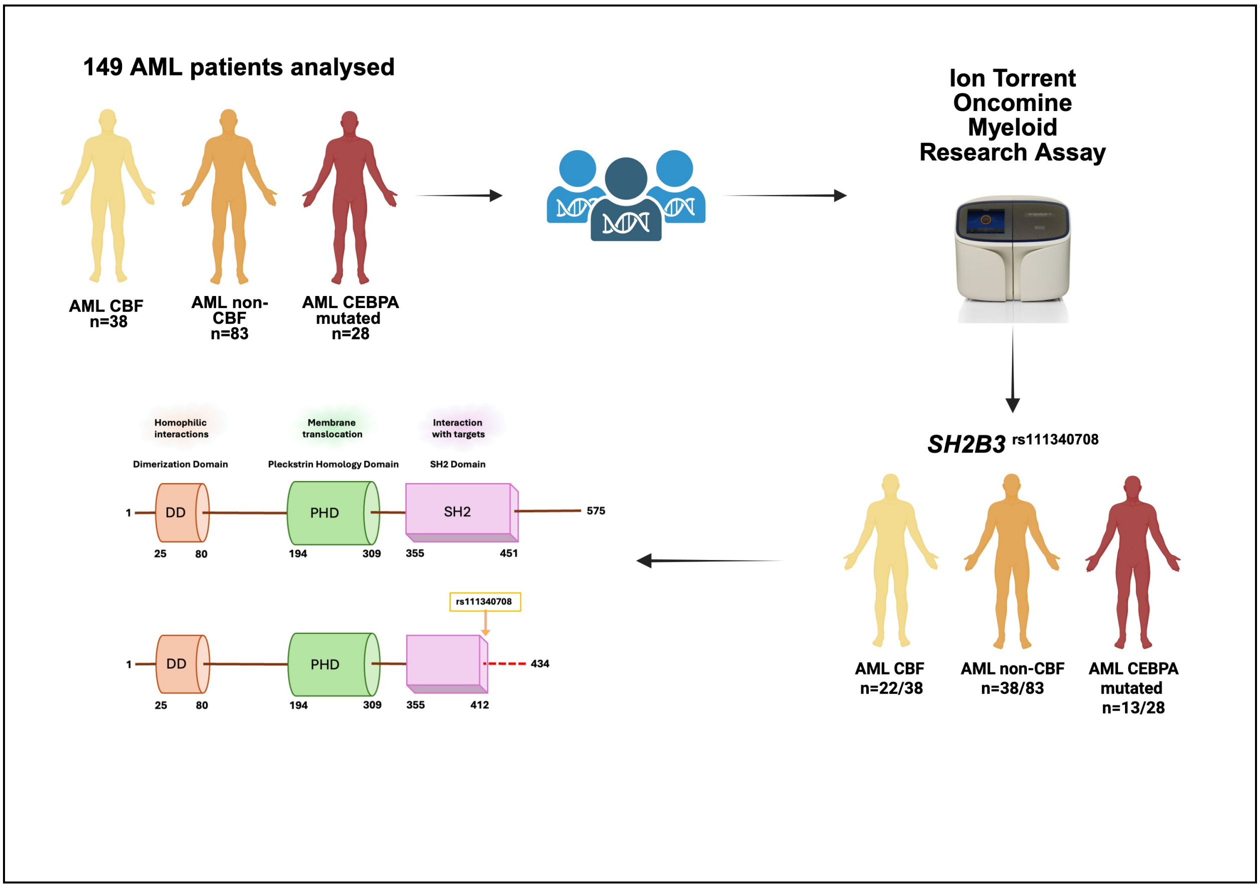

Ghaphical abstract of the experimental workflow. A total of 149 acute myeloid leukemia (AML) patients were categorized into three molecular groups: core-binding factor AML (CBF, n = 38), non-CBF AML (n = 83), and CEBPA-mutated AML (n = 28). Samples were analyzed using the Ion Torrent Oncomine Myeloid Research Assay, which enabled the identification of a polymorphism in the SH2B3 gene (rs111340708). Based on this finding, further analyses were performed, including RT-PCR to assess transcript levels and immunoblotting to evaluate protein expression across the three patient groups (AML CBF n = 22/38; AML non-CBF n = 38/83; CEBPA-mutated AML n = 13/28). Structural representation of wild-type SH2B3 protein and the predicted variant is shown at the end of the workflow. The upper scheme illustrates the domain organization of the wild-type SH2B3 protein, including the dimerization domain (DD), the pleckstrin homology domain (PH), and the Src homology 2 domain (SH2). The lower scheme shows the SH2B3 predicted by the variant carrying the polymorphic intron 6 retention event. This aberrant splicing introduces a premature termination codon (PTC), resulting in a truncated protein lacking the C-terminal portion of the SH2 domain, which is essential for proper inhibitory signaling.

**Copyright Information:** (Online) ISSN: 2572-9241
©2026 The Author(s). HemaSphere is published by John Wiley & Sons Ltd on behalf of European Hematology Association. This is an open access Abstract Book distributed under the Attribution-NonCommercial-NoDerivs (CC BY-NC-ND), which allows third parties to download the articles and share them with others as long as they credit the author and the Abstract Book, but they cannot change the content in any way or use them commercially.
**Abstract Book Citations:** Authors, Title, HemaSphere, 2026;10:(S1):pages. The abstract book DOIs can be found on the “Author Information” page.
**Disclaimer:** Articles published in the journal HemaSphere exclusively reflect the opinions of the authors. The authors are responsible for all content in their abstracts including accuracy of the facts, statements, citing resources, etc.

### (PB2667) GFI1B LOSS IMPAIRS DNA DAMAGE REPAIR KINETICS IN ACUTE MYELOID LEUKEMIA

**Topic:** 03. Acute myeloid leukemia - Biology & translational research

Xuntao Lai[1], Donghua Zhang[2], Anqiao Li[1], Xinliang Mao[3, 4], Jia Wei[2], Longlong Liu[1], Zhenqian Huang*[1]

*[1]The First Affiliated Hospital of Guangzhou Medical University, Hematology, Guangzhou, China;[2]Tongji Hospital, Tongji Medical College, Huazhong University of Science and Technology, Hematology, Wuhan, China; [3]The First Affiliated Hospital of Guangzhou Medical University, Key Laboratory of Advanced Interdisciplinary Studies, Guangzhou, China; [4]School of Basic Medical Sciences, Guangzhou Medical University, Guangdong Provincial Key Laboratory of Protein Modification and Diseases, Guangzhou, China;*

### Background

Acute myeloid leukemia (AML) is characterized by pronounced genomic instability that contributes to disease progression and therapeutic resistance. Growth factor independence 1B (GFI1B) is a hematopoietic zinc-finger transcriptional repressor that recruits chromatin-modifying complexes and functions as a tumor suppressor in myelodysplastic syndromes and AML. Reduced GFI1B expression promotes leukemogenesis and leukemia stem cell–associated phenotypes and has been linked to altered reactive oxygen species (ROS) signaling and metabolic rewiring. Emerging evidence further implicates GFI1B in the regulation of mitochondrial metabolic programs such as oxidative phosphorylation and fatty acid oxidation. Given the close interplay among cellular metabolism, chromatin regulation, oxidative stress, and DNA damage responses (DDR), we hypothesized that GFI1B contributes to genome maintenance by regulating DNA repair capacity in AML cells.

### Aims

To explore whether GFI1B contributes to genome maintenance by regulating DNA repair capacity in AML cells.

### Methods

Publicly available RNA-seq and ChIP-seq datasets were analyzed to explore associations between GFI1B and DDR-related pathways. For functional validation, GFI1B was silenced using two independent shRNAs in HEL AML cells. Following ionizing radiation exposure (5 Gy), DNA damage and repair kinetics were assessed using alkaline comet assays at 0, 0.5, 2, and 8 hours post-irradiation and γH2AX immunofluorescence at 0, 1, and 4 hours. Quantitative comparisons were performed against shRNA control cells.

### Results

Transcriptomic analyses of Gfi1b-deficient murine models revealed significant enrichment of DNA damage and repair–associated signatures, suggesting impaired genome maintenance upon GFI1B loss. Functionally, GFI1B knockdown delayed the resolution of irradiation-induced DNA lesions. Comet assays demonstrated significantly increased tail moments in GFI1B-silenced cells during early repair phases (0.5–2 h), with residual DNA damage persisting at 8 hours. Consistently, γH2AX immunofluorescence showed a higher proportion of cells harboring prominent DNA damage foci after irradiation and sustained γH2AX signals at 4 hours, indicative of defective double-strand break repair kinetics. Integration with ChIP-seq datasets identified candidate DDR-associated genes potentially under direct GFI1B regulatory control.

**Copyright Information:** (Online) ISSN: 2572-9241
©2026 The Author(s). HemaSphere is published by John Wiley & Sons Ltd on behalf of European Hematology Association. This is an open access Abstract Book distributed under the Attribution-NonCommercial-NoDerivs (CC BY-NC-ND), which allows third parties to download the articles and share them with others as long as they credit the author and the Abstract Book, but they cannot change the content in any way or use them commercially.
**Abstract Book Citations:** Authors, Title, HemaSphere, 2026;10:(S1):pages. The abstract book DOIs can be found on the "Author Information" page.
**Disclaimer:** Articles published in the journal HemaSphere exclusively reflect the opinions of the authors. The authors are responsible for all content in their abstracts including accuracy of the facts, statements, citing resources, etc.

**Summary/Conclusion**

These findings identify GFI1B as a previously underappreciated regulator of DNA damage repair dynamics in AML. Loss of GFI1B defines a biologically distinct AML context characterized by impaired genome maintenance, suggesting increased dependence on compensatory DDR pathways. This work provides a mechanistic rationale for exploring DDR-targeted therapeutic strategies in GFI1B-low AML.

**Copyright Information:** (Online) ISSN: 2572-9241
©2026 The Author(s). HemaSphere is published by John Wiley & Sons Ltd on behalf of European Hematology Association. This is an open access Abstract Book distributed under the Attribution-NonCommercial-NoDerivs (CC BY-NC-ND), which allows third parties to download the articles and share them with others as long as they credit the author and the Abstract Book, but they cannot change the content in any way or use them commercially.
**Abstract Book Citations:** Authors, Title, HemaSphere, 2026;10:(S1):pages. The abstract book DOIs can be found on the "Author Information" page.
**Disclaimer:** Articles published in the journal HemaSphere exclusively reflect the opinions of the authors. The authors are responsible for all content in their abstracts including accuracy of the facts, statements, citing resources, etc.

### (PB2668) CLINICAL IMPACT OF HEMODILUTION ON MEASURABLE RESIDUAL DISEASE ASSESSED BY FLOW CYTOMETRY

**Topic:** 03. Acute myeloid leukemia - Biology & translational research

Lukas Haaksma*[1, 2], Tom Reuvekamp[1, 2], H. Gout[1], Kasper Croese[1, 2], Glenn Fransen[1, 2], Daphne den Hartog[1, 2], Angèle Kelder[1, 2], Dimitri Breems[3], Thomas Fischer[4], Bjørn T. Gjertsen[5], Laimonas Griškevičius[6], Gunnar Juliusson[7], Johan Maertens[8], Markus G. Manz[9, 10], Thomas Pabst[10, 11], Jakob Passweg[10, 12], Kimmo Porkka[13], Patrycja Gradowska[14, 15], Bob Löwenberg[14, 16], Gert Ossenkoppele[1, 2], Francesco Buccisano[17], Costa Bachas[1, 2], Jesse Tettero[1, 2], Jacqueline Cloos[1, 2], David de Leeuw[1, 2]

*[1]Amsterdam UMC, Department of Hematology, Amsterdam, The Netherlands;[2]Cancer Center Amsterdam, Amsterdam, The Netherlands; [3]Ziekenhuis aan de Stroom, Antwerpen, Belgium;[4]Otto von Guericke University Hospital Magdeburg, Magdeburg, Germany; [5]Haukeland University Hospital, Bergen, Norway;[6]Vilnius University Hospital Santaros Klinikos and Vilnius University, Vilnius, Lithuania; [7]Skanes University Hospital, Lund, Sweden;[8]University Hospital Gasthuisberg, Leuven, Belgium;[9]University Hospital, Zurich, Switzerland; [10]Swiss Group for Clinical Cancer Research, Bern, Switzerland;[11]University Hospital Inselspital, Bern, Switzerland, Bern, Switzerland; [12]University Hospital Basel, Basel, Switzerland; [13]Helsinki University Hospital Cancer Center, Helsinki, Finland; [14]Erasmus Medical Center Cancer Institute, Rotterdam, The Netherlands; [15]HOVON Foundation, Rotterdam, The Netherlands; [16]Erasmus MC Cancer Institute, Rotterdam, The Netherlands; [17]University of Rome "Tor Vergata", Rome, Italy;*

### Background

In acute myeloid leukemia (AML), measurable residual disease (MRD) detected by flow cytometry after intensive chemotherapy is an established prognostic biomarker. MRD is reported as the percentage of leukemia cells among white blood cells (WBC-MRD) in bone marrow (BM) samples. We previously showed that hemodilution, the admixture of peripheral blood (PB) during BM aspiration, reduced WBC-MRD levels, potentially causing false-negative results (Tettero *Leukemia* 2024). Quantifying MRD as a percentage of the primitive cell compartment (PM-MRD) could circumvent this effect.

### Aims

To investigate the occurrence and prognostic relevance of hemodilution on MRD measurements in patients with AML.

### Methods

We first evaluated four cell subsets as surrogate markers for hemodilution, using our 5-tube 8-color MRD and leukemia stem cell assay, in paired PB and first-pull BM samples from a previously published study (Tettero 2024): plasma cells (PC), mast cells (MC), granulocytes, and pre-B cells (pBC). We performed receiver operating characteristic (ROC) analysis to establish optimal cutoffs for differentiating PB and BM. We then applied these cutoffs to assess hemodilution in samples of AML patients treated in the HOVON132 trial. We included patients in complete remission after two cycles of induction, with an MRD measurement with ≥500,000 WBC per tube. MRD-positivity was defined as ≥ 0.1% leukemia cells of WBC and PM-MRD positivity as ≥ 10% leukemia cells of the primitive compartment. We used the Wilcoxon rank-sum test for between-group comparisons, and Kaplan Meier estimates for survival analysis.

**Copyright Information:** (Online) ISSN: 2572-9241
©2026 The Author(s). HemaSphere is published by John Wiley & Sons Ltd on behalf of European Hematology Association. This is an open access Abstract Book distributed under the Attribution-NonCommercial-NoDerivs (CC BY-NC-ND), which allows third parties to download the articles and share them with others as long as they credit the author and the Abstract Book, but they cannot change the content in any way or use them commercially.
**Abstract Book Citations:** Authors, Title, HemaSphere, 2026;10:(S1):pages. The abstract book DOIs can be found on the "Author Information" page.
**Disclaimer:** Articles published in the journal HemaSphere exclusively reflect the opinions of the authors. The authors are responsible for all content in their abstracts including accuracy of the facts, statements, citing resources, etc.

**Results**

ROC analysis identified PC, MC and pBC as potential surrogate markers for hemodilution (Figure 1A). Among the 276 patients from the HOVON132 trial who met the inclusion criteria, median WBC-MRD percentages were significantly lower in samples classified as hemodiluted based on PC (0.008% vs 0.021%, p<0.01) and MC (0.008% vs 0.014%, p<0.01) individually, but not pBC (0.01% vs 0.012%, p=0.48). In contrast, PM-MRD percentages remained similar regardless of hemodilution status across all surrogate cell subsets (2.2% vs 2.5%, p=0.38; 2.2% vs 2.3%, p=0.78; and 2.5% vs 2.2%, p=0.44, respectively). Combined classification based on PC and MC together further refined hemodilution assessment: samples classified as hemodiluted by both markers (43%) showed significantly lower WBC-MRD compared to those by either PC or MC (33%) and neither marker (24%), with correspondingly lower rates of WBC-MRD-positivity (Figure 1B). Despite these differences, we found similar prognostic value of WBC-MRD when stratified by hemodilution status (Figure 1C). While PM-MRD and WBC-MRD status showed high concordance, patients positive by either WBC-MRD or PM-MRD seemed to have intermediate outcomes (Figure 1D). Herein, WBC-MRDnegative/PM-MRDpositive cases were rare (4.3%), consistent with the expectation that likely only a limited number of patients with residual disease near the 0.1% the cutoff is susceptible to misclassification due to hemodilution.

**Summary/Conclusion**

We classified 43% of MRD samples as hemodiluted based on their PC and MC percentage. However, despite significantly lower WBC-MRD percentages in hemodiluted samples, the limited impact on prognostic stratification might reflect that clinically relevant hemodilution, leading to misclassification, is likely rare and supports the robustness of the 0.1% cutoff which was established under similar sampling conditions.

**Copyright Information:** (Online) ISSN: 2572-9241
©2026 The Author(s). HemaSphere is published by John Wiley & Sons Ltd on behalf of European Hematology Association. This is an open access Abstract Book distributed under the Attribution-NonCommercial-NoDerivs (CC BY-NC-ND), which allows third parties to download the articles and share them with others as long as they credit the author and the Abstract Book, but they cannot change the content in any way or use them commercially.
**Abstract Book Citations:** Authors, Title, HemaSphere, 2026;10:(S1):pages. The abstract book DOIs can be found on the "Author Information" page.
**Disclaimer:** Articles published in the journal HemaSphere exclusively reflect the opinions of the authors. The authors are responsible for all content in their abstracts including accuracy of the facts, statements, citing resources, etc.

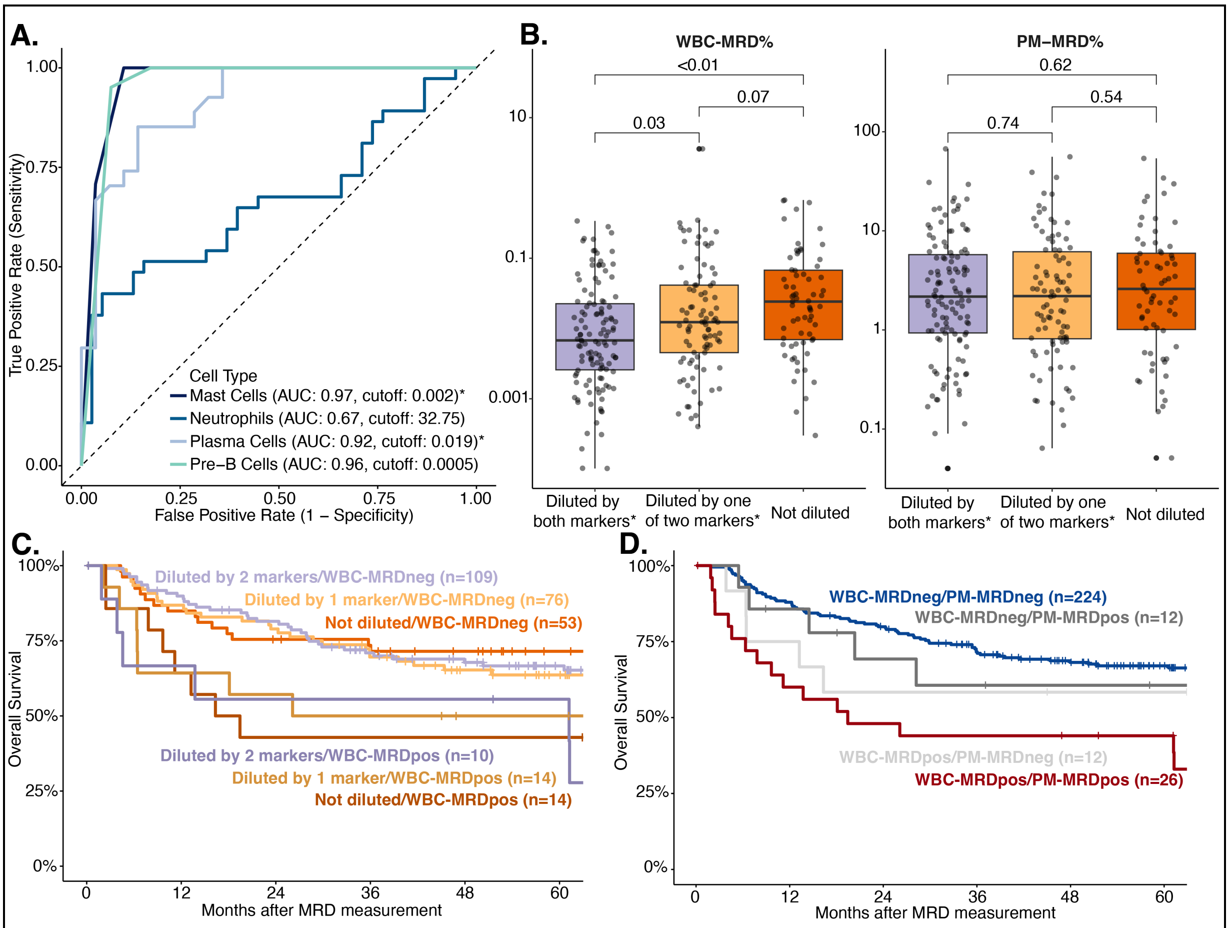

Figure 1. **(A.)** Receiver operating characteristics for candidate hemodilution surrogate markers based on their respective prevalence in first-pull 2mL bone marrow compared to peripheral blood, which was analyzed in previously described samples (Tettero, Leukemia 2024). (*) Cell subsets used to define of hemodilution in Figure 1B-D **(B.)** Left: Measurable residual disease (MRD) as a percentage of white blood cells (WBC-MRD) and right: as a percentage of the primitive cell compartment (PM-MRD). Patients are stratified based on a combined hemodilution status using both mast cells* and plasma cells*: diluted by both cell subsets (purple), by either one of the two (yellow) or by none (orange). **(C.)** Overall survival stratified by WBC-MRD and hemodilution status. Time in months after MRD measurement. **(D.)** Overall survival stratified by combined classification of MRD status by WBC-MRD and PM-MRD. Time in months after MRD measurement.

**Copyright Information:** (Online) ISSN: 2572-9241
©2026 The Author(s). HemaSphere is published by John Wiley & Sons Ltd on behalf of European Hematology Association. This is an open access Abstract Book distributed under the Attribution-NonCommercial-NoDerivs (CC BY-NC-ND), which allows third parties to download the articles and share them with others as long as they credit the author and the Abstract Book, but they cannot change the content in any way or use them commercially.
**Abstract Book Citations:** Authors, Title, HemaSphere, 2026;10:(S1):pages. The abstract book DOIs can be found on the "Author Information" page.
**Disclaimer:** Articles published in the journal HemaSphere exclusively reflect the opinions of the authors. The authors are responsible for all content in their abstracts including accuracy of the facts, statements, citing resources, etc.

### (PB2669) MALT1 INHIBITION SUPPRESSES AML CELL GROWTH BY MODULATING THE KMT2D/NCOR2/C-MYC AXIS VIA MCPIP1 INDUCTION AND SYNERGIZES WITH VENETOCLAX.

**Topic:** 03. Acute myeloid leukemia - Biology & translational research

Weiguang Cui*[1], Liyuan Li[1], Zhenzhen Wang[1], Nan Zhou[1], Fan Yang[1], Mengying Zheng[1], Li Sun[1], Xiaoyan Zhang[1], Lin Yang[1], Jianmin Luo[1]

[1]*Department of Hematology, The Second Hospital of Hebei Medical University, shijiazhuang, China;*

### Background

Acute myeloid leukemia (AML) is the most prevalent acute leukemia in adults. Despite significant pharmacological advancements over the past decade, clinical outcomes remain suboptimal due to high relapse rates following remission. MALT1 (mucosa-associated lymphoid tissue lymphoma translocation protein 1), a paracaspase known to drive oncogenic signaling in ABC-DLBCL and MALT lymphoma, has emerged as a potential therapeutic target. While database screening (TCGA, Beat-AML) indicates high MALT1 expression in AML cell lines, its systematic role and therapeutic potential in AML remain poorly characterized.

### Aims

This study aimed to evaluate the anti-leukemic effects of MALT1 inhibitors, both as monotherapy and in combination with the BCL-2 inhibitor venetoclax, and to elucidate the underlying molecular mechanisms in AML cell lines.

### Methods

AML cell lines (THP-1, Kasumi-1, and MV4-11) were treated with MALT1 inhibitors (MI-2 and mepazine), venetoclax, or their combination. Cell viability and $IC_{50}$ values were determined via CCK-8 assays across various time points (4–72 h). Apoptosis and cell cycle distribution were analyzed using flow cytometry. Protein expression levels of anti-apoptotic factors (MCL-1, BCL-XL, BCL-2), apoptotic markers (cleaved PARP1), NF-κB components (p65, c-Rel), MCPIP1, and c-MYC were quantified via Western blot. To investigate mechanistic pathways, MCPIP1-overexpressing cell lines were generated via lentiviral transduction, and *MALT1*-knockout (KO) lines were established using the CRISPR/Cas9 system. Whole-transcriptome sequencing (RNA-seq) was performed on these engineered lines, and Co-Immunoprecipitation (Co-IP) assays were utilized to validate protein-level interactions between MALT1 and MCPIP1.

### Results

MALT1 inhibition(MI-2 mepazine) induced a dose-dependent decrease in cell viability and significant cell cycle arrest at the G1 phase. Molecular analysis revealed significant upregulation of MCPIP1 and downregulation of c-MYC following treatment. When combined with venetoclax, MALT1 inhibition showed potent synergistic lethality, evidenced by a marked reduction in cell activity and enhanced expression of apoptotic markers compared to monotherapy. Co-IP assays confirmed that MALT1 physically binds to and facilitates the degradation of MCPIP1. Furthermore, RNA-seq identified the KMT2D/NCOR2 axis as a shared downstream pathway in both *MALT1*-KO and MCPIP1-overexpressing cells.

**Copyright Information:** (Online) ISSN: 2572-9241
©2026 The Author(s). HemaSphere is published by John Wiley & Sons Ltd on behalf of European Hematology Association. This is an open access Abstract Book distributed under the Attribution-NonCommercial-NoDerivs (CC BY-NC-ND), which allows third parties to download the articles and share them with others as long as they credit the author and the Abstract Book, but they cannot change the content in any way or use them commercially.
**Abstract Book Citations:** Authors, Title, HemaSphere, 2026;10:(S1):pages. The abstract book DOIs can be found on the "Author Information" page.
**Disclaimer:** Articles published in the journal HemaSphere exclusively reflect the opinions of the authors. The authors are responsible for all content in their abstracts including accuracy of the facts, statements, citing resources, etc.

### Summary/Conclusion

This study demonstrates that MALT1 inhibitors significantly suppress AML cell proliferation. Beyond inhibiting the NF-κB pathway and inducing cell cycle arrest, MALT1 inhibition stabilizes MCPIP1 by preventing its degradation. The resulting upregulation of MCPIP1 subsequently suppresses c-MYC expression via the KMT2D/NCOR2 axis. Moreover, the synergistic effect with venetoclax underscores MALT1 as a promising novel therapeutic target for AML.

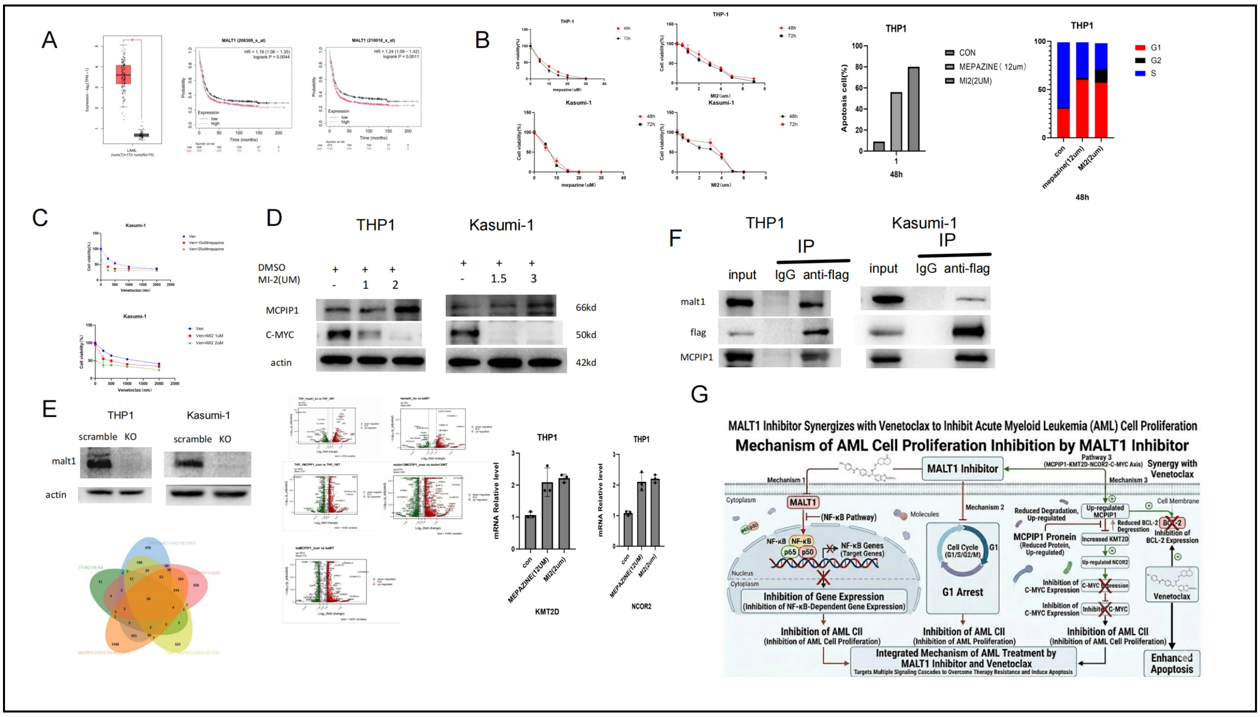

**Copyright Information:** (Online) ISSN: 2572-9241
©2026 The Author(s). HemaSphere is published by John Wiley & Sons Ltd on behalf of European Hematology Association. This is an open access Abstract Book distributed under the Attribution-NonCommercial-NoDerivs (CC BY-NC-ND), which allows third parties to download the articles and share them with others as long as they credit the author and the Abstract Book, but they cannot change the content in any way or use them commercially.
**Abstract Book Citations:** Authors, Title, HemaSphere, 2026;10:(S1):pages. The abstract book DOIs can be found on the "Author Information" page.
**Disclaimer:** Articles published in the journal HemaSphere exclusively reflect the opinions of the authors. The authors are responsible for all content in their abstracts including accuracy of the facts, statements, citing resources, etc.

### (PB2670) DUAL-LAYER MOLECULAR SIGNATURES OF CEBPA AND DNMT3A IN AML SURVIVAL STRATIFICATION

**Topic:** 03. Acute myeloid leukemia - Biology & translational research

Jairo Jattin-Balcázar*[1]

[1]*Universidad Nacional de Colombia, Facultad de Medicina, Bogotá, Colombia;*

### Background

Acute Myeloid Leukemia (AML) is a genetically and epigenetically heterogeneous malignancy with variable clinical outcomes. Current prognostic models based on cytogenetics and mutational status often fail to capture the layered molecular complexity that drives disease progression. DNA methylation and gene expression profiles may provide complementary information for survival stratification.

### Aims

This study aimed to evaluate whether integrating gene expression and methylation profiles across key AML-associated genes could support the development of a composite molecular framework for prognostic assessment.

### Methods

A cohort of 200 adult AML patients was curated from the TCGA LAML dataset. Expression and methylation data for seven genes (CEBPA, DNMT3A, HDAC1, IDH1, IDH2, NPM1, RUNX1) were analyzed. Survival associations were first evaluated independently using Kaplan-Meier and Cox models. Genes with significant prognostic value were combined to assess additive effects. A composite score integrating scaled CEBPA methylation and DNMT3A expression was constructed, and patients were stratified into low, intermediate, and high-risk groups. Validation was performed in an independent cohort of 115 patients.

### Results

DNMT3A expression was significantly associated with favorable survival (HR = 0.65, p = 0.0036), while HDAC1 expression predicted adverse outcomes (HR = 2.75, p = 0.0068; figure 1). Methylation analysis identified CEBPA (p = 0.0154), DNMT3A (p = 0.0417), and NPM1 (p = 0.0243) as significant markers. Composite scoring showed that the integration of DNMT3A expression and CEBPA methylation stratified patients into distinct risk groups with significant survival differences (log-rank p = 0.0013). NPM1 methylation did not improve model performance. Temporal analysis showed concentrated early mortality in the high-risk group, while low-risk patients demonstrated sustained survival. In the validation cohort, the two-layer score confirmed survival separation (p = 0.0035).

### Summary/Conclusion

This preliminary study suggests that combining DNMT3A expression and CEBPA methylation provides a dual-layer molecular score with prognostic relevance in AML. The model identifies patients at higher risk of early mortality and highlights the potential of targeted multi-omic integration for personalized risk stratification. Further validation in prospective cohorts is warranted to confirm its clinical utility.

**Copyright Information:** (Online) ISSN: 2572-9241
©2026 The Author(s). HemaSphere is published by John Wiley & Sons Ltd on behalf of European Hematology Association. This is an open access Abstract Book distributed under the Attribution-NonCommercial-NoDerivs (CC BY-NC-ND), which allows third parties to download the articles and share them with others as long as they credit the author and the Abstract Book, but they cannot change the content in any way or use them commercially.
**Abstract Book Citations:** Authors, Title, HemaSphere, 2026;10:(S1):pages. The abstract book DOIs can be found on the “Author Information” page.
**Disclaimer:** Articles published in the journal HemaSphere exclusively reflect the opinions of the authors. The authors are responsible for all content in their abstracts including accuracy of the facts, statements, citing resources, etc.

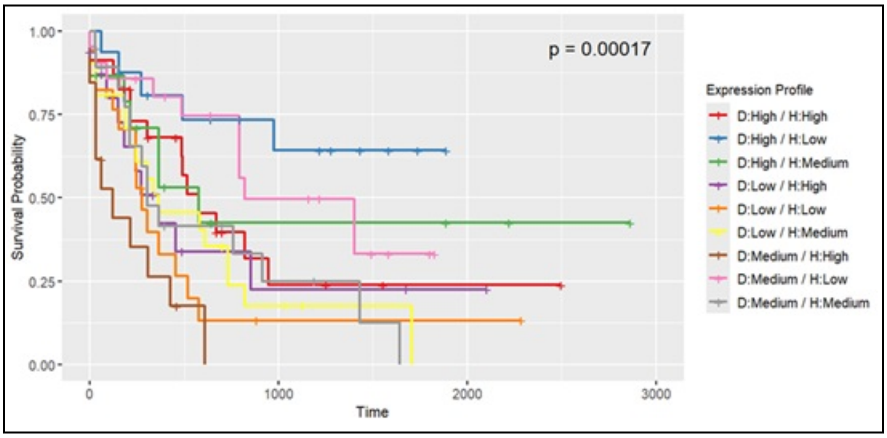

Figure 1. Significant survival differences when combining DNMT3A and HDAC1 expression levels, categorized as low, medium, or high. Patients were stratified into nine composite profiles, each reflecting a unique combination of expression levels. The group with high DNMT3A and high HDAC1 expression showed the poorest survival outcomes, whereas the profile with high DNMT3A and low HDAC1 was associated with the most favorable trajectory. Overall, the separation between curves was statistically significant (log-rank p = 0.00017), indicating a complementary effect of both genes in survival stratification

**Copyright Information:** (Online) ISSN: 2572-9241
©2026 The Author(s). HemaSphere is published by John Wiley & Sons Ltd on behalf of European Hematology Association. This is an open access Abstract Book distributed under the Attribution-NonCommercial-NoDerivs (CC BY-NC-ND), which allows third parties to download the articles and share them with others as long as they credit the author and the Abstract Book, but they cannot change the content in any way or use them commercially.
**Abstract Book Citations:** Authors, Title, HemaSphere, 2026;10:(S1):pages. The abstract book DOIs can be found on the “Author Information” page.
**Disclaimer:** Articles published in the journal HemaSphere exclusively reflect the opinions of the authors. The authors are responsible for all content in their abstracts including accuracy of the facts, statements, citing resources, etc.

### (PB2671) ADVANCING MINIMAL RESIDUAL DISEASE (MRD) ASSESSMENT IN ACUTE MYELOID LEUKEMIA (AML): FROM CELLS TO VESICLES THROUGH NEXT GENERATION SEQUENCING (NGS) AND DIGITAL PCR (DPCR)

**Topic:** 03. Acute myeloid leukemia - Biology & translational research

Alessandro Leoni*[1, 2], Mirko Farina[2], Erika Borlenghi[2], Luca Garuffo[1], Besjana Xhahysa[1], Alessia Cavalleri[1], Silvia Mutti[1], Enrico Morello[2], Daniele Avenoso[1, 2], Domenico Russo[1, 2], Michele Malagola[1, 2], Simona Bernardi[1]

*[1]University of Brescia, Brescia, Italy; [2]ASST Spedali Civili, Brescia, Italy;*

### Background

Acute myeloid leukemia (AML), is a biologically complex and heterogeneous hematologic malignancy characterized by clonal evolution and several genetic and epigenetic alterations. Despite achieving complete remission, long-term survival remains poor, with relapse occurring in 50 to 80% of patients (pts). Minimal Residual Disease (MRD), defined as the persistence of leukemic cells, is a key predictor of relapse and survival. Current MRD assessment relies on bone marrow (BM) or peripheral blood (PB) analysis using molecular assays and multiparameter flow cytometry, but these methods are limited by restricted applicability, invasiveness, and variable sensitivity. Our combined NGS-dPCR approach overcomes several of these limitations. In this context, we propose the use of Extracellular Vesicles (EVs), released by leukemic cells into the bloodstream, as an alternative matrix. EVs carry nucleic acids reflecting the leukemia-specific mutational profile, and their stability and molecular cargo make them a promising, minimally invasive tool for MRD monitoring.

### Aims

We conducted a feasibility study to evaluate an integrated NGS-driven dPCR approach for detecting and longitudinally monitoring AML-associated mutations across BM, PB and EV derived DNA, aiming  to expand MRD applicability and improve sensitivity.

### Methods

A targeted NGS Myeloid panel (SOPHiA Genetics) was performed at diagnosis to identify patient-specific AML-associated mutations. These mutation were subsequently tracked using digital PCR (dPCR) with custom-designed (78%) or commercial (22%) TaqMan assays. Mutations related to clonal hematopoiesis were excluded. Feasibility was predefined as the ability to longitudinally monitor ≥70% of pts with this workflow.

### Results

NGS identified AML-related mutation in 22 of 25 pts (88%), all of whom were successfully monitored by dPCR, exceeding the predefined feasibility threshold. Of the 22 pts monitored, 5 had no conventional markers, while 17 had markers. Of these, 12 showed results consistent with conventional techniques, while 5 were inconsistent. dPCR achieved high analytical sensitivity (up to $10^{-5}$), and showed strong concordance across BM, PB, and EV derived DNA. Compared with conventional method, the NGS-driven dPCR approach significantly expanded MRD coverage and sensitivity, enabling monitoring even in pts lacking standard molecular markers. dPCR-MRD positivity in BM, PB, and EVs anticipated hematologic relapse in 9 out of 10 cases (Fig. 1).

**Copyright Information:** (Online) ISSN: 2572-9241
©2026 The Author(s). HemaSphere is published by John Wiley & Sons Ltd on behalf of European Hematology Association. This is an open access Abstract Book distributed under the Attribution-NonCommercial-NoDerivs (CC BY-NC-ND), which allows third parties to download the articles and share them with others as long as they credit the author and the Abstract Book, but they cannot change the content in any way or use them commercially.
**Abstract Book Citations:** Authors, Title, HemaSphere, 2026;10:(S1):pages. The abstract book DOIs can be found on the "Author Information" page.
**Disclaimer:** Articles published in the journal HemaSphere exclusively reflect the opinions of the authors. The authors are responsible for all content in their abstracts including accuracy of the facts, statements, citing resources, etc.

### Summary/Conclusion

Given the biological complexity of the AML and the high relapse rate, even among MRD-negative pts, more sensitive and informative monitoring strategies are needed.

Our combined NGS-dPCR approach extend MRD assessment from cellular compartments to EVs. By integrating the broad mutational profiling of NGS with the quantitative precision of dPCR, and using EV-derived DNA as a liquid biopsy source, this strategy provides a highly sensitive, minimally invasive, and widely applicable platform for next-gen MRD monitoring. EV-MRD showed strong concordance with BM and PB, sometimes with greater signal clarity and lower background noise. Overall, this EV-based, NGS-driven dPCR approach proved feasible, sensitive, and clinically promising, with potential to improve risk stratification and enable earlier, more personalized therapeutic interventions.

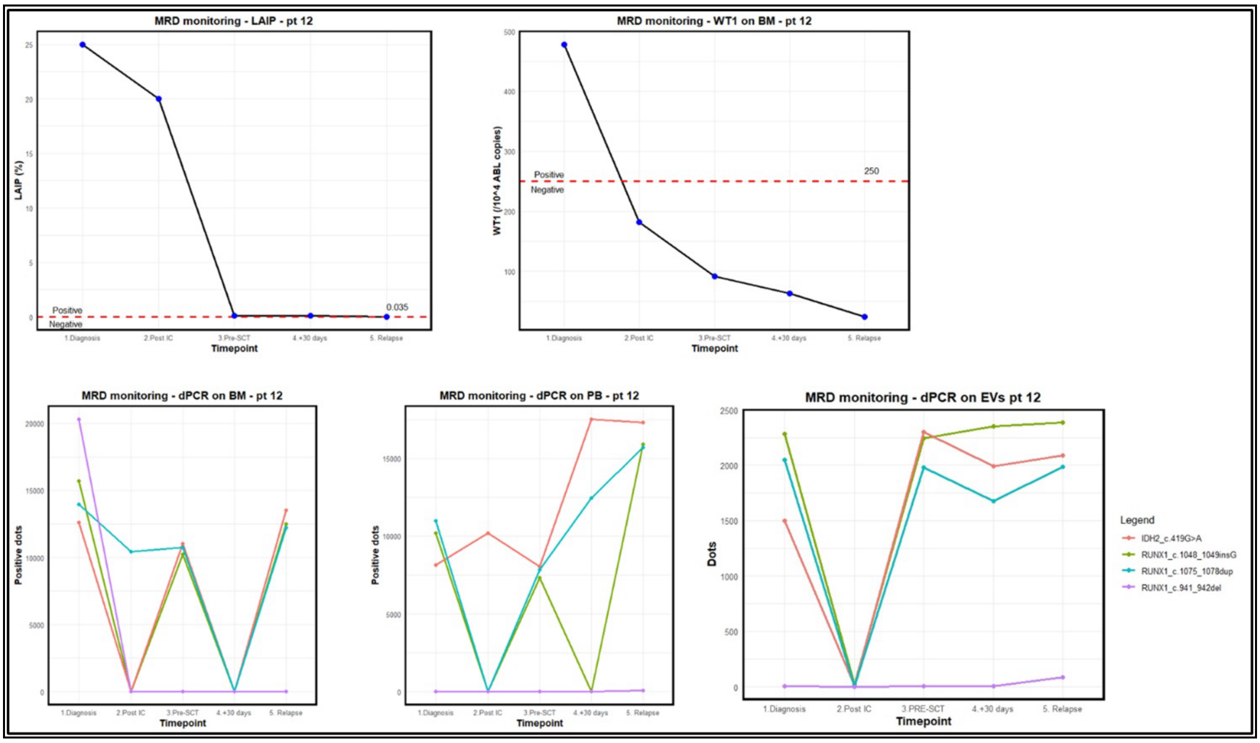

Comparision between Bone Marrow (lower left), Peripheral Blood (lower middle) and Extracellular Vesicles (lower right) by NGS-dPCR approach and conventional methods (upper left: LAIP upper right: Molecular test - on WT1 )

**Copyright Information:** (Online) ISSN: 2572-9241
©2026 The Author(s). HemaSphere is published by John Wiley & Sons Ltd on behalf of European Hematology Association. This is an open access Abstract Book distributed under the Attribution-NonCommercial-NoDerivs (CC BY-NC-ND), which allows third parties to download the articles and share them with others as long as they credit the author and the Abstract Book, but they cannot change the content in any way or use them commercially.
**Abstract Book Citations:** Authors, Title, HemaSphere, 2026;10:(S1):pages. The abstract book DOIs can be found on the "Author Information" page.
**Disclaimer:** Articles published in the journal HemaSphere exclusively reflect the opinions of the authors. The authors are responsible for all content in their abstracts including accuracy of the facts, statements, citing resources, etc.

### (PB2672) METABOLOMIC SIGNATURES IN UNTREATED AML SERUM ASSOCIATED WITH IN VITRO SENSITIVITY TO SYK INHIBITION

**Topic:** 03. Acute myeloid leukemia - Biology & translational research

Marte Brattås*[1, 2], Kristin Rye[1], Kimberley Hatfield[3], Hakon Reikvam[1, 3]

*[1]University of Bergen, K.G. Jebsen Center of Myeloid Malignancies, Department of Clinical Science, Bergen, Norway; [2]Haukeland University Hospital, Medicine, Bergen, Norway; [3]Haukeland University Hospital, Department of Immunology and Transfusion Medicine, Bergen, Norway;*

### Background

Acute myeloid leukemia (AML) remains challenging due to high relapse rates and treatment resistance driven by genetic and phenotypic heterogeneity. Spleen tyrosine kinase (SYK) promotes leukemic cell survival and proliferation, making it an emerging therapeutic target. However, patient responses to SYK inhibition vary considerably, and predictive biomarkers are lacking. Metabolomic profiling of patient serum may offer a non-invasive approach to identify baseline signatures predictive of in vitro drug sensitivity.

### Aims

To determine whether baseline serum metabolic profiles distinguish AML patients with high versus low in vitro sensitivity to SYK-targeted inhibitors.

### Methods

Untreated serum from primary AML patients (n=49, median age 71 years, 36% FLT3-mutated) underwent untargeted metabolomics using the Metabolon platform with Liquid Chromatography-Mass Spectrometry (LC-MS). In vitro sensitivity to SYK-selective (Entospletinib, RO9021) and dual SYK/FLT3 inhibitors (Fostamatinib, TAK-659) was assessed by thymidine incorporation assay. Hierarchical clustering identified three sensitivity groups (high, intermediate, low); subsequent analyses compared the high (n=21) and low (n=15) response groups for SYK-selective inhibitors, and high (n=18) and low (n=8) for dual SYK/FLT3 inhibitors. Differentially abundant metabolites were identified using the limma package in R. Pathway and enrichment analyses were performed using MetaboAnalyst. Exploratory Random Forest (RF) classification in R, incorporating limma-based feature selection and SMOTE resampling, was applied to evaluate predictive accuarcy.

### Results

No significant association was found between FLT3 or NPM1 mutation status and drug sensitivity (Mann-Whitney U and Fisher's exact tests). Untargeted metabolomics identified 1,204 metabolites across major pathways. Pathway and enrichment analyses revealed consistent alterations in amino acid, lipid, and xenobiotic metabolism between sensitivity groups for both inhibitor classes. Among top-ranked RF features, lipid metabolites dominated for dual SYK/FLT3 inhibitors, particularly acylcarnitines and fatty acid derivatives. For SYK-selective inhibitors, the metabolic signature was more diverse, with lipids, carotenoid/antioxidant compounds, and proline-related amino acids among the most discriminating features. Following feature selection and SMOTE resampling, RF classification improved sensitivity for the low-response group from 36% to 75.6% (AUC=0.833) for SYK-selective inhibitors, and from 0% to 41.7% (AUC=0.590) for dual SYK/FLT3 inhibitors, likely reflecting the limited sample size (n=8) in the low-response group.

**Copyright Information:** (Online) ISSN: 2572-9241
©2026 The Author(s). HemaSphere is published by John Wiley & Sons Ltd on behalf of European Hematology Association. This is an open access Abstract Book distributed under the Attribution-NonCommercial-NoDerivs (CC BY-NC-ND), which allows third parties to download the articles and share them with others as long as they credit the author and the Abstract Book, but they cannot change the content in any way or use them commercially.
**Abstract Book Citations:** Authors, Title, HemaSphere, 2026;10:(S1):pages. The abstract book DOIs can be found on the "Author Information" page.
**Disclaimer:** Articles published in the journal HemaSphere exclusively reflect the opinions of the authors. The authors are responsible for all content in their abstracts including accuracy of the facts, statements, citing resources, etc.

**Summary/Conclusion**

Baseline serum metabolic signatures may inform in vitro sensitivity to SYK-targeted therapy in AML, though the metabolic features associated with response appeared to differ between inhibitor classes. Serum metabolomics, being both non-invasive and accessible, may offer valuable insights that help inform individualized therapeutic approaches. These findings are hypothesis-generating and require validation in larger, independent cohorts.

**Copyright Information:** (Online) ISSN: 2572-9241
©2026 The Author(s). HemaSphere is published by John Wiley & Sons Ltd on behalf of European Hematology Association. This is an open access Abstract Book distributed under the Attribution-NonCommercial-NoDerivs (CC BY-NC-ND), which allows third parties to download the articles and share them with others as long as they credit the author and the Abstract Book, but they cannot change the content in any way or use them commercially.
**Abstract Book Citations:** Authors, Title, HemaSphere, 2026;10:(S1):pages. The abstract book DOIs can be found on the "Author Information" page.
**Disclaimer:** Articles published in the journal HemaSphere exclusively reflect the opinions of the authors. The authors are responsible for all content in their abstracts including accuracy of the facts, statements, citing resources, etc.

### (PB2673) REVEALING METABOLITES MODULATING IMMUNOSUPPRESSION IN AML-TME

**Topic:** 03. Acute myeloid leukemia - Biology & translational research

Natividad Alquezar Artieda*[1], Lucas Pontel[1]

[1]*Josep Carreras Leukaemia Research Institute, BADALONA, Spain;*

### Background

Acute Myeloid Leukemia (AML) remains a major therapeutic challenge, with overall survival below 30%. While chemotherapy resistance mechanisms have been extensively investigated, why immune surveillance fails to control AML progression remains poorly defined. Metabolic reprogramming is a recognized hallmark of cancer, also observed in AML; thus, we hypothesize that dysregulated AML metabolism modifies the tumor microenvironment (TME) metabolome, promoting immune dysfunction.

### Aims

To determine whether AML-induced metabolite fluxes within the TME impact leukemia progression by addressing the role of specific solute transporters in (i) modulating intrinsic AML properties and/or (ii) exerting extrinsic effects on immune cells.

### Methods

Prognostic relevance and expression of genes coding for solute transporters were assessed by averaging hazard ratios (HRs) derived from the KM Plotter and GEPIA databases, both based on TCGA AML cohorts. Functional studies were conducted in MonoMac-6, HL-60, and U937 cells following CRISPR/Cas9-mediated knockout (KO) or overexpression (OE) of selected transporters. Peripheral blood mononuclear cells (PBMCs) were used as a source of naïve T cells, and flow cytometry was used to determine T-cell activation dynamics and cytotoxicity.

### Results

We analyzed 412 genes coding for solute transporters, obtaining 24 that met our selection criteria. The cystine/glutamate antiporter SLC7A11 ranked in the top five (1.95 HR), correlating high expression with poor prognosis. Given the role of SLC7A11 in cystine/glutamate flux, we explored whether this transporter affects AML growth in standard culture conditions. To this end, we generated U937 and HL-60 AML cell models with dysregulated expression of SLC7A11 and found no significant differences in proliferation over 7 days. We noted that SLC7A11-deficient cells presented reduced levels of the cellular antioxidant glutathione (GSH). Accordingly, U937 cells lacking SLC7A11 were more dependent on GSH for growth (L-BSO $IC_{50}$: KO 1 µM vs. WT 3.6 µM), whereas HL60 OE cells were less dependent on GSH for proliferation ($IC_{50}$: OE 7.5 µM vs. WT 0.4 µM). Accordingly, cells lacking SLC7A11 were more sensitive to metabolic toxins like formaldehyde ($IC_{50}$: KO 19.3 µM vs. WT 28.6 µM), which are usually detoxified by GSH. These data suggest that while SLC7A11 may not alter the intrinsic properties of AML in normal cell growth conditions, its function may be necessary in conditions of cellular homeostasis disturbance.
To gain insight into the role of SLC7A11 in the TME, we are optimizing a co-culture system with AML, mesenchymal stromal and T cells, evaluating whether the dysregulation of SLC7A11 in AML cells can impair the activation or cytotoxicity of T-cells.

**Copyright Information:** (Online) ISSN: 2572-9241
©2026 The Author(s). HemaSphere is published by John Wiley & Sons Ltd on behalf of European Hematology Association. This is an open access Abstract Book distributed under the Attribution-NonCommercial-NoDerivs (CC BY-NC-ND), which allows third parties to download the articles and share them with others as long as they credit the author and the Abstract Book, but they cannot change the content in any way or use them commercially.
**Abstract Book Citations:** Authors, Title, HemaSphere, 2026;10:(S1):pages. The abstract book DOIs can be found on the "Author Information" page.
**Disclaimer:** Articles published in the journal HemaSphere exclusively reflect the opinions of the authors. The authors are responsible for all content in their abstracts including accuracy of the facts, statements, citing resources, etc.

**Summary/Conclusion**

We identified 24 solute transporters that may impact AML outcomes. Among them, we initially focused on SLC7A11 showing that it does not significantly affect growth in standard culture conditions, but it is required for proliferation under conditions of GSH homeostasis impairment. Ongoing work will determine whether SLC7A11-driven solute mobilization remodels the bone marrow immune landscape, revealing potential metabolic vulnerabilities in AML.

**Copyright Information:** (Online) ISSN: 2572-9241
©2026 The Author(s). HemaSphere is published by John Wiley & Sons Ltd on behalf of European Hematology Association. This is an open access Abstract Book distributed under the Attribution-NonCommercial-NoDerivs (CC BY-NC-ND), which allows third parties to download the articles and share them with others as long as they credit the author and the Abstract Book, but they cannot change the content in any way or use them commercially.
**Abstract Book Citations:** Authors, Title, HemaSphere, 2026;10:(S1):pages. The abstract book DOIs can be found on the "Author Information" page.
**Disclaimer:** Articles published in the journal HemaSphere exclusively reflect the opinions of the authors. The authors are responsible for all content in their abstracts including accuracy of the facts, statements, citing resources, etc.

### (PB2674) SCREENING MITOCHONDRIAL-TARGETING COMPOUNDS IN AML IDENTIFIES MITOTAM AS A BROAD VENETOCLAX-SENSITIZER

**Topic:** 03. Acute myeloid leukemia - Biology & translational research

Juan Coelho-Silva*[1], Diego A Pereira-Martins[2], Gerwin Huls[2], Jan Jacob Schuringa[2], João Agostinho MacHado Neto[1]

*[1]University of Sao Paulo, Department of Pharmacology, Institute of Biomedical Sciences, Sao Paulo, Brazil;[2]University Medical Centre Groningen, Groningen, The Netherlands;*

### Background

Venetoclax resistance is a major barrier in acute myeloid leukemia (AML) and is often driven by adaptive programs that preserve mitochondrial bioenergetics and integrity. As venetoclax priming and apoptotic commitment are tightly coupled to mitochondrial state, disrupting mitochondrial fitness, using mitochondria-targeted agents such as MitoTam, or targeting mitochondrial-supporting pathways (e.g., DHODH and SPHK1), may re-sensitize resistant AML.

### Aims

To assess single-agent activity and venetoclax-combination effects of mitochondrial-targeting compounds across a molecularly diverse AML cell-line panel.

### Methods

Sixteen AML cell line models covering prevalent genetic subtypes were screened, including two in-house venetoclax-resistant models (MV4;11VR and MOLM-13VR) generated by intermittent, stepwise venetoclax escalation and characterized by loss of venetoclax-induced apoptosis with distinct metabolic rewiring (MV4;11VR: glycolytic shift; MOLM-13VR: increased oxidative phosphorylation). Cells were exposed (72h) to MitoTam (TPP-conjugated, mitochondria-targeted tamoxifen derivative), brequinar (DHODH inhibitor) or SK1-In-1 (SPHK1 inhibitor). Combinations with venetoclax were assessed using a mitochondrial metabolic activity readout; interactions were quantified by ZIP (ZIP > +10, synergistic; ZIP < −10, antagonistic). CCLE baseline transcriptomes were integrated with drug-response metrics and correlated pathways were identified by gene set enrichment of ranked gene–response correlations. Mechanistic follow-up of MitoTam used flow cytometry (Annexin V; JC-1 for mitochondrial membrane potential, MMP).

### Results

Brequinar and SK1-In-1 showed heterogeneous single-agent activity and variable interactions with venetoclax (ZIP spanning antagonism to synergy). MitoTam showed consistent activity (72h IC50 0.18–1.83µM), with potency maintained in resistant derivatives (MOLM-13VR 0.23µM vs MOLM-13 0.28µM; MV4;11VR 0.56µM vs MV4;11 0.27µM) and high sensitivity in Kasumi-1 (0.18 µM). Time-course analyses showed increasing potency from 24/48/72h: MOLM-13 0.82/0.68/0.28 µM and MOLM-13VR 0.91/0.69/0.23µM; MV4;11 0.60/0.48/0.27µM and MV4;11VR 1.3/0.83/0.56µM. MitoTam yielded uniformly positive ZIP scores with venetoclax across all models (range 2.76–16.37; median 10.82), including MOLM-13VR (13.77) and MV4;11VR (8.59). CCLE baseline transcriptomics revealed that MitoTam resistance tracked with a mitochondrial “fitness”/stress-adaptation phenotype, including stemness, PRC2-linked epigenetic repression programs, and HIF2-associated hypoxia signaling, consistent with adaptive survival states. MitoTam sensitivity aligned with more differentiated myeloid states and oncogenic drivers, including GMP-like programs, MLL-rearranged leukemia signatures, and MYC target, supporting a vulnerability in proliferative, GMP/MLL-driven contexts. Functionally, MitoTam increased Annexin V positivity after 48h in both parental and resistant contexts, and JC-1 revealed a dose-dependent loss of MMP, reaching 90–95% at 5 µM in MV4;11 and Kasumi-1 (P<0.001).

**Copyright Information:** (Online) ISSN: 2572-9241
©2026 The Author(s). HemaSphere is published by John Wiley & Sons Ltd on behalf of European Hematology Association. This is an open access Abstract Book distributed under the Attribution-NonCommercial-NoDerivs (CC BY-NC-ND), which allows third parties to download the articles and share them with others as long as they credit the author and the Abstract Book, but they cannot change the content in any way or use them commercially.
**Abstract Book Citations:** Authors, Title, HemaSphere, 2026;10:(S1):pages. The abstract book DOIs can be found on the “Author Information” page.
**Disclaimer:** Articles published in the journal HemaSphere exclusively reflect the opinions of the authors. The authors are responsible for all content in their abstracts including accuracy of the facts, statements, citing resources, etc.

### Summary/Conclusion

In a heterogeneous AML panel including acquired venetoclax-resistant models, MitoTam demonstrated time-dependent cytotoxicity and uniformly positive synergy with venetoclax by ZIP scoring. Annexin V and JC-1 assays support mitochondrial dysfunction and apoptosis as associated features. These findings support mitochondrial targeting with MitoTam as a strategy to overcome venetoclax resistance in AML.

**Copyright Information:** (Online) ISSN: 2572-9241
©2026 The Author(s). HemaSphere is published by John Wiley & Sons Ltd on behalf of European Hematology Association. This is an open access Abstract Book distributed under the Attribution-NonCommercial-NoDerivs (CC BY-NC-ND), which allows third parties to download the articles and share them with others as long as they credit the author and the Abstract Book, but they cannot change the content in any way or use them commercially.
**Abstract Book Citations:** Authors, Title, HemaSphere, 2026;10:(S1):pages. The abstract book DOIs can be found on the "Author Information" page.
**Disclaimer:** Articles published in the journal HemaSphere exclusively reflect the opinions of the authors. The authors are responsible for all content in their abstracts including accuracy of the facts, statements, citing resources, etc.

### (PB2675) ALKBH1 EXPRESSION MARKS RNA/TRANSLATION AND MITOCHONDRIAL GENE PROGRAMS IN AML AND SUPPORTS A THERAPEUTICALLY TRACTABLE VULNERABILITY

**Topic:** 03. Acute myeloid leukemia - Biology & translational research

Juan Coelho-Silva*[1], Isabelle Santos[1], Diego A Pereira-Martins[2], Gerwin Huls[2], Jan Jacob Schuringa[2], João Agostinho MacHado Neto[1]

*[1]University of Sao Paulo, Department of Pharmacology, Institute of Biomedical Sciences, Sao Paulo, Brazil;[2]University Medical Centre Groningen, Groningen, The Netherlands;*

### Background

ALKBH1 (AlkB homolog 1), an Fe(II)/α-ketoglutarate–dependent dioxygenase of the AlkB family, mediates nucleic acid demethylation and has emerged as a leukemia-promoting RNA-modifying enzyme that regulates tRNA decoding, codon-biased translation, leukemia-initiating cell self-renewal, mitochondrial fitness, and venetoclax resistance, suggesting a potentially druggable dependency in acute myeloid leukemia (AML).

### Aims

To integrate clinical transcriptomic evidence with functional pharmacology to determine whether ALKBH1 represents a therapeutically actionable vulnerability in AML and to evaluate the pre-clinical activity of ALKBH1-In-1, a selective small-molecule ALKBH1 inhibitor.

### Methods

ALKBH1 expression was analyzed in TCGA and BeatAML cohorts. Overall survival was assessed by Kaplan–Meier analysis. Differential expression compared ALKBH1-high versus low cases, and correlated genes were identified (Pearson r>0.5, p<0.001). ALKBH1-In-1 was evaluated across 16 AML cell-line models representing prevalent genetic subtypes; 72h IC50 values were determined using mitochondrial metabolic activity readouts, and venetoclax interactions were quantified using the ZIP model (ZIP > +10 synergistic; ZIP < −10 antagonistic). CCLE baseline transcriptomes were integrated with drug-response metrics, and associated pathways were identified by gene set enrichment of ranked correlations. Apoptosis (Annexin V/PI) and mitochondrial membrane potential (JC-1) were assessed by flow cytometry.

### Results

ALKBH1 expression was not associated with overall survival in TCGA or BeatAML (p=0.163 and p=0.334). However, ALKBH1-high cases consistently mapped to RNA/translation-centered transcriptional programs, including enrichment of RNA-processing and mRNA metabolism factors (SART3, MFAP1, CSTF1, SMG8/UPF1), translation regulators (EIF1, EIF4A1), and stress-responsive transcription factors (JUN, ATF3, KLF10). Correlated genes included mitochondrial expression and maintenance components (PARS2, MRPL35, C12orf65, YRDC, DNM1L), linking ALKBH1 to mitochondrial homeostasis in patient-derived datasets. ALKBH1-In-1 showed single-agent activity across most AML models (72h IC50 0.18–1.87 µM), with limited response in BCR::ABL1 models (K562, MEG-01). Venetoclax combinations displayed heterogeneous interactions (ZIP −12.43 to 9.09), indicating context-dependent combinability. Transcriptomic correlates associated resistance with mitochondrial and bioenergetic programs, including TCA cycle, oxidative phosphorylation, mitochondrial protein processing, and cholesterol homeostasis, whereas sensitivity aligned with GMP/MLL-fusion signatures, E2F-driven cell-cycle activity, and RNA-processing programs. Functionally, ALKBH1-In-1 induced dose-dependent apoptosis after 48h (P<0.001) and reduced mitochondrial membrane potential, consistent with engagement of mitochondrial vulnerability.

**Copyright Information:** (Online) ISSN: 2572-9241
©2026 The Author(s). HemaSphere is published by John Wiley & Sons Ltd on behalf of European Hematology Association. This is an open access Abstract Book distributed under the Attribution-NonCommercial-NoDerivs (CC BY-NC-ND), which allows third parties to download the articles and share them with others as long as they credit the author and the Abstract Book, but they cannot change the content in any way or use them commercially.
**Abstract Book Citations:** Authors, Title, HemaSphere, 2026;10:(S1):pages. The abstract book DOIs can be found on the “Author Information” page.
**Disclaimer:** Articles published in the journal HemaSphere exclusively reflect the opinions of the authors. The authors are responsible for all content in their abstracts including accuracy of the facts, statements, citing resources, etc.

**Summary/Conclusion**

ALKBH1 defines an RNA-processing and translation-linked transcriptional state enriched for mitochondrial gene-expression and homeostasis programs. Pre-clinical data demonstrate activity of the selective inhibitor ALKBH1-In-1 across AML models, including venetoclax-resistant contexts, supporting ALKBH1 as a mechanistically grounded and biomarker-informable therapeutic vulnerability in AML.

**Copyright Information:** (Online) ISSN: 2572-9241
©2026 The Author(s). HemaSphere is published by John Wiley & Sons Ltd on behalf of European Hematology Association. This is an open access Abstract Book distributed under the Attribution-NonCommercial-NoDerivs (CC BY-NC-ND), which allows third parties to download the articles and share them with others as long as they credit the author and the Abstract Book, but they cannot change the content in any way or use them commercially.
**Abstract Book Citations:** Authors, Title, HemaSphere, 2026;10:(S1):pages. The abstract book DOIs can be found on the "Author Information" page.
**Disclaimer:** Articles published in the journal HemaSphere exclusively reflect the opinions of the authors. The authors are responsible for all content in their abstracts including accuracy of the facts, statements, citing resources, etc.

### (PB2676) RESVERATROL SYNERGISTICALLY POTENTIATES VENETOCLAX-INDUCED APOPTOSIS IN ACUTE MYELOID LEUKEMIA BY CO-TARGETING THE C-MYC/MCL-1 SIGNALING AXIS

**Topic:** 03. Acute myeloid leukemia - Biology & translational research

Liyuan Li[1], Weiguang Cui*[1], Zhenzhen Wang[1], Zhuofeng Mao[2], Xiaoyan Zhang[1], Lin Yang[1, 3], Jianmin Luo[1, 3]

*[1]The Second Hospital of Hebei Medical University, Department of Hematology, Shijiazhuang, China;[2]The Second Hospital of Hebei Medical University, Department of Neurology, Shijiazhuang, China; [3]Hebei Institute of Hematology, Hebei Key Laboratory of Hematology, Shijiazhuang, China;*

### Background

The clinical introduction of the Bcl-2 inhibitor Venetoclax (VEN) has fundamentally shifted the therapeutic landscape for acute myeloid leukemia (AML). However, the emergence of acquired resistance—frequently characterized by the compensatory upregulation of MCL-1—critically limits its long-term efficacy. Resveratrol (RSV), a natural polyphenolic compound, has garnered attention for its multifaceted anti-tumor properties, including the modulation of pro-survival pathways. This study aims to investigate whether RSV can augment the anti-leukemic potency of VEN and to decipher the molecular underpinnings of this synergy, specifically focusing on the c-myc/MCL-1 regulatory loop.

### Aims

This study aims to investigate whether RSV can sensitize AML cells to VEN and to elucidate the molecular mechanism, specifically focusing on whether the synergy is achieved through co-targeting the c-myc/MCL-1 signaling axis.

### Methods

The synergistic potential of RSV and VEN was evaluated in human AML cell lines (THP-1 and MOLM-13). Cell proliferative capacity was assessed via CCK-8 assays, and the nature of the pharmacological interaction was rigorously quantified using the Combination Index (CI) based on the Chou-Talalay method. Apoptotic induction was characterized through Annexin V/PI dual-staining flow cytometry. To further elucidate the underlying mechanism, Western blotting was employed to examine the expression profiles of c-myc and its downstream effector MCL-1, alongside apoptotic execution markers including cleaved caspase-3 and PARP-1.

### Results

Both RSV and VEN as monotherapies exhibited dose- and time-dependent attenuation of AML cell viability. The integration of RSV significantly sensitized AML cells to VEN, yielding CI values markedly below 1.0, indicative of potent synergism across a broad range of concentrations. This combinatorial approach led to a profound escalation in apoptotic rates compared to single-agent treatments. Mechanistically, RSV-mediated sensitization was associated with a significant downregulation of c-myc expression, which subsequently abrogated the expression of the anti-apoptotic protein MCL-1. This targeted depletion of MCL-1 effectively lowered the mitochondrial apoptotic threshold, thereby facilitating the robust activation of the caspase cascade triggered by VEN-mediated Bcl-2 inhibition.

**Copyright Information:** (Online) ISSN: 2572-9241
©2026 The Author(s). HemaSphere is published by John Wiley & Sons Ltd on behalf of European Hematology Association. This is an open access Abstract Book distributed under the Attribution-NonCommercial-NoDerivs (CC BY-NC-ND), which allows third parties to download the articles and share them with others as long as they credit the author and the Abstract Book, but they cannot change the content in any way or use them commercially.
**Abstract Book Citations:** Authors, Title, HemaSphere, 2026;10:(S1):pages. The abstract book DOIs can be found on the "Author Information" page.
**Disclaimer:** Articles published in the journal HemaSphere exclusively reflect the opinions of the authors. The authors are responsible for all content in their abstracts including accuracy of the facts, statements, citing resources, etc.

### Summary/Conclusion

Our findings demonstrate that Resveratrol exerts a potent synergistic effect with Venetoclax in AML by disrupting the c-myc/MCL-1 signaling axis. By simultaneously targeting Bcl-2 and MCL-1-dependent survival pathways, this combination strategy provides a compelling biochemical rationale for overcoming Venetoclax resistance and warrants further clinical exploration as a novel therapeutic intervention for AML.

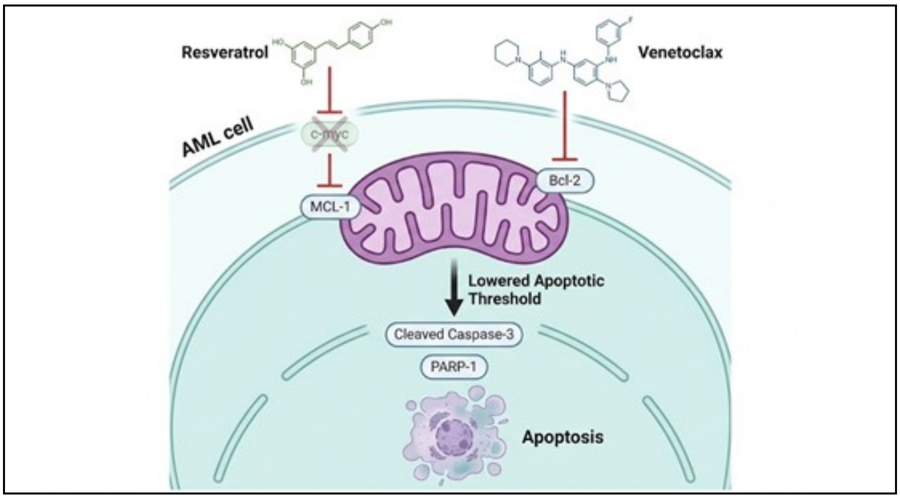

Copyright Information: (Online) ISSN: 2572-9241
©2026 The Author(s). HemaSphere is published by John Wiley & Sons Ltd on behalf of European Hematology Association. This is an open access Abstract Book distributed under the Attribution-NonCommercial-NoDerivs (CC BY-NC-ND), which allows third parties to download the articles and share them with others as long as they credit the author and the Abstract Book, but they cannot change the content in any way or use them commercially.
Abstract Book Citations: Authors, Title, HemaSphere, 2026;10:(S1):pages. The abstract book DOIs can be found on the “Author Information” page.
Disclaimer: Articles published in the journal HemaSphere exclusively reflect the opinions of the authors. The authors are responsible for all content in their abstracts including accuracy of the facts, statements, citing resources, etc.

### (PB2677) FAULT TREE ANALYSIS IDENTIFIES HIGH-RISK PATTERNS OF INDUCTION FAILURE AND EARLY MORTALITY IN AML

**Topic:** 03. Acute myeloid leukemia - Biology & translational research

Mobina Shrestha*[1], Salina Dahal[2, 2], Vishal Mandal[3, 3]

*[1]Sentara Albemarle Medical Center, Elizabeth City, United States of America;[2]The Ohio State University, Columbus, United States of America; [3]University of Missouri, Columbia, United States of America;*

### Background

Risk assessment in acute myeloid leukemia (AML) relies primarily on cytogenetic and molecular classification. However, early mortality and induction failure remain frequent, particularly among older patients. In clinical practice, the worst outcomes are often seen when aggressive leukemia occurs in patients who are already physically weak.

### Aims

In this study, we aimed to determine whether constellation-based modeling could identify extreme-risk subgroups in AML and refine prognostic assessment alongside ELN classification.

### Methods

We analyzed 942 patients from the BeatAML2 dataset with available genomic, clinical, and survival data. Using a constellation-based modeling approach derived from fault tree methodology, we identified specific combinations of baseline mutations and clinical variables associated with induction failure and 60-day mortality. Extreme-risk status was defined by the presence of at least one high-impact constellation. Multivariable Cox regression models adjusted for cytogenetic-molecular risk classification.

### Results

Among 688 evaluable patients, 38.7% failed to achieve remission. RUNX1 and TP53 mutations were strongly associated with induction failure, particularly when combined with low platelet counts. Among 910 patients with known early outcomes, 60-day mortality was 10%. Advanced age together with low albumin or renal dysfunction identified subgroups with significantly elevated early mortality. An extreme-risk constellation was present in 32.6% of patients and was associated with significantly inferior overall survival (median 297 vs 861 days, $p<0.0001$). After adjustment for cytogenetic-molecular risk classification, extreme-risk status remained independently associated with survival (Hazard Ratio: 2.03, $p<0.0001$) and improved model discrimination.

### Summary/Conclusion

We found that in AML, the poorest outcomes are often seen when high-risk genetic features and patient frailty are present together. Using constellation-based modeling, we identified a distinct extreme-vulnerability state affecting about one-third of patients. This approach provides additional prognostic insight when considered alongside ELN risk groups. It may help refine risk assessment at diagnosis and highlight patients who may benefit from closer monitoring, alternative treatment strategies, or earlier palliative care planning.

**Copyright Information:** (Online) ISSN: 2572-9241
©2026 The Author(s). HemaSphere is published by John Wiley & Sons Ltd on behalf of European Hematology Association. This is an open access Abstract Book distributed under the Attribution-NonCommercial-NoDerivs (CC BY-NC-ND), which allows third parties to download the articles and share them with others as long as they credit the author and the Abstract Book, but they cannot change the content in any way or use them commercially.
**Abstract Book Citations:** Authors, Title, HemaSphere, 2026;10:(S1):pages. The abstract book DOIs can be found on the “Author Information” page.
**Disclaimer:** Articles published in the journal HemaSphere exclusively reflect the opinions of the authors. The authors are responsible for all content in their abstracts including accuracy of the facts, statements, citing resources, etc.

### (PB2679) CELL TYPE AWARE MULTIPLE INSTANCE LEARNING REVEALS ASSOCIATIONS BETWEEN GENETIC ALTERATIONS AND BONE MARROW CYTOMORPHOLOGY IN AML

**Topic:** 03. Acute myeloid leukemia - Biology & translational research

Mikko Purhonen*[1, 2, 3], Stas Tatun[1, 2, 3], Lotta Joutsi-Korhonen[3], Anna Lempiäinen[3], Sanna Siitonen[3], Oscar Brück[1, 2, 3]

*[1]Hematoscope Lab, Comprehensive Cancer Center, Helsinki University Hospital, Helsinki, Finland;[2]University of Helsinki, Department of Oncology, Helsinki, Finland; [3]University of Helsinki and Helsinki University Hospital, Department of Clinical Chemistry, Helsinki, Finland;*

### Background

Cytogenetic and molecular abnormalities are central for disease classification and patient risk stratification in acute myeloid leukemia (AML). Advances in digital pathology have permitted to study bone marrow (BM) and peripheral blood (PB) morphology in greater detail. This may provide novel insights to understand genetic abnormalities in AML.

### Aims

We aimed to learn the BM cytomorphological fingerprints associated with common genetic alterations at the cell level.

### Methods

We included 565 diagnostic *de novo* and secondary AML patients with BM aspirate slide from Helsinki University Hospital, Finland. We digitized representative areas of BM slides at 100x magnification and oil immersion. We analyzed images with the Cellbytes application performing cell detection and cell and dysplasia classification, utilizing Vision Transformer (ViT)-backbone, trained on 27 million unlabeled single-cell images.
We employed cell type aware multiple instance learning (MIL) with single-cell image embeddings to predict genomic and cytogenetic alterations. We evaluated the model performance across 5-fold cross-validation and report AUROC as mean ± SD across folds and analyzed the cell type importance based on the model logits. We studied the association of interpretable cytomorphological features such as cell differentials, occurrence of dysplasia, and measures of cell size and shape with genetic alterations (Wilcoxon rank-sum test).

### Results

The models demonstrated high performance for several alterations. Consistent with prior studies, we could predict occurrence of *PML::RARA* (AUROC 0.99 ± 0.02), *CBFB::MYH11* (0.97 ± 0.03), *NPM1*mut (0.90 ± 0.03), *TP53*mut (0.81 ± 0.08), *RUNX1::RUNX1T1* (0.77 ± 0.04) and *FLT3-ITD/TKD* (0.67 ± 0.06). However, we demonstrate reliable performance for monosomy 5 / del 5q (0.84 ± 0.04), *KIT* (0.83 ± 0.13), *IDH2* (0.79 ± 0.08), and complex karyotype (0.77 ± 0.06). Cell type importance analysis revealed high attention in the granulocytic and monocytic lineages (Fig. 1A).
Next, we decoded molecular-morphologic correlations. As expected, *PML::RARA* was associated with higher proportion of promyelocytes (median 32.6% vs. 0.85%, $p<0.001$) and increased dysplastic features (hypergranularity, bilobed nucleus, and Auer rods) in blasts, promyelocytes and myelocytes. The mutation model focused on similar features (Fig 1B). *NPM1*mut was associated with cup-shaped blasts (Fig. 1B), increased proportion of hypergranulated immature myeloid cells, and increased nuclear-cytoplasmic (N:C)-ratio in promyelocytes, myelocytes and metamyelocytes. In contrary, *NPM1*wt was associated with blasts with prominent nuclei (Fig. 1B) and increased proportion of vacuolated immature myeloid cells. *TP53*mut was associated with cytoplasmic vacuolization in blasts and across granulocyte maturation (Fig. 1B). *IDH2* was associated with blasts with bilobed nucleus and increased N:C-ratio in immature and maturing granulocytes (Fig 1B).

**Copyright Information:** (Online) ISSN: 2572-9241
©2026 The Author(s). HemaSphere is published by John Wiley & Sons Ltd on behalf of European Hematology Association. This is an open access Abstract Book distributed under the Attribution-NonCommercial-NoDerivs (CC BY-NC-ND), which allows third parties to download the articles and share them with others as long as they credit the author and the Abstract Book, but they cannot change the content in any way or use them commercially.
**Abstract Book Citations:** Authors, Title, HemaSphere, 2026;10:(S1):pages. The abstract book DOIs can be found on the "Author Information" page.
**Disclaimer:** Articles published in the journal HemaSphere exclusively reflect the opinions of the authors. The authors are responsible for all content in their abstracts including accuracy of the facts, statements, citing resources, etc.

### Summary/Conclusion

The results reflect how the interaction of common genetic alterations and BM cytomorphology may be better understood by applying advanced modelling. While we do not believe that image-based prediction should replace sequencing, rapid identification of *PML::RARA* or other key genetic changes could affect treatment decisions given its superior speed. Future work will aim to validate the results in an additional dataset and extend analyses to PB smears and granulocyte dysplasia.

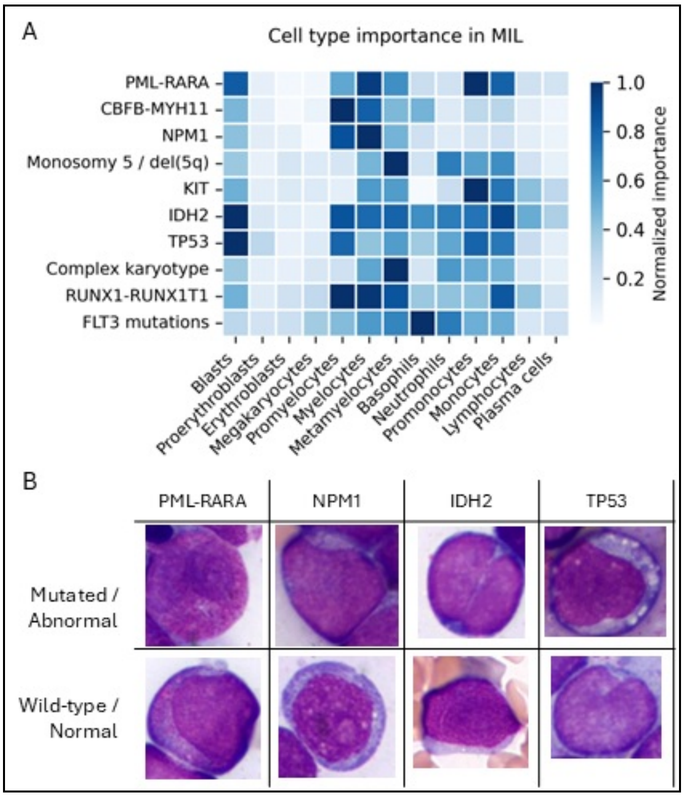

Figure 1. (A) Relative cell type importance, normalized independently for each predicted outcome. (B) Representative cell images corresponding to high attention scores for the predicted outcome.

**Copyright Information:** (Online) ISSN: 2572-9241
©2026 The Author(s). HemaSphere is published by John Wiley & Sons Ltd on behalf of European Hematology Association. This is an open access Abstract Book distributed under the Attribution-NonCommercial-NoDerivs (CC BY-NC-ND), which allows third parties to download the articles and share them with others as long as they credit the author and the Abstract Book, but they cannot change the content in any way or use them commercially.
**Abstract Book Citations:** Authors, Title, HemaSphere, 2026;10:(S1):pages. The abstract book DOIs can be found on the "Author Information" page.
**Disclaimer:** Articles published in the journal HemaSphere exclusively reflect the opinions of the authors. The authors are responsible for all content in their abstracts including accuracy of the facts, statements, citing resources, etc.

### (PB2680) GUT MICROBIOME TRAJECTORIES DURING HIGH- AND LOW-INTENSITY FRONTLINE AML THERAPY AND ASSOCIATED OUTCOMES

**Topic:** 03. Acute myeloid leukemia - Biology & translational research

Kuo-Kai Chin*[1], Kenton Wu[2], Zoe Katsamakis[2], Anqi Dai[2], Karen Zhao[3], Mirae Baichoo[2], Christopher Ford[4], Mary-Jane Lombardo[4], Elizabeth Halvorsen[4], Lindsay Rutte[4], Eytan Stein[1], Natalie Smith[5], Aaron Goldberg[1], Jonathan Peled[2]

*[1]Memorial Sloan Kettering Cancer Center, Leukemia Service, Division of Hematologic Malignancies, New York, United States of America; [2]Memorial Sloan Kettering Cancer Center, Bone Marrow Transplantation Service, Division of Hematologic Malignancies, New York, United States of America; [3]Zucker School of Medicine at Hofstra/Northwell, Hempstead, United States of America; [4]Seres Therapeutics, Cambridge, United States of America; [5]City of Hope, Division of Leukemia, Department of Hematology & Hematopoietic Cell Transplantation, Duarte, United States of America;*

### Background

Gut microbiome features predict outcomes in patients with AML undergoing alloHCT, including bloodstream infections. In contrast, the role of the microbiome during frontline AML therapy prior to alloHCT is less well-defined, particularly in the setting of low-intensity induction regimens. Prior microbiome studies have largely focused on high-intensity (HI) chemotherapy (e.g. 7+3), whose sequelae include profound neutropenia, mucosal barrier injury, microbial domination, and consequent bloodstream infections. However, low-intensity (LI) regimens (e.g. azacitidine and venetoclax) are increasingly used, and their effect on microbiome disruption and infectious outcomes have not been reported.

### Aims

To characterize intestinal microbiome trajectories during HI and LI AML therapy and evaluate associations with infectious outcomes.

### Methods

We prospectively collected fecal samples from patients with newly diagnosed AML receiving HI or LI therapy at our center (2023-2025). Fecal microbiota composition was profiled by whole metagenomic shotgun sequencing (mean 12M reads/sample). Alpha-diversity was quantified using the inverse Simpson index. Beta-diversity was assessed using Bray-Curtis dissimilarity. Longitudinal changes were modeled using generalized estimating equations.

**Copyright Information:** (Online) ISSN: 2572-9241
©2026 The Author(s). HemaSphere is published by John Wiley & Sons Ltd on behalf of European Hematology Association. This is an open access Abstract Book distributed under the Attribution-NonCommercial-NoDerivs (CC BY-NC-ND), which allows third parties to download the articles and share them with others as long as they credit the author and the Abstract Book, but they cannot change the content in any way or use them commercially.
**Abstract Book Citations:** Authors, Title, HemaSphere, 2026;10:(S1):pages. The abstract book DOIs can be found on the "Author Information" page.
**Disclaimer:** Articles published in the journal HemaSphere exclusively reflect the opinions of the authors. The authors are responsible for all content in their abstracts including accuracy of the facts, statements, citing resources, etc.

**Results**

Forty-seven patients contributed 99 samples in the analysis window (D-7 to D+50 relative to induction, HI n=27; LI n=20). Mean age was 57.2 years; LI patients were older (67.2 vs 49.8 years, p<0.001). Response rates did not differ significantly between groups (CR/CRi: HI n=18, 67%; LI n=12, 60%; p=0.08).
As expected, HI was associated with more fever (n=27, 100% vs n=9, 45%), febrile neutropenia (FN; n=27, 100% vs n=8, 40%), and documented infections (composite of bloodstream infection and stool PCR positivity, n=15, 56% vs n=2, 10%) (fever/FN p<0.001, infection p=0.002). All of the ICU admissions occurred in the HI group (n=4, 17%).
At baseline (D-7 to D+3), α-diversity did not differ between HI and LI (p=1.0). α-diversity declined in both HI and LI patients comparably (Figure 1, p=0.91). Higher α-diversity was associated with fewer fever and FN days in LI patients in a stratified analysis (IRR 0.90, p=0.01 for both).
Global β-diversity did not differ between HI and LI groups by PERMANOVA before or after baseline (F=1.37, F=1.42, p=1 both). However, HI patients exhibited greater within-patient compositional displacement from baseline (median Bray-Curtis 0.51 vs 0.01, p=0.01) suggesting greater disruption of microbiome composition.
Domination events (relative abundance ≥30%) were numerically more frequent in HI patients (14/27, 52% vs 5/20, 20%, p=0.08). The most commonly dominating genera were *Bacteroides* (n=6, 4 HI, 2 LI), *Enterococcus* (n=6, 3 HI, 3 LI), and *Streptococcus* (n=6, all HI). Microbiota domination was associated with increased FN days (median 5 vs 2 days, p=0.026) and numerically higher infection rates (44.1% vs 32.1%, p=0.55). In most cases, FN and/or infection preceded documented domination, coinciding with initiation of broad-spectrum antibiotics.

**Summary/Conclusion**

During frontline AML therapy, both HI and LI regimens are accompanied by declining α-diversity. However, HI therapy is associated with greater microbiome displacement from baseline and more frequent domination events, which correlate with FN, infection and exposure to broad-spectrum antibiotics. These findings support investigation of microbiome-targeted therapeutic strategies to mitigate toxicity in AML, including with LI treatment.

**Copyright Information:** (Online) ISSN: 2572-9241
©2026 The Author(s). HemaSphere is published by John Wiley & Sons Ltd on behalf of European Hematology Association. This is an open access Abstract Book distributed under the Attribution-NonCommercial-NoDerivs (CC BY-NC-ND), which allows third parties to download the articles and share them with others as long as they credit the author and the Abstract Book, but they cannot change the content in any way or use them commercially.
**Abstract Book Citations:** Authors, Title, HemaSphere, 2026;10:(S1):pages. The abstract book DOIs can be found on the “Author Information” page.
**Disclaimer:** Articles published in the journal HemaSphere exclusively reflect the opinions of the authors. The authors are responsible for all content in their abstracts including accuracy of the facts, statements, citing resources, etc.

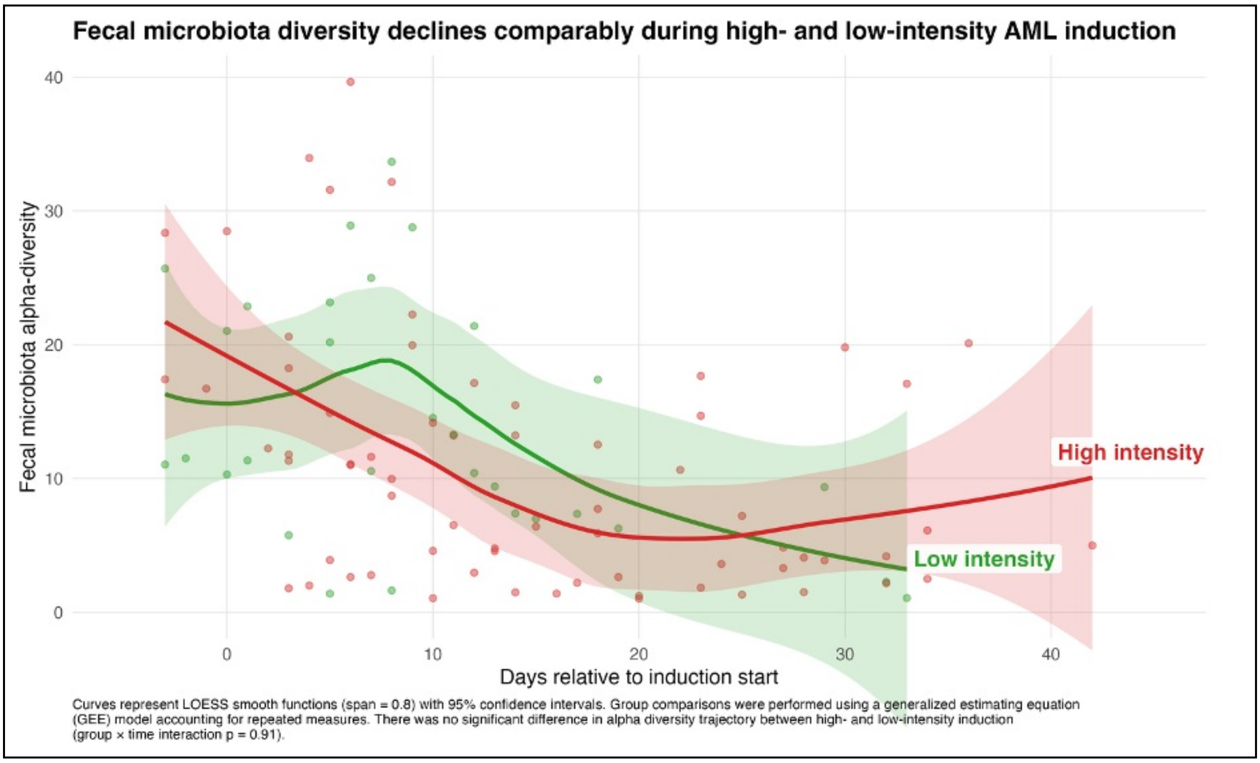

**Copyright Information:** (Online) ISSN: 2572-9241
©2026 The Author(s). HemaSphere is published by John Wiley & Sons Ltd on behalf of European Hematology Association. This is an open access Abstract Book distributed under the Attribution-NonCommercial-NoDerivs (CC BY-NC-ND), which allows third parties to download the articles and share them with others as long as they credit the author and the Abstract Book, but they cannot change the content in any way or use them commercially.
**Abstract Book Citations:** Authors, Title, HemaSphere, 2026;10:(S1):pages. The abstract book DOIs can be found on the "Author Information" page.
**Disclaimer:** Articles published in the journal HemaSphere exclusively reflect the opinions of the authors. The authors are responsible for all content in their abstracts including accuracy of the facts, statements, citing resources, etc.

### (PB2681) CPX-351 INHIBITS FUNGAL GROWTH AND PREVENTS INFECTIONS VIA A HEALTHY INTER-KINGDOM NETWORK

**Topic:** 03. Acute myeloid leukemia - Biology & translational research

Luigina Romani*[1], Marilena Pariano[1], Emilia Nunzi[1], Luana Fianchi[2], Livio Pagano[2]

*[1]University of Perugia, Department of Medicine and Surgery, Perugia, Italy; [2]Fondazione Policlinico Universitario A. Gemelli-IRCCS- Università Cattolica del Sacro Cuore-, Roma, Italy;*

### Background

CPX-351 (also known as VYXEOS® or Vyxeos liposomal), a dual-drug liposomal encapsulation of cytarabine and daunorubicin, significantly improves survival and remission rates compared with “7+3” combination with a good safety profile in AML patients with high-risk features (t-AML and AML-MRC) and facilitates allo-HCT for the majority of patients independent of age. Emerging evidence indicates that CPX-351 affords additional benefits compared with conventional combination with cytarabine plus daunorubicin (3+7), namely prevention of intestinal damage and microbial dysbiosis

### Aims

The lower incidence of fungal infections and the infection-related mortality observed in AML patients treated with CPX-351, prompted us to investigate whether CPX-351 prevents infectious complications by modulating the mycobiota and the host response to fungi

### Methods

We have addressed this issue by comparing the fungal composition in the lung and feces of mice treated with CPX-351 or the "7+3" combination and its relationship with bacterial dysbiosis and resistance to infection.

### Results

On average, 66,988 reads of the Internal Transcribed Spacer unit (ITS)2 of the rDNA operon were generated for both samples by next-generation sequencing and quantitative PCR. At the phylum level, Ascomycota (*Candida, Aspergillus, Penicillium and Fusarium* genera) were less abundant after CPX-351 as compared to “7+3” treatment (P≤ 0.05, *Candida* spp. in both samples; P=0.01 *Penicillium* in both samples; P≤ 0.05, *Aspergillus* spp. in both samples; P= 0.05 *Fusarium* in the feces, CPX-351 vs “7+3”). Overall, combined with the decreased *Candida* and *Aspergillus* 18S rDNA expression levels observed by quantitative PCR in CPX-351-treated animals, these results indicate that different from the "7+3" combination, CPX-351 reduced the fungal biomass and prevented the overgrowth of fungal pathobionts leading to infections and dissemination, an effect to which the promotion of bacterial eubiosis, the activation of innate immune resistance and a direct antifungal activity contributed

### Summary/Conclusion

Treatment with CPX-351 appears to be associated with a healthy microbial ecosystem at different mucosal sites that relies on a healthy inter-kingdom signaling network whose ingredients are controlled inflammation, barrier integrity, immune tolerance, and microbial community stability.

**Copyright Information:** (Online) ISSN: 2572-9241
©2026 The Author(s). HemaSphere is published by John Wiley & Sons Ltd on behalf of European Hematology Association. This is an open access Abstract Book distributed under the Attribution-NonCommercial-NoDerivs (CC BY-NC-ND), which allows third parties to download the articles and share them with others as long as they credit the author and the Abstract Book, but they cannot change the content in any way or use them commercially.
**Abstract Book Citations:** Authors, Title, HemaSphere, 2026;10:(S1):pages. The abstract book DOIs can be found on the “Author Information” page.
**Disclaimer:** Articles published in the journal HemaSphere exclusively reflect the opinions of the authors. The authors are responsible for all content in their abstracts including accuracy of the facts, statements, citing resources, etc.

### (PB2682) INTEGRATION OF MULTIPLE RNA-SEQ DATASETS ENHANCES DEEP PROFILING OF NPM1-MUTATED FLT3-ITD NEGATIVE AML

**Topic:** 03. Acute myeloid leukemia - Biology & translational research

Matteo Caridi*[1, 2], Gaetano Cimino[3], William Villiers[4], Christian Rausch[5], Francesca Milano[3], Samaneh Boroumand[3, 6], Roberta Ranieri[3], Michael Lim[4], Sara De Santis[7], Valeria Cardinali[3], Phoebe Aucken[4], Maja Rothenberg-Thurley[5], Annika Dufour[5], Michaela Neusser[5, 8], Stephanie Schneider[5, 8], Maria Cristina Sauerland[9], Dennis Görlich[9], Utz Krug[10], Wolfgang E. Berdel[11], Bernhard Wörmann[12], Wolfgang Hiddemann[8, 13, 14], Jan Braess[15], Karsten Spiekermann[5, 13, 14, 16], Klaus Metzeler[17], Tobias Herold[5, 18], Richard Dillon[4], Maria Paola Martelli[3]

*[1]Hematology Unit-ASL Viterbo, Santa Rosa Hospital, Viterbo, Italy; [2]Department of Physiology and Pharmacology, La Sapienza University of Rome, Rome, Italy; [3]Hematology and Clinical Immunology section, Center for Hemato-Oncology Research, Department of Medicine and Surgery, Perugia, Italy; [4]Department of Medical and Molecular Genetics, King's College, London, United Kingdom; [5]Department of Medicine III, University Hospital, LMU Munich, Germany; [6]Department of hematology and blood banking, Faculty of Medicine, Mashhad University of Medical Sciences, Mashhad, Iran; [7]Immunohematology and Transfusion Medicine Service (SIT), Santa Maria Hospital, Terni, Italy; [8]Institute of Human Genetics, University Hospital, LMU Munich, Germany; [9]Institute of Biostatistics and Clinical Research, University of Münster, Münster, Germany;[10]DKMS Collection Center, Cologne, Germany; [11]Department of Medicine A, University Hospital Münster, Münster, Germany; [12]German Society of Hematology and Oncology, Berlin, Germany; [13]German Cancer Consortium (DKTK), Partner Site Munich, Munich, Germany; [14]German Cancer Research Center (DKFZ), Heidelberg, Germany; [15]Department of Oncology and Hematology, Hospital Barmherzige Brüder, Regensburg, Germany; [16]Bavarian Cancer Research Center (BZKF), Munich, Germany; [17]Department of Hematology, Cellular Therapy, Hemostaseology and Infectious Diseases, University Hospital Leipzig, Germany; [18]Department of Hematology, Oncology, and Stem-Cell Transplantation, Munich Hospital Bogenhausen, Munich Municipal Hospital Group, Munich, Germany;*

### Background

Acute Myeloid Leukemia (AML) is an aggressive malignancy with limited survival despite advances in its characterization and treatment. Nucleophosmin (*NPM1*) mutations without *FLT3*-ITD (*NPM1*$^{mut}$ / *FLT3*-ITD$^{neg}$) define a subgroup with favorable outcomes when treated with chemotherapy alone. However, up to 40% of these patients relapse. We analyzed the biological landscape of this clinically relevant subgroup using an integrated RNA-seq dataset to identify novel prognostic and biological biomarkers and therapeutic insights.

### Aims

Three public and one unpublished RNA-seq datasets were included in the study. We created the 'Gu' algorithm (EHA2025 - PS1463) to generate an n = 894 unified RNA-seq dataset with strong biological coherence. Several bioinformatic analyses were performed to investigate the clinically relevant *NPM1*$^{mut}$ / *FLT3*-ITD$^{neg}$ AML subgroup, including drug sensitivity which was inferred using the pRRophetic algorithm. As a validation cohort, gene expression profiling by microarray of 562 patients with AML treated in the German AMLCG 1999 trial was used (GSE37642).

**Copyright Information:** (Online) ISSN: 2572-9241
©2026 The Author(s). HemaSphere is published by John Wiley & Sons Ltd on behalf of European Hematology Association. This is an open access Abstract Book distributed under the Attribution-NonCommercial-NoDerivs (CC BY-NC-ND), which allows third parties to download the articles and share them with others as long as they credit the author and the Abstract Book, but they cannot change the content in any way or use them commercially.
**Abstract Book Citations:** Authors, Title, HemaSphere, 2026;10:(S1):pages. The abstract book DOIs can be found on the "Author Information" page.
**Disclaimer:** Articles published in the journal HemaSphere exclusively reflect the opinions of the authors. The authors are responsible for all content in their abstracts including accuracy of the facts, statements, citing resources, etc.

### Methods

The unified dataset demonstrated correct data integration and biological coherence (Fig. 1a), also reproducing the ELN 2022 risk stratification (Fig. 1b). Within *NPM1*$^{mut}$ AML (n = 308, 187 FLT3-wt), the transcriptomic profile was heterogeneous contrasting with other homogeneous entities. The archetypal *NPM1*$^{mut}$ signature, with *HOX* overexpression and *CD34*/*CD133* downregulation was confirmed while novel genes linked to the NPM1$^{mut}$ phenotype were uncovered (Fig. 1c). Within *NPM1*$^{mut}$, we identified a distinct transcriptomic program stratified by *FLT3*-ITD mutational status (Fig. 1d), alongside a set of genes consistently upregulated across all *NPM1*$^{mut}$ cases irrespective of *FLT3*, defining a stable *NPM1*$^{mut}$ "core" signature predominantly driven by *HOX* genes.

### Results

Transcriptomic dissection revealed two subgroups (Fig. 1e): a "*FLT3*$^{LIKE}$" cluster resembling *FLT3*-ITD AML, and a pure *NPM1*$^{mut}$ / *FLT3*-ITD$^{neg}$ cluster enriched in inflammatory/immune genes. *IDH2* mutations prevailed in *FLT3*$^{LIKE}$ and *RAS* in the pure cases.
Further analysis highlighted a markedly "hotter" immune signature profile within the pure *NPM1*$^{mut}$ / *FLT3*-ITD$^{neg}$, whereas *FLT3*$^{LIKE}$ samples appeared relatively "cold", suggesting not only a distinct tumor microenvironment but also a potential divergence in their response to allo-HSC and future immunotherapeutic strategies. Consistent with a transcriptional program partially overlapping with *FLT3*-ITD AML, *NPM1*$^{mut}$/*FLT3*$^{LIKE}$ AML samples showed a predicted drug sensitivity to *FLT3* inhibitors (including quizartinib, midostaurin, and sorafenib) compared with "*NPM1*-pure" AML. These results were confirmed in the independent validation cohort. No survival difference emerged under standard therapy. Finally, genes with prognostic value were identified, including genes related to immune regulation. A preliminary scoring system based on RNA-seq 4-gene panel expression levels could potentially stratify prognosis (Fig. 1f); however, validation is ongoing.

### Summary/Conclusion

Our work opens an innovative window into the biology of *NPM1*$^{mut}$ *FLT3*-ITD$^{neg}$ AML, revealing a distinctive transcriptomic signature that had never emerged from previous analyses, as limited sample size had so far precluded deep investigations on this subgroup. Several potential biomarkers for further prognostic stratification and new targets for therapy have emerged. Biological validations are ongoing in our lab.

**Copyright Information:** (Online) ISSN: 2572-9241
©2026 The Author(s). HemaSphere is published by John Wiley & Sons Ltd on behalf of European Hematology Association. This is an open access Abstract Book distributed under the Attribution-NonCommercial-NoDerivs (CC BY-NC-ND), which allows third parties to download the articles and share them with others as long as they credit the author and the Abstract Book, but they cannot change the content in any way or use them commercially.
**Abstract Book Citations:** Authors, Title, HemaSphere, 2026;10:(S1):pages. The abstract book DOIs can be found on the "Author Information" page.
**Disclaimer:** Articles published in the journal HemaSphere exclusively reflect the opinions of the authors. The authors are responsible for all content in their abstracts including accuracy of the facts, statements, citing resources, etc.

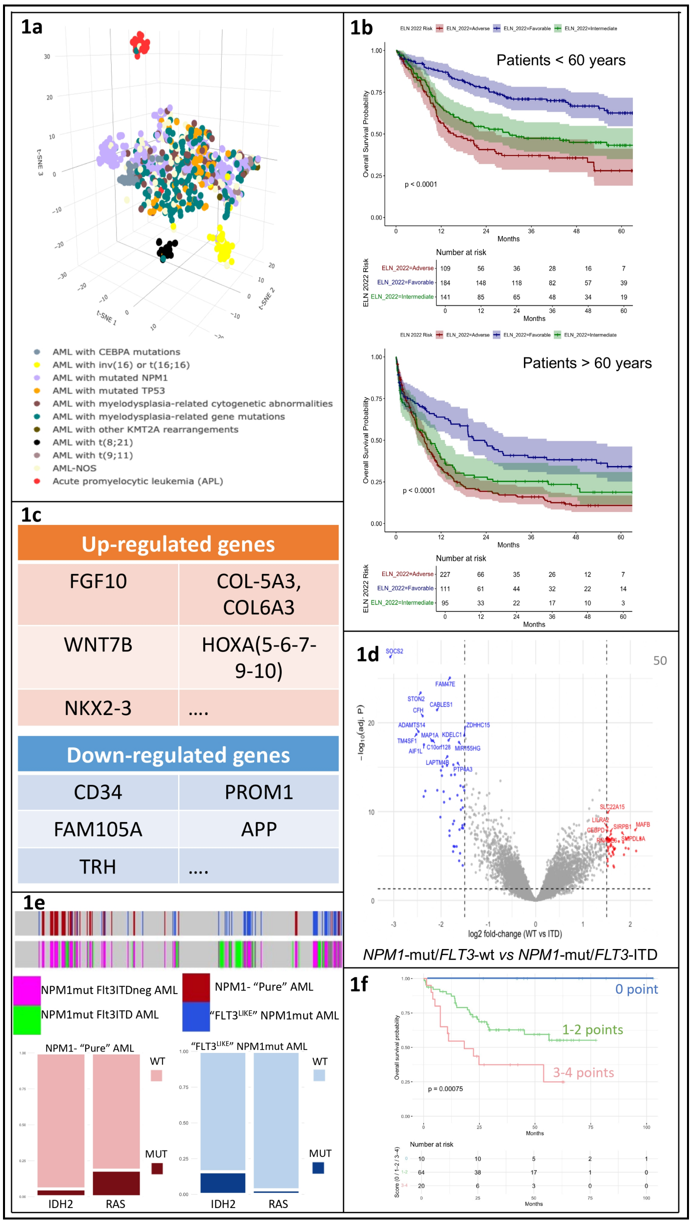

**Copyright Information:** (Online) ISSN: 2572-9241
©2026 The Author(s). HemaSphere is published by John Wiley & Sons Ltd on behalf of European Hematology Association. This is an open access Abstract Book distributed under the Attribution-NonCommercial-NoDerivs (CC BY-NC-ND), which allows third parties to download the articles and share them with others as long as they credit the author and the Abstract Book, but they cannot change the content in any way or use them commercially.
**Abstract Book Citations:** Authors, Title, HemaSphere, 2026;10:(S1):pages. The abstract book DOIs can be found on the "Author Information" page.
**Disclaimer:** Articles published in the journal HemaSphere exclusively reflect the opinions of the authors. The authors are responsible for all content in their abstracts including accuracy of the facts, statements, citing resources, etc.

### (PB2683) DIFFERENTIAL NLRP3 INFLAMMASOME ACTIVATION IN LEUKEMIC BLASTS AND NON-BLAST BONE MARROW CELLS IN ACUTE MYELOID LEUKEMIA

**Topic:** 03. Acute myeloid leukemia - Biology & translational research

Dimitri Bulté*[1], Sara Crespi[1, 2], Emanuela Pettinato[1], Alessia Lai[3], Vincenzo Mercurio[3], Elisa Cantarelli[4], Cristina Tresoldi[4], Annalisa Sgarlata[5], Massimo Vincenzo Martino[5], Maurilio Ponzoni[2, 6], Sara Mastaglio[3], Alessandra Mortellaro[1]

*[1]San Raffaele Telethon Institute for Gene Therapy (SR-Tiget), IRCCS San Raffaele Scientific Institute, Mechanisms of Inflammation in Health and Disease Unit, Milan, Italy; [2]Università Vita-Salute San Raffaele, milan, Italy; [3]IRCCS Ospedale San Raffaele, Hematology and Bone Marrow Transplantation Unit, Milan, Italy; [4]IRCCS Ospedale San Raffaele, Centro Risorse Biologiche (CRB-OSR), Milan, Italy; [5]Grande Ospedale Metropolitano "Bianchi Melacrino Morelli" di Reggio Calabria, Department of Oncology and Hematology, Reggio Calabria, Italy; [6]IRCCS San Raffaele Scientific Institute, Pathology Unit, Milan, Italy;*

### Background

**Background:** Acute myeloid leukemia (AML) is an aggressive hematologic malignancy characterized by clonal expansion of immature myeloid blasts and profound disruption of normal hematopoiesis. Beyond genetic alterations, AML progression is increasingly linked to inflammatory remodeling of the bone marrow (BM) microenvironment. NLRP3 inflammasome activation and downstream interleukin-1β (IL-1β) signaling have been implicated in leukemic progression and therapy resistance. However, the relative contribution of leukemic blasts versus non-blast BM cells to inflammasome activity, and its association with treatment response, remain poorly defined.

### Aims

**Aims:** To dissect cell-type-specific NLRP3 inflammasome expression and functional activation in AML BM and to determine their association with therapeutic response.

### Methods

**Methods:** BM samples from 25 AML patients were analyzed at diagnosis and, when available, post-treatment. Patients were classified as responders or non-responders. Leukemic blasts were purified from BM mononuclear cells using CD34/CD117 magnetic separation. Inflammasome-related gene expression (NLRP3, IL1B and associated components) was quantified by RT-qPCR. Functional activation was evaluated using a two-step priming/activation protocol, followed by IL-1β quantification by ELISA.

### Results

**Results:** Both leukemic blasts and non-blast BM fractions constitutively expressed inflammasome-related genes. TLR2 or TLR4 priming enhanced inflammasome gene expression in both compartments, with stronger induction in non-blast cells. Upon activation, robust IL-1β secretion was detected predominantly in the non-blast BM fraction. In contrast, leukemic blasts displayed limited IL-1β secretion despite preserved inflammasome gene expression and inducibility, indicating functional uncoupling between inflammasome transcriptional priming and cytokine output in blasts. At diagnosis, basal inflammasome activity did not significantly differ between responders and non-responders. However, longitudinal analysis revealed heterogeneous patient-specific changes. Notably, responder patients showed increased inflammasome-induced IL-1β secretion in the blast-negative BM fraction post-treatment, a pattern absent in non-responders.

**Copyright Information:** (Online) ISSN: 2572-9241
©2026 The Author(s). HemaSphere is published by John Wiley & Sons Ltd on behalf of European Hematology Association. This is an open access Abstract Book distributed under the Attribution-NonCommercial-NoDerivs (CC BY-NC-ND), which allows third parties to download the articles and share them with others as long as they credit the author and the Abstract Book, but they cannot change the content in any way or use them commercially.
**Abstract Book Citations:** Authors, Title, HemaSphere, 2026;10:(S1):pages. The abstract book DOIs can be found on the "Author Information" page.
**Disclaimer:** Articles published in the journal HemaSphere exclusively reflect the opinions of the authors. The authors are responsible for all content in their abstracts including accuracy of the facts, statements, citing resources, etc.

**Summary/Conclusion**

**Summary:** Our data reveal a functional dissociation between inflammasome expression and cytokine release in AML blasts and identify non-blast BM cells as the dominant source of IL-1β production. Treatment-associated changes in inflammasome responsiveness selectively observed in responders suggest that microenvironmental inflammasome activity may reflect or contribute to therapeutic outcome. These findings support cell-specific inflammasome activity pathways in AML.

**Copyright Information:** (Online) ISSN: 2572-9241
©2026 The Author(s). HemaSphere is published by John Wiley & Sons Ltd on behalf of European Hematology Association. This is an open access Abstract Book distributed under the Attribution-NonCommercial-NoDerivs (CC BY-NC-ND), which allows third parties to download the articles and share them with others as long as they credit the author and the Abstract Book, but they cannot change the content in any way or use them commercially.
**Abstract Book Citations:** Authors, Title, HemaSphere, 2026;10:(S1):pages. The abstract book DOIs can be found on the "Author Information" page.
**Disclaimer:** Articles published in the journal HemaSphere exclusively reflect the opinions of the authors. The authors are responsible for all content in their abstracts including accuracy of the facts, statements, citing resources, etc.

### (PB2684) MONOCYTE-BASED TRANSPORT OF HSV-1 ONCOLYTIC VIRUSES FOR ENHANCED ACUTE MYELOID LEUKEMIA TARGETING

**Topic:** 03. Acute myeloid leukemia - Biology & translational research

Alessandra Rossetto*[1], Alberto Reale[1], Sara Micheli[2], Beatrice Zombini[2], Alessandra Sperotto[3], Giorgia Simonetti[4], Elisabetta Calistri[3], Elisa Cimetta[2, 5], Michele Gottardi[3], Arianna Calistri[1]

*[1]University of Padua, Department of Molecular Medicine, Padova, Italy;[2]University of Padua, Department of Industrial Engineering, Padova, Italy; [3]Istituto oncologico Veneto, Castelfranco, Padova, Italy; [4]Istituto Romagnolo per lo studio dei tumori, Meldola, Italy; [5]Istituto di Ricerca Pediatrica, Padova, Italy;*

### Background

In light of the urgent need for innovative therapies against acute myeloid leukemia (AML), where current treatments fail to target the protective bone marrow niche effectively, our research investigates monocyte-mediated delivery of oncolytic Herpes Simplex Virus type 1 (HSV-1) as a novel virotherapy approach.
Among innovative cancer treatments, oncolytic viruses (OV) represent an emerging field due to their ability to bridge cytotoxicity, immunotherapy, and gene therapy. Despite the growing number of viruses proposed as oncolytic agents, HSV-1-based OV are among the most promising, thanks to their large genome, lack of insertional mutagenesis, broad tropism, and induction of immunogenic cell death. AML should be an optimal target for HSV-1 vectors, due to the expression of key receptors (nectin-1 and HVEM) for viral entry on malignant blasts surface; consistent with this, we confirmed the ability of an engineered HSV-1 oncolytic vector to infect various AML cell lines and induce their killing (data to be presented at “Viruses 2026 – New Horizons in Virology” Conference, Barcelona, Spain, 11-13 March 2026).
However, a big issue in oncolytic virotherapy is represented by the strategy of delivery of the selected OV that must address the disease’s intravascular dissemination, bone marrow niche and pre-existing neutralizing antibodies against HSV-1 in at least 50% of the population.
To overcome these limitations, we propose using autologous monocytes as carrier cells for HSV-1 OVs in AML. This approach leverages their ease of recovery from peripheral blood, natural tumor tropism (as precursors of tumor-associated macrophages)—making cancers less likely to develop resistance to monocyte-based therapy—and ability to access compartmentalized body sites.

### Aims

To overcome these limitations, we propose using autologous monocytes as carrier cells for HSV-1 OVs in AML. This approach leverages their ease of recovery from peripheral blood, natural tumor tropism (as precursors of tumor-associated macrophages)—making cancers less likely to develop resistance to monocyte-based therapy—and ability to access compartmentalized body sites.

### Methods

To test monocyte-mediated delivery in an AML context, we performed a migration assay and assessed monocytes' capacity to transmit HSV-1 infection to leukemia cells. We also developed and validated an innovative bone marrow-on-chip platform comprising two distinct compartments: (i) a single channel lined with endothelial cells, mimicking blood vessels, and (ii) an adjacent layer composed of Gelma 7%, replicating the fibrous and porous extracellular matrix (ECM), populated with AML cells.

**Copyright Information:** (Online) ISSN: 2572-9241
©2026 The Author(s). HemaSphere is published by John Wiley & Sons Ltd on behalf of European Hematology Association. This is an open access Abstract Book distributed under the Attribution-NonCommercial-NoDerivs (CC BY-NC-ND), which allows third parties to download the articles and share them with others as long as they credit the author and the Abstract Book, but they cannot change the content in any way or use them commercially.
**Abstract Book Citations:** Authors, Title, HemaSphere, 2026;10:(S1):pages. The abstract book DOIs can be found on the “Author Information” page.
**Disclaimer:** Articles published in the journal HemaSphere exclusively reflect the opinions of the authors. The authors are responsible for all content in their abstracts including accuracy of the facts, statements, citing resources, etc.

**Results**

The migration assay confirmed monocytes' tropism for leukemia cells and their capacity to transmit HSV-1 infection, inducing a significant reduction in leukemia cell viability (Viruses 2026). In the bone marrow-on-chip model, monocytes crossed the endothelial barrier and became actively recruited into the bone marrow microenvironment, confirming their strong natural tropism for AML cells. Subsequent transmission of infection to leukemia cells resulted in progressive disappearance of cellular aggregates, thus confirming monocytes' ability to transmit viral infection even under dynamic perfusion conditions.

**Summary/Conclusion**

In summary, our preliminary data on monocyte-mediated HSV-1 delivery to AML cells, coupled with advanced bone marrow-on-chip modeling, are promising for the future use of monocytes as carrier cells for oHSV-1 virotherapy in AML treatment.

**Copyright Information:** (Online) ISSN: 2572-9241
©2026 The Author(s). HemaSphere is published by John Wiley & Sons Ltd on behalf of European Hematology Association. This is an open access Abstract Book distributed under the Attribution-NonCommercial-NoDerivs (CC BY-NC-ND), which allows third parties to download the articles and share them with others as long as they credit the author and the Abstract Book, but they cannot change the content in any way or use them commercially.
**Abstract Book Citations:** Authors, Title, HemaSphere, 2026;10:(S1):pages. The abstract book DOIs can be found on the “Author Information” page.
**Disclaimer:** Articles published in the journal HemaSphere exclusively reflect the opinions of the authors. The authors are responsible for all content in their abstracts including accuracy of the facts, statements, citing resources, etc.

### (PB2685) SINGLE-CELL ANALYSIS REVEALS HETEROGENEOUS HEMATOPOIETIC HIERARCHIES IN NPM1-MUTATED AML

**Topic:** 03. Acute myeloid leukemia - Biology & translational research

Anna Giné-Vilalta*[1], Guillermo Ramil[1, 2], Guadalupe Oñate[1, 2], Helena Castellet[1], Laura Ranera[2], Montserrat Arnan Sangerman[3], Antonia Sampol[4], Josep Nomdedeu[1, 2], Jordi Esteve Reyner[5], Jorge Sierra Gil[1, 2], Marta Pratcorona[1, 2]

*[1]Institut de Recerca Sant Pau (IR Sant Pau), Barcelona, Spain;[2]Hospital de la Santa Creu i Sant Pau, Barcelona, Spain;[3]Institut Catala d'Oncologia-Institut d'Investigació Biomèdica de Bellvitge (IDIBELL),, L'Hospitalet de Llobregat, Spain; [4]Hospital Son Espases, Palma, Spain; [5]Institute of Cancer & Blood Disorders (ICAMS), IDIBAPS, Hospital Clinic, Barcelona, Spain;*

### Background

Acute myeloid leukemia (AML) is a heterogeneous neoplastic disease characterized by an aberrant proliferation and differentiation of hematopoietic stem cells, resulting in impaired hematopoiesis. This heterogeneity arises from a complex cytogenetic and molecular landscape and has been progressively shaped by advances in genomic technologies.
One of the molecular entities defined to reduce this heterogeneity is AML with *NPM1* mutation (*NPM1*mut). Nevertheless, even within this subgroup, patients have different responses to treatment, highlighting the need for a deeper understanding of the underlying cellular biology of this disease.

### Aims

To determine the hematopoietic stem cell hierarchies within the *NPM1*mut subgroup.

### Methods

Cryopreserved mononuclear cells from bone marrow samples from the biobank of the CETLAM group were processed for single-cell RNA sequencing using the GEM-X Universal 3′ Gene Expression v4 and the Chromium GEM-X Single Cell 3′ Chip v4 (10x Genomics), and were run on the Chromium X instrument. Sequencing was performed using a NovaSeq X platform (Illumina). *CellRanger* v10 was run on all samples with the human reference genome GRCh38 2024-A and the obtained data was analyzed using *Seurat* v5, *scanpy* and *scvi-tools.*

**Copyright Information:** (Online) ISSN: 2572-9241
©2026 The Author(s). HemaSphere is published by John Wiley & Sons Ltd on behalf of European Hematology Association. This is an open access Abstract Book distributed under the Attribution-NonCommercial-NoDerivs (CC BY-NC-ND), which allows third parties to download the articles and share them with others as long as they credit the author and the Abstract Book, but they cannot change the content in any way or use them commercially.
**Abstract Book Citations:** Authors, Title, HemaSphere, 2026;10:(S1):pages. The abstract book DOIs can be found on the "Author Information" page.
**Disclaimer:** Articles published in the journal HemaSphere exclusively reflect the opinions of the authors. The authors are responsible for all content in their abstracts including accuracy of the facts, statements, citing resources, etc.

### Results

Nine samples containing between 45 and 95% blasts were obtained from *NPM1*mut patients at diagnosis. Two samples from healthy donors were also included in this study. Approximately, 10,000 cells were sequenced per sample.
First, all samples were projected onto a healthy bone marrow atlas (Azimuth) to annotate our cells. In healthy controls samples, we found that all cell types were well represented, with comparable proportions across both samples. In contrast, *NPM1*mut samples showed marked differences in cell type composition, reflecting disruption of hematopoiesis at different progenitor stages.
Second, integration of all eleven samples showed that, except for the B and NK/T subsets, the vast majority of *NPM1*mut cells were different from the healthy control ones and they clustered into three subsets (*NPM1*mut-1, 2 and 3). All samples contained cells within the *NPM1*mut-1 and *NPM1*mut-2 subsets except for one sample (sample 8), which clustered exclusively within the *NPM1*mut-3 group. B and NK/T cells were detected in all *NPM1*mut samples and clustered separately from the myeloid cells (Figure A).
Hierarchical clustering analysis revealed that the integrated *NPM1*mut samples segregated into three groups according to co-mutations detected by NGS: *NPM1*mut with concurrent *IDH1* mutation (n=4), without *IDH1* mutation (n=4), and with myelodysplasia-related gene mutations (n=1) (Figure B). This latter group corresponded to sample 8, the only case clustering within the *NPM1*mut-3 subset. When cell type composition was analyzed according to these groups, a different distribution of cell subpopulations was observed across *NPM1*mut samples, although a clear pattern was not defined (Figure C).

### Summary/Conclusion

In summary, our data shows that *NPM1*mut is characterized by a disrupted hematopoiesis at different progenitor stages, with leukemic cells forming distinct clusters separated from healthy counterparts. Although co-mutational status may influence sample clustering and cellular composition, further studies are needed to better define these associations.
Overall, our data reinforce that *NPM1*mut is still a very heterogeneous subgroup that requires further investigation.

**Copyright Information:** (Online) ISSN: 2572-9241
©2026 The Author(s). HemaSphere is published by John Wiley & Sons Ltd on behalf of European Hematology Association. This is an open access Abstract Book distributed under the Attribution-NonCommercial-NoDerivs (CC BY-NC-ND), which allows third parties to download the articles and share them with others as long as they credit the author and the Abstract Book, but they cannot change the content in any way or use them commercially.
**Abstract Book Citations:** Authors, Title, HemaSphere, 2026;10:(S1):pages. The abstract book DOIs can be found on the “Author Information” page.
**Disclaimer:** Articles published in the journal HemaSphere exclusively reflect the opinions of the authors. The authors are responsible for all content in their abstracts including accuracy of the facts, statements, citing resources, etc.

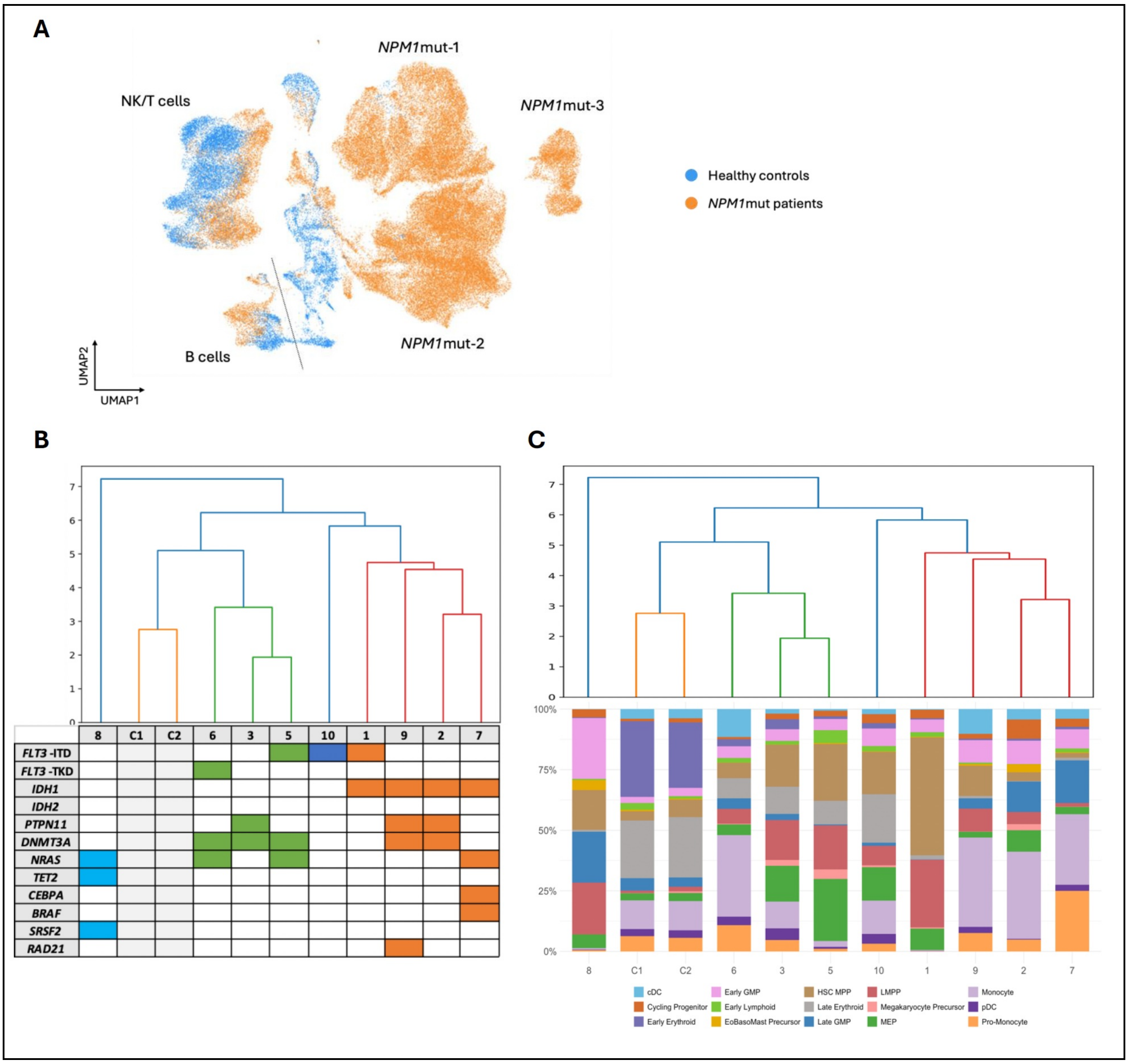

(A) UMAP plot showing cells (n=95,965) from 9 AML NPM1mut patients and 2 healthy controls. (B) Gene mutations detected by NGS at diagnosis in our cohort (besides NPM1 mutation), sorted by hierarchical clustering. (C) Bar plot illustrating the myeloid cell type composition of each patient and healthy control, sorted by hierarchical clustering.

Copyright Information: (Online) ISSN: 2572-9241
©2026 The Author(s). HemaSphere is published by John Wiley & Sons Ltd on behalf of European Hematology Association. This is an open access Abstract Book distributed under the Attribution-NonCommercial-NoDerivs (CC BY-NC-ND), which allows third parties to download the articles and share them with others as long as they credit the author and the Abstract Book, but they cannot change the content in any way or use them commercially.
Abstract Book Citations: Authors, Title, HemaSphere, 2026;10:(S1):pages. The abstract book DOIs can be found on the "Author Information" page.
Disclaimer: Articles published in the journal HemaSphere exclusively reflect the opinions of the authors. The authors are responsible for all content in their abstracts including accuracy of the facts, statements, citing resources, etc.

### (PB2686) PREDOMINANCE OF THE PML-RARA BCR3 ISOFORM AND ITS PROGNOSTIC IMPACT IN A TUNISIAN APL COHORT: IMPLICATIONS FOR RISK-ADAPTED THERAPY

**Topic:** 03. Acute myeloid leukemia - Biology & translational research

Manel Kasdallah*[1], Emna Sahli[1], Rym Cherni[2], Ahlem Farrah[1], Raihane Ben Lakhal[2], Samia Menif[1]

*[1]Pasteur Institute of Tunis, Laboratory of cellular an molecular Hematology, Tunis, Tunisia;[2]Aziza Othmana Hospital, Hematology Clinic Department, Tunis, Tunisia;*

### Background

Risk stratification in Acute Promyelocytic Leukemia (APL) relies on the identification of *PML-RARA* isoforms (bcr1/2/3), with bcr3 typically linked to inferior outcomes and hyperleukocytosis. However, the impact of these molecular subtypes varies globally, and their role as independent predictors in diverse populations is not fully established. In North Africa, the absence of large-scale molecular studies limits the implementation of optimized, risk-adapted strategies. We describe here the molecular and clinical landscape of a Tunisian APL cohort.

### Aims

This study aims to **investigate the prognostic impact** of *PML-RARA* transcript subtypes within a North African context. We specifically focused on **correlating isoform distribution** with biological markers of severity and **validating their independent predictive value** for clinical outcomes, aiming to refine risk-adapted therapeutic strategies in real-world settings.

### Methods

We performed a retrospective analysis of 51 APL patients (2018–2024). *PML-RARA* isoforms were identified by RT-PCR at diagnosis. Clinical features, coagulopathy (DIC), and survival outcomes (median follow-up: 27 months) were correlated with molecular findings.

### Results

**Distinct Molecular Landscape:** The distribution of *PML-RARA* isoforms revealed a unique epidemiological signature. The **bcr3 (short) isoform was the most prevalent, occurring in 43.1% of cases**, a rate significantly higher than the 25–30% typically reported in European registries. This was followed by bcr1 (35.3%) and bcr2 (15.7%). Interestingly, **double transcripts** (bcr1/3 or bcr1/2+bcr3) were identified in 5.9% of the cohort, suggesting complex clonal architecture in some patients.
**Aggressive Clinical Phenotype:** The bcr3 isoform was not merely a molecular marker but a driver of clinical severity. Patients harboring bcr3 were significantly more likely to present with **high-risk features (Sanz score >2, p<0.05)**. This was characterized by profound hyperleukocytosis and a markedly higher incidence of **clinical Disseminated Intravascular Coagulation (DIC)** at admission compared to bcr1/bcr2 carriers.
**Robust Prognostic Impact:** Survival analysis demonstrated that bcr3 is a major determinant of outcome. It was strongly associated with **unfavorable clinical evolution**—encompassing early death, resistance, or relapse—with a **Hazard Ratio (HR) of 3.04 (p=0.036)**. The median overall survival for bcr3 patients was 37.2 months, highlighting a continuous risk of adverse events. **Independent Predictor Status (Multivariate Analysis):** In a multivariate Cox regression model, the bcr3 isoform remained an **independent predictor of mortality**, alongside a White Blood Cell (WBC) count >16.96 × $10^9$/L. This critical finding proves that the bcr3 transcript confers an **intrinsic biological aggressiveness** that is independent of the initial tumor burden (WBC count).

**Copyright Information:** (Online) ISSN: 2572-9241
©2026 The Author(s). HemaSphere is published by John Wiley & Sons Ltd on behalf of European Hematology Association. This is an open access Abstract Book distributed under the Attribution-NonCommercial-NoDerivs (CC BY-NC-ND), which allows third parties to download the articles and share them with others as long as they credit the author and the Abstract Book, but they cannot change the content in any way or use them commercially.
**Abstract Book Citations:** Authors, Title, HemaSphere, 2026;10:(S1):pages. The abstract book DOIs can be found on the "Author Information" page.
**Disclaimer:** Articles published in the journal HemaSphere exclusively reflect the opinions of the authors. The authors are responsible for all content in their abstracts including accuracy of the facts, statements, citing resources, etc.

### Summary/Conclusion

Our results identify the bcr3 isoform as a **major independent determinant of poor outcomes** in North African APL patients. The unusually high frequency of this aggressive transcript underscores the **limitation of universal risk models** and the need for **region-specific clinical guidelines**. Implementing routine molecular subtyping at diagnosis is essential to identify this vulnerable subset early, justifying a **proactive escalation of supportive and targeted care** to bridge the survival gap observed in real-world North African cohorts.

**Copyright Information:** (Online) ISSN: 2572-9241
©2026 The Author(s). HemaSphere is published by John Wiley & Sons Ltd on behalf of European Hematology Association. This is an open access Abstract Book distributed under the Attribution-NonCommercial-NoDerivs (CC BY-NC-ND), which allows third parties to download the articles and share them with others as long as they credit the author and the Abstract Book, but they cannot change the content in any way or use them commercially.
**Abstract Book Citations:** Authors, Title, HemaSphere, 2026;10:(S1):pages. The abstract book DOIs can be found on the "Author Information" page.
**Disclaimer:** Articles published in the journal HemaSphere exclusively reflect the opinions of the authors. The authors are responsible for all content in their abstracts including accuracy of the facts, statements, citing resources, etc.

### (PB2688) DYNAMIC BLOOD COUNT TRAJECTORIES IMPROVE SURVIVAL PREDICTION OF INTENSIVELY-TREATED AML PATIENTS OVER STANDARD ELN CLASSIFICATION

**Topic:** 03. Acute myeloid leukemia - Biology & translational research

Katariina Luukkainen*[1, 2, 3], Anna Mervaala-Muroke[1, 2, 3], Oscar Brück[1, 2, 3]

*[1]Hematoscope Lab, Comprehensive Cancer Center, Helsinki University Hospital, Helsinki, Finland;[2]Department of Oncology, University of Helsinki, Helsinki, Finland; [3]Department of Clinical Chemistry, HUS Diagnostic Center, Helsinki University Hospital and University of Helsinki, Helsinki, Finland;*

### Background

Risk stratification in acute myeloid leukemia (AML) is primarily based on genetic and chromosomal abnormalities at diagnosis, as defined by the European LeukemiaNet (ELN) classification. While this framework guides treatment decisions, it does not incorporate early treatment dynamics. Early blast clearance and blood count recovery during induction therapy are associated with clinical outcomes beyond baseline cytogenetic and molecular features. We therefore hypothesized that dynamic laboratory trajectories during the first induction cycle could improve risk stratification in AML.

### Aims

To evaluate whether incorporating early laboratory changes during induction therapy with baseline ELN classification improves risk stratification and prediction of overall survival (OS) compared with baseline ELN classification alone.

### Methods

We retrospectively analyzed 373 adult AML patients treated with first induction therapy. A total of 389 baseline and longitudinal laboratory features were collected, including blast kinetics, cytopenia depth, and blood count recovery. We trained a XGBoost survival model using a Cox proportional hazards objective function. The dataset was divided into training (80 %) and independent test sets (20 %). Hyperparameters were optimized using 5-fold cross-validation within the training set. Feature importance was assessed using SHAP values, which guided forward feature selection. Model performance was evaluated using the concordance index (C-index) and time-dependent area under the curve (AUC). Model-derived risk scores were used to reclassify patients within ELN risk groups, and survival differences were assessed using Kaplan-Meier analysis.

**Copyright Information:** (Online) ISSN: 2572-9241
©2026 The Author(s). HemaSphere is published by John Wiley & Sons Ltd on behalf of European Hematology Association. This is an open access Abstract Book distributed under the Attribution-NonCommercial-NoDerivs (CC BY-NC-ND), which allows third parties to download the articles and share them with others as long as they credit the author and the Abstract Book, but they cannot change the content in any way or use them commercially.
**Abstract Book Citations:** Authors, Title, HemaSphere, 2026;10:(S1):pages. The abstract book DOIs can be found on the "Author Information" page.
**Disclaimer:** Articles published in the journal HemaSphere exclusively reflect the opinions of the authors. The authors are responsible for all content in their abstracts including accuracy of the facts, statements, citing resources, etc.

### Results

In univariate Cox regression analyses, 63 laboratory and clinical features were significantly associated with OS ($P < 0.05$), including age, comorbidity index, leukocyte nadir, blast clearance, and ELN risk group. The final XGBoost model achieved a test-set C-index of 0.75 compared to 0.66 using ELN classification alone. The mean time-dependent AUC was 0.85. SHAP analysis identified the most important predictors and their directionality of OS risk. Older age, ELN adverse risk, higher comorbidity index, and elevated RDW at nadir were associated with increased mortality risk, whereas ELN favorable risk, greater blast reduction during the first three days, higher platelet count at day 28, greater lymphocyte count at day 28, and *de novo* AML were associated with reduced mortality risk. Of note, these laboratory features capture longitudinal changes occurring across the entire induction cycle.

Dynamic modeling revealed significant outcome heterogeneity within ELN intermediate and adverse risk groups. Model-defined high-risk patients had significantly worse survival than low-risk patients in the intermediate (HR = 7.91, P = 0.0017) and adverse groups (HR = 7.52, P = 0.0027). In contrast, no significant survival difference was observed within the ELN favorable group (HR = 0.98, P = 0.9870), suggesting that dynamic laboratory changes during induction provide additional prognostic value primarily in intermediate- and adverse-risk disease.

### Summary/Conclusion

Integrating early laboratory changes during induction therapy with baseline ELN classification allows dynamic risk assessment in AML. Together, these could enhance the identification of high-risk patients, enabling timely treatment escalation to mitigate the risk of relapse. External validation is currently underway in an independent large-scale hospital cohort.

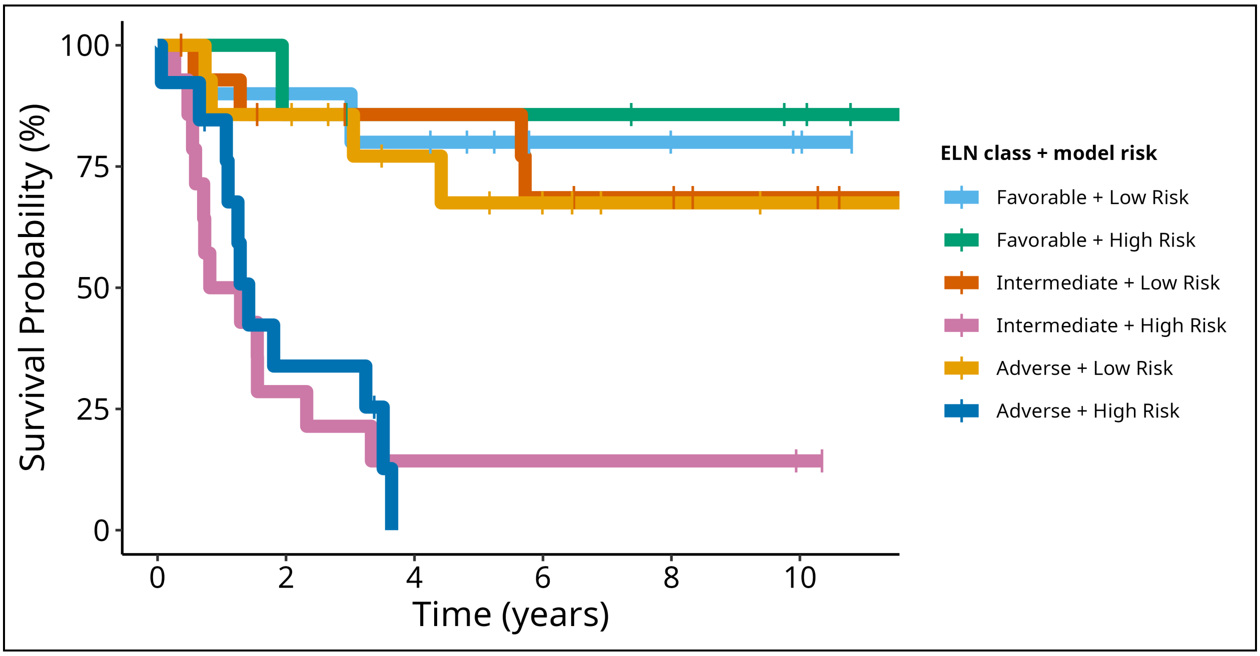

**Copyright Information:** (Online) ISSN: 2572-9241
©2026 The Author(s). HemaSphere is published by John Wiley & Sons Ltd on behalf of European Hematology Association. This is an open access Abstract Book distributed under the Attribution-NonCommercial-NoDerivs (CC BY-NC-ND), which allows third parties to download the articles and share them with others as long as they credit the author and the Abstract Book, but they cannot change the content in any way or use them commercially.
**Abstract Book Citations:** Authors, Title, HemaSphere, 2026;10:(S1):pages. The abstract book DOIs can be found on the "Author Information" page.
**Disclaimer:** Articles published in the journal HemaSphere exclusively reflect the opinions of the authors. The authors are responsible for all content in their abstracts including accuracy of the facts, statements, citing resources, etc.

### (PB2689) FOURIER-TRANSFORM INFRARED (FT-IR) SPECTROSCOPY OF PLASMA AS A LIQUID BIOPSY APPROACH FOR DISEASE MONITORING IN ACUTE MYELOID LEUKEMIA (AML) POST ALLOGENIC HEMATOPOIETIC STEM CELL TRANSPLANTATION

**Topic:** 03. Acute myeloid leukemia - Biology & translational research

Alessandro Leoni*[1, 2], Besjana Xhahysa[1], Luca Garuffo[1], Alessia Cavalleri[1], Silvia Mutti[1], Daniele Avenoso[1, 2], Mirko Farina[2], Enrico Morello[2], Domenico Russo[1, 2], Michele Malagola[1, 2], Simona Bernardi[1]

*[1]University of Brescia, Brescia, Italy; [2]ASST Spedali Civili, Brescia, Italy;*

**Background**

Acute myeloid leukemia (AML), is a biologically complex hematologic malignancy marked by heterogeneity, clonal evolution and several genetic and epigenetic alterations. Relapse after allogeneic hematopoietic stem cell transplantation (Allo-HSCT) remains the major cause of treatment failure. Minimal Residual Disease (MRD), defined as the persistence of leukemic cells below conventional detection the threshold, is a key predictor of relapse. However, early detection of post-transplant relapse remain challenging, especially in cases with low tumor burden or atypical clinical presentation, such as Myeloid Sarcoma or during immune and metabolic reconstitution. Therefore, minimally invasive approaches capable of capturing global biochemical changes could represent the future in terms of disease monitoring.

**Aims**

The study aims to evaluate plasma Fourier-Transform Infrared (FT-IR) spectroscopy as a liquid biopsy tool for disease monitoring AML patients undergoing allo-HSCT, focusing on early detection of post-transplant relapse and characterization of relapse patterns.

**Methods**

In this retrospective study, we includes healthy subjects as a controls and AML patients. For AML patients, plasma samples were collected at multiple timepoints: (i) diagnosis, (ii) pre-Allo-HSCT, (iii) post-transplant and (iv) at relapse. Spectral data were pre-processed using standard normal variate normalization and analyzed with multivariate statistical methods and machine learning algorithms, with leave-one-patient-out cross-validation.

**Results**

Twelve pathological subjects and four healthy subject, matched for age and sex, were included. All twelve transplanted patients underwent Allo-HSCT in first complete remission. Of these, 8 patients relapsed (4 as medullary and 4 as extramedullary).FT-IR analysis showed a strong ability to discriminate between healthy subjects and AML patients at diagnosis (AUC=0.866; Accuracy=0.826), supporting a disease-associated spectral fingerprint. Notably, post-HSCT samples from patients who relapsed where clearly distinguishable from those who remained in remission, indicating that plasma FT-IR profiles capture biochemical changes linked to relapse. Longitudinal analysis showed progressive normalization of the spectral signature after transplantation, with approximately 95% of samples at +90 days resembling healthy controls, whereas this trend was less evident at +30 days, suggesting gradual metabolic recovery.

**Copyright Information:** (Online) ISSN: 2572-9241
©2026 The Author(s). HemaSphere is published by John Wiley & Sons Ltd on behalf of European Hematology Association. This is an open access Abstract Book distributed under the Attribution-NonCommercial-NoDerivs (CC BY-NC-ND), which allows third parties to download the articles and share them with others as long as they credit the author and the Abstract Book, but they cannot change the content in any way or use them commercially.
**Abstract Book Citations:** Authors, Title, HemaSphere, 2026;10:(S1):pages. The abstract book DOIs can be found on the "Author Information" page.
**Disclaimer:** Articles published in the journal HemaSphere exclusively reflect the opinions of the authors. The authors are responsible for all content in their abstracts including accuracy of the facts, statements, citing resources, etc.

**Summary/Conclusion**

Plasma FT-IR spectroscopy is a promising non-invasive liquid biopsy approach for post-Allo-HSCT monitoring in AML. It discriminates between healthy and diseased states, tracks progressive metabolic normalization after transplantation, and detects relapse-associated biochemical alterations, including distinct relapse patterns. Notably, extramedullary relapse, often presenting as myeloid sarcoma, remains clinically challenging due to its asymptomatic course, heterogeneous localization and possible lack of bone marrow involvement. Overall, FT-IR spectroscopy support precision monitoring strategies although validation in larger cohorts and comparison with standard MRD methods are needed. Novel matrices such as extracellular vesicles are under investigation to further enhance signal clarity.

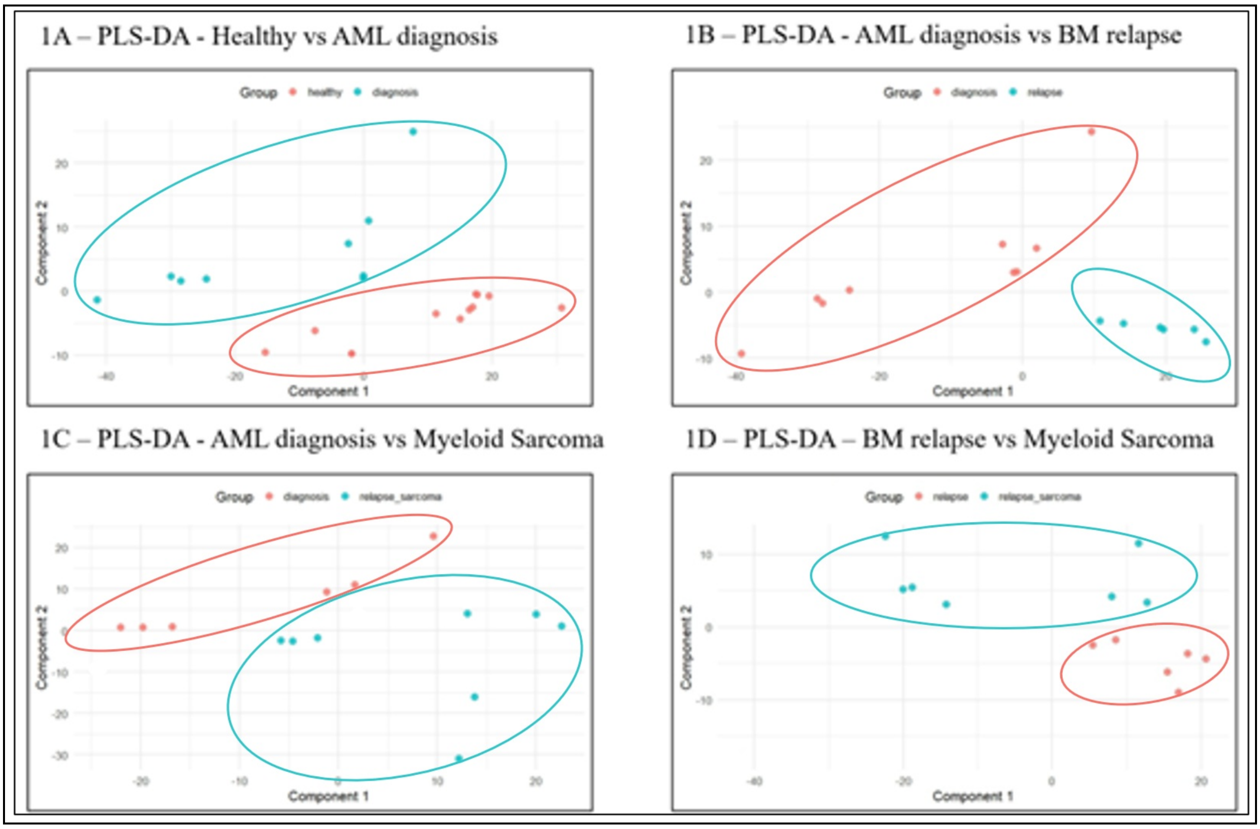

Comparision between healthy and AML patients at diagnosis (A), AML at diagnosis and medullary relapse (B), AML diagnosis and extramedullary relapse (C) and medullary and extramedullary relapse (D)

**Copyright Information:** (Online) ISSN: 2572-9241
©2026 The Author(s). HemaSphere is published by John Wiley & Sons Ltd on behalf of European Hematology Association. This is an open access Abstract Book distributed under the Attribution-NonCommercial-NoDerivs (CC BY-NC-ND), which allows third parties to download the articles and share them with others as long as they credit the author and the Abstract Book, but they cannot change the content in any way or use them commercially.
**Abstract Book Citations:** Authors, Title, HemaSphere, 2026;10:(S1):pages. The abstract book DOIs can be found on the “Author Information” page.
**Disclaimer:** Articles published in the journal HemaSphere exclusively reflect the opinions of the authors. The authors are responsible for all content in their abstracts including accuracy of the facts, statements, citing resources, etc.

### (PB2690) METABOLIC AND INFLAMMATORY SIGNATURES ASSOCIATED WITH NAMPT AND SCD EXPRESSION IN ACUTE MYELOID LEUKEMIA

**Topic:** 03. Acute myeloid leukemia - Biology & translational research

Pedro Antonio Gonçalves Dos Santos*[1], Diego Antonio Pereira-Martins[2], Fabiola Traina[1]

*[1]University of São Paulo at Ribeirão Preto Medical School, Department of Medical Imaging, Hematology, and Oncology, Ribeirão Preto, Brazil; [2]University of Groningen, Department of Hematology, Groningen, The Netherlands;*

### Background

Acute myeloid leukemia (AML) is sustained by leukemic stem cells (LSCs), which exhibit resistance to chemotherapy and display increased metabolic dependency, particularly on NAD salvage and lipid biosynthesis pathways. Nicotinamide phosphoribosyltransferase (NAMPT), the rate-limiting enzyme of the NAD salvage pathway, supports cellular bioenergetics and survival under metabolic stress. Stearoyl-CoA desaturase (SCD), a key enzyme in monounsaturated fatty acid synthesis, has been implicated in adaptive resistance to NAMPT inhibition. The clinical and transcriptional relevance of NAMPT and SCD expression in AML remains incompletely defined.

### Aims

To evaluate the prognostic impact of NAMPT and SCD expression in adult AML and to characterize transcriptional programs associated with NAMPT overexpression.

### Methods

Gene expression and clinical data from a transcriptomic study enrolling 200 patients were analyzed. Patients with acute promyelocytic leukemia and cases with incomplete data were excluded, leaving 162 pacientes (93 males, median age of 60, range 18-88). Expression values were log2-transformed when appropriate. NAMPT and SCD gene expressions were dichotomized by median values. Overall survival (OS) was evaluated using Kaplan–Meier analysis and compared with the log-rank test. Cox proportional hazards regression was performed for univariate and multivariate analyses, adjusting for age, sex, leukocyte count, and blast percentage. Functional enrichment was assessed using GSEA (Hallmark gene sets, MSigDB). Gene sets with FDR q-value < 0.25 and p&lt < 0.05 were considered significant.

### Results

NAMPT expression did not significantly differ across ELN 2022 molecular risk groups and did not independently predict OS in multivariable analysis. Age and leukocyte count remained the strongest prognostic factors. In contrast, high SCD expression was associated with inferior OS in univariate analysis (HR = 1.65, p = 0,009), although this association did not remain significant after adjustment. Combined stratification based on NAMPT and SCD expression identified significant survival differences between patients with low expression of both genes versus high expression of both genes (HR = 1.91, p = 0.03), although subgroup sizes were limited (37 e 32, respectively). Transcriptional profiling revealed significant enrichment of inflammatory and immune-related hallmark gene sets in NAMPT-high samples, including TNFα signaling via NF-κB (NES = 1.89, FDR = 0.028), complement activation (NES = 1.85, FDR = 0.023), and inflammatory response signatures (NES = 1.81, FDR = 0.028). These findings support a role for NAMPT beyond intracellular NAD metabolism, linking metabolic activity with inflammatory transcriptional programs.

**Copyright Information:** (Online) ISSN: 2572-9241
©2026 The Author(s). HemaSphere is published by John Wiley & Sons Ltd on behalf of European Hematology Association. This is an open access Abstract Book distributed under the Attribution-NonCommercial-NoDerivs (CC BY-NC-ND), which allows third parties to download the articles and share them with others as long as they credit the author and the Abstract Book, but they cannot change the content in any way or use them commercially.
**Abstract Book Citations:** Authors, Title, HemaSphere, 2026;10:(S1):pages. The abstract book DOIs can be found on the “Author Information” page.
**Disclaimer:** Articles published in the journal HemaSphere exclusively reflect the opinions of the authors. The authors are responsible for all content in their abstracts including accuracy of the facts, statements, citing resources, etc.

**Summary/Conclusion**

While NAMPT expression alone does not independently predict survival in AML, it is associated with inflammatory transcriptional signatures consistent with immunometabolic activation. SCD expression correlates with adverse survival and may represent a metabolically actionable vulnerability. These data highlight lipid metabolic pathways as potential therapeutic targets in AML and support further investigation of metabolic-inflammation crosstalk in leukemic stem cell biology.

**Copyright Information:** (Online) ISSN: 2572-9241
©2026 The Author(s). HemaSphere is published by John Wiley & Sons Ltd on behalf of European Hematology Association. This is an open access Abstract Book distributed under the Attribution-NonCommercial-NoDerivs (CC BY-NC-ND), which allows third parties to download the articles and share them with others as long as they credit the author and the Abstract Book, but they cannot change the content in any way or use them commercially.
**Abstract Book Citations:** Authors, Title, HemaSphere, 2026;10:(S1):pages. The abstract book DOIs can be found on the "Author Information" page.
**Disclaimer:** Articles published in the journal HemaSphere exclusively reflect the opinions of the authors. The authors are responsible for all content in their abstracts including accuracy of the facts, statements, citing resources, etc.

### (PB2691) IDENTIFICATION OF IMMUNE MODULATORS OF CAR-T CELLS TARGETING CD33 IN ACUTE MYELOID LEUKEMIA

**Topic:** 03. Acute myeloid leukemia - Biology & translational research

Hugo Legenne*[1, 2, 3], Martina Franchini[1, 2, 3], Maxime Boy[1, 3], Adrian Ochsenbein[1, 3], Carsten Riether[1, 3]

*[1]Inselspital, Bern University Hospital; University of Bern, Department of Medical Oncology, Bern, Switzerland;[2]University of Bern, Graduate School of Cellular and Biomedical Sciences, Bern, Switzerland; [3]University of Bern, Tumor Immunology, Department of Biomedical Research, Bern, Switzerland;*

### Background

Acute myeloid leukemia (AML) is a malignant bone marrow disease arising from epigenetic/genetic alterations in hematopoietic stem cells (HSCs), leading to their transformation into leukemia stem cells (LSCs) that drive relapse and frequently resist treatment. Treatment with the antibody-drug conjugate Gemtuzumab Ozogamicin (GO), directed against CD33 (Siglec-3), is approved in relapsed/refractory AML. In B-cell acute lymphoblastic leukemia (B-ALL), chimeric antigen receptor (CAR)-T cell therapy has shown high remission rates; however, clinically approved products such as CD19- or BCMA-targeting CAR-T cells often exhibit limited persistence upon repeated antigen exposure.

### Aims

To systematically identify intrinsic immune modulators regulating CD33 CAR-T cell function and persistence in AML and to evaluate their translatability across hematologic malignancies.

### Methods

We performed a pooled CRISPR enrichment screen targeting 1,204 validated T cell surface proteins, using four optimized sgRNAs per gene. Neutral (non-targeting) and positive (TCR signaling) controls were included. CD33 CAR-T cells were screened in parallel with CD19 CAR-T cells (FMC63-based construct) to distinguish AML-specific from general CAR signaling modulators. Screening was conducted in an allogeneic serial co-culture system with primary AML LSCs under repeated antigen challenge.
As proof-of-concept, CRISPR-mediated CD4 disruption achieved ~80% knockout efficiency while preserving CAR expression, antigen specificity, and cytotoxic function.

### Results

Both CD33 and CD19 CAR-T cells showed stable expression and robust antigen-specific killing. The screen identified candidate surface immune modulators associated with enhanced persistence and functional fitness during serial AML challenge. Hits demonstrated reproducible enrichment patterns and differential behavior between CD33 and CD19 contexts, suggesting AML-specific regulatory programs.

### Summary/Conclusion

This focused surfaceome CRISPR screen establishes a scalable platform to dissect intrinsic regulators of CAR-T cell persistence in AML. Key targets will be validated individually across multiple AML models, including diverse patient-derived xenografts (PDX) and cell lines available in our laboratory. To assess translational breadth, selected modulators will be tested across additional CAR constructs and disease settings, including B-ALL and multiple myeloma. In vivo AML PDX studies are planned to evaluate the impact of prioritized immune modulators on CAR-T cell persistence and therapeutic efficacy.

**Copyright Information:** (Online) ISSN: 2572-9241
©2026 The Author(s). HemaSphere is published by John Wiley & Sons Ltd on behalf of European Hematology Association. This is an open access Abstract Book distributed under the Attribution-NonCommercial-NoDerivs (CC BY-NC-ND), which allows third parties to download the articles and share them with others as long as they credit the author and the Abstract Book, but they cannot change the content in any way or use them commercially.
**Abstract Book Citations:** Authors, Title, HemaSphere, 2026;10:(S1):pages. The abstract book DOIs can be found on the “Author Information” page.
**Disclaimer:** Articles published in the journal HemaSphere exclusively reflect the opinions of the authors. The authors are responsible for all content in their abstracts including accuracy of the facts, statements, citing resources, etc.

### (PB2692) OVEREXPRESSION OF MCPIP1 INDUCES APOPTOSIS IN ACUTE MYELOID LEUKEMIA CELLS VIA ACTIVATION OF THE ENDOPLASMIC RETICULUM STRESS PATHWAY

**Topic:** 03. Acute myeloid leukemia - Biology & translational research

Liyuan Li[1], Weiguang Cui*[1], Xiaoyan Zhang[1], Nan Zhou[1], Zhenzhen Wang[1], Mengying Zheng[1], Fan Yang[1], Li Sun[1], Lin Yang[1, 2], Jianmin Luo[1, 2]

*[1]The Second Hospital of Hebei Medical University, Department of Hematology, Shijiazhuang, China;[2]Hebei Institute of Hematology, Hebei Key Laboratory of Hematology, Shijiazhuang, China;*

### Background

Acute myeloid leukemia (AML) is characterized by a poor prognosis. Despite advances in targeted therapies, clinical outcomes are often compromised by limited efficacy and persistent relapse, underscoring the urgent need for novel therapeutic targets. Endoplasmic reticulum (ER) stress is a crucial determinant of cell fate, whereby its hyperactivation initiates apoptotic cascades. MCPIP1, a zinc finger RNA-binding protein exhibiting ribonuclease activity, predominantly mediates inflammatory responses and plays context-dependent roles in various malignancies. Although recent evidence links MCPIP1 to ER stress, its specific biological function and underlying mechanisms in AML remain largely elusive.

### Aims

This study aims to elucidate the functional role and underlying molecular mechanisms of MCPIP1 in AML, specifically focusing on its capacity to induce apoptosis via the modulation of the ER stress signaling pathway.

### Methods

MCPIP1 expression was exogenously upregulated in human AML cell lines (Kasumi-1 and THP-1) to evaluate its impact on cell viability and ER stress. Cell proliferation was assessed using the CCK-8 assay, and apoptosis was quantified via flow cytometry. Western blotting was utilized to analyze the protein expression levels of MCPIP1, the universal apoptosis marker PARP-1 (and its specific cleavage), and key components of the ER stress pathway (total PERK, p-PERK, eIF2α, p-eIF2α, and ATF4). Furthermore, pharmacological rescue experiments utilizing a specific PERK inhibitor were performed to mechanistically validate whether ER stress strictly mediates MCPIP1-induced apoptosis.

### Results

Initial evaluation revealed that MCPIP1 expression was significantly downregulated in primary samples from AML patients and in established AML cell lines compared to healthy controls. Functionally, the overexpression of MCPIP1 in Kasumi-1 and THP-1 cells profoundly suppressed cell viability and induced robust apoptosis, as demonstrated by CCK-8 assays and flow cytometry. Concordantly, Western blot analysis confirmed a time- and dose-dependent increase in apoptotic markers. Mechanistically, MCPIP1 overexpression potently triggered the ER stress pathway, characterized by the marked upregulation and phosphorylation of the PERK/eIF2α/ATF4 axis components. Crucially, targeted blockade of ER stress via a specific PERK inhibitor effectively abrogated the apoptosis induced by MCPIP1 overexpression. These comprehensive findings confirm that the robust pro-apoptotic effect exerted by MCPIP1 in AML is primarily mediated through the activation of the ER stress cascade.

**Copyright Information:** (Online) ISSN: 2572-9241
©2026 The Author(s). HemaSphere is published by John Wiley & Sons Ltd on behalf of European Hematology Association. This is an open access Abstract Book distributed under the Attribution-NonCommercial-NoDerivs (CC BY-NC-ND), which allows third parties to download the articles and share them with others as long as they credit the author and the Abstract Book, but they cannot change the content in any way or use them commercially.
**Abstract Book Citations:** Authors, Title, HemaSphere, 2026;10:(S1):pages. The abstract book DOIs can be found on the "Author Information" page.
**Disclaimer:** Articles published in the journal HemaSphere exclusively reflect the opinions of the authors. The authors are responsible for all content in their abstracts including accuracy of the facts, statements, citing resources, etc.

### Summary/Conclusion

Our findings definitively demonstrate that MCPIP1 exerts a potent tumor-suppressive effect in AML by activating the PERK-mediated ER stress pathway, culminating in cellular apoptosis. This study not only elucidates a novel regulatory mechanism of leukemia cell fate but also highlights the restoration of MCPIP1 function or the targeted engagement of the ER stress network as a promising translational therapeutic strategy for AML.

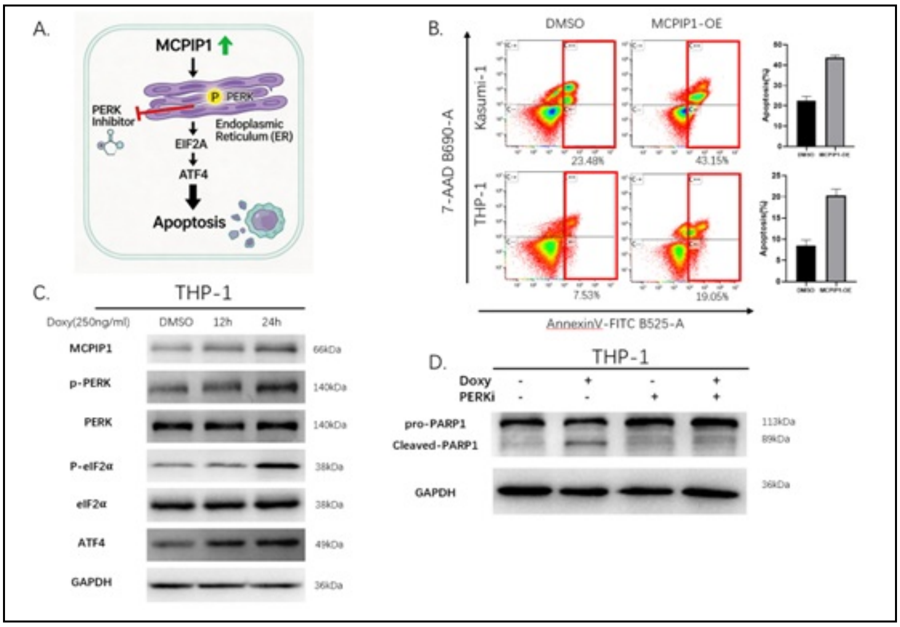

Figure 1. MCPIP1 induces AML cell apoptosis via the PERK-mediated ER stress pathway. (A) Schematic of the MCPIP1/PERK/eIF2α/ATF4 signaling axis. (B) Flow cytometry showing significantly increased apoptosis in Kasumi-1 and THP-1 cells upon MCPIP1 overexpression. (C) Western blot analysis of time-dependent upregulation of ER stress markers (p-PERK, p-eIF2α, and ATF4) following MCPIP1 induction. (D) Rescue experiment demonstrating that a PERK inhibitor (PERKi) abrogates MCPIP1-induced PARP1 cleavage, confirming ER stress-mediated apoptosis.

**Copyright Information:** (Online) ISSN: 2572-9241
©2026 The Author(s). HemaSphere is published by John Wiley & Sons Ltd on behalf of European Hematology Association. This is an open access Abstract Book distributed under the Attribution-NonCommercial-NoDerivs (CC BY-NC-ND), which allows third parties to download the articles and share them with others as long as they credit the author and the Abstract Book, but they cannot change the content in any way or use them commercially.
**Abstract Book Citations:** Authors, Title, HemaSphere, 2026;10:(S1):pages. The abstract book DOIs can be found on the "Author Information" page.
**Disclaimer:** Articles published in the journal HemaSphere exclusively reflect the opinions of the authors. The authors are responsible for all content in their abstracts including accuracy of the facts, statements, citing resources, etc.

### (PB2693) ASSOCIATIONS OF RS55829688 AND RS145204276 PROMOTER VARIANTS WITH LNCRNA GAS5 EXPRESSION IN AML: PROGNOSTIC SIGNIFICANCE AND FUNCTIONAL ANALYSIS

**Topic:** 03. Acute myeloid leukemia - Biology & translational research

Đorđe Pavlović*[1], Nataša Tošić[1], Isidora Ćurić[1], Bojan Ristivojević[1], Teodora Karan-Djurasevic[1], Ivana Grubiša[1], Marina Jelovac[1], Zlatko Pravdic[2], Branka Zukić[1], Vladimir Gašić[1]

*[1]Institute of Molecular Genetics and Genetic Engineering, Laboratory for Molecular Biomedicine, Belgrade, Serbia;[2]Clinical Center of Serbia, Clinic of Hematology, Belgrade, Serbia;*

### Background

Acute myeloid leukemia is a genetically diverse hematological malignancy where patient outcomes vary significantly. Long non-coding RNA (lncRNA) GAS5 acts as a tumor suppressor and is frequently downregulated in various cancers, as well as in AML.

### Aims

In the current study, we aimed to explore the effects of GAS5 promoter variants on its expression levels in AML patients, their prognostic significance, and to investigate their functional effects.

### Methods

The GAS5 promoter region containing rs55829688 and rs145204276 was sequenced in 75 AML patients. Statistical analyses were performed to assess their associations with GAS5 expression and outcomes. An in vitro functional study in K562 cells evaluated the effects of these variants on the transcriptional activity of constructs containing each variant. In silico analysis was used to predict changes to transcription factor binding sites.

### Results

Patients carrying the rs55829688 TC/CC genotype exhibited lower GAS5 expression and were more frequently categorized into the adverse risk group. In intermediate-risk patients, this genotype trended toward lower overall survival and higher bone marrow blast percentages. In vitro, the construct harboring the rs55829688 C allele showed a two-fold decrease in reporter gene activity compared to the construct bearing both wild type alleles. In silico analysis identified RUNX3 as the most likely transcription factor affected by this variant. The variant rs145204276 was considered for the first time in AML; however, no significant clinical associations or transcriptional effects were found.

### Summary/Conclusion

Taken together, our findings provide evidence that the rs55829688 promoter variant reduces GAS5 expression in AML and could potentially be a prognostic marker.

**Copyright Information:** (Online) ISSN: 2572-9241
©2026 The Author(s). HemaSphere is published by John Wiley & Sons Ltd on behalf of European Hematology Association. This is an open access Abstract Book distributed under the Attribution-NonCommercial-NoDerivs (CC BY-NC-ND), which allows third parties to download the articles and share them with others as long as they credit the author and the Abstract Book, but they cannot change the content in any way or use them commercially.
**Abstract Book Citations:** Authors, Title, HemaSphere, 2026;10:(S1):pages. The abstract book DOIs can be found on the "Author Information" page.
**Disclaimer:** Articles published in the journal HemaSphere exclusively reflect the opinions of the authors. The authors are responsible for all content in their abstracts including accuracy of the facts, statements, citing resources, etc.

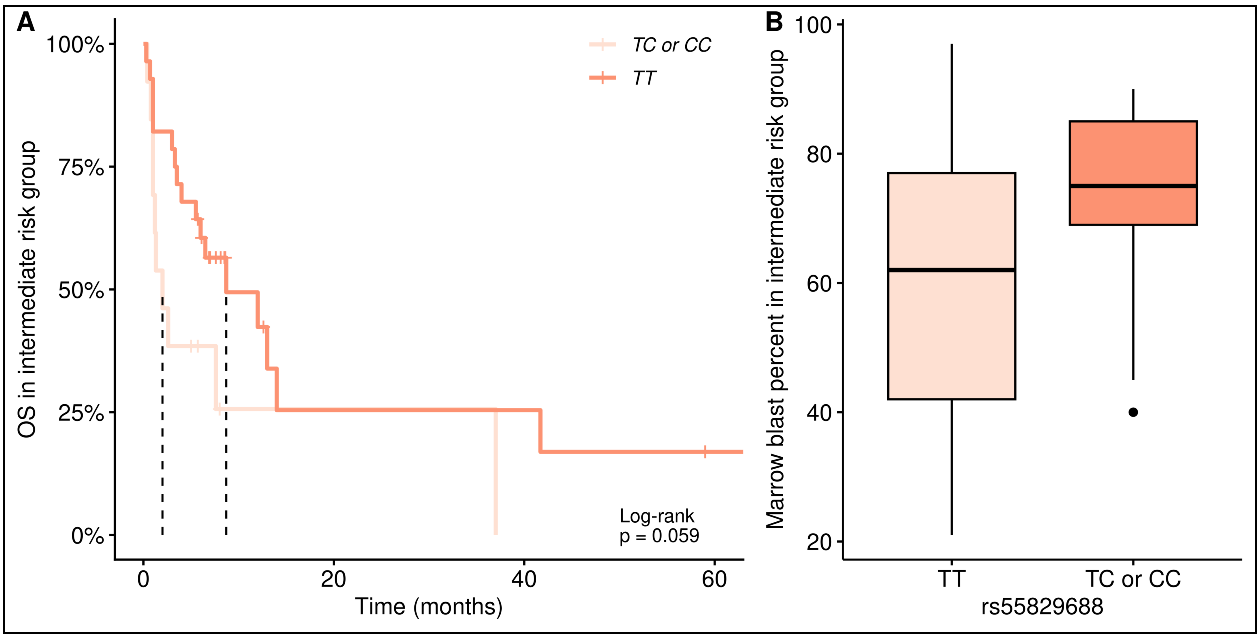

**Copyright Information:** (Online) ISSN: 2572-9241
©2026 The Author(s). HemaSphere is published by John Wiley & Sons Ltd on behalf of European Hematology Association. This is an open access Abstract Book distributed under the Attribution-NonCommercial-NoDerivs (CC BY-NC-ND), which allows third parties to download the articles and share them with others as long as they credit the author and the Abstract Book, but they cannot change the content in any way or use them commercially.
**Abstract Book Citations:** Authors, Title, HemaSphere, 2026;10:(S1):pages. The abstract book DOIs can be found on the "Author Information" page.
**Disclaimer:** Articles published in the journal HemaSphere exclusively reflect the opinions of the authors. The authors are responsible for all content in their abstracts including accuracy of the facts, statements, citing resources, etc.

### (PB2694) INHIBITION OF LYSOSOMAL DETOXIFICATION AND RECYCLING PATHWAYS IN ACUTE MYELOBLASTIC LEUKAEMIA

**Topic:** 03. Acute myeloid leukemia - Biology & translational research

Anaïs Dubiau[1], Celine Baier[2], Lamia Madaci[1], Erwann Collomb[1], Eloine Bestion[3], Sonia Mezouar[3], Julien Colle[1], Guillaume Martin[2], Ismail Ceylan[2], Geoffroy Venton[1], Spyridon Vlachopoulos[4], Mileidys Perez[2], Philippe Halfon[3], Régis Costello*[1]

*[1]TAGC/INSERM U1090, Marseille, France; [2]AdvancedBioDesign, Marseille, France; [3]Genoscience, Marseille, France; [4]University of Athens, Athens, Greece;*

### Background

Acute myeloid leukaemia (AML) remains an aggressive malignant blood disorder, with a 5-year survival rate of less than 30% and frequent relapses due to treatment-resistant leukaemic stem or progenitor cells. These cells survive chemotherapy, hypomethylating agents and targeted therapies by neutralising oxidative and aldehyde stress and recycling damaged components via cytoprotective autophagy. Although ALDHA1 inhibition affects a protective mechanism essential for cell survival, escape mechanisms exist, as evidenced by the possible development of resistant cell lines, the survival of cell populations identified in single-cell studies, and the existence of genes involved in resistance identified by the CRISPRcas9 approach, despite pharmacological inhibition of ALDHA1. the possible development of resistant cell lines, the survival of cell populations identified in single-cell studies, and the existence of genes involved in resistance identified by the CRISPRcas9 approach (unpublished data).

### Aims

This invites us to evaluate the combination of ALDHA1 blockade with autophagy blockade.

### Methods

We studied a series of AML cell lines (HL-60, KASUMI1, KG1, MONOMAC 6, MOLM14, MV-4-11, K562), cultured in the presence of an ALDHA1 inhibitor (Dimate), in the presence of an autophagy inhibitor (GNS561-Ezurpimtrostat), as well as the Azacytidine-Venetoclax combination, and by combining these various inhibitors

### Results

Most of the cell lines tested proved to be sensitive to the autophagy inhibitor at concentrations compatible with in vivo use, as they were relatively comparable to the results obtained in clinical trials in hepatocellular carcinoma. A single-cell RNAseq study of 12 patient samples treated in vitro with the azacitidine-venetoclax combination shows a very significant increase in the number of cells expressing the PPT1, LC3, STINGn, P62, and MTOR genes, which are central elements of the autophagy mechanism. These data suggest that one of the mechanisms of escape from the azacytidine-ventoclax combination is the increase in the autophagy pathway, allowing both the elimination of toxic waste and the supply of energy to the leukemia cell. Preliminary data show synergy between GNS561 and both the ALDH inhibitor and the azacytidine-venetoclax combination, suggesting the possibility of blocking this therapeutic escape pathway.

**Copyright Information:** (Online) ISSN: 2572-9241
©2026 The Author(s). HemaSphere is published by John Wiley & Sons Ltd on behalf of European Hematology Association. This is an open access Abstract Book distributed under the Attribution-NonCommercial-NoDerivs (CC BY-NC-ND), which allows third parties to download the articles and share them with others as long as they credit the author and the Abstract Book, but they cannot change the content in any way or use them commercially.
**Abstract Book Citations:** Authors, Title, HemaSphere, 2026;10:(S1):pages. The abstract book DOIs can be found on the “Author Information” page.
**Disclaimer:** Articles published in the journal HemaSphere exclusively reflect the opinions of the authors. The authors are responsible for all content in their abstracts including accuracy of the facts, statements, citing resources, etc.

**Summary/Conclusion**

Our preliminary data suggest that blocking the autophagy pathway suppresses a basic mechanism of leukemia cell survival as well as a mechanism of tumor escape from chemotherapy. These results will be supplemented by a study focusing on leukemia stem cells and identifying mechanisms of tumor escape from autophagy blockade, in particular by studying the effects of GNS561 on blasts from relapsed or refractory patients (SCALA clinical research protocol, Assistance Publique Hôpitaux de Marseille).

This study aims to lead to a phase II trial to demonstrate the efficacy of autophagy inhibition in AML using a combination of GNS561 with current treatments, particularly the azacytidine-venetoclax combination, while also evaluating the synergy of blocking the ALDH pathway in combination with the autophagy pathway.

Bestion E, Costello R, Mezouar S, Halfon P. Autophagy in cancer resistance: New combinatorial strategy for cancer therapy. Leukemia. 2024 Oct;38(10):2289-2290.

Costello R, Dancik GM, Dubiau A, Madaci L, Vlahopoulos S. Expression of Aldehyde Dehydrogenase 1A1 in Relapse-Associated Cells in Acute Myeloid Leukemia. Cells. 2025 Jul 7;14(13):1038.

**Copyright Information:** (Online) ISSN: 2572-9241
©2026 The Author(s). HemaSphere is published by John Wiley & Sons Ltd on behalf of European Hematology Association. This is an open access Abstract Book distributed under the Attribution-NonCommercial-NoDerivs (CC BY-NC-ND), which allows third parties to download the articles and share them with others as long as they credit the author and the Abstract Book, but they cannot change the content in any way or use them commercially.
**Abstract Book Citations:** Authors, Title, HemaSphere, 2026;10:(S1):pages. The abstract book DOIs can be found on the "Author Information" page.
**Disclaimer:** Articles published in the journal HemaSphere exclusively reflect the opinions of the authors. The authors are responsible for all content in their abstracts including accuracy of the facts, statements, citing resources, etc.

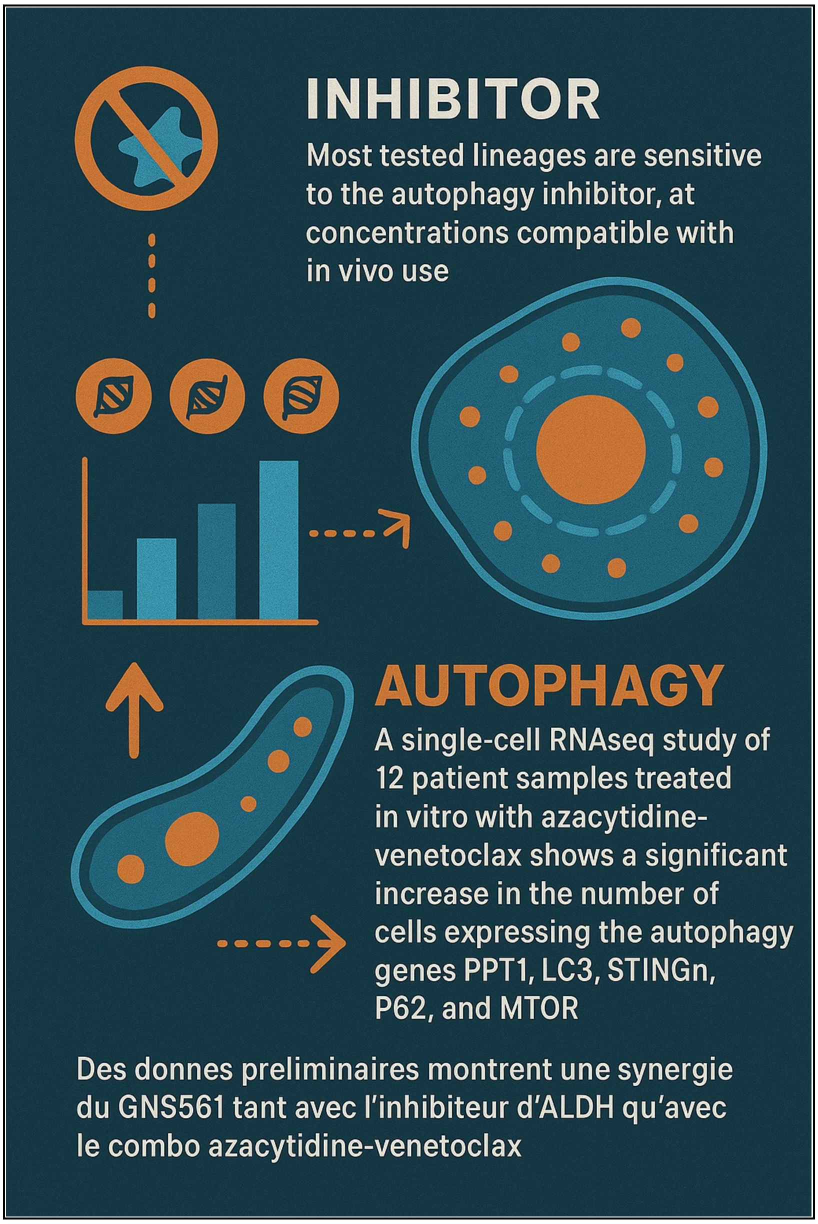

**Copyright Information:** (Online) ISSN: 2572-9241
©2026 The Author(s). HemaSphere is published by John Wiley & Sons Ltd on behalf of European Hematology Association. This is an open access Abstract Book distributed under the Attribution-NonCommercial-NoDerivs (CC BY-NC-ND), which allows third parties to download the articles and share them with others as long as they credit the author and the Abstract Book, but they cannot change the content in any way or use them commercially.
**Abstract Book Citations:** Authors, Title, HemaSphere, 2026;10:(S1):pages. The abstract book DOIs can be found on the "Author Information" page.
**Disclaimer:** Articles published in the journal HemaSphere exclusively reflect the opinions of the authors. The authors are responsible for all content in their abstracts including accuracy of the facts, statements, citing resources, etc.

### (PB2695) NGS-BASED MIRNA EXPRESSION ANALYSIS IN PATIENTS CLASSIFIED AS MDS/AML VS AML ACCORDING TO ICC 2022

**Topic:** 03. Acute myeloid leukemia - Biology & translational research

Claudia Núñez-Torron Stock*[1, 2, 3], Clara Lopez Hernandez[4], Carlos Jimenez-Chillon[5], Alba Sara Vilches Moreno[1, 3], Maria Jose Penalva Moreno[1, 3], Alicia Roldan Perez[1, 3], Juan Andres Vazquez Paganini[1, 3], Eugenio Jimenez Mesa[6], Miguel Piris-Villaespesa[2, 4], Leticia Olavarrieta Scappini[7], Val Fernandez Lanza[7], Javier Lopez Jimenez[2, 4], Pilar Herrera Puente[2, 4], Maria Regina Herraez Garcia[1, 3]

*[1]Hospital Universitario Infanta Sofía, San Sebastian de los Reyes, Spain;[2]Hematology and Hemotherapy Service, Instituto Ramón y Cajal de Investigación Sanitaria (IRYCIS), Hospital Universitario Ramón y Cajal, Madrid, Spain. Madrid, Spain, MADRID, Spain; [3]Universidad Europea, Departamento de Medicina, MADRID, Spain;[4]Hospital Universitario Ramón y Cajal, Hemathology and Hemotherapy, Madrid, Spain; [5]Hospital Universitario Gregorio Marañon, Hemathology and Hemotherapy, Madrid, Spain; [6]Hospital Universitario Alcalá de Henares, Hemathology and Hemotherapy, Alcalá de Henares, Spain;[7]Translational Genomics and Bioinformatics Unit, Instituto Ramón y Cajal de Investigación Sanitaria (IRYCIS), Hospital Universitario Ramón y Cajal, Madrid, Spain. Madrid, Spain, MADRID, Spain;*

### Background

In patients with acute myeloid leukemia (AML), the International Consensus Classification (ICC) 2022 has included cases with 10–19% blasts (previously classified as myelodysplastic syndrome with excess of blast 2 [MDS-EB2]) within the AML category. It remains unclear whether these two groups represent distinct biological entities. MicroRNAs (miRNAs) are powerful cancer biomarkers and provide a valuable tool to investigate these differences.

### Aims

To evaluate the miRNA expression profile by next-generation sequencing (NGS) in the mononuclear fraction of bone marrow aspirates at diagnosis and compare patients previously classified as MDS-EB2 (10–19% blasts) with those with ≥20% blasts.

### Methods

Samples were prospectively collected from a dedicated miRNA biobank including patients ≥18 years with suspected or confirmed MDS-EB2 or AML. Mononuclear cells were isolated by Ficoll density gradient centrifugation, and miRNA-enriched RNA was extracted (NORGEN). miRNA expression profiling was performed by NGS on an Illumina platform (NovaXPLus). following quality control. Differential expression analysis was conducted in R using DESeq2; miRNAs with FDR < 0.05 and |log2FC| > 1 were considered significant. Validated targets were retrieved from multiMiR. Exploratory functional enrichment (GO, KEGG, Reactome) was performed in R. Tables and graphical representations were generated in Python. A total of 27 samples were analyzed.

**Copyright Information:** (Online) ISSN: 2572-9241
©2026 The Author(s). HemaSphere is published by John Wiley & Sons Ltd on behalf of European Hematology Association. This is an open access Abstract Book distributed under the Attribution-NonCommercial-NoDerivs (CC BY-NC-ND), which allows third parties to download the articles and share them with others as long as they credit the author and the Abstract Book, but they cannot change the content in any way or use them commercially.
**Abstract Book Citations:** Authors, Title, HemaSphere, 2026;10:(S1):pages. The abstract book DOIs can be found on the “Author Information” page.
**Disclaimer:** Articles published in the journal HemaSphere exclusively reflect the opinions of the authors. The authors are responsible for all content in their abstracts including accuracy of the facts, statements, citing resources, etc.

**Results**

The cohort was divided in AML (n =20) and MDS-EB2 patients (n =7). Median age at diagnosis was 71.5 years in AML vs 73 years in MDS-EB2, and 55.0% and 57.1% were males respectively. The median percentage of blast at diagnosis was 71.0% (27-87) vs 16.0% (11-18). In both groups most patients belonged to adverse risk by European LeukemiaNet 2022 risk classification ( 50.0% in AML vs 87.5% in MDS-EB2). According to ICC 2022 patients were classified as: AML group, mutated NPM1 8 (40%), mutated TP53 (TP53mut) 4 (20%), myelodysplasia-related gene mutations (MDS-gene mut) 4 (20%), myelodysplasia-related cytogenetic abnormalities 1 (5%), Other rare recurring translocations 1 (5%), not otherwise specified (NOS) 2 (10%); MDS-EB2 group, TP53mut 3 (42.9%), MDS-gene mut 2 (28.6%), NOS 2 (28.6%). A total of 1315 miRNAs were detected, of which 31 were differentially expressed between the two groups (22 upregulated and 9 downregulated in AML patients). Figure 1 presents the volcano plot and the top 10 differentially expressed miRNAs.The most significantly deregulated miRNAs included known leukemogenesis-associated candidates (e.g., miR-181a-3p or miR-181b-5p) and potentially novel findings in this diagnostic setting (e.g., miR-885-5p) Together, the 31 differentially expressed miRNAs target more than 13000 genes validated experimentally. Enriched GO biological processes included regulation of apoptotic signaling and myeloid cell differentiation. Key molecular pathways identified by KEGG comprised the PI3K-Akt and Wnt signaling pathways, while Reactome analysis highlighted the RHO GTPase cycle and transcriptional regulation by RUNX1 as additional relevant mechanisms.

**Summary/Conclusion**

The miRNA profile differed between MDS-EB2 and AML, suggesting potential biological differences beyond blast count. We identified previously reported AML-associated miRNAs as well as additional candidates. These findings are exploratory and warrant validation in larger cohorts.

**Copyright Information:** (Online) ISSN: 2572-9241
©2026 The Author(s). HemaSphere is published by John Wiley & Sons Ltd on behalf of European Hematology Association. This is an open access Abstract Book distributed under the Attribution-NonCommercial-NoDerivs (CC BY-NC-ND), which allows third parties to download the articles and share them with others as long as they credit the author and the Abstract Book, but they cannot change the content in any way or use them commercially.
**Abstract Book Citations:** Authors, Title, HemaSphere, 2026;10:(S1):pages. The abstract book DOIs can be found on the "Author Information" page.
**Disclaimer:** Articles published in the journal HemaSphere exclusively reflect the opinions of the authors. The authors are responsible for all content in their abstracts including accuracy of the facts, statements, citing resources, etc.

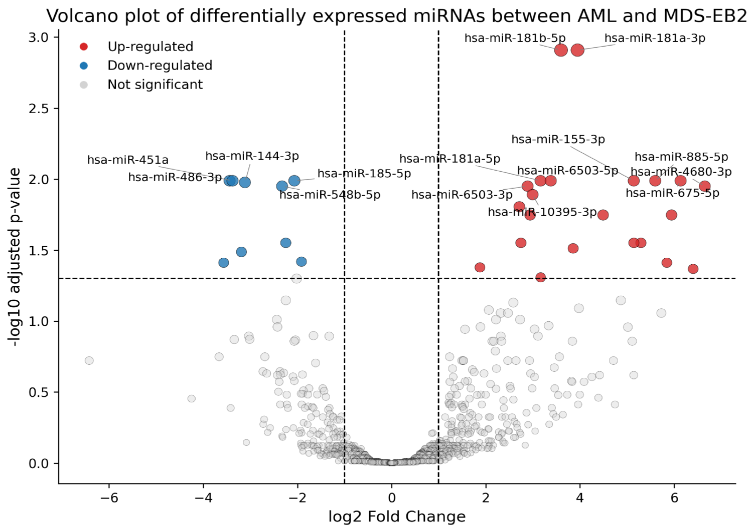

| miRNA | log2FoldChange | padj | Regulation |
|---|---|---|---|
| hsa-miR-181a-3p | 3.95 | 0.001 | Up-regulated |
| hsa-miR-181b-5p | 3.60 | 0.001 | Up-regulated |
| hsa-miR-181a-5p | 3.16 | 0.010 | Up-regulated |
| hsa-miR-486-3p | -3.44 | 0.010 | Down-regulated |
| hsa-miR-185-5p | -2.07 | 0.010 | Down-regulated |
| hsa-miR-155-3p | 5.14 | 0.010 | Up-regulated |
| hsa-miR-451a | -3.38 | 0.010 | Down-regulated |
| hsa-miR-6503-5p | 3.38 | 0.010 | Up-regulated |
| hsa-miR-675-5p | 6.14 | 0.010 | Up-regulated |
| hsa-miR-885-5p | 5.60 | 0.010 | Up-regulated |

Figure 1. Volcano plot of miRNA expression and table of the 10 most differentially expressed miRNAs between AML and MDS-EB2.

**Copyright Information:** (Online) ISSN: 2572-9241
©2026 The Author(s). HemaSphere is published by John Wiley & Sons Ltd on behalf of European Hematology Association. This is an open access Abstract Book distributed under the Attribution-NonCommercial-NoDerivs (CC BY-NC-ND), which allows third parties to download the articles and share them with others as long as they credit the author and the Abstract Book, but they cannot change the content in any way or use them commercially.
**Abstract Book Citations:** Authors, Title, HemaSphere, 2026;10:(S1):pages. The abstract book DOIs can be found on the "Author Information" page.
**Disclaimer:** Articles published in the journal HemaSphere exclusively reflect the opinions of the authors. The authors are responsible for all content in their abstracts including accuracy of the facts, statements, citing resources, etc.

### (PB2696) LEVELS OF 91 CIRCULATING INFLAMMATORY PROTEINS AND RISK OF ACUTE MYELOID LEUKEMIA : A MENDELIAN RANDOMIZATION STUDY

**Topic:** 03. Acute myeloid leukemia - Biology & translational research

Huihui An*[1], Wenhui Liu[1], Tao Wu[1]

[1]*The 940th Hospital of Joint Logistics Support force of Chinese People's Liberation Army, hematology, Gansu, China;*

### Background

Acute Myeloid Leukemia (AML) is a malignant disease of the blood system caused by the abnormal differentiation and proliferation of hematopoietic stem cells, with common symptoms
including anemia and infections. Mendelian Randomization (MR) is a method that uses genetic variants as instrumental variables to infer causal relationships between diseases and their potential causes. When randomized controlled trials are not feasible or observational studies are biased due to confounding factors, MR provides a reliable solution[8].With the advancement of Genome-Wide Association Studies (GWAS), proteomics has become a new avenue for exploring the pathogenesis and treatment of diseases such as cancer, inflammatory bowel disease, and multiple myeloma. Although epidemiological studies have shown an association between levels of inflammatory proteins and the risk of developing AML, the causal relationship remains unclear. Therefore, by integrating high-throughput proteomics and genetic data, we identified 91 circulating inflammatory proteins in the recently published GWAS database. Through two-sample Mendelian randomization analysis, we investigated the causal relationship between these 91 circulating inflammatory proteins and the incidence of AML.

### Aims

Previous cohort studies have observed a relationship between inflammatory proteins and acute myeloid leukemia (AML). However, it is currently unclear which circulating inflammatory proteins can affect the risk of developing AML.

### Methods

We utilized the summary results of Genome-Wide Association Studies (GWAS) for 91 circulating inflammatory proteins, conducting Mendelian Randomization (MR) analysis to assess their causal impact on AML. The genetic variation data for AML were sourced from the FinnGen consortium. As the primary analysis method, we employed the Inverse Variance Weighted (IVW) method, and further strengthened the final results through MR-Egger and weighted median methods. Additionally, we conducted sensitivity analyses to evaluate the robustness and reliability of our results.

### Results

Our study found that 8 circulating inflammatory proteins have a causal effect with the occurrence of AML ($p<0.05$). Among them, increased levels of ARTN, IL-2RB, SIRT2, and STAMPB are associated with a decreased risk of developing AML: [(OR: 0.4583, 95%CI: 0.2190-0.9591); (OR: 0.2347, 95%CI: 0.0941-0.5853); (OR: 0.3104, 95%CI: 0.1380-0.6982); (OR: 0.2890, 95%CI: 0.1049-0.7961)]. Conversely, increased levels of CD6, CXCL5, IL-15RA, and MMP-10 are associated with an increased risk of developing AML: [(OR: 3.2693, 95%CI: 1.2853-8.3159); (OR: 1.6946, 95%CI: 1.0134-2.8336); (OR: 1.5729, 95%CI: 1.0500-2.3448); (OR: 1.8820, 95%CI: 1.0614-3.3371)].

**Copyright Information:** (Online) ISSN: 2572-9241
©2026 The Author(s). HemaSphere is published by John Wiley & Sons Ltd on behalf of European Hematology Association. This is an open access Abstract Book distributed under the Attribution-NonCommercial-NoDerivs (CC BY-NC-ND), which allows third parties to download the articles and share them with others as long as they credit the author and the Abstract Book, but they cannot change the content in any way or use them commercially.
**Abstract Book Citations:** Authors, Title, HemaSphere, 2026;10:(S1):pages. The abstract book DOIs can be found on the "Author Information" page.
**Disclaimer:** Articles published in the journal HemaSphere exclusively reflect the opinions of the authors. The authors are responsible for all content in their abstracts including accuracy of the facts, statements, citing resources, etc.

**Summary/Conclusion**

Our study has provided new insights into the relationship between circulating inflammatory proteins and AML using MR analysis study.

**Copyright Information:** (Online) ISSN: 2572-9241
©2026 The Author(s). HemaSphere is published by John Wiley & Sons Ltd on behalf of European Hematology Association. This is an open access Abstract Book distributed under the Attribution-NonCommercial-NoDerivs (CC BY-NC-ND), which allows third parties to download the articles and share them with others as long as they credit the author and the Abstract Book, but they cannot change the content in any way or use them commercially.
**Abstract Book Citations:** Authors, Title, HemaSphere, 2026;10:(S1):pages. The abstract book DOIs can be found on the "Author Information" page.
**Disclaimer:** Articles published in the journal HemaSphere exclusively reflect the opinions of the authors. The authors are responsible for all content in their abstracts including accuracy of the facts, statements, citing resources, etc.

### (PB2697) EFFECT OF IRRADIATION ON CLONAL HETEROGENEITY OF AML IN NSG MICE

**Topic:** 03. Acute myeloid leukemia - Biology & translational research

Jörg Cammenga*[1], Emma Rorby[2], Gunnar Juliusson[1], Erik Hultin[2], Tatjana Pandzic[3], Claes Ladenvall[3], Panagiotis Baliakas[3]

*[1]Lund University, Lund, Sweden; [2]Linköping University, Linköping, Sweden; [3]Uppsala University, Uppsala, Sweden;*

### Background

Patient-derived xenograft (PDX) models are essential tools for studying AML biology. Whether human AMLs preserves the genetic and phenotypic features of the leukemia cells in PDX models has not been studied for all genetic AML subgroups . Additionally, the effect of pre-transplantation conditioning with irradiation on clonality has also not be rigorously studied. To address this gap, we investigated the clonal genetic and phenotypic evolution of normal-karyotype FLT3-ITD–mutated AML after transplantation into NSG mice, with a specific focus on assessing the impact of pre-transplant irradiation.

### Aims

No comparative studies transplanting the same human AML sample, of a specific genetic subgroup from blood or bone marrow, into irradiated and non-irradiated mice have been performed in a controlled fashion This complicates the interpretation of the conflicting findings regarding whether the dominant AML clone or a more aggressive subclone preferentially engrafts in PDX models.
In this study we investigate if irradiation prior to transplantation of human AML cells into NSG mice could influence human engraftment levels and clonal composition of the AML or both. We use the common subgroup of FLT-3 ITD mutated AML (with the majority being also mutated for NPM1c) to investigate clonal evolution in NSG mice and the effect of irradiation prior to transplantation.

### Methods

Human FLT-3 ITD positive AML cells from blood or bone marrow were transplanted into either irradiated or non-irradiated NSG mice. Engraftment levels were compared and also the phenotypic and molecular phenotype of the engrafted human cells by advanced flow cytometry and panel sequencing.

**Copyright Information:** (Online) ISSN: 2572-9241
©2026 The Author(s). HemaSphere is published by John Wiley & Sons Ltd on behalf of European Hematology Association. This is an open access Abstract Book distributed under the Attribution-NonCommercial-NoDerivs (CC BY-NC-ND), which allows third parties to download the articles and share them with others as long as they credit the author and the Abstract Book, but they cannot change the content in any way or use them commercially.
**Abstract Book Citations:** Authors, Title, HemaSphere, 2026;10:(S1):pages. The abstract book DOIs can be found on the "Author Information" page.
**Disclaimer:** Articles published in the journal HemaSphere exclusively reflect the opinions of the authors. The authors are responsible for all content in their abstracts including accuracy of the facts, statements, citing resources, etc.

**Results**

Engraftment level of human cells in the bone marrow was investigated 15 weeks post transplantation.
To investigate the phenotypic status of human AML cells we analysed cell surface markers followed by t-VSNE analysis. Similar engraftment levels were seen regardless of source of human cells or conditioning prior to transplantation. For two of the originating BM AML samples the phenotypic composition was similar between de novo patient sample and the transplanted cells.
To better understand which surface markers were differentially expressed between theclusters of input de novo AML and the cells obtained from the irradiated or non-irradiated mice we created heatmaps illustrating the expression of cell surface markers in the identified clusters.

We wanted to address whether irradiation prior to transplantation or cell source infuences the genetic composition of the human AML when transplanted into NSG mice. For this reason, human CD45 enriched AML cells from transplanted mice were subjected to panel sequencing and compared to the input *de novo* AML cells.
This analysis revealed that in the majority of cases the dominant clone in the input AML samples was also the dominant clone in both the irradiated and the non-irradiated mice showing that non-irradiated condition did not affect engraftment or genetic composition.
In one AML sample the FLT-3 ITD VAF increased caused by uniparental disomy.
Interestingly, in one other case a novel Gata-2 mutation occurred in two out of three non-irradiated mice.

**Summary/Conclusion**

Across the majority of AML samples analyzed, neither cell source (blood versus bone marrow) nor pre-transplant conditioning did influence engraftment levels or genetic composition of the human AML cells.

**Copyright Information:** (Online) ISSN: 2572-9241
©2026 The Author(s). HemaSphere is published by John Wiley & Sons Ltd on behalf of European Hematology Association. This is an open access Abstract Book distributed under the Attribution-NonCommercial-NoDerivs (CC BY-NC-ND), which allows third parties to download the articles and share them with others as long as they credit the author and the Abstract Book, but they cannot change the content in any way or use them commercially.
**Abstract Book Citations:** Authors, Title, HemaSphere, 2026;10:(S1):pages. The abstract book DOIs can be found on the “Author Information” page.
**Disclaimer:** Articles published in the journal HemaSphere exclusively reflect the opinions of the authors. The authors are responsible for all content in their abstracts including accuracy of the facts, statements, citing resources, etc.

### (PB2698) ACUTE MYELOID LEUKEMIA DRIVEN BY RARE HNRNPH1::ERG FUSION IN A YOUNG ADULT, WITH DISTINCT RNASEQ-DETECTED TRANSCRIPTS AT DIAGNOSIS AND RELAPSE.

**Topic:** 03. Acute myeloid leukemia - Biology & translational research

Pablo Prieto-Martinez*[1], Hannah Al-Yousuf[2], Baker Robert[2], Ke Xu[2], Andrew Wilson[2], Jiexin Zheng[2], Gupta Rajeev[2], Rob Sellar[2], Jenny O'nions[2]

*[1]Hospital Universitario Morales Meseguer, Hematology, Murcia, Spain;[2]University College of London Hospitals, Haematology, London, United Kingdom;*

### Background

Fusion genes are present in 30–40% of acute myeloid leukemia (AML) and underpin current diagnostic and prognostic frameworks, particularly for canonical entities such as BCR::ABL, RUNX1::RUNX1T1, CBFB::MYH11 and PML::RARA. Rarer fusions including those which involve genes such as ERG on 21q22 occur in less than 1% of AML, and are classified as "AML with other rare recurring translocations". Occasionally, these fusions may be cryptic by conventional cytogenetic testing but detectable by RNA sequencing (RNAseq) techniques, being increasingly identified as genomic platforms improve and are more widely applied in cancer diagnostics The prognostic significance of these rare fusion genes is still unclear, and their use in clinical decision-making and MRD monitoring is not standardized. HNRNPH1::ERG rearrangement is exceptional, more often reported in paediatrics and solid tumours, with only five AML cases described to date and generally poor outcomes.

### Aims

To describe an adult AML case with a novel HNRNPH1::ERG fusion at diagnosis and a distinct HNRNPH1::ERG transcript at relapse, and to explore implications for measurable residual disease (MRD) monitoring.

### Methods

Diagnostic work-up included morphology, multiparameter flow cytometry, fluorescence in situ hybridization, chromosomal microarray, and targeted RNAseq using the Archer Pan-Heme FusionPlex panel, with parallel VariantPlex DNA sequencing. Patient-specific quantitative RT-PCR assays were designed to each HNRNPH1::ERG breakpoint for serial MRD assessment in bone marrow samples taken during therapy and follow up. Stored end-of-treatment and post-treatment samples were tested once the relapse fusion transcript had been identified.

### Results

A 19-year-old female presented with pancytopenia and 89% myeloid blasts; immunophenotype was CD34+, CD117+, CD33+, CD13+, CD7+, CD56w, MPO+. Conventional karyotype and microarray were normal. RNAseq detected an HNRNPH1::ERG fusion (exon 14::ERG exon 11; chr5:179614341–chr21:38383923) with no additional pathogenic variants. Induction with DA (3+10) plus gemtuzumab ozogamicin, followed by DA (3+8) and two cycles of high-dose cytarabine, achieved complete remission with undetectable MRD by qRT-PCR at all interim assessments. At 6 months post end-of-treatment the patient relapsed with 28%. The original MRD assay remained negative, but RNAseq identified a second fusion (exon 4::exon 11; chr5:179621034–chr21:38383923). Performed in retrospect, a new qPCR assay demonstrated low-level exon 4::exon 11 transcripts at end-of-treatment and rising levels at 3 months post end-of-treatment, indicating outgrowth of this clone before morphologic relapse (Figure 1). Salvage FLA-IDA induced a second complete remission with MRD reduction, and the patient underwent myeloablative matched unrelated donor allogeneic stem cell transplantation, remaining in remission at 12 months.

**Copyright Information:** (Online) ISSN: 2572-9241
©2026 The Author(s). HemaSphere is published by John Wiley & Sons Ltd on behalf of European Hematology Association. This is an open access Abstract Book distributed under the Attribution-NonCommercial-NoDerivs (CC BY-NC-ND), which allows third parties to download the articles and share them with others as long as they credit the author and the Abstract Book, but they cannot change the content in any way or use them commercially.
**Abstract Book Citations:** Authors, Title, HemaSphere, 2026;10:(S1):pages. The abstract book DOIs can be found on the "Author Information" page.
**Disclaimer:** Articles published in the journal HemaSphere exclusively reflect the opinions of the authors. The authors are responsible for all content in their abstracts including accuracy of the facts, statements, citing resources, etc.

### Summary/Conclusion

This AML case represents only the third adult HNRNPH1::ERG-positive case and the first to document acquisition or expansion of a distinct HNRNPH1::ERG transcript at relapse. The failure of a single fusion-specific assay to detect emerging disease, despite molecularly trackable transcripts, underscores that rare fusions involving highly spliced partners may generate multiple breakpoints within the same patient. Comprehensive RNAseq at both diagnosis and relapse, coupled with flexible assay design, may be essential to optimise MRD monitoring and to understand clonal dynamics in fusion-driven, high-risk AML subsets.

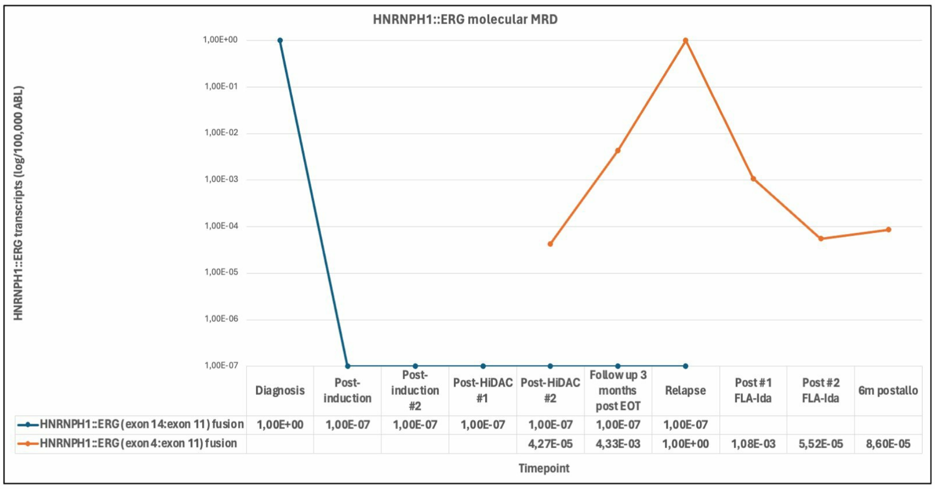

| | Diagnosis | Post-induction | Post-induction #2 | Post-HiDAC #1 | Post-HiDAC #2 | Follow up 3 months post EOT | Relapse | Post #1 FLA-Ida | Post #2 FLA-Ida | 6m postallo |
|---|---|---|---|---|---|---|---|---|---|---|
| HNRNPH1::ERG (exon 14:exon 11) fusion | 1,00E+00 | 1,00E-07 | 1,00E-07 | 1,00E-07 | 1,00E-07 | 1,00E-07 | 1,00E-07 | | | |
| HNRNPH1::ERG (exon 4:exon 11) fusion | | | | | 4,27E-05 | 4,33E-03 | 1,00E+00 | 1,08E-03 | 5,52E-05 | 8,60E-05 |

**Figure 1:** Molecular MRD measurement of *HNRNPH1::ERG* fusion transcripts by quantitative RT-PCR over the disease course. *HNRNPH1::ERG* fusion (exons 14:11) transcripts detected at diagnosis and *HNRNPH1::ERG* (exons 4:11) detected at relapse and retrospectively tested are shown as log-fold changes from baseline.

**Copyright Information:** (Online) ISSN: 2572-9241
©2026 The Author(s). HemaSphere is published by John Wiley & Sons Ltd on behalf of European Hematology Association. This is an open access Abstract Book distributed under the Attribution-NonCommercial-NoDerivs (CC BY-NC-ND), which allows third parties to download the articles and share them with others as long as they credit the author and the Abstract Book, but they cannot change the content in any way or use them commercially.
**Abstract Book Citations:** Authors, Title, HemaSphere, 2026;10:(S1):pages. The abstract book DOIs can be found on the "Author Information" page.
**Disclaimer:** Articles published in the journal HemaSphere exclusively reflect the opinions of the authors. The authors are responsible for all content in their abstracts including accuracy of the facts, statements, citing resources, etc.

### (PB2700) TRANSCRIPTIONAL CHARACTERIZATION OF CELLULAR COMPONENTS OF THE IMMUNE SYSTEM IN REMISSION OF ACUTE MYELOID LEUKEMIA: PERIPHERAL BLOOD SINGLE-CELL RNA-SEQ DATA

**Topic:** 03. Acute myeloid leukemia - Biology & translational research

Irina Baratova*[1], Ivan Abramov[1], Sergey Nikolaev[1], Galina Dudina[1], Anzhelika Chegodar[1], Dariya Mineeva[1], Svetlana Dmitrieva[1], Maria Blinova[1], Nikolay Pozdnyakov[1], Natalia Bodunova[1]

[1]*The Loginov Moscow Clinical Scientific Center, Moscow, Russia;*

### Background

Acute myeloid leukemia (AML) is a clonal hematologic malignancy characterized by accumulation of myeloid blasts in the bone marrow. Risk stratification is mainly based on cytogenetic and molecular features of the leukemic clone, whereas the systemic immune landscape during remission remains insufficiently characterized. Single-cell RNA sequencing (scRNA-seq) enables high-resolution analysis of immune cell composition and functional transcriptional states.

### Aims

To characterize peripheral blood immune composition and transcriptional states in a patient with AML during clinical remission using scRNA-seq.

### Methods

Peripheral blood was collected three months after diagnosis during confirmed remission. Single-cell 3′ gene expression libraries were generated using MobiDrop reagents and sequenced on the SURFSeq 5000 platform. Reads were aligned to GRCh38 followed by UMI-based quantification and quality control filtering.
A total of 1,405 high-quality cells were analyzed. Data processing included normalization, Louvain clustering, and UMAP visualization. Clusters were annotated using canonical lineage markers. Functional programs assessed comprised cytotoxicity, exhaustion, proliferation, inflammation, and antigen presentation. AML-associated genes (HOXA9, MEIS1, FLT3, KIT, CEBPA, RUNX1) were evaluated to exclude residual leukemic transcriptional signatures.

### Results

Seven transcriptionally distinct clusters were identified. CD8⁺ T cells were the predominant population (24.3%), followed by cytotoxic T/NK cells (20.1%), NK cells (16.4%), CD4⁺ T cells (15.1%), inflammatory monocytes (9.5%), megakaryocytes (9.4%), and a proliferating cluster (4.7%).
The cytotoxic activity index was highest in cytotoxic T/NK cells (1.70) and NK cells (1.35), with moderate activity in CD8⁺ T cells (0.53). Exhaustion markers (PDCD1, CTLA4) remained low (mean <0.12) and did not define a distinct exhausted population. Cell cycle distribution was G1 — 44.9%, S — 32.4%, and G2M — 22.7%, with proliferation not enriched in the myeloid compartment.
At diagnosis, bone marrow immunophenotyping identified a myeloid blast clone with phenotype MPO+CD13+CD33+CD15+CD11c+CD64+CD34-CD117+CD38+HLA-DR+CD123+CD7+CD45+, used as the reference for monitoring. Corresponding transcriptomic markers (PTPRC, CD33, ANPEP, CD7, KIT, HLA-DRA) were assessed; IL3RA (CD123) was not detected. Expression frequencies were PTPRC — 91.99%, HLA-DRA — 16.72%, CD7 — 11.54%, CD33 — 3.42%, ANPEP — 1.71%, and KIT — 0.16%. Cells co-expressing at least four markers comprised <1% of the population (15 cells), while none fulfilled strict co-expression criteria. MPO and KIT expression was sporadic and did not form a distinct cluster.

**Copyright Information:** (Online) ISSN: 2572-9241
©2026 The Author(s). HemaSphere is published by John Wiley & Sons Ltd on behalf of European Hematology Association. This is an open access Abstract Book distributed under the Attribution-NonCommercial-NoDerivs (CC BY-NC-ND), which allows third parties to download the articles and share them with others as long as they credit the author and the Abstract Book, but they cannot change the content in any way or use them commercially.
**Abstract Book Citations:** Authors, Title, HemaSphere, 2026;10:(S1):pages. The abstract book DOIs can be found on the “Author Information” page.
**Disclaimer:** Articles published in the journal HemaSphere exclusively reflect the opinions of the authors. The authors are responsible for all content in their abstracts including accuracy of the facts, statements, citing resources, etc.

**Summary/Conclusion**

scRNA-seq demonstrated preserved cytotoxic immunity, moderate myeloid activation, and absence of detectable leukemic transcriptional populations in peripheral blood during remission, supporting immune reconstitution and the complementary value of single-cell analysis for remission assessment.

**Copyright Information:** (Online) ISSN: 2572-9241
©2026 The Author(s). HemaSphere is published by John Wiley & Sons Ltd on behalf of European Hematology Association. This is an open access Abstract Book distributed under the Attribution-NonCommercial-NoDerivs (CC BY-NC-ND), which allows third parties to download the articles and share them with others as long as they credit the author and the Abstract Book, but they cannot change the content in any way or use them commercially.
**Abstract Book Citations:** Authors, Title, HemaSphere, 2026;10:(S1):pages. The abstract book DOIs can be found on the "Author Information" page.
**Disclaimer:** Articles published in the journal HemaSphere exclusively reflect the opinions of the authors. The authors are responsible for all content in their abstracts including accuracy of the facts, statements, citing resources, etc.

### (PB2701) IMMUNOPHENOTYPIC ABERRATIONS IN ACUTE MYELOID LEUKEMIA AND THEIR IMPACT ON CLINICAL OUTCOMES AND PATIENT SURVIVAL

**Topic:** 03. Acute myeloid leukemia - Biology & translational research

Reyhan Aliyeva*[1], Azer Kerimov[1]

[1]*National Center of Hematology and Transfusiology, Oncohematology, Baku, Azerbaijan;*

### Background

Acute myeloid leukemia (AML) is a hematopoietic disorder characterized by clonal proliferation of myeloid blasts and disruption of normal hematopoiesis. Immunophenotyping is a key diagnostic tool. Assessing the prognostic value of immunological markers allows for more accurate prognosis of the disease's course and optimization of treatment strategies.

### Aims

To determine the frequency of early differentiation and cross-lineage marker expression in AML and to assess their impact on response to induction therapy, resistance, and overall survival.

### Methods

The study included 172 patients with newly diagnosed AML treated between 2020 and 2023. The diagnosis was confirmed by morphological and immunophenotypic evaluation. Flow cytometric analysis was performed using BD FACS Canto II. Surface antigen expression ≥20% and cytoplasmic antigen expression ≥10% were considered positive. Complete remission (CR), resistant disease, early mortality, and overall survival (OS) were analyzed. Statistical evaluation included calculation of relative risk (RR), odds ratios (OR), and Kaplan–Meier survival analysis with log-rank testing

### Results

The most frequently expressed myeloid antigens were CD13 (91.9%) and CD33 (91.3%), consistent with a typical myeloid phenotype. High expression rates of CD117 (80.2%) and CD123 (77.9%) reflected active blast proliferation. CD34 was expressed in 50% of patients and was associated with a more immature leukemic clone.
Cross-lineage antigen expression was identified in 41.9% of patients. The most common aberrant markers were CD9 (27.3%), CD4 (26.7%), and CD7 (20.3%), whereas CD56 (12.8%) and CD19 (4.7%) were less frequent. Co-expression of two or more aberrant markers was observed in 20.3% of cases, highlighting marked immunophenotypic heterogeneity.
CD34 expression was significantly associated with adverse outcomes. The CR rate was 38.4% in CD34-positive patients compared with 53.5% in CD34-negative patients (OR=0.54; 95% CI 0.30–0.99). Resistant disease occurred more frequently in the CD34-positive group (24.4% vs 8.1%; OR=3.65; 95% CI 1.46–9.11). Median OS was 129 days in CD34-positive patients versus 236 days in CD34-negative patients (p=0.010). Kaplan–Meier curves demonstrated statistically significant survival separation. CD123 expression alone was not independently associated with CR or OS; however, combined assessment with CD34 revealed prognostic relevance. The worst survival was observed in the CD34-positive/CD123-negative subgroup (median 32 days), whereas patients with a CD34-negative/CD123-negative phenotype demonstrated the most favorable prognosis (median 601 days; p<0.0001). CD9 expression was associated with improved outcomes. The CR rate reached 61.7% in CD9-positive patients compared with 40.0% in CD9-negative patients (OR=2.42; 95% CI 1.21–4.81). Median OS was 432 days versus 149 days, respectively (p=0.0005). CD7, CD56, CD4, and CD117 did not demonstrate independent prognostic impact (p>0.05). However, CD34-positive/CD123-positive and CD34-positive/CD117-positive phenotypes were associated with higher resistance rates.

**Copyright Information:** (Online) ISSN: 2572-9241
©2026 The Author(s). HemaSphere is published by John Wiley & Sons Ltd on behalf of European Hematology Association. This is an open access Abstract Book distributed under the Attribution-NonCommercial-NoDerivs (CC BY-NC-ND), which allows third parties to download the articles and share them with others as long as they credit the author and the Abstract Book, but they cannot change the content in any way or use them commercially.
**Abstract Book Citations:** Authors, Title, HemaSphere, 2026;10:(S1):pages. The abstract book DOIs can be found on the "Author Information" page.
**Disclaimer:** Articles published in the journal HemaSphere exclusively reflect the opinions of the authors. The authors are responsible for all content in their abstracts including accuracy of the facts, statements, citing resources, etc.

**Summary/Conclusion**

AML demonstrates substantial immunophenotypic heterogeneity. CD34 expression is associated with adverse prognosis, whereas CD9 correlates with improved clinical outcomes. Combined evaluation of CD34 and CD123 enhances risk stratification and may support therapeutic decision-making .

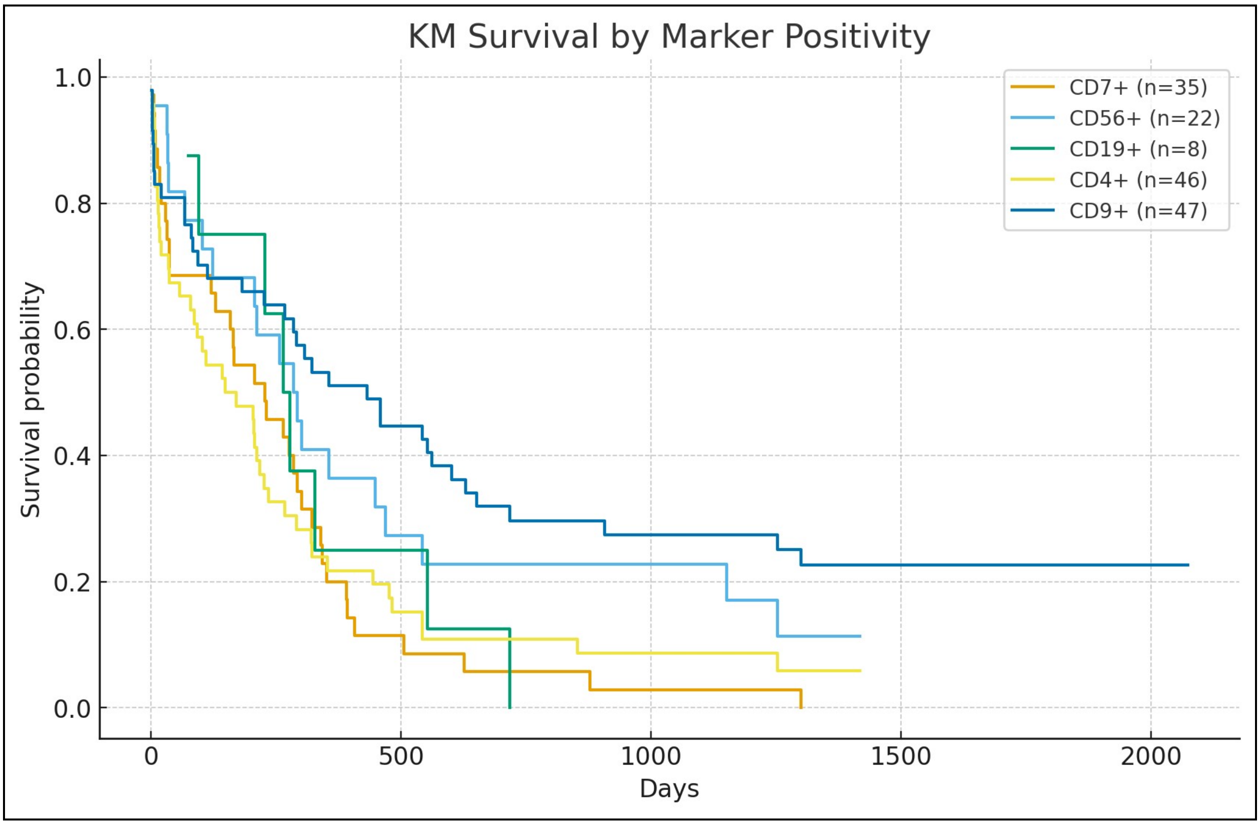

Kaplan–Meier overall survival curves stratified by marker expression

**Copyright Information:** (Online) ISSN: 2572-9241
©2026 The Author(s). HemaSphere is published by John Wiley & Sons Ltd on behalf of European Hematology Association. This is an open access Abstract Book distributed under the Attribution-NonCommercial-NoDerivs (CC BY-NC-ND), which allows third parties to download the articles and share them with others as long as they credit the author and the Abstract Book, but they cannot change the content in any way or use them commercially.
**Abstract Book Citations:** Authors, Title, HemaSphere, 2026;10:(S1):pages. The abstract book DOIs can be found on the "Author Information" page.
**Disclaimer:** Articles published in the journal HemaSphere exclusively reflect the opinions of the authors. The authors are responsible for all content in their abstracts including accuracy of the facts, statements, citing resources, etc.

### (PB2702) DONOR-DERIVED LEUKEMIA FOLLOWING ALLOGENEIC HEMATOPOIETIC STEM CELL TRANSPLANTATION: TEMPORAL GENOMIC EVOLUTION AND THE PUTATIVE CONTRIBUTION OF DONOR CLONAL HEMATOPOIESIS (CASE REPORT)

**Topic:** 03. Acute myeloid leukemia - Biology & translational research

Ildar Barkhatov*[1], Victoria Arzamasova[1, 1], Dmitrii Bug[1], Tatiana Gindina[1], Nikita Pastukhov[1], Anna Smirnova[1], Ludmila Zubarovskaya[1], Alexander Kulagin[1]

[1]*First Pavlov St.Petersburg State Medical University, RM Gorbacheva Research Institute (EBMT CIC 725), St.Petersburg, Russia;*

### Background

Donor cell leukemia (DCL) represents a rare yet clinically consequential complication of allogeneic hematopoietic stem cell transplantation (allo-HSCT). Its recognition relies on an integrated interpretation of chimerism dynamics, cytogenetic findings, and molecular markers, since DCL may clinically and laboratory-wise resemble relapse of the recipient's original malignancy.

### Aims

The main aim is to characterize and substantiate molecular and cytogenetic criteria for the differential diagnosis of donor cell leukemia (DCL) versus "classic" relapse of NPM1-mutated AML after allo-HSCT in a patient whose donor harbored clonal hematopoiesis (ASXL1 mutation), and to assess the contribution of donor-derived CH (ASXL1) to clonal evolution and the pathogenesis of donor-associated myeloid neoplasia.

### Methods

A patient with NPM1-mutated acute myeloid leukemia (AML) underwent allo-HSCT from a related donor (brother), achieving engraftment on day +20. Conditioning consisted of FluBu10, and graft-versus-host disease prophylaxis included CyBeMMF. Post-transplant monitoring comprised serial donor chimerism testing, conventional karyotyping, WT1 expression analysis, disease-specific molecular surveillance (including NPM1 MRD), and NGS-based mutation profiling of donor and recipient samples. Donor genotyping demonstrated clonal hematopoiesis characterized by an ASXL1 mutation (VAF 28%), whereas the recipient's leukemic clone was defined by an NPM1 mutation.

### Results

Early post-transplant evaluation was favorable, with sustained donor chimerism >95% and a donor-consistent karyotype (46,XY). Four months after transplantation, relapse was documented and initially interpreted as recurrence of the original AML: NPM1-positive molecular activity (VAF 13.25%) was detected concomitantly with a decline in donor chimerism (mixed chimerism ~80–90%), consistent with loss of complete donor engraftment and re-expansion of recipient-derived hematopoiesis. Subsequent therapy led to molecular remission.
Approximately three years later, a second relapse developed with a clearly divergent biological signature: karyotype 45,XY, −7, inv(3); loss of detectable NPM1 mutation; WT1 overexpression; and restoration of full donor chimerism. The constellation of complete donor chimerism, donor-type XY karyotype, and NPM1 negativity supported a donor-derived myeloid neoplasm (donor-origin relapse/DCL) rather than classical relapse of the recipient's initial NPM1-positive AML.
NGS findings were consistent with clonal evolution at the time of the second relapse, revealing mutations affecting signaling pathways, including NRAS p.Gly13Arg, alongside alterations in PTPN11 and BCOR. Importantly, the donor-associated ASXL1 c.2385del (p.Trp796Glyfs*22) variant did not emerge as a dominant lesion: it was present only at low abundance (VAF ~2%) and was undetectable following FACS enrichment of the blast population.

**Copyright Information:** (Online) ISSN: 2572-9241
©2026 The Author(s). HemaSphere is published by John Wiley & Sons Ltd on behalf of European Hematology Association. This is an open access Abstract Book distributed under the Attribution-NonCommercial-NoDerivs (CC BY-NC-ND), which allows third parties to download the articles and share them with others as long as they credit the author and the Abstract Book, but they cannot change the content in any way or use them commercially.
**Abstract Book Citations:** Authors, Title, HemaSphere, 2026;10:(S1):pages. The abstract book DOIs can be found on the "Author Information" page.
**Disclaimer:** Articles published in the journal HemaSphere exclusively reflect the opinions of the authors. The authors are responsible for all content in their abstracts including accuracy of the facts, statements, citing resources, etc.

**Summary/Conclusion**

These data underscore that donor clonal hematopoiesis (CH) with an ASXL1 mutation is not inherently equivalent to an inevitable post–allo-HSCT donor-derived relapse and, in this case, did not constitute the principal molecular driver of the secondary neoplasm. Instead, donor CH may have served as a permissive background increasing clonal susceptibility, with subsequent leukemogenic evolution proceeding through alternative genetic pathways.

**Copyright Information:** (Online) ISSN: 2572-9241
©2026 The Author(s). HemaSphere is published by John Wiley & Sons Ltd on behalf of European Hematology Association. This is an open access Abstract Book distributed under the Attribution-NonCommercial-NoDerivs (CC BY-NC-ND), which allows third parties to download the articles and share them with others as long as they credit the author and the Abstract Book, but they cannot change the content in any way or use them commercially.
**Abstract Book Citations:** Authors, Title, HemaSphere, 2026;10:(S1):pages. The abstract book DOIs can be found on the "Author Information" page.
**Disclaimer:** Articles published in the journal HemaSphere exclusively reflect the opinions of the authors. The authors are responsible for all content in their abstracts including accuracy of the facts, statements, citing resources, etc.

### (PB2703) DETECTION OF PROGNOSTIC AND ACTIONABLE BIOMARKERS IN AML ON A SINGLE NEXT GENERATION SEQUENCING (NGS) ASSAY

**Topic:** 03. Acute myeloid leukemia - Biology & translational research

Ghayas Issa*[1], Jessica Lee[2], Alex Bataller[1], Natalie Danziger[2], Caleb Ho[2], Bernard Fendler[2], Megan Friedrichsen[2], Richard Huang[2], Amaya Gasco[2], Lincoln Pasquina[2], Russell Madison[2]

*[1]The University of Texas MD Anderson Cancer Center, Department of Leukemia, Houston, United States of America;[2]Foundation Medicine, Inc., Boston, United States of America;*

### Background

Treatment of AML, a heterogeneous disease, relies on the identification of genomic alterations (alts) for risk stratification, monitoring, and therapy selection, which is routinely done through multiple PCR or FISH-based companion diagnostics (CDx) in addition to conventional cytogenetics (CG) with a limited yield and a risk of missing critical alts.

### Aims

We aimed to demonstrate the clinical validity of detecting actionable and prognostic alts with a single NGS assay.

### Methods

We queried results from bone marrow or blood from 1189 AML patients (pts) profiled with FoundationOne Heme, a DNA and RNA-based NGS assay, in the Foundation Medicine [FMI] genomic database. Clinical annotation was available for a subset of 198 pts in the US-based deidentified Flatiron Health-FMI Clinico-Genomic Database (CGDB) with a biopsy collected <8 weeks after diagnosis. Real-world overall survival (rwOS) from diagnosis was assessed.

**Copyright Information:** (Online) ISSN: 2572-9241
©2026 The Author(s). HemaSphere is published by John Wiley & Sons Ltd on behalf of European Hematology Association. This is an open access Abstract Book distributed under the Attribution-NonCommercial-NoDerivs (CC BY-NC-ND), which allows third parties to download the articles and share them with others as long as they credit the author and the Abstract Book, but they cannot change the content in any way or use them commercially.
**Abstract Book Citations:** Authors, Title, HemaSphere, 2026;10:(S1):pages. The abstract book DOIs can be found on the “Author Information” page.
**Disclaimer:** Articles published in the journal HemaSphere exclusively reflect the opinions of the authors. The authors are responsible for all content in their abstracts including accuracy of the facts, statements, citing resources, etc.

### Results

Of 1,189 AML pts, 18%, 17%, and 55% were classified as favorable (fav), intermediate (int), and adverse (adv) risk by ELN 2022 (11% not classified). 432 (36%) pts were positive for an alt associated with an approved CDx: 157 (13%) pts had alts in *FLT3* (115 internal tandem duplications, 42 tyrosine kinase domain mutations [muts]), 142 (12%) pts had *IDH1/2* muts, and 63 (5.3%) had a *KMT2A* rearrangement (*KMT2A*r) with 22 unique fusion partners identified. *MLLT3* (27%), *MLLT10* (23%), and *ELL* (9.5%) were most frequent, with potentially novel partners *SEPT7* and *MAD1L1* identified in 1 case each.
176 (15%) pts harbored an *NPM1* mut (97% W288fs*12, 2.3% W290fs*10). The type A (c.863_864insTCTG), B (c.863_864insCATG), and D (c.863_864insCCTG) variants comprised 74.9%, 5.3%, and 9.9% of insertions resulting in W288fs*12, with 9.9% of pts harboring a non-A/B/D variant.
Additional alts that may be susceptible to menin inhibition were also observed, including *KMT2A* partial tandem duplications (4.5%), *NPM1-MLF1* (0.4%), *NUP98*r (2.8%), *NUP214-SET* (0.1%), *RUNX1-MECOM* (0.3%), *KAT6A-CREBBP* (0.2%), *PICALM-MLLT10* (0.6%), and *MN1-ETV6* (0.3%).
Prognostic fusions were also observed: *RUNX1-RUNX1T1* (4.3%), *CBFB-MYH11* (2.9%), other *CBFB*r (0.1%) and *RUNX1*r (2.3%), *BCR-ABL1* (1.3%), *MECOM*r (1.0%), and *DEK-NUP214* (0.5%).
478 (40%) pts who were negative for a CDx or menin inhibitor biomarker were positive for an alt associated with an enrolling trial, most frequently *TP53* (22%), *RUNX1* (11%), and *TET2* (8.2%), with 184 (38%; 15% overall) pts harboring >1 trial-associated alt.
In the CGDB, abstracted CG risk was available for 71 pts. Potential cryptic rearrangements which changed risk were identified by NGS in 3 pts (1 *CBF-AML* pt with adv CG risk, 1 *BCR-ABL1*r with int CG risk, 1 *KMT2A-MLLT10*r with int CG risk) and 9 pts had MDS-associated muts which changed risk from fav/int to adv.
rwOS was assessed for these 71 pts by CG risk (5 fav, 15 int, 51 adv) and NGS-based ELN risk (7 fav, 11 int, 53 adv). CG risk did not differentiate rwOS between int and adv (HR 0.9 [0.4-1.7]) but was prognostic for pts with fav risk (HR 0.2 [0.02-1.1]) (C-index=0.55). ELN by NGS showed inferior rwOS for int (HR 10 [1.3-86]) and adv (HR 13 [1.8-94] compared to fav risk (C-index=0.61).

### Summary/Conclusion

Actionable and prognostic alts were identified in 86% and 78% of AML profiled by NGS. Potentially cryptic fusions were detected in pts that resulted in a reclassification of the CG risk. NGS offers a comprehensive approach to capture rare and diverse alts for AML pt risk stratification and therapy selection.

**Copyright Information:** (Online) ISSN: 2572-9241
©2026 The Author(s). HemaSphere is published by John Wiley & Sons Ltd on behalf of European Hematology Association. This is an open access Abstract Book distributed under the Attribution-NonCommercial-NoDerivs (CC BY-NC-ND), which allows third parties to download the articles and share them with others as long as they credit the author and the Abstract Book, but they cannot change the content in any way or use them commercially.
**Abstract Book Citations:** Authors, Title, HemaSphere, 2026;10:(S1):pages. The abstract book DOIs can be found on the "Author Information" page.
**Disclaimer:** Articles published in the journal HemaSphere exclusively reflect the opinions of the authors. The authors are responsible for all content in their abstracts including accuracy of the facts, statements, citing resources, etc.

### (PB2704) EXPLORING THE ANTI-AML MECHANISMS OF COMPOUND ZHEBEI GRANULE VIA INTEGRATED NETWORK PHARMACOLOGY, TRANSCRIPTOMIC SEQUENCING, AND EXPERIMENTAL VALIDATION

**Topic:** 03. Acute myeloid leukemia - Biology & translational research

Yuping Si[1], Yu Zhang[1], Chengyuan Xue[1], Li Hou*[1]

[1]*Dongzhimen Hospital, Beijing University of Chinese Medicine, Beijing, China;*

### Background

Acute myeloid leukemia (AML) is a hematologic malignancy with a poor prognosis. In recent years, the clinical application of the Bcl-2 inhibitor Venetoclax (VEN) has significantly improved remission rates and short-term survival in AML patients. However, the increasing incidence of drug resistance has severely limited its long-term efficacy. Compound Zhebei Granule (CZBG), a traditional Chinese herbal formula, has demonstrated promising clinical potential in the treatment of relapsed and refractory (R/R) AML. Nevertheless, its underlying therapeutic mechanisms remain unclear and warrant further investigation.

### Aims

To investigate the therapeutic effects and potential molecular mechanisms of CZBG in the treatment of AML, with a particular focus on its efficacy in overcoming VEN resistance.

### Methods

The active components of CZBG were identified using UHPLC-MS/MS. Network pharmacology was employed to predict potential anti-AML targets, followed by Gene Ontology (GO) and Kyoto Encyclopedia of Genes and Genomes (KEGG) enrichment analyses to screen for key signaling pathways. An AML zebrafish model was established with three groups: a control group, a VEN-treated group, and a VEN combined with CZBG group, to evaluate tumor growth inhibition. Transcriptomic sequencing of zebrafish tissues was performed to identify differentially expressed genes and enriched core pathways, with selected results validated by qPCR. In the cellular experiments, a VEN-resistant AML cell line (THP-1R) was established by low-dose VEN induction. The resistance index was determined using the CCK-8 assay, and the expression of resistance-related genes and proteins was analyzed by qPCR and western blot. THP-1 R cells were then treated with VEN, CZBG, or their combination to assess in vitro efficacy, including cell viability (AO/PI double staining), apoptosis (flow cytometry), and the expression changes of key signaling molecules, verified by qPCR and western blot.

### Results

CZBG may exert its anti-AML effects by modulating the FOXO signaling pathway, thereby influencing cell proliferation and apoptosis. In the zebrafish in vivo model, the tumor fluorescence intensity in the CZBG combined with VEN group was significantly lower than that in both the model and VEN groups ($P < 0.05$), with reduced leukemic cell infiltration observed in tissues. The THP-1 R cells exhibited a 77.44-fold increase in resistance to VEN, accompanied by a marked upregulation of MCL-1 and significant downregulation of FOXO1 and FOXO3 expression (all $P < 0.05$). Following combination treatment, apoptosis levels increased, and the expression of FOXO1 and FOXO3 was significantly restored.

**Copyright Information:** (Online) ISSN: 2572-9241
©2026 The Author(s). HemaSphere is published by John Wiley & Sons Ltd on behalf of European Hematology Association. This is an open access Abstract Book distributed under the Attribution-NonCommercial-NoDerivs (CC BY-NC-ND), which allows third parties to download the articles and share them with others as long as they credit the author and the Abstract Book, but they cannot change the content in any way or use them commercially.
**Abstract Book Citations:** Authors, Title, HemaSphere, 2026;10:(S1):pages. The abstract book DOIs can be found on the "Author Information" page.
**Disclaimer:** Articles published in the journal HemaSphere exclusively reflect the opinions of the authors. The authors are responsible for all content in their abstracts including accuracy of the facts, statements, citing resources, etc.

**Summary/Conclusion**

CZBG exhibits significant potential in overcoming VEN resistance in AML, and its mechanism may be closely associated with activation of the FOXO signaling pathway and induction of apoptosis. This
study provides experimental evidence and new insights into the use of traditional Chinese medicine in combination with targeted therapies for the treatment of AML.

**Copyright Information:** (Online) ISSN: 2572-9241
©2026 The Author(s). HemaSphere is published by John Wiley & Sons Ltd on behalf of European Hematology Association. This is an open access Abstract Book distributed under the Attribution-NonCommercial-NoDerivs (CC BY-NC-ND), which allows third parties to download the articles and share them with others as long as they credit the author and the Abstract Book, but they cannot change the content in any way or use them commercially.
**Abstract Book Citations:** Authors, Title, HemaSphere, 2026;10:(S1):pages. The abstract book DOIs can be found on the "Author Information" page.
**Disclaimer:** Articles published in the journal HemaSphere exclusively reflect the opinions of the authors. The authors are responsible for all content in their abstracts including accuracy of the facts, statements, citing resources, etc.

### (PB2705) SYNERGISTIC ANTI-LEUKEMIC ACTIVITY OF HOMOHARRINGTONINE AND SELINEXOR IN ACUTE MYELOID LEUKEMIA VIA DUAL INHIBITION OF THE C-MYC/MCL-1 PATHWAY

**Topic:** 03. Acute myeloid leukemia - Biology & translational research

Weiguang Cui*[1], Liyuan Li[1], Mengying Zheng[1], Fan Yang[1], Lin Yang[1], Jianmin Luo[1]

[1]*Department of Hematology, The Second Hospital of Hebei Medical University, shijiazhuang, China;*

### Background

Current therapeutic strategies for AML are hindered by drug resistance; although the HHT-selinexor regimen has been integrated into clinical practice for relapsed/refractory cases, its specific anti-tumor mechanisms have not been fully elucidated."

### Aims

This study investigated the synergistic anti-leukemic potential of Homoharringtonine (HHT) in combination with the XPO1 inhibitor selinexor and elucidated the underlying molecular mechanisms

### Methods

The effects of HHT and selinexor, as monotherapies and in combination, were evaluated in THP-1 and MOLM-13 AML cell lines. Cell viability was assessed using the CCK-8 assay, and synergistic effects were quantified by calculating the Combination Index (CI). Apoptosis was analyzed via flow cytometry (Annexin V/PI staining). The expression of key signaling proteins, including apoptotic markers (cleaved caspase-3, PARP-1), anti-apoptotic regulators (Bcl-xL, MCL-1), and the oncogene c-Myc, was determined by Western blotting.

### Results

Both HHT and selinexor exhibited dose- and time-dependent anti-proliferative effects against AML cell lines as monotherapies. The combination of HHT and selinexor demonstrated potent synergy in inhibiting cell proliferation, with efficacy progressively enhanced by increasing drug concentrations and exposure duration. While both agents individually induced apoptosis, the combinatorial treatment significantly amplified apoptotic activation. At the molecular level, HHT and selinexor both targeted the c-Myc pathway; their combination resulted in a more pronounced suppression of c-Myc expression compared to either monotherapy, subsequently downregulating the downstream anti-apoptotic protein MCL-1. This was accompanied by increased cleavage of caspase-3 and PARP-1 and decreased expression of Bcl-xL.

### Summary/Conclusion

The combination of Homoharringtonine and selinexor synergistically promotes apoptosis in AML cells by co-suppressing the c-Myc/MCL-1 axis and inhibiting anti-apoptotic regulators. These findings provide a mechanistic rationale for evaluating this combinatorial strategy as a promising therapeutic approach for patients with AML.

**Copyright Information:** (Online) ISSN: 2572-9241
©2026 The Author(s). HemaSphere is published by John Wiley & Sons Ltd on behalf of European Hematology Association. This is an open access Abstract Book distributed under the Attribution-NonCommercial-NoDerivs (CC BY-NC-ND), which allows third parties to download the articles and share them with others as long as they credit the author and the Abstract Book, but they cannot change the content in any way or use them commercially.
**Abstract Book Citations:** Authors, Title, HemaSphere, 2026;10:(S1):pages. The abstract book DOIs can be found on the "Author Information" page.
**Disclaimer:** Articles published in the journal HemaSphere exclusively reflect the opinions of the authors. The authors are responsible for all content in their abstracts including accuracy of the facts, statements, citing resources, etc.

### (PB2706) IDENTIFICATION OF NEW SURFACE TARGETS ON LEUKEMIC STEM CELLS IN ACUTE MYELOID LEUKEMIA BY RNA-SEQ PROFILING

**Topic:** 03. Acute myeloid leukemia - Biology & translational research

Karin Bauer*[1], Daniel Ivanov[1], Jelena D. Milosevic Feenstra[1], Michael Schuster[2], Lina Degenfeld-Schonburg[1], Narges Aghaallaei[1], Gregor Eisenwort[1], Irina Sadovnik[1], Harald Hermann[1], Georg Greiner[3], Gregor Hörmann[4], Karoline Gleixner[1], Elisabteh Koller[5], Wolfgang Sperr[1], Felix Keil[5], Christoph Bock[2], Peter Valent[1]

*[1]Medical University of Vienna, Vienna, Austria; [2]CeMM Research Center for Molecular Medicine of the Austrian Academy of Sciences, Vienna, Austria; [3]Ihr Labor, Vienna, Austria; [4]MLL Munich Leukemia Laboratory, Munich, Germany; [5]Hanusch Hospital, Vienna, Austria;*

**Background**

Acute myeloid leukemia (AML) is a life-threatening malignancy of clonal myeloid stem and progenitor cells. Despite the availability of novel targeted drugs and the efficacy of conventional chemotherapy and stem cell transplantation, the prognosis is still poor, as many patients with AML relapse due to the resistance of leukemic stem cells (LSC). Therefore, considerable efforts have been made to develop more specific and potent LSC-targeting treatment approaches in AML. However, most druggable targets on LSC, such as CD33 or CD123, are also expressed on normal CD34+ hematopoietic stem cells. Moreover, AML LSC reside in various fractions of the malignant clone, including CD34+/CD38– and CD34+/CD38+ cells.

**Aims**

The aim of this study was to identify immunological surface targets that are expressed on both LSC-containing AML fractions but not on normal hematopoietic stem cells.

**Methods**

We applied RNA-sequencing (RNA-seq) to screen for novel LSC targets that are expressed abundantly in both LSC-containing AML cell fractions (CD34+/CD38– and CD34+/CD38+ cells) but not in normal CD34+/CD38– stem cells. Multicolor flow cytometry was applied to validate RNA-seq results and to identify surface membrane antigens that are expressed specifically on CD34+/CD38– and CD34+/CD38+ AML cell-fractions.

**Results**

Apart from well-known surface targets, including CD25, CD123, CLL-1, and IL-1RAP, we identified several additional cell surface target antigens on AML LSC, including CD36, CD115, CD119, and CD312. Flow cytometry validation confirmed expression of these antigens on AML LSC in most donors and most AML variants. In contrast to other markers, CD36 was expressed in distinct sub-fractions of CD34+/CD38– and CD34+/CD38+ cells in all patient-cohorts, including patients with FLT3 ITD+ AML and FLT3 ITD- AML.

**Summary/Conclusion**

Whether CD36 or other LSC markers identified in this study are useful to quantify and isolate LSC in AML and to develop LSC-eradicating therapies, remains to be elucidated in clinical studies.

**Copyright Information:** (Online) ISSN: 2572-9241
©2026 The Author(s). HemaSphere is published by John Wiley & Sons Ltd on behalf of European Hematology Association. This is an open access Abstract Book distributed under the Attribution-NonCommercial-NoDerivs (CC BY-NC-ND), which allows third parties to download the articles and share them with others as long as they credit the author and the Abstract Book, but they cannot change the content in any way or use them commercially.
**Abstract Book Citations:** Authors, Title, HemaSphere, 2026;10:(S1):pages. The abstract book DOIs can be found on the "Author Information" page.
**Disclaimer:** Articles published in the journal HemaSphere exclusively reflect the opinions of the authors. The authors are responsible for all content in their abstracts including accuracy of the facts, statements, citing resources, etc.

### (PB2707) FEASIBILITY AND CLINICAL IMPACT OF FLT3-ITD ULTRA-HIGH-SENSITIVE (UHS) NGS MRD ASSESSMENT IN A MULTICENTRIC CLINICAL ROUTINE SETTING.

**Topic:** 03. Acute myeloid leukemia - Biology & translational research

Eleni Linskens*[1, 2], Emanuelle Kabongo Kanjinga[1], Anton Evenepoel[1, 2], Friedel Nollet[3], Zan Janssen[3], Helena Devos[3], Barbara Depreter[4, 5], Alexander Schauwvlieghe[6], Dries Deeren[7], Marleen Bakkus[1, 2], Ann Debecker[2, 8]

*[1]Universitair Ziekenhuis Brussel (UZ Brussel), Vrije Universiteit Brussel (VUB), Department of Clinical Biology, Molecular diagnostics and Hematology, Brussels, Belgium; [2]Universitair Ziekenhuis Brussel (UZ Brussel), Vrije Universiteit Brussel (VUB), Research Group Hematology-Immunology, Brussels, Belgium; [3]AZ Sint-Jan General Hospital, Department of Clinical Biology, Molecular diagnostics and Hematology, Brugges, Belgium; [4]AZ Delta General Hospital, Department of Laboratory Medicine, Roeselare, Belgium; [5]Vrije Universiteit Brussel (VUB), Department of Biomedical Sciences, Brussels, Belgium;[6]AZ Sint-Jan General Hospital, Department of Clinical Hematology, Brugges, Belgium; [7]AZ Delta General Hospital, Department of Clinical Hematology, Roeselare, Belgium; [8]Universitair Ziekenhuis Brussel (UZ Brussel), Vrije Universiteit Brussel (VUB), Department of Clinical Hematology, Brussels, Belgium;*

### Background

Recent studies illustrate the prognostic value of *FLT3*-ITD measurable residual disease (MRD) detection via NGS-based assays, leading to its inclusion in the updated ELN AML MRD 2025 recommendations as MRD marker. However, the feasibility of sequencing-based *FLT3*-ITD MRD in routine practice and the value for therapeutic decision-making require further evaluation.

### Aims

This pilot study aimed to explore the feasibility of UHS-NGS-based *FLT3*-ITD MRD monitoring in a clinical routine setting and assess its clinical impact.

### Methods

Thirty-eight follow-up (FU) samples of fifteen *FLT3*-ITD positive AML patients in complete morphologic remission (median age: 56y, range 35–70y; M/F: 9/6) from three hospitals are included in this analysis. Samples were divided into three different timepoint (TP) categories: 1) post two cycles of intensive chemotherapy/pre-alloHSCT (n=10), 2) end of treatment (post consolidation chemotherapy/post-alloHSCT; n=8), 3) other FU timepoint (n=20). Median FU of the patients was 18 months (range: 2 – 31m).
An in-house optimized UHS-NGS method for FLT3-ITD MRD assessment was performed using a one-step PCR to amplify *FLT3* exon 14-15 with sample indices incorporated. Mass parallel sequencing was performed using Illumina MiSeq. Sequence analysis was evaluated using the bio-informatic tool Pindel. *FLT3*-ITD variant allelic frequency (VAF) was calculated with a minimum of 2 mutant reads required. Sample dependent sensitivity was calculated based on DNA-input and total read count.

**Copyright Information:** (Online) ISSN: 2572-9241
©2026 The Author(s). HemaSphere is published by John Wiley & Sons Ltd on behalf of European Hematology Association. This is an open access Abstract Book distributed under the Attribution-NonCommercial-NoDerivs (CC BY-NC-ND), which allows third parties to download the articles and share them with others as long as they credit the author and the Abstract Book, but they cannot change the content in any way or use them commercially.
**Abstract Book Citations:** Authors, Title, HemaSphere, 2026;10:(S1):pages. The abstract book DOIs can be found on the “Author Information” page.
**Disclaimer:** Articles published in the journal HemaSphere exclusively reflect the opinions of the authors. The authors are responsible for all content in their abstracts including accuracy of the facts, statements, citing resources, etc.

### Results

MRD could not be performed for 4/38 (10.5%) samples due to very low DNA quantity/quality. For 34 FU samples (6 blood, 28 bone marrow), median technical characteristics are detailed in Table 1. 10/34 (29.4%) samples (of six patients) were *FLT3*-ITD MRD positive, with VAF ranging from 0.0006% to 0.9%. A higher proportion of MRD positive samples was observed for TP1 compared to the other two TP categories (7/9 (77.8%) vs. 1/9 and 2/18 (11.1%), respectively). For all six MRD positive patients at TP1, *FLT3*-ITD MRD results had a therapeutic impact: continuation of FLT3-inhibitor (FLT3-I) maintenance until alloHSCT (n=4), continuation of FLT3-I maintenance post consolidation (n=1) and FLT3-I maintenance post-alloHSCT (n=1). In two of these six patients, rising MRD kinetics were observed in two consecutive FU samples (0.3% to 0.9%; 0.004% to 0.03%, respectively). Both patients died 2 and 5 months post-alloHSCT, due to multi-organ failure and AML progression, respectively. Overall, MRD negativity was achieved in 11/15 (73.3%) patients (sensitivity ≤2E-05). To date, only one of these patients showed an *FLT3*-ITD molecular relapse (10m post-alloHSCT) for which FLT3-I maintenance was initiated. All other patients remained MRD negative and are to date alive and in remission.

**Table 1: Technical characteristics**

| | Median | IQR | Aim |
|---|---|---|---|
| *DNA input (ng) (albumin corrected)* | 1257 | 896-1883 | ≥700 |
| *Cell input (x10$^{3}$)* | 210 | 149-314 | ≥200 |
| *Total reads (x10$^{6}$)* | 2.17 | 1.43-3.15 | ≥1.0 |
| *Sensitivity* | 1E-05 | 7E-06-1E-05 | 1E-05 |

### Summary/Conclusion

Our study shows that UHS NGS MRD assessment for *FLT3*-ITD is feasible in a routine clinical laboratory setting with high quality technical assay characteristics and sensitivity.
In the small population of our pilot study, we confirm an unfavorable prognostic value for patients that remain*FLT3*-ITD MRD positive as shown in several large studies. Furthermore, our results indicate that this assay can be implemented in daily clinical practice to better inform clinical decision making in the treatment of FLT3-ITD positive AML.

**Copyright Information:** (Online) ISSN: 2572-9241
©2026 The Author(s). HemaSphere is published by John Wiley & Sons Ltd on behalf of European Hematology Association. This is an open access Abstract Book distributed under the Attribution-NonCommercial-NoDerivs (CC BY-NC-ND), which allows third parties to download the articles and share them with others as long as they credit the author and the Abstract Book, but they cannot change the content in any way or use them commercially.
**Abstract Book Citations:** Authors, Title, HemaSphere, 2026;10:(S1):pages. The abstract book DOIs can be found on the “Author Information” page.
**Disclaimer:** Articles published in the journal HemaSphere exclusively reflect the opinions of the authors. The authors are responsible for all content in their abstracts including accuracy of the facts, statements, citing resources, etc.

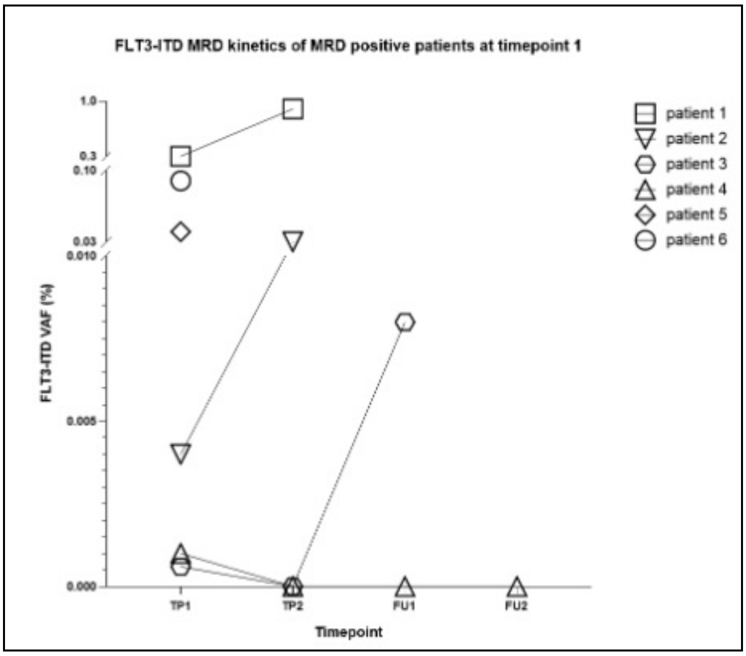

Figure 1: FLT3-ITD MRD kinetics of MRD positive patients at timepoint 1.

**Copyright Information:** (Online) ISSN: 2572-9241
©2026 The Author(s). HemaSphere is published by John Wiley & Sons Ltd on behalf of European Hematology Association. This is an open access Abstract Book distributed under the Attribution-NonCommercial-NoDerivs (CC BY-NC-ND), which allows third parties to download the articles and share them with others as long as they credit the author and the Abstract Book, but they cannot change the content in any way or use them commercially.
**Abstract Book Citations:** Authors, Title, HemaSphere, 2026;10:(S1):pages. The abstract book DOIs can be found on the "Author Information" page.
**Disclaimer:** Articles published in the journal HemaSphere exclusively reflect the opinions of the authors. The authors are responsible for all content in their abstracts including accuracy of the facts, statements, citing resources, etc.

### (PB2708) PROGNOSTIC VALUE OF MEASURING LEUKEMIA STEM CELL FREQUENCY IN ACUTE MYELOID LEUKEMIA: A SYSTEMATIC REVIEW AND INDIVIDUAL PATIENT DATA META-ANALYSIS

**Topic:** 03. Acute myeloid leukemia - Biology & translational research

Tom Reuvekamp*[1, 2, 3], Marry Lin[1, 2], Lok Lam Ngai[1, 2], Kasper Croese[1, 2], Kirsten Ziesemer[4], Mitra Nekouei Shahraki[5], Vera von Pickartz[1, 2], Lukas Haaksma[1, 2], Arjan Van de Loosdrecht[1, 2], Sylvie Freeman[6], David de Leeuw[1, 2], Jacqueline Cloos[1, 2]

*[1]Amsterdam UMC location Vrije Universiteit Amsterdam, Department of Hematology, Amsterdam, The Netherlands;[2]Cancer Center Amsterdam, Imaging and Biomarkers, Amsterdam, The Netherlands; [3]Amsterdam UMC location Universiteit van Amsterdam, Department of Hematology, Amsterdam, The Netherlands; [4]Vrije Universiteit Amsterdam, University Library, Amsterdam, The Netherlands; [5]Amsterdam UMC location Universiteit van Amsterdam, Department of Epidemiology and Data Science, Amsterdam, The Netherlands; [6]University of Birmingham College of Medicine and Health, School of Infection, Inflammation and Immunology, Birmingham, United Kingdom;*

### Background

In acute myeloid leukemia (AML), leukemia stem cells (LSC) are considered responsible for initiating the disease and because of their self-renewal and resistance to therapy also responsible for relapse after treatment. LSC can be quantified by flow cytometry focusing on CD34+CD38- cells, or by gene scores, such as the LSC17 gene score. In individual studies, these methods have prognostic relevance with patients with higher LSC burden having shorter survival and higher relapse rates both at diagnosis and during follow-up in the context of measurable residual disease (MRD) detection.

### Aims

To determine and aggregate the evidence on the prognostic relevance of LSC assessment at diagnosis and during follow-up in the context of MRD in patients with AML.

### Methods

We conducted a systematic review, reconstructed individual patient data, and performed meta-analysis. We searched three bibliographic databases combing LSC and AML search strings. We performed blind selection based on study type, LSC definition, study population, and availability of survival analysis. We reconstructed individual patient data based on reported Kaplan Meier curves (IPDfromKM). Study characteristics, LSC assessment technique (flow cytometry, LSC gene score, or single gene/miRNA), univariable and multivariable hazard ratios (HRs) were extracted from the included studies using a four-eye principle, where all extracted data were checked by a second reviewer. Bias assessment was performed using the PROBAST tool and similarly evaluated by the four-eye principle. Extracted individual patient data were analyzed using Kaplan Meier analysis and Cox regression. As sensitivity analysis, univariable HR were pooled.

**Copyright Information:** (Online) ISSN: 2572-9241
©2026 The Author(s). HemaSphere is published by John Wiley & Sons Ltd on behalf of European Hematology Association. This is an open access Abstract Book distributed under the Attribution-NonCommercial-NoDerivs (CC BY-NC-ND), which allows third parties to download the articles and share them with others as long as they credit the author and the Abstract Book, but they cannot change the content in any way or use them commercially.
**Abstract Book Citations:** Authors, Title, HemaSphere, 2026;10:(S1):pages. The abstract book DOIs can be found on the "Author Information" page.
**Disclaimer:** Articles published in the journal HemaSphere exclusively reflect the opinions of the authors. The authors are responsible for all content in their abstracts including accuracy of the facts, statements, citing resources, etc.

**Results**

A total of 8851 abstracts were screened, of which 707 full texts were screened. We included 56 articles, with a total of 44 cohorts (n=7781) for overall survival (OS) and 37 cohorts (n=5032) for event-free survival (EFS) at diagnosis, and 19 cohorts (n=2006) for OS and 22 cohorts (n=1342) for EFS in the context of MRD. Bias was deemed low in the participants, predictors and outcome domains, whereas in the analysis domain bias was unclear in 38%, low in 36% and high in 27% of studies due to the strict criteria of the PROBAST tool. Overall bias was low, with low applicability concerns. At diagnosis, LSC was most used technique was multiparameter flow cytometry (51% and 59% of OS and EFS cohorts respectively). The most used LSC gene score was the LSC17 gene score. In the MRD setting, almost all studies performed flow cytometry (95% and 91% of OS and EFS cohorts respectively). LSC in the MRD setting was measured most frequently post-induction, followed by post-consolidation. At diagnosis, LSC status had prognostic relevance (OS: HR 1.96 (95%CI 1.85-2.08); EFS: HR 2.32 (95%CI 2.15-2.49); Figure 1A&B), as well as during follow-up (OS: HR 2.59 (95%CI 2.23 -3.03); EFS: HR 2.53 (95%CI 2.18 -2.94); Figure 1C&D). In subgroup analyses, the prognostic significance of LSC was apparent across methodologies and within pediatric and adult patients. Later timepoints of MRD measurements had the highest HR.

**Summary/Conclusion**

To conclude, in this comprehensive meta-analysis, we found that LSC status by different detection methods has prognostic relevance both at diagnosis and follow-up. This study underscores the valuable contribution of LSC frequency as prognostic factor and warrants further implementation in clinical practice.

**Copyright Information:** (Online) ISSN: 2572-9241
©2026 The Author(s). HemaSphere is published by John Wiley & Sons Ltd on behalf of European Hematology Association. This is an open access Abstract Book distributed under the Attribution-NonCommercial-NoDerivs (CC BY-NC-ND), which allows third parties to download the articles and share them with others as long as they credit the author and the Abstract Book, but they cannot change the content in any way or use them commercially.
**Abstract Book Citations:** Authors, Title, HemaSphere, 2026;10:(S1):pages. The abstract book DOIs can be found on the “Author Information” page.
**Disclaimer:** Articles published in the journal HemaSphere exclusively reflect the opinions of the authors. The authors are responsible for all content in their abstracts including accuracy of the facts, statements, citing resources, etc.

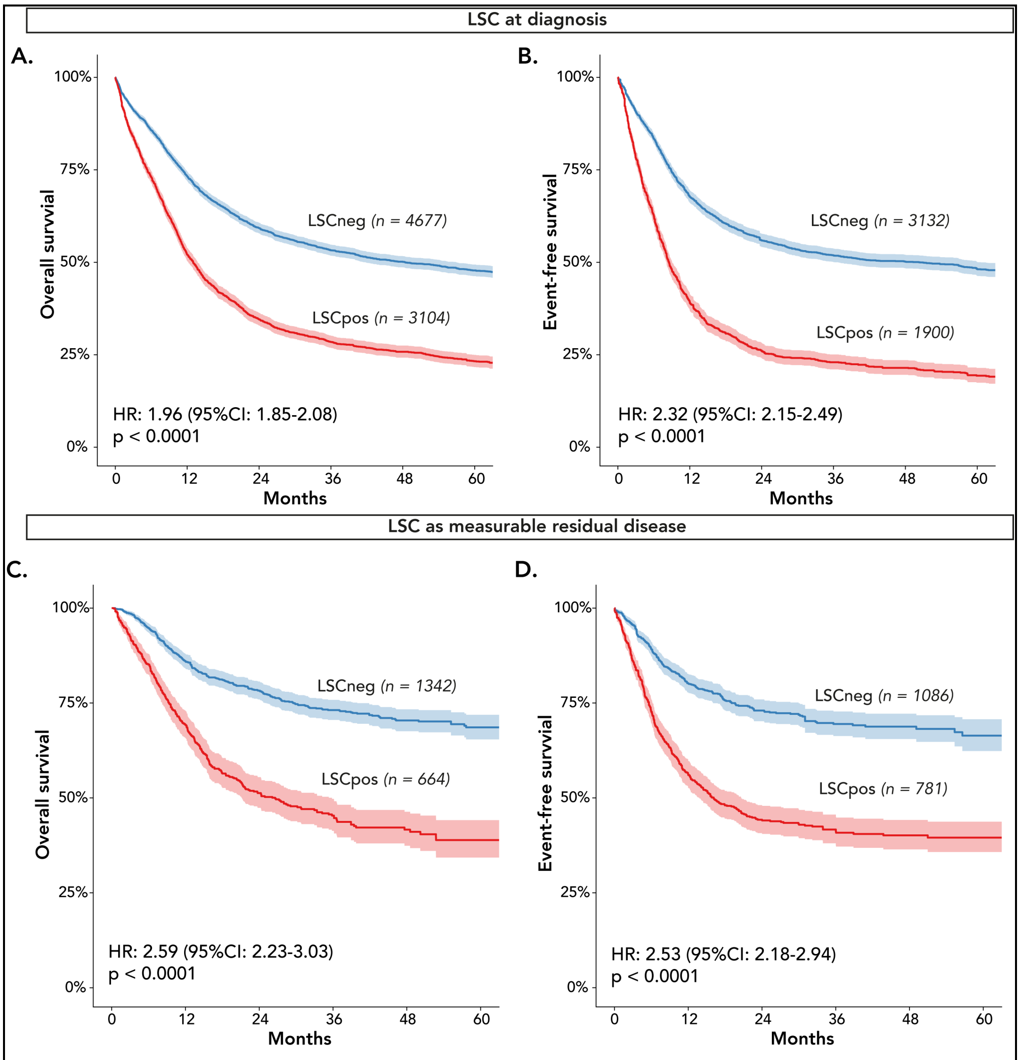

**Figure 1.** Kaplan Meier survival analysis based on extracted individual patient data for **A)** overall survival based on leukemia stem cell status at diagnosis; **B)** event-free survival based on leukemia stem cell status at diagnosis; **C)** overall survival based on leukemia stem cell status during follow-up; **D)** event-free survival based on leukemia stem cell status during follow-up.

**Copyright Information:** (Online) ISSN: 2572-9241
©2026 The Author(s). HemaSphere is published by John Wiley & Sons Ltd on behalf of European Hematology Association. This is an open access Abstract Book distributed under the Attribution-NonCommercial-NoDerivs (CC BY-NC-ND), which allows third parties to download the articles and share them with others as long as they credit the author and the Abstract Book, but they cannot change the content in any way or use them commercially.
**Abstract Book Citations:** Authors, Title, HemaSphere, 2026;10:(S1):pages. The abstract book DOIs can be found on the “Author Information” page.
**Disclaimer:** Articles published in the journal HemaSphere exclusively reflect the opinions of the authors. The authors are responsible for all content in their abstracts including accuracy of the facts, statements, citing resources, etc.

### (PB2709) BRIDGING THE GAP IN ACUTE MYELOID LEUKEMIA (AML): LONG-READ SEQUENCING (LRS) NANOPORE FOR COMPREHENSIVE SOMATIC GENOMIC CHARACTERIZATION AND METHYLATION PROFILING IN 4 PATIENTS

**Topic:** 03. Acute myeloid leukemia - Biology & translational research

Sergiu Moraru*[1], Odile Léopold[1], Chahinez Dehri[1], Martine Becker[1], Veronique Saada[1], Véronique Vergé[1], Ahmadreza Arbab[1], Romain Loyaux[1], Mohamad Hachem[1], Florence Pasquier[1], Christophe Willekens[1], Jean Baptiste Micol[1], Stéphane De Botton[1], Nathalie Auger[1], Marzac Christophe[1], Thomas Steimlé[1]

[1]*Gustave Roussy, Villejuif, France;*

### Background

Conventional karyotyping and FISH (CK&F) stratify only 50% of AML cases, while Optical Genome Mapping adds clinical value in 15% [PMID:39016111]. To date CK&F coupled to whole genome NGS is revealing ≥2 co-occurring driver mutations in 86% of cases [27276561]. While combined whole genome and transcriptome sequencing achieves ≈ 96% concordance for variants with VAF ≥ 5% [PMID:41057686], high costs limit routine use. Nanopore Long-Read Sequencing (LRS) offers a unified workflow for SVs, SNVs, methylation profiling, and variant phasing. In myeloid cancers, LRS demonstrate perfect recall for key driver SVs and ≈ 96% SNV sensitivity, though small indel accuracy remains more limited (≈69%) [PMID:41016628].

### Aims

We want to share our experience using Nanopore LRS as a potential *all-in-one* diagnostic tool to characterize novel fusions, cryptic SVs and methylation data.

### Methods

We performed a blinded retrospective analysis of 4 AML cases at diagnostic, previously characterized by cytology, CK&F, NGS, bulk RNAseq and WGS. LRS was done on the P2i platform (Oxford Nanopore Technologies), with data aligned to the T2T assembly. SVs, SNVs, and DNA methylation were identified via *in-house* pipeline and curated manually in IGV (Broad Institute) after comparison with the non-tumoral counterpart.

**Copyright Information:** (Online) ISSN: 2572-9241
©2026 The Author(s). HemaSphere is published by John Wiley & Sons Ltd on behalf of European Hematology Association. This is an open access Abstract Book distributed under the Attribution-NonCommercial-NoDerivs (CC BY-NC-ND), which allows third parties to download the articles and share them with others as long as they credit the author and the Abstract Book, but they cannot change the content in any way or use them commercially.
**Abstract Book Citations:** Authors, Title, HemaSphere, 2026;10:(S1):pages. The abstract book DOIs can be found on the "Author Information" page.
**Disclaimer:** Articles published in the journal HemaSphere exclusively reflect the opinions of the authors. The authors are responsible for all content in their abstracts including accuracy of the facts, statements, citing resources, etc.

**Results**

Patients received intensive chemotherapy (3+7) and targeted agents when available. **P1** harbored a *KMT2A::TNS3* fusion on der(11) (ELN 2022 adverse [PMID:35797463], **Fig A**), showing severe refractoriness post-transplant. *KMT2A* amplification flagged by FISH was resolved by LRS to the reciprocal derivative, sparing the oncogenic *KMT2A::TNS3* allele from amplification **(Fig B)**, furthermore this amplification was absent at relapse. The *KMT2A::TNS3* fusion provided the rationale for Revumenib [PMID:38905635], the initial CR by flow cytometry was not sustained, with follow-up showing cytological response. **P2** presented a cryptic *BCR::ABL1* duplication associated with TKIs resistance [PMID:20809971], along with a novel fusion *NUP98::SMARCA5* **(Fig C)**. Asciminib treatment achieved major molecular response prior to transplantation.
Crucially, LRS DNA analysis in both **P1** and **P2** identified significantly hypomethylated *HOXA7/HOXA9* locus **(Fig. D)**. This epigenetic signature is a well-known adverse biomarker [PMID:39920624] and reflecting *KMT2A/NUP98* deregulation. These findings have theranostic relevance, as the Menin-KMT2A complex occupies these intergenic positions [PMID:20541448, 30270123] – the precise target of recently approved Revumenib, as clinically evidenced in **P1**.
**P3**, a myeloid sarcoma with karyotype failure and negative bulk RNAseq presented only a *FLT3* A680V mutation on NGS, revealed chromothripsis, and many deletions via LRS. This molecular equivalent of a complex karyotype might explain resistance to anti-FLT3, anti-BCL2, and anti-CD33 therapies. **P4** confirmed a *CBFB::MYH11* rearrangement detected by FISH and *KRAS* mutation, achieving complete molecular remission at 1-year.

**Summary/Conclusion**

Nanopore LRS provides an *all-in-one* tool that can identify any SVs (translocations, chromotripsis, cryptic SV) and resolve complex cases where standard methods find their limits. By integrating SV with HOXA7/HOXA9 methylation, LRS offer a rational and a theranostic biomarker for Menin-inhibitor therapy. This dual approach refines risk stratification and uncovers actionable pathways in refractory AML. Despite its significant bioinformatic challenge, Nanopore LRS is emerging as the *Holy Grail* of contemporary genetic diagnostics.

**Copyright Information:** (Online) ISSN: 2572-9241
©2026 The Author(s). HemaSphere is published by John Wiley & Sons Ltd on behalf of European Hematology Association. This is an open access Abstract Book distributed under the Attribution-NonCommercial-NoDerivs (CC BY-NC-ND), which allows third parties to download the articles and share them with others as long as they credit the author and the Abstract Book, but they cannot change the content in any way or use them commercially.
**Abstract Book Citations:** Authors, Title, HemaSphere, 2026;10:(S1):pages. The abstract book DOIs can be found on the “Author Information” page.
**Disclaimer:** Articles published in the journal HemaSphere exclusively reflect the opinions of the authors. The authors are responsible for all content in their abstracts including accuracy of the facts, statements, citing resources, etc.

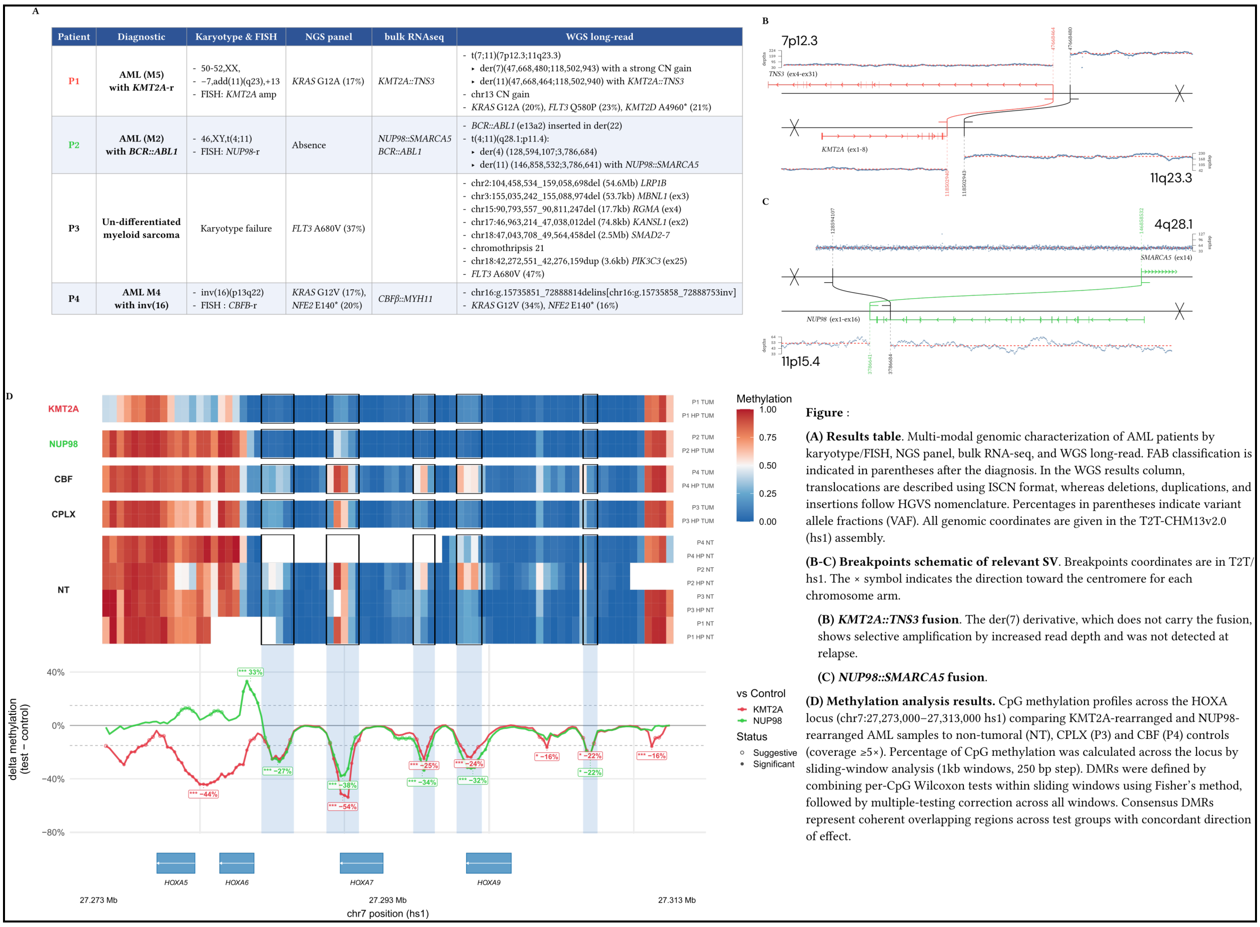

| Patient | Diagnostic | Karyotype & FISH | NGS panel | bulk RNAseq | WGS long-read |
|---|---|---|---|---|---|
| P1 | **AML (M5) with *KMT2A*-r** | - 50-52,XX,<br>- −7,add(11)(q23),+13<br>- FISH: *KMT2A* amp | *KRAS* G12A (17%) | *KMT2A::TNS3* | - t(7;11)(7p12.3;11q23.3)<br>‣ der(7)(47,668,480;118,502,943) with a strong CN gain<br>‣ der(11)(47,668,464;118,502,940) with *KMT2A::TNS3*<br>- chr13 CN gain<br>- *KRAS* G12A (20%), *FLT3* Q580P (23%), *KMT2D* A4960* (21%) |
| P2 | **AML (M2) with *BCR::ABL1*** | - 46,XY,t(4;11)<br>- FISH: *NUP98*-r | Absence | *NUP98::SMARCA5*<br>*BCR::ABL1* | - *BCR::ABL1* (e13a2) inserted in der(22)<br>- t(4;11)(q28.1;p11.4):<br>‣ der(4) (128,594,107;3,786,684)<br>‣ der(11) (146,858,532;3,786,641) with *NUP98::SMARCA5* |
| P3 | **Un-differentiated myeloid sarcoma** | Karyotype failure | *FLT3* A680V (37%) | | - chr2:104,458,534_159,058,698del (54.6Mb) *LRP1B*<br>- chr3:155,035,242_155,088,974del (53.7kb) *MBNL1* (ex3)<br>- chr15:90,793,557_90,811,247del (17.7kb) *RGMA* (ex4)<br>- chr17:46,963,214_47,038,012del (74.8kb) *KANSL1* (ex2)<br>- chr18:47,043,708_49,564,458del (2.5Mb) *SMAD2-7*<br>- chromothripsis 21<br>- chr18:42,272,551_42,276,159dup (3.6kb) *PIK3C3* (ex25)<br>- *FLT3* A680V (47%) |
| P4 | **AML M4 with inv(16)** | - inv(16)(p13q22)<br>- FISH : *CBFB*-r | *KRAS* G12V (17%), *NFE2* E140* (20%) | *CBFβ::MYH11* | - chr16:g.15735851_72888814delins[chr16:g.15735858_72888753inv]<br>- *KRAS* G12V (34%), *NFE2* E140* (16%) |

**Figure** :

**(A) Results table**. Multi-modal genomic characterization of AML patients by karyotype/FISH, NGS panel, bulk RNA-seq, and WGS long-read. FAB classification is indicated in parentheses after the diagnosis. In the WGS results column, translocations are described using ISCN format, whereas deletions, duplications, and insertions follow HGVS nomenclature. Percentages in parentheses indicate variant allele fractions (VAF). All genomic coordinates are given in the T2T-CHM13v2.0 (hs1) assembly.

**(B-C) Breakpoints schematic of relevant SV**. Breakpoints coordinates are in T2T/hs1. The × symbol indicates the direction toward the centromere for each chromosome arm.

**(B) *KMT2A::TNS3* fusion.** The der(7) derivative, which does not carry the fusion, shows selective amplification by increased read depth and was not detected at relapse.

**(C) *NUP98::SMARCA5* fusion.**

**(D) Methylation analysis results.** CpG methylation profiles across the HOXA locus (chr7:27,273,000–27,313,000 hs1) comparing KMT2A-rearranged and NUP98-rearranged AML samples to non-tumoral (NT), CPLX (P3) and CBF (P4) controls (coverage ≥5×). Percentage of CpG methylation was calculated across the locus by sliding-window analysis (1kb windows, 250 bp step). DMRs were defined by combining per-CpG Wilcoxon tests within sliding windows using Fisher's method, followed by multiple-testing correction across all windows. Consensus DMRs represent coherent overlapping regions across test groups with concordant direction of effect.

**Copyright Information:** (Online) ISSN: 2572-9241
©2026 The Author(s). HemaSphere is published by John Wiley & Sons Ltd on behalf of European Hematology Association. This is an open access Abstract Book distributed under the Attribution-NonCommercial-NoDerivs (CC BY-NC-ND), which allows third parties to download the articles and share them with others as long as they credit the author and the Abstract Book, but they cannot change the content in any way or use them commercially.
**Abstract Book Citations:** Authors, Title, HemaSphere, 2026;10:(S1):pages. The abstract book DOIs can be found on the "Author Information" page.
**Disclaimer:** Articles published in the journal HemaSphere exclusively reflect the opinions of the authors. The authors are responsible for all content in their abstracts including accuracy of the facts, statements, citing resources, etc.

### (PB2711) THE IMPACT OF RNA SPLICEOSOME MUTATIONS ON THE TREATMENT EFFICACY AND SURVIVAL OF PATIENTS WITH ACUTE MYELOID LEUKEMIA

**Topic:** 03. Acute myeloid leukemia - Biology & translational research

Miao He*[1], Tao Wu[1]

[1] *The 940th Hospital of Joint Logistics Support force of Chinese People's Liberation Army, hematology, Gansu, China;*

### Background

Mutations in RNA spliceosome genes (SF3B1, U2AF1, SRSF2 and ZRSR2) have been extensively investigated as important driver factors for hematologic malignancies [1]. Acute myeloid leukemia (AML), a hematologic malignancy, is primarily caused by the uncontrolled proliferation and maturation arrest of myeloid blasts, among which the dysregulation of splicing machinery may serve as one of the crucial mechanisms promoting AML development. The incidence of RNA spliceosome gene mutations is approximately 10% in de novo AML, thus mutations in distinct types of RNA spliceosome genes and genome-wide aberrant splicing have become prominent hallmarks of leukemia [2]. The 2022 WHO Classification has identified RNA spliceosome gene mutations as an independent indicator of poor prognosis [3]. In this study, next-generation sequencing was employed to detect the relevant genes. By elucidating the correlations between RNA spliceosome mutations and clinical efficacy as well as patient prognosis, we further clarified the pivotal role of these mutations in the progression of AML, thereby providing a scientific basis for optimizing clinical therapeutic strategies and improving the long-term survival of patients.

### Aims

To analyze the genetic testing by second-generation sequencing in patients with acute myeloid leukemia (AML) at first diagnosis, and to explore the impact of RNA spliceosome mutations on the efficacy and survival of patients with acute myeloid leukemia (AML).

### Methods

The clinical data of 164 patients with acute myeloid leukemia (non-APL) who were initially diagnosed in our hospital from January 2018 to June 2024 were retrospectively analyzed, of which there were 23 cases of RNA spliceosome mutations and 141 cases of non-RNA spliceosome mutations. AML-related gene detection was performed based on second-generation sequencing and statistically analyzed using SPSS 27.0 software to study the effect of RNA spliceosomes on survival time of AML patients and the mutation characteristics of RNA spliceosomes in AML.

### Results

Compared with patients in the non-RNA spliceosome mutation group, patients in the RNA spliceosome mutation group were characterized by≥60 years of age, low leukocytes ($P<0.05$), and had shorter overall survival ($P<0.05$). Multifactorial COX retrospective analysis revealed that age and bone marrow primitive cell count were the main factors affecting the prognostic survival of the patients ($P<0.05$).Among the 23 patients with RNA spliceosome mutations, the mutation rates of SF3B1, U2AF1, and SRSF2 were 39.1% (9/23), 30.4% (7/23), and 17.4% (4/23) in the respective order. Overall survival analysis of patients with SF3B1, U2AF1 and SRSF2 mutations did not show significant differences ($P>0.5$), but patients with SF3B1 mutations had lower median survival and survival rates compared with patients in the U2AF1 and SRSF2 mutation groups ($P>0.5$).

**Copyright Information:** (Online) ISSN: 2572-9241
©2026 The Author(s). HemaSphere is published by John Wiley & Sons Ltd on behalf of European Hematology Association. This is an open access Abstract Book distributed under the Attribution-NonCommercial-NoDerivs (CC BY-NC-ND), which allows third parties to download the articles and share them with others as long as they credit the author and the Abstract Book, but they cannot change the content in any way or use them commercially.
**Abstract Book Citations:** Authors, Title, HemaSphere, 2026;10:(S1):pages. The abstract book DOIs can be found on the "Author Information" page.
**Disclaimer:** Articles published in the journal HemaSphere exclusively reflect the opinions of the authors. The authors are responsible for all content in their abstracts including accuracy of the facts, statements, citing resources, etc.

### Summary/Conclusion

The onset of patients in the group with RNA spliceosome mutations was characterized by age≥60 years and low leukocytes. Overall survival was shorter than that of patients in the non-RNA spliceosome group (*P*<0.05). Patient age and bone marrow primitive cell count were factors affecting patient survival, and patients with SF3B1 mutation had a high mutation rate, poor prognosis, and shorter overall survival than patients with other RNA spliceosome mutations (*P*>0.5).

**Copyright Information:** (Online) ISSN: 2572-9241
©2026 The Author(s). HemaSphere is published by John Wiley & Sons Ltd on behalf of European Hematology Association. This is an open access Abstract Book distributed under the Attribution-NonCommercial-NoDerivs (CC BY-NC-ND), which allows third parties to download the articles and share them with others as long as they credit the author and the Abstract Book, but they cannot change the content in any way or use them commercially.
**Abstract Book Citations:** Authors, Title, HemaSphere, 2026;10:(S1):pages. The abstract book DOIs can be found on the "Author Information" page.
**Disclaimer:** Articles published in the journal HemaSphere exclusively reflect the opinions of the authors. The authors are responsible for all content in their abstracts including accuracy of the facts, statements, citing resources, etc.

### (PB2712) PATHWAY-LEVEL SUPPRESSION OF PURINERGIC, CALCIUM, AND MATRIX SIGNALING IN ACUTE MYELOID LEUKEMIA IDENTIFIED BY PRE-RANKED GSEA META-ANALYSIS

**Topic:** 03. Acute myeloid leukemia - Biology & translational research

Anzhelika Chegodar*[1], Ivan Abramov[1], Dariya Mineeva[1], Irina Baratova[1], Natalia Bodunova[1], Irina Martynkevich[2], Galina Dudina[1]

*[1]The Loginov Moscow Clinical Scientific Center, Moscow, Russia;[2]Russian Research Institute of Hematology and Transfusiology of the Federal Medical and Biological Agency, St-Petersburg, Russia;*

**Background**

Acute myeloid leukemia (AML) is characterized by extensive transcriptional reprogramming. Although numerous differentially expressed genes have been described, the coordinated biological pathways that reproducibly distinguish AML from non-leukemic controls remain incompletely defined. Gene Set Enrichment Analysis (GSEA) applied to ranked gene lists enables detection of coordinated perturbations in biological processes beyond single-gene differential expression.

**Aims**

To identify reproducible and biologically coherent transcriptional programs distinguishing AML from non-leukemic controls through integrative meta-analysis of whole-transcriptome sequencing data followed by pre-ranked GSEA.

**Methods**

Whole-transcriptome sequencing was performed using long-read RNA sequencing on the Oxford Nanopore Technologies (ONT) platform. Libraries were prepared using the ONT cDNA-PCR Sequencing Kit (V14) with barcoding (SQK-PCB114.24). Reverse transcription was primed from the poly(A) tail using the kit-provided strand-specific RT primer. Sequencing was conducted on R9.4.1 flow cells. Basecalling was performed using Guppy (ONT), and reads were aligned to the human reference genome (GRCh38) using minimap2 with splice-aware parameters optimized for long-read data. Gene-level expression quantification was derived from aligned reads following quality filtering.
Whole-transcriptome profiles from five patients with AML were analyzed against non-leukemic controls. Differential gene expression analysis was performed independently for each comparison. A unified ranked gene list was generated using Stouffer's method for meta-analysis, incorporating both statistical significance and directionality of effect.
Pre-ranked GSEA was conducted using KEGG and Reactome pathway collections. Statistically significant gene sets were visualized using enrichment map analysis, accounting for gene overlap and pathway interconnectivity.

**Copyright Information:** (Online) ISSN: 2572-9241
©2026 The Author(s). HemaSphere is published by John Wiley & Sons Ltd on behalf of European Hematology Association. This is an open access Abstract Book distributed under the Attribution-NonCommercial-NoDerivs (CC BY-NC-ND), which allows third parties to download the articles and share them with others as long as they credit the author and the Abstract Book, but they cannot change the content in any way or use them commercially.
**Abstract Book Citations:** Authors, Title, HemaSphere, 2026;10:(S1):pages. The abstract book DOIs can be found on the "Author Information" page.
**Disclaimer:** Articles published in the journal HemaSphere exclusively reflect the opinions of the authors. The authors are responsible for all content in their abstracts including accuracy of the facts, statements, citing resources, etc.

**Results**

Across all five AML-versus-control comparisons, 175 genes were consistently downregulated in AML (adjusted p-value < 0.05 where multiple-testing correction was available; otherwise nominal p < 0.05). No genes demonstrated uniform upregulation across all samples.
Pre-ranked GSEA revealed coordinated downregulation of pathways involved in:

- calcium-dependent and ion-mediated signaling (Calcium signaling pathway);
- purinergic signaling (including P2RX7 and P2RY6);
- ion channel transport and regulation;
- cell–extracellular matrix interactions (ECM–receptor interaction, including FN1).

Leading-edge genes contributing to pathway enrichment included P2RX7, P2RY6, MCOLN3, RYR2, RYR3, members of the CACNA gene family, and FN1.
Comparison with the validated leukemic stem cell (LSC17) transcriptional signature demonstrated no significant gene overlap, suggesting that the identified pathway-level suppression is not solely attributable to leukemic stem cell–associated transcriptional programs but reflects broader molecular reorganization in AML.

**Summary/Conclusion**

AML is characterized by reproducible and coordinated suppression of purinergic, calcium-dependent, and extracellular matrix–associated signaling pathways relative to non-leukemic controls. Integrative pre-ranked GSEA meta-analysis of long-read transcriptomic data identifies pathway-level transcriptional perturbations extending beyond established leukemic stem cell signatures and may reflect fundamental mechanisms of leukemogenesis and bone marrow microenvironment remodeling.

**Copyright Information:** (Online) ISSN: 2572-9241
©2026 The Author(s). HemaSphere is published by John Wiley & Sons Ltd on behalf of European Hematology Association. This is an open access Abstract Book distributed under the Attribution-NonCommercial-NoDerivs (CC BY-NC-ND), which allows third parties to download the articles and share them with others as long as they credit the author and the Abstract Book, but they cannot change the content in any way or use them commercially.
**Abstract Book Citations:** Authors, Title, HemaSphere, 2026;10:(S1):pages. The abstract book DOIs can be found on the “Author Information” page.
**Disclaimer:** Articles published in the journal HemaSphere exclusively reflect the opinions of the authors. The authors are responsible for all content in their abstracts including accuracy of the facts, statements, citing resources, etc.

### (PB2713) THE U2AF1–EZH2–LNCRNA REGULATORY AXIS CONTROLS THE EXPRESSION OF GENES ASSOCIATED WITH MALIGNANT PHENOTYPES IN K562 CELLS VIA THE ALTERNATIVE SPLICING PATHWAY

**Topic:** 03. Acute myeloid leukemia - Biology & translational research

Xiangli Chen*[1], Yuzu Zang[1], Wenyi Lu[1], Pingfan Lu[1], Fei Ge[1], Xiaona Niu[1]

*[1]Henan Provincial People's Hospital, Department of Hematology, Zhengzhou City, China;*

### Background

U2AF1 is a spliceosome core factor whose abnormality is associated with hematopoietic malignancies. However, the molecular mechanism by which it regulates long non-coding RNA (lncRNA) through alternative splicing remains to be elucidated.

### Aims

To explore the molecular mechanism by which U2AF1 regulates the transcription and expression of lncRNA through alternative splicing, with a focus on its regulation of transcription factor EZH2.

### Methods

K562 cells were used as a model to construct U2AF1 knockdown (shU2AF1) and control (ctrl) cell lines. Differentially expressed genes (DEG) and differential alternative splicing events (RAS) were analyzed by transcriptome sequencing. This was combined with eCLIP-seq technology to clarify the RNA binding signature of U2AF1. Bioinformatics tools (GO, KEGG, Metascape, etc.) were used to perform functional enrichment analysis. The regulatory associations of U2AF1, transcription factors (TFs) and differentially expressed lncRNA (DE lncRNA) were analyzed through database mining and network construction.

### Results

After U2AF1 knockdown, the expression of lncRNA and mRNA in K562 cells was significantly remodeled. A total of 141 DElncRNA and thousands of differentially expressed mRNA (DEmRNA) were identified. These DEGs were enriched in cancer-related biological processes such as inflammatory response, cell migration, cell cycle regulation, and TP53 signaling pathway. At the same time, knockdown of U2AF1 elicited 1450 significant RAS. Differential alternative splicing of lncRNA predominantly involved exon hopping, 3 '-terminal alternative splicing sites and 5'-terminal alternative splicing sites, while exon hopping and intron retention? remained the main types of mRNAs. eCLIP-seq analysis showed that the binding sites of U2AF1 are mainly concentrated in intron and coding regions, preferring to bind AG-rich and UC-rich sequences. Gene bonnd by U2AF1 overlapped with 140 RAS genes, which are involved in RNA splicing and gene expression regulation. The key finding is that U2AF1 can directly bind and regulate the alternative splicing of TF-EZH2 (specifically occurring in the ES region). These directly bound TFs (including AFF4, RBPJ, etc.) regulate the expression of 126 DElncRNAs through target genes, constituting the U2AF1-TF-DE lncRNA regulatory network.

### Summary/Conclusion

U2AF1 regulatess gene alternative splicing through specific RNA-binding sequences. It not only directly affects the alternative splicing and expression of lncRNA but also indirectly regulates the expression of downstream DE lncRNA through targeting the alternative splicing of EZH2 and other transcription factors, thereby participating in cancer-related biological processes. This study provides a new molecular mechanism for elucidating the regulatory network of U2AF1 in hematopoietic malignancies.

**Copyright Information:** (Online) ISSN: 2572-9241
©2026 The Author(s). HemaSphere is published by John Wiley & Sons Ltd on behalf of European Hematology Association. This is an open access Abstract Book distributed under the Attribution-NonCommercial-NoDerivs (CC BY-NC-ND), which allows third parties to download the articles and share them with others as long as they credit the author and the Abstract Book, but they cannot change the content in any way or use them commercially.
**Abstract Book Citations:** Authors, Title, HemaSphere, 2026;10:(S1):pages. The abstract book DOIs can be found on the "Author Information" page.
**Disclaimer:** Articles published in the journal HemaSphere exclusively reflect the opinions of the authors. The authors are responsible for all content in their abstracts including accuracy of the facts, statements, citing resources, etc.

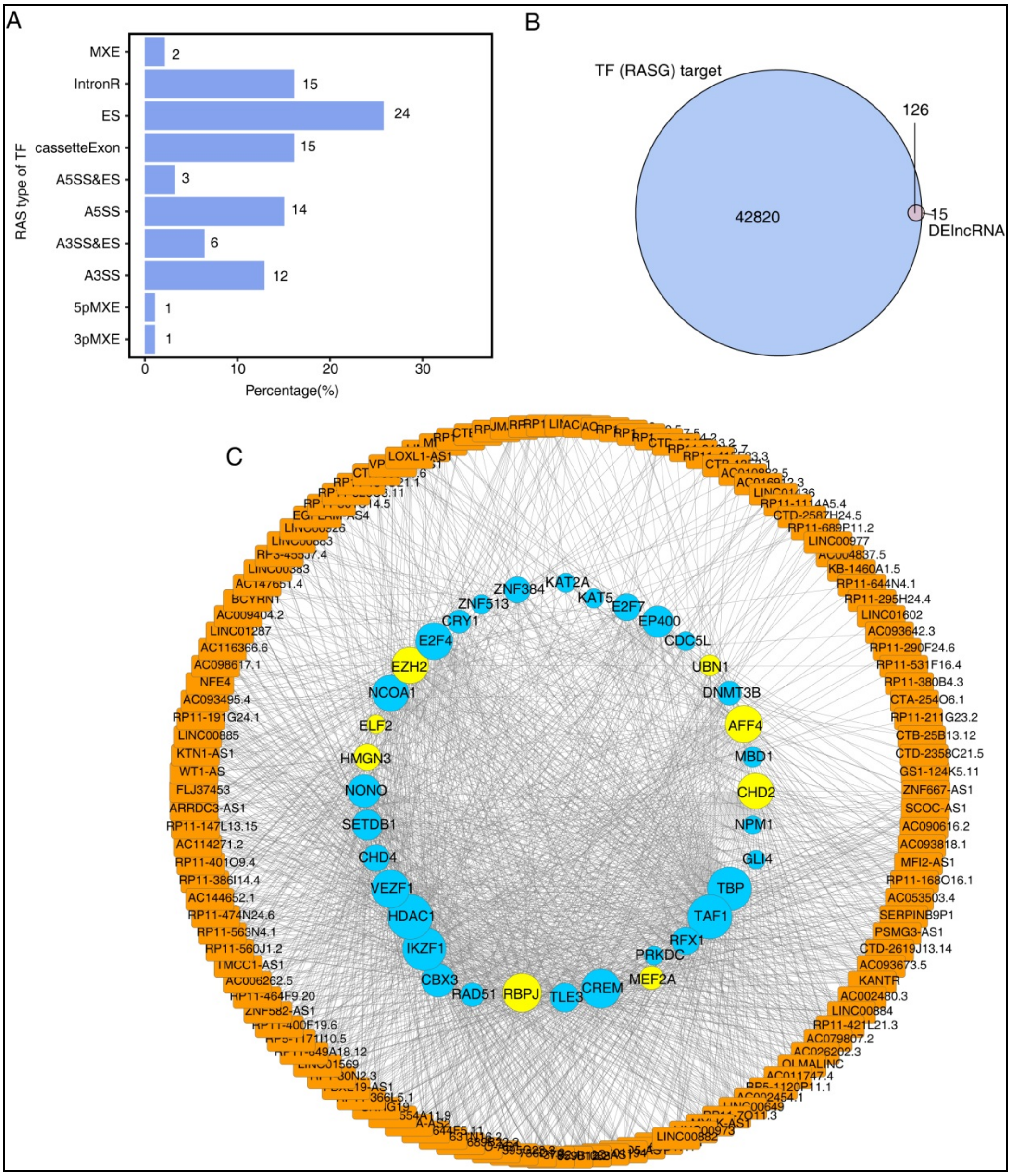

Figure. The regulatory network of transcription factors with RAS and U2AF1-regulated DEGs. A. Classification of RAS from TFs. X-axis: RASE number. B. Venn diagram of targets of TFs with RAS and up-/downregulated lncRNAs. The targets of TFs were screened from the Gene Transcription Regulation Database (GTRD, http://gtrd.biouml.org/). C. The TF-DElncRNA network. The circles in centre indicate TFs with RAS, and the blue circles indicate TFs with U2AF1 bound around the RAS. The circle size indicates the number of targeted DElncRNAs. The orange rectangles indicate DE lncRNAs.

**Copyright Information:** (Online) ISSN: 2572-9241
©2026 The Author(s). HemaSphere is published by John Wiley & Sons Ltd on behalf of European Hematology Association. This is an open access Abstract Book distributed under the Attribution-NonCommercial-NoDerivs (CC BY-NC-ND), which allows third parties to download the articles and share them with others as long as they credit the author and the Abstract Book, but they cannot change the content in any way or use them commercially.
**Abstract Book Citations:** Authors, Title, HemaSphere, 2026;10:(S1):pages. The abstract book DOIs can be found on the “Author Information” page.
**Disclaimer:** Articles published in the journal HemaSphere exclusively reflect the opinions of the authors. The authors are responsible for all content in their abstracts including accuracy of the facts, statements, citing resources, etc.

### (PB2714) TARGETING MIR551B-3P ELIMINATES LEUKEMIC STEM- AND PROGENITOR CELLS IN CD34-POSITIVE ACUTE MYELOID LEUKEMIA

**Topic:** 03. Acute myeloid leukemia - Biology & translational research

Noortje van Gils[1], Tania Martiañez Canales[1], Eline Vermue[1], Floortje Kessler[1], Anna Wojtuszkiewicz[1], Esmée Dekens[1], Arjo Rutten[1], Fedor Denkers[1], Gert Ossenkoppele[1], David de Leeuw[1], Linda Smit*[1]

*[1]Amsterdam UMC, Cancer Center Amsterdam, Adore, Hematology, Amsterdam, The Netherlands;*

### Background

Although most acute myeloid leukemia (AML) patients initially achieve complete remission after intensive chemotherapy or treatment with hypomethylating agents combined with BCL2 inhibitors, long-term outcomes remain poor. Five-year overall survival rates are approximately 35–40% in adults younger than 60 years and decline to 5–15% in older patients.
In AML, *microRNA551b-3p (miR551b-3p)* is higher expressed in cases with a stem cell–like immature phenotype, co-expressed with leukemic stem cell (LSC)-associated genes, and is an independent prognostic marker predicting relapse (De Leeuw et al. Leukemia 2016:30(3):742-6). Given that miRNAs simultaneously regulate multiple genes and pathways, we hypothesized that modulation of miR551b-3p may represent a powerful strategy to eliminate LSCs and prevent disease recurrence.

### Aims

To investigate the therapeutic potential of targeting miR551b-3p for the selective elimination of AML stem- and progenitor cells.

### Methods

To further elucidate the link between miR551b-3p and AML "stemness," and to determine whether downregulation of miR551b-3p can eradicate AML stem- and progenitor cells, we modulated miRNA551b expression in AML cell lines and primary AML samples using lentiviral transduction, locked nucleic acid (LNA) oligonucleotides or mimics. We subsequently assessed proliferation, apoptosis, and survival of leukemic stem- and progenitor cells using *ex vivo* stem/progenitor assays and an *in vivo* AML mouse model. In addition, to identify miR551b-3p target genes, we downregulated its expression in the immature AML cell line KG1a and in primary AML samples, followed by transcriptome analysis using RNA sequencing.

### Results

*MiR551b-3p* is absent or expressed at low levels in favorable-prognosis, CD34-negative AML, but highly expressed in CD34-positive AML. Its expression is elevated in AML cases with EVI-1 overexpression or RUNX1 or TP53 mutations, and it is enriched in LSCs displaying a hematopoietic stem cell (HSC) or multipotent progenitor (MPP) phenotype, commonly referred to as MPP-like LSCs. Functionally, miR551b-3p supports proliferation and self-renewal of primary AML cells, and its upregulation reduces sensitivity to the anthracycline doxorubicin. Targeting miR551b-3p inhibits proliferation, promotes differentiation, and selectively eliminates LSCs and leukemic progenitors in CD34-positive AML (Figure 1) while sparing normal HSCs. Furthermore, our *in silico* and transcriptomic data identified *FOXO3A* and *CEBPα* as key gene targets of miR551b-3p.

### Summary/Conclusion

Together, our results suggest that inhibition of miR551b-3p could be a promising therapeutic approach to eliminate LSCs and leukemic progenitors from CD34-positive AML, thereby decreasing relapse rates and improving AML treatment outcome.

**Copyright Information:** (Online) ISSN: 2572-9241
©2026 The Author(s). HemaSphere is published by John Wiley & Sons Ltd on behalf of European Hematology Association. This is an open access Abstract Book distributed under the Attribution-NonCommercial-NoDerivs (CC BY-NC-ND), which allows third parties to download the articles and share them with others as long as they credit the author and the Abstract Book, but they cannot change the content in any way or use them commercially.
**Abstract Book Citations:** Authors, Title, HemaSphere, 2026;10:(S1):pages. The abstract book DOIs can be found on the "Author Information" page.
**Disclaimer:** Articles published in the journal HemaSphere exclusively reflect the opinions of the authors. The authors are responsible for all content in their abstracts including accuracy of the facts, statements, citing resources, etc.

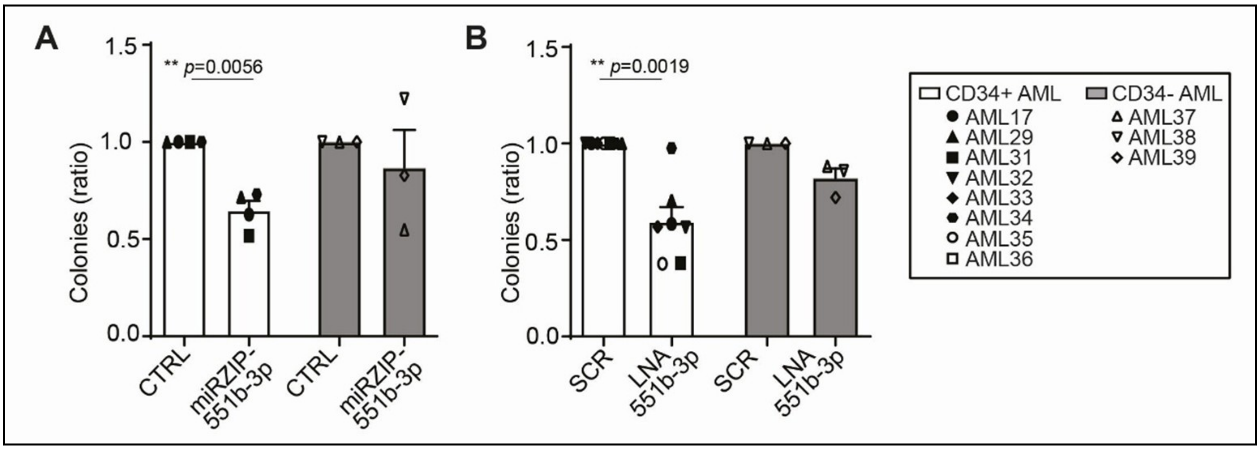

Figure 1: Colony forming unit (CFU) progenitor liquid culture assays of primary AML samples incubated (in duplicate) for 1 week. Colonies were quantified using an Axiovert 25 bright field microscope and normalized against controls. AML samples (A) lentivirally transduced with miRZIP000-GFP (CTRL) or miRZIP551b-3p-GFP vectors, and (B) treated with locked nuclei acid (LNA) anti-scrambled-GFP (SCR) or anti-miR551b-3p-GFP (LNA551b-3p) oligonucleotides.

**Copyright Information:** (Online) ISSN: 2572-9241
©2026 The Author(s). HemaSphere is published by John Wiley & Sons Ltd on behalf of European Hematology Association. This is an open access Abstract Book distributed under the Attribution-NonCommercial-NoDerivs (CC BY-NC-ND), which allows third parties to download the articles and share them with others as long as they credit the author and the Abstract Book, but they cannot change the content in any way or use them commercially.
**Abstract Book Citations:** Authors, Title, HemaSphere, 2026;10:(S1):pages. The abstract book DOIs can be found on the "Author Information" page.
**Disclaimer:** Articles published in the journal HemaSphere exclusively reflect the opinions of the authors. The authors are responsible for all content in their abstracts including accuracy of the facts, statements, citing resources, etc.

### (PB2715) CIRCULATING HISTONE PROFILING IDENTIFIES A DISTINCT PLASMA SIGNATURE IN NEWLY DIAGNOSED ACUTE MYELOID LEUKEMIA

**Topic:** 03. Acute myeloid leukemia - Biology & translational research

Nikol Daskalova*[1], Desislava Tsoneva[1], Manlio Vinciguerra[1], Diana Buzova[2], Jan Cerveny[2], Igor Resnick[1], Ilina Micheva[1]

*[1]Medical University of Varna, Second Department of Internal Diseases, Varna, Bulgaria;[2]Global Change Research Institute CAS, Brno, Czech Republic, Department of Adaptive Biotechnologies, Brno, Czechia;*

### Background

Histones are nuclear proteins that maintain chromatin structure and regulate gene expression. Acute myeloid leukemia (AML) is characterized by profound epigenetic dysregulation and chromatin instability that contribute to leukemogenesis and disease progression. During cellular death and chromatin disruption, histones may be released into circulation, where they function as damage-associated molecular patterns (DAMPs) with pro-inflammatory, cytotoxic, and endothelial-damaging effects. Circulating histones may therefore serve as measurable indicators of leukemic cell turnover and chromatin disruption. Circulating histones have been investigated in solid tumors and hematologic malignancies, predominantly in the context of circulating nucleosomes and cell-free DNA rather than quantitative profiling of individual histone variants. However, quantitative characterization of individual circulating histone variants in AML remains limited. We hypothesized that AML is associated with a distinct circulating histone profile reflecting leukemic chromatin disruption and representing a potential source of novel translational biomarkers.

### Aims

To quantify plasma levels of individual core histones and histone variants in newly diagnosed AML patients and to determine whether AML is associated with a distinct circulating histone profile compared with healthy controls.

### Methods

We analyzed plasma samples from healthy controls (n=30) and treatment-naïve AML patients at diagnosis (n=15). Individual histone variants (H2A, H2B, H3, H4, macroH2A1.1, macroH2A1.2), histone dimers, and nucleosomes were quantified using ImageStreamX imaging flow cytometry. Histone levels were expressed as relative abundance (% of gated events). Statistical comparisons between groups were performed to assess differences in mean values and variance, with statistical significance defined as $p<0.05$.

### Results

AML patients demonstrated significantly elevated circulating histone levels compared with healthy controls. H2A levels were markedly increased (1.971% vs 0.023%, p=0.044), as were H2B levels (0.123% vs 0.000%, p=0.031). The most pronounced differences were observed for histone variants. MacroH2A1.1 and macroH2A1.2 levels were substantially elevated in AML patients compared with controls (8.002% vs 0.396%, p<0.001 and 11.892% vs 0.572%, p<0.001, respectively), representing the strongest discriminatory signal among all measured histones. Total circulating histone levels were also significantly higher in AML patients (8.058% vs 2.527%, p=0.006). Collectively, circulating histone profiling revealed significant quantitative disruption of histone composition in AML.

**Copyright Information:** (Online) ISSN: 2572-9241
©2026 The Author(s). HemaSphere is published by John Wiley & Sons Ltd on behalf of European Hematology Association. This is an open access Abstract Book distributed under the Attribution-NonCommercial-NoDerivs (CC BY-NC-ND), which allows third parties to download the articles and share them with others as long as they credit the author and the Abstract Book, but they cannot change the content in any way or use them commercially.
**Abstract Book Citations:** Authors, Title, HemaSphere, 2026;10:(S1):pages. The abstract book DOIs can be found on the "Author Information" page.
**Disclaimer:** Articles published in the journal HemaSphere exclusively reflect the opinions of the authors. The authors are responsible for all content in their abstracts including accuracy of the facts, statements, citing resources, etc.

**Summary/Conclusion**

This pilot study identifies a distinct circulating histone signature in AML, with histone variants emerging as prominent disease-associated alterations. Circulating histone profiling represents a biologically informed and minimally invasive biomarker platform with potential applications in disease characterization and risk stratification. The present study will be expanded to include patients with myelodysplastic syndromes and a larger cohort to further define the clinical relevance of circulating histone profiles.

**Copyright Information:** (Online) ISSN: 2572-9241
©2026 The Author(s). HemaSphere is published by John Wiley & Sons Ltd on behalf of European Hematology Association. This is an open access Abstract Book distributed under the Attribution-NonCommercial-NoDerivs (CC BY-NC-ND), which allows third parties to download the articles and share them with others as long as they credit the author and the Abstract Book, but they cannot change the content in any way or use them commercially.
**Abstract Book Citations:** Authors, Title, HemaSphere, 2026;10:(S1):pages. The abstract book DOIs can be found on the “Author Information” page.
**Disclaimer:** Articles published in the journal HemaSphere exclusively reflect the opinions of the authors. The authors are responsible for all content in their abstracts including accuracy of the facts, statements, citing resources, etc.

### (PB2716) NGS-BASED COMPREHENSIVE GENOMIC PROFILING IMPROVES RISK STRATIFICATION AND THERAPEUTIC STRATEGY OF ACUTE MYELOID LEUKEMIA ACROSS AGE GROUPS

**Topic:** 03. Acute myeloid leukemia - Biology & translational research

Alessandra Galdiero[1], Alessandra Potenza[1], Roberta Visconti[1], Santa Errichiello[1], Angelo Zanniti*[1], Fabrizio Quarantelli[1], Ciro Del Prete[1], Teresa Femiano[1], Cosimo Barbato[1], Giuseppina Esposito[1], Francesco Grimaldi[2], Barbara Izzo[1, 3]

*[1]CEINGE Biotecnologie Avanzate Franco Salvatore, Naples, Italy; [2]University of Naples "Federico II", Clinical Medicine and Surgery, Hematology Section, Naples, Italy; [3]University of Naples "Federico II", Molecular Medicine and Medical Biotechnology, Naples, Italy;*

### Background

Acute myeloid leukemia (AML) is an aggressive bone marrow malignancy characterized by a heterogeneous genomic landscape associated with variable clinical outcomes, generally poorer in elderly patients. With the global aging of the population, it has become increasingly important to capture molecular mechanism underlying age-related differences in AML. Recent advances in molecular diagnostics and the implementation of Next-Generation Sequencing (NGS) have enhanced the understanding of AML oncogenic mutation profiles, refined prognostic stratification according to the European LeukemiaNet (ELN) 2022 guidelines and expanded therapeutic options.

### Aims

To define the genetic differences between younger and older AML patients using an NGS-based approach, in order to establish proper risk category and guide therapeutic decision.

### Methods

Throughout 2025, NGS data were collected from 402 AML patients. DNA and RNA were extracted from bone marrow samples using the Maxwell CSC 48 system. Sequencing was performed with the Oncomine Myeloid Assay GX V2 on the Ion Torrent Genexus platform, providing a comprehensive myeloid mutational profile—including point mutations and gene rearrangements—within 24 hours. All variants with a coverage >1000× were evaluated using the ClinVar, COSMIC, and Franklin Genoox databases, and only pathogenic ones were included in the study. All analysis were performed at the CEINGE Onco-Hematology Laboratory.

### Results

After NGS analysis of 402 newly diagnosed AML patients, this population was divided into two age groups (cut-off: 60 years): 299 (74.4%) patients were older than 60 years, in agreement with literature-reported data. No genetic alterations were detected in 38 (12.7%) older patients and 19 (18.4%) younger ones. Frequencies of gene mutations and rearrangements according to age are shown respectively in Figure 1A and 1B. Mutations affecting epigenetic regulators (*ASXL1*, *TET2*, *IDH1/2*), RNA splicing factors (*SRSF2*, *SF3B1*, *U2AF1*), transcriptional regulators (*RUNX1*, *BCOR*), tumor suppressor/genomic stability genes (*TP53*, *STAG2*), and signaling pathway components (*JAK2*) were more frequent in older patients; conversely, mutations in *FLT3, NPM1, NRAS, KRAS,* and *WT1* genes exhibited higher frequencies in younger patients (Figure 1A). In particular, significant differences ($p < 0.05$) were observed for *ASXL1, TET2, IDH1, SRSF2, FLT3, NPM1* and *NRAS* genes. Furthermore, analysis of chromosomal rearrangements revealed that *KMT2A* Partial Tandem Duplication (PTD) was more frequently observed in older patients, whereas inv (16) was more frequent in younger patients (Figure 1B), even if a statistically significant difference was observed only for the latter ($p = 0.003869$). The median number of genetic alterations was 3 in older patients and 2 in younger patients. Finally, these patients were stratified into three prognostic risk groups according to the ELN 2022 guidelines (Figure 1C).

**Copyright Information:** (Online) ISSN: 2572-9241
©2026 The Author(s). HemaSphere is published by John Wiley & Sons Ltd on behalf of European Hematology Association. This is an open access Abstract Book distributed under the Attribution-NonCommercial-NoDerivs (CC BY-NC-ND), which allows third parties to download the articles and share them with others as long as they credit the author and the Abstract Book, but they cannot change the content in any way or use them commercially.
**Abstract Book Citations:** Authors, Title, HemaSphere, 2026;10:(S1):pages. The abstract book DOIs can be found on the "Author Information" page.
**Disclaimer:** Articles published in the journal HemaSphere exclusively reflect the opinions of the authors. The authors are responsible for all content in their abstracts including accuracy of the facts, statements, citing resources, etc.

### Summary/Conclusion

NGS analysis of 402 AML patients at diagnosis revealed significant age-related differences in their genomic landscape. ELN 2022 risk stratification showed that older patients carry mutations more commonly associated with the adverse risk category, whereas younger patients were more evenly distributed across all prognostic categories. These findings highlight the influence of age on AML molecular profiles, underscore the value of age-tailored genetic assessment for prognosis and molecular oriented therapy.

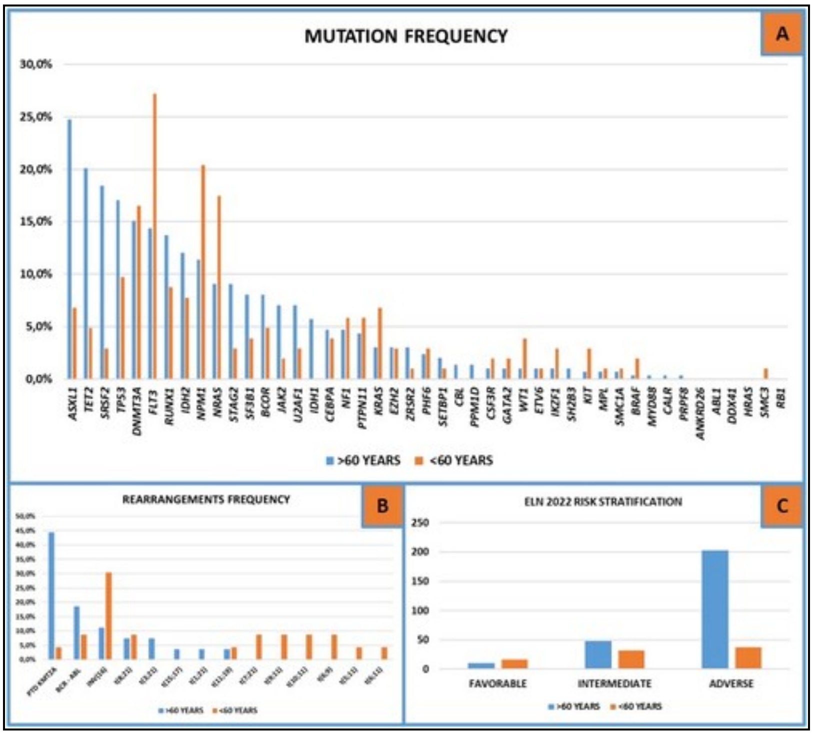

Figure 1. (A) Mutation frequencies of genes included in the Oncomine Myeloid panel across the two patient groups enrolled in this study. (B) Frequencies of gene rearrangements observed in the two age groups. (C) ELN 2022 risk stratification of patients among the three prognostic categories, based on mutational profiles and gene rearrangements. (blue: >60 years; orange <60 years).

**Copyright Information:** (Online) ISSN: 2572-9241
©2026 The Author(s). HemaSphere is published by John Wiley & Sons Ltd on behalf of European Hematology Association. This is an open access Abstract Book distributed under the Attribution-NonCommercial-NoDerivs (CC BY-NC-ND), which allows third parties to download the articles and share them with others as long as they credit the author and the Abstract Book, but they cannot change the content in any way or use them commercially.
**Abstract Book Citations:** Authors, Title, HemaSphere, 2026;10:(S1):pages. The abstract book DOIs can be found on the “Author Information” page.
**Disclaimer:** Articles published in the journal HemaSphere exclusively reflect the opinions of the authors. The authors are responsible for all content in their abstracts including accuracy of the facts, statements, citing resources, etc.

### (PB2717) HIGH-RISK UBTF TANDEM DUPLICATIONS IN PEDIATRIC AML: DETECTION CHALLENGES AND INTEGRATED CHARACTERIZATION IN A SINGLE-CENTER COHORT

**Topic:** 03. Acute myeloid leukemia - Biology & translational research

Alba Crespo-Carrasco*[1, 2, 3], Clara Vicente Garcés[1, 2], Mercè Richarte-Franqués[1, 2, 4], Nidia Barco-Armengol[5, 6], Guerau Fernández[7, 8], Giancarlo Castellano[9], Edurne Sarrate[10], María Antonia Ruiz Cobo[11], Mireia Camos Guijosa[1, 8, 10], Jose Luis Dapena[11], Susana Rives[1, 2, 8, 11], Albert Català[1, 8, 11], Montserrat Torrebadell[1, 8, 10], Nerea Vega-Garcia[1, 2, 10], Elena Esperanza Cebollada[1, 2]

*[1]Institut de Recerca Sant Joan de Déu (IRSJD), Leukemia and Pediatric Hematology Disorders, Developmental Tumors Biology Group, Santa Rosa 39-57, 08950 Esplugues de Llobregat, Spain; [2]Institut de Recerca Sant Joan de Déu (IRSJD)-Josep Carreras Leukemia Research Institute (IJC), Leukemia and Pediatric Hematology Disorders, Pediatric Hematologic malignancies Research program, Esplugues de Llobregat, Spain; [3]Universitat de Barcelona (UB), Facultat de Medicina i Ciències de la Salut, c. Casanova, 143, 08036 Barcelona, Spain; [4]Universitat de Barcelona (UB), Facultat de Biologia, Av. Diagonal, 643, 08028 Barcelona, Spain; [5]Institut de Recerca Sant Joan de Déu (IRSJD), Laboratory of Neurogenetics and Molecular Medicine, Center for Genomic Sciences in Medicine, Esplugues de Llobregat, Spain; [6]Universitat Politècnica de Catalunya - BarcelonaTech (UPC), Barcelona, Spain; [7]Hospital Sant Joan de Déu, Department of Genetic and Molecular Medicine-IPER, Passeig Sant Joan de Déu 2, 08950 Esplugues de Llobregat, Spain; [8]Instituto de Salud Carlos III, Centro de Investigación Biomédica en Red de Enfermedades Raras (CIBERER), Madrid, Spain; [9]SJD Pediatric Cancer Center Barcelona, Institut de Recerca Sant Joan de Déu (IRSJD), Bioinformatics Unit, Barcelona, Spain; [10]Hospital Sant Joan de Déu Barcelona, Hematology Laboratory, Passeig Sant Joan de Déu 2 08950 Esplugues de Llobregat, Spain; [11]Pediatric Cancer Center Barcelona (PCCB), Hospital Sant Joan de Déu Barcelona, Leukemia and Lymphoma Department, Barcelona, Spain;*

### Background

Tandem duplications in the upstream binding transcription factor gene (*UBTF*-TDs) represent a recurrent genetic alteration occurring in approximately 4% of patients with *de novo* pediatric acute myeloid leukemia (pAML). They have recently been proposed as a distinct high-risk subtype associated with poor outcomes and increased relapse risk, underscoring the importance of their accurate identification for prognostic assessment and potential targeted therapies. *UBTF*-TDs occur within exons 9 and 13, and in some cases, complex secondary insertion-deletions (indels) may arise adjacent to the duplications, complicating their detection.

### Aims

To assess the presence of *UBTF*-TDs and to describe the genetic, biological, and clinical features of the identified cases in a cohort of patients with pAML at Hospital Sant Joan de Déu.

### Methods

RNA-sequencing (RNA-seq) was performed using the *Illumina® TruSeq™ stranded mRNA kit* in a cohort of 57 pAML patients. RNA-seq data was analyzed by the Fusion InPipe algorithm. Given the reported limitations in their detection due to secondary indels, exons 9 and 13 of *UBTF* were also visually inspected using the Integrative Genomics Viewer (IGV) to further assess their status.

**Copyright Information:** (Online) ISSN: 2572-9241
©2026 The Author(s). HemaSphere is published by John Wiley & Sons Ltd on behalf of European Hematology Association. This is an open access Abstract Book distributed under the Attribution-NonCommercial-NoDerivs (CC BY-NC-ND), which allows third parties to download the articles and share them with others as long as they credit the author and the Abstract Book, but they cannot change the content in any way or use them commercially.
**Abstract Book Citations:** Authors, Title, HemaSphere, 2026;10:(S1):pages. The abstract book DOIs can be found on the "Author Information" page.
**Disclaimer:** Articles published in the journal HemaSphere exclusively reflect the opinions of the authors. The authors are responsible for all content in their abstracts including accuracy of the facts, statements, citing resources, etc.

### Results

The median age was 6.6 years (range, 0.2-16.4), and 38.6% of patients were female. The application of Fusion InPipe did not detect any *UBTF*-TD in our cohort. Considering the estimated frequency of these alterations, further manual inspection with IGV was performed, which allowed the identification of soft-clipped nucleotide reads at the 3' end of exon 13 in two patients (n=2/57, 3.5%), suggesting potential *UBTF*-TDs. The frequency of potential cases in our cohort was consistent with that reported in the literature. One patient was a 13-year-old female who presented with leukocytosis, myelomonocytic AML (FAB M4), and trisomy 8. The other patient, a 6-year-old female with leukopenia and AML with maturation (FAB M2), had a normal karyotype. Both showed trilineage dysplasia at diagnosis. Fusion calling with Fusion InPipe identified co-occurring alterations: an *FLT3*-ITD in the first patient and a *PRDM16::SKI* fusion in the second (Figure 1). Clinically, both patients had poor responses. One exhibited primary refractoriness to the first and second lines of therapy, including the *FLT3* inhibitor gilteritinib. She finally achieved complete remission after treatment with venetoclax-gemtuzumab followed by allogeneic hematopoietic stem cell transplantation, and remains in remission after 3 years of follow-up. The other patient experienced an early relapse after initial remission and later died due to disease progression.

### Summary/Conclusion

Complementary manual inspection using a genome viewer was required to detect the aberration in exon 13 in two patients, highlighting the complexity of identifying these alterations. Patients exhibited different features typical of *UBTF*-TD cases, including biological characteristics and an unsatisfactory clinical course. Although upregulation of the *PRDM16* gene has been described in *UBTF*-TD patients, we report the first case with a co-occurring *PRDM16::SKI* fusion. Next steps include confirming *UBTF*-TD cases by PCR and implementing bioinformatics algorithms for indel detection. RNA-seq data will also be used to validate the gene expression patterns previously described in this subgroup of patients. Altogether, the identification of these alterations could enable accurate patient classification and may guide personalized therapeutic decisions.

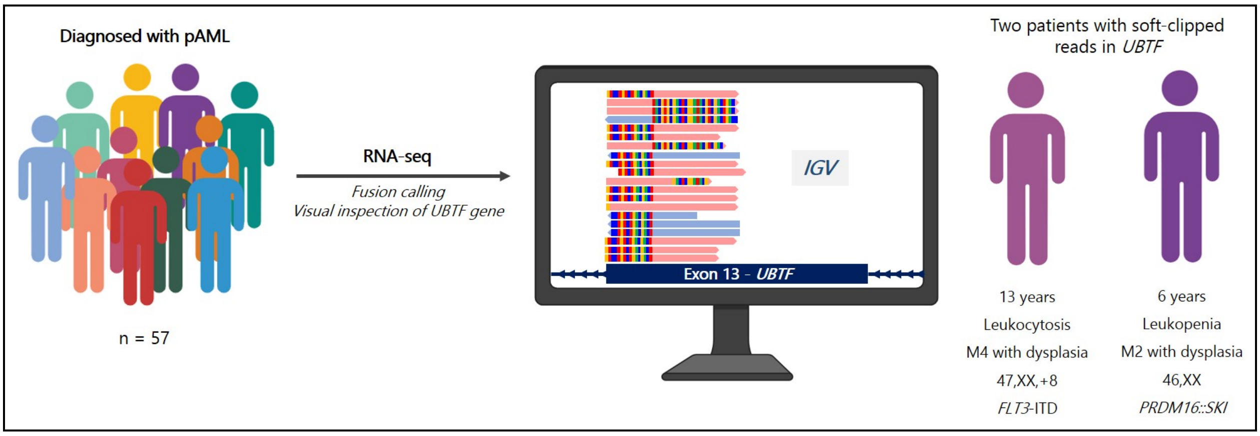

Figure 1. Graphical abstract. Created with BioRender.com

**Copyright Information:** (Online) ISSN: 2572-9241
©2026 The Author(s). HemaSphere is published by John Wiley & Sons Ltd on behalf of European Hematology Association. This is an open access Abstract Book distributed under the Attribution-NonCommercial-NoDerivs (CC BY-NC-ND), which allows third parties to download the articles and share them with others as long as they credit the author and the Abstract Book, but they cannot change the content in any way or use them commercially.
**Abstract Book Citations:** Authors, Title, HemaSphere, 2026;10:(S1):pages. The abstract book DOIs can be found on the "Author Information" page.
**Disclaimer:** Articles published in the journal HemaSphere exclusively reflect the opinions of the authors. The authors are responsible for all content in their abstracts including accuracy of the facts, statements, citing resources, etc.

### (PB2718) COMPARATIVE CHARACTERISTICS OF THE FORMATION AND EVOLUTION OF CHROMOSOMAL CLONAL ABNORMALITIES AT DIAGNOSIS OF ACUTE MYELOID LEUKEMIA

**Topic:** 03. Acute myeloid leukemia - Biology & translational research

Kateryna Korets*[1], Svitlana Andreieva[1], Nadiya Goryainova[1]

[1]*State institution "National Research Center for Radiation Medicine, Hematology and Oncology of NAMS of Ukraine", Diseases of blood system, Kyiv, Ukraine;*

### Background

The determination of the stages of karyotype formation with chromosomal instability, as well as the analysis of the stages of clonal chromosomal abnormality evolution compared to the presence of karyotypes with one or two chromosomal abnormalities are priorities in understanding the pathways of chemotherapy resistance development before acute myeloid leukemia (AML) treatment.

### Aims

To compare the development of clonal quantitative and/or structural chromosomal abnormalities in karyotypes with 1-2 abnormalities and in complex karyotypes at diagnosis of AML.

### Methods

The study was successfully conducted on bone marrow cells from 159 patients with AML. Karyotyping was performed on metaphase chromosomes stained using the GTG method. A complex karyotype was defined as the presence of ≥3 unrelated chromosomal abnormalities simultaneously. All patients were divided into two groups: group I with 1-2 quantitative and structural abnormalities included 128 patients. The average age was (45.0±1.3) years (ranging from 20 to 79 years). Group II, with a complex karyotype, included 33 patients. The average age was (48.2±2.5) years (ranging from 19 to 66 years).

### Results

Karyotypes with the evolution of clonal chromosomal abnormalities in groups I and II were identified in 10 (7.8%) and 8 cases (24.2%), respectively. Correlation with morphological subtypes of AML did not reveal any specific patterns in the formation of clonal chromosomal abnormality evolution. In the stemlines, only balanced or unbalanced chromosomal abnormalities (5 and 13 cases, respectively) were detected. Based on the mechanisms of abnormal karyotype formation, all cases of evolution were grouped as follows: 1) duplication of quantitative and structural chromosome abnormalities in sidelines (No. 1–3) and in only one case did duplication of a structural abnormality occur in a hyperdiploid clone (No. 4); 2) stemlines with balanced abnormalities and formation of imbalance in sideline (No. 5–7) or the appearance of an additional unbalanced structural rearrangement (No. 8, 9). The largest group was that with an intensification of genome imbalance, which was divided into two parts: part 1 – stemlines with imbalanced quantitative and structural abnormalities and deepening due to unbalanced structural chromosome abnormalities in sideline (No. 10–15); part 2 – stemlines with imbalanced structural abnormalities and deepening due to quantitative chromosomal abnormalities (No. 16–18).

### Summary/Conclusion

Patterns were identified in the evolution of clonal chromosomal abnormalities both in karyotypes with 1–2 chromosomal abnormalities and in complex karyotypes, indicating the universality of the mechanisms leading to complications regardless of the subtype of AML. This universality did not depend on the presence of balanced or unbalanced rearrangements in the base clones.

**Copyright Information:** (Online) ISSN: 2572-9241
©2026 The Author(s). HemaSphere is published by John Wiley & Sons Ltd on behalf of European Hematology Association. This is an open access Abstract Book distributed under the Attribution-NonCommercial-NoDerivs (CC BY-NC-ND), which allows third parties to download the articles and share them with others as long as they credit the author and the Abstract Book, but they cannot change the content in any way or use them commercially.
**Abstract Book Citations:** Authors, Title, HemaSphere, 2026;10:(S1):pages. The abstract book DOIs can be found on the "Author Information" page.
**Disclaimer:** Articles published in the journal HemaSphere exclusively reflect the opinions of the authors. The authors are responsible for all content in their abstracts including accuracy of the facts, statements, citing resources, etc.

| № | Groups | Stemline | Sideline |
|---|---|---|---|
| Duplication of quantitative and structural chromosome abnormalities in sidelines | | | |
| 1 | I | +A | 4n±,(+A)×2 |
| 2 | I | del(A) | 4n±,del(A)×2 |
| 3 | II | del(A),t(B;C),der(C)<br>t(B;C),add(D),add(E) | 4n±,idem×2 |
| 4 | I | r | r×2 |
| Stemlines with balanced abnormalities and formation of imbalance in sideline | | | |
| 5 | I | inv(A) | inv(A),del(A) |
| 6 | II | inv(A) | -X,inv(A),t(B;C) |
| 7 | II | t(A;B) | -Y,t(A;B),+der(B)t(A;B) |
| 8 | I | t(A;B) | -Y,t(A;B) |
| 9 | I | t(A;B) | t(A;B),add(C) |
| Stemlines with imbalanced quantitative and structural abnormalities and deepening due to unbalanced structural chromosome abnormalities in sideline | | | |
| 10 | II | t(A;B)del(A) | t(A;B)del(A),-C,-D,+mar1, +mar2, +r |
| 11 | II | -A,-B,del(C) | -A,del(B),del(C) |
| 12 | II | t(A;B),t(C;D;E),-F | t(A;B),t(C;D;E),- F,del(G) |
| 13 | I | r(A) | r(A),i(B) |
| 14 | I | +A | +A,add(B) |
| 15 | II | +A,del(B),dup(C) | del(B),dup(C),dup(D),dup(E) |
| Stemlines with imbalanced structural abnormalities and deepening due to quantitative chromosomal abnormalities | | | |
| 16 | I | del(A) | del(A),+B |
| 17 | II | del(A) | del(A),+B,+C,+mar |
| 18 | I | dup(A) | dup(A),+B |

Copyright Information: (Online) ISSN: 2572-9241
©2026 The Author(s). HemaSphere is published by John Wiley & Sons Ltd on behalf of European Hematology Association. This is an open access Abstract Book distributed under the Attribution-NonCommercial-NoDerivs (CC BY-NC-ND), which allows third parties to download the articles and share them with others as long as they credit the author and the Abstract Book, but they cannot change the content in any way or use them commercially.
Abstract Book Citations: Authors, Title, HemaSphere, 2026;10:(S1):pages. The abstract book DOIs can be found on the "Author Information" page.
Disclaimer: Articles published in the journal HemaSphere exclusively reflect the opinions of the authors. The authors are responsible for all content in their abstracts including accuracy of the facts, statements, citing resources, etc.

### (PB2719) PRESENCE OF COMUTATIONS AND OTHER PROGNOSTIC FACTORS IN ACUTE MYELOID LEUKEMIA WITH NPM1 MUTATION. EXPERIENCE AT HOSPITAL UNIVERSITARIO DONOSTIA.

**Topic:** 03. Acute myeloid leukemia - Biology & translational research

Marina Aranguren Ostolaza*[1], Nerea Caminos[1], Julia Santa Catalina Nieto[1], Teresa Artola[1], Clara Diez Sanz[1], Alasne Uranga Aguirregomezcorta[1], Sheila Carrasco MacEda[1], Sara Sanchez Matias[1], Esther Pérez[1], Izaskun Zeberio Etxetxipia[1], Carlos Panizo[1], Maria Teresa Iglesias[1]

[1]*Hospital Universitario Donostia, Hematology, Donostia, Spain;*

### Background

Acute myeloid leukemia with NPM1 mutation (NPM1 AML) is one of the most common molecular subgroups in adults, with high rates of complete remission following intensive chemotherapy. In this context, national therapeutic protocols consider consolidation with autologous transplantation (ASCT) or chemotherapy, reserving allogeneic transplantation (allo-SCT) for selected cases. Nevertheless, the molecular heterogeneity within NPM1 AML and the presence of comutations could influence its evolution and may be associated with an adverse prognosis.

### Aims

To clinically and molecularly characterize patients with mutated NPM1 AML treated at our center, describing the therapeutic strategies used and clinical outcomes.
To analyze the association between molecular variables at diagnosis and unfavorable prognosis.
To explore the relationship between non-classical NPM1 variants and CNS involvement.

### Methods

Retrospective observational study including patients diagnosed with NPM1 AML treated at Hospital Universitario Donostia between January 2017 and December 2025. Clinical variables, molecular characteristics, administered treatment, and clinical outcomes were collected. A statistical analysis was performed to evaluate the association between variants other than the classical types A, B, and D; presence of comutations FLT3-ITD, FLT3-TKD, DNMT3A, IDH1/2, TET2, DDX41; and the co-occurrence of NPM1, FLT3-ITD, and DNMT3A, with an unfavorable prognosis. Unfavorable prognosis was defined as disease relapse at any time, performance of allo-SCT, or death within the first 5 years. The association between non-classical NPM1 variants and CNS infiltration was also analyzed.
Chi-square or Fisher's exact test were used for statistical analysis. Survival curves were generated using the Kaplan-Meier method and compared with the log-rank test.

**Copyright Information:** (Online) ISSN: 2572-9241
©2026 The Author(s). HemaSphere is published by John Wiley & Sons Ltd on behalf of European Hematology Association. This is an open access Abstract Book distributed under the Attribution-NonCommercial-NoDerivs (CC BY-NC-ND), which allows third parties to download the articles and share them with others as long as they credit the author and the Abstract Book, but they cannot change the content in any way or use them commercially.
**Abstract Book Citations:** Authors, Title, HemaSphere, 2026;10:(S1):pages. The abstract book DOIs can be found on the “Author Information” page.
**Disclaimer:** Articles published in the journal HemaSphere exclusively reflect the opinions of the authors. The authors are responsible for all content in their abstracts including accuracy of the facts, statements, citing resources, etc.

**Results**

A total of 35 patients were analyzed. The median age at diagnosis was 57 years. 29 were treated with an intensive regimen according to the PETHEMA-2017 protocol and 6 with non-intensive regimens using hypomethylating agents and venetoclax. Of the intensive patients, 14 were consolidated with ASCT (48.3%), 6 with high-dose cytarabine-based chemotherapy (20.7%), and 6 with allo-SCT in first line (20.7%). 2 died before consolidation and in 1 case allo-SCT could not be performed. There were 10 relapses (34.9%) and 7 patients received allo-SCT in second line. Progression-free survival was 60% and overall survival was 77% at 7 years, with the median follow-up not yet reached. The combination of NPM1+FLT3-ITD+DNMT3A mutations was associated with adverse prognosis ($p = 0.045$), with no statistical association found with the remaining comutations. No statistical association was demonstrated between variants other than A, B, D — probably due to sample size — however, this variant was associated with CNS infiltration ($p = 0.003$).

**Summary/Conclusion**

AML with NPM1 mutation generally presents a favorable prognosis; however, the presence of concomitant mutations can modify its evolution.
The combination NPM1 + FLT3-ITD + DNMT3A was associated with adverse risk and worse outcomes ($p < 0.05$).
NPM1 variant mutations other than A, B, D are associated with a higher incidence of CNS infiltration. In this regard, no statistical significance was found for adverse prognosis, but the results may have been influenced by sample size.

**Copyright Information:** (Online) ISSN: 2572-9241
©2026 The Author(s). HemaSphere is published by John Wiley & Sons Ltd on behalf of European Hematology Association. This is an open access Abstract Book distributed under the Attribution-NonCommercial-NoDerivs (CC BY-NC-ND), which allows third parties to download the articles and share them with others as long as they credit the author and the Abstract Book, but they cannot change the content in any way or use them commercially.
**Abstract Book Citations:** Authors, Title, HemaSphere, 2026;10:(S1):pages. The abstract book DOIs can be found on the "Author Information" page.
**Disclaimer:** Articles published in the journal HemaSphere exclusively reflect the opinions of the authors. The authors are responsible for all content in their abstracts including accuracy of the facts, statements, citing resources, etc.

### (PB2721) TP53-POSITIVE ACUTE MYELOID LEUKEMIA: A SINGLE-CENTER RETROSPECTIVE CASE SERIES

**Topic:** 03. Acute myeloid leukemia - Biology & translational research

Gokhan Burul*[1], Isa Yalcinkaya[1], Istemi Serin[1], Mesut Ayer[1]

[1]*Başakşehir Çam and Sakura City Hospital, Hematology, Istanbul, Türkiye;*

### Background

The aim of this study was to evaluate the clinical features, cytogenetic and molecular characteristics, treatment patterns, and overall survival outcomes of patients with TP53-positive acute myeloid leukemia treated at a single center, and to explore the impact of TP53 hit status on prognosis.

### Aims

This study aimed to describe the clinical characteristics, molecular profiles, treatment approaches, and survival outcomes of patients with TP53-positive acute myeloid leukemia in a real-world setting at a single tertiary center.

### Methods

This single-center retrospective study included 20 adults with TP53-positive AML. TP53 abnormalities were detected by NGS, PCR, or FISH, and classified as single- or multi-hit per ELN 2022. Data collected included demographics, lab/genetic findings, treatments, hospitalization, post-induction MRD, and OS. OS was analyzed by Kaplan–Meier; MRD reported as median (IQR).

### Results

The median age was 64 years (range 25–75), and 70% were male. Fourteen patients (70%) had de novo AML and six (30%) had secondary AML. At diagnosis, median leukocytes were $12.67\times10^9$/L, hemoglobin 7.8 g/dL, and platelets $53\times10^9$/L.
TP53 status: 12 patients (60%) were multi-hit, 3 (15%) single-hit, and 5 unclassified due to missing NGS. Multi-hit TP53 occurred in 66.7% of secondary and 57.1% of de novo AML. VAF ranged 5–86%. Complex karyotype was seen in 50%, mostly with del(5q) and del(7q). Common TP53 hotspots were p.Arg248, p.Arg249, p.Arg273, and p.Arg181. Co-mutations were rare (NPM1, TET2, SRSF2, ZRSR2, RUNX1, KRAS, PTPN11, JAK2, PPM1D). Induction therapy: 55% received azacitidine+venetoclax, 45% 7+3; three underwent allogeneic SCT. Median hospitalization was 25 days (IQR 11–32), longer for 7+3 (29 vs 11 days); three azacitidine+venetoclax patients did not require admission.
Post-induction MRD ranged 0.01–76.8; median MRD was 1.97 (IQR 1.11–9.58) for azacitidine+venetoclax and 3.1 (IQR 0.59–30.17) for 7+3, with lower MRD in some azacitidine+venetoclax patients.
Overall survival was 1.0–36.3 months, median 3.5 months. By study end, 55% had died. Mortality was 50% in both secondary and de novo AML, higher in multi-hit TP53 (58.3%) than single-hit (33.3%). Most deaths were infection-related, followed by typhlitis and other causes.

**Copyright Information:** (Online) ISSN: 2572-9241
©2026 The Author(s). HemaSphere is published by John Wiley & Sons Ltd on behalf of European Hematology Association. This is an open access Abstract Book distributed under the Attribution-NonCommercial-NoDerivs (CC BY-NC-ND), which allows third parties to download the articles and share them with others as long as they credit the author and the Abstract Book, but they cannot change the content in any way or use them commercially.
**Abstract Book Citations:** Authors, Title, HemaSphere, 2026;10:(S1):pages. The abstract book DOIs can be found on the "Author Information" page.
**Disclaimer:** Articles published in the journal HemaSphere exclusively reflect the opinions of the authors. The authors are responsible for all content in their abstracts including accuracy of the facts, statements, citing resources, etc.

**Summary/Conclusion**

In this single-center real-world cohort, TP53-positive AML showed extremely poor outcomes, confirming its aggressive biology. Patients were mostly older males, with a majority harboring multi-hit TP53, which carried higher mortality than single-hit cases. Half had complex karyotypes, often with del(5q) and del(7q)/monosomy 7, linking TP53 alterations to genomic instability. TP53 VAFs were highly variable, reflecting clonal heterogeneity. Multi-hit cases generally had high VAF and adverse cytogenetics, highlighting their high-risk nature. Azacitidine plus venetoclax led to lower median post-induction MRD and shorter hospital stays than 7+3, suggesting better tolerability, though MRD responses were heterogeneous in both groups and no durable survival benefit was observed. Median overall survival was only 3.5 months, with mortality largely from infections, emphasizing the impact of prolonged cytopenias, treatment-related immunosuppression, and the need for optimized supportive care. These data reinforce the limited efficacy of standard therapies and the urgent need for TP53-targeted and innovative treatments.

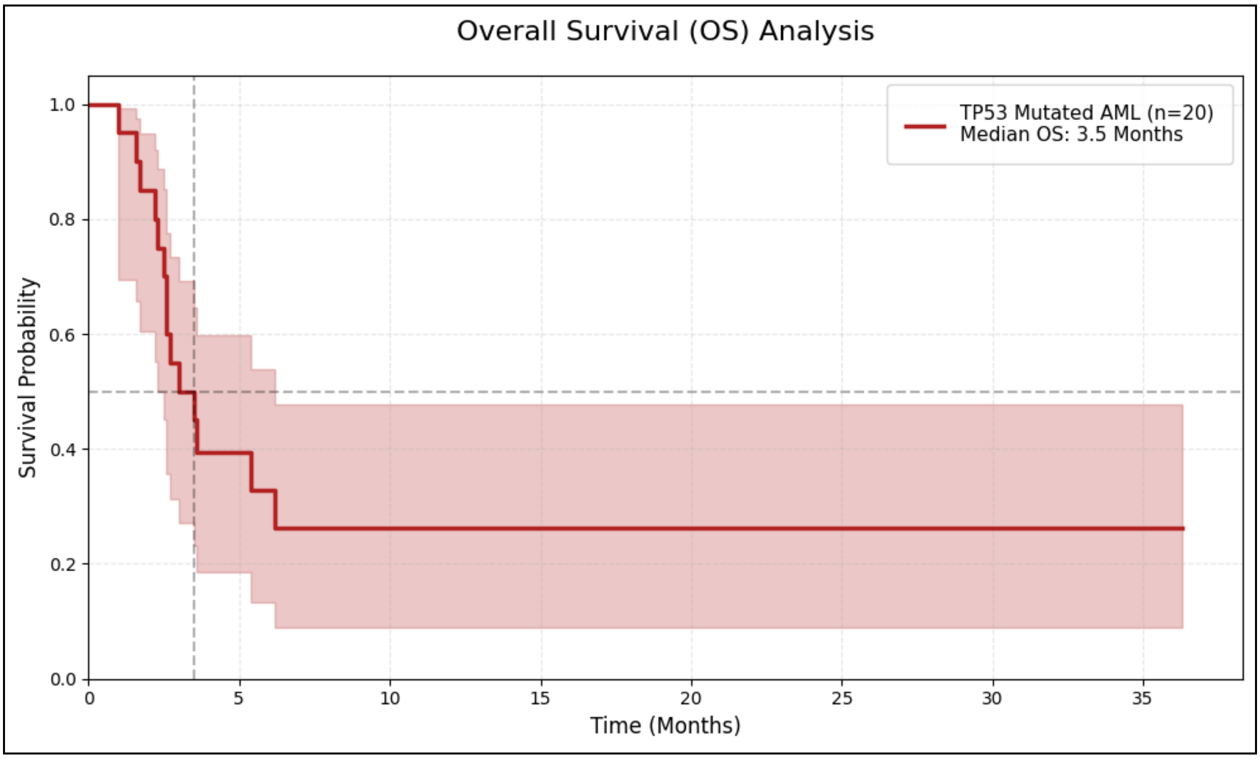

**Copyright Information:** (Online) ISSN: 2572-9241
©2026 The Author(s). HemaSphere is published by John Wiley & Sons Ltd on behalf of European Hematology Association. This is an open access Abstract Book distributed under the Attribution-NonCommercial-NoDerivs (CC BY-NC-ND), which allows third parties to download the articles and share them with others as long as they credit the author and the Abstract Book, but they cannot change the content in any way or use them commercially.
**Abstract Book Citations:** Authors, Title, HemaSphere, 2026;10:(S1):pages. The abstract book DOIs can be found on the "Author Information" page.
**Disclaimer:** Articles published in the journal HemaSphere exclusively reflect the opinions of the authors. The authors are responsible for all content in their abstracts including accuracy of the facts, statements, citing resources, etc.

### (PB2722) BCR::ABL1-POSITIVE ACUTE MYELOID LEUKEMIA: FROM CLASSICAL TO RARE TRANSCRIPTS IN UKRAINE PATIENTS

**Topic:** 03. Acute myeloid leukemia - Biology & translational research

Olha Kravets*[1], Natalia Trofimova[1, 2], Nataliia Levkovych[1, 2], Uliana Lykhova[1], Svitlana Cherniuk[1], Anna Chaikovska[1], Arsenii Lykhovyi[1], Mariana Kharko[1], Nataliia Olkhovich[1, 2]

*[1]National Children's Specialized Hospital "Ohmatdyt," Ministry of Health of Ukraine, Kyiv, Ukraine;[2]State Institution «National scientific center «Institute of Cardiology, Clinical and Regenerative Medicine named after academician M.D. Strazhesko», Department of Genetic Diagnostics, Kyiv, Ukraine;*

### Background

*BCR::ABL1*-positive acute myeloid leukemia (Ph+AML) is recognized as a distinct high-risk subgroup characterized by an aggressive clinical course and a poor prognosis. Various types of Philadelphia chromosome (Ph) transcripts modulate the genomic landscape of leukemia in specific ways, making their study a key focus for developing personalized targeted therapy approaches.

### Aims

To characterize the cytogenetic and molecular profiles of *de novo* Ph+AML cases, with an accent on identifying rare transcript variants.

### Methods

A total of 98 adult patients with *de novo* AML were examined at the National Specialized Children's Hospital "Okhmatdyt" between 2023 and 2025. The study group included 56 men and 42 women. RNA isolated from sample of bone marrow aspirate was used for molecular-genetic analysis. The diagnostic algorithm involved standard karyotyping (GTG-banding) and next-generation sequencing (NGS). NGS results were verified using real-time PCR (RT-PCR).

### Results

The cytogenetics study showed that the Ph chromosome t(9;22)(q34;q11) was detected in 5 of 98 patients (5.1%) and NGS confirmed that. This frequency consistent with other authors. Despite the homogeneous cytogenetic marker, molecular profiling revealed significant transcript heterogeneity. The most common isoform was e14a2 (p210), identified in 3 patients, corresponding to the classical rearrangement for the myeloid lineage. The e1a2 (p190) isoform, typically associated with lymphoid lineage or undifferentiated phenotypes, was found in one patient. Of particular scientific interest is a rare case of the double isoform e18a2/e19a2 (p230) (n=1). Such a transcript occurs in less than 1% of cases in global clinical practice.

### Summary/Conclusion

The study results confirm the molecular heterogeneity within the Ph+AML subgroup. The identification of atypical isoforms and rare genetic events, specifically the double transcript e18a2/e19a2, underscores the critical necessity of implementing comprehensive molecular genetic testing.

**Copyright Information:** (Online) ISSN: 2572-9241
©2026 The Author(s). HemaSphere is published by John Wiley & Sons Ltd on behalf of European Hematology Association. This is an open access Abstract Book distributed under the Attribution-NonCommercial-NoDerivs (CC BY-NC-ND), which allows third parties to download the articles and share them with others as long as they credit the author and the Abstract Book, but they cannot change the content in any way or use them commercially.
**Abstract Book Citations:** Authors, Title, HemaSphere, 2026;10:(S1):pages. The abstract book DOIs can be found on the "Author Information" page.
**Disclaimer:** Articles published in the journal HemaSphere exclusively reflect the opinions of the authors. The authors are responsible for all content in their abstracts including accuracy of the facts, statements, citing resources, etc.

### (PB2723) NON-CODING RNA-GUIDED REGULATORY LANDSCAPES IN ACUTE MYELOID LEUKAEMIA

**Topic:** 03. Acute myeloid leukemia - Biology & translational research

Aonghus Naughton*[1], Masaki Kato[2], Takeya Kasukawa[2, 2], Chi Wai Yip[3], Christine Wells[4], Chung-Chau Hon[2], Hazuki Takahashi[2], Piero Carninci[2], Andreas Lennartsson[1]

*[1]Department of Medicine, Center for Hematology and Regenerative Medicine Karolinska Institutet, Stockholm, Sweden;[2]RIKEN Center for Integrative Medical Sciences, Yokohama, Japan; [3]Functional Genomics, Human Technopole, Milan, Italy;[4]Institut de Génétique Moléculaire de Montpellier, Université de Montpellier, Montpellier, France;*

### Background

Non-coding RNAs (ncRNAs) are emerging regulators of gene expression and chromatin organisation, and are frequently dysregulated in acute myeloid leukaemia (AML). Despite extensive transcriptional profiling in AML, the spatial organisation of RNA-chromatin interactions in the leukemic state remains largely unexplored.

### Aims

Identify key ncRNAs that drive leukemogenesis.

### Methods

We performed RADICL-seq, a proximity ligation-based approach that maps physical RNA-chromtin interactions across the genome, in leukemic THP-1 cells and healthy iPSC-derived myeloid progenitors, and integrated these data with CAGE-based transcriptional profiling of *cis*-regulatory element (CRE) activity.

### Results

Comparative analysis revealed widespread rewiring of RNA-chromatin contacts in the leukemic state. By jointly modeling changes in interaction frequency and regulatory activity, we defined a set of dual differential RNA-chromatin interactions linking differentially active RNAs to dynamic CREs. These coordinated changes suggest coupling between RNA binding and local transcriptional remodeling. Network analysis further identified candidate ncRNAs occupying central positions within chromatin interaction networks.

### Summary/Conclusion

Our findings support a model in which ncRNAs contribute to reshaping regulatory lanscapes in AML and provide a framework for prioritising candidates for functional validation.

**Copyright Information:** (Online) ISSN: 2572-9241
©2026 The Author(s). HemaSphere is published by John Wiley & Sons Ltd on behalf of European Hematology Association. This is an open access Abstract Book distributed under the Attribution-NonCommercial-NoDerivs (CC BY-NC-ND), which allows third parties to download the articles and share them with others as long as they credit the author and the Abstract Book, but they cannot change the content in any way or use them commercially.
**Abstract Book Citations:** Authors, Title, HemaSphere, 2026;10:(S1):pages. The abstract book DOIs can be found on the "Author Information" page.
**Disclaimer:** Articles published in the journal HemaSphere exclusively reflect the opinions of the authors. The authors are responsible for all content in their abstracts including accuracy of the facts, statements, citing resources, etc.

### (PB2724) SPLENIC INFARCTION AS INITIAL PRESENTATION OF ACUTE PROMYELOCYTIC LEUKEMIA COMPLICATED BY EARLY DIFFERENTIATION SYNDROME UNDER ATRA

**Topic:** 03. Acute myeloid leukemia - Biology & translational research

Ekram Laghmari*[1], Samia Sabri[1], Zahida Aqodad[1], Ikram Sadki[1], Houda Bachir[1], Siham Hamaz[1], Habiba Bennesser Alaoui[1], Khalid Serraj Andaloussi[1]

[1]*Mohammed VI University Hospital Center (CHU Mohammed VI), Faculty of Medicine and Pharmacy, Mohammed First University,, Department of Internal Medicine and Immuno-Hematology and Cellular Therapy Laboratory,, Oujda, Morocco;*

### Background

Acute promyelocytic leukemia (APL), a distinct subtype of acute myeloid leukemia characterized by the t(15;17)(q24;q21) translocation, is a hematologic emergency due to its high risk of early hemorrhagic complications related to disseminated intravascular coagulation (DIC). The introduction of all-trans retinoic acid (ATRA) has dramatically improved survival outcomes, transforming APL into a highly curable leukemia when treated promptly.

Although hemorrhagic manifestations are classical at presentation, thrombotic events may also occur and remain under-recognized. Arterial thrombosis, including splenic infarction, is uncommon and may obscure the initial diagnosis. Furthermore, differentiation syndrome (DS), a potentially life-threatening complication of ATRA therapy, is characterized by leukocytosis, capillary leak, pulmonary infiltrates, and organ dysfunction, requiring early recognition and corticosteroid treatment.

We report a case of APL revealed by splenic infarction and complicated by early severe differentiation syndrome, highlighting the complex hemostatic and inflammatory imbalance in this disease.

### Aims

To describe an uncommon thrombotic presentation of acute promyelocytic leukemia revealed by splenic infarction.
To highlight the coexistence of thrombotic and hemorrhagic risks in APL at diagnosis.
To emphasize the importance of early recognition and management of differentiation syndrome following ATRA initiation.
To underline the need for prompt multidisciplinary management in atypical presentations of APL.

### Methods

This is a descriptive single-patient case report conducted in a tertiary care university hospital. Clinical data were collected retrospectively from medical records.

**Copyright Information:** (Online) ISSN: 2572-9241
©2026 The Author(s). HemaSphere is published by John Wiley & Sons Ltd on behalf of European Hematology Association. This is an open access Abstract Book distributed under the Attribution-NonCommercial-NoDerivs (CC BY-NC-ND), which allows third parties to download the articles and share them with others as long as they credit the author and the Abstract Book, but they cannot change the content in any way or use them commercially.
**Abstract Book Citations:** Authors, Title, HemaSphere, 2026;10:(S1):pages. The abstract book DOIs can be found on the "Author Information" page.
**Disclaimer:** Articles published in the journal HemaSphere exclusively reflect the opinions of the authors. The authors are responsible for all content in their abstracts including accuracy of the facts, statements, citing resources, etc.

**Results**

Initial laboratory evaluation revealed leukocytosis (26,880/mm³) with 68% circulating blasts and presence of Auer rods. Bone marrow examination and cytogenetic analysis confirmed acute promyelocytic leukemia with t(15;17)(q24;q21).

Thoraco-abdomino-pelvic CT scan identified a mid and upper pole splenic infarction as the initial clinical manifestation.

ATRA therapy was initiated immediately after diagnosis. Within 8 hours, the patient developed acute respiratory distress associated with bilateral pleural effusions and pulmonary infiltrates. Leukocytosis rapidly increased to 147,410/mm³. Coagulation tests demonstrated disseminated intravascular coagulation (DIC), along with progressive pancytopenia.

Pleural fluid analysis revealed hemorrhagic effusion with high blast infiltration. Based on hyperleukocytosis, pleuro-pulmonary involvement, and edema, a diagnosis of severe differentiation syndrome was established.

ATRA was temporarily discontinued, hydroxyurea was intensified, and dexamethasone was initiated. Clinical and biological improvement was observed, with progressive reduction of leukocytosis and resolution of respiratory symptoms. ATRA was successfully reintroduced under corticosteroid coverage without recurrence of differentiation syndrome.

**Summary/Conclusion**

Acute promyelocytic leukemia may exceptionally present with thrombotic manifestations such as splenic infarction, highlighting the complex hemostatic imbalance at diagnosis.
This case underscores that APL should not be considered solely a hemorrhagic disease, as thrombotic complications may precede classical bleeding features.
Early recognition and prompt management of differentiation syndrome following ATRA initiation are critical to prevent life-threatening complications and to allow safe continuation of curative therapy.

**Copyright Information:** (Online) ISSN: 2572-9241
©2026 The Author(s). HemaSphere is published by John Wiley & Sons Ltd on behalf of European Hematology Association. This is an open access Abstract Book distributed under the Attribution-NonCommercial-NoDerivs (CC BY-NC-ND), which allows third parties to download the articles and share them with others as long as they credit the author and the Abstract Book, but they cannot change the content in any way or use them commercially.
**Abstract Book Citations:** Authors, Title, HemaSphere, 2026;10:(S1):pages. The abstract book DOIs can be found on the “Author Information” page.
**Disclaimer:** Articles published in the journal HemaSphere exclusively reflect the opinions of the authors. The authors are responsible for all content in their abstracts including accuracy of the facts, statements, citing resources, etc.

### (PB2726) CYTOGENETIC CHARACTERISTICS OF THE DISTRIBUTION OF ABNORMAL KARYOTYPES IN DIFFERENT AGE GROUPS IN ACUTE MYELOID LEUKEMIA

**Topic:** 03. Acute myeloid leukemia - Biology & translational research

Kateryna Korets*[1], Svitlana Andreieva[1], Nadiya Goryainova[1], Halyna Starodub[1]

*[1]SI "National Research Center for Radiation Medicine, Hematology and Oncology of NAMS of Ukraine", Diseases of Blood System, Kyiv, Ukraine;*

**Background**

Chromosomal abnormalities and age are an important predictive factors in acute myeloid leukemia. However, the percentage distribution of the frequency and spectrum abnormal karyotypes in relation to age have not been performed in Ukraine.

**Aims**

To compare features of abnormal karyotypes in different age groups of patients at diagnosis of acute myeloid leukemia.

**Methods**

Cytogenetic studies were performed on bone marrow cells from 263 patients at diagnosis of acute myeloid leukemia, who were admitted to the laboratory from 2015 to 2025. The female-to-male ratio was 1.0:1.0. The mean age was 45.9±1.3 for women and 46.8±1.2 years for men (range 18 to 79 years). The patients were divided into four age groups: I) 18 – 35 years; II) 36 – 50 years; III) 51 – 65 years; IV) patients older than 66 years. Metaphase chromosome preparations were performed according to generally accepted methodology and stained for G-banding. At least 20 metaphase spreads were analyzed for each patient.

**Results**

Abnormal karyotypes were detected in 61.2% of patients and prevailed in all age groups (69.3%, 51.7%, 62.1% and 65.4%, respectively). The frequency of unbalanced chromosomal abnormalities increased with age (from 47.5% in group I to 73.7% in group IV). Balanced translocations were identitified in all age groups (33.9%, 41.2%, 16.0%, and 15.7%, respectively). The highest frequency of marker and ring chromosomes was observed in group IV (26.3% and 10.5%, respectively). Abnormal karyotypes with 1-2 chromosomal abnormalities dominated in groups I and IV (57.3% and 57.7%, respectively). In turn, the highest percentage of complex karyotypes was founded in the age group III (51-65 years) (19.5%). The average number of chromosomal abnormalities involved in complex karyotypes were from five to six abnormalities. In all age groups, marker chromosomes were involved in the formation of complex karyotypes (33.3%, 33.3%, 52.9% and 50.0%, respectively). The evolution of clonal chromosomal abnormalities in complex karyotypes was detected in age groups I, II and III (11.1%, 55.5%, and 17.6%, respectevely).

**Summary/Conclusion**

The frequency and characteristics structural of complex karyotypes in acute myeloid leukemia vary across age groups. The highest incidence was observe in patients aged 51-65 years, indicating a potential age-related increase in cytogenetic complexity. The frequency of unbalanced chromosomal abnormalities increased with patient age. These findings highlight the importance of age-stratified cytogenetic assessment at diagnosis for improved risk evaluation.

**Copyright Information:** (Online) ISSN: 2572-9241
©2026 The Author(s). HemaSphere is published by John Wiley & Sons Ltd on behalf of European Hematology Association. This is an open access Abstract Book distributed under the Attribution-NonCommercial-NoDerivs (CC BY-NC-ND), which allows third parties to download the articles and share them with others as long as they credit the author and the Abstract Book, but they cannot change the content in any way or use them commercially.
**Abstract Book Citations:** Authors, Title, HemaSphere, 2026;10:(S1):pages. The abstract book DOIs can be found on the "Author Information" page.
**Disclaimer:** Articles published in the journal HemaSphere exclusively reflect the opinions of the authors. The authors are responsible for all content in their abstracts including accuracy of the facts, statements, citing resources, etc.

### (PB2727) PROGNOSTIC ROLE OF MINIMAL RESIDUAL DISEASE IN THE TREATMENT OF PATIENTS WITH ACUTE MYELOID LEUKEMIA WITH NPM1 MUTATION.

**Topic:** 04. Acute myeloid leukemia - Clinical

Anna Drobinina*[1], Irina Lukianova[2], Andrey Sudarikov[3], Yulia Sidorova[4], Nataliya Severina[4], Irina Galtseva[5], Yulia Chabaeva[6], Elena Parovichnikova[7]

*[1]National Medical Research Center for Hematology, Department of Chemotherapy of Hemoblastosis and Hematopoietic Depressions with a Day In-patient Facility, Moscow, Russia; [2]National Medical Research Center for Hematology, Head of the Department of Chemotherapy of Hemoblastosis and Hematopoietic Depressions with a Day In-patient Facility, Moscow, Russia; [3]National Medical Research Center for Hematology, Head of the Laboratory of Molecular Hematology,, Moscow, Russia; [4]National Medical Research Center for Hematology, Laboratory of Molecular Hematology,, Moscow, Russia;[5]National Medical Research Center for Hematology, Head of the Laboratory for Immunophenotyping of Blood and Bone Marrow Cells, Moscow, Russia; [6]National Medical Research Center for Hematology, Informational Analytical Department, Moscow, Russia;[7]National Medical Research Center for Hematology, CEO of the National Medical Research Center for Hematology, Moscow, Russia;*

### Background

Detection of a mutation in the nucleophosmin 1 gene (NPM1mut) is a favorable prognostic factor for patients with acute myeloid leukemia (AML). However, this group is highly heterogeneous. Prognosis depends on the clearance of minimal residual disease (MRD). According to most studies, failure to achieve molecular remission after two courses of treatment increases the risk of relapse.

### Aims

To evaluate the prognostic significance of persistent MRD after completion of two courses of standard treatment according to the "AML-21" protocol in patients with NPM1mut AML.

### Methods

A prospective multicenter randomized clinical trial "AML-21" enrolled 64 patients with NPM1mut AML from January 2021 to August 2025. The analysis included patients whose MRD status was assessed by allele-specific polymerase chain reaction (PCR) in bone marrow after two intensive courses (57 patients). MRD negativity (MRD(PCR)"–") was defined as the complete absence of NPM1mut copies. There were 26 males and 31 females, with a median age of 31 (19-58). Normal karyotype was confirmed by standard cytogenetic analysis in 53 patients, and chromosomal abnormalities belonging to the intermediate prognosis group were identified in 4. NPM1 mutation was combined with FLT3-ITD mutation in 25/57 patients. The impact of co-mutations identified at AML diagnosis on prognosis was analyzed. A comparative assessment of two MRD detection methods was also performed: allele-specific PCR and multiparameter flow cytometry (FCM) with simultaneous determination of leukemia-associated immunophenotype and abnormal patterns of differentiation.

**Copyright Information:** (Online) ISSN: 2572-9241
©2026 The Author(s). HemaSphere is published by John Wiley & Sons Ltd on behalf of European Hematology Association. This is an open access Abstract Book distributed under the Attribution-NonCommercial-NoDerivs (CC BY-NC-ND), which allows third parties to download the articles and share them with others as long as they credit the author and the Abstract Book, but they cannot change the content in any way or use them commercially.
**Abstract Book Citations:** Authors, Title, HemaSphere, 2026;10:(S1):pages. The abstract book DOIs can be found on the "Author Information" page.
**Disclaimer:** Articles published in the journal HemaSphere exclusively reflect the opinions of the authors. The authors are responsible for all content in their abstracts including accuracy of the facts, statements, citing resources, etc.

### Results

33/57 patients (58%) achieved molecular remission (MRD(PCR)“–”) after two courses of induction therapy. Allogeneic hematopoietic stem cell transplantation (allo-HSCT) was performed in first complete remission (CR1) in 6/33 (18%). In the MRD(PCR)“+” group, allo-HSCT was performed in 8/24 (33%) patients in CR1, and in 2/24 after confirmation of MRD(FCM)-relapse. At a follow-up of 18 months, relapse-free survival (RFS) in the MRD(PCR)“–” group was 80% versus 55% in the MRD(PCR)“+” group (p=0.04) (Fig. 2). No differences were observed in overall survival (OS) (85% versus 80%, respectively, p=0.04) (Fig. 1). RFS at 18 months in the FLT3+MRD(PCR)“+” group was 30%, FLT3–MRD(PCR)“+” = 81%, FLT3+MRD(PCR)“–” = 91%, FLT3–MRD(PCR)“–” = 72% (p=0.01) (Fig. 3). Analysis of prognosis based on MRD status after two courses, determined by two methods (FCM and PCR), revealed that in the case of FCM+ PCR+, the risk of an adverse event was 11 times higher compared to FCM-PCR- (p=0.0007). In the case of FCM-PCR+, we did not observe significant differences from the FCM-PCR- group (p=0.5) (Fig. 4).

### Summary/Conclusion

When analyzing RFS according to FLT3 mutation status and flow cytometry data, we did not observe a significant impact of MRD-PCR on prognosis in patients with NPM1-mutated AML (p = 0.5).The prognostic value of combining flow cytometry and quantitative PCR in MRD diagnosis was noted, as well as the need to determine an appropriate threshold of NPM1mut copies on a larger sample size before completion of the intensive phase of therapy. The highest risk of relapse was observed in combination with FLT3 mutation and persistent MRD.

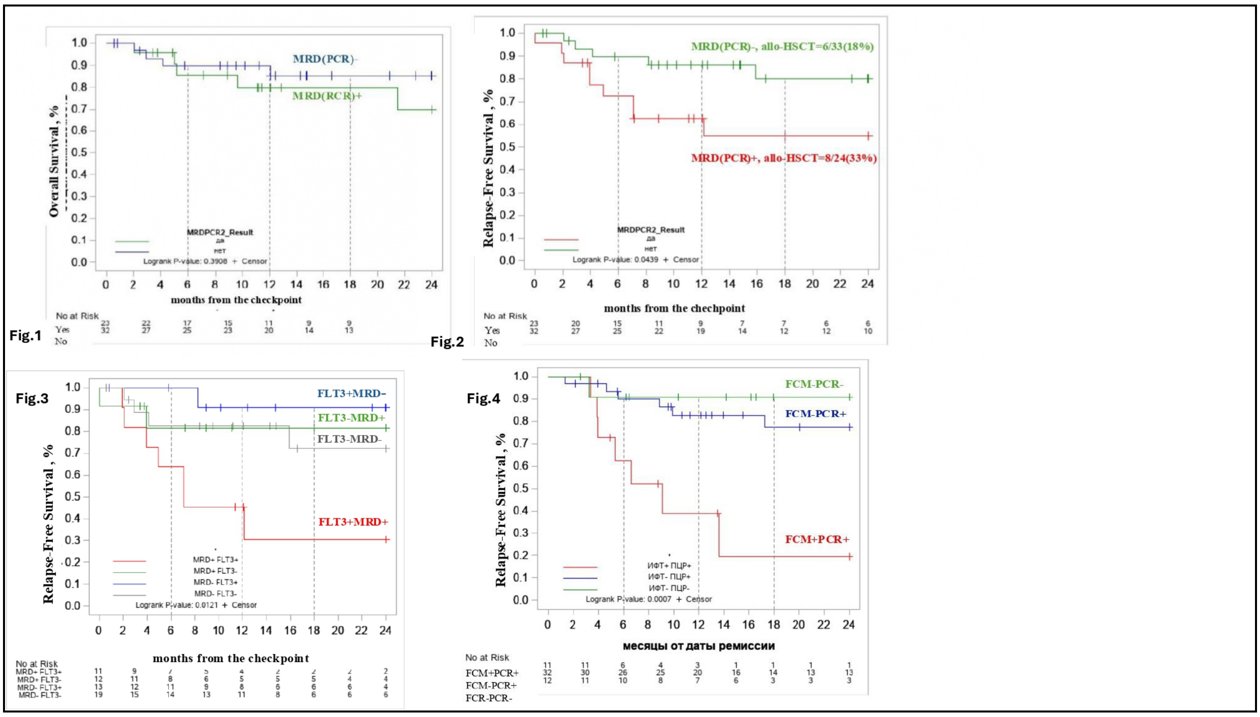

**Copyright Information:** (Online) ISSN: 2572-9241
©2026 The Author(s). HemaSphere is published by John Wiley & Sons Ltd on behalf of European Hematology Association. This is an open access Abstract Book distributed under the Attribution-NonCommercial-NoDerivs (CC BY-NC-ND), which allows third parties to download the articles and share them with others as long as they credit the author and the Abstract Book, but they cannot change the content in any way or use them commercially.
**Abstract Book Citations:** Authors, Title, HemaSphere, 2026;10:(S1):pages. The abstract book DOIs can be found on the “Author Information” page.
**Disclaimer:** Articles published in the journal HemaSphere exclusively reflect the opinions of the authors. The authors are responsible for all content in their abstracts including accuracy of the facts, statements, citing resources, etc.

### (PB2728) EFFICACY AND SAFETY OF GILTERITINIB-BASED COMBINATION THERAPY IN RELAPSED/REFRACTORY FLT3-MUTATED ACUTE MYELOID LEUKEMIA: A REAL-WORLD MULTICENTER STUDY

**Topic:** 04. Acute myeloid leukemia - Clinical

Zhiying He*[1], Yunqian Yao[1], Sijian Yu[1], Yiqing Li[2], Xiaoli Xu[3], Yu Zhan[4], Dan Xiong[5], Xiaomin Niu[6], Jieyu Ye[1], Hui Liu[1], Yu Zhang[1], Qifa Liu[1], Pengcheng Shi[1]

*[1]Nanfang Hospital, Southern medical University, Department of Hematology, Guangzhou, China;[2]Sun Yat-Sen Memorial Hospital, Sun Yat-Sen University, Department of Hematology, Guangzhou, China; [3]the First People Hospital of Foshan, Foshan, China; [4]the Twelfth People Hospital of Guangzhou, Guangzhou, China;[5]the Eighth Affiliated Hospital of Southern Medical University, Department of Hematology, Foshan, China; [6]Zhongshan City People's Hospital, Department of Hematology, Zhongshan, China;*

### Background

Relapsed or refractory acute myeloid leukemia with fms-like tyrosine kinase 3 mutations (R/R FLT3$^{mut+}$AML) remains a major clinical challenge. Although gilteritinib monotherapy resulted in improved overall survival (OS) compared to salvage chemotherapy for R/R FLT3$^{mut+}$AML patients, the remission rates and survival with gilteritinib monotherapy are still unsatisfactory. Gilteritinib-based combination therapy demonstrated increased response rates, but the survival did not improve because of increased toxicities. Thus, the optimal regimen for R/R FLT3 mutated AML remained unclear.

### Aims

To compare the efficacy and safety of gilteritinib-based combination therapy for R/R FLT3$^{mut+}$AML.

### Methods

Patients with R/R FLT3$^{mut+}$AML in six hospitals of China were screened from July 2019 to July 2025. Eligible criteria include: (1) aged ≥18 years with confirmed FLT3$^{mut+}$AML; (2) relapse or refractory (R/R) to first or greater lines of AML treatment (3) received gilteritinib-based therapy: gilteritinib plus venetoclax (Gil+Ven), gilteritinib plus venetoclax and "X" (gilteritinib plus venetoclax and azacitidine, gilteritinib plus venetoclax, azacitidine and homoharringtonine), or gilteritinib combined with intensive chemotherapy (Gil+Chemo). The primary endpoint was the composite complete remission (CRc) rate after 1-2 cycle of treatment. The secondary endpoints were safety, OS, event-free survival (EFS) and relapse.

**Copyright Information:** (Online) ISSN: 2572-9241
©2026 The Author(s). HemaSphere is published by John Wiley & Sons Ltd on behalf of European Hematology Association. This is an open access Abstract Book distributed under the Attribution-NonCommercial-NoDerivs (CC BY-NC-ND), which allows third parties to download the articles and share them with others as long as they credit the author and the Abstract Book, but they cannot change the content in any way or use them commercially.
**Abstract Book Citations:** Authors, Title, HemaSphere, 2026;10:(S1):pages. The abstract book DOIs can be found on the "Author Information" page.
**Disclaimer:** Articles published in the journal HemaSphere exclusively reflect the opinions of the authors. The authors are responsible for all content in their abstracts including accuracy of the facts, statements, citing resources, etc.

**Results**

54 patients were enrolled with a median age of 44.8 years old. 42 patients had FLT3-ITD mutation, 11 had FLT3-TKD mutation and 1 had both mutations. 14 received Gil+Ven, 29 received Gil+Ven+X and 11 received Gil+Chemo. There were no significant differences among the three groups in baseline patient characteristics. Gil+Ven group showed a trend toward higher CRc (85.7%) compared to Gil+Ven+X (65.6%) and Gil+Chemo (63.6%) groups (p=0.341). The CR rate of Gil+Ven group was 71.4%, numerically higher than Gil+Ven+X (41.4%) and Gil+Chemo (54.5%) groups (p=0.127). The MRD-negative rate in the Gil+Ven group (50%) was comparable with the Gil+Ven+X group (56%, p=0.749) and higher than the Gil+Chemo group (28.6%, p=0.401). The proportion of bridging to allogeneic hematopoietic stem cell transplantation were comparable across the Gil+Ven, Gil+Ven+X, and Gil+Chemo groups (57.1% vs. 55.2% vs. 63.6%, respectively, p=0.894). With a median follow-up of 18 months, the median OS was longer in Gil+Ven group (not reached) than that in Gil+Ven+X (7.0 months) or Gil+Chemo (19.0 months) group (p=0.19). Gil+Ven group had a higher 2-year OS (74.48%) than Gil+Ven+X group (31.78%, p=0.009) and Gil+Chemo group (38.35%, p=0.083), respectively. The 2-years EFS were also higher in Gil+Ven group (68.18%) than Gil+Ven+X (24.52%, p=0.006) and Gil+Chemo group (34.09%, p=0.090). Furthermore, the 2-year cumulative incidence of relapse (CIR) was lower in Gil+Ven group (12.5%) than Gil+Ven+X (36.91%, p=0.147) and Gil+Chemo group (46.43%, p=0.144). Gil+Ven group was associated with fewer grades 3 or higher hematologic and non-hematologic adverse events (AEs) compared to the other two groups. In Gil+Ven group, neutropenia (p=0.023), anemia (p=0.048) and BLOOD infection (p=0.045) were significantly less frequent than the other two groups.

**Summary/Conclusion**

Gilteritinib plus venetoclax demonstrated a trend toward better efficacy and fewer adverse events than other gilteritinib-based combination therapy, suggesting it might be the optimal salvage strategy for R/R FLT3$^{mut+}$AML.

**Copyright Information:** (Online) ISSN: 2572-9241
©2026 The Author(s). HemaSphere is published by John Wiley & Sons Ltd on behalf of European Hematology Association. This is an open access Abstract Book distributed under the Attribution-NonCommercial-NoDerivs (CC BY-NC-ND), which allows third parties to download the articles and share them with others as long as they credit the author and the Abstract Book, but they cannot change the content in any way or use them commercially.
**Abstract Book Citations:** Authors, Title, HemaSphere, 2026;10:(S1):pages. The abstract book DOIs can be found on the "Author Information" page.
**Disclaimer:** Articles published in the journal HemaSphere exclusively reflect the opinions of the authors. The authors are responsible for all content in their abstracts including accuracy of the facts, statements, citing resources, etc.

### (PB2729) SPLICING FACTOR MUTATION CLEARANCE AND TREATMENT OUTCOMES IN CLONAL MYELOID DISORDERS: A REFERRAL CENTER EXPERIENCE

**Topic:** 04. Acute myeloid leukemia - Clinical

Kittika Poonsombudlert*[1], Manasawee Tanariyakul[1], Sarah Mott[1], Bethany Daniels[1], Avani Yaganti[1], Chalothorn Wannaphut[2], Sarah Hornberg[1], Grerk Sutamtewagul[1]

*[1]University of Iowa Health Care, Iowa City, United States of America;[2]University of Hawaii, Internal Medicine Residency Program, Honolulu, United States of America;*

### Background

Splicing factor (SF) mutations (*SF3B1*, *SRSF2*, *U2AF1*, and *ZRSR2*) have been implicated in the pathogenesis of acute myeloid leukemia (AML), and Myelodysplastic Syndrome/Myeloproliferative Neoplasm overlap syndrome (MDS/MPN), but the significance of the clearance of SF mutations in the era of measurable residual disease (MRD)-directed management and the association with clinical outcomes remain to be characterized.

### Aims

To evaluate the outcomes of AML and MDS/MPN patients who received treatment of cytoreductive potential, including chemotherapy, hypomethylating agent (HMA) +/- venetoclax (ven), and allogeneic stem cell transplant (HSCT), and whether clearance of the SF mutations after initial treatment is associated with the odds of achieving a complete remission (CR), and overall survival (OS).

### Methods

We performed a retrospective chart review of adult patients treated at UIHC between 2016-2025. SF mutations were detected on the UIHC institutional  panel using a reporting threshold of variant allelic frequency (VAF) ≥ 2%. Patients with measured SF mutations at diagnosis and after initial treatment were included. Logistic and Cox regression models were used to estimate the effect of patient, disease, and treatment characteristics on achieving CR, proceeding to HSCT, and OS, respectively.

### Results

In the AML cohort (N=44, median age 72 years old, 59% male). The initial treatment included chemotherapy (N=16), HMA/ven (N=25), and HMA (N=3), resulting in a 75% CR rate. SF mutation clearance was achieved in 18 patients but was not associated with CR (OR 2.22, 95% CI 0.50-9.89). Ten patients later proceeded to HSCT. On univariate analysis, increasing age, treatment with HMA +/- ven, and persistent SF mutation were associated with decreased odds of proceeding to HSCT. OS was not statistically different between patients with clearance vs persistence of SF mutations after initial treatment (median OS 27.2 vs 18.7 months). In the multivariable model, co-mutation with RAS pathway mutation (HR 2.84, 95% CI 1.15-6.98) and not achieving CR (HR 3.35, 95% CI 1.40-8.01) were associated with decreased OS.
In the MDS/MPN cohort (N=31, median age 71 years old, 84% male), all patients initially received HMA, resulting in a 35% CR rate. SF mutation clearance was achieved in 6 patients, but was not associated with CR (OR 5.14, 95% CI 0.76-34.69). Five patients progressed to AML despite treatment, and 11 patients ultimately received consolidative HSCT. Only increasing age was found to be associated with decreased odds of proceeding to HSCT. OS was not statistically different between patients with clearance vs persistence of SF mutations after initial treatment (median OS 16.7 vs 23.7 months). No factors were found to be significantly associated with OS.

**Copyright Information:** (Online) ISSN: 2572-9241
©2026 The Author(s). HemaSphere is published by John Wiley & Sons Ltd on behalf of European Hematology Association. This is an open access Abstract Book distributed under the Attribution-NonCommercial-NoDerivs (CC BY-NC-ND), which allows third parties to download the articles and share them with others as long as they credit the author and the Abstract Book, but they cannot change the content in any way or use them commercially.
**Abstract Book Citations:** Authors, Title, HemaSphere, 2026;10:(S1):pages. The abstract book DOIs can be found on the "Author Information" page.
**Disclaimer:** Articles published in the journal HemaSphere exclusively reflect the opinions of the authors. The authors are responsible for all content in their abstracts including accuracy of the facts, statements, citing resources, etc.

**Summary/Conclusion**

Our data suggests that the clearance of SF mutations does not statistically impact the odds of CR and OS of MDS/AML patients and may not be a suitable surrogate for MRD. With the relatively small cohort, we were not able to stratify the outcomes among patients with each SF mutation, which can be biologically diverse. The impact of initial SF mutation VAF in relation to other co-mutations/cytogenetic abnormalities and clonal dynamics in response to each treatment modality also deserves further investigation in a large multi-center study.

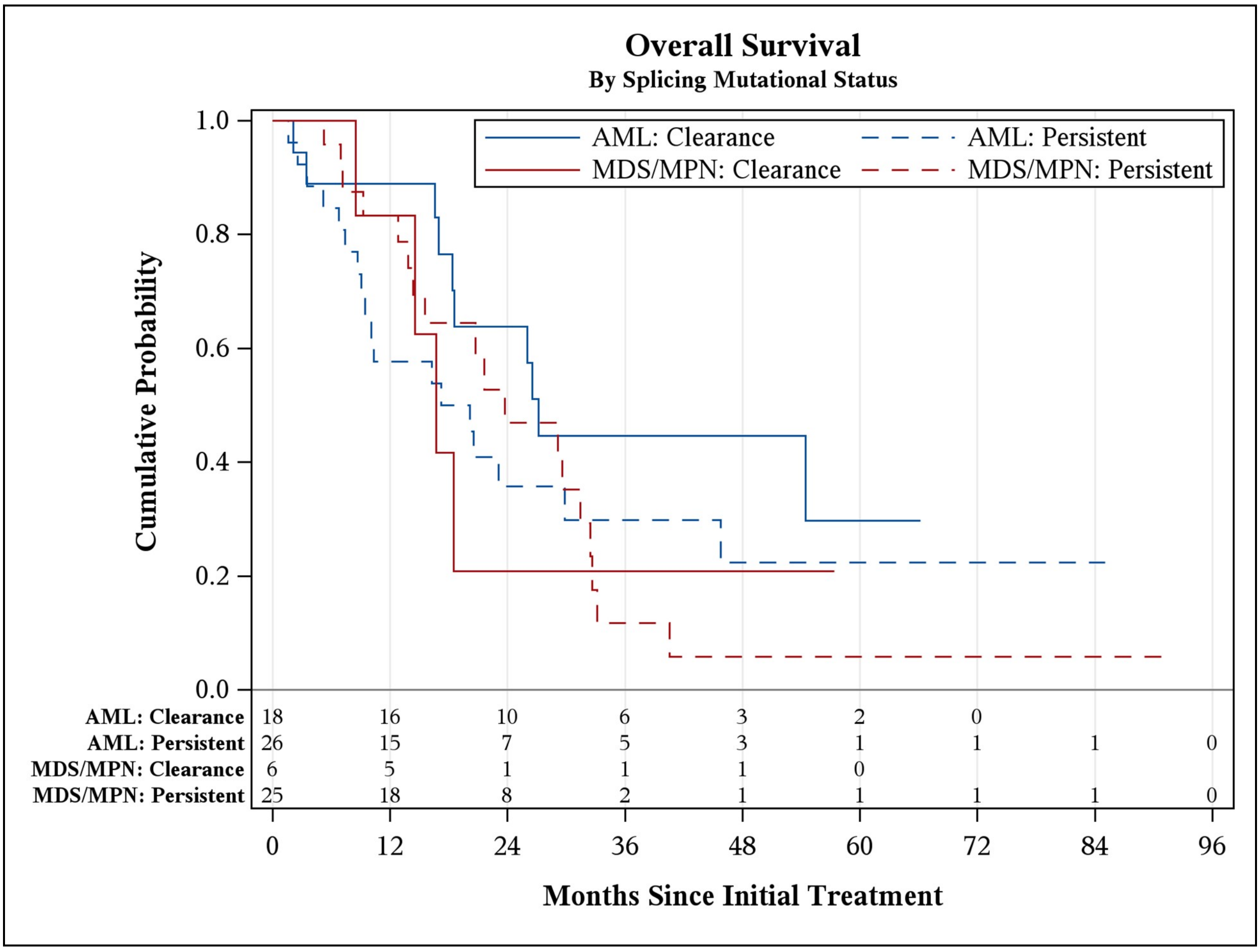

**Copyright Information:** (Online) ISSN: 2572-9241
©2026 The Author(s). HemaSphere is published by John Wiley & Sons Ltd on behalf of European Hematology Association. This is an open access Abstract Book distributed under the Attribution-NonCommercial-NoDerivs (CC BY-NC-ND), which allows third parties to download the articles and share them with others as long as they credit the author and the Abstract Book, but they cannot change the content in any way or use them commercially.
**Abstract Book Citations:** Authors, Title, HemaSphere, 2026;10:(S1):pages. The abstract book DOIs can be found on the "Author Information" page.
**Disclaimer:** Articles published in the journal HemaSphere exclusively reflect the opinions of the authors. The authors are responsible for all content in their abstracts including accuracy of the facts, statements, citing resources, etc.

### (PB2730) CLINICAL CONCORDANCE AND DECISION SUPPORT UTILITY OF FUNCTIONAL PRECISION MEDICINE TESTING IN RELAPSED/REFRACTORY ACUTE MYELOID LEUKEMIA

**Topic:** 04. Acute myeloid leukemia - Clinical

Weng Tong Ho*[1], Abdul Halim Nurul Aidah[2], Zhentang Lao[2], William Ying Khee Hwang[2], Andreas Voss[1], Edward Chow[1]

*[1]KYAN Technologies Pte Ltd, Medical Affairs, Singapore, Singapore;[2]Singapore General Hospital, Hematology, Singapore, Singapore;*

### Background

The management of refractory and relapsed Acute Myeloid Leukemia (AML) remains highly challenging due to the heterogeneity of the disease, limited targeted therapies, and absence of reliable predictive markers for cytotoxic treatments. Functional precision medicine platforms like Optim.AI, assess ex vivo drug sensitivity across potential drug combinations, offers an new approach for prioritizing therapies and exploring drug repurposing opportunities. This retrospective real-world study evaluated the clinical utility of Optim.AI for relapsed and refractory Acute Myeloid Leukemia (AML) at Singapore General Hospital (SGH).

### Methods

Bone marrow–derived AML blasts from 22 R/R AML patients at Singapore General Hospital underwent high-throughput drug sensitivity testing against a customized panel of chemotherapeutic and targeted agents, including standard-of-care treatments. All two- and three-drug combinations were analyzed and ranked by Optim.AI using normalised cell viability (NCV), with sensitivity prespecified as NCV <0.3. Reports were made available to treating physicians in routine practice, with both prospective and retrospective use. Concordance analysis was performed in 10 consented cases with evaluable clinical outcomes. Recurrent sensitive doublets were summarised across 26 reports using a mean NCV threshold of <0.5.

### Results

Marked inter-patient variability was observed in sensitivity to standard salvage regimens, reinforcing the limitations of uniform empiric therapy in R/R AML. Ex vivo predictions demonstrated strong alignment with observed clinical response, with an overall accuracy of 82.4%, sensitivity of 75%, specificity of 84.6%, positive predictive value of 60%, and negative predictive value of 91.7%. The high negative predictive value is particularly relevant in time-critical settings, suggesting utility in deprioritising ineffective regimens. Frequency analysis demonstrated convergence on repurposable doublets enriched for proteostasis targeting, epigenetic modulation, and nuclear export inhibition. The most recurrent combinations included selinexor plus romidepsin, venetoclax plus bortezomib, and venetoclax plus romidepsin. Across all sensitive doublets, the most frequently represented individual agents were bortezomib, romidepsin, venetoclax, idarubicin, and selinexor. These recurrent patterns suggest shared adaptive dependencies in R/R AML involving proteostasis stress responses and epigenetic vulnerability states that may not be apparent from genomic profiling alone.

### Summary/Conclusion

In routine clinical practice, functional precision profiling using Optim.AI demonstrated high concordance with observed treatment response and reproducibly identified biologically convergent, repurposable combination strategies. Beyond genomic classification, FPM offers a pragmatic framework for regimen prioritisation, avoidance of ineffective therapy, and rational drug repurposing in heavily pretreated AML. Prospective validation integrating turnaround time, toxicity, depth of response, and survival endpoints is warranted.

**Copyright Information:** (Online) ISSN: 2572-9241
©2026 The Author(s). HemaSphere is published by John Wiley & Sons Ltd on behalf of European Hematology Association. This is an open access Abstract Book distributed under the Attribution-NonCommercial-NoDerivs (CC BY-NC-ND), which allows third parties to download the articles and share them with others as long as they credit the author and the Abstract Book, but they cannot change the content in any way or use them commercially.
**Abstract Book Citations:** Authors, Title, HemaSphere, 2026;10:(S1):pages. The abstract book DOIs can be found on the “Author Information” page.
**Disclaimer:** Articles published in the journal HemaSphere exclusively reflect the opinions of the authors. The authors are responsible for all content in their abstracts including accuracy of the facts, statements, citing resources, etc.

### (PB2731) CHARACTERISTICS AND OUTCOMES OF ACUTE MYELOID LEUKEMIA PATIENTS ADMITTED TO INTENSIVE CARE UNIT AT DIAGNOSIS AND INDUCTION CHEMOTHERAPY: A RETROSPECTIVE SINGLE CENTRE ANALYSIS.

**Topic:** 04. Acute myeloid leukemia - Clinical

Luisa Hofmann[1], Katjana Schwab[1], Michael Serries[1], Roberto Passera[2], Peter Brossart[1], Semra Aydin*[1]

[1]*University Hospital of Bonn, Department of Oncology, Hematology, Immuno-Oncology and Rheumatology, Bonn, Germany;*
[2]*University of Torino, Department of Medical Sciences, Turin, Italy;*

### Background

The initial management of acute myeloid leukemia (AML) patients in the intensive care unit (ICU) may be associated with complications arising from the nature of the disease itself as well as the anti-leukemic treatment administered.

### Aims

The **aim** of the present study was to evaluate the association of clinical and ICU parameters at the time of AML diagnosis and initial induction chemotherapy with patient survival.

### Methods

Patients diagnosed with AML and admitted to the University Hospital of Bonn's ICU, between January 2017 and December 2025 at the time of diagnosis and during their first induction chemotherapy cycle were included in the analysis. The baseline characteristics of the patients, including their hematological parameters and the scores obtained from the ICU, were collected retrospectively and analyzed with regard to their association with the patients' overall survival (OS).

### Results

A total of 109 consecutive adult AML patients, with a median age of 66.8 years, were included in the study. The median OS for the entire cohort was 1.0 month (interquartile range [IQR]: 0.3–8.2 months). The three primary admission indications were as follows: respiratory insufficiency (35.8%), leukostasis (24.9%), and infections (29.4%). Cardiological indications (14.7%), neurological disorders (11.0%), and electrolyte imbalance (6.4%) were observed less frequently.  Of note, 28.4% of patients presented with multiple indications for ICU admission.
In the present study, 28.4% of patients required invasive ventilation, while 54.2% required non-invasive ventilation. The non-invasive ventilation treatment included continuous positive airway pressure (CPAP), high-flow nasal-, or nasal cannula. Leukapheresis was performed in 27.7% of patients, vasopressin used in 48.6% and renal replacement therapy required in 27.5%. Extracorporeal membrane oxygenation was performed in 18% of patients, all of them surviving.
With regard to the ICU scores, a significant association was observed between the APACHE II score and the survival probability. Patients exhibiting an APACHE II score <19 demonstrated a median OS that was significantly higher than that of patients with a score of ≥19, 29 months (IQR: 12.2–72.6 months) versus 11.3 months (IQR: 2–36.1 months), p = 0.042.  Furthermore, the Hematopoietic Cell Transplantation Comorbidity Index (HCT-CI) seperated patients with regard to OS. Patients with an HCT-CI of ≤4 had a significantly longer overall survival of 36.1 months (IQR: 18–86.3 months) when compared to patients with an HCT-CI of >4, with 28.3 months (IQR: 13.5–72.5 months), p = 0.029. Univariate analysis confirmed their statistical significance. Interestingly, at this early stage of the diesease standard, European Leukemia Network (ELN)-based prognostic parameters, as well as performed ICU interventions, such as invasive ventilation, leukapheresis or vasopressin use, respectively, were not associated with survival in this chemotherapy-naïve patient cohort. A total of 29.4% high-risk patients were able to proceed to allogeneic transplant.

**Copyright Information:** (Online) ISSN: 2572-9241
©2026 The Author(s). HemaSphere is published by John Wiley & Sons Ltd on behalf of European Hematology Association. This is an open access Abstract Book distributed under the Attribution-NonCommercial-NoDerivs (CC BY-NC-ND), which allows third parties to download the articles and share them with others as long as they credit the author and the Abstract Book, but they cannot change the content in any way or use them commercially.
**Abstract Book Citations:** Authors, Title, HemaSphere, 2026;10:(S1):pages. The abstract book DOIs can be found on the "Author Information" page.
**Disclaimer:** Articles published in the journal HemaSphere exclusively reflect the opinions of the authors. The authors are responsible for all content in their abstracts including accuracy of the facts, statements, citing resources, etc.

### Summary/Conclusion

In the present cohort, there was a significant association between higher APACHE II- and HCT-CI scores and impaired OS. None of the hematological risk-stratification parameters nor the ICU resources or treatments resulted to be associated with OS, underlining the efficacy and importance of a maximal treatment approach in these particularly high-risk AML patients.

**Copyright Information:** (Online) ISSN: 2572-9241
©2026 The Author(s). HemaSphere is published by John Wiley & Sons Ltd on behalf of European Hematology Association. This is an open access Abstract Book distributed under the Attribution-NonCommercial-NoDerivs (CC BY-NC-ND), which allows third parties to download the articles and share them with others as long as they credit the author and the Abstract Book, but they cannot change the content in any way or use them commercially.
**Abstract Book Citations:** Authors, Title, HemaSphere, 2026;10:(S1):pages. The abstract book DOIs can be found on the "Author Information" page.
**Disclaimer:** Articles published in the journal HemaSphere exclusively reflect the opinions of the authors. The authors are responsible for all content in their abstracts including accuracy of the facts, statements, citing resources, etc.

### (PB2732) SAFETY AND EFFICACY OF MITOXANTRONE LIPOSOME COMBINED CHEMOTHERAPY IN THE TREATMENT OF MIXED PHENOTYPE ACUTE LEUKEMIA

**Topic:** 04. Acute myeloid leukemia - Clinical

Huiwen Jiang*[1], Cong Lu[1], Qiuzhe Wei[1], Junbin Hu[1], Yaohui Wu[1]

[1]*Institute of Hematology, Union Hospital, Tongji Medical College, Huazhong University of Science and Technology, Wuhan, China;*

### Aims

To evaluate the safety and efficacy of mitoxantrone liposome (MIT-LIP) combined chemotherapy regimen in the treatment of mixed phenotype acute leukemia (MPAL).

### Methods

This is a retrospective and prospective clinical trial ([2024](0992)), which enrolls MPAL patients who received the MAED (MIT-LIP, cytarabine, etoposide, dexamethasone) ± venetoclax combined chemotherapy regimen. The clinical characteristics, treatment regimens, adverse effects, and therapeutic effects of the patients were collected.

### Results

From December 2021 to February 2026, a total of 16 MPAL patients who received MIT-LIP combined chemotherapy were admitted to multiple clinical centers. Among them, two patients were initially diagnosed with T-ALL or B-ALL, respectively, and transformed into AML after treatment, so the diagnosis were revised to MPAL. Six patients were initially diagnosed as MPAL (myeloid /B), seven patients were initially diagnosed as MPAL (myeloid /T), and one patient was initially diagnosed as MPAL (myeloid/plasmacytoid dendritic cell). There were 9 male patients (56.3%) and 7 female patients (43.7%). The median age was 33 years (ranging from 14 to 65 years). Six cases (6/11, 54.5%) of chromosomal abnormalities were detected. Fifteen cases (93.8%) of genetic abnormalities were detected, among which 3 cases were accompanied by the BCR-ABL fusion gene. Sixteen patients underwent MIT-LIP combined chemotherapy for 34 times, and no definite drug related allergy occurred. The main adverse effects were hematological toxicities. The incidence of agranulocytosis was 100%, the median duration time was 9 days (ranging from 2 to 18 days). The incidences of grade 4, grade 3, and grade 2 thrombocytopenia were 85.3%, 8.8%, and 5.9%, respectively. Only 4 cases (11.8%) of grade 3 transaminase elevation were observed, and no other obvious organ toxicity was observed. After induced chemotherapy with MIT-LIP combined regimen, 14 patients achieved complete remission (CR), 2 patients did not achieve remission, so the CR rate was 87.5%. Eight patients achieved MRD- CR and then received allo-HSCT. Among them, six patients maintained MRD- CR, one patient relapsed and achieved MRD+ CR again through MIT-LIP combined chemotherapy and secondary transplantation, and one patient died after recurrence. Two patients received allo-HSCT after achieving MRD+ CR. Among them, one received and maintained MRD- CR, one loss to follow-up after recurrence. Two patients are still in consolidation treatment and planning to receive allo-HSCT subsequently. Among them, one maintained MRD- CR, one achieved MRD+ CR. One patient lost follow-up after achieving MRD+ CR through MIT-LIP combined chemotherapy. One patient achieved MRD- CR through MIT-LIP combined chemotherapy but couldn't receive regular treatment due to financial reasons, and the patient finally died after recurrence. As for now, the median follow-up time for 14 CR patients is 13.4 months (ranging from 2.7 to 42.6 months), with an OS rate of 85.7% and an RFS rate of 78.6%. The median OS time and median RFS time have not yet been reached.

**Copyright Information:** (Online) ISSN: 2572-9241
©2026 The Author(s). HemaSphere is published by John Wiley & Sons Ltd on behalf of European Hematology Association. This is an open access Abstract Book distributed under the Attribution-NonCommercial-NoDerivs (CC BY-NC-ND), which allows third parties to download the articles and share them with others as long as they credit the author and the Abstract Book, but they cannot change the content in any way or use them commercially.
**Abstract Book Citations:** Authors, Title, HemaSphere, 2026;10:(S1):pages. The abstract book DOIs can be found on the "Author Information" page.
**Disclaimer:** Articles published in the journal HemaSphere exclusively reflect the opinions of the authors. The authors are responsible for all content in their abstracts including accuracy of the facts, statements, citing resources, etc.

**Summary/Conclusion**

According to the long-term follow-up results, the MAED ± venetoclax regimen has good safety and a relatively high induction remission rate in the treatment of MPAL.

**Copyright Information:** (Online) ISSN: 2572-9241
©2026 The Author(s). HemaSphere is published by John Wiley & Sons Ltd on behalf of European Hematology Association. This is an open access Abstract Book distributed under the Attribution-NonCommercial-NoDerivs (CC BY-NC-ND), which allows third parties to download the articles and share them with others as long as they credit the author and the Abstract Book, but they cannot change the content in any way or use them commercially.
**Abstract Book Citations:** Authors, Title, HemaSphere, 2026;10:(S1):pages. The abstract book DOIs can be found on the “Author Information” page.
**Disclaimer:** Articles published in the journal HemaSphere exclusively reflect the opinions of the authors. The authors are responsible for all content in their abstracts including accuracy of the facts, statements, citing resources, etc.

### (PB2733) RADIOTHERAPY AS A STRATEGY FOR LOCAL DISEASE CONTROL IN EXTRAMEDULLARY AML – A RETROSPECTIVE COHORT STUDY

**Topic:** 04. Acute myeloid leukemia - Clinical

Louisa Fredrikson*[1, 2], Anna Embring[3, 4], Christer Nilsson[1, 2], Lotta Hansson[1, 4], Marzia Palma[1, 4], Martin Jädersten[1, 2]

*[1]Karolinska University Hospital, Department of Hematology, Stockholm, Sweden;[2]Karolinska Institutet, Department of Medicine, Center for Hematology and Regenerative Medicine, Stockholm, Sweden; [3]Karolinska University Hospital, Department of Oncology, Stockholm, Sweden; [4]Karolinska Institutet, Department of Oncology-Pathology, Sweden, Sweden;*

### Background

Extramedullary acute myeloid leukemia (eAML) is a rare presentation of AML, in which leukemic blasts infiltrate tissues outside the bone marrow, forming a tumorous mass. eAML is commonly referred to as myeloid sarcoma when occurring in the absence of medullary disease. Extramedullary disease is an underdiagnosed condition at the time of AML diagnosis. It may be more frequently apparent at time of relapse, particularly after allogenic stem cell transplantation (HSCT).
The primary treatment for eAML is systemic chemotherapy, according to the therapeutic regimens for medullary disease. Radiotherapy (RT) is occasionally used in conjunction with chemotherapy, to improve local disease control. Since the published data on outcomes following RT for eAML are limited, we conducted a retrospective analysis to explore this further.

### Aims

The primary aim of this study was to assess the risk of local relapse at irradiated sites following RT for eAML. Additionally, we aimed to characterize the clinical features of patients receiving RT and evaluate event-free survival (EFS) and overall survival (OS).

### Methods

Patients treated with RT for eAML during the years 2009-2021 at Karolinska University Hospital in Stockholm, Sweden, were included in the analysis. Patients treated with total body irradiation were excluded. Radiation doses were recalculated into equivalent dose in 2 Gy fractions (EQD2) using $\alpha/\beta$=10 Gy. Clinical characteristics and treatment outcomes were retrospectively collected from medical records.
In patients with suspected local relapses, individual RT treatment plans were compared to diagnostic radiological images to determine whether recurrences had occurred within the irradiated volumes.

**Copyright Information:** (Online) ISSN: 2572-9241
©2026 The Author(s). HemaSphere is published by John Wiley & Sons Ltd on behalf of European Hematology Association. This is an open access Abstract Book distributed under the Attribution-NonCommercial-NoDerivs (CC BY-NC-ND), which allows third parties to download the articles and share them with others as long as they credit the author and the Abstract Book, but they cannot change the content in any way or use them commercially.
**Abstract Book Citations:** Authors, Title, HemaSphere, 2026;10:(S1):pages. The abstract book DOIs can be found on the “Author Information” page.
**Disclaimer:** Articles published in the journal HemaSphere exclusively reflect the opinions of the authors. The authors are responsible for all content in their abstracts including accuracy of the facts, statements, citing resources, etc.

**Results**

The study cohort consisted of 32 patients (male, n=20; female, n=12). The median age was 56 years (range 23-91). At the time of the first RT course, 15 patients (46.8%) had eAML also at other anatomical sites than the irradiated lesions. The median time from primary diagnosis of AML to initiation of RT was 9.7 months (range 0.2 - 110), and 14 (43.7%) patients had previously undergone a HSCT. The median EFS for patients from the first RT course to disease progression was 5.8 months, and the median OS was 18.2 months.

Ten patients (31.2%) received additional RT courses for subsequent extramedullary relapses, and seven patients (21.8 %) underwent irradiation at two or more sites concurrently. This resulted in a total of 66 separate lesions treated with RT. The median total RT dose was 24 Gy (range 4.7-40).
Local relapse occurred within the irradiated fields in 12 lesions (18.2 %). The median RT dose in lesions with local relapses was 24 Gy (4.7-39), which was not significantly different from the dose administered to lesions for which local recurrence did not occur (p=0.5).

**Summary/Conclusion**

Recurrence within irradiated volumes occurred in less than a fifth of the lesions, with no significant correlation between the dose level of RT and subsequent local disease control, indicating that the risk of local relapse is partly driven by the underlying disease characteristics.
We conclude that RT is a valuable strategy to improve local disease control in eAML, and may be used in conjunction with both chemotherapy and HSCT.

**Copyright Information:** (Online) ISSN: 2572-9241
©2026 The Author(s). HemaSphere is published by John Wiley & Sons Ltd on behalf of European Hematology Association. This is an open access Abstract Book distributed under the Attribution-NonCommercial-NoDerivs (CC BY-NC-ND), which allows third parties to download the articles and share them with others as long as they credit the author and the Abstract Book, but they cannot change the content in any way or use them commercially.
**Abstract Book Citations:** Authors, Title, HemaSphere, 2026;10:(S1):pages. The abstract book DOIs can be found on the “Author Information” page.
**Disclaimer:** Articles published in the journal HemaSphere exclusively reflect the opinions of the authors. The authors are responsible for all content in their abstracts including accuracy of the facts, statements, citing resources, etc.

### (PB2734) EFFECT OF CONCOMITANT WT1 OR DNMT3A MUTATIONS ON THE PROGNOSIS OF PATIENTS WITH CEBPA-MUTATED ACUTE MYELOID LEUKEMIA

**Topic:** 04. Acute myeloid leukemia - Clinical

Yifan Jia[1], Xin Yan[1], Zheng Ge*[1]

[1]*Department of Hematology, Zhongda Hospital, School of Medicine, Southeast University, Institute of Hematology Southeast University, Nanjing, China;*

**Background**

CEBPA is a frequently mutated gene in acute myeloid leukemia (AML). While double-mutant CEBPA (CEBPA dm) was traditionally linked to favorable prognosis, recent studies indicate that prognostic impact depends primarily on mutation type and location. Specifically, in-frame mutations in the bZIP region (bZIP-inf) are the true favorable markers, independent of allelic state. CEBPA mutations often co-occur with other mutations (e.g., GATA2, WT1, DNMT3A, NPM1), which may further influence prognosis. Yet, systematic evaluations of these interactions in real-world cohorts remain limited.

**Aims**

This study aims to determine the prognostic impact of CEBPA mutation types and co-occurring mutations in 42 AML patients to inform precise risk stratification and treatment decisions.

**Methods**

This single-center retrospective study enrolled 42 newly diagnosed AML patients with CEBPA mutations. All received standard induction chemotherapy; three underwent allo-HSCT in first remission. Mutations in CEBPA and 54 other AML-related genes were detected using next-generation sequencing (NGS). Patients were stratified by CEBPA mutation type (bZIP-inf vs. non-bZIP-inf) and analyzed according to co-mutation profiles. Primary endpoints were RFS and OS. Survival curves were estimated using the Kaplan-Meier method, and multivariate Cox regression analysis was performed to adjust for confounders including sex and age. All statistical analyses were conducted using SPSS version 27.0.

**Results**

Among the 42 patients, 28 had definitive CEBPA bZIP mutation status, including 15 (35.7%) in the bZIP-inf group and 13 (30.9%) in the non-bZIP-inf group. Patients in the bZIP-inf group had a significantly lower median age compared to the non-bZIP-inf group (54 years vs. 70 years, $P < 0.01$). Co-mutation analysis revealed that GATA2 (38.1%), DNMT3A (30.9%), and WT1 (19.0%) mutations were relatively common, while NPM1 mutations were less frequent (16.6%). Survival analysis within the bZIP-inf group showed no significant difference in prognosis between patients with single or double mutations (5-year OS: 75.2% vs. 77.6%, $P = 0.68$). Further analysis demonstrated that the presence of accompanying WT1 or DNMT3A mutations significantly impacted the prognosis of patients with CEBPA mutations ($P = 0.019$). Patients without DNMT3A or WT1 mutations ($n = 28$) had a better prognosis, with a median OS (mOS) of 21 months, whereas patients harboring DNMT3A or WT1 mutations ($n = 14$) had a dismal prognosis, with an mOS of only 10 months. Multivariate analysis confirmed that co-occurring WT1 or DNMT3A mutations were independent adverse prognostic factors in patients with CEBPA mutations ($P = 0.034$). Co-mutations in GATA2 (16 patients, 38.1%) and NPM1 (7 patients, 16.6%) did not significantly affect prognosis ($P = 0.34$ and $P = 0.39$).

**Copyright Information:** (Online) ISSN: 2572-9241
©2026 The Author(s). HemaSphere is published by John Wiley & Sons Ltd on behalf of European Hematology Association. This is an open access Abstract Book distributed under the Attribution-NonCommercial-NoDerivs (CC BY-NC-ND), which allows third parties to download the articles and share them with others as long as they credit the author and the Abstract Book, but they cannot change the content in any way or use them commercially.
**Abstract Book Citations:** Authors, Title, HemaSphere, 2026;10:(S1):pages. The abstract book DOIs can be found on the "Author Information" page.
**Disclaimer:** Articles published in the journal HemaSphere exclusively reflect the opinions of the authors. The authors are responsible for all content in their abstracts including accuracy of the facts, statements, citing resources, etc.

### Summary/Conclusion

The presence of accompanying WT1 or DNMT3A mutations is a critical adverse factor influencing the outcome of patients with CEBPA-mutated AML. Patients harboring these co-mutations have a significantly shortened median survival (10 months vs. 21 months), which serves as an independent poor prognostic factor, potentially warranting consideration of early transplant intervention.

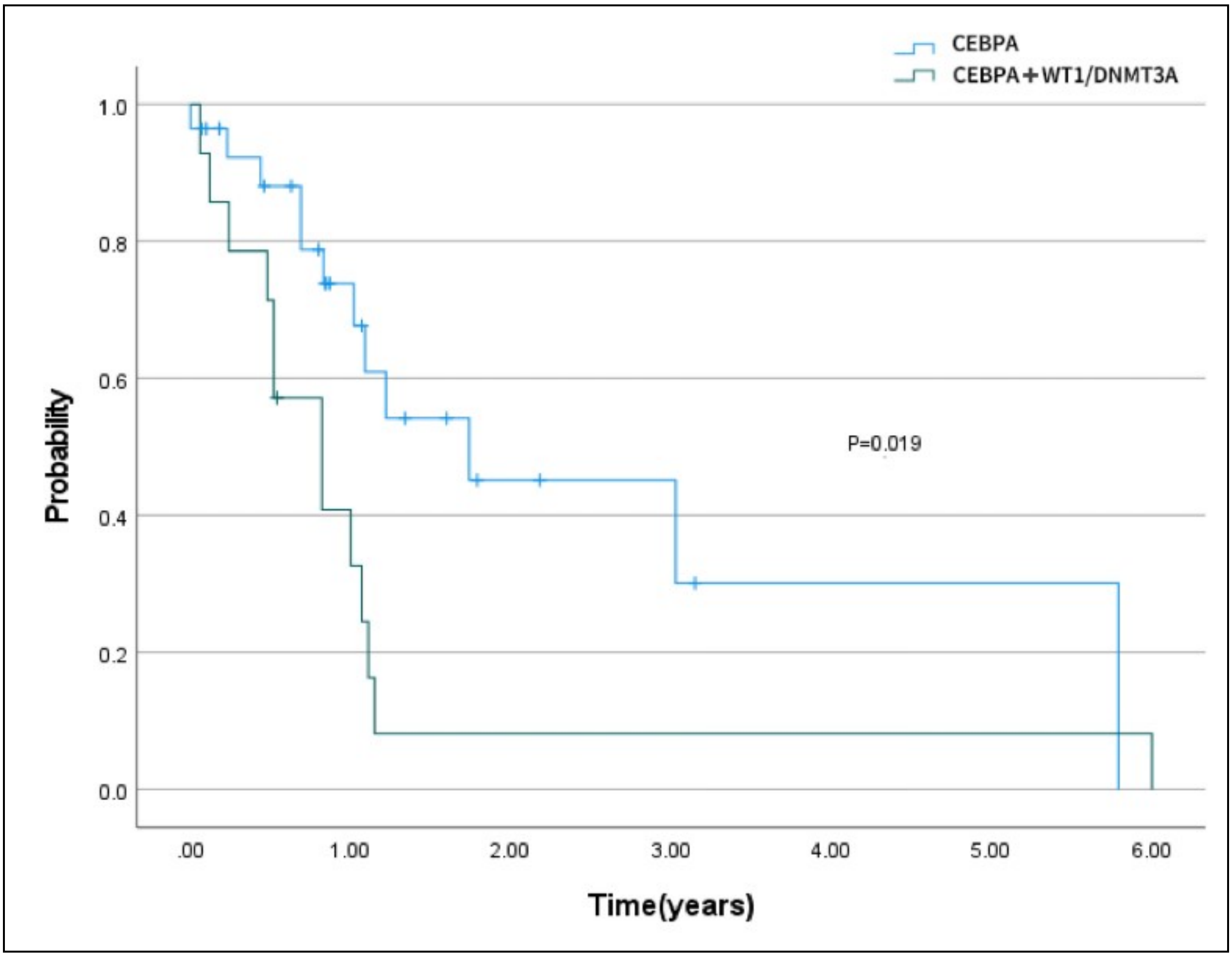

**Copyright Information:** (Online) ISSN: 2572-9241
©2026 The Author(s). HemaSphere is published by John Wiley & Sons Ltd on behalf of European Hematology Association. This is an open access Abstract Book distributed under the Attribution-NonCommercial-NoDerivs (CC BY-NC-ND), which allows third parties to download the articles and share them with others as long as they credit the author and the Abstract Book, but they cannot change the content in any way or use them commercially.
**Abstract Book Citations:** Authors, Title, HemaSphere, 2026;10:(S1):pages. The abstract book DOIs can be found on the "Author Information" page.
**Disclaimer:** Articles published in the journal HemaSphere exclusively reflect the opinions of the authors. The authors are responsible for all content in their abstracts including accuracy of the facts, statements, citing resources, etc.

### (PB2735) MACHINE LEARNING-GUIDED RISK STRATIFICATION IN ELDERLY AML BASED ON GENOMIC, IMMUNOPHENOTYPIC AND THERAPEUTIC PROFILES

**Topic:** 04. Acute myeloid leukemia - Clinical

Ling Zhang*[1], Jiang Liu[2], Jingjing Liang[2], Xialin Zhang[3], Jialong Xin[4], Mingxuan Wei[5], Lina Wang[2], Jiaxin Huo[6], Chunxia Dong[7], Yan Qiang[8], Junyan Zhang[9], Ruijuan Zhang[3]

*[1]Third Hospital of Shanxi Medical University, Shanxi Bethune Hospital, Shanxi Academy of Medical Sciences,Tongji Shanxi Hospital, Department of Hematology, Taiyuan, China; [2]Third Hospital of Shanxi Medical University, Shanxi Bethune Hospital, Shanxi Academy of Medical Sciences,Tongji Shanxi Hospital, Taiyuan, China; [3]Department of Hematology, First Hospital of Shanxi Medical University, Taiyuan, 030001, China., Taiyuan, China; [4]College of Computer Science and Technology(College of Data Science ), Taiyuan University of Technology,Taiyuan, 030600, China., Taiyuan, China; [5]Xi'an Jiaotong-Liverpool University,Suzhou, 215123,China., Suzhou, China; [6]School of Computer Science and Technology (Big Data School), North University of China, Taiyuan, 030051,China., Taiyuan, China; [7]Department of Hematology, The Second Hospital of Shanxi Medical University,Taiyuan, 030001, China., Taiyuan, China; [8]School of Software, North University of China, Taiyuan, 030051[illegible]China., Taiyuan, China; [9]Department of Clinical Epidemiology and Evidence-Based Medicine, Shanxi Bethune Hospital, Academy of Medical Sciences, Tongji Shanxi Hospital, Taiyuan, 030032, China., Taiyuan, China;*

### Background

**Elderly patients with acute myeloid leukemia (AML) exhibit considerable biological and clinical heterogeneity, hindering precise prognosis. Existing prognostic systems inadequately capture the complexity of elderly AML due to their reliance on data from younger cohorts and omission of key factors like immunophenotypic markers and therapeutic profiles.**

### Aims

**This study aimed to develop and internally validate a machine learning-based prognostic model specifically tailored to elderly AML patients.**

### Methods

**A total of 156 patients were analyzed using a two-stage modeling strategy. Clinical and genomic variables were modeled first, followed by independent analysis of immunophenotypic features. Feature selection was performed using multilayer perceptron (MLP) and random forest (RF), while multivariate Cox regression used for final model construction. Internal validation was conducted using 1000 bootstrap iterations to assess model stability and performance.**

**Copyright Information:** (Online) ISSN: 2572-9241
©2026 The Author(s). HemaSphere is published by John Wiley & Sons Ltd on behalf of European Hematology Association. This is an open access Abstract Book distributed under the Attribution-NonCommercial-NoDerivs (CC BY-NC-ND), which allows third parties to download the articles and share them with others as long as they credit the author and the Abstract Book, but they cannot change the content in any way or use them commercially.
**Abstract Book Citations:** Authors, Title, HemaSphere, 2026;10:(S1):pages. The abstract book DOIs can be found on the "Author Information" page.
**Disclaimer:** Articles published in the journal HemaSphere exclusively reflect the opinions of the authors. The authors are responsible for all content in their abstracts including accuracy of the facts, statements, citing resources, etc.

### Results

The model demonstrated strong predictive performance, with a concordance index (C-index) of 0.702. Time-dependent area under the curve (AUC) and calibration plots confirmed accurate prediction of 1-, 3-, and 5-year overall survival. Decision curve analysis indicated favorable net benefit across a range of threshold probabilities. Key independent prognostic factors identified included TP53 mutations, high CD13 expression, and IDH2 mutations.

### Summary/Conclusion

This model provides a robust and interpretable tool for individualized risk stratification in elderly AML. By integrating genomic, immunophenotypic, and therapeutic variables, it may help optimize treatment decisions and improve outcomes for this vulnerable population. Future efforts should focus on external validation and integration of dynamic biomarkers.

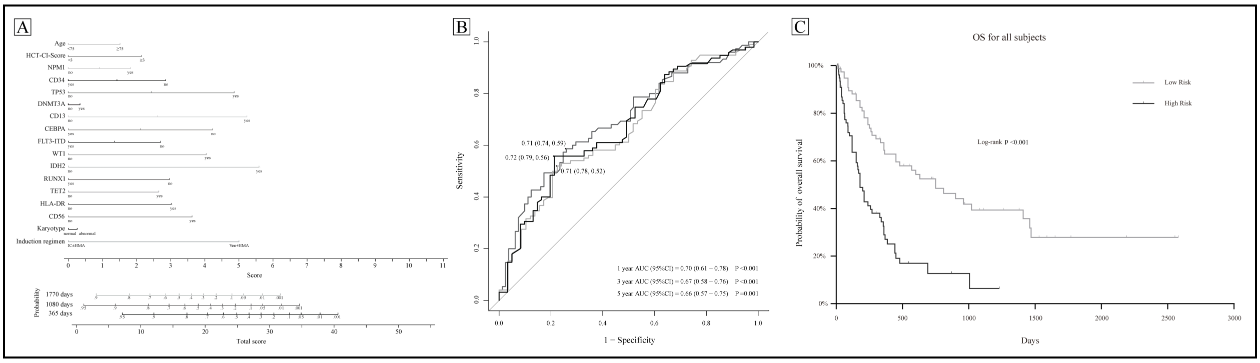

Figure.(A)Nomogram Scoring Table for Prediction of 1-, 3-, and 5-Year Survival. (B)The ROC curves of the nomograms for 1-, 2- and 3-year OS prediction ofelderly patients with AML.(C) Kaplan-Meier Survival Curves Stratified by the Nomogram-Derived Risk Groups.

**Copyright Information:** (Online) ISSN: 2572-9241
©2026 The Author(s). HemaSphere is published by John Wiley & Sons Ltd on behalf of European Hematology Association. This is an open access Abstract Book distributed under the Attribution-NonCommercial-NoDerivs (CC BY-NC-ND), which allows third parties to download the articles and share them with others as long as they credit the author and the Abstract Book, but they cannot change the content in any way or use them commercially.
**Abstract Book Citations:** Authors, Title, HemaSphere, 2026;10:(S1):pages. The abstract book DOIs can be found on the "Author Information" page.
**Disclaimer:** Articles published in the journal HemaSphere exclusively reflect the opinions of the authors. The authors are responsible for all content in their abstracts including accuracy of the facts, statements, citing resources, etc.

### (PB2736) TREATMENT LANDSCAPE OF PATIENTS WITH ACUTE MYELOID LEUKEMIA OVER 70 YEARS OF AGE IN A SINGLE-CENTER COHORT BETWEEN 2011 AND 2025: ARE WE DOING BETTER?

**Topic:** 04. Acute myeloid leukemia - Clinical

Tomas Rivero Equiza*[1, 2], Maria Izquierdo Montagut[1], Pablo Castillo Jimenez[1, 2], Daniel Esteban Corredera[1, 2], Georgina Gener-Ricos[1, 2], Blanca Xicoy Cirici[1, 2], Marta Gomez Hernando[1, 2], Mireia Morgades[1, 2], Rebeca Jurado[1, 2], Neus Ruiz-Xivillé[1, 2], Marta Canet Maldonado[1, 2], Marta Cabezon Marco[1, 2], Ulises Ferrandíz[1, 2], Lurdes Zamora Plana[1, 2], Juan-Manuel Sancho Cia[1, 2], Susana Vives Polo[1, 2]

*[1]Institut Català d'Oncologia Badalona- Hospital Germans Trias i Pujol, Badalona, Spain;[2]Josep Carreras Research Institute., Badalona, Spain;*

### Background

Acute myeloid leukemia (AML) in patients (pts) ≥ 70 years old (yrs) represents a major clinical challenge with poor prognosis. While intensive therapy (IT) has remained unchanged, the emergence of venetoclax-azacytidine (VENAZA) has transformed non-IT.

### Aims

To analyze clinical characteristics and treatment efficacy (overall survival [OS] and response rate) according to diagnostic period and therapeutic strategy in AML pts ≥70 yrs at a single institution.

### Methods

Pts ≥70 yrs diagnosed with AML between January 2011-January 2025 were included in this retrospective analysis. Pts were classified in three groups according to the first therapeutic decision: IT, non-IT and best supportive care (BSC). Clinical trial (CT) participation was recorded and stratified as IT and non-IT. Geriatric assessment was performed using the G8 and GAH scales. Three study cohorts were defined: 2011- 2017 (C1), 2018-2021 (C2) and 2022-2025 (C3).

### Results

A total of 191 pts (median age: 77 yrs, 59% male) were included. Seventy-two pts (38%) were in C1; 58 (30%) in C2; and 61 (32%) in C3. Baseline characteristics were comparable across cohorts. Cytogenetic risk was adverse in 55/172 pts (32%); 19/172 pts (11%) harbored *FLT3-ITD* and 42/174 pts (24%) *NPM1* mutations, without inter-cohort differences.

Overall, 126 pts (66%) received non-IT, 14 (7%) IT and 51 (27%) BSC, without differences across periods. CT participation decreased over time in parallel with VENAZA implementation (p=0.001). Significant differences in comorbidity burden and geriatric assessment were observed according to treatment type. Median HCT-CI scores were 2, 1, and 3; median G8 scores 14,13, and 11; and median GAH scores 2, 46, and 82 in the IT, non-IT, and BSC groups, respectively.

The CR rate was 47% overall and improved over time (33% in C1, 48% in C2 and 59% in C3, p<0.001). Although higher in IT (71% vs 44% non-IT), differences were not statistically significant.

Due to collinearity between geriatric scales, separate multivariable models were constructed and the G8-based model was retained as the main analysis. In multivariable logistic regression (n=136), younger age (OR 0.85 per year; p=0.001), later diagnostic period (C2: OR 3.63, p=0.018; C3: OR 4.23, p=0.008) and ELN 2022 favorable/intermediate risk (OR 5.35 and 3.10, p=0.004 and 0.02 respectively) were independently associated with higher response rates. G8 was not independently associated with probability of response.

Two-year global OS probability was 13.6% (95%CI 9.3–20%). OS probability across period cohorts and treatment modalities are shown in Fig 1.

In univariate analysis, G8 and GAH were associated with superior OS (both p<0.001.In multivariable Cox analysis (n=136), only older age (HR 1.08; p=0.006) and G8 index (HR 0.89, p: 0.032) remained independently associated with OS.

No significant differences in CR rate or OS were observed according to clinical trial participation.

**Copyright Information:** (Online) ISSN: 2572-9241
©2026 The Author(s). HemaSphere is published by John Wiley & Sons Ltd on behalf of European Hematology Association. This is an open access Abstract Book distributed under the Attribution-NonCommercial-NoDerivs (CC BY-NC-ND), which allows third parties to download the articles and share them with others as long as they credit the author and the Abstract Book, but they cannot change the content in any way or use them commercially.
**Abstract Book Citations:** Authors, Title, HemaSphere, 2026;10:(S1):pages. The abstract book DOIs can be found on the "Author Information" page.
**Disclaimer:** Articles published in the journal HemaSphere exclusively reflect the opinions of the authors. The authors are responsible for all content in their abstracts including accuracy of the facts, statements, citing resources, etc.

**Summary/Conclusion**

Despite therapeutic advances, AML in pts ≥70 years continues to confer poor survival. Age and ELN risk remain the main determinants of outcome. Although IT was associated with longer survival in unadjusted analyses, this effect was not independent after adjustment, suggesting selection bias. However, selected elderly pts may benefit from intensive approaches. Importantly, G8 demonstrated independent prognostic value for overall survival. Integrating G8 into routine clinical assessment may improve risk stratification and support treatment allocation in this vulnerable population.

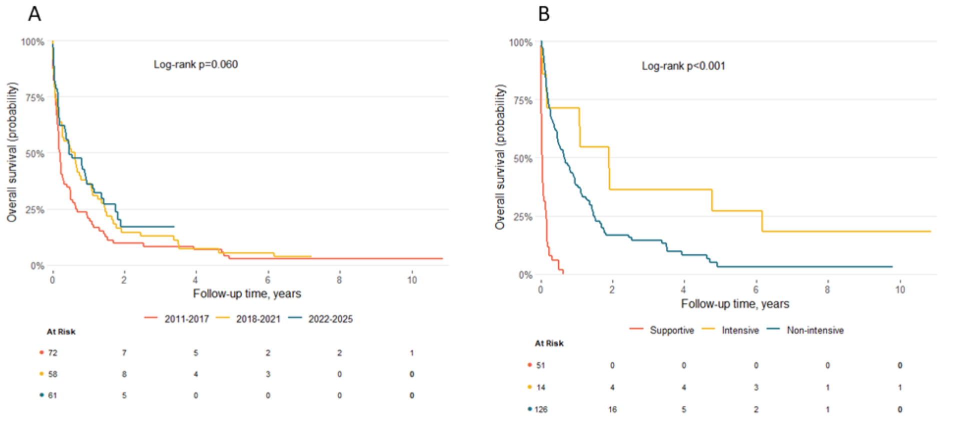

| | Median survival time[1] | 24-months OS prob.[2] |
|---|---|---|
| 2011-2017 | 2.4 (1.9; 3.7) | 9.72% (4.81%; 19.7%) |
| 2018-2021 | 6.8 (3.2; 13) | 14.6% (7.75%; 27.5%) |
| 2022-2025 | 5.6 (3.9; 13) | 16.9% (8.44%; 33.9%) |

[1]Median overall survival, months (95%CI).
[2]Overall survival probability.

| | Median survival time[1] | 24-months OS prob.[2] |
|---|---|---|
| Supportive | 0.53 (0.23; 0.79) | NE |
| Intensive | 23 (13; —) | 36.3% (17.0%; 77.4%) |
| Non-intensive | 7.9 (5.6; 11) | 16.7% (11.0%; 25.4%) |

[1]Median overall survival, months (95%CI).
[2]Overall survival probability. NE: not estimated.

**Copyright Information:** (Online) ISSN: 2572-9241
©2026 The Author(s). HemaSphere is published by John Wiley & Sons Ltd on behalf of European Hematology Association. This is an open access Abstract Book distributed under the Attribution-NonCommercial-NoDerivs (CC BY-NC-ND), which allows third parties to download the articles and share them with others as long as they credit the author and the Abstract Book, but they cannot change the content in any way or use them commercially.
**Abstract Book Citations:** Authors, Title, HemaSphere, 2026;10:(S1):pages. The abstract book DOIs can be found on the “Author Information” page.
**Disclaimer:** Articles published in the journal HemaSphere exclusively reflect the opinions of the authors. The authors are responsible for all content in their abstracts including accuracy of the facts, statements, citing resources, etc.

### (PB2737) DIVERGENT PROGNOSTIC ROLES OF CLL-1/CD123 LEUKEMIA-ASSOCIATED COMBINATIONS, REGULATORY T CELLS (TREGS) PERCENTAGE AND PD-1-EXPRESSING TREGS IN ADULT AMLS: A SINGLE CENTRE EXPERIENCE

**Topic:** 04. Acute myeloid leukemia - Clinical

Neha Singh*[1, 2], Deepali Saxena[1, 2], Anil Singh[2, 3], Pradeep Arumugam[1, 2], Ankita Pal[2, 4], Nilesh Dhole[1, 2], Rohit Kori[1, 2], Snehal Jaiswar[1, 2], Anil Yadav[2, 5], Seema Biswas[1, 2], Bal Mishra[2, 3]

*[1]TATA MEMORIAL CENTRE (HBCH & MPMMCC), HEMATOPATHOLOGY, VARANASI, India;[2]HOMI BHABHA NATIONAL INSTITUTE, MUMBAI, Mumbai, India; [3]TATA MEMORIAL CENTRE (HBCH & MPMMCC), ADULT HEMATOLYMPHOID UNIT, VARANASI, India; [4]TATA MEMORIAL CENTRE (HBCH & MPMMCC), BIOSTATISTICS & EPIDEMIOLOGY, VARANASI, India; [5]TATA MEMORIAL CENTRE (HBCH & MPMMCC), CANCER CYTOGENETICS, VARANASI, India;*

### Background

Regulatory T cells (TREGs) and PD1 have emerged as central contributors to immune evasion and leukemic persistence in AML. Concurrently, the expression of CLL-1 and CD123 on AML blasts serve as important targets for novel immunotherapies. Despite growing evidence, the interplay between these immune and leukemic markers, ELN risk classification, and measurable residual disease (MRD) status remains incompletely characterised.

### Aims

This study aimed to evaluate the prognostic role of Tregs percentage and PD-1-Expressing Tregs as well as CLL-1/CD123 Leukemia-Associated Combinations [LAC] in a prospective cohort of 128 adult AML patients.

### Methods

Immunophenotyping was performed to assess Tregs percentage, PD-1 expression on TREGs (CD4+CD25+CD127dim to neg) in peripheral blood, and expression of CLL-1/CD123/CD25/CD32 on leukemic blasts in bone marrow at baseline. ELN 2022 risk stratification was applied based on cytogenetic and next generation sequencing data. MRD was assessed after two cycles of intensive chemotherapy.

**Copyright Information:** (Online) ISSN: 2572-9241
©2026 The Author(s). HemaSphere is published by John Wiley & Sons Ltd on behalf of European Hematology Association. This is an open access Abstract Book distributed under the Attribution-NonCommercial-NoDerivs (CC BY-NC-ND), which allows third parties to download the articles and share them with others as long as they credit the author and the Abstract Book, but they cannot change the content in any way or use them commercially.
**Abstract Book Citations:** Authors, Title, HemaSphere, 2026;10:(S1):pages. The abstract book DOIs can be found on the “Author Information” page.
**Disclaimer:** Articles published in the journal HemaSphere exclusively reflect the opinions of the authors. The authors are responsible for all content in their abstracts including accuracy of the facts, statements, citing resources, etc.

**Results**

The median age at presentation was 45 years (IQR: 32.25–56.75). The median TREG percentage was 2.5% (Range: 0-13.3). PD-1 was expressed on TREGs in 62.5% of patients, suggesting high prevalence of immune checkpoint expression in AML. CLL-1, CD25, CD32 and CD123 were expressed in 84.4%, 6.3%, 6.3% and 91.4% of patients respectively. The LAC combination of CLL-1+CD123 was found in 76.6% of patients. ELN risk distribution was good 43.8%, intermediate 37.5%, and adverse 18.8% respectively. High mutation burden (≥3) was seen in 25.8% patients.  Our study demonstrates that specific LAC combination patterns (CLL-1 only, CD123 only, or co-expression) carry different ELN risk distributions (p=0.03) and mutation burden profiles (p=0.02), with CD123-only patients over-represented in adverse risk and CLL-1-only in good risk. CLL-1-only cases carry the highest mutation burden ≥3 (60%). MRD positivity was observed in 22.7% of patients after two cycles of intensive chemotherapy. Survival status demonstrated a highly significant association with MRD status (p<0.001). 3.1% patients relapsed while 16.4% were deceased within 1-year. In contrast, neither PD-1 expression on TREGs (p=0.96) nor TREG percentage (p=0.76) showed any significant association with MRD status or ELN risk grouping or mutation burden, aligning with a recent real-world study by Callis et al on PD1/PDL1 expression in AML. CD123 showed a borderline association with MRD positivity (p=0.06), with universal CD123 positivity in the MRD-positive as well as deceased patients (p=0.005). Elevated total leukocyte count (TLC) was significantly associated with mortality (p=0.007). CLL-1 median expression was significantly lower in MRD-positive patients compared to MRD-negative patients (37.0 vs 52.3, p=0.02), a seemingly paradoxical finding that may reflect a more immature, progenitor-like blast coinciding with greater chemoresistance. CLL-1 expression was significantly lower in deceased patients (median 25.3 vs 49.3 in alive patients; p=0.03).

**Summary/Conclusion**

CD123 positivity and lower CLL-1 expression were associated with inferior outcomes. PD-1 expression on TREGs and TREG percentage demonstrated no significant independent association with either ELN risk or MRD status, suggesting that TREG-mediated immune suppression via PD-1, while biologically relevant, does not independently stratify treatment response in newly diagnosed AML by currently employed metrics.

**Copyright Information:** (Online) ISSN: 2572-9241
©2026 The Author(s). HemaSphere is published by John Wiley & Sons Ltd on behalf of European Hematology Association. This is an open access Abstract Book distributed under the Attribution-NonCommercial-NoDerivs (CC BY-NC-ND), which allows third parties to download the articles and share them with others as long as they credit the author and the Abstract Book, but they cannot change the content in any way or use them commercially.
**Abstract Book Citations:** Authors, Title, HemaSphere, 2026;10:(S1):pages. The abstract book DOIs can be found on the “Author Information” page.
**Disclaimer:** Articles published in the journal HemaSphere exclusively reflect the opinions of the authors. The authors are responsible for all content in their abstracts including accuracy of the facts, statements, citing resources, etc.

### (PB2739) VENETOCLAX PLUS GILTERITINIB FOR FLT3-MUTATED RELAPSED/REFRACTORY ACUTE MYELOID LEUKEMIA: A SINGLE-CENTER EXPERIENCE

**Topic:** 04. Acute myeloid leukemia - Clinical

Francesca Crupi*[1, 2, 3], Gaia Ciolli[1, 2], Matteo Piccini[2], Barbara Scappini[2], Laura Fasano[1], Elisa Quinti[1], Andrea Pasquini[1, 2], Jessica Caroprese[1, 2, 3], Giacomo Gianfaldoni[2], Giovanna Motta[4], Raffaella Santi[5], Paola Guglielmelli[1, 2, 3], Alessandro Maria Vannucchi[1, 2, 3], Francesco Mannelli[1, 2, 3]

*[1]Università di Firenze, Medicina Sperimentale e Clinica, Florence, Italy;[2]AOU Careggi, Hematology, Florence, Italy;[3]AOU Careggi, Centro di Ricerca e Innovazione Malattie Mieloproliferative (CRIMM), Florence, Italy; [4]IRCCS Azienda Ospedaliero-Universitaria di Bologna, Haematopathology Unit, Bologna, Italy; [5]Università di Firenze, AOU Careggi, Unità di Ematopatologia, Florence, Italy;*

**Background**

FLT3 inhibition represents a key strategy in *FLT3*-mutated (mut) relapsed refractory (R/R) acute myeloid leukemia (AML). Gilteritinib proved superior over conventional chemotherapy in the ADMIRAL trial. However, its use has limitations: a relatively long time to response, infrequent minimal redisual disease (MRD) negativity, and reduced efficacy in patients (pts) with low *FLT3*-ITD allelic ratios or adverse cytogenetics. Recently, a study by Daver et al. reported high response rates with the venetoclax–gilteritinib (VenGilt) combination in R/R $FLT3^{mut}$ AML, including heavily pretreated and FLT3 inhibitor–exposed pts.

**Aims**

To explore whether the addition of venetoclax may improve the management of R/R$FLT3^{mut}$ AML pts with suboptimal responses to gilteritinib in a real-world setting.

**Methods**

Single-centre retrospective analysis of R/R $FLT3^{mut}$ AML pts treated with off-label VenGilt. Notably, all but one pt had previously received gilteritinib monotherapy and venetoclax was later added, differently from the simultaneous administration by Daver et al.

**Copyright Information:** (Online) ISSN: 2572-9241
©2026 The Author(s). HemaSphere is published by John Wiley & Sons Ltd on behalf of European Hematology Association. This is an open access Abstract Book distributed under the Attribution-NonCommercial-NoDerivs (CC BY-NC-ND), which allows third parties to download the articles and share them with others as long as they credit the author and the Abstract Book, but they cannot change the content in any way or use them commercially.
**Abstract Book Citations:** Authors, Title, HemaSphere, 2026;10:(S1):pages. The abstract book DOIs can be found on the "Author Information" page.
**Disclaimer:** Articles published in the journal HemaSphere exclusively reflect the opinions of the authors. The authors are responsible for all content in their abstracts including accuracy of the facts, statements, citing resources, etc.

**Results**

From November 2023 to December 2025, 12 R/R *FLT3*$^{mut}$ pts received VenGilt, as showed in Figure 1. The median age was 59 (range 18–78) years. ELN 2022 risk stratification at diagnosis was favourable in 3 (25.0%), intermediate in 6 (50.0%), and adverse in 3 (25.0%) pts. *NPM1* co-mutation was detected in 7 (58.3%) pts. All but one patient carried a *FLT3* mutation at disease onset, including one patient with *FLT3*-TKD. The median number of previously therapy lines was 3 (range 1-3), including gilteritinib monotherapy for a median of 42 days (range 28–616). 7 (58.3%) pts had already received midostaurin as FLT3 inhibitor in induction and consolidation therapy. Venetoclax was given for 28 (n=2), 21 (n=8) and 15 (n=2) days per cycle, based on clinical conditions and blood counts during treatment. Gilteritinib was administered continuously. Median number of cycles was 1.5 (range 1-8). A complete composite response (CCR) was achieved in 8 pts (66.7%), including 5 CR and 3 CRi. MRD negativity, was achieved in 3 responding pts (37.5%). Moreover, among pts eligible for allogenic steam cell transplantation (HSCT) (n = 9), 7 (77.8%) proceeded to transplantation, of whom 5 were in CR. Three pts resumed gilteritinib after HSCT and remain alive in CR. Median OS was not reached, with a median follow-up of 19.2 months (95% CI, 8.8–36.4). At 30 and 60 days from VenGilt start, mortality rates were 8.3% and 16.7%, respectively, due to disease progression (n=2) and transplant-related mortality (n=1); no deaths due to treatment-related toxicities. In pts achieving a response, the median time to neutrophil and platelet recovery (ANC > 1,000/µL and Plt > 50,000/µL) was 16 days (range 0–68) and 22 days (range 0–67), respectively. No QTc prolongation and grade ≥3 adverse events were reported. Notably, one pt (#3) experienced differentiation syndrome while on gilteritinib monotherapy, with transient improvement after treatment interruption and steroids but recurrence upon rechallenge. In this case the addition of venetoclax led to complete resolution.

**Summary/Conclusion**

Despite the limited numbers, the results of our real world series align with prior reports and show that adding venetoclax can induce deep responses even after prior exposure to gilteritinib. This combination may offer deeper responses without adding substantial toxicity, making it a valuable bridge to HSCT in eligible pts. Further studies are needed to confirm its efficacy and safety.

**Copyright Information:** (Online) ISSN: 2572-9241
©2026 The Author(s). HemaSphere is published by John Wiley & Sons Ltd on behalf of European Hematology Association. This is an open access Abstract Book distributed under the Attribution-NonCommercial-NoDerivs (CC BY-NC-ND), which allows third parties to download the articles and share them with others as long as they credit the author and the Abstract Book, but they cannot change the content in any way or use them commercially.
**Abstract Book Citations:** Authors, Title, HemaSphere, 2026;10:(S1):pages. The abstract book DOIs can be found on the "Author Information" page.
**Disclaimer:** Articles published in the journal HemaSphere exclusively reflect the opinions of the authors. The authors are responsible for all content in their abstracts including accuracy of the facts, statements, citing resources, etc.

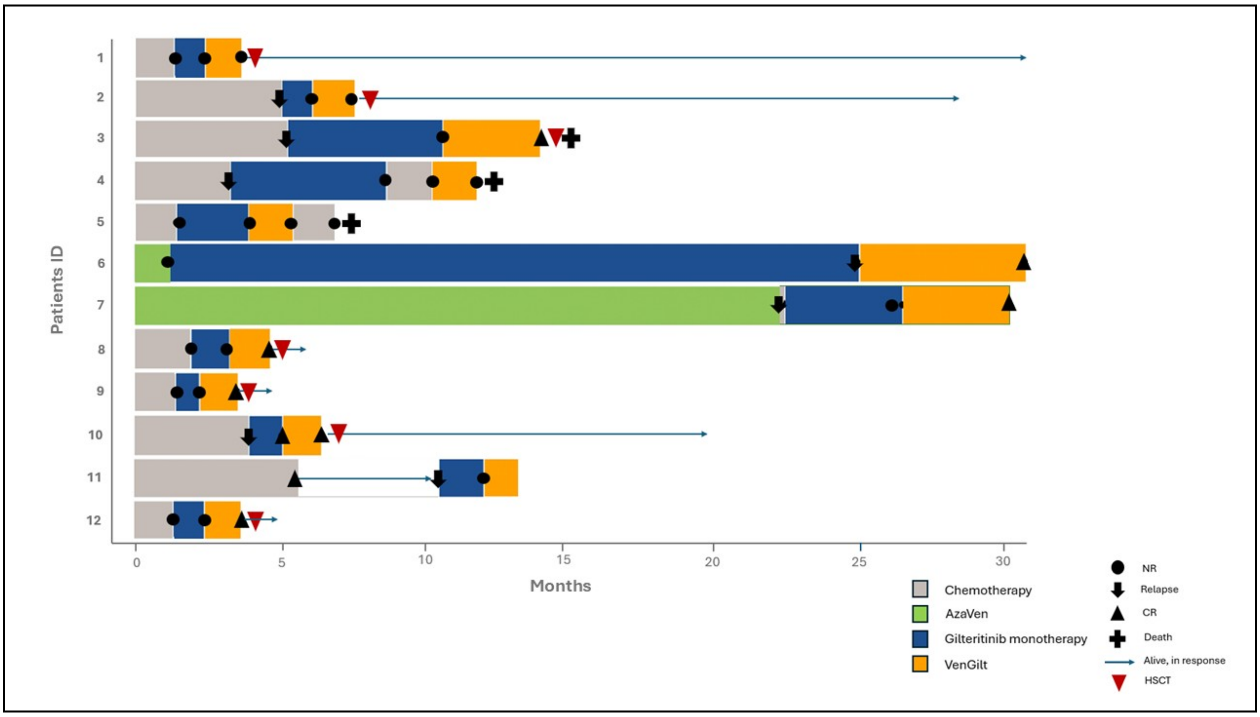

Figure 1. Swimmer plot illustrating treatment duration of gilteritinib monotherapy (blue) and combination venetoclax-gilteritinib (orange), response status, and follow-up for individual patients over time.

**Copyright Information:** (Online) ISSN: 2572-9241
©2026 The Author(s). HemaSphere is published by John Wiley & Sons Ltd on behalf of European Hematology Association. This is an open access Abstract Book distributed under the Attribution-NonCommercial-NoDerivs (CC BY-NC-ND), which allows third parties to download the articles and share them with others as long as they credit the author and the Abstract Book, but they cannot change the content in any way or use them commercially.
**Abstract Book Citations:** Authors, Title, HemaSphere, 2026;10:(S1):pages. The abstract book DOIs can be found on the "Author Information" page.
**Disclaimer:** Articles published in the journal HemaSphere exclusively reflect the opinions of the authors. The authors are responsible for all content in their abstracts including accuracy of the facts, statements, citing resources, etc.

### (PB2740) LISAFTOCLAX AS A NOVEL BCL-2 INHIBITOR: EFFICACY AND SAFETY IN INDUCTION OR RE-INDUCTION COMBINED REGIMENS FOR HEMATOLOGIC MALIGNANCIES

**Topic:** 04. Acute myeloid leukemia - Clinical

Mengze Hao*[1], Jialin Wei[1], Rongli Zhang[1], Erlie Jiang[1]

[1]*Institute of Hematology & Blood Diseases Hospital, Chinese Academy of Medical Sciences & Peking Union Medical College, Tianjin, China;*

### Background

Lisaftoclax (Lisa) is an investigational oral small-molecule Bcl-2 inhibitor with high selectivity, thereby inducing apoptosis in tumor cells. However, its therapeutic efficacy in induction or re-induction regimens for patients with hematologic malignancies (HM) remains to be elucidated.

### Methods

A total of 13 eligible patients with HM were enrolled in this study. All patients received induction or re-induction therapy comprising chemotherapy or immunotherapy combined with Lisa. Patients received one of two lisa dosing schedules in combination with backbone therapies. The 14-day schedule (Lisa-14) consisted of 200 mg on day 1, 400 mg on day 2, followed by a ramp-up to 600 mg daily from day 3 to day 14. The 7-day schedule (Lisa-7) consisted of 200 mg on day 1, 400 mg on day 2, followed by a ramp-up to 600 mg daily from day 3 to day 7. The primary endpoint was the overall response rate (ORR), assessed to evaluate the efficacy and safety of the regimen. Responses were categorized according to the 2022 ELN criteria as complete remission (CR), complete remission with incomplete hematologic recovery (CRi), stable disease (SD), no response, or unevaluable.

### Results

As of February 26, 2026, 13 patients with HM receiving a Lisa-based regimen as induction or re-induction therapy were enrolled. The median age was 38 years (range: 15–61), and 53.8% (7/13) were male. Genetic mutational data were available for all patients. Detected mutations included *TP53* in three patients with cute lymphoblastic leukemia (ALL) (23.1%) and *U2AF1* in three patients with myelodysplastic syndromes (MDS)/relapsed/refractory (R/R) acute myeloid leukemia (AML) (23.1%). Cytogenetically, nine patients exhibited normal karyotypes and three had complex karyotypes. Three patients carried high-risk fusions: *KMT2A*::*KMT2A* (R/R AML), *MLL*::*AF4* (ALL), and *MEF2D*::*HNRNPUL1* (ALL). The ORR (CR + CRi) was 69.2% (9/13). Responses by disease subtype were as follows: relapsed MDS, 100% (2/2); R/R AML, 50.0% (2/4); mixed phenotype acute leukemia (MPAL), 100% (1/1); and ALL, 83.3% (5/6), including 100% (2/2) in *de novo* ALL. Among the six relapsed patients, the ORR was 50% (3/6). Both patients with post-transplant relapsed MDS achieved CR (100%, 2/2). One patient with AML relapsed after autologous HSCT (auto-HSCT) received re-induction with the Lisa-7 regimen combined with chemotherapy and a donor lymphocyte infusion (DLI), achieving CR with minimal residual disease (MRD) negativity. Of the two patients with relapsed AML, one had no response, and the other was not evaluable due to severe pneumonia leading to discharge against medical advice. Among the three patients with TP53-mutant ALL, all of whom exhibited multiple hits, two achieved CR following Lisa-based combination therapy, yielding an ORR of 66.7% (2/3).Treatment-emergent adverse events were generally manageable. The most common hematologic toxicities included neutropenia, thrombocytopenia, and anemia. No severe infections occurred during the treatment period. Non-hematologic toxicities were predominantly mild to moderate and included nausea, vomiting, and one case of diarrhea. Notably, no episodes of tumor lysis syndrome (TLS) were observed.

**Copyright Information:** (Online) ISSN: 2572-9241
©2026 The Author(s). HemaSphere is published by John Wiley & Sons Ltd on behalf of European Hematology Association. This is an open access Abstract Book distributed under the Attribution-NonCommercial-NoDerivs (CC BY-NC-ND), which allows third parties to download the articles and share them with others as long as they credit the author and the Abstract Book, but they cannot change the content in any way or use them commercially.
**Abstract Book Citations:** Authors, Title, HemaSphere, 2026;10:(S1):pages. The abstract book DOIs can be found on the "Author Information" page.
**Disclaimer:** Articles published in the journal HemaSphere exclusively reflect the opinions of the authors. The authors are responsible for all content in their abstracts including accuracy of the facts, statements, citing resources, etc.

**Summary/Conclusion**

The combination regimens incorporating Lisa demonstrated promising clinical efficacy and a favorable safety profile in patients with newly diagnosed or relapsed/refractory HM. The combination therapy was well tolerated, as evidenced by low infection rates and the absence of early mortality.

**Copyright Information:** (Online) ISSN: 2572-9241
©2026 The Author(s). HemaSphere is published by John Wiley & Sons Ltd on behalf of European Hematology Association. This is an open access Abstract Book distributed under the Attribution-NonCommercial-NoDerivs (CC BY-NC-ND), which allows third parties to download the articles and share them with others as long as they credit the author and the Abstract Book, but they cannot change the content in any way or use them commercially.
**Abstract Book Citations:** Authors, Title, HemaSphere, 2026;10:(S1):pages. The abstract book DOIs can be found on the "Author Information" page.
**Disclaimer:** Articles published in the journal HemaSphere exclusively reflect the opinions of the authors. The authors are responsible for all content in their abstracts including accuracy of the facts, statements, citing resources, etc.

### (PB2741) VENETOCLAX PLUS MITOXANTRONE HYDROCHLORIDE LIPOSOME AND AZACITIDINE (VMA) IN RELAPSED/REFRACTORY ACUTE MYELOID LEUKEMIA: A RETROSPECTIVE REAL-WORLD ANALYSIS

**Topic:** 04. Acute myeloid leukemia - Clinical

Zhuo Wan*[1], Dian Lou[1], Yueru Ji[1], Huaipeng Guo[1], Jingyu Hu[1], Yi Chen[1], Xixi Wang[1], Li Liu[1], Weiwei Qin[1]

[1]*Tangdu hospital of Fourth military medical university, Hematology, Xi'an, China;*

### Background

Relapsed or refractory (R/R) acute myeloid leukemia (AML) remains associated with poor prognosis and limited therapeutic options. Venetoclax-based combinations have improved outcomes in unfit newly diagnosed AML, yet optimal regimens in the R/R setting remain undefined. Liposomal mitoxantrone offers sustained drug exposure with potentially reduced cardiotoxicity. We previously explored the VMA regimen in newly diagnosed AML ; however, its activity in R/R AML has not been systematically evaluated.

### Aims

To evaluate the efficacy, survival outcomes, and safety of venetoclax plus mitoxantrone hydrochloride liposome and azacitidine (VMA) in patients with relapsed or refractory AML in a real-world setting.

### Methods

This retrospective single-center analysis included adult patients with R/R AML treated with the VMA regimen. Treatment consisted of intravenous mitoxantrone hydrochloride liposome (20 mg/m², day 1), oral venetoclax (dose ramp-up to 400 mg daily, days 1–14), and subcutaneous azacitidine (100 mg/m², days 1–7) in 28–35-day cycles. Primary endpoints included composite complete remission (CRc: CR + CRi) and overall response rate (ORR). Secondary endpoints included overall survival (OS), relapse-free survival (RFS), and safety. Survival was estimated using Kaplan–Meier methodology.

### Results

A total of 23 patients with R/R AML were included. Patients had received at least one prior line of therapy, and several had adverse cytogenetic or molecular features.The overall response rate was 65.2%, including CR/CRi in 56.5% of patients. Partial remission was achieved in 8.7%, while 21.7% had no response. Early mortality was limited. At a median follow-up of approximately 6 months, the median overall survival was not reached. The estimated 6-month OS rate exceeded 70%. Patients achieving CR/CRi demonstrated durable responses, and several proceeded to allogeneic hematopoietic stem cell transplantation. The regimen was generally well tolerated. Hematologic toxicities were expected but manageable. No unexpected organ toxicity or treatment-related cardiotoxicity was observed. Treatment discontinuation due to toxicity was uncommon.

### Summary/Conclusion

In this retrospective real-world analysis, the VMA regimen demonstrated encouraging remission rates and favorable early survival in patients with relapsed or refractory AML. Median overall survival has not yet been reached. The regimen showed manageable toxicity and may represent a viable salvage strategy. Prospective studies are warranted to confirm these findings.

**Copyright Information:** (Online) ISSN: 2572-9241
©2026 The Author(s). HemaSphere is published by John Wiley & Sons Ltd on behalf of European Hematology Association. This is an open access Abstract Book distributed under the Attribution-NonCommercial-NoDerivs (CC BY-NC-ND), which allows third parties to download the articles and share them with others as long as they credit the author and the Abstract Book, but they cannot change the content in any way or use them commercially.
**Abstract Book Citations:** Authors, Title, HemaSphere, 2026;10:(S1):pages. The abstract book DOIs can be found on the "Author Information" page.
**Disclaimer:** Articles published in the journal HemaSphere exclusively reflect the opinions of the authors. The authors are responsible for all content in their abstracts including accuracy of the facts, statements, citing resources, etc.

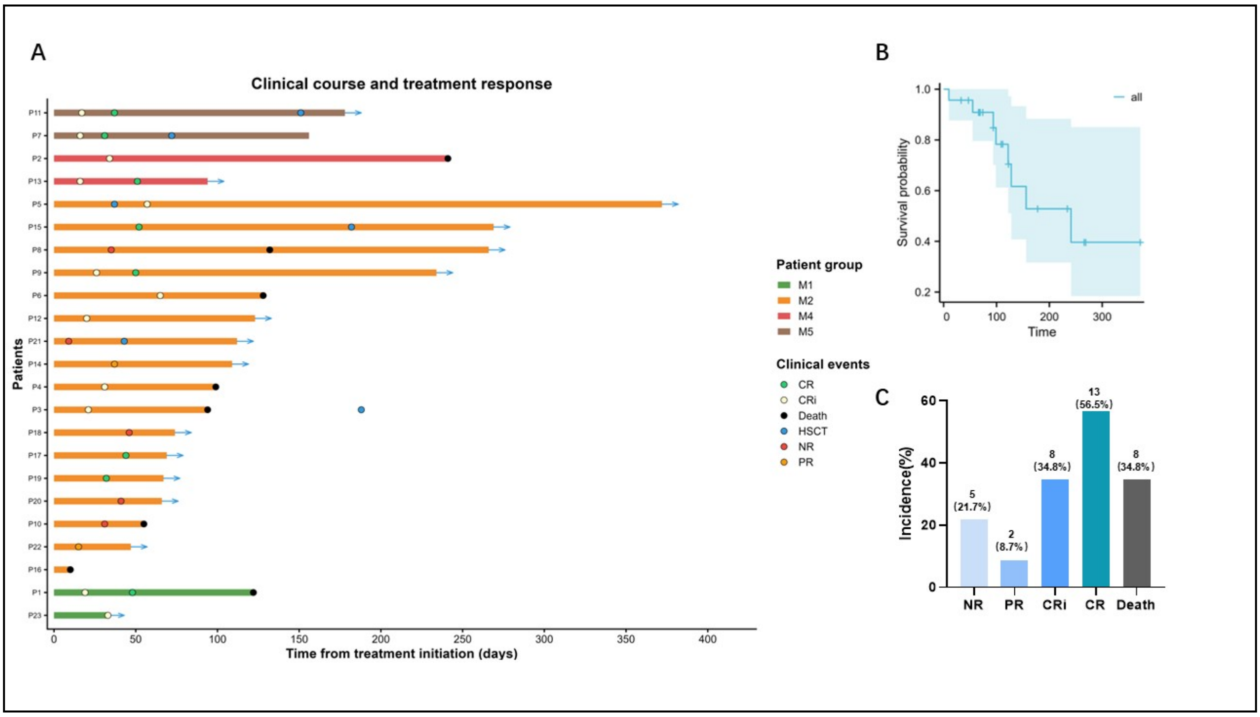

**Copyright Information:** (Online) ISSN: 2572-9241
©2026 The Author(s). HemaSphere is published by John Wiley & Sons Ltd on behalf of European Hematology Association. This is an open access Abstract Book distributed under the Attribution-NonCommercial-NoDerivs (CC BY-NC-ND), which allows third parties to download the articles and share them with others as long as they credit the author and the Abstract Book, but they cannot change the content in any way or use them commercially.
**Abstract Book Citations:** Authors, Title, HemaSphere, 2026;10:(S1):pages. The abstract book DOIs can be found on the "Author Information" page.
**Disclaimer:** Articles published in the journal HemaSphere exclusively reflect the opinions of the authors. The authors are responsible for all content in their abstracts including accuracy of the facts, statements, citing resources, etc.

### (PB2742) ELTROMBOPAG PLUS VENETOCLAX AND AZACITIDINE IN NEWLY DIAGNOSED ACUTE MYELOID LEUKEMIA: A RETROSPECTIVE STUDY

**Topic:** 04. Acute myeloid leukemia - Clinical

Haifeng Zheng[1], Zhenqing Tan[1], Wenqun Yang[1], Lu Zhang[1], Fang Chen[1], Juan Hong[1], Ping Liu[1], Juan Jin[1], Zhuo Wen[1], Lian Cai[1], Wei Chen[1], Kaibo Zhu*[1]

[1] *The Central Hospital of Xiangtan(The affiliated hospital of Hunan University), Xiangtan, China;*

### Background

Eltrombopag, a first-generation thrombopoietin receptor agonist (TPO-RA), has demonstrated anti-leukaemic activity in preclinical models and early clinical studies. While eltrombopag has been shown to reduce platelet transfusion requirements when added to intensive daunorubicin and cytarabine (DA) chemotherapy in eligible elderly AML patients, clinical data on its use in combination with venetoclax and azacitidine (VA) induction are currently lacking.

### Aims

To investigate the efficacy and safety of eltrombopag plus VA in patients with newly diagnosed AML.

### Methods

As of October 2025, a total of 60 newly diagnosed patients with AML treated with the VA induction regimen were retrospectively enrolled. 9 of these patients were excluded because they never experienced grade 3 or higher thrombocytopenia during the induction regimen.51 patients received induction therapy with either eltrombopag plus VA(n=26) or VA alone (n=25). The median time to add eltrombopag was 13 days (range 11–17), with an initial median dose of 50 mg /d (range 25–100). Eltrombopag was increased by 25mg every 2 weeks to a maximum of 150 mg/d if no platelet response was observed(platelet response defined per IWG criteria:if baseline < $20\times10^9$/L,increase to >$20\times10^9$/L and ≥2× baseline;or if baseline ≥ $20\times10^9$/L,increase to ≥$50\times10^9$/L and ≥2× baseline). Eltrombopag was continued until the platelet counts exceeded 50 x $10^9$ /L or the AML response was assessed (maximum d45).VA regimen: venetoclax PO 100mg d1, 200mg d2, 400mg d3-28; azacitidine SC 75mg/m² d1-7. Primary objectives: incidence of ≥2 bleeding events(according to WHO scale);platelet transfusion dose;duration of platelet count <10 × $10^9$/L ;and time to platelet response during induction. Secondary goals were disease response (according to CRC criteria) and adverse events (graded by CTCAE v5.0) .

### Results

The median age of patients was 67 years. Eltrombopag had a lower rate of ≥2 bleeding events than the VA group, with15.4% (4/26, 95% CI: 4.4%–34.9%)vs 36.0% (9/25, 95% CI: 18.0%–57.5%) , $P$= 0.091(10% risk)(Figure A). Of these, the VA group experienced two bleeding-related deaths (n=1 for gastrointestinal hemorrhage and n=1 for intracranial hemorrhage), while the eltrombopag group experienced no bleeding-related deaths. Platelet transfusion requirements did not differ significantly between the two groups(5.30 ± 3.59 vs 3.65 ± 1.94 units, $P$= 0.128)(Figure B).The duration of platelet counts < 10 × $10^9$/L in the eltrombopag group was shorter than the VA group(14.80 ± 16.87 vs 21.36 ± 13.56 days,$P$<0.05)(Figure C)and likewise took less time to platelet response(20.23 ± 17.11 vs 28.59 ± 11.15 days,$P$<0.05)(Figure D).Following induction,the eltrombopag and VA groups showed comparable rates of CR/CRi (53.8% vs 44.0%) and grade ≥3 adverse events (26.9% vs 44.0%), with infection being the most frequent. Three patients experienced adverse events attributed to eltrombopag (hepatic impairment, n=2; diarrhoea, n=1), all of which resolved following treatment discontinuation and supportive care.

**Copyright Information:** (Online) ISSN: 2572-9241
©2026 The Author(s). HemaSphere is published by John Wiley & Sons Ltd on behalf of European Hematology Association. This is an open access Abstract Book distributed under the Attribution-NonCommercial-NoDerivs (CC BY-NC-ND), which allows third parties to download the articles and share them with others as long as they credit the author and the Abstract Book, but they cannot change the content in any way or use them commercially.
**Abstract Book Citations:** Authors, Title, HemaSphere, 2026;10:(S1):pages. The abstract book DOIs can be found on the "Author Information" page.
**Disclaimer:** Articles published in the journal HemaSphere exclusively reflect the opinions of the authors. The authors are responsible for all content in their abstracts including accuracy of the facts, statements, citing resources, etc.

### Summary/Conclusion

For newly diagnosed elderly/unfit patients with AML , the addition of eltrombopag to VA induction regimen reduced the risk of bleeding.CRc rate was comparable to the VA regimen alone, and overall safety was under control.
(This work was supported by Hunan Provincial Natural Science Foundation Project[2025JJ81126]; Municipal Science and Technology Innovation Project of Xiangtan[SF-YB20240011]; Xiangtan Medical Research Project[2022-xtyx-33])

Characteristics of patients

| | EPAG+VA (n=26) | VA (n=25) | All patients (n=51) |
|---|---|---|---|
| Age, y | | | |
| Median (IQR) | 68 (58-73) | 67 (56.5-71) | 67 (58-73) |
| Sex, n (%) | | | |
| Male | 15 (58) | 16 (64) | 31 (61) |
| Female | 11 (42) | 9 (36) | 20 (39) |
| ELN 2024 Less-Intensive (%) * | | | |
| Favorable | 11 (42) | 18 (72) | 29 (57) |
| Intermediate | 10 (38) | 3 (12) | 13 (25) |
| Adverse | 2 (8) | 4 (16) | 6 (12) |
| Unkown | 3 (12) | 0 (0) | 3 (6) |
| BM blast count, n (%) * | | | |
| ≥20 to <50% | 5 (19) | 12 (48) | 11 (22) |
| ≥50 | 21 (81) | 13 (52) | 40 (78) |
| Platelet count * $10^9$/L | | | |
| Median (IQR) | 17 (8.5-22) | 18 (9-26) | 18 (9-23) |
| Platelet count, n (%) | | | |
| <10 *$10^9$/L | 9 (35) | 6 (24) | 15 (30) |
| ≥10–20 *$10^9$/L | 7 (27) | 7 (28) | 14 (28) |
| ≥20 *$10^9$/L | 10 (38) | 12 (48) | 22 (43) |
| Platelet transfusion dependence, n (%)[a] | | | |
| Yes | 6 (23) | 8 (32) | 14 (27) |
| No | 20 (77) | 17 (68) | 37 (73) |
| CR+CRi, n(%) | | | |
| One cycle | 14 (54) | 11(44)) | 25 (49) |
| MRD negativity,n(%) | | | |
| One cycle | 3 (12) | 5 (20) | 8 (16) |

[a]Platelet transfusion dependency was defined as having received at least two platelet transfusions in the 4 weeks before randomisation

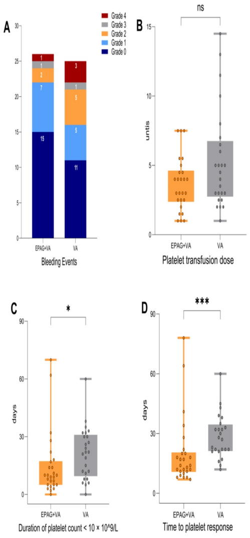

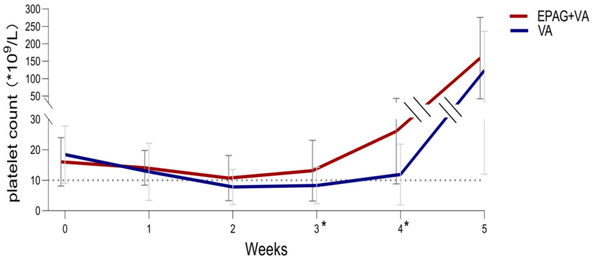

| Median | 0 | 1 | 2 | 3* | 4* | 5 |
|---|---|---|---|---|---|---|
| EPAG+VA | 17 | 14 | 9.5 | 9.5 | 27.5 | 153.5 |
| VA | 18 | 10 | 6 | 7 | 9.5 | 109 |

*The difference between the two groups was statistically significant ($P < 0.05$)

**Copyright Information:** (Online) ISSN: 2572-9241
©2026 The Author(s). HemaSphere is published by John Wiley & Sons Ltd on behalf of European Hematology Association. This is an open access Abstract Book distributed under the Attribution-NonCommercial-NoDerivs (CC BY-NC-ND), which allows third parties to download the articles and share them with others as long as they credit the author and the Abstract Book, but they cannot change the content in any way or use them commercially.
**Abstract Book Citations:** Authors, Title, HemaSphere, 2026;10:(S1):pages. The abstract book DOIs can be found on the "Author Information" page.
**Disclaimer:** Articles published in the journal HemaSphere exclusively reflect the opinions of the authors. The authors are responsible for all content in their abstracts including accuracy of the facts, statements, citing resources, etc.

### (PB2743) REAL-WORLD EXPERIENCE OF CLADRIBINE PLUS LOW-DOSE CYTARABINE AND VENETOCLAX IN PATIENTS WITH NEWLY DIAGNOSED AND RELAPSED/REFRACTORY ACUTE MYELOID LEUKEMIA

**Topic:** 04. Acute myeloid leukemia - Clinical

Sarah Marini*[1], Himachandana Atluri[1], Rafael Marroquin[2], Mahesh Kumar[3], Alexander Coltoff[4], Nepheli Raptis[1], Anand Patel[2], Talha Badar[3], Jessica K. Altman[1], Yasmin Abaza[1]

*[1]Robert H. Lurie Comprehensive Cancer Center, Northwestern University, Division of Hematology and Oncology, Chicago, IL, United States of America; [2]The University of Chicago Medicine, Section of Hematology/Oncology, Department of Medicine, Chicago, IL, United States of America; [3]Mayo Clinic Florida, Division of Hematology, Jacksonville, FL, United States of America; [4]Medical University of South Carolina, Hollings Cancer Center, Charleston, SC, United States of America;*

### Background

Venetoclax (VEN) combined with hypomethylating agents (HMA) or low-dose cytarabine (LDAC) are established as the standard of care for older, unfit adults with newly diagnosed (ND) acute myeloid leukemia (AML) ineligible for intensive chemotherapy with improved overall survival (OS) compared with HMA alone. Recent studies have demonstrated that the addition of Cladribine (Clad) to LDAC + VEN has resulted in complete remission or complete remission with incomplete hematologic recovery (CR/CRi) rates of 86% with many patients attaining MRD negativity.

### Aims

We aim to analyze real-world outcomes in a multicenter, retrospective study of Clad/LDAC + VEN in ND and relapsed/refractory (R/R) AML.

### Methods

This was a multicenter, retrospective chart review across four academic institutions evaluating patients with AML who received Clad/LDAC + VEN for ND or R/R AML between October 2021 and October 2025. Patients with mixed phenotypic leukemias were excluded. Time to event analysis was performed using the Kaplan-Meier method. OS was calculated from C1D1 to time of last follow up or death for both cohorts.

**Copyright Information:** (Online) ISSN: 2572-9241
©2026 The Author(s). HemaSphere is published by John Wiley & Sons Ltd on behalf of European Hematology Association. This is an open access Abstract Book distributed under the Attribution-NonCommercial-NoDerivs (CC BY-NC-ND), which allows third parties to download the articles and share them with others as long as they credit the author and the Abstract Book, but they cannot change the content in any way or use them commercially.
**Abstract Book Citations:** Authors, Title, HemaSphere, 2026;10:(S1):pages. The abstract book DOIs can be found on the "Author Information" page.
**Disclaimer:** Articles published in the journal HemaSphere exclusively reflect the opinions of the authors. The authors are responsible for all content in their abstracts including accuracy of the facts, statements, citing resources, etc.

**Results**

A total of 64 patients (24 ND, 40 R/R) were included in the data analysis. Baseline characteristics are outlined in Table 1. Among the ND cohort (n = 24), the overall response rate (ORR) was 79%. 42% of patients attained a CR (n=10), 17% (n=4) attained a CRi and 17% (n=4) achieved a morphologic leukemia-free state (MLFS). One patient (4%) attained partial remission (PR), while 5 patients (21%) had no response (NR). Of the non-responders, 3 patients had a complex karyotype and 2 had TP53-mutated disease. Five ND patients (21%) underwent allogeneic stem cell transplantation (alloSCT) after attaining first complete remission (CR1).

At a median follow-up of 14.9 months (mos), the median overall survival (mOS) among ND patients was 9.44 mos (95% CI, 4.51–NA). Among patients with RAS-mutated AML (n=7), mOS was 10 mos (95% CI, 1.38–NA), while TP53-mutated AML (n=6) had a mOS of 4.08 months (95% CI, 2.27–NA). Patients with secondary AML demonstrated a mOS of 12.83 months (95% CI, 1.51–NA). When stratified by ELN 2024, mOS was 11.58 mos (95% CI, 4.51–NA) in patients with favorable risk disease, NR (95% CI, 10–NA) for intermediate risk, and 5.03 mos (95% CI, 3.88–NA) for adverse risk.

In the R/R cohort (n = 40), the ORR was 43% (n=17) with including with 8 patients (20%) achieving CR, 3 (8%) achieving CRi, 4 (10%) achieving complete remission with partial hematologic recovery (CRh), and 2 (5%) achieving MLFS. One patient (2.5%) attained PR, while 22 patients (55%) had NR. One R/R patient (2.5%) underwent alloSCT after attaining second complete remission (CR2).

With a median follow-up of 13.8 months, the mOS for R/R was 4.84 mos (95% CI, 3.91–7.17). Among RAS-mutated R/R AML (n=8), mOS was 2.88 months, while patients with TP53-mutated disease had a mOS of 4.84 mos.

**Table 1. Baseline Characteristics**

| | Newly Diagnosed (n=24) | Relapsed/Refractory (n=40) |
|---|---|---|
| Median Age (years) | 70 | 70 |
| Gender (n (%)) | | |
| *Male* | 17 (71%) | 25 (63%) |
| *Female* | 7 (29%) | 15 (38%) |
| AML Subtype (n (%)) | | |
| *de novo AML* | 10 (42%) | 22 (55%) |
| *sAML* | 8 (33%) | 13 (33%) |
| *stAML* | 5 (21%) | 3 (8%) |
| *tAML* | 1 (4%) | 2 (5%) |
| Mutation(s) (n (%)) | | |
| *RAS* | 7 (29%) | 8 (20%) |
| *TP53* | 6 (25%) | 13 (33%) |
| ELN 2022 Risk (n (%)) | | |
| *Favorable* | 1 (4%) | 2 (5%) |
| *Intermediate* | 3 (13%) | 12 (30%) |
| *Adverse* | 20 (83%) | 26 (65%) |

**Summary/Conclusion**

Clad/LDAC + Ven is an active treatment option in patients with ND and R/R AML. Our analysis included a very high-risk subset of patients with promising response rates comparable to previously published data.

**Copyright Information:** (Online) ISSN: 2572-9241
©2026 The Author(s). HemaSphere is published by John Wiley & Sons Ltd on behalf of European Hematology Association. This is an open access Abstract Book distributed under the Attribution-NonCommercial-NoDerivs (CC BY-NC-ND), which allows third parties to download the articles and share them with others as long as they credit the author and the Abstract Book, but they cannot change the content in any way or use them commercially.
**Abstract Book Citations:** Authors, Title, HemaSphere, 2026;10:(S1):pages. The abstract book DOIs can be found on the "Author Information" page.
**Disclaimer:** Articles published in the journal HemaSphere exclusively reflect the opinions of the authors. The authors are responsible for all content in their abstracts including accuracy of the facts, statements, citing resources, etc.

### (PB2744) SALVAGE THERAPIES IN RELAPSED ACUTE MYELOID LEUKEMIA POST VENETOCLAX-BASED THERAPY: A MULTICENTER, REAL-WORLD STUDY

**Topic:** 04. Acute myeloid leukemia - Clinical

Yuxin Li*[1], Dan Wang[1], Danchen Meng[1], Dinghui Zhao[1], Zhengfeng Hou[1], Jichun Yang[2], Xiaofeng Shi[3], Jian Ge[1], Zhangbiao Long[1]

*[1]The First Affiliated Hospital of Anhui Medical University, Hefei, China;[2]Tianjin University of Traditional Chinese Medicine, Tianjin, China; [3]The Second Affiliated Hospital of Nanjing Medical University, Nanjing, China;*

### Background

Venetoclax (VEN)-based therapy represents a revolutionary advancement for elderly, unfit for intensive chemotherapy AML patients, yielding superior response rates and survival outcomes. Despite impressive initial responses, some patients inevitably develop resistance or experience relapse. Treatment options after relapse are limited, and survival is poor. Determining how to select optimal salvage therapy and improve survival outcomes for these patients remains an unresolved clinical challenge.

### Aims

This real-world multicenter study aimed to evaluate the clinical outcomes of salvage therapies in patients with relapsed acute myeloid leukemia (AML) exposed to venetoclax (VEN)-based therapy.

### Methods

This real-world retrospective study included patients with relapsed AML (non-M3 subtype) who had received prior venetoclax-based therapy. The analysis focused on evaluating patients' performance status post-relapse, treatment regimens administered, therapeutic responses, adverse events, and survival outcomes. We classified the salvage treatment regimens into the following four categories: VEN-based regimens, "3+7" regimens, high-dose cytarabine (HD-Ara-C)-based regimens, and low-dose cytarabine (LD-Ara-C)-based regimens. Categorical variables were compared using *Fisher*'s exact test. Overall survival (OS) after relapse was compared using the *Kaplan-Meier* method, with between-group differences assessed by *log-rank* test. Adverse event frequencies and severity grades were analyzed descriptively, with calculation of incidence rates and distribution across severity grades.

### Results

Among 106 patients analyzed, 15 received best supportive care (BSC) and 91 salvage therapy, and the latter was further subdivided into four treatment-based cohorts: VEN-based, "3+7", high-dose cytarabine (HD-Ara-C)-based, and low-dose cytarabine (LD-Ara-C)-based regimens. The median overall survival (OS) post-relapse for the entire cohort (N=106) was 3.4 months. Salvage therapy significantly prolonged survival compared to BSC (median OS: 4.8 *vs.* 1.0 months, $P < 0.0001$). Responders (ORR-achievers) demonstrated superior survival to non-responders (median OS: 10.6 *vs.* 2.6 months, $P < 0.0001$). Among responders, patients achieving composite complete remission (CRc: CR + CRi) had a median OS of 12.7 months, partial responders (PR) 5.05 months, and non-responders (NR) 2.6 months. The VEN-based regimen yielded the highest ORR (36.4%), followed by HD-Ara-C (35.3%), while "3+7" and LD-Ara-C regimens showed lower efficacy (20.8% and 17.2%, respectively). Notably, VEN-based therapy combined with targeted agents achieved an ORR of 55.0%. The most frequent hematologic adverse events were anemia, neutropenia, and thrombocytopenia; pneumonia and febrile infections predominated among non-hematologic toxicities. Treatment-related mortality was highest with HD-Ara-C (47.1%), followed by LD-Ara-C (37.1%), whereas VEN-based and "3+7" regimens exhibited lower rates (35.1% and 29.2%, respectively).

**Copyright Information:** (Online) ISSN: 2572-9241
©2026 The Author(s). HemaSphere is published by John Wiley & Sons Ltd on behalf of European Hematology Association. This is an open access Abstract Book distributed under the Attribution-NonCommercial-NoDerivs (CC BY-NC-ND), which allows third parties to download the articles and share them with others as long as they credit the author and the Abstract Book, but they cannot change the content in any way or use them commercially.
**Abstract Book Citations:** Authors, Title, HemaSphere, 2026;10:(S1):pages. The abstract book DOIs can be found on the "Author Information" page.
**Disclaimer:** Articles published in the journal HemaSphere exclusively reflect the opinions of the authors. The authors are responsible for all content in their abstracts including accuracy of the facts, statements, citing resources, etc.

**Summary/Conclusion**

In real-world practice, VEN-based salvage regimens demonstrate optimal efficacy and tolerability for relapsed AML after VEN-AZA therapy. The addition of targeted agents to VEN-based regimens further enhance response rates.

**Copyright Information:** (Online) ISSN: 2572-9241
©2026 The Author(s). HemaSphere is published by John Wiley & Sons Ltd on behalf of European Hematology Association. This is an open access Abstract Book distributed under the Attribution-NonCommercial-NoDerivs (CC BY-NC-ND), which allows third parties to download the articles and share them with others as long as they credit the author and the Abstract Book, but they cannot change the content in any way or use them commercially.
**Abstract Book Citations:** Authors, Title, HemaSphere, 2026;10:(S1):pages. The abstract book DOIs can be found on the "Author Information" page.
**Disclaimer:** Articles published in the journal HemaSphere exclusively reflect the opinions of the authors. The authors are responsible for all content in their abstracts including accuracy of the facts, statements, citing resources, etc.

### (PB2745) WT1 2 LOG-REDUCTION AFTER INDUCTION CHEMOTHERAPY IN ACUTE MYELOID LEUKEMIA PATIENTS: AN UNEXPECTED PREDICTOR OF OUTCOME

**Topic:** 04. Acute myeloid leukemia - Clinical

Mariachiara Cuccaro*[1], Margherita Malchiodi[2], Giovanni Marconi[3], Michela Rondoni[3], Anna Sicuranza[2], Adele Santoni[2], Alessandra Cartocci[2], Karim Palombo[2], Sara Monti[4], Habiba Ben Chelbi[2], Anna Zannoli[2], Giulia Beffa[2], Francesco Lanza[4], Monica Bocchia[2, 2, 2]

*[1]Azienda Ospedaliero Universitaria Senese, University of Siena, siena, Italy;[2]Azienda Ospedaliero Universitaria Senese, University of Siena, Siena, Italy; [3]Ospedale S. Maria delle Croci, University of Bologna, Ravenna, Italy;[4]Ospedale S. Maria delle Croci, University of Bologna, Siena, Italy;*

### Background

Wilms tumor 1 (WT1) is a tumor suppressor gene involved in several malignancies, which affects key transcription factors in hematopoiesis differentiation. In Acute Myeloid Leukemia (AML) WT1 is frequently overexpressed, therefore it could represent an attractive minimal residual disease (MRD) marker, especially in absence of leukemia-related abnormalities suitable for qPCR or flow cytometry monitoring. However, its prognostic role in this setting is controversial and the lack of clinical trial causes insufficient evidence to support its use.

### Aims

The aim of our study is to assess the role of WT1 as a MRD marker in non-M3 AML.

### Methods

This is a retrospective observational dual-center analysis which involves 120 AML patients (pts) from Siena and Ravenna Hematology Units, treated with high dose chemotherapy, from January 2008 to December 2024.
WT1 expression was detected by Real-Time Quantitative-Polymerase Chain Reaction (RT-qPCR) at diagnosis and after induction chemotherapy.
All pts presented WT1 bone marrow value at diagnosis greater than 250 copies (cps).

**Copyright Information:** (Online) ISSN: 2572-9241
©2026 The Author(s). HemaSphere is published by John Wiley & Sons Ltd on behalf of European Hematology Association. This is an open access Abstract Book distributed under the Attribution-NonCommercial-NoDerivs (CC BY-NC-ND), which allows third parties to download the articles and share them with others as long as they credit the author and the Abstract Book, but they cannot change the content in any way or use them commercially.
**Abstract Book Citations:** Authors, Title, HemaSphere, 2026;10:(S1):pages. The abstract book DOIs can be found on the "Author Information" page.
**Disclaimer:** Articles published in the journal HemaSphere exclusively reflect the opinions of the authors. The authors are responsible for all content in their abstracts including accuracy of the facts, statements, citing resources, etc.

### Results

We enrolled 120 AML pts, 61/120 (50.8%) male and 52/120 (49.2%) female. The median age at diagnosis was 59 years (49-66.5 years). 88/120 pts (73.3%) had de novo AML, whereas 32/120 pts (26.7%) were diagnosed with secondary AML.
According to European Leukemia Net (ELN) 2022 guidelines, 44/120 (52.8%) pts were classified as adverse risk, 50/120 (60%) as intermediate risk, 26/120 (31.2%) as favorable risk. Notably, FLT3 mutations were detected in 37/120 (30.8%) pts and NPM1 mutations in 38/120 (31.6%) pts.
LDH serum levels were abnormal in 62/120 (51.6%%) pts, with median LDH value of 413 UI/L.
The median WT1 value at diagnosis was 4559 (257-45224) cps. WT1 level at diagnosis had no significant statistical correlation with de novo/secondary AML (p=0.058), karyotype (p=0.265), FISH (p=0.653), NPM1 mutation (p=0.064) and FLT3 mutation (p=0.111).
To investigate the WT1 role as a prognostic marker we collected WT1 value after induction chemotherapy in 107 pts. We used a ROC curve to evaluate and compare WT1 at diagnosis, WT1 after induction chemotherapy and logarithms (log) reduction as response predictors, discovering that log-reduction has the strongest predictive value. However, all the variables are not strong predictors, because their areas under the curve are below 0.7.
Indeed, classifying pts according to WT1 level reduction, 28/107 (26.1%) pts achieved a WT1 level decrease greater than two log, while 79/107 (73.8%) pts had a WT1 decline less than two log. Evaluating clinical outcomes, our data showed that WT1 level reduction greater than two log correlated with a better Overall Survival (OS) and better Event Free Survival (EFS) (p=0.0024 and p=0.00092, respectively) (Figure 1).

### Summary/Conclusion

In conclusion, despite the limitations of a small sample size, our study documented a potential role for WT1 as a prognostic marker, indeed the greater than two-log reduction after induction chemotherapy correlates with OS and EFS. Thus, it could be useful to value WT1 both at diagnosis and after induction chemotherapy, suggesting an alternative opportunity to monitor disease and maybe MRD in AML patients, especially when immunophenotypic or molecular markers are lacking.

**Copyright Information:** (Online) ISSN: 2572-9241
©2026 The Author(s). HemaSphere is published by John Wiley & Sons Ltd on behalf of European Hematology Association. This is an open access Abstract Book distributed under the Attribution-NonCommercial-NoDerivs (CC BY-NC-ND), which allows third parties to download the articles and share them with others as long as they credit the author and the Abstract Book, but they cannot change the content in any way or use them commercially.
**Abstract Book Citations:** Authors, Title, HemaSphere, 2026;10:(S1):pages. The abstract book DOIs can be found on the "Author Information" page.
**Disclaimer:** Articles published in the journal HemaSphere exclusively reflect the opinions of the authors. The authors are responsible for all content in their abstracts including accuracy of the facts, statements, citing resources, etc.

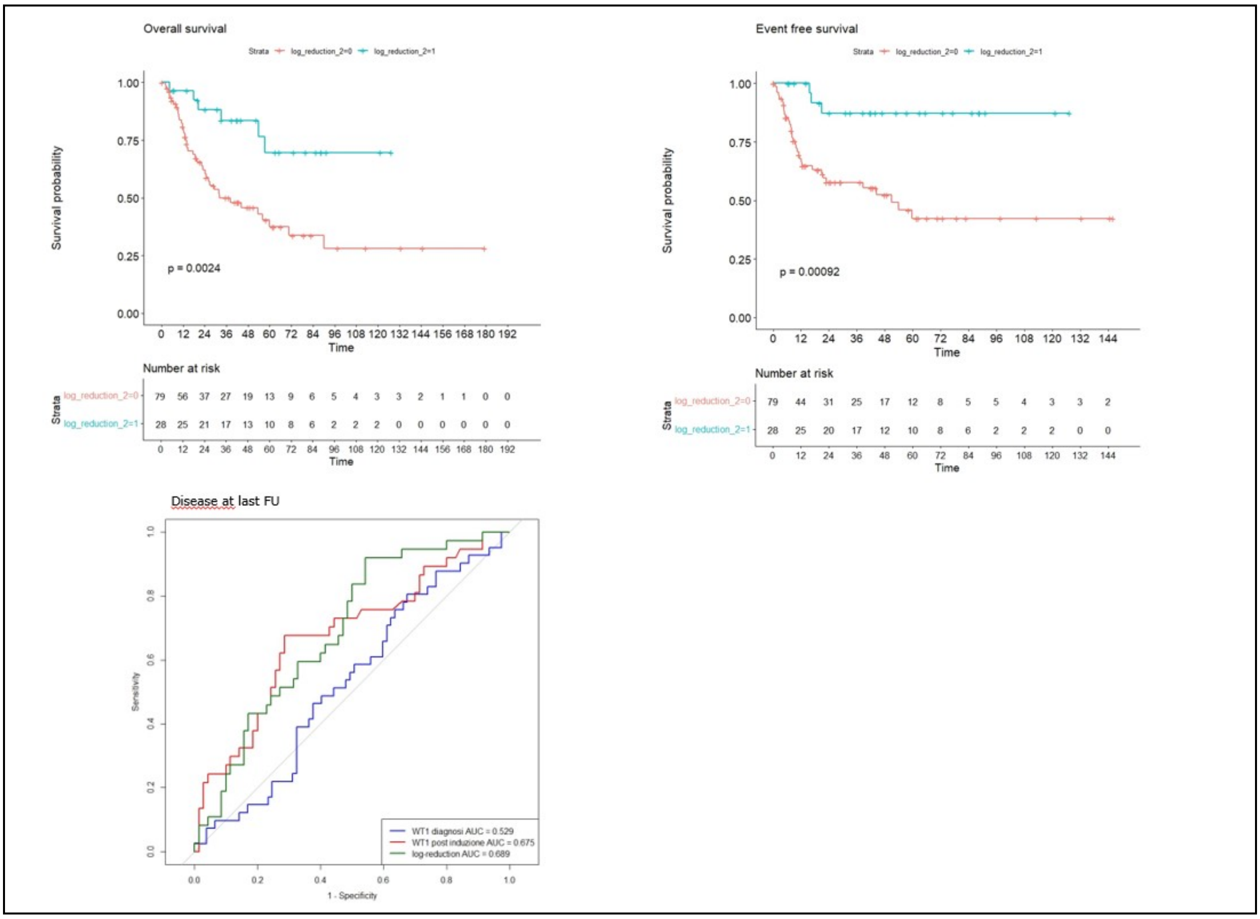

Figure 1. OS, EFS and ROC curves.

**Copyright Information:** (Online) ISSN: 2572-9241
©2026 The Author(s). HemaSphere is published by John Wiley & Sons Ltd on behalf of European Hematology Association. This is an open access Abstract Book distributed under the Attribution-NonCommercial-NoDerivs (CC BY-NC-ND), which allows third parties to download the articles and share them with others as long as they credit the author and the Abstract Book, but they cannot change the content in any way or use them commercially.
**Abstract Book Citations:** Authors, Title, HemaSphere, 2026;10:(S1):pages. The abstract book DOIs can be found on the "Author Information" page.
**Disclaimer:** Articles published in the journal HemaSphere exclusively reflect the opinions of the authors. The authors are responsible for all content in their abstracts including accuracy of the facts, statements, citing resources, etc.

### (PB2746) SURVIVAL HETEROGENEITY ACCORDING TO CYTOGENETICS AND CO-MUTATION PATTERNS IN AML TREATED WITH AZACITIDINE–VENETOCLAX

**Topic:** 04. Acute myeloid leukemia - Clinical

Cristiano MacHado de Freitas*[1], Zahra Alhaj Issa[1], Aarya Murali[1], Aniket Bankar[1], Marta Davidson[1], Maria Agustina Perusini[1], Dawn Maze[1], Steven M Chan[1], Aaron Schimmer[1], Hassan, Sibai[1], Karen Yee[1], Andre Schuh[1], Vikas Gupta[1], Guillaume Richard-Carpentier[1]

[1]*Princess Margaret Cancer Centre, University of Toronto, Medicine, Toronto, Canada;*

### Background

Azacitidine plus venetoclax (Aza-Ven) is standard frontline therapy for older or unfit patients (pts) with acute myeloid leukemia (AML). Although response rates are high, survival outcomes are heterogeneous and the prognostic impact of specific molecular and cytogenetic subgroups is incompletely defined.

### Aims

We aimed to evaluate responses and survival in pts with AML treated with frontline Aza-Ven and to identify genomic subgroups associated with differential prognosis.

### Methods

We retrospectively analyzed 175 AML pts treated with frontline Aza-Ven at Princess Margaret Cancer Centre (2017–2024). Clinical, cytogenetic, and molecular variables were collected. Composite complete remission (CRc) included CR + CRi/CRh; overall response rate (ORR) included CRc, morphological leukemia-free state (MLFS), and partial remission (PR). Overall survival (OS) was assessed using Kaplan–Meier and Cox regression models.

**Copyright Information:** (Online) ISSN: 2572-9241
©2026 The Author(s). HemaSphere is published by John Wiley & Sons Ltd on behalf of European Hematology Association. This is an open access Abstract Book distributed under the Attribution-NonCommercial-NoDerivs (CC BY-NC-ND), which allows third parties to download the articles and share them with others as long as they credit the author and the Abstract Book, but they cannot change the content in any way or use them commercially.
**Abstract Book Citations:** Authors, Title, HemaSphere, 2026;10:(S1):pages. The abstract book DOIs can be found on the "Author Information" page.
**Disclaimer:** Articles published in the journal HemaSphere exclusively reflect the opinions of the authors. The authors are responsible for all content in their abstracts including accuracy of the facts, statements, citing resources, etc.

### Results

Median age was 74 years (range 31–96); 65% were male and 74% had ECOG 0–1. Prior MDS/MPN was observedin 21% and 31% had prior cytotoxic exposure. Complex karyotype (CK) was present in 30%. Most frequent mutations (mut) included *ASXL1* (24%), *SRSF2* (22%), *TP53* (21%), *TET2* (19%), *NPM1* (17%), RUNX1 (17%), *DNMT3A* (16%), *FLT3-ITD* (14%), and *IDH2* (14%). Among 154 evaluable pts after cycle 1, CRc was 46% (CR 21%, CRi 22%, CRh 3%) and ORR was 78% (MLFS 21%, PR 10%). With a median follow-up of 31.4 months in the global cohort, the median OS was 13.2 months (95% CI, 11.1 – 18.8) and the 2-year OS rate was 36% (95% CI 29 – 44%). Pts with *NPM1* mut (n=29) had a median OS of 16.0 months (95% CI, 9.9 – not reached [NR]) and 2-year OS rate of 40% (95% CI, 25 – 63) which was not different from pts without *NPM1* mut (median OS 13.1 months; 2-year OS 35%; p=0.47). Prognosis varied substantially according to co-mutations with favorable OS with *IDH1/2* mutand adverse OS with *TET2* mut. Interestingly, *NRAS/KRAS/FLT3* comutations did not have a significant prognostic impact in presence of *NPM1* mut (HR 1.39, p=0.49).Among *IDH1/2*-mutated pts (n=35), the median OS was 20.7 months (95% CI, 18.8 – not reached) and the 2-year OS rate was 49% (95% CI, 34 – 71). Presence of *NPM1* co-mutation had no prognostic impact (median OS, 20.5 vs 20.7 months), but co-occurring *NRAS/KRAS/FLT3* mut were associated with inferior OS with median OS of 12.3 vs 31.3 months, and 2-year OS rate of 20% vs 62% (HR 3.09, 95% CI 1.21–7.90; p=0.02). Altogether, mutually exclusive *IDH1/2, TET2*, and other-mutated groups had significantly different survival with median OS of 20.7, 12.4 and 7.2 months, respectively (p=0.02). CK was the only cytogenetic subgroup independently associated with inferior OS (HR 2.33, 95% CI 1.43–3.79; p<0.001), and remained significant after adjustment for *TP53* mut status (HR 2.19, 95% CI 1.21–3.97; p<0.01). Pts with CK had a median OS of 8.8 months (95% CI, 7.7 – 11.9) and 2-year OS rate of 10% (95% CI, 4 – 27) In contrast, *TP53* mut was not prognostic after adjustment for CK (HR 1.03; p=0.92), noting only three pts had isolated *TP53* without CK.

### Summary/Conclusion

Frontline Aza-Ven achieves high response rates with substantial heterogeneity in survival according to co-mutation patterns. NPM1 mut does not appear as an independent prognostic factor and survival mostly depend on comutations in NPM1-mutated AML. Complex karyotype is anindependent adverse prognostic factor, supporting itsincorporation into risk classification for AML treated with Aza-Ven.

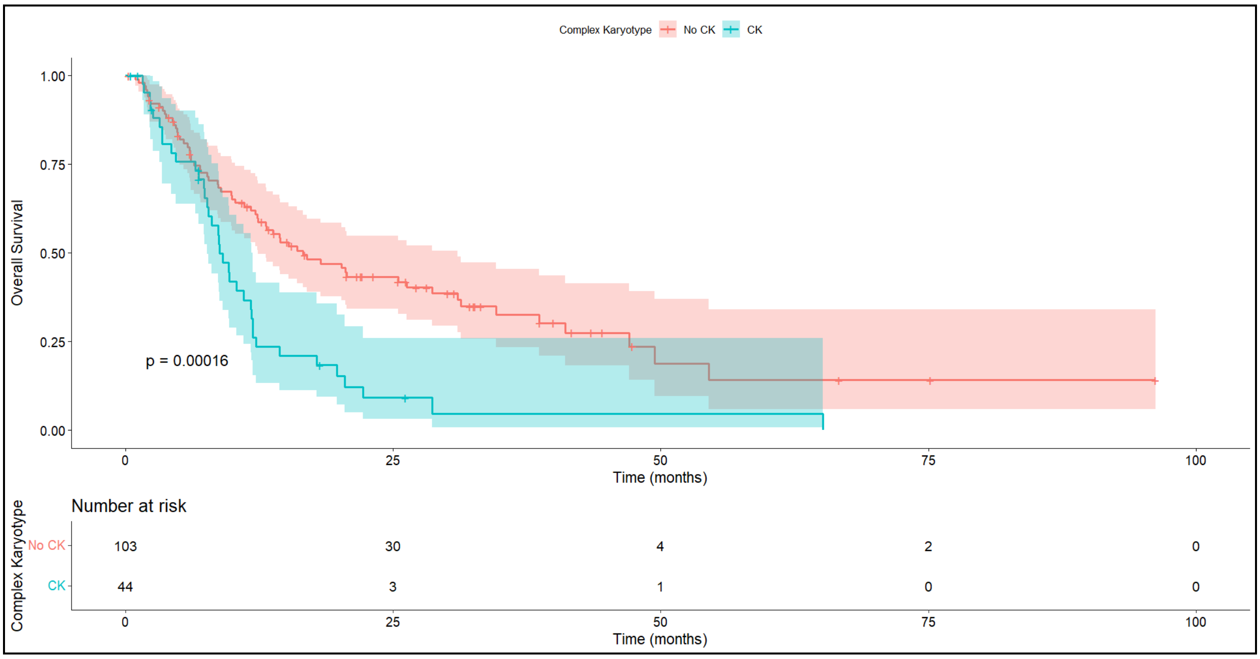

**Copyright Information:** (Online) ISSN: 2572-9241
©2026 The Author(s). HemaSphere is published by John Wiley & Sons Ltd on behalf of European Hematology Association. This is an open access Abstract Book distributed under the Attribution-NonCommercial-NoDerivs (CC BY-NC-ND), which allows third parties to download the articles and share them with others as long as they credit the author and the Abstract Book, but they cannot change the content in any way or use them commercially.
**Abstract Book Citations:** Authors, Title, HemaSphere, 2026;10:(S1):pages. The abstract book DOIs can be found on the "Author Information" page.
**Disclaimer:** Articles published in the journal HemaSphere exclusively reflect the opinions of the authors. The authors are responsible for all content in their abstracts including accuracy of the facts, statements, citing resources, etc.

### (PB2747) FLT3 INHIBITORS PLUS INTENSIVE CHEMOTHERAPY IN NEWLY DIAGNOSED FLT3-MUTATED AML: A SYSTEMATIC REVIEW AND META-ANALYSIS

**Topic:** 04. Acute myeloid leukemia - Clinical

Shankar Biswas*[1], Yashasvi Srivastava[1], Kridhay Jindal[2], Raghabendra Kumar Mahato[3]

*[1]Ivano-Frankivsk National Medical University, Internal Medicine, Ivano-Frankivsk, Ukraine; [2]Government Medical College, Internal Medicine, Patiala, India; [3]Gandaki Medical College Teaching Hospital and Research Center, Internal Medicine, Pokhara, Nepal;*

### Background

FLT3 mutations occur in approximately 30% of adults with acute myeloid leukemia (AML) and are associated with high relapse rates and poor survival. Two FLT3 inhibitors including midostaurin and quizartinib  are now approved in combination with intensive chemotherapy for newly diagnosed FLT3-mutated AML based on the RATIFY and QuANTUM-First trials, respectively. Sorafenib has also been studied in this setting. While individual trials demonstrated survival benefits, no pairwise meta-analysis has quantified the pooled class-level effect of adding a FLT3 inhibitor to intensive chemotherapy versus placebo in this population.

### Aims

To perform a systematic review and meta-analysis of randomized controlled trials (RCTs) evaluating FLT3 inhibitors combined with intensive chemotherapy versus placebo in adults with newly diagnosed FLT3-mutated AML, and to assess the certainty of evidence using the GRADE framework.

### Methods

PubMed, Embase, and Scopus were searched through February 2026. Eligible studies were double-blind, placebo-controlled RCTs enrolling adults with newly diagnosed FLT3-mutated AML receiving intensive chemotherapy plus a FLT3 inhibitor. The primary outcome was overall survival (OS). Secondary outcomes included event-free survival (EFS) and composite complete remission (CRc). Hazard ratios (HRs) and risk ratios (RRs) were pooled using inverse-variance common-effect models. Risk of bias was assessed using Cochrane RoB 2.0.

### Results

Three RCTs (1,355 patients) were included: QuANTUM-First (quizartinib, n=539), RATIFY (midostaurin, n=717), and ALLG AMLM16 (sorafenib, n=99). All were judged at low risk of bias. FLT3 inhibitor therapy significantly improved OS (HR 0.78, 95% CI 0.67-0.90; p=0.001; $I^2$=0%) and EFS (2 trials, 815 patients; HR 0.79, 95% CI 0.67-0.93; p=0.004; $I^2$=0%). CRc rates were not significantly different (RR 1.05, 95% CI 0.97-1.12; p=0.22; $I^2$=56%). Results were robust in leave-one-out sensitivity analysis and consistent across first- and second-generation inhibitors (subgroup interaction p=0.99). GRADE certainty was high for OS, moderate for EFS, and low for CRc.

### Summary/Conclusion

Adding a FLT3 inhibitor to intensive chemotherapy provides a consistent and statistically significant 22% reduction in the risk of death across three placebo-controlled RCTs, with high-certainty evidence. This class-level survival benefit was independent of inhibitor generation or study phase. These findings reinforce the role of FLT3 inhibition as a standard component of frontline intensive therapy for FLT3-mutated AML.

**Copyright Information:** (Online) ISSN: 2572-9241
©2026 The Author(s). HemaSphere is published by John Wiley & Sons Ltd on behalf of European Hematology Association. This is an open access Abstract Book distributed under the Attribution-NonCommercial-NoDerivs (CC BY-NC-ND), which allows third parties to download the articles and share them with others as long as they credit the author and the Abstract Book, but they cannot change the content in any way or use them commercially.
**Abstract Book Citations:** Authors, Title, HemaSphere, 2026;10:(S1):pages. The abstract book DOIs can be found on the “Author Information” page.
**Disclaimer:** Articles published in the journal HemaSphere exclusively reflect the opinions of the authors. The authors are responsible for all content in their abstracts including accuracy of the facts, statements, citing resources, etc.

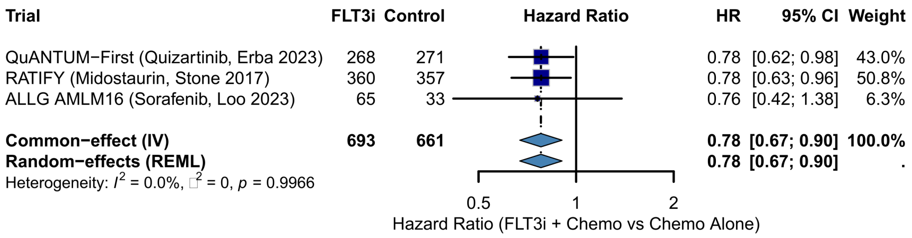

Abbreviations: AML, acute myeloid leukemia; Chemo, chemotherapy; CI, confidence interval; FLT3i, FLT3 inhibitor; HR, hazard ratio; IV, inverse variance; OS, overall survival; REML, restricted maximum likelihood. Pooled estimate represents FLT3i (quizartinib, midostaurin, sorafenib) plus chemotherapy versus chemotherapy alone in FLT3-mutated AML; $I^2$=0.0% and p=0.9966 indicate no statistically significant heterogeneity across trials, with identical common-effect and random-effects estimates confirming result robustness.

**Copyright Information:** (Online) ISSN: 2572-9241
©2026 The Author(s). HemaSphere is published by John Wiley & Sons Ltd on behalf of European Hematology Association. This is an open access Abstract Book distributed under the Attribution-NonCommercial-NoDerivs (CC BY-NC-ND), which allows third parties to download the articles and share them with others as long as they credit the author and the Abstract Book, but they cannot change the content in any way or use them commercially.
**Abstract Book Citations:** Authors, Title, HemaSphere, 2026;10:(S1):pages. The abstract book DOIs can be found on the "Author Information" page.
**Disclaimer:** Articles published in the journal HemaSphere exclusively reflect the opinions of the authors. The authors are responsible for all content in their abstracts including accuracy of the facts, statements, citing resources, etc.

### (PB2748) ADDING FLT3 INHIBITORS TO HMA+VEN IMPROVES OUTCOMES IN UNFIT FLT3-ITD+ AML: A MULTI-CENTER REAL-WORLD LANDMARK ANALYSIS

**Topic:** 04. Acute myeloid leukemia - Clinical

Hui Zhou*[1, 2], Lixia Zhu[1], Yuanyuan Zhu[1], Ming Hong[3], Lixin Wang[4], Li Yang[5], Jingyi Huang[6], Huifang Jiang[7], Diehong Tao[7], Zuyi Yang[2], Wanting Feng[6], Ninghong Zhao[1], Weijia Huang[1], Weiyan Zheng[1], Jie Sun[1]

*[1]The First Affiliated Hospital, Zhejiang University School of Medicine, Hangzhou, China;[2]the Affiliated Hospital of Hangzhou Normal University, Hangzhou, China; [3]Jiangsu Province Hospital,, Nanjing, China;[4]Shenzhen University General Hospital, Shenzhen, China; [5]Tongren Hospital Hospital, Shanghai Jiao Tong University School of Medicine, Shanghai, China;[6]Zhejiang University School of Medicine, Hangzhou, China; [7]Tongde Hospital of Zhejiang Province, Hangzhou, China;*

### Background

The combination of venetoclax and hypomethylating agents (HMA+VEN) has achieved remarkable therapeutic success in patients（pts） with acute myeloid leukemia (AML) but has shown only intermediate sensitivity in *FLT3*-ITD-mutated AML. Studies, including those in relapsed/refractory (R/R) and newly diagnosed (ND) patients, suggest that adding a FLT3 inhibitor (FLT3i) to HMA+VEN may offer superior efficacy compared to HMA+VEN alone. However, robust real-world clinical data for ND AML patients remain limited.

### Aims

To evaluate the efficacy and safety of HMA+VEN plus FLT3i in unfit patients with ND*FLT3*-ITD-mutated AML, comparing outcomes against those who received HMA+VEN alone.

### Methods

This multi-center retrospective study enrolled newly diagnosed AML patients with*FLT3*-ITD mutations (with no VAF value requirement) who were unsuitable for intensive chemotherapy across six medical centers in China. Patients were stratified into two groups based on frontline treatment: the Triplet group (HMA+VEN+FLT3i) and the Doublet group (HMA+VEN). The primary endpoint was the composite complete remission (cCR) rate. Secondary endpoints included the rate of measurable residual disease (MRD) negativity by flow cytometry, overall survival (OS), disease-free survival (DFS), and cumulative relapse rate (CRR). Molecular profiling, including fragment count and variant allele frequency for *FLT3*-ITD, was performed via next-generation sequencing (NGS) at diagnosis. FLT3 inhibitors used were sorafenib (200mg bid) and gilteritinib (80mg qd). Statistical methods included Kaplan-Meier estimation with log-rank tests for survival comparisons. A 6-month landmark analysis was conducted to assess the long-term benefit of the Triplet regimen. Informed consent was obtained from all patients in accordance with the Declaration of Helsinki.

**Copyright Information:** (Online) ISSN: 2572-9241
©2026 The Author(s). HemaSphere is published by John Wiley & Sons Ltd on behalf of European Hematology Association. This is an open access Abstract Book distributed under the Attribution-NonCommercial-NoDerivs (CC BY-NC-ND), which allows third parties to download the articles and share them with others as long as they credit the author and the Abstract Book, but they cannot change the content in any way or use them commercially.
**Abstract Book Citations:** Authors, Title, HemaSphere, 2026;10:(S1):pages. The abstract book DOIs can be found on the "Author Information" page.
**Disclaimer:** Articles published in the journal HemaSphere exclusively reflect the opinions of the authors. The authors are responsible for all content in their abstracts including accuracy of the facts, statements, citing resources, etc.

**Results**

From 2020 to 2024, 145 patients were enrolled, with 88 in the Doublet group and 57 in the Triplet group. The median age was 68 years (range 25-86); 69 patients (47.6%) were male. Baseline characteristics, including age (P=0.604), gender (P=0.215), ELN 2024 risk stratification (P=0.584), and hematopoietic stem cell transplant (HSCT) rates (19.3% in Triplet vs 14.8% in Doublet, P=0.763), were well-balanced between the two groups.
The Triplet group demonstrated significantly higher cCR rates (82.1% vs 43.7%, P < 0.001) and MRD negativity rates (68.4% vs 27.3%, P < 0.001) compared to the Doublet group. With a median follow-up of 15.2 months, a 6-month landmark analysis revealed a significant long-term survival advantage for the Triplet group, with an estimated 2-year OS of 67.1% vs. 44.2% (P=0.041). Subgroup analysis based on *FLT3*-ITD fragment counts showed that the Triplet therapy improved the estimated 2-year OS in patients with single-fragment mutations (74.9% vs 57.9%, P=0.048); however, no significant benefit was observed in patients with two or more fragments (47.5% vs 33.4%, P=0.26).
The median times to neutrophil (24.5 days) and platelet (20.0 days) recovery in the Triplet group were similar to those in the Doublet group (P=0.960; P=1.000). The 30-day mortality rates were comparable (3.5% vs 3.4%, P=1.000).

**Summary/Conclusion**

The addition of a FLT3 inhibitor to the HMA+VEN backbone is well-tolerated and significantly improves clinical response and survival in unfit AML patients with *FLT3*-ITD mutations, particularly those with single-fragment mutations. These findings support the triplet regimen as a preferable first-line treatment option for this patient population.

**Copyright Information:** (Online) ISSN: 2572-9241
©2026 The Author(s). HemaSphere is published by John Wiley & Sons Ltd on behalf of European Hematology Association. This is an open access Abstract Book distributed under the Attribution-NonCommercial-NoDerivs (CC BY-NC-ND), which allows third parties to download the articles and share them with others as long as they credit the author and the Abstract Book, but they cannot change the content in any way or use them commercially.
**Abstract Book Citations:** Authors, Title, HemaSphere, 2026;10:(S1):pages. The abstract book DOIs can be found on the "Author Information" page.
**Disclaimer:** Articles published in the journal HemaSphere exclusively reflect the opinions of the authors. The authors are responsible for all content in their abstracts including accuracy of the facts, statements, citing resources, etc.

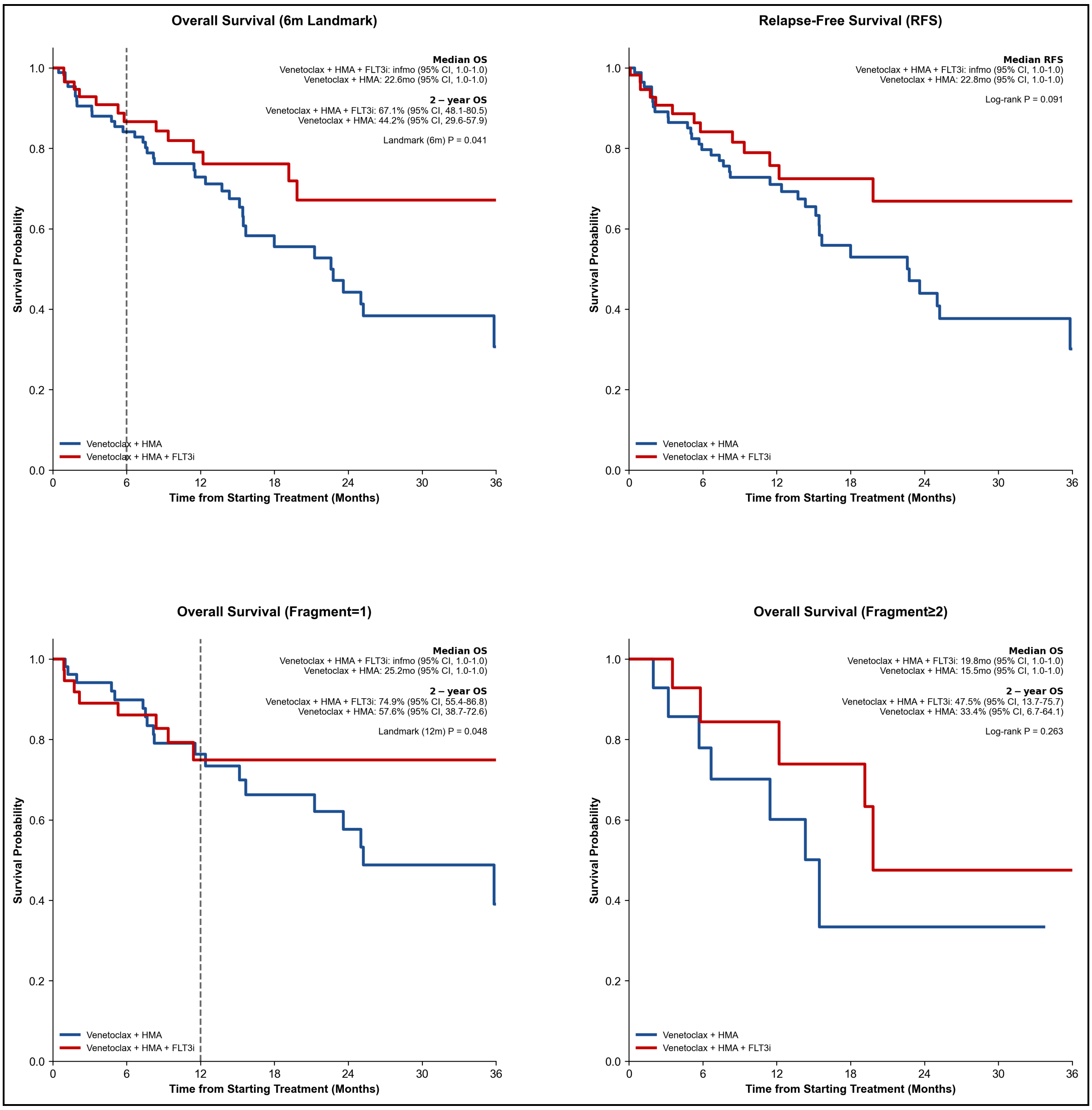

**Copyright Information:** (Online) ISSN: 2572-9241
©2026 The Author(s). HemaSphere is published by John Wiley & Sons Ltd on behalf of European Hematology Association. This is an open access Abstract Book distributed under the Attribution-NonCommercial-NoDerivs (CC BY-NC-ND), which allows third parties to download the articles and share them with others as long as they credit the author and the Abstract Book, but they cannot change the content in any way or use them commercially.
**Abstract Book Citations:** Authors, Title, HemaSphere, 2026;10:(S1):pages. The abstract book DOIs can be found on the "Author Information" page.
**Disclaimer:** Articles published in the journal HemaSphere exclusively reflect the opinions of the authors. The authors are responsible for all content in their abstracts including accuracy of the facts, statements, citing resources, etc.

### (PB2750) ASSOCIATION BETWEEN PML::RARA ISOFORM AND CD34 EXPRESSION IN ACUTE PROMYELOCYTIC LEUKEMIA AND ITS CORRELATION WITH MORPHOLOGICAL SUBTYPE

**Topic:** 04. Acute myeloid leukemia - Clinical

María Florencia Sanchez*[1], Encina Tania Mayra[1], María Paula Iommi[1], Cecilia Lang[1], Andrea Bender[1], Marisa Sandoval[2], Evangelina Agriello[1]

*[1]LEB, Bahía Blanca, Buenos Aires, Argentina; [2]Universidad Nacional del Sur, Hematología Clínica, Bahía Blanca, Buenos Aires, Argentina;*

### Background

Acute promyelocytic leukemia (APL) is characterized by the PML::RARA fusion gene and shows biological heterogeneity related to different transcript isoforms (bcr1, bcr2, bcr3). Previous reports have suggested a potential association between CD34 expression and specific molecular or morphological features, although results remain heterogeneous. Integrated molecular and immunophenotypic characterization may provide further insight into APL biology.

### Aims

1) To evaluate the association between PML::RARA fusion isoform and CD34 expression.
2) To analyze the relationship of this combined biological profile with clinical and morphological variables, including age, leukocytosis, coagulopathy, and early death.

### Methods

We retrospectively analyzed patients diagnosed with APL over the last five years, confirmed by detection of PML::RARA. Fusion isoforms (bcr1, bcr2, bcr3) were determined by RT-PCR (Biomed-1). CD34 expression was assessed by multiparametric flow cytometry according to EuroFlow recommendations using FACSCanto II and FACSLyric cytometers (BD). Early death was defined as death within 30 days from diagnosis. Associations were evaluated using $\chi^2$ or Fisher's exact test; non-parametric tests were applied when appropriate. A p-value <0.05 was considered statistically significant.

**Copyright Information:** (Online) ISSN: 2572-9241
©2026 The Author(s). HemaSphere is published by John Wiley & Sons Ltd on behalf of European Hematology Association. This is an open access Abstract Book distributed under the Attribution-NonCommercial-NoDerivs (CC BY-NC-ND), which allows third parties to download the articles and share them with others as long as they credit the author and the Abstract Book, but they cannot change the content in any way or use them commercially.
**Abstract Book Citations:** Authors, Title, HemaSphere, 2026;10:(S1):pages. The abstract book DOIs can be found on the "Author Information" page.
**Disclaimer:** Articles published in the journal HemaSphere exclusively reflect the opinions of the authors. The authors are responsible for all content in their abstracts including accuracy of the facts, statements, citing resources, etc.

### Results

Among 88 patients, 76 had known isoform; 12 were excluded due to unavailable molecular data, several because of early death at diagnosis precluding characterization. The final cohort included 73 patients (median age 36 years, range 5–93; F:M ratio 1:0,9). To reduce potential confounding, bcr2 cases (n=3) were excluded. Among the analyzed patients, 43 were bcr1 and 30 bcr3. A significant association between isoform and CD34 expression was observed ($\chi^2$=7.92; p=0.005; OR≈4.7), with higher CD34 positivity in bcr3 cases (Table 1).

**Table 1. Isoform vs CD34**

| Isoform | CD34+ | CD34− |
|---|---|---|
| bcr3 | 13 | 17 |
| bcr1 | 6 | 37 |

Table 2 presents the clinical and morphological characteristics of each analyzed group.
No significant associations were found between the combined biological profile and age, leukocytosis, coagulopathy, or early death, although leukocytosis and coagulopathy showed a trend toward higher early mortality.

**Table 2. Clinical and morphological characteristics by biological group**

| Group (n) | Age median (range) | Leukocytosis (%) | Hypogranular morphology (%) | Coagulopathy (%) | Early death (%) |
|---|---|---|---|---|---|
| bcr1/CD34− (37) | 39 (7–93) | 4 (15) | 1 (4) | 20 (69) | 9 (24) |
| bcr1/CD34+ (6) | 28 (10–64) | 1 (17) | 2 (40) | 4 (80) | 0 (0) |
| bcr3/CD34− (17) | 42 (5–61) | 3 (21) | 2 (15) | 13 (93) | 3 (18) |
| bcr3/CD34+ (13) | 30 (5–58) | 3 (23) | 4 (40) | 9 (82) | 2 (15) |
| p value | 0.301 | 0.881 | 0.030 | 0.350 | 0.531 |

Hypogranular morphology was significantly associated with CD34 expression alone (40% in CD34+ vs 7.7% in CD34−; $\chi^2$=8.14; p=0.004; Fisher p=0.010).

### Summary/Conclusion

The bcr3 isoform is significantly associated with increased CD34 expression in our cohort. CD34 positivity correlates with hypogranular morphology, suggesting a specific biological phenotype. No independent association with early mortality was demonstrated, possibly limited by sample size and exclusion of patients lacking isoform data. These findings underscore the importance of integrating molecular and phenotypic characterization in APL diagnosis and emphasize that CD34 expression does not rule out promyelocytic leukemia when clinical and immunophenotypic findings are suggestive, especially in cases with hyperleukocytosis or hypogranular morphology.

**Copyright Information:** (Online) ISSN: 2572-9241
©2026 The Author(s). HemaSphere is published by John Wiley & Sons Ltd on behalf of European Hematology Association. This is an open access Abstract Book distributed under the Attribution-NonCommercial-NoDerivs (CC BY-NC-ND), which allows third parties to download the articles and share them with others as long as they credit the author and the Abstract Book, but they cannot change the content in any way or use them commercially.
**Abstract Book Citations:** Authors, Title, HemaSphere, 2026;10:(S1):pages. The abstract book DOIs can be found on the “Author Information” page.
**Disclaimer:** Articles published in the journal HemaSphere exclusively reflect the opinions of the authors. The authors are responsible for all content in their abstracts including accuracy of the facts, statements, citing resources, etc.

### (PB2751) ANALYTICAL RELIABILITY OF LARGE LANGUAGE MODELS IN NETWORK META-ANALYSIS: A VALIDATION STUDY IN NEWLY DIAGNOSED ACUTE MYELOID LEUKEMIA INELIGIBLE FOR INTENSIVE CHEMOTHERAPY

**Topic:** 04. Acute myeloid leukemia - Clinical

Sameena Parveen[1], Darko Medin[2], Bruno Larvol[1], Mark Gramling[1], Judith Pérez-Granado*[1]

*[1]The Larvol Group LLC, San Francisco, United States of America;[2]University of Montenegro, Podgorica, Montenegro;*

### Background

In newly diagnosed (ND) acute myeloid leukemia (AML) patients unfit for intensive chemotherapy, venetoclax plus azacitidine is the established standard of care (SOC). However, most alternative regimens lack direct head-to-head comparisons. Comparative efficacy can be inferred through network meta-analyses (NMAs). Large language models (LLMs) are increasingly being explored to support evidence synthesis, but their methodological reliability remains uncertain.

### Aims

This study evaluates LLMs' ability to assist with trial selection, assess transitivity, and conduct a frequentist NMA, benchmarking outputs against standard statistical software to assess analytical agreement, reliability, and reproducibility.

### Methods

Randomized clinical trials enrolling ND AML patients ineligible for intensive chemotherapy without actionable mutations were identified using the LARVOL CLIN outcomes database. Independent LLMs (GPT-5.2, Claude 4.6 Opus, Gemini 3 Pro, and Grok 4) assisted in trial selection and transitivity assessment. Outputs were used to establish a consensus set of eligible studies. The resulting overall survival dataset was analyzed using a random-effects frequentist NMA with the R package netmeta, with cytarabine as the reference. The LLMs analyzed the same dataset, and the outputs were benchmarked for concordance in P-score-based treatment ranking and alignment with established SOC.

**Copyright Information:** (Online) ISSN: 2572-9241
©2026 The Author(s). HemaSphere is published by John Wiley & Sons Ltd on behalf of European Hematology Association. This is an open access Abstract Book distributed under the Attribution-NonCommercial-NoDerivs (CC BY-NC-ND), which allows third parties to download the articles and share them with others as long as they credit the author and the Abstract Book, but they cannot change the content in any way or use them commercially.
**Abstract Book Citations:** Authors, Title, HemaSphere, 2026;10:(S1):pages. The abstract book DOIs can be found on the "Author Information" page.
**Disclaimer:** Articles published in the journal HemaSphere exclusively reflect the opinions of the authors. The authors are responsible for all content in their abstracts including accuracy of the facts, statements, citing resources, etc.

### Results

LLMs showed high concordance with the expert-curated trial set (Table 1). Selection discrepancies were limited to trials with ambiguous fitness criteria. The final dataset comprised 10 trials evaluating 10 regimens.
P-score estimates showed minor variations preserving order relative to the reference frequentist NMA. Venetoclax-based combinations ranked highest and hypomethylating monotherapy intermediate-to-low, consistent with established treatment paradigms (Figure 1). ChatGPT API outputs showed reduced reproducibility compared with browser-based execution, with variability in p-scores and ranking.
The network formed a cytarabine-centered star topology with one study per comparison, precluding formal heterogeneity and inconsistency testing. Results varied across models. In browser-based outputs, ChatGPT correctly identified heterogeneity as non-estimable, whereas other models generated non-zero estimates despite structural constraints. In API-based analyses, Grok and Gemini were accurate and reproducible; Claude was consistent but inaccurate, and ChatGPT showed both inaccuracies and reduced reproducibility. Similar patterns were observed for inconsistency.

**Table 1. LLM trial selection accuracy versus expert consensus**

| NCT ID | ChatGPT | Gemini | Grok | Claude |
|---|---|---|---|---|
| AML-19 | ✓ | ✓ | ✓ | ✓ |
| POLO-AML-2 | ✓ | ✓ | ✓ | ✓ |
| Viale-a | ✓ | ✓ | ✓ | ✓ |
| VIALE-C | ✓ | ✓ | ✓ | ✓ |
| NCT02348489 | ✓ | ✓ | ✓ | ✓ |
| NCT02446145 | ✓ | ✓ | ✓ | ✓ |
| NCT05079230 | ✓ | ✓ | ✓ | ✓ |
| NCT00260832 | ✗ | ✓ | ✓ | ✓ |
| NCT02472145 | ✗ | ✓ | ✗ | ✓ |
| AZA-AML-001 | ✓ | ✗ | ✓ | ✓ |
| Accuracy | **80%** | **90%** | **90%** | **100%** |

### Summary/Conclusion

LLMs demonstrated high accuracy in trial selection and reproduced treatment hierarchies consistent with the established SOC. However, the reliability of LLM-generated NMA outputs was limited by inconsistent diagnostic handling and a lack of reproducibility. While LLMs may assist structured evidence synthesis tasks, NMA estimation and validation should remain grounded in established statistical software with expert oversight.

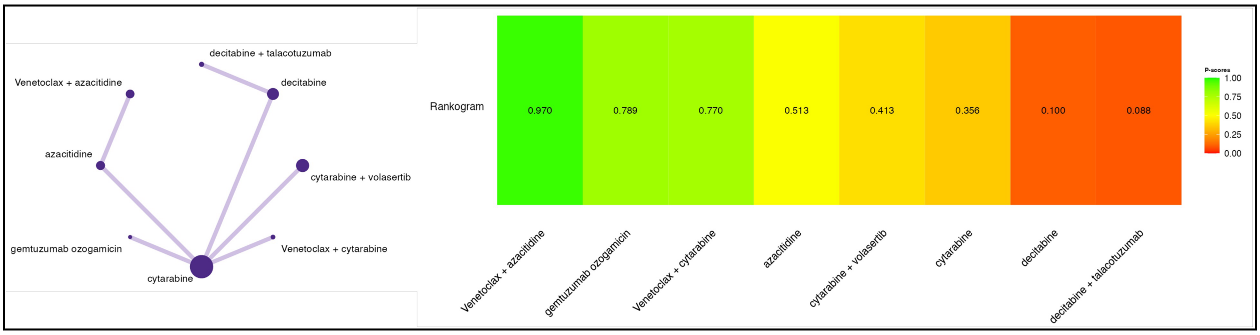

Image 1. NMA network plot and P-score rankogram

**Copyright Information:** (Online) ISSN: 2572-9241
©2026 The Author(s). HemaSphere is published by John Wiley & Sons Ltd on behalf of European Hematology Association. This is an open access Abstract Book distributed under the Attribution-NonCommercial-NoDerivs (CC BY-NC-ND), which allows third parties to download the articles and share them with others as long as they credit the author and the Abstract Book, but they cannot change the content in any way or use them commercially.
**Abstract Book Citations:** Authors, Title, HemaSphere, 2026;10:(S1):pages. The abstract book DOIs can be found on the "Author Information" page.
**Disclaimer:** Articles published in the journal HemaSphere exclusively reflect the opinions of the authors. The authors are responsible for all content in their abstracts including accuracy of the facts, statements, citing resources, etc.

### (PB2752) VARIANT TOPOGRAPHY OF NPM1 MUTATIONS IN ACUTE MYELOID LEUKEMIA

**Topic:** 04. Acute myeloid leukemia - Clinical

Markie Zimmer*[1], Vikram Dhillon[2], Jeff Aguilar[3], Dakshin Padmanabhan[3], Asmita Shukla[3], Sushmitha Nanja Reddy[3], Sikander Cohan[3], Nagasai Yalavarthi[3], Yang Shi[3], Jay Yang[3], Suresh Balasubramanian[3]

*[1]Henry Ford Hospital, Detroit, United States of America;[2]Neal Cancer Center, Houston, United States of America;[3]Karmanos Cancer Institute, Detroit, United States of America;*

### Background

Despite its favorable prognosis in the absence of *FLT3* mutations, *NPM1* mutated AML frequently relapses. Current risk stratification models approach all *NPM1* subtypes (ST) uniformly. Evaluating Type A, B, D and non-ABD *NPM1* variants may elucidate the biological heterogeneity driving the discordance between predicted risk and clinical outcomes.

### Aims

To evaluate the molecular intricacies of *NPM1* variants and their co-mutational profiles in AML.

### Methods

Patient data from Karmanos Cancer Institute, published meta-analytic cohorts (Awada et al., 2021; Kewan et al., 2023), cBioPortal and AACR GENIE (v17) (Cerami et al., 2012; Gao et al., 2013; de Bruijn et al., 2023) were evaluated. Clinical and genomic characteristics of *NPM1*$^{MT}$ AML were reviewed. *NPM1* mutations were classified as Type A, B, D or non-ABD according to nucleotide insertion sequencing. Statistical analysis was performed via GraphPad Prism.

### Results

Of 7653 patients, 1540 had *NPM1* mutated AML. Type A was most common (74%), followed by non-ABD (18%), B (4.8%) and D (4.7%). Type A was the most proliferative, with a median bone marrow blast count of 79%, compared to 54% in non-ABD (p<0.0001). B had the highest *NPM1* VAF (37.6 vs 30.9% in non-ABD, p <0.0001).

The most common co-mutation with *NPM1* was *DNMT3A* (48%). Type A was enriched for *DNMT3A* compared to other ST (54% vs 34%, p<0.00001). Type A and D had more *IDH1*, *IDH2*, and *NRAS* mutations than B and non-ABD (17% vs 10%, p=0.01; 23% vs 9%, p<0.00001; 16% vs 10%, p=0.03, respectively). B and non-ABD were more enriched in *WT1* compared to A and D (12% vs 4%, p=0.00002). Non-ABD was more enriched for *FLT3* mutations than other ST (39% vs 24%, p=0.00012). *DNMT3A*$^{R882}$ was enriched in high VAF (HV) compared to low VAF (LV) *NPM1* (54% vs 46%, p=0.01). *FLT3* was more common in HV *NPM1* (34% vs 17%, p<0.0001). *NPM1* was often a subclone (82%), but was more likely to be dominant in B (29%, p=0.001).

The median OS of *NPM1*$^{MT}$ AML was 19 mo. OS was numerically higher in D (26 mo) and non-ABD (25 mo). LV *NPM1* (Q1 ≤22.8%) had a significantly better OS than HV (Q3 ≥38%): 21 vs 10 mo (p<0.0001). This trend was observed across ST and was most pronounced in D and non-ABD (D: 58 vs 10 mo, p=0.009; non-ABD: 29 vs 7 mo, p=0.005).

### Summary/Conclusion

While *NPM1* variants are distinct in disease presentation, outcomes appear to be shaped by complex interactions with co-mutational patterns and clonal hierarchy rather than subtype alone. Future work will evaluate whether variant topography actively shapes permissive co-mutational landscapes and disease ontogeny, thereby driving clinical behavior and MRD dynamics.

**Copyright Information:** (Online) ISSN: 2572-9241
©2026 The Author(s). HemaSphere is published by John Wiley & Sons Ltd on behalf of European Hematology Association. This is an open access Abstract Book distributed under the Attribution-NonCommercial-NoDerivs (CC BY-NC-ND), which allows third parties to download the articles and share them with others as long as they credit the author and the Abstract Book, but they cannot change the content in any way or use them commercially.
**Abstract Book Citations:** Authors, Title, HemaSphere, 2026;10:(S1):pages. The abstract book DOIs can be found on the “Author Information” page.
**Disclaimer:** Articles published in the journal HemaSphere exclusively reflect the opinions of the authors. The authors are responsible for all content in their abstracts including accuracy of the facts, statements, citing resources, etc.

### (PB2753) REAL-WORLD OUTCOMES OF VENETOCLAX–AZACITIDINE IN RELAPSED/REFRACTORY ACUTE MYELOID LEUKEMIA

**Topic:** 04. Acute myeloid leukemia - Clinical

Amjad Althobaiti*[1], Yasser Abou Mourad[1, 2], Reyna Altook[1, 2], Hannah Cherniawsky[1, 2], Shanee Chung[1, 2], Donna Forrest[1, 2], Florian Kuchenbauer[1, 2], Deepesh Lad[1, 2], Stephen Nantel[1, 2], Sujaatha Bhatt[1, 2], Thomas Nevill[1, 2], Judith Rodrigo[1, 2], Claudie Roy[1, 2], Kevin Song[1, 2], Ryan Stubbins[1, 2], Cynthia Toze[1, 2], Jennifer White[1, 2], Eric Zhao[1, 2], David Sanford[1, 2]

*[1]Leukemia/BMT Program of BC, BC Cancer and Vancouver Coastal Health, Leukemia/BMT, Vancouver, Canada;[2]Division of Hematology, Department of Medicine, University of British Columbia, Vancouver, Canada;*

### Background

Relapsed/refractory acute myeloid leukemia  is a significant therapeutic challenge, with limited treatment options and a poor overall prognosis. Venetoclax combined with azacitidine has demonstrated efficacy in treatment-naïve AML. Ven-Aza is also used off-label in R/R patients; real-world data supporting this approach remain limited.

### Aims

To evaluate efficacy and safety outcomes of Ven-Aza treatment in patients with R/R AML.

### Methods

We performed a retrospective single-center analysis of consecutive patients with R/R AML treated with Ven-Aza from 2020 to 2025. Data was collected from a chart review. The primary efficacy outcomes for the study were response and overall survival (OS), measured from the time of treatment initiation with Ven-Aza to death or last follow-up. Response was assessed after 1 cycle of therapy and classified according to the European LeukemiaNet (ELN) 2022 criteria, with PR considered equivalent to no response. Overall survival was estimated using Kaplan–Meier methodology, and survival distributions were compared using log-rank tests. Outcomes were also assessed according to the ELN 2024 prognostic model, initial response, and receipt of allogeneic stem cell transplant (allo-HSCT). Safety outcomes of interest included rates of hospitalization and infection. The study was approved by the University of British Columbia Research Ethics Board.

### Results

We identified 66 patients with R/R AML treated with Ven-Aza. Twenty-nine (44%) were relapsed, and 37 (56%) had refractory disease following their initial induction. All patients received at least 1 line of intensive chemotherapy prior to receiving Ven-Aza. The composite response rate (CR/CRi/MLFS) for the entire group was 53.0% (35/66 patients), comprising CR 18.2% (n=12), CRi 25.8% (n=17), and MLFS 9.1% (n=6). Refractory disease occurred in 43.9% (n=29). Response rates by ELN 2024 risk were: favorable 66.7% (4/6), intermediate 51.9% (27/52), and adverse 42.9% (3/7), with no significant difference across risk groups. Median OS for the entire cohort was 7.4 months (95% CI 3.56-11.16). Responders (CR/CRi/MLFS) had a significantly longer median OS of 24.4 months (95% CI 16.15-32.67) compared to 3.6 months (95% CI 0.32-6.94) in patients without response ($p<0.001$). Notably, 28.8% (19/66) of patients were successfully bridged to allo-HSCT after Ven-Aza therapy.  Among responders, the median OS in patients undergoing transplant after Ven-Aza was 29.9 months (95% CI 25.2-44.5) compared to 13.5 months (95% CI 4.9-22.1) in those who did not receive alloHSCT ($p<0.001$) (Figure 1). The ELN 2024 risk classification did not significantly predict OS ($p=0.682$). During the first treatment cycle, 12% of patients were hospitalized at treatment initiation, and 30% required hospitalization at any time during Cycle 1. Infections occurred in 48.5% of patients, including bacterial (37.9%), viral (7.6%), and fungal (18.2%).

**Copyright Information:** (Online) ISSN: 2572-9241
©2026 The Author(s). HemaSphere is published by John Wiley & Sons Ltd on behalf of European Hematology Association. This is an open access Abstract Book distributed under the Attribution-NonCommercial-NoDerivs (CC BY-NC-ND), which allows third parties to download the articles and share them with others as long as they credit the author and the Abstract Book, but they cannot change the content in any way or use them commercially.
**Abstract Book Citations:** Authors, Title, HemaSphere, 2026;10:(S1):pages. The abstract book DOIs can be found on the "Author Information" page.
**Disclaimer:** Articles published in the journal HemaSphere exclusively reflect the opinions of the authors. The authors are responsible for all content in their abstracts including accuracy of the facts, statements, citing resources, etc.

**Summary/Conclusion**

This real-world cohort study reports that a significant proportion of patients with R/R AML respond to Ven-Aza. Response and transplantation after Ven-Aza were determinants of OS, supporting Ven-Aza as an effective bridge-to-transplant strategy. Larger cohorts are needed to identify baseline predictors of response and long-term outcome with Ven-Aza in R/R AML.

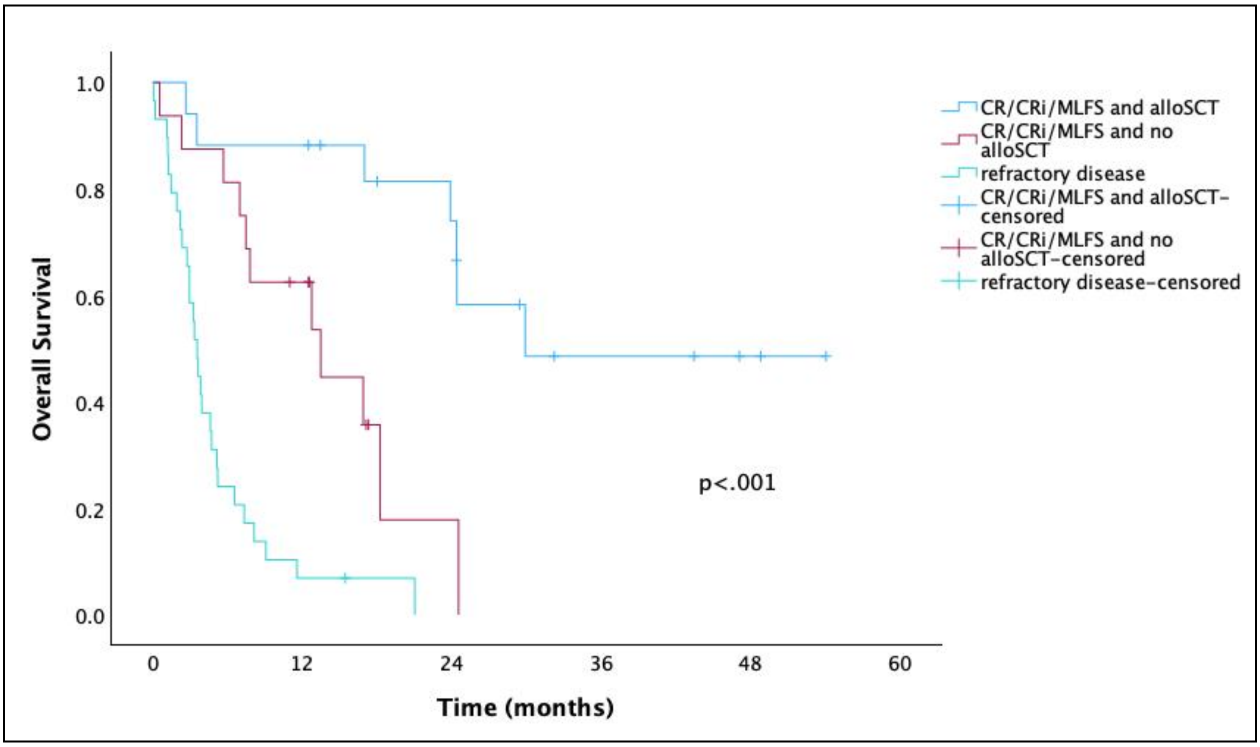

Overall survival by response and receipt of allogeneic stem cell transplant

**Copyright Information:** (Online) ISSN: 2572-9241
©2026 The Author(s). HemaSphere is published by John Wiley & Sons Ltd on behalf of European Hematology Association. This is an open access Abstract Book distributed under the Attribution-NonCommercial-NoDerivs (CC BY-NC-ND), which allows third parties to download the articles and share them with others as long as they credit the author and the Abstract Book, but they cannot change the content in any way or use them commercially.
**Abstract Book Citations:** Authors, Title, HemaSphere, 2026;10:(S1):pages. The abstract book DOIs can be found on the "Author Information" page.
**Disclaimer:** Articles published in the journal HemaSphere exclusively reflect the opinions of the authors. The authors are responsible for all content in their abstracts including accuracy of the facts, statements, citing resources, etc.

### (PB2754) CPX-351 LEADS TO HIGH RESPONSE RATES AND DURABLE OUTCOMES IN NPM1 MUTATED AML: AN ITALIAN MULTICENTRIC REAL-LIFE STUDY

**Topic:** 04. Acute myeloid leukemia - Clinical

Francesco Grimaldi*[1], Calogero Vetro[2], Paola Minetto[3], Luana Fianchi[4], Patrizia Chiusolo[5], Carola Riva[6], Caterina Alati[7], Selenia Vitiello[8], Lucia Ammirati[9], Bianca Serio[10], Valeria Cardinali[11], Vera Carluccio[12], Michele Cimminiello[13], Matteo Molica[14], Crescenza Pasciolla[15], Maria Gabriella Rascato[16], Marina Aurora Urbano[17], Ernesto Vigna[18], Mara Memoli[19], Mario Annunziata[8], Elisabetta Todisco[20], Fabrizio Pane[19], Roberto M. Lemoli[21], Fabio Guolo[21]

*[1]Hematology Division, Department of Clinical Medicine and Surgery, Università Federico II di Napoli, Clinical Medicine and Surgery, Napoli, Italy; [2]Hematology and Bone Marrow Transplantation Unit, Hospital of Bolzano (SABES-Azienda Sanitaria dell'Alto Adige), Teaching Hospital of Paracelsus Medical University, Bolzano, Italy; [3]IRCCS Ospedale Policlinico San Martino, Genova, Italy; [4]Dipartimento di Scienze Radiologiche Radioterapiche ed Ematologiche, Fondazione Policlinico Universitario A. Gemelli, IRCCS Università Cattolica del Sacro Cuore, Roma, Italy; [5]Fondazione Policlinico Universitario Agostino Gemelli IRCCS, Roma, Italy; [6]IRCCS Ospedale Policlinico San Martino di Genova, Genova, Italy; [7]UOC di Ematologia, Grande Ospedale Metropolitano Bianchi Melacrino Morelli, Reggio Calabria, Italy; [8]UOC Ematologia, Azienda Ospedaliera di Rilievo Nazionale Cardarelli, Napoli, Italy; [9]Onco-Ematologia, Ospedale A. Tortora di Pagani, Pagani (SA), Italy; [10]UOC Ematologia e Trapianto di cellule staminali, Azienda Ospedaliero Universitaria San Giovanni di Dio e Ruggi d'Aragona, Salerno, Italy; [11]Dipartimento di Medicina e Chirurgia, Università di Perugia, Ospedale Santa Maria della Misericordia, Perugia, Italy; [12]UOC di Ematologia, Ospedale Dimiccoli, Barletta, Italy; [13]UOC di Ematologia e trapianto di Midollo osseo, AORN S. Carlo, Potenza, Italy; [14]Dipartimento di Ematologia Oncologia, AOU Renato Dulbecco, Catanzaro, Italy;[15]IRCCS Istituto Tumori Giovanni Paolo II, Bari, Italy; [16]UOC di Ematologia, Ospedale, P.O. Moscati di Aversa, Aversa, Italy;[17]UOC di Ematologia, Ospedale A. Perrino, Brindisi, Italy; [18]UOC di Ematologia, Azienda Ospedaliera Annunziata di Cosenza, Cosenza, Italy;[19]Hematology Division, Department of Clinical Medicine and Surgery, Università Federico II di Napoli, Napoli, Italy; [20]S.C. di Ematologia e Trapianto di Cellule Staminali Emopoietiche, Ospedale di Busto Arsizio, ASST Valle Olona, Busto Arsizio, Italy; [21]Dipartimento di Medicina Interna (DiMI), Clinica Ematologica, Università di Genova, Genova, Italy;*

### Background

CPX-351 has been approved for treatment of Acute Myeloid Leukemia (AML) with Myelodysplastic Related Changes (AML-MRC) or therapy-related AML (t-AML) as per former WHO 2016 classification. Italian Real-World Experience confirmed benefit of CPX-351 in high-risk AML, and CPX-351 is now being explored as frontline treatment for de novo AML. However, data on the efficacy in NPM1 mutated AML remain limited, given rarity of this subgroup among t-AML, and results still immature from frontline trials.

### Aims to analyze efficacy and outcome of CPX-351 in NPM1-mutated AML.

### Methods

59 patients (median age 64 years, range 46-74, 52,5% aged≥65) (28/31, male/female) from 29 Italian Centers were retrospectively included in this study. Complete diagnostic testing were performed locally, and HSCT given as per local policy. Diagnosis of t-AML or AML-MRC were 19(32,2%) and 40(67,8%), respectively. Type A was most prevalent (88%) NPM1 mutation, while FLT3 was mutated in 12 patients (20%). Cytogenetics showed complex karyotype (>3 abnormalities) in 14 patients (23,7%). NGS data at diagnosis were available in 20 patients, with DNMT3a and IDH1/2 as prevalent mutations (19% respectively). Median number of comorbidities was 1 (Range 0-4).

**Copyright Information:** (Online) ISSN: 2572-9241
©2026 The Author(s). HemaSphere is published by John Wiley & Sons Ltd on behalf of European Hematology Association. This is an open access Abstract Book distributed under the Attribution-NonCommercial-NoDerivs (CC BY-NC-ND), which allows third parties to download the articles and share them with others as long as they credit the author and the Abstract Book, but they cannot change the content in any way or use them commercially.
**Abstract Book Citations:** Authors, Title, HemaSphere, 2026;10:(S1):pages. The abstract book DOIs can be found on the "Author Information" page.
**Disclaimer:** Articles published in the journal HemaSphere exclusively reflect the opinions of the authors. The authors are responsible for all content in their abstracts including accuracy of the facts, statements, citing resources, etc.

### Results

After induction 1, 47/59 patients (79%) achieved complete remission (CR); 2 achieved CR after induction 2, with a final CR rate of 83% (63% achieving MRD negativity). After inductions, 10 patients resulted refractory (16%). Complex karyotype was more frequent in refractory patients (p<0,005) with an OR of 3.12 (p=NS in a logistic stepwise regression analysis). Among responding patients, 8 (16,3%), 17 (34,8%) and 24 (48,9%) received none, one or two consolidation cycles, respectively. HSCT consolidation in first CR was performed in 17/49 responding patients (34,6%). Median RFS was of 19.6 months (CI 95% 14.8 - NA), and median of OS was of 20.2 months (CI 95% 17.21 – NA). In a Cox multivariate analysis RFS was significantly influenced by t-AML diagnosis (HR 48.5, p=0.026), FLT3 status (HR 32.1, p=0.035), number of consolidation (2 vs other)(HR 0,07, p=0.021) and HSCT in CR1 (HR 0.01, p=0.032), while OS was significantly influenced only by age (HR 1,34, p=0.012), with HSCT not significantly impacting on survival (Fig1).

### Summary/Conclusion

In this large cohort of NPM1-mutated AML, CPX-351 was associated with good response and optimal survival outcome. Combinatory strategies with newer agents may further improve these results.

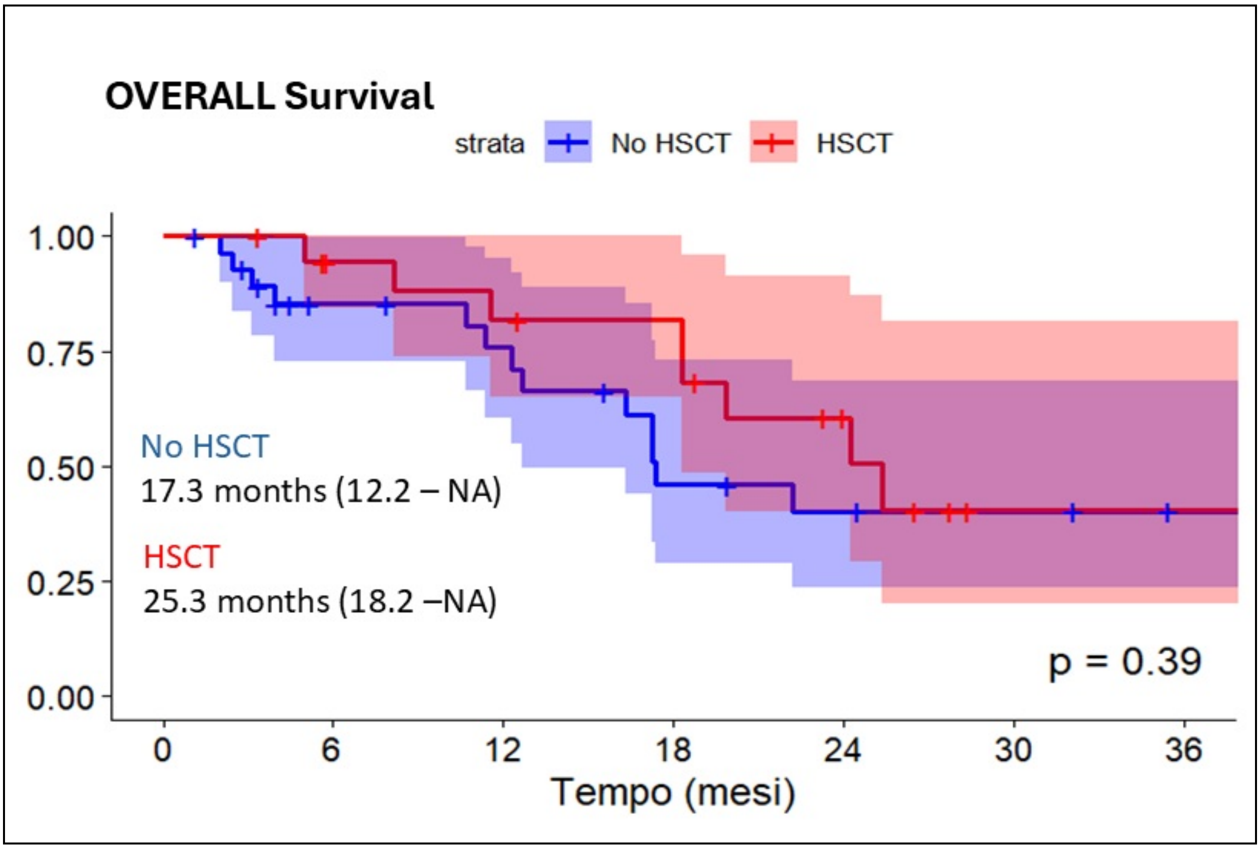

**Copyright Information:** (Online) ISSN: 2572-9241
©2026 The Author(s). HemaSphere is published by John Wiley & Sons Ltd on behalf of European Hematology Association. This is an open access Abstract Book distributed under the Attribution-NonCommercial-NoDerivs (CC BY-NC-ND), which allows third parties to download the articles and share them with others as long as they credit the author and the Abstract Book, but they cannot change the content in any way or use them commercially.
**Abstract Book Citations:** Authors, Title, HemaSphere, 2026;10:(S1):pages. The abstract book DOIs can be found on the “Author Information” page.
**Disclaimer:** Articles published in the journal HemaSphere exclusively reflect the opinions of the authors. The authors are responsible for all content in their abstracts including accuracy of the facts, statements, citing resources, etc.

### (PB2755) ACUTE MYELOID LEUKEMIA IN THE TARGETED-THERAPY ERA: UNITED STATES VS HIGH-SDI TRENDS, 1990–2021

**Topic:** 04. Acute myeloid leukemia - Clinical

Tapan Giri*[1], Zeal Soni[2]

*[1]University of Nevada, Reno, NV, Reno, United States of America;[2]Smt NHL Municipal Medical College, Ahmedabad, Gujarat, Ahmedabad, India;*

### Background

Acute myeloid leukemia (AML) predominantly affects older adults and carries high mortality. Recent approvals of targeted and venetoclax-based therapies have expanded treatment options for patients ineligible for intensive chemotherapy. However, population-level trends in AML burden across high-resource settings remain incompletely characterized. We evaluated long-term AML incidence, mortality, and disability trends comparing the United States with high Socio-demographic Index (SDI) regions.

### Aims

To evaluate long-term trends in the incidence, mortality, and disability burden of acute myeloid leukemia in the United States compared with high Socio-demographic Index regions from 1990–2021, and to identify temporal inflections in mortality patterns during the targeted-therapy era.

### Methods

Global Burden of Disease (GBD) 2021 data for AML (1990–2021) were analyzed. Age-standardized incidence (ASIR), mortality (ASMR), and disability-adjusted life year rates (ASDR) per 100,000 population (95% uncertainty intervals) were extracted for the United States and high-SDI regions. Temporal trends were assessed using estimated annual percentage change (EAPC) and joinpoint regression to identify inflection periods.

### Results

Across high-SDI regions, incident AML cases increased from 27,830 (95% UI 26,575–28,799) in 1990 to 56,646 (51,611–59,717) in 2021, largely reflecting demographic aging. ASIR rose modestly from 2.65 to 2.88 per 100,000 (EAPC +0.41%). Absolute deaths increased, whereas ASMR remained broadly stable with modest declines in recent years.
In the United States, 21,533 incident cases were recorded in 2021, with consistently higher ASIR than the high-SDI aggregate (EAPC +0.37%). Joinpoint analysis identified a post-2015 inflection toward declining ASMR in the United States. DALY burden remained substantial, with high body mass index and occupational carcinogen exposure among leading modifiable contributors.

**Copyright Information:** (Online) ISSN: 2572-9241
©2026 The Author(s). HemaSphere is published by John Wiley & Sons Ltd on behalf of European Hematology Association. This is an open access Abstract Book distributed under the Attribution-NonCommercial-NoDerivs (CC BY-NC-ND), which allows third parties to download the articles and share them with others as long as they credit the author and the Abstract Book, but they cannot change the content in any way or use them commercially.
**Abstract Book Citations:** Authors, Title, HemaSphere, 2026;10:(S1):pages. The abstract book DOIs can be found on the "Author Information" page.
**Disclaimer:** Articles published in the journal HemaSphere exclusively reflect the opinions of the authors. The authors are responsible for all content in their abstracts including accuracy of the facts, statements, citing resources, etc.

**Summary/Conclusion**

Acute myeloid leukemia (AML) remains a high-mortality hematologic malignancy with a disease burden that increases with population aging. Over the past decade, the therapeutic landscape has evolved with the introduction of mutation-directed agents and venetoclax-based regimens, expanding treatment options for older and medically unfit patients. Using Global Burden of Disease 2021 estimates, we evaluated long-term population trends in AML incidence, mortality, and disability burden from 1990 to 2021, comparing the United States with high Socio-demographic Index (SDI) regions. Age-standardized metrics and joinpoint analyses were used to identify temporal changes in disease patterns. While absolute incidence and deaths increased across high-resource settings, age-standardized mortality stabilized with emerging declines in recent years, particularly in the United States following mid-2010s inflection points. These findings highlight the growing demographic burden of AML alongside signals of improving outcomes, underscoring the importance of molecular diagnostics, equitable access to novel therapies, and supportive care optimization to sustain progress.

**Copyright Information:** (Online) ISSN: 2572-9241
©2026 The Author(s). HemaSphere is published by John Wiley & Sons Ltd on behalf of European Hematology Association. This is an open access Abstract Book distributed under the Attribution-NonCommercial-NoDerivs (CC BY-NC-ND), which allows third parties to download the articles and share them with others as long as they credit the author and the Abstract Book, but they cannot change the content in any way or use them commercially.
**Abstract Book Citations:** Authors, Title, HemaSphere, 2026;10:(S1):pages. The abstract book DOIs can be found on the "Author Information" page.
**Disclaimer:** Articles published in the journal HemaSphere exclusively reflect the opinions of the authors. The authors are responsible for all content in their abstracts including accuracy of the facts, statements, citing resources, etc.

### (PB2756) WT1 MUTATION PREDICTED A HIGHER RISK OF RELAPSE IN ACUTE MYELOID LEUKEMIA PATIENTS WITH INV(16)(P13Q22)

**Topic:** 04. Acute myeloid leukemia - Clinical

Shunjie Yu*[1], Zongru Li[2], Lijuan Hu[1], Lu Yu[1], Xiao-Jun Huang[1], Qian Jiang[1]

*[1]Peking University Institute of Hematology, Beijing, China;[2]Peking University Institute of Hematology, Peking University Institute of Hematology, Beijing, China;*

### Background

Acute myeloid leukemia (AML) with inv(16)(p13q22) is associated with favorable outcomes. To date, the impact of genomic features on outcomes has not been elucidated.

### Aims

To explore the genomic abnormalities associated with the outcomes in AML patients with inv(16)(p13q22).

### Methods

Data of consecutive AML patients with inv(16)(p13q22) including clinical and genomic information were reviewed. The effects of genes mutation on relapse-free survival (RFS) and survival were evaluated by using the Cox proportional hazards regression model, and data were adjusted for clinical factors that were significantly associated with RFS and survival in the univariate analysis.

### Results

155 AML patients with inv(16)(p13q22) receiving intensive or less intensive induction chemotherapy followed by consolidation chemotherapy were included in this study. 93 (60%) patients were male. Median age was 43 years (IQR, 33-55 years). The most frequently mutated genes (frequency ≥5%) were *NRAS* (n = 85, 55%), followed by *KIT* (n = 68, 44%), *KRAS* (n = 45, 29%), *FLT3*-TKD (n = 22, 14%) , *FLT3*-ITD (n = 10, 6%), *WT1* (n = 9, 6%), *NF1* (n = 9, 6%), *PTPN11* (n = 9, 6%) and *CBL* (n = 8, 5%). 142 (92%) patients achieved complete remission (CR) after the first induction therapy with a final CR rate of 99%. With a median follow-up of 23 (IQR, 15-40) months for all patients and 25 (IQR, 15-41) months for survivors. The 3-year probabilities of RFS and survival were 78% (95% confidential interval [CI], 72, 88%) and 91% (86, 97%), respectively. After performing adjustment for clinical factors to correct for the possible effects of an association between the clinical and genetic factors, RAS pathway related genes mutations (*NRAS/KRAS/NF1/PTPN11/CBL*) showed significant association with favorable RFS (HR = 0.2 [0.1, 0.7], $p$ = 0.014); *WT1* mutation, inferior RFS (HR = 6.9 [1.6, 29.8], $p$ = 0.010), but both had no impact on survival. In multivariate analyses *WT1* mutation was the sole gene mutation identified significantly-associated with inferior RFS (HR = 7.1 [1.4, 34.9], $p$ = 0.016), but had no impact on survival. In addition, lower platelet count and higher bone marrow blasts at diagnosis were associated with inferior RFS; male sex, inferior survival.

### Summary/Conclusion

Harboring *WT1* mutation independently predicted a high likelyhood of relapse in AML patients with inv(16)(p13q22). However, harboring RAS pathway related genes mutations predicted favorable RFS after adjusting for clinical factors.

**Copyright Information:** (Online) ISSN: 2572-9241
©2026 The Author(s). HemaSphere is published by John Wiley & Sons Ltd on behalf of European Hematology Association. This is an open access Abstract Book distributed under the Attribution-NonCommercial-NoDerivs (CC BY-NC-ND), which allows third parties to download the articles and share them with others as long as they credit the author and the Abstract Book, but they cannot change the content in any way or use them commercially.
**Abstract Book Citations:** Authors, Title, HemaSphere, 2026;10:(S1):pages. The abstract book DOIs can be found on the "Author Information" page.
**Disclaimer:** Articles published in the journal HemaSphere exclusively reflect the opinions of the authors. The authors are responsible for all content in their abstracts including accuracy of the facts, statements, citing resources, etc.

### (PB2757) PRAGMATIC GERIATRIC FITNESS STRATIFICATION IN OLDER ACUTE MYELOID LEUKEMIA PATIENTS RECEIVING NON-INTENSIVE THERAPY: A RETROSPECTIVE SINGLE-CENTER STUDY

**Topic:** 04. Acute myeloid leukemia - Clinical

Lucía Gómez-Pérez*[1], Sara García Ávila[1, 2], Clara Nadal Pellise[1], Ramón Diez-Feijóo[1, 2], Maria Alicia Senin Magan[1], Patricia Vélez[1], Christelle Ferrà[1]

*[1]Hospital del Mar Research Institute, Department of Hematology, Barcelona, Spain;[2]Pompeu Fabra University, Barcelona, Spain;*

### Background

Acute myeloid leukemia (AML) predominantly affects older adults. Geriatric assessment (GA)—covering physical function, comorbidity burden, cognition, polypharmacy, nutrition, and social factors—has emerged as a relevant tool because it is associated with treatment tolerance and survival, while classical scores like ECOG PS or Ferrara criteria may be insufficient as they mainly rely on age and comorbidities.

### Aims

To retrospectively evaluate a pragmatic GA in an older AML cohort receiving non-intensive therapy at a tertiary center and to explore its association with overall survival (OS).

### Methods

Consecutive patients diagnosed with AML between 2017 and 2025 and treated with non-intensive regimens (hypomethylating agents [HMA] alone or combined with venetoclax) were included. A pragmatic retrospective GA was constructed using domains most consistently associated with outcomes in elderly AML: physical function (mobility, basic and instrumental activities of daily life), comorbidity burden (HCT-CI), cognition, and nutritional status. Frailty was defined by any major vulnerability (ADL or mobility impairment, ECOG ≥3, HCT-CI ≥3, or documented cognitive dysfunction) or ≥2 minor deficits (impaired instrumental ADLs, ECOG 2, depression, or nutritional deficit defined as albumin <3.5 g/dL and/or unintentional weight loss). Vulnerable patients had only one minor deficit and did not meet frailty criteria; robust patients had no deficits.

### Results

Forty-four AML patients (median age 76 years [65–93]; 57% male) were included. The most frequent diagnosis (ICC 2022) was AML with myelodysplasia-related gene mutations (n=21, 47.7%), followed by AML with mutated NPM1 (n=8, 18.2%), and AML with mutated TP53 (n=7, 15.9%). According to ELN2024, 18.2% (n=8) were favorable, 59.1% (n=26) intermediate, and 22.7% (n= 10) adverse risk. Previous myeloid malignancy was present in 38.6% (n=17) and 6 patients (13.6%) were qualified as therapy-related. GA classified patients as robust (18.2%, n=8), vulnerable (27.3%, n=12), and as frail (54.5%, n=24). Treatment type did not show significant differences between ELN2024 risk category or frailty distribution. ELN2024 retained prognostic discrimination in this real- world cohort, with median overall survival (mOS) of 17.6, 9.6, and 4.4 months for favorable, intermediate, and adverse risk groups, respectively (HR 3.75, 95% CI 1.64–8.56; p=0.0038). Figure 1A. When only analyzing the patients considered as frail, ELN2024 prognostic discrimination was reduced (p=0.12).
Frail patients showed a marked reduction in mOS compared with robust (5.8 months vs 14.7 months, p=0.18). When frailty was dichotomized (robust vs vulnerable+frail), mOS was 14.7 vs 7.7 months (HR 0.56, 95% CI 0.29–1.06; p=0.07), suggesting the presence of a trend despite the limited sample size (Figure 1B). Treatment type did not significantly impact OS.

**Copyright Information:** (Online) ISSN: 2572-9241
©2026 The Author(s). HemaSphere is published by John Wiley & Sons Ltd on behalf of European Hematology Association. This is an open access Abstract Book distributed under the Attribution-NonCommercial-NoDerivs (CC BY-NC-ND), which allows third parties to download the articles and share them with others as long as they credit the author and the Abstract Book, but they cannot change the content in any way or use them commercially.
**Abstract Book Citations:** Authors, Title, HemaSphere, 2026;10:(S1):pages. The abstract book DOIs can be found on the “Author Information” page.
**Disclaimer:** Articles published in the journal HemaSphere exclusively reflect the opinions of the authors. The authors are responsible for all content in their abstracts including accuracy of the facts, statements, citing resources, etc.

**Summary/Conclusion**

In older AML patients receiving non-intensive therapy, ELN2024 retained prognostic discrimination. Frailty is suggested as an independent factor besides the biological risk through a trend towards inferior survival. Prospectively integrating GA with biological stratification may refine risk assessment in this population and condition treatment approach.

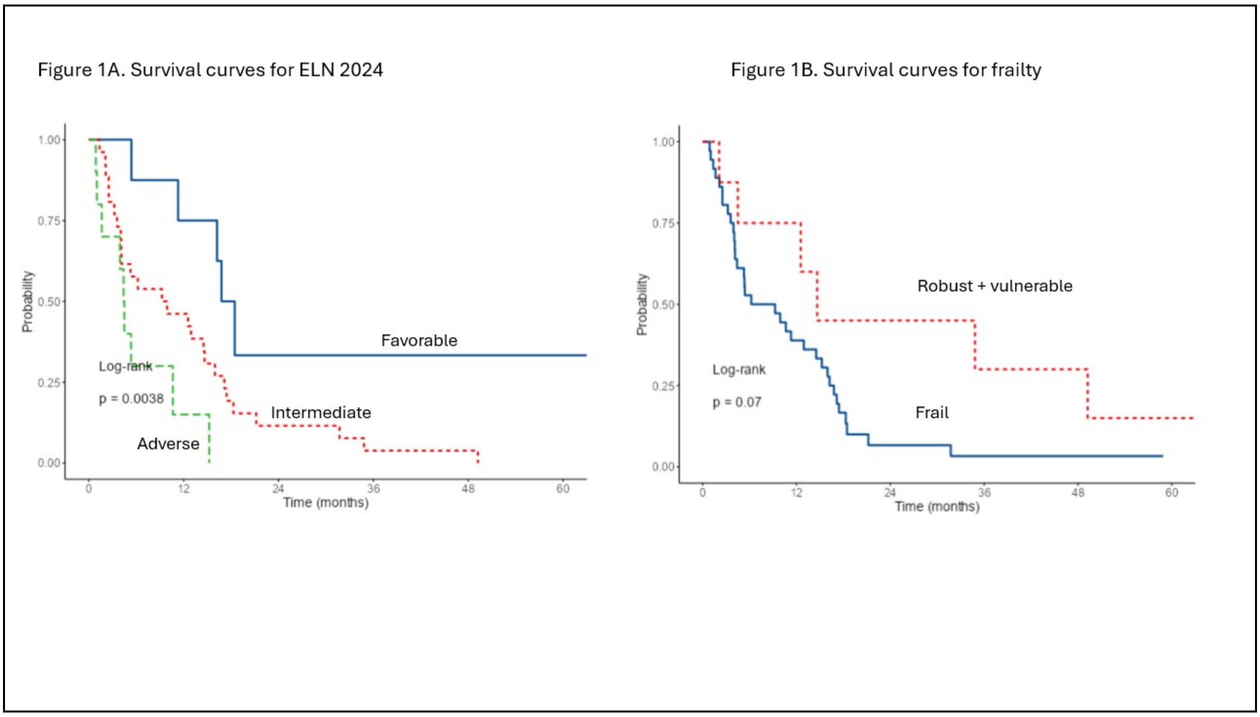

Survival curves for ELN2024 and for frailty

**Copyright Information:** (Online) ISSN: 2572-9241
©2026 The Author(s). HemaSphere is published by John Wiley & Sons Ltd on behalf of European Hematology Association. This is an open access Abstract Book distributed under the Attribution-NonCommercial-NoDerivs (CC BY-NC-ND), which allows third parties to download the articles and share them with others as long as they credit the author and the Abstract Book, but they cannot change the content in any way or use them commercially.
**Abstract Book Citations:** Authors, Title, HemaSphere, 2026;10:(S1):pages. The abstract book DOIs can be found on the "Author Information" page.
**Disclaimer:** Articles published in the journal HemaSphere exclusively reflect the opinions of the authors. The authors are responsible for all content in their abstracts including accuracy of the facts, statements, citing resources, etc.

### (PB2758) CLINICAL CHARACTERISTICS OF PEDIATRIC ACUTE MYELOID LEUKEMIA WITH MYELODYSPLASIA-RELATED CHANGES: A RETROSPECTIVE COHORT STUDY

**Topic:** 04. Acute myeloid leukemia - Clinical

Xinling Tu*[1], Yang Wan[2], Wenyu Yang[2], Xiaofan Zhu[3, 3]

*[1]Haihe Laboratory of Cell Ecosystem, Tianjin Medical University, Tianjin, China., Tianjin, China;[2] 1. State Key Laboratory of Experimental Hematology, National Clinical Research Center for Blood Diseases, Haihe Laboratory of Cell Ecosystem, Institute of Hematology & Blood Diseases Hospital, Chinese Academy of Medical Sciences & Peking Union Medical College, Tianjin 300020, China 2. Tianjin Institutes of Health Science Tianjin 301600, China., Tianjin, China; [3] 1. State Key Laboratory of Experimental Hematology, National Clinical Research Center for Blood Diseases, Haihe Laboratory of Cell Ecosystem, Institute of Hematology & Blood Diseases Hospital, Chinese Academy of Medical Sciences & Peking Union Medical College, Tianjin 300020, China 2. Tianjin Institutes of Health Science Tianjin 301600, China., TianJin, China;*

### Background

Acute myeloid leukemia (AML) is a common hematologic malignancy in adults. Acute myeloid leukemia with myelodysplasia-related changes (AML-MR) accounts for approximately 25-30% of all AML cases. Compared with AML patients without myelodysplasia-related features, AML-MR patients exhibit significantly lower overall treatment response rates and higher relapse rates following chemotherapy, resulting in markedly inferior overall survival (OS). Currently, no unified diagnostic and prognostic criteria exist for pediatric AML-MR/myelodysplasia-related changes (AML-MRC), and the definitions of AML-MR differ among the WHO-HAEM4, WHO-HAEM5, and ICC classifications.

### Aims

Therefore, this study aimed to analyze a pediatric AML cohort to elucidate the clinical characteristics of AML-MR/MRC under different classification systems and determine which classification is most appropriate for sporadic pediatric AML with myelodysplasia-related features.

### Methods

We conducted a retrospective analysis of pediatric patients with newly diagnosed AML at the Institute of Hematology & Blood Diseases Hospital, Chinese Academy of Medical Sciences, between January 2009 and December 2023. Patients were stratified into AML-MRC/AML-MR and AML-not otherwise specified (AML-NOS) groups according to the WHO-HAEM4, WHO-HAEM5, and ICC classification criteria. Clinical and laboratory characteristics were compared between groups. Kaplan-Meier survival analysis was performed to evaluate outcomes under hematopoietic stem cell transplantation (HSCT) and conventional chemotherapy regimens.

**Copyright Information:** (Online) ISSN: 2572-9241
©2026 The Author(s). HemaSphere is published by John Wiley & Sons Ltd on behalf of European Hematology Association. This is an open access Abstract Book distributed under the Attribution-NonCommercial-NoDerivs (CC BY-NC-ND), which allows third parties to download the articles and share them with others as long as they credit the author and the Abstract Book, but they cannot change the content in any way or use them commercially.
**Abstract Book Citations:** Authors, Title, HemaSphere, 2026;10:(S1):pages. The abstract book DOIs can be found on the “Author Information” page.
**Disclaimer:** Articles published in the journal HemaSphere exclusively reflect the opinions of the authors. The authors are responsible for all content in their abstracts including accuracy of the facts, statements, citing resources, etc.

**Results**

A total of 791 patients with newly diagnosed AML were screened, and 166 eligible pediatric AML patients were enrolled. Of these, 106 patients met the WHO-HAEM4 criteria for AML-MRC, 110 met the WHO-HAEM5 criteria for AML-MR, and 109 met the ICC criteria for AML-MR. Additionally, 11 patients were classified as TP53-mutated AML under the ICC criteria. Regardless of the classification system employed, clinical and laboratory characteristics were generally similar; however, compared with AML-NOS, the AML-MRC/AML-MR groups demonstrated higher frequencies of complex karyotypes, monosomy 17, and TP53 mutations. TP53 mutations were associated with distinct clinicopathological features and dismal outcomes (HR, 2.218; 95% CI, 1.143-4.305; P=0.0162). Among intensively treated patients, ICC-AML-MR exhibited marginally better outcomes than WHO-AML-MR, likely attributable to the separate categorization of TP53-mutated AML as a distinct entity in the ICC classification. Survival analyses revealed that, under the WHO2016 criteria, AML with mutated RUNX1 demonstrated the most favorable prognosis, whereas AML-MR showed the poorest outcomes. At 1-year follow-up, the event-free survival (EFS) rate was merely 20.0% in the TP53-AML group compared with 49.3% in the AML-MR group. Overall, patients with TP53-AML had a significantly shorter median EFS (7.17 months) than those with AML-MR (11.73 months).

**Summary/Conclusion**

Pediatric AML-MR/MRC exhibits distinct clinical phenotypes and universally poor prognosis. Compared with the WHO2016 and WHO2022 classifications, the ICC system enables superior prognostic stratification of pediatric AML-MR patients, facilitating early identification and risk-adapted intervention in clinical practice.

**Copyright Information:** (Online) ISSN: 2572-9241
©2026 The Author(s). HemaSphere is published by John Wiley & Sons Ltd on behalf of European Hematology Association. This is an open access Abstract Book distributed under the Attribution-NonCommercial-NoDerivs (CC BY-NC-ND), which allows third parties to download the articles and share them with others as long as they credit the author and the Abstract Book, but they cannot change the content in any way or use them commercially.
**Abstract Book Citations:** Authors, Title, HemaSphere, 2026;10:(S1):pages. The abstract book DOIs can be found on the “Author Information” page.
**Disclaimer:** Articles published in the journal HemaSphere exclusively reflect the opinions of the authors. The authors are responsible for all content in their abstracts including accuracy of the facts, statements, citing resources, etc.

### (PB2759) THE EFFECT OF TREATMENT INTENSITY AND THERAPY-RELATED STATUS ON SURVIVAL IN KMT2A-REARRANGED AML

**Topic:** 04. Acute myeloid leukemia - Clinical

Jack Shapiro*[1], Madina Sukhanova[2], Yasmin Abaza[2], Shira Dinner[2], Irum Khan[2], Himachandana Atluri[2], Jessica K. Altman[2], Yuchen Liu[2]

*[1]Northwestern Feinberg School of Medicine, Medicine, Chicago, United States of America;[2]Robert H. Lurie Comprehensive Cancer Center, Northwestern University, Chicago, United States of America;*

### Background

Acute myeloid leukemia (AML) with KMT2A rearrangements represents a high-risk subtype with poor outcomes. This subtype of AML can be associated with either de novo disease or therapy-related. KMT2A fusions are classified based on ELN 2022 with t(9;11) considered intermediate risk and the remainder adverse risk.

### Aims

We aim to characterize real-world outcomes of KMT2A-rearranged AML stratified by KMT2A fusion partner, therapy-related disease, and treatment regimen intensity.

### Methods

We performed a retrospective single-center cohort study of patients with KMT2A rearranged AML identified from December 2008 to October 2025 based on in house cytogenetic testing. Overall survival (OS) was conducted using Kaplan Meier curves with hazard ratios estimated based on Cox Proportional Hazard models.

### Results

63 patients were included. 28 had t(9;11) fusion and 21 were therapy-related AML. A higher number of patients with t(9;11) fusion was seen in therapy related disease compared to de novo disease (62% vs 36%). 49 (78%) received intensive therapy and 13 (21%) lower-intensity therapy, including 9 (14%) treated with venetoclax/hypomethylating agent. 1 patient received no anti-leukemia therapy. 44 in total (70%) underwent hematopoietic stem cell transplant (HSCT).

Median overall survival (mOS) for the entire cohort was 15.1 months (95% CI 12.6-35.1), with 1-year OS of 62% (95% CI 51-75%). There was no difference in mOS between patients with t(9;11) and t(v;11) despite higher HSCT utilization in variant cases (82% vs 57%, OR 3.43, 95% CI 0.97–13.47, p=0.048). Therapy-related disease was associated with inferior OS compared to de novo AML (mOS 9.0 months vs 17.6 months, HR 1.96, 95% CI 1.06-3.64, p=0.033). This difference was not explained by age, complete response (CR) rate, relapse-free survival (RFS), or HSCT. Early before 142 days (median time to HSCT) was markedly higher in therapy-related AML (33% vs 2%; OR 19.4, 95% CI 2.2–940, p=0.0013), with a majority of these deaths (5/7) due to progressive disease.

Lower-intensity induction therapy was associated with inferior OS compared to intensive therapy (mOS 5.9 months vs 23.2 months, HR 5.63, 95% CI 2.72-11.66, p<0.001). A significant interaction between therapy-related status and treatment intensity was observed for overall survival (p=0.006). In the lower-intensity cohort, therapy-related AML was associated with worse OS (HR 9.47, 95% CI 1.14–78.9 p=0.038), whereas no significant differences in CR, RFS, or OS were observed among intensively treated patients.

**Table 1.** Baseline characteristics of patients with de novo and therapy-related AML.FLT3 denominators reflect comprehensive NGS and PCR testing. KRAS and TP53 denominators reflect comprehensive NGS.

**Copyright Information:** (Online) ISSN: 2572-9241
©2026 The Author(s). HemaSphere is published by John Wiley & Sons Ltd on behalf of European Hematology Association. This is an open access Abstract Book distributed under the Attribution-NonCommercial-NoDerivs (CC BY-NC-ND), which allows third parties to download the articles and share them with others as long as they credit the author and the Abstract Book, but they cannot change the content in any way or use them commercially.
**Abstract Book Citations:** Authors, Title, HemaSphere, 2026;10:(S1):pages. The abstract book DOIs can be found on the "Author Information" page.
**Disclaimer:** Articles published in the journal HemaSphere exclusively reflect the opinions of the authors. The authors are responsible for all content in their abstracts including accuracy of the facts, statements, citing resources, etc.

| | De Novo (n = 42) | Therapy-related (n = 21) |
|---|---|---|
| Median Age (range) | 42 (18 - 79) | 62 (32 - 82) |
| Treatment intensity (%) | | |
| Intensive | 37/42 (88) | 12/21 (57) |
| Lower intensity | 4/42 (10) | 9/21 (43) |
| Fusion partner (%) | | |
| t(9;11) | 15/42 (36) | 13/21 (62) |
| t(v;11) | 27/42 (64) | 8/21 (38) |
| FLT3-ITD (%) | 2/29 (7) | 1/18 (8) |
| FLT3-TKD (%) | 2/29 (7) | 2/18 (11) |
| TP53 (%) | 1/24 (4) | 1/17 (6) |
| KRAS mutation (%) | 7/24 (29) | 4/17 (24) |
| Complex karyotype | 14/42 (33) | 8/21 (38) |

**Summary/Conclusion**

In this real-world cohort of patients with KMT2A rearranged AML, survival did not differ by fusion partner. Therapy-related disease was associated with inferior survival, which appears to be driven by early mortality and confined to patients receiving lower-intensity therapy, while outcomes remain similar for patients treated intensively.

**Copyright Information:** (Online) ISSN: 2572-9241
©2026 The Author(s). HemaSphere is published by John Wiley & Sons Ltd on behalf of European Hematology Association. This is an open access Abstract Book distributed under the Attribution-NonCommercial-NoDerivs (CC BY-NC-ND), which allows third parties to download the articles and share them with others as long as they credit the author and the Abstract Book, but they cannot change the content in any way or use them commercially.
**Abstract Book Citations:** Authors, Title, HemaSphere, 2026;10:(S1):pages. The abstract book DOIs can be found on the "Author Information" page.
**Disclaimer:** Articles published in the journal HemaSphere exclusively reflect the opinions of the authors. The authors are responsible for all content in their abstracts including accuracy of the facts, statements, citing resources, etc.

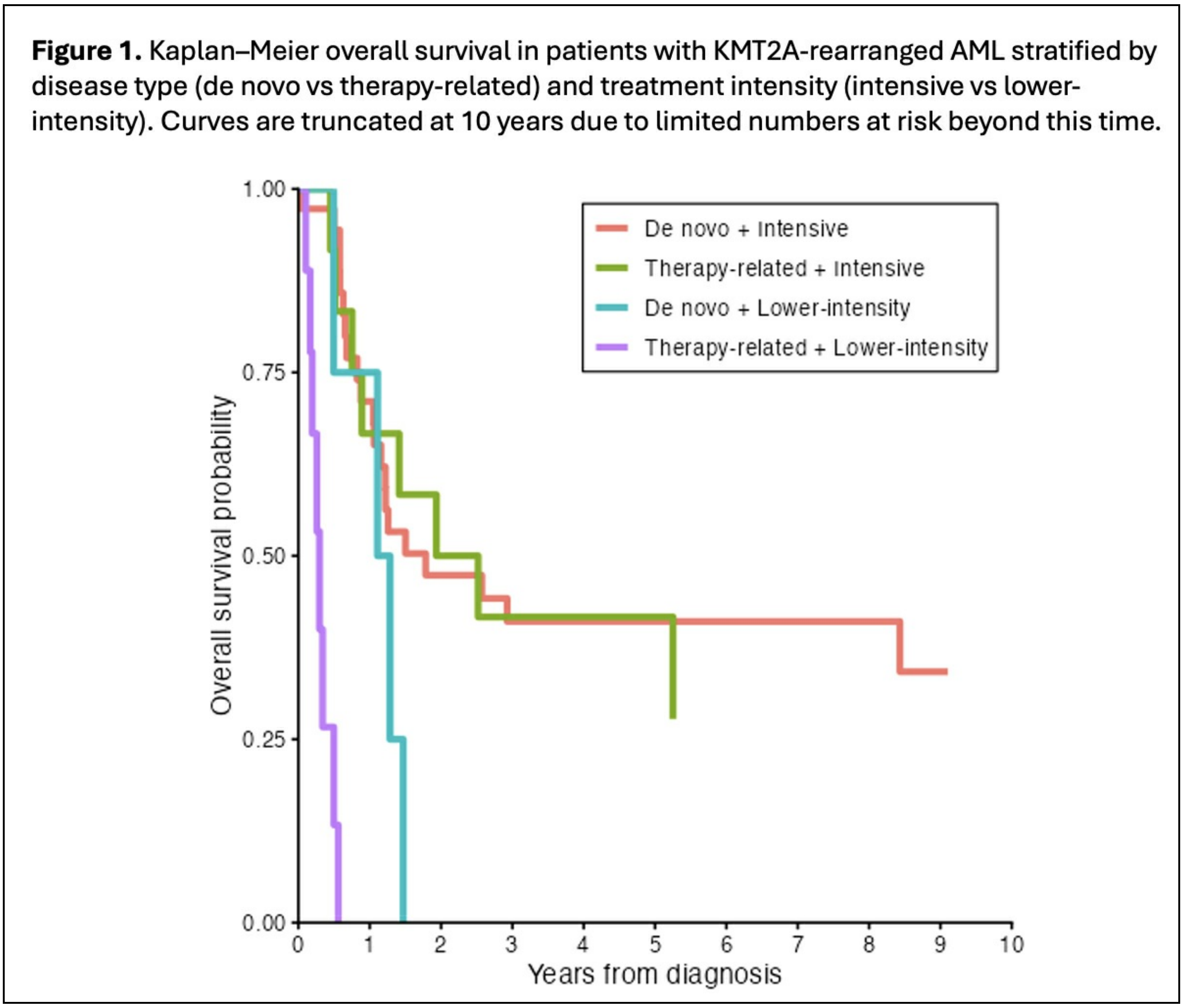

Figure 1. Kaplan–Meier overall survival in patients with KMT2A-rearranged AML stratified by disease type (de novo vs therapy-related) and treatment intensity (intensive vs lower-intensity). Curves are truncated at 10 years due to limited numbers at risk beyond this time.

**Copyright Information:** (Online) ISSN: 2572-9241
©2026 The Author(s). HemaSphere is published by John Wiley & Sons Ltd on behalf of European Hematology Association. This is an open access Abstract Book distributed under the Attribution-NonCommercial-NoDerivs (CC BY-NC-ND), which allows third parties to download the articles and share them with others as long as they credit the author and the Abstract Book, but they cannot change the content in any way or use them commercially.
**Abstract Book Citations:** Authors, Title, HemaSphere, 2026;10:(S1):pages. The abstract book DOIs can be found on the "Author Information" page.
**Disclaimer:** Articles published in the journal HemaSphere exclusively reflect the opinions of the authors. The authors are responsible for all content in their abstracts including accuracy of the facts, statements, citing resources, etc.

### (PB2760) CPX-351 IS ASSOCIATED WITH LOWER SEVERITY OF CUTANEOUS ADVERSE EVENTS DESPITE HIGHER REPORTING PROPORTIONS COMPARED WITH CYTARABINE OR DAUNORUBICIN: A DISPROPORTIONALITY ANALYSIS OF EUDRAVIGILANCE

**Topic:** 04. Acute myeloid leukemia - Clinical

Calogero Vetro*[1], Arianna Scala[2], Teresa Angela Trunfio[2], Fanny Erika Palumbo[3], Giovanni Enrico Lombardo[4], Cecilia Gozzo[5], Giovanni Improta[2], Mario Damiano Toro[2], Giovanni Luca Romano[4], Paola De Luca[6], Sara Marino[6], Salvatore Leotta[6], Andrea Duminuco[6], Anna Elisa Verzì[7], Lucia Gozzo[8]

*[1]Hematology and Bone Marrow Transplantation Unit, Hospital of Bolzano (SABES-Azienda Sanitaria dell'Alto Adige), Teaching Hospital of Paracelsus Medical University,, Bolzano, Italy; [2]Department of Public Health, University of Naples Federico II, Napoli, Italy; [3]Hematology Department, Ospedale di Trento, Trento, Italy; [4]Department of Medicine and Surgery, University of Enna "Kore", Enna, Italy; [5]Radiology Unit, Humanitas Istituto Clinico Catanese, Catania, Italy; [6]Hematology with BMT Unit, A.O.U. Policlinico "G. Rodolico-S. Marco", Catania, Italy; [7]Dermatology Clinic, University of Catania, Catania, Italy; [8]Clinical Trial Unit, Azienda Ospedaliero Universitaria Policlinico "G. Rodolico–S. Marco",, Catania, Italy;*

### Background

Cutaneous adverse events are a relevant complication of intensive AML chemotherapy. While cytarabine (CYT) and daunorubicin (DAUNO) are associated with inflammatory eruptions and tissue necrosis, the cutaneous safety profile of CPX-351 remains incompletely characterized.

### Aims

We assessed cutaneous toxicity patterns with CPX-351 compared with CYT and DAUNO.

### Methods

Individual Case Safety Reports (ICSRs) listing CPX-351, CYT, or DAUNO as suspected drugs were retrieved from EudraVigilance up to July 2025. Reports including skin and subcutaneous tissue disorders were analyzed descriptively and by disproportionality analysis, calculating reporting odds ratios (ROR) with 95% confidence intervals (95% CI). Analyses were conducted irrespective of indication, as reactions were not stratified by underlying disease.

**Copyright Information:** (Online) ISSN: 2572-9241
©2026 The Author(s). HemaSphere is published by John Wiley & Sons Ltd on behalf of European Hematology Association. This is an open access Abstract Book distributed under the Attribution-NonCommercial-NoDerivs (CC BY-NC-ND), which allows third parties to download the articles and share them with others as long as they credit the author and the Abstract Book, but they cannot change the content in any way or use them commercially.
**Abstract Book Citations:** Authors, Title, HemaSphere, 2026;10:(S1):pages. The abstract book DOIs can be found on the "Author Information" page.
**Disclaimer:** Articles published in the journal HemaSphere exclusively reflect the opinions of the authors. The authors are responsible for all content in their abstracts including accuracy of the facts, statements, citing resources, etc.

**Results**

Overall, 38,549 ICSRs were identified (32,842 CYT; 4,672 DAUNO; 1,035 CPX-351). Of these, 2,785 (7.2%) reported skin disorders: 2,268 CYT (81.4%), 327 DAUNO (11.7%), and 190 CPX-351 (6.8%).
With CPX-351 (n=190), the most frequent events were rash (n=95, 44.2%), maculopapular rash (n=18, 8.4%), erythema (n=15, 7%), drug eruption (n=14, 6.5%), toxic skin eruption (n=10, 4.7%), alopecia and purpura (n=6 each, 2.8%), and pruritus and urticaria (n=5 each, 2.3%).
With CYT (n=2,268), predominant reactions were rash (n=704, 21%), pruritus (n=438, 13.1%), erythema (n=200, 6%), erythematous rash (n=190, 5.7%), maculopapular rash (n=142, 4.2%), and palmar–plantar erythrodysesthesia (n=91, 2.7%). Severe reactions included toxic epidermal necrolysis (n=39, 1.2%) and Stevens–Johnson syndrome (n=19, 0.6%).
With DAUNO (n=327), the most frequent events were rash (n=79, 16.6%), erythema (n=31, 6.5%), maculopapular rash (n=25, 5.2%), hidradenitis (n=23, 4.8%), pruritus and skin lesion (n=21 each, 4.4%), and palmar–plantar erythrodysesthesia (n=11, 2.3%), toxic epidermal necrolysis (n=5, 1%), Stevens–Johnson syndrome (n=4, 0.8%), skin necrosis (n=5, 1%), and skin ulceration (n=7, 1.5%).
Overall reporting of skin disorders was higher for CPX-351 (ROR 2.96, 95% CI 2.92–3.00). CPX-351 showed disproportionate reporting for rash (3.08, 2.96–3.20), maculopapular rash (2.00, 1.76–2.28), drug eruption (3.39, 2.84–4.04), toxic skin eruption (2.28, 1.81–2.88), purpura (2.59, 1.76–3.79), urticaria (2.00, 1.28–3.12), alopecia (2.08, 1.43–3.03), and skin necrosis (2.56, 1.20–5.46). CYT was associated with pruritus (3.86, 3.55–4.19), erythematous rash (3.73, 3.07–4.51), palmar–plantar erythrodysesthesia (1.73, 1.41–2.12), and toxic epidermal necrolysis (1.62, 1.04–2.53). DAUNO showed disproportionate reporting for acute febrile neutrophilic dermatosis (3.37, 2.78–4.09), hidradenitis (2.86, 2.53–3.23), skin ulcer (2.78, 1.89–4.09), skin necrosis (1.98, 1.20–3.26), psoriasis (6.02, 2.48–14.61), and transient acantholytic dermatosis (6.47, 3.51–11.93).
Serious cutaneous reactions were reported in 90% of CYT/DAUNO ICSRs versus 73.2% with CPX-351 (p=0.001). Recovery was more frequent with CPX-351 (n=83, 42.6%; p=0.0017), while fatal outcomes were more common with CYT (n=57, 2.4%; p=0.07).

**Summary/Conclusion**

CPX-351 displayed fewer serious reports and higher recovery rates compared with CYT and DAUNO, supporting a comparatively more favorable severity pattern in real-world practice. Underreporting inherent to spontaneous pharmacovigilance systems should also be considered when interpreting these results.

**Copyright Information:** (Online) ISSN: 2572-9241
©2026 The Author(s). HemaSphere is published by John Wiley & Sons Ltd on behalf of European Hematology Association. This is an open access Abstract Book distributed under the Attribution-NonCommercial-NoDerivs (CC BY-NC-ND), which allows third parties to download the articles and share them with others as long as they credit the author and the Abstract Book, but they cannot change the content in any way or use them commercially.
**Abstract Book Citations:** Authors, Title, HemaSphere, 2026;10:(S1):pages. The abstract book DOIs can be found on the "Author Information" page.
**Disclaimer:** Articles published in the journal HemaSphere exclusively reflect the opinions of the authors. The authors are responsible for all content in their abstracts including accuracy of the facts, statements, citing resources, etc.

### (PB2761) NEXT GENERATION SEQUENCING BASED UBTF TANDEM DUPLICATION MEASURABLE RESIDUAL DISEASE (UBTF-MRD) IN PATIENTS WITH MDS AND AML

**Topic:** 04. Acute myeloid leukemia - Clinical

Tamia Nguyen*[1, 2, 3], William Nicol[4, 5], Noel Djitro[1, 2, 3], Lachlin Vaughan[6, 7, 8], Seong L. Khaw[9], Courtney Tate[10], Jad Othman[11], Sushmitha Kannan[1, 3], Annabelle Yap[1, 3], Sally Hunter[1, 3], Andrew H. Wei[4, 5, 12, 13], Piers Blombery[1, 2, 3], Ing Soo Tiong[1, 2, 3]

*[1]Peter MacCallum Cancer Centre, Department of Pathology, Melbourne, Australia;[2]University of Melbourne, Sir Peter MacCallum Department of Oncology, Melbourne, Australia; [3]Collaborative Centre for Genomic Cancer Medicine, Melbourne, Australia; [4]Peter MacCallum Cancer Centre, Department of Haematology, Melbourne, Australia;[5]Royal Melbourne Hospital, Department of Haematology, Melbourne, Australia; [6]Westmead Hospital, Crown Princess Mary Cancer Centre, Sydney, Australia;[7]University of New South Wales, School of Clinical Medicine, Sydney, Australia; [8]Westmead Hospital, ICPMR, Department of Haematology, Sydney, Australia; [9]Royal Children's Hospital, Melbourne, Australia; [10]Pathology Queensland, Brisbane, Australia; [11]Royal North Shore Hospital, Department of Haematology, Sydney, Australia; [12]The Walter and Eliza Hall Institute of Medical Research, Melbourne, Australia; [13]University of Melbourne, Department of Medical Biology, Melbourne, Australia;*

### Background

*UBTF* tandem duplications (*UBTF*-TD) define a molecular-distinct, adverse-risk subset of myelodysplastic syndrome/acute myeloid leukaemia (MDS/AML) occurring predominantly in children and young adults. The clinical utility of molecular measurable residual disease (MRD) assessment of *UBTF*-TD is currently unknown.

### Aims

To develop a next generation sequencing (NGS) based *UBTF*-TD MRD assay, and assess its clinical utility in a retrospective cohort of patients with *UBTF*-TD MDS/AML.

### Methods

A DNA-based NGS amplicon assay targeting *UBTF* exon 13 (NM_014233.3) was designed to capture duplication breakpoints and non-template insertions, enabling patient-specific tracking. Analytical sensitivity was assessed using 48, 72, and 310 bp *UBTF*-TD across a range of variant allele frequency (VAF).

Patients (pts) with *UBTF*-TD MDS/AML and available stored bone marrow aspirate (BMA) DNA were identified from institutional databases. Remission samples underwent *UBTF*-MRD testing, diagnostic and relapse samples underwent targeted NGS panel for co-mutation profiling. Paired multiparameter flow cytometry (MFC) MRD ($10^{-3}$-$10^{-5}$) and *FLT3*-ITD MRD ($10^{-5}$ VAF) were analysed where available. Molecular lead time was defined from first timepoint demonstrating a rise in *UBTF*-TD to morphologic relapse.

**Copyright Information:** (Online) ISSN: 2572-9241
©2026 The Author(s). HemaSphere is published by John Wiley & Sons Ltd on behalf of European Hematology Association. This is an open access Abstract Book distributed under the Attribution-NonCommercial-NoDerivs (CC BY-NC-ND), which allows third parties to download the articles and share them with others as long as they credit the author and the Abstract Book, but they cannot change the content in any way or use them commercially.
**Abstract Book Citations:** Authors, Title, HemaSphere, 2026;10:(S1):pages. The abstract book DOIs can be found on the "Author Information" page.
**Disclaimer:** Articles published in the journal HemaSphere exclusively reflect the opinions of the authors. The authors are responsible for all content in their abstracts including accuracy of the facts, statements, citing resources, etc.

**Results**

Eleven pts were identified (median age 25, range 6-72; 9 AML, 2 MDS-EB2; 48-197bp UBTF-TD). Ten underwent allogeneic stem cell transplant (alloSCT): four relapsed at 6-22mo, two developed MFC-MRD relapse at 3 and 8mo, and four remained in remission with 3.5-7y follow-up.

A *UBTF*-MRD assay was developed with an analytical sensitivity of $10^{-6}$ with high linearity ($R^2$=0.99) at mean sequencing depth 1.2M reads. Across 54 morphologic-remission BMA post therapy, *UBTF*-MRD was positive in 40 (74%; VAF 0.0003-45%). Discordant MFC-MRD-negative/*UBTF*-MRD-positive results occurred in 13/38 (34%) paired samples, including two with >30% *UBTF*-TD VAF, consistent with persistence of a dominant preleukemic clone. No MFC-MRD-positive/ *UBTF*-MRD-negative results were observed. Of five samples with paired *FLT3*-ITD MRD, four were *FLT3*-MRD-negative/*UBTF*-MRD-positive.

Given the presence of *UBTF*-TD in the preleukemic compartment limiting potential use as an MRD biomarker, we went on to evaluate the use of *UBTF*-MRD after alloSCT, where eradication of the preleukemic compartment is expected. Of seven pts evaluated post-alloSCT, three had detectable *UBTF*-MRD at D+30 (0.0004-0.18% VAF). Rising *UBTF*-MRD post-alloSCT consistently preceded relapse (**Figure 1**). In three morphologic relapses with serial sampling, *UBTF*-MRD rose 3-7 months before relapse, at a rate of 0.51-0.94 log10 rise/month, and preceded MFC positivity in one pt with paired assessment. Two additional pts showed rising *UBTF*-MRD concurrent with MFC-MRD relapse, whereas pts with durable remission showed molecular clearance or sustained negativity.

One additional pt treated with non-intensive therapy (IDH2 inhibitor) demonstrated a decline in peripheral blood *UBTF*-MRD with therapy, before subsequent rise (0.002% to 0.01% VAF) five months before morphologic relapse.

**Summary/Conclusion**

We developed a highly sensitive NGS-MRD assay for *UBTF*-TD and evaluated its utility as an MRD biomarker in MDS/AML. While *UBTF*-TD clonal haematopoiesis may confound interpretation in some remission contexts, serial monitoring of *UBTF*-MRD post-alloSCT enabled early detection of impending relapse. These data support further evaluation of *UBTF*-MRD to determine the prognostic and predictive value in larger cohorts.

**Copyright Information:** (Online) ISSN: 2572-9241
©2026 The Author(s). HemaSphere is published by John Wiley & Sons Ltd on behalf of European Hematology Association. This is an open access Abstract Book distributed under the Attribution-NonCommercial-NoDerivs (CC BY-NC-ND), which allows third parties to download the articles and share them with others as long as they credit the author and the Abstract Book, but they cannot change the content in any way or use them commercially.
**Abstract Book Citations:** Authors, Title, HemaSphere, 2026;10:(S1):pages. The abstract book DOIs can be found on the "Author Information" page.
**Disclaimer:** Articles published in the journal HemaSphere exclusively reflect the opinions of the authors. The authors are responsible for all content in their abstracts including accuracy of the facts, statements, citing resources, etc.

**Figure 1. Serial UBTF-TD MRD kinetics after allo-SCT**

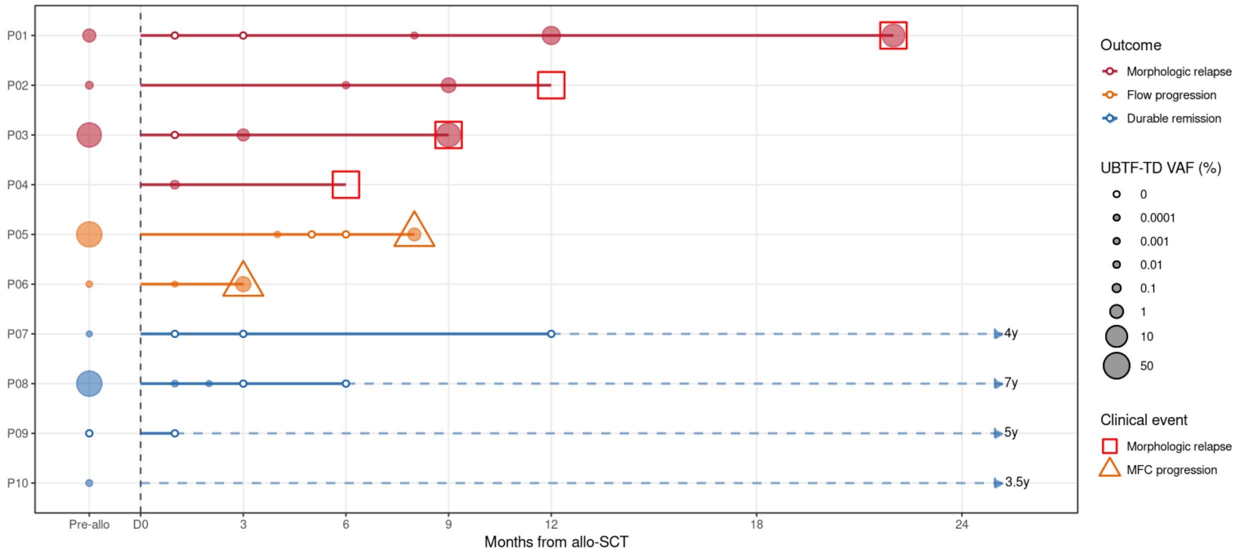

**Copyright Information:** (Online) ISSN: 2572-9241
©2026 The Author(s). HemaSphere is published by John Wiley & Sons Ltd on behalf of European Hematology Association. This is an open access Abstract Book distributed under the Attribution-NonCommercial-NoDerivs (CC BY-NC-ND), which allows third parties to download the articles and share them with others as long as they credit the author and the Abstract Book, but they cannot change the content in any way or use them commercially.
**Abstract Book Citations:** Authors, Title, HemaSphere, 2026;10:(S1):pages. The abstract book DOIs can be found on the “Author Information” page.
**Disclaimer:** Articles published in the journal HemaSphere exclusively reflect the opinions of the authors. The authors are responsible for all content in their abstracts including accuracy of the facts, statements, citing resources, etc.

### (PB2762) SAFETY OF EARLY G-CSF ADMINISTRATION DURING FIRST-CYCLE VENETOCLAX + AZACITIDINE IN AML: NO IMPACT ON SURVIVAL IN A REAL-WORLD EVIDENCE

**Topic:** 04. Acute myeloid leukemia - Clinical

Lubomir Minarik*[1, 2], Slavka Sotakova[1], Jana Karolová[1], Tomas Stopka[1], Zuzana Zemanova[3], Anna Jonášová[1]

*[1]First Department of Internal Medicine – Hematology, General University Hospital and First Faculty of Medicine, Charles University, Prague, Czechia; [2]BIOCEV - 1st Faculty of Medicine, Charles University, Prague, Czechia;[3]Center of Oncocytogenomics, General University Hospital and First Faculty of Medicine, Charles University, Prague, Czechia;*

### Background

Venetoclax plus azacitidine (Ven + Aza) is the standard first-line therapy for older or unfit patients with acute myeloid leukemia (AML). This regimen frequently induces profound and prolonged neutropenia due to rapid leukemic clearance and myelosuppression. The use of granulocyte colony-stimulating factor (G-CSF) during early treatment remains controversial because of concerns regarding potential stimulation of leukemic progenitors. However, infectious complications represent a major cause of early mortality in this population. Long-term follow-up of VIALE-A shows superior overall survival (OS) in the subgroup of patients exposed to G-CSF after achieving complete remission (CR). The safety of G-CSF administration during the first cycle of Ven + Aza remains unclear.

### Aims

To evaluate safety and survival impact of G-CSF administration during the first cycle of Ven + Aza in AML patients.

### Methods

We performed a retrospective analysis of 125 AML patients treated with Ven + Aza. Overall survival (OS) was defined from treatment initiation to death from any cause and estimated using the Kaplan–Meier method. Cox proportional hazards regression included baseline absolute neutrophile count (ANC), ECOG performance status (≥2 vs <2), age, cumulative Ven dose and G-CSF exposure.

### Results

Based on retrospective data of 125 patients treated with Ven + Aza regimen with median follow-up of 41.7 months, 92 (73.6 %) patients received G-CSF at the median dose of 240 IU within the first cycle of therapy due to grade 4 neutropenia with or without presence of infection. Median of ANC level on D1C1 of Ven + Aza was significantly lower in the group of patients exposed to G-CSF compared to the patients without any G-CSF (1.2 vs. 2.02x10e9/l; p=0.0048). Both groups had similar age (74.5 vs 75.8 years) and the same ECOG (median = 1). Patients that were not exposed to G-CSF showed elevated level of circulating myeloblasts in peripheral blood (17.1 vs 5.5 %; p=0.0027) before administration of Ven + Aza. Median overall survival was 9.6 months in the G-CSF group and 12.1 months in the non–G-CSF group. G-CSF exposure was not associated with overall survival in Cox regression analysis (HR 1.33, 95% CI 0.79–2.25; p=0.28) (Kaplan-Meier curve shown in Figure). G-CSF exposure was not associated with inferior overall composite rates in univariable analysis (56.5% vs 57.6%; p=1.00). Rates of infections were similar between groups (35% vs. 19%; p=0.122). Thirty-day mortality rate was 6.4%, without significant difference between G-CSF–exposed and non-exposed patients.

**Copyright Information:** (Online) ISSN: 2572-9241
©2026 The Author(s). HemaSphere is published by John Wiley & Sons Ltd on behalf of European Hematology Association. This is an open access Abstract Book distributed under the Attribution-NonCommercial-NoDerivs (CC BY-NC-ND), which allows third parties to download the articles and share them with others as long as they credit the author and the Abstract Book, but they cannot change the content in any way or use them commercially.
**Abstract Book Citations:** Authors, Title, HemaSphere, 2026;10:(S1):pages. The abstract book DOIs can be found on the "Author Information" page.
**Disclaimer:** Articles published in the journal HemaSphere exclusively reflect the opinions of the authors. The authors are responsible for all content in their abstracts including accuracy of the facts, statements, citing resources, etc.

### Summary/Conclusion

Our data supports that the early use of G-CSF within the first cycle of Ven + Aza therapy was not associated with inferior survival or response rates in AML patients, despite its use in individuals with more profound neutropenia. Importantly, even though G-CSF was predominantly used in patients with more profound neutropenia, 30-day mortality remained comparable between exposed and non-exposed groups, underscoring the clinical safety of G-CSF in this high-risk population.

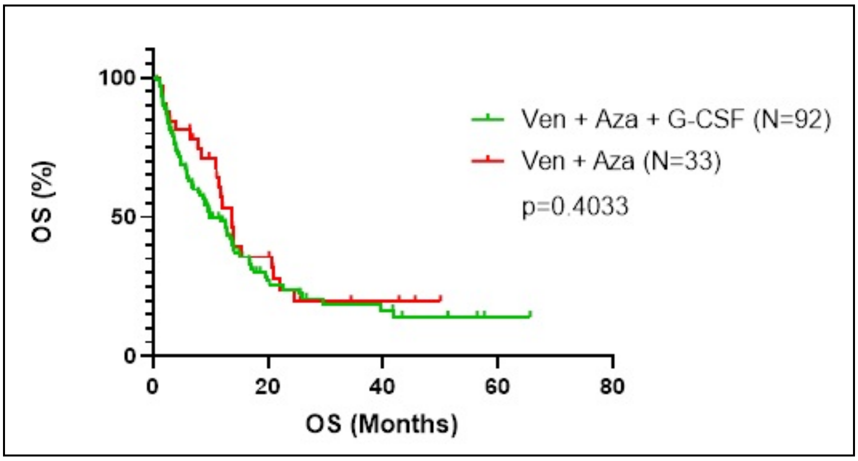

Copyright Information: (Online) ISSN: 2572-9241
©2026 The Author(s). HemaSphere is published by John Wiley & Sons Ltd on behalf of European Hematology Association. This is an open access Abstract Book distributed under the Attribution-NonCommercial-NoDerivs (CC BY-NC-ND), which allows third parties to download the articles and share them with others as long as they credit the author and the Abstract Book, but they cannot change the content in any way or use them commercially.
**Abstract Book Citations:** Authors, Title, HemaSphere, 2026;10:(S1):pages. The abstract book DOIs can be found on the “Author Information” page.
**Disclaimer:** Articles published in the journal HemaSphere exclusively reflect the opinions of the authors. The authors are responsible for all content in their abstracts including accuracy of the facts, statements, citing resources, etc.

### (PB2763) AMONG S-AML PATIENTS RECEIVING CPX-351 WITHOUT HSCT CONSOLIDATION, IDH2 BUT NOT IDH1 MUTATIONS ARE RELATED WITH A LONGER SURVIVAL

**Topic:** 04. Acute myeloid leukemia - Clinical

Carola Riva*[1], Paola Minetto.[1], Alessandro Cacioppo[2], Stefano Nicoletta[2], Clara Nurra[2], Enrico Carminati[1], Nicoletta Colombo[1], Sara Rosellini[1], Elisabetta Tedone[1], Paolo Nozza[1], Michele Cea[1, 2], Roberto M. Lemoli[1, 2], Fabio Guolo[1, 2]

[1]*IRCCS Azienda Ospedaliera Metropolitana, Genoa, Italy;* [2]*University of Genoa, Genoa, Italy;*

### Background

Secondary Acute Myeloid Leukemia (s-AML) is characterized by a dismal outcome, mainly due to older age at diagnosis and high frequency of high risk mutations. In this setting, the best option in order to achieve a cure is to perform an allogeneic stem cell transplantation (HSCT) after a complete remission (CR) is achieved. Recently, CPX-351 showed better activity among s-AML patients if compared to conventional chemotherapy, both in patients receiving or not HSCT consolidation. However, as conventional AML risk score such as ELN 2022 are inadequate as most s-AML patients fall in the adverse risk category, there are no reliable tools to predict outcome in s-AML patients receiving CPX-351.
Among elderly patients receiving less intensive treatment based on hypomethylating agents and venetoclax (HMA-VEN), newer risk score have been developed and in particular the presence of IDH mutations is generally related to a better outcome.

### Aims

The aim of this study was to evaluate if IDH mutations could predict outcome also among s-AML patients receiving CPX-351 induction with or without HSCT consolidation

### Methods

A total of 96 s-AML patients (median age 69, range 38-77) received CPX-351 in our center.
NGS was performed on bone marrow with the Myeloid Solution panel by SOPHiA Genetics, targeting 34 critical genes, using Illumina MiSeq platform and SOPHiA DDM® Software.
Minimal residual disease (MRD) was performed with multicolour flow cytometry (MFC) in all patients.

**Copyright Information:** (Online) ISSN: 2572-9241
©2026 The Author(s). HemaSphere is published by John Wiley & Sons Ltd on behalf of European Hematology Association. This is an open access Abstract Book distributed under the Attribution-NonCommercial-NoDerivs (CC BY-NC-ND), which allows third parties to download the articles and share them with others as long as they credit the author and the Abstract Book, but they cannot change the content in any way or use them commercially.
**Abstract Book Citations:** Authors, Title, HemaSphere, 2026;10:(S1):pages. The abstract book DOIs can be found on the “Author Information” page.
**Disclaimer:** Articles published in the journal HemaSphere exclusively reflect the opinions of the authors. The authors are responsible for all content in their abstracts including accuracy of the facts, statements, citing resources, etc.

**Results**

As expected, according to ELN 2022 risk score, the majority of patients fell in the adverse risk group (79/96, 82.3%), whereas 10 (10.4%) and 7 (7.3%) were assigned to the favourable and intermediate risk group, respectively. IDH1 mutations were found in 9 patients (9.3%), whereas IDH2 mutations were demonstrated in 13 patients (13.5%).
After cycle 1, 77/96 patients (80.2%) achieved CR. Among them, MFC MRD was negative in 44 (57%). The presence of IDH1 or 2 mutations did not impact CR rate or negative MRD probability.
Median follow-up was 43 months and the median overall survival (OS) was 14 months.
In the whole cohort, only achieving a MRD negative CR was related to a longer OS (median OS 29 and 10 months, for MRD negative and positive CR patients, respectively, p<0.001). IDH1 and 2 mutations were not related to survival.
Allogeneic hematopoietic stem cell transplantation (HSCT) was performed in 29/77 CR patients (37.7%) and was the strongest predictor of survival in a landmark model (p<0.05).
Among transplanted patient, none of the analysed variables impacted survival.
On the contrary, among non-transplanted patients, the presence of IDH2 mutations were related to a significantly longer survival (median OS 45 months and 15 months, for CR patients not receiving HSCT with or without IDH2 mutations, respectively, Fig.1). IDH1 mutations did not impact survival.

**Summary/Conclusion**

The best chance of cure for s-AML patients remains HSCT. However, due to age and comorbidity, some patients may not be eligible to this procedure. Among them, CPX-351 seems to be more effective if an IDH2 mutation is present. In this view, if confirmed, future trials exploring the combination of CPX-351 and IDH2 inhibitors may further improve the results. Moreover, the high sensitivity of IDH2 mutated AML to HMA-VEN combination allows non transplanted patient to receive an effective second line treatment, if needed.

**Copyright Information:** (Online) ISSN: 2572-9241
©2026 The Author(s). HemaSphere is published by John Wiley & Sons Ltd on behalf of European Hematology Association. This is an open access Abstract Book distributed under the Attribution-NonCommercial-NoDerivs (CC BY-NC-ND), which allows third parties to download the articles and share them with others as long as they credit the author and the Abstract Book, but they cannot change the content in any way or use them commercially.
**Abstract Book Citations:** Authors, Title, HemaSphere, 2026;10:(S1):pages. The abstract book DOIs can be found on the "Author Information" page.
**Disclaimer:** Articles published in the journal HemaSphere exclusively reflect the opinions of the authors. The authors are responsible for all content in their abstracts including accuracy of the facts, statements, citing resources, etc.

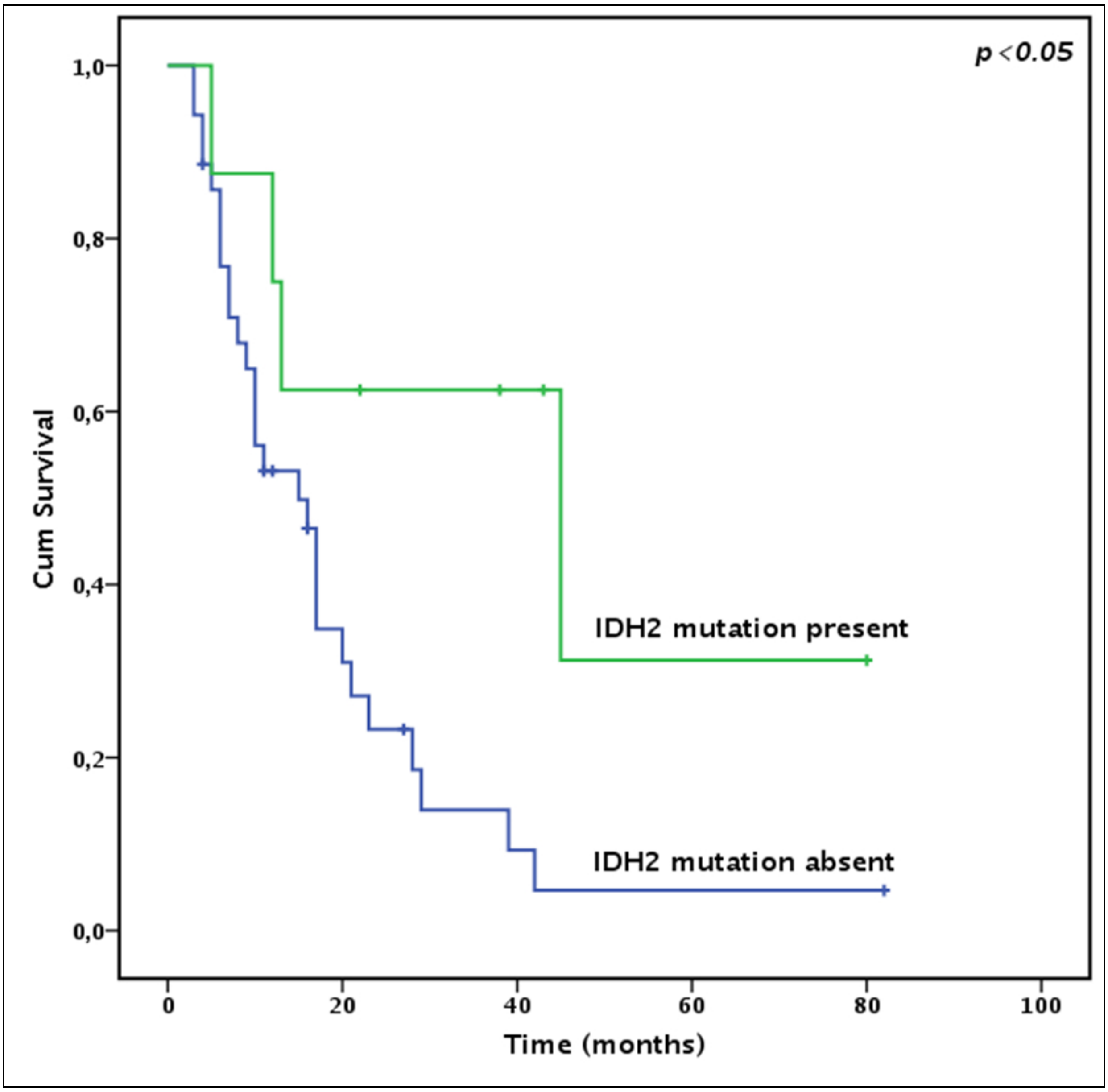

Figure 1: Overall Survival according to IDH2 mutational status in CR patients not receiving HSCT consolidation

**Copyright Information:** (Online) ISSN: 2572-9241
©2026 The Author(s). HemaSphere is published by John Wiley & Sons Ltd on behalf of European Hematology Association. This is an open access Abstract Book distributed under the Attribution-NonCommercial-NoDerivs (CC BY-NC-ND), which allows third parties to download the articles and share them with others as long as they credit the author and the Abstract Book, but they cannot change the content in any way or use them commercially.
**Abstract Book Citations:** Authors, Title, HemaSphere, 2026;10:(S1):pages. The abstract book DOIs can be found on the "Author Information" page.
**Disclaimer:** Articles published in the journal HemaSphere exclusively reflect the opinions of the authors. The authors are responsible for all content in their abstracts including accuracy of the facts, statements, citing resources, etc.

### (PB2764) SEX-STRATIFIED SINGLE-YEAR AML INCIDENCE: PERSISTENT MALE PREDOMINANCE AND FASTER ELDERLY RISE – SEER 2000–2022

**Topic:** 04. Acute myeloid leukemia - Clinical

Tapan Giri*[1], Zeal Soni[2]

*[1]University of Nevada, Reno school of medicine, Department of Internal medicine, Reno, United States of America;[2]Smt. NHL municipal medical college, Department of Internal medicine, Ahmedabad, India;*

### Background

Acute myeloid leukemia (AML) is a strongly age-associated malignancy with established sex disparities. The latest SEER data release (November 2024 submission, diagnoses 2000–2022) enables detailed single-year age-specific and sex-stratified incidence analysis to better characterize burden, patterns, and evolving trends.

### Aims

To compute sex-stratified, single-year age-specific AML incidence rates; quantify temporal trends; model exponential age-related increases (ages ≥40); and highlight sex differences in elderly peaks and overall predominance using the most recent SEER dataset.

### Methods

We analyzed SEER 17 Registries data (November 2024 submission, 2000–2022 diagnoses) to calculate age-adjusted incidence rates per 100,000 (2000 U.S. standard population, single ages to 89) for AML (AYA site recode 1.2). Analyses included overall and sex-stratified rates, annual temporal trends, and exponential modeling via log-linear regression of rate vs. age for ages ≥40.

### Results

Overall age-adjusted incidence was 4.3 per 100,000 (82,738 cases). Males had consistently higher rates (5.2, 45,200 cases) than females (3.6, 37,538 cases; male:female ratio ≈1.44, range 1.37–1.59 across years), with persistent predominance throughout. Rates were minimal in youth (mean 0.84 under 20 years, similar by sex) and diverged after age 40. For ages ≥40, incidence showed strong exponential growth ($R^2$ >0.98): doubling every ~9.4 years in males (slope 0.0740) vs. 11.9 years in females (slope 0.0583; both $p < 10^{-43}$). Elderly burden was markedly higher in males: mean 26.3 per 100,000 (ages 60–89) vs. 15.1 in females. Absolute peak at age 86 overall (30.6), but 41.8 in males vs. 24.0 in females. Temporal trends indicated modest increase (+0.0315/year, p<0.001) driven by both sexes, followed by plateau in the targeted therapy era (2018–2022 mean 4.44, stable by sex).

### Summary/Conclusion

AML incidence shows persistent male predominance that widens dramatically with age, driven by faster exponential acceleration and substantially higher peak rates in elderly males. These granular insights from the latest SEER data underscore the need for sex- and age-stratified strategies in AML prevention, clinical trial design, and management, particularly in the very elderly.

**Copyright Information:** (Online) ISSN: 2572-9241
©2026 The Author(s). HemaSphere is published by John Wiley & Sons Ltd on behalf of European Hematology Association. This is an open access Abstract Book distributed under the Attribution-NonCommercial-NoDerivs (CC BY-NC-ND), which allows third parties to download the articles and share them with others as long as they credit the author and the Abstract Book, but they cannot change the content in any way or use them commercially.
**Abstract Book Citations:** Authors, Title, HemaSphere, 2026;10:(S1):pages. The abstract book DOIs can be found on the "Author Information" page.
**Disclaimer:** Articles published in the journal HemaSphere exclusively reflect the opinions of the authors. The authors are responsible for all content in their abstracts including accuracy of the facts, statements, citing resources, etc.

### (PB2765) EARLY LOW-LEVEL PERIPHERAL BLOOD WT1 AT DAY +30 IDENTIFIES RELAPSE RISK AFTER ALLOGENEIC TRANSPLANTATION IN AML: A PROSPECTIVE MULTICENTER STUDY

**Topic:** 04. Acute myeloid leukemia - Clinical

Viktor Blaslov*[1], Margareta Radić Antolic[2], Ivana Horvat[2], Tamara Vasilj[3], Zeljko Prka[3], Antonija Miljak[1], Alen Ostojic[2], Pavle Roncevic[2], Iva Ivanko[4], Goran Rincic[4], Radovan Vrhovac[2], Lana Desnica[2], Ranka Serventi Seiwerth[2], Ante Vulić[2], Mirta Mikulic[2], Nadira Duraković[5], Ivan Krecak[6], Davor Galusic[1], Lučana Vicelić Čutura[1], Marija Petrić[1], Zinaida Perić[7]

*[1]University Hospital of Split, Split, Croatia;[2]University Hospital Centre Zagreb, Zagreb, Croatia;[3]Clinical Hospital Dubrava, Zagreb, Croatia; [4]University Hospital Centre Sisters of Mercy Zagreb, Zagreb, Croatia;[5]Clinical Hospital Centre Zagreb, Zagreb, Croatia; [6]General Hospital of Sibenik-Knin County, Šibenik, Croatia;[7]University Hospital Centre Rijeka, Rijeka, Croatia;*

### Background

Relapse remains the leading cause of treatment failure after allogeneic hematopoietic stem cell transplantation (allo-HSCT) in acute myeloid leukemia (AML). Early identification of patients at increased relapse risk may enable intensified monitoring and pre-emptive therapeutic interventions. WT1 overexpression represents a broadly applicable molecular marker, but the role of peripheral blood (PB) WT1 monitoring early after allo-HSCT remains incompletely defined.

### Aims

To evaluate the prognostic value of PB WT1 expression before transplantation and at day +30 for relapse risk and survival outcomes in AML patients undergoing allo-HSCT.

### Methods

In this prospective multicenter cohort study conducted across six hematology centers in Croatia, 43 adults with AML in complete remission underwent allo-HSCT between 2021 and 2023. WT1 expression in PB was quantified using standardized real-time quantitative PCR before transplantation (WT1_pre) and at day +30 (WT1_30). Receiver operating characteristic analysis was used to define optimal cutoffs for relapse prediction. Survival outcomes were assessed using Kaplan–Meier and Cox regression models, while cumulative incidence of relapse (CIR) was analyzed using competing-risk methodology.

### Results

ROC analysis identified a WT1 threshold of ≥3 copies/$10^4$ ABL as optimal for relapse prediction. WT1_30 demonstrated strong discriminative performance (AUC 0.79, p=0.005), whereas WT1_pre showed more modest predictive value (AUC 0.69, p=0.037). Among patients with evaluable WT1_30 (n=28), those with WT1_30 ≥3 had inferior 12-month PFS compared with patients <3 (52.9% vs 90.9%, p=0.0059) and higher 12-month CIR (31% vs 9%, p=0.054). In multivariable analysis adjusting for ELN 2022 risk, WT1_30 ≥3 remained independently associated with inferior PFS (HR 10.5, 95% CI 1.32–78.4, p=0.026). Relapsed patients had significantly higher WT1_30 levels compared with non-relapsed patients (median 5.0 vs 2.0 copies/$10^4$ ABL, p=0.018).

### Summary/Conclusion

Peripheral blood WT1 expression, particularly at day +30 after allo-HSCT, is associated with increased relapse risk in AML and may support early post-transplant risk stratification. Day +30 peripheral blood WT1 provides an early and minimally invasive marker of relapse risk after allo-HSCT in AML.

**Copyright Information:** (Online) ISSN: 2572-9241
©2026 The Author(s). HemaSphere is published by John Wiley & Sons Ltd on behalf of European Hematology Association. This is an open access Abstract Book distributed under the Attribution-NonCommercial-NoDerivs (CC BY-NC-ND), which allows third parties to download the articles and share them with others as long as they credit the author and the Abstract Book, but they cannot change the content in any way or use them commercially.
**Abstract Book Citations:** Authors, Title, HemaSphere, 2026;10:(S1):pages. The abstract book DOIs can be found on the "Author Information" page.
**Disclaimer:** Articles published in the journal HemaSphere exclusively reflect the opinions of the authors. The authors are responsible for all content in their abstracts including accuracy of the facts, statements, citing resources, etc.

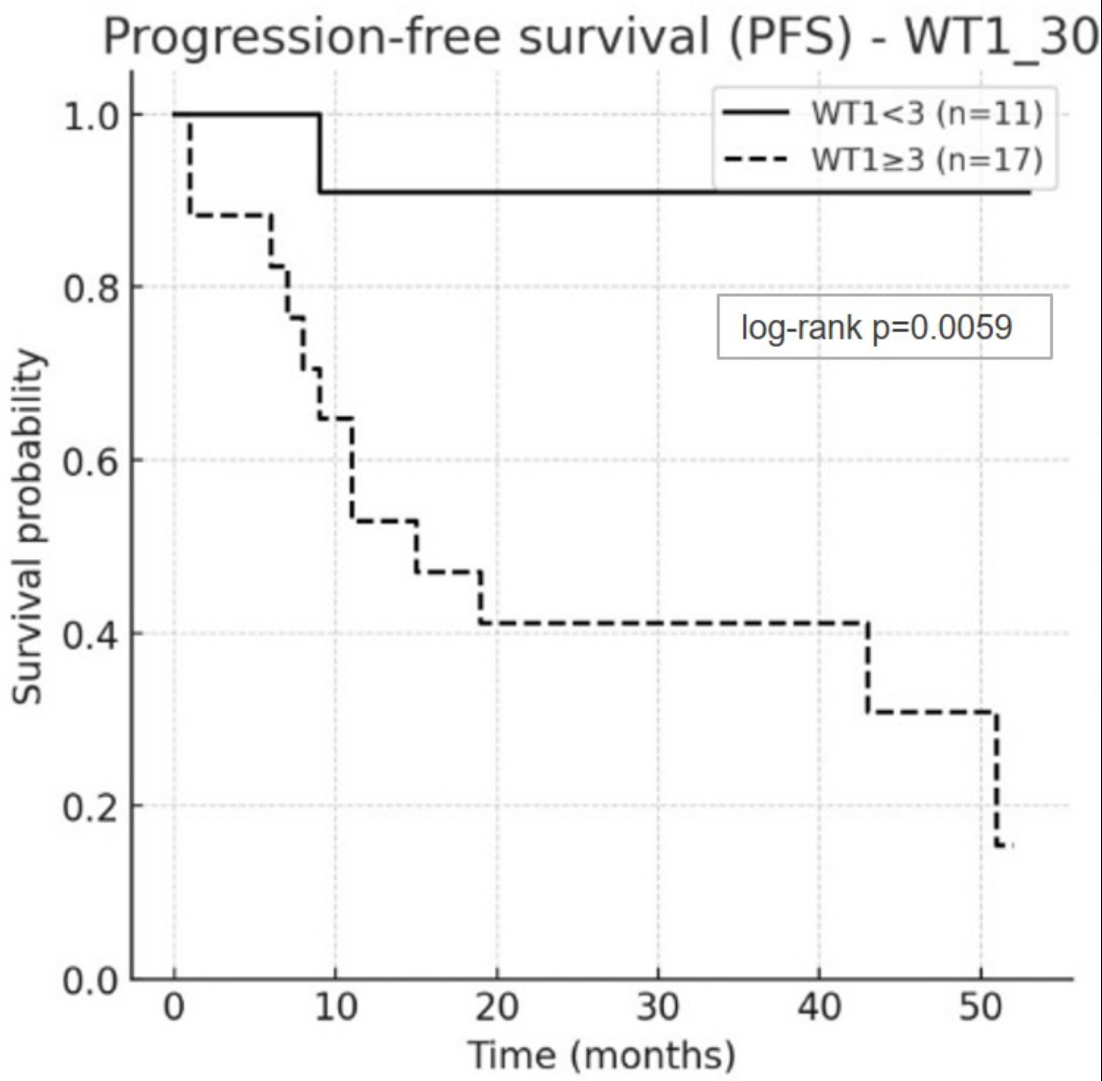

Kaplan–Meier curves for progression-free survival according to day +30 peripheral blood WT1 expression (WT1<3 vs ≥3 copies/$10^4$ ABL)

**Copyright Information:** (Online) ISSN: 2572-9241
©2026 The Author(s). HemaSphere is published by John Wiley & Sons Ltd on behalf of European Hematology Association. This is an open access Abstract Book distributed under the Attribution-NonCommercial-NoDerivs (CC BY-NC-ND), which allows third parties to download the articles and share them with others as long as they credit the author and the Abstract Book, but they cannot change the content in any way or use them commercially.
**Abstract Book Citations:** Authors, Title, HemaSphere, 2026;10:(S1):pages. The abstract book DOIs can be found on the "Author Information" page.
**Disclaimer:** Articles published in the journal HemaSphere exclusively reflect the opinions of the authors. The authors are responsible for all content in their abstracts including accuracy of the facts, statements, citing resources, etc.

### (PB2766) REGISTRATIONAL PHASE 3 STUDIES OF ZIFTOMENIB IN COMBINATION WITH NONINTENSIVE OR INTENSIVE CHEMOTHERAPY FOR NEWLY DIAGNOSED NPM1-M OR KMT2A-R ACUTE MYELOID LEUKEMIA (AML): THE KOMET-017 TRIAL

**Topic:** 04. Acute myeloid leukemia - Clinical

Amer M. Zeidan*[1], Eunice Wang[2], Richard Dillon[3], Lionel Ades[4], Jessica K. Altman[5], Eduardo Rodríguez-Arbolí[6], Suresh Balasubramanian[7], Uma Borate[8], Harry Erba[9], Yazan F. Madanat[10], Christine McMahon[11], Pau Montesinos[12], Cristina Papayannidis[13], Stephen Strickland[14], Tianle Chen[15], Ying Tian[15], Marcie Riches[15], Erica Warlick[15], Daniel Corum[15], Mollie Leoni[15], Ghayas Issa[16], Amir Fathi[17]

*[1]Department of Internal Medicine, Section of Hematology, Yale University, New Haven, United States of America;[2]Roswell Park Comprehensive Cancer Center, Buffalo, United States of America; [3]Department of Medical and Molecular Genetics, King's College London, London, United Kingdom; [4]Hôpital Saint-Louis (AP-HP) and Université Paris Cité and Centre d'Investigations Cliniques-Inserm CIC-1427, Paris, France; [5]Northwestern University-Robert H Lurie Comprehensive Cancer Center, Chicago, United States of America; [6]Department of Hematology, Hospital Universitario Virgen del Rocío, Instituto de Biomedicina de Sevilla (IBiS/CSIC), University of Seville, Seville, Spain; [7]Department of Oncology, Karmanos Cancer Institute, Wayne State University, Detroit, United States of America; [8]The Ohio State University Comprehensive Cancer Center, Columbus, United States of America; [9]Duke Cancer Institute, Durham, United States of America; [10]The University of Texas Southwestern Medical Center, Dallas, United States of America; [11]Anschutz Medical Campus, Division of Hematology, University of Colorado School of Medicine, Aurora, United States of America; [12]Hospital Universitario y Politécnico La Fe, Valencia, Spain; [13]IRCCS Azienda Ospedaliero-Universitaria di Bologna, Istituto di Ematologia "Seràgnoli", Bologna, Italy; [14]SCRI at TriStar Centennial, Nashville, United States of America; [15]Kura Oncology, Inc., San Diego, United States of America; [16]Department of Leukemia, The University of Texas MD Anderson Cancer Center, Houston, United States of America; [17]Massachusetts General Hospital, Harvard Medical School, Boston, United States of America;*

### Background

*NPM1* mutations (*NPM1*-m) and *KMT2A* rearrangements (*KMT2A*-r) drive leukemogenesis in ~35–40% of AML. Standards-of-care for *NPM1*-m and *KMT2A*-r AML include venetoclax/azacitidine (Ven/Aza; nonintensive) for unfit patients (pts) and cytarabine/daunorubicin (7+3; intensive) for fit pts. Ziftomenib – a potent, selective oral menin inhibitor – is U.S. FDA-approved as monotherapy for adults with relapsed/refractory *NPM1*-m AML. It has also shown clinically meaningful activity and tolerability in combination for newly diagnosed *NPM1*-m or *KMT2A*-r AML.

### Aims

Here we present the design of KOMET-017 (NCT07007312) evaluating ziftomenib vs placebo combined with standard-of-care regimens in newly diagnosed *NPM1*-m or *KMT2A*-r AML.

**Copyright Information:** (Online) ISSN: 2572-9241
©2026 The Author(s). HemaSphere is published by John Wiley & Sons Ltd on behalf of European Hematology Association. This is an open access Abstract Book distributed under the Attribution-NonCommercial-NoDerivs (CC BY-NC-ND), which allows third parties to download the articles and share them with others as long as they credit the author and the Abstract Book, but they cannot change the content in any way or use them commercially.
**Abstract Book Citations:** Authors, Title, HemaSphere, 2026;10:(S1):pages. The abstract book DOIs can be found on the "Author Information" page.
**Disclaimer:** Articles published in the journal HemaSphere exclusively reflect the opinions of the authors. The authors are responsible for all content in their abstracts including accuracy of the facts, statements, citing resources, etc.

### Methods

KOMET-017 is a single protocol comprising two independent, global, randomized, double-blind, placebo-controlled, registrational phase 3 studies of ziftomenib combined with nonintensive (KOMET-017-NIC) or intensive (KOMET-017-IC) chemotherapy in newly diagnosed *NPM1*-m or *KMT2A*-r AML (**Figure**). Both studies include adults (≥18y) with ECOG PS 0–2 and adequate liver and kidney function. Pts with prior AML treatment, acute promyelocytic leukemia, blast phase chronic myeloid leukemia, isolated myeloid sarcoma, *BCR-ABL* mutation history, or CNS involvement are excluded. AEs are assessed per CTCAE v5.0. All pts provide written informed consent.

KOMET-017-NIC is enrolling pts with *NPM1*-m AML, either age ≥75y or <75y with ECOG PS 2 or cardiac, renal or hepatic impairment. Pts are randomized 1:1 to ziftomenib with Ven/Aza (Arm A) or placebo with Ven/Aza (Arm B). Prescribed modified Ven dosing is included to incorporate real-world practice. Dual primary endpoints are complete remission (CR; US only; investigator-assessed per ELN 2022) and overall survival.

KOMET-017-IC is enrolling pts with *NPM1*-m or *KMT2A*-r AML who have wild-type *FLT3* or *FLT3*-ITD ratio <0.05, or are otherwise ineligible for FLT3-targeted therapy, and LVEF ≥50%. Pts are randomized 1:1:1 to continuous ziftomenib during 7+3 induction, cytarabine consolidation, and maintenance (Arm A); ziftomenib during 7+3 induction and cytarabine consolidation, and placebo during maintenance (Arm B); or continuous placebo during 7+3 induction, cytarabine consolidation and maintenance (Arm C). CR with measurable residual disease negativity ($CR_{MRD–}$) is a key potentially clinically meaningful surrogate endpoint, as published data suggest $CR_{MRD–}$ may correlate better with long-term survival than morphologic CR alone. Dual primary endpoints are centrally assessed $CR_{MRD–}$ (CR per ELN 2022) by the end of 2 cycles for *NPM1*-m pts, and event-free survival for combined *NPM1*-m/*KMT2A*-r pts. The 3-arm design enables the ability to isolate ziftomenib's impact in maintenance.

### Results

KOMET-017 is currently enrolling pts as of Sep 2025.

### Summary/Conclusion

KOMET-017 evaluates the addition of ziftomenib to backbone standard-of-care therapies for *NPM1*-m and/or *KMT2A*-r AML. $CR_{MRD–}$ will be assessed as a potential new surrogate endpoint in *NPM1*-m AML pts undergoing intensive therapy. KOMET-017 allows pts to enroll under a single protocol but be treated within one of the independent registrational studies based on fitness for intensive chemotherapy. The study design is intended to be pt-centric, facilitate rapid enrollment, offer operational advantages to study sites, and potentially allow for accelerated approval based on accepted surrogates for meaningful clinical benefit. Details on statistical assumptions, including sample sizes, will be presented.

**Copyright Information:** (Online) ISSN: 2572-9241
©2026 The Author(s). HemaSphere is published by John Wiley & Sons Ltd on behalf of European Hematology Association. This is an open access Abstract Book distributed under the Attribution-NonCommercial-NoDerivs (CC BY-NC-ND), which allows third parties to download the articles and share them with others as long as they credit the author and the Abstract Book, but they cannot change the content in any way or use them commercially.
**Abstract Book Citations:** Authors, Title, HemaSphere, 2026;10:(S1):pages. The abstract book DOIs can be found on the "Author Information" page.
**Disclaimer:** Articles published in the journal HemaSphere exclusively reflect the opinions of the authors. The authors are responsible for all content in their abstracts including accuracy of the facts, statements, citing resources, etc.

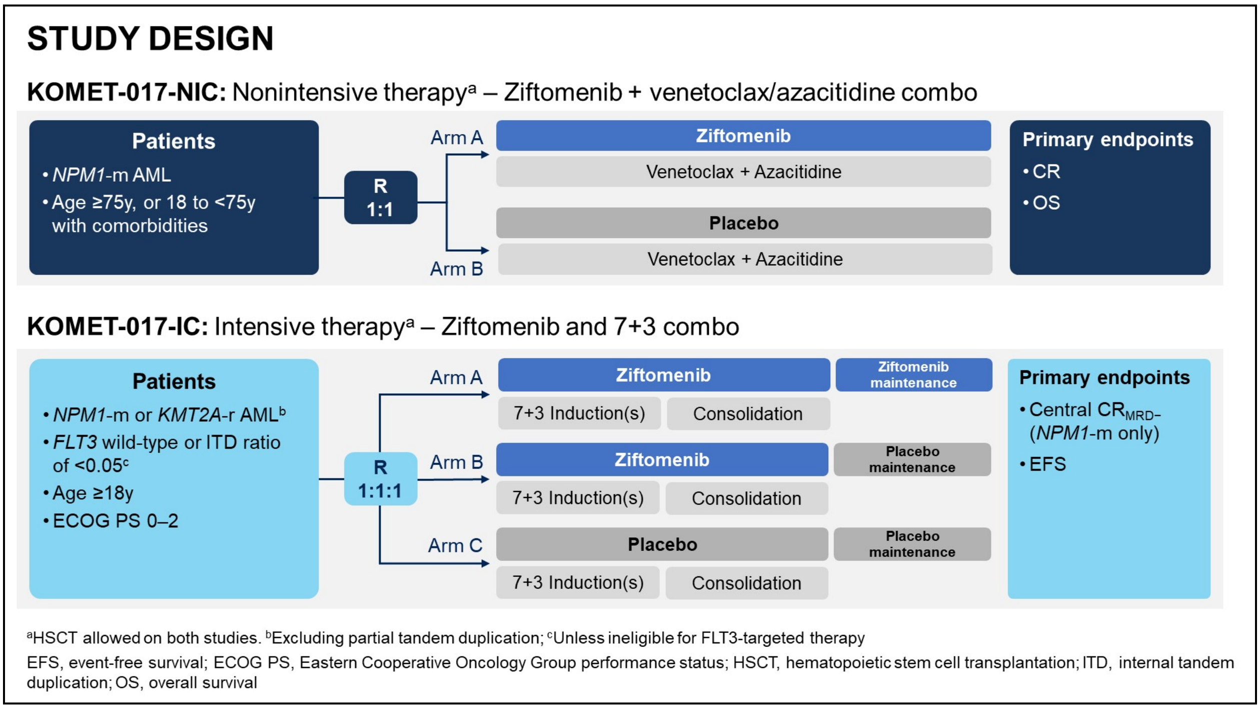

**Copyright Information:** (Online) ISSN: 2572-9241
©2026 The Author(s). HemaSphere is published by John Wiley & Sons Ltd on behalf of European Hematology Association. This is an open access Abstract Book distributed under the Attribution-NonCommercial-NoDerivs (CC BY-NC-ND), which allows third parties to download the articles and share them with others as long as they credit the author and the Abstract Book, but they cannot change the content in any way or use them commercially.
**Abstract Book Citations:** Authors, Title, HemaSphere, 2026;10:(S1):pages. The abstract book DOIs can be found on the "Author Information" page.
**Disclaimer:** Articles published in the journal HemaSphere exclusively reflect the opinions of the authors. The authors are responsible for all content in their abstracts including accuracy of the facts, statements, citing resources, etc.

### (PB2767) HAS BONE MARROW CORE BIOPSY A CRUCIAL ROLE AT DIAGNOSIS AND AT POST-INDUCTION EVALUATION IN PATIENTS WITH ACUTE MYELOID LEUKEMIA TREATED WITH HYPOMETHYLATING AGENTS PLUS VENETOCLAX?

**Topic:** 04. Acute myeloid leukemia - Clinical

Gianluca Martini*[1], Claudia Patricia Tobar Cabrera[1], Emanuela Boveri[2], Cristina Picone[3], Marianna Rossi[1], Elisa Roncoroni[1], Giacomo Riccaboni[4], Yury Yudin[4], Luca Arcaini[1, 4], Patrizia Zappasodi[1]

*[1]SC Ematologia 1, Fondazione IRCCS Policlinico San Matteo, Pavia, Italy;[2]Anatomic Pathology Unit, Fondazione IRCCS Policlinico San Matteo, Pavia, Italy; [3]SC Ematologia Molecolare e Medicina di Precisione, Fondazione IRCCS Policlinico San Matteo, Pavia, Italy; [4]Department of Molecular Medicine, University of Pavia, Pavia, Italy;*

### Background

Diagnostic assessment for Acute Myeloid Leukemia (AML) integrates morphology, multiparameter flow cytometry (MFC), bone marrow core biopsy (BMCb), cytogenetics, and broad molecular testing. Both the WHO 5th edition and ICC2022 classifications require a blast cut-off as a necessary parameter to discriminate acute leukemia from dysplastic conditions, in the absence of AML-defining genetic abnormalities. Moreover, quantifying residual blasts is crucial at post-induction marrow evaluation, but can be challenging through morphologic evaluation and MFC; indeed, counts could be underestimated due to the hypocellular marrow frequently occurring after the first cycle of hypomethylating agents (HMA) and venetoclax (VEN). The role of BMCb compared to MFC regarding blast count remains not fully standardized.

### Aims

To determine if BMCb provides reliable and useful information regarding blast counts – compared to MFC – both at diagnosis and at response assessment in AML patients treated with HMA-VEN.

### Methods

In this single-center, retrospective observational study, we collected data on bone marrow assessment in 93 adult AML patients treated with HMA–VEN between November 2018 and December 2024. Diagnosis was established according to ICC 2022, and responses were defined according to European Leukaemia Network (ELN) 2022 criteria. Data regarding blast count through MFC and BMCb, were compared both at diagnosis and at post-induction response evaluation.

**Copyright Information:** (Online) ISSN: 2572-9241
©2026 The Author(s). HemaSphere is published by John Wiley & Sons Ltd on behalf of European Hematology Association. This is an open access Abstract Book distributed under the Attribution-NonCommercial-NoDerivs (CC BY-NC-ND), which allows third parties to download the articles and share them with others as long as they credit the author and the Abstract Book, but they cannot change the content in any way or use them commercially.
**Abstract Book Citations:** Authors, Title, HemaSphere, 2026;10:(S1):pages. The abstract book DOIs can be found on the "Author Information" page.
**Disclaimer:** Articles published in the journal HemaSphere exclusively reflect the opinions of the authors. The authors are responsible for all content in their abstracts including accuracy of the facts, statements, citing resources, etc.

### Results

Among the 55 newly diagnosed (ND) patients, 3 (5.4%) presented with a dry tap, and diagnosis was established solely via BMCb; another 17 (31%) were diagnosed through MFC alone. Both tests were available in 35 patients (63.6%) and a discordance in blast count was observed in 12/35 (34.3%) cases. Notably, in 10 of these 12 patients, blast count assessed via BMCb - but not by MFC - was conclusive for a diagnosis of AML. Among the 38 relapsed/refractory (R/R) patients 5 (13.2%) presented with a dry tap, and diagnosis was established solely via BMCb; another 20 (52.6%) were diagnosed through MFC alone. Both tests were available in 13 patients (34.2%) and a discordance in blast count was observed in 2/13 (15.4%) cases. In both cases, the BMCb blast count confirmed the diagnosis of relapsed/refractory AML.
Response evaluation after cycle 1 was performed in 75/93 (80.6%) cases, with both MFC and BMCb data available in 52/75 (69.3%) patients. Although cellularity was very low in all cases, discordance was observed in one patient only, where BMCb blast count was consistent with morphologic complete remission, while MFC blast count was higher than 5%.

| Patient Cohort | Total (n) | Dry Tap (BMCb only) | MFC only | Both Tests (n) | Discordance (n/N) |
|---|---|---|---|---|---|
| ND AML | 55 | 3 (5.4%) | 17 (31.0%) | 35 (63.6%) | **12/35 (34.3%)** |
| R/R AML | 38 | 5 (13.2%) | 20 (52.6%) | 13 (34.2%) | **2/13 (15.4%)** |
| Post-C1 Evaluation | 75 | 12 (16%) | 11 (14.6%) | 52 (69.3%) | **1/52 (1.9%)** |

**Table 1.** Comparison of Diagnostic Performance and Concordance Between BMCb and MFC.

### Summary/Conclusion

Our study confirms that BMCb is crucial in the diagnostic process to accurately define blasts count, ensuring a correct diagnosis and, ultimately, guaranteeing access to AML-specific therapies.
Conversely, the utility of BMCb was not confirmed for response assessment. Even in the presence of hypocellular material, MFC proved to be adequate to discriminate remission from persistent disease, thereby guiding the choice to interrupt or continue VEN administration.

**Copyright Information:** (Online) ISSN: 2572-9241
©2026 The Author(s). HemaSphere is published by John Wiley & Sons Ltd on behalf of European Hematology Association. This is an open access Abstract Book distributed under the Attribution-NonCommercial-NoDerivs (CC BY-NC-ND), which allows third parties to download the articles and share them with others as long as they credit the author and the Abstract Book, but they cannot change the content in any way or use them commercially.
**Abstract Book Citations:** Authors, Title, HemaSphere, 2026;10:(S1):pages. The abstract book DOIs can be found on the "Author Information" page.
**Disclaimer:** Articles published in the journal HemaSphere exclusively reflect the opinions of the authors. The authors are responsible for all content in their abstracts including accuracy of the facts, statements, citing resources, etc.

### (PB2769) QUATRO-APL: A PIVOTAL OPEN-LABEL PHASE 3 TRIAL EVALUATING THE EFFICACY AND SAFETY OF QTX-2101 IN COMBINATION WITH ALL-TRANS RETINOIC ACID IN NEWLY DIAGNOSED, LOW-RISK ACUTE PROMYELOCYTIC LEUKEMIA

**Topic:** 04. Acute myeloid leukemia - Clinical

Pau Montesinos*[1], Uwe Platzbecker[2], Maria Teresa Voso[3], Jessica K. Altman[4], Uma Borate[5], Gabriel Ghiaur[6], Farhad Ravandi[7], Shaad Abedin[8], Chabha Allek[9], Sridhar Desikan[8], Howard Goodall[10], Sara Mathews[8], Swaminathan Murugappan[8], Pierre Fenaux[11]

*[1]Hospital Universitari i Politècnic La Fe, Department of Hematology, Valencia, Spain;[2]University Hospital Carl Gustav Carus, TU Dresden, National Center for Tumor Disease (NCT), Dresden, Germany; [3]Tor Vergata University, Department of Biomedicine and Prevention, Rome, Italy; [4]Robert H. Lurie Comprehensive Cancer Center of Northwestern University, Division of Hematology and Oncology, Chicago, United States of America; [5]The Ohio State University Comprehensive Cancer Center, Columbus, United States of America; [6]Johns Hopkins University, Sidney Kimmel Comprehensive Cancer Center, Baltimore, United States of America; [7]The University Of Texas MD Anderson Cancer Center, Leukemia, Houston, United States of America;[8]Quetzal Therapeutics, LLC, Chicago, United States of America; [9]Quetzal Therapeutics, LLC, Nice, France; [10]Quetzal Therapeutics, LLC, London, United Kingdom; [11]Hôpital Saint-Louis, APHP, Université Paris Cité, Service d'Hématologie Séniors, Paris, France;*

### Background

Intravenous (IV) arsenic trioxide (ATO) combined with all-trans retinoic acid (ATRA) has transformed outcomes in all risk acute promyelocytic leukemia, leading to >90% molecular complete remission (mCR) rates and favorable long-term survival. However, while survival rates are favorable, treatment-related burden remains a significant challenge. Standard IV ATO requires prolonged hospitalization during induction, frequent outpatient infusions during consolidation, central venous catheter (CVC) placement, and substantial burden on patients and healthcare systems. QTX-2101 is an investigational ATO developed as an oral capsule formulation to overcome limitations of IV ATO by delivering comparable systemic arsenic exposure while eliminating infusion-related burden. In Phase 1 studies, QTX-2101 15 mg once daily achieved arsenic exposure comparable to IV ATO 0.15 mg/kg with oral bioavailability ~100%, a manageable safety profile and a lower Cmax (32.22 +/- 10.10 ng/mL vs 24.95 +/- 9.76 ng/mL), supporting the potential for reduced peak related toxicities, including QTc prolongation, while preserving therapeutic exposure.

### Aims

This is a randomized, open-label, multicenter Phase 3 study evaluating the efficacy and safety of oral QTX-2101 plus ATRA compared with IV ATO plus ATRA in adults with newly diagnosed low-risk acute promyelocytic leukemia (LR-APL).

### Methods

QUATRO-APL (QTX-2101-301) consists of two parallel parts, including a pharmacokinetic evaluation and a pivotal efficacy study. The pivotal component will enroll approximately 150 adults with newly diagnosed LR-APL (WBC ≤$10\times10^9$/L, as defined by NCCN criteria and corresponding to the ELN non–high-risk group), with locally confirmed PML/RARA fusion, across approximately 70 sites in Europe and North America. Patients will be randomized 2:1 to receive oral QTX-2101 once daily plus ATRA (Arm 1) or IV ATO once daily plus ATRA (Arm 2), delivered over one induction and four consolidation cycles. Primary endpoint: mCR rate at end of consolidation cycle 3, defined as the absence of PML/RARA transcripts in bone marrow by RT-qPCR, as assessed by centralized testing. Key secondary endpoints will include EFS at the time of primary endpoint analysis, OS, CR/CRi rate, safety (AEs, lab abnormalities, ECG/QTc changes, vital signs). Additional endpoints: PK parameters (AUC, Cmax) from population PK analyses, concentration-QTc analysis, and patient reported outcomes.

**Copyright Information:** (Online) ISSN: 2572-9241
©2026 The Author(s). HemaSphere is published by John Wiley & Sons Ltd on behalf of European Hematology Association. This is an open access Abstract Book distributed under the Attribution-NonCommercial-NoDerivs (CC BY-NC-ND), which allows third parties to download the articles and share them with others as long as they credit the author and the Abstract Book, but they cannot change the content in any way or use them commercially.
**Abstract Book Citations:** Authors, Title, HemaSphere, 2026;10:(S1):pages. The abstract book DOIs can be found on the "Author Information" page.
**Disclaimer:** Articles published in the journal HemaSphere exclusively reflect the opinions of the authors. The authors are responsible for all content in their abstracts including accuracy of the facts, statements, citing resources, etc.

### Results

The study is currently open and recruiting in the US and Europe.

### Summary/Conclusion

QUATRO-APL is a pivotal Phase 3 study designed to demonstrate comparable efficacy and safety of oral QTX-2101 relative to standard IV ATO, while evaluating potential improvements in patient quality of life. Based on prior clinical experience, the lower Cmax associated with oral QTX-2101 may support improved tolerability versus IV ATO; effects on QTc and other safety parameters will be assessed in this study. With comparable systemic exposure, QTX-2101 is intended to maintain the established efficacy of IV ATO while reducing treatment burden, enabling a fully oral approach for patients with LR-APL with the potential to establish a new frontline standard of care.

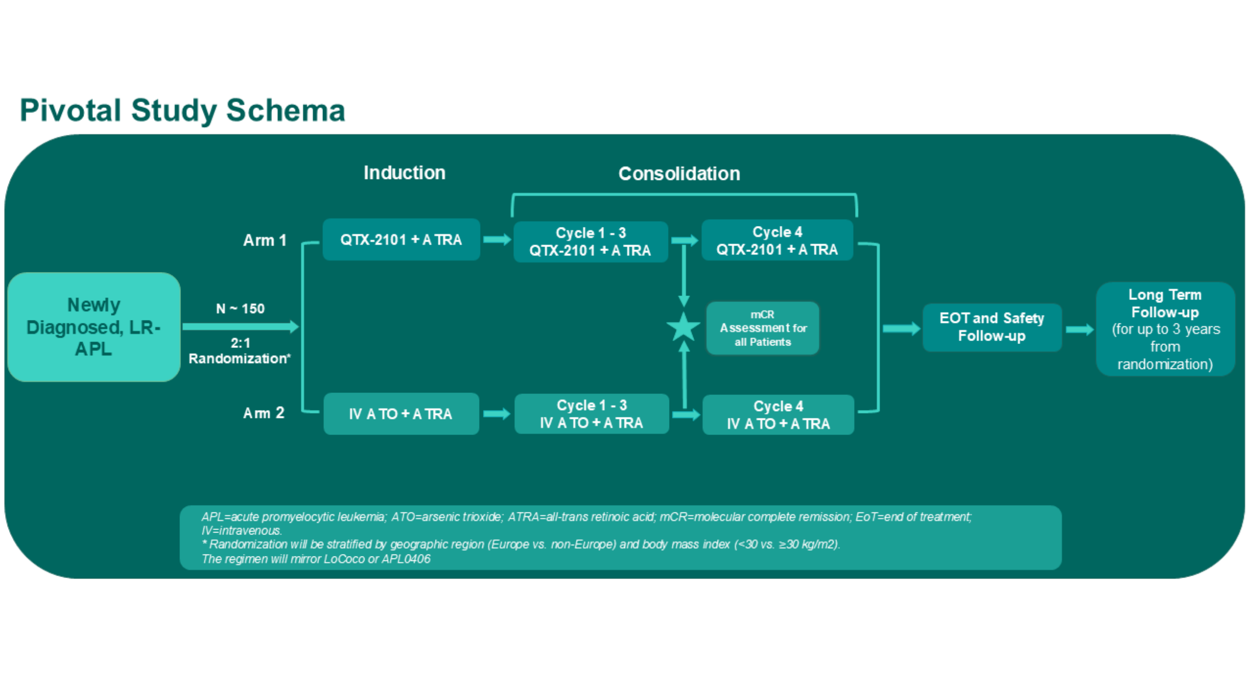

**Copyright Information:** (Online) ISSN: 2572-9241
©2026 The Author(s). HemaSphere is published by John Wiley & Sons Ltd on behalf of European Hematology Association. This is an open access Abstract Book distributed under the Attribution-NonCommercial-NoDerivs (CC BY-NC-ND), which allows third parties to download the articles and share them with others as long as they credit the author and the Abstract Book, but they cannot change the content in any way or use them commercially.
**Abstract Book Citations:** Authors, Title, HemaSphere, 2026;10:(S1):pages. The abstract book DOIs can be found on the "Author Information" page.
**Disclaimer:** Articles published in the journal HemaSphere exclusively reflect the opinions of the authors. The authors are responsible for all content in their abstracts including accuracy of the facts, statements, citing resources, etc.

### (PB2770) CLOSING THE GAPS FOR PERSONALISED THERAPY IN NEWLY DIAGNOSED AML: IMPACT OF A MICRO-LEARNING ONLINE EDUCATION INITIATIVE ON THE PRACTICALITIES OF CARE

**Topic:** 04. Acute myeloid leukemia - Clinical

Ben Johnson*[1], Michael Beyer[2], Rianna Hanif[1], Kadrin Wilfong[2], Jessica Massey[2], Carmine Deluca[2]

*[1]PeerView Institute for Medical Education, London, United Kingdom;[2]PeerView Institute for Medical Education, New York, United States of America;*

### Background

The availability of targeted treatments has elevated clinical care for eligible patients with newly diagnosed acute myeloid leukaemia (ND AML) unfit for intensive induction therapy. However, given the rarity of these mutations, clinical gaps persist in integrating early and accurate mutational analysis and ensuring the right patient is given the right treatment at the right time and appropriately managed during throughout treatment.

### Aims

The objective of this initiative was to develop an online micro-learning educational activity aimed at empowering healthcare professionals to improve the practical application of precision approaches in the management of ND AML.

### Methods

PeerView deployed a CME-certified online micro-learning video-based activity that blended a clinical journey of an elderly patient with ND AML with expert perspectives, associated clinical evidence, and practical applications. Assessment questions were administered to learners after the education and to a demographically matched group of nonparticipants to evaluate differences in knowledge and competence. Here, we report the findings 3 months after launching this initiative.

### Results

From 26 November 2025 to 13 February 2026, this initiative reached 1,671 physicians, including haematologists (38%), oncologists (19%), pathologists (10%), nurses and nurse practitioners (9%) and other HCPs (24%) in community (70%) and academic (30%) settings globally. Compared with nonparticipants, learners showed a post-activity increase of 28% in knowledge of the appropriate genomic testing strategies to guide diagnosis and treatment selection in ND AML. Learners also demonstrated gains of 40% in the ability to align treatment selection to the individual based upon clinical presentation of the patient and 42% in skills needed to monitor for and manage adverse events in patients undergoing treatment. Polling of nonparticipants (n = 50) on ongoing challenges/barriers in utilising personalised approaches in ND AML revealed three key themes that should be addressed in future educational initiatives: sequencing of available therapeutic options, adverse event management and future directions including novel combinations and approaches.

**Copyright Information:** (Online) ISSN: 2572-9241
©2026 The Author(s). HemaSphere is published by John Wiley & Sons Ltd on behalf of European Hematology Association. This is an open access Abstract Book distributed under the Attribution-NonCommercial-NoDerivs (CC BY-NC-ND), which allows third parties to download the articles and share them with others as long as they credit the author and the Abstract Book, but they cannot change the content in any way or use them commercially.
**Abstract Book Citations:** Authors, Title, HemaSphere, 2026;10:(S1):pages. The abstract book DOIs can be found on the "Author Information" page.
**Disclaimer:** Articles published in the journal HemaSphere exclusively reflect the opinions of the authors. The authors are responsible for all content in their abstracts including accuracy of the facts, statements, citing resources, etc.

**Summary/Conclusion**

The presence of actionable mutations in ND AML can lead to favourable outcomes when patients are appropriately treated with targeted therapies; however, the application of this in practice is suboptimal. Enduring online education has the ability to improve awareness of the global treating community regarding the associated evidence and reinforce existing guideline recommendations. Improvements in knowledge and competence were noted following education in practically applying this information to case scenarios and ensuring appropriate management during treatment. Despite this, challenges persist and new questions arise with the emergence of future therapeutic approaches, warranting further exploration and guidance on who to treat with what and how.

**Copyright Information:** (Online) ISSN: 2572-9241
©2026 The Author(s). HemaSphere is published by John Wiley & Sons Ltd on behalf of European Hematology Association. This is an open access Abstract Book distributed under the Attribution-NonCommercial-NoDerivs (CC BY-NC-ND), which allows third parties to download the articles and share them with others as long as they credit the author and the Abstract Book, but they cannot change the content in any way or use them commercially.
**Abstract Book Citations:** Authors, Title, HemaSphere, 2026;10:(S1):pages. The abstract book DOIs can be found on the "Author Information" page.
**Disclaimer:** Articles published in the journal HemaSphere exclusively reflect the opinions of the authors. The authors are responsible for all content in their abstracts including accuracy of the facts, statements, citing resources, etc.

### (PB2771) PROGNOSTIC VALUE OF MEASURABLE RESIDUAL DISEASE BY FLOW CYTOMETRY BEFORE ALLOGENEIC HEMATOPOIETIC CELL TRANSPLANTATION FOR PATIENTSWITH AML OR MDS

**Topic:** 04. Acute myeloid leukemia - Clinical

Huijuan Tian*[1], Yu Wang[1]

[1]*Peking University People's Hospital, Beijing, China;*

**Background**

Multiparameter flow cytometry (MFC)-based measurable residual disease (MRD) prior to allogeneic hematopoietic cell transplantation (HCT) is a known predictor of poor outcomes in acute myeloid leukemia (AML). However, its prognostic relevance in myelodysplastic syndromes (MDS) remains unclear.

**Aims**

This study aimed to investigate the association between pre-HCT MFC MRD and post-transplant outcomes in patients with AML versus MDS, and to assess whether disease type modifies this relationship.

**Methods**

We conducted a retrospective analysis of 605 patients with AML (n = 444) or MDS (n = 161) who underwent first allogeneic HCT in first or second morphologic remission between October 2018 and December 2021. Cox proportional hazard regression models were built for time-to-event outcomes.

**Results**

At two years post-HCT, no statistically significant differences were observed between MDS and AML patients in relapse rates (11.8% vs. 15.1%, P = 0.153), OS (85.1% vs. 84.4%, P = 0.7883), or NRM (8.1% vs. 3.8%, P = 0.034). However, a significant interaction was found between pre-HCT MRD and disease type for relapse (P < 0.001), NRM (P = 0.008), and OS (P < 0.001). Hazard ratios for MRD-associated risks were markedly lower in MDS than in AML: for relapse, HR = 3.43 (0.789–14.917) in MDS vs. 4.447 (2.735–7.23) in AML; for OS, HR = 2.773 (0.824–9.332) vs. 4.874 (3.018–7.871); and for NRM, HR = 2.231 (0.490–10.158) vs. 2.355 (0.892–6.214)

**Summary/Conclusion**

Our findings indicate that the utility of pre-HCT MFC-based MRD testing for prognostication is limited in MDS relative to AML.

**Copyright Information:** (Online) ISSN: 2572-9241
©2026 The Author(s). HemaSphere is published by John Wiley & Sons Ltd on behalf of European Hematology Association. This is an open access Abstract Book distributed under the Attribution-NonCommercial-NoDerivs (CC BY-NC-ND), which allows third parties to download the articles and share them with others as long as they credit the author and the Abstract Book, but they cannot change the content in any way or use them commercially.
**Abstract Book Citations:** Authors, Title, HemaSphere, 2026;10:(S1):pages. The abstract book DOIs can be found on the "Author Information" page.
**Disclaimer:** Articles published in the journal HemaSphere exclusively reflect the opinions of the authors. The authors are responsible for all content in their abstracts including accuracy of the facts, statements, citing resources, etc.

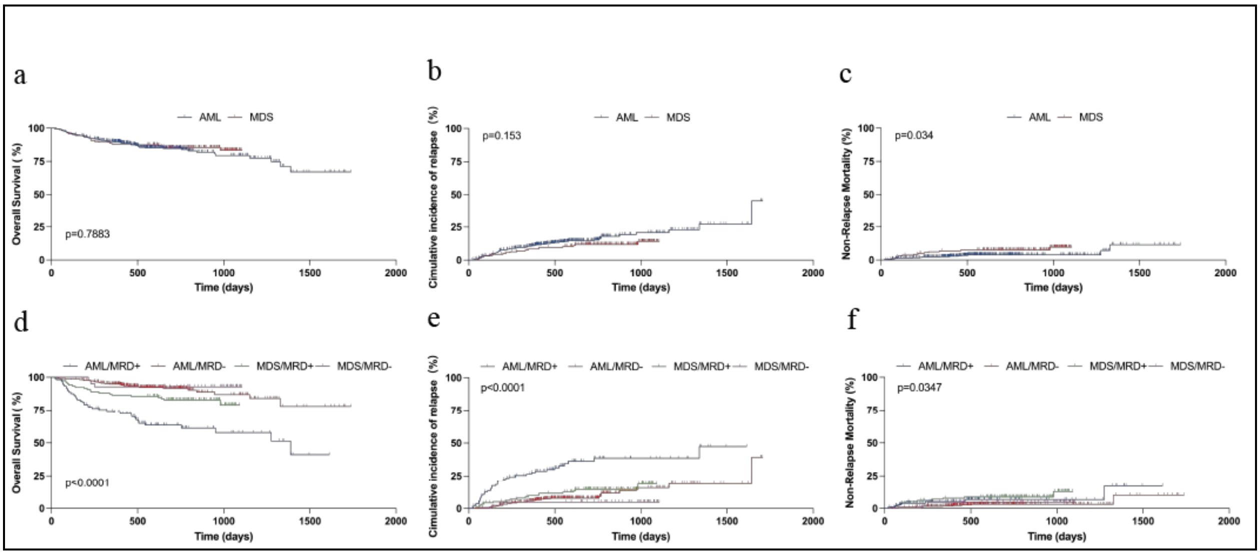

Post-HCT outcomes for 605 adults with MDS and AML undergoing allogeneic HCT

**Copyright Information:** (Online) ISSN: 2572-9241
©2026 The Author(s). HemaSphere is published by John Wiley & Sons Ltd on behalf of European Hematology Association. This is an open access Abstract Book distributed under the Attribution-NonCommercial-NoDerivs (CC BY-NC-ND), which allows third parties to download the articles and share them with others as long as they credit the author and the Abstract Book, but they cannot change the content in any way or use them commercially.
**Abstract Book Citations:** Authors, Title, HemaSphere, 2026;10:(S1):pages. The abstract book DOIs can be found on the "Author Information" page.
**Disclaimer:** Articles published in the journal HemaSphere exclusively reflect the opinions of the authors. The authors are responsible for all content in their abstracts including accuracy of the facts, statements, citing resources, etc.

### (PB2772) FRAILTY RISK ASSESSMENT IN OLDER PATIENTS WITH AML TREATED WITH VEN-AZA REGIMEN. SINGLE-CENTER EXPERIENCE.

**Topic:** 04. Acute myeloid leukemia - Clinical

Justyna Rybka*[1], Tomasz Wróbel[1], Donata Szymczak[1], Aleksandra Mroczkowska-Bekarciak[1]

[1]*Wroclaw Medical University, Department of Hematology, Cellular Therapies and Internal Medicine, Wroclaw, Poland;*

### Background

Older patients with acute myeloid leukemia (AML) represent a heterogeneous population.Frailty is defined as a state of deterioration across multiple physiological and functional parameters that can significantly affect quality of life. In Europe and North America, approximately 15% of individuals over the age of 65 are classified as frail. Among older patients with AML, this proportion is considerably higher, ranging from 17% to 68% . The frailty status of patients with hematologic malignancies influences treatment efficacy, therapy-related toxicity, and overall survival . Various assessment scales are used to evaluate the condition of patients with hematologic proliferative disorders.

### Aims

The aim of the study was to assess the frailty status in AML patients over the age of 65 with AML and treated with the azacitidine plus venetoclax regimen (Aza-Ven) at the Department of Hematology, Cellular Therapies, and Internal Medicine of the Wroclaw Medical University

### Methods

The analysis included 56 patients (25 women and 31 men). The mean age was 74 years. All patients were diagnosed with AML according to the 2022 ELN criteria. Forty-one patients were classified as intermediate risk, 12 as high risk, and 3 as low risk. All patients were enrolled in treatment with azacitidine combined with venetoclax. The median number of treatment cycles was 7. Treatment efficacy was assessed after 1 or 2 cycles of therapy. Frailty was assessed using the cumulative frailty index (FI). Patients were divided into four groups according to FI: fit, mildly frail, moderately frail, and severely frail. For fit patients, the FI score was <0.12; for mildly frail patients, the score ranged from 0.12 to 0.24; for moderately frail patients, from 0.24 to 0.36; and for severely frail patients, >0.36.Statistical analyses were performed using R software (version 4.4.2). Categorical variables were presented as n (%). Continuous variables were expressed as mean ± standard deviation (SD) or median (interquartile range, IQR). Overall survival was analyzed using the Kaplan–Meier method.

### Results

Complete remission (CR), after 1 or 2 cycles, was confirmed in 73% of patients, including 23 (41%) from the fit group, 10 (18%) from the mildly frail group, 3 (5%) from the moderately frail group, and 5 (9%) from the severely frail group. CR was confirmed in 92% of fit patients and 77% of mildly frail patients. Fifteen patients (27%) did not achieve response to the treatment (NR). Of the patients with NR, 10 belonged to the moderately frail and severely frail groups. The median overall survival time in the entire study group was 16.3 months. In the VIALE-A study, the median overall survival was 14,7 months.One-year survival in CR group was achieved in 62% of patients, and two-year survival in 39.5%. In the VIALE-A study, the 1-year overall survival rate was 66%, while the 2-year overall survival rate was 31%.

**Copyright Information:** (Online) ISSN: 2572-9241
©2026 The Author(s). HemaSphere is published by John Wiley & Sons Ltd on behalf of European Hematology Association. This is an open access Abstract Book distributed under the Attribution-NonCommercial-NoDerivs (CC BY-NC-ND), which allows third parties to download the articles and share them with others as long as they credit the author and the Abstract Book, but they cannot change the content in any way or use them commercially.
**Abstract Book Citations:** Authors, Title, HemaSphere, 2026;10:(S1):pages. The abstract book DOIs can be found on the "Author Information" page.
**Disclaimer:** Articles published in the journal HemaSphere exclusively reflect the opinions of the authors. The authors are responsible for all content in their abstracts including accuracy of the facts, statements, citing resources, etc.

### Summary/Conclusion

Frailty status in older AML patients affects the effectiveness of the applied therapy. The response rate was significantly higher in the fit and mildly frail groups compared to the moderately frail and severely frail groups. The efficacy of Aza-Ven therapy significantly decreases with increasing frailty. Frailty assessment may be a stronger predictor of treatment response than age alone. The analysis is preliminary and needs to be conducted in a larger group of patients.

**Overall survival analysis in total study group**

**Figure 1.** Overall survival curve in total study group

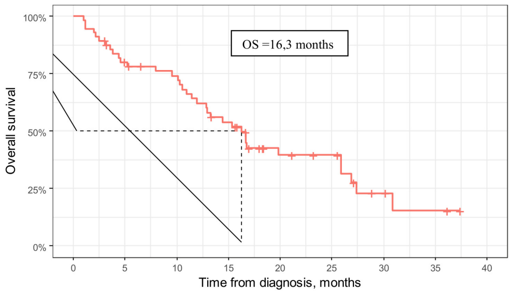

**Overall survival analysis among patients with CR**

**Figure 2.** Overall survival curve among patients with CR

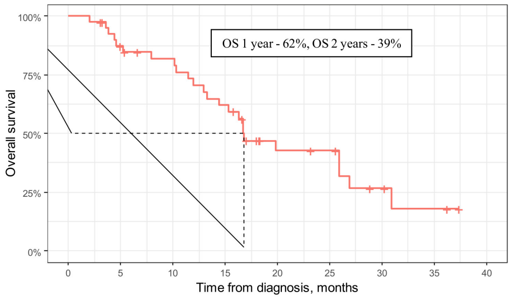

**Copyright Information:** (Online) ISSN: 2572-9241
©2026 The Author(s). HemaSphere is published by John Wiley & Sons Ltd on behalf of European Hematology Association. This is an open access Abstract Book distributed under the Attribution-NonCommercial-NoDerivs (CC BY-NC-ND), which allows third parties to download the articles and share them with others as long as they credit the author and the Abstract Book, but they cannot change the content in any way or use them commercially.
**Abstract Book Citations:** Authors, Title, HemaSphere, 2026;10:(S1):pages. The abstract book DOIs can be found on the "Author Information" page.
**Disclaimer:** Articles published in the journal HemaSphere exclusively reflect the opinions of the authors. The authors are responsible for all content in their abstracts including accuracy of the facts, statements, citing resources, etc.

### (PB2773) REFINING RELAPSE RISK IN NON-TRANSPLANTED AML IN FIRST REMISSION: SURVIVAL BENCHMARKS AND THE IMPACT OF RUNX1 MUTATION

**Topic:** 04. Acute myeloid leukemia - Clinical

Eitan Kugler*[1], Farhad Ravandi[1], Alexandre Bazinet[1], Courtney Dinardo[1], Naval Daver[1], Musa Yilmaz[1], Nicholas Short[1], Wei-Ying Jen[1], Jayastu Senapati[1], Abhishek Maiti[1], Gautam Borthakur[1], Guillermo Garcia-Manero[1], Hagop Kantarjian[1], Tapan Kadia[1]

*[1]MD Anderson Cancer Center, Leukemia, Houston, Tx, United States of America;*

### Background

Relapse remains a primary cause of treatment failure and mortality in acute myeloid leukemia (AML). While allogeneic stem cell transplantation (allo-SCT) improves cure rates, it is feasible for only a subset of patients. For those not proceeding to transplant, post-remission maintenance strategies are essential to prolonging survival, yet robust survival benchmarks to inform their development remain largely undefined.

### Aims

To establish relapse-free survival (RFS) and overall survival (OS) benchmarks across clinically relevant subgroups in a real-world analysis and to characterize clinical and molecular determinants of relapse risk in non-transplanted AML patients in first complete remission (CR1).

### Methods

We retrospectively analyzed 362 adults with newly diagnosed de novo AML treated at our institution since 2016 who achieved CR or CR with incomplete hematologic recovery (CRi) and did not undergo allo-SCT. Acute promyelocytic leukemia and core-binding factor AML were excluded. The cohort was stratified by low-intensity (LIT, n=257) and intensive therapy (IT, n=105). European LeukemiaNet (ELN) 2022 and ELN 2024 risk classifications were applied to IT and LIT cohorts, respectively. Measurable residual disease (MRD) was assessed by multiparameter flow cytometry. An exploratory random survival forest (RSF) analysis followed by multivariable Cox regression (MVA) was performed in the venetoclax-treated LIT cohort.

### Results

Median age was 69 years (19–94); 74 and 52 years in LIT and IT, respectively. *NPM1*, *FLT3*-ITD, and IDH1/2 were enriched in IT, whereas *TP53* and myelodysplasia-related (MR) gene mutations were more prevalent in LIT (45% vs. 27%, p=0.001), along with a higher frequency of complex karyotype (27% vs. 11%, p=0.002). Overall median RFS and OS were 11 and 19 months, respectively. Venetoclax reduced 24-month relapse rates in LIT (68% to 45%, p<0.01) and IT (49% to 27%, p<0.01). ELN risk stratification demonstrated inferior RFS in adverse- vs. favorable/intermediate-risk groups (no significant difference between the latter two), with median RFS of 58.0, 25.5, and 9.6 months in IT (p=0.01) and 13.7, 10.2, and 5.3 months in LIT (p<0.01) among FAV, INT, and ADV, respectively. *TP53*-mutated patients in LIT had median RFS of 5.3 and OS of 10.6 months; IT outcomes were not analyzed due to the small number of TP53-mutated patients in IT (n=6). MR-gene mutations were not associated with survival in either cohort. Flow-based MRD was more strongly associated with outcomes in LIT (MRD-negative vs. positive: RFS 12.2 vs. 5.9 months, p<0.01; OS 18.8 vs. 13.5 months, p=0.01) than in IT. On MVA, ELN 2024, *TP53* mutation, and MRD positivity were independently associated with RFS in venetoclax-treated LIT patients, while ELN 2022 was most strongly associated with survival in IT.
RSF analysis (n=198) identified ELN 2024, *RUNX1* mutation, karyotype, age, and MRD as top RFS predictors. A subsequent RSF-informed MVA identified *RUNX1* mutation as independently associated with inferior RFS (HR 1.83, p=0.013), most pronounced in the ELN 2024 favorable-risk subgroup (*RUNX1*-mutated vs. wild-type: median RFS 10.8 vs. 32.3 months, p=0.006).

**Copyright Information:** (Online) ISSN: 2572-9241
©2026 The Author(s). HemaSphere is published by John Wiley & Sons Ltd on behalf of European Hematology Association. This is an open access Abstract Book distributed under the Attribution-NonCommercial-NoDerivs (CC BY-NC-ND), which allows third parties to download the articles and share them with others as long as they credit the author and the Abstract Book, but they cannot change the content in any way or use them commercially.
**Abstract Book Citations:** Authors, Title, HemaSphere, 2026;10:(S1):pages. The abstract book DOIs can be found on the "Author Information" page.
**Disclaimer:** Articles published in the journal HemaSphere exclusively reflect the opinions of the authors. The authors are responsible for all content in their abstracts including accuracy of the facts, statements, citing resources, etc.

### Summary/Conclusion

This analysis establishes contemporary survival benchmarks for non-transplanted AML patients in CR1 across diverse clinically relevant subgroups. *RUNX1* mutation was independently associated with early relapse in the ELN 2024 favorable-risk group treated with venetoclax-based LIT.

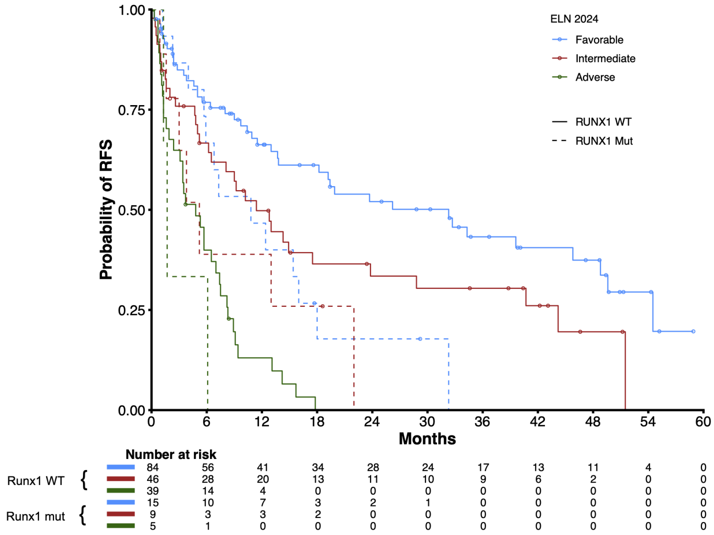

**Copyright Information:** (Online) ISSN: 2572-9241
©2026 The Author(s). HemaSphere is published by John Wiley & Sons Ltd on behalf of European Hematology Association. This is an open access Abstract Book distributed under the Attribution-NonCommercial-NoDerivs (CC BY-NC-ND), which allows third parties to download the articles and share them with others as long as they credit the author and the Abstract Book, but they cannot change the content in any way or use them commercially.
**Abstract Book Citations:** Authors, Title, HemaSphere, 2026;10:(S1):pages. The abstract book DOIs can be found on the "Author Information" page.
**Disclaimer:** Articles published in the journal HemaSphere exclusively reflect the opinions of the authors. The authors are responsible for all content in their abstracts including accuracy of the facts, statements, citing resources, etc.

### (PB2774) REAL-WORLD EFFICACY AND MOLECULAR RESPONSE ANALYSIS OF IVOSIDENIB-BASED REGIMENS VERSUS VENETOCLAX PLUS AZACITIDINE IN IDH1-MUTATED AML

**Topic:** 04. Acute myeloid leukemia - Clinical

Qingli Ji*[1], Xinwen Jiang[1], Xiaoqing Li[1], Xinrui Zhang[1], Chen Cao[1], Minran Zhou[1], Sai MA[1], Chunyan Chen[1]

*[1]Qilu Hospital of Shandong University, Department of Hematology, Jinan, China;*

### Background

Isocitrate dehydrogenase 1 (IDH1) mutations occur in approximately 7-14% of acute myeloid leukemia (AML) patients. Ivosidenib is an oral targeted IDH1 inhibitor. However, real-world data directly comparing its efficacy with the standard low-intensity regimen of venetoclax plus azacitidine (VA), particularly regarding deep molecular responses, remain limited.

### Aims

To compare the real-world efficacy and safety of ivosidenib-based regimens versus VA regimens in patients with IDH1-mutated AML.

### Methods

This single-center retrospective study screened 48 patients diagnosed with IDH1-mutated AML at Qilu Hospital of Shandong University from January 2021 to January 2025. To focus on low-intensity treatment comparisons, patients receiving intensive chemotherapy were excluded. A total of 23 patients were analyzed and stratified into two groups: the ivosidenib-based group (ivosidenib group, n=8; including 5 with azacitidine and 3 with VA) and the VA group (n=15). Endpoints included complete remission/complete remission with incomplete hematologic recovery (CR/CRi) rate, IDH1 mutation clearance, minimal residual disease (MRD) status, event-free survival (EFS), and adverse events.

### Results

Baseline characteristics were balanced: median age (62 vs 64 years), ELN 2022 adverse-risk proportion (62.5% vs 60.0%), and IDH1 R132C as the predominant subtype. In the ivosidenib group, 87.5% (7/8) received first-line treatment. CR/CRi rates were 75.0% in the ivosidenib group and 86.7% in the VA group. However, the ivosidenib group demonstrated significantly superior molecular responses: a higher IDH1 mutation clearance rate (87.5% vs 20.0%). Notably, all patients achieving IDH1 clearance in the ivosidenib group remained relapse-free, whereas all cleared patients in the VA group subsequently experienced molecular relapse. Furthermore, the ivosidenib group achieved shorter median times to first CR/CRi, MRD negativity, and IDH1 clearance: 1.2, 1.4, and 1.7 months, respectively. At a median follow-up of 4.7 months (ivosidenib group) and 12.2 months (VA group), the 6-month EFS rate was significantly higher in the ivosidenib group (87.5% vs 53.3%). Both regimens were well-tolerated, with grade 1-3 hematologic toxicities being the main adverse events, and no discontinuations due to toxicity.

### Summary/Conclusion

This real-world study suggests that, compared to VA, ivosidenib-based therapy induces deeper, faster, and more durable molecular responses in IDH1-mutated AML, with a trend towards improved early survival. Larger studies with longer follow-up are warranted to confirm long-term survival benefits. Furthermore, exploring triplet regimens combining ivosidenib, hypomethylating agents, and venetoclax may represent a promising strategy to further improve outcomes in this patient population.

**Copyright Information:** (Online) ISSN: 2572-9241
©2026 The Author(s). HemaSphere is published by John Wiley & Sons Ltd on behalf of European Hematology Association. This is an open access Abstract Book distributed under the Attribution-NonCommercial-NoDerivs (CC BY-NC-ND), which allows third parties to download the articles and share them with others as long as they credit the author and the Abstract Book, but they cannot change the content in any way or use them commercially.
**Abstract Book Citations:** Authors, Title, HemaSphere, 2026;10:(S1):pages. The abstract book DOIs can be found on the “Author Information” page.
**Disclaimer:** Articles published in the journal HemaSphere exclusively reflect the opinions of the authors. The authors are responsible for all content in their abstracts including accuracy of the facts, statements, citing resources, etc.

### (PB2775) VENETOCLAX-BASED LOW-INTENSITY COMBINATIONS BEYOND FIRST LINE IN AML: REAL-WORLD OUTCOMES FROM THE MULTICENTER VENERA COHORT

**Topic:** 04. Acute myeloid leukemia - Clinical

Maksim Granatkin*[1], Nikita Pastukhov[2], Natalia Persianova[3], Efim Pozharsky[4], Marina Kaden[5], Maret Murzabekova[6], Elena Starikova[7], Varvara Bahtina[8], Rada Ebzeeva[9], Elena Zinina[10], Yuliya Gammershmidt[11], Ksenia Deduhina[12], Elena Komartceva[13], Aigul Latipova[14], Ilya Usankin[15], Mikhail Lubchenko[16], Tatiana Mitina[17], Vitaly Dubov[18], Irina Kudinova[19], Olga Serdyuk[3], Mikhail Kunst[20], Natalya Kosocheva[21], Irina Nechunaeva[22], Anastasia Vinokurova[23], Victoria Abramova[24], Zalina Kharasova[25], Anna Kopylova[26], Vadim Ptushkin[1], Eugene Nikitin[1], Sergey Bondarenko[2]

*[1]S.P. Botkin City Clinical Hospital, Moscow Healthcare Department, Moscow, Russia; [2]Raisa Gorbacheva Memorial Research Institute for Pediatric Oncology, Hematology and Transplantation, I. P. Pavlov First Saint Petersburg State Medical University, Ministry of Health of Russia, Saint Petersburg, Russia; [3]Clinical Oncology Dispensary No. 1 of the Krasnodar region, Krasnodar, Russia; [4]Moscow Multidisciplinary Clinical Center "Kommunarka", Moscow, Russia; [5]Volgograd Regional Clinical Oncology Center, Volgograd, Russia; [6]Stavropol Regional Clinical Oncology Center, Stavropol, Russia, Stavropol, Russia;[7]Clinical Oncology Dispensary No. 2 of the Krasnodar region, Krasnodar, Russia; [8]Professor VF Voyno-Yasenetsky Krasnoyarsk State Medical University, Krasnoyarsk, Russia; [9]Karachay-Cherkessia Oncology Dispensary named after S.P. Butov, Cherkessk, Russia; [10]Surgut Regional Clinical Hospital, Surgut, Russia; [11]Tomsk Regional Clinical Hospital, Tomsk, Russia; [12]Trans-Baikal Regional Cancer Center, Chita, Chita, Russia; [13]Regional Clinical Hospital, Rostov, Russia; [14]State Clinical Hospital № 2, Novosibirsk, Russia, Novosibirsk, Russia; [15]Central City Hospital No. 7, Ekaterinburg, Russia; [16]Chelyabinsk Regional Clinical Hospital, Chelyabinsk, Russia; [17]Moscow Regional Research and Clinical Institute ("MONIKI"), Moscow, Russia; [18]Regional Clinical Hospital No. 2, Vladivostok, Russia; [19]Altai Regional Clinical Hospital, Barnaul, Russia; [20]Republican Clinical Hospital, Kazan, Russia; [21]regional clinical hospital, Permian, Russia; [22]State Clinical Hospital № 2, Novosibirsk, Russia; [23]Tyumen Regional Clinical Hospital No. 1, Tyumen, Russia; [24]Regional Clinical Hospital, Vladimir, Russia; [25]M.F. Vladimirskiy Moscow Regional Research Clinical Institute, Moscow, Russia; [26]Lipetsk Municipal Hospital № 3 "Svobodnyi Sokol", Lipetsk, Russia;*

### Background

Outcomes after frontline failure in acute myeloid leukemia (AML) remain poor, and salvage options are limited. Venetoclax-based low-intensity combinations are widely used in routine practice, yet multicenter real-world evidence beyond first line remains scarce.

### Aims

To retrospectively assess the real-world effectiveness and tolerability of venetoclax-based low-intensity combinations administered beyond first line in AML (multicenter VENERA cohort).

### Methods

We conducted a multicenter retrospective cohort study across 23 centers in the Russian Federation, including 215 AML patients who received venetoclax-based low-intensity therapy beyond first line between 2019 and 2025. Venetoclax was combined with a hypomethylating agent (HMA) or low-dose cytarabine (LDAC). The primary (mutually exclusive) indication for venetoclax initiation was classified as early relapse (<12 months), late relapse (≥12 months), primary refractory AML, lack of response to first induction, prior-line toxicity, or rising measurable residual disease (MRD).

**Copyright Information:** (Online) ISSN: 2572-9241
©2026 The Author(s). HemaSphere is published by John Wiley & Sons Ltd on behalf of European Hematology Association. This is an open access Abstract Book distributed under the Attribution-NonCommercial-NoDerivs (CC BY-NC-ND), which allows third parties to download the articles and share them with others as long as they credit the author and the Abstract Book, but they cannot change the content in any way or use them commercially.
**Abstract Book Citations:** Authors, Title, HemaSphere, 2026;10:(S1):pages. The abstract book DOIs can be found on the "Author Information" page.
**Disclaimer:** Articles published in the journal HemaSphere exclusively reflect the opinions of the authors. The authors are responsible for all content in their abstracts including accuracy of the facts, statements, citing resources, etc.

**Results**

Data completeness varied across variables, consistent with the multicenter real-world design. Among patients with available data on prior therapy lines (n=175), venetoclax-based therapy was administered as first, second, and ≥third salvage line in 93 (53.1%), 61 (34.9%), and 21 (12.0%) patients, respectively; the median number of venetoclax-based cycles was 3 (range, 1–29). Allogeneic HSCT was performed in 53 patients (CR1 n=18; ≥CR2 n=18; outside remission n=16; status unknown n=1). Clinically significant comorbidities were present in 80/104 patients (77%); baseline grade 4 neutropenia and fever were observed in 21/84 (25%) and 18/89 (20%), respectively.
CRc was 132/215 (61.4%); 69/215 (32.1%) were non-responders and response status was missing for 14/215 (6.5%). Among responders with time-to-response available (n=131), responses occurred mainly after cycle 1 (107; 81.7%), followed by cycle 2 (16; 12.2%) and cycle 3 (5; 3.8%); delayed responses were rare (cycle 4 n=2; cycle 5 n=1). Among patients with a documented start date (n=214), 30-, 60-, and 90-day all-cause mortality was 3.7% (n=8), 9.3% (n=20), and 13.1% (n=28), respectively.
Progression was documented in 127/215 (59.1%) and absent in 84/215 (39.1%) (missing n=4). Primary refractory was reported in 49/215 (22.8%), typically defined as no response after two cycles. In the time-to-event cohort (n=211), 92 deaths occurred; median OS was 14.1 months (95% CI, 11.8–26.1) and median EFS was 7.4 months (95% CI, 6.0–10.7) (Fig. 1).
OS by primary indication excluded rare categories (rising MRD; prior-line toxicity). Median OS did not differ significantly (log-rank p=0.14): 23.1 months (95% CI, 16.8–NR) for lack of response to first induction, 13.7 months (95% CI, 9.4–NR) for primary refractory AML, 8.7 months (95% CI, 7.6–NR) for early relapse (<12 months), and 47.6 months (95% CI, 13.4–NR) for late relapse (≥12 months) (Fig. 2). Upper confidence limits were not reached for several subgroups, reflecting limited follow-up and/or a low number of events.

**Summary/Conclusion**

In this multicenter real-world cohort, venetoclax-based low-intensity combinations beyond first line achieved a CRc of 61%, with most responses occurring after cycle 1. Early mortality was modest, while EFS remained shorter than OS, underscoring the need to deepen and sustain remissions. These data support the feasibility and real-world activity of venetoclax-based low-intensity salvage therapy in AML.

**Copyright Information:** (Online) ISSN: 2572-9241
©2026 The Author(s). HemaSphere is published by John Wiley & Sons Ltd on behalf of European Hematology Association. This is an open access Abstract Book distributed under the Attribution-NonCommercial-NoDerivs (CC BY-NC-ND), which allows third parties to download the articles and share them with others as long as they credit the author and the Abstract Book, but they cannot change the content in any way or use them commercially.
**Abstract Book Citations:** Authors, Title, HemaSphere, 2026;10:(S1):pages. The abstract book DOIs can be found on the "Author Information" page.
**Disclaimer:** Articles published in the journal HemaSphere exclusively reflect the opinions of the authors. The authors are responsible for all content in their abstracts including accuracy of the facts, statements, citing resources, etc.

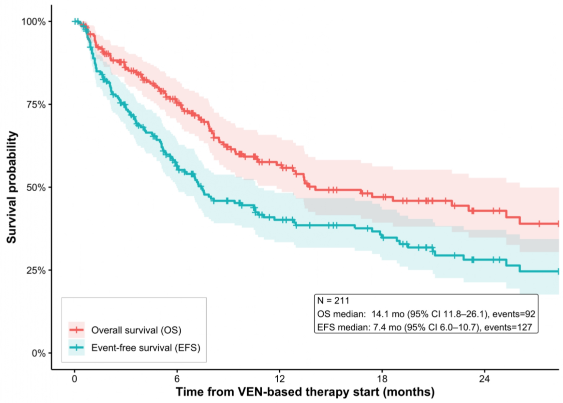

**Figure 1.** Overall survival and failure-/progression-free survival from the start of venetoclax-based

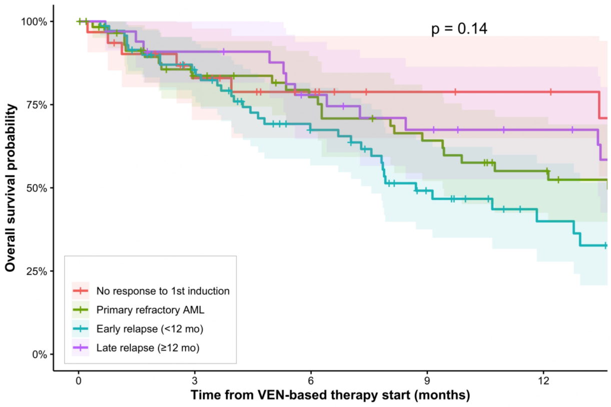

**Figure 2.** Overall survival by indication for venetoclax-based therapy

**Copyright Information:** (Online) ISSN: 2572-9241
©2026 The Author(s). HemaSphere is published by John Wiley & Sons Ltd on behalf of European Hematology Association. This is an open access Abstract Book distributed under the Attribution-NonCommercial-NoDerivs (CC BY-NC-ND), which allows third parties to download the articles and share them with others as long as they credit the author and the Abstract Book, but they cannot change the content in any way or use them commercially.
**Abstract Book Citations:** Authors, Title, HemaSphere, 2026;10:(S1):pages. The abstract book DOIs can be found on the "Author Information" page.
**Disclaimer:** Articles published in the journal HemaSphere exclusively reflect the opinions of the authors. The authors are responsible for all content in their abstracts including accuracy of the facts, statements, citing resources, etc.

### (PB2776) AGE-ASSOCIATED DIFFERENCES IN PREDICTORS OF SURVIVAL AND EARLY MORTALITY IN ACUTE MYELOID LEUKEMIA: A RETROSPECTIVE ANALYSIS

**Topic:** 04. Acute myeloid leukemia - Clinical

Evgenii Kunevich*[1], Sergey Voloshin[1, 2], Victor Solovyov[1], Asiyat Radzhabova[3], Olga Uspenskaya[1], Alexey Kuvshinov[3], Elena Karyagina[4], Sergey Alekseev[1, 5]

*[1]Leningrad Regional Clinical Hospital, Saint-Petersburg, Russia;[2]S.M. Kirov Military Medical Academy, Saint-Petersburg, Russia; [3]Russian Research Institute of Hematology and Transfusiology, Saint-Petersburg, Russia;[4]City Hospital No. 15, Saint-Petersburg, Russia; [5]N.N. Petrov National Medical Research Center of Oncology, Saint-Petersburg, Russia;*

### Background

Despite advances in targeted therapy, the overall prognosis of acute myeloid leukemia (AML) remains unsatisfactory, highlighting the importance of identifying predictors of mortality, particularly across different age groups.

### Aims

To identify key predictors of survival and early mortality (within 4 weeks) in patients with AML across different age groups using real-world clinical data.

### Methods

This retrospective analysis included 484 patients with AML treated between 2009 and November 1, 2025. Patients were stratified into two age groups: younger than 60 years (median age 49 years, n=261) and 60 years or older (median age 68 years, n=223). Regression models included age as well as cytogenetic and molecular genetic parameters as predictors. Results are presented as hazard ratios (HR) or odds ratios (OR). All variables were tested for multicollinearity (VIF range: 1.017–1.198). Non-significant covariates were excluded from multivariable models based on stepwise selection (p>0.05). Statistical analyses were performed using Jamovi v2.4.8.0 and R v4.4.2.

**Copyright Information:** (Online) ISSN: 2572-9241
©2026 The Author(s). HemaSphere is published by John Wiley & Sons Ltd on behalf of European Hematology Association. This is an open access Abstract Book distributed under the Attribution-NonCommercial-NoDerivs (CC BY-NC-ND), which allows third parties to download the articles and share them with others as long as they credit the author and the Abstract Book, but they cannot change the content in any way or use them commercially.
**Abstract Book Citations:** Authors, Title, HemaSphere, 2026;10:(S1):pages. The abstract book DOIs can be found on the "Author Information" page.
**Disclaimer:** Articles published in the journal HemaSphere exclusively reflect the opinions of the authors. The authors are responsible for all content in their abstracts including accuracy of the facts, statements, citing resources, etc.

**Results**

In patients younger than 60 years, univariable analysis identified the following significant predictors of mortality: age (HR=1.03, p=0.003), 11q23/*KMT2A* rearrangements (HR=2.58, p=0.016), del(17p) (HR=4.43, p=0.001), chromosome 5 abnormalities (del(5q)/t(5q)/add(5q)) (HR=2.58, p=0.026), chromosome 7 abnormalities (−7/del(7q)) (HR=1.97, p=0.044), and *FLT3*-ITD mutation (HR=1.58, p=0.029).
After inclusion of significant univariable predictors into the multivariable Cox proportional hazards model and confirmation of the proportional hazards assumption (global p=0.3516), three predictors remained independently associated with all-cause mortality: age (HR=1.02, p=0.034), del(17p) (HR=5.90, p<0.001), and *FLT3*-ITD (HR=1.72, p=0.013). Thus, in patients younger than 60 years, each 10-year increase in age was associated with a 22% increase in mortality risk (HR=$1.02^{10}$=1.22).
In patients aged 60 years or older, the only significant predictor of mortality in univariable analysis was *FLT3*-ITD mutation (HR=1.95, p=0.002). However, in the multivariable model, two markers retained independent prognostic significance: *FLT3*-ITD (HR=2.08, p=0.002) and del(5q) (HR=2.14, p=0.031). The lack of age among significant predictors in this group is likely attributable to non-linearity of the age effect in patients older than 60 years (Figure B).
No significant predictors associated with early mortality were identified in patients younger than 60 years. In contrast, in patients aged 60 years or older, early mortality was associated with age (OR=1.08, p=0.028) and del(5q) (OR=3.49, p=0.022). In the multivariable logistic regression model, significant predictors of early mortality included age (OR=1.09, p=0.029), del(5q) (OR=4.42, p=0.016), and *FLT3*-ITD (OR=2.70, p=0.040). The model demonstrated acceptable calibration (Hosmer–Lemeshow $\chi^2$=12.16; df=8; p=0.1441), moderate discrimination (AUC=0.673), and absence of multicollinearity (VIF range: 1.027–1.045).

**Summary/Conclusion**

*FLT3*-ITD mutations and deletions of chromosomes 5q and 17p have a significant impact on AML prognosis, with distinct patterns of prognostic predictors across age groups. In younger patients, early mortality is not associated with tumor biology, whereas older patients with *FLT3*-ITD and/or del(5q) represent a high-risk population requiring the earliest possible use of targeted agents and alternative treatment strategies based on these therapies.

**Copyright Information:** (Online) ISSN: 2572-9241
©2026 The Author(s). HemaSphere is published by John Wiley & Sons Ltd on behalf of European Hematology Association. This is an open access Abstract Book distributed under the Attribution-NonCommercial-NoDerivs (CC BY-NC-ND), which allows third parties to download the articles and share them with others as long as they credit the author and the Abstract Book, but they cannot change the content in any way or use them commercially.
**Abstract Book Citations:** Authors, Title, HemaSphere, 2026;10:(S1):pages. The abstract book DOIs can be found on the “Author Information” page.
**Disclaimer:** Articles published in the journal HemaSphere exclusively reflect the opinions of the authors. The authors are responsible for all content in their abstracts including accuracy of the facts, statements, citing resources, etc.

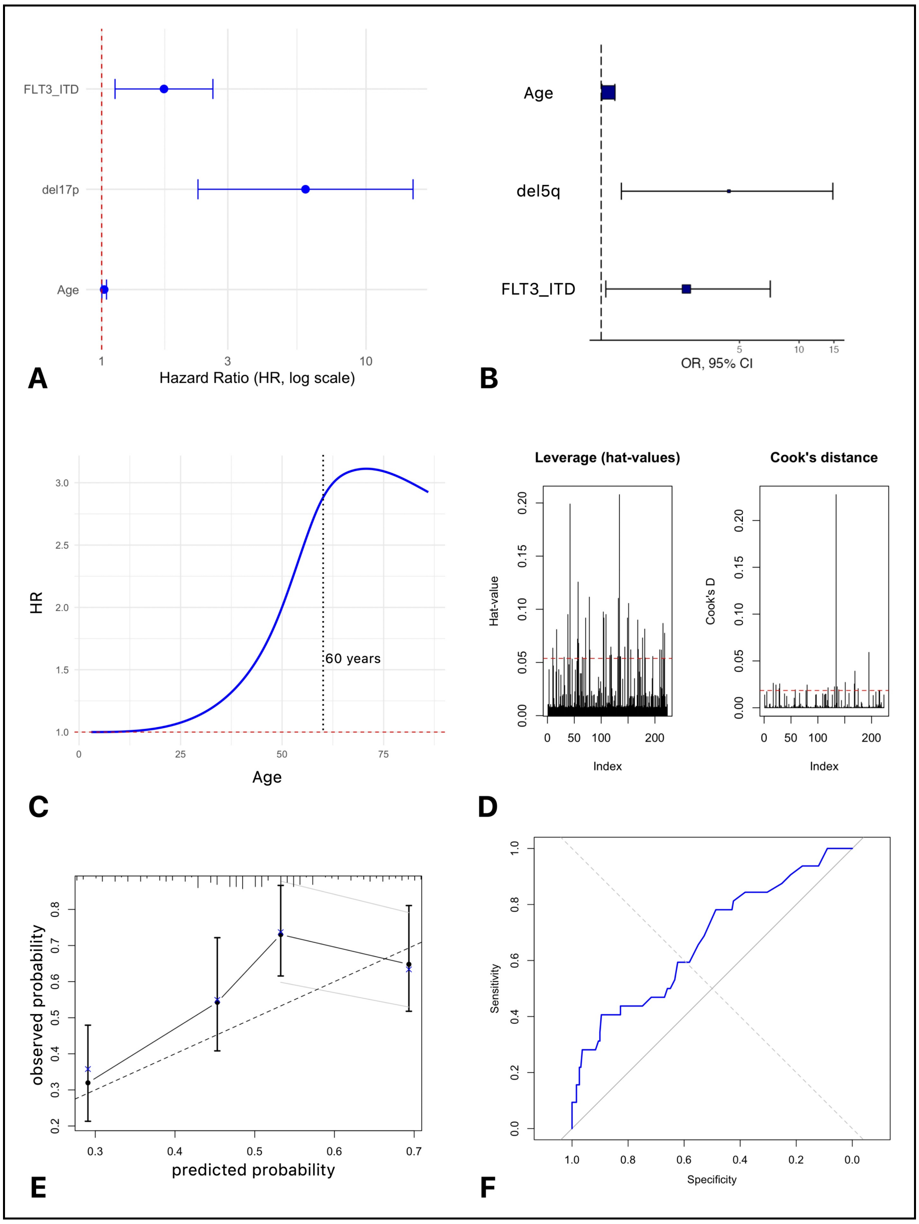

Multivariable Cox Proportional Hazards Model (Dependent variable: all-cause mortality) A) Forest plot of the multivariable Cox model showing hazard ratios (HRs) for each predictor with 95% confidence intervals. B) Relationship between age and hazard ratio for all-cause mortality. D) Comparison of model-predicted survival probabilities with observed outcomes (perfect calibration corresponds to the y=x line). Multivariable Logistic Regression Model (Dependent variable: mortality within the first 4 weeks after initiation of disease-specific therapy) C) Forest plot of the multivariable logistic regression model showing odds ratios (ORs) for early mortality with 95% confidence intervals. E) Influence diagnostics plots (hat-values and Cook's distance) used to identify influential observations and assess model robustness. F) Receiver operating characteristic (ROC) curve with area under the curve (AUC) assessing the discriminative performance of the model.

**Copyright Information:** (Online) ISSN: 2572-9241
©2026 The Author(s). HemaSphere is published by John Wiley & Sons Ltd on behalf of European Hematology Association. This is an open access Abstract Book distributed under the Attribution-NonCommercial-NoDerivs (CC BY-NC-ND), which allows third parties to download the articles and share them with others as long as they credit the author and the Abstract Book, but they cannot change the content in any way or use them commercially.
**Abstract Book Citations:** Authors, Title, HemaSphere, 2026;10:(S1):pages. The abstract book DOIs can be found on the "Author Information" page.
**Disclaimer:** Articles published in the journal HemaSphere exclusively reflect the opinions of the authors. The authors are responsible for all content in their abstracts including accuracy of the facts, statements, citing resources, etc.

### (PB2777) VENERA. A MULTICENTER EXPERIENCE OF LOW-INTENSITY TREATMENT BASED ON VENETOCLAX IN PATIENTS WITH NEWLY DIAGNOSED ACUTE MYELOID LEUKEMIA

**Topic:** 04. Acute myeloid leukemia - Clinical

Nikita Pastukhov*[1], Maksim Granatkin[2], Natalia Persianova[3], Olga Lagunova[4], Efim Pozharsky[5], Marina Kaden[6], Maret Murzabekova[7], Elena Starikova[8], Varvara Bahtina[9], Rada Ebzeeva[10], Irina Kudinova[11], Ksenia Deduhina[12], Elena Zinina[13], Ilya Usankin[14], Vitaly Dubov[15], Yuliya Gammershmidt[16], Mikhail Kunst[17], Victoria Abramova[18], Mikhail Lubchenko[19], Tatiana Mitina[20], Anastasia Vinokurova[21], Irina Nechunaeva[22], Aigul Latipova[23], Olga Serdyuk[3], Sergey Bondarenko[1], Alexander Kulagin[1], Eugene Nikitin[2], Vadim Ptushkin[2]

*[1]Raisa Gorbacheva Memorial Research Institute for Pediatric Oncology, Hematology and Transplantation, I. P. Pavlov First Saint Petersburg State Medical University, Ministry of Health of Russia, Saint Petersburg, Russia; [2]S.P. Botkin City Clinical Hospital, Moscow Healthcare Department, Moscow, Russia; [3]Clinical Oncology Dispensary No. 1 of the Krasnodar region, Krasnodar, Russia; [4]Kirov Research Institute of Hematology and Blood Transfusion, Federal Medical and Biological Agency, Kirov, Russia; [5]Moscow Multidisciplinary Clinical Center "Kommunarka", Moscow, Russia; [6]Volgograd Regional Clinical Oncology Center, Volgograd, Russia; [7]Stavropol Regional Clinical Oncology Center, Stavropol, Russia;[8]State Budgetary Healthcare Organisation "Oncology Dispensary No.2", Sochi, Russia; [9]Krasnoyarsk Regional Clinical Hospital, Krasnoyarsk, Russia; [10]Karachay-Cherkessia Oncology Dispensary named after S.P. Butov, Cherkessk, Russia; [11]Altai Regional Clinical Hospital, Barnaul, Russia; [12]Zabaikalsky Regional Oncological Dispensary, Chita, Russia; [13]Surgut Regional Clinical Hospital, Surgut, Russia; [14]Central City Hospital №7, Ekaterinburg, Russia; [15]Regional Clinical Hospital No. 2, Vladivostok, Russia; [16]Tomsk Regional Clinical Hospital, Tomsk, Russia; [17]Republican Clinical Hospital, Kazan, Russia; [18]Regional Clinical Hospital, Vladimir, Russia; [19]Chelyabinsk Regional Clinical Hospital, Chelyabinsk, Russia; [20]Moscow Regional Research and Clinical Institute ("MONIKI"), Moscow, Russia; [21]Tyumen Regional Clinical Hospital No. 1, Tyumen, Russia;[22]State Clinical Hospital № 2, Novosibirsk, Russia;[23]Republican Clinical Hospital named after G.G. Kuvatova, Ufa, Russia;*

### Background

The combination of venetoclax with hypomethylating agents or low-dose cytarabine is the standard of care for patients with acute myeloid leukemia (AML) who are unfit for intensive chemotherapy. Despite its widespread use in clinical practice, published data on the use of this treatment regimen in the Russian Federation are fragmented and limited to the experience of individual centers and a small sample size of patients.

### Aims

To evaluate real-world outcomes of venetoclax-based therapy in AML within a multicenter study.

### Methods

This retrospective analysis included 304 patients with newly diagnosed AML from 23 centers across the Russian Federation. The patients received first-line therapy with venetoclax combined with hypomethylating agents or low-dose cytarabine between 2019 and 2025. The median follow-up, calculated using the reverse Kaplan-Meier method, was 14 (range 0-63) months. The median age was 66 (range 19-88) years. 219 (72%) patients had de novo AML, and 71 (23%) had secondary AML.

**Copyright Information:** (Online) ISSN: 2572-9241
©2026 The Author(s). HemaSphere is published by John Wiley & Sons Ltd on behalf of European Hematology Association. This is an open access Abstract Book distributed under the Attribution-NonCommercial-NoDerivs (CC BY-NC-ND), which allows third parties to download the articles and share them with others as long as they credit the author and the Abstract Book, but they cannot change the content in any way or use them commercially.
**Abstract Book Citations:** Authors, Title, HemaSphere, 2026;10:(S1):pages. The abstract book DOIs can be found on the "Author Information" page.
**Disclaimer:** Articles published in the journal HemaSphere exclusively reflect the opinions of the authors. The authors are responsible for all content in their abstracts including accuracy of the facts, statements, citing resources, etc.

**Results**

Response was assessed in 300 patients. An overall response was achieved in 72% (N=217) of patients, refractory disease was documented in 16% (N=47), and 12% (N=36) died before response evaluation. The need for supportive care was assessed in 135 patients. During the first cycle of therapy, 81% of patients required red cell transfusions, 73% required platelet transfusions, and 75% required intravenous antibiotics. However, starting from the second cycle, the proportion of patients requiring supportive care significantly decreased, reaching 8%, 14%, and 0%, respectively, by the fourth cycle (Fig. 1C). The 30- and 60-day mortality rates were 7.7% (95% CI 5.2-11.3) and 14.9% (95% CI 11.3-19.5), respectively. Median overall survival (OS) was 15.9 months (95% CI, 11.9–20.0), and the 2-year OS rate was 38.5% (95% CI 30.8-46.0). Median relapse-free survival (EFS) was 9.3 months (95% CI, 7.3–12.8), and the 2-year EFS rate was 30.2% (95% CI 23.4-37.3) (Fig. 1A). The 2-year cumulative incidence of relapse was 46% (95% CI 37-55), with a non-relapse mortality of 13% (95% CI 8-19), (Fig. 1B). Allogeneic hematopoietic stem cell transplantation was performed in 39 (13%) patients, with a median age of 57 (range 20-75) years. Of these, 32 (82%) patients underwent transplantation in remission, while 7 (18%) underwent it after loss of response. The 2-year OS after transplantation in remission was 65.2% (95% CI 32.8-84.9), whereas for patients not in remission it was 21.4% (95% CI 17.8-58.6), p=0.002.

**Summary/Conclusion**

This study presents Russian experience with the use of venetoclax in first-line AML therapy. The response rate, survival outcomes and mortality are comparable with global data.

**Copyright Information:** (Online) ISSN: 2572-9241
©2026 The Author(s). HemaSphere is published by John Wiley & Sons Ltd on behalf of European Hematology Association. This is an open access Abstract Book distributed under the Attribution-NonCommercial-NoDerivs (CC BY-NC-ND), which allows third parties to download the articles and share them with others as long as they credit the author and the Abstract Book, but they cannot change the content in any way or use them commercially.
**Abstract Book Citations:** Authors, Title, HemaSphere, 2026;10:(S1):pages. The abstract book DOIs can be found on the "Author Information" page.
**Disclaimer:** Articles published in the journal HemaSphere exclusively reflect the opinions of the authors. The authors are responsible for all content in their abstracts including accuracy of the facts, statements, citing resources, etc.

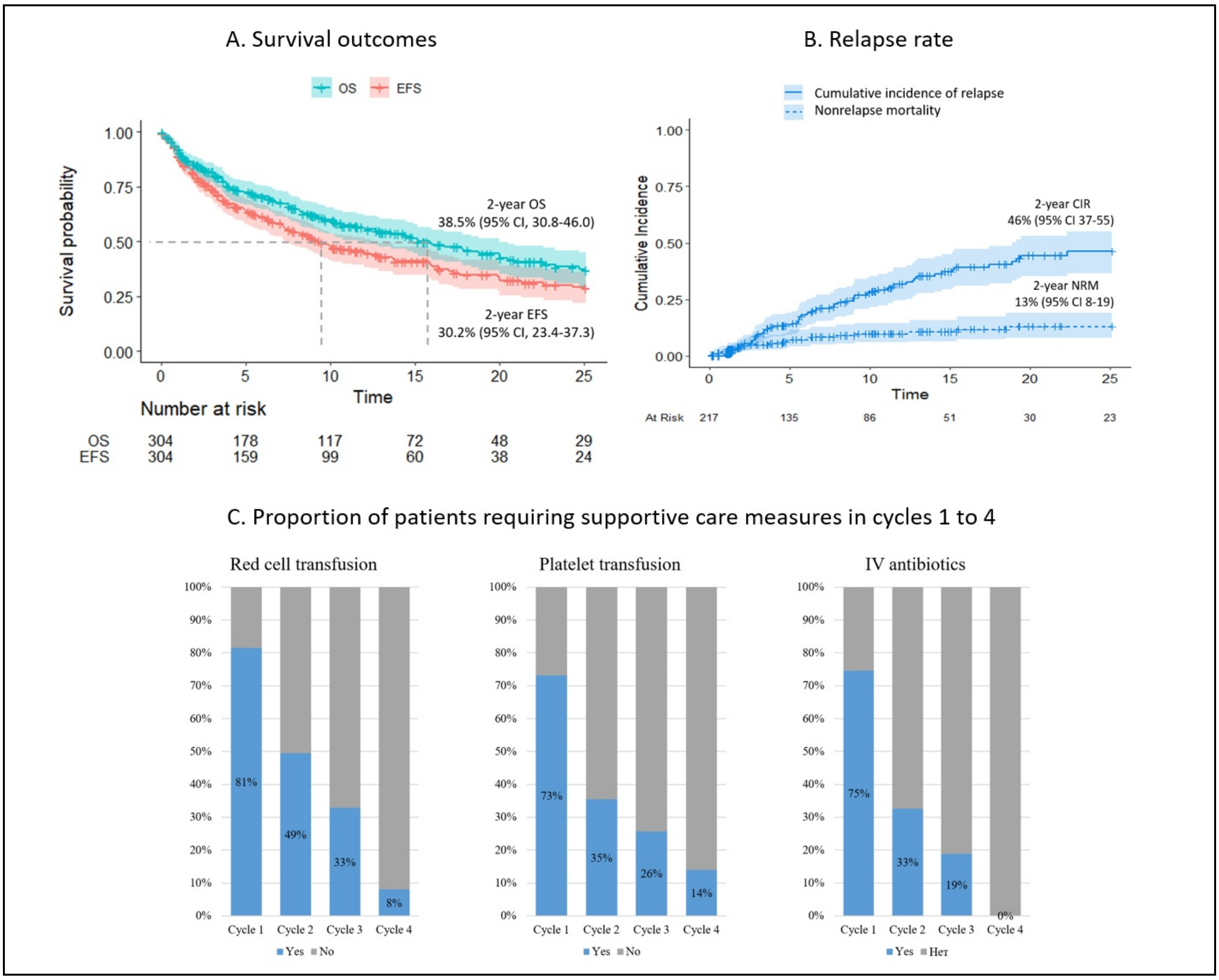

Figure 1. Survival outcomes (A). Relapse rate (B). Proportion of patients requiring supportive care measures in cycles 1 to 4 (C).

**Copyright Information:** (Online) ISSN: 2572-9241
©2026 The Author(s). HemaSphere is published by John Wiley & Sons Ltd on behalf of European Hematology Association. This is an open access Abstract Book distributed under the Attribution-NonCommercial-NoDerivs (CC BY-NC-ND), which allows third parties to download the articles and share them with others as long as they credit the author and the Abstract Book, but they cannot change the content in any way or use them commercially.
**Abstract Book Citations:** Authors, Title, HemaSphere, 2026;10:(S1):pages. The abstract book DOIs can be found on the "Author Information" page.
**Disclaimer:** Articles published in the journal HemaSphere exclusively reflect the opinions of the authors. The authors are responsible for all content in their abstracts including accuracy of the facts, statements, citing resources, etc.

### (PB2778) DOES MODERATE CYP3A4 INHIBITION REQUIRE VENETOCLAX DOSE REDUCTION IN ACUTE MYELOID LEUKEMIA? REAL-WORLD EVIDENCE WITH ISAVUCONAZOLE.

**Topic:** 04. Acute myeloid leukemia - Clinical

María Ibáñez Lacalle*[1], Rodrigo Cantera Estefanía[1], Nerea Elorza Odriozola[1], Amaia Aldea García de Vicuña[1], María Elena Sola Aparicio[1], Beatriz Larrayoz Sola[1], Maria Piva Sanchez Anton[1], Mª Ángeles Goñi Hernández[1], Blai Signes Fort[1], Ernesto Pérez Persona[1], José María Arguiñano[1], Ana Gorena Nicuesa[1], Amaya Zabalza San Martín[1], Maite Zudaire Ripa[1], Maria Carmen Mateos Rodriguez[1], Ana Margarita Redondo Izal[1]

[1]*Hospital Universitario de Navarra, Hematology, Pamplona, Spain;*

### Background

Isavuconazole, a moderate CYP3A4 inhibitor, is used for antifungal prophylaxis in patients receiving venetoclax-based therapy for AML. Venetoclax dose reduction is frequently performed to prevent toxicity, although its impact on treatment efficacy remains uncertain.

### Aims

To compare toxicity and clinical outcomes of venetoclax 200 mg vs. 400 mg in AML patients receiving concomitant isavuconazole.

### Methods

Retrospective single-center cohort study including adults with AML/myeloid sarcoma treated with venetoclax and isavuconazole (2015–2026). Baseline characteristics, early toxicities, response (CR/MLFS), OS, and PFS were analyzed using Fisher's exact test, Mann–Whitney U test, and Kaplan–Meier estimates.

### Results

Thirty-nine patients were included (median age 74 years, IQR 64–81); 22 received venetoclax 200 mg and 17 received 400 mg. Baseline characteristics are shown in Table 1. The 200 mg group was older (77 vs 64 years, p=0.006) with worse ECOG (p=0.003). Genetic risk and comorbidity index were comparable. Early toxicities are summarized in Table 2. TLS parameters were similar. Renal TLS occurred in 5/22 patients receiving 200 mg vs 0/17 with 400 mg (p=0.056), with no cardiac or neurologic events. The 200 mg group had longer severe anemia duration (Hb <8 g/dL; p=0.004), while neutropenia and thrombocytopenia duration did not differ. Infections were more frequent with 200 mg (17/22 vs 3/17, p<0.001). Given baseline imbalances, these differences likely reflect patient frailty rather than a dose effect. No invasive fungal infections were observed, and hepatotoxicity was similar. Responses were numerically higher with 400 mg (10/15 vs 7/21; p=0.090), with no significant differences in OS (1.12 vs 1.41 years; p=0.424) or PFS (p=0.336).

Table 1. **Baseline characteristics**

**Copyright Information:** (Online) ISSN: 2572-9241
©2026 The Author(s). HemaSphere is published by John Wiley & Sons Ltd on behalf of European Hematology Association. This is an open access Abstract Book distributed under the Attribution-NonCommercial-NoDerivs (CC BY-NC-ND), which allows third parties to download the articles and share them with others as long as they credit the author and the Abstract Book, but they cannot change the content in any way or use them commercially.
**Abstract Book Citations:** Authors, Title, HemaSphere, 2026;10:(S1):pages. The abstract book DOIs can be found on the "Author Information" page.
**Disclaimer:** Articles published in the journal HemaSphere exclusively reflect the opinions of the authors. The authors are responsible for all content in their abstracts including accuracy of the facts, statements, citing resources, etc.

| | | Venetoclax dose | | | | |
|---|---|---|---|---|---|---|
| | | 200 mg (n= 22) | | 400 mg (n=17) | | p |
| | | n | % | n | % | |
| Age (median, [IQR]) | | 77 [74-83] | | 64 [54-78] | | 0.006 |
| Women | | 9 | 40.9 | 4 | 23.5 | 0.318 |
| Diagnosis | AML | 6 | 27.3 | 14 | 82.4 | 0.001 |
| | AML-MDr | 16 | 72.2 | 2 | 11.8 | |
| | Myeloid sarcoma | 0 | 0 | 1 | 5.9 | |
| Genetic risk (ELN_2022) | High | 18 | 81.8 | 11 | 64.7 | 0.455 |
| | Intermediate | 3 | 13.6 | 5 | 29.4 | |
| | Low | 1 | 4.5 | 1 | 5.9 | |
| ECOG | 0 | 5 | 22.7 | 13 | 76.5 | 0.003 |
| | 1 | 16 | 72.7 | 3 | 17.6 | |
| | 2 | 0 | 0% | 1 | 5.9 | |
| | 3 | 1 | 4.5% | 0 | 0 | |
| Charlson index (median [IQR]) | | 6 [5-7] | | 5 [4-9] | | 0.585 |
| Line of treatment | 1 | 15 | 68.2% | 8 | 47.1 | 0.095 |
| | 2 | 5 | 22.7% | 9 | 52.9 | |
| | 3 | 2 | 9.1% | 0 | 0 | |
| Previous allo-SCT | | 1 | 4,5% | 6 | 35.3 | 0.030 |
| Bridge to allo-SCT (transplant delay) | | 0 | 0% | 4 | 23.5 | 0.029 |

Table 2. **Toxicities**

| | | Venetoclax Dose | | | | |
|---|---|---|---|---|---|---|
| | | 200 mg (n= 22) | | 400 mg (n=17) | | p |
| | | Median | IQR | Median | IQR | |
| TLS | Max uric acid | 5.1 | 4.0 – 6.5 | 5.5 | 4.8 – 6.1 | 0.591 |
| | Max potassium | 4.7 | 4.4 – 5.1 | 4.5 | 4.3 – 4.7 | 0.232 |
| | Max phosphate | 4.6 | 3.3 – 4.9 | 3.8 | 3.5 – 4.2 | 0.172 |
| | Min calcium | 8.0 | 7.7 – 8.1 | 8.7 | 8.0 – 8.8 | 0.012 |
| | Max Creatinine | 1.08 | 0.79 – 1.86 | 1.01 | 0.82 – 1.26 | 0.457 |
| Aplasia | Days Hb <8 g/dL | 2 | 1 - 5 | 0 | 0 - 1 | 0.004 |
| | Days neutrophils <500/µL | 26 | 14 - 30 | 16 | 3 - 33 | 0.644 |
| | Days platelets <20.000/µL | 1 | 0 - 12 | 0 | 0 - 2 | 0.347 |
| | RBC units transfused | 3 | 1 - 5 | 1 | 0 - 3 | 0.092 |
| | Platelet pools transfused | 0.5 | 0 - 3 | 0 | 0 - 0 | 0.081 |
| Hepatotoxicity | Max ALP level | 116 | 80 – 153 | 87 | 70 - 131 | 0.311 |
| | Max GGT level | 43 | 29 - 163 | 49 | 35 - 199 | 0.798 |
| | Max AST level | 29 | 21 - 42 | 29 | 23 - 40 | 0.908 |
| | Max ALT level | 33 | 18.0 - 61 | 35 | 19 - 57 | 0.931 |
| | Max bilirrubine level | 0.80 | 0.61 – 0.9 | 0.70 | 0.60 – 1.10 | 0.581 |

**Copyright Information:** (Online) ISSN: 2572-9241
©2026 The Author(s). HemaSphere is published by John Wiley & Sons Ltd on behalf of European Hematology Association. This is an open access Abstract Book distributed under the Attribution-NonCommercial-NoDerivs (CC BY-NC-ND), which allows third parties to download the articles and share them with others as long as they credit the author and the Abstract Book, but they cannot change the content in any way or use them commercially.
**Abstract Book Citations:** Authors, Title, HemaSphere, 2026;10:(S1):pages. The abstract book DOIs can be found on the "Author Information" page.
**Disclaimer:** Articles published in the journal HemaSphere exclusively reflect the opinions of the authors. The authors are responsible for all content in their abstracts including accuracy of the facts, statements, citing resources, etc.

### Summary/Conclusion

In this real-world cohort, venetoclax 400 mg administered with isavuconazole did not increase early toxicity compared with 200 mg. Although infections were less frequent in the 400 mg group, baseline differences likely influenced this result. A numerical improvement in response to 400 mg suggests that routine dose reduction of venetoclax in the setting of moderate CYP3A4 inhibition may not be universally required. These findings are hypothesis-generating and warrant validation in larger prospective studies.

Figure 1. Kaplan-Meier analysis of overall survival and progression free survival.

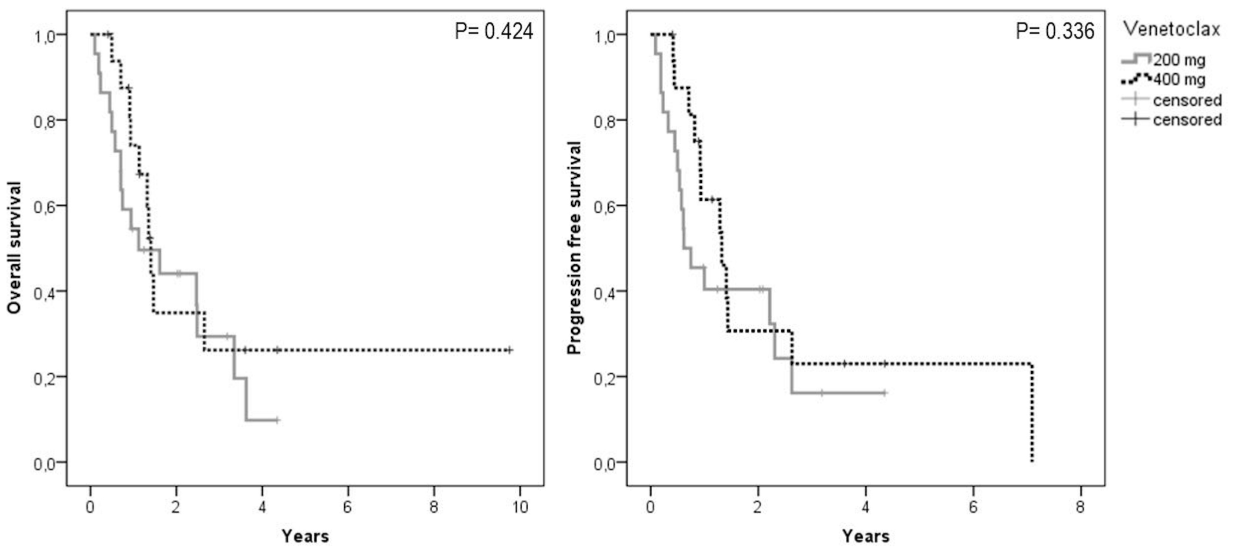

Abbreviations: OS, overall survival; PFS, progression-free survival. Survival curves estimated by the Kaplan–Meier method and compared using the log-rank test. Tick marks represent censored patients.

Copyright Information: (Online) ISSN: 2572-9241
©2026 The Author(s). HemaSphere is published by John Wiley & Sons Ltd on behalf of European Hematology Association. This is an open access Abstract Book distributed under the Attribution-NonCommercial-NoDerivs (CC BY-NC-ND), which allows third parties to download the articles and share them with others as long as they credit the author and the Abstract Book, but they cannot change the content in any way or use them commercially.
Abstract Book Citations: Authors, Title, HemaSphere, 2026;10:(S1):pages. The abstract book DOIs can be found on the "Author Information" page.
Disclaimer: Articles published in the journal HemaSphere exclusively reflect the opinions of the authors. The authors are responsible for all content in their abstracts including accuracy of the facts, statements, citing resources, etc.

### (PB2779) REMAIN-CR STUDY : REMISSION MAINTENANCE WITH ORAL AZACITIDINE IN AML PATIENTS IN COMPLETE RESPONSE AFTER VENETOCLAX + AZACITIDINE IN FIRST LINE

**Topic:** 04. Acute myeloid leukemia - Clinical

Helene Gardeney*[1], Jose Miguel Torregrosa Diaz[1], Maud Brechet[1], Anna Raimbault[1], Maria Pilar Gallego Hernanz[1]

[1]*CHU de POITIERS, POITIERS, France;*

**Background**

The combination of VENETOCLAX and AZACITIDINE (AZA-VEN) has resulted in an increase in the duration of first remission (CR1) in many older patients with newly diagnosed acute myeloid leukemia (AML). However, it came at the cost of increased toxicity. Thus, it can be challenging to maintain it over months of treatment due to adverse events (AEs). Meanwhile, prevention of AML relapse is an important goal, as it is known that the longer the duration of CR1 is, the better the survival is. So, there is an unmet need for de-escalation and a maintenance strategy after AZA-VEN induction treatment to sustain therapeutic activity while lowering toxicity.

**Methods**

We conducted a retrospective study in our institution (Poitiers' University Hospital) of using oral azacitidine (ONUREG®), as maintenance therapy in patients with AML in first remission after frontline treatment with AZA-VEN.
Patients included were 65 years old or older, not candidates for hematopoietic stem-cell transplantation or intensive chemotherapy, and in first cytologic response with (CR) or without (CRi) complete blood count recovery after a minimum of 6 cycles of AZA-VEN.
Patients started ONUREG® at 300mg daily for 14 days over a 28-day cycle.
The primary endpoint was the overall survival. .
Secondary endpoints included progression-free survival and tolerance.

**Results**

A total of 21 patients were included. The median age was 74 years old (range 59 to 87). Noticeably, 10 patients (48%) had an adverse prognostic according to 2022 ELN score, and on a molecular level, 5 (26%) patients were *NPM1* mutated, and 6 (32%) patients presented an arg140 *IDH2* mutation. The median number of AZA-VEN cycles before ONUREG® was 7 (range, 6 to 20), and 10 (48%) patients had a CRi at the start of ONUREG®.
After a median follow-up of 33,8 months (95% CI : 26-48.1) from diagnosis and 23,5 months on ONUREG® (95% CI : 17,8 -NA), Median overall is not reached and median PFS is 32,2 months (26-NA). At the bone marrow assessment performed after a minimum of 3 cycles of ONUREG® (or sooner if relapse was feared), 11 (91%) patients were still in cytologic response. Moreover, all NPM1 mutated patients who achieved MRD negativity by RT-qPCR on AZA VEN remained MRD- on ONUREG. Unforunately, 7 (33%) of patients experienced cytologic relapse with a median time to relapse of 8 months (1-19). However, among 5 recored deaths, only one is related to AML progression.

The most common grade 3 and 4 adverse events (AEs) were neutropenia (in 33% of patients), thrombopenia (in 42 %), and gastrointestinal events (in 0.8 %). Dose adjustments were necessary for 14 (66%) patients but no patients stopped treatement due to toxicity. No febrile neutropenia or hospitalization related to ONUREG® treatment was noticed.

Two of three patients who were still reliant on transfusion during AZA-VEN treatment acquired transfusion independence after starting ONUREG® maintenance after two and three cycles, respectively.

**Copyright Information:** (Online) ISSN: 2572-9241
©2026 The Author(s). HemaSphere is published by John Wiley & Sons Ltd on behalf of European Hematology Association. This is an open access Abstract Book distributed under the Attribution-NonCommercial-NoDerivs (CC BY-NC-ND), which allows third parties to download the articles and share them with others as long as they credit the author and the Abstract Book, but they cannot change the content in any way or use them commercially.
**Abstract Book Citations:** Authors, Title, HemaSphere, 2026;10:(S1):pages. The abstract book DOIs can be found on the "Author Information" page.
**Disclaimer:** Articles published in the journal HemaSphere exclusively reflect the opinions of the authors. The authors are responsible for all content in their abstracts including accuracy of the facts, statements, citing resources, etc.

**Summary/Conclusion**

ONUREG® maintenance therapy is effective and well tolerated after AZA-VEN in older AML patients. Once obtained, cytologic response does not seem to be impact by switching to ONUREG® maintenance. As previously reported, side effects were mainly gastrointestinal and neutropenia, both easily manageable. This approach can improve the quality of life for patients, with fewer hospital visits and no life-threatening events.

**Copyright Information:** (Online) ISSN: 2572-9241
©2026 The Author(s). HemaSphere is published by John Wiley & Sons Ltd on behalf of European Hematology Association. This is an open access Abstract Book distributed under the Attribution-NonCommercial-NoDerivs (CC BY-NC-ND), which allows third parties to download the articles and share them with others as long as they credit the author and the Abstract Book, but they cannot change the content in any way or use them commercially.
**Abstract Book Citations:** Authors, Title, HemaSphere, 2026;10:(S1):pages. The abstract book DOIs can be found on the "Author Information" page.
**Disclaimer:** Articles published in the journal HemaSphere exclusively reflect the opinions of the authors. The authors are responsible for all content in their abstracts including accuracy of the facts, statements, citing resources, etc.

### (PB2781) GENOMIC DNA-BASED QUANTITATIVE PCR IS A USEFUL METHOD FOR MRD ASSESSMENT IN NPM1-MUTATED AML

**Topic:** 04. Acute myeloid leukemia - Clinical

Pia-Sofia Ettala*[1, 2], Kari Remes[1], Vesa Juvonen[1], Mirkka Montonen[1], Maija Itala-Remes[1, 2], Helena Ollila[2]

*[1]Turku University hospital, Department of Clinical hematology and stem cells transplantation, Turku, Finland;[2]University of Turku, Turku, Finland;*

### Background

Measurable residual disease (MRD) monitoring is clinically important in acute myeloid leukemia (AML) as a prognostic tool and therapy guide. NPM1 mutations occur in ~30% of adult AML and include >100 variants. NPM1 mutations are excellent MRD targets. Large prospective trials in intensively treated NPM1-mutated ($NPM1^{mut}$) AML have shown that MRD after induction and at the end of treatment strongly predicts relapse and survival. MRD is most often assessed by RNA-based reverse-transcriptase quantitative PCR (RT-qPCR), but limitations of this technic include RNA instability, expression-based quantification rather than an exact leukemic cell number, restricted coverage of rarer variants, and possibility of false positives. Mutated NPM1 (mNPM1) MRD can also be assessed by genomic DNA-based (gDNA-based) assays. However, the data regarding the prognostic significance of gDNA-based qPCR MRD-results in $NPM1^{mut}$AML are missing.

### Aims

This retrospective study assessed the prognostic impact of MRD assessment by gDNA-based qPCR in a real-world cohort of $NPM1^{mut}$ AML patients treated with intensive chemotherapy.

### Methods

Patients treated for $NPM1^{mut}$ AML at Turku University hospital in Finland during years 2010-2023 in first remission (CR1) after first-line intensive chemotherapy and with existing mNPM1 MRD evaluations assessed by gDNA-based qPCR were retrospectively analysed. The impact of bone marrow (BM) MRD levels after the first two chemotherapy cycles and at the end of therapy on overall survival (OS) and cumulative incidence of relapse  (CIR) according to the 2022 ELN criteria was evaluated. Survival analyses were performed using Kaplan-Meier method, and significance was assessed using the log-rank test. P values of <0.05 were considered statistically significant. ROC analysis was used to determine the optimal MRD cutoffs for OS and CIR after the first and second cycles of intensive chemotherapy.

**Copyright Information:** (Online) ISSN: 2572-9241
©2026 The Author(s). HemaSphere is published by John Wiley & Sons Ltd on behalf of European Hematology Association. This is an open access Abstract Book distributed under the Attribution-NonCommercial-NoDerivs (CC BY-NC-ND), which allows third parties to download the articles and share them with others as long as they credit the author and the Abstract Book, but they cannot change the content in any way or use them commercially.
**Abstract Book Citations:** Authors, Title, HemaSphere, 2026;10:(S1):pages. The abstract book DOIs can be found on the "Author Information" page.
**Disclaimer:** Articles published in the journal HemaSphere exclusively reflect the opinions of the authors. The authors are responsible for all content in their abstracts including accuracy of the facts, statements, citing resources, etc.

### Results

A total of 65 patients with a median age of 60 years carrying 15 different NPM1 mutation subtypes, of which the type A mutation was the most common (48%), were included. One half (51,5%) of the patients had a non-favourable genetic risk (ELN2022). With a median follow-up time of 3,6 years, the median OS and CIR (counting only hematological relapses) of all patients were 13,3 years and 2,6 years respectively. Early high MRD-positivity, post-cycle1 (post-C1) BM MRD >/=1%, was detected in 16 (27%) of 59 evaluable patients. Achieving post-C1 MRD <1% had a statistically significant association with better OS, with a median OS of 13.3 years vs 3.9 years for patients with post-cycle1 MRD level >/=1% (p=0.025), with a trend to decreased CIR (p=0.18). Not even allogeneic stem cells transplantation (HSCT) performed in first remission (CR1) could obliterate the prognostic significance of the post-C1 MRD. Achieving mNPM1 MRD negativity in bone marrow after the second chemotherapy cycle had no significant impact on OS or CIR. The MRD level at the end of treatment (EOT) in the 23 patients not proceeding to HSCT in CR1 had a significant impact on OS; the median-OS being 13,3 years in patients with EOT-MRD undetectable to <0.001% in opposite to 2.0 years for those with EOT-MRD >/=0,001% (p=0.006).

### Summary/Conclusion

We found gDNA-based qPCR to be a highly useful method in MRD assessment in patients with $NPM1^{mut}$ AML receiving first-line intensive chemotherapy . MRD values post-cycle1 and at the end of therapy served as significant prognostic markers for survival.

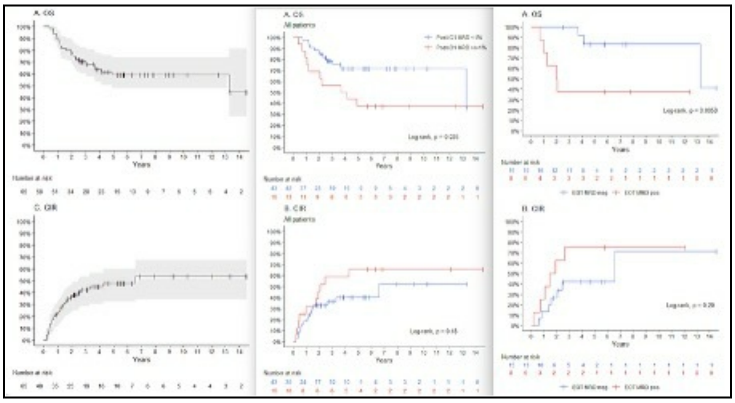

Figure 1. left) OS and CIR in all NPM1mut AML patients, middle) Effect of post-C1 MRD on OS and CIR, Right) Effect of EOT MRD on OS and CIR

**Copyright Information:** (Online) ISSN: 2572-9241
©2026 The Author(s). HemaSphere is published by John Wiley & Sons Ltd on behalf of European Hematology Association. This is an open access Abstract Book distributed under the Attribution-NonCommercial-NoDerivs (CC BY-NC-ND), which allows third parties to download the articles and share them with others as long as they credit the author and the Abstract Book, but they cannot change the content in any way or use them commercially.
**Abstract Book Citations:** Authors, Title, HemaSphere, 2026;10:(S1):pages. The abstract book DOIs can be found on the “Author Information” page.
**Disclaimer:** Articles published in the journal HemaSphere exclusively reflect the opinions of the authors. The authors are responsible for all content in their abstracts including accuracy of the facts, statements, citing resources, etc.

### (PB2782) THIS REPORT PROVIDES A COMPREHENSIVE ASSESSMENT OF THE BASELINE CHARACTERISTICS, THERAPEUTIC PATTERNS, EFFICACY, AND TOLERABILITY OF ORAL-AZA IN THE POLISH POPULATION WITH AML.

**Topic:** 04. Acute myeloid leukemia - Clinical

Łukasz Bołkun*[1], Agnieszka Pluta[2], Bożena Budziszewska[3], Kamila Kruczkowska-Tarantowicz[4], Edyta Cichocka[5], Marta Sobas[6], Szczepaniak Andrzej[7], Jarosław Piszcz[1], Kacper Czachor[8], Karolina Bełdzińska-Gądek[9], Pawel Kuczia[10], Agnieszka Podgórska[11], Sebastian Giebel[12], Agnieszka Wierzbowska[2]

*[1]Medical University, Bialystok, Poland,, Hematology Department, Białystok, Poland;[2]Department of Hematology, Medical University of Lodz, Multidisciplinary Provincial Centre of Traumatology and Oncology Nicolas Copernicus in Lodz, Lodz, Poland., Lodz, Poland; [3]Department of Hematology, Institute of Hematology and Transfusion Medicine, Warsaw, Poland., Warsaw, Poland; [4]Department of Internal Medicine and Hematology, Military Institute of Medicine, Warsaw, Poland., Warsaw, Poland; [5]Department of Hematology, Rydygier Hospital Torun, Torun, Poland., Torun, Poland;[6]Department of Hematology, Collegium Medicum in Bydgoszcz, Nicolaus Copernicus University in Toruń, Bydgoszcz, Poland., Bydgoszcz, Poland; [7]Department of Hematology Transplantation and Cellular Therapy, Poznan University of Medical Sciences, Poznan, Poland., Poznan, Poland; [8]Department of Haematology and Bone Marrow Transplantation, Andrzej Mielęcki Independent Public Clinical Hospital, Katowice, Poland., Katowice, Poland; [9]Department of Haematology and Transplantology, Medical University of Gdańsk, Gdańsk, Poland., Gdansk, Poland; [10]Jagiellonian University Medical College, Department of Hematology, Krakow, Poland., Krakow, Poland; [11]Clinical Hospital of the Ministry of Internal Affairs and Administration with the Warmia-Mazury Oncology Centre in Olsztyn, Department of Hematology and BMT, Olsztyn, Poland., Olsztyn, Poland; [12]Department of Bone Marrow Transplantation and Onco-Hematology, Maria Sklodowska-Curie National Research Institute of Oncology, Gliwice, Poland., Gliwice, Poland;*

### Background

Allogeneic stem cell transplantation (allo-SCT) is a standard of care for post-remission therapy in acute myeloid leukemia (AML), significantly reducing relapse rate in eligible patients who achieve composite complete remission (cCR). For those deemed ineligible for transplant, effective maintenance therapies are critically needed. Oral azacitidine (Oral-Aza) is the only non-targeted agent approved by authorities. Limited data are available on clinical outcomes with Oral-Aza maintenance for AML in real-world practice, particularly across different induction regimens, including CPX-351 or with FLT3 inhibitors.

### Aims

This report provides a comprehensive assessment of the baseline characteristics, therapeutic patterns, efficacy, and tolerability of Oral-Aza in the Polish population with AML.

### Methods

This retrospective, observational study included 90 pts with AML from 14 Polish Hematology Departments who were in cCR after at least induction therapy and received Oral-Aza maintenance.

**Copyright Information:** (Online) ISSN: 2572-9241
©2026 The Author(s). HemaSphere is published by John Wiley & Sons Ltd on behalf of European Hematology Association. This is an open access Abstract Book distributed under the Attribution-NonCommercial-NoDerivs (CC BY-NC-ND), which allows third parties to download the articles and share them with others as long as they credit the author and the Abstract Book, but they cannot change the content in any way or use them commercially.
**Abstract Book Citations:** Authors, Title, HemaSphere, 2026;10:(S1):pages. The abstract book DOIs can be found on the “Author Information” page.
**Disclaimer:** Articles published in the journal HemaSphere exclusively reflect the opinions of the authors. The authors are responsible for all content in their abstracts including accuracy of the facts, statements, citing resources, etc.

**Results**

The characteristics of pts. are reported in Table 1, Figure 1.
All patients received cytarabine- and anthracyclin-based induction (IC), followed by consolidation in 71% of pts (n=69), with a median of 2 cycles.
At the initiation of maintenance therapy, the most frequent reasons for non-eligibility for allo-SCT in 1st cCR were severe comorbidities (n=31), patient preference (n=18), an unavailable donor (n=11), advanced age (n=9), and favorable-risk genetics (n=21).
The median duration of Oral-Aza with a median follow-up of 14 months was 7 cycles (1-32 cycles), and 30% remained on the drug for ≥12 months. Notably, 14 out of 90 patients (16%) in our cohort received allo-SCT at different times of Oral-Aza maintenance (median 3 cycles).
Analysis of progression-free survival (PFS) censored on allo-SCT demonstrated the best outcome in pts treated with DA plus midostaurin, for which the median PFS was not reached, compared to the DA, CPX-351, and DAC regimens (median PFS: 6.5 and 5,5 months respectively, Fig. 1A). Furthermore, patients initiating Oral-Aza directly after induction experienced significantly shorter median PFS (6 months) than those who received at least one consolidation cycle (edian PFS: 10 months, Fig. 1B). Importantly, the number of preceding consolidation cycles did not have a significant impact on PFS.
At the baseline of Oral-Aza treatment, measurable residual disease (MRD) by MFC was positive in 40% (36/90) of pts. Among these MRD-positive pts, six converted to MRD negativity during Oral-Aza treatment; all had a baseline MRD level ≤0.5%.
Surprisingly, the presence of MRD at baseline (≥0.1%) was not associated with a shorter PFS, Fig. 1C.
Pts with adverse cytogenetics had shorter PFS than those in the favourable- and intermediate-risk groups (median PFS: 6 months*vs* 12 months, Fig. 1D).
All pts began Oral-AZA at 300 mg for 14 days/cycle, with 94% remaining on this schedule at discontinuation/last visit.
Approximately, half (55%) of pts had ≥1 AE during Oral-Aza treatment. Six pts (7%) discontinued treatment due to cytopenias, and 28 (31%) due to disease progression.

**Summary/Conclusion**

The real-world outcomes of patients with AML in remission who received Oral-Aza were consistent with those reported in the pivotal Quazar AML-001 trial.
Our findings indicate that Oral-Aza can serve as an effective bridging therapy to allo-SCT for patients with temporary contraindications related to complications of prior intensive therapy or prolonged donor search.

**Copyright Information:** (Online) ISSN: 2572-9241
©2026 The Author(s). HemaSphere is published by John Wiley & Sons Ltd on behalf of European Hematology Association. This is an open access Abstract Book distributed under the Attribution-NonCommercial-NoDerivs (CC BY-NC-ND), which allows third parties to download the articles and share them with others as long as they credit the author and the Abstract Book, but they cannot change the content in any way or use them commercially.
**Abstract Book Citations:** Authors, Title, HemaSphere, 2026;10:(S1):pages. The abstract book DOIs can be found on the “Author Information” page.
**Disclaimer:** Articles published in the journal HemaSphere exclusively reflect the opinions of the authors. The authors are responsible for all content in their abstracts including accuracy of the facts, statements, citing resources, etc.

**Table 1**

| Patients' characteristics (N = 90) | |
|---|---|
| Age median (range) | 62 years (16-75) |
| ≤65 years | 48/90 (53%) |
| > 65 years | 42/90 (47%) |
| Female / Male [no. (%)] | 47 (52%) / 43 (48%) |
| **AML type [no./total (%)]** | |
| De novo | 37/90 (41%) |
| Secondary | 53/90 (59%) |
| **Cytogenetic risk (MRC) [no./total (%)]** | |
| Favorable | 17/90 (19%) |
| Intermediate | 48/90 (53%) |
| Adverse | 25/90 (28%) |
| **Genetic mutations [no./total (%)]** | |
| NPM1 | 21/90 (23%) |
| FLT3-ITD/FLT3-TKD | 15/90 (17%) |
| **Treatment regimens for induction, n (%)** | |
| Cytarabine + anthracycline (3+7) | 41/90 (45%) |
| DNR+AraC (DA) + midostaurin | 14/90 (16%) |
| DA+ clardribine (DAC) | 19/90 (21%) |
| CPX-351 | 16/90 (18%) |
| **Received consolidation, [no./total (%)]** | |
| No | 26/90 (29%) |
| Yes | 64/90 (71%) |
| 1 consolidation | 10/64 (16%) |
| 2 consolidation | 28/64 (44%) |
| 3 consolidation | 26/64 (40%) |
| **MRD status at oral-AZA initiation, [no./total (%)]** | |
| cCR MRD Negative (<0.1%) | 54/90 (60%) |
| cCR MRD Positive (≥0.1%) | 36/90 (40%) |
| **The reason for discontinuation of Oral-AZA, [no./total ]** | |
| Progression of disease | 28/90 (31%) |
| Allo-HSCT | 14/90 (16%) |
| AEs or SAEs | 6/90 (7%) |

AML -Acute Myeloid Leukemia; IC - Intensive Chemotherapy; ITD - Internal Tandem Duplication; MDS - Myelodysplastic Syndrome; TKD -Tyrosine Kinase Domain. CPX-351-liposomal cytarabine-daunorubicin, AEs- Adverse Events, SAEs- Serious Adverse Events, cCR-composite complete remission, MRD-Minimal residual disease, allo-SCT-Allogeneic Stem CellTransplantation,

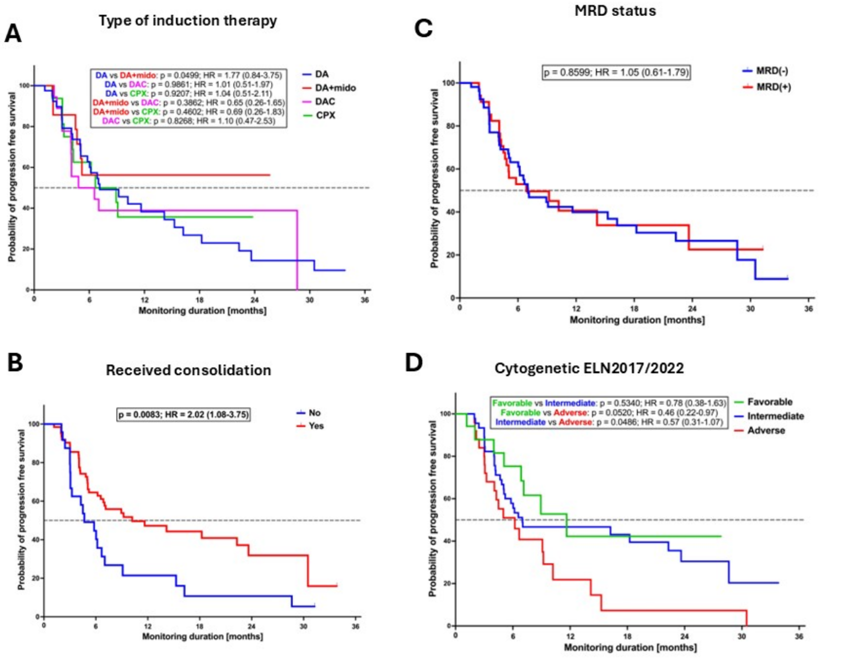

**Copyright Information:** (Online) ISSN: 2572-9241
©2026 The Author(s). HemaSphere is published by John Wiley & Sons Ltd on behalf of European Hematology Association. This is an open access Abstract Book distributed under the Attribution-NonCommercial-NoDerivs (CC BY-NC-ND), which allows third parties to download the articles and share them with others as long as they credit the author and the Abstract Book, but they cannot change the content in any way or use them commercially.
**Abstract Book Citations:** Authors, Title, HemaSphere, 2026;10:(S1):pages. The abstract book DOIs can be found on the "Author Information" page.
**Disclaimer:** Articles published in the journal HemaSphere exclusively reflect the opinions of the authors. The authors are responsible for all content in their abstracts including accuracy of the facts, statements, citing resources, etc.

### (PB2783) ANALYSIS OF CLINICAL CHARACTERISTICS AND PROGNOSTIC FACTORS FOR INFANT ACUTE MYELOID LEUKEMIA

**Topic:** 04. Acute myeloid leukemia - Clinical

[illegible][1], Shaoyan Hu*[1]

[1]*Suzhou Children's Hospital, SUZHOU, China;*

### Aims

This study investigated the clinical characteristics of infant acute myeloid leukemia (AML), analyzed the risk and protective factors affecting the prognosis of affected children, and laid a foundation for further optimizing clinical treatment strategies and improving the prognostic evaluation system.

### Methods

A total of 43 infants with acute myeloid leukemia (AML) who were admitted and received complete treatment at Children's Hospital of Soochow University from 2014 to 2025 were enrolled in this retrospective study. General data at initial diagnosis were collected, and clinical characteristics, treatment regimens, therapeutic efficacy, relapse patterns, time to relapse, and prognosis were analyzed and discussed.

### Results

1. A total of 43 infants with acute myeloid leukemia AML were enrolled between 2014 and 2025, including 25 males (58.1%, n=25/43) and 18 females (41.9%, n=18/43), with a male-to-female ratio of 1.4:1. The age at initial diagnosis ranged from 1 day to 365 days (12 months), with a median age of 216 days (7.2months). Stratified by age, 8 cases (18.6%, n=8/43) were 0–3 months old, 12 cases (27.9%, n=12/43) were 3–6 months old, 8 cases (18.6%, n=8/43) were 6–9 months old, and 15 cases (34.9%, n=15/43) were 9–12 months old.
2. Among the 43 infants, the most common initial symptom was fever, accounting for 62.8% (n=27/43). Gastrointestinal symptoms and respiratory symptoms each accounted for 11.6% (n=5/43), anemia for 4.7% (n=2/43), bleeding for 7.0% (n=3/43), and lymphadenopathy for 2.3% (n=1/43). Fever was the most prevalent initial symptom, while lymphadenopathy was the least common.
3. Among the 43 AML infants, the families of 14 cases chose to abandon treatment after a clear diagnosis. All 29 infants who received induction therapy achieved complete remission (CR), of which 28 cases (96.6%) achieved CR after 1 course of induction therapy, and 1 case (3.4%) achieved CR after 2 courses. Among these 29 infants, 21 cases survived (72.4%) and 8 cases died (27.6%). Specifically, 7 deaths were due to the primary disease, and 1 death was caused by cardiotoxicity after chemotherapy. Additionally, 9 infants experienced relapse, with a relapse rate of 31.0%.
4. Univariate prognostic analysis showed that NRAS/KRAS mutations, hemoglobin (Hb) < 90 g/L at initial diagnosis, and failure to receive hematopoietic stem cell transplantation (HSCT) were risk factors affecting the overall survival (OS) of the infants. Similarly, NRAS/KRAS mutations, Hb < 90 g/L at initial diagnosis, and non-receipt of HSCT were identified as risk factors for event-free survival (EFS) in the infants. Results of Cox multivariate analysis indicated that Hb < 90 g/L at initial diagnosis and failure to undergo HSCT were independent risk factors influencing both EFS and OS of the patients.

**Copyright Information:** (Online) ISSN: 2572-9241
©2026 The Author(s). HemaSphere is published by John Wiley & Sons Ltd on behalf of European Hematology Association. This is an open access Abstract Book distributed under the Attribution-NonCommercial-NoDerivs (CC BY-NC-ND), which allows third parties to download the articles and share them with others as long as they credit the author and the Abstract Book, but they cannot change the content in any way or use them commercially.
**Abstract Book Citations:** Authors, Title, HemaSphere, 2026;10:(S1):pages. The abstract book DOIs can be found on the "Author Information" page.
**Disclaimer:** Articles published in the journal HemaSphere exclusively reflect the opinions of the authors. The authors are responsible for all content in their abstracts including accuracy of the facts, statements, citing resources, etc.

**Summary/Conclusion**

1、Infant acute myeloid leukemia (AML) is characterized by high relapse and treatment abandonment rates, highlighting an urgent need for effective therapeutic regimens to improve patient prognosis.
2、Infant acute myeloid leukemia (AML) exhibits a relatively unique gene expression profile and high heterogeneity.
3、Hb < 90 g/L at initial diagnosis and failure to receive hematopoietic stem cell transplantation were independent risk factors for both overall survival (OS) and event-free survival (EFS) in infants with acute myeloid leukemia (AML)
4、Hematopoietic stem cell transplantation (HSCT) can effectively improve the clinical prognosis of infants with acute myeloid leukemia (AML).

**Copyright Information:** (Online) ISSN: 2572-9241
©2026 The Author(s). HemaSphere is published by John Wiley & Sons Ltd on behalf of European Hematology Association. This is an open access Abstract Book distributed under the Attribution-NonCommercial-NoDerivs (CC BY-NC-ND), which allows third parties to download the articles and share them with others as long as they credit the author and the Abstract Book, but they cannot change the content in any way or use them commercially.
**Abstract Book Citations:** Authors, Title, HemaSphere, 2026;10:(S1):pages. The abstract book DOIs can be found on the “Author Information” page.
**Disclaimer:** Articles published in the journal HemaSphere exclusively reflect the opinions of the authors. The authors are responsible for all content in their abstracts including accuracy of the facts, statements, citing resources, etc.

### (PB2784) «AZACITIDINE+VENETOCLAX» IN REFRACTORY AML: REAL-WORLD DATA FROM UKRAINE DURING WAR

**Topic:** 04. Acute myeloid leukemia - Clinical

Bohdana Kuiavovych*[1], Nadiya Goryainova[1], Zinaida Stupakova[1], Halyna Starodub[1], Olga Basova[1], Anna Sergeieva[1], Kateryna Khurdepa[1]

[1]*State institution «National Research Center for Radiation Medicine, Hematology and Oncology of the National Academy of Medical Sciences of Ukraine», Kyiv, Ukraine;*

### Background

The «7+3» regimen is still considered to be the backbone of therapy for patients with acute myeloid leukemia (AML) who are eligible for intensive induction. However, some patients develop refractory/relapsed disease, requiring salvage strategies and timely transition to alloHCT. In the context of the war in Ukraine, intensive polychemotherapy is associated with significant non-medical challenges, including shortages of blood components, limited medical personnel, recurrent electricity disruptions. Frequent air raid alerts necessitate evacuation to shelters, which poses considerable risks to patients with severe neutropenia and thrombocytopenia, including the risk of infection and bleeding, physical instability and physical discomfort, particularly in patients with existing AML-related complications or treatment-associated toxicities. Given these constraints, the combination of azacitidine and venetoclax was considered a more adaptable therapeutic alternative.

### Aims

To evaluate the efficacy, hematologic recovery, and short-term outcomes of «Azacitidine+Venetoclax» in primary refractory AML after two failed «7+3» inductions.

### Methods

The study included 11 patients with intermediate-risk AML according to ELN criteria who had primary refractory disease after two induction courses of standard «7+3». Subsequent treatment was «Azacitidine+Venetoclax». Treatment response was evaluated according to the ELN response criteria. Measurable residual disease (MRD) was assessed using flow cytometry (FC) and molecular techniques (PCR).

### Results

The median age was 58 years (range 28–72). All 11 patients achieved complete remission (CR), yielding an overall response rate of 100%. CR was achieved after one cycle of «Azacitidine+Venetoclax» in 2 patients (18.2%), after two cycles in 5 patients (45.5%), after three cycles in 3 patients (27.3%), and after four cycles in 1 patient (9.1%). The median number of cycles required to achieve CR was 2, with 7 of 11 patients (63.6%) achieving CR within the first two cycles. According to ELN response criteria, CR with full hematologic recovery was observed in 2 patients (18.2%), CR with incomplete hematologic recovery in 5 patients (45.5%), and CR with partial hematologic recovery in 4 patients (36.4%). At the final assessment, FC-MRD negativity was achieved in 8 patients (72.7%), while molecular MRD negativity was documented in 5 patients (45.5%). Neutrophil recovery (ANC ≥1.0 $\times 10^9$/L) was achieved in all patients with a median time of 40 days (range 29–62; IQR 37–51). Platelet recovery to ≥50 $\times 10^9$/L occurred in all patients at a median of 28 days (range 14–40); to ≥100 $\times 10^9$/L - in 7 patients (63.6%) with a median time of 25 days (range 21–38). At a median follow-up of 6 months, all patients were alive and no relapses were observed. Three patients have been identified with suitable donors and are planned to proceed to alloHCT.

**Copyright Information:** (Online) ISSN: 2572-9241
©2026 The Author(s). HemaSphere is published by John Wiley & Sons Ltd on behalf of European Hematology Association. This is an open access Abstract Book distributed under the Attribution-NonCommercial-NoDerivs (CC BY-NC-ND), which allows third parties to download the articles and share them with others as long as they credit the author and the Abstract Book, but they cannot change the content in any way or use them commercially.
**Abstract Book Citations:** Authors, Title, HemaSphere, 2026;10:(S1):pages. The abstract book DOIs can be found on the "Author Information" page.
**Disclaimer:** Articles published in the journal HemaSphere exclusively reflect the opinions of the authors. The authors are responsible for all content in their abstracts including accuracy of the facts, statements, citing resources, etc.

**Summary/Conclusion**

«Azacitidine+Venetoclax» demonstrated antileukemic activity in patients with intermediate-risk refractory AML following two unsuccessful intensive induction courses, achieving a 100% CR rate with a high proportion of MRD-negative responses. In the setting of the war in Ukraine, this regimen represents a clinically rational and logistically feasible alternative to high-intensity chemotherapy, given its favorable tolerability and reduced need for transfusions and prolonged hospitalization. The depth of response suggest that this approach may serve both as an effective salvage strategy and as a bridge to alloHCT.

**Copyright Information:** (Online) ISSN: 2572-9241
©2026 The Author(s). HemaSphere is published by John Wiley & Sons Ltd on behalf of European Hematology Association. This is an open access Abstract Book distributed under the Attribution-NonCommercial-NoDerivs (CC BY-NC-ND), which allows third parties to download the articles and share them with others as long as they credit the author and the Abstract Book, but they cannot change the content in any way or use them commercially.
**Abstract Book Citations:** Authors, Title, HemaSphere, 2026;10:(S1):pages. The abstract book DOIs can be found on the "Author Information" page.
**Disclaimer:** Articles published in the journal HemaSphere exclusively reflect the opinions of the authors. The authors are responsible for all content in their abstracts including accuracy of the facts, statements, citing resources, etc.

### (PB2785) CARDIAC SAFETY OF L-ANNAMYCIN ACROSS HIGH CUMULATIVE ANTHRACYCLINE EXPOSURE: IMPLICATIONS FOR RELAPSED/REFRACTORY AML

**Topic:** 04. Acute myeloid leukemia - Clinical

Angela Dewar*[1], Ziad Zalaquett[1], Stuart Waymack[2], Patrick Collier[1]

*[1]Cleveland Clinic, Cleveland, United States of America;[2]Virginia Tech, Blacksburg, United States of America;*

### Background

Anthracyclines are foundational agents in acute myeloid leukemia (AML), but cumulative dose limits due to cardiotoxicity frequently limit further anthracycline therapy. In relapsed/refractory (R/R) AML, outcomes following venetoclax-based regimens remain poor, with reported CR/CRi rates of approximately 12.5% using available salvage therapies. Preliminary clinical data have demonstrated encouraging responses with L-Annamycin-based therapy in venetoclax-exposed R/R AML. L-Annamycin is an investigational anthracycline in late-stage clinical development for AML, developed to address limitations associated with conventional anthracyclines, including cardiotoxicity and multidrug resistance.

### Aims

To evaluate the cardiac safety of L-Annamycin using pooled clinical trial data with comprehensive cardiac monitoring and independent cardio-oncology review.

### Methods

Cardiac safety was evaluated across sponsor- and investigator-initiated clinical trials of L-Annamycin and included serial 12-lead ECGs, cardiac biomarkers (troponin I and T), and transthoracic echocardiography with centralized global longitudinal strain (GLS) analysis. All available cardiac data underwent independent review by a cardio-oncology laboratory at the Cleveland Clinic.

### Results

An independent cardio-oncology review was performed for 90 patients treated with L-Annamycin across five completed clinical trials. Among those, 78 had source-data verified pre- and post-treatment ejection fraction (EF) measurements. Median cumulative dose was 660 mg/m$^2$ (95% CI, 645-690; range 210-2,970), exceeding conventional lifetime anthracycline limits. No patients met criteria for clinically significant left ventricular dysfunction (≥10% absolute EF decline to <50%). Mean EF change from baseline to final assessment was −0.12% (95% CI, −1.34 to 1.09; p=0.84). Linear regression demonstrated no correlation between cumulative dose and EF change (p=0.12). Independent review of serial ECGs, serum troponins, and available GLS values also demonstrated no evidence of drug-induced cardiotoxicity.

### Summary/Conclusion

Across cumulative doses exceeding conventional lifetime anthracycline limits, no clinically significant left ventricular dysfunction was observed, and comprehensive cardiac review revealed no treatment-related cardiac abnormalities. These findings support continued evaluation of L-Annamycin as a second-generation anthracycline in R/R AML, including in patients previously treated with anthracycline-based therapy and venetoclax-based regimens, where prior cumulative exposure and limited salvage efficacy remain significant clinical challenges.

**Copyright Information:** (Online) ISSN: 2572-9241
©2026 The Author(s). HemaSphere is published by John Wiley & Sons Ltd on behalf of European Hematology Association. This is an open access Abstract Book distributed under the Attribution-NonCommercial-NoDerivs (CC BY-NC-ND), which allows third parties to download the articles and share them with others as long as they credit the author and the Abstract Book, but they cannot change the content in any way or use them commercially.
**Abstract Book Citations:** Authors, Title, HemaSphere, 2026;10:(S1):pages. The abstract book DOIs can be found on the "Author Information" page.
**Disclaimer:** Articles published in the journal HemaSphere exclusively reflect the opinions of the authors. The authors are responsible for all content in their abstracts including accuracy of the facts, statements, citing resources, etc.

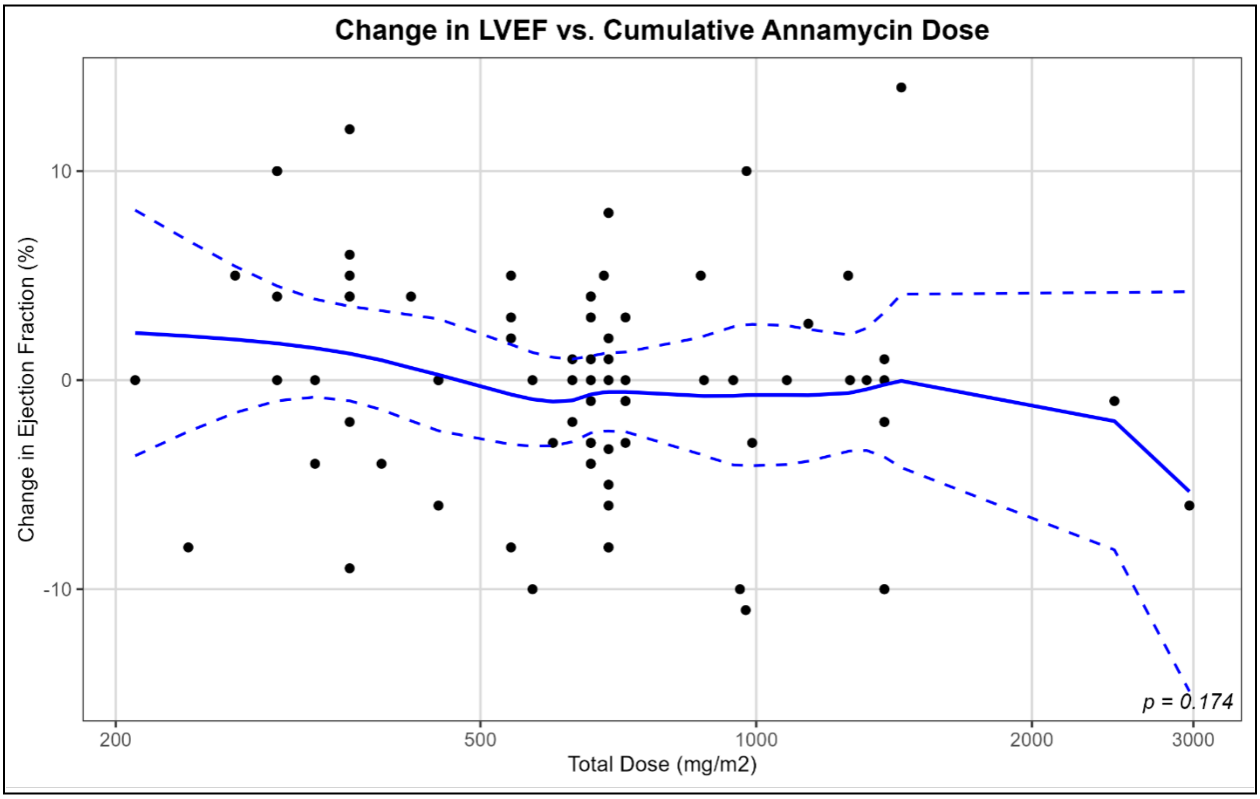

**Copyright Information:** (Online) ISSN: 2572-9241
©2026 The Author(s). HemaSphere is published by John Wiley & Sons Ltd on behalf of European Hematology Association. This is an open access Abstract Book distributed under the Attribution-NonCommercial-NoDerivs (CC BY-NC-ND), which allows third parties to download the articles and share them with others as long as they credit the author and the Abstract Book, but they cannot change the content in any way or use them commercially.
**Abstract Book Citations:** Authors, Title, HemaSphere, 2026;10:(S1):pages. The abstract book DOIs can be found on the "Author Information" page.
**Disclaimer:** Articles published in the journal HemaSphere exclusively reflect the opinions of the authors. The authors are responsible for all content in their abstracts including accuracy of the facts, statements, citing resources, etc.

### (PB2786) TWENTY YEARS OF EXPERIENCE WITH THE MRC-10 PROTOCOL FOR THE TREATMENT OF ADULT ACUTE MYELOID LEUKEMIA IN THE SOUTH OF TUNISIA

**Topic:** 04. Acute myeloid leukemia - Clinical

Nour Siala*[1], Fakhfekh Yousra[1], Imen Ben Amor[1], Hanen Charfi[1], Ikram Dammak[2], Nour Louati[3], Imen Frikha[1], Maha Charfi[1], Faten Kallel[1], Taycir Rekik[3], Rahma Mallek[1], Ines Turki[1], Asma Koubaa[1], Aicha Ben Kahla[1], Moez Medhaffar[1], Mourad Chaari[2], Ikram Ben Amor[3], Nour Ben Abdeljelil[4], Moez Elloumi[1]

*[1]Hematology department Hedi Chaker Hospital, Sfax, Tunisia; [2]Hematology laboratory Hedi Chaker Hospital, Sfax, Tunisia; [3]Blood transfusion center, Sfax, Tunisia; [4]National bone marrow transplant center, Tunis, Tunisia;*

### Background

Acute myeloid leukemia (AML) is an aggressive but potentially curable hematologic malignancy. Despite significant therapeutic advances over recent decades, long-term survival remains limited and is associated with a substantial risk of relapse in the absence of allogeneic hematopoietic stem cell transplantation (allo-HSCT).

### Aims

We aimed to evaluate the therapeutic outcomes of the national AML protocol inspired by the MRC-10 study in young adults.

### Methods

We retrospectively analyzed adults (20–60 years) with de novo AML treated between 2005 and 2024 at the Hematology Department of Sfax-Tunisia. Secondary AML and FAB M3 were excluded. AML were stratified into favorable and unfavorable therapeutic groups according to cytogenetic, molecular features, and chemosensitivity. Allo-HSCT was indicated for eligible patients in the unfavorable group and favorable group with delayed molecular remission with an HLA-matched related donor. Outcomes assessed included complete remission rate (CRR), induction failure, treatment-related mortality (TRM), relapse rate, overall survival (OS), event-free survival (EFS), and disease-free survival (DFS). Survival was analyzed using the Kaplan–Meier method.

### Results

We collected 222 patients. Medium age was 42 years (range: 20 to 59 years). M/F ratio was 1.15. The distribution of our patients according to the cytogenetic risk group was as follows: 18% low-risk abnormalities, 69% intermediate-risk, and 13% high-risk. Molecular study was feasible only for 60% of cases concluding CBF-myh11, AML-ETO, and FLT3 in 15%, 10% and 1 % respectively. Eighty-two % of patients received Idarubicin during induction and 18% received Daunorubicin. The CRR was 67%; it is only correlated with age **(P=0.004)** and cytogenetic risk group **(P<0.0001).** The failure induction rate was 17%. The mortality rate during induction therapy was 20%. The main causes of death were severe infections (60%). Therapeutic group was favorable in 36 cases and unfavorable in 77 Cases . Seventy-two % of patients had consolidation by chemotherapy alone. Among the 137 patients in CR and treated according to the protocol, an allograft was indicated for 80 patients (58%): 73 belonged to the unfavorable group and 7 patients belonged to the favorable group. Among the 75 patients who had an HLA study (94%), 52 patients (65%) had an HLA-compatible family donor. The allograft was performed only for 38 patients: 73% of patients with an HLA-compatible donor and 48% of patients in CR and with an indication for allograft. The relapse rate was 33%. Relapses correlated only with the feasibility allograft in CR1 **(P=0.006).** After a mean follow-up of 126 months, the rates of OS, EFS, and DFS at 5 years were 37%,34%, and 60%, respectively. Both OS and EFS were correlated with age at diagnosis (**p=0.01** and **p= 0.005** respectively), cytogenetic group (**p=0.001** and **p=0.007** respectively) and allograft in CR1 (**p<0.0001** respectively) while DFS was correlated only with allograft in CR1 **(p<0.0001).**

**Copyright Information:** (Online) ISSN: 2572-9241
©2026 The Author(s). HemaSphere is published by John Wiley & Sons Ltd on behalf of European Hematology Association. This is an open access Abstract Book distributed under the Attribution-NonCommercial-NoDerivs (CC BY-NC-ND), which allows third parties to download the articles and share them with others as long as they credit the author and the Abstract Book, but they cannot change the content in any way or use them commercially.
**Abstract Book Citations:** Authors, Title, HemaSphere, 2026;10:(S1):pages. The abstract book DOIs can be found on the "Author Information" page.
**Disclaimer:** Articles published in the journal HemaSphere exclusively reflect the opinions of the authors. The authors are responsible for all content in their abstracts including accuracy of the facts, statements, citing resources, etc.

**Summary/Conclusion**

The therapeutic results of our patients are similar to the literature in terms of initial failure (17% vs 10%) and survival rate (OS at 5 years was 37% vs 40%). However, our population is characterized by a very high rate of induction death (20% vs 5-10%) and a lower rate of CR after induction (67% vs >80%). Our results can be improved by reducing the death rate by developing of hematological reanimation and by adapting the consolidation treatment according to the prognostic group and the rationalization of the allograft

**Copyright Information:** (Online) ISSN: 2572-9241
©2026 The Author(s). HemaSphere is published by John Wiley & Sons Ltd on behalf of European Hematology Association. This is an open access Abstract Book distributed under the Attribution-NonCommercial-NoDerivs (CC BY-NC-ND), which allows third parties to download the articles and share them with others as long as they credit the author and the Abstract Book, but they cannot change the content in any way or use them commercially.
**Abstract Book Citations:** Authors, Title, HemaSphere, 2026;10:(S1):pages. The abstract book DOIs can be found on the "Author Information" page.
**Disclaimer:** Articles published in the journal HemaSphere exclusively reflect the opinions of the authors. The authors are responsible for all content in their abstracts including accuracy of the facts, statements, citing resources, etc.

### (PB2787) CLINICOPATHOLOGIC AND CYTOGENETIC CHARACTERISTICS, PROGNOSTIC PREDICTORS IN T-MN PATIENTS: A SINGLE-CENTER RETROSPECTIVE ANALYSIS

**Topic:** 04. Acute myeloid leukemia - Clinical

Huawei Jiang*[1], Xi Qiu[1], Zhaoxing Wu[1], Yang Xu[1]

[1]*Department of Hematology, The Second Affiliated Hospital of Zhejiang University School of Medicine, Hangzhou, China;*

### Background

Therapy-related myeloproliferative neoplasms (t-MN), which includes therapy-related acute myeloid leukemia (t-AML) and therapy-related myelodysplastic syndrome (t-MDS), are severe complications following chemotherapy or radiation therapy for prior malignancies. Understanding the clinicopathologic features and prognostic factors associated with t-MN is critical to improving patient outcomes and optimizing treatment strategies.

### Aims

This study aimed to analyze the clinicopathologic and cytogenetic characteristics of t-MN patients, with a focus on identifying key prognostic predictors of overall survival (OS) through both univariate and multivariate analyses.

### Methods

This single-center, retrospective study included t-MN patients diagnosed between May 2020 and May 2025. A total of 45 patients were enrolled, with 29 diagnosed with t-AML and 16 with t-MDS. Data on clinicopathologic features, genetic mutations, and prior malignancies were collected. Prognostic factors affecting OS were analyzed using univariate and multivariate Cox regression models.

### Results

The median age at diagnosis was 63 years (range 25–83 years), and the median latency interval was 6 years (range 0–21 years). The most frequent prior malignancies included colorectal cancer, lung cancer, breast cancer, and B-cell lymphoma. TP53 mutations were the most common genetic abnormality (10 cases), followed by ASXL1 mutations (6 cases).
Univariate analysis revealed that in t-AML patients, achieving complete remission (CR) after one course of treatment ($P < 0.01$) was significantly associated with improved OS. In t-MDS patients, older age ($P = 0.02$) and the presence of a complex karyotype ($P = 0.02$) were associated with worse OS. In t-MN patients, CR after one course ($P < 0.01$) was associated with significantly better OS, while complex karyotype ($P = 0.02$) was a poor prognostic factor.
Multivariable analysis revealed that for t-AML patients, hemoglobin (HR = 1.03, $P = 0.02$) was an unfavorable prognostic factor, while CR after one course (HR = 0.39, $P = 0.01$) was a favorable prognostic factor. In t-MDS patients, complex karyotype was identified as a poor prognostic factor (HR = 13.60, $P = 0.04$). For t-MN patients, CR after one course (HR = 0.45, $P < 0.01$) and a latency period >5 years (HR = 0.48, $P < 0.01$) were associated with improved OS.

### Summary/Conclusion

This study identifies critical prognostic factors for t-MN patients, including hemoglobin levels, CR achievement, and complex karyotype. These factors provide valuable insights for clinicians in predicting survival outcomes and tailoring treatment strategies. The findings highlight the importance of incorporating these predictors into clinical practice to improve management and outcomes in this high-risk patient population.

**Copyright Information:** (Online) ISSN: 2572-9241
©2026 The Author(s). HemaSphere is published by John Wiley & Sons Ltd on behalf of European Hematology Association. This is an open access Abstract Book distributed under the Attribution-NonCommercial-NoDerivs (CC BY-NC-ND), which allows third parties to download the articles and share them with others as long as they credit the author and the Abstract Book, but they cannot change the content in any way or use them commercially.
**Abstract Book Citations:** Authors, Title, HemaSphere, 2026;10:(S1):pages. The abstract book DOIs can be found on the “Author Information” page.
**Disclaimer:** Articles published in the journal HemaSphere exclusively reflect the opinions of the authors. The authors are responsible for all content in their abstracts including accuracy of the facts, statements, citing resources, etc.

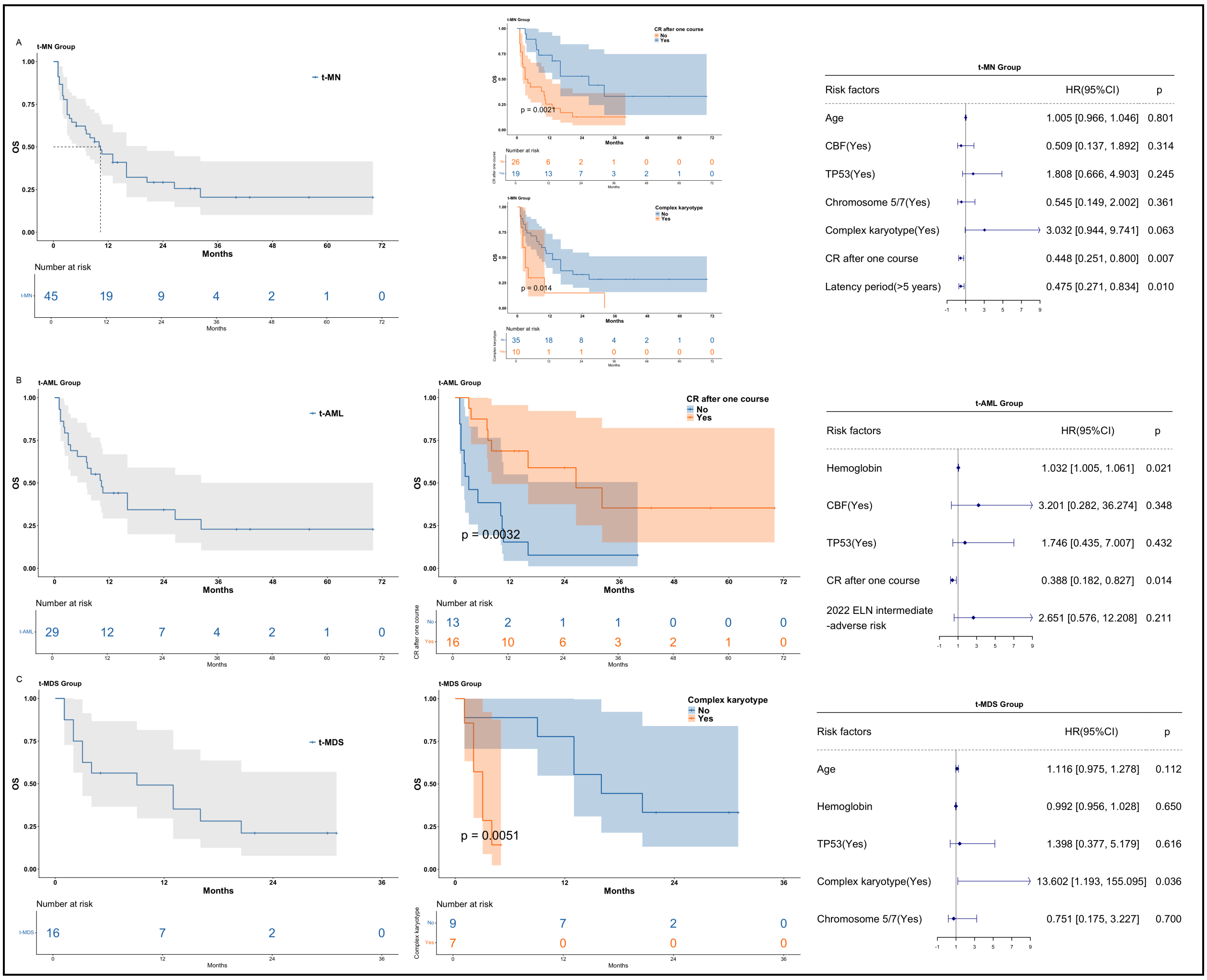

Figure 1. Survival Analysis and Prognostic Predictors in t-MN and Subgroups. (A) t-MN Cohort: Kaplan-Meier curves (left/middle) demonstrate significantly improved OS for patients achieving CR after one course (p=0.0021) and those without complex karyotypes (p=0.014). Multivariate forest plot (right) identifies CR after one course and latency >5 years as independent favorable factors. (B) t-AML Subgroup: Survival advantage was significant for patients achieving CR after one course (p=0.0032). Hemoglobin and CR after one course were confirmed as key prognostic variables. (C) t-MDS Subgroup: Complex karyotype was associated with significantly inferior OS (p=0.0051) and identified as the primary independent risk factor (HR 13.60).

**Copyright Information:** (Online) ISSN: 2572-9241
©2026 The Author(s). HemaSphere is published by John Wiley & Sons Ltd on behalf of European Hematology Association. This is an open access Abstract Book distributed under the Attribution-NonCommercial-NoDerivs (CC BY-NC-ND), which allows third parties to download the articles and share them with others as long as they credit the author and the Abstract Book, but they cannot change the content in any way or use them commercially.
**Abstract Book Citations:** Authors, Title, HemaSphere, 2026;10:(S1):pages. The abstract book DOIs can be found on the "Author Information" page.
**Disclaimer:** Articles published in the journal HemaSphere exclusively reflect the opinions of the authors. The authors are responsible for all content in their abstracts including accuracy of the facts, statements, citing resources, etc.

### (PB2788) GENOMIC LANDSCAPE OF AML PATIENTS IN UKRAINE

**Topic:** 04. Acute myeloid leukemia - Clinical

Nataliia Levkovych*[1, 2], Natalia Trofimova[1], Uliana Lykhova[3], Svitlana Cherniuk[3], Olha Kravets[3], Anna Chaikovska[3], Arsenii Lykhovyi[3], Mariana Kharko[3], Nataliia Olkhovich[1, 4], Nataliia Gorovenko[2]

*[1]State Institution «National scientific center «Institute of Cardiology, Clinical and Regenerative Medicine named after academician M.D. Strazhesko», Department of Genetic Diagnostics, Kyiv, Ukraine; [2]Shupyk National Healthcare University of Ukraine, Department Medical and Laboratory Genetics, Kyiv, Ukraine; [3]National Children's Specialized Hospital "Ohmatdyt," Ministry of Health of Ukraine, Department of Oncogenetic Research in the Medical Genetics Laboratory, Kyiv, Ukraine; [4]National Children's Specialized Hospital "Ohmatdyt," Ministry of Health of Ukraine, Kyiv, Ukraine;*

### Background

Acute myeloid leukemia (AML) is a group of hematological diseases, phenotypically and genetically heterogeneous. AML is a very complex, heterogeneous, and intricate hematological malignancy in which classification is based on genetic and cytogenetic abnormalities as assigned to the current WHO and ICC classification systems. The introduction of NGS techniques into diagnostics and increased awareness of the genetic complexity of AML have enabled the transition from studying individual genes to evaluating a comprehensive panel of genomic loci.

### Aims

**The aim** was to describe the genetic heterogeneity of AML in Ukrainian patients using the capabilities of next-generation sequencing (NGS) technology.

### Methods

Bone marrow (BM) or peripheral blood (PB) samples were collected from 235 patients with AML. The age distribution was as follows: 124 (52.77%) children and 111 (47.23%) adult patients.The Ion Torrent NGS technology of targeted gene sequencing (Oncomine™ Myeloid Research Assay) was applied to detect the genetic alterations based on 40 key DNA genes and a broad fusion of 29 driver genes.

### Results

We identified 21 different types of fusions in 97 patients (41.28%). It should be noted that fusions in children group occurred 2 times more often than in adult patients ($p<0.05$). The most frequent alterations were KMT2A rearrangements (27 cases in children group and 8 – in adult) and *RUNX1::RUNX1T1* fusion (12, 9.68%). No fusions were identified in 58 cases of pediatric AML and in 78 of adult.
In all patients, a total of 355 pathogenic variants were identified in genes included in the panel of myeloid diseases. In children group there were 140 mutation and in group of adults – 215 mutations. Moreover, there were statistically significantly more mutations in DNA in adult patients than in children ($p<0.05$). The most common genetic variants in children group were in signaling genes such *NRAS*, *PTPN11*, *KRAS* and also in *FLT* gene *(FLT3-ITD* and *FLT3-TKD).* Adult patient had another distribution of pathogenic variants. They had most frequent alterations in *NPM1* gene, genes of epigenetic regulation (such *DNMT3A*, *IDH1*/2) and genes of transcription factors ( *WT1*, *RUNX1*). At the same time, a higher number of mutations per person was noted in the adult group.
The data we obtained agree with The European LeukemiaNet (ELN) risk classification system and emphasizes the role of genetic alterations in AML.

**Copyright Information:** (Online) ISSN: 2572-9241
©2026 The Author(s). HemaSphere is published by John Wiley & Sons Ltd on behalf of European Hematology Association. This is an open access Abstract Book distributed under the Attribution-NonCommercial-NoDerivs (CC BY-NC-ND), which allows third parties to download the articles and share them with others as long as they credit the author and the Abstract Book, but they cannot change the content in any way or use them commercially.
**Abstract Book Citations:** Authors, Title, HemaSphere, 2026;10:(S1):pages. The abstract book DOIs can be found on the "Author Information" page.
**Disclaimer:** Articles published in the journal HemaSphere exclusively reflect the opinions of the authors. The authors are responsible for all content in their abstracts including accuracy of the facts, statements, citing resources, etc.

**Summary/Conclusion**

As follows, the study of spectrum of genetic changes in a cohort of patients with AML is a tool that allows doctors to stratify patients by risk groups and make a prognosis.

**Copyright Information:** (Online) ISSN: 2572-9241
©2026 The Author(s). HemaSphere is published by John Wiley & Sons Ltd on behalf of European Hematology Association. This is an open access Abstract Book distributed under the Attribution-NonCommercial-NoDerivs (CC BY-NC-ND), which allows third parties to download the articles and share them with others as long as they credit the author and the Abstract Book, but they cannot change the content in any way or use them commercially.
**Abstract Book Citations:** Authors, Title, HemaSphere, 2026;10:(S1):pages. The abstract book DOIs can be found on the "Author Information" page.
**Disclaimer:** Articles published in the journal HemaSphere exclusively reflect the opinions of the authors. The authors are responsible for all content in their abstracts including accuracy of the facts, statements, citing resources, etc.

### (PB2789) TREATMENT OUTCOMES AND PROGNOSTIC FACTORS OF PAEDIATRIC ACUTE MYELOID LEUKAEMIA: A SINGLE-CENTER STUDY FORM A MIDDLE-INCOME COUNTRY

**Topic:** 04. Acute myeloid leukemia - Clinical

Hanen Charfi*[1], Yousra Fakhfakh Derbel[1], Maha Charfi[1], Nour Siala[1], Faten Kallel[1], Imen Ben Amor[1], Rahma Mallek[1], Ines Turki[1], Asma Koubaa[1], Aicha Ben Kahla[1], Moez Medhaffar[1], Imen Frikha[1], Moez Elloumi[1]

[1]*Hedi Chaker Hospital, Heamatology, Sfax, Tunisia;*

### Background

Paediatric acute myeloid leukaemia (AML) is a rare and heterogenous disease that represents 15–20% of paediatric leukaemia. In recent years, the prognosis of childhood AML has significantly improved in developed countries, mainly due to risk-adapted intensive chemotherapy, hematopoietic stem cell transplantation (HSCT), and advances in supportive care.

### Aims

Our aim is to characterize paediatric AML in Tunisia by evaluating clinical outcomes and identifying prognostic factors for remission, mortality, and survival.

### Methods

The study was conducted at the haematology department of the Sfax University Hospital and included all medical records of patients under 18 years newly diagnosed with de novo AML between January 2005 and December 2024.
Induction therapy consisted of standard-dose cytarabine combined with either daunorubicin (180 mg/m² cumulative dose) or novantrone (300 mg/m² cumulative dose), followed by 3–4 cycles of high-dose cytarabine–based consolidation. Endpoints used for this study included, complete remission rate (CR), overall survival (OS), disease-free survival (DFS) and relapse rate (RR)

### Results

We analysed 70 cases of paediatric AML diagnosed over a 19-year period. The mean age was 13 years [range: 1–18 years], with a sex ratio of 1.12. Genetic analysis was conclusive in 56 cases (80%), showing intermediate-risk abnormalities in 58%, favourable in 28%, and unfavourable in 12.5%.
Of the 70 patients, 43 (61%) received daunorubicin. Induction mortality occurred in 12 cases (17%) during the first course. Among 58 evaluable patients, 43 (74%) achieved complete remission. Age below 3 years, a higher White blood cells (WBC) count at diagnosis, and M0/M7/M6 subtypes according to the FAB classification were identified as predictors of failure at the univariate analysis. Multivariate analysis showed that a WBC count > 50 × $10^9$/L was associated with a higher failure rate.
HSCT was indicated for 44 of 50 patients in CR (80%), including 26 with an HLA-identical sibling, but was performed in only 11 cases (25%) during first remission. Relapse occurred in 16 cases (32%), with half of them being early relapses.
After a median follow-up of 126 months, the 5-year OS and DFS were 48% ± 6% and 67.5% ± 6.9% respectively. Age under 3 years was associated with significantly poorer OS compared to older children (14% vs. 60%, p=0.01), whereas favourable cytogenetic/molecular features, HSCT in CR1, and achievement of CR after one induction course were associated with improved OS The multivariate analysis of OS concluded that achievement of CR after one induction course was the most predictive factor (HR: 4, 95% CI [1.39–11.8], p=0.01).

**Copyright Information:** (Online) ISSN: 2572-9241
©2026 The Author(s). HemaSphere is published by John Wiley & Sons Ltd on behalf of European Hematology Association. This is an open access Abstract Book distributed under the Attribution-NonCommercial-NoDerivs (CC BY-NC-ND), which allows third parties to download the articles and share them with others as long as they credit the author and the Abstract Book, but they cannot change the content in any way or use them commercially.
**Abstract Book Citations:** Authors, Title, HemaSphere, 2026;10:(S1):pages. The abstract book DOIs can be found on the "Author Information" page.
**Disclaimer:** Articles published in the journal HemaSphere exclusively reflect the opinions of the authors. The authors are responsible for all content in their abstracts including accuracy of the facts, statements, citing resources, etc.

**Summary/Conclusion**

The CR rate in our series (74%), while comparable to reports from other middle-income countries, remains lower than in developed countries, where it can exceed 90%. This can be explained by a higher mortality rate and failure rate at induction. In our study, relapse rate (32%) and disease-free survival (67.5%) align with literature, highlighting relapse as a major challenge in pediatric AML. Both the response to induction and molecular/cytogenetic classification remain crucial prognostic factors, as demonstrated in our findings. To further refine prognostic stratification, we emphasize the importance of identifying key molecular features such as FLT3, NPM1, and CEBPA mutations.

**Copyright Information:** (Online) ISSN: 2572-9241
©2026 The Author(s). HemaSphere is published by John Wiley & Sons Ltd on behalf of European Hematology Association. This is an open access Abstract Book distributed under the Attribution-NonCommercial-NoDerivs (CC BY-NC-ND), which allows third parties to download the articles and share them with others as long as they credit the author and the Abstract Book, but they cannot change the content in any way or use them commercially.
**Abstract Book Citations:** Authors, Title, HemaSphere, 2026;10:(S1):pages. The abstract book DOIs can be found on the “Author Information” page.
**Disclaimer:** Articles published in the journal HemaSphere exclusively reflect the opinions of the authors. The authors are responsible for all content in their abstracts including accuracy of the facts, statements, citing resources, etc.

### (PB2790) COMPARATIVE ANALYSIS OF T-CELL AND BONE MARROW CHIMERISM FOR RELAPSE PREDICTION IN ACUTE LEUKAEMIA POST-TRANSPLANTATION

**Topic:** 04. Acute myeloid leukemia - Clinical

Liting Chen*[1], Lifang Huang[1], Tongyuan Liu[1]

*[1]Tongji Hospital, Tongji Medical College, Huazhong University of Science and Technology, Department of Hematology, Wuhan, China;*

### Background

Allogeneic hematopoietic stem cell transplantation is a critical treatment for acute myeloid leukemia (AML) and acute lymphoblastic leukemia (ALL), yet the risk of treatment failure still persists. Chimerism analysis serves as a potential tool for predicting disease recurrence and survival rates, but its specific role has not yet been clearly defined. This study aimed to explore the role of decreased T-cell and bone marrow (BM) chimerism in predicting posttransplant relapse and survival in patients with acute myeloid leukemia (AML) and acute lymphoblastic leukemia (ALL).

### Aims

This study aimed to evaluate the predictive value of post-transplant T-cell versus bone marrow chimerism for relapse and survival in AML and ALL patients. By analyzing 305 patients, the study sought to determine which chimerism parameter better predicts disease recurrence and whether the order of chimerism decrease (BM first vs. T-cell first) correlates with clinical outcomes, ultimately guiding early intervention strategies.

### Methods

The study subjects were 305 AML and ALL patients who underwent allogeneic hematopoietic stem cell transplantation at Tongji Hospital, TongjiM edical College, Huazhong University of Science and Technology, from January 1, 2018, to September 1, 2023. We monitored the chimerism rate at monthly intervals after transplantation until either patient relapse or the end of follow-up. T-cell and BM chimerism were tested on the same time points. Relapse probabilities were estimated by the Kaplan-Meier method and compared with the log-rank test. Gehan-Breslow-Wilcoxon test was used to assess the impact of T-cell and BM chimerism levels on survival probabilities.

### Results

In AML patients, our analysis revealed no significant correlation between the presence of initial mixed chimerism (at Day +30 posttransplantation) and the relapse rate. Among patients with ALL, the number with initial mixed chimerism was insufficient for statistical analysis. In AML Patients whose bone marrow chimerism rate decreased first (n=12) had a higher relapse rate and a lower survival rate than those whose T-cell chimerism rate decreased first (n=8) ($P < 0.01$). In patients with ALL, there was no significant difference in the relapse or survival rates between patients whose bone marrow chimerism rate decreased first (n=9) and those whose T-cell chimerism rate decreased first (n=6) ($P > 0.05$).

**Copyright Information:** (Online) ISSN: 2572-9241
©2026 The Author(s). HemaSphere is published by John Wiley & Sons Ltd on behalf of European Hematology Association. This is an open access Abstract Book distributed under the Attribution-NonCommercial-NoDerivs (CC BY-NC-ND), which allows third parties to download the articles and share them with others as long as they credit the author and the Abstract Book, but they cannot change the content in any way or use them commercially.
**Abstract Book Citations:** Authors, Title, HemaSphere, 2026;10:(S1):pages. The abstract book DOIs can be found on the "Author Information" page.
**Disclaimer:** Articles published in the journal HemaSphere exclusively reflect the opinions of the authors. The authors are responsible for all content in their abstracts including accuracy of the facts, statements, citing resources, etc.

**Summary/Conclusion**

While a decrease in bone marrow chimerism effectively predicts AML relapse, T-cell chimerism demonstrates lower predictive efficacy.Further research is necessary to identify reliable predictors for relapse in ALL patients. The integration of chimerism analysis with other prognostic indicators, along with early monitoring and preemptive intervention, may enhance patient survival and quality of life.

**Copyright Information:** (Online) ISSN: 2572-9241
©2026 The Author(s). HemaSphere is published by John Wiley & Sons Ltd on behalf of European Hematology Association. This is an open access Abstract Book distributed under the Attribution-NonCommercial-NoDerivs (CC BY-NC-ND), which allows third parties to download the articles and share them with others as long as they credit the author and the Abstract Book, but they cannot change the content in any way or use them commercially.
**Abstract Book Citations:** Authors, Title, HemaSphere, 2026;10:(S1):pages. The abstract book DOIs can be found on the "Author Information" page.
**Disclaimer:** Articles published in the journal HemaSphere exclusively reflect the opinions of the authors. The authors are responsible for all content in their abstracts including accuracy of the facts, statements, citing resources, etc.

### (PB2791) PHASE 1/1B TRIAL OF IMETELSTAT AND AZACITIDINE WITH OR WITHOUT VENETOCLAX RELAPSED ACUTE MYELOID LEUKEMIA (IMAGINE TRIAL)

**Topic:** 04. Acute myeloid leukemia - Clinical

Douglas Tremblay*[1], Alan Shih[1], Jonathan Feld[2], Erin Moshier[1], Carly Sullivan[1], Kathryn Johnson[1], Khadiza Bhuiyan[1], Christopher Swierczek[1], Hannah Levavi[1], Marina Kremyanskaya[1], John Mascarenhas[1]

*[1]Icahn School of Medicine at Mount Sinai, New York, United States of America;[2]Moffitt Malignant Hematology & Cellular Therapy at Memorial Healthcare System, Pembroke Pines, United States of America;*

### Background

Patients with acute myeloid leukemia (AML) who are relapsed or refractory to standard therapies have a dismal survival of 2-3 months. Treatment options that can meaningfully impact the course of disease and produce clinical responses are urgently needed. Imetelstat is a first-in-class telomerase inhibitor approved in 2024 for patients with transfusion-dependent lower-risk myelodysplastic syndromes who are relapsed or refractory (R/R) or ineligible for erythropoiesis-stimulating agents. Importantly, telomerase is upregulated in AML blasts and associated with disease progression making it a rational target. Imetelstat decreases AML disease burden in preclinical models, regulated by ferroptosis (Bruedigam et al Nat Cancer 2023). However, imetelstat alone failed to produce meaningful responses in R/R myelodysplastic syndrome and AML (Ades et al ASH 2025). Combination approaches of imetelstat with azacitidine (AZA) or venetoclax (VEN) have demonstrated synergy in AML cell lines and xenograft models and may significantly enhance clinical activity of imetelstat. In addition, imetelstat is active in several leukemia cell lines that are BCL-2 inhibitor resistant suggesting a role in AML that is relapsed to hypomethylating agent and venetoclax combination.

### Methods

IMAGINE is a two-part single institution trial to evaluate the safety and preliminary efficacy of imetelstat in combination with AZA with or without VEN in patients with relapsed or refractory AML. The trial will consist of a safety run-in phase (Part A) enrolling 9-12 patients employing a '3+3' design to monitor dose-limiting toxicities at the 5.6 mg/kg and 7.1 mg/kg dose levels of imetelstat when administered in a 28-day cycle in combination with a fixed dose of AZA (75mg/m$^2$ days 1-7). Part B will consist of a phase 1b trial enrolling an additional 21 patients employing a BOIN12 design to determine the optimal biological dose of imetelstat, starting at a lower dose level of 4.4mg/kg and escalating to 5.6mg/kg and 7.1mg/kg, in combination with a fixed dose of AZA and VEN (14 days). Response assessments will occur after C2, C4 and C6.

Eligible patients have relapsed AML without available standard treatment options and inclusion is mutation agnostic. The primary objective for Part A is to determine the safety of the doublet (AZA+imetelstat) before moving on to the Part B triplet therapy (AZA+VEN+imetelstat), with a primary objective of identifying the optimal biological dose (OBD) of imetelstat with azacitidine and venetoclax. Secondary objectives include efficacy metrics including composite complete response rates, duration of response, and overall survival. Exploratory analysis will assess the mutational burden with treatment as well as measure ferroptosis during treatment.

The trial is open to accrual at Mount Sinai (NCT07320235) and enrollment is ongoing.

### Summary/Conclusion

**Copyright Information:** (Online) ISSN: 2572-9241
©2026 The Author(s). HemaSphere is published by John Wiley & Sons Ltd on behalf of European Hematology Association. This is an open access Abstract Book distributed under the Attribution-NonCommercial-NoDerivs (CC BY-NC-ND), which allows third parties to download the articles and share them with others as long as they credit the author and the Abstract Book, but they cannot change the content in any way or use them commercially.
**Abstract Book Citations:** Authors, Title, HemaSphere, 2026;10:(S1):pages. The abstract book DOIs can be found on the "Author Information" page.
**Disclaimer:** Articles published in the journal HemaSphere exclusively reflect the opinions of the authors. The authors are responsible for all content in their abstracts including accuracy of the facts, statements, citing resources, etc.

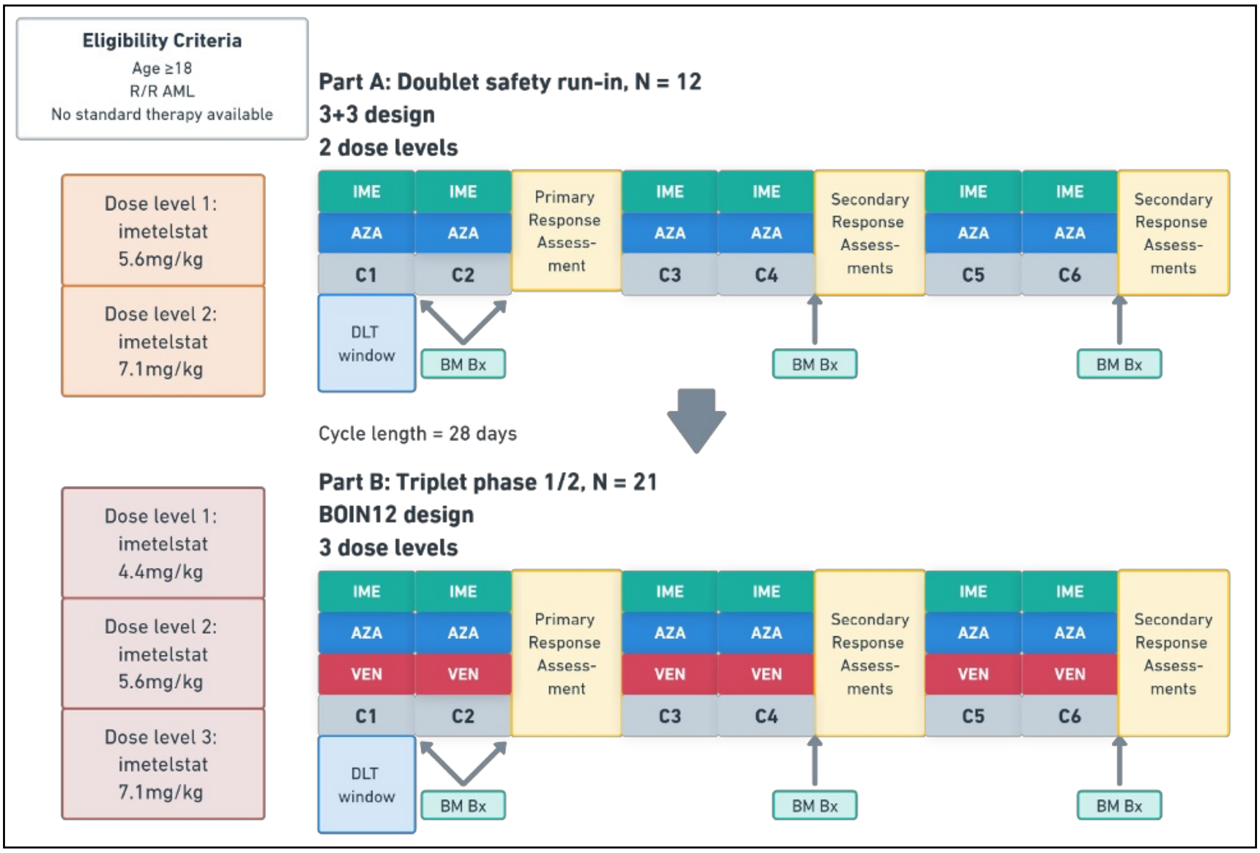

**Copyright Information:** (Online) ISSN: 2572-9241
©2026 The Author(s). HemaSphere is published by John Wiley & Sons Ltd on behalf of European Hematology Association. This is an open access Abstract Book distributed under the Attribution-NonCommercial-NoDerivs (CC BY-NC-ND), which allows third parties to download the articles and share them with others as long as they credit the author and the Abstract Book, but they cannot change the content in any way or use them commercially.
**Abstract Book Citations:** Authors, Title, HemaSphere, 2026;10:(S1):pages. The abstract book DOIs can be found on the "Author Information" page.
**Disclaimer:** Articles published in the journal HemaSphere exclusively reflect the opinions of the authors. The authors are responsible for all content in their abstracts including accuracy of the facts, statements, citing resources, etc.

### (PB2792) POST-TRANSPLANTATION CYCLOPHOSPHAMIDE-BASED GRAFT VERSUS HOST DISEASE PROPHYLAXIS FOR MATCHED SIBLING DONOR ALLOGENEIC HEMATOPOIETIC CELL TRANSPLANTATION IN ACUTE MYELOID LEUKEMIA

**Topic:** 04. Acute myeloid leukemia - Clinical

Sabrina Akhrouf[1], Farih Mehdid[1], Baazizi Mounira[1], Hanane Bouarab[1], Ait Ouali Dina[1], Nadia Rahmoune[1], Sarah Zerkout[1], Fouzia Louar[1], Mounira Bitam[1], Amira Fahem[1], Farida Harieche[1], Rose Marie Hamladji[1], Redhouane Ahmed Nacer[1], Malek Benakli*[1]

*[1]Hematology and Bone Marrow Transplantation department, Pierre and Marie Curie Center, Algiers, Algeria;*

### Background

The Post-Transplant Cyclophosphamide (PTCy) approach was first studied in the haploidentical transplant setting and resulted in high percentages of engraftment and reduced frequency of acute and chronic Graft versus Host Disease (GvHD). Numerous studies have shown that PTCy used in GVHD prophylaxis reduces the incidence of severe forms of acute GVHD even after use of peripheral blood stem cell transplantation in the HLA-matched donor transplant setting.

### Methods

We retrospectively analyzed 38 pts with acute myeloid leukemia (AML) who underwent allogeneic stem cell transplantation (allo-HSCT), between May 2023 and May 2024, with HLA identical sibling donors who received GVHD prophylaxis with PTCy. The median age is 43 years (18 -59) and the sex ratio is 1.37 (22 M/16 F). At the transplant: 34 pts are in 1st complete remission (CR), 03 pts in 2nd CR and 01 pt in blast progression. Myeloablative conditioning associated Fludarabine 160 mg/m², Busilvex 12.8 mg/kg. GVHD prophylaxis combined Cyclosporin up to D100, Mycophenolate Mofetil (MMF) and PTCy (40 to 50 mg/kg/d on D3 and D4). A peripheral blood stem cell (PBSC) graft was used in all pts, with a median CD34+ cell count: 5.9x10*6/kg (2.08-10.76). At November 2024, the maximum follow-up is 18 months and minimum 06 months.

### Results

Neutropenia occured in all pts with an average duration of 11 days (6-20). The median day of neutrophils engraftment was 17 days (14-25) with an average duration of hospitalization of 28 days (15-47). Sixteen pts (42%) required transfusion of red blood cells with an average of 2 units/pt (1-5) and 34 pts (89%) needed platelet transfusions with an average of 1 unit/pt (0.5-2.5). Thirty pts (79%) presented grade III-IV mucositis. A resolving VOD was observed in one pt (2.6%) and 1 pt presented grade 2 hemorrhagic cystitis on day 43. Acute GVHD was observed in 05 pts (13.5%) and chronic GVHD occured in 07 pts (19.4%). Three pts (8%) relapsed after an average delay of 4.6 months (2-7). At November 2024, after a median follow-up of 12 months (06-18), 30 pts are still alive (79%) and 8 pts (21%) are died (21%) of whom 5 pts (13%) related to the procedure (TRM: septic shock: 01, aGVHD: 01, invasive pulmonary aspergillosis: 03) and 03 pts (7.8%) from relapse. The overall survival (OS) and relapse-free survival (EFS) are 77% and 74.3% respectively and the GVHD-free and relapse-free survival (GRFS) is 60%.

### Summary/Conclusion

Our study demonstrates the efficacy of PTCy-based GvHD prophylaxis strategy in matched allo-HSCT in a contemporary setting and shows reduce rate of acute GVHD without increasing the infectious risk, less TRM and a significant improvement in clinically meaningful outcomes

**Copyright Information:** (Online) ISSN: 2572-9241
©2026 The Author(s). HemaSphere is published by John Wiley & Sons Ltd on behalf of European Hematology Association. This is an open access Abstract Book distributed under the Attribution-NonCommercial-NoDerivs (CC BY-NC-ND), which allows third parties to download the articles and share them with others as long as they credit the author and the Abstract Book, but they cannot change the content in any way or use them commercially.
**Abstract Book Citations:** Authors, Title, HemaSphere, 2026;10:(S1):pages. The abstract book DOIs can be found on the "Author Information" page.
**Disclaimer:** Articles published in the journal HemaSphere exclusively reflect the opinions of the authors. The authors are responsible for all content in their abstracts including accuracy of the facts, statements, citing resources, etc.

### (PB2793) THERAPEUTIC EFFICACY ANALYSIS OF NEWLY DIAGNOSEDACUTE MYELOID LEUKEMIA(AML) BASED ON MINIMAL RESIDUAL DISEASE

**Topic:** 04. Acute myeloid leukemia - Clinical

Shun Yao Qi[1], Ye Hui Tan*[1], Yan Ping Yang[1], Yu Ying Li[1], Sujun Gao[1]

*[1]The First Hospital of Jilin University, Changchun, China;*

### Background

Studies have shown that even minimal levels of MRD positivity significantly increase the risk of disease relapse and adversely affect overall patient survival.

### Aims

This study explores the clinical value of using multi-parameter flow cytometry (MFC) to detect early minimal residual disease (MRD) in newly diagnosed acute myeloid leukemia (AML) patients after their first induction chemotherapy. It evaluates the impact of MRD detection on the assessment of treatment response following the initial therapeutic course and its significance for predicting disease outcomes.

### Methods

This study retrospectively analyzed data from newly diagnosed AML（non-M3) patients treated at the Hematology Department of The First Hospital of Jilin University Tumor Center between January 2022 and December 2025. We collected general clinical characteristics of eligible subjects and MRD status on day 6 after the first induction chemotherapy, remission status, and survival outcomes. Through MFC detection of MRD on day 6 following the first induction therapy, this research aimed to explore how early intervention based on MRD status affects the prognosis of AML patients.

**Copyright Information:** (Online) ISSN: 2572-9241
©2026 The Author(s). HemaSphere is published by John Wiley & Sons Ltd on behalf of European Hematology Association. This is an open access Abstract Book distributed under the Attribution-NonCommercial-NoDerivs (CC BY-NC-ND), which allows third parties to download the articles and share them with others as long as they credit the author and the Abstract Book, but they cannot change the content in any way or use them commercially.
**Abstract Book Citations:** Authors, Title, HemaSphere, 2026;10:(S1):pages. The abstract book DOIs can be found on the “Author Information” page.
**Disclaimer:** Articles published in the journal HemaSphere exclusively reflect the opinions of the authors. The authors are responsible for all content in their abstracts including accuracy of the facts, statements, citing resources, etc.

### Results

1.Among the 102 patients, 58 had MRD <1% on day 6 of induction therapy, while 44 had MRD ≥1%. Baseline characteristics were compared between the two groups, and it was found that the risk stratification differed significantly between MRD ≥1% patients and MRD <1% patients (P=0.008). No other baseline characteristics showed significant differences.3.Based on the MRD status in peripheral blood on Day 6, patients with MRD levels between 1% and 10% will receive oral venetoclax (100 mg on Day 8, 200 mg on Day 9, and 400 mg from Day 10 to Day 14). Patients with MRD levels ≥10% will receive combination therapy with venetoclax and azacitidine (venetoclax: 100 mg on Day 8, 200 mg on Day 9, and 400 mg from Day 10 to Day 21; azacitidine: 75 mg/m$^2$, administered via subcutaneous injection from Day 8 to Day 14).In the MRD ≥1% group, 22 patients received early intervention therapy, while 22 did not. Baseline characteristics were similar between the two subgroups. Among the intervention group, 15 patients achieved a compound complete remission (CRc) (68.1%), 4 achieved partial remission (PR) (18.1%), and 3 had no response (NR) (13.8%). In the non-intervention group, 7 patients achieved CRc (32%), 6 achieved PR (27%), and 9 had NR (41%). A significant difference in treatment efficacy was observed between the two groups after the first course of therapy (P=0.043). In the MRD <1% group, 50 patients achieved CRc (86.2%), 4 achieved PR (6.9%), and 4 had NR (6.9%). No significant difference in treatment efficacy was observed between the intervention group and the MRD <1% group (P=0.097).
2.Among patients who received regular treatment for three or more cycles, overall survival (OS) differed significantly between the MRD ≥1% patients who received early intervention and those who did not (79% vs 50%, P=0.047). The median survival time was not reached in the intervention group, while it was 16 months in the non-intervention group. No significant difference in disease-free survival (DFS) was observed between the two groups (P=0.036[illegible]. Survival outcomes for patients who received early intervention were similar to those in the MRD <1% group (P=0.6539).

### Summary/Conclusion

MRD detection by MFC after initial induction therapy in newly diagnosed AML patients is of significant importance for prognostic evaluation and guiding treatment regimens.

**Copyright Information:** (Online) ISSN: 2572-9241
©2026 The Author(s). HemaSphere is published by John Wiley & Sons Ltd on behalf of European Hematology Association. This is an open access Abstract Book distributed under the Attribution-NonCommercial-NoDerivs (CC BY-NC-ND), which allows third parties to download the articles and share them with others as long as they credit the author and the Abstract Book, but they cannot change the content in any way or use them commercially.
**Abstract Book Citations:** Authors, Title, HemaSphere, 2026;10:(S1):pages. The abstract book DOIs can be found on the "Author Information" page.
**Disclaimer:** Articles published in the journal HemaSphere exclusively reflect the opinions of the authors. The authors are responsible for all content in their abstracts including accuracy of the facts, statements, citing resources, etc.

### (PB2794) REAL-WORLD EARLY MORTALITY WITH CPX-351: AN INSTITUTIONAL EXPERIENCE COMPARED WITH PUBLISHED OUTCOMES

**Topic:** 04. Acute myeloid leukemia - Clinical

Adetomilola Essem[1], Sheenam Sheenam*[1], Moeed Ahmad[1], Osama Ali[1], Charlotte Kallmeyer[1]

[1]*United Lincolnshire Hospital NHS trust, Haematology, Lincolnshire, United Kingdom;*

### Background

Acute Myeloid Leukaemia with myelodysplasia-related changes or therapy-related AML remains a clinical challenge. CPX-351 demonstrates better outcomes and tolerability compared to DA 7+3 in clinical trials in these patients. Though our institution was part of database for Real-world experience with CPX 351 treatment for AML in England 2023 (NRAS STUDY). This retrospective study shows higher mortality in first 30 days than national average

### Aims

To Identify independent risk factors for early mortality in patients treated with CPX-351 in our institution, compared to published real-world data and validating known published risk factors

### Methods

A retrospective study of 22 AML patients ≥ 18 years who received induction chemotherapy with CPX-351 in our institution up to 2024. Primary aim was to identify risk factors for early mortality by day 30. Comparing local outcomes with the CREST UK and NRAS studies. Most of these patients would have been part of these dataset

### Results

The study cohort consisted of 22 patients aged 55-77. 30-day mortality was 22.7% compared with 6% in the NRAS study. Most patients were over 60. Median age 67 years compared with 62 in NRAS study, this difference was statistically significant (p =0.02). Males comprised 59% of the cohort, male sex was found to be associated with poorer outcomes within this group (p-value =0.04) . Platelet recovery time ranged from 12 to 35 days in the local cohort. A delay in platelet recovery of >28 days showed association with poorer outcomes (p-value =0.01) . Adverse Risk Factors using ELN2017 risk stratification (as used in CREST study), showed 68.2% adverse risk compared to 47.6% in CREST study. This trend towards higher risk, it did not reach statistical significance (p-value =0.07), likely due to sample size. A rise in blood pressure of >20 mmHg at day 14 was associated with poorer outcomes (p-value = 0.04) and cardiotoxicity. Despite CPX-351 being associated with lower cardiotoxicity in trials, local experience showed that cardiotoxicity after cycle 1 was associated with poorer outcomes (p-value =0.03). Prolonged neutropenia (>28 days), sepsis, tachycardia-a published indicator of cardiotoxic risk with CPX- 351 did not demonstrate risk for poorer outcomes in our cohort. Bacterial agents documented in the study were like those reported in the SEIFEM experience, a distinct feature of this cohort showed 22.8% of the patients harboured oral cavity microbes.

**Copyright Information:** (Online) ISSN: 2572-9241
©2026 The Author(s). HemaSphere is published by John Wiley & Sons Ltd on behalf of European Hematology Association. This is an open access Abstract Book distributed under the Attribution-NonCommercial-NoDerivs (CC BY-NC-ND), which allows third parties to download the articles and share them with others as long as they credit the author and the Abstract Book, but they cannot change the content in any way or use them commercially.
**Abstract Book Citations:** Authors, Title, HemaSphere, 2026;10:(S1):pages. The abstract book DOIs can be found on the "Author Information" page.
**Disclaimer:** Articles published in the journal HemaSphere exclusively reflect the opinions of the authors. The authors are responsible for all content in their abstracts including accuracy of the facts, statements, citing resources, etc.

**Summary/Conclusion**

The study highlights a poorer early outcome at day 30, significantly higher than the 30-day mortality observed in the phase III pivotal trial 5.9% and NRAS study 6%.
This disparity may be attributed to a higher incidence of conventional risk factors like age, adverse cytogenetics risk and delay in platelet recovery. Other independent factors identified in this cohort include male gender and a rise in systolic blood pressure (>20 mmHg) at day 14 unexpectedly, as at Day 14, most patients would be septic and have a drop in blood pressure. Although sepsis was not identified as a risk factor, finding of oropharyngeal microbes, along with other potentially modifiable factors linked to early mortality in patients receiving CPX-351 in this cohort, warrants future consideration, including incorporating a more comprehensive approach to assessing cardiovascular risk and a robust frailty assessment in the older age cohort. Validating this data in bigger cohorts would be essential.

**Copyright Information:** (Online) ISSN: 2572-9241
©2026 The Author(s). HemaSphere is published by John Wiley & Sons Ltd on behalf of European Hematology Association. This is an open access Abstract Book distributed under the Attribution-NonCommercial-NoDerivs (CC BY-NC-ND), which allows third parties to download the articles and share them with others as long as they credit the author and the Abstract Book, but they cannot change the content in any way or use them commercially.
**Abstract Book Citations:** Authors, Title, HemaSphere, 2026;10:(S1):pages. The abstract book DOIs can be found on the "Author Information" page.
**Disclaimer:** Articles published in the journal HemaSphere exclusively reflect the opinions of the authors. The authors are responsible for all content in their abstracts including accuracy of the facts, statements, citing resources, etc.

### (PB2795) DELPHI CONSENSUS ON OPTIMAL TREATMENT STRATEGIES USING IDH1 INHIBITORS IN PATIENTS WITH R/R MIDH1 AML

**Topic:** 04. Acute myeloid leukemia - Clinical

Justin Watts[1], Jorge Cortes*[2], Amer M. Zeidan[3], Ashwin Kishtagari[4], Brian A. Jonas[5], Curtis Lachowiez[6], Hetty Carraway[7], Joshua Zeidner[8], Keri Maher[9], Yasmin Abaza[10], Michaela Spence[11], Megan Bourque[11], Courtney Dinardo[12]

*[1]University of Miami Sylvester Comprehensive Cancer Center, Department of Hematology, Miami, United States of America; [2]Georgia Cancer Center, Augusta, United States of America;[3]Yale School of Medicine and Yale Comprehensive Cancer Center, Section of Medical Oncology and Hematology, Department of Internal Medicine, New Haven, United States of America; [4]Vanderbilt University Medical Center, Department of Medicine, Nashville, United States of America;[5]UC Davis School of Medicine and UC Davis Comprehensive Cancer Center, Division of Malignant Hematology/Cellular Therapy and Transplantation, Department of Internal Medicine, Sacramento, United States of America; [6]Knight Cancer Institute, Oregon Health and Science University, Division of Hematology/Medical Oncology, Portland, United States of America; [7]Taussig Cancer Institute, Leukemia Program, Department of Hematology and Medical Oncology, Cleveland, United States of America; [8]University of North Carolina, Lineberger Comprehensive Cancer Center, Division of Hematology, Chapel Hill, United States of America; [9]VCU Medical Center, Oncology Department, Richmond, United States of America; [10]Robert H. Lurie Comprehensive Cancer Center, Division of Hematology, Chicago, United States of America; [11]EVERSANA LifeSciences Services, Value & Evidence, Burlington, Canada; [12]The University of Texas MD Anderson Cancer Center, Department of Leukemia, Houston, United States of America;*

### Background

The isocitrate dehydrogenase 1 gene mutation (m*IDH1*) occurs in up to 14% of acute myeloid leukemia (AML). The introduction of novel IDH1 inhibitors (IDH1i) such as ivosidenib (IVO) and olutasidenib (OLU) have altered the landscape of AML treatment for these patients, especially those who are relapsed or refractory (R/R) to traditional therapies. IVO and OLU have not been compared head-to-head. The optimal IDH1i treatment strategy within the R/R m*IDH1* AML treatment paradigm is unknown.

### Aims

To develop evidence and consensus-based recommendations for treatment of patients with R/R m*IDH1* AML.

### Methods

A panel of 17 clinicians [including a three-person steering committee (SC)] with expertise in the treatment of patients with R/R m*IDH1* AML participated in a Delphi panel. Current published data along with SC insights were used to draft consensus statements (CSs). Electronic surveys were distributed allowing for anonymous completion of scoping questions and Likert-scale based CSs ranked from strongly agree to strongly disagree. Consensus was defined as ≥75% of panelists agreeing with a CS. If consensus was not reached, initial CSs were revised according to panelist feedback and re-polled in a second round of asynchronous electronic survey.

**Copyright Information:** (Online) ISSN: 2572-9241
©2026 The Author(s). HemaSphere is published by John Wiley & Sons Ltd on behalf of European Hematology Association. This is an open access Abstract Book distributed under the Attribution-NonCommercial-NoDerivs (CC BY-NC-ND), which allows third parties to download the articles and share them with others as long as they credit the author and the Abstract Book, but they cannot change the content in any way or use them commercially.
**Abstract Book Citations:** Authors, Title, HemaSphere, 2026;10:(S1):pages. The abstract book DOIs can be found on the "Author Information" page.
**Disclaimer:** Articles published in the journal HemaSphere exclusively reflect the opinions of the authors. The authors are responsible for all content in their abstracts including accuracy of the facts, statements, citing resources, etc.

### Results

After two rounds, 29/34 CSs reached consensus, with at least 15 panelist responses for each CS. When considering treatment of R/R AML patients, acknowledging limited evidence, panelists confirmed the need for further genetic testing to determine personalized therapeutic options. When selecting preferred initial treatments in R/R m*IDH1* AML patients, consensus established prioritizing OLU over IVO for patients previously treated with venetoclax-based treatment pending prospective comparative data. Additionally, panelists agreed that venetoclax-based therapy for patients previously treated with IVO-based treatment would be preferred. Due to variability in patient and disease characteristics, consensus was not achieved on the preferred treatment option for patients previously treated with intensive chemotherapy.
When evaluating the impact of IDH1i, there was consensus that olutasidenib and ivosidenib provide benefit in R/R m*IDH1* AML patients and that treatment should continue for a minimum of six months, unless there is disease progression, unacceptable toxicity, stem cell transplant, or if dictated by patient-driven factors. Consensus was achieved that both drugs had a well-tolerated safety profile with manageable adverse events. Panelists agreed that the safety concerns of QT interval prolongation, hepatotoxicity, and potential drug interactions should be considered when selecting an IDH1i.
Consensus was achieved that evidence supports the clinical efficacy of both OLU and IVO in patients with R/R m*IDH1* AML. Panelists reached agreement that in the absence of head-to-head comparative data, indirect evidence may indicate that OLU shows favorable effectiveness with a higher proportion of R/R mIDH1 AML patients achieving a CR and longer duration of response versus IVO, including in those with prior venetoclax therapy. There was consensus agreement that OLU's distinct structure and unique binding kinetics could provide a potential rationale for its longer duration of response versus IVO.

### Summary/Conclusion

IDH1i demonstrate clinical efficacy with adequate tolerability in patients with R/R m*IDH1* AML. In patients with prior venetoclax therapy, OLU may be the preferred treatment option.

**Copyright Information:** (Online) ISSN: 2572-9241
©2026 The Author(s). HemaSphere is published by John Wiley & Sons Ltd on behalf of European Hematology Association. This is an open access Abstract Book distributed under the Attribution-NonCommercial-NoDerivs (CC BY-NC-ND), which allows third parties to download the articles and share them with others as long as they credit the author and the Abstract Book, but they cannot change the content in any way or use them commercially.
**Abstract Book Citations:** Authors, Title, HemaSphere, 2026;10:(S1):pages. The abstract book DOIs can be found on the "Author Information" page.
**Disclaimer:** Articles published in the journal HemaSphere exclusively reflect the opinions of the authors. The authors are responsible for all content in their abstracts including accuracy of the facts, statements, citing resources, etc.

### (PB2796) REVUMENIB + VENETOCLAX/AZACITIDINE IN ADULTS WITH NEWLY DIAGNOSED NPM1M OR KMT2AR ACUTE LEUKEMIA INELIGIBLE FOR INTENSIVE CHEMOTHERAPY (EVOLVE-2/HO177/AMLSG35-24/ACT-HOV-AML-002): TRIAL IN PROGRESS

**Topic:** 04. Acute myeloid leukemia - Clinical

Gerwin Huls*[1], Michael Kühn[2], Paresh Vyas[3], Hartmut Döhner[4], Bob Löwenberg[5, 6], Patrycja Gradowska[6], Samiyeh J. Price[7], Angela Smith[7], Stéphane De Botton[8], Christian Recher[9], Jordi Esteve Reyner[10], Adriano Venditti[11], Thomas Pabst[12], Vladimir Lazarevic[13], Kimmo Porkka[14], Dimitri Breems[15], Andrius Žučenka[16], Elisabteh Koller[17], Andrew H. Wei[18], Joshua Zeidner[19]

*[1]University Medical Center Groningen, University of Groningen, Groningen, The Netherlands; [2]University Medical Center, Johannes Gutenberg University, Mainz, Germany; [3]University of Oxford, Oxford, United Kingdom; [4]Ulm University Hospital, Ulm, Germany; [5]Erasmus University Medical Center, Rotterdam, The Netherlands; [6]Erasmus MC Cancer Institute, Rotterdam, The Netherlands; [7]Syndax Pharmaceuticals, Inc., New York, NY, United States of America; [8]Institute Gustave Roussy, Villejiuf, France; [9]CHU de Toulouse, Toulouse, France; [10]Hospital Clínic of Barcelona, Barcelona, Spain; [11]University Tor Vergata, Rome, Italy; [12]Inselspital-Universitätsspital, Bern, Switzerland; [13]Skåne University Hospital, Lund, Sweden; [14]Helsinki University Hospital Cancer Center, Helsinki, Finland; [15]Ziekenhuis aan de Stroom, Antwerp, Belgium; [16]Vilnius University, Faculty of Medicine and Vilnius University Hospital Santaros Klinikos, National Cancer Center, Hematology, Oncology, and Transfusion Medicine Center, Vilnius, Lithuania; [17]Hanusch Hospital, Vienna, Austria; [18]The Alfred Hospital, Melbourne, Australia; [19]Lineberger Comprehensive Cancer Center, University of North Carolina, Chapel Hill, NC, United States of America;*

### Background

Optimal treatment for patients with acute myeloid leukemia (AML) unfit for intensive chemotherapy (IC) remains challenging and depends on patient and disease characteristics and patient preference. In AML with a nucleophosmin-1 mutation (*NPM1*m) or lysine methyltransferase-2A (*KMT2A*) rearrangement (*KMT2A*r), menin-KMT2A fusion proteins upregulate *HOX/MEIS* gene expression, resulting in hematopoietic differentiation arrest and leukemogenesis. Revumenib is a first-in-class, oral, potent, and selective inhibitor of the menin-KMT2A interaction, which disrupts multiple key drivers of leukemogenesis (*KMT2A*r, *NPM1*m, and *NUP98* rearrangement). In the phase 1/2 AUGMENT-101 study (NCT04065399), revumenib monotherapy demonstrated clinically meaningful response rates and was generally well tolerated in patients with relapsed/refractory *NPM1*m AML or *KMT2A*r acute leukemia. To further improve outcomes, combination regimens are being studied as front-line therapy for newly diagnosed (ND) patients. In the phase 1b Beat AML study (NCT03013998), revumenib plus venetoclax/azacitidine (VEN/AZA) demonstrated deep responses in patients ≥60 years (y) of age with ND *NPM1*m or *KMT2A*r AML.

### Aims

EVOLVE-2 will assess whether revumenib plus VEN/AZA prolongs overall survival (OS) and improves complete remission (CR) rates in patients with ND *NPM1*m or *KMT2A*r AML ineligible for IC.

**Copyright Information:** (Online) ISSN: 2572-9241
©2026 The Author(s). HemaSphere is published by John Wiley & Sons Ltd on behalf of European Hematology Association. This is an open access Abstract Book distributed under the Attribution-NonCommercial-NoDerivs (CC BY-NC-ND), which allows third parties to download the articles and share them with others as long as they credit the author and the Abstract Book, but they cannot change the content in any way or use them commercially.
**Abstract Book Citations:** Authors, Title, HemaSphere, 2026;10:(S1):pages. The abstract book DOIs can be found on the “Author Information” page.
**Disclaimer:** Articles published in the journal HemaSphere exclusively reflect the opinions of the authors. The authors are responsible for all content in their abstracts including accuracy of the facts, statements, citing resources, etc.

### Methods

EVOLVE-2 is a phase 3, multicenter, randomized, double-blind, placebo-controlled trial (NCT06652438/EU-CT 2024-512733-32-00; **Figure**). Eligible patients are ≥75 y of age (18–74 y with comorbidities), have ND AML with centrally confirmed *NPM1*m or *KMT2A*r (excluding *KMT2A* partial tandem duplications/deletions), are ineligible for IC, and are AML treatment-naive. Patients will be randomized 1:1 to revumenib or placebo in combination with VEN/AZA and stratified by age (<75 vs ≥75 y), genotype (*NPM1*m vs *KMT2A*r), and region (Europe vs Australia vs United States). In Cycle (C) 1, VEN is given daily on Days (D) 1–28 and AZA daily on D1–7, with or without revumenib twice daily on D1–28. In C2+, responders (per 2022 European LeukemiaNet criteria) may adjust VEN based on remission status/count recovery. Treatment continues until disease progression, unacceptable toxicity, patient withdrawal, or death.

### Results

The dual primary endpoints are OS and CR rate. Key secondary endpoints include event-free survival and rate of CR/CR with partial hematologic recovery (CRh). Other endpoints include rates of CRh and CR/CR with incomplete hematologic recovery (CRi), measurable residual disease (MRD) negativity (CR, CR/CRh, CR/CRi with MRD negativity), time to and duration of response, adverse events, time to hematopoietic recovery, and incidence of platelet and red blood cell transfusions. Overall, 448 patients will be enrolled. As of 1 March 2026, the study is open to enrollment.

### Summary/Conclusion

This phase 3 study will provide insight into the role of revumenib plus VEN/AZA in improving efficacy and survival outcomes in patients with ND *NPM1*m or *KMT2A*r AML ineligible for IC and inform the development of combination treatment approaches for this patient population.

© 2026 American Society of Clinical Oncology, Inc. Reused with permission. This abstract was accepted and previously presented at the 2026 ASCO Annual Meeting. All rights reserved.

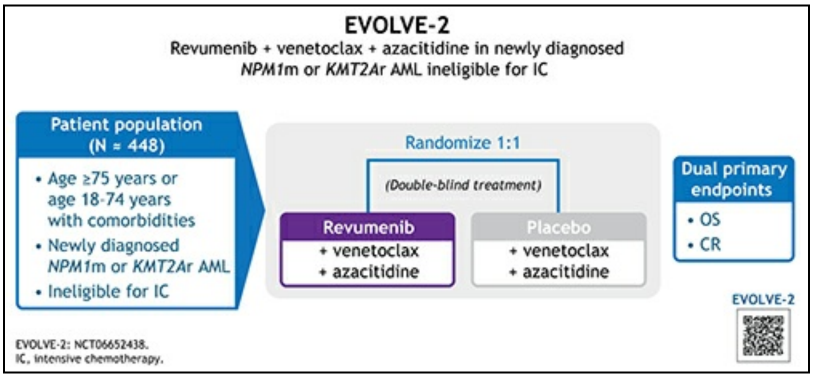

**Copyright Information:** (Online) ISSN: 2572-9241
©2026 The Author(s). HemaSphere is published by John Wiley & Sons Ltd on behalf of European Hematology Association. This is an open access Abstract Book distributed under the Attribution-NonCommercial-NoDerivs (CC BY-NC-ND), which allows third parties to download the articles and share them with others as long as they credit the author and the Abstract Book, but they cannot change the content in any way or use them commercially.
**Abstract Book Citations:** Authors, Title, HemaSphere, 2026;10:(S1):pages. The abstract book DOIs can be found on the "Author Information" page.
**Disclaimer:** Articles published in the journal HemaSphere exclusively reflect the opinions of the authors. The authors are responsible for all content in their abstracts including accuracy of the facts, statements, citing resources, etc.

### (PB2797) MUTATIONAL LANDSCAPE AND SURVIVAL OUTCOMES OF ACUTE MYELOID LEUKEMIA IN CENTRAL KAZAKHSTAN: A REAL-WORLD ANALYSIS

**Topic:** 04. Acute myeloid leukemia - Clinical

Maxim Selivanov*[1], Anton Klodzinsky[2], Aliya Baimurzina[1], Alyona Zinchenko[1], Ayimzhan Anafina[1]

[1]*Hematology Center LLP, Karaganda, Kazakhstan;* [2]*Hematology Center LLP, Astana, Kazakhstan;*

### Background

Acute myeloid leukemia (AML) predominantly affects elderly patients and is characterized by marked molecular heterogeneity. Molecular profiling and ELN-based risk stratification are essential for prognostication and treatment selection; however, access to comprehensive diagnostics and modern therapies remains limited in many regions.

### Aims

To analyze the mutational spectrum, clinical characteristics, and survival outcomes of patients with AML treated in Central Kazakhstan and to evaluate the prognostic impact of ELN 2022 risk stratification, treatment intensity, and transplantation in a real-world setting.

### Methods

A retrospective analysis of 246 adult AML patients treated between August 2018 and September 2025 was performed. Molecular genetic testing was conducted when feasible. Risk stratification followed ELN 2022 criteria. Overall survival (OS) and relapse-free survival (RFS) were analyzed using the Kaplan–Meier method. Statistical analysis was performed using SPSS Statistics version 14.

### Results

The median age was 65 years (range 18–91), with a male-to-female ratio of 1.1:1. Primary AML predominated over secondary AML (ratio 3.5:1). According to ELN 2022 classification, 14.6% of patients were assigned to favorable risk, 40.7% to intermediate risk, and 22.4% to adverse risk; ELN risk could not be assessed in 22.4% of cases. Intensive therapy was administered in 44.7% of patients, non-intensive therapy in 36.5%, while 18.6% received no antileukemic treatment.
Molecular genetic testing was performed in 77% of patients. The most frequent genetic alterations were *NPM1* (25%), t(8;21) (20%), and *NPM1+FLT3* (18%), followed by *FLT3-ITD* (14%). *CBF::MYH11* rearrangements were detected in 6% of cases, with an additional 3% showing *CBF::MYH11* with co-mutations (*c-KIT/FLT3*). *CEBPA* mutations were identified in 3%, while other genetic abnormalities accounted for 6%.
Overall survival differed significantly across ELN risk groups (log-rank p=0.005). Median OS was 26 months in the favorable-risk group, 6.8 months in the intermediate-risk group, 4.8 months in the adverse-risk group, and 3.5 months in patients without ELN stratification. Among patients aged ≤65 years, median OS was not reached in the favorable-risk group, but remained poor in intermediate-risk (9 months), adverse-risk (7 months), and non-stratified patients (1 month) (log-rank p=0.005).Survival of *NPM1*-mutated patients was inferior to expected, and no statistically significant difference in OS was observed between patients with *NPM1* and *NPM1+FLT3* mutations. Treatment intensity and allogeneic hematopoietic stem cell transplantation significantly improved survival, with transplanted patients demonstrating more than a twofold increase in median OS.

**Copyright Information:** (Online) ISSN: 2572-9241
©2026 The Author(s). HemaSphere is published by John Wiley & Sons Ltd on behalf of European Hematology Association. This is an open access Abstract Book distributed under the Attribution-NonCommercial-NoDerivs (CC BY-NC-ND), which allows third parties to download the articles and share them with others as long as they credit the author and the Abstract Book, but they cannot change the content in any way or use them commercially.
**Abstract Book Citations:** Authors, Title, HemaSphere, 2026;10:(S1):pages. The abstract book DOIs can be found on the “Author Information” page.
**Disclaimer:** Articles published in the journal HemaSphere exclusively reflect the opinions of the authors. The authors are responsible for all content in their abstracts including accuracy of the facts, statements, citing resources, etc.

**Summary/Conclusion**

The mutational spectrum of AML in Kazakhstan aligns with global trends; however, the prevalence of *NPM1* mutations was lower than in global registries. According to ELN 2022 classification, most patients belonged to intermediate and adverse risk groups, consistent with data from Eastern Europe. Treatment intensity and allogeneic hematopoietic stem cell transplantation significantly improved survival, with more than a twofold increase in median OS in transplanted patients. These findings highlight the need to implement routine molecular profiling at AML diagnosis and to expand access to targeted therapies in Kazakhstan.

**Copyright Information:** (Online) ISSN: 2572-9241
©2026 The Author(s). HemaSphere is published by John Wiley & Sons Ltd on behalf of European Hematology Association. This is an open access Abstract Book distributed under the Attribution-NonCommercial-NoDerivs (CC BY-NC-ND), which allows third parties to download the articles and share them with others as long as they credit the author and the Abstract Book, but they cannot change the content in any way or use them commercially.
**Abstract Book Citations:** Authors, Title, HemaSphere, 2026;10:(S1):pages. The abstract book DOIs can be found on the "Author Information" page.
**Disclaimer:** Articles published in the journal HemaSphere exclusively reflect the opinions of the authors. The authors are responsible for all content in their abstracts including accuracy of the facts, statements, citing resources, etc.

### (PB2798) IMMUNOPHENOTYPE, CYTOGENETICS, AND MOLECULAR GENETICS OF ACUTE LEUKAEMIAS IN LAGOS, NIGERIA

**Topic:** 04. Acute myeloid leukemia - Clinical

Oluwafemi Akinwunmi*[1], Samson Adegbe[1], Oluyemisi Osakwe[1], Chiazom Arubaleze[1], Imoh Essien[1], Anu Matthew[1], Modupe Elebute-Odunsi[1]

[1]*Marcelle Ruth Cancer Centre & Specialist Hospital, Lagos, Nigeria;*

### Background

Acute leukaemias are clonal haematopoietic malignancies characterized by uncontrolled proliferation of immature precursor cells and marked biological heterogeneity. Accurate classification requires integration of morphology, immunophenotyping, cytogenetics, and molecular genetics per World Health Organization (WHO) and International Consensus Classification (ICC) of Myeloid and Lymphoid Neoplasms criteria. However, data from sub-Saharan Africa remain limited due to restricted access to specialist diagnostic laboratories.

### Aims

This study aims to characterize the immunophenotypic, cytogenetic, and molecular profiles of cases diagnosed at a specialist centre in Lagos, Nigeria.

### Methods

A retrospective registry-based study included all patients evaluated for suspected acute leukaemia at Marcelle Ruth Cancer Centre, Lagos, between July 2020 and December, 2025. Demographic variables were summarized using descriptive statistics. Diagnostic evaluation comprised multiparameter flow cytometry, FISH, conventional karyotyping, and RT-qPCR for recurrent fusion transcripts, assessing rearrangements involving PML::RARA, KMT2A, PDGFRA, PDGFRB, ABL1, and ABL2.

### Results

A total of 28 patients with acute leukaemia were treated during the study period, with a male predominance (57%) and peak age distribution in the 0–14 years and 35–44 years groups (24% each). The most common presenting symptoms were pain (25%), fever (22.7%), weakness (15.9%), night sweats (13.6%), bleeding manifestations (11.4%), and weight loss (11.4%). Acute myeloid leukaemia (AML) accounted for 17 cases (60.7%) and acute lymphoblastic leukaemia (ALL) for 11 cases (39.3%). Seventeen patients underwent at least one advanced diagnostic modality (immunophenotyping, cytogenetics, or molecular analysis), of whom 15 had evaluable reports, comprising 10 AML and 5 ALL cases (3 B-cell ALL and 2 T-ALL). AML immunophenotyping demonstrated frequent expression of CD13 (87.5%) and CD33 (87.5%), followed by CD34 (75%), CD117 (62.5%), and HLA-DR, CD45, and MPO (50% each). Cytogenetic findings ranged from normal karyotypes to abnormalities including t(12;13)(p13;q12) involving ETV6, KMT2A rearrangement, and PML::RARA fusion (variant t(15;17)(q24;q21)). Molecular analysis revealed the absence of pathogenic variants in some patients, while others harboured mutations involving NPM1, FLT3-ITD, IDH2, ASXL1, BCOR, PML::RARA, and KMT2A rearrangements. In the ALL cohort, B-cell ALL expressed CD19, CD81, CD10, CD123, CD73, and CD22, whereas T-ALL showed cytCD3, TdT, CD7, membrane CD3, CD8, CD2, CD13, CD33, CD45, and CD56 expression, with negativity for CD1a and other myeloid and B-cell lineage markers. Cytogenetic abnormalities in B-cell ALL included t(9;22) and t(1;19), while one T-ALL case demonstrated complex abnormalities (12p-, 16p-, 14q-) and the other had normal cytogenetics. Molecular findings included BCR-ABL1 transcript and TCF-PBX1 fusion gene, with no pathogenic lesions detected in remaining cases.

**Copyright Information:** (Online) ISSN: 2572-9241
©2026 The Author(s). HemaSphere is published by John Wiley & Sons Ltd on behalf of European Hematology Association. This is an open access Abstract Book distributed under the Attribution-NonCommercial-NoDerivs (CC BY-NC-ND), which allows third parties to download the articles and share them with others as long as they credit the author and the Abstract Book, but they cannot change the content in any way or use them commercially.
**Abstract Book Citations:** Authors, Title, HemaSphere, 2026;10:(S1):pages. The abstract book DOIs can be found on the "Author Information" page.
**Disclaimer:** Articles published in the journal HemaSphere exclusively reflect the opinions of the authors. The authors are responsible for all content in their abstracts including accuracy of the facts, statements, citing resources, etc.

**Summary/Conclusion**

This study demonstrates the biological heterogeneity of acute leukaemias diagnosed in Lagos, Nigeria, with immunophenotypic, cytogenetic, and molecular profiles comparable to high-income settings. Detection of actionable alterations, including PML-RARA, BCR-ABL1, TCF-PBX1, KMT2A rearrangements, and mutations in NPM1 and FLT3-ITD, underscores the feasibility of advanced diagnostics in resource-limited settings and calls for the development of local diagnostic capability and expanded genomic research in West Africa.

**Copyright Information:** (Online) ISSN: 2572-9241
©2026 The Author(s). HemaSphere is published by John Wiley & Sons Ltd on behalf of European Hematology Association. This is an open access Abstract Book distributed under the Attribution-NonCommercial-NoDerivs (CC BY-NC-ND), which allows third parties to download the articles and share them with others as long as they credit the author and the Abstract Book, but they cannot change the content in any way or use them commercially.
**Abstract Book Citations:** Authors, Title, HemaSphere, 2026;10:(S1):pages. The abstract book DOIs can be found on the “Author Information” page.
**Disclaimer:** Articles published in the journal HemaSphere exclusively reflect the opinions of the authors. The authors are responsible for all content in their abstracts including accuracy of the facts, statements, citing resources, etc.

### (PB2799) AGE-DEPENDENT SURVIVAL DISPARITIES BETWEEN PUBLIC AND PRIVATE HEALTHCARE SETTINGS IN FLT3-ITD AML: DATA FROM THE BRAZILIAN AML REGISTRY

**Topic:** 04. Acute myeloid leukemia - Clinical

Abel Costa[1], Luiza Perruso*[1], Wellington Fernandes Da Silva Júnior[2], Andre Americo[3], Marco Sartori[4], Verena Pfister[4], Maria Daniela Perico[5], Flavia Xavier[6], Yana Rabelo[7], Renata Lyrio Rafael Baptista[8], Ederson Roberto De Mattos[9], Marina Nascimento[10], Eduardo M. Rego[1, 2, 11]

*[1]D'Or Institute for Research and Education, São Paulo, Brazil; [2]Cancer Institute of São Paulo - ICESP/HCFMUSP, São Paulo, Brazil; [3]Hospital Beneficiência Portuguesa São Paulo, São Paulo, Brazil; [4]Federal University of São Paulo - UNIFESP, São Paulo, Brazil; [5]Centro de Hematologia e Hemoterapia de Santa Catarina, Santa Catarina, Brazil;[6]D'Or Institute for Research and Education, Brasília, Brazil; [7]Hospital das Clínicas da Universidade de Goiânia, Goiânia, Brazil;[8]D'Or Institute for Research and Education, Rio de Janeiro, Brazil; [9]Hospital Amaral Carvalho, Jaú, Brazil; [10]A.C. Camargo Cancer Center, São Paulo, Brazil; [11]University of São Paulo Medical School, Hematology Division, São Paulo, Brazil;*

### Background

FLT3-ITD is a key actionable mutation in AML. Adding FLT3 inhibitors (FLT3i) to first-line intensive chemotherapy (IC) or venetoclax-based regimens is standard of care, but these agents are not covered by the Brazilian public healthcare system (SUS). Private insurance reimburses FLT3i and venetoclax, and this access gap allows evaluation of the survival impact of targeted therapy in a middle-income country.

### Aims

To compare overall survival between public and private healthcare settings in FLT3-ITD AML, with prespecified subgroup analysis by age (<60 vs ≥60 years).

### Methods

We retrospectively analyzed a FLT3-mutated dataset from the Brazilian AML Registry (N=93; data cutoff February 2026). Analysis was restricted to FLT3-ITD-positive patients (76/93); after excluding 2 with invalid survival data, the final cohort included 74 patients (44 public, 30 private). OS was estimated by Kaplan-Meier and compared using log-rank test and Cox regression, with prespecified age-stratified subgroup analyses (<60 vs ≥60 years) and setting-by-age interaction testing. Sensitivity analyses included IPTW (inverse probability of treatment weighting) to balance age between settings and Cox regression with HSCT as a time-dependent covariate.

**Copyright Information:** (Online) ISSN: 2572-9241
©2026 The Author(s). HemaSphere is published by John Wiley & Sons Ltd on behalf of European Hematology Association. This is an open access Abstract Book distributed under the Attribution-NonCommercial-NoDerivs (CC BY-NC-ND), which allows third parties to download the articles and share them with others as long as they credit the author and the Abstract Book, but they cannot change the content in any way or use them commercially.
**Abstract Book Citations:** Authors, Title, HemaSphere, 2026;10:(S1):pages. The abstract book DOIs can be found on the “Author Information” page.
**Disclaimer:** Articles published in the journal HemaSphere exclusively reflect the opinions of the authors. The authors are responsible for all content in their abstracts including accuracy of the facts, statements, citing resources, etc.

### Results

Median age was 53.8 years. Public centers relied on IC without FLT3i (75.0%), while private centers favored AZA-VEN (53.3%) and IC+FLT3i (33.3%) — IC+FLT3i in younger and AZA-VEN in older private patients. First-line FLT3i was less frequent in public vs private (11.4% vs 36.7%; p=0.019), but HSCT rates were similar (43.2% vs 50.0%; p=0.638). Median OS was 9.2 months in public vs 21.1 in private (log-rank p=0.027); 12-month OS was 40.5% vs 68.0% (Figure 1); six-month mortality was 36.4% (16/44) vs 10.0% (3/30; p=0.014).

Subgroup analysis showed age-dependent effect modification. In patients <60, HSCT rates were similar between settings (59% vs 71%; p=0.520) and OS did not differ significantly (HR 0.81, 95%CI 0.32–2.08; p=0.660). In ≥60, where public centers used IC (75%) and private AZA-VEN (81%), 30-day mortality was 33% vs 0% and OS differed markedly (median 1.3 vs 40.4 months; HR 0.04, 95%CI 0.01–0.20; p<0.001; interaction p<0.001). In multivariable Cox regression (n=67), private setting remained associated with better OS (HR 0.19, 95%CI 0.07–0.53; p=0.002). The healthcare setting effect persisted after adjusting for HSCT as a time-dependent covariate (HR 0.16, p<0.001) and in IPTW analysis (HR 0.39, 95%CI 0.20–0.73; p=0.003).

### Summary/Conclusion

In this Brazilian FLT3-ITD AML cohort, public-private survival disparities were driven by patients ≥60 years, where access to venetoclax-based regimens was associated with lower early mortality in private settings. In younger patients, similar HSCT rates across settings may explain comparable survival. These findings highlight the need for incorporation of targeted therapies into the Brazilian public healthcare system.

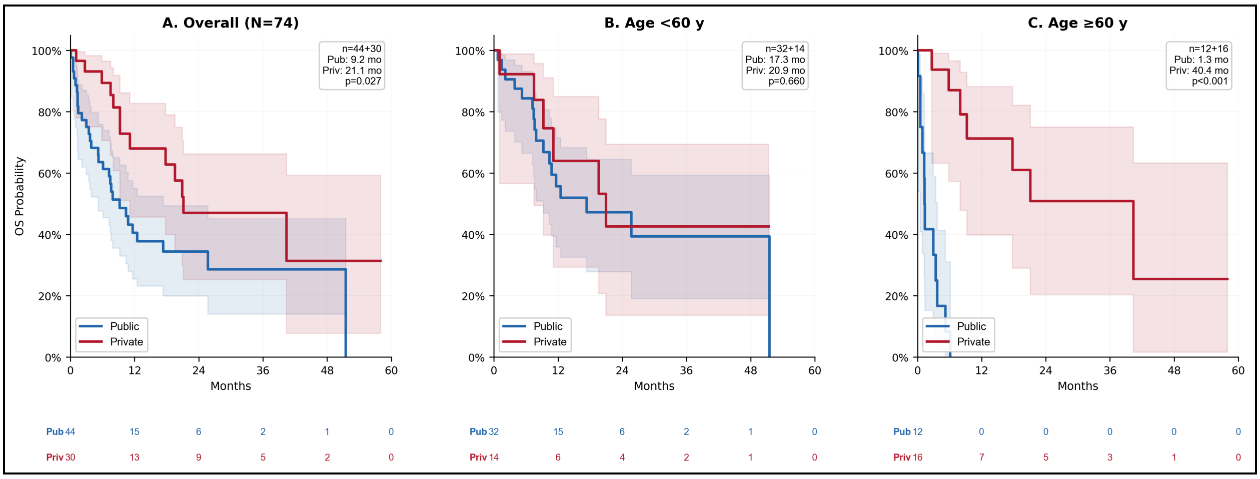

Figure 1. Overall survival by healthcare setting in FLT3-ITD AML, overall and by age strata (N=74). Kaplan-Meier curves comparing public versus private care settings. Shaded areas: 95% CI. Panel A: Overall ITD cohort (public n=44, private n=30; log-rank p=0.027). Panel B: <60 years (public n=32, private n=14; p=0.660). Panel C: ≥60 years (public n=12, private n=16; p<0.001).

**Copyright Information:** (Online) ISSN: 2572-9241
©2026 The Author(s). HemaSphere is published by John Wiley & Sons Ltd on behalf of European Hematology Association. This is an open access Abstract Book distributed under the Attribution-NonCommercial-NoDerivs (CC BY-NC-ND), which allows third parties to download the articles and share them with others as long as they credit the author and the Abstract Book, but they cannot change the content in any way or use them commercially.
**Abstract Book Citations:** Authors, Title, HemaSphere, 2026;10:(S1):pages. The abstract book DOIs can be found on the "Author Information" page.
**Disclaimer:** Articles published in the journal HemaSphere exclusively reflect the opinions of the authors. The authors are responsible for all content in their abstracts including accuracy of the facts, statements, citing resources, etc.

### (PB2800) EFFICACY AND SAFETY OF LIPOSOMAL MITOXANTRONE COMBINED WITH VENETOCLAX, AZACITIDINE, AND CHIDAMIDE (VACM) IN ACUTE MONOCYTIC LEUKEMIA

**Topic:** 04. Acute myeloid leukemia - Clinical

Dairong Xie*[1], Qiuling Wu[2]

*[1]Union Hospital, Tongji Medical College, Huazhong University of Science and Technology, Wuhan, China., Institute of Hematology, Wuhan, China; [2]Union Hospital, Tongji Medical College, Huazhong University of Science and Technology, Wuhan, China., Institute of Hematology,, Wuhan, China;*

### Background

Acute monocytic leukemia (AML-M5) is a distinct and clinically challenging subtype of acute myeloid leukemia (AML). It is characterized by inherent resistance to standard azacitidine plus venetoclax (Aza+Ven) therapy, manifesting as lower response rates and a higher propensity for relapse. Liposomal mitoxantrone offers advantages through enhanced drug delivery, extended circulation time for better tumor accumulation, and reduced systemic toxicity, making it suitable for combination therapy in AML-M5.

### Aims

This study aimed to evaluate the efficacy and safety of Liposomal Mitoxantrone combined with Venetoclax, Azacitidine, and Chidamide (VACM) regimen in treating acute monocytic leukemia, with primary focus on complete remission rate (CR), MRD negativity, and treatment response.

### Methods

This prospective, multicenter clinical study was conducted across two centers including Union Hospital, Tongji Medical College, Huazhong University of Science and Technology and another participating center. The study included AML-M5 patients treated with VACM regimen between 2025-2026. The treatment protocol consisted of Liposomal Mitoxantrone (20-24 mg/m², ivgtt, d1), azacitidine (75 mg/m², i.v. or i.h., d1-7), venetoclax (100 mg d1, 200 mg d2, 400 mg d3-7, p.o.), and chidamide (30 mg d3, d7, p.o.). Response assessment included morphological evaluation and MRD detection by flow cytometry. Primary endpoints were CR rate and MRD negativity, with secondary endpoints including overall response rate (ORR) and treatment safety.

### Results

Thirteen AML-M5 patients were analyzed (10 males, 3 females; median age 50.5 years, range 15–66). With a median follow-up of 12 months (IQR 6–18), the CR rate was 92.31% (12/13), and the ORR was 92.31% (12/13). Notably, 84.62% (11/13) of evaluable patients achieved MRD negativity. Among CR patients, the median time to MRD negativity was 14 days (range 5–39). The safety profile was manageable, characterized by predictable and reversible hematological toxicity. The primary adverse event was Grade 3/4 neutropenia, with a median duration of bone marrow suppression of 18 days (range 14–21). Crucially, no treatment-related cardiotoxicity or unexpected Grade 3/4 non-hematological toxicities were observed, confirming the safety advantage of the liposomal formulation.

### Summary/Conclusion

The VACM regimen demonstrates promising efficacy in AML-M5, achieving high CR and MRD negativity rates. These preliminary findings suggest that VACM may represent a viable therapeutic alternative for AML-M5 patients with poor response to standard Aza+Ven regimens, warranting validation in larger prospective studies.

**Copyright Information:** (Online) ISSN: 2572-9241
©2026 The Author(s). HemaSphere is published by John Wiley & Sons Ltd on behalf of European Hematology Association. This is an open access Abstract Book distributed under the Attribution-NonCommercial-NoDerivs (CC BY-NC-ND), which allows third parties to download the articles and share them with others as long as they credit the author and the Abstract Book, but they cannot change the content in any way or use them commercially.
**Abstract Book Citations:** Authors, Title, HemaSphere, 2026;10:(S1):pages. The abstract book DOIs can be found on the "Author Information" page.
**Disclaimer:** Articles published in the journal HemaSphere exclusively reflect the opinions of the authors. The authors are responsible for all content in their abstracts including accuracy of the facts, statements, citing resources, etc.

### (PB2801) VALIDATING PIVOTAL TRIAL OUTCOMES: SINGLE-CENTER EXPERIENCE SHOWING MRD-NEGATIVITY AS THE PRIMARY DRIVER OF LONG-TERM OUTCOMES IN HIGH-RISK AML TREATED WITH CPX-351

**Topic:** 04. Acute myeloid leukemia - Clinical

Alba Manasa Gonzalez Arias*[1], Rosalia Riaza Grau[1], Lucia Rico Taverna[1], Alvaro Gonzalo Sanchez[1], Veronica Martinez Navarro[1], Irene Hervas Gonzalez[1], Maria Mar Diaz Goizueta[1], María Luisa Bengochea-Casado[1], Ramon Rodriguez Gonzalez[1], Maria Jose Requena Rodriguez[1], Margarita Berberana Fernandez DE Murias[1], Sandra Garcia Lazaro[1]

[1]*HOSPITAL UNIVERSITARIO SEVERO OCHOA, HEMATHOLOGY AND HEMOTHERAPY, Madrid, Spain;*

### Background

Real-world evidence is essential to confirm CPX-351 outcomes in high-risk AML and to evaluate the prognostic impact of deep molecular responses, particularly measurable residual disease (MRD) negativity, in routine clinical practice.

### Aims

We aimed to assess the efficacy and safety of CPX-351 in all patients treated at our center and to determine the impact of MRD status on survival outcomes.

### Methods

We retrospectively analyzed 22 patients (median age 67, range 45–78). The cohort was predominantly high-risk: 68% adverse ELN 2022 risk, 32% TP53 mutations, and 27% complex karyotypes. Indications included AML-MRC (55%), t-AML (27%), and bridge to transplant (18%). Measurable residual disease (MRD) was monitored using multiparametric flow cytometry and molecular techniques where available. Survival analysis utilized Kaplan-Meier and Cox proportional hazards models.

### Results

The impact of MRD negativity on survival was immediately evident: after a median follow-up of 7.2 months, the median overall survival (OS) for the entire cohort was 10.2 months (95% CI: 5.6–NC), closely aligning with the 9.6 months reported in the pivotal phase 3 trial. OS was strongly driven by the quality of response: patients achieving complete remission with MRD negativity (CR MRD-) reached a median OS of 24.97 months (n=11), compared to 6.34 months for MRD+ responders and 4.66 months for non-responders, with a log-rank p < 0.0001. In the Cox regression model, CR MRD- emerged as an independent predictor of reduced mortality (HR 0.22; 95% CI: 0.05–0.89; p=0.033). Median progression-free survival (PFS) for the cohort was 10.25 months (95% CI: 4.99–NE), with a 12-month PFS rate of 43%. Although the difference between MRD- and MRD+ patients did not reach statistical significance (19.94 vs. 10.25 months; p=0.31; HR 0.32; p=0.076), a clear trend toward reduced progression risk in MRD-negative patients was observed. Event-free survival (EFS), analyzed among patients achieving CR (n=10), reached a median of 35.9 months (95% CI: 19.9–NC), with a pronounced benefit for MRD-negative patients (48.2 months vs. 9.6 months for MRD+; log-rank p=0.014). The observed global response rate of 63.6% closely mirrors the 63.7% reported in the Spanish multicenter real-world registry.

**Copyright Information:** (Online) ISSN: 2572-9241
©2026 The Author(s). HemaSphere is published by John Wiley & Sons Ltd on behalf of European Hematology Association. This is an open access Abstract Book distributed under the Attribution-NonCommercial-NoDerivs (CC BY-NC-ND), which allows third parties to download the articles and share them with others as long as they credit the author and the Abstract Book, but they cannot change the content in any way or use them commercially.
**Abstract Book Citations:** Authors, Title, HemaSphere, 2026;10:(S1):pages. The abstract book DOIs can be found on the "Author Information" page.
**Disclaimer:** Articles published in the journal HemaSphere exclusively reflect the opinions of the authors. The authors are responsible for all content in their abstracts including accuracy of the facts, statements, citing resources, etc.

**Summary/Conclusion**

Our single-center real-world experience confirms that CPX-351 efficacy in high-risk AML is primarily driven by deep therapeutic responses, with MRD-negativity serving as the key predictor of long-term survival. These results validate the outcomes reported in pivotal trials and real-world registries, reinforcing MRD negativity as a critical therapeutic and prognostic milestone in clinical practice.

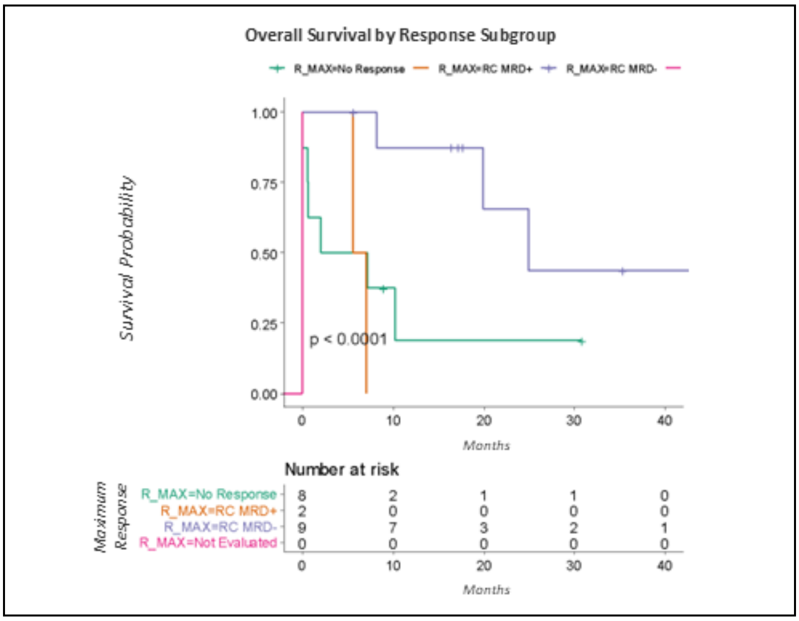

This figure presents Kaplan-Meier curves of OS stratified by maximum response (Non-Responders vs. CR MRD+ vs. CR MRD-), demonstrating a significant survival advantage for the MRD-negative group, which reached a median OS of 24.97 months, compared to the other subgroups (log-rank p < 0.0001).

**Copyright Information:** (Online) ISSN: 2572-9241
©2026 The Author(s). HemaSphere is published by John Wiley & Sons Ltd on behalf of European Hematology Association. This is an open access Abstract Book distributed under the Attribution-NonCommercial-NoDerivs (CC BY-NC-ND), which allows third parties to download the articles and share them with others as long as they credit the author and the Abstract Book, but they cannot change the content in any way or use them commercially.
**Abstract Book Citations:** Authors, Title, HemaSphere, 2026;10:(S1):pages. The abstract book DOIs can be found on the "Author Information" page.
**Disclaimer:** Articles published in the journal HemaSphere exclusively reflect the opinions of the authors. The authors are responsible for all content in their abstracts including accuracy of the facts, statements, citing resources, etc.

### (PB2802) EFFICACY OF LIPOSOMAL MITOXANTRONE COMBINED WITH VENETOCLAX AND AZACITIDINE FOR RELAPSED/REFRACTORY ACUTE MYELOID LEUKEMIA

**Topic:** 04. Acute myeloid leukemia - Clinical

Jiaxin Zhao[1], Yan Ping Yang[1], Yu Ying Li[1], Ye Hui Tan*[1], Sujun Gao[1]

[1]*The First Hospital of Jilin University, Depertment of hematology, Changchun, China;*

### Background

Adult patients with acute myeloid leukemia (AML) achieve high initial remission rates; however, most eventually experience relapse. The prognosis for relapsed/refractory AML (R/R AML) remains extremely poor, with a 5-year overall survival rate of less than 10%. Although venetoclax combined with azacitidine (the VA regimen) has improved outcomes in this setting, approximately one-third of patients develop resistance, resulting in treatment failure, and subsequent salvage options are severely limited. Liposomal mitoxantrone, a novel nano-formulation, demonstrates potentiated anti-leukemic activity while mitigating systemic toxicities compared with conventional mitoxantrone, particularly the risk of cardiotoxicity. Its therapeutic potential in AML has been validated by multiple studies. Here, we present updated real-world data on the use of liposomal mitoxantrone in patients with R/R AML.

### Aims

To assess the efficacy and safety of the liposomal mitoxantrone, venetoclax, and azacitidine (MVA) regimen in relapsed/refractory acute myeloid leukemia (R/R AML).

### Methods

This retrospective analysis enrolled 16 patients with R/R AML or those who failed to achieve CR after one cycle of chemotherapy ± targeted therapy at the First Hospital of Jilin University.Patients received the MVA regimen: liposomal mitoxantrone 24 mg/m² on day 1, venetoclax escalated to 100–400 mg daily on days 1–14 (dose reduction when combined with posaconazole), and azacitidine 75 mg/m²/day on days 1–7. The primary endpoint was composite complete remission rate (CRc). Secondary endpoints included objective response rate (ORR), event-free survival (EFS), overall survival (OS), and safety.

### Results

A total of 16 patients were enrolled, with a median age of 58.5 years. Among them, 8 had relapsed AML, 5 had refractory AML, and 3 experienced early induction failure. After a median follow-up of 9.8 months, the composite complete remission rate (CRc) was 62.5% (10/16), and the objective response rate (ORR) was 87.5% (14/16). In patients with prior venetoclax exposure, the CRc reached 77.8% (7/9). Notably, CRc was achieved in 50% (2/2) of patients with complex karyotype, 83.3% (5/6) of patients with ASXL1 mutations, and 50% (2/4) of patients with RAS mutations. Grade 3–4 hematologic toxicities were common (neutropenia 93.8%, thrombocytopenia 87.5%) but manageable, with no treatment-related mortality.

### Summary/Conclusion

The MVA regimen demonstrates high response rates with a manageable safety profile in R/R AML. It exhibits substantial activity even in patients with prior venetoclax exposure or high-risk genetic abnormalities, representing a promising salvage therapeutic option for this challenging population.

**Copyright Information:** (Online) ISSN: 2572-9241
©2026 The Author(s). HemaSphere is published by John Wiley & Sons Ltd on behalf of European Hematology Association. This is an open access Abstract Book distributed under the Attribution-NonCommercial-NoDerivs (CC BY-NC-ND), which allows third parties to download the articles and share them with others as long as they credit the author and the Abstract Book, but they cannot change the content in any way or use them commercially.
**Abstract Book Citations:** Authors, Title, HemaSphere, 2026;10:(S1):pages. The abstract book DOIs can be found on the "Author Information" page.
**Disclaimer:** Articles published in the journal HemaSphere exclusively reflect the opinions of the authors. The authors are responsible for all content in their abstracts including accuracy of the facts, statements, citing resources, etc.

| Clinical features | |
|---|---|
| **Median age** | 58.5 (20-73) |
| **Gender, no. (%)** | |
| **Male** | 7 (43.8%) |
| **Female** | 9 (56.2%) |
| **AML type, no. (%)** | |
| **De novo AML** | 13 (81.3%) |
| **Secondary AML** | 3 (18.8%) |
| **Pre-treatment status for MVA, no. (%)** | |
| **Refractory** | 5 (31.3%) |
| **Relapse** | 8 (50.0%) |
| **Early Induction Failure** | 3 (18.8%) |
| **Molecular mutation, no. (%)** | |
| **FLT3(FLT3-TKD/FLT3-ITD)** | 4（25.0%） |
| **RAS（KRAS/NRAS）** | 4（25.0%） |
| **ASXL1** | 6（37.5%） |
| **IDH2** | 2 (12.5%) |
| **U2AF1** | 3 (18.8%) |
| **CEBPA** | 2 (12.5%) |
| **RUNX1** | 2 (12.5%) |
| **Chromosomal abnormalities, no. (%)** | |
| **Normal karyotype** | 12（75.0%） |
| **Complex karyotype** | 2（12.5%） |
| **Other** | 2（12.5%） |

Table 1: Baseline Characteristics of Patients

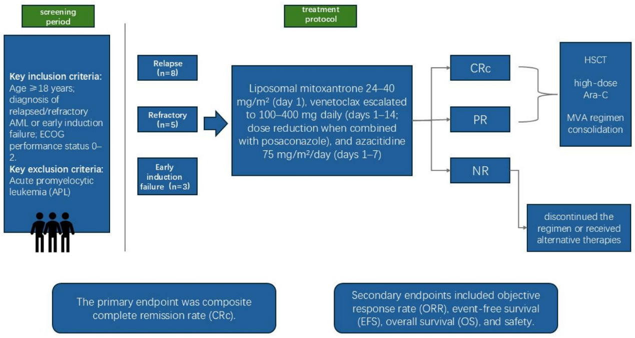

Figure 1. Flowchart of the study methodology.

Table 1: Baseline Characteristics of Patients , Figure 1. Flowchart of the study methodology.

**Copyright Information:** (Online) ISSN: 2572-9241
©2026 The Author(s). HemaSphere is published by John Wiley & Sons Ltd on behalf of European Hematology Association. This is an open access Abstract Book distributed under the Attribution-NonCommercial-NoDerivs (CC BY-NC-ND), which allows third parties to download the articles and share them with others as long as they credit the author and the Abstract Book, but they cannot change the content in any way or use them commercially.
**Abstract Book Citations:** Authors, Title, HemaSphere, 2026;10:(S1):pages. The abstract book DOIs can be found on the "Author Information" page.
**Disclaimer:** Articles published in the journal HemaSphere exclusively reflect the opinions of the authors. The authors are responsible for all content in their abstracts including accuracy of the facts, statements, citing resources, etc.

### (PB2803) REDUCED-DOSE VENETOCLAX WITH POSACONAZOLE SHOWS COMPARABLE EFFICACY TO STANDARD-DOSE IN FIRST-LINE TREATMENT OF ACUTE MYELOID LEUKEMIA

**Topic:** 04. Acute myeloid leukemia - Clinical

Sang-A Kim*[1], Ji Yun Lee[1], Jeong-Ok Lee[1], Soo Mee Bang[1]

*[1]Seoul National University Bundang Hospital, Internal Medicine, SeongNam, South Korea;*

### Background

Venetoclax (VEN) combined with hypomethylating agents (HMA) is the standard first-line treatment for older or unfit patients with acute myeloid leukemia (AML). When co-administered with posaconazole, a strong CYP3A4 inhibitor commonly used for antifungal prophylaxis, VEN dose reduction to 50–100 mg is recommended due to significantly increased drug exposure. However, the optimal reduced dose of VEN with posaconazole remains unclear, and real-world data comparing different dose levels are limited, particularly in Asian populations.

### Aims

To compare the efficacy and safety of reduced-dose VEN (≤50 mg) versus standard-dose VEN (70–100 mg) with posaconazole in first-line VEN/HMA treatment for AML.

### Methods

We conducted a single-center retrospective study of treatment-naïve AML patients who received first-line VEN/HMA with concomitant posaconazole between January 2021 and May 2025. Patients were classified into two groups based on cycle 1 target VEN dose after ramp-up: ≤50 mg (reduced-dose) and 70–100 mg (standard-dose). The primary endpoint was composite complete remission rate (CR/CRi). Secondary endpoints included overall response rate (ORR; CR/CRi/MLFS), overall survival (OS), and cycle 1 myelosuppression (nadir ANC and platelet count). Fisher's exact test, Mann-Whitney U test, and Kaplan-Meier analysis with log-rank test were used.

### Results

Sixty patients were included (≤50 mg, n=19; 70–100 mg, n=41). Baseline characteristics were generally balanced between groups: median age 77 vs 77 years, male 52.6% vs 73.2%, ECOG ≥2 36.8% vs 46.3%. Decitabine was the most common HMA in both groups (42.1% vs 53.7%).
The CR/CRi rate was 57.9% in the ≤50 mg group versus 48.8% in the 70–100 mg group (p=0.585). The ORR (CR/CRi/MLFS) was 63.2% versus 58.5% (p=0.784). Median OS was 17.3 months in the ≤50 mg group versus not reached in the 70–100 mg group (log-rank p=0.741). Thirty-day mortality was 5.3% versus 7.3%, and 60-day mortality was 15.8% versus 12.2%, respectively. Cycle 1 nadir ANC (median 76 vs 68/µL, p=0.489) and nadir platelet count (median 12 vs 15 $\times 10^3$/µL, p=0.697) were comparable.

**Copyright Information:** (Online) ISSN: 2572-9241
©2026 The Author(s). HemaSphere is published by John Wiley & Sons Ltd on behalf of European Hematology Association. This is an open access Abstract Book distributed under the Attribution-NonCommercial-NoDerivs (CC BY-NC-ND), which allows third parties to download the articles and share them with others as long as they credit the author and the Abstract Book, but they cannot change the content in any way or use them commercially.
**Abstract Book Citations:** Authors, Title, HemaSphere, 2026;10:(S1):pages. The abstract book DOIs can be found on the "Author Information" page.
**Disclaimer:** Articles published in the journal HemaSphere exclusively reflect the opinions of the authors. The authors are responsible for all content in their abstracts including accuracy of the facts, statements, citing resources, etc.

### Summary/Conclusion

In this real-world cohort of AML patients receiving first-line VEN/HMA with posaconazole, reduced-dose VEN (≤50 mg) demonstrated comparable CR/CRi rates and similar myelosuppression profiles to standard-dose VEN (70–100 mg). These findings suggest that VEN 50 mg with posaconazole provides adequate antileukemic efficacy, supporting dose reduction in clinical practice when strong CYP3A4 inhibitors are co-administered. The numerically shorter OS in the ≤50 mg group warrants further investigation in larger studies. A prospective pharmacokinetic study to measure actual venetoclax exposure in this population is currently being designed.

Table 1. Efficacy and safety outcomes by VEN dose group

| Outcome | ≤50 mg (n=19) | 70–100 mg (n=41) | p-value |
|---|---|---|---|
| CR/CRi, n (%) | 11 (57.9) | 20 (48.8) | 0.585 |
| ORR (CR/CRi/MLFS), n (%) | 12 (63.2) | 24 (58.5) | 0.784 |
| Median OS, months | 17.3 | NR | 0.130 |
| 30-day mortality, n (%) | 1 (5.3) | 3 (7.3) | – |
| 60-day mortality, n (%) | 3 (15.8) | 5 (12.2) | – |
| Nadir ANC, median (/μL) | 76 | 68 | 0.489 |
| Nadir PLT, median (×10³/μL) | 12 | 15 | 0.697 |

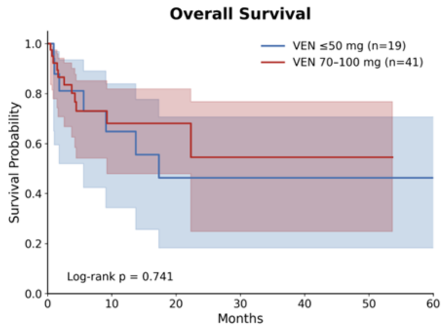

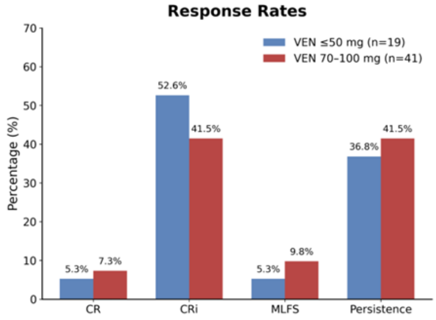

Table 1. Efficacy and safety outcomes by VEN dose group. CR, complete remission; CRi, complete remission with incomplete hematologic recovery; ORR, overall response rate; MLFS, morphologic leukemia-free state; OS, overall survival; ANC, absolute neutrophil count; PLT, platelet count; NR, not reached. P-values were calculated using Fisher's exact test for categorical variables and Mann-Whitney U test for continuous variables. OS was compared using the log-rank test. Figure 1. Kaplan-Meier estimates of overall survival according to cycle 1 venetoclax target dose in patients receiving concomitant posaconazole. Shaded areas represent 95% confidence intervals. Tick marks indicate censored observations. Figure 2. Response rates after first-line venetoclax/HMA treatment according to cycle 1 venetoclax target dose. CR, complete remission; CRi, complete remission with incomplete hematologic recovery; MLFS, morphologic leukemia-free state.

Copyright Information: (Online) ISSN: 2572-9241
©2026 The Author(s). HemaSphere is published by John Wiley & Sons Ltd on behalf of European Hematology Association. This is an open access Abstract Book distributed under the Attribution-NonCommercial-NoDerivs (CC BY-NC-ND), which allows third parties to download the articles and share them with others as long as they credit the author and the Abstract Book, but they cannot change the content in any way or use them commercially.
**Abstract Book Citations:** Authors, Title, HemaSphere, 2026;10:(S1):pages. The abstract book DOIs can be found on the "Author Information" page.
**Disclaimer:** Articles published in the journal HemaSphere exclusively reflect the opinions of the authors. The authors are responsible for all content in their abstracts including accuracy of the facts, statements, citing resources, etc.

### (PB2804) VALIDATION OF THE ELN RISK CLASSIFICATIONS FOR AML IN A 971 PATIENTS UNICENTRIC COHORT

**Topic:** 04. Acute myeloid leukemia - Clinical

Diana Tholen*[1], Felicitas Schulz[1], Annika Kasprzak[1], Corinna Strupp[1], Barbara Hildebrandt[2], Beate Betz[2], Sascha Dietrich[1], Kathrin Nachtkamp[1], Guido Kobbe[1], Ulrich Germing[1], Paul Jaeger[1]

*[1]Heinrich Heine University Düsseldorf, Department of Haematology, Oncology and Clinical Immunology, Düsseldorf, Germany; [2]Heinrich Heine University Düsseldorf, Institute of Human Genetics, Düsseldorf, Germany;*

### Background

AML risk stratification can be assessed by ELN classifications (2017, 2022).

### Aims

To validate ELN classifications in a real-world AML population.

### Methods

971 patients (Pts) diagnosed with AML in our department (2007-2025) including ELN categories, age, comorbidities, and type of treatment were analyzed. Overall survival (OS) was assessed using Kaplan-Meier analyses and multivariable Cox regression. Pts were separated based on the maximal intensive treatment that was administered from best supportive care only (BSC), non-intensive cytoreduction (NIC), treatment with hypomethylating agents (HMA), treatment with HMA+Venetoclax (HMAven), induction chemotherapy, and allogeneic transplantation (allo-SCT). Pts were followed up until february 15, 2026.

**Copyright Information:** (Online) ISSN: 2572-9241
©2026 The Author(s). HemaSphere is published by John Wiley & Sons Ltd on behalf of European Hematology Association. This is an open access Abstract Book distributed under the Attribution-NonCommercial-NoDerivs (CC BY-NC-ND), which allows third parties to download the articles and share them with others as long as they credit the author and the Abstract Book, but they cannot change the content in any way or use them commercially.
**Abstract Book Citations:** Authors, Title, HemaSphere, 2026;10:(S1):pages. The abstract book DOIs can be found on the "Author Information" page.
**Disclaimer:** Articles published in the journal HemaSphere exclusively reflect the opinions of the authors. The authors are responsible for all content in their abstracts including accuracy of the facts, statements, citing resources, etc.

### Results

Median age at diagnosis was 61 years (19–91). BSC was given to 15 pts(1.5%), NIC to 43 (4.4%), HMA to 99 (10.2%), HMAven to 96 (9.9%), induction chemotherapy to 231 (23.8%), and allo-SCT to 486 (50.1%).
Therapies have evolved over years, justifying stratification into pre-2017, 2017–2022, and post-2022 eras. Before 2017 (n=401): 42% allo-SCT, 32% induction, 16.5% HMA, 8% NIC, and 2% BSC. Between 2017–2022 (n=295): 52% allo-SCT, 21.4% induction, 11.2% HMA, 13.6% HMAven, 1% NIC, and 1% BSC. Since 2022 (n=275): 60% allo-SCT, 14.5% induction, 20.4% HMAven, 2.9% NIC, and 2.2% BSC.
According to ELN17 264 pts (27.1%) were classified as favorable, 282 (29%) as intermediate, and 425 (43.8%) as adverse risk. In ELN22, 221 pts (22.8%) were assigned to the favorable-, 303 (31.2%) to the intermediate-, and 447 (46%) to the adverse-risk group. Using ELN22 43 of 264 pts (16.3%) previously classified as favorable by ELN17 were classified to intermediate- (n=40) or high-risk (n=3) category, mainly due to FLT3-ITD low, and non-bZIP biallelic CEBPA as intermediate risk group and inclusion of additional adverse mutations such as EZH2, SF3B1 to high-risk group.
OS of the entire group was 18 months (m) (0-313). OS was 271 m for pts undergoing allo-SCT, 185 m for induction therapy, and 10 m for HMAven, followed by 5 m for HMA only, NIC or BSC had an OS of 1 m (p<0.001).
OS with HMAven was significantly superior to HMA only (10 vs 5 ms; p<0.001).
OS was 24 m pre-2017, 80 m in 2017–2022, and n.r. post-2022 (p<0.001).
Clinical factors associated with inferior OS were age >70 years, OS (8 m vs. 133m), and the presence of any comorbidity, OS (28 m vs. n.r.) (p<0.001).
According to ELN17, OS in the favorable-, intermediate-, and adverse-risk groups was 188, 60, and 16 m, respectively (p<0.001). Based on the ELN22, OS was 188, 64, and 16 m accordingly (p<0.001).
OS was 103 m for pts reclassified from low to intermediate/high vs. 188, 60, and 16 m for low-, intermediate-, and high-risk groups, respectively (p<0.001).
When focusing on pts who underwent allo-SCT, ELN22 still seperated risk-groups.
Multivariable analyses showed that choice of treatment followed by ELN22 category, and presence of comorbidities were the most important independent prognostic parameters.

### Summary/Conclusion

Over time, use of allo-SCT and HMAven increased, while induction chemotherapy and HMA only decreased, reflecting evolving treatment strategies in a transplant-focused tertiary AML center.
ELN22 reclassified 16% of previously favorable pts to higher-risk categories and provided refined prognostic discrimination compared to ELN17.
In multivariable analysis, treatment intensity, ELN22 risk category, and comorbidities independently predicted overall survival.

**Copyright Information:** (Online) ISSN: 2572-9241
©2026 The Author(s). HemaSphere is published by John Wiley & Sons Ltd on behalf of European Hematology Association. This is an open access Abstract Book distributed under the Attribution-NonCommercial-NoDerivs (CC BY-NC-ND), which allows third parties to download the articles and share them with others as long as they credit the author and the Abstract Book, but they cannot change the content in any way or use them commercially.
**Abstract Book Citations:** Authors, Title, HemaSphere, 2026;10:(S1):pages. The abstract book DOIs can be found on the "Author Information" page.
**Disclaimer:** Articles published in the journal HemaSphere exclusively reflect the opinions of the authors. The authors are responsible for all content in their abstracts including accuracy of the facts, statements, citing resources, etc.

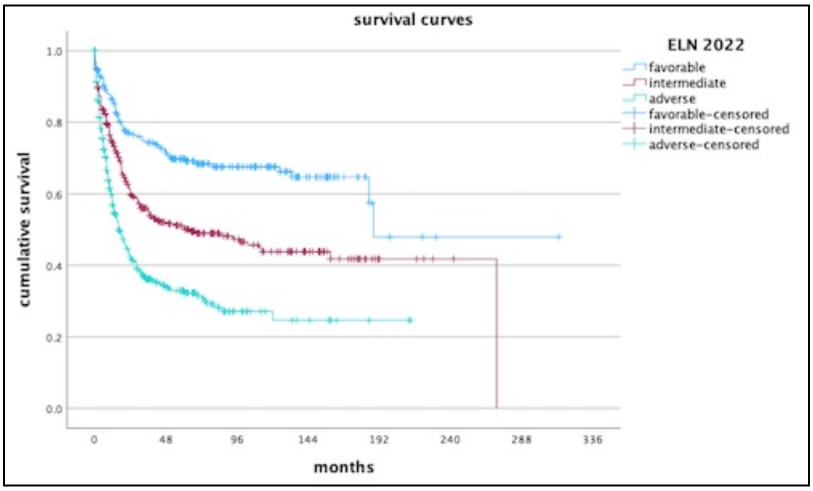

overall survival (OS) according to ELN 2022 risk stratification

**Copyright Information:** (Online) ISSN: 2572-9241
©2026 The Author(s). HemaSphere is published by John Wiley & Sons Ltd on behalf of European Hematology Association. This is an open access Abstract Book distributed under the Attribution-NonCommercial-NoDerivs (CC BY-NC-ND), which allows third parties to download the articles and share them with others as long as they credit the author and the Abstract Book, but they cannot change the content in any way or use them commercially.
**Abstract Book Citations:** Authors, Title, HemaSphere, 2026;10:(S1):pages. The abstract book DOIs can be found on the "Author Information" page.
**Disclaimer:** Articles published in the journal HemaSphere exclusively reflect the opinions of the authors. The authors are responsible for all content in their abstracts including accuracy of the facts, statements, citing resources, etc.

### (PB2805) LONG-COURSE (≥4 CYCLES) VENETOCLAX-AZACITIDINE IMPROVES OUTCOMES IN RAS-MUTATED AML: A REAL-WORLD STUDY

**Topic:** 04. Acute myeloid leukemia - Clinical

Tingjun Zhu*[1, 2], Yuanyuan Zhu[3], Lixia Zhu[1], Jingyi Huang[1], Ninghong Zhao[1], Wanting Feng[4], Jia Hu[1], Hui Zhou[4], Weijia Huang[1], Jie Sun[1]

*[1]The First Affiliated Hospital, Zhejiang University School of Medicine, Hangzhou, China;[2]ted Jinhua Hospital, Zhejiang University School of Medicine Hangzhou, China Edit Delete Affiliation The Fir, Jinhua, China; [3]Affiliated Jinhua Hospital, Zhejiang University School of Medicine, Hangzhou, China; [4]Zhejiang University School of Medicine, Hangzhou, China;*

### Background

The venetoclax-azacitidine (VA) regimen is a first-line therapy not only for unfit AML patients but is now also considered for fit patients. Multiple studies have confirmed that AML with RAS pathway mutations exhibits a poor response to VA therapy. Furthermore, AML with RAS pathway mutations lacks targeted agents. The optimal treatment duration of the VA regimen in this population remains undefined.

### Aims

We sought to describe the outcomes of patients with RAS-pathway-mutated AML treated with a VA regimen, comparing those who received short-course versus long-course therapy. The goal was to explore the optimal VA treatment duration for this high-risk population.

### Methods

RAS-pathway-related gene mutations were defined as mutations in one or more of the following genes: *BRAF*, *CBL*, *CBLC*, *KRAS*, *NRAS*, *PTPN11*, and *NF1*. These mutations were detected by next-generation sequencing using a panel that includes 475 genes associated with hematopoietic and lymphoid neoplasms, encompassing all seven RAS-pathway-related genes listed above. We conducted a retrospective study of newly diagnosed AML patients with RAS-pathway mutations at The First Affiliated Hospital of Zhejiang University School of Medicine. All enrolled patients were treated with a VA regimen. Patients were stratified into two groups based on treatment duration: a short-course group (1 cycle) and a long-course group (≥4 cycles). The primary endpoint was the complete remission (CR) rate. Secondary endpoints included the minimal residual disease (MRD) negativity rate, mortality, overall survival (OS), disease-free survival (DFS), and cumulative relapse rate (CRR).

### Results

From 2021 to 2025, 66 patients were enrolled, including 43 in the short-course group and 23 in the long-course group. The median age for all patients was 70.5 years, and 35 ⌧53%⌧were male. There were no significant differences in age, sex, blast cell percentage, or risk levels between the two groups (all $p > 0.05$). The median follow-up time was 15.6 months, with a maximum follow-up of 46.7 months. The long-course group had a significantly higher CR rate (96.7% vs 74.4%, $p < 0.05$) and a higher MRD negativity rate (100% vs 79.1%, $p < 0.05$) assessed after 4-cycles of VA compared to the short-course group assessed after a single VA cycle. Moreover, mortality was significantly lower in the long-course group than in the short-course group (17.4% vs. 32.6%, $p < 0.05$). Median overall survival (OS) was 19.09 months in the long-course group versus 15.21 months in the short-course group, and median disease-free survival (DFS) was 17.08 months versus 12.75 months, respectively. However, neither OS nor DFS differed significantly between groups (both $p > 0.05$). The 1-year cumulative relapse rate was 5.3% in the long-course group and 14.8% in the short-course group, with a difference that did not reach statistical significance ($p = 0.387$).

**Copyright Information:** (Online) ISSN: 2572-9241
©2026 The Author(s). HemaSphere is published by John Wiley & Sons Ltd on behalf of European Hematology Association. This is an open access Abstract Book distributed under the Attribution-NonCommercial-NoDerivs (CC BY-NC-ND), which allows third parties to download the articles and share them with others as long as they credit the author and the Abstract Book, but they cannot change the content in any way or use them commercially.
**Abstract Book Citations:** Authors, Title, HemaSphere, 2026;10:(S1):pages. The abstract book DOIs can be found on the "Author Information" page.
**Disclaimer:** Articles published in the journal HemaSphere exclusively reflect the opinions of the authors. The authors are responsible for all content in their abstracts including accuracy of the facts, statements, citing resources, etc.

**Summary/Conclusion**

Long-course VA therapy provides superior efficacy and induces deeper remissions in RAS-pathway-mutated AML. Prolonging VA treatment to ≥4 cycles is recommended for this high-risk population.

**Copyright Information:** (Online) ISSN: 2572-9241
©2026 The Author(s). HemaSphere is published by John Wiley & Sons Ltd on behalf of European Hematology Association. This is an open access Abstract Book distributed under the Attribution-NonCommercial-NoDerivs (CC BY-NC-ND), which allows third parties to download the articles and share them with others as long as they credit the author and the Abstract Book, but they cannot change the content in any way or use them commercially.
**Abstract Book Citations:** Authors, Title, HemaSphere, 2026;10:(S1):pages. The abstract book DOIs can be found on the "Author Information" page.
**Disclaimer:** Articles published in the journal HemaSphere exclusively reflect the opinions of the authors. The authors are responsible for all content in their abstracts including accuracy of the facts, statements, citing resources, etc.

### (PB2806) PHASE 1, OPEN-LABEL, MULTI-CENTER TRIAL OF RPT1G IN PATIENTS WITH RELAPSED/REFRACTORY ACUTE MYELOID LEUKEMIA AND HIGHER-RISK MYELODYSPLASTIC SYNDROMES/NEOPLASMS

**Topic:** 04. Acute myeloid leukemia - Clinical

Dennise De Jesus Diaz*[1], Aaron Goldberg[2], Nicholas Short[3], Steve Abella[1], Caroline Robb[1], Michael Schelle[1], Gregory Crimmins[1]

*[1]Remedy Plan Therapeutics, Gaithersburg, MD, United States of America;[2]Memorial Sloan Kettering Cancer Center, New York, NY, United States of America; [3]MD Anderson Cancer Center, Houston, TX, United States of America;*

### Background

Nicotinamide phosphoribosyltransferase (NAMPT) is the rate limiting enzyme in the NAD salvage pathway and plays a central role in cellular energy regulation. It is frequently overexpressed in hematologic malignancies, where increased NAD flux supports tumor cell survival and proliferation. As a result, cancer cells are particularly vulnerable to NAMPT inhibition. However, because NAD is also essential for normal cell function, prior clinical trials of complete NAMPT inhibitors were limited by significant dose-limiting on-target toxicities. RPT1G is a novel first-in-class hyperbolic NAMPT inhibitor with an improved therapeutic window that enables effective NAD depletion in malignant cells while sparing normal tissues (Crimmins, ASH 2023).
Orally administered RPT1G was safe, well-tolerated, and demonstrated favorable pharmacokinetics (PK) in a first-in-human, randomized, double-blind, placebo-controlled phase 1 study that evaluated single-ascending and multiple-ascending doses in healthy volunteers (NCT06667765). Pharmacodynamic assessments confirmed target engagement and showed that RPT1G inhibits NAMPT at doses predicted to be therapeutically relevant (Crimmins, ASH 2025 ). Together with supportive preclinical safety data and *in vitro* and *in vivo* acute myeloid leukemia (AML) models demonstrating sensitivity to RPT1G, these findings support clinical evaluation in patients with relapsed/refractory acute myeloid leukemia (R/R AML) and higher-risk myelodysplastic syndromes/neoplasms (HR-MDS).

### Aims

Primary objectives include: 1) defining safety and tolerability; 2) determining the recommended phase 2 dose (RP2D), optimal schedule and/or biologically effective dose (BED). Key secondary objectives include: 1) evaluating PK; 2) assessing preliminary efficacy by European LeukemiaNet 2022 AML response criteria and International Working Group 2023 HR-MDS response criteria, and assessing clinical benefit by transfusion independence.

### Methods

RPT1G is being investigated in a phase 1, multi-center, open-label clinical trial (NCT07107126) for treatment of R/R AML and HR-MDS.
This study explores escalating oral doses of RPT1G, given twice daily in 28-day cycles, using a standard 3+3 design. Other dosing schedules will be studied based on observed PK and pharmacodynamic relationships, safety, and tolerability to identify the maximum tolerated dose or presumptive BED and select the RP2D. The starting dose of 120 mg BID demonstrated target engagement and was well tolerated in a previous study in healthy adult volunteers.
Inclusion criteria include: adults $\geq$ 18 years old, with histological confirmation of R/R AML or HR-MDS that have received appropriate standard of care therapy(s) or declined receipt of these. Adequate organ function is required. Exclusion criteria include: ongoing adverse events from prior therapies; radiation therapy $\leq$ 14 days from the first RPT1G dose; known active infections; uncontrolled cardiac disease, or other medical comorbidities that preclude safety evaluation.

**Copyright Information:** (Online) ISSN: 2572-9241
©2026 The Author(s). HemaSphere is published by John Wiley & Sons Ltd on behalf of European Hematology Association. This is an open access Abstract Book distributed under the Attribution-NonCommercial-NoDerivs (CC BY-NC-ND), which allows third parties to download the articles and share them with others as long as they credit the author and the Abstract Book, but they cannot change the content in any way or use them commercially.
**Abstract Book Citations:** Authors, Title, HemaSphere, 2026;10:(S1):pages. The abstract book DOIs can be found on the "Author Information" page.
**Disclaimer:** Articles published in the journal HemaSphere exclusively reflect the opinions of the authors. The authors are responsible for all content in their abstracts including accuracy of the facts, statements, citing resources, etc.

**Results**

The study is currently open and recruiting at U.S. sites. Dosing will commence in Q1 2026.

**Summary/Conclusion**

RPT1G is the first hyperbolic NAMPT inhibitor to enter clinical development in R/R AML and HR-MDS and is designed to overcome the dose-limiting toxicities observed with complete NAMPT inhibitors while preserving antileukemic activity. This ongoing Phase 1 trial will define the safety, optimal dosing strategy, and preliminary clinical activity of RPT1G in these high-risk patient populations.

**Copyright Information:** (Online) ISSN: 2572-9241
©2026 The Author(s). HemaSphere is published by John Wiley & Sons Ltd on behalf of European Hematology Association. This is an open access Abstract Book distributed under the Attribution-NonCommercial-NoDerivs (CC BY-NC-ND), which allows third parties to download the articles and share them with others as long as they credit the author and the Abstract Book, but they cannot change the content in any way or use them commercially.
**Abstract Book Citations:** Authors, Title, HemaSphere, 2026;10:(S1):pages. The abstract book DOIs can be found on the "Author Information" page.
**Disclaimer:** Articles published in the journal HemaSphere exclusively reflect the opinions of the authors. The authors are responsible for all content in their abstracts including accuracy of the facts, statements, citing resources, etc.

### (PB2807) TRANSCRIPTOMICS-BASED MULTI-OMICS APPROACH FOR IMPROVED RISK STRATIFICATION IN ACUTE MYELOID LEUKEMIA

**Topic:** 04. Acute myeloid leukemia - Clinical

Yang Yang*[1]

[1]*State Key Laboratory of Trauma and Chemical Poisoning, chongqing, China;*

### Background

Acute myeloid leukemia (AML) is a subtype of hematopoietic neoplasm affecting myeloid cells in blood and bone marrow. Molecular subtyping of AML provides in-depth insights into disease pathogenesis, facilitating clinical diagnostics and therapeutic management. While current molecular screening is mainly established on DNA-based approaches, including targeted sequencing of gene panels and PCR-based fusion gene detection, transcriptome-based RNA-req has gained increasing attention for its potential in improving molecular subtyping as well as in facilitating prognostic prediction and therapeutic selection.

### Aims

By constructing a multi-omics predictive model based on transcriptomics to improve risk stratification in AML patients.

### Methods

The study included 125 patients with PML-RARA-negative AML. Bone marrow and blood samples were collected for targeted DNA sequencing, fusion gene RT-qPCR detection, and RNA-seq. Differential expression genes were identified using 30 normal control samples as a reference. Molecular subtyping was then performed via unsupervised hierarchical clustering. Furthermore, key pathways were identified through GO enrichment analysis and KEGG pathway analysis. Finally, a two-step prognostic prediction model was constructed using Cox regression and Lasso regression analysis, with model performance evaluated via ROC curves.

### Results

A total of 921 key genes were screened from 6303 differentially expressed genes. The patients were classified into 8 molecular subtypes (C1-C8), further integrated into 3 superclusters (SC1-SC3), and subsequently optimized into 2 prognostic groups (PG1/PG2), with a statistically significant difference in progression-free survival (PFS) between the two groups (p=3.734e-10). In addition, this study systematically revealed the significant enrichment of olfactory receptors (ORs) and G protein-coupled receptors (GPCRs) signaling pathways in AML for the first time. Eventually, a prediction model containing 10 core genes (Formula A) was constructed, achieving 100% specificity and 100% sensitivity in distinguishing PG1 from PG2, and a prognostic stratification formula comprising 6 genes (Formula B) was established specifically for patients in PG1.

### Summary/Conclusion

Multi-omics approaches based on RNA-seq can effectively improve risk stratification of patients with AML, providing a new tool for disease diagnosis, treatment selection, and prognosis prediction. However, its potential value requires further validation in studies with larger sample sizes.

**Copyright Information:** (Online) ISSN: 2572-9241
©2026 The Author(s). HemaSphere is published by John Wiley & Sons Ltd on behalf of European Hematology Association. This is an open access Abstract Book distributed under the Attribution-NonCommercial-NoDerivs (CC BY-NC-ND), which allows third parties to download the articles and share them with others as long as they credit the author and the Abstract Book, but they cannot change the content in any way or use them commercially.
**Abstract Book Citations:** Authors, Title, HemaSphere, 2026;10:(S1):pages. The abstract book DOIs can be found on the "Author Information" page.
**Disclaimer:** Articles published in the journal HemaSphere exclusively reflect the opinions of the authors. The authors are responsible for all content in their abstracts including accuracy of the facts, statements, citing resources, etc.

### (PB2808) PRE-INDUCTION WITH AZACITIDINE AND VENETOCLAX IN NEWLY DIAGNOSED PATIENTS WITH ACUTE MYELOGENOUS LEUKEMIA (AML): TWO YEARS SURVIVAL UPDATE OF A COMPARATIVE STUDY

**Topic:** 04. Acute myeloid leukemia - Clinical

Maher Salamoon*[1]

[1]*al bairouni university cancer center, hematology/oncology, Damascus, Syrian Arab Republic;*

### Background

The standard upfront treatment for acute myeloid leukemia (AML) among fit patients (pts) has been intensive induction chemotherapy (IC), typically with cytarabine and anthracycline as (7+3) or (5+3) depending on the aggressiveness of disease, age and genetic complexities. However, long-term outcomes remain poor, and IC continues to cause a high percentage of mortality rate especially in the first inductive cycle. azacitidine and venetoclax are a combination that confers a good response rate, elimination a high percentage of leukemic cells without posing an acute serious neutropenia in newly diagnosed AML patients. It can be taken as an outpatient schedule and has shown to improve response rate and event free survival.

### Aims

The combination of Azactidine and venetoclax is used in a pre-induction setting to purge bone marrow and decrease the percentage of leukemic cells making it a background for further induction and intensive treatment with serious chemotherapy.

### Methods

30 patients were included with ages between 60 and 72 (66 in median) a male to female ratio of 4:1 diagnosed with AML other than M3. They were treated with a pre-induction phase of two month including Azacitidine at a dose of 75mg/m² for five consecutive days every month for two months accompanied with Venetoclax at a fixed dose of 200 mg daily for two months. Bone marrow aspiration was taken at the end of every month, and the sample was compared with a similar group counting 30 patients treated with the conventional induction treatment. Aspiration thereafter every 3 months until disease progression. The primary endpoint was event free survival (EFS), with events defined as progressive disease, persistent disease prompting therapy change, relapse, hospice, or death. Among secondary endpoints were response rates, OS, toxicity, measurable residual disease (MRD), hospitalization metrics, and quality of life (QOL)

### Results

The event free survival after 3 years was 56% (95% CI: 47.5-61.6), hazard ratio of 72% and p value of .01 compared with 22% in patients treated with the induction arm. an improved quality of life p value .018 and decrease the need to enter the intensive care unite 2 patients in the pre-induction setting compared to 11 in the conventional treatment arm. Grades of grade 3 and 4 hematologic toxicities documented in 4patients of pre-induction setting compared with 25 in the conventional induction arm. Mortality rate was zero in the first 3 months compared with 5 patients in the induction arm, p value .009. symptom burden and depression were less likely to be documented in the pre-induction patients.

**Copyright Information:** (Online) ISSN: 2572-9241
©2026 The Author(s). HemaSphere is published by John Wiley & Sons Ltd on behalf of European Hematology Association. This is an open access Abstract Book distributed under the Attribution-NonCommercial-NoDerivs (CC BY-NC-ND), which allows third parties to download the articles and share them with others as long as they credit the author and the Abstract Book, but they cannot change the content in any way or use them commercially.
**Abstract Book Citations:** Authors, Title, HemaSphere, 2026;10:(S1):pages. The abstract book DOIs can be found on the “Author Information” page.
**Disclaimer:** Articles published in the journal HemaSphere exclusively reflect the opinions of the authors. The authors are responsible for all content in their abstracts including accuracy of the facts, statements, citing resources, etc.

**Summary/Conclusion**

After three years of follow-up, Azacitidine and Venetoclax showed to improve the event free survival with patients with good quality of life and less toxicity profile compared with patients under treatment with the conventional treatment of AML. Studies to be expanded to gather more patients taking into consideration genetic factors and chromosomal abnormalities and whether the patient is fit or unfit to allogeneic bone marrow transplant.

**Copyright Information:** (Online) ISSN: 2572-9241
©2026 The Author(s). HemaSphere is published by John Wiley & Sons Ltd on behalf of European Hematology Association. This is an open access Abstract Book distributed under the Attribution-NonCommercial-NoDerivs (CC BY-NC-ND), which allows third parties to download the articles and share them with others as long as they credit the author and the Abstract Book, but they cannot change the content in any way or use them commercially.
**Abstract Book Citations:** Authors, Title, HemaSphere, 2026;10:(S1):pages. The abstract book DOIs can be found on the "Author Information" page.
**Disclaimer:** Articles published in the journal HemaSphere exclusively reflect the opinions of the authors. The authors are responsible for all content in their abstracts including accuracy of the facts, statements, citing resources, etc.

### (PB2809) REAL-WORLD OUTCOMES OF ACUTE MYELOID LEUKEMIA IN PATIENTS AGED ≥60 YEARS TREATED SINCE 2015: COHORT COMPARISON INCLUDING VENETOCLAX-BASED LESS-INTENSITY THERAPY

**Topic:** 04. Acute myeloid leukemia - Clinical

Alexander Wild*[1], Klaudia Mesikova[1], Alzbeta Pucatova[1], Anna Vallova[1], Juliana Holasova[1], Ľubica Majerová[2], Martin Čermák[3], Eva Králiková[1]

*[1]Teaching F.D.Roosevelt Hospital, Haematology department, Banská Bystrica, Slovakia;[2]Medirex, Genetic laboratory, Bratislava, Slovakia; [3]National oncology institute, Department of Genetics, Bratislava, Slovakia;*

### Background

Treatment regimens of older adults with acute myeloid leukemia (AML) have evolved from sollely chemotherapy-based to nowadays possibilities included also less-intensive chemotherapy-free therapies.

### Aims

We assessed real-world outcomes in AML patients aged ≥60 years treated since 2015, including various-intensity chemotherapy and venetoclax-based less-intensity therapy.

### Methods

We retrospectively analyzed AML patients aged ≥60 years diagnosed from 2015 onward. Patients were grouped according to first-line therapy into daunorubicin+cytarabine (DA) day 3+7 30+100mg (D), 45+200mg (E), 60+200mg (Y), venetoclax plus azacytidine (A+V; pooled cohorts of various intensity), low-dose cytarabine (L), and symptomatic care (S). Baseline characteristics, complete remission (CR), hospitalization duration, progression-free survival (PFS), and overall survival (OS) were evaluated. OS and PFS were estimated by Kaplan–Meier methods and compared using log-rank tests; administrative censoring was applied at 31 December 2025.

### Results

A total of 136 patients were included: D *n*=49 (36.0%), A+V *n*=15 (11.0%), E *n*=15 (11.0%), Y *n*=5 (3.7%), L *n*=9 (6,6%), and symptomatic care *n*=43 (31.6%). Baseline age differed significantly across cohorts (p<0.001) as the only one of basic characteristics. CR rates also differed significantly (p<0.001): D 45.8% (22/48), A+V 50.0% (7/14), E 60.0% (9/15), Y 100% (5/5; very small sample), and L 22,2% (2/9). Hospitalization duration varied markedly (p<0.001); median stay was 10 days [IQR 3–24.8] in A+V versus 64.5 days [33.8–121.5] in D. Across all cohorts, OS and PFS differed significantly (overall log-rank p<0.001 for both). In the primary comparison, median OS was 250 days (8.21 months) in D versus 182 days (5.98 months) in A+V (log-rank p=0.185). Median PFS was 217 days (7.13 months) in D versus 166 days (5.45 months) in A+V (log-rank p=0.299). Two out of five patients with mutated NPM1 and favorable prognosis treated with D have lived long without relapse for 56 and 89 months.

### Summary/Conclusion

In this real-world cohort of AML patients aged ≥60 years treated since 2015, outcomes varied substantially across treatment cohorts. Venetoclax-based low-intensity therapy achieved remission rates comparable to intensive chemotherapy with substantially shorter hospitalization. While survival differed across cohorts overall, OS and PFS were not significantly different between D and A+V in this dataset, underscoring the impact of patient selection, small subgroup sizes, and incomplete biomarker/response data. Patients with mutated NPM1 and favorable prognosis treated with reduced intensity chemotherapy have a chance of living long without relapse.

**Copyright Information:** (Online) ISSN: 2572-9241
©2026 The Author(s). HemaSphere is published by John Wiley & Sons Ltd on behalf of European Hematology Association. This is an open access Abstract Book distributed under the Attribution-NonCommercial-NoDerivs (CC BY-NC-ND), which allows third parties to download the articles and share them with others as long as they credit the author and the Abstract Book, but they cannot change the content in any way or use them commercially.
**Abstract Book Citations:** Authors, Title, HemaSphere, 2026;10:(S1):pages. The abstract book DOIs can be found on the “Author Information” page.
**Disclaimer:** Articles published in the journal HemaSphere exclusively reflect the opinions of the authors. The authors are responsible for all content in their abstracts including accuracy of the facts, statements, citing resources, etc.

### (PB2810) REAL-WORLD OUTCOMES OF ACUTE MYELOID LEUKEMIA IN A RESOURCE-LIMITED, CONFLICT-AFFECTED SETTING: A SINGLE-CENTER COHORT FROM SYRIA

**Topic:** 04. Acute myeloid leukemia - Clinical

Mahmoud Alhamadeh Alswij*[1], Jose Tinajero[2], Mais Musleh[1], Monzr Al Malki[2], Qossay Alhusein[1]

*[1]Al Mouwassat University Hospital, Faculty of Medicine, Damascus, Syrian Arab Republic;[2]City of Hope National Medical Center, Duarte, United States of America;*

### Background

Acute myeloid leukemia (AML) is common in older patients and has been associated with high early mortality and poor long-term survival.  Advances in less-intensive chemotherapy regimens, supportive care, emerging targeted agents, and consolidation with allogeneic hematopoietic cell transplantation have all improved outcomes in high income countries. However, in low- and middle-income countries (LMICs), survival remains inferior due to delayed diagnosis, treatment interruptions, restricted access to potentially curative but expensive therapies, and limited supportive care infrastructure. Data from conflict-affected regions in the Middle East—particularly Syria—are scarce, and real-world outcomes in this setting remain poorly characterized.

### Aims we describe the clinical characteristics, treatment patterns, response rates to available therapy, and survival outcomes of adults with AML treated at a tertiary center in Damascus, Syria.

### Methods

This retrospective cohort study included all consecutive patients diagnosed with AML at  Al Mouwassat University Hospital (Damascus, Syria) between January 1, 2019, and December 31, 2024. Eligible patients were ≥13 years old with a confirmed AML diagnosis and received treatment or follow-up at the center. Patients lost to follow-up before treatment initiation or with diagnoses inconsistent with AML were excluded. Demographic, clinical, cytogenetic, treatment, and outcome data were collected from medical records.

**Copyright Information:** (Online) ISSN: 2572-9241
©2026 The Author(s). HemaSphere is published by John Wiley & Sons Ltd on behalf of European Hematology Association. This is an open access Abstract Book distributed under the Attribution-NonCommercial-NoDerivs (CC BY-NC-ND), which allows third parties to download the articles and share them with others as long as they credit the author and the Abstract Book, but they cannot change the content in any way or use them commercially.
**Abstract Book Citations:** Authors, Title, HemaSphere, 2026;10:(S1):pages. The abstract book DOIs can be found on the "Author Information" page.
**Disclaimer:** Articles published in the journal HemaSphere exclusively reflect the opinions of the authors. The authors are responsible for all content in their abstracts including accuracy of the facts, statements, citing resources, etc.

**Results**

A total of 198 patients were included. The median age was 43.5 years (range, 13–81), and 51% were male. Most patients had a Charlson Comorbidity Index (CCI) of 0 (60.6%). According to cytogenetic- based ELN 2022 classification, 77.3% were intermediate risk, 11.6% adverse, and 11.1% favorable. Acute promyelocytic leukemia accounted for 8.6%of cases. Median baseline WBC was $7.8\times10^9$/L (range, 0.3–300), and median bone marrow blast percentage was 70% (range, 20–95). Most patients (n=182, 91.9%) received intensive induction therapy, predominantly 7+3–based regimens. The overall response rate was 38.4%, with CR/CRh achieved in 37.8%. CR/CRh occurred in 40.6% of intensively treated patients and in none of those receiving non-intensive HMA-based
therapy.

Early mortality was high, with 30-day and 60-day mortality of 30% and 40%, respectively. Infection was the leading cause of death (71.2%), followed by organ failure (23.8%). Among responders, 24.3% relapsed. The most used salvage regimens were FLAG (44.4%) and HAM (55.6%). With a median follow-up for surviving patients of 50.4 months, 3-year overall survival (OS) was 23.8% (95% CI, 18.4-30.9). ELN risk stratification significantly predicted OS, with median OS of not reached, 3.08 months, and 4.49 months in the favorable, intermediate, and adverse groups,
respectively (p=0.01).

**Summary/Conclusion**

In this AML cohort from Syria (comparable to reports from other LMICs), response rates were modest and early mortality was markedly high despite a relatively young population with low comorbidity
burden. High infection-related mortality underscoring the impact of limited supportive care resources and restricted access to alternative treatment options. Improving diagnostic capacity, infection
prevention and management, transfusion support, and access to lower-intensity targeted therapies will be essential to improving AML outcomes in resource-limited and conflict-affected settings.

**Copyright Information:** (Online) ISSN: 2572-9241
©2026 The Author(s). HemaSphere is published by John Wiley & Sons Ltd on behalf of European Hematology Association. This is an open access Abstract Book distributed under the Attribution-NonCommercial-NoDerivs (CC BY-NC-ND), which allows third parties to download the articles and share them with others as long as they credit the author and the Abstract Book, but they cannot change the content in any way or use them commercially.
**Abstract Book Citations:** Authors, Title, HemaSphere, 2026;10:(S1):pages. The abstract book DOIs can be found on the "Author Information" page.
**Disclaimer:** Articles published in the journal HemaSphere exclusively reflect the opinions of the authors. The authors are responsible for all content in their abstracts including accuracy of the facts, statements, citing resources, etc.

### (PB2811) ANALYSIS OF CLINICAL FEATURES AND RISK FACTORS OF NEUROLOGICAL COMPLICATIONS AFTER ALLOGENEIC HEMATOPOIETIC STEM CELL TRANSPLANTATION

**Topic:** 04. Acute myeloid leukemia - Clinical

Yajun Shi*[1], Tao Wu[1]

[1] *The 940th Hospital of Joint Logistics Support force of Chinese People's Liberation Army, hematology, Gansu, China;*

### Background

The increasing clinical adoption of allogeneic hematopoietic stem cell transplantation (allo-HSCT) has heightened awareness among transplant specialists regarding the clinical significance and potential severity of neurological complications (NCs). Existing literature indicates that NCs may arise from diverse infectious and non-infectious etiologies, including pharmacologic agents, metabolic disturbances, cerebrovascular pathology, and immune-mediated neurotoxicity. Several patient- and procedure-related factors influence both the incidence and severity of post-transplant NCs, such as human leukocyte antigen (HLA) mismatch, myeloablative conditioning regimens incorporating total-body irradiation, and the development of graft-versus-host disease (GVHD)—a well-established high-risk predictor.

### Aims

The complication of nervous system after allogeneic hematopoietic stem cell transplantation (allo-HSCT) affects the success rate of transplantation and seriously threatens the life of patients. The types, clinical characteristics and risk factors of allo-HSCT patients with neurological complications (NCs) in the hematology Department of 940th Hospital of Joint Logistics Support Force in the past 20 years are analyzed.

### Methods

The clinical data of allo-HSCT patients with NCs from January 2000 to December 2023 in our center were analyzed retrospectively.

### Results

A total of 30 allo-HSCT patients developed NCs, including cerebral hemorrhage in 6 cases, intracranial infection in 8 cases, reversible posterior encephalopathy syndrome in 6 cases, myasthenia gravis in 2 cases, Guillanbarre syndrome in 1 case, central tumor in 1 case, and epilepsy in 8 cases (**Table 1**). Among them, intracranial infection included viral infection in 4 cases, bacterial infection in 3 cases and fungal infection in 1 case. The prognosis of patients with reversible posterior encephalopathy syndrome, myasthenia gravis and Guillain-Barre syndrome after transplantation is good, while the prognosis of other types of complications is poor. Univariate analysis showed that delayed platelet implantation, severe infection and acute GVHD were risk factors for NCs in allo-HSCT patients.

### Summary/Conclusion

Epilepsy and intracranial infection are the most common risk factors for NCs in allo-HSCT patients. Delayed platelet implantation, severe infection and acute GVHD are the risk factors for NCs in allo-HSCT patients.

**Copyright Information:** (Online) ISSN: 2572-9241
©2026 The Author(s). HemaSphere is published by John Wiley & Sons Ltd on behalf of European Hematology Association. This is an open access Abstract Book distributed under the Attribution-NonCommercial-NoDerivs (CC BY-NC-ND), which allows third parties to download the articles and share them with others as long as they credit the author and the Abstract Book, but they cannot change the content in any way or use them commercially.
**Abstract Book Citations:** Authors, Title, HemaSphere, 2026;10:(S1):pages. The abstract book DOIs can be found on the "Author Information" page.
**Disclaimer:** Articles published in the journal HemaSphere exclusively reflect the opinions of the authors. The authors are responsible for all content in their abstracts including accuracy of the facts, statements, citing resources, etc.

### (PB2812) GENOMIC AND CLINICAL DETERMINANTS OF OUTCOME IN IRISH AML: A REAL-WORLD ANALYSIS (2007–2025), SINGLE CENTRE EXPERIENCE.

**Topic:** 04. Acute myeloid leukemia - Clinical

Ezzat El Hassadi*[1], Ellen O'rourke[1], Ahmed Bannaga[1], Senthil Kumar[1], Brian Hennessy[1]

[1]*University Hospital Waterford, haematology, Waterford, Ireland;*

### Background

Acute myeloid leukaemia (AML) is a genetically heterogeneous malignancy in which cytogenetic and molecular features critically inform diagnosis, prognosis, and therapeutic decision-making. While traditional karyotyping has long guided risk stratification, high-throughput sequencing has revealed extensive molecular diversity, particularly within cytogenetically normal AML, reshaping AML classification and management.

### Aims

This study characterises the genomic landscape and clinical outcomes of an Irish AML cohort using integrated cytogenetic and molecular profiling. Mutation frequencies were compared with published literature, and genomic subgroups were examined for prognostic impact.

### Methods

We retrospectively analysed 166 adult AML patients diagnosed at our cancer centre between 2007–2025. Demographic, clinical, cytogenetic, molecular, and treatment data were collected. Karyotype-based and ELN 2022/2024 molecular classifications were applied. Survival analyses were performed using Prism v10.06, with statistical significance assessed using the log-rank (Mantel–Cox) test.

### Results

The median age at diagnosis was 70 years (range 24–92), with 68% de novo AML, 19% secondary AML, 10% therapy-related AML, and 3% APL. Of 126 treated patients, 72 received intensive therapy and 53 received less-intensive regimens. CR rates were 75% in the intensive cohort versus 36% in the less-intensive cohort. Karyotyping (available in 87%) revealed 47% with normal cytogenetics, favourable risk in 5% and 30% with adverse-risk features. Molecular profiling identified ELN 2022 adverse-, intermediate-, and favourable-risk categories in 42%, 47%, and 7% of intensively treated patients; in the less-intensive cohort, adverse risk predominated (87%). TP53 mutations occurred in 8% of the entire cohort.

| Risk Group | UHW Cohort (%) | Typical Published Range | Interpretation |
|---|---|---|---|
| Favourable | 5% | 10-15% | Lower than expected |
| Intermediate | 56% | 40-50% | Slightly higher but consistent |
| Normal karyotype subset | 41% | 40-50% | Well-aligned |
| Adverse | 40% | 20-30% | Higher than expected |

**Copyright Information:** (Online) ISSN: 2572-9241
©2026 The Author(s). HemaSphere is published by John Wiley & Sons Ltd on behalf of European Hematology Association. This is an open access Abstract Book distributed under the Attribution-NonCommercial-NoDerivs (CC BY-NC-ND), which allows third parties to download the articles and share them with others as long as they credit the author and the Abstract Book, but they cannot change the content in any way or use them commercially.
**Abstract Book Citations:** Authors, Title, HemaSphere, 2026;10:(S1):pages. The abstract book DOIs can be found on the “Author Information” page.
**Disclaimer:** Articles published in the journal HemaSphere exclusively reflect the opinions of the authors. The authors are responsible for all content in their abstracts including accuracy of the facts, statements, citing resources, etc.

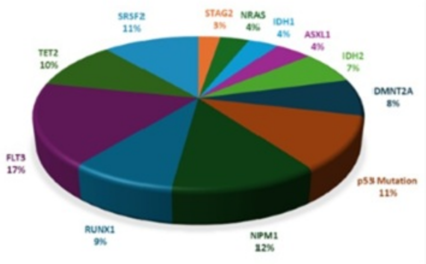

Frequency of the common Genetic alteration on the study Cohort

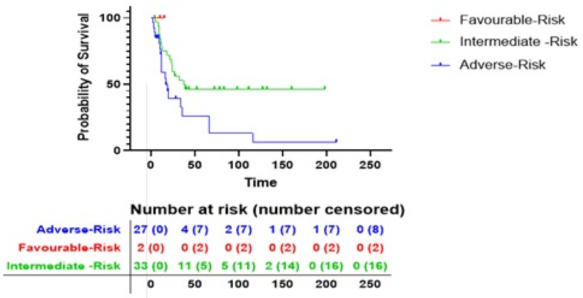

OS KM Curves for treated AML Cohort per ELN 2022 Risk stratification

Median OS and PFS were 19 and 11 months, respectively. Younger age (<65 years), favourable / intermediate cytogenetics, de novo AML, favourable / intermediate-risk ELN 2022 classification, and TP53 wild-type status were associated with significantly superior survival (all $P < 0.05$). Intensive therapy conferred marked survival benefit (median OS 32 vs 9 months; HR 0.35, $P < 0.0001$). ELN 2024 risk groups did not discriminate survival in the less-intensive cohort. TP53 mutations were associated with significantly inferior OS (median 12 vs 23 months; $P = 0.008$).

**Summary/Conclusion**

This single-centre study demonstrates a heterogeneous genomic landscape in Irish AML patients, with mutation frequencies broadly consistent with international datasets but with notable enrichment of adverse-risk disease in older and less-intensively treated populations. Integrated cytogenetic and molecular profiling strongly predicted outcomes, while age, treatment intensity, and TP53 status emerged as dominant prognostic factors. These findings highlight the critical value of comprehensive genomic assessment to guide stratified AML management within the Irish population.

**Copyright Information:** (Online) ISSN: 2572-9241
©2026 The Author(s). HemaSphere is published by John Wiley & Sons Ltd on behalf of European Hematology Association. This is an open access Abstract Book distributed under the Attribution-NonCommercial-NoDerivs (CC BY-NC-ND), which allows third parties to download the articles and share them with others as long as they credit the author and the Abstract Book, but they cannot change the content in any way or use them commercially.
**Abstract Book Citations:** Authors, Title, HemaSphere, 2026;10:(S1):pages. The abstract book DOIs can be found on the “Author Information” page.
**Disclaimer:** Articles published in the journal HemaSphere exclusively reflect the opinions of the authors. The authors are responsible for all content in their abstracts including accuracy of the facts, statements, citing resources, etc.

### (PB2813) VENETOCLAX PLUS AZACITIDINE AND LENALIDOMIDE (AVL) FOR NEWLY DIAGNOSED ELDERLY PATIENTS WITH ACUTE MYELOID LEUKEMIA/MYELODYSPLASTIC SYNDROMES: A SINGLE-CENTER, PROSPECTIVE, SINGLE-ARM CLINICAL STUDY

**Topic:** 04. Acute myeloid leukemia - Clinical

Fanqiao Meng[1], Maoyuan Xiang[2], Qian Dong[2], Yu Liu[3], Dongfeng Zeng*[2]

*[1]Daping Hospital, Third Military Medical University (Army Medical University), Department of Hematology, Chongqing, China; [2]Daping Hospital, Third Military Medical University (Army Medical University), Chongqing, China;[3]Daping Hospital, Third Military Medical University (Army Medical University),, Chongqing, China;*

### Background

Elderly patients with acute myeloid leukemia/myelodysplastic syndromes (AML/MDS) remains a particularly adverse population necessitating improved therapeutic strategies.

### Aims

In this study, we evaluated the efficacy and safety of venetoclax plus azacitidine and lenalidomide (AVL) for newly diagnosed elderly patients with AML/MDS.

### Methods

We conducted a single-center, prospective, single-arm clinical study (ChiCTR2500103716) to evaluate the efficacy and safety of venetoclax plus azacitidine and lenalidomide (VAL) for newly diagnosed elderly AML/MDS.All patients received induction treatment with venetoclax (100 mg on day 1, 200 mg on day 2, 400 mg on days 3-14), azacitidine (75 mg/m2/d on days 1-7), azacitidine (75 mg/m2 on days 1-7), and lenalidomide (15 mg on day 1-10). The primary endpoint was the treatment-related adverse events and overall response rate (ORR) following one cycle of the VAL regimen.

### Results

Fifteen patients were enrolled, with AML (n = 9) and MDS (n = 7). 11 patients achieved ORR (73.3%), with 7 achieving complete response (46.7%) and 4 achieving partial response (26.7%). 5 patients had TP53 gene mutations, among whom 4 achieved ORR (80%) and 1 had no response to treatment. The most common grade 3 to 4 toxicities were hematologic toxicities and febrile neutropenia. No treatment-related deaths occurred.

### Summary/Conclusion

In conclusion, VAL was effective and well tolerated in elderly patients with AML/MDS.

**Copyright Information:** (Online) ISSN: 2572-9241
©2026 The Author(s). HemaSphere is published by John Wiley & Sons Ltd on behalf of European Hematology Association. This is an open access Abstract Book distributed under the Attribution-NonCommercial-NoDerivs (CC BY-NC-ND), which allows third parties to download the articles and share them with others as long as they credit the author and the Abstract Book, but they cannot change the content in any way or use them commercially.
**Abstract Book Citations:** Authors, Title, HemaSphere, 2026;10:(S1):pages. The abstract book DOIs can be found on the "Author Information" page.
**Disclaimer:** Articles published in the journal HemaSphere exclusively reflect the opinions of the authors. The authors are responsible for all content in their abstracts including accuracy of the facts, statements, citing resources, etc.

### (PB2814) OUTCOMES IN YOUNG PATIENTS WITH ACUTE MYELOID LEUKEMIA: REAL-WORLD PRACTICE

**Topic:** 04. Acute myeloid leukemia - Clinical

Victor Solovyov*[1], Evgenii Kunevich[1], Sergey Voloshin[1, 2], Asiyat Radzhabova[3], Olga Uspenskaya[1], Ruslan Bekoev[1], Alexey Kuvshinov[3], Elena Karyagina[4], Mariia Penkova[1], Albina Aidemirova[1], Olga Ruzhinskaya[1], Olga Ryabykina[1], Natalia Sokolova[1], Svetlana Tolstova[1], Sergey Alekseev[1, 5]

*[1]Leningrad Regional Clinical Hospital, Saint-Petersburg, Russia;[2]S.M. Kirov Military Medical Academy, Saint-Petersburg, Russia; [3]Russian Research Institute of Hematology and Transfusiology, Saint-Petersburg, Russia;[4]City Hospital No. 15, Saint-Petersburg, Russia; [5]N.N. Petrov National Medical Research Center of Oncology, Saint-Petersburg, Russia;*

### Background

Despite the use of standard induction therapy, treatment outcomes in a substantial proportion of young patients with acute myeloid leukemia (AML) remain unsatisfactory. This underscores the need for detailed molecular profiling and broader implementation of targeted therapies to improve survival.

### Aims

To assess the impact of biological markers on prognosis and survival in young patients with AML and to evaluate the effect of FLT3 inhibitors (FLT3i) on treatment outcomes.

### Methods

A retrospective analysis was conducted on 267 patients with AML younger than 60 years (median age 48 years) treated between 2009 and November 1, 2025. Only 16 patients (6.0%) received low-intensity induction regimens other than standard "7+3" ± FLT3i. Median follow-up was 17.5 months.

**Copyright Information:** (Online) ISSN: 2572-9241
©2026 The Author(s). HemaSphere is published by John Wiley & Sons Ltd on behalf of European Hematology Association. This is an open access Abstract Book distributed under the Attribution-NonCommercial-NoDerivs (CC BY-NC-ND), which allows third parties to download the articles and share them with others as long as they credit the author and the Abstract Book, but they cannot change the content in any way or use them commercially.
**Abstract Book Citations:** Authors, Title, HemaSphere, 2026;10:(S1):pages. The abstract book DOIs can be found on the "Author Information" page.
**Disclaimer:** Articles published in the journal HemaSphere exclusively reflect the opinions of the authors. The authors are responsible for all content in their abstracts including accuracy of the facts, statements, citing resources, etc.

### Results

Overall survival (OS) analysis identified several cytogenetic and molecular prognostic factors with corresponding hazard ratios (HR): complex karyotype (CK) 1.90 (p=0.011), 11q23/*KMT2A* rearrangements 2.20 (p=0.025), del(5q) 3.22 (p=0.023), del(17p) 4.37 (p=0.002), *RUNX1::RUNX1T1* 0.25 (p=0.059), and *FLT3* mutations 1.62 (p=0.017). Patients with CK (≥3 abnormalities) had similarly poor outcomes regardless of the number of aberrations (p=0.9329). Young patients treated with low-intensity induction therapy had inferior 2-year OS (16.1% vs 57.5%, p=0.095) and event-free survival (EFS) (12.0% vs 34.4%, p=0.043).

No significant differences in OS (p=0.88) or relapse-free survival (p=0.64) were observed when comparing patients treated before and after 2022. However, the introduction of targeted agents and improvements in supportive care resulted in a significantly higher 1-year EFS (56.1% vs 38.6%, p=0.0072). Since 2022, the incorporation of *FLT3* inhibitors has markedly improved outcomes in *FLT3*-mutated AML. Prior to 2022, 2-year OS in *FLT3*-mutated patients was 37.8%. After 2022, only two *FLT3*-mutated patients did not receive FLT3i; OS in *FLT3*-mutated patients reached 93.3% (95% CI 81.5–100) compared with 48.6% in *FLT3* wild-type patients (p=0.039). No intergroup differences in EFS were observed (p=0.89).

Achievement of measurable residual disease (MRD) negativity was a strong predictor of improved OS and reduced relapse risk. Two-year OS and relapse-free survival were 93.8% (median not reached) and 50% (median 23.3 months), respectively, in the MRD-negative group, compared with 33.8% (p=0.013) and 27.0% (p=0.018) in MRD-positive patients.

Allogeneic hematopoietic stem cell transplantation (allo-HSCT) was performed in 23% of patients (53 of 229 eligible). Allo-HSCT reduced the risk of death from any cause by 49% (HR=0.57; 95% CI 0.33–0.96; p=0.035). Two-year OS was 70.6% (95% CI 56.9–85.1) in transplanted patients versus 55.4% in non-transplanted patients (p=0.032).

Factors associated with early refractoriness in univariate analysis included ELN 2022 high-risk group (OR=5.52, p=0.001), CK (OR=8.80, p=0.001), prior MDS or MDS/MPN (OR=3.44, p=0.044), del(5q) (OR=9.27, p=0.052), del(7q) (OR=9.21, p=0.009), and *NPM1* mutation (OR=0.10, p=0.030). Results of multivariate analysis of mortality risk are presented in Figure E.

### Summary/Conclusion

In young patients with AML, complex karyotype, deletions of 5q and 17p, and *FLT3* mutations are major adverse prognostic factors, whereas achievement of MRD negativity and allo-HSCT significantly improve survival. The introduction of *FLT3* inhibitors into routine clinical practice since 2022 has substantially improved outcomes in *FLT3*-mutated AML.

**Copyright Information:** (Online) ISSN: 2572-9241
©2026 The Author(s). HemaSphere is published by John Wiley & Sons Ltd on behalf of European Hematology Association. This is an open access Abstract Book distributed under the Attribution-NonCommercial-NoDerivs (CC BY-NC-ND), which allows third parties to download the articles and share them with others as long as they credit the author and the Abstract Book, but they cannot change the content in any way or use them commercially.
**Abstract Book Citations:** Authors, Title, HemaSphere, 2026;10:(S1):pages. The abstract book DOIs can be found on the "Author Information" page.
**Disclaimer:** Articles published in the journal HemaSphere exclusively reflect the opinions of the authors. The authors are responsible for all content in their abstracts including accuracy of the facts, statements, citing resources, etc.

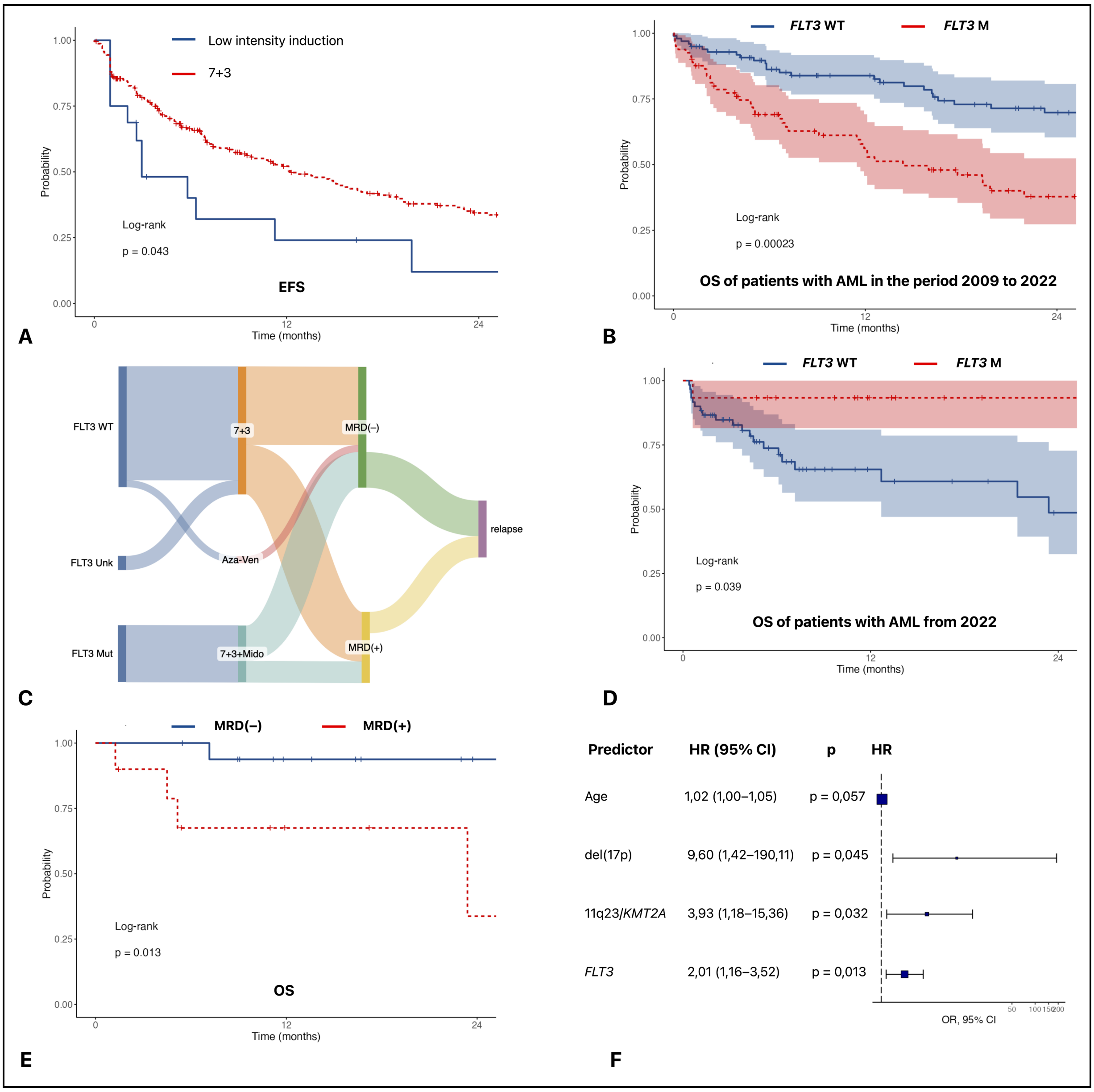

A) Event-free survival in patients with acute myeloid leukemia according to the intensity of induction therapy regimens. B) Overall survival of patients with acute myeloid leukemia from 2009 to 2022 according to FLT3 mutation status. C) Sankey plot illustrating measurable residual disease (MRD) status in patients with acute myeloid leukemia according to FLT3 mutation status. D) Overall survival of patients with acute myeloid leukemia after 2022 according to FLT3 mutation status. E) Prognostic significance of achieving MRD negativity in patients with acute myeloid leukemia based on overall survival analysis. F) Factors associated with the risk of death identified by multivariable logistic regression analysis.

**Copyright Information:** (Online) ISSN: 2572-9241
©2026 The Author(s). HemaSphere is published by John Wiley & Sons Ltd on behalf of European Hematology Association. This is an open access Abstract Book distributed under the Attribution-NonCommercial-NoDerivs (CC BY-NC-ND), which allows third parties to download the articles and share them with others as long as they credit the author and the Abstract Book, but they cannot change the content in any way or use them commercially.
**Abstract Book Citations:** Authors, Title, HemaSphere, 2026;10:(S1):pages. The abstract book DOIs can be found on the "Author Information" page.
**Disclaimer:** Articles published in the journal HemaSphere exclusively reflect the opinions of the authors. The authors are responsible for all content in their abstracts including accuracy of the facts, statements, citing resources, etc.

### (PB2815) EARLY PERIPHERAL BLAST CLEARANCE AS A PREDICTOR OF COMPLETE REMISSION AND OVERALL SURVIVAL

**Topic:** 04. Acute myeloid leukemia - Clinical

Chihaoui N*[1], Kharrat R[1], Selmi A[1], Rabeb Berred[1], Ben Neji H[1], Bchir M[1], Achour M[1], Cherni R[1], Ben Lakhel F[2], Feki S[2], Gouider E[2], Ben Lakhel R[1]

*[1]Aziza othemena hospital, adult clinical hematology, Tunis, Tunisia; [2]Aziza othemena hospital, Laboratory of Hematological Biology, Tunis, Tunisia;*

### Background

Hyperleukocytic acute myeloid leukemia (AML) is associated with high early mortality and inferior long-term survival. In acute lymphoblastic leukemia, early peripheral blast clearance (PBC) after prephase therapy is a validated marker of chemosensitivity. However, the prognostic value of early peripheral blast clearance in AML has not been specifically evaluated in hyperleukocytic AML.

### Aims

We aim to assess blast clearance in patients with AML and evaluate its impact on clinical outcomes, particularly complete remission (CR) and overall survival (OS).

### Methods

We conducted a retrospective study including adult patients newly diagnosed with de novo or secondary AML treated with induction course in the adult clinical hematology department of Aziza othemana hospital.
AML with initial white blood count (WBC) greater than 50.000 is considered hyperleukocytic.
We analysed (WBC) before treatment and at day 5 (D5) of the cure.
Early WBC clearance (WBCC) at D5 was evaluated as a surrogate marker for PBC, calculated as the percentage reduction in total WBC count at day 5 compared to baseline.. A threshold of **≥97.56% reduction** was identified using ROC analysis (Youden index = 0.389).

### Results

Among 296 patients diagnosed with AML, 95 patients had hyperleukocytosis. The median age was 43 years, the mean WBC count was $139 \times 10^9$/L [range:50 –454]. cytogenetic risk was : favorable in 8.4%, intermediate in 68.4%, and adverse in 23.2% of cases. D5 leukocyte data were available for 53 patients.
In univariate analysis, a higher CR rate was observed in patients with rapid early PBC (OR=5.4, 95% CI 1.10-26.46, p=0.037). Rapid clearance was independently associated with improved OS after adjustment for age and cytogenetic risk ( median OS 32 vs 1 month HR 2.96, 95% CI 1.42–6.20, p=0.004) . Multivariate analysis did not identify independent predictors of CR, likely due to the limited number of induction failures.

**Copyright Information:** (Online) ISSN: 2572-9241
©2026 The Author(s). HemaSphere is published by John Wiley & Sons Ltd on behalf of European Hematology Association. This is an open access Abstract Book distributed under the Attribution-NonCommercial-NoDerivs (CC BY-NC-ND), which allows third parties to download the articles and share them with others as long as they credit the author and the Abstract Book, but they cannot change the content in any way or use them commercially.
**Abstract Book Citations:** Authors, Title, HemaSphere, 2026;10:(S1):pages. The abstract book DOIs can be found on the “Author Information” page.
**Disclaimer:** Articles published in the journal HemaSphere exclusively reflect the opinions of the authors. The authors are responsible for all content in their abstracts including accuracy of the facts, statements, citing resources, etc.

**Summary/Conclusion**

Early blast clearance may be an early prognostic marker in hyperleukocytic AML. However, the limited availability of day 5 data and the small sample size restrict the generalizability of these findings Further  larger  studies are needed to validate this finding and assess its impact on relapse-free survival.

**Copyright Information:** (Online) ISSN: 2572-9241
©2026 The Author(s). HemaSphere is published by John Wiley & Sons Ltd on behalf of European Hematology Association. This is an open access Abstract Book distributed under the Attribution-NonCommercial-NoDerivs (CC BY-NC-ND), which allows third parties to download the articles and share them with others as long as they credit the author and the Abstract Book, but they cannot change the content in any way or use them commercially.
**Abstract Book Citations:** Authors, Title, HemaSphere, 2026;10:(S1):pages. The abstract book DOIs can be found on the “Author Information” page.
**Disclaimer:** Articles published in the journal HemaSphere exclusively reflect the opinions of the authors. The authors are responsible for all content in their abstracts including accuracy of the facts, statements, citing resources, etc.

### (PB2816) OBSERVATIONAL STUDY OF ACUTE LEUKEMIA CASES TRANSFERRED FROM GUADELOUPE TO MAINLAND FRANCE

**Topic:** 04. Acute myeloid leukemia - Clinical

Louis Guézet*[1], Leila Cysique-Foinlan[1], Jean Baptiste Micol[2], Mohamad Hachem[2], Ludovic Janaud[3], Marie Laruelle-Henaux[4], Emmanuel Raffoux[5], Jean-Edouard Martin[5], Jean Valere Malfuson[6], Veronique Baccini[1], Philippe Helias[1]

*[1]General Hospital of the Guadeloupe, Hematolology, Les Abymes, Guadeloupe;[2]Gustave Roussy Institut, Clinic Hematolology, Ville-Juif, France; [3]General Hospital of Basse-Terre, Clinic Hematolology, Basse-Terre, Guadeloupe; [4]General Hospital of Saint-Martin, Clinic Hematolology, SAINT-MARTIN, Guadeloupe; [5]Saint-Louis Hospital, Clinic Hematolology, PARIS, France; [6]Inter army Hospital, Clinic Hematolology, Clamart, France;*

### Background

Acute leukemia (AL) requires urgent specialized treatment. When patients are eligible for intensive curative treatment, hospitalization in a highly specialized facility with a “sterile” environment is necessary from the outset. This type of service does not exist in the Lesser Antilles, both due to logistical constraints and a lack of sufficient expert staff. Initial care therefore requires urgent, long-distance medical evacuation, involving an 8-hour commercial flight to mainland France, before the usual intensive care can be provided.

### Aims

The aim of our work was to describe the population eligible for intensive treatment, regardless of AL diagnosis, and to assess the impact of transfer on survival.

### Methods

This retrospective study included all acute leukemia diagnoses made in Guadeloupe and the Northern Islands (Saint Martin and Saint Barthélemy) from January 1, 2019, to December 31, 2024. We studied the records of patients who had undergone emergency medical evacuation to perform a descriptive analysis of their clinical and biological characteristics and a survival analysis.

### Results

Over the six-year period, 111 new cases of ALL were recorded, of which 64 were evacuated to mainland France. These included 50 cases of acute myeloid leukemia (AML), 7 cases of acute promyelocytic leukemia (APL), and 7 cases of acute lymphoblastic leukemia (ALL). The median age at diagnosis for AML, APL, and ALL was 58, 39, and 50 years, respectively. The median evacuation time was 2 days. However, evacuations took longer than 5 days for a quarter of the patients due to waiting for results or opinions, complications, or logistical or social difficulties. Prior to transfer, 39% of patients underwent cytoreduction with hydroxycarbamide and 20% received corticosteroids. There were no deaths during transatlantic flights, nor there were any complications requiring diversion. The overall mortality rate was 33% (AML 36%, APL 14%, and ALL 28%). The median survival for AML was 66 months, for ALL 24 months, and for APL was not reached with a median follow-up of 22 months.

Analysis based on transfer times showed a significant reduction in survival as the delay increased (p=0.015). Eight of the 15 patients transferred after more than 5 days died.

**Copyright Information:** (Online) ISSN: 2572-9241
©2026 The Author(s). HemaSphere is published by John Wiley & Sons Ltd on behalf of European Hematology Association. This is an open access Abstract Book distributed under the Attribution-NonCommercial-NoDerivs (CC BY-NC-ND), which allows third parties to download the articles and share them with others as long as they credit the author and the Abstract Book, but they cannot change the content in any way or use them commercially.
**Abstract Book Citations:** Authors, Title, HemaSphere, 2026;10:(S1):pages. The abstract book DOIs can be found on the “Author Information” page.
**Disclaimer:** Articles published in the journal HemaSphere exclusively reflect the opinions of the authors. The authors are responsible for all content in their abstracts including accuracy of the facts, statements, citing resources, etc.

**Summary/Conclusion**

Despite the relatively small sample size, the prognosis is broadly comparable to that reported in the literature for acute leukemias of the same type. Our criteria for selecting patients eligible for intensive treatment and transfer appear to be relevant in light of these results. However, evacuation delay appears to be an unfavorable prognostic factor, particularly when it exceeds 5 days.

To our knowledge, no study has ever been conducted on the management of acute leukemia requiring long-distance air medical transport, and our work is the first to demonstrate its value and feasibility in routine practice.

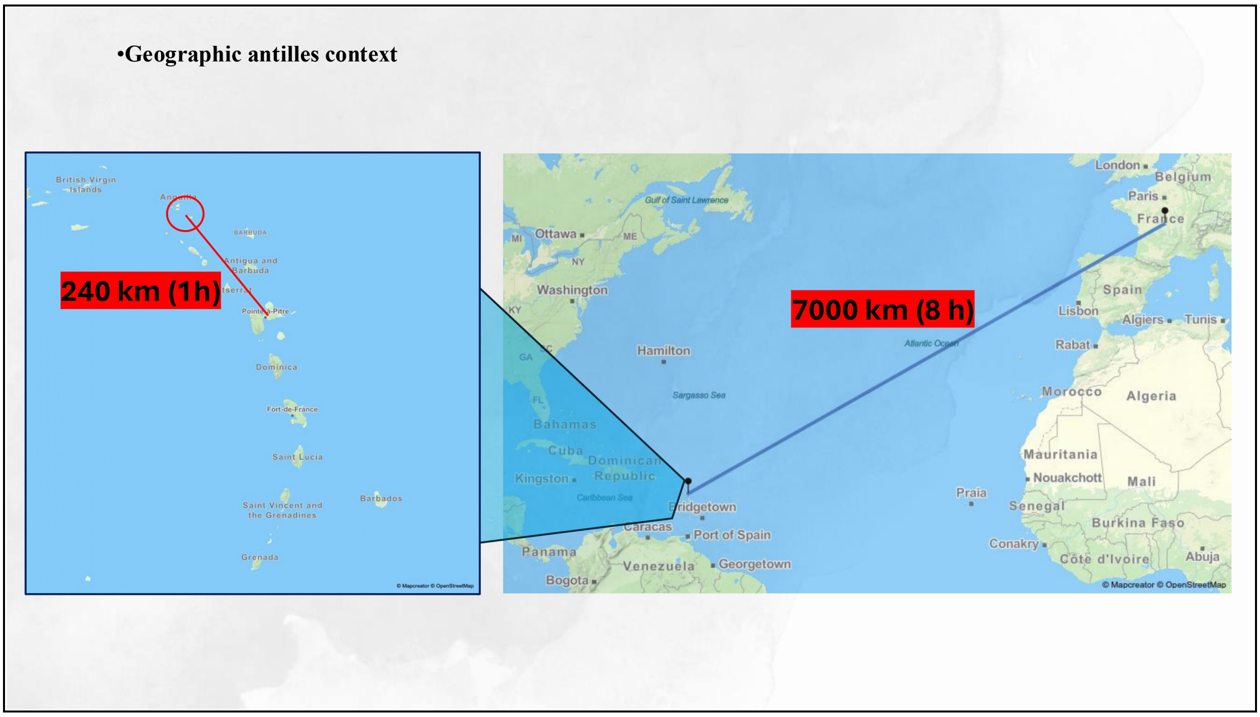

Geographic Antilles context

**Copyright Information:** (Online) ISSN: 2572-9241
©2026 The Author(s). HemaSphere is published by John Wiley & Sons Ltd on behalf of European Hematology Association. This is an open access Abstract Book distributed under the Attribution-NonCommercial-NoDerivs (CC BY-NC-ND), which allows third parties to download the articles and share them with others as long as they credit the author and the Abstract Book, but they cannot change the content in any way or use them commercially.
**Abstract Book Citations:** Authors, Title, HemaSphere, 2026;10:(S1):pages. The abstract book DOIs can be found on the "Author Information" page.
**Disclaimer:** Articles published in the journal HemaSphere exclusively reflect the opinions of the authors. The authors are responsible for all content in their abstracts including accuracy of the facts, statements, citing resources, etc.

### (PB2817) EVALUATION OF THE RELATIONSHIP BETWEEN CYTOGENETIC / MOLECULAR RISK FACTORS AND TREATMENT RESPONSE, DISEASE COURSE AND SURVIVAL IN PATIENTS WITH ACUTE MYELOID LEUKEMIA

**Topic:** 04. Acute myeloid leukemia - Clinical

Seda Şenyurt[1], Pınar Tığlıoğlu*[2], Işil Erdoğan Özünal[3], Tayfun Elibol[3], Mesut Tiglioglu[3], Özkan Kaya[3], Tahacan Aydin[3], Erman Öztürk[4]

*[1]Istanbul Medeniyet Univercity, Internal Medicine, Istanbul, Türkiye;[2]Istanbul Medeniyet Univercity, Goztepe Prof. Dr. Suleyman Yalcın City Hospital Hematology Clinic, Istanbul, Türkiye; [3]Göztepe Prof. Dr. Süleyman Yalçın City Hospital, Hematology, Istanbul, Türkiye; [4]Istanbul Medeniyet Univercity, Hematology, Istanbul, Türkiye;*

### Background

Acute myeloid leukemia  is a clonal hematologic malignancy characterized by the uncontrolled proliferation of hematopoietic progenitor cells, leading to the infiltration of the bone marrow, peripheral blood and other tissues. The prognosis of acute myeloid leukemia  is influenced by numerous factors, including cytogenetic alterations, molecular mutation profile, patient age, and comorbidities.

### Aims

We aim to review our molecular-based findings, which we frequently use in diagnosis, risk categorization, and treatment planning in AML, and to observe the relationship of this data to the clinical course of our patients.

### Methods

A total of 126 patients diagnosed with acute myeloid leukemia and followed between January 1, 2020, and June 30, 2025, at the Hematology Department of Göztepe Prof. Dr. Süleyman Yalçın City Hospital were included in this study. This retrospective analysis recorded patients' baseline laboratory findings, cytogenetic and next-generation sequencing (NGS) results, and treatment modalities, evaluating their associations with treatment response, relapse risk, early mortality, progression-free survival  and overall survival.

**Copyright Information:** (Online) ISSN: 2572-9241
©2026 The Author(s). HemaSphere is published by John Wiley & Sons Ltd on behalf of European Hematology Association. This is an open access Abstract Book distributed under the Attribution-NonCommercial-NoDerivs (CC BY-NC-ND), which allows third parties to download the articles and share them with others as long as they credit the author and the Abstract Book, but they cannot change the content in any way or use them commercially.
**Abstract Book Citations:** Authors, Title, HemaSphere, 2026;10:(S1):pages. The abstract book DOIs can be found on the "Author Information" page.
**Disclaimer:** Articles published in the journal HemaSphere exclusively reflect the opinions of the authors. The authors are responsible for all content in their abstracts including accuracy of the facts, statements, citing resources, etc.

**Results**

The mean follow-up period was 18.3 months, and the median overall survival for the entire cohort was 15 months. The early mortality rate was 19.8%. Median overall survival and progression-free survival were not reached in patients receiving intensive induction chemotherapy, while in those treated with low-intensity regimens, median overall survival and progression-free survival were 12.7 and 11 months, respectively. The combination of a hypomethylating agent with venetoclax provided significantly longer progression-free survival compared to hypomethylating agent monotherapy (17 vs. 5 months; p=0.006). Early mortality was associated with advanced age and higher baseline levels of leukocytes, creatinine, and LDH. In cytogenetic analyses, abnormalities were detected in the FISH panel in 57.1% (n=72) of patients. The most frequently observed cytogenetic abnormalities were 5q31 deletion (15.2%), trisomy 8 (11.1%), and 7q deletion (8.3%). Karyotype analysis revealed a complex karyotype in 16.7% (n=21) of patients, whereas 83.3% (n=105) did not exhibit a complex karyotype. Molecular analysis performed using NGS for the detection of genetic mutations revealed that the majority of patients (81%; n=102) harbored at least one mutation, whereas 19% (n=24) showed no detectable mutations

The most frequently identified mutations were NPM1 (27%) and FLT3 (24.6%). Concurrent NPM1 and FLT3 mutations were detected in 14.3% of patients. Other commonly observed mutations included TET2, RUNX1, ASXL1 and NRAS, DNMT3A, and IDH2. Less frequently detected mutations were SRSF2, IDH1, KRAS, STAG2, CEBPA, SF3B1, and TP53. Cytogenetically, del(5q), del(7q), complex karyotype, t(9;22) and MLL rearrangement were associated with poor prognosis, whereas t(8;21) and inv(16) correlated with better survival outcomes. Among molecular findings, FLT3 and NPM1 mutations were associated with higher remission rates but also increased early mortality risk. ASXL1 mutations were significantly associated with shorter progression-free survival, particularly in patients receiving intensive therapy.

**Summary/Conclusion**

Genetic markers remain among the most critical determinants of survival in acute myeloid leukemia. Performing comprehensive molecular and cytogenetic testing at diagnosis enables accurate risk stratification and facilitates individualized treatment planning.

**Copyright Information:** (Online) ISSN: 2572-9241
©2026 The Author(s). HemaSphere is published by John Wiley & Sons Ltd on behalf of European Hematology Association. This is an open access Abstract Book distributed under the Attribution-NonCommercial-NoDerivs (CC BY-NC-ND), which allows third parties to download the articles and share them with others as long as they credit the author and the Abstract Book, but they cannot change the content in any way or use them commercially.
**Abstract Book Citations:** Authors, Title, HemaSphere, 2026;10:(S1):pages. The abstract book DOIs can be found on the “Author Information” page.
**Disclaimer:** Articles published in the journal HemaSphere exclusively reflect the opinions of the authors. The authors are responsible for all content in their abstracts including accuracy of the facts, statements, citing resources, etc.

### (PB2818) A NOVEL RISK STRATIFICATION MODEL FOR ACUTE MYELOID LEUKEMIA TREATED WITH VENETOCLAX PLUS HYPOMETHYLATIC AGENTS: A MULTICENTER RETROSPECTIVE STUDY

**Topic:** 04. Acute myeloid leukemia - Clinical

Sang-A Kim*[1], Ji Yun Lee[1], Jeong-Ok Lee[1], Soo Mee Bang[1], Dong-Yeop Shin[2]

*[1]Seoul National University Bundang Hospital, Internal Medicine, SeongNam, South Korea;[2]Seoul National University Hospital, Seoul, South Korea;*

### Background

Venetoclax combined with hypomethylating agents (Ven+HMA) has become a standard low-intensity regimen for acute myeloid leukemia (AML) patients ineligible for intensive chemotherapy. However, current risk stratification systems, including ELN2022 and ELN2024, were developed based on intensively treated patients. Their prognostic performance in the Ven+HMA-treated population remains suboptimal, highlighting the need for a treatment-specific risk model.

### Aims

Venetoclax combined with hypomethylating agents (Ven+HMA) has become a standard low-intensity regimen for acute myeloid leukemia (AML) patients ineligible for intensive chemotherapy. However, current risk stratification systems, including ELN2022 and ELN2024, were developed based on intensively treated patients. Their prognostic performance in the Ven+HMA-treated population remains suboptimal, highlighting the need for a treatment-specific risk model.

### Methods

We retrospectively analyzed 138 AML patients treated with Ven+HMA at two academic hospitals in South Korea (Seoul National University Bundang Hospital and Seoul National University Hospital). The prognostic performance of ELN2022, ELN2024, and Beat-AML 2024 mutation score was assessed using Harrell's C-index. Univariable and multivariable Cox regression analyses were performed to identify independent prognostic factors. A point-based scoring model was constructed and evaluated by bootstrap internal validation (1,000 iterations).

### Results

The median age was 74.7 years (range 20–89), and 68.1% were male. Prior hematologic diseases were present in 31.9%. The CR/CRi rate was 59.4%, with 1-year OS and PFS rates of 50.3% and 41.3%, respectively. Existing risk models showed limited discrimination: C-indices were 0.535 (ELN2022), 0.599 (ELN2024), and 0.587 (Beat-AML 2024), with poor separation between favorable and intermediate groups. Multivariable analysis identified complex karyotype (CK), TP53 mutation, and SH2B3 mutation as independent adverse prognostic factors (C-index 0.647). A scoring system assigning 1 point each for CK and TP53 and 2 points for SH2B3 stratified patients into low-risk (0 points; n=105, 76%), intermediate-risk (1 point; n=12, 9%), and high-risk (≥2 points; n=21, 15%) groups. Median OS was 14.3, 10.6, and 2.5 months, respectively, with significant pairwise separations (low vs. intermediate, P=0.008; low vs. high, P<0.001; intermediate vs. high, P=0.044; C-index 0.635). Bootstrap validation confirmed model stability with minimal optimism (OS bias −0.001) and consistent significance (98.8% of iterations with P<0.001).

**Copyright Information:** (Online) ISSN: 2572-9241
©2026 The Author(s). HemaSphere is published by John Wiley & Sons Ltd on behalf of European Hematology Association. This is an open access Abstract Book distributed under the Attribution-NonCommercial-NoDerivs (CC BY-NC-ND), which allows third parties to download the articles and share them with others as long as they credit the author and the Abstract Book, but they cannot change the content in any way or use them commercially.
**Abstract Book Citations:** Authors, Title, HemaSphere, 2026;10:(S1):pages. The abstract book DOIs can be found on the "Author Information" page.
**Disclaimer:** Articles published in the journal HemaSphere exclusively reflect the opinions of the authors. The authors are responsible for all content in their abstracts including accuracy of the facts, statements, citing resources, etc.

### Summary/Conclusion

Current ELN-based and Beat-AML risk stratification systems have limited prognostic utility in Ven+HMA-treated AML patients. We propose a simple, three-variable scoring model incorporating CK, TP53, and SH2B3 that provides improved risk discrimination. This model is easy to implement and may help guide treatment decisions including allogeneic transplantation candidacy. External validation in independent cohorts is warranted.

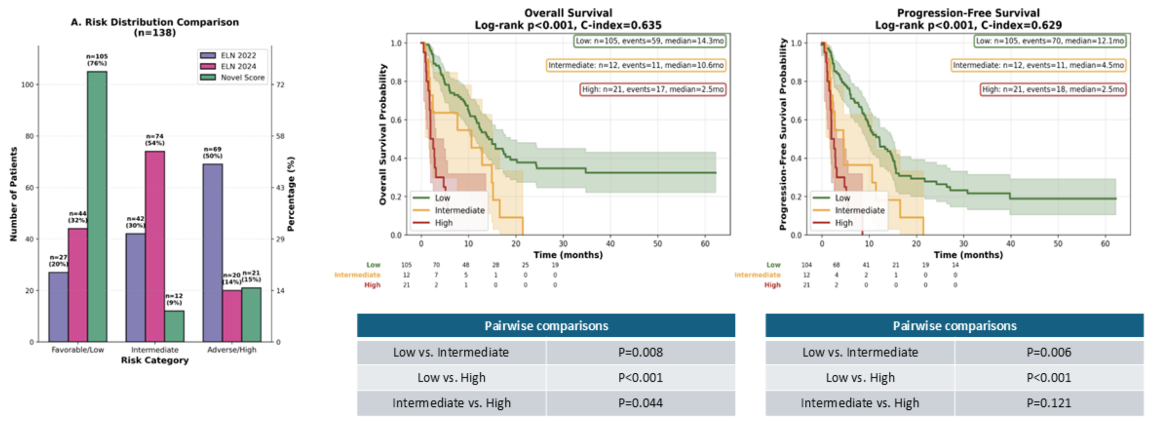

Left: Oncoplot of somatic mutations with ELN2022/2024 classifications. (A) Risk distribution comparison across ELN2022, ELN2024, and the novel score. (B) Reclassification flow diagram. Bottom: Kaplan-Meier curves for OS (C-index=0.635) and PFS (C-index=0.629) by the novel score, with pairwise comparisons.

**Copyright Information:** (Online) ISSN: 2572-9241
©2026 The Author(s). HemaSphere is published by John Wiley & Sons Ltd on behalf of European Hematology Association. This is an open access Abstract Book distributed under the Attribution-NonCommercial-NoDerivs (CC BY-NC-ND), which allows third parties to download the articles and share them with others as long as they credit the author and the Abstract Book, but they cannot change the content in any way or use them commercially.
**Abstract Book Citations:** Authors, Title, HemaSphere, 2026;10:(S1):pages. The abstract book DOIs can be found on the "Author Information" page.
**Disclaimer:** Articles published in the journal HemaSphere exclusively reflect the opinions of the authors. The authors are responsible for all content in their abstracts including accuracy of the facts, statements, citing resources, etc.

### (PB2819) CLINICAL CHARACTERISTICS AND THERAPEUTIC OUTCOMES OF ASYMPTOMATIC NEWLY DIAGNOSED ACUTE MYELOID LEUKEMIA

**Topic:** 04. Acute myeloid leukemia - Clinical

Xinwen Jiang[1], Minran Zhou[1], Chunyan Chen*[1]

[1]*Qilu Hospital of Shandong University, Department of Hematology, Jinan, China;*

### Background

Acute myeloid leukemia (AML), a common hematologic malignancy, arises from uncontrolled proliferation and impaired differentiation of hematopoietic progenitors, suppressing normal hematopoiesis and leading to life-threatening cytopenias. In real-world practice, some patients are diagnosed incidentally without symptoms; their heterogeneous clinical and genetic profiles contribute to variable treatment responses and outcomes.

### Aims

This study compares clinical characteristics, treatment responses, and survival between asymptomatic and symptomatic newly diagnosed non-M3 AML patients, and identifies factors influencing outcomes.

### Methods

This study initially screened a total of 962 patients with AML who presented to our center between October 1, 2018, and October 1, 2025, comprising 221 asymptomatic and 741 symptomatic cases. After applying stringent exclusion criteria, 409 patients with newly diagnosed non-M3 AML were ultimately enrolled in the final analysis.

### Results

No significant differences were observed in complete remission (CR) rates and minimal residual disease (MRD) negativity between asymptomatic and symptomatic AML patients, regardless of whether they received intensive or non-intensive chemotherapy. Multivariate analysis revealed that advanced age was an adverse factor for CR in the asymptomatic group, while male and 2022 ELN high-risk stratification were unfavorable factors for CR in the symptomatic group. At the molecular level, U2AF1 mutations emerged as an independent adverse predictor for both CR and MRD negativity in asymptomatic patients. In the symptomatic group, IDH1 and NRAS mutations were favorable factors for CR, and CEBPA-bZIP mutations conferred advantage for both CR and MRD negativity, whereas DNMT3A, KIT, TP53, and U2AF1 mutations were associated with lower CR rates.
The symptomatic group exhibited significantly higher relapse rates compared to the asymptomatic group (37.8% vs 27.4%), with relapsed patients more frequently characterized by advanced age, complex karyotype, and failure to achieve MRD negativity. The asymptomatic group demonstrated superior median overall survival (OS) and progression-free survival (PFS) compared to the symptomatic group, with consistent trends across all ELN risk subgroups. Undergo intensive chemotherapy, achievement of CR, and transplantation were protective factors for OS and PFS in asymptomatic patients. In the symptomatic group, advanced age was identified as a risk factor, while CR achievement and transplantation served as protective factors. Regarding molecular predictors, DNMT3A and U2AF1 mutations indicated poor prognosis in asymptomatic patients; in the symptomatic group, CEBPA-bZIP and NPM1 mutations were protective, whereas DNMT3A, U2AF1, and TP53 mutations predicted unfavorable outcomes.

**Copyright Information:** (Online) ISSN: 2572-9241
©2026 The Author(s). HemaSphere is published by John Wiley & Sons Ltd on behalf of European Hematology Association. This is an open access Abstract Book distributed under the Attribution-NonCommercial-NoDerivs (CC BY-NC-ND), which allows third parties to download the articles and share them with others as long as they credit the author and the Abstract Book, but they cannot change the content in any way or use them commercially.
**Abstract Book Citations:** Authors, Title, HemaSphere, 2026;10:(S1):pages. The abstract book DOIs can be found on the “Author Information” page.
**Disclaimer:** Articles published in the journal HemaSphere exclusively reflect the opinions of the authors. The authors are responsible for all content in their abstracts including accuracy of the facts, statements, citing resources, etc.

**Summary/Conclusion**

While no significant differences were observed in induction therapeutic efficacy between asymptomatic and symptomatic AML patients, intensive chemotherapy was associated with improved CR and MRD negativity rates. Symptomatic patients demonstrated higher relapse rates and were characterized by advanced age, increased frequency of complex/monosomal karyotypes, and lower MRD negativity rates. Asymptomatic patients exhibited significantly longer PFS, and although OS did not reach statistical significance, a favorable trend was observed. Furthermore, genetic mutations significantly influenced therapeutic outcomes and prognosis, with the 2022 ELN risk stratification effectively discriminating patient prognosis.

**Copyright Information:** (Online) ISSN: 2572-9241
©2026 The Author(s). HemaSphere is published by John Wiley & Sons Ltd on behalf of European Hematology Association. This is an open access Abstract Book distributed under the Attribution-NonCommercial-NoDerivs (CC BY-NC-ND), which allows third parties to download the articles and share them with others as long as they credit the author and the Abstract Book, but they cannot change the content in any way or use them commercially.
**Abstract Book Citations:** Authors, Title, HemaSphere, 2026;10:(S1):pages. The abstract book DOIs can be found on the "Author Information" page.
**Disclaimer:** Articles published in the journal HemaSphere exclusively reflect the opinions of the authors. The authors are responsible for all content in their abstracts including accuracy of the facts, statements, citing resources, etc.

### (PB2820) OPTIMIZING MAINTENANCE STRATEGIES FOR UNFIT OR ELDERLY PATIENTS WITH ACUTE MYELOID LEUKEMIA: INSIGHTS FROM A META-ANALYSIS AND A RETROSPECTIVE STUDY OF VENETOCLAX PLUS AZACITIDINE

**Topic:** 04. Acute myeloid leukemia - Clinical

Liyuan Li[1], Weiguang Cui*[1], Xin Gao[1], Nan Zhou[1], Zhenzhen Wang[1], Mengying Zheng[1], Fan Yang[1], Xiaoyan Zhang[1], Li Sun[1], Lin Yang[1, 2], Jianmin Luo[1, 2]

*[1]The Second Hospital of Hebei Medical University, Department of Hematology, Shijiazhuang, China;[2]Hebei Institute of Hematology, Hebei Key Laboratory of Hematology, Shijiazhuang, China;*

### Background

Despite advances in AML therapy, many patients relapse after initial remission. Maintenance therapy remains controversial. This study evaluates maintenance strategies via meta-analysis and real-world venetoclax plus azacitidine (VA) data.

### Aims

This study aims to: (1) systematically evaluate the efficacy and safety of various maintenance therapies in AML through a meta-analysis of published clinical trials; (2) validate these findings in a real-world setting by retrospectively analyzing the efficacy of venetoclax plus azacitidine (VA) as maintenance therapy in unfit or elderly patients at our institution.

### Methods

Meta-analysis: Literature search (up to Dec 2024) for AML maintenance trials; pooled HRs for OS/RFS calculated using RevMan/Stata. Retrospective study: Unfit/elderly AML patients receiving VA maintenance (Aug 2022-Dec 2024) were analyzed. Survival estimated by Kaplan-Meier, stratified by pre-maintenance MRD and ELN risk.

### Results

The meta-analysis incorporated 9 studies comprising 5,178 patients (follow-up range: 15.4 to 43.8 months), evaluating interventions such as CC-486, sorafenib, gilteritinib, FLT3 inhibitors, azacitidine, and decitabine. Maintenance therapy significantly prolonged OS (HR=0.49, P=0.000) and RFS (HR=0.42, P=0.000) compared to observation. Subgroup analyses revealed that these survival benefits were most pronounced in patients with MRD+ status (excluding post-transplant) and those with intermediate-risk cytogenetics (P<0.05), though the maintenance cohorts exhibited higher incidences of cytopenias, infections, and skin toxicity. In our institutional retrospective cohort of 24 unfit/elderly patients (median age: 66 years), 25.0% were MRD+ and 75.0% were MRD- prior to maintenance. Based on ELN criteria, the cohort comprised 12.5% low-risk, 50.0% intermediate-risk, and 37.5% high-risk patients. Over a median follow-up of 25.5 months, the 2-year OS and RFS rates were 72.5% and 55.3%, respectively. While survival differences among ELN risk groups lacked statistical significance (P>0.05), MRD- patients demonstrated significantly superior OS and RFS compared to their MRD+ counterparts (P<0.05). This highlights the intrinsic prognostic advantage of deep molecular remission (MRD-), which contextualizes our meta-analysis finding that high-risk MRD+ patients derive the greatest relative magnitude of benefit from the maintenance intervention. The VA maintenance regimen exhibited an acceptable safety profile, characterized primarily by manageable neutropenia, thrombocytopenia, and infections.

**Copyright Information:** (Online) ISSN: 2572-9241
©2026 The Author(s). HemaSphere is published by John Wiley & Sons Ltd on behalf of European Hematology Association. This is an open access Abstract Book distributed under the Attribution-NonCommercial-NoDerivs (CC BY-NC-ND), which allows third parties to download the articles and share them with others as long as they credit the author and the Abstract Book, but they cannot change the content in any way or use them commercially.
**Abstract Book Citations:** Authors, Title, HemaSphere, 2026;10:(S1):pages. The abstract book DOIs can be found on the "Author Information" page.
**Disclaimer:** Articles published in the journal HemaSphere exclusively reflect the opinions of the authors. The authors are responsible for all content in their abstracts including accuracy of the facts, statements, citing resources, etc.

### Summary/Conclusion

The meta-analysis confirms that maintenance therapy significantly improves OS, DFS, and RFS in AML, particularly benefiting MRD+ and intermediate-risk subgroups. Furthermore, our retrospective analysis indicates that the VA regimen offers a promising, well-tolerated maintenance strategy for unfit or elderly AML patients, providing effective disease control with manageable cytopenias. However, findings are limited by the heterogeneity of regimens in the meta-analysis and the small sample size and retrospective nature of our single-center cohort. Future large-scale, prospective randomized controlled trials are warranted to standardize the role of the VA regimen in AML maintenance.

**Copyright Information:** (Online) ISSN: 2572-9241
©2026 The Author(s). HemaSphere is published by John Wiley & Sons Ltd on behalf of European Hematology Association. This is an open access Abstract Book distributed under the Attribution-NonCommercial-NoDerivs (CC BY-NC-ND), which allows third parties to download the articles and share them with others as long as they credit the author and the Abstract Book, but they cannot change the content in any way or use them commercially.
**Abstract Book Citations:** Authors, Title, HemaSphere, 2026;10:(S1):pages. The abstract book DOIs can be found on the "Author Information" page.
**Disclaimer:** Articles published in the journal HemaSphere exclusively reflect the opinions of the authors. The authors are responsible for all content in their abstracts including accuracy of the facts, statements, citing resources, etc.

### (PB2821) A PHASE 3 STUDY OF REVUMENIB IN COMBINATION WITH INTENSIVE CHEMOTHERAPY IN PATIENTS WITH NEWLY DIAGNOSED NPM1-MUTATED ACUTE MYELOID LEUKEMIA (REVEAL-ND NPM1): TRIAL IN PROGRESS

**Topic:** 04. Acute myeloid leukemia - Clinical

Eytan Stein*[1], Ghayas Issa[2], Eduardo Magalhães Rego[3], Kentaro Fukushima[4], Michael Kühn[5], Shaun Fleming[6], Angela Smith[7], Paresh Vyas[8]

*[1]Memorial Sloan Kettering Cancer Center, New York, NY, United States of America;[2]The University of Texas MD Anderson Cancer Center, Houston, TX, United States of America; [3]University of São Paulo, São Paulo, Brazil; [4]University of Osaka, Osaka, Japan; [5]University Medical Center, Johannes Gutenberg-University, Mainz, Germany;[6]The Alfred Hospital, Melbourne, Australia; [7]Syndax Pharmaceuticals, Inc., New York, NY, United States of America;[8]University of Oxford, Oxford, United Kingdom;*

### Background

There are currently no approved targeted therapies for newly diagnosed (ND) acute myeloid leukemia (AML) harboring a nucleophosmin-1 mutation (*NPM1*m), which occurs in ~30% of ND adult AML cases. In *NPM1*m AML, the NPM1m/XPO1 protein complex binds to DNA to sustain the interaction of menin and wild-type KMT2A that drives upregulation of *HOX*/*MEIS* gene expression, resulting in hematopoietic differentiation arrest and leukemogenesis. Revumenib is a first-in-class, oral, potent, and selective inhibitor of the menin-KMT2A interaction, which disrupts multiple key drivers of leukemogenesis (*KMT2A* rearrangement, *NPM1*m, and *NUP98* rearrangement). In the phase 1/2 AUGMENT-101 study (NCT04065399), revumenib monotherapy demonstrated clinically meaningful response rates and was generally well tolerated in relapsed/refractory *NPM1*m AML, leading to US Food and Drug Administration approval for that patient population on October 24, 2025. Standard AML treatment for younger, non-frail adults is based on intensive chemotherapy (IC) regimens and hematopoietic stem cell transplant (HSCT). The addition of revumenib to standard IC may further improve treatment responses specifically in the ND setting.

### Aims

This study is designed to assess the safety and efficacy of revumenib in combination with IC in patients with ND*NPM1*m AML.

### Methods

REVEAL-ND NPM1 is a phase 3, multicenter, randomized, double-blind, placebo-controlled trial (NCT07211958). Eligible patients are ≥12 years of age, weigh ≥40 kg, and have treatment-naive ND AML with locally determined (centrally confirmed) *NPM1*m. Patients will be randomized 1:1 to revumenib or placebo in combination with IC. Induction consists of 1 to 2 cycles of revumenib or placebo (dosed orally) alongside IC with cytarabine and daunorubicin or idarubicin (dosed intravenously). Consolidation consists of 1 to 3 cycles of revumenib or placebo plus cytarabine. HSCT may be performed after initial induction or consolidation. Treatment with revumenib or placebo monotherapy will continue for up to 2 years, with long-term follow-up until death, withdrawal of consent, or study closure.

### Results

The dual primary endpoints are event-free survival and measurable residual disease-negative complete remission (CR) in the bone marrow, both assessed by independent reviewers. A key secondary endpoint is overall survival. Additional investigator-assessed endpoints include CR rate, composite complete remission (CRc) rate, overall response rate, duration of response (CR, CRc), safety, and quality of life. Overall, ~468 patients will be enrolled. As of 1 March 2026, the study is open to enrollment.

**Copyright Information:** (Online) ISSN: 2572-9241
©2026 The Author(s). HemaSphere is published by John Wiley & Sons Ltd on behalf of European Hematology Association. This is an open access Abstract Book distributed under the Attribution-NonCommercial-NoDerivs (CC BY-NC-ND), which allows third parties to download the articles and share them with others as long as they credit the author and the Abstract Book, but they cannot change the content in any way or use them commercially.
**Abstract Book Citations:** Authors, Title, HemaSphere, 2026;10:(S1):pages. The abstract book DOIs can be found on the “Author Information” page.
**Disclaimer:** Articles published in the journal HemaSphere exclusively reflect the opinions of the authors. The authors are responsible for all content in their abstracts including accuracy of the facts, statements, citing resources, etc.

**Summary/Conclusion**

This phase 3 study will provide insight into the efficacy and safety of revumenib versus placebo in combination with IC in patients with ND *NPM1*m AML.

© 2026 American Society of Clinical Oncology, Inc. Reused with permission. This abstract was accepted and previously presented at the 2026 ASCO Annual Meeting. All rights reserved.

**Copyright Information:** (Online) ISSN: 2572-9241
©2026 The Author(s). HemaSphere is published by John Wiley & Sons Ltd on behalf of European Hematology Association. This is an open access Abstract Book distributed under the Attribution-NonCommercial-NoDerivs (CC BY-NC-ND), which allows third parties to download the articles and share them with others as long as they credit the author and the Abstract Book, but they cannot change the content in any way or use them commercially.
**Abstract Book Citations:** Authors, Title, HemaSphere, 2026;10:(S1):pages. The abstract book DOIs can be found on the "Author Information" page.
**Disclaimer:** Articles published in the journal HemaSphere exclusively reflect the opinions of the authors. The authors are responsible for all content in their abstracts including accuracy of the facts, statements, citing resources, etc.

### (PB2822) PROGNOSTIC IMPACT OF MOLECULAR-GENETIC RISK, COMORBIDITY BURDEN, AND ECOG PERFORMANCE STATUS IN ACUTE MYELOID LEUKEMIA: A PROSPECTIVE SINGLE-CENTER STUDY

**Topic:** 04. Acute myeloid leukemia - Clinical

Kateryna Khurdepa*[1], Zinaida Stupakova[1], Anna Sergeieva[1], Bohdana Kuiavovych[1]

[1]*National Research Center for Radiation Medicine, Oncology and Hematology of NAMS of Ukraine, Blood Pathology Department, Kyiv, Ukraine;*

### Background

Acute myeloid leukemia (AML) is a biologically heterogeneous hematologic malignancy characterized by substantial variability in clinical outcomes. Molecular- genetic abnormalities represent the cornerstone of contemporary risk stratification; however, baseline functional status and comorbidity burden may also significantly influence treatment tolerance and survival, particularly in real-world clinical settings.

### Aims

To evaluate survival outcomes and treatment response in patients with AML according to ELN 2022 molecular risk stratification, Charlson Comorbidity Index (CCI), and ECOG performance status in a prospective single-center cohort.

### Methods

This prospective observational study included 55 consecutive adult patients with newly diagnosed AML diagnosed between January 2023 and March 2025. Baseline assessment included cytogenetic and molecular profiling in accordance with ELN 2022 recommendations, ECOG performance status evaluation, and comorbidity assessment using the Charlson Comorbidity Index (CCI). Patients were stratified into low (CCI 0–2), moderate (CCI 3–4), and high (CCI ≥5) comorbidity burden groups. Treatment strategy (intensive induction chemotherapy or lower-intensity therapy) was determined according to patient age, ECOG performance status, and comorbidity profile. Treatment response was assessed according to standard AML response criteria (CR, CRi , PR, and PD). Survival outcomes were analyzed according to molecular risk category, ECOG performance status, and CCI stratification.

### Results

A total of 55 AML patients were included, with a median age of 61 years; 27 patients were male and 28 female. At diagnosis, ECOG performance status was ≥2 in all patients, including ECOG 3–4 in 15 cases (27%), reflecting substantial baseline functional impairment. According to ELN 2022 classification, 23 patients (42%) were categorized as adverse risk, 8 (15%) as favorable risk, and 22 (40%) as intermediate risk, while molecular risk data were unavailable in 2 patients (3%). Comorbidity burden was considerable, with a median CCI of 4 (range 2–9). Low comorbidity (CCI 0–2) was observed in 12 patients (22%), moderate comorbidity (CCI 3–4) in 20 (36%), and high comorbidity burden (CCI ≥5) in 23 patients (42%). Following induction therapy, complete remission (CR) was achieved in 22 patients (40%), CRi in 3 patients (5%), and partial remission in 10 patients (18%), while progressive disease was observed in 9 patients (16%). During follow-up, 24 deaths (44%) were recorded, predominantly due to infectious complications and disease progression. Higher ELN risk category, ECOG ≥3, and elevated CCI were associated with inferior treatment response and higher mortality rates.

**Copyright Information:** (Online) ISSN: 2572-9241
©2026 The Author(s). HemaSphere is published by John Wiley & Sons Ltd on behalf of European Hematology Association. This is an open access Abstract Book distributed under the Attribution-NonCommercial-NoDerivs (CC BY-NC-ND), which allows third parties to download the articles and share them with others as long as they credit the author and the Abstract Book, but they cannot change the content in any way or use them commercially.
**Abstract Book Citations:** Authors, Title, HemaSphere, 2026;10:(S1):pages. The abstract book DOIs can be found on the "Author Information" page.
**Disclaimer:** Articles published in the journal HemaSphere exclusively reflect the opinions of the authors. The authors are responsible for all content in their abstracts including accuracy of the facts, statements, citing resources, etc.

**Summary/Conclusion**

In this prospective real-world cohort, molecular-genetic risk stratification, comorbidity burden, and baseline functional status were important prognostic factors influencing treatment response and survival in AML patients. A high proportion of patients presented with adverse ELN risk, impaired ECOG performance status, and substantial comorbidity burden, highlighting the importance of comprehensive baseline assessment for individualized risk-adapted treatment strategies.

**Copyright Information:** (Online) ISSN: 2572-9241
©2026 The Author(s). HemaSphere is published by John Wiley & Sons Ltd on behalf of European Hematology Association. This is an open access Abstract Book distributed under the Attribution-NonCommercial-NoDerivs (CC BY-NC-ND), which allows third parties to download the articles and share them with others as long as they credit the author and the Abstract Book, but they cannot change the content in any way or use them commercially.
**Abstract Book Citations:** Authors, Title, HemaSphere, 2026;10:(S1):pages. The abstract book DOIs can be found on the "Author Information" page.
**Disclaimer:** Articles published in the journal HemaSphere exclusively reflect the opinions of the authors. The authors are responsible for all content in their abstracts including accuracy of the facts, statements, citing resources, etc.

### (PB2823) BLASTOID PLASMACYTOID DENDRITIC CELL NEOPLASM – 15 YEAR UNICENTER EXPERIENCE IN BULGARIA

**Topic:** 04. Acute myeloid leukemia - Clinical

Yanitsa Davidkova*[1], Naum Simonoski[1], Tanya Yankova[1], Martin Donchev[1]

*[1]SHATHD, Sofia, Bulgaria;*

### Background

Blastic plasmacytoid dendritic cell neoplasm (BPDCN) is a rare and aggressive clonal hematological malignancy. The disease is distinguished by involvement of the skin, bone marrow and/or peripheral blood, lymph nodes, including the central nervous system. The neoplasm primarily affects elderly patients, among whom the frequency of comorbidities is higher.

### Aims

To analyze the main characteristics of patients diagnosed with BPDCN in a 15-year follow-up period at the Specialized Hospital for Active Treatment of Hematological Diseases in Sofia, Bulgaria.

### Methods

The retrospective study includes 13 patients diagnosed at SHATHD, Sofia for the period 2010-2025. The average age of the analyzed group was 69.1 years, with a predominance of the male gender (77% men compared to 23% women).

### Results

The patients aged above 60 years predominated (85%). The most common extramedullary location was the skin (69%), lymphadenomegaly was found in 31%, and splenomegaly and hepatomegaly occurred in 23% and 15%, respectively. Initial hematological disease of the patients was presented in 62%. The bone marrow involvement ranged from 40% to total infiltration. While no significant leukocytosis appeared - mean WBC 5.6 x$10^9$/l (range 1.3-12.1 x$10^9$/l), with mean hemoglobin level was 107.3 g/l (range 56-150 g/l) and mean platelet count 124.5 x$10^9$/l (range 32-250 x$10^9$/l). In three of the patients, other organs such as the testis, bronchial wall, and stomach mucosa were affected at the onset of the disease without any leukemic involvement being observed. In one case, the patient had a 70% immature blast cells in the peripheral blood, with a specific phenotype and CNS involvement. In three patients we encountered complex karyotype, and in one – FLT3-TKD mutation.
Therapeutic regimens for lymphoma treatment (such as CHOP, hyper-CVAD, deVIC) were applied to 54% of the patients, while one (8%) received AML induction therapy. CR was assessed in 3 patients who received intensive therapy. However, the mean overall survival was only 7.6 months (ranging from early mortality before therapy initiation to 29 months), median OS was 10 months (95% CI 7.5-12.5 months)(Figure1).

### Summary/Conclusion

BPDCN is an aggressive hematological neoplasm with an unfavourable clinical course. It is necessary to establish prognostic markers for determining new therapeutic approaches that can influence the flow of the disease. In recent years, hope lies in the implementation of an anti-CD123 antibody, but its use is still not authorized in Bulgaria.

**Copyright Information:** (Online) ISSN: 2572-9241
©2026 The Author(s). HemaSphere is published by John Wiley & Sons Ltd on behalf of European Hematology Association. This is an open access Abstract Book distributed under the Attribution-NonCommercial-NoDerivs (CC BY-NC-ND), which allows third parties to download the articles and share them with others as long as they credit the author and the Abstract Book, but they cannot change the content in any way or use them commercially.
**Abstract Book Citations:** Authors, Title, HemaSphere, 2026;10:(S1):pages. The abstract book DOIs can be found on the “Author Information” page.
**Disclaimer:** Articles published in the journal HemaSphere exclusively reflect the opinions of the authors. The authors are responsible for all content in their abstracts including accuracy of the facts, statements, citing resources, etc.

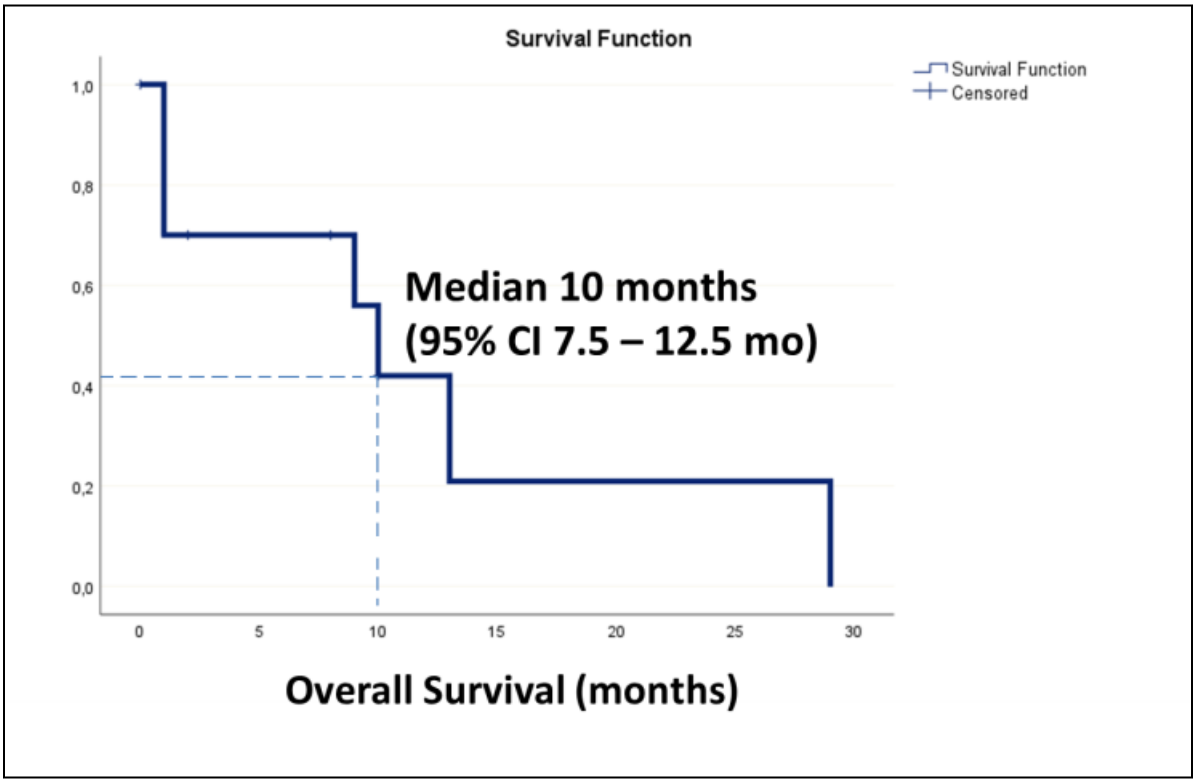

Figure 1. Median OS for the BPDCN patients

**Copyright Information:** (Online) ISSN: 2572-9241
©2026 The Author(s). HemaSphere is published by John Wiley & Sons Ltd on behalf of European Hematology Association. This is an open access Abstract Book distributed under the Attribution-NonCommercial-NoDerivs (CC BY-NC-ND), which allows third parties to download the articles and share them with others as long as they credit the author and the Abstract Book, but they cannot change the content in any way or use them commercially.
**Abstract Book Citations:** Authors, Title, HemaSphere, 2026;10:(S1):pages. The abstract book DOIs can be found on the "Author Information" page.
**Disclaimer:** Articles published in the journal HemaSphere exclusively reflect the opinions of the authors. The authors are responsible for all content in their abstracts including accuracy of the facts, statements, citing resources, etc.

### (PB2824) THERAPY-RELATED AML: DISTINCT GENETIC PROFILE AND INFERIOR OUTCOMES COMPARED WITH DE NOVO AML

**Topic:** 04. Acute myeloid leukemia - Clinical

Denis Cetin[1], Alp Peker[2], Ajda Gunes*[1], Fatma Keklik Karadag[1], Derya Demir[3], Nur Soyer[1], Filiz Vural[1], Mahmut Töbü[1], Fahri Sahin[1], Emin Karaca[4], Güray Saydam[1]

*[1]Ege University, Hematology, İzmir, Türkiye; [2]Ege University, Department of Medical Genetics, İzmir, Türkiye; [3]Ege University, Pathology, İzmir, Türkiye; [4]Ege University, Genetics, İzmir, Türkiye;*

### Background

Therapy-related acute myeloid leukemia (t-AML) is a biologically and clinically distinct form of AML that arises as a late complication of prior exposure to cytotoxic chemotherapy or radiotherapy. Its pathogenesis and latency depend on the type of prior exposure, with alkylating agents typically associated with prolonged latency and chromosome 5 and 7 abnormalities, and topoisomerase II inhibitors linked to shorter latency and KMT2A rearrangements. Biologically, t-AML is characterized by a higher prevalence of adverse-risk features such as complex karyotypes and TP53 mutations, which contribute to treatment resistance, lower remission rates, and inferior survival, rendering management challenging and leaving allogeneic stem cell transplantation as the only potentially curative option.

### Aims

To compare the clinical characteristics, treatment patterns, and survival outcomes between patients with therapy-related AML and de novo AML.

### Methods

A retrospective analysis included 139 patients treated in our center between 2015 and 2025, comprising 55 with t-AML and 86 with de novo AML; the median follow-up was 7 mo in de novo AML (0–120), and 2 mo in t-AML (0–60).

### Results

The median age at diagnosis was 63 years in t-AML and 56 years in de novo AML. Breast cancer was the most frequent primary malignancy (27.3%), followed by endometrium–ovarian cancers (10.9%) and lung cancer and sarcoma (7.3% each), and 45.5% of patients had received prior radiotherapy. The median interval from primary malignancy to AML diagnosis was 39.5 months (range, 3–721), and 46.3% had active disease at AML diagnosis; among evaluable t-AML cases, complex karyotypes and monosomy 5 and/or 7 were the most frequent cytogenetic abnormalities, with occasional del(17p). In the de novo AML cohort, 10 of 86 patients (11.6%) were classified as favorable risk according to ELN criteria, none had APL, and 17 (19.8%) had prior MDS transformation; hypomethylating agent ± venetoclax–based therapies were administered in 25.5% of t-AML and 18.6% of de novo AML patients, while the remainder received conventional intensive chemotherapy. Median overall survival (OS) was 3 months in the t-AML group and 10 months in the de novo AML group. Age >60 years at diagnosis was independently associated with higher 30-day mortality ($p = 0.008$), and as expected remission rates after the first two induction cycles were significantly higher in de novo AML. In the t-AML cohort, only five patients underwent allo-SCT, largely due to factors such as treatment-refractory disease, active primary malignancy, treatment-related mortality, and poor performance status limiting eligibility in the remaining patients and median OS was markedly longer in transplanted patients (16 mo) than in non-transplanted patients (4 mo).

**Copyright Information:** (Online) ISSN: 2572-9241
©2026 The Author(s). HemaSphere is published by John Wiley & Sons Ltd on behalf of European Hematology Association. This is an open access Abstract Book distributed under the Attribution-NonCommercial-NoDerivs (CC BY-NC-ND), which allows third parties to download the articles and share them with others as long as they credit the author and the Abstract Book, but they cannot change the content in any way or use them commercially.
**Abstract Book Citations:** Authors, Title, HemaSphere, 2026;10:(S1):pages. The abstract book DOIs can be found on the "Author Information" page.
**Disclaimer:** Articles published in the journal HemaSphere exclusively reflect the opinions of the authors. The authors are responsible for all content in their abstracts including accuracy of the facts, statements, citing resources, etc.

**Summary/Conclusion**

Despite the limitations of a small cohort and incomplete genetic data, our findings suggest that t-AML is a biologically aggressive and clinically vulnerable AML subtype characterized by adverse cytogenetics, restricted eligibility for intensive therapy and transplantation, and significantly inferior survival compared with de novo AML.The limited use of HMA–venetoclax–based therapy in our cohort, largely due to its later incorporation into clinical practice, may have contributed to the higher treatment-related mortality observed, particularly in the t-AML group; nevertheless, allo-SCT was associated with a meaningful survival advantage and remains the only potentially curative option in carefully selected patients.

**Copyright Information:** (Online) ISSN: 2572-9241
©2026 The Author(s). HemaSphere is published by John Wiley & Sons Ltd on behalf of European Hematology Association. This is an open access Abstract Book distributed under the Attribution-NonCommercial-NoDerivs (CC BY-NC-ND), which allows third parties to download the articles and share them with others as long as they credit the author and the Abstract Book, but they cannot change the content in any way or use them commercially.
**Abstract Book Citations:** Authors, Title, HemaSphere, 2026;10:(S1):pages. The abstract book DOIs can be found on the "Author Information" page.
**Disclaimer:** Articles published in the journal HemaSphere exclusively reflect the opinions of the authors. The authors are responsible for all content in their abstracts including accuracy of the facts, statements, citing resources, etc.

### (PB2825) ACUTE MYELOID LEUKEMIA REAL DATA OVER 5 YEARS IN ALBANIA (2020-2024)

**Topic:** 04. Acute myeloid leukemia - Clinical

Ana Qose*[1], Eneo Bixheku[2], Elsuarta Calliku[3], Adela Perolla[3], Valentina Semanaj[3], Teuta Dedej[3], Arben Ivanaj[3]

*[1]Regional Hospital Center of Berat, Berat, Albania;[2]Regional Hospital of Gjirokastra, Gjirokaster, Albania;[3]UHC Tirana, Tirane, Albania;*

### Background

Acute Myeloid Leukemia (AML) is a malignant hematologic disease characterized by abnormal proliferation and differentiation of myeloid precursors in the bone marrow.

### Aims

This study aims to assess the incidence of AML in Albania over a 5 year period, as well as to analyze the epidemiological, clinical, and diagnostic characteristics of patients diagnosed and managed at the Hematology Service of UHC Tirana from January 2020- December 2024.

### Methods

This is a retrospective cohort study conducted at the Hematology Service of UHC Tirana over the period from January 2020 to December 2024. Data were collected from the medical records documented by the Hematology Service for all cases diagnosed with AML, and treated at the Hematology Service during this time period.

### Results

During the time period from January 2020 to December 2024, a total of 160 cases of AML were diagnosed and treated in the Hematology Service, UHC Tirana. The age-adjusted incidence is 1.9/100,000 inhabitants per year. The median age of patients included in our study is 63.5 years, ranging from 16-92 years. It is observed that the most represented age groups are 60-69 years and 70-79 years, with 28.5% of patients in each group. The male-female ratio is 1.3:1. The average age of the women was 61.78 (14.67) years, while the average age of the men was 64.35 (13.00) years, with no significant difference between them (p=0.220). In our study, 80% of the patients were AML2, and 19.3% of the patients were AML4, while 0.6% of the patients were AML5. Primary AML was present in 85% of patients, while secondary AML was present in 15% of patients. The most frequently observed comorbidities were cardiovascular diseases 48.58%, followed by endocrine disorders 14.68% and neoplastic conditions 7.34%. The mean age of patients with comorbidities is 67.07 years, which is significantly higher compared to patients without comorbidities, whose mean age is 56.65 years (p = 0.01). In the results of the induction treatment, it was found that 22% of the patients achieved complete remission, 49.3% of the patients were refractory to induction and 28.7% of the patients died during the induction. At the time of analysis, 11.25% of patients who achieved complete remission relapsed. The mean PFS was 270.25 days (95% CI 270. 216- 323.57). The mean OS was 384.34 days (95% CI 270.77 - 497.92). Correlation analysis show that the median overall survival is lower for patients in the age group >56 years with a significant difference with other age groups(Log rank test p=0.034). Low PLT is a significant risk factor for death (p=0.01). Relaps <366 days is an unfavorable prognostic factor for survival (K-M p=0.013). The median overall survival in patients who achieved complete remission after induction is greater than the median overall survival in patients who had refractory disease or died during induction (K-M p<0.001).

**Copyright Information:** (Online) ISSN: 2572-9241
©2026 The Author(s). HemaSphere is published by John Wiley & Sons Ltd on behalf of European Hematology Association. This is an open access Abstract Book distributed under the Attribution-NonCommercial-NoDerivs (CC BY-NC-ND), which allows third parties to download the articles and share them with others as long as they credit the author and the Abstract Book, but they cannot change the content in any way or use them commercially.
**Abstract Book Citations:** Authors, Title, HemaSphere, 2026;10:(S1):pages. The abstract book DOIs can be found on the “Author Information” page.
**Disclaimer:** Articles published in the journal HemaSphere exclusively reflect the opinions of the authors. The authors are responsible for all content in their abstracts including accuracy of the facts, statements, citing resources, etc.

**Summary/Conclusion**

The average age-adjusted incidence is 1.9/100000 inhabitants. AML occurs less often in men than in women. AML2 is the predominating type. In the results of the induction treatment, 22% of patients achieved complete remission. 11.25% of patients who achieved complete remission relapsed. The mean PFS was 270.25 days (95% CI 270. 216- 323.57). The mean OS was 384.34 days (95% CI 270.77 - 497.92). Age group >56 years, male gender, low PLT value and relaps <366 days are unfavorable prognostic factors for survival. The results of induction treatment affect OS.

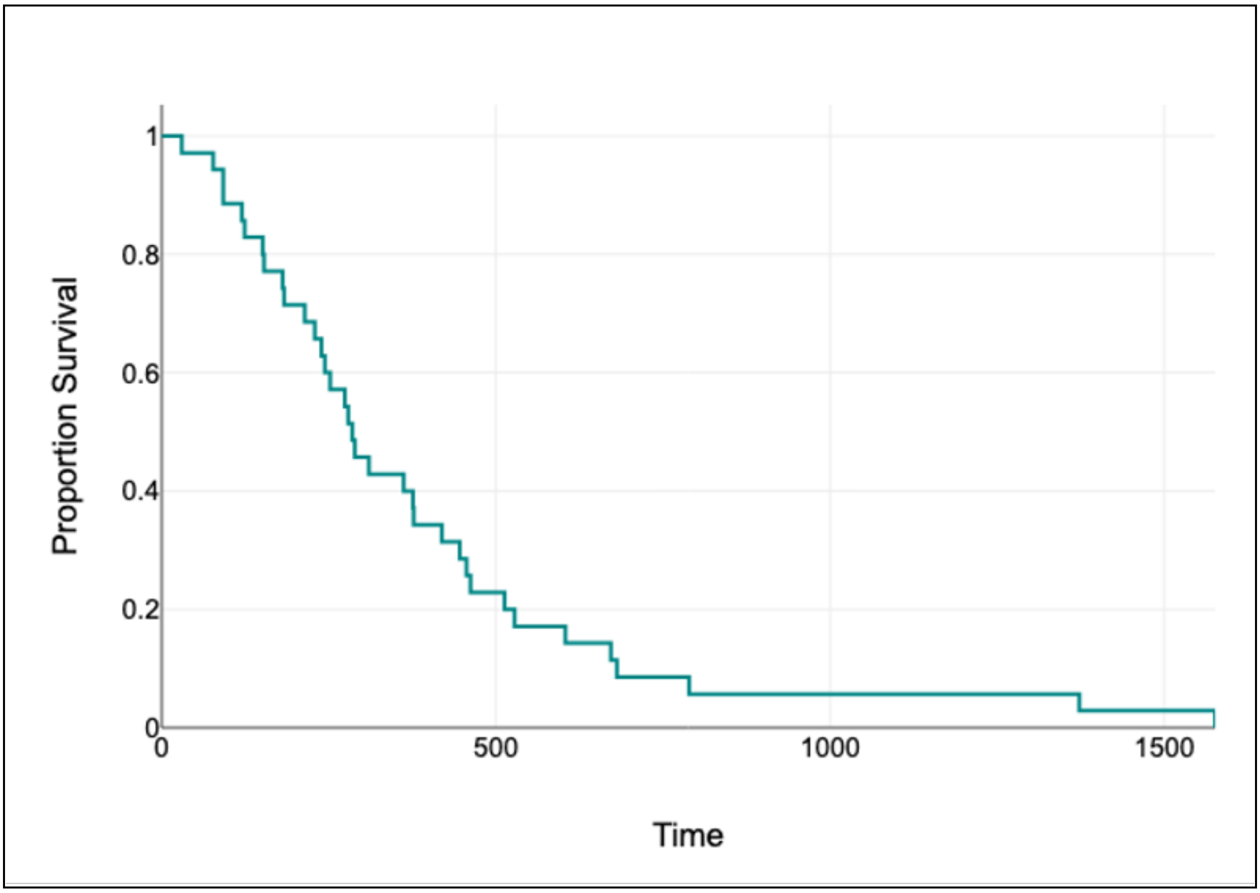

Overall Survival (OS) for patients with AML

**Copyright Information:** (Online) ISSN: 2572-9241
©2026 The Author(s). HemaSphere is published by John Wiley & Sons Ltd on behalf of European Hematology Association. This is an open access Abstract Book distributed under the Attribution-NonCommercial-NoDerivs (CC BY-NC-ND), which allows third parties to download the articles and share them with others as long as they credit the author and the Abstract Book, but they cannot change the content in any way or use them commercially.
**Abstract Book Citations:** Authors, Title, HemaSphere, 2026;10:(S1):pages. The abstract book DOIs can be found on the “Author Information” page.
**Disclaimer:** Articles published in the journal HemaSphere exclusively reflect the opinions of the authors. The authors are responsible for all content in their abstracts including accuracy of the facts, statements, citing resources, etc.

### (PB2826) FIVE-YEAR RETROSPECTIVE ANALYSIS OF ACUTE LEUKEMIA MORTALITY IN ARMENIA

**Topic:** 04. Acute myeloid leukemia - Clinical

Lusine Harutyunyan*[1], Nerses Ghahramanyan[1], Heghine Khachatryan[1], Nare Martirosyan[1], Iveta Mosesyan[1], Hasmik Maghakyan[1], Lusine Sahakyan[1], Anna Movsisyan[1], Anna Sevoyan[1], Naira Simonyan[1], Astghik Voskanyan[1]

[1]*Yeolyan Hematology and Oncology Center, Yerevan, Armenia;*

### Background

Leukemia represents a heterogeneous group of malignancies driven by the uncontrolled proliferation of immature leukocytes. In the United States, 61090 new leukemia cases were estimated in 2021 (3.2% of all cancers; SEER). Acute lymphoblastic leukemia (ALL) predominates in childhood (about 80%), whereas acute myeloid leukemia (AML) is the most common acute leukemia (AL) in adults and is associated with an aggressive course and molecularly determined prognosis. The 5-year relative survival is 32.9% for AML and 72.6% (90% in children, 30-40% in adults) for ALL (2015–2021; SEER). In Armenia, previous 2016-2020 AL outcome analysis showed a 72% case fatality rate (CFR) with 5-year overall survival (OS) of 26% for ALL, 21% for AML, and 22% overall, lower than reported in high-income countries (60% overall).

### Aims

This study updates our previous analysis to report recent mortality outcomes of AL in Armenia to support local treatment policy development.

### Methods

Study involved 480 adult newly diagnosed AL cases treated between 1 January 2021 and 31 December 2025 at the Yeolyan Hematology and Oncology Center, covering all national cases. Clinical data were retrospectively collected from paper records and entered into an electronic database (Excel), with mortality obtained from the national electronic healthcare system. Outcomes were compared with the 2016–2020 cohort from the same center (follow-up until 31 December 2024). Follow-up for the current cohort was shorter due to recent data.

### Results

Over 5 years, 480 adults were diagnosed with AL, of which 224 deaths with CFR 46.7%: AML 163 (72.8%), ALL 39 (17.4%), and 22 (9.8%) AL-unidentified. Female patients numbered 106 (47.3%), and median age was 64 years (IQR 54-71; range 18-86). Complete morphological remission after first-line therapy was achieved in 61 patients (27.2%; ALL n=20, AML n=41), of whom 44 subsequently relapsed.

During 2021–2025, the population-based crude mortality rate (CMR) for AL in Armenia was 1.48:100000 per year and 7.38:100000 over 5 years. Median time from diagnosis to death was 118 days (IQR 26.5-351.8, range 0-7842). Death before initial therapy occurred in 61 (27.2%) patients due to critical condition or treatment refusal, mainly from financial or psychological reasons. Death within 30 days faced 55 (27.2%) patients, and within 90 days 95 (47%) patients.

The leading causes of death were disease progression (n=167), infections/sepsis/pneumonia (n=42), septic shock (n=18), COVID19 (n=6), cerebrovascular events (n=8), thromboembolism (n=6), cardiac complications (n=12), renal failure (n=6), DIC (n=4), tumor intoxication/tumor lysis syndrome (n=7), acute respiratory failure (n=24), and unknown causes (n=9) (Figure 1).

**Copyright Information:** (Online) ISSN: 2572-9241
©2026 The Author(s). HemaSphere is published by John Wiley & Sons Ltd on behalf of European Hematology Association. This is an open access Abstract Book distributed under the Attribution-NonCommercial-NoDerivs (CC BY-NC-ND), which allows third parties to download the articles and share them with others as long as they credit the author and the Abstract Book, but they cannot change the content in any way or use them commercially.
**Abstract Book Citations:** Authors, Title, HemaSphere, 2026;10:(S1):pages. The abstract book DOIs can be found on the "Author Information" page.
**Disclaimer:** Articles published in the journal HemaSphere exclusively reflect the opinions of the authors. The authors are responsible for all content in their abstracts including accuracy of the facts, statements, citing resources, etc.

### Summary/Conclusion

Although the CMR in the general population was low, the CFR among diagnosed adults was relatively high, reflecting the aggressive nature of AL despite its rarity. Compared with the previous cohort, the improved outcomes with a decreased CFR was assessed during recent years in Armenia. The leading cause of death remains disease progression. Further local improvements in access to diagnostics, therapy, and supportive care are required.

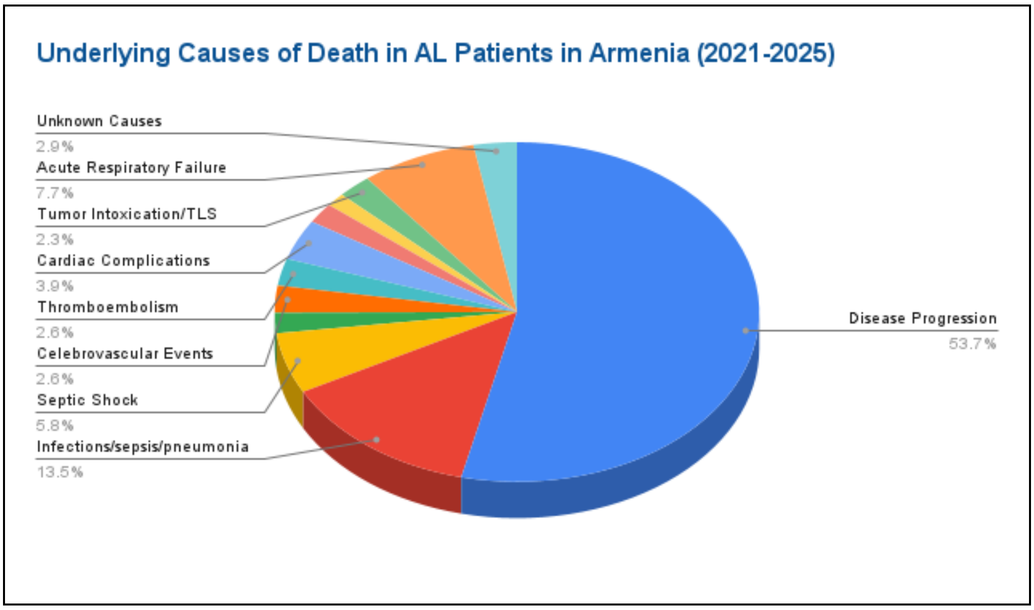

Figure 1. Underlying Causes of Death in AL Patients in Armenia (2021-2025).

**Copyright Information:** (Online) ISSN: 2572-9241
©2026 The Author(s). HemaSphere is published by John Wiley & Sons Ltd on behalf of European Hematology Association. This is an open access Abstract Book distributed under the Attribution-NonCommercial-NoDerivs (CC BY-NC-ND), which allows third parties to download the articles and share them with others as long as they credit the author and the Abstract Book, but they cannot change the content in any way or use them commercially.
**Abstract Book Citations:** Authors, Title, HemaSphere, 2026;10:(S1):pages. The abstract book DOIs can be found on the "Author Information" page.
**Disclaimer:** Articles published in the journal HemaSphere exclusively reflect the opinions of the authors. The authors are responsible for all content in their abstracts including accuracy of the facts, statements, citing resources, etc.

### (PB2827) REAL-WORLD OUTCOMES OF VENETOCLAX PLUS CHEMOTHERAPY AS SALVAGE BRIDGE TO ALLOGENEIC STEM CELL TRANSPLANTATION IN RELAPSED / REFRACTORY ACUTE MYELOID LEUKEMIA: A MULTICENTER PERUVIAN COHORT

**Topic:** 04. Acute myeloid leukemia - Clinical

Cesar Samanez*[1], Sergio Murillo[2], Jorge Cotito[1], Saul Ferreyra[3], Lourdes David[2], Claudio Flores[1], Shirley Quintana[3], Jose Untama[2], Karina Rojas[4], Joaquin Villalobos[5], Ana Alfonso Pierola[6], Jose Juan Rifon Roca[6], Antonio Carrasco[7]

*[1]Oncosalud - AUNA, Lima, Peru; [2]Hospital Edgardo Rebaglati Martins, Lima, Peru; [3]Instituto Nacional de Enfermedades Neoplasicas, Lima, Peru; [4]Clínica San Borja, Lima, Peru; [5]Clinica Angloamericana, Lima, Peru; [6]Clinica Universidad de Navarra, Pamplona, Spain; [7]Universidad Nacional Mayor de San Marcos, Lima, Peru;*

### Background

The treatment of patients (pts) with relapsed or refractory acute myeloid leukemia (r/r AML) remains challenging. In fit patients, several salvage chemotherapy (CT) options based on high-dose- cytarabine (HDC) could achieve complete remission (CR) rates ranging from 40-55%. The BCL2 inhibitor Venetoclax (VEN) has demonstrated synergistic anti-leukemic activity when combined with CT and could induce higher rates of CR including incomplete hematologic recovery (CRi) in this setting and becomes a bridge for allogeneic hematopoietic cell transplantation (Allo-HCT).

### Aims

This retrospective multicenter real-world study aimed to evaluate the clinical efficacy and safety of VEN + CT as a salvage strategy and bridge to Allo-HCT in patients with r/r AML.

### Methods

We collected clinicopathological data and outcomes of 57 pts with r/r AML from 5 hemato-oncology centers in Lima, Peru who received at least one course of VEN + CT as salvage bridge therapy for Allo-HCT. Treatment included VEN doses from 100 to 400 mg PO QD in combination with HAM (HDC and Mitoxantrone), FLAG-based CT (Fludarabine, Cytarabine and Filgrastim) or Azacitidine (AZA).

**Copyright Information:** (Online) ISSN: 2572-9241
©2026 The Author(s). HemaSphere is published by John Wiley & Sons Ltd on behalf of European Hematology Association. This is an open access Abstract Book distributed under the Attribution-NonCommercial-NoDerivs (CC BY-NC-ND), which allows third parties to download the articles and share them with others as long as they credit the author and the Abstract Book, but they cannot change the content in any way or use them commercially.
**Abstract Book Citations:** Authors, Title, HemaSphere, 2026;10:(S1):pages. The abstract book DOIs can be found on the “Author Information” page.
**Disclaimer:** Articles published in the journal HemaSphere exclusively reflect the opinions of the authors. The authors are responsible for all content in their abstracts including accuracy of the facts, statements, citing resources, etc.

### Results

Between 2020 and 2025, 57 pts with r/r AML with a median age of 44 (15-69) years received VEN in combination mainly with HAM (31.5%), FLAG-based CT (31.5%) or AZA (22%). Thirty-one (54.3%) pts had relapsed AML and 26 (45.7%) had refractory disease. In evaluable pts (15), genetic risk according to ELN Risk 2017 was intermediate (66.7%) or adverse (33.3%). Eighty percent of pts received only 1 prior line of therapy, mainly based of 7+3 (Cytarabine + Daunorubicin). The median daily VEN dose was 100 mg because of the concomitant use of azoles as prophylaxis for high incidence of invasive fungal infections.
Forty-two (73.7%) pts achieved CR/CRi with MRD-negative in 90.4% of them, after 1 cycle (95.2%) or ≥2 (4.8%) of VEN + CT. Grade 3 and 4 adverse events occurred in most pts including febrile neutropenia (52.6%), sepsis (19.3%), gastrointestinal disorders (14.0%) and pneumonia (10.5%). There were no deaths during the treatment.
Twenty-six (61.9%) of the pts who achieved CR/CRi underwent Allo-HCT and 6 (14.3%) are still in process at the moment of this analysis. Fourteen pts (53.8%) had haploidentical donor, 10 (38.4%) identical donor and 2 (7.7%) non-related donor. Ten (23.8%) pts could not proceed Allo-HCT because of early relapse (9.5%), unavailable donor (9.5%) or death for infection in CR (4.7%).
At a median follow-up of 16.4 (11.5-21.3) months, pts who proceeded to Allo-HCT achieved 1-year Relapse-free Survival (RFS) and Overall Survival (OS) of 63.8% and 78.6% respectively. By contrast, pts who did not respond to VEN + CT had poor median OS (4.2 months).

### Summary/Conclusion

In this Peruvian multicenter real-world study on fit pts with r/r AML, the combination of Venetoclax with CT demonstrated high CR/CRi rates and enabled a substantial proportion of patients to proceed to Allo-HCT. Post-transplant survival outcomes were encouraging despite the relatively short follow-up. These findings support VEN + CT as a feasible and active salvage and transplant-bridging strategy in this challenging patient population.

**Copyright Information:** (Online) ISSN: 2572-9241
©2026 The Author(s). HemaSphere is published by John Wiley & Sons Ltd on behalf of European Hematology Association. This is an open access Abstract Book distributed under the Attribution-NonCommercial-NoDerivs (CC BY-NC-ND), which allows third parties to download the articles and share them with others as long as they credit the author and the Abstract Book, but they cannot change the content in any way or use them commercially.
**Abstract Book Citations:** Authors, Title, HemaSphere, 2026;10:(S1):pages. The abstract book DOIs can be found on the "Author Information" page.
**Disclaimer:** Articles published in the journal HemaSphere exclusively reflect the opinions of the authors. The authors are responsible for all content in their abstracts including accuracy of the facts, statements, citing resources, etc.

### (PB2828) VALIDATION AND COMPARISON OF ELN 2024 REVISED AND MAYO GENETIC RISK MODELS IN PATIENTS WITH AML RECEIVING VENETOCLAX-AZACITIDINE A REAL-WORLD STUDY FROM CHINA

**Topic:** 04. Acute myeloid leukemia - Clinical

Weiguang Cui*[1], Yaxuan Lv[1], Liyuan Li[1], Zhenzhen Wang[1], Nan Zhou[1], Fan Yang[1], Mengying Zheng[1], Xiaoyan Zhang[1], Li Sun[1], Lin Yang[1], Jianmin Luo[1]

[1]*Department of Hematology, The Second Hospital of Hebei Medical University, shijiazhuang, China;*

### Background

While several prognostic systems, including ELN 2024, the Revised ELN 2024, and the Mayo models, have been established for risk stratification in newly diagnosed AML patients receiving less-intensive therapy, a systematic validation of these scoring systems remains lacking in Asian populations.

### Aims

We conducted a retrospective study to validate and compare the prognostic accuracy of the ELN 2024, ELN 2024 Revised, and Mayo models in a Chinese cohort of patients with newly diagnosed AML

### Methods

In this single-center, retrospective study,we retrospectively analyzed 53 treatment-naive patients with acute myeloid leukemia (AML) who were ineligible for intensive chemotherapy and received venetoclax plus azacitidine (VA) at our center between August 2020 and June 2024. The VA regimen consisted of azacitidine (75 mg/m² daily for 7 days) and continuous venetoclax (400 mg daily for 28 days) in 28-day cycles. Patient risk was stratified according to the European LeukemiaNet (ELN) 2022, ELN 2024, Revised ELN 2024, and the Mayo Clinic prognostic models. The primary endpoints were overall survival (OS) and relapse-free survival (RFS). Secondary endpoints included composite complete remission (cCR) rates, measurable residual disease (MRD) clearance, and the impact of specific molecular mutations on clinical outcomes.

### Results

With a median follow-up of 27.6 months, the median age of the cohort was 65 years (range, 20–78 years). The four risk models demonstrated varying degrees of prognostic power in this real-world setting. Under the **ELN 2022** criteria, 6 (11.3%), 24 (45.3%), and 23 (43.4%) patients were categorized into favorable, intermediate, and adverse groups, respectively; however, this model failed to achieve significant OS discrimination (median OS: 39.8, 33.9, and 25.4 months, respectively; log-rank $P = .539$). In contrast, the **ELN 2024** system significantly differentiated outcomes among favorable (n=15, 28.3%), intermediate (n=27, 50.9%), and adverse (n=11, 20.8%) groups, with median OS of 39.8, 25.4, and 6.0 months, respectively (log-rank $P = .033$). Notably, the **Revised ELN 2024** and **Mayo Score** exhibited superior predictive value, both yielding highly significant prognostic stratification ($P < .001$). Specifically, the Revised ELN 2024 model identified 18 (34.0%) favorable, 19 (35.8%) intermediate, and 16 (30.2%) adverse patients, with median OS of 39.8 months, not reached (NR), and 3.6 months, respectively. Similarly, the Mayo Score partitioned the cohort into favorable (n=12, 22.6%), intermediate (n=19, 35.8%), and adverse (n=22, 41.5%) risks, with corresponding median OS of 39.8 months, NR, and 7.9 months. *PTPN11* mutations appeared to confer an adverse prognosis in this cohort. Notably, five of the six patients (83.3%) harboring *PTPN11* mutations experienced disease relapse within six months of achieving initial remission, underscoring the aggressive nature of this molecular subgroup under the VA regimen.

**Copyright Information:** (Online) ISSN: 2572-9241
©2026 The Author(s). HemaSphere is published by John Wiley & Sons Ltd on behalf of European Hematology Association. This is an open access Abstract Book distributed under the Attribution-NonCommercial-NoDerivs (CC BY-NC-ND), which allows third parties to download the articles and share them with others as long as they credit the author and the Abstract Book, but they cannot change the content in any way or use them commercially.
**Abstract Book Citations:** Authors, Title, HemaSphere, 2026;10:(S1):pages. The abstract book DOIs can be found on the "Author Information" page.
**Disclaimer:** Articles published in the journal HemaSphere exclusively reflect the opinions of the authors. The authors are responsible for all content in their abstracts including accuracy of the facts, statements, citing resources, etc.

### Summary/Conclusion

Our findings demonstrate a significant evolution in predictive accuracy across these models. While the VA regimen has shifted the prognostic landscape, the Revised ELN 2024 and Mayo models are superior in identifying patients with true refractory disease, thereby providing a more reliable framework for early clinical intervention. However, the present study is limited by its relatively small sample size. Further validation in larger, prospective multicenter cohorts is warranted to definitively establish the optimal prognostic framework for this patient population.

A

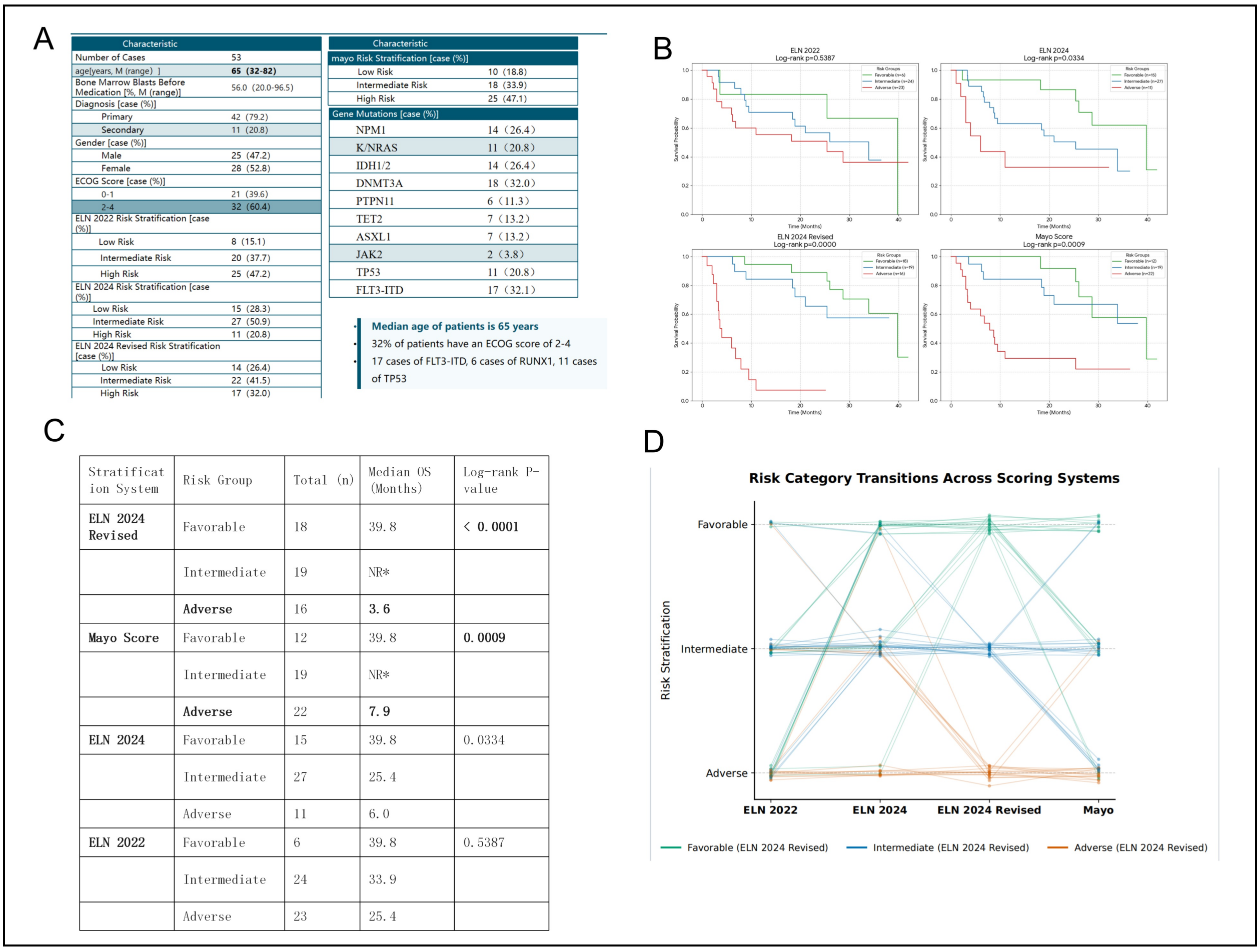

| Characteristic | |
|---|---|
| Number of Cases | 53 |
| age[years, M (range) ] | **65 (32-82)** |
| Bone Marrow Blasts Before Medication [%, M (range)] | 56.0 (20.0-96.5) |
| Diagnosis [case (%)] | |
| Primary | 42 (79.2) |
| Secondary | 11 (20.8) |
| Gender [case (%)] | |
| Male | 25 (47.2) |
| Female | 28 (52.8) |
| ECOG Score [case (%)] | |
| 0-1 | 21 (39.6) |
| 2-4 | 32 (60.4) |
| ELN 2022 Risk Stratification [case (%)] | |
| Low Risk | 8 (15.1) |
| Intermediate Risk | 20 (37.7) |
| High Risk | 25 (47.2) |
| ELN 2024 Risk Stratification [case (%)] | |
| Low Risk | 15 (28.3) |
| Intermediate Risk | 27 (50.9) |
| High Risk | 11 (20.8) |
| ELN 2024 Revised Risk Stratification [case (%)] | |
| Low Risk | 14 (26.4) |
| Intermediate Risk | 22 (41.5) |
| High Risk | 17 (32.0) |

| Characteristic | |
|---|---|
| mayo Risk Stratification [case (%)] | |
| Low Risk | 10 (18.8) |
| Intermediate Risk | 18 (33.9) |
| High Risk | 25 (47.1) |
| Gene Mutations [case (%)] | |
| NPM1 | 14 (26.4) |
| K/NRAS | 11 (20.8) |
| IDH1/2 | 14 (26.4) |
| DNMT3A | 18 (32.0) |
| PTPN11 | 6 (11.3) |
| TET2 | 7 (13.2) |
| ASXL1 | 7 (13.2) |
| JAK2 | 2 (3.8) |
| TP53 | 11 (20.8) |
| FLT3-ITD | 17 (32.1) |

- **Median age of patients is 65 years**
- 32% of patients have an ECOG score of 2-4
- 17 cases of FLT3-ITD, 6 cases of RUNX1, 11 cases of TP53

B

C

| Stratification System | Risk Group | Total (n) | Median OS (Months) | Log-rank P-value |
|---|---|---|---|---|
| ELN 2024 Revised | Favorable | 18 | 39.8 | < 0.0001 |
| | Intermediate | 19 | NR* | |
| | **Adverse** | 16 | **3.6** | |
| Mayo Score | Favorable | 12 | 39.8 | 0.0009 |
| | Intermediate | 19 | NR* | |
| | **Adverse** | 22 | **7.9** | |
| ELN 2024 | Favorable | 15 | 39.8 | 0.0334 |
| | Intermediate | 27 | 25.4 | |
| | Adverse | 11 | 6.0 | |
| ELN 2022 | Favorable | 6 | 39.8 | 0.5387 |
| | Intermediate | 24 | 33.9 | |
| | Adverse | 23 | 25.4 | |

D

**Copyright Information:** (Online) ISSN: 2572-9241
©2026 The Author(s). HemaSphere is published by John Wiley & Sons Ltd on behalf of European Hematology Association. This is an open access Abstract Book distributed under the Attribution-NonCommercial-NoDerivs (CC BY-NC-ND), which allows third parties to download the articles and share them with others as long as they credit the author and the Abstract Book, but they cannot change the content in any way or use them commercially.
**Abstract Book Citations:** Authors, Title, HemaSphere, 2026;10:(S1):pages. The abstract book DOIs can be found on the “Author Information” page.
**Disclaimer:** Articles published in the journal HemaSphere exclusively reflect the opinions of the authors. The authors are responsible for all content in their abstracts including accuracy of the facts, statements, citing resources, etc.

### (PB2829) CLINICAL AND BIOLOGICAL CHARACTERISTICS OF ACUTE MYELOID LEUKEMIA IN PATIENTS FROM THE LENINGRAD REGION AND SAINT PETERSBURG

**Topic:** 04. Acute myeloid leukemia - Clinical

Sergey Voloshin*[1, 2], Evgenii Kunevich[1], Victor Solovyov[1], Asiyat Radzhabova[3], Olga Uspenskaya[1], Ruslan Bekoev[1], Alexey Kuvshinov[3], Elena Karyagina[4], Mariia Penkova[1], Albina Aidemirova[1], Olga Ruzhinskaya[1], Olga Ryabykina[1], Natalia Sokolova[1], Svetlana Tolstova[1], Sergey Alekseev[1, 5]

*[1]Leningrad Regional Clinical Hospital, Saint-Petersburg, Russia;[2]S.M. Kirov Military Medical Academy, Saint-Petersburg, Russia; [3]Russian Research Institute of Hematology and Transfusiology, Saint-Petersburg, Russia;[4]City Hospital No. 15, Saint-Petersburg, Russia; [5]N.N. Petrov National Medical Research Center of Oncology, Saint-Petersburg, Russia;*

### Background

Acute myeloid leukemia (AML) is characterized by marked clinical and biological heterogeneity, necessitating the analysis of regional disease features to optimize diagnostic and therapeutic strategies.

### Aims

To assess the prevalence and prognostic impact of clinical and biological characteristics of AML.

### Methods

The study included 497 patients with AML (excluding acute promyelocytic leukemia) with a mean age of 56.3 years (range 18–93), treated between 2009 and November 1, 2025. The cohort comprised 418 patients with de novo AML (84.1%) and 79 patients (15.9%) with secondary AML arising after myelodysplastic syndrome (n=58), MDS/MPN (n=17), or prior therapy (n=4). At diagnosis, hyperleukocytosis was detected in 13.8% of cases and central nervous system involvement in 5.6%. Mean follow-up duration was 12.2 months. Data were analyzed using Jamovi v2.4.8.0 and R v4.2.2.

**Copyright Information:** (Online) ISSN: 2572-9241
©2026 The Author(s). HemaSphere is published by John Wiley & Sons Ltd on behalf of European Hematology Association. This is an open access Abstract Book distributed under the Attribution-NonCommercial-NoDerivs (CC BY-NC-ND), which allows third parties to download the articles and share them with others as long as they credit the author and the Abstract Book, but they cannot change the content in any way or use them commercially.
**Abstract Book Citations:** Authors, Title, HemaSphere, 2026;10:(S1):pages. The abstract book DOIs can be found on the “Author Information” page.
**Disclaimer:** Articles published in the journal HemaSphere exclusively reflect the opinions of the authors. The authors are responsible for all content in their abstracts including accuracy of the facts, statements, citing resources, etc.

### Results

Age had a significant impact on overall survival (OS): HR=1.60 (95% CI 1.22–2.21), p=0.001. Two-year OS was 41.8% (median 12.3 months) in patients older than 60 years and 56.0% (median 23.4 months) in those younger than 60 years. Two-year OS in secondary AML was 31.7% compared with 52.0% in de novo AML (p=0.15).

Significant differences were observed in relapse-free survival (RFS): 2-year RFS was 34.0% (median 13.1 months) in de novo AML and 11.0% (median 8.7 months) in secondary AML (p=0.0032; HR=1.58 [95% CI 1.16–2.17], p=0.004). Compared with de novo AML, the risk of death and relapse increased 1.50-fold in AML transformed from MDS (p=0.021) and 2.53-fold in AML arising from MDS/MPN (p=0.003).

ELN 2022 risk stratification was a strong prognostic factor for both OS and RFS. Two-year OS rates were 73.3% in the favorable-risk group, 49.4% in the intermediate-risk group (HR=2.10, p=0.011), and 38.9% in the adverse-risk group (HR=3.44, p<0.001). Hyperleukocytosis at diagnosis increased the risk of death by 1.5-fold (HR=1.50, p=0.026).

Patients with a complex karyotype (CK) had inferior 2-year OS compared with those without CK (40.9% vs 53.2%; median 16.2 vs 29.8 months; p=0.0006; HR=1.82 [95% CI 1.29–2.58], p=0.001). The prognostic impact of specific cytogenetic abnormalities on OS was as follows: t(8;21) HR=0.46 (p=0.086), monosomy 7 HR=1.98 (p=0.037), del(5q) HR=2.51 (p=0.001), del(17p) HR=2.59 (p=0.009), and 11q23/*KMT2A* rearrangements HR=2.05 (p=0.013). Molecular markers were also prognostically relevant: *RUNX1::RUNX1T1* HR=0.30 (p=0.041) and *FLT3* (TKD+ITD) HR=1.50 (p=0.005).

The impact of combined *NPM1* and *FLT3* mutation status on 2-year OS was as follows: WT+WT 51.4%; mutated *NPM1*/WT *FLT3* 82.1% (HR=0.39, p=0.024); double-mutant 43.0% (HR=1.21, p=0.383); WT *NPM1*/mutated FLT3 30.0% (HR=1.68, p=0.002), all compared with the WT+WT subgroup.

Early death occurred in 44 patients (8.9%). Biological factors associated with early mortality included CK (p=0.0298), del(5q) (p=0.0129), and *FLT3*-TKD mutations (p=0.0296). Older patients more frequently had secondary AML, ELN 2022 high-risk disease, and cytogenetic abnormalities associated with myelodysplasia.

### Summary/Conclusion

AML outcomes are determined by multiple factors, with age, secondary disease origin, ELN 2022 risk group, and specific cytogenetic and molecular abnormalities playing key prognostic roles.

**Copyright Information:** (Online) ISSN: 2572-9241
©2026 The Author(s). HemaSphere is published by John Wiley & Sons Ltd on behalf of European Hematology Association. This is an open access Abstract Book distributed under the Attribution-NonCommercial-NoDerivs (CC BY-NC-ND), which allows third parties to download the articles and share them with others as long as they credit the author and the Abstract Book, but they cannot change the content in any way or use them commercially.
**Abstract Book Citations:** Authors, Title, HemaSphere, 2026;10:(S1):pages. The abstract book DOIs can be found on the "Author Information" page.
**Disclaimer:** Articles published in the journal HemaSphere exclusively reflect the opinions of the authors. The authors are responsible for all content in their abstracts including accuracy of the facts, statements, citing resources, etc.

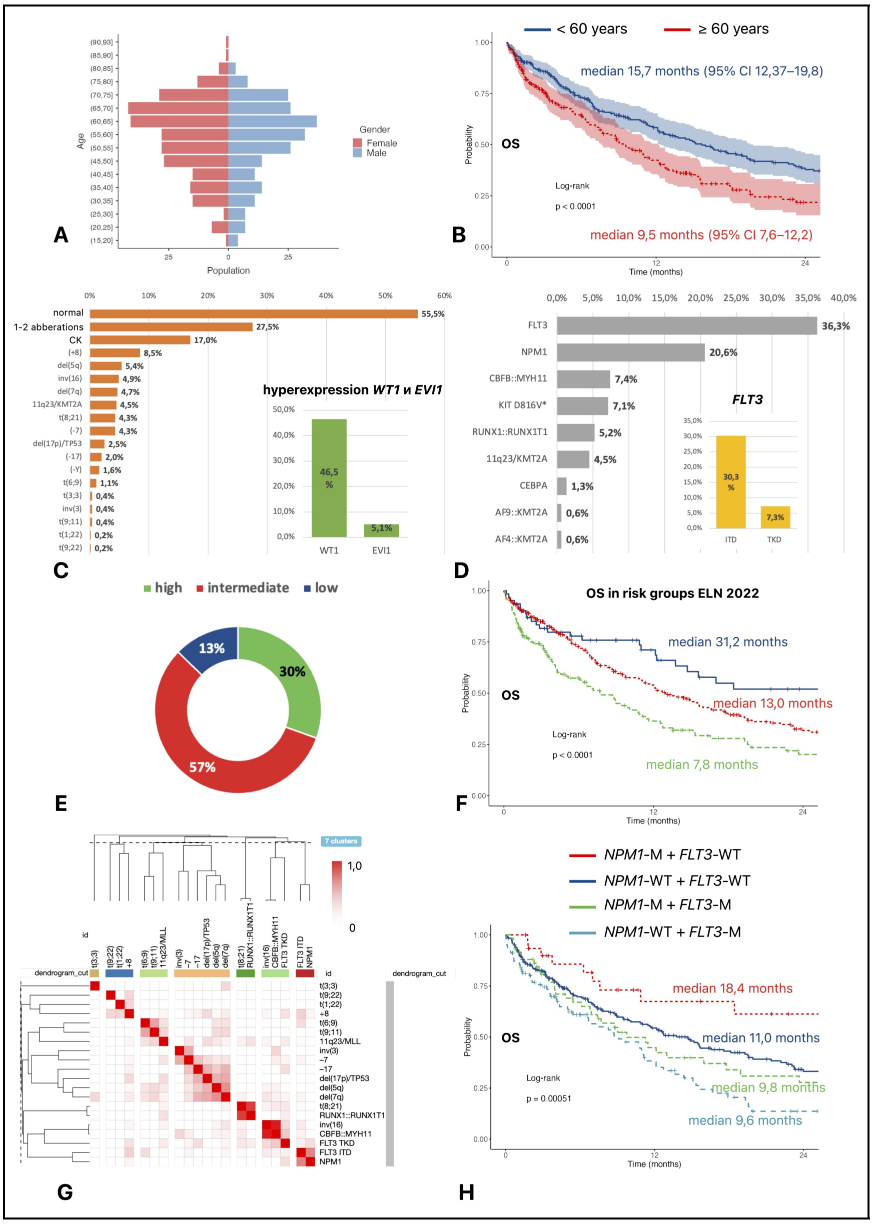

A) Age–sex pyramid. B) Overall survival of AML patients according to age. C) Frequency of cytogenetic aberrations; inset histogram showing WT1 and EVI1 gene expression levels. D) Frequency of molecular genetic abnormalities; inset histogram showing the distribution of FLT3 mutations by ITD and TKD variants. E) Distribution of ELN 2022 risk groups. F) Overall survival of AML patients according to ELN 2022 risk group. G) Heatmap with hierarchical clustering demonstrating co-occurrence and mutual exclusivity of cytogenetic and molecular genetic AML markers. H) Overall survival of AML patients according to FLT3 and NPM1 mutation status.

**Copyright Information:** (Online) ISSN: 2572-9241
©2026 The Author(s). HemaSphere is published by John Wiley & Sons Ltd on behalf of European Hematology Association. This is an open access Abstract Book distributed under the Attribution-NonCommercial-NoDerivs (CC BY-NC-ND), which allows third parties to download the articles and share them with others as long as they credit the author and the Abstract Book, but they cannot change the content in any way or use them commercially.
**Abstract Book Citations:** Authors, Title, HemaSphere, 2026;10:(S1):pages. The abstract book DOIs can be found on the "Author Information" page.
**Disclaimer:** Articles published in the journal HemaSphere exclusively reflect the opinions of the authors. The authors are responsible for all content in their abstracts including accuracy of the facts, statements, citing resources, etc.

### (PB2830) NEUTROPENIC ENTEROCOLITIS IN ADULT PATIENTS DIAGNOSED WITH ACUTE LEUKEMIA TREATED WITH INTENSIVE CHEMOTHERAPY IN A SINGLE CENTER: DESCRIPTION OF CLINICAL CHARACTERISTICS AND MANAGEMENT RESULTS.

**Topic:** 04. Acute myeloid leukemia - Clinical

Monica Alejandra Romero Riquelme*[1, 1], Gonzalez Daniel[1], Alarcon Francesca[1]

[1]*Hospital Guillermo Grant Benavente, Haematology, Concepcion, Chile;*

### Background

Neutropenic enterocolitis (NEC) is a serious and life-threatening complication that affects immunocompromised patients, especially those with acute leukemias undergoing intensive chemotherapy. Despite its high morbidity and mortality, there are few studies that characterize its clinical, microbiological and imaging behavior, which makes it difficult to recognize and manage it in a timely manner, especially in this group of patients

### Aims

To describe the clinical, microbiological, and imaging features of NEC and management outcomes in adult  patients with acute leukemias treated with intensive chemotherapy in a chilean center

### Methods

Retrospective observational study of patients ≥15 years of age diagnosed with acute leukemia, treated with intensive chemotherapy and who developed NEC between 2022 and 2024. Clinical, microbiological, and imaging variables were collected in an anonymized database in REDCap platform. NEC was established according de Nesher´s criteria This study has the approval of the local scientific ethics committee

### Results

Data from 42 patients were analyzed, with a median age of 39 years at diagnosis of acute leukemia, 50% of whom were women. 42% had  a diagnosis of Acute Myeloid Leukemia (AML) on induction chemotherapy. 47.6% had comorbidities, the most frequent being diabetes mellitus, high blood pressure and smoking. The median number of days between the start of chemotherapy and the diagnosis of NEC was 10 (1–30). The symptoms associated with NEC were: fever (81%), abdominal pain (78.6%), diarrhea (59.5%), abdominal distension (16.7%). The most affected intestinal segments were: cecum (70.3%) and right colon (51.4%) and the main imaging findings were: mucosal enhancement (91.7%), adipose tissue attenuation (52.8%), mesenteric tissue striation (50%), abdominal free fluid (41.7%). A microbiological agent was documented in 43% of the cases, with Gram-negative bacilli being the most frequent (72.7% E.Coli ESBL). All patients received intravenous antibiotics (59.6% carbapenemics; 33.3% vancomycin; 21.4% methronidazole). 90.5% received total parenteral nutrition. Only 7.1% required surgical intervention. Complications were present in 33% of patients, mainly septic shock. 23.8% of this patients required admission to a critical care unit and 60% received support with vasoactive drugs. In-hospital mortality was 19%

**Copyright Information:** (Online) ISSN: 2572-9241
©2026 The Author(s). HemaSphere is published by John Wiley & Sons Ltd on behalf of European Hematology Association. This is an open access Abstract Book distributed under the Attribution-NonCommercial-NoDerivs (CC BY-NC-ND), which allows third parties to download the articles and share them with others as long as they credit the author and the Abstract Book, but they cannot change the content in any way or use them commercially.
**Abstract Book Citations:** Authors, Title, HemaSphere, 2026;10:(S1):pages. The abstract book DOIs can be found on the “Author Information” page.
**Disclaimer:** Articles published in the journal HemaSphere exclusively reflect the opinions of the authors. The authors are responsible for all content in their abstracts including accuracy of the facts, statements, citing resources, etc.

**Summary/Conclusion**

NEC is a very serious complication of patients undergoing intensive chemotherapy for acute leukemias. This study presents the first cohort in our knowledge from adult patients from a Chilean center in this pathology. A high frequency of complications and mortality was observed. Mucosal enhancement and adipose tissue attenuation on computed tomography were associated with increased risk of morbidity and mortality. Clinical, microbiological, and imaging characterization would allow the identification of patients at higher risk of presenting NEC and associated complications, which could guide more timely therapeutic decisions and generate local management guidelines that would impact the improvement of morbidity and mortality in this setting of patients

**Copyright Information:** (Online) ISSN: 2572-9241
©2026 The Author(s). HemaSphere is published by John Wiley & Sons Ltd on behalf of European Hematology Association. This is an open access Abstract Book distributed under the Attribution-NonCommercial-NoDerivs (CC BY-NC-ND), which allows third parties to download the articles and share them with others as long as they credit the author and the Abstract Book, but they cannot change the content in any way or use them commercially.
**Abstract Book Citations:** Authors, Title, HemaSphere, 2026;10:(S1):pages. The abstract book DOIs can be found on the "Author Information" page.
**Disclaimer:** Articles published in the journal HemaSphere exclusively reflect the opinions of the authors. The authors are responsible for all content in their abstracts including accuracy of the facts, statements, citing resources, etc.

### (PB2831) HYDROCARBON EXPOSURE AND THE RISK OF MYELOID MALIGNANCIES: A SYSTEMATIC REVIEW WITH TRANSLATIONAL IMPLICATIONS FOR OIL PRODUCING REGIONS, INCLUDING RIAU, INDONESIA

**Topic:** 04. Acute myeloid leukemia - Clinical

Nanda Noor Muhammad Nasution*[1], Annes Waren[2]

*[1]RS. Awal Bros Pekanbaru, Indonesia, Internal Medicine, Hematologist-Oncologists, Pekanbaru, Indonesia;[2]Padjajaran University, Indonesia, Public Health, Occupational Medicine, Bandung, Indonesia;*

### Background

Hydrocarbon and petrochemical exposure, particularly to benzene containing compounds, is a recognized cause of bone marrow toxicity and leukemogenesis. Occupational activities in oil producing regions involve sustained exposure during extraction, refining, and related industrial processes. Although the association between hydrocarbon exposure and myeloid malignancies has been reported, heterogeneity in exposure assessment and study design has led to variability in risk estimates. This issue remains relevant for contemporary oil producing regions, including provinces such as Riau, Indonesia, where substantial workforce populations are engaged in petroleum related industries.

### Aims

To systematically evaluate the association between occupational and environmental hydrocarbon exposure and the risk of myeloid malignancies, including acute myeloid leukemia (AML) and myelodysplastic syndromes (MDS).

### Methods

This systematic review was conducted in accordance with PRISMA guidelines. PubMed, Embase, Scopus, and Web of Science were searched from database inception through December 31, 2025. Eligible studies included cohort and case control investigations evaluating defined hydrocarbon or petrochemical exposure in relation to AML, MDS, or persistent bone marrow cytopenias. Studies lacking exposure characterization or clearly defined hematologic outcomes were excluded.
Two independent reviewers screened titles, abstracts, and full texts. Data extracted included exposure metrics, duration and intensity categories, adjustment variables, and reported effect estimates (OR, RR, HR). Study quality was assessed using the Newcastle Ottawa Scale. Where studies were methodologically comparable, effect estimates were synthesized using a random-effects model; otherwise, findings were integrated qualitatively with emphasis on exposure response relationships and consistency across study designs.

### Results

The search identified 1,247 records, of which 318 remained after duplicate removal. Following title and abstract screening, 69 full-text articles were assessed for eligibility. Thirty one studies met inclusion criteria, comprising 17 cohort studies and 14 case control investigations.
Across included studies, chronic hydrocarbon exposure was consistently associated with increased risk of AML and MDS. Elevated risk estimates were most pronounced among individuals with prolonged or high intensity exposure. Several studies demonstrated exposure response gradients. Where quantitative synthesis was feasible, pooled estimates indicated a significantly increased risk of myeloid malignancies among exposed workers compared with non-exposed populations. Mechanistic investigations supported leukemogenic pathways involving genotoxicity, chromosomal instability, and disruption of hematopoietic stem cell function. Evidence from emerging oil-producing regions remains limited, indicating underrepresentation of contemporary industrial settings outside high income countries.

**Copyright Information:** (Online) ISSN: 2572-9241
©2026 The Author(s). HemaSphere is published by John Wiley & Sons Ltd on behalf of European Hematology Association. This is an open access Abstract Book distributed under the Attribution-NonCommercial-NoDerivs (CC BY-NC-ND), which allows third parties to download the articles and share them with others as long as they credit the author and the Abstract Book, but they cannot change the content in any way or use them commercially.
**Abstract Book Citations:** Authors, Title, HemaSphere, 2026;10:(S1):pages. The abstract book DOIs can be found on the “Author Information” page.
**Disclaimer:** Articles published in the journal HemaSphere exclusively reflect the opinions of the authors. The authors are responsible for all content in their abstracts including accuracy of the facts, statements, citing resources, etc.

**Summary/Conclusion**

The cumulative evidence supports a consistent association between hydrocarbon exposure and increased risk of myeloid malignancies. These findings reinforce the need for strengthened occupational exposure monitoring and structured hematologic surveillance in oil producing regions. In provinces such as Riau, Indonesia, integration of systematic exposure assessment with early detection strategies for bone marrow disorders may contribute to improved workforce health protection and risk mitigation.

**Copyright Information:** (Online) ISSN: 2572-9241
©2026 The Author(s). HemaSphere is published by John Wiley & Sons Ltd on behalf of European Hematology Association. This is an open access Abstract Book distributed under the Attribution-NonCommercial-NoDerivs (CC BY-NC-ND), which allows third parties to download the articles and share them with others as long as they credit the author and the Abstract Book, but they cannot change the content in any way or use them commercially.
**Abstract Book Citations:** Authors, Title, HemaSphere, 2026;10:(S1):pages. The abstract book DOIs can be found on the "Author Information" page.
**Disclaimer:** Articles published in the journal HemaSphere exclusively reflect the opinions of the authors. The authors are responsible for all content in their abstracts including accuracy of the facts, statements, citing resources, etc.

### (PB2832) THE PROGNOSTIC EFFECT OF BONE MARROW FIBROSIS AT DIAGNOSIS IN ACUTE MYELOID LEUKEMIA (AML) PATIENTS ASSESSED WITH OTHER LABORATORY PARAMETERS: SINGLE CENTER DATA

**Topic:** 04. Acute myeloid leukemia - Clinical

Omer Aydın Yıldırım[1], Mehmet Yilmaz*[1], Onuralp Tekin[1], Salih Sertac Durusoy[1]

[1]*Sanko University Faculty of Medicine, Internal Medicine Division of Hematology, Gaziantep, Türkiye;*

**Background**

The heterogeneous nature of AML encompasses complex variables that play a role at every step of diagnosis, classification, treatment, monitoring, risk scoring, and prognosis. The prognostic impact of bone marrow fibrosis (BMF) in AML remains unclear. The question of whether fibrosis should be incorporated into ELN scoring requires further evidence.

**Aims**

This single-center study aimed to investigate the prognostic impact of bone marrow fibrosis at diagnosis in de novo AML patients in conjunction with laboratory, cytogenetic, immunophenotypic, and clinical parameters.

**Methods**

A retrospective archive review was conducted on 61 de novo AML patients followed between January 1 2017, and April 1 2025, at the Department of Hematology, SANKO University Practice and Research Hospital. Fibrosis was graded using the EUMNET (2005) classification. Hemogram, biochemistry, flow cytometry, and genetic analyses were evaluated alongside treatment response and survival data. Statistical analyses included Kaplan-Meier, Log-Rank, and multivariate logistic regression tests (SPSS 25.0; $p<0.05$).

**Results**

The mean age was 55.5±17.2 years; 63.9% were male. Fibrosis grade 0-1 was present in 57.4% and grade 2-3 in 42.6% of patients. Mortality was observed in 55.7%. BMF did not show a statistically significant effect on overall survival (OS) ($p=0.411$). However, CD34 positivity, t(15;17), intensive chemotherapy, and induction response significantly influenced survival. In multivariate analysis, CD34 expression (OR=3.808, $p=0.041$) and intensive chemotherapy (OR=0.118, $p=0.011$) were independent predictors of mortality. Additionally, elevated urea, albumin level, and direct bilirubin were associated with survival outcomes.

***Table 1. Comparison of mean survival times by selected prognostic factors and treatment status***

**Copyright Information:** (Online) ISSN: 2572-9241
©2026 The Author(s). HemaSphere is published by John Wiley & Sons Ltd on behalf of European Hematology Association. This is an open access Abstract Book distributed under the Attribution-NonCommercial-NoDerivs (CC BY-NC-ND), which allows third parties to download the articles and share them with others as long as they credit the author and the Abstract Book, but they cannot change the content in any way or use them commercially.
**Abstract Book Citations:** Authors, Title, HemaSphere, 2026;10:(S1):pages. The abstract book DOIs can be found on the "Author Information" page.
**Disclaimer:** Articles published in the journal HemaSphere exclusively reflect the opinions of the authors. The authors are responsible for all content in their abstracts including accuracy of the facts, statements, citing resources, etc.

| Factor / Group | Mean (mo) | SE | 95% CI Lower | 95% CI Upper | p | 1-yr OS % | 3-yr OS % | 5-yr OS % |
|---|---|---|---|---|---|---|---|---|
| **Overall Survival** | | | | | | | | |
| **All patients** | 70.5 | 9.8 | 51.2 | 89.8 | - | 67.1 | 49.8 | 38.9 |
| **CD34 Expression** | | | | | | | | |
| Negative | 108.8 | 14.9 | 79.7 | 137.9 | 0.004** | 80.8 | 66.2 | 66.2 |
| Positive | 27.7 | 4.9 | 18.1 | 37.2 | - | 56.8 | 38.4 | 20.9 |
| **t(15;17) Translocation** | | | | | | | | |
| Absent | 32.4 | 4.6 | 23.4 | 41.4 | 0.012* | 61.8 | 45.5 | 29.4 |
| Present | 131.3 | 18.1 | 95.8 | 166.8 | - | 90.9 | 80.8 | 80.8 |
| **Bone Marrow Fibrosis** | | | | | | | | |
| Absent (Grade 0-1) | 74.2 | 12.8 | 49.0 | 99.4 | 0.411 | 74.3 | 55.4 | 40.4 |
| Present (Grade 2-3) | 48.1 | 10.4 | 27.6 | 68.5 | - | 57.2 | 47.7 | 37.1 |
| **Intensive Chemotherapy (IT)** | | | | | | | | |
| No | 14.6 | 4.9 | 4.9 | 24.2 | <0.001** | 31.6 | 10.5 | 10.5 |
| Yes | 92.6 | 12.1 | 68.9 | 116.3 | - | 83.2 | 71.9 | 52.3 |
| **Induction Response (Rm)** | | | | | | | | |
| No response (Rm=0) | 23.4 | 5.5 | 12.6 | 34.2 | <0.001** | 60.0 | 32.7 | 16.4 |
| Response (Rm=1) | 112.7 | 12.9 | 87.4 | 138.0 | - | 90.4 | 79.2 | 65.5 |
| Not assessable (Rm=2) | 2.2 | 0.6 | 1.01 | 3.3 | - | - | - | - |

**p<0.05, **p<0.01. Kaplan-Meier Log-Rank test. OS: Overall Survival; SE: Standard Error; CI: Confidence Interval; IT: Intensive Therapy; Rm: Remission.*

***Table 2. Multivariate logistic regression analysis of factors affecting mortality***

| Factor | p | OR | 95% CI Lower | 95% CI Upper |
|---|---|---|---|---|
| **CD34 Expression** | | | | |
| Negative (ref) | 0.041* | 3.808 | 1.057 | 13.712 |
| Positive | - | - | - | - |
| **t(15;17) Translocation** | | | | |
| Absent (ref) | 0.175 | 0.260 | 0.037 | 1.818 |
| Present | - | - | - | - |
| **Intensive Chemotherapy (IT)** | | | | |
| No (ref) | 0.011* | 0.118 | 0.023 | 0.613 |
| Yes | - | - | - | - |

### Summary/Conclusion

BMF at diagnosis did not reach statistical significance as a prognostic factor in this single-center cohort. The well-established prognostic factors — CD34 expression, t(15;17), intensive chemotherapy eligibility, and induction response — were confirmed. Additionally, urea, creatinine, and direct bilirubin demonstrated significant associations with survival, findings not widely reported in the literature. Further multicenter studies with larger samples are needed to clarify the role of BMF in AML prognosis.

**Copyright Information:** (Online) ISSN: 2572-9241
©2026 The Author(s). HemaSphere is published by John Wiley & Sons Ltd on behalf of European Hematology Association. This is an open access Abstract Book distributed under the Attribution-NonCommercial-NoDerivs (CC BY-NC-ND), which allows third parties to download the articles and share them with others as long as they credit the author and the Abstract Book, but they cannot change the content in any way or use them commercially.
**Abstract Book Citations:** Authors, Title, HemaSphere, 2026;10:(S1):pages. The abstract book DOIs can be found on the "Author Information" page.
**Disclaimer:** Articles published in the journal HemaSphere exclusively reflect the opinions of the authors. The authors are responsible for all content in their abstracts including accuracy of the facts, statements, citing resources, etc.

### (PB2833) NEXT GENERATION SEQUENCING, CLASSICAL CYTOGENETICS AND MOLECULAR TECHNIQUES AS COMPLEMENTARY TOOLS IN THE CHARACTERIZATION OF ACUTE MYELOID LEUKEMIA (AML): A REAL-WORLD ANALYSIS IN LATIN AMERICA

**Topic:** 04. Acute myeloid leukemia - Clinical

Cecilia Lang*[1], Andrea Bender[1], Nehuen Gasparini[1], Maria Bender[1], Federico Torreguitart[1], Pablo Pombo[1], María Paula Iommi[1], Encina Tania Mayra[1], María Florencia Sanchez[1], Antonela Castiglia[1], MacArena Giuliani[1], Julieta Miranda Zanetti[1], Marisa Sandoval[1, 2], Evangelina Agriello[1, 2]

*[1]LABORATORIO LEB, CYTOGENETICS, BAHIA BLANCA, Argentina; [2]universidad nacional del sur, catedra de hematologia, bahia blanca, Argentina;*

### Background

**Background**: genetic profiling is essencial for the diagnosis, prognosis, and treatment of myeloid neoplasms, mostly for AML. Next-generation sequencing (NGS) is a massively parallel sequencing technology which has overcome the limitations of previous techniques, enabling the detection of CNVs, indels, and gene fusions. Chromosome banding analysis (CBA), FISH, classical molecular biology techniques (CMBT), and NGS are complementary approaches; understanding their strengths and limitations will allow their optimal use for genetic characterization in AML.

### Aims

**Aims:** 1) To characterize the genetic profile of a cohort of AML patients and compare them with previously reported. 2) To demonstrate how CBA, FISH, CMBT and NGS complement each other, and to devise the most appropriate strategy for the accurate genetic characterization of patients with AML.

### Methods

**Methods:** this analysis includes AML patients studied from January 2019 up today. All patients underwent NGS to identify sequence variants and fusion genes using DNA and RNA samples, respectively. CBA was used to study copy number abnormalities (CNAs) and SVs (balance and unbalanced rearrangements); FISH, RT-PCR, PCR by fragment analysis, and Sanger sequencing were used to evaluate specific alterations. NGS was also used to evaluate copy number variations (CNVs) when available.

### Results

**Results:** this cohort includes 205 AML patients (M:F=1.2:1), 181 adult (52 yrs (42.0–63.0)), and 24 children (11.5 yrs (1.0-15.2)). Combined results defined genetic subtype (WHO and ICC 2022) in 180/205 (88%) pts and risk categorization (ELN 2022) in 174/205 (85%) pts: 57 (32.8%) favorable, 50 (28.7%) intermediate and 67 (38.5%) high risk. Pathogenic variants (PV) were found by NGS in 186/205 (91%) (25 suspected germline origin, confirmed in 3 cases (GATA1, DDX41 and TP53)). The most frequent variants: FLT3 (23%), NPM1 (22%), DNMT3A (16%), NRAS (14%) and ASXL1 (13%). TP53 variants were found in 22 (11%) ptes, isolated in 56% of them. Fusion genes were detected by NGS but not CBA in 18/170 pts (3 complex karyotype, 2 cryptic abnormalities, and 5 KMT2A-PTD), however 8 were confirmed by either FISH or PCR. Eleven translocations seen by CBA had no counterpart gene fusion by NGS, probably because not clinically relevant. CNVs were evaluable in 149 patients, with positive results observed in 67 of them. Relevant numerical abnormalities seen by CBA were also detected as CNVs by NGS in 100% of the cases: -7, -5/del5q, -17/del17p, del20q; excepting +8 in one single case. Some rare and non-canonical variants were seen by NGS but not detected by PCR-Sanger sequencing: FLT3-TKD, NPM1, TP53.

**Copyright Information:** (Online) ISSN: 2572-9241
©2026 The Author(s). HemaSphere is published by John Wiley & Sons Ltd on behalf of European Hematology Association. This is an open access Abstract Book distributed under the Attribution-NonCommercial-NoDerivs (CC BY-NC-ND), which allows third parties to download the articles and share them with others as long as they credit the author and the Abstract Book, but they cannot change the content in any way or use them commercially.
**Abstract Book Citations:** Authors, Title, HemaSphere, 2026;10:(S1):pages. The abstract book DOIs can be found on the “Author Information” page.
**Disclaimer:** Articles published in the journal HemaSphere exclusively reflect the opinions of the authors. The authors are responsible for all content in their abstracts including accuracy of the facts, statements, citing resources, etc.

**Summary/Conclusion**

**Conclusions:** The genetic profile and frequency of alterations in this analysis are similar to other series of AML patients. The high concordance between NGS and CBA results allowed us to confidently rely on CNVs detection. Besides, NGS allowed the discovery of genetic abnormalities not detected, or that could not even be evaluated, by other classical techniques. The combination of CBA, PCR-Sanger analysis to rapidly detect FLT3 mutations and NGS allows the most accurate genetic characterization and risk stratification of patients with AML, optimizing the use of samples and reducing the time to results for therapeutic decision-making.

**Copyright Information:** (Online) ISSN: 2572-9241
©2026 The Author(s). HemaSphere is published by John Wiley & Sons Ltd on behalf of European Hematology Association. This is an open access Abstract Book distributed under the Attribution-NonCommercial-NoDerivs (CC BY-NC-ND), which allows third parties to download the articles and share them with others as long as they credit the author and the Abstract Book, but they cannot change the content in any way or use them commercially.
**Abstract Book Citations:** Authors, Title, HemaSphere, 2026;10:(S1):pages. The abstract book DOIs can be found on the “Author Information” page.
**Disclaimer:** Articles published in the journal HemaSphere exclusively reflect the opinions of the authors. The authors are responsible for all content in their abstracts including accuracy of the facts, statements, citing resources, etc.

### (PB2834) RETROSPECTIVE ANALYSIS OF SURVIVAL OUTCOME OF AUTOLOGOUS SCT IN ACUTE MYELOID LEUKAEMIA PATIENTS IN CR1 VERSUS AML IN CR1 WITHOUT AUTOSCT ACROSS ALL RISK GROUPS, A SINGLE CENTRE EXPERIENCE.

**Topic:** 04. Acute myeloid leukemia - Clinical

Adilah Zaki Morad*[1], Veena Selvaratnam[1], Tee Chuan Ong[1]

[1]*HOSPITAL AMPANG, HAEMATOLOGY DEPARTMENT, AMPANG, Malaysia;*

### Background

Acute Myeloid Leukaemia is a heterogenous condition with multiple different biological entities and mutations that carry their own risks of relapse and prognosis. For patients with Acute Myeloid Leukaemia (especially Intermediate Risk AML), who do not have a HLA Matched donor for Allogeneic HSCT, the role of Autologous Stem Cell Transplant as a post remission consolidative treatment remains unclear especially in the intermediate and unfavourable risk groups.

### Aims

This retrospective analysis intends to look at the progression free survival and overall survival of Acute Myeloid Leukaemia patients who were subjected to Autologous Stem Cell Transplant as a consolidative and curative measure for their disease in a local setting as compared to those who did not undergo the stem cell transplant process and establishing if there is survival advantage between the former and the latter cohorts.

### Methods

This is a retrospective analysis of patients diagnosed with AML across all risks as outlined in the ELN 2022. This analysis is performed on patients treated by the Department of Haematology at Hospital Ampang from 2000-2023, who were subjected to Autologous Stem Cell transplant post intensive chemotherapy as a curative measure for their AML. The comparison group is patients treated only with intensive chemotherapy from year 2017-2021 at Hospital Ampang, also through a retrospective analysis approach.

Analysis is performed on of their progression free survival and overall survival between these 2 cohorts. Non statistical analysis is also performed on the survival and causes of death between these two groups based on their ELN 2022 risk classification.

**Copyright Information:** (Online) ISSN: 2572-9241
©2026 The Author(s). HemaSphere is published by John Wiley & Sons Ltd on behalf of European Hematology Association. This is an open access Abstract Book distributed under the Attribution-NonCommercial-NoDerivs (CC BY-NC-ND), which allows third parties to download the articles and share them with others as long as they credit the author and the Abstract Book, but they cannot change the content in any way or use them commercially.
**Abstract Book Citations:** Authors, Title, HemaSphere, 2026;10:(S1):pages. The abstract book DOIs can be found on the "Author Information" page.
**Disclaimer:** Articles published in the journal HemaSphere exclusively reflect the opinions of the authors. The authors are responsible for all content in their abstracts including accuracy of the facts, statements, citing resources, etc.

**Results**

169 patients were analysed. 61 from the Auto-SCT group and 108 from the Non-AutoSCT (chemotherapy only). The primary outcomes are progression free survival , defined as time to disease relapse or death from the date of diagnosis and overall survival, is defined as the time from diagnosis to death from any cause or last follow-up. There was almost equal distribution of male and females between both groups.
Within the AutoSCT cohort, 30 patients died ( 49%) within 2 years from diagnosis whilst 31 survived (51%) beyond that period of time. Within the Non-AutoSCT group, 50 patients died (46%) within 2 years from diagnosis and 58 patients were alive (54%) beyond 2 years from diagnosis.

As for progression free survival, the AutoSCT group did slightly better than their Non AutoASCT cohort, 63% were event free versus 54% at 2 years. In terms of overall survival, the Auto-SCT seems to do a little worse, albeit at different time frames when compared to Non-AutoSCT cohort. The Auto-SCT group showing a steep initial decline in survival, with the median survival time recorded at 6.16 months (95% CI: 1.07 – not available), indicating that half of the transplanted patients died within approximately six months of diagnosis. In contrast, the Non-Auto SCT group experienced a more gradual decline initially, followed by a more notable drop, with a longer median survival time of 25.50 months (95% CI: 21.10 – 28.66).

**Summary/Conclusion**

There is progression free survival advantage in AML patients who undergo AutoSCT when compared to those who did not undergo the transplant. However, there is no statistically significant overall survival advantage of AutoSCT for AML in CR1 compared to those who only received Intensive Chemotherapy (Non-AutoSCT) in CR1, when all risk groups of AML were compared.

**Copyright Information:** (Online) ISSN: 2572-9241
©2026 The Author(s). HemaSphere is published by John Wiley & Sons Ltd on behalf of European Hematology Association. This is an open access Abstract Book distributed under the Attribution-NonCommercial-NoDerivs (CC BY-NC-ND), which allows third parties to download the articles and share them with others as long as they credit the author and the Abstract Book, but they cannot change the content in any way or use them commercially.
**Abstract Book Citations:** Authors, Title, HemaSphere, 2026;10:(S1):pages. The abstract book DOIs can be found on the “Author Information” page.
**Disclaimer:** Articles published in the journal HemaSphere exclusively reflect the opinions of the authors. The authors are responsible for all content in their abstracts including accuracy of the facts, statements, citing resources, etc.

### (PB2835) REGULATORY DIVERGENCE IN R/R AML: CONTRASTING FDA AND EMA APPROACHES TO REAL-WORLD EVIDENCE AND TRIAL DESIGN

**Topic:** 04. Acute myeloid leukemia - Clinical

Sinan Bardakci Sarac*[1], Mike D'ambrosio[2]

*[1]Parexel, Gentofte, Denmark; [2]Parexel, London, United Kingdom;*

### Background

Global development of therapies for relapsed/refractory acute myeloid leukaemia (R/R AML) increasingly requires alignment with both FDA and EMA expectations. However, significant divergence remains in the acceptance and application of real-world evidence (RWE) and single-arm trial designs, impacting regulatory strategy and patient access.

### Aims

To analyse the operational and regulatory implications of differing FDA and EMA standards for RWE and trial design in R/R AML, and to propose strategies for sponsors navigating these divergent requirements.

### Methods

We reviewed regulatory precedents, focusing on how each agency evaluates RWE, single-arm data, and comparator selection. The analysis included consequences for trial feasibility, data interpretation, and regulatory timelines.

### Results

The FDA has demonstrated increasing flexibility in accepting RWE and single-arm studies, particularly in rare and high-need oncology indications, often allowing RWE as supportive evidence. The FDA has repeatedly acknowledged that randomized trials are often infeasible in late-line AML due to small patient populations, rapid clinical deterioration, and ethical concerns. This is reflected across multiple reviews, e.g., gilteritinib, ivosidenib, enasidenib, etc. FDA's practical stance is that it tends to accept single-arm trials in R/R AML, when response rates are high, duration of response is meaningful, safety is manageable and RWE contextualises expected outcomes.
In contrast, the EMA maintains a more conservative stance, emphasizing prospective planning, robust comparators, and methodological rigor. Applications relying on retrospective endpoints or lacking control arms face greater scrutiny in Europe, with higher demands for data quality and pre-specified analyses. Across haematologic malignancies, including AML, EMA has been more conservative than FDA in accepting responses rates as surrogacy for efficacy, uncontrolled study designs and approving therapies based on single-arm data. This has contributed to delayed approvals, requests for confirmatory randomised trials and more restrictive labels.

**Copyright Information:** (Online) ISSN: 2572-9241
©2026 The Author(s). HemaSphere is published by John Wiley & Sons Ltd on behalf of European Hematology Association. This is an open access Abstract Book distributed under the Attribution-NonCommercial-NoDerivs (CC BY-NC-ND), which allows third parties to download the articles and share them with others as long as they credit the author and the Abstract Book, but they cannot change the content in any way or use them commercially.
**Abstract Book Citations:** Authors, Title, HemaSphere, 2026;10:(S1):pages. The abstract book DOIs can be found on the "Author Information" page.
**Disclaimer:** Articles published in the journal HemaSphere exclusively reflect the opinions of the authors. The authors are responsible for all content in their abstracts including accuracy of the facts, statements, citing resources, etc.

**Summary/Conclusion**

The divergence between FDA and EMA expectations for real-world evidence and trial design creates scientific, operational, and strategic challenges for global development in relapsed/refractory AML. While the FDA has increasingly adopted a pragmatic approach, recognizing feasibility constraints, ethical considerations, and the urgent unmet need in late-line AML, the EMA continues to emphasize methodological rigor, prospective planning, and controlled evidence generation. These differing evidentiary thresholds affect regulatory outcomes, trial feasibility, timelines, and ultimately patient access.
For sponsors, these discrepancies require early, proactive, and iterative engagement with both agencies to define viable global pathways. High-quality, fit-for-purpose real-world evidence, planned prospectively, methodologically transparent, and aligned with regulatory expectations, can help contextualize single-arm efficacy results and address feasibility barriers to randomized trials. Looking forward, greater regulatory convergence around RWE, surrogate endpoints, and flexible trial designs would meaningfully support innovation in high-need areas like R/R AML. Harmonized standards would streamline global programs, reduce duplicative evidence requirements, and accelerate patient access.

**Copyright Information:** (Online) ISSN: 2572-9241
©2026 The Author(s). HemaSphere is published by John Wiley & Sons Ltd on behalf of European Hematology Association. This is an open access Abstract Book distributed under the Attribution-NonCommercial-NoDerivs (CC BY-NC-ND), which allows third parties to download the articles and share them with others as long as they credit the author and the Abstract Book, but they cannot change the content in any way or use them commercially.
**Abstract Book Citations:** Authors, Title, HemaSphere, 2026;10:(S1):pages. The abstract book DOIs can be found on the “Author Information” page.
**Disclaimer:** Articles published in the journal HemaSphere exclusively reflect the opinions of the authors. The authors are responsible for all content in their abstracts including accuracy of the facts, statements, citing resources, etc.

### (PB2836) AZA-VEN-GILTERITINIB COMBINATION AS SALVAGE THERAPY IN RELAPSED AND REFRACTORY FLT3-ITD MUTANT AML: SINGLE-CENTRE REAL-WORLD DATA

**Topic:** 04. Acute myeloid leukemia - Clinical

Denis Cetin*[1], Ajda Gunes[1], Nur Soyer[1], Fatma Keklik Karadag[1], Fahri Sahin[1], Mahmut Töbü[1], Filiz Vural[1], Derya Demir[2], Nazan Özsan[2], Mine Hekimgil[2, 2, 2], Mehmet Burak Durmaz[3], Emin Karaca[3], Haluk Akın[3], Alp Peker[3], Ufuk Demirci[4], Güray Saydam[1]

*[1]Ege University Hospital, Hematology, Izmir, Türkiye; [2]Ege University Hospital, Pathology, Izmir, Türkiye; [3]Ege University Hospital, Genetics, Izmir, Türkiye; [4]Celal Bayar University Medical Faculty, Hematology, Manisa, Türkiye;*

### Background

FLT3 mutations (ITD and TKD) are among the most frequently observed genetic abnormalities in acute myeloid leukemia (AML) and are associated with a high risk of relapse and a poor prognosis. Although the combination of azacitidine and venetoclax (AZA+VEN) is currently the standard first-line treatment for AML patients ineligible for intensive chemotherapy, the presence of FLT3 mutations constitutes a primary mechanism of resistance to this regimen. In relapsed/refractory (R/R) FLT3-mutant AML, the standard of care is monotherapy with the potent oral FLT3 inhibitor gilteritinib, which demonstrated a survival advantage over salvage chemotherapy (median 9.3 months vs. 5.6 months) in the Phase 3 ADMIRAL trial. However, responses achieved with gilteritinib monotherapy are generally short-lived; the rates of durable and deep remissions (measurable residual disease [MRD] negativity) are low, and the majority of patients ultimately progress. Current clinical trials evaluating the efficacy of the triplet combination in R/R FLT3-mutant AML have shown that the regimen yields high molecular response rates and is highly successful in bridging patients to allogeneic hematopoietic stem cell transplantation (allo-HSCT).

### Aims

In this report, the characteristics of relapsed/refractory four AML patients who received the azacitidine-venetoclax-gilteritinib combination at our institution are presented.

### Methods

A retrospective review of patient records from 2024 to 2026 was conducted and the findings were reported.

### Results

The median age at diagnosis was 67.6 years (range, 48–72), and all patients harbored an *FLT3*-ITD mutation. Two patients were *FLT3*-ITD positive at the time of diagnosis, whereas *FLT3*-ITD positivity emerged at relapse in the remaining two patients. Three patients had received three prior lines of therapy, while one patient had received two, resulting in a median of three prior lines of therapy. As first-line treatment, two patients had received intensive chemotherapy combined with midostaurin, and the other two had received azacitidine monotherapy. Three of the four patients were refractory to their first-line treatment, whereas one patient had achieved a complete remission with partial hematologic recovery (CRh). Upon response evaluation following the triplet combination therapy, one patient achieved CRh, two patients achieved complete remission with incomplete hematologic recovery (CRi), and one patient achieved complete remission (CR). All patients derived clinical benefit from the triplet regimen. One patient was successfully bridged to allogeneic hematopoietic stem cell transplantation (allo-HSCT) owing to this response; however, the patient succumbed to sepsis secondary to opportunistic infections during the second week post-transplant. The longest post-remission survival was observed as 14.4 months, and this patient is currently alive. The median overall survival was calculated to be 2.4 months.

**Copyright Information:** (Online) ISSN: 2572-9241
©2026 The Author(s). HemaSphere is published by John Wiley & Sons Ltd on behalf of European Hematology Association. This is an open access Abstract Book distributed under the Attribution-NonCommercial-NoDerivs (CC BY-NC-ND), which allows third parties to download the articles and share them with others as long as they credit the author and the Abstract Book, but they cannot change the content in any way or use them commercially.
**Abstract Book Citations:** Authors, Title, HemaSphere, 2026;10:(S1):pages. The abstract book DOIs can be found on the "Author Information" page.
**Disclaimer:** Articles published in the journal HemaSphere exclusively reflect the opinions of the authors. The authors are responsible for all content in their abstracts including accuracy of the facts, statements, citing resources, etc.

**Summary/Conclusion**

Although the azacitidine-venetoclax-gilteritinib combination is associated with highly favorable remission rates, it can induce prolonged, severe cytopenias and predispose patients to opportunistic infections. In heavily pretreated patients, this regimen can serve as an effective bridge to transplantation for eligible candidates, while offering the potential for long-term remission in transplant-ineligible individuals.

**Copyright Information:** (Online) ISSN: 2572-9241
©2026 The Author(s). HemaSphere is published by John Wiley & Sons Ltd on behalf of European Hematology Association. This is an open access Abstract Book distributed under the Attribution-NonCommercial-NoDerivs (CC BY-NC-ND), which allows third parties to download the articles and share them with others as long as they credit the author and the Abstract Book, but they cannot change the content in any way or use them commercially.
**Abstract Book Citations:** Authors, Title, HemaSphere, 2026;10:(S1):pages. The abstract book DOIs can be found on the "Author Information" page.
**Disclaimer:** Articles published in the journal HemaSphere exclusively reflect the opinions of the authors. The authors are responsible for all content in their abstracts including accuracy of the facts, statements, citing resources, etc.

### (PB2837) SEASONAL DISTRIBUTION OF ACUTE PROMYELOCYTIC LEUKAEMIA IN TURKİYE

**Topic:** 04. Acute myeloid leukemia - Clinical

Ali Turunç*[1], Yusuf Kemal Arslan[1], Müzeyyen Aslaner Ak[2], Sinem Ayhan[3], Tuba Ersal[4], Aslı Kara[5], Esra Pirinççi[6], İlhami Kiki[5], Tuğba Karaçamlı[7], Ünal Ataş[7], Fatma Temiz[8], Metban Mastanzade[9], Yazgulu Cansu Ozkan[10], Zehra Narlı Özdemir[10], Işıl Erdoğan Özünal[11], Erman Öztürk[11], Melda Cömert[12], Fehmi Hindilerden[13], Emine Gültürk[13], Eren Arslan Davulcu[13], Esin Oguz Kozan[14], Meral Ilgaz[15], Gülcan Kökcü[16], Berkin Anıl Çiçin[17], Hakkı Onur Kırkızlar[17], Esra Terzi Demirsoy[18], Osman Can Öztürk[19], Kübra Yel Uygun[20], Abdulkadir Basturk[20], Sinan Demircioglu[21], Mesut Ayer[22], Nurhilal Buyukkurt[23], Gülşah Polat Akyol[24], Şerife Emre Ünsal[24], Rafiye Çiftçiler[25], Hilal Nur Yaldız[25], Filiz Yavaşoğlu[26], Ramazan Esen[27], Huseyin Saffet Bekoz[28], Ebru Pekgüç[29], Osman Kara[30], Mine Miskioğlu[31], Işık Atagündüz[8], Meltem Kurt Yüksel[16], Yahya Büyükaşık[15], İnci Alacacıoğlu[19], Selami Toprak[16], Mehmet Yilmaz[32], Emin Kaya[33], Eyüp Naci Tiftik[14], Mehmet Orhan Ayyildiz[6], Mehmet Sönmez[3], Emel Gürkan[1], Funda Tanrıkulu[1]

*[1]Çukurova University, Faculty of Medicine, Adana, Türkiye;[2]Zonguldak Bülent Ecevit University, Faculty of Medicine, Hematology Department, Zonguldak, Türkiye; [3]Karadeniz Technical University, Faculty of Medicine, Hematology Department, Trabzon, Türkiye; [4]Bursa Uludağ University, Faculty of Medicine, Bursa, Türkiye;[5]Atatürk University, Faculty of Medicine, Erzurum, Türkiye; [6]Dicle University, Faculty of Medicine, Diyarbakır, Türkiye;[7]Akdeniz University, Faculty of Medicine, Antalya, Türkiye;[8]Marmara University, Faculty of Medicine, İstanbul, Türkiye; [9]Istanbul University Istanbul Faculty of Medicine, İstanbul, Türkiye;[10]İzmir City Hospital, İzmir, Türkiye; [11]Göztepe Prof. Dr. Süleyman Yalçın City Hospital, İstanbul, Türkiye; [12]Gülhane Training and Research Hospital, Ankara, Türkiye; [13]Bakırkoy Dr. Sadi Konuk Training and Research Hospital, İstanbul, Türkiye;[14]Mersin University, Faculty of Medicine, Mersin, Türkiye; [15]Hacettepe University, Faculty of Medicine, Ankara, Türkiye; [16]Ankara University, Faculty of Medicine, Ankara, Türkiye; [17]Trakya University, Faculty of Medicine, Edirne, Türkiye;[18]Kocaeli University, Faculty of Medicine, Kocaeli, Türkiye; [19]Dokuz Eylül University, Faculty of Medicine, İzmir, Türkiye;[20]Konya City Hospital, Konya, Türkiye;[21]Necmettin Erbakan University, Faculty of Medicine, Konya, Türkiye; [22]Başakşehir Çam ve Sakura City Hospital, İstanbul, Türkiye;[23]Adana City Training and Research Hospital, Adana, Türkiye; [24]Erciyes University, Faculty of Medicine, Kayseri, Türkiye;[25]Selçuk University, Faculty of Medicine, Konya, Türkiye; [26]Eskişehir Osmangazi University, Faculty of Medicine, Eskişehir, Türkiye;[27]Van Yüzüncü Yıl University, Faculty of Medicine, Van, Türkiye; [28]Medipol University Hospital, İstanbul, Türkiye;[29]Koç University, Faculty of Medicine, İstanbul, Türkiye; [30]Medicana Ataşehir Hospital, İstanbul, Türkiye;[31]Manisa Celal Bayar University, Faculty of Medicine, Manisa, Türkiye; [32]Sanko University, Faculty of Medicine, Gaziantep, Türkiye;[33]İnönü University, Faculty of Medicine, Malatya, Türkiye;*

### Background

Acute promyelocytic leukaemia (APL) is a haematological malignancy characterized by t(15;17)(q24.1;q21.2); PML::RARA fusion and is now recognized as a distinct entity from acute myeloid leukaemia due to its unique genetic and therapeutic profile [1,2]. Several studies have investigated whether APL incidence shows seasonal variation, with some suggesting possible links to viral infections, sunlight exposure and vitamin D levels [3,4]. A study from Iraq reported peak incidence in March [5], while a single-centre Turkish study suggested winter-autumn predominance possibly related to vitamin D deficiency [9]. However, no significant seasonal pattern was found in Saudi Arabia [6], Ireland [7] and other studies [8]. The evidence remains conflicting.

### Aims

To evaluate seasonal and monthly distribution of APL diagnosis across Turkey using multicentre data selected according to NUTS-1 regional classification system, and to assess whether geographical representation effects the findings.

**Copyright Information:** (Online) ISSN: 2572-9241
©2026 The Author(s). HemaSphere is published by John Wiley & Sons Ltd on behalf of European Hematology Association. This is an open access Abstract Book distributed under the Attribution-NonCommercial-NoDerivs (CC BY-NC-ND), which allows third parties to download the articles and share them with others as long as they credit the author and the Abstract Book, but they cannot change the content in any way or use them commercially.
**Abstract Book Citations:** Authors, Title, HemaSphere, 2026;10:(S1):pages. The abstract book DOIs can be found on the "Author Information" page.
**Disclaimer:** Articles published in the journal HemaSphere exclusively reflect the opinions of the authors. The authors are responsible for all content in their abstracts including accuracy of the facts, statements, citing resources, etc.

### Methods

This was a multicentre retrospective epidemiological study including adult patients (≥18 years) diagnosed with APL between 2010 and 2025. Centres were selected to ensure representation from all 12 NUTS-1 regions of Turkey. Age, gender, diagnosis date and centre informations were collected. Categorical variables were expressed as counts and percentages; age was reported as mean ± SD, median and interquartile range. Seasonal and monthly distributions were compared using the one-sample chi-square goodness-of-fit test, with $p<0.05$ considered statistically significant.

### Results

A total of 1,022 APL cases from all 12 NUTS-1 regions were analysed. Of these, 524 (51.3%) were male and 498 (48.7%) female (M/F: 1.05). Mean age at diagnosis was 44.33 ± 15.35 years (median 43; IQR 32–56; range 18–91). The seasonal distribution was: winter 263 (25.7%), spring 273 (26.7%), summer 237 (23.2%) and autumn 249 (24.4%). No statistically significant difference was found across seasons (p=0.404) or months (p=0.320).

### Summary/Conclusion

This study represents the largest multicentre APL series in Turkey at the NUTS-1 level, encompassing 1,022 patients across all 12 regions over 15 years. Our findings demonstrate no significant seasonal or monthly peak in APL diagnosis. The seasonal increase reported in single-centre studies from Iraq [5] and Turkey [9] may be attributable to limited sample sizes or regional climatic differences. Turkey encompasses diverse climate zones from Mediterranean to continental, yet no seasonal pattern emerged. When geriatric patients (≥65 years) were analysed seperately, no seasonal difference was observed either (p=0.451), suggesting that age does not modify this relationship. With 1,022 cases, our study had sufficient power to detect a clinically meaningful seasonal difference if one truly existed. These results suggest that the seasonality hypothesis may not be generalizable across different geographies and populations.

### References

1. Arber DA, et al. Blood. 2022;140(11):1200-1228.
2. Khoury JD, et al. Leukemia. 2022;36(7):1703-1719.
3. Vickers M, et al. Leukemia. 2000;14(4):722-6.
4. Kashanian SM, et al. EJHaem. 2021;2(1):33-39.
5. Mohammed AS, et al. Iraqi J Hematol. 2020;9(2):113-117.
6. Al-Mashdali AF, et al. Int J Blood Res Disord. 2022;9:087.
7. O'Connell N, et al. EXCLI J. 2022;21:1042-1052.
8. Salooja N, et al. Br J Haematol. 2005;131(1):90-94.
9. Cömert M, et al. Clin Lymphoma Myeloma Leuk. 2021;21(Suppl 2):S272.

**Copyright Information:** (Online) ISSN: 2572-9241
©2026 The Author(s). HemaSphere is published by John Wiley & Sons Ltd on behalf of European Hematology Association. This is an open access Abstract Book distributed under the Attribution-NonCommercial-NoDerivs (CC BY-NC-ND), which allows third parties to download the articles and share them with others as long as they credit the author and the Abstract Book, but they cannot change the content in any way or use them commercially.
**Abstract Book Citations:** Authors, Title, HemaSphere, 2026;10:(S1):pages. The abstract book DOIs can be found on the "Author Information" page.
**Disclaimer:** Articles published in the journal HemaSphere exclusively reflect the opinions of the authors. The authors are responsible for all content in their abstracts including accuracy of the facts, statements, citing resources, etc.

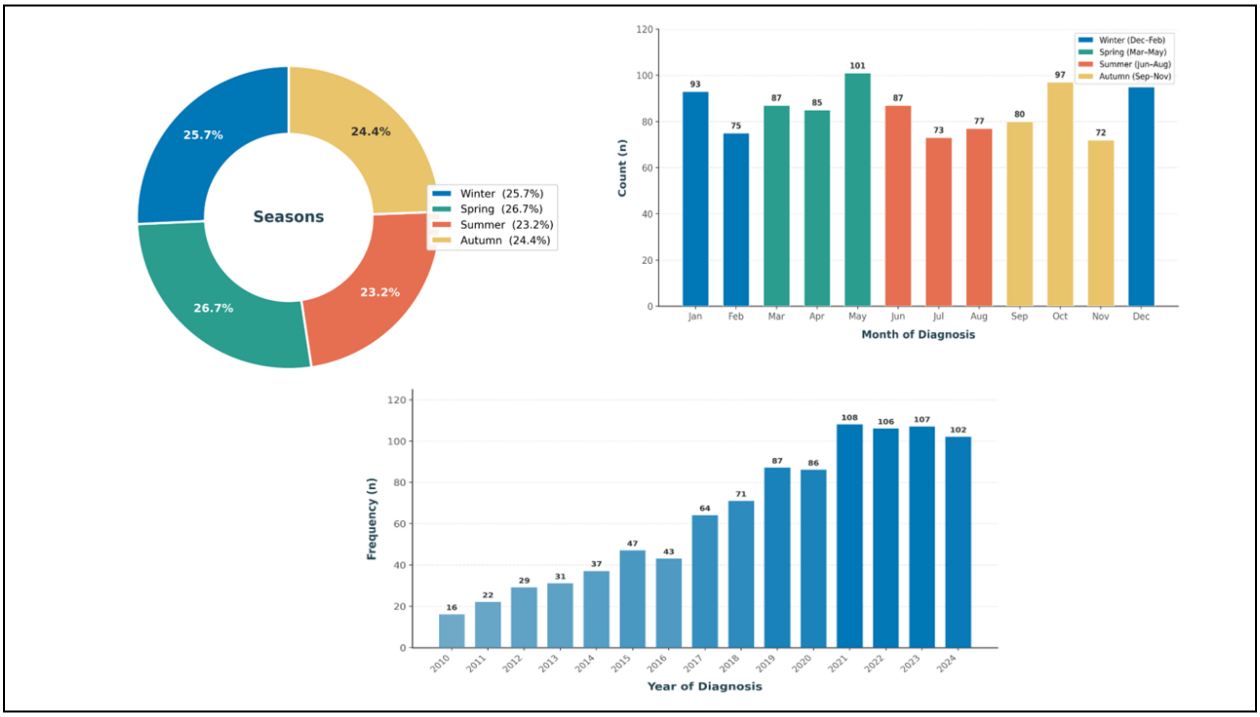

Figure 1. Distribution of 1,022 APL cases diagnosed between 2010 and 2025 across Turkey. Top-left: Seasonal distribution of APL diagnoses; no statistically significant difference was observed between seasons (p=0.404). Top-right: Monthly distribution of APL diagnoses, color-coded by season; no significant monthly variation was detected (p=0.320). Bottom: Annual frequency of APL cases by year of diagnosis.

**Copyright Information:** (Online) ISSN: 2572-9241
©2026 The Author(s). HemaSphere is published by John Wiley & Sons Ltd on behalf of European Hematology Association. This is an open access Abstract Book distributed under the Attribution-NonCommercial-NoDerivs (CC BY-NC-ND), which allows third parties to download the articles and share them with others as long as they credit the author and the Abstract Book, but they cannot change the content in any way or use them commercially.
**Abstract Book Citations:** Authors, Title, HemaSphere, 2026;10:(S1):pages. The abstract book DOIs can be found on the "Author Information" page.
**Disclaimer:** Articles published in the journal HemaSphere exclusively reflect the opinions of the authors. The authors are responsible for all content in their abstracts including accuracy of the facts, statements, citing resources, etc.

### (PB2838) TRIPLE THERAPY WITH AZACITIDINE, VENETOCLAX AND GILTERITINIB AS A THERAPEUTIC OPTION FOR ADULTS PATIENTS WITH REFRACTORY/RELAPSED FLT3 MUTATED ACUTE MYELOID LEUKEMIA – A SINGLE CENTER EXPERIENCE

**Topic:** 04. Acute myeloid leukemia - Clinical

Bianca Constantin*[1, 2], Dana Frangu[1], Andreea Andrunache[1], Isabela Roxana Hîrjan[1], Mihaela Cîrstea[1, 2], Bogdan Ionescu[1], Maria Camelia Stancioaica[1, 2], Alexandra Elena Ghiaur[1], Aurelia Tatic[1, 2], Daniel Coriu[1, 2]

*[1]Fundeni Clinical Institute, Hematology, Bucharest, Romania;[2]Carol Davila University of Medicine and Pharmacy, Bucharest, Romania;*

### Background

*FLT3 mutations occur in approximately 30% of acute myeloid leukemia (AML) cases and are associated with high relapse rates and poor prognosis. While second-generation inhibitors like gilteritinib have improved outcomes in the relapsed/refractory (R/R) setting, therapeutic resistance remains a significant challenge. This study evaluates the real-world experience of a single center using an off-label triplet regimen (Azacitidine, Venetoclax and Gilteritinib) as a bridge to allogeneic hematopoietic stem cell transplantation (allo-HSCT).*

### Aims

*Given the lack of published national data, there is a clear need to document and evaluate the real-world experience of centers administering this triplet therapy off-label, particularly in Romania, in order to inform and optimize future therapeutic strategies in FLT3-mutated AML.*

### Methods

*We conducted a retrospective, observational study between March 2022 and September 2025. Out of 78 identified FLT3-positive AML patients, 8 patients with R/R disease received the triplet therapy following partial response or disease progression on gilteritinib monotherapy.*

### Results

*Within the subgroup who experienced disease progression under gilteritinib (n = 4 of 8), all patients achieved complete remission (CR) after receiving triple therapy and successfully underwent allo-HSCT. Regarding the safety profile, 63% of patients developed grade 3–4 hematologic toxicities (febrile neutropenia, anemia, thrombocytopenia), while hepatic cytolysis remained mild (grade 1–2), although it was observed in all patients (n = 8). At a median follow-up of 9 months, 75% of patients who underwent allo-HSCT post-triplet therapy remained alive.We conducted a retrospective, observational study between March 2022 and September 2025. Out of 78 identified FLT3-positive AML patients, 8 patients with R/R disease received the triplet therapy following partial response or disease progression on gilteritinib monotherapy.*

### Summary/Conclusion

*Our findings suggest that the triplet combination of azacitidine, venetoclax and gilteritinib is a highly effective salvage strategy. Although careful monitoring is required due to moderate toxicities, particularly hematologic adverse events, it is a viable bridge to transplant for R/R FLT3-mutated AML patients who fail standard monotherapy.*

**Copyright Information:** (Online) ISSN: 2572-9241
©2026 The Author(s). HemaSphere is published by John Wiley & Sons Ltd on behalf of European Hematology Association. This is an open access Abstract Book distributed under the Attribution-NonCommercial-NoDerivs (CC BY-NC-ND), which allows third parties to download the articles and share them with others as long as they credit the author and the Abstract Book, but they cannot change the content in any way or use them commercially.
**Abstract Book Citations:** Authors, Title, HemaSphere, 2026;10:(S1):pages. The abstract book DOIs can be found on the "Author Information" page.
**Disclaimer:** Articles published in the journal HemaSphere exclusively reflect the opinions of the authors. The authors are responsible for all content in their abstracts including accuracy of the facts, statements, citing resources, etc.

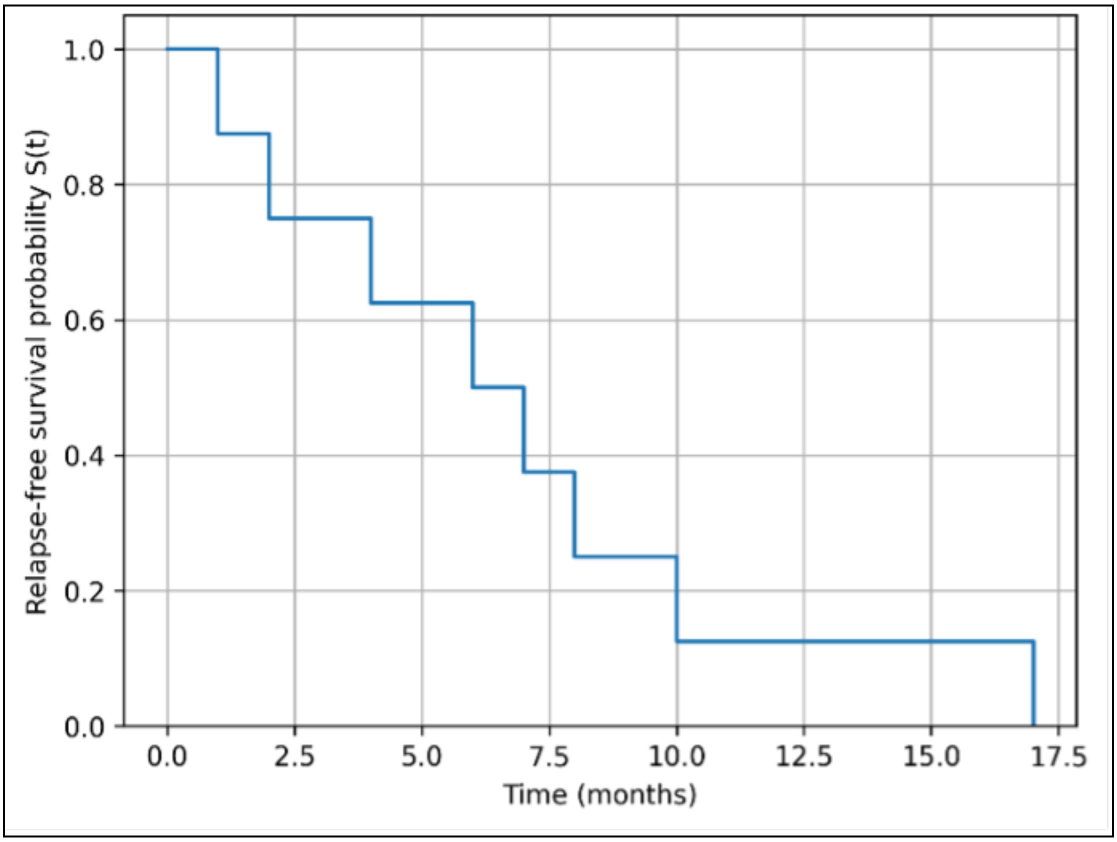

Figure 1. Kaplan–Meier curve for relapse-free survival for first remission

**Copyright Information:** (Online) ISSN: 2572-9241
©2026 The Author(s). HemaSphere is published by John Wiley & Sons Ltd on behalf of European Hematology Association. This is an open access Abstract Book distributed under the Attribution-NonCommercial-NoDerivs (CC BY-NC-ND), which allows third parties to download the articles and share them with others as long as they credit the author and the Abstract Book, but they cannot change the content in any way or use them commercially.
**Abstract Book Citations:** Authors, Title, HemaSphere, 2026;10:(S1):pages. The abstract book DOIs can be found on the "Author Information" page.
**Disclaimer:** Articles published in the journal HemaSphere exclusively reflect the opinions of the authors. The authors are responsible for all content in their abstracts including accuracy of the facts, statements, citing resources, etc.

### (PB2839) ALLOGENEIC HEMATOPOIETIC STEM CELL TRANSPLANTATION OUTCOMES IN SECONDARY ACUTE MYELOID LEUKEMIA: A SINGLE-CENTER RETROSPECTIVE ANALYSIS

**Topic:** 04. Acute myeloid leukemia - Clinical

Melis Bektaş*[1], Serhat Çelik[1], Güldane Cengiz Seval[1], Selami Kocak Toprak[1], Pervin Topcuoglu[1], Meltem Kurt Yuksel[1]

[1]*Ankara Universiy School of Medicine, Department of Hematology, ANKARA, Türkiye;*

### Background

Secondary acute myeloid leukemia (s-AML) is a group of diseases that due to pre-existing myeloid neoplasia or cytotoxic treatments, characterized by poor prognosis compared to de novo AML (1,2). Allogeneic hematopoietic stem cell transplantation (allo-HSCT) is the only treatment option with curative potential for this patient group; however, relapse and non-relapse mortality (NRM) significantly limit survival (2,3).
WHO 2022 and ICC classifications have defined s-AML as a distinct group by highlighting myelodysplasia-related genetic changes (4,5). Especially complex cytogenetic and high-risk ELN categories negatively affect post-transplant survival (1,2).

### Aims

This study aims to evaluate the clinical and cytogenetic factors affecting survival in patients with secondary AML diagnosed at a single center are eligible for allo-HSCT.

### Methods

Fifty-seven adult patients diagnosed with secondary AML at our center between 2015- 2025 were retrospectively analyzed. Allo-HSCT was applied to 53 patients (93%). Their diagnoses were confirmed the ELN 2022 and WHO 2022 criteria. Clinical, cytogenetic, molecular, and transplant-related variables have been recorded.
Overall survival (OS) is defined as the time from the date of transplantation to death or the last follow-up. $p<0.05$ was statistically significant.

### Results

A total of 57 s-AML patients with an allo-HSCT were included. 93% of these underwent allo-HSCT. The median age is 58.4 years (23–74), with 36 patients (63%) male and 21 patients (37%) are female. In 78.9% of patients, there is a precursor disease associated with myelodysplasia. 21.1% are in the t-AML.
Complex karyotype was detected in 10.5%. TP53 mutation rate was 5.3%. In the NGS, the most observed mutations were ASXL1 (12.3%), U2AF1 (10.5%), and IDH2 (8.8%).
When examining the transplant regimens were MAC 52.6%, RIC 40.4%.
The median OS was found to be 17.2 ± 2.35 months (95% CI 12.59–21.81). In patients with complex karyotypes, the median OS was 6 months, without this was 17.3 months (p=0.04). The median OS was found to be 19.3 months in male patients and 12.7 months in female (p=0.011).
In the univariate analysis, age (HR 1.039, p=0.007), male gender (HR 0.437, p=0.014), and complex karyotype (HR 2.68, p=0.049) were found to be significant. The causes of death, infection is the most common cause (28.1%). The mortality rate is 64.9%. No significant difference in survival was found between t-AML and myelodysplasia related s-AML (p=0.312).

**Copyright Information:** (Online) ISSN: 2572-9241
©2026 The Author(s). HemaSphere is published by John Wiley & Sons Ltd on behalf of European Hematology Association. This is an open access Abstract Book distributed under the Attribution-NonCommercial-NoDerivs (CC BY-NC-ND), which allows third parties to download the articles and share them with others as long as they credit the author and the Abstract Book, but they cannot change the content in any way or use them commercially.
**Abstract Book Citations:** Authors, Title, HemaSphere, 2026;10:(S1):pages. The abstract book DOIs can be found on the "Author Information" page.
**Disclaimer:** Articles published in the journal HemaSphere exclusively reflect the opinions of the authors. The authors are responsible for all content in their abstracts including accuracy of the facts, statements, citing resources, etc.

**Summary/Conclusion**

Our study confirms s-AML remains a high-risk even after allo-HSCT. The median OS of 17.2 months is consistent with the reported outcomes of allo-HSCT for s-AML (2). Complex cytogenetics has been identified as the strongest disease-related prognostic factor (1,2).
It is important that age remains an independent prognostic factor. Each year of age increased the risk of mortality by approximately 3%.
In our study, the relationship between male gender has longer survival is differing from the literature. Most of the solid tumor-related cases (breast cancer) being predominantly seen in female patients may have contributed to a higher risk distribution. No OS difference was found between t-AML and other s-AML subgroups (p=0.312). While allo-HSCT increases survival in s-AML. Prospective randomized multicenter studies on secondary AML are needed.

1. Döhner H Blood. 2022.
2. Núñez-Torrón Stock C Blood. 2019
3. Metheny L J Clin Oncol. 2020
4. WHO Classification of Haematolymphoid Tumors 2022
5. Arber DA Blood 2022.

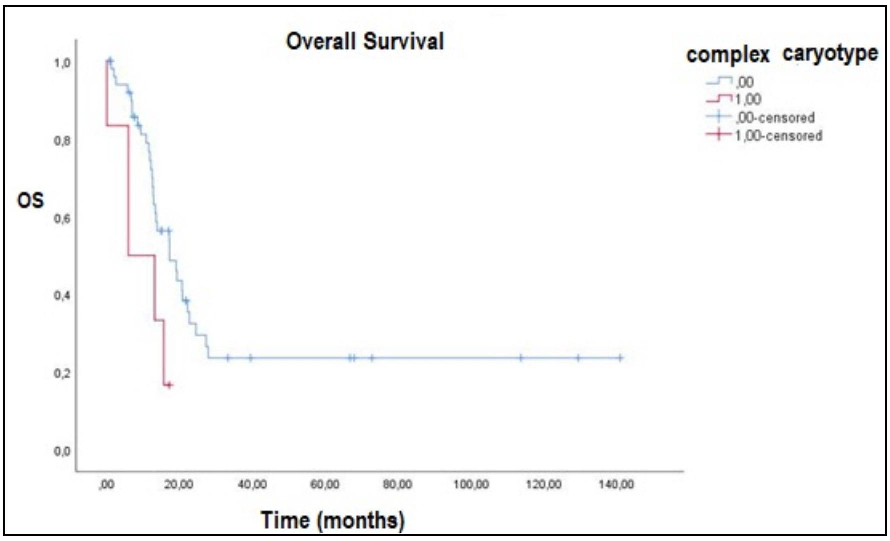

The median survival time for secondary AML patients with complex karyotype was 6±5.3 months (0.1-16.48), compared to 17.3±3.2 months (11.03-23.57) in those without complex karyotype, p=0.04 (log-rank).

**Copyright Information:** (Online) ISSN: 2572-9241
©2026 The Author(s). HemaSphere is published by John Wiley & Sons Ltd on behalf of European Hematology Association. This is an open access Abstract Book distributed under the Attribution-NonCommercial-NoDerivs (CC BY-NC-ND), which allows third parties to download the articles and share them with others as long as they credit the author and the Abstract Book, but they cannot change the content in any way or use them commercially.
**Abstract Book Citations:** Authors, Title, HemaSphere, 2026;10:(S1):pages. The abstract book DOIs can be found on the "Author Information" page.
**Disclaimer:** Articles published in the journal HemaSphere exclusively reflect the opinions of the authors. The authors are responsible for all content in their abstracts including accuracy of the facts, statements, citing resources, etc.

### (PB2840) COMPLETE REMISSION AND IMPROVED SURVIVAL OF PATIENTS WITH NUP214 FUSION GENES TREATED WITH VENETOCLAX-BASED CHEMOTHERAPY

**Topic:** 04. Acute myeloid leukemia - Clinical

Liya MA*[1], Shanshan Suo[1], Wei Lang[1], Hongyan Tong[1]

[1] *The First Affiliated Hospital, Zhejiang University School of Medicine, Hangzhou, China;*

### Background

NUP214 plays a key role in protein and mRNA export between the nucleus and cytoplasm. In acute leukemia, recurrent chromosomal translocations at the NUP214 locus generate fusion proteins that link its C-terminal domain to either SET or DEK, resulting in SET::NUP214 and DEK::NUP214.

### Aims

Patients with DEK:: NUP214 and SET:: NUP214 fusion show poor outcomes with conventional chemotherapy, prompting the need for novel therapeutic approaches.

### Methods

We depicted the clinical and genetic characteristics of 10 cases including 6 with DEK::NUP214 fusion and 4 with SET::NUP214 fusion. We  also analysed the clinical and laboratory data, survial of the patients.

### Results

All cases except one (n=9) received induction therapy with venetoclax based chemotherapy, all evaluable patients (100%) achieved CR or CRi and MRD-negativity by flow cytometry. Among seven evaluable patients reexamined DEK::NUP214 fusion, five (71.4%) achieved MRD-negativity and five patients with abnormal karyotypes achieved a complete cytogenetic response. Six patients (60%) underwent allo-HSCT in CR1 and 3 patients (30%) were scheduled to proceed allo-HSCT. The 3-year LFS of the cohort was 73%, and the 3-year OS was 88%.

### Summary/Conclusion

Most patients with NUP214 fusion achieved morphologic complete remission, MRD-negativity, molecular and cytogenetic remission, and a long-term survival via venetoclax based chemotherapy and allo-HSCT, making it a promising treatment strategy for these diseases.

**Copyright Information:** (Online) ISSN: 2572-9241
©2026 The Author(s). HemaSphere is published by John Wiley & Sons Ltd on behalf of European Hematology Association. This is an open access Abstract Book distributed under the Attribution-NonCommercial-NoDerivs (CC BY-NC-ND), which allows third parties to download the articles and share them with others as long as they credit the author and the Abstract Book, but they cannot change the content in any way or use them commercially.
**Abstract Book Citations:** Authors, Title, HemaSphere, 2026;10:(S1):pages. The abstract book DOIs can be found on the "Author Information" page.
**Disclaimer:** Articles published in the journal HemaSphere exclusively reflect the opinions of the authors. The authors are responsible for all content in their abstracts including accuracy of the facts, statements, citing resources, etc.

### (PB2841) THE OUTCOME OF A SUBSIDIZED COMPREHENSIVE FINANCIAL PACKAGE FOR THE TREATMENT OF AML IN THE ABSENCE OF UNIVERSAL HEALTH CARE IN THE LOW AND MIDDLE INCOME SETTING

**Topic:** 04. Acute myeloid leukemia - Clinical

Anne Mwirigi*[1], Josephine Mbithe[1], Laura Kirui[1], Rohini Radia[1], Riyat Malkit[2]

*[1]AGA KHAN UNIVERSITY HOSPITAL NAIROBI, Nairobi, Kenya; [2]AGA KHAN UNIVERSITY HOSPITAL NAIROBI, Haematology, Nairobi, Kenya;*

### Background

Induction chemotherapy with daunorubicin and cytarabine (DA) for acute myeloid leukaemia (AML) is expensive and difficult to deliver outside of dedicated units and universal healthcare financing (UHF) and is inaccessible to patients in low and middle-income countries.
A financially subsidised AML DA Induction Package paired with multidisciplinary capacity building of nursing staff, anti-microbial stewardship team and blood bank was introduced at Aga Khan University Hospital, Nairobi (AKUH,N) in June 2023, in response to the number of new AML diagnoses.

In the preceding 5 years, only 13% diagnosed with AML at AKUH,N had sought treatment at AKUH,N, and less than 20% of all patients diagnosed received induction chemotherapy.

### Aims

We describe the impact of the AML package on the access to treatment, uptake of genetic and molecular testing, management of neutropenic infection and overall patient outcome.

### Methods

We included all patients who were treated for AML at AKUH,N since the AML package was first introduced in June 2023 until December 2025.
We recorded demographic data, cytogenetic and molecular results, neutropenic fever outcomes, remission rates and overall patient outcome

**Copyright Information:** (Online) ISSN: 2572-9241
©2026 The Author(s). HemaSphere is published by John Wiley & Sons Ltd on behalf of European Hematology Association. This is an open access Abstract Book distributed under the Attribution-NonCommercial-NoDerivs (CC BY-NC-ND), which allows third parties to download the articles and share them with others as long as they credit the author and the Abstract Book, but they cannot change the content in any way or use them commercially.
**Abstract Book Citations:** Authors, Title, HemaSphere, 2026;10:(S1):pages. The abstract book DOIs can be found on the “Author Information” page.
**Disclaimer:** Articles published in the journal HemaSphere exclusively reflect the opinions of the authors. The authors are responsible for all content in their abstracts including accuracy of the facts, statements, citing resources, etc.

**Results**

60 patients (16y-88y; 29 women) received treatment for AML at AKUH,N between June 2023 and December 2025, a 2.7 fold increase on the composite for the preceding 3 years.

Majority had de novo AML; 6 transformed MPN and 2 therapy-related.
Cytogenetics and molecular markers were available for 25/60 (41.6%): 8 favourable,14 unfavourable, 3 intermediate risk (32%, 56% and 12% respectively).
*DA*:
28/60 were eligible for DA chemotherapy (median age 50y (16y-64y)).
All patients had febrile neutropenia. Majority posted at least 1 positive blood culture; multi-drug resistant bacteria were cultured in approximately 30%.
There were 2 deaths due to neutropenic sepsis with *Escherichia Coli in induction* and the third, due to hyperleukocytosis in CML in blast crisis, within 36h of admission.
12/28 patients (42%) achieved DA-remission, an additional 6 achieving remission with salvage strategies (FLAG-Ida [1]; Aza-Ven [2]; allogeneic HSCT [3])
All patients with poor risk profile were either refractory to DA or experienced early relapse.
13/18 are still alive today (46.4% of all DA patients); 5 died after disease relapse and one with grade IV GVHD.
Two additional patients have recently relapsed and are undergoing salvage therapy.
*Aza-Ven:*
32 patients were ineligible for DA and received aza-ven (median 66y (22y-94y).
The 9 patients younger than 60y were ineligible for DA because of: severe infection in 4 patients, therapy-related AML, predicted DA failure in TP53 AML, CML in blast crisis and previous MDS post-transplant with no allogeneic HSCT prospects.
11 achieved a complete remission (34%), 4 relapsed and one died
10 patients are still alive, as are four other patients who achieved a partial response (14/32 of all aza-ven patients; 43.75%).

**Summary/Conclusion**

A financially subsidised AML package can safely deliver AML induction in LMIC in the absence of UHF and result in increased uptake of risk stratification.

Majority of patients tested were poor risk, which might account for the lower remission induction rates for DA when compared to the standard.
The rate of neutropenic sepsis death, 2/28 (7%) demonstrates the effect of implementation of robust training and team working.
The rate of remission in the aza-ven group was lower than that in the DA group but survival rate was matched, for the limited time period reported.

**Copyright Information:** (Online) ISSN: 2572-9241
©2026 The Author(s). HemaSphere is published by John Wiley & Sons Ltd on behalf of European Hematology Association. This is an open access Abstract Book distributed under the Attribution-NonCommercial-NoDerivs (CC BY-NC-ND), which allows third parties to download the articles and share them with others as long as they credit the author and the Abstract Book, but they cannot change the content in any way or use them commercially.
**Abstract Book Citations:** Authors, Title, HemaSphere, 2026;10:(S1):pages. The abstract book DOIs can be found on the “Author Information” page.
**Disclaimer:** Articles published in the journal HemaSphere exclusively reflect the opinions of the authors. The authors are responsible for all content in their abstracts including accuracy of the facts, statements, citing resources, etc.

**OVERVIEW** Combined 2023–2025 · n=60 patients

| | DA (n= 28) | | AzaVEN (n= 3) |
|---|---|---|---|
| Total Patients | 28 | vs | 32 |
| Alive | 13 (46.4%) | vs | 14 (43.7%) |
| Deceased | 15 (52%) | vs | 18 (58%) |
| Survival Rate | 46.4% | vs | 43.7% |

**Copyright Information:** (Online) ISSN: 2572-9241
©2026 The Author(s). HemaSphere is published by John Wiley & Sons Ltd on behalf of European Hematology Association. This is an open access Abstract Book distributed under the Attribution-NonCommercial-NoDerivs (CC BY-NC-ND), which allows third parties to download the articles and share them with others as long as they credit the author and the Abstract Book, but they cannot change the content in any way or use them commercially.
**Abstract Book Citations:** Authors, Title, HemaSphere, 2026;10:(S1):pages. The abstract book DOIs can be found on the "Author Information" page.
**Disclaimer:** Articles published in the journal HemaSphere exclusively reflect the opinions of the authors. The authors are responsible for all content in their abstracts including accuracy of the facts, statements, citing resources, etc.

### (PB2842) FACTORS ASSOCIATED WITH EARLY MORTALITY DURING INTENSIVE INDUCTION CHEMOTHERAPY IN YOUNGER ADULTS WITH ACUTE MYELOID LEUKEMIA

**Topic:** 04. Acute myeloid leukemia - Clinical

Chihaoui N*[1], Kharrat R[1], Selmi A[1], Rabeb Berred[1], Ben Neji H[1], Bchir M[1], Achour M[1], Cherni R[1], Ben Lakhel F[2], Feki S[2], Ben Ayed W[3], Gouider E[2], Ben Lakhel R[1]

*[1]Aziza othemena hospital, adult clinical hematology, Tunis, Tunisia; [2]Aziza othemena hospital, Laboratory of Hematological Biology, Tunis, Tunisia; [3]Pasteur Institute of Tunis, Department of Histology and Medical Cytogenetics, Tunis, Tunisia;*

### Background

Early mortality (EM) continues to be a major challenge in younger patients with acute myeloid leukemia (AML), despite improvements in therapy and risk stratification. Reported rates range from 5% to 15% and are even higher in resource-limited countries, emphasizing the necessity of identifying factors associated with early death.

### Aims

We aim to identify factors associated with early death in order to improve survival.

### Methods

This retrospective study included patients aged between 18–60 years diagnosed with AML and followed in the adult clinical Hematology Department at Aziza Othmana Hospital between January 2013 and December 2022. Early death during induction was defined as the time to death from the start of induction until day 30. predictive factors was analyzed using Cox proportional hazards regression.

### Results

We collected data on 296 patients with a median age of 43 years and a male-to-female ratio of 1.13. Cytogenetic risk classification was dominated by intermediate risk (63%), adverse risk (21%) and favorable risk (16%). Eighty-eight percent of the patients received an intensive induction with 7+3 regimen.

In this cohort, Early mortality reached 20% (one in five patients). In univariate analysis, age ≥45 (HR 1.86, 95% CI 1.11–3.12, p=0.018), hyperleukocytosis with leucocyte count ≥ 100.000 (HR 1.82, 95% CI 1.01–3.26, p=0.045), and the presence of tumor lysis syndrome (TLS) (HR 3.67, 95% CI 1.67–8.08, p=0.001), were significantly associated with early mortality with TLS as the strongest predictor of EM. However, in multivariate analysis, its effect was attenuated after adjustment and only age remained an independant predictor (HR 2.12, 95% CI 1.11–4.11, p=0.023).

### Summary/Conclusion

Our results of EM remains alarmingly high in a young cohort. Early identifying and prevention of TLS may represent a solution to reduce induction related-mortality.

**Copyright Information:** (Online) ISSN: 2572-9241
©2026 The Author(s). HemaSphere is published by John Wiley & Sons Ltd on behalf of European Hematology Association. This is an open access Abstract Book distributed under the Attribution-NonCommercial-NoDerivs (CC BY-NC-ND), which allows third parties to download the articles and share them with others as long as they credit the author and the Abstract Book, but they cannot change the content in any way or use them commercially.
**Abstract Book Citations:** Authors, Title, HemaSphere, 2026;10:(S1):pages. The abstract book DOIs can be found on the "Author Information" page.
**Disclaimer:** Articles published in the journal HemaSphere exclusively reflect the opinions of the authors. The authors are responsible for all content in their abstracts including accuracy of the facts, statements, citing resources, etc.

### (PB2843) FRONTLINE AZACITIDINE–VENETOCLAX IN NEWLY DIAGNOSED ACUTE MYELOID LEUKEMIA: REAL-WORLD DATA FROM A SINGLE TERTIARY CARE CENTRE IN INDIA

**Topic:** 04. Acute myeloid leukemia - Clinical

Chaitanya H Balakrishnan*[1, 1], Sanjukta S Rao[1], Veronica Lobo[1], Muhammad Mohsin[2], Michael Pais[1], Nisarga Mj[1], Cecil Ross[1], Seetharam Anandram[1]

*[1]St. John's Medical College, Clinical Hematology, Bangalore, India;[2]Barnsley Hospital NHS Foundation Trust, Clinical Hematology, Barnsley, United Kingdom;*

### Background

Azacitidine–venetoclax (AZA–VEN) is the standard frontline therapy for newly diagnosed acute myeloid leukemia (AML) in older or unfit patients. However, real-world outcome data stratified by the ICC 2022 genomic classification remains limited, particularly in AML cohorts with adverse risk and MDS-related biology. We evaluated frontline AZA–VEN therapy in a consecutive AML cohort, with emphasis on induction outcomes, consolidation/maintenance strategies, and long-term outcomes.

### Methods

We conducted a retrospective single-center study of 66 consecutive adults with newly diagnosed AML treated upfront with AZA–VEN. Patients were classified using the ICC 2022 hierarchical risk framework. Induction therapy consisted of three cycles, followed by assessment of measurable residual disease (MRD) using multiparameter flow cytometry. Primary endpoints were complete remission (CR) or complete remission with incomplete count recovery (CRi) rates and MRD negativity at the end of induction. Secondary endpoints included induction mortality, infectious and bleeding complications, allogeneic hematopoietic stem cell transplantation (HSCT), relapse-free survival (RFS), overall survival (OS), and maintenance therapy.

### Results

The median age was 63 years (range 19–84). The cohort had 18 patients with favorable risk(27%), 11 with intermediate risk(17%) and 37 patients with adverse risk(56%) AML. Induction mortality was 12.1% (8/66), primary non-response or progressive disease occurred in 13.6% (9/66) and 3 patients were lost to follow-up. Neutropenic sepsis occurred in 18.2%(12/66), and major bleeding in 7.6%(5/66) of the cohort.

80.4% of the patients who underwent assessment at the end of induction(37/46) had CR/CRI while the intention-to-treat (ITT) CR/CRi rate was 56.1% (37/66). The median time to CR/CRi was 63 days. MRD negativity was achieved in 56.8% (21/37) of these patients.

Among adverse-risk AML (37/66), CR/CRi was 51% (ITT, 19/37), with 53%(10/19) of responders achieving MRD negativity. Of the patients with MDS-related AML as per ICC (34/66), patients with MDS-related cytogenetic abnormalities (17/34) demonstrated higher induction mortality (29% versus 17.6%), greater neutropenic sepsis burden (41% versus 29%), and inferior MRD negativity(17.6% versus 41%) compared with MDS-related gene mutation associated AML.

The median OS for the ITT cohort was 16.6 months, with 12-month OS of 56%. Adverse-risk AML demonstrated inferior OS (median 6.4 months), Intermediate-risk AML had a median OS of 11 months while Favorable-risk AML did not reach the median OS.

Among those who achieved CR/CRi (37/66), relapse occurred in 24%(8/37) on long term follow-up. Six patients underwent HSCT, and three additional patients are planned for transplant. A durable remission was observed, with 10 patients remaining on maintenance after 12 months and 5 after 24 months

**Copyright Information:** (Online) ISSN: 2572-9241
©2026 The Author(s). HemaSphere is published by John Wiley & Sons Ltd on behalf of European Hematology Association. This is an open access Abstract Book distributed under the Attribution-NonCommercial-NoDerivs (CC BY-NC-ND), which allows third parties to download the articles and share them with others as long as they credit the author and the Abstract Book, but they cannot change the content in any way or use them commercially.
**Abstract Book Citations:** Authors, Title, HemaSphere, 2026;10:(S1):pages. The abstract book DOIs can be found on the "Author Information" page.
**Disclaimer:** Articles published in the journal HemaSphere exclusively reflect the opinions of the authors. The authors are responsible for all content in their abstracts including accuracy of the facts, statements, citing resources, etc.

**Summary/Conclusion**

In this real-world AML cohort with adverse-risk and MDS-related biology, frontline AZA–VEN achieved meaningful remission rates and substantial MRD-negative responses. However, induction mortality and infection-related morbidity remain, particularly in MDS-related cytogenetic AML.
A subset of patients demonstrated durable disease control beyond two years, and transplant bridging was feasible in selected high-risk cases. These findings underscore both the therapeutic efficacy of AZA–VEN across genomic risk groups and the continued impact of infectious and biological vulnerability in our population group.

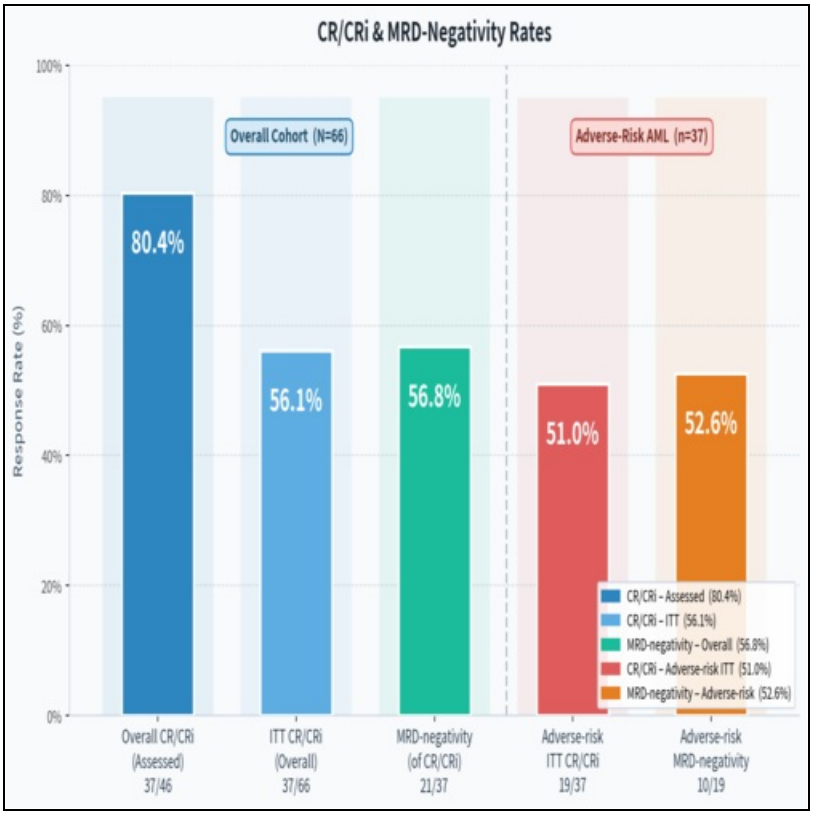

**Copyright Information:** (Online) ISSN: 2572-9241
©2026 The Author(s). HemaSphere is published by John Wiley & Sons Ltd on behalf of European Hematology Association. This is an open access Abstract Book distributed under the Attribution-NonCommercial-NoDerivs (CC BY-NC-ND), which allows third parties to download the articles and share them with others as long as they credit the author and the Abstract Book, but they cannot change the content in any way or use them commercially.
**Abstract Book Citations:** Authors, Title, HemaSphere, 2026;10:(S1):pages. The abstract book DOIs can be found on the "Author Information" page.
**Disclaimer:** Articles published in the journal HemaSphere exclusively reflect the opinions of the authors. The authors are responsible for all content in their abstracts including accuracy of the facts, statements, citing resources, etc.

### (PB2844) REAL-WORLD OUTCOMES OF HYPOMETHYLATING AGENTS AND VENETOCLAX IN ACUTE MYELOID LEUKEMIA: A MONOCENTRIC EXPERIENCE.

**Topic:** 04. Acute myeloid leukemia - Clinical

Martina Quattrone[1], Antonio Giordano[1], Marianna Criscuolo[1], Alessia Di Pilla[1], Sara Caterina Brunetti[1], Patrizia Chiusolo[1], Silvia Bellesi[1], Pagano Livio[1], Luana Fianchi*[1]

*[1]Fondazione Policlinico Universitario A. Gemelli IRCCS, Dipartimento di Scienze di Laboratorio ed Ematologiche, Roma, Italy;*

### Background

The combination of Hypomethylating Agents (HMAs) and Venetoclax has proven to be a valid therapeutic strategy for unfit patients with Acute Myeloid Leukemia (AML) who are not eligible for intensive chemotherapy. Despite the encouraging outcomes reported in clinical trials, real-world data are essential to better define effectiveness and safety in heterogeneous and complex patients.

### Aims

To evaluate the clinical outcomes of HMAs in combination with Venetoclax in a cohort of patients aged >60 years with AML

### Methods

We retrospectively reviewed the medical records of 58 patients treated with HMAs in combination with Venetoclax at our institution. We collected data on patients' clinical characteristics and AML-related features, antifungal prophylaxis, infectious events and response to treatment. Univariate and multivariate analyses were performed to identify clinical and laboratory variables significantly associated with treatment response.

### Results

Male to female ratio was 0.62, with a median age of 71.5 years (range 61-82). According to the ELN 2022 classification, 42 of 58 pts (72%) were classified as intermediate risk, 15 (26%) as adverse risk and only 1 as a favorable risk. Thirty-four pts (59%) were treated in the frontline setting, while 24 at relapse (R/R). Overall, 41 pts (71%) were treated with Azacitidine and 17 with Decitabine. Most pts (49/58, 84%) received antifungal prophylaxis (48/49 with posaconazole). No patient received antibacterial prophylaxis. Infectious events occurred in 25 patients (43%), mainly of bacterial origin, with only 2 fungal infections.
The overall complete response (CR) rate was 50% (18/35 pts in the first-line setting and 11/23 in the R/R group), while 6/58 (10%) had a partial response and 20 (34%) were resistant to treatment. Twelve patients (21%) underwent allogeneic hematopoietic stem cell transplantation (alloHCT) (3 as a first line and 9 in R/R). The median overall survival (OS) of the population was 10.4 months (7.7-28.4). Among pts treated at diagnosis, median OS was 9.1 months, compared with 7.4 months in R/R pts.
Disease-free survival (DFS) was 12.3 months in the frontline setting and 9 months in R/R pts. Overall, 40/58 pts (69%) experienced relapse and 34 (59%) died during follow-up.

On univariate analysis, achievement of CR after induction was the only variable significantly associated with both OS and DFS in both frontline and relapsed pts, whereas antifungal prophylaxis did not significantly impact treatment response. In multivariate analysis, among pts treated at diagnosis, both achievement of CR and subsequent alloHCT were independently associated with improved OS and DFS. In the R/R setting, achievement of CR remained significantly associated with improved OS, and treatment with decitabine (compared with azacitidine) was also associated with superior OS. Although alloHCT was associated with a trend toward improved OS, this difference did not reach statistical significance (p=0.08). Notably, pts treated with decitabine were more likely to undergo alloHCT (p=0.04).

**Copyright Information:** (Online) ISSN: 2572-9241
©2026 The Author(s). HemaSphere is published by John Wiley & Sons Ltd on behalf of European Hematology Association. This is an open access Abstract Book distributed under the Attribution-NonCommercial-NoDerivs (CC BY-NC-ND), which allows third parties to download the articles and share them with others as long as they credit the author and the Abstract Book, but they cannot change the content in any way or use them commercially.
**Abstract Book Citations:** Authors, Title, HemaSphere, 2026;10:(S1):pages. The abstract book DOIs can be found on the "Author Information" page.
**Disclaimer:** Articles published in the journal HemaSphere exclusively reflect the opinions of the authors. The authors are responsible for all content in their abstracts including accuracy of the facts, statements, citing resources, etc.

### Summary/Conclusion

We report a real-world experience with HMAs in combination with venetoclax in a heterogeneous cohort of AML pts. Achievement of CR and subsequent alloHCT emerged as the main factors associated with prolonged OS. In contrast, antifungal prophylaxis did not significantly impact treatment response, despite the administration of reduced-dose venetoclax.

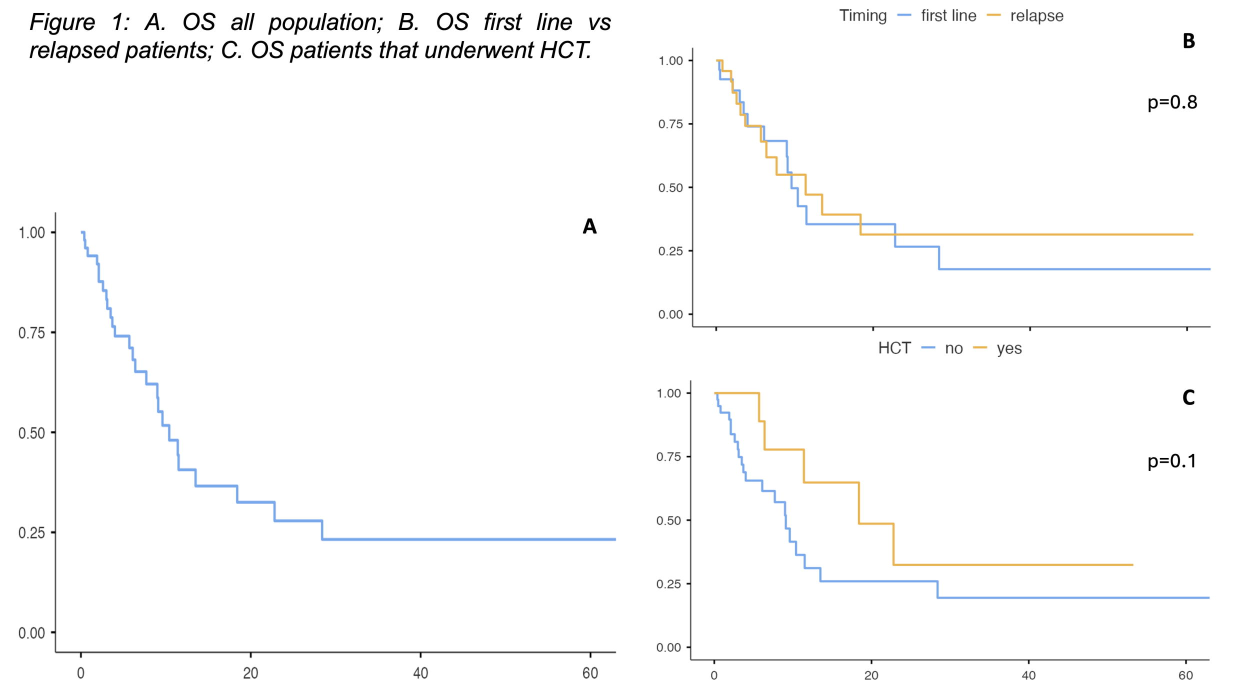

*Figure 1: A. OS all population; B. OS first line vs relapsed patients; C. OS patients that underwent HCT.*

**Copyright Information:** (Online) ISSN: 2572-9241
©2026 The Author(s). HemaSphere is published by John Wiley & Sons Ltd on behalf of European Hematology Association. This is an open access Abstract Book distributed under the Attribution-NonCommercial-NoDerivs (CC BY-NC-ND), which allows third parties to download the articles and share them with others as long as they credit the author and the Abstract Book, but they cannot change the content in any way or use them commercially.
**Abstract Book Citations:** Authors, Title, HemaSphere, 2026;10:(S1):pages. The abstract book DOIs can be found on the "Author Information" page.
**Disclaimer:** Articles published in the journal HemaSphere exclusively reflect the opinions of the authors. The authors are responsible for all content in their abstracts including accuracy of the facts, statements, citing resources, etc.

### (PB2845) CLINICAL IMPACT OF NEXT-GENERATION SEQUENCING ON RE-CLASSIFICATION AND POST-REMISSION TREATMENT STRATEGY IN ACUTE MYELOID LEUKEMIA – A SINGLE CENTER STUDY

**Topic:** 04. Acute myeloid leukemia - Clinical

Mirjana Cucakovic*[1], Marija Dencic Fekete[2], Jovan Rajic[1], Nikica Sabljic[1], Zlatko Pravdic[1, 3], Lazar Trajković[1], Nikola Pantic[1], Nevena Bešević[1], Andrej Pešić[1], Violeta Milosevic[1], Marijana Virijevic[1, 3], Mirjana Mitrovic[1, 3], Nada Suvajdzic Vukovic[1, 3], Vesna Djordjevic[1], Nada Kraguljac-Kurtovic[1], Ljubomir Jakovic[1, 3], Andrija Bogdanovic[1, 3], Milena Todorovic Balint[1, 3], Darko Antic[1, 3], Ana Vidovic[1, 3]

*[1]Clinic of Hematology, University Clinical Centre of Serbia, Belgrade, Serbia;[2]Institute of Pathology, Faculty of medicine, University of Belgrade, Belgrade, Serbia; [3]Medical Faculty, University of Belgrade, Belgrade, Serbia;*

### Background

Genetic analyses have become essential in diagnostic stratification of acute myeloid leukemia (AML). Traditional approaches, including cytogenetics, fluorescence in situ hybridization (FISH) and polymerase chain reaction (PCR) remain crucial in the initial diagnostic workup. However, these methods provide limited insight into the full molecular landscape of AML. The implementation of next-generation sequencing (NGS) enables comprehensive mutational profiling, that refines risk stratification and may directly influence post-remission therapeutic strategies.

### Aims

To evaluate clinical impact of molecular re-stratification on post-remission treatment planning.

### Methods

A retrospective study included de novo AML patients >18 years, diagnosed and treated at Clinic of Hematology, University Clinical Centre of Serbia between 2025-2026, in whom NGS analysis was performed at diagnosis. The diagnosis of AML was based on 2022 World Health Organization (WHO) criteria and risk stratification of AML patients was carried out in keeping with European LeukemiaNet (ELN) 2022 recommendations. Genomic DNA was isolated from bone marrow (BM) samples of 46 patients. Library preparation was performed using 200ng of input DNA according to the manufacturer's protocol for the Myeloid Solutions™ Panel (MYS) (SOPHiA Genetics, Saint-Sulpice, Switzerland).

**Copyright Information:** (Online) ISSN: 2572-9241
©2026 The Author(s). HemaSphere is published by John Wiley & Sons Ltd on behalf of European Hematology Association. This is an open access Abstract Book distributed under the Attribution-NonCommercial-NoDerivs (CC BY-NC-ND), which allows third parties to download the articles and share them with others as long as they credit the author and the Abstract Book, but they cannot change the content in any way or use them commercially.
**Abstract Book Citations:** Authors, Title, HemaSphere, 2026;10:(S1):pages. The abstract book DOIs can be found on the "Author Information" page.
**Disclaimer:** Articles published in the journal HemaSphere exclusively reflect the opinions of the authors. The authors are responsible for all content in their abstracts including accuracy of the facts, statements, citing resources, etc.

### Results

We have identified a total of 46 patients with *de novo* AML with available molecular profiling, with a median age of 52 years (range, 23-76), overall male predominance (M:F=1.25), and median follow up time of 5 months (range, 1-15). According to pre-NGS ELN classification, 8 patients (17.4%) were classified as low risk, 31 (67.4%) as intermediate risk, and 7 (15.2%) as high risk. After including NGS findings, risk distribution shifted to 7 (15.2%) low risk, 26 (56.5%) intermediate risk and 13 (28.3%) high risk patients **(Figure 1)**. Overall, risk re-classification occurred in 7 patients (15.2%), predominantly resulting in upstaging from intermediate to high risk category, which almost doubled the adverse risk group (15.2% to 28.3%) and subsequently lead to the identification of new candidates for allo-SCT. Most frequent mutations responsible for upstaging were TP53, ASXL1, SRSF2 and SF3B1. Importantly, molecular re-stratification influenced post-remission therapeutic strategy. Majority of the patients (40/46, 87%) were treated with intensive chemotherapy induction and complete remission was achieved in 28 patients (60.9%), while 10 patients (21.7%) were primary refractory. MRD negativity after first consolidation was observed in 20 patients (43.5%), while 5 (10.9%) remained MRD positive. Relapse occurred in 7 patients (15.2%) during follow-up. Among 7 reclassified patients, all were eligible for allo-SCT after NGS analysis. At the time of analysis, 1 patient had undergone allo-SCT and remains in remission, 2 died during induction therapy and 4 are awaiting allo-SCT.

### Summary/Conclusion

In our cohort, incorporation of NGS findings led to clinically meaningful risk re-classification in 15% of the cases, predominantly upstaging from intermediate to adverse risk category. This shift had direct therapeutical implications, influencing post-remission treatment planning, including consideration for allo-SCT. Comprehensive genomic profiling expands our understanding of AML molecular heterogeneity and supports more individualized, risk-adapted treatment strategies.

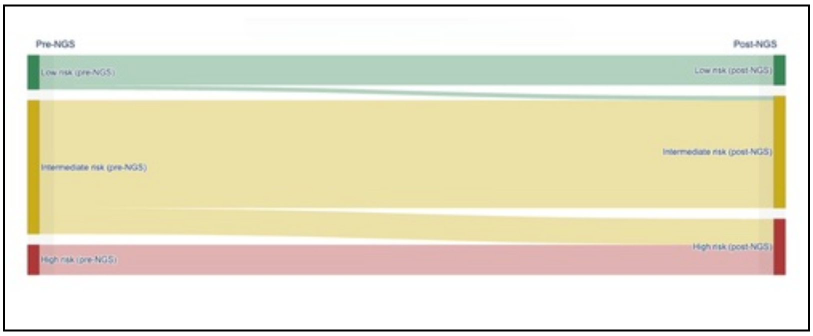

Figure 1. Risk re-classification with NGS

**Copyright Information:** (Online) ISSN: 2572-9241
©2026 The Author(s). HemaSphere is published by John Wiley & Sons Ltd on behalf of European Hematology Association. This is an open access Abstract Book distributed under the Attribution-NonCommercial-NoDerivs (CC BY-NC-ND), which allows third parties to download the articles and share them with others as long as they credit the author and the Abstract Book, but they cannot change the content in any way or use them commercially.
**Abstract Book Citations:** Authors, Title, HemaSphere, 2026;10:(S1):pages. The abstract book DOIs can be found on the "Author Information" page.
**Disclaimer:** Articles published in the journal HemaSphere exclusively reflect the opinions of the authors. The authors are responsible for all content in their abstracts including accuracy of the facts, statements, citing resources, etc.

### (PB2846) IVORY-MAST: STUDY DESIGN OF A PHASE 2 TRIAL EVALUATING IVOSIDENIB AS MAINTENANCE THERAPY IN IDH1-MUTATED AML OR HIGH-RISK MDS INELIGIBLE FOR TRANSPLANT

**Topic:** 04. Acute myeloid leukemia - Clinical

Ying Wang*[1], Yubao Chen[1], Li Chen[1], Xiaoqian Xu[1], Zhiyin Liu[1], Haimin Sun[1], Ran An[1], Jie Tian[1], Xing-Ying Li[1], Sujiang Zhang[1]

*[1]Ruijin Hospital Affiliated to Shanghai Jiao Tong University School of Medicine, Department of Hematology, Shanghai, China;*

### Background

IDH1 mutations drive leukemogenesis through accumulation of the oncometabolite D-2-hydroxyglutarate, resulting in DNA and histone hypermethylation, epigenetic dysregulation, and impaired cellular differentiation. Patients with IDH1-mutated AML and high-risk MDS face poor outcomes, particularly those ineligible for allogeneic hematopoietic stem cell transplantation (allo-HSCT), where no effective maintenance strategies currently exist. Ivosidenib, an oral IDH1 inhibitor, has demonstrated favorable efficacy and safety in relapsed/refractory (R/R) and newly diagnosed IDH1-mutated AML, and is approved in China for adults with R/R AML. However, its role as maintenance therapy in transplant-ineligible patients with IDH1-mutated AML or high-risk MDS who have responded to induction therapy has not yet been investigated. Given its targeted mechanism and favorable tolerability, ivosidenib represents a promising candidate for long-term maintenance in this population.

### Aims

This study aims to evaluate the efficacy and safety of ivosidenib as maintenance therapy in patients with IDH1-mutated AML or high-risk MDS who are ineligible for allo-HSCT.

### Methods

IVORY-MAST is a prospective, single-arm, exploratory phase 2 trial. A total of 20 patients with IDH1-mutated AML or high-risk MDS will be enrolled in China. Eligible patients must have achieved complete remission (CR) or partial remission (PR) following induction therapy, be considered ineligible for allo-HSCT per investigator assessment, have an ECOG performance status ≤2, and provide written informed consent. Patients with acute promyelocytic leukemia (M3) or prior exposure to IDH1 inhibitors are excluded. Enrolled patients will receive ivosidenib monotherapy (500 mg once daily in continuous 28-day cycles) until disease progression, unacceptable toxicity, or withdrawal. The primary endpoint is 1-year disease-free survival (DFS). Secondary endpoints include CR, overall survival (OS), and safety. Exploratory endpoint includes correlation of molecular biomarkers (e.g., IDH1 mutation clearance, co-mutation profile) with clinical outcomes. Assuming a null hypothesis 1-year DFS rate of 0.4 and a target rate of 0.74, with a one-sided alpha of 0.025 and 80% power, and accounting for 10% dropout, 20 patients are required. Patient enrollment is anticipated to commence in March 2026, with expected completion of enrollment by December 2026.

### Results

N/A (Trial in progress)

### Summary/Conclusion

N/A (Trial in progress)

**Copyright Information:** (Online) ISSN: 2572-9241
©2026 The Author(s). HemaSphere is published by John Wiley & Sons Ltd on behalf of European Hematology Association. This is an open access Abstract Book distributed under the Attribution-NonCommercial-NoDerivs (CC BY-NC-ND), which allows third parties to download the articles and share them with others as long as they credit the author and the Abstract Book, but they cannot change the content in any way or use them commercially.
**Abstract Book Citations:** Authors, Title, HemaSphere, 2026;10:(S1):pages. The abstract book DOIs can be found on the "Author Information" page.
**Disclaimer:** Articles published in the journal HemaSphere exclusively reflect the opinions of the authors. The authors are responsible for all content in their abstracts including accuracy of the facts, statements, citing resources, etc.

### (PB2847) ACUTE MYELOID LEUKEMIA ASSOCIATED WITH BARDET-BIEDL SYNDROME:A CASE REPORT

**Topic:** 04. Acute myeloid leukemia - Clinical

Belgacem Raoudha*[1], Wiem Boufrikha[1], Daami Mayssa[1], Inaam Bizid[1], Zaineb Mlayah[1], Nader Slama[1], Sarra Boukhriss[1], Med.Adnene Laatiri[1]

[1]*CHU Fattouma Bourguiba Monastir, Hematology Clinic Department, Monastir, Tunisia;*

### Background

Bardet-Biedl Syndrome (BBS) is a rare autosomal recessive ciliopathy characterized by multisystem involvement,mainly obesity,polydactyly,retinal dystrophy, and renal impairment.To date,no association with hematologic malignancies has been described.We report a case of acute myeloid leukemia (AML) occurring in a patient with BBS.

### Results

We report the case of a 6-year-old girl diagnosedwith BBS since the age of 2 years.The diagnosis was established based on suggestive clinical features,including obesity,hexadactyly,retinitis pigmentosa,intellectual disability and situs abdominis withpolysplenia.
In October 2025,the patient was admitted in our Hematology Department witha tumor syndrome and an infectious process (pleuropneumonia). The complete blood count revealed a white cell count of 19,8 G/Lwith71% circulating blasts,normochromic normocytic anemia (hemoglobin4,2 g/dL)and thrombocytopenia (50 G/L).Bone narrow aspiration confirmed the diagnosis of AML showing 76 % medullary blasts consisting of promonocytes and dysplastic monoblasts.Immunophenotyping supported the diagnosis with the expression of CD13,CD33,MPO,CD64 and CD14.Cytogenetic analysis identified a structural abnormality on chromosome 7 within a single clone.
Given the patient's polymorbid status, induction was adapted using the ELAM 02 (5+2)protocol.Treatment was complicated by S.aureus bacteremia and pulmonary-induced septic shock.The patient required intensive care managementincluding intubation and mechanical ventilation with good evolution. The patient achieved complete cytologic and cytogenetic remission following induction therapy. After that, she received a first course of consolidation therapy with high dose Cytarabine which was complicated by febrile neutropenia, documented as an ESBL-producing E.coli urinary tract infection.This was successfully managed with dual intravenous antibiotic therapy (Piperacillin/Tazobactam and Gentamicin). Subsequent evaluation confirmed the persistence of cytological complete remission, though minimal residual disease remained positive at 10-4.

### Summary/Conclusion

The occurrence of AML in this patient may be a coincidental finding.However,the hypothesis of underlying genetic susceptibility of cellular instability linked to signalling pathway aberrations warrants exploration. This case also raises questions regarding the specific therapeutic management of such medically complex patients.

**Copyright Information:** (Online) ISSN: 2572-9241
©2026 The Author(s). HemaSphere is published by John Wiley & Sons Ltd on behalf of European Hematology Association. This is an open access Abstract Book distributed under the Attribution-NonCommercial-NoDerivs (CC BY-NC-ND), which allows third parties to download the articles and share them with others as long as they credit the author and the Abstract Book, but they cannot change the content in any way or use them commercially.
**Abstract Book Citations:** Authors, Title, HemaSphere, 2026;10:(S1):pages. The abstract book DOIs can be found on the "Author Information" page.
**Disclaimer:** Articles published in the journal HemaSphere exclusively reflect the opinions of the authors. The authors are responsible for all content in their abstracts including accuracy of the facts, statements, citing resources, etc.

### (PB2848) PREDICTIVE VALUE OF THE NEUTROPHIL-TO-LYMPHOCYTE RATIO FOR 30-DAY MORTALITY IN PATIENTS WITH ACUTE MYELOID LEUKEMIA

**Topic:** 04. Acute myeloid leukemia - Clinical

Andreea Vasile*[1], Maria Bacanu[1, 2], Meilin Omer[1], Lidia Felicia Mihai[1], Mihaela Popescu[1], Florina Oana Gianina Pătrînoiu[1], Oana-Diana Dragoi-Cozmaciuc[1], Geanina Ofiteru[1], Viola Maria Popov[1], Mihaela Andreescu[1, 3]

*[1]Clinical Hospital Colentina, Hematology 1, Bucharest, Romania;[2]Carol Davila University, Bucharest, Romania; [3]Titu Maiorescu University, Bucharest, Romania;*

### Background

Early mortality remains a major challenge in acute myeloid leukemia (AML), with most deaths occurring during the first weeks after diagnosis and frequently related to infectious or hemorrhagic complications. Rapid identification of patients at high risk at admission is clinically important. The neutrophil-to-lymphocyte ratio (NLR), an inexpensive parameter derived from the complete blood count, may serve as a simple marker for early risk stratification.

### Aims

To assess whether the neutrophil-to-lymphocyte ratio at diagnosis can identify AML patients at increased risk of early death.

### Methods

We retrospectively analyzed 36 consecutive adults with newly diagnosed non-APL AML, including both de novo and secondary (post-MDS) cases, diagnosed and treated at a single tertiary center between January 2022 and January 2026. Diagnosis was established according to WHO 2022 criteria and patients received standard institutional therapy (intensive or non-intensive). Early death was defined as mortality within 30 days from diagnosis. Baseline NLR was calculated from peripheral blood counts at diagnosis. NLR values were compared between patients with early death and survivors. Receiver operating characteristic (ROC) analysis was performed to evaluate the predictive performance of NLR and to determine the optimal cut-off value.

### Results

Early death within 30 days occurred in 6 patients (16.7%), while 30 survived beyond 30 days. Using the ROC-derived cut-off (NLR ≥1), 12 patients were classified as high NLR and 24 as low NLR.
Median NLR was significantly higher in patients with early death compared with survivors [1.40 (95% CI 0.9-5.1) vs 0.30 (95% 0.35-0.80), p=0.009]. ROC analysis demonstrated good predictive performance of NLR for early mortality (AUC 0.844, 95% CI 0.700–0.989, p<0.001). At the optimal cut-off, sensitivity was 83.3% and specificity 76.7%.

### Summary/Conclusion

Baseline NLR was significantly associated with early mortality in patients with AML and may represent a simple and readily available parameter for risk stratification at diagnosis. In this cohort, low NLR identified patients with a very low probability of early death, suggesting potential clinical utility for initial assessment and closer monitoring. NLR likely reflects early inflammatory complications rather than intrinsic leukemia biology.
The small sample size and the low number of early death events limit statistical robustness, and the results should be interpreted cautiously. Larger prospective multicenter studies are required to validate these findings and to determine the role of NLR within established AML risk models.

**Copyright Information:** (Online) ISSN: 2572-9241
©2026 The Author(s). HemaSphere is published by John Wiley & Sons Ltd on behalf of European Hematology Association. This is an open access Abstract Book distributed under the Attribution-NonCommercial-NoDerivs (CC BY-NC-ND), which allows third parties to download the articles and share them with others as long as they credit the author and the Abstract Book, but they cannot change the content in any way or use them commercially.
**Abstract Book Citations:** Authors, Title, HemaSphere, 2026;10:(S1):pages. The abstract book DOIs can be found on the "Author Information" page.
**Disclaimer:** Articles published in the journal HemaSphere exclusively reflect the opinions of the authors. The authors are responsible for all content in their abstracts including accuracy of the facts, statements, citing resources, etc.

### (PB2849) SUSPECTED TRANSIENT HEPATOSPLENIC PELIOSIS IN AML-M2 AFTER INDUCTION COMPLICATED BY SEVERE SARS-COV-2 INFECTION: « CASE REPORT »

**Topic:** 04. Acute myeloid leukemia - Clinical

Rihab Bentahar*[1], Imane El Boutahiri[1, 2], Oumayma Hari[1, 2], Safae Regragui[1, 2]

[1]*university hospital center Mohamed VI Tangier, Tanger, Morocco;*[2]*Faculty of Medicine and Pharmacy of Tangier, Tanger, Morocco;*

### Background

Hepatosplenic peliosis is a rare vascular disorder characterized by multiple blood-filled cystic spaces within the liver parenchyma. While it may remain asymptomatic, it can lead to severe complications, including non-cirrhotic intrahepatic portal hypertension and life-threatening haemorrhage. It has been described in association with chronic infections, immunosuppression, and hematologic malignancies, as well as in patients exposed to cytotoxic therapies.

### Aims

To describe a case of suspected transient hepatosplenic peliosis in AML-M2 after induction complicated by severe SARS-COV-2 infection .

### Methods

We present a case of 43-year-old man presented with gingival bleeding and constitutional symptoms (10-kg weight loss in 1 month, asthenia, anorexia). He was cachectic with marked mucocutaneous pallor, without fever, palpable lymphadenopathy, or other infectious signs. Bone marrow aspiration showed a hypercellular marrow infiltrated by 62% blasts with myeloperoxidase positivity, consistent with acute myeloid leukemia (FAB M2). Baseline work-up revealed no disseminated intravascular coagulation (DIC) or spontaneous tumour lysis syndrome, preserved renal and hepatic function, negative serologies, and normal left ventricular ejection fraction. Induction chemotherapy with daunorubicin plus cytarabine (7+3) was initiated.

### Results

On day 15, he developed febrile cough and dyspnea with oxygen desaturation. Chest CT demonstrated CO-RADS 5 lesions with ~30% pulmonary involvement, highly suggestive of SARS-CoV-2 infection, requiring ICU admission and invasive mechanical ventilation. Recovery from aplasia occurred by day 35; however, bicytopenia persisted (haemoglobin ~5 g/dL despite regular packed red blood cell transfusions; platelets ~60,000/mm³ without bleeding). End-of-induction marrow confirmed complete morphological remission, with predominant granulopoiesis and marked erythroid hypoplasia (erythroblastopenia) with mild multilineage dysplasia. Schistocytes were absent; coagulation studies showed decreased prothrombin time (43%) with fibrinogen 1.43 g/L and normal aPTT, excluding DIC.

Abdominal CT angiography revealed hepatosplenomegaly with a micronodular hepatic and splenic pattern suggestive of hepatosplenic peliosis; abdominal vessels were patent. Etiologic investigations were negative, including GeneXpert for tuberculosis, and the patient declined liver biopsy. After 10 months lost to follow-up, he returned with sustained complete remission, normalization of blood counts and prothrombin time, and homogeneous liver imaging, supporting a secondary, reversible hepatosplenic peliosis in this setting.

**Copyright Information:** (Online) ISSN: 2572-9241
©2026 The Author(s). HemaSphere is published by John Wiley & Sons Ltd on behalf of European Hematology Association. This is an open access Abstract Book distributed under the Attribution-NonCommercial-NoDerivs (CC BY-NC-ND), which allows third parties to download the articles and share them with others as long as they credit the author and the Abstract Book, but they cannot change the content in any way or use them commercially.
**Abstract Book Citations:** Authors, Title, HemaSphere, 2026;10:(S1):pages. The abstract book DOIs can be found on the “Author Information” page.
**Disclaimer:** Articles published in the journal HemaSphere exclusively reflect the opinions of the authors. The authors are responsible for all content in their abstracts including accuracy of the facts, statements, citing resources, etc.

**Summary/Conclusion**

This case suggests a rare, likely secondary and transient hepatosplenic peliosis occurring in an AML patient after induction chemotherapy complicated by severe SARS-CoV-2 infection. Recognition of this entity is important in hematologic patients with unexplained hepatosplenomegaly and cytopenias, given potential haemorrhagic complications and the possibility of spontaneous resolution once triggering factors subside.

**Copyright Information:** (Online) ISSN: 2572-9241
©2026 The Author(s). HemaSphere is published by John Wiley & Sons Ltd on behalf of European Hematology Association. This is an open access Abstract Book distributed under the Attribution-NonCommercial-NoDerivs (CC BY-NC-ND), which allows third parties to download the articles and share them with others as long as they credit the author and the Abstract Book, but they cannot change the content in any way or use them commercially.
**Abstract Book Citations:** Authors, Title, HemaSphere, 2026;10:(S1):pages. The abstract book DOIs can be found on the "Author Information" page.
**Disclaimer:** Articles published in the journal HemaSphere exclusively reflect the opinions of the authors. The authors are responsible for all content in their abstracts including accuracy of the facts, statements, citing resources, etc.

### (PB2850) BLUEBERRY MUFFIN RASH AS THE INITIAL PRESENTATION OF CONGENITAL ACUTE MYELOID LEUKEMIA: DIAGNOSTIC PATHWAYS IN TWO INFANTS WITH KMT2A-REARRANGED AML

**Topic:** 04. Acute myeloid leukemia - Clinical

Maria Kourti[1], Athanasios Tragiannidis*[1], Athina Ditsiou[1], Paraskevi Panagopoulou[2], Anastasia Athanasiadou[3], Apostolia Papalexandri[3], Emmanouil Hatzipantelis[1]

*[1]Faculty of Health Sciences, Aristotle University of Thessaloniki, Pediatric & Adolescent Hematology Oncology Unit, 2nd Pediatric Department Faculty of Health Sciences, Aristotle University of Thessaloniki, AHEPA Hospital,, Thessaloniki, Greece; [2]Faculty of Health Sciences, Aristotle University of Thessaloniki, 4th Department of Pediatrics, Thessaloniki, Greece; [3]Hematology Department and HCT Unit, G. Papanicolaou Hospital, Thessaloniki, Greece;*

### Background

Congenital acute myeloid leukemia (AML) is an extremely rare and highly aggressive malignancy of the neonatal period. Blueberry muffin–like skin lesions, reflecting leukemia cutis may represent the first manifestation of disease and require prompt diagnostic evaluation.

### Aims

To present two infants with congenital AML in whom cutaneous lesions constituted the initial clinical presentation, highlighting different diagnostic pathways.

### Methods

Two cases are described: a 20-day-old male neonate and a 48-day-old female infant, both presenting with bluish–violaceous infiltrated skin lesions from the first days of life. In the first infant, diagnosis was initially established through skin biopsy with immunohistochemical analysis, whereas in the second through bone marrow aspiration and immunophenotypic evaluation. Comprehensive cytogenetic and molecular analyses were subsequently performed in both cases.

### Results

In the first case, skin biopsy demonstrated leukemia cutis with immunohistochemical features consistent with monocytic AML. In the second case, ecchymoses from birth, gingival involvement, and hepatomegaly were observed, accompanied by laboratory findings of leukocytosis, anemia, and elevated lactate dehydrogenase; bone marrow examination confirmed acute monocytic leukemia (AML M5). In both infants, cytogenetic analysis revealed t(9;11)(p21;q23), indicative of KMT2A rearrangement, with negative FLT3 and NPM1 mutation status. Intensive chemotherapy according to the AML-BFM 2019 protocol was initiated. Following induction therapy, both infants achieved complete remission, with resolution of skin lesions and disappearance of the abnormal cytogenetic clone.

### Summary/Conclusion

Blueberry muffin–like lesions in neonates may represent an early manifestation of congenital AML. These cases underscore the importance of maintaining a high index of suspicion and adopting an individualized diagnostic approach (skin biopsy or bone marrow examination) to facilitate timely diagnosis and prompt initiation of therapy.

**Copyright Information:** (Online) ISSN: 2572-9241
©2026 The Author(s). HemaSphere is published by John Wiley & Sons Ltd on behalf of European Hematology Association. This is an open access Abstract Book distributed under the Attribution-NonCommercial-NoDerivs (CC BY-NC-ND), which allows third parties to download the articles and share them with others as long as they credit the author and the Abstract Book, but they cannot change the content in any way or use them commercially.
**Abstract Book Citations:** Authors, Title, HemaSphere, 2026;10:(S1):pages. The abstract book DOIs can be found on the “Author Information” page.
**Disclaimer:** Articles published in the journal HemaSphere exclusively reflect the opinions of the authors. The authors are responsible for all content in their abstracts including accuracy of the facts, statements, citing resources, etc.

### (PB2851) EFFICACY AND SAFETY OF IVOSIDENIB-BASED THERAPY IN IDH1-MUTATED ACUTE MYELOID LEUKEMIA: A SINGLE-CENTER REAL-WORLD STUDY

**Topic:** 04. Acute myeloid leukemia - Clinical

Guopan Yu*[1, 2], Dandan Chen[1], Jiacheng Xu[1], Zhao Yin[1], Pengcheng Shi[1], Dan Xu[1], Jieyu Ye[1], Hui Liu[1], Li Xuan[1], Yu Zhang[1], Qifa Liu[1]

*[1]Nanfang Hospital, Southern Medical University, Guangzhou, China;[2]The Affiliated Guangdong Second Provincial General Hospital of Jinan University, Guangzhou, China;*

### Background

Ivosidenib (IVO), the first targeted therapy for IDH1-mutated acute myeloid leukemia (AML), has been recommended by clinical guidelines for the treatment of both newly diagnosed and relapsed/refractory AML. However, real-world evidence in Chinese patient populations remain scarce.

### Aims

To evaluate the real-world application of IVO in patients with IDH1-mutated AML, as well as its efficacy and safety.

### Methods

We retrospectively analyzed clinical data from 16 patients with IDH1-mutated AML who received IVO based therapy at a single center between September 2021 and May 2025. Treatment responses were assessed according to the 2025 European Leukemia Net (ELN) criteria, and safety was evaluated using the National Cancer Institute Common Terminology Criteria for Adverse Events (NCI CTCAE) version 5.0.

### Results

The median age was 57 years (range, 25–80), with 7 males and 9 females. Six patients (37.5%) had an ECOG performance status of 3–4, and 14 patients (87.5%) were classified as intermediate- or high-risk according to the ELN criteria. Among five patients (31.2%) receiving venetoclax (VEN) plus azacitidine (AZA) combined with IVO for induction, three (60%) achieved complete remission (CR), including two (40%) with measurable residual disease (MRD) negative and IDH1 clone clearance. Two patients (12.5%) received VEN+AZA plus IVO for consolidation, both maintained CR, with one achieving MRD negative and IDH1 clone clearance. Four relapsed/refractory patients (25.0%) received VEN plus hypomethylating agents (HMA) combined with IVO (n=3) or IVO alone (n=1) for salvage therapy, all achieved CR, of whom three (75.0%) obtained MRD-negative, and one (25%) had IDH1 clone clearance. Five patients (31.2%) received IVO as maintenance therapy, with a duration of response (DOR) exceeding 12 months. The most common ≥Grade 3 adverse events were neutropenia (56.3%), anemia (43.8%), and thrombocytopenia (37.5%), with no treatment-related deaths.

### Summary/Conclusion

IVO-based regimens demonstrated favorable efficacy and a manageable safety profile across all treatment phases in patients with IDH1-mutated AML, supporting their broader clinical application.

**Copyright Information:** (Online) ISSN: 2572-9241
©2026 The Author(s). HemaSphere is published by John Wiley & Sons Ltd on behalf of European Hematology Association. This is an open access Abstract Book distributed under the Attribution-NonCommercial-NoDerivs (CC BY-NC-ND), which allows third parties to download the articles and share them with others as long as they credit the author and the Abstract Book, but they cannot change the content in any way or use them commercially.
**Abstract Book Citations:** Authors, Title, HemaSphere, 2026;10:(S1):pages. The abstract book DOIs can be found on the "Author Information" page.
**Disclaimer:** Articles published in the journal HemaSphere exclusively reflect the opinions of the authors. The authors are responsible for all content in their abstracts including accuracy of the facts, statements, citing resources, etc.

### (PB2852) IVOSIDENIB AS LONG-TERM MAINTENANCE TREATMENT FOR PATIENTS WITH IDH1-MUTATED ACUTE MYELOID LEUKEMIA (AML): A RETROSPECTIVE CASE SERIES

**Topic:** 04. Acute myeloid leukemia - Clinical

Ligen Liu*[1, 2], Limin Zhao[1], Li Yang[1], Fangli Chen[1, 2], Xuewei Jiang[1], Tinghua Zhang[1], Zhizhi Zhang[1], Xiang Zhou[1], Li Chang[1], Xinying Zhu[1], Chao Du[1], Chunrong Yin[1]

*[1]Department of Hematology, Tongren Hospital, Shanghai Jiao Tong University School of Medicine, Shanghai, China;[2]Laboratory of Targeted Therapy and Clinical Translation, Shanghai Institute of Hematology, Shanghai Jiao Tong University School of Medicine, Laboratory of Targeted Therapy and Clinical Translation, Shanghai, China;*

### Background

Patients with IDH1-mutated acute myeloid leukemia (AML) generally have a poor prognosis. Although the IDH1 inhibitor ivosidenib has demonstrated efficacy and a manageable safety profile in clinical trials, its real-world data on long-term effectiveness as maintenance therapy remain limited.

### Aims

This analysis aims to evaluate the real-world efficacy and safety of ivosidenib as a long-term maintenance therapy in patients with IDH1-mutated AML.

### Methods

We retrospectively analyzed data from 4 Chinese patients with IDH1-mutated AML who received long-term maintenance therapy with ivosidenib between May 2018 and January 2026 across various clinical settings. Prognostic stratification was performed according to the ELN 2022 genetic risk guidelines. Adverse events (AEs) were sourced from electronic medical records, and treatment responses were assessed based on standard clinical guidelines.

**Copyright Information:** (Online) ISSN: 2572-9241
©2026 The Author(s). HemaSphere is published by John Wiley & Sons Ltd on behalf of European Hematology Association. This is an open access Abstract Book distributed under the Attribution-NonCommercial-NoDerivs (CC BY-NC-ND), which allows third parties to download the articles and share them with others as long as they credit the author and the Abstract Book, but they cannot change the content in any way or use them commercially.
**Abstract Book Citations:** Authors, Title, HemaSphere, 2026;10:(S1):pages. The abstract book DOIs can be found on the “Author Information” page.
**Disclaimer:** Articles published in the journal HemaSphere exclusively reflect the opinions of the authors. The authors are responsible for all content in their abstracts including accuracy of the facts, statements, citing resources, etc.

### Results

Two newly diagnosed patients (Pt 1 and Pt 2) were classified with intermediate cytogenetic risk, with IDH1 variant allele frequencies (VAF) of 15.7% and 42.5%, respectively.
**Pt 1:** A 65-year-old female with newly diagnosed AML, harboring DNMT3A and NPM1 mutations alongside IDH1. Following one cycle of "7+3" induction, the patient achieved CRi. Ivosidenib was introduced on day 3 of the second consolidation cycle (intermediate-dose cytarabine), followed by ivosidenib maintenance. The patient achieved MRD-negativity at 5 months and has maintained CR for over 38 months. A temporary dose reduction due to florid lymphoid follicular hyperplasia led to a transient increase in VAF, which resolved upon resuming the full dose.
**Pt 2:** A 59-year-old female with newly diagnosed AML and a co-occurring FLT3-TKD mutation. Due to a concurrent pulmonary infection at diagnosis, the patient received ivosidenib combined with azacitidine. Blast counts dropped to 4% within 14 days, and the patient achieved a CR that persisted for 24 months.
**Pt 3:** A 67-year-old female with FLT3, NPM1, KRAS, and DNMT3A co-mutations. The disease was primarily refractory to a regimen of selinexor, azacitidine, and venetoclax (SAV), with bone marrow blasts remaining at 78%. The addition of gilteritinib reduced blasts to 19%. Subsequently, the patient switched to ivosidenib combined with the SAV regimen, achieving CRi after 30 days and MRD-negative CR 35 days later. As of January 2026, the patient remains in CR (>45 months) with an overall survival (OS) exceeding 51 months.
**Pt 4:** A 61-year-old female with relapsed/refractory (R/R) AML and multiple mutations (FLT3-TKD, DNMT3A, PHF6, and TP53). Following a CRi achieved via DLAAG plus venetoclax, the patient switched to ivosidenib maintenance due to recurrent severe infections. Neutrophil counts normalized within one week, the infection resolved, and the patient achieved CR. The patient remains on maintenance therapy, with a CR duration of >85 months and an OS from diagnosis exceeding 114 months.
Pt 1 experienced grade 4 neutropenia and a grade 3 rash during consolidation, as well as rare florid lymphoid follicular hyperplasia requiring a temporary treatment interruption. No further AEs occurred after therapy resumed. Patients 2, 3, and 4 reported no significant adverse events.

### Summary/Conclusion

Ivosidenib-based regimens yielded favorable outcomes in this 4 patients with IDH1-mutated AML. All patients achieved or maintained CR with significantly prolonged survival. The treatment was well-tolerated, demonstrating manageable toxicity even during long-term maintenance.

**Copyright Information:** (Online) ISSN: 2572-9241
©2026 The Author(s). HemaSphere is published by John Wiley & Sons Ltd on behalf of European Hematology Association. This is an open access Abstract Book distributed under the Attribution-NonCommercial-NoDerivs (CC BY-NC-ND), which allows third parties to download the articles and share them with others as long as they credit the author and the Abstract Book, but they cannot change the content in any way or use them commercially.
**Abstract Book Citations:** Authors, Title, HemaSphere, 2026;10:(S1):pages. The abstract book DOIs can be found on the “Author Information” page.
**Disclaimer:** Articles published in the journal HemaSphere exclusively reflect the opinions of the authors. The authors are responsible for all content in their abstracts including accuracy of the facts, statements, citing resources, etc.

### (PB2853) MULTI-MODULE TARGETED NURSING PRACTICE AND OUTCOME ANALYSIS IN ACUTE MYELOID LEUKEMIA PATIENTS WITH PRE-EXISTING INTESTINAL INFECTIONS UNDERGOING DONOR-DERIVED CD33 CAR-T THERAPY

**Topic:** 04. Acute myeloid leukemia - Clinical

Xiaoyuan Wu*[1], Lanzhu Su[1], Guanyi Li[1], Zelin Wang[1], Sha Li[1], Jing Pan[1]

[1]*Beijing Gobroad Hospital, Beijing, China;*

### Background

Donor-derived CD33 chimeric antigen receptor T-cell (CAR-T) therapy is a key bridging strategy for relapsed/refractory acute myeloid leukemia (AML) prior to hematopoietic stem cell transplantation (HSCT). However, it is accompanied by risks of prolonged cytopenia and graft-versus-host disease. If patients have a pre-existing intestinal infection before infusion, the coexistence of an infectious focus and the therapy-induced profound immunosuppression can readily lead to uncontrolled infection and even treatment interruption. Currently, there is a lack of systematic summarization regarding refined nursing practices for this high-risk population characterized by "infection superimposed on immunosuppression."

### Aims

To develop and validate a structured multi-module care protocol for AML patients with underlying intestinal infections undergoing donor-derived CD33 CAR-T therapy, aiming to control infections, manage treatment toxicity, and ensure transplant continuity. This will provide an evidence-based foundation for the care of such high-risk populations.

### Methods

This study was a retrospective case series analysis. Three relapsed/refractory AML patients (2 males, 1 female, aged 10–14 years) with pre-existing intestinal infections were included. All patients received donor-derived CD33 CAR-T cell therapy and were planned for subsequent HSCT. A structured, multi-module nursing strategy was implemented. This involved placing patients in protective isolation with laminar airflow beds and medical air disinfection machines, conducting daily whole-body chlorhexidine baths, and providing intensified oral and perianal mucosal care to manage infection risks (oral care three times daily and perianal care twice daily). Concurrently, a digital nursing follow-up platform was utilized to obtain real-time patient feedback on symptoms, enabling dynamic assessment of CRS and ICANS according to ASTCT criteria and facilitating prompt clinical intervention. To ensure a smooth transition, patients also received standardized transplant diet education and nutritional management in preparation for hematopoietic stem cell transplantation.

### Results

During the treatment period, the pre-existing intestinal infections in all patients did not worsen or spread, and oral mucosa remained intact without the occurrence of ≥ Grade 2 oral mucositis. Regarding toxicity management, all patients developed CRS(two with Grade 2 and one with Grade 3), with only one patient experiencing mild ICANS (Grade 1). These toxicities were effectively controlled through real-time monitoring and intervention. Ultimately, all patients successfully bridged to hematopoietic stem cell transplantation. At the end of this follow-up, two patients survived, while one patient died due to a concurrent intracranial infection following transplantation.

**Copyright Information:** (Online) ISSN: 2572-9241
©2026 The Author(s). HemaSphere is published by John Wiley & Sons Ltd on behalf of European Hematology Association. This is an open access Abstract Book distributed under the Attribution-NonCommercial-NoDerivs (CC BY-NC-ND), which allows third parties to download the articles and share them with others as long as they credit the author and the Abstract Book, but they cannot change the content in any way or use them commercially.
**Abstract Book Citations:** Authors, Title, HemaSphere, 2026;10:(S1):pages. The abstract book DOIs can be found on the "Author Information" page.
**Disclaimer:** Articles published in the journal HemaSphere exclusively reflect the opinions of the authors. The authors are responsible for all content in their abstracts including accuracy of the facts, statements, citing resources, etc.

**Summary/Conclusion**

This study demonstrates that, for AML patients with pre-existing intestinal infections undergoing donor-derived CD33 CAR-T therapy, a structured multi-module targeted nursing strategy enables stable control of the baseline infection, safe management of treatment-related toxicities (e.g., CRS/ICANS), and ultimately ensures a smooth bridge to hematopoietic stem cell transplantation. It provides a concrete and feasible practical framework for the refined nursing care of such patients and offers important evidence to support its clinical dissemination and application.

**Copyright Information:** (Online) ISSN: 2572-9241
©2026 The Author(s). HemaSphere is published by John Wiley & Sons Ltd on behalf of European Hematology Association. This is an open access Abstract Book distributed under the Attribution-NonCommercial-NoDerivs (CC BY-NC-ND), which allows third parties to download the articles and share them with others as long as they credit the author and the Abstract Book, but they cannot change the content in any way or use them commercially.
**Abstract Book Citations:** Authors, Title, HemaSphere, 2026;10:(S1):pages. The abstract book DOIs can be found on the "Author Information" page.
**Disclaimer:** Articles published in the journal HemaSphere exclusively reflect the opinions of the authors. The authors are responsible for all content in their abstracts including accuracy of the facts, statements, citing resources, etc.

### (PB2854) IS EXRAMEDULLARY INVOLVEMENT A DISTINICT ENTITY IN NPM1-MUTATED AML WITH NORMAL CYTOGENETICS ?A CASE REPORT AND LITERATURE REVIEW

**Topic:** 04. Acute myeloid leukemia - Clinical

Jack Seki[1], Hassan Sibai*[2]

*[1]University of Toronto, pharmacy, Toronto, Canada;[2]Princess Margaret Cancer Centre, Toronto, Canada;*

### Background

NPM1 mutations occur in 25–30% of adult acute myeloid leukemia (AML), typically in the setting of normal cytogenetics, and may be associated with extramedullary (EM) involvement. Myeloid sarcoma (MS) is uncommon, affecting a small proportion of newly diagnosed AML and a higher proportion of relapsed cases. Testicular MS is exceptionally rare and may mimic primary testicular malignancy. The clinical and molecular characteristics of NPM1-mutated MS remain incompletely defined, and recent studies suggest that EM disease may demonstrate distinct clonal evolution and co-mutation patterns.

### Aims

To evaluate whether extramedullary involvement represents a distinct clinical entity in newly NPM1-mutated cytogenetically normal AML

### Methods

A literature review of PubMed, Embase, and Google Scholar (1993–2026) identified adult patients with NPM1-mutated AML, normal cytogenetics, and EM involvement. Eligible publications included case reports, case series, and molecular studies reporting patient-level data. Extracted variables included age, sex, EM site, cytogenetics, co-mutations, treatment, response, relapse, transplantation status, and survival. Findings were integrated with a representative case of bilateral testicular MS.

### Results

Across published cohorts, NPM1 mutations were identified in approximately 25–40% of MS/eAML cases and were most frequently associated with normal karyotype. Across all reported cases, 196 of 683 myeloid sarcoma patients (28.7%) harbored NPM1 mutations, with a (95% CI: 25.3% – 32.1%). Median age ranged from 50–60 years. The most common EM sites included skin, lymph nodes, soft tissue, bone, and central nervous system; testicular involvement was rare.
Recurrent co-mutations included FLT3-ITD, DNMT3A, IDH1/2, and RAS/MAPK pathway alterations. Recent genomic analyses demonstrated frequent MAPK pathway cooperation and molecular discordance between bone marrow and EM sites, consistent with clonal branching and compartment-specific evolution.
Most patients received high-intensity AML-type induction chemotherapy. Complete remission rates ranged from 60–80%; however, EM relapse was common. Median overall survival in larger series was approximately 8–24 months. Allogeneic hematopoietic stem cell transplantation was performed in 30–50% of eligible patients and was associated with improved disease control in high-risk cases.

**Copyright Information:** (Online) ISSN: 2572-9241
©2026 The Author(s). HemaSphere is published by John Wiley & Sons Ltd on behalf of European Hematology Association. This is an open access Abstract Book distributed under the Attribution-NonCommercial-NoDerivs (CC BY-NC-ND), which allows third parties to download the articles and share them with others as long as they credit the author and the Abstract Book, but they cannot change the content in any way or use them commercially.
**Abstract Book Citations:** Authors, Title, HemaSphere, 2026;10:(S1):pages. The abstract book DOIs can be found on the “Author Information” page.
**Disclaimer:** Articles published in the journal HemaSphere exclusively reflect the opinions of the authors. The authors are responsible for all content in their abstracts including accuracy of the facts, statements, citing resources, etc.

**Summary/Conclusion**

NPM1-mutated MS represents a distinct clinicopathologic subset characterized by normal cytogenetics, recurrent cooperating mutations, and frequent clonal divergence between marrow and extramedullary compartments. Despite high remission rates with intensive induction therapy, relapse remains common, supporting consideration of early transplant consolidation. Testicular MS, although rare, should be recognized as a potential manifestation of NPM1-mutated AML and may require integrated molecular evaluation to guide risk stratification and management.

**Copyright Information:** (Online) ISSN: 2572-9241
©2026 The Author(s). HemaSphere is published by John Wiley & Sons Ltd on behalf of European Hematology Association. This is an open access Abstract Book distributed under the Attribution-NonCommercial-NoDerivs (CC BY-NC-ND), which allows third parties to download the articles and share them with others as long as they credit the author and the Abstract Book, but they cannot change the content in any way or use them commercially.
**Abstract Book Citations:** Authors, Title, HemaSphere, 2026;10:(S1):pages. The abstract book DOIs can be found on the "Author Information" page.
**Disclaimer:** Articles published in the journal HemaSphere exclusively reflect the opinions of the authors. The authors are responsible for all content in their abstracts including accuracy of the facts, statements, citing resources, etc.

### (PB2855) PREDICTORS OF REDUCED RELAPSE-FREE SURVIVAL IN YOUNG ADULTS WITH ACUTE MYELOID LEUKEMIA

**Topic:** 04. Acute myeloid leukemia - Clinical

Chihaoui N*[1], Kharrat R[1], Selmi A[1], Rabeb Berred[1], Ben Neji H[1], Bchir M[1], Achour M[1], Cherni R[1], Ben Lakhel F[2], Feki S[2], Ayed W[3], Gouider E[2], Ben Lakhel R[1]

*[1]Aziza othemena hospital, adult clinical hematology, Tunis, Tunisia; [2]Aziza othemena hospital, Laboratory of Hematological Biology, Tunis, Tunisia; [3]Pasteur Institute of Tunis, Department of Histology and Medical Cytogenetics, Tunis, Tunisia;*

### Background

Despite advances in cytogenetics and therapeutic strategies, relapse remains the leading cause of treatment failure and poor long-term outcomes in acute myeloid leukemia (AML). In younger adults treated with intensive chemotherapy, relapse occurs in approximately 40–60% of cases despite achieving complete remission. Therefore identifying factors associated with reduced relapse free survival (RFS) remains crucial to refine risk stratification and optimize post-remission strategies

### Aims

To identify predictive factors of reduced relapse free survival in young adults with AML.

### Methods

We conducted a retrospective, single center study including patients aged 18-60 years treated for AML in adult department of clinical hematology in Aziza Othemena hospital for 10 years between 2013-2022. Cytogenetic risk stratification was based on cytogenetic abnormalities due to the absence of molecular analysis.
RFS was estimated using Kaplan–Meier method. Cox regression was applied for univariate and multivariate analyses.

### Results

We collected data for 296 patients with a median age was 43 years. Risk stratification was favorable risk 16%, intermediate risk 63%, adverse risk 21%. Complete remission was achieved in 203 patients (68%). Thirty-one patients underwent stem cell transplantation while the remaining patients received chemotherapy consolidation. During follow-up, 112 patients (55%) relapsed. Five-year RFS was 30% with a median RFS of 13 months.
In univariate analysis, high LDH level (HR 2.02, 95% CI 1.34-3.04, p<0.001), intermediate (HR 2.37, 95% CI 1.36-4.13, p 0.002), adverse (HR=3.14, 95% CI 1.6-6.19, p<0.001), consolidation chemotherapy (HR=1.52, 95% CI 1.01-2.28, p=0.041) and the absence of transplantation (HR=1.51, 95% CI 1.34-6.39, p=0.007) Were significantly associated with reduced RFS.
After adjustment, only elevated LDH (HR 2.57, 95% CI 1.46-4.53, p=0.001) remains independently predictive.

### Summary/Conclusion

Elevated LDH at diagnosis emerged as an independent predictor of reduced RFS. Limited access to molecular profiling may have affected risk stratification accuracy. Improved biological characterization is essential to optimize relapse prevention strategies in resource-limited countries.

**Copyright Information:** (Online) ISSN: 2572-9241
©2026 The Author(s). HemaSphere is published by John Wiley & Sons Ltd on behalf of European Hematology Association. This is an open access Abstract Book distributed under the Attribution-NonCommercial-NoDerivs (CC BY-NC-ND), which allows third parties to download the articles and share them with others as long as they credit the author and the Abstract Book, but they cannot change the content in any way or use them commercially.
**Abstract Book Citations:** Authors, Title, HemaSphere, 2026;10:(S1):pages. The abstract book DOIs can be found on the "Author Information" page.
**Disclaimer:** Articles published in the journal HemaSphere exclusively reflect the opinions of the authors. The authors are responsible for all content in their abstracts including accuracy of the facts, statements, citing resources, etc.

### (PB2856) POST-INDUCTION FAILURE IN ADULT ACUTE MYELOID LEUKEMIA: TOWARD PREDICTIVE STRATIFICATION OF HIGH-RISK PATIENTS

**Topic:** 04. Acute myeloid leukemia - Clinical

Ahlem Slama*[1], Zaineb Mlayah[1], Kmar Mrad[1], Ameni Yahia[1], Amira Guizeni[1], Inaam Bizid[1], Nader Slama[1], Wiem Boufrikha[1], Med.Adnene Laatiri[1], Sarra Boukhriss[1]

[1]*Fattouma Bourguiba University Hospital, Clinical Hematology Department, Monastir, Tunisia;*

### Background

Post-induction failure in adult acute myeloid leukemia (AML) remains a major clinical challenge, associated with poor prognosis and a high risk of complications. Understanding the prognostic factors associated with induction failure is essential to tailor therapeutic strategies, optimize reinduction, and improve patient survival. In resource-limited settings, early identification of high-risk patients is critical to adapt treatment and reinforce clinical monitoring, ultimately improving outcomes.

### Methods

We retrospectively analyzed 43 adult AML patients (excluding APL) treated at the Department of Clinical Hematology of Monastir between 2019 and 2024. Induction failure was defined as the absence of complete remission (CR) following the first induction cycle. Clinical, biological, cytogenetic, and treatment data were reviewed. Univariate and multivariate analyses using Cox regression were performed to identify prognostic factors associated with induction failure. Reinduction regimens and outcomes were also assessed.

**Copyright Information:** (Online) ISSN: 2572-9241
©2026 The Author(s). HemaSphere is published by John Wiley & Sons Ltd on behalf of European Hematology Association. This is an open access Abstract Book distributed under the Attribution-NonCommercial-NoDerivs (CC BY-NC-ND), which allows third parties to download the articles and share them with others as long as they credit the author and the Abstract Book, but they cannot change the content in any way or use them commercially.
**Abstract Book Citations:** Authors, Title, HemaSphere, 2026;10:(S1):pages. The abstract book DOIs can be found on the “Author Information” page.
**Disclaimer:** Articles published in the journal HemaSphere exclusively reflect the opinions of the authors. The authors are responsible for all content in their abstracts including accuracy of the facts, statements, citing resources, etc.

**Results**

Among the 43 patients, 14 (32%) failed to achieve CR after the first induction cycle. Reinduction was administered to 10 patients, achieving CR in 4 cases (40%). The remaining four patients did not receive reinduction due to poor performance status (n = 1) or delayed cytogenetic results (n = 3).
Induction failures were distributed as follows: isolated cytological failure (5 patients, 35.7%), isolated cytogenetic failure (3 patients, 21.5%), and combined cytological + cytogenetic failure (6 patients, 42.8%). Among isolated cytological failures, three had a normal karyotype, while two had initial abnormalities that disappeared post-induction.
Univariate analysis showed no significant association between induction failure and demographic, clinical, or laboratory variables including age, sex, tumor syndrome, hyperleukocytosis, anemia, thrombocytopenia, DIC, or AML subtype except for the type of induction chemotherapy: daunorubicin-based regimens were associated with lower failure rates ($p = 0.03$). Multivariate Cox regression identified absence of CD34 expression as the only factor significantly associated with a reduced risk of induction failure (HR = 0.233; 95% CI = 0.05–1.087; $p = 0.046$). Daunorubicin-based therapy was no longer significant after adjustment (HR = 1.245; 95% CI = 0.408–3.803; $p = 0.698$).

Reinduction regimens included HDAC combined with an anthracycline: daunorubicin (n = 5), idarubicin (n = 3), doxorubicin (n = 1), and mitoxantrone (n = 1). CR was achieved in 40% of reinduced patients. The remaining patients received alternative regimens (ADE, MIDAC, or Vidaza-Venetoclax with anti-FLT3) or were unable to undergo further therapy due to poor performance status.

**Summary/Conclusion**

Post-induction failure remains frequent (32%) with limited response to reinduction. Absence of CD34 expression appears protective, suggesting a potential prognostic role. Early risk stratification integrating CD34 and additional biological factors may allow adaptation of induction protocols or consideration of intensified or targeted therapies to improve outcomes in high-risk adult AML patients.

**Copyright Information:** (Online) ISSN: 2572-9241
©2026 The Author(s). HemaSphere is published by John Wiley & Sons Ltd on behalf of European Hematology Association. This is an open access Abstract Book distributed under the Attribution-NonCommercial-NoDerivs (CC BY-NC-ND), which allows third parties to download the articles and share them with others as long as they credit the author and the Abstract Book, but they cannot change the content in any way or use them commercially.
**Abstract Book Citations:** Authors, Title, HemaSphere, 2026;10:(S1):pages. The abstract book DOIs can be found on the "Author Information" page.
**Disclaimer:** Articles published in the journal HemaSphere exclusively reflect the opinions of the authors. The authors are responsible for all content in their abstracts including accuracy of the facts, statements, citing resources, etc.

### (PB2857) CONCURRENT LANGERHANS CELL HISTIOCYTOSIS AND KMT2A-REARRANGED ACUTE MYELOID LEUKEMIA SHOWING DUAL RESPONSE TO AZACITIDINE

**Topic:** 04. Acute myeloid leukemia - Clinical

Derya Demirtas*[1], Fatma Arikan[1], Yucel Erbilgin[2], Mehmet Fatih Tekin[3], Ozlem Candan[1], Ahmet Mert Yanik[1], Asu Fergun Yilmaz[1], Işik Atagunduz[1], Suheyla Bozkurt[3], Ayse Tulin Tuglular[1], Tayfur Toptas[1, 1]

*[1]Marmara University Medical Faculty, Hematology, Istanbul, Türkiye;[2]Istanbul University, Genetics, Istanbul, Türkiye;[3]Marmara University Medical Faculty, Pathology, Istanbul, Türkiye;*

### Background

Langerhans cell histiocytosis (LCH) is currently classified as a myeloid neoplasm that may occasionally coexist with other hematologic malignancies; however, its association with KMT2A-rearranged acute myeloid leukemia (AML) is extremely rare.

### Aims

We report a 76-year-old man with BRAF-mutant multisystem LCH involving the skin, lymph nodes, and spleen, who concurrently presented with acute myeloid leukemia characterized by t(9;11)(p21.3;q23.3) and an MLLT3–KMT2A fusion. Given his advanced age, significant comorbidities, and frailty, azacitidine was initiated as frontline therapy. After two treatment cycles, the patient achieved complete metabolic remission of LCH lesions and complete hematologic remission of AML, as confirmed by positron emission tomography/computed tomography and bone marrow evaluation.

### Summary/Conclusion

This case highlights a dual therapeutic response of concurrent LCH and KMT2A-rearranged AML to hypomethylating therapy and supports the concept of a shared, epigenetically vulnerable myeloid precursor. Hypomethylating agents may represent a feasible treatment option in selected patients with concomitant LCH and myeloid neoplasms.

**Copyright Information:** (Online) ISSN: 2572-9241
©2026 The Author(s). HemaSphere is published by John Wiley & Sons Ltd on behalf of European Hematology Association. This is an open access Abstract Book distributed under the Attribution-NonCommercial-NoDerivs (CC BY-NC-ND), which allows third parties to download the articles and share them with others as long as they credit the author and the Abstract Book, but they cannot change the content in any way or use them commercially.
**Abstract Book Citations:** Authors, Title, HemaSphere, 2026;10:(S1):pages. The abstract book DOIs can be found on the "Author Information" page.
**Disclaimer:** Articles published in the journal HemaSphere exclusively reflect the opinions of the authors. The authors are responsible for all content in their abstracts including accuracy of the facts, statements, citing resources, etc.

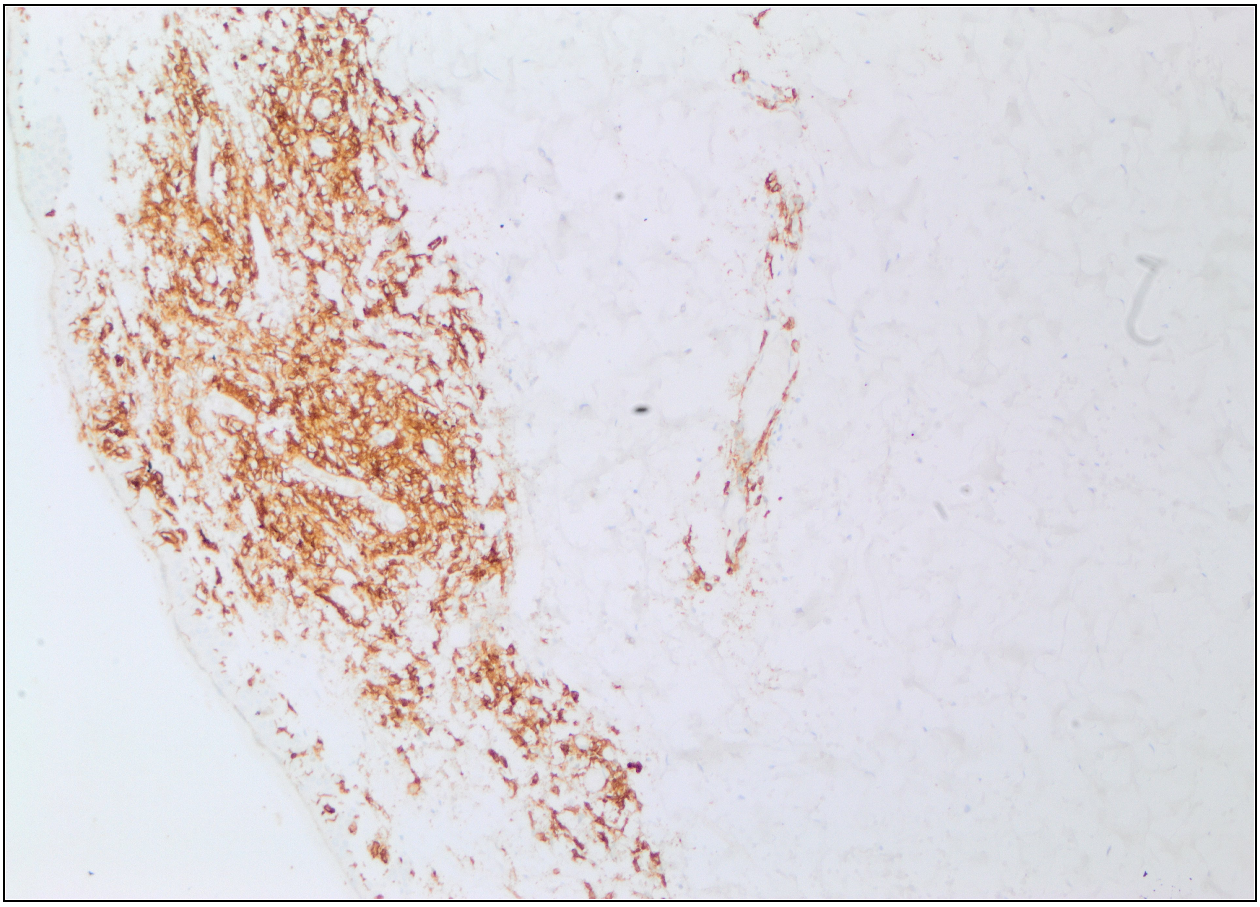

Immunohistochemical staining demonstrating diffuse membranous CD1a positivity of infiltrating Langerhans cells

**Copyright Information:** (Online) ISSN: 2572-9241
©2026 The Author(s). HemaSphere is published by John Wiley & Sons Ltd on behalf of European Hematology Association. This is an open access Abstract Book distributed under the Attribution-NonCommercial-NoDerivs (CC BY-NC-ND), which allows third parties to download the articles and share them with others as long as they credit the author and the Abstract Book, but they cannot change the content in any way or use them commercially.
**Abstract Book Citations:** Authors, Title, HemaSphere, 2026;10:(S1):pages. The abstract book DOIs can be found on the "Author Information" page.
**Disclaimer:** Articles published in the journal HemaSphere exclusively reflect the opinions of the authors. The authors are responsible for all content in their abstracts including accuracy of the facts, statements, citing resources, etc.

### (PB2858) PRIMARY ISOLATED MYELOID SARCOMA WITHOUT MARROW INVOLVEMENT: PARTIAL RESPONSE TO 7+3 FOLLOWED BY FURTHER RADIOLOGIC REGRESSION ON VENETOCLAX IN A NON-ELIGIBLE PATIENT

**Topic:** 04. Acute myeloid leukemia - Clinical

Anna Sergeieva*[1], Zinaida Stupakova[1]

*[1]National Research Center for Radiation Medicine, Oncology and Hematology of NAMS of Ukraine, Blood pathology department, Kyiv, Ukraine;*

### Background

Primary isolated myeloid sarcoma without bone marrow involvement is a rare extramedullary myeloid neoplasm. Management generally follows AML-based treatment principles, but data on post-induction non-intensive strategies are particularly limited in patients who become ineligible for further intensive consolidation.

### Aims

To describe a patient with primary isolated retroperitoneal myeloid sarcoma without bone marrow involvement who achieved partial response to AML-type induction and subsequent further radiologic regression during venetoclax therapy after severe infectious complications precluded standard intensive consolidation.

### Methods

We analyzed the clinical course, pathology, bone marrow studies, imaging response, infectious complications, and subsequent venetoclax-based management in a 65-year-old man with primary retroperitoneal myeloid sarcoma.

### Results

A 65-year-old man was diagnosed with primary left retroperitoneal myeloid sarcoma based on histopathology and immunohistochemistry, without bone marrow involvement at diagnosis (blasts 1%; no evidence of systemic hematologic disease by flow cytometry). Baseline MRI on 20 May 2025 showed a 22.0×12.0×10.5 cm retroperitoneal mass associated with left grade III hydronephrosis. The patient received two cycles of 7+3 (29 May-4 June 2025 and 3-9 July 2025). After the second cycle, MRI on 28 July 2025 demonstrated a partial response, with tumor shrinkage to 16.3×8.5×6.3 cm. However, further intensive treatment was precluded by severe toxicity, including profound cytopenias and multiple infectious complications: Sphingomonas paucimobilis sepsis after the first cycle, followed after the second by E. coli sepsis, Klebsiella pneumoniae sepsis, and invasive pulmonary mycosis. Because these complications made standard intensive consolidation unsuitable, venetoclax monotherapy was initiated in September 2025. Follow-up MRI after 2 months of venetoclax monotherapy (10 November 2025) demonstrated further regression compared with post-induction imaging, with only residual left retroperitoneal infiltrative thickening measuring approximately 22×50 mm, reduced extension along the left psoas/perirenal region, and no renewed progression of hydronephrosis. Overall, these findings were interpreted as a deepening partial radiologic response. At the time of reporting, the patient remains alive, continues venetoclax with stable blood counts, and is scheduled for repeat imaging.

**Copyright Information:** (Online) ISSN: 2572-9241
©2026 The Author(s). HemaSphere is published by John Wiley & Sons Ltd on behalf of European Hematology Association. This is an open access Abstract Book distributed under the Attribution-NonCommercial-NoDerivs (CC BY-NC-ND), which allows third parties to download the articles and share them with others as long as they credit the author and the Abstract Book, but they cannot change the content in any way or use them commercially.
**Abstract Book Citations:** Authors, Title, HemaSphere, 2026;10:(S1):pages. The abstract book DOIs can be found on the "Author Information" page.
**Disclaimer:** Articles published in the journal HemaSphere exclusively reflect the opinions of the authors. The authors are responsible for all content in their abstracts including accuracy of the facts, statements, citing resources, etc.

**Summary/Conclusion**

This case illustrates that primary isolated myeloid sarcoma without bone marrow involvement can achieve meaningful response to AML-type induction, while subsequent therapy may be limited by severe infectious toxicity. In a patient no longer suitable for further intensive consolidation, venetoclax monotherapy was associated with ongoing radiologic regression and may represent a feasible disease-control strategy in selected non-eligible cases, a setting for which evidence remains limited.

**Copyright Information:** (Online) ISSN: 2572-9241
©2026 The Author(s). HemaSphere is published by John Wiley & Sons Ltd on behalf of European Hematology Association. This is an open access Abstract Book distributed under the Attribution-NonCommercial-NoDerivs (CC BY-NC-ND), which allows third parties to download the articles and share them with others as long as they credit the author and the Abstract Book, but they cannot change the content in any way or use them commercially.
**Abstract Book Citations:** Authors, Title, HemaSphere, 2026;10:(S1):pages. The abstract book DOIs can be found on the "Author Information" page.
**Disclaimer:** Articles published in the journal HemaSphere exclusively reflect the opinions of the authors. The authors are responsible for all content in their abstracts including accuracy of the facts, statements, citing resources, etc.

### (PB2859) PATIENT OUTCOMES FOR ACUTE MYELOID LEUKAEMIA AND MYELODYSPLASTIC SYNDROMES WITH MUTATED TP53 AT CORK UNIVERSITY HOSPITAL, IRELAND

**Topic:** 04. Acute myeloid leukemia - Clinical

David Parfrey*[1, 1, 1], James Beazley[1], Conor Browne[1], Vitaliy Mykytiv[1]

[1]*Cork University Hospital, Department of Haematology, Cork, Ireland;*

### Background

The TP53 gene encodes the p53 protein, which functions as a critical regulatory transcription factor in healthy humans. In both acute myeloid leukaemia (AML) and myelodysplastic syndromes (MDS), mutations in TP53 are associated with an unfavourable prognosis and poorer responses to conventional treatments. In the *European LeukaemiaNet* (ELN) risk stratification system, patients with AML with mutated TP53 are categorised as having adverse risk disease. In younger patients with this sub-type of AML with good performance status, intensive induction chemotherapy followed by consolidation with allogeneic stem cell transplantation is recommended.

### Aims

Our aim was to establish disease outcomes for those treated by our department with AML and MDS with increased blasts (MDS-IB) (WHO 2022) with mutated TP53, as well as MDS and MDS/MPN with mutated TP53, who have been treatment by our department.

### Methods

We conducted a retrospective review of patients with AML, MDS-IB, MDS and MDS/MPN with mutated TP53 who had been treated by the Department of Haematology at Cork University Hospital over a 3.5-year period between 2020 to 2023.

### Results

We identified 23 patients with AML and MDS-IB with mutated TP53 in the study period. Median age at diagnosis was 71 years (range 43-89). 8 were female (35%). 17 (74%) had additional molecular mutations identified.
For treatment of AML and MDS-IB, 7 patients received induction treatment with intensive chemotherapy, of whom 4 received Fludarabine-Idarubicin-Cytarabine-GCSF-Venetoclax (FLAG-IDA-Ven) and 3 received Daunorubicin-Cytarabine (DA) or liposomal DA. 5 patients (22%) received consolidation with allogeneic stem cell transplant. 12 patients received non-intensive chemotherapy as first-line treatment (Azacitidine or Azacitidine-Venetoclax).
21 of 23 patients (91%) with AML or MDS-IB had died in the time following initial diagnosis. 1 patient's status was unknown as they were lost to follow-up. 1 patient was known to be alive. The one known survivor in this group had received intensive chemotherapy with FLAG-IDA-Ven and had received consolidation with allogeneic stem cell transplant. Median overall survival was 6 months (range 1 month to 37 months).
We identified 8 patients with MDS and MDS/MPN with mutated TP53 in the study period. Median age at diagnosis was 68 years (range 59 to 79). 3 were female (38%).
4 patients (50%) with MDS and MDS/MPN received treatment with non-intensive chemotherapy as first-line treatment (Azacitidine or Azacitidine-Venetoclax). 1 patient received treatment with intensive chemotherapy following progression to AML.
6 patients with MDS and MDS/MPN had died since diagnosis – overall survival rate was 25%. Median overall survival was 5 months (range 1 month to 44 months).

**Copyright Information:** (Online) ISSN: 2572-9241
©2026 The Author(s). HemaSphere is published by John Wiley & Sons Ltd on behalf of European Hematology Association. This is an open access Abstract Book distributed under the Attribution-NonCommercial-NoDerivs (CC BY-NC-ND), which allows third parties to download the articles and share them with others as long as they credit the author and the Abstract Book, but they cannot change the content in any way or use them commercially.
**Abstract Book Citations:** Authors, Title, HemaSphere, 2026;10:(S1):pages. The abstract book DOIs can be found on the "Author Information" page.
**Disclaimer:** Articles published in the journal HemaSphere exclusively reflect the opinions of the authors. The authors are responsible for all content in their abstracts including accuracy of the facts, statements, citing resources, etc.

**Summary/Conclusion**

AML and MDS with mutated TP53 are associated with a poor prognosis. However, in fit patients with AML and TP53 mutation, treatment with intensive chemotherapy and consolidation with allogeneic transplant offers the possibility of long-term survival.

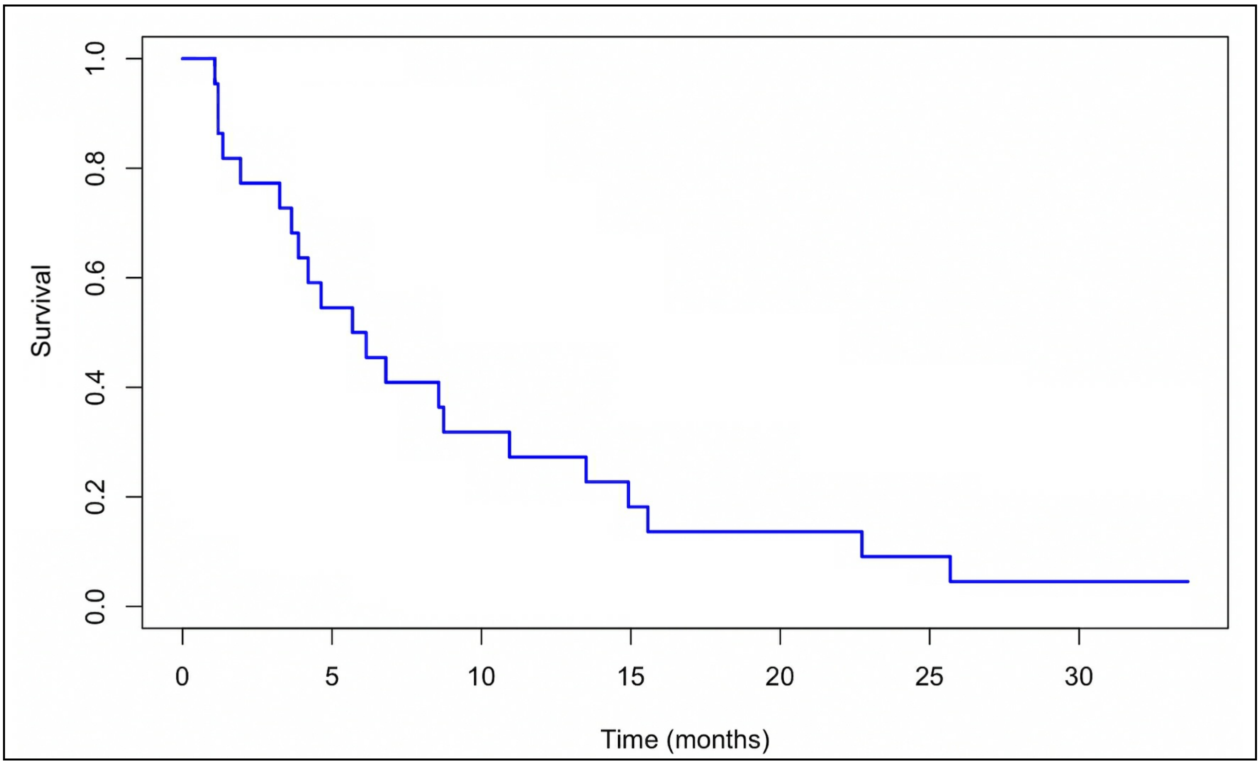

Kaplan-Meier curve for overall survival for patients with AML and MDS-IB with mutated TP53

**Copyright Information:** (Online) ISSN: 2572-9241
©2026 The Author(s). HemaSphere is published by John Wiley & Sons Ltd on behalf of European Hematology Association. This is an open access Abstract Book distributed under the Attribution-NonCommercial-NoDerivs (CC BY-NC-ND), which allows third parties to download the articles and share them with others as long as they credit the author and the Abstract Book, but they cannot change the content in any way or use them commercially.
**Abstract Book Citations:** Authors, Title, HemaSphere, 2026;10:(S1):pages. The abstract book DOIs can be found on the "Author Information" page.
**Disclaimer:** Articles published in the journal HemaSphere exclusively reflect the opinions of the authors. The authors are responsible for all content in their abstracts including accuracy of the facts, statements, citing resources, etc.

### (PB2860) REAL-WORLD PRACTICE WITH GENERIC VENETOCLAX AND GENERIC HYPOMETHYLATING (HMA) AGENTS IN NEWLY DIAGNOSED ACUTE MYELOGENOUS LEUKEMIA (AML) WITH DIFFERENT RISK GROUPS: A STUDY ON A SYRIAN COHORT

**Topic:** 04. Acute myeloid leukemia - Clinical

Maher Salamoon*[1]

*[1]al bairouni university cancer center, hematology/oncology, Damascus, Syrian Arab Republic;*

**Background**

AML is treated traditionally by induction chemotherapy (7+3) or (5+2) regimens demonstrating a high percentage of mortality rates especially with those with high-risk presentation. The emergence of Venetoclax and Hypomethylating agents has improved the tolerance of treatment and improved outcome in different risk groups, however; there are still patients showing poor compliance to treatment, further, the best Venetoclax dosing has not been reached yet.

**Aims**

Syria is a developing country with limited medical resources. The market of generic new drugs is growing overtime. In our study, we used generic sources for both Venetoclax and Hypomethylating agents (Azacitidine) given with the standard doses until disease progression in different risk groups of newly diagnosed AML patients. Our cohort was not eligible for intensive induction chemotherapy.

**Methods**

We have included 30 newly diagnosed patients with AML, given Azacitidine at a dose of 75mg/m² for 5 days every 28 days and Venetoclax at a dose ranging between 100-400mg per day in continuous according to patients' tolerance accompanied with azole antifungal therapy as prophylaxis. Those with high white blood cells received subcutaneous Cytarabine for cytoreduction before starting our treatment. Bone marrow aspiration performed after each cycle and flowcytometry was employed whenever needed to evaluate residual disease. treatment continued until disease progression.

**Results**

We have recruited newly diagnosed 30 patients with ages ranging between 18-66 years (46 in median) and a male to female ratio of 1.8:1. Documented genetic abnormalities are FLT-3 in 12%, NPM1 in 8% and AML1: ETO in 5%. 8% presented with complex Karyotype. Mean baseline total leucocyte count, platelet, & hemoglobin was 34.300/L, 36000/L. After the first month, 10 patients (33.3%) demonstrated complete morphologic remission and a residual disease of less than 5% by flowcytometry. After the second cycle, 13 patients (43/3%) demonstrated complete response on both morphology and flowcytometry. After the 4$^{th}$ cycle, 15 patients are in complete remission (50%) while 12 patients (40%) switched ti intensive chemotherapy. We lost 5 patients (16.6%) after the first cycle due to infections related to profound neutropenia. The percentage of febrile neutropenia was 86.6% after the 1$^{st}$ cycle and 26.6% after the 2$^{nd}$ one. Toxicities were acute renal failure, tumor lysis syndrome, pulmonary and urinary infections.
After a median follow-up of 14 months the overall survival documented and censored in 18 patients (60%).

**Copyright Information:** (Online) ISSN: 2572-9241
©2026 The Author(s). HemaSphere is published by John Wiley & Sons Ltd on behalf of European Hematology Association. This is an open access Abstract Book distributed under the Attribution-NonCommercial-NoDerivs (CC BY-NC-ND), which allows third parties to download the articles and share them with others as long as they credit the author and the Abstract Book, but they cannot change the content in any way or use them commercially.
**Abstract Book Citations:** Authors, Title, HemaSphere, 2026;10:(S1):pages. The abstract book DOIs can be found on the "Author Information" page.
**Disclaimer:** Articles published in the journal HemaSphere exclusively reflect the opinions of the authors. The authors are responsible for all content in their abstracts including accuracy of the facts, statements, citing resources, etc.

**Summary/Conclusion**

In our study, we have shown that generic new drugs can be employed in newly diagnosed AML patients with evident response and survival advantage in low-income countries. The study results are comparable to other studies using the brands or original versions. However, we need extra time to correlate between response rate and other factors such as genetic abnormalities, sex and karyotype status.

**Copyright Information:** (Online) ISSN: 2572-9241
©2026 The Author(s). HemaSphere is published by John Wiley & Sons Ltd on behalf of European Hematology Association. This is an open access Abstract Book distributed under the Attribution-NonCommercial-NoDerivs (CC BY-NC-ND), which allows third parties to download the articles and share them with others as long as they credit the author and the Abstract Book, but they cannot change the content in any way or use them commercially.
**Abstract Book Citations:** Authors, Title, HemaSphere, 2026;10:(S1):pages. The abstract book DOIs can be found on the "Author Information" page.
**Disclaimer:** Articles published in the journal HemaSphere exclusively reflect the opinions of the authors. The authors are responsible for all content in their abstracts including accuracy of the facts, statements, citing resources, etc.

### (PB2861) A CLINICAL CASE OF AML WITH FLT3-ITD FRAMESHIFT MUTATION

**Topic:** 04. Acute myeloid leukemia - Clinical

Aleksandra Shalyova[1, 2], Tatiana Subbotina*[1, 2], Yulia Samoylova[1], Nikita Evseev[3]

*[1]Siberian Federal University, Krasnoyarsk, Russia; [2]The Federal State-Financed Institution «Federal Siberian Research Clinical Centre under the Federal Medical Biological Agency», Krasnoyarsk, Russia; [3]Prof. V.F. Voino-Yasenetsky Krasnoyarsk State Medical University, Krasnoyarsk, Russia;*

### Background

*FLT*3-ITD mutations are usually represented by in-frame insertions that are multiples of three, causing elongation of the mutant protein while preserving the reading frame. ITD mutations that are not multiples of three and disrupt the reading frame are very rare (~0.8% of cases).

### Aims

Description of AML with *FLT3*-ITD frameshift mutation.

### Methods

*FLT3*-ITD mutations were analysed using the fragment analysis. Identification was performed using the Sanger sequencing.

**Copyright Information:** (Online) ISSN: 2572-9241
©2026 The Author(s). HemaSphere is published by John Wiley & Sons Ltd on behalf of European Hematology Association. This is an open access Abstract Book distributed under the Attribution-NonCommercial-NoDerivs (CC BY-NC-ND), which allows third parties to download the articles and share them with others as long as they credit the author and the Abstract Book, but they cannot change the content in any way or use them commercially.
**Abstract Book Citations:** Authors, Title, HemaSphere, 2026;10:(S1):pages. The abstract book DOIs can be found on the "Author Information" page.
**Disclaimer:** Articles published in the journal HemaSphere exclusively reflect the opinions of the authors. The authors are responsible for all content in their abstracts including accuracy of the facts, statements, citing resources, etc.

### Results

During the screening of patients with AML for the presence of mutations in the *FLT3* gene, one patient was found to have an *FLT3*-ITD frameshift mutation (65 bp, AF=85.7%) (Fig. 1).
The onset of the disease was in Dec. 2021 (the patient was 63 years old). Upon admission, G2 metaplastic anaemia, G4 thrombocytopenia, hyperleukocytosis 181x10*9/L due to blasts of 86%, myelogram showing subtotal blast metaplasia (90%), positive (60%) lipids and MPO, and according to the immunophenotyping of bone marrow (BM), a phenotype characteristic of AML M4 according to FAB was identified. On Dec. 20, 2021, cytoreductive therapy with HU was initiated, followed by remission induction according to the '7+3 dauno' protocol from Dec. 30, 2021, to Jan. 5, 2022. On Feb. 1, 2022, a sternal puncture was performed: subtotal BM blastosis (88%) persisted. On Feb. 08, 2022, the patient was re-admitted with symptoms of tumour intoxication. On Feb. 10, 2022, remission reinduction was initiated according to the '5-AZA-Ida-AraC' protocol. On day +6 of the course, an *FLT3*-ITD mutation was detected and Midostaurin (Mido) was added to the therapy at the standard dosage. Administration of the FLT3 inhibitor was continued until Mar. 2, 2022. Further, by 28 Mar. 2022, blasts accounted for 75% of peripheral blood and 84% of BM. A decision was made to conduct two courses of '5-AZA-Ida-AraC+Mido'. Since Apr. 17, 2022, episodes of hyperthermia occurred, CRP–131, PCT–10.4, according to chest X-ray data: presence of right upper lobe pneumonia. By Apr. 19: further negative dynamics in the form of respiratory failure, continuing episodes of fever, and episodes of epistaxis. Multiple organ failure syndrome developed on May 1, 2022, requiring inotropic support and mechanical ventilation. Biological death was confirmed on May 3, 2022.
Sanger sequencing revealed that the identified *FLT3*-ITD mutation is represented by a tandem duplication c.1761_1825dup. This leads to a change in part of the FLT3 protein sequence at the end of exon 14 and the formation of an early stop codon at the B1-sheet locus, at the beginning of the TKD1 domain, and shortening of the protein from 993 amino acids to 631. The inhibitory effect of Mido lies in its interaction with the ATP-binding site of the FLT3 protein activation loop, which is located in the TKD2 region of the tyrosine kinase domain, and its subsequent inactivation. Due to the identified mutation, the protein completely loses the TKD2 domain, as a result of which Mido cannot bind to mutant FLT3 and inhibit it. However, also due to the loss of the TKD2 domain by mutant FLT3, the protein must completely lose its kinase activity.

### Summary/Conclusion

It is likely that the identified *FLT3*-ITD frameshift mutation affected the structure and function of the FLT3 protein, as well as its ability to bind to Mido. At the same time, the rapid progression of the disease in the patient and the lack of response to induction therapy are most likely due to the presence of other mutations from the high-risk progression group according to ELN 2022.

**Copyright Information:** (Online) ISSN: 2572-9241
©2026 The Author(s). HemaSphere is published by John Wiley & Sons Ltd on behalf of European Hematology Association. This is an open access Abstract Book distributed under the Attribution-NonCommercial-NoDerivs (CC BY-NC-ND), which allows third parties to download the articles and share them with others as long as they credit the author and the Abstract Book, but they cannot change the content in any way or use them commercially.
**Abstract Book Citations:** Authors, Title, HemaSphere, 2026;10:(S1):pages. The abstract book DOIs can be found on the "Author Information" page.
**Disclaimer:** Articles published in the journal HemaSphere exclusively reflect the opinions of the authors. The authors are responsible for all content in their abstracts including accuracy of the facts, statements, citing resources, etc.

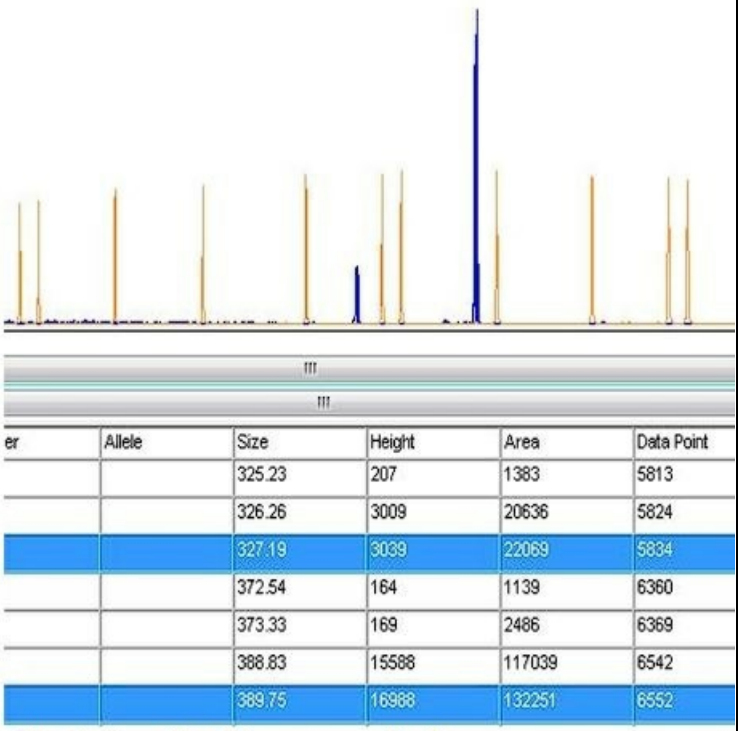

| er | Allele | Size | Height | Area | Data Point |
|---|---|---|---|---|---|
| | | 325.23 | 207 | 1383 | 5813 |
| | | 326.26 | 3009 | 20636 | 5824 |
| | | 327.19 | 3039 | 22069 | 5834 |
| | | 372.54 | 164 | 1139 | 6360 |
| | | 373.33 | 169 | 2486 | 6369 |
| | | 388.83 | 15588 | 117039 | 6542 |
| | | 389.75 | 16988 | 132251 | 6552 |

Fig.1 - Result of fragment analysis.

**Copyright Information:** (Online) ISSN: 2572-9241
©2026 The Author(s). HemaSphere is published by John Wiley & Sons Ltd on behalf of European Hematology Association. This is an open access Abstract Book distributed under the Attribution-NonCommercial-NoDerivs (CC BY-NC-ND), which allows third parties to download the articles and share them with others as long as they credit the author and the Abstract Book, but they cannot change the content in any way or use them commercially.
**Abstract Book Citations:** Authors, Title, HemaSphere, 2026;10:(S1):pages. The abstract book DOIs can be found on the “Author Information” page.
**Disclaimer:** Articles published in the journal HemaSphere exclusively reflect the opinions of the authors. The authors are responsible for all content in their abstracts including accuracy of the facts, statements, citing resources, etc.

### (PB2862) TARGETED THERAPIES MODULATE ANTIFUNGAL HOST DEFENSE IN CHRONIC LYMPHOCYTIC LEUKEMIA

**Topic:** 05. Chronic lymphocytic leukemia and related disorders - Biology & translational research

Daniele Sorcini[1], Sara Alhihi[1], Filomena De Falco[1], Luigi Mari[2], Laura Mariano[1], Gianluca Vascelli[1], Alessia Sulla[1], Martina Bordoni[1], Gandino Mencarelli[1], Francesco Grignani[1], Chiara Brachelente[1], Giorgia Renga[1], Teresa Zelante[1], Paolo Sportoletti[1], Vasileios Oikonomou*[1]

*[1]University of Perugia, Medicine and Surgery, Perugia, Italy; [2]St. Jude Children's Research Hospital, Department of Immunology, Memphis, United States of America;*

### Background

Patients with chronic lymphocytic leukemia (CLL) are at increased risk of invasive fungal infections, particularly invasive pulmonary aspergillosis. Targeted therapies have improved CLL outcomes; however, some treatments, including the Bruton tyrosine kinase (BTK) inhibitor ibrutinib, may interfere with immune pathways involved in antifungal defense, increasing risk of invasive fungal infections. LC3-associated phagocytosis (LAP), a form of non-canonical autophagy, is critical for antifungal host defense against *Aspergillus fumigatus*. The impact of BTK inhibitors on LAP-mediated antifungal immunity in CLL remains incompletely understood.

### Aims

We investigated the effects of ibrutinib on LAP activation and antifungal host defense in CLL, with a particular focus on its impact on *Aspergillus fumigatus* clearance in preclinical murine models and primary CLL patient samples.

### Methods

LAP activation was assessed in vitro following exposure to ibrutinib by measuring specific markers of the LAP pathway and reactive oxygen species production. Antifungal responses were evaluated via fungal growth, germination, and fungus-induced cellular damage. In vivo studies used TCL-1 transgenic and adoptive transfer CLL mouse models infected with *Aspergillus fumigatus* and treated with ibrutinib; lung fungal burden and histopathology were quantified. RNA-seq analysis assessed transcriptional changes in LAP-related and antifungal pathways. Translational analyses were performed on primary CLL samples from patients receiving ibrutinib.

### Results

Ibrutinib impaired LAP activation, resulting in reduced fungal clearance and increased pulmonary inflammation in adoptive transfer mouse models of CLL. RNA-seq revealed downregulation of key LAP-associated genes and antifungal pathways, reflecting impaired fungal clearance. Leukemia control was achieved, but treated mice had higher *Aspergillus* burden than untreated controls. Primary CLL samples confirmed therapy-associated alterations in LAP-related pathways, supporting translational relevance.

### Summary/Conclusion

Ibrutinib modulates LAP-mediated antifungal host defense in CLL, increasing susceptibility to invasive fungal infections. Transcriptional profiling suggests downregulation of LAP-associated genes as a potential mechanism, providing mechanistic insight into infection risk during BTK inhibitor therapy. LAP represents a potential target to preserve antifungal host defense, with ongoing studies in our lab evaluating other BTK inhibitors.

**Copyright Information:** (Online) ISSN: 2572-9241
©2026 The Author(s). HemaSphere is published by John Wiley & Sons Ltd on behalf of European Hematology Association. This is an open access Abstract Book distributed under the Attribution-NonCommercial-NoDerivs (CC BY-NC-ND), which allows third parties to download the articles and share them with others as long as they credit the author and the Abstract Book, but they cannot change the content in any way or use them commercially.
**Abstract Book Citations:** Authors, Title, HemaSphere, 2026;10:(S1):pages. The abstract book DOIs can be found on the "Author Information" page.
**Disclaimer:** Articles published in the journal HemaSphere exclusively reflect the opinions of the authors. The authors are responsible for all content in their abstracts including accuracy of the facts, statements, citing resources, etc.

### (PB2863) QUANTITATIVE AND HIGH-DIMENSIONAL PROFILING REVEALS PROGRESSIVE CD8+ T CELL REMODELING AND THERAPY SPECIFIC IMMUNE SIGNATURES IN CLL

**Topic:** 05. Chronic lymphocytic leukemia and related disorders - Biology & translational research

Roberta Arcaleni[1], Fabio Gurrieri*[1], Daniele Sorcini[1], Filomena De Falco[1], Angela Esposito[1], Andrea Atzeni[1], Gabriele Astolfi[1], Miriam Pugliese[1], Valentina Puglisi[1], Letizia Valmarini[1], Lorenzo Moretti[1], Maria Grazia Mameli[1], Paolo Sportoletti[1]

*[1]Institute of Hematology and Center for Hemato-Oncology Research (CREO), University of Perugia, Santa Maria della Misericordia Hospital, Department of Medicine and Surgery, Perugia, Italy;*

### Background

Chronic Lymphocytic Leukemia (CLL) is the most common leukemia in Western countries, predominantly affecting the elderly. It arises from pre-malignant Monoclonal B Lymphocytosis (MBL) and may evolve into Richter syndrome (Landau et al. 2015). Despite targeted therapies, CLL remains incurable. Increasing evidence highlights the pivotal role of the tumor microenvironment (TME) in disease pathogenesis and progression (López-Guerra et al. 2020; Friedman et al. 2024).

### Aims

The goals of this study were: 1) identify new populations or mechanisms involved in disease evolution along the MBL-CLL continuum; 2) identify how different targeted therapies used in CLL affect the T cell compartment.

### Methods

Peripheral blood (PB) of patients were collected from Healthy Controls (HC), MBL and CLL diagnosis; in the PB of CLL patients we measured T populations after treatments. Multiparametric flow cytometry was used to define and analyze the CD4+ and CD8+ T cell compartments, including naïve, $T_{CM}$, $T_{EM}$, and $T_{EMRA}$ cells. Within the CD8+ compartment, we defined early differentiated T cells and terminally differentiated T cells. We also applied the mass cytometry–based algorithms t-SNE and v-SNE to evaluate the clustering patterns of different T cell subsets.

**Copyright Information:** (Online) ISSN: 2572-9241
©2026 The Author(s). HemaSphere is published by John Wiley & Sons Ltd on behalf of European Hematology Association. This is an open access Abstract Book distributed under the Attribution-NonCommercial-NoDerivs (CC BY-NC-ND), which allows third parties to download the articles and share them with others as long as they credit the author and the Abstract Book, but they cannot change the content in any way or use them commercially.
**Abstract Book Citations:** Authors, Title, HemaSphere, 2026;10:(S1):pages. The abstract book DOIs can be found on the "Author Information" page.
**Disclaimer:** Articles published in the journal HemaSphere exclusively reflect the opinions of the authors. The authors are responsible for all content in their abstracts including accuracy of the facts, statements, citing resources, etc.

**Results**

Among the CLL patients, age was associated with T-cell alterations, with patients ≥75 years showing reduced total CD3+ T cells, particularly CD8+ naïve (respectively <65y=131.5 cells/µl n=18, 65≤y<75=121.5 cells/µl n=18, ≥75y=36.5 cells/ul n=24) and $T_{EMRA}$ subsets (respectively <65y=133.5 cells/µl n=18, 65≤y<75=161 cells/µl n=18, ≥75y=75 cells/ul n=24). The comparison between HC, MBL and CLL revealed an early T cell dysregulation in MBL, including loss of CD8+ naïve cells (respectively HC=238 cells/µl n=6, MBL=116 cells/µl n=15, CLL=88 cells/ul n=60) with progressive changes in CD4+ and CD8+ memory and effector subsets in CLL, accompanied by increased T cell activation. Clustering analysis with t-SNE and v-SNE among HC, MBL and CLL revealed that there are no appreciable differences in CD4+, while on CD8+ there is a progressive increase of the subset CD45RA+, CD57-, CD27- (respectively HC=0%, MBL=3.6%, CLL=6.3%). Targeted therapies differently modulated T cell homeostasis: Venetoclax reduced CD4+ (respectively Untreated CLL (U-CLL)=1478 cells/ul n=16, BTKi=1567 cells/ul n=8, BCL2i=528,5 cells/ul n=4, MBL=1087 cells/ul n=15) and CD8+ subsets, whereas BTK inhibitors restored CD4+ levels and expanded CD8+ T cells toward pre-leukemic (MBL) profiles (respectively U-CLL=876 cells/ul n=16, BTKi=1326 cells/ul n=8, BCL2i=563,5 cells/ul n=4, MBL=398 cells/ul n=15). Among treatments, clustering analysis in CD4+ showed that both BTKi and BCL2i have more CD57+CD45RA-CD27- (respectively U-CLL=40%, BTKi=49.2%, BCL2i=59.6%), while in U-CLL there are more CD57+CD45RA+CD27-, compatible with an exhausted T subset (respectively U-CLL=32.9%, BTKi=20.7%, BCL2i=16.8%). While clustering analysis in CD8+ showed that both BTKi and BCL2i have more CD57+CD45RA+CD27+, with a terminally differentiated phenotype (respectively U-CLL=19.9%, BTKi=29.4%, BCL2i=36%); in BCL2i treatment there is a decrease of CD8+CD45RA+CD57- T population (respectively U-CLL=35%, BTKi=34%, BCL2i=12%).

**Summary/Conclusion**

T cell dysregulation progressively increases from HC to MBL and finally to CLL. Targeted therapies partially reverse the exhaustion phenotype observed in CLL.

**Copyright Information:** (Online) ISSN: 2572-9241
©2026 The Author(s). HemaSphere is published by John Wiley & Sons Ltd on behalf of European Hematology Association. This is an open access Abstract Book distributed under the Attribution-NonCommercial-NoDerivs (CC BY-NC-ND), which allows third parties to download the articles and share them with others as long as they credit the author and the Abstract Book, but they cannot change the content in any way or use them commercially.
**Abstract Book Citations:** Authors, Title, HemaSphere, 2026;10:(S1):pages. The abstract book DOIs can be found on the "Author Information" page.
**Disclaimer:** Articles published in the journal HemaSphere exclusively reflect the opinions of the authors. The authors are responsible for all content in their abstracts including accuracy of the facts, statements, citing resources, etc.

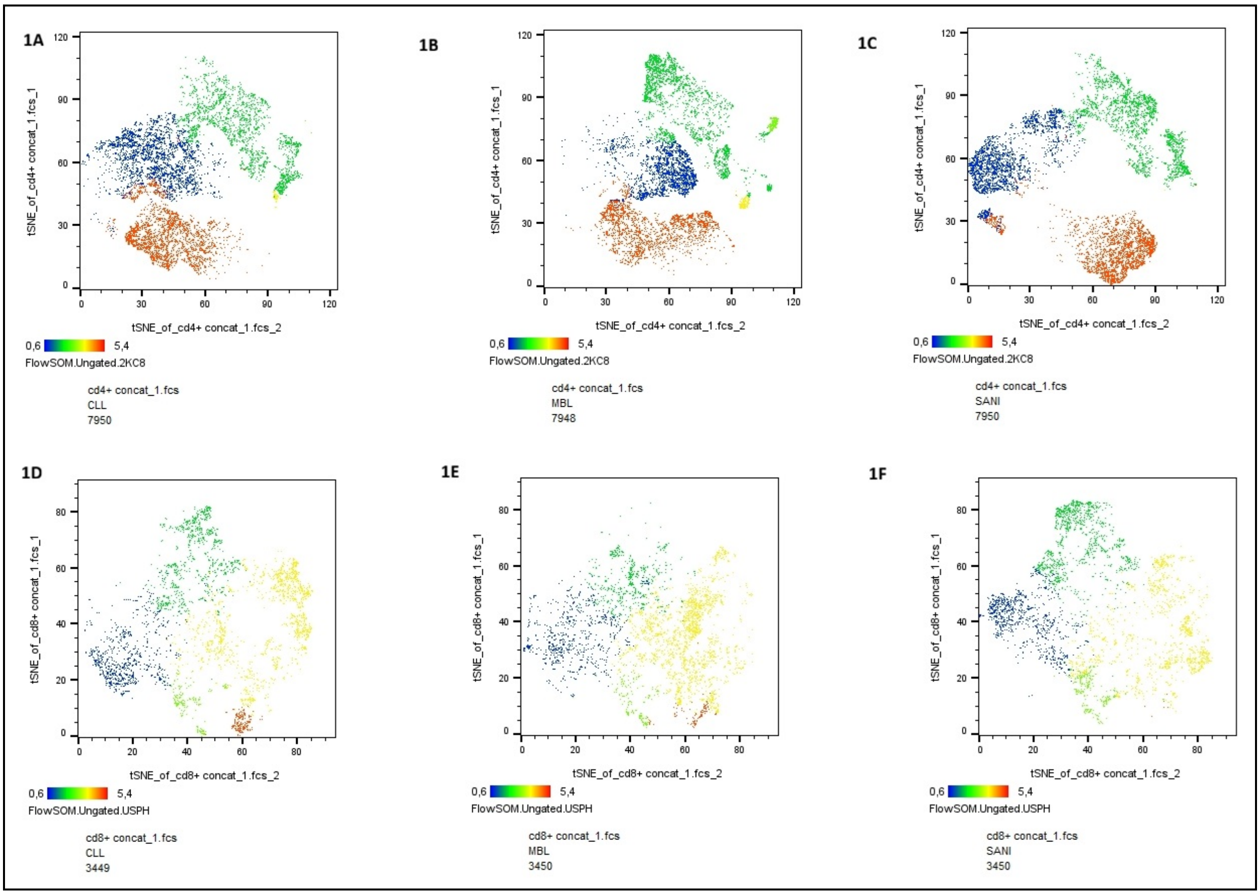

Figure 1. FlowSOM analysis in CLL, MBL and HC, representing the different clustering analysis of CD4+ (1A-1C) and CD8+ T cells (1D-1F).

**Copyright Information:** (Online) ISSN: 2572-9241
©2026 The Author(s). HemaSphere is published by John Wiley & Sons Ltd on behalf of European Hematology Association. This is an open access Abstract Book distributed under the Attribution-NonCommercial-NoDerivs (CC BY-NC-ND), which allows third parties to download the articles and share them with others as long as they credit the author and the Abstract Book, but they cannot change the content in any way or use them commercially.
**Abstract Book Citations:** Authors, Title, HemaSphere, 2026;10:(S1):pages. The abstract book DOIs can be found on the "Author Information" page.
**Disclaimer:** Articles published in the journal HemaSphere exclusively reflect the opinions of the authors. The authors are responsible for all content in their abstracts including accuracy of the facts, statements, citing resources, etc.

### (PB2864) METABOLIC HETEROGENEITY IN TREATMENT-NAIVE CHRONIC LYMPHOCYTIC LEUKEMIA DEFINED BY MULTI-PARAMETRIC MITOCHONDRIAL PROFILING

**Topic:** 05. Chronic lymphocytic leukemia and related disorders - Biology & translational research

William Wierda*[1], Nitin Jain[1], Alessandra Ferrajoli[1], Mahesh Swaminathan[1], Jan Burger[1], Mariela Sivina[1], Rong Chen[1], Kaitlyn Maffuid Quebbemann[2], Sierra Simpson[2, 3], Hemal Patel[2, 3, 4]

*[1]MD Anderson Cancer Center, Houston, United States of America;[2]Versea Discovery Inc., San Diego, United States of America; [3]University of California, San Diego, United States of America;[4]VA San Diego Healthcare Systems, San Diego, United States of America;*

### Background

Chronic lymphocytic leukemia (CLL) exhibits marked clinical and biological heterogeneity driven by cytogenetic and molecular features. Increasing evidence implicates metabolic reprogramming as a determinant of disease behavior. We applied a next-generation mitochondrial and metabolic efficiency platform (mescreen, Versea, Inc.) to characterize bioenergetic phenotypes in patients with untreated CLL and correlated metabolic signatures with established prognostic markers.

### Methods

Fifty treatment-naïve plasma samples from patients with CLL were analyzed. Clinical and genomic annotation included Rai stage, absolute lymphocyte count, IGHV mutation status (mutated [M-CLL], n=25; unmutated [U-CLL], n=25), hierarchical FISH cytogenetics (del(13q), n=32; normal, n=9; del(11q), n=4; trisomy 12, n=4; del(17p), n=1), and 162-gene next-generation sequencing (EndLymphoma panel). mescreen, a series of adoptive plasma transfer assays, quantified nine functional metabolic parameters: baseline respiration, mitochondrial efficiency, mitochondrial potential, glycolytic score, mitochondrial score, baseline reactive oxygen species (ROS), stressed ROS, baseline mitochondrial network, and stressed mitochondrial network. Associations were evaluated using multivariate modeling and clustering approaches.

### Results

Trisomy 12 CLL emerged as the most metabolically distinct subgroup, demonstrating significantly elevated glycolytic ($p<0.0001$) and mitochondrial scores ($p=0.0009$) compared with deletion-associated subgroups, together with pronounced mitochondrial network fragmentation under stress ($p=0.0003$). This concurrent upregulation of glycolysis and oxidative phosphorylation suggests a bioenergetically flexible phenotype potentially supporting proliferation under metabolic stress.
U-CLL exhibited increased basal ROS and glycolytic activity relative to M-CLL, consistent with enhanced tonic B-cell receptor signaling and PI3K/AKT pathway activation. In
contrast, M-CLL demonstrated higher stressed mitochondrial network scores, indicating greater adaptive mitochondrial remodeling capacity. Partial least squares discriminant analysis identified basal ROS (VIP=1.70) and stressed mitochondrial network (VIP=1.43) as principal discriminators of IGHV-defined subgroups. Unsupervised hierarchical clustering independently resolved biologically coherent clusters aligned with cytogenetic and mutational features.
Mutation-specific metabolic perturbations were observed. NOTCH1 and ATM mutations were associated with elevated ROS, while MYD88 and CHD2 mutations correlated with innate immune–linked oxidative stress signatures. SF3B1 mutations were associated with altered mitochondrial efficiency. Increasing somatic mutation burden (≥3 mutations) correlated with cumulative oxidative stress and enhanced glycolysis.

**Copyright Information:** (Online) ISSN: 2572-9241
©2026 The Author(s). HemaSphere is published by John Wiley & Sons Ltd on behalf of European Hematology Association. This is an open access Abstract Book distributed under the Attribution-NonCommercial-NoDerivs (CC BY-NC-ND), which allows third parties to download the articles and share them with others as long as they credit the author and the Abstract Book, but they cannot change the content in any way or use them commercially.
**Abstract Book Citations:** Authors, Title, HemaSphere, 2026;10:(S1):pages. The abstract book DOIs can be found on the "Author Information" page.
**Disclaimer:** Articles published in the journal HemaSphere exclusively reflect the opinions of the authors. The authors are responsible for all content in their abstracts including accuracy of the facts, statements, citing resources, etc.

**Summary/Conclusion**

Multi-dimensional metabolic profiling using the mescreen platform identifies distinct bioenergetic phenotypes in patients with CLL that align with cytogenetic and molecular heterogeneity. These data provide a framework for integrating metabolic state with treatment responder and risk stratification, and therapeutic development, including strategies that exploit disease-specific metabolic vulnerabilities. Additional patient numbers are needed to more fully characterize metabolic profiling by disease characteristics, such as for del(17p) CLL. Ongoing longitudinal studies will evaluate dynamic metabolic remodeling during targeted therapy.

**Copyright Information:** (Online) ISSN: 2572-9241
©2026 The Author(s). HemaSphere is published by John Wiley & Sons Ltd on behalf of European Hematology Association. This is an open access Abstract Book distributed under the Attribution-NonCommercial-NoDerivs (CC BY-NC-ND), which allows third parties to download the articles and share them with others as long as they credit the author and the Abstract Book, but they cannot change the content in any way or use them commercially.
**Abstract Book Citations:** Authors, Title, HemaSphere, 2026;10:(S1):pages. The abstract book DOIs can be found on the "Author Information" page.
**Disclaimer:** Articles published in the journal HemaSphere exclusively reflect the opinions of the authors. The authors are responsible for all content in their abstracts including accuracy of the facts, statements, citing resources, etc.

### (PB2865) VENETOCLAX IN COMBINATION WITH THE DNA-PK INHIBITOR M3814 (PEPOSERTIB) AS A NOVEL THERAPEUTIC STRATEGY FOR RESISTANT CHRONIC LYMPHOCYTIC LEUKEMIA

**Topic:** 05. Chronic lymphocytic leukemia and related disorders - Biology & translational research

Clare Hibbert*[1, 2], Ali Saleh[1, 2], Sara Kost[2], Evgeniia Maksimova[1, 2], Marcelo Ninalaya[1, 2], Abdo-Jose Tomajian[1, 2], Yongqiang Chen[2], Dana Henderson[3], James Thliveris[3], Bozena Kuzio[1, 2], Rene Zahedi[4], Versha Banerji[2, 4, 5], Lin Yang[2, 4], Spencer Gibson[6], James Johnston[2, 4], Sachin Katyal[1, 2]

*[1]University of Manitoba, Pharmacology and Therapeutics, Winnipeg, Canada; [2]Paul Albrechtsen Research Institute, CancerCare Manitoba, Winnipeg, Canada; [3]University of Manitoba, Human Anatomy and Cell Science, Winnipeg, Canada;[4]University of Manitoba, Internal Medicine, Winnipeg, Canada; [5]University of Manitoba, Biochemistry and Medical Genetics, Winnipeg, Canada; [6]University of Alberta, Oncology, Edmonton, Canada;*

### Background

Venetoclax (VEN) is a BH3 mimetic preventing the mitochondrial interactions of BCL2 with BAX/BAK, leading to apoptosis. Additionally, VEN may also directly affect mitochondrial function. In chronic lymphocytic leukemia (CLL), VEN with an anti-CD20 antibody is a standard first- or second-line therapy, but relapse occurs, more rapidly in patients with *TP53* dysfunction, unmutated *IGHV* and bulky lymph nodes. Moreover, these drug combinations cause myelo- and immune-suppression. Resistance to VEN may be reversed by altering the levels of the BCL2 family of proteins or inhibiting mitochondrial function.

Activated (phosphorylated) DNA-dependent protein kinase (pDNA-PK$^{S2056}$) is primarily involved in DNA repair but may also influence metabolism and mitochondrial function. M3814, a new well-tolerated oral inhibitor of DNA-PK, is not myelosuppressive and presently in phase II clinical trials. We previously found that M3814 is synergistic with chemotherapy, but not ibrutinib, in CLL cells (Kost, S. et al 2023, Blood Cancer J).

### Aims

1. Determine if M3814 is cytotoxic to CLL cells *ex vivo* and can potentiate the activity of VEN.
2. Determine if M3814 affects mitochondrial function and whether this underpins M3814 and VEN synergism.

### Methods

The activities of M3814 and/or VEN were evaluated in 31 patient samples obtained through the Manitoba CLL Clinic and Tumour Bank. Cells were incubated for 72h +/- CD40L to simulate the protective CLL microenvironment. Cell death/synergy was measured by 7AAD/Annexin V staining and Combenefit analysis. Two acute myeloid leukemia (AML) cell lines and one CLL cell line (MEC1) were also evaluated. Metabolism of CLL cells was analyzed by Seahorse while mitochondria structure via electron microscopy. The Comet assay assessed for DNA breaks. To validate changes in levels of pDNA-PK +/- M3814, pDNA-PK/DNA-PK ratios and BCL2 family members, protein levels were measured via Western analysis.

**Copyright Information:** (Online) ISSN: 2572-9241
©2026 The Author(s). HemaSphere is published by John Wiley & Sons Ltd on behalf of European Hematology Association. This is an open access Abstract Book distributed under the Attribution-NonCommercial-NoDerivs (CC BY-NC-ND), which allows third parties to download the articles and share them with others as long as they credit the author and the Abstract Book, but they cannot change the content in any way or use them commercially.
**Abstract Book Citations:** Authors, Title, HemaSphere, 2026;10:(S1):pages. The abstract book DOIs can be found on the "Author Information" page.
**Disclaimer:** Articles published in the journal HemaSphere exclusively reflect the opinions of the authors. The authors are responsible for all content in their abstracts including accuracy of the facts, statements, citing resources, etc.

### Results

There was marked variation in CLL cell sensitivity to M3814 and VEN with the median inhibitory concentrations for cell viability ($IC_{50}$) being 2.4 µM (range, 0.27-20 µM) and 1.7 nM (range, 0.21-20 nM) for M3814 and VEN, respectively. No significant correlation was found between cell sensitivities to M3814 and VEN. M3814-resistant cells were more likely to have been exposed to prior therapy, be *IGHV* unmutated and/or had p53 dysfunction. These cells also showed increased levels of anti-apoptotic proteins (pMCL1, pBCL2, BCL2, pBAX), but not increased pDNA-PK. Synergy was seen between M3814 and VEN, particularly in VEN-resistant cells and those treated with CD40L. However, synergy was also seen in AML cell lines indicating this is not a tumour-specific phenomenon. The Comet assay demonstrated that VEN did not induce DNA breaks. In contrast, metabolic studies using Seahorse showed that M3814 significantly decreased maximal respiration, and in MEC1 cells M3814 was toxic to mitochondria, causing swelling and disruption of cristae.  Ongoing studies with proteomics are evaluating the mitochondrial protein changes induced by M3814 and whether M3814 influences the effect of VEN on normal lymphocytes and marrow.

### Summary/Conclusion

These novel results indicate that DNA-PK inhibition by M3814 is cytotoxic to CLL cells and enhances the activity of VEN particularly in VEN-resistant cells, possibly due to M3814 toxicity to mitochondria. As M3814 is not myelosuppressive it may clinically improve the therapeutic efficacy of VEN, in addition to potentially reversing drug resistance.

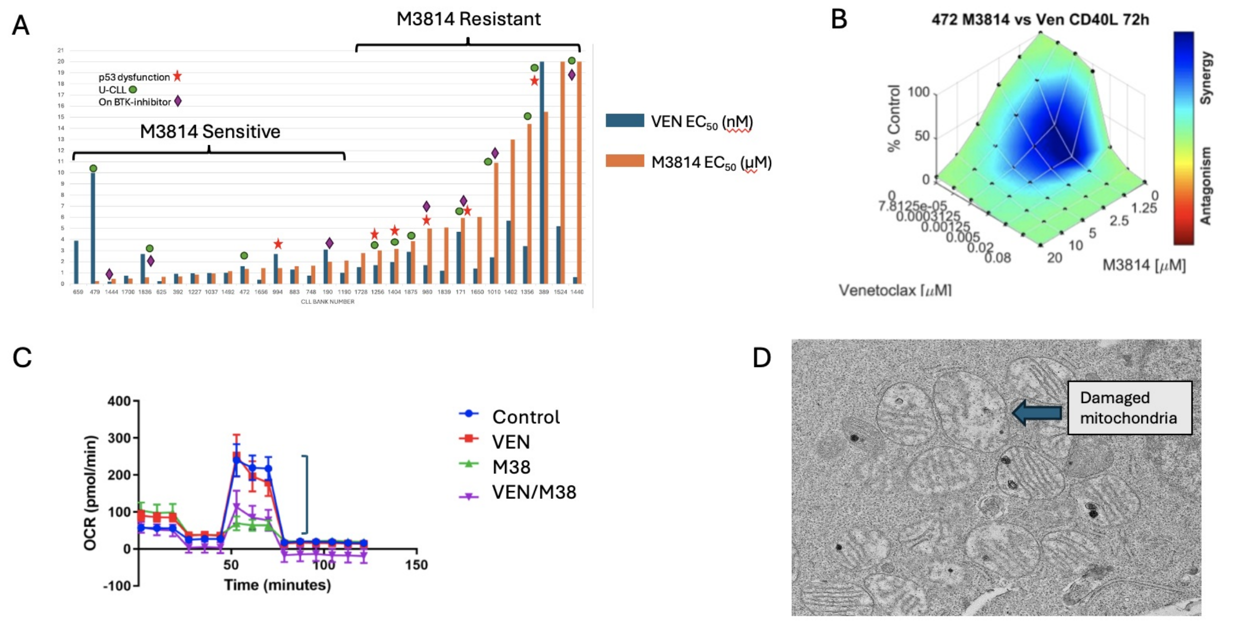

A) $EC_{50}$ values for Venetoclax (VEN) and M3814 in 31 CLL patient samples. B) **Synergistic cell death** caused by the **combination of VEN and M3814** in primary CLL patient cells. C) Seahorse XF Cell Mito Stress Test in CLL cells shows that **M3814 reduces maximal respiration**. D) Electron microscopy of M3814-treated MEC-1 cells display **swollen mitochondria and disrupted cristae**.

**Copyright Information:** (Online) ISSN: 2572-9241
©2026 The Author(s). HemaSphere is published by John Wiley & Sons Ltd on behalf of European Hematology Association. This is an open access Abstract Book distributed under the Attribution-NonCommercial-NoDerivs (CC BY-NC-ND), which allows third parties to download the articles and share them with others as long as they credit the author and the Abstract Book, but they cannot change the content in any way or use them commercially.
**Abstract Book Citations:** Authors, Title, HemaSphere, 2026;10:(S1):pages. The abstract book DOIs can be found on the "Author Information" page.
**Disclaimer:** Articles published in the journal HemaSphere exclusively reflect the opinions of the authors. The authors are responsible for all content in their abstracts including accuracy of the facts, statements, citing resources, etc.

### (PB2866) A COMBINED IMMUNOPHENOTYPIC, TCR, AND MOLECULAR APPROACH TO DEFINE THE SPECTRUM OF CLONAL T-LGL DISORDERS

**Topic:** 05. Chronic lymphocytic leukemia and related disorders - Biology & translational research

Elena Buson*[1, 2], Valentina Trimarco[1], Luca Massarotti[1], Giulia Calabretto[1, 2], Elisa Rampazzo[1, 2], Rebecca Crivellari[1, 2], Giulia Pertile[1, 2], Monica Facco[1], Livio Trentin[1], Gianpietro Semenzato[2], Renato Zambello[1, 2], Antonella Teramo[1, 2]

*[1]University of Padova, Department of Medicine, Hematology division, Padova, Italy;[2]Veneto Institute of Molecular Medicine, Padova, Italy;*

### Background

T-LGLL is a chronic clonal disorder of cytotoxic T lymphocytes often associated with neutropenia and activating STAT mutations. From a biological standpoint, T-LGLL is classified according to T-cell receptor (TCR) expression into αβ and γδ variants, which differ in immunophenotype and possibly in clinical behavior. The identification of small T-cell clones sharing overlapping immunophenotypic and molecular features has blurred the distinction among LGL disorders, including T-cell Clones of Uncertain Significance (T-CUS), Low-Count (LC) T-cell Large Granular Lymphocyte Leukemia (T-LGLL) and full blown T-LGLL. However, current classification largely relies on arbitrary numerical thresholds (500/1,000 LGL/µL), without fully integrating clinical and molecular features into disease stratification.

### Aims

This study challenges the use of numerical cut-offs and proposes an integrated approach combining immunophenotypic, molecular and clinical features to better define T-LGL proliferations.

### Methods

A cohort of 175 patients was consecutively enrolled and classified according to clinical features and LGL count as full blown T-LGLL (34%), LC T-LGLL (20%) and T-CUS (46%). Patients were stratified based on immunophenotype, TCR expression (αβ and γδ) and STAT3/STAT5B mutational status. TCR (Vβ, Vγ/Vδ repertoire) and TRBC1/2 expression were evaluated (positivity ≥85% or ≤15%).

**Copyright Information:** (Online) ISSN: 2572-9241
©2026 The Author(s). HemaSphere is published by John Wiley & Sons Ltd on behalf of European Hematology Association. This is an open access Abstract Book distributed under the Attribution-NonCommercial-NoDerivs (CC BY-NC-ND), which allows third parties to download the articles and share them with others as long as they credit the author and the Abstract Book, but they cannot change the content in any way or use them commercially.
**Abstract Book Citations:** Authors, Title, HemaSphere, 2026;10:(S1):pages. The abstract book DOIs can be found on the "Author Information" page.
**Disclaimer:** Articles published in the journal HemaSphere exclusively reflect the opinions of the authors. The authors are responsible for all content in their abstracts including accuracy of the facts, statements, citing resources, etc.

**Results**

Within full blown T-LGLL we predominantly identified Tαβ-LGLL subtype (84%), while 15% were Tγδ-LGLL cases. In contrast, Tαβ and Tγδ subsets were more evenly distributed in LC T-LGLL patients and in T-CUS. STAT3 mutations were mainly detected in CD8+ Tαβ-LGLL (29%) and Tγδ-LGLL (56%), and in a minority of LC CD8+ Tαβ-LGLL (23%). Only CD4+ Tαβ-LGLL cases (8%) harboured STAT5B mutations. No *STAT* mutations were identified in T-CUS patients. Immunophenotypic analysis revealed distinct patterns among patient subgroups ($p<0.05$). In detail, symptomatic CD8+ Tαβ STAT3-mutated (MUT) T-LGLL cases displayed a terminal effector memory phenotype (CD28-/CD62L-/CD45RA+/CD45RO-). Differently, Tγδ-LGLL exhibited CD28-/CD62L+ expression and LC T-LGLL, including symptomatic patients, were similarly identified by CD28-/CD62L+ phenotype, independently on TCR subtype. In contrast, most T-CUS cases exhibited a less differentiated phenotype, marked by CD28+/CD62L+ (63% Tαβ and 82% Tγδ). All Tγδ-LGLL patients and asymptomatic Tαβ-LGLL cases were CD45RA+/CD45RO+. This intermediate memory-to-effector phenotype was also detected in LC T-LGLL (52% Tαβ and 31% Tγδ) and T-CUS (64% Tαβ and 38% Tγδ) patients. CD57 is almost universally expressed in T-LGLL; whereas, CD56- cell expansions characterizes symptomatic patients. Similarly, 64% of LC T-LGLL cases were CD56-. Conversely, a CD56+ phenotype was observed in 80% of T-CUS patients, suggesting an association with indolent disease. Regarding TCR repertoire, VB2 was frequently expressed in CD8+ T-CUS and VB13.1 was significant enriched in CD4+ Tαβ-T-LGLL and CD8+ T-CUS, compared with other patient subgroups. Vδ2+ clones were prevalent in Tγδ-CUS cases, whereas LC Tγδ-LGL were Vδ2-. TRBC1/2 restriction confirmed clonality across all Tab disease subsets.

**Summary/Conclusion**

Beyond the recognized heterogeneity of T-LGLL, we identified distinct immunophenotypic and molecular patterns that help distinguish indolent T-CUS from LC T-LGLL and full blown T-LGLL, dissecting their biological features and contributing to a more refined patient classification.

**Copyright Information:** (Online) ISSN: 2572-9241
©2026 The Author(s). HemaSphere is published by John Wiley & Sons Ltd on behalf of European Hematology Association. This is an open access Abstract Book distributed under the Attribution-NonCommercial-NoDerivs (CC BY-NC-ND), which allows third parties to download the articles and share them with others as long as they credit the author and the Abstract Book, but they cannot change the content in any way or use them commercially.
**Abstract Book Citations:** Authors, Title, HemaSphere, 2026;10:(S1):pages. The abstract book DOIs can be found on the "Author Information" page.
**Disclaimer:** Articles published in the journal HemaSphere exclusively reflect the opinions of the authors. The authors are responsible for all content in their abstracts including accuracy of the facts, statements, citing resources, etc.

### (PB2867) STAT3-DEPENDENT CIRCRNAS IN T-LGLL: CIRCPVT1, CIRCZBTB46, AND CIRCSETBP1 AS POTENTIAL CLINICAL BIOMARKERS

**Topic:** 05. Chronic lymphocytic leukemia and related disorders - Biology & translational research

Rebecca Crivellari*[1, 2], Elisa Rampazzo[1, 2], Cristina Vicenzetto[1, 2], Alessia Buratin[3, 4], Eleonora Roncaglia[4, 5], Vanessa Rebecca Gasparini[1, 2], Enrico Gaffo[5], Elena Buson[1, 2], Giulia Calabretto[1, 2], Gregorio Barilà[6], Luca Massarotti[2], Giulia Pertile[1, 2], Livio Trentin[2], Valentina Trimarco[2], Monica Facco[2], Gianpietro Semenzato[1], Renato Zambello[1, 2], Antonella Teramo[1, 2], Stefania Bortoluzzi[5]

*[1]Veneto Institute of Molecular Medicine (VIMM), Padova, Italy; [2]Department of Medicine, Hematology Branch, University of Padova, Padova, Italy; [3]Department of Molecular Medicine, University of Padova, Padova, Italy; [4]Department of Biology, University of Padova, Padova, Italy; [5]Department of Surgery, Oncology and Gastroenterology, University of Padova, Padova, Italy; [6]Hematology Unit, San Bortolo Hospital, Vicenza, Italy;*

### Background

T-Large Granular Lymphocyte Leukemia (T-LGLL) is a rare chronic lymphoproliferative disease, whose pathogenesis is only partially elucidated. Clonal lymphocytes expansion is promoted by pro-inflammatory cytokines that trigger JAK/STAT signaling. This activation may also result from gain-of-function mutation of *STAT* genes, with *STAT3* being typical of CD8+ T-LGLL and associated with symptomatic disease, particularly in patients with cytopenias. Somatic mutations drive disease progression through complex regulatory networks that include non-coding RNAs.

### Aims

This study aims to clarify the role of circular RNAs (circRNAs) in T-LGLL, a class of single-stranded ncRNAs whose involvement in T-LGLL remains largely unexplored.

### Methods

RNA-sequencing (seq) (GEO GSE228868) was performed in a pilot cohort of 20 T-LGLL patients and 5 healthy donors (HD), followed by circRNA identification using CircCompara2/edgeR, and validation by Sanger sequencing and RT-qPCR in an independent cohort (n=26). STAT3 binding sites were obtained from publicly available ChIP-seq datasets (GEO GSE117164). Patients' cells were treated with IL-6 (20 ng/µL) or the STAT3 inhibitor STATTIC (ST) (3.5 µM) and after 24 hours, cell viability via Annexin V staining, STAT3 activation via Western blot and circRNAs expression via RT-qPCR were assessed. CircRNA expression levels were evaluated by RT-qPCR on 3 patients' samples collected pre- and post-therapy.

**Copyright Information:** (Online) ISSN: 2572-9241
©2026 The Author(s). HemaSphere is published by John Wiley & Sons Ltd on behalf of European Hematology Association. This is an open access Abstract Book distributed under the Attribution-NonCommercial-NoDerivs (CC BY-NC-ND), which allows third parties to download the articles and share them with others as long as they credit the author and the Abstract Book, but they cannot change the content in any way or use them commercially.
**Abstract Book Citations:** Authors, Title, HemaSphere, 2026;10:(S1):pages. The abstract book DOIs can be found on the "Author Information" page.
**Disclaimer:** Articles published in the journal HemaSphere exclusively reflect the opinions of the authors. The authors are responsible for all content in their abstracts including accuracy of the facts, statements, citing resources, etc.

**Results**

Nearly 6,000 circRNAs (p-adj<0.05) were detected through RNA-seq. Principal component analysis revealed distinct circRNA expression patterns between T-LGLL patients and HD, with 358 circRNAs differentially expressed in neutropenic *STAT3*-mutated patients compared to asymptomatic ones. The altered expression of 11 circRNAs was validated in an independent cohort. Of these, 5 circRNAs were specifically altered in *STAT3*-mutated CD8+ patients: 4 were up-regulated, and circZC3H12B was the only down-regulated one. Of these circRNAs, we also assessed the subcellular localization in Jurkat cells, identifying circZBTB46, circPVT1 and circZC3H12B as predominantly cytoplasmic, while circSETPB1 displayed a mainly nuclear localization. We next investigated the potential correlation between STAT3 activation and circRNAs dysregulation; notably, according to ChIP-seq datasets, circSETBP1, circPVT1 and circZBTB46 are under the direct STAT3 transcriptional control (p-adj<0.05). This evidence is further supported by *ex vivo* experiments on primary cells obtained from asymptomatic and neutropenic T-LGLL patients treated with IL-6 and ST, respectively. IL-6 protein activation (p<0.01) modulated circRNA expression to levels comparable to those observed in symptomatic cases (p<0.05), while STAT3 inhibition with ST (p<0.05) induced opposite circRNA expression patterns. Finally, the expression of the three STAT3-driven circRNAs was evaluated in T-LGLL patients pre- and post-therapy achieving complete clinical response, revealing a significant post-treatment reduction to levels comparable to HD.

**Summary/Conclusion**

This study delineates a dysregulated circRNA landscape in T-LGLL, with expression profiles that distinguish patients from HD and, notably, symptomatic from indolent cases, supporting a role for these molecules in disease heterogeneity and severity. Importantly, we demonstrate the STAT3-dependent regulation of 3 circRNAs, circSETBP1, circPVT1 and circZBTB46, which emerge as potential clinical biomarkers in symptomatic T-LGLL patients undergoing treatment.

**Copyright Information:** (Online) ISSN: 2572-9241
©2026 The Author(s). HemaSphere is published by John Wiley & Sons Ltd on behalf of European Hematology Association. This is an open access Abstract Book distributed under the Attribution-NonCommercial-NoDerivs (CC BY-NC-ND), which allows third parties to download the articles and share them with others as long as they credit the author and the Abstract Book, but they cannot change the content in any way or use them commercially.
**Abstract Book Citations:** Authors, Title, HemaSphere, 2026;10:(S1):pages. The abstract book DOIs can be found on the "Author Information" page.
**Disclaimer:** Articles published in the journal HemaSphere exclusively reflect the opinions of the authors. The authors are responsible for all content in their abstracts including accuracy of the facts, statements, citing resources, etc.

### (PB2868) EARLY IMMUNOLOGICAL CHANGES DURING VENETOCLAX MONOTHERAPY IN PATIENTS WITH CHRONIC LYMPHOCYTIC LEUKEMIA

**Topic:** 05. Chronic lymphocytic leukemia and related disorders - Biology & translational research

Olina Lind*[1], Maria Andersson[1, 2], Kia Heimersson[1], Lotta Hansson[1, 2], Anders Osterborg[1, 2], Marzia Palma[1, 2]

*[1]Department of Oncology-Pathology, Karolinska Institutet, Stockholm, Sweden;[2]Lymphoma Unit, Department of Hematology, Karolinska University Hospital, Stockholm, Sweden;*

### Background

Chronic lymphocytic leukemia (CLL) is associated with immune dysfunction leading to increased risk of infections, suboptimal vaccine response and lower response to T-cell based therapies. Another defining feature of the disease is impaired apoptosis, driven by overexpression of the pro-survival protein B-cell lymphoma 2 (BCL-2). Venetoclax, a selective BCL-2 inhibitor, targets this pathway and has significantly improved outcomes in patients with CLL. Although venetoclax rapidly induces apoptosis in CLL cells within hours of treatment initiation, its early effects on the immune cell profile are yet to be defined.

### Aims

This study aims to evaluate changes in immune cell populations from 4 hours up to 4 weeks after initiating venetoclax monotherapy in patients with CLL.

### Methods

Peripheral blood (PB) samples were collected from 12 patients with CLL at the Dept. of Hematology, Karolinska University Hospital. Samples were obtained prior to treatment initiation and at 4-6 hours, 24 hours and after 4 weeks of treatment. The starting dose was 20 mg, followed by weekly dose escalation to a maximum of 400 mg if tolerated. Six patients had relapsed refractory (R/R) disease and six patients were treatment-naïve. Eight healthy individuals were included as controls. PB cells were analyzed by flow-cytometry.

### Results

At week 4 of treatment, 7/12 patients had reached the full dose of 400 mg. The absolute number of $CD19^+$ cells decreased significantly at 24 hours compared to baseline, and at week 4 levels had normalized ($p \le 0.001$). $CD4^+$ T cells were at baseline in the range of controls and declined at week 4 ($p \le 0.01$). $CD8^+$ T cells, which were elevated compared to controls at baseline, decreased significantly and normalized at week 4 ($p \le 0.01$). Baseline levels of NK cells were increased and showed a significant reduction by week 4 ($p \le 0.05$).

A reduction was observed for Th1 cells while Th2 cells remained unchanged throughout treatment, resulting in a normalization of the Th1/Th2 ratio ($p \le 0.05$). Regulatory T cells (Tregs) were significantly higher compared to controls at early timepoints and decreased at week 4 ($p \le 0.01$). A reduction was evident across multiple Treg subsets, comprising naïve, memory and activated Tregs, along with Tregs expressing Programmed Death-1 (PD-1) ($p \le 0.01$). $CD8^+$ and $CD4^+$ T cells co-expressing exhaustion markers PD-1 and T-cell immunoglobulin and mucin-domain containing-3 (TIM-3), and cells expressing PD-1 or T-cell immunoglobulin and ITIM domain (TIGIT), were significantly elevated compared to controls at baseline, showing reduced numbers and restoration to normal levels at week 4 ($p \le 0.01$).

**Copyright Information:** (Online) ISSN: 2572-9241
©2026 The Author(s). HemaSphere is published by John Wiley & Sons Ltd on behalf of European Hematology Association. This is an open access Abstract Book distributed under the Attribution-NonCommercial-NoDerivs (CC BY-NC-ND), which allows third parties to download the articles and share them with others as long as they credit the author and the Abstract Book, but they cannot change the content in any way or use them commercially.
**Abstract Book Citations:** Authors, Title, HemaSphere, 2026;10:(S1):pages. The abstract book DOIs can be found on the "Author Information" page.
**Disclaimer:** Articles published in the journal HemaSphere exclusively reflect the opinions of the authors. The authors are responsible for all content in their abstracts including accuracy of the facts, statements, citing resources, etc.

**Summary/Conclusion**

In the present study, we show that venetoclax monotherapy induces early changes across multiple immune cell subsets. A significant decrease in CLL cells was evident within 24 hours, while major T-cell alterations occurred at week 4. With few exceptions, all T-cell populations decreased and subsets initially elevated were restored to normal levels by week 4.

Collectively, we demonstrate that treatment with venetoclax reduces multiple immune cell populations including immunosuppressive features in T cells, potentially restoring immune function as the leukemic burden declines. Indirect and direct mechanisms likely contribute, as depletion of CLL cells could influence the number of normal lymphocytes but other immune cells might also depend on BCL-2 for survival, and are thus affected by treatment. Olink analysis is ongoing to reveal early proteomic alterations during treatment.

**Copyright Information:** (Online) ISSN: 2572-9241
©2026 The Author(s). HemaSphere is published by John Wiley & Sons Ltd on behalf of European Hematology Association. This is an open access Abstract Book distributed under the Attribution-NonCommercial-NoDerivs (CC BY-NC-ND), which allows third parties to download the articles and share them with others as long as they credit the author and the Abstract Book, but they cannot change the content in any way or use them commercially.
**Abstract Book Citations:** Authors, Title, HemaSphere, 2026;10:(S1):pages. The abstract book DOIs can be found on the "Author Information" page.
**Disclaimer:** Articles published in the journal HemaSphere exclusively reflect the opinions of the authors. The authors are responsible for all content in their abstracts including accuracy of the facts, statements, citing resources, etc.

### Figure 1

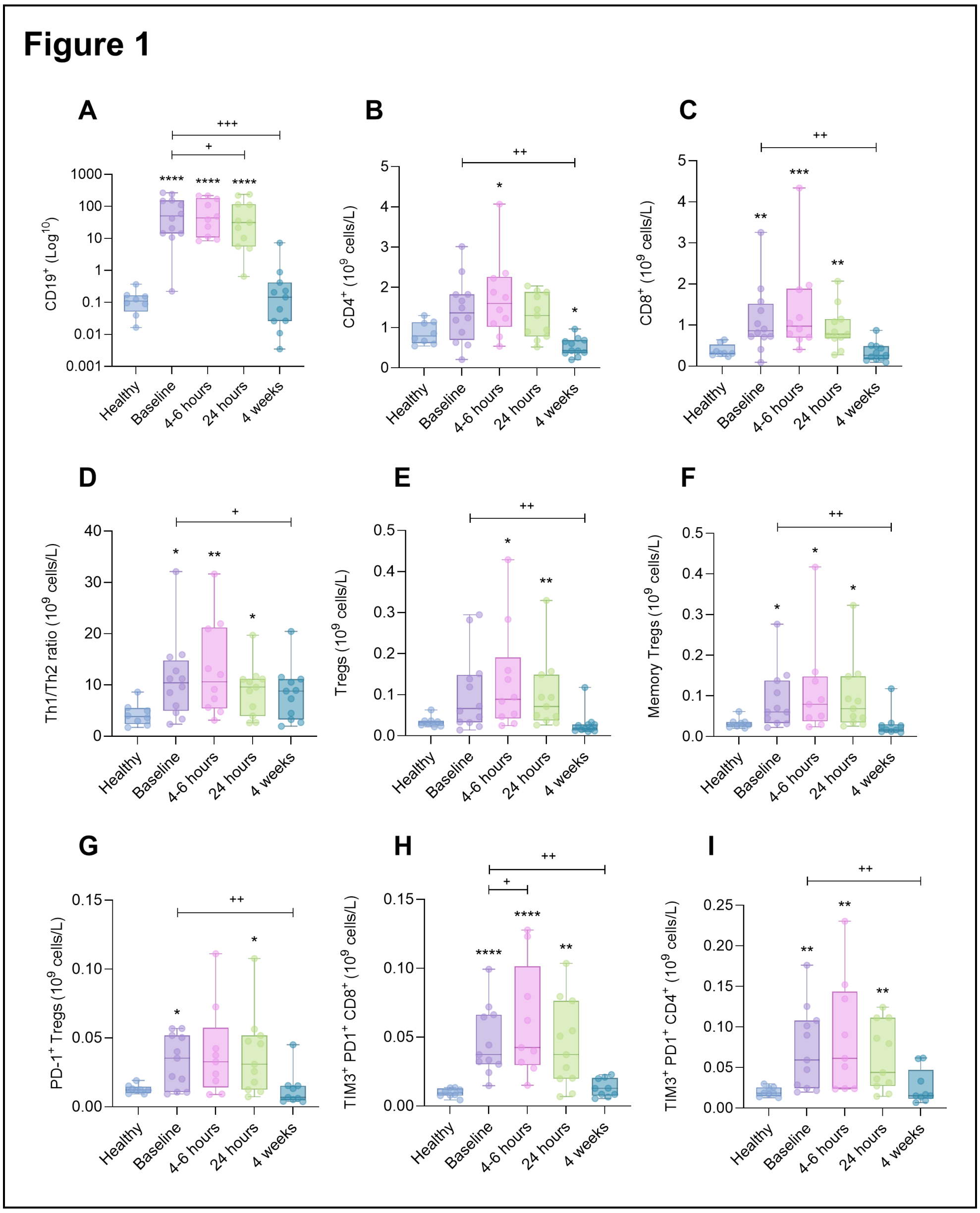

Treatment with venetoclax lead to significant immunological changes at early timepoints. Plus signs indicate a statistically significant difference between baseline and each respective time point as analyzed by Wilcoxon signed-rank tests. Stars indicate a statistically significant difference between healthy controls and each respective timepoint as analyzed by Mann-Whitney U-tests. +/* = p≤0.05, ++/** = p≤0.01, +++/*** = p≤0.001, ++++/**** = p≤0.0001.

**Copyright Information:** (Online) ISSN: 2572-9241
©2026 The Author(s). HemaSphere is published by John Wiley & Sons Ltd on behalf of European Hematology Association. This is an open access Abstract Book distributed under the Attribution-NonCommercial-NoDerivs (CC BY-NC-ND), which allows third parties to download the articles and share them with others as long as they credit the author and the Abstract Book, but they cannot change the content in any way or use them commercially.
**Abstract Book Citations:** Authors, Title, HemaSphere, 2026;10:(S1):pages. The abstract book DOIs can be found on the “Author Information” page.
**Disclaimer:** Articles published in the journal HemaSphere exclusively reflect the opinions of the authors. The authors are responsible for all content in their abstracts including accuracy of the facts, statements, citing resources, etc.

### (PB2869) THE SUPERRCA ASSAY FOR ULTRA-SENSITIVE DETECTION OF DRUG RESISTANCE MUTATIONS IN PATIENTS WITH CHRONIC LYMPHOCYTIC LEUKEMIA (CLL)

**Topic:** 05. Chronic lymphocytic leukemia and related disorders - Biology & translational research

Jan Burger*[1], Ekaterina Kim[1], Feri Shahin[2], Marie Engvall[2], Sofia Nordin[2], Johanna Lindstrom[2], Irene Golán Cancela[2], Lei Chen[2], Alessandra Ferrajoli[1], Nitin Jain[1], Keyur Patel[3], Fatima Zahra Jelloul[3], Mahesh Swaminathan[1], William Wierda[1], Gitanjali Jayachandran[1]

*[1]The University of Texas MD Anderson Cancer Center, Department of Leukemia, Houston, United States of America;[2]Rarity Bioscience AB, Uppsala, Sweden; [3]The University of Texas MD Anderson Cancer Center, Department of Hematopathology, Houston, United States of America;*

### Background

With wider and longer use of Bruton's tyrosine kinase inhibitors (BTKi) and the BCL2 inhibitor venetoclax for treatment of chronic lymphocytic leukemia (CLL) we are challenged with increasing numbers of patients developing resistance to these targeted agents. C481 mutations are common in patients resistant to covalent BTKi (ibrutinib, acalabrutinib, and zanubrutinib). Noncovalent BTKi, such as pirtobrutinib, are associated with non-C481 *BTK* mutations, including T474 and L528W *BTK* mutations, which also confer resistance to acalabrutinib and zanubrutinib. *BCL2* mutations are commonly found in venetoclax-resistant CLL patients. Next-generation sequencing (NGS), the current standard methodology for detecting CLL resistance mutations, has relatively low sensitivity (1–5% variant allele frequency/VAF). SuperRCA, based on rolling circle amplification (RCA), is a novel technology for detection and quantification of exceedingly rare variant DNA sequences, with a sensitivity of detecting mutations at frequencies as low as 1 in 100,000. SuperRCA allows for multiplexing, performs well with challenging high GC-content sequences, and has a short turnaround time.

### Aims

To develop the superRCA technology for detection of drug resistance mutations in CLL patients.

### Methods

Seven different *BTK* mutations and the G101V mutation in *BCL2* were targeted by our initial superRCA panel. The sequences of interest were amplified with a super high-fidelity DNA polymerase, followed by ligase-mediated circularization and target sequence amplification by RCA. Then, padlock probes were hybridized to first-generation RCA products and ligated in a sequence-specific manner and amplified in a secondary RCA reaction. The resulting superRCA products were mixed with fluorophore-labeled oligonucleotide probes specific for the different superRCA products and analyzed by flow cytometry. Samples were also tested by EndLymphoma, an NGS-based mutation profiling assay at MD Anderson.

### Results

We initially tested samples from 10 patients with CLL on long-term BTKi +/- venetoclax treatment, 7 with NGS confirmed resistance mutations. Samples from 2 untreated patients served as controls. We were able to detect and quantify *BTK* and *BCL2* mutations in all 7 patient samples with NGS-confirmed mutations. No mutations were detected in the negative controls. We furthermore detected additional low frequency mutations by superRCA in 6 samples, in 3 samples where NGS was negative, and in 3 samples where NGS detected only a single *BTK* mutation. Currently, we are testing serial samples from CLL patients who developed drug resistance, there additional data will be presented at the meeting.

**Copyright Information:** (Online) ISSN: 2572-9241
©2026 The Author(s). HemaSphere is published by John Wiley & Sons Ltd on behalf of European Hematology Association. This is an open access Abstract Book distributed under the Attribution-NonCommercial-NoDerivs (CC BY-NC-ND), which allows third parties to download the articles and share them with others as long as they credit the author and the Abstract Book, but they cannot change the content in any way or use them commercially.
**Abstract Book Citations:** Authors, Title, HemaSphere, 2026;10:(S1):pages. The abstract book DOIs can be found on the "Author Information" page.
**Disclaimer:** Articles published in the journal HemaSphere exclusively reflect the opinions of the authors. The authors are responsible for all content in their abstracts including accuracy of the facts, statements, citing resources, etc.

### Summary/Conclusion

Our initial experience with superRCA showed complete concordance between superRCA and NGS in the detection of resistance mutations. SuperRCA testing was able to detect additional low frequency *BTK* mutations that were not detected by NGS. Ongoing serial sample testing will determine if superRCA can detect resistance mutations at early time points or even before start of targeted therapy. Future plans are to establish superRCA as a method for early identification of resistance to targeted therapies, monitoring of subclonal mutations during therapy, assessment of resistance risk across different treatment regimens (e.g., time-limited versus long-term therapy), and as an ultra-sensitive detection of measurable residual disease (MRD).

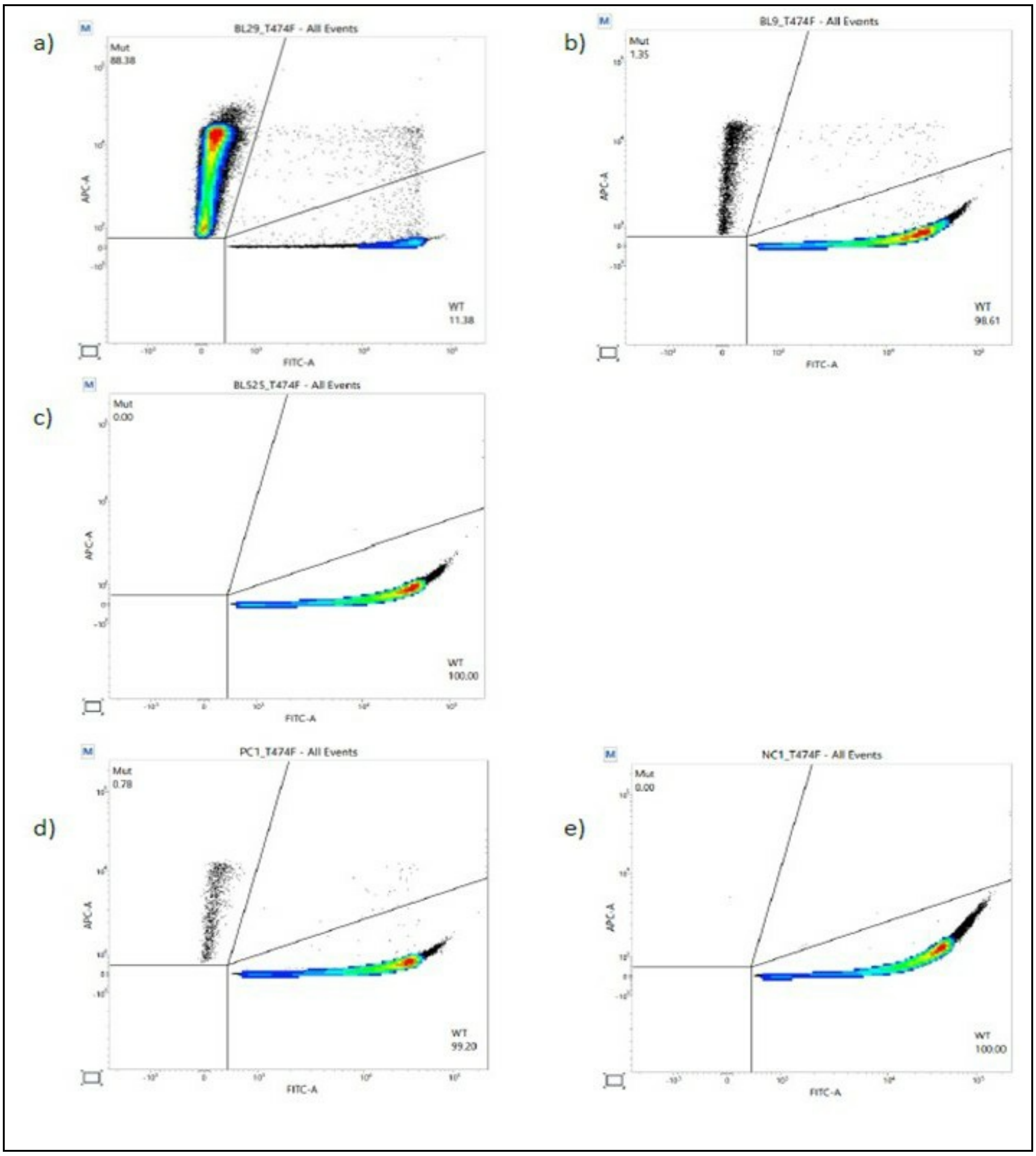

SuperRCA analysis of target BTK p.T474F in different samples. a) positive result with a VAF of 89%. b) positive result with a VAF of 1.4%. c) negative result. d) gDNA positive control e) gDNA negative control.

**Copyright Information:** (Online) ISSN: 2572-9241
©2026 The Author(s). HemaSphere is published by John Wiley & Sons Ltd on behalf of European Hematology Association. This is an open access Abstract Book distributed under the Attribution-NonCommercial-NoDerivs (CC BY-NC-ND), which allows third parties to download the articles and share them with others as long as they credit the author and the Abstract Book, but they cannot change the content in any way or use them commercially.
**Abstract Book Citations:** Authors, Title, HemaSphere, 2026;10:(S1):pages. The abstract book DOIs can be found on the "Author Information" page.
**Disclaimer:** Articles published in the journal HemaSphere exclusively reflect the opinions of the authors. The authors are responsible for all content in their abstracts including accuracy of the facts, statements, citing resources, etc.

### (PB2870) BASELINE IMMUNE PHENOTYPING IN OLDER (≥80 YEARS) AND FRAIL PATIENTS WITH CLL: ASSOCIATIONS WITH INFECTIONS, SPMS, AND IGHV STATUS

**Topic:** 05. Chronic lymphocytic leukemia and related disorders - Biology & translational research

Ben Mechtel*[1, 2, 3], Rudy Ligtvoet[1], Eloísa Felipe Fumero[1, 3], Sandra Robrecht[1], Androniki Kolovou[1, 3], Oliver A. Cornely[1, 2, 3], Michael Hallek[1], Jan Rybniker[1, 2], Martin Thelen[4, 5], Rosanne Sprute[1, 2, 3], Barbara Eichhorst[1], Paula Cramer[1], Hans A. Schlösser[4, 5], Sebastian Theobald[1, 2], Joachim Steiner[6, 7], Kirsten Fischer[1], Eugen Tausch[8], Christof Schneider[8], Stephan Stilgenbauer[8], Florian Simon[1], Petra Langerbeins[1], Sibylle Mellinghoff[1, 2, 3]

*[1]University Hospital Cologne, Department I of Internal Medicine, Cologne, Germany; [2]German Centre for Infection Research (DZIF), Cologne, Germany; [3]Institute of Translational Research, Cologne Excellence Cluster on Cellular Stress Responses in Aging-Associated Diseases (CECAD), Cologne, Germany; [4]University Hospital Cologne, Center for Molecular Medicine Cologne, Cologne, Germany; [5]University Hospital Cologne, Department of General, Visceral, Thoracic, and Transplantation Surgery, Cologne, Germany; [6]University Hospital Cologne, Department II of Internal Medicine, Cologne, Germany; [7]Max Planck Institute for Biology of Ageing, Cologne, Germany; [8]Ulm University, Department of Internal Medicine III, Ulm, Germany;*

### Background

Infections are a major cause of morbidity and mortality in patients with CLL, particularly in those aged ≥80 years, who represent ~20% of the CLL population.

### Aims

We aimed to identify immune patterns associated with infection susceptibility and secondary primary malignancies (SPM) in elderly or frail patients.

### Methods

Pre-treatment peripheral blood samples (n=47) originated from the CLL-Frail phase II trial investigating efficacy and safety of acalabrutinib monotherapy over two years in old (≥80y) or frail patients (). Flow cytometry was used to characterise B-, T-cell and monocyte subsets. Patients were categorised by severe infections, SPM, frailty, IGHV status and immunoglobulin levels. Markers were selected using Storey-adjusted q-values (<0.1) and rank-biserial correlation (|r|≥0.2).

**Copyright Information:** (Online) ISSN: 2572-9241
©2026 The Author(s). HemaSphere is published by John Wiley & Sons Ltd on behalf of European Hematology Association. This is an open access Abstract Book distributed under the Attribution-NonCommercial-NoDerivs (CC BY-NC-ND), which allows third parties to download the articles and share them with others as long as they credit the author and the Abstract Book, but they cannot change the content in any way or use them commercially.
**Abstract Book Citations:** Authors, Title, HemaSphere, 2026;10:(S1):pages. The abstract book DOIs can be found on the "Author Information" page.
**Disclaimer:** Articles published in the journal HemaSphere exclusively reflect the opinions of the authors. The authors are responsible for all content in their abstracts including accuracy of the facts, statements, citing resources, etc.

### Results

Median observation time was 34.7 months (0.7–44.2); median age 81 years (54–91); 33 (70.2%) were male and 22 (46.8%) frail (FRAIL score ≥3). A total of 32 (68.1%) patients were treatment naïve, 19 (40.4%) had Binet C disease, unmutated IGHV was present in 29 (61.7%) and TP53 aberrations/del(17p) in 10 (21.3%) patients. CTCAE grade ≥3 AEs were observed in 33 patients (70.2%), including 25 patients (53.2%) with serious AEs (SAEs) and 1 (2.1%) with SPM. The median number of AEs and SAEs (irrespective of CTCAE grade) was 12 (1–44) and 1 (0–5), respectively. In total, 102 infections and 15 SPMs were documented in 40 (85.1%) and 10 (21.3%) patients, respectively. Patients exhibited T-cell exhaustion compared to healthy controls (HC), characterised by upregulation of co-inhibitory (median PD-1 expression: HC = 11.4% vs CLL = 23.2%) and downregulation of co-stimulatory markers on T-cells (median ICOS expression: HC = 47.1% vs CLL = 28.4%). Patients with grade ≥3 infections expressed lower levels of PD-L1 and ICOS on T-cells, whereas patients with SPMs showed reduced BTLA and LAIR1 expression and an increase of CD66a/c/e and 2B4. Mutated IGHV status was associated with lower expression of DNAM1, TOX, ICOS, and CTLA-4 on T-cells, as well as reduced CD69 expression on regulatory T-cells (Tregs) and higher proportions of classical monocytes and Th17/Th1 cells. Several T-cell markers showed associations with clinical variables, including LAIR1, BTLA, CD66a/c/e and 2B4 with SPMs, and TOX, ICOS and Th17/Th1 cells with IGHV mutation status. (**Fig.1A**). Overall, patients without infections exhibited a more coherent immune pattern with higher absolute inter-marker Spearman correlation coefficients and a higher number of positive correlations (**Fig.1B**). Patients experiencing infections displayed a more heterogeneous expression profile with clusters of tightly correlated markers. ICOS expression clustered with BTLA and LAIR1, while CD66a/c/e aligned with PD-1 expression on central memory T-cells and LAG3 on Tregs. The proportion of Th17/Th1 cells correlated with LAG3 and CD73 expression on Tregs.

### Summary/Conclusion

Immunophenotyping revealed distinct patterns of T-cell exhaustion in this elderly, comorbid and predominantly high-risk CLL patient cohort. Notably, patients with severe infections exhibited distinct T-cell profiles, suggesting prognostic immune signatures. IGHV mutational status and SPMs were associated with differential T-cell activation and monocyte subsets, highlighting the interplay between disease biology and immune competence. Observed checkpoint expression differences were modest and exploratory, requiring validation in larger high-risk CLL cohorts.

**Copyright Information:** (Online) ISSN: 2572-9241
©2026 The Author(s). HemaSphere is published by John Wiley & Sons Ltd on behalf of European Hematology Association. This is an open access Abstract Book distributed under the Attribution-NonCommercial-NoDerivs (CC BY-NC-ND), which allows third parties to download the articles and share them with others as long as they credit the author and the Abstract Book, but they cannot change the content in any way or use them commercially.
**Abstract Book Citations:** Authors, Title, HemaSphere, 2026;10:(S1):pages. The abstract book DOIs can be found on the “Author Information” page.
**Disclaimer:** Articles published in the journal HemaSphere exclusively reflect the opinions of the authors. The authors are responsible for all content in their abstracts including accuracy of the facts, statements, citing resources, etc.

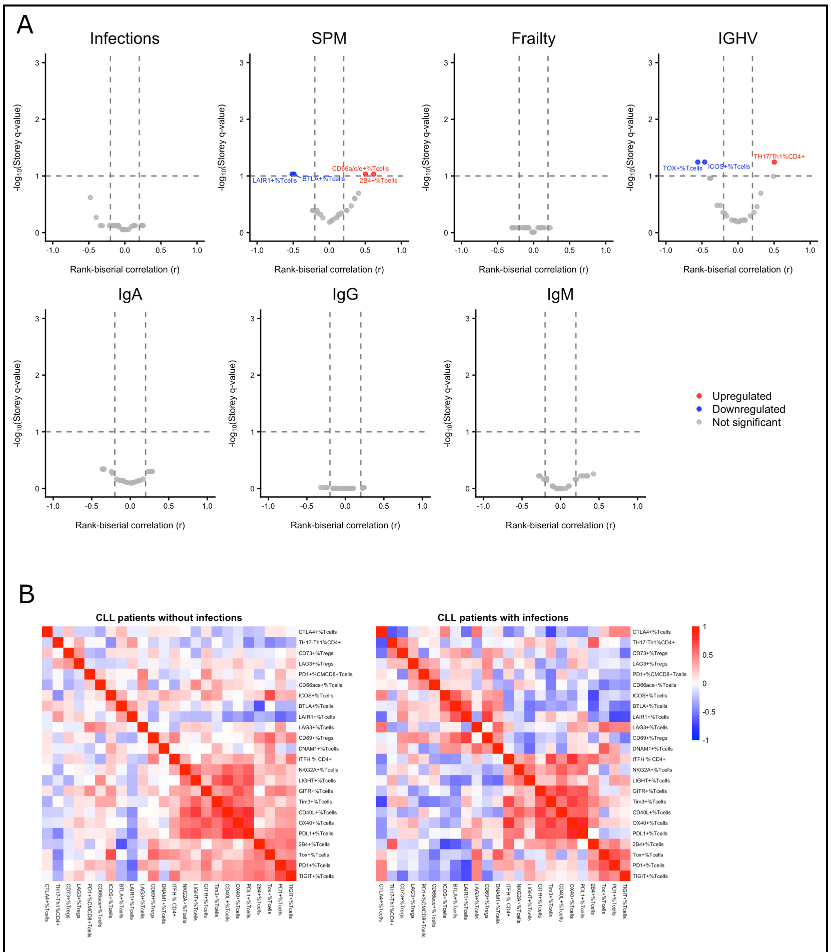

Figure 1.
Baseline immune marker associations with clinical variables in CLL patients.
(A) Volcano plots display effect sizes (rank-biserial correlation) and Storey-adjusted significance.
(B) Similarity matrices of baseline immune checkpoint molecule expression on T cells in CLL patients without (left) and with ≥1 CTCAE grade ≥3 infection (right), based on pairwise Spearman rank correlation coefficients (ρ). Hierarchical clustering was performed using 1 − ρ with single linkage.

**Copyright Information:** (Online) ISSN: 2572-9241
©2026 The Author(s). HemaSphere is published by John Wiley & Sons Ltd on behalf of European Hematology Association. This is an open access Abstract Book distributed under the Attribution-NonCommercial-NoDerivs (CC BY-NC-ND), which allows third parties to download the articles and share them with others as long as they credit the author and the Abstract Book, but they cannot change the content in any way or use them commercially.
**Abstract Book Citations:** Authors, Title, HemaSphere, 2026;10:(S1):pages. The abstract book DOIs can be found on the "Author Information" page.
**Disclaimer:** Articles published in the journal HemaSphere exclusively reflect the opinions of the authors. The authors are responsible for all content in their abstracts including accuracy of the facts, statements, citing resources, etc.

### (PB2871) HIGH-CONTENT IMAGING AND HIGH-THROUGHPUT SCREENING TO DISRUPT HIGH GRADE B-CELL LYMPHOMA EXTRACELLULAR VESICLE – TUMOUR ASSOCIATED MACROPHAGE COMMUNICATION

**Topic:** 05. Chronic lymphocytic leukemia and related disorders - Biology & translational research

Jing Zhang*[1]

[1]*Uniklinik Köln, Köln, Germany;*

### Background

The tumour microenvironment (TME) is shaped by dynamic interactions between malignant cells and immune components. Tumour-associated macrophages (TAMs) play a central role in disease progression and therapeutic response in high-grade B-cell lymphoma. Extracellular vesicles (EVs) are key mediators of tumour–immune communication by transferring bioactive cargo that can reprogram recipient immune cells. TP53 loss is a hallmark of aggressive lymphoma biology; however, its impact on EV-associated communication within the TME remains unclear.

### Aims

We aimed to determine whether B cell–specific TP53 loss reshapes EV-related gene regulation within the TME and to identify compounds capable of modulating EV-driven macrophage responses.

### Methods

Eμ-TCL1 and Eμ-TCL1/Trp53^fl/fl^ mice, in which Trp53 is conditionally deleted in B cells, were analyzed by single-cell RNA sequencing to characterize immune transcriptional programs within the TME. EV-related functional activity was assessed using mouse GO Biological Process gene sets (MSigDB C5) filtered for vesicle-associated pathways. Single-sample Gene Set Enrichment Analysis (ssGSEA, ESCAPE framework) generated pathway activity scores at single-cell resolution. Core EV pathways, including EV biogenesis and endocytosis, were evaluated across immune cell types and biological replicates.
To functionally assess EV–macrophage interactions, Exoria-labelled EVs derived from TP53 wild-type (GRANTA-452) or TP53-deficient (CARNAVAL) lymphoma cells were co-cultured with murine C57BL/6J macrophages. A focused Exosome Secretion–Related Compound Library (51 compounds) was screened under pre-, co-, and post-treatment conditions. EV uptake and macrophage phenotypes were quantified by automated multiparametric imaging and analyzed using principal factor analysis, Euclidean distance profiling, and statistical filtering.

### Results

Single-cell transcriptomic analysis revealed that B cell–specific TP53 loss was associated with globally increased EV-related pathway activity in the TME compared to wild-type and Eμ-TCL1 controls. This effect was particularly pronounced in monocyte populations, which exhibited elevated activity in pathways related to endocytosis and vesicle trafficking.
Functional screening demonstrated that macrophage responses were strongly influenced by the TP53 status of the EV-producing lymphoma cells. EVs derived from TP53-deficient CARNAVAL cells induced distinct and more drug-sensitive phenotypic alterations compared to GRANTA-452-derived EVs. Multivariate profiling identified selective modulators of macrophage responses to TP53-deficient EVs. Omeprazole, Ketotifen fumarate, and Pantoprazole consistently induced significant shifts in EV-associated phenotypic signatures across experimental conditions.

**Copyright Information:** (Online) ISSN: 2572-9241
©2026 The Author(s). HemaSphere is published by John Wiley & Sons Ltd on behalf of European Hematology Association. This is an open access Abstract Book distributed under the Attribution-NonCommercial-NoDerivs (CC BY-NC-ND), which allows third parties to download the articles and share them with others as long as they credit the author and the Abstract Book, but they cannot change the content in any way or use them commercially.
**Abstract Book Citations:** Authors, Title, HemaSphere, 2026;10:(S1):pages. The abstract book DOIs can be found on the "Author Information" page.
**Disclaimer:** Articles published in the journal HemaSphere exclusively reflect the opinions of the authors. The authors are responsible for all content in their abstracts including accuracy of the facts, statements, citing resources, etc.

**Summary/Conclusion**

B cell–intrinsic TP53 loss reshapes EV-related transcriptional programs within the TME and enhances EV-driven reprogramming of tumour-associated monocytes. Moreover, macrophage responses to TP53-deficient EVs are pharmacologically modulatable, highlighting EV–immune communication as a potential therapeutic axis in high-grade B-cell lymphoma.

**Copyright Information:** (Online) ISSN: 2572-9241
©2026 The Author(s). HemaSphere is published by John Wiley & Sons Ltd on behalf of European Hematology Association. This is an open access Abstract Book distributed under the Attribution-NonCommercial-NoDerivs (CC BY-NC-ND), which allows third parties to download the articles and share them with others as long as they credit the author and the Abstract Book, but they cannot change the content in any way or use them commercially.
**Abstract Book Citations:** Authors, Title, HemaSphere, 2026;10:(S1):pages. The abstract book DOIs can be found on the "Author Information" page.
**Disclaimer:** Articles published in the journal HemaSphere exclusively reflect the opinions of the authors. The authors are responsible for all content in their abstracts including accuracy of the facts, statements, citing resources, etc.

### (PB2873) GUT FUNGAL MYCOBIOME IN PATIENTS WITH CHRONIC LYMPHOCYTIC LEUKEMIA

**Topic:** 05. Chronic lymphocytic leukemia and related disorders - Biology & translational research

Yajuan Gao*[1], Jun Chen[1], Purna Kashyap[1], Stephen Johnson[1], Kari Rabe[1], Paul Hampel[1], Lindsey Roeker*[1], Saad Kenderian[1], Eli Muchtar[1], Amber Koehler[1], Amy Behnken[1], Catherine Wagner[1], Susan Schwager[1], Matthew Holets[1], Wei Ding[1], Jose Leis[2], Talal Hilal[2], Mazie Tsang[2], Ricardo D. Parrondo[3], Susan Slager[1], Neil Kay[1], Thomas Witzig[1], Sameer Parikh[1], Yucai Wang[1]

*[1]Mayo Clinic, Rochester, United States of America; [2]Mayo Clinic, Phoenix, United States of America; [3]Mayo Clinic, Jacksonville, United States of America;*

### Background

Fungi are important components in gut microbiota, and fungal dysbiosis may contribute to cancer development. However, little is known about gut fungal composition among patients with chronic lymphocytic leukemia (CLL) or how CLL treatment may affect the gut fungal mycobiome.

### Aims

To investigate the differences in gut fungal diversity and richness among patients with untreated CLL compared to healthy controls (HC) and between patients with untreated CLL and treated CLL.

### Methods

Fecal samples were collected from patients with untreated CLL (n=55), treated CLL (after prior treatment or still on treatmentn=18), and age- and sex- matched HC (n=48). Clinical information was abstracted from the Mayo Clinic CLL database. Microbial DNA was extracted using DNeasy PowerSoil Pro Kit. Fungal ITS2 regions were amplified and sequenced using the Illumina MiSeq platform. Mycobiome sequence reads were processed using DADA2. Taxonomy was assigned using the RDP classifier. Differences between groups were evaluated using beta- and alpha-diversity analyses by PERMANOVA and linear models, respectively. Differential abundance analysis was performed by the LinDA method with log2 fold-change as coefficients.

**Copyright Information:** (Online) ISSN: 2572-9241
©2026 The Author(s). HemaSphere is published by John Wiley & Sons Ltd on behalf of European Hematology Association. This is an open access Abstract Book distributed under the Attribution-NonCommercial-NoDerivs (CC BY-NC-ND), which allows third parties to download the articles and share them with others as long as they credit the author and the Abstract Book, but they cannot change the content in any way or use them commercially.
**Abstract Book Citations:** Authors, Title, HemaSphere, 2026;10:(S1):pages. The abstract book DOIs can be found on the “Author Information” page.
**Disclaimer:** Articles published in the journal HemaSphere exclusively reflect the opinions of the authors. The authors are responsible for all content in their abstracts including accuracy of the facts, statements, citing resources, etc.

### Results

The median age (range) was 66 (33-82) for patients with untreated CLL and 69 (49-81) for patients with treated CLL. Male patients were 39/55 (71%) in untreated and 14/18 (78%) in treated patients. Among untreated CLL patients, the Rai stage distribution was 21 in stage 0, 10 in stage I, 9 in stage II, 3 in stage III and 2 in stage IV. 23/50 (46%) patients had unmutated *IGHV*, 5/37(14%) had del11q and 5/37(14%) had del17p. 7/38 (18%) had *TP53* mutation. Among patients with treated CLL, the treatment regimens included chemoimmunotherapy (CIT) and BTK inhibitor (BTKi) in 8, BTKi alone in 6, BTKi plus anti-CD20 antibody in 3, and CIT alone in 1.
For fungal diversity, untreated CLL patients exhibited higher alpha diversity than HC (Shannon index 0.380, p=0.002; Simpson index 0.170, p=0.001; observed species 1.708, p=0.013), but there was no difference in beta diversity (Figure 1). No significant differences in fungal diversity were observed between patients with untreated and treated CLL.
For fungal abundance, *Saccharomycetales incertae sedis* family (coefficient=2.658, p=0.051), *Candida* genus (coefficient=2.651, p=0.055), and *Candida sake* species (coefficient=1.125, p=0.085) showed trends toward higher abundance in untreated CLL patients compared with HC.
Compared with untreated CLL patients, patients with treated CLL showed higher abundance of *Basidiomycota* phylum (coefficient=1.174, p=0.033), *Bulleribasidiaceae* family (coefficient=1.567, p=0.001), and *Malasseziaceae* family (coefficient=1.513, p=0.001). *Cladosporium* genus (coefficient=-1.593, p=0.081), *Rhodotorula* genus (coefficient=-1.341, p=0.081), *Pichia* genus (coefficient=-0.957, p=0.084), and *Candida* genus (coefficient=-2.382, p=0.090) all tended to have lower abundance in patients with treated CLL.

### Summary/Conclusion

Our results suggest an association between CLL and fungal dysbiosis marked by enrichment of yeast and yeast-associated taxa, which appeared to decrease following CLL treatment, accompanied by a relative increase in *Basidiomycota*-associated taxa. This may reflect underlying immune dysfunction in CLL and gut fungal profiles could potentially serve as marker of immune status or treatment-related immune remodeling in the future studies.

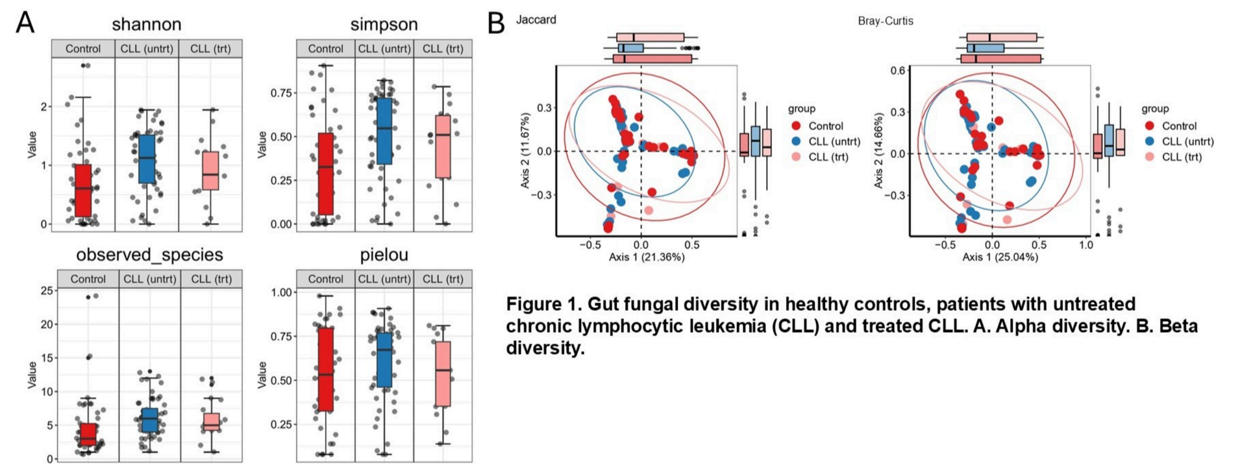

**Figure 1. Gut fungal diversity in healthy controls, patients with untreated chronic lymphocytic leukemia (CLL) and treated CLL. A. Alpha diversity. B. Beta diversity.**

**Copyright Information:** (Online) ISSN: 2572-9241
©2026 The Author(s). HemaSphere is published by John Wiley & Sons Ltd on behalf of European Hematology Association. This is an open access Abstract Book distributed under the Attribution-NonCommercial-NoDerivs (CC BY-NC-ND), which allows third parties to download the articles and share them with others as long as they credit the author and the Abstract Book, but they cannot change the content in any way or use them commercially.
**Abstract Book Citations:** Authors, Title, HemaSphere, 2026;10:(S1):pages. The abstract book DOIs can be found on the "Author Information" page.
**Disclaimer:** Articles published in the journal HemaSphere exclusively reflect the opinions of the authors. The authors are responsible for all content in their abstracts including accuracy of the facts, statements, citing resources, etc.

### (PB2874) SINGLE-CELL SEQUENCING ANALYSIS OF THE CELLULAR LANDSCAPE AND INTER-CLONAL CROSSTALK IN CONCURRENT CLL/SLL AND CML

**Topic:** 05. Chronic lymphocytic leukemia and related disorders - Biology & translational research

Jiaojiao Zhang*[1, 2], Xiaozhong Shen[1], Lu Jiang[1], Jin Wang[1, 2], Jian-qing Mi[1, 2]

*[1]Shanghai Institute of Hematology, State Key Laboratory of Medical Genomics, National Research Center for Translational Medicine at Shanghai, Ruijin Hospital, Shanghai Jiao Tong University School of Medicine, Shanghai, China; [2]Shanghai Key Laboratory of Gene Editing and Cell-based Immunotherapy for Hematological Diseases, Ruijin Hospital, Shanghai jiao Tong University School of Medicine, Shanghai, China;*

### Background

Concurrent CLL/SLL and CML is exceptionally rare. The molecular interplay between these two clonal malignancies and their response to sequential targeted therapies remain poorly understood. We employed longitudinal single-cell RNA sequencing (scRNA-seq) to dissect the cellular composition and inter-clonal communication in this complex case.

### Aims

To characterize the longitudinal cellular landscape and investigate the potential crosstalk between CML cells and the CLL tumor microenvironment (TME).

### Methods

Serial samples (peripheral blood, bone marrow, and lymph nodes) were collected at five timepoints. The scRNA-seq analysis utilized Seurat and Harmony for data integration. SciCSR and scRepertoire were applied to identify B-cell clonality and class-switching. CML cells were defined by specific transcriptomic signatures. GSEA and CellChat were used to identify enriched pathways and infer intercellular communication networks.

### Results

Integration of the five scRNA-seq samples yielded a comprehensive, multi-lineage cellular atlas. CLL cells constituted the dominant population across all specimens; however, bone marrow biopsies exhibited the greatest cellular diversity, likely reflecting tissue architecture and sampling methods. Two distinct B-cell subpopulations—memory B cells and plasma cells—were annotated, distinguished by marker co-expression (MS4A1, CD27, CD38, SDC1), B-cell receptor (BCR) monoclonality, and IGH class-switching patterns (predominantly IGHG1). CML cells were successfully identified via transcriptomic signatures, and their estimated proportions were highly concordant with contemporaneous BCR::ABL1 International Scale (IS) measurements, validating the annotation strategy.

GSEA of CML cells compared to all other populations revealed a strong enrichment of inflammatory granulocyte gene sets—including chemotaxis, cell migration, and degranulation pathways—alongside canonical CML/myeloid tumor signatures. Furthermore, CellChat analysis of bone marrow biopsies containing CML cells demonstrated that these cells mediated not only conventional myeloid and granulocyte signaling but also prominent MMP9 ligand-receptor interactions directed toward CLL cells. This suggests a paracrine mechanism by which CML-derived MMP9 signaling may modulate the CLL microenvironment.

**Copyright Information:** (Online) ISSN: 2572-9241
©2026 The Author(s). HemaSphere is published by John Wiley & Sons Ltd on behalf of European Hematology Association. This is an open access Abstract Book distributed under the Attribution-NonCommercial-NoDerivs (CC BY-NC-ND), which allows third parties to download the articles and share them with others as long as they credit the author and the Abstract Book, but they cannot change the content in any way or use them commercially.
**Abstract Book Citations:** Authors, Title, HemaSphere, 2026;10:(S1):pages. The abstract book DOIs can be found on the "Author Information" page.
**Disclaimer:** Articles published in the journal HemaSphere exclusively reflect the opinions of the authors. The authors are responsible for all content in their abstracts including accuracy of the facts, statements, citing resources, etc.

### Summary/Conclusion

This study provides the first longitudinal single-cell resolution of the co-evolving CLL and CML landscape. Our findings suggest that CML cells function as active modulators of the CLL tumor microenvironment, utilizing MMP9-mediated signaling to potentially influence CLL cell behavior and disease progression. These results provide novel mechanistic insights into inter-clonal crosstalk and demonstrate the power of single-cell multi-omics in resolving the complexity of synchronous hematological malignancies, offering a foundation for future studies on how co-existing clones shape the immune landscape.

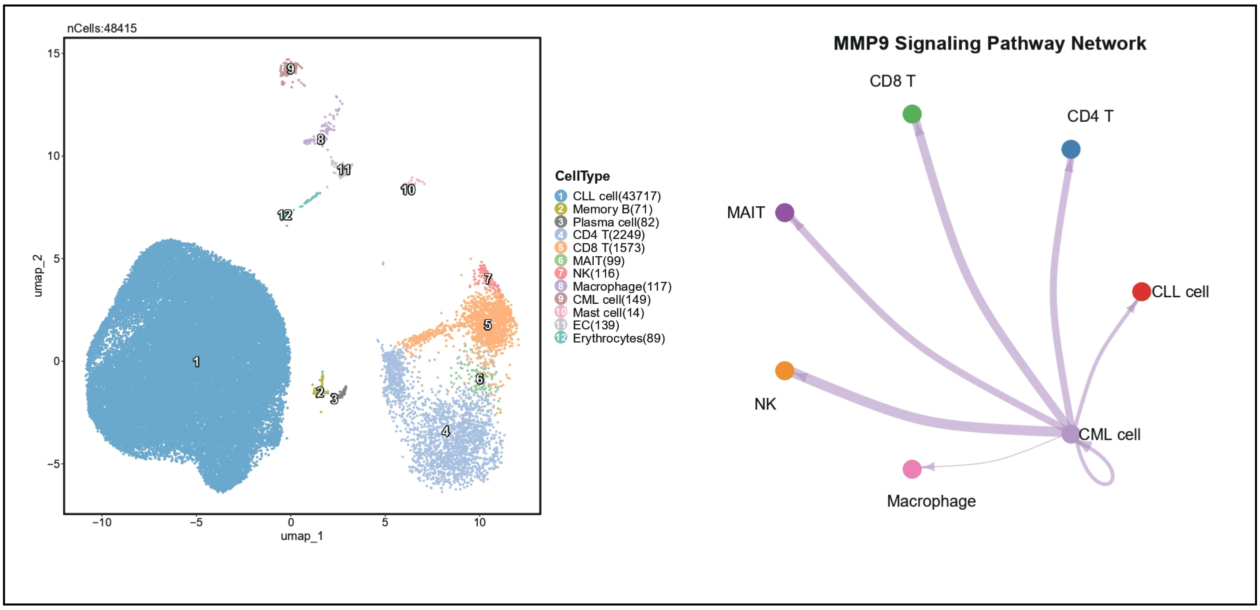

(Left) UMAP plot showing the distribution of 48,415 cells where CLL cells (Cluster 1) and CML cells (Cluster 9) represent the two co-existing malignant clones. (Right) CellChat analysis of the MMP9 signaling network identifying the malignant CML clone as the primary signaling source mediating paracrine crosstalk directed toward CLL cells and the microenvironment.

**Copyright Information:** (Online) ISSN: 2572-9241
©2026 The Author(s). HemaSphere is published by John Wiley & Sons Ltd on behalf of European Hematology Association. This is an open access Abstract Book distributed under the Attribution-NonCommercial-NoDerivs (CC BY-NC-ND), which allows third parties to download the articles and share them with others as long as they credit the author and the Abstract Book, but they cannot change the content in any way or use them commercially.
**Abstract Book Citations:** Authors, Title, HemaSphere, 2026;10:(S1):pages. The abstract book DOIs can be found on the "Author Information" page.
**Disclaimer:** Articles published in the journal HemaSphere exclusively reflect the opinions of the authors. The authors are responsible for all content in their abstracts including accuracy of the facts, statements, citing resources, etc.

### (PB2875) GUT MICROBIOME IN PATIENTS WITH UNTREATED CHRONIC LYMPHOCYTIC LEUKEMIA AND ASSOCIATIONS WITH BASELINE CLINICAL AND MOLECULAR FEATURES

**Topic:** 05. Chronic lymphocytic leukemia and related disorders - Biology & translational research

Yajuan Gao*[1], Jun Chen[1], Purna Kashyap[1], Stephen Johnson[1], Kari Rabe[1], Paul Hampel[1], Lindsey Roeker*[1], Saad Kenderian[1], Eli Muchtar[1], Amber Koehler[1], Amy Behnken[1], Catherine Wagner[1], Susan Schwager[1], Matthew Holets[1], Wei Ding[1], Jose Leis[2], Talal Hilal[2], Mazie Tsang[2], Ricardo D. Parrondo[3], Susan Slager[1], Neil Kay[1], Thomas Witzig[1], Sameer Parikh[1], Yucai Wang[1]

*[1]Mayo Clinic, Rochester, United States of America; [2]Mayo Clinic, Phoenix, United States of America; [3]Mayo Clinic, Jacksonville, United States of America;*

### Background

Gut microbiome (GM) is associated with host immune status. Chronic lymphocytic leukemia (CLL) features immune dysregulation with increased infection risks. However, the understanding of GM profiles in CLL remains very limited.

### Aims

To characterize GM in patients with untreated CLL and evaluate its association with baseline prognostic features.

### Methods

Feces samples were collected from 78 untreated CLL patients and 82 age- and sex-matched healthy controls (HC). Clinical information was abstracted, and prior antibiotic exposure was reviewed. Stool DNA was extracted and the 16S rRNA V4 region sequenced. Reads were processed with DADA2, and taxonomy was assigned using RDP/SILVA (v128). We assessed group differences by alpha/beta diversity (linear models, PERMANOVA) and differential abundance by LinDA (log2 fold-change as coefficients).

### Results

The median age of CLL patients was 67 years (range 33-86), and 25 (32%) were female. Rai stage was 0 in 34, I–II in 25, and III–IV in 6 patients. *IGHV* was unmutated in 32/67 (48%) patients. The CLL FISH profile was del17p in 6/50 (12%), del11q in 5/50 (10%), trisomy 12 in 9/50 (18%), del13q in 15/50 (30%), and no abnormalities detected in 14/50 (28%). The CLL-International Prognostic Index (CLL-IPI) was low risk in 13/42 (31%), intermediate risk in 15/42 (36%), and high/very-high risk in 14/42 (33%).
Compared with HC, the GM of untreated CLL patients showed no difference in richness or diversity. Among untreated CLL patients, higher GM richness was observed in those with Rai stage I–IV vs 0 [mean difference in the number of observed species between groups (MD)=21.5, p=0.011, Figure 1A], either del11q or del17p vs not (MD=20.1, p=0.083, Figure 1B), and intermediate-risk (MD=22.6, p=0.065) or high/very high-risk (MD=33.3, p=0.009) compared with low-risk CLL-IPI. GM richness increased with higher Rai stage (increase of 8.2 observed species per stage, p=0.031) and higher CLL-IPI (increase of 14.5 observed species per risk category, p=0.006, Figure 1C). No associations were observed between GM richness and *IGHV* or *TP53* status.
Beta-diversity analysis showed significant differences in GM composition between patients with either del11q or del17p and those without (Jaccard $R^2$=0.025, p=0.059; Bray-Curtis $R^2$=0.028, p=0.059; UniFrac $R^2$=0.043, p=0.033; WUniFrac $R^2$=0.035, p=0.032, Figure 1D). No associations were observed between GM diversity and Rai stage, CLL-IPI, *IGHV*, or *TP53* status.
*Euryarchaeota* phylum showed an increasing trend with Rai stage (coefficient =1.39, p=0.084) and was higher in patients with del11q or del17p vs those without (coefficient =4.80, p=0.008). *Methanobacteriaceae* family showed a trend toward higher abundance in patients with either del11q or 17p than others (coefficient =1.53, p=0.060). The genera *Christensenellaceae R-7 group* (coefficient =1.32), *Clostridium sensu stricto 1* (coefficient =1.49), *Klebsiella* (coefficient =1.41), and *Ruminococcus 1* (coefficient =0.82) demonstrated a consistent tendency to be more abundant in patients with higher CLL-IPI (p=0.089 for all).

**Copyright Information:** (Online) ISSN: 2572-9241
©2026 The Author(s). HemaSphere is published by John Wiley & Sons Ltd on behalf of European Hematology Association. This is an open access Abstract Book distributed under the Attribution-NonCommercial-NoDerivs (CC BY-NC-ND), which allows third parties to download the articles and share them with others as long as they credit the author and the Abstract Book, but they cannot change the content in any way or use them commercially.
**Abstract Book Citations:** Authors, Title, HemaSphere, 2026;10:(S1):pages. The abstract book DOIs can be found on the "Author Information" page.
**Disclaimer:** Articles published in the journal HemaSphere exclusively reflect the opinions of the authors. The authors are responsible for all content in their abstracts including accuracy of the facts, statements, citing resources, etc.

### Summary/Conclusion

Increased GM diversity was associated with adverse baseline prognostic features in untreated CLL. Higher levels of methane-producing species (*Euryarchaeota* and *Methanobacteriaceae*), short-chain fatty acid-producing species (*Clostridium sensu stricto 1* and *Ruminococcus 1*), and dysbiosis-related *Klebsiella* suggest disease-related alterations in the GM of untreated high-risk CLL.

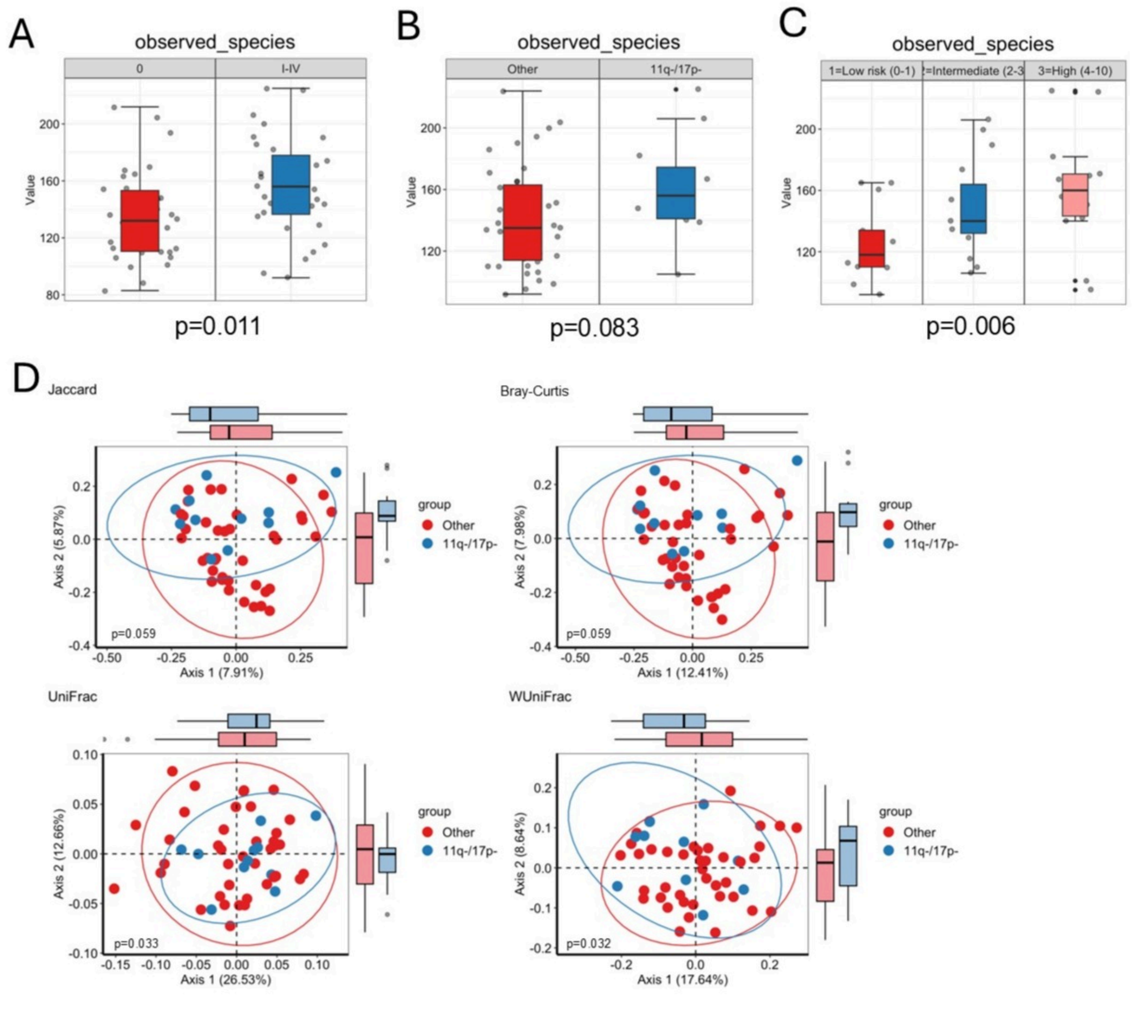

**Figure 1. Gut microbiome diversity in untreated chronic lymphocytic leukemia.**
**A)** Alpha diversity (observed species) by Rai stage (0 vs I-IV). **B)** Alpha diversity (observed species) in patients with del(11q) or del(17p) vs without. **C)** Alpha diversity (observed species) by CLL-IPI scores (low, intermediate, high/very high). **D)** Beta diversity in patients with del(11q) or del(17p) vs without.

**Copyright Information:** (Online) ISSN: 2572-9241
©2026 The Author(s). HemaSphere is published by John Wiley & Sons Ltd on behalf of European Hematology Association. This is an open access Abstract Book distributed under the Attribution-NonCommercial-NoDerivs (CC BY-NC-ND), which allows third parties to download the articles and share them with others as long as they credit the author and the Abstract Book, but they cannot change the content in any way or use them commercially.
**Abstract Book Citations:** Authors, Title, HemaSphere, 2026;10:(S1):pages. The abstract book DOIs can be found on the “Author Information” page.
**Disclaimer:** Articles published in the journal HemaSphere exclusively reflect the opinions of the authors. The authors are responsible for all content in their abstracts including accuracy of the facts, statements, citing resources, etc.

### (PB2876) LONG-READ NANOPORE SEQUENCING: A RAPID AND HIGHLY ACCURATE APPROACH FOR IGHV GENE ANALYSIS IN CHRONIC LYMPHOCYTIC LEUKEMIA

**Topic:** 05. Chronic lymphocytic leukemia and related disorders - Biology & translational research

Leily Rabbani*[1], Teresa del Peso Santos[1], Alexis Ioannou[2], Daniel Hägerstrand[1], Larry Mansouri[1], Richard Rosenquist[1, 3]

*[1]Karolinska Institutet, Dept. of Molecular Medicine and Surgery, Stockholm, Sweden; [2]Institute of Genetics and Cancer, University of Edinburgh, 2MRC Human Genetics Unit, Edinburgh, United Kingdom; [3]Karolinska University Hospital, Dept. of Clinical Genetics and Genomics, Stockholm, Sweden;*

### Background

The somatic hypermutation (SHM) status of the immunoglobulin heavy variable (IGHV) gene is a key prognostic and predictive biomarker in chronic lymphocytic leukemia (CLL). Traditionally, IGHV gene analysis has relied on Sanger sequencing, a labor-intensive method with limited scalability. More recently, short-read next-generation sequencing (NGS) has offered comparable accuracy, improved detection of multiple IGH gene rearrangements, and allowed higher throughput. However, NGS often requires large sample batches to remain cost-effective, potentially delaying turnaround times in routine diagnostics. Long-read Nanopore sequencing has previously been explored for IGHV analysis, but early approaches used first-generation platforms with higher error rates and employed framework region 1 primers, failing to capture full-length IGH gene rearrangements and thereby limiting clinical applicability in CLL prognostication.

### Aims

This study aimed to develop an integrated workflow for multiplexed long-read Nanopore IGHV-IGHD-IGJH sequencing, enabling an accurate, rapid and cost-efficient full-length assessment of IGH gene rearrangements in CLL. This approach utilizes the latest Nanopore improvements in chemistry and base-calling, which substantially improve accuracy.

### Methods

The study included peripheral blood samples from 48 patients with CLL, 12 in an initial pilot cohort and 36 additional cases, representing a diverse repertoire of IGHV gene subgroups (IGHV1-7) and a broad spectrum of SHM levels, as previously defined by Illumina sequencing (MiSeq). Nanopore sequencing was performed using custom EuroClonality-based primers enabling single-step PCR amplification of clonal IGH gene rearrangements, with simultaneous sample barcoding allowing multiplexing of 12 samples per run. Libraries were prepared with the Ligation Sequencing Kit V14 and sequenced on a MinION Mk1B (ONT) using R10.4.1 flow cells. Data were analyzed via an in-house open-source pipeline from raw POD5 files to clonotype assignment, while germline identity was assessed with IMGT/V-QUEST.

**Copyright Information:** (Online) ISSN: 2572-9241
©2026 The Author(s). HemaSphere is published by John Wiley & Sons Ltd on behalf of European Hematology Association. This is an open access Abstract Book distributed under the Attribution-NonCommercial-NoDerivs (CC BY-NC-ND), which allows third parties to download the articles and share them with others as long as they credit the author and the Abstract Book, but they cannot change the content in any way or use them commercially.
**Abstract Book Citations:** Authors, Title, HemaSphere, 2026;10:(S1):pages. The abstract book DOIs can be found on the "Author Information" page.
**Disclaimer:** Articles published in the journal HemaSphere exclusively reflect the opinions of the authors. The authors are responsible for all content in their abstracts including accuracy of the facts, statements, citing resources, etc.

### Results

Sequencing runs generated, on average, >8 million reads and >4 Gbps per 9-hour run. After quality filtering (Q30), approximately 200,000 reads per sample and barcode were retained. In all samples, a dominant productive clone was identified, accounting for a mean of 78% of total reads (range, 38-93%). When comparing results for the major productive clone obtained from the Nanopore sequencing runs with previous data, we found complete concordance for IGHV-IGHD-IGHJ gene usage across all 48 samples. Additionally, SHM levels were identical in 46 of 48 cases, while the two remaining samples showed only minimal differences (96.84% vs. 97.19% and 94.38% vs. 94.44%). In 8 samples, Nanopore sequencing validated the presence of a second, unproductive rearrangement (range, 14-40%). In an additional sample, a subclone exhibiting additional somatic hypermutation was confirmed, demonstrating highly concordant frequencies between Nanopore and MiSeq. The total cost (based on 12 samples per run) was estimated to €70/sample, with a turnaround time of approximately 24 hours.

### Summary/Conclusion

Long-read Nanopore sequencing using the MinION platform enables rapid, accurate, and cost-effective full-length analysis of IGHV gene SHM status in patients with CLL. The method requires minimal upfront investment and offers a scalable, clinically applicable alternative for routine prognostic assessment.

**Copyright Information:** (Online) ISSN: 2572-9241
©2026 The Author(s). HemaSphere is published by John Wiley & Sons Ltd on behalf of European Hematology Association. This is an open access Abstract Book distributed under the Attribution-NonCommercial-NoDerivs (CC BY-NC-ND), which allows third parties to download the articles and share them with others as long as they credit the author and the Abstract Book, but they cannot change the content in any way or use them commercially.
**Abstract Book Citations:** Authors, Title, HemaSphere, 2026;10:(S1):pages. The abstract book DOIs can be found on the "Author Information" page.
**Disclaimer:** Articles published in the journal HemaSphere exclusively reflect the opinions of the authors. The authors are responsible for all content in their abstracts including accuracy of the facts, statements, citing resources, etc.

### (PB2879) CIRCULATING MIRNA SIGNATURE TRACKS DISEASE PROGRESSION AND TARGETED THERAPY RESPONSE IN CHRONIC LYMPHOCYTIC LEUKEMIA

**Topic:** 05. Chronic lymphocytic leukemia and related disorders - Biology & translational research

Rocío García-Serra[1], Antonio José Cabrera-Serrano[2], Mariam Ibáñez Company*[1], Mª José Lis[1], Carmen Amorós[1], Deborah Pérez[1], María Monera[1], Karla Javier[1], Josefa Marco Buades[1], Juan Sainz[3], Rosa Collado Nieto[1]

*[1]Hematology and Hemotherapy Department. Consorcio Hospital General Universitario de Valencia. Hematology and Hemotherapy Research Group. Fundación Investigación Hospital General de Valencia., Valencia, Spain; [2]Genomic Oncology Area, GENYO. Centre for Genomics and Oncological Research: Pfizer / University of Granada / Andalusian Regional Government, PTS, Granada, Spain Instituto de Investigación Biosanataria IBs.Granada, Granada, Spain, GRANADA, Spain; [3]Genomic Oncology Area, GENYO. Centre for Genomics and Oncological Research: Pfizer / University of Granada / Andalusian Regional Government, PTS, Granada, Spain. Instituto de Investigación Biosanataria IBs.Granada, Granada, Spain. Consortium for Biomedical Research in Epidemiology and Public Health (CIBERESP), University of Barcelona, 08908 Barcelona, Spain. Department of Biochemistry and Molecular Biology I, University of Granada, Granada, Spain, GRANADA, Spain;*

### Background

Chronic lymphocytic leukaemia (CLL) is a lymphoproliferative entity clinically heterogeneous. Circulating microRNAs (miRNAs) have emerged as minimally invasive biomarkers with potential prognostic and therapeutic relevance.

### Aims

We aimed to identify plasma miRNA signatures associated with disease progression that could enhance current risk stratification strategies and serve as potential prognostic biomarkers.

**Copyright Information:** (Online) ISSN: 2572-9241
©2026 The Author(s). HemaSphere is published by John Wiley & Sons Ltd on behalf of European Hematology Association. This is an open access Abstract Book distributed under the Attribution-NonCommercial-NoDerivs (CC BY-NC-ND), which allows third parties to download the articles and share them with others as long as they credit the author and the Abstract Book, but they cannot change the content in any way or use them commercially.
**Abstract Book Citations:** Authors, Title, HemaSphere, 2026;10:(S1):pages. The abstract book DOIs can be found on the "Author Information" page.
**Disclaimer:** Articles published in the journal HemaSphere exclusively reflect the opinions of the authors. The authors are responsible for all content in their abstracts including accuracy of the facts, statements, citing resources, etc.

### Methods

We analyzed 46 plasma samples from 17 CLL patients enrolled at disease progression (DP) (Table 1) with paired longitudinal follow-up at 24 and 36 months after treatment initiation (n=17, and n=12, respectively). Total small RNA was extracted from plasma and libraries were prepared using NEXTFLEX Small RNA-Seq kit v4 (Revvity), sequenced on an Illumina NextSeq 550. Data was processed using miRge3.0 and annotated with MirGeneDB2.1. Differential expression analysis was performed with DESeq2 (v1.36.0), applying a false discovery rate <0.05 and |log2 fold change| >1. Clinical response (partial: PR; complete: CR) was classified according to the International Workshop on CLL (iwCLL) criteria. Three comparisons were evaluated [DP *vs* last available follow-up (LFU) (24 or 36 months), DP *vs* PR/CR] adjusting for age, sex, IGHV status, Rai/Binet stage, treatment, and karyotypic alterations.

**Table 1.** Clinical characteristics of the study population.

| **Characteristics** | **N=17 (%; median; range)** |
|---|---|
| Age, years | 69 (46-84) |
| Male | 12 (71%) |
| Advanced Binet Stage (B/C) | 15 (88%) |
| Lymphocyte count ($\times 10^9$/L) | 70.0 (6.1-348.6) |
| Altered Karyotype | 11 (65%) |
| Complex Karyotype | 6 (35%) |
| Altered FISH | 15 (88%) |
| Döhner hierarchical classification | |
| del(13q) alone | 7 (41%) |
| Trisomy 12 | 2 (12%) |
| del(11q)/*ATM* | 4 (24%) |
| del(17p)/ *TP53* | 2 (12%) |
| Unmutated IGHV | 12 (71%) |
| Mutated cases | |
| *TP53* | 5 (29%) |
| *NOTCH1* | 5 (29%) |
| *XPO1* | 3 (18%) |
| *ATM* | 2 (12%) |
| *POT1* | 2 (12%) |
| *SF3B1* | 1 (6%) |
| Treatment received | |
| BTK-inhibitor | 14 (82%) |
| antiCD20+BCL2-inhibitor | 2 (12%) |
| Chemoinmunotherapy | 1 (6%) |
| Follow-up, months | 96 (39-259) |
| TTFT, months | 37 (1-147) |

### Results

We identified a core signature of 15 miRNAs consistently dysregulated across comparisons. Among these, 14 miRNAs were downregulated after treatment onset and remained persistently decreased at both 24 and 36 months, whereas only one miRNA was upregulated. (Figure 1). The stability of this signature over time provides intra-study validation and indicates that targeted therapies induce durable remodeling of circulating miRNA profiles. Key downregulated miRNAs included miR-128-2, miR-34a, and members of the miR-29 family, implicating *TP53*-related pathways, apoptosis resistance, immune modulation, and epigenetic regulation. Among the differentially expressed miRNAs, miR-375 was uniquely overexpressed following treatment and is known to regulate pathways involved in cellular adaptation to metabolic or therapeutic stress. Notably, no additional miRNAs differentiated CR from PR at later timepoints, suggesting an early molecular remodeling after therapy onset that is subsequently maintained over time.

**Copyright Information:** (Online) ISSN: 2572-9241
©2026 The Author(s). HemaSphere is published by John Wiley & Sons Ltd on behalf of European Hematology Association. This is an open access Abstract Book distributed under the Attribution-NonCommercial-NoDerivs (CC BY-NC-ND), which allows third parties to download the articles and share them with others as long as they credit the author and the Abstract Book, but they cannot change the content in any way or use them commercially.
**Abstract Book Citations:** Authors, Title, HemaSphere, 2026;10:(S1):pages. The abstract book DOIs can be found on the "Author Information" page.
**Disclaimer:** Articles published in the journal HemaSphere exclusively reflect the opinions of the authors. The authors are responsible for all content in their abstracts including accuracy of the facts, statements, citing resources, etc.

### Summary/Conclusion

We identify a circulating miRNA signature linked to disease progression and long-term molecular response to targeted therapy in CLL. Their non-invasive detection in plasma of these circulating miRNAs enables real-time monitoring of disease dynamics and therapeutic impact. These findings provide a biological framework for miRNA-based biomarkers and support further validation in independent cohorts, alongside functional studies to clarify their mechanistic relevance and potential clinical applicability.

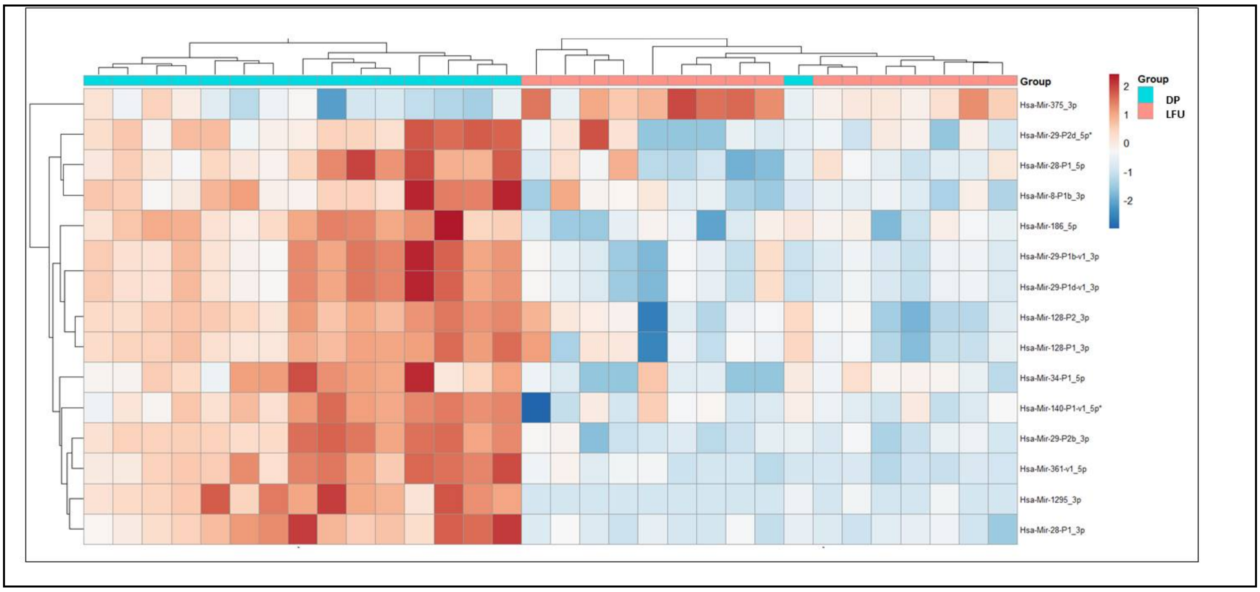

Figure 1. Heatmap of differential miRNA expression between disease progression (DP) and long-term follow-up (LFU) (24–36 Months).

**Copyright Information:** (Online) ISSN: 2572-9241
©2026 The Author(s). HemaSphere is published by John Wiley & Sons Ltd on behalf of European Hematology Association. This is an open access Abstract Book distributed under the Attribution-NonCommercial-NoDerivs (CC BY-NC-ND), which allows third parties to download the articles and share them with others as long as they credit the author and the Abstract Book, but they cannot change the content in any way or use them commercially.
**Abstract Book Citations:** Authors, Title, HemaSphere, 2026;10:(S1):pages. The abstract book DOIs can be found on the "Author Information" page.
**Disclaimer:** Articles published in the journal HemaSphere exclusively reflect the opinions of the authors. The authors are responsible for all content in their abstracts including accuracy of the facts, statements, citing resources, etc.

### (PB2880) ADVANCED MRD ANALYSIS AND IMMUNOLOGICAL MONITORING IN RELAPSED CLL PATIENTS RECEIVING RITUXIMAB-VENETOCLAX: RESULTS FROM THE "DEDALUS" STUDY

**Topic:** 05. Chronic lymphocytic leukemia and related disorders - Biology & translational research

Maria D'amato*[1], Gabriele Bolognesi[1], Claudia Barate'[2], Clara Bono[1], Sara Galimberti[1]

*[1]Department of Clinical and Experimental Medicine, Haematology Unit, University of Pisa, Pisa, Italy;[2]Department of Hematology and Oncology, Hematology Unit, Niguarda Metropolitan Hospital, Pisa, Italy;*

### Background

The implementation of time-limited regimens combining Rituximab (R) and Venetoclax (Ven) has significantly transformed the management of chronic lymphocytic leukemia (CLL), demonstrating a 7-year overall survival rate of 69.6% alongside deep clearance of minimal residual disease (MRD). Given its prognostic value, precise MRD quantification is vital for evaluating therapy success. The "Dedalus" trial was designed to evaluate the concordance between multiparametric flow cytometry (MFC) and next-generation sequencing (NGS) for MRD detection in the relapsed/refractory setting, while also investigating the therapy's impact on the immune system.

### Aims

The primary objective of the "Dedalus" protocol was to assess the efficacy of Rituximab-Venetoclax in relapsed CLL by comparing the diagnostic precision of ERIC-compliant flow cytometry against next-generation sequencing for MRD detection. Furthermore, the study sought to correlate these molecular findings with long-term clinical outcomes and to characterize the specific immunomodulatory changes, such as T-cell fluctuations and B-cell depletion, induced by the treatment.

### Methods

A cohort of 22 patients was monitored using ERIC-standardized MFC and high-sensitivity Lymphotrack NGS (threshold $10^{-5}$) Treatment efficacy was further assessed via advanced ultrasonography. MRD levels and comprehensive immune profiles were analyzed at specific intervals (months 1, 6, 12, and 18) or at the point of therapy completion.

### Results

Clinical data showed that 20 patients reached a complete remission (CR). At the initial post-treatment evaluation, 15 patients were classified as MRD-negative by both diagnostic platforms. Notably, in two instances of partial response, NGS identified low-level IgH clonality (ranging from 3 to 45 cells per $10^{6}$) that remained undetected by MFC. The analytical sensitivities were confirmed at $10^{-4}$ for MFC and $10^{-5}$ for NGS.
Furthermore, the R-Ven combination led to a persistent lack of B cells and a decline in CD4+ T-lymphocyte counts, resulting in a shifted CD4/CD8 ratio in 25% of the subjects. Overall, NGS demonstrated a stronger predictive value for clinical outcomes, particularly in cases with very low disease burden.

**Copyright Information:** (Online) ISSN: 2572-9241
©2026 The Author(s). HemaSphere is published by John Wiley & Sons Ltd on behalf of European Hematology Association. This is an open access Abstract Book distributed under the Attribution-NonCommercial-NoDerivs (CC BY-NC-ND), which allows third parties to download the articles and share them with others as long as they credit the author and the Abstract Book, but they cannot change the content in any way or use them commercially.
**Abstract Book Citations:** Authors, Title, HemaSphere, 2026;10:(S1):pages. The abstract book DOIs can be found on the "Author Information" page.
**Disclaimer:** Articles published in the journal HemaSphere exclusively reflect the opinions of the authors. The authors are responsible for all content in their abstracts including accuracy of the facts, statements, citing resources, etc.

**Summary/Conclusion**

The R-Ven protocol confirms its high efficacy in inducing long-term remissions in relapsed CLL. Although MFC remains a practical and affordable diagnostic tool, NGS provides a higher degree of sensitivity and superior prognostic accuracy. These findings suggest that future research should focus on digital PCR for IgH monitoring as a potentially less invasive biomarker and continue to explore the complex immunomodulatory effects of Venetoclax.

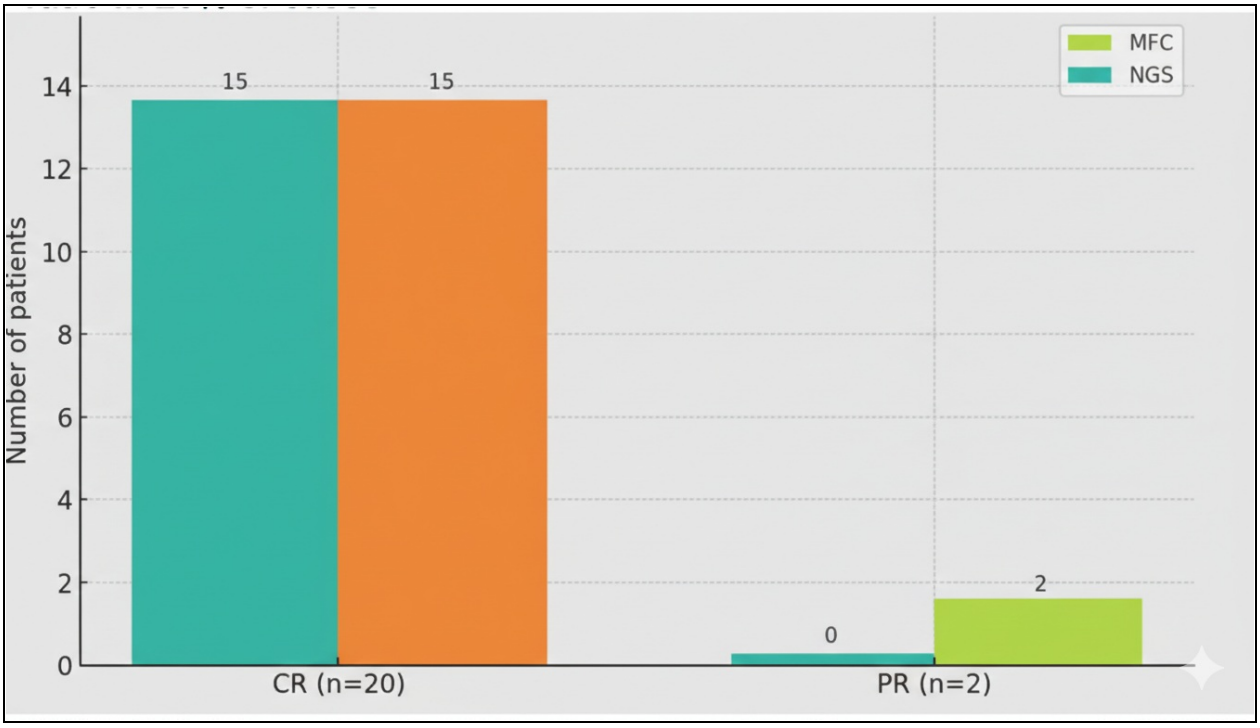

**Copyright Information:** (Online) ISSN: 2572-9241
©2026 The Author(s). HemaSphere is published by John Wiley & Sons Ltd on behalf of European Hematology Association. This is an open access Abstract Book distributed under the Attribution-NonCommercial-NoDerivs (CC BY-NC-ND), which allows third parties to download the articles and share them with others as long as they credit the author and the Abstract Book, but they cannot change the content in any way or use them commercially.
**Abstract Book Citations:** Authors, Title, HemaSphere, 2026;10:(S1):pages. The abstract book DOIs can be found on the "Author Information" page.
**Disclaimer:** Articles published in the journal HemaSphere exclusively reflect the opinions of the authors. The authors are responsible for all content in their abstracts including accuracy of the facts, statements, citing resources, etc.

### (PB2881) INTEGRATING EPIDEMIOLOGICAL INTELLIGENCE AND MULTI-OMICS PERSPECTIVES: ADVANCING BIOMARKER-DRIVEN STRATEGIES AGAINST THE GLOBAL BURDEN OF CHRONIC LYMPHOCYTIC LEUKEMIA

**Topic:** 05. Chronic lymphocytic leukemia and related disorders - Biology & translational research

Zi Wang*[1], Wei Zhang[1], Dao-Bin Zhou[1]

[1]*Peking Union Medical College Hospital, Chinese Academy of Medical Science and Peking Union Medical College, Beijing, China;*

### Background

The global epidemiological trends of chronic lymphocytic leukemia (CLL) provide a crucial macro-level foundation for guiding precision medicine. However, translating broad risk factors and burden disparities into actionable clinical strategies requires a bridge to molecular pathogenesis.

### Aims

This study analyzes the global CLL burden to not only delineate its public health landscape but also to inform the development of biomarker-based prevention and early detection frameworks.

### Methods

Leveraging data from the Global Burden of Disease Study 2021, we evaluated CLL incidence, mortality, and disability-adjusted life years (DALYs) from 1990 to 2021 using estimated annual percentage change (EAPC), Joinpoint regression, and decomposition analysis to identify key drivers of trends and inequalities. Predictive modeling (ARIMA) was employed to project future burden. Critically, the epidemiological insights—particularly regarding identified risk factors (e.g., smoking, high BMI) and high-burden populations—were framed as a strategic map to prioritize hypotheses for subsequent multi-omics and biomarker discovery research in CLL etiology and progression.We additionally analyzed three independent Gene Expression Omnibus (GEO) transcriptomic cohorts including CLL patients and non-leukemic controls. Differential expression analysis across cohorts identified 67 overlapping CLL-related genes, which underwent Gene Ontology (GO) and Kyoto Encyclopedia of Genes and Genomes (KEGG) enrichment. Using least absolute shrinkage and selection operator (LASSO)-regularized logistic regression in a training cohort, we derived a 14-gene predictive signature and validated its discriminative performance in independent datasets.

### Results

The analysis revealed a stable global age-standardized incidence but significant declines in mortality (33.7%) and DALYs (34.8%), alongside marked socioeconomic disparities. Decomposition analysis quantified the dominant role of population aging in driving DALY increases in Western Europe (+11403.53%) and epidemiological factors in Central Asia (+633.72%). Smoking and high body-mass index were reaffirmed as modifiable risk factors. Projections indicate declining incidence and mortality but rising prevalence through 2040. These findings pinpoint specific demographics and risk exposures as high-priority targets for biomarker investigation and stratified screening. At the molecular level, differential expression analyses across three GEO cohorts identified 67 overlapping CLL-related genes, which were significantly enriched in immune cell differentiation, leukocyte adhesion, T/B cell receptor signaling, and cytokine/growth factor pathways. A LASSO-based model further distilled these into a 14-gene signature that effectively discriminated CLL from non-CLL samples in the training cohort and retained stable predictive performance in independent validation sets.

**Copyright Information:** (Online) ISSN: 2572-9241
©2026 The Author(s). HemaSphere is published by John Wiley & Sons Ltd on behalf of European Hematology Association. This is an open access Abstract Book distributed under the Attribution-NonCommercial-NoDerivs (CC BY-NC-ND), which allows third parties to download the articles and share them with others as long as they credit the author and the Abstract Book, but they cannot change the content in any way or use them commercially.
**Abstract Book Citations:** Authors, Title, HemaSphere, 2026;10:(S1):pages. The abstract book DOIs can be found on the "Author Information" page.
**Disclaimer:** Articles published in the journal HemaSphere exclusively reflect the opinions of the authors. The authors are responsible for all content in their abstracts including accuracy of the facts, statements, citing resources, etc.

### Summary/Conclusion

This study synthesizes the global epidemiology of CLL into an intelligence framework that directly informs clinical and translational research. By identifying populations with the greatest burden shift and confirmed risk factors, it creates a rationale for targeted multi-omics studies to uncover underlying molecular biomarkers. Integrating such biomarkers with population-level data is essential for evolving current practices towards precision screening, risk-stratified monitoring, and ultimately, reducing the global burden of CLL through more personalized public health and clinical interventions.

**Copyright Information:** (Online) ISSN: 2572-9241
©2026 The Author(s). HemaSphere is published by John Wiley & Sons Ltd on behalf of European Hematology Association. This is an open access Abstract Book distributed under the Attribution-NonCommercial-NoDerivs (CC BY-NC-ND), which allows third parties to download the articles and share them with others as long as they credit the author and the Abstract Book, but they cannot change the content in any way or use them commercially.
**Abstract Book Citations:** Authors, Title, HemaSphere, 2026;10:(S1):pages. The abstract book DOIs can be found on the "Author Information" page.
**Disclaimer:** Articles published in the journal HemaSphere exclusively reflect the opinions of the authors. The authors are responsible for all content in their abstracts including accuracy of the facts, statements, citing resources, etc.

### (PB2883) A COMPARISON OF FULL-LENGTH VDJ AND CONVENTIONAL CDR3-BASED CLONAL CLUSTERING METHODS FOR EVALUATING SUB-CLONAL COMPOSITION IN CLL

**Topic:** 05. Chronic lymphocytic leukemia and related disorders - Biology & translational research

Loubna Boutkhil*[1, 2], Valérie Rimelen[3], Ines Schultz[3], Laurent Vallat[1, 2, 3]

*[1]CNRS-UMR-7242, Strasbourg, France;[2]Strasbourg University, Strasbourg, France;[3]University hospital of Strasbourg, Department of Molecular Genetics of Cancer, Strasbourg, France;*

**Background**

In Chronic lymphocytic leukemia (CLL), analyzing the B-cell receptor (BCR) repertoire is important to understand CLL pathogenesis, especially by studying somatic hypermutations (SHMs) in the immunoglobulin heavy chain variable gene (IGHV).

Generally, clonal and sub-clonal architecture in CLL is measured using clustering methods based on the most variable region (CDR3). However, these approaches could miss all mutations outside the CDR3, that preclude characterization of the sub-clonal complexity of the CLL BCR repertoire.

**Aims**

To evaluate sub-clonal composition in CLL, based on the analysis of SHMs in the full-length VDJ sequence compared to the conventional CDR3-based clustering method.

**Methods**

In this study, we reanalysed NGS data generated using IGH-VDJ amplicon approach. The resulting sequences were grouped into clonotypes using two different clustering approaches.

The first method consists of CDR3/ID50-based clustering using the Vidjil algorithm, which clusters sequences that share a 50nt window overlapping the CDR3 region.
The second method involves a VDJ-Full-length-based clustering using an adapted MiXCR preset. Briefly, MiXCR first clusters CDR1 to FR4 reads, then extends the sequence to the start of FR1 and separates sub-clonotypes if SHMs are detected.

A cutoff of 1% of the total number of reads was applied to eliminate the normal B cell background and poor quality/non-reproducible clonotypes.

**Copyright Information:** (Online) ISSN: 2572-9241
©2026 The Author(s). HemaSphere is published by John Wiley & Sons Ltd on behalf of European Hematology Association. This is an open access Abstract Book distributed under the Attribution-NonCommercial-NoDerivs (CC BY-NC-ND), which allows third parties to download the articles and share them with others as long as they credit the author and the Abstract Book, but they cannot change the content in any way or use them commercially.
**Abstract Book Citations:** Authors, Title, HemaSphere, 2026;10:(S1):pages. The abstract book DOIs can be found on the "Author Information" page.
**Disclaimer:** Articles published in the journal HemaSphere exclusively reflect the opinions of the authors. The authors are responsible for all content in their abstracts including accuracy of the facts, statements, citing resources, etc.

**Results**

For the first CLL-01 patient, CDR3/ID50 clustering revealed the presence of a single major clonotype, IGHV3-30-5, which represented the predominant CLL clone. However, full-length-VDJ clustering revealed a major clonotype IGHV3-30-5, as well as a subclonotype with a frequency of 33.6%, characterized by an N>S replacement mutation in the FR3 region.
For the second patient (CLL-02), the CDR3/ID50 clustering analysis revealed a major IGHV3-23 clonotype and six subclonotypes with frequencies ranging from 1.73% to 19.48%. Performing MiXCR full-length-VDJ clustering identified a major IGHV3-23 clonotype and 13 subclonotypes with percentages ranging from 1.16% to 18.58%. All subclonotypes expressed distinct amino acid sequences, indicating that BCR reactivity among the different subclonotypes may be significantly impacted by SHMs occurring outside the CDR3 region.
Interestingly, for a CLL-03 patient classified as 100% unmutated (UM-CLL), the CDR3/ID50 clustering analysis revealed only a major IGHV3-30-5 clonotype. However, the MiXCR full-length-VDJ clustering identified also a sub-clonotype with a frequency of 13.65% that differ from the major clone by a mutation in the CDR1 region.

**Summary/Conclusion**

In this comparative study, we demonstrate that different sub-clonal compositions can be observed in CLL depending on the clonal clustering method used. We reveal that the approach used can influence the number of CLL subclones detected, especially when using full-length VDJ clustering, which provides a more accurate visualization of the BCR repertoire complexity in CLL.

**Copyright Information:** (Online) ISSN: 2572-9241
©2026 The Author(s). HemaSphere is published by John Wiley & Sons Ltd on behalf of European Hematology Association. This is an open access Abstract Book distributed under the Attribution-NonCommercial-NoDerivs (CC BY-NC-ND), which allows third parties to download the articles and share them with others as long as they credit the author and the Abstract Book, but they cannot change the content in any way or use them commercially.
**Abstract Book Citations:** Authors, Title, HemaSphere, 2026;10:(S1):pages. The abstract book DOIs can be found on the “Author Information” page.
**Disclaimer:** Articles published in the journal HemaSphere exclusively reflect the opinions of the authors. The authors are responsible for all content in their abstracts including accuracy of the facts, statements, citing resources, etc.

### (PB2884) TARGETED NGS REVEALS THE MUTATIONAL LANDSCAPE OF UNTREATED WATCH-AND-WAIT CLL PATIENTS IN A REAL-WORLD BRAZILIAN COHORT

**Topic:** 05. Chronic lymphocytic leukemia and related disorders - Biology & translational research

Pedro Henrique Fernandes Do Carmo Las Casas*[1, 2, 3], Thamiris Silva Soares[2, 3], Javier Marco[4, 5], Julia Fernandes Do Carmo Las Casas[2], Vanessa Lucas Oliveira[3], Maria Lucia Ferraz de Carvalho[2, 3], Carolina Pereira de Souza Melo[2, 3], Leticia Conceição Braga[2, 3], Frederico Lisboa Nogueira[2, 3]

*[1]Saint Antoine University Hospital Assistance Publique Hôpitaux de Paris (AP-HP), Sorbonne University, INSERM UMR_S_938, CRSA, Research Department "Cancer, Vessels, Biology and Therapeutics" (CaVITE), Research Group "Cancer-Angiogenesis-Thrombosis and Haemostasis," Thrombosis Center, University Institute of Cancerology (UIC),, Paris, France; [2]Federal University of Minas Gerais, Faculty of Medicine, Belo Horizonte, Brazil; [3]Luxemburgo Hospital, Instituto Mario Penna, Belo Horizonte, Brazil; [4]University Hospital Morales Meseguer, Hematology Department,, Murcia, Spain; [5]Biomedical Research Institute of Murcia Pascual Parrilla-IMIB, CIBERER, Murcia, Spain;*

### Background

Recurrent mutations in *TP53, NOTCH1, SF3B1, ATM* and *BIRC3* are established adverse prognostic markers in chronic lymphocytic leukemia (CLL) and are frequently enriched in treatment-requiring and relapsed cohorts. However, the genomic architecture of patients managed with a watch-and-wait (WW) strategy remains incompletely characterized in real-world populations.

### Aims

To characterize the epidemiological incidence and prognostic distribution of gene mutations in untreated WW CLL patients from a real-world Brazilian cohort.

### Methods

We performed a descriptive mutational analysis of 23 consecutive treatment-naïve WW CLL patients from a single-center Brazilian cohort. Targeted next-generation sequencing was conducted using the SOPHiA GENETICS CCLL_A_v3 panel (23 CLL-associated genes). Variants classified as pathogenic or likely pathogenic were included in the primary analysis. Benign or likely benign variants were analyzed separately. Mutation incidence per gene and mutational burden per patient were calculated.

**Copyright Information:** (Online) ISSN: 2572-9241
©2026 The Author(s). HemaSphere is published by John Wiley & Sons Ltd on behalf of European Hematology Association. This is an open access Abstract Book distributed under the Attribution-NonCommercial-NoDerivs (CC BY-NC-ND), which allows third parties to download the articles and share them with others as long as they credit the author and the Abstract Book, but they cannot change the content in any way or use them commercially.
**Abstract Book Citations:** Authors, Title, HemaSphere, 2026;10:(S1):pages. The abstract book DOIs can be found on the "Author Information" page.
**Disclaimer:** Articles published in the journal HemaSphere exclusively reflect the opinions of the authors. The authors are responsible for all content in their abstracts including accuracy of the facts, statements, citing resources, etc.

### Results

Fifteen of 23 patients (65.2%) harbored at least one pathogenic or likely pathogenic alteration, while 8 (34.8%) had no detectable pathogenic variants within the panel. The median number of pathogenic mutations per patient was 3 (range 1–6; mean 2.67). The mutational landscape is summarized in Figure 1.
Sixteen distinct genes were affected. The most frequent pathogenic alterations involved *ATM* (21.7%), *BIRC3* (17.4%), and *SF3B1* (13.0%). *TP53* and *NOTCH1* mutations were each identified in 8.7% of cases.
When compared with treatment-requiring cohorts such as CLL4 and CLL8, in which *TP53*, *NOTCH1* and *SF3B1* mutations were reported in approximately 7–12%, 10% and 18–20% of patients respectively, our WW cohort demonstrated lower enrichment of adverse prognostic drivers, particularly for *SF3B1*. Importantly, over one-third of patients lacked any pathogenic alteration within the 23-gene panel, suggesting a subset with limited detectable high-risk genomic features.
In parallel, benign or likely benign variants were identified in 65.2% of patients, most frequently involving *TP53* (60.9%), consistent with common polymorphic variants. This finding underscores the importance of pathogenicity-based filtering to avoid overestimation of clinically relevant *TP53* disruption.
Taken together, the cohort demonstrates detectable genomic complexity but limited enrichment of mutations consistently associated with inferior prognosis, while a substantial fraction of patients show either no pathogenic variants or alterations not traditionally linked to aggressive disease biology.

### Summary/Conclusion

This real-world WW cohort demonstrates that early-stage CLL is not genomically silent, yet its mutational spectrum appears quantitatively and qualitatively distinct from treatment-enriched populations. The combination of limited *TP53/NOTCH1* disruption and a substantial proportion of patients without pathogenic variants supports the concept that WW patients may represent a biologically less genomically unstable subset at diagnosis. These findings reinforce the importance of stage-specific genomic characterization and careful interpretation of pathogenicity in early CLL.

**Copyright Information:** (Online) ISSN: 2572-9241
©2026 The Author(s). HemaSphere is published by John Wiley & Sons Ltd on behalf of European Hematology Association. This is an open access Abstract Book distributed under the Attribution-NonCommercial-NoDerivs (CC BY-NC-ND), which allows third parties to download the articles and share them with others as long as they credit the author and the Abstract Book, but they cannot change the content in any way or use them commercially.
**Abstract Book Citations:** Authors, Title, HemaSphere, 2026;10:(S1):pages. The abstract book DOIs can be found on the “Author Information” page.
**Disclaimer:** Articles published in the journal HemaSphere exclusively reflect the opinions of the authors. The authors are responsible for all content in their abstracts including accuracy of the facts, statements, citing resources, etc.

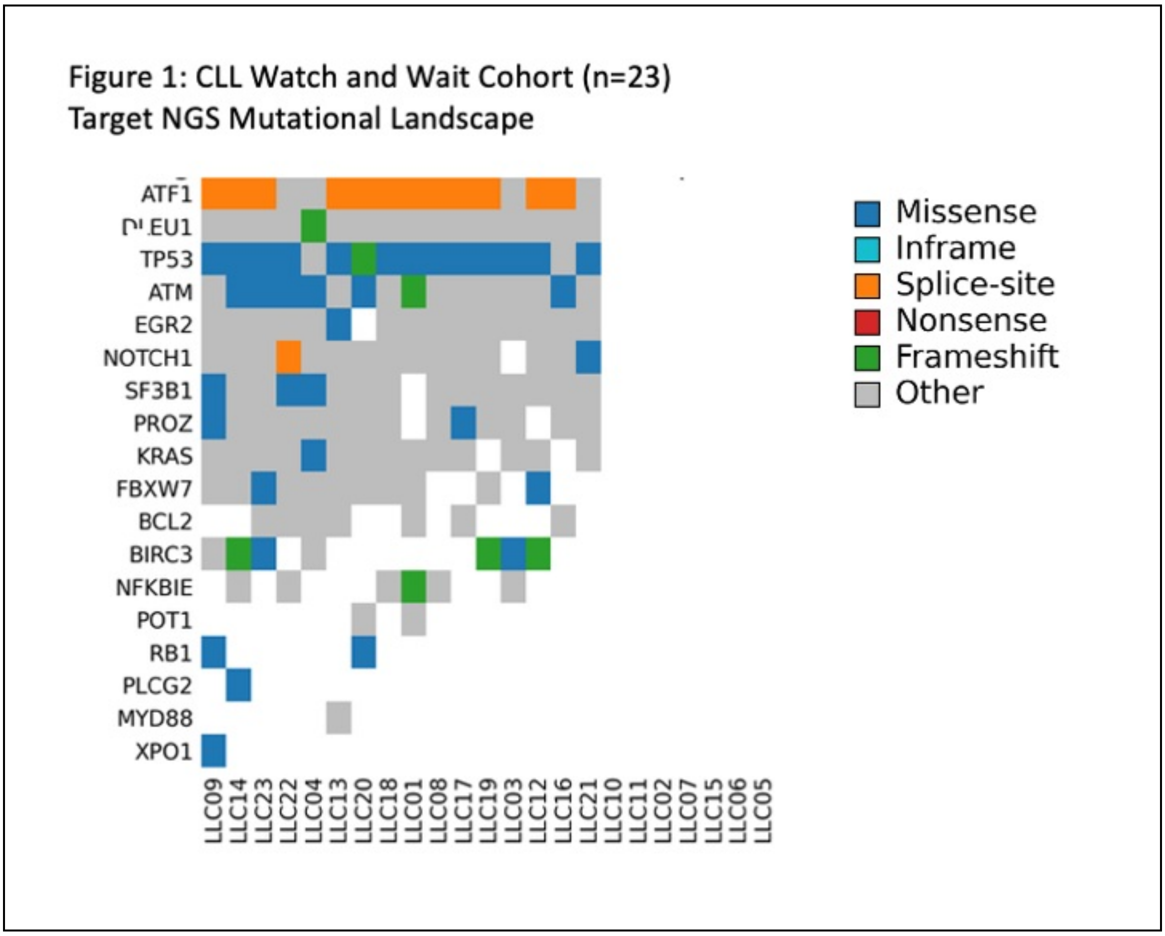

Figure 1. Mutational landscape of untreated watch-and-wait CLL patients (n=23). Oncoplot depicting the distribution of gene alterations identified by targeted next-generation sequencing using the SOPHiA GENETICS CCLL_A_v3 panel. Genes (rows) are ordered by decreasing incidence across the cohort, and patients (columns) are ordered by mutational burden. Variants classified as pathogenic or likely pathogenic were prioritized for visualization; when absent, other detected variants are shown. Colors indicate mutation type: missense (blue), in-frame indel (cyan), splice-site (orange), nonsense/stop-gain (red), frameshift (green), and other/low-impact consequences (grey).

**Copyright Information:** (Online) ISSN: 2572-9241
©2026 The Author(s). HemaSphere is published by John Wiley & Sons Ltd on behalf of European Hematology Association. This is an open access Abstract Book distributed under the Attribution-NonCommercial-NoDerivs (CC BY-NC-ND), which allows third parties to download the articles and share them with others as long as they credit the author and the Abstract Book, but they cannot change the content in any way or use them commercially.
**Abstract Book Citations:** Authors, Title, HemaSphere, 2026;10:(S1):pages. The abstract book DOIs can be found on the “Author Information” page.
**Disclaimer:** Articles published in the journal HemaSphere exclusively reflect the opinions of the authors. The authors are responsible for all content in their abstracts including accuracy of the facts, statements, citing resources, etc.

### (PB2885) NEXT-GENERATION SEQUENCING-BASED ASSESSMENT OF GENE DIVERSITY IN CHRONIC LYMPHOCYTIC LEUKEMIA: A SINGLE-CENTER EXPERIENCE

**Topic:** 05. Chronic lymphocytic leukemia and related disorders - Biology & translational research

Sofija Kozarac[1], Vojin Vukovic*[1, 2], Marija Dencic Fekete[3], Jelica Jovanović[1], Nada Kraguljac-Kurtovic[1], Tamara Bibic[1], Kristina Tomic[1], Jelena Ivanović[1], Darko Aleksic[1], Darko Antic[1, 2]

[1]*Clinic of Hematology, University Clinical Centre of Serbia, Belgrade, Serbia, Lymphoma and CLL unit, Belgrade, Serbia;* [2]*Faculty of Medicine, University of Belgrade, Serbia, Belgrade, Serbia;*[3]*Institute of Pathology, Faculty of Medicine, University of Belgrade, Belgrade, Serbia., Belgrade, Serbia;*

### Background

Next-generation sequencing (NGS) provides insight in diverse biology of chronic lymphocytic leukemia (CLL). Commercial NGS panels include detection of numerous gene aberrations whose routine clinical practice use is undefined.

### Aims

To explore genes diversity in CLL patients with NGS and its association with aberrations detected by fluorescence in situ hybridization (FISH) and with IGHV mutational status.

### Methods

We assessed medical records of 140 CLL patients in whom NGS (custom CLL genes panel by Sophia Genetics) and FISH analyses were performed at our institution between 2023 and 2025. We evaluated the spectrum of NGS-detected gene aberrations and their correlation with results of FISH analyses and IGHV mutational status.

### Results

NGS detected aberrations in 131 (93.6%) patients with following frequency: *TP53* mutation in 35 (25%), *ATM* mutation in 32 (22.9%), *ATM* deletion in 14 (10%), *DLEU* mutation in 1 (0.7%), *DLEU* deletion in 61 (43.6%), *RB1* mutation in 3 (2.1%), *RB1* deletion in 34 (24.3%), *SF3B1* mutation in 19 (13.6%), *BIRC3* mutation in 8 (5.7%), *BIRC3* deletion in 8 (5.7%), *PROZ* mutation in 3 (2.1%), *PROZ* deletion in 1 (0.7%), *PROZ* amplification in 4 (2.9%), *CUL4A* mutation in 1 (0.7%), *CUL4A* deletion in 1 (0.7%), *CUL4A* amplification in 4 (2.9%), *KLF5* mutation in 1 (0.7%), *KLF5* deletion in 1 (0.7%), *KLF5* amplification in 3 (2.1%), *XPO1* mutation in 15 (10.7%), *XPO1* deletion in 1 (0.7%), *XPO1* amplification in 2 (1.4%), *KRAS* mutation in 5 (3.6%), *KRAS* gain in 1 (0.7%), *KRAS* amplification in 11 (7.9%), *ATF1* deletion in 1 (0.7%), *ATF1* gain in 2 (1.4%), *ATF1* amplification in 13 (9.3%), *CDK4* mutation in 1 (0.7%), *CDK4* deletion in 1 (0.7%), *CDK4* amplification in 13 (9.3%), *CDK4* gain in 2 (1.4%), *NOTCH1* mutation in 14 (10%), *NOTCH1* deletion in 1 (0.7%), *NFKBIE1* mutation in 17 (12.1%), *NFKBIE1* deletion in 1 (0.7%), *POT1* mutation in 6 (4.3%), *POT1* deletion in 3 (2.1%), *POT1* amplification in 2 (1.4%), *POT1* gain in 1 (0.7%), *CXCR4* mutation in 2 (1.4%), *FBXW7* mutation in 5 (3.6%), *FBXW7* gain in 1 (0.7%), *BTK* mutation in 3 (2.1%), *MYD88* mutation in 1 (0.7%), *EGR2* mutation in 3 (2.1%), *BCL2* mutation in 2 (1.4%), *BCL2* amplification in 2 (1.4%), *PLCG2* mutation in 1 (0.7%) patients. There was a statistically significant association between negative FISH result and absence of NGS-detected aberrations (p=0.016); FISH-detected deletion of 13q (del13q) or 13q nulisomy and absence of NGS mutations (p=0.041); FISH-detected del13q or 13q nulisomy and *NFKBIE1* mutation (p=0.021); FISH-detected del13q or 13q nulisomy and *DLEU* deletion (p<0.001). There was a statistically significant association between FISH-detected deletion of 17p (del17p) and *TP53* mutation (p<0.001). There was a statistically significant association between mutated IGHV and *SF3B1* mutation (p=0.024). No other statistically significant associations were observed between NGS-detected aberrations and FISH results or IGHV mutational status.

**Copyright Information:** (Online) ISSN: 2572-9241
©2026 The Author(s). HemaSphere is published by John Wiley & Sons Ltd on behalf of European Hematology Association. This is an open access Abstract Book distributed under the Attribution-NonCommercial-NoDerivs (CC BY-NC-ND), which allows third parties to download the articles and share them with others as long as they credit the author and the Abstract Book, but they cannot change the content in any way or use them commercially.
**Abstract Book Citations:** Authors, Title, HemaSphere, 2026;10:(S1):pages. The abstract book DOIs can be found on the "Author Information" page.
**Disclaimer:** Articles published in the journal HemaSphere exclusively reflect the opinions of the authors. The authors are responsible for all content in their abstracts including accuracy of the facts, statements, citing resources, etc.

**Summary/Conclusion**

Majority of CLL patients had NGS-detected aberration (93.6%), with most frequent: *DLEU* deletion (43.6%), *TP53* mutation (25%), *RB1* deletion (24.3%) and *ATM* mutation (22.9%). Statistically significant associations were observed between FISH-detected del13q or 13q nulisomy and *NFKBIE1* mutation (p=0.021); FISH-detected del13q or 13q nulisomy and *DLEU* deletion (p<0.001); and mutated IGHV and *SF3B1* mutation (p=0.024). Further analyses are needed to determine the impact of NGS-detected abberations on clinical outcome.

**Copyright Information:** (Online) ISSN: 2572-9241
©2026 The Author(s). HemaSphere is published by John Wiley & Sons Ltd on behalf of European Hematology Association. This is an open access Abstract Book distributed under the Attribution-NonCommercial-NoDerivs (CC BY-NC-ND), which allows third parties to download the articles and share them with others as long as they credit the author and the Abstract Book, but they cannot change the content in any way or use them commercially.
**Abstract Book Citations:** Authors, Title, HemaSphere, 2026;10:(S1):pages. The abstract book DOIs can be found on the "Author Information" page.
**Disclaimer:** Articles published in the journal HemaSphere exclusively reflect the opinions of the authors. The authors are responsible for all content in their abstracts including accuracy of the facts, statements, citing resources, etc.

### (PB2886) PERSISTENT LYMPHOCYTOSIS AS A DIAGNOSTIC CHALLENGE: TWO CLINICAL CASES OF T- AND B-CELL LYMPHOID PROLIFERATION

**Topic:** 05. Chronic lymphocytic leukemia and related disorders - Biology & translational research

Raquel Lopes*[1], Fernando Miguel[1]

[1]*ULS REGIAO DE LEIRIA, SERVIÇO DE SANGUE, LEIRIA, Portugal;*

### Background

Persistent lymphocytosis in adults represents a diagnostic challenge, ranging from indolent conditions to aggressive lymphoid neoplasms. A systematic approach and close clinical monitoring are essential for the timely identification of treatment criteria. We present two illustrative cases of T- and B-cell lymphoid proliferation with distinct clinical courses but potential severity.

### Aims

To characterize the clinical and laboratory features of patients presenting with extreme lymphocytosis in lymphoproliferative syndromes of different lineages (B and T).

### Methods

Analysis of the patients' clinical and laboratory data obtained from medical records, along with a review of the relevant literature on the topic.

### Results

**Case 1 –** A 66-year-old man with a history of obesity, hypertension, and dyslipidemia was found to have mild lymphocytosis in June 2023 (white blood cells 13,100/µL; lymphocytes 5,200/µL). He was asymptomatic, with no organomegaly and normal LDH levels. Peripheral blood immunophenotyping revealed an aberrant CD4+ T-cell population suggestive of a T-cell lymphoproliferative disorder. Initial imaging and bone marrow studies showed no systemic involvement. He remained under surveillance until May 2025, when a bimonthly lymphocyte doubling time and rising LDH were observed. By November 2025, laboratory results showed white blood cells 267,600/µL (lymphocytes 246,000/µL), hemoglobin 12.2 g/dL, platelets 87,000/µL, LDH 790 U/L, and β2-microglobulin 5 mg/dL. CT scan revealed generalized lymphadenopathy and splenomegaly (15.5 cm). Repeat immunophenotyping confirmed **T-Prolymphocytic Leukemia**, with 82% pathological T cells (CD4+, CD2+, CD5+, CD7+, CD26+, CD45RA+, CCR7+, cytoplasmic TCL1+, absence of surface CD3 and CD8). Despite the absence of constitutional symptoms, treatment criteria were met due to rapidly progressive lymphocytosis and thrombocytopenia. Alemtuzumab was proposed, followed by allogeneic stem cell transplantation. **Case 2 –** A 60-year-old man with previously diagnosed **B-Cell Chronic Lymphocytic Leukemia** (2020), under watchful waiting, presented to the Emergency Department with presyncope and progressive fatigue. On admission: hemoglobin 3.4 g/dL, MCV 126.9 fL, white blood cells 252,900/µL (lymphocytes 241,300/µL), platelets 175,000/µL. Direct antiglobulin test (DAT) IgG 4++, without unequivocal laboratory markers of hemolysis; clinical improvement followed transfusion. CT imaging showed cervical, mediastinal, and abdominal lymphadenopathy without significant splenomegaly. Molecular study revealed unmutated IGHV; del17p/TP53 negative. Disease progression complicated by autoimmune hemolytic anemia was assumed. Corticosteroids were initiated, followed by ibrutinib, with sustained complete response.

**Copyright Information:** (Online) ISSN: 2572-9241
©2026 The Author(s). HemaSphere is published by John Wiley & Sons Ltd on behalf of European Hematology Association. This is an open access Abstract Book distributed under the Attribution-NonCommercial-NoDerivs (CC BY-NC-ND), which allows third parties to download the articles and share them with others as long as they credit the author and the Abstract Book, but they cannot change the content in any way or use them commercially.
**Abstract Book Citations:** Authors, Title, HemaSphere, 2026;10:(S1):pages. The abstract book DOIs can be found on the "Author Information" page.
**Disclaimer:** Articles published in the journal HemaSphere exclusively reflect the opinions of the authors. The authors are responsible for all content in their abstracts including accuracy of the facts, statements, citing resources, etc.

**Summary/Conclusion**

These cases illustrate two ends of the lymphoproliferative spectrum presenting with lymphocytosis. In T-PLL, an initially indolent phase evolved into rapidly progressive disease, highlighting the importance of close laboratory monitoring even in asymptomatic patients. In B-CLL, severe anemia was the first manifestation of progression, emphasizing autoimmune cytopenias as a significant complication. Both cases underscore the importance of recognizing persistent lymphocytosis, appropriate prognostic stratification, and early collaboration between Internal Medicine and Hematology to ensure timely therapeutic intervention and improved outcomes.

**Copyright Information:** (Online) ISSN: 2572-9241
©2026 The Author(s). HemaSphere is published by John Wiley & Sons Ltd on behalf of European Hematology Association. This is an open access Abstract Book distributed under the Attribution-NonCommercial-NoDerivs (CC BY-NC-ND), which allows third parties to download the articles and share them with others as long as they credit the author and the Abstract Book, but they cannot change the content in any way or use them commercially.
**Abstract Book Citations:** Authors, Title, HemaSphere, 2026;10:(S1):pages. The abstract book DOIs can be found on the “Author Information” page.
**Disclaimer:** Articles published in the journal HemaSphere exclusively reflect the opinions of the authors. The authors are responsible for all content in their abstracts including accuracy of the facts, statements, citing resources, etc.

### (PB2887) ORELABRUTINIB MONOTHERAPY IN CHINESE PATIENTS WITH CHRONIC LYMPHOCYTIC LEUKEMIA: A MULTI-CENTER REAL-WORLD RETROSPECTIVE STUDY

**Topic:** 06. Chronic lymphocytic leukemia and related disorders - Clinical

Huayuan Zhu*[1], Hanyi Ouyang[1], Jing Li[2], Zhuxia Jia[3], Xiaoyan Feng[4], Heng Chen[5], Jingjing Ye[6], Yifei Chen[7], Ling Gao[8], Jianyong Li[9]

*[1]The First Affiliated Hospital with Nanjing Medical University, Department of Hematology, Nanjing, China;[2]The First Affiliated Hospital of Guangxi Medical University, Department of Hematology, Nanning, China; [3]The Second people's hospital of Changzhou, The Third affiliated hospital of Nanjing medical university, Department of Hematology, Changzhou, China; [4]The First Affiliated Hospital of Zhengzhou University, Department of Oncology, Zhengzhou, China; [5]Wuxi People's Hospital Affiliated to Nanjing Medical University, Department of Hematology, Wuxi, China; [6]Qilu Hospital of Shandong University, Department of Hematology, Jinan, China; [7]Jiangdu People's Hospital Affiliated to Yangzhou University, Department of Hematology, Yangzhou, China; [8]Jiangsu Provincial (Suqian) Hospital, Department of Hematology, Suqian, China;[9]The First Affiliated Hospital of Nanjing Medical University, Jiangsu Province Hospital, Department of Hematology, Nanjing, China;*

### Background

Bruton tyrosine kinase inhibitors (BTKi) have been established as treatment options for patients (pts) with chronic lymphocytic leukaemia (CLL), both in the first-line and relapsed or refractory (R/R) setting. Orelabrutinib is a novel, oral, potent, highly selective irreversible BTKi approved in China for pts with newly diagnosed and R/R CLL.

### Aims

To assess the clinical outcomes of orelabrutinib monotherapy in CLL pts.

### Methods

This real-world study retrospectively reviewed CLL pts treated with orelabrutinib in 8 centers of China. Baseline peripheral blood or bone marrow samples were profiled for genetic mutations using targeted next-generation sequencing. Progression-free (PFS) and overall survival (OS) analyses were conducted in the overall population and stratified by treatment lines. PFS was also evaluated according to *IGHV* status, cytogenetic abnormalities (del17p, del13q, Trisomy 12), and complex karyotype.

**Copyright Information:** (Online) ISSN: 2572-9241
©2026 The Author(s). HemaSphere is published by John Wiley & Sons Ltd on behalf of European Hematology Association. This is an open access Abstract Book distributed under the Attribution-NonCommercial-NoDerivs (CC BY-NC-ND), which allows third parties to download the articles and share them with others as long as they credit the author and the Abstract Book, but they cannot change the content in any way or use them commercially.
**Abstract Book Citations:** Authors, Title, HemaSphere, 2026;10:(S1):pages. The abstract book DOIs can be found on the "Author Information" page.
**Disclaimer:** Articles published in the journal HemaSphere exclusively reflect the opinions of the authors. The authors are responsible for all content in their abstracts including accuracy of the facts, statements, citing resources, etc.

### Results

Between March 2022, and September 2025, 119 pts were included, with a median age of 58 years (range 30–87) and 69.7% males. Of evaluable pts, 47.9% (57/119) had Binet stage C disease, 21.4% (21/98) bulky disease. Based on CLL-IPI, 60.3% (35/58) of pts were classified as high-risk or very high-risk. Unmutated *IGHV*, complex karyotype (CK), del(17p), del(13q), del (11q), and trisomy 12 were detected in 45.2%, 28.1%, 9.6%, 36.1%, 21.3%, and 32.4% of tested pts, respectively (Figure 1). Of 93 available samples, the mutation frequencies of *TP53*, *IGLL5*, *NOTCH1*, *ATM*, *SF3B1* were 17.2%, 16.1%, 9.7%, 8.6%, and 8.6%, respectively. 21 pts received prior BTKi, all of whom switched to orelabrutinib primarily due to adverse events (AEs; 52.4% [11/21]). Orelabrutinib was administered in 49 pts as first-line, 45 as second-line, and 25 as third- or later-line therapy. Of 56 pts who discontinued treatment, the most common reason was AEs (41.1% [23/56], including 4 hematologic and 19 nonhematologic events), and 11 pts later resumed orelabrutinib. With a median follow-up of 25.9 months (*mo*; range 21.4–34.8), median PFS and overall survival (OS) in 119 pts were respectively 49.8 *mo* (95% CI 42.3–not reached [NR]) and 82.3 *mo* (95% CI 81.1–NR). When stratified by line of therapy, no significant differences in PFS (NR *vs* 49.9 *vs* 44.8 *mo*; p=0.496) or OS (NR *vs* NR *vs* 82.3 *mo*; p=0.988) were observed. The survival benefits observed in the first-line and later-line settings were numerically comparable to those reported in prospective studies of orelabrutinib in treatment-naïve (12-*mo* PFS/OS 96.7%) and relapsed/refractory pts (24-*mo* PFS 73.5%; 24-*mo* OS 81.3%). When stratified by genetic factors, PFS was associated with the absence of trisomy 12 in the overall (49.9 *vs* 35.5 *mo*; p=0.036) and first-line populations (NR *vs* 35.5 *mo*; p=0.041), but not associated with CK, del(17p), del(13q), del (11q), or *IGHV* status. In the later-line setting, no factors, including trisomy 12 status, were found to be associated with PFS.

### Summary/Conclusion

This study provided real-world evidence, supporting orelabrutinib as both first-line and subsequent monotherapy for CLL, including in pts harboring high-risk genetic features.

**Baseline characteristics and treatment**

| Characteristics | Patients (N=119) |
|---|---|
| **Age, median (range), years** | 58.0 (30.0-87.0) |
| **Male sex, n (%)** | 83 (69.7) |
| **CLL-IPI, n (%)** | **N=58** |
| 0–1 | 7 (12.1) |
| 2–3 | 16 (27.6) |
| 4–6 | 24 (41.4) |
| 7–10 | 11 (19.0) |
| **Bulky diease, n/N (%)** | 21/98 (21.4) |
| **B symptoms, n/N (%)** | 47/110 (42.7) |
| **ECOG performance status, n (%)** | **N=97** |
| 0 | 38(39.2) |
| 1 | 50 (51.5) |
| 2 | 8 (8.2) |
| 3 | 1 (1.0) |
| **Binet stage C, n (%)** | 57 (47.9) |
| **Complex karyotype, n/N (%)** | 25/89 (28.1) |
| **17p deletion, n/N (%)** | 8/83 (9.6) |
| **13q deletion, n/N (%)** | 26/72 (36.1) |
| **11q deletion, n/N (%)** | 16/75 (21.3) |
| **Trisomy 12, n/N (%)** | 23/71 (32.4) |
| **Unmutated *IGHV*, n/N (%)** | 42/93 (45.2) |
| **Number of lines of orelabrutinib therapy** | |
| 1 | 49 (41.2) |
| 2 | 45 (37.8) |
| ≥3 | 25 (21.0) |
| **Prior therapy, n (%)** | **N=70** |
| B/BR/FCR/R-CHOP/Clb like | 37 (52.9) |
| B/BR/FCR/R-CHOP/Clb like+BTKi | 8 (11.4) |
| BTKi | 13 (18.6) |
| R | 5 (7.1) |
| Others | 7 (10.0) |
| **Reasons for prior BTKi switching to orelabrutinib, n (%)** | **N=21** |
| Adverse events | 11 (52.4) |
| Disease progression | 2 (9.5) |
| Patient decision | 4 (19.0) |
| Unknown | 4 (19.0) |
| **Initial dose of orelabrutinib, n (%)** | **N=105** |
| 150 mg once daily | 88 (83.8) |
| 100 mg once daily | 5 (4.8) |
| 50 mg once daily | 12 (11.4) |
| **Reasons for orelabrutinib discontinuation, n (%)** | **N=56** |
| Adverse events | 23 (41.1) |
| Disease progression | 17 (30.4) |
| Death of unknown cause | 8 (14.3) |
| Patient decision | 8 (14.3) |
| **Resume orelabrutinib after discontinuation, n/N (%)** | 11/105 (10.5) |

**PFS and OS analyzed in the overall population and stratified by treatment lines**

| Population | Overall | First-line | Second-line | Third- or later-line |
|---|---|---|---|---|
| Median PFS | 49.8 (42.3-NA) | NA (NA-NA) | 49.9 (40.9-NA) | 44.8 (27.5-NA) |
| 12-month PFS | 94.5 (90.3-98.9) | 93.3 (86.2-100.0) | 95.1 (88.6-100.0) | 96.0 (88.6-100.0) |
| 24-month PFS | 83.1 (75.9-91.1) | 84.3 (73.4-96.9) | 83.8 (72.7-96.7) | 80.8 (65.5-99.8) |
| Median OS | 82.3 (81.1-NA) | NA (NA-NA) | NA (81.1-NA) | 82.3 (65.0-NA) |
| 12-month OS | 98.3 (95.9-100.0) | 97.9 (94.0-100.0) | 97.7 (93.3-100.0) | 100.0 (100.0-100.0) |
| 24-month OS | 94.2 (89.3-99.4) | 94.4 (87.0-100.0) | 94.2 (86.5-100.0) | 94.4 (84.4-100.0) |

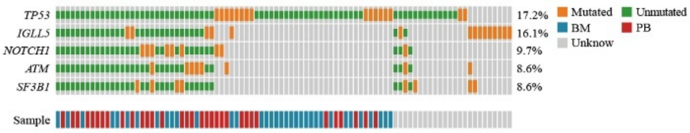

**Copyright Information:** (Online) ISSN: 2572-9241
©2026 The Author(s). HemaSphere is published by John Wiley & Sons Ltd on behalf of European Hematology Association. This is an open access Abstract Book distributed under the Attribution-NonCommercial-NoDerivs (CC BY-NC-ND), which allows third parties to download the articles and share them with others as long as they credit the author and the Abstract Book, but they cannot change the content in any way or use them commercially.
**Abstract Book Citations:** Authors, Title, HemaSphere, 2026;10:(S1):pages. The abstract book DOIs can be found on the "Author Information" page.
**Disclaimer:** Articles published in the journal HemaSphere exclusively reflect the opinions of the authors. The authors are responsible for all content in their abstracts including accuracy of the facts, statements, citing resources, etc.

### (PB2889) DURATION OF TREATMENT-FREE REMISSION AFTER FIXED DURATION THERAPY IN CHRONIC LYMPHOCYTIC LEUKEMIA: A REAL-WORLD ANALYSIS

**Topic:** 06. Chronic lymphocytic leukemia and related disorders - Clinical

Mattia D'antiga*[1], Andrea Serafin[1], Francesco Angotzi[1], Alessandro Cellini[1], Arianna Bevilacqua[1], Giovanni Leone[1], Nicolò Danesin[1], Annalisa Martines[2], Laura Bonaldi[2], Elisa Pagnin[1], Irene Olimpo[1], Valentina Trimarco[1], Sara Teolato[1], Federica Frezzato[1], Guido Capasso[1], Allison Beltrame[1], Sabrina Manni[1], Monica Facco[1], Francesco Piazza[1], Livio Trentin[1], Andrea Visentin[1]

*[1]University of Padua, Hematology Unit, Department of Medicine, Padova, Italy;[2]Veneto Institute of Oncology IOV-IRCSS, Immunology and Molecular Oncology Unit, Padova, Italy;*

**Background**

Fixed-duration therapy (FDT) represents a paradigm shift in chronic lymphocytic leukemia (CLL) management, offering sustained treatment-free remission periods compared to continuous regimens. The duration of off-therapy remission following FDT is a critical endpoint that impacts patients' quality of life, reduces cumulative toxicities, and minimizes selective pressure for resistance mutations. However, real-world data on treatment-free remission durability remain limited, particularly in high-risk subgroups such as those with TP53 abnormalities, NOTCH1 mutations, and complex karyotype (CK)

**Aims**

To asses the duration of TFR after FDT in a real life cohort, focusing on high risk subgroups

**Methods**

We retrospectively analyzed 103 consecutive CLL patients treated with FDT at our center till October 2025 (68 treatment-naïve [TN], 35 relapsed/refractory [RR]). TN patients received ibrutinib-venetoclax (38.2%), BTKi plus anti-CD20 monoclonal antibody (26.5%), or venetoclax plus anti-CD20 (35.3%); all RR patients received venetoclax plus rituximab.
Complex karyotype (CK) was defined as ≥3 chromosomal aberrations; high CK (hCK) as ≥5 aberrations. Primary outcome was Treatment Free Remission (TFR), measured from FDT completion to death or disease progression. Median follow-up was calculated using reverse Kaplan-Meier method.

**Results**

Among 103 patients the overall response rates were 95% (TN) and 94% (RR), with complete remissions in 56% and 62%, respectively. For the whole population 50% showed IGHV unmutated status, 18% TP53 abnormalities, 9.7% NOTCH1 mutations, 26% CK and 16.5% hCK. Ninety-three (90.3%) patients completed FDT and were evaluable for TFR analysis.
After median follow-up of 30 and 60 months for TN and RR patients, the median TFR was not reached in TN patients with 2-year TFR estimate of 90.6% (95%CI 82-100), while RR patients achieved median TFR of 24 months with 2-year estimate of 44.4% (95%CI 26.9-73.2).
In TN hCK significantly reduced TFR (44 months vs not reached, HR 4.87, p=0.035), while CK showed a trend towards a worse outcome (HR 3.78, p=0.05).
Peripheral blood MRD-negativity at end of therapy in TN was associated with superior TFR (HR 0.14, 95%CI 0.03-0.69, p=0.016).
In RR patients, neither CK, hCK nor TP53 abnormalities significantly affected TFR (median TFR 25 months). Remarkably, TP53 abnormalities and NOTCH1 mutations showed no significant impact on TFR duration in either TN or RR cohorts. Safety data will be reported.

**Copyright Information:** (Online) ISSN: 2572-9241
©2026 The Author(s). HemaSphere is published by John Wiley & Sons Ltd on behalf of European Hematology Association. This is an open access Abstract Book distributed under the Attribution-NonCommercial-NoDerivs (CC BY-NC-ND), which allows third parties to download the articles and share them with others as long as they credit the author and the Abstract Book, but they cannot change the content in any way or use them commercially.
**Abstract Book Citations:** Authors, Title, HemaSphere, 2026;10:(S1):pages. The abstract book DOIs can be found on the "Author Information" page.
**Disclaimer:** Articles published in the journal HemaSphere exclusively reflect the opinions of the authors. The authors are responsible for all content in their abstracts including accuracy of the facts, statements, citing resources, etc.

**Summary/Conclusion**

This real-world analysis demonstrates that FDT provides clinically meaningful TFR in CLL patients across different risk categories.TN patients achieved prolonged off-therapy periods with a 2-year remission rate exceeding 90%, while RR patients maintained disease control for almost 24 months. hCK emerged as the primary factor impinging TFR, though this subset of patients still achieved substantial drug-free periods. The lack of significant TFR impact from TP53 or NOTCH1 mutations suggests that combos of targeted agents may effectively overcome these traditionally high-risk features, although further investigation is warranted. The lack of impact in the RR may reflect limited numerosity rather than true biological differences.
These findings strongly support FDT implementation even in TN high-risk populations, offering patients extended periods off treatment with acceptable safety profiles. Future MRD-guided strategies or novel combinations may further extend treatment-free intervals, particularly optimizing outcomes for hCK patients.

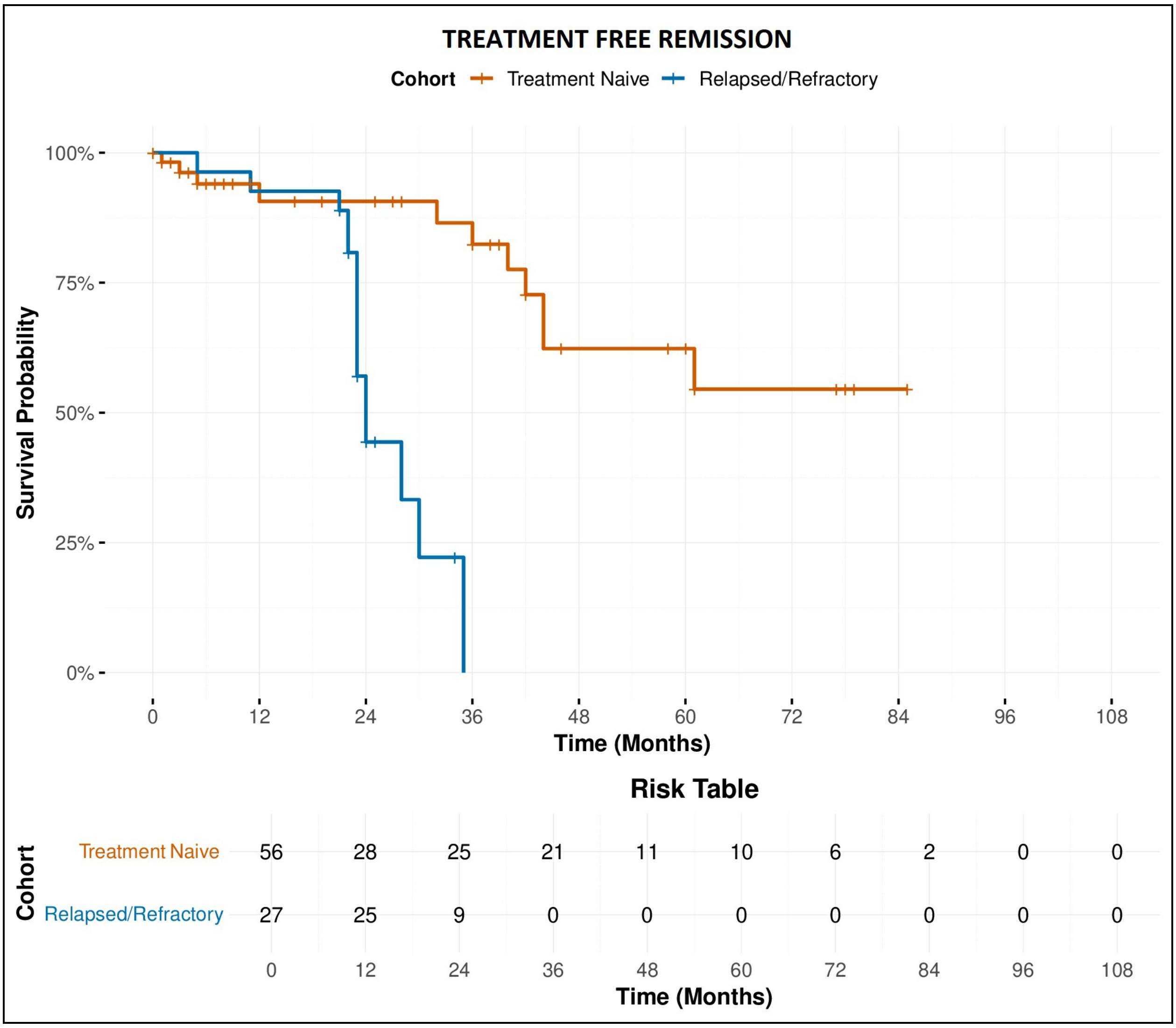

**Copyright Information:** (Online) ISSN: 2572-9241
©2026 The Author(s). HemaSphere is published by John Wiley & Sons Ltd on behalf of European Hematology Association. This is an open access Abstract Book distributed under the Attribution-NonCommercial-NoDerivs (CC BY-NC-ND), which allows third parties to download the articles and share them with others as long as they credit the author and the Abstract Book, but they cannot change the content in any way or use them commercially.
**Abstract Book Citations:** Authors, Title, HemaSphere, 2026;10:(S1):pages. The abstract book DOIs can be found on the "Author Information" page.
**Disclaimer:** Articles published in the journal HemaSphere exclusively reflect the opinions of the authors. The authors are responsible for all content in their abstracts including accuracy of the facts, statements, citing resources, etc.

### (PB2890) RETROSPECTIVE EVALUATION OF THE G8 SCORE IN CLL PATIENTS TREATED WITH TARGETED THERAPIES: A MULTI-CENTER EXPERIENCE

**Topic:** 06. Chronic lymphocytic leukemia and related disorders - Clinical

Alessandro Sanna*[1], Maria D'amato[2], Roberta Giachetti[3], Sara Ciofini[4], Giacomo Maestrini[5], Serena Rosati[6], Gabriella Carlomagno[7], Francesca Guerrini[4], Maria Teresa Pirrotta[8], Fabrizio Mavilia[9], Chiara Maria Rapolla[7], Giulia Papini[10], Federico Simonetti[9], Edoardo Benedetti[2], Simone Santini[6], Alessandro Gozzetti[4], Enrico Capochiani[9], Sabrina Moretti[5], Monica Bocchia[4], Sara Galimberti[2]

*[1]AOU Careggi, Hematology Unit, Department of Oncology, Florence, Italy;[2]University of Pisa, Hematology Unit, Department of Clinical and Experimental Medicine, Pisa, Italy; [3]Azienda USL Toscana Centro, SOC Oncohematology, Pescia and Pistoia, Italy; [4]University of Siena, Hematology, AOUS, Department of Medicine, Surgery and Neurosciences, Siena, Italy;[5]ASL Toscana Centro, Onco-hematology Unit, Department of Oncology, Florence, Italy; [6]Azienda USL Toscana Centro, Unit of Onco Hematology, Department of Oncology, Prato, Italy; [7]University of Florence, Hematology Unit, Department of Experimental and Clinical Medicine, Florence, Italy; [8]ASL Toscana Centro, Onco Hematology Unit, Department of Oncology, Empoli, Italy;[9]Azienda Toscana NordOvest, Lido di Camaiore, Italy; [10]San Donato Hospital, Hematology Unit, Department of Oncology, Arezzo, Italy;*

### Background

Assessment of fitness status is crucial for treatment selection in chronic lymphocytic leukemia (CLL), although no universally accepted definition exists. In clinical trials, fitness has commonly been evaluated using CIRS combined with creatinine clearance. Comprehensive Geriatric Assessment provides a multidimensional evaluation to support treatment decisions and toxicity management, but its complexity limits routine use. The Geriatric-8 (G8) screening tool represents a rapid and validated alternative to identify frailty in older cancer patients. A score ≤14 indicates frailty and may help guide treatment intensity.

### Aims

The aim was to evaluate the feasibility of G8 assessment and its utility in predicting overall survival (OS), progression-free survival (PFS), and adverse events.

### Methods

In this retrospective multicentre study, we assessed the G8 score in 584 consecutive patients with CLL treated with targeted therapies in 9 Tuscan hematological centres between 2016 and 2026, including both treatment-naïve (TN) and relapsed/refractory (R/R). Treatment regimens included: ibrutinib (n=215), acalabrutinib (n=80), zanubrutinib (n=83), venetoclax monotherapy (n=36), venetoclax plus rituximab (VR, n=64), venetoclax plus obinutuzumab (VO, n=52), and ibrutinib plus venetoclax (I+V, n=54).

**Copyright Information:** (Online) ISSN: 2572-9241
©2026 The Author(s). HemaSphere is published by John Wiley & Sons Ltd on behalf of European Hematology Association. This is an open access Abstract Book distributed under the Attribution-NonCommercial-NoDerivs (CC BY-NC-ND), which allows third parties to download the articles and share them with others as long as they credit the author and the Abstract Book, but they cannot change the content in any way or use them commercially.
**Abstract Book Citations:** Authors, Title, HemaSphere, 2026;10:(S1):pages. The abstract book DOIs can be found on the "Author Information" page.
**Disclaimer:** Articles published in the journal HemaSphere exclusively reflect the opinions of the authors. The authors are responsible for all content in their abstracts including accuracy of the facts, statements, citing resources, etc.

### Results

The mean age at treatment initiation was 73 years (range 29–92); 299 patients were TN, and 284 were R/R. The median number of prior treatments was 2 (range 1–6), and median follow-up was 25 months (range 0.2–182).
A G8 score ≤14 (unfit) was found in 39.7% of patients: with no difference between TN and R/R setting. Distribution by treatment type showed score ≤14 in: ibrutinib 38.5%, acalabrutinib 56.5%, zanubrutinib 57.7%, venetoclax monotherapy 54.5%, VO 8.5%, VR 32.2%, and I+V 9.3%.
Median OS for fit patients was not reached versus 68.5 months for unfit patients (p<0.001). In treatment-naïve patients, median OS was not reached at 120 months for fit patients vs 79.7 months for unfit (p<0.001); in the R/R cohort, median OS was not reached at 120 months for TN and 58.9 months for R/R (p<0.001).
Median PFS was significantly longer in fit patients (59.9 vs 42.9 months, p<0.001). In the naïve cohort, median PFS at 50 months was 83% for fit patients vs 55.2% (p=0.002) In the R/R group, median PFS was 54.6 vs 40.9 months in fit vs unfit patients (p=0.002).
Median OS for patients receiving continuous therapy (CT) was not reached in fit patients and was 66 months in unfit patients (p<0.001). Median PFS for CT was 67.9 months in fit patients vs 42.3 months for unfit.
We did not observe significant difference in OS and PFS for patients receiving fixed duration, but the follow up is still shorter.
Clinically significant adverse events were more frequent in unfit group vs fit (p.0.040)

### Summary/Conclusion

Defining patient fitness is essential in guiding treatment strategies for CLL. The G8 score is a rapid and practical tool for identifying patients at higher risk, as it was predictive of both overall survival (OS) and progression-free survival (PFS) in our cohort. Interestingly, even in the absence of formal scoring, patients subjectively judged as “fit” were more frequently treated with fixed-duration regimens, whereas unfit patients more often received continuous therapies. Future studies should prospectively evaluate the integration of the G8 score into routine clinical decision-making to further validate its role in optimizing treatment selection.

**Copyright Information:** (Online) ISSN: 2572-9241
©2026 The Author(s). HemaSphere is published by John Wiley & Sons Ltd on behalf of European Hematology Association. This is an open access Abstract Book distributed under the Attribution-NonCommercial-NoDerivs (CC BY-NC-ND), which allows third parties to download the articles and share them with others as long as they credit the author and the Abstract Book, but they cannot change the content in any way or use them commercially.
**Abstract Book Citations:** Authors, Title, HemaSphere, 2026;10:(S1):pages. The abstract book DOIs can be found on the “Author Information” page.
**Disclaimer:** Articles published in the journal HemaSphere exclusively reflect the opinions of the authors. The authors are responsible for all content in their abstracts including accuracy of the facts, statements, citing resources, etc.

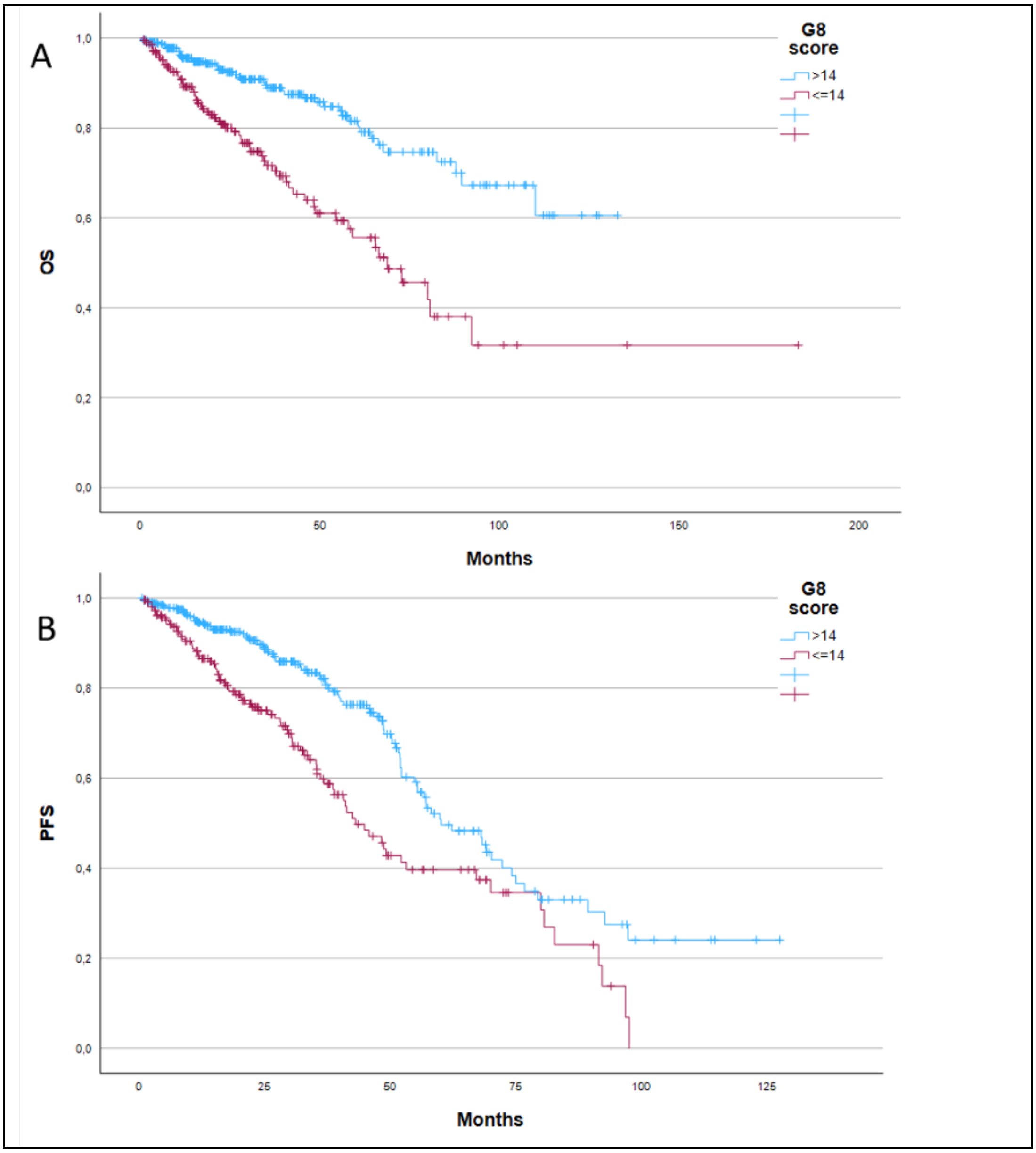

FIG1 A. Overall survival for entire cohort. B. PFS for the entire cohort

**Copyright Information:** (Online) ISSN: 2572-9241
©2026 The Author(s). HemaSphere is published by John Wiley & Sons Ltd on behalf of European Hematology Association. This is an open access Abstract Book distributed under the Attribution-NonCommercial-NoDerivs (CC BY-NC-ND), which allows third parties to download the articles and share them with others as long as they credit the author and the Abstract Book, but they cannot change the content in any way or use them commercially.
**Abstract Book Citations:** Authors, Title, HemaSphere, 2026;10:(S1):pages. The abstract book DOIs can be found on the "Author Information" page.
**Disclaimer:** Articles published in the journal HemaSphere exclusively reflect the opinions of the authors. The authors are responsible for all content in their abstracts including accuracy of the facts, statements, citing resources, etc.

### (PB2891) LANDSCAPE OF LATENT TUBERCOLOSIS INFECTION (LTBI) IN CHRONIC LYMPHOCYTIC LEUKEMIA. AN ITALIAN CLL CAMPUS STUDY.

**Topic:** 06. Chronic lymphocytic leukemia and related disorders - Clinical

Andrea Visentin*[1], Ilaria Angeletti[2], Laura Arletti[3], Laura Ballotta[4], Edoardo Benedetti[5], Alessandro Bosi[6], Gabriella Cancemi[7], Martina Cantelli[8], Maria Costanza Caparello[5], Gioacchino Catania[9], Maurizio Cavallari[10], Sara Ciofini[11], Marianna Criscuolo[12], Laura De Padua[13], Alessandra Di Francesco[14], Lucia Farina[15], Isacco Ferrarini[16], Annamaria Giordano[17], Idanna Innocenti[18], Ester Lovato[19], Monica MacCaferri[20], Alessandro Mancini[21], Salvatrice Mancuso[22], Enrica Antonia Martino[23], Nilla Maschio[24], Lucia Morello[25], Marina Motta[26], Roberta Murru[27], Maura Nicolosi[28], Andrea Nunzi[29], Jacopo Olivieri[30], Raffaella Pasquale[30], Sara Pepe[31], Francesca Perutelli[32], Paola Picardi[33], Barbara Pocali[34], Marika Porrazzo[35], Giulia Quaresmini[36], Costantino Riemma[37], Ilaria Romano[38], Filomena Russo[39], Chiara Salvetti[40], Alessandro Sanna[41], Gabriella Santuccio[42], Luana Schiattone[43], Ilaria Scortechini[44], Anna Tamburini[45], Marzia Varettoni[46], Federico Vozella[47], Sabrina Zoli[48], Francesco Angotzi[1], Alessandro Cellini[1], Andrea Serafin[1], Arianna Bevilacqua[1], Mattia D'antiga[1], Nicolò Danesin[1], Anna Maria Frustaci[49], Luca Laurenti[50], Massimo Gentile[51], Alessandro Broccoli[52], Gian Matteo Rigolin[53], Riccardo Moia[54], Candida Vitale[32], Paolo Sportoletti[21], Roberto Marasca[55], Valter Gattei[56], Gianluca Gaidano[54], Paolo Ghia[57], Antonio Cuneo[53], Livio Trentin[1]

*[1]Hematology unit, Department of Medicine, University of Padova, Padova, Italy;[2]Ematologia - azienda ospedaliera "S.Maria" Terni, Terni, Italy; [3]Hematology, Azienda USL - IRCCS di Reggio Emilia, Reggio Emilia, Italy;[4]UCO di Ematologia - Ospedale Maggiore di Trieste, Trieste, Italy; [5]Ematologia presso azienda ospedaliera di Pisa, Pisa, Italy;[6]Fondazione IRCCS Ca' Granda - Ospedale Maggiore Policlinico, Milano, Italy; [7]Divisione Ematologia presso A.O. Papardo di Messina, Messina, Italy; [8]U.O.C. Ematologia Ospedale di Ravenna, Ravenna, Italy; [9]Azienda Ospedaliera Universitaria SCDU di Ematologia, SS Antonio e Biagio e C. Arrigo, Alessandria, Italy; [10]U.O. di Ematologia - ULSS3 Serenissima, Ospedale dell'Angelo, Mestre, Italy; [11]Azienda ospedaliera Universitaria Senese, Siena, Italy; [12]Dipartimento Scienze di Laboratorio ed Ematologiche, Fondazione Policlinico Universitario A. Gemelli IRCCS, Padova, Italy; [13]UOC di Ematologia, Ospedale F. Spaziani, Frosinone, Italy; [14]Hematology and Bone Marrow Transplant Unit, University Hospital of Sassari, Sassari, Italy; [15]Hematology Department, Fondazione IRCCS Istituto Nazionale dei Tumori, Milano, Italy; [16]Hematology Unit, Section of Biomedicine of Innovation, Department of Engineering for Innovative Medicine, University of Verona, Verona, Italy; [17]Dipartimento di Medicina di Precisione e Rigenerativa e Area Ionica, Struttura Complessa di Ematologia con Trapianto, Azienda Ospedaliero Universitaria Consorziale Policlinico di Bari, Bari, Italy; [18]Dipartimento di Scienze di Laboratorio ed Ematologiche, Area Ematologia, presso Fondazione Policlinico Universitario A. Gemelli, IRCCS, Roma, Italy; [19]SC di Ematologia ASST Spedali Civili di Brescia, Brescia, Italy; [20]Unità Ematologia, AOU di Modena, Policlinico, Modena, Italy; [21]Institute of Hematology, University and Hospital of Perugia, Perugia, Italy;[22]University of Palermo, U.O. di Ematologia, AOUP P. Giaccone di Palermo, Palermo, Italy; [23]Divisione di ematologia, Azienda Ospedaliera Annunziata, Cosenza, Italy; [24]Onco Hematology Unit, Veneto Institute of Oncology IOV-IRCCS, Castelfranco Veneto, Italy; [25]UO Ematologia e CTMO Ospedale Guglielmo da Saliceto, Piacenza, Italy; [26]SC di Ematologia ASST Spedali Civili di Brescia, Brescia, Italy; [27]Hematology and Stem Cell Transplantation Unit, Ospedale Oncologico A. Businco, ARNAS G. Brotzu, Cagliari, Italy; [28]AOU Città della salute e della Scienza, SC Ematologia, Torino, Italy;[29]UO Ematologia, Dipartimento di Biomedicina e Prevenzione, Università di Roma Tor Vergata, Roma, Italy; [30]Clinica Ematologica, Azienda Sanitaria Universitaria Friuli Centrale (ASUFC), Udine, Italy; [31]Dipartimento di Medicina traslazionale e di precisione - Università Sapienza, Roma, Italy;[32]Dipartimento di Biotecnologie Molecolari e Scienze per la Salute, Università di Torino, Ematologia Universitaria, AOU "Città della Salute e della Scienza di Torino", Torino, Italy; [33]Ematologia presso ospedale di Ascoli Piceno, Ascoli Piceno, Italy;[34]UOC di Ematologia, ospedale Cardarelli, Napoli, Italy; [35]"Divisione di Oncoematologia presso Azienda Ospedaliera Ospedali Riuniti Villa Sofia Cervello di Palermo ", Palermo, Italy; [36]Divisione di Ematologia presso Ospedale Papa Giovanni XXIII di Bergamo, Bergamo, Italy; [37]Istituto Romagnolo per lo Studio dei Tumori "Dino Amadori" - IRST, Meldola, Italy;[38]Divisione di Ematologia presso Oncology Institute of Southern Switzerland - Ente Ospedaliero Cantonale, Bellinzona, Switzerland; [39]Divisone di Ematologia pressso Ospedale Maggiore di Parma, Parma, Italy; [40]Clinica Ematologica, IRCCS Azienda Ospedaliera Metropolitana di Genova, Genova, Italy; [41]SOD Ematologia - AOU Careggi, Firenze, Italy;[42]Divisione di ematologia c/o POLICLINICO OSPEDALIEREO di CATANIA, Catania, Italy; [43]UOC Ematologia, ospedale civile S. Spirito, Pescara, Italy;[44]Clinica di Ematologia – Ospedali Riuniti,*

**Copyright Information:** (Online) ISSN: 2572-9241
©2026 The Author(s). HemaSphere is published by John Wiley & Sons Ltd on behalf of European Hematology Association. This is an open access Abstract Book distributed under the Attribution-NonCommercial-NoDerivs (CC BY-NC-ND), which allows third parties to download the articles and share them with others as long as they credit the author and the Abstract Book, but they cannot change the content in any way or use them commercially.
**Abstract Book Citations:** Authors, Title, HemaSphere, 2026;10:(S1):pages. The abstract book DOIs can be found on the "Author Information" page.
**Disclaimer:** Articles published in the journal HemaSphere exclusively reflect the opinions of the authors. The authors are responsible for all content in their abstracts including accuracy of the facts, statements, citing resources, etc.

*Ancona, Italy; [45]Divisione di Ematologia presso Az. Osp. San Giovanni Addolorata, Roma, Italy; [46]Divisione di Ematologia, Fondazione IRCCS Policlinico San Matteo, Pavia, Italy; [47]Divisione di ematologia presso FONDAZIONE POLICLINICO UNIVERSITARIO CAMPUS BIO MEDICO, Roma, Italy; [48]U.O.C. Ematologia dell'Ospedale di Rimini, Rimini, Italy; [49]SC Ematologia, ASST Grande Ospedale Metropolitano Niguarda, Milano, Italy; [50]Dipartimento di Scienze di Laboratorio ed Ematologiche, Area Ematologia, Fondazione Policlinico Universitario A. Gemelli, IRCCS, Roma, Italy - Università Cattolica del Sacro Cuore, Roma, Italy; [51]UOC di Ematologia, Azienda Ospedaliera di Cosenza, University of Calabria, Cosenza, Italy; [52]Ematologia, Dipartimento di Scienze Mediche e Chirurgiche (DIMEC), Università degli Studi di Bologna, Bologna, Italy;[53]UO Ematologia A.O.U. Ferrara, University of Ferrara, Ferrara, Italy; [54]Division of Hematology, Department of Translational Medicine, Università del Piemonte Orientale and AOU Maggiore della Carità di Novara, Novara, Italy; [55]Università di Modena e Reggio Emilia - Dipartimento di Scienze Mediche e Chirurgiche Materno Infantili e dell'Adulto, sezione di Ematologia, Policlinico di Modena, Modena, Italy; [56]SOC Onco-ematologia Clinico-sperimentale, Centro di Riferimento Oncologico di Aviano IRCCS, Aviano, Italy; [57]Medical school, Università Vita-Salute San Raffaele; Programma strategico di ricerca sulla LLC, Comprehensive cancer center, IRCCS Ospedale San Raffaele, Milano, Italy;*

**Background**

Chronic lymphocytic leukemia (CLL) determines profound cellular and humoral immunodeficiency, increasing susceptibility to infections. Latent tuberculosis infection (LTBI) affects up to 15% of the general population in Europe and less commonly in Italy (<5%), but patients with hematologic malignancies are at increased risk of developing active tuberculosis (TB). Despite this, LTBI screening is not currently recommended in CLL

**Aims**

To evaluate the relevance of LTBI and TB in CLL by integrating data from clinical trials, pharmacovigilance databases, and real-world clinical practice

**Methods**

The Italian CLL Campus conducted i) a random effects meta-analysis of clinical trials published in PubMed up to January 2026, that treated at least 50 CLL patients with BTK inhibitors (BTKi), venetoclax (VEN) or combinations in any lines of therapy, assessing LTBI screening (tuberculin skin test or interferon-gamma release assay, IGRA) among baseline assessments; ii) rate of TB events in the same identified trials; iii) an analysis of TB cases associated with BTKi (ibrutinib, acalabrutinib, zanubrutinib, pirtobrutinib) and VEN reported in EudraVigilance (January 31, 2026); iv) a survey among CLL Campus members on LTBI assessment and management

**Copyright Information:** (Online) ISSN: 2572-9241
©2026 The Author(s). HemaSphere is published by John Wiley & Sons Ltd on behalf of European Hematology Association. This is an open access Abstract Book distributed under the Attribution-NonCommercial-NoDerivs (CC BY-NC-ND), which allows third parties to download the articles and share them with others as long as they credit the author and the Abstract Book, but they cannot change the content in any way or use them commercially.
**Abstract Book Citations:** Authors, Title, HemaSphere, 2026;10:(S1):pages. The abstract book DOIs can be found on the "Author Information" page.
**Disclaimer:** Articles published in the journal HemaSphere exclusively reflect the opinions of the authors. The authors are responsible for all content in their abstracts including accuracy of the facts, statements, citing resources, etc.

### Results

We analyzed 46 trials enrolling 9,404 patients (6,065 treatment naive, 3,339 relapsed/refractory RR) since 2014. No clinical trial included LTBI screening as baseline procedure. Only 2 (0.02%) active TB cases were reported, both in RR patients (1 ibrutinib, 1 venetoclax–rituximab) (Figure 1A, I2 0%, p=0.98)
In EudraVigilance, 57 TB cases were identified among 43,096 adverse drug reaction (ADR) reports (0.13%). Sixty percent occurred in male and half being ≥65 years old. TB was reported as not otherwise specified in 51%, pulmonary in 30%, disseminated in 12% and exclusively extrapulmonary in 7%. Outcomes were resolved in 8.8%, recovering in 14%, not recovered in 17.5%, fatal in 7% but unknown in 52.6%. TB events strongly correlated with time since EMA authorization ($R^2$=0.91) and number of ADRs ($R^2$=0.99, Figure 1B). TB incidence was higher with ibrutinib and VEN (0.14%), lower with second-generation BTKi (0.07%), and absent with non-covalent BTKi. Normalized TB events per ADR per 12 months were comparable across drugs
Among 32 Italian referral centers, LTBI screening is routinely performed at diagnosis or before treatment initiation by 12% and 31% of the physicians, respectively. Among IGRA-tested patients, a mean of 4% were positive, with center-specific rates ranging 0–20%. In such cases, 25% delayed CLL therapy until prophylaxis completion, while 72% initiated both concomitantly. Prophylaxis consisted mainly of isoniazid alone (44%) or combined with rifampicin (47%) for 6-9 months. Among 5.700 CLL patients treated outside of clinical trials, 216 had IGRA positivity. While no active TB occurred in patients who received LTBI prophylaxis, 4 patients developed active TB among 7 who did not receive prophylaxis (p<0.0001)

### Summary/Conclusion

Despite our data shows a high mortality rate, LTBI screening is systematically neglected in CLL clinical trials and inconsistently applied in real-world practice. Normalized TB rates across agents and their correlation with drug exposure duration argue against a drug-specific risk. Given relevant drug–drug interactions between TB prophylaxis and CLL therapies, our data challange current recommendations and support routine LTBI screening at CLL diagnosis as a preventive strategy before exposure to targeted therapies.

Figure 1

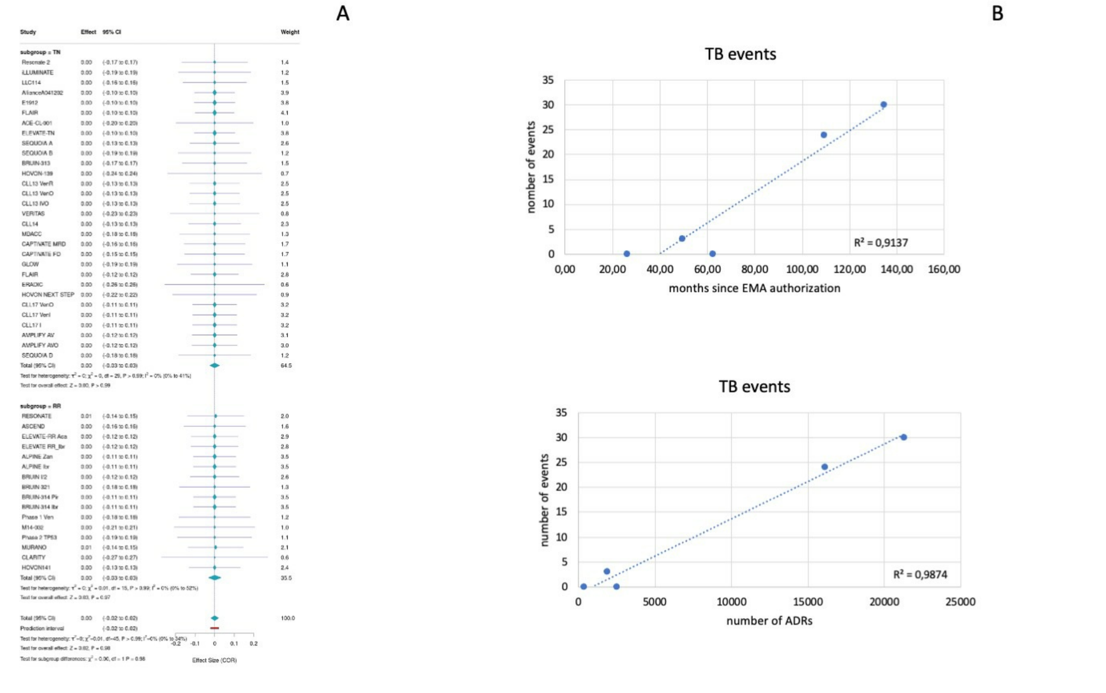

**Copyright Information:** (Online) ISSN: 2572-9241
©2026 The Author(s). HemaSphere is published by John Wiley & Sons Ltd on behalf of European Hematology Association. This is an open access Abstract Book distributed under the Attribution-NonCommercial-NoDerivs (CC BY-NC-ND), which allows third parties to download the articles and share them with others as long as they credit the author and the Abstract Book, but they cannot change the content in any way or use them commercially.
**Abstract Book Citations:** Authors, Title, HemaSphere, 2026;10:(S1):pages. The abstract book DOIs can be found on the "Author Information" page.
**Disclaimer:** Articles published in the journal HemaSphere exclusively reflect the opinions of the authors. The authors are responsible for all content in their abstracts including accuracy of the facts, statements, citing resources, etc.

### (PB2892) BASELINE CHOLESTATIC LIVER ENZYME ELEVATION INDEPENDENTLY PREDICTS INFERIOR OVERALL SURVIVAL IN PATIENTS WITH CHRONIC LYMPHOCYTIC LEUKEMIA: A NATIONWIDE REAL-WORLD COHORT STUDY

**Topic:** 06. Chronic lymphocytic leukemia and related disorders - Clinical

Yotam Bronstein*[1], Lior Rokach[2], Yair Herishanu[1], Ronen Arbel[3], Talish Razi[3], Noy Nachmias[4]

*[1]Tel Aviv Sourasky Medical Center, Hematology, Tel Aviv, Israel;[2]Ben-Gurion University of the Negev, Department of Software and Information Systems Engineering, Beer-Sheva, Israel; [3]Clalit Health Services, Community Medical Services Division, Tel Aviv, Israel; [4]Tel Aviv Sourasky Medical Center, Gastroenterology, Tel Aviv, Israel;*

### Background

Liver enzyme abnormalities may be present at chronic lymphocytic leukemia (CLL) diagnosis; however, their prognostic significance remains uncertain, and the relative impact of cholestatic versus hepatocellular enzyme elevation is not well defined.

### Aims

To evaluate the association between liver enzyme abnormalities at CLL diagnosis and overall survival (OS) and time to first treatment (TTFT).

### Methods

We analyzed a nationwide real-world cohort of patients diagnosed with CLL between 2004-2025 within Clalit Health Services (Israel) who had liver function tests available within ±3 months of diagnosis. Patients with pre-existing liver disease were excluded. Enzyme patterns were categorized as cholestatic (alkaline phosphatase [ALP] and/or gamma-glutamyl transferase [GGT]) or hepatocellular (alanine aminotransferase [ALT] and/or aspartate aminotransferase [AST]). OS and TTFT were estimated using Kaplan–Meier methods and compared by log-rank testing. Multivariable Cox proportional hazards models were constructed to evaluate independent associations with OS and TTFT, adjusting for confounders. All tests were two-sided, with $p<0.05$ considered statistically significant. Analyses were performed using R version 4.5.2.

**Copyright Information:** (Online) ISSN: 2572-9241
©2026 The Author(s). HemaSphere is published by John Wiley & Sons Ltd on behalf of European Hematology Association. This is an open access Abstract Book distributed under the Attribution-NonCommercial-NoDerivs (CC BY-NC-ND), which allows third parties to download the articles and share them with others as long as they credit the author and the Abstract Book, but they cannot change the content in any way or use them commercially.
**Abstract Book Citations:** Authors, Title, HemaSphere, 2026;10:(S1):pages. The abstract book DOIs can be found on the "Author Information" page.
**Disclaimer:** Articles published in the journal HemaSphere exclusively reflect the opinions of the authors. The authors are responsible for all content in their abstracts including accuracy of the facts, statements, citing resources, etc.

**Results**

A total of 3,448 patients were included. The median age at CLL diagnosis was 69.0 (IQR 62.0-77.0) and 55% were male. The median follow-up time was 74.9 months (IQR 37.6-125.3). In the cohort, 567 (16.4%) had baseline cholestatic enzyme elevation (ALP and/or GGT) and 119 (3.5%) had baseline hepatocellular enzyme elevation (ALT and/or AST).
Among patients with cholestatic enzyme elevation, median ALP was 129 U/L (IQR 121–142; ULN 120) compared with 75 U/L (IQR 62–87) in those without cholestatic elevation (p<0.001). Median GGT was 57 U/L (IQR 48–80; ULN 45) vs. 21 U/L (IQR 17–23), respectively (p<0.001). Among patients with hepatocellular enzyme elevation, median ALT was 44 U/L (IQR 42–50; ULN 34) compared with 17 U/L (IQR 13–21) in those without hepatocellular elevation (p<0.001), and median AST was 45 U/L (IQR 42–49; ULN 31) vs. 21 U/L (IQR 18–24), respectively (p<0.001).
At diagnosis, patients with cholestatic elevation (vs no cholestatic elevation) had higher CCI, higher WBC count and β2-microglobulin levels, and lower hemoglobin (all p≤0.01), while age and sex distribution were similar. Patients with hepatocellular elevation (vs no hepatocellular elevation) were more frequently male and had slightly higher CCI, lower hemoglobin and platelet counts, without significant differences in WBC count or β2-microglobulin.
In multivariable analysis, cholestatic enzyme elevation was independently associated with inferior OS (HR 1.21, 95% CI 1.05–1.39; p=0.008) after adjustment for sex, CCI, WBC, hemoglobin and platelet count, whereas hepatocellular enzyme elevation was not (HR 1.22, 95% CI 0.93–1.59; p=0.145). OS differed significantly across ALP/GGT subgroups (log-rank p<0.001), with the poorest survival in patients with concurrent ALP and GGT elevation (Figure 1). Neither cholestatic nor hepatocellular enzyme elevation was independently associated with TTFT (HR 1.15, 95% CI 0.95–1.40; p=0.150 and HR 1.13, 95% CI 0.77–1.66; p=0.528, respectively).

**Summary/Conclusion**

Baseline cholestatic, but not hepatocellular liver enzyme elevation independently predicts inferior OS in CLL without influencing TTFT. Routine liver biochemistry may provide a simple adjunct to baseline risk stratification and warrants external validation.

**Copyright Information:** (Online) ISSN: 2572-9241
©2026 The Author(s). HemaSphere is published by John Wiley & Sons Ltd on behalf of European Hematology Association. This is an open access Abstract Book distributed under the Attribution-NonCommercial-NoDerivs (CC BY-NC-ND), which allows third parties to download the articles and share them with others as long as they credit the author and the Abstract Book, but they cannot change the content in any way or use them commercially.
**Abstract Book Citations:** Authors, Title, HemaSphere, 2026;10:(S1):pages. The abstract book DOIs can be found on the "Author Information" page.
**Disclaimer:** Articles published in the journal HemaSphere exclusively reflect the opinions of the authors. The authors are responsible for all content in their abstracts including accuracy of the facts, statements, citing resources, etc.

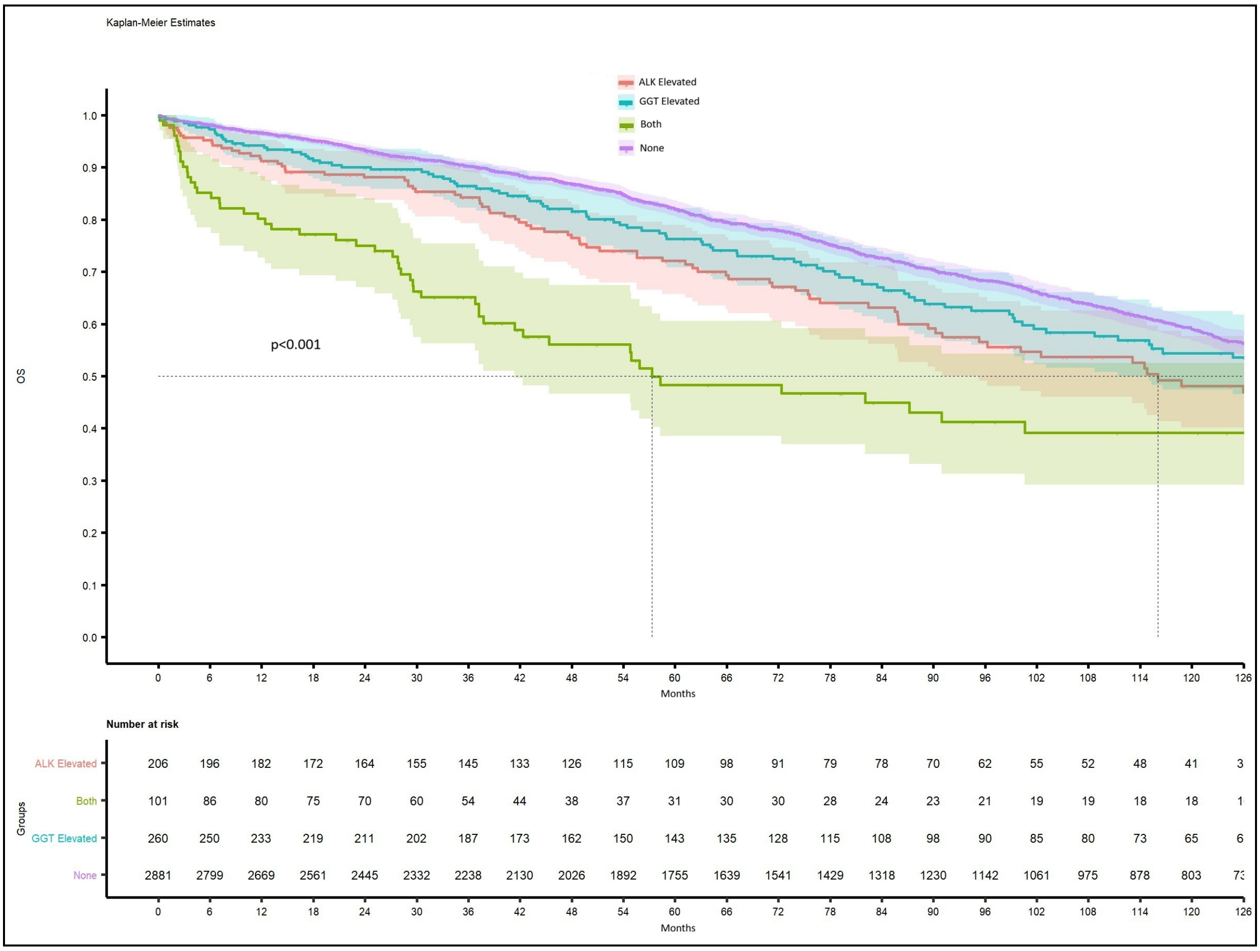

Figure 1: Overall Survival Among Patients with CLL According to Baseline Cholestatic Liver Enzyme Pattern

**Copyright Information:** (Online) ISSN: 2572-9241
©2026 The Author(s). HemaSphere is published by John Wiley & Sons Ltd on behalf of European Hematology Association. This is an open access Abstract Book distributed under the Attribution-NonCommercial-NoDerivs (CC BY-NC-ND), which allows third parties to download the articles and share them with others as long as they credit the author and the Abstract Book, but they cannot change the content in any way or use them commercially.
**Abstract Book Citations:** Authors, Title, HemaSphere, 2026;10:(S1):pages. The abstract book DOIs can be found on the "Author Information" page.
**Disclaimer:** Articles published in the journal HemaSphere exclusively reflect the opinions of the authors. The authors are responsible for all content in their abstracts including accuracy of the facts, statements, citing resources, etc.

### (PB2894) REAL-WORLD EFFECTIVENESS AND TOLERABILITY OF ACALABRUTINIB IN CHRONIC LYMPHOCYTIC LEUKEMIA: A MULTICENTER REAL-LIFE EXPERIENCE

**Topic:** 06. Chronic lymphocytic leukemia and related disorders - Clinical

Andrea Duminuco*[1], Dario Leotta[1], Alessandro Allegra[2], Daniele Caracciolo[3], Laura Caruso[1], Annalisa Chiarenza[1], Vittorio Del Fabro[4], Valentina Di Martina[5], Amalia Figuera[1], Paolo Fabio Fiumara[1], Massimo Gentile[6], Vanessa Innao[7], Salvatrice Mancuso[8], Antonio Mosca[9], Giovanna Motta[1], Francesca Pedalino[8], Marco Rossi[9], Fabio Stagno[2], Giorgio Tona[10], Francesco Di Raimondo[1], Giuseppe Alberto Maria Palumbo[1]

*[1]AOU Policlinico "G.Rodolico-San Marco", Catania, Italy; [2]Azienda Ospedaliera Universitaria Gaetano Martino, Messina, Italy; [3]Università di Catanzaro, Catanzaro, Italy; [4]Università degli Studi di Enna "Kore", Enna, Italy; [5]Ospedale "San Vincenzo", Taormina, Italy; [6]GRANDE OSPEDALE METROPOLITANO - BIANCHI MELACRINO MORELLI, Cosenza, Italy; [7]ARNAS Garibaldi, Catania, Italy; [8]Policlinico Universitario "P. Giaccone", Palermo, Italy; [9]Azienda Ospedaliero Universitaria "Renato Dulbecco", Catanzaro, Italy; [10]Ospedale "Sant'Elia", Caltanissetta, Italy;*

### Background

Acalabrutinib is a next-generation BTK inhibitor approved for CLL treatment, demonstrating high efficacy and a favorable safety profile in clinical trials; however, comprehensive real-world evidence on the durability of treatment and hematologic recovery remains limited.

### Aims

We aimed to evaluate the real-world effectiveness of acalabrutinib monotherapy in CLL patients across multiple Italian centers, focusing on time-to-discontinuation (TTD) as the primary endpoint and evaluating overall response rate (ORR), overall survival (OS), hematological parameter evolution, safety profile, and subgroup analyses.

### Methods

This retrospective, observational multicenter study enrolled adult CLL patients who initiated acalabrutinib monotherapy across 10 Italian centers. Patients were included if they received ≥1 month of treatment; treatment indications and ORR followed iwCLL criteria. TTD (assessed through Kaplan-Meier) was defined as time from initiation to permanent discontinuation (≥28 days interruption, progression, Richter transformation, or death); OS from initiation to death. Hematological parameters were assessed at baseline, 6, 12, and 24 months.

**Copyright Information:** (Online) ISSN: 2572-9241
©2026 The Author(s). HemaSphere is published by John Wiley & Sons Ltd on behalf of European Hematology Association. This is an open access Abstract Book distributed under the Attribution-NonCommercial-NoDerivs (CC BY-NC-ND), which allows third parties to download the articles and share them with others as long as they credit the author and the Abstract Book, but they cannot change the content in any way or use them commercially.
**Abstract Book Citations:** Authors, Title, HemaSphere, 2026;10:(S1):pages. The abstract book DOIs can be found on the "Author Information" page.
**Disclaimer:** Articles published in the journal HemaSphere exclusively reflect the opinions of the authors. The authors are responsible for all content in their abstracts including accuracy of the facts, statements, citing resources, etc.

**Results**

The cohort included 155 CLL patients (median age 74 years [45-89], 58.7% male), 64.5% treatment-naïve, and a median time from diagnosis to acalabrutinib initiation of 4 years [1-21]. Regarding high-risk biology, del(17p) was found in 15.3% (21/137), TP53 mutation in 20.2% (31/153), and unmutated IGHV in 65.7% (98/149). Cardiovascular comorbidities affected 59.4% (hypertension 50.3%, arrhythmias 3.9%), higher in ≥75 years (65.2% vs 51.2%). Median treatment duration was 16 months [1-61]; median TTD not reached, with 80% (124/155) ongoing and 78.7% (122/155) alive (median OS 18 months). ORR reached 84.5% (32.9% CR, 51.6% PR) after a median of 12 months; stable/progressive disease in 9.1%, unevaluable in 6.5%.
Responders (CR/PR) were less frequently aged ≥75 years compared with non-responders (37.4% vs 83.3%; p<0.001), and trended toward fewer cytopenias (47.3% vs 66.7%; p=0.119), but similar biology (IGHV, del(17p)/TP53), staging, prior lines, or comorbidities.
Subgroup TTD analyses showed favorable trends: younger age (<75 years; p=0.068), mutated IGHV (p=0.021, initial advantage, but later decline), borderline shorter in del(17p)/TP53 (p=0.063), no differences by line (1L vs R/R; p=0.93) or cardiovascular risk (p=0.365).
Hematological recovery was reported: hemoglobin rose from 11.1 g/dL (baseline) to 12.6 (6 mo), 13.1 (12 mo), 13.6 (24 mo); platelets from 146 to 160, 160, 189 $\times 10^9$/L; lymphocytes fell from 57.46 to 12.3, 5.37, 3.95 $\times 10^9$/L; WBC from 63.73 to 15.4, 10.32, 8.36 $\times 10^9$/L (all p<0.001); neutrophils stable at ~4 $\times 10^9$/L (p=0.48).
The safety profile was acceptable. Hematological 15.8% (9.7% G3/4 neutropenia needing G-CSF, rare G2/3 anemia/thrombocytopenia); non-hematological G3 events in 3.2% (pneumonia, hematomas, AF); common G1/2 included infections (4.5%), musculoskeletal/cutaneous (4.5%), GI (1.9%), headaches (4.5%). Dose reductions occurred in 18.7% (for tolerability, allowing continuation); discontinuations occurred in 20% (primarily AEs, followed by 10% progression/resistance). Post-discontinuation, 15 patients received additional lines.

**Summary/Conclusion**

Acalabrutinib demonstrated robust real-world effectiveness in a heterogeneous CLL cohort, with high ORR, prolonged TTD, hematological improvements, and manageable toxicity with no severe CV AEs (also for patients with prior risk), across ages, lines, and risk groups.

**Copyright Information:** (Online) ISSN: 2572-9241
©2026 The Author(s). HemaSphere is published by John Wiley & Sons Ltd on behalf of European Hematology Association. This is an open access Abstract Book distributed under the Attribution-NonCommercial-NoDerivs (CC BY-NC-ND), which allows third parties to download the articles and share them with others as long as they credit the author and the Abstract Book, but they cannot change the content in any way or use them commercially.
**Abstract Book Citations:** Authors, Title, HemaSphere, 2026;10:(S1):pages. The abstract book DOIs can be found on the "Author Information" page.
**Disclaimer:** Articles published in the journal HemaSphere exclusively reflect the opinions of the authors. The authors are responsible for all content in their abstracts including accuracy of the facts, statements, citing resources, etc.

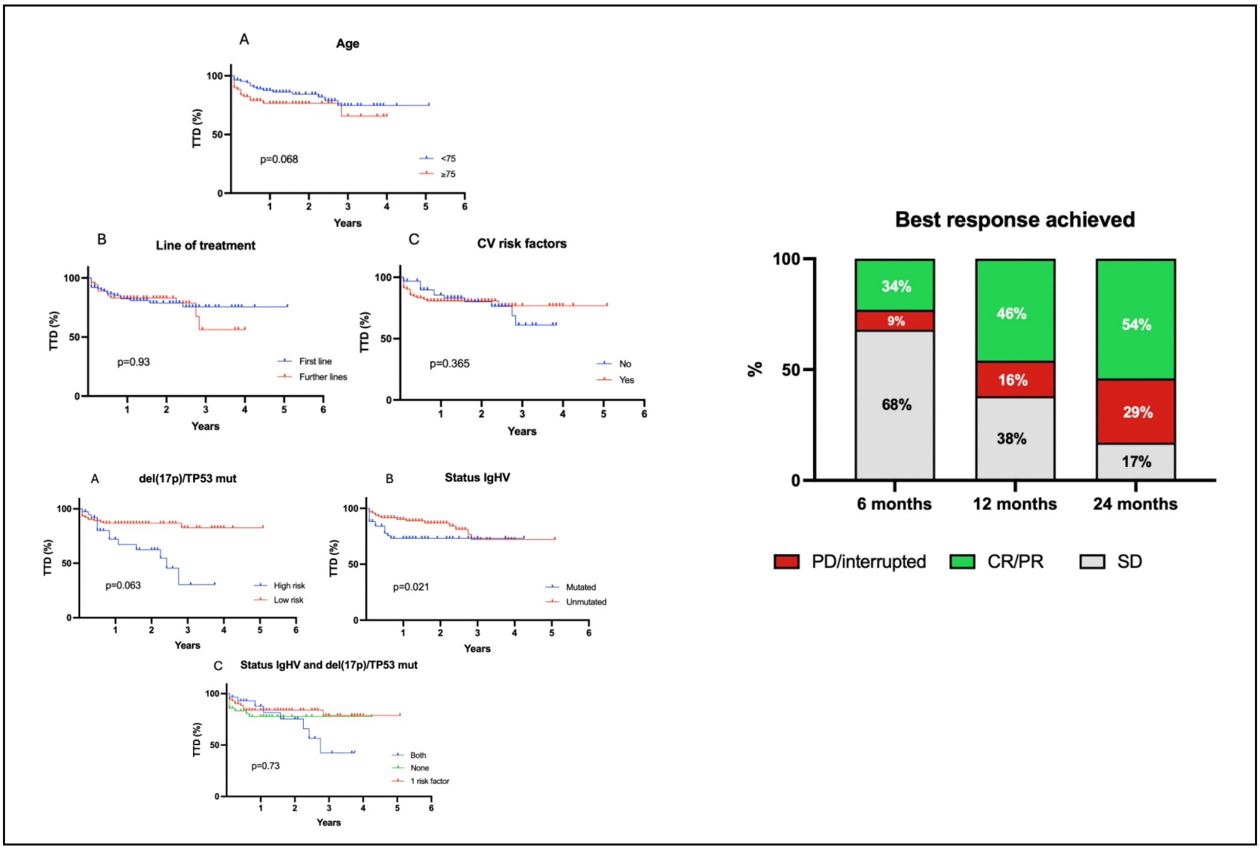

**Copyright Information:** (Online) ISSN: 2572-9241
©2026 The Author(s). HemaSphere is published by John Wiley & Sons Ltd on behalf of European Hematology Association. This is an open access Abstract Book distributed under the Attribution-NonCommercial-NoDerivs (CC BY-NC-ND), which allows third parties to download the articles and share them with others as long as they credit the author and the Abstract Book, but they cannot change the content in any way or use them commercially.
**Abstract Book Citations:** Authors, Title, HemaSphere, 2026;10:(S1):pages. The abstract book DOIs can be found on the "Author Information" page.
**Disclaimer:** Articles published in the journal HemaSphere exclusively reflect the opinions of the authors. The authors are responsible for all content in their abstracts including accuracy of the facts, statements, citing resources, etc.

### (PB2895) INDIRECT COMPARISON OF ZANUBRUTINIB VS ACALABRUTINIB AND IBRUTINIB EFFICACY IN PATIENTS WITH TREATMENT-NAIVE CHRONIC LYMPHOCYTIC LEUKEMIA

**Topic:** 06. Chronic lymphocytic leukemia and related disorders - Clinical

Mazyar Shadman*[1, 2], Talha Munir[3], Sheng Xu[4], Keri Yang[5], Leyla Mohseninejad[6], Grace Guthrie[5], Jamie Hirata[5], Marcus Lefebure[7], G. Rhys Williams[5], Constantine S. Tam[8]

*[1]Fred Hutchinson Cancer Center, Seattle, WA, United States of America;[2]University of Washington, Seattle, WA, United States of America; [3]Leeds Teaching Hospitals NHS Trust, Leeds, United Kingdom;[4]BeOne Medicines, Ltd, Shanghai, China;[5]BeOne Medicines, Ltd, San Carlos, CA, United States of America; [6]BeOne Medicines, Ltd, Schiphol, The Netherlands; [7]BeOne Medicines, Ltd, London, United Kingdom; [8]Alfred Hospital and Monash University, Melbourne, VIC, Australia;*

### Background

In treatment-naive (TN) chronic lymphocytic leukemia (CLL), zanubrutinib (SEQUOIA [NCT03336333]), acalabrutinib (ELEVATE-TN; [NCT02475681]), and ibrutinib (RESONATE-2; [NCT01722487]) were evaluated in separate trials. This study compared zanubrutinib vs acalabrutinib and zanubrutinib vs ibrutinib via indirect naive comparison analysis.

### Methods

The zanubrutinib vs acalabrutinib comparison comprised zanubrutinib-treated patients without del(17p) and/or *TP53* mutation (mut) from arm A of SEQUOIA (n=215; median follow-up, 72.8 months) and acalabrutinib monotherapy-treated patients from ELEVATE-TN, (n=156; median follow-up, 74.5 months). SEQUOIA arm A and RESONATE-2 enrolled patients without del(17p), but patients could have *TP53*mut. The zanubrutinib vs ibrutinib comparison comprised zanubrutinib-treated patients from SEQUOIA arm A (n=241; median follow-up, 72.8 months) and ibrutinib-treated patients from RESONATE-2 (n=136; median follow-up, 82.7 months); the proportion of patients with *TP53*mut were comparable for zanubrutinib vs ibrutinib. For the zanubrutinib vs ibrutinib analysis, COVID-19–adjusted progression-free survival (PFS) and overall survival (OS) were used from SEQUOIA due to impact of the COVID-19 pandemic on SEQUOIA. Pseudo-individual patient data for patients treated with acalabrutinib and ibrutinib were reconstructed from digitized Kaplan-Meier (KM) curves of PFS per investigator assessment, and OS. Hazard ratios (HRs) and 95% CIs were estimated using a Cox proportional hazards model, and landmark rates and rate differences were estimated using the KM method; 95% CIs for rate differences were calculated using a Wald-type statistic.

### Results

Baseline clinical characteristics between groups were relatively well balanced for each comparison. PFS and OS rates are shown (**Table**). The HRs for zanubrutinib vs acalabrutinib for PFS and OS were 0.76 (95% CI, 0.52-1.11; *P*=.1553) and 0.66 (95% CI, 0.41-1.06; *P*=.0836), respectively. For zanubrutinib vs ibrutinib, these were 0.65 (95% CI, 0.44-0.97; *P*=.0330) for PFS and 0.60 (95% CI, 0.36-1.01; *P*=.0532) for OS. At the 72-month landmark, the rate difference in PFS between zanubrutinib vs acalabrutinib was 9.9% (95% CI, 3.0%-16.9%); for OS, the rate difference was 8.8% (95% CI, 2.0%-15.5%). For zanubrutinib vs ibrutinib, the rate difference was 15.3% (95% CI, 8.3%-22.2%) for PFS and 9.9% (95% CI, 3.0%-16.7%) for OS.

**Copyright Information:** (Online) ISSN: 2572-9241
©2026 The Author(s). HemaSphere is published by John Wiley & Sons Ltd on behalf of European Hematology Association. This is an open access Abstract Book distributed under the Attribution-NonCommercial-NoDerivs (CC BY-NC-ND), which allows third parties to download the articles and share them with others as long as they credit the author and the Abstract Book, but they cannot change the content in any way or use them commercially.
**Abstract Book Citations:** Authors, Title, HemaSphere, 2026;10:(S1):pages. The abstract book DOIs can be found on the "Author Information" page.
**Disclaimer:** Articles published in the journal HemaSphere exclusively reflect the opinions of the authors. The authors are responsible for all content in their abstracts including accuracy of the facts, statements, citing resources, etc.

**Summary/Conclusion**

In this indirect naive comparison analysis of patients with TN CLL, zanubrutinib demonstrated higher PFS and OS than ibrutinib, and higher PFS and OS than acalabrutinib at 72 months. The analysis included a large population with comparable risk features and similar median follow-up across SEQUOIA, ELEVATE-TN, and RESONATE-2, though remaining differences in patient characteristics should be considered when interpreting these results.

**Table: PFS and OS rates**

| Time (months) | SEQUOIA vs ELEVATE-TN | | | | SEQUOIA vs RESONATE-2[a] | | | |
|---|---|---|---|---|---|---|---|---|
| | PFS, % | | OS, % | | PFS, % | | OS, % | |
| | Zanu w/o del(17p) and/or *TP53*mut n=215 | Acala w/o del(17p) and/or *TP53*mut n=156 | Zanu w/o del(17p) and/or *TP53*mut n=215 | Acala w/o del(17p) and/or *TP53*mut n=156 | Zanu n=241 | Ibru n=136 | Zanu n=241 | Ibru n=136 |
| **12** | 95.8 | 94 | 98.6 | 98 | 95.8 | 95 | 98.3 | 98 |
| **24** | 90.6 | 90 | 95.7 | 95 | 90.7 | 88 | 96.2 | 95 |
| **36** | 85.8 | 83 | 92.4 | 93 | 87.1 | 82 | 94.0 | 88 |
| **48** | 83.4 | 80 | 90.4 | 88 | 85.3 | 77 | 92.7 | 86 |
| **60** | 78.0 | 73 | 87.0 | 84 | 80.3 | 72 | 89.4 | 83 |
| **72** | 74.0 | 64 | 85.0 | 76 | 76.5 | 61 | 87.6 | 78 |

[a]Includes *TP53*mut
**Abbreviations:** acala, acalabrutinib; ibru, ibrutinib; mut, mutated; OS, overall survival; PFS, progression-free survival; w/o, without; zanu, zanubrutinib.

**Copyright Information:** (Online) ISSN: 2572-9241
©2026 The Author(s). HemaSphere is published by John Wiley & Sons Ltd on behalf of European Hematology Association. This is an open access Abstract Book distributed under the Attribution-NonCommercial-NoDerivs (CC BY-NC-ND), which allows third parties to download the articles and share them with others as long as they credit the author and the Abstract Book, but they cannot change the content in any way or use them commercially.
**Abstract Book Citations:** Authors, Title, HemaSphere, 2026;10:(S1):pages. The abstract book DOIs can be found on the "Author Information" page.
**Disclaimer:** Articles published in the journal HemaSphere exclusively reflect the opinions of the authors. The authors are responsible for all content in their abstracts including accuracy of the facts, statements, citing resources, etc.

### (PB2896) RISK OF TUMOR LYSIS SYNDROME AMONG PATIENTS WITH CHRONIC LYMPHOCYTIC LEUKEMIA OR MANTLE CELL LYMPHOMA TREATED WITH VENETOCLAX: A REAL-WORLD STUDY

**Topic:** 06. Chronic lymphocytic leukemia and related disorders - Clinical

Alessandra Ferrajoli*[1], Lili Zhou[2], Ayad Ali[2], Gabriel Man[2], Catherine Fu[2], Wassim Aldairy[2]

*[1]The University of Texas MD Anderson Cancer Center, Houston, TX, United States of America;[2]BeOne Medicines, Ltd, San Carlos, CA, United States of America;*

### Background

Tumor lysis syndrome (TLS) is a life-threatening oncological emergency caused by the rapid destruction of malignant cells that can result in renal failure, cardiac arrhythmias, or death. Venetoclax, a B-cell lymphoma 2 inhibitor, has been associated with TLS in clinical trials. The incidence of TLS events ranges from 1.8% to 3.0% across clinical trials evaluating the efficacy and safety of venetoclax in patients with B-cell malignancies. However, data on TLS among patients treated with venetoclax in routine clinical practice are limited. This study aimed to describe the incidence of TLS among patients with chronic lymphocytic leukemia (CLL) or mantle cell lymphoma (MCL) treated with venetoclax in routine clinical practice in the United States.

### Methods

Deidentified data from patients with CLL or MCL who initiated venetoclax from April 2016 to December 2024 were identified from the TriNetX Dataworks-USA Network electronic health record database. Patient demographics, comorbidities, and concomitant medications were described. The primary outcome was the 3-month TLS risk following venetoclax initiation. A 3-month follow-up was selected to encompass the dose ramp-up period in patients with CLL or MCL. TLS was identified using two definitions: (1) the presence of the *International Classification of Diseases, Tenth Revision, Clinical Modification* diagnosis code E88.3 and (2) receipt of rasburicase treatment. The secondary outcome was the incidence of all-cause mortality in patients experiencing TLS, defined as the presence of TLS diagnosis within the same month as death or the month prior, with no trauma-related diagnosis recorded during the same period.

### Results

A total of 4747 patients with CLL and 408 patients with MCL initiated venetoclax therapy and were included in this analysis; 68.7% of patients with CLL and 67.2% of patients with MCL were aged ≥65 years. Approximately 54.1% of patients with CLL and 55.9% of patients with MCL had at least 1 comorbidity. Venetoclax was administered in combination with either rituximab or obinutuzumab in 44.3% and 40.7% of patients with CLL and MCL, respectively. Antihyperuricemic prophylaxis with either allopurinol or febuxostat at baseline was observed in 58.2% of patients with CLL and 63.7% of patients with MCL. Using the *ICD-10* diagnosis code-based definition, TLS occurred within 3 months of venetoclax initiation in 5.6% (n=265) of patients with CLL and 8.6% (n=35) of patients with MCL, with median time to onset of 17 and 16 days, respectively. Using the definition based on rasburicase use, TLS incidence was 3.9% (n=186) among patients with CLL and 5.9% (n=24) among patients with MCL, with a median time to onset of 14 and 11 days, respectively. All-cause mortality occurred in 0.3% (n=12) of patients with CLL and 2.2% (n=9) of patients with MCL who were identified as having TLS; median time to mortality was 47 and 43 days after the start of therapy with venetoclax, respectively.

**Copyright Information:** (Online) ISSN: 2572-9241
©2026 The Author(s). HemaSphere is published by John Wiley & Sons Ltd on behalf of European Hematology Association. This is an open access Abstract Book distributed under the Attribution-NonCommercial-NoDerivs (CC BY-NC-ND), which allows third parties to download the articles and share them with others as long as they credit the author and the Abstract Book, but they cannot change the content in any way or use them commercially.
**Abstract Book Citations:** Authors, Title, HemaSphere, 2026;10:(S1):pages. The abstract book DOIs can be found on the "Author Information" page.
**Disclaimer:** Articles published in the journal HemaSphere exclusively reflect the opinions of the authors. The authors are responsible for all content in their abstracts including accuracy of the facts, statements, citing resources, etc.

### Summary/Conclusion

This real-world study demonstrated that the risk of TLS was common among patients treated with venetoclax and generally occurred shortly after treatment initiation. The risk appeared to be relatively higher among patients with MCL than in those with CLL. These findings underscore the importance of close monitoring during venetoclax therapy, particularly during the dose ramp-up period.

**Table.** TLS Risk in Patients With CLL or MCL Receiving Treatment With Venetoclax

| 3-month TLS risk | | CLL (n=4747) | MCL (n=408) |
|---|---|---|---|
| *ICD-10* diagnosis code-based definition | n (%) | 265 (5.6) | 35 (8.6) |
| | Median (IQR) time to onset, days | 17 (7-37) | 16 (8-31) |
| Rasburicase use-based definition | n (%) | 186 (3.9) | 24 (5.9) |
| | Median (IQR) time to onset, days | 14 (7-26) | 11 (7-30) |
| Incidence of mortality in patient with TLS[a] | n (%) | 12 (0.3) | 9 (2.2) |
| | Median (IQR) time to onset, days | 47 (36-60) | 43 (29-50) |

[a]Includes all-cause mortality; cause of death was not available in the data source.

CLL, chronic lymphocytic leukemia; *ICD-10*, *International Classification of Diseases, Tenth Revision, Clinical Modification*; IQR, interquartile range; MCL, mantle cell lymphoma; TLS, tumor lysis syndrome.

**Copyright Information:** (Online) ISSN: 2572-9241
©2026 The Author(s). HemaSphere is published by John Wiley & Sons Ltd on behalf of European Hematology Association. This is an open access Abstract Book distributed under the Attribution-NonCommercial-NoDerivs (CC BY-NC-ND), which allows third parties to download the articles and share them with others as long as they credit the author and the Abstract Book, but they cannot change the content in any way or use them commercially.
**Abstract Book Citations:** Authors, Title, HemaSphere, 2026;10:(S1):pages. The abstract book DOIs can be found on the "Author Information" page.
**Disclaimer:** Articles published in the journal HemaSphere exclusively reflect the opinions of the authors. The authors are responsible for all content in their abstracts including accuracy of the facts, statements, citing resources, etc.

### (PB2897) LOW-NORMAL IMMUNOGLOBULIN LEVELS SUGGEST MONOCLONAL B-CELL LYMPHOCYTOSIS AND CONSTITUTIONAL CLL SUSCEPTIBILITY: A DUAL-MECHANISM ANALYSIS

**Topic:** 06. Chronic lymphocytic leukemia and related disorders - Clinical

Ramon Cohen*[1, 2, 3], Shay Nemet[1, 3], Shira Bezalel-Rosenberg[1, 3], Ilan Asher[1, 3], Keren Mahlab-Guri[1, 3], Pia Raanani[4, 5, 6], Daniel Elbirt Daniel Elbirt[1, 3], Liron Hofstetter[4, 5]

*[1]Kaplan Medical Center, Department of Clinical Immunology, Allergy and AIDS,, Rehovot, Israel;[2]Kaplan Medical Center and Faculty of Medicine, Department of Internal Medicine B, Rehovot, Israel; [3]Faculty of Medicine, Hebrew University of Jerusalem, Israel, Jerusalem, Israel; [4]Davidoff Cancer Centre, Rabin Medical Centre, Institute of Hematology, Petah Tikva, Israel;[5]Tel Aviv University, The Gray Faculty of Medical and Health Sciences, Tel Aviv, Israel; [6]Felsenstein Medical Research centre, Rabin Medical Centre, Petah Tikva, Israel;*

### Background

Hypogammaglobulinemia is common at diagnosis of chronic lymphocytic leukemia (CLL), yet the significance of immunoglobulin variation within the normal range years before disease onset is unclear. Monoclonal B-cell lymphocytosis (MBL), a precursor state, may be present decades before diagnosis and subtly impair humoral immunity. We hypothesized that low-normal immunoglobulin levels identify individuals with either occult clonal B-cell expansion or intrinsic immune susceptibility that predisposes to CLL.

### Aims

To determine whether low-normal immunoglobulin levels predict future CLL and to distinguish whether associations reflect tumor burden from undiagnosed MBL or lymphocyte-independent constitutional susceptibility.

### Methods

We performed a population-based retrospective cohort study using Clalit Health Services data- the largest HMO in Israel (2004–2024), including 294,712 adults aged 40–80 with IgG, IgA, and IgM measurements. Immunoglobulin levels were assessed 10 years before diagnosis of CLL and dichotomized based on the median value of the cohort. We used multivariate Cox proportional hazards models, adjusted for age and sex, with sensitivity analyses stratified by baseline lymphocyte counts.

**Copyright Information:** (Online) ISSN: 2572-9241
©2026 The Author(s). HemaSphere is published by John Wiley & Sons Ltd on behalf of European Hematology Association. This is an open access Abstract Book distributed under the Attribution-NonCommercial-NoDerivs (CC BY-NC-ND), which allows third parties to download the articles and share them with others as long as they credit the author and the Abstract Book, but they cannot change the content in any way or use them commercially.
**Abstract Book Citations:** Authors, Title, HemaSphere, 2026;10:(S1):pages. The abstract book DOIs can be found on the “Author Information” page.
**Disclaimer:** Articles published in the journal HemaSphere exclusively reflect the opinions of the authors. The authors are responsible for all content in their abstracts including accuracy of the facts, statements, citing resources, etc.

### Results

At a follow-up of 10 years, 1,571 CLL cases occurred. CLL cases had markedly lower median and mean IgM, IgG, and IgA levels compared with controls (all p<0.01). They also displayed higher absolute lymphocyte counts at baseline, which was significantly elevated in future CLL cases (p<0.01).
We found consistent differences in cumulative 10-year risk of developing CLL according to baseline immunoglobulin levels. IgA <187 mg/dL showed Hazard Ratio [HR] of 2.62 (97.5% CI: 2.33-2.96), IgM <70.5 mg/dL showed HR 1.77 (CI 1.57–1.99), and IgG <1120 mg/dL; HR 1.43 (CI 1.26–1.62) (Figure 1). Analyses were repeated excluding diagnoses within two years of measurement, with all associations remaining statistically significant.
Among individuals with lymphocyte <2.2 × $10^9$/L at index date, low IgA remained significantly associated with CLL risk (HR 1.76, CI 1.26–2.47), whereas low IgM and low IgG showed no significant associations. In contrast, among patients with lymphocytes of 3.0–4.8 × $10^9$/L, all three immunoglobulins showed significant associations (Table 1).

Table 1: Cox regression of immunoglobulins according to lymphocyte count

| | **#Lym< 2.2 (×$10^9$/L)** | | **#Lym 3-4.8 (×$10^9$/L)** | |
|---|---|---|---|---|
| | **0–10 years HR (97.5% CI)** | **2–10 years HR (97.5% CI)** | **0–10 years HR (97.5% CI)** | **2–10 years HR (97.5% CI)** |
| **Age** | **1.04 (1.02–1.06)** | **1.03 (1.02–1.05)** | **1.05 (1.04–1.07)** | **1.04 (1.02–1.06)** |
| **Male** | **1.44 (1.04–2.00)** | 1.32 (0.90–1.95) | **1.95 (1.38–2.75)** | **1.83 (1.15–2.90)** |
| **IgM < 70.5 mg/dL** | 0.89 (0.63–1.27) | 0.83 (0.54–1.26) | **1.78 (1.25–2.52)** | 1.51 (0.95–2.41) |
| **IgG < 1120 mg/dL** | 0.90 (0.64–1.26) | 1.00 (0.67–1.49) | **1.50 (1.04–2.17)** | 1.55 (0.95–2.53) |
| **IgA < 187 mg/dL** | **1.76 (1.26–2.47)** | **1.49 (1.00–2.24)** | **2.51 (1.76–3.58)** | **1.85 (1.15–2.99)** |

### Summary/Conclusion

Low-normal immunoglobulin levels may serve as early biomarkers for CLL through a dual mechanism. While low IgM and IgG may reflect tumor burden from prevalent undiagnosed MBL, low IgA identifies a high-risk phenotype even in low lymphocyte counts. This suggests IgA captures a constitutional susceptibility trait in addition to tumor-related suppression.

**Copyright Information:** (Online) ISSN: 2572-9241
©2026 The Author(s). HemaSphere is published by John Wiley & Sons Ltd on behalf of European Hematology Association. This is an open access Abstract Book distributed under the Attribution-NonCommercial-NoDerivs (CC BY-NC-ND), which allows third parties to download the articles and share them with others as long as they credit the author and the Abstract Book, but they cannot change the content in any way or use them commercially.
**Abstract Book Citations:** Authors, Title, HemaSphere, 2026;10:(S1):pages. The abstract book DOIs can be found on the “Author Information” page.
**Disclaimer:** Articles published in the journal HemaSphere exclusively reflect the opinions of the authors. The authors are responsible for all content in their abstracts including accuracy of the facts, statements, citing resources, etc.

Figure 1: 10-year risk of developing CLL according to baseline immunoglobulin levels

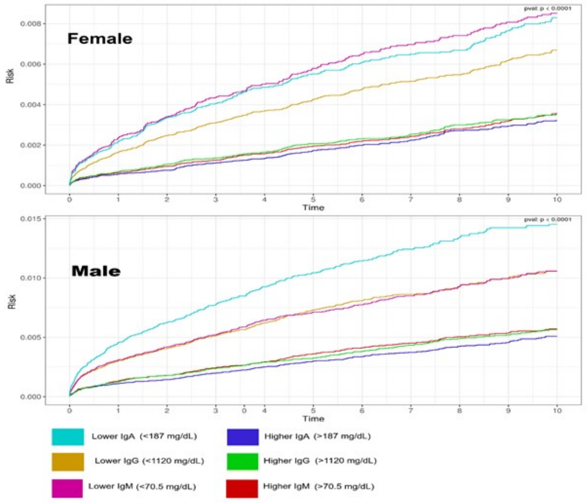

**Copyright Information:** (Online) ISSN: 2572-9241
©2026 The Author(s). HemaSphere is published by John Wiley & Sons Ltd on behalf of European Hematology Association. This is an open access Abstract Book distributed under the Attribution-NonCommercial-NoDerivs (CC BY-NC-ND), which allows third parties to download the articles and share them with others as long as they credit the author and the Abstract Book, but they cannot change the content in any way or use them commercially.
**Abstract Book Citations:** Authors, Title, HemaSphere, 2026;10:(S1):pages. The abstract book DOIs can be found on the "Author Information" page.
**Disclaimer:** Articles published in the journal HemaSphere exclusively reflect the opinions of the authors. The authors are responsible for all content in their abstracts including accuracy of the facts, statements, citing resources, etc.

### (PB2898) REAL-WORLD EXPERIENCE WITH ZANUBRUTINIB IN CHRONIC LYMPHOCYTIC LEUKAEMIA – PATIENT PROFILE, TREATMENT PATTERNS, AND SAFETY: PRELIMINARY ANALYSIS OF FRENCH ROZALY STUDY

**Topic:** 06. Chronic lymphocytic leukemia and related disorders - Clinical

Florian Bouclet*[1], Borhane Slama[2], Sara Burcheri[3], Cecile Tomowiak[4], Lauris Gastaud[5], Pierre Feugier[6], Anne-Sophie Michallet[7], Vincent Levy[8], Marion Willaime[9], Laura Dima[10], Aude Ominetti[10], Anne Quinquenel[11], Stephane Lepretre[1]

*[1]Centre Henri Becquerel, Rouen, France; [2]CH Avignon, Avignon, France; [3]CH Perpignan, Perpignan, France; [4]CHU de Poitiers, Poitiers, France; [5]CH Princesse Grace, Monaco, Monaco; [6]CHRU de Nancy, Nancy, France;[7]Centre Léon Bérard, Lyon, France; [8]Hôpital Avicenne, Bobigny, France; [9]Kappa Santé (CRO), Paris, France; [10]BeOne Medicines, Paris, France; [11]CHU de Reims, Reims, France;*

### Background

Zanubrutinib, a next-generation Bruton tyrosine kinase (BTK) inhibitor, has shown high efficacy and favourable safety in chronic lymphocytic leukaemia (CLL) in the phase 3 SEQUOIA and ALPINE trials, and is approved in Europe for treatment naïve (TN) and relapsed/refractory (R/R) disease. ROZALY was designed to generate real-world data on its use in France in a rapidly evolving treatment landscape.

### Aims

To describe patient and disease characteristics and treatment use in patients initiated with zanubrutinib for CLL in both TN and R/R real-world settings.

### Methods

ROZALY is a prospective, longitudinal, non-interventional, multicentre study conducted in 55 sites in France. Patients were prospectively enrolled at treatment start (M0) and followed up to 5 years. This preliminary analysis was performed with 121 patients recruited in the first inclusion year (Dec 2024–Dec 2025) and depicts demographic and clinical characteristics and preliminary data.

**Copyright Information:** (Online) ISSN: 2572-9241
©2026 The Author(s). HemaSphere is published by John Wiley & Sons Ltd on behalf of European Hematology Association. This is an open access Abstract Book distributed under the Attribution-NonCommercial-NoDerivs (CC BY-NC-ND), which allows third parties to download the articles and share them with others as long as they credit the author and the Abstract Book, but they cannot change the content in any way or use them commercially.
**Abstract Book Citations:** Authors, Title, HemaSphere, 2026;10:(S1):pages. The abstract book DOIs can be found on the "Author Information" page.
**Disclaimer:** Articles published in the journal HemaSphere exclusively reflect the opinions of the authors. The authors are responsible for all content in their abstracts including accuracy of the facts, statements, citing resources, etc.

**Results**

Of the 121 patients treated with zanubrutinib, 59 were TN (48.8%) and 62 were R/R (51.2%). The median age was 76 years (range, 50.6-93.4), with 27 patients aged ≥80 years. Sixty-two percent of patients were male. *TP53* mutation (mut) or del(17p) was present in 45.8% of TN and 35.5% of R/R patients (**Table**). Complex karyotype (>3) was observed in 22.3% of overall patients. IGHV was unmutated in 52.5% of TN and 45.2% of R/R patients. Among 72 patients without *TP53* mut and del(17p), IGHV was unmutated in 51.4% (TN: 65.6%; R/R: 40.0%).

Among patients with prior treatment (n=56), 33.9% had received FCR, 35.7% venetoclax, and 41.1% a BTK inhibitor (ibrutinib, 32.1%; acalabrutinib, 10.7%). A direct change from a prior BTK inhibitor to zanubrutinib occurred in 14 patients, mostly due to adverse event (AE; 35.7%) or physician decision (28.6%). Zanubrutinib was started at the labeled dose in 84.3% of patients; the remaining 15.7%, with a median age of 82.6 years, received reduced doses. At the time of analysis (median treatment exposure, 2.1 months), 3 patients discontinued zanubrutinib (2 AEs and 1 patient inconvenience).

Most patients (74.4%) had comorbidities, including 22.3 % with cardiovascular (CV) disorders (6.6% with atrial fibrillation) and 37.2% with hypertension. Of 80 patients with follow-up, treatment-related (TR) AEs were reported in 23 patients (28.8%), Grade ≥3 treatment-related AEs (TRAEs) occurred in 12.5% of patients, and TRAEs leading to discontinuation in 3.8%. Most frequent TRAEs were haemorrhagic events (10%), diarrhoea (5.0%), and neutropenia (3.8%). CV TRAEs occurred in 3 patients (3.8%); 2 patients had grade ≥3 cardiac failures and 1 had grade 2 hypertension.

**Summary/Conclusion**

ROZALY is the first prospective real-world European study of zanubrutinib in CLL, describing patient characteristics and treatment use in routine practice. The cohort reflects an older population with more comorbidities than phase 3 trials. Nearly half of first-line patients had high-risk disease (*TP53* mut/del(17p)), consistent with French guideline recommendations and clinical trial evidence supporting continuous BTK inhibitor therapy. Longer follow-up is needed to assess real-world effectiveness and tolerability.

**Copyright Information:** (Online) ISSN: 2572-9241
©2026 The Author(s). HemaSphere is published by John Wiley & Sons Ltd on behalf of European Hematology Association. This is an open access Abstract Book distributed under the Attribution-NonCommercial-NoDerivs (CC BY-NC-ND), which allows third parties to download the articles and share them with others as long as they credit the author and the Abstract Book, but they cannot change the content in any way or use them commercially.
**Abstract Book Citations:** Authors, Title, HemaSphere, 2026;10:(S1):pages. The abstract book DOIs can be found on the “Author Information” page.
**Disclaimer:** Articles published in the journal HemaSphere exclusively reflect the opinions of the authors. The authors are responsible for all content in their abstracts including accuracy of the facts, statements, citing resources, etc.

**Table. Patient and Disease Characteristics at M0**

| Characteristic | TN CLL (n=59) | R/R CLL (n=62) | Overall (N=121) |
|---|---|---|---|
| Age, median (range), year | 76.3 (50.6-90.7) | 75.9 (50.8-93.4) | 76 (50.6-93.4) |
| Sex | | | |
| Male, n (%) | 39 (66.1) | 36 (58.1) | 75 (62.0) |
| Female, n (%) | 20 (33.9) | 26 (41.9) | 46 (38.0) |
| ECOG performance status, n (%) | | | |
| 0 | 20 (33.9) | 24 (38.7) | 44 (36.4) |
| 1 | 23 (39.0) | 19 (30.6) | 42 (34.7) |
| 2 | 2 (3.4) | 5 (8.1) | 7 (5.8) |
| 3 | 1 (1.7) | 4 (6.5) | 5 (4.1) |
| Missing | 13 (22.0) | 10 (16.1) | 23 (19.0) |
| *TP53* mutation or del(17p) status, n (%) | | | |
| *TP53* mutation or del(17p) | 27 (45.8) | 22 (35.5) | 49 (40.5) |
| Del(17p) | 19 (42.2)[a] | 6 (14.3)[a] | 25 (28.7)[a] |
| *TP53* mutation | 23 (41.8)[a] | 22 (43.1)[a] | 45 (42.5)[a] |
| Del(11q) status, n (%) | | | |
| Del(11q) | 6 (10.2) | 12 (19.4) | 18 (14.9) |
| Not tested | 16 (27.1) | 22 (35.5) | 38 (31.4) |
| IGHV mutation status, n (%) | | | |
| Mutated | 12 (20.3) | 13 (21.0) | 25 (20.7) |
| Unmutated | 31 (52.5) | 28 (45.2) | 59 (48.8) |
| Not tested | 16 (27.1) | 21 (33.9) | 37 (30.6) |
| Unmutated IGHV without *TP53* mutation and del(17p) | 21 (35.6%) | 16 (25.8%) | 37 (30.6%) |
| Complex karyotype, n (%) | | | |
| >3 abnormalities | 14 (23.7) | 13 (21.0) | 27 (22.3) |
| Not tested | 17 (28.8) | 22 (35.5) | 39 (32.2) |
| Bulky disease, n (%) | | | |
| Yes | 3 (5.1) | 6 (9.7) | 9 (7.4) |
| No | 52 (88.1) | 51 (82.3) | 103 (85.1) |
| Unknown | 4 (6.8) | 5 (8.1) | 9 (7.4) |

[a]Percentage of patients with deletion or mutation divided by number patients tested.

Copyright Information: (Online) ISSN: 2572-9241
©2026 The Author(s). HemaSphere is published by John Wiley & Sons Ltd on behalf of European Hematology Association. This is an open access Abstract Book distributed under the Attribution-NonCommercial-NoDerivs (CC BY-NC-ND), which allows third parties to download the articles and share them with others as long as they credit the author and the Abstract Book, but they cannot change the content in any way or use them commercially.
Abstract Book Citations: Authors, Title, HemaSphere, 2026;10:(S1):pages. The abstract book DOIs can be found on the "Author Information" page.
Disclaimer: Articles published in the journal HemaSphere exclusively reflect the opinions of the authors. The authors are responsible for all content in their abstracts including accuracy of the facts, statements, citing resources, etc.

### (PB2899) AN INTERPRETABLE MACHINE LEARNING MODEL FOR THERAPY FAILURE IN PATIENTS WITH CHRONIC LYMPHOCYTIC LEUKEMIA RECEIVING BRUTON'S TYROSINE KINASE INHIBITORS

**Topic:** 06. Chronic lymphocytic leukemia and related disorders - Clinical

Yutang Li*[1], Xiaoqian Liu[2], Ling Wang[3], Yupiao Feng[1], Kang Lu[4], Mei Ding[4], Xiaohui Sui[4], Xinting Hu[4], Xiangxiang Zhou[4]

*[1]Department of Hematology, Shandong Provincial Hospital, Cheeloo College of Medicine, Shandong University, Jinan, China; [2]Department of Hematology, The Affiliated Yantai Yuhuangding Hospital of Qingdao University, Yantai, China;[3]Department of Hematology, The Affiliated Taian City Central Hospital of Qingdao University, Taian, China; [4]Department of Hematology, Shandong Provincial Hospital Affiliated to Shandong First Medical University, Jinan, China;*

### Background

Although Bruton's tyrosine kinase inhibitors (BTKi) have markedly improved the survival of patients with chronic lymphocytic leukemia/small lymphocytic lymphoma (CLL/SLL), 10% to 20% of patients still experienced therapy failure, including lack of response, disease progression, or Richter transformation.

### Aims

The present study was aimed to develop a clinical prediction model for BTKi-therapy failure.

### Methods

We conducted a multicenter retrospective study of 139 CLL patients receiving BTKi therapy from three centers. Predictor screening was performed within a leave-one-center-out cross-validation (LOCO-CV) framework by combining univariable Cox and LASSO regression. Four survival algorithms- Cox, LASSO-Cox, random survival forest (RSF), and XGBoost- were compared using C-index, time-dependent AUC, and Uno's integrated AUC. The best-performing model (XGBoost) was interpreted with SHAP and benchmarked against simplified Rai staging and Chronic Lymphocytic Leukemia International Prognostic Index (CLL-IPI) for progression-free survival (PFS) and overall survival (OS).

### Results

Among 139 patients (center 1: 54, center 2: 25, center 3: 60), LASSO-Cox identified three robust predictors (TP53 alteration, Albumin < 35 g/L, ECOG-PS ≥ 2) in at least two of the three training folds within the LOCO-CV framework (**Figure 1A**). XGBoost outperformed other models in six of nine LOCO-CV comparisons, followed by RSF. In LOCO-CV, the XGBoost model achieved a 24-month time-dependent AUC of 0.647 for PFS, surpassing both simplified Rai staging (AUC = 0.57) and CLL-IPI (AUC = 0.565) (**Figure 1B**), as well as OS. Using the final model trained on the entire cohort, SHAP analysis identified TP53 as the dominant contributor to risk prediction (mean |SHAP| = 0.088), followed by Albumin < 35 g/L (0.056) and ECOG-PS ≥ 2 (0.046) (**Figure 1C**). The alluvial diagram displayed systematic comparison of the discriminative ability among the Rai staging, CLL-IPI and novel models. Kaplan-Meier curves confirmed that patients stratified by the XGBoost model had significantly different PFS outcomes (log-rank p = 0.003; **Figure 1D**). Furthermore, to facilitate clinical application, we have developed an online risk assessment tool based on the aforementioned risk stratification model.

**Copyright Information:** (Online) ISSN: 2572-9241
©2026 The Author(s). HemaSphere is published by John Wiley & Sons Ltd on behalf of European Hematology Association. This is an open access Abstract Book distributed under the Attribution-NonCommercial-NoDerivs (CC BY-NC-ND), which allows third parties to download the articles and share them with others as long as they credit the author and the Abstract Book, but they cannot change the content in any way or use them commercially.
**Abstract Book Citations:** Authors, Title, HemaSphere, 2026;10:(S1):pages. The abstract book DOIs can be found on the "Author Information" page.
**Disclaimer:** Articles published in the journal HemaSphere exclusively reflect the opinions of the authors. The authors are responsible for all content in their abstracts including accuracy of the facts, statements, citing resources, etc.

### Summary/Conclusion

Taken together, our investigation provides an explainable machine learning model improving prediction of initial BTKi-therapy failure in CLL patients, offering superior discrimination compared to conventional risk scores including Rai staging and CLL-IPI scores. This approach may support clinicians to estimate the likelihood of initial BTKi-therapy failure and identify high-risk CLL patients.

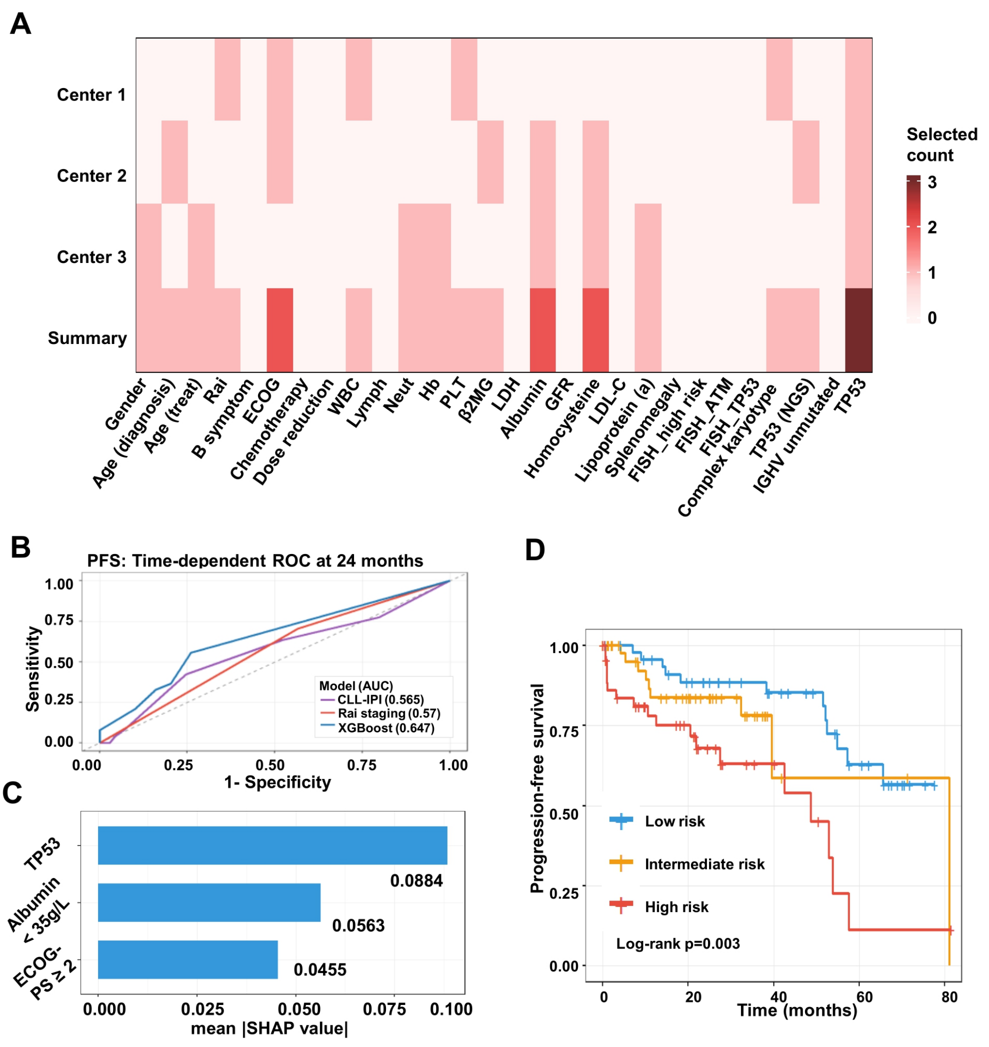

**Copyright Information:** (Online) ISSN: 2572-9241
©2026 The Author(s). HemaSphere is published by John Wiley & Sons Ltd on behalf of European Hematology Association. This is an open access Abstract Book distributed under the Attribution-NonCommercial-NoDerivs (CC BY-NC-ND), which allows third parties to download the articles and share them with others as long as they credit the author and the Abstract Book, but they cannot change the content in any way or use them commercially.
**Abstract Book Citations:** Authors, Title, HemaSphere, 2026;10:(S1):pages. The abstract book DOIs can be found on the "Author Information" page.
**Disclaimer:** Articles published in the journal HemaSphere exclusively reflect the opinions of the authors. The authors are responsible for all content in their abstracts including accuracy of the facts, statements, citing resources, etc.

### (PB2900) COMORBIDITIES AND TREATMENT PATTERNS IN PATIENTS WITH CHRONIC LYMPHOCYTIC LEUKEMIA IN LITHUANIA, 2021–2024

**Topic:** 06. Chronic lymphocytic leukemia and related disorders - Clinical

Regina Pileckyte[1], Migle Kulbokė*[2], Ligita Kasnauskiene[3], Inga Legė[4], Rolandas Gerbutavicius[2]

*[1]Vilnius University Hospital Santaros Klinikos, Hematology, Oncology and Transfusion Medicine Center, Vilnius, Lithuania; [2]Lithuanian University of Health Sciences (LSMU) Kauno klinikos, Department of Oncology and Hematology, Kaunas, Lithuania; [3]Klaipėda University Hospital, Hematology Center, Klaipeda, Lithuania; [4]AstraZeneca Lietuva, Vilnius, Lithuania;*

### Background

Chronic lymphocytic leukemia (CLL) is the most common leukemia in Western countries, with an incidence of approximately 3.8 per 100,000 population (Villavicencio *et al.*, 2021) and a 5-year relative survival rate ranging from 82% to 90% (Shadman, 2023; Hemminki *et al.,* 2024). Comorbidities are highly prevalent among CLL patients, emphasizing the importance of incorporating these factors into clinical decision-making to achieve personalized medicine (Stamatopoulos *et al.,* 2024). Analysis of CLL patients data can provide valuable insights to optimize individualized therapeutic strategies and improve outcomes.

### Aims

This analysis aimed to evaluate the prevalence of comorbidities and treatment practices among CLL patients in Lithuania during 2021-2024, focusing on hypertension (I10–I15), ischemic heart disease (I20–I25), cardiac conduction and rhythm disorders (I44–I49), heart failure (I50), diabetes mellitus (E10–E14), and chronic kidney disease (N18).

### Methods

Secondary health data (2021-2024) were analyzed using the Lithuanian State Data Agency's data reuse platform, specifically the Contour analytic tool in Palantir Foundry, and Microsoft® Excel® for Microsoft 365.

### Results

Among 3,518 unique patients with CLL, hypertension was observed in 81.1%, ischemic heart disease in 40.6 %, conduction and rhythm disorders in 37.7%, heart failure in 38.5%, diabetes mellitus in 17.2%, and chronic kidney disease in 17.7%.
From 2021 to 2024, 2,768 patients (78.7%) were alive, and 750 (21.3%) had died. The leading causes of death were CLL (35.6%), ischemic heart disease (18.5%), and COVID-19 (11.5%).
In the subset of 1,076 patients who received CLL-specific treatment, 80.1% had hypertension, 38.9% had ischemic heart disease, 40.3% had conduction and rhythm disorders, 41.0% had heart failure, 15.8% had diabetes mellitus, and 18.7% had chronic kidney disease.

Among the 337 newly diagnosed patients who initiated 1st line treatment between 2021 and 2024, 273 individuals (81.0%) had at least one comorbidity: 75.7% had hypertensive diseases, 32.9% had ischemic heart disease, 36.8% had conduction and rhythm disorders, 34.7% had heart failure, 13.4% had diabetes mellitus, and 13.1% had chronic kidney disease. BTK inhibitors (BTKis) were the dominant treatment choice for CLL in Lithuania from 2021 to 2024, with second-generation BTKis becoming the most frequently used agents. The use of BCL2 inhibitor increased over time, while chemoimmunotherapy (CIT/CT) declined substantially.

**Copyright Information:** (Online) ISSN: 2572-9241
©2026 The Author(s). HemaSphere is published by John Wiley & Sons Ltd on behalf of European Hematology Association. This is an open access Abstract Book distributed under the Attribution-NonCommercial-NoDerivs (CC BY-NC-ND), which allows third parties to download the articles and share them with others as long as they credit the author and the Abstract Book, but they cannot change the content in any way or use them commercially.
**Abstract Book Citations:** Authors, Title, HemaSphere, 2026;10:(S1):pages. The abstract book DOIs can be found on the "Author Information" page.
**Disclaimer:** Articles published in the journal HemaSphere exclusively reflect the opinions of the authors. The authors are responsible for all content in their abstracts including accuracy of the facts, statements, citing resources, etc.

**Summary/Conclusion**

Cardiovascular comorbidities are highly prevalent among Lithuanian patients with CLL. In the management of CLL the second-generation BTKis and BCL2 inhibitor increasingly favored, and CIT/CT declining. These findings highlight the importance of considering comorbid conditions when planning personalized CLL therapy.

**Financial disclosures:**
Medical writing support is provided by GuruMA, Kaunas, Lithuania, and funded by AstraZeneca.
Funding for this work was provided by AstraZeneca.

**Copyright Information:** (Online) ISSN: 2572-9241
©2026 The Author(s). HemaSphere is published by John Wiley & Sons Ltd on behalf of European Hematology Association. This is an open access Abstract Book distributed under the Attribution-NonCommercial-NoDerivs (CC BY-NC-ND), which allows third parties to download the articles and share them with others as long as they credit the author and the Abstract Book, but they cannot change the content in any way or use them commercially.
**Abstract Book Citations:** Authors, Title, HemaSphere, 2026;10:(S1):pages. The abstract book DOIs can be found on the "Author Information" page.
**Disclaimer:** Articles published in the journal HemaSphere exclusively reflect the opinions of the authors. The authors are responsible for all content in their abstracts including accuracy of the facts, statements, citing resources, etc.

### (PB2901) REAL-WORLD OUTCOMES AMONG MEDICARE BENEFICIARIES TREATED WITH FIRST-LINE (1L) BRUTON TYROSINE KINASE INHIBITORS (BTKIS) FOR CHRONIC LYMPHOCYTIC LEUKEMIA (CLL)

**Topic:** 06. Chronic lymphocytic leukemia and related disorders - Clinical

Daniel Ermann*[1], Deborah Stephens[2], Xiaoliang Wang[3], Irene Varghese[4], Heidi De Souza[4], Caitlin Sheetz[4], Catherine Fu[3], Gregory Maglinte[3], Erlene Seymour[3], Derrick Van Beuge[3], Mazyar Shadman[5, 6], Ryan Jacobs[7]

*[1]Huntsman Cancer Institute, University of Utah School of Medicine, Salt Lake City, UT, United States of America;[2]University of North Carolina School of Medicine, Chapel Hill, NC, United States of America; [3]BeOne Medicines, Ltd, San Carlos, CA, United States of America; [4]ADVI Health LLC, Washington, DC, United States of America;[5]Fred Hutchinson Cancer Center, Seattle, WA, United States of America; [6]University of Washington, Seattle, WA, United States of America;[7]Atrium Health Levine Cancer Institute, Wake Forest University School of Medicine, Charlotte, NC, United States of America;*

### Background

Covalent BTKi monotherapies are standard of care for 1L CLL in the US. However, there is a lack of head-to-head BTKi trials in the 1L setting, and previous real-world studies had limited sample size and follow-up time. We used a large US database to evaluate clinical outcomes among patients treated with BTKis for 1L CLL.

### Methods

This retrospective cohort study utilized the de-identified Medicare Fee-For-Services database. Eligible patients included those with a CLL/SLL diagnosis at age ≥65 years who started 1L BTKi monotherapy between 01/01/2020 and 06/30/2025 and had ≥12 months enrollment pre-1L. Outcomes included real-world overall survival (OS), time to next treatment or death (TTNT-D), and time to treatment discontinuation or death (TTD-D) from 1L start. Landmark treatment and survival probabilities at 12 and 24 months were estimated using Kaplan–Meier methods. Adjusted hazard ratios (aHRs) and 95% CIs were estimated using Cox proportional hazard models, adjusting for age, sex, race/ethnicity, Charlson Comorbidity Index (CCI) and year of 1L start. Subgroup analysis was performed by age groups (65-74, 75-84, ≥85 years).

### Results

A total of 10,523 patients were included (zanubrutinib: 3006; acalabrutinib: 4309; ibrutinib: 3208). Median age at 1L was 77 years for zanubrutinib and acalabrutinib, and 76 years for ibrutinib. Most patients were male (58%), non-Hispanic White (91%), and resided in urban areas (78%). Median CCI score was 4 for zanubrutinib and acalabrutinib, and 3 for ibrutinib. Overall, 65% of patients had hypertension at baseline. Baseline atrial fibrillation was 17% for zanubrutinib and acalabrutinib, and 12% for ibrutinib. Median follow-up was 16 months (range, 0-61) for zanubrutinib, 21 months (0-69) for acalabrutinib and 35 months (0-69) for ibrutinib. Median OS was not reached (NR) for all groups. Median TTNT-D was NR for zanubrutinib (95% CI, 45-NR), 40 months (38-42) for acalabrutinib, and 30 months (29-32) for ibrutinib. Median TTD-D was NR (NR-NR) for zanubrutinib, 24 months (22-25) for acalabrutinib, and 14 months (13-15) for ibrutinib. Patients on zanubrutinib had higher probability of survival, not advancing to next line of therapy and not discontinuing treatment at 12 and 24 months, than those receiving acalabrutinib and ibrutinib (**Table**; with 95% CIs). After adjusting for baseline factors and year of 1L, patients on zanubrutinib had a statistically significantly lower risk of death, advancing to next line, or discontinuing treatment, than those on ibrutinib or acalabrutinib. Similar results were observed across age subgroups.

**Copyright Information:** (Online) ISSN: 2572-9241
©2026 The Author(s). HemaSphere is published by John Wiley & Sons Ltd on behalf of European Hematology Association. This is an open access Abstract Book distributed under the Attribution-NonCommercial-NoDerivs (CC BY-NC-ND), which allows third parties to download the articles and share them with others as long as they credit the author and the Abstract Book, but they cannot change the content in any way or use them commercially.
**Abstract Book Citations:** Authors, Title, HemaSphere, 2026;10:(S1):pages. The abstract book DOIs can be found on the "Author Information" page.
**Disclaimer:** Articles published in the journal HemaSphere exclusively reflect the opinions of the authors. The authors are responsible for all content in their abstracts including accuracy of the facts, statements, citing resources, etc.

### Summary/Conclusion

In this large cohort of patients aged ≥65 years with longest follow up to date, zanubrutinib monotherapy was associated with better survival and treatment outcomes, compared to ibrutinib and acalabrutinib.

| Treatment | OS | | | TTNT-D | | | TTD-D | | |
|---|---|---|---|---|---|---|---|---|---|
| | 12 months % | 24 months % | aHR | 12 months % | 24 months % | aHR | 12 months % | 24 months % | aHR |
| Zanubrutinib | 91 (90-92) | 86 (84-87) | | 82 (81-94) | 71 (69-74) | | 72 (71-74) | 63 (61-65) | |
| Ibrutinib (reference) | 85 (84-86) | 75 (74-77) | 0.64 (0.54-0.77) | 74 (72-75) | 56 (55-58) | 0.63 (0.55-0.71) | 53 (51-55) | 35 (33-37) | 0.57 (0.51-0.64) |
| Acalabrutinib (reference) | 87 (86-88) | 80 (78-81) | 0.77 (0.66-0.88) | 78 (77-80) | 67 (65-68) | 0.87 (0.78-0.96) | 63 (61-64) | 49 (47-51) | 0.86 (0.78-0.94) |

aHR, adjusted hazard ratio; OS, overall survival; TTD-D, time to treatment discontinuation or death; TTNT-D, time to next treatment or death.

**Copyright Information:** (Online) ISSN: 2572-9241
©2026 The Author(s). HemaSphere is published by John Wiley & Sons Ltd on behalf of European Hematology Association. This is an open access Abstract Book distributed under the Attribution-NonCommercial-NoDerivs (CC BY-NC-ND), which allows third parties to download the articles and share them with others as long as they credit the author and the Abstract Book, but they cannot change the content in any way or use them commercially.
**Abstract Book Citations:** Authors, Title, HemaSphere, 2026;10:(S1):pages. The abstract book DOIs can be found on the "Author Information" page.
**Disclaimer:** Articles published in the journal HemaSphere exclusively reflect the opinions of the authors. The authors are responsible for all content in their abstracts including accuracy of the facts, statements, citing resources, etc.

### (PB2902) FROM INNATE RESERVE TO HUMORAL COLLAPSE: DETERMINANTS OF SEVERE INFECTION IN CHRONIC LYMPHOCYTIC LEUKEMIA

**Topic:** 06. Chronic lymphocytic leukemia and related disorders - Clinical

Carmen Dbouk Y Jaudenes*[1], María Argüello Marina[1], Rocío López Martínez[1], Dunia De Miguel Llorente[1]

[1]*Hospital Universitario de Guadalajara, Guadalajara, Spain;*

### Background

Infections are the leading cause of morbidity and mortality in chronic lymphocytic leukemia (CLL). Despite well-established tumor biological markers, the role of dynamic immune remodeling in shaping severe infectious risk remains poorly characterized.

### Aims

To characterize the infectious burden in CLL and identify dynamic humoral and innate immune determinants associated with severe infections (SI) and profound hypogammaglobulinemia (PH).

### Methods

Single-centre retrospective study including CLL patients diagnosed between 2011 and 2025. Infectious episodes were graded per CTCAE criteria. Immunological parameters (IgG, NK cells, T-cell subsets, polyclonal B cells) were assessed at baseline and nadir. Correlation analyses, paired comparisons, and multivariable logistic regression were performed.

### Results

66 patients were included (Table 1). Overall, 81.8% developed ≥1 infection, accounting for 253 episodes (median 3 per patient). SI occurred in 53.0% and infection-related mortality was 6.1%. Bacterial respiratory infections predominated, followed by viral reactivations. Treated patients showed significantly higher total infectious burden (TIB) and SI rates than untreated patients (p=0.027 and p=0.042).

Classical tumor markers (IGHV status, FISH abnormalities) were not associated with SI. CD38 positivity correlated with SI frequency (p=0.014) but not TIB (p=0.743), suggesting a link with severity rather than overall burden. CD38-positive patients also exhibited lower CD4 nadir levels (p=0.010).

Innate immune parameters showed the strongest associations. Lower baseline NK counts correlated with SI and higher TIB, with stronger correlations observed at NK nadir (ρ up to −0.507; p<0.001). Baseline NK levels correlated positively with IgG nadir (p≤0.003), suggesting that preserved innate immunity associates with more stable humoral profiles.

IgG nadir—but not baseline IgG—was significantly associated with SI (ρ=−0.411; p<0.001), underscoring the relevance of dynamic immune decline over static measurements. Polyclonal B-cell depletion at both baseline and nadir correlated with infectious risk (p=0.048 and p=0.004), whereas monoclonal B-cell burden did not, suggesting that residual adaptive immune competence rather than tumor load may influence susceptibility. In parallel, treatment was associated with CD4 decline. Together, this results reflect treatment-driven remodeling of acquired immunity.

Treatment exposure was the main predictor of PH (IgG <400 mg/dL), remaining independently associated in multivariable analysis (adjusted OR 32.78; p=0.0017), while higher baseline NK counts were independently associated with reduced PH risk and lower TIB (adjusted OR 0.29; p=0.048). Paired analyses confirmed a significant increase in infections after treatment initiation (p=0.048), alongside reductions in CD4 counts (p=0.012) and CD4/CD8 ratio (p=0.018). Excess infectious burden was primarily driven by chemotherapy exposure (p=0.022).

**Copyright Information:** (Online) ISSN: 2572-9241
©2026 The Author(s). HemaSphere is published by John Wiley & Sons Ltd on behalf of European Hematology Association. This is an open access Abstract Book distributed under the Attribution-NonCommercial-NoDerivs (CC BY-NC-ND), which allows third parties to download the articles and share them with others as long as they credit the author and the Abstract Book, but they cannot change the content in any way or use them commercially.
**Abstract Book Citations:** Authors, Title, HemaSphere, 2026;10:(S1):pages. The abstract book DOIs can be found on the “Author Information” page.
**Disclaimer:** Articles published in the journal HemaSphere exclusively reflect the opinions of the authors. The authors are responsible for all content in their abstracts including accuracy of the facts, statements, citing resources, etc.

### Summary/Conclusion

Infectious vulnerability in CLL was driven by dynamic immune remodeling rather than classical tumor biology. PH was predominantly treatment-induced, while preserving baseline NK levels independently associated with reduced hypogammaglobulinemia risk and lower infectious burden. SI emerged in patients combining impaired innate reserve with treatment-related humoral deterioration. These findings support an integrated immune-reserve model in which innate integrity and dynamic humoral collapse, jointly, determine infection risk in CLL.

**Table 1.a) Patient Characteristics (N = 66)**

| Variable | Category | n | % / Value |
|---|---|---|---|
| Sex | Female | 28 | 42.4% |
| Age at diagnosis (years) | Mean (SD) | 66 | 66.5 years |

**Table 1.b) Infectious Burden**

| Variable | Result |
|---|---|
| Patients with =1 infection | 81.8% (54/66) |
| Total infectious episodes | 253 |
| Median infections per patient | 3 (IQR 1–5) |
| Patients with grade 3–4 infections | 53.0% (35/66) |
| Infection-related mortality | 6.1% (4/66) |
| =1 infection in first year | 30.3% (20/66) |
| Older age associated with infection | p = 0.018 |

**Table 1.b.2) Infection Type Distribution (253 Episodes)**

| Infection Type | N (%) |
|---|---|
| Bacterial pneumonia | 52 (20.6%) |
| Urinary tract infection | 36 (14.2%) |
| Bacteremia | 24 (9.5%) |
| HSV reactivation | 23 (9.1%) |
| Lower respiratory tract (PPBB) | 23 (9.1%) |
| URTI (non-COVID/influenza) | 32 (12.6%) |
| COVID-19 | 13 (5.1%) |
| Sepsis | 7 (2.8%) |
| Febrile neutropenia | 5 (2.0%) |

**Table 1.c) Immunological Correlates of Infectious Outcomes**

| Parameter | Grade 3–4 Infections | Total Infections |
|---|---|---|
| IgG baseline | NS | NS |
| **IgG nadir** | ρ = –0.411 (p<0.001) | ρ = –0.479 (p<0.001) |
| **Baseline NK** | ρ = –0.438 (p<0.001) | ρ = –0.389 (p=0.001) |
| **NK nadir** | ρ = –0.507 (p<0.001) | ρ = –0.464 (p<0.001) |
| Baseline polyclonal B cells | p=0.048 | ρ = –0.365 (p=0.009) |
| Nadir polyclonal B cells | ρ = –0.399 (p=0.004) | p=0.003 |
| Monoclonal B cells | NS | NS |
| CD4 (baseline/nadir) | NS | NS |
| CD4/CD8 ratio | NS | NS |

**Table 1.d) Treatment Impact on Infectious Burden**

| Variable | Category | n | % |
|---|---|---|---|
| Treatment | Yes | 39 | 59.1% |
| | No | 27 | 40.9% |

**Table 1.e) Multivariable Predictors of Severe Hypogammaglobulinemia**

| Variable | Adjusted OR (95% CI) | p-value |
|---|---|---|
| **Treatment exposure** | 32.78 (3.71–289.41) | 0.0017 |
| **Baseline NK (per SD increase)** | 0.29 (0.08–0.99) | 0.048 |

**Copyright Information:** (Online) ISSN: 2572-9241
©2026 The Author(s). HemaSphere is published by John Wiley & Sons Ltd on behalf of European Hematology Association. This is an open access Abstract Book distributed under the Attribution-NonCommercial-NoDerivs (CC BY-NC-ND), which allows third parties to download the articles and share them with others as long as they credit the author and the Abstract Book, but they cannot change the content in any way or use them commercially.
**Abstract Book Citations:** Authors, Title, HemaSphere, 2026;10:(S1):pages. The abstract book DOIs can be found on the "Author Information" page.
**Disclaimer:** Articles published in the journal HemaSphere exclusively reflect the opinions of the authors. The authors are responsible for all content in their abstracts including accuracy of the facts, statements, citing resources, etc.

### (PB2904) MINIMAL RESIDUAL DISEASE TRENDS AMONG CLL PATIENTS TREATED WITH BRUTON'S TYROSINE KINASE INHIBITORS

**Topic:** 06. Chronic lymphocytic leukemia and related disorders - Clinical

Laura Samples*[1], Pia Nierman[1, 1], Clare Sun[2], Victoria Rogness[1], Mohammed Farooqui[1], Susan Soto[1], Ingrid Frey[1], Jeanine Superata[1], Constance Yuan[3], Hao-Wei Wang[3], Adrian Wiestner[1]

*[1]National Heart, Lung, and Blood Institute, Hematology Branch, Bethesda, United States of America;[2]Memorial Sloan Kettering Cancer Center, Basking Ridge, NJ, United States of America; [3]National Cancer Institute, Laboratory of Pathology, Bethesda, MD, United States of America;*

### Background

Minimal residual disease (MRD) testing is increasingly used in patients with chronic lymphocytic leukemia (CLL) as a therapeutic endpoint and to monitor ongoing responses to treatment. MRD stratification into low versus high levels and time to undetectable MRD (uMRD) have become important concepts within the context of fixed-duration combination therapy; however, many patients continue to receive indefinite treatment with a single-agent Bruton's tyrosine kinase inhibitor (BTKi) with good disease control, but not uMRD. We report MRD outcomes among patients treated with ibrutinib or acalabrutinib.

### Methods

MRD data from four phase II BTKi trials were combined. Two of these trials involved a short course of fludarabine or fludarabine plus pembrolizumab prior to the continuation of ibrutinib monotherapy. One trial administered acalabrutinib monotherapy. All MRD testing was conducted via peripheral blood flow cytometry with a limit of detection of 0.002% neoplastic cells. uMRD was defined as <1 CLL cell per 10,000 leukocytes ($10^{-4}$), low MRD was defined as ≥ $10^{-4}$ to < $10^{-2}$, and high MRD was defined as ≥ $10^{-2}$.

### Results

A total of 178 patients with a median age of 65 were treated with acalabrutinib (27%) or ibrutinib (73%) for a median of 5.29 years. Of these patients, 114 (64%) were male, 67 (37.6%) had relapsed/refractory disease, 123 (69%) were classified as IGHV unmutated, and 61 (34%) harbored del(17p).

At best response, 30 patients (17%) achieved uMRD after a median of 5.21 years (range 0.5 - 10.1). Six (20%) of these 30 patients had del(17p) and 17 (54.8%) had unmutated IGHV. All patients who achieved low MRD at some point during treatment did so after a median of 4.04 years (range 0.45 – 10.1). The time to low MRD was also 4.04 years for 42 patients (24%) with a best response of low MRD (range 0.46 – 10.1). A high degree of variability in MRD changes over time was observed for patients who ultimately achieved uMRD, as demonstrated in Figure 1.

### Summary/Conclusion

Although BTKi monotherapy is not expected to yield high rates of uMRD, a proportion of patients on BTKis, including those with adverse risk features, achieve low MRD with or without uMRD. These patients demonstrate a diversity of patterns in achieving and maintaining their MRD status.

**Copyright Information:** (Online) ISSN: 2572-9241
©2026 The Author(s). HemaSphere is published by John Wiley & Sons Ltd on behalf of European Hematology Association. This is an open access Abstract Book distributed under the Attribution-NonCommercial-NoDerivs (CC BY-NC-ND), which allows third parties to download the articles and share them with others as long as they credit the author and the Abstract Book, but they cannot change the content in any way or use them commercially.
**Abstract Book Citations:** Authors, Title, HemaSphere, 2026;10:(S1):pages. The abstract book DOIs can be found on the "Author Information" page.
**Disclaimer:** Articles published in the journal HemaSphere exclusively reflect the opinions of the authors. The authors are responsible for all content in their abstracts including accuracy of the facts, statements, citing resources, etc.

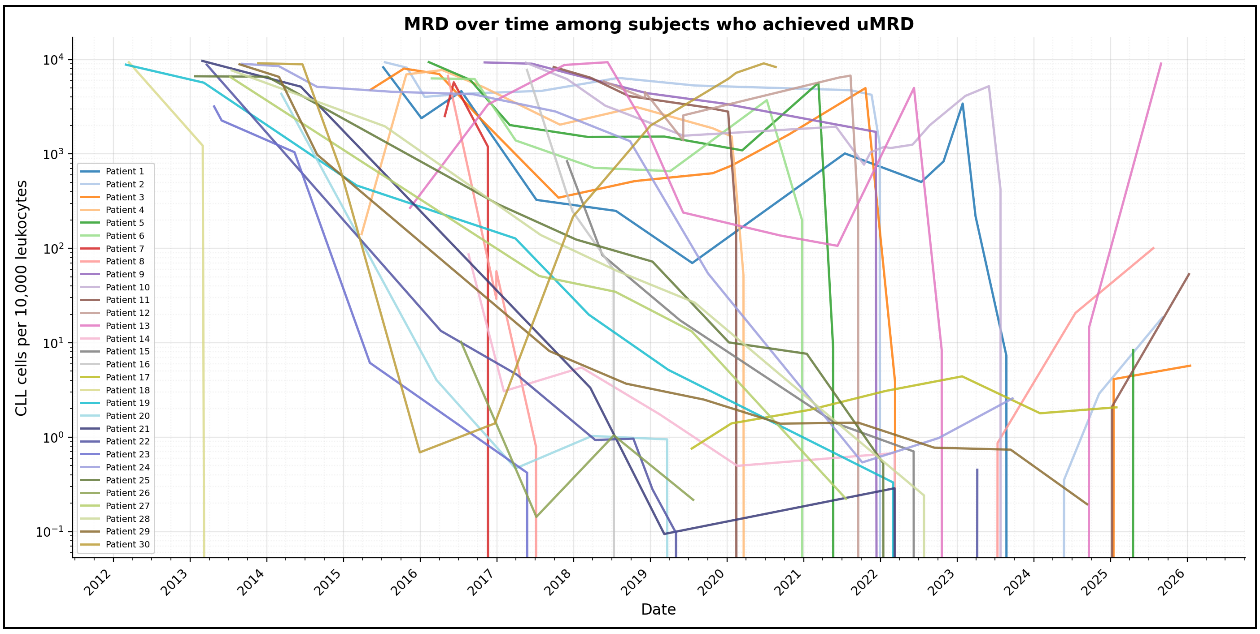

Figure 1

**Copyright Information:** (Online) ISSN: 2572-9241
©2026 The Author(s). HemaSphere is published by John Wiley & Sons Ltd on behalf of European Hematology Association. This is an open access Abstract Book distributed under the Attribution-NonCommercial-NoDerivs (CC BY-NC-ND), which allows third parties to download the articles and share them with others as long as they credit the author and the Abstract Book, but they cannot change the content in any way or use them commercially.
**Abstract Book Citations:** Authors, Title, HemaSphere, 2026;10:(S1):pages. The abstract book DOIs can be found on the "Author Information" page.
**Disclaimer:** Articles published in the journal HemaSphere exclusively reflect the opinions of the authors. The authors are responsible for all content in their abstracts including accuracy of the facts, statements, citing resources, etc.

### (PB2905) REAL-WORLD OUTCOMES OF CONTINUOUSLY REDUCED-DOSE ZANUBRUTINIB MONOTHERAPY IN CLL/SLL: A MULTI-CENTER RETROSPECTIVE STUDY

**Topic:** 06. Chronic lymphocytic leukemia and related disorders - Clinical

Kai Shen*[1, 2], Hongyun Xing[3], Fang Xu[4], Liangliang MA[5], Xiaohong Kuang[6], Shihua Huang[7], Xingli Zou[8], Tao Wei[9], Li Zhang[10], Li Wei[11], Wenrong Zou[12], Lin Wang[13], Ting Niu[1, 2]

*[1]West China Hospital, Sichuan University, Department of Hematology, Institute of Hematology, Chengdu, China;[2]Sichuan Workshop On CLL (scwCLL), Chengdu, China; [3]The Affiliated Hospital of Southwest Medical University, Yibing, China;[4]Mianyang Central Hospital, Mianyang, China; [5]Meishan People's Hospital, Meishan, China; [6]The Third Hospital of Mianyang, Mianyang, China; [7]The Second People's Hospital of Yibin, Yibing, China;[8]The Affiliated Hospital of North Sichuan Medical College, Department of Hematology, Nanchong, China; [9]Jianyang People's Hospital, Jianyang, China; [10]Chengdu Seventh People's Hospital, Hematology, Chengdu, China; [11]Chengdu Fifth People`s Hospital, Chengdu, China; [12]Mianyang 404 Hospital, Mianyang, China; [13]The First People's Hospital of Guangyuan, Guangyuan, China;*

### Background

Chronic lymphocytic leukemia/small lymphocytic lymphoma (CLL/SLL) is an indolent B-cell malignancy. Zanubrutinib, a second-generation covalent Bruton tyrosine kinase inhibitor (BTKi), has emerged as a standard-of-care therapy. However, long-term continuous BTKi treatment may lead to adverse effect(AE) and significant economic burden, often resulting in treatment discontinuation or dose reduction. To date, the outcomes of CLL patients receiving continuously reduced-dose zanubrutinib remain unclear.

### Aims

This study aimed to describe outcomes of CLL patients receiving continuously reduced-dose zanubrutinib in the real-world setting.

### Methods

This was a multi-center retrospective study of adult CLL patients who received continuously reduced-dose zanubrutinib monotherapy between January 2021 and October 2025 in 12 sites from Sichuan Province, China. The follow-up ended on January 1, 2026. Baseline characteristics, reasons for dose reduction, and survival data were collected and analyzed.

**Copyright Information:** (Online) ISSN: 2572-9241
©2026 The Author(s). HemaSphere is published by John Wiley & Sons Ltd on behalf of European Hematology Association. This is an open access Abstract Book distributed under the Attribution-NonCommercial-NoDerivs (CC BY-NC-ND), which allows third parties to download the articles and share them with others as long as they credit the author and the Abstract Book, but they cannot change the content in any way or use them commercially.
**Abstract Book Citations:** Authors, Title, HemaSphere, 2026;10:(S1):pages. The abstract book DOIs can be found on the "Author Information" page.
**Disclaimer:** Articles published in the journal HemaSphere exclusively reflect the opinions of the authors. The authors are responsible for all content in their abstracts including accuracy of the facts, statements, citing resources, etc.

### Results

A total of 92 CLL/SLL patients on reduced-dose zanubrutinib monotherapy were included. The median age was 64 years (range, 42-87), with a male-to-female ratio of 1.14:1. According to Binet staging, 23.9% and 38.0% of patients were in stage B and C, respectively. Most patients (78.3%) received zanubrutinib as first-line therapy. The cytogenetic profiles were as follows: mutated *IGHV* was present in 66.7% (20/30) of patients, unmutated IGHV in 33.3% (10/30), del17p in 23.3% (7/30), TP53 aberration in 30.4% (7/23), and complex karyotype (defined as ≥3 abnormalities) in 16.2% (6/37). The median duration of full-dose zanubrutinib prior to dose reduction was 4.2 months (range, 0-51.2). 53(57.5%) patients started dose reduction within the first 6 months of treatment. Following reduction, the daily zanubrutinib dose groups were: 160 mg (80 mg twice daily) in 66 (71.7%) patients, 80 mg in 19(20.7%) , and 240 mg (160 mg + 80 mg) in 7(7.6%).Reasons for dose reduction were primarily personal (69.5%), including economic burden or fear of future toxicity. The remaining 30.5% were attributed to AEs. The most common AEs were infection (23.3%), thrombocytopenia (18.6%), and bleeding (16.3%). After a median follow-up of 21.0 months (95% CI: 16.7-25.3), the median progression-free survival (PFS) was not reached. The estimated 3-year PFS rate was 80% while the 4-year PFS rate dropped to 56% (Figure 1A). Although statistically non-significant, patients receiving zanubrutinib as second-line therapy showed a lower 3-year PFS rate compared with first-line patients (67% vs 83%, p=0.872). In different dosage subgroups (240 mg vs 160 mg vs 80 mg), the estimated 3-year PFS rates were 100%, 92% and 42%, respectively (p=0.054) (Figure 1B). Lower dose intensity was significantly associated with an inferior PFS in the first-line treatment subgroup(p=0.01). Notably, the 160 mg daily dose subgroup still exhibited an unfavorable 4-year PFS rate of 56% in the first-line setting.

### Summary/Conclusion

Continuous dose reduction of zanubrutinib is a common practice in underdeveloped regions of China, primarily driven by economic constraints and concerns over drug toxicity during long-term BTKi treatment. Sustained treatment with reduced dose zanubrutinib, particularly when the daily dose was reduced to 160mg or below, is associated with inferior outcomes compared with historical data. Our multi-center real-world data support maintaining the standard, full-dose zanubrutinib monotherapy for CLL whenever feasible.

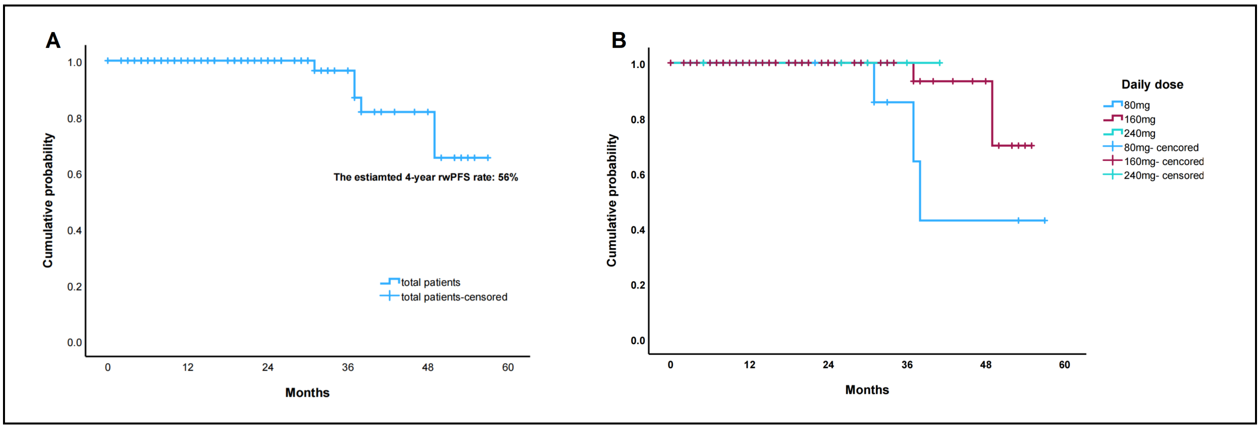

Copyright Information: (Online) ISSN: 2572-9241
©2026 The Author(s). HemaSphere is published by John Wiley & Sons Ltd on behalf of European Hematology Association. This is an open access Abstract Book distributed under the Attribution-NonCommercial-NoDerivs (CC BY-NC-ND), which allows third parties to download the articles and share them with others as long as they credit the author and the Abstract Book, but they cannot change the content in any way or use them commercially.
Abstract Book Citations: Authors, Title, HemaSphere, 2026;10:(S1):pages. The abstract book DOIs can be found on the "Author Information" page.
Disclaimer: Articles published in the journal HemaSphere exclusively reflect the opinions of the authors. The authors are responsible for all content in their abstracts including accuracy of the facts, statements, citing resources, etc.

### (PB2906) PROFILE OF PATIENTS USING ACALABRUTINIB FOR CHRONIC LYMPHOCYTIC LEUKEMIA IN REAL-LIFE SETTINGS IN ROMANIA: TREATMENT-NAÏVE AND RELAPSE/REFRACTORY COHORTS FROM THE NON-INTERVENTIONAL STUDY REACH

**Topic:** 06. Chronic lymphocytic leukemia and related disorders - Clinical

Ciprian Tomuleasa*[1], Georgeta Daniela Georgescu[2], Bianca Vladutiu[3], Beatrice Costea[3]

*[1]Institutul Oncologic Prof. Dr.i. Chiricuta, Cluj-Napoca, Romania;[2]Colentina Clinical Hospital, Bucharest, Romania;[3]AstraZeneca, Bucharest, Romania;*

### Background

The advances of the last decades led to the approval of highly effective new molecules that changed the treatment paradigm of Chronic Lymphocytic Leukemia (CLL), among which the Bruton tyrosine kinase inhibitors (BTKi). Acalabrutinib is a second generation, highly selective BTKi that was approved in Europe in 2020 and reimbursed in Romania in 2023. Real-world evidence is required to confirm effectiveness and safety of a treatment after registration, in broader patient populations and across various geographies. To cover these knowledge gaps, the non-interventional study REACH is collecting data from representative hematology centers in Romania, over a 3-year follow-up since acalabrutinib initiation.

### Aims

To describe the use of acalabrutinib for the treatment of CLL in real-life settings. Here we present the baseline characteristics of the study cohorts by prior CLL treatment.

### Methods

REACH is a physician-led medical chart review ongoing in 31 public and private hematology centers. Adult patients with confirmed diagnosis of CLL with acalabrutinib initiated in front-line or subsequent lines according to the national therapeutic protocol between Jan'23 – Jan'25 were eligible for enrolment. Date of acalabrutinib initiation was defined as index. Enrolment in less than 30 days before index or acalabrutinib administered in a clinical trial were exclusion criteria. All patients signed informed consent before any data collection. Descriptive statistics methods were used.

### Results

The full analysis set (FAS) included 243 patients: 142 (58%) treatment-naïve (TN) and 101 (42%) relapse/refractory (R/R). In R/R patients, up to 5 prior lines (L) were reported: 1L 101 (42%) patients, 2L 33 (14%), 3L 16 (7%), 4L 6 (2%), 5L 3 (1%) patients. Main patient characteristics at baseline (index) are shown in **Table 1.**
At index, acalabrutinib was used as monotherapy in most cases: 133 (94%) of TN patients and all (100%) of R/R patients; in the remaining 9 (6%) patients from the TN group, acalabrutinib was administered in combination with obinutuzumab. In 1 (<1%) TN patient, and 2 (2%) R/R patients, the initial total daily dose of acalabrutinib was 100 mg, and for the rest 200 mg.
In the R/R group, prior ibrutinib was used by 40 (40%) patients, distributed between lines as 1L in 22 (22%) patients, 2L in 13 (40%) patients, 3L in 4 (25%) patients and 4L in 1 (16%) patient. Prior venetoclax was overall used by 18 (18%) patients: as 1L by 6 (6%) patients, 2L by 3 (9%) patients, 3L by 8 (50%) patients, and 4L by 1 (16%) patient. Among frequent chemotherapy regimens, fludarabine, cyclophosphamide, rituximab (FCR) was used as 1L by 25 (25%) patients, 2L by 7 (21%) patients and 5L by 1 (33%) patient; whereas rituximab, cyclophosphamide, vincristine, and prednisone (R-CVP) was used as 1L by 10 (10%) patients, and as 2L by 1 (3%) patients.

**Copyright Information:** (Online) ISSN: 2572-9241
©2026 The Author(s). HemaSphere is published by John Wiley & Sons Ltd on behalf of European Hematology Association. This is an open access Abstract Book distributed under the Attribution-NonCommercial-NoDerivs (CC BY-NC-ND), which allows third parties to download the articles and share them with others as long as they credit the author and the Abstract Book, but they cannot change the content in any way or use them commercially.
**Abstract Book Citations:** Authors, Title, HemaSphere, 2026;10:(S1):pages. The abstract book DOIs can be found on the "Author Information" page.
**Disclaimer:** Articles published in the journal HemaSphere exclusively reflect the opinions of the authors. The authors are responsible for all content in their abstracts including accuracy of the facts, statements, citing resources, etc.

**Summary/Conclusion**

To our knowledge, REACH is probably the first real-world study in Romania to characterize the CLL patient population initiated on acalabrutinib. Acalabrutinib was used in general in frail patients, with ECOG $^3$2 and multiple comorbidities (2 or 3 categories of interest involved for 49% of patients), mostly cardio-vascular, irrespective of prior exposure to CLL treatment. Access to diagnostics to identify high-risk patients remains suboptimal and requires improvement. The forthcoming 3-year follow-up will further elucidate the effectiveness and safety of acalabrutinib in this population.

**Funding:** AstraZeneca

**Table 1.** Baseline characteristics of study cohorts

| Characteristics | TN n=142 | R/R n=101 | FAS N=243 |
|---|---|---|---|
| Age, median (IQR), years | | | |
| At CLL diagnosis | 70 (63-68) | 65 (58-72) | 68 (60-75) |
| At index | 73 (65-79) | 71 (65-77) | 72 (65-78) |
| Male, n (%) | 76 (54) | 64 (63) | 140 (58) |
| Disease duration, median (IQR), years | 0 (0-2) | 5 (2-8) | 1 (0-5) |
| Binet stage, n (%) | | | |
| Stage A | 12 (9) | 9 (9) | 21 (9) |
| Stage B | 73 (51) | 59 (58) | 132 (54) |
| Stage C | 57 (40) | 33 (33) | 90 (37) |
| ECOG performance status, n (%) | | | |
| 0 | 19 (13) | 12 (12) | 31 (13) |
| 1 | 37 (26) | 23 (23) | 60 (25) |
| ≥2 | 57 (40) | 41 (41) | 98 (40) |
| Unknown | 29 (20) | 25 (25) | 54 (22) |
| Comorbidities by type of major class of interest, n (%) | 116 (82) | 83 (82) | 199 (82) |
| Cardiovascular | 88 (62) | 69 (68) | 157 (65) |
| Hypertension | 76 (54) | 58 (57) | 134 (55) |
| Atrial fibrillation | 12 (9) | 17 (17) | 29 (12) |
| Heart failure | 16 (11) | 16 (16) | 32 (13) |
| Hepatic | 19 (13) | 13 (13) | 32 (13) |
| Pulmonary | 14 (10) | 11 (11) | 25 (10) |
| Renal | 11 (8) | 4 (4) | 15 (6) |
| Other hemato-oncological diseases | 19 (13) | 12 (12) | 31 (13) |
| Comorbidities by number of major classes of interest involved*, n (%) | | | |
| 1 class | 41 (29) | 25 (25) | 66 (27) |
| 2 classes | 54 (38) | 37 (37) | 91 (37) |
| 3 classes | 15 (11) | 14 (14) | 29 (12) |
| 4 classes | 4 (3) | 6 (6) | 10 (4) |
| 5 classes | 2 (1) | 1 (1) | 3 (1) |
| Cytogenetic abnormalities | | | |
| Del(17p) test performed, N | N=95 | N=58 | N=153 |
| Deletion detected, n (%) | 9 (10) | 12 (21) | 21 (14) |
| Del(11q) test performed, N | N=53 | N=23 | N=76 |
| Deletion detected, n (%) | 10 (19) | 5 (22) | 15 (20) |
| *TP53* status performed, N | N=96 | N=69 | N=165 |
| Mutation detected, n (%) | 19 (20) | 15 (22) | 34 (21) |
| *IGHV* status performed, N | N=94 | N=58 | N=152 |
| Unmutated status, n (%) | 61 (65) | 39 (67) | 100 (66) |
| Borderline status, n (%) | 2 (2) | 1 (2) | 3 (3) |

Note: due to rounding, percentages may not sum up exactly 100%; *includes also a class of other non-hematological – non-oncological, beyond those of interest listed.
Abbreviations: CLL, Chronic Lymphocytic Leukemia; Del=deletion; ECOG=Eastern Cooperative Oncology Group; FAS=full analysis set; IQR=interquartile; N=number of patients overall; n=number of patients in a subgroup; R/R=relapse/refractory; TN=treatment-naive.

**Copyright Information:** (Online) ISSN: 2572-9241
©2026 The Author(s). HemaSphere is published by John Wiley & Sons Ltd on behalf of European Hematology Association. This is an open access Abstract Book distributed under the Attribution-NonCommercial-NoDerivs (CC BY-NC-ND), which allows third parties to download the articles and share them with others as long as they credit the author and the Abstract Book, but they cannot change the content in any way or use them commercially.
**Abstract Book Citations:** Authors, Title, HemaSphere, 2026;10:(S1):pages. The abstract book DOIs can be found on the "Author Information" page.
**Disclaimer:** Articles published in the journal HemaSphere exclusively reflect the opinions of the authors. The authors are responsible for all content in their abstracts including accuracy of the facts, statements, citing resources, etc.

### (PB2907) REVACCINATION WITH CONJUGATED PNEUMOCOCCAL VACCINE IMPROVES FUNCTIONALITY OF VACCINE MEDIATED ANTIBODIES IN CHRONIC LYMPHOCYTIC LEUKEMIA.

**Topic:** 06. Chronic lymphocytic leukemia and related disorders - Clinical

Simon Golowin*[1], Camilla Virta[2], Merit Melin[2], Nina Ekström[2], Anders Magnusson[3], Per-Ola Andersson[4], Ylva Hammarlund[5], Sandra Lockmer[6], Ingmar Nilsson[7], Daniel Roth[8], Tobias Tolf[9], Eva Kimby[6], Torbjörn Norén[10], Bertil Uggla[1], Simon Athlin[11], Magdalena Kättström[1]

*[1]Örebro University, Section of Hematology, Department of Medicine, Faculty of Medicine and Health, Örebro, Sweden;[2]Finnish Institute for Health and Welfare, Department of Public Health, Helsinki, Finland; [3]Örebro University, Clinical Epidemiology and Biostatistics, School of Medical Sciences, Faculty of Medicine and Health, Örebro, Sweden; [4]Sahlgrenska University Hospital, Section of Hematology and Coagulation, Department of Specialist Medicine, Göteborg, Sweden; [5]Falun Hospital, Department of Medicine,, Falun, Sweden; [6]Karolinska Institutet, Division of Hematology, Department of Medicine Huddinge, Huddinge, Sweden; [7]Karlstad Hospital, Department of Medicine, Karlstad, Sweden;[8]Lund University Hospital, Institution of Clinical Sciences, Faculty of Medicine, Lund, Sweden; [9]Uppsala University, Department of Medical Sciences, Section of Hematology, Uppsala, Sweden; [10]Örebro University, Department of Laboratory Medicine, Clinical Microbiology, Faculty of Medicine and Health, Örebro, Sweden; [11]Örebro University, School of Medical Sciences, Faculty of Medicine and Health, Örebro, Sweden;*

### Background

Patients with Chronic Lymphocytic Leukemia (CLL) have an increased risk of invasive pneumococcal disease and poor response to vaccination. Pneumococcal vaccination is recommended for all CLL patients however recommendations on revaccination strategies are lacking. In a previous study, we randomized treatment-naïve CLL patients (n=126) for primary immunization with a conjugated vaccine (PCV13) or a polysaccharide vaccine (PPSV23), showing a superior immune response to PCV13. Median five years after primary immunization, a revaccination study was conducted, and a control group was recruited. Concentration of serotype-specific pneumococcal IgG antibodies against 12 common serotypes increased after PCV13 revaccination but with no additive effect after PPSV23. Long term antibody functionality after primary immunization with PCV13 or PPSV23 and the effect of revaccination in CLL patients are not known.

### Aims

We aim to evaluate long term antibody functionality in CLL patients after primary immunization with PCV13 or PPSV23 followed by revaccination with PCV13+PPSV23 or PCV13+PCV13.

### Methods

Seventy-seven CLL patients were recruited from the cohort of our previous multi-center randomized vaccination trial, median five years after primary immunization with PCV13 (Group A, n=37) or PPSV23 (Group B, n=40). An immunocompetent control group (n=31), previously immunized with PCV13 (n=9) or PPSV23 (n=22), was recruited. All patients and controls were revaccinated with PCV13. After eight weeks, the CLL patients received a second revaccination with either PPSV23 (Group A) or PCV13 (Group B). Serum samples were collected before and after each revaccination and after 12 months. Antibody functionality of eight of the vaccine specific serotypes (4, 6B, 9V, 14, 18C, 19A, 19F, 23F) was analyzed by the Finnish Institute for Health and Welfare in Helsinki, Finland, using a multiplex opsonophagocytosis assay. Serological Protection (SP) was defined as titers ≥1:8 in >70% of the analyzed serotypes (6/8) and was statistically evaluated with mixed Poisson regression.

**Copyright Information:** (Online) ISSN: 2572-9241
©2026 The Author(s). HemaSphere is published by John Wiley & Sons Ltd on behalf of European Hematology Association. This is an open access Abstract Book distributed under the Attribution-NonCommercial-NoDerivs (CC BY-NC-ND), which allows third parties to download the articles and share them with others as long as they credit the author and the Abstract Book, but they cannot change the content in any way or use them commercially.
**Abstract Book Citations:** Authors, Title, HemaSphere, 2026;10:(S1):pages. The abstract book DOIs can be found on the "Author Information" page.
**Disclaimer:** Articles published in the journal HemaSphere exclusively reflect the opinions of the authors. The authors are responsible for all content in their abstracts including accuracy of the facts, statements, citing resources, etc.

### Results

Before revaccination, five years after primary immunization, 41% in Group A achieved SP compared to 20% in Group B (RR=2.0; 95% CI 0.9-4.2). After the first revaccination with PCV13, significantly increased proportions of patients with SP were seen in both groups (Group A: 76% Group B: 61% RR=1.2 CI 0.9-1.7). A second revaccination with PPSV23 or PCV13 did not increase the response further. However, after 12 months, Group B (PCV13+PCV13), showed an improved long-term antibody functionality compared to Group A (PCV13+PPSV23) (RR=0.4; CI 0.2-0.9).
The proportion of participants with SP was significantly lower before and after first revaccination in CLL patients compared to controls (30% vs. 58%; RR=0.4; CI 0.3-0.7). At 12 months, after a second revaccination in the CLL group, the difference decreased (66% vs. 81%; RR=0.8; CI 0.6-1.1).

### Summary/Conclusion

Antibody functionality was lower in CLL patients compared to controls five years after primary pneumococcal immunization. Revaccination with a conjugated vaccine improved immunity. In CLL patients, a closely spaced second revaccination did not further enhance antibody functionality. However, enhanced long term immunity was observed following a conjugated vaccine compared to a polysaccharide vaccine. Our findings support revaccination with pneumococcal conjugated vaccine in CLL patients to enhance protection against severe pneumococcal disease.

**Copyright Information:** (Online) ISSN: 2572-9241
©2026 The Author(s). HemaSphere is published by John Wiley & Sons Ltd on behalf of European Hematology Association. This is an open access Abstract Book distributed under the Attribution-NonCommercial-NoDerivs (CC BY-NC-ND), which allows third parties to download the articles and share them with others as long as they credit the author and the Abstract Book, but they cannot change the content in any way or use them commercially.
**Abstract Book Citations:** Authors, Title, HemaSphere, 2026;10:(S1):pages. The abstract book DOIs can be found on the "Author Information" page.
**Disclaimer:** Articles published in the journal HemaSphere exclusively reflect the opinions of the authors. The authors are responsible for all content in their abstracts including accuracy of the facts, statements, citing resources, etc.

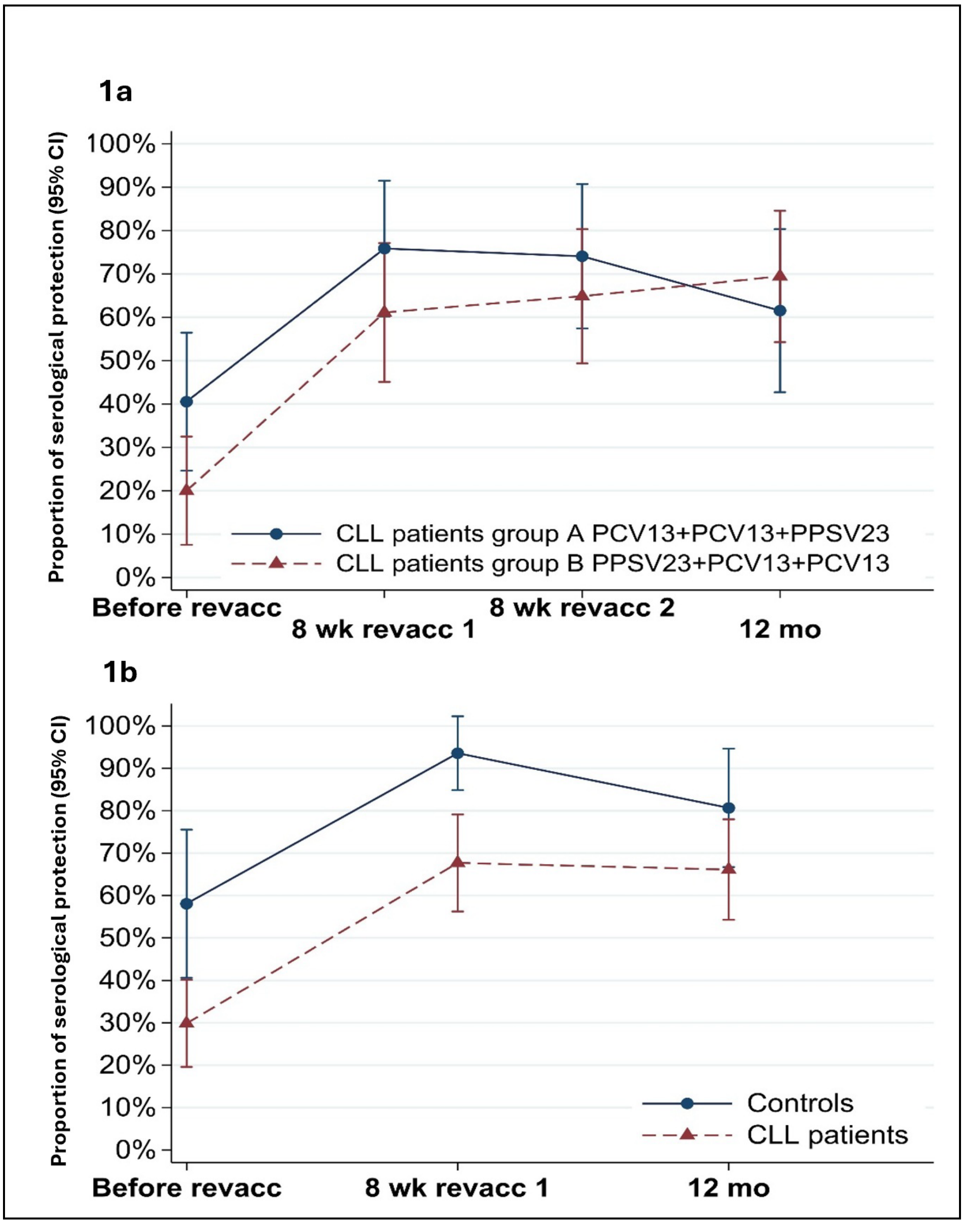

Figure 1a; Percentage of CLL patients achieving SP, defined as a MOPA-titer of ≥1:8 in 70% (6/8) of the investigated pneumococcal serotypes (4, 6B, 9V, 14, 18C, 19A, 19F, 23F) before revaccination, eight weeks after each revaccination and 12 months after first revaccination. Presented with 95% confidence interval. Group A (n=37), Group B (n=40). 1b; Percentage of CLL-patients and controls achieving SP before revaccination with PCV13, eight weeks after and 12 months after revaccination. CLL-Patients (n=77), Controls (n=31). CLL= Chronic Lymphocytic Leukemia, SP=Serological Protection, MOPA=Multiplex Opsonophagocytosis Assay

**Copyright Information:** (Online) ISSN: 2572-9241
©2026 The Author(s). HemaSphere is published by John Wiley & Sons Ltd on behalf of European Hematology Association. This is an open access Abstract Book distributed under the Attribution-NonCommercial-NoDerivs (CC BY-NC-ND), which allows third parties to download the articles and share them with others as long as they credit the author and the Abstract Book, but they cannot change the content in any way or use them commercially.
**Abstract Book Citations:** Authors, Title, HemaSphere, 2026;10:(S1):pages. The abstract book DOIs can be found on the “Author Information” page.
**Disclaimer:** Articles published in the journal HemaSphere exclusively reflect the opinions of the authors. The authors are responsible for all content in their abstracts including accuracy of the facts, statements, citing resources, etc.

### (PB2908) REAL-WORLD OUTCOMES OF ACALABRUTINIB MONOTHERAPY FOR CHRONIC LYMPHOCYTIC LEUKAEMIA IN THE WEST OF SCOTLAND

**Topic:** 06. Chronic lymphocytic leukemia and related disorders - Clinical

Sidhant Seth*[1, 2], Katrina Farrell[3], Alistair Hart[4], Mark Rafferty[1]

*[1]Beatson West of Scotland Cancer Centre, Glasgow, United Kingdom;[2]School of Cancer Sciences, University of Glasgow, Glasgow, United Kingdom; [3]Forth Valley Royal Hospital, Larbert, United Kingdom;[4]Queen Elizabeth University Hospital, Glasgow, United Kingdom;*

### Background

Acalabrutinib is a second-generation Bruton tyrosine kinase (BTK) inhibitor approved for the treatment of chronic lymphocytic leukaemia/small lymphocytic lymphoma (CLL/SLL). In Scotland, it was approved in 2021 following results from the ELEVATE-TN study, which demonstrated superior progression-free survival (PFS) compared with obinutuzumab–chlorambucil. The West of Scotland has a diverse population with a high burden of comorbidities, particularly cardiovascular disease, which may influence real-world outcomes.

### Aims

To evaluate the efficacy and safety of acalabrutinib monotherapy for CLL/SLL in a real-world clinical setting in a patient group with a high comorbidity burden.

### Methods

Consecutive patients with CLL/SLL treated with acalabrutinib monotherapy across six centres in the West of Scotland were identified from electronic prescribing systems. Retrospective data collection included baseline characteristics, pre-specified adverse events of interest, treatment response according to iwCLL 2018 criteria (without mandatory bone marrow assessment and based on clinical examination findings/laboratory parameters) and clinical outcomes. Kaplan–Meier methods were used to estimate real-world PFS and overall survival (OS), with multivariate Cox proportional hazards models to identify factors associated with PFS. Fine–Gray analysis was used to model CLL-related events, treating non-CLL death as a competing risk. Analyses were conducted using R version 4.5.1.

### Results

A total of 154 patients treated between May 2020 and January 2025 were identified, with a median follow-up of 29.6 months. Of these, 102 (66%) were treatment-naïve and 52 (34%) had received prior CLL-directed therapy. Among patients with evaluable response (n=152), the best overall response rate (ORR) was 94.1%, exceeding that reported in ELEVATE-TN (89%). However, 2-year PFS (77.2% vs 87%) and OS (80.4% vs 95%) were inferior in first-line treated patients compared with ELEVATE-TN.
On multivariate analysis, age >75 years (HR 2.80, p<0.001) and ECOG performance status ≥1 (HR 1.94, p=0.03) were independently associated with shorter PFS, while *TP53* aberrancy was not (HR 0.85, p=0.70). Our cohort was older than ELEVATE-TN (median age 75 vs 70 years) and had a substantial comorbidity burden (72.7% with ≥1 comorbidity). Only 21 of 55 (38.2%) PFS events were attributable to CLL/SLL progression or treatment, with a 2-year cumulative incidence of CLL-related events of 7.3%, reflecting competing risks from non-CLL mortality.
73 patients (47.4%) experienced at least one adverse event, most commonly bruising (17.5%) and gastrointestinal toxicity (16.2%). 42 patients (27.3%) discontinued treatment early, of which 24 (15.6%) were due to adverse events. Dose reductions occurred in 21 patients (13.6%), most frequently due to neutropenia (n=6). 50 patients (32.5%) required hospitalisation for infection during treatment, predominantly due to respiratory infections (88.9%). Infection was the recorded cause of death in 13 patients (8.4%).

**Copyright Information:** (Online) ISSN: 2572-9241
©2026 The Author(s). HemaSphere is published by John Wiley & Sons Ltd on behalf of European Hematology Association. This is an open access Abstract Book distributed under the Attribution-NonCommercial-NoDerivs (CC BY-NC-ND), which allows third parties to download the articles and share them with others as long as they credit the author and the Abstract Book, but they cannot change the content in any way or use them commercially.
**Abstract Book Citations:** Authors, Title, HemaSphere, 2026;10:(S1):pages. The abstract book DOIs can be found on the "Author Information" page.
**Disclaimer:** Articles published in the journal HemaSphere exclusively reflect the opinions of the authors. The authors are responsible for all content in their abstracts including accuracy of the facts, statements, citing resources, etc.

**Summary/Conclusion**

In this large real-world cohort, acalabrutinib monotherapy achieved real-world ORR comparable to the ELEVATE-TN clinical trial, but with inferior 2-year PFS and OS, likely reflecting older age, high comorbidity burden and competing non-CLL mortality. Toxicity was generally manageable in this older population. These findings support the effectiveness and tolerability of acalabrutinib monotherapy in routine clinical practice while highlighting the impact of patient frailty on real-world outcomes.

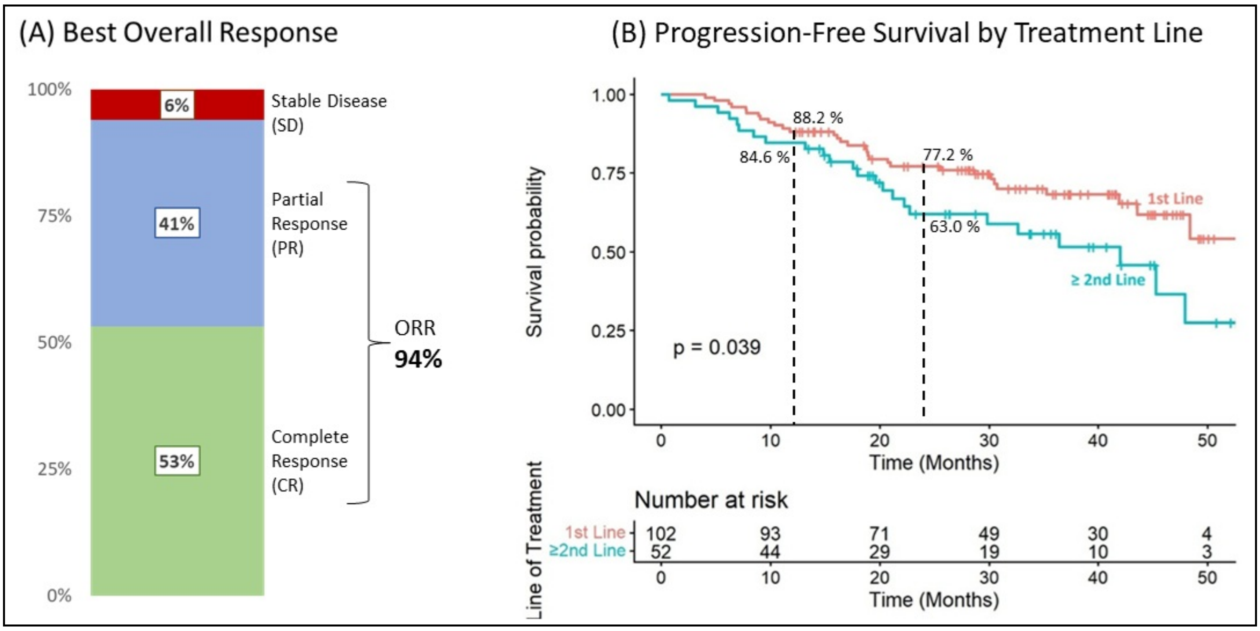

**Copyright Information:** (Online) ISSN: 2572-9241
©2026 The Author(s). HemaSphere is published by John Wiley & Sons Ltd on behalf of European Hematology Association. This is an open access Abstract Book distributed under the Attribution-NonCommercial-NoDerivs (CC BY-NC-ND), which allows third parties to download the articles and share them with others as long as they credit the author and the Abstract Book, but they cannot change the content in any way or use them commercially.
**Abstract Book Citations:** Authors, Title, HemaSphere, 2026;10:(S1):pages. The abstract book DOIs can be found on the "Author Information" page.
**Disclaimer:** Articles published in the journal HemaSphere exclusively reflect the opinions of the authors. The authors are responsible for all content in their abstracts including accuracy of the facts, statements, citing resources, etc.

### (PB2909) PATIENT-REPORTED OUTCOMES AND DISEASE PROGRESSION IN CHRONIC LYMPHOCYTIC LEUKEMIA AND SMALL LYMPHOCYTIC LYMPHOMA: A SYSTEMATIC LITERATURE REVIEW AND GAP ANALYSIS

**Topic:** 06. Chronic lymphocytic leukemia and related disorders - Clinical

Loic Ysebaert*[1], Lianne Barnieh[2], Gisoo Barnes[3], Rasika Korde[3], Atara Laor[4], Shanti Neff-Baro[5], Leyla Mohseninejad[6]

*[1]University of Toulouse, CHU Toulouse, IUCT-Oncopole, Toulouse, France;[2]BeOne Medicines Ltd, Paris, France; [3]BeOne Medicines Ltd, San Carlos, CA, United States of America; [4]Amaris Consulting, Montreal, QC, Canada; [5]Amaris Consulting, Paris, France; [6]BeOne Medicines Ltd, Schiphol, The Netherlands;*

### Background

While progression-free survival is considered a key endpoint in oncology, the German Federal Joint Committee (G-BA) and other health technology assessment (HTA) bodies have raised concerns about its direct patient relevance as a standalone endpoint. Other therapy areas have developed patient-relevant endpoints that combine disease progression with symptoms. To inform the development of a patient-focused progression endpoint in hematology trials, it is necessary to understand how patients are impacted by progression.

### Aims

This study aimed to identify patient-reported disease symptoms related to disease progression in chronic lymphocytic leukemia (CLL) and small lymphocytic lymphoma (SLL).

### Methods

A systematic literature review (SLR) was conducted to identify evidence on CLL/SLL-related symptoms and patient-reported outcomes (PROs). Searches were conducted in Embase, MEDLINE, MEDLINE In-Process, and the Cochrane Library for publications from January 2015 to October 2025. Targeted supplementary searches were also performed through December 2025, including Google Scholar, conference proceedings, clinical trial registries, and relevant professional society, government, and regulatory agency websites, as well as reference lists of identified SLRs. Only studies published in English were included. Eligible studies were required to either report an association between symptoms and disease progression or use CLL/SLL-validated PRO instruments assessing symptoms or clinically relevant events.

**Copyright Information:** (Online) ISSN: 2572-9241
©2026 The Author(s). HemaSphere is published by John Wiley & Sons Ltd on behalf of European Hematology Association. This is an open access Abstract Book distributed under the Attribution-NonCommercial-NoDerivs (CC BY-NC-ND), which allows third parties to download the articles and share them with others as long as they credit the author and the Abstract Book, but they cannot change the content in any way or use them commercially.
**Abstract Book Citations:** Authors, Title, HemaSphere, 2026;10:(S1):pages. The abstract book DOIs can be found on the “Author Information” page.
**Disclaimer:** Articles published in the journal HemaSphere exclusively reflect the opinions of the authors. The authors are responsible for all content in their abstracts including accuracy of the facts, statements, citing resources, etc.

**Results**

A total of 3510 unique publications were identified, of which 3420 were excluded at title and abstract screening. Of the 90 publications screened in full, 40 met at least one of the inclusion criteria; an additional conference abstract was identified through hand searching.
Two publications explicitly assessed the relationship between a CLL/SLL PRO measure and disease progression as part of randomized controlled trials. The first publication reported global health status and fatigue before and after disease progression among 428 patients randomized to chlorambucil or chlorambucil with ofatumumab who had at least one PRO measurement. No changes were observed at progression compared with the pre-progression assessment in either arm. The second publication reported a post-hoc analysis among the 560 patients with relapsed/refractory CLL/SLL with at least one evaluable PRO who were randomized to zanubrutinib or ibrutinib. Results demonstrated that longitudinal deterioration in patient-reported fatigue, insomnia, and nausea/vomiting was significantly associated with increased risk of disease progression, irrespective of treatment received. While pain deterioration (worsening of pain) was associated with increased risk of disease progression, the association did not reach statistical significance.
The remaining 39 publications reported on 88 distinct PROs assessing 38 distinct symptoms or domains. The most frequently reported symptoms and domains included fatigue, physical functioning, pain, gastrointestinal symptoms, emotional and social functioning, and overall symptom burden. None of these 39 publications explored an association between symptoms and progressive disease.

**Summary/Conclusion**

This study highlights a gap in evidence regarding patient-reported symptoms and disease progression in CLL/SLL. Further work is needed to develop a consensus-driven symptomatic progression composite endpoint in CLL/SLL to support future clinical and HTA decision-making.

**Copyright Information:** (Online) ISSN: 2572-9241
©2026 The Author(s). HemaSphere is published by John Wiley & Sons Ltd on behalf of European Hematology Association. This is an open access Abstract Book distributed under the Attribution-NonCommercial-NoDerivs (CC BY-NC-ND), which allows third parties to download the articles and share them with others as long as they credit the author and the Abstract Book, but they cannot change the content in any way or use them commercially.
**Abstract Book Citations:** Authors, Title, HemaSphere, 2026;10:(S1):pages. The abstract book DOIs can be found on the "Author Information" page.
**Disclaimer:** Articles published in the journal HemaSphere exclusively reflect the opinions of the authors. The authors are responsible for all content in their abstracts including accuracy of the facts, statements, citing resources, etc.

### (PB2910) PIRTOBRUTINIB VERSUS SECOND-GENERATION BTK INHIBITORS IN RELAPSED/REFRACTORY CLL/SLL: A BAYESIAN NETWORK META-ANALYSIS.

**Topic:** 06. Chronic lymphocytic leukemia and related disorders - Clinical

Stefano Molica*[1], Diana Giannarelli[2], Luciano Levato[3], Talha Munir[4], David Allsup[5, 6]

*[1]University of Hull, Hematology, Hull, United Kingdom;[2]Biostatistics Unit, Scientific Directorate Fondazione Policlinico Universitario Agostino Gemelli IRCCS, Rome,Italy, Rome, Italy; [3]Azienda Ospedaliera-Universitaria, Dulbecco, Catanzaro, Hematology, Catanzaro, Italy; [4]St James's Hospital, Hematology, Leeds, United Kingdom;[5]3Centre for Biomedicine, Hull York Medical School, Hull, UK, Hematology, Hull, United Kingdom; [6]Centre for Biomedicine, Hull York Medical School, Hull, UK Hull, United Kingdom, Hematology, Hull, United Kingdom;*

### Background

The BRUIN CLL-314 trial demonstrated noninferiority of pirtobrutinib versus ibrutinib for overall response rate (ORR) in TN and R/R CLL/SLL pts without prior BTKi exposure.  However, how pirtobrutinib compares in terms of efficacy or safety with second-generation BTKis is not known.

### Aims

Given guideline preferences for second-generation covalent BTKis (acalabrutinib, zanubrutinib) over ibrutinib and the absence of direct head-to-head trials, we conducted a network meta-analysis (NMA) to contextualize pirtobrutinib within the landscape of novel BTKis for R/R CLL.

### Methods

This Bayesian NMA synthesized randomized data from three trials in R/R CLL/SLL: BRUIN CLL-314 (pirtobrutinib), ELEVATE-RR (acalabrutinib), and ALPINE (zanubrutinib), each versus ibrutinib. Unlike matching-adjusted indirect comparisons (MAICs), this approach preserved randomization integrity without post-hoc covariate adjustments, reducing bias from reweighting. We analyzed hazard ratios (HRs) for PFS and OS, and odds ratios (ORs) for ORR. Safety endpoints—including treatment discontinuation due to adverse events (AEs), infections grade ≥3, and cardiovascular AEs—were evaluated as ORs. Baseline imbalances in high-risk cytogenetics (TP53 aberrations; complex karyotype [KC], >3 abnormalities) prompted subgroup analyses of ORR.

**Copyright Information:** (Online) ISSN: 2572-9241
©2026 The Author(s). HemaSphere is published by John Wiley & Sons Ltd on behalf of European Hematology Association. This is an open access Abstract Book distributed under the Attribution-NonCommercial-NoDerivs (CC BY-NC-ND), which allows third parties to download the articles and share them with others as long as they credit the author and the Abstract Book, but they cannot change the content in any way or use them commercially.
**Abstract Book Citations:** Authors, Title, HemaSphere, 2026;10:(S1):pages. The abstract book DOIs can be found on the "Author Information" page.
**Disclaimer:** Articles published in the journal HemaSphere exclusively reflect the opinions of the authors. The authors are responsible for all content in their abstracts including accuracy of the facts, statements, citing resources, etc.

### Results

Compared with acalabrutinib, pirtobrutinib showed a favorable but non-significant PFS trend (HR 0.73, 95% CrI 0.44–1.20), with no corresponding benefit in ORR (OR 1.39, 95% CrI 0.74–2.61) or OS (HR 1.17, 95% CrI 0.59–2.33). None achieved statistical significance.
Similar patterns were observed versus zanubrutinib: PFS (HR 1.12, 95% CrI 0.67–1.89), ORR (OR 1.01, 95% CrI 0.55–1.85), and OS (HR 1.26, 95% CrI 0.62–2.58). The higher baseline prevalence of high-risk features in the pirtobrutinib cohort compared with the zanubrutinib cohort (TP53 aberrations 42.9% vs 23.0%,, KC,45.2% vs 17.1%) prompted subgroup analyses stratified by the presence or absence of high-risk features.  In high-risk subgroups, the ORR tended to favor zanubrutinib over pirtobrutinib (TP53 aberration: OR 0.46, 95% Crl, 0.16–1.36; KC: OR 0.74, 95%; Crl, 0.22–2.42). By contrast, among patients without TP53 aberrations and among those without KC pirtobrutinib showed a favorable trend relative to zanubrutinib (TP53 wild-type: OR 1.55, 95% CrI 0.73-3.28; KC absence:1.04, 95% Crl 0.42–2.56).
Safety outcomes showed no significant differences in AE-related discontinuation (vs. acalabrutinib: OR 1.34, 95% CrI 0.68–2.66; vs. zanubrutinib: OR 1.34, 95% CrI 0.71–2.55) or grade ≥3 infections (vs. acalabrutinib: OR 0.99, 95% CrI 0.57–1.72; vs. zanubrutinib: OR 1.12, 95% CrI 0.65–1.88). However, cardiovascular AEs were significantly less frequent with pirtobrutinib versus zanubrutinib (OR 0.52, 95% CrI 0.31–0.88).

### Summary/Conclusion

This Bayesian NMA identified no statistically significant differences in efficacy between pirtobrutinib and second-generation BTKis (acalabrutinib, zanubrutinib) across PFS, OS, and ORR in R/R CLL. In predefined high-risk subgroups, zanubrutinib yielded ORR ORs >1 versus pirtobrutinib. This trend did not extend to patients without TP53 aberrations or CK, where ORs >1 favored pirtobrutinib over zanubrutinib. These immature data necessitate longer follow-up and confirmatory evidence. Importantly, pirtobrutinib demonstrated significantly superior cardiovascular tolerability relative to zanubrutinib. This finding warrants validation in populations enriched for cardiovascular comorbidities

**Copyright Information:** (Online) ISSN: 2572-9241
©2026 The Author(s). HemaSphere is published by John Wiley & Sons Ltd on behalf of European Hematology Association. This is an open access Abstract Book distributed under the Attribution-NonCommercial-NoDerivs (CC BY-NC-ND), which allows third parties to download the articles and share them with others as long as they credit the author and the Abstract Book, but they cannot change the content in any way or use them commercially.
**Abstract Book Citations:** Authors, Title, HemaSphere, 2026;10:(S1):pages. The abstract book DOIs can be found on the "Author Information" page.
**Disclaimer:** Articles published in the journal HemaSphere exclusively reflect the opinions of the authors. The authors are responsible for all content in their abstracts including accuracy of the facts, statements, citing resources, etc.

### (PB2911) CONCORDANCE OF MEASURABLE RESIDUAL DISEASE (MRD) RATES IN THE PERIPHERAL BLOOD AND BONE MARROW AFTER FIRST-LINE TREATMENT FOR CLL: A SYSTEMATIC REVIEW AND META-ANALYSIS

**Topic:** 06. Chronic lymphocytic leukemia and related disorders - Clinical

Graham Wehmeyer*[1], Guiseppe Fiorica[2], Claire Yee[3], Diana Segovia[3], Fausto Alfredo Rios-Olais[4], Talal Hilal[3], Adam Kittai[1]

*[1]Tisch Cancer Center, Icahn School of Medicine at Mount Sinai, Department of Medicine (Hematology/Oncology), New York, United States of America; [2]Icahn School of Medicine, Department of Medicine, New York, United States of America;[3]Mayo Clinic, Phoenix, Arizona, United States of America; [4]Instituto Nacional de Ciencias Medicas y Nutrición Salvador Zubiran, Mexico City, Mexico;*

### Background

Undetectable measurable residual disease (uMRD) is associated with prolonged progression free survival in patients with chronic lymphocytic leukemia (CLL).(Rios-Olais et al., 2024) Peripheral blood (PB) MRD testing is less invasive and allows more frequent MRD monitoring than bone marrow (BM) MRD testing. As MRD-guided time-limited therapies become more common, there is a need to determine the concordance between rates of uMRD in the PB and BM.

### Aims

To determine the concordance between uMRD rates in PB and BM in patients with CLL receiving first-line, time-limited, chemotherapy-free treatment.

### Methods

We performed a systematic review and meta-analysis of studies in CLL to determine the concordance between uMRD rates in the PB and BM. Prospective trials evaluating first-line, time-limited, combination treatment of CLL with targeted agents or anti-CD20 monoclonal antibodies (mAbs) were identified via searches of PubMed, Embase, Scopus, and Web of Science from inception until December 15, 2025. Studies that reported rates of PB and BM uMRD at the same timepoint using the same MRD testing method were included. Study sample size, number of patients receiving MRD testing, uMRD rates in PB and BM, method and depth of MRD testing, and treatment arm were extracted. The agreement of uMRD rates between PB and BM was assessed using intraclass correlation coefficients (ICC).

### Results

3128 studies were identified on initial search. After study selection criteria were applied, 24 timepoints with 3121 PB and 2782 BM MRD measurements across 12 clinical trials were analyzed (Table 1). All timepoints defined uMRD at a depth of under 0.01% CLL cells (10^-4). The MRD testing method was flow cytometry in 20 timepoints, ASO-PCR in 3 timepoints, and next-generation sequencing in 1 timepoint. Patients were treated with venetoclax + an anti-CD20 mAb in 10 timepoints, a BTKi + venetoclax in 8 timepoints, and triplet therapy in 6 timepoints.
The ICC between rates of uMRD in PB and BM was 0.93 (95% CI: 0.89, 0.95), indicating an excellent degree of agreement.(Koo & Li, 2016) The mean uMRD rates in PB and BM were 70.1% and 64.3%, respectively. The mean uMRD rate in PB was 5.9% (95% CI: -7.1, 18.8%) higher than in BM; indicating a consistent slight bias of higher PB uMRD rates across timepoints (Figure 1).

**Copyright Information:** (Online) ISSN: 2572-9241
©2026 The Author(s). HemaSphere is published by John Wiley & Sons Ltd on behalf of European Hematology Association. This is an open access Abstract Book distributed under the Attribution-NonCommercial-NoDerivs (CC BY-NC-ND), which allows third parties to download the articles and share them with others as long as they credit the author and the Abstract Book, but they cannot change the content in any way or use them commercially.
**Abstract Book Citations:** Authors, Title, HemaSphere, 2026;10:(S1):pages. The abstract book DOIs can be found on the "Author Information" page.
**Disclaimer:** Articles published in the journal HemaSphere exclusively reflect the opinions of the authors. The authors are responsible for all content in their abstracts including accuracy of the facts, statements, citing resources, etc.

### Summary/Conclusion

We demonstrate that in patients with CLL treated with modern, time-limited therapy, there is high concordance in uMRD rates between PB and BM. Rates of uMRD in PB are typically slightly higher than in BM.

**Table 1.** Characteristics of included timepoints

| Trial Name | Treatment | MRD Testing Timepoint | MRD Testing Method | % uMRD (PB) | % uMRD (BM) |
|---|---|---|---|---|---|
| AMPLIFY | A + V | 3 months post-EOT | Flow | 38 | 40 |
| | A + V + O | 12 weeks post-EOT | Flow | 94 | 93 |
| CAPTIVATE | CAPTIVATE-MRD (I + V) | C16 | Flow | 74 | 72 |
| | CAPTIVATE-FD (I + V) | 12 weeks post-EOT | Flow | 57 | 52 |
| GLOW | I + V | 12 weeks post-EOT | Flow | 61 | 57 |
| | | 12 weeks post-EOT | NGS | 55 | 52 |
| CLL13 | V + R | Month 15 | Flow | 61 | 53 |
| | V + O | Month 15 | Flow | 90 | 83 |
| | I + V + O | Month 15 | Flow | 94 | 92 |
| FLAIR | I + V | Month 12 (PB), Month 9 (BM) | Flow | 58 | 43 |
| CLL14 | V + O | 12 weeks post-EOT | ASO-PCR | 90 | 83 |
| CLL17 | V + O | Final Restaging | Flow | 88 | 85 |
| | I + V | Final Restaging | Flow | 55 | 52 |
| HOVON 139 | V + O | C12 | Flow | 95 | 88 |
| | V + O (V consolidation) | 3 months post-EOT | Flow | 77 | 76 |
| | V + O (MRD-guided V consolidation) | 3 months post-EOT | Flow | 61 | 63 |
| CLL2-GIVe | I + V + O | C15 | Flow | 89 | 87 |
| AVO in a population enriched for high-risk disease | A + V + O | C4 | Flow | 13 | 3 |
| | | C8 | Flow | 71 | 55 |
| | | C16 | Flow | 90 | 82 |
| VERITAS | V + R | Month 7 | ASO-PCR | 71 | 47 |
| | | Month 15 | ASO-PCR | 69 | 59 |
| HOVON 158 | I + V | C15 | Flow | 42 | 28 |
| | I + O | Month 9 | Flow | 92 | 98 |

A: Acalabrutinib, EOT: End of Treatment, I: Ibrutinib, O: Obinutuzumab, R: Rituximab, V: Venetoclax

**Copyright Information:** (Online) ISSN: 2572-9241
©2026 The Author(s). HemaSphere is published by John Wiley & Sons Ltd on behalf of European Hematology Association. This is an open access Abstract Book distributed under the Attribution-NonCommercial-NoDerivs (CC BY-NC-ND), which allows third parties to download the articles and share them with others as long as they credit the author and the Abstract Book, but they cannot change the content in any way or use them commercially.
**Abstract Book Citations:** Authors, Title, HemaSphere, 2026;10:(S1):pages. The abstract book DOIs can be found on the “Author Information” page.
**Disclaimer:** Articles published in the journal HemaSphere exclusively reflect the opinions of the authors. The authors are responsible for all content in their abstracts including accuracy of the facts, statements, citing resources, etc.

### (PB2912) SHORTENED OBINUTUZUMAB INFUSION IN FRONTLINE CLL: REDUCED CHAIR TIME WITHOUT COMPROMISING SAFETY

**Topic:** 06. Chronic lymphocytic leukemia and related disorders - Clinical

Eva Vejrazkova*[1, 2], Dominika Ecsiova[1, 3], Pavel Vodarek[1, 3], Lukas Smolej[1, 3], Pavel Zak[1, 3], Martin Simkovic[1, 3]

*[1]University Hospital, 4th Department of Internal Medicine – Hematology, Hradec Králové, Czechia; [2]University Hospital, Tissue Bank, Hradec Králové, Czechia; [3]Charles University Faculty of Medicine in Hradec Králové, Hradec Králové, Czechia;*

### Background

Obinutuzumab-based regimens represent a standard treatment option for previously untreated patients with chronic lymphocytic leukemia. However, duration of infusion substantially increases outpatient unit burden. Real-world evidence on shortened infusion strategies remains limited.

### Aims

To evaluate whether shortened obinutuzumab infusion in treatment-naïve CLL patients reduces chair time while maintaining safety and disease control compared with standard-duration administration.

### Methods

We conducted a retrospective unicentric analysis of 114 treatment-naïve patients with chronic lymphocytic leukemia (CLL) receiving obinutuzumab in combination with venetoclax or chlorambucil. All patients received standard 4-hour obinutuzumab infusions on cycle 1 day 1 (C1D1) and day 2 (C1D2). From C1D8 onward, infusion duration was reduced to 2 hours in patients who did not experience clinically significant infusion-related reactions (IRRs), defined as Grade ≥1 events per CTCAE v5.0, forming the reduced-duration infusion group (n=64). Patients maintained on 4-hour infusions throughout treatment constituted the standard-duration infusion group (n=50).
Infusion duration was recorded for each administration. The primary endpoint was reduction in chair time from C1D8 onward. Safety was also assessed by incidence of IRR per patient and per infusion. Follow-up and progression-free survival (PFS) were analyzed descriptively. Baseline clinical and biological characteristics at C1D1 are summarized in Table 1.

**Copyright Information:** (Online) ISSN: 2572-9241
©2026 The Author(s). HemaSphere is published by John Wiley & Sons Ltd on behalf of European Hematology Association. This is an open access Abstract Book distributed under the Attribution-NonCommercial-NoDerivs (CC BY-NC-ND), which allows third parties to download the articles and share them with others as long as they credit the author and the Abstract Book, but they cannot change the content in any way or use them commercially.
**Abstract Book Citations:** Authors, Title, HemaSphere, 2026;10:(S1):pages. The abstract book DOIs can be found on the "Author Information" page.
**Disclaimer:** Articles published in the journal HemaSphere exclusively reflect the opinions of the authors. The authors are responsible for all content in their abstracts including accuracy of the facts, statements, citing resources, etc.

| Characteristic | All Patients (N = 114) |
|---|---|
| Age, years, median (IQR) | 68.5 (63.2–73.0) |
| Sex, n (%) | |
| Male | 72 (63.2%) |
| ECOG performance status, n (%) | |
| 0-1 | 109 (95.6%) |
| 2-3 | 5 (4,4%) |
| Rai risk group | |
| Low | 7 (6.1%) |
| Intermediate | 25 (21.9%) |
| High | 82 (71.9%) |
| Creatinine clearance, mL/min, median (IQR) | 67 (57–82.5) |
| Elevated lactate dehydrogenase (LDH), n (%) | 93 (81.6%) |
| Tumor lysis syndrome (TLS) risk category, n (%) | |
| Low | 23 (20.2%) |
| Intermediate | 86 (75.4%) |
| High | 5 (4.4%) |
| Absolute lymphocyte count at C1D1, $\times 10^9$/L, median (IQR) | 72.9 (16.9–159.2) |
| ALC ≥25 $\times 10^9$/L, n (%) | 78 (68.4%) |
| ALC <25 $\times 10^9$/L, n (%) | 36 (31.6%) |
| Lymphadenopathy (largest node), n (%) | |
| <2 cm | 35 (30.7%) |
| ≥2 cm to <5 cm | 52 (45.6%) |
| ≥5 cm to <10 cm | 23 (20.2%) |
| ≥10 cm | 4 (3.5%) |
| Splenomegaly, n (%) | 67 (58.8%) |
| Massive (>10 cm below costal margin) | 16 (14.0%) |
| Unmutated IGHV, n (%) | 75 (65.8%) |
| *TP53* mutation and/or del(17p), n (%) | 7 (6.1%) |
| Complex karyotype, n (%) | 9 (7.9%) |

### Results

From C1D8 onward, median cumulative infusion time per patient was 16.3 hours (IQR 14.9–16.6) in the reduced-duration group versus 27.5 hours (23.0–30.3) in the standard-duration group, corresponding to a median reduction of 11.2 hours (95% CI 8.6–13.1; p<0.001).
IRR occurred predominantly during C1D1 (68.8% vs 80.0%), with rapidly declining incidence at C1D2 (6.5% vs 8.2%) and C1D8 (3.1% vs 4.3%). No IRR were observed beyond C1D8 in either group. Overall IRR incidence was comparable between the reduced-duration group (71.9%) and the standard-duration group (82.0%). Median follow-up was 38.0 months in the reduced-duration group and 37.3 months in the standard-duration group.

### Summary/Conclusion

Accelerated obinutuzumab infusions substantially reduced chair time while maintaining a comparable safety profile and disease control. These findings support implementation of shortened infusion protocols in routine frontline CLL care to optimize outpatient resource utilization.

Supported by COOPERATIO (research area ONCO), RVO MH CZ (UHHK, 00179906).

**Copyright Information:** (Online) ISSN: 2572-9241
©2026 The Author(s). HemaSphere is published by John Wiley & Sons Ltd on behalf of European Hematology Association. This is an open access Abstract Book distributed under the Attribution-NonCommercial-NoDerivs (CC BY-NC-ND), which allows third parties to download the articles and share them with others as long as they credit the author and the Abstract Book, but they cannot change the content in any way or use them commercially.
**Abstract Book Citations:** Authors, Title, HemaSphere, 2026;10:(S1):pages. The abstract book DOIs can be found on the "Author Information" page.
**Disclaimer:** Articles published in the journal HemaSphere exclusively reflect the opinions of the authors. The authors are responsible for all content in their abstracts including accuracy of the facts, statements, citing resources, etc.

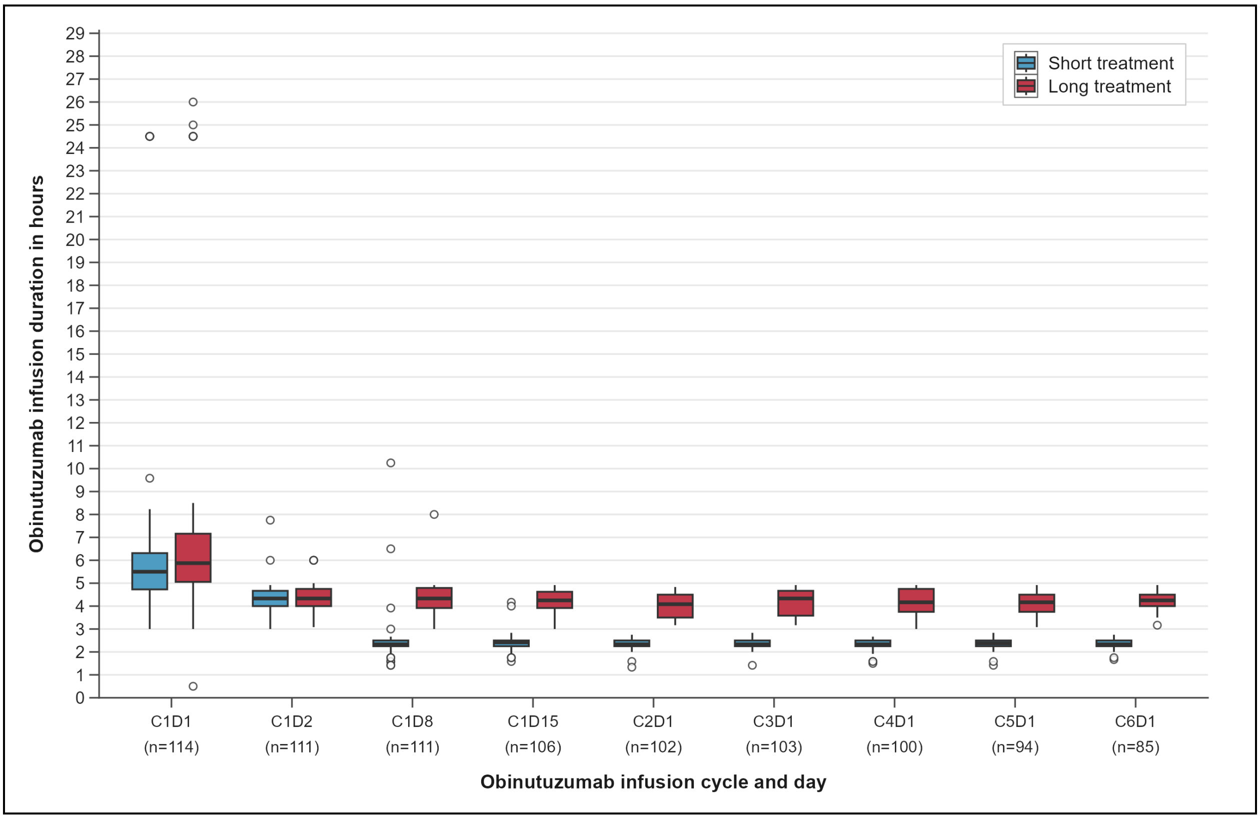

**Copyright Information:** (Online) ISSN: 2572-9241
©2026 The Author(s). HemaSphere is published by John Wiley & Sons Ltd on behalf of European Hematology Association. This is an open access Abstract Book distributed under the Attribution-NonCommercial-NoDerivs (CC BY-NC-ND), which allows third parties to download the articles and share them with others as long as they credit the author and the Abstract Book, but they cannot change the content in any way or use them commercially.
**Abstract Book Citations:** Authors, Title, HemaSphere, 2026;10:(S1):pages. The abstract book DOIs can be found on the "Author Information" page.
**Disclaimer:** Articles published in the journal HemaSphere exclusively reflect the opinions of the authors. The authors are responsible for all content in their abstracts including accuracy of the facts, statements, citing resources, etc.

### (PB2915) REAL-WORLD INTRAVENOUS IMMUNOGLOBULIN (IVIG) UTILIZATION IN PATIENTS WITH CHRONIC LYMPHOCYTIC LEUKEMIA/SMALL LYMPHOCYTIC LYMPHOMA (CLL/SLL) TREATED WITH VENETOCLAX PLUS OBINUTUZUMAB VS ZANUBRUTINIB

**Topic:** 06. Chronic lymphocytic leukemia and related disorders - Clinical

Nicole Lamanna*[1], Erin Mulvey[2], Keri Yang[3], Jaspreet Jaggi[3], Catherine Fu[3], Abby Wu[3], G. Rhys Williams[3], Nakhle S. Saba[4]

*[1]Herbert Irving Comprehensive Cancer Center, Columbia University, New York, NY, United States of America;[2]Weill Cornell Medicine, New York, NY, United States of America; [3]BeOne Medicines, Ltd, San Carlos, CA, United States of America;[4]Our Lady of the Lake Blood Cancer & Cell Therapy Institute, Baton Rouge, LA, United States of America;*

### Background

Patients with CLL/SLL often develop secondary immunodeficiency and remain at elevated risk for serious infections, for which IVIG is commonly used as supportive care. However, real-world data on IVIG use patterns across targeted regimens and timing relative to infections remain limited.

### Aims

To characterize real-world IVIG utilization patterns, including frequency and timing relative to treatment initiation, completion, and occurrence of serious infections in patients with CLL/SLL treated with fixed-duration combination venetoclax plus obinutuzumab (VO) or continuous zanubrutinib (zanu) monotherapy.

### Methods

This retrospective observational study used US Symphony Integrated Dataverse database to identify adults with ≥2 CLL/SLL diagnoses who initiated VO (04/2016-12/2025) or zanu monotherapy (01/2020-12/2025). IVIG use was assessed for VO and zanu after treatment initiation through end of follow-up, with additional analysis of IVIG use after VO completion and zanu discontinuation. Subgroup analysis was conducted by recorded serious infections, and timing of IVIG use (before /after) relative to first serious infection, defined as receipt of injectable antimicrobials ≤15 days of hospitalization. Treatment utilization patterns were examined overall, by first line (1L) vs second or later line (2L+), and baseline characteristics between the cohorts.

### Results

A total of 851 VO patients and 365 zanu patients received IVIG after treatment initiation (median follow-up: VO, 36.5 months; zanu, 22.6 months). Baseline characteristics were generally comparable, with zanu patients older than VO patients (median, 74 vs 69 years; $P$<.0001). Within 1 year prior to treatment initiation, 40% of VO and 59% of zanu patients had ≥1 IVIG use. Overall IVIG use after treatment initiation was higher with VO vs zanu (17.4% vs 5.2%; $P$<.0001) and increased in 2L+ more than 1L in both cohorts (VO: 27.2% vs 9.0%; zanu: 7.5% vs 2.1%, respectively; $P$<.0001). In IVIG recipients, mean number of IVIG administrations after treatment initiation was significantly higher with VO vs zanu (13.4 vs 9.8; $P$<.0001), driven primarily by use in 2L+. In patients with serious infections, first IVIG use before first serious infection occurred more commonly with VO than zanu (12.0% vs 6.3%), particularly in 2L+ (12.9% vs 6.7%). These patients also demonstrated higher frequency of IVIG use (mean: VO, 19.4 vs zanu, 13.6). First IVIG after first serious infection was observed in 8.7% of VO and 5.2% of zanu patients, with lower subsequent IVIG use intensity (mean use: 9.0 and 6.3, respectively). While 20.7% of VO patients and 11.5% of zanu patients had serious infections, most IVIG recipients had no recorded serious infection (VO, 79.3%; zanu, 88.5%), yet IVIG use remained substantial (mean use: 13.0 vs 9.7, respectively). Across all categories, IVIG use increased with later lines of therapy. Notably, 11.5% of VO patients continued IVIG use after VO completion and 2.3% of zanu patients continued IVIG use after treatment discontinuation.

**Copyright Information:** (Online) ISSN: 2572-9241
©2026 The Author(s). HemaSphere is published by John Wiley & Sons Ltd on behalf of European Hematology Association. This is an open access Abstract Book distributed under the Attribution-NonCommercial-NoDerivs (CC BY-NC-ND), which allows third parties to download the articles and share them with others as long as they credit the author and the Abstract Book, but they cannot change the content in any way or use them commercially.
**Abstract Book Citations:** Authors, Title, HemaSphere, 2026;10:(S1):pages. The abstract book DOIs can be found on the "Author Information" page.
**Disclaimer:** Articles published in the journal HemaSphere exclusively reflect the opinions of the authors. The authors are responsible for all content in their abstracts including accuracy of the facts, statements, citing resources, etc.

**Summary/Conclusion**

This study characterizes real-world IVIG utilization patterns, highlighting use prior to serious infection, suggesting proactive immune support. IVIG use was significantly more frequent and intensive in patients treated with VO than zanu. Findings underscore variability in supportive care and potential associated financial burden. Future prospective studies are needed to further define optimal IVIG strategies in patients with CLL.

**Copyright Information:** (Online) ISSN: 2572-9241
©2026 The Author(s). HemaSphere is published by John Wiley & Sons Ltd on behalf of European Hematology Association. This is an open access Abstract Book distributed under the Attribution-NonCommercial-NoDerivs (CC BY-NC-ND), which allows third parties to download the articles and share them with others as long as they credit the author and the Abstract Book, but they cannot change the content in any way or use them commercially.
**Abstract Book Citations:** Authors, Title, HemaSphere, 2026;10:(S1):pages. The abstract book DOIs can be found on the “Author Information” page.
**Disclaimer:** Articles published in the journal HemaSphere exclusively reflect the opinions of the authors. The authors are responsible for all content in their abstracts including accuracy of the facts, statements, citing resources, etc.

### (PB2916) A GLOBAL MULTICENTER, OPEN-LABEL, RANDOMIZED, PHASE 3 REGISTRATIONAL STUDY OF LISAFTOCLAX (APG-2575) IN PREVIOUSLY TREATED CLL/SLL: GLORA TRIAL IN PROGRESS

**Topic:** 06. Chronic lymphocytic leukemia and related disorders - Clinical

Matthew Davids*[1], Sikander Ailawadhi[2], Gerardo Musuraca[3], Wojciech Jurczak[4], Martin Simkovic[5], Antonio Gutierrez Garcia[6], Vladimir Ivanov[7], Nitin Jain[8], Jacqueline Barrientos[9], Ronald Tang[10], Eugene Nikitin[11], Elena Misyurina[12], Gulnara Khusainova[13], Gayane Tumyan[14], Cecile Tomowiak[15], Katell Le Du[16], Andrea Visentin[17], Monica Tani[18], Ciprian Tomuleasa[19], Eva Mikuskova[20], Ricardo D. Parrondo[2], Maksim Aksenov[7], Zi Chen[21], Tommy Fu[22], Mingyu Li[22], Boyd Mudenda[22], Shanthi Marur[22], Vallari Shah[22], Mohammad Ahmad[22], Eric Liang[22], Divya Mekala[22], Edward Lee[22], Dajun Yang[21, 23], Yifan Zhai[21, 22]

*[1]Dana-Farber Cancer Institute, Boston, MA, United States of America; [2]Mayo Clinic, Jacksonville, FL, United States of America; [3]Istituto Romagnolo per lo Studio dei Tumori "Dino Amadori", Meldola, Italy; [4]Maria Sklodowska-Curie National Research Institute of Oncology, Krakow, Poland; [5]Faculty of Medicine in Hradec Králové, University Hospital and Charles University in Prague, Department of Internal Medicine - Haematology, Hradec Kralove, Czechia; [6]Hospital Universitari Son Espases, Palma de Mallorca, Spain; [7]National Medical Research Center named after V.A. Almazov, Saint Petersburg, Russia; [8]Department of Leukemia, The University of Texas MD Anderson Cancer Center, Houston, TX, United States of America; [9]Mount Sinai Comprehensive Cancer Center, Miami Beach, FL, United States of America; [10]Los Angeles Cancer Network, Los Angeles, CA, United States of America; [11]City Clinical Hospital named after S.P. Botkin, Moscow, Russia; [12]City Clinical Hospital No. 52, Moscow, Russia; [13]Republican Clinical Oncology Dispensary named after Professor M.Z. Sigal, Kazan, Russia; [14]N.N. Blokhin National Medical Research Center of Oncology, Moscow, Russia; [15]University Hospital, Department of Hematology, Poitiers, France; [16]L'Hôpital Privé du Confluent, Nantes, France; [17]Hematology Unit, Department of Medicine, University of Padova, Azienda Ospedale - Università Padova, Padova, Italy; [18]Azienda USL di Ravenna, Ravenna, Italy; [19]Medfuture Institute for Biomedical Research, Hematology, Iuliu Hațieganu University of Medicine and Pharmacy, Department of Personalized Medicine and Rare Diseases, Cluj-Napoca, Romania; [20]Národný Onkologický Ústav, Bratislava, Slovakia; [21]Ascentage Pharma (Suzhou) Co., Ltd., Suzhou, China; [22]Ascentage Pharma Group Inc., Rockville, MD, United States of America; [23]Department of Experimental Research, State Key Laboratory of Oncology in South China Collaborative Innovation Center for Cancer Medicine, Sun Yat-sen University Cancer Center, Guangzhou, China;*

### Background

Patients with high-risk chronic lymphocytic leukemia/small lymphocytic lymphoma (CLL/SLL) not achieving a complete response (CR) after ≥ 12 months of BTK inhibitor (BTKi) monotherapy remain at risk for early progression due to residual disease and BTKi resistance. A BCL-2 inhibitor (BCL-2i) introduces a complementary, BCR-independent apoptotic mechanism that may deepen responses and prolong progression-free survival (PFS). Lisaftoclax, a selective oral BCL-2i with a short half-life (4-6 h), enables once-daily dosing and is approved in China as second-line CLL therapy. Lisaftoclax monotherapy has demonstrated overall response rates (ORRs) of 67% in relapsed/refractory CLL and 62.5% in BTKi-refractory disease, including del(17p)/*TP53*-mutated cases. Lisaftoclax combined with acalabrutinib achieved ORRs >97% with favorable tolerability.

### Aims

This study evaluates whether lisaftoclax plus BTKi (acalabrutinib, zanubrutinib, or ibrutinib) improves PFS and/or overall survival (OS) (vs continued BTKi monotherapy) in patients with CLL/SLL and residual disease after prolonged BTKi treatment.

**Copyright Information:** (Online) ISSN: 2572-9241
©2026 The Author(s). HemaSphere is published by John Wiley & Sons Ltd on behalf of European Hematology Association. This is an open access Abstract Book distributed under the Attribution-NonCommercial-NoDerivs (CC BY-NC-ND), which allows third parties to download the articles and share them with others as long as they credit the author and the Abstract Book, but they cannot change the content in any way or use them commercially.
**Abstract Book Citations:** Authors, Title, HemaSphere, 2026;10:(S1):pages. The abstract book DOIs can be found on the "Author Information" page.
**Disclaimer:** Articles published in the journal HemaSphere exclusively reflect the opinions of the authors. The authors are responsible for all content in their abstracts including accuracy of the facts, statements, citing resources, etc.

**Methods**

This study will enroll approximately 440 patients randomly allocated to receive lisaftoclax plus BTKi or BTKi alone. Eligible patients have CLL/SLL and, after ≥12 months of BTKi monotherapy (lines 1-3), have achieved neither CR nor progressive disease (PD). Randomization is stratified by BTKi, del(17p)/*TP53* status, line of therapy, response status before enrollment, and time on lead-in BTKi. Key inclusion criteria include age ≥18 years, ECOG PS 0-2, and residual disease with either high-risk features (del(17p)/*TP53* mutation, unmutated IGHV, or complex karyotype [≥5 abnormalities]), or measurable residual disease with lymph nodes ≥2.5 cm in the absence of high-risk features. Exclusion criteria include prior BCL-2i therapy or Richter transformation. Patients randomly allocated to the investigational arm undergo a 5-day oral lisaftoclax ramp-up to the target 400-mg clinical dose without interrupting the patients' current BTKi treatment. Once daily lisaftoclax combined with the patient's current BTKi is administered in 28-day cycles until progression or toxicity. Patients randomly allocated to the control arm continue their current BTKi monotherapy. Safety and disease assessments (CT/MRI) occur regularly. The primary endpoint is PFS by independent review committee (iwCLL 2018). Key secondary endpoints are OS, PFS by investigator, ORR, duration of response, MRD negativity, and safety. Enrollment is ongoing across 126 sites in 18 countries (ClinicalTrials.gov ID: NCT06104566, EU CT# 2023-508005-24).

**Copyright Information:** (Online) ISSN: 2572-9241
©2026 The Author(s). HemaSphere is published by John Wiley & Sons Ltd on behalf of European Hematology Association. This is an open access Abstract Book distributed under the Attribution-NonCommercial-NoDerivs (CC BY-NC-ND), which allows third parties to download the articles and share them with others as long as they credit the author and the Abstract Book, but they cannot change the content in any way or use them commercially.
**Abstract Book Citations:** Authors, Title, HemaSphere, 2026;10:(S1):pages. The abstract book DOIs can be found on the "Author Information" page.
**Disclaimer:** Articles published in the journal HemaSphere exclusively reflect the opinions of the authors. The authors are responsible for all content in their abstracts including accuracy of the facts, statements, citing resources, etc.

### (PB2918) EFFECTIVENESS AND SAFETY OF FIXED-DURATION VERSUS CONTINUOUS THERAPY IN PATIENTS WITH CHRONIC LYMPHOCYTIC LEUKEMIA: A SINGLE-TERTIARY-CENTER RETROSPECTIVE ANALYSIS

**Topic:** 06. Chronic lymphocytic leukemia and related disorders - Clinical

Arianna Bevilacqua*[1], Andrea Serafin[1], Serena De Santo[1], Alessandro Cellini[1], Francesco Angotzi[1], Nicolò Danesin[1], Giovanni Leone[1], Mattia D'antiga[1], Martina De Ferrari[1], Laura Bonaldi[2], Annalisa Martines[2], Valentina Trimarco[1], Sara Teolato[1], Federica Frezzato[1], Monica Facco[1], Francesco Piazza[1], Livio Trentin[1], Andrea Visentin[1]

*[1]Hematology Unit, Department of Medicine, University of Padova, Padova, Italy;[2]Immunology and Molecular Oncology Unit, Veneto Institute of Oncology IOV-IRCSS, Padova, Italy;*

### Background

Fixed-duration therapy (FDT) and continuous therapy (CT) are approved treatment strategies for patients with chronic lymphocytic leukemia (CLL) in both treatment-naïve (TN) and relapsed/refractory (RR) settings. Recently, after a median follow up of 3 years, the CLL17 trial showed the non-inferiority of FDT vs CT in TN patients. In the RR setting, however, randomized comparisons and real-world data remain limited.

### Aims

The aim of this study is to compare the effectiveness and safety of FDT versus CT in TN and RR CLL patients in a real-world setting.

### Methods

We retrospectively analysed 327 CLL patients treated at the University Hospital of Padua, Italy, classified as TN FDT, TN CT, RR FDT, or RR CT. Baseline characteristics were compared using one-way ANOVA for continuous variables and chi-square or Fisher's test for categorical variables. Overall survival (OS) was evaluated with Kaplan–Meier and log-rank methods. Major bleeding and cardiovascular events were assessed using Fine–Gray competing-risk models, and recurrent grade ≥3 infections with an Andersen–Gill Cox model.

### Results

Baseline characteristics are summarized in Table 1. TN FDT patients were significantly younger than other groups. High complex karyotype (hCK, $^3$ 5 cytogenetic abnormalities) and immunoglobulin replacement therapy (IgRT) were more common in RR patients. With a median follow-up of 53 months, complete response (CR) rates were higher in TN versus RR patients (p 0.003) and higher with FDT versus CT in both settings (p<0.001) [Figure 1]. OS did not significantly differ between FDT and CT in either TN [HR 1.24 (0.83–1.86)] or RR [HR 1.42 (0.91–2.21)] cohorts. In multivariable analysis, only hCK and CR independently predicted OS. Bleeding risk did not differ significantly between FDT and CT in TN patients, while in the RR cohort, CT was associated with a higher risk compared with FDT [HR 12.6 (3.1–50.5)]. Cardiovascular events were similar in TN patients but more frequent with CT in RR patients [HR 5.83 (2.08–16.8)].
Grade ≥3 infection rates were comparable between strategies (8.9 vs 9.9 events per 100 patient-years). CT was not associated with increased infectious risk [HR 0.66 (0.36–1.23)]. Infections were predominantly pneumonia (58.9%), mainly bacterial and grade 3–4. Other events included fever without microbiological documentation (22.2%), urinary tract infections (6.7%), and gastrointestinal infections (6.7%). Grade 5 events accounted for 16.4% of cases, with no significant differences between treatment groups (p 0.25). Notably, although limited by small numbers, no grade 5 infectious events occurred in the TN FDT group, which was also the youngest cohort. Age was the only independent predictor of infection [HR 1.07 per year (1.03–1.12)], moreover IgRT was more frequently administered to RR patients but not independently associated with infection.

**Copyright Information:** (Online) ISSN: 2572-9241
©2026 The Author(s). HemaSphere is published by John Wiley & Sons Ltd on behalf of European Hematology Association. This is an open access Abstract Book distributed under the Attribution-NonCommercial-NoDerivs (CC BY-NC-ND), which allows third parties to download the articles and share them with others as long as they credit the author and the Abstract Book, but they cannot change the content in any way or use them commercially.
**Abstract Book Citations:** Authors, Title, HemaSphere, 2026;10:(S1):pages. The abstract book DOIs can be found on the "Author Information" page.
**Disclaimer:** Articles published in the journal HemaSphere exclusively reflect the opinions of the authors. The authors are responsible for all content in their abstracts including accuracy of the facts, statements, citing resources, etc.

### Summary/Conclusion

In this real-world cohort, FDT and CT demonstrated comparable survival outcomes in both TN and RR CLL. Prognosis was primarily driven by hCK and achievement of CR, which emerged as the only independent prognostic factors. Notably, CR rates were consistently higher with FDT than with CT in both TN and RR settings. No significant differences in infectious risk were observed between strategies across disease settings. However, in RR patients, CT was associated with increased bleeding and cardiovascular toxicity, underscoring the importance of safety considerations in therapeutic decision-making.

***Table 1:*** *Baseline characteristics.* uIGHV=unmutated Immunoglobulin Heavy-Chain Variable region; CK=complex karyotype; hCK=high complex karyotype; IgRT=immunoglobulin replacement therapy; ATT=anti-thrombotic therapy

| | *TN FDT n = 41* | *TN CT n = 113* | *RR FDT n = 45* | *RR CT n = 128* | *p value* |
|---|---|---|---|---|---|
| Age (yr) | 63±7,9 | 73±9,7 | 71±9,6 | 73±9,5 | **p <0,001** |
| Sex, male | 30 (73,2%) | 65 (57,5%) | 29 (64,4%) | 67 (52,3%) | 0,097 |
| uIGHV | 30 (73,2%) | 59 (52,2%) | 36 (80,0%) | 86 (67,8%) | 0,094 |
| del(11q) | 5 (12,2%) | 18 (15,9%) | 12 (26,7%) | 29 (22,7%) | 0,200 |
| Trisomy 12 | 11 (26,8%) | 29 (25,7%) | 6 (13,3%) | 20 (15,6%) | 0,107 |
| del(13q) | 17 (41,5%) | 57 (50,4%) | 21 (46,7%) | 64 (50,0%) | 0,717 |
| del(17p) | 6 (14,6%) | 19 (16,8%) | 13 (28,9%) | 34 (26,6%) | 0,113 |
| Karyotype | | | | | **0,003** |
| CK | 3 (7,3%) | 11 (9,7%) | 13 (28,9%) | 33 (25,8%) | |
| hCK | 2 (4,9%) | 13 (11,5%) | 8 (17,8%) | 23 (18,0%) | |
| CV comorbidities | 23 (10,1%) | 68 (60,2%) | 34 (75,6%) | 76 (59,4%) | 0,205 |
| IgRT | 2 (4,9%) | 13 (11,5%) | 13 (28,9%) | 40 (31,3%) | **0,001** |
| ATT | 6 (14,6%) | 26 (23,0%) | 19 (42,2%) | 36 (28,1%) | 0,023 |
| Therapy | | | | | |
| I | NA | 58 (51,3%) | NA | 72 (56,3%) | |
| A | NA | 26 (23,0%) | NA | 13 (10,2%) | |
| Z | NA | 18 (15,9%) | NA | 13 (10,2%) | |
| V | NA | 7 (6,2%) | NA | 12 (9,4%) | |
| VI | 27 (65,9%) | NA | 1 (2,2%) | NA | |
| VO | 7 (17,1%) | NA | 0 (0%) | NA | |
| VR | 4 (9,8%) | NA | 44 (97,8%) | NA | |
| Other | 3 (7,3%) | 4 (3,5%) | 0 (0%) | 18 (14,1%) | |

***Figure 1:*** *Percentage rates of best overall response achieved in patient subgroups according to treatment strategy and line of therapy.* CR=complete response; PR=partial response; SD=stable disease; PD=progressive disease

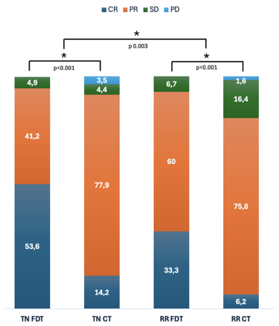

**Copyright Information:** (Online) ISSN: 2572-9241
©2026 The Author(s). HemaSphere is published by John Wiley & Sons Ltd on behalf of European Hematology Association. This is an open access Abstract Book distributed under the Attribution-NonCommercial-NoDerivs (CC BY-NC-ND), which allows third parties to download the articles and share them with others as long as they credit the author and the Abstract Book, but they cannot change the content in any way or use them commercially.
**Abstract Book Citations:** Authors, Title, HemaSphere, 2026;10:(S1):pages. The abstract book DOIs can be found on the "Author Information" page.
**Disclaimer:** Articles published in the journal HemaSphere exclusively reflect the opinions of the authors. The authors are responsible for all content in their abstracts including accuracy of the facts, statements, citing resources, etc.

### (PB2919) SPECTRUM OF AUTO- AND ALLOANTIBODIES IN PATIENTS WITH CHRONIC LYMPHOCYTIC LEUKEMIA AND THEIR IMPACT ON TRANSFUSION THERAPY TACTICS (PRELIMINARY DATA)

**Topic:** 06. Chronic lymphocytic leukemia and related disorders - Clinical

Karina Manucharova*[1], Irina Krobinets[1], Angelina Shchapova[1], Aleksei Koviazin[1]

[1]*Federal State Budgetary Institution Russian Scientific Research Institute of Hematology and Transfusiology of the Federal Medical-Biological Agency of Russia, Department of Hematology, Chemotherapy and Bone Marrow Transplantation, Saint Petersburg, Russia;*

### Background

Autoimmune cytopenias (AIHA, ITP) are common complications of CLL, required specialized transfusion strategies. Data on antibodies and their associations with markers are limited.

### Aims

To characterize the spectrum of antibodies, influence on transfusions, and their associations with immunogenetic markers in CLL.

### Methods

Retrospective analysis of 31 patients with CLL (median age 70 years, 59% male). Assessments included: direct antiglobulin test (DAT), antibody specificity, CD38 expression, 11q/13q deletions, IGHV mutation status, IgG, IgA, IgM levels, β2-microglobulin (β2-MG), and transfusion data (doses of red blood cell [RBC] concentrates/platelet concentrates, hemoglobin [Hb] increment, individualized matching). Statistical analyses: Mann-Whitney U-test, Fisher's exact test, logistic regression (n=28).

**Copyright Information:** (Online) ISSN: 2572-9241
©2026 The Author(s). HemaSphere is published by John Wiley & Sons Ltd on behalf of European Hematology Association. This is an open access Abstract Book distributed under the Attribution-NonCommercial-NoDerivs (CC BY-NC-ND), which allows third parties to download the articles and share them with others as long as they credit the author and the Abstract Book, but they cannot change the content in any way or use them commercially.
**Abstract Book Citations:** Authors, Title, HemaSphere, 2026;10:(S1):pages. The abstract book DOIs can be found on the “Author Information” page.
**Disclaimer:** Articles published in the journal HemaSphere exclusively reflect the opinions of the authors. The authors are responsible for all content in their abstracts including accuracy of the facts, statements, citing resources, etc.

### Results

Autoantibodies were detected in 8 (25%) patients. Alloantibodies: anti-C (n=1), anti-E (n=1), cold anti-M (n=1) on a DAT-positive background, anti-Jka in 2 patients. Combined cytopenias were observed in 5 patients. Hemolysis (n=11) vs no hemolysis (n=20): trends included CD38 positivity (40% vs 10.5%, p=0.143), IgM levels (0.345 vs 0.300 g/L, p=0.266), unmutated IGHV (72.7% vs 52.6%, p=0.442), and β2-MG (4.77 vs 6.04 mg/L, p=0.195). Extremely elevated β2-MG was noted in 2 patients with hemolysis (132 and 9230 mg/L). The multifactorial model (CD38, β2-MG, IgM) was significant (p=0.009), with sensitivity of 40% and specificity of 94.4%; individual predictors were nonsignificant (p>0.2). Genetic factors were not associated with hemolysis (p>0.2).
Transfusions were administered to 12 (38.7%) patients: in those with hemolysis, 63.6% vs 25% (p=0.02). Individualized matching was required for 3 patients (anti-C, -E, -Jka), with no reactions (mean Hb increment 8.5 g/L). Hb increment per RBC dose: 8.0 [5.1; 16.0] g/L (hemolysis) vs 17.25 [8.7; 20.5] g/L (p=0.12). In 2 patients receiving ibrutinib/acalabrutinib, DAT seroconversion occurred.
Table 2. Characteristics of Transfusion Therapy in Patients with CLL Depending on the Presence of Hemolytic Complications.

| Indicator | All Patients (n=31) | Presence of Hemolytic Complications (n=11) | Absence of Hemolytic Complications (n=20) | p |
|---|---|---|---|---|
| Received transfusions, n (%) | 12 (38.7%) | 7 (63.6%) | 5 (25%) | 0.02* |
| Individualized matching, n (%) | 3 (9.7%) | 3 (27.3%) | 0 | |
| RBC transfusions, total doses | 43 | 28 | 15 | |
| Number of RBC doses per patient, median [Q1; Q3] | - | 3 [2; 6] | 3 [3; 3] | 0.28** |
| Platelet transfusions, total doses | 16 | 16 | 0 | |
| Hb increment per RBC dose, g/L, median [Q1; Q3] | - | 8.0 [5.1; 16.0] | 17.25 [8.7; 20.5] | 0.12** |

*Fisher's exact test **Mann-Whitney U-test

### Summary/Conclusion

Autoantibodies were detected in 25% of patients with CLL, including rare ones (anti-M); additionally, alloantibodies (anti-C, -E, -Jka) were identified in 2 patients on a background of autoimmunization, justifying the need for a comprehensive approach to such patients. Patients with hemolysis required significantly more transfusions; individualized matching was effective. The significance of the multifactorial model (CD38, β2-MG, IgM; p=0.009) aligns with global data and indicates a potential role of these factors in the development of hemolytic complications in CLL. Targeted therapy with BTK inhibitors may eliminate autoantibodies, thereby reducing the risk of transfusion complications. The obtained data require confirmation in an expanded cohort.

**Copyright Information:** (Online) ISSN: 2572-9241
©2026 The Author(s). HemaSphere is published by John Wiley & Sons Ltd on behalf of European Hematology Association. This is an open access Abstract Book distributed under the Attribution-NonCommercial-NoDerivs (CC BY-NC-ND), which allows third parties to download the articles and share them with others as long as they credit the author and the Abstract Book, but they cannot change the content in any way or use them commercially.
**Abstract Book Citations:** Authors, Title, HemaSphere, 2026;10:(S1):pages. The abstract book DOIs can be found on the "Author Information" page.
**Disclaimer:** Articles published in the journal HemaSphere exclusively reflect the opinions of the authors. The authors are responsible for all content in their abstracts including accuracy of the facts, statements, citing resources, etc.

### (PB2920) CONCOMITANT USE OF VENETOCLAX 100MG AND KETOCONAZOLE IS CLINICALLY EQUIVALENT TO VENETOCLAX 400MG FOR TREATING RELAPSED OR REFRACTORY CHRONIC LYMPHOCYTIC LEUKEMIA (CLL).

**Topic:** 06. Chronic lymphocytic leukemia and related disorders - Clinical

Gabriel Cunha[1], Fernanda Marques[1], Verena Pfister[1], Rodrigo Santucci[2], Talita Silveira[3], Jayr Schmidt Filho[3], Danielle Leão[4], Valeria Buccheri[5], Roberto Luiz Da Silva[6], Leila Perobelli[7], Glaciano Ribeiro[8], Maura Ikoma[9], Nelson Hamerschlak[10], Pedro Amoedo[1], Fernanda Santos Azevedo[1], Carlos Sergio Chiattone[11], Milton Alexandre Ferreira Aranha[2], Celso Arrais-Rodrigues*[1]

*[1]Universidade Federal de São Paulo (UNIFESP), São Paulo, Brazil; [2]Instituto Hemomed de Oncologia e Hematologia, São Paulo, Brazil; [3]AC Camargo Cancer Center, São Paulo, Brazil; [4]Bp - A Beneficência Portuguesa De São Paulo, São Paulo, Brazil; [5]Hospital das Clínicas da Faculdade de Medicina da Universidade de São Paulo, São Paulo, Brazil;[6]Instituto Brasileiro de Controle do Câncer (IBCC), São Paulo, Brazil; [7]Hospital de Transplantes Euryclides de Jesus Zerbini – Hospital Brigadeiro, São Paulo, Brazil; [8]Universidade Federal de Minas Gerais., Belo Horizonte, Brazil;[9]Hospital Amaral Carvalho, Jaú, Brazil; [10]Hospital Israelita Albert Einstein, São Paulo, Brazil; [11]Hospital Samaritano, São Paulo, Brazil;*

### Background

Venetoclax is primarily metabolized by CYP3A4. Co-administration with strong CYP3A4 inhibitors increases systemic exposure by two to three times. Current prescribing recommendations advise reducing the dose when strong CYP3A4 inhibitors are used concomitantly. In settings with heterogeneous drug access, a pharmacokinetically rational strategy may be to administer dose-adjusted venetoclax with a strong CYP3A4 inhibitor. In Brazilian public healthcare institutions, access constraints have led to the implementation of this dose-adjustment strategy under strong CYP3A4 inhibition in order to preserve treatment continuity while adhering to pharmacologic guidance.

### Aims

We evaluated the effectiveness and safety of 100 mg of venetoclax combined with ketoconazole versus the standard dose of 400 mg of venetoclax in patients with relapsed or refractory chronic lymphocytic leukemia (CLL).

### Methods

This retrospective, multicenter study examined 118 patients with relapsed or refractory CLL who were treated within the Brazilian CLL Registry. A total of 124 venetoclax treatment courses were analyzed, as six patients received venetoclax in two separate treatment lines.
All patients completed the standard five-week ramp-up period (20–200 mg). Thereafter, patients received one of the following:

- Venetoclax 100 mg daily plus ketoconazole 200 mg daily (n = 27) or
- Venetoclax 400 mg daily (n = 97).

Rituximab was administered in combination in 86 cases (69%).
The primary endpoint was time to next treatment (TTNT). The secondary endpoints included the overall response rate (ORR), overall survival (OS), and safety. Multivariable Cox models were adjusted for line of therapy and TP53 aberrations.

**Copyright Information:** (Online) ISSN: 2572-9241
©2026 The Author(s). HemaSphere is published by John Wiley & Sons Ltd on behalf of European Hematology Association. This is an open access Abstract Book distributed under the Attribution-NonCommercial-NoDerivs (CC BY-NC-ND), which allows third parties to download the articles and share them with others as long as they credit the author and the Abstract Book, but they cannot change the content in any way or use them commercially.
**Abstract Book Citations:** Authors, Title, HemaSphere, 2026;10:(S1):pages. The abstract book DOIs can be found on the “Author Information” page.
**Disclaimer:** Articles published in the journal HemaSphere exclusively reflect the opinions of the authors. The authors are responsible for all content in their abstracts including accuracy of the facts, statements, citing resources, etc.

**Results**

Median age was 66 years; 61% were male. Venetoclax was administered as second-line therapy in 49%, third-line in 27%, fourth-line in 21%, and ≥fifth-line in 3%. Overall response rate was 96%, with comparable rates between dose-adjusted and standard-dose groups (96% vs 97%). After a median follow-up of 21 months (range 6–102), median TTNT was not reached. TTNT at 2 years was 70% overall and did not differ between groups (69% vs 74%; p=0.95). In multivariable analysis, TTNT remained similar between strategies. TTNT at 2 years was inferior in later-line therapy compared with second-line use (50% vs 87%; p=0.01), and a trend toward inferior TTNT was observed in patients with del(17p)/TP53 aberrations (50% vs 72%; p=0.056). Median OS was not reached; OS was 76% at 2 years, with no significant difference between groups (82% vs 75%). No grade ≥2 hepatotoxicity attributable to ketoconazole was observed. Rates of tumor lysis syndrome and grade ≥3 neutropenia were comparable between groups.

**Summary/Conclusion**

In this real-world cohort, dose-adjusted venetoclax administered under strong CYP3A4 inhibition demonstrated comparable short-term effectiveness and safety to standard-dose venetoclax in relapsed/refractory CLL. These findings may be particularly relevant in resource-constrained public healthcare settings, suggesting that pharmacokinetically guided dose adjustment can preserve clinical effectiveness without compromising safety. This approach reflects real-world adaptation to health system constraints while remaining consistent with established dose-reduction recommendations.

**Copyright Information:** (Online) ISSN: 2572-9241
©2026 The Author(s). HemaSphere is published by John Wiley & Sons Ltd on behalf of European Hematology Association. This is an open access Abstract Book distributed under the Attribution-NonCommercial-NoDerivs (CC BY-NC-ND), which allows third parties to download the articles and share them with others as long as they credit the author and the Abstract Book, but they cannot change the content in any way or use them commercially.
**Abstract Book Citations:** Authors, Title, HemaSphere, 2026;10:(S1):pages. The abstract book DOIs can be found on the “Author Information” page.
**Disclaimer:** Articles published in the journal HemaSphere exclusively reflect the opinions of the authors. The authors are responsible for all content in their abstracts including accuracy of the facts, statements, citing resources, etc.

### (PB2922) A TIME-LIMITED STRATEGY COMBINING ORELABRUTINIB WITH SHORT-COURSE BENDAMUSTINE-RITUXIMAB AS FIRST-LINE THERAPY FOR CHRONIC LYMPHOCYTIC LEUKEMIA: A PROSPECTIVE MULTICENTER STUDY

**Topic:** 06. Chronic lymphocytic leukemia and related disorders - Clinical

Linlin Cao*[1], Kaiyang Ding[1], Hao Song[1], Cuicui Wang[1], Qiang Xu[1], Chenyang Zhou[1], Maogui Hu[1], Jing Ming[1], Guolin Wu[1], Dandan Fan[1], Yuanyuan Feng[1], Xinchen Wang[1], Youzhi Wu[2], Long Zhong[2], Meiqi Ding[3]

*[1]Anhui Provincial Cancer Hospital, Hefei, Anhui, China;[2]Anqing Municipal Hospital, Anqing, Anhui, China;[3]The People's Hospital of Lu'an City, Lu'an, Anhui, China;*

### Background

While combination therapy with BTK inhibitors (BTKi) and 6 cycles of FCR (fludarabine, cyclophosphamide, rituximab) yields high rates of undetected minimal residual disease (uMRD) and durable treatment-free remission, its utility is limited by significant hematologic toxicity, infection risks, and secondary malignancies. The bendamustine-rituximab (BR) regimen offers a more favorable safety profile. Orelabrutinib, a highly selective covalent BTKi, demonstrates potent efficacy with low incidence of off-target toxicities. We hypothesized that combining orelabrutinib with short-course BR would deepen therapeutic responses while maintaining a favorable safety profile, and enable a time-limited therapeutic strategy. Here, we present interim results from the prospective, multicenter study evaluating this approach in treatment-naïve chronic lymphocytic leukemia (CLL) patients.

### Aims

We present interim results from the prospective, multicenter study evaluating this approach in treatment-naïve chronic lymphocytic leukemia (CLL) patients.

### Methods

This study enrolled previously untreated CLL patients characterized by age ≥ 65 years, CrCl 30-69 mL/min, or a CIRS score > 6. Patients received induction therapy consisting of one cycle of BR followed by three cycles of orelabrutinib plus BR (OBR). Post-induction management allowed patients to proceed to maintenance orelabrutinib (up to a total of 18 cycles) or enter observation based on investigator judgment and patient preference. The primary endpoint was the rate of complete response with uMRD (CR & uMRD). Secondary endpoints included overall response rate (ORR), CR rate, uMRD rate, progression-free survival (PFS), overall survival (OS), and safety. MRD was assessed in peripheral blood (sensitivity threshold $<10^{-4}$).

### Results

As of February 6, 2026, 10 patients (pts) were enrolled, with a median age of 62 years (range: 47-76). Disease staging included Binet stage B (n=4) and C (n=4), and Rai stages I–IV (n=2, 1, 3, 2, respectively). Among 8 evaluable patients, 3 harbored TP53 abnormalities, and 2 exhibited unmutated IGHV status. At a median follow-up of 16.8 months (range: 13.3–20.7) for the 5 efficacy-evaluable patients (**Figure 1**), the ORR was 80% (3 CR, 2 PR). Notably, deep remissions were observed in high-risk subgroups, with 2 of 3 patients with TP53 abnormalities achieving CR & uMRD. Dynamic MRD assessment showed uMRD rates of 40% (2/5) at the end of Cycle 3, increasing to 80% (4/5) by Cycle 9. The composite primary endpoint (CR & uMRD) was achieved in 60% (3/5) of evaluable patients. All enrolled patients remain on study treatment. In the safety-evaluable population (n=9; median follow-up: 13.3 months), grade ≥3 hematologic adverse events (AEs) included lymphopenia (n=4), anemia (n=3), and neutropenia (n=2). All grade ≥3 hematologic AEs resolved with standard supportive care. No treatment-related deaths occurred.

**Copyright Information:** (Online) ISSN: 2572-9241
©2026 The Author(s). HemaSphere is published by John Wiley & Sons Ltd on behalf of European Hematology Association. This is an open access Abstract Book distributed under the Attribution-NonCommercial-NoDerivs (CC BY-NC-ND), which allows third parties to download the articles and share them with others as long as they credit the author and the Abstract Book, but they cannot change the content in any way or use them commercially.
**Abstract Book Citations:** Authors, Title, HemaSphere, 2026;10:(S1):pages. The abstract book DOIs can be found on the “Author Information” page.
**Disclaimer:** Articles published in the journal HemaSphere exclusively reflect the opinions of the authors. The authors are responsible for all content in their abstracts including accuracy of the facts, statements, citing resources, etc.

### Summary/Conclusion

The combination of orelabrutinib and short-course BR induces deep and durable clinical responses in treatment-naïve CLL patients, achieving high rates of CR (60%) and uMRD (80%), even in patients with TP53 abnormalities. The regimen exhibits a manageable safety profile with no unexpected toxicities. These data support the potential of this time-limited strategy to facilitate prolonged treatment-free intervals.

**Figure 1. Response of efficacy-evaluable patients**

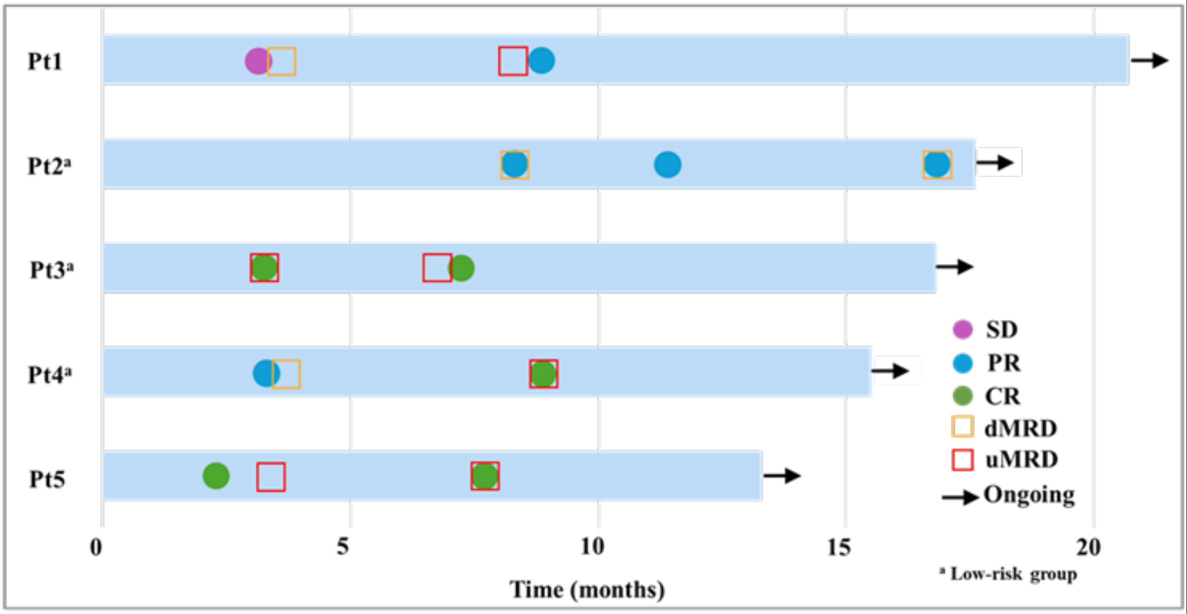

**Copyright Information:** (Online) ISSN: 2572-9241
©2026 The Author(s). HemaSphere is published by John Wiley & Sons Ltd on behalf of European Hematology Association. This is an open access Abstract Book distributed under the Attribution-NonCommercial-NoDerivs (CC BY-NC-ND), which allows third parties to download the articles and share them with others as long as they credit the author and the Abstract Book, but they cannot change the content in any way or use them commercially.
**Abstract Book Citations:** Authors, Title, HemaSphere, 2026;10:(S1):pages. The abstract book DOIs can be found on the "Author Information" page.
**Disclaimer:** Articles published in the journal HemaSphere exclusively reflect the opinions of the authors. The authors are responsible for all content in their abstracts including accuracy of the facts, statements, citing resources, etc.

### (PB2923) DISCRETE CHOICE EXPERIMENT FOR TREATMENT PREFERENCES IN CHRONIC LYMPHOCYTIC LEUKEMIA

**Topic:** 06. Chronic lymphocytic leukemia and related disorders - Clinical

Alex Cornelisse*[1], Pythia Nieuwkerk[2], Sabina Kersting[1]

*[1]Haga Ziekenhuis, Den Haag, The Netherlands; [2]Amsterdam UMC, Amsterdam, The Netherlands;*

### Background

In recent years various new treatment strategies have been developed for the treatment of chronic lymphocytic leukemia (CLL). Given the differences between these novel approaches—such as treatment duration, route of administration, and adverse event profiles—gaining insight into patient treatment preferences has become increasingly important. Only few studies have explored patient preferences in treatment of CLL. To address this, we conducted a discrete choice experiment (DCE) to assess which treatment attributes are most valued by patients.

### Aims

The aim of this study is to is to assess treatment preferences among patients with chronic lymphocytic leukemia using a discrete choice experiment.

### Methods

In this study a mixed-method approach was used for the selection of attributes and their corresponding levels. For the DCE questionnaire six attributes were selected: administration route, treatment duration, adverse events, long term effects, time to next treatment and possible subsequent therapies after relapse. Participants could take part through an anonymous online survey. Patients, caregivers and hematologists could take part. An orthogonal design and a mixed logit panel data model were used for statistical analysis.

### Results

396 patients filled in the DCE questionnaire. The most important attributes in the choice for making treatment choices were time to next treatment (efficacy), long term effects and administration route. For older patients (>76 years) administration route was more important than for younger patients. Treatment status did not alter the treatment preferences. Patients preferred an oral treatment method over IV administration. Regarding long term effects patients chose to avoid risk of secondary cancer over the risk of cardiac arrythmias or unknown long-term effects. Patients were willing to trade 3.9 years of time to next treatment to avoid the risk of secondary cancer.

### Summary/Conclusion

This study identified patient preferences for Dutch CLL patients. The most important aspect of treatment is the efficacy, however patients also highly value long term effects and administration route. The results of this study can be used by caregivers in discussing treatment options with CLL patients and for future studies.

**Copyright Information:** (Online) ISSN: 2572-9241
©2026 The Author(s). HemaSphere is published by John Wiley & Sons Ltd on behalf of European Hematology Association. This is an open access Abstract Book distributed under the Attribution-NonCommercial-NoDerivs (CC BY-NC-ND), which allows third parties to download the articles and share them with others as long as they credit the author and the Abstract Book, but they cannot change the content in any way or use them commercially.
**Abstract Book Citations:** Authors, Title, HemaSphere, 2026;10:(S1):pages. The abstract book DOIs can be found on the “Author Information” page.
**Disclaimer:** Articles published in the journal HemaSphere exclusively reflect the opinions of the authors. The authors are responsible for all content in their abstracts including accuracy of the facts, statements, citing resources, etc.

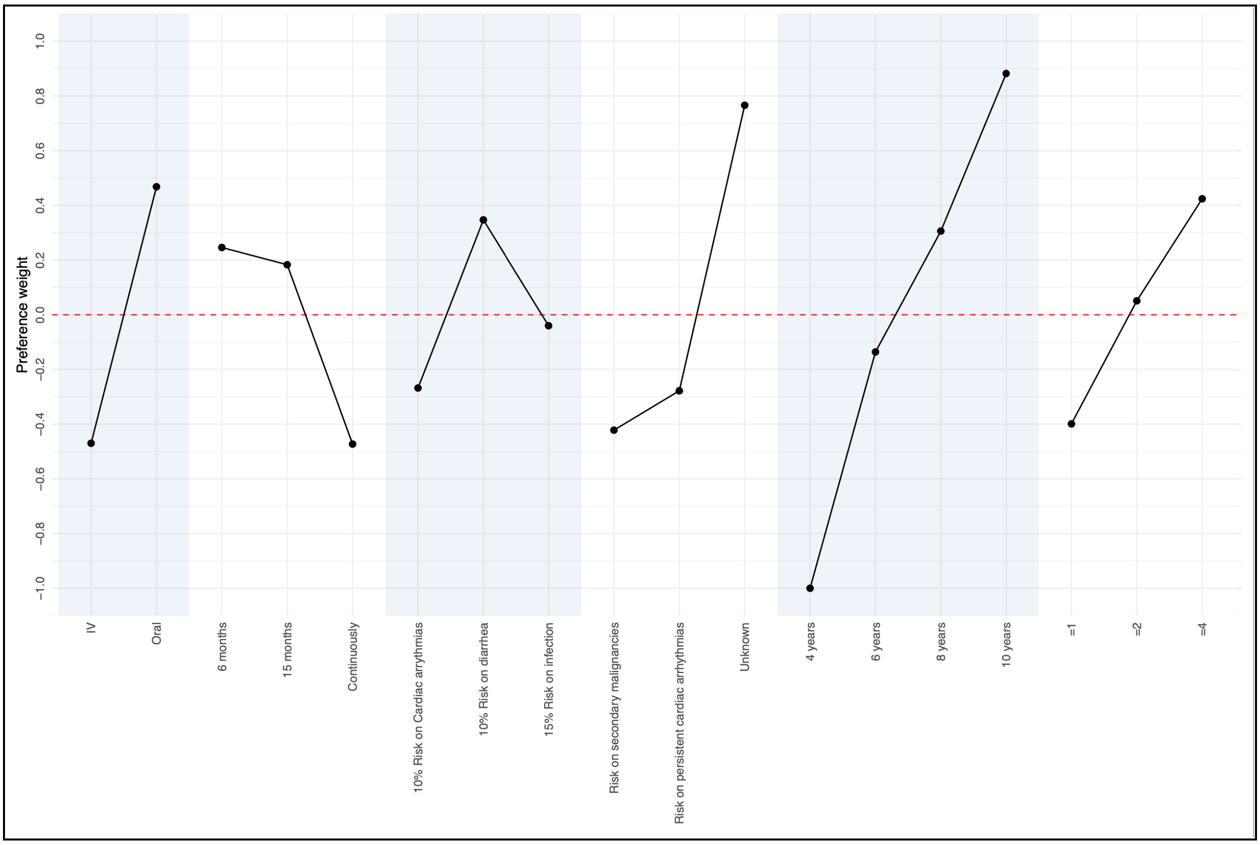

Figure 1 shows the preference weight for the different levels within each attribute

**Copyright Information:** (Online) ISSN: 2572-9241
©2026 The Author(s). HemaSphere is published by John Wiley & Sons Ltd on behalf of European Hematology Association. This is an open access Abstract Book distributed under the Attribution-NonCommercial-NoDerivs (CC BY-NC-ND), which allows third parties to download the articles and share them with others as long as they credit the author and the Abstract Book, but they cannot change the content in any way or use them commercially.
**Abstract Book Citations:** Authors, Title, HemaSphere, 2026;10:(S1):pages. The abstract book DOIs can be found on the “Author Information” page.
**Disclaimer:** Articles published in the journal HemaSphere exclusively reflect the opinions of the authors. The authors are responsible for all content in their abstracts including accuracy of the facts, statements, citing resources, etc.

### (PB2924) INCIDENCE OF SECOND PRIMARY MALIGNANCIES IN CHRONIC LYMPHOCYTIC LEUKEMIA

**Topic:** 06. Chronic lymphocytic leukemia and related disorders - Clinical

Regina Zsigmond*[1], Előd Zsigmond[2], Zita Borbényi[1], Adrienn Sulák[1], Blanka Godza[1], Tímea Gurbity Pálfi[1]

*[1]Centre of Hematology, University of Szeged, Szeged, Hungary; [2]North-Pest Central Hospital-Honvéd Hospital, Department of Cardiology, Budapest, Hungary;*

**Background**

Chronic lymphocytic leukemia (CLL) involves a broad spectrum of genetic mutations and is treated with multiple therapeutic approaches. This disorder may be associated with second primary malignancies (SPM), including solid tumors (ST) and other hematologic neoplasms (HN), potentially due to therapy side effect and genetic predisposition.

**Aims**

This study aimed to evaluate the incidence and long-term prognostic value of SPM in CLL patients.

**Methods**

We conducted a retrospective analysis at the Hematology Department of the University of Szeged including consecutive CLL patients from January 2012 to December 2025. The study assessed the prevalence of SPM, the influence of therapy and genetic background on SPM development, and the long-term prognostic significance of SPM.

**Results**

CLL was confirmed in 491 patients and therapy was initiated in 358 cases, 296 were male, the average age at diagnosis was 67±10 years. SPM was diagnosed in 146 (29.7%) cases, of which 123 (25%) were ST and 16 (3.2%) were HN.
The following therapies were administered: Rituximab, Cyclophosphamide, Vincristine, Prednisolon (R-CVP) in 70 (14.2%) patients, Rituximab, Fludarabine, Cytoxan (R-FC) in 66 (13.4%), Rituximab, Bendamustine in 79 (16%), Rituximab, Chlorambucil in 132 (26.8%), Venetoclax, Obinutuzumab in 31 (6.3%), Chlorambucil, Obinutuzumab in 14 (2.8%), Cyclophosphamide, Obinutuzumab in 2 (0.4%), Obinutuzumab in 4 (0.8%), Bruton tyrosine kinase-inhibitor (BTK-inhibitor) in 127 (25.8%) patients. The use of chemotherapy was not found to be a significant risk factor (OR 1.19, 95% CI 0.80-1.78, p=0.388) for the development of SPM, while significantly fewer SPM occurred with obinutuzumab containing therapy (OR 0.42, 95%CI: 0.19-0.92, p=0.029).
In patients with known IGHV status (52 mutated, 92 unmutated) and TP53 mutation status (13 positive, 122 negative) did not influenced the SPM developement (OR 0.77, 95% CI 0.38-1.57, p=0.479 and OR 1.74, 95% CI 0.72-4.21, p=0.221, respectively).
The mean follow-up time was 7±4.9 years, during this period 273 (56%) patients died. The presence of SPM did not influenced the long-term survival in CLL patients (aHR 0.91, 95% CI 0.71-1.18, p=0.485).

**Summary/Conclusion**

In our cohort, 29.7% CLL patients were diagnosed with SPM, emphasizing the importance of regular screening. The quality of therapy and follow-up time may play a role in the development of SPM. Interestingly, SPM did not influence survival outcomes in the studied population.

**Copyright Information:** (Online) ISSN: 2572-9241
©2026 The Author(s). HemaSphere is published by John Wiley & Sons Ltd on behalf of European Hematology Association. This is an open access Abstract Book distributed under the Attribution-NonCommercial-NoDerivs (CC BY-NC-ND), which allows third parties to download the articles and share them with others as long as they credit the author and the Abstract Book, but they cannot change the content in any way or use them commercially.
**Abstract Book Citations:** Authors, Title, HemaSphere, 2026;10:(S1):pages. The abstract book DOIs can be found on the "Author Information" page.
**Disclaimer:** Articles published in the journal HemaSphere exclusively reflect the opinions of the authors. The authors are responsible for all content in their abstracts including accuracy of the facts, statements, citing resources, etc.

### (PB2925) BGB-16673 VERSUS IDELALISIB + RITUXIMAB (R), BENDAMUSTINE + R, OR VENETOCLAX + R RE-TREATMENT IN PATIENTS WITH CHRONIC LYMPHOCYTIC LEUKEMIA/SMALL LYMPHOCYTIC LYMPHOMA: THE PHASE 3 CADANCE-302 TRIAL

**Topic:** 06. Chronic lymphocytic leukemia and related disorders - Clinical

Paolo Ghia*[1, 2], Constantine S. Tam[3], John F. Seymour[4], Jiayi Shen[5], Jan Michel[6], Maryam Sarraf Yazdy[5], Patrick Brown[5], Shelonitda Rose[5], Motohisa Takai[5], Meghan C. Thompson[7], Mark-David Levin[8], Talha Munir[9], Stephan Stilgenbauer[10]

*[1]Università Vita-Salute San Raffaele, Milano, Italy; [2]Comprehensive Cancer Center, IRCCS Ospedale San Raffaele, Milano, Italy; [3]Alfred Hospital and Monash University, Melbourne, VIC, Australia; [4]Peter MacCallum Cancer Centre, Royal Melbourne Hospital, and University of Melbourne, Melbourne, VIC, Australia; [5]BeOne Medicines, Ltd, San Carlos, CA, United States of America; [6]BeOne Medicines, Ltd, Basel, Switzerland; [7]Memorial Sloan Kettering Cancer Center, New York, NY, United States of America; [8]Albert Schweitzer Hospital, Dordrecht, The Netherlands; [9]Leeds Teaching Hospitals NHS Trust, Leeds, United Kingdom; [10]Ulm University, Ulm, Germany;*

### Background

Novel therapies, including Bruton tyrosine kinase inhibitors (BTKis) and B-cell lymphoma 2 inhibitors (BCL2is), have meaningfully improved patient outcomes in chronic lymphocytic leukemia/small lymphocytic lymphoma (CLL/SLL). However, CLL/SLL remains incurable, and most patients will experience disease relapse following a BTKi or BCL2i. BGB-16673 is a BTK degrader that blocks signaling by tagging BTK for degradation through the cell's proteasome pathway, leading to tumor regression. Data from CaDAnCe-101 (BGB-16673-101, NCT05006716), an ongoing phase 1/2 study, demonstrate that BGB-16673 has a tolerable safety profile and can achieve responses in heavily pretreated patients with relapsed/refractory (R/R) CLL/SLL, including those with prior BTKi treatment and BTK resistance mutations. Here, CaDAnCe-302, an ongoing phase 3 study that will evaluate the efficacy and safety of BGB-16673 monotherapy vs investigator’s choice (IC; idelalisib + rituximab [R], bendamustine + R, or venetoclax + R re-treatment) in patients with CLL/SLL previously exposed to BTK and BCL2 inhibitors, is described.

**Copyright Information:** (Online) ISSN: 2572-9241
©2026 The Author(s). HemaSphere is published by John Wiley & Sons Ltd on behalf of European Hematology Association. This is an open access Abstract Book distributed under the Attribution-NonCommercial-NoDerivs (CC BY-NC-ND), which allows third parties to download the articles and share them with others as long as they credit the author and the Abstract Book, but they cannot change the content in any way or use them commercially.
**Abstract Book Citations:** Authors, Title, HemaSphere, 2026;10:(S1):pages. The abstract book DOIs can be found on the “Author Information” page.
**Disclaimer:** Articles published in the journal HemaSphere exclusively reflect the opinions of the authors. The authors are responsible for all content in their abstracts including accuracy of the facts, statements, citing resources, etc.

**Methods**

CaDAnCe-302 (BGB-16673-302, NCT06846671) is a global, open-label, randomized, phase 3 clinical study (Figure) conducted across approximately 137 sites in 14 countries. Eligible patients are ≥18 years of age with a confirmed diagnosis of CLL/SLL that has relapsed after treatment with a covalent BTKi and a BCL2i or is refractory to these treatments. Prior treatment with a noncovalent BTKi is also required in regions where they are approved and available. Patients are excluded if they have prolymphocytic leukemia or history of, or currently suspected, Richter transformation, have received an autologous stem cell transplant or chimeric antigen receptor T-cell therapy in the last 3 months, or had prior exposure to any BTK protein degraders. Approximately 250 patients are planned to be enrolled and randomized 3:2 to receive either BGB-16673 once daily (arm A) or IC of idelalisib + R (for CLL only), bendamustine + R [for CLL/SLL without del(17p) or *TP53* mutation], or venetoclax + R re-treatment (arm B) until disease progression or intolerance. For patients to be considered for venetoclax + R re-treatment, the best overall response of the last BCL2i-based regimen should be partial response or better, with the last BCL2i dose being ≥1 year prior to the most recent disease progression, and the patient should have been able to tolerate previous BCL2i treatment. Patients in arm B will be given the option to cross over to BGB-16673 treatment after confirmation of progressive disease by both the investigator and independent review committee (IRC). The primary endpoint is progression-free survival (PFS), as determined by IRC per modified 2018 International Workshop on CLL criteria (for CLL) or Lugano classification (for SLL). Overall survival is the key secondary endpoint; additional secondary endpoints include PFS in patients with prior exposure to noncovalent BTKis by IRC, PFS by investigator, overall response rate by IRC and investigator, rate of partial response with lymphocytosis or higher by IRC and investigator, duration of response by IRC and investigator, time to next treatment, and safety/tolerability per National Cancer Institute Common Terminology Criteria for Adverse Events v5.0. Recruitment is ongoing. © American Society of Hematology (2026). Reused with permission.

**Results**

N/A

**Summary/Conclusion**

N/A

**Copyright Information:** (Online) ISSN: 2572-9241
©2026 The Author(s). HemaSphere is published by John Wiley & Sons Ltd on behalf of European Hematology Association. This is an open access Abstract Book distributed under the Attribution-NonCommercial-NoDerivs (CC BY-NC-ND), which allows third parties to download the articles and share them with others as long as they credit the author and the Abstract Book, but they cannot change the content in any way or use them commercially.
**Abstract Book Citations:** Authors, Title, HemaSphere, 2026;10:(S1):pages. The abstract book DOIs can be found on the "Author Information" page.
**Disclaimer:** Articles published in the journal HemaSphere exclusively reflect the opinions of the authors. The authors are responsible for all content in their abstracts including accuracy of the facts, statements, citing resources, etc.

**Figure. CaDAnCe-302 Study Design**

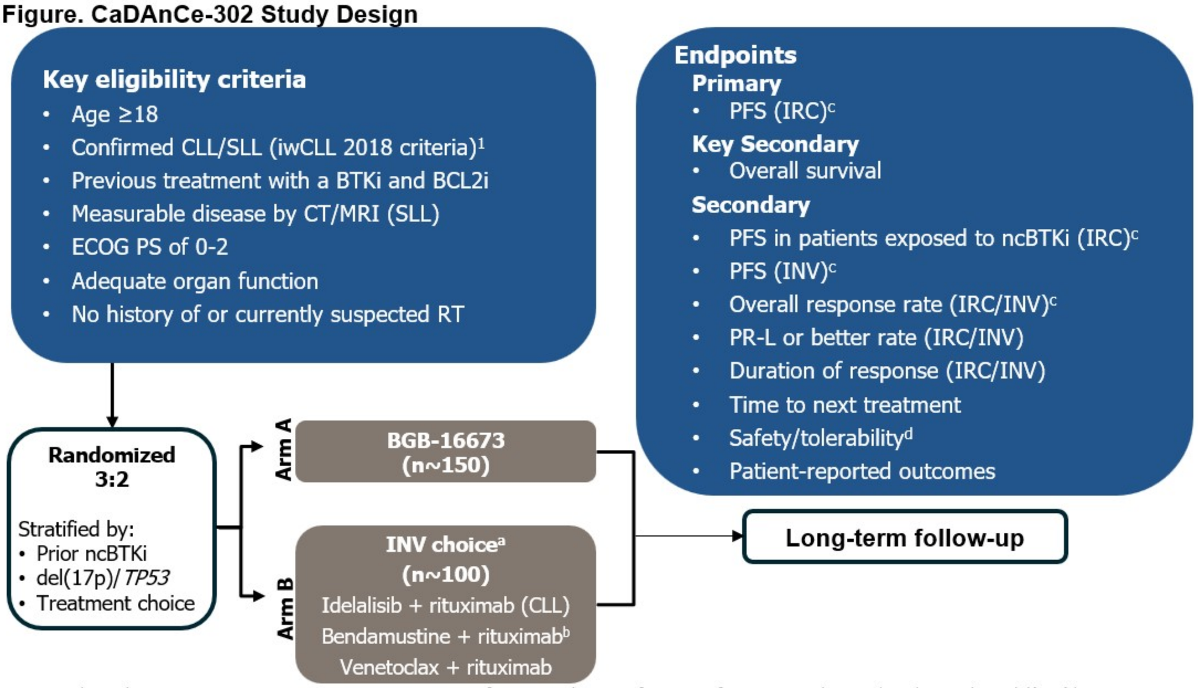

[a]Patients have the option to crossover to BGB-16673 treatment after INV and IRC confirmation of progressive disease. [b]CLL/SLL without del(17p)/*TP53* mutation. [c]Per modified 2018 iwCLL criteria[1] for CLL and Lugano classification[2] for SLL. [d]Per National Cancer Institute Common Terminology Criteria for Adverse Events v5.0.

**Abbreviations:** BCL2i, B-cell lymphoma-2 inhibitor; BTK, Bruton tyrosine kinase; BTK, Bruton tyrosine kinase inhibitor; CLL/SLL, chronic lymphocytic leukemia/small lymphocytic lymphoma; CT, computed tomography; ECOG PS, Eastern Cooperative Oncology Group performance status; INV, investigator; IRC, independent review committee; iwCLL, International Workshop on Chronic Lymphocytic Leukemia; MRI, magnetic resonance imaging; ncBTKi, noncovalent BTK inhibitor; PFS, progression-free survival; PR-L, partial response with lymphocytosis; R/R, relapsed/refractory; RT, Richter transformation.

1. Hallek M, et al. *Blood.* 2018;131(25):2745-60. 2. Cheson BD, et al. *J Clin Oncol.* 2014;32(27):3059-68.

**Copyright Information:** (Online) ISSN: 2572-9241
©2026 The Author(s). HemaSphere is published by John Wiley & Sons Ltd on behalf of European Hematology Association. This is an open access Abstract Book distributed under the Attribution-NonCommercial-NoDerivs (CC BY-NC-ND), which allows third parties to download the articles and share them with others as long as they credit the author and the Abstract Book, but they cannot change the content in any way or use them commercially.
**Abstract Book Citations:** Authors, Title, HemaSphere, 2026;10:(S1):pages. The abstract book DOIs can be found on the "Author Information" page.
**Disclaimer:** Articles published in the journal HemaSphere exclusively reflect the opinions of the authors. The authors are responsible for all content in their abstracts including accuracy of the facts, statements, citing resources, etc.

### (PB2926) BEYOND CLINICAL TRIALS: CARDIOVASCULAR RISK FACTORS AND ADVERSE EVENTS IN PATIENTS WITH CLL TREATED WITH BTK INHIBITORS IN A MULTICENTRE REAL-LIFE ITALIAN COHORT

**Topic:** 06. Chronic lymphocytic leukemia and related disorders - Clinical

Andrea Serafin*[1], Arianna Bevilacqua[1], Serena De Santo[1], Raffaella Pasquale[2], Massimo Moratti[2], Francesco Angotzi[1], Alessandro Cellini[1], Martina De Ferrari[1], Nicolò Danesin[1], Giovanni Leone[1], Francesca Martini[3], Elisa Albi[3], Emanuela Sant'antonio[3], Lydia Scarfò[3, 4], Paolo Ghia[3, 4], Gian Matteo Rigolin[5], Antonio Cuneo[5], Francesco Piazza[1], Livio Trentin[1], Andrea Visentin[1]

*[1]Hematology Unit, University of Padua, Department of Medicine, Padua, Italy;[2]Hematology Unit, Azienda Sanitaria Universitaria Friuli Centrale (ASUFC), Udine, Italy; [3]Strategic Research Program on CLL, Comprehensive Cancer Center, IRCCS Ospedale San Raffaele, Milan, Italy; [4]Medical School, Università Vita-Salute San Raffaele, Milan, Italy; [5]Division of Hematology, University of Ferrara, Ferrara, Italy;*

### Background

Bruton tyrosine kinase inhibitors (BTKi) are a cornerstone of treatment in chronic lymphocytic leukemia (CLL), delivered as continuous or fixed-duration strategies (ibrutinib+venetoclax, I+V). Second-generation BTKi (acalabrutinib, zanubrutinib, 2gBTKi) appear to have a more favourable cardiovascular (CV) profile than I, yet real-life multicentre data on risk factors and event timing remain limited.

### Aims

To compare the incidence of atrial fibrillation (AF), hypertension (HTN), congestive heart failure (CHF) and bleeding in patients treated with I-based therapy and 2gBTKi as well as the impact of starting at full vs lower doses.

### Methods

We retrospectively analysed 382 patients (pts) with CLL treated with I based regimens or 2gBTKi across three Italian centres. Baseline CV risk factors (HTN, AF, other arrythmias, valvular diseases, smoking, dyslipidemia, diabetes, antiplatelet and anticoagulant therapy) and echocardiographic features (e.g. atrial dilatation, left ventricular remodelling), treatment characteristics, and dose modifications were recorded. Cumulative incidence of CV events was evaluated using competing-risk analysis (Fine Gray models). Overall survival (OS) and progression-free survival (PFS) were assessed using Kaplan-Meier estimates and multivariable Cox models.

**Copyright Information:** (Online) ISSN: 2572-9241
©2026 The Author(s). HemaSphere is published by John Wiley & Sons Ltd on behalf of European Hematology Association. This is an open access Abstract Book distributed under the Attribution-NonCommercial-NoDerivs (CC BY-NC-ND), which allows third parties to download the articles and share them with others as long as they credit the author and the Abstract Book, but they cannot change the content in any way or use them commercially.
**Abstract Book Citations:** Authors, Title, HemaSphere, 2026;10:(S1):pages. The abstract book DOIs can be found on the “Author Information” page.
**Disclaimer:** Articles published in the journal HemaSphere exclusively reflect the opinions of the authors. The authors are responsible for all content in their abstracts including accuracy of the facts, statements, citing resources, etc.

**Results**

Baseline data are summarized in table 1. Median follow-up was 32 months (mo) (range 1-118). At 30 mo, OS/PFS were 84.2%/75.4% for I, 86.7%/72.9% for 2gBTKi, and 94.1%/85% for I+V. In survival analyses, age >80 yr (OS: HR 1.77, p=0.038; PFS: HR 1.22, p<0.001) and starting with a lower dose of drug (OS: HR 2.21, p=0.002; PFS: HR 1.66, p=0.046) were independent predictors. In addition, complex karyotype impacted on PFS (HR 1.30, p=0.03).
CV adverse events are detailed in table 1. Two CV grade 5 events occurred (1 major bleeding with I, 1 Ventricular Arrythmia with I+V). Median time-to-event differed: bleeding was an early event (99 days, IQR 43–341), AF and HTN developed later (median 12.0 mo, IQR 4.5–22.5, and 14.5 mo, IQR 9.0–26.8) and CHF after a median of 11.0 mo (IQR 5.0–33.0). In multivariable analysis, AF was independently associated with older age (p=0.003), baseline AF (HR 3.68, p<0.001), smoking (HR 2.66, p=0.008), and left atrial dilatation (HR 2.0, p=0.034), while 2gBTKi reduced AF risk (HR 0.26, p<0.001). HTN was associated with left ventricular (LV) remodelling (HR 2.3, p=0.001) and was less frequent with 2gBTKi (HR 0.54, p=0.045). CHF correlated with baseline other arrhythmias at baseline (HR 4.81, p=0.019) and tobacco smoking (HR 5.44, p=0.004). Baseline antiplatelet or anticoagulant therapy did not increase bleeding risk. During the follow-up, 16% (n=61) of pts discontinued treatment (n=10 due to CV events) and 13% (n=50) reduced drug dosage (n=11 due to CV events).

**Summary/Conclusion**

2gBTKi showed a safer CV profile including fewer rates of HTN and AF. Starting treatment at a reduced dose was associated with inferior OS and PFS, likely reflecting a significantly fragile population rather than reduced efficacy, whereas dose reduction during therapy did not adversely impact survival. Baseline clinical and echocardiographic features (age, smoking, atrial dilatation, LV remodelling, and pre-existing arrhythmias), identified pts at increased CV risk. These findings support baseline CV assessment and tailored monitoring strategies, including the use of proactive dose modifications, rather than therapeutic exclusion, in routine clinical practice.

**Copyright Information:** (Online) ISSN: 2572-9241
©2026 The Author(s). HemaSphere is published by John Wiley & Sons Ltd on behalf of European Hematology Association. This is an open access Abstract Book distributed under the Attribution-NonCommercial-NoDerivs (CC BY-NC-ND), which allows third parties to download the articles and share them with others as long as they credit the author and the Abstract Book, but they cannot change the content in any way or use them commercially.
**Abstract Book Citations:** Authors, Title, HemaSphere, 2026;10:(S1):pages. The abstract book DOIs can be found on the “Author Information” page.
**Disclaimer:** Articles published in the journal HemaSphere exclusively reflect the opinions of the authors. The authors are responsible for all content in their abstracts including accuracy of the facts, statements, citing resources, etc.

| Characteristics | N (%) N=382 |
|---|---|
| **Therapy** | |
| Ibrutinib monotherapy | 166 (43.4) |
| Ibrutinib + venetoclax | 56 (14.6) |
| Second generation BTKi | 160 (42) |
| - Acalabrutinib | 79 (20.7) |
| - Zanubrutinib | 81 (21.2) |
| Age | 71 years (range 39-93) |
| Sex F/M | 143 (37)/239 (63) |
| Treatment naïve | 240 (63) |
| Relapsed/refractory | 142 (37) |
| Low dose of drug at baseline | 62 (16) |
| - Ibrutinib | 36 (9.4) |
| - Second generation BTKi | 26 (6.6) |
| IGHV um | 248 (71) |
| Unknown | 34 (8.9) |
| TP53 mut | 114 (32) |
| Unknown | 22 (5.7) |
| Del(17p) | 81 (21) |
| Unknown | 3 (0.8) |
| Complex Karyotype | 83 (29) |
| Unknown | 95 (24.9) |
| Echocardiography available at baseline | 303 (80) |
| **Baseline CV comorbidities** | |
| - Hypertension | 187 (49) |
| - Atrial fibrillation | 34 (9) |
| - Other arrythmias | 21 (5.5) |
| - Valvular heart diseases | 52 (13.6) |
| - Tobacco smoking | 44 (12) |
| - Dyslipidaemia | 102 (27) |
| - Diabetes | 57 (15) |
| - Atrial dilatation | 117 (39) |
| - Left ventricular remodelling | 113 (37) |
| - Antiplatelets therapy | 53 (14) |
| - Anticoagulants | 47 (12) |
| **CV Adverse Events** | **N=382, I=166, I+V=56, 2gBTKi=160 N (%)** |
| **Bleeding** | 136 (35.5), G3-4 10 (2.7), G5 1 (0.3) |
| - Ibrutinib | 83 (50), G3-4 3 (1.8) |
| - I+V | 12 (21.4), G3-4 0 (0) |
| - 2gBTKi | 41 (25.6), G3-4 7 (4.4) |
| **Hypertension** | 70 (28.3), G3-4 15 (3.9) |
| - Ibrutinib | 50 (30.1), G3-4 10 (6) |
| - I+V | 7 (12.5), G3-4 2 (3.6) |
| - 2gBTKi | 13 (8.1), G3-4 3 (1.9) |
| **Atrial fibrillation** | 51 (13.4), G3-4 13 (3.4) |
| - Ibrutinib | 38 (22.9). G3-4 10 (6) |
| - I+V | 4 (7.1), G3-4 1 (1.8) |
| - 2gBTKi | 9 (5.6), G3-4 2 (1.3) |
| **Congestive Heart Failure** | 16 (4.2), G3-4 3 5 (1.3) |
| - Ibrutinib | 9 (5.4), G3-4 2 (1.2) |
| - I+V | 1 (0.3), G3-4 1 (1.8) |
| - 2gBTKi | 6 (3.8), G3-4 2 (1.3) |

Table 1. Baseline characteristics and cardiovascular adverse events of our cohort population.

Copyright Information: (Online) ISSN: 2572-9241
©2026 The Author(s). HemaSphere is published by John Wiley & Sons Ltd on behalf of European Hematology Association. This is an open access Abstract Book distributed under the Attribution-NonCommercial-NoDerivs (CC BY-NC-ND), which allows third parties to download the articles and share them with others as long as they credit the author and the Abstract Book, but they cannot change the content in any way or use them commercially.
Abstract Book Citations: Authors, Title, HemaSphere, 2026;10:(S1):pages. The abstract book DOIs can be found on the "Author Information" page.
Disclaimer: Articles published in the journal HemaSphere exclusively reflect the opinions of the authors. The authors are responsible for all content in their abstracts including accuracy of the facts, statements, citing resources, etc.

### (PB2927) IMPACT OF MEASURABLE RESIDUAL DISEASE IN HAIRY CELL LEUKEMIA: A SYSTEMATIC REVIEW AND META-ANALYSIS

**Topic:** 06. Chronic lymphocytic leukemia and related disorders - Clinical

Francesco Angotzi*[1], Arianna Bevilacqua[1], Alessandro Cellini[1], Nicolò Danesin[1], Andrea Serafin[1], Giovanni Leone[1], Anna Giordano[1], Federica Frezzato[1], Elisa Pagnin[1], Francesco Piazza[1], Livio Trentin[1], Andrea Visentin[1]

*[1]Hematology Unit, University of Padova, Department of Medicine (DIMED), Padova, Italy;*

### Background

Despite high response rates, hairy cell leukemia (HCL) remains incurable, with many patients eventually experiencing disease relapse and often progressively shorter response durations upon retreatment. Early identification of those at higher risk is therefore crucial. Measurable residual disease (MRD) may predict remission duration, but its prognostic value in HCL remains uncertain due to small cohorts, methodological heterogeneity, and the long follow-up needed to capture relapse. Moreover, higher level evidence for the association of MRD with response duration in HCL is currently missing.

### Aims

To assess the prognostic impact of MRD in HCL through a systematic review and meta-analysis, in order to provide clinicians with an up-to-date synthesis of available evidence and identify grey areas which will benefit from further research.

### Methods

The primary outcome of the analysis was relapse-free survival (RFS) in patients with detectable MRD (dMRD) vs undetectable MRD (uMRD) assessed by hazard ratio (HR). The literature search was conducted across 3 databases (PubMed, Scopus, Web of Science). Eligible articles needed enough information to conduct analysis for the primary outcome, MRD measured at the time of response assessment after any therapy and by any technique. Literature search, data extraction and synthesis were performed by two reviewers with conflicts resolved by a third. This random effects meta-analysis was prospectively registered in PROSPERO (CRD420251186626).

### Results

A total 837 studies were identified, and after removal of duplicates (n=306), 531 were assessed for eligibility. Nine studies met the inclusion criteria, 4 prospective trials and 5 retrospective cohort studies, for a total of 426 patients (n=166 dMRD; n=260 uMRD). Administered treatments were purine analogues (PAs) in 4 (44%), PAs + rituximab (R) in 1 (12%), moxetumomab pasudotox (MP) in 2 (22%), and R-Vemurafenib in 2 (22%). MRD was measured by multiparameter flow-cytometry (MFC) in 5 studies (55%), immunohistochemistry (IHC) in 2 (22%) and RTq-PCR for BRAF-V600E mutation in 2 (22%). One study investigated MRD after first-line treatment (12%), 4 after therapy for relapsed/refractory disease (R/R) (44%) and 4 in mixed populations of newly diagnosed and R/R patients (44%). dMRD at the time of response evaluation was associated with shorter RFS (HR: 4.71; 95% CI: 2.20–10.09; p <0.0001; $I^2$: 64.5%) (Figure). Estimated median RFS was 5.87 years for dMRD vs not reached for uMRD. In subgroup analysis the prognostic impact of MRD varied by treatment type (p=0.042), with higher HR observed in patients treated with R-Vemurafenib (HR: 28.2) and MP (HR: 8.86) than with PAs (HR: 2.71), and also based on whether it was evaluated after first-line therapy or in the R/R setting (p=0.023, HR: 2.7 and 12.4 respectively). There were instead no statistically significant differences in terms of prognostic impact depending on site (bone marrow, peripheral blood or both; p=0.94), or technique of MRD evaluation (IHC, MFC or RTq-PCR; p=0.08).

**Copyright Information:** (Online) ISSN: 2572-9241
©2026 The Author(s). HemaSphere is published by John Wiley & Sons Ltd on behalf of European Hematology Association. This is an open access Abstract Book distributed under the Attribution-NonCommercial-NoDerivs (CC BY-NC-ND), which allows third parties to download the articles and share them with others as long as they credit the author and the Abstract Book, but they cannot change the content in any way or use them commercially.
**Abstract Book Citations:** Authors, Title, HemaSphere, 2026;10:(S1):pages. The abstract book DOIs can be found on the “Author Information” page.
**Disclaimer:** Articles published in the journal HemaSphere exclusively reflect the opinions of the authors. The authors are responsible for all content in their abstracts including accuracy of the facts, statements, citing resources, etc.

**Summary/Conclusion**

We reported the first formal meta-analysis of MRD in HCL, confirming its association with RFS independently of the employed technique and site of evaluation. However, most of this prognostic impact was observed after therapy with novel agents (R-Vemurafenib or MP) and in the R/R setting. Further studies should be directed at confirming the prognostic value of MRD after first-line therapy and its correlation with overall survival.

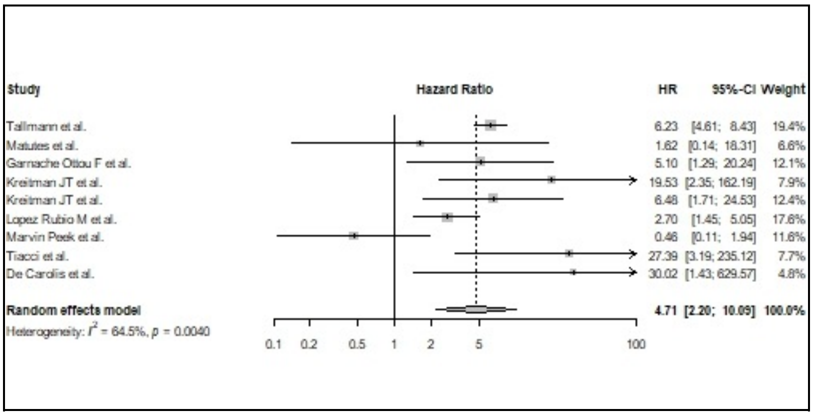

Forest-plot for the primary outcome analysis showing each study HR and the pooled HR for RFS based on MRD status

**Copyright Information:** (Online) ISSN: 2572-9241
©2026 The Author(s). HemaSphere is published by John Wiley & Sons Ltd on behalf of European Hematology Association. This is an open access Abstract Book distributed under the Attribution-NonCommercial-NoDerivs (CC BY-NC-ND), which allows third parties to download the articles and share them with others as long as they credit the author and the Abstract Book, but they cannot change the content in any way or use them commercially.
**Abstract Book Citations:** Authors, Title, HemaSphere, 2026;10:(S1):pages. The abstract book DOIs can be found on the "Author Information" page.
**Disclaimer:** Articles published in the journal HemaSphere exclusively reflect the opinions of the authors. The authors are responsible for all content in their abstracts including accuracy of the facts, statements, citing resources, etc.

### (PB2928) FINAL ANALYSIS OF AUTOIMMUNE CYTOPENIAS IN A SERIES OF PATIENTS AFFECTED BY CHRONIC LYMPHOCYTIC LEUKEMIA TREATED WITH ACALABRUTINIB

**Topic:** 06. Chronic lymphocytic leukemia and related disorders - Clinical

Andrea Ferrario*[1], Giulia Zamprogna[1], Benedetta Bianchi[1], Francesca Perutelli[2], Gloria Camagni[3], Lorenza Bertu[4], Idanna Innocenti[5], Valeria Oggianu[6], Alessandro Cellini[7], Michele Merli[8], Riccardo Moia[9], Francesca Martini[10], Andrea Galitzia[11], Elia Boccellato[12], Filomena De Falco[13], Marina Deodato[14], Enrica Antonia Martino[15], Massimo Gentile[15], Anna Maria Frustaci[14], Paolo Sportoletti[13], Lydia Scarfò[16], Gianluca Gaidano[9], Candida Vitale[2], Andrea Visentin[7], Roberta Murru[6], Luca Laurenti[5], Marta Coscia[3]

*[1]Division of Hematology, ASST Sette Laghi, Ospedale di Circolo, Varese, Italy; [2]Department of Molecular Biotechnology and Health Sciences, University of Torino & University Division of Hematology, A.O.U. Città della Salute e della Scienza, Torino, Italy; [3]Department of Medicine and Surgery, University of Insubria and Division of Hematology, ASST Sette Laghi, Ospedale di Circolo, Varese, Italy; [4]Department of Medicine and Surgery, University of Insubria, Varese, Italy; [5]Department of Scienze di Laboratorio ed Ematologiche, Area Ematologia, Fondazione Policlinico Universitario A. Gemelli, IRCCS, Roma, Italy; [6]Hematology and Stem Cell Transplantation Unit, Ospedale Oncologico A. Businco, ARNAS G. Brotzu, Cagliari, Italy; [7]Division of Hematology, Department of Medicine-DIMED, University of Padova, Padova, Italy; [8]Division of Hematology, Fondazione IRCCS Ca' Granda, Ospedale Maggiore Policlinico, Milano, Italy; [9]Division of Hematology, Department of Translational Medicine, Università del Piemonte Orientale, Novara, Italy; [10]Strategic Research Program on CLL, Comprehensive Cancer Center, IRCCS Ospedale San Raffaele, Milano, Italy; [11]Hematology and Stem Cell Transplantation Unit, Ospedale San Francesco, Nuoro, Italy; [12]Department of Hematology, Santa Croce e Carle Hospital, Cuneo, Italy; [13]Department of Medicine and Surgery, Institute of Hematology and Center for Hemato-Oncology Research (CREO), University of Perugia and Santa Maria della Misericordia Hospital, Perugia, Italy; [14]Department of Hematology, Niguarda Cancer Center, ASST Grande Ospedale Metropolitano Niguarda, Milano, Italy; [15]Department of Onco-hematology, Hematology Unit, Azienda Ospedaliera Annunziata, Cosenza, Italy; [16]Strategic Research Program on CLL, Comprehensive Cancer Center, IRCCS Ospedale San Raffaele; Università Vita Salute San Raffaele, Milano, Italy;*

### Background

Chronic lymphocytic leukemia (CLL) is the most common leukaemia in adults in Western countries, with an estimated incidence of 30%. Autoimmune cytopenias (AIC) in CLL involve about 5-9% of patients. The impact of acalabrutinib, a second-generation covalent BTK inhibitor, on both pre-existing and newly occurring AIC have not been comprehensively studied. Evidence is currently limited to case reports describing its use in immune thrombocytopenia (ITP) and pure red cell aplasia (PRCA).

### Aims

This study aimed to evaluate the clinical characteristics and outcomes of pre-existing AIC, as well as the incidence, presentation, clinical course and outcomes of treatment-emergent AIC, in patients with CLL receiving acalabrutinib monotherapy.

### Methods

We conducted a retrospective multicenter analysis including consecutive patients diagnosed with CLL and treated with acalabrutinib across 13 Italian centers.

**Copyright Information:** (Online) ISSN: 2572-9241
©2026 The Author(s). HemaSphere is published by John Wiley & Sons Ltd on behalf of European Hematology Association. This is an open access Abstract Book distributed under the Attribution-NonCommercial-NoDerivs (CC BY-NC-ND), which allows third parties to download the articles and share them with others as long as they credit the author and the Abstract Book, but they cannot change the content in any way or use them commercially.
**Abstract Book Citations:** Authors, Title, HemaSphere, 2026;10:(S1):pages. The abstract book DOIs can be found on the "Author Information" page.
**Disclaimer:** Articles published in the journal HemaSphere exclusively reflect the opinions of the authors. The authors are responsible for all content in their abstracts including accuracy of the facts, statements, citing resources, etc.

**Results**

The study cohort comprised 285 patients treated with acalabrutinib. Median age at treatment initiation was 73 years (range, 44–93), and 77% were treatment-naïve. Among evaluable patients, 71% (192/271) exhibited unmutated IGHV status, 13% (36/278) had TP53 mutations, and 14% (37/271) presents del(17p). In terms of response to treatment, the overall response rate (ORR) was 90%, including 14% complete responses (CR) and 76% partial responses (PR). The median follow-up was 23.5 months.
Pre-existing AIC were identified in 21 patients (7.4%), including AIHA in 52% (11/21), ITP in 43% (9/21), and PRCA in 5% (1/21). The great majority of patients with pre-existing AIC (95%) had received AIC-directed therapy, most commonly corticosteroids alone (35%) or in combination with rituximab (25%). At acalabrutinib initiation, AIC were active in 38% of patients (8/21), controlled in 38% (8/21), and resolved in 24% (5/21).
During treatment, 5 patients (1.8%) developed treatment-emergent AIC, including three cases of ITP and two of AIHA. Acalabrutinib was continued in 80% of these cases. Among patients with ITP, one required no specific therapy, one was managed with corticosteroids, and one required sequential treatment with corticosteroids, rituximab, and intravenous immunoglobulins, ultimately necessitating temporary acalabrutinib interruption due to inadequate control. Of the two AIHA cases, only one received corticosteroids while continuing acalabrutinib. Overall, treatment-emergent AIC were resolved or adequately controlled in 62% of cases.
Survival analysis showed no significant differences in progression-free survival or overall survival between patients with and without AIC, including both pre-existing and treatment-emergent events.

**Summary/Conclusion**

In this real-word multicenter retrospective study, pre-existing AIC were generally manageable in patients with CLL receiving acalabrutinib. Treatment-emergent AIC were uncommon and effectively treated. Importantly, the presence of AIC did not adversely affect survival outcomes in this population.

**Copyright Information:** (Online) ISSN: 2572-9241
©2026 The Author(s). HemaSphere is published by John Wiley & Sons Ltd on behalf of European Hematology Association. This is an open access Abstract Book distributed under the Attribution-NonCommercial-NoDerivs (CC BY-NC-ND), which allows third parties to download the articles and share them with others as long as they credit the author and the Abstract Book, but they cannot change the content in any way or use them commercially.
**Abstract Book Citations:** Authors, Title, HemaSphere, 2026;10:(S1):pages. The abstract book DOIs can be found on the “Author Information” page.
**Disclaimer:** Articles published in the journal HemaSphere exclusively reflect the opinions of the authors. The authors are responsible for all content in their abstracts including accuracy of the facts, statements, citing resources, etc.

### (PB2930) A RETROSPECTIVE ANALYSIS OF ORELABRUTINIB MONOTHERAPY AFTER OTHER BRUTON'S TYROSINE KINASE INHIBITORS EXPOSURE IN CHRONIC LYMPHOCYTIC LEUKEMIA/SMALL LYMPHOCYTIC LYMPHOMA

**Topic:** 06. Chronic lymphocytic leukemia and related disorders - Clinical

Kai Shen*[1], Jie Wang[1], Jinrong Yang[1], Chenlu Yang[1], He Li[1], Zhengyu Yu[1], Jiazhuo Liu[1], Yun Tang[1], Chuan He[1], Xiang Bing[1], Ting Niu[1]

[1]*West China Hospital, Sichuan University, Department of Hematology, Institute of Hematology, Chengdu, China;*

**Background**

Chronic lymphocytic leukemia/small lymphocytic lymphoma (CLL/SLL) is one of the most common B-cell hematological malignancies. With advances in targeted therapy, bruton's tyrosine kinase inhibitor (BTKi) has become the standard of care for CLL/SLL. However, off-target effects and clinically significant adverse events (AEs) limit their clinical application. Orelabrutinib is a novel and potent BTKi with higher selectivity and fewer off-target effects than other BTKis. It has been approved by the National Medical Products Administration in China for the treatment of patients with relapsed or refractory CLL/SLL. However, real-world evidence of orelabrutinib for patients with CLL/SLL remains limited, especially for those previously treated with BTKi.

**Aims**

To evaluate the clinical outcomes and safety of orelabrutinib monotherapy in CLL/SLL switched from previous BTKi.

**Methods**

This retrospective study included 22 patients with CLL/SLL who switched to orelabrutinib after other BTKis therapy between January 1, 2022 and November 1, 2025. Data on demographics and characteristics, treatment, outcomes, and safety were collected and analyzed.

**Results**

Among 22 patients, 21 had CLL and 1 had SLL. The median age was 60.5 years (range, 30.0-84.0), and 50.0% (11/22) were male. All (100.0%) patients had an Eastern Cooperative Oncology Group performance status of 0-1 and bone marrow involvement. According to CLL-IPI, 85.7% (18/21) of CLL patients were low-risk. No patients with confirmed *TP53* mutation, and 3 (13.6%) patients carried an *IGHV* unmutated status. Five (22.7%) patients presented splenomegaly. In the overall patients, 3 (13.6%) patients had a complete response (CR), 18 (81.8%) had a partial response (PR), and 1 (4.5%) had stable disease (SD) as the best response, resulting in an objective response rate (ORR) of 95.5% (95% CI, 77.2-99.9) and a disease control rate (DCR) of 100.0% (95% CI, 84.6-100.0) (Figure 1). As of February 11, 2026, the median follow-up was 25.8 months (range 0.8-48.7). The 36-month PFS and OS rates were 93.8% (95% CI, 82.6-100.0) and 100% (95% CI, 100.0-100.0), respectively. Subgroup analyses showed that among patients previously treated with ibrutinib (12), 1 (8.3%) had CR and 11 (91.7%) PR, with both ORR and DCR of 100.0% (95% CI, 73.5-100.0). In patients previously treated with zanubrutinib (n=6), 5 (83.3%) had PR and 1 (16.7%) SD, with an ORR of 83.3% (95% CI, 35.9-99.6) and a DCR of 100.0% (95% CI, 54.1-100.0). In patients previously exposed to both agents (n=4), both ORR and DCR were 100.0% (95% CI, 39.8-100.0), with 2 (50.0%) CR and 2 (50.0%) PR. The 12-month PFS rates were 100.0% (95% CI, 100.0-100.0), 66.7% (95% CI, 30.0-100.0), and 100.0% (95% CI, 100.0-100.0), respectively. The 12-month OS rate was 100.0% (95% CI, 100.0-100.0) across all groups. One patient (4.5%) experienced a treatment-related AE (grade 1 cutaneous petechiae). No serious AEs or treatment-related deaths occurred.

**Copyright Information:** (Online) ISSN: 2572-9241
©2026 The Author(s). HemaSphere is published by John Wiley & Sons Ltd on behalf of European Hematology Association. This is an open access Abstract Book distributed under the Attribution-NonCommercial-NoDerivs (CC BY-NC-ND), which allows third parties to download the articles and share them with others as long as they credit the author and the Abstract Book, but they cannot change the content in any way or use them commercially.
**Abstract Book Citations:** Authors, Title, HemaSphere, 2026;10:(S1):pages. The abstract book DOIs can be found on the "Author Information" page.
**Disclaimer:** Articles published in the journal HemaSphere exclusively reflect the opinions of the authors. The authors are responsible for all content in their abstracts including accuracy of the facts, statements, citing resources, etc.

**Summary/Conclusion**

Orelabrutinib monotherapy demonstrated promising clinical outcomes and a favorable safety profile in BTKi-pretreated CLL/SLL. Further studies with larger cohorts are warranted to validate our findings.

Figure 1. Sankey diagram for tumor response transition

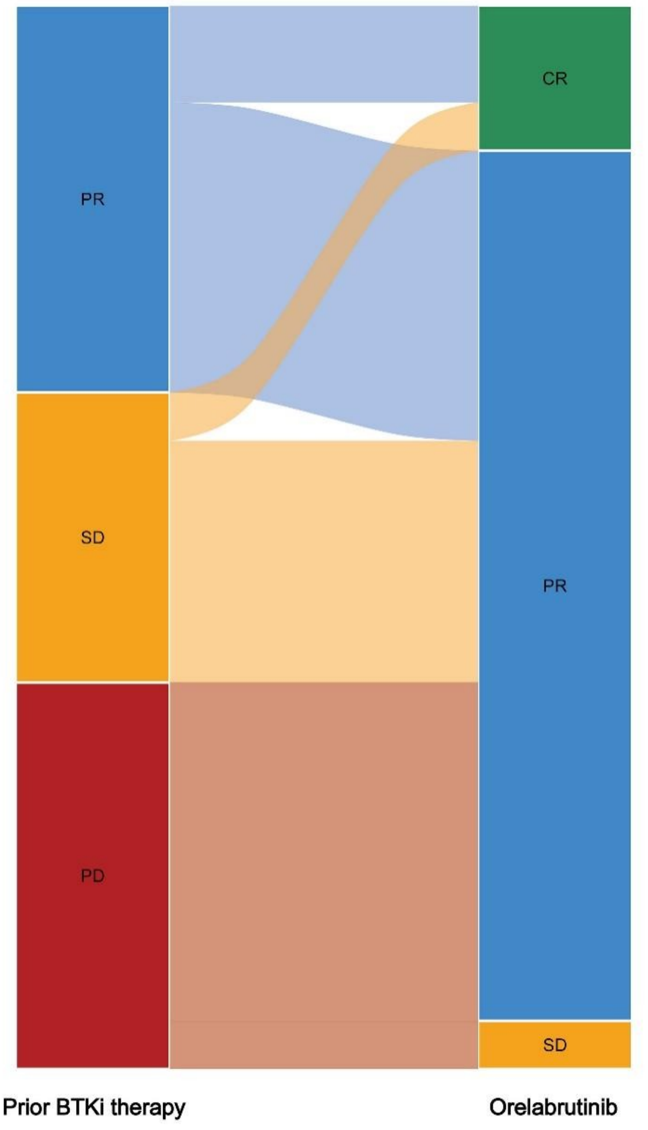

**Copyright Information:** (Online) ISSN: 2572-9241
©2026 The Author(s). HemaSphere is published by John Wiley & Sons Ltd on behalf of European Hematology Association. This is an open access Abstract Book distributed under the Attribution-NonCommercial-NoDerivs (CC BY-NC-ND), which allows third parties to download the articles and share them with others as long as they credit the author and the Abstract Book, but they cannot change the content in any way or use them commercially.
**Abstract Book Citations:** Authors, Title, HemaSphere, 2026;10:(S1):pages. The abstract book DOIs can be found on the "Author Information" page.
**Disclaimer:** Articles published in the journal HemaSphere exclusively reflect the opinions of the authors. The authors are responsible for all content in their abstracts including accuracy of the facts, statements, citing resources, etc.

### (PB2931) REAL-WORLD EFFICACY AND SAFETY DATA OF ORELABRUTINIB MONOTHERAPY IN PATIENTS WITH CHRONIC LYMPHOCYTICLEUKEMIA: A SINGLE-CENTER RETROSPECTIVE STUDY

**Topic:** 06. Chronic lymphocytic leukemia and related disorders - Clinical

Kai Shen*[1], Jie Wang[1], Chenlu Yang[1], He Li[1], Jinrong Yang[1], Zhengyu Yu[1], Yun Tang[1], Chuan He[1], Xiang Bing[1], Ting Niu[1]

[1] *West China Hospital, Sichuan University, Department of Hematology, Institute of Hematology, Chengdu, China;*

**Background**

Chronic lymphocytic leukaemia (CLL), a hematologic malignancy of B cells, is one of the most frequent types of leukemia. Recently, Bruton's tyrosine kinase inhibitors (BTKis) have become a standard first-line treatment for patients (pts) with CLL, showing remarkable clinical efficacy compared to traditional chemoimmunotherapy. However, first-generation BTKis are associated with limited target selectivity, which may result in off-target effects and serious adverse events. Given the safety concerns, there is an urgent need for next-generation BTKis with enhanced selectivity and improved safety profiles. Orelabrutinib is a novel, orally administered, and highly selective irreversible BTKi. Its improved target selectivity contributes to a more favorable safety profile. Orelabrutinib has been approved in China for the treatment of pts with relapsed or refractory CLL.

**Aims**

To assess the clinical efficacy and safety of orelabrutinib in pts with CLL in real-world setting.

**Methods**

Between November 1, 2021, and November 1, 2025we retrospectively reviewed CLL pts who received orelabrutinib monotherapy (150 mg orally once daily). Patient baseline characteristics, response data (overall response rate [ORR]), survival outcomes (progression-free survival [PFS] and overall survival [OS]), and safety data (incidence and severity of adverse events [AEs]) were descriptively analyzed.

**Results**

As of the cut-off date (November 1, 2025 ), 68 pts were included,55 had CLL, 13 had SLL; 59 were still receiving treatment, and9 had discontinued. The median age was 64.5 years (IQR 54.0-71.0), with 37 (54.4%) males. Of these pts, 67(98.5%) pts had an ECOG performance status of ≤1; 19 (34.5%) Binet stage C; 38 (69.1%) Rai stage I-IV. Based on CLL-IPI, 72.7% (40/55) of pts were classified as low-risk, 12.7% (7/55) intermediate-risk, 10.9% (6/55) high-risk,and 3.6%(2/55) very high-risk. IGHV mutation was detected in 61.2% (19/31) of tested pts, while TP53 mutation was identified in 8.3% (5/24) of tested pts. 22 pts had a history of BTKi treatment (ibrutinib, n=12; zanubrutinib, n=6;ibrutinib and zanubrutinib n=4 ), all of whom switched due to inadequate efficacy or AEs. Orelabrutinib was administered to 33 pts in first-line and 35 pts in second- or later-line settings.
Among all pts, 67 (98.5%,95% Cl:92.1%-100.0%) achieved an objective response, including 12 (17.6%) complete responses (CR) and 55 (80.9%) partial responses (PR). One patient (3.3%) had stable disease (SD), resulting in a disease control rate (DCR) of 100%.In the first-line cohort (n=33), CRR was 9.1% (3/33) and ORR 100%; in the ≥2L cohort (n=35), CRR was 25.7% (9/35) and ORR 97.1%. In the TP53-mutated subgroup, ORR was 100% (5/5) in mutated pts versus 100.0% in wild-type (19/19). ORR was 100% (5/5) in bulky disease subgroup. At a median follow-up of 25.8 months (range, 0.8–48.7), median PFS and OS were not reached. The 48-month PFS and OS rates were 96% and 97.7% (Figure 1A and B). Kaplan-Meier plots of PFS and OS for different risk groups were summarized in Figure 1 C to H. TP53 mutation subgroup had an inferior OS (p=0.02). In the total of 30 pts, any-grade AEs were reported in 4 (5.9%), all with grade 1~2. AEs related to BTKi off-target activity were not observed.

**Copyright Information:** (Online) ISSN: 2572-9241
©2026 The Author(s). HemaSphere is published by John Wiley & Sons Ltd on behalf of European Hematology Association. This is an open access Abstract Book distributed under the Attribution-NonCommercial-NoDerivs (CC BY-NC-ND), which allows third parties to download the articles and share them with others as long as they credit the author and the Abstract Book, but they cannot change the content in any way or use them commercially.
**Abstract Book Citations:** Authors, Title, HemaSphere, 2026;10:(S1):pages. The abstract book DOIs can be found on the "Author Information" page.
**Disclaimer:** Articles published in the journal HemaSphere exclusively reflect the opinions of the authors. The authors are responsible for all content in their abstracts including accuracy of the facts, statements, citing resources, etc.

### Summary/Conclusion

This real-world study suggested that orelabrutinib monotherapy showed promising efficacy and a favorable safety profile in pts with CLL, both as first-line and subsequent therapy. Further prospective investigations are needed to validate the observations.

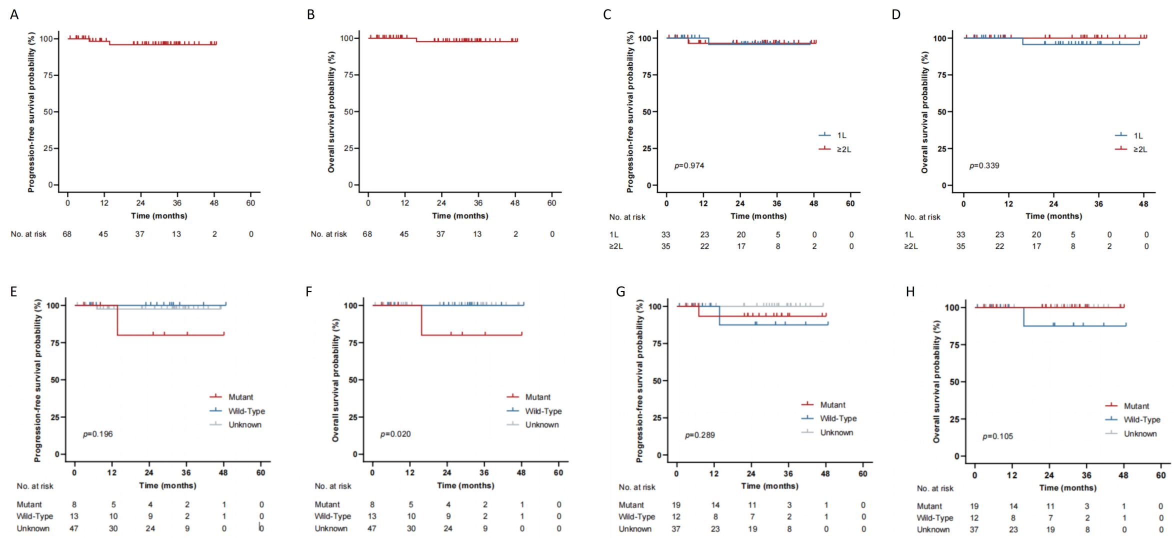

Figure 1. Kaplan-Meier plots for the PFS and OS of total paitents and different risk groups. A & B, the total patients; C & D, 1L vs ≥2L ; E & F, TP53 mutated vs wild type; G & H, mIGHV vs uIGHV

**Copyright Information:** (Online) ISSN: 2572-9241
©2026 The Author(s). HemaSphere is published by John Wiley & Sons Ltd on behalf of European Hematology Association. This is an open access Abstract Book distributed under the Attribution-NonCommercial-NoDerivs (CC BY-NC-ND), which allows third parties to download the articles and share them with others as long as they credit the author and the Abstract Book, but they cannot change the content in any way or use them commercially.
**Abstract Book Citations:** Authors, Title, HemaSphere, 2026;10:(S1):pages. The abstract book DOIs can be found on the “Author Information” page.
**Disclaimer:** Articles published in the journal HemaSphere exclusively reflect the opinions of the authors. The authors are responsible for all content in their abstracts including accuracy of the facts, statements, citing resources, etc.

### (PB2933) PRIMARY CARE MANAGEMENT PATHWAYS TO REDUCE WAIT TIMES IN HEMATOLOGY: LYMPHOCYTOSIS AND LYMPHOPENIA

**Topic:** 06. Chronic lymphocytic leukemia and related disorders - Clinical

Sarah Ryan*[1, 2], Benjamin Ott[1], Janet Lee[1], Pallavi Ganguli[2], Annette Hay[1, 3], Janique Dyba[1], Karine Gauthier[1], Bethany Monteith[1]

*[1]Queen's University, Department of Medicine, Kingston, Canada;[2]University of Ottawa, Department of Medicine, Ottawa, Canada; [3]Canadian Cancer Trials Group, Kingston, Canada;*

### Background

Wait times for specialist care in Canada are high. At Kingston Health Sciences Centre, we developed primary care management pathways (PCMPs) to support management of low-risk conditions by primary care providers (PCPs). A PCMP for lymphocytosis and lymphopenia was implemented in April 2023 (https://kingstonhsc.ca/hematology).

### Aims

This study aims to evaluate the effectiveness and safety of the PCMP in reducing specialist waitlist burden, while ensuring appropriate patient care.

### Methods

We examined consecutive hematology referrals received from July 2021 to October 2025. Primary outcomes were waitlist volume and wait times; number of referrals deferred to the PCMP was a process measure; re-referrals and deaths on the waitlist were balancing measures. A T-test or Wilcoxon-Mann-Whitney test was used for continuous variables, proportion or Fisher Exact test for discrete variables, and an Interrupted Time Series (ITS) model to assess the impact on monthly waitlist volume. Post-PCMP referrals were sub-classified as backlog (referred pre-PCMP, seen post-PCMP) or new referrals (referred and seen post-PCMP) to account for their distinct wait time profiles. PCP satisfaction was assessed through a survey.

### Results

Over the study period 12,634 hematology referrals were received, 287 (2.3%) for lymphocytosis (Table 1). Monthly lymphocytosis referrals remained stable post-PCMP launch, while monthly waitlist volume decreased, though ITS modelling indicates it has not yet reached a new equilibrium (Figure 1). Among new post-PCMP referrals, median wait time decreased compared to pre-PCMP (-24 days, 95% CI -2,-60, p=0.03), and the proportion seen within their triage target increased (+18.2%, 95% CI +1.1,+35.3, p=0.04).

**Copyright Information:** (Online) ISSN: 2572-9241
©2026 The Author(s). HemaSphere is published by John Wiley & Sons Ltd on behalf of European Hematology Association. This is an open access Abstract Book distributed under the Attribution-NonCommercial-NoDerivs (CC BY-NC-ND), which allows third parties to download the articles and share them with others as long as they credit the author and the Abstract Book, but they cannot change the content in any way or use them commercially.
**Abstract Book Citations:** Authors, Title, HemaSphere, 2026;10:(S1):pages. The abstract book DOIs can be found on the “Author Information” page.
**Disclaimer:** Articles published in the journal HemaSphere exclusively reflect the opinions of the authors. The authors are responsible for all content in their abstracts including accuracy of the facts, statements, citing resources, etc.

**Table 1.** Pre- and post-PCMP outcomes for hematology and lymphocytosis referrals

| | **Pre-PCMP** (Jul 2021-Mar 2023) | **Post-PCMP** (Apr 2023-Oct 2025) | **p-value** |
|---|---|---|---|
| **Hematology** | | | |
| Referrals Received | 4309 | 8325 | - |
| Referrals Seen or Closed | 3254 | 8007 | - |
| Monthly Referrals *(mean±SD)* | 205.2±34.2 | 266.0±23.6 | <0.001 |
| Monthly Waitlist Volume *(mean±SD)* | 1211.5±304.6 | 1901.8±73.3 | <0.001 |
| **Lymphocytosis** | | | |
| Referrals Received | 112 | 175 | - |
| Referrals Seen or Closed | 65 seen, 6 closed | 127 seen, 100 closed | - |
| Monthly Referrals *(mean±SD)* | 5.3±2.3 | 5.6±2.4 | 0.64 |
| Monthly Waitlist Volume *(mean±SD)* | 65.3±10.6 | 42.6±24.1 | <0.001 |
| New Referral Wait Time in Days *(median [IQR])* | 84.0 [167.3] | 65.0 [63.6]* | 0.03 |
| New Referrals Seen in Target Time | 35.4% | 53.6%* | 0.04 |

*Excludes 44 backlog cases (referred pre-PCMP, seen post-PCMP)

Of the 79 referrals deferred to the PCMP (68 for lymphocytosis and 11 for lymphopenia), 8 were re-referred due to not having a PCP (n=2), or with new flow cytometry (n=4), imaging (n=1) or monoclonal gammopathy (n=1) test results. There were 5 deaths of unknown cause among patients waiting to be seen for lymphocytosis, all identified post-PCMP implementation, with median age 90. Among 28 respondents to the PCP survey, 19 (67.9%) reported using PCMPs. Median overall satisfaction was 4/5; 7 respondents (25%) cited downloading work to primary care as their main concern.

**Copyright Information:** (Online) ISSN: 2572-9241
©2026 The Author(s). HemaSphere is published by John Wiley & Sons Ltd on behalf of European Hematology Association. This is an open access Abstract Book distributed under the Attribution-NonCommercial-NoDerivs (CC BY-NC-ND), which allows third parties to download the articles and share them with others as long as they credit the author and the Abstract Book, but they cannot change the content in any way or use them commercially.
**Abstract Book Citations:** Authors, Title, HemaSphere, 2026;10:(S1):pages. The abstract book DOIs can be found on the "Author Information" page.
**Disclaimer:** Articles published in the journal HemaSphere exclusively reflect the opinions of the authors. The authors are responsible for all content in their abstracts including accuracy of the facts, statements, citing resources, etc.

### Summary/Conclusion

Implementation of a PCMP for lymphocyte abnormalities was associated with reduced waitlist volume and improved wait times of new referrals for lymphocytosis. Limitations include unaccounted concurrent waitlist reduction interventions (e.g. staffing increase, other PCMPs) and potential undetected harms that warrant future study. By supporting PCPs to manage low-risk cases, this pathway optimizes specialist resources and could be expanded to other hematologic conditions.

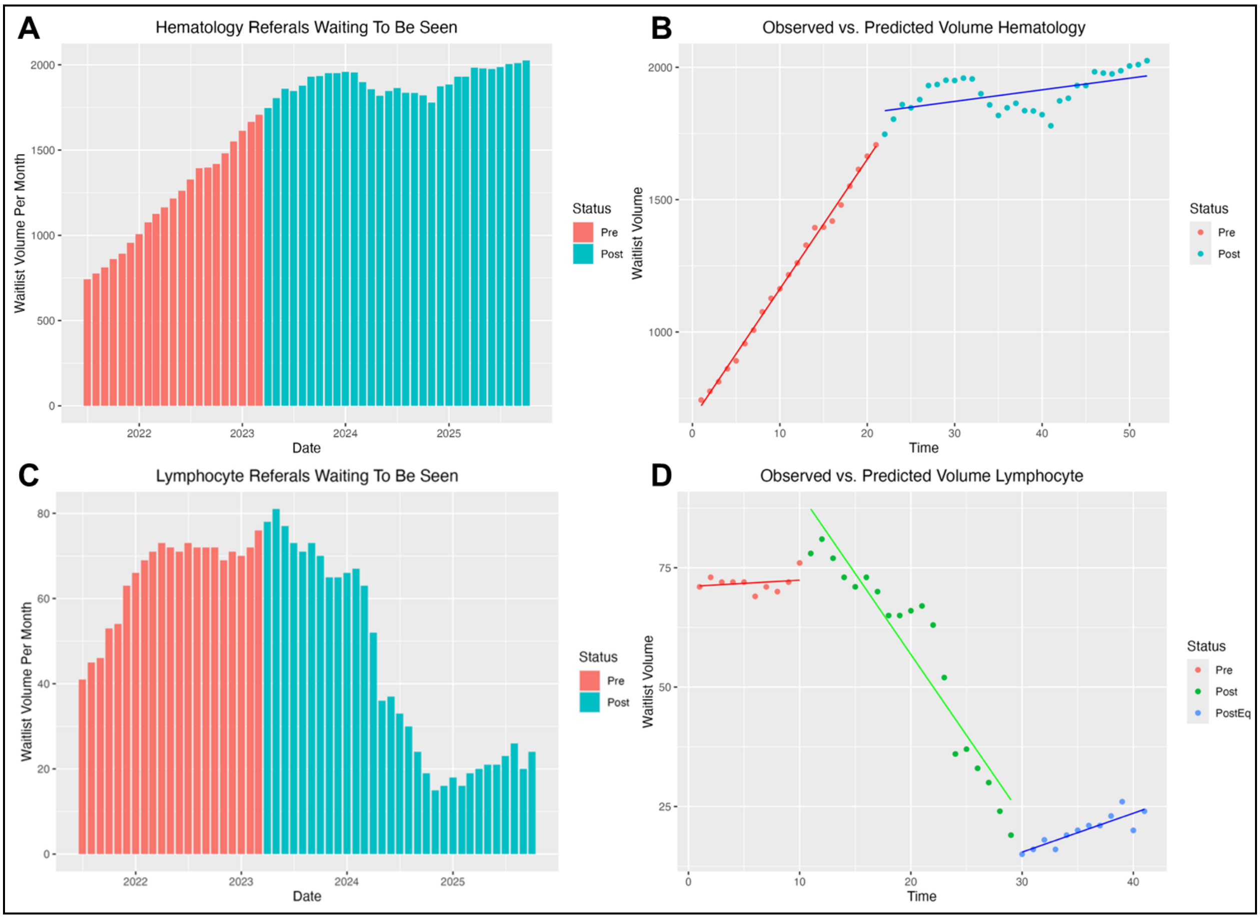

Figure 1. Monthly lymphocytosis waitlist volume pre- and post-PCMP implementation for (A) hematology and (C) lymphocytosis (waitlist data collection started Jan 2021). Interrupted Time Series model for (B) hematology and (D) lymphocytosis. For lymphocytosis, a significant reduction in slope was observed post-implementation (slope difference -3.52 [95% CI -4.35, -2.69]/month, p<0.001); however, a significantly positive late post-pathway slope (0.81 [95% CI 0.69, 0.94]/month, p<0.001) indicates the waitlist has not yet reached a new equilibrium.

**Copyright Information:** (Online) ISSN: 2572-9241
©2026 The Author(s). HemaSphere is published by John Wiley & Sons Ltd on behalf of European Hematology Association. This is an open access Abstract Book distributed under the Attribution-NonCommercial-NoDerivs (CC BY-NC-ND), which allows third parties to download the articles and share them with others as long as they credit the author and the Abstract Book, but they cannot change the content in any way or use them commercially.
**Abstract Book Citations:** Authors, Title, HemaSphere, 2026;10:(S1):pages. The abstract book DOIs can be found on the "Author Information" page.
**Disclaimer:** Articles published in the journal HemaSphere exclusively reflect the opinions of the authors. The authors are responsible for all content in their abstracts including accuracy of the facts, statements, citing resources, etc.

### (PB2934) UNDERSTANDING PATIENTS' PERSPECTIVES AND PREFERENCES REGARDING FIRST-LINE CHRONIC LYMPHOCYTIC LEUKEMIA (CLL) TREATMENT ACROSS EUROPE

**Topic:** 06. Chronic lymphocytic leukemia and related disorders - Clinical

Lydia Scarfò*[1, 2], Anne-Sophie Michallet[3], Lianne Barnieh[4], Jennifer Fitzgerald[5], Silvy Mardiguian[6], Claudia Rigamonti[5], Robert Olie[5], Alyssa Sutormina[7], Anna Vartanian-Paleeva[7], Talha Munir[8]

*[1]University San Raffaele, Milan, Italy; [2]Strategic Research Program on CLL, IRCCS Ospedale San Raffaele, Milan, Italy; [3]Centre Léon Bérard, Lyon, France; [4]BeOne Medicines, Paris, France; [5]BeOne Medicines, Basel, Switzerland; [6]BeOne Medicines, London, United Kingdom; [7]Semantic Hub, Lausanne, Switzerland; [8]Leeds Teaching Hospital, Leeds, United Kingdom;*

### Background

Following a cancer diagnosis, patients may turn to digital social community platforms to seek information and share their personal experiences, needs, and concerns. Valuable insights can be generated by systematically collecting and analyzing the patient voice on social media and online patient platforms. Artificial intelligence now enables the processing and extraction of meaningful insights from large volumes of unstructured online data. Digital listening leverages real-world insights by highlighting common themes and trends over time of topics important to patients.

### Aims

This analysis aimed to understand patients' perspectives on the factors that most influenced treatment choice and the extent to which patients were involved in their treatment decision-making for first-line CLL using AI-based Semantic-NLP technology.

### Methods

A multilingual ontology-based AI information extraction system (Semantic Hub) was used to identify the patient "voice" from online posts in France, Germany, Italy, Spain, and the United Kingdom (UK). Data related to patients and first-line treatments in CLL were collected from social community platforms and other online patient platforms (i.e. Facebook, Instagram, HealthUnlocked.com) between January 1, 2020 and December 31, 2025. Key medical concepts, including factors influencing treatment and how treatment decisions were made, were extracted from the data, grouped into thematic categories, and analyzed using both qualitative and quantitative approaches.

### Results

A total of 44,451 messages from 2,699 individuals were detected across the five countries. The semantic analysis revealed that treatment decisions were generally guided by hematologists. In the UK and Germany, out of 1,090 and 629 authors, respectively, only 7% and 11% explicitly reported that they were involved in the decision-making for their choice of treatment. Of note, in Germany, patients posted that they were rarely asked about their personal priorities by their doctor. In terms of the factors that matter most to patients with CLL, safety and effectiveness were the primary drivers of treatment choice across the five countries (**Figure**); these factors remained stable over time as the primary drivers of treatment choice. Treatment duration was the lowest ranking criteria influencing treatment choice across the five countries. Notably, in the UK, patients mentioned that those who chose a fixed-duration treatment were influenced by their hematologists' recommendation and the expectation of achieving a deep remission.

**Copyright Information:** (Online) ISSN: 2572-9241
©2026 The Author(s). HemaSphere is published by John Wiley & Sons Ltd on behalf of European Hematology Association. This is an open access Abstract Book distributed under the Attribution-NonCommercial-NoDerivs (CC BY-NC-ND), which allows third parties to download the articles and share them with others as long as they credit the author and the Abstract Book, but they cannot change the content in any way or use them commercially.
**Abstract Book Citations:** Authors, Title, HemaSphere, 2026;10:(S1):pages. The abstract book DOIs can be found on the "Author Information" page.
**Disclaimer:** Articles published in the journal HemaSphere exclusively reflect the opinions of the authors. The authors are responsible for all content in their abstracts including accuracy of the facts, statements, citing resources, etc.

### Summary/Conclusion

This analysis demonstrated that the safety and effectiveness of a treatment were the top priorities for patients, with treatment duration being the lowest priority, when discussing treatment choices for first-line CLL. Insights also revealed that shared decision-making between patients and their healthcare providers remains limited. Further research warrants validating these findings across a wide range of CLL patients beyond social community platforms as there may be a reporting bias from patients who are more motivated to share their experiences online.

**Figure. Insights from patients on factors influencing treatment choice, %**

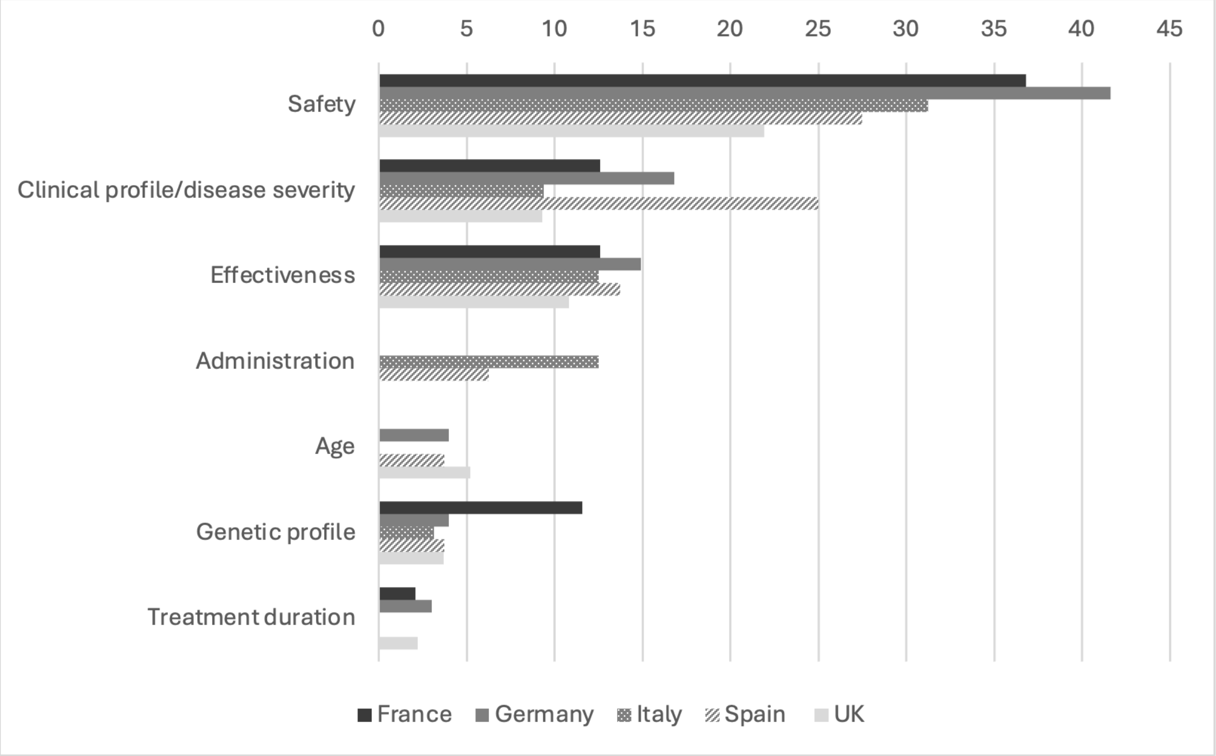

Copyright Information: (Online) ISSN: 2572-9241
©2026 The Author(s). HemaSphere is published by John Wiley & Sons Ltd on behalf of European Hematology Association. This is an open access Abstract Book distributed under the Attribution-NonCommercial-NoDerivs (CC BY-NC-ND), which allows third parties to download the articles and share them with others as long as they credit the author and the Abstract Book, but they cannot change the content in any way or use them commercially.
Abstract Book Citations: Authors, Title, HemaSphere, 2026;10:(S1):pages. The abstract book DOIs can be found on the "Author Information" page.
Disclaimer: Articles published in the journal HemaSphere exclusively reflect the opinions of the authors. The authors are responsible for all content in their abstracts including accuracy of the facts, statements, citing resources, etc.

### (PB2936) EFFICACY OF VENETOCLAX-BASED REGIMENS IN PATIENTS WITH RELAPSED CHRONIC LYMPHOCYTIC LEUKEMIA AFTER CHEMOIMMUNOTHERAPY: A MULTICENTER STUDY IN RUSSIA

**Topic:** 06. Chronic lymphocytic leukemia and related disorders - Clinical

Maria Kislova*[1], Maria Ivanova[2], Ekaterina Salaheeva[3], Efim Pozharsky[4], Tatiana Mitina[5], Olga Berezina[6], Svetlana Volkova[7], Galina Kuchma[8], Svetlana Samarina[9], Gulnara Khusainova[10], Yuliya Gammershmidt[11], Tatyana Tsiganok[12], Aigul Latipova[13], Andrei Zhuravkov[14], Daria Alekseeva[15], Vitaly Dubov[16], Irina Kudinova[17], Ksenia Deduhina[18], Elena Zinina[19], Varvara Bahtina[20], Mikhail Lubchenko[21], Ilya Usankin[22], Maret Murzabekova[23], Ylia Martynova[24], Marina Kaden[25], Elena Komartceva[26], Elena Starikova[27], Tatyana Byalik[28], Inga Piskunova[29], Vadim Ptushkin[30], Eugene Nikitin[1, 31]

*[1]Botkin hospital, Moscow, Russia; [2]Federal State Budgetary Educational Institution of Higher Education Academician I P. Pavlov First St. Petersburg State Medical University of the Ministry of Healthcare of Russian Federation, St. Petersburg, Russia; [3]Moscow City Clinical Hospital 52, Moscow, Russia; [4]Moscow Multidisciplinary Clinical Center "Kommunarka", Moscow, Russia; [5]Moscow Regional Research and Clinical Institute ("MONIKI"), Moscow, Russia; [6]epartment of Therapy, Hematology and Transfusiology, Novosibirsk State Medical University, Novosibirsk, Russia; [7]Departments of Hospital Therapy and General Medical Practice, Privolzhsk Research Medical University, Niznii Novgorod, Russia; [8]Orenburg Medical State University, Orenburg, Russia; [9]The Federal State-Financed Scientific Institution Kirov Research Institute of Hematology and Blood Transfusion under the Federal Medical Biological Agency, Kirov, Russia; [10]Republican Clinical Oncology Dispensary named after Professor M.Z. Sigal, Kazan, Russia; [11]Tomsk Regional Clinical Hospital, Tomsk, Russia; [12]Sakhalin Regional Clinical Hospital, Yuzhno-Sakhalinsk, Russia; [13]Republican Clinical Hospital named after G.G. Kuvatova, Ufa, Russia; [14]Bryansk City Hospital №1, Bryansk, Russia; [15]The State Budgetary Health Care Institution of the Yaroslavl Region «Regional Clinical Hospital», Yaroslavl, Russia; [16]Regional Clinical Hospital No. 2, Vladivostok, Russia; [17]Altai Regional Clinical Hospital, Barnaul, Russia; [18]Zabaikalsky Regional Oncological Dispensary, Chita, Russia; [19]Surgut Regional Clinical Hospital, Surgut, Russia; [20]Krasnoyarsk Regional Clinical Hospital, Krasnoyarsk, Russia; [21]Chelyabinsk Regional Clinical Hospital, Chelyabinsk, Russia; [22]Central City Hospital №7, Ekaterinburg, Russia; [23]Stavropol Regional Oncological Center, Stavropol, Russia; [24]Clinical Oncology Dispensary №1, Krasnodar, Russia; [25]Volgograd Regional Clinical Oncology Center, Volgograd, Russia; [26]Regional Clinical Hospital, Rostov on Don, Russia; [27]State Budgetary Healthcare Organisation "Oncology Dispensary No.2", Sochi, Russia; [28]N.N. Blokhin Medical Research Center of Oncology, Moscow, Russia; [29]Hematology Research Center of Russia, Moscow, Russia; [30]Botkin Hospital, Mocow, Russia; [31]Russian Medical Academy of Continuous Professional Education, Moscow, Russia;*

### Background

Venetoclax-based regimens represent an effective therapeutic option for patients with chronic lymphocytic leukemia (CLL) relapsing after chemoimmunotherapy (CIT), including those with high-risk biological features. However, real-world data on long-term outcomes and comparative effectiveness of different venetoclax-containing regimens remain limited.

### Aims

To evaluate the effectiveness of venetoclax-based regimens in patients with relapsed CLL after CIT in real-world clinical practice in Russia.

**Copyright Information:** (Online) ISSN: 2572-9241
©2026 The Author(s). HemaSphere is published by John Wiley & Sons Ltd on behalf of European Hematology Association. This is an open access Abstract Book distributed under the Attribution-NonCommercial-NoDerivs (CC BY-NC-ND), which allows third parties to download the articles and share them with others as long as they credit the author and the Abstract Book, but they cannot change the content in any way or use them commercially.
**Abstract Book Citations:** Authors, Title, HemaSphere, 2026;10:(S1):pages. The abstract book DOIs can be found on the "Author Information" page.
**Disclaimer:** Articles published in the journal HemaSphere exclusively reflect the opinions of the authors. The authors are responsible for all content in their abstracts including accuracy of the facts, statements, citing resources, etc.

### Methods

This retrospective multicenter study included 248 patients with relapsed CLL treated with venetoclax-based regimens after prior CIT. Data were collected from 32 centers across 29 regions of Russia. Venetoclax was administered as monotherapy (VenMONO, n=70), in combination with obinutuzumab (GVen, n=72), or rituximab (RVen, n=104). Endpoints included overall survival (OS), progression-free survival (PFS), and time to treatment discontinuation (TTD). The impact of clinical and biological factors on PFS was analyzed.

### Results

The median age was 64 years (range 26–86); 71% were male. Most patients had advanced disease, Binet B: 90 pts, 60%; C: 51 pts, 35%. High-risk biological features were: del(17p) in 26.2% and unmutated IGHV in 60.2% of patients. The median number of prior therapy lines was 2 (range 1–10). Baseline clinical characteristics were generally balanced across treatment groups, although differences in certain cytogenetic parameters were observed.

After a median follow-up of 24.9 months, median OS and PFS were not reached in the overall cohort. Median TTD was 24.7 months, reflecting predominantly fixed-duration therapy and planned venetoclax discontinuation. In subgroup analyses by regimen, median OS and PFS were not reached in any treatment group. PFS were significantly superior in patients treated with GVen compared with RVen (p=0.021) and VenMONO (p=0.021) and OS (p=0.043) was better in GVen treated patients compared to RVen (p=0.011), whereas no statistically significant differences were observed in other pairwise comparisons (Figure 1).

In subgroup analyses, PFS did not differ according to del(17p) status, IGHV mutational status, relapse after CIT <24 months, Binet stage, or ECOG performance status. In contrast, bulky lymphadenopathy (>10 cm) (p=0.03), more than one prior therapy line before venetoclax (p=0.0024), and complex karyotype ≥3 abnormalities (p=0.0017) were associated with inferior PFS.

### Summary/Conclusion

Venetoclax-based regimens demonstrate high effectiveness in patients with relapsed CLL after CIT, including those with adverse cytogenetic and molecular characteristics. In regimen subgroup analyses, GVen was associated with superior PFS and OS compared with RVen and VenMONO. These real-world data confirm the durable activity of venetoclax in routine clinical practice and highlight the need for further studies to optimize regimen selection according to clinical context.

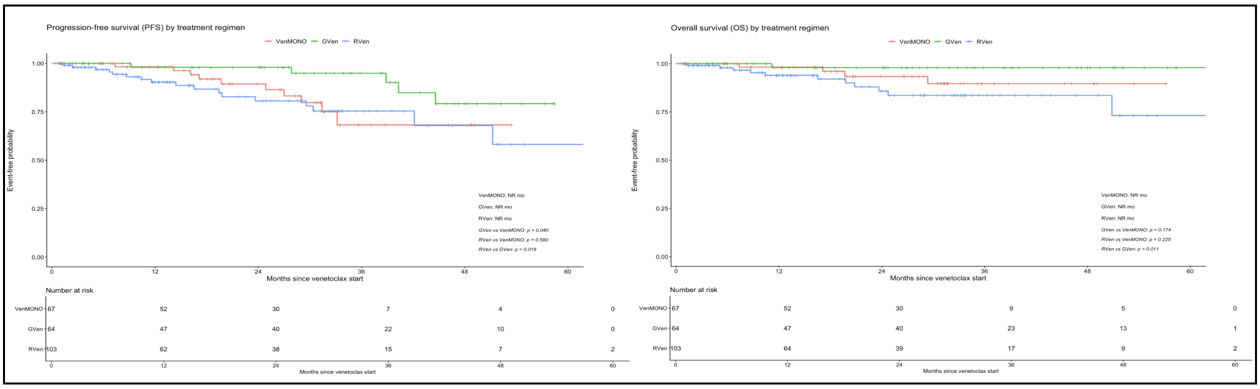

**Copyright Information:** (Online) ISSN: 2572-9241
©2026 The Author(s). HemaSphere is published by John Wiley & Sons Ltd on behalf of European Hematology Association. This is an open access Abstract Book distributed under the Attribution-NonCommercial-NoDerivs (CC BY-NC-ND), which allows third parties to download the articles and share them with others as long as they credit the author and the Abstract Book, but they cannot change the content in any way or use them commercially.
**Abstract Book Citations:** Authors, Title, HemaSphere, 2026;10:(S1):pages. The abstract book DOIs can be found on the "Author Information" page.
**Disclaimer:** Articles published in the journal HemaSphere exclusively reflect the opinions of the authors. The authors are responsible for all content in their abstracts including accuracy of the facts, statements, citing resources, etc.

### (PB2937) REAL-WORLD CARDIOVASCULAR EVALUATION AND BURDEN IN PATIENTS WITH CHRONIC LYMPHOCYTIC LEUKEMIA (CLL) INITIATED ON ACALABRUTINIB IN ROMANIA: RESULTS OF THE NON-INTERVENTIONAL STUDY REACH

**Topic:** 06. Chronic lymphocytic leukemia and related disorders - Clinical

Mihaela Andreescu*[1], Nicoleta Mariana Berbec[2], Bianca Vladutiu[3], Beatrice Costea[3]

*[1]Colentina Clinical Hospital, Bucharest, Romania;[2]Coltea Clinical Hospital, Bucharest, Romania;[3]AstraZeneca, Bucharest, Romania;*

### Background

Advances in understanding the CLL biology led to revolutionary treatments that significantly improved survival. Inhibitors of Bruton tyrosine kinase (BTK) and B-cell lymphoma 2(BCL-2) are currently the standard of care in CLL, replacing the traditional chemoimmunotherapy (CIT). BTK inhibitors (BTKi) are associated with cardiotoxicity, yet the true incidence of cardiovascular (CV) events under BTKi is not known. CLL is typically a disease of elderly, and pre-existing CV diseases (CVD), other comorbidities and treatments increasing CVD risk are highly prevalent in the CLL population. In Romania, information on baseline CV risk assessment and CVD prevalence in CLL patients initiated on the new generation BTKi acalabrutinib is scarce.

### Aims

To describe the use of acalabrutinib for the treatment of CLL in real-world settings over a 3-year follow-up, including safety outcomes and concomitant treatment patterns. Here we present the baseline characteristics of the study cohort, focusing on the CVD risk factors and history.

### Methods

REACH is a physician-led medical chart review ongoing in 31 hematology centers. Eligibility required age at least 18 years, confirmed CLL diagnosis, initiation of acalabrutinib in real-life settings between Jan'23-Jan'25 and provision of written informed consent for data collection. Patients were excluded if acalabrutinib was initiated in a clinical trial or enrolment was performed in <30 days before acalabrutinib initiation. Information on CV risk factors, electrocardiogram (ECG) tests, CVD and CV treatments at acalabrutinib start were collected. Descriptive statistical methods were used.

### Results

This study included 243 patients, 140 (58%) male, median (interquartile [IQR]) age 68 (60-75) years at CLL diagnosis and 72 (65-78) years at acalabrutinib initiation, 142 (58%) patients treatment-naïve (TN) and 101 (42%) relapse/refractory (R/R). Pre-existing CVD were documented for 157 (65%) patients (62% TN and 68% R/R). In patients with CVD, hypertension was reported in 85% of patients, and cardiac rhythm disorders in 27%. Atrial fibrillation (AF) was reported more R/R patients (17 [25%]) vs TN (12 [14%]).

Electrocardiogram (ECG) in the prior 12 months before acalabrutinib initiation was available in medical charts for 140 (58%) patients, more in the TN vs R/R subgroup (89 [63%] vs 51 [51%]). ECG abnormalities were described for 29 (12%) patients, numerically more in the TN subgroup, affecting mostly the heart rhythm and conduction system. More CV characteristics are described in **Table 1**. Prior cardiotoxic CLL medications included ibrutinib in 40 (40%) R/R patients, with discontinuation due to CV events (including bleeding) reported for 13 (33%) of them. CIT containing anthracycline (doxorubicin) was described in 13 (13%) of the R/R patients, with no CV-related discontinuation.

**Copyright Information:** (Online) ISSN: 2572-9241
©2026 The Author(s). HemaSphere is published by John Wiley & Sons Ltd on behalf of European Hematology Association. This is an open access Abstract Book distributed under the Attribution-NonCommercial-NoDerivs (CC BY-NC-ND), which allows third parties to download the articles and share them with others as long as they credit the author and the Abstract Book, but they cannot change the content in any way or use them commercially.
**Abstract Book Citations:** Authors, Title, HemaSphere, 2026;10:(S1):pages. The abstract book DOIs can be found on the "Author Information" page.
**Disclaimer:** Articles published in the journal HemaSphere exclusively reflect the opinions of the authors. The authors are responsible for all content in their abstracts including accuracy of the facts, statements, citing resources, etc.

**Summary/Conclusion**

To our knowledge, REACH is the first real-world study conducted in Romania exploring the CVD assessments and prevalence at the time of initiating the BTKi acalabrutinib. This cohort showed a high CV burden at acalabrutinib start, with a slightly higher percentage of AF and heart failure in R/R patients. Availability in medical charts of ECG test results before acalabrutinib start seemed higher for the TN subgroup, yet suboptimal. Identifying patients at high CV risk or with established CVD becomes crucial in the era of long-term BTKi therapy for implementing adequate surveillance after treatment initiation.

**Funding:** AstraZeneca

**Table 1**. Baseline CVD risk assessment, comorbidities and concomitant treatments in the full analysis set and cohorts stratified by prior exposure to CLL therapies

| Characteristics | FAS N=243 | TN N=142 | R/R N=101 |
|---|---|---|---|
| **CV risk factors** | | | |
| Age ≥65 years, n (%) | 183 (75) | 107 (75) | 76 (75) |
| Age 50-64 years, n (%) | 52 (21) | 31 (22) | 21 (21) |
| Smoking, n (%) | 45 (19) | 27 (19) | 18 (18) |
| BMI ≥30 kg/m², n (%) | 34 (14) | 20 (14) | 14 (14) |
| **Concomitant CVD, n (%)** | | | |
| Any CVD | 157 (65) | 88 (62) | 69 (68) |
| Hypertension | 134 (55) | 76 (54) | 58 (57) |
| Heart failure | 32 (13) | 16 (11) | 16 (16) |
| Atrial fibrillation | 29 (12) | 12 (9) | 17 (17) |
| Atrial flutter | 2 (<1) | 1 (<1) | 1 (1) |
| Ventricular arrhythmias | 5 (2) | 1 (<1) | 4 (4) |
| Other cardiac rhythm disorders | 7 (3) | 4 (3) | 3 (3) |
| Myocardial infarction | 6 (3) | 4 (3) | 2 (2) |
| Other CVD | | | |
| Heart valve disorders | 9 (4) | 3 (2) | 6 (6) |
| Ischemic heart disease | 21 (9) | 12 (9) | 9 (9) |
| Vascular disorders* | 13 (5) | 5 (4) | 8 (8) |
| **Concomitant non-CVD, n (%)** | | | |
| Diabetes | 44 (18) | 24 (17) | 20 (20) |
| Dyslipidemia | 19 (8) | 10 (7) | 9 (9) |
| **Cancer history, n (%)** | | | |
| Solid tumors | 23 (10) | 15 (11) | 8 (8) |
| **Measurements (n=240)** | | | |
| Heart rate, bpm, mean (SD) | 77 (11) | 77 (12) | 76 (10) |
| Systolic blood pressure >130 mmHg, n (%) | 92 (38) | 50 (36) | 42 (42) |
| Diastolic blood pressure >80 mmHg, n (%) | 33 (14) | 18 (13) | 15 (15) |
| **ECG test in the prior 12 months, n (%)** | | | |
| Yes | 140 (58) | 89 (63) | 51 (51) |
| ECG normal findings | 111 (79) | 69 (78) | 42 (82) |
| ECG abnormal findings | 29 (21) | 20 (22) | 9 (18) |
| Atrial fibrillation | 8 (6) | 4 (5) | 4 (8) |
| Other rhythm abnormalities** | 10 (7) | 8 (9) | 2 (4) |
| Conduction defects | 15 (11) | 10 (11) | 5 (10) |
| QTc>420 ms | 13 (9) | 9 (10) | 4 (8) |
| QTc≥480 ms | 1 (<1) | - | 1 (2) |
| **Concomitant CV medications** | | | |
| Hypertensive medication | 113 (47) | 63 (44) | 50 (50) |
| Beta-blockers | 54 (48) | 26 (41) | 28 (56) |
| Angiotensin-converting enzyme inhibitors | 30 (27) | 18 (29) | 12 (24) |
| Atrial fibrillation treatment | 22 (9) | 7 (5) | 15 (15) |
| Direct oral anticoagulants | 15 (68) | 4 (57) | 11 (73) |
| Coumarins | 3 (14) | 1 (14) | 2 (13) |
| Direct thrombin inhibitors | 1 (5) | 1 (14) | - |
| Other CVD medication | 51 (21) | 25 (18) | 26 (26) |

**Notes:** *Includes peripheral occlusive artery diseases, aortic atheromatosis, pulmonary hypertension, atherosclerotic disease, venous disease; **Includes sinus tachycardia, bradycardia, ectopic atrial rhythm, arrhythmia unspecified, supraventricular extrasystoles. **Abbreviations**: BMI, body mass index; bpm, beats per minute; CV(D), cardiovascular (disease); ECG, electrocardiogram; FAS, full analysis set; ms, millisecond; R/R, relapse/refractory; SD, standard deviation, TN, treatment-naïve.

**Copyright Information:** (Online) ISSN: 2572-9241
©2026 The Author(s). HemaSphere is published by John Wiley & Sons Ltd on behalf of European Hematology Association. This is an open access Abstract Book distributed under the Attribution-NonCommercial-NoDerivs (CC BY-NC-ND), which allows third parties to download the articles and share them with others as long as they credit the author and the Abstract Book, but they cannot change the content in any way or use them commercially.
**Abstract Book Citations:** Authors, Title, HemaSphere, 2026;10:(S1):pages. The abstract book DOIs can be found on the "Author Information" page.
**Disclaimer:** Articles published in the journal HemaSphere exclusively reflect the opinions of the authors. The authors are responsible for all content in their abstracts including accuracy of the facts, statements, citing resources, etc.

### (PB2938) TREATMENT PATTERNS, AND SURVIVAL OUTCOMES OF PATIENTS AGED ≥80 YEARS WITH CLL: DATA FROM THE BRAZILIAN REGISTRY OF CLL (RBLLC).

**Topic:** 06. Chronic lymphocytic leukemia and related disorders - Clinical

Thiago Carneiro*[1], Caio Justino[2], Leila Perobelli[3], Maura Ikoma[4], Valeria Buccheri[5], Alita Azevedo[6], Vinicius Molla[2, 7], Rodrigo Santucci Silva[8], Danielle Leão[9], Laura Fogliato[10], Fernando Barroso[11], Cristiana Solza[12], Vera Lucia Piratininga de Figueiredo[13], Glaciano Ribeiro[14], Gislaine B. Oliveira[15], Henrique Nunes Pêcego[16], Talita Maira Bueno Da Silveira[17], Pedro Amoedo[18], Nelson Hamerschlak[19], Abel Costa[20], Patricia Giacon[21], Eduardo Flavio Oliveira Ribeiro[22], Adriana Alves De Souza Scheliga[23], Carlos Sergio Chiattone[24], Celso Arrais-Rodrigues[2, 7]

*[1]Hospital Ophir Loyola / UEPA, Belém, Brazil; [2]UNIFESP, São Paulo, Brazil; [3]Hospital Brigadeiro / Hospital de Transplantes Euryclides de J. Zerbini, São Paulo, Brazil; [4]Hospital Amaral Carvalho, Jaú, Brazil; [5]ICESP, Faculdade de Medicina da USP, São Paulo, Brazil; [6]HEMOPE, Recife, Brazil; [7]Hospital Nove de Julho / Rede Américas, São Paulo, Brazil; [8]Instituto Hemomed, São Paulo, Brazil; [9]Beneficiência Portuguesa de São Paulo, São Paulo, Brazil; [10]Hospital de Clínicas de Porto Alegre, Porto Alegre, Brazil; [11]Universidade Federal do Ceará, Fortaleza, Brazil; [12]UERJ - Hospital Universitário Pedro Ernesto, Rio de Janeiro, Brazil; [13]Hospital do Servidor Público Estadual, São Paulo, Brazil; [14]Universidade Federal de Minas Gerais, Belo Horizonte, Brazil; [15]UNICAMP, Campinas, Brazil; [16]Instituto D'Or de Pesquisa e Ensino, Rio de Janeiro, Brazil; [17]AC Camargo Cancer Center, São Paulo, Brazil; [18]Clinica AMO, Salvador, Brazil; [19]Hospital Israelita Albert Einstein, São Paulo, Brazil; [20]Instituto D'or de Pesquisa e Ensino, São Paulo, Brazil; [21]Hospital Santa Marcelina, São Paulo, Brazil; [22]Hospital Santa Lúcia Sul, Brasília, Brazil; [23]Grupo Oncoclínicas, Rio de Janeiro, Brazil; [24]Santa Casa de São Paulo, São Paulo, Brazil;*

### Background

Very elderly patients (≥80 years) with chronic lymphocytic leukemia (CLL) are underrepresented in prospective trials, and real-world data in this population—particularly in middle-income countries—remain scarce.

### Aims

To describe characteristics, treatment patterns, and survival of patients starting first-line therapy at age ≥80 years in the Brazilian CLL Registry.

### Methods

We conducted a retrospective analysis of patients enrolled in the Brazilian CLL Registry who were diagnosed between 1999 and 2022. Individuals aged ≥80 years at diagnosis were identified, and those who initiated first-line therapy were included in outcome analyses. Demographics, clinical stage, comorbidity burden, biological markers, time to treatment, treatment regimens, and overall survival (OS) from treatment initiation were evaluated.

**Copyright Information:** (Online) ISSN: 2572-9241
©2026 The Author(s). HemaSphere is published by John Wiley & Sons Ltd on behalf of European Hematology Association. This is an open access Abstract Book distributed under the Attribution-NonCommercial-NoDerivs (CC BY-NC-ND), which allows third parties to download the articles and share them with others as long as they credit the author and the Abstract Book, but they cannot change the content in any way or use them commercially.
**Abstract Book Citations:** Authors, Title, HemaSphere, 2026;10:(S1):pages. The abstract book DOIs can be found on the "Author Information" page.
**Disclaimer:** Articles published in the journal HemaSphere exclusively reflect the opinions of the authors. The authors are responsible for all content in their abstracts including accuracy of the facts, statements, citing resources, etc.

**Results**

Among 5,130 registered patients, 560 (10.9%) were ≥80 years at diagnosis; 468 initiated first-line therapy and were included in survival analyses.
Median age at treatment initiation was 84 years (IQR 82–87), and 52.1% were male. At therapy start, 60.9% were aged 80–84 years, 26.9% 85–89 years, and 12.2% ≥90 years. Rai stage was available in 76.1%, with 21.1% classified as high-risk (III–IV). ECOG performance status was reported in 44.4%, of whom 25.5% had ECOG ≥2.
CIRS was documented in 22.2%, with 72.1% scoring ≥6, indicating substantial comorbidity burden. Among tested patients, del(17p) was detected in 14.9% and TP53 mutation in 11.1%.
Median time from diagnosis to treatment was 12 months (IQR 1.6–37.9); 49.8% initiated therapy more than 12 months after diagnosis, and 45.1% received ≥2 treatment lines.
First-line therapy was predominantly chlorambucil-based (70.9%), including chlorambucil monotherapy in 56.2% and chlorambucil combined with an anti-CD20 antibody in 14.7%. Uptake of targeted therapies was limited: BTK inhibitor–based regimens were administered in 5.8%, primarily ibrutinib (3.8%) and less frequently acalabrutinib (1.9%), while venetoclax-based therapy accounted for 3.2%. Anti-CD20 monotherapy was used in 2.1% of patients, whereas FCR-like and bendamustine-based regimens were uncommon (2.1% and 0.6%, respectively). The adoption of targeted agents was confined to the contemporary era; 21.8% of patients initiating therapy after 2015 received BTK inhibitor– or venetoclax-based regimens, although chemotherapy-based approaches remained the predominant strategy.
Early mortality (≤6 months) was 5.8%. Median overall survival from first-line initiation was 104.5 months (95% CI 85.1–115.1). Estimated OS at 12, 24, and 36 months was 89.8%, 82.6%, and 74.4%, respectively. ECOG ≥2 was independently associated with inferior OS (HR 2.22, 95% CI 1.42–3.49; p<0.001).

**Summary/Conclusion**

In this large Brazilian real-world cohort, 11% of patients with CLL were ≥80 years at diagnosis. Biological and comorbidity data were available for a limited subset of patients, reflecting real-world documentation constraints over the study period. Despite advanced age and substantial comorbidity burden among evaluable cases, early mortality was low and median OS was 8.7 years. Treatment remained predominantly chlorambucil-based, with limited use of targeted agents. These findings provide meaningful real-world evidence and strengthen the global understanding of CLL in very elderly patients.

**Copyright Information:** (Online) ISSN: 2572-9241
©2026 The Author(s). HemaSphere is published by John Wiley & Sons Ltd on behalf of European Hematology Association. This is an open access Abstract Book distributed under the Attribution-NonCommercial-NoDerivs (CC BY-NC-ND), which allows third parties to download the articles and share them with others as long as they credit the author and the Abstract Book, but they cannot change the content in any way or use them commercially.
**Abstract Book Citations:** Authors, Title, HemaSphere, 2026;10:(S1):pages. The abstract book DOIs can be found on the "Author Information" page.
**Disclaimer:** Articles published in the journal HemaSphere exclusively reflect the opinions of the authors. The authors are responsible for all content in their abstracts including accuracy of the facts, statements, citing resources, etc.

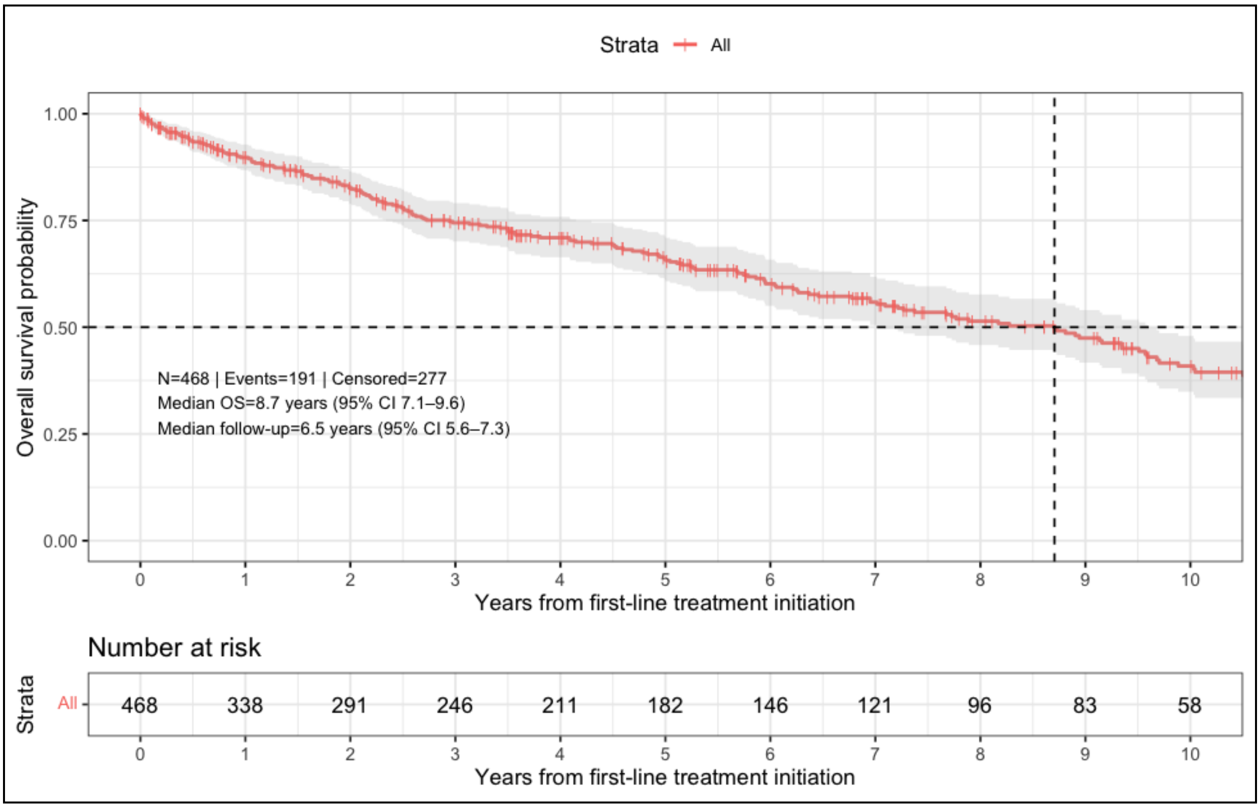

Figure 1. Kaplan–Meier estimate of overall survival from first-line treatment initiation in CLL patients aged ≥80 years (n=468). Median OS was 8.7 years (95% CI 7.1–9.6). Median follow-up was 6.5 years (95% CI 5.6–7.3). Number at risk is shown below the curve. Dashed horizontal line indicates 50% survival probability; dashed vertical line indicates median OS.

**Copyright Information:** (Online) ISSN: 2572-9241
©2026 The Author(s). HemaSphere is published by John Wiley & Sons Ltd on behalf of European Hematology Association. This is an open access Abstract Book distributed under the Attribution-NonCommercial-NoDerivs (CC BY-NC-ND), which allows third parties to download the articles and share them with others as long as they credit the author and the Abstract Book, but they cannot change the content in any way or use them commercially.
**Abstract Book Citations:** Authors, Title, HemaSphere, 2026;10:(S1):pages. The abstract book DOIs can be found on the "Author Information" page.
**Disclaimer:** Articles published in the journal HemaSphere exclusively reflect the opinions of the authors. The authors are responsible for all content in their abstracts including accuracy of the facts, statements, citing resources, etc.

### (PB2939) ANCHOR: FIXED-DURATION SONROTOCLAX AND ZANUBRUTINIB IN CLL/SLL PATIENTS PREVIOUSLY TREATED WITH BTKI MONOTHERAPY, A MULTICENTER, PHASE II STUDY

**Topic:** 06. Chronic lymphocytic leukemia and related disorders - Clinical

Haiwen Huang*[1], Hao Guo[2], Tingyu Wang[3], Xiaobing Huang[4], Chenghao Jin[5], Keshu Zhou[2], Depei Wu[1]

*[1]The First Affiliated Hospital Of Soochow University, Hematology, Suzhou, China;[2]The Affiliated Cancer Hospital of Zhengzhou University & Henan Cancer Hospital, Hematology, Zhengzhou, China; [3]State Key Laboratory of Experimental Haematology, National Clinical Research Centre for Blood Diseases, Haihe Laboratory of Cell Ecosystem, Institute of Haematology & Blood Diseases Hospital, Chinese Academy of Medical Sciences & Peking Union Medical College, Tianjin, China; [4]Sichuan Academy of Medical Sciences & Sichuan Provincial People's Hospital, Hematology, Chengdu, China; [5]Jiangxi Provincial People's Hospital, Nanchang, China;*

### Background

Bruton tyrosine kinase inhibitors (BTKi) have significantly improved outcomes for chronic lymphocytic leukemia (CLL) and small lymphocytic lymphoma (SLL), becoming a standard of care in China. However, the remission depth of BTKi monotherapy is often limited, necessitating continuous treatment. Such prolonged treatment increases the risk of acquired resistance, accumulated toxicity and other challenges, such as economic burden and patient's adherence. Consequently, there remains an unmet need for therapies that achieve deeper remissions and allow for treatment discontinuation, especially for patients currently in continuous treatment mode and would like to have a "treatment holiday". Time-limited therapy is not a regular choice for CLL patients in China, since B-cell lymphoma 2 inhibitor (BCL2i) based fixed duration treatment was not approved for CLL. Regimens of BTKi in combination with BCL2i for a limited treatment duration are being explored. A Phase II study examining this strategy by adding venetoclax to ongoing ibrutinib demonstrated that 71% of patients achieved undetectable minimal residual disease (uMRD) and successfully discontinued both drugs (Thompson et al. *Leukemia.* 2023).
Sonrotoclax, a next-generation BCL2i, is more selective and more pharmacologically potent than venetoclax, featuring a shorter half-life and no drug accumulation. Zanubrutinib, a next-generation BTKi, has shown improved PFS with fewer cardiac adverse events compared to ibrutinib in patients with CLL/SLL (Brown et al. *N Engl J Med.* 2023). The combination of sonrotoclax and zanubrutinib as frontline treatment for CLL/SLL has demonstrated a high MRD clearance rate of 91% by week 48 with favorable tolerability (Soumerai, et al. *Blood.* 2024).

### Aims

This study aims to evaluate the efficacy and safety of fixed-duration of sonrotoclax and zanubrutinib in CLL/SLL patients who have received continuous BTKi therapy for at least 12 months without disease progression.

**Copyright Information:** (Online) ISSN: 2572-9241
©2026 The Author(s). HemaSphere is published by John Wiley & Sons Ltd on behalf of European Hematology Association. This is an open access Abstract Book distributed under the Attribution-NonCommercial-NoDerivs (CC BY-NC-ND), which allows third parties to download the articles and share them with others as long as they credit the author and the Abstract Book, but they cannot change the content in any way or use them commercially.
**Abstract Book Citations:** Authors, Title, HemaSphere, 2026;10:(S1):pages. The abstract book DOIs can be found on the "Author Information" page.
**Disclaimer:** Articles published in the journal HemaSphere exclusively reflect the opinions of the authors. The authors are responsible for all content in their abstracts including accuracy of the facts, statements, citing resources, etc.

**Methods**

ANCHOR is an open-label, single-arm, multicenter, phase II study. Approximately 50 patients with CLL/SLL will be enrolled. Eligible patients were aged ≥18 years and diagnosed according to the iwCLL 2018 criteria for CLL or the NHL Lugano 2014 criteria for SLL. Key inclusion criteria include prior treatment with BTKi monotherapy for at least 12 months without disease progression, failure to achieve uMRD with a sensitivity of $10^{-4}$ (uMRD4), and a willingness to discontinue treatment. Other eligible criteria include Eastern Cooperative Oncology Group performance status ≤2, adequate organ function, and a life expectancy >6 months. Patients who have received prior systemic therapy for CLL/SLL other than BTKi monotherapy will be excluded.
Patients will receive sonrotoclax combined with zanubrutinib (160 mg twice daily) for 12 cycles (28 day per cycle), or until disease progression, unacceptable toxicity, death, withdrawal of consent, or study termination by the sponsor, whichever occurs first. Sonrotoclax will be initiated on cycle 1 day 1 with a ramp-up schedule (4 weeks) to the target dosage of 320 mg daily.
The primary endpoint is the rate of uMRD4 in peripheral blood and bone marrow at the end of 12 cycles of treatment, assessed by flow cytometry. Secondary endpoints include overall response rate, complete response rate, response improvement rate, duration of response, duration of improved response, progression-free survival, overall survival, time to next treatment, and safety.

**Copyright Information:** (Online) ISSN: 2572-9241
©2026 The Author(s). HemaSphere is published by John Wiley & Sons Ltd on behalf of European Hematology Association. This is an open access Abstract Book distributed under the Attribution-NonCommercial-NoDerivs (CC BY-NC-ND), which allows third parties to download the articles and share them with others as long as they credit the author and the Abstract Book, but they cannot change the content in any way or use them commercially.
**Abstract Book Citations:** Authors, Title, HemaSphere, 2026;10:(S1):pages. The abstract book DOIs can be found on the “Author Information” page.
**Disclaimer:** Articles published in the journal HemaSphere exclusively reflect the opinions of the authors. The authors are responsible for all content in their abstracts including accuracy of the facts, statements, citing resources, etc.

### (PB2940) RICHTER TRANSFORMATION IN CHRONIC LYMPHOCYTIC LEUKEMIA: CHARACTERISTICS, TREATMENT AND OUTCOMES IN A SINGLE-CENTER COHORT OF 57 PATIENTS OVER 25 YEARS

**Topic:** 06. Chronic lymphocytic leukemia and related disorders - Clinical

Maria Angelopoulou[1], Alexandros MacHairas*[1], Iliana Konstantinou[1], Anastasia Kopsaftopoulou[1], Maria Dimitrakoudi[1], Eleni Siavou[1], Alexia Piperidou[1], Athanasios Liaskas[1], Aikaterini Kolotsiou[1], Maria Anna Papadopoulou[1], Grigorios Karianakis[1], Rodanthi Fioretzaki[1], Georgios Petros Barakos[1], Stavriana Diavati[1], Konstantina Zikou[1], Panagiota Maria Arapaki[1], Ioannis Asimakopoulos[1], Marina Belia[1], Aikaterini Bitsani[1], Panagiota Petsa[1], Aikaterini Benekou[1], Fotios Panitsas[1], Maria Dimopoulou[2], Helen Plata[1], Panagiotis Tsaftaridis[1], Marina Siakantaris[1], Panayiotis Panayiotidis[1], Maria Dimou[1], Theodoros Vassilakopoulos[1]

*[1]Department of Haematology and Bone Marrow Transplantation, Laiko General Hospital, National and Kapodistrian University of Athens, Athens, Greece; [2]Thalassemia Unit, Laiko General Hospital, Athens, Greece;*

### Background

Richter transformation (RT) represents a rare clonal evolution of chronic lymphocytic leukemia (CLL) into aggressive lymphoma, most commonly diffuse large B-cell lymphoma (DLBCL) or Hodgkin lymphoma (HL) and is associated with poor prognosis. In the evolving era of novel agents, treatment options and prognostic factors are not well established.

### Aims

To evaluate clinical characteristics, treatment patterns, and outcomes of RT patients at a single center.

### Methods

We retrospectively analyzed 960 consecutive CLL patients followed between 2000–2024; 57 developed RT, histologically confirmed in 47.

**Copyright Information:** (Online) ISSN: 2572-9241
©2026 The Author(s). HemaSphere is published by John Wiley & Sons Ltd on behalf of European Hematology Association. This is an open access Abstract Book distributed under the Attribution-NonCommercial-NoDerivs (CC BY-NC-ND), which allows third parties to download the articles and share them with others as long as they credit the author and the Abstract Book, but they cannot change the content in any way or use them commercially.
**Abstract Book Citations:** Authors, Title, HemaSphere, 2026;10:(S1):pages. The abstract book DOIs can be found on the "Author Information" page.
**Disclaimer:** Articles published in the journal HemaSphere exclusively reflect the opinions of the authors. The authors are responsible for all content in their abstracts including accuracy of the facts, statements, citing resources, etc.

### Results

Median age was 62 years at CLL diagnosis and 69.7 years at RT. Three groups of patients were identified: 41 (72 %) developed RT after prior treatment for CLL, 10 (17.5%) after watch and wait- with, while 6 (10.5%) experienced de novo RT. Median time from diagnosis to first CLL treatment was 12.7 months and from diagnosis to RT 84.6 months. Median time from diagnosis to RT was significantly shorter in watch-and-wait versus previously treated patients (30.7 vs 113.6 months, p<0.001) and in SLL versus CLL/atypical CLL (16.7 vs 93.2 months, p=0.017). Among 41 patients treated for CLL prior to RT, 19 had received BTKi and/or BCL2i. Histologic transformation occurred mainly to aggressive B-cell lymphoma [38/47, 80.9%, including DLBCL/high-grade lymphoma (74.5%) and Burkitt lymphoma (6.4%], and HL (12.8%). Bone marrow involvement was present in 35%, cytogenetic clonal evolution in 7/13 evaluable cases and hypercalcemia occurred in 30%. Treatment was histology-driven. Patients with aggressive B-cell lymphoma primarily received R-CHOP–based immunochemotherapy, while salvage regimens included platinum-based combinations. Subsequent therapies included BTKi (n=12), venetoclax-based combinations (n=6), PD-1 inhibitors (n=5), bispecific antibodies (n=3), and polatuzumab-based regimens (n=3). One patient underwent allogeneic stem cell transplantation. Overall response rate to first-line RT therapy was 33.4% (CR 24.6%, PR 8.8%), with higher CR rates in de novo RT (p=0.04). No patient exposed to both BTKi and BCL2i achieved CR, and prior novel agent exposure was associated with lower CR rates (p=0.022). Patients with ≤2 prior therapy lines had higher CR rates (33.3% vs 5.9%, p=0.043).
Median OS was 116.4 months from CLL diagnosis and 5.3 months from RT; 21% were alive at last follow-up. Prior novel agent exposure predicted inferior OS (2.6 vs 8.16 months, p=0.029, Figure 1A). OS was superior in HL versus B-NHL (not reached vs 3.1 months, p=0.012) and without bone marrow involvement (15.2 vs 1.8 months, p=0.023). Achieving CR/PR after first-line RT therapy was the strongest prognostic factor (54.98 vs 1.57 months, p<0.001, Figure 1B).

### Summary/Conclusion

RT occurred in 5.9% of CLL patients and conferred poor prognosis. Prior novel agent exposure, B-cell non-Hodgkin lymphoma histologic subtypes, and bone marrow involvement predicted for inferior OS, while achieving CR/PR after first-line RT therapy was the strongest favorable prognostic factor. Patients receiving ≤2 prior therapy lines demonstrated improved treatment responsiveness.

Author contribution: Angelopoulou M and Machairas A contributed equally to this work and share first authorship. Dimou M and Vassilakopoulos TP contributed equally and share senior authorship.

**Copyright Information:** (Online) ISSN: 2572-9241
©2026 The Author(s). HemaSphere is published by John Wiley & Sons Ltd on behalf of European Hematology Association. This is an open access Abstract Book distributed under the Attribution-NonCommercial-NoDerivs (CC BY-NC-ND), which allows third parties to download the articles and share them with others as long as they credit the author and the Abstract Book, but they cannot change the content in any way or use them commercially.
**Abstract Book Citations:** Authors, Title, HemaSphere, 2026;10:(S1):pages. The abstract book DOIs can be found on the “Author Information” page.
**Disclaimer:** Articles published in the journal HemaSphere exclusively reflect the opinions of the authors. The authors are responsible for all content in their abstracts including accuracy of the facts, statements, citing resources, etc.

### (PB2941) EFFICACY AND TOLERABILITY OF VENETOCLAX PLUS RITUXIMAB IN RELAPSED/REFRACTORY CHRONIC LYMPHOCYTIC LEUKEMIA – REAL-WORLD ANALYSIS OF THE POLISH ADULT LEUKEMIA STUDY GROUP AND THE CZECH CLL STUDY GROUP

**Topic:** 06. Chronic lymphocytic leukemia and related disorders - Clinical

Anita Soboń[1], Anna Panovská[2], Kacper Kościelny[3, 4], Tomas Arpas[2], Elzbieta Iskierka-Jażdżewska[3, 5], Jitka Malcikova[2], Aleksandra Gołos[3, 4], Sarka Pavlova[2], Joanna Drozd-Sokołowska[6], Małgorzata Gajewska[7], Michał Danecki[8], Łukasz Bołkun[9], Agata Ogłoza-Puchowska[10], Marta Morawska[11], Janusz Halka[12], Jagoda Tryc-Szponder[13], Ewa Paszkiewicz-Kozik[14], Leszek Czernik[14], Anna Kopinska[15], Justyna Rybka[16], Agnieszka Szymczyk[17], Kamil Krzysztof Wdowiak[18], Paweł Steckiewicz[19], Iwona Hus[17], Krzysztof Jamroziak[6], Tadeusz Robak[3, 5], Ewa Lech-Marańda[1], Marie Jarosova[2], Jana Kotaskova[2], Sarka Pospisilova[2], Michael Doubek[2], Bartosz Puła*[3, 5]

*[1]Institute of Hematology and Transfusion Medicine, Department of Hematology, Warsaw, Poland;[2]Masaryk University and University Hospital, Department of Internal Medicine – Hematology and Oncology, Faculty of Medicine, Brno, Czechia; [3]Medical University of Lodz, Department of Hematology, Lodz, Poland; [4]Copernicus Memorial Hospital, Department of Hematooncology and Internal Medicine, Lodz, Poland; [5]Copernicus Memorial Hospital, Department of General Hematology and Internal Medicine, Lodz, Poland; [6]Medical University of Warsaw, Department of Hematology, Transplantation and Internal Medicine, Warsaw, Poland; [7]Military Institute of Medicine – National Research Institute, Department of Internal Medicine and Hematology, Warsaw, Poland; [8]Maria Skłodowska-Curie National Research Institute of Oncology, Department of Clinical Oncology, Cracow, Poland; [9]Medical University of Białystok, Department of Hematology, Bialystok, Poland;[10]Medical University of Gdansk, Faculty of Medicine, Department of Hematology, Transplantology and Cellular Therapies, Gdansk, Poland; [11]Medical University of Lublin, Experimental Hematooncology Department, Lublin, Poland; [12]Clinical Hospital of the Ministry of Internal Affairs and Administration with the Warmia-Mazury Oncology Centre, Department of Hematology, Internal Medicine and Bone Marrow Transplantation, Olsztyn, Poland; [13]Poznan University of Medical Sciences, Department of Hematology, Transplantation and Cellular Therapy, Poznan, Poland; [14]Maria Skłodowska-Curie National Research Institute of Oncology, Department of Lymphoid Malignancies, Warsaw, Poland; [15]Medical University of Silesia, Katowice, Poland; [16]Wroclaw Medical University, Department and Clinic of Hematology, Cellular Therapies and Internal Medicine, Wroclaw, Poland; [17]National Medical Institute of the Ministry of Interior and Administration, Department of Hematology, Warsaw, Poland; [18]Medical University of Silesia, Faculty of Medical Sciences, Department of Internal Diseases and Oncological Chemotherapy, Katowice, Poland; [19]Holy Cross Cancer Center, Department of Hematology, Kielce, Poland;*

### Background

Venetoclax-rituximab (VR) has become a standard treatment for relapsed/refractory chronic lymphocytic leukemia (R/R CLL). Nevertheless, large real-world datasets that reflect routine clinical practice and outcomes remain limited.

### Aims

To assess the real-world effectiveness and safety of VR in patients with R/R CLL and to identify clinical and biological prognostic factors linked to outcomes.

### Methods

We conducted a retrospective multicenter study involving 670 patients with R/R CLL treated with VR. Baseline characteristics, previous therapies, cytogenetic and molecular abnormalities, comorbidity burden (CIRS), treatment responses, adverse events, and survival outcomes were analyzed. Overall survival (OS) and progression-free survival (PFS) were estimated using the Kaplan–Meier method, and prognostic factors were evaluated with Cox proportional hazards models.

**Copyright Information:** (Online) ISSN: 2572-9241
©2026 The Author(s). HemaSphere is published by John Wiley & Sons Ltd on behalf of European Hematology Association. This is an open access Abstract Book distributed under the Attribution-NonCommercial-NoDerivs (CC BY-NC-ND), which allows third parties to download the articles and share them with others as long as they credit the author and the Abstract Book, but they cannot change the content in any way or use them commercially.
**Abstract Book Citations:** Authors, Title, HemaSphere, 2026;10:(S1):pages. The abstract book DOIs can be found on the “Author Information” page.
**Disclaimer:** Articles published in the journal HemaSphere exclusively reflect the opinions of the authors. The authors are responsible for all content in their abstracts including accuracy of the facts, statements, citing resources, etc.

### Results

The median age at VR initiation was 69 years. 84.8% of patients had ECOG performance status 0–1. High-risk features, such as del(17p) and *TP53* mutations (Sanger sequencing), were noted in 13.9% and 13.0% of evaluable patients, respectively. At 6 months, the overall response rate (ORR) was 51% (CR 20%, PR 31%). The best ORR during treatment was 78% (CR 36%, PR 42%). Among patients previously treated with BTK inhibitors (22.1% of patients), complete responses were less common (CR 14% at 6 months and 29% as the best response). The estimated 1-, 3-, and 5-year OS rates were 86.5%, 72.7%, and 58.5%, respectively (median OS 6.7 years, 95% CI 5.1–9.5), while the corresponding PFS rates were 84.4%, 65.3%, and 33.3% (median PFS 4.1 years, 95% CI 3.8–4.6). Presence of del(17p) (OS: HR 2.31, 95% CI 1.60–3.34; PFS: HR 2.09, 95% CI 1.50–2.91), combined del(17p)/*TP53* (OS: HR 2.24, 95% CI 1.44–3.50; PFS: HR 1.99, 95% CI 1.36–2.91), *TP53* mutation (OS: HR 2.21, 95% CI 1.40–3.49; PFS: HR 1.86, 95% CI 1.25–2.78), prior BTK inhibitor exposure (OS: HR 1.93, 95% CI 1.35–2.77; PFS: HR 2.01, 95% CI 1.45–2.78) and high comorbidity burden (CIRS >6; OS: HR 1.92, 95% CI 1.35–2.71; PFS: HR 1.45, 95% CI 1.08–1.95) all associated with worse OS and PFS. Clinical tumor lysis syndrome was uncommon (0.9%). Grade ≥3 neutropenia affected 43.5% of patients, pneumonia occurred in 14.8%, and febrile neutropenia occurred in 4.0%.

### Summary/Conclusion

In routine clinical practice, VR provides sustained disease control with an acceptable safety profile in patients with R/R CLL. Outcomes are strongly affected by TP53 pathway abnormalities, comorbidity burden, and prior BTK inhibitor use.

**Copyright Information:** (Online) ISSN: 2572-9241
©2026 The Author(s). HemaSphere is published by John Wiley & Sons Ltd on behalf of European Hematology Association. This is an open access Abstract Book distributed under the Attribution-NonCommercial-NoDerivs (CC BY-NC-ND), which allows third parties to download the articles and share them with others as long as they credit the author and the Abstract Book, but they cannot change the content in any way or use them commercially.
**Abstract Book Citations:** Authors, Title, HemaSphere, 2026;10:(S1):pages. The abstract book DOIs can be found on the "Author Information" page.
**Disclaimer:** Articles published in the journal HemaSphere exclusively reflect the opinions of the authors. The authors are responsible for all content in their abstracts including accuracy of the facts, statements, citing resources, etc.

### (PB2942) LONGITUDINAL SHIFTS IN TREATMENT SELECTION AND SEQUENCING IN CLL: EVIDENCE ALIGNMENT AND INTEGRATION OF PATIENT PREFERENCES

**Topic:** 06. Chronic lymphocytic leukemia and related disorders - Clinical

Carmine Deluca*[1], Michael Beyer[1], Marina Wanes[1], Jessica Massey[1], Meghan C. Thompson[2], Talha Munir[3], Wojciech Jurczak[4], Lydia Scarfò[5], Nicole Lamanna[6], Matthew Davids[7], Jennifer Woyach[8]

*[1]PeerView Institute for Medical Education, New York, United States of America;[2]Memorial Sloan Kettering Cancer Center, New York, United States of America; [3]St James Hospital, Leeds, United Kingdom; [4]Maria Sklodowska-Curie National Research Institute of Oncology, Krakow, Poland; [5]Università Vita Salute and IRCCS Ospedale San Raffaele, Milano, Italy; [6]Columbia University, New York, United States of America; [7]Dana-Farber Cancer Institute, Boston, United States of America; [8]The Ohio State University, Columbus, United States of America;*

### Background

The management of chronic lymphocytic leukaemia (CLL) has undergone a decisive shift from chemoimmunotherapy to targeted strategies including continuous covalent BTK inhibitors (cBTKi), time-limited (TL) BCL2 inhibitor (BCL2i) regimens, BTKi–BCL2i combinations, MRD-guided approaches, and non-covalent BTK inhibitors (ncBTKi). Increasing attention to patient preferences has further influenced treatment selection. This evolution has increased the complexity of first-line (1L) and relapsed/refractory (R/R) decision-making.

### Aims

To assess the impact of these changes on modern CLL treatment selection and sequencing in the United States and Europe, we analysed data captured in a series of educational interventions targeting clinicians over a three-year period.

### Methods

A global initiative evaluating 1L treatment patterns, including 8,215 unique clinicians in active practice across programmes, was conducted from 2023–2025. Participants included haematologist-oncologists and other clinicians from North America, Europe, and Rest of World. Pre- and post-activity assessments measured evidence-aligned 1L treatment selection. Over the same time period, polling of clinicians (N = 1,817) participating in educational initiatives focused on sequential R/R CLL management was analysed to assess shifts in second-line (2L) and third-line (3L) strategy selection. A global patient survey (N = 219) was conducted in 2025 to evaluate patient preferences and perceptions regarding 1L and sequential care.

### Results

Selection of evidence-aligned 1L strategies increased by 33% (50% vs 83%) across all programmes, with the largest gains observed outside the United States and Europe. In multivariable modelling, learners in the highest engagement group (5–8 programmes) had 44% higher odds of selecting evidence-supported time-limited cBTKi–BCL2i therapy compared with those in the lowest engagement group (1–2 programmes) ($p<0.001$; Table and Figure).
In the R/R CLL assessment, adoption of sequential ncBTKi strategies increased across both 2L and 3L settings, with more pronounced gains in 2L selection (+24% in Europe; +47% in the United States) compared with 3L use (+8% and +36%, respectively).
Analysis of the global patient survey (N = 219) showed that 35% of respondents reported uneven provision of guidance on 1L and sequential treatment choices, while 21% noted they had not received adequate counselling regarding time-limited versus continuous therapy. Following structured dissemination of patient-reported findings, clinicians demonstrated increased intention to prioritise structured counselling and proactive patient engagement.

**Copyright Information:** (Online) ISSN: 2572-9241
©2026 The Author(s). HemaSphere is published by John Wiley & Sons Ltd on behalf of European Hematology Association. This is an open access Abstract Book distributed under the Attribution-NonCommercial-NoDerivs (CC BY-NC-ND), which allows third parties to download the articles and share them with others as long as they credit the author and the Abstract Book, but they cannot change the content in any way or use them commercially.
**Abstract Book Citations:** Authors, Title, HemaSphere, 2026;10:(S1):pages. The abstract book DOIs can be found on the "Author Information" page.
**Disclaimer:** Articles published in the journal HemaSphere exclusively reflect the opinions of the authors. The authors are responsible for all content in their abstracts including accuracy of the facts, statements, citing resources, etc.

**Summary/Conclusion**

During a period of rapid therapeutic diversification in CLL, longitudinal education was associated with substantial shifts in evidence-aligned 1L selection and earlier integration of sequential strategies. Higher levels of engagement correlated with greater alignment to contemporary data, while patient-reported gaps in counselling highlighted ongoing needs in shared decision-making. These findings underscore the dynamic interplay between emerging evidence, patient preferences, and clinical implementation, and support continued educational efforts to harmonise modern CLL sequencing strategies across regions.

**Table. Logistic regression model assessing the likelihood of correct responses based on pre- versus post-education status and the number of programs in which each learner participated.**

| Predictor | Odds Ratio [95% CI] | p-value |
|---|---|---|
| Pre vs. Post | 4.46 [3.52, 5.68] | <.001 |
| 3-4 Engagements | 1.23 [1.07, 1.42] | 0.005 |
| 5-8 Engagements | 1.44 [1.22, 1.71] | <.001 |

**Figure. Shift in knowledge and skills related to use of 1L time-limited therapy according to number of engagements**

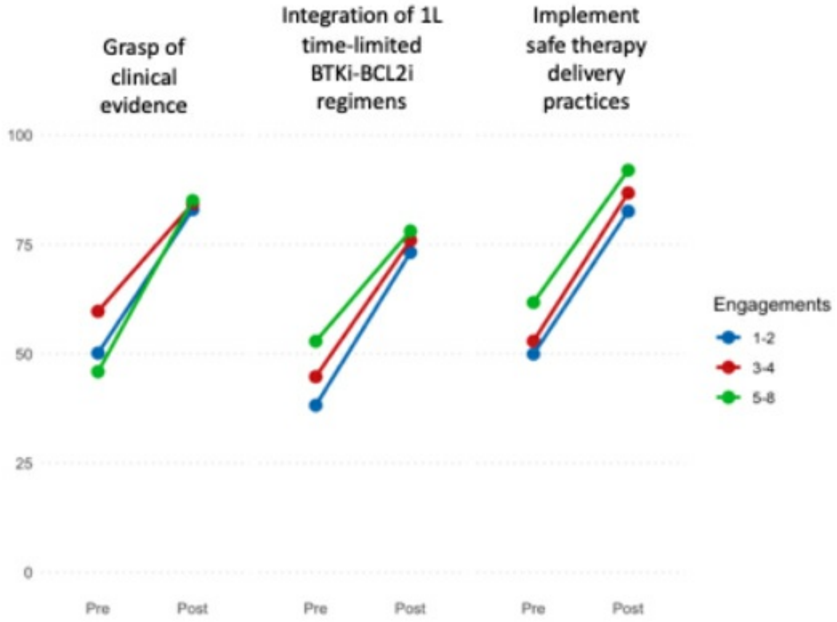

**Copyright Information:** (Online) ISSN: 2572-9241
©2026 The Author(s). HemaSphere is published by John Wiley & Sons Ltd on behalf of European Hematology Association. This is an open access Abstract Book distributed under the Attribution-NonCommercial-NoDerivs (CC BY-NC-ND), which allows third parties to download the articles and share them with others as long as they credit the author and the Abstract Book, but they cannot change the content in any way or use them commercially.
**Abstract Book Citations:** Authors, Title, HemaSphere, 2026;10:(S1):pages. The abstract book DOIs can be found on the "Author Information" page.
**Disclaimer:** Articles published in the journal HemaSphere exclusively reflect the opinions of the authors. The authors are responsible for all content in their abstracts including accuracy of the facts, statements, citing resources, etc.

### (PB2944) NUMBER OF DEATHS AVOIDED WITH USE OF ZANUBRUTINIB VERSUS IBRUTINIB FOR THE TREATMENT OF CHRONIC LYMPHOCYTIC LEUKEMIA (CLL) IN EUROPE

**Topic:** 06. Chronic lymphocytic leukemia and related disorders - Clinical

Talha Munir*[1], Paolo Sportoletti[2], Andrea Visentin[3], Leyla Mohseninejad[4], Lianne Barnieh[5], Keri Yang[6], Wassim Aldairy[6], Shijie Ren[7], Raju Gautam[7], Anurag Gupta[8], G. Rhys Williams[6], Raul Diez Fernandez[9], Francisco Javier Díaz Gálvez[10], Vincent Levy[11]

*[1]Leeds Teaching Hospital, Leeds, United Kingdom; [2]Center for Hemato-Oncological Research (CREO), Universita Delgi Studi di Perugia, Perugia, Italy; [3]University of Padova, Padova, Italy; [4]BeOne Medicines Ltd, Schiphol, The Netherlands; [5]BeOne Medicines Ltd, Paris, France; [6]BeOne Medicines Ltd, San Carlos, CA, United States of America; [7]ConnectHEOR Ltd., London, United Kingdom; [8]ConnectHEOR Ltd., Delhi, India; [9]Hospital Universitario de Getafe, Getafe, Spain; [10]University Hospital of Burgos, Burgos, Spain; [11]Hôpital Avicenne, APHP, Bobigny, France;*

### Background

Bruton tyrosine kinase inhibitors (BTKis), including first-generation ibrutinib (ibru) and next-generation zanubrutinib (zanu), are approved for CLL in Europe. Both have demonstrated efficacy as monotherapy for first-line (1L) treatment versus other agents (zanu in SEQUOIA [NCT03336333]; ibru in RESONATE-2 [NCT01722487]). In the second-line or later (2L+) setting, zanu demonstrated superiority over ibru in preventing progression, relapse or death (ALPINE [NCT03734016]); in ALPINE, ibru was associated with more cardiac deaths than zanu. However, real-world comparative data on CLL cardiac deaths in Europe remain limited.

### Aims

This study estimated the number of cardiac deaths avoided if patients were treated with zanu instead of ibru based on real-world patient numbers from France, Germany, Italy, Spain and the UK.

### Methods

The number of cardiac deaths avoided by treating with zanu was calculated using a number-needed-to-treat (NNT) model for both the 2L+ (base case) and 1L settings (sensitivity analysis). The NNT was calculated as the reciprocal of absolute cardiac death risk increase for ibru versus zanu (1/ |cardiac death risk with ibru – cardiac death risk with zanu|). To estimate the potential total number of avoidable cardiac deaths, the NNT was applied to the number of real-world CLL patients receiving ibru (number of avoidable deaths = number of patients starting on ibru/NNT). To reflect treatment uptake, new patient starts on ibru were calculated starting from the date of availability of zanu for CLL in each respective country to the end of 2025 (derived from IQVIA Oncology Dynamics database). For the base-case analysis, risk of cardiac death was obtained from the ALPINE study; for the sensitivity analysis, risk of cardiac death for zanu and ibru were obtained from SEQUOIA and RESONATE-2, respectively.

### Results

In the base case, the NNT to avoid one cardiac death with treatment with zanu was 54. When considering the time period starting from availability of zanu, new patient starts on ibru across the five countries were estimated to total 1743 in the 2L+ setting and thus a total of 32 deaths would have been avoided if patients were treated with zanu instead of ibru (**Table**). In the sensitivity analysis in the 1L setting, the NNT to avoid one cardiac death with zanu treatment was 46. An estimated total of 52 deaths may have been avoided if these patients were treated with zanu instead of ibru, based on 2428 new patient starts on ibru.

### Summary/Conclusion

**Copyright Information:** (Online) ISSN: 2572-9241
©2026 The Author(s). HemaSphere is published by John Wiley & Sons Ltd on behalf of European Hematology Association. This is an open access Abstract Book distributed under the Attribution-NonCommercial-NoDerivs (CC BY-NC-ND), which allows third parties to download the articles and share them with others as long as they credit the author and the Abstract Book, but they cannot change the content in any way or use them commercially.
**Abstract Book Citations:** Authors, Title, HemaSphere, 2026;10:(S1):pages. The abstract book DOIs can be found on the "Author Information" page.
**Disclaimer:** Articles published in the journal HemaSphere exclusively reflect the opinions of the authors. The authors are responsible for all content in their abstracts including accuracy of the facts, statements, citing resources, etc.

This NNT analysis demonstrated that treatment with zanu instead of ibru monotherapy could have prevented ~84 cardiac deaths across both 2L+ and 1L CLL settings. The study findings should be interpreted within the context of the model assumptions and data inputs. Further real-world studies using European registry data are needed to confirm these findings.

**Table. Number of cardiac deaths avoided by country**

| Outcome | Country | | | | |
|---|---|---|---|---|---|
| | France | Germany | Italy | Spain | UK |
| **Time period*** | **Feb 2024–Dec 2025** | **Nov 2022–Dec 2025** | **Nov 2023–Dec 2025** | **Jun 2023–Dec 2025** | **Oct 2023–Dec 2025** |
| | **Base case (2L+)** | | | | |
| Cardiac death risk with zanu | 0% | | | | |
| Cardiac death risk with ibru | 1.852% | | | | |
| NNT | 54 | | | | |
| Number of patients | 109 | 602 | 728 | 142 | 162 |
| Number of deaths avoided | 2 | 11 | 13 | 3 | 3 |
| **Total deaths avoided** | **32** | | | | |
| | **Sensitivity analysis (1L)** | | | | |
| Cardiac death risk with zanu | 0.833% | | | | |
| Cardiac death risk with ibru | 2.963% | | | | |
| NNT | 46 | | | | |
| Number of patients | 337 | 710 | 775 | 267 | 339 |
| Number of deaths avoided | 7 | 15 | 17 | 6 | 7 |
| **Total deaths avoided** | **52** | | | | |

*Considering the date of zanu availability.

**Copyright Information:** (Online) ISSN: 2572-9241
©2026 The Author(s). HemaSphere is published by John Wiley & Sons Ltd on behalf of European Hematology Association. This is an open access Abstract Book distributed under the Attribution-NonCommercial-NoDerivs (CC BY-NC-ND), which allows third parties to download the articles and share them with others as long as they credit the author and the Abstract Book, but they cannot change the content in any way or use them commercially.
**Abstract Book Citations:** Authors, Title, HemaSphere, 2026;10:(S1):pages. The abstract book DOIs can be found on the "Author Information" page.
**Disclaimer:** Articles published in the journal HemaSphere exclusively reflect the opinions of the authors. The authors are responsible for all content in their abstracts including accuracy of the facts, statements, citing resources, etc.

### (PB2945) PATIENT CHARACTERISTICS, GENETIC PROFILING, AND TREATMENT PATTERNS IN CHRONIC LYMPHOCYTIC LEUKEMIA (CLL) IN LITHUANIA, 2021–2024

**Topic:** 06. Chronic lymphocytic leukemia and related disorders - Clinical

Regina Pileckyte*[1], Rasa Ugenskiene[2], Mindaugas Stoškus[3], Ligita Kasnauskiene[4], Migle Kulbokė[5], Inga Legė[6], Rolandas Gerbutavicius[5]

*[1]Vilnius University Hospital Santaros Klinikos, Hematology, Oncology and Transfusion Medicine Center, Vilnius, Lithuania; [2]Lithuanian University of Health Sciences (LSMU), Department of Genetics and Molecular Medicine, Kaunas, Lithuania;[3]Vilnius University Hospital Santaros Klinikos, Department of Molecular Medicine; Hematology, Oncology and Transfusion Medicine Center, Vilnius, Lithuania; [4]Klaipėda University Hospital, Hematology Center, Klaipeda, Lithuania; [5]Lithuanian University of Health Sciences (LSMU) Kauno klinikos, Department of Oncology and Hematology, Kaunas, Lithuania; [6]AstraZeneca Lietuva, Vilnius, Lithuania;*

### Background

CLL is the most common adult leukemia in Western countries, characterized by the accumulation of mature $CD5^+$ B lymphocytes in the blood, bone marrow, and lymphoid tissues (Hallek, 2025). Disease progression and treatment response are influenced by genetic factors, including del(17p), *TP53* mutations, and *IGHV* mutation status (van der Straten, 2024). Personalized treatment strategies integrating genetic, clinical, and patient-specific factors are essential to optimize outcomes (Stamatopoulos *et al.*, 2024). Analysis of secondary Data from CLL patients provides insights to guide individualized therapy.

### Aims

This analysis evaluated the demographic characteristics, diagnosis, and treatment practices among CLL patients in Lithuania, focusing on genetic prognostic testing from 2021 to 2024.

### Methods

Secondary health data (2021-2024) were analyzed using the Lithuanian State Data Agency's data reuse platform, specifically the Contour analytic tool in Palantir Foundry, and Microsoft® Excel® for Microsoft 365.

**Copyright Information:** (Online) ISSN: 2572-9241
©2026 The Author(s). HemaSphere is published by John Wiley & Sons Ltd on behalf of European Hematology Association. This is an open access Abstract Book distributed under the Attribution-NonCommercial-NoDerivs (CC BY-NC-ND), which allows third parties to download the articles and share them with others as long as they credit the author and the Abstract Book, but they cannot change the content in any way or use them commercially.
**Abstract Book Citations:** Authors, Title, HemaSphere, 2026;10:(S1):pages. The abstract book DOIs can be found on the "Author Information" page.
**Disclaimer:** Articles published in the journal HemaSphere exclusively reflect the opinions of the authors. The authors are responsible for all content in their abstracts including accuracy of the facts, statements, citing resources, etc.

### Results

Between 2021 and 2024, 3,518 unique patients with CLL were recorded. Most patients (n = 2,617; 74.4%) had healthcare encounters in 2024, while 901 had their last visit in 2021–2023. Overall, 1,076 patients (30.6%) received at least one CLL-specific treatment line.
In the overall cohort, the sex distribution was 51.1% men and 48.9% women. Among patients treated for CLL, men accounted for 56.8% and women for 43.2%. Most diagnoses were made in patients aged 61–90 years (82.3%), particularly 71–80 years (31.5%). Men were more often diagnosed at 41–70 years (42.1% vs. 30.9% of women), while women predominated in older age groups (71–100 years: 67.7% vs. 56.6% of men). The median age was 75 years (range: 12-100 years), with a median of 76 years for women and 73 years for men.
Genetic prognostic testing was performed in 1,049 patients. Among these, 17p13 (*TP53*) deletion was detected in 75 patients (7.6%), *TP53* exon 2–11 mutations in 65 (9.9%). In this cohort, 209 patients (29.7%) had mutated *IGHV*, and 453 (64.4%) had unmutated *IGHV*. *IGHV* testing was unsuccessful in 5.8% of patients.
Treatment trends showed increasing use of Bruton's tyrosine kinase inhibitors (BTKis), particularly second-generation agents, and growing use of BCL2 inhibitor, while chemoimmunotherapy (CIT/CT) usage declined. Second-generation BTKis were preferred as first-line therapy for patients with *TP53* mutations, 17p deletions, or unmutated *IGHV*. First-generation BTKi use declined. BCL2 inhibitor was not used as first-line for *TP53*-mutated patients, but was prescribed for those with mutated or unmutated *IGHV*.

### Summary/Conclusion

In Lithuania, CLL predominantly affects older adults. Genetic profiling is a key factor for therapy selection, supporting personalized treatment strategies, particularly with second-generation BTKis and BCL2 inhibitor. Data from 2021–2024 demonstrate the integration of genetic testing into routine CLL management, enabling tailored therapies.

**Financial disclosures:**
Medical writing support was provided by GuruMA, Kaunas, Lithuania, funded by AstraZeneca. Funding for this work was provided by AstraZeneca.

**Copyright Information:** (Online) ISSN: 2572-9241
©2026 The Author(s). HemaSphere is published by John Wiley & Sons Ltd on behalf of European Hematology Association. This is an open access Abstract Book distributed under the Attribution-NonCommercial-NoDerivs (CC BY-NC-ND), which allows third parties to download the articles and share them with others as long as they credit the author and the Abstract Book, but they cannot change the content in any way or use them commercially.
**Abstract Book Citations:** Authors, Title, HemaSphere, 2026;10:(S1):pages. The abstract book DOIs can be found on the "Author Information" page.
**Disclaimer:** Articles published in the journal HemaSphere exclusively reflect the opinions of the authors. The authors are responsible for all content in their abstracts including accuracy of the facts, statements, citing resources, etc.

### (PB2946) CLINICAL CHARACTERISTICS AND TREATMENT OUTCOMES OF RICHTER TRANSFORMATION AND CLL ACCELERATION: A 15-YEAR SINGLE-CENTER EXPERIENCE

**Topic:** 06. Chronic lymphocytic leukemia and related disorders - Clinical

Maria Kislova*[1], Timur Yusupov[1], Elena Dmitrieva[1, 2], Alla Loban'[1], Maria Gladysheva[3], Tatiana Obukhova[3], Bella Biderman[3], Andrey Sudarikov[3], Ilya Sidorov[1], Dmitry Abramov[1], Alla Kovrigina[3], Vadim Ptushkin[1], Eugene Nikitin[1, 2]

*[1]Botkin hospital, Moscow, Russia; [2]Russian Medical Academy of Continuous Professional Education, Moscow, Russia; [3]Hematology Research Center of Russia, Moscow, Russia;*

### Background

Richter transformation (RT) and chronic lymphocytic leukemia (CLL) acceleration represent distinct forms of disease progression in CLL with potentially different biology and outcomes.

### Aims

To characterize clinical profile of patients with RT, treatment options and outcomes in Botkin hospital, Moscow, Russia.

### Methods

A retrospective single-center study included 54 patients: 42 with histologically confirmed RT and 12 with CLL acceleration treated at Botkin Hospital from 2010 to 2025. Overall survival (OS) was calculated from the date of progression diagnosis to death or last contact. Targeted agents (TA) were defined as regimens containing BTK inhibitors, venetoclax, checkpoint inhibitors, or obinutuzumab.

### Results

Richter transformation cohort (n=42): The cohort included 29 men and 13 women; median age was 64 years (range 40–91). Six patients (14%) had not received CLL therapy prior to the RT diagnosis. Histological sybtypes of RT were DLBCL in 42, Hodgkin lymphoma variant in 2, and EBER-negative extranodal plasmablastic lymphoma in 1. The median number of prior CLL therapy lines before transformation was 4 (range 0–10), and the median number of transformation therapy lines was 1 (range 0–7). The median time from CLL diagnosis to RT was 59 months (range 0–227). The median time from the start of 1st-line CLL therapy to RT was 37.5 months (range 0–153). High-risk genetic features included del17p in 11/28 (39%), del11q in 7/20 (35%), and TP53 mutations in 7/11 (64%) evaluated patients. Unmutated IGHV status was determined in 100% of patients. Clonal relationship of the RT and CLL was performed in 5 patients, of whom it was established in 4. Regarding first-line treatment, TA were used in 14 (33%) patients and chemoimmunotherapy (CIT) in 20 (48%). Median OS from RT diagnosis was 5.0 months. The number of prior CLL therapy lines and age at RT diagnosis did not impact outcomes (p>0.05). Comparing TA versus CIT, median OS was 8.5 and 4.0 months, respectively (p=0.8; Figure 1). Allo-HSCT was performed in 2 patients (both achieved CR); CAR-T in 1 patient.
CLL acceleration cohort (n=12): The cohort included 8 men and 4 women; median age was 64 years (range 51–77). High-risk genetic features were less frequent than in RT: del17p was detected in 3/7 (42.9%) and TP53 mutations in 1/5 (20%) evaluated patients. Regarding first-line treatment, TA were used in 5 (41.7%) patients (G-VEN n=2, venetoclax n=1, acalabrutinib n=1, VIPOR n=1) and CIT in 5 (41.7%) patients (R-B n=2, R-CHOP/CHOP n=3). Two patients did not receive immediate therapy or had missing treatment data. Median OS was not reached; 8/12 (67%) patients remain alive (follow-up range 0.5–22 months, 4 deaths).

**Copyright Information:** (Online) ISSN: 2572-9241
©2026 The Author(s). HemaSphere is published by John Wiley & Sons Ltd on behalf of European Hematology Association. This is an open access Abstract Book distributed under the Attribution-NonCommercial-NoDerivs (CC BY-NC-ND), which allows third parties to download the articles and share them with others as long as they credit the author and the Abstract Book, but they cannot change the content in any way or use them commercially.
**Abstract Book Citations:** Authors, Title, HemaSphere, 2026;10:(S1):pages. The abstract book DOIs can be found on the "Author Information" page.
**Disclaimer:** Articles published in the journal HemaSphere exclusively reflect the opinions of the authors. The authors are responsible for all content in their abstracts including accuracy of the facts, statements, citing resources, etc.

**Summary/Conclusion**

CLL acceleration demonstrated a distinct clinical course with a high rate of short-term survival (median not reached) compared to the poor prognosis of RT (median 5.6 months). In the RT cohort, TA and CIT yielded equivalent outcomes, suggesting that prior TA exposure and patient performance status might be more critical determinants of outcome than the specific class of RT therapy. The small sample size of the acceleration cohort precludes definitive statistical comparison but highlights a trend toward better outcomes. Harmonizing histological and clonality evaluations remains a diagnostic challenge. Further prospective studies are needed to better understand the clinical trajectory of both forms of progression and to define the optimal use of targeted agents.

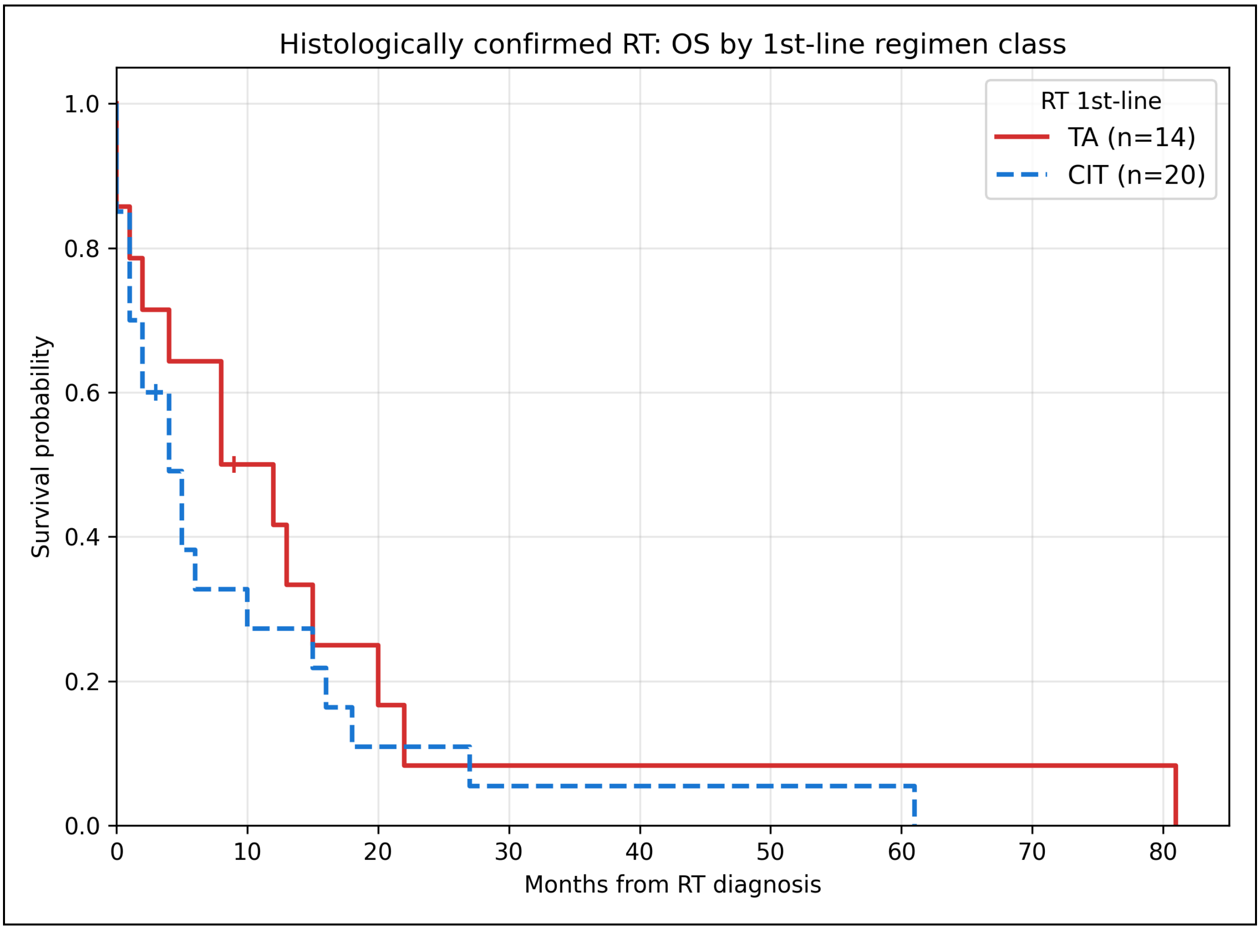

**Copyright Information:** (Online) ISSN: 2572-9241
©2026 The Author(s). HemaSphere is published by John Wiley & Sons Ltd on behalf of European Hematology Association. This is an open access Abstract Book distributed under the Attribution-NonCommercial-NoDerivs (CC BY-NC-ND), which allows third parties to download the articles and share them with others as long as they credit the author and the Abstract Book, but they cannot change the content in any way or use them commercially.
**Abstract Book Citations:** Authors, Title, HemaSphere, 2026;10:(S1):pages. The abstract book DOIs can be found on the "Author Information" page.
**Disclaimer:** Articles published in the journal HemaSphere exclusively reflect the opinions of the authors. The authors are responsible for all content in their abstracts including accuracy of the facts, statements, citing resources, etc.

### (PB2947) SIGNIFICANT IMPROVEMENT OF DISEASE-SPECIFIC OVERALL SURVIVAL IN PATIENTS WITH CHRONIC LYMPHOCYTIC LEUKEMIA: ANALYSIS OF AN UNSELECTED COHORT FROM THE HRADEC KRÁLOVÉ DISTRICT, CZECH REPUBLIC.

**Topic:** 06. Chronic lymphocytic leukemia and related disorders - Clinical

Lukáš Smolej*[1], Pavel Vodarek[1], Dominika Écsiová[1], Kateřina MacHálková[2], Martin Šimkovič[1]

*[1]University Hospital and Charles University Faculty of Medicine, 4th Department of Internal Medicine - Hematology, Hradec Králové, Czechia; [2]Hematology outpatient clinic Medika HK, Hradec Králové, Czechia;*

### Background

One of the key characteristics of chronic lymphocytic leukemia (CLL) is the marked variability of its clinical course; approximately half of patients never require treatment. Over the past two decades, the therapeutic approach to CLL has undergone a profound transformation-from chemotherapy to chemoimmunotherapy and, more recently, to oral targeted agents (Bruton tyrosine kinase inhibitors and the BCL-2 inhibitor venetoclax). However, analyses of the natural history and overall survival at large specialized centers are often hampered by referral bias, as regional hospitals typically refer only patients who require therapy and therefore have a poorer prognosis.

### Aims

To evaluate evolution of time to first-line therapy (TTFT) and CLL-specific overall survival (OS) over time in an unselected cohort of CLL patients (pts) with long-term follow-up from a well-defined geographical region (Hradec Králové District, Czech Republic; population approximately 168,000).

### Methods

Between January 1, 1999, and December 31, 2023, a total of 371 pts with the place of permanent residence in the Hradec Králové District were diagnosed with CLL. The median age was 69 years (IQR 67–73); 58.0% were male, 94.6% had an ECOG performance status of 0–1, and Binet stage A/B/C was present in 84.6%/6.5%/8.1%. Unmutated IGHV status was identified in 42.7% and TP53 aberrations (mutation or deletion) in 6.8%. At a median follow-up of 152 months (12.7 years), 162 pts (43.6%) required first-line (1L) therapy and 210 (56.6%) had died. Data on cause of death were available for 166 of 210 patients (79.0%). To compare baseline characteristics and clinical outcomes, the cohort was divided according to years of diagnosis into Group 1 (1999–2010; n=168; median follow-up 225 months) and Group 2 (2011–2023; n=203; median follow-up 113 months).

**Copyright Information:** (Online) ISSN: 2572-9241
©2026 The Author(s). HemaSphere is published by John Wiley & Sons Ltd on behalf of European Hematology Association. This is an open access Abstract Book distributed under the Attribution-NonCommercial-NoDerivs (CC BY-NC-ND), which allows third parties to download the articles and share them with others as long as they credit the author and the Abstract Book, but they cannot change the content in any way or use them commercially.
**Abstract Book Citations:** Authors, Title, HemaSphere, 2026;10:(S1):pages. The abstract book DOIs can be found on the “Author Information” page.
**Disclaimer:** Articles published in the journal HemaSphere exclusively reflect the opinions of the authors. The authors are responsible for all content in their abstracts including accuracy of the facts, statements, citing resources, etc.

**Results**

Baseline characteristics did not differ significantly between Groups 1 and 2. Similarly, TTFT was not significantly different (median 113 vs. 80 months; HR 1.13; 95% CI 0.86–1.56; p=0.46). In contrast, there was a statistically and clinically significant improvement of CLL-specific OS in Group 2 (10-year OS 79.8% vs. 62.9%; HR 0.58; 95% CI 0.38–0.90; p=0.015; Fig. 1A). Survival from initiation of 1L therapy was likewise significantly prolonged in Group 2 (median 133 vs. 74 months; HR 0.60; 95% CI 0.37–0.98; p=0.042; Fig. 1B). Patients treated with oral targeted agents in the 1L setting achieved superior OS compared with those receiving chemoimmunotherapy, while chemotherapy alone was associated with the poorest outcomes (median not reached vs. 129 vs. 42 months, respectively; p<0.0001; Fig. 1C). In multivariable analysis of CLL-specific OS, diagnosis in Group 2 retained independent favorable prognostic significance (HR 0.94; 95% CI 0.92–0.97; p=0.0006), along with younger age as a continuous variable (HR 0.46; 95% CI 0.29–0.71; p<0.0001), mutated IGHV status (HR 0.56; 95% CI 0.36–0.89; p=0.014), ECOG performance status 0–1 (HR 0.25; 95% CI 0.10–0.61; p=0.0022), Binet stage A/B (HR 0.29; 95% CI 0.15–0.58; p=0.0005), and absence of del(17p) (HR 0.26; 95% CI 0.11–0.60; p=0.0014).

**Summary/Conclusion**

This retrospective analysis of a large, unselected cohort of patients with CLL and long-term follow-up demonstrated a significant improvement in CLL-specific OS over time, likely reflecting advances in therapeutic strategies. The most favorable outcomes were observed in patients treated with oral targeted agents in the first-line setting.

**Copyright Information:** (Online) ISSN: 2572-9241
©2026 The Author(s). HemaSphere is published by John Wiley & Sons Ltd on behalf of European Hematology Association. This is an open access Abstract Book distributed under the Attribution-NonCommercial-NoDerivs (CC BY-NC-ND), which allows third parties to download the articles and share them with others as long as they credit the author and the Abstract Book, but they cannot change the content in any way or use them commercially.
**Abstract Book Citations:** Authors, Title, HemaSphere, 2026;10:(S1):pages. The abstract book DOIs can be found on the “Author Information” page.
**Disclaimer:** Articles published in the journal HemaSphere exclusively reflect the opinions of the authors. The authors are responsible for all content in their abstracts including accuracy of the facts, statements, citing resources, etc.

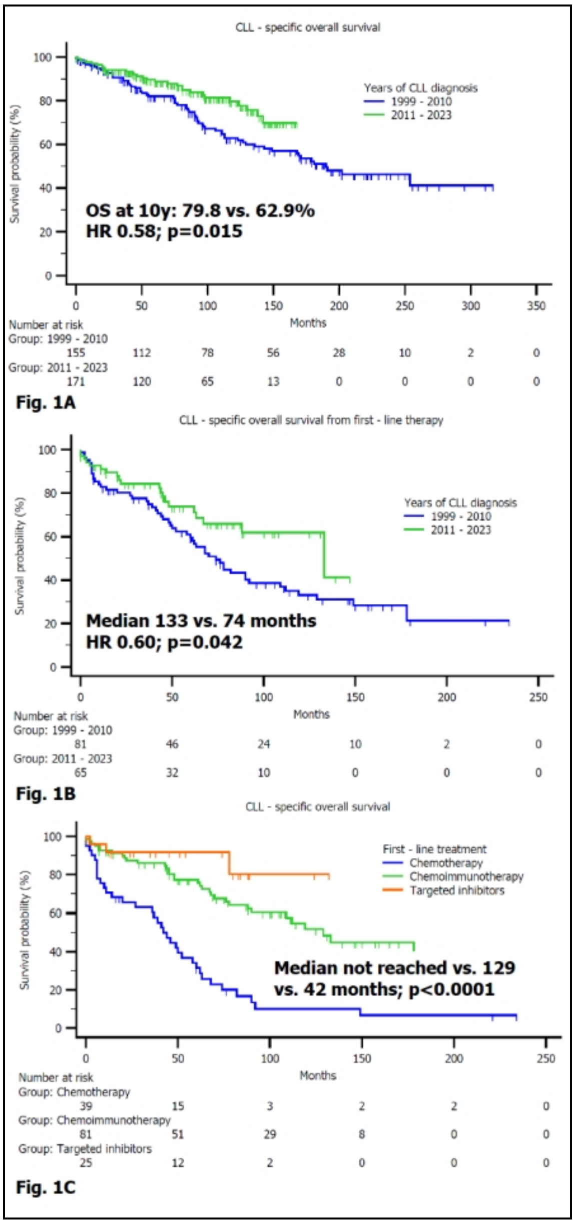

Fig 1A: Significant improvement of CLL-specific OS in patients diagnosed between 2011-2023 vs 1999-2010. Fig. 1B: Significantly superior CLL-specific OS from initiation of 1L therapy in patients diagnosed between 2011-2023. Fig. 1C: Significantly better CLL-specific OS from the start of 1L therapy in patients treated with oral targeted agents vs. chemoimmunotherapy vs. chemotherapy.

**Copyright Information:** (Online) ISSN: 2572-9241
©2026 The Author(s). HemaSphere is published by John Wiley & Sons Ltd on behalf of European Hematology Association. This is an open access Abstract Book distributed under the Attribution-NonCommercial-NoDerivs (CC BY-NC-ND), which allows third parties to download the articles and share them with others as long as they credit the author and the Abstract Book, but they cannot change the content in any way or use them commercially.
**Abstract Book Citations:** Authors, Title, HemaSphere, 2026;10:(S1):pages. The abstract book DOIs can be found on the “Author Information” page.
**Disclaimer:** Articles published in the journal HemaSphere exclusively reflect the opinions of the authors. The authors are responsible for all content in their abstracts including accuracy of the facts, statements, citing resources, etc.

### (PB2948) TIME TO NEXT TREATMENT ACCORDING TO SECOND-LINE REGIMEN FOR CHRONIC LYMPHOCYTIC LEUKEMIA: A RETROSPECTIVE ANALYSIS OF THE BRAZILIAN REGISTRY OF CLL

**Topic:** 06. Chronic lymphocytic leukemia and related disorders - Clinical

Marcelo Pitombeira De Lacerda*[1], Vinicius Molla[2], Caio Cesar Justino de Oliveira[3], Fernanda Santos Azevedo[3], Fernanda Marques[3], Leila Perobelli[4], Valeria Buccheri[5], Alita Azevedo[6], Rodrigo Santucci[7], Danielle Leão[8], Talita Maira Bueno Da Silveira[9], Laura Fogliatto[10], Fernando Barroso[11], Cristiana Solza[12], Vera Lucia Piratininga de Figueiredo[13], Glaciano Ribeiro[14], Gislaine B. Oliveira[15], Henrique Nunes Pêcego[16], Pedro Amoedo[17], Nelson Hamerschlak[18], Abel Costa Neto[19], Adriana Alves De Souza Scheliga[20], Thiago Carneiro[21], Patricia Giacon[22], Eduardo Flavio Oliveira Ribeiro[23], Wolney Gois Barreto[24], Rafael Dezen Gaiolla[25], Carlos Sergio Chiattone[26], Celso Arrais-Rodrigues[2, 3]

*[1]Universidade da Regiao de Joinville - UNIIVLLE, Joinville, Brazil;[2]Hospital Nove de Julho, DASA, São Paulo, Brazil; [3]Hemocentro Unifesp, São Paulo, Brazil; [4]Hospital de Transplantes Euryclides Zerbini, Sao Paulo, Brazil; [5]ICESP, Sao Paulo, Brazil; [6]Fundação Hemope, Recife, Brazil; [7]Instituto Hemomed, São Paulo, Brazil; [8]Hospital Beneficência Portuguesa de São Paulo, São Paulo, Brazil; [9]AC Camargo Câncer Center P7, São Paulo, Brazil; [10]Hospital de Clínicas de Porto Alegre, Porto Alegre, Brazil; [11]Universidade Federal do Ceará, Fortaleza, Brazil; [12]Uerj, Rio de Janeiro, Brazil; [13]IAMSPE, São Paulo, Brazil; [14]UFMG - Universidade Federal de Minas Gerais, Belo Horizonte, Brazil; [15]UNICAMP, Campinas, Brazil; [16]Instituto D'Or de Pesquisa e Ensino, Rio de Janeiro, Brazil; [17]Clinica Amo Assistencia Multidisciplinar Em Oncologia, Salvador, Brazil; [18]Hospital Israelita Albert Einstein, São Paulo, Brazil; [19]Instituto D'or de Pesquisa E Ensino, São Paulo, Brazil;[20]Grupo Oncoclinicas, Rio de Janeiro, Brazil; [21]Hospital Ophir Loyola, Belém, Brazil; [22]Hospital Santa Marcelina, São Paulo, Brazil; [23]Hospital Santa Lucia, Brasilia, Brazil; [24]Unisalesiano, Aracatuba, Brazil; [25]UNESP, Botucatu, Brazil; [26]Hospital Samaritano, São Paulo, Brazil;*

### Background

Targeted therapies have significantly improved outcomes in relapsed/refractory chronic lymphocytic leukemia (CLL). However, delayed adoption and unequal access to these agents remain major challenges in low- and middle-income countries. Real-world data are essential to evaluate treatment patterns and outcomes in such settings.

### Aims

To evaluate time to next treatment (TTNT) and overall survival (OS) according to second-line regimen in patients enrolled in the Brazilian CLL Registry.

### Methods

This observational study included patients who initiated second-line therapy between 2015 and 2025. To account for temporal changes in drug availability, analyses were stratified by treatment era. TTNT and OS were estimated using Kaplan–Meier methods and compared using the log-rank test. Multivariable Cox proportional hazards models adjusted for age, TP53 aberrations, and year of treatment initiation. Second-line regimens were categorized as: Targeted therapy (continuous BTK inhibitors or venetoclax-based regimens), CLL-directed chemo(immunotherapy) (chlorambucil or FC ± anti-CD20), Alternative lymphoma-based chemotherapy.

**Copyright Information:** (Online) ISSN: 2572-9241
©2026 The Author(s). HemaSphere is published by John Wiley & Sons Ltd on behalf of European Hematology Association. This is an open access Abstract Book distributed under the Attribution-NonCommercial-NoDerivs (CC BY-NC-ND), which allows third parties to download the articles and share them with others as long as they credit the author and the Abstract Book, but they cannot change the content in any way or use them commercially.
**Abstract Book Citations:** Authors, Title, HemaSphere, 2026;10:(S1):pages. The abstract book DOIs can be found on the "Author Information" page.
**Disclaimer:** Articles published in the journal HemaSphere exclusively reflect the opinions of the authors. The authors are responsible for all content in their abstracts including accuracy of the facts, statements, citing resources, etc.

**Results**

A total of 629 patients were included. Median age at second-line initiation was 62 years. Targeted therapies were administered in 224 patients (36%), including continuous BTK inhibitors in 167 (27%) and venetoclax-based regimens in 57 (9%). CLL-directed chemo(immunotherapy) was used in 269 patients (44%), and lymphoma-based chemotherapy in 123 (20%).
Median follow-up after second-line initiation was 36 months. Median TTNT was 31 months overall. TTNT was significantly longer for targeted therapies (60 months; 95% CI 49–69) compared with CLL-directed chemo(immunotherapy) (24 months; 95% CI 20–29) and lymphoma-based chemotherapy (12 months; 95% CI 8–17; p<0.0001).
In multivariable analysis, targeted therapy was independently associated with longer TTNT compared with CLL-directed chemo(immunotherapy) (HR 0.42; 95% CI 0.29–0.60) and lymphoma-based chemotherapy (HR 0.27; 95% CI 0.17–0.41). Three-year OS was 86–95% for targeted therapies versus 59% for chemotherapy-based regimens (p=0.00017). In adjusted analysis, targeted therapy remained independently associated with improved OS (HR 0.35; 95% CI 0.25–0.49).

**Summary/Conclusion**

In this large real-world Brazilian cohort, targeted therapies were associated with significantly longer TTNT and improved OS compared with chemotherapy-based approaches in second-line CLL. Despite regulatory approval of novel agents, chemotherapy-only regimens remain frequently used, highlighting persistent disparities in treatment access. These findings illustrate the impact of therapeutic adoption on real-world outcomes in middle-income settings.

**Copyright Information:** (Online) ISSN: 2572-9241
©2026 The Author(s). HemaSphere is published by John Wiley & Sons Ltd on behalf of European Hematology Association. This is an open access Abstract Book distributed under the Attribution-NonCommercial-NoDerivs (CC BY-NC-ND), which allows third parties to download the articles and share them with others as long as they credit the author and the Abstract Book, but they cannot change the content in any way or use them commercially.
**Abstract Book Citations:** Authors, Title, HemaSphere, 2026;10:(S1):pages. The abstract book DOIs can be found on the “Author Information” page.
**Disclaimer:** Articles published in the journal HemaSphere exclusively reflect the opinions of the authors. The authors are responsible for all content in their abstracts including accuracy of the facts, statements, citing resources, etc.

### (PB2949) EARLY TREATMENT-PHASE CARE DELIVERY BURDEN WITH VENETOCLAX-BASED VERSUS BTK INHIBITOR–BASED REGIMENS IN TREATMENT-NAÏVE CLL

**Topic:** 06. Chronic lymphocytic leukemia and related disorders - Clinical

Truman Koh*[1], Farrukh Awan[1]

*[1]University of Texas Southwestern Medical Center at Dallas, Department of Internal Medicine, Division of Hematology and Oncology, Dallas, United States of America;*

### Background

Targeted therapies have transformed the first-line treatment of chronic lymphocytic leukemia (CLL), yet regimens differ in duration, monitoring intensity, and combination phases. These differences may generate distinct care delivery demands that are not captured by traditional utilization metrics.

### Aims

To compare early treatment-phase care delivery burden between venetoclax-based and Bruton tyrosine kinase inhibitor (BTKi)–based first-line regimens in treatment-naïve CLL.

### Methods

We conducted a retrospective single health system study of treatment-naïve CLL patients receiving acalabrutinib (A), acalabrutinib plus obinutuzumab (AO), or venetoclax plus obinutuzumab (VO). Patients were identified using structured queries and manual chart review. Regimen-specific timepoints were defined relative to treatment initiation. The baseline period represented the 180-day pre-treatment window. The early phase represented the first post-initiation monotherapy treatment window, and the obinutuzumab phase corresponded to active combination therapy for AO and VO.
Primary outcomes included healthcare encounter rates, electronic patient portal message thread rates, and physician one-to-one encounter rates. Rates were calculated as total events divided by total patient-days within each regimen phase and expressed as events per patient-month (30.44 days).

### Results

The analytic cohort included 134 patients (VO n=35; AO n=63; A n=36), contributing 22,865 analyzed encounters across all regimen phases after filtering (VO 7,278; AO 11,951; A 3,636). Baseline encounter rates were similar across regimens (VO 4.0; AO 4.7; A 4.2 events/month).
During the early treatment phase, encounter rates increased across all regimens but were highest with VO (17.2 events/month) compared with AO (10.4) and A (8.6). This corresponded to baseline-adjusted increases of +13.3, +5.7, and +4.4 events/month, respectively.
Portal message thread burden demonstrated an even greater early divergence. Early-phase message thread rates were 24.1, 8.4, and 8.1 threads/month for VO, AO, and A, respectively, compared with baseline rates of 5.6, 4.6, and 6.3 threads/month.
Early-phase physician one-to-one encounter rates were 1.1, 1.0, and 0.7 events/month for VO, AO, and A, respectively, compared with baseline rates of 0.4, 0.4, and 0.4 events/month, representing a smaller proportion of total workload.
Encounter and message rates declined after the early phase but remained higher for VO relative to BTKi-based regimens during active combination therapy.
Additional analyses examined encounter composition, including nursing/triage and administrative coordination domains, with further qualitative and quantitative findings to be presented at the meeting

**Copyright Information:** (Online) ISSN: 2572-9241
©2026 The Author(s). HemaSphere is published by John Wiley & Sons Ltd on behalf of European Hematology Association. This is an open access Abstract Book distributed under the Attribution-NonCommercial-NoDerivs (CC BY-NC-ND), which allows third parties to download the articles and share them with others as long as they credit the author and the Abstract Book, but they cannot change the content in any way or use them commercially.
**Abstract Book Citations:** Authors, Title, HemaSphere, 2026;10:(S1):pages. The abstract book DOIs can be found on the "Author Information" page.
**Disclaimer:** Articles published in the journal HemaSphere exclusively reflect the opinions of the authors. The authors are responsible for all content in their abstracts including accuracy of the facts, statements, citing resources, etc.

### Summary/Conclusion

First-line regimen selection in treatment-naïve CLL is associated with distinct early care delivery demands. Venetoclax-based therapy demonstrated substantially greater early increases in encounter and portal messaging burden compared with BTK inhibitor–based regimens. These findings highlight meaningful differences during treatment initiation that may inform resource planning and shared decision-making when multiple first-line options are clinically appropriate.

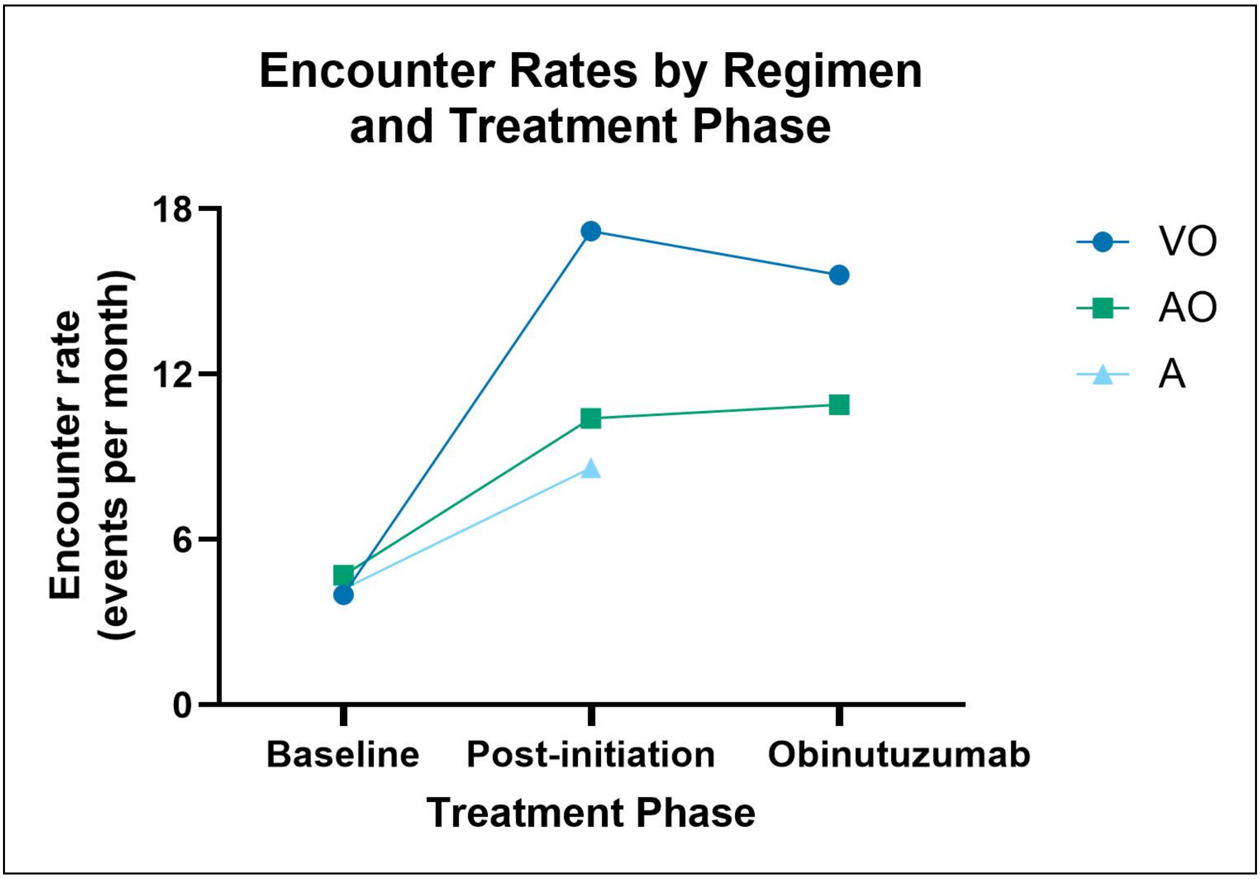

Encounter rates (events per patient-month) by regimen and treatment phase. Baseline = 180-day pre-treatment period; Post-initiation = first on-treatment phase; Obinutuzumab = active combination phase for AO and VO. Rates were calculated per patient-month. VO (n=35), AO (n=63), A (n=36).

Copyright Information: (Online) ISSN: 2572-9241
©2026 The Author(s). HemaSphere is published by John Wiley & Sons Ltd on behalf of European Hematology Association. This is an open access Abstract Book distributed under the Attribution-NonCommercial-NoDerivs (CC BY-NC-ND), which allows third parties to download the articles and share them with others as long as they credit the author and the Abstract Book, but they cannot change the content in any way or use them commercially.
Abstract Book Citations: Authors, Title, HemaSphere, 2026;10:(S1):pages. The abstract book DOIs can be found on the "Author Information" page.
Disclaimer: Articles published in the journal HemaSphere exclusively reflect the opinions of the authors. The authors are responsible for all content in their abstracts including accuracy of the facts, statements, citing resources, etc.

### (PB2950) SURVIVAL IN VERY ELDERLY (≥75 YEARS) PATIENTS WITH CHRONIC LYMPHOCYTIC LEUKEMIA: MULTINATIONAL REAL-WORLD ANALYSIS FROM LATIN AMERICA

**Topic:** 06. Chronic lymphocytic leukemia and related disorders - Clinical

Thiago Carneiro*[1], Celso Arrais-Rodrigues[2, 3], Ana Oliver[4, 5], María Silvana Cugliari[6], Luis Villela[7], MacArena Roa[8], Lorena Cardozo[9], Maria A. Torres Viera[9], Denisse Castro Uriol[10], Daniela Trujillo[11], Leila Perobelli[12], Anelys Garcia Salgado[13], Victoria Irigoin[5], Sofia Rivarola[14], Virginia Lema[15], Jose Alvarez[16], Valeria Buccheri[17], Caio Justino[2], Gislaine B. Oliveira[18], Maura Ikoma[19], Laura Fogliatto[20], Ana Ines Landoni[21], Sabrina Ranero[22], Augusto Miroli[23], Marta Elisa Zerga[24], Miguel Arturo Pavlovsky[25], Nancy Soledad Cristaldo[26], María del Rosario Custidiano[27], Fernando Pérez Jacobo[28], Danielle Leão[29], Maria Orlova[26], Julio Pose Caabarcos[30], Fernando Barroso[31], Etelvina MacHiavello[32], Sergio Costa Fortier[33], Julia Laviano[34], Clarisa Pagano Vilar[35], Nelson Hamerschlak[36], Vera Lucia Piratininga de Figueiredo[37], Rosa Oliday Rios[38], Arianna Robles Rodriguez[39], Melani Otañez[40], German Stemmelin[14], Jorge Castillo[41], Luis Malpica[42], Carlos Sergio Chiattone[43]

*[1]Hospital Ophir Loyola / UEPA, Belém, Brazil; [2]UNIFESP, São Paulo, Brazil; [3]Hospital Nove de Julho / Rede Américas, São Paulo, Brazil; [4]British Hospital, Montevideo, Uruguay; [5]CASMU, Montevideo, Uruguay; [6]Instituto de Oncología A. Roffo – Facultad de Medicina UBA-CABA, Buenos Aires, Argentina; [7]Escuela de Medicina del Tecnológico de Monterrey, Servicio de Hematología, Centro Médico Dr. Ignacio Chávez del ISSSTESON, Hermosillo, Mexico; [8]Hospital del Salvador, Santiago, Chile; [9]Universidad Central de Venezuela, Clínica Santa Sofía, Caracas, Venezuela; [10]Hospital Edgardo Rebagliati Martins, Lima, Peru; [11]Universidad Tecnológica de Pereira, Pereira, Colombia; [12]Hospital Brigadeiro / Hospital de Transplantes Euryclides de J. Zerbini, São Paulo, Brazil; [13]Ivan Portuondo General Teaching Hospital, Artemisa, Cuba; [14]British Hospital, Buenos Aires, Argentina; [15]Hospital Militar, Montevideo, Uruguay; [16]Centro Medico Nacional "20 de Noviembre", Ciudad de Mexico, Mexico; [17]ICESP, Faculdade de Medicina da USP, São Paulo, Brazil; [18]UNICAMP, Campinas, Brazil; [19]Hospital Amaral Carvalho, Jaú, Brazil; [20]Hospital de Clínicas de Porto Alegre, Porto Alegre, Brazil; [21]Hospital Maciel, Montevideo, Uruguay; [22]Hospital de Clínicas, Montevideo, Uruguay; [23]Complejo Médico Churruca-Visca, Buenos Aires, Argentina; [24]Hospital Alemán, Buenos Aires, Argentina; [25]FUNDALEU, Buenos Aires, Argentina; [26]Hospital Italiano, Buenos Aires, Argentina; [27]Instituto A. Fleming, Buenos Aires, Argentina; [28]Hospital Central Norte, Ciudad de Mexico, Mexico; [29]Beneficiência Portuguesa de São Paulo, São Paulo, Brazil; [30]Sanatorio Otamendi, Buenos Aires, Argentina; [31]Universidade Federal do Ceará, Fortaleza, Brazil; [32]Clínica La Pequeña Familia, Junín, Argentina; [33]Santa Casa de São Paulo, São Paulo, Brazil; [34]Clínica Centro, Chacabuco, Argentina; [35]Hospital Dr. I. Pirovano, Buenos Aires, Argentina; [36]Hospital Israelita Albert Einstein, São Paulo, Brazil; [37]Hospital do Servidor Público Estadual, São Paulo, Brazil; [38]Hospital Dr. Luis Díaz Soto, La Habana, Cuba; [39]Universidad de Guadalajara, Guadalajara, Mexico; [40]Hospital General del Estado de Sonora, Hermosillo, Brazil; [41]Dana-Farber Cancer Institute, Boston, United States of America; [42]MD Anderson Cancer Center, Houston, United States of America; [43]Hospital Samaritano, São Paulo, Brazil, São Paulo, Brazil;*

### Background

Very elderly patients (≥75 years) with chronic lymphocytic leukemia (CLL) represent a growing yet persistently underrepresented population in randomized trials evaluating targeted therapies. Real-world data focused specifically on this age group remain scarce, and multinational evidence from Latin America is lacking. Consequently, survival outcomes and the effectiveness of modern therapeutic strategies in very elderly patients within this regional context remain insufficiently characterized.

### Aims

To evaluate overall survival (OS) and treatment patterns in very elderly (≥75 years) patients with CLL treated in clinical practice across Latin America, and to assess whether modern therapeutic strategies are associated with improved survival in this population.

**Copyright Information:** (Online) ISSN: 2572-9241
©2026 The Author(s). HemaSphere is published by John Wiley & Sons Ltd on behalf of European Hematology Association. This is an open access Abstract Book distributed under the Attribution-NonCommercial-NoDerivs (CC BY-NC-ND), which allows third parties to download the articles and share them with others as long as they credit the author and the Abstract Book, but they cannot change the content in any way or use them commercially.
**Abstract Book Citations:** Authors, Title, HemaSphere, 2026;10:(S1):pages. The abstract book DOIs can be found on the "Author Information" page.
**Disclaimer:** Articles published in the journal HemaSphere exclusively reflect the opinions of the authors. The authors are responsible for all content in their abstracts including accuracy of the facts, statements, citing resources, etc.

### Methods

We analyzed patients aged ≥75 years who initiated first-line therapy between 2010 and 2024 within the multinational GELL registry, including centers from 10 Latin American countries. Kaplan–Meier methods and log-rank testing were used for survival comparisons. Restricted mean survival time (RMST) at 36 months was calculated to mitigate bias related to differential follow-up. Multivariable Cox proportional hazards models adjusted for age, ECOG performance status, and year of treatment initiation. Given treatment heterogeneity, three mutually exclusive groups were defined for comparative survival analyses: chlorambucil monotherapy, chlorambucil plus anti-CD20 antibody, and continuous Bruton tyrosine kinase inhibitor (iBTK) monotherapy.

### Results

A total of 365 patients were included. Median age was 80.3 years (range 75–102), 57.1% were female, and 25.5% had Binet stage C disease. ECOG ≥2 was documented in 24% of evaluable patients. Median follow-up was 42 months (95% CI 36.2–47.1), and median OS in the overall cohort was 62.0 months (95% CI 51.7–NA). Most common treatments were chlorambucil monotherapy (39.5%), chlorambucil plus anti-CD20 (19.2%), and iBTK monotherapy (15.7%).
Among 271 patients, 101 deaths occurred. At 36 months, OS was 78.2% for chlorambucil plus anti-CD20, 59.5% for iBTK, and 55.4% for chlorambucil monotherapy (log-rank p=0.03). In the iBTK monotherapy group, ibrutinib accounted for 84.2% of cases and acalabrutinib for 15.8%. del(17p) was numerically more frequent with iBTK (7/38, 18.4%) than with chlorambucil plus anti-CD20 (2/32, 6.2%) or chlorambucil alone (0/19, 0%). Median OS was 76.9 months for chlorambucil plus anti-CD20 versus approximately 42 months for chlorambucil monotherapy and iBTK monotherapy. In multivariable analysis (n=182), chlorambucil plus anti-CD20 remained independently associated with improved OS compared with chlorambucil monotherapy (HR 0.44; 95% CI 0.24–0.79; p=0.007), while iBTK monotherapy was not significantly associated with improved survival (HR 0.71; 95% CI 0.39–1.31; p=0.27). ECOG performance status was independently prognostic (HR 0.43 for ECOG 0–1; p=0.001). Sensitivity analyses yielded consistent estimates.

### Summary/Conclusion

In this multinational Latin American real-world cohort of very elderly CLL patients, chlorambucil plus anti-CD20 was associated with improved overall survival versus chlorambucil alone, even after adjustment for age, performance status, and treatment era. Continuous iBTK monotherapy showed no significant survival benefit. Given the retrospective design and the potential for treatment-selection bias and residual confounding, these findings should be interpreted cautiously and warrant prospective validation in very elderly patients.

**Copyright Information:** (Online) ISSN: 2572-9241
©2026 The Author(s). HemaSphere is published by John Wiley & Sons Ltd on behalf of European Hematology Association. This is an open access Abstract Book distributed under the Attribution-NonCommercial-NoDerivs (CC BY-NC-ND), which allows third parties to download the articles and share them with others as long as they credit the author and the Abstract Book, but they cannot change the content in any way or use them commercially.
**Abstract Book Citations:** Authors, Title, HemaSphere, 2026;10:(S1):pages. The abstract book DOIs can be found on the "Author Information" page.
**Disclaimer:** Articles published in the journal HemaSphere exclusively reflect the opinions of the authors. The authors are responsible for all content in their abstracts including accuracy of the facts, statements, citing resources, etc.

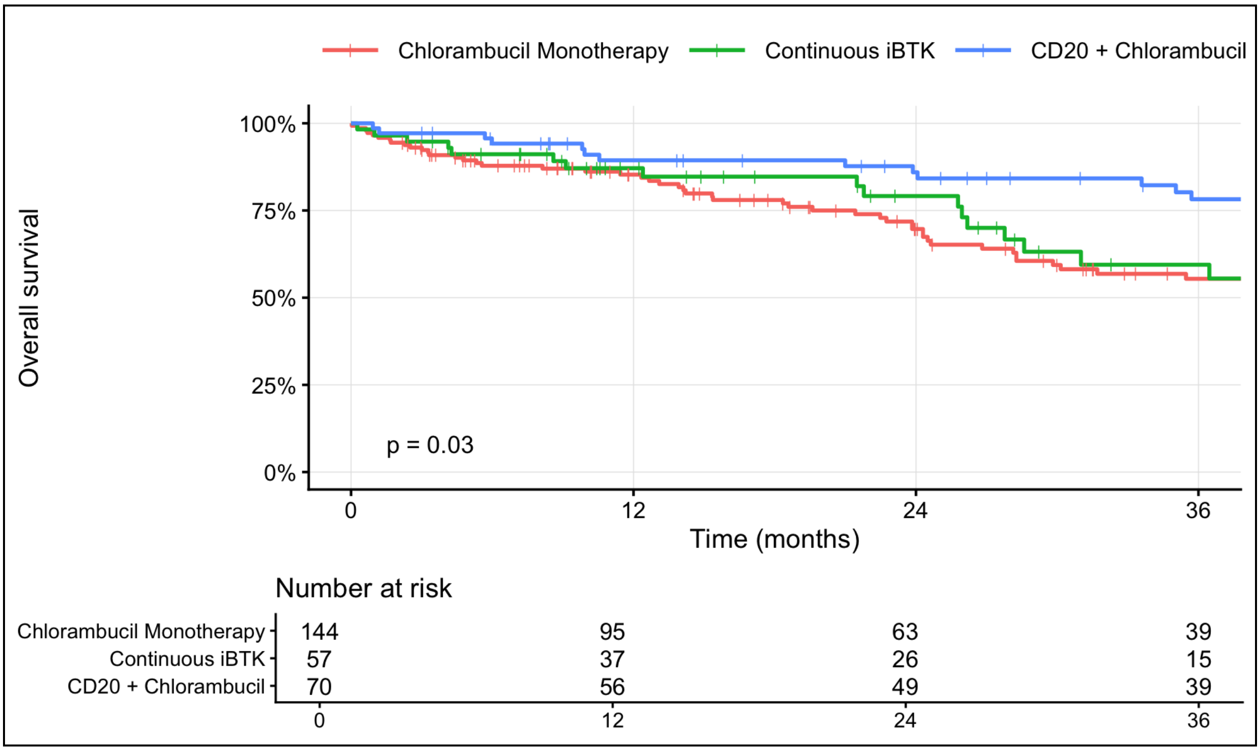

Overall survival by treatment group (chlorambucil monotherapy, continuous iBTK, and chlorambucil + anti-CD20) in patients ≥75 years. Log-rank p=0.03. Numbers at risk are displayed below.

**Copyright Information:** (Online) ISSN: 2572-9241
©2026 The Author(s). HemaSphere is published by John Wiley & Sons Ltd on behalf of European Hematology Association. This is an open access Abstract Book distributed under the Attribution-NonCommercial-NoDerivs (CC BY-NC-ND), which allows third parties to download the articles and share them with others as long as they credit the author and the Abstract Book, but they cannot change the content in any way or use them commercially.
**Abstract Book Citations:** Authors, Title, HemaSphere, 2026;10:(S1):pages. The abstract book DOIs can be found on the “Author Information” page.
**Disclaimer:** Articles published in the journal HemaSphere exclusively reflect the opinions of the authors. The authors are responsible for all content in their abstracts including accuracy of the facts, statements, citing resources, etc.

### (PB2951) A STUDY OF BEXOBRUTIDEG (NX-5948), A BTK DEGRADER, IN PATIENTS WITH CLL/SLL PREVIOUSLY TREATED WITH A COVALENT BTK INHIBITOR, A NON-COVALENT BTK INHIBITOR AND A BCL2 INHIBITOR: DAYBREAK CLL-201

**Topic:** 06. Chronic lymphocytic leukemia and related disorders - Clinical

Dima El-Sharkawi*[1], Michal Kwiatek[2], Sebastian Grosicki[3], Anne Lok[4], Jane Robertson[5], Krish Patel[6], Michael Tees[7], Iwona Hus[8], Lucy Yan[9], Srinand Nandakumar[9], Kelly O'brien[9], Wojciech Jurczak[10]

*[1]Royal Marsden NHS Foundation Trust, Sutton, United Kingdom;[2]AidPort Hospital, Skórzewo (Poznan), Poland;[3]Pratia Onkologia, Katowice, Poland;[4]Centre Hospitalier Universitaire de Nantes, Nantes, France;[5]The Christie Hospital and Manchester Cancer Research Centre, Division of Cancer Sciences, Manchester, United Kingdom;[6]Sarah Cannon Research Institute, Nashville, United States of America;[7]Colorado Blood Cancer Institute, Denver, United States of America;[8]Medical University of Lublin, Lublin, Poland;[9]Nurix Therapeutics, Inc., Brisbane, United States of America;[10]Maria Sklodowska-Curie National Research Institute of Oncology, Krakow, Poland;*

### Background

Bruton's tyrosine kinase (BTK) plays a central role in the B-cell receptor (BCR) signaling pathway and is involved in the pathogenesis of several B-cell malignancies. Although BTK inhibition has proven to be a successful treatment modality in chronic lymphocytic leukemia (CLL), the emergence of BTK inhibitor (BTKi) resistance mutations and identification of kinase-independent signaling via the scaffolding function of BTK underscore the need for alternative approaches to target the totality of BTK protein functions. Bexobrutideg (NX-5948) is a novel, highly selective, orally administered small molecule protein degrader that induces the removal of wild-type and mutant forms of BTK through ubiquitination by the cereblon E3 ligase complex and subsequent proteasomal degradation. In a previous Phase 1 trial in relapsed/refractory B-cell malignancies including CLL (NX-5948-301; NCT05131022), bexobrutideg was well tolerated and showed rapid and durable responses, and a recommended Phase 2 dose of 600 mg once daily was identified.

### Methods

NX-5948-201 (DAYBreak CLL-201; NCT07221500) is a single-arm, open-label Phase 2 study designed to evaluate the efficacy and safety of bexobrutideg in adult patients with relapsed/refractory (R/R) CLL or small lymphocytic lymphoma (SLL) previously exposed to a covalent BTKi, a non-covalent BTKi and a B-cell lymphoma-2 inhibitor (BCL2i). Key eligibility criteria include: histologically confirmed R/R CLL/SLL per the International Workshop on Chronic Lymphocytic Leukemia (iwCLL; Hallek 2018); an Eastern Cooperative Oncology Group score of 0–2; prior exposure to a covalent BTKi, a non-covalent BTKi and a BCL2i (separate lines or in combination); measurable disease by radiographic assessment per iwCLL (≥1 lymph node with longest diameter >1.5 cm); adequate organ and bone-marrow function. Exclusion criteria include: known or suspected prolymphocytic leukemia or Richter's transformation; previous treatment with a BTK degrader; active second malignancy; and known central nervous system lymphoma or leukemia. Bexobrutideg 600 mg is given orally once daily, with response assessment every 8−12 weeks. The primary endpoint is the objective response rate (ORR) without partial response with lymphocytosis (PR-L), per 2018 iwCLL criteria (as assessed by an independent review committee [IRC]). Secondary endpoints include the following additional efficacy outcomes: ORR with PR-L (as determined by IRC); ORR with/without PR-L (as determined by investigator); duration of response (IRC and investigator); progression-free survival (IRC and investigator); complete response rate (IRC and investigator); time to response (IRC and investigator); and overall survival. Safety, pharmacokinetics and health-related quality of life will also be evaluated.

**Copyright Information:** (Online) ISSN: 2572-9241
©2026 The Author(s). HemaSphere is published by John Wiley & Sons Ltd on behalf of European Hematology Association. This is an open access Abstract Book distributed under the Attribution-NonCommercial-NoDerivs (CC BY-NC-ND), which allows third parties to download the articles and share them with others as long as they credit the author and the Abstract Book, but they cannot change the content in any way or use them commercially.
**Abstract Book Citations:** Authors, Title, HemaSphere, 2026;10:(S1):pages. The abstract book DOIs can be found on the "Author Information" page.
**Disclaimer:** Articles published in the journal HemaSphere exclusively reflect the opinions of the authors. The authors are responsible for all content in their abstracts including accuracy of the facts, statements, citing resources, etc.

**Summary/Conclusion**

Enrollment is underway and the study is expected to recruit ~100 patients globally.

**Figure.** DAYBreak CLL-201 trial design

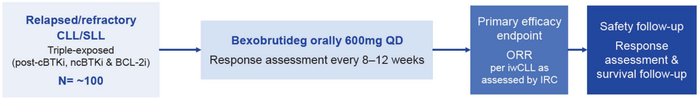

| Primary endpoint | Secondary endpoints | Exploratory endpoints |
|---|---|---|
| **Objective response rate (ORR)** as assessed by an independent review committee | **Efficacy:** ORR as assessed by investigator, DOR, PFS, CR rate, TTR, OS<br><br>**Safety:** incidence of TEAEs, SAEs, TEAEs leading to treatment discontinuation, changes from baseline in lab parameters and vitals<br><br>**Pharmacokinetics:** NX-5948 plasma concentration | **Patient reported outcomes** (EORTC QLQ C30 and EQ-5D-5L)<br><br>**Pharmacodynamics:** Biomarkers of BTK signaling and resistance pathways<br><br>**Minimal residual disease:** time to uMRD, time to MRD relapse, change in MRD from baseline |

**Copyright Information:** (Online) ISSN: 2572-9241
©2026 The Author(s). HemaSphere is published by John Wiley & Sons Ltd on behalf of European Hematology Association. This is an open access Abstract Book distributed under the Attribution-NonCommercial-NoDerivs (CC BY-NC-ND), which allows third parties to download the articles and share them with others as long as they credit the author and the Abstract Book, but they cannot change the content in any way or use them commercially.
**Abstract Book Citations:** Authors, Title, HemaSphere, 2026;10:(S1):pages. The abstract book DOIs can be found on the "Author Information" page.
**Disclaimer:** Articles published in the journal HemaSphere exclusively reflect the opinions of the authors. The authors are responsible for all content in their abstracts including accuracy of the facts, statements, citing resources, etc.

### (PB2952) CHRONIC LYMPHOCYTIC LEUKEMIA IN A RESOURCE-LIMITED SETTING: EXPERIENCE FROM NORTHERN MOROCCO

**Topic:** 06. Chronic lymphocytic leukemia and related disorders - Clinical

Tarik Arahou*[1], Oumayma Hari[1], Dina Rachidi[1], Imane El Boutahiri[1], Safae Regragui[1]

[1] *University Hospital Center Mohamed VI Tangier, Clinical Hematology, Tangier, Morocco;*

### Background

Chronic lymphocytic leukemia (CLL) is a low-grade B-cell malignancy with a highly heterogeneous clinical course, ranging from indolent disease to aggressive forms. Prognosis and therapeutic decisions are strongly influenced by biological factors, particularly TP53 disruption, whose assessment remains challenging in resource-limited settings.

### Aims

To describe the clinical, biological, therapeutic, and outcome characteristics of patients diagnosed with CLL in northern Morocco.

### Methods

We conducted a retrospective descriptive study, including patients diagnosed with CLL according to iwCLL 2018 criteria, managed at the Clinical Hematology Department of the University Hospital Center Mohamed VI of Tangier, between July 2021 and April 2025. Clinical, laboratory, treatment, and follow-up data were extracted from medical records and analyzed descriptively.

### Results

Forty-three patients were included, with a mean age of 66.5 years and a male predominance (male-to-female ratio 2.3). Patients originated equally from urban and rural areas. Hypertension (12%), smoking (14%), and cardiovascular disease (9%) were the most frequent comorbidities. Diagnosis was incidental in nearly half of the cases. Peripheral lymphadenopathy was observed in 84% of patients, splenomegaly in 47%, and hepatomegaly in 9%. Most patients had preserved performance status (WHO 0–1: 90%).
The mean lymphocyte count was 120 $\times 10^9$/L. Anemia was present in 35% of patients, mainly related to bone marrow infiltration, and thrombocytopenia in 14%. Binet stages were evenly distributed (A: 33%, B: 30%, C: 37%). A Matutes score ≥4 was found in 41 patients. TP53 mutation was detected in 3 of the 17 patients tested.
Fourteen patients were initially managed with active surveillance, with subsequent treatment required in more than half. First-line therapy consisted mainly of Bendamustine–Rituximab in patients over 65 years and Fludarabine–Cyclophosphamide–Rituximab in younger patients. Responses included complete remission in 9 patients, partial remission in 3, and disease progression in 5. Second-line treatment was administered to 4 patients with heterogeneous outcomes. Loss to follow-up was frequent, occurring in 19 patients overall. Richter transformation developed in 4 patients. Mortality rate was 14%, with a mean time from diagnosis to death of 18 months. Infectious and hematological toxicities were the most common complications.

### Summary/Conclusion

This real-world study highlights the low prevalence of CLL in northern Morocco and underscores the marked clinical and biological heterogeneity of the disease. Limited access to cytogenetic and molecular testing significantly influences therapeutic strategies. These findings emphasize the need for resource-adapted management approaches and improved access to prognostic testing to optimize CLL care in low- and middle-income settings.

**Copyright Information:** (Online) ISSN: 2572-9241
©2026 The Author(s). HemaSphere is published by John Wiley & Sons Ltd on behalf of European Hematology Association. This is an open access Abstract Book distributed under the Attribution-NonCommercial-NoDerivs (CC BY-NC-ND), which allows third parties to download the articles and share them with others as long as they credit the author and the Abstract Book, but they cannot change the content in any way or use them commercially.
**Abstract Book Citations:** Authors, Title, HemaSphere, 2026;10:(S1):pages. The abstract book DOIs can be found on the “Author Information” page.
**Disclaimer:** Articles published in the journal HemaSphere exclusively reflect the opinions of the authors. The authors are responsible for all content in their abstracts including accuracy of the facts, statements, citing resources, etc.

### (PB2953) REAL-WORLD EFFICACY AND CARDIOVASCULAR SAFETY OF ACALABRUTINIB IN B-CELL MALIGNANCIES: A TURKISH MULTICENTER EXPERIENCE COMPARING FRONTLINE INITIATION VS. TRANSITION AFTER IBRUTINIB INTOLERANCE

**Topic:** 06. Chronic lymphocytic leukemia and related disorders - Clinical

Vildan Gursoy*[1], Vildan Özkocaman[1], Fehmi Hindilerden[2], Ahmet Emre Eşkazan[3], Tuba Ozkan[3], Osman Ilhan[4], İbrahim Halil Açar[5], Mehmet Orhan Ayyıldız[6], Ebru Kavak Yavuz[6], Aysun Şentürk Yikilmaz[7], Seval Akpınar[8], Mehmet Baysal[8], Mehmet Can Ugur[9], Mehmet Günhan Tekin[9], Guray Saydam[10], Fatma Keklik Karadag[10], Eren Arslan Davulcu[2], Yildiz Ipek[11], Tuba Güllü Koca[12], Beyza Oluk[13], Nil Güler[14], Nevin Alayvaz Aslan[14], Atakan Tekinalp[15], Ufuk Demirci[16], Mine Miskioğlu[16], Tuba Hacibekiroglu[17], Berkay Özdenoğlu[17], Taha Ulutan Kars[18], Serhat Çelik[19], Esra Turan Erkek[11], Tarik Onur Tiryaki[20], Meltem Aylı[21], Selim Sayin[21], Murat Yıldırım[21], Emel Gürkan[22], Bahar Sevgili[23], Idris Ince[5], Serkan Güven[24], Seda Aydin[25], Muhammet Kürşat Kaptan[26], Damla Ortaboz[26], Tuba Ersal[1], Ibrahim Ethem Pinar[1], Fahir Özkalemkaş[1]

*[1]Bursa Uludağ University Faculty of Medicine, Hematology, bursa, Türkiye;[2]Bakırköy Dr. Sadi Konuk Training and Research Hospital, Hematology, istanbul, Türkiye; [3]Istanbul University Cerrahpasa Faculty of Medicine, Hematology, istanbul, Türkiye; [4]Lokman Hekim Akay Hospital, ankara, Türkiye;[5]Gaziantep City Hospital, Hematology, gaziantep, Türkiye; [6]Dicle University Faculty of Medicine Department of Hematology, diyarbakır, Türkiye; [7]Denizli State Hospital, denizli, Türkiye;[8]Tekirdağ Namık Kemal University, Hematology, tekirdağ, Türkiye; [9]Tepecik Training and Research Hospital, Hematology, izmir, Türkiye; [10]Ege University Faculty of Medicine, Hematology, izmir, Türkiye; [11]Kartal Dr. Lütfi Kırdar City Hospital, istanbul, Türkiye; [12]Bursa City Hospital, Bursa, Türkiye; [13]kocaeli city hospital, kocaeli, Türkiye; [14]Pamukkale University, denizli, Türkiye; [15]Konya Necmettin Erbakan University Meram Faculty Of Medicine Hospital, Hematology, konya, Türkiye; [16]Celal Bayar University Medical Faculty, manisa, Türkiye; [17]Sakarya University Training and Research Hospital, sakarya, Türkiye; [18]Konya City Hospital, Konya, Türkiye; [19]Yıldırım Beyazıt University, Yenimahalle Training and Research Hospital, Ankara, Ankara, Türkiye;[20]University of Health Sciences Türkiye, Hamidiye Etfal Training and Research Hospital, Department of Internal Medicine, Division of Hematology, İstanbul, Türkiye, istanbul, Türkiye; [21]Gülhane Educational and Research Hospital, istanbul, Türkiye; [22]cukurova university, adana, Türkiye; [23]Iskenderun State Hospital, iskendurun, Türkiye;[24]Mehmet Akif Ersoy Hospital, Hematology, çanakkale, Türkiye; [25]Ankara Bilkent City Hospital, Hematology, ankara, Türkiye;[26]Health Sciences University Sultan Abdülhamid Han Training and Research Hospital, Hematology, istanbul, Türkiye;*

### Background

Bruton tyrosine kinase inhibitors (BTKis) have transformed the therapeutic landscape for B-cell malignancies, particularly chronic lymphocytic leukemia (CLL). However, first-generation BTKis like ibrutinib are associated with significant off-target effects, including atrial fibrillation (AF) and hypertension, often leading to treatment discontinuation. Acalabrutinib, a more selective second-generation BTKi, was designed to minimize these toxicities while maintaining efficacy.

### Aims

This study evaluates the real-world clinical outcomes and cardiovascular safety of acalabrutinib in Turkey, comparing patients initiated on frontline therapy versus those transitioning after ibrutinib intolerance.

### Methods

This retrospective, multicenter study analyzed data from **126 patients** (CLL 87.2%, MCL 4.8%) treated with acalabrutinib. The cohort was stratified into acalabrutinib-naive patients (**n=108**) and those with prior ibrutinib exposure who switched due to intolerance (**n=18**). Independent predictors of response and adverse events (AEs) were identified using multivariable logistic regression. Survival analysis was performed using Kaplan-Meier and univariable Cox regression models.

**Copyright Information:** (Online) ISSN: 2572-9241
©2026 The Author(s). HemaSphere is published by John Wiley & Sons Ltd on behalf of European Hematology Association. This is an open access Abstract Book distributed under the Attribution-NonCommercial-NoDerivs (CC BY-NC-ND), which allows third parties to download the articles and share them with others as long as they credit the author and the Abstract Book, but they cannot change the content in any way or use them commercially.
**Abstract Book Citations:** Authors, Title, HemaSphere, 2026;10:(S1):pages. The abstract book DOIs can be found on the “Author Information” page.
**Disclaimer:** Articles published in the journal HemaSphere exclusively reflect the opinions of the authors. The authors are responsible for all content in their abstracts including accuracy of the facts, statements, citing resources, etc.

### Results

**Patient Profile and Baseline Burden:** The mean age at diagnosis was 66.0 ± 11.1 years. Notably, the prevalence of cerebrovascular disease (CVD) was significantly higher in the prior ibrutinib group compared to the naive group (16.7% vs. 1.9%; p=0.021).
**Efficacy and Persistence:** The overall response rate (ORR) was 83.1%, with a complete response (CR) rate of 33.9%. Multivariable analysis identified negative TP53 mutation / del(17p) status (OR: 0.199; p=0.034) and lower baseline serum creatinine (OR: 0.025; p=0.030) as independent predictors of achieving CR. The rate of treatment continuation was significantly higher in the naive cohort (83.2% vs. 55.6%; p=0.013).

### Acalabrutinib Treatment Outcomes and Persistence

| Outcome Parameter | Naive (n=108) | Prior Ibrutinib (n=18) | p-value |
|---|---|---|---|
| **Partial Response (PR)**, n (%) | **56 (52.83%)** | **5 (27.78%)** | **0.032** |
| **Complete Response (CR)**, n (%) | 36 (33.96%) | 6 (33.33%) | 1.000 |
| **Treatment Continuation**, n (%) | **89 (83.18%)** | **10 (55.56%)** | **0.013** |
| **Acalabrutinib related AE**, n (%) | 19 (17.76%) | 4 (22.22%) | 0.742 |

**Cardiovascular Safety:** Acalabrutinib-related AEs occurred in 18.3% of patients. Strikingly, the incidence of new-onset atrial fibrillation under acalabrutinib was only 1.6% (n=2). Multivariable analysis revealed that the absence of baseline hypertension was independently associated with an increased likelihood of reporting AEs (OR: 0.176 for present HTN; p=0.007).
**Survival Outcomes:** Kaplan-Meier analysis demonstrated that frontline acalabrutinib initiation provided significantly superior progression-free survival (PFS) compared to the switch strategy (p=0.023). Prior ibrutinib exposure was associated with a nearly three-fold increase in the risk of progression (HR: 2.99; p=0.051).

### Summary/Conclusion

In this Turkish real-world cohort, acalabrutinib demonstrated an excellent cardiovascular safety profile with a remarkably low incidence of new-onset AF (1.6%), consistent with or superior to international real-world data (2.1%). While acalabrutinib is a highly effective and tolerable "salvage" option for ibrutinib-intolerant patients, initiating acalabrutinib as a frontline strategy is associated with significantly better clinical persistence and progression-free survival. These findings support the early use of more selective BTKis to minimize cumulative cardiovascular risk and optimize long-term disease control.

**Copyright Information:** (Online) ISSN: 2572-9241
©2026 The Author(s). HemaSphere is published by John Wiley & Sons Ltd on behalf of European Hematology Association. This is an open access Abstract Book distributed under the Attribution-NonCommercial-NoDerivs (CC BY-NC-ND), which allows third parties to download the articles and share them with others as long as they credit the author and the Abstract Book, but they cannot change the content in any way or use them commercially.
**Abstract Book Citations:** Authors, Title, HemaSphere, 2026;10:(S1):pages. The abstract book DOIs can be found on the "Author Information" page.
**Disclaimer:** Articles published in the journal HemaSphere exclusively reflect the opinions of the authors. The authors are responsible for all content in their abstracts including accuracy of the facts, statements, citing resources, etc.

### (PB2954) CLL2000: A PROSPECTIVE PLATFORM FOR LONG-TERM OUTCOMES AND TREATMENT SEQUENCING OF PATIENTS WITH CHRONIC LYMPHOCYTIC LEUKEMIA AFTER FIRST-LINE THERAPY WITHIN GCLLSG STUDIES

**Topic:** 06. Chronic lymphocytic leukemia and related disorders - Clinical

Kirsten Fischer*[1], Léa Mazot[1], Valerie Von Maydell[1], Arianna Zappaterra[1], Sandra Robrecht[1], Anna-Maria Fink[1], Nadine Kutsch[1], Moritz Fürstenau[1], Karl-Anton Kreuzer[1], Aline Zey[1], Emily Holmes[1], Ronald D'brot[1], Sinja Kohlmeyer[1], Sebastian Böttcher[2], Andrea Stoltefuß[3], Tobias Gaska[4], Manfred Hensel[5], Ingo Schwaner[6], Francisco Bosch Albareda[7], Ruth Clifford[8], Paula Cramer[1], Emadoldin Feyzi[9], Paolo Ghia[10, 11], Michael Gregor[12], Henriette Huber[13], Arnon Kater[14], Mark-David Levin[15], Mattias Mattsson[16], Carsten Niemann[17], Philipp Staber[18, 19], Tamar Tadmor[20, 21], Tuija Tapaninen[22], Patrick Thornton[23], Julia von Tresckow[24], Robert Weinkove[25], Matthias Ritgen[26], Anke Schilhabel[26], Eugen Tausch[27], Othman Al-Sawaf[1], Stephan Stilgenbauer[27], Michael Hallek[1], Barbara Eichhorst[1]

*[1]University Hospital of Cologne, Department I of Internal Medicine; German CLL Study Group, Cologne, Germany;[2]University Medical Center Rostock, Department III – Hematology, Oncology, Palliative Medicine, Rostock, Germany; [3]Evangelical Hospital Hamm, Department of Internal Medicine, Hematology, Hamm, Germany; [4]Brüderkrankenhaus St Josef Paderborn, Clinic for Hematology and Oncology, Paderborn, Germany; [5]Mannheimer Oncology Practice, Mannheim, Germany; [6]Oncology Practice Kurfürstendamm, Berlin, Germany; [7]Vall d'Hebron University Hospital, Department of Hematology, Barcelona, Spain;[8]University Hospital Limerick, Department of Haematology, Limerick Cancer Trials, Mid West Cancer Centre, Limerick, Ireland; [9]St. Olavs University Hospital, Department of Hematology, Trondheim, Norway; [10]IRCCS Ospedale San Raffaele, Comprehensive Cancer Center, Milan, Italy; [11]Università Vita-Salute San Raffaele, Medical Scool, Milan, Italy; [12]Luzerner Kantonsspital, Department of Hematology, Lucerne, Switzerland; [13]Karlsruhe Municipal Hospital, Department of Hematology/Oncology, Karlsruhe, Germany; [14]University Medical Centers Amsterdam, Amsterdam, The Netherlands; [15]Albert Schweitzer Hospital, Department of Internal Medicine, Dordrecht, The Netherlands; [16]University Hospital Uppsala, Department of Hematology, Uppsala, Sweden; [17]Rigshospitalet, Copenhagen University Hospital, Copenhagen, Denmark; [18]Medical University Vienna, Vienna, Austria; [19]Saarland University Medical School, Homburg, Germany; [20]Bnai Zion Medical Center, Hematology Unit, Haifa, Israel; [21]The Ruth and Bruce Rappaport Faculty of Medicine, Technion, Haifa, Israel; [22]Meilahti Triangle University Hospital, Comprehensive Cancer Center, Department of Hematology, Helsinki, Finland; [23]Beaumont Hospital, Cancer Clinical Trials Unit, Dublin, Ireland; [24]University Hospital St. Josef-Hospital, Ruhr-University, Department of Hematology and Oncology with Palliative Care, Bochum, Germany; [25]Malaghan Institute of Medical Research, Department of Hematology, Wellington, New Zealand;[26]Universitätsklinikum Schleswig-Holstein, Campus Kiel, Kiel, Germany; [27]University Hospital Ulm, Division of CLL, Department III of Internal Medicine, Ulm, Germany;*

### Background

Fixed-duration therapy is a standard-of-care for patients (pts) with previously untreated chronic lymphocytic leukemia (CLL). The safety and efficacy of fixed-duration regimens, also in comparison with continuous therapy, was characterized and studied within the randomized clinical trials CLL13, CLL14 and CLL17. However, due to the increasing effectiveness of therapies, clinical endpoints such as progression-free survival (PFS), time to next treatment (TTNT), overall survival (OS) and patient-reported outcome measures (PROMs) are not followed-up for a sufficient time period within the respective trial. Consequently, we need to extend post-treatment follow-up to gain further insight into durability of response and value of prognostic factors. To address this gap in clinical data, we designed the CLL2000 observational trial to generate long-term evidence of the therapeutic first-line international GCLLSG studies CLL2-GIVe, CLL13, CLL14, and CLL17. Within a large international collaborative effort, we will be collecting clinical data on survival, long-term responses, treatment sequencing, second primary malignancies (SPM), Richter transformation (RT) incidence, and PROMs. In addition, we will be performing exploratory analyses on biomarkers to identify potential characteristics of long-term responders and to study the potential treatment effects on clonal evolution.

**Copyright Information:** (Online) ISSN: 2572-9241
©2026 The Author(s). HemaSphere is published by John Wiley & Sons Ltd on behalf of European Hematology Association. This is an open access Abstract Book distributed under the Attribution-NonCommercial-NoDerivs (CC BY-NC-ND), which allows third parties to download the articles and share them with others as long as they credit the author and the Abstract Book, but they cannot change the content in any way or use them commercially.
**Abstract Book Citations:** Authors, Title, HemaSphere, 2026;10:(S1):pages. The abstract book DOIs can be found on the "Author Information" page.
**Disclaimer:** Articles published in the journal HemaSphere exclusively reflect the opinions of the authors. The authors are responsible for all content in their abstracts including accuracy of the facts, statements, citing resources, etc.

### Aims

The aim of CLL2000 is to assess prospective longitudinal survival data and biomarker of well annotated and predefined patient populations after they had been treated within the large international phase-III first-line trials of the German CLL Study Group.

### Methods

CLL2000 is a prospective, multicenter observational study enrolling patients previously treated in CLL2-GIVe, CLL13, CLL14, or CLL17. The final sample size is estimated at up to 2000 patients and enrollment will be performed at approximately 200 sites across 20 countries. No study-specific interventions are administered. Clinical data from the end of the first-line trial to CLL2000 enrollment will be collected retrospectively; thereafter, data will be collected prospectively as part of routine care. Annual documentation includes survival status, progression, subsequent CLL and RT treatments, SPMs, and patient-reported quality of life (EORTC QLQ-C30). Blood sampling (at registration, annually, and at progression) is performed during clinical routine to enable biomarker analyses. The primary endpoint is OS. Secondary endpoints include time-to-event outcomes after both first- and second-line therapy. SPM and RT incidence will be reported as follow-up–adjusted rates. No type I error control is applied. Data cut-offs are planned at 20, 40, and 60 months after first patient enrollment on CLL2000.

### Results

At the time of abstract submission, the study is approved in Germany and Finland with further approvals and start of recruitment expected soon.

### Summary/Conclusion

The CLL2000 trial provides a new platform to address the data gap of comprehensive long-term outcome assessment in CLL after first-line therapy within GCLLSG trials. This platform will provide novel longitudinal data on overall survival, treatment sequences, onset of second primary malignancies or Richter transformation. We expect the findings to help inform future treatment decisions, refine risk stratification, and guide personalized therapeutic strategies in CLL.

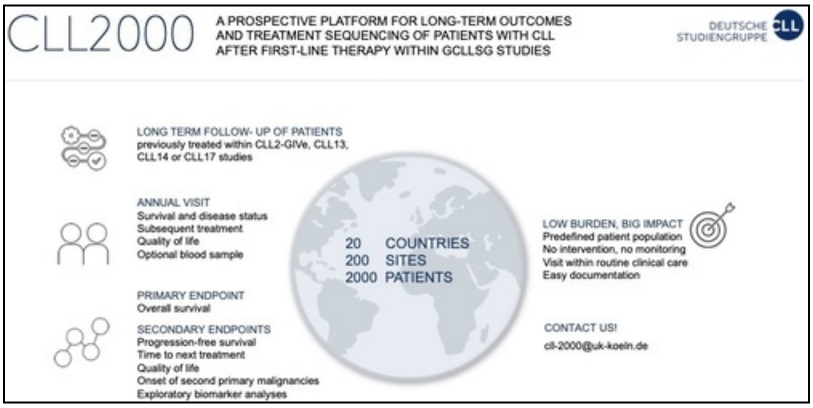

Study Overview

**Copyright Information:** (Online) ISSN: 2572-9241
©2026 The Author(s). HemaSphere is published by John Wiley & Sons Ltd on behalf of European Hematology Association. This is an open access Abstract Book distributed under the Attribution-NonCommercial-NoDerivs (CC BY-NC-ND), which allows third parties to download the articles and share them with others as long as they credit the author and the Abstract Book, but they cannot change the content in any way or use them commercially.
**Abstract Book Citations:** Authors, Title, HemaSphere, 2026;10:(S1):pages. The abstract book DOIs can be found on the “Author Information” page.
**Disclaimer:** Articles published in the journal HemaSphere exclusively reflect the opinions of the authors. The authors are responsible for all content in their abstracts including accuracy of the facts, statements, citing resources, etc.

### (PB2955) CD20 EXPRESSION LEVEL AS A FACTOR IN THE SELECTION OF MONOCLONAL ANTIBODIES FOR THE TREATMENT OF PATIENTS WITH CHRONIC LYMPHOCYTIC LEUKEMIA.

**Topic:** 06. Chronic lymphocytic leukemia and related disorders - Clinical

Morozova Oksana*[1], Natalya Milanovich[1], Tatsiana Talako[1], Oksana Nechay[1], Kuzmich Alena[1], Strongin Yuri[1], Dziuba Alena[1], Uss Anatoly[1], Viktirya Smolnikova[1]

[1]*Hematology and bone marrow, Minsk, Belarus;*

### Background

The incidence of chronic lymphocytic leukemia (CLL) in the Republic of Belarus is 5.5 cases per 100,000 population. Patients under 50 years of age account for 5.7–7.5% (20–25 patients per year with a new diagnosis). The success of targeted agents in the treatment of CLL is impressive, but in real-world clinical practice, immunochemotherapy (ICT) is the most common treatment method. The advent of the second-generation anti-CD20 monoclonal antibody (mAb), obinutuzumab, has not completely replaced its predecessor, rituximab.

### Aims

To determine which patients should be given priority for obinutuzumab treatment.

### Methods

We retrospectively assessed the efficacy of ICT depending on the level of CD20 expression by tumor cells in 85 patients with CLL who had not previously received therapy: 30 patients each with CD20 expression levels <65% received rituximab-containing and obinutuzumab-containing protocols, and 25 patients with CD20 expression levels >65% received rituximab-containing protocols (Table 1).

### Results

The obtained data indicate the effectiveness of anti-CD20 mAbs administration depending on the CD20 expression level: in patients with CD 20 expression level <65%, when using rituximab, we achieved a complete response in 20 out of 30 patients (MRD negative - 10 (33.3%) patients, MRD positive - 10 (33.3%) patients); in patients with CD20 expression level>65%, when using rituximab, we achieved a complete response in 22 out of 25 patients (MRD negative - 17 (68%) patients, MRD positive - 5 (20%) patients), which is significantly better than in patients with CD20 expression levels <65% (p <0.01).
The use of obinutuzumab-containing ICT protocols in patients with CD20 expression levels <65% allowed achieving a complete response in all patients (negative MRD response - 16 (53.3%) patients, positive MRD in 14 (46.7%) patients), which is significantly better than when using rituximab in patients with CD20 expression levels <65% (p <0.01).

### Summary/Conclusion

Therefore, when choosing an ICT regimen in previously untreated patients with CLL, it makes sense to consider the level of CD20 expression. For tumor cells with low CD20 expression, we prefer obinutuzumab-based treatment protocols. For CD20 expression levels >65%, in the absence of unfavorable prognostic factors, RFC or RB protocols may be used.

**Copyright Information:** (Online) ISSN: 2572-9241
©2026 The Author(s). HemaSphere is published by John Wiley & Sons Ltd on behalf of European Hematology Association. This is an open access Abstract Book distributed under the Attribution-NonCommercial-NoDerivs (CC BY-NC-ND), which allows third parties to download the articles and share them with others as long as they credit the author and the Abstract Book, but they cannot change the content in any way or use them commercially.
**Abstract Book Citations:** Authors, Title, HemaSphere, 2026;10:(S1):pages. The abstract book DOIs can be found on the "Author Information" page.
**Disclaimer:** Articles published in the journal HemaSphere exclusively reflect the opinions of the authors. The authors are responsible for all content in their abstracts including accuracy of the facts, statements, citing resources, etc.

### (PB2956) CHARACTERISTICS AND 5-YEAR OUTCOMES IN PATIENTS WITH CHRONIC LYMPHOCYTIC LEUKEMIA RECEIVING IBRUTINIB (A REAL-WORLD STUDY)

**Topic:** 06. Chronic lymphocytic leukemia and related disorders - Clinical

Umida Ergasheva*[1], Elizaveta Panchenko[1], Elena Yarovaya[2], Ekaterina Chernyshenko[2], Elena Dmitrieva[3], Julia Fedotkina[1], Maria Kislova[3], Eugene Nikitin[3]

*[1]Chazov National Medical Research Center for Cardiology, Moscow, Russia;[2]Lomonosov Moscow State University, Moscow, Russia; [3]S.P.Botkin Moscow multidisciplinary scientific and clinical center, Moscow, Russia;*

### Background

Ibrutinib has changed the management of patients with chronic lymphocytic leukemia (CLL). Despite efficacy in the treatment of CLL, cardiovascular complications, particularly atrial fibrillation (AF), remain one of the reasons for discontinuation of ibrutinib. Currently, the ESC guidelines for cardio-oncology 2022 do not fully address the management of patients with ibrutinib.

### Aims

To evaluate cardiovascular complications and characterize patients with new-onset AF during ibrutinib therapy, assessing clinical outcomes in a real-world practice.

### Methods

This retrospective study analyzed 641 patients with CLL treated with ibrutinib at the Botkin's Hospital (2013–2024). The primary endpoint was the incidence of AF. Secondary outcomes included thrombotic and hemorrhagic complications, as well as all-cause and cardiovascular mortality. To assess the impact of AF, cohorts were balanced for age and sex using propensity score matching.

**Copyright Information:** (Online) ISSN: 2572-9241
©2026 The Author(s). HemaSphere is published by John Wiley & Sons Ltd on behalf of European Hematology Association. This is an open access Abstract Book distributed under the Attribution-NonCommercial-NoDerivs (CC BY-NC-ND), which allows third parties to download the articles and share them with others as long as they credit the author and the Abstract Book, but they cannot change the content in any way or use them commercially.
**Abstract Book Citations:** Authors, Title, HemaSphere, 2026;10:(S1):pages. The abstract book DOIs can be found on the "Author Information" page.
**Disclaimer:** Articles published in the journal HemaSphere exclusively reflect the opinions of the authors. The authors are responsible for all content in their abstracts including accuracy of the facts, statements, citing resources, etc.

### Results

The incidence of new-onset AF in patients receiving ibrutinib therapy during the five-year was about 15%. All complications during ibrutinib therapy are demonstrated on Table 1. After a median follow-up of 39 [23; 57] months, 94 patients (about 15%) developed new-onset AF during ibrutinib therapy. Patients with new-onset AF were significantly older (median 70 vs. 64 years; p<0.001) and exhibited a higher prevalence of cardiovascular comorbidities: arterial hypertension (78% vs. 55%; p=0.004), coronary artery disease (28% vs. 13%; p=0.002), chronic heart failure (11% vs. 2%; p=0.001), diabetes mellitus (24.5% vs. 13%; p=0.019), and stroke/TIA (6% vs. 2%; p=0.048). The majority of patients with new-onset AF had pre-existing chronic kidney disease stage 3–5 (51% vs. 30%; p = 0.042). The AF group exhibited a significantly higher overall comorbidity burden (median CIRS: 11 [8; 15] vs.4 [2; 7]; p < 0.001). Conversely, no significant differences were observed regarding CLL disease burden, prior lines of therapy, or TP53 mutation status (all p > 0.05).

Table 1. Complications during ibrutinib therapy (n=641).

| Complication | Frequency |
|---|---|
| New-onset AF, n (%) | 94 (14,7%) |
| Worsening of pre-existing AF (recurrence of paroxysmal AF, progression to permanent AF), n (%) | 27 (4,2%) |
| Thrombotic complications | 75 (11,7%) |
| Ischemic stroke, n (%) | 31 (4,8%) |
| Venous thromboembolism, n (%) | 31 (4,8%) |
| Myocardial infarction, n (%) | 13 (2,1%) |
| Hemorrhagic complications | |
| All BARC-type bleeding events, n (%) | 229 (35,7%) |
| Minor bleeding (BARC type 1), n (%) | 115 (17,9%) |
| Major bleeding (BARC types 3–4), n (%) | 29 (4,5%) |
| Fatal bleeding (BARC type 5), n (%) | 13 (2,0%) |
| Clinically significant bleeding (BARC types 2–5), n (%) | 114 (17,8%) |
| Heart failure decompensation, n (%) | 41 (6,4%) |

Ibrutinib-induced AF was associated with a fourfold increased risk of thrombotic complications (OR 4.071; 95% CI 1.837–9.024; p < 0.001), including a higher incidence of fatal pulmonary embolism (p = 0.035). Notably, the incidence of fatal bleeding remained comparable between groups (Figure 1).

Figure 1. Thrombosis-free survival in age- and sex-standardized cohorts of ibrutinib-treated patients with and without atrial fibrillation.

### Summary/Conclusion

The incidence of AF in CLL patients receiving ibrutinib significantly exceeds that of the elderly population without CLL. New-onset AF during ibrutinib therapy is associated with a higher risk of thrombotic complications, including fatal events, whereas the incidence of fatal hemorrhagic complications remains comparable.

**Copyright Information:** (Online) ISSN: 2572-9241
©2026 The Author(s). HemaSphere is published by John Wiley & Sons Ltd on behalf of European Hematology Association. This is an open access Abstract Book distributed under the Attribution-NonCommercial-NoDerivs (CC BY-NC-ND), which allows third parties to download the articles and share them with others as long as they credit the author and the Abstract Book, but they cannot change the content in any way or use them commercially.
**Abstract Book Citations:** Authors, Title, HemaSphere, 2026;10:(S1):pages. The abstract book DOIs can be found on the "Author Information" page.
**Disclaimer:** Articles published in the journal HemaSphere exclusively reflect the opinions of the authors. The authors are responsible for all content in their abstracts including accuracy of the facts, statements, citing resources, etc.

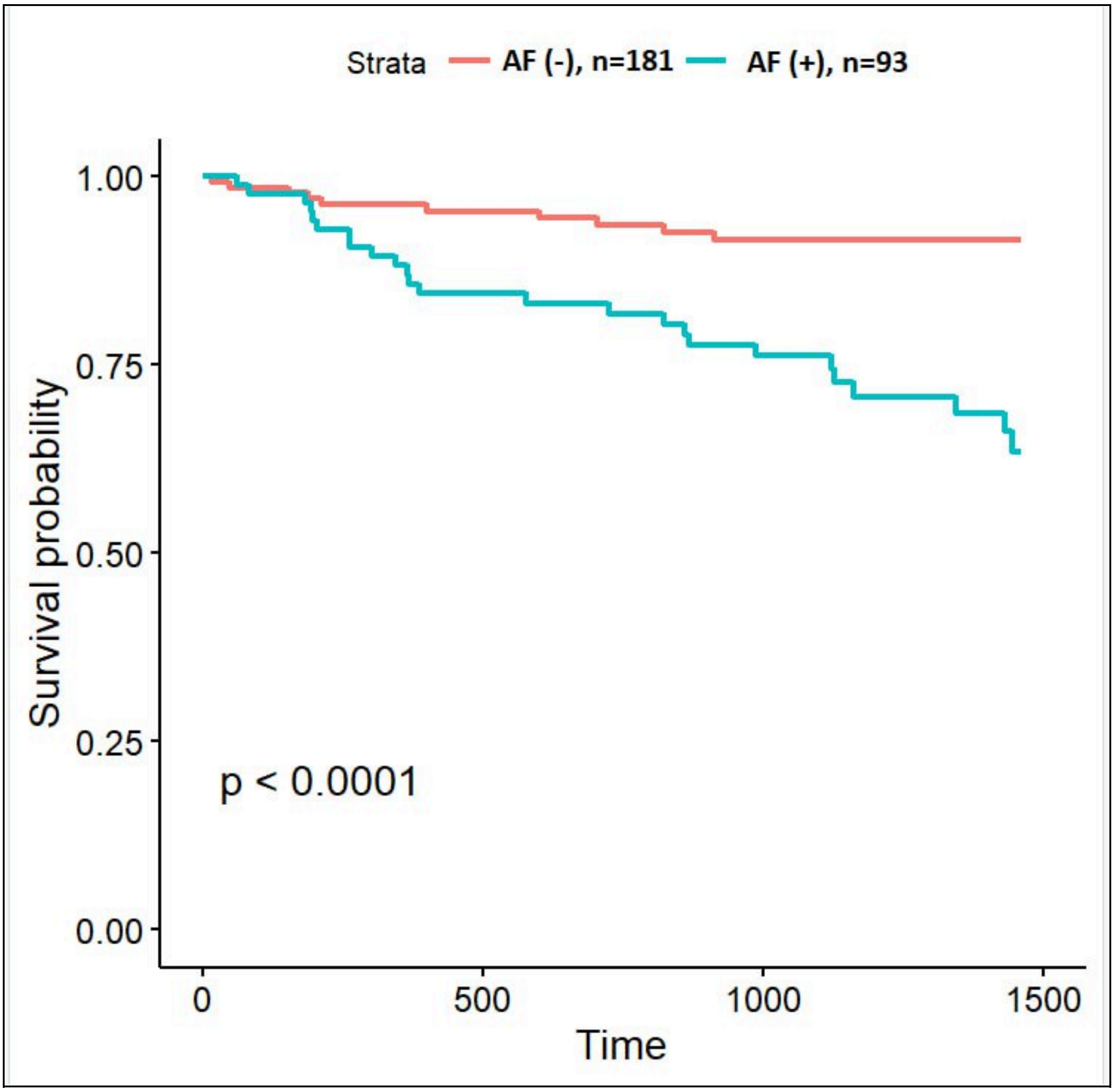

**Copyright Information:** (Online) ISSN: 2572-9241
©2026 The Author(s). HemaSphere is published by John Wiley & Sons Ltd on behalf of European Hematology Association. This is an open access Abstract Book distributed under the Attribution-NonCommercial-NoDerivs (CC BY-NC-ND), which allows third parties to download the articles and share them with others as long as they credit the author and the Abstract Book, but they cannot change the content in any way or use them commercially.
**Abstract Book Citations:** Authors, Title, HemaSphere, 2026;10:(S1):pages. The abstract book DOIs can be found on the "Author Information" page.
**Disclaimer:** Articles published in the journal HemaSphere exclusively reflect the opinions of the authors. The authors are responsible for all content in their abstracts including accuracy of the facts, statements, citing resources, etc.

### (PB2957) REAL-WORLD OUTCOMES OF FIRST-LINE CHEMOIMMUNOTHERAPY FOR CLL IN KAZAKHSTAN: IMPACT OF IGHV STATUS AND TP53 ABERRATIONS

**Topic:** 06. Chronic lymphocytic leukemia and related disorders - Clinical

Anton Klodzinskiy[1], Maria Kahanova[1], Mariya Butyugina[1, 2], Lyudmila Turgunova*[1, 2], Saule Gabbassova[3], Akmaral Jazyltayeva[3], Azat Karabekov[4], Meiramgul' Yussupova[4], Raigul Ramazanova[5]

*[1]Hematology Center LLP, Hematology, Astana, Kazakhstan; [2]Karaganda Medical University NPJSC, Hematology, Karaganda, Kazakhstan; [3]Kazakh Institute Oncology and Radiology, Hematology, Almaty, Kazakhstan; [4]National Research Oncology Center LLP, Lymphoproliferative disorders, Astana, Kazakhstan; [5]Kazakh National Medical University Named After S.d. Asfendiyarov, Clinic, Almaty, Kazakhstan;*

### Background

Access to targeted agents for chronic lymphocytic leukemia (CLL) remains limited in Kazakhstan; consequently, first-line care in routine practice is still dominated by chemoimmunotherapy (CIT). Whether CIT outcomes are inferior in biologically higher-risk but TP53-intact patients (unmutated IGHV) under real-world conditions is insufficiently documented in Central Asia.

### Aims

To evaluate real-world outcomes of first-line CIT in Kazakhstan and quantify the impact of IGHV mutational status and TP53 aberrations on treatment patterns and survival.

### Methods

Retrospective cohort study of adult CLL patients managed in a tertiary hematology network (Kazakhstan). IGHV status was determined by sequencing and classified per ERIC criteria. TP53 aberrations were defined as del(17p) by FISH and/or TP53 mutation by sequencing when available. Treatment patterns were described for first-line therapy. Overall survival (OS) was measured from diagnosis to death from any cause and censored at last contact. Time to first treatment (TTFT) was measured from diagnosis to start of first-line therapy. Survival was estimated by Kaplan–Meier and compared with log-rank tests; hazard ratios (HR) are presented for key comparisons.

### Results

Among 248 patients, median follow-up from diagnosis to last contact was 26.3 months (IQR 8.3–63.4); 94/248 (37.9%) deaths were recorded. IGHV status was available in 162/248 (65.3%): 99/162 (61.1%) unmutated, 58/162 (35.8%) mutated, and 5/162 (3.1%) dual. TP53 aberrations were assessed in 180/248 (72.6%) and detected in 31/180 (17.2%). First-line therapy was initiated in 173/248 (69.8%). CIT predominated: 167/173 (96.5%) received CIT, whereas only 6/173 (3.5%) received targeted therapy in first line. In the TP53-intact CIT-treated subgroup with known IGHV (n=87), unmutated IGHV was associated with earlier treatment requirement (median TTFT 1.0 vs 5.0 months for mutated IGHV) and inferior OS despite absence of TP53 aberrations (log-rank p=0.025; HR 2.48, 95% CI 1.12–5.47 for unmutated vs mutated IGHV).

**Copyright Information:** (Online) ISSN: 2572-9241
©2026 The Author(s). HemaSphere is published by John Wiley & Sons Ltd on behalf of European Hematology Association. This is an open access Abstract Book distributed under the Attribution-NonCommercial-NoDerivs (CC BY-NC-ND), which allows third parties to download the articles and share them with others as long as they credit the author and the Abstract Book, but they cannot change the content in any way or use them commercially.
**Abstract Book Citations:** Authors, Title, HemaSphere, 2026;10:(S1):pages. The abstract book DOIs can be found on the "Author Information" page.
**Disclaimer:** Articles published in the journal HemaSphere exclusively reflect the opinions of the authors. The authors are responsible for all content in their abstracts including accuracy of the facts, statements, citing resources, etc.

**Summary/Conclusion**

In Kazakhstan real-world practice, first-line CLL treatment is overwhelmingly CIT, reflecting restricted access to novel agents. Within TP53-intact patients treated with CIT, unmutated IGHV identifies a subgroup with earlier need for therapy and significantly worse OS. These findings support routine baseline biomarker testing (IGHV and TP53) and prioritization of early access to targeted therapies for biologically high-risk patients.

**Copyright Information:** (Online) ISSN: 2572-9241
©2026 The Author(s). HemaSphere is published by John Wiley & Sons Ltd on behalf of European Hematology Association. This is an open access Abstract Book distributed under the Attribution-NonCommercial-NoDerivs (CC BY-NC-ND), which allows third parties to download the articles and share them with others as long as they credit the author and the Abstract Book, but they cannot change the content in any way or use them commercially.
**Abstract Book Citations:** Authors, Title, HemaSphere, 2026;10:(S1):pages. The abstract book DOIs can be found on the "Author Information" page.
**Disclaimer:** Articles published in the journal HemaSphere exclusively reflect the opinions of the authors. The authors are responsible for all content in their abstracts including accuracy of the facts, statements, citing resources, etc.

### (PB2958) EFFECTIVENESS OF SUBCUTANEOUS CLADRIBINE IN HAIRY CELL LEUKEMIA: A MULTICENTER REAL-WORLD STUDY FROM BRAZIL

**Topic:** 06. Chronic lymphocytic leukemia and related disorders - Clinical

Fernanda Santos Azevedo[1, 2], Caio Justino[1], Gabriel Cunha[1], Maria Cristina Seiwald[1, 3], Ana Paula Tavares[1], Leila Perobelli[4], Fernanda Marques[1], Danielle Leão[5], Talita Silveira[6], Celso Arrais-Rodrigues*[1, 2]

*[1]Federal University of São Paulo, São Paulo, Brazil; [2]Hospital Nove de Julho/ Rede Américas, São Paulo, Brazil; [3]Hospital Sírio Libanes, São Paulo, Brazil; [4]Hospital de Transplantes Euryclides de Jesus Zerbini, São Paulo, Brazil; [5]A Beneficência Portuguesa de São Paulo, São Paulo, Brazil; [6]A C Camargo Cancer Center, São Paulo, Brazil;*

### Background

Hairy cell leukemia (HCL) is an indolent B-cell lymphoproliferative disorder, characterized by B lymphocytes that infiltrate the bone marrow, spleen, and peripheral blood. Common clinical manifestations include splenomegaly, cytopenias and increased susceptibility to infections. Purine analogues remain the standard first-line therapy and achieve high complete response (CR) rates. Cladribine can be administered intravenously or subcutaneously with comparable efficacy and safety.

### Aims

The aim of this study is to evaluate the efficacy and safety of subcutaneous cladribine in patients (pts) with HCL.

### Methods

This retrospective, multicenter study included pts diagnosed with HCL between July 1998 and February 2026 at five institutions that participate in the Brazilian CLL Database. Response was assessed according to established HCL criteria. Overall survival (OS) and time to next treatment (TTNT) were estimated using the Kaplan–Meier method and compared using the log-rank test. Cox regression analysis was performed to identify factors associated with poorer survival.

### Results

A total of 116 pts were included in this cohort. Classical HCL was diagnosed in 115 pts (99%), and one had variant HCL. Eighty percent (n= 92) were male. The median age at diagnosis was 51 years (range, 29–90).
The median hemoglobin level was 10.1 g/dL (range, 3.5–15.1) and the median platelet count was 64 $\times 10^9$/L (range, 12–346 $\times 10^9$/L). Monocytopenia was present in 98%. After a median follow-up of 59 months (range, 3–334), five-year OS was 92%. Age greater than 75 years and hemoglobin less than 10 g/dL were independently associated with poorer survival.
By the data cutoff, 94 pts (81%) required first-line therapy. Cladribine was the most frequently used regimen (95%), administered intravenously (IV) in 61 cases (68%) and subcutaneously (SC) in 28 cases (32%). Rituximab was combined with cladribine in four cases; all of these pts received IV administration. SC cladribine was administered entirely in the outpatient setting. Alternative first-line approaches included fludarabine-based therapy (n = 3), splenectomy (n = 1), and rituximab monotherapy (n = 1).
Treatment was generally well tolerated. Overall response rate was 98% and complete remission was achieved in 88%. Among pts who received cladribine, the median follow-up period after first-line therapy was 58 months (range, 3–332 months). Five-year OS was 94%, with no significant difference between IV and SC administration (95% vs. 93%, respectively). Median TTNT was not reached, with no difference according to route of administration. Only 20 of the 89 patients treated with cladribine required second-line therapy, with a median time to second-line treatment of 106 months. Retreatment with cladribine, with or without rituximab, was performed in 13 cases (65%). Rituximab monotherapy was administered in four pts, chemotherapy and splenectomy in one case each, and allogeneic hematopoietic stem cell transplantation in one patient.

**Copyright Information:** (Online) ISSN: 2572-9241
©2026 The Author(s). HemaSphere is published by John Wiley & Sons Ltd on behalf of European Hematology Association. This is an open access Abstract Book distributed under the Attribution-NonCommercial-NoDerivs (CC BY-NC-ND), which allows third parties to download the articles and share them with others as long as they credit the author and the Abstract Book, but they cannot change the content in any way or use them commercially.
**Abstract Book Citations:** Authors, Title, HemaSphere, 2026;10:(S1):pages. The abstract book DOIs can be found on the “Author Information” page.
**Disclaimer:** Articles published in the journal HemaSphere exclusively reflect the opinions of the authors. The authors are responsible for all content in their abstracts including accuracy of the facts, statements, citing resources, etc.

**Summary/Conclusion**

This multicenter, real-world study represents the largest reported HCL cohort in Latin America. First-line cladribine treatment produced high complete remission rates, SC cladribine demonstrated efficacy comparable to IV administration. Importantly, SC cladribine enables full outpatient treatment. This provides a substantial logistical and economic advantage with limited resources. These findings support the use of ambulatory subcutaneous cladribine as an effective, safe, and pragmatic first-line treatment strategy.

**Copyright Information:** (Online) ISSN: 2572-9241
©2026 The Author(s). HemaSphere is published by John Wiley & Sons Ltd on behalf of European Hematology Association. This is an open access Abstract Book distributed under the Attribution-NonCommercial-NoDerivs (CC BY-NC-ND), which allows third parties to download the articles and share them with others as long as they credit the author and the Abstract Book, but they cannot change the content in any way or use them commercially.
**Abstract Book Citations:** Authors, Title, HemaSphere, 2026;10:(S1):pages. The abstract book DOIs can be found on the "Author Information" page.
**Disclaimer:** Articles published in the journal HemaSphere exclusively reflect the opinions of the authors. The authors are responsible for all content in their abstracts including accuracy of the facts, statements, citing resources, etc.

### (PB2959) SECONDARY BREAST INVOLVEMENT BY CHRONIC LYMPHOCYTIC LEUKEMIA IN A MALE PATIENT: A RARE PRESENTATION WITH MOLECULAR CORRELATES

**Topic:** 06. Chronic lymphocytic leukemia and related disorders - Clinical

Karla Fernanda Rodriguez Estrada*[1]

[1] *Salvadoran Social Security Institute, San Salvador, El Salvador;*

### Background

Breast involvement by lymphoma is rare, particularly in male patients, and represents a diagnostic challenge due to its nonspecific clinical and imaging features. It may mimic primary breast carcinoma, potentially leading to delayed or inappropriate management. Recognition of this entity and correlation with histopathology and immunophenotyping are essential for accurate diagnosis.

### Aims

To report a rare case of secondary breast involvement by small B-cell lymphoma in a male patient and to highlight the role of breast ultrasound, histopathology, immunohistochemistry, and next-generation sequencing (NGS) in the diagnostic workup.

### Methods

Clinical, imaging, histological, and immunophenotypic data were retrospectively reviewed. Breast ultrasound findings, core needle biopsy results, and immunohistochemical profiles were analyzed. A focused literature review was conducted to summarize key diagnostic features of breast involvement by small B-cell lymphomas.

### Results

A 79-year-old male patient presented with a one-year history of fatigue and weight loss and a four-month history of a painless solid mass in the right breast. Physical examination revealed a 1-cm axillary lymph node, a 2-cm non-mobile, painless solid nodule in the upper outer quadrant of the right breast, and 1–2-cm inguinal lymphadenopathies. No hepatomegaly or splenomegaly was detected. Laboratory evaluation showed marked leukocytosis (385,500/µL) due to lymphocytosis and anemia (hemoglobin 105 g/L). Peripheral blood flow cytometry identified a clonal population of kappa light chain–restricted B cells, representing 92% of lymphoid cells and expressing CD20, CD5, and CD23. Computed tomography revealed cervical, axillary, and inguinal lymphadenopathy, as well as a retroperitoneal mass measuring 11.1 × 10.2 × 5.2 cm. Breast ultrasound identified two solid nodular lesions with suspicious features, classified as BI-RADS 4B (Figure 1). Core needle biopsy demonstrated diffuse infiltration by a small- to medium-sized lymphoid neoplasm with a diffuse growth pattern. Immunohistochemistry was positive for CD20, CD45, CD79a, and CD23, with a low proliferation index (Ki-67 approximately 5%), consistent with small B-cell lymphocytic lymphoma involving the breast. The disease was classified as Rai stage III, Binet stage C, and high-risk CLL-IPI. Due to limited access to targeted therapies, first-line treatment with R-COP achieved a partial hematologic and nodal response but was followed by disease progression, manifested as a pleural effusion with cytological confirmation of leukemic involvement. Second-line therapy with Rituximab–Bendamustine resulted in clinical improvement and PET CT end of treatment in complete response. NGS revealed a pathogenic ATM frameshift mutation and an unmutated IGHV status, while no mutations in TP53, NOTCH1, SF3B1, or BIRC3 were detected, supporting an aggressive disease biology.

**Copyright Information:** (Online) ISSN: 2572-9241
©2026 The Author(s). HemaSphere is published by John Wiley & Sons Ltd on behalf of European Hematology Association. This is an open access Abstract Book distributed under the Attribution-NonCommercial-NoDerivs (CC BY-NC-ND), which allows third parties to download the articles and share them with others as long as they credit the author and the Abstract Book, but they cannot change the content in any way or use them commercially.
**Abstract Book Citations:** Authors, Title, HemaSphere, 2026;10:(S1):pages. The abstract book DOIs can be found on the "Author Information" page.
**Disclaimer:** Articles published in the journal HemaSphere exclusively reflect the opinions of the authors. The authors are responsible for all content in their abstracts including accuracy of the facts, statements, citing resources, etc.

**Summary/Conclusion**

Breast lymphoma should be considered in the differential diagnosis of breast masses. Flow cytometry plays a critical role in confirming clonality and establishing the diagnosis. This case underscores the importance of integrating different studies for accurate diagnosis. The presence of an unmutated IGHV status and a pathogenic ATM mutation correlated with an aggressive clinical course and limited response to chemoimmunotherapy, highlighting the prognostic value of molecular characterization, especially in settings with restricted access to targeted therapies.

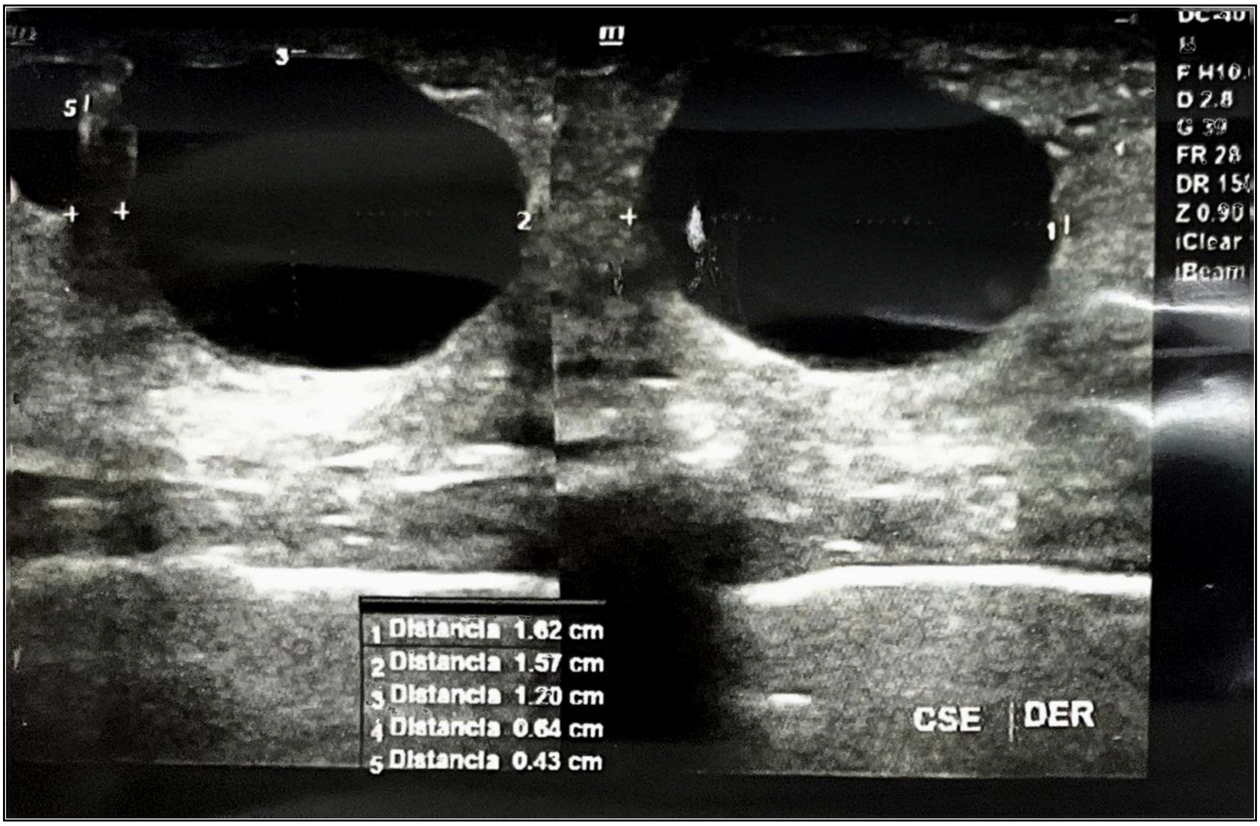

**Copyright Information:** (Online) ISSN: 2572-9241
©2026 The Author(s). HemaSphere is published by John Wiley & Sons Ltd on behalf of European Hematology Association. This is an open access Abstract Book distributed under the Attribution-NonCommercial-NoDerivs (CC BY-NC-ND), which allows third parties to download the articles and share them with others as long as they credit the author and the Abstract Book, but they cannot change the content in any way or use them commercially.
**Abstract Book Citations:** Authors, Title, HemaSphere, 2026;10:(S1):pages. The abstract book DOIs can be found on the "Author Information" page.
**Disclaimer:** Articles published in the journal HemaSphere exclusively reflect the opinions of the authors. The authors are responsible for all content in their abstracts including accuracy of the facts, statements, citing resources, etc.

### (PB2960) FDG PET-CT IN HAIRY CELL LEUKEMIA: DEFINING STANDARD METABOLIC UPTAKE ACROSS DISEASE COMPARTMENTS

**Topic:** 06. Chronic lymphocytic leukemia and related disorders - Clinical

Tamar Tadmor*[1, 2], Ilana Levy[2, 3], Natalia Kreiniz[2, 3], Miguel Gorenberg[4, 5], Konstantin Gurevich[4], Gilad Itchaki[6, 7]

*[1]Bnai Zion medical center, Hematology, Haifa, Israel; [2]The Ruth and Bruce Rappaport Faculty of Medicine, Technion,, Haifa, Israel; [3]Bnai Zion medical center, Haifa, Israel; [4]Bnai Zion medical center, Nuclera medicine, Haifa, Israel; [5]The Ruth and Bruce Rappaport Faculty of Medicine, Technion,, Nuclera medicine, Haifa, Israel; [6]meir medical center, Hematology, KFAR SABA, Israel; [7]The Gray Faculty of Medical and Health Sciences, Tel Aviv University, Tel Aviv, Israel, TEL AVIV, Israel;*

### Background

Hairy Cell Leukaemia (HCL) is an indolent B-cell neoplasm with predominant bone marrow and splenic involvement.
Currently, Hairy Cell Leukemia guidelines recommend the routine use of chest X-ray and abdominal ultrasound as imaging workup of a patient with HCL. The use of computed tomography of abdomen to evaluate organomegaly, and lymphadenopathy is only indicated for the minority of patients in a clinical trial or those with associated symptoms referable to these systems(
PET- CT / CT has become a key tool in assessing disease spread and treatment response of several types of cancer in general and B-cell malignancies in particular
In HCL, the use of PET-CT has been reported mainly in patients who present with unusual clinical sites of involvement or manifestation and are available in the literature only as case reports

### Aims

This was a retrospective study evaluating the role of ^18F-FDG PET-CT/CT in patients with classical Hairy Cell Leukemia (HCL)

### Methods

Eligibility criteria were: Adult patients (≥18 years) with a confirmed diagnosis of classical HCL between 2000 and 2020 and with at least one available PET-CT scan suitable for central review were included.
Clinical, laboratory and imaging data were collected retrospectively from institutional databases including demographic and baseline variables: disease characteristics at diagnosis and treatment and outcome data.
For scan evaluation we collected: PET-CT variables: date and indication for PET-CT, sites and pattern of FDG uptake, maximum standardized uptake value (SUVmax) of pathological sites and SUVmax of bone marrow involvement.
Visual assessment was performed using the Deauville 5-point scale, with mediastinal blood pool and liver uptake as reference. Semi-quantitative analysis included recording SUVmax for all FDG-avid sites and for bone marrow involvement.

**Copyright Information:** (Online) ISSN: 2572-9241
©2026 The Author(s). HemaSphere is published by John Wiley & Sons Ltd on behalf of European Hematology Association. This is an open access Abstract Book distributed under the Attribution-NonCommercial-NoDerivs (CC BY-NC-ND), which allows third parties to download the articles and share them with others as long as they credit the author and the Abstract Book, but they cannot change the content in any way or use them commercially.
**Abstract Book Citations:** Authors, Title, HemaSphere, 2026;10:(S1):pages. The abstract book DOIs can be found on the “Author Information” page.
**Disclaimer:** Articles published in the journal HemaSphere exclusively reflect the opinions of the authors. The authors are responsible for all content in their abstracts including accuracy of the facts, statements, citing resources, etc.

### Results

Eight patients with classical Hairy Cell Leukemia (HCL) were included in the analysis. The median age at diagnosis was 47 years (range 34–84), and the cohort was predominantly male (6/8, 75%). All patients were Jewish, including 6 Ashkenazi (75%) and 2 Sephardic (25%).
The median time from diagnosis to the first PET-CT was 8 months (range 0–275 months). Patients underwent a median of 2 PET-CT scans per patient (range 1–5). Indications for PET-CT included evaluation of extramedullary disease, unusual clinical presentations, suspected transformation, assessment of other malignancies, and systemic symptoms.
PET-CT demonstrated FDG-avid extramedullary disease in several patients, including lytic bone lesions, renal, adrenal, and nodal involvement (lymphadenopathy in 3/8 patients).
Unusual presentations included vasculitis and seronegative arthritis (one case each). PET-CT also performed due other malignancies in three patients (CLL, melanoma, and a lung mass). One patient presented with B symptoms, including weight loss.
Regarding metabolic activity, the average SUVmax of active disease was 5.4 in bone marrow (range 3.6–8.8), 2.9 in spleen (range 2.1–3.6), 3.1 in liver (range 2.2–3.5), 3.7 in lymph nodes (range 1.4–5.9), and 7.8 in extramedullary sites (range 5.3–14)

### Summary/Conclusion

In HCL, FDG uptake in bone marrow, spleen, liver and lymph nodes is typically low, whereas extramedullary disease demonstrates distinctly higher SUV values. PET-CT is therefore most useful in selected patients with suspected extramedullary involvement, atypical features or concern for transformation or second malignancy, rather than as a routine imaging tool.

**Copyright Information:** (Online) ISSN: 2572-9241
©2026 The Author(s). HemaSphere is published by John Wiley & Sons Ltd on behalf of European Hematology Association. This is an open access Abstract Book distributed under the Attribution-NonCommercial-NoDerivs (CC BY-NC-ND), which allows third parties to download the articles and share them with others as long as they credit the author and the Abstract Book, but they cannot change the content in any way or use them commercially.
**Abstract Book Citations:** Authors, Title, HemaSphere, 2026;10:(S1):pages. The abstract book DOIs can be found on the "Author Information" page.
**Disclaimer:** Articles published in the journal HemaSphere exclusively reflect the opinions of the authors. The authors are responsible for all content in their abstracts including accuracy of the facts, statements, citing resources, etc.

### (PB2961) CONCOMITANT DIAGNOSIS OF CHRONIC LYMPHOCYTIC LEUKEMIA AND B-LYMPHOBLASTIC LEUKEMIA IN A 68-YEAR-OLD MAN: AN EXTREMELY RARE CASE OF BILINEAL ACUTE TRANSFORMATION

**Topic:** 06. Chronic lymphocytic leukemia and related disorders - Clinical

Marsela Shani*[1]

[1]*Mother Theresa University Hospital, Hematology, Tirana, Albania;*

### Background

We report the exceptionally rare occurrence of simultaneous chronic lymphocytic leukemia (CLL) and de novo B-lymphoblastic leukemia (B-ALL) in a 68-year-old man. Flow cytometry and molecular studies demonstrated two distinct neoplastic B-cell populations with different immunophenotypes and genotypes, confirming true bilineal leukemia rather than blastic transformation of CLL.

### Aims

Presenting a case of bilineal acute trasformation

### Methods

A 68-year-old man presented with fatigue, night sweats, and cervical lymphadenopathy. Physical examination revealed generalized lymphadenopathy and splenomegaly. Complete blood count showed WBC 78 × $10^9$/L with 82% lymphocytes, hemoglobin 10.8 g/dL, and platelets 112 × $10^9$/L..Peripheral blood smear revealed two morphologically distinct lymphocyte populations:
-Abundant small mature lymphocytes
-Occasional larger cells with high nuclear/cytoplasmic ratio, dispersed chromatin, and prominent nucleoli (lymphoblasts 8%)
Bone marrow aspirate confirmed hypercellular marrow with 68% small mature lymphocytes and 22% lymphoblasts.
Immunophenotyping (Flow Cytometry)
Two distinct abnormal B-cell populations were identified:
CLL population (65% of lymphocytes):CD19+, CD20dim, CD5+, CD23+, FMC7–, CD10–, sIg dim (kappa restricted), CD38–, ZAP70–
Separate B-ALL population (20% of total cells):CD19+, CD20–, TdT+, CD34+, CD10+, CD5–, CD23–, sIg–, cytoplasmic CD79a+, kappa light chain restricted (different intensity and pattern from CLL)
Cytogenetics and Molecular Studies
CLL clone: del(13q14) monoallelic, trisomy 12 absent, del(11q) absent, del(17p) absent, IGHV mutated
B-ALL clone: normal karyotype 46,XY; no Ph chromosome; no MLL, ETV6-RUNX1, or BCR-ABL rearrangements
Separate next-generation sequencing (NGS) panels on flow-sorted populations confirmed no shared mutations (CLL: no TP53, NOTCH1, SF3B1, or BIRC3 mutations; B-ALL: no pathogenic variants detected)Clonality assessment by IGH gene rearrangement PCR on sorted populations demonstrated completely different rearrangement patterns, confirming two independent neoplastic clones.
Differential
Richter transformation of CLL to B-ALL – excluded by different immunophenotype and unrelated clonality
Prolymphocytic transformation of CLL – excluded by TdT and CD34 positivity
Mixed-phenotype acute leukemia (MPAL) – excluded by two distinct populations
Therapy-related B-ALL

**Copyright Information:** (Online) ISSN: 2572-9241
©2026 The Author(s). HemaSphere is published by John Wiley & Sons Ltd on behalf of European Hematology Association. This is an open access Abstract Book distributed under the Attribution-NonCommercial-NoDerivs (CC BY-NC-ND), which allows third parties to download the articles and share them with others as long as they credit the author and the Abstract Book, but they cannot change the content in any way or use them commercially.
**Abstract Book Citations:** Authors, Title, HemaSphere, 2026;10:(S1):pages. The abstract book DOIs can be found on the "Author Information" page.
**Disclaimer:** Articles published in the journal HemaSphere exclusively reflect the opinions of the authors. The authors are responsible for all content in their abstracts including accuracy of the facts, statements, citing resources, etc.

**Results**

The patient was treated according to hyper-CVAD regimen . Intrathecal prophylaxis was administered.
After cycle 1A: lymphoblasts cleared from peripheral blood and bone marrow (<1% by flow)
After cycle 4: bone marrow showed persistent CLL (52%) but no detectable B-ALL (MRD-negative by flow cytometry at $10^{-4}$ sensitivity)
CNS evaluation remained negative throughout
Consolidation with blinatumomab was administered for B-ALL, followed by maintenance POMP for 24 months. The CLL remained Rai stage II, TP53 wild-type, and did not require therapy (iwCLL criteria). At last follow-up (48 months from diagnosis), the patient remains in complete remission for B-ALL with stable low-bulk CLL (lymphocytes 18 × $10^9$/L).

**Summary/Conclusion**

True bilineal CLL + de novo B-ALL is one of the rarest combinations in hematology. Only seven prior cases with proven clonal unrelatedness have been reported.[3–6] The prognosis appears driven primarily by the acute leukemia component. In our patient, aggressive ALL-directed therapy successfully eradicated the B-ALL clone while the indolent CLL remained controlled without specific treatment.

**Copyright Information:** (Online) ISSN: 2572-9241
©2026 The Author(s). HemaSphere is published by John Wiley & Sons Ltd on behalf of European Hematology Association. This is an open access Abstract Book distributed under the Attribution-NonCommercial-NoDerivs (CC BY-NC-ND), which allows third parties to download the articles and share them with others as long as they credit the author and the Abstract Book, but they cannot change the content in any way or use them commercially.
**Abstract Book Citations:** Authors, Title, HemaSphere, 2026;10:(S1):pages. The abstract book DOIs can be found on the “Author Information” page.
**Disclaimer:** Articles published in the journal HemaSphere exclusively reflect the opinions of the authors. The authors are responsible for all content in their abstracts including accuracy of the facts, statements, citing resources, etc.

### (PB2962) RICHTER SYNDROME IN ROUTINE CLINICAL PRACTICE: A SINGLE TERTIARY-CENTER EXPERIENCE

**Topic:** 06. Chronic lymphocytic leukemia and related disorders - Clinical

Bernardo Soto Román*[1], Luis Miguel Juarez-Salcedo[1], Maria Jimena Cannata Ortiz[1], Ana Maria Garcia-Noblejas Moya[1], Eva Arranz[2], Magdalena Adrados de Llano[3], Adrián Alegre Amor[1], Javier Loscertales Pueyo[1]

*[1]La Princesa University Hospital, Hematology and Hemotherapy, Madrid, Spain; [2]La Princesa University Hospital, Oncohematologic genetics, Madrid, Spain; [3]La Princesa University Hospital, Pathology, Madrid, Spain;*

### Background

Richter syndrome(RS)is defined as the aggressive transformation of chronic lymphocytic leukemia(CLL)into a high-grade lymphoma,most commonly diffuse large B-cell lymphoma(DLBCL),and is associated with a dismal prognosis.Despite therapeutic advances in CLL,the incidence of RS has remained stable,affecting approximately 2–10%of patients throughout the course of the disease,with historically limited overall survival.Most available evidence derives from retrospective series;therefore,real-world studies provide key insights into its clinical presentation,therapeutic management,and survival outcomes.

### Aims

To describe the clinical characteristics,therapeutic management,and survival outcomes of patients diagnosed with Richter syndrome in a real-world setting at a tertiary care hospital.

### Methods

We performed a retrospective study including 13 patients diagnosed with Richter syndrome at a tertiary care hospital.Baseline clinical and biological characteristics,as well as prior treatments,are summarized in ***Table 1***.OS and PFS were assessed using the Kaplan–Meier method.

**Copyright Information:** (Online) ISSN: 2572-9241
©2026 The Author(s). HemaSphere is published by John Wiley & Sons Ltd on behalf of European Hematology Association. This is an open access Abstract Book distributed under the Attribution-NonCommercial-NoDerivs (CC BY-NC-ND), which allows third parties to download the articles and share them with others as long as they credit the author and the Abstract Book, but they cannot change the content in any way or use them commercially.
**Abstract Book Citations:** Authors, Title, HemaSphere, 2026;10:(S1):pages. The abstract book DOIs can be found on the "Author Information" page.
**Disclaimer:** Articles published in the journal HemaSphere exclusively reflect the opinions of the authors. The authors are responsible for all content in their abstracts including accuracy of the facts, statements, citing resources, etc.

### Results

| Variable | Distribution |
|---|---|
| Age at RS diagnosis (median [range]) | 67 (40–87) |
| Time from CLL diagnosis to RS (years) | 4.5 (0.31–14) |
| Histological subtype (DLBCL / HL), n (%) | 12 (92%) / 1 (8%) |
| ECOG >1, n (%) | 2 (15.3%) |
| Bulky disease, n (%) | 2 (15.3%) |
| Extranodal involvement, n (%) | 6 (46.1%) |
| LDH >1.5 × ULN, n (%) | 4 (30.7%) |
| β2-microglobulin >2.2 mg/L, n (%) | 6 (46.1%) |
| TP53 mutation and/or del(17p), n (%) | 7 (53.8%) |
| ≥2 prior lines of therapy, n (%) | 5 (38.4%) |
| Prior exposure to BTK inhibitors and/or venetoclax, n (%) | 6 (46.1%) |

Regarding biological features and prior therapeutic exposure,more than half of the patients harbored TP53 mutations and/or del(17p).Additionally,38.4%had received prior treatment for CLL,including exposure to BTK inhibitors and/or venetoclax,reflecting a clinically complex and partially pretreated population.Among previously treated patients,CLL had been refractory in 7 of 9 cases.Conventional karyotyping was performed in only two patients;both exhibited a complex karyotype,suggesting a high prevalence of complex cytogenetic abnormalities within the constraints of routine real-world practice.An Anderson score ≥2 was observed in 6/13 patients(46.1%).

Most patients received R-CHOP–like regimens(7/13;53.8%).Other approaches included VR-DA-EPOCH(2/13;15.4%)and mosunetuzumab plus CHOP within a clinical trial(2/13;15.4%).The patient with Hodgkin-variant RS was treated with ABVD followed by radiotherapy,achieving partial response and prolonged survival.

Response to first-line treatment was limited,with disease progression in 6/13 patients(46.2%).Three complete responses(23.1%)and two partial responses(15.4%)were documented.In two patients(15.4%),response was not evaluable due to early death or pending reassessment.During follow-up,8 deaths(61.5%)were recorded.Median OS was 22 months,with substantial early mortality.PFS,assessed in 12 treated patients,showed 5 progression events with early curve declines and a median not reached,reflecting early relapses and a small subgroup with durable disease control.Kaplan–Meier curves for OS and PFS are shown in **Figure 1**.

### Summary/Conclusion

In this real-world series,Richter syndrome was associated with adverse biological features,limited treatment response,and substantial early mortality,confirming its poor prognosis even in the era of novel agents.The marked clinical heterogeneity highlights the need for more effective therapies and improved biological characterization in routine practice.

**Copyright Information:** (Online) ISSN: 2572-9241
©2026 The Author(s). HemaSphere is published by John Wiley & Sons Ltd on behalf of European Hematology Association. This is an open access Abstract Book distributed under the Attribution-NonCommercial-NoDerivs (CC BY-NC-ND), which allows third parties to download the articles and share them with others as long as they credit the author and the Abstract Book, but they cannot change the content in any way or use them commercially.
**Abstract Book Citations:** Authors, Title, HemaSphere, 2026;10:(S1):pages. The abstract book DOIs can be found on the “Author Information” page.
**Disclaimer:** Articles published in the journal HemaSphere exclusively reflect the opinions of the authors. The authors are responsible for all content in their abstracts including accuracy of the facts, statements, citing resources, etc.

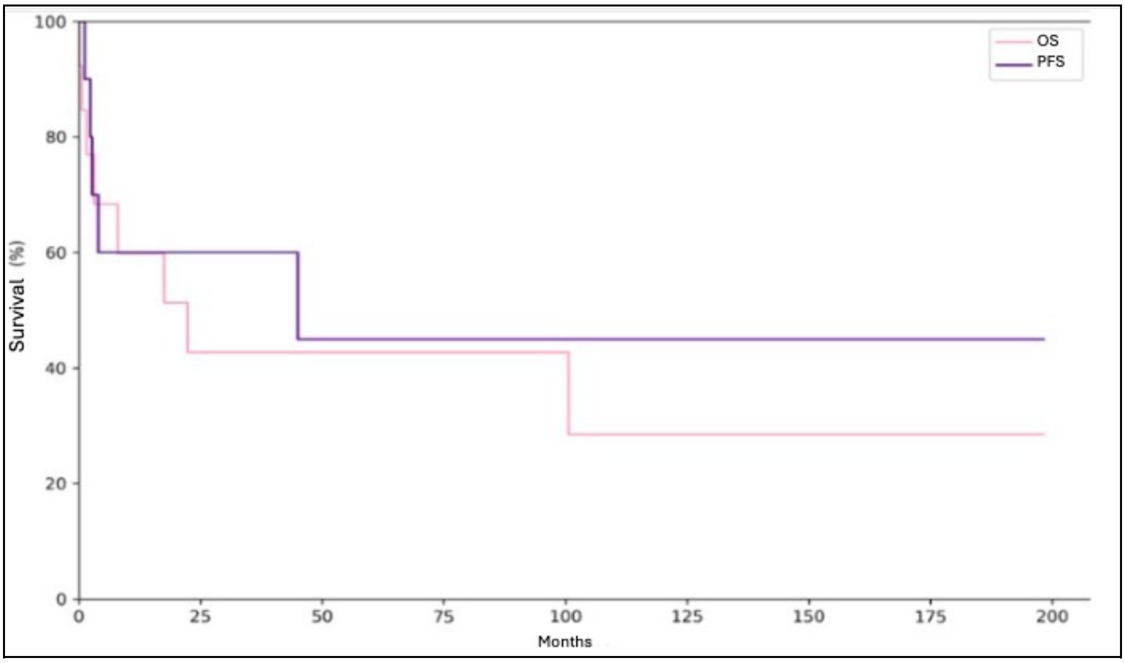

Figure 1: Overall survival and progression-free survival (N=13). OS calculated from the diagnosis of RS and PFS from initiation of first-line therapy.

**Copyright Information:** (Online) ISSN: 2572-9241
©2026 The Author(s). HemaSphere is published by John Wiley & Sons Ltd on behalf of European Hematology Association. This is an open access Abstract Book distributed under the Attribution-NonCommercial-NoDerivs (CC BY-NC-ND), which allows third parties to download the articles and share them with others as long as they credit the author and the Abstract Book, but they cannot change the content in any way or use them commercially.
**Abstract Book Citations:** Authors, Title, HemaSphere, 2026;10:(S1):pages. The abstract book DOIs can be found on the "Author Information" page.
**Disclaimer:** Articles published in the journal HemaSphere exclusively reflect the opinions of the authors. The authors are responsible for all content in their abstracts including accuracy of the facts, statements, citing resources, etc.

### (PB2963) EFFICACY AND SAFETY OF VENETOCLAX IN PREDOMINANTLY HEAVILY PRETREATED CLL PATIENTS: A REAL-WORLD SINGLE-CENTER EXPERIENCE

**Topic:** 06. Chronic lymphocytic leukemia and related disorders - Clinical

Darko Aleksic*[1], Vojin Vukovic[1], Tamara Bibic[1], Jelena Ivanović[1], Sofija Kozarac[1], Kristina Tomic[1], Darko Antic[1]

[1]*Clinic of hematology, Univeristy Clinical Centre of Serbia,, Belgrade, Serbia;*

### Background

The transition from standard chemoimmunotherapy to targeted therapies, such as the BCL-2 inhibitor venetoclax has improved response rates and overall survival in patients with chronic lymphocytic leukemia (CLL). Despite this, real-world data on outcomes and prognostic factors remain limited.

### Aims

To assess the efficacy and safety of venetoclax in real-world CLL patients, as well as to explore factors associated with PFS and OS.

### Methods

In this retrospective single-center study, we included 21 CLL patients treated with venetoclax as monotherapy or in combination with rituximab, between 2019 and 2026 predominantly in the relapsed/refractory (R/R) setting. Data were collected using medical records. PFS and OS were estimated using the Kaplan–Meier method and compared using the log-rank test.

### Results

Median age at treatment initiation was 68 years (range 47–81). Del(17p) and/or TP53 mutation was present in 47.6% (10/21) of patients. Two-thirds of patients were heavily pretreated, having received more than two prior therapy lines, with only one patient receiving venetoclax as first-line therapy. Twelve patients (57.1%) had received prior Bruton's tyrosine kinase inhibitor (BTKi) therapy, specifically ibrutinib. Fourteen patients (66.7%) received venetoclax in combination with rituximab, while the remainder were treated with venetoclax monotherapy. The 6-month overall response rate (ORR) was 88.2% [complete response (CR) 10 (58.8%), partial response (PR) 5 (29.4%)]. At a median follow-up of 24 months (range 3-56) the median PFS and OS, have not been reached. At the time of analysis, 7 patients (33.3%) were still receiving venetoclax. Numerically longer PFS and OS were observed in ibrutinib-naive patients compared to those previously exposed to ibrutinib (mean PFS 47.0 vs 21.1 months; mean OS 37.0 vs 17.6 months), although these differences did not reach statistical significance (p=0.332 and p=0.463, respectively). Interestingly, del(17p)/TP53 mutation was not associated with inferior PFS in our cohort (mean PFS 33.8 vs 36.4 months in non-del(17p)/TP53; p=0.235). This may reflect the earlier introduction of targeted therapies in higher-risk patients. No statistically significant differences in survival outcomes were observed between heavily pretreated patients (> 2 prior lines) and those treated in earlier lines (0–2 prior lines). The addition of rituximab was associated with significantly improved OS (44.1 vs 12.9 months; p=0.004) and longer PFS, although it did not reach statistical significance (46.6 vs 17.6 months; p=0.066). Frequent hematologic toxicity and treatment modifications were observed. Grade ≥3 neutropenia (76.2%) and infections (42.8%) were the most frequent adverse events. Dose reduction was necessary in 57.1% of patients but without a statistically significant impact on PFS or OS. In total, ten patients discontinued therapy prematurely, including one Richter transformation, two secondary malignancies, four cases of progressive disease, and the remainder due to toxicity.

**Copyright Information:** (Online) ISSN: 2572-9241
©2026 The Author(s). HemaSphere is published by John Wiley & Sons Ltd on behalf of European Hematology Association. This is an open access Abstract Book distributed under the Attribution-NonCommercial-NoDerivs (CC BY-NC-ND), which allows third parties to download the articles and share them with others as long as they credit the author and the Abstract Book, but they cannot change the content in any way or use them commercially.
**Abstract Book Citations:** Authors, Title, HemaSphere, 2026;10:(S1):pages. The abstract book DOIs can be found on the "Author Information" page.
**Disclaimer:** Articles published in the journal HemaSphere exclusively reflect the opinions of the authors. The authors are responsible for all content in their abstracts including accuracy of the facts, statements, citing resources, etc.

**Summary/Conclusion**

Venetoclax demonstrated high response rates and good disease control in this real-world cohort. Prior ibrutinib exposure was associated with numerically shorter survival, while rituximab improved OS. Despite frequent toxicity, dose reductions did not adversely affect outcomes, supporting the effectiveness and manageable safety profile of venetoclax in routine practice.

**Copyright Information:** (Online) ISSN: 2572-9241
©2026 The Author(s). HemaSphere is published by John Wiley & Sons Ltd on behalf of European Hematology Association. This is an open access Abstract Book distributed under the Attribution-NonCommercial-NoDerivs (CC BY-NC-ND), which allows third parties to download the articles and share them with others as long as they credit the author and the Abstract Book, but they cannot change the content in any way or use them commercially.
**Abstract Book Citations:** Authors, Title, HemaSphere, 2026;10:(S1):pages. The abstract book DOIs can be found on the "Author Information" page.
**Disclaimer:** Articles published in the journal HemaSphere exclusively reflect the opinions of the authors. The authors are responsible for all content in their abstracts including accuracy of the facts, statements, citing resources, etc.

### (PB2964) IBRUTINIB MAINTENANCE AFTER FIRST-LINE CHEMOIMMUNOTHERAPY IN CLL: A PROSPECTIVE MULTICENTER STUDY

**Topic:** 06. Chronic lymphocytic leukemia and related disorders - Clinical

Nayara Guedes*[1], Gabriel Cunha[1], Ana Rita Fonseca[1, 2], Thiago Carneiro[3], Rodrigo Santucci Silva[4], Sergio Fortier[5], Vera Lucia Piratininga de Figueiredo[6], Larissa Ommati[7], Valeria Buccheri[8], Gislaine Oliveira[9], Leila Perobelli[10], Fernando Ferreira[11], Larissa Gandolpho[1, 12], Caio Justino[1], Vinicius Molla[1, 12], Celso Arrais-Rodrigues[1, 12]

*[1]Universidade Federal de São Paulo, Hematology, São Paulo, Brazil; [2]Hospital Sírio-Libanês, hematology, São Paulo, Brazil; [3]Universidade Estadual do Pará, Hematology, Belém, Brazil; [4]HEMOMED Instituto de Oncologia e Hematologia, SAO PAULO, Brazil; [5]Santa Casa de São Paulo, SAO PAULO, Brazil; [6]Hospital Servidor Publico Estadual, São Paulo, Brazil; [7]Hospital Santa Marcelina, SAO PAULO, Brazil; [8]Instituto do Câncer do Estado de São Paulo, São Paulo, Brazil; [9]UNICAMP, CAMPINAS, Brazil; [10]Hospital Brigadeiro, SAO PAULO, Brazil; [11]Instituto D'Or de Pesquisa, BRASILIA, Brazil; [12]Hospital Nove de Julho, Hematology, São Paulo, Brazil;*

### Background

Although targeted, fixed-duration regimens have reshaped frontline chronic lymphocytic leukemia (CLL) management, chemoimmunotherapy (CIT) remains widely used in several regions due to heterogeneous access to novel agents. A substantial proportion of patients treated with CIT achieve clinical remission but remain measurable residual disease (MRD)-positive. The potential role of maintenance therapy to deepen response and prolong disease control in this setting remains uncertain.

### Methods

Although targeted, fixed-duration regimens have reshaped frontline chronic lymphocytic leukemia (CLL) management, chemoimmunotherapy (CIT) remains widely used in several regions due to heterogeneous access to novel agents. A substantial proportion of patients treated with CIT achieve clinical remission but remain measurable residual disease (MRD)-positive. The potential role of maintenance therapy to deepen response and prolong disease control in this setting remains uncertain.

### Results

Forty patients from nine centers were enrolled. Median age was 59 years; 62.5% were in CR and 37.5% in PR at maintenance initiation. Among MRD-positive patients at baseline, 23.5% achieved undetectable MRD and 85% experienced at least a 1-log reduction in MRD levels. All patients who initiated maintenance in PR converted to CR.
After a median follow-up of 100 months, 48-month PFS was 65%, OS was 78%, and TTNT was 76%. Adverse events were predominantly grade 1–2; grade ≥3 toxicities were infrequent. Asymptomatic atrial fibrillation occurred in 8% of patients.

### Summary/Conclusion

Ibrutinib maintenance following first-line CIT resulted in MRD reduction, response deepening, and durable disease control in this prospective cohort. While contemporary frontline strategies increasingly favor fixed-duration targeted combinations, these findings suggest that maintenance BTK inhibition may represent a pragmatic consolidation approach in settings where access to novel frontline regimens remains limited. Further comparative studies are warranted.

**Copyright Information:** (Online) ISSN: 2572-9241
©2026 The Author(s). HemaSphere is published by John Wiley & Sons Ltd on behalf of European Hematology Association. This is an open access Abstract Book distributed under the Attribution-NonCommercial-NoDerivs (CC BY-NC-ND), which allows third parties to download the articles and share them with others as long as they credit the author and the Abstract Book, but they cannot change the content in any way or use them commercially.
**Abstract Book Citations:** Authors, Title, HemaSphere, 2026;10:(S1):pages. The abstract book DOIs can be found on the "Author Information" page.
**Disclaimer:** Articles published in the journal HemaSphere exclusively reflect the opinions of the authors. The authors are responsible for all content in their abstracts including accuracy of the facts, statements, citing resources, etc.

### (PB2965) ROCKET-CLL: A RANDOMIZED, OPEN-LABEL, MULTICENTER, PHASE 3 STUDY OF ROCBRUTINIB (LP-168) VERSUS PIRTOBRUTINIB IN COVALENT BTK INHIBITOR (CBTKI) PRETREATED RELAPSED OR REFRACTORY (R/R) CLL/SLL

**Topic:** 06. Chronic lymphocytic leukemia and related disorders - Clinical

Yu Chen*[1], Jennifer Woyach[2], John F. Seymour[3], Danielle M. Brander[4], Deborah Stephens[5], Neil Kay[6], Fenlai Tan[7], Stephen Anthony[7], Yi Chen[7], Kewei Shang[7], Yue Shen[8], Yejiang Lou[8], John Byrd[9]

*[1]Newave Pharmaceutical Inc., Pleasanton, United States of America;[2]The Ohio State University, Columbus, United States of America; [3]Peter Mac Callum, Melbourne, Australia; [4]Duke University, Durham, United States of America;[5]The University of North Carolina at Chapel Hill, Chapel Hill, United States of America; [6]Mayo Clinic, Rochester, United States of America; [7]Newave Pharmaceutical Inc, Pleasanton, United States of America; [8]Guangzhou Lupeng Pharmaceutical, Guangzhou, China;[9]UMPC Hillman Cancer Center, Pittsburgh, United States of America;*

### Background

Bruton tyrosine kinase covalent inhibitors (cBTKi) have been transformative in CLL treatment. Despite achieving extended remissions, in most patients disease still relapses despite cBTKi therapy, often associated with *BTK* C481 mutations. Non-covalent BTKi (ncBTKi), including pirtobrutinib, can overcome this resistance (Sharman et al. *ASCO.* 2025). Pirtobrutinib received FDA approval in Dec 2025, for R/R CLL/CLL patients pretreated with cBTKi. Rocbrutinib (LP-168) is a highly selective next-generation BTKi that can covalently bind wild-type and gatekeeper mutations (T474X, commonly seen in patients relapsed after pirtobrutinib) and non-covalently target C481-mutated *BTK*, with preclinical efficacy in treatment-naïve and BTKi-resistant CLL (Gordon et al. *IWCLL.* 2025). In the rocbrutinib phase 1 trial (NCT04775745), encouraging safety and efficacy have been observed in CLL patients with prior exposure to BTKi and/or BCL2 inhibitor (BCL2i) (Woyach et al. *Blood.* 2025). Presented here is the design of a phase 3 trial aiming to compare the efficacy of rocbrutinib versus pirtobrutinib in cBTKi-pretreated R/R CLL/SLL patients.

### Methods

ROCKET-CLL (NCT07342478) is a randomized, open-label, multicenter, phase 3 study comparing rocbrutinib (Arm 1) to pirtobrutinib (Arm 2) in R/R CLL/SLL subjects who previously received treatments including covalent BTKi. Eligible subjects must require treatment per 2018 iwCLL criteria, have measurable lesions by CT, an ECOG performance score of 0-2, and adequate hematologic and other organ functions. About 306 subjects will be randomized 1:1 based on stratification factors such as 17p deletion/*TP53* mutation presence, reasons for discontinuing prior cBTKi, prior BCL2i treatment, and geographic region. Participants assigned to each arm will receive rocbrutinib (200 mg QD) or pirtobrutinib (200 mg QD) tablets continuously until disease progression, unacceptable toxicity, withdrawal, or other study discontinuation criteria are met. The primary endpoint is progression-free survival (PFS), assessed by an independent review committee (IRC) per iwCLL 2018 criteria with CLL (Hallek et al. 2018) and Lugano 2014 criteria with SLL (Cheson et al. 2014). Important secondary endpoints include overall survival (OS), time to next treatment (TTNT), event-free survival (EFS), overall response rate (ORR), duration of response (DOR), PFS assessed by investigator (INV), concordance between IRC and INV PFS, safety and tolerability, and population PK. Exploratory endpoints include health-related quality of life and biomarker assessment. Recruitment is ongoing.

**Copyright Information:** (Online) ISSN: 2572-9241
©2026 The Author(s). HemaSphere is published by John Wiley & Sons Ltd on behalf of European Hematology Association. This is an open access Abstract Book distributed under the Attribution-NonCommercial-NoDerivs (CC BY-NC-ND), which allows third parties to download the articles and share them with others as long as they credit the author and the Abstract Book, but they cannot change the content in any way or use them commercially.
**Abstract Book Citations:** Authors, Title, HemaSphere, 2026;10:(S1):pages. The abstract book DOIs can be found on the "Author Information" page.
**Disclaimer:** Articles published in the journal HemaSphere exclusively reflect the opinions of the authors. The authors are responsible for all content in their abstracts including accuracy of the facts, statements, citing resources, etc.

### (PB2966) SONROTOCLAX (BGB-11417) + ZANUBRUTINIB VS VENETOCLAX + ACALABRUTINIB IN TREATMENT-NAIVE CHRONIC LYMPHOCYTIC LEUKEMIA: A PHASE 3 RANDOMIZED TRIAL DESIGN (CELESTIAL-TNCLL-2)

**Topic:** 06. Chronic lymphocytic leukemia and related disorders - Clinical

Mazyar Shadman*[1, 2], Jennifer R. Brown[3], Tanya Siddiqi[4], Loic Ysebaert[5], Paolo Ghia[6, 7], Jianyong Li[8], Constantine S. Tam[9], Alicia Palomino Mosquera[10], Wai Y. Chan[10], Zaixing Shi[11], Arnon Philip Kater[12]

*[1]Fred Hutchinson Cancer Center, Seattle, WA, United States of America;[2]University of Washington, Seattle, WA, United States of America; [3]Dana-Farber Cancer Institute, Boston, MA, United States of America; [4]City of Hope National Medical Center, Duarte, CA, United States of America; [5]University Cancer Institute of Toulouse Oncopole, Toulouse, France;[6]Università Vita-Salute San Raffaele, Milano, Italy; [7]Comprehensive Cancer Center, IRCCS Ospedale San Raffaele, Milano, Italy; [8]The First Affiliated Hospital of Nanjing Medical University, Jiangsu Province Hospital, Nanjing, China; [9]Alfred Hospital and Monash University, Melbourne, VIC, Australia; [10]BeOne Medicines, Ltd, San Carlos, CA, United States of America; [11]BeOne Medicines, Ltd, Shanghai, China; [12]Lymphoma and Myeloma Research Amsterdam, Amsterdam, The Netherlands;*

### Background

Inhibition of B-cell lymphoma 2 (BCL2) and Bruton tyrosine kinase (BTK) has emerged as an effective fixed-duration treatment strategy that can induce high rates of undetectable minimal residual disease (uMRD) in patients with treatment-naive chronic lymphocytic leukemia (TN CLL). Venetoclax + acalabrutinib (AV) is approved in the EU as a first-line fixed-duration treatment for TN CLL. Sonrotoclax, a next-generation BCL2 inhibitor, is a more selective and pharmacologically potent inhibitor of BCL2 than venetoclax, with a shorter half-life and no drug accumulation. Zanubrutinib is a highly potent next-generation BTK inhibitor that is approved in the US and EU for CLL. In an ongoing phase 1/1b trial (NCT04277637), sonrotoclax + zanubrutinib (SZ) has had promising efficacy, with a 100% overall response rate (ORR; n=135) and high rates of blood uMRD at $10^{-4}$ sensitivity (uMRD4) in patients with TN CLL, including those with high-risk disease features. SZ is generally well tolerated, with neutropenia as the most common grade ≥3 treatment-emergent adverse event  and no laboratory or clinical tumor lysis syndrome events occurred.

### Aims

The phase 3 trial BGB-11417-304 (NCT07277231) was designed to directly compare fixed-duration SZ vs AV in TN CLL to investigate whether SZ compared with AV, may improve efficacy as assessed by uMRD rate after treatment completion and progression-free survival (PFS) and potentially improve tolerability and safety.

**Copyright Information:** (Online) ISSN: 2572-9241
©2026 The Author(s). HemaSphere is published by John Wiley & Sons Ltd on behalf of European Hematology Association. This is an open access Abstract Book distributed under the Attribution-NonCommercial-NoDerivs (CC BY-NC-ND), which allows third parties to download the articles and share them with others as long as they credit the author and the Abstract Book, but they cannot change the content in any way or use them commercially.
**Abstract Book Citations:** Authors, Title, HemaSphere, 2026;10:(S1):pages. The abstract book DOIs can be found on the "Author Information" page.
**Disclaimer:** Articles published in the journal HemaSphere exclusively reflect the opinions of the authors. The authors are responsible for all content in their abstracts including accuracy of the facts, statements, citing resources, etc.

**Methods**

BGB-11417-304 is a global phase 3, open-label, randomized study. Eligible adults have a confirmed diagnosis of previously untreated CLL requiring treatment per iwCLL 2018 criteria, adequate hematologic and organ function, an ECOG PS of 0-2, and measurable disease confirmed by CT or MRI. Exclusion criteria include prior systemic treatment for CLL; diagnosis of prolymphocytic leukemia or Richter transformation; known central nervous system involvement; history of confirmed progressive multifocal leukoencephalopathy; or uncontrolled hypertension or clinically significant cardiovascular disease. Approximately 500 patients will be enrolled and randomized 1:1 to arm A (3 lead-in cycles of oral zanubrutinib monotherapy followed by 12 cycles of oral SZ) or arm B (2 lead-in cycles of oral acalabrutinib monotherapy followed by 12 cycles of oral AV). Randomization will be stratified by age (<65 years vs ≥65 years), IGHV mutation status, and presence of del(17p) and/or *TP53* mutations. The primary endpoint is PFS in arm A vs arm B, as determined by independent review committee (IRC), with an intermediate endpoint of uMRD4 rate in blood and bone marrow in arm A vs arm B, assessed by next-generation sequencing (clonoSEQ) at the first post-treatment follow-up visit. Key secondary endpoints are PFS-IRC in high-risk subgroups and overall survival (OS). Other secondary endpoints are ORR and complete response assessed by IRC and investigator (INV), uMRD5 rate (clonoSEQ), PFS-INV, duration of response by IRC and INV, time to next treatment, patient-reported outcomes, and safety/tolerability. Enrollment in BGB-11417-304 is currently ongoing.

**Copyright Information:** (Online) ISSN: 2572-9241
©2026 The Author(s). HemaSphere is published by John Wiley & Sons Ltd on behalf of European Hematology Association. This is an open access Abstract Book distributed under the Attribution-NonCommercial-NoDerivs (CC BY-NC-ND), which allows third parties to download the articles and share them with others as long as they credit the author and the Abstract Book, but they cannot change the content in any way or use them commercially.
**Abstract Book Citations:** Authors, Title, HemaSphere, 2026;10:(S1):pages. The abstract book DOIs can be found on the "Author Information" page.
**Disclaimer:** Articles published in the journal HemaSphere exclusively reflect the opinions of the authors. The authors are responsible for all content in their abstracts including accuracy of the facts, statements, citing resources, etc.

### (PB2967) CLINICAL CHARACTERISTICS AND PROGNOSIS ANALYSIS OF PATIENTS WITH CHRONIC LYMPHOCYTIC LEUKEMIA

**Topic:** 06. Chronic lymphocytic leukemia and related disorders - Clinical

Xiangli Wang*[1], Tao Wu[1]

*[1]The 940th Hospital of Joint Logistics Support force of Chinese People's Liberation Army, hematology, Gansu, China;*

### Background

Chronic lymphocytic leukemia (CLL) is a malignant tumor originating from hematopoietic tissue, accounting for 30% of all leukemia cases in Europe and the United States, while its incidence in China is significantly lower than that in Western countries. The clinical course and survival of CLL are highly heterogeneous, primarily driven by its biological characteristics. In recent years, with the development of novel therapeutic agents and molecular biology detection methods, the prognosis of CLL has been greatly improved. This study conducted a retrospective analysis of 90 patients diagnosed and treated in the hematology department of our center over the past decade, aiming to analyze their clinical features and prognostic factors, so as to provide reference for optimizing the management of CLL patients.

### Aims

To analyze the clinical characteristics and prognostic factors of patients with chronic lymphocytic leukemia (CLL).

### Methods

A retrospective analysis was conducted on the medical records of 90 CLL patients admitted to the Hematology Department of the 940th Hospital of the Joint Logistics Support Force from January 2015 to December 2024. The Kaplan-Meier survival analysis method and the Cox multivariate regression model were used to analyze the factors influencing the time to first treatment (TTFT), overall survival (OS), and progression-free survival (PFS) of the patients.

### Results

A total of 90 CLL patients were included in this study. The ratio of male to female was 2.33:1. The median age at onset was 69 (39-93) years. The median TTFT was 29 (18-39) months. The 1-year, 3-year and 5-year TTFT rates were 74.4%, 44.4% and 34.8% respectively. The median OS was 105 (100-109) months. The 5-year and 9-year OS rates were 78.1% and 47.6% respectively. The median PFS was 87 (70-103) months. The 3-year and 5-year PFS rates were 88.0% and 68.1% respectively. Univariate prognostic analysis showed that absolute lymphocyte count (ALC), lactate dehydrogenase (LDH) level, Binet stage, TP53 mutation, and initial treatment indication were factors influencing TTFT□$P$<0.05□; age, β2-microglobulin (β2-MG), cystatin C (CYS), ECOG score and whether receiving treatment were factors influencing OS□$P$<0.05□; age, β2-MG, C-reactive protein (CRP), ECOG score were factors influencing PFS□$P$<0.05□. Cox multivariate analysis showed that LDH level, *TP53* mutation and initial treatment indication were independent risk factors for TTFT□$P$<0.05□;age, CYS were independent risk factors for OS□$P$<0.05□;age, CRP and ECOG score were independent risk factors for PFS□$P$<0.05□.

### Summary/Conclusion

This study investigated the factors influencing TTFT, OS and PFS in CLL patients, which to some extent reflects the characteristics of CLL in China. In clinical practice, it is necessary to comprehensively assess the risk of disease progression based on these indicators and optimize treatment decisions.

**Copyright Information:** (Online) ISSN: 2572-9241
©2026 The Author(s). HemaSphere is published by John Wiley & Sons Ltd on behalf of European Hematology Association. This is an open access Abstract Book distributed under the Attribution-NonCommercial-NoDerivs (CC BY-NC-ND), which allows third parties to download the articles and share them with others as long as they credit the author and the Abstract Book, but they cannot change the content in any way or use them commercially.
**Abstract Book Citations:** Authors, Title, HemaSphere, 2026;10:(S1):pages. The abstract book DOIs can be found on the "Author Information" page.
**Disclaimer:** Articles published in the journal HemaSphere exclusively reflect the opinions of the authors. The authors are responsible for all content in their abstracts including accuracy of the facts, statements, citing resources, etc.

### (PB2968) EFFICACY OF TARGETED THERAPY IN CHRONIC LYMPHOCYTIC LEUKEMIA ACROSS DIFFERENT LINES OF TREATMENT: A REAL-WORLD PRACTICE ANALYSIS

**Topic:** 06. Chronic lymphocytic leukemia and related disorders - Clinical

Dmitrii Voroshin[1], Evgenii Kunevich*[1], Sergey Voloshin[1, 2], George Pavlinov[1], Ruslan Bekoev[1], Olga Uspenskaya[1], Mariia Penkova[1], Maria Bashkova[1], Mariia Mikhaleva[3], Alexey Kuvshinov[3], Sergey Alekseev[1, 4]

*[1]Leningrad Regional Clinical Hospital, Saint-Petersburg, Russia;[2]S.M. Kirov Military Medical Academy, Saint-Petersburg, Russia; [3]Russian Research Institute of Hematology and Transfusiology, Saint-Petersburg, Russia;[4]N.N. Petrov National Medical Research Center of Oncology, Saint-Petersburg, Russia;*

### Background

Over the past five years, the treatment landscape for chronic lymphocytic leukemia (CLL) has undergone profound changes. While targeted agents were previously administered only to a subset of patients, Bruton tyrosine kinase inhibitors (BTKi) and BCL2 inhibitors (BCL2i) have now largely replaced conventional immunochemotherapy (ICT). They have become the standard of care in the first-line setting and have enabled fixed-duration treatment strategies.

### Aims

To evaluate the efficacy of targeted therapy across different lines of treatment in patients with CLL.

### Methods

A total of 73 patients with CLL who received targeted therapy were analyzed. The median age at diagnosis was 62.5 years (range 38–79), and the median follow-up was 55.0 months (range 3.8–266). Outcomes were compared between patients treated with targeted agents (BTKi or BCL2i) and those treated with ICT across the first three lines of therapy: first line (L1, n=23 [31.5%] vs n=50 [68.5%]), second line (L2, n=31 [62%] vs n=19 [38%]), and third line (L3, n=15 [75%] vs n=5 [25%]), respectively. Statistical analysis was performed using Jamovi version 2.4.8.0.

**Copyright Information:** (Online) ISSN: 2572-9241
©2026 The Author(s). HemaSphere is published by John Wiley & Sons Ltd on behalf of European Hematology Association. This is an open access Abstract Book distributed under the Attribution-NonCommercial-NoDerivs (CC BY-NC-ND), which allows third parties to download the articles and share them with others as long as they credit the author and the Abstract Book, but they cannot change the content in any way or use them commercially.
**Abstract Book Citations:** Authors, Title, HemaSphere, 2026;10:(S1):pages. The abstract book DOIs can be found on the "Author Information" page.
**Disclaimer:** Articles published in the journal HemaSphere exclusively reflect the opinions of the authors. The authors are responsible for all content in their abstracts including accuracy of the facts, statements, citing resources, etc.

### Results

In the L1 cohort, median progression-free survival (PFS) was significantly longer in the targeted therapy group compared with ICT (70.0 vs 28.3 months, p=0.002). Targeted therapy in L1 was associated with a higher overall response rate (ORR): 95.7% (22/23) versus 56.0% (28/50), p=0.0004. Treatment with targeted agents reduced the risk of progression or death by 81% compared with ICT (HR=0.19; 95% CI 0.06–0.62; p=0.006). Age did not affect treatment efficacy in the targeted therapy group (3-year PFS 100%), in contrast to the ICT group, where 3-year PFS was 22.2% in patients older than 65 years and 51.6% in those younger than 65 years (p=0.034). IGHV status had no impact on PFS in the targeted therapy group (3-year PFS 100%), whereas in the ICT group, 3-year PFS was 75.0% for mutated and 40.4% for unmutated IGHV.
In the L2 cohort, median PFS was 94.9 months with targeted therapy versus 9.0 months with ICT (p<0.0001). ORR was also higher with targeted therapy: 74.2% (23/31) versus 42.1% (8/19), p=0.0233. Three-year PFS was significantly superior in the targeted therapy group (71.1%, 95% CI 43.3–100.0) compared with ICT (12.5%, 95% CI 3.5–45.4), p<0.0001. Similar results were observed in L3, with 3-year PFS of 88.9% (95% CI 70.6–100) for targeted therapy and 0% for ICT (p=0.0047); all patients (n=5) in the ICT subgroup experienced disease progression within 30 months.
Regardless of treatment line or targeted agent used, survival curves were comparable. A trend toward better outcomes was observed in L1, reflected by 3-year PFS rates of 100% in L1, 71.1% in L2, and 88.9% in L3 (p=0.077).
Fixed-duration therapy with BCL2i demonstrated excellent outcomes during the first two years (PFS 100%). However, two deaths (15.4%) due to secondary malignancies were recorded in this subgroup, significantly impacting 3-year PFS (37.5%) compared with continuous therapy with BTKi (97.1%).

### Summary/Conclusion

Targeted therapy provides superior PFS and ORR compared with immunochemotherapy in CLL. Targeted agents demonstrate consistently high efficacy irrespective of treatment line or specific drug used. These real-world data support the use of targeted therapy as the preferred treatment strategy for CLL in routine clinical practice.

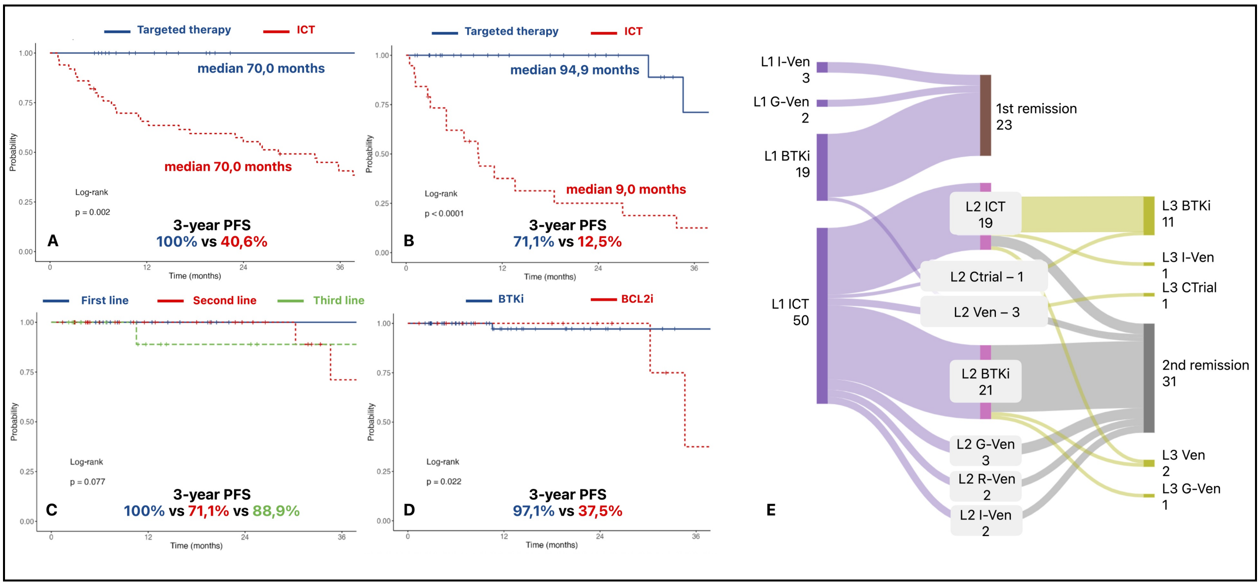

A) Progression-free survival in first-line therapy. B) Progression-free survival in second-line therapy. C) Progression-free survival with targeted therapy. D) Progression-free survival comparing Bruton tyrosine kinase inhibitors (BTKi) and BCL2 inhibitors (BCL2i). E) Treatment sequence for chronic lymphocytic leukemia.

**Copyright Information:** (Online) ISSN: 2572-9241
©2026 The Author(s). HemaSphere is published by John Wiley & Sons Ltd on behalf of European Hematology Association. This is an open access Abstract Book distributed under the Attribution-NonCommercial-NoDerivs (CC BY-NC-ND), which allows third parties to download the articles and share them with others as long as they credit the author and the Abstract Book, but they cannot change the content in any way or use them commercially.
**Abstract Book Citations:** Authors, Title, HemaSphere, 2026;10:(S1):pages. The abstract book DOIs can be found on the "Author Information" page.
**Disclaimer:** Articles published in the journal HemaSphere exclusively reflect the opinions of the authors. The authors are responsible for all content in their abstracts including accuracy of the facts, statements, citing resources, etc.

### (PB2969) LEPTOMENINGEAL INVOLVEMENT IN NEWLY DIAGNOSED CHRONIC LYMPHOCYTIC LEUKEMIA

**Topic:** 06. Chronic lymphocytic leukemia and related disorders - Clinical

Gabriela Diana Cantor*[1], Bumbea Horia[1], Ana Maria Ilinescu[1], Daiana Andreea Tunaru[1], Ion Dumitru[1], Georgiana Elena Ene[1]

[1]*Emergency University Hospital Bucharest, Stem cell transplant unit, Bucharest, Romania;*

### Background

Central nervous system (CNS) involvement in chronic lymphocytic leukemia (CLL) is rare, particularly in treatment-naïve patients with favorable biological features. Leptomeningeal localization is often underdiagnosed due to nonspecific neurological symptoms and limited sensitivity of imaging. Concomitant inherited thrombophilia further complicates clinical presentation and therapeutic decision-making. Evidence guiding management in this setting remains scarce.

### Aims

To describe a rare case of leptomeningeal CLL involvement in a treatment-naïve patient with previously undiagnosed high-risk inherited thrombophilia and to correlate clinical management with available literature.

### Methods

We report the case of a 54-year-old male with treatment-naïve CLL (mutated IGHV, absence of high-risk cytogenetic or molecular abnormalities) who presented with acute pulmonary thromboembolism. Thrombophilia screening revealed combined Factor V Leiden and prothrombin (Factor II) mutations. Neurological symptoms during hospitalization prompted cerebrospinal fluid (CSF) analysis, including flow cytometry, confirming leptomeningeal CLL involvement. A focused review of published cases of CNS CLL was performed to contextualize diagnostic and therapeutic strategies.

### Results

Despite favorable leukemia biology, the patient developed severe thrombotic and neurological manifestations. Family history revealed premature fatal cerebrovascular events in first-degree relatives. Treatment consisted of fixed-duration obinutuzumab–venetoclax combined with intrathecal chemotherapy, alongside therapeutic anticoagulation. CSF flow cytometry demonstrated complete clearance of leukemic cells at the second lumbar puncture. Systemic therapy was well tolerated, and neurological symptoms improved without hemorrhagic complications.

### Summary/Conclusion

This case demonstrates that leptomeningeal involvement can occur in biologically favorable, treatment-naïve CLL and may coexist with unrecognized high-risk inherited thrombophilia. Early CSF evaluation is essential in patients with unexplained neurological symptoms. Fixed-duration venetoclax–obinutuzumab combined with intrathecal therapy represents a feasible therapeutic approach, though evidence remains limited. Reporting such cases is crucial to inform future diagnostic algorithms and management strategies.

**Copyright Information:** (Online) ISSN: 2572-9241
©2026 The Author(s). HemaSphere is published by John Wiley & Sons Ltd on behalf of European Hematology Association. This is an open access Abstract Book distributed under the Attribution-NonCommercial-NoDerivs (CC BY-NC-ND), which allows third parties to download the articles and share them with others as long as they credit the author and the Abstract Book, but they cannot change the content in any way or use them commercially.
**Abstract Book Citations:** Authors, Title, HemaSphere, 2026;10:(S1):pages. The abstract book DOIs can be found on the "Author Information" page.
**Disclaimer:** Articles published in the journal HemaSphere exclusively reflect the opinions of the authors. The authors are responsible for all content in their abstracts including accuracy of the facts, statements, citing resources, etc.

### (PB2970) IMMUNOPHENOTYPIC AND MOLECULAR BIOLOGICAL MARKERS BASED RISK STRATIFICATION OF PATIENTS WITH CHRONIC LYMPHOCYTIC LEUKEMIA,

**Topic:** 06. Chronic lymphocytic leukemia and related disorders - Clinical

Morozova Oksana*[1], Uss Anatoly[1], Natalya Milanovich[1], Tatiana Lebedeva[1], Viktirya Smolnikova[1], Viktoryia Hrynevich[1]

[1]*Hematology and bone marrow, Minsk, Belarus;*

### Background

The CLL International Prognostic Index (CLL-IPI) was destricted in 2016, based on data from 3,472 patients in eight clinical trials between 1997 and 2007 (Table 1). Some testing mathods, such as sequencing, can only be performed in large clinical centers. The search for new diagnostic and prognostic factors that could be used in routine practice continues.

### Aims

To identify the relationship between immunophenotypic and molecular genetic markers for risk stratification of CLL

### Methods

Markers necessary for prognosis assessment and monitoring of the CLL tumor population include CD38, CD49d, CD11c, CD25 and ZAP-70 were determined using multicolor flow cytometry. FISH was used to detect del13q, del11q, del17p and trisomy 12. The number of patients examined was 115.

### Results

We identified cytogenetic aberration in 82% of pqtients with CLL: isolated deletion 13q14 was detected in 21,7% of case,conbination of 13q14 deletion with deletion ATM gene (11q22-q23) and p53 gene in 17,4% of case, p53 gene mutation - 4,3%, trisomy 12 - 8,7%, deletion 11q22-q23 - 11,3%. Thus, a favorable prognosis was observed in 67 (58,2%) patients: 42 (36,5%) patients had a normal karyotype, 25 (21,7%) patients had del13q; an intermediate prognosis was odserved in 23 (20%) patients: del11q was detected in 13 (11,3%) patients, trisomy 12 in 10(8,7%) patients; an unfavorable prognosis was observed in 25 (21,7%) patients: del17p - 5 (4,3%) patients, complex karyotype was detected in 20 (17,4%) patients.
Analysus of the immunophenotype of tumor B lymphocytes with varios cytogenetic aberrations revealed the follow:- a significant increase ($p=0.03$) in the expression level of ZAP-70 in the group of patients with del17p .- a relationship between a significant increase in CD38 levels and del11q22-q23 and trisomy 12 chromosomes ($p=0.0003$; $p<0.02$ respectively)- an increase in CD25 expression is associated with del17p ($H=3.6$; $p<0.02$) - in the group of patients with CD23 expression levels >70%, the mediane time of treatment initiation was 35 months, which is significantly ($p=0.017$) higher than in group with reduced CD23 expression (less then 70%)? with a median time of 4 months

### Summary/Conclusion

Variability in the expression of surface antigens CD38, CD25, Cd23 and ZAP is associated with intermediate and High-risk cytogenetic aberration and is a prognostic factors for the disease.

**Copyright Information:** (Online) ISSN: 2572-9241
©2026 The Author(s). HemaSphere is published by John Wiley & Sons Ltd on behalf of European Hematology Association. This is an open access Abstract Book distributed under the Attribution-NonCommercial-NoDerivs (CC BY-NC-ND), which allows third parties to download the articles and share them with others as long as they credit the author and the Abstract Book, but they cannot change the content in any way or use them commercially.
**Abstract Book Citations:** Authors, Title, HemaSphere, 2026;10:(S1):pages. The abstract book DOIs can be found on the "Author Information" page.
**Disclaimer:** Articles published in the journal HemaSphere exclusively reflect the opinions of the authors. The authors are responsible for all content in their abstracts including accuracy of the facts, statements, citing resources, etc.

| Prognostic factor | Points |
|---|---|
| Del17p on FISH or *TP53* mutation | 4 |
| Unmutated *IGHV* genes | 2 |
| Serum β2 microglobulin >3.5 mg/L | 2 |
| Rai stage I–IV | 1 |
| Age >65 years | 1 |

| Cumulative CLL-IPI score | Risk category | 5-year TFS[a] |
|---|---|---|
| 0–1 | Low risk | 78% |
| 2–3 | Intermediate risk | 54% |
| 4–6 | High risk | 32% |
| 7–10 | Very high risk | 0% |

*FISH* fluorescence in situ hybridization, *IGHV* immunoglobulin heavy chain gene, *TFS* treatment-free survival
[a]For the Mayo validation cohort

**Copyright Information:** (Online) ISSN: 2572-9241
©2026 The Author(s). HemaSphere is published by John Wiley & Sons Ltd on behalf of European Hematology Association. This is an open access Abstract Book distributed under the Attribution-NonCommercial-NoDerivs (CC BY-NC-ND), which allows third parties to download the articles and share them with others as long as they credit the author and the Abstract Book, but they cannot change the content in any way or use them commercially.
**Abstract Book Citations:** Authors, Title, HemaSphere, 2026;10:(S1):pages. The abstract book DOIs can be found on the "Author Information" page.
**Disclaimer:** Articles published in the journal HemaSphere exclusively reflect the opinions of the authors. The authors are responsible for all content in their abstracts including accuracy of the facts, statements, citing resources, etc.

### (PB2971) IGHV STATUS DEFINES A MARROW-DOMINANT PROGRESSION PHENOTYPE IN CHRONIC LYMPHOCYTIC LEUKEMIA

**Topic:** 06. Chronic lymphocytic leukemia and related disorders - Clinical

Maria Bacanu*[1, 2, 3], Meilin Omer[1], Diana Grasu[1, 2], Andreea Vasile[1, 2], Elena Pirvu[1, 2], Alexandra-Diana Malenda[1, 2], Viola Maria Popov[1], Mihaela Andreescu[1, 2]

*[1]Colentina Clinical Hospital, Hematology, Bucharest, Romania;[2]Titu Maiorescu University, Faculty of Medicine, Bucharest, Romania; [3]Carol Davila University, Faculty of Medicine and Pharmacy, Bucharest, Romania;*

### Background

Chronic lymphocytic leukemia (CLL) is characterized by marked biological heterogeneity and variable progression kinetics. IGHV mutational status is a well-established prognostic determinant, with unmutated IGHV associated with inferior outcomes and shorter time to first treatment (TTFT). Experimental data suggest that IGHV-unmutated CLL shows stronger interaction with the bone marrow microenvironment, which may contribute to distinct clinical progression patterns. While its impact on disease kinetics is well documented, whether IGHV-defined biological subtypes translate into specific IWCLL-defined clinical mechanisms remains insufficiently explored.

### Aims

To determine whether IGHV mutational status defines distinct progression phenotypes reflected by specific IWCLL criteria and early treatment requirement in a real-world setting.

### Methods

We conducted a retrospective real-world analysis of 92 consecutive CLL patients with available IGHV status managed at a tertiary referral center in Eastern Europe. Treatment timing was categorized as early (<90 days from diagnosis) or non-early. IWCLL 2018 criteria were grouped as bone marrow failure, tumor burden, B symptoms, or rapid lymphocytosis. Associations were evaluated using chi-square testing, and multivariable logistic regression was performed to identify independent predictors of early treatment.

### Results

Among the 92 patients, 59.8% had unmutated IGHV and 40.2% had mutated IGHV; 72 patients (78.3%) required therapy. Median age was 65 years (range 40–87). Consistent with established literature, unmutated IGHV was strongly associated with early treatment (OR≈5.7, p<0.001).
Within unmutated IGHV cases progressing early, bone marrow failure was the predominant driver (48.4%) and was significantly linked to early therapy compared to other treated patients (OR≈2.4, p=0.041).
In multivariable analysis including IGHV status and IWCLL category, unmutated IGHV remained independently associated with early treatment (adjusted OR≈4.9, p=0.002), whereas marrow failure lost statistical significance.
Across the treated cohort, bone marrow failure was the most frequent indication overall (38.9%). Stratification by IGHV status revealed divergent clinical patterns: unmutated cases more commonly exhibited marrow failure (44.9%), whereas mutated cases more often presented tumor burden (39.1%) or B symptoms (34.8%) (p=0.058).

**Copyright Information:** (Online) ISSN: 2572-9241
©2026 The Author(s). HemaSphere is published by John Wiley & Sons Ltd on behalf of European Hematology Association. This is an open access Abstract Book distributed under the Attribution-NonCommercial-NoDerivs (CC BY-NC-ND), which allows third parties to download the articles and share them with others as long as they credit the author and the Abstract Book, but they cannot change the content in any way or use them commercially.
**Abstract Book Citations:** Authors, Title, HemaSphere, 2026;10:(S1):pages. The abstract book DOIs can be found on the "Author Information" page.
**Disclaimer:** Articles published in the journal HemaSphere exclusively reflect the opinions of the authors. The authors are responsible for all content in their abstracts including accuracy of the facts, statements, citing resources, etc.

**Summary/Conclusion**

In this real-world cohort, unmutated IGHV status was independently associated with early treatment requirement and defines a marrow-dominant progression phenotype. These findings suggest that IGHV-defined biological subtypes influence not only disease kinetics but also the clinical pathway leading to therapy in CLL and may reflect stronger interaction with the bone marrow microenvironment.

**Copyright Information:** (Online) ISSN: 2572-9241
©2026 The Author(s). HemaSphere is published by John Wiley & Sons Ltd on behalf of European Hematology Association. This is an open access Abstract Book distributed under the Attribution-NonCommercial-NoDerivs (CC BY-NC-ND), which allows third parties to download the articles and share them with others as long as they credit the author and the Abstract Book, but they cannot change the content in any way or use them commercially.
**Abstract Book Citations:** Authors, Title, HemaSphere, 2026;10:(S1):pages. The abstract book DOIs can be found on the "Author Information" page.
**Disclaimer:** Articles published in the journal HemaSphere exclusively reflect the opinions of the authors. The authors are responsible for all content in their abstracts including accuracy of the facts, statements, citing resources, etc.

### (PB2972) FACTORS PREDICTING PULMONARY INFECTIONS IN CHRONIC LYMPHOCYTIC LEUKEMIA (CLL) TREATED WITH TARGETED THERAPIES WITH A HISTORY OF LUNG DISEASES: FACTORS ARE IGHV, TP53 (LOW AND HIGH BURDEN)

**Topic:** 06. Chronic lymphocytic leukemia and related disorders - Clinical

Maher Salamoon*[1]

*[1]al bairouni university cancer center, hematology/oncology, Damascus, Syrian Arab Republic;*

### Background

CLL patients complain from defected T-cell mediated immunity as well as a depleted B-cell due to B-cell targeted agents such as Rituximab, Obinutuzumab and Ibrutinib. This de novo immunodeficiency predisposes those patients to several bacterial and opportunistic pathogens in the light of accompanying decrease in immunoglobulins

### Aims

It is a prospective observational study including newly diagnosed patients with CLL treated with anti-CD20 (first and second generation) as well as Ibrutinib. The study documents the frequency of pulmonary infection with a 2-year follow-up correlating infection with the basal genetic status at presentation.

### Methods

We have included 40 newly diagnosed patients with CLL (ages between 62-85 years), 10 of which showed IGHV mutant vs 30 non-mutants. TP53 mutation documented in 15 patients (9 with FAV more than 10% and 6 with less than 10 %) while COBBLL1 was mutant in 12 patients. 10 patients received Rituximab upfront, 10 received Obinutuzumab while the remaining 20 received Ibrutinib. 24 patients had a history of COPD, 16 with COVID-19. IGs level ranged between 580-1120 mg/dl at presentation. The last follow-up of all patients was in December.15.2025.

### Results

Pulmonary infection documented in 16 patients started between 3-9 months after starting treatment with main pathogens: streptococcus pneumoniae (8 patients), streptococcus jirovecii (2 patients) and 6 patients with staphylococcus aureus. CMV reactivation was documented in 3 patients receiving Ibrutinib and 2 patients receiving Obinutuzumab. There was a between high-burden TP53 and pulmonary infection (p value .04). COBBLL1 has no effect on developing infection (p value .08). non mutated IGHV correlated with increased risk of infection (p value .05). low levels of IGs at presentation were not correlated with increased risk of infection (p value .06). in multivariate analysis, factors correlated with higher risk of infection were high burden TP53 p value .04, COPD p value .05 and patients with poor adherence to treatment p value .05. in univariate analysis the main factor associated with infection was TP53 with FAV more than 10% with a p value of .02.

### Summary/Conclusion

The genetic identity of newly diagnosed CLL patients might play a role in predicting infection in high-risk group with a history of lung diseases. This sample should contain a bigger cohort with several risk groups to obtain more information about the future of our patients

**Copyright Information:** (Online) ISSN: 2572-9241
©2026 The Author(s). HemaSphere is published by John Wiley & Sons Ltd on behalf of European Hematology Association. This is an open access Abstract Book distributed under the Attribution-NonCommercial-NoDerivs (CC BY-NC-ND), which allows third parties to download the articles and share them with others as long as they credit the author and the Abstract Book, but they cannot change the content in any way or use them commercially.
**Abstract Book Citations:** Authors, Title, HemaSphere, 2026;10:(S1):pages. The abstract book DOIs can be found on the "Author Information" page.
**Disclaimer:** Articles published in the journal HemaSphere exclusively reflect the opinions of the authors. The authors are responsible for all content in their abstracts including accuracy of the facts, statements, citing resources, etc.

### (PB2973) OUTCOME OF FIRST-LINE VENETOCLAX THERAPY IN PATIENTS WITH CHRONIC LYMPHOCYTIC LEUKEMIA. SINGLE-CENTER EXPERIENCE.

**Topic:** 06. Chronic lymphocytic leukemia and related disorders - Clinical

Morozova Oksana[1], Natalya Milanovich[1], Uss Anatoly[1], Tatsiana Talako[1], Kuzmich Alena[1], Natalia Antipenko[1], Oksana Nechay[1], Strongin Yuri[1], Dziuba Alena[1]

[1]*Hematology and bone marrow, Minsk, Belarus;*

### Background

The use of targeted therapy for the treatment of patients with CLL makes it possible to achieve deep and long-term remission [Eyre TA, Hori S, Munir T. Treatment strategies for a rapidly evolving landscape in chronic lymphocytic leukemia management. *Hematol Oncol.*2022;40(2):129-159]. Venetoclax combined with anti-CD20 monoclonal antibody can induce high disease response rate, lead to durable remission in patients with CLL and may be an effective treatment for patients with high-risk cytogenetic factors [ Eichhorst B., Niemann C.U., Kater, A. P., et al. First-line venetoclax combinations in chronic lymphocytic leukemia. *New England Journal of Medicine*, 2023;*388*(19):1739-1754.].

### Aims

To estimate the efficacy of Venetoclax+Obinutuzumab treatmenr in previously untreated CLL patients at a single-center in Belarus/

### Methods

A retrospective analysis of data from 12 previously untreated patients with CLL treated with venetoclax+obinutuzzumab regimens at our center from 2022 to 2025 was performed. The midian follow-up after completion of therapy was 24 months (renge, 4048 months). Treatment response was assessed 3 months after the end of therapy, with chest and abdominal CT scans and bone marrow minimal residual desease (MRD) determinations. MRD in peripheral blood was subsequently determined every 3 months. The median follow-up was 24 month (range, 7-48 months).

### Results

8 males and 4 females were included in the analysis with a median age of 55 (34-71) years. Deletion 17p was detected in 1 patient, deletion 11q - 3 patients, normal karyotype - 3 patients, deletion 13 - 2 patients, a combination of deletions 13, 11 and trisomy 12 - 1 patient, deletion 13 and 17 - 1 patient, trisomy 12 and deletion 13 - 1 patient, and 5 patients had IGHV-unmutated status (umIGHV). An unfavorable prognosis for CLL was established in 10 of 12 patients. A complite remission (CR) was achieved in all patients, and an MRD response was achieved in 8 (66.7%) of 12 patients; the MRD rate in 5 patients ranged from 0.03 to 0.05%. Disease progression was observed in one of these patients over the course of one year; Car-T therapy was performed.

Among patients with an MRD response of bone marrow, MRD-negative status in peripheral blood was maintained throughout the entire observation period.

### Summary/Conclusion

Venetoclax-based regimens are effective treatment strategies in CLL, including patients with del (17p) and umIGHV if therapy with other targeted drugs is not possible, .

Early relapse after venetoclax therapy requires either the administration of other targeted drugs or the use of cell therapy (CAR-T therapy) in such patients.

**Copyright Information:** (Online) ISSN: 2572-9241
©2026 The Author(s). HemaSphere is published by John Wiley & Sons Ltd on behalf of European Hematology Association. This is an open access Abstract Book distributed under the Attribution-NonCommercial-NoDerivs (CC BY-NC-ND), which allows third parties to download the articles and share them with others as long as they credit the author and the Abstract Book, but they cannot change the content in any way or use them commercially.
**Abstract Book Citations:** Authors, Title, HemaSphere, 2026;10:(S1):pages. The abstract book DOIs can be found on the "Author Information" page.
**Disclaimer:** Articles published in the journal HemaSphere exclusively reflect the opinions of the authors. The authors are responsible for all content in their abstracts including accuracy of the facts, statements, citing resources, etc.

### (PB2975) TREATMENT-FREE REMISSION (TFR) FOR NEWLY DIAGNOSED ACCELERATED PHASE CML (AP-CML)

**Topic:** 07. Chronic myeloid leukemia - Biology & translational research

Franck Emmanuel Nicolini*[1], Gabriel Etienne[2], Vincent Alcazer[3], Julien Bollard[1], Julie Hildt[1], Christophe Bouvier[1], François-Xavier Mahon[2], Sara Dellasala[4], Stéphane Morisset[1], Ghayas C. Issa[4], Elias Jabbour[4], Hagop Kantarjian[4], Fadi Haddad[4]

*[1]Centre Léon Bérard, Lyon, France; [2]Institut Bergonié, Bordeaux, France; [3]CHU Lyon, Lyon, France; [4]MD Anderson Cancer Center, Houston, United States of America;*

### Background

AP-CML according to the ELN 2013 classification (Baccarani M. et al., Blood 2013) is a difficult therapeutic challenge, even nowadays with the availability of second and third generation tyrosine kinase inhibitors (TKIs). However, some patients with AP-CML might respond well and obtain sustained deep molecular response (DMR). To date, TFR strategies are currently not recommended outside the first CP-CML setting (Apperley J. et al. Leukemia 2025).

### Aims

To assess the feasibility of TFR in a series of patients in AP-CML according to ELN 2013 at onset, obtaining subsequent sustained DMR on TKI.

### Methods

Retrospective observational international study of patients with AP-CML at onset, who fulfilled TFR criteria (i.e. ≥MR4.5 2 years) and discontinued TKI therapy. Loss of MMR was the criteria triggering TKI resumption. All patients have expressed their non-opposition to their involvement in this study in the 3 centers (MDACC, Centre Léon Bérard and Institut Bergonié).

### Results

Thirty-five patients were included, with a median age of 43 years (23-76), with 18 males (51.43%), median WBC at diagnosis were 41.8 G/l (3.6-375), median hemoglobin 12 g/dl (8.6-15.8), median platelets 398 G/l (239-514.5), median PB basophils 6.5% (1-38), median PB promyelocytes 1 % (0-1.5), median PB blasts 1.5 % (0-4.25).median BM promyelocytes 7% (2-8), median BM blasts 3% (0-19, 3 missing data). Seventeen (48.5%) patients had clonal evolution only. Two patients harbored an atypical *BCR::ABL1* transcript (e19a2).

The first TKI was commenced shortly after diagnosis [0.46 (0.03-3.15) months], with imatinib first-line in 19 (54.3%) patients, dasatinib in 8 (22.86%), nilotinib in 7 (20%), and ponatinib in 1 (2.9%) patient. Overall, 23 patients (65.71%) received only one TKI since diagnosis. At the time of TKI discontinuation, 10 (28.57%) patients were on imatinib, 11 (31.43%) on dasatinib, 12 (34.29%) on nilotinib, and 2 (5.71%) on ponatinib. Median sustained MR4 duration was 105 (63-118.5) months, and median sustained MR4.5 duration 78 (0-291) months, prior to cessation.

The median follow-up after cessation was 59.2 months (1.48-211). The molecular relapse-free survival was 91.43% (82.61-100) at 3 months, 82.86% (71.27-96.33) at 6 months, 80% (67.79-96.41) at 12 months and 24 months, 77% (64.26-92.36) at 36 and 48 months and 69.5% (55.18-87.56) at 51 months and thereafter until last follow-up. Nine patients lost MMR after a median of 4.34 months (1.48-48.99) following TKI discontinuation (Figure 1).

Two patients died in DMR and MMR from a truck accident and from a liver cirrhosis and one died in hematological relapse (CP) from end-stage kidney disease, (i. e. competitive events).

**Copyright Information:** (Online) ISSN: 2572-9241
©2026 The Author(s). HemaSphere is published by John Wiley & Sons Ltd on behalf of European Hematology Association. This is an open access Abstract Book distributed under the Attribution-NonCommercial-NoDerivs (CC BY-NC-ND), which allows third parties to download the articles and share them with others as long as they credit the author and the Abstract Book, but they cannot change the content in any way or use them commercially.
**Abstract Book Citations:** Authors, Title, HemaSphere, 2026;10:(S1):pages. The abstract book DOIs can be found on the "Author Information" page.
**Disclaimer:** Articles published in the journal HemaSphere exclusively reflect the opinions of the authors. The authors are responsible for all content in their abstracts including accuracy of the facts, statements, citing resources, etc.

### Summary/Conclusion

These preliminary results are the first to demonstrate the feasibility of TFR strategies including in patients in AP-CML at diagnosis.

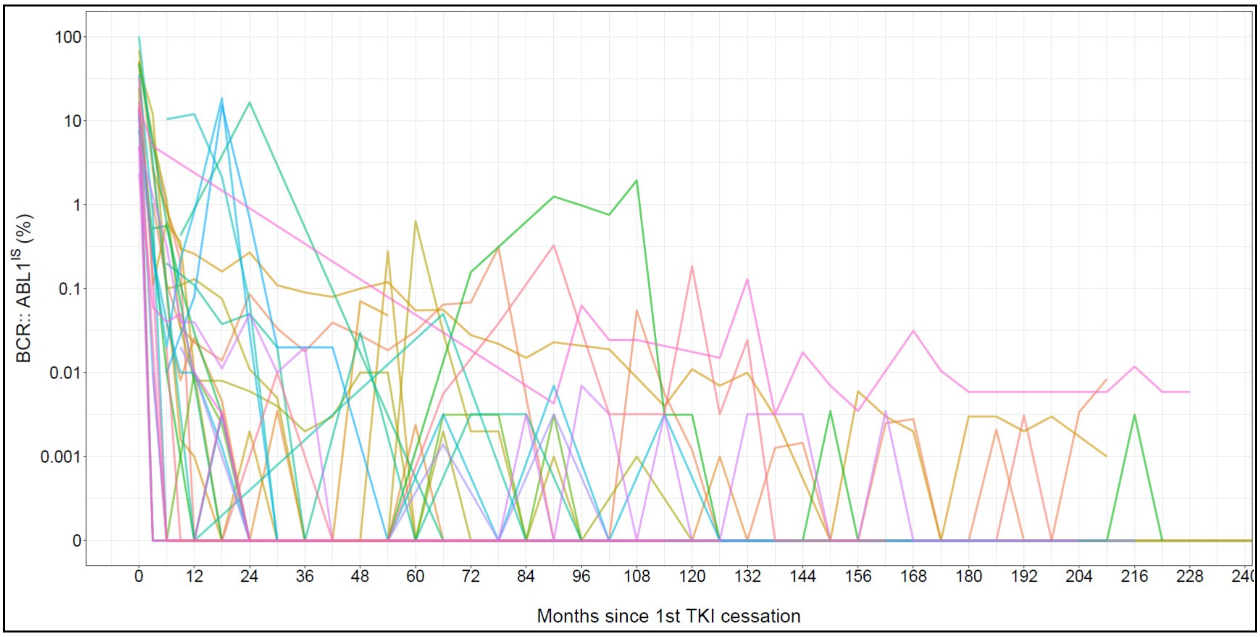

Figure 1. Individual evolution of BCR::ABL1 transcripts (%IS) after TKI cessation.

**Copyright Information:** (Online) ISSN: 2572-9241
©2026 The Author(s). HemaSphere is published by John Wiley & Sons Ltd on behalf of European Hematology Association. This is an open access Abstract Book distributed under the Attribution-NonCommercial-NoDerivs (CC BY-NC-ND), which allows third parties to download the articles and share them with others as long as they credit the author and the Abstract Book, but they cannot change the content in any way or use them commercially.
**Abstract Book Citations:** Authors, Title, HemaSphere, 2026;10:(S1):pages. The abstract book DOIs can be found on the “Author Information” page.
**Disclaimer:** Articles published in the journal HemaSphere exclusively reflect the opinions of the authors. The authors are responsible for all content in their abstracts including accuracy of the facts, statements, citing resources, etc.

### (PB2976) SINGLE-CELL TRANSCRIPTOMIC AND CYTOGENETIC INSIGHTS IN A RARE VARIANT OF CHRONIC MYELOID LEUKEMIA SUGGEST A METABOLICALLY "PROTECTED" PHENOTYPE

**Topic:** 07. Chronic myeloid leukemia - Biology & translational research

Pilar Giraldo[1], Irene Serrano-Gonzalo*[2, 2], Eduardo Aranda-Canada[3], Rebeca Sanz-Pamplona[4], Isidro Arévalo-Vargas[2], Sonia Roca Esteve[2], Luis Felipe Casado Montero[1], Pilar Mozas[2], Juan Luis Steegmann[1]

*[1]Fundacion ZeroLMC, Zaragoza, Spain; [2]FEETEG, Zaragoza, Spain; [3]Aragon Health Research Institute, Zaragoza, Spain; [4]Aragonese Foundation for Research and Development (ARAID), Zaragoza, Spain;*

### Background

Most CML patients achieve deep molecular responses with TKIs and allosteric BCR-ABL inhibitors therapy. We report a unique case of a 64-year-old female diagnosed at age 28 with variant CML [46,XX,t(1;9;22)(q32;q34;q11)] expressing BCR-ABL1 p210 b2a2. Despite more tan 30 years of therapy—including α-IFN, all TKIs, and allosteric inhibitors—molecular remission was never achieved (BCR/ABL: 46,047), no variants in ABL1 were developed and disease progression has not occurred.

### Aims

To explore transcriptomic and immune mechanisms underlying this prolonged indolent course by comparing her PBMC single-cell RNA profiles with those three chronic-phase CML patients at different TKI response stages (two in deep molecular response and one in molecular relapse).

### Methods

Peripheral blood mononuclear cells were analyzed using 10x Genomics Chromium 3' scRNA-seq plattform. Data were processed with Cell Ranger and Seurat R package. After initial quality control filtering, data were normalized using SCTransform. Cells were then clustered based on their gene expression profiles. Automated cell annotation was performed using SingleR, with references hematopoietic datasets, identifying major immune populations: dendritic cells, monocytes, granulocytes, CD4+, and CD8+ T cells, B lymphocytes and NK cells. Finally, differential gene expression profiling was conducted across these populations.

### Results

When comparing the patient who never achieved molecular remission with the other three patients, it was observed that the monocytes and dendritic cells exhibited consistent downregulation of antigen presentation and activation genes, including ***CD74, B2M, HLA***, and inhibitory or signaling regulators such as ***TYROBP, FGL2, VSIR, MARCKS***. Genes associated with metabolism and cytoskeleton function (***EEF2, CFL1, ACTG1, FLNA, CD44***) were also reduced, indicating a basal hypometabolic and moderately activated state. CD4+ and CD8+ T lymphocytes displayed decreased expression of activation and proliferation markers (***CD74, B2M, KANSL1, CFL1, CD44, S100A4***), reflecting functional equilibrium without overt exhaustion. NK cells demonstrated marked downregulation of cytotoxic genes (***PRF1, CD247, KLRF1, IL2RB, S1PR5***) and adhesion/signaling genes (***CFL1, FLNA, ACTB, ITGB2***), while stress-related genes (***TMSB4X, SAT1***) were slightly upregulated, suggesting homeostatic adaptation. Collectively, these data reveal an immune landscape characterized by moderate cytotoxicity and controlled activation, potentially restricting leukemic proliferation without systemic toxicity in target patient. This signature contrasts with profiles of patients achieving deep molecular response or experiencing relapse, suggesting lower basal metabolic and activation states.

**Copyright Information:** (Online) ISSN: 2572-9241
©2026 The Author(s). HemaSphere is published by John Wiley & Sons Ltd on behalf of European Hematology Association. This is an open access Abstract Book distributed under the Attribution-NonCommercial-NoDerivs (CC BY-NC-ND), which allows third parties to download the articles and share them with others as long as they credit the author and the Abstract Book, but they cannot change the content in any way or use them commercially.
**Abstract Book Citations:** Authors, Title, HemaSphere, 2026;10:(S1):pages. The abstract book DOIs can be found on the "Author Information" page.
**Disclaimer:** Articles published in the journal HemaSphere exclusively reflect the opinions of the authors. The authors are responsible for all content in their abstracts including accuracy of the facts, statements, citing resources, etc.

### Summary/Conclusion

Single-cell transcriptomics identifies a distinct immune and metabolic landscape in this rare CML case, potentially explaining decades-long disease stability without molecular remission. These findings provide a reference for other rare, slowly progressive CML variants and highlight the relevance of natural immune control in disease modulation.

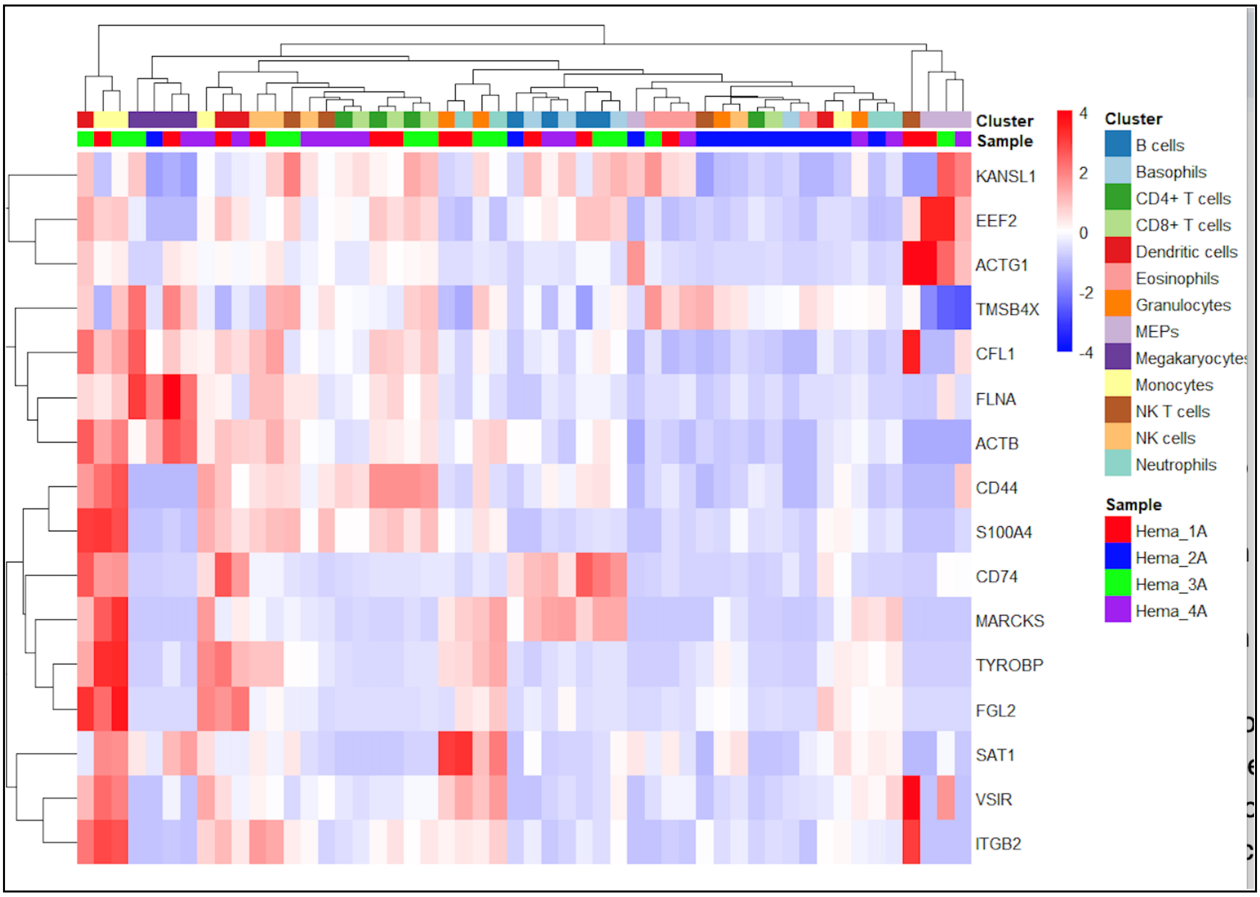

Heatmap of gene expression in different cell types and four samples

**Copyright Information:** (Online) ISSN: 2572-9241
©2026 The Author(s). HemaSphere is published by John Wiley & Sons Ltd on behalf of European Hematology Association. This is an open access Abstract Book distributed under the Attribution-NonCommercial-NoDerivs (CC BY-NC-ND), which allows third parties to download the articles and share them with others as long as they credit the author and the Abstract Book, but they cannot change the content in any way or use them commercially.
**Abstract Book Citations:** Authors, Title, HemaSphere, 2026;10:(S1):pages. The abstract book DOIs can be found on the "Author Information" page.
**Disclaimer:** Articles published in the journal HemaSphere exclusively reflect the opinions of the authors. The authors are responsible for all content in their abstracts including accuracy of the facts, statements, citing resources, etc.

### (PB2977) MOLECULAR REMISSION AFTER TYROSINE KINASE INHIBITOR CESSATION IN CHRONIC MYELOID LEUKEMIA (CML): A SYSTEMATIC REVIEW AND META-ANALYSIS

**Topic:** 07. Chronic myeloid leukemia - Biology & translational research

Songphol Tungjitviboonkun*[1], Brian Schult[1], Paige Bracci[1], Christopher Seaman[1]

[1] *University of California San Francisco, San Francisco, United States of America;*

### Background

Tyrosine kinase inhibitors (TKIs) have transformed the management of chronic myeloid leukemia (CML), enabling many patients to achieve deep molecular responses. Discontinuation of TKIs with the goal of treatment-free remission (TFR) is now an important therapeutic consideration, but relapse rates remain variable across studies.

### Aims

This review aims to summarize the rates and durability of molecular remission after TKI cessation in chronic-phase CML, and describe the timing and patterns of molecular relapse.

### Methods

We systematically searched PubMed, Embase, and Scopus from inception to August 31, 2025, for prospective and observational studies reporting TFR outcomes in adults with chronic-phase CML following TKI cessation. Proportions of patients in TFR at 6, 12, 24, and 36 months were pooled using random-effects meta-analysis. Heterogeneity was assessed with the $I^2$ statistic. Risk of bias was evaluated with the Newcastle–Ottawa Scale (NOS), and certainty of evidence was graded using GRADE. Meta-regression was performed to examine the association between TKI duration and TFR outcomes. The review protocol was registered on PROSPERO (CRD420251143733).

### Results

Sixteen studies comprising 2,156 patients were included. The pooled TFR rates were 60% (95% CI, 54–65%) at 6 months, 55% (95% CI, 49–60%) at 12 months, 54% (95% CI, 48–59%) at 24 months, and 52% (95% CI, 45–59%) at 36 months, with substantial heterogeneity ($I^2$ >80%). Most relapses occurred within the first 12 months. Meta-regression showed a positive association between longer TKI duration and higher 6-month TFR rates (**TFR6mo = 0.0835 + 0.2744×log10[median TKI duration, months]**). The certainty of evidence for all outcomes was rated as moderate.

### Summary/Conclusion

Approximately half of patients with chronic-phase CML who discontinue TKI therapy sustain long-term TFR. Longer TKI exposure is associated with improved success, supporting guideline recommendations to ensure durable deep molecular remission before discontinuation. These findings reinforce the feasibility of TFR as a treatment goal while highlighting the importance of careful patient selection and close monitoring.

**Copyright Information:** (Online) ISSN: 2572-9241
©2026 The Author(s). HemaSphere is published by John Wiley & Sons Ltd on behalf of European Hematology Association. This is an open access Abstract Book distributed under the Attribution-NonCommercial-NoDerivs (CC BY-NC-ND), which allows third parties to download the articles and share them with others as long as they credit the author and the Abstract Book, but they cannot change the content in any way or use them commercially.
**Abstract Book Citations:** Authors, Title, HemaSphere, 2026;10:(S1):pages. The abstract book DOIs can be found on the "Author Information" page.
**Disclaimer:** Articles published in the journal HemaSphere exclusively reflect the opinions of the authors. The authors are responsible for all content in their abstracts including accuracy of the facts, statements, citing resources, etc.

### (PB2978) COMPLEX VARIANT TRANSLOCATIONS IN PATIENTS WITH CHRONIC MYELOID LEUKEMIA

**Topic:** 07. Chronic myeloid leukemia - Biology & translational research

Olena Zotova*[1], Solomiia Shmyhelska[1], Maria Valchuk[2], Olha Shalay[1], Vasyl Novak[1]

*[1]SI «Institute of Blood Pathology and Transfusion Medicine, NAMS of Ukraine», Lviv, Ukraine;[2]Laboratory of DNA research «LeoGEN», Lviv, Ukraine;*

### Background

Chronic myeloid leukemia (CML) is characterized by the present of Philadelphia (Ph) chromosome as a result of reciprocal translocation between chromosomes 9 and 22: t(9;22)(q34;q11). This translocation is found in 90-95% of CML cases at diagnosis. The remaining 5-10% patients have the complex variant translocations involving additional chromosomes or have an unknown rearrangement, usually an insertion, resulting in the presence of a *BCR-ABL* gene fusion.

### Aims

Aim of this study was to determine frequency of complex variant translocations in patients with CML and to detect the most common additional chromosomes involved in complex translocations.

### Methods

Cytogenetic investigations of bone marrow cells from 102 adult patients with CML were performed. The methods of conventional cytogenetics (GTG) and fluorescence *in situ* hybridization (FISH) were used. Cytogenetic methods were performed using standard techniques and karyotypes were described according to the International System for Human Cytogenomic Nomenclature.

### Results

Of 102 patients with cytogenetically confirmed diagnosis of CML, 92 (90%) had standard translocation t(9;22)(q34;q11), whereas other 10 (10%) – had complex variant translocations, namely t(9;22;5)(q34;q11;q35), t(9;22;6)(q34;q11;p22), t(9;22;7)(q34;q11;q31), t(9;22;13)(q34;q11;p13), t(9;22;13)(q34;q11;q13), t(9;22;13)(q34;q11;q14), t(9;22;17)(q34;q11;p12), t(9;22;17)(q34;q11;p13), t(9;22;19)(q34;q11;q13) and (9;22;12;5)(q34;q11;q15;q11). Except for the typical chromosomes 9 and 22, in 9 cases of complex variant translocations only 1 additional chromosome was involved, in 1 case – 2 chromosomes. The most common additional chromosomes involved in complex translocations were: 13 (3 cases) (30%), 5 (2 cases) (20%) and 17 (2 cases) (20%). The presence of the t(9;22)(q34;q11) and complex translocations was confirmed using the FISH method, namely, the chimeric *BCR-ABL* gene was detected.

### Summary/Conclusion

In our study the frequency of complex variant translocations in patients with CML was 10%. The most common additional chromosomes involved in complex translocations were: 13 (30%), 5 and 17 (20% each).

**Copyright Information:** (Online) ISSN: 2572-9241
©2026 The Author(s). HemaSphere is published by John Wiley & Sons Ltd on behalf of European Hematology Association. This is an open access Abstract Book distributed under the Attribution-NonCommercial-NoDerivs (CC BY-NC-ND), which allows third parties to download the articles and share them with others as long as they credit the author and the Abstract Book, but they cannot change the content in any way or use them commercially.
**Abstract Book Citations:** Authors, Title, HemaSphere, 2026;10:(S1):pages. The abstract book DOIs can be found on the "Author Information" page.
**Disclaimer:** Articles published in the journal HemaSphere exclusively reflect the opinions of the authors. The authors are responsible for all content in their abstracts including accuracy of the facts, statements, citing resources, etc.

### (PB2979) BIOCHEMICAL AND MOLECULAR-GENETIC ARRAYS OF CHRONIC MYELOID LEUKEMIA: MANAGEMENT AND PROGNOSIS IMPACT

**Topic:** 07. Chronic myeloid leukemia - Biology & translational research

Vasile Musteata*[1]

[1]*N. Testemitanu State University of Medicine and Pharmacy, Institute of Oncology, Hematology discipline, Chisinau, Moldova;*

### Background

Management approaches in chronic myeloid leukemia (CML) continue to be of both scientific and practical issues, especially after COVID-19 pandemic, due to complexity of biochemical and molecular-genetic changes. It is suggested that BCR-ABL1 gene may not be the only genetic aberration required for the development of CML. The assessment of lactate dehydrogenase (LDH) and BCR-ABL1 transcripts during the follow-up of CML patients could be considered the markers for therapy decision-making continuation. The biological significance of LDH levels, BCR-ABL1 transcripts and T315I mutation remains under ongoing research in order to optimize therapeutic strategies, commonly in relapsed and refractory cases.

### Aims

The study objectives were the assessments of impact of LDH levels, BCR-ABL gene transcripts and T315I mutation on short- and long-term outcomes of CML patients' management.

### Methods

This prospective and cohort study included 136 patients with CML, who were treated and followed up at the Oncologic Institute from Moldova between 2018–2025. The age range was 14–82 years (average age - 47.91±2.13 years). Venous blood specimens were collected for biochemical tests (alkaline phosphatase (ALP), LDH) and molecular-genetic detection of BCR-ABL1 gene expression and T315I mutation with the aim to evaluate the biological significance of these biochemical and molecular-genetic markers. Molecular-cytogenetic research was performed by FISH test on the bone marrow samples. Real time quantitative RT-PCR was performed in all patients in order to determine the expression of the BCR-ABL p210 and p190 transcripts. Five transcription products (b2a2, b3a2, b2a3, b3a3 si e1a2) were analyzed by the usage of the PCR test.

### Results

The diagnosis was established in the chronic phase (CP) in 124 (91.18±2.32%) cases, in the accelerated and acute phases – in 12 (8.82±2.03%) cases. The increased plasmatic values of LDH were found at diagnosis In the overwhelming majority of CML patients (78.5%),. The statistically significant elevation was assigned to LDH (<0.01) and ALP (<0.05) levels, which rose from 212 U/L and 460.07 U/l in the early CP up to 282 U/L and 1069 U/L in the acute phase, respectiively. Overall survival (OS) rates were significantly higher in CP (Figure 1) in the patients' cohorts with LDH levels ≤225 U/L and 226-500 U/L (36 months – 91.7% and 96.4%, 120 months – 83.3% and 84.0%, 180 months – 83.3.7% and 66.5%, M=221.090 and 198.346 months, 95% CI 187.788-254.392). In most cases (69.8%), the expression of the BCR-ABL gene transcripts exceeded 65%. The b3a2 transcript proved to be the most common (68 cases/56.7%) (<0.001). The b3a3 transcript was identified as the seldom one (2 patients/1.7%) (<0.01). OS rates proved to be superior in the patients' cohort with b3a2 transcript (12 months – 97.1%, 36 months – 92.9%, 60 months – 90.0%, 120 months – 81.1%, M=195,124 months, 95% CI 176,768-213,481, Mdn not reached). Relapses emerged in 62.1% of cases with initially elevated LDH levels, and in 64.3% of cases with T315I mutation.

**Copyright Information:** (Online) ISSN: 2572-9241
©2026 The Author(s). HemaSphere is published by John Wiley & Sons Ltd on behalf of European Hematology Association. This is an open access Abstract Book distributed under the Attribution-NonCommercial-NoDerivs (CC BY-NC-ND), which allows third parties to download the articles and share them with others as long as they credit the author and the Abstract Book, but they cannot change the content in any way or use them commercially.
**Abstract Book Citations:** Authors, Title, HemaSphere, 2026;10:(S1):pages. The abstract book DOIs can be found on the "Author Information" page.
**Disclaimer:** Articles published in the journal HemaSphere exclusively reflect the opinions of the authors. The authors are responsible for all content in their abstracts including accuracy of the facts, statements, citing resources, etc.

**Summary/Conclusion**

LDH and BCR-ABL1 gene expression values exhibit elevation during the CML relapse or progression. The increased LDH and ALP levels notify the activity of CML, and may be used for phase asserting at diagnosis confirmation and during patients' follow-up. Positive correlations were evidenced between BCR-ABL1 transcripts expression and LDH values. Low LDH and BCR-ABL gene expression (≤25%) levels, b3a2 transcript at diagnosis were associated with the higher OS rates.

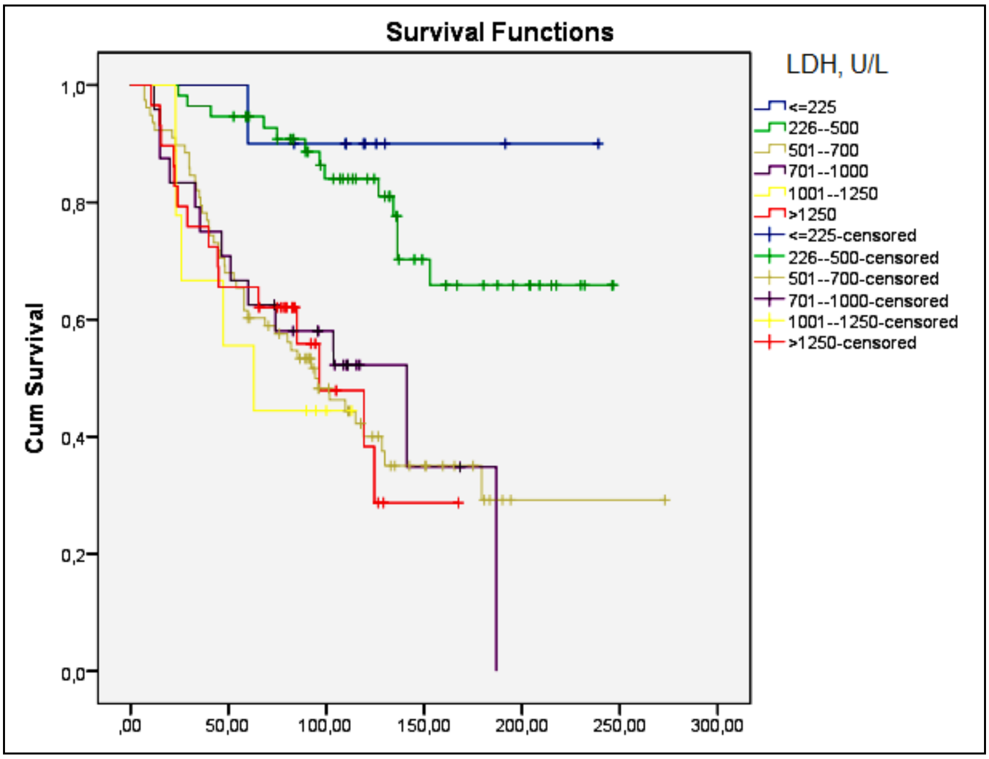

Figure 1. LDH-related overall survival of patients with chronic myeloid leukemia.

**Copyright Information:** (Online) ISSN: 2572-9241
©2026 The Author(s). HemaSphere is published by John Wiley & Sons Ltd on behalf of European Hematology Association. This is an open access Abstract Book distributed under the Attribution-NonCommercial-NoDerivs (CC BY-NC-ND), which allows third parties to download the articles and share them with others as long as they credit the author and the Abstract Book, but they cannot change the content in any way or use them commercially.
**Abstract Book Citations:** Authors, Title, HemaSphere, 2026;10:(S1):pages. The abstract book DOIs can be found on the "Author Information" page.
**Disclaimer:** Articles published in the journal HemaSphere exclusively reflect the opinions of the authors. The authors are responsible for all content in their abstracts including accuracy of the facts, statements, citing resources, etc.

### (PB2980) DEVELOPMENT AND EVALUATION OF A NOVEL EXOSOME–SULFORAPHANE FORMULATION FOR ANTI-CANCER ACTIVITY IN CHRONIC MYELOID LEUKAEMIA

**Topic:** 07. Chronic myeloid leukemia - Biology & translational research

Banan Alkhataybeh*[1], Shohreh .Jafarinejad[1], Adi Baumgartner[2], Mohammad Isreb[1], Mojgan Najafzadeh1[1]

*[1]University of Bradford, BRADFORD, United Kingdom;[2]York St John University, York, United Kingdom., york, United Kingdom;*

### Background

Despite the promising advances in the diagnostics and therapeutics of chronic myeloid leukaemia (CML) in recent years, this remains a disease of the era. Conventional therapeutic approaches are often associated with toxicity and severe side effects. Recent research has highlighted the potential of human umbilical cord blood stem cells (hCBSC)-derived exosomes as promising therapeutic agents due to their roles in intercellular communication and regenerative processes. In addition, sulforaphane (SFN), an antioxidant compound, has demonstrated potent anti-cancer properties.
This study aims to investigate the anti-cancer effect of hCBSC-derived exosomes and SFN, individually and in combination, against haematological malignancies in general and CML in particular, compared to healthy controls. The results revealed that both individual and combined treatments significantly induced DNA damage in haematological malignancies and LAMA-87 cells with no significant genotoxic effects in healthy controls.

### Aims

This study was conducted to investigate the anti-cancer effects of hCBSC-derived exosomes and sulforaphane and their combined application on haematological malignancies, with a particular focus on chronic myeloid leukaemia (CML). This aim will be achieved through the following objectives:

### Methods

CCK-8 assay
COMET ASSAY
qPCR
Western blotting.

### Results

SFN-exosomes co-treatment has significantly increased the cell survival of PBMCs from healthy individuals, while demonstrating a significant reduction in cell viability of CML cells (***p ˂ 0.001). qPCR and Western Blotting techniques were used to investigate the influence of SFN, hCBSC-derived exosomes and their combination on apoptosis-related gene and protein expression in LAMA-87 cells. This study focused on key apoptotic markers such as *P53, P21, BCL-2 and CASP3,* alongside the oncogenic *BCR/ABL1* transcript. The results showed that all three treatments promoted apoptotic pathways and suppressed oncogenic BCR/ABL1 signalling in CML cells, compared to the untreated controls (**p ˂ 0.01). This indicates their potential application as novel therapies for haematological malignancies and future leukaemia treatment strategies.

**Copyright Information:** (Online) ISSN: 2572-9241
©2026 The Author(s). HemaSphere is published by John Wiley & Sons Ltd on behalf of European Hematology Association. This is an open access Abstract Book distributed under the Attribution-NonCommercial-NoDerivs (CC BY-NC-ND), which allows third parties to download the articles and share them with others as long as they credit the author and the Abstract Book, but they cannot change the content in any way or use them commercially.
**Abstract Book Citations:** Authors, Title, HemaSphere, 2026;10:(S1):pages. The abstract book DOIs can be found on the “Author Information” page.
**Disclaimer:** Articles published in the journal HemaSphere exclusively reflect the opinions of the authors. The authors are responsible for all content in their abstracts including accuracy of the facts, statements, citing resources, etc.

### Summary/Conclusion

The study showed novel insights into the action of SFN, exosomes and SFN-exosomes in their anti-cancer activity in haematological cancers and LAMA-87 chronic myeloid leukaemia cells. Overall, the results support the potential of SFN, exosomes, and SFN-exosomes to be further researched as promising compounds for the treatment of haematological cancers, including CML. The administration of two concentrations of SFN (10 and 15 µM), exosomes and SFN-exosomes treatment, did not cause significant DNA damage in healthy individual cells, in contrast to haematological cancer patients and LAMA-87 cells, when these treatments were co-supplemented with 80 µM $H_2O_2$, DNA damage caused by oxidative stress was reduced in healthy cells. Based on these results, the proliferation inhibition induced by these treatments in CML cells might be mediated through their modulation of the apoptosis pathway by upregulating p53, p21, and caspase-3, as well as downregulating Bcl-2, which subsequently affects the cell proliferation and apoptosis process of leukaemia cells. Interestingly, it directly affects the *BCR/ABL1* tumourigenic gene.

**Copyright Information:** (Online) ISSN: 2572-9241
©2026 The Author(s). HemaSphere is published by John Wiley & Sons Ltd on behalf of European Hematology Association. This is an open access Abstract Book distributed under the Attribution-NonCommercial-NoDerivs (CC BY-NC-ND), which allows third parties to download the articles and share them with others as long as they credit the author and the Abstract Book, but they cannot change the content in any way or use them commercially.
**Abstract Book Citations:** Authors, Title, HemaSphere, 2026;10:(S1):pages. The abstract book DOIs can be found on the “Author Information” page.
**Disclaimer:** Articles published in the journal HemaSphere exclusively reflect the opinions of the authors. The authors are responsible for all content in their abstracts including accuracy of the facts, statements, citing resources, etc.

### (PB2981) IMPACT OF BASOPHILIA AND EOSINOPHILIA ON IMATINIB RESISTANCE IN CHRONIC MYELOID LEUKEMIA

**Topic:** 07. Chronic myeloid leukemia - Biology & translational research

Siham Belkadi*[1], Oumayma Hari[1], Imane El Boutahiri[1], Karima Sammoud[2], Adil Najdi[2], Safae Regragui[1]

*[1]University Hospital Center Mohammed VI, Clinical Hematology Service, Tangier, Morocco;[2]University Hospital Center Mohammed VI, Epidemiology Department, Tangier, Morocco;*

### Background

Imatinib, a tyrosine kinase inhibitor (TKI), is recognized as a revolutionary treatment for chronic myeloid leukemia (CML). However, despite its efficacy, a non-negligible number of patients resist or escape.
The underlying mechanisms of this resistance remain partially unclear, highlighting the need for in-depth exploration of prognostic factors.
Among these factors, basophil and eosinophil counts stand out due to their inclusion in several prognostic scores such as SOKAL (1984), HASFORD (1998), and EUTOS (2011). However, it is crucial to note that the first two scores were developed before the TKI era, raising questions about their current relevance. The EUTOS score is the only score designed in the imatinib era and incorporates basophil percentage and spleen size.
However, although these scores all include basophil and eosinophil counts, data independently evaluating the specific impact of each remain limited.

### Aims

This study aims to independently analyze the impact of basophil and eosinophil counts on imatinib resistance and to assess whether these two parameters can serve as early biological indicators of treatment resistance.

### Methods

We conducted a retrospective analytical study in the hematology department of the Tangier University Hospital Center, covering the period from January 2019 to January 2025 with a median follow-up of 49 months (13-73 months).
**Inclusion criteria:** Patients with CML in chronic phase according to the WHO 2022 diagnostic criteria, treated with Imatinib.
**Exclusion criteria:** Patients whose initial basophil and eosinophil counts were not recorded in the medical record before treatment initiation, as well as patients who were lost to follow-up.
Responses to imatinib were evaluated according to ELN 2020 criteria.
The independent impact of basophil and eosinophil on therapeutic response was analyzed separately using univariate tests with SPSS version 27 software, supplemented by ROC curves to assess their discriminatory power. The significance threshold was set at 0.05.

### Results

Among 79 patients diagnosed with CML and treated with imatinib, 45 patients were included (58% women [n=26], 42% men [n=19]), with a median age of 48 years. The response rate to first-line imatinib was 34.1% (n=15); 65.9% failures (n=30).
Basophil and eosinophil counts were distributed identically between responders and non-responders to imatinib. Indeed, for basophils, the area under the ROC curve was 0.536 and was not statistically different from 0.5 (p = 0.717). The eosinophil levels also showed no discriminatory capacity.
Statistical analysis thus demonstrates no statistically significant association between basophil and eosinophil levels and resistance to imatinib.

**Copyright Information:** (Online) ISSN: 2572-9241
©2026 The Author(s). HemaSphere is published by John Wiley & Sons Ltd on behalf of European Hematology Association. This is an open access Abstract Book distributed under the Attribution-NonCommercial-NoDerivs (CC BY-NC-ND), which allows third parties to download the articles and share them with others as long as they credit the author and the Abstract Book, but they cannot change the content in any way or use them commercially.
**Abstract Book Citations:** Authors, Title, HemaSphere, 2026;10:(S1):pages. The abstract book DOIs can be found on the "Author Information" page.
**Disclaimer:** Articles published in the journal HemaSphere exclusively reflect the opinions of the authors. The authors are responsible for all content in their abstracts including accuracy of the facts, statements, citing resources, etc.

**Summary/Conclusion**

In the imatinib era, the prognostic implications of basophilia and eosinophilia require reevaluation. Our analysis demonstrates that therapeutic response is independent of these counts, calling into question their inclusion in current CML prognostic scores and advocating for the identification of new biomarkers.
A better understanding of biological factors associated with therapeutic response could enable a more personalized therapeutic approach, facilitating the selection of the appropriate first-line TKI.

**Copyright Information:** (Online) ISSN: 2572-9241
©2026 The Author(s). HemaSphere is published by John Wiley & Sons Ltd on behalf of European Hematology Association. This is an open access Abstract Book distributed under the Attribution-NonCommercial-NoDerivs (CC BY-NC-ND), which allows third parties to download the articles and share them with others as long as they credit the author and the Abstract Book, but they cannot change the content in any way or use them commercially.
**Abstract Book Citations:** Authors, Title, HemaSphere, 2026;10:(S1):pages. The abstract book DOIs can be found on the "Author Information" page.
**Disclaimer:** Articles published in the journal HemaSphere exclusively reflect the opinions of the authors. The authors are responsible for all content in their abstracts including accuracy of the facts, statements, citing resources, etc.

### (PB2982) RESULTS OF TWO-STEP REDUCED-DOSE TKI THERAPY FOLLOWED BY TREATMENT-FREE OBSERVATION IN CML PATIENTS IN READIT (STUDY OF REDUCTION AND DISCONTINUATION TREATMENT OF TKI) AND POST-STUDY FOLLOW-UP

**Topic:** 08. Chronic myeloid leukemia - Clinical

Anna Petrova[1], Ekaterina Chelysheva*[1], Margarita Gurianova[1], Oleg Shukhov[1], Elena Kuzmina[1], Anastasia Bykova[1], Irina Nemchenko[1], Lubov' Gavrilova[2], Azaliya Valitova[1], Andrey Sudarikov[1], Alina Kohno[1], Anna Turkina[1]

*[1]National Medical Research Center for Hematology, Moscow, Russia;[2]Federal State Budgetary Institution of Mordovia republic, Republican City Hospital 4, Saransk, Russia;*

### Background

The feasibility of dose reduction of tyrosine kinase inhibitors (TKI) prior to observation in treatment-free remission (TFR) in patients (pts) with chronic myeloid leukemia (CML) and a deep molecular response (DMR) has been confirmed in several studies. However, the effectiveness of resuming reduced dose TKI therapy remains unevaluated.

### Aims

To determine the probability of re-achieving MMR and DMR using reduced-dose TKI in CML pts with prior molecular relapse during TFR in the READIT trial (NCT 04578847).

### Methods

The study included adult CML pts in chronic phase on TKI therapy for >3 years (y) with DMR (*BCR::ABL1*≤0.01%) >1y.
The trial had 2 phases: (1) two-step TKI dose reduction with each step lasting for at least 6 months (mo). Step 1 TKI daily doses: imatinib 300 mg, nilotinib 400 mg, dasatinib 50 mg, bosutinib 300 mg, step 2 doses: dasatinib 25 mg (50 mg every other day), others TKI 200 mg QD. (2) TFR observation (DMR ≥2y, MR4.5 (*BCR::ABL1*≤0.0032%) confirmation before cessation). In case of MMR loss the dose was re-escalated. If MMR was lost during TFR the 2nd step reduced dose was restarted.

### Results

Of 103 included pts, 78 (76%) pts entered TFR; 33 (42%) maintained MMR after TKI cessation with a median (Me) follow-up (FU) of 56 months (mo) (41 – 65 mo). The survival without TKI resumption was 47%, 43% and 40% at 24, 36 and 48 mo, respectively. Therapy was resumed in 45 (58%) pts: with MMR loss (n=41) and without MMR loss (withdrawal syndrome – 2 pts, patient's choice – 2 pts). All pts with MMR loss restarted TKI at the doses of 2nd step.
Among 39 pts analyzed for response recovery 38 (97%) restored MMR and DMR (1 pt had <3 mo FU after TKI restart). The probability of MMR recovery was 58%, 84%, and 100% at 3, 6, and 7.5 mo, respectively. Me time to MMR recovery was 3 mo (3-7.5 mo). The probability of MR4.5 recovery was 26%, 58%, 79% and 100% at 3, 6, 12 and 28 mo, respectively. Me time to MR4.5 recovery was 6 mo (3-28 mo).
Dose re-escalation to 1st step level was required in 13 (33%) pts (failure to restore MMR n=9, failure to restore MR4 n=4), 2 pts required full dose TKI (failure to restore MMR -1pt, patient's choice -1 pt). One pt had a change of TKI due to failure to restore MR4. Me time to dose increase/TKI change was 3.7 мес (2.8 – 36.4 мес).
Therapy at last FU of the pts who resumed TKI was as follows: 2nd step doses -22 pts, 1st step doses -11 pts, full (standard) doses -2 pts, 2nd TFR observation – 3 pts.
Of 25 pts not entering TFR in READIT trial (mainly due to MR4.5/MR4 loss) 20(80%) regained DMR at Me FU duration of 15 mo (6-36 mo), 16 of 20 pts used reduced doses of TKI

**Copyright Information:** (Online) ISSN: 2572-9241
©2026 The Author(s). HemaSphere is published by John Wiley & Sons Ltd on behalf of European Hematology Association. This is an open access Abstract Book distributed under the Attribution-NonCommercial-NoDerivs (CC BY-NC-ND), which allows third parties to download the articles and share them with others as long as they credit the author and the Abstract Book, but they cannot change the content in any way or use them commercially.
**Abstract Book Citations:** Authors, Title, HemaSphere, 2026;10:(S1):pages. The abstract book DOIs can be found on the "Author Information" page.
**Disclaimer:** Articles published in the journal HemaSphere exclusively reflect the opinions of the authors. The authors are responsible for all content in their abstracts including accuracy of the facts, statements, citing resources, etc.

**Summary/Conclusion**

Gradual TKI dose reduction followed by TFR and dose re-escalation in the case of molecular relapse in the READIT study proved safe and effective. Although 33% of pts did not restore MMR/DMR with step 2 reduced doses, most of them (87%) avoided to restart the full-dose therapy, with 58% maintaining minimal therapeutic doses. Therapy scenarios in pts not entering TFR phase require additional evaluation.

**Copyright Information:** (Online) ISSN: 2572-9241
©2026 The Author(s). HemaSphere is published by John Wiley & Sons Ltd on behalf of European Hematology Association. This is an open access Abstract Book distributed under the Attribution-NonCommercial-NoDerivs (CC BY-NC-ND), which allows third parties to download the articles and share them with others as long as they credit the author and the Abstract Book, but they cannot change the content in any way or use them commercially.
**Abstract Book Citations:** Authors, Title, HemaSphere, 2026;10:(S1):pages. The abstract book DOIs can be found on the "Author Information" page.
**Disclaimer:** Articles published in the journal HemaSphere exclusively reflect the opinions of the authors. The authors are responsible for all content in their abstracts including accuracy of the facts, statements, citing resources, etc.

### (PB2983) MANAGEMENT OF CML AND SECOND PRIMARY MALIGNANCIES IN THE ERA OF MODERN THERAPIES: REAL-WORLD STRATEGIES AND IMPACT ON CLINICAL OUTCOMES.

**Topic:** 08. Chronic myeloid leukemia - Clinical

Gianni Binotto*[1], Andrea Bernardelli[2], Alda Strazimiri[3], Luca Tosoni[4], Gianluca Morelli[4], Alessandro Atanasio[5], Rita Bresciani[6], Elena De March[7], Stefania Fortuna[8], Ilaria Gianesello[1], Marisa Calleri[1], Nicola Franzoni[3], Luca Frison[9], Davide Griguolo[10], Cristina Ancora[10], Rossella Stella[10], Gessica Marchesini[11], Davide Facchinelli[11], Lara Crosera[12], Rosanna Ciancia[13], Laura Dorotea[14], Stefania Cavallin[15], Michele Dell'eva[16], Lara Furlani[17], Giuseppe Carli[11], Elisabetta Calistri[5], Mario Tiribelli[4], Massimiliano Bonifacio[2]

*[1]PADUA UNIVERSITY CITY HOSPITAL, HEMATOLOGY UNIT, PADUA, Italy; [2]University of Verona and Azienda Ospedaliera Universitaria Integrata, Department of Engineering for Innovation Medicine, Section of Innovation Biomedicine, Hematology Area, VERONA, Italy; [3]Onco-hematology Unit, Santa Maria della Misericordia Hospital, Rovigo, Italy; [4]Division of Hematology and Stem Cell Transplantation, University Hospital ASUFC, Udine, Italy; [5]Onco-hematology Unit, Department of Oncology, Veneto Institute of Oncology, IOV-IRCCS, Padova, Italy; [6]Hematology Unit, Ca' Foncello Hospital, Treviso, Italy; [7]Hematology Unit, San Martino Hospital, Belluno, Italy; [8]Onco-hematology Unit, Alto Vicentino Hospital, Santorso, Italy; [9]Hematology Unit, Dell'Angelo Hospital, Mestre, Italy; [10]Hematology Unit, Azienda Sanitaria Universitaria Giuliano-Isontina, Trieste, Italy; [11]Hematology Unit, Ospedale San Bortolo, Vicenza, Italy; [12]Department of Medicine, Oncology Unit, San Donà di Piave, Italy; [13]Onco-hematology, Stem Cell Transplantation and Cellular Therapies, Department of Medical Oncology, CRO Aviano National Cancer Institute IRCCS, Aviano, Italy; [14]Oncology Unit, Ospedali Riuniti Padova Sud-Schiavonia, Monselice, Italy; [15]Oncology Unit, Vittorio Veneto Hospital, Vittorio Veneto, Italy; [16]Hematology Unit, San Maurizio Hospital, Bolzano, Italy; [17]Oncology Unit, Ospedale Mater Salutis, Legnago, Italy;*

### Background

BCR::ABL1 tyrosine kinase inhibitors have transformed chronic myeloid leukemia into a chronic, largely controllable condition, substantially extending life expectancy. However, improved long-term survival may increase cumulative exposure to age-related and treatment-related oncogenic risks, potentially leading to a higher incidence of second primary malignancies (SPM). This evolving scenario adds complexity to the concurrent management of CML and subsequent cancers, particularly with regard to therapeutic sequencing, safety, and drug–drug interactions.

### Aims

To comprehensively characterize real-world management strategies, patterns of TKI modification, and survival outcomes among CML patients who develop a SPM in the era of targeted and immunomodulatory therapies.

### Methods

We retrospectively reviewed 51 cases of CML complicated by a second primary malignancy (SPM) within an 820-patient cohort from several centers. De-identified datasets included age at CML, initial TKI choice, treatment line when the SPM occurred, molecular response depth, SPM pathology and stage, SPM treatment modalities, any variations in TKI therapy, and follow-up outcomes.

**Copyright Information:** (Online) ISSN: 2572-9241
©2026 The Author(s). HemaSphere is published by John Wiley & Sons Ltd on behalf of European Hematology Association. This is an open access Abstract Book distributed under the Attribution-NonCommercial-NoDerivs (CC BY-NC-ND), which allows third parties to download the articles and share them with others as long as they credit the author and the Abstract Book, but they cannot change the content in any way or use them commercially.
**Abstract Book Citations:** Authors, Title, HemaSphere, 2026;10:(S1):pages. The abstract book DOIs can be found on the "Author Information" page.
**Disclaimer:** Articles published in the journal HemaSphere exclusively reflect the opinions of the authors. The authors are responsible for all content in their abstracts including accuracy of the facts, statements, citing resources, etc.

### Results

The median age at CML diagnosis was 64 years (range, 27–81). First-line therapy consisted predominantly of imatinib (92.1%), followed by nilotinib (5.9%) and interferon (2%). Second primary malignancies (SPMs) occurred after a median latency of 88 months (interquartile range [IQR], 40–151). At the time of SPM diagnosis, 60% of patients had achieved a deep molecular response. The most frequent SPM categories were genitourinary (30.4%), gastrointestinal (21.7%), respiratory (13.0%), and hematologic (13.0%) neoplasms; notably, 56% were diagnosed at an advanced stage. Management strategies for SPMs included chemotherapy (34%), combination systemic regimens (17%), targeted therapies (17%), radiotherapy (7%), surgery (7%), palliative approaches (7%), and other treatments (9%). At SPM onset, 50% of patients were already receiving reduced-dose TKI therapy. Additional dose reductions were implemented in 6.5% of cases due to toxicity and in 15.2% due to potential drug–drug interactions. TKI switching was required in 6.5% of patients, whereas permanent discontinuation occurred in 17.4%, primarily driven by poor SPM prognosis. Treatment-free remission (TFR) was attempted in 28.3% of cases. Although loss of major molecular response (MR3) was documented in 7.8% of patients, no progression to blast phase or CML-related mortality was observed. Median overall survival (OS) was 191 months from CML diagnosis and 73 months from SPM diagnosis. The estimated OS was 89.5% at 5 years (95% CI: 76.5–95.5) and 68.8% at 10 years (95% CI: 53.0–80.3) among patients who developed an SPM.

### Summary/Conclusion

The interval between CML diagnosis and the onset of a second malignancy often allows achievement of a sustained deep molecular response, enabling TKI dose de-escalation and, in selected patients, TFR attempts. Given the adverse prognostic impact of second malignancies, therapeutic priorities should focus primarily on their optimal management, as they frequently drive overall outcome. The expanding use of innovative therapies—including monoclonal antibodies, immune checkpoint inhibitors, antibody–drug conjugates, targeted agents, and cellular therapies—is expected to further improve survival, increasingly shifting many cancers toward a chronic disease model and reinforcing the need for structured multidisciplinary management.

**Copyright Information:** (Online) ISSN: 2572-9241
©2026 The Author(s). HemaSphere is published by John Wiley & Sons Ltd on behalf of European Hematology Association. This is an open access Abstract Book distributed under the Attribution-NonCommercial-NoDerivs (CC BY-NC-ND), which allows third parties to download the articles and share them with others as long as they credit the author and the Abstract Book, but they cannot change the content in any way or use them commercially.
**Abstract Book Citations:** Authors, Title, HemaSphere, 2026;10:(S1):pages. The abstract book DOIs can be found on the "Author Information" page.
**Disclaimer:** Articles published in the journal HemaSphere exclusively reflect the opinions of the authors. The authors are responsible for all content in their abstracts including accuracy of the facts, statements, citing resources, etc.

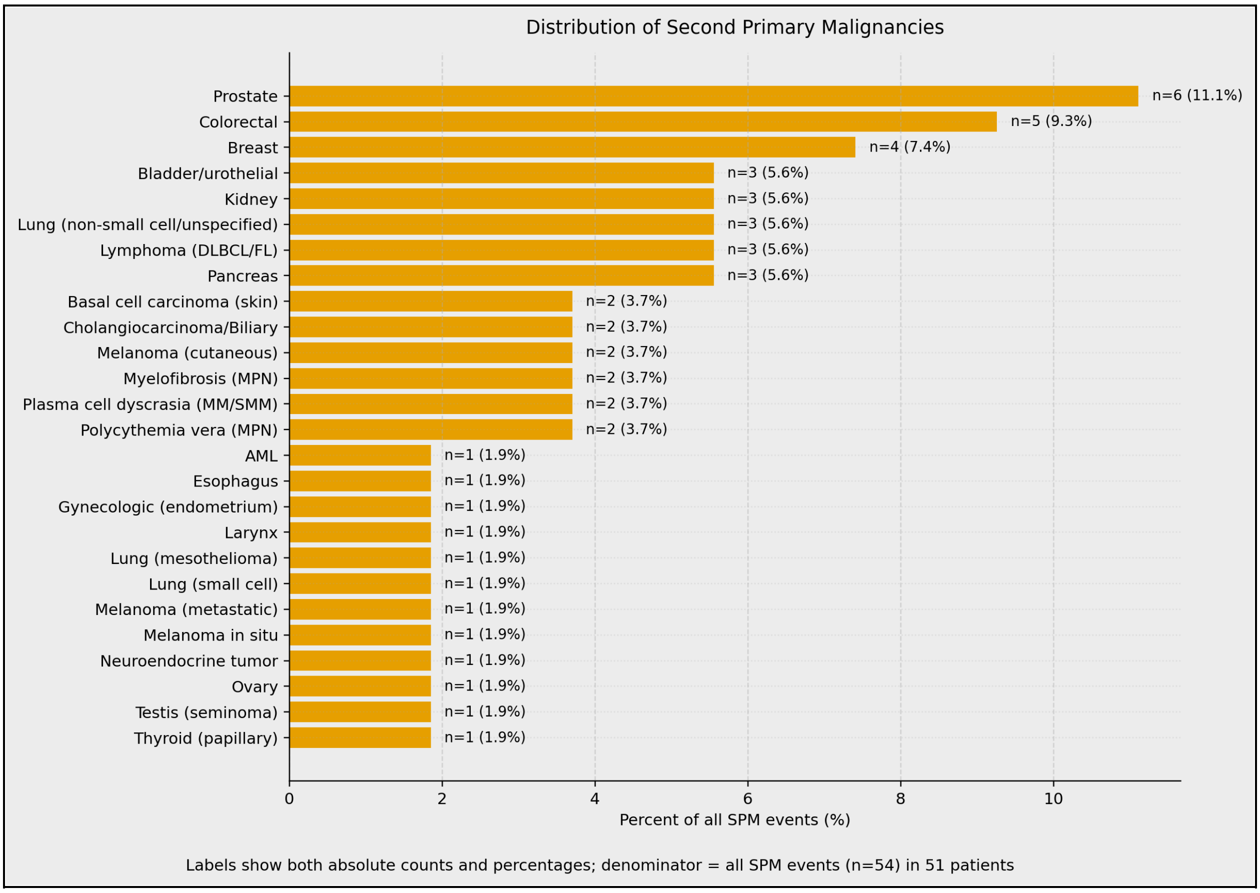

Figure 1. Tumor-Type Distribution of Second Primary Malignancies in CML patients

**Copyright Information:** (Online) ISSN: 2572-9241
©2026 The Author(s). HemaSphere is published by John Wiley & Sons Ltd on behalf of European Hematology Association. This is an open access Abstract Book distributed under the Attribution-NonCommercial-NoDerivs (CC BY-NC-ND), which allows third parties to download the articles and share them with others as long as they credit the author and the Abstract Book, but they cannot change the content in any way or use them commercially.
**Abstract Book Citations:** Authors, Title, HemaSphere, 2026;10:(S1):pages. The abstract book DOIs can be found on the "Author Information" page.
**Disclaimer:** Articles published in the journal HemaSphere exclusively reflect the opinions of the authors. The authors are responsible for all content in their abstracts including accuracy of the facts, statements, citing resources, etc.

### (PB2984) TKI DOSE INTENSITY AND TREATMENT DISCONTINUATION IN CHRONIC MYELOID LEUKEMIA: A SINGLE CENTER ANALYSIS

**Topic:** 08. Chronic myeloid leukemia - Clinical

Daniele Sannipoli*[1], Lucia Iaquinta[2], Maria Helena Parrinello[1, 2], Marta Ricci[1, 2], Francesca Lunghi[1], Annalisa Ruggeri[1], Fabio Ciceri[1, 2]

*[1]IRCCS San Raffaele Hospital, Hematology and Bone Marrow Transplantation Unit, Milan, Italy; [2]Vita-Salute San Raffaele University, Milan, Italy;*

### Background

Survival in chronic myeloid leukemia (CML) is now excellent in the TKI era, prompting a paradigm shift toward optimization of long-term quality of life (QoL). Beyond efficacy, treatment tolerability, treatment discontinuation leading to treatment-free remission (TFR), and dose-reduction strategies—with potential pharmacoeconomic implications—are emerging priorities in clinical practice. However, the real-life prevalence of these strategies and their relationship with tolerability and molecular response depth remain incompletely characterized.

### Aims

We aimed to quantify real-life TKI dose intensity and treatment discontinuation strategies in a single-center CML cohort and to explore their association with intolerance, resistance, and molecular response depth according to ELN 2025.

### Methods

We performed a retrospective single-center analysis of consecutive patients diagnosed with chronic myeloid leukemia (CML) from 2002 to 2025. Blast-phase cases were excluded. Patients were classified as treatment-free remission (TFR), enrolled in reduced-dose programs, or receiving routine TKI therapy. In treated patients, the current line of treatment and dose were defined as the last recorded therapy and the most recent dose. Dose intensity was categorized as full or reduced according to label recommendations. Molecular response was assessed per ELN 2025, using MR4+ as a surrogate for deep molecular response (DMR). Intolerance and resistance were recorded as binary variables. Associations were tested using Fisher's exact test in evaluable patients.

### Results

A total of 115 patients were included (median age 64.6 years), with a median time from diagnosis of 8.45 years; at last follow-up, 106/115 (92.2%) were alive. Thirty-one patients (27.0%) were in TFR and 10 (8.7%) were enrolled in reduced-dose programs. Among 65 alive patients on active therapy, 37/65 (56.9%) received full-label dosing and 28/65 (43.1%) reduced dosing. The most frequently used TKI was imatinib (43/65, 66.2%), followed by dasatinib (n=9), nilotinib (n=6), bosutinib (n=3), asciminib (n=3), and ponatinib (n=1). Notably, no patients receiving second- or later-line dasatinib, bosutinib, nilotinib, or ponatinib were treated at full label dose. Dose reduction was mainly driven by intolerance (13/28, 46.4%) or achievement of deep molecular response (15/28, 53.6%). MR4+ rates were comparable between reduced- and full-dose groups (45.8% vs 51.4%; p=0.79) and were high in TFR (93.3%) and reduced-dose program patients (87.5%). Age >65 years was not associated with dose reduction (p=0.62). When considering imatinib-exposed patients and including TFR and dose-optimization strategies, treatment de-escalation was numerically more frequent in patients aged ≥70 years, although not statistically significant (OR 1.78, p=0.27).

**Copyright Information:** (Online) ISSN: 2572-9241
©2026 The Author(s). HemaSphere is published by John Wiley & Sons Ltd on behalf of European Hematology Association. This is an open access Abstract Book distributed under the Attribution-NonCommercial-NoDerivs (CC BY-NC-ND), which allows third parties to download the articles and share them with others as long as they credit the author and the Abstract Book, but they cannot change the content in any way or use them commercially.
**Abstract Book Citations:** Authors, Title, HemaSphere, 2026;10:(S1):pages. The abstract book DOIs can be found on the "Author Information" page.
**Disclaimer:** Articles published in the journal HemaSphere exclusively reflect the opinions of the authors. The authors are responsible for all content in their abstracts including accuracy of the facts, statements, citing resources, etc.

**Summary/Conclusion**

In this real-life single-center CML cohort, a substantial proportion of patients were managed through TFR or dose-optimization strategies, and dose reductions were common in routine clinical practice. Dose reduction was strongly associated with treatment intolerance, without an apparent compromise in deep molecular response, supporting the clinical relevance of individualized TKI dosing in long-term CML management. Further studies integrating quality-of-life and pharmacoeconomic data are warranted to better evaluate the benefits of these strategies.

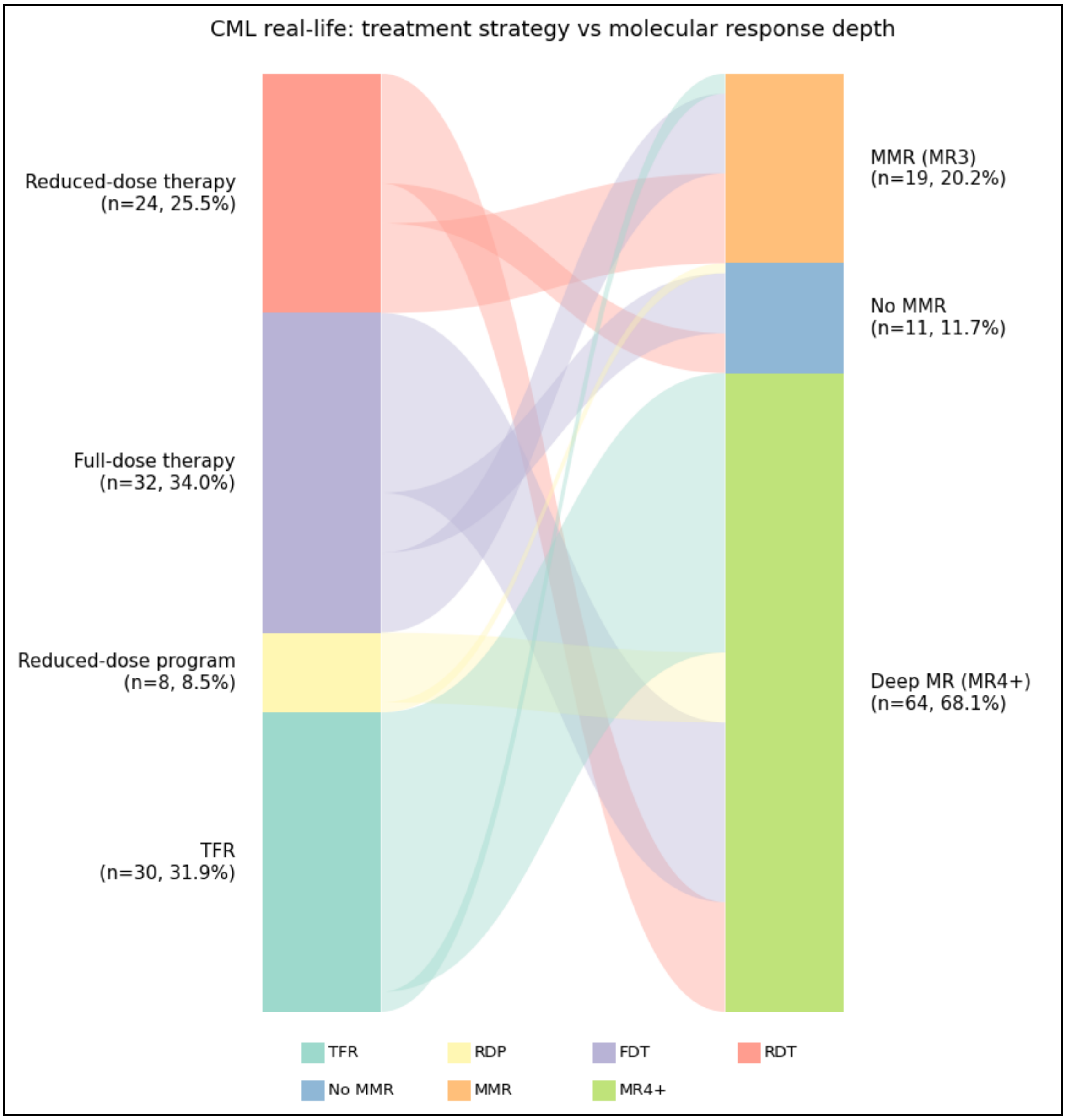

**Copyright Information:** (Online) ISSN: 2572-9241
©2026 The Author(s). HemaSphere is published by John Wiley & Sons Ltd on behalf of European Hematology Association. This is an open access Abstract Book distributed under the Attribution-NonCommercial-NoDerivs (CC BY-NC-ND), which allows third parties to download the articles and share them with others as long as they credit the author and the Abstract Book, but they cannot change the content in any way or use them commercially.
**Abstract Book Citations:** Authors, Title, HemaSphere, 2026;10:(S1):pages. The abstract book DOIs can be found on the "Author Information" page.
**Disclaimer:** Articles published in the journal HemaSphere exclusively reflect the opinions of the authors. The authors are responsible for all content in their abstracts including accuracy of the facts, statements, citing resources, etc.

### (PB2985) NINE-YEARS FOLLOW-UP OF THE PHASE I/II TRIAL OF IMATINIB DISCONTINUATION AFTER PIOGLITAZONE (EDI-PIO) IN CHRONIC MYELOID LEUKEMIA PATIENTS IN DEEP MOLECULAR RESPONSE

**Topic:** 08. Chronic myeloid leukemia - Clinical

Katia Pagnano*[1], Ana Beatriz Lopes[1], Gislaine Borba[1], Guilherme Duffles[1], Christopher Cralcev[1], Graziele Furlin[1], Isabella Toni[1], Erich De Paula[1], Carmino Antonio de Souza[1]

[1] *Universidade Estadual de Campinas (UNICAMP), Centro de Hematologia e Hemoterapia, Campinas, Brazil;*

### Background

Pioglitazone (PIO) is a PPARγ agonist that reduces STAT5 activity and its downstream targets, HIF2α and CITED2, key guardians of the quiescent residual leukemic stem cells (LSC) responsible for relapse after treatment-free remission (TFR) attempts in chronic myeloid leukemia (CML) patients.

### Aims

To evaluate the long-term follow-up of a cohort of CML patients who participated in the EDI-PIO trial. *STAT5, HIF2, PPARγ* gamma and *CITED* expression were analyzed before and after pioglitazone, and 6 months after IM discontinuation.

### Methods

EDI-PIO is a prospective, single-arm, phase I/II trial. Inclusion criteria: CML in chronic phase, treated with IM for at least 3 years, with MR4.5 reported by the International Scale (IS) for 2 years. Patients received PIO 30 mg/day orally for three months before IM discontinuation. After IM discontinuation, BCR::ABL1 levels were measured by real-time quantitative RT-PCR monthly for 12 months, every two months in the second year, every three months until 5 years and then after every 3-6 months. IM was re-initiated at molecular relapse (MR) (single sample with a value of >0.1% or two consecutive samples >0.01%). Other gene expression was calculated by real time quantitative RT-PCR. TFR was calculated from IM discontinuation until MR, progression, or death to causes related to CML. Overall survival (OS) was calculated from the date of IM discontinuation until the date of last follow-up or death from any cause. Multivariate was done by Cox regression. ClinicalTrials.gov register: NCT02852486. The cut-off date for this analysis was January 2026.

### Results

Between Jun/2016 and Jan/2019, we recruited 32 CML patients (53% male), with a median age of 54 years (29-77), treated with IM for a median of 9.5 years (3-16). Sokal score: 64% of patients were low risk, 24% intermediate, and 12% high risk. Six patients (18.8%) had previously received IFN-α. The median duration of IM treatment was 9.5 years (3-16). The MR4 and MR4.5 durations were 106 and 93 months, respectively. The median follow-up of the patients who discontinued IM was 82.2 (2.7-111.5) months.STAT5, HIF2, PPAR gamma and CITED expression were not significantly different before and after PIO and 3 and 6 months after IM and PIO discontinuation. There were no grade 3-4 events related to PIO. Twelve patients relapsed after a median time of 4.3 months (1.9-28.4). Nine relapses occurred within the first six months, and three at 7, 13, and 30 months after stopping IM. All patients recovered MMR in a median time of 3 months after IM reintroduction (1.8-4.1). 48% presented withdrawal syndrome (grade 1-2). TFR was 62.5% at 9 years and was higher for patients with low Sokal risk vs Intermediate/high risk (81% vs. 33%; P=0.018), but not statistically different among sex, IFN use or BCR::ABL1 transcript type. OS was 90%. There were 3 deaths, two caused by COVID-19 and one by a gastrointestinal tumor, in a patient with a previous autologous hematopoietic stem cell transplant. In the multivariate analysis, the independent factors for MR were an intermediate/high Sokal score (HR=16.053; 95% CI=2.307-111.715; P=0.005) and shorter duration of TKI treatment (HR=0.962; 95% CI=0.935-0.991; P=0.0091).

**Copyright Information:** (Online) ISSN: 2572-9241
©2026 The Author(s). HemaSphere is published by John Wiley & Sons Ltd on behalf of European Hematology Association. This is an open access Abstract Book distributed under the Attribution-NonCommercial-NoDerivs (CC BY-NC-ND), which allows third parties to download the articles and share them with others as long as they credit the author and the Abstract Book, but they cannot change the content in any way or use them commercially.
**Abstract Book Citations:** Authors, Title, HemaSphere, 2026;10:(S1):pages. The abstract book DOIs can be found on the “Author Information” page.
**Disclaimer:** Articles published in the journal HemaSphere exclusively reflect the opinions of the authors. The authors are responsible for all content in their abstracts including accuracy of the facts, statements, citing resources, etc.

**Summary/Conclusion**

After 9 years of IM discontinuation, no relapses after 30 months were observed in this cohort. There were no significant changes in gene expression profile after PIO.

**Copyright Information:** (Online) ISSN: 2572-9241
©2026 The Author(s). HemaSphere is published by John Wiley & Sons Ltd on behalf of European Hematology Association. This is an open access Abstract Book distributed under the Attribution-NonCommercial-NoDerivs (CC BY-NC-ND), which allows third parties to download the articles and share them with others as long as they credit the author and the Abstract Book, but they cannot change the content in any way or use them commercially.
**Abstract Book Citations:** Authors, Title, HemaSphere, 2026;10:(S1):pages. The abstract book DOIs can be found on the "Author Information" page.
**Disclaimer:** Articles published in the journal HemaSphere exclusively reflect the opinions of the authors. The authors are responsible for all content in their abstracts including accuracy of the facts, statements, citing resources, etc.

### (PB2986) ULTRA-EARLY MOLECULAR RESPONSE AT 6 WEEKS OF ASCIMINIB THERAPY FOR PATIENTS WITH NEWLY DIAGNOSED CHRONIC PHASE CHRONIC MYELOID LEUKEMIA

**Topic:** 08. Chronic myeloid leukemia - Clinical

Bingbing Wen*[1], Junchong Peng[1], Xiaoliang Li[2], Xiaoman Chen[3], Na Xu[4], Xin Du[1]

*[1]The Second People's Hospital of Shenzhen, The First Affiliated Hospital of Shenzhen University, Shenzhen, China;[2]Zhuhai People's Hospital, Zhuhai, China; [3]Affiliated Hospital of Guangdong Medical University, Guangzhou, China;[4]Nanfang Hospital, Southern Medical University, Guangzhou, China;*

### Background

Asciminib, the first BCR::ABL1 inhibitor that specifically targets the ABL Myristoyl Pocket (STAMP), has been reported for patients with chronic myeloid leukemia (CML). Ultra-early molecular response (u-EMR) is associated with subsequent depth of response and long-term outcomes in patients with CML. However, the ultra-early response at 6 weeks of asciminib in patients with newly diagnosed chronic-phase CML (CML-CP) patients remains been detected.

### Aims

This study aimed to evaluate ultra-early molecular responses and safety at 6 weeks of asciminib in patients with newly diagnosed CML-CP.

### Methods

This is a multicenter, prospective, phase IV study. Patients with newly diagnosed Ph+ or BCR/ABL1-positive CML-CP were enrolled. Patients will receive asciminib (80 mg orally daily). The primary endpoint is the ultra-early molecular response (u-EMR) rate at 6 weeks (BCR:: $ABL1^{IS}$ <10%). The key secondary endpoint is the proportion of patients achieving a ≥50% reduction in BCR:: $ABL1^{IS}$ from baseline at 6 weeks. Baseline risk stratification (Sokal and ELTS) was performed, and comorbidities and ABL kinase-domain mutations were recorded. Safety was assessed per NCI CTCAE v6.0.

### Results

Between July 9, 2025 and February 26, 2026, 15 patients were assessed and enrolled in this multicenter, prospective study. At baseline, the median age was 42 years (rang, 18-63 years), and male were 1/15 (73.3.%) totally. All patients expressed P210 transcript type. The median WBC was $61.1\times10^9$ /L (range 11.7–445.6), and the median PLT was $362\times10^9$ /L (range 107–3941). Besides, high-risk states of CML-CP patients were observed with 4/15 (26.7%) of Sokal, 1/15 (6.7%) of ELTS, and 4/15 (26.7%) of EUTOS. Complications were reported in 7/15 (46.7%), and an ABL kinase-domain mutation was detected in 1/15 (6.7%) (**Figure A**). At 6 week, 9/15 (60.0%) of CML-CP patients achieved u-EMR, and 14/15 (93.3%) of CML-CP patients achieved a ≥50% reduction in BCR:: $ABL1^{IS}$ from baseline with treatment of asciminib (**Figure B-E**). At 3 month, 12/15 (80.0%) of CML-CP patients achieved EMR. The most common treatment-emergent adverse events (TEAEs) of asciminib were primarily grade 1-2 and manageable. Only 3 patients were observed in thrombocytopenia (3/15, 20.0%). The non-hematological events were observed with fever(1/15, 6.7%), hyperuricemia ( 1/15, 6.7%), and increased serum creatinine (1/15 ,6.7%). All AEs were improved and resolved after dose reduction with a 1-week adjustment.

**Copyright Information:** (Online) ISSN: 2572-9241
©2026 The Author(s). HemaSphere is published by John Wiley & Sons Ltd on behalf of European Hematology Association. This is an open access Abstract Book distributed under the Attribution-NonCommercial-NoDerivs (CC BY-NC-ND), which allows third parties to download the articles and share them with others as long as they credit the author and the Abstract Book, but they cannot change the content in any way or use them commercially.
**Abstract Book Citations:** Authors, Title, HemaSphere, 2026;10:(S1):pages. The abstract book DOIs can be found on the "Author Information" page.
**Disclaimer:** Articles published in the journal HemaSphere exclusively reflect the opinions of the authors. The authors are responsible for all content in their abstracts including accuracy of the facts, statements, citing resources, etc.

### Summary/Conclusion

Asciminib can quickly reduce the transcript of BCR:: ABL, with a high rate of ultra-early molecular response and ≥50% reduction at 6 weeks from baseline in patients with newly diagnosed CML-CP. Theses findings maybe as an important value to early identify CML-CP patients with Deep Molecular Response (DMR) and Treatment-Free Remission (TFR) in the future.

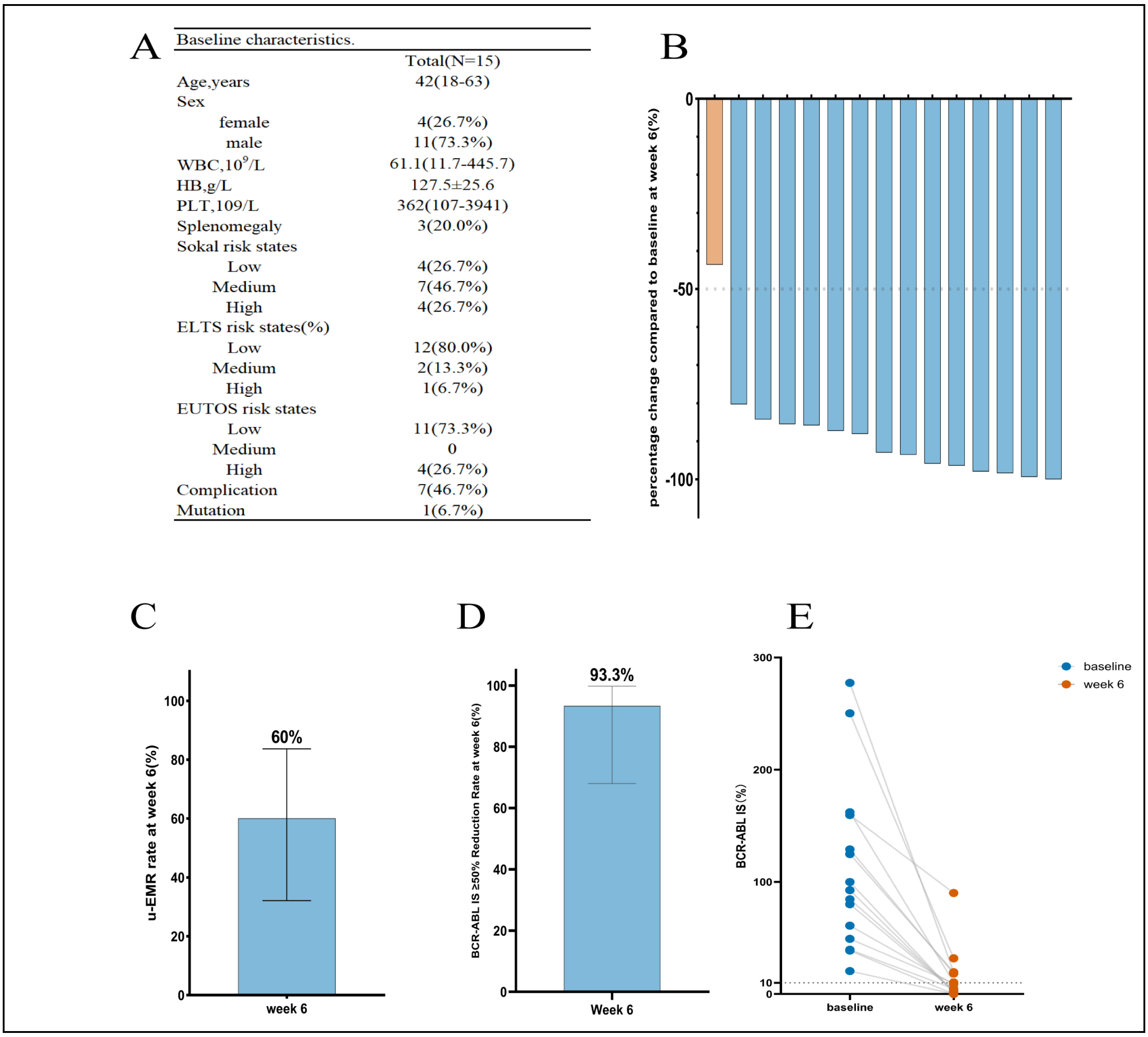

| Baseline characteristics. | Total(N=15) |
|---|---|
| Age,years | 42(18-63) |
| Sex | |
| female | 4(26.7%) |
| male | 11(73.3%) |
| WBC,$10^9$/L | 61.1(11.7-445.7) |
| HB,g/L | 127.5±25.6 |
| PLT,109/L | 362(107-3941) |
| Splenomegaly | 3(20.0%) |
| Sokal risk states | |
| Low | 4(26.7%) |
| Medium | 7(46.7%) |
| High | 4(26.7%) |
| ELTS risk states(%) | |
| Low | 12(80.0%) |
| Medium | 2(13.3%) |
| High | 1(6.7%) |
| EUTOS risk states | |
| Low | 11(73.3%) |
| Medium | 0 |
| High | 4(26.7%) |
| Complication | 7(46.7%) |
| Mutation | 1(6.7%) |

**Copyright Information:** (Online) ISSN: 2572-9241
©2026 The Author(s). HemaSphere is published by John Wiley & Sons Ltd on behalf of European Hematology Association. This is an open access Abstract Book distributed under the Attribution-NonCommercial-NoDerivs (CC BY-NC-ND), which allows third parties to download the articles and share them with others as long as they credit the author and the Abstract Book, but they cannot change the content in any way or use them commercially.
**Abstract Book Citations:** Authors, Title, HemaSphere, 2026;10:(S1):pages. The abstract book DOIs can be found on the “Author Information” page.
**Disclaimer:** Articles published in the journal HemaSphere exclusively reflect the opinions of the authors. The authors are responsible for all content in their abstracts including accuracy of the facts, statements, citing resources, etc.

### (PB2987) PREDICTORS OF OUTCOMES IN PATIENTS WITH CHRONIC MYELOID LEUKEMIA IN LYMPHOID BLAST PHASE IN THE MODERN ERA

**Topic:** 08. Chronic myeloid leukemia - Clinical

Omer Karrar[1], Manuel Maroun[1], Maria Hachem[1], Ghayas Issa[1], Fadi Haddad[1], Nitin Jain[1], Farhad Ravandi[1], Jayastu Senapati[1], Hussein Abbas[1], Sherry Pierce[1], Sara Dellasala[1], Elias Jabbour[1], Hagop Kantarjian[1], Nicholas Short*[1]

[1]*University of Texas MD Anderson Cancer Center, Leukemia, Houston, United States of America;*

### Background

Despite improvements in outcomes following the introduction of tyrosine kinase inhibitors (TKIs) in chronic myeloid leukemia (CML), CML in lymphoid blast phase (CML-LBP) remains a therapeutic challenge. Current consensus guidelines recommend acute lymphoblastic leukemia (ALL)-type induction chemotherapy in combination with a TKI. However, real-world predictors of outcomes of patients (pts) with CML-LBP in the modern TKI era are not well-established.

### Aims

This is a retrospective study to evaluate the predictors of response and long-term outcomes of pts with newly diagnosed CML-LBP treated with TKI-based therapy.

### Methods

The outcomes of adult pts ≥18 years of age with newly diagnosed CML-LBP were analyzed. Only pts treated with a TKI-based combination therapy as first CML-LBP therapy were included.

**Copyright Information:** (Online) ISSN: 2572-9241
©2026 The Author(s). HemaSphere is published by John Wiley & Sons Ltd on behalf of European Hematology Association. This is an open access Abstract Book distributed under the Attribution-NonCommercial-NoDerivs (CC BY-NC-ND), which allows third parties to download the articles and share them with others as long as they credit the author and the Abstract Book, but they cannot change the content in any way or use them commercially.
**Abstract Book Citations:** Authors, Title, HemaSphere, 2026;10:(S1):pages. The abstract book DOIs can be found on the "Author Information" page.
**Disclaimer:** Articles published in the journal HemaSphere exclusively reflect the opinions of the authors. The authors are responsible for all content in their abstracts including accuracy of the facts, statements, citing resources, etc.

### Results

Between 2001 and 2025, we identified 80 pts with CML-LBP treated with TKI-based combination therapy at our institution. The median age at CML-LBP diagnosis was 53 years (range, 23-82 years). 22 pts (28%) had *de novo* CML-LBP and 58 (72%) were transformed from previously treated earlier phase CML. 53% of transformed pts had ≥1 *ABL1* KD mutation, including 6 (15%) with *T315I*, 4 (10%) with *F317L* and 3 (8%) with *E255K*. The TKI used for first CML-LBP therapy was imatinib in 21 pts (26%), a 2nd generation TKI in 30 (38%), ponatinib in 28 (35%) and asciminib in 1 (1%). Backbone therapy was intensive chemotherapy in 55 pts (69%), lower-intensity chemotherapy in 14 (18%), and a chemotherapy-free regimen in 11 (14%; blinatumomab [n=10], inotuzumab ozogamicin [n=1]).
The overall response rate (ORR: defined as CR+CRi+MLFS per ALL criteria) was 81%, including CR in 71%, CRi in 8%, and MLFS in 3%. 79% of pts achieved CCyR and 69% achieved MMR or better. ORR was significantly higher in pts who received a 2nd or later-generation TKI vs imatinib (92% and 52%, respectively; P<0.001). ORR rates were similar in pts with *de novo* vs transformed CML-LBP. Among the 65 responders, 27 (42%) were bridged to allogeneic stem cell transplant (ASCT).
The median follow-up was 116.6 months. The 3-year CIR, RFS and OS rates were 22%, 53% and 45%, respectively. The only variables that significantly impacted survival outcomes were age, TKI generation, *de novo* + early transformed (<1 year from CP) vs. late transformed (≥1 year from CP) and molecular response. Outcomes were superior in pts <60 years of age (3-year OS 56% vs 28% in those ≥60 years P=0.008), those who received a 2nd-generation TKI, ponatinib, or asciminib (3-year OS 52% vs 24% with imatinib; P=0.002), and *de novo* or transformed within 1 year of CP (3-year OS 55% vs. 34% for late transformed; P=0.01). 3-year OS rates for pts who achieved CMR/$MR^5$ vs $MR^{4.5}$ to MMR vs. less than MMR were 73%, 48% and 32%, respectively (P=0.01). Neither type of backbone therapy nor presence of *ABL1* KD mutation impacted survival.
In a landmark analysis, responding pts who underwent ASCT had similar outcomes to those who did not (3-year OS 60% vs. 59%, respectively; P=0.41), including when the analysis was restricted to pts with transformed CML-LBP (P=0.47).

### Summary/Conclusion

TKI-based combination therapy achieves high response rates in CML-LBP, with a 3-year OS of 45%. Younger age, use of 2nd or later generation TKI and deeper molecular response are associated with superior survival. Pts with newly diagnosed CML-LBP, including those who transformed after previously treated earlier phase CML, do not appear to benefit from routine ASCT.

**Copyright Information:** (Online) ISSN: 2572-9241
©2026 The Author(s). HemaSphere is published by John Wiley & Sons Ltd on behalf of European Hematology Association. This is an open access Abstract Book distributed under the Attribution-NonCommercial-NoDerivs (CC BY-NC-ND), which allows third parties to download the articles and share them with others as long as they credit the author and the Abstract Book, but they cannot change the content in any way or use them commercially.
**Abstract Book Citations:** Authors, Title, HemaSphere, 2026;10:(S1):pages. The abstract book DOIs can be found on the "Author Information" page.
**Disclaimer:** Articles published in the journal HemaSphere exclusively reflect the opinions of the authors. The authors are responsible for all content in their abstracts including accuracy of the facts, statements, citing resources, etc.

### (PB2988) PROSPECTIVE VALIDATION AND CLINICAL FOLLOW-UP OF DIGITAL PCR FOR BCR::ABL1 QUANTIFICATION IN CHRONIC MYELOID LEUKAEMIA IN CHRONIC PHASE (CML-CP)

**Topic:** 08. Chronic myeloid leukemia - Clinical

Trung Ngo*[1], Matthew Reed[2], Adrian Choy[1], Baker Robert[2], Shaila Chinchole[2], Claire Nicholas[1], Chiara Bond[1], Jonathan Lambert[1], Donal McLornan[1], Rajeev Gupta[1, 2, 3], Samah Alimam[1]

*[1]University College London Hospitals NHS Trust, Haematology, London, United Kingdom;[2]Health Services Laboratories, London, United Kingdom; [3]UCL Cancer Institute, London, United Kingdom;*

### Background

*BCR::ABL1* is a molecular marker that tracks the measurable residual disease (MRD) status of chronic myeloid leukaemia (CML). Traditionally, MRD has been quantified using international standard real-time quantitative PCR (qPCR) assays. Digital PCR (dPCR) is a novel technology that partitions samples for endpoint amplification. It has advantages in MRD monitoring by directly counting transcripts to improve reproducibility, eliminate variability and achieve deeper levels of MRD sensitivity. However, it remains unclear whether these technical advantages translate into meaningful improvements in clinical outcomes for patients undergoing CML monitoring.

### Aims

To validate dPCR for *BCR::ABL1* quantification in CML-CP by comparing with qPCR, and to correlate discrepant results with 24 months of clinical follow-up data.

### Methods

Paired qPCR and dPCR analyses were prospectively performed on consecutive peripheral blood *BCR::ABL1* samples from patients with CML-CP between October 2022 to January 2024. *ABL1* was used as the housekeeping gene in both assays. Quantitative agreement between methods was assessed using Bland-Altman analysis and Spearman correlation. Patients were followed for 24 months to determine whether the increased sensitivity of dPCR correlated with differences in treatment outcomes.

### Results

131 peripheral blood samples from 78 patients were available for paired *BCR::ABL1* quantification by qPCR and dPCR. 78% of patients had a single sample, 4% had two samples and 18% had three or more samples analysed. Among patients with three or more samples, Fisher's exact test demonstrated that dPCR exhibited fewer transcript level oscillations than qPCR in those patients responding to tyrosine kinase inhibitors (TKI). However, performance was comparable between the two platforms in patients who lost response to TKI (p=0.324) or had persistently low-level disease (p=0.468).

Bland-Altman analysis demonstrated excellent correlation between qPCR and dPCR across all 131 samples, especially at *BCR::ABL1* levels below 10% (Figure 1). This concordance was further supported by Spearman analysis (r = 0.872, p<0.0001). Of note, dPCR demonstrated greater sensitivity than qPCR; among 32 patients with qPCR results below the limit of quantification, 69% of samples had detectable low-level positivity by dPCR assay. Clinical follow-up showed that 95% and 86% of patients with negative qPCR but positive dPCR maintained at least a major molecular response after 12 and 24 months, respectively. All four patients who were in treatment-free remission (TFR) at the study entry, remained in TFR at 24 months. Four further patients successfully attempted TFR during the follow-up period. Only one patient with discrepant results experienced imminent disease progression, which was detected by qPCR three months after initial dPCR positivity.

**Copyright Information:** (Online) ISSN: 2572-9241
©2026 The Author(s). HemaSphere is published by John Wiley & Sons Ltd on behalf of European Hematology Association. This is an open access Abstract Book distributed under the Attribution-NonCommercial-NoDerivs (CC BY-NC-ND), which allows third parties to download the articles and share them with others as long as they credit the author and the Abstract Book, but they cannot change the content in any way or use them commercially.
**Abstract Book Citations:** Authors, Title, HemaSphere, 2026;10:(S1):pages. The abstract book DOIs can be found on the "Author Information" page.
**Disclaimer:** Articles published in the journal HemaSphere exclusively reflect the opinions of the authors. The authors are responsible for all content in their abstracts including accuracy of the facts, statements, citing resources, etc.

**Summary/Conclusion**

This validation study has shown that digital PCR correlates well with real-time quantitative PCR for *BCR::ABL1* quantification and appears to exhibit advantages in serial monitoring with fewer transcript level oscillations. Although dPCR provides greater sensitivity than qPCR, this does not translate into differences in clinical outcomes over 24 months of follow-up. Further, real world studies are required to determine whether enhanced molecular detection by dPCR influences clinical decision making, particularly with TKI switching and attempts at TFR.

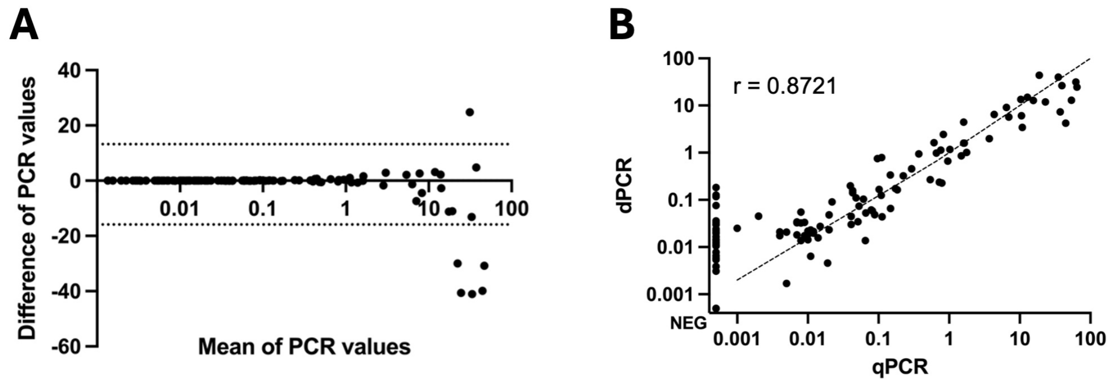

**Figure 1 (A)** Bland-Altman plot of difference in levels between quantitative PCR (qPCR) and digital PCR (dPCR). (**B**) Spearman correlation analysis between qPCR and dPCR.

**Copyright Information:** (Online) ISSN: 2572-9241
©2026 The Author(s). HemaSphere is published by John Wiley & Sons Ltd on behalf of European Hematology Association. This is an open access Abstract Book distributed under the Attribution-NonCommercial-NoDerivs (CC BY-NC-ND), which allows third parties to download the articles and share them with others as long as they credit the author and the Abstract Book, but they cannot change the content in any way or use them commercially.
**Abstract Book Citations:** Authors, Title, HemaSphere, 2026;10:(S1):pages. The abstract book DOIs can be found on the "Author Information" page.
**Disclaimer:** Articles published in the journal HemaSphere exclusively reflect the opinions of the authors. The authors are responsible for all content in their abstracts including accuracy of the facts, statements, citing resources, etc.

### (PB2990) LONG-TERM OUTCOMES OF PONATINIB TREATMENT IN CHRONIC PHASE CHRONIC MYELOID LEUKEMIA WITH OR WITHOUT THE T315 MUTATION

**Topic:** 08. Chronic myeloid leukemia - Clinical

Olga Kulemina[1], Ekaterina Chelysheva[2], Sergey Voloshin[3], Anna Petrova[2], Irina Martynkevich[4], Elizaveta Efremova[4], Oleg Shukhov[2], Vasiliy Shuvaev[5, 6], Anna Turkina[2, 7], Elza Lomaia*[1]

*[1]Almazov National medical research centre, St. Petersburg, Russia;[2]National Medical Research Centre for Hematology, Moscow, Russia; [3]Leningrad Regional Clinical Hospital, St. Petersburg, Russia;[4]Russian Research Institute of Hematology and Transfusiology, Federal Medical and Biological Agency, St. Petersburg, Russia; [5]Tsyb Medical Radiological Research Center – branch of the National Medical Research Radiological Centre, Obninsk, Russia; [6]Russian Medical Academy of Continuing Professional Education, Moscow, Russia; [7]I.M. Sechenov First Moscow State Medical University, Moscow, Russia;*

### Background

Resistance to tyrosine kinase inhibitor (TKI) therapy is observed in 50% of patients already in 2L therapy for chronic-phase (CP) chronic myeloid leukemia (CML). BCR::ABL1 point mutations are a leading cause of resistance, particularly in the presence of the T315I mutation. Ponatinib (PONA) demonstrated high efficacy in the OPTIC study in patients with TKI resistance, including those with the T315I mutation.This study conducted a subanalysis of a cohort of patients from the OPTIC study enrolled at three Russian centers.

### Aims

Analysis of long-term results of PONA in $1^{st}$ CP CML without any mutations (NM) or with the T315I mutation.

### Methods

A retrospective study included 85 patients who received PONA in ≥3L of therapy in the NM group or in any line in the presence of T315I. The T315I group included 31 patients (36.5%), including 3/31 (9.6%) with compound mutations (T315I/G250E, T315I/E499E, T315I/M244V). The NM group included 40 patients (47.1%). Patients with mutations other than T315I (n=11 (12.9%), as well as patients with unknown mutation status (n=3 (3.5%) were excluded from the analysis. The NM and T315I mutation groups were comparable in median age, CML duration at the time of initiation of PONA and number of previous TKIs. The probability of achieving a molecular response 2 (MR2) and major molecular response (MMR) was estimated by the cumulative incidence function and the Gray's test. Overall (OS), progression-free (PFS), and event-free (EFS) survival rates were calculated using the Kaplan-Meier method, and comparative analyses were performed using the log-rank test.

**Copyright Information:** (Online) ISSN: 2572-9241
©2026 The Author(s). HemaSphere is published by John Wiley & Sons Ltd on behalf of European Hematology Association. This is an open access Abstract Book distributed under the Attribution-NonCommercial-NoDerivs (CC BY-NC-ND), which allows third parties to download the articles and share them with others as long as they credit the author and the Abstract Book, but they cannot change the content in any way or use them commercially.
**Abstract Book Citations:** Authors, Title, HemaSphere, 2026;10:(S1):pages. The abstract book DOIs can be found on the "Author Information" page.
**Disclaimer:** Articles published in the journal HemaSphere exclusively reflect the opinions of the authors. The authors are responsible for all content in their abstracts including accuracy of the facts, statements, citing resources, etc.

### Results

The median duration of PONA for T315I was to 24.2 (1.7-84.3) months, while 46.0 (0.5-88.5) months for NM. There were no significant differences in the probability of achieving molecular responses between the T315I and NM groups: 44.6% versus 53.3% for MR2 at 12 months; 44.6% versus 75.1% for MR2 at 24 months, and the 24.2% versus 25.0% for MMR at 12 months, 50.0 and 36.7% for MMR at 24 months respectively. The efficacy of the 45 mg dose was assessed separately for the NM and T315 groups: the cumulative incidence of MR2 by 12 months in both groups was comparable - 59% versus 70%, respectively.
5 – year OS, PFS, and EFS for patients with T315I and NM were 70,5% [95% CI 43.9-79.8%] versus 97.4% [95% CI 82.7-99.6%] (p=0.0032); 71.3% [95% CI 50.4-84.6%] versus 94.4% [95% CI 79.4-98.6%] (p=0.0106); 22.6% [95% CI 10.0-28.3%] versus 45.0% [95% CI 29.3-59.5%] (p=0.022), respectively (Fig.1). Median time of follow-up for NM was 70.2 months (1.4-88.5 months) and for T315I was 67.7 months (1.7 – 87.4 months).
In the T315I group, 10 patients (32%) died, 5 of whom (50%) died from CML-related causes; disease progression was observed in 7 patients (22.6%). Among T315I patients, 5 (16%) patients continue treatment with a median duration of 71.9 (61.6-84.3) months. In the NM group, 2 (5%) patients died, both from causes unrelated to CML; disease progression was observed in 1 patient (2.5%). A total of 17 (42.5%) NM patients continue therapy with PONA with a median duration of 77.4 (60.7-88.5) months.

### Summary/Conclusion

A cohort of patients with CP CML without any mutations and carrying the T315I mutation who received PONA therapy was analyzed. Despite comparable molecular response rates in patients in both groups, the presence of the T315I mutation seemed be an unfavorable factor in assessing long-term survival outcomes.

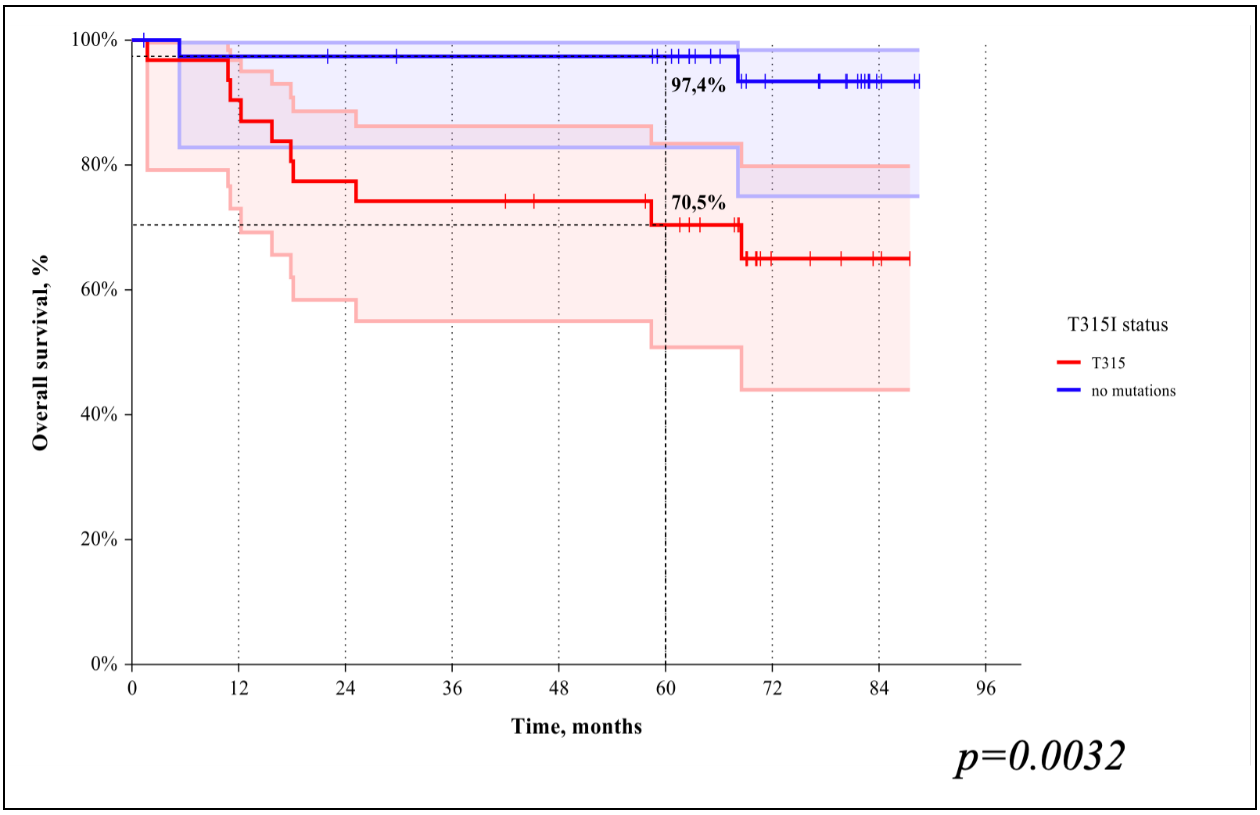

Fig. 1. Overall survival for unmutated (NM) and T315I-mutated patients in CP CML

**Copyright Information:** (Online) ISSN: 2572-9241
©2026 The Author(s). HemaSphere is published by John Wiley & Sons Ltd on behalf of European Hematology Association. This is an open access Abstract Book distributed under the Attribution-NonCommercial-NoDerivs (CC BY-NC-ND), which allows third parties to download the articles and share them with others as long as they credit the author and the Abstract Book, but they cannot change the content in any way or use them commercially.
**Abstract Book Citations:** Authors, Title, HemaSphere, 2026;10:(S1):pages. The abstract book DOIs can be found on the "Author Information" page.
**Disclaimer:** Articles published in the journal HemaSphere exclusively reflect the opinions of the authors. The authors are responsible for all content in their abstracts including accuracy of the facts, statements, citing resources, etc.

### (PB2991) XS003 ELIMINATES NILOTINIB FOOD INTERACTIONS ENABLING OPTIMIZED DOSE REDUCTIONS, RESULTING IN MITIGATED QT-PROLONGATION RISK

**Topic:** 08. Chronic myeloid leukemia - Clinical

Börje Darpö[1], Claes-Henrik Andersson[2], Mikael von Euler[2], Charlotta Liljebris[2], Magnus Brisander[2], Per Andersson[2], Hans Lennernäs[3], Leif Stenke*[4], Jorge Cortes[5]

*[1]Clario, Stockholm, Sweden; [2]Xspray, Solna, Sweden; [3]Uppsala University, Uppsala, Sweden; [4]Karolinska University Hospital and Karolinska Institutet, Solna, Sweden; [5]University of Alabama at Birmingham, Birmingham, United States of America;*

### Background

Crystalline nilotinib is an established therapy for chronic myeloid leukemia (CML). Nilotinib can however cause concentration-dependent QT-prolongation, which may trigger ventricular arrhythmias and sudden death. The effect is directly associated with nilotinib serum exposure, estimated at 9ms per 1000ng/mL [1]. Crystalline nilotinib exhibits weak dose-exposure linearity; doubling the dose from 200mg to 400mg increases systemic exposure by ~30%. Consequently, dose reductions to manage toxicity yield disproportionately small reductions in exposure. Furthermore, $C_{max}$ increases >2-fold following a high-fat meal, necessitating strict fasting to prevent life-threatening cardiac events. XS003 is a novel amorphous solid dispersion nilotinib formulation that is bioequivalent to crystalline nilotinib at 52% lower doses.

### Aims

Demonstrate that XS003 mitigates the risk of concentration-dependent QTc-prolongation by eliminating food-induced overexposure and providing improved dose-linearity.

### Methods

The food effect was evaluated in 48 healthy subjects in a randomized, single-dose, crossover study receiving XS003 (192mg nilotinib) under fasted and high-fat fed conditions. Dose-linearity was assessed in an open-label, 4-period study of 128 subjects comparing XS003 (96mg and 192mg) to crystalline nilotinib (200mg and 400mg). Subjects were monitored for AEs and safety parameters. Steady-state were predicted using population PK (PopPK) modeling and QTc-time impact was estimated based on plasma exposure at steady state and the known 9ms/1000ng/mL relationship [1].

**Copyright Information:** (Online) ISSN: 2572-9241
©2026 The Author(s). HemaSphere is published by John Wiley & Sons Ltd on behalf of European Hematology Association. This is an open access Abstract Book distributed under the Attribution-NonCommercial-NoDerivs (CC BY-NC-ND), which allows third parties to download the articles and share them with others as long as they credit the author and the Abstract Book, but they cannot change the content in any way or use them commercially.
**Abstract Book Citations:** Authors, Title, HemaSphere, 2026;10:(S1):pages. The abstract book DOIs can be found on the "Author Information" page.
**Disclaimer:** Articles published in the journal HemaSphere exclusively reflect the opinions of the authors. The authors are responsible for all content in their abstracts including accuracy of the facts, statements, citing resources, etc.

### Results

XS003 demonstrated improved dose-linearity: doubling the dose (96mg to 192mg) increased systemic exposure by ~70%, compared to only ~30% for crystalline nilotinib (200mg to 400mg). $C_{max}$ (least square geometric mean) in the fed and fasted states were 880ng/mL and 1053ng/mL (90%CI: 73.0–95.7), a decrease of 16% in the fed state (Table). This demonstrates absence of a clinically relevant food effect. Variability (CV) ranged from 40–46% to 28–36% in fasted and fed conditions (Table).

At predicted steady-state and at fasted and fed conditions, XS003 (192mg) $C_{max}$ was 1960ng/mL and 1838ng/mL, respectively, as compared to 1746ng/mL and 2869ng/mL for crystalline nilotinib (400mg) (Figure). The mean max QTc-prolongation for crystalline nilotinib was estimated to increase from 16ms (fasted) to 26ms (fed) and remained unaffected with XS003 at 18ms (fasted) and 16ms (fed).

| Parameters | XS003 Fasted | | | XS003 Fed | | |
|---|---|---|---|---|---|---|
| | Mean±SD | CV% | n | Mean±SD | CV% | n |
| $C_{max}$ (ng/mL) | 1171±470 | 40 | 43 | 938±263 | 28 | 43 |
| $AUC_{0\text{-}inf}$ (h*ng/mL) | 19319±8809 | 46 | 43 | 24120±8788 | 36 | 43 |

*AUC: area under the curve; Mean: arithmetic mean; SD: standard deviation; n=number of observations*

### Summary/Conclusion

The impact of food on nilotinib exposure when administered as XS003 was not clinically significant. Consequently, and in contrast to crystalline nilotinib, the risk of QT-prolongation with XS003 remains similar when taken at fasted and fed conditions. The improved dose-proportionality of XS003 (~70% vs ~30% exposure scaling) means that dose reductions translate into proportional exposure decreases-a property largely absent with crystalline nilotinib. Combined with a 52% lower administered dose, XS003 may provide hematologists with an optimized nilotinib dosing possibility, and a reduced risk of cardiovascular adverse events.

### Reference

1. CDER. Clinical Pharmacology and Biopharmaceutic review(s), IRT QT study review Section 4.2.7 (Tasigna).2012. 203585Orig1s000CrossR.pdf

**Copyright Information:** (Online) ISSN: 2572-9241
©2026 The Author(s). HemaSphere is published by John Wiley & Sons Ltd on behalf of European Hematology Association. This is an open access Abstract Book distributed under the Attribution-NonCommercial-NoDerivs (CC BY-NC-ND), which allows third parties to download the articles and share them with others as long as they credit the author and the Abstract Book, but they cannot change the content in any way or use them commercially.
**Abstract Book Citations:** Authors, Title, HemaSphere, 2026;10:(S1):pages. The abstract book DOIs can be found on the "Author Information" page.
**Disclaimer:** Articles published in the journal HemaSphere exclusively reflect the opinions of the authors. The authors are responsible for all content in their abstracts including accuracy of the facts, statements, citing resources, etc.

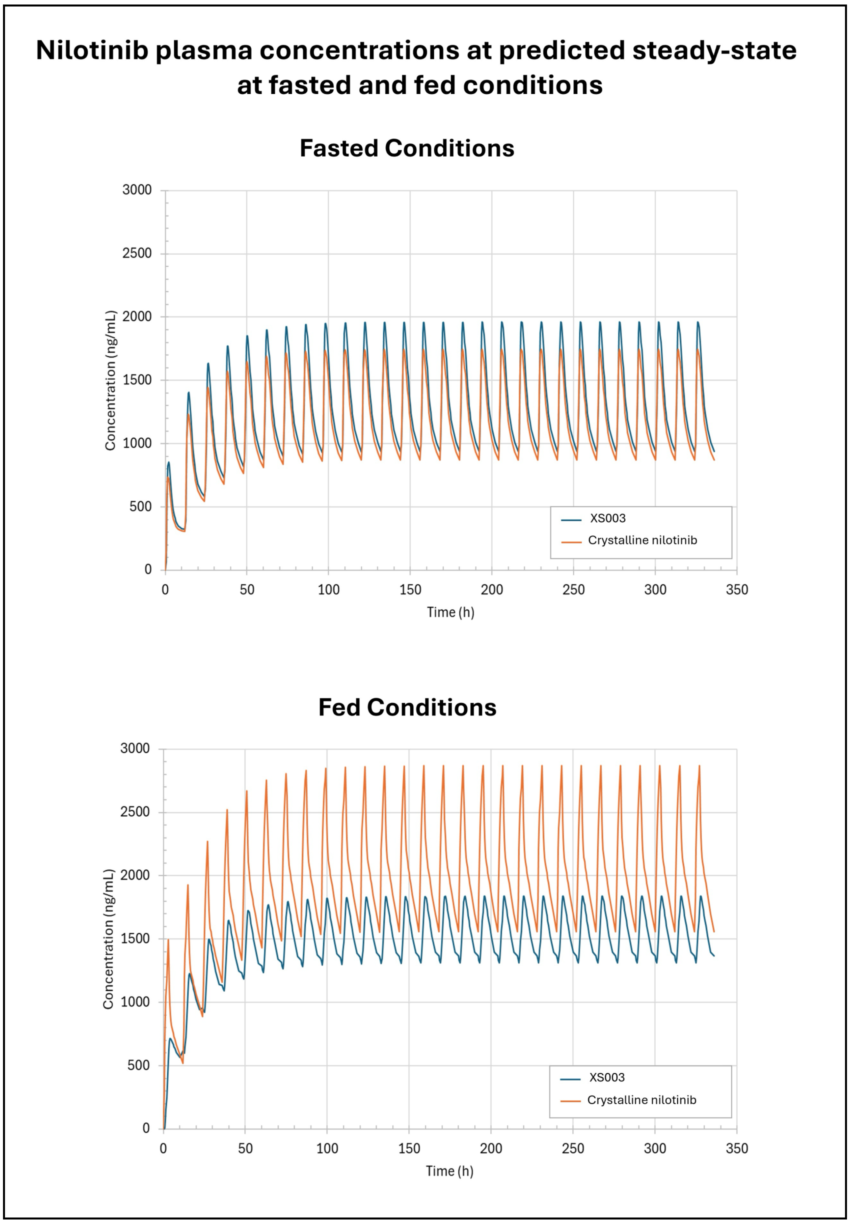

**Copyright Information:** (Online) ISSN: 2572-9241
©2026 The Author(s). HemaSphere is published by John Wiley & Sons Ltd on behalf of European Hematology Association. This is an open access Abstract Book distributed under the Attribution-NonCommercial-NoDerivs (CC BY-NC-ND), which allows third parties to download the articles and share them with others as long as they credit the author and the Abstract Book, but they cannot change the content in any way or use them commercially.
**Abstract Book Citations:** Authors, Title, HemaSphere, 2026;10:(S1):pages. The abstract book DOIs can be found on the "Author Information" page.
**Disclaimer:** Articles published in the journal HemaSphere exclusively reflect the opinions of the authors. The authors are responsible for all content in their abstracts including accuracy of the facts, statements, citing resources, etc.

### (PB2992) REAL-WORLD ANALYSIS OF FIRST-LINE TKI USE IN CML-CP: A 16-YEAR MULTICENTER EXPERIENCE

**Topic:** 08. Chronic myeloid leukemia - Clinical

Xiaoyan MA[1], Tian Zeng[1], Qingmei Han[1], Rumeng Li[2], Zhikang Zheng[3], Bingbing Wen[4], Xiaojing Bao[3], Jia Li[1], Jian Huang*[1]

*[1]The First Affiliated Hospital, Zhejiang University School of Medicine, Hangzhou, China;[2]The First Affiliated Hospital, Zhejiang University School of Medicine, Shenzhen, China; [3]The Fourth Affiliated Hospital, Zhejiang University School of Medicine, Hangzhou, China; [4]The second hospital of shenzhen, Shenzhen, China;*

### Background

The introduction of tyrosine kinase inhibitors (TKIs) has dramatically improved the prognosis of patients with chronic myeloid leukemia (CML). Pivotal head-to-head clinical trials have demonstrated the efficacy of next-generation TKIs in the first-line setting. However, in routine practice, poor tolerability, inadequate efficacy, access limitations, and comorbidities frequently prompt TKI switching—aspects underrepresented in controlled trial settings.Direct comparative evidence from real-world studies evaluating multiple TKIs is still lacking.

### Aims

To address this evidence gap, we conducted a large-scale, long-term multicenter real-world cohort study evaluating the effectiveness of all approved TKIs as first-line therapy for patients with CML.

### Methods

This retrospective study enrolled patients with CML-CP who were diagnosed and regularly followed at 48 centers across Zhejiang Province since January, 2010. Data were collected longitudinally from diagnosis until death or last follow-up, with the final data update completed in January, 2026. Baseline variables included demographic characteristics, cytogenetic findings, and hematologic parameters. Treatment response was assessed based on BCR-ABL transcript levels. Outcome measures included overall survival (OS), progression-free survival (PFS), and event-free survival (EFS) defined according to NCCN 2024 criteria, which specifically reflects treatment sustainability.

### Results

A total of 3210 patients were enrolled in this study, including 2652 treated with first-line imatinib, 81 with dasatinib, 144 with nilotinib, and 333 with flumatinib. For landmark efficacy assessments, the rates of achieving BCR-ABL ≤1% at 6 months were 67.9%�73.2%�84.1% and 83.6% for imatinib, dasatinib, nilotinib, and flumatinib, respectively ($p<0.001$), and the rates of achieving BCR-ABL ≤0.1% at 12 months were 66.2%�66.7%�84.3% and 67.4% ,respectively ($p=0.066$). The median treatment duration before first-line therapy switch was 16 months for imatinib, 21 months for dasatinib, 12 months for nilotinib, and 3 months for flumatinib. Regarding reasons for discontinuation, intolerance was the primary cause in 21.2% of patients on imatinib, 57.1% on dasatinib, 44.0% on nilotinib, and 35.0% on flumatinib. The 5-year PFS rates and HRs: imatinib 87.4% (reference), dasatinib 71.7% (HR 0.948 [0.505-1.781], $p=0.868$), nilotinib 97.6% (HR 0.273[0.102-0.731], $p=0.010$), and flumatinib 95.1% (HR 0.286 [0.135-0.605], $p=0.001$). The 5-year EFS rates and HRs: imatinib 57.1% (reference), dasatinib 41.4% (HR 1.426 [1.032-1.972], $p=0.032$), nilotinib 65.1% (HR 0.731 [0.522-1.024], $p=0.068$), and flumatinib 32.9% (HR 0.946[0.717-1.249], $p=0.698$).

**Copyright Information:** (Online) ISSN: 2572-9241
©2026 The Author(s). HemaSphere is published by John Wiley & Sons Ltd on behalf of European Hematology Association. This is an open access Abstract Book distributed under the Attribution-NonCommercial-NoDerivs (CC BY-NC-ND), which allows third parties to download the articles and share them with others as long as they credit the author and the Abstract Book, but they cannot change the content in any way or use them commercially.
**Abstract Book Citations:** Authors, Title, HemaSphere, 2026;10:(S1):pages. The abstract book DOIs can be found on the "Author Information" page.
**Disclaimer:** Articles published in the journal HemaSphere exclusively reflect the opinions of the authors. The authors are responsible for all content in their abstracts including accuracy of the facts, statements, citing resources, etc.

**Summary/Conclusion**

This real-world analysis demonstrates that flumatinib and nilotinib showed better performance in improving 5-year PFS. However, in real-world practice, treatment discontinuation limited the sustainability of second-generation TKI therapy, resulting in no significant improvement in 5-year EFS compared with imatinib. These findings highlight the dissociation between efficacy and treatment persistence with second-generation TKIs in routine clinical settings, underscoring the need for novel agents that integrate high efficacy with improved safety and tolerability profiles.

This research was supported by the National Natural Science Foundation of China (No. 82570191) and the Zhejiang Provincial Health Highlevel Innovative Talent Project (2022-2026).
Correspondence to: Prof Jian Huang, E-mail: househuang@zju.edu.cn.

**Copyright Information:** (Online) ISSN: 2572-9241
©2026 The Author(s). HemaSphere is published by John Wiley & Sons Ltd on behalf of European Hematology Association. This is an open access Abstract Book distributed under the Attribution-NonCommercial-NoDerivs (CC BY-NC-ND), which allows third parties to download the articles and share them with others as long as they credit the author and the Abstract Book, but they cannot change the content in any way or use them commercially.
**Abstract Book Citations:** Authors, Title, HemaSphere, 2026;10:(S1):pages. The abstract book DOIs can be found on the “Author Information” page.
**Disclaimer:** Articles published in the journal HemaSphere exclusively reflect the opinions of the authors. The authors are responsible for all content in their abstracts including accuracy of the facts, statements, citing resources, etc.

### (PB2993) VERY LONG-TERM RESULTS OF AUTOLOGOUS PERIPHERAL BLOOD STEM CELL TRANSPLANTATION FOLLOWED BY TYROSINE KINASE INHIBITORS (TKI) FOR CP-CML

**Topic:** 08. Chronic myeloid leukemia - Clinical

Mauricette Michallet*[1], Romain Buono[1], Eric Deconinck[2], Valerie Coiteux[3], Emilie Cayssials[4], Julie Hildt[1], Julien Bollard[1], Francoise Huguet[5], Antoine MacHet[6], Vincent Alcazer[7], François-Xavier Mahon[8], Gabriel Etienne[8], Franck Emmanuel Nicolini[1]

*[1]Centre Léon Bérard, Lyon, France; [2]CHU Besancon, Besoncon, France; [3]CHU Lille, Lille, France; [4]CHU Poitiers, Poitiers, France; [5]IUCT, Toulouse, France; [6]CHU Tours, Tours, France; [7]CHU Lyon, Lyon, France; [8]Institut Bergonié, Bordeaux, France;*

### Background

In the mid-nineties, autologous PBSCT with unselected cells harvested at diagnosis has been used as a debulking strategy for CP-CML pts, eventually preceded, and usually followed by interferon-a (IFN-a). Most pts were later exposed to TKI, however, whether this early strategy confers any long-term advantage compared to non-transplanted pts remains uncertain.

### Aims

Analysis of the very long-term outcome of a national cohort of newly diagnosed CP-CML pts who underwent autologous PBSCT as initial therapy between 1989 and 2004, ± TKI.

### Methods

Retrospective analysis of the Société Francophone de Greffe de Moelle et de Thérapie Cellulaire registry undergoing autologous PBSCT for CP-CML. All pts have consented in the capture of PBSCT data and follow-up.

**Copyright Information:** (Online) ISSN: 2572-9241
©2026 The Author(s). HemaSphere is published by John Wiley & Sons Ltd on behalf of European Hematology Association. This is an open access Abstract Book distributed under the Attribution-NonCommercial-NoDerivs (CC BY-NC-ND), which allows third parties to download the articles and share them with others as long as they credit the author and the Abstract Book, but they cannot change the content in any way or use them commercially.
**Abstract Book Citations:** Authors, Title, HemaSphere, 2026;10:(S1):pages. The abstract book DOIs can be found on the "Author Information" page.
**Disclaimer:** Articles published in the journal HemaSphere exclusively reflect the opinions of the authors. The authors are responsible for all content in their abstracts including accuracy of the facts, statements, citing resources, etc.

### Results

Seventy-two pts with confirmed Philadelphia positive CP-CML were enrolled. The median age at diagnosis was 46 (26-66) years, with 44 males (66%). Sokal scores were low for 24 (45%), intermediate for 19 (36%), and high for 10 (19%) [unavailable for 19 pts]. Sixty-seven pts (93%) received treatment prior to PBSCT: 56 (84%) IFN-a ± HU ± sc Cytarabine, 9 HU (13%) alone, 1 (1.5%) Cytarabine alone, 1 (1.5%) HU + 6-Mercaptopurine. Best responses observed prior to PBSCT were PCyR in 23 (52%), MCyR in 6 (14%), CCyR in 2 (4.5%), MMR in 1 (2.3%) and CHR or no response in 12 (27%) among 44 evaluable pts. Pts were transplanted at a median of 18 (2-100) months after diagnosis with unmanipulated autologous PBSC harvested at diagnosis, after a conditioning regimen consisting mainly in Busulfan/Melphalan. The median follow-up after PBSCT was 216 (35-395) months. Best responses after PBSCT and before TKI initiation were PCyR in 19 (31%), MCyR in 6 (9.7%), CCyR in 8 (13%) and CHR only or no response in 29 (47%) on 62 evaluable pts. Patients received their first TKI after a median of 39 (2-205) months after PBSCT post hematopoietic recovery according to their response status and availability of TKIs. Nine (13%) pts never received a TKI ever, while 31 (43%) patients received 1 TKI, 13 (18%) 2, 8 (11%) 3, 7 (9.7%) 4, 2 (2.8%) and 2, 5 and 6 lines of TKI after PBSCT. Imatinib was the first-line TKI of choice in 62 (98% of evaluable pts), and 21 of those (40%) obtained deep molecular response (DMR, i.e. ≥MR4). Thirty-one (43%) pts never switched to another TKI. Because of intolerance of insufficient response, 16 (50%) pts received Dasatinib and 16 (50%) received Nilotinib as 2nd-line TKI. At last follow-up, in living pts, overall best responses were CCyR in 2 (3.3%), MMR in 9 (15%), DMR in 36 (60%), and CHR only or no response in 13 (22%) among 60 evaluable pts. At last follow-up, 47 (65%) pts were alive and the median OS after PBSCT was 27.6 (22.4-NR) years. The median OS since diagnosis was 33.18 (22.69-NR) years (Figure 1).

In log-rank analyses, age at diagnosis and deeper response at last follow-up were associated with improved OS ($p=0.0021$ and $p<0.0001$ respectively). Multivariate analysis, restricted to age, Sokal score, free of immortal bias, showed that age at diagnosis was the only independent factor related to OS [HR=1.077/year, 95% CI: 1.03-1.128), $p=0.003$], each additional year increasing the risk of death by ~8%.

### Summary/Conclusion

Autologous PBSCT followed or not by TKI resulted in substantial OS rates, whether or not the same rates would have been obtained by TKI alone remains questionable and under ongoing analyses.

**Copyright Information:** (Online) ISSN: 2572-9241
©2026 The Author(s). HemaSphere is published by John Wiley & Sons Ltd on behalf of European Hematology Association. This is an open access Abstract Book distributed under the Attribution-NonCommercial-NoDerivs (CC BY-NC-ND), which allows third parties to download the articles and share them with others as long as they credit the author and the Abstract Book, but they cannot change the content in any way or use them commercially.
**Abstract Book Citations:** Authors, Title, HemaSphere, 2026;10:(S1):pages. The abstract book DOIs can be found on the “Author Information” page.
**Disclaimer:** Articles published in the journal HemaSphere exclusively reflect the opinions of the authors. The authors are responsible for all content in their abstracts including accuracy of the facts, statements, citing resources, etc.

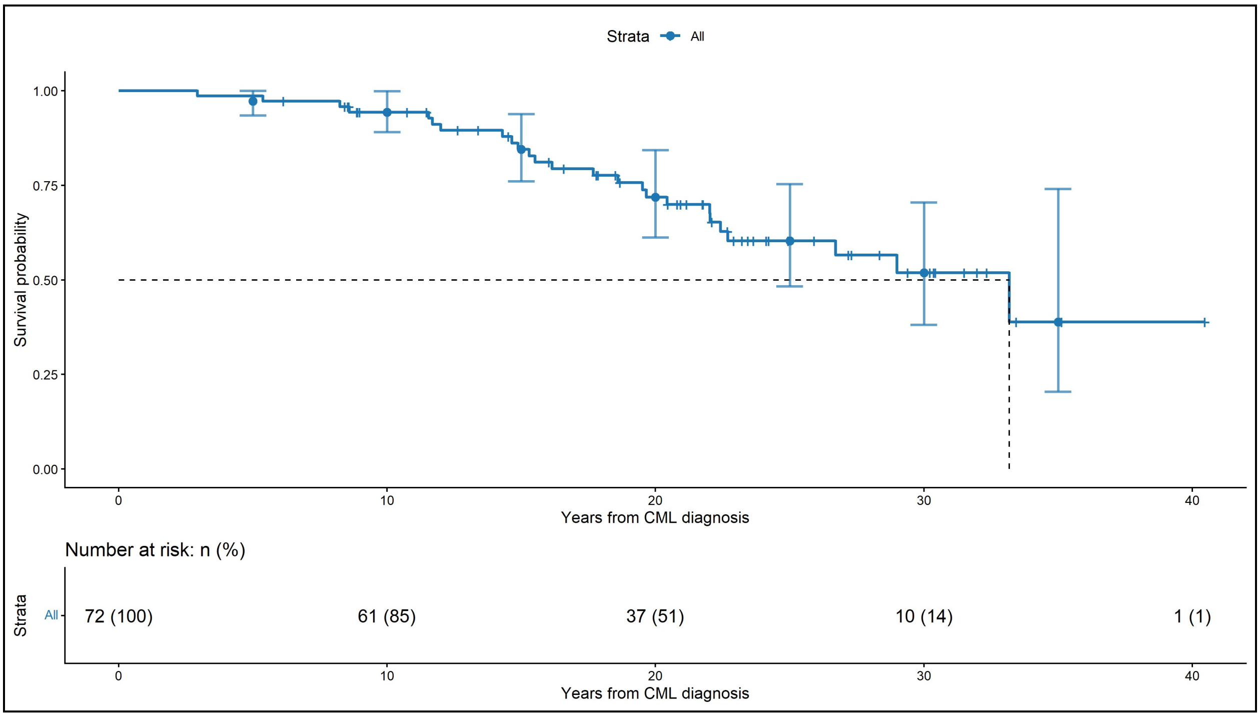

Figure 1. Overall survival of CP-CML pts since CML diagnosis.

**Copyright Information:** (Online) ISSN: 2572-9241
©2026 The Author(s). HemaSphere is published by John Wiley & Sons Ltd on behalf of European Hematology Association. This is an open access Abstract Book distributed under the Attribution-NonCommercial-NoDerivs (CC BY-NC-ND), which allows third parties to download the articles and share them with others as long as they credit the author and the Abstract Book, but they cannot change the content in any way or use them commercially.
**Abstract Book Citations:** Authors, Title, HemaSphere, 2026;10:(S1):pages. The abstract book DOIs can be found on the "Author Information" page.
**Disclaimer:** Articles published in the journal HemaSphere exclusively reflect the opinions of the authors. The authors are responsible for all content in their abstracts including accuracy of the facts, statements, citing resources, etc.

### (PB2994) INCIDENCE OF THE DEVELOPMENT OF PROTEINURIA FOLLOWING DASATINIB THERAPY IN CML PATIENTS AND ITS ASSOCIATION WITH THE OCCURRENCE OF PLEURAL EFFUSION

**Topic:** 08. Chronic myeloid leukemia - Clinical

Eleanore Musick*[1, 2, 3], Yael Morgenstern[1, 3], Ali Alahmari[4], Hassan, Sibai[3], Maria Agustina Perusini[1, 3], Dennis Kim[1, 3]

*[1]Princess Margaret Hospital, Malignant Haematology, Toronto, Canada;[2]University of Toronto, Temerty Faculty of Medicine, Toronto, Canada; [3]University Health Network, Haematology, Toronto, Canada;[4]King Faisal Specialist Hospital & Research Centre, Haematology, Riyadh, Saudi Arabia;*

### Background

In patients with chronic myeloid leukemia (CML), Dasatinib is one of the most widely used ATP-competitive tyrosine kinase inhibitors (TKIs). Proteinuria, reported sporadically, is one of the adverse effects affecting the management of patients on Dasatinib.

### Aims

We reviewed our database of 1100 CML patients to determine the incidence of and conditions associated with Dasatinib-induced proteinuria.

### Methods

Retrospective chart review was performed using a database of 1100 patients diagnosed with CML at Princess Margaret Cancer Centre. Patients receiving Dasatinib for CML management between 1995 and 2025 were included. Analysis was performed in R. Dasatinib exposure was defined as time from start of dasatinib to last follow up date or date dasatinib was stopped. Associations between proteinuria and pleural effusion were assessed using Fisher's exact test and logistic regression. Odds ratio and 95% confidence interval (CI) were reported; statistical significance was defined as $p < 0.05$.

**Copyright Information:** (Online) ISSN: 2572-9241
©2026 The Author(s). HemaSphere is published by John Wiley & Sons Ltd on behalf of European Hematology Association. This is an open access Abstract Book distributed under the Attribution-NonCommercial-NoDerivs (CC BY-NC-ND), which allows third parties to download the articles and share them with others as long as they credit the author and the Abstract Book, but they cannot change the content in any way or use them commercially.
**Abstract Book Citations:** Authors, Title, HemaSphere, 2026;10:(S1):pages. The abstract book DOIs can be found on the "Author Information" page.
**Disclaimer:** Articles published in the journal HemaSphere exclusively reflect the opinions of the authors. The authors are responsible for all content in their abstracts including accuracy of the facts, statements, citing resources, etc.

**Results**

We identified a total of 165 patients who received dasatinib as first line (n=79, 48%), 2nd line (n=57, 35%) or beyond (n=10, 6%). The remaining 11% of patients were placed on Dasatinib following transplantation or were otherwise lost to follow up. Patient characteristics were: male, 89 pts (54%), median age at diagnosis: 44 years, stage at diagnosis: chronic vs accelerated vs blastic phase, 93%:5%:2%.
With a median follow-up duration of 14 years after starting dasatinib, during 624.44 person-years dasatinib exposure, 7 patients were found to have proteinuria, confirmed to be associated with dasatinib after excluding secondary causes such as hypertension or diabetic nephropathy. This resulted in 11.2 events of Dasatinib-induced proteinuria per 1000 person-years. Proteinuria was measured with a median of 2g/L and range (1-3g/L). Proteinuria was detected at a median of 3 years (48 mo) following start of Dasatinib.
The characteristics of the 7 patients who developed proteinuria were: 5/7 (71%) male with a median age of 58 years old. Dasatinib was given as 1st line (n=3, 43%), 2nd line (n=2, 29%) or 3rd line (n=2, 29%). This occurred independently of dose. At the time proteinuria was confirmed, all cases had achieved at least MR4 response with dasatinib. All cases of proteinuria resolved after discontinuation/switch to other TKIs.
In the entire cohort, pleural effusion was detected in 31 pts (19%). 4/7 (57%) of patients with proteinuria developed pleural effusions during their Dasatinib therapy. In a Fisher's exact test, pleural effusion was shown to occur more frequently among patients with proteinuria, but this did not reach statistical significance (p = 0.051.) The estimated OR was 5.02 (95% CI 0.761-38.03). Proteinuria was associated with a statistically significant increased odds of pleural effusion: logistic regression showed an OR of 5.16 (95% CI 1.033-28.63).
Incidence of other toxicities related to dasatinib such as pleural effusion and pulmonary arterial hypertension (PAH) was calculated. The incidence of pleural effusion was 49.6 per 1000 person years (95% CI 0.033 to 0.070). The incidence of PAH was 16 per 1000 person years (95% CI 0.008 to 0.029).

**Summary/Conclusion**

The occurrence of dasatinib-induced proteinuria appears non-trivial with 11.4 events per 1000 person-years, which is similar to the incidence of PAH (16 per 1000 person years). This warrants more regular monitoring of urinalysis in patients with CML.

**Copyright Information:** (Online) ISSN: 2572-9241
©2026 The Author(s). HemaSphere is published by John Wiley & Sons Ltd on behalf of European Hematology Association. This is an open access Abstract Book distributed under the Attribution-NonCommercial-NoDerivs (CC BY-NC-ND), which allows third parties to download the articles and share them with others as long as they credit the author and the Abstract Book, but they cannot change the content in any way or use them commercially.
**Abstract Book Citations:** Authors, Title, HemaSphere, 2026;10:(S1):pages. The abstract book DOIs can be found on the "Author Information" page.
**Disclaimer:** Articles published in the journal HemaSphere exclusively reflect the opinions of the authors. The authors are responsible for all content in their abstracts including accuracy of the facts, statements, citing resources, etc.

### (PB2995) ELN RECOMMENDED RESPONSE MILESTONES PREDICT SURVIVAL AND DEEP MOLECULAR RESPONSE IN CHRONIC PHASE CHRONIC MYELOID LEUKEMIA RECEIVING SECOND-GENERATION TYROSINE KINASE INHIBITOR

**Topic:** 08. Chronic myeloid leukemia - Clinical

Guorong Chai*[1], Zongru Li[1], Qian Jiang[1]

[1]*Peking University People's Hospital, Beijing, China;*

### Background

The European LeukemiaNet (ELN) recommended response milestones have become a cornerstone for guiding therapeutic decisions in chronic phase chronic myeloid leukemia (CML-CP) patients receiving tyrosine kinase inhibitor (TKI) therapy. The recent studies have focused on the patients treated with imatinib. However, the significance of the ELN recommended response milestones in the context of (2G) TKI remains poorly defined.

### Aims

A retrospective study was designed to analyze the impact of the ELN recommended response milestones on survival outcomes and deep molecular responses (DMR) in patients with CML-CP receiving 2G TKI.

### Methods

Patients aged ≥18 years with CML-CP receiving nilotinib, dasatinib or flumatinib as the first-line therapy at Peking University People's Hospital between 2002 and 2025 were retrospectively reviewed. *BCR::ABL1* transcript levels at 3, 6 and 12 months following TKI initiation were analyzed by quantitative real-time polymerase chain reaction. Cox regression and Fine-Gray models were applied for multivariate analyses to identify co-variates associated with transformation-free survival (TFS), CML-related overall survival (CML-OS) and DMR.

### Results

636 patients receiving 2G-TKI were included in the study, including nilotinib (n = 369, 58%), dasatinib (n = 70, 11%), and flumatinib (n = 197, 31%). The median age was 37 years (interquartile range [IQR], 29-47 years). The median follow-up period was 32 months (IQR, 17-59 months). The 5-year TFS and CML related-OS was 92% (95% confidence interval [CI], 91-93%) and 96% (95% CI, 95-97%), respectively. The 5-year cumulative incidence of DMR was 51% (95% CI, 48-54%). TFS, CML related-OS and cumulative incidence of DMR differed significantly between subgroups stratified by ELN-recommended response milestones (**Table 1**). In multivariate analysis, higher *BCR::ABL1* per the international scales (IS) at 3 months (ref: >1–10%; >10%, HR = 2.2 [1.0, 4.8], *P*=0.04; ≤1%, 0.3 [0.1, 0.8], *P*=0.02), at 6 months (ref: ≤1%; >1%, 6.6 [2.6, 16.8], *P*<0.001), and at 12 months (ref: ≤1%; >1%, 9.1 [1.8, 45.7], *P*=0.007) were significantly associated with lower TFS; higher *BCR::ABL1*$^{IS}$ at 3 months (ref: >1–10%; >10%, HR = 3.8 [0.8, 18.8], *P*=0.10; ≤1%, 0.5 [0.1, 3.4], *P*=0.46; overall *P*=0.025) were significantly associated with lower CML-related OS. Higher *BCR::ABL1*$^{IS}$ at 3 months (ref: >0.1–1%; >10%, HR = 0.3 [0.2, 0.6], *P* < 0.001; >1–10%, 0.5 [0.4, 0.8], *P* = 0.002; ≤0.1%, 2.0 (1.4, 2.8], *P* < 0.001), at 6 months (ref: >0.1–1%; >10%, 0.1 [0.02, 0.4], *P* = 0.001; >1–10%, 0.5 [0.3, 1.1], *P* =0.07; ≤0.1%, 3.0 [2.0, 4.5], *P* < 0.001), and at 12 months (ref: >0.1–1%; >1%, 0.3 (0.1, 1.4], *P* = 0.11; ≤0.1%, 2.6 [1.5, 4.6], *P* < 0.001) were significantly associated with lower cumulative incidences of DMR.

**Copyright Information:** (Online) ISSN: 2572-9241
©2026 The Author(s). HemaSphere is published by John Wiley & Sons Ltd on behalf of European Hematology Association. This is an open access Abstract Book distributed under the Attribution-NonCommercial-NoDerivs (CC BY-NC-ND), which allows third parties to download the articles and share them with others as long as they credit the author and the Abstract Book, but they cannot change the content in any way or use them commercially.
**Abstract Book Citations:** Authors, Title, HemaSphere, 2026;10:(S1):pages. The abstract book DOIs can be found on the "Author Information" page.
**Disclaimer:** Articles published in the journal HemaSphere exclusively reflect the opinions of the authors. The authors are responsible for all content in their abstracts including accuracy of the facts, statements, citing resources, etc.

**Summary/Conclusion**

For patients receiving 2G-TKI, the ELN 3-month treatment milestone is an robust predictor of CML-related OS, whereas the ELN milestones at 3, 6, and 12 months all serve as predictive factors for TFS and DMR.

Table 1 The impact of the milestone on survival outcomes and DMR

| ELN recommended response milestones | TFS | | CML-related OS | | DMR | |
|---|---|---|---|---|---|---|
| | 5-year rate,% | *P*-Value | 5-year rate,% | *P*-Value | 5-year rate,% | *P*-Value |
| 3 months | | <0.001 | | <0.001 | | <0.001 |
| ≤0.1 | 98 | | 100 | | 77 | |
| >0.1-1 | 97 | | 99 | | 58 | |
| >1-10 | 91 | | 98 | | 39 | |
| >10 | 78 | | 87 | | 20 | |
| 6 months | | <0.001 | | <0.001 | | <0.001 |
| ≤0.1 | 99 | | 100 | | 71 | |
| >0.1-1 | 99 | | 100 | | 47 | |
| >1-10 | 85 | | 92 | | 24 | |
| >10 | 74 | | 84 | | 0 | |
| 12 months | | <0.001 | | <0.001 | | <0.001 |
| ≤0.1 | 99 | | 99 | | 63 | |
| >0.1-1 | 99 | | 99 | | 25 | |
| >1-10 | 82 | | 100 | | 10 | |
| >10 | 79 | | 83 | | 0 | |

**Copyright Information:** (Online) ISSN: 2572-9241
©2026 The Author(s). HemaSphere is published by John Wiley & Sons Ltd on behalf of European Hematology Association. This is an open access Abstract Book distributed under the Attribution-NonCommercial-NoDerivs (CC BY-NC-ND), which allows third parties to download the articles and share them with others as long as they credit the author and the Abstract Book, but they cannot change the content in any way or use them commercially.
**Abstract Book Citations:** Authors, Title, HemaSphere, 2026;10:(S1):pages. The abstract book DOIs can be found on the "Author Information" page.
**Disclaimer:** Articles published in the journal HemaSphere exclusively reflect the opinions of the authors. The authors are responsible for all content in their abstracts including accuracy of the facts, statements, citing resources, etc.

### (PB2996) IMPACT OF BETA-THALASSEMIA TRAIT ON RISK STRATIFICATION AND OUTCOMES IN CHRONIC MYELOID LEUKEMIA.

**Topic:** 08. Chronic myeloid leukemia - Clinical

Chingiz Asadov[1], Aytan Shirinova*[1], Aypara Hasanova[1], Zohra Alimirzoyeva[1]

*[1]National Center of Hematology and Transfusiology, Baku, Azerbaijan;*

### Background

The coexistence of beta-thalassemia trait (BTT) and chronic myeloid leukemia (CML) is rare, and its influence on prognostic scores, tyrosine kinase inhibitor (TKI) response, and survival is unclear. Hematologic features of BTT (microcytosis, reactive thrombocytosis, baseline anemia) may distort CML risk assessment and tolerance to therapy.

### Aims

To evaluate (1) the prevalence of BTT among CML patients, (2) its impact on baseline hematologic parameters and risk scores, and (3) differences in TKI response, transfusion needs, progression, and survival between CML patients with and without BTT.

### Methods

This single-center retrospective cohort included all adults with CML diagnosed 2013-2024 at the National Hematology and Blood Transfusion Center, Azerbaijan (n=848). BTT was defined by $HbA_2$ ≥3.5% on electrophoresis/HPLC. For detailed comparison, 15 CML+BTT patients were matched 1:2 by age and sex to 30 CML patients without hemoglobinopathy. Baseline counts, spleen size, Sokal/Hasford/EUTOS scores, TKI use and modification, transfusion requirement, time to major molecular response (MMR), progression-free survival (PFS), and overall survival (OS) were analyzed using chi-square/Fisher tests, Mann-Whitney U tests, and Kaplan-Meier estimates.

### Results

BTT prevalence among CML patients was 1.77% vs ~4% in the general Azerbaijani population (OR 0.43; p=0.0005), suggesting a possible protective association. At diagnosis, BTT patients had lower MCV (63.6 vs 86.2 fL; p<0.0001) and higher platelets ($687\times10^9$/L vs $331\times10^9$/L; p=0.0003), with similar hemoglobin, WBC, and spleen size. Sokal low-risk classification was more frequent in BTT (46.7% vs 13.3%; p=0.041), with trends toward lower Hasford/EUTOS risk. Time to MMR was comparable (15.3 vs 16.3 months; p=0.291). Rates of TKI dose reduction (40.0% vs 50.0%; p=0.751) and TKI switching (46.7% vs 66.7%; p=0.218) did not differ significantly. Transfusions were more frequent in BTT (40.0% vs 13.3%; p=0.099). Progression to accelerated/blast phase occurred in 33.3% vs 50.0% (p=0.458). OS was significantly better in BTT (death 6.7% vs 46.7%; p=0.019), with Kaplan-Meier curves showing sustained survival >90% in BTT up to 240 months.

### Summary/Conclusion

Beta-thalassemia trait modifies baseline hematologic profile and shifts CML patients toward lower Sokal risk, potentially underestimating true disease risk. Despite higher transfusion needs, BTT does not impair TKI response and may be associated with improved long-term survival. In regions with prevalent hemoglobinopathies, CML risk scores should be interpreted cautiously, with closer anemia monitoring in BTT carriers. Prospective multicenter studies are warranted to validate a possible protective effect of BTT on CML development and outcomes and to refine prognostic tools for this subgroup.

**Copyright Information:** (Online) ISSN: 2572-9241
©2026 The Author(s). HemaSphere is published by John Wiley & Sons Ltd on behalf of European Hematology Association. This is an open access Abstract Book distributed under the Attribution-NonCommercial-NoDerivs (CC BY-NC-ND), which allows third parties to download the articles and share them with others as long as they credit the author and the Abstract Book, but they cannot change the content in any way or use them commercially.
**Abstract Book Citations:** Authors, Title, HemaSphere, 2026;10:(S1):pages. The abstract book DOIs can be found on the "Author Information" page.
**Disclaimer:** Articles published in the journal HemaSphere exclusively reflect the opinions of the authors. The authors are responsible for all content in their abstracts including accuracy of the facts, statements, citing resources, etc.

### (PB2997) NONLINEAR ASSOCIATION BETWEEN PRE-DISCONTINUATION DOSE INTENSITY AND TREATMENT-FREE REMISSION OUTCOMES IN CHRONIC MYELOID LEUKEMIA: A REAL-WORLD ANALYSIS

**Topic:** 08. Chronic myeloid leukemia - Clinical

Dae-Ho Choi*[1], Jun Ho Jang[1], Chul Won Jung[1]

*[1]Samsung Medical Center, Division of Hematology-Oncology, Department of Internal Medicine, Gangnam-gu, Seoul, South Korea;*

### Background

To investigate whether the dose intensity of TKI therapy during the 2-year window period before discontinuation is associated with TFR outcome, and to characterize the shape of this association using restricted cubic spline modeling.

### Aims

To investigate whether the dose intensity of TKI therapy during the 2-year window period before discontinuation is associated with TFR outcome, and to characterize the shape of this association using restricted cubic spline modeling.

### Methods

We retrospectively analyzed 123 chronic-phase CML patients who attempted TFR after achieving sustained DMR (MR4 or deeper for ≥2 years). DI was calculated as the ratio of actual to standard dose during a 2-year window before TFR. All documented dose reductions (n=88, 71.5%) were attributed to clinically significant intolerance. Restricted cubic splines (RCS, 4 knots) were used to model the nonlinear association between DI and the hazard of molecular relapse. Patients were categorized into Low (≤0.35), Intermediate (0.35–0.80), and High (≥0.80) DI groups based on RCS-derived inflection points.

### Results

Among 123 patients (imatinib 50, dasatinib 38, nilotinib 35), DI group distribution was Low (n=35), Intermediate (n=51), and High (n=37). During a median follow-up of 21.3 months, 43 patients (35.0%) experienced molecular relapse (Low 9 [25.7%], Intermediate 25 [49.0%], High 9 [24.3%]). RCS analysis confirmed a significant nonlinear relationship between DI and relapse hazard (nonlinearity LRT P=0.023). In univariable analysis, the Intermediate DI group had a significantly higher risk of TFR failure compared to the High DI group (HR 2.21, 95% CI 1.03–4.74, P=0.042), whereas the Low DI group did not differ from the High group (HR 1.29, 95% CI 0.51–3.26, P=0.591). The Low DI group was predominantly composed of dasatinib-treated patients (26/35, 74.3%). After adjustment for TKI type, the Intermediate DI group maintained a clinically meaningful elevated risk (aHR 2.07, 95% CI 0.96–4.47, P=0.064), and the nonlinear trend persisted (TKI-adjusted nonlinearity LRT P=0.023). This remained significant after further adjustment for Sokal risk (nonlinearity LRT P=0.033). In imatinib-treated patients (n=50, events=23), the nonlinear association was similarly observed (nonlinearity LRT P=0.024). The 4-year molecular relapse-free survival rates were 73.2% (95% CI 59.5–90.1) for Low, 46.4% (95% CI 33.3–64.5) for Intermediate, and 73.9% (95% CI 60.5–90.1) for High DI groups (log-rank P=0.08).

**Copyright Information:** (Online) ISSN: 2572-9241
©2026 The Author(s). HemaSphere is published by John Wiley & Sons Ltd on behalf of European Hematology Association. This is an open access Abstract Book distributed under the Attribution-NonCommercial-NoDerivs (CC BY-NC-ND), which allows third parties to download the articles and share them with others as long as they credit the author and the Abstract Book, but they cannot change the content in any way or use them commercially.
**Abstract Book Citations:** Authors, Title, HemaSphere, 2026;10:(S1):pages. The abstract book DOIs can be found on the "Author Information" page.
**Disclaimer:** Articles published in the journal HemaSphere exclusively reflect the opinions of the authors. The authors are responsible for all content in their abstracts including accuracy of the facts, statements, citing resources, etc.

**Summary/Conclusion**

Pre-discontinuation DI demonstrates a significant nonlinear, inverted U-shaped association with TFR outcome. Patients with intermediate DI (0.35–0.80) had the highest relapse risk, while both low and high DI groups showed favorable TFR rates. The favorable outcome in the Low DI group should be interpreted cautiously given the predominance of dasatinib-treated patients. These findings suggest that intermediate DI—reflecting ongoing dose instability rather than dose reduction per se—may serve as a clinically relevant marker of TFR risk.

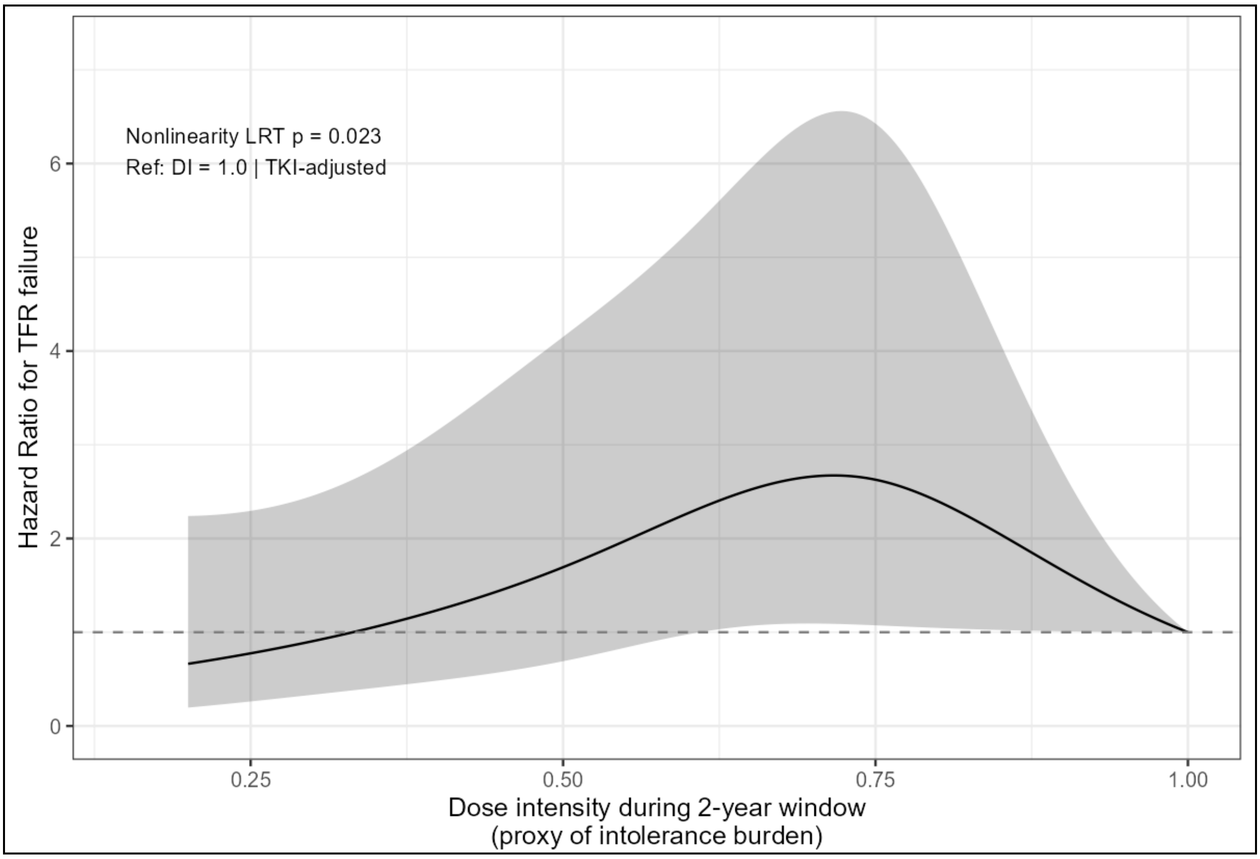

Restricted cubic spline analysis showing the nonlinear association between pre-discontinuation dose intensity and TFR failure hazard ratio, adjusted for TKI type (reference: DI=1.0, imatinib). Shaded area represents 95% confidence interval. Nonlinearity LRT P=0.023.

**Copyright Information:** (Online) ISSN: 2572-9241
©2026 The Author(s). HemaSphere is published by John Wiley & Sons Ltd on behalf of European Hematology Association. This is an open access Abstract Book distributed under the Attribution-NonCommercial-NoDerivs (CC BY-NC-ND), which allows third parties to download the articles and share them with others as long as they credit the author and the Abstract Book, but they cannot change the content in any way or use them commercially.
**Abstract Book Citations:** Authors, Title, HemaSphere, 2026;10:(S1):pages. The abstract book DOIs can be found on the "Author Information" page.
**Disclaimer:** Articles published in the journal HemaSphere exclusively reflect the opinions of the authors. The authors are responsible for all content in their abstracts including accuracy of the facts, statements, citing resources, etc.

### (PB2998) CLINICAL CHARACTERISTICS AND OUTCOMES OF PATIENTS WITH CHRONIC MYELOID LEUKEMIA AND SOLID ORGAN TRANSPLANT: A 15-PATIENT CASE SERIES

**Topic:** 08. Chronic myeloid leukemia - Clinical

Mohammed Al Kurnas*[1], Muluken Megiso[2], Ayalew Tefferi[3], Kebede Begna[3]

*[1]Mayo Clinic, Department of Internal Medicine, Rochester, United States of America;[2]Jefferson Einstein Philadelphia Hospital, Department of Internal Medicine, Philadelphia, United States of America; [3]Mayo Clinic, Department of Internal Medicine, Division of Hematology, Rochester, United States of America;*

### Background

Solid organ transplantation in patients with chronic myeloid leukemia (CML) has historically been approached with caution, as the presence of a hematologic malignancy has traditionally been viewed as a relative contraindication. Advances in CML management and improved long term disease control with tyrosine kinase inhibitors (TKIs) have renewed interest in understanding transplant outcomes in this unique patient population.

### Aims

To describe baseline characteristics and clinical outcomes of patients with CML and solid organ transplant.

### Methods

We conducted a retrospective descriptive case series of adults with CML who had a history of solid organ transplantation between 1985-2020. Extracted variables included age, sex, type of solid organ transplant, alive/deceased status, BCR-ABL transcript type, therapies used for CML, and remission status. Time intervals included the duration from CML diagnosis to transplant and from transplant to CML diagnosis where applicable. Descriptive statistics were summarized as median with interquartile range (Q1- Q3), or n (%). Survival status was assessed using the most recent follow-up or date of death.

### Results

Median age at diagnosis of CML was 54 years (45.5 – 65.0), and 9 cases were male (60%). Six (40%) received solid organ transplantation after diagnosis of CML. Twelve (80%) patients underwent renal transplantation, 1 (6.7%) received a liver transplant, and 2 (13.3%) underwent multiorgan transplantation, including one case of repeat heart transplantation with a renal graft and one case of combined liver and renal transplantation. Four (26.67%) patients were found to be deceased. Eleven (73.33%) patients had BCR/ABL transcript type performed of which 10 (91%) were found to be p210 and one (9%) was found to be p190. Of patients who received solid organ transplantation after CML diagnosis, 3 (50%) were on TKI therapy at the time of transplant, and 3 (50%) were treated in the pre-TKI era with bone marrow transplantation. Three (50%) achieve major molecular response (MMR) at the time of solid organ transplant, and three (50%) achieved cytogenetic complete response on bone marrow (CyCR) at time of solid organ transplant. Of patients who were diagnosed with CML after solid organ transplant, 9 (100%) were on TKI therapy. Six (66.7%) achieved MMR, 2 patients (22.2%) attained CyCR, and 1 patient (11.1%) failed to achieve either response and died within one year of diagnosis. Median time from CML diagnosis to transplant was 13.66 years (10.94 -16.92). Median time from transplant to CML diagnosis was 8.79 years (8.08 -11.90). Median survival from time of CML diagnosis to death or last follow up in all patients was 8.08 years (4.79–19.71). Median survival from time of CML diagnosis to death or last follow up in patients with CML followed by transplant was 24.92 years (16.33–29.42). Median survival from time of CML diagnosis to death or last follow up in patients with transplant followed by diagnosis of CML was 5.08 years (4.50–8.08).

**Copyright Information:** (Online) ISSN: 2572-9241
©2026 The Author(s). HemaSphere is published by John Wiley & Sons Ltd on behalf of European Hematology Association. This is an open access Abstract Book distributed under the Attribution-NonCommercial-NoDerivs (CC BY-NC-ND), which allows third parties to download the articles and share them with others as long as they credit the author and the Abstract Book, but they cannot change the content in any way or use them commercially.
**Abstract Book Citations:** Authors, Title, HemaSphere, 2026;10:(S1):pages. The abstract book DOIs can be found on the "Author Information" page.
**Disclaimer:** Articles published in the journal HemaSphere exclusively reflect the opinions of the authors. The authors are responsible for all content in their abstracts including accuracy of the facts, statements, citing resources, etc.

**Summary/Conclusion**

This case series adds supportive evidence that solid organ transplantation can be safely pursued in patients with chronic myeloid leukemia. Our findings strengthen the emerging trend that as outcomes in CML continue to improve with modern therapy, CML may no longer be viewed as a contraindication to transplantation.

**Copyright Information:** (Online) ISSN: 2572-9241
©2026 The Author(s). HemaSphere is published by John Wiley & Sons Ltd on behalf of European Hematology Association. This is an open access Abstract Book distributed under the Attribution-NonCommercial-NoDerivs (CC BY-NC-ND), which allows third parties to download the articles and share them with others as long as they credit the author and the Abstract Book, but they cannot change the content in any way or use them commercially.
**Abstract Book Citations:** Authors, Title, HemaSphere, 2026;10:(S1):pages. The abstract book DOIs can be found on the "Author Information" page.
**Disclaimer:** Articles published in the journal HemaSphere exclusively reflect the opinions of the authors. The authors are responsible for all content in their abstracts including accuracy of the facts, statements, citing resources, etc.

### (PB2999) EFFICACY AND SAFETY OF DIFFERENT NILOTINIB DOSES AS SECOND-LINE THERAPY IN PATIENTS WITH CML RECEIVED FIRST-LINE IMATINIB: A MULTICENTER RETROSPECTIVE STUDY

**Topic:** 08. Chronic myeloid leukemia - Clinical

Hongju Yan*[1], Yanli Zhang[2], Weiming Li[3], Yu Zhu[4], Weng Jianyu[5], Na Xu[6], Huanling Zhu[7], Qin Wen[8], Xi Zhang[9]

*[1]Xinqiao Hospital, Army Medical University, Chongqing, China;[2]Henan Cancer Hospital, Zhengzhou, China;[3]Union Hospital,Tongji Medical College,Huazhong University of Science and Technology, Wuhan, China; [4]Department of Hematology, Jiangsu Province Hospital, The First Affiliated Hospital of Nanjing Medical University, Jiangsu, China; [5]Southern Medical University, Guangzhou, China; [6]Nanfang Hospital, Southern Medical University, Guangzhou, China;[7]West China Hospital Of Sichuan University, sichuan, China; [8]Hematology Medical Center, Xinqiao Hospital, Army Medical University, chongqin, China; [9]Xinqiao Hospital of Army Medical University, Chongqing, China;*

### Background

Imatinib is the first-line standard therapy for chronic myeloid leukemia (CML). A proportion of patients need to switch to second-line nilotinib due to imatinib resistance, intolerance, or the pursuit of superior therapeutic efficacy. The currently recommended clinical dose of second-line nilotinib is 600-800 mg daily in two divided doses, yet there is no published evidence comparing the efficacy and safety between 300 mg twice daily (bid) and 400 mg bid nilotinib in this setting.

### Aims

To compare the 2-year efficacy and safety of 300 mg bid versus 400 mg bid nilotinib as second-line therapy in CML patients switched from first-line imatinib, and to provide evidence-based reference for clinical decision-making.

### Methods

This was a multicenter retrospective study. A total of 197 eligible CML patients from 7 medical centers in China, who were switched from first-line imatinib to second-line nilotinib (300 mg bid or 400 mg bid) before January 2023, were enrolled. Patients were divided into the 300 mg bid group (n=116) and the 400 mg bid group (n=81). Efficacy was assessed in accordance with the 2020 European LeukemiaNet (ELN) guidelines for CML. The primary endpoints were the best treatment response at 3 and 6 months, as well as the major molecular response (MMR) and deep molecular response (DMR) rates at 1 and 2 years after treatment switch. The secondary endpoint was the difference in the incidence of treatment-related adverse events between the two groups.

### Results

There were no statistically significant differences in baseline characteristics including age, gender, duration of prior imatinib therapy, and reasons for treatment switch between the two groups (all P>0.05), indicating good comparability. For efficacy, no statistically significant differences were observed between the two groups in the best response at 3 and 6 months, MMR and DMR rates at 1 and 2 years, regardless of whether patients switched due to imatinib resistance, intolerance, or pursuit of better efficacy (all P>0.05). For safety, adverse events including rash, bilirubin elevation, transaminase abnormality, and hematological toxicity occurred in both groups. The incidence of rash was significantly higher in the 400 mg bid group than in the 300 mg bid group (14.81% vs 4.31%, P=0.009). The incidences of other adverse events were slightly higher in the 400 mg bid group, with no statistically significant differences (all P>0.05).

**Copyright Information:** (Online) ISSN: 2572-9241
©2026 The Author(s). HemaSphere is published by John Wiley & Sons Ltd on behalf of European Hematology Association. This is an open access Abstract Book distributed under the Attribution-NonCommercial-NoDerivs (CC BY-NC-ND), which allows third parties to download the articles and share them with others as long as they credit the author and the Abstract Book, but they cannot change the content in any way or use them commercially.
**Abstract Book Citations:** Authors, Title, HemaSphere, 2026;10:(S1):pages. The abstract book DOIs can be found on the “Author Information” page.
**Disclaimer:** Articles published in the journal HemaSphere exclusively reflect the opinions of the authors. The authors are responsible for all content in their abstracts including accuracy of the facts, statements, citing resources, etc.

### Summary/Conclusion

For CML patients requiring a switch to second-line nilotinib after first-line imatinib therapy, the 400 mg bid regimen does not confer a significant efficacy advantage, but is associated with a higher incidence of adverse events, especially a significantly elevated risk of rash. The 300 mg bid nilotinib regimen is recommended as the first-line choice for this patient population in clinical practice.

Table 1 Baseline characteristics of the enrolled patients

| Characteristics | Nilotinib 300 mg bid (n=116) | Nilotinib 400 mg bid (n=81) | P value |
|---|---|---|---|
| Male/Female, n | 71/45 | 53/28 | 0.54 |
| Median age, years (range) | 37 (8-73) | 38 (14-74) | 0.98 |
| Median duration of prior imatinib therapy, months (range) | 12 (1-238) | 18 (1-182) | 0.10 |
| Prior TKI therapy ≤12 months, n | 58 | 31 | - |
| Prior TKI therapy >12 months, n | 58 | 50 | - |
| Reasons for treatment switch, n (%) | | | 0.38 |
| Imatinib resistance | 46 (39.66) | 42 (51.85) | - |
| Imatinib intolerance | 28 (24.14) | 9 (11.11) | - |
| Pursuit of better efficacy | 42 (36.21) | 30 (37.04) | - |
| Baseline BCR/ABL levels in patients with imatinib resistance, n (%) | | | - |
| BCR/ABL >10% | 28 (60.87) | 29 (69.05) | - |
| BCR/ABL >1% | 18 (39.13) | 13 (30.95) | - |
| Baseline BCR/ABL levels in patients with imatinib intolerance, n (%) | | | - |
| BCR/ABL >10% | 13 (46.43) | 4 (57.14) | - |
| BCR/ABL >1% | 3 (10.71) | - | - |
| BCR/ABL >0.1% | 3 (10.71) | - | - |
| MMR | 2 (7.14) | - | - |
| DMR | 7 (25.00) | 3 (42.86) | - |
| Baseline BCR/ABL levels in patients for better efficacy, n (%) | | | - |
| BCR/ABL >0.1% | 24 (57.14) | 26 (86.67) | - |
| MMR | 17 (40.48) | 4 (13.33) | - |
| DMR | 1 (2.38) | - | - |

Table 2 Efficacy outcomes in patients with imatinib resistance after switching to nilotinib

| Efficacy measures | 300 mg bid (n=46) | 400 mg bid (n=42) | P value |
|---|---|---|---|
| Response at 3 months | | | 0.10 |
| BCR/ABL >10%, n(%) | 6/41 (14.63) | 10/33 (30.30) | - |
| Best response (BCR/ABL ≤10%), n(%) | 35/41 (85.37) | 23/33 (69.70) | - |
| Response at 6 months | | | 0.34 |
| BCR/ABL >1%, n(%) | 6/39 (15.38) | 9/33 (26.47) | - |
| Best response (BCR/ABL ≤1%), n(%) | 33/39 (84.62) | 25/33 (73.53) | - |
| Response at 1 year | | | |
| MMR, n(%) | 28/44 (63.64) | 26/42 (61.90) | 0.86 |
| DMR, n(%) | 10/44 (22.73) | 14/42 (33.33) | 0.27 |
| Response at 2 years | | | |
| MMR, n(%) | 28/41 (68.29) | 26/38 (61.90) | 0.99 |
| DMR, n(%) | 16/41 (39.02) | 16/38 (38.10) | 0.78 |

Table 3 Efficacy outcomes in patients with imatinib intolerance after switching to nilotinib

| Efficacy measures | 300 mg bid (n=28) | 400 mg bid (n=9) | P value |
|---|---|---|---|
| Response at 3 months | | | 0.62 |
| BCR/ABL >10%, n(%) | 4/25 (16.00) | 1/7 (14.29) | - |
| Best response (BCR/ABL ≤10%), n(%) | 21/25 (84.00) | 6/7 (85.71) | - |
| Response at 6 months | | | 0.55 |
| BCR/ABL >1%, n(%) | 4/24 (16.67) | 0/9 (0.00) | - |
| Best response (BCR/ABL ≤1%), n(%) | 20/24 (83.33) | 9/9 (100.00) | - |
| Response at 1 year | | | |
| MMR, n(%) | 16/23 (69.57) | 6/9 (66.67) | 0.79 |
| DMR, n(%) | 10/23 (43.80) | 4/9 (44.44) | 0.72 |
| Response at 2 years | | | |
| MMR, n(%) | 20/26 (76.92) | 6/9 (66.67) | 0.86 |
| DMR, n(%) | 16/26 (61.54) | 5/9 (55.56) | 0.93 |

Table 4 Efficacy outcomes in patients switching for better therapeutic efficacy

| Efficacy measures | 300 mg bid (n=42) | 400 mg bid (n=30) | P value |
|---|---|---|---|
| Response at 1 year | | | |
| MMR, n(%) | 31/42 (73.81) | 19/28 (67.86) | 0.58 |
| DMR, n(%) | 21/42 (50.00) | 13/28 (44.83) | 0.76 |
| Response at 2 years | | | |
| MMR, n(%) | 28/36 (77.78) | 22/28 (78.57) | 0.93 |
| DMR, n(%) | 23/36 (63.89) | 16/28 (57.14) | 0.58 |

Table 5 Safety profile of the two treatment groups [n (%)]

| Adverse events | 300 mg bid (n=116) | 400 mg bid (n=81) | P value |
|---|---|---|---|
| Rash | 5 (4.31) | 12 (14.81) | 0.009 |
| Hyperbilirubinemia | 14 (12.07) | 9 (11.11) | 0.84 |
| Transaminase elevation | 13 (11.21) | 10 (12.25) | 0.81 |
| Hyperlipidemia | 4 (3.45) | 4 (4.94) | 0.87 |
| Hematological abnormalities | 11 (9.48) | 7 (8.64) | 0.84 |
| Renal dysfunction | 1 (0.86) | 1 (1.23) | 0.64 |
| Hypertension | 1 (0.86) | 3 (3.70) | 0.37 |
| Alopecia | 0 (0.00) | 2 (2.47) | 0.16 |
| Liver fibrosis | 0 (0.00) | 1 (1.23) | 0.41 |
| Arteriosclerosis | 0 (0.00) | 2 (2.47) | 0.16 |
| Hyperglycemia | 4 (3.45) | 3 (3.70) | 0.76 |
| Cerebral infarction | 5 (5.17) | 5 (7.41) | 0.76 |
| Osteoarthralgia | 2 (1.72) | 4 (4.94) | 0.38 |
| Thyroid dysfunction | 2 (1.72) | 2 (2.47) | 0.88 |

tables

**Copyright Information:** (Online) ISSN: 2572-9241
©2026 The Author(s). HemaSphere is published by John Wiley & Sons Ltd on behalf of European Hematology Association. This is an open access Abstract Book distributed under the Attribution-NonCommercial-NoDerivs (CC BY-NC-ND), which allows third parties to download the articles and share them with others as long as they credit the author and the Abstract Book, but they cannot change the content in any way or use them commercially.
**Abstract Book Citations:** Authors, Title, HemaSphere, 2026;10:(S1):pages. The abstract book DOIs can be found on the “Author Information” page.
**Disclaimer:** Articles published in the journal HemaSphere exclusively reflect the opinions of the authors. The authors are responsible for all content in their abstracts including accuracy of the facts, statements, citing resources, etc.

### (PB3000) ALLOGENEIC HEMATOPOIETIC STEM CELL TRANSPLANTATION IN PATIENTS UNDER 18 YEARS OF AGE WITH CHRONIC MYELOID LEUKEMIA; LONG-TERM OUTCOMES

**Topic:** 08. Chronic myeloid leukemia - Clinical

Fouzia Louar[1], Farih Mehdid[1], Baazizi Mounira[1], Nadia Rahmoune[1], Ait Ouali Dina[1], Sarah Zerkout[1], Sabrina Akhrouf[1], Hanane Bouarab[1], Hanane Moussaoui[1], Belkis Dekhili[1], Amira Fahem[1], Farida Harieche[1], Rose Marie Hamladji[1], Redhouane Ahmed Nacer[1], Malek Benakli*[1]

*[1]Hematology and Bone Marrow Transplantation department, Pierre and Marie Curie Center, Algiers, Algeria;*

### Background

The therapeutic paradigm of chronic myeloid leukemia (CML) has changed significantly following the introduction of tyrosine kinase inhibitors (TKIs). The indication for allogeneic hematopoietic stem cell transplantation (HSCT) is limited to patients (pts) in advanced phase (accelerated or blast phase) or those resistant/intolerant to TKIs (ELN 2013 to 2025). This single-center retrospective study reports the outcomes of a series of patients under 18 years of age who underwent this procedure before and after the era of tyrosine kinase inhibitors.

### Methods

From December 1998 to December 2021, 35 children with CML underwent allogeneic HSCT, including 34 (97%) HLA-identical (geno-identical) and one haplo-identical transplant. The median age was 13 years (5–18); M/F sex ratio was 0.75. Disease status at transplant: chronic phase: 26 pts (74.2%), accelerated phase: 6 pts (17%), blast crisis: 3 pts (8.5%). Only 4 pts (11.4%) received a transplant due to TKI resistance. All pts received chemotherapy-only conditioning regimens, consisting of Busulfan combined with Cyclophosphamide in 29 pts (82.8%), Etoposide (4 pts), Fludarabine (1 pt), and one haplo-identical transplant with TBF conditioning (1 pt). GVHD prophylaxis consisted of Cyclosporine-Methotrexate +/- Cellcept. Peripheral blood stem cell were used in 31 pts (88.5%) with a mean CD34+ cell count of 8.57 x $10^6$/kg (1.6–41.5); bone marrow in 3 pts with a mean nucleated cell count of 5.25 x $10^7$/kg (4–6.7); and a cord blood graft in 1 pt.

### Results

Aplasia was observed in all pts, with a mean duration of 13 days (4–26). The median time to engraftment was 20 days (11–30). Eleven pts (31.4%) developed acute GVHD, including 5 (14.2%) with grade III–IV, at a median of 28 days (16–42). Chronic GVHD was observed in 18 pts (58%), including 12 (38.7%) with extensive form, occurring after a median of 17 months (4–73). Relapse occurred in 9 pts (18.7%), 4 of whom are in molecular remission following treatment with TKIs +/- DLI. As of June 2025, after a median follow-up of 239 months (42–318), 21 pts (60%) are alive in CR and 14 pts (40%) have died, including 9 (25.7%) due to TRM (GVHD: 6, Infection: 2, transplant-associated microangiopathy: 1) and 5 (14.2%) due to relapse. Actuarial overall survival and event-free survival rates are 58.7% and 37%, respectively.

### Summary/Conclusion

For a long time, allogeneic HSCT was the only curative treatment for CML prior to the TKI era. Since then, the number of pts undergoing transplantation has significantly decreased. However, despite the advent of several generations of TKIs, it remains indicated for a minority of CML pts. The major challenge lies in reducing procedure-related morbidity and mortality, particularly GVHD.

**Copyright Information:** (Online) ISSN: 2572-9241
©2026 The Author(s). HemaSphere is published by John Wiley & Sons Ltd on behalf of European Hematology Association. This is an open access Abstract Book distributed under the Attribution-NonCommercial-NoDerivs (CC BY-NC-ND), which allows third parties to download the articles and share them with others as long as they credit the author and the Abstract Book, but they cannot change the content in any way or use them commercially.
**Abstract Book Citations:** Authors, Title, HemaSphere, 2026;10:(S1):pages. The abstract book DOIs can be found on the “Author Information” page.
**Disclaimer:** Articles published in the journal HemaSphere exclusively reflect the opinions of the authors. The authors are responsible for all content in their abstracts including accuracy of the facts, statements, citing resources, etc.

### (PB3001) FRONTLINE TREATMENT APPROACHES AND THEIR IMPACT ON THE OUTCOMES OF PATIENTS WITH CHRONIC PHASE CHRONIC MYELOID LEUKAEMIA WITH HYPERLEUKOCYTOSIS: A SINGLE CENTRE CHART REVIEW IN THE UNITED KINGDOM

**Topic:** 08. Chronic myeloid leukemia - Clinical

Adrian Choy*[1], Trung Ngo[1], Claire Nicholas[1], Chiara Bond[1], Chloe Jenkins[1], Jonathan Lambert[1], Donal McLornan[1], Samah Alimam[1]

*[1]University College London Hospitals NHS Foundation Trust, Haematology, London, United Kingdom;*

### Background

Hyperleukocytosis is an established adverse prognostic factor in acute leukaemia but its impact on outcomes in chronic phase chronic myeloid leukaemia (CP-CML) remains less well-defined. Widely used prognostic scores including Sokal and ELTS do not directly incorporate leukocyte count as a criteria for risk stratification. However, a number of recent studies have explored integrating leukocyte count into emerging prognostic models.

### Aims

We aimed to evaluate the impact of frontline tyrosine kinase inhibitor (TKI) selection choice on the clinical outcomes in patients with CP-CML presenting with hyperleukocytosis at University College London Hospitals (UCLH).

### Methods

From our local database, 57 CP-CML patients had hyperleukocytosis at diagnosis between January 2015 to December 2025. Hyperleukocytosis was defined as leukocyte count of >100 x $10^9$/L. Retrospective chart reviews was performed using electronic patient records. Baseline demographics & frontline treatment choices were analysed. Event-free survival (EFS) was assessed across subgroups. Events were defined as TKI switch due to resistance, disease progression or death while receiving TKI.

**Copyright Information:** (Online) ISSN: 2572-9241
©2026 The Author(s). HemaSphere is published by John Wiley & Sons Ltd on behalf of European Hematology Association. This is an open access Abstract Book distributed under the Attribution-NonCommercial-NoDerivs (CC BY-NC-ND), which allows third parties to download the articles and share them with others as long as they credit the author and the Abstract Book, but they cannot change the content in any way or use them commercially.
**Abstract Book Citations:** Authors, Title, HemaSphere, 2026;10:(S1):pages. The abstract book DOIs can be found on the "Author Information" page.
**Disclaimer:** Articles published in the journal HemaSphere exclusively reflect the opinions of the authors. The authors are responsible for all content in their abstracts including accuracy of the facts, statements, citing resources, etc.

**Results**

Median age at diagnosis was 37 years (range [r] 15 – 72). Baseline characteristics are summarised in ***Figure 1a***. BCR::ABL1 transcript subtypes e13a2 or/and e14a2 were identified in 98.2% (56/57) while one patient expressed an e8a2 transcript. 22 patients underwent next-generation sequencing myeloid gene panel (NGS-MGP) testing. Four (18.2%) had pathogenic *ASXL1* variants. Additional somatic mutations were identified in 5 patients (*DNMT3A*, *SH2B3*, *TET2* and *IKZF1*).
Amongst 56 evaluable patients, major molecular remission (MMR) rates were 31.8% (7/22), 65.2% (15/23) and 54.5% (6/11) in patients treated with frontline imatinib, nilotinib and dasatinib respectively (p=0.0899) (***Figure 1b***). Pairwise comparison showed significantly higher MMR rates with nilotinib compared to imatinib (65.2% vs 31.8%; p=0.0377). With a median follow up time of 54 months, median EFS was 18 months in the imatinib group (95% CI: 11 months to undefined) while median EFS was not reached in the nilotinib & dasatinib groups (p=0.0505). Pairwise comparison showed clinically significant improved median EFS with nilotinib compared to the imatinib group (p=0.0236). Four patients attempted treatment-free remission (TFR) while on a frontline TKI with all subsequently losing MMR.
Leukapheresis was performed at presentation in 31.6% (18/57) of patients, of whom 83.3% (15/18) had leukostasis symptoms. One patient underwent leukapheresis due to presenting in pregnancy, while two patients had no documented indication. MMR rates were similar between leukapheresis & non-leukapheresis groups (50% vs 50%). Median EFS did not differ significantly between these groups (66 vs 44 months, p=0.8768).
Among four patients with pathogenic *ASXL1* variants, none achieved MMR with frontline therapy. Two received dasatinib and two were on imatinib. Three of these patients switched TKIs due to resistance within 6 months of treatment initiation.

**Summary/Conclusion**

In our cohort of CP-CML patients presenting with hyperleukocytosis, frontline nilotinib was associated with superior molecular and survival outcomes compared with imatinib. Leukapheresis at presentation did not appear to influence clinical outcomes with no significant differences seen when compared to those without leukapheresis. Though, we acknowledge that our patient cohort is relatively small.

**Copyright Information:** (Online) ISSN: 2572-9241
©2026 The Author(s). HemaSphere is published by John Wiley & Sons Ltd on behalf of European Hematology Association. This is an open access Abstract Book distributed under the Attribution-NonCommercial-NoDerivs (CC BY-NC-ND), which allows third parties to download the articles and share them with others as long as they credit the author and the Abstract Book, but they cannot change the content in any way or use them commercially.
**Abstract Book Citations:** Authors, Title, HemaSphere, 2026;10:(S1):pages. The abstract book DOIs can be found on the "Author Information" page.
**Disclaimer:** Articles published in the journal HemaSphere exclusively reflect the opinions of the authors. The authors are responsible for all content in their abstracts including accuracy of the facts, statements, citing resources, etc.

| | n (N=57) | Proportion, % |
|---|---|---|
| **Male Sex** | 34 | 59.6% |
| **Leucocyte Count** | | |
| 100 – 200 x 10⁹/L | 14 | 24.6% |
| 200 – 300 x 10⁹/L | 21 | 36.8% |
| >300 x 10⁹/L | 22 | 38.6% |
| **ETLS Score at Diagnosis** Low / Intermediate / High / Unknown | 22 / 24 / 10 / 1 | 38.6% / 42.1% / 17.5% / 1.8% |
| **Additional Chromosomal Abnormalities (ACAs) Present** | 14 | 24.6% |
| **Choice of 1st Frontline TKI** Imatinib / Nilotinib / Dasatinib | 22 / 24 / 11 | 38.6% / 42.1% / 19.3% |
| **Hydroxycarbamide for Cytoreduction** | 51 | 89.5% |
| **Leukapheresis at Presentation** | 18 | 31.6% |

***Figure 1a: Baseline demographics and initial frontline treatment at presentation***

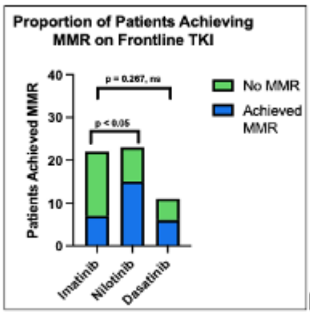

***Figure 1b: Proportion of patients achieving MMR on frontline TKI***

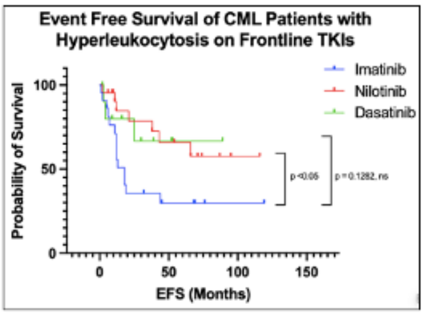

***Figure 1c: Kaplan-Meier curves of EFS for our cohort of CML patients with hyperleukocytosis on frontline TKIs***

**Copyright Information:** (Online) ISSN: 2572-9241
©2026 The Author(s). HemaSphere is published by John Wiley & Sons Ltd on behalf of European Hematology Association. This is an open access Abstract Book distributed under the Attribution-NonCommercial-NoDerivs (CC BY-NC-ND), which allows third parties to download the articles and share them with others as long as they credit the author and the Abstract Book, but they cannot change the content in any way or use them commercially.
**Abstract Book Citations:** Authors, Title, HemaSphere, 2026;10:(S1):pages. The abstract book DOIs can be found on the "Author Information" page.
**Disclaimer:** Articles published in the journal HemaSphere exclusively reflect the opinions of the authors. The authors are responsible for all content in their abstracts including accuracy of the facts, statements, citing resources, etc.

### (PB3002) IMPLEMENTATION OF A THERAPEUTIC DRUG MONITORING PROGRAM FOR IMATINIB DOSAGE INDIVIDUALIZATION IN PATIENTS WITH CML AND GIST: A PILOT STUDY.

**Topic:** 08. Chronic myeloid leukemia - Clinical

Monica Alejandra Romero Riquelme*[1], Salvador Cabrera[2], Hector Morales[3]

*[1]Hospital Guillermo Grant Benavente, Haematology, Concepcion, Chile;[2]Hospital Guillermo Grant Benavente, personalized therapies, concepcion, Chile; [3]Hospital Intercultural de Nueva Imperial, pharmacology, Temuco, Chile;*

### Background

Imatinib is a first-line tyrosine kinase inhibitor (TKI) for the treatment of Chronic Myeloid Leukemia (CML) and Gastrointestinal Stromal Tumors (GIST). Despite its demonstrated efficacy, it presents high interindividual pharmacokinetic variability that conditions therapeutic response and safety. Therapeutic drug monitoring (TDM) allows for treatment optimization through dose adjustment based on plasma levels, where it has been evidenced that achieving steady-state trough concentrations ($C^{SS}_{mín}$) ≥1000 ng/mL in CML and ≥1100 ng/mL in GIST patients are associated with effectiveness, while values > 3000 ng/mL are related to higher toxicity

### Aims

To develop a program for imatinib dosage individualization through Bayesian analysis. Specific objectives included validating an HPLC-UV/Vis analytical technique, determining efficacy, toxicity, and optimization outcomes; in addition to analyzing the relationship of $C^{SS}_{mín}$ with alpha1-acid glycoprotein (AGP) and treatment adherence

### Methods

A quasi-experimental, longitudinal, and prospective study was conducted including 31 patients (CML: 20; GIST: 11) who where in active treatment with imatinib at the standard recommended dose. An HPLC-UV/Vis analytical technique was validated according to FDA M10 guidelines. Pharmacokinetic analysis was performed using Abbott PKS System® software, utilizing one-compartment population models for each pathology. Toxicity was evaluated using NCI-CTCAE v.5.0 criteria and treatment adherence through the SMAQ questionnaire. For CML patients, molecular response status was evaluated with the hematology team in case of requering an imatinib dose modification regarding the results of the pharmacokinetic study

### Results

49 pharmacokinetic analyses were performed, with a medical acceptance rate > 90% of the modified imatinib dose recommendations. After the dose was adjusted, a statistically significant reduction in the average number of toxicities per patient was observed, decreasing from 5.8 ± 2.4 to 3.4 ± 2.8 ($p = 0.004$), specifically reducing gastrointestinal and musculoskeletal toxicity. Furthermore, 82.4% reached imatinib concentrations within the therapeutic range after the study. A moderate and significant positive correlation was confirmed between AGP levels and $C^{SS}_{mín}$ ($\rho = 0.614$, $p < 0.001$). Finally, TDM proved to be a useful tool for evaluating treatment adherence. In CML patients who required and accept an imatinib dose modification, special attention will be recommended regarding the molecular response status for the future molecular evaluation

**Copyright Information:** (Online) ISSN: 2572-9241
©2026 The Author(s). HemaSphere is published by John Wiley & Sons Ltd on behalf of European Hematology Association. This is an open access Abstract Book distributed under the Attribution-NonCommercial-NoDerivs (CC BY-NC-ND), which allows third parties to download the articles and share them with others as long as they credit the author and the Abstract Book, but they cannot change the content in any way or use them commercially.
**Abstract Book Citations:** Authors, Title, HemaSphere, 2026;10:(S1):pages. The abstract book DOIs can be found on the “Author Information” page.
**Disclaimer:** Articles published in the journal HemaSphere exclusively reflect the opinions of the authors. The authors are responsible for all content in their abstracts including accuracy of the facts, statements, citing resources, etc.

**Summary/Conclusion**

The implementation of a TDM program is a clinical tool that significantly improves the safety profile of imatinib. Treatment individualization allows for a significant reduction in toxicity and guarantees the achievement of therapeutic objectives, as optimal molecular response for CML patients. Likewise, TDM stands as the most appropriate method to verify treatment adherence compared to indirect instruments such as the SMAQ

**Copyright Information:** (Online) ISSN: 2572-9241
©2026 The Author(s). HemaSphere is published by John Wiley & Sons Ltd on behalf of European Hematology Association. This is an open access Abstract Book distributed under the Attribution-NonCommercial-NoDerivs (CC BY-NC-ND), which allows third parties to download the articles and share them with others as long as they credit the author and the Abstract Book, but they cannot change the content in any way or use them commercially.
**Abstract Book Citations:** Authors, Title, HemaSphere, 2026;10:(S1):pages. The abstract book DOIs can be found on the "Author Information" page.
**Disclaimer:** Articles published in the journal HemaSphere exclusively reflect the opinions of the authors. The authors are responsible for all content in their abstracts including accuracy of the facts, statements, citing resources, etc.

### (PB3003) ANALYSIS OF THE IMPACT OF TYROSINE KINASE INHIBITORS ON RENAL FUNCTION IN PATIENTS WITH CHRONIC-PHASE CHRONIC MYELOID LEUKEMIA

**Topic:** 08. Chronic myeloid leukemia - Clinical

Yan Li[1], Huanling Zhu[1], Ling Pan[1], Yunfan Yang*[1]

[1]*West China Hospital Of Sichuan University, Chengdu, China;*

### Background

Multiple tyrosine kinase inhibitors (TKIs) are currently approved for the treatment of chronic myeloid leukemia (CML). Among the long-term adverse effects associated with TKI therapy, renal function impairment is relatively common and clinically significant.

### Aims

This study aims to evaluate the effects of different types of TKIs on renal function in patients with chronic-phase CML, thereby providing evidence to assist clinicians in selecting the most appropriate TKI therapy.

### Methods

We conducted a retrospective analysis of patients with chronic-phase CML who received first-line and/or second-line TKI therapy at West China Hospital, Sichuan University. Renal function parameters were collected at various time points over a five-year period following TKI treatment. The primary analysis compared the estimated glomerular filtration rate (eGFR) at different time points with the baseline eGFR (pre-treatment) to assess changes in renal function.

### Results

A total of 503 patients receiving first-line TKI therapy and 111 receiving second-line TKI therapy were included in the analysis. In the first-line setting, 263 patients were treated with imatinib, 128 with flumatinib, 87 with nilotinib, 21 with dasatinib, and 4 with other TKIs (2 asciminib, 2 radotinib). Longitudinal analysis of eGFR revealed distinct patterns among different TKIs. In patients treated with imatinib, eGFR showed a progressive decline over time. In contrast, patients receiving flumatinib experienced a rapid decline in eGFR during the first six months of treatment. At the 6-month time point, 22 patients (12.8%) in the flumatinib group had progressed by at least one stage in their chronic kidney disease (CKD) classification compared to baseline. Thereafter, eGFR levels in this group stabilized with no further significant decline. For patients treated with nilotinib or dasatinib in the first-line setting, no statistically significant changes in eGFR were observed at any of the time points evaluated (Figure 1). In the second-line setting, the most frequently used TKI was dasatinib (n=88), followed by nilotinib (n=11), flumatinib (n=11), and olverembatinib (n=1). Subgroup analysis of patients treated with dasatinib as second-line therapy revealed that eGFR levels at 24, 36, 48, and 60 months were significantly lower compared to the pre-second-line treatment baseline ($p < 0.05$).

### Summary/Conclusion

Among first-line TKIs, imatinib and flumatinib demonstrated a more pronounced impact on renal function in patients with CML. For patients receiving flumatinib, close monitoring of renal function is recommended, particularly during the first six months of treatment. While dasatinib showed no significant effect on eGFR in the first-line setting, long-term dasatinib therapy in the second-line setting was associated with a significant decline in eGFR. This discrepancy may be attributable to the relatively small sample size of dasatinib-treated patients in the first-line group.

**Copyright Information:** (Online) ISSN: 2572-9241
©2026 The Author(s). HemaSphere is published by John Wiley & Sons Ltd on behalf of European Hematology Association. This is an open access Abstract Book distributed under the Attribution-NonCommercial-NoDerivs (CC BY-NC-ND), which allows third parties to download the articles and share them with others as long as they credit the author and the Abstract Book, but they cannot change the content in any way or use them commercially.
**Abstract Book Citations:** Authors, Title, HemaSphere, 2026;10:(S1):pages. The abstract book DOIs can be found on the "Author Information" page.
**Disclaimer:** Articles published in the journal HemaSphere exclusively reflect the opinions of the authors. The authors are responsible for all content in their abstracts including accuracy of the facts, statements, citing resources, etc.

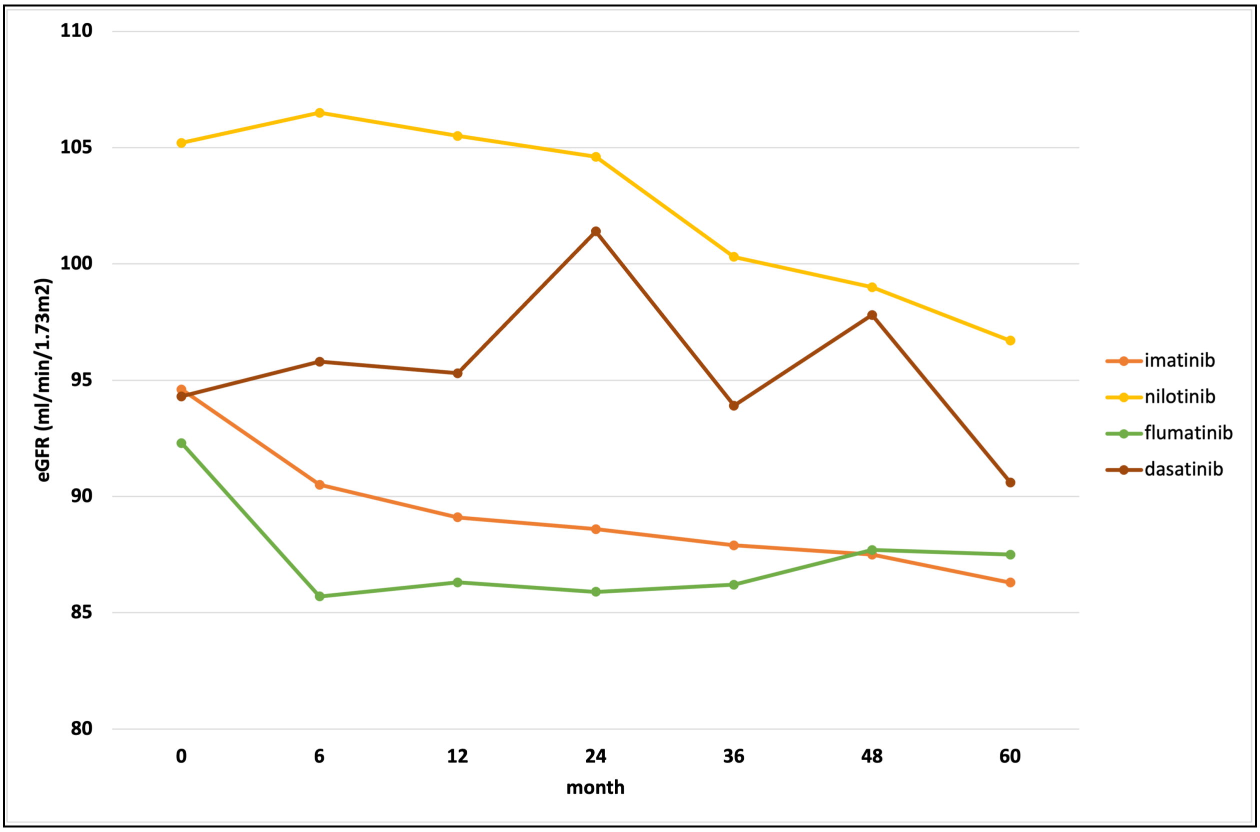

Longitudinal changes in eGFR over time in patients receiving first-line TKI therapies

**Copyright Information:** (Online) ISSN: 2572-9241
©2026 The Author(s). HemaSphere is published by John Wiley & Sons Ltd on behalf of European Hematology Association. This is an open access Abstract Book distributed under the Attribution-NonCommercial-NoDerivs (CC BY-NC-ND), which allows third parties to download the articles and share them with others as long as they credit the author and the Abstract Book, but they cannot change the content in any way or use them commercially.
**Abstract Book Citations:** Authors, Title, HemaSphere, 2026;10:(S1):pages. The abstract book DOIs can be found on the "Author Information" page.
**Disclaimer:** Articles published in the journal HemaSphere exclusively reflect the opinions of the authors. The authors are responsible for all content in their abstracts including accuracy of the facts, statements, citing resources, etc.

### (PB3004) REAL-WORLD ASSESSMENT OF SYMPTOM BURDEN USING ESAS SCORE IN CHRONIC MYELOID LEUKEMIA PATIENTS TREATED WITH ASCIMINIB

**Topic:** 08. Chronic myeloid leukemia - Clinical

Ana Flavia Patiño*[1], Hassan, Sibai[1], Noora Obaidallah[2], Jessica Danquah[1], Dennis Kim[1], Maria Agustina Perusini[1]

*[1]Princess Margaret Hospital, Hematology-Leukemia, Toronto, Canada;[2]Sheikh Shakhbout Medical City, Abu Dhabi, United Arab Emirates;*

### Background

Long-term tyrosine kinase inhibitor (TKI) therapy in chronic myeloid leukemia (CML) is effective but frequently associated with adverse events impacting quality of life and adherence. Asciminib (ASC) has shown favorable efficacy and tolerability; however, real-world data on patient-reported symptom burden remain limited.

### Aims

To describe real-world symptom burden assessed by the Edmonton Symptom Assessment System (ESAS) in patients with CML treated with ASC, evaluate longitudinal changes after treatment initiation, and explore associations with molecular response.

### Methods

We conducted a retrospective single-center study of patients with CML treated with ASC in second or later line before February 1, 2025. Patients with ≥1 ESAS assessment were included. ESAS evaluates nine symptoms scored 0–10, generating a total score from 0 to 90. Pre-treatment ESAS was defined as the most recent assessment before ASC initiation; post-treatment ESAS as the first assessment after initiation. Clinically meaningful change was defined as a ≥5-point difference. Continuous variables are reported as medians (IQR) and categorical variables as n (%). Fisher exact test was used for subgroup comparisons. Major molecular response (MMR; BCR-ABL1 ≤0.1% IS) was assessed per institutional monitoring.

### Results

Of 94 ASC-treated patients, 47 met inclusion criteria; 37 had baseline ESAS, 39 post-treatment ESAS, and 32 paired assessments. Median age was 61.8 years (IQR 49.3–71.4); 62% were male. ASC was initiated for intolerance in 47% and resistance in 53%. Median follow-up was 29.4 months (IQR 20.2–38). Median time from baseline ESAS to ASC initiation was 23 days (IQR 2–63), and from ASC initiation to post-treatment ESAS was 159 days (IQR 69–249).
Baseline ESAS total score was 18 (IQR 6–31). Fatigue and impaired well-being were the most prominent symptoms (median 3 [2–6] and 3 [2–5]). Moderate-to-severe fatigue (16/37, 43%), impaired well-being (17/37, 46%), and depression (10/37, 27%) were common. Baseline ESAS did not differ between intolerance and resistance groups (p=0.727).
Post-treatment ESAS (n=39) had a median of 11 (IQR 5–23). Among paired patients (n=32), 47% improved, 44% worsened, and 9% remained stable.Clinically meaningful improvement, defined as a reduction of at least 5 points, was observed in 41% (n=13), whereas 22% (n=7) experienced a clinically meaningful worsening. The median paired change in ESAS total score was −2 (IQR −9 to 4), which was not statistically significant (p=0.187). The magnitude of change in total ESAS score was similar between intolerance and resistance groups (median change −2 [IQR −9 to 4] vs −2 [IQR −10.5 to 3], p=0.88), and the proportion achieving clinically meaningful improvement did not differ between groups (41% vs 40%, p=1.00).
Among 25 patients not in MMR at ASC initiation, 17 (68%) achieved MMR. Of these, 16 (94%) achieved MMR within 12 months. MMR occurred in 13/15 (87%) patients without clinically meaningful ESAS improvement compared with 4/10 (40%) of those with improvement (p=0.028).

**Copyright Information:** (Online) ISSN: 2572-9241
©2026 The Author(s). HemaSphere is published by John Wiley & Sons Ltd on behalf of European Hematology Association. This is an open access Abstract Book distributed under the Attribution-NonCommercial-NoDerivs (CC BY-NC-ND), which allows third parties to download the articles and share them with others as long as they credit the author and the Abstract Book, but they cannot change the content in any way or use them commercially.
**Abstract Book Citations:** Authors, Title, HemaSphere, 2026;10:(S1):pages. The abstract book DOIs can be found on the "Author Information" page.
**Disclaimer:** Articles published in the journal HemaSphere exclusively reflect the opinions of the authors. The authors are responsible for all content in their abstracts including accuracy of the facts, statements, citing resources, etc.

**Summary/Conclusion**

In routine clinical practice, ASC is associated with clinically meaningful changes in patient-reported symptom burden in a substantial proportion of patients with CML treated beyond first line. The discordance observed between symptom improvement and molecular response underscores the complexity of treatment benefit assessment and supports systematic integration of patient-reported outcomes into CML management strategies.

**Table 1. Baseline ESAS symptom scores prior to ASC initiation (n = 37) Scores range from 0 (no symptom) to 10 (worst severity).**

| Symptom | Median (IQR) |
|---|---|
| Fatigue | 3 (2-6) |
| Well-being | 3 (2-5) |
| Drowsiness | 1 (0-5) |
| Anxiety | 1 (0-3) |
| Depression | 1 (0-4) |
| Pain | 1 (0-3) |
| Dyspnea | 1 (0-3) |
| Appetite | 1 (0-3) |
| Nausea | 0 (0-2) |

**Copyright Information:** (Online) ISSN: 2572-9241
©2026 The Author(s). HemaSphere is published by John Wiley & Sons Ltd on behalf of European Hematology Association. This is an open access Abstract Book distributed under the Attribution-NonCommercial-NoDerivs (CC BY-NC-ND), which allows third parties to download the articles and share them with others as long as they credit the author and the Abstract Book, but they cannot change the content in any way or use them commercially.
**Abstract Book Citations:** Authors, Title, HemaSphere, 2026;10:(S1):pages. The abstract book DOIs can be found on the "Author Information" page.
**Disclaimer:** Articles published in the journal HemaSphere exclusively reflect the opinions of the authors. The authors are responsible for all content in their abstracts including accuracy of the facts, statements, citing resources, etc.

### (PB3005) REAL-WORLD OUTCOMES OF ASCIMINIB IN PATIENTS WITH CHRONIC MYELOID LEUKEMIA RESISTANT OR INTOLERANT TO AT LEAST TWO TYROSINE KINASE INHIBITORS: A SINGLE-CENTER RETROSPECTIVE STUDY IN POLAND

**Topic:** 08. Chronic myeloid leukemia - Clinical

Joanna Wącław-Koziara*[1], Tomasz Sacha[2], Zuzanna Piątek[3], Katarzyna Zięba[3]

*[1]The University Hospital in Krakow,, Department of Hematology, Krakow, Poland;[2]The University Hospital in Krakow,, Department of Hematology, KRAKOW, Poland; [3]The University Hospital in Krakow,, KRAKOW, Poland;*

### Background

Asciminib (ASC) is a first-in-class Specifically Targeting the ABL Myristoyl Pocket (STAMP) inhibitor that binds to the ABL myristoyl pocket. In Poland, it is approved for adults with Philadelphia chromosome–positive chronic-phase chronic myeloid leukemia (CML-CP) previously treated with ≥ 2 tyrosine kinase inhibitors (TKIs). It provides a treatment option for those resistant or intolerant to ATP-competitive TKIs.

### Aims

We aim to describe our real-world experiences and clinical outcomes of CML patients on asciminib at a single-center in Poland: The University Hospital in Krakow.

### Methods

Retrospective chart reviews were conducted using local electronic records. The patients' demographic, efficacy, and safety data were analysed and descriptively reported.

### Results

Fifty-nine patients were initiated on asciminib between December 2021 to December 2025, all in CP. 32 patients (54%) were male and 27 (46%) were female. The median age was 58 years (range [r] 22 – 86). Median follow-up duration was 20 months (r 2 – 52). ASC was intiated due to intolerance (46%), resistance (32%) or both (22%) to prior TKIs. 23 (39%) patients received ASC as third-line therapy, while the remaining patients received it in the fourth line and beyond. 9 patients had detectable tyrosine kinase domain mutations before asciminib initiation. The median dose of ASC was 80 mg daily (r 40-80 mg). 9 patients received combined treatment, 4 with imatinib, and 5 with dasatinib.
Asciminib was discontinued in 8.5% (5/59) of patients due to resistance, including one patient who progressed to blast phase shortly after asciminib introduction. One patient died from non-CML causes. Among the patients not in major molecular remission (MMR) before starting asciminib (38/59), 58% achieved at least MMR. Overall, 91,5% of patients improved or maintained their previous response during ASC therapy. 13 patients (22%) developed hematological toxicity, of which 4 (6,7%) developed grade 3 or 4 toxicity, specifically neutropenia and thrombocytopenia. Non-hematological adverse events (AEs) were observed in 22 (37,2%) of patients, of which 9 (15,3%) developed grade 3 or 4 toxicity, comprising of lipase increase, ALT/AST increase and hypertension.

### Summary/Conclusion

In our cohort, asciminib demonstrated efficacy in maintaining as well as deepening molecular responses in third line and beyond showing a favorable safety profile with no discontinuations due to treatment intolerance. We recognise our cohort size is small, undermining the strength of statistical analysis.

**Copyright Information:** (Online) ISSN: 2572-9241
©2026 The Author(s). HemaSphere is published by John Wiley & Sons Ltd on behalf of European Hematology Association. This is an open access Abstract Book distributed under the Attribution-NonCommercial-NoDerivs (CC BY-NC-ND), which allows third parties to download the articles and share them with others as long as they credit the author and the Abstract Book, but they cannot change the content in any way or use them commercially.
**Abstract Book Citations:** Authors, Title, HemaSphere, 2026;10:(S1):pages. The abstract book DOIs can be found on the "Author Information" page.
**Disclaimer:** Articles published in the journal HemaSphere exclusively reflect the opinions of the authors. The authors are responsible for all content in their abstracts including accuracy of the facts, statements, citing resources, etc.

### (PB3006) PRELIMINARY GLOBAL TRENDS CAPTURED THROUGH A DIGITAL QUALITY OF LIFE (QOL) TRACKER TOOL THAT HAVE AN IMPACT ON DAILY LIFE IN PATIENTS (PTS) WITH CHRONIC MYELOID LEUKEMIA (CML)

**Topic:** 08. Chronic myeloid leukemia - Clinical

Susanne Saussele*[1], Catherine Moura[2], Sarunas Narbutas[3], Gerald Raymond Clements[4], Alexander Schulte[5], Anurag Agarwal[5], Tomoiku Takaku[6], Daniela Žáčková[7]

*[1]Universitätsmedizin Mannheim, III. Medizinische Klinik, Mannheim, Germany; [2]Abrale - Brazilian Lymphoma and Leukemia Association, São Paulo, Brazil; [3]Lithuanian Cancer Patient Coalition, Vilnius, Lithuania; [4]CML Buster Foundation, Costa Mesa, CA, United States of America; [5]Novartis International AG, Basel, Switzerland; [6]Saitama Medical University, Saitama, Japan; [7]University Hospital Brno and Masaryk University, Brno, Czechia;*

### Background

QOL is central to long-term disease management yet is not consistently captured or communicated in routine clinical care.
A QOL tracker tool was developed from validated instruments to allow pts with CML to monitor daily life impact and facilitate proactive conversations with healthcare providers (HCPs). After prototype testing with a small group, the tool was launched globally in Sep 2025 as the first CML-specific QOL tool that also enables systematic data collection from registered users. We report results from users (pts and/or caregivers) worldwide who have tested this tool.

### Aims

Describe preliminary trends that impact daily life captured through a QOL tracker tool in pts with CML.

### Methods

A 2-minute QOL tracker tool was rolled out in the Gulf (Kuwait, the United Arab Emirates, Qatar, Bahrain, and Oman), Republic of Korea, Italy, Lithuania, and Japan between Sep and Nov; "global" refers to any other country where the website was in English. Users were asked to assess the impact on their life (improved, stayed the same, got worse) across 7 domains (day-to-day routine, family and social life, mental wellbeing, hobbies and leisure activities, sexual health and activity, concentration/remembering things, and work), with the option to request discussion with their HCP if it "got worse." Users could then opt to identify where on their body (head, stomach and digestion, muscles and bones, mental wellbeing, sexual health and activity, and whole body) they experienced symptoms with greatest negative impact on QOL and select associated symptoms graded mild, moderate, or severe. Multiple completed submissions per user were eligible for inclusion.

**Copyright Information:** (Online) ISSN: 2572-9241
©2026 The Author(s). HemaSphere is published by John Wiley & Sons Ltd on behalf of European Hematology Association. This is an open access Abstract Book distributed under the Attribution-NonCommercial-NoDerivs (CC BY-NC-ND), which allows third parties to download the articles and share them with others as long as they credit the author and the Abstract Book, but they cannot change the content in any way or use them commercially.
**Abstract Book Citations:** Authors, Title, HemaSphere, 2026;10:(S1):pages. The abstract book DOIs can be found on the "Author Information" page.
**Disclaimer:** Articles published in the journal HemaSphere exclusively reflect the opinions of the authors. The authors are responsible for all content in their abstracts including accuracy of the facts, statements, citing resources, etc.

**Results**

At the time of data collection, the webpage was accessed 2765 times, 45 accounts were created, 424 questionnaires were completed, and 103 requests for HCP discussions were made. On average, 20% of users reported improvement in impact on their life, 55% reported no change, and 25% reported worsening. The domains in which users most frequently selected “got worse” were concentration/remembering things (30%), mental wellbeing (27%), and hobbies and leisure activities (27%); HCP discussion requests were 24%, 35%, and 16%, respectively (**Figure**). Rate of HCP discussion requests per region was 26% (global), 33% (Italy), 24% (Lithuania), 23% (Republic of Korea), and 15% (Japan) (average: 24%). Domains with the most HCP discussion requests were work and mental wellbeing (39% each) globally, mental wellbeing (62%) in Italy, day-to-day routine (40%) in Lithuania, day-to-day routine (60%) in Republic of Korea, and family and social life and day-to-day routine (21% each) in Japan.
Of 485 category selections made when asked about impact on the body, muscles and bones, head, and mental wellbeing were selected by 21% of users each, stomach and digestion by 20%, whole body by 20%, and sexual health by 11%.
Users reported 7 symptoms (range, 1-34) on average; top 4 were emotional ups and downs and muscle pain (21% each), feeling worried (11%), and joint pain (11%).

**Summary/Conclusion**

Cognitive concerns were the most frequently reported QOL impact; concentration/remembering things was the key driver of HCP discussion requests. The QOL tracker tool may empower pts and caregivers to take an active role in managing life with CML and has the potential to identify real-world QOL concerns and facilitate discussions that improve symptom management. Regional variation highlights the importance of tailored communication strategies and data interpretation.

**Copyright Information:** (Online) ISSN: 2572-9241
©2026 The Author(s). HemaSphere is published by John Wiley & Sons Ltd on behalf of European Hematology Association. This is an open access Abstract Book distributed under the Attribution-NonCommercial-NoDerivs (CC BY-NC-ND), which allows third parties to download the articles and share them with others as long as they credit the author and the Abstract Book, but they cannot change the content in any way or use them commercially.
**Abstract Book Citations:** Authors, Title, HemaSphere, 2026;10:(S1):pages. The abstract book DOIs can be found on the “Author Information” page.
**Disclaimer:** Articles published in the journal HemaSphere exclusively reflect the opinions of the authors. The authors are responsible for all content in their abstracts including accuracy of the facts, statements, citing resources, etc.

**Figure. Impact on daily life in patients with CML**

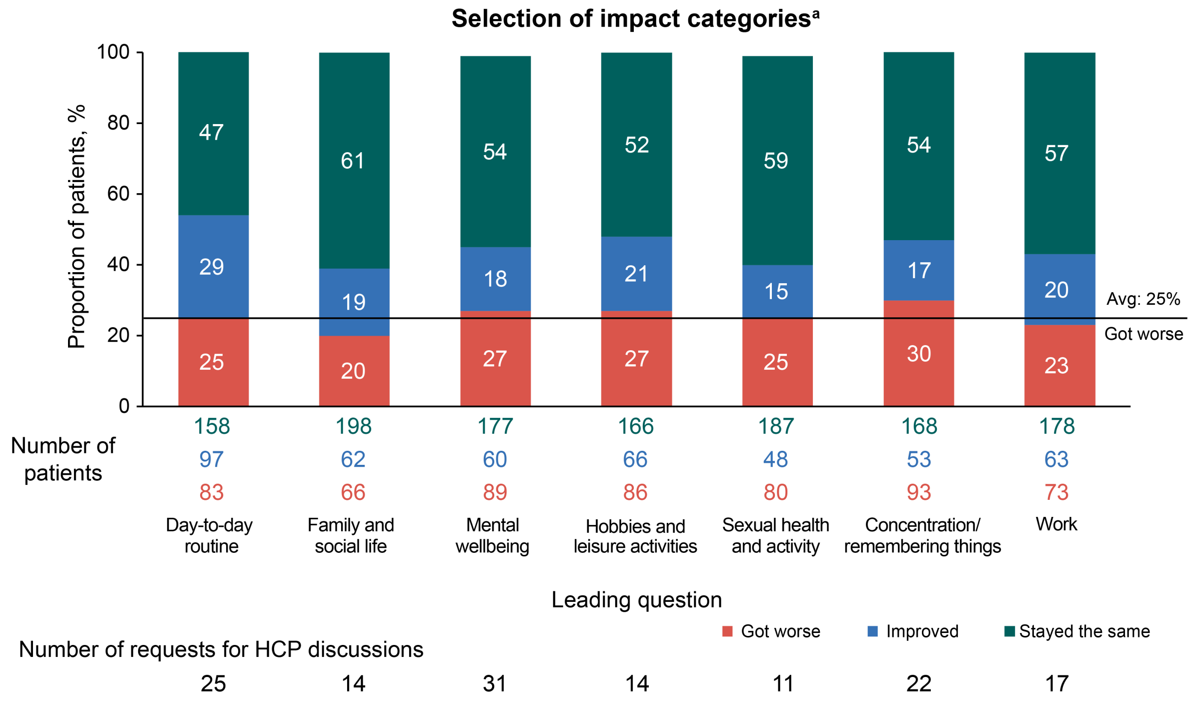

Avg, average; CML, chronic myeloid leukemia; HCP, healthcare provider.
[a] Counts are based on recorded entries and may include multiple submissions from individual users.

**Copyright Information:** (Online) ISSN: 2572-9241
©2026 The Author(s). HemaSphere is published by John Wiley & Sons Ltd on behalf of European Hematology Association. This is an open access Abstract Book distributed under the Attribution-NonCommercial-NoDerivs (CC BY-NC-ND), which allows third parties to download the articles and share them with others as long as they credit the author and the Abstract Book, but they cannot change the content in any way or use them commercially.
**Abstract Book Citations:** Authors, Title, HemaSphere, 2026;10:(S1):pages. The abstract book DOIs can be found on the "Author Information" page.
**Disclaimer:** Articles published in the journal HemaSphere exclusively reflect the opinions of the authors. The authors are responsible for all content in their abstracts including accuracy of the facts, statements, citing resources, etc.

### (PB3007) TREATMENT FREE REMISSION IN CHRONIC MYELOID LEUKAEMIA: A SURVEY OF PRACTICE OF HEALTHCARE PROFESSIONALS IN THE UNITED KINGDOM

**Topic:** 08. Chronic myeloid leukemia - Clinical

Nia Evans*[1], Nick Duncan[2], Jess Pealing[3]

*[1]Hywel Dda Health Board, Haematology, Carmarthen, United Kingdom;[2]University Hospitals Birmingham, Pharmacy, Birmingham, United Kingdom; [3]The Christie, Manchester, United Kingdom;*

### Background

Treatment Free Remission (TFR) has been demonstrated to be a viable strategy in selected Chronic Myeloid Leukaemia (CML) patients[1]. However, uncertainties exist as to how TFR is being delivered in real-world practice.

### Aims

The aims of the survey were to investigate if practice in the UK reflected published guidelines and whether TFR was being offered to eligible patients.

### Methods

We devised an anonymous survey of practice using Microsoft Forms, and circulated to Healthcare Professionals (HCPs) working in the area of CML in the UK.

**Copyright Information:** (Online) ISSN: 2572-9241
©2026 The Author(s). HemaSphere is published by John Wiley & Sons Ltd on behalf of European Hematology Association. This is an open access Abstract Book distributed under the Attribution-NonCommercial-NoDerivs (CC BY-NC-ND), which allows third parties to download the articles and share them with others as long as they credit the author and the Abstract Book, but they cannot change the content in any way or use them commercially.
**Abstract Book Citations:** Authors, Title, HemaSphere, 2026;10:(S1):pages. The abstract book DOIs can be found on the "Author Information" page.
**Disclaimer:** Articles published in the journal HemaSphere exclusively reflect the opinions of the authors. The authors are responsible for all content in their abstracts including accuracy of the facts, statements, citing resources, etc.

**Results**

29 HCPs looking after patients with CML answered the survey, 72% worked at a Tertiary centre,28% at a District General Hospital. The favoured guideline for TFR, used by 55%, was the European LeukaemiaNet recommendations (ELN)[2] with 31% using the British Society of Haematology (BSH) Guideline [3], 7% using both and 7% using other (DESTINY trial [4]).
The ELN provides guidance for treatment discontinuation stipulating duration of TKI therapy, line of therapy, transcript type and duration of Deep molecular Response according to 'Minimal (stop allowed)' and 'Optimal (stop recommended for consideration)' criteria. Respondents were asked whether they offer TFR according to 'optimal' or 'minimal' patient eligibility criteria in the ELN guidelines or whether they do not always follow ELN criteria – see table 1
Table 1 Criteria for TFR (ELN guideline)

| | |
|---|---|
| Always Follow Optimal Criteria | 10% |
| Always Follow Minimum Criteria | 38% |
| Do not always follow ELN criteria | 52% |

Two thirds of respondents reported offering TFR in 75-100% of eligible patients but 24% of respondents reported offering in <50% of eligible patients. Reasons for not offering included lack of capacity to handle the extra monitoring, not identifying patients as being eligible, physician hesitancy and lack of confidence in managing the requirements of TFR.
Respondents were asked what percentage of eligible patients in their experience do not want to proceed to TFR. The median "refusal rate" was 30% (range 0-99%). Reasons for not wanting to proceed included "reluctance to undertake extra monitoring", "fear of relapse", "happy on meds, nervous, worried re joints". 34% of respondents did not discuss patients at an MDT before proceeding to TFR with 24% always discussing and 41% sometimes discussing at an MDT. 66% of respondents always halved the dose of TKI for 1 year before discontinuation [4] with another 21% sometimes doing so. Results for monitoring frequency are shown in table 2.
Table 2 Post TFR BCR-ABL monitoring frequency

| | |
|---|---|
| 6-8 weekly for 6 months, 8 weekly for 6 months, 3-6 monthly thereafter (ELN) | 45% |
| Monthly for 6 months, 6 weekly for 6 months, 8 weekly for 2 years, 3 monthly thereafter (BSH) | 45% |
| Other | 10% |

**Summary/Conclusion**

This survey has demonstrated marked heterogeneity in practice amongst UK HCPs with regard to the TFR process. Given the advantages of TFR (e.g. reduced drug spend, tablet burden and long term side effects), it was surprising that almost a quarter of respondents would only offer TFR to <50% of eligible patients. For those offered TFR, there was significant variation in patient numbers agreeing to proceed which may reflect enthusiasm of the practitioner for TFR. Interestingly, >50% of those offering TFR were sometimes prepared to do so outside of ELN criteria. We suggest that increased education should be provided to staff in regards to TFR. There should also be a stronger focus on identification and targeting of eligible patients.

**Copyright Information:** (Online) ISSN: 2572-9241
©2026 The Author(s). HemaSphere is published by John Wiley & Sons Ltd on behalf of European Hematology Association. This is an open access Abstract Book distributed under the Attribution-NonCommercial-NoDerivs (CC BY-NC-ND), which allows third parties to download the articles and share them with others as long as they credit the author and the Abstract Book, but they cannot change the content in any way or use them commercially.
**Abstract Book Citations:** Authors, Title, HemaSphere, 2026;10:(S1):pages. The abstract book DOIs can be found on the "Author Information" page.
**Disclaimer:** Articles published in the journal HemaSphere exclusively reflect the opinions of the authors. The authors are responsible for all content in their abstracts including accuracy of the facts, statements, citing resources, etc.

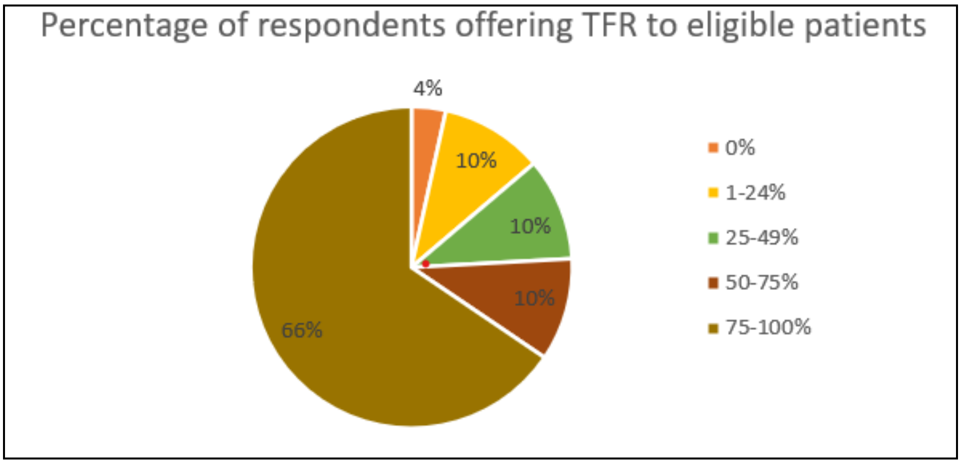

Copyright Information: (Online) ISSN: 2572-9241
©2026 The Author(s). HemaSphere is published by John Wiley & Sons Ltd on behalf of European Hematology Association. This is an open access Abstract Book distributed under the Attribution-NonCommercial-NoDerivs (CC BY-NC-ND), which allows third parties to download the articles and share them with others as long as they credit the author and the Abstract Book, but they cannot change the content in any way or use them commercially.
**Abstract Book Citations:** Authors, Title, HemaSphere, 2026;10:(S1):pages. The abstract book DOIs can be found on the "Author Information" page.
**Disclaimer:** Articles published in the journal HemaSphere exclusively reflect the opinions of the authors. The authors are responsible for all content in their abstracts including accuracy of the facts, statements, citing resources, etc.

### (PB3008) REAL-WORLD PEDIATRIC CML IN A LOWER-MIDDLE-INCOME COUNTRY: LONG-TERM SURVIVAL, DEEP MOLECULAR RESPONSE, GROWTH IMPAIRMENT, AND QUALITY-OF-LIFE OUTCOMES OVER A DECADE

**Topic:** 08. Chronic myeloid leukemia - Clinical

Syed Yasar*[1], Raghunadharao Digumarti[1], Krishna Chaitanya Puligundla[1], Radhika Parimkayala[1], Vishal Toka[1]

*[1]MNJ INSTITUTE OF ONCOLOGY & REGIONAL CANCER CENTRE, MEDICAL ONCOLOGY, Hyderabad, India;*

### Background

Pediatric chronic myeloid leukemia (CML) is rare and biologically distinct from adult CML. While tyrosine kinase inhibitors (TKIs) have transformed survival, prolonged exposure during critical growth periods raises concerns regarding linear growth suppression and health-related quality of life (HRQoL). Data from lower-middle-income countries (LMICs) remain limited. We report long-term real-world outcomes from a decade-long pediatric CML cohort.

### Aims

To evaluate long-term molecular response, survival outcomes, growth parameters, and health-related quality of life in pediatric patients with chronic-phase chronic myeloid leukemia treated with tyrosine kinase inhibitors in a real-world lower-middle-income country setting, and to identify clinical predictors of growth impairment and quality-of-life outcomes.

### Methods

We retrospectively analyzed 42 consecutive pediatric patients diagnosed with chronic-phase CML between 2014 and 2023. All received first-line imatinib (n=26) or dasatinib (n=16) and had ≥24 months follow-up. Molecular monitoring was performed using standardized quantitative RT-PCR and reported on the International Scale according to European LeukemiaNet recommendations. Deep molecular response (DMR) was defined as MR4.5 (BCR-ABL1 ≤0.0032%). Event-free survival (EFS) and overall survival (OS) were estimated using Kaplan–Meier methodology. Growth outcomes were assessed using serial height standard deviation scores (SDS). HRQoL was measured using validated PedsQL instruments. Multivariable regression evaluated predictors of HRQoL and growth decline.

### Results

Median age at diagnosis was 12.4 years (range 4–17), with median follow-up of 6.8 years. MMR was achieved in 95% of patients. A high cumulative incidence of sustained DMR was observed during longitudinal follow-up, with shorter median time to DMR in dasatinib-treated patients compared to imatinib (12 vs 20 months). Five-year OS was 97.6% (95% CI: 87.1–99.6), and 5-year EFS was 88.1% (95% CI: 73.2–95.1). No progression to accelerated or blast phase occurred during follow-up. Growth impairment was observed in 42.8% of patients, with a mean decline in height SDS of −0.84 among affected individuals. Younger age at TKI initiation independently predicted greater height SDS reduction ($p=0.02$). Patients achieving MMR reported significantly higher HRQoL scores ($p=0.0013$). Duration since diagnosis independently correlated with improved HRQoL ($\beta=0.62$, $p<0.0001$). TKI-related adverse events led to therapy modification in 28.5%.

**Copyright Information:** (Online) ISSN: 2572-9241
©2026 The Author(s). HemaSphere is published by John Wiley & Sons Ltd on behalf of European Hematology Association. This is an open access Abstract Book distributed under the Attribution-NonCommercial-NoDerivs (CC BY-NC-ND), which allows third parties to download the articles and share them with others as long as they credit the author and the Abstract Book, but they cannot change the content in any way or use them commercially.
**Abstract Book Citations:** Authors, Title, HemaSphere, 2026;10:(S1):pages. The abstract book DOIs can be found on the "Author Information" page.
**Disclaimer:** Articles published in the journal HemaSphere exclusively reflect the opinions of the authors. The authors are responsible for all content in their abstracts including accuracy of the facts, statements, citing resources, etc.

**Summary/Conclusion**

This LMIC real-world cohort demonstrates survival and molecular outcomes comparable to high-income pediatric series, with high rates of sustained deep molecular response and excellent 5-year OS. However, clinically meaningful growth suppression and measurable HRQoL impact were observed, particularly in younger children. These findings highlight the need for structured endocrine surveillance and pediatric-adapted long-term management strategies globally, especially in resource-constrained settings.

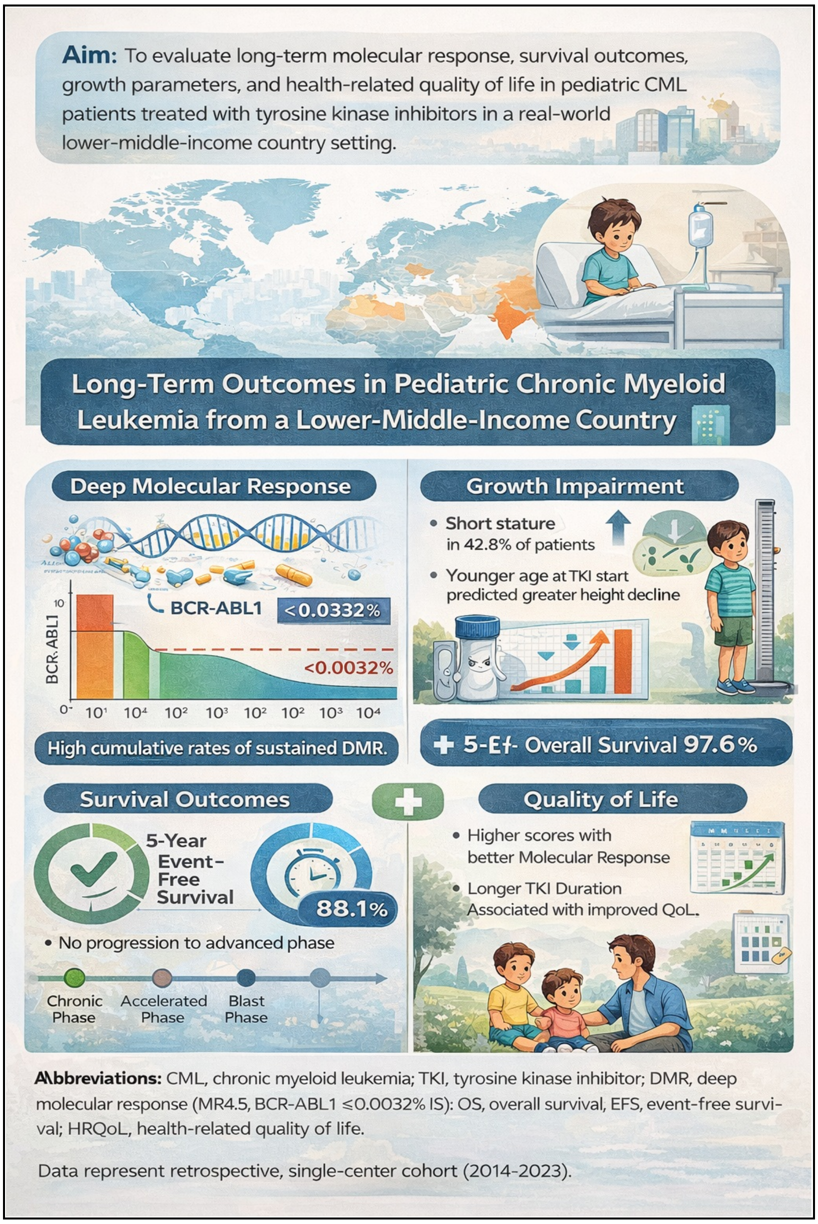

CML, chronic myeloid leukemia; TKI, tyrosine kinase inhibitor; MMR, major molecular response; DMR, deep molecular response; MR4.5, BCR-ABL1 ≤0.0032% (International Scale); OS, overall survival; EFS, event-free survival; HRQoL, health-related quality of life; SDS, standard deviation score.

**Copyright Information:** (Online) ISSN: 2572-9241
©2026 The Author(s). HemaSphere is published by John Wiley & Sons Ltd on behalf of European Hematology Association. This is an open access Abstract Book distributed under the Attribution-NonCommercial-NoDerivs (CC BY-NC-ND), which allows third parties to download the articles and share them with others as long as they credit the author and the Abstract Book, but they cannot change the content in any way or use them commercially.
**Abstract Book Citations:** Authors, Title, HemaSphere, 2026;10:(S1):pages. The abstract book DOIs can be found on the "Author Information" page.
**Disclaimer:** Articles published in the journal HemaSphere exclusively reflect the opinions of the authors. The authors are responsible for all content in their abstracts including accuracy of the facts, statements, citing resources, etc.

### (PB3009) REAL-WORLD EFFECTIVENESS AND SAFETY OF OLVEREMBATINIB IN CHRONIC MYELOID LEUKEMIA: A SINGLE-CENTER RETROSPECTIVE ANALYSIS

**Topic:** 08. Chronic myeloid leukemia - Clinical

Tian Dong[1], Yunfan Yang*[1], Qiushi Liang[1], Yan Li[1], Huanling Zhu[1]

[1]*West China Hospital, Sichuan University, Department of Hematology, Institute of Hematology, Chengdu, China;*

### Background

20-30% of patients with chronic myeloid leukemia(CML) in the chronic phase(CP) develop resistance or intolerance to 1st- and 2nd-generation TKIs each year, and subsequent treatment options are limited. The efficacy of switching to another TKI after failure of 2nd-generation TKI treatment is poor. Olverembatinib (OLV) is a new 3rd-generation BCR::ABL inhibitor that exhibits high inhibitory activity against almost all mutant BCR::ABL1 kinases, including T315I, and has shown preliminary good efficacy and safety in clinical trials, but there are few real world data reports.

### Aims

To observe the efficacy and safety of olverembatinib in the treatment of CML in the real world, so as to better guide clinical practice.

### Methods

This was a single-center retrospective study included adult patients (≥18 years) with CML who received OLV monotherapy or combination therapy in West China Hospital of Sichuan University, China. The demographic, efficacy, and safety data of the patients were collected from the hospital HIS system and follow-up by telephone and questionnaire.

### Results

Between Jan. 2020 and Dec. 2025, a total of 60 CML patients were enrolled for OLV-based therapy, including 39 patients in CP, 7 patients in accelerated phase(AP), and 14 patients in blast phase(BP). 33 patients (55%) were male and 27 patients (45%) were female. The median age was 52 years (range: 25- 83), and the median number of previous treatment lines was 2 (range 1-4). The median disease duration was 65 months (range: 11-317), and the median follow-up time after initiation of OLV treatment was 12 months (range: 3-71). The median BCR::ABL fusion gene level before OLV was 33.9% (range: 0%-202.6%), and 50 patients (83.3%) were resistant to the most recent TKI. 29 patients had kinase domain mutations, including 17 T315I, 8 E255K/V, 3 F317L/V, 2 Y253H mutation and 5 other mutations, with 7 patients had multi-mutations. 6 patients received combined treatment. 55 (91.7%) patients achieved CHR within 3 months. 30(50%) patients achieved CCyR. 22(36.7%) patients achieved MMR. 9(15%) patients achieved DMR in whom 4 underwent transplantation. 21 patients (35%) developed grade 3 or higher hematological toxicity. Non-hematological toxicity with an incidence of more than 10 % included 24(45%) pigmented nevus, 10 (13.9%) infections, 6(10%) rash, 6(10%) myoarthritis. 4(6.7%) patients developed cardia-cerebrovascular events(1 angina, 1 cerebral infarction and 2 arrhythmias). Dose adjustments were required in 38 patients, of whom 24 (40%) had their dose reduced to <30 mg every other day (including treatment discontinuation), all due to hematologic intolerance. 5(8.3%) patients received allogeneic hematopoietic stem cell transplantation, of which one died of extramedullary relapse after transplantation, while the other 4 were all in DMR survival status. A total of 9 (15%) patient deaths were recorded and 6 (67%) of them took the drug for less than 3 months.

**Copyright Information:** (Online) ISSN: 2572-9241
©2026 The Author(s). HemaSphere is published by John Wiley & Sons Ltd on behalf of European Hematology Association. This is an open access Abstract Book distributed under the Attribution-NonCommercial-NoDerivs (CC BY-NC-ND), which allows third parties to download the articles and share them with others as long as they credit the author and the Abstract Book, but they cannot change the content in any way or use them commercially.
**Abstract Book Citations:** Authors, Title, HemaSphere, 2026;10:(S1):pages. The abstract book DOIs can be found on the “Author Information” page.
**Disclaimer:** Articles published in the journal HemaSphere exclusively reflect the opinions of the authors. The authors are responsible for all content in their abstracts including accuracy of the facts, statements, citing resources, etc.

**Summary/Conclusion**

Compared with clinical trials, the baseline conditions of patients treated with OLV in the real world vary greatly. Under different baseline conditions, more patients can still achieve a good treatment response, but thrombocytopenia is still a hematological adverse event that needs to be vigilant and may be the main reason for dose adjustment. The occurrence of pigmented nevus is relatively common but rarely has serious consequences. The ability to take allogeneic hematopoietic stem cell transplantation may currently be associated with a better prognosis.

**Copyright Information:** (Online) ISSN: 2572-9241
©2026 The Author(s). HemaSphere is published by John Wiley & Sons Ltd on behalf of European Hematology Association. This is an open access Abstract Book distributed under the Attribution-NonCommercial-NoDerivs (CC BY-NC-ND), which allows third parties to download the articles and share them with others as long as they credit the author and the Abstract Book, but they cannot change the content in any way or use them commercially.
**Abstract Book Citations:** Authors, Title, HemaSphere, 2026;10:(S1):pages. The abstract book DOIs can be found on the “Author Information” page.
**Disclaimer:** Articles published in the journal HemaSphere exclusively reflect the opinions of the authors. The authors are responsible for all content in their abstracts including accuracy of the facts, statements, citing resources, etc.

### (PB3010) PRE-TREATMENT COMORBIDITIES AND MANAGEMENT OF TYROSINE KINASE INHIBITOR THERAPY IN PATIENTS WITH CHRONIC-PHASE CHRONIC MYELOID LEUKEMIA

**Topic:** 08. Chronic myeloid leukemia - Clinical

Tugcan Alp Kirkizlar*[1], Oguz Ilarslan[1], Hakki Onur Kirkizlar[1], Ahmet Muzaffer Demir[1]

*[1]Trakya University, Hematology, Edirne, Türkiye;*

### Background

CML patients with a low comorbidity burden have overall survival (OS) approaching that of the general population, whereas survival decreases significantly as the number of comorbidities increases. Comorbidities influence treatment selection, drug tolerability, adverse event (AE) & long-term outcomes.

### Aims

This study aimed to evaluate the prevalence and distribution of baseline comorbidities in patients with chronic-phase CML (CP-CML) and to assess their impact on treatment course, AE & OS.

### Methods

This retrospective study included 121 pts with CP-CML between 2005 and 2024. Demographic, laboratory, genetic, comorbidity, treatment, AE & outcomes were analyzed. Comorbidities were assessed using the Charlson Comorbidity Index (CCI) & AEs were graded according to CTCAE v5.0. Given the high prevalence and multiplicity of comorbidities a system-based classification approach was applied.

**Copyright Information:** (Online) ISSN: 2572-9241
©2026 The Author(s). HemaSphere is published by John Wiley & Sons Ltd on behalf of European Hematology Association. This is an open access Abstract Book distributed under the Attribution-NonCommercial-NoDerivs (CC BY-NC-ND), which allows third parties to download the articles and share them with others as long as they credit the author and the Abstract Book, but they cannot change the content in any way or use them commercially.
**Abstract Book Citations:** Authors, Title, HemaSphere, 2026;10:(S1):pages. The abstract book DOIs can be found on the "Author Information" page.
**Disclaimer:** Articles published in the journal HemaSphere exclusively reflect the opinions of the authors. The authors are responsible for all content in their abstracts including accuracy of the facts, statements, citing resources, etc.

**Results**

The median age at diagnosis was 57y and 57.9% were male. At diagnosis, 80.2% had at least 1 comorbidity; the median CCI score was 2 and the median number of comorbidities was 2. Cardiovascular (63.6%), endocrine (38.8%) and respiratory (28.9%) systems were most frequently affected. HT was the most prevalent condition, present in 50.4% of the cohort, 79.2% of those with cardiovascular disease and 62.9% of patients with any comorbidity. Coronary artery disease and DM were observed in 18.2% and 26.4% of patients, respectively.
All patients initiated imatinib as first-line therapy due to regulatory requirements. At last follow-up , 62.8% remained on imatinib, 16.5% were receiving nilotinib, 5.8% dasatinib, 11.6% bosutinib, and 3.3% ponatinib. The transition rate to second-line therapy was 37.2%, mainly due to resistance (82.2%). More than 91% of patients requiring second- or third-line therapy had at least one comorbidity.
The median follow-up was 82 months. Mortality rate was 19%, significantly higher in patients with comorbidities (p=0.043). CML-related causes accounted for 21.7% of deaths. Mean OS was 190.8 months and was significantly shorter in patients with comorbidities (181.8 vs. 200 months; p=0.040, Fig1). 5-year OS rate was approximately 90%, decreasing to 75% at 10 years. 5-year OS was 100% in patients without comorbidities versus 87.2% in those with comorbidities (p=0.040). Cardiovascular (p=0.003), respiratory (p=0.017), and urinary (p=0.035) comorbidities were associated with inferior survival. In Cox regression analysis, the presence and number of comorbidities, higher CCI scores, shorter first-line treatment duration and the need for second-line therapy were independent predictors of mortality.
With imatinib, hematologic toxicities were most frequent (44/123), predominantly mild (71.5% G1); neutropenia was the most severe event. Peripheral edema and rash were also observed. With nilotinib, hematologic events were most common, followed by cardiovascular and gastrointestinal toxicities. 1 case of pancreatitis and 1 cardiovascular death due to A-V block occurred. Dasatinib-related pleural effusion rates were comparable to the literature, whereas bosutinib-associated AEs were mainly gastrointestinal and dermatologic.

**Summary/Conclusion**

Comorbidities were highly prevalent in CP-CML patients & were associated with inferior survival. The presence & number of comorbidities, higher CCI scores,were adverse prognostic factors. AEs may further increase mortality risk and complicate optimal TKI selection & long-term management.

**Copyright Information:** (Online) ISSN: 2572-9241
©2026 The Author(s). HemaSphere is published by John Wiley & Sons Ltd on behalf of European Hematology Association. This is an open access Abstract Book distributed under the Attribution-NonCommercial-NoDerivs (CC BY-NC-ND), which allows third parties to download the articles and share them with others as long as they credit the author and the Abstract Book, but they cannot change the content in any way or use them commercially.
**Abstract Book Citations:** Authors, Title, HemaSphere, 2026;10:(S1):pages. The abstract book DOIs can be found on the “Author Information” page.
**Disclaimer:** Articles published in the journal HemaSphere exclusively reflect the opinions of the authors. The authors are responsible for all content in their abstracts including accuracy of the facts, statements, citing resources, etc.

Table 1. Demographic and Clinical Characteristics of Patients with Chronic-Phase Chronic Myeloid Leukemia Included in the Study

| Parameters | Values |
|---|---|
| Age (n=121) | Mean: 55.5 years (±14.72)<br>Median: 57 years (22–84) |
| Sex (n=121) | Female: 42.1%; Male: 57.9% |
| Charlson Comorbidity Index (CCI) score (n=121) | Median: 2 (0–6) |
| Presence of comorbidities (n=121) | 80.17% |
| Number of comorbidities (n=121) | Median: 2 (0–11) |
| Duration on first-line therapy (n=121) | Mean: 72.9 months (±67.27)<br>Median: 46 months (2–230) |
| Transition to second-line therapy (n=121) | 37.2% |
| Reason for second-line therapy (n=45) | Resistance: 82.2%<br>Intolerance: 17.8% |
| Transition to third-line therapy (n=121) | 19.8% |
| Reason for third-line therapy (n=24) | Resistance: 50%<br>Intolerance: 50% |
| Follow-up duration (n=121) | Mean: 98.5 months (±67.53)<br>Median: 82 months (9–240) |
| Mortality rate (n=121) | 19% |

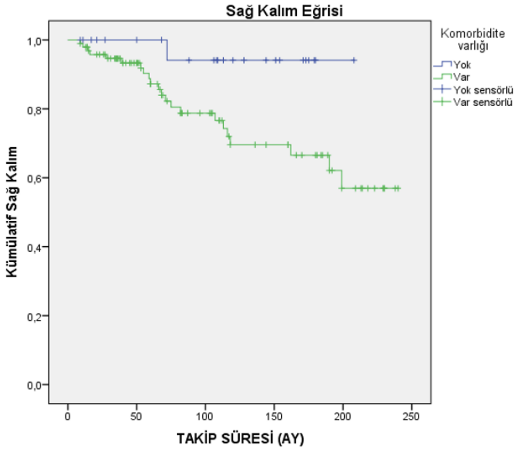

Fig 1. Kaplan–Meier (Log-Rank) Survival Curve According to the Presence of Comorbidity

Table 1 and Figure 1

Copyright Information: (Online) ISSN: 2572-9241
©2026 The Author(s). HemaSphere is published by John Wiley & Sons Ltd on behalf of European Hematology Association. This is an open access Abstract Book distributed under the Attribution-NonCommercial-NoDerivs (CC BY-NC-ND), which allows third parties to download the articles and share them with others as long as they credit the author and the Abstract Book, but they cannot change the content in any way or use them commercially.
Abstract Book Citations: Authors, Title, HemaSphere, 2026;10:(S1):pages. The abstract book DOIs can be found on the "Author Information" page.
Disclaimer: Articles published in the journal HemaSphere exclusively reflect the opinions of the authors. The authors are responsible for all content in their abstracts including accuracy of the facts, statements, citing resources, etc.

### (PB3011) TIMING TO MAJOR MOLECULAR RESPONSE (MMR) DOES NOT IMPACT OVERALL SURVIVAL IN CHRONIC PHASE CML: A MULTICENTRE REAL-WORLD PERUVIAN STUDY

**Topic:** 08. Chronic myeloid leukemia - Clinical

Naty López Córdova*[1], Cesar Samanez[2], Victor Mallma Soto[1], Mariana Barrantes Hadzich[3], Susy Bazán Ruiz[4], Cindy Calle Carreño[1], Claudia Castillo Chilet[1], Laura Chavez Jimenez[1], Mirtha Flores Arenas[5], Gledy Jimenez Tornero[6], Jessica Luque Guevara[1], Mayra Mariscal Huamaní[6], Estefany Menacho Medina[6], Marlies Pizarro Perea[4], Victor Seminario Marcelo[3], Guillermo Valladares Zevallos[6], Jesus Olga Vargas Castro[3], María Alejandra Vera Vasquez[6], Jose Untama[4]

*[1]Instituto Nacional de Enfermedades Neoplásicas, Medical Oncology, Lima, Peru;[2]Oncosalud-AUNA, Lima, Peru; [3]Hospital Nacional Guillermo Almenara Irigoyen, Lima, Peru; [4]Hospital Nacional Edgardo Rebagliati Martins, Lima, Peru;[5]Hospital Nacional Carlos Alberto Seguin Escobedo, Arequipa, Peru; [6]Hospital Nacional Alberto Sabogal Sologuren, Lima, Peru;*

### Background

Tyrosine kinase inhibitors (TKIs) have transformed chronic phase chronic myeloid leukaemia (CML-CP) into a condition with near-normal life expectancy. Current guidelines define achievement of major molecular response (MMR, BCR::ABL1 ≤0.1% IS) within 12 months as an optimal milestone associated with improved long-term outcomes. However, the prognostic impact of achieving MMR beyond 12 months (late responders) remains uncertain, particularly in developing countries, where treatment optimisation may be limited.

### Aims

To evaluate the impact of MMR timing on survival and response duration in CML-CP treated with first-line TKI in a multicentre, real-world Peruvian cohort.

### Methods

A retrospective cohort study was conducted, including 261 evaluable patients from 6 Peruvian institutions with newly diagnosed CML-CP treated with first-line TKI. Data were collected from each centre's physical and electronic records. Early responders (ER) were defined as those who achieved an MMR within 12 months of the start of treatment, and late responders (LR) as those who achieved it beyond 12 months. Overall survival (OS) was defined as the time from diagnosis to death from any cause or last follow-up. Chi-square test was used for dichotomous variables, Kaplan-Meier method and log-rank test for survival curves.

### Results

Median age at diagnosis was 44.7 years (range 14-90), and 147 (56.3%) patients were male. The most frequently used TKIs were Imatinib (94.3%), Dasatinib (3.5%), and Nilotinib (2.3%). ER were 110 (42.2%), and LR were 151 (57.8%).
After analysis, 50% of ER and 19.7% of LR achieved MR4.5 or deeper ($p < 0.001$). In relation to the length of response, 9.1% (median: 9.6 months, range: 2.1 – 50.2) of ER, and 25.8% (median: 15 months, range: 2.1 – 117.7) of LR lost MMR ($p < 0.001$). After a median follow-up of 73 months, the estimated 10 and 15-year OS was 96.5% and 93.5%, respectively. No statistical significance was found in estimated OS between ER and LR group (15-year OS 98.6% for ER and 91.7% for LR; $p = 0.6$).

**Copyright Information:** (Online) ISSN: 2572-9241
©2026 The Author(s). HemaSphere is published by John Wiley & Sons Ltd on behalf of European Hematology Association. This is an open access Abstract Book distributed under the Attribution-NonCommercial-NoDerivs (CC BY-NC-ND), which allows third parties to download the articles and share them with others as long as they credit the author and the Abstract Book, but they cannot change the content in any way or use them commercially.
**Abstract Book Citations:** Authors, Title, HemaSphere, 2026;10:(S1):pages. The abstract book DOIs can be found on the "Author Information" page.
**Disclaimer:** Articles published in the journal HemaSphere exclusively reflect the opinions of the authors. The authors are responsible for all content in their abstracts including accuracy of the facts, statements, citing resources, etc.

**Summary/Conclusion**

In this real-world Peruvian cohort, time to MMR was not associated with overall survival. However, early responders (ER) achieved deeper and more durable molecular responses. In contrast, the outcomes observed in the late responders (LR) may limit the future feasibility of treatment-free remission.
In Latin American countries with limited access to second- and third-generation TKIs, these findings are particularly relevant and may help to adapt CML treatment guidelines in low-income settings.

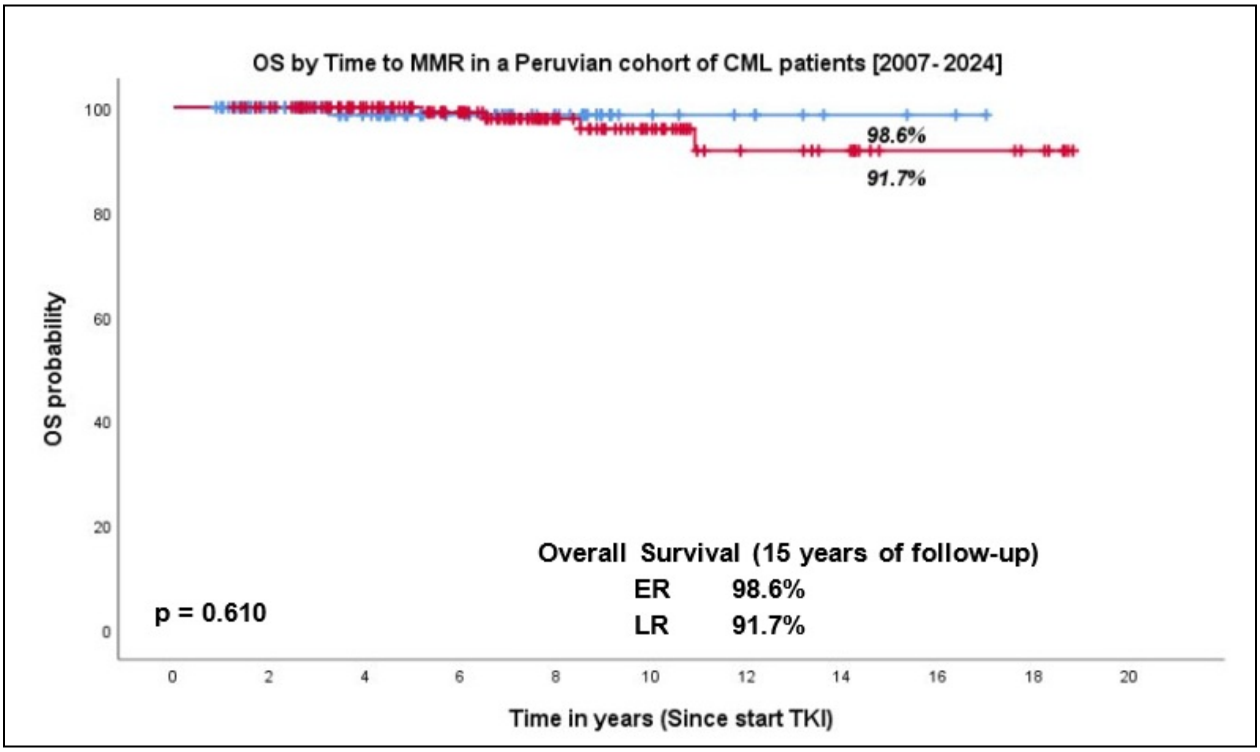

OS by time to MMR

**Copyright Information:** (Online) ISSN: 2572-9241
©2026 The Author(s). HemaSphere is published by John Wiley & Sons Ltd on behalf of European Hematology Association. This is an open access Abstract Book distributed under the Attribution-NonCommercial-NoDerivs (CC BY-NC-ND), which allows third parties to download the articles and share them with others as long as they credit the author and the Abstract Book, but they cannot change the content in any way or use them commercially.
**Abstract Book Citations:** Authors, Title, HemaSphere, 2026;10:(S1):pages. The abstract book DOIs can be found on the “Author Information” page.
**Disclaimer:** Articles published in the journal HemaSphere exclusively reflect the opinions of the authors. The authors are responsible for all content in their abstracts including accuracy of the facts, statements, citing resources, etc.

### (PB3012) GENERIC VERSUS BRANDED IMATINIB IN CHRONIC MYELOID LEUKEMIA: A SYSTEMATIC REVIEW AND META-ANALYSIS OF 21 OBSERVATIONAL STUDIES

**Topic:** 08. Chronic myeloid leukemia - Clinical

Shankar Biswas*[1], Yashasvi Srivastava[1]

[1] *Ivano-Frankivsk National Medical University, Internal Medicine, Ivano-Frankivsk, Ukraine;*

### Background

Generic imatinib is endorsed by the European LeukemiaNet (ELN) 2020 recommendations as the most cost-effective frontline therapy for chronic-phase chronic myeloid leukemia (CML-CP). However, conflicting real-world data on efficacy and safety have fueled ongoing uncertainty. The only prior systematic review (Erçalışkan et al., Blood Adv 2021) narratively summarized 36 papers but did not perform quantitative pooling, leaving a critical evidence gap.

### Aims

To conduct the first meta-analysis quantitatively comparing clinical outcomes of generic versus branded imatinib in CML-CP patients, including both frontline and switch settings.

### Methods

We systematically searched PubMed, Embase, Cochrane Library, and Web of Science (inception–January 2025) per PRISMA 2020 guidelines. Eligible studies were comparative observational studies reporting efficacy or safety of generic versus branded imatinib in adult CML-CP. Two reviewers independently extracted data and assessed quality using the Newcastle-Ottawa Scale (NOS). Random-effects meta-analyses (Mantel-Haenszel, DerSimonian-Laird) pooled odds ratios (OR) for complete cytogenetic response (CCyR), major molecular response (MMR), overall survival (OS), early molecular response (EMR), and treatment failure. Hazard ratios (HR) were pooled for event-free survival (EFS). Proportions were pooled for switch response maintenance. Subgroup analyses by region and study quality, leave-one-out sensitivity analyses, and meta-regression were performed. Heterogeneity was assessed with $I^2$ and Cochran's Q.

### Results

Twenty-one studies (13 countries; ~5,500 patients) were included: 13 frontline comparisons, 6 switch studies, and 2 mixed designs. Median NOS was 7 (range 2–9). No significant differences were found for CCyR (OR 1.05, 95%CI 0.84–1.31; k=7; $I^2$=0%), MMR (OR 0.82, 95%CI 0.56–1.18; k=8; $I^2$=53%), or OS (OR 1.12, 95%CI 0.79–1.58; k=4; $I^2$=0%). However, generic imatinib was associated with significantly higher treatment failure (OR 1.52, 95%CI 1.08–2.12; k=3; $I^2$=1%) and worse EFS (HR 1.56, 95%CI 1.05–2.32; k=3). EMR showed no difference after excluding one outlier study (OR 1.00; $I^2$=0%). Among 6 switch studies, 91.5% (95%CI 87.3–94.4%) of patients maintained or improved molecular response, with only 8.1% worsening. Meta-regression revealed that publication year explained 90% of MMR heterogeneity (p=0.007), with later studies showing diminishing differences. Subgroup analysis demonstrated significant regional variation for MMR (p=0.038). Sensitivity analyses excluding studies with overlap concerns or low quality did not alter primary conclusions. Trim-and-fill analysis detected no meaningful publication bias.

**Copyright Information:** (Online) ISSN: 2572-9241
©2026 The Author(s). HemaSphere is published by John Wiley & Sons Ltd on behalf of European Hematology Association. This is an open access Abstract Book distributed under the Attribution-NonCommercial-NoDerivs (CC BY-NC-ND), which allows third parties to download the articles and share them with others as long as they credit the author and the Abstract Book, but they cannot change the content in any way or use them commercially.
**Abstract Book Citations:** Authors, Title, HemaSphere, 2026;10:(S1):pages. The abstract book DOIs can be found on the "Author Information" page.
**Disclaimer:** Articles published in the journal HemaSphere exclusively reflect the opinions of the authors. The authors are responsible for all content in their abstracts including accuracy of the facts, statements, citing resources, etc.

### Summary/Conclusion

This first quantitative meta-analysis demonstrates that generic imatinib achieves comparable CCyR, MMR, and OS to branded imatinib in CML-CP. Higher treatment failure and worse EFS with generics warrant monitoring but may reflect confounding in lower-quality studies, as meta-regression showed convergence in recent, higher-quality evidence. Switching from branded to generic imatinib is safe, with >90% maintaining response. These findings support the ELN recommendation of generic imatinib as cost-effective frontline CML-CP therapy.

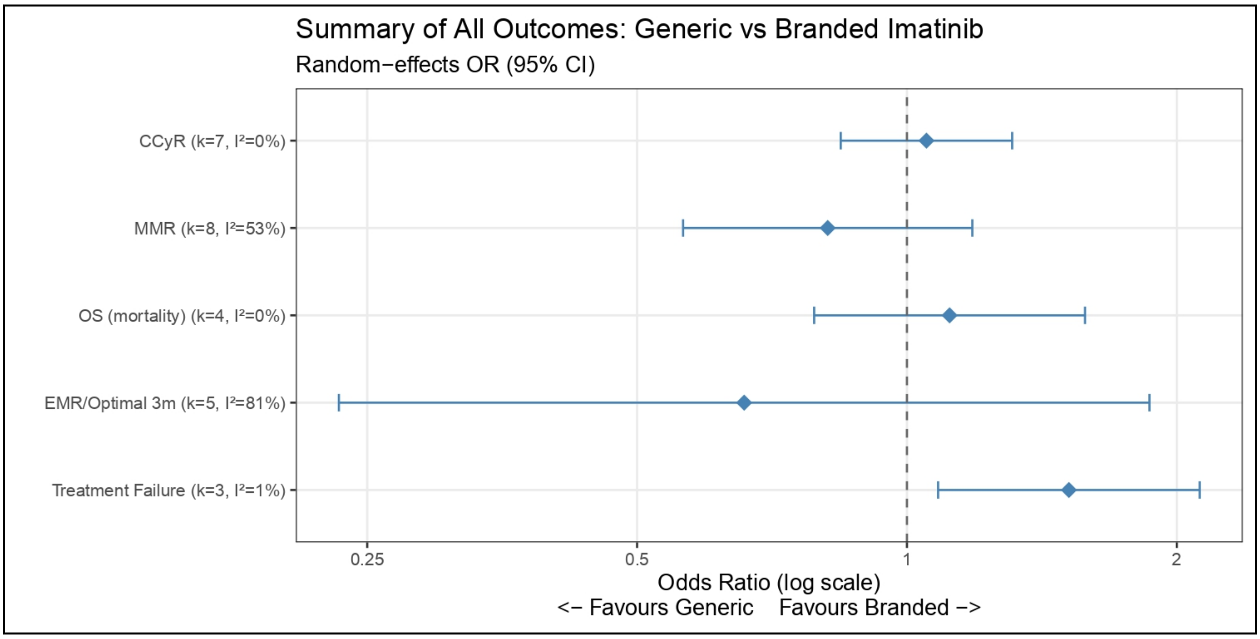

Forest plot

**Copyright Information:** (Online) ISSN: 2572-9241
©2026 The Author(s). HemaSphere is published by John Wiley & Sons Ltd on behalf of European Hematology Association. This is an open access Abstract Book distributed under the Attribution-NonCommercial-NoDerivs (CC BY-NC-ND), which allows third parties to download the articles and share them with others as long as they credit the author and the Abstract Book, but they cannot change the content in any way or use them commercially.
**Abstract Book Citations:** Authors, Title, HemaSphere, 2026;10:(S1):pages. The abstract book DOIs can be found on the "Author Information" page.
**Disclaimer:** Articles published in the journal HemaSphere exclusively reflect the opinions of the authors. The authors are responsible for all content in their abstracts including accuracy of the facts, statements, citing resources, etc.

### (PB3013) EVALUATION OF THE EFFICACY OF IMATINIB IN NEWLY DIAGNOSED CML PATIENTS WITH T(9;22;?)(Q34;Q11.2;?) THREE-WAY TRANSLOCATION

**Topic:** 08. Chronic myeloid leukemia - Clinical

Wusheng Wang*[1, 1], Xiangui Peng[1, 1], Hao Wang[1, 1], Xi Zhang[1]

[1]*Xinqiao Hospital of Army Medical University, CHONGQING, China;*

### Aims

To evaluate the efficacy of imatinib in newly diagnosed chronic myeloid leukemia (CML) patients with t(9;22;?)(q34;q11.2;?) three-way translocation.

### Methods

A total of 15 newly diagnosed chronic myeloid leukemia (CML) patients with t(9;22;?)(q34;q11.2;?) three-way translocation and 15 newly diagnosed CML patients with t(9;22)(q34;q11.2) canonical translocation were enrolled. Bone marrow morphology, bone marrow biopsy, flow cytometry immunophenotyping, chromosome analysis (CA), fluorescence in situ hybridization (FISH), quantitative real-time polymerase chain reaction (qPCR), and clinical data were comprehensively analyzed.The inclusion criteria were as follows: positive BCR::ABL1 P210 fusion gene detected by qPCR; 1F2R2G single-fusion signal pattern of BCR::ABL1 observed by FISH; t(9;22;?)(q34;q11.2;?) three-way translocation identified by karyotyping and confirmed by metaphase FISH.Treatment responses to imatinib (first-generation tyrosine kinase inhibitor, TKI) were monitored in all the above patients. Follow-up and comprehensive prognostic analyses were performed, including complete remission, non-remission, partial remission, and progression to acute leukemia.

### Results

Among the 15 newly diagnosed CML patients with three-way translocation, 5 patients achieved remission after oral administration of imatinib. 8 patients showed poor response to imatinib, with persistent BCR::ABL1 fusion gene detected by PCR, indicating molecular non-remission. After switching to third-generation TKI treatment, BCR::ABL1 became negative by PCR, indicating molecular remission accompanied by thrombocytopenia. 2 patients developed blast crisis of CML and received salvage chemotherapy with the IA regimen (idarubicin [IDA] combined with cytarabine [Ara-C]), followed by allogeneic hematopoietic stem cell transplantation.Among the control group, 13 achieved remission after imatinib treatment, and the remaining 2 achieved remission after switching to third-generation TKI, accompanied by thrombocytopenia.

### Summary/Conclusion

Newly diagnosed BCR::ABL1-positive chronic myeloid leukemia (CML) patients with t(9;22;?)(q34;q11.2;?) three-way translocation exhibit inferior response to imatinib compared with those carrying the typical t(9;22)(q34;q11.2) translocation. Switching to third-generation tyrosine kinase inhibitors (T/KIs) might improve their prognosis.

**Copyright Information:** (Online) ISSN: 2572-9241
©2026 The Author(s). HemaSphere is published by John Wiley & Sons Ltd on behalf of European Hematology Association. This is an open access Abstract Book distributed under the Attribution-NonCommercial-NoDerivs (CC BY-NC-ND), which allows third parties to download the articles and share them with others as long as they credit the author and the Abstract Book, but they cannot change the content in any way or use them commercially.
**Abstract Book Citations:** Authors, Title, HemaSphere, 2026;10:(S1):pages. The abstract book DOIs can be found on the "Author Information" page.
**Disclaimer:** Articles published in the journal HemaSphere exclusively reflect the opinions of the authors. The authors are responsible for all content in their abstracts including accuracy of the facts, statements, citing resources, etc.

### (PB3014) ANALYSIS OF SECONDARY MALIGNANCIES IN PATIENTS WITH CHRONIC MYELOID LEUKEMIA TREATED WITH TYROSINE KINASE INHIBITORS – A SINGLE-CENTER EXPERIENCE FROM POLAND

**Topic:** 08. Chronic myeloid leukemia - Clinical

Ewa Mędraś[1], Monika M. Biernat*[1], Tomasz Wróbel[1]

[1] *Wroclaw Medical University, Department and Clinic of Hematology, Cellular Therapies and Internal Medicine, Wrocław, Poland;*

### Background

The introduction of tyrosine kinase inhibitors (TKIs) has revolutionized the treatment of chronic myeloid leukemia (CML), leading to life expectancies for patients that now approach those of the general population. This prolonged survival, however, brings with it an increased risk of developing secondary malignancies. Data from international registries indicate a slightly elevated relative risk of second cancers in this patient population compared to the general population (standardized incidence ratio [SIR] = 1.204) [1,2]. In Poland, the annual incidence of malignant tumors ranges from 185,000 to 193,000 cases, corresponding to an incidence rate of approximately 463 per 100,000 inhabitants. The most prevalent cancers in the Polish population are lung, prostate, and colorectal cancer in men, and breast and colorectal cancer in women.

### Aims

This study aimed to assess the incidence and characterize the types of secondary malignancies occurring in CML patients treated at a single hematology center in Poland. To the best of our knowledge, this is the first such analysis conducted in a Polish population of CML patients in the TKI era.

### Methods

The analysis included 180 patients diagnosed with CML (105 men, 75 women) who were treated at a hematology outpatient clinic between 2000 and 2025. The median age of the study group was 49.5 years (range 18–82 years). A retrospective analysis of the incidence and characteristics of secondary malignancies in this population was performed.

### Results

Secondary malignancies were identified in 16 out of 180 patients (8.9%). In four cases (25%), CML was diagnosed as a secondary malignancy following another primary cancer. In 12 patients (75%), the secondary malignancy was diagnosed during the course of CML treatment. Among men, prostate cancer was the most prevalent (6 cases). In women, a diverse range of diagnoses was observed, including breast cancer, ovarian cancer, lung cancer, and follicular lymphoma. In two cases, the secondary malignancy was lymphoma.

**Copyright Information:** (Online) ISSN: 2572-9241
©2026 The Author(s). HemaSphere is published by John Wiley & Sons Ltd on behalf of European Hematology Association. This is an open access Abstract Book distributed under the Attribution-NonCommercial-NoDerivs (CC BY-NC-ND), which allows third parties to download the articles and share them with others as long as they credit the author and the Abstract Book, but they cannot change the content in any way or use them commercially.
**Abstract Book Citations:** Authors, Title, HemaSphere, 2026;10:(S1):pages. The abstract book DOIs can be found on the "Author Information" page.
**Disclaimer:** Articles published in the journal HemaSphere exclusively reflect the opinions of the authors. The authors are responsible for all content in their abstracts including accuracy of the facts, statements, citing resources, etc.

**Summary/Conclusion**

1. In the studied population of CML patients, the incidence of secondary malignancies (8.9%) is higher than that reported by other authors (4–5% according to SEER registry data) [1], which may suggest the existence of population-based or environmental differences.
2. The spectrum of secondary malignancy diagnoses in the study group is consistent with international observations, with a predominance of prostate cancer in men.
3. The observed incidence of secondary malignancies in the Polish population of CML patients requires confirmation through larger, population-based, multicenter analyses to determine whether this phenomenon is localized or reflects a nationwide trend.
4. It is essential to conduct systematic surveillance for secondary malignancies in CML patients, in accordance with general population screening recommendations.

**Copyright Information:** (Online) ISSN: 2572-9241
©2026 The Author(s). HemaSphere is published by John Wiley & Sons Ltd on behalf of European Hematology Association. This is an open access Abstract Book distributed under the Attribution-NonCommercial-NoDerivs (CC BY-NC-ND), which allows third parties to download the articles and share them with others as long as they credit the author and the Abstract Book, but they cannot change the content in any way or use them commercially.
**Abstract Book Citations:** Authors, Title, HemaSphere, 2026;10:(S1):pages. The abstract book DOIs can be found on the "Author Information" page.
**Disclaimer:** Articles published in the journal HemaSphere exclusively reflect the opinions of the authors. The authors are responsible for all content in their abstracts including accuracy of the facts, statements, citing resources, etc.

### (PB3015) SINGLE-TUBE MULTIPLEX ABSOLUTE QUANTIFICATION OF BCR::ABL1 TRANSCRIPTS FOR SENSITIVE MRD MONITORING IN CHRONIC MYELOID LEUKEMIA

**Topic:** 08. Chronic myeloid leukemia - Clinical

Jung Won Keum[1], Pamela Ward[2], Phillip Lai[2], Mihaela Campan[2], Ibrahim Hajjali[2], Dries Van Hemelen*[1], Eleen Shum[1], Christina Fan[1]

*[1]Countable Labs, Inc., Palo Alto, CA, United States of America;[2]USC Clinical Laboratories, Molecular Pathology, Los Angeles, CA, United States of America;*

### Background

Quantitative monitoring of *BCR-ABL1* transcripts is central to minimal residual disease (MRD) assessment in chronic myeloid leukemia (CML). Current clinical workflows rely on RT-qPCR or digital PCR (dPCR), which require multi-step processing and, for qPCR, calibration to external standards for quantification. In addition, only a subset of isoforms are measured, making routine, cost-effective monitoring of rare isoforms challenging. A simplified approach enabling direct, absolute quantification across clinically relevant *BCR-ABL1* transcripts without standard curves could offer an alternative paradigm for MRD monitoring.

### Aims

To evaluate the analytical performance and clinical feasibility of a one-step, single-tube multiplex RNA assay enabling direct absolute molecular counting *of BCR::ABL1* fusion transcripts for MRD monitoring in CML.

### Methods

We developed a one-step, single-tube multiplex RNA assay for direct detection and absolute molecular counting of *BCR-ABL1* fusion transcripts(e14a2, e13a2, e1a2, and e19a2), encoding p210, p190, and p230 protein isoforms. Reverse transcription, amplification, and counting occur in a closed 50 µL reaction with individual molecules quantified without calibration curves by 3D light-sheet microscopy of the full reaction volume, eliminating dead volume and partitioning statistics.
Analytical performance was evaluated using BCR-ABL1–positive cell line RNA serially diluted into wild-type background. Accuracy was benchmarked using dilutions traceable to the international scale (%IS) , with dilutions detectable down to 0.003% IS (MR4.5). Clinical feasibility was assessed using RNA from diagnostic p210 and longitudinal p230 patient samples (USC Clinical Laboratories, Molecular Pathology).

### Results

Multiplex detection of p210, p190, and p230 isoforms was achieved within a single tube in the presence of approximately $10^6$ wild-type *ABL1* molecules. Absolute *BCR-ABL1* molecular counts decreased proportionally with %IS dilution, while wild-type*ABL1* counts remained stable, demonstrating linear quantification across a six-log dynamic range. The assay showed low variance and analytical sensitivity detectable down to 0.003% IS (MR4.5).

### Summary/Conclusion

This single-tube, PCR-based single-molecule counting approach enables direct, absolute quantification across four *BCR-ABL1* fusion transcripts without reliance on calibration standards during measurement. Concordance with %IS-referenced cell line dilutions and detection of diagnostic and longitudinal clinical samples demonstrate this as a more comprehensive and streamlined approach for *BCR-ABL1* quantification and MRD monitoring in CML.

**Copyright Information:** (Online) ISSN: 2572-9241
©2026 The Author(s). HemaSphere is published by John Wiley & Sons Ltd on behalf of European Hematology Association. This is an open access Abstract Book distributed under the Attribution-NonCommercial-NoDerivs (CC BY-NC-ND), which allows third parties to download the articles and share them with others as long as they credit the author and the Abstract Book, but they cannot change the content in any way or use them commercially.
**Abstract Book Citations:** Authors, Title, HemaSphere, 2026;10:(S1):pages. The abstract book DOIs can be found on the “Author Information” page.
**Disclaimer:** Articles published in the journal HemaSphere exclusively reflect the opinions of the authors. The authors are responsible for all content in their abstracts including accuracy of the facts, statements, citing resources, etc.

### (PB3016) RAPID MOLECULAR RESPONSES AND PRESERVED QUALITY OF LIFE WITH ASCIMINIB IN CHRONIC MYELOID LEUKEMIA: A REAL-WORLD STUDY

**Topic:** 08. Chronic myeloid leukemia - Clinical

Bingyu Lin*[1], Xin Du[2], Qiang Wang[1], Xuan Zhou[1], Bingbing Wen[2], Zhi Liu[3], Xiaoli Liu[1], Na Xu[1]

*[1]Nanfang Hospital, Southern Medical University CN, Hematology, Guangzhou, China;[2]The Second People's Hospital of Shenzhen, the First Affiliated Hospital of Shenzhen University, shenzhen, China; [3]Guangdong Second Provincial General Hospital (Guangdong Provincial Health Emergency Hospital), Guangzhou, China;*

### Background

With the introduction of tyrosine kinase inhibitors (TKIs), chronic myeloid leukemia (CML) has evolved into a manageable chronic disease, with disease control and preservation of quality of life (QoL) as major treatment goals. However, adverse events and cumulative toxicities of conventional ATP-competitive TKIs frequently lead to dose adjustments or discontinuations, compromising treatment adherence. Asciminib, a first-in-class STAMP inhibitor, has shown promising efficacy and favorable safety in clinical trials, yet real-world evidence on its effectiveness and QoL in Chinese CML patients remains limited.

### Aims

To evaluate the real-world effectiveness and QoL outcomes in CML patients receiving front-line (1L) or later-line (≥2L) asciminib in a real-world multicenter setting.

### Methods

This observational cohort study stratified CML patients treated with asciminib into 1L and relapsed/refractory ≥2L cohorts. Primary endpoints were Early Molecular Response (EMR) for the 1L cohort and Complete Cytogenetic Response (CCyR) equivalence for the ≥2L cohort. In the 1L cohort, longitudinal HRQoL and patient-reported outcomes were assessed using the EORTC QLQ-CML24 questionnaire.

### Results

**1L Cohort**: A total of 23 patients with CML-CP received front-line asciminib at 80 mg once daily. Among them, 10 were in the low-risk EUTOS Long-Term Survival (ELTS) group, 5 in the intermediate-risk ELTS group, and 8 in the high-risk ELTS group. 86.9% (20/23) achieved EMR (BCR/ABL<10%) at 3 months. Overall, 69.6% (16/23) attained Major Molecular Response (MMR), and 39.1% (9/23) achieved Deep Molecular Response (DMR) at 6 months. No arterial occlusive events (AOEs) or severe non-hematologic toxicities were observed. The main adverse event was manageable thrombocytopenia (17.4%, n=4; Grade 1, 1, 2, 3, n=1 each) without treatment interruption. Consistent with earlier trends, the expanded data show that by week 12, asciminib significantly improves QLQ-CML24 outcomes by reducing symptom burden, daily life impact, and worry.
**≥2L Cohort**: Eighteen heavily pretreated patients (median age 53.5 years; 1-4 prior TKIs) received asciminib, including 10 switched due to TKI intolerance and 8 due to TKI resistance (including ponatinib/olverembatinib failure and E255K mutations). Among them, 10 patients were switched to asciminib due to TKI intolerance (1 with hematologic adverse event, 9 with non-hematologic adverse events). Asciminib dosages: 6 received 80 mg once daily, 3 received 40 mg once daily, and 1 received 40 mg every other day. All 10 patients with TKI intolerance maintained CCyR efficacy. Among 8 patients with TKI resistance, 62.5% (5/8) achieved CCyR within 6 months. No AOEs were reported. The most common AEs were Grade 1 decreased appetite and mild edema (n=1 each). No patients discontinued asciminib due to AEs. Consistent with previous findings, the updated data demonstrate that by week 12, asciminib significantly improves QLQ-CML24 scores by reducing symptom burden, impairment in daily functioning, and disease-related worry.

**Copyright Information:** (Online) ISSN: 2572-9241
©2026 The Author(s). HemaSphere is published by John Wiley & Sons Ltd on behalf of European Hematology Association. This is an open access Abstract Book distributed under the Attribution-NonCommercial-NoDerivs (CC BY-NC-ND), which allows third parties to download the articles and share them with others as long as they credit the author and the Abstract Book, but they cannot change the content in any way or use them commercially.
**Abstract Book Citations:** Authors, Title, HemaSphere, 2026;10:(S1):pages. The abstract book DOIs can be found on the "Author Information" page.
**Disclaimer:** Articles published in the journal HemaSphere exclusively reflect the opinions of the authors. The authors are responsible for all content in their abstracts including accuracy of the facts, statements, citing resources, etc.

**Summary/Conclusion**

In this multicenter real-world cohort, asciminib demonstrated favorable clinical benefits in both front-line and later-line CML therapy, with excellent tolerability and no evidence of cardiovascular toxicity.

**Copyright Information:** (Online) ISSN: 2572-9241
©2026 The Author(s). HemaSphere is published by John Wiley & Sons Ltd on behalf of European Hematology Association. This is an open access Abstract Book distributed under the Attribution-NonCommercial-NoDerivs (CC BY-NC-ND), which allows third parties to download the articles and share them with others as long as they credit the author and the Abstract Book, but they cannot change the content in any way or use them commercially.
**Abstract Book Citations:** Authors, Title, HemaSphere, 2026;10:(S1):pages. The abstract book DOIs can be found on the "Author Information" page.
**Disclaimer:** Articles published in the journal HemaSphere exclusively reflect the opinions of the authors. The authors are responsible for all content in their abstracts including accuracy of the facts, statements, citing resources, etc.

### (PB3017) TOWARDS INDIVIDUALIZED TREATMENT STEWARDSHIP IN CHRONIC MYELOID LEUKEMIA BASED ON COMBINATION OF MOLECULAR DATA AND MATHEMATICAL MODELING

**Topic:** 08. Chronic myeloid leukemia - Clinical

Sebastian Gerdes[1], Vaidehi Krishnan[2], Naranie Shanmuganathan[3], Matthias Kuhn[1], Timothy Hughes[3], Tiong Ong[2, 4], Ingo Roeder*[1]

*[1]Faculty of Medicine, Technische Universität Dresden, Institute for Medical Informatics and Biometry, Dresden, Germany;[2]Duke-NUS Medical School, Cancer & Stem Cell Biology Signature Research Programme, Singapore, Singapore; [3]Precision Cancer Medicine Theme, South Australian Health and Medical Research Institute, Adelaide Medical School, University of Adelaide, Adelaide, Australia; [4]Singapore General Hospital, Department of Haematology, Singapore, Singapore;*

### Background

Chronic myeloid leukemia (CML) can be effectively controlled in the majority of patients by lifelong treatment with tyrosine kinase inhibitors (TKI). Although generally well tolerated, TKI therapy is associated with side effects and considerable costs. Clinical studies show that treatment-free remission (TFR) can be achieved in a substantial proportion of patients after treatment discontinuation. However, reliable criteria to identify suitable candidates and optimal timing remain limited. Previously developed mathematical models of CML show that disease dynamics after TKI dose alterations are predictive for TFR success [1] and that successful TKI discontinuation can in principle be predicted once patient-specific parameters are identified [2, 3]. However, parameter identification requires long observation periods and informative treatment perturbations, such as dose modifications. On the other hand, a recent study analyzing single-cell (sc)RNA data shortly after diagnosis suggests that early molecular signatures may contain predictive information [4].

### Aims

We investigate whether incorporating proliferation estimates derived from early scRNA-seq data can be integrated with established mathematical models of CML to enhance accurate estimation of patient-specific parameters, already early during therapy. This approach aims to support individualized predictions of treatment response, effects of dose adaptation, and optimal timing for safe TKI discontinuation.

### Methods

We combine longitudinal BCR::ABL1 measurements and a measure of proliferative activity inferred from early scRNA profiling [4] into a mathematical model of CML [2,3]. scRNA-based classifications for TFR success will be incorporated as prior information and constraints within an existing model-parameter estimation framework. Using the patient-specific parametrizations, we will build digital twins to simulate potential courses of treatment, including dose alterations and treatment cessation at different time points, prospectively, with the ultimate goal to support treatment optimization. Predictive performance is evaluated by comparing model-based relapse predictions with observed molecular disease trajectories.

### Results

Preliminary results suggest that integrating early molecular data into model calibration may substantially reduce the observation time required for reliable parameter identification, because uncertainties with respect to general individual immune response or the proliferative capacity of leukemic stem cells can be reduced by the molecular characterization. Furthermore, initial analyses indicate improved early relapse prediction compared with model fitting based solely on longitudinal monitoring data.

**Copyright Information:** (Online) ISSN: 2572-9241
©2026 The Author(s). HemaSphere is published by John Wiley & Sons Ltd on behalf of European Hematology Association. This is an open access Abstract Book distributed under the Attribution-NonCommercial-NoDerivs (CC BY-NC-ND), which allows third parties to download the articles and share them with others as long as they credit the author and the Abstract Book, but they cannot change the content in any way or use them commercially.
**Abstract Book Citations:** Authors, Title, HemaSphere, 2026;10:(S1):pages. The abstract book DOIs can be found on the “Author Information” page.
**Disclaimer:** Articles published in the journal HemaSphere exclusively reflect the opinions of the authors. The authors are responsible for all content in their abstracts including accuracy of the facts, statements, citing resources, etc.

**Summary/Conclusion**

Mechanistic mathematical models of CML could be strengthened by integrating biologically meaningful features derived from scRNA-seq data potentially paving the way for patient-specific digital twins. This approach would allow early prediction of disease dynamics and may support personalized decisions regarding TKI dose adaptation and treatment discontinuation.

References

1. Patterson et al. *Leukemia* (2026): 1-5.
2. Karg et al. *Frontiers in Oncology* 12 (2022): 1028871.
3. Fassoni et al. *npj Systems Biology and Applications* 11.1 (2025): 115.
4. Krishnan et al. *Blood* 146 (2025): 74.

**Copyright Information:** (Online) ISSN: 2572-9241
©2026 The Author(s). HemaSphere is published by John Wiley & Sons Ltd on behalf of European Hematology Association. This is an open access Abstract Book distributed under the Attribution-NonCommercial-NoDerivs (CC BY-NC-ND), which allows third parties to download the articles and share them with others as long as they credit the author and the Abstract Book, but they cannot change the content in any way or use them commercially.
**Abstract Book Citations:** Authors, Title, HemaSphere, 2026;10:(S1):pages. The abstract book DOIs can be found on the "Author Information" page.
**Disclaimer:** Articles published in the journal HemaSphere exclusively reflect the opinions of the authors. The authors are responsible for all content in their abstracts including accuracy of the facts, statements, citing resources, etc.

### (PB3018) PAX5::MLLT3 FUSION DEFINES A HIGH-RISK SHIFT TOWARD LYMPHOID BLAST EVOLUTION IN CML

**Topic:** 08. Chronic myeloid leukemia - Clinical

Matteo Caridi*[1, 2], Michela Tarnani[1], Giulio Trapè[1], Caterina Mercanti[1], Silvia Quattromani[3], Gloria Pessina[3], Gioia De Angelis[1], Ambra Di Veroli[1], Valentina Panichi[4], Giuseppe Topini[4], Loredana Bassi[3], Monica Poscente[3], Raffaella Isidori[3], Roberto La Tagliata[1], Paolo Cercola[3]

*[1]Hematology Unit-ASL Viterbo, Santa Rosa Hospital, Viterbo, Italy; [2]Department of Physiology and Pharmacology, La Sapienza University of Rome, Rome, Italy; [3]Medical Genetics and Molecular Biology - ASL Viterbo, Santa Rosa Hospital, Viterbo, Italy; [4]Laboratory of Immunophenotyping-ASL Viterbo, Belcolle Hospital, Viterbo, Italy., Viterbo, Italy;*

### Background

Tyrosine kinase inhibitors (TKIs) have profoundly modified the natural history of chronic myeloid leukemia (CML). The use of next-generation sequencing (NGS) to detect additional mutations at diagnosis currently has no formally established clinical indication; however, it is under investigation to identify alterations that may serve as prognostic factors capable of predicting the disease's natural course.

### Aims

To describe a case in which diagnostic NGS identified a rare PAX5::MLLT3 subclone that anticipated lymphoid blast-phase transformation and TKI resistance, and to explore its potential biological and clinical implications.

### Methods

Peripheral blood (PB) morphology, immunophenotyping, cytogenetics, and NGS (Illumina MiSeq, QIAseq Targeted DNA Custom Panel) were performed at diagnosis and at progression. An AlphaFold-based structural model of the PAX5::MLLT3 fusion protein was generated for the first time from the inferred junction sequence.

**Copyright Information:** (Online) ISSN: 2572-9241
©2026 The Author(s). HemaSphere is published by John Wiley & Sons Ltd on behalf of European Hematology Association. This is an open access Abstract Book distributed under the Attribution-NonCommercial-NoDerivs (CC BY-NC-ND), which allows third parties to download the articles and share them with others as long as they credit the author and the Abstract Book, but they cannot change the content in any way or use them commercially.
**Abstract Book Citations:** Authors, Title, HemaSphere, 2026;10:(S1):pages. The abstract book DOIs can be found on the "Author Information" page.
**Disclaimer:** Articles published in the journal HemaSphere exclusively reflect the opinions of the authors. The authors are responsible for all content in their abstracts including accuracy of the facts, statements, citing resources, etc.

### Results

A 48-year-old woman presented with marked leukocytosis (WBC 198×$10^9$/L), anemia, and splenomegaly. PB morphology showed neutrophilia with 5–10% myeloid blasts. NGS detected BCR::ABL1 (17.1%) and IKZF1 D252Efs*19 (26.5%). CML (WHO 2022) was diagnosed and imatinib was initiated, achieving complete hematologic response at 1 month.
After 5 months, abrupt progression occurred with lymphocytosis and severe thrombocytopenia. PB showed 90% lymphoid blasts. Cytogenetics, not evaluable at diagnosis, revealed trisomy 21. NGS identified ABL1 Y253H, ABL1 H396R, EZH2 T573A, and PAX5::MLLT3, alongside baseline alterations. Reanalysis of the diagnostic sample revealed a minor PAX5::MLLT3 subclone (0.5%), while ABL1 and EZH2 mutations were absent.
Dasatinib induced a transient hematologic and molecular response (BCR::ABL1/ABL1 0.52%), followed by relapse after 3 months.
PAX5::MLLT3 (PAX5-AF9) is extremely rare (≈6 reported cases) and enriched in B-ALL and lymphoid blast-phase CML. To the best of our knowledge, five patients with PAX5::MLLT3 rearrangement have been reported in the literature. Interestingly, including our patient, 3/6 patients were represented by lymphoid blast-phase CML, and the other three were B-ALL. Among the three B-ALL cases, one was a Ph+ ALL and one was a Ph-like (CRLF2::P2RY8) case. No additional information was available on the last patient.

The fusion retains the PAX5 paired DNA-binding domain and MLLT3 regulatory domains (Fig. 1), likely impairing B-cell differentiation. To facilitate translational investigation, we generated an AlphaFold-predicted 3D structure of the fusion protein from the inferred junction sequence, providing a tractable framework for hypothesis-driven functional studies and, ultimately, improving therapeutic strategies in this rare but clinically significant poor-prognosis setting.

### Summary/Conclusion

This case demonstrates that a micro-clone harboring an ultra–low VAF PAX5::MLLT3 fusion was already present at CML diagnosis and preceded both ABL1 kinase-domain mutations and lymphoid blast transformation. The coexistence of BCR::ABL1 and PAX5::MLLT3 may create a permissive environment for clonal evolution and TKI resistance. Early comprehensive NGS profiling may identify rare high-risk subsets and inform therapeutic strategies in CML.

**Copyright Information:** (Online) ISSN: 2572-9241
©2026 The Author(s). HemaSphere is published by John Wiley & Sons Ltd on behalf of European Hematology Association. This is an open access Abstract Book distributed under the Attribution-NonCommercial-NoDerivs (CC BY-NC-ND), which allows third parties to download the articles and share them with others as long as they credit the author and the Abstract Book, but they cannot change the content in any way or use them commercially.
**Abstract Book Citations:** Authors, Title, HemaSphere, 2026;10:(S1):pages. The abstract book DOIs can be found on the “Author Information” page.
**Disclaimer:** Articles published in the journal HemaSphere exclusively reflect the opinions of the authors. The authors are responsible for all content in their abstracts including accuracy of the facts, statements, citing resources, etc.

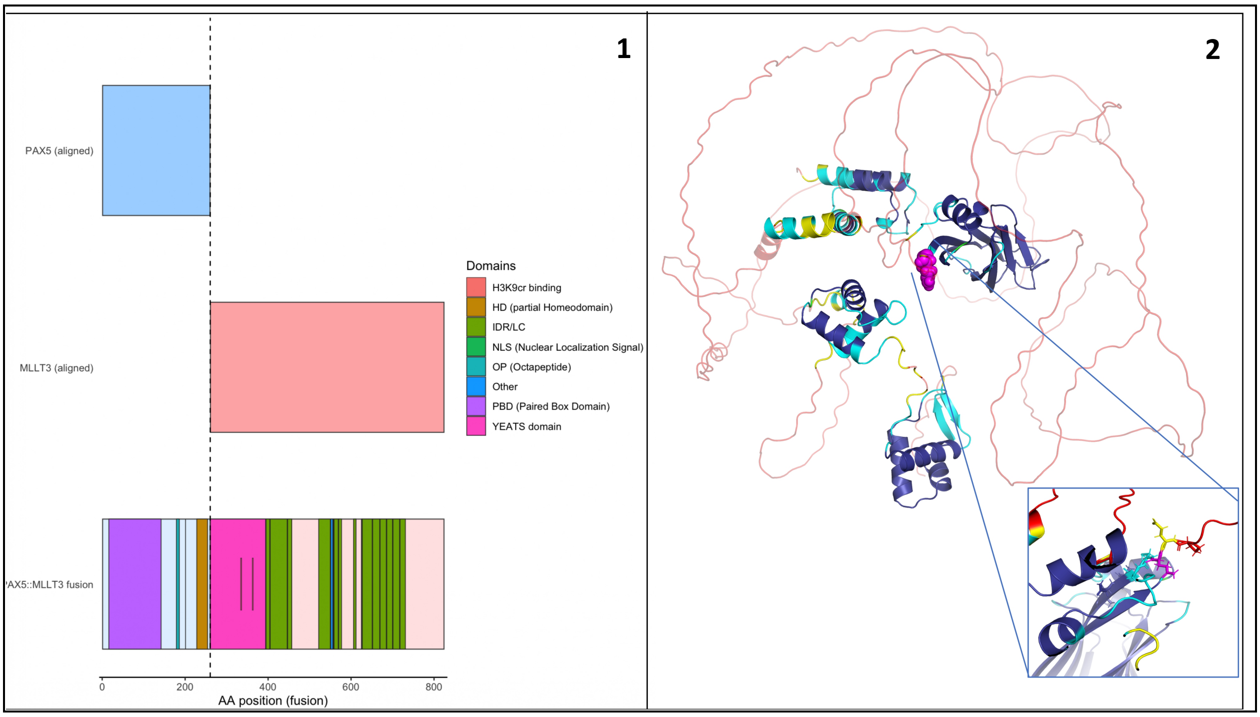

**Copyright Information:** (Online) ISSN: 2572-9241
©2026 The Author(s). HemaSphere is published by John Wiley & Sons Ltd on behalf of European Hematology Association. This is an open access Abstract Book distributed under the Attribution-NonCommercial-NoDerivs (CC BY-NC-ND), which allows third parties to download the articles and share them with others as long as they credit the author and the Abstract Book, but they cannot change the content in any way or use them commercially.
**Abstract Book Citations:** Authors, Title, HemaSphere, 2026;10:(S1):pages. The abstract book DOIs can be found on the "Author Information" page.
**Disclaimer:** Articles published in the journal HemaSphere exclusively reflect the opinions of the authors. The authors are responsible for all content in their abstracts including accuracy of the facts, statements, citing resources, etc.

### (PB3019) DIFFERENT BCR::ABL1 TRANSCRIPT LEVELS @ 3 AND 6 MONTHS MIGHT BE USEFUL IN IDENTIFYING WARNING/RESISTANT CML PATIENTS WHEN USING 2G-TKIS AS FRONTLINE TREATMENT

**Topic:** 08. Chronic myeloid leukemia - Clinical

Fabio Stagno*[1], Stefania Stella[2], Uros Markovic[3], Sabina Russo[1], Cristina Tomarchio[4], Silvia Vitale[5], Giuseppe Mirabile[1], Manlio Fazio[1], Maria Nasso[1], Veronica Vecchio[3], Michele Massimino[6], Livia Manzella[7], Francesco Di Raimondo[3], Alessandro Allegra[1]

*[1]Hematology Section, AOU Policlinico "G. Martino", University of Messina, Messina, Italy;[2]Department of Clinical and Experimental Medicine, University of Catania, 95123 Catania, Italy., Catania, Italy; [3]Hematology Unit & BMT, AOU Policlinico "Rodolico - San Marco", Catania, Catania, Italy; [4]Center of Experimental Oncology and Hematology, A.O.U. Policlinico "Rodolico-San Marco", Catania, Catania, Italy; [5]Center of Experimental Oncology and Hematology, A.O.U. Policlinico "Rodolico-San Marco", Catania, Messina, Italy; [6]Department of General Surgery and Medical-Surgical Specialties, University of Catania, Catania, Catania, Italy; [7]Department of Clinical and Experimental Medicine, University of Catania, Catania, Catania, Italy;*

### Background

**Background**: Current ELN recommendations suggest the achievement of a BCR::ABL1$^{IS}$ transcript level ≤10% at 3 months as mandatory for optimal response to initial TKIs-therapy. Nevertheless, this cut-off level was made equal for both 1G- and 2G-TKIs. Therefore, no molecular data at 3 and 6 months, except for Marin D. findings (Blood 2012), may help to identify those CML patients (pts) treated frontline with 2G-TKIs who both might have a low probability of achieving an optimal response and be candidates for a 3G-TKIs treatment.

### Aims

**Aim and Methods:** With this aim we investigated at diagnosis 104 CP-CML pts, evaluated in two hematological centers, who received 2G-TKIs as frontline therapy at standard dose. Clinical and molecular features are depicted in Table. All 104 pts received 2G-TKI as first line therapy and all had a valid sample for quantitative RT-PCR for BCR::ABL1$^{IS}$ analysis. CML pts were monitored for molecular response (MR) in the same laboratory according to ELN 2025.

### Results

**Results:** Our findings showed a median BCR::ABL1$^{IS}$ transcript level of 109,8% at diagnosis. CML pts were then stratified according to their MR to treatment. At 3 months 86/104 pts achieved an optimal response (OP: BCR::ABL1$^{IS}$ ≤10%) with a median BCR::ABL1$^{IS}$ level of 1,65%, whereas 18/104 displayed a warning/failure (W/F: BCR::ABL1$^{IS}$ ≥10%) feature with a BCR::ABL1$^{IS}$ level of 25,38%. At 6 months 81/104 showed an OP (BCR::ABL1$^{IS}$ ≤1%) with a median BCR::ABL1$^{IS}$ level of 0,2%, 12/104 had a W (BCR::ABL1$^{IS}$ >1-10%) with a median BCR::ABL1$^{IS}$ level of 3,2%, 5/104 had a F (BCR::ABL1$^{IS}$ >10%) with a median BCR::ABL1$^{IS}$ level of 23,1%, 6/104 not reached the follow-up.

**Copyright Information:** (Online) ISSN: 2572-9241
©2026 The Author(s). HemaSphere is published by John Wiley & Sons Ltd on behalf of European Hematology Association. This is an open access Abstract Book distributed under the Attribution-NonCommercial-NoDerivs (CC BY-NC-ND), which allows third parties to download the articles and share them with others as long as they credit the author and the Abstract Book, but they cannot change the content in any way or use them commercially.
**Abstract Book Citations:** Authors, Title, HemaSphere, 2026;10:(S1):pages. The abstract book DOIs can be found on the "Author Information" page.
**Disclaimer:** Articles published in the journal HemaSphere exclusively reflect the opinions of the authors. The authors are responsible for all content in their abstracts including accuracy of the facts, statements, citing resources, etc.

### Summary/Conclusion

**Summary:** In conclusion, these results are consistent with those of Marin D. et al and are significantly different from those already reported for 1G-TKI and highlight the need to revise the frontline MR kinetics of 2G-TKIs in the light of the possible sequential use of both the new 3G-TKIs and allosteric inhibitors.

### Table

| | | |
|---|---|---|
| Patients (N) | | 104 |
| Sex (M/F) | | 58/46 |
| Age (median, years) | | 50.3 |
| Sokal risk | Low | 40 |
| | Intermediate | 41 |
| | High | 18 |
| | missing | 5 |
| ELTS score | Low | 62 |
| | Intermediate | 28 |
| | High | 8 |
| | missing | 6 |
| Hb (median, g/dl) | | 11.5 |
| WBC x $10^9$/l(median) | | 150,0 |
| PLT x $10^9$/L (median) | | 492,3 |
| Ph-positive (%) | | 100 |
| Complex Kariotype and ACA (N) | | 13/104 |
| Transcript Type | e13a2 (b2a2) | 49 |
| | e14a2 (b3a2) | 54 |
| | e1a2 (p190) | 1 |
| Frontline 2G-TKI | DAS | 32 |
| | NIL | 70 |
| | BOS | 2 |

**Copyright Information:** (Online) ISSN: 2572-9241
©2026 The Author(s). HemaSphere is published by John Wiley & Sons Ltd on behalf of European Hematology Association. This is an open access Abstract Book distributed under the Attribution-NonCommercial-NoDerivs (CC BY-NC-ND), which allows third parties to download the articles and share them with others as long as they credit the author and the Abstract Book, but they cannot change the content in any way or use them commercially.
**Abstract Book Citations:** Authors, Title, HemaSphere, 2026;10:(S1):pages. The abstract book DOIs can be found on the “Author Information” page.
**Disclaimer:** Articles published in the journal HemaSphere exclusively reflect the opinions of the authors. The authors are responsible for all content in their abstracts including accuracy of the facts, statements, citing resources, etc.

### (PB3020) LONG-TERM OUTCOMES OF CHRONIC MYELOID LEUKEMIA IN CHILDREN AND ADOLESCENTS TREATED WITH GENERIC IMATINIB AS FIRST-LINE AND ITK2 AS SECOND-LINE THERAPY : A RETROSPECTIVE STUDY.

**Topic:** 08. Chronic myeloid leukemia - Clinical

Yamina Bouchakour Moussa[1], Souad Taoussi[2], Salima Oukid[2], Fatima Lamraoui[2], Nabila Rekab*[2], Khadoudj Mounira Benlabiod[2], Hamida Brahimi[2], Zineb Ferdi[2], Amina Rebouh[3], Ahmed Amine Ziani[2], Bradai Mohamed[2]

*[1]SERVICE D'HEMATOLOGIE EHS ELCC, FACULTY OF MEDECINE, BLIDA, Algeria; [2]SERVICE HEMATOLOGY EHS ELCC ZABANA, FACULTY OF MEDECINE, BLIDA, Algeria; [3]SERVICE HEMETOLOGY EHS ELCC ZABANA, FACULTY OF MEDECINE, BLIDA, Algeria;*

### Background

Chronic myeloid leukemia (CML) in children and adolescents is rare ,, accounts for less than 10% of pediatric leukemias.The management has been profoundly transformed by the introduction of tyrosine kinase inhibitors (TKIs), which have become the standard first-line treatment. These targeted therapies achieve high rates of , cytogenetic, and molecular responses, radically changing the prognosis. The current challenge goes beyond simply controlling the disease and concerns optimizing long-term follow-up, managing adverse effects, and improving the quality of life of patients undergoing prolonged treatment.

### Aims

Among 653 patients followed for CML in all phases, 29(4.4%) were aged ≤ 20 years. W e analyzed clinical, hematological, cytogenetic, and evolutionary characteristics, as well as response to first-line generic imatinib (IMg) and second-generation TKIs in cases of failure or intolérance, with a mean follow-up of 104 months (3–228).

### Results

The cohort included 12 children,mean age 10.5years(2-14), and16 adolescents mean age18 years ,with a predominance of males.The mean time to diagnosis was 5 months. Splenomegaly was present in 83% of patients, hyperleukocytosis in 100%, anemia in 83%, and thrombocytosis in 48%. The mean myeloid percentage was 39%, with basophilia >5% in 34%. Medullary blasts were <5% in 69% of cases.Philadelphia chromosome was present in 100%. At diagnosis,86% were in the chronic phase, 4% in the accelerated phase, and 10% in the blast phase.48% were at high risk Sokal.1$^{st}$-line treatment was IMg in 28 patients and nilotinib in one patient. 3 blast phases received IMg combined with chemotherapy followed by allogeneic transplantation.**Under IMg**, at 3 months, complete hematologic response (CHR) was achieved in 96%. Complete cytogenetic response (CCyR) was observed in 27%, and early molecular response in 61.5%. At 6–12 months, 69% had an optimal response. With longer follow-up,CHR was maintained in 92%, and major molecular response (MMR) was achieved in 77%, including deep molecular responses (DMR) (2MR4/11MR4.5) .
Progression occurred in 32% of patients :2 early transformations, 2 post-transplant relapses, and 5losses of CCyR. 2$^{nd}$-line , dasatinib (7 patients) resulted in 2DMR, where as 5 patients experienced treatment failure, including AP and BP case ; Nilotinib (2 patients) induced one MMR; one patient required third-line switching.**Tolerance** was marked by frequent early effects (edema 24%, bone pain 34%, grade 3–4 cytopenias) but these were generally controllable. No growth or pubertal delays were observed. One case of gynecomastia and one case of ovarian cancer occurring after 8 years of IMg were reported. At the end of follow-up, 86% of patients were alive,13 were still on IMg, with a median OS NR,04 on dasatinib, 02 on nilotinib, and one transplant recipient (OS of 192 months). 5 deaths occurred after acute transformation. After a DMR under IMg confirmed for more than7 years ,5 patients discontinued treatment (TFR), with a maintenance rate of 100% at 36 months ; secondary,one loss of MMR at 60months, was recovered after resumption of IMG.

**Copyright Information:** (Online) ISSN: 2572-9241
©2026 The Author(s). HemaSphere is published by John Wiley & Sons Ltd on behalf of European Hematology Association. This is an open access Abstract Book distributed under the Attribution-NonCommercial-NoDerivs (CC BY-NC-ND), which allows third parties to download the articles and share them with others as long as they credit the author and the Abstract Book, but they cannot change the content in any way or use them commercially.
**Abstract Book Citations:** Authors, Title, HemaSphere, 2026;10:(S1):pages. The abstract book DOIs can be found on the "Author Information" page.
**Disclaimer:** Articles published in the journal HemaSphere exclusively reflect the opinions of the authors. The authors are responsible for all content in their abstracts including accuracy of the facts, statements, citing resources, etc.

**Summary/Conclusion**

Frontline generic imatinib provides durable molecular responses and favorable long-term survival in pediatric CML. Second-generation TKIs offer effective salvage therapy. Nevertheless, blast transformation remains associated with poor prognosis. Prospective studies are needed to define optimal frontline strategies in high-risk patients and refine TFR approaches in the pediatric population.

**Copyright Information:** (Online) ISSN: 2572-9241
©2026 The Author(s). HemaSphere is published by John Wiley & Sons Ltd on behalf of European Hematology Association. This is an open access Abstract Book distributed under the Attribution-NonCommercial-NoDerivs (CC BY-NC-ND), which allows third parties to download the articles and share them with others as long as they credit the author and the Abstract Book, but they cannot change the content in any way or use them commercially.
**Abstract Book Citations:** Authors, Title, HemaSphere, 2026;10:(S1):pages. The abstract book DOIs can be found on the "Author Information" page.
**Disclaimer:** Articles published in the journal HemaSphere exclusively reflect the opinions of the authors. The authors are responsible for all content in their abstracts including accuracy of the facts, statements, citing resources, etc.

### (PB3021) A PHASE 3 STUDY OF OLVEREMBATINIB (HQP1351) IN PATIENTS WITH CHRONIC-PHASE CHRONIC MYELOID LEUKEMIA: POLARIS-2 TRIAL IN PROGRESS

**Topic:** 08. Chronic myeloid leukemia - Clinical

Elias Jabbour*[1], Hagop Kantarjian[1], Anna Turkina[2], Elza Lomaia[3], Dennis Kim[4], Celeste Bremer[5], Vivian G. Oehler[6], Nicholas Viiala[7], Alexey Kuvshinov[8], María José Fernández Llavador[9], Dong-Yeop Shin[10], Paul Koller[11], Sebastian Grosicki[12], Ilisse Minto[13], Taryn Losch-beridon[13], Agnes Yong[14], Adriano Salaroli[15], Santhosh Ambika[16], Elena Crisà[17], Aleksandra Butrym[18], Dominik Chraniuk[19], Sergey Voloshin[20], Gonzalo Caballero Navarro[21], Jasson Andres Villarreal Hernandez[22], Blanca Xicoy Cirici[23], Dragana Milojkovic[24], Vallari Shah[13], Lynda Beaupin[13], Wanxing Chai-Ho[25], Jun Choi[26], Shaker Dakhil[27], Omer Jamy[28], Kathryn Shantz[13], Miguel Rosado Cadena[13], Lixin Jiang[13], Zhihong Yang[13], Ximena Jordan-Bruno[29], Jamie Koprivnikar[30], Reinhold Munker[31], Joshua Zeidner[32], Huanshan Guo[33], Zi Chen[34], Dajun Yang[34, 35], Yifan Zhai[13, 33, 34]

*[1]Department of Leukemia, The University of Texas MD Anderson Cancer Center, Houston, TX, United States of America; [2]National Medical Research Center for Hematology, Moscow, Russia; [3]National Medical Research Center named after V.A. Almazov, Saint Petersburg, Russia; [4]Princess Margaret Cancer Centre, University Health Network, University of Toronto, Toronto, Canada; [5]Virginia Oncology Associates, Norfolk, VA, United States of America; [6]Translational Science and Therapeutics, Fred Hutchinson Cancer Center, Seattle, WA, United States of America; [7]Liverpool Hospital – South Western Sydney Local Health District, Sydney, Australia; [8]Russian Research Institute of Hematology and Transfusiology of the Federal Medical and Biological Agency, Saint Petersburg, Russia; [9]Hospital Universitario Dr. Peset, Valencia, Spain; [10]Seoul National University Hospital, Seoul, South Korea; [11]Department of Hematology and Hematopoietic Cell Transplantation in the Division of Leukemia, City of Hope National Medical Center, Duarte, CA, United States of America; [12]Department of Cancer Prevention, Medical University of Silesia, Katowice, Poland; [13]Ascentage Pharma Group Inc., Rockville, MD, United States of America; [14]Royal Perth Hospital, Perth, Australia; [15]Institut Jules Bordet, Brussels, Belgium; [16]Cancer Care Specialists, Reno, NV, United States of America; [17]Fondazione del Piemonte per l'Oncologia IRCC Candiolo, Candiolo, Italy; [18]Wroclaw Medical University, Wroclaw, Poland, Specjalistyczny Szpital im. Dr A. Sokołowskiego, Wrocław, Poland; [19]MICS Centrum Medyczne, Toruń, Poland; [20]Leningrad Regional Clinical Hospital, Saint Petersburg, Russia; [21]Fundación para la Investigación Biomédica del Hospital Universitario 12 de Octubre, Madrid, Spain; [22]ICO L'Hospitalet – Hospital Duran i Reynals, Barcelona, Spain; [23]ICO Badalona – Hospital Universitari Germans Trias i Pujol, Badalona, Spain; [24]Hammersmith Hospital, London, United Kingdom; [25]University of California Los Angeles, Los Angeles, CA, United States of America; [26]NYU Langone Health Perlmutter Cancer Center, New York, NY, United States of America; [27]Cancer Center of Kansas, Wichita, KS, United States of America; [28]Division of Hematology and Oncology, Department of Medicine, University of Alabama at Birmingham, Birmingham, AL, United States of America; [29]University of Pennsylvania, Philadelphia, PA, United States of America; [30]Hackensack University Medical Center, Hackensack, NJ, United States of America; [31]University of Kentucky, Lexington, KY, United States of America; [32]University of North Carolina Chapel Hill, Chapel Hill, NC, United States of America; [33]Guangzhou Healthquest Pharma Co., Ltd., Guangzhou, China; [34]Ascentage Pharma (Suzhou) Co., Ltd., Suzhou, China; [35]Department of Experimental Research, State Key Laboratory of Oncology in South China Collaborative Innovation Center for Cancer Medicine, Sun Yat-sen University Cancer Center, Guangzhou, China;*

### Background

Despite advances in the management of chronic myeloid leukemia (CML), effective treatment options in later lines are limited. Treatment failure rates increase with each line of treatment, and sequential tyrosine kinase inhibitor (TKI) therapy is associated with increased resistance. Olverembatinib is a novel third-generation BCR::ABL1 TKI with activity against wild-type BCR::ABL1, multiple resistance-conferring mutations including T315I, and challenging compound mutations. It has also demonstrated activity in heavily pretreated patients with CML who received prior third-generation TKIs (i.e., asciminib and ponatinib) in phase 1 and 2 trials.

**Copyright Information:** (Online) ISSN: 2572-9241
©2026 The Author(s). HemaSphere is published by John Wiley & Sons Ltd on behalf of European Hematology Association. This is an open access Abstract Book distributed under the Attribution-NonCommercial-NoDerivs (CC BY-NC-ND), which allows third parties to download the articles and share them with others as long as they credit the author and the Abstract Book, but they cannot change the content in any way or use them commercially.
**Abstract Book Citations:** Authors, Title, HemaSphere, 2026;10:(S1):pages. The abstract book DOIs can be found on the "Author Information" page.
**Disclaimer:** Articles published in the journal HemaSphere exclusively reflect the opinions of the authors. The authors are responsible for all content in their abstracts including accuracy of the facts, statements, citing resources, etc.

### Aims

This study evaluates the efficacy and safety of olverembatinib in patients with relapsed/refractory chronic phase CML (CP-CML).

### Methods

POLARIS-2 (HQP1351CG301; NCT06423911) is an ongoing global, phase 3, multicenter, open-label, randomized registrational study that includes two patient cohorts. Part A randomly allocates patients with CP-CML previously treated with at least two approved TKIs to receive either olverembatinib or bosutinib (2:1 randomization). The primary endpoint is major molecular response (MMR) rate at 24 weeks, and the key secondary endpoint is MMR at 96 weeks. Allocation is stratified by the BCR::ABL1 ratio. Patients in Part A receiving bosutinib with treatment failure are allowed to cross over to olverembatinib.
Part B is a single-arm study in patients with CP-CML and the T315I mutation at screening; all patients receive olverembatinib. The primary endpoint is MMR rate by 24 weeks, and the key secondary endpoint is MMR by 96 weeks. Key inclusion criteria include age ≥18 years, diagnosis of CP-CML, Eastern Cooperative Oncology Group performance status ≤2, and adequate organ function. Patients in Part A must have had prior treatment with at least two approved TKIs. Part B patients must have the T315I mutation. Key exclusion criteria include any active infection requiring systemic therapy, hypersensitivity to study drugs, and pregnancy or lactation. The study hypothesis posits that olverembatinib will demonstrate superior MMR compared to bosutinib in Part A and provide clinical benefit in T315I-positive patients in Part B.
An estimated 333 patients are expected to be enrolled in the study, with 285 in part A and 48 in part B.

### Summary/Conclusion

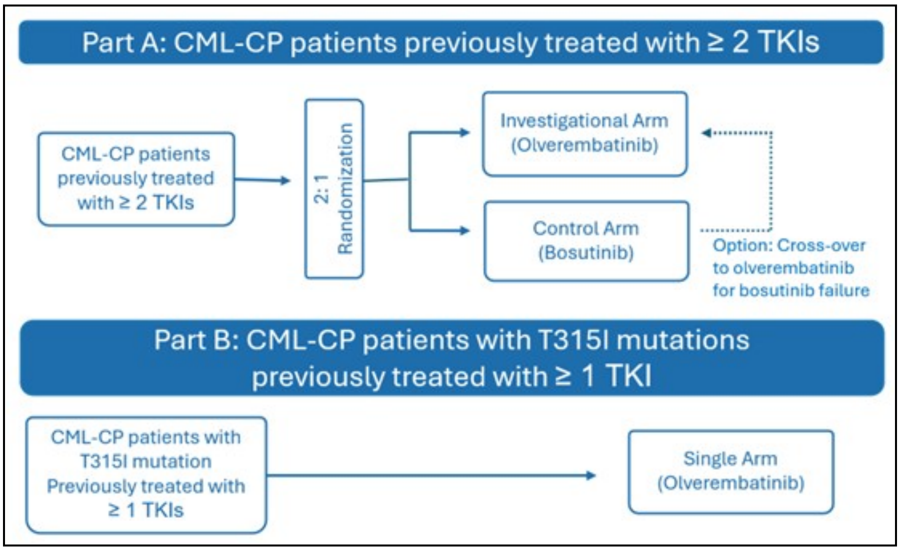

Figure. POLARIS-2 study design

**Copyright Information:** (Online) ISSN: 2572-9241
©2026 The Author(s). HemaSphere is published by John Wiley & Sons Ltd on behalf of European Hematology Association. This is an open access Abstract Book distributed under the Attribution-NonCommercial-NoDerivs (CC BY-NC-ND), which allows third parties to download the articles and share them with others as long as they credit the author and the Abstract Book, but they cannot change the content in any way or use them commercially.
**Abstract Book Citations:** Authors, Title, HemaSphere, 2026;10:(S1):pages. The abstract book DOIs can be found on the "Author Information" page.
**Disclaimer:** Articles published in the journal HemaSphere exclusively reflect the opinions of the authors. The authors are responsible for all content in their abstracts including accuracy of the facts, statements, citing resources, etc.

### (PB3022) ASC4KIDS-2 TRIAL-IN-PROGRESS: ASCIMINIB IN PEDIATRIC CHRONIC MYELOID LEUKEMIA WITH OR WITHOUT T315I

**Topic:** 08. Chronic myeloid leukemia - Clinical

Markus Metzler*[1], Andrew Place[2], Cornelia Garnitz[3], Ana Paula Torres Cardoso[4], Matthias Hoch[5], Sara Quenet[5], C. Michel Zwaan[6], Nobuko Hijiya[7]

*[1]University Hospital Erlangen, Erlangen, Germany; [2]Dana-Farber/Boston Children's Cancer and Blood Disorders Center, Boston, United States of America; [3]CML Patient Advocates Network and Leukämie-Online/LeukaNET e.V., Hohenbrunn, Germany; [4]Novartis Pharmaceuticals Corporation, East Hanover, United States of America; [5]Novartis Pharma AG, Basel, Switzerland; [6]Princess Máxima Center for Pediatric Oncology, Utrecht and Erasmus MC Sophia Children's Hospital, Utrecht, The Netherlands; [7]Columbia University Irving Medical Center, New York, United States of America;*

### Background

Adenosine triphosphate (ATP)-competitive tyrosine kinase inhibitors (TKIs), including imatinib, dasatinib, nilotinib, and bosutinib, are the standard of care for pediatric patients with chronic myeloid leukemia in chronic phase (CML-CP), with efficacy and safety profiles generally comparable to those observed in adults. However, TKIs can cause long-term adverse effects in pediatric patients, including delayed growth and development. Effective therapies that minimize long-term adverse effects in pediatric patients are needed. No TKI is approved for pediatric patients with the T315I mutation, which is linked with lower progression-free and overall survival.

Asciminib represents an alternative to ATP-competitive TKIs that works by Specifically Targeting the ABL Myristoyl Pocket (STAMP). Asciminib is approved for adults with newly diagnosed and previously treated CML-CP (at 80 mg daily), and those harboring T315I (at 200 mg twice daily). In ASC4Kids (NCT04925479), asciminib was well-tolerated and efficacious in pediatric patients resistant or intolerant to 1 prior TKI without T315I.

Here, we describe ASC4Kids-2 (NCT07354074), a phase 2 study evaluating asciminib in pediatric patients with CML-CP.

### Aims

The primary objective is to evaluate asciminib efficacy via major molecular response (MMR) at week 48 in pediatric patients with CML-CP who are (1) newly diagnosed without known T315I, (2) resistant or intolerant to ≥1 previous TKI without known T315I, or (3) with known T315I irrespective of prior TKIs.

**Copyright Information:** (Online) ISSN: 2572-9241
©2026 The Author(s). HemaSphere is published by John Wiley & Sons Ltd on behalf of European Hematology Association. This is an open access Abstract Book distributed under the Attribution-NonCommercial-NoDerivs (CC BY-NC-ND), which allows third parties to download the articles and share them with others as long as they credit the author and the Abstract Book, but they cannot change the content in any way or use them commercially.
**Abstract Book Citations:** Authors, Title, HemaSphere, 2026;10:(S1):pages. The abstract book DOIs can be found on the "Author Information" page.
**Disclaimer:** Articles published in the journal HemaSphere exclusively reflect the opinions of the authors. The authors are responsible for all content in their abstracts including accuracy of the facts, statements, citing resources, etc.

**Methods**

ASC4Kids-2 is a multicenter, open-label, single-arm study evaluating asciminib in approximately 50 pediatric patients aged 1 to <18 years with newly diagnosed or previously treated Ph+ CML-CP with or without known T315I, who provide informed consent. Asciminib will be administered at 80 mg once daily, or 200 mg twice daily for patients with T315I, using the adult formulation under fasting conditions or at an equivalent body weight–based dose of the pediatric formulation (2.6 mg/kg once daily or 6.5 mg/kg twice daily for patients with T315I, rounded to the nearest 5 mg) in the fed state.
ASC4Kids-2 includes a 22-day screening period followed by a treatment period with visits at weeks 1, 2, 4, 8, 12, 24, 36, and 48, then every 12 weeks. A posttreatment follow-up period includes a 30-day safety follow-up call and survival assessments every 12 weeks until study end, which is 240 weeks after the last patient receives the first dose.
The primary endpoint is MMR at week 48 (defined as the proportion of patients who meet MMR criteria at week 48; those who discontinue treatment before or lose response before/at week 48 will be considered nonresponders). Secondary endpoints include MMR at week 96; hematologic, cytogenetic, and molecular responses at and by scheduled time points; time to response, progression, or treatment failure; duration of response; event-free and overall survival; growth and sexual maturation (assessed via height, weight, bone age measured by x-ray, and Tanner staging); safety; and pharmacokinetic parameters.

**Results**

First-patient-first-visit is planned for Q2/2026.

**Summary/Conclusion**

ASC4Kids-2 will evaluate safety and efficacy of asciminib in pediatric patients with Ph+ CML-CP.

**Copyright Information:** (Online) ISSN: 2572-9241
©2026 The Author(s). HemaSphere is published by John Wiley & Sons Ltd on behalf of European Hematology Association. This is an open access Abstract Book distributed under the Attribution-NonCommercial-NoDerivs (CC BY-NC-ND), which allows third parties to download the articles and share them with others as long as they credit the author and the Abstract Book, but they cannot change the content in any way or use them commercially.
**Abstract Book Citations:** Authors, Title, HemaSphere, 2026;10:(S1):pages. The abstract book DOIs can be found on the “Author Information” page.
**Disclaimer:** Articles published in the journal HemaSphere exclusively reflect the opinions of the authors. The authors are responsible for all content in their abstracts including accuracy of the facts, statements, citing resources, etc.

### (PB3024) REAL-WORLD EXPERIENCE OF TREATMENT-FREE REMISSION AFTER TKI DISCONTINUATION IN CHRONIC MYELOID LEUKEMIA: WITH REDUCED FREQUENCY OF MOLECULAR MONITORING OF BCR::ABL1 QPCR TEST

**Topic:** 08. Chronic myeloid leukemia - Clinical

Maria Agustina Perusini*[1, 2], Jessica Danquah[1, 2], Ana Flavia Patino[1], Hassan, Sibai[1, 2], Dennis Kim[1, 2]

*[1]University Health Network, Princess Margaret Cancer Centre, Toronto, Canada;[2]Faculty of Medicine, University of Toronto, Toronto, Canada;*

### Background

Approximately 95% of patients with chronic myeloid leukemia (CML) are diagnosed in chronic phase (CP), which typically follows an indolent course and is highly responsive to tyrosine kinase inhibitor (TKI) therapy. Patients with CML-CP who achieve an optimal molecular response have a life expectancy comparable to that of age-matched individuals in the general population. Consequently, the therapeutic goal in CML has evolved toward treatment-free remission (TFR), defined as sustained leukemia control after discontinuation (DISC) of TKI therapy, without any active leukemia therapy. In our clinical practice, we implemented a pragmatic molecular monitoring schedule consisting of BCR::ABL1 assessment every 6–8 weeks during the first 6 months after TKI discontinuation, followed by testing every 3 months from months 6 to 18.

### Aims

We conducted a retrospective real-world analysis to evaluate outcomes following discontinuation of TKI therapy.

### Methods

We retrospectively included all patients who attempted TFR at the discretion of the treating physician. Molecular relapse-free survival (mRFS) was calculated from the date of TKI discontinuation to the date of confirmed loss of major molecular response (MMR; BCR::ABL1 ≥0.1% on the International Scale). Predictors of molecular relapse were evaluated using Cox proportional hazards regression models.

### Results

Seventy-nine patients who attempted TFR were included. The discontinued TKIs were imatinib in 33 patients (42%), dasatinib in 23 (29%), nilotinib in 19 (24%), bosutinib in 2 (3%), and asciminib in 2 (3%). Most of the patients were previously treated with 1-line TKI therapy, median 1 (range, 1–3). Thirty-one patients (40%) decided on TKI discontinuation mainly because of drug-related toxicity leading to intolerance, while 46 (58%) elected to stop attempting TFR without having any issue of tolerability.
Sixty-six patients (83%) had achieved MR4 prior to discontinuation, with a median MR4 duration of 2,633 days (IQR, 1,322–3,054). The median follow-up after TKI discontinuation was 342 days (range, 121–2,137). The 6-month mRFS was 77.4% (95% CI, 64.8–86.0), and the 1-year mRFS was 75.3% (95% CI, 62.3–84.4). The Kaplan–Meier curve demonstrated a clear plateau after 6 months, indicating that patients who maintained MMR beyond the first 6 months were unlikely to subsequently lose response. Patients previously treated with first-generation TKI (imatinib) had a numerically higher 1-year mRFS compared with those treated with second-generation TKIs and asciminib (84.1% vs 66.3%), although this difference was not statistically significant (p=0.16).
In multivariable Cox regression adjusting for MR4 duration and reason for discontinuation (intolerance vs elective), the generation of TKI was not independently associated with relapse risk (HR 2.14, 95% CI 0.65–7.01; p=0.21). Longer MR4 duration was independently associated with improved TFR success (HR 0.77 per additional year of MR4, 95% CI 0.62–0.93; p=0.009), whereas reason for discontinuation was not predictive.

**Copyright Information:** (Online) ISSN: 2572-9241
©2026 The Author(s). HemaSphere is published by John Wiley & Sons Ltd on behalf of European Hematology Association. This is an open access Abstract Book distributed under the Attribution-NonCommercial-NoDerivs (CC BY-NC-ND), which allows third parties to download the articles and share them with others as long as they credit the author and the Abstract Book, but they cannot change the content in any way or use them commercially.
**Abstract Book Citations:** Authors, Title, HemaSphere, 2026;10:(S1):pages. The abstract book DOIs can be found on the “Author Information” page.
**Disclaimer:** Articles published in the journal HemaSphere exclusively reflect the opinions of the authors. The authors are responsible for all content in their abstracts including accuracy of the facts, statements, citing resources, etc.

**Summary/Conclusion**

In this real-world cohort, TFR rates were encouraging even with reduced frequency of molecular monitoring  Molecular relapse occurred predominantly within the first 6 months, after which outcomes plateaued. TKI generation did not independently influence TFR outcomes. Duration of sustained MR4 was significantly associated with successful TFR, underscoring the importance of prolonged molecular response before TKI discontinuation.

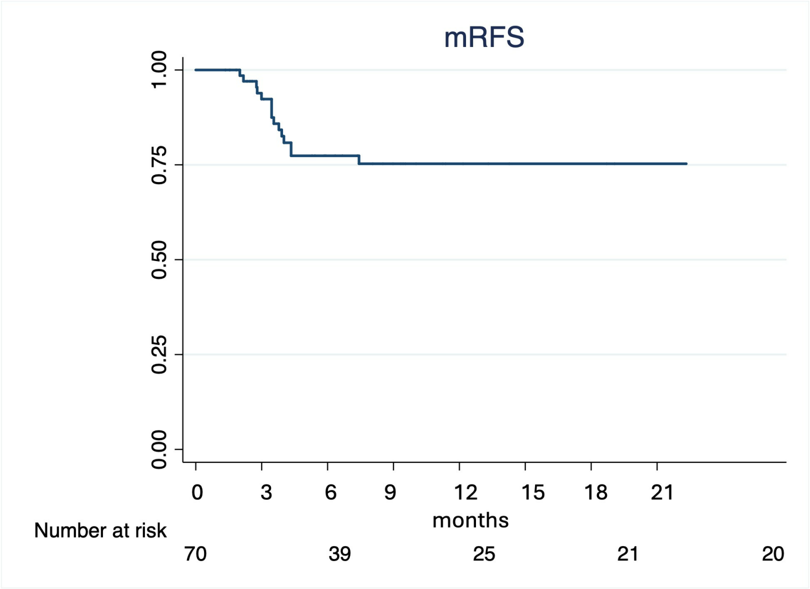

Molecular Relapse free survival since TKI discontinuation

**Copyright Information:** (Online) ISSN: 2572-9241
©2026 The Author(s). HemaSphere is published by John Wiley & Sons Ltd on behalf of European Hematology Association. This is an open access Abstract Book distributed under the Attribution-NonCommercial-NoDerivs (CC BY-NC-ND), which allows third parties to download the articles and share them with others as long as they credit the author and the Abstract Book, but they cannot change the content in any way or use them commercially.
**Abstract Book Citations:** Authors, Title, HemaSphere, 2026;10:(S1):pages. The abstract book DOIs can be found on the “Author Information” page.
**Disclaimer:** Articles published in the journal HemaSphere exclusively reflect the opinions of the authors. The authors are responsible for all content in their abstracts including accuracy of the facts, statements, citing resources, etc.

### (PB3025) STAMP INHIBITORS IMPROVE EARLY MOLECULAR RESPONSE (BCR::ABL1 <5% AT 3 MONTHS) IN CHRONIC-PHASE CML: A MULTICENTER REAL-WORLD COHORT FROM SOUTHERN CHINA

**Topic:** 08. Chronic myeloid leukemia - Clinical

Lisi Huang*[1], Na Xu[2], Xin Du[3], Xiaoliang Li[4], Maoquan Zou[5], Suxia Geng[1], Yulian Wang[1], Lingji Zeng[1], Zesheng Lu[1], Xiaoli Liu[2], Xin Du[1], Jianyu Weng[1]

*[1]Guangdong Provincial People's Hospital, Guanghzou, China;[2]Nanfang Hospital, Southern Medical University, Guanghzou, China; [3]The Second People's Hospital of Shenzhen, The First Affiliated Hospital of Shenzhen University, Guanghzou, China; [4]Zhuhai People's Hospital, Zhuhai, China;[5]Jiangmen Central Hospital, Jiangmen, China;*

### Background

Treatment-free remission (TFR) has become an important therapeutic goal in chronic myeloid leukemia (CML). Achieving deep molecular response (DMR) and sustaining DMR are prerequisites for successful TFR, and, critically earlier attainment of DMR may reduce cumulative tyrosine kinase inhibitor (TKI) exposure. In our previous work from Guangdong Provincial People's Hospital (GDPH), BCR::ABL1 <5% at 3 months (early molecular response, EMR) independently predicted DMR at 24 months. However, with conventional first-line TKIs, fewer than 50% of patients achieve EMR <5% at 3 months. STAMP inhibitor (STAMPi), specifically target the ABL1 myristoyl pocket, the novel therapeutic for CML, have demonstrated a 1.8-fold higher DMR rate at 96 weeks compared with conventional TKIs. Nevertheless, real-world data on early molecular responses to STAMPi in patients with CML remain limited. We therefore conducted a multicenter study to evaluate early responses to STAMPi in real-world CML-CP patients in Southern China.

### Aims

To evaluate early responses (BCR::ABL1 <5% at 3 months) to STAMPi in real-world CML-CP patients

### Methods

This multicenter real-world cohort study from five centers in Southern China, included adult patients with CML-CP treated prospectively with STAMP inhibitor and matched historical controls treated with 1G/2G -TKI. Quantitative RT-PCR for BCR::ABL1 mRNA was performed monthly during the first 3 months and every 3 months thereafter. Follow-up was cut off on February 28, 2026.

**Copyright Information:** (Online) ISSN: 2572-9241
©2026 The Author(s). HemaSphere is published by John Wiley & Sons Ltd on behalf of European Hematology Association. This is an open access Abstract Book distributed under the Attribution-NonCommercial-NoDerivs (CC BY-NC-ND), which allows third parties to download the articles and share them with others as long as they credit the author and the Abstract Book, but they cannot change the content in any way or use them commercially.
**Abstract Book Citations:** Authors, Title, HemaSphere, 2026;10:(S1):pages. The abstract book DOIs can be found on the "Author Information" page.
**Disclaimer:** Articles published in the journal HemaSphere exclusively reflect the opinions of the authors. The authors are responsible for all content in their abstracts including accuracy of the facts, statements, citing resources, etc.

### Results

A total of 36 patients received STAMP inhibitor (27 first-line, 4 second-line, 5 third-line or beyond), The median age of the STAMPi group was 43 years (range 28–65). Overall, the 3-month complete cytogenetic response (CCyR), BCR::ABL1⊠5%, and major molecular response (MMR) rates were 83.3%, 88.9%, and 36.7%, respectively. Subgroup analysis showed that in patients treated in ≥ second-line settings (N= 9), the 3-month CCyR, BCR::ABL1 < 5%, and MMR rates were 66.7%, 77.8%, and 44.4%, respectively. Baseline characteristics of patients receiving first-line treatment are presented in the Table below. For comparison, 287 patients received a first-generation TKI (imatinib) and 27 received a second-generation TKI (Nilotinib n = 23; Dasatinib n = 4). Among first-line STAMP-treated patients (Asciminib n = 26; TGRX-678 n = 1), 96.3% (26/27) achieved BCR::ABL1 < 5% at 3 months (CCyR 88.9%, MMR 33.3%), significantly higher than those treated with 1G TKI (45%, 130/287; P < 0.001) and 2G TKI (74%, 20/27; P = 0.022). The median time to reach BCR::ABL1 < 5% was 3 months (range 0.5–3), shorter than with 1G TKI (6 months, range 0.5–72) and 2G TKIs (3 months, range 1–21) (both P < 0.001).
At 12 weeks, the probability of achieving BCR::ABL1 < 5% with a STAMP inhibitor was 1.3-fold higher compared with 2G TKI and 2.2-fold higher compared with 1G TKI (P=0.022, P=0.004, respectively, Figure).

### Summary/Conclusion

Compared with conventional TKIs, STAMPi enabled a greater proportion of patients with CML-CP to achieve EMR <5% at an earlier time point. Given that EMR <5% predicts DMR at 24 months, these findings suggest that STAMPi-treated patients may have a higher likelihood of becoming eligible for treatment discontinuation and achieving TFR.

Table. Baseline Characteristics

| Characteristic | TKI IMA | TKI 2G | STAMPi（1L） | P value |
|---|---|---|---|---|
| **No. of patients** | 287 | 27 | 27 | 0.058 |
| **Age, years** | | | | |
| Median (Range) | 40.5 (18-76) | 43 (22-66) | 42 (28-65) | |
| **Sex, n (%)** | | | | 0.183 |
| Male | 174 (40.8%) | 23 (88.9%) | 17 (63%) | |
| Female | 113 (59.2%) | 4 (11.1%) | 10 (37%) | |
| **Median time from diagnosis to TKI treatment, months** Median (Range) | 0.2 (0.1-113) | 0.7(0.1-29) | 0.2 (0.1-10) | 0.035 |
| **ELTS risk group** | | | | 0.064 |
| Low | 120 | 9 | 23 | |
| Intermediate | 13 | 1 | 0 | |
| High | 1 | 0 | 2 | |

Copyright Information: (Online) ISSN: 2572-9241
©2026 The Author(s). HemaSphere is published by John Wiley & Sons Ltd on behalf of European Hematology Association. This is an open access Abstract Book distributed under the Attribution-NonCommercial-NoDerivs (CC BY-NC-ND), which allows third parties to download the articles and share them with others as long as they credit the author and the Abstract Book, but they cannot change the content in any way or use them commercially.
Abstract Book Citations: Authors, Title, HemaSphere, 2026;10:(S1):pages. The abstract book DOIs can be found on the "Author Information" page.
Disclaimer: Articles published in the journal HemaSphere exclusively reflect the opinions of the authors. The authors are responsible for all content in their abstracts including accuracy of the facts, statements, citing resources, etc.

### (PB3026) COMPARISON OF THE INCIDENCE OF CARDIOVASCULAR ADVERSE EVENTS IN PATIENTS WITH CHRONIC MYELOID LEUKAEMIA RECEIVING TYROSINE KINASE INHIBITORS WITH REPRESENTATIVES OF THE GENERAL POPULATION

**Topic:** 08. Chronic myeloid leukemia - Clinical

Nadezhda Shnalieva*[1, 1], Oxana Rotar[1], Elza Lomaia[1]

[1]*Almazov National Medical Research Centre, Saint Petersburg, Russia;*

### Background

ABL tyrosine kinase inhibitors (TKI) might lead to cardiovascular toxicity, especially in patients with preexisting cardiovascular (CV) risk factors including CV disease (CVD) history. At the same time, in this group of patients, it is difficult to determine the contribution of TKI or premorbid background to their CV outcomes

### Aims

To compare the frequency and profile of CV outcomes in patients with chronic myeloid leukemia (CML) receiving TKI therapy with those in the general population with similar anthropometric parameters, CV risk factors, and history of CV disease

### Methods

The study included patients with CML in the chronic phase on TKI therapy as a CML-group and representatives of the general population (included in the epidemiological study ESSE-RF, residents of St. Petersburg, Russia) as the control group. Members of the CML group and the control group were comparable in terms of gender, age (+-5 years), body mass index (BMI) (+-3 kg/m2), history of CV disease or diabetes mellitus (DM), and statin use.
CML-group: 112 patients (44 men (39%)), median age was 49.5 years. At baseline - arterial hypertension (AH) was present in 35 patients (31%). 17 (15%) patients were smokers at present , and 22 (20%) were former smokers. The mean total cholesterol (chol) level was 4.44 mmol/L, and the mean low-density lipoprotein (LDL) level was 2.58 mmol/L. The average BMI was 27.38 kg/m2. 3 patients (3%) had DM, and 16 patients (14%) had a history of CV disease. Overall 22 patients (20%) were taking statins.
TKI treatment at baseline: imatinib – 22 pts (20%), nilotinib – 31 pts (28%), dasatinib – 17 pts (15%), bosutinib – 3 pts (3%), ponatinib – 33 pts (29%), asciminib – 5 pts (4%), PF-114 – 1 pt (1%).
Control group: 224 people (88 men (39%)), median age was 49 years. AH was present in 115 people (51%). At the time of inclusion in the study, 73 (33%) people were smokers, 46 (21%) were former smokers. The average total chol was 5.42 mmol/L, the average LDL level was 3.46 mmol/L. The average BMI was 27.64 kg/m2. 6 people (3%) had DM, and 32 people (14%) had a history of CV disease. 44 people (20%) were taking statins

**Copyright Information:** (Online) ISSN: 2572-9241
©2026 The Author(s). HemaSphere is published by John Wiley & Sons Ltd on behalf of European Hematology Association. This is an open access Abstract Book distributed under the Attribution-NonCommercial-NoDerivs (CC BY-NC-ND), which allows third parties to download the articles and share them with others as long as they credit the author and the Abstract Book, but they cannot change the content in any way or use them commercially.
**Abstract Book Citations:** Authors, Title, HemaSphere, 2026;10:(S1):pages. The abstract book DOIs can be found on the "Author Information" page.
**Disclaimer:** Articles published in the journal HemaSphere exclusively reflect the opinions of the authors. The authors are responsible for all content in their abstracts including accuracy of the facts, statements, citing resources, etc.

**Results**

CV outcomes were analyzed for the CML group and the control group with a median follow-up of 80 months (CML group) and 72 months (control group).
CV adverse events (CVAE) were defined as: stable angina pectoris/other types of stable ischaemic heart disease, unstable angina pectoris, myocardial infarction (MI), peripheral artery (PA) atherosclerosis, visceral artery (VA) atherosclerosis, acute stroke/TIA, pulmonary embolism (PE), sudden cardiac death, chronic heart failure (CHF), venous thrombosis.
CVAE developed in 18 CML patients (16%): stable angina pectoris – 1, MI – 1, PA atherosclerosis– 5, VA atherosclerosis – 1, acute stroke – 3, PE – 1, CHF – 2, venous thrombosis – 1. At the time of CVAE development, patients were receiving the following TKIs: 1 patient (5.9%) was on stop therapy, 4 (23.5%) - imatinib, 4 (23.5%) - nilotinib, 2 11.8%) - dasatinib, 5 (29.4%) - ponatinib, 1 (5.9%) - PF-114. The median time from the start of TKI, on which CVAE developed, to CVAE was 45 months.
CVAE in the control group: MI in 4 people (2%), stroke in 2 people (1%), while 1 person developing stroke and MI sequentially.
Median time to CVAE was 50.9 months

**Summary/Conclusion**

When comparing the group of CML patients with the control group, assuming comparable anthropometric characteristics and CV history, a significantly higher number of CVAE was observed in patients with CML under TKI therapy

**Copyright Information:** (Online) ISSN: 2572-9241
©2026 The Author(s). HemaSphere is published by John Wiley & Sons Ltd on behalf of European Hematology Association. This is an open access Abstract Book distributed under the Attribution-NonCommercial-NoDerivs (CC BY-NC-ND), which allows third parties to download the articles and share them with others as long as they credit the author and the Abstract Book, but they cannot change the content in any way or use them commercially.
**Abstract Book Citations:** Authors, Title, HemaSphere, 2026;10:(S1):pages. The abstract book DOIs can be found on the "Author Information" page.
**Disclaimer:** Articles published in the journal HemaSphere exclusively reflect the opinions of the authors. The authors are responsible for all content in their abstracts including accuracy of the facts, statements, citing resources, etc.

### (PB3027) CHRONIC MYELOID LEUKAEMIA IN THE PAEDIATRIC AND YOUNG ADULT POPULATION – A MULTI-CENTRE RETROSPECTIVE STUDY

**Topic:** 08. Chronic myeloid leukemia - Clinical

Abbie Wilson*[1, 2], Katherine Stark[3], Victoria Campbell[4], Anthony Bench[4], Claire McKeeve[5], Gillian Horne[1, 2]

*[1]Beatson West of Scotland Cancer Centre, Glasgow, United Kingdom;[2]Paul O'Gorman Leukaemia Research Centre, Glasgow, United Kingdom; [3]Royal Infirmary of Edinburgh, Edinburgh, United Kingdom;[4]Western General Hospital, Edinburgh, United Kingdom; [5]West of Scotland Genomics Centre, Glasgow, United Kingdom;*

### Background

Chronic Myeloid Leukaemia (CML) is increasingly recognised as a biologically and clinically distinct entity in children, adolescents and young adults. Although rare in this age group, treatment burden, implications for fertility, and psychological impact make it uniquely significant. Recent evidence has suggested that younger patients with CML may present with higher disease burden and may have poorer response to Tyrosine Kinase Inhibitors (TKIs) compared with older adults. Moreover, CML in younger people presents unique challenges, such as the impact on fertility and psychological sequelae. Furthermore, published prognostic scoring systems have not been validated in children and young people, creating uncertainty in risk stratification and optimal first-line therapy selection.

### Aims

To better characterise disease presentation, treatment response, and molecular features of CML in children and young adults aged 0–30 years within a Scottish population.

### Methods

We conducted a multicentre, retrospective study of patients aged 0-30 diagnosed with CML across two of Scotland's largest paediatric centres. Patients aged 0-30 years with molecularly confirmed CML were identified.  Details collected included basic demographics, genetic testing, pathology reports and access to specialist services. Data was anonymised and analysed centrally.

### Results

Thirty-four children and young people with CML were included, with a median age of 23 years (range 6 – 30 years). Of those with information available, 86.2% had splenomegaly and 96.6% had basophilia. Median presenting white cell count was 188.5 x $10^9$/L.  87.1% of patients were symptomatic at presentation, with abdominal pain, fatigue and light-headedness/syncope most common. The most commonly used 1$^{st}$ line TKI was Imatinib (61.8%), followed by Dasatinib (32.4%). However, only 26.3% of patients had an optimal response to first-line Imatinib and over half of patients required two or more lines of treatment. Eight patients underwent TKD testing and five next-generation sequencing due to sub-optimal response however no significant variants were detected.

**Copyright Information:** (Online) ISSN: 2572-9241
©2026 The Author(s). HemaSphere is published by John Wiley & Sons Ltd on behalf of European Hematology Association. This is an open access Abstract Book distributed under the Attribution-NonCommercial-NoDerivs (CC BY-NC-ND), which allows third parties to download the articles and share them with others as long as they credit the author and the Abstract Book, but they cannot change the content in any way or use them commercially.
**Abstract Book Citations:** Authors, Title, HemaSphere, 2026;10:(S1):pages. The abstract book DOIs can be found on the "Author Information" page.
**Disclaimer:** Articles published in the journal HemaSphere exclusively reflect the opinions of the authors. The authors are responsible for all content in their abstracts including accuracy of the facts, statements, citing resources, etc.

**Summary/Conclusion**

Children and young adults with CML are more likely to present symptomatically and with extreme leukocytosis, splenomegaly and basophilia compared to older adults. Younger people appear to have a much poorer response to TKI therapy, especially first-line Imatinib. These findings raise important questions regarding optimal frontline TKI selection and whether younger patients represent a biologically distict subgroup. Ongoing expansion of this cohort to include patients aged 30-40 years (anticipated n>80) will provide our largest Scottish dataset describing CML in children and young adults. This work aims to inform age-adapted risk stratification and treatment strategies for this underserved population.

**Copyright Information:** (Online) ISSN: 2572-9241
©2026 The Author(s). HemaSphere is published by John Wiley & Sons Ltd on behalf of European Hematology Association. This is an open access Abstract Book distributed under the Attribution-NonCommercial-NoDerivs (CC BY-NC-ND), which allows third parties to download the articles and share them with others as long as they credit the author and the Abstract Book, but they cannot change the content in any way or use them commercially.
**Abstract Book Citations:** Authors, Title, HemaSphere, 2026;10:(S1):pages. The abstract book DOIs can be found on the "Author Information" page.
**Disclaimer:** Articles published in the journal HemaSphere exclusively reflect the opinions of the authors. The authors are responsible for all content in their abstracts including accuracy of the facts, statements, citing resources, etc.

### (PB3028) REAL WORLD MOLECULAR RESPONSE TO INHIBITOR ALLOSTERIC IN ADULT CHRONIC MYELOID LEUKEMIA: A MEXICAN MULTICENTER RETROSPECTIVE COHORT

**Topic:** 08. Chronic myeloid leukemia - Clinical

Álvaro Cabrera García*[1], Christian Ramos[1], Jose Alvarez[2], Gilberto Israel Barranco Lampon[3], Rafael Hurtado Monroy[4], Raul Martinez[5], Dafne Itzel Campa Monroy[6], Maria Luisa Alcivas[7], Josue Ruiz[6], Patricia Zagoya[8], Elena Juventina Tuna Aguilar[9], Carmen Lopez[10], Carlos Chavez-Trillo[11], Andrea Mirelly Lopez[6], Erendira Pacheco Zavala Ere[12], Angelica Romo[13], Alva Nora Zaragoza Vazquez[14], Auandar Mendoza Gamez[15]

*[1]Helipuerto Hospital de Alta Especialidad Ixtapaluca, Hematology, Ixtapaluca, Mexico;[2]ISSSTE Centro Médico Nacional 20 de Noviembre, Hematology, Ciudad de México, Mexico; [3]Hospital General de México Doctor Eduardo Liceaga, Hematology, Ciudad de México, Mexico; [4]Hospital Ángeles del Pedregal, Hematology, Ciudad de México, Mexico;[5]IMSS, Hematology, Ciudad de México, Mexico; [6]IMSS, Pediatric hematology, Ciudad de México, Mexico;[7]Tultitlan de Mariano Escobedo, Hematology, Tultitlán, Mexico; [8]Puebla State Delegation IMSS, Hematology, Puebla, Mexico;[9]Incmnsz.edu.mx, Hematology, Ciudad de México, Mexico; [10]One Life Oncology, Hematology, Guadalajara, Mexico; [11]Civil State Pensions of Chihuahua, Chihuahua, Mexico; [12]Imss Umae 1, León, Mexico; [13]Andador November 20, Hematology, Zapopan, Mexico; [14]El Saucito Industrial Park, Chihuahua, Mexico; [15]UNAM Palacio de la Escuela de Medicina Museo de la Medicina, Medicine, Ciudad de México, Mexico;*

### Background

The development of tyrosine kinase inhibitors (TKIs) has transformed the treatment of chronic myeloid leukemia (CML); however, some patients develop resistance or intolerance to these therapies. Asciminib, an allosteric inhibitor targeting the ABL1 domain, represents an alternative therapeutic strategy with a differentiated mechanism of action and favorable safety profileThe development of tyrosine kinase inhibitors (TKIs) has transformed the treatment of chronic myeloid leukemia (CML); however, some patients develop resistance or intolerance to these therapies. Asciminib, an allosteric inhibitor targeting the ABL1 domain, represents an alternative therapeutic strategy with a differentiated mechanism of action and favorable safety profile

### Aims

To evaluate the efficacy and safety of asciminib in Mexican patients with CML resistant or intolerant to prior TKIs in a real-world clinical setting and explore factors associated with molecular response

### Methods

This multicenter retrospective study included adult patients with CML treated with asciminib and followed by serial molecular monitoring using BCR::ABL1 quantification on the International Scale (IS). Major molecular response (MMR) and deep molecular response (DMR) were evaluated as the primary outcomes. Exploratory analyses were performed using odds ratios (OR) with 95% confidence intervals (95% CI).

**Copyright Information:** (Online) ISSN: 2572-9241
©2026 The Author(s). HemaSphere is published by John Wiley & Sons Ltd on behalf of European Hematology Association. This is an open access Abstract Book distributed under the Attribution-NonCommercial-NoDerivs (CC BY-NC-ND), which allows third parties to download the articles and share them with others as long as they credit the author and the Abstract Book, but they cannot change the content in any way or use them commercially.
**Abstract Book Citations:** Authors, Title, HemaSphere, 2026;10:(S1):pages. The abstract book DOIs can be found on the "Author Information" page.
**Disclaimer:** Articles published in the journal HemaSphere exclusively reflect the opinions of the authors. The authors are responsible for all content in their abstracts including accuracy of the facts, statements, citing resources, etc.

**Results**

A total of 32 patients were included, with a mean age of 50 years, female predominance (56.3%), and chronic phase disease in 87.5% of patients. Nearly half of the patients (46.9%) had received three or more prior TKIs, and toxicity or intolerance was the main reason for the treatment change (71.9%). During follow-up, 80.6% achieved a complete hematologic response, 40% achieved a major molecular response, and 35.5% achieved a deep molecular response. A reduction in BCR::ABL1 transcript levels was observed in 59.4% of the evaluable patients. Treatment demonstrated favorable tolerability, as 93.5% of patients did not require dose modification or treatment discontinuation.In exploratory analyses, the presence of comorbidities was not associated with a lack of MMR (OR 1.40; 95% CI 0.31–6.33) but showed a trend toward a higher probability of not achieving DMR (OR 6.43; 95% CI 1.05–39.33; p=0.054). Exposure to ≥2 prior TKIs was not associated with the absence of DMR (OR 0.64; 95% CI 0.14–3.04). Treatment change due to progression or lack of response was not significantly associated with the absence of MMR (OR 1.17; 95% CI 0.28–4.87) or DMR (OR 2.67; 95% CI 0.54–13.08). Similarly, dose modification was not associated with the absence of MMR (OR 0.93; 95% CI 0.05–16.39) or DMR (OR 0.53; 95% CI 0.03–9.34). Treatment discontinuation showed a trend toward lower molecular response probability, although without statistical significance for MMR (OR 3.23; 95% CI, 0.30–35.11) or DMR (OR 1.76; 95% CI, 0.16–19.18).

**Summary/Conclusion**

In this first real-world experience in a Mexican population, asciminib demonstrated efficacy and good tolerability in patients with CML who were resistant or intolerant to prior TKIs. Molecular efficacy appeared to be independent of prior treatment burden and therapy modifications, suggesting that baseline patient-related factors, particularly comorbidities, may play a greater role in determining the depth of molecular response.

**Copyright Information:** (Online) ISSN: 2572-9241
©2026 The Author(s). HemaSphere is published by John Wiley & Sons Ltd on behalf of European Hematology Association. This is an open access Abstract Book distributed under the Attribution-NonCommercial-NoDerivs (CC BY-NC-ND), which allows third parties to download the articles and share them with others as long as they credit the author and the Abstract Book, but they cannot change the content in any way or use them commercially.
**Abstract Book Citations:** Authors, Title, HemaSphere, 2026;10:(S1):pages. The abstract book DOIs can be found on the “Author Information” page.
**Disclaimer:** Articles published in the journal HemaSphere exclusively reflect the opinions of the authors. The authors are responsible for all content in their abstracts including accuracy of the facts, statements, citing resources, etc.

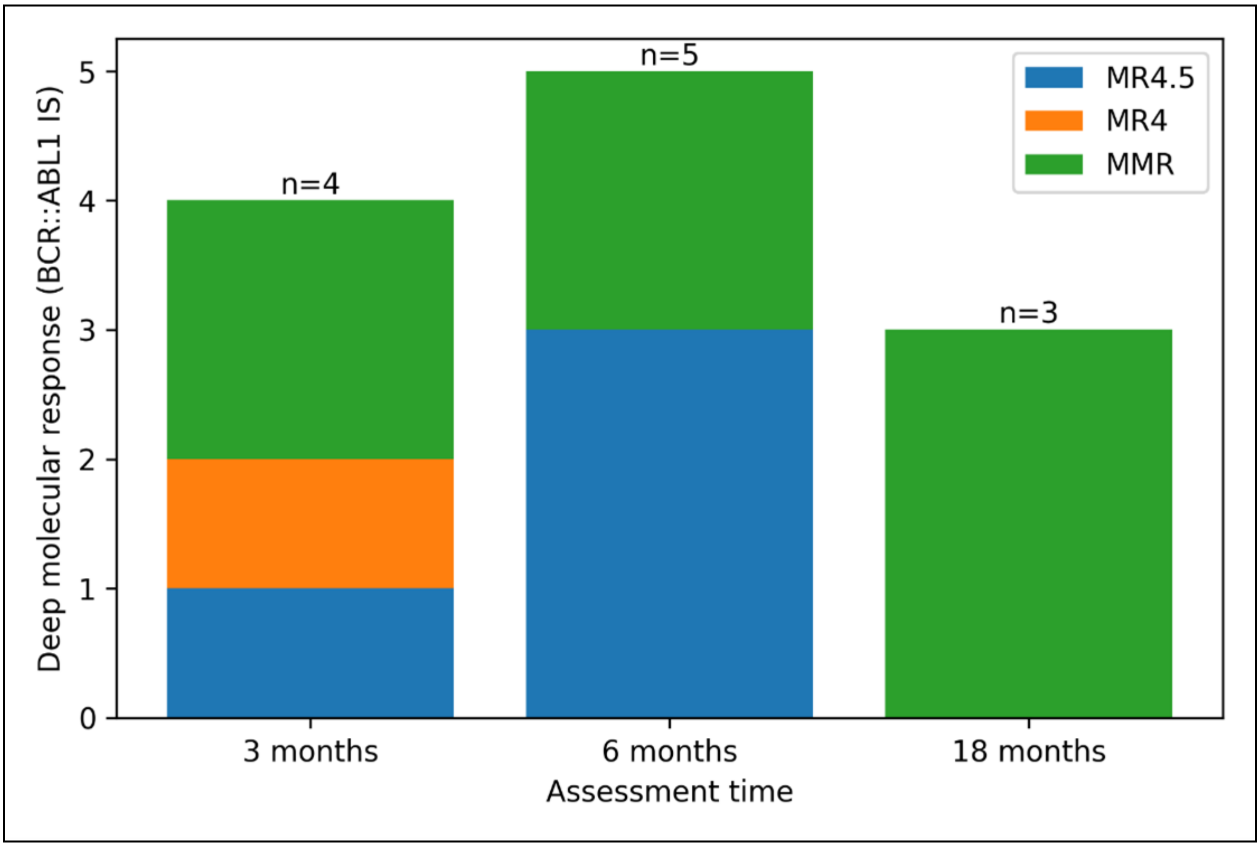

deep molecular response

Copyright Information: (Online) ISSN: 2572-9241
©2026 The Author(s). HemaSphere is published by John Wiley & Sons Ltd on behalf of European Hematology Association. This is an open access Abstract Book distributed under the Attribution-NonCommercial-NoDerivs (CC BY-NC-ND), which allows third parties to download the articles and share them with others as long as they credit the author and the Abstract Book, but they cannot change the content in any way or use them commercially.
Abstract Book Citations: Authors, Title, HemaSphere, 2026;10:(S1):pages. The abstract book DOIs can be found on the "Author Information" page.
Disclaimer: Articles published in the journal HemaSphere exclusively reflect the opinions of the authors. The authors are responsible for all content in their abstracts including accuracy of the facts, statements, citing resources, etc.

### (PB3029) CURRENT STATUS SURVEY OF TOLERABILITY BURDEN OF FIRST- OR SECOND-LINE TKI THERAPY IN CHINESE PATIENTS WITH CHRONIC MYELOID LEUKEMIA (CML)

**Topic:** 08. Chronic myeloid leukemia - Clinical

Fang Cheng[1], Zhenya Hong[2], Yanli Zhang[3], Yunfan Yang[4], Qin Wen[5], Li Zhou[6], Guohui Li[7], Minghui Duan[8], Zhijian Cao[9], Jian Huang[10], Jianyu Weng[11], Yu Zhu[12], Na Xu[13], Zhenfang Liu[14], Suning Chen[15], Weiming Li*[1], Yu Hu[1]

*[1]Union Hospital, Tongji Medical College, Huazhong University of Science and Technology, Department of Hematology, Wuhan, China; [2]Tongji Hospital, Tongji Medical College, Huazhong University of Science and Technology, Department of Hematology, Wuhan, China; [3]The Affiliated Cancer Hospital of Zhengzhou University & Henan Cancer Hospital, Department of Hematology, Zhengzhou, China; [4]West China hospital of Sichuan university, Hematology department, Chengdu, China;[5]Xinqiao Hospital, Army Medical University, Department of Hematology, Chongqing, China; [6]National Research Center for Translational Medicine at Shanghai, Ruijin Hospital affiliated to Shanghai Jiao Tong University School of Medicine, Department of Hematology, Shanghai, China; [7]Hospital of Hematology, Xi'an International Medical Center, Department of Hematology, Xi'an, China;[8]Peking Union Medical College Hospital, Chinese Academy of Medical Sciences and Peking Union Medical College, Department of Hematology, Beijing, China; [9]The first hospital of Harbin, Harbin Institute of Hematology & Oncology, Harbin, China;[10]The First Affiliated Hospital, College of Medicine, Zhejiang University, Department of Hematology, Zhejiang, China; [11]Guangdong Provincial People's Hospital, Guangzhou, China; [12]The First Affiliated Hospital of Nanjing Medical University, Jiangsu Province Hospital, Nanjing, China; [13]Nanfang Hospital, Southern Medical University, Guangzhou, China;[14]The First Affiliated Hospital of Guangxi Medical University, Department of Hematology, Nanning, China; [15]The First Affiliated Hospital of Soochow University, Suzhou, China;*

### Background

CML has evolved into a manageable chronic disease, with patients experiencing prolonged survival due to long-term treatment. Consequently, treatment tolerability has become a critical factor in disease management. Tyrosine kinase inhibitor (TKI)-related adverse events (AEs) often lead to reduced adherence, treatment modifications, or discontinuation, ultimately affecting patients' quality of life (QoL). However, real-world data on the impact of TKI-related AEs in Chinese patients remain limited.

### Aims

To comprehensively evaluate the impact of TKI-related AEs on QoL, economic burden and work productivity in Chinese patients with CML using patient-reported outcomes in real-world clinical settings.

### Methods

This cross-sectional, questionnaire-based study enrolled adult patients with CML receiving first- or second-line TKIs across 15 sites in China from August to December 2025. Data was collected via online surveys and structured interviews conducted by uniformly trained personnel. Patient-reported outcomes included QoL, AE-related economic burden and work productivity.

**Copyright Information:** (Online) ISSN: 2572-9241
©2026 The Author(s). HemaSphere is published by John Wiley & Sons Ltd on behalf of European Hematology Association. This is an open access Abstract Book distributed under the Attribution-NonCommercial-NoDerivs (CC BY-NC-ND), which allows third parties to download the articles and share them with others as long as they credit the author and the Abstract Book, but they cannot change the content in any way or use them commercially.
**Abstract Book Citations:** Authors, Title, HemaSphere, 2026;10:(S1):pages. The abstract book DOIs can be found on the "Author Information" page.
**Disclaimer:** Articles published in the journal HemaSphere exclusively reflect the opinions of the authors. The authors are responsible for all content in their abstracts including accuracy of the facts, statements, citing resources, etc.

### Results

Among the 200 patients analyzed, 94% experienced at least one TKI-related AE (median: 3; range: 0–13), with 81.5% reporting two or more AEs. The most common AEs were fatigue (59%), rash/skin irritation (40%), diarrhea (35%), and nausea/vomiting (32%). In the three months prior to the survey, 78% of patients reported that AEs negatively impacted their QoL, and 28% perceived the burden as severe. AEs led to additional outpatient visits in 61% of patients and hospitalization in 14%. Based on patient reports, the mean annual out-of-pocket cost for these additional visits was RMB 1,106/patient. Among employed patients (n=128), 56% reduced workload or work stoppage due to their disease or AEs, and 48% experienced varying degrees of work productivity loss. In 13% of patients, treatment efficacy was reduced due to AEs. Baseline characteristics are summarized in Table 1.

### Summary/Conclusion

Findings from this study demonstrate a substantial tolerability burden among Chinese patients with CML receiving TKIs. The high prevalence of multiple, persistent AEs not only compromised patients' QoL and work productivity but also drove increased healthcare utilization, resulting in considerable clinical and economic burden. These findings highlight an urgent need for treatment options with favorable tolerability profiles to mitigate the impact of AEs and safeguard quality of life in patients with CML.

**Table 1. Patient characteristics**

| Characteristics | N=200 |
|---|---|
| **Age [years, median (range)]** | 43.5(19-76) |
| **Age at diagnosis [years, median (range)]** | 38(10-76) |
| **Disease course [years, median (range)]** | 4.9(0.4-24.3) |
| **Gender (%)** | |
| Male | 108(54%) |
| Female | 92(46%) |
| **Baseline ECOG score (%)** | |
| 0 | 95(47%) |
| 1 | 86(43%) |
| 2 | 17(9%) |
| 3 | 1(1%) |

**Copyright Information:** (Online) ISSN: 2572-9241
©2026 The Author(s). HemaSphere is published by John Wiley & Sons Ltd on behalf of European Hematology Association. This is an open access Abstract Book distributed under the Attribution-NonCommercial-NoDerivs (CC BY-NC-ND), which allows third parties to download the articles and share them with others as long as they credit the author and the Abstract Book, but they cannot change the content in any way or use them commercially.
**Abstract Book Citations:** Authors, Title, HemaSphere, 2026;10:(S1):pages. The abstract book DOIs can be found on the "Author Information" page.
**Disclaimer:** Articles published in the journal HemaSphere exclusively reflect the opinions of the authors. The authors are responsible for all content in their abstracts including accuracy of the facts, statements, citing resources, etc.

| | |
|---|---|
| 4 | 0 |
| **Current employment status (%)** | |
| Employed | 119(60%) |
| Unemployed | 81(40%) |
| **TKI treatment duration [months, median (range)]** | 48.5(3-241) |
| **Current line of therapy (%)** | |
| First line | 120(60%) |
| Second line | 80(40%) |
| **Current generation of TKI (%)** | |
| $1^{st}$ TKI (Imatinib) | 70(35%) |
| $2^{nd}$ TKI (Nilotinib/Dasatinib/Flumatinib) | 125(63%) |
| $3^{rd}$ TKI (Olverembatinib/Ponatinib) | 5(2%) |
| **Comorbidities (%)** | |
| Diabetes mellitus | 16(8%) |
| Hypertension | 23(12%) |
| Coronary heart disease | 11(6%) |
| Gastrointestinal disorders | 18(9%) |
| Liver disease | 6(3%) |
| Kidney disease | 7(4%) |

**Copyright Information:** (Online) ISSN: 2572-9241
©2026 The Author(s). HemaSphere is published by John Wiley & Sons Ltd on behalf of European Hematology Association. This is an open access Abstract Book distributed under the Attribution-NonCommercial-NoDerivs (CC BY-NC-ND), which allows third parties to download the articles and share them with others as long as they credit the author and the Abstract Book, but they cannot change the content in any way or use them commercially.
**Abstract Book Citations:** Authors, Title, HemaSphere, 2026;10:(S1):pages. The abstract book DOIs can be found on the "Author Information" page.
**Disclaimer:** Articles published in the journal HemaSphere exclusively reflect the opinions of the authors. The authors are responsible for all content in their abstracts including accuracy of the facts, statements, citing resources, etc.

| | |
|---|---|
| Cerebrovascular disease | 5(3%) |
| Pulmonary disease | 3(2%) |
| Thrombus | 0 |
| None of the above | 107(54%) |

**Copyright Information:** (Online) ISSN: 2572-9241
©2026 The Author(s). HemaSphere is published by John Wiley & Sons Ltd on behalf of European Hematology Association. This is an open access Abstract Book distributed under the Attribution-NonCommercial-NoDerivs (CC BY-NC-ND), which allows third parties to download the articles and share them with others as long as they credit the author and the Abstract Book, but they cannot change the content in any way or use them commercially.
**Abstract Book Citations:** Authors, Title, HemaSphere, 2026;10:(S1):pages. The abstract book DOIs can be found on the "Author Information" page.
**Disclaimer:** Articles published in the journal HemaSphere exclusively reflect the opinions of the authors. The authors are responsible for all content in their abstracts including accuracy of the facts, statements, citing resources, etc.

### (PB3030) MOLECULAR AND CLINICAL OUTCOMES OF FIRST-LINE TKIS IN A MULTIETHNIC CML COHORT UNDER UNIVERSAL ACCESS IN MALAYSIA: INTERIM ANALYSIS OF THE MYMPN RWE STUDY

**Topic:** 08. Chronic myeloid leukemia - Clinical

Lee Ping Chew*[1], Christopher Liam Chin Keong[2], Kar Poh Wong[3], Meriell Leeza Gayongala[4], Ahlam Naila Kori[5], Ahmad Zakiyy Mohamed[6], Carol Goh Chew Yuen[7], Ganesh Kasinathan[8], Hong Keng Liew[7], Kam Hoo Yip[9], Kar Ying Yong[10], Kuldeep Kaur Sarban Singh[11], Lily Wong Lee Lee[12], Nur Aini Syakirah Ahmad Shuyuti[13], See Guan Toh[14], Sharifah Suryani Syed Rahim Shah[15], Si Yuan Ng[16], Ai Sim Goh[7]

*[1]Sarawak General Hospital, Kuching, Malaysia;[2]Hospital Sultanah Aminah, Johor, Malaysia; [3]Novartis Corporation (Malaysia) Sdn. Bhd., Selangor, Malaysia; [4]Novartis Corporation (Malaysia) Sdn. Bhd., Medical Affairs, Selangor, Malaysia;[5]Hospital Tengku Ampuan Afzan, Kuantan, Malaysia; [6]Hospital Tengku Ampuan Rahimah, Selangor, Malaysia; [7]Hospital Pulau Pinang, Pulau Pinang, Malaysia; [8]Hospital Ampang, Selangor, Malaysia; [9]Sultanah Nur Zahirah Hospital, Terengganu, Malaysia; [10]Hospital Miri, Miri, Malaysia; [11]Hospital Taiping, Taiping, Malaysia; [12]Hospital Queen Elizabeth, Sabah, Malaysia; [13]Hospital Raja Perempuan Zainab II, Kelantan, Malaysia; [14]Hospital Tuanku Jaafar, Seremban, Malaysia; [15]Hospital Kuala Lumpur, Kuala Lumpur, Malaysia; [16]Hospital Melaka, Melaka, Malaysia;*

### Background

Real-world data on Chronic myeloid leukemia (CML) in Southeast Asia remain limited and are largely derived from population-based registries, with heterogeneity in molecular monitoring and treatment patterns. In Malaysia, patients treated within the Ministry of Health (MOH) system have access to tyrosine kinase inhibitors (TKIs) imatinib and nilotinib, via the Malaysian Patient Assistance Program (MyPAP)[1]. However, real-world national data on outcomes with universal TKI access remains limited. This study provides comprehensive real-world evidence on the epidemiology, treatment patterns, and survival outcomes of CML patients in Malaysia.

### Aims

This interim analysis of the Malaysia MPN Real-World Evidence (MyMPN RWE) study characterizes baseline demographics, treatment patterns, and clinical outcomes among Malaysian CML patients stratified by first-line TKI therapy (1-LOT).

### Methods

MyMPN RWE is a real-world, multicenter, observational, retrospective study. This interim analysis included 608 Philadelphia-positive chronic-phase CML patients (aged ≥18 years) diagnosed between January 2018 and December 2023, followed up for at least 24 months. Secondary data on demographics, disease characteristics, treatment patterns, and outcomes were retrieved from manual and electronic medical records.

**Copyright Information:** (Online) ISSN: 2572-9241
©2026 The Author(s). HemaSphere is published by John Wiley & Sons Ltd on behalf of European Hematology Association. This is an open access Abstract Book distributed under the Attribution-NonCommercial-NoDerivs (CC BY-NC-ND), which allows third parties to download the articles and share them with others as long as they credit the author and the Abstract Book, but they cannot change the content in any way or use them commercially.
**Abstract Book Citations:** Authors, Title, HemaSphere, 2026;10:(S1):pages. The abstract book DOIs can be found on the “Author Information” page.
**Disclaimer:** Articles published in the journal HemaSphere exclusively reflect the opinions of the authors. The authors are responsible for all content in their abstracts including accuracy of the facts, statements, citing resources, etc.

**Results**

Median age was 44.0 (range 18 – 87) years; 57% males. Ethnic Malays accounted for 51%, Chinese 26%, and Native Bumiputera 10%. Most of the patients were symptomatic at diagnosis; unintentional weight loss (34%) was the most frequent symptom. Hypertension (23%), dyslipidemia (15%), and diabetes (14%) were the most common comorbidities. The 1-LOT included imatinib (61.7%), nilotinib (36%), asciminib (0.7%), and dasatinib (0.2%). Major molecular response (MMR) rates were higher with nilotinib vs. imatinib at 12 months (35% vs. 26%) and 24 months (36% vs. 32%). Deep molecular response (DMR) rates were also higher with nilotinib at 12 months (21% vs. 13%) and 24 months (31% vs. 23%). At end of observation period, 34.4% of imatinib patients discontinued and switched TKI (24% resistance, 6% intolerance), vs 21.5% on nilotinib (9% resistance, 11% intolerance). Overall, disease progression was 4% and the mortality rate was 5%, majority unrelated to CML progression (infections 33%, secondary malignancy 6%). Adverse events occurred in 30% of patients (number of events: imatinib, 136; nilotinib, 100; asciminib, 5; bosutinib, 5; ponatinib, 3; dasatinib, 2), mostly hematologic (46%) and grade 1 - 2 (68%).

**Summary/Conclusion**

This study provides the first real-world characterization of multiethnic Malaysian CML patients under a universal TKI access program. Although nilotinib achieved higher MMR and DMR rates than imatinib, overall molecular responses were comparatively lower than those reported in international real-world cohorts[2-4], with a substantial proportion of patients requiring treatment switches due to resistance or intolerance. Real-world factors such as disease burden at presentation, comorbidities and variability in molecular monitoring continue to impact treatment outcomes. These gaps between therapy access and optimal disease control highlight the need to strengthen molecular monitoring, earlier treatment optimization, and refinement of national treatment pathways to improve long-term outcomes in real-world CML care.

**Copyright Information:** (Online) ISSN: 2572-9241
©2026 The Author(s). HemaSphere is published by John Wiley & Sons Ltd on behalf of European Hematology Association. This is an open access Abstract Book distributed under the Attribution-NonCommercial-NoDerivs (CC BY-NC-ND), which allows third parties to download the articles and share them with others as long as they credit the author and the Abstract Book, but they cannot change the content in any way or use them commercially.
**Abstract Book Citations:** Authors, Title, HemaSphere, 2026;10:(S1):pages. The abstract book DOIs can be found on the “Author Information” page.
**Disclaimer:** Articles published in the journal HemaSphere exclusively reflect the opinions of the authors. The authors are responsible for all content in their abstracts including accuracy of the facts, statements, citing resources, etc.

### (PB3031) ASCIMINIB IN CHRONIC-PHASE CML AFTER PRIOR TKI INTOLERANCE OR RESISTANCE: REAL-WORLD OUTCOMES FROM A SINGLE-CENTER COHORT

**Topic:** 08. Chronic myeloid leukemia - Clinical

Maria Dimou[1], Aikaterini Kolotsiou*[1], Maria Dimitrakoudi[1], Athanasios Liaskas[1], Aikaterini Bitsani[1], Anastasia Kopsaftopoulou[1], Ioannis Asimakopoulos[1], Theodoros Vassilakopoulos[1], Panagiotis Panagiotidis[1], Maria Angelopoulou[1]

*[1]Haematology Department and Bone Marrow Transplantation Unit, National and Kapodistrian University of Athens, Laikon General Hospital, Athens, Greece, ATHENS, Greece;*

### Background

Asciminib(ASC) is a first-in-class STAMP inhibitor that has demonstrated superior efficacy and an excellent safety profile compared with standard tyrosine kinase inhibitors (TKIs) in the frontline setting (Hochhaus A, et al. N Engl J Med. 2024) as well as in previously pretreated chronic myeloid leukemia patients in chronic phase(CP-CML) (Hochhaus A, et al. Leukemia. 2023). However, real-world data on ASC remain limited.

### Aims

The current study evaluates the safety and efficacy of ASC in a real-world, single-center cohort of patients with previously treated CP-CML.

### Methods

We retrospectively analyzed consecutive adult CP-CML patients in our Center treated with ASC following its EMA approval in August 2022. Baseline characteristics, prior TKI exposure, molecular response according to ELN 2020 criteria, adverse events according to CTCAE v.5, and treatment (trm) outcomes were recorded.

**Copyright Information:** (Online) ISSN: 2572-9241
©2026 The Author(s). HemaSphere is published by John Wiley & Sons Ltd on behalf of European Hematology Association. This is an open access Abstract Book distributed under the Attribution-NonCommercial-NoDerivs (CC BY-NC-ND), which allows third parties to download the articles and share them with others as long as they credit the author and the Abstract Book, but they cannot change the content in any way or use them commercially.
**Abstract Book Citations:** Authors, Title, HemaSphere, 2026;10:(S1):pages. The abstract book DOIs can be found on the “Author Information” page.
**Disclaimer:** Articles published in the journal HemaSphere exclusively reflect the opinions of the authors. The authors are responsible for all content in their abstracts including accuracy of the facts, statements, citing resources, etc.

**Results**

A total of 15 patients (60% female) with CP-CML were treated in our Center with ASC between 1/2023 and 2/2026. Median age at trm initiation was 66 (20–80) years. The median number of prior TKI lines was 2 (1–4). Twelve patients (80 %) had previously received imatinib, 6 (40 %) dasatinib, 6 (40%) nilotinib, 1(6.7%) bosutinib, and 2 (13.3%) ponatinib. Eleven patients (73%) had at least one comorbidity at baseline with the most common in 6 (40%) being cardiovascular disease.
ASC was initiated due to intolerance to the most recent TKI in 12 (80%) patients, failure to the most recent TKI in 2 (13%), and pregnancy considerations following unsuccessful TFR after nilotinib in 1 (7%).
Initially, 7 (46.6%) patients received ASC 40 mg BID, 7 (46.6%) 80 mg QD, and 1 (7%) 200 mg BID due to T315I mutation. Median follow-up was 6.9 months (0.5–33.4), and median ASC duration was 6.5 months(0.5–33.1).
Molecular response improved in 4 (27%), was stable in 8 (53%), worsened in 1 (7%), and was not evaluable in 2(13%) patients. At data cutoff, 9 (60%) patients achieved ≥MMR, including MR4.5 in 3(20%) and MR4 in 2(13%).
ASC was discontinued in 6(40%) patients. Discontinuation was due to intolerance in 5(33%) and progression in 1(7%) patient, the latter harboring T315I mutation and progressing to blastic phase. Among those discontinuing for intolerance, 2 (13%) patients had new grade 3 toxicities (hypertension, pancreatic enzyme elevation) and 2 (13%) recurrence of prior toxicity (grade 3 arthralgia and pericardial effusion), all persisting despite dose reduction; 1 (7%) patient died from heart failure having severe cardiovascular disease aquired during prior nilotinib exposure.
At data cutoff, among the 9 patients remaining on ASC, 5 (55.6%) maintained baseline dosing, 2(22.2%) switched from 40 mg BID to 80 mg QD, and 1 (11.1%) reduced to 40 mg QD due to grade 3 pancreatic enzyme elevation recurring from prior nilotinib toxicity; no grade ≥3 hematologic toxicities occurred.

**Summary/Conclusion**

ASC showed favorable tolerability and meaningful molecular responses in this heavily pretreated CP-CML cohort with significant comorbidities. Discontinuation due to newly emergent or recurring toxicity was at low rates, supporting ASC as an effective and safe option in patients requiring therapy change due to standard TKI intolerance or resistance.

**Copyright Information:** (Online) ISSN: 2572-9241
©2026 The Author(s). HemaSphere is published by John Wiley & Sons Ltd on behalf of European Hematology Association. This is an open access Abstract Book distributed under the Attribution-NonCommercial-NoDerivs (CC BY-NC-ND), which allows third parties to download the articles and share them with others as long as they credit the author and the Abstract Book, but they cannot change the content in any way or use them commercially.
**Abstract Book Citations:** Authors, Title, HemaSphere, 2026;10:(S1):pages. The abstract book DOIs can be found on the “Author Information” page.
**Disclaimer:** Articles published in the journal HemaSphere exclusively reflect the opinions of the authors. The authors are responsible for all content in their abstracts including accuracy of the facts, statements, citing resources, etc.

### (PB3032) ADOLESCENT AND YOUNG ADULT CHRONIC MYELOID LEUKEMIA IN MALAYSIA: 25-YEAR EPIDEMIOLOGICAL PROFILE AND TREATMENT OUTCOMES FROM A MALAYSIAN SINGLE-CENTRE ANALYSIS

**Topic:** 08. Chronic myeloid leukemia - Clinical

Nur Syazwani Jamhuri*[1], Tshering Pem[1], Nor Rafeah Tumian[1], Chong Guang Yong[1], Nor Saaidah Kamal Rodin[1], Azhreen Fuad Mohamad Azudin[1], Siti Nur Hakimah Hashim[1], Lailatulema Abbas[1], Hany Haqimi Wan Hanafi[1]

[1]*Hospital Canselor Tuanku Muhriz, Kuala Lumpur, Malaysia;*

### Background

Chronic myeloid leukemia (CML) is a myeloproliferative neoplasm and typically affects older adults, but attention to adolescents and young adults (AYA), age 15 to 39 years is increasing. Epidemiologic and outcome data for AYA CML in Malaysia are scarce.

### Aims

To describe demographics, clinical presentation, laboratory and bone marrow features, treatments, cytogenetic findings, and disease outcome at 12-month among AYA CML treated in middle-income country from 2000–2025.

### Methods

This retrospective single-centre study reviewed electronic medical records and folders for AYA CML from 2000 to 2025. Extracted data at diagnosis included age, gender, ethnicity, disease phase, spleen size, Sokal and EOTUS long-term survival (ELTS) risk scores, hematologic and bone marrow morphology (white cell and platelet counts, blast percentage, bone marrow reticulin fibrosis grade and megakaryocyte clusters) and cytogenetics. Further data were first-line tyrosine kinase inhibitor (TKI), tyrosine kinase domain (TKD) mutations, receipt of stem cell transplantation (SCT), and response to first-line TKI at 12-month. Data were analysed with SPSS v30.

### Results

Over 25 years, 39 AYA CML cases were identified; 17 were excluded for missing data, transfer of care, or default, leaving 22 patients for analysis. The median age was 30.5 year. Median total white cell count was 143.5 × 10^9/L and median platelet count of 339 × 10^9/L. 68.2%, had no blasts. Presenting symptoms most commonly were fever (20.8%), abdominal discomfort (18.8%), and weight loss (14.6%). Hyperviscosity symptoms (blurred vision, dyspnoea) were uncommon. Notably, 36.4% had no splenomegaly, a finding that differs from many published series. Bone marrow and cytogenetics data availability varied due to cost and test access. Megakaryocyte clusters were present in 31.8% (n=22). Reticulin stain (n=16) showed fibrosis (Grade 1 n=7; Grade 2–3 n= 3). Most cytogenetic analysis (n=19), had isolated BCR-ABL, while two had complex abnormalities (a three-way t(6;9;22) and trisomy 8 with additional del(22)). Mean time to complete hematologic response was 2.4 months (±2.7). Majority (59.1%) had not achieved MMR response at 12-month, with four patients had TKD mutations (F359V, F359I, V289F, and ASXL1). Two patients underwent allogeneic SCT for TKI resistance and blast crisis each; both were in complete remission at analysis. Other data are depicted in Table 1. In Malaysia, access to TKI, BCR-ABL quantitative, and mutation analysis are not widely available.
Table 1: Clinical characteristics, treatment and response at 12-month

**Copyright Information:** (Online) ISSN: 2572-9241
©2026 The Author(s). HemaSphere is published by John Wiley & Sons Ltd on behalf of European Hematology Association. This is an open access Abstract Book distributed under the Attribution-NonCommercial-NoDerivs (CC BY-NC-ND), which allows third parties to download the articles and share them with others as long as they credit the author and the Abstract Book, but they cannot change the content in any way or use them commercially.
**Abstract Book Citations:** Authors, Title, HemaSphere, 2026;10:(S1):pages. The abstract book DOIs can be found on the “Author Information” page.
**Disclaimer:** Articles published in the journal HemaSphere exclusively reflect the opinions of the authors. The authors are responsible for all content in their abstracts including accuracy of the facts, statements, citing resources, etc.

| | Frequency (n) | Percentage (%) |
|---|---|---|
| N=22 | | |
| Gender | | |
| Male | 17 | 77.3 |
| Female | 5 | 22.7 |
| Race | | |
| Malay | 20 | 90.9 |
| Chinese | 2 | 9.1 |
| Disease phase | | |
| Chronic | 22 | 100 |
| Blast | - | - |
| Risk stratification | | |
| ELTS | | |
| Low | 14 | 63.6 |
| Intermediate | 7 | 31.8 |
| High | 1 | 4.5 |
| Sokal | | |
| Low | 13 | 59.1 |
| Intermediate | 7 | 31.8 |
| High | 2 | 9.1 |
| First-line TKI | | |
| $1^{st}$ generation | 15 | 68.2 |
| $2^{nd}$ generation | 7 | 31.8 |
| $3^{rd}$ generation | - | |
| Stamp inhibitor | - | |
| Deepest response at 12 months | | |
| Less than MMR | 13 | 59.1 |
| MMR | 5 | 22.7 |
| DMR | 2 | 9.1 |
| Blast | 1 | 4.5 |
| Death | 1 | 4.5 |

### Summary/Conclusion

Little is known on clinico-pathological characteristics of AYA CML with analysed data reported poor treatment response. The retrospective, single-centre design limit representation for Malaysian cohort. Missing data due to cost and variable test and treatment availability reflect real-world constraints in middle-income country. There are gaps in access to therapies, and monitoring, which translates to outcomes. Larger multicentre studies and health system interventions are needed to improve adherence, monitoring, and access to effective TKIs.

**Copyright Information:** (Online) ISSN: 2572-9241
©2026 The Author(s). HemaSphere is published by John Wiley & Sons Ltd on behalf of European Hematology Association. This is an open access Abstract Book distributed under the Attribution-NonCommercial-NoDerivs (CC BY-NC-ND), which allows third parties to download the articles and share them with others as long as they credit the author and the Abstract Book, but they cannot change the content in any way or use them commercially.
**Abstract Book Citations:** Authors, Title, HemaSphere, 2026;10:(S1):pages. The abstract book DOIs can be found on the "Author Information" page.
**Disclaimer:** Articles published in the journal HemaSphere exclusively reflect the opinions of the authors. The authors are responsible for all content in their abstracts including accuracy of the facts, statements, citing resources, etc.

### (PB3033) REAL-WORLD PERFORMANCE OF ELTS AND SOKAL FOR PREDICTION OF EARLY MOLECULAR RESPONSE IN CHRONIC-PHASE CML TREATED WITH MIXED FRONTLINE TKIS

**Topic:** 08. Chronic myeloid leukemia - Clinical

Diana Grasu*[1, 2], Andreea Farcas[1, 2], Maria Bacanu[1, 2], Elena Pirvu[1, 2], Alexandra-Diana Malenda[1, 2], Viola Maria Popov[2], Mihaela Andreescu[1, 2]

*[1]Universitatea Titu Maiorescu, București, Romania;[2]Colentina Clinical Hospital, Hematology I, Bucharest, Romania;*

### Background

Early molecular response (EMR) at 3 months is a validated therapeutic milestone in chronic-phase chronic myeloid leukemia (CML) and correlates with long-term survival. Although the ELTS score was developed to predict CML-related mortality, its ability to discriminate early molecular response in contemporary real-world cohorts treated with heterogeneous frontline tyrosine kinase inhibitors (TKIs) remains clinically relevant.

### Aims

To compare the predictive performance of ELTS and SOKAL for EMR in a real-world single-center cohort of chronic-phase CML patients treated with mixed frontline TKIs.

### Methods

We retrospectively analyzed 33 consecutive patients with newly diagnosed chronic-phase CML receiving frontline TKIs (imatinib or second-generation TKIs). EMR was defined as BCR-ABL ≤10% at 3 months. Baseline risk stratification was performed using ELTS, SOKAL, Hasford, and EUTOS scores. Each score was evaluated as an independent predictor of EMR using univariate logistic regression. Discrimination was assessed using receiver operating characteristic (ROC) analysis and area under the curve (AUC) with 95% confidence intervals. Internal validation was performed by bootstrap resampling. Calibration was evaluated using Brier score and calibration slope. Exploratory analyses assessed frontline TKI generation and inflammatory markers.

### Results

EMR was achieved in 66.7% of patients (22/33). Frontline therapy consisted of imatinib in 16 patients and second-generation TKIs in 15 patients; EMR rates did not significantly differ between groups (68.8% vs 60.0%, p=0.716).
ELTS was significantly associated with EMR (OR 6.37, 95% CI 1.20–33.81, p=0.030) and demonstrated moderate discrimination (AUC 0.73, 95% CI 0.56–0.90). SOKAL showed comparable performance (AUC 0.74, 95% CI 0.58–0.91). Hasford demonstrated weaker association, while EUTOS was not predictive. Bootstrap validation suggested limited optimism, and calibration metrics indicated acceptable agreement between predicted and observed probabilities. Frontline TKI generation and exploratory inflammatory markers did not provide incremental predictive value.

### Summary/Conclusion

In this real-world Eastern European cohort treated with mixed frontline TKIs, ELTS demonstrated moderate discrimination for early molecular response, comparable to SOKAL. These findings provide real-world support for established baseline risk scores in early response stratification, while highlighting the need for validation in larger prospective cohorts.

**Copyright Information:** (Online) ISSN: 2572-9241
©2026 The Author(s). HemaSphere is published by John Wiley & Sons Ltd on behalf of European Hematology Association. This is an open access Abstract Book distributed under the Attribution-NonCommercial-NoDerivs (CC BY-NC-ND), which allows third parties to download the articles and share them with others as long as they credit the author and the Abstract Book, but they cannot change the content in any way or use them commercially.
**Abstract Book Citations:** Authors, Title, HemaSphere, 2026;10:(S1):pages. The abstract book DOIs can be found on the "Author Information" page.
**Disclaimer:** Articles published in the journal HemaSphere exclusively reflect the opinions of the authors. The authors are responsible for all content in their abstracts including accuracy of the facts, statements, citing resources, etc.

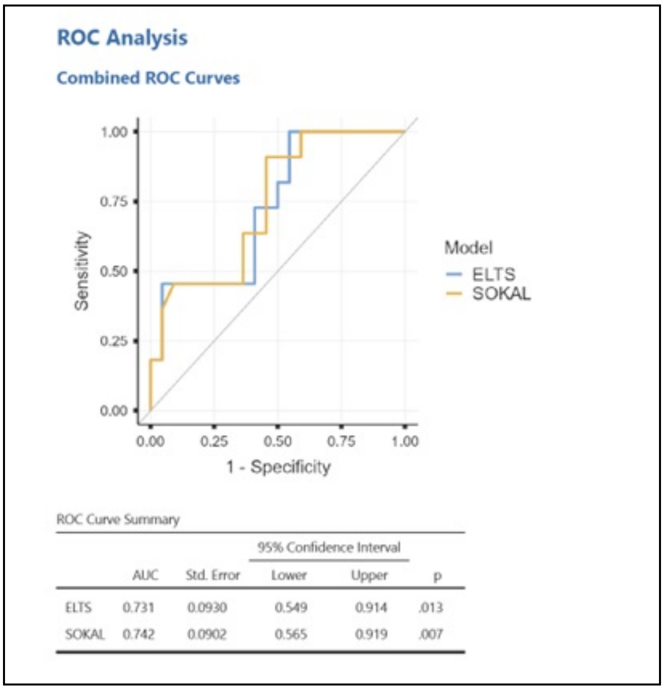

| | AUC | Std. Error | 95% Confidence Interval Lower | 95% Confidence Interval Upper | p |
|---|---|---|---|---|---|
| ELTS | 0.731 | 0.0930 | 0.549 | 0.914 | .013 |
| SOKAL | 0.742 | 0.0902 | 0.565 | 0.919 | .007 |

ROC Curves for ELTS and SOKAL in Predicting Early Molecular Response at 3 Months

**Copyright Information:** (Online) ISSN: 2572-9241
©2026 The Author(s). HemaSphere is published by John Wiley & Sons Ltd on behalf of European Hematology Association. This is an open access Abstract Book distributed under the Attribution-NonCommercial-NoDerivs (CC BY-NC-ND), which allows third parties to download the articles and share them with others as long as they credit the author and the Abstract Book, but they cannot change the content in any way or use them commercially.
**Abstract Book Citations:** Authors, Title, HemaSphere, 2026;10:(S1):pages. The abstract book DOIs can be found on the "Author Information" page.
**Disclaimer:** Articles published in the journal HemaSphere exclusively reflect the opinions of the authors. The authors are responsible for all content in their abstracts including accuracy of the facts, statements, citing resources, etc.

### (PB3034) THERAPEUTIC RESULTS OF SECOND-GENERATION TKI FOR THE TREATMENT OF CHRONIC MYELOID LEUKEMIA

**Topic:** 08. Chronic myeloid leukemia - Clinical

Yousra Fakhfakh Derbel*[1], Hanen Charfi[1], Fatima Kaddour[1], Rahma Mallek[1], Asma Koubaa[1], Aicha Ben Kahla[1], Rim Frikha[2], Fatma Turki[2], Maha Charfi[1], Faten Kallel[1], Imen Ben Amor[1], Imen Frikha[1, 1], Nour Siala[1, 1], Moez Medhaffar[1], Hassen Kamoun[2], Olfa Kassar[1], Moez Elloumi[1]

*[1]Hematology, University Hospital Hedi Chaker,university of Sfax, Hematology,, Sfax, Tunisia;[2]Genetics Department, University Hospital Hedi Chaker-Sfax, Genetics Department, Sfax, Tunisia;*

### Background

The introduction of first-line Tyrosine Kinase Inhibitor (TKI) therapy (Imatinib) dramatically changed the treatment and natural history of chronic myeloid leukemia (CML). However, approximately 20–40% of CML patients do not benefit from the first-generation TKI due to intolerance or resistance.

### Aims

We report our experience with second-generation (2G) TKI for the treatment of CML in southern Tunisia.

### Methods

Our study is retrospective. We included patients with CML in chronic or accelerated phase treated with 2G TKI over the period January 2011, December 2024. Patients received either oral Dasatinib (100 mg once daily in the chronic phase and 140 mg in the accelerated phase) or oral Nilotinib (400 mg X 2/day). Through the analysis of hematological response, cytogenetic response, and molecular response, we classified the cases according to the criteria of ELN 2020 on optimal response, suboptimal, and failure.

### Results

We collected 84 cases of CML treated with 2G TKI: 39 Dasatinib (46%) and 46 Nilotinb (54%). The sex ratio was 1.1, and the median age was 50 years (19-77 years). Eighty-one patients were in the chronic phase (96%) and 3 in the accelerated phase (4%). According to the ELTS score, patients were classified as low, intermediate, and high risk in 30%, 38%, and 32% of cases, respectively. Seventy-two patients (86%) switched from due to treatment resistance, and twelve (14%) switched because of Imatinib intolerance. The 12-month major molecular response was 78%. According to the ELN criteria 2020, an optimal response, suboptimal, and failure at 6 months were respectively 81%, 16%, and 3% out of evaluable cases. Sixty-four percent of patients had side effects from 2G TKI

### Summary/Conclusion

The use of 2G TKI has salvaged the two third of Imatinib resistant patients in our series. Our preliminary results are encouraging and similar to those reported in the literature, with an acceptable safety profile. Better management of 2G TKI may improve CML outcomes (early TKI switch and even 2G TKI in first-line therapy).

**Copyright Information:** (Online) ISSN: 2572-9241
©2026 The Author(s). HemaSphere is published by John Wiley & Sons Ltd on behalf of European Hematology Association. This is an open access Abstract Book distributed under the Attribution-NonCommercial-NoDerivs (CC BY-NC-ND), which allows third parties to download the articles and share them with others as long as they credit the author and the Abstract Book, but they cannot change the content in any way or use them commercially.
**Abstract Book Citations:** Authors, Title, HemaSphere, 2026;10:(S1):pages. The abstract book DOIs can be found on the “Author Information” page.
**Disclaimer:** Articles published in the journal HemaSphere exclusively reflect the opinions of the authors. The authors are responsible for all content in their abstracts including accuracy of the facts, statements, citing resources, etc.

### (PB3035) ONE VS. 2 VS. ≥3 TET2 MUTATIONS IN CHRONIC MYELOMONOCYTIC LEUKEMIA: CO-MUTATION PATTERNS AND PROGNOSTIC CORRELATES

**Topic:** 08. Chronic myeloid leukemia - Clinical

Yousuf Muhammad*[1], Saubia Fathima[1], Samuel Wu[2], Maymona Abdelmajid[1], Priyansh Faldu[1], Rania Abdelaziz[1], Clifford Csizmar[1], Abhishek Mangaonkar[1], Cinthya Mendoza[2], Kaaren Reichard[2], David Viswanatha[2], Rong He[2], Animesh Pardanani[1], Naseema Gangat[1], Mrinal Patnaik[1], Ayalew Tefferi[1]

*[1]Mayo Clinic, Hematology, Rochester, United States of America; [2]Mayo Clinic, Pathology and Laboratory Medicine, Rochester, United States of America;*

### Background

*TET2* is the most frequently mutated gene in chronic myelomonocytic leukemia (CMML) and has been generally associated with favorable prognosis. Furthermore, recent studies have suggested a more pronounced prognostic impact in the presence of ≥2 *TET2* mutations.

### Aims

The objective of the current study was to obtain additional information on the relationship between the number of *TET2*$^{\mathrm{MUT}}$ and prognosis in CMML, in the context of contemporary risk models.

### Methods

The current study was conducted under institutional review board approved minimum risk protocols. CLIA-approved NGS testing used to curate mutational data. Conventional statistics were employed.

**Copyright Information:** (Online) ISSN: 2572-9241
©2026 The Author(s). HemaSphere is published by John Wiley & Sons Ltd on behalf of European Hematology Association. This is an open access Abstract Book distributed under the Attribution-NonCommercial-NoDerivs (CC BY-NC-ND), which allows third parties to download the articles and share them with others as long as they credit the author and the Abstract Book, but they cannot change the content in any way or use them commercially.
**Abstract Book Citations:** Authors, Title, HemaSphere, 2026;10:(S1):pages. The abstract book DOIs can be found on the "Author Information" page.
**Disclaimer:** Articles published in the journal HemaSphere exclusively reflect the opinions of the authors. The authors are responsible for all content in their abstracts including accuracy of the facts, statements, citing resources, etc.

**Results**

536 CMML patients were stratified according to the number of *TET2*$^{MUT}$: 221 (41%) wild-type, 148 (28%) with one, 153 (29%) 2, and 14 (2%) ≥3 *TET2*$^{MUT}$. Co-mutation frequencies with significant differences between one vs. two *TET2*$^{MUT}$ included *ASXL1* (46% vs. 29%; p<0.01), *KRAS* (7% vs. 14%; p=0.02) and *SETBP1* (5% vs. 0.6%; p=0.02). Phenotypic comparisons revealed preponderance of prognostically favorable traits associated with wild-type *TET2* but no significant differences between one vs. two *TET2*$^{MUT}$. In age-adjusted univariate analysis, transplant-censored overall survival (TCOS*)*, calculated from the time of mutation detection, was superior in patients with one (p<0.01; HR 0.7) or 2 (p<0.01; HR 0.4) but not ≥3 (p=0.4) *TET2*$^{MUT}$, compared to those with wild-type *TET2.* TCOS was also superior with 2 vs. 1 (p=0.02; HR 0.7) but not with ≥3 vs. 1 (p=0.7)*TET2*$^{MUT}$; the results were similar when TCOS was calculated from the time of diagnosis. The survival advantage of CMML patients with two *TET2*$^{MUT}$, compared to those with wild-type *TET2* (p<0.01; HR 0.5), one *TET2*$^{MUT}$ (p<0.01; HR 0.6), or ≥3 *TET2*$^{MUT}$ (p<0.01; HR 0.4) was confirmed in multivariable analysis that included age, sex, sex- and severity-adjusted anemia, circulating blasts ≥2%, and leukocyte count ≥13 x $10^9$/L; in the particular analysis, there was no significant difference between wild-type *TET2* and one *TET2*$^{MUT}$ (p=0.08) or between wild-type *TET2* and ≥3 *TET2*$^{MUT}$ (p=0.6); similar favorable impact of exactly 2 *TET2*$^{MUT}$ in CMML-MD and CMML-MP was also observed. Additional multivariable analyses considered the presence or absence of two *TET2*$^{MUT}$ against *TET2* VAF (i.e., sum of individual VAFs ≥55% vs. <55%; dominant allele frequency ≥55% vs. <55%), type of mutation (i.e., truncating vs. non-truncating; C-terminal vs. N-terminal), karyotype, and other mutations previously identified as being prognostically relevant in CMML, including *TP53*, *BCOR*, *RUNX1*, *SETBP1*, *NRAS*, *ASXL1*, *PTPN11*, *U2AF1* and *DNMT3A*; the analysis confirmed the independent association of exactly two *TET2*$^{MUT}$ and significantly longer TCOS (p<0.01). While the prognostic relevance of two *TET2*$^{MUT}$ was sustained in the context of the BLAST (p<0.01; HR 0.5) and CPSS-mol (p=0.02; HR 0.7) risk models, it lost significance when accounting for the BLAST-mol risk model (p=0.12). Our observations were largely similar in regard to blast transformation free survival.

**Summary/Conclusion**

A mechanistic explanation for this novel observation includes the possibility that *TET2* mutational categories based on mutation count reflect distinct biological states rather than having a simple linear effect. The persistence of this signal despite adjustment for established molecular and genetic risk factors supports its consideration in the development of future CMML prognostic models.

**Copyright Information:** (Online) ISSN: 2572-9241
©2026 The Author(s). HemaSphere is published by John Wiley & Sons Ltd on behalf of European Hematology Association. This is an open access Abstract Book distributed under the Attribution-NonCommercial-NoDerivs (CC BY-NC-ND), which allows third parties to download the articles and share them with others as long as they credit the author and the Abstract Book, but they cannot change the content in any way or use them commercially.
**Abstract Book Citations:** Authors, Title, HemaSphere, 2026;10:(S1):pages. The abstract book DOIs can be found on the “Author Information” page.
**Disclaimer:** Articles published in the journal HemaSphere exclusively reflect the opinions of the authors. The authors are responsible for all content in their abstracts including accuracy of the facts, statements, citing resources, etc.

**Figure 1. Overall survival data in 536 Mayo Clinic patients with chronic myelomonocytic leukemia, stratified by the number of *TET2* mutations - calculated from the time of mutation detection and censored for allogeneic stem cell transplantation**

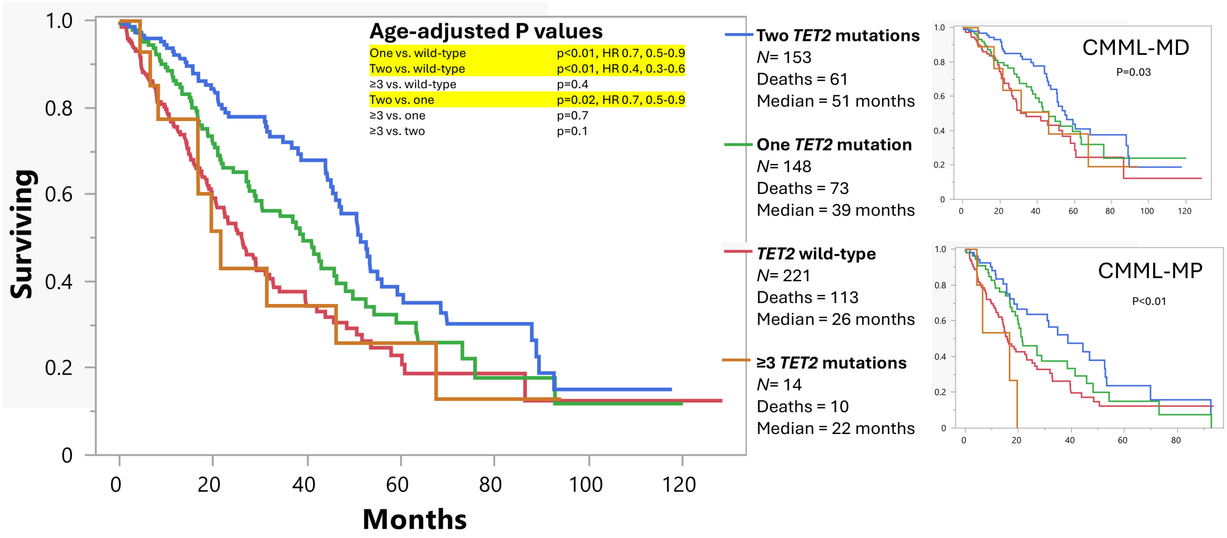

**Copyright Information:** (Online) ISSN: 2572-9241
©2026 The Author(s). HemaSphere is published by John Wiley & Sons Ltd on behalf of European Hematology Association. This is an open access Abstract Book distributed under the Attribution-NonCommercial-NoDerivs (CC BY-NC-ND), which allows third parties to download the articles and share them with others as long as they credit the author and the Abstract Book, but they cannot change the content in any way or use them commercially.
**Abstract Book Citations:** Authors, Title, HemaSphere, 2026;10:(S1):pages. The abstract book DOIs can be found on the "Author Information" page.
**Disclaimer:** Articles published in the journal HemaSphere exclusively reflect the opinions of the authors. The authors are responsible for all content in their abstracts including accuracy of the facts, statements, citing resources, etc.

### (PB3036) EXPERIENCE WITH PONATINIB IN CHRONIC MYELOID LEUKAEMIA IN PARAGUAY

**Topic:** 08. Chronic myeloid leukemia - Clinical

Ruth López[1], Carolina Barreto[2], Carlos Díaz[3], Rodrigo Santacruz[4], José Zarza*[4]

*[1]Clinicas Hospital, Haematology, San Lorenzo, Paraguay;[2]Central Hospital of the Social Security Institute, Haematology, Asunción, Paraguay; [3]National Hospital of Itauguá, Haematology, Itauguá, Paraguay;[4]Clínicas Hospital, Haematology, San Lorenzo, Paraguay;*

### Background

Ponatinib is a third-generation tyrosine kinase inhibitor (TKI) indicated for patients with Chronic Myeloid Leukaemia (CML) who are resistant to first- or second-generation TKIs, or in cases where the BCR::ABL T315I mutation is present.

### Aims

The primary objective was to assess treatment response at 12 months, together with tolerance and adverse events associated with ponatinib.

### Methods

We conducted a retrospective study of adult patients (>18 years) diagnosed with Philadelphia chromosome-positive CML who received ponatinib in three reference centres in Paraguay. Variables analysed included age, disease phase, number of prior TKI therapies, baseline and post-treatment molecular response, presence of the T315I mutation, ponatinib dosage, comorbidities, previous exposure to other TKIs and adverse events.

### Results

A total of 43 patients (28 men and 15 women) were treated with ponatinib between June 2016 and February 2026 across three centres in Paraguay. The median age at diagnosis was 41 years (range, 20–79 years). Forty-two per cent (18 patients) had received two prior TKIs, while 56% (25 patients) had received three or more. Imatinib was consistently used as first-line therapy, followed by dasatinib or nilotinib as second-line agents depending on the centre. Median exposure time with TKI previous to ponatinib was 96 months (range, 14-
219 months). Median follow-up was 20 months (range, 4–72 months). At the time of ponatinib initiation, 84% were in chronic phase, 5% in accelerated phase, and 12% in blast crisis.

**Copyright Information:** (Online) ISSN: 2572-9241
©2026 The Author(s). HemaSphere is published by John Wiley & Sons Ltd on behalf of European Hematology Association. This is an open access Abstract Book distributed under the Attribution-NonCommercial-NoDerivs (CC BY-NC-ND), which allows third parties to download the articles and share them with others as long as they credit the author and the Abstract Book, but they cannot change the content in any way or use them commercially.
**Abstract Book Citations:** Authors, Title, HemaSphere, 2026;10:(S1):pages. The abstract book DOIs can be found on the "Author Information" page.
**Disclaimer:** Articles published in the journal HemaSphere exclusively reflect the opinions of the authors. The authors are responsible for all content in their abstracts including accuracy of the facts, statements, citing resources, etc.

| Experience with the use of ponatinib in Chronic Myeloid Leukemia in Paraguay<br><br>Baseline Date Table | |
|---|---|
| Number of patients (N) | 43 (100%) |
| Study Period | June 2016 to February 2026 (10 years) |
| Median age | 41 years (20 - 79) |
| Sex | Male: 28 (65%)<br>Female: 15 (35%) |
| TKI previus | 2 TKIs (Imatinib + Dasatinib): 19 (44%)<br>2 TKIs (Imatinib + Nilotinib): 3 (7%)<br>3 ITKs (Imatinib + Dasatinib + Nilotinib): 4 (9%)<br>3 TKIs (Imatinib + Dasatinib + Bosutinib): 17 (40%) |
| Ponatinib Dose | 45 mg/día: 18 (42%)<br>30 mg/día: 16 (37%)<br>15 mg/día: 9 (21%) |
| Median Follow-up | 20 months (4 - 72) |
| Response to ponatinib at 12 months | Major Molecular Response - MR (IS < 0.1%): 16 (37%)<br>Deep Molecular Response (IS < 0,01%) : 6 (14%)<br>No response/not achieved: 14 (32%)<br>No evaluable: 11 (25%) |
| Progression-free survival at 24 months | 76% |

At baseline, 41 patients (95%) exhibited BCR::ABL1 transcript levels >10% on the International Scale (IS). At 12 months, 47% (n=32) achieved a molecular response (MR, BCR::ABL1 IS <1%), and 16% (n=32) achieved a deep molecular response.
Testing for the BCR::ABL T315I mutation was performed in 11 patients, with positivity confirmed in 3 patients. Ponatinib dosing regimens were 45 mg/day (42%), 30 mg/day (37%), and 15 mg/day (21%).
Thirty-four patients completed 2 years of follow-up with a progression-free survival (PFS) of 76%. Eleven patients (26%) experienced cardiovascular events, the most frequent being hypertension (21%). One patient suffered myocardial infarction (2%), one developed arrhythmia (2%), and one reported erectile dysfunction (2%). Overall, tolerance was favourable, with no gastrointestinal adverse effects or haematological toxicity necessitating treatment discontinuation.

### Summary/Conclusion

Ponatinib demonstrates substantial efficacy as a therapeutic option in patients refractory or intolerant to other TKIs. Nevertheless, careful consideration of cardiovascular risk factors - particularly age, diabetes mellitus, and arterial hypertension - is essential prior to treatment initiation.

**Copyright Information:** (Online) ISSN: 2572-9241
©2026 The Author(s). HemaSphere is published by John Wiley & Sons Ltd on behalf of European Hematology Association. This is an open access Abstract Book distributed under the Attribution-NonCommercial-NoDerivs (CC BY-NC-ND), which allows third parties to download the articles and share them with others as long as they credit the author and the Abstract Book, but they cannot change the content in any way or use them commercially.
**Abstract Book Citations:** Authors, Title, HemaSphere, 2026;10:(S1):pages. The abstract book DOIs can be found on the “Author Information” page.
**Disclaimer:** Articles published in the journal HemaSphere exclusively reflect the opinions of the authors. The authors are responsible for all content in their abstracts including accuracy of the facts, statements, citing resources, etc.

### (PB3037) TRIAL IN PROGRESS: REAL-WORLD STUDY OF ASCIMINIB IN THE TREATMENT OF CHRONIC MYELOID LEUKEMIA IN CHINA (ASC4CN)

**Topic:** 08. Chronic myeloid leukemia - Clinical

Weiming Li*[1], Shengji Gu[2]

*[1]Union Hospital, Tongji Medical College, Huazhong University of Science and Technology, Department of Hematology, Wuhan, China; [2]Novartis China, On behalf of the ASC4CN study team, Shanghai, China;*

### Background

Chronic myeloid leukemia (CML) is a myeloproliferative neoplasm characterized by the BCR::ABL1 fusion gene. Asciminib, 1st BCR::ABL1 inhibitor/only BCR::ABL1 inhibitor that Specifically Targets the ABL Myristoyl Pocket (STAMP), has demonstrated superior efficacy and favorable safety and tolerability versus all investigator selected TKIs in the phase III ASC4FIRST trial for newly diagnosed Philadelphia chromosome-positive CML in chronic phase (Ph+ CML-CP). Based on these results, asciminib was approved in China for first-line treatment of adult Ph+ CML-CP in May 2025. Randomized controlled trial (RCT) data may not fully reflect real-world clinical practice due to strict selection criteria and controlled settings. Notably, Chinese CML patients are younger (median age 45-50 years) than Western populations, and real-world evidence on asciminib's performance in this broader, more heterogeneous population is lacking. The ASC4CN study (NCT07375355) was designed to address this evidence gap.

### Aims

This multicenter, non-interventional, prospective real-world study aims to evaluate the efficacy, safety, tolerability, and disease burden of asciminib in newly diagnosed adult patients with Ph+ CML-CP in China.

### Methods

Approximately 200 patients from 20 sites across China will be enrolled. Eligible patients are adults (≥18 years) with newly diagnosed (within 3 months) Ph+ CML-CP, with ≤2 weeks of prior TKI therapy, and for whom a decision to initiate asciminib has been made prior to enrollment. The primary endpoint is the cumulative rate of major molecular response (MMR; BCR::ABL1 ≤0.1% $^{IS}$) by 12 months. Secondary endpoints include MMR rates at 3, 6, 9, 18, and 24 months; deep molecular response rates (MR4, MR4.5); time to and duration of responses; event-free, progression-free, and overall survival; safety profile (adverse events [AEs] per CTCAE v5.0); tolerability measures (treatment discontinuation/modification rates, patient-reported outcomes, medication possession ratio); and healthcare resource utilization. Data will be collected from medical records and patient reports during routine clinical visits over a 24-month follow-up period. All analyses will be descriptive, with point estimates and 95% confidence intervals provided for the primary endpoint.

### Results

ASC4CN will begin recruitment from February 2026 across 20 centers. Approximately 210 patients will be screened; at least 200 will be enrolled. The primary endpoint results are expected in 2028. As of February 26, 2026, the first patient has been enrolled in the study. No efficacy or safety data are available currently.

### Summary/Conclusion

This study will provide insight into the real world effectiveness and safety of asciminib in the diverse Chinese CML patient population encountered in clinical practice, to further support optimal care for patients with newly diagnosed CML-CP in China.

**Copyright Information:** (Online) ISSN: 2572-9241
©2026 The Author(s). HemaSphere is published by John Wiley & Sons Ltd on behalf of European Hematology Association. This is an open access Abstract Book distributed under the Attribution-NonCommercial-NoDerivs (CC BY-NC-ND), which allows third parties to download the articles and share them with others as long as they credit the author and the Abstract Book, but they cannot change the content in any way or use them commercially.
**Abstract Book Citations:** Authors, Title, HemaSphere, 2026;10:(S1):pages. The abstract book DOIs can be found on the “Author Information” page.
**Disclaimer:** Articles published in the journal HemaSphere exclusively reflect the opinions of the authors. The authors are responsible for all content in their abstracts including accuracy of the facts, statements, citing resources, etc.

### (PB3038) FLUMATINIB IN CHRONIC MYELOID LEUKEMIA WITH RARE BCR-ABL TRANSCRIPTS: A REPORT OF 4 CASES AND ANALYSIS OF EFFICACY CHARACTERISTICS

**Topic:** 08. Chronic myeloid leukemia - Clinical

Qiguo Zhang*[1, 2], Wenjing Fu[1], Chun Ling[1], Wenqiang Bao[1], Yin Wang[1], Changzhi Zhao[1], Wenyu Gong[1], Shiyun Xu[1], Jing Wu[1], Qichuan Jin[1], Bing Chen[2]

*[1]Chuzhou Hospital Affiliated to Anhui Medical University, Department of Hematology, Chuzhou, China;[2]Nanjing Drum Tower Hospital, Affiliated Hospital of Medical School, Nanjing University, Department of Hematology, Nanjing, China;*

### Background

The intrinsic tyrosine kinase activity of BCR-ABL variants differs, with p190 being the strongest (approximately 10-fold that of c-ABL1), followed by p210 (6-fold) and p230 (3-fold). Furthermore, p190 and p210 activate distinct signaling pathways. The transcript type can influence the clinical phenotype and treatment response in Chronic Myeloid Leukemia (CML).

### Aims

This study aims to enhance the understanding of the clinical and laboratory characteristics of CML with rare transcripts.

### Methods

We screened for rare BCR-ABL transcripts among 95 CML patients treated at the outpatient clinics of Chuzhou First People's Hospital and Nanjing Drum Tower Hospital from September 1, 2017, to February 28, 2026. Clinical data were analyzed, and the literature was reviewed.

### Results

Four patients (3 males, 1 female; median age 62 years) with rare transcripts were identified. This included one case each of isolated p190 and isolated p230, and two cases with dual positivity for both p190 and p210 transcripts.

Isolated p190 CML: The patient presented with elevated blood counts in two lineages without monocytosis. After 3 months of flumatinib, complete cytogenetic response (CCR) was achieved (p190 1.27%), reaching 0.544% at 6 months. At 9 months (1.039%), treatment was interrupted for one month due to gastric discomfort, leading to a rise in p190 to 3.472% without detectable ABL kinase mutations. Interferon was added, after which transcript levels gradually declined. After >4 years of follow-up, the patient finally achieved deep molecular response (DMR, 0.0064%).

Isolated p230 CML: The patient had a 2-year history of mild leukocytosis and thrombocytosis without seeking medical attention, and no neutrophilia. After diagnosis, flumatinib led to a complete molecular response (CMR, p230 0%) at 3 months and 0.126% at 6 months. Liver function abnormalities occurred; dose reduction to two tablets improved liver enzymes but resulted in p230 fluctuation (0.508%-0.575%). The patient was switched to dasatinib and currently has a p230 level of 0.005%.

Dual-positive CML (Case 1): Initially treated with imatinib, the patient achieved molecular negativity at 12 months but lost major molecular response (MMR, P210 0.217%) at 31 months, with detection of an M244V mutation. Nilotinib for 3 months re-induced negativity but was switched to flumatinib due to cardiac side effects. The patient remains stable on flumatinib for over 2 years.

Dual-positive CML (Case 2): Treated with first-line flumatinib. CCR (P210 0.35%) was achieved at 3 months, and MMR (P210 0.086%) at 6 months. Liver function abnormalities were managed with hepatoprotective therapy and dose reduction.

**Copyright Information:** (Online) ISSN: 2572-9241
©2026 The Author(s). HemaSphere is published by John Wiley & Sons Ltd on behalf of European Hematology Association. This is an open access Abstract Book distributed under the Attribution-NonCommercial-NoDerivs (CC BY-NC-ND), which allows third parties to download the articles and share them with others as long as they credit the author and the Abstract Book, but they cannot change the content in any way or use them commercially.
**Abstract Book Citations:** Authors, Title, HemaSphere, 2026;10:(S1):pages. The abstract book DOIs can be found on the "Author Information" page.
**Disclaimer:** Articles published in the journal HemaSphere exclusively reflect the opinions of the authors. The authors are responsible for all content in their abstracts including accuracy of the facts, statements, citing resources, etc.

### Summary/Conclusion

Flumatinib demonstrates good efficacy in CML patients with rare BCR-ABL transcripts. For cases with suboptimal response, combining with interferon may be beneficial. The speed and depth of response to flumatinib appear to correlate with the biological characteristics of the specific transcript. Close monitoring and management of flumatinib-associated hepatotoxicity are warranted.

**Copyright Information:** (Online) ISSN: 2572-9241
©2026 The Author(s). HemaSphere is published by John Wiley & Sons Ltd on behalf of European Hematology Association. This is an open access Abstract Book distributed under the Attribution-NonCommercial-NoDerivs (CC BY-NC-ND), which allows third parties to download the articles and share them with others as long as they credit the author and the Abstract Book, but they cannot change the content in any way or use them commercially.
**Abstract Book Citations:** Authors, Title, HemaSphere, 2026;10:(S1):pages. The abstract book DOIs can be found on the "Author Information" page.
**Disclaimer:** Articles published in the journal HemaSphere exclusively reflect the opinions of the authors. The authors are responsible for all content in their abstracts including accuracy of the facts, statements, citing resources, etc.

### (PB3039) REAL-WORLD CLINICAL CHARACTERISTICS AND TREATMENT PATTERNS IN PEDIATRIC CHRONIC MYELOID LEUKEMIA: A MEXICAN COHORT STUDY

**Topic:** 08. Chronic myeloid leukemia - Clinical

Emmanuel Rolando Rodríguez Cedeño*[1], Susana Gabriel[1], Angel Rodolfo Rivas Peyrano[1, 1], Angelica Romo[1, 1, 2], Camila Terreros[3], Auandar Mendoza Gamez[3]

[1]*IMSS, Pediatric hematology, Ciudad de México, Mexico;*[2]*Andador November 20, Hematology, Zapopan, Mexico;*[3]*UNAM Palacio de la Escuela de Medicina Museo de la Medicina, Medicine, Ciudad de México, Mexico;*

### Background

Pediatric CML is a very rare disease. Incidence of CML is low, accounting for ~2% to 3% of all leukemias in children aged in the world.  There is some evidence that CML in children may differ from CML in adults and real-world data from Latin America remain limited. In Mexico mirrors this trend as well; although clinical guidelines and recommendations for its management exist, the lack of systematic, robust registries restricts accurate estimation of its incidence and prevalence and with scarce regional data describing clinical characteristics and treatment patterns in children and adolescents. Multiple TKIs have been approved for the treatment of pediatric patients with Ph-positive CML, but despite this, the role of HSCT in pediatrics remains fundamental for these patients. Therefore, our objective is to share the experience of national referral centers regarding this disease, the clinical resources for its treatment, and their outcomes over a 4-year follow-up period.

### Aims

In this review of two of the most important reference centers at the national level, of the IMSS institution, we want to describe the clinical characteristics, treatment patterns, and outcomes of pediatric patients with CML treated in two Mexican centers.

### Methods

We performed a retrospective descriptive analysis of pediatric patients diagnosed with CML and treated with tyrosine kinase inhibitors (TKIs). Demographic data, treatment history, TKI sequencing, hematologic parameters, molecular responses, resistance and intolerance to sequential treatments, and referral to transplantation were collected. Follow-up was calculated from diagnosis to date, showing that patients are still alive and with the necessary resources to improve treatment and have a good quality of life

### Results

In a cohort of 28 patients (100%), the mean age at diagnosis was 8 years 7 months. The predominant BCR-ABL1 transcript was p210 in 25 patients (89%), followed by p190 in 2 (7%) and T315I in 1 (3.5%). All patients received first-line imatinib; however, 18 (64%) developed TKI resistance or intolerance, causes included nausea, neuropathy, gastrointestinal symptoms, myelosuppression, weight loss, and Dress syndrome. Managed by imatinib dose modification in 55%, switch to dasatinib in 39%, or nilotinib in 6%. Under TKI therapy, thrombocytopenia and neutropenia were observed in a minority (14% and 18% respectively). Hematopoietic stem cell transplantation was performed in 12 patients (43%). Molecular response could not be assessed in most cases due to lack of PCR availability at referral centers.

**Copyright Information:** (Online) ISSN: 2572-9241
©2026 The Author(s). HemaSphere is published by John Wiley & Sons Ltd on behalf of European Hematology Association. This is an open access Abstract Book distributed under the Attribution-NonCommercial-NoDerivs (CC BY-NC-ND), which allows third parties to download the articles and share them with others as long as they credit the author and the Abstract Book, but they cannot change the content in any way or use them commercially.
**Abstract Book Citations:** Authors, Title, HemaSphere, 2026;10:(S1):pages. The abstract book DOIs can be found on the "Author Information" page.
**Disclaimer:** Articles published in the journal HemaSphere exclusively reflect the opinions of the authors. The authors are responsible for all content in their abstracts including accuracy of the facts, statements, citing resources, etc.

**Summary/Conclusion**

The cohort described above present with resistance/intolerance in 64%, although initial therapy with imatinib is indicated, patients require modifications of the 1L, up to 55% the main causes are gastrointestinal problems and myelosuppression, requiring dose change or TKI modification. From this analysis of 28 patients, it is of importance to highlight that 9 of the 28 patients did not present with molecular responses, and one had progression of the diseases. 43% of the patients require a HSCT. We know that the need for more specific therapies with good tolerance is necessary in the pediatric patient. As knowledge about pediatric CML increases, recommendations in our country should be updated to optimize care and outcomes in children with chronic-phase CML. Hematopoietic stem cell transplantation continues to be a good option for those patients with low tolerance, lack of molecular response, and disease progression.

Table 1. Characteristics and results

| **Characteristics** | |
|---|---|
| N (%) | 28 (100) |
| Average age of diagnosis | 8 years 7 months |
| p210 mutation (%) | 25 (89) |
| p190 mutation (%) | 2 (7) |
| T315i mutation (%) | 1 (3.5) |
| **TKI selection 1L** | |
| Imatinib (%) | 28 (100) |
| TKI resistance/intolerance (%) | 18 (64) |
| **Choice of TKI in case of intolerance or resistance** | |
| Imatinib dose modification | 10 (55) |
| Dasatinib (%) | 7 (39) |
| Nilotinib (%) | 1 (6) |
| **Hematologic response to TKI** | |
| Hb >10g/dL (%) | 27 (96) |
| Leukocytes <10^$10^6$/uL (%) | 27 (96) |
| Platelets <450^$10^6$/uL (%) | 1 (3.5) |
| Neutrophils <1.5^$10^6$/uL (%) | 5 (18) |
| Platelets <100^$10^6$/uL (%) | 4 (14) |
| **Hematopoietic stem cell transplantation** | |
| HSCT (%) | 12 (43) |

Characteristic and results

**Copyright Information:** (Online) ISSN: 2572-9241
©2026 The Author(s). HemaSphere is published by John Wiley & Sons Ltd on behalf of European Hematology Association. This is an open access Abstract Book distributed under the Attribution-NonCommercial-NoDerivs (CC BY-NC-ND), which allows third parties to download the articles and share them with others as long as they credit the author and the Abstract Book, but they cannot change the content in any way or use them commercially.
**Abstract Book Citations:** Authors, Title, HemaSphere, 2026;10:(S1):pages. The abstract book DOIs can be found on the "Author Information" page.
**Disclaimer:** Articles published in the journal HemaSphere exclusively reflect the opinions of the authors. The authors are responsible for all content in their abstracts including accuracy of the facts, statements, citing resources, etc.

### (PB3040) ADDITIONAL CHROMOSOMAL ABNORMALITIES AT DIAGNOSIS PREDICT INFERIOR EARLY MOLECULAR RESPONSE IN CHRONIC MYELOID LEUKEMIA: A SINGLE-CENTER STUDY

**Topic:** 08. Chronic myeloid leukemia - Clinical

Sanjukta S Rao*[1]

[1]*St Johns Medical College, Clinical Hematology, Bengaluru, India;*

### Background

CML is defined by the presence of the reciprocal translocation t(9;22)(q34;q11), the Philadelphia chromosome resulting in the generation of the BCR-ABL1 fusion oncogene. Despite excellent overall outcomes, heterogeneity in treatment response persists. Additional chromosomal abnormalities (ACA), represent cytogenetic changes beyond the Philadelphia chromosome and occur in approximately 5–10% of patients. ACA have been associated with adverse prognosis. However, the prognostic impact of ACA at diagnosis in the TKI era, remains incompletely characterized.Early molecular response (EMR), defined as BCR-ABL1 ≤10% at 3 months, is a validated prognostic milestone strongly associated with long-term outcomes. Patients failing to achieve EMR have increased risk of disease progression and inferior survival.

### Aims

This study aimed to evaluate the prevalence of ACA at diagnosis and assess their association with EMR and clinical characteristics in a cohort of patients with newly diagnosed CML.

### Methods

Retrospective observational study included patients with CML between November 2023 and November 2025. Patients with available cytogenetic analysis at diagnosis and molecular response assessment at 3 months were included. Conventional karyotyping analysis was performed at diagnosis using GTG-banding of bone marrow metaphases. ACA were defined as cytogenetic abnormalities in addition to the Philadelphia chromosome. Molecular response was assessed using quantitative real-time PCR standardized to the International Scale. EMR was defined as BCR-ABL1 ≤10% at 3 months of therapy initiation. Risk assessment was performed using the ELTS score.

### Results

68 patients were included. ACA were identified in 17 of 62 patients. See Figure 1.
Table 1 Baseline Demographics

| | Overall (n=68) | ACA Present (n=17) | ACA Absent (n=45) |
|---|---|---|---|
| **Age (years), Median (IQR)** | 41.5 (33.0–49.0) | 42.5 (33.8–52.8) | 41.5 (33.8–48.8) |
| **Total leukocyte count (×10⁹/L), Median (IQR)** | 138.0 (85.5–242.9) | 129.0 (53.7–196.0) | 140.0 (82.5–274.8) |

Patients with ACA had significantly lower baseline hemoglobin levels compared to those without ACA (median 6.7 g/dL vs 9.2 g/dL, p=0.011). EMR data was available in 60 patients. Among patients with ACA, 5 of 15 (33.3%) achieved EMR compared to 33 of 45 patients without ACA (73.3%) (p=0.011). Patients with ACA had significantly lower probability of achieving EMR. ELTS risk score demonstrated a strong association with EMR.Response rates were highest in the low risk category; Low risk: 82.8% achieved EMR; Intermediate risk: 47.4%; High risk: 31.3%. This association was statistically significant (p=0.0015).

**Copyright Information:** (Online) ISSN: 2572-9241
©2026 The Author(s). HemaSphere is published by John Wiley & Sons Ltd on behalf of European Hematology Association. This is an open access Abstract Book distributed under the Attribution-NonCommercial-NoDerivs (CC BY-NC-ND), which allows third parties to download the articles and share them with others as long as they credit the author and the Abstract Book, but they cannot change the content in any way or use them commercially.
**Abstract Book Citations:** Authors, Title, HemaSphere, 2026;10:(S1):pages. The abstract book DOIs can be found on the “Author Information” page.
**Disclaimer:** Articles published in the journal HemaSphere exclusively reflect the opinions of the authors. The authors are responsible for all content in their abstracts including accuracy of the facts, statements, citing resources, etc.

**Summary/Conclusion**

This study demonstrates that ACA at diagnosis was associated with inferior EMR in patients with CML treated with TKIs. The prevalence of ACA in this cohort was 27.4%, which is higher than historically reported rates. Patients with ACA were significantly less likely to achieve EMR, supporting the adverse prognostic significance of cytogenetic complexity at diagnosis. Importantly, ACA were not associated with leukocyte count, platelet count, or spleen size, suggesting that their prognostic impact reflects underlying disease biology rather than disease burden alone. ELTS risk score also showed strong prognostic discrimination, consistent with previous studies. These findings reinforce the importance of cytogenetic analysis at diagnosis for risk stratification.
ACA at diagnosis are associated with inferior EMR in CML. Cytogenetic risk assessment remains an essential component of baseline evaluation and prognostication

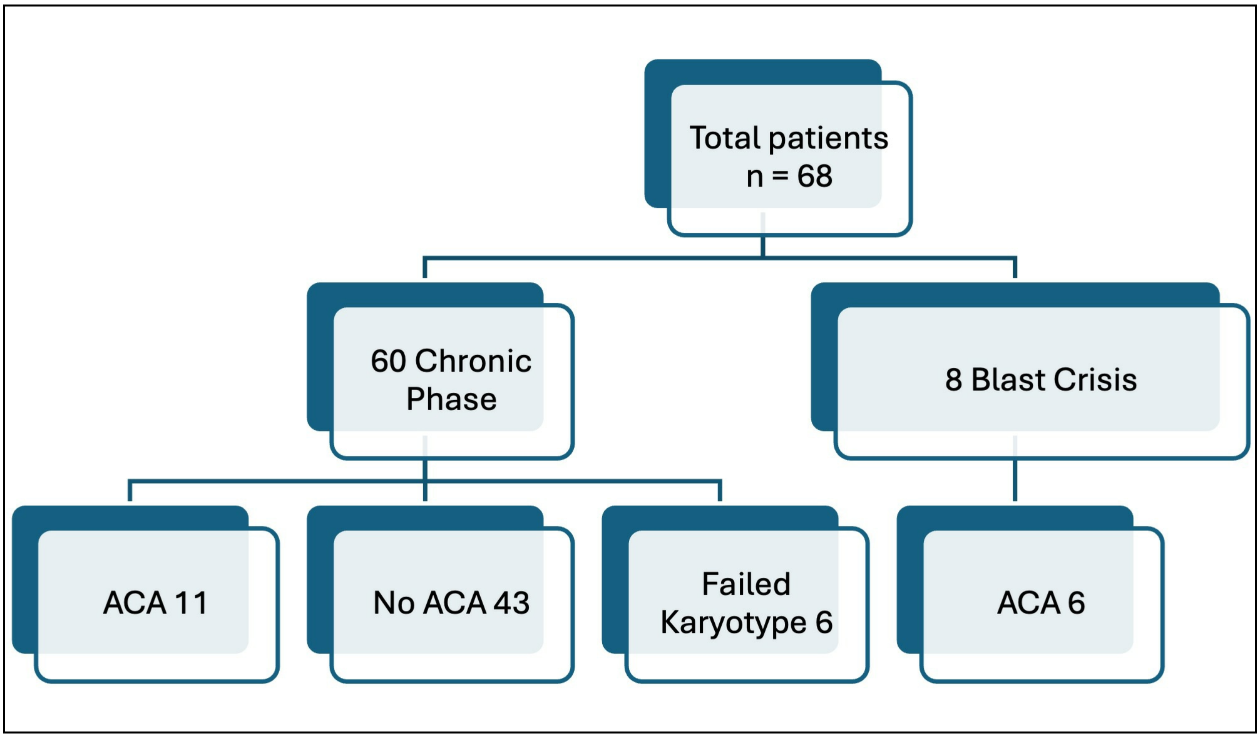

Figure 1 – the presence of ACA and distribution of CML phase

Copyright Information: (Online) ISSN: 2572-9241
©2026 The Author(s). HemaSphere is published by John Wiley & Sons Ltd on behalf of European Hematology Association. This is an open access Abstract Book distributed under the Attribution-NonCommercial-NoDerivs (CC BY-NC-ND), which allows third parties to download the articles and share them with others as long as they credit the author and the Abstract Book, but they cannot change the content in any way or use them commercially.
Abstract Book Citations: Authors, Title, HemaSphere, 2026;10:(S1):pages. The abstract book DOIs can be found on the "Author Information" page.
Disclaimer: Articles published in the journal HemaSphere exclusively reflect the opinions of the authors. The authors are responsible for all content in their abstracts including accuracy of the facts, statements, citing resources, etc.

### (PB3041) ASCIMINIB IN THE TREATMENT OF CHRONIC MYELOID LEUKEMIA: A REAL-WORLD ANALYSIS OF EFFICACY AND SAFETY

**Topic:** 08. Chronic myeloid leukemia - Clinical

Xinrui Zhang*[1], Xinwen Jiang[1], Qingli Ji[1], Xiaoqing Li[1], Chen Cao[1], Lei Chen[2], Wenbo Zhao[3], Yuanyuan Zhang[4], Yan Wang[5], Yan Wang[6], Sai MA[1], Minran Zhou[1], Chunyan Chen[1]

*[1]Qilu Hospital of Shandong University, Hematology, Jinan, China;[2]Liaocheng People's Hospital, Liaocheng, China;[3]Shandong Provincial Hospital, Jinan, China; [4]Linyi People's Hospital, Linyi, China;[5]Jining No.1 People's Hospital, Jining, China;[6]Shandong Provincial Qianfoshan Hospital, Jinan, China;*

**Background**

Tyrosine kinase inhibitors (TKIs) have significantly improved the prognosis of patients with chronic myeloid leukemia (CML); however, off-target effects and long-term adverse reactions continue to affect quality of life in a subset of patients. Asciminib, a novel specifically targeting the ABL myristoyl pocket (STAMP) inhibitor, has garnered recommendations in multiple clinical practice guidelines owing to its favorable efficacy and safety profile.

**Aims**

This retrospective study aims to evaluate the efficacy and safety of asciminib in Chinese patients with CML through real-world data analysis.

**Methods**

The study retrospectively collected clinic data from 13 adult CML patients who received asciminib treatment across six centers in Shandong Province between January 2025 and February 2026. Efficacy was assessed by quantitative polymerase chain reaction (qPCR) detection of BCR-ABL1 international standard (IS) values. Major molecular response (MMR) was defined as BCR-ABL1 IS ≤ 0.1%. Early hematologic response was defined as achievement of complete hematologic response (CHR) within one month of treatment initiation. Safety was graded according to the Common Terminology Criteria for Adverse Events (CTCAE) version 5.0.

**Results**

Among the 13 patients, the median age was 52.7 years, and 84.6% were male. Five patients (38.5%) received asciminib as first-line therapy, while eight patients (61.5%) received it as multi-line (≥2 prior lines) therapy, with a median of two prior treatment lines. Eleven patients (84.6%) received asciminib as monotherapy. All patients received asciminib at a dosage of 80 mg once daily, with a median treatment duration of 175 days. In the first-line treatment cohort, all five patients achieved CHR within three months, of whom three (60.0%) achieved CHR within one month. Among four evaluable patients at the three-month timepoint, three (75.0%) achieved MMR or deeper molecular response. In the multi-line treatment cohort, four patients switched to asciminib due to inadequate response to prior TKIs, including two who had progressed to accelerated or blast phase. Two patients with high tumor burden (BCR-ABL1 >90%) demonstrated >60% reduction in transcript levels within one month of switching; four patients who had achieved CHR and MR2 at baseline showed steady deepening of molecular response. One patient with post-TKI relapse (BCR-ABL1 72.76%) exhibited a reduction to 0.39% at one month post-switch, but rebounded to 65.84% at two months and succumbed to disease at five months, suggestive of disease progression or clonal evolution. Regarding safety, the most common adverse events were grade ≥2 thrombocytopenia (38.5%) and leukopenia (15.4%); no patient discontinued treatment due to adverse events. Notably, TKI-related toxicities (hepatic dysfunction, fatigue, etc.) present prior to asciminib initiation showed amelioration following the switch in several patients.

**Copyright Information:** (Online) ISSN: 2572-9241
©2026 The Author(s). HemaSphere is published by John Wiley & Sons Ltd on behalf of European Hematology Association. This is an open access Abstract Book distributed under the Attribution-NonCommercial-NoDerivs (CC BY-NC-ND), which allows third parties to download the articles and share them with others as long as they credit the author and the Abstract Book, but they cannot change the content in any way or use them commercially.
**Abstract Book Citations:** Authors, Title, HemaSphere, 2026;10:(S1):pages. The abstract book DOIs can be found on the "Author Information" page.
**Disclaimer:** Articles published in the journal HemaSphere exclusively reflect the opinions of the authors. The authors are responsible for all content in their abstracts including accuracy of the facts, statements, citing resources, etc.

**Summary/Conclusion**

This real-world study demonstrates that asciminib, whether administered as first-line or subsequent-line therapy, elicits rapid and substantial hematologic and molecular responses in Chinese patients with CML, with an overall favorable tolerability profile. For patients with TKI intolerance or suboptimal response, asciminib represents an effective therapeutic option. Long-term efficacy, treatment-free remission strategies, and combination regimens warrant validation in larger cohort studies.

**Copyright Information:** (Online) ISSN: 2572-9241
©2026 The Author(s). HemaSphere is published by John Wiley & Sons Ltd on behalf of European Hematology Association. This is an open access Abstract Book distributed under the Attribution-NonCommercial-NoDerivs (CC BY-NC-ND), which allows third parties to download the articles and share them with others as long as they credit the author and the Abstract Book, but they cannot change the content in any way or use them commercially.
**Abstract Book Citations:** Authors, Title, HemaSphere, 2026;10:(S1):pages. The abstract book DOIs can be found on the "Author Information" page.
**Disclaimer:** Articles published in the journal HemaSphere exclusively reflect the opinions of the authors. The authors are responsible for all content in their abstracts including accuracy of the facts, statements, citing resources, etc.

### (PB3042) COMPARATIVE REVIEW OF EMERGING TYROSINE KINASE INHIBITORS IN THE PIPELINE FOR CHRONIC MYELOID LEUKEMIA IN CHRONIC PHASE

**Topic:** 08. Chronic myeloid leukemia - Clinical

Mohammad Riyaz Beg*[1]

[1]*Institute of Chemical Technology, Mumbai, Department of Pharmaceutical Sciences and Technology, Mumbai, India;*

### Background

Despite the availability of multiple approved tyrosine kinase inhibitors (TKIs), unmet needs remain in chronic myeloid leukemia in chronic phase (CML-CP), including intolerance, resistance to earlier-generation TKIs, and long-term safety considerations. Several next-generation TKIs with distinct binding modes and target profiles are currently in clinical development, with the potential to further refine treatment sequencing and personalize therapy in CML-CP.

### Aims

To systematically review and comparatively analyze the clinical profile of emerging TKIs in development for CML-CP, focusing on mechanism of action, efficacy signals, safety/tolerability, and potential positioning relative to existing therapies.

### Methods

A structured literature review was conducted using publicly available sources, including peer-reviewed publications, major hematology congress abstracts, clinical trial registries, and company disclosures. TKIs in clinical development for CML-CP were evaluated across predefined dimensions: kinase selectivity and binding characteristics, clinical efficacy endpoints (e.g., MMR, MR4/4.5), activity in resistant or previously treated populations, and reported safety profiles. Comparative analysis was qualitative in nature, given heterogeneity in trial design, patient populations, and follow-up duration.

### Results

Several investigational TKIs demonstrate differentiated profiles compared with approved agents. Novel ATP-competitive and allosteric inhibitors show promising molecular response rates, including in patients with prior TKI exposure. Select agents exhibit activity against resistance-associated BCR::ABL1 mutations, while others emphasize improved selectivity with the aim of minimizing off-target toxicities. Safety data to date suggest variability across compounds, particularly with respect to cardiovascular, hematologic, and gastrointestinal adverse events. Cross-trial comparison highlights important trade-offs between depth of response, speed of molecular remission, and tolerability, underscoring the challenge of direct efficacy comparisons across non-randomized studies.

### Summary/Conclusion

The evolving CML-CP pipeline reflects a shift toward more targeted, mutation-aware, and tolerability-focused TKI development. While early clinical data are encouraging, differences in study populations and endpoints limit direct comparison. Ongoing and future trials will be critical to define the optimal role of these emerging TKIs within the CML treatment landscape, including their potential use in specific patient subsets and treatment lines.

**Copyright Information:** (Online) ISSN: 2572-9241
©2026 The Author(s). HemaSphere is published by John Wiley & Sons Ltd on behalf of European Hematology Association. This is an open access Abstract Book distributed under the Attribution-NonCommercial-NoDerivs (CC BY-NC-ND), which allows third parties to download the articles and share them with others as long as they credit the author and the Abstract Book, but they cannot change the content in any way or use them commercially.
**Abstract Book Citations:** Authors, Title, HemaSphere, 2026;10:(S1):pages. The abstract book DOIs can be found on the "Author Information" page.
**Disclaimer:** Articles published in the journal HemaSphere exclusively reflect the opinions of the authors. The authors are responsible for all content in their abstracts including accuracy of the facts, statements, citing resources, etc.

| | TERN-701 (Allosteric) | | ELVN-001 (Next Gen Active Site) | | | Olverembatinib (Next Gen Active site) | TGRX-678 (Allosteric) |
|---|---|---|---|---|---|---|---|
| Dose | 160mg - 500mg QD | | 10 mg–120 mg QD; 60 mg–80 mg BID | 80mg QD | 60/120mg QD | 30-50mg Q2D | 10-80 mg BID; 40-240 mg QD |
| Trial | CARDINAL Ph1 | | Ph1 (ENABLE) | Ph1b (ENABLE) | Ph1b (ENABLE) | Ph 1b (NCT04260022) | Chinese Ph1 (NCT05434312) |
| N | 63 | | 90 | 19 | 41 | 62 | 108 |
| Median Age | 57 | | 58 | -- | -- | 51 | 46 |
| Baseline BCR::ABL1 | | | | | | | |
| >10% | 44% | | -- | -- | -- | 69% | 84% |
| >1% to 10% | 13% | | -- | -- | -- | 21% | |
| >0.1% to 1% | 25% | | -- | -- | -- | -- | |
| ≤0.1% (i.e., in MMR) | 18% | | 18% | -- | -- | 8% | |
| Discontinuation to last TKI | | | | | | | |
| Lack of efficacy | 64% | | 72% | -- | -- | -- | |
| Lack of tolerability | 29% | | 23% | -- | -- | -- | |
| Other | 8% | | 3% | -- | -- | -- | |
| Median # Prior TKIs | 3 | | 3 | 42% ≥4 Prior TKIs | 59% ≥4 Prior TKIs | ≥4 Prior TKIs | ≥3 Prior TKIs |
| Prior asciminib | 38% | | 58% | 47% | 76% | 27% | -- |
| Prior ponatinib | 22% | | 43% | 37% | 29% | 50% | -- |
| BCR::ABL1 mutations | | | 46% | -- | -- | -- | |
| T315I | 10% | | 9% | -- | -- | 29% | 23% |
| F317L | 3% | | -- | -- | -- | -- | |
| E255K | 2% | | -- | -- | -- | -- | |
| **Efficacy** | **All Doses at 24 Wks** | **RP2D (≥320 mg QD) at 24 wks** | **All Doses at 24 Wks** | **24 Wks** | **24 Wks** | **Median 48 Wks** | **18 mo** |
| MMR | 74% (28/38) | 80% (24/30) | 47% (25/53) | 47% (9/19) | 69% (18/26) | 42% (25/59) | 26% (13/49) |
| Achieved MMR | 64% (18/28) | 75% (18/24) | 32% (13/41) | 38% (6/16) | 53% (9/17) | -- | -- |
| Maintained MMR | 100% (10/10) | 100% (6/6) | 100% (12/12) | 3/3 (100%) | 100% (9/9) | -- | -- |
| In Pts w/ lack of efficacy to Prior TKI | 63% (12/19) | -- | 41% (14/34) | -- | -- | -- | -- |
| In Pts w/ lack of tol to Prior TKI | 71% (5/7) | -- | 53% (9/17) | -- | -- | -- | -- |
| In Pts w/ Prior asciminib | 43% (3/7) | -- | 32% (9/28) | -- | -- | -- | -- |
| In Pts w/ Prior ponatinib | -- | -- | 35% (7/20) | -- | -- | -- | -- |
| In Pts w/ Prior asciminib / ponatinib / investigational TKI | 50% (4/8) | -- | -- | -- | -- | -- | -- |
| With T315I | -- | -- | -- | -- | -- | 44% (8/18) | 50% (25/49) |
| Time to MMR (median) | -- | -- | -- | -- | -- | -- | -- |
| DMR | 29% (10/34) | 36% (10/34) | -- | 16% (3/19) | 35% (9/26) | -- | |
| **Safety** | | | | | | *Both CML and ALL* | |
| Gr ≥3 | 32% | | 23% | -- | -- | 63% | |
| Discontinuation Due to AEs | 2% | | 5% | -- | -- | 24% | 3% |
| **TEAEs (All Grade / Gr >3)** | | | | | | | *TRAEs* |
| Hypertension | 0% / 0% | | 0% / 0% | -- | -- | 10% / 5% | |
| Leukopenia | NR / 3% | | 0% / 0% | -- | -- | 12% / 9% | NR / 28% |
| Thrombocytopenia | 13% / 8% | | 14% / 7% | -- | -- | 28% / 18% | NR / 46% |
| Neutropenia | 13% / 8% | | 13% / 6% | -- | -- | 19% / 13% | NR / 44% |
| Anemia | 10% / 2% | | 0% / 0% | -- | -- | 10% / 6% | NR / 27% |
| Rash | 11% / 2% | | 0% / 0% | -- | -- | 0% / 0% | -- |
| Fatigue | 14% / 2% | | 10% / 0% | -- | -- | 25% / 1% | -- |
| Increased Lipase | 0% / 0% | | 18% / 1% | -- | -- | 20% / 8% | -- |
| Pancreatitis | 0% / 0% | | 0% / 0% | -- | -- | -- | -- |
| Diarrhea | 21% / 0% | | 15% / 0% | -- | -- | 12% / 0% | -- |
| Increased AST | -- | | 0% / 0% | -- | -- | 21% / 5% | -- |
| Increased ALT | 10% / 0% | | 0% / 0% | -- | -- | 24% / 3% | -- |
| CV Toxicity | 0% / 0% | | 0% / 2%**** | -- | -- | -- | -- |
| Fluid Retention | -- | | 0% / 0% | -- | -- | -- | -- |
| Pleural effusion | -- | | 0% / 0% | -- | -- | -- | -- |
| Hemorrhage | 0% / 0% | | 0% / 0% | -- | -- | -- | -- |
| Source | ASH 2025 | | EHA 2025 | Enliven Jan 2026 Corporate Deck | Enliven Jan 2026 Corporate Deck | Jabbour, JAMA 2024 | ASH 2024 Abstract |

Key AEs for Each Drug (per UpToDate)

*Note: FDA labels were used for safety information where available*

*Per label, across trials (1L and 2L)

**Heart failure occurred in 20% of patients (2% Gr 3/4); AOEs in 13% (2% of which were suddent death)

***Pooled, per label

****2 AOE events, unrelated to treatment

*Source: Company presentations*

**Copyright Information:** (Online) ISSN: 2572-9241
©2026 The Author(s). HemaSphere is published by John Wiley & Sons Ltd on behalf of European Hematology Association. This is an open access Abstract Book distributed under the Attribution-NonCommercial-NoDerivs (CC BY-NC-ND), which allows third parties to download the articles and share them with others as long as they credit the author and the Abstract Book, but they cannot change the content in any way or use them commercially.
**Abstract Book Citations:** Authors, Title, HemaSphere, 2026;10:(S1):pages. The abstract book DOIs can be found on the "Author Information" page.
**Disclaimer:** Articles published in the journal HemaSphere exclusively reflect the opinions of the authors. The authors are responsible for all content in their abstracts including accuracy of the facts, statements, citing resources, etc.

### (PB3043) IMPACT OF ADDITIONAL CYTOGENETIC ABNORMALITIES ON THE EVOLUTION OF CHRONIC MYELOID LEUKEMIA: A RETROSPECTIVE STUDY FROM NORTHERN MOROCCO.

**Topic:** 08. Chronic myeloid leukemia - Clinical

Tarik Arahou*[1], Oumayma Hari[1], Siham Belkadi[1], Imane El Boutahiri[1], Safae Regragui[1], Karima Sammoud[2], Adil Najdi[2]

*[1]University Hospital Center Mohamed VI Tangier, Clinical Hematology, Tangier, Morocco;[2]Faculty of Medicine and Pharmacy of Tangier, Laboratory of Epidemiology and Public Health, Tangier, Morocco;*

### Background

Additional cytogenetic abnormalities (ACAs) in chronic myeloid leukemia (CML) are considered potential indicators of genomic instability, but their impact on treatment response to first-line tyrosine kinase inhibitors (TKIs), such as Imatinib, is not fully understood.

### Aims

The primary objective of this study was to compare the response to first-line Imatinib between patients with and without ACAs, while the secondary objective was to describe the baseline clinical and biological features of both groups.

### Methods

We conducted a retrospective study of 44 patients diagnosed with chronic-phase CML between January 2019 and December 2023 at the University Hospital of Tangier. Clinical, hematologic, and cytogenetic parameters were analyzed. We categorized patients into two groups: with ACAs (n=7) and without ACAs (n=37). All patients received Imatinib as first-line therapy, and we monitored treatment response by RT-qPCR at 12 months.

### Results

The median age was 50 years (15-94); 64% were females. Splenomegaly was present in 86.4% of cases. Seven patients (16%) exhibited ACAs at diagnosis. The hemoglobin level was significantly lower in patients with ACAs compared to those without ACAs ($p = 0.011$). The rate of molecular response (MR) at 12 months was 36.4%. However, no significant difference in overall treatment response was found between groups ($p = 1.0$). Progression to blast phase occurred in 2 patients (4.5%), both in the ACAs group ($p = 0.022$).

### Summary/Conclusion

While anemia was more prevalent in patients with ACAs, the response to first-line Imatinib therapy was similar between the two groups. The presence of ACAs was associated with a higher risk of leukemic transformation. Larger studies are needed to confirm these findings.

**Copyright Information:** (Online) ISSN: 2572-9241
©2026 The Author(s). HemaSphere is published by John Wiley & Sons Ltd on behalf of European Hematology Association. This is an open access Abstract Book distributed under the Attribution-NonCommercial-NoDerivs (CC BY-NC-ND), which allows third parties to download the articles and share them with others as long as they credit the author and the Abstract Book, but they cannot change the content in any way or use them commercially.
**Abstract Book Citations:** Authors, Title, HemaSphere, 2026;10:(S1):pages. The abstract book DOIs can be found on the "Author Information" page.
**Disclaimer:** Articles published in the journal HemaSphere exclusively reflect the opinions of the authors. The authors are responsible for all content in their abstracts including accuracy of the facts, statements, citing resources, etc.

### (PB3044) MYELOID BLAST TRANSFORMATION OF CHRONIC MYELOID LEUKEMIA INTO ACUTE MYELOID LEUKEMIA: TWO DISTINCT EVOLUTIONARY PATHWAYS IN THE TKI ERA

**Topic:** 08. Chronic myeloid leukemia - Clinical

Daami Mayssa*[1], Zaineb Mlayah[1], Belgacem Raoudha[1], Wiem Boufrikha[1], Bizid Inaam[1], Slama Nader[1], Sarra Boukhriss[1], Mohamed Adnene Laatiri[1]

*[1]Fattouma Bourguiba University Hospital, clinical hematology, MONASTIR, Tunisia;*

### Background

Chronic myeloid leukemia (CML) is a BCR::ABL1-driven myeloproliferative neoplasm with excellent long-term survival under tyrosine kinase inhibitors (TKIs). However, progression to blast phase remains the leading cause of CML-related mortality and most commonly manifests as acute myeloid leukemia (AML). In the TKI era, transformation has become uncommon, with an estimated annual incidence of 1–2% and <5% at 5 years in optimally treated patients. Blast phase reflects genomic instability driven by ABL1 kinase domain mutations and/or additional cytogenetic abnormalities.

### Aims

To report two cases of CML transforming into AML through distinct molecular and cytogenetic evolutionary pathways.

### Methods

Both patients were evaluated according to WHO 2022 criteria. Diagnostic assessment included complete blood count, peripheral smear, bone marrow morphology, multiparameter flow cytometry, conventional karyotyping, quantitative BCR::ABL1 RT-PCR, and ABL1 kinase domain mutation analysis.

### Results

**Case 1 – Mutation-driven transformation:**
A 33-year-old woman presented with dyspnea and massive splenomegaly (23 cm), hyperleukocytosis ($442\times10^9$/L), severe anemia (Hb 5.8 g/dL), and normal platelets. Bone marrow confirmed chronic phase CML with t(9;22) in all metaphases and b3a2 transcript. Imatinib 400 mg daily was initiated. After treatment interruption at her own discretion, she re-presented five months later with cytopenias and 95% circulating blasts. Bone marrow showed 83% myeloid blasts. Flow cytometry demonstrated CD34+, CD117+, MPO+, CD33+, CD7+, HLA-DR+ phenotype. Cytogenetics revealed persistent Philadelphia chromosome with hyperdiploidy (92 chromosomes). ABL1 mutation analysis identified T315I. She received 3+7 induction chemotherapy combined with ponatinib and achieved morphological complete remission. End-of-treatment marrow confirmed sustained CR, but molecular evaluation showed persistent minimal residual disease (BCR::ABL1 0.86%).
**Case 2 – Cytogenetic-driven transformation:**
A 64-year-old woman presented with leukocytosis ($47\times10^9$/L), anemia, thrombocytopenia, and 11% circulating blasts. Bone marrow revealed hypercellularity with dyserythropoiesis and increased blasts. Cytogenetics identified t(9;22) with additional t(9;11). Progressive cytopenias and rising blasts fulfilled criteria for myeloid blast phase CML. She was treated with azacitidine plus venetoclax, achieving hematologic improvement with currently normalized blood counts.

**Copyright Information:** (Online) ISSN: 2572-9241
©2026 The Author(s). HemaSphere is published by John Wiley & Sons Ltd on behalf of European Hematology Association. This is an open access Abstract Book distributed under the Attribution-NonCommercial-NoDerivs (CC BY-NC-ND), which allows third parties to download the articles and share them with others as long as they credit the author and the Abstract Book, but they cannot change the content in any way or use them commercially.
**Abstract Book Citations:** Authors, Title, HemaSphere, 2026;10:(S1):pages. The abstract book DOIs can be found on the "Author Information" page.
**Disclaimer:** Articles published in the journal HemaSphere exclusively reflect the opinions of the authors. The authors are responsible for all content in their abstracts including accuracy of the facts, statements, citing resources, etc.

**Summary/Conclusion**

These cases illustrate two distinct mechanisms of CML progression in the TKI era: mutation-driven resistance through T315I acquisition and cytogenetic-driven clonal evolution. Although morphological remission can be achieved, persistent molecular disease highlights the aggressive biology of blast phase. Early molecular monitoring and strict therapeutic adherence remain essential to prevent AML transformation and improve outcomes.

**Copyright Information:** (Online) ISSN: 2572-9241
©2026 The Author(s). HemaSphere is published by John Wiley & Sons Ltd on behalf of European Hematology Association. This is an open access Abstract Book distributed under the Attribution-NonCommercial-NoDerivs (CC BY-NC-ND), which allows third parties to download the articles and share them with others as long as they credit the author and the Abstract Book, but they cannot change the content in any way or use them commercially.
**Abstract Book Citations:** Authors, Title, HemaSphere, 2026;10:(S1):pages. The abstract book DOIs can be found on the "Author Information" page.
**Disclaimer:** Articles published in the journal HemaSphere exclusively reflect the opinions of the authors. The authors are responsible for all content in their abstracts including accuracy of the facts, statements, citing resources, etc.

### (PB3045) A STUDY ON PREGNANCY SUCCESS, BIRTHS, AND MISCARRIAGES IN CML-CP PATIENTS DURING TYROSINE KINASE INHIBITOR THERAPY: A SINGLE-CENTER ANALYSIS.

**Topic:** 08. Chronic myeloid leukemia - Clinical

Eriko Sato*[1]

[1]*Juntendo University Nerima Hospital, Hematology, Tokyo, Japan;*

### Background

Considering pregnancy during tyrosine kinase inhibitor (TKI) treatment, the 1st line treatment for chronic myeloid leukemia in chronic phase (CML-CP), is an extremely important and delicate issue for patients with CML who have childbearing potentials. CML also affects men and women of reproductive age, the timing of pregnancy during treatment is critically important in the life plan. While TKIs have known teratogenic potential, the effects of TKIs on fertility are not yet fully understood, sufficient real-world clinical data are still lacking.

### Aims

To summarize the actual circumstances of pregnancy and childbirth in patients with CML-CP who are currently receiving or have previously received TKI therapy in clinical settings, as well as the effects of treatment on pregnancy and childbirth, and the effects of pregnancy and childbirth on the disease and treatment.

### Methods

Data were retrospectively collected from the patients diagnosed with CML-CP between 2005 and 2015 who conceived, gave birth or having children while taking TKIs or after discontinuing treatment at Juntendo University Nerima Hospital.

### Results

Among the patients, 4 patients became pregnant, resulting in a total of 7 pregnant cases. The median age of CML diagnosis was 21.0 (18-29) years, the median age at pregnancy was 25.0 (22-43) years, the median time from diagnosis to conception was 7.0 (2-17) years, and the median TKI treatment duration was 7.0 (2-9) years. At the time of pregnancy, 3 cases were undergoing TKI therapy, 4 cases had discontinued TKI. One dasatinib case elected abortion. One dasatinib case resulted in miscarriage. One case with dasatinib treatment who were willing to continue pregnant, switched to interferon, had normal live infant. Three patients had 4 pregnant cases after TKI discontinuation, who had live infants with 3 low birth weight infants and 1 heavy-for-dates infant. Hypertensive disorders of pregnancy (HDP) occurred 4 out of 7 pregnant cases. Two men on TKIs treatment who desire to bear children successfully had child respectively. No recurrence of CML was observed during pregnancy or after childbirth.

**Copyright Information:** (Online) ISSN: 2572-9241
©2026 The Author(s). HemaSphere is published by John Wiley & Sons Ltd on behalf of European Hematology Association. This is an open access Abstract Book distributed under the Attribution-NonCommercial-NoDerivs (CC BY-NC-ND), which allows third parties to download the articles and share them with others as long as they credit the author and the Abstract Book, but they cannot change the content in any way or use them commercially.
**Abstract Book Citations:** Authors, Title, HemaSphere, 2026;10:(S1):pages. The abstract book DOIs can be found on the "Author Information" page.
**Disclaimer:** Articles published in the journal HemaSphere exclusively reflect the opinions of the authors. The authors are responsible for all content in their abstracts including accuracy of the facts, statements, citing resources, etc.

**Summary/Conclusion**

Teratogenicity is known as an effect on the fetus during TKI therapy for CML. In this study, among the three cases undergoing treatment, one resulted in spontaneous abortion, and one resulted in a normal delivery with no congenital malformations observed in the newborn. Regarding maternal complications, HDS was observed in all four cases after discontinuation of TKI therapy. Reports of maternal complications are scarce, and the observed frequency appeared higher than the previously reported general HDS incidence rate (7.2%). Inhibition of c-KIT or PDGFR prior to vascular placental barrier formation is known to cause abnormalities in placental formation and angiogenesis. Several reports suggest effects following TKI discontinuation, necessitating further data accumulation. While teratogenic effects are not observed in male patients, some reports indicate impacts on reproductive capacity, such as decreased testosterone levels and oligospermia. However, analyzing these factors was difficult in this study. Further collection and accumulation of clinical knowledge is required.

**Copyright Information:** (Online) ISSN: 2572-9241
©2026 The Author(s). HemaSphere is published by John Wiley & Sons Ltd on behalf of European Hematology Association. This is an open access Abstract Book distributed under the Attribution-NonCommercial-NoDerivs (CC BY-NC-ND), which allows third parties to download the articles and share them with others as long as they credit the author and the Abstract Book, but they cannot change the content in any way or use them commercially.
**Abstract Book Citations:** Authors, Title, HemaSphere, 2026;10:(S1):pages. The abstract book DOIs can be found on the “Author Information” page.
**Disclaimer:** Articles published in the journal HemaSphere exclusively reflect the opinions of the authors. The authors are responsible for all content in their abstracts including accuracy of the facts, statements, citing resources, etc.

### (PB3046) REAL-WORLD OUTCOMES OF FRONTLINE IMATINIB IN CHRONIC MYELOID LEUKEMIA: A RETROSPECTIVE MONOCENTRIC SURVIVAL ANALYSIS

**Topic:** 08. Chronic myeloid leukemia - Clinical

Imane Chajai*[1], Amal Terfai[1], Otman Abdelmoula[1], Souad Fennan[1], Zineb Afroukh[1], Maha EL Amani[1], Othman Doghmi[1], Elmaachi Nora[1], Naoufal Benlachgar[1], Soukaina Haidouri[1], Zoubida Tazi Mezalek[1]

*[1]Ibn Sina University Hospital, Clinical Hematology, Rabat, Morocco;*

### Background

Tyrosine kinase inhibitors (TKIs) have transformed the prognosis of chronic myeloid leukemia (CML), with survival approaching that of the general population. Real-world monocentric data remain essential to validate long-term molecular responses and survival outcomes outside clinical trials.

### Methods

We conducted a retrospective monocentric study including adult patients diagnosed with CML between 2002 and 2025 in Ibn Sina University hospital of Rabat. Survivals were estimated using the Kaplan–Meier method.

### Results

The median age of patients was 47 years with a range of 17 to 80 years.The male-to-female ratio was 1.12. At diagnosis 97.2% of patients were in the chronic phase and 2.8% in the accelerated phase. First-line imatinib intolerance occurred in 10.2% of patients. Major molecular response or deeper was achieved in 64.8% of patients, whereas 27.8% failed to achieve an optimal response. Poor treatment adherence was documented in 8.3% of cases. A total of 25.0% of patients were switched to another TKI due to absence or loss of optimal response.
With a median follow-up of 65 months, among 102 evaluable patients, twenty-five progression events were observed. Median progression-free survival (PFS) was 192 months (95% CI, 124–NA). The 1-, 3-, and 5-year PFS rates were 94.1% (95% CI, 89.6–98.8), 84.7% (95% CI, 77.8–92.1), and 78.4% (95% CI, 70.4–87.4), respectively. Only one death was recorded. The 1-, 3-, and 5-year overall survival (OS) rates were 100%, with median OS not reached.
Treatment-free remission was attempted in 6.5% of patients; only one patient lost molecular response and required re-initiation of imatinib therapy.

### Summary/Conclusion

Frontline imatinib achieved durable molecular responses and exceptional overall survival in routine clinical practice. These real-world data confirm its long-term effectiveness while underscoring the importance of early response optimization and adherence to further improve disease control.

**Copyright Information:** (Online) ISSN: 2572-9241
©2026 The Author(s). HemaSphere is published by John Wiley & Sons Ltd on behalf of European Hematology Association. This is an open access Abstract Book distributed under the Attribution-NonCommercial-NoDerivs (CC BY-NC-ND), which allows third parties to download the articles and share them with others as long as they credit the author and the Abstract Book, but they cannot change the content in any way or use them commercially.
**Abstract Book Citations:** Authors, Title, HemaSphere, 2026;10:(S1):pages. The abstract book DOIs can be found on the "Author Information" page.
**Disclaimer:** Articles published in the journal HemaSphere exclusively reflect the opinions of the authors. The authors are responsible for all content in their abstracts including accuracy of the facts, statements, citing resources, etc.

### (PB3047) CLONAL HYPEREOSINOPHILIA IN A 20-YEAR-OLD MALE PRESENTING WITH NEUROPATHY AND VISUAL DISTURBANCE: CASE REPORT

**Topic:** 08. Chronic myeloid leukemia - Clinical

Jan Francis Alpay*[1]

[1]*Perpetual Help Medical Center Binan, Internal Medicine, Binan City, Philippines;*

### Background

*Hypereosinophilic syndrome (HES) is a rare hematologic condition marked by sustained eosinophilia (with an absolute eosinophil count [AEC] >1.5 × $10^9$/L) and organ involvement in the absence of a secondary cause. Among its clonal forms, PDGFRB rearrangements are rare and predominantly seen in older males, making diagnosis in young individuals challenging.*

### Aims

*We report a case of a 20-year-old male who presented with left leg pain, numbness, and intermittent visual loss. Initial workup revealed severe eosinophilia (AEC 18,396) and peripheral neuropathy. Secondary causes were ruled out. Treatment with corticosteroids resulted in symptom improvement and normalization of eosinophil count. Fluorescence in situ hybridization (FISH) testing later confirmed a PDGFRB rearrangement, and the patient was initiated on Imatinib therapy. Clinical remission was sustained on follow-up.*

### Results

*This case highlights an atypical presentation of clonal hypereosinophilia in a young adult, underlining the importance of early consideration of primary causes when eosinophilia is persistent and accompanied by systemic symptoms. The favorable response to corticosteroids and Imatinib emphasizes the need for timely molecular diagnostics to guide targeted therapy. Ocular involvement, as seen in this case, remains exceedingly rare in PDGFRB-positive disease.*

### Summary/Conclusion

Clonal hypereosinophilia should be considered in young patients with unexplained eosinophilia and systemic manifestations. Early identification and targeted treatment with tyrosine kinase inhibitors like Imatinib are essential to prevent irreversible organ damage.

**Copyright Information:** (Online) ISSN: 2572-9241
©2026 The Author(s). HemaSphere is published by John Wiley & Sons Ltd on behalf of European Hematology Association. This is an open access Abstract Book distributed under the Attribution-NonCommercial-NoDerivs (CC BY-NC-ND), which allows third parties to download the articles and share them with others as long as they credit the author and the Abstract Book, but they cannot change the content in any way or use them commercially.
**Abstract Book Citations:** Authors, Title, HemaSphere, 2026;10:(S1):pages. The abstract book DOIs can be found on the "Author Information" page.
**Disclaimer:** Articles published in the journal HemaSphere exclusively reflect the opinions of the authors. The authors are responsible for all content in their abstracts including accuracy of the facts, statements, citing resources, etc.

### (PB3048) DIFFUSE LARGE B-CELL LYMPHOMA DURING DEEP MOLECULAR RESPONSE IN CML: BLAST CRISIS OR SECOND MALIGNANCY?

**Topic:** 08. Chronic myeloid leukemia - Clinical

Wafa Chenbah*[1], Oumeyma Touati[2], Nadia Sassi[1], Guermazi Monia[2], Rihab Ben Sghaier[1], Wided Cherif[1], Haifa Regaieg[1], Bouteraa Walid[1], Kmira Zahra[1], Monia Zaier[1], Nesrine Ben Sayed[1], Yosra Ben Youssef[1]

*[1]Hôpital Farhat Hached, hematolgy, Sousse, Tunisia;[2]Hôpital Farhat Hached, hematology, Sousse, Tunisia;*

### Background

In the tyrosine kinase inhibitor (TKI) era, chronic myeloid leukemia (CML) has become a chronic condition with excellent long-term outcomes. Progression typically occurs as myeloid or lymphoid blast crisis. However, the emergence of an aggressive B-cell lymphoma during sustained deep molecular response is exceptional and raises a critical diagnostic challenge: clonal evolution versus independent secondary malignancy.

### Aims

To describe a case of diffuse large B-cell lymphoma (DLBCL) arising during sustained MR4 in chronic-phase CML and to discuss the diagnostic and pathogenic implications in the TKI era.

### Methods

Clinical, hematological, cytogenetic, molecular, imaging, and histopathological data were retrospectively analyzed.

### Results

A 63-year-old man was diagnosed in November 2023 with chronic-phase CML, presenting with left upper quadrant discomfort, splenomegaly (6 cm below the costal margin), and hyperleukocytosis (159 $\times 10^9$/L). Cytogenetic analysis revealed t(9;22) with a BCR-ABL1 b2a2 transcript. The Sokal score was intermediate (0.9).
Imatinib therapy achieved major molecular response at 6 months. Due to drug-related lower limb edema, treatment was switched to dasatinib in November 2024, with sustained deep molecular response (MR4).
In December 2025, isolated cervical lymphadenopathy developed. Excisional biopsy demonstrated CD20-positive, CD3-negative diffuse large B-cell lymphoma. The patient had no B symptoms, ECOG performance status was 1, and complete blood count remained normal (WBC 4.5 $\times 10^9$/L, hemoglobin 13.9 g/dL, platelets 194 $\times 10^9$/L). No circulating blasts were detected. Molecular monitoring confirmed persistent MR4. PET-CT staging is ongoing. Dasatinib was maintained.

### Summary/Conclusion

The occurrence of DLBCL during sustained deep molecular remission of CML, in the absence of hematologic progression or circulating blasts, strongly argues against lymphoid blast transformation and favors an independent secondary lymphoma. Although rare, similar associations have been reported in the TKI era. Potential mechanisms include long-term immune modulation, genomic instability, or stochastic secondary oncogenesis. Molecular analysis of lymphoma tissue for BCR-ABL1 rearrangement would be crucial to definitively exclude clonal relatedness.
This case highlights the importance of maintaining diagnostic vigilance for secondary malignancies in well-controlled CML patients and underscores the need for long-term surveillance strategies in the TKI era.

**Copyright Information:** (Online) ISSN: 2572-9241
©2026 The Author(s). HemaSphere is published by John Wiley & Sons Ltd on behalf of European Hematology Association. This is an open access Abstract Book distributed under the Attribution-NonCommercial-NoDerivs (CC BY-NC-ND), which allows third parties to download the articles and share them with others as long as they credit the author and the Abstract Book, but they cannot change the content in any way or use them commercially.
**Abstract Book Citations:** Authors, Title, HemaSphere, 2026;10:(S1):pages. The abstract book DOIs can be found on the "Author Information" page.
**Disclaimer:** Articles published in the journal HemaSphere exclusively reflect the opinions of the authors. The authors are responsible for all content in their abstracts including accuracy of the facts, statements, citing resources, etc.

### (PB3049) EXTRAMEDULLARY T-LYMPHOBLASTIC TRANSFORMATION DURING CHRONIC-PHASE CHRONIC MYELOID LEUKEMIA: A DIAGNOSTIC AND THERAPEUTIC CHALLENGE

**Topic:** 08. Chronic myeloid leukemia - Clinical

Ameni Yahia*[1], Slama Nader[1], Kmar Mrad[1], Amira Guizeni[1], Bizid Inaam[1], Zaineb Mlayah[1], Wiem Boufrikha[1], Mohamed Adnene Laatiri[1], Sarra Boukhriss[1]

[1]*Fattouma Bourguiba University Hospital, Hematology Department, Monastir, Tunisia;*

### Background

Chronic Myeloid Leukemia (CML) classically progresses to blast crisis of either myeloid or B-lymphoid phenotype. Transformation into T-lymphoblastic lymphoma is a rare entity, particularly when it occurs in a dissociated manner with a bone marrow remaining in chronic phase.

### Results

We report the case of a 44-year-old man with no significant medical history who was diagnosed with chronic-phase CML following bilateral visual blurring revealing leukemic retinopathy (Roth spots). Initial laboratory workup showed marked hyperleukocytosis (286,480/mm$^3$) and the presence of the BCR-ABL1 fusion transcript (b2a2 type).
A complete hematologic response was achieved after one month of treatment. However, at month 3 (M3), the patient developed left cervical and supraclavicular lymphadenopathy. Lymph node biopsy revealed infiltration by T-lymphoblastic lymphoma (IHC profile: CD3+, CD5+, CD10+, CD99+, TdT+, CD20−, CD23−). Imaging (contrast-enhanced CT scan of the chest, abdomen, and pelvis) classified the lymphoma as Ann Arbor stage III.
Paradoxically, concomitant bone marrow evaluation showed no excess blasts (76% granulocytic lineage cells, blasts <1%), whereas molecular analysis revealed a very high BCR-ABL1/ABL ratio of 80%, confirming early cytogenetic and molecular failure. The patient declined intensive chemotherapy.

T-lymphoblastic transformation of CML is a rare entity. The literature emphasizes the importance of confirming the presence of BCR-ABL1 rearrangement within lymph node tumor cells, ideally by fluorescence in situ hybridization (FISH), in order to distinguish true extramedullary blast crisis from a de novo T-cell lymphoma—a distinction that is crucial for therapeutic decision-making.
In our case, the persistence of a high BCR-ABL1 ratio (80%) despite morphologically normal bone marrow suggests either a predominant extramedullary tumor burden or the emergence of a resistant clone with proliferative advantage within a nodal sanctuary site.
The prognosis of T-lymphoid blast crisis remains particularly poor. Current recommendations advocate for an aggressive therapeutic strategy combining second- or third-generation tyrosine kinase inhibitors (TKIs) with intensive acute lymphoblastic leukemia (ALL)-type chemotherapy. In the absence of such treatment intensification, as illustrated by the patient's refusal of chemotherapy, the clinical outcome is inevitably unfavorable, highlighting the need for rapid multidisciplinary management at the time of transformation diagnosis.

### Summary/Conclusion

This case illustrates a rare extramedullary T-lymphoblastic transformation occurring during chronic-phase CML, underscoring the necessity of an integrated diagnostic approach based on clinical, histological, immunophenotypic, and molecular data. Particular vigilance is required when lymphadenopathy develops in a patient with CML, even in the presence of apparent hematologic response.

**Copyright Information:** (Online) ISSN: 2572-9241
©2026 The Author(s). HemaSphere is published by John Wiley & Sons Ltd on behalf of European Hematology Association. This is an open access Abstract Book distributed under the Attribution-NonCommercial-NoDerivs (CC BY-NC-ND), which allows third parties to download the articles and share them with others as long as they credit the author and the Abstract Book, but they cannot change the content in any way or use them commercially.
**Abstract Book Citations:** Authors, Title, HemaSphere, 2026;10:(S1):pages. The abstract book DOIs can be found on the "Author Information" page.
**Disclaimer:** Articles published in the journal HemaSphere exclusively reflect the opinions of the authors. The authors are responsible for all content in their abstracts including accuracy of the facts, statements, citing resources, etc.

### (PB3050) CHRONIC MYELOID LEUKEMIA DEVELOPING AFTER BREAST CANCER REMISSION

**Topic:** 08. Chronic myeloid leukemia - Clinical

Tsira Gogokhia*[1], Irine Datikashvili-David[1]

[1]*Aladashvili's clinic, Hematology, Tbilisi, Georgia;*

### Background

Secondary hematologic malignancies are a recognized long-term risk in cancer survivors, though chronic myeloid leukemia (CML) following breast cancer remains poorly characterized. While therapy-related acute myeloid leukemia is well-described after cytotoxic exposure, the emergence of CML in breast cancer survivors—even without radiotherapy—suggests possible shared predisposition or microenvironmental influences. The co-management of CML and recurrent solid tumors presents unique therapeutic challenges, particularly regarding tyrosine kinase inhibitor (TKI) timing during myelosuppressive chemotherapy.
We present a clinical case of Philadelphia chromosome–positive CML emerging in a middle-aged woman with a history of breast cancer in remission, outlining diagnostic findings, therapeutic decisions, and outcomes

### Aims

To describe the diagnostic findings, therapeutic decisions, and clinical outcomes of Philadelphia chromosome-positive CML diagnosed in a breast cancer survivor with subsequent cancer relapse.

### Methods

To describe the diagnostic findings, therapeutic decisions, and clinical outcomes of Philadelphia chromosome-positive CML diagnosed in a breast cancer survivor with subsequent cancer relapse.

### Results

A 45-year-old woman with hormone receptor-positive breast cancer in remission (diagnosed 2018, treated surgically, maintained on tamoxifen) presented in November 2023 with weakness, night sweats, and bone pain. Evaluation revealed leukocytosis with left shift (5% blasts), and bone marrow examination confirmed chronic phase CML with Philadelphia chromosome positivity (BCR-ABL 40% IS). Imatinib 400 mg daily was initiated, achieving complete hematologic remission within one month and partial molecular response by May 2024 (BCR-ABL 19.05% IS).

In March 2024, biopsy-confirmed breast cancer relapse required surgical resection followed by adjuvant docetaxel/cyclophosphamide chemotherapy. During cycles 1-6, the patient developed grade ≥3 neutropenia. Imatinib was temporarily interrupted . Throughout this period, serial BCR-ABL monitoring was performed, with transcript levels remaining detectable (range 18-22% IS) without loss of hematologic remission. Supportive care included growth factor support and antimicrobial prophylaxis, with hematologic recovery following chemotherapy completion. Imatinib was subsequently resumed at 400 mg daily, with the patient currently maintaining complete hematologic remission and ongoing partial molecular response.

**Copyright Information:** (Online) ISSN: 2572-9241
©2026 The Author(s). HemaSphere is published by John Wiley & Sons Ltd on behalf of European Hematology Association. This is an open access Abstract Book distributed under the Attribution-NonCommercial-NoDerivs (CC BY-NC-ND), which allows third parties to download the articles and share them with others as long as they credit the author and the Abstract Book, but they cannot change the content in any way or use them commercially.
**Abstract Book Citations:** Authors, Title, HemaSphere, 2026;10:(S1):pages. The abstract book DOIs can be found on the “Author Information” page.
**Disclaimer:** Articles published in the journal HemaSphere exclusively reflect the opinions of the authors. The authors are responsible for all content in their abstracts including accuracy of the facts, statements, citing resources, etc.

**Summary/Conclusion**

This case illustrates the complexity of managing Philadelphia chromosome–positive CML in the context of concurrent solid tumor therapy. While therapy-related leukemogenesis is well described for alkylators and topoisomerase II inhibitors, the appearance of CML after breast cancer remission — including in patients without extensive radiotherapy exposure — suggests possible underlying predispositions or microenvironmental influences that warrant further investigation.
Managing dual malignancies requires multidisciplinary coordination to minimize overlapping toxicities, particularly when TKI therapy is interrupted during cytotoxic chemotherapy. Continuous molecular monitoring of BCR-ABL transcripts is crucial for timely assessment of disease control and therapeutic adjustment. Prospective studies and registry analyses are needed to clarify incidence, risk factors, and optimal management strategies for secondary CML in cancer survivors .

**Copyright Information:** (Online) ISSN: 2572-9241
©2026 The Author(s). HemaSphere is published by John Wiley & Sons Ltd on behalf of European Hematology Association. This is an open access Abstract Book distributed under the Attribution-NonCommercial-NoDerivs (CC BY-NC-ND), which allows third parties to download the articles and share them with others as long as they credit the author and the Abstract Book, but they cannot change the content in any way or use them commercially.
**Abstract Book Citations:** Authors, Title, HemaSphere, 2026;10:(S1):pages. The abstract book DOIs can be found on the "Author Information" page.
**Disclaimer:** Articles published in the journal HemaSphere exclusively reflect the opinions of the authors. The authors are responsible for all content in their abstracts including accuracy of the facts, statements, citing resources, etc.

### (PB3051) INVESTIGATION OF THE REGULATORY MECHANISM BY WHICH THE HMGA1/RACK1 AXIS MEDIATES STAT3 ABERRATION TO DRIVE MALIGNANT CLONES IN MDS

**Topic:** 09. Myelodysplastic syndromes - Biology & translational research

Lijuan Li*[1], Xinyu Guo[1], Mengjie Yuan[1]

[1] *Tianjin Medical University General Hospital, Tianjin, China;*

### Background

Myelodysplastic syndromes (MDS) are characterized by malignant clonal proliferation of hematopoietic stem and progenitor cells and dysregulation of the immune microenvironment. Single-cell RNA sequencing reveals that high mobility group protein A1 (HMGA1) is highly expressed in MDS patients, with RACK1 predicted as its downstream target gene; both are closely associated with malignant clonal proliferation, apoptosis, and patient prognosis in MDS. Previous studies have demonstrated that RACK1 enhances STAT3 protein stability and promotes its signaling activation, while STAT3 plays a pivotal regulatory role in tumor proliferation, immune evasion, and drug resistance.

### Aims

Taking HMGA1 as the central focus, this study investigates its regulatory interplay with RACK1 and subsequent modulation of the STAT3 signaling pathway. Using integrated experimental approaches, we aim to elucidate the role of the HMGA1/RACK1/STAT3 axis in driving malignant clonal proliferation in MDS and assess its impact on disease progression and therapeutic response. These findings will provide potential targets for molecular subtyping and personalized treatment of MDS, thereby advancing precision diagnostic and therapeutic strategies.

### Methods

1. Expression of HMGA1 was analyzed in public/previous single-cell RNA sequencing data from unselected bone marrow cells of MDS patients and healthy controls. Validation was performed by qRT-PCR in CD34+ cells from newly diagnosed MDS patients (stratified by IPSS-R) and healthy donors, and its correlation with STAT3, RACK1, and clinical indices was analyzed.
2. HMGA1 was silenced using siRNA in SKM-1, MDS-L, and KG-1 cells; cell proliferation (EdU), apoptosis (Annexin V/PI), and cell cycle (PI) were assessed. Rescue assays were performed using shHMGA1 combined with RACK1 overexpression.
3. A cell line overexpressing HMGA1 was constructed, and its interaction with RACK1 was validated by co-immunoprecipitation.A cell line overexpressing RACK1 was constructed, and its interaction with STAT3 was validated by co-immunoprecipitation.
4. The effect of an HMGA1 inhibitor was tested in MDS-L/SKM-1/KG-1 cells and primary MDS CD34+ cells.

### Results

1. Single-cell RNA sequencing revealed increased HMGA1 expression in the HSPC compartment of the MDS group compared to the control group. In CD34+ cells, HMGA1 mRNA levels were higher in higher-risk MDS patients than in healthy donors and lower-risk MDS patients, while no significant difference was observed between lower-risk MDS patients and controls. HMGA1 expression was positively correlated with IPSS-R scores.
2. Knockdown of HMGA1 reduced cell proliferation, increased apoptosis, and induced G0/G1 phase arrest. These effects were partially rescued by HMGA1 knockdown combined with RACK1 overexpression.
3. RACK1 was identified as an interacting protein of HMGA1 and STAT3.
4. An HMGA1 inhibitor promoted apoptosis and inhibited proliferation in both cell lines and primary MDS CD34+ cells.

**Copyright Information:** (Online) ISSN: 2572-9241
©2026 The Author(s). HemaSphere is published by John Wiley & Sons Ltd on behalf of European Hematology Association. This is an open access Abstract Book distributed under the Attribution-NonCommercial-NoDerivs (CC BY-NC-ND), which allows third parties to download the articles and share them with others as long as they credit the author and the Abstract Book, but they cannot change the content in any way or use them commercially.
**Abstract Book Citations:** Authors, Title, HemaSphere, 2026;10:(S1):pages. The abstract book DOIs can be found on the "Author Information" page.
**Disclaimer:** Articles published in the journal HemaSphere exclusively reflect the opinions of the authors. The authors are responsible for all content in their abstracts including accuracy of the facts, statements, citing resources, etc.

**Summary/Conclusion**

The HMGA1/RACK1 axis mediates STAT3 aberration to drive malignant clones in MDS. The development of HMGA1 inhibitors holds promise for clinical translation and offers potential for identifying novel therapeutic targets for MDS treatment.

**Copyright Information:** (Online) ISSN: 2572-9241
©2026 The Author(s). HemaSphere is published by John Wiley & Sons Ltd on behalf of European Hematology Association. This is an open access Abstract Book distributed under the Attribution-NonCommercial-NoDerivs (CC BY-NC-ND), which allows third parties to download the articles and share them with others as long as they credit the author and the Abstract Book, but they cannot change the content in any way or use them commercially.
**Abstract Book Citations:** Authors, Title, HemaSphere, 2026;10:(S1):pages. The abstract book DOIs can be found on the "Author Information" page.
**Disclaimer:** Articles published in the journal HemaSphere exclusively reflect the opinions of the authors. The authors are responsible for all content in their abstracts including accuracy of the facts, statements, citing resources, etc.

### (PB3052) REFINING GERMLINE TESTING IN MYELOID NEOPLASMS: CLINICAL VARIABLES IMPROVE DIAGNOSTIC YIELD IN A REAL-WORLD COHORT

**Topic:** 09. Myelodysplastic syndromes - Biology & translational research

Guillermo Ramil*[1], Guadalupe Oñate[1], Laura Ranera[1], Benjamín Rodríguez-Santiago[1], Alícia Artigas-Baleri[1, 2], Ana Garrido[1], Irene Garcia Cadenas[1], Rodrigo Martino[1], Iván López-Torija[1], Montserrat Torrent[1], Lucía Gómez-Pérez[2], Sara García Ávila[2], Maria Laura Blanco[1], Anna Giné-Vilalta[1], Helena Castellet[1], Josep Nomdedeu[1], Marta Pratcorona[1]

*[1]Hospital de la Santa Creu i Sant Pau (IR Sant Pau), Barcelona, Spain;[2]Hospital del Mar, Barcelona, Spain;*

**Background**

Germline predisposition syndromes (GPS) are increasingly recognized in myeloid neoplasms (MN) and bone marrow failure (BMF) and are being incorporated into routine diagnostics. However, current testing criteria rely on broad clinical and genetic features, leading to heterogeneous detection rates and significant laboratory burden.

**Aims**

To evaluate the real-world performance of current germline testing criteria and to identify clinical variables associated with a higher likelihood of GPS.

**Methods**

Patients diagnosed with MN or BMF were consecutively screened for germline testing based on predefined genetic and clinical criteria, including: (i) detection of a pathogenic or likely pathogenic (P/LP) variant in *CEBPA*, *DDX41*, *ETV6*, *GATA2*, or *RUNX1* with VAF ≥30% at diagnosis; (ii) MN diagnosed before 50 years of age; (iii) BMF regardless of age; (iv) personal or family history of oncologic malignancy; and (v) phenotypic features suggestive of GPS.
A stepwise workflow was implemented. Targeted NGS on bone marrow or peripheral blood was used as an initial screening approach. Variants suspected to be germline were confirmed by Sanger sequencing on non-hematopoietic DNA. Patients with clinical suspicion but no candidate variant identified underwent clinical exome sequencing.

**Copyright Information:** (Online) ISSN: 2572-9241
©2026 The Author(s). HemaSphere is published by John Wiley & Sons Ltd on behalf of European Hematology Association. This is an open access Abstract Book distributed under the Attribution-NonCommercial-NoDerivs (CC BY-NC-ND), which allows third parties to download the articles and share them with others as long as they credit the author and the Abstract Book, but they cannot change the content in any way or use them commercially.
**Abstract Book Citations:** Authors, Title, HemaSphere, 2026;10:(S1):pages. The abstract book DOIs can be found on the "Author Information" page.
**Disclaimer:** Articles published in the journal HemaSphere exclusively reflect the opinions of the authors. The authors are responsible for all content in their abstracts including accuracy of the facts, statements, citing resources, etc.

### Results

Among 492 consecutively diagnosed patients between 2022 and 2025, 86 (17%) met criteria for germline testing. The overall diagnostic yield was 36% (31/86), but the probability of GPS confirmation varied markedly across clinical scenarios.
Variants identified by somatic NGS were confirmed as germline in 41%, with significant differences across genes (p < 0.001; **Figure 1**). In patients without a candidate variant on NGS, exome sequencing identified P/LP variants in 26% and VUS in 28%.
Age at diagnosis emerged as a major discriminator. GPS diagnosis was significantly more frequent in patients diagnosed under 50 years. Non-*DDX41* variants predominated in younger individuals (50.0% vs 6.7%; OR 12.4; p = 0.022), whereas all confirmed *DDX41* germline variants occurred in patients ≥50 years (**Figure 2**).
Differences were also observed according to disease phenotype (p = 0.050). MDS was the most frequent diagnosis among GPS cases (56%), followed by BMF (36%) and AML (26%). Notably, germline variants were identified in 67% of young patients (<50 y.o.) with high-risk MNs, including BMF-related genes such as *ERCC6L2* and *SBDS*.
A family history of malignancy was more common in patients with GPS (48% vs 17%; p = 0.004), particularly hematological malignancies (29% vs 8%; p = 0.013), while family history of solid tumors was not associated with GPS. Personal history of solid cancer was also not associated with GPS diagnosis.
Finally, among patients tested for thrombocytopenia-related genes (*RUNX1*, *ETV6*), a previously normal platelet count was associated with a very low likelihood of germline confirmation (6%; p = 0.004).

### Summary/Conclusion

Approximately one in six newly diagnosed patients met current criteria for germline testing. Despite a relatively high diagnostic yield, the probability of GPS varied substantially across clinical scenarios. Our findings support a more risk-adapted approach to germline testing in hematology. Integration of simple clinical variables—particularly age at diagnosis, disease phenotype and family history of hematologic malignancy—may improve patient selection and optimize genetic testing.

**Copyright Information:** (Online) ISSN: 2572-9241
©2026 The Author(s). HemaSphere is published by John Wiley & Sons Ltd on behalf of European Hematology Association. This is an open access Abstract Book distributed under the Attribution-NonCommercial-NoDerivs (CC BY-NC-ND), which allows third parties to download the articles and share them with others as long as they credit the author and the Abstract Book, but they cannot change the content in any way or use them commercially.
**Abstract Book Citations:** Authors, Title, HemaSphere, 2026;10:(S1):pages. The abstract book DOIs can be found on the "Author Information" page.
**Disclaimer:** Articles published in the journal HemaSphere exclusively reflect the opinions of the authors. The authors are responsible for all content in their abstracts including accuracy of the facts, statements, citing resources, etc.

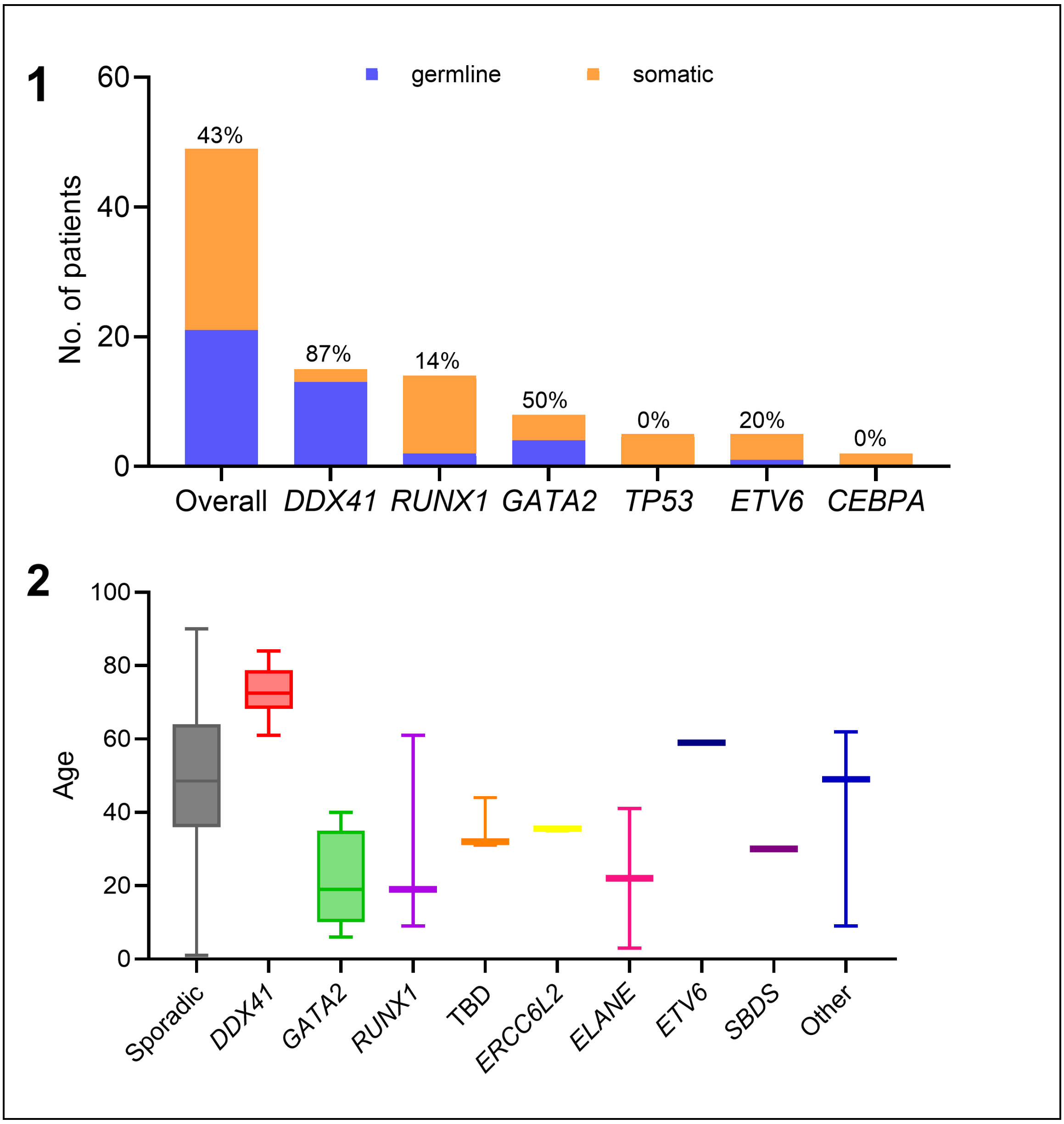

Figure 1. Confirmation rates of variants detected by somatic NGS. Figure 2. Age at disease onset according to gene affected. TBD: telomere biology disorders.

**Copyright Information:** (Online) ISSN: 2572-9241
©2026 The Author(s). HemaSphere is published by John Wiley & Sons Ltd on behalf of European Hematology Association. This is an open access Abstract Book distributed under the Attribution-NonCommercial-NoDerivs (CC BY-NC-ND), which allows third parties to download the articles and share them with others as long as they credit the author and the Abstract Book, but they cannot change the content in any way or use them commercially.
**Abstract Book Citations:** Authors, Title, HemaSphere, 2026;10:(S1):pages. The abstract book DOIs can be found on the "Author Information" page.
**Disclaimer:** Articles published in the journal HemaSphere exclusively reflect the opinions of the authors. The authors are responsible for all content in their abstracts including accuracy of the facts, statements, citing resources, etc.

### (PB3053) CLINICAL AND MOLECULAR FEATURES OF CLONAL CYTOPENIA OF UNDETERMINED SIGNIFICANCE (CCUS): IMPACT OF THERAPY-RELATED CASES IN A SINGLE-CENTER COHORT.

**Topic:** 09. Myelodysplastic syndromes - Biology & translational research

Clara Nadal Pellise*[1], Juan José Rodríguez-Sevilla[2], Xavier Calvo[3, 4], Leonor Arenillas[5], Ana Ferrer[3, 4], Beatriz Bellosillo[3, 4, 6], Blanca Espinet Sola[3, 4], Silvia Saumell[3, 4], Lucía Gómez-Pérez[1, 7], Aina Cardús[2], Maria Alicia Senin Magan[1, 7], Thalia Guiulfo Knell[1], Patricia Vélez[1, 7], Eva Gimeno[1, 7], Ramón Diez-Feijóo[1, 6, 7], Christelle Ferrà[1, 7], Sara García Ávila[1, 6, 7]

*[1]Hospital del Mar, Hematology, Barcelona, Spain; [2]Hematology Department, Hospital Clínic de Barcelona (HCB) Fundació de Recerca Clínic Barcelona. Institut d'Investigacions Biomèdiques August Pi i Sunyer (IDIBAPS), Barcelona, Spain; [3]Hospital del Mar, Pathology, Barcelona, Spain; [4]Hospital Del Mar Research Institute, Translational research group on hematological neoplasms, Barcelona, Spain; [5]Hospital Clinic, Pathology, Barcelona, Spain; [6]Pompeu Fabra University, Barcelona, Spain; [7]Hospital Del Mar Research Institute, Group of Applied Clinical Research in Hematology, Barcelona, Spain;*

### Background

CCUS is a precursor condition with variable risk of progression to myeloid neoplasms. Distinction from low-risk myelodysplastic syndromes (LR-MDS) remains challenging, particularly in older patients with comorbidities contributing to subtle dysplasia. Clinical–molecular risk models, including the Clonal Hematopoiesis Risk Score (CHRS) and the Clonal Cytopenia Risk Score (CCRS), have been developed to refine prognostic stratification. In parallel, clonal cytopenia arising after cytotoxic exposure (t-CCUS) has been proposed as a biologically distinct subgroup, but its characteristics within CCUS cohorts remain insufficiently characterized.

### Aims

To characterize the clinical and molecular heterogeneity of CCUS in a single-center cohort diagnosed between 2019 and 2025.

### Methods

We retrospectively analyzed 41 CCUS patients diagnosed between 2019 and 2025 at a single center after double morphological review. All cases underwent flow cytometry (Ogata score), conventional cytogenetics, and targeted myeloid next-generation sequencing. CHRS and CCRS were applied, and prior cytotoxic exposure was recorded.

**Copyright Information:** (Online) ISSN: 2572-9241
©2026 The Author(s). HemaSphere is published by John Wiley & Sons Ltd on behalf of European Hematology Association. This is an open access Abstract Book distributed under the Attribution-NonCommercial-NoDerivs (CC BY-NC-ND), which allows third parties to download the articles and share them with others as long as they credit the author and the Abstract Book, but they cannot change the content in any way or use them commercially.
**Abstract Book Citations:** Authors, Title, HemaSphere, 2026;10:(S1):pages. The abstract book DOIs can be found on the "Author Information" page.
**Disclaimer:** Articles published in the journal HemaSphere exclusively reflect the opinions of the authors. The authors are responsible for all content in their abstracts including accuracy of the facts, statements, citing resources, etc.

**Results**

Forty-one patients were included: median age 76 years (range 53–89), 24(59%) male. Nine (22%) were t-CCUS: all had received prior chemotherapy, 4 also radiotherapy.
Median Hb 11.7 g/dL (IQR 10.4–12.5), ANC 2.38×$10^9$/L (IQR 1.2–4.2), platelets 144×$10^9$/L (IQR 114–222). Anemia was the most frequent cytopenia, isolated (n=12, 29%) or combined (n=15, 37%).
Nonspecific dysgranulopoiesis was the predominant morphological finding. Median bone marrow blasts 1% (range 0–3); no ring sideroblasts; Ogata score <2 in most evaluable cases. Karyotype was normal in most patients; isolated −Y was the only recurrent abnormality.
The most frequently mutated genes were TET2(n=16, 39%), DNMT3A(n=10, 24%), SRSF2(n=7, 17%), PPM1D (n=5, 12%) and SF3B1 (n=5, 12%) (Figure 1). Median number of mutations was 1 (range 1–5) with a median VAF of 13% (IQR 6.5–30). No significant differences in clinical, laboratory, or morphological parameters were observed between t-CCUS and de novo cases. However, t-CCUS was significantly enriched for DDR gene mutations (PPM1D and/or TP53; 56% vs. 6%; p<0.01), whereas de novo CCUS more frequently harbored DTA gene mutations (72% vs. 33%; p=0.03)
By CHRS, 8 (20%) patients were low-risk, 22 (54%) intermediate-risk, and 11 (27%) high-risk. By CCRS, 23 (56%) were low-risk, 6 (15%) intermediate-risk, and 12 (29%) high-risk. With a median follow-up of 29.7 months (95% CI 22.0–35.7), 7 patients (17%) progressed to myeloid neoplasia (4 MDS, 3 CMML) including two t-CCUS. CCRS was significantly associated with progression and showed good discriminatory capacity in ROC analysis (AUC 0.830; 95% CI 0.704–0.955; p<0.001), whereas CHRS did not reach statistical significance (AUC 0.653; 95% CI 0.448–0.859; p=0.143). Notably, t-CCUS cases scored lower on CCRS (median 0.0 vs. 2.25; p=0.057), and none were classified as high-risk by either CHRS or CCRS.

**Summary/Conclusion**

CCUS is a biologically heterogeneous condition requiring integrated morphological and molecular assessment. While DTA mutations predominated in de novo cases, t-CCUS displayed a distinct profile enriched for DNA damage–response (DDR) gene alterations. CCRS showed significant predictive discrimination for progression, whereas CHRS did not. Notably, therapy-related cases scored lower in existing risk models despite enrichment for DDR mutations, suggesting they may be underrepresented within current prognostic frameworks.

**Copyright Information:** (Online) ISSN: 2572-9241
©2026 The Author(s). HemaSphere is published by John Wiley & Sons Ltd on behalf of European Hematology Association. This is an open access Abstract Book distributed under the Attribution-NonCommercial-NoDerivs (CC BY-NC-ND), which allows third parties to download the articles and share them with others as long as they credit the author and the Abstract Book, but they cannot change the content in any way or use them commercially.
**Abstract Book Citations:** Authors, Title, HemaSphere, 2026;10:(S1):pages. The abstract book DOIs can be found on the "Author Information" page.
**Disclaimer:** Articles published in the journal HemaSphere exclusively reflect the opinions of the authors. The authors are responsible for all content in their abstracts including accuracy of the facts, statements, citing resources, etc.

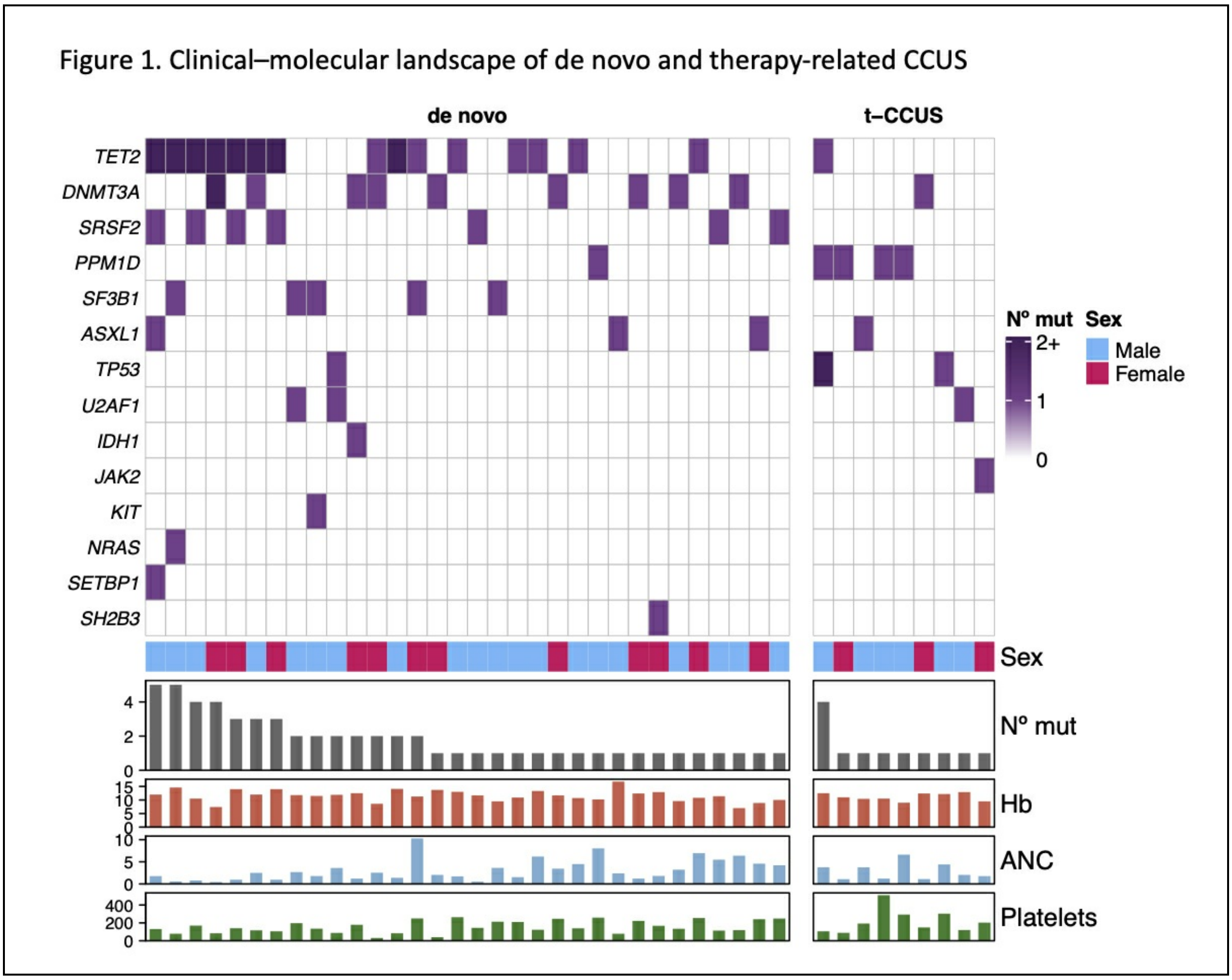

Figure 1. Clinical–molecular landscape of de novo and therapy-related CCUS

**Copyright Information:** (Online) ISSN: 2572-9241
©2026 The Author(s). HemaSphere is published by John Wiley & Sons Ltd on behalf of European Hematology Association. This is an open access Abstract Book distributed under the Attribution-NonCommercial-NoDerivs (CC BY-NC-ND), which allows third parties to download the articles and share them with others as long as they credit the author and the Abstract Book, but they cannot change the content in any way or use them commercially.
**Abstract Book Citations:** Authors, Title, HemaSphere, 2026;10:(S1):pages. The abstract book DOIs can be found on the "Author Information" page.
**Disclaimer:** Articles published in the journal HemaSphere exclusively reflect the opinions of the authors. The authors are responsible for all content in their abstracts including accuracy of the facts, statements, citing resources, etc.

### (PB3054) CRISPRI SCREENING IDENTIFIES CANDIDATE MONOSOMY 7-ASSOCIATED GENES CONTRIBUTING TO MALIGNANT TRANSFORMATION IN GATA2 DEFICIENCY

**Topic:** 09. Myelodysplastic syndromes - Biology & translational research

Maria Magallon-Mosella*[1], Damia Romero Moya[1], Eric Torralba-Sales[1], Joan Pera[1], Maximiliano Distefano[2], Albert Català[2], Miriam Erlacher[3, 4], Alessandra Giorgetti[1, 5]

*[1]Regenerative Medicine Program, Bellvitge Institute for Biomedical Research (IDIBELL), Barcelona, Spain;[2]Department of Hematology and Oncology, Institut de Recerca Sant Joan de Déu, Hospital Sant Joan de Déu, Barcelona, Spain; [3]Department of Pediatrics and Adolescent Medicine, University Medical Center Ulm, Ulm, Germany; [4]Department of Pediatrics and Adolescent Medicine, Division of Pediatric Hematology and Oncology, University of Freiburg, Freiburg, Germany; [5]Department of Pathology and Experimental Therapeutics, Faculty of Medicine and Health Sciences, Barcelona University, Barcelona, Spain;*

### Background

While the majority of MDS and AML are sporadic, rare germline predisposition syndromes have been delineated. GATA2 deficiency is one of the most common hereditary causes of MDS/AML in children, with a very high prevalence in those with monosomy 7 (-7). There remain important unanswered questions in GATA2 deficiency: Which are the epigenetic/transcriptomic changes cooperating with -7 in leukemic progression of GATA2 deficiency? Which are the genes lost in chromosome 7 (chr7) aneuploidy of GATA2-deficient related to clonal transformation? Answering these questions has been thus far hampered by the absence of trustable human disease models.

### Aims

Here we attempt to identify chr7 genes cooperating with GATA2 haploinsufficiency to promote malignant transformation, utilizing a CRISPRi library targeting chr7 genes in K562 cell lines engineered to harbor GATA2 mutations (via CRISPR/Cas9). This approach will ultimately be applied to human hematopoietic stem and progenitor cells (HSPCs).

**Copyright Information:** (Online) ISSN: 2572-9241
©2026 The Author(s). HemaSphere is published by John Wiley & Sons Ltd on behalf of European Hematology Association. This is an open access Abstract Book distributed under the Attribution-NonCommercial-NoDerivs (CC BY-NC-ND), which allows third parties to download the articles and share them with others as long as they credit the author and the Abstract Book, but they cannot change the content in any way or use them commercially.
**Abstract Book Citations:** Authors, Title, HemaSphere, 2026;10:(S1):pages. The abstract book DOIs can be found on the "Author Information" page.
**Disclaimer:** Articles published in the journal HemaSphere exclusively reflect the opinions of the authors. The authors are responsible for all content in their abstracts including accuracy of the facts, statements, citing resources, etc.

### Results

To address this, we started by generating two K562 pro-myelocytic lines carrying GATA2 R396Q and R398W mutations, creating both heterozygous and homozygous clones. Functional analyses were performed to assess the fidelity of the model generated and its utility as a disease model of GATA2 deficiency. Proliferation assays revealed a significantly reduction of proliferation cells in GATA2 mutant lines compared to the isogenic control. Upon megakaryocyte differentiation we observed a decrease in differentiation potential of GATA2 mutant cell lines compared to control. Our data are in line with previous literature and resemble some aspects of the complex phenotype observed in GATA2-related MDS patients. Next, K562 GATA2-mutant cell lines were engineered to express idCas9-KRAB and used to screen 604 selected chr7 genes (5 sgRNAs each). A multiplicity of infection (MOI) of 0,3 enabled single gene knockdown, to investigate the cooperative effect of the GATA2 mutations with chr7 gene inhibitions. After selection and doxycycline induction, cells were cultured for 31 days, showing rescue of the GATA2 mutant phenotype. Proliferation assays revealed that K562 GATA2-mutant cells treated with doxycycline and infected with the CRISPRi library had a significant increase in proliferation compared to control. To identify chr7 genes responsible for the increased proliferation, we performed deep sequencing analyses. Using this approach, together with an exhaustive bioinformatic analysis, we identified 15 candidate genes on chr7 (including CUX1, ACHE, HOXA9, CDK13, KMT2E, and RASA4B) that may be responsible for the increased proliferation of GATA2-mutant K562 cells. These candidates are currently being validated in a second series of experiments using a 15-gene CRISPRi library in HSPCs derived from an iPSC-based cellular platform. Specifically, we are using iPSC lines engineered to express inducible dCas9-KRAB, including lines carrying the GATA2 R396Q mutation alone as well as lines harboring additional recurrent ontogenetic somatic mutations in SETBP1 and ASXL1 associated with disease progression.

### Summary/Conclusion

In conclusion, we established CRISPRi K562 and iPSC models harboring GATA2 mutations that recapitulate key features of GATA2 deficiency, enabling functional screening of chromosome 7 genes. This integrated platform uncovered candidate drivers of malignant transformation and provides a versatile, human disease-relevant system for dissecting the genetic mechanisms underlying GATA2-deficient MDS/AML.

**Copyright Information:** (Online) ISSN: 2572-9241
©2026 The Author(s). HemaSphere is published by John Wiley & Sons Ltd on behalf of European Hematology Association. This is an open access Abstract Book distributed under the Attribution-NonCommercial-NoDerivs (CC BY-NC-ND), which allows third parties to download the articles and share them with others as long as they credit the author and the Abstract Book, but they cannot change the content in any way or use them commercially.
**Abstract Book Citations:** Authors, Title, HemaSphere, 2026;10:(S1):pages. The abstract book DOIs can be found on the "Author Information" page.
**Disclaimer:** Articles published in the journal HemaSphere exclusively reflect the opinions of the authors. The authors are responsible for all content in their abstracts including accuracy of the facts, statements, citing resources, etc.

### (PB3056) THE MECHANISM OF LOW-DOSE DESLANOSIDE IN ENHANCING THE THERAPEUTIC EFFICACY OF AZACITIDINE IN MYELODYSPLASTIC SYNDROMES

**Topic:** 09. Myelodysplastic syndromes - Biology & translational research

Jiarun Li*[1], Xioalong Tian[1], Guancui Yang[1], Xueqing Yang[1], Jinyi Liu[1], Xi Zhang[1]

[1]*Xinqiao Hospital of Army Medical University, Medical Center of Hematology, Chongqing, China;*

### Background

Myelodysplastic Syndromes (MDS) are a heterogenous clonal disorder of hemopoietic stem cells and have the risk of progression to acute myeloid leukemia (AML).The management of MDS typically included hypomethylating agents and allogeneic hematopoietic stem cell transplant. However, the constrained response and limited duration of efficacy have motivated the pursuit of novel therapies to optimize clinical outcomes for MDS patients.

### Aims

The study aims to explore novel drugs and improve the therapy efficacy of azacitidine in MDS patients.

### Methods

Novel drug were discovered through drug screening.CCK-8, EdU and soft agar assay were used to assess the anti-tumor effect of the candidate drug combined with azacitidine.RNA-seq, DAPGreen staining and Western blot were employed to confirm the occurrence of autophagy in cells after combination treatment.ROS, MitoSOX, TMRE and mitochondrial complex activity assay were employed to verify oxidative stress of mitochondria.Additionally, a xenograft model was performed to explore the efficacy and safety of the candidate drug in vivo.

### Results

Deslanoside(Des) holds potential as an anti-tumor agent to be combined with azacitidine for inhibiting the proliferation of MDS cells.Low-dose Des enhance therapeutic efficacy of Azacitidine in MDS cell lines by inhibiting cell proliferation, DNA synthesis and colony formation.Compared with single-drug treatment, the combination of two drugs achieves better efficacy and exerts a distinct synergistic effect.Low-dose Des combined with Azacitidine could induce autophagy by triggering oxidative stress of mitochondria.Mechanistically, combination of the two drugs induced autophagy by targeting phospholipid metabolic disorder.Similar results were found in a xenograft model in NOG mice with a good safety profile.

### Summary/Conclusion

Our study demonstrated that Des may serve as a therapeutic drug for MDS.Low-dose Des could enhance therapeutic efficacy of Azacitidine in MDS cell lines by inducing cell autophagy in vitro and in vivo.Thus,the combination of Des and Azacitidine may be much more favorable and safer in the treatment with MDS.

**Copyright Information:** (Online) ISSN: 2572-9241
©2026 The Author(s). HemaSphere is published by John Wiley & Sons Ltd on behalf of European Hematology Association. This is an open access Abstract Book distributed under the Attribution-NonCommercial-NoDerivs (CC BY-NC-ND), which allows third parties to download the articles and share them with others as long as they credit the author and the Abstract Book, but they cannot change the content in any way or use them commercially.
**Abstract Book Citations:** Authors, Title, HemaSphere, 2026;10:(S1):pages. The abstract book DOIs can be found on the "Author Information" page.
**Disclaimer:** Articles published in the journal HemaSphere exclusively reflect the opinions of the authors. The authors are responsible for all content in their abstracts including accuracy of the facts, statements, citing resources, etc.

### (PB3057) HEMATOPOIETIC PROGENITOR HETEROGENEITY ACROSS RISK CATEGORIES IN MYELODYSPLASTIC SYNDROMES

**Topic:** 09. Myelodysplastic syndromes - Biology & translational research

Aglaia Kavidopoulou[1], Antigoni Linardaki[1], Athina Damianaki[1], Ioannis Moysis Skianis[1], Eirinaios Pappas[1], Aikaterini Aliki Keller[1], Angelo Matheakakis[2], Panagiotis Verginis[1, 3], Christina Kalpadaki*[1]

*[1]Laboratory of Hematology, School of Medicine, University of Crete and University General Hospital of Heraklion, Heraklion, Greece; [2]Department of Hematology, School of Medicine, University of Crete and University General Hospital of Heraklion, Heraklion, Greece; [3]Laboratory of Immune Regulation and Tolerance, School of Medicine, University of Crete, Heraklion, Greece;*

### Background

Myelodysplastic syndromes (MDS) comprise a heterogeneous group of clonal hematopoietic disorders characterized by diverse biological features, clinical courses, and outcomes. Current prognostic stratification relies primarily on clinical and cytogenetic parameters, including the Revised International Prognostic Scoring System. However, the contribution of hematopoietic stem and progenitor cell dysfunction to disease biology and prognosis remains incompletely defined and may provide additional prognostic refinement.

### Aims

To characterize the hematopoietic progenitor compartment in MDS and evaluate the association between disrupted hematopoiesis, established disease features, and clinical outcome.

### Methods

Twenty-seven fresh bone marrow (BM) samples from MDS patients and ten age-matched healthy BM controls were analyzed. The study was conducted in accordance with the Declaration of Helsinki and approved by the Medical Ethics Committee of the University Hospital of Crete. Multiparametric flow cytometry was performed using a 13-antibody panel (CD45RA, CD38, CD34, CD33, CD123, CD90, CLEC12A, TIM3, CD56, CD11b, CD19, CD44, CD45). The CD34+ compartment was divided into CD38– and CD38+ cells, the latter including common myeloid progenitors (CMPs), granulocyte-monocyte progenitors (GMPs), and megakaryocyte-erythroid progenitors. Normal hematopoietic stem cells (HSCs) are defined as CD34+CD38– CD45RA–CLEAC12A-CD90+. Immunophenotypic aberrant hematopoietic stem cells (IA-HSCs) are defined as CD34+CD38– and CD33++ and/or CD44+/++ and CD45RA+ and/or CD123++. IA-HSC positivity (IA-HSC$^{+}$) was defined as ≥10 aberrant HSC events. Perturbed hematopoiesis was defined as <10% normal HSCs within the $CD34^{+}CD38^{-}$ compartment.

**Copyright Information:** (Online) ISSN: 2572-9241
©2026 The Author(s). HemaSphere is published by John Wiley & Sons Ltd on behalf of European Hematology Association. This is an open access Abstract Book distributed under the Attribution-NonCommercial-NoDerivs (CC BY-NC-ND), which allows third parties to download the articles and share them with others as long as they credit the author and the Abstract Book, but they cannot change the content in any way or use them commercially.
**Abstract Book Citations:** Authors, Title, HemaSphere, 2026;10:(S1):pages. The abstract book DOIs can be found on the “Author Information” page.
**Disclaimer:** Articles published in the journal HemaSphere exclusively reflect the opinions of the authors. The authors are responsible for all content in their abstracts including accuracy of the facts, statements, citing resources, etc.

**Results**

The median age of MDS patients was 72 years, with a male predominance (56%). According to the IPSS-R, patients were classified as: very low risk (2), low risk (7), intermediate risk (6), high risk (6), and very high risk (6). IA-HSCs were detected in 67% of MDS patients, whereas none were identified in normal BM samples. MDS patients demonstrated increased total CD34+ cells and GMP frequency, with a significant reduction in normal HSCs and common lymphoid progenitors (CLPs) compared to healthy controls (Figure 1A). Perturbed hematopoiesis (<10% normal HSCs) was associated with higher IA-HSC and GMP frequencies, lower platelet counts, higher IPSS-R scores, and increased risk of leukemic progression (Table 1) (Figure 1B). High and very high IPSS-R categories exhibited increased IA-HSCs and GMPs and a profound reduction in normal HSCs (Figure 1C).

Table 1: Comparison between MDS patients with >10% and <10% normal HSCs.

| MDS | HSCs <10% on CD34+CD38- | HSCs >10% on CD34+CD38- | *P-values* |
|---|---|---|---|
| **Median Hb** | 9,7 | 8,15 | *0.04* |
| **Median PLTs** | 75.000 | 120.000 | *0.05* |
| **IPSS-R Classification** | | | |
| Very low-risk/Low Risk | 2 | 7 | |
| Intermediate-risk | 4 | 2 | |
| High-risk /Very High Risk | 11 | 1 | |
| **Evolution to AML** | 15/17 | 1/10 | |

**Copyright Information:** (Online) ISSN: 2572-9241
©2026 The Author(s). HemaSphere is published by John Wiley & Sons Ltd on behalf of European Hematology Association. This is an open access Abstract Book distributed under the Attribution-NonCommercial-NoDerivs (CC BY-NC-ND), which allows third parties to download the articles and share them with others as long as they credit the author and the Abstract Book, but they cannot change the content in any way or use them commercially.
**Abstract Book Citations:** Authors, Title, HemaSphere, 2026;10:(S1):pages. The abstract book DOIs can be found on the "Author Information" page.
**Disclaimer:** Articles published in the journal HemaSphere exclusively reflect the opinions of the authors. The authors are responsible for all content in their abstracts including accuracy of the facts, statements, citing resources, etc.

### Summary/Conclusion

Disrupted hematopoiesis characterized by depletion of normal HSCs and expansion of IA-HSCs identifies a biologically aggressive MDS subset associated with higher-risk disease and increased leukemic transformation. Quantitative assessment of the hemopoietic stem cell compartment may serve as a clinically applicable tool to refine prognostic stratification beyond conventional scoring systems.

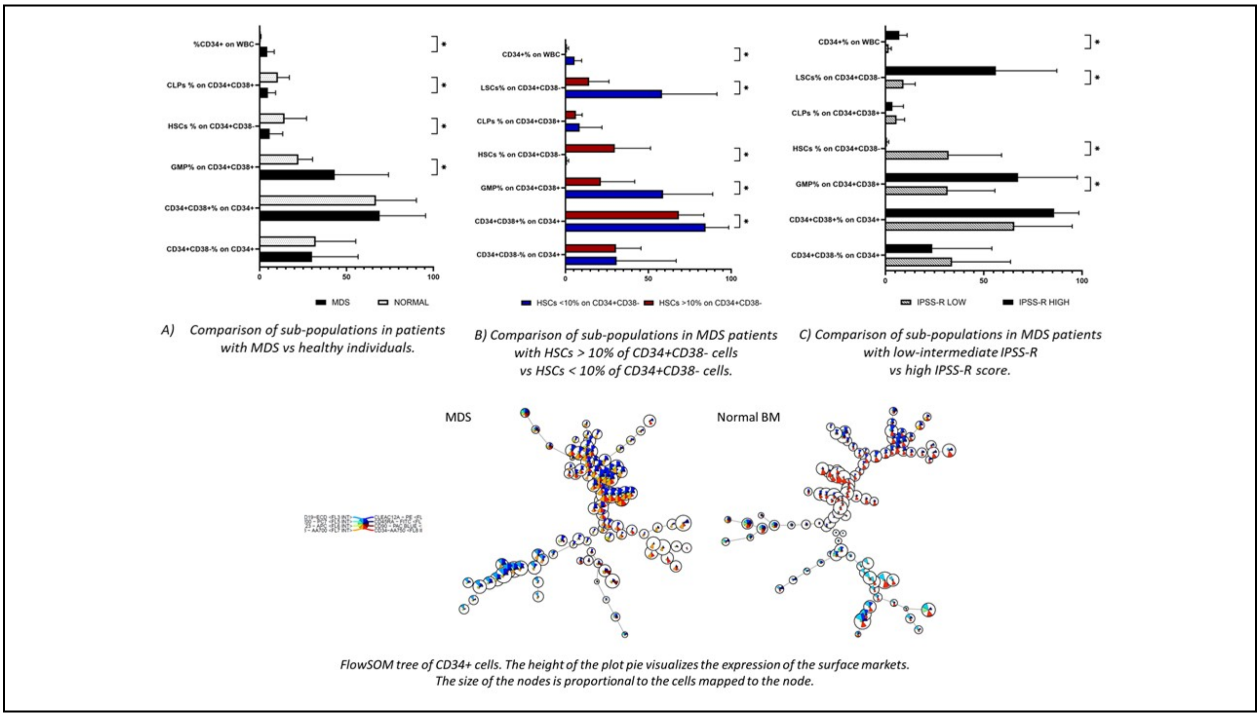

Figure 1

**Copyright Information:** (Online) ISSN: 2572-9241
©2026 The Author(s). HemaSphere is published by John Wiley & Sons Ltd on behalf of European Hematology Association. This is an open access Abstract Book distributed under the Attribution-NonCommercial-NoDerivs (CC BY-NC-ND), which allows third parties to download the articles and share them with others as long as they credit the author and the Abstract Book, but they cannot change the content in any way or use them commercially.
**Abstract Book Citations:** Authors, Title, HemaSphere, 2026;10:(S1):pages. The abstract book DOIs can be found on the "Author Information" page.
**Disclaimer:** Articles published in the journal HemaSphere exclusively reflect the opinions of the authors. The authors are responsible for all content in their abstracts including accuracy of the facts, statements, citing resources, etc.

### (PB3058) EPIGENETICS OF VEXAS AND MDS REVEALS AGE-DEPENDENT SIGNALS AND INVARIANT LONGITUDINAL METHYLATION PATTERNS AFTER HYPOMETHYLATING AGENTS THERAPY

**Topic:** 09. Myelodysplastic syndromes - Biology & translational research

Francesca Romano*[1], Emiliano Fabiani[1], Eleonora Lumia[1], Giulia Falconi[1], Giorgia Ranucci[1], Elisa Meddi[1], Tiziana Ottone[1], Mariadomenica Divona[1], Elisa Casciani[1], Elisa Diral[2], Corrado Campochiaro[3], Giorgia Battipaglia[4], Paola Triggianese[1], Nicola Toschi[1], Maria Teresa Voso[1], Carmelo Gurnari[1, 5]

*[1]University of Rome Tor Vergata, Department of Biomedicine and Prevention, Rome, Italy;[2]Unit of Hematology and Bone Marrow Transplantation, IRCCS San Raffaele Hospital, Milan, Italy; [3]IRCCS Ospedale San Raffaele & Vita-Salute, San Raffaele University, Unit of Immunology, Rheumatology, Allergy and Rare Diseases (UnIRAR), Milan, Italy; [4]Hematology, Department of Clinical Medicine and Surgery, University of Naples "Federico II", Naples, Italy, Napels, Italy; [5]Taussig Cancer Institute, Cleveland Clinic, Department of Translational Hematology and Oncology Research, Cleveland, United States of America;*

### Background

VEXAS is an adult-onset autoinflammatory syndrome driven by somatic *UBA1* mutations, frequently associated with myelodysplastic syndromes (MDS). Despite the clinical success of hypomethylating agents (HMA) in VEXAS, its fine interface with MDS, risk stratification and mutational status remain incompletely characterized. Herein, we studied VEXAS/MDS epigenetics with the goal of identifying disease-specific signatures and clarify associations with baseline clinico-biological characteristics and HMA interactions.

### Methods

We analyzed data from 10 patients with VEXAS, 38 MDS (low risk, MDS-LR, n=15; high risk, MDS-HR, n=23), and 7 healthy donors (HD). Promoter methylation was assessed by pyrosequencing for *OTP, PRRX2, ANXA4,* and *BCL2L10* genes, including mean methylation and individual CpG-level measurements.

**Copyright Information:** (Online) ISSN: 2572-9241
©2026 The Author(s). HemaSphere is published by John Wiley & Sons Ltd on behalf of European Hematology Association. This is an open access Abstract Book distributed under the Attribution-NonCommercial-NoDerivs (CC BY-NC-ND), which allows third parties to download the articles and share them with others as long as they credit the author and the Abstract Book, but they cannot change the content in any way or use them commercially.
**Abstract Book Citations:** Authors, Title, HemaSphere, 2026;10:(S1):pages. The abstract book DOIs can be found on the "Author Information" page.
**Disclaimer:** Articles published in the journal HemaSphere exclusively reflect the opinions of the authors. The authors are responsible for all content in their abstracts including accuracy of the facts, statements, citing resources, etc.

### Results

In VEXAS, *ANXA4* promoter methylation correlated positively with age (*ANXA4* mean: Spearman ρ=0.84, q=0.03; CpG1: ρ=0.85, Benjamini–Hochberg False Discovery Rate, BH-FDR, q=0.03; similar direction for CpG3). Importantly, the age–*ANXA4* relationship differed significantly between VEXAS and MDS: in the latter group, *ANXA4* mean decreased with age (slope ≈ −0.86% per year), whereas increased in the former (slope ≈ +2.18% per year), with a significant age×VEXAS interaction ($p=2.75\times10^{-6}$).
In between-groups, age/sex/HD-adjusted models, *ANXA4* methylation appeared lower in MDS-HR compared with VEXAS across all CpGs (e.g., *ANXA4* mean −27.8%, p=0.01; CpG1 −30.8%, p<0.01), remaining significant within the *ANXA4* promoter family (within *ANXA4* BH-FDR, q≤0.02). When looking at potential explanation of such divergence of methylation patterns between VEXAS and MDS-HR, we noticed that this contrast was largely driven by genotype composition: within MDS-HR, *RUNX1* mutation was strongly associated with profound *ANXA4* hypomethylation (mean 16.7% in *RUNX1*-mutant vs 53.7% in *RUNX1*-wt; p<0.01; promoter-level FDR q<0.01). Indeed, *RUNX1* mutation status remained independently associated with lower *ANXA4* methylation (−32.5%, p<0.01) when adjusting for the disease-context.
While no signal was identified with regards to *OTP* and *BCL2L10*, *PRRX2* mean methylation and CpGs-level values were higher in MDS-HR than MDS-LR (*PRRX2* mean 59.9% vs 42.8%; p=0.02; within-*PRRX2* BH-FDR q=0.04), whereas VEXAS showed intermediate values, not significantly differing from MDS groups after adjustment.
Finally, we looked at longitudinal methylation profiles of VEXAS (n=5) and MDS-HR (n=6) undergoing HMA.*PRRX2* methylation analysis showed a consistent decrease after HMA in all VEXAS cases with an 18% mean reduction (p=0.07) paralleled by *UBA1* mutational clearance and clinical reversion of disease's manifestations. Conversely, no differences were noticed in *OTP*, *ANXA4*, and *BCL2L10* in VEXAS, while HR-MDS longitudinal samples did not reveal meaningful changes for any of the 4 tested genes.

### Summary/Conclusion

Our results reveal a VEXAS-specific age dependency for *ANXA4* methylation, directionally opposite to that seen in MDS, suggesting that inflammatory context and/or clonal architecture (specifically *RUNX1* mutations) may shape epigenetic regulation differently from canonical MDS. *PRRX2* methylation remains a robust marker of MDS risk stratification, also correlating in VEXAS with HMA therapy exposure, a finding not replicated in general MDS, further corroborating VEXAS-specific epigenetic dynamics.

**Copyright Information:** (Online) ISSN: 2572-9241
©2026 The Author(s). HemaSphere is published by John Wiley & Sons Ltd on behalf of European Hematology Association. This is an open access Abstract Book distributed under the Attribution-NonCommercial-NoDerivs (CC BY-NC-ND), which allows third parties to download the articles and share them with others as long as they credit the author and the Abstract Book, but they cannot change the content in any way or use them commercially.
**Abstract Book Citations:** Authors, Title, HemaSphere, 2026;10:(S1):pages. The abstract book DOIs can be found on the "Author Information" page.
**Disclaimer:** Articles published in the journal HemaSphere exclusively reflect the opinions of the authors. The authors are responsible for all content in their abstracts including accuracy of the facts, statements, citing resources, etc.

### (PB3059) STRATIFICATION OF PATIENTS PER TP53 CLONAL BURDEN REFINES PROGNOSIS BEYOND SINGLE- AND MULTI-HIT ALLELIC STATE IN MYELOID NEOPLASMS

**Topic:** 09. Myelodysplastic syndromes - Biology & translational research

Oriol Calvete*[1], Julia Mestre[1], Lucia Ruiz Pérez-Hita[1], Lorea Chaparro[1], Emili Cid[1], Angela Llop[2], Juan Jose Rodriguez Sevilla[2], Blanca Xicoy Cirici[3], Claudia Lozano[1], Estefania Mancini[1], Mar Mallo[1], María J. Larráyoz[4], Pamela Acha[5], Raúl Azibeiro-Melchor[6], Sandra Muntion[6], Teresa Bernal[7], Juan Carlos Caballero[8], Ana Alfonso Pierola[4], María José Calasanz Abinzano[4], Maria Julia Montoro Gomez[5], Mónica del Rey[6], José Cervera[9], Felipe Prosper[4], David Valcárcel[5], María Díez Campelo[6], Francesc Sole Ristol[1]

*[1]Josep Carreras Leukaemia Research Institute, ICO-Hospital Germans Trias i Pujol, Universitat Autònoma de Barcelona, MDS Research Group, Barcelona, Spain; [2]Hospital Clinic, Department of Hematology, Barcelona, Spain;[3]Hospital Germans Trias i Pujol-Catalan Institute of Oncology, Haematology Department, Badalona, Spain; [4]Clínica Universidad de Navarra, Instituto de Investigación Sanitaria de Navarra (IDISNA), Department of Hematology, Pamplona, Spain; [5]Vall d'Hebron Institute of Oncology (VHIO), Hospital Universitari Vall d'Hebron, Experimental Hematology, Barcelona, Spain; [6]Hospital Universitario de Salamanca-IBSAL, Universidad de Salamanca, Department of Hematology, Salamanca, Spain; [7]Instituto Universitario de Oncología del Principado de Asturias, Oviedo, Spain; [8]Hospital Clínico Universitario de Valladolid, Hematology Service, Valladolid, Spain; [9]Hospital Universitario y Politécnico La Fe, Hematology Service and Genetics Unit, Valencia, Spain;*

### Background

According to the ICC classification, *TP53* mutations in myeloid neoplasms (MNs) are categorized as single-hit (monoallelic), or multi-hit when involving biallelic inactivation through multiple mutations or a mutation accompanied by deletion or loss of heterozygosity. Recent studies have applied a 20% variant allele frequency (VAF) cutoff to distinguish low- from high-clonality monoallelic *TP53* mutations. High-VAF monoallelic mutations involved outcomes comparable to multi-hit disease, which is associated with complex karyotypes and inferior overall survival (OS). In addition, both monoallelic and multi-hit *TP53* mutations in therapy-related MNs (t-MNs) have been associated with aggressive clinical behaviour. Comprehensive models integrating allelic state must be performed to improve prognostic stratification of MN patients.

### Aims

To investigate the prognostic impact of *TP53* mutations according to their clonal burden (VAF) and to assess their value in refining risk stratification in MNs.

### Methods

We retrospectively analysed 208 patients with *TP53*-mutated MNs (63.8% of AML and 36.2% of MDS) including 175 primary MN and 33 t-MN cases. Among the cohort, 53.5% were classified as single-hit and 46.5% as multi-hit patients based on next-generation sequencing (NGS) and cytogenetic analysis. Patients were further stratified based on *TP53* allelic state and VAF to evaluate the prognostic impact of clonal burden.

**Copyright Information:** (Online) ISSN: 2572-9241
©2026 The Author(s). HemaSphere is published by John Wiley & Sons Ltd on behalf of European Hematology Association. This is an open access Abstract Book distributed under the Attribution-NonCommercial-NoDerivs (CC BY-NC-ND), which allows third parties to download the articles and share them with others as long as they credit the author and the Abstract Book, but they cannot change the content in any way or use them commercially.
**Abstract Book Citations:** Authors, Title, HemaSphere, 2026;10:(S1):pages. The abstract book DOIs can be found on the "Author Information" page.
**Disclaimer:** Articles published in the journal HemaSphere exclusively reflect the opinions of the authors. The authors are responsible for all content in their abstracts including accuracy of the facts, statements, citing resources, etc.

### Results

Significantly higher *TP53* VAFs were observed in patients with complex karyotypes compared to those with altered (1–2 abnormalities) or normal karyotypes, not only in multi-hit but also among single-hit patients (Figure 1A). Single-hit patients with low clonal burden had significantly better OS compared to high-clonality single-hit cases when applying a 20% VAF threshold (p=0.0122). However, a 20% cutoff failed to discriminate high-clonality single-hit from multi-hit TP53 cases (p=0.138). When applying a higher threshold based on the cohort's mean VAF (30%), no significant differences were observed between low- and high-clonality single-hit patients (p=0.108). In contrast, all pairwise comparisons showed statistically significant differences when using a 10% VAF threshold as an intermediate discriminator between subclonal and dominant *TP53* mutations (Figure 1B).
The distribution of mutation types did not differ between groups. However, normal karyotypes were more frequent among low-clonality single-hit patients using 10% (44.1%), 20% (40.0%), or 30% (38.8%) cutoffs, compared to multi-hit patients (5.3%) (Figure 1C). Low-clonality monoallelic patients also exhibited a significantly higher frequency of co-mutations (68.8%) compared to high-clonality single-hit (41.7%) and multi-hit patients (36.3%).
Furthermore, the OS of t-MN patients with single-hit *TP53* mutations resembled that of multi-hit *TP53* patients (Figure 1D). t-MN patients displayed higher *TP53* VAFs (Figure 1E) and prevalence of complex karyotypes (Figure 1F) compared to primary *TP53*-mutated MN cases. Thus, cytotoxic exposure in t-MN appears to promote clonal expansion of *TP53*-mutated cells rather than increasing overall mutational burden.

### Summary/Conclusion

These findings contribute to the ongoing debate regarding the relevance of integrating clonal burden into risk stratification models to refine the prognostic discrimination of *TP53* allelic states in MNs.

**Funding:** This work was supported by the ISC III (PI23/01103, PI23/00007, PI23/00518, PI25/01629 and PMP24/00025), 2021 SGR00560 (GRC).

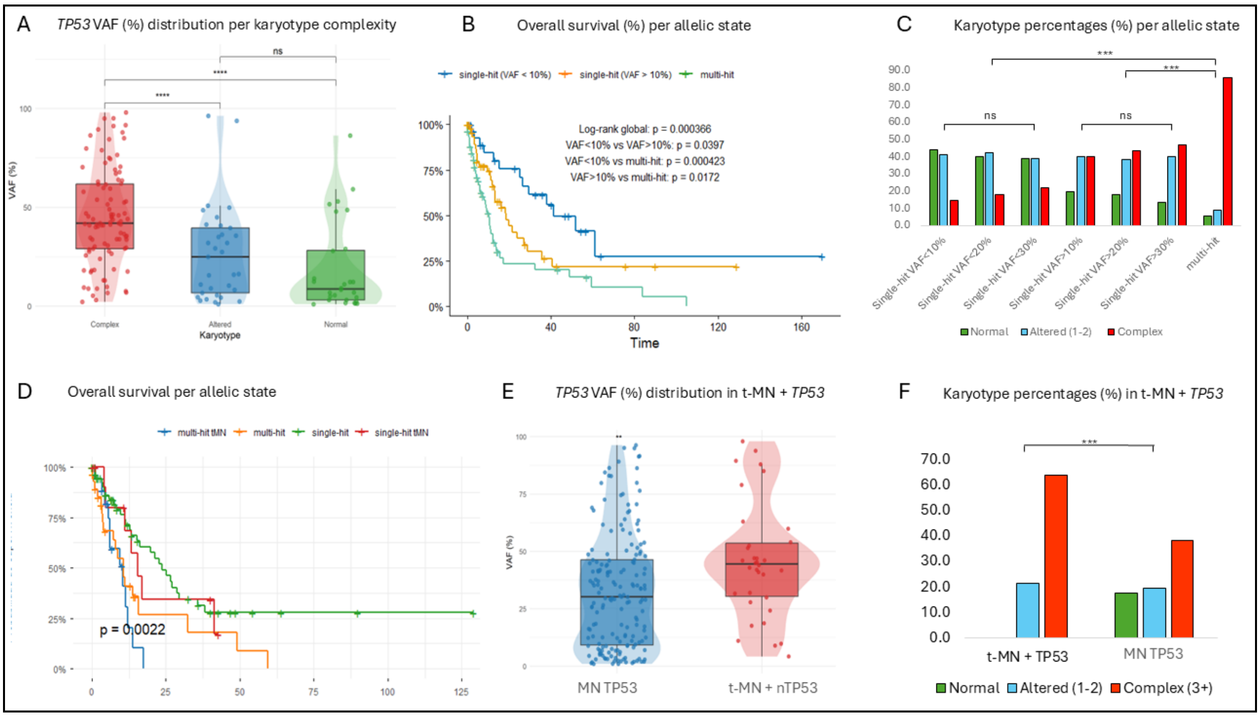

**Copyright Information:** (Online) ISSN: 2572-9241
©2026 The Author(s). HemaSphere is published by John Wiley & Sons Ltd on behalf of European Hematology Association. This is an open access Abstract Book distributed under the Attribution-NonCommercial-NoDerivs (CC BY-NC-ND), which allows third parties to download the articles and share them with others as long as they credit the author and the Abstract Book, but they cannot change the content in any way or use them commercially.
**Abstract Book Citations:** Authors, Title, HemaSphere, 2026;10:(S1):pages. The abstract book DOIs can be found on the "Author Information" page.
**Disclaimer:** Articles published in the journal HemaSphere exclusively reflect the opinions of the authors. The authors are responsible for all content in their abstracts including accuracy of the facts, statements, citing resources, etc.

### (PB3060) CROSS-COHORT SINGLE-CELL TRANSCRIPTOMIC INTEGRATION REVEALS SUBTYPE-ASSOCIATED TRANSCRIPTIONAL REMODELING IN MYELODYSPLASTIC SYNDROMES

**Topic:** 09. Myelodysplastic syndromes - Biology & translational research

Yan Liu*[1], Yuhan Zhang[1], Jun Peng[1]

[1]*Qilu Hospital Of Shandong University, Department Of Hematology, Jinan, China;*

### Background

Myelodysplastic syndromes (MDS) are clonal hematopoietic disorders characterized by ineffective hematopoiesis and diverse genetic alterations. Subtypes defined by SF3B1 mutation, GATA2 deficiency, or del(5q) display distinct clinical phenotypes, but how these lesions reshape bone marrow hematopoietic stem and progenitor cell (HSPC) states remains incompletely understood. Previous single-cell studies have largely focused on individual cohorts, limiting systematic comparison across molecular subtypes.

### Aims

To define subtype-associated alterations in hematopoietic composition and transcriptional programs through cross-cohort integration of publicly available single-cell RNA sequencing datasets.

### Methods

We integrated scRNA-seq datasets from independent cohorts encompassing major MDS molecular subtypes and healthy donors. A harmonized healthy donor reference atlas was constructed to enable projection of MDS samples into the integrated transcriptional space, facilitating unified cell-type annotation and comparative analyses across studies. Subtype-associated transcriptional programs were evaluated using cohort-adjusted pseudobulk modeling.

### Results

Our analysis revealed marked variability in cell-type proportions across datasets, likely reflecting differences in HSPC enrichment and sample processing across studies. When analyzed relative to matched controls within each cohort, lineage-associated shifts emerged as subtype-specific trends, particularly within early progenitor compartments. However, compositional alterations were not consistently reproducible across all studies.

In contrast, transcriptional analyses revealed more robust and recurrent subtype-associated remodeling within conserved progenitor populations. SF3B1-mutant samples exhibited enhanced erythroid differentiation signatures and stress-response pathway activation, whereas GATA2-deficient and del(5q) cases demonstrated distinct perturbations in inflammatory and progenitor maintenance programs. Across cohorts, transcriptional changes were more reproducible than shifts in lineage abundance, indicating that molecular drivers primarily reprogram regulatory states rather than uniformly altering cellular composition.

### Summary/Conclusion

This integrative single-cell analysis delineates subtype-associated transcriptional remodeling in MDS and provides a comparative framework for understanding molecularly stratified hematopoietic dysregulation. Our findings suggest that transcriptional reprogramming within progenitor compartments represents a central and reproducible feature of MDS subtypes.

**Copyright Information:** (Online) ISSN: 2572-9241
©2026 The Author(s). HemaSphere is published by John Wiley & Sons Ltd on behalf of European Hematology Association. This is an open access Abstract Book distributed under the Attribution-NonCommercial-NoDerivs (CC BY-NC-ND), which allows third parties to download the articles and share them with others as long as they credit the author and the Abstract Book, but they cannot change the content in any way or use them commercially.
**Abstract Book Citations:** Authors, Title, HemaSphere, 2026;10:(S1):pages. The abstract book DOIs can be found on the "Author Information" page.
**Disclaimer:** Articles published in the journal HemaSphere exclusively reflect the opinions of the authors. The authors are responsible for all content in their abstracts including accuracy of the facts, statements, citing resources, etc.

### (PB3061) SINGLE-CELL FULL-LENGTH TP53 CDNA SEQUENCING TO RESOLVE CLONAL AND ALLELIC DISTRIBUTION IN HEMATOLOGIC MALIGNANCIES

**Topic:** 09. Myelodysplastic syndromes - Biology & translational research

Emili Cid*[1], Alejandro Negro[2], Lídia López[2], Lorea Chaparro[1, 3], Julia Mestre[1, 3], Lucía Ruíz-Pérez-Hita[1], Oriol Calvete[1], Claudia Lozano[1], Estefania Mancini[1], Mar Mallo[1, 4], Laura Palomo[5], Lurdes Zamora Plana[5, 6], Blanca Xicoy Cirici[6, 7], Mónica del Rey[8], Ana Alfonso Pierola[9], Felipe Prosper[9], David Valcárcel[10], María Díez Campelo[8], Caterina Mata[2], Francesc Sole Ristol[1, 3, 4]

*[1]Josep Carreras Leukaemia Research Institute, ICO-Hospital Germans Trias i Pujol, Universitat Autònoma de Barcelona, MDS Research Group, Badalona, Spain; [2]Josep Carreras Leukaemia Research Institute, ICO-Hospital Germans Trias i Pujol, Universitat Autònoma de Barcelona, Single Cell Unit, Badalona, Spain; [3]Universitat Autònoma de Barcelona, Facultat de Biociències, Barcelona, Spain; [4]Josep Carreras Leukaemia Research Institute, ICO-Hospital Germans Trias i Pujol, Universitat Autònoma de Barcelona, Microarrays Unit, Badalona, Spain; [5]Hospital Germans Trias i Pujol-Catalan Institute of Oncology, Laboratory of Hematology, Badalona, Spain; [6]Josep Carreras Leukaemia Research Institute, ICO-Hospital Germans Trias i Pujol, Universitat Autònoma de Barcelona, Myeloid Neoplasm Group, Badalona, Spain; [7]Hospital Germans Trias i Pujol-Catalan Institute of Oncology, Hematology Department, Badalona, Spain; [8]Hospital Universitario de Salamanca-IBSAL, Universidad de Salamanca, Department of Hematology, Salamanca, Spain; [9]Clínica Universidad de Navarra, Instituto de Investigación Sanitaria de Navarra (IDISNA), Department of Hematology, Pamplona, Spain; [10]Vall d'Hebrón Institute of Oncology (VHIO), Hospital Universitari Vall d'Hebrón, Experimental Hematology. Hematology Department, Barcelona, Spain;*

### Background

Mutations in *TP53* are key prognostic markers in myelodysplastic syndromes (MDS) as clinical outcomes are dictated by the *TP53* allelic state and, moreover, clinical guidelines recognize a specific MDS entity based on biallelic inactivation of *TP53*. Patients with a biallelic/multi-hit configuration —defined by multiple mutations or a mutation combined with 17p loss or copy-neutral loss of heterozygosity— exhibit marked genomic instability, complex karyotypes, and very poor prognosis. In contrast, patients with monoallelic *TP53* mutations often display outcomes and hematologic features comparable to *TP53* wild-type cases. Despite this divergence, standard bulk next-generation sequencing frequently fails to resolve the clonal architecture required to distinguish true biallelic targeting within a single clone from independent monoallelic clones. Cell-specific approaches are therefore needed to definitively characterize *TP53* allelic distribution at the single-cell level.

### Aims

We aimed to develop and validate an affordable single-cell, full-length *TP53* cDNA sequencing workflow to precisely resolve *TP53* allelic distribution, that is, to definitively classify cases as *biallelic TP53* versus multiple clone *monoallelic TP53.*

### Methods

Single CCRF-CEM cells were isolated by FACS, using Bigfoot Spectral Cell Sorter into 96-well LoBind plates containing lysis buffer. Full-length cDNA synthesis was achieved using a template-switching reverse transcription strategy SMART-seq2. Two reverse transcriptases, SuperScript IV and Maxima H Minus were evaluated, followed by KAPA HiFi HotStart pre-amplification optimized for ultra-low RNA input. cDNA quality was assessed by TapeStation analysis. Next, nested PCR was used to amplify the *TP53* full length canonical coding sequence using Platinum Taq. Amplicons were confirmed by agarose gel electrophoresis, cloned into the pCR4 vector using TOPO cloning, and transformed into TOP10 competent cells. Multiple colonies per amplicon were grown, plasmid DNA was extracted using the ChargeSwitch miniprep kit, and *TP53* sequences were determined by Sanger sequencing.

**Copyright Information:** (Online) ISSN: 2572-9241
©2026 The Author(s). HemaSphere is published by John Wiley & Sons Ltd on behalf of European Hematology Association. This is an open access Abstract Book distributed under the Attribution-NonCommercial-NoDerivs (CC BY-NC-ND), which allows third parties to download the articles and share them with others as long as they credit the author and the Abstract Book, but they cannot change the content in any way or use them commercially.
**Abstract Book Citations:** Authors, Title, HemaSphere, 2026;10:(S1):pages. The abstract book DOIs can be found on the "Author Information" page.
**Disclaimer:** Articles published in the journal HemaSphere exclusively reflect the opinions of the authors. The authors are responsible for all content in their abstracts including accuracy of the facts, statements, citing resources, etc.

### Results

Validation was performed using the CCRF-CEM cell line harboring two distinct *TP53* mutations on separate alleles (c.743G>A, p.Arg248Gln and c.524G>A, p.Arg175His). Both reverse transcriptases (SSIV, MHM) produced viable templates and nested PCR consistently yielded a single band corresponding to full-length *TP53* coding sequence (1182 bp), as seen in Fig. 1. Sequencing of 3 clones per cell revealed mutually exclusive detection of either mutation, demonstrating resolution of the biallelic configuration at the single-cell level.

### Summary/Conclusion

We have developed a single-cell, full-length *TP53* cDNA sequencing workflow that resolves allelic distribution in compound heterozygous models. Ongoing experiments are applying it to primary myeloid samples with multiple *TP53* mutations identified by bulk sequencing to further validate its utility for characterizing clonal architecture and confirm true biallelic status in a relatively quick and economical manner. In the long term, the integration of this approach into routine clinical workflows could contribute to a more precise classification of MDS patients within the *TP53* with biallelic inactivation category.

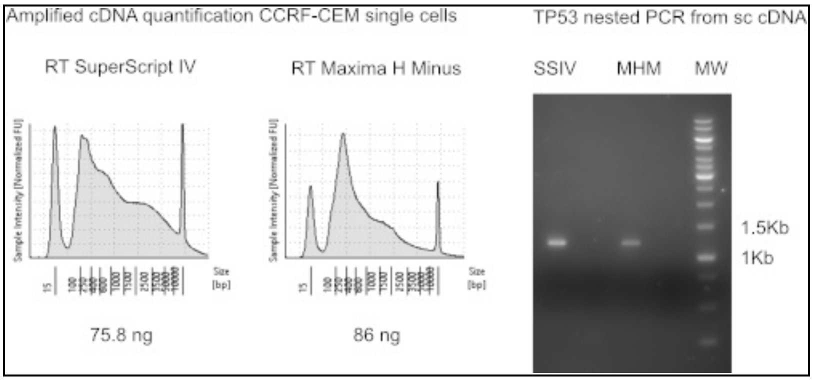

Figure 1. Comparison of cDNA synthesis efficiency from single CCRF-CEM cells and nested PCR amplification of TP53 from single cell cDNA. (Left) Representative TapeStation electropherograms showing cDNA size distribution and total yield (ng) using SuperScript IV (SSIV) or Maxima H Minus (MHM) reverse transcriptases following the SMART-seq2 protocol. (Right) Agarose gel electrophoresis of TP53 nested PCR products (approx. 1.2 kb) amplified from the respective single-cell cDNA. MW: molecular weight ladder.

**Copyright Information:** (Online) ISSN: 2572-9241
©2026 The Author(s). HemaSphere is published by John Wiley & Sons Ltd on behalf of European Hematology Association. This is an open access Abstract Book distributed under the Attribution-NonCommercial-NoDerivs (CC BY-NC-ND), which allows third parties to download the articles and share them with others as long as they credit the author and the Abstract Book, but they cannot change the content in any way or use them commercially.
**Abstract Book Citations:** Authors, Title, HemaSphere, 2026;10:(S1):pages. The abstract book DOIs can be found on the “Author Information” page.
**Disclaimer:** Articles published in the journal HemaSphere exclusively reflect the opinions of the authors. The authors are responsible for all content in their abstracts including accuracy of the facts, statements, citing resources, etc.

### (PB3062) CORRELATION BETWEEN FLOW CYTOMETRY ERYTHROID MARKERS (CD71 AND CD36) AND CYTOMORPHOLOGICAL ASSESSMENT OF ERYTHROBLAST DYSPLASIA IN BONE MARROW

**Topic:** 09. Myelodysplastic syndromes - Biology & translational research

Alicja Dudzik*[1], Agata Raniszewska[1], Elżbieta Rutkowska[1], Piotr Rzepecki[2], Iwona Kwiecień[1]

*[1]Military Institute of Medicine - National Research Institute, Laboratory of Hematology and Flow Cytometry, Department of Internal Medicine and Hematology, Warsaw, Poland; [2]Military Institute of Medicine - National Research Institute, Department of Internal Medicine and Hematology, Warsaw, Poland;*

### Background

Assessment of erythroid dysplasia in bone marrow is a key component of myelodysplastic syndromes (MDS) diagnostics, but remains subjective and prone to interobserver variability. Flow cytometry offers a potential objective tool for the evaluation of erythroid abnormalities; however the clinical significance of the expression of erythroid markers such as CD71 and CD36 in relation to cytomorphologically assessed dysplasia severity is still insufficiently defined.

### Aims

To evaluate the usefulness of flow cytometric erythroid markers CD71 and CD36 and their correlation with the severity of erythroblast dysplasia in the bone marrow of patients undergoing diagnostic work-up for suspected MDS.

### Methods

The study included 91 consecutive patients undergoing bone marrow examination due to suspected MDS (41 MDS patients diagnosed according to WHO 2022 criteria and 50 non-MDS patients). Erythroid cells were analyzed by flow cytometry (DxFlex, Beckman Coulter), and the geometric mean fluorescence intensity (GeoMean) of CD71 and CD36 was assessed using Kaluza software. Morphological evaluation of erythroid dysplasia was performed on bone marrow smears by microscopic examination of 200 erythroblasts per patient. Dysplasia severity was graded on a 0-6 scale based on the percentage of dysplastic erythroblasts and the number of dysplastic features, such as abnormal hemoglobinization, nuclear abnormalities, chromatin irregularities, basophilic stippling, multinuclearity, and vacuolization. Statistical analyses, including Mann–Whitney U testing and Spearman correlation between flow cytometric parameters and dysplasia indices, were performed.

### Results

CD71 expression was markedly reduced in bone marrow erythroblasts from MDS patients compared with non-MDS individuals (median GeoMean: 167,406 vs 285,725; $p = 0.008$), whereas CD36 showed a similar but non-significant decreasing trend. CD71 expression correlated negatively with MDS diagnosis ($r = -0.281$, $p = 0.007$). Notably, CD71 GeoMean expression demonstrated a moderate negative correlation with the severity of erythroid dysplasia ($r = -0.437$, $p < 0.001$) and a strong negative correlation with the number of dysplastic features ($r = -0.514$, $p < 0.001$). CD36 showed a weaker but significant inverse relationship with the number of dysplastic features ($r = -0.298$, $p = 0.004$) and severity of dysplasia ($r = -0.216$, $p = 0.041$). CD71 and CD36 expression levels were positively linked ($r = 0.333$, $p = 0.001$). A progressive decrease in both CD71 and CD36 GeoMean values was observed with increasing grades of dysplasia severity.

**Copyright Information:** (Online) ISSN: 2572-9241
©2026 The Author(s). HemaSphere is published by John Wiley & Sons Ltd on behalf of European Hematology Association. This is an open access Abstract Book distributed under the Attribution-NonCommercial-NoDerivs (CC BY-NC-ND), which allows third parties to download the articles and share them with others as long as they credit the author and the Abstract Book, but they cannot change the content in any way or use them commercially.
**Abstract Book Citations:** Authors, Title, HemaSphere, 2026;10:(S1):pages. The abstract book DOIs can be found on the “Author Information” page.
**Disclaimer:** Articles published in the journal HemaSphere exclusively reflect the opinions of the authors. The authors are responsible for all content in their abstracts including accuracy of the facts, statements, citing resources, etc.

**Summary/Conclusion**

Flow cytometry assessment of CD71, and to a lesser extent CD36, reflects the severity of erythroid dysplasia in bone marrow. CD71 expression shows strong negative correlations with both the severity of morphological dysplasia and the number of dysplastic features, outperforming CD36 as a marker of dyserythropoiesis. These findings suggest that CD71 GeoMean may represent the biological burden of erythroid dysplasia and could serve as an objective and quantitative parameter supporting morphological assessment in routine hematological diagnostics.

**Copyright Information:** (Online) ISSN: 2572-9241
©2026 The Author(s). HemaSphere is published by John Wiley & Sons Ltd on behalf of European Hematology Association. This is an open access Abstract Book distributed under the Attribution-NonCommercial-NoDerivs (CC BY-NC-ND), which allows third parties to download the articles and share them with others as long as they credit the author and the Abstract Book, but they cannot change the content in any way or use them commercially.
**Abstract Book Citations:** Authors, Title, HemaSphere, 2026;10:(S1):pages. The abstract book DOIs can be found on the "Author Information" page.
**Disclaimer:** Articles published in the journal HemaSphere exclusively reflect the opinions of the authors. The authors are responsible for all content in their abstracts including accuracy of the facts, statements, citing resources, etc.

### (PB3063) S100A12 DRIVES MACROPHAGE DIFFERENTIATION DEFECT AND ER STRESS CROSSTALK IN MYELODYSPLASTIC SYNDROMES: MOLECULAR MECHANISMS AND THERAPEUTIC EXPLORATION

**Topic:** 09. Myelodysplastic syndromes - Biology & translational research

Li Zhang[1], Lijuan Li[1], Liansheng Zhang*[1]

[1]*Lanzhou University Second Hospital, Lanzhou, China;*

### Background

The immune pathogenesis of myelodysplastic syndromes (MDS) and the limited efficacy of azacitidine-based combination immunotherapy remain unresolved.

### Aims

This study aimed to elucidate the role of S100A12 in macrophage (Mφ) differentiation and functional reprogramming, and to explore azacitidine-induced immune alterations, thereby providing a theoretical foundation for combining hypomethylating agents with immunotherapy in MDS.

### Methods

Integrated single-cell and bulk transcriptomic analyses were performed to identify key regulators of impaired monocyte/macrophage lineage differentiation in MDS. Peripheral blood mononuclear cells (PBMCs) from MDS patients were treated ex vivo with azacitidine, followed by assessment of dynamic changes in S100A12 expression and immune parameters. A MUTZ-1–macrophage co-culture system was established to assess the role of S100A12 in endoplasmic reticulum (ER) stress signaling and macrophage polarization.

### Results

This study revealed monocyte/macrophage lineage differentiation impairment in MDS patients.S100A12 as a master regulator of this defect. The inverse correlation between low S100A12 expression and differentiation blockade supports its role in impairing differentiation via the non-canonical ER stress axis. Further investigations characterized S100A12 expression relative to macrophage differentiation trajectories and elucidated molecular switches involving epigenetic (DNA methylation) or transcriptional (NF-κB/STAT3) regulation.
Azacitidine bidirectionally regulated S100A12 expression: downregulation in cells with high pre-treatment expression and upregulation in those exhibiting low baseline expression. Integrated analysis revealed an inverse correlation between modulation extent and monocyte levels ($r = -0.549$, $p<0.01$), with positive correlation to Treg frequencies ($r = 0.589$, $p<0.01$) and IL-6 concentrations ($r = 0.454$, $p<0.05$). This heterogeneity stratified intermediate/high-risk MDS patients into distinct immunosuppressive subsets, with the upregulation group showing enhanced immunosuppression.
Exogenous supplementation of recombinant S100A12 protein in the MUTZ-1–macrophage coculture system strongly activated core ER stress-sensing pathways (IRE1–XBP1 and PERK–ATF4) in Mφ, concurrently enhancing M2-like polarization and elevating immunosuppressive cytokines (IL-10, TGF-β). S100A12 functions as a molecular bridge that facilitates bidirectional ER stress propagation between cell types, transmits inhibitory signals from tumor cells to macrophages (Mφ), thus maintaining them in an immunosuppressive state.

**Copyright Information:** (Online) ISSN: 2572-9241
©2026 The Author(s). HemaSphere is published by John Wiley & Sons Ltd on behalf of European Hematology Association. This is an open access Abstract Book distributed under the Attribution-NonCommercial-NoDerivs (CC BY-NC-ND), which allows third parties to download the articles and share them with others as long as they credit the author and the Abstract Book, but they cannot change the content in any way or use them commercially.
**Abstract Book Citations:** Authors, Title, HemaSphere, 2026;10:(S1):pages. The abstract book DOIs can be found on the “Author Information” page.
**Disclaimer:** Articles published in the journal HemaSphere exclusively reflect the opinions of the authors. The authors are responsible for all content in their abstracts including accuracy of the facts, statements, citing resources, etc.

### Summary/Conclusion

Our findings elucidate a pivotal role for S100A12 in MDS pathobiology: 1. Low expression at least partially promotes disease progression by inhibiting Mφ differentiation; 2. Heterogeneity of this gene influences azacitidine-induced immune remodeling; 3. As an ER stress signaling hub, S100A12 orchestrates tumor-macrophage crosstalk to maintain an immunosuppressive microenvironment. Future work will 1) decode S100A12-regulated molecular networks controlling Mφ polarization (IL-10/TGF-β) and ER stress (IRE1-XBP1/PERK-ATF4), 2) validate the impact of S100A12 heterogeneity on azacitidine efficacy, and 3) establish its central role in MDS immunometabolic imbalance. Collectively, these insights will facilitate precise azacitidine-based combination immunotherapy development.

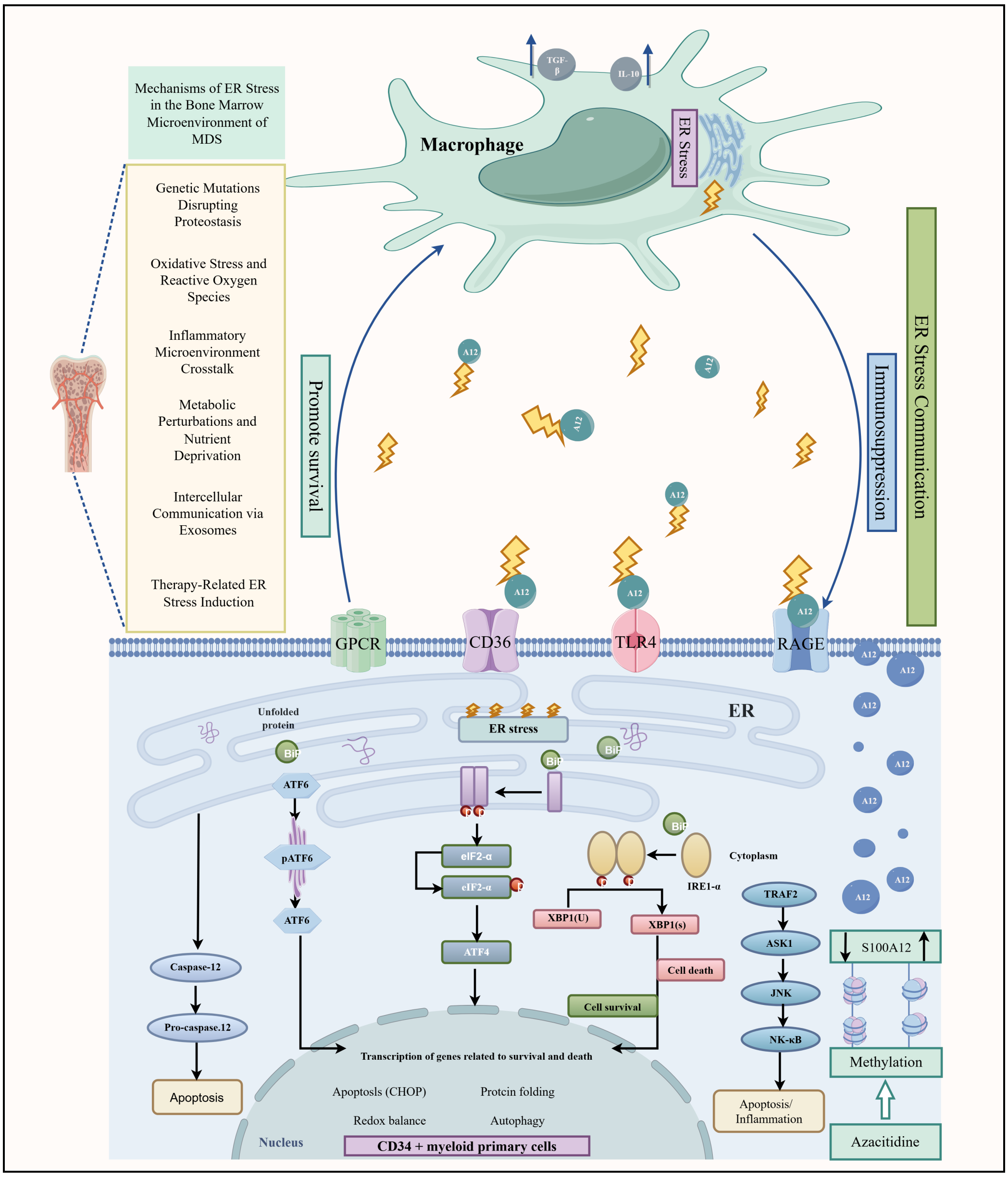

Mechanism diagram

**Copyright Information:** (Online) ISSN: 2572-9241
©2026 The Author(s). HemaSphere is published by John Wiley & Sons Ltd on behalf of European Hematology Association. This is an open access Abstract Book distributed under the Attribution-NonCommercial-NoDerivs (CC BY-NC-ND), which allows third parties to download the articles and share them with others as long as they credit the author and the Abstract Book, but they cannot change the content in any way or use them commercially.
**Abstract Book Citations:** Authors, Title, HemaSphere, 2026;10:(S1):pages. The abstract book DOIs can be found on the "Author Information" page.
**Disclaimer:** Articles published in the journal HemaSphere exclusively reflect the opinions of the authors. The authors are responsible for all content in their abstracts including accuracy of the facts, statements, citing resources, etc.

### (PB3064) BONE MARROW STROMAL MODULATION IN MYELODYSPLASTIC SYNDROMES

**Topic:** 09. Myelodysplastic syndromes - Biology & translational research

Matilde Boada Burutaran*[1], Carolina Ottati[1], Pablo Straneo[1], Lucia Vazquez[1], Lucia Spangenberg[1], Daniela Lens[1], Sofia Grille[1]

[1]*Hospital de Clínicas. Dr. Manuel QUintela, Montevideo, Uruguay;*

### Background

Myelodysplastic syndromes (MDS) are clonal hematologic disorders in which pathogenesis involves not only intrinsic abnormalities of hematopoietic stem and progenitor cells but also alterations in the bone marrow microenvironment (BMM) and immune response (IR). Our group previously demonstrated that mesenchymal stromal cells (MSCs) derived from MDS patients exhibit a pro-inflammatory cytokine production profile.

### Aims

The aim of the present study was to characterize gene expression patterns in MSCs focusing on key genes involved in immune regulation and hematopoietic support.

### Methods

Patients aged ≥18 years with a confirmed diagnosis of myelodysplastic syndrome (MDS), prior to receiving disease-modifying therapy, were eligible for inclusion. Cardiac surgery patients with normal complete blood counts (CBC) were included as controls.
Mesenchymal stromal cells (MSCs) were cultured in accordance with the International Society for Cellular Therapy (ISCT) criteria.
RNA was extracted between passages 1 and 3, and both RNA sequencing (RNA-seq) and quantitative real-time PCR analyses were performed.
RNA-seq was used as an exploratory approach to identify differentially expressed genes, which were subsequently validated by quantitative real-time PCR. The expression of 15 selected genes of interest was assessed using β-actin as the reference gene.
The study protocol was approved by the Institutional Ethics Committee of the Hospital de Clínicas. Statistical analyses were conducted using parametric or non-parametric tests according to data distribution.

### Results

Twenty-seven patients and nine healthy controls were included. The median age was 66 years for patients and 59 years for controls. Among patients, 59.3% were classified as low-risk MDS and 40.7% as high-risk MDS.
RNA sequencing was performed in five samples (two low-risk MDS patients and three controls), revealing differential expression of 41 genes (25 upregulated and 17 downregulated).
Notably, IL27, IL32, and IL6 were upregulated, whereas WNT, CDK1, and TGF-β were downregulated. Figure 1 illustrates the relative over- or under-expression of the analyzed genes in patients compared with controls.
Statistically significant differences were observed for four genes: IL27 (overexpressed; p=0.005), caspase-1 (p=0.005), TGF-β (p=0.007), and WNT (p=0.027), with the latter three showing reduced expression. Additionally, differential expression patterns were identified between low-risk and high-risk MDS patients compared with controls (Figure 2).

**Copyright Information:** (Online) ISSN: 2572-9241
©2026 The Author(s). HemaSphere is published by John Wiley & Sons Ltd on behalf of European Hematology Association. This is an open access Abstract Book distributed under the Attribution-NonCommercial-NoDerivs (CC BY-NC-ND), which allows third parties to download the articles and share them with others as long as they credit the author and the Abstract Book, but they cannot change the content in any way or use them commercially.
**Abstract Book Citations:** Authors, Title, HemaSphere, 2026;10:(S1):pages. The abstract book DOIs can be found on the "Author Information" page.
**Disclaimer:** Articles published in the journal HemaSphere exclusively reflect the opinions of the authors. The authors are responsible for all content in their abstracts including accuracy of the facts, statements, citing resources, etc.

**Summary/Conclusion**

MSCs derived from MDS patients display an altered gene expression profile characterized by upregulation of pro-inflammatory cytokines and downregulation of key genes involved in hematopoietic support, immune regulation, and inflammasome activation.These findings further support the role of stromal cells in immune dysregulation and disease pathogenesis in MDS.

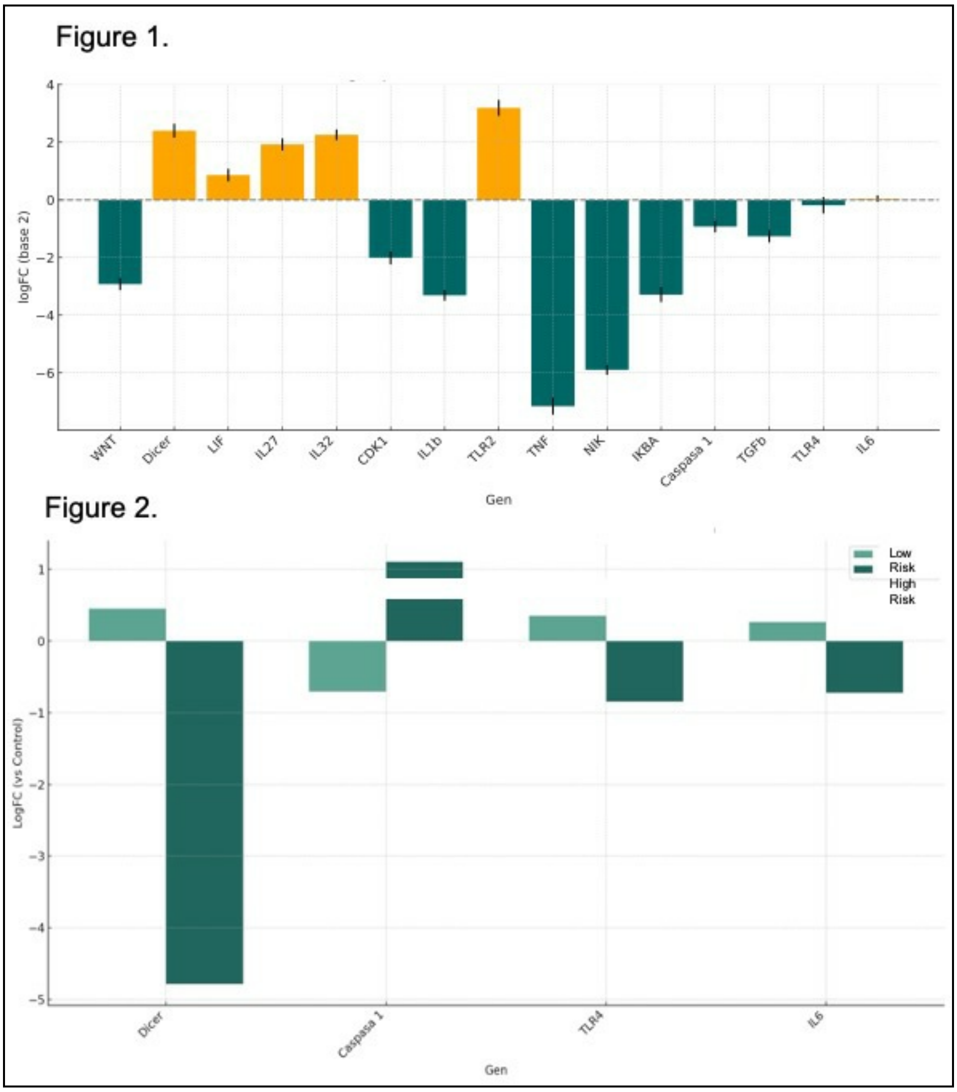

FIGURE 1 Gene expression of selected genes of interest in patients compared with controls, expressed as LogFC. Upregulated genes are shown in orange and downregulated genes in green. FIGURE 2 Gene expression in high-risk MDS (HR-MDS) and low-risk MDS (LR-MDS) compared with controls. Light green represents LR-MDS and dark green represents HR-MDS.

**Copyright Information:** (Online) ISSN: 2572-9241
©2026 The Author(s). HemaSphere is published by John Wiley & Sons Ltd on behalf of European Hematology Association. This is an open access Abstract Book distributed under the Attribution-NonCommercial-NoDerivs (CC BY-NC-ND), which allows third parties to download the articles and share them with others as long as they credit the author and the Abstract Book, but they cannot change the content in any way or use them commercially.
**Abstract Book Citations:** Authors, Title, HemaSphere, 2026;10:(S1):pages. The abstract book DOIs can be found on the "Author Information" page.
**Disclaimer:** Articles published in the journal HemaSphere exclusively reflect the opinions of the authors. The authors are responsible for all content in their abstracts including accuracy of the facts, statements, citing resources, etc.

### (PB3065) SELF-REPORTED FATIGUE IN PATIENTS WITH MYELODYSPLASTIC NEOPLASMS IS LINKED TO INNATE IMMUNE AND MITOCHONDRIAL DYSFUNCTION

**Topic:** 09. Myelodysplastic syndromes - Biology & translational research

Verena Petzer*[1], Friederike Schmitz[2], Anne Sophie Platzbecker[3, 4, 5], Sieghart Sopper[1], Tobias A.W. Holderried[2, 6], Uwe Platzbecker[7], Klaus Metzeler[7], Kai Zimmer[1], Janine Nathalie Steichen[1], Chiara Volani[8], Eicke Latz[9], Dominik Zboril[1], Johanna Mascherbauer[1], Lorin Loacker[10], Josia Fauser[1], Melanie Nuesch-Germano[9], Karin Koinig[1], Piotr Tymoszuk[11], Reinhard Stauder[1], Dominik Wolf[1]

*[1]Department of Internal Medicine V (Hematology and Oncology), Comprehensive Cancer Center Innsbruck (CCCI), Austrian Comprehensive Cancer Network (ACCN), Medical University Innsbruck (MUI), Innsbruck, Austria, Innsbruck, Austria; [2]University of Bonn, University Hospital Bonn, Medical Clinic and Polyclinic III Internal Medicine with a focus on Oncology, Hematology, and Rheumatology, Bonn, Germany; [3]Else Kroener Fresenius Center for Digital Health, Faculty of Medicine and University Hospital Carl Gustav Carus, Dresden University of Technology, Germany, Dresden, Germany; [4]Medizinische Universität Lausitz – Carl Thiem, Cottbus, Germany; [5]Digital Health Cluster, Department for Digital Health, Economics & Policy, Hasso-Plattner Institute, University of Potsdam, Potsdam, Germany; [6]Division of Hematology, Medical University of Graz, Graz, Austria; [7]Department of Hematology, Cellular Therapy and Hemostaseology, University Hospital of Leipzig, Leipzig, Germany; [8]Department of Internal Medicine II (Infectious Diseases, Immunology, Rheumatology, Pneumology), Medical University of Innsbruck, Innsbruck, Austria; [9]Deutsches Rheuma-Forschungszentrum (DRFZ), Berlin, Germany; [10]Central Institute of Clinical and Chemical Laboratory Diagnostics, University Hospital of Innsbruck, Innsbruck, Austria; [11]Data Analysis as a Service, Wörgl, Austria;*

### Background

Fatigue (FA) is a leading symptom in patients with Myelodysplastic Neoplasms (MDS), which impairs quality of life, daily functioning, and overall survival. FA is a multifaceted phenomenon, which is yet not well understood in MDS.

### Aims

The cross-sectional, prospective, multi-center InflamGen study was set up to better understand the biological background of FA in MDS. Therefore, we collected in parallel quality of life data, clinical, genetic, transcriptomic, inflammatory and immunological parameters at high resolution in MDS patients.

### Methods

Self-reported FA was rated by the EORTC Fatigue (EORTC QLQ-FA12) tool in MDS patients (n = 85) not receiving immune modulatory therapy. Mutations and gene expression in whole blood samples was assessed by DNA and RNA sequencing, inflammatory/immune features were investigated by flow cytometry and cytokine profiling. An age-matched healthy control group was also analyzed (n=13). The study protocol was approved by the local Ethics Committee (1088/2019). All patients gave written informed consent. $FA^{low}$ and $FA^{hi}$ clusters were defined by KMEANS clustering with EORTC FA domain scores. Elastic Net and Random Forest algorithms were used to model the $FA^{low}$ and $FA^{hi}$ cluster assignment with transcriptomic and flow cytometry predictors. Differential gene expression was assessed by negative binomial modeling and weighted gene co-expression networks. Metabolism was modeled with differential gene expression by Monte Carlo simulations. Activity of transcription factors was predicted with decoupleR linear regression.

**Copyright Information:** (Online) ISSN: 2572-9241
©2026 The Author(s). HemaSphere is published by John Wiley & Sons Ltd on behalf of European Hematology Association. This is an open access Abstract Book distributed under the Attribution-NonCommercial-NoDerivs (CC BY-NC-ND), which allows third parties to download the articles and share them with others as long as they credit the author and the Abstract Book, but they cannot change the content in any way or use them commercially.
**Abstract Book Citations:** Authors, Title, HemaSphere, 2026;10:(S1):pages. The abstract book DOIs can be found on the "Author Information" page.
**Disclaimer:** Articles published in the journal HemaSphere exclusively reflect the opinions of the authors. The authors are responsible for all content in their abstracts including accuracy of the facts, statements, citing resources, etc.

### Results

Two fatigue clusters [FA$^{low}$ (n = 67), FA$^{hi}$ (n = 18)] were assigned based on domain scores of EORTC FA tools by KMEANS unsupervised clustering. MDS-associated parameters (blood counts, IPSS-R, molecular profile) including hemoglobin (median 99 g/l vs. 98 g/l) did not significantly differ between the clusters. An overall higher inflammatory tonus (IL-8, TNF-α, IL-1β) was seen in MDS compared to healthy controls. However, cytokine levels were comparable in FA$^{low}$ vs. FA$^{hi}$. Significantly lower levels of NK cells were detected in FA$^{hi}$ patients (median 67 cells/µl vs. 160 cells/µl; p= 0.0044), which is supported by reduced expression of Transcription Factor 7, which transcriptionally regulates NK cells. Differential gene expression and co-expression network analyses revealed suppression of genes related to mitochondrial and mitochondrial iron metabolism in FA$^{hi}$ patients. FA$^{hi}$ had reduced oxidative phosphorylation and heme synthesis but higher fatty acid synthesis, suggestive of anaerobic energy metabolism. Finally, we performed machine learning analyses based on expression of genes related to 1) mitochondria, 2) mitochondrial iron metabolism, and 3) immunity features. Independently of the algorithm, all three sets of explanatory factors accurately identified FA$^{hi}$ and FA$^{low}$ patients (cross-validation, Elastic Net: accuracy 0.85-0.92, Cohen's  0.69-0.85, Random Forest: accuracy 0.9-0.94,  0.79- 0.87) (Figure 1).

### Summary/Conclusion

Self-reported FA in MDS is tightly linked to aberrant expression of mitochondria- and mitochondrial iron metabolism-related genes, metabolic reprogramming, and reduced counts of circulating NK cells. The functional significance of these findings and the potential for their modulation are currently being investigated in greater detail.

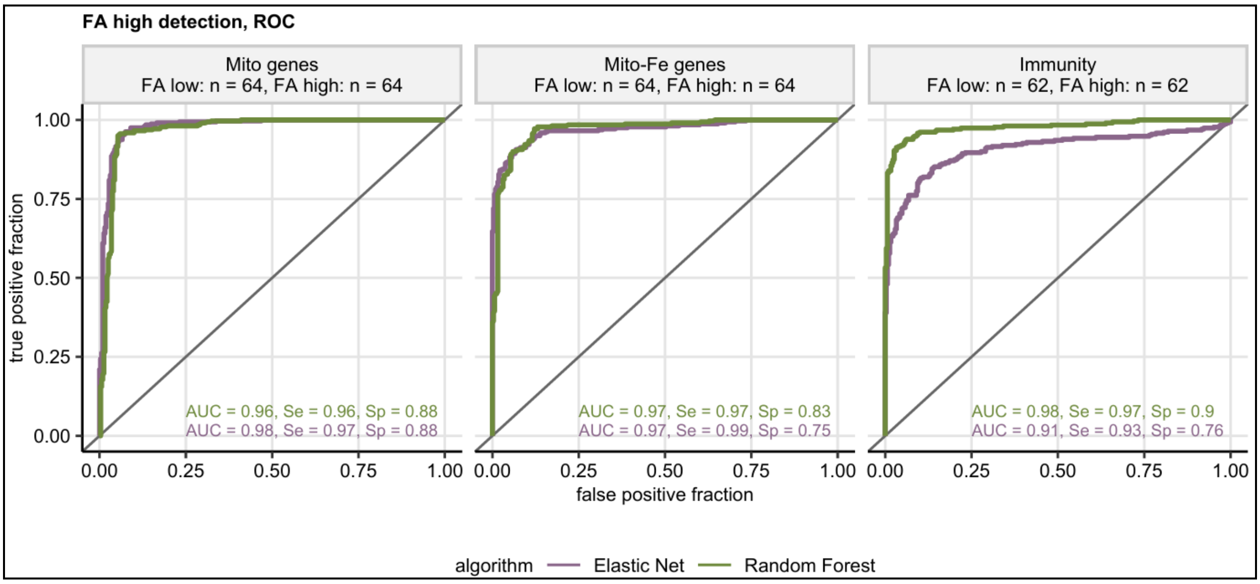

Machine learning approach.

Copyright Information: (Online) ISSN: 2572-9241
©2026 The Author(s). HemaSphere is published by John Wiley & Sons Ltd on behalf of European Hematology Association. This is an open access Abstract Book distributed under the Attribution-NonCommercial-NoDerivs (CC BY-NC-ND), which allows third parties to download the articles and share them with others as long as they credit the author and the Abstract Book, but they cannot change the content in any way or use them commercially.
Abstract Book Citations: Authors, Title, HemaSphere, 2026;10:(S1):pages. The abstract book DOIs can be found on the "Author Information" page.
Disclaimer: Articles published in the journal HemaSphere exclusively reflect the opinions of the authors. The authors are responsible for all content in their abstracts including accuracy of the facts, statements, citing resources, etc.

### (PB3066) MOLECULAR ARCHITECTURE OF TP53-MUTATED MYELOID NEOPLASMS IN A SINGLE-CENTER COHORT: CO-MUTATION PATTERNS, MULTI-HIT STATUS, AND CYTOGENOMIC PROFILING

**Topic:** 09. Myelodysplastic syndromes - Biology & translational research

Lucía Gómez-Pérez*[1, 2], Sara García Ávila[1, 3], Concepción Fernández-Rodriguez[2, 4], Barbara Ymaine[2, 4], Clara Nadal Pellise[1], Patricia Vélez[1, 2], Maria Alicia Senin Magan[1, 2], Ramón Diez-Feijóo[1, 3], Silvia Saumell[4], Anna Puiggros[4], Marta Salido[4], Blanca Espinet Sola[4], Ana Ferrer[4], Xavier Calvo[4], Christelle Ferrà[1], Beatriz Bellosillo[2, 4]

*[1]Hospital del Mar Research Institute, Department of Hematology, Barcelona, Spain;[2]Hospital del Mar Research Institute, Group of Applied Clinical Research in Hematology, Barcelona, Spain; [3]Pompeu Fabra University, Barcelona, Spain; [4]Hospital del Mar Research Institute, Department of Patology, Barcelona, Spain;*

### Background

*TP53* mutations in myeloid neoplasms (MN), including high-risk myelodysplastic neoplasms (MDS) and acute myeloid leukemia (AML), are associated with complex karyotypes (CK), treatment resistance, and poor outcomes. *TP53* inactivation typically requires biallelic disruption (multi-hit), through two pathogenic mutations or a single mutation accompanied by 17p deletion or copy-neutral loss of heterozygosity. Differences in how single versus multi-hit *TP53* alterations are defined across recent classifications (WHO 2022 and ICC 2022), together with variable allele frequency (VAF) thresholds, may lead to diagnostic discordance and ultimately to heterogeneous clinical management.

### Aims

To describe the molecular architecture of *TP53*-mutated MN in a single-center cohort.

### Methods

We retrospectively analyzed patients diagnosed from 2017 to 2025 with MN harboring pathogenic/probably pathogenic *TP53* variants. Mutational profiling was performed by targeted next-generation sequencing (NGS): Oncomine Myeloid Assay GX v2 (ThermoFisher Scientific) and QIASeq custom panel including 26 myeloid-related genes (QIAGEN). Cytogenetic analysis included conventional karyotyping, FISH, and optical genome mapping (OGM, Bionano Genomics) when available.

**Copyright Information:** (Online) ISSN: 2572-9241
©2026 The Author(s). HemaSphere is published by John Wiley & Sons Ltd on behalf of European Hematology Association. This is an open access Abstract Book distributed under the Attribution-NonCommercial-NoDerivs (CC BY-NC-ND), which allows third parties to download the articles and share them with others as long as they credit the author and the Abstract Book, but they cannot change the content in any way or use them commercially.
**Abstract Book Citations:** Authors, Title, HemaSphere, 2026;10:(S1):pages. The abstract book DOIs can be found on the “Author Information” page.
**Disclaimer:** Articles published in the journal HemaSphere exclusively reflect the opinions of the authors. The authors are responsible for all content in their abstracts including accuracy of the facts, statements, citing resources, etc.

### Results

Thirty-seven patients with *TP53*-mutated MNs were analyzed (median age 75 years, range 23–97; 51% female). According to WHO 2022, the most frequent category was MDS with biallelic *TP53* inactivation (40.5%), followed by AML with myelodysplasia-related changes (32.4%). As per ICC 2022, AML with *TP53* mutation accounted for 29.7%, while MDS with *TP53* mutation represented 37.8%. Diagnostic discordance was more frequent in AML (p=0.032).
At the molecular level, median *TP53* VAF was 29% (range 1.6%-95%). Co-mutations were present in 59.5%: 35.1% in genes involved in epigenetic regulation (*DNMT3A*, *TET2*, *ASXL1* and *EZH2*), 10.8% in splicing factor genes (*SF3B1, SRSF2* and *U2AF1*), and 27% in genes involved in signaling pathways (*KRAS, NRAS, CBL, JAK2, NF1* and *CSF3R*). Figure 1. Patients with VAF>50% (n=12) more frequently harbored isolated *TP53* mutations, whereas co-mutations predominated in the low-VAF group (48.6% vs 13.5%, p=0.07). Detection of CK was associated with a high *TP53* VAF (p=0.03).
Notably, *PPM1D* co-mutations were exclusively observed in patients with VAF≤50% (n=4,16%). The majority of *TP53* mutations (n=42, 84%) occurred outside classical hotspots, with 5 patients presenting substitutions V272M/L/G suggesting an alternative hotspot within the DNA-binding domain.
At the cytogenetic study, 58.3% cases had CK, 33.3% del(17p), and 58.3% harbored del(5q)/del(7q). OGM was available in 16 cases, detecting more genomic complexity and chromothripsis/cromoanasynthesis-like events (n=3).
AML cases were enriched for CK (p=0.03), del(17p) (p<0.01), and del(5q)/del(7) (p=0.03) compared to MDS. Median overall survival was 5.35 months in AML vs 23.69 months in MDS patients (HR 2.51, 95% CI 0.93–6.76; p=0.068). CK status alone did not significantly impact survival in this cohort (p=0.453).

### Summary/Conclusion

*TP53*-mutated MN display heterogeneous genomic profiles with recurrent co-mutation patterns across epigenetic, splicing and signaling pathways. V272M/L/G emerges as a potential alternative hotspot. Integrating *TP53* VAF with cytogenetics and OGM improves detection of structural complexity, while classifications discordance supports the need for harmonized *TP53* criteria.

**Copyright Information:** (Online) ISSN: 2572-9241
©2026 The Author(s). HemaSphere is published by John Wiley & Sons Ltd on behalf of European Hematology Association. This is an open access Abstract Book distributed under the Attribution-NonCommercial-NoDerivs (CC BY-NC-ND), which allows third parties to download the articles and share them with others as long as they credit the author and the Abstract Book, but they cannot change the content in any way or use them commercially.
**Abstract Book Citations:** Authors, Title, HemaSphere, 2026;10:(S1):pages. The abstract book DOIs can be found on the “Author Information” page.
**Disclaimer:** Articles published in the journal HemaSphere exclusively reflect the opinions of the authors. The authors are responsible for all content in their abstracts including accuracy of the facts, statements, citing resources, etc.

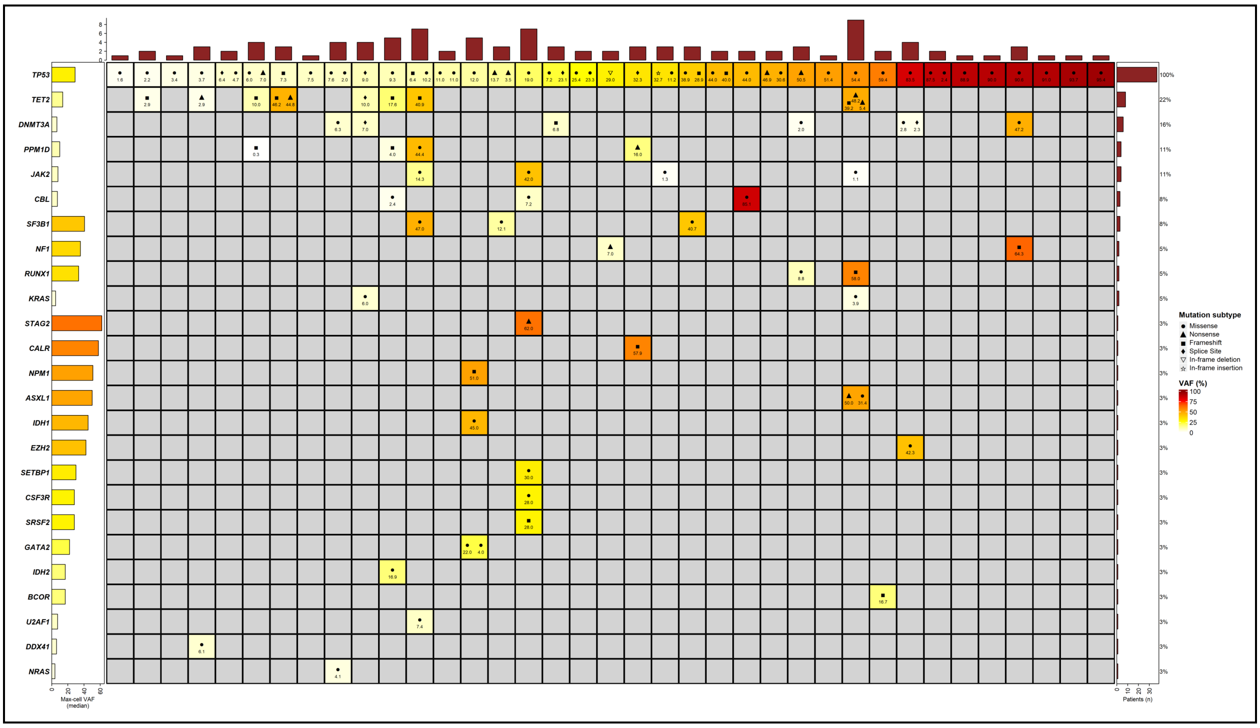

Figure 1. Oncoprint with TP53 mutations ordered by VAF

**Copyright Information:** (Online) ISSN: 2572-9241
©2026 The Author(s). HemaSphere is published by John Wiley & Sons Ltd on behalf of European Hematology Association. This is an open access Abstract Book distributed under the Attribution-NonCommercial-NoDerivs (CC BY-NC-ND), which allows third parties to download the articles and share them with others as long as they credit the author and the Abstract Book, but they cannot change the content in any way or use them commercially.
**Abstract Book Citations:** Authors, Title, HemaSphere, 2026;10:(S1):pages. The abstract book DOIs can be found on the "Author Information" page.
**Disclaimer:** Articles published in the journal HemaSphere exclusively reflect the opinions of the authors. The authors are responsible for all content in their abstracts including accuracy of the facts, statements, citing resources, etc.

### (PB3067) RNA PSEUDOURIDYLATION-DRIVEN TRANSLATION AND GENOME REGULATION IN HEMATOPOIETIC STEM CELLS AND MYELODYSPLASTIC SYNDROMES

**Topic:** 09. Myelodysplastic syndromes - Biology & translational research

Gaia Menichincheri*[1], Mariel Kleer[1], Phuong Cao Thi Ngoc[2], Silvia Tucciarone[1], Sowndarya Muthukumar[1], Cristian Bellodi[1, 2]

*[1]University of Copenhagen, Biotech Research & Innovation Centre, BRIC, Copenhagen, Denmark;[2]Lund University, Division of Molecular Hematology, Department of Laboratory Medicine, Lund Stem Cell Center, Lund, Sweden;*

**Background**

Exciting research indicates that pseudouridine (Ψ), a widespread RNA modification type, is newly emerging as a central epitranscriptomic regulator in normal and malignant hematopoiesis. However, how Ψ adapts the immune response to stress remains poorly understood. Prior work from our lab has identified the Ψ synthase PUS10 as a key modulator of autoinflammation. Loss of PUS10 alters the cellular pool of tRNA-derived RNAs (tDRs), dysregulating transposable elements (TE), which lead to the accumulation of DNA hybrids and cGAS-STING driven inflammation *in vitro* and *in vivo*. Significantly, PUS10 deficiency aberrantly enhances *de novo* protein synthesis and genomic instability in various cells, including hematopoietic stem cells (HSC).

**Aims**

Here we aim to define how PUS10 impacts translation and DNA repair in HSC upon pathophysiological stress, given the importance of a tight regulation of these processes, which are altered both in aging and disease.

**Methods**

We are currently combining PUS10 depleted mice and human cells and an *in vivo* model with molecular and sequencing approaches to identify pathways aberrantly deregulated in unperturbed and stress condition. We will investigate translation changes and the contribution of tDRs and Ψ modifications toward translatome rewiring in HSC upon inflammatory stress. In addition, we will analyze how translational changes interplay with DNA repair mechanisms in the maintenance of genome stability. Specifically, we will examine PUS10 contribution to myelodysplastic syndrome (MDS), a clonal disease characterized by HSC with aberrant translation, accumulation of DNA damage and inflammation.

**Results**

Our preliminary data suggests that not only does PUS10 loss increase protein synthesis in a Ψ dependent manner, but it also increases DNA damage levels. Furthermore, PUS10 is upregulated in MDS hinting to a role in disease development and progression.

**Summary/Conclusion**

Collectively, our findings suggest that PUS10 has a function in regulating translation and genome stability, these insights may reveal novel therapeutic targets and biomarkers for age-related hematological disorders.

**Copyright Information:** (Online) ISSN: 2572-9241
©2026 The Author(s). HemaSphere is published by John Wiley & Sons Ltd on behalf of European Hematology Association. This is an open access Abstract Book distributed under the Attribution-NonCommercial-NoDerivs (CC BY-NC-ND), which allows third parties to download the articles and share them with others as long as they credit the author and the Abstract Book, but they cannot change the content in any way or use them commercially.
**Abstract Book Citations:** Authors, Title, HemaSphere, 2026;10:(S1):pages. The abstract book DOIs can be found on the "Author Information" page.
**Disclaimer:** Articles published in the journal HemaSphere exclusively reflect the opinions of the authors. The authors are responsible for all content in their abstracts including accuracy of the facts, statements, citing resources, etc.

### (PB3068) FAMILIAL CHARACTERIZATION AND VARIANT INTERPRETATION OF A GERMLINE VARIANT IN DDX41 IN THE CONTEXT OF MYELOID NEOPLASM PREDISPOSITION

**Topic:** 09. Myelodysplastic syndromes - Biology & translational research

Ada Esteban-Figuerola*[1], Alicia Calvo Ferrer[2], Diana Alegre González[3], Ana Bastida Saenz[2], Samuel Martín Rodríguez[2], Andrea Campeny-Najara[1], Montserrat Hernandez-Perez[1], Javier Larreina-Perez[1], Jesus Feliu Sanchez[1], Sofia Rincon-Lopez[1], Johelys Atencio-Matos[1], Olalla Pereda-Sainz[1], Ricardo Francisco Garcia Muñoz[1]

*[1]Hospital San Pedro, Hematology, Logroño (La Rioja), Spain; [2]Hospital San Pedro, Clinical Analysis Department, Genetics Section., Logroño (La Rioja), Spain; [3]Hospital San Pedro, Internal Medicine, Logroño (La Rioja), Spain;*

### Background

Myeloid neoplasms with germline predisposition comprise a heterogeneous group of disorders caused by deleterious inherited variants in genes conferring susceptibility to hematologic malignancies. Germline variants in DDX41, located on chromosome 5 and inherited in an autosomal-dominant pattern, account for ~3% of myeloid malignancies and typically present in the sixth decade (range 44–70 years) as apparently sporadic myelodysplastic syndrome or acute myeloid leukemia. Clinical features include cytopenia, macrocytosis, and bone-marrow dysplasia with or without blast excess, usually with normal karyotype and immunophenotype. International guidelines recommend identification of pathogenic/likely pathogenic variants, genetic counselling, and family screening. Although hundreds of germline DDX41 variants have been described, the clinical significance of many missense variants remains uncertain.

### Aims

To investigate the clinical significance of the DDX41 variant c.1079C>T/p.(Thr360Ile) (NM_016222.4) through familial segregation analysis in a carrier family.

### Results

A 32-year-old woman with IgA deficiency and persistent eosinophilia (>1500/µL) associated with monocytosis, without cytopenias or blasts, underwent bone-marrow evaluation showing no dysplasia or blast excess. Secondary and clonal causes of eosinophilia were excluded, including PDGFRA, PDGFRB, FGFR1, JAK2, and BCR-ABL rearrangements; karyotype was 46,XX.
Targeted NGS identified the DDX41 variant c.1079C>T/p.(Thr360Ile) with a variant allele frequency of 51%, and germline origin was confirmed in peripheral-blood $CD3^+$ lymphocytes. The variant is reported in GnomAD with a population frequency of 0.0032% (rs1284223764) and in ClinVar (ID1337724), where it is classified as a variant of uncertain significance. In silico predictors are inconclusive, although the substitution affects a moderately conserved residue within the DEAD-box domain. According to ACMG/AMP criteria, it meets PM2_supporting and PM1_moderate evidence and was therefore classified as class 3 (VUS).
The variant had previously been described as a somatic mutation in one patient with acute myeloid leukemia[9], prompting a segregation study. Familial testing identified the variant in the patient's mother and maternal aunt (66 and 70 years), neither of whom shows cytopenias, dysplasia, or blast excess; the variant was absent in her sister. Germline pathogenic/likely pathogenic DDX41 variants are established drivers of inherited susceptibility to myeloid neoplasms. Although penetrance is incomplete, clinically overt disease—characterized by cytopenias, dysplasia, or increased blasts—typically manifests between 44 and 70 years. The absence of hematologic abnormalities in relatives at the upper end of this age range suggests limited clinical impact or reduced penetrance of the p.Thr360Ile substitution. Accordingly, the variant remains classified as a VUS, and further functional and segregation studies are required to clarify its pathogenic role.

**Copyright Information:** (Online) ISSN: 2572-9241
©2026 The Author(s). HemaSphere is published by John Wiley & Sons Ltd on behalf of European Hematology Association. This is an open access Abstract Book distributed under the Attribution-NonCommercial-NoDerivs (CC BY-NC-ND), which allows third parties to download the articles and share them with others as long as they credit the author and the Abstract Book, but they cannot change the content in any way or use them commercially.
**Abstract Book Citations:** Authors, Title, HemaSphere, 2026;10:(S1):pages. The abstract book DOIs can be found on the "Author Information" page.
**Disclaimer:** Articles published in the journal HemaSphere exclusively reflect the opinions of the authors. The authors are responsible for all content in their abstracts including accuracy of the facts, statements, citing resources, etc.

### Summary/Conclusion

In this family, the germline DDX41 p.Thr360Ile variant lacks evidence of a demonstrated pathogenic role in inherited predisposition to myeloid neoplasms. This case underscores the diagnostic and counselling challenges posed by rare germline missense variants in DDX41 and highlights the need for functional studies, epidemiologic validation, and extended familial co-segregation analyses to enable definitive reclassification.

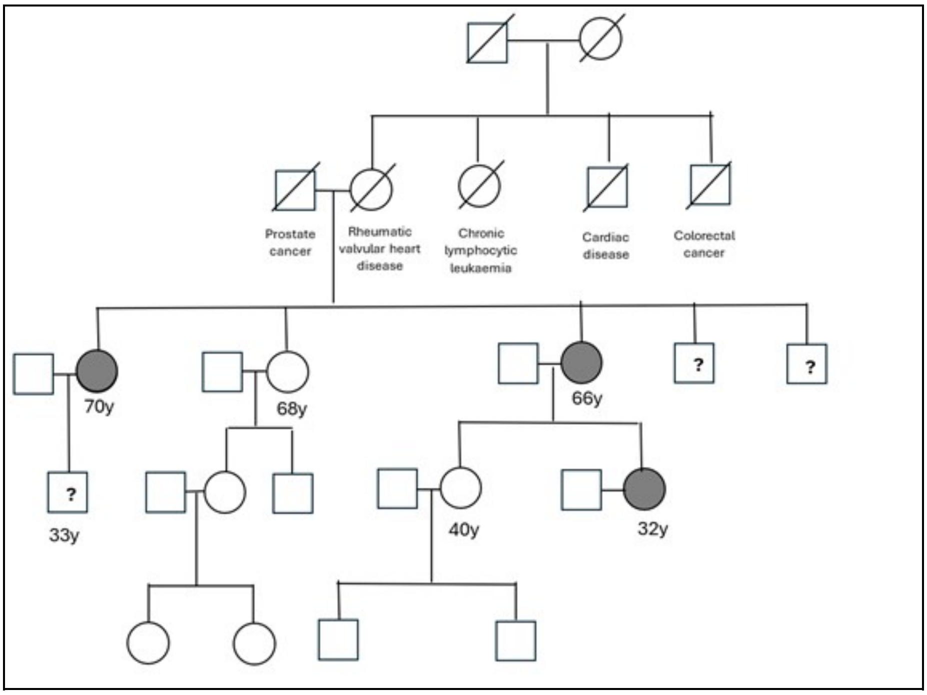

Figure. 32-year-old female; ages and causes of death are provided where applicable. Question marks denote living, untested individuals.

**Copyright Information:** (Online) ISSN: 2572-9241
©2026 The Author(s). HemaSphere is published by John Wiley & Sons Ltd on behalf of European Hematology Association. This is an open access Abstract Book distributed under the Attribution-NonCommercial-NoDerivs (CC BY-NC-ND), which allows third parties to download the articles and share them with others as long as they credit the author and the Abstract Book, but they cannot change the content in any way or use them commercially.
**Abstract Book Citations:** Authors, Title, HemaSphere, 2026;10:(S1):pages. The abstract book DOIs can be found on the "Author Information" page.
**Disclaimer:** Articles published in the journal HemaSphere exclusively reflect the opinions of the authors. The authors are responsible for all content in their abstracts including accuracy of the facts, statements, citing resources, etc.

### (PB3069) CLINICAL AND MOLECULAR HETEROGENEITY OF SF3B1-MUTATED MYELODYSPLASTIC SYNDROMES:IMPACT OF VARIANT ALLELE FREQUENCY, MUTATION SITES, AND CO-MUTATION PATTERNS

**Topic:** 09. Myelodysplastic syndromes - Biology & translational research

Kainan Zhang*[1], Xiaosu Zhao[1, 2]

*[1]Peking University People's Hospital, Peking University Institute of Hematology, National Clinical Research Center for Hematologic Disease, Beijing Key Laboratory of Cell and Gene Therapy for Hematologic Malignan-cies, Peking University, Beijing, China; [2]Collaborative Innovation Center of Hematology, Peking University, Beijing, China;*

### Background

SF3B1 is one of the most frequently mutated splicing factor genes in myelodysplastic syndromes and defines a distinct disease subtype. The 5th edition of the WHO classification formally categorizes MDS with SF3B1 mutation as "MDS with SF3B1 mutation". However, SF3B1 mutations exhibit considerable heterogeneity, and the impact of different variant allele frequencies, mutation sites, and co-mutation patterns on clinical phenotype and prognosis remains incompletely understood.

### Aims

This study aims to systematically characterize the clinical and molecular features of newly diagnosed MDS patients harboring SF3B1 mutations, with a focus on the role of VAF, mutation sites, and co-mutation patterns in patient stratification and prognosis.

### Methods

A total of 132 newly diagnosed MDS patients with SF3B1 mutations were enrolled. Clinical characteristics, laboratory parameters, IPSS-R scores, and molecular genetic data were collected. Differences in clinical and molecular features were analyzed by mutation type, VAF and site.

### Results

Among 132 patients(**Fig 1A**), 93 met the WHO 5th edition criteria for MDS-SF3B1. MDS-SF3B1 patients exhibited higher Hb levels (81g/L vs. 68g/L, p=0.03), and a higher proportion of low-to mediate-risk IPSS-R categories (70% vs. 33%, p<0.0001)(**Table 1**). When stratified by median VAF(38%), the low VAF group showed younger age (59y vs.68y, p=0.0002), male predominance (58% vs. 46%, p=0.05), lower WBC counts ($3.2\times10^9$/L vs. $4.1\times10^9$/L,p=0.0003) and PLT counts ($129\times10^9$/L vs. $216\times10^9$/L, p=0.008), and a lower proportion of low-to-mediate-risk IPSS-R categories (52% vs. 70%, p=0.06)(**Table 2**). The most common mutation hotspots was K700E(**Fig 1B**). Patients with K700E mutation had lower bone marrow blast percentages (2.0% vs. 3.8%, p=0.02)but no significant differences in other clinical features. Co-mutation analysis revealed that MDS-SF3B1 patients had higher frequencies of TET2 and CUX1 mutations and lower NRAS mutation rates. The K700E subgroup retained enrichment for CUX1 mutations but showed significantly lower frequencies of ASXL1 and U2AF1 mutations(**Fig 1C**).
Table1 Characteristics Stratified by SF3B1 Mutation Type

**Copyright Information:** (Online) ISSN: 2572-9241
©2026 The Author(s). HemaSphere is published by John Wiley & Sons Ltd on behalf of European Hematology Association. This is an open access Abstract Book distributed under the Attribution-NonCommercial-NoDerivs (CC BY-NC-ND), which allows third parties to download the articles and share them with others as long as they credit the author and the Abstract Book, but they cannot change the content in any way or use them commercially.
**Abstract Book Citations:** Authors, Title, HemaSphere, 2026;10:(S1):pages. The abstract book DOIs can be found on the "Author Information" page.
**Disclaimer:** Articles published in the journal HemaSphere exclusively reflect the opinions of the authors. The authors are responsible for all content in their abstracts including accuracy of the facts, statements, citing resources, etc.

| | MDS-SF3B1 | Others | *p* |
|---|---|---|---|
| n (%) | 93(71%) | 39(29%) | |
| Age(year), median (IQR) | 64(55-72) | 64(50-71) | 0.57 |
| Sex, n(%) | | | 0.08 |
| Male | 43(46%) | 25(64%) | |
| Female | 50(54%) | 14(36%) | |
| Blast %, median (IQR) | 2(1-3) | 7(6-10) | 0.0001 |
| White Blood Cell($\times10^9$/L), median (IQR) | 3.6(2.6-4.9) | 3.4(2.6-6.4) | 0.84 |
| Hemoglobin (g/L), median (IQR) | 81(66-94) | 68(52-87) | 0.03 |
| Platelet($\times10^9$/L), median (IQR) | 199(86-307) | 124(71-253) | 0.25 |
| IPSS-R, n(%) | | | 0.0001 |
| Low to mediate | 65(70%) | 13(33%) | |
| High | 5(5%) | 13(33%) | |

Table2 Characteristics Stratified by SF3B1 Mutation VAF

| | VAF Low | VAF High | *p* |
|---|---|---|---|
| n (%) | 66(50%) | 66(50%) | |
| Age(year), median (IQR) | 59(47-69) | 68(59-72) | 0.0002 |
| Sex, n(%) | | | 0.05 |
| Male | 38(58%) | 30(46%) | |
| Female | 28(42%) | 36(54%) | |
| Blast %, median (IQR) | 3(2-6) | 2(1-5) | 0.08 |
| White Blood Cell($\times10^9$/L), median (IQR) | 3.2(2.3-3.9) | 4.1(2.8-6.9) | 0.0003 |
| Hemoglobin (g/L), median (IQR) | 77(62-90) | 80(63-93) | 0.64 |
| Platelet($\times10^9$/L), median (IQR) | 129(59-215) | 216(92-345) | 0.008 |
| IPSS-R, n(%) | | | 0.06 |
| Low to mediate | 34(52%) | 46(70%) | |
| High | 11(17%) | 5(8%) | |

### Summary/Conclusion

Heterogeneity in SF3B1 mutations is closely associated with clinical phenotype and prognosis. The MDS-SF3B1 subtype exhibits a distinct clinical and co-mutation profile with a favorable prognostic tendency. Low VAF and non-K700E mutations may correlate with adverse clinical features. Integrating VAF, mutation sites, and co-mutation patterns may enable more precise risk stratification in MDS patients with SF3B1 mutations.

**Copyright Information:** (Online) ISSN: 2572-9241
©2026 The Author(s). HemaSphere is published by John Wiley & Sons Ltd on behalf of European Hematology Association. This is an open access Abstract Book distributed under the Attribution-NonCommercial-NoDerivs (CC BY-NC-ND), which allows third parties to download the articles and share them with others as long as they credit the author and the Abstract Book, but they cannot change the content in any way or use them commercially.
**Abstract Book Citations:** Authors, Title, HemaSphere, 2026;10:(S1):pages. The abstract book DOIs can be found on the "Author Information" page.
**Disclaimer:** Articles published in the journal HemaSphere exclusively reflect the opinions of the authors. The authors are responsible for all content in their abstracts including accuracy of the facts, statements, citing resources, etc.

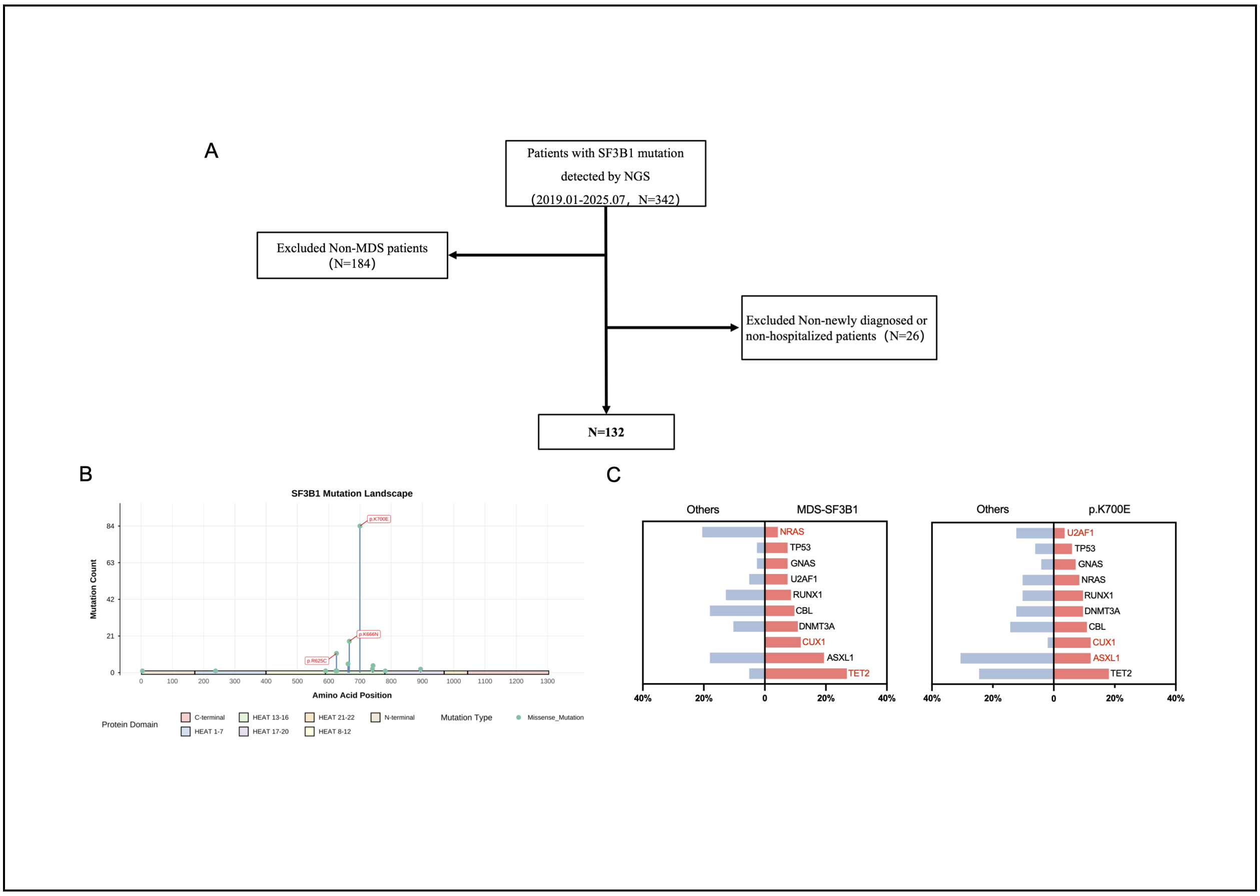

Figure 1. Screening, Mutation Site Distribution, and Co-mutation Patterns in MDS Patients with SF3B1 Mutations (A) Screening flowchart of MDS patients with SF3B1 mutations. (B) Distribution of SF3B1 mutation sites, with the three most frequent sites labeled. (C) Distribution of co-mutation frequencies in SF3B1-mutated subgroups. Co-mutations with statistical significance between groups ($p < 0.05$) are highlighted in red.

**Copyright Information:** (Online) ISSN: 2572-9241
©2026 The Author(s). HemaSphere is published by John Wiley & Sons Ltd on behalf of European Hematology Association. This is an open access Abstract Book distributed under the Attribution-NonCommercial-NoDerivs (CC BY-NC-ND), which allows third parties to download the articles and share them with others as long as they credit the author and the Abstract Book, but they cannot change the content in any way or use them commercially.
**Abstract Book Citations:** Authors, Title, HemaSphere, 2026;10:(S1):pages. The abstract book DOIs can be found on the “Author Information” page.
**Disclaimer:** Articles published in the journal HemaSphere exclusively reflect the opinions of the authors. The authors are responsible for all content in their abstracts including accuracy of the facts, statements, citing resources, etc.

### (PB3070) ISOLATED PPM1D MUTATIONS IN BONE MARROW: CLINICOPATHOLOGIC FEATURES IN A 52-PATIENT COHORT

**Topic:** 09. Myelodysplastic syndromes - Biology & translational research

April Chiu*[1], Kaaren Reichard[1]

[1]*Mayo Clinic, Laboratory Medicine and Pathology, Rochester, United States of America;*

### Background

PPM1D encodes a p53-regulating serine/threonine phosphatase that negatively regulates the DNA damage response pathway. Truncating mutations result in a stabilized, gain-of-function protein that confers resistance to genotoxic stress and promotes clonal expansion, particularly following cytotoxic therapy. While PPM1D is more commonly seen as a co-mutation in overt myeloid neoplasms, it is otherwise more commonly isolated, typically interpretted as clonal hematopoiesis. However, the clinicopathologic significance of isolated PPM1D mutations remains incompletely defined.

### Aims

To characterize the clinicopathologic features of bone marrow specimens in patients harboring isolated pathogenic PPM1D mutations.

### Methods

We retrospectively analyzed 52 patients with isolated pathogenic PPM1D mutations identified by next-generation sequencing (NGS) from 10/2022 to 10/2024. Cases with additional pathogenic mutations were excluded. Demographics, CBC parameters, bone marrow findings (including cellularity, degree of dysplasia, blast percentage), karyotype, and PPM1D variant allele frequency (VAF) were evaluated.

### Results

The patient median age was 70 (range 45-89) years with equal sex distribution (M:F 1:1). The indications were as follows: cytopenia(s) and/or rule out MDS (N=45; including N=2 also with leukocytosis), AML or myeloid neoplasms (N=5), red cell aplasia (N=1), and lymphoproliferative disorder (N=1). The CBC showed median hemoglobin 9.6 g/dL; WBC 4.65 K/uL; platelets 122.5 K/uL. Of the 52 patients, 42 had least one previous cancer; including solid tumor (N=24), lymphoma (N=16), MDS or AML (N=7), and multiple myeloma (N=2); 10 patients had 2 or more previous cancer diagnoses. 42 patients received prior cytotoxic therapy (chemotherapy and/or radiation); with 1 (N=14), 2 (N=11), 3 (N=7), and >3 (N=10) prior courses. Bone marrow cellularity was normal or only slightly increased/decreased (N=37), hypocellular (N=10), and hypercellular (N=5) for age. Bone marrow blast percentage was generally not increased (<5%; N=49). Dysplasia was not significant (N=33), subtle (N=7), significant (N=10; unilineage N=5, bilineage N=2), or not assessable (N=2). Karyotype was normal in 41/49 (84%) biopsies. Abnormal karyotypes (N=8) included monosomy 7 (N=3) and complex abnormalities (N=3). There were 1 (N=35), 2 (N=10), and 3 or more (N=7) PPM1D mutations; with VAF ≥10% in at least one mutation in 24/52 cases. There was no significant difference in CBC, blast percentage, incidence of abnormal karyotype, or PPM1D VAF or mutation numbers between patients with and without prior cytotoxic therapy. The number of prior cytotoxic regimens also did not correlate with VAF. Of the 52 patients, 27 were alive and 25 were dead at the time of this analysis. Higher PPM1D VAF was significantly associated with mortality (median 21.5% vs 4.0%, p=0.008), whereas mutation number was not.

**Copyright Information:** (Online) ISSN: 2572-9241
©2026 The Author(s). HemaSphere is published by John Wiley & Sons Ltd on behalf of European Hematology Association. This is an open access Abstract Book distributed under the Attribution-NonCommercial-NoDerivs (CC BY-NC-ND), which allows third parties to download the articles and share them with others as long as they credit the author and the Abstract Book, but they cannot change the content in any way or use them commercially.
**Abstract Book Citations:** Authors, Title, HemaSphere, 2026;10:(S1):pages. The abstract book DOIs can be found on the "Author Information" page.
**Disclaimer:** Articles published in the journal HemaSphere exclusively reflect the opinions of the authors. The authors are responsible for all content in their abstracts including accuracy of the facts, statements, citing resources, etc.

**Summary/Conclusion**

Isolated PPM1D mutations are frequently associated with low blast percentages, normal karyotype, and often normal morphology to subtle dysplasia in bone marrow specimens evaluated for cytopenias or myeloid neoplasms. These findings support that isolated PPM1D mutations often represent clonal hematopoiesis or therapy-related clonal selection rather than overt myeloid neoplasm. A subset with abnormal karyotype highlights biologic heterogeneity. While higher PPM1D VAF was significantly associated with mortality, longitudinal studies are required to determine progression risk.

**Copyright Information:** (Online) ISSN: 2572-9241
©2026 The Author(s). HemaSphere is published by John Wiley & Sons Ltd on behalf of European Hematology Association. This is an open access Abstract Book distributed under the Attribution-NonCommercial-NoDerivs (CC BY-NC-ND), which allows third parties to download the articles and share them with others as long as they credit the author and the Abstract Book, but they cannot change the content in any way or use them commercially.
**Abstract Book Citations:** Authors, Title, HemaSphere, 2026;10:(S1):pages. The abstract book DOIs can be found on the "Author Information" page.
**Disclaimer:** Articles published in the journal HemaSphere exclusively reflect the opinions of the authors. The authors are responsible for all content in their abstracts including accuracy of the facts, statements, citing resources, etc.

### (PB3071) THE EFFECTS OF BLOOD SERA FROM PATIENTS WITH MDS WITH DIFFERENT KARYOTYPES ON THE GROWTH OF MULTIPOTENT MESENCHYMAL STROMAL CELLS FROM HEALTHY DONORS

**Topic:** 09. Myelodysplastic syndromes - Biology & translational research

Nataliya Petinati*[1], Aleksandra Sadovskaya[1], Nina Drize[1], Mariya Domanitskaya[1], Alina Kohno[1], Tatyana Obukhova[1], Valentina Dvirnik[1], Elena Parovichnikova[1]

[1]*National Medical Research Center for Hematology, Moscow, Russia;*

### Background

Myelodysplastic syndrome (MDS) is a heterogeneous group of diseases, and MDS pathogenesis is not sufficiently studied. In MDS, quantity of blood cells produced by the bone marrow is not enough to subsist. It is known that hematopoietic progenitors are impaired in MDS, and stromal progenitors (including multipotent mesenchymal stromal cells, or MSCs) can be altered as well. Bone marrow stroma regulates hematopoiesis, and some cases of cytopenia may be caused or exacerbated by the stromal niche damage. This damage may result from MSC genetic alterations, or be caused by the blood composition.

### Aims

To analyze the impact of sera of patients suffering from MDS with various karyotypes (normal, 5q deletion, 7q deletion/monosomy, complex) on the growth of healthy donors' MSCs.

### Methods

The sera were obtained from blood samples collected at the same time as diagnostic blood tests after the donors and patients gave their informed consent, and stored at -40°C. Sera from 16 healthy donors were used as control. MDS karyotype was characterized by standard methods and FISH.
MSCs were derived from bone marrow samples of 8 healthy donors obtained at the time of exfusion after the donors' informed consent, and cultivated for 2-3 passages. Then, MSCs were seeded into a 96-well plate at 1000 cells per well in αMEM with 10% of donor or patient serum added, and cultivated for 7 days. Each sample was present in triplicate.
MTT-test was performed to quantify cell growth. Optical density (OD) was used as a measure. In order to compare data obtained from cultivating different MSCs, OD was normalized to mean donor value.

**Copyright Information:** (Online) ISSN: 2572-9241
©2026 The Author(s). HemaSphere is published by John Wiley & Sons Ltd on behalf of European Hematology Association. This is an open access Abstract Book distributed under the Attribution-NonCommercial-NoDerivs (CC BY-NC-ND), which allows third parties to download the articles and share them with others as long as they credit the author and the Abstract Book, but they cannot change the content in any way or use them commercially.
**Abstract Book Citations:** Authors, Title, HemaSphere, 2026;10:(S1):pages. The abstract book DOIs can be found on the "Author Information" page.
**Disclaimer:** Articles published in the journal HemaSphere exclusively reflect the opinions of the authors. The authors are responsible for all content in their abstracts including accuracy of the facts, statements, citing resources, etc.

### Results

Sera from patients with MDS-5q- support the growth of MSCs from healthy donors as well as sera from healthy donors, but differ significantly (p<0.0001) in this capacity from sera of patients with MDS with other karyotypes (Table). 5q contains not only genes necessary for erythropoiesis but also thrombopoiesis inhibitors (including microRNAs miR-145 and miR-146a). As a result, erythrocytic cell lineage is suppressed while platelet counts, unlike in other MDS subtypes, tends to be normal or even elevated.
It is known that MSC growth depends, among other things, on factors secreted by platelets. The presence of these factors may be the reason
of the observed differences in MSC growth. The serum samples from MDS patients with monosomy 7 or 7q deletion, as well as those with a complex karyotype, on the other hand, were the least capable of supporting MSC growth. Among patients with a normal karyotype, both OD values and platelet counts varied widely, and did not correlate.
Table OD and platelet count in patients with MDS with different karyotyes

| **Karyotype** | **N** | **OD mean** | **OD min** | **OD max** | **OD comparison with 5q-, *p*-value** | **PLT mean** | **PLT min** | **PLT max** |
|---|---|---|---|---|---|---|---|---|
| 5q- | 19 | **1.06** | 0.75 | 1.56 | | **336** | 73 | 710 |
| -7/7q- | 9 | **0.45** | 0.2 | 0.86 | 8.72E-06 | **82** | 20 | 254 |
| **complex** | 19 | **0.41** | 0.12 | 0.72 | 1.87E-12 | **87** | 7 | 330 |
| **normal** | 75 | **0.58** | 0.12 | 1.87 | 1.24E-09 | **145** | 13 | 584 |
| **other** | 21 | **0.66** | 0.12 | 1.63 | 1.24E-05 | **286** | 19 | 1474 |
| **normal (donors)** | 42 | **1** | 0.31 | 1.57 | 0.486 | **221** | 146 | 292 |

### Summary/Conclusion

Sera of MDS patients with 5q-deletion subtype, in contrary to other MDS subtypes, support MSC growth as well as healthy donors' sera do. 7q monosomy/deletion and complex karyotype MDS subtypes produce sera with the lowest capacity for MSC growth support. This highlights the heterogeneity of MDS and implies differences in pathogeneses of MDS subtypes.
The study was supported by a grant from Russian Science Foundation project №25-25-00663, https://rscf.ru/en/project/25-25-00663.

**Copyright Information:** (Online) ISSN: 2572-9241
©2026 The Author(s). HemaSphere is published by John Wiley & Sons Ltd on behalf of European Hematology Association. This is an open access Abstract Book distributed under the Attribution-NonCommercial-NoDerivs (CC BY-NC-ND), which allows third parties to download the articles and share them with others as long as they credit the author and the Abstract Book, but they cannot change the content in any way or use them commercially.
**Abstract Book Citations:** Authors, Title, HemaSphere, 2026;10:(S1):pages. The abstract book DOIs can be found on the "Author Information" page.
**Disclaimer:** Articles published in the journal HemaSphere exclusively reflect the opinions of the authors. The authors are responsible for all content in their abstracts including accuracy of the facts, statements, citing resources, etc.

### (PB3072) THERMODYNAMIC CHARACTERISATION OF DDX41 MISSENSE VARIANTS OF UNCERTAIN SIGNIFICANCE

**Topic:** 09. Myelodysplastic syndromes - Biology & translational research

Natsuki Omori Rison*[1], Wendy Stewart[1]

[1]*University Hospitals Dorset NHS Foundation Trust, Bournemouth, United Kingdom;*

### Background

The DDX41 mutation is the most common germline predisposition in adult myelodysplastic syndrome (MDS) and acute myeloid leukaemia (AML). However, next-generation sequencing frequently identifies missense variants of uncertain significance (VUS), which creates diagnostic ambiguity and limits clinical interpretation for surveillance and risk stratification.

### Aims

This study aims to characterise DDX41 missense VUS through in-silico thermodynamic structural profiling.

### Methods

A list of DDX41 missense variants was extracted from ClinVar, and the full-length wild-type protein structure was obtained from AlphaFold Protein Structure Database (AF-Q9UJV9-F1-v6). FoldX was used to analyse the stability of the protein in-silico, running on macOS Sequoia Version 15.6.1.
The AlphaFold protein structure was first pre-processed with RepairPDB to remove steric clashes and optimise rotamers. The change in Gibbs Free Energy ($\Delta\Delta G$) for each missense variant was then calculated based on the FoldX 5.0 force field to estimate the impact of the missense variation on protein structural stability. Variants annotated as Pathogenic/Likely Pathogenic (P/LP) and Benign/Likely Benign (B/LB) on ClinVar were analysed alongside VUS as positive and negative controls.
$\Delta\Delta G > 1.58$ kcal/mol was used as the threshold for the protein instability predictor based on the FoldX force field standard error margin and previous literature.

### Results

A total of 403 unique missense VUS were extracted from ClinVar and used in the analysis. Internal validation using P/LP and B/LB variants showed a statistically significant increase in $\Delta\Delta G$ in P/LP variants (median 2.67 vs 0.18 kcal/mol, Mann–Whitney U, $p = 0.004$). Analysis of the VUS cohort showed a subset of variants with $\Delta\Delta G > 1.58$ kcal/mol suggestive of a significant structural destabilisation. These variants were enriched in the helicase ATP-binding domain (residue 212-396), which suggests functional domain sensitivity to missense variation.

### Summary/Conclusion

Thermodynamic structural profiling enables systematic characterisation of DDX41 missense VUS. This framework supports the functional interpretation of germline variants in MDS/AML and may refine risk stratification to predisposition.

**Copyright Information:** (Online) ISSN: 2572-9241
©2026 The Author(s). HemaSphere is published by John Wiley & Sons Ltd on behalf of European Hematology Association. This is an open access Abstract Book distributed under the Attribution-NonCommercial-NoDerivs (CC BY-NC-ND), which allows third parties to download the articles and share them with others as long as they credit the author and the Abstract Book, but they cannot change the content in any way or use them commercially.
**Abstract Book Citations:** Authors, Title, HemaSphere, 2026;10:(S1):pages. The abstract book DOIs can be found on the "Author Information" page.
**Disclaimer:** Articles published in the journal HemaSphere exclusively reflect the opinions of the authors. The authors are responsible for all content in their abstracts including accuracy of the facts, statements, citing resources, etc.

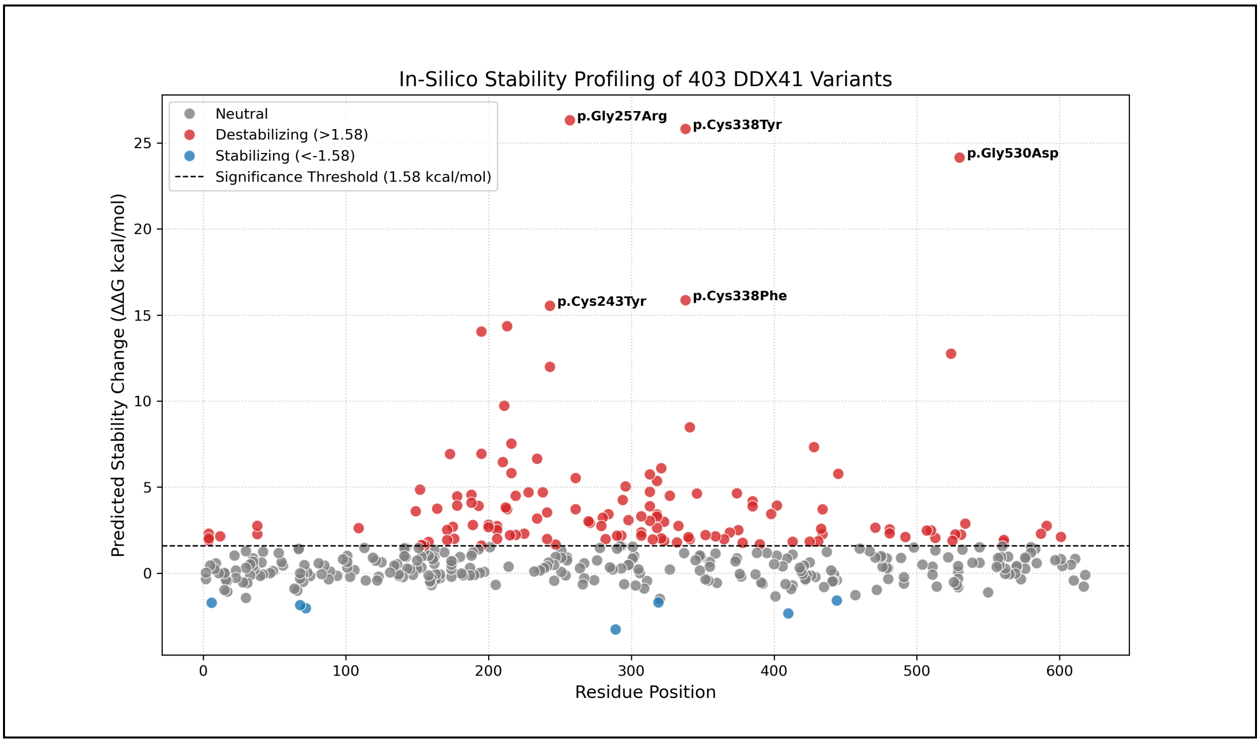

In-silico stability profiling of DDX41 Variants. Whilst the majority of VUS are structurally neutral (Grey), a high-risk cluster (Red) exhibits a significant destabilisation (ΔΔG > 1.58 kcal/mol).

**Copyright Information:** (Online) ISSN: 2572-9241
©2026 The Author(s). HemaSphere is published by John Wiley & Sons Ltd on behalf of European Hematology Association. This is an open access Abstract Book distributed under the Attribution-NonCommercial-NoDerivs (CC BY-NC-ND), which allows third parties to download the articles and share them with others as long as they credit the author and the Abstract Book, but they cannot change the content in any way or use them commercially.
**Abstract Book Citations:** Authors, Title, HemaSphere, 2026;10:(S1):pages. The abstract book DOIs can be found on the "Author Information" page.
**Disclaimer:** Articles published in the journal HemaSphere exclusively reflect the opinions of the authors. The authors are responsible for all content in their abstracts including accuracy of the facts, statements, citing resources, etc.

### (PB3073) RED BLOOD CELL PHENOTYPES IN LOWER RISK MYELODYSPLASTIC SYNDROMES : ASSOCIATION WITH DISEASE RISK

**Topic:** 09. Myelodysplastic syndromes - Biology & translational research

Pinelopi Vryttia*[1], Konstantina Theocharaki[2], Christina Apostolopoulou[1], Georgia Tzafa[2], Aikaterini Ntzoura-Kani[2], Ioannis Kostopoulos[2], Thomas Thomopoulos[1], Papageorgiou Sotirios[1], Anthi Bouchla[1], Vasiliki Pappa[1], Marianna Antonelou[2]

*[1]"Attikon" University General Hospital, National and Kapodistrian University of Athens, Second Department of Internal Medicine and Research Unit, Hematology Unit, Athens, Greece; [2]National and Kapodistrian University of Athens, Department of Biology, School of Science, Athens, Greece;*

### Background

Oxidative stress and impaired protein quality control, which contribute to genomic instability and BM inflammation in myelodysplastic syndromes (MDS),are also reflected in circulating red blood cells (RBCs). As shown by our group, treatment with recombinant erythropoietin (rEPO)has been associated with an induction of an RBC aging phenotype, characterized by PS exposure, membrane-bound hemichromes, and calcium overload.The disease-associated marker basigin (CD147) is overexpressed not only in erythroid progenitors of del(5q) MDS and other hematologic malignancies, but also in RBCs from treated and untreated low-risk (LR) MDS patients.

### Aims

In this cross-sectional study, we investigated whether these RBC phenotypes correlate with disease risk as it is determined byIPSS-M and IPSS-R scores in a cohort of non-transfused, LR MDS patients.

### Methods

We analyzed 48 transfusion-independent patients (26 untreated and 22 receiving rEPO) and 22 sex- and age-matched healthy controls. Disease progression, genetic profiling, cytogenetic status, serum biochemistry, and RBC physiological characteristics were assessed by NGS, BM morphology and cytogenetics, flow cytometry, and spectrofluorometry respectively.

### Results

The patients' clinical characteristics are shown in Table 1. Hb oxidation was directly correlated with the severity of anemia. In untreated patients, adverse outcomes (AML or death) were associated with increased proteasome activity. In addition, susceptibility to hemolysis correlated with serum ferritin levels (r 0.694, p<0.001). In contrast, in the EPO-group, it was positively correlated with *SRSF2* mutations and negatively with the more favorable *SF3B1* and *TET2* mutations.
Untreated patients with higher IPSS-M scores exhibited increased RBC expression of CD147 and CD47, and higher PS exposure. RBC calcium levels showed a strong discriminatory effect between IPSS-M risk groups according to Cohen's *d* effect size. Higher IPSS-R score patients had elevated ICAM4 expression in RBCs and a trend towards intracellular calcium stress (ANCOVA, F 3.58, p<0.10).In the EPO-group, higher IPSS-M and IPSS-R scores were associated with a statistical trend for increased hemolysis and had a positive correlation with met-Hb levels.

**Copyright Information:** (Online) ISSN: 2572-9241
©2026 The Author(s). HemaSphere is published by John Wiley & Sons Ltd on behalf of European Hematology Association. This is an open access Abstract Book distributed under the Attribution-NonCommercial-NoDerivs (CC BY-NC-ND), which allows third parties to download the articles and share them with others as long as they credit the author and the Abstract Book, but they cannot change the content in any way or use them commercially.
**Abstract Book Citations:** Authors, Title, HemaSphere, 2026;10:(S1):pages. The abstract book DOIs can be found on the "Author Information" page.
**Disclaimer:** Articles published in the journal HemaSphere exclusively reflect the opinions of the authors. The authors are responsible for all content in their abstracts including accuracy of the facts, statements, citing resources, etc.

### Summary/Conclusion

RBC phenotypes, including proteasome activity and susceptibility to hemolysis, were strongly associated with key disease variables such as IPSS-R and IPSS-M scores, mutations and clinical outcomes. Pathophysiological characteristics of RBCs in MDS were linked not only to dyserythropoiesis, but also to systemic iron homeostasis.

| | Controls | LR-MDS Untreated | LR-MDS EPO-treated |
|---|---|---|---|
| Age (Average) | 69.6 | 77.6 | 77.8 |
| Hb (Average)* g/dL | 13.5 | 11.0 | 10.6 |
| NEUT (Average)* $x10^3/\mu L$ | 3.52 | 2.53 | 3.45 |
| PLT (Average)* $x10^3/\mu L$ | 228.6 | 183.0 | 206.0 |
| N (Population) | 22 | 26 | 22 |
| IPSS-R Very Low (n) | | 13 | 4 |
| IPSS-R Low (n) | | 10 | 18 |
| IPSS-M Very Low (n) | | 3 | 0 |
| IPSS-M Low (n) | | 15 | 17 |
| IPSS-M Moderate Low (n) | | 5 | 2 |
| IPSS-M High (n) | | 0 | 1 |

Table1: Patient and control characteristics (* lab values at sample collection)

**Copyright Information:** (Online) ISSN: 2572-9241
©2026 The Author(s). HemaSphere is published by John Wiley & Sons Ltd on behalf of European Hematology Association. This is an open access Abstract Book distributed under the Attribution-NonCommercial-NoDerivs (CC BY-NC-ND), which allows third parties to download the articles and share them with others as long as they credit the author and the Abstract Book, but they cannot change the content in any way or use them commercially.
**Abstract Book Citations:** Authors, Title, HemaSphere, 2026;10:(S1):pages. The abstract book DOIs can be found on the "Author Information" page.
**Disclaimer:** Articles published in the journal HemaSphere exclusively reflect the opinions of the authors. The authors are responsible for all content in their abstracts including accuracy of the facts, statements, citing resources, etc.

### (PB3074) ESTABLISHING A VITAMIN B5–COENZYME A METABOLIC SCORE TO CHARACTERIZE HEME BIOSYNTHESIS AND ENABLE CLINICAL MONITORING IN SF3B1-MUTATED MYELODYSPLASTIC NEOPLASMS.

**Topic:** 09. Myelodysplastic syndromes - Biology & translational research

Shoshana Burke*[1], Oni Chowdhury[2, 3], Angelo D'alessandro[4], Kevin Rouault-Pierre[1, 5]

*[1]Centre for Haemato-Oncology, Barts Cancer Institute, Queen Mary University of London, London, United Kingdom;[2]Oxford University Hospitals NHS Foundation Trust, Oxford, United Kingdom; [3]Molecular Haematology Unit, Weatherall institute of Molecular Medicine NHR, Biomedical Research Centre University of Oxford, Oxford, United Kingdom; [4]University of Colorado Anschutz Medical Campus, Department of Biochemistry and Molecular Genetics, Aurora, Colorado, United States of America; [5]INSERM UMR 1342, IRSL, Saint-Louis Hospital, Paris Cité University, Paris, France;*

### Background

Vitamin B5 (pantothenate) has recently emerged as an essential metabolite for heme biosynthesis and erythroid maturation. Coenzyme A synthase (*COASY*) catalyses the conversion of vitamin B5 into coenzyme A (CoA) metabolites required for heme synthesis and red blood cell maturation. In *SF3B1*-mutated myelodysplastic neoplasms (MDS), *COASY* is aberrantly regulated downstream of *SF3B1* mutations, leading to impaired heme synthesis and contributing to anemia, the most common clinical manifestation of the disease. Supplementation with vitamin B5 has been shown to rescue ineffective erythropoiesis in primary patient-derived cultures, supporting its potential as a therapeutic strategy for patients with *SF3B1*-mutated MDS, who currently have limited treatment options. Our research team is initiating a clinical trial evaluating vitamin B5 supplementation in *SF3B1*-mutated MDS; however, the absence of reference measurements for vitamin B5 and CoA metabolites currently limits understanding of its pharmacokinetics and pharmacodynamics in erythropoiesis.

### Aims

To establish a quantitative vitamin B5 metabolic scoring system based on ratios between vitamin B5, coenzyme A (CoA) derivatives, and downstream heme metabolites in peripheral blood. This approach aims to define physiological reference standards in population >50 years old, that will enable assessment of vitamin B5 pathway activity and determine whether this metabolic axis is dysregulated in *SF3B1*-mutated MDS and related myeloid malignancies, including leukemia. Ultimately, this framework will support biological interpretation of vitamin B5 metabolism in disease and may inform patient stratification, pharmacodynamic monitoring, and the development of targeted therapeutic strategies.

### Methods

Peripheral blood samples were obtained from 14 healthy donors (mean age 53.4 years; range 52–61). Samples were fractionated into whole blood, plasma, packed red blood cells (pRBCs), and peripheral blood mononuclear cells (PBMCs). All samples were flash-frozen and subjected to global metabolomic analysis at the University of Colorado.

**Copyright Information:** (Online) ISSN: 2572-9241
©2026 The Author(s). HemaSphere is published by John Wiley & Sons Ltd on behalf of European Hematology Association. This is an open access Abstract Book distributed under the Attribution-NonCommercial-NoDerivs (CC BY-NC-ND), which allows third parties to download the articles and share them with others as long as they credit the author and the Abstract Book, but they cannot change the content in any way or use them commercially.
**Abstract Book Citations:** Authors, Title, HemaSphere, 2026;10:(S1):pages. The abstract book DOIs can be found on the "Author Information" page.
**Disclaimer:** Articles published in the journal HemaSphere exclusively reflect the opinions of the authors. The authors are responsible for all content in their abstracts including accuracy of the facts, statements, citing resources, etc.

**Results**

- Plasma contained negligible levels of vitamin B5 and CoA species, indicating that this compartment is unsuitable for their measurement.
- PBMC metabolite levels showed high inter-donor variability and did not correlate with other blood compartments.
- Spearman rank correlation analysis demonstrated strong correlations ($\rho > 0.5$) between whole blood and pRBCs for heme, heme intermediates, vitamin B5, and CoA metabolites.
- In pRBCs, the mean vitamin B5/CoA metabolite ratio was 0.7 with minimal variability (SEM 0.09).
- The vitamin B5/heme ratio in pRBCs was 0.007 (SEM 0.0013).
- The CoA/heme ratio was 0.02 (SEM 0.003).

**Summary/Conclusion**

Vitamin B5 and CoA metabolites are predominantly detected within red blood cells in peripheral blood. Given the strong correlation between whole blood and pRBC measurements, either compartment represents a suitable matrix for clinical assessment. The low inter-individual variability observed in the vitamin B5/CoA ratio suggests its potential utility as a metabolic biomarker in health and disease. As vitamin B5 is not routinely measured in clinical practice, these data provide essential reference values to support pharmacodynamic monitoring in the forthcoming B5forMDS clinical trial evaluating vitamin B5 as a non-toxic therapeutic strategy for patients with *SF3B1*-mutated MDS.

**Copyright Information:** (Online) ISSN: 2572-9241
©2026 The Author(s). HemaSphere is published by John Wiley & Sons Ltd on behalf of European Hematology Association. This is an open access Abstract Book distributed under the Attribution-NonCommercial-NoDerivs (CC BY-NC-ND), which allows third parties to download the articles and share them with others as long as they credit the author and the Abstract Book, but they cannot change the content in any way or use them commercially.
**Abstract Book Citations:** Authors, Title, HemaSphere, 2026;10:(S1):pages. The abstract book DOIs can be found on the "Author Information" page.
**Disclaimer:** Articles published in the journal HemaSphere exclusively reflect the opinions of the authors. The authors are responsible for all content in their abstracts including accuracy of the facts, statements, citing resources, etc.

### (PB3075) PERIPHERAL BLOOD NK CELLS AND LMR: INDEPENDENT IMMUNE PROGNOSTIC BIOMARKERS IN NEWLY DIAGNOSED MDS

**Topic:** 10. Myelodysplastic syndromes - Clinical

Tiantian Dai[1], Xiangli Chen*[2], Yijie Wang[3], Yaru Wang[3], Xiaoting Xu[3], Wenyi Lu[2], Pingfan Lu[2], Fei Ge[2], Xiaona Niu[2]

*[1]People's Hospital of Henan University, Department of Hematology, Zhengzhou, China;[2]Henan Provincial People's Hospital, Department of Hematology, Zhengzhou, China; [3]Henan Provincial People's Clinical Medical School of Zhengzhou University, Department of Hematology, Zhengzhou, China;*

### Background

The host immune-inflammatory status significantly impacts myelodysplastic syndromes (MDS) outcomes but is not captured by the IPSS-R. Peripheral blood natural killer (NK) cell percentage and lymphocyte-to-monocyte ratio (LMR) reflect innate immunity and systemic inflammation, respectively.

### Aims

To evaluate the independent and additive prognostic value of NK cell percentage and LMR beyond IPSS-R in MDS.

### Methods

This retrospective study included 209 treatment-naïve MDS patients. IPSS-R was assessed. NK cell percentage (flow cytometry) and LMR (complete blood count) were measured at diagnosis. Patients were stratified by median values (NK: 8.79%; LMR: 5.79). Overall survival (OS) was analyzed using Kaplan-Meier and Cox regression, adjusting for age, sex, and IPSS-R.

### Results

The cohort had a median age of 65 years. After a median follow-up of 50.0 months, high NK cell percentage and high LMR were each associated with significantly superior OS (P<0.001 and P=0.009, respectively). In multivariate analysis, both high NK cell percentage (HR=0.116, 95% CI 0.057–0.234, P<0.001) and high LMR (HR=0.633, 95% CI 0.407–0.986, P=0.043) remained independent favorable prognostic factors for OS after adjusting for IPSS-R, age, and sex. Notably, a high NK cell percentage conferred a substantial survival benefit, with a median OS extended by 31.5 months compared to the low group. Age was inversely correlated with NK cell percentage (r=-0.342, P<0.001), suggesting a link to immunosenescence. The prognostic power of these immune markers was consistent across IPSS-R subgroups.

### Summary/Conclusion

Baseline peripheral blood NK cell percentage and LMR are potent, independent prognostic biomarkers in MDS, providing significant risk stratification beyond the IPSS-R. They quantify critical biological dimensions of host anti-tumor immunity and inflammatory state. Incorporating these readily available metrics can refine prognostic models, particularly for intermediate-risk patients, and inform personalized management strategies.

**Copyright Information:** (Online) ISSN: 2572-9241
©2026 The Author(s). HemaSphere is published by John Wiley & Sons Ltd on behalf of European Hematology Association. This is an open access Abstract Book distributed under the Attribution-NonCommercial-NoDerivs (CC BY-NC-ND), which allows third parties to download the articles and share them with others as long as they credit the author and the Abstract Book, but they cannot change the content in any way or use them commercially.
**Abstract Book Citations:** Authors, Title, HemaSphere, 2026;10:(S1):pages. The abstract book DOIs can be found on the "Author Information" page.
**Disclaimer:** Articles published in the journal HemaSphere exclusively reflect the opinions of the authors. The authors are responsible for all content in their abstracts including accuracy of the facts, statements, citing resources, etc.

### (PB3076) INTERNATIONAL MULTICENTER EXTERNAL VALIDATION OF THE JAKPOT PREDICTION RULE TO GUIDE JAK2 MUTATION TESTING IN PATIENTS WITH ERYTHROCYTOSIS

**Topic:** 10. Myelodysplastic syndromes - Clinical

Benjamin Chin-Yee*[1], Cristhiam Rojas Hernandez[2], Pierre Sujobert[3], Vincent Alcazer[3], Ilyès Benhalima[3], Pascale Flandrin Gresta[4], Shreyash Dalmia[5], Christopher Hillis[5], Mayte Martinez Garza[6], Mariam Shenouda[7], Cyrus Hsia[1], Alejandro Lazo-Langner[1]

*[1]Western University, London, Canada; [2]University of Texas MD Anderson Cancer Center, Houston, United States of America; [3]Hospices Civils de Lyon, Lyon, France; [4]Centre Hospitalier Universitaire de Saint-Étienne, Saint-Étienne, France; [5]Hamilton Health Sciences, Hamilton, Canada; [6]Instituto Tecnológico de Monterrey, Monterrey, Mexico; [7]University of Toronto, Toronto, Canada;*

### Background

Erythrocytosis is a common reason for referral to hematology, frequently prompting *JAK2* mutation testing to exclude polycythemia vera. The JAKPOT study (Chin-Yee et al. *EHA* 2022; *JGIM* 2023) derived and validated a simple complete blood count (CBC)-based prediction rule to exclude *JAK2* mutation positivity with a high negative predictive value (NPV) in patients with elevated hemoglobin.

### Aims

To conduct a multicenter external validation of the JAKPOT rule and evaluate its diagnostic performance across independent cohorts.

### Methods

We conducted a retrospective validation study across 5 centres in Canada (London Health Sciences Centre [LHSC], Hamilton Health Sciences [HHS]), France (Hospices Civils de Lyon [HCL], Centre Hospitalier Universitaire de Saint-Étienne [CHU-STE]), and the US (MD Anderson Cancer Centre [MDACC]). The LHSC cohort comprised patients evaluated after the original JAKPOT study. Inclusion criteria were adults newly referred for erythrocytosis (hemoglobin ≥160 g/L in females; ≥165 g/L in males) who underwent *JAK2* testing by NGS or targeted PCR. *JAK2* positivity was defined as presence of *JAK2 V617F* or exon 12 mutations with variant allele frequency (VAF)≥1%; cases with indeterminate results or VAF<1% were excluded. The JAKPOT rule was applied using CBC parameters from the initial visit: patients meeting any one of three criteria (RBC>$6.45\times10^{12}$/L, platelets>$350\times10^{9}$/L, or neutrophils>$6.2\times10^{9}$/L) were classified JAKPOT-positive; those meeting none were classified JAKPOT-negative. Sensitivity, specificity, NPV, PPV, false negative rate (FNR), and potential reduction in *JAK2* testing were calculated per cohort and for pooled datasets. The primary analysis included pooled results for treatment-naïve patients (no prior cytoreductive therapy or phlebotomy) across all centres. Secondary analyses included per-center performance and, at MDACC, a pre-specified analysis in previously treated patients.

**Copyright Information:** (Online) ISSN: 2572-9241
©2026 The Author(s). HemaSphere is published by John Wiley & Sons Ltd on behalf of European Hematology Association. This is an open access Abstract Book distributed under the Attribution-NonCommercial-NoDerivs (CC BY-NC-ND), which allows third parties to download the articles and share them with others as long as they credit the author and the Abstract Book, but they cannot change the content in any way or use them commercially.
**Abstract Book Citations:** Authors, Title, HemaSphere, 2026;10:(S1):pages. The abstract book DOIs can be found on the “Author Information” page.
**Disclaimer:** Articles published in the journal HemaSphere exclusively reflect the opinions of the authors. The authors are responsible for all content in their abstracts including accuracy of the facts, statements, citing resources, etc.

### Results

The LHSC, HCL, CHU-STE, and MDACC cohorts included 207 (66.7% male), 504 (82.5% male), 137 (83.9% male), and 75 patients (70.7% male), with *JAK2* mutation prevalence of 21.3%, 10.7%, 10.2%, and 33.3%, respectively. At MDACC, 44 patients (58.7%) were treatment-naïve; 31 (41.3%) had received prior cytoreductive therapy or phlebotomy. In the primary analysis, among pooled treatment-naïve patients from LHSC, HCL, CHU-STE, and MDACC (n=892), the JAKPOT rule demonstrated sensitivity of 98.3%, NPV 99.6%, and FNR 1.7%, with potential to reduce *JAK2* testing by 60% (535 fewer tests). The JAKPOT rule demonstrated sensitivity of 97.7%, 98.1%, and 100%, NPV of 99.0%, 99.7%, and 100%, and potential testing reductions of 47.8%, 64.1%, and 60.6% at LHSC, HCL, and CHU-STE, respectively. In the MDACC treatment-naïve subgroup (n=44), sensitivity was 100% and NPV 100%. In previously treated patients at MDACC (n=31), sensitivity was 83.3% and NPV 62.5%. Data from the HHS cohort will be presented at EHA.

### Summary/Conclusion

The JAKPOT rule demonstrated consistently high sensitivity and NPV in treatment-naïve patients across 4 independent cohorts in 3 countries. This simple rule, requiring no additional investigations beyond the CBC, could safely guide *JAK2* mutation testing in newly referred, treatment-naïve patients, reducing low-yield *JAK2* testing by approximately 60%. NPV is lower in previously treated patients where the rule should be applied with caution, and clinical judgment should always guide final testing decisions. Prospective validation is currently underway.

**Table 1A. Patient characteristics.**

| Characteristic | LHSC | HCL | CHU-STE | MDACC (overall) | MDACC (treatment-naïve) | MDACC (previously treated) | Pooled treatment-naïve# |
|---|---|---|---|---|---|---|---|
| N | 207 | 504 | 137 | 75 | 44 | 31 | 892 |
| Male, n (%) | 138 (66.7%) | 416 (82.5%) | 115 (83.9%) | 53 (70.7%) | 30 (68.2%) | 23 (74.2%) | 699 (78.4%) |
| Age, median (IQR), years | 61.7 (52.1–71.5) | 61.0 (58.0–73.0) | 65.0 (54.0–75.0) | 59.9 (48.9–67.5) | 57.3 (48.9–66.1) | 61.2 (51.1–68.9) | 61.7 (51.0–72.8) |
| **Laboratory parameters at first visit** | | | | | | | |
| Hemoglobin, mean (SD), g/L | 178.9 (13.2) | 177.2 (10.4) | 179.1 (12.1) | 175.5 (11.3) | 176.0 (10.6) | 174.9 (12.2) | 177.8 (11.4) |
| Hematocrit, mean (SD), L/L | 0.536 (0.043) | 0.522 (0.038) | 0.524 (0.040) | 0.517 (0.038) | 0.511 (0.036) | 0.526 (0.039) | 0.525 (0.040) |
| RBC, mean (SD), $\times 10^{12}$/L | 5.85 (0.99) | 5.84 (0.58) | 5.90 (0.59) | 5.94 (0.83) | 5.75 (0.60) | 6.20 (1.02) | 5.85 (0.70) |
| Platelets, mean (SD), $\times 10^{9}$/L | 299 (183) | 264 (145) | 265 (112) | 288 (191) | 261 (200) | 326 (175) | 272 (154) |
| Leukocytes, mean (SD), $\times 10^{9}$/L | 9.4 (5.1) | 8.3 (3.2) | 8.3 (2.4) | 11.6 (13.5) | 10.7 (16.3) | 12.8 (8.4) | 8.7 (5.1) |
| Neutrophils, mean (SD), $\times 10^{9}$/L | 6.1 (3.3) | 5.3 (2.4) | 5.4 (2.3) | 7.5 (9.3) | 6.7 (11.1) | 8.7 (5.9) | 5.6 (3.6) |
| MCV, mean (SD), fL | 90.4 (6.7) | 89.6 (5.6) | 89.3 (5.3) | N/A | N/A | N/A | 89.7 (5.8) [n=844] |
| *JAK2**-positive, n (%) | 44 (21.3%) | 54 (10.7%) | 14 (10.2%) | 25 (33.3%) | 7 (15.9%) | 18 (58.1%) | 119 (13.3%) |

*Includes *JAK2 V617F* and exon 12 mutations with VAF ≥1%. N/A, not available. #Pooled LHSC, HCL, CHU-STE, and MDACC treatment-naïve cohorts (primary analysis cohort).

**Table 1B. Diagnostic performance of the JAKPOT prediction rule.**

| | LHSC | HCL | CHU-STE | MDACC (treatment-naïve) | MDACC (previously treated) | Pooled treatment-naïve* |
|---|---|---|---|---|---|---|
| N | 207 | 504 | 137 | 44 | 31 | 892 |
| *JAK2*-positive prevalence, % | 21.3 | 10.7 | 10.2 | 15.9 | 58.1 | 13.3 |
| **Diagnostic performance** | | | | | | |
| Sensitivity, % | 97.7 | 98.1 | 100 | 100 | 83.3 | 98.3 |
| Specificity, % | 60.1 | 71.6 | 67.5 | 81.1 | 38.5 | 69.0 |
| NPV, % | 99.0 | 99.7 | 100 | 100 | 62.5 | 99.6 |
| PPV, % | 39.8 | 29.3 | 25.9 | 50.0 | 65.2 | 32.8 |
| False negative rate, % | 2.3 | 1.9 | 0 | 0.0 | 16.7 | 1.7 |
| Testing reduction, % (n) | 47.8% (99) | 64.1% (323) | 60.6% (83) | 68.2% (30) | 25.8% (8) | 60.0% (535) |

*Pooled LHSC, HCL, CHU-STE, and MDACC treatment-naïve cohorts (primary analysis cohort). NPV, negative predictive value; PPV, positive predictive value; LHSC, London Health Sciences Centre; HCL, Hospices Civils de Lyon; CHU-STE, Centre Hospitalier Universitaire de Saint-Étienne; MDACC, MD Anderson Cancer Centre.

Table 1A. Patient characteristics. Table 1B. Diagnostic performance of the JAKPOT prediction rule.

**Copyright Information:** (Online) ISSN: 2572-9241
©2026 The Author(s). HemaSphere is published by John Wiley & Sons Ltd on behalf of European Hematology Association. This is an open access Abstract Book distributed under the Attribution-NonCommercial-NoDerivs (CC BY-NC-ND), which allows third parties to download the articles and share them with others as long as they credit the author and the Abstract Book, but they cannot change the content in any way or use them commercially.
**Abstract Book Citations:** Authors, Title, HemaSphere, 2026;10:(S1):pages. The abstract book DOIs can be found on the "Author Information" page.
**Disclaimer:** Articles published in the journal HemaSphere exclusively reflect the opinions of the authors. The authors are responsible for all content in their abstracts including accuracy of the facts, statements, citing resources, etc.

### (PB3077) A PROGNOSTIC NOMOGRAM FOR SURVIVAL STRATIFICATION IN MYELODYSPLASTIC NEOPLASMS FOLLOWING ALLOGENEIC STEM CELL TRANSPLANTATION

**Topic:** 10. Myelodysplastic syndromes - Clinical

Kaili Min*[1, 2], Lei Xu[1], Xu Zhang[3], Yalei Hu[1, 2], Fei Li[4], Liangding Hu[1], Chun-Ji Gao[1], Dai-Hong Liu[4], Yao Sun[3], Xiao-Ning Gao[1]

*[1]Department of Hematology, The Fifth Medical Center of Chinese PLA General Hospital, Beijing, China;[2]Medical School of Chinese PLA, Beijing, China; [3]The Fifth Medical Center of Chinese PLA General Hospital, Beijing, China;[4]The First Medical Center of Chinese PLA General Hospital, Beijing, China;*

### Background

Allogeneic hematopoietic stem cell transplantation (allo-HSCT) remains the only curative potential treatment for myelodysplastic neoplasms (MDS). The Molecular International Prognostic Scoring System (IPSS-M) has advanced risk stratification by incorporating molecular, cytogenetic, and clinical data. However, its clinical utility is limited by inherent complexity. Additionally, it is not designed to predict outcomes specifically in the transplant setting. Although transplant-specific prognostic models have been developed, they are primarily derived from non-Chinese populations and leave room for further optimization in discriminative accuracy.

### Aims

This study aims to develop and validate a novel, transplant-specific prognostic model for Chinese MDS patients undergoing allo-HSCT, providing a more refined tool for outcome prediction and post-transplant management.

### Methods

This retrospective study enrolled consecutive patients with MDS who underwent allo-HSCT at the Senior Department of Hematology, Medical Center of PLA General Hospital, between 2014 and 2024. Eligible patients were required to have complete transplant-essential data available, and those with evidence of transformation to acute myeloid leukemia before transplantation were excluded. Overall survival (OS) was defined as the time from the first day of transplantation until death from any cause; survivors were censored at the last follow-up before the data-cutoff date of June 20, 2025. Prognostic predictors were first screened using the least absolute shrinkage and selection operator (LASSO) regression method, followed by the construction of a Cox proportional hazards regression model for multivariable analysis. Internal validation was performed using 1,000 bootstrap resamples to evaluate optimism-corrected discrimination and calibration. A nomogram was constructed, and patients were stratified into **risk tertiles** based on total points. Written informed consent was obtained from all enrolled patients.

### Results

The development set included 214 consecutive patients with a median age of 46.5 years. 61.7% were male, and most patients (53.7%) were classified as very-high risk by IPSS-M, 66.8% had received hypomethylating agents during conditioning. Median follow-up was 56.3 months. LASSO regression identified five independent prognostic factors for OS: age, ECOG performance status, *TP53* mutation status, gender and the use of hypomethylating agents in conditioning. The factors were used to build a multivariable Cox model, presented as a nomogram. The concordance index of the model was 0.704 (95% CI: 0.652-0.756), with an optimism-corrected concordance index of 0.691. The overall calibration slope was 0.910. The 1-year calibration curve aligned with the 45-degree reference line, whereas the 3- and 5-year curves positioned below the reference line, with a larger downward deviation at 5 years.The areas under the time-dependent receiver operating characteristic curves for predicting 1-, 3-, and 5-year overall survival were 0.707, 0.776, and 0.782, respectively. Decision curve analysis indicated clinical net benefit between threshold probabilities of 0.20 and 0.60. Overall survival differed significantly among the three risk groups stratified by total points ($P < 0.001$).

**Copyright Information:** (Online) ISSN: 2572-9241
©2026 The Author(s). HemaSphere is published by John Wiley & Sons Ltd on behalf of European Hematology Association. This is an open access Abstract Book distributed under the Attribution-NonCommercial-NoDerivs (CC BY-NC-ND), which allows third parties to download the articles and share them with others as long as they credit the author and the Abstract Book, but they cannot change the content in any way or use them commercially.
**Abstract Book Citations:** Authors, Title, HemaSphere, 2026;10:(S1):pages. The abstract book DOIs can be found on the "Author Information" page.
**Disclaimer:** Articles published in the journal HemaSphere exclusively reflect the opinions of the authors. The authors are responsible for all content in their abstracts including accuracy of the facts, statements, citing resources, etc.

### Summary/Conclusion

The nomogram provides a quantitative reference for survival stratification. While long-term outcomes are overestimated, the model may inform risk-adapted clinical decisions.

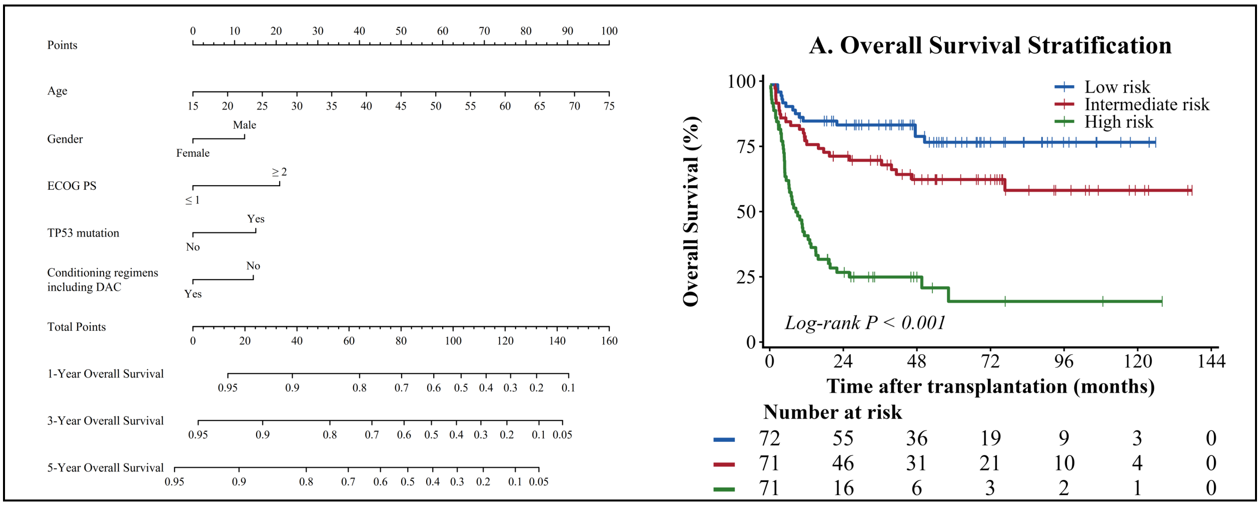

**Copyright Information:** (Online) ISSN: 2572-9241
©2026 The Author(s). HemaSphere is published by John Wiley & Sons Ltd on behalf of European Hematology Association. This is an open access Abstract Book distributed under the Attribution-NonCommercial-NoDerivs (CC BY-NC-ND), which allows third parties to download the articles and share them with others as long as they credit the author and the Abstract Book, but they cannot change the content in any way or use them commercially.
**Abstract Book Citations:** Authors, Title, HemaSphere, 2026;10:(S1):pages. The abstract book DOIs can be found on the "Author Information" page.
**Disclaimer:** Articles published in the journal HemaSphere exclusively reflect the opinions of the authors. The authors are responsible for all content in their abstracts including accuracy of the facts, statements, citing resources, etc.

### (PB3078) EFFICACY AND SAFETY OF TISLELIZUMAB COMBINED WITH AZACITIDINE AND VENETOCLAX IN MYELODYSPLASTIC NEOPLASMS WITH INCREASED BLASTS (MDS-IB): A SINGLE-CENTER RETROSPECTIVE STUDY

**Topic:** 10. Myelodysplastic syndromes - Clinical

Zi-Yuan Nie*[1], Zhipeng Xu[2], Liwei Fang[3]

*[1]Department of Hematology, The Second Hospital of Hebei Medical University, Shijiazhuang, China;[2]The Second People's Hospital of Datong, Datong, China; [3]State Key Laboratory of Experimental Hematology, National Clinical Research Center for Blood Diseases, Haihe Laboratory of Cell Ecosystem, Institute of Hematology & Blood Diseases Hospital, Chinese Academy of Medical Sciences & Peking Union Medical College, Tianjin, China;*

### Background

Myelodysplastic neoplasms with increased blasts (MDS-IB) are associated with poor prognosis, and current therapies, including the combination of hypomethylating agents with venetoclax (VA regimen), remain suboptimal. Preclinical and clinical evidence suggests that PD-1/PD-L1 blockade may enhance hematopoiesis in MDS and synergize with HMAs. However, data on PD-1 inhibitors combined with the VA backbone in MDS-IB are limited.

### Aims

This study aims to preliminarily evaluate the efficacy and safety of tislelizumab combined with azacitidine and venetoclax (TVA regimen) in patients with MDS-IB.

### Methods

We retrospectively analyzed 7 patients with MDS-IB who received the TVA regimen, consisting of tislelizumab (200 mg on day 1), azacitidine (75 mg/m² on days 1-7), and venetoclax (400 mg on days 1-14). Cycles were repeated every 28-42 days until allogeneic hematopoietic stem cell transplantation or disease progression. Responses were assessed according to IWG 2023 criteria. Primary endpoints included complete remission (CR) rate and overall survival (OS).

### Results

The median patient age was 54 years. Among the 7 patients, 3 had MDS-IB1, 4 had MDS-IB2, and 2 had MDS with biallelic TP53 inactivation. The median bone marrow blast count was 10.5% (range, 5-19%). According to IPSS-R, 6 patients were classified as high-risk and 1 as very high-risk. The median number of gene mutations per patient was 3 (range, 1-6). After one cycle of treatment, the composite complete remission rate was 58% (4/7), with CR and CRL rates of 29% (2/7) each. With a median follow-up of 26 months (range, 3-42 months), the median OS was 26 months (95% CI: 8.7-43.3 months). The 1-year, 2-year, and 3-year OS rates were 85.7% (95% CI: 42.1%-99.6%), 57.1% (95% CI: 18.4%-90.1%), and 42.9% (95% CI: 9.9%-81.6%), respectively. The regimen was generally well-tolerated, with grade ≥3 hematologic toxicities being the most common adverse events (occurring in 5 patients, 71.4%).

### Summary/Conclusion

These preliminary findings suggest that the TVA triplet regimen exhibits promising efficacy and a manageable safety profile in patients with MDS-IB. Compared to historical outcomes with the VA doublet, this regimen may offer improved CR rates and OS in this high-risk population, warranting further prospective validation.

**Copyright Information:** (Online) ISSN: 2572-9241
©2026 The Author(s). HemaSphere is published by John Wiley & Sons Ltd on behalf of European Hematology Association. This is an open access Abstract Book distributed under the Attribution-NonCommercial-NoDerivs (CC BY-NC-ND), which allows third parties to download the articles and share them with others as long as they credit the author and the Abstract Book, but they cannot change the content in any way or use them commercially.
**Abstract Book Citations:** Authors, Title, HemaSphere, 2026;10:(S1):pages. The abstract book DOIs can be found on the "Author Information" page.
**Disclaimer:** Articles published in the journal HemaSphere exclusively reflect the opinions of the authors. The authors are responsible for all content in their abstracts including accuracy of the facts, statements, citing resources, etc.

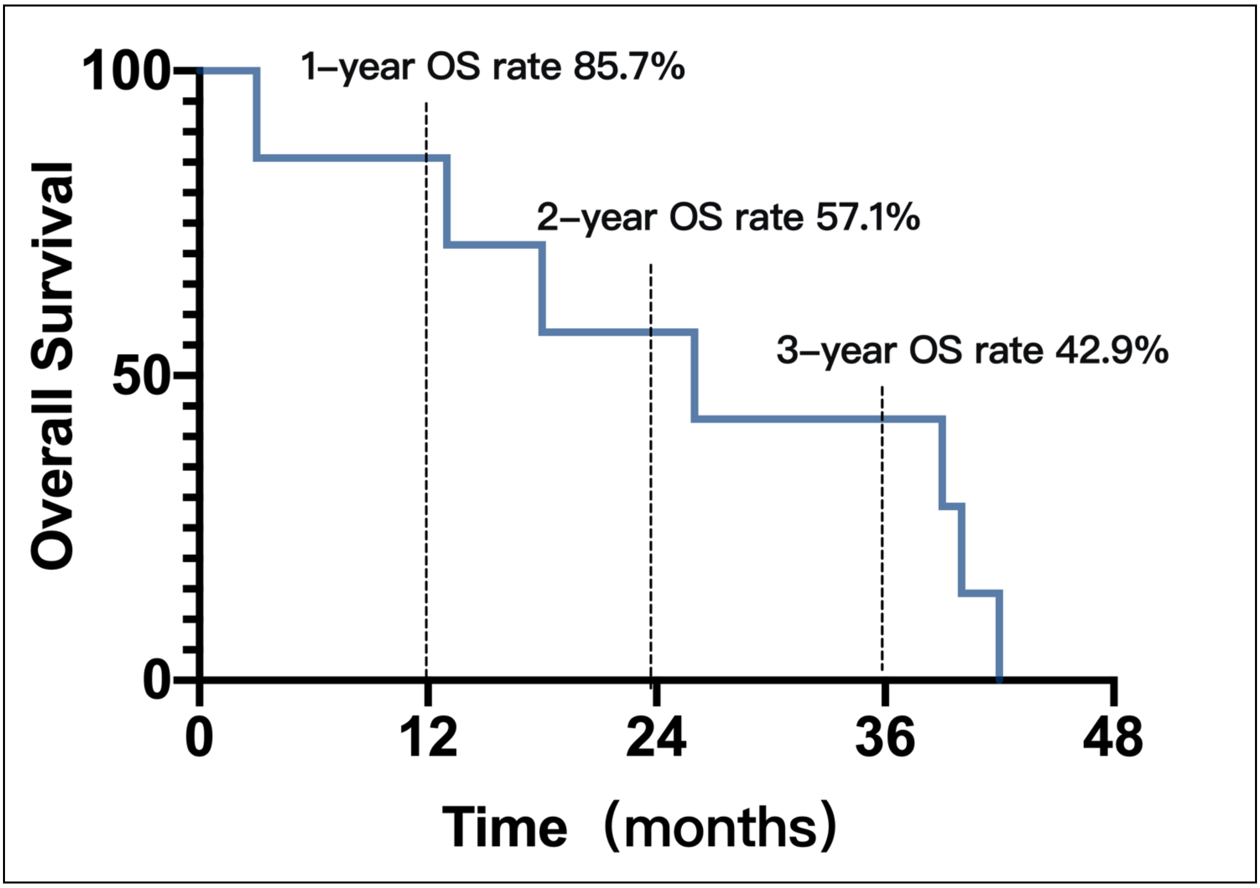

**Copyright Information:** (Online) ISSN: 2572-9241
©2026 The Author(s). HemaSphere is published by John Wiley & Sons Ltd on behalf of European Hematology Association. This is an open access Abstract Book distributed under the Attribution-NonCommercial-NoDerivs (CC BY-NC-ND), which allows third parties to download the articles and share them with others as long as they credit the author and the Abstract Book, but they cannot change the content in any way or use them commercially.
**Abstract Book Citations:** Authors, Title, HemaSphere, 2026;10:(S1):pages. The abstract book DOIs can be found on the "Author Information" page.
**Disclaimer:** Articles published in the journal HemaSphere exclusively reflect the opinions of the authors. The authors are responsible for all content in their abstracts including accuracy of the facts, statements, citing resources, etc.

### (PB3079) A PRECISE DIFFERENTIAL DIAGNOSTIC MODEL CONSTRUCTED BASED ON COMPARATIVE ANALYSIS OF CLINICAL CHARACTERISTICS BETWEEN APLASTIC ANEMIA AND MYELODYSPLASTIC SYNDROMES PATIENTS

**Topic:** 10. Myelodysplastic syndromes - Clinical

Lei Huang[1], Yinxing Wang[1], Xiangyu Li[2], Xintong Xu[1], Ziqi Zhang[1], Xinru Wu[1], Lele Ou[1], Shengxuan Zhu[1], Chunyan Liu[1], Rong Fu*[1]

*[1]Tianjin Medical University General Hospital, Tianjin, China;[2]Tianjin Medical University, Tianjin, China;*

### Background

Both aplastic anemia (AA) and myelodysplastic syndromes (MDS) patients may experience with cytopenia. However, their differential diagnosis remains challenging.

### Aims

We aim to establish a predictive differential diagnostic model based on the differential indicators between AA and MDS patients, thereby providing clinical evidence for the differential diagnosis of AA and lower-risk MDS patients.

### Methods

The clinical characteristics of 154 AA patients and 218 MDS patients newly diagnosed between January 2019 and December 2023 were analyzed in our study. MDS patients were stratified into a lower-risk MDS group (IPSS-R ≤ 3.5, n=60) and a higher-risk MDS group (IPSS-R > 3.5, n=158) according to IPSS-R score.

### Results

AA patients were younger with better performance status. Significant differences in triglyceride and peripheral blood cells were observed between AA and MDS patients. Furthermore, AA patients manifested as significant decreased hemopoietic cells, whereas MDS patients displayed significantly increased immature cells and ring sideroblasts. Compared with AA patients, the ratio of mDC1/mDC in lower-risk MDS patients was significantly increased, while the level of C3, the ratio of $CD4^{+}/CD8^{+}T$, mDC/DC and $CD16^{+}DC/mDC$ were significantly decreased. Meanwhile, in higher-risk MDS patients, the proportion of $CD16^{+}CD56^{+}NK$ and the ratio of mDC1/mDC were significantly increased, while the level of C3, the ratio of mDC/DC and $CD16^{+}DC/mDC$ were significantly decreased. There were significant differences in gene mutations and chromosomal abnormalities between AA and MDS patients. AA patients frequently harbored gene mutations such as BCOR/BCORL1, ASXL1, DNMT3A, and PIGA, while MDS patients commonly carried gene mutations such as ASXL1, SF3B1, TET2, TP53, U2AF1, and DNMT3A. The most common chromosomal abnormalities in AA patients are +8, -Y and 20q-, while lower-risk MDS patients are +8, 5q-, 20q-, while higher-risk MDS patients are +8, 5q-, 7q-, -7, 20q- and -5. By screening the indicators that are significantly different between AA and lower-risk MDS patients, a precise diagnostic model for differentiating AA and lower-risk MDS patients was established through Logistic regression analysis: Logit(P)=-6.3345+0.0597*age +0.4896*Neutrophil-1.5972*Lymphocyte+0.0280*PLT+0.0201*sternal megakaryocytes.Through internal data validation, the AUC values were all above 0.9, indicating good discriminative performance of the model.

**Copyright Information:** (Online) ISSN: 2572-9241
©2026 The Author(s). HemaSphere is published by John Wiley & Sons Ltd on behalf of European Hematology Association. This is an open access Abstract Book distributed under the Attribution-NonCommercial-NoDerivs (CC BY-NC-ND), which allows third parties to download the articles and share them with others as long as they credit the author and the Abstract Book, but they cannot change the content in any way or use them commercially.
**Abstract Book Citations:** Authors, Title, HemaSphere, 2026;10:(S1):pages. The abstract book DOIs can be found on the "Author Information" page.
**Disclaimer:** Articles published in the journal HemaSphere exclusively reflect the opinions of the authors. The authors are responsible for all content in their abstracts including accuracy of the facts, statements, citing resources, etc.

### Summary/Conclusion

In conclusion, significant differences between AA and MDS patients have shown in bone marrow hematopoiesis, immune functions, gene mutations and chromosomes. And the differential diagnostic model constructed based on age, the counts of neutrophils, lymphocytes, platelets and sternum megakaryocytes can effectively distinguish AA and lower-risk MDS patients.

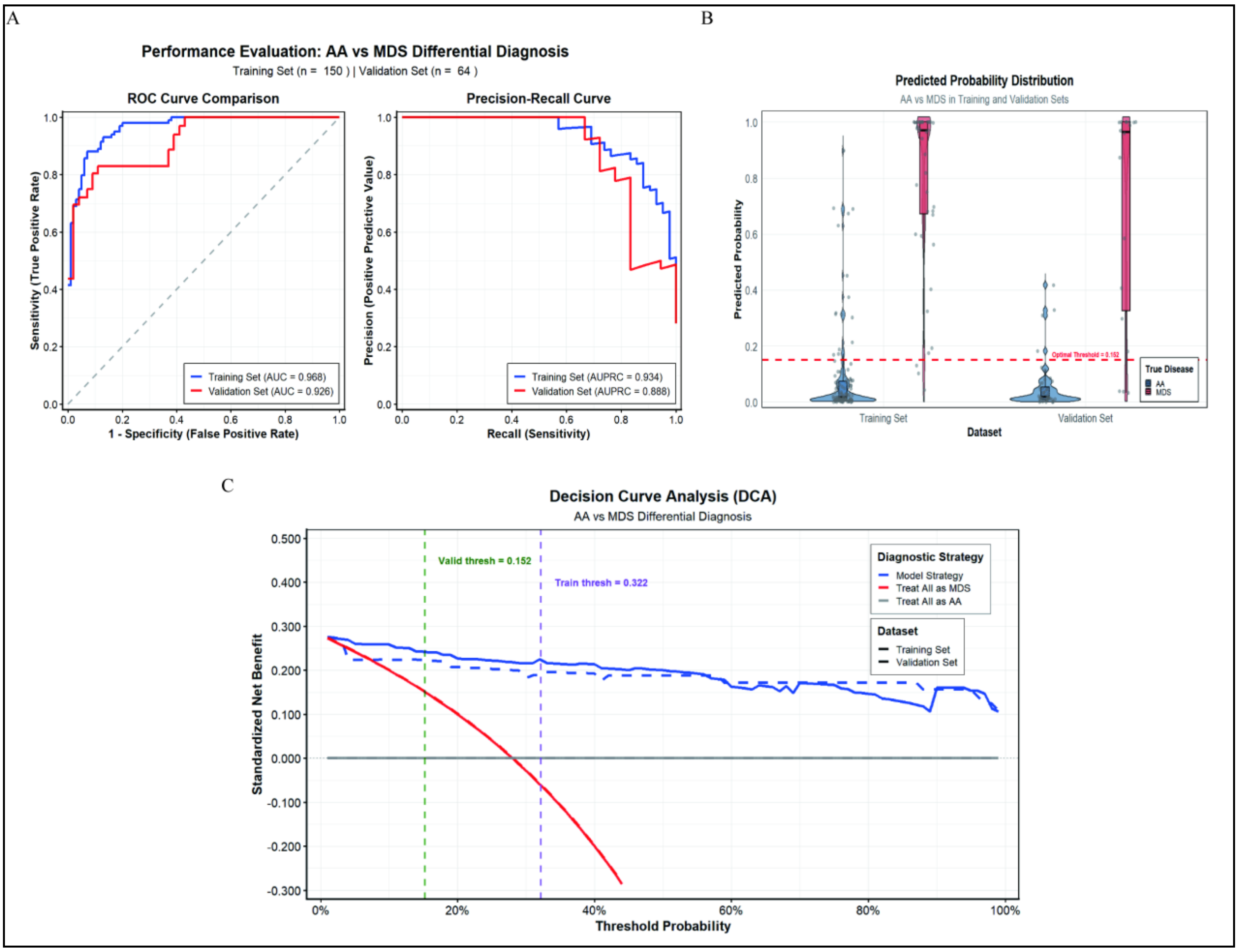

**Copyright Information:** (Online) ISSN: 2572-9241
©2026 The Author(s). HemaSphere is published by John Wiley & Sons Ltd on behalf of European Hematology Association. This is an open access Abstract Book distributed under the Attribution-NonCommercial-NoDerivs (CC BY-NC-ND), which allows third parties to download the articles and share them with others as long as they credit the author and the Abstract Book, but they cannot change the content in any way or use them commercially.
**Abstract Book Citations:** Authors, Title, HemaSphere, 2026;10:(S1):pages. The abstract book DOIs can be found on the "Author Information" page.
**Disclaimer:** Articles published in the journal HemaSphere exclusively reflect the opinions of the authors. The authors are responsible for all content in their abstracts including accuracy of the facts, statements, citing resources, etc.

### (PB3080) CHARACTERISTICS OF PATIENTS WITH MDS AND MARROW FIBROSIS: SINGLE INSTITUTION EXPERIENCE

**Topic:** 10. Myelodysplastic syndromes - Clinical

Ahmed Shaban*[1], Mohamad Sheikh Najeeb[1], Dragan Jevremovic[1], Patricia Greipp[1], Rong He[1], David Viswanatha[1], Kathy Alvarez-Wurderman[1], Aasiya Matin[1], Mehrdad Hefazi[1], Naseema Gangat[1], Abhishek Mangaonkar[1], William Hogan[1], Kebede Begna[1], Mark Litzow[1], Mrinal Patnaik[1], Antoine Saliba[1], Mithun Shah[1], Hassan Alkhateeb[1], Tariq Kewan[1], Aref Al-Kali[1]

[1]*Mayo Clinic, Hematology, Rochester, United States of America;*

### Background

Bone marrow fibrosis has been identified in up to 10–30% of myelodysplastic syndrome (MDS) patients and is associated with higher-risk features, lower overall survival and increased transfusion dependence. Recent studies have demonstrated that fibrotic MDS patients exhibit higher rates of adverse cytogenetics and a greater risk for leukemic transformation compared with non-fibrotic patients.

### Aims

To compare prognostic factors and survival outcomes between fibrotic and non-fibrotic MDS patients, focusing on clinical and cytogenic determinants of prognosis.

### Methods

After obtaining IRB approval, we retrospectively reviewed patients diagnosed with MDS (2016 – 2024) who had next-generation sequencing (NGS) at diagnosis. Clinical data, baseline hematologic parameters, bone marrow characteristics (cellularity, blasts, ring sideroblasts), cytogenetics, and molecular findings were collected. Patients were classified as fibrotic (1-3) or non-fibrotic based on bone marrow biopsy at diagnosis. Fibrotic MDS cases meeting WHO definition were MDS-f.

### Results

A total of 300 patients with MDS were included (median age 74 years, 63.7% male), of whom 68 (23%) were identified as fibrotic MDS, only 8 (2.7%) patients met the WHO definition MDS-f. At baseline, hematologic parameters in fibrotic versus non-fibrotic MDS were as follows: hemoglobin 8.9 vs 9.6 g/dL ($p = 0.08$), mean corpuscular volume 97 vs 101 fL ($p = 0.004$), and platelet count 115 vs 123 $\times 10^9$/L ($p = 0.63$). LDH levels were higher in fibrotic group (263.5 vs 224.5, p= 0.008). Bone marrow biopsy demonstrated significantly higher cellularity in the fibrotic group (73.6% vs 55.6%, $p = 0.001$), whereas ring sideroblasts (6.6% vs 8.1%, $p = 0.4$) and bone marrow blasts (5.2% vs 5.4%, $p = 0.7$) were similar between groups. Abnormal cytogenetics were identified in 55% of patients overall, with similar rates in the fibrotic and non-fibrotic groups (54.4% vs 55.2%, $p = 0.9$). Genetic mutations were detected in 251 (83.7%) patients with no differences between the groups (87.5% vs 80% vs 84.5, P= 0.67). *SRSF2* was the most common mutation among MDS-f group (3 cases), followed by *DNMT3* and *ASXL1* (2 cases each). While *ASXL1* was the most common mutation (12 cases) followed by *TP53* (11 cases) then *TET2* and *SRSF2* (9 cases each) in fibrotic MDS group. In non-fibrotic group the most common mutation was *ASXL1* (50 cases) followed by *TP53* and *TET2* (48 cases each). After a median follow-up of 64 months, 193 patients (64%) had deceased. Median OS did not differ significantly between patients with fibrotic and non-fibrotic MDS (21 vs 30.6 months, $p = 0.3$). However, lower survival rate was detected in the MDS-f group compared with fibrotic MDS and non-fibrotic MDS (18.3 vs 24.4 vs 30.6 months, $p = 0.58$). Progression to AML differed significantly among the three groups (62.5% vs 25% vs 23.7%, $p = 0.04$). Platelet transfusion dependence also varied significantly across groups (20% vs 38.3% vs 24.1%, $p = 0.03$). RBC transfusion dependence did not differ significantly among the three groups ($p = 0.06$). Response to therapy was similar across groups (33.3% vs 45.2% vs 44.6%, $p = 0.08$).

**Copyright Information:** (Online) ISSN: 2572-9241
©2026 The Author(s). HemaSphere is published by John Wiley & Sons Ltd on behalf of European Hematology Association. This is an open access Abstract Book distributed under the Attribution-NonCommercial-NoDerivs (CC BY-NC-ND), which allows third parties to download the articles and share them with others as long as they credit the author and the Abstract Book, but they cannot change the content in any way or use them commercially.
**Abstract Book Citations:** Authors, Title, HemaSphere, 2026;10:(S1):pages. The abstract book DOIs can be found on the "Author Information" page.
**Disclaimer:** Articles published in the journal HemaSphere exclusively reflect the opinions of the authors. The authors are responsible for all content in their abstracts including accuracy of the facts, statements, citing resources, etc.

**Summary/Conclusion**

Bone marrow fibrosis in MDS is associated with higher marrow cellularity, lower MCV but did not significantly impact overall survival or treatment response, with various distinct patterns in leukemic transformation risk and transfusion dependence, in this cohort. A larger study is needed to confirm our findings.

**Copyright Information:** (Online) ISSN: 2572-9241
©2026 The Author(s). HemaSphere is published by John Wiley & Sons Ltd on behalf of European Hematology Association. This is an open access Abstract Book distributed under the Attribution-NonCommercial-NoDerivs (CC BY-NC-ND), which allows third parties to download the articles and share them with others as long as they credit the author and the Abstract Book, but they cannot change the content in any way or use them commercially.
**Abstract Book Citations:** Authors, Title, HemaSphere, 2026;10:(S1):pages. The abstract book DOIs can be found on the "Author Information" page.
**Disclaimer:** Articles published in the journal HemaSphere exclusively reflect the opinions of the authors. The authors are responsible for all content in their abstracts including accuracy of the facts, statements, citing resources, etc.

### (PB3081) EFFICACY AND SAFETY OF LISAFTOCLAX COMBINED WITH AZACITIDINE IN TREATMENT OF PATIENTS WITH MYELODYSPLASTIC SYNDROME: A MULTICENTER RETROSPECTIVE STUDY

**Topic:** 10. Myelodysplastic syndromes - Clinical

Mengying Gao[1], Lina Hu[1], Huijun Li[1], Jing Luo[1], Xiaohua He[1], Peng Ke[1], Guangyang Weng[2], Suyun Wang[3], Qiuxia Wan[3], Heng Wang[3], Jihao Zhou*[1]

*[1]Shenzhen People's Hospital, Department of Hematology, Shenzhen, China;[2]Shenzhen Second People's Hospital, Department of Hematology, Shenzhen, China; [3]Shenzhen Longhua District Central Hospital, Department of Hematology, Shenzhen, China;*

### Background

Myelodysplastic Syndrome (MDS) is a clonal hematopoietic disorder with poor prognosis. B-cell lymphoma 2 (*BCL2*) serves as a pivotal regulator of apoptotic pathways and is aberrantly overexpressed across diverse hematological malignancies. In a phase 1b study in high-risk MDS patients, the BCL2 inhibitor venetoclax plus azacitidine (AZA) induced a complete remission (CR) rate of 29.9%, and a modified OR (mOR) rate of 80.4%. However, in the subsequent phase 3 VERONA study, venetoclax plus AZA (VA) failed to improve overall survival (OS) compared to placebo plus AZA in newly diagnosed high-risk MDS patients. Lisaftoclax is a new oral BCL2 selective inhibitor. In a phase 1b study, lisaftoclax plus AZA (LA) induced a CR rate of 29% and a ORR rate of 64% in high-risk MDS patients. But the efficacy and safety of LA for MDS patients have not been evaluated in real-world practice.

### Aims

To retrospectively observe the efficacy and safety of LA for MDS patients in the real-world practice.

### Methods

Patients with intermediate to high-risk MDS, including newly-diagnosed or relapsed/refractory disease, were enrolled. LA was administered as lisaftoclax once daily orally from days 1 to 14 and AZA (75 mg/m2/day) from days 1 to 7 in repeated 28-day cycles.

### Results

A total of 10 MDS patients were enrolled. 5 patients were newly diagnosed, the other 5 were relapsed or refractory to first-line treatment. 3 patients had *TP53* mutation. The median age is 67 (40-75) years. Baseline clinical characteristics of enrolled patients were shown in table 1. Treatment response was evaluable in 10 patients. The ORR was 70% of the total cohort, with 60% in the first-line and 80% in the second-line. 3 patients achieved CR, 1 in the first-line and 2 in the second-line. Partial remission (PR) was observed in 2 patients in the first-line. 2 patients achieved hematologic improvement (HI) in the second-line. 1 patient died of severe pneumonia in the first-line. The median time to achieve initial treatment response is 1.8 months. In the 4 patients previously received VA treatment, 1 patient achieved CR by LA as second-line treatment, which indicates that lisaftoclx may overcome venetoclax resistance. The preliminary treatment response of the total cohort is as shown in figure 1. Reported adverse effects in our cohort were generally manageable under active supportive treatment.

### Summary/Conclusion

In our study, LA demonstrates promising therapeutic activity and acceptable toxicity in MDS patients in the real-world practice, in accordance with results reported in previous phase 1b Study. Considering that lisaftoclax exhibited similar efficacy but less toxicity than venetoclax, we believe LA regimen deserve further study in the field of MDS treatment.

**Copyright Information:** (Online) ISSN: 2572-9241
©2026 The Author(s). HemaSphere is published by John Wiley & Sons Ltd on behalf of European Hematology Association. This is an open access Abstract Book distributed under the Attribution-NonCommercial-NoDerivs (CC BY-NC-ND), which allows third parties to download the articles and share them with others as long as they credit the author and the Abstract Book, but they cannot change the content in any way or use them commercially.
**Abstract Book Citations:** Authors, Title, HemaSphere, 2026;10:(S1):pages. The abstract book DOIs can be found on the "Author Information" page.
**Disclaimer:** Articles published in the journal HemaSphere exclusively reflect the opinions of the authors. The authors are responsible for all content in their abstracts including accuracy of the facts, statements, citing resources, etc.

Table 1. Patients' Clinical Characteristics and Treatment Responses

| Variables | First-line (n=5) | Second-line (n=5) | Total (N=10) |
|---|---|---|---|
| Demographics | | | |
| Age, median (range), years | 65 (44-69) | 74 (40-75) | 67 (40-75) |
| Gender, male, n (%) | 2 (40.0%) | 5 (100.0%) | 7 (70.0%) |
| BM Blast, n (%) | | | |
| Median (range), % | 11.0 (1.5-19.0) | 4.9 (1.0-11.5) | 5.8 (1.0-19.0) |
| ≤5% | 1 (20.0%) | 4 (80.0%) | 5 (50.0%) |
| >5% to ≤10% | 1 (20.0%) | 0 (0.0%) | 1 (10.0%) |
| >10% | 3 (60.0%) | 1 (20.0%) | 4 (40.0%) |
| Molecular Profile, n (%) | | | |
| TP53 mutation | 3 (60.0%) | 0 (0.0%) | 3 (30.0%) |
| ASXL1 mutation | 0 (0.0%) | 3 (60.0%) | 3 (30.0%) |
| TET2 mutation | 1 (20.0%) | 3 (60.0%) | 4 (40.0%) |
| Clinical Efficacy, n (%) | | | |
| CR | 1 (20.0%) | 2 (40.0%) | 3 (30.0%) |
| PR | 2 (40.0%) | 0 (0.0%) | 2 (20.0%) |
| HI | 0 (0.0%) | 2 (40.0%) | 2 (20.0%) |
| ORR | 3 (60.0%) | 4 (80.0%) | 7 (70.0%) |

**Figure. Swimmer Plot of 10 MDS Patients Receiving Lisaftoclax combined with Azacitidine Treatment**

*Bars indicate duration of LA therapy. Sorted by treatment duration (longest to shortest).*
*X-axis: months from LA initiation. Data cutoff: February 26, 2026.*

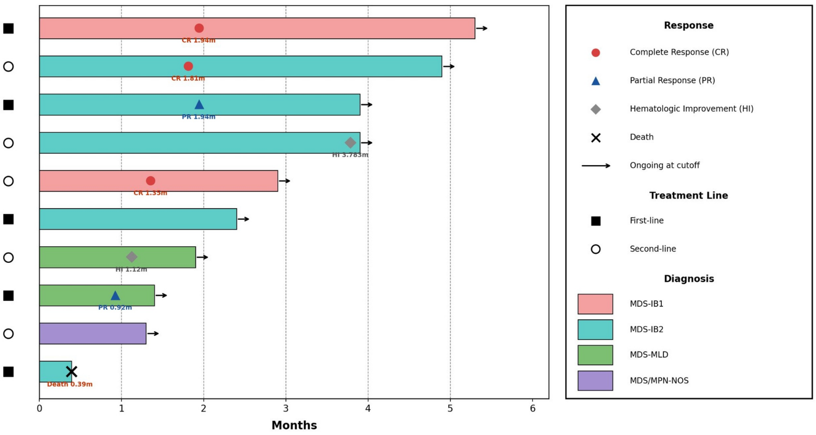

**Copyright Information:** (Online) ISSN: 2572-9241
©2026 The Author(s). HemaSphere is published by John Wiley & Sons Ltd on behalf of European Hematology Association. This is an open access Abstract Book distributed under the Attribution-NonCommercial-NoDerivs (CC BY-NC-ND), which allows third parties to download the articles and share them with others as long as they credit the author and the Abstract Book, but they cannot change the content in any way or use them commercially.
**Abstract Book Citations:** Authors, Title, HemaSphere, 2026;10:(S1):pages. The abstract book DOIs can be found on the "Author Information" page.
**Disclaimer:** Articles published in the journal HemaSphere exclusively reflect the opinions of the authors. The authors are responsible for all content in their abstracts including accuracy of the facts, statements, citing resources, etc.

### (PB3083) TOBACCO SMOKING IS ASSOCIATED WITH WORSE PROGNOSIS IN MYELODYSPLASTIC SYNDROMES PATIENTS - RESULTS FROM THE POLISH ADULT LEUKEMIA GROUP MDS REGISTRY

**Topic:** 10. Myelodysplastic syndromes - Clinical

Klaudia Ziółek*[1], Michał Dąbrowski[2], Paweł Szwedyk[3], Oktawia Sosnia[4], Anna Kopinska[5], Małgorzata Gajewska[6], Magdalena Olszewska-Szopa[7], Rafal MacHowicz[1], Karol Lis[1], Jagoda Rytel[1], Joanna Drozd-Sokołowska[1], Grzegorz Basak[1], Krzysztof Madry[1]

*[1]Department of Hematology, Transplantation and Internal Medicine, Medical University of Warsaw, Warsaw, Poland; [2]Computational Biology Group, Institute of Computer Science of the Polish Academy of Sciences, Warsaw, Poland;[3]Department of Hematology, Rydygier Specialistic Hospital, Krakow, Poland; [4]Department of Hematology, Institute of Hematology and Transfusion Medicine, Warsaw, Poland; [5]Department of Hematology and Bone Marrow Transplantation, Medical University of Silesia, Katowice, Poland; [6]Department of Hematology, Military Institute of Medicine, Warsaw, Poland;[7]Department of Hematology, Blood Neoplasms and Bone Marrow Transplantation, Wroclaw Medical University, Wroclaw, Poland;*

### Background

Tobacco smoking is a recognized risk factor for developing myelodysplastic syndrome (MDS). However, its impact on the disease phenotype, specific laboratory parameters, and long-term clinical complications remains insufficiently characterized.

### Aims

The aim of the study was to comprehensively compare the disease phenotype, laboratory characteristics, and clinical outcomes between smoking and never-smoking patients diagnosed with MDS.

### Methods

Data from 2,246 MDS patients (2009–2025) were analyzed from the Polish MDS Registry. Continuous variables were compared using the Wilcoxon rank-sum test (due to non-normal distribution, Shapiro-Wilk $p < 0.05$). Categorical variables were assessed using Fisher's exact or Chi-square tests. Overall survival (OS) was estimated using the Kaplan-Meier method and compared via the log-rank test. Median follow-up was calculated using the reverse Kaplan-Meier method. Hazard ratios (HR) were determined using a univariable Cox proportional hazards model (UVA) to assess the impact of smoking status on mortality.

**Copyright Information:** (Online) ISSN: 2572-9241
©2026 The Author(s). HemaSphere is published by John Wiley & Sons Ltd on behalf of European Hematology Association. This is an open access Abstract Book distributed under the Attribution-NonCommercial-NoDerivs (CC BY-NC-ND), which allows third parties to download the articles and share them with others as long as they credit the author and the Abstract Book, but they cannot change the content in any way or use them commercially.
**Abstract Book Citations:** Authors, Title, HemaSphere, 2026;10:(S1):pages. The abstract book DOIs can be found on the "Author Information" page.
**Disclaimer:** Articles published in the journal HemaSphere exclusively reflect the opinions of the authors. The authors are responsible for all content in their abstracts including accuracy of the facts, statements, citing resources, etc.

**Results**

We included 2,246 patients: ever-smokers (n = 718) and never-smokers (n = 1,528). Ever-smokers were predominantly male (73.0% vs. 45.5%; p < 0.001) and were diagnosed at a significantly younger age (median 69 vs. 72 years; p < 0.001).
At diagnosis, ever-smokers presented with a more aggressive phenotype: lower hemoglobin (9.0 vs. 9.2 g/dL; p = 0.025), higher MCV (96.2 vs. 95.5 fL; p = 0.031), and higher bone marrow blast percentages (5.4% vs. 4.6%; p = 0.026). Subtype distribution differed significantly (p = 0.012); ever-smokers more frequently presented with RAEB-1 (20.9% vs. 14.3%), while del(5q) was more prevalent in never-smokers (8.4% vs. 4.6%).
Survival data were available for 1,933 patients. The death rate was significantly higher in the ever-smoker group (68.0% vs. 59.4%; p < 0.001). Median OS was 27.7 months (95% CI: 24.0–30.5) for ever-smokers versus 37.1 months (95% CI: 33.0–43.8) for never-smokers (p < 0.001) (Figure 1). In the UVA Cox model, smoking was a significant risk factor for death (HR = 1.27; 95% CI: 1.12–1.44; p < 0.001).
Ever-smokers experienced a more complicated clinical course, with a higher incidence of severe infections (39.2% vs. 32.0%; p = 0.002), most notably bacterial pneumonia. Interestingly, while ever-smokers had a higher overall rate of RBC transfusion dependency since diagnosis (p = 0.001), their monthly transfusion intensity during follow-up was lower (median 2 vs. 3 units/month; p = 0.014), potentially reflecting shorter survival or earlier progression.

**Summary/Conclusion**

Smoking significantly modifies the MDS landscape, leading to an earlier onset of more aggressive disease subtypes and a 27% increase in the risk of death. The higher rate of severe infections and earlier mortality in smokers underscore the need for stringent smoking cessation counseling and tailored clinical monitoring in this population.

**Copyright Information:** (Online) ISSN: 2572-9241
©2026 The Author(s). HemaSphere is published by John Wiley & Sons Ltd on behalf of European Hematology Association. This is an open access Abstract Book distributed under the Attribution-NonCommercial-NoDerivs (CC BY-NC-ND), which allows third parties to download the articles and share them with others as long as they credit the author and the Abstract Book, but they cannot change the content in any way or use them commercially.
**Abstract Book Citations:** Authors, Title, HemaSphere, 2026;10:(S1):pages. The abstract book DOIs can be found on the "Author Information" page.
**Disclaimer:** Articles published in the journal HemaSphere exclusively reflect the opinions of the authors. The authors are responsible for all content in their abstracts including accuracy of the facts, statements, citing resources, etc.

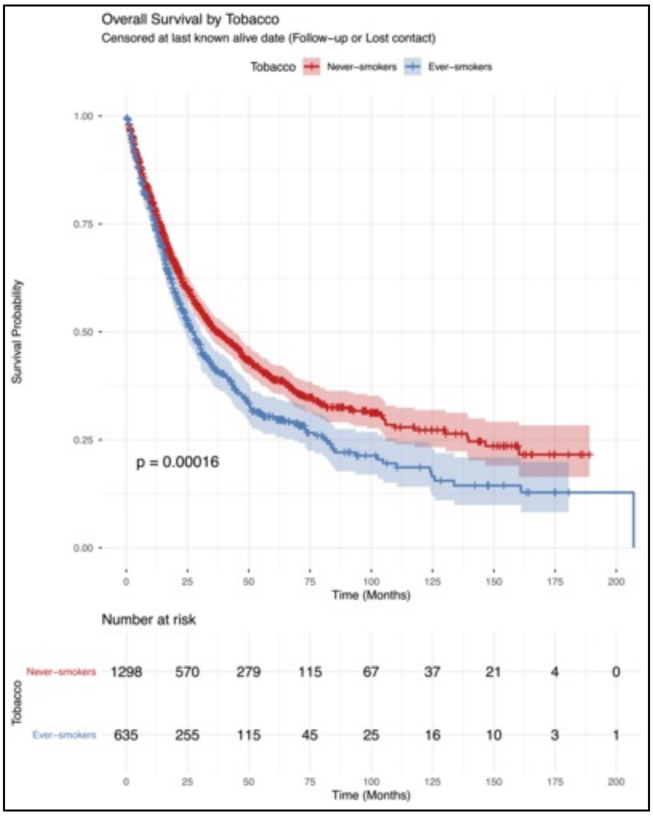

Fig 1. Kaplan-Meier survival curves comparing overall survival (OS) between never-smokers and ever-smokers with Myelodysplastic Syndromes (MDS). The log-rank test indicates a significant survival advantage for never-smokers (p < 0.001).

**Copyright Information:** (Online) ISSN: 2572-9241
©2026 The Author(s). HemaSphere is published by John Wiley & Sons Ltd on behalf of European Hematology Association. This is an open access Abstract Book distributed under the Attribution-NonCommercial-NoDerivs (CC BY-NC-ND), which allows third parties to download the articles and share them with others as long as they credit the author and the Abstract Book, but they cannot change the content in any way or use them commercially.
**Abstract Book Citations:** Authors, Title, HemaSphere, 2026;10:(S1):pages. The abstract book DOIs can be found on the "Author Information" page.
**Disclaimer:** Articles published in the journal HemaSphere exclusively reflect the opinions of the authors. The authors are responsible for all content in their abstracts including accuracy of the facts, statements, citing resources, etc.

### (PB3084) BASELINE TRANSFUSION BURDEN AND HB LEVEL, BUT NOT IPSS-R, OR IPSS-M WERE ASSOCIATED WITH LUSPATERCEPT EFFICACY IN MYELODYSPLASTIC SYNDROMES IN REAL WORLD

**Topic:** 10. Myelodysplastic syndromes - Clinical

Chengtao Qin[1], Ting Zhang[2], Yawen Zhang[1], Yuemin Gong[1], Guangsheng He*[1]

*[1]The First Affiliated Hospital with Nanjing Medical University, Nanjing, China;[2]Nanjing Jiangning Hospital, affiliated to Nanjing Medical University, Nanjing, China;*

### Background

Luspatercept is approved for treatment of refractory anemia in myelodysplastic syndromes (MDS) patients with lower IPSS-R risk, based on the results from MEDALIST and COMMANDS trials. About 7% of patients with lower IPSS-R risk were reclassified as IPSS-Molecular (IPSS-M) high/very high, with significantly shorter OS and LFS (Bernard et al. NEJM Evid 2022;1(7)). Whether the efficacy of luspatercept would be affected by IPSS-M restratification and other factors remains unknown.

### Aims

This study aimed to evaluate the efficacy of luspatercept in RBC transfusion-dependent (TD) MDS patients with different categories of IPSS-R, IPSS-M and other factors in a prospective multicenter registry study (ChiCTR2300071857).

### Methods

Eligible patients were ≥ 18 years old and were RBC TD (defined as ≥2 units of RBC transfusion per 8 weeks). Primary endpoint was RBC transfusion independence (RBC-TI) for ≥8 weeks. Secondary endpoints are hematological improvement-erythroid (HI-E, defined as >50% reduction in RBC transfusion).

### Results

From June 2022 to January 2026, 55 patients were treated with luspatercept for a median of 10 cycles. The median follow-up time was 30 weeks. The rate of RBC-TI was 33%. The median duration of RBC-TI was 159 [IQR: 123-324] days.
Compared to lower-risk patients, those with higher-risk IPSS-R scores show a difference in the rate of TI, but it was not statistically significant. (46% vs 22%, $p$=0.089) (**Table 1**). Uni-variant analysis of the whole cohort showed no significant correlation between IPSS-M categories with RBC-TI (41% vs 36%, $p$=0.701). Either in the patients with lower IPSS-R risk or in those with higher IPSS-R risk, the IPSS-M high/very high subgroup did not have significantly different incidence of TI than IPSS-M lower risk ($p$=0.529 and 0.156, respectively). Patients with lower proportion of erythroid precursors in bone morrow and those who had been failed to prior hypomethylating agent (HMA) therapy tended to response poorly to luspatercept, and with statistical significance ($p$=0.045 and 0.033, respectively). Multi-variant analysis revealed that lower transfusion burden ([illegible]6 u/8w) and higher hemoglobin levels before treatment favored higher RBC-TI rate ($p$=0.034 and 0.042, respectively) (**Table 1**).

### Summary/Conclusion

The efficacy of luspatercept in treating transfusion-dependent might not be affected by IPSS-R or IPSS-M risk. But it is correlated with the lower transfusion burden, higher hemoglobin levels before treatment, higher proportion of erythroid precursors in bone morrow, and prior HMA failed.

**Copyright Information:** (Online) ISSN: 2572-9241
©2026 The Author(s). HemaSphere is published by John Wiley & Sons Ltd on behalf of European Hematology Association. This is an open access Abstract Book distributed under the Attribution-NonCommercial-NoDerivs (CC BY-NC-ND), which allows third parties to download the articles and share them with others as long as they credit the author and the Abstract Book, but they cannot change the content in any way or use them commercially.
**Abstract Book Citations:** Authors, Title, HemaSphere, 2026;10:(S1):pages. The abstract book DOIs can be found on the “Author Information” page.
**Disclaimer:** Articles published in the journal HemaSphere exclusively reflect the opinions of the authors. The authors are responsible for all content in their abstracts including accuracy of the facts, statements, citing resources, etc.

Table 1 Baseline Clinical Characteristics Associated With Efficacy of Luspatercept

| Characteristics | TI-RBC | TD-RBC | *Uni-p* | *Multi-p* |
|---|---|---|---|---|
| **Age（yrs）** | 66 | 63 | 0.470 | |
| **Gender** | | | | |
| Male | 10/32 | 17/32 | 0.869 | |
| Female | 8/23 | 15/23 | | |
| **RBC transfusion burden(unitsper8wks)** | 5 | 9 | 0.004 | 0.034 |
| **SF3B1** | | | | |
| mutated | 14/28 | 14/28 | 0.123 | |
| wild-type | 8/27 | 19/27 | | |
| **Ring sideroblasts** | | | | |
| ≥5% | 12/24 | 12/24 | 0.112 | |
| <5% | 9/31 | 22/31 | | |
| **IPSS-R** | | | | |
| lower risk | 17/37 | 20/37 | 0.089 | 0.332 |
| higher risk | 4/18 | 14/18 | | |
| **IPSS-M** | | | | |
| lower risk | 11/27 | 16/27 | 0.701 | |
| higher risk | 10/28 | 18/28 | | |
| **Erythropoiesis-stimulating agent therapy** | | | | |
| failed | 10/30 | 20/30 | 0.418 | |
| naive | 11/25 | 14/25 | | |
| Hypomethylating agent therapy | | | | |
| failed | 7/25 | 18/25 | 0.033 | 0.145 |
| naive | 17/30 | 13/30 | | |
| HB（g/L） | 62 | 54 | 0.022 | 0.042 |
| Neutrophil（×109/L） | 2.1 | 2.0 | 0.986 | |
| Platelet（×109/L） | 112 | 137 | 0.541 | |
| ErythropoieticprecursorsinBM(（%） | 48 | 34.2 | 0.045 | 0.141 |

**Copyright Information:** (Online) ISSN: 2572-9241
©2026 The Author(s). HemaSphere is published by John Wiley & Sons Ltd on behalf of European Hematology Association. This is an open access Abstract Book distributed under the Attribution-NonCommercial-NoDerivs (CC BY-NC-ND), which allows third parties to download the articles and share them with others as long as they credit the author and the Abstract Book, but they cannot change the content in any way or use them commercially.
**Abstract Book Citations:** Authors, Title, HemaSphere, 2026;10:(S1):pages. The abstract book DOIs can be found on the “Author Information” page.
**Disclaimer:** Articles published in the journal HemaSphere exclusively reflect the opinions of the authors. The authors are responsible for all content in their abstracts including accuracy of the facts, statements, citing resources, etc.

### (PB3085) ELRISE: A PHASE 3 STUDY OF ELRITERCEPT VS EPOETIN ALFA IN ERYTHROPOIESIS-STIMULATING AGENT (ESA)-NAÏVE ADULTS WITH LOWER-RISK MYELODYSPLASTIC SYNDROMES (MDS) AND TRANSFUSION-DEPENDENT ANEMIA

**Topic:** 10. Myelodysplastic syndromes - Clinical

Mikkael A. Sekeres*[1], Rami S. Komrokji[2], Guillermo Garcia-Manero[3], Hetty Carraway[4], Matteo Giovanni Della Porta[5], Thomas Cluzeau[6], Aristoteles Giagounidis[7], María Díez Campelo[8], John F. Seymour[9], Phillip Scheinberg[10], Cong Li[11], Meliessa Hennessy[11], Leopold Sellner[11], Alexander Vorog[11], Valeria Santini[12]

*[1]Divison of Hematology, Sylvester Comprehensive Cancer Center, Miller School of Medicine, University of Miami, Miami, United States of America; [2]Department of Malignant Hematology, Moffitt Cancer Center, Tampa, United States of America;[3]Department of Leukemia, Division of Cancer Medicine, MD Anderson Cancer Center, University of Texas, Houston, United States of America; [4]Leukemia Program, Taussig Cancer Institute, Cleveland Clinic, Cleveland, United States of America;[5]Humanitas Clinical and Research Center, IRCCS, Rozzano, Milan, Italy & Humanitas University Pieve Emanuele, Milan, Italy; [6]Hematology Department, Côte d'Azur University, Centre Hospitalier Universitaire de Nice, Nice, France; [7]Hematology Oncology and Palliative Medicine Clinic, Marien Hospital Düsseldorf, Düsseldorf, Germany; [8]Department of Hematology, Hospital Universitario de Salamanca, Salamanca, Spain; [9]Clinical Haematology Peter MacCallum Cancer Centre and Royal Melbourne Hospital, Melbourne, Australia; [10]Division of Hematology, Hospital A Beneficencia Portuguesa, Sao Paulo, Brazil; [11]Takeda Development Center Americas, Inc. (TDCA), Cambridge, United States of America; [12]MDS Unit, AOU Careggi, DMSC, University of Florence, Florence, Italy;*

### Background

Anemia is a key clinical consequence of MDS. Long-term red blood cell (RBC) transfusions can cause iron overload, leading to end-organ complications, reduced quality of life (QoL) and decreased survival. ESAs can lessen transfusion needs but most patients (pts) either do not respond or become unresponsive over time. Identifying new treatments for pts with MDS that can decrease anemia symptoms, lessen transfusion burden, and provide rapid and durable responses while maintaining QoL remains a crucial unmet need. Elritercept (TAK-226), an investigational, modified activin receptor type IIA ligand trap that rebalances transforming growth factor-β superfamily ligands, is being evaluated in diseases with dysfunctional hematopoiesis, such as MDS. In phase 2 studies, elritercept resulted in durable transfusion independence (TI) and clinically meaningful improvements in QoL, including decreasing fatigue, in pts with very low-, low- and intermediate-risk (lower risk) MDS (LR-MDS), including those with packed RBC transfusion dependence (TD).

### Aims

This open-label, randomized phase 3 study will evaluate the efficacy and safety of elritercept vs epoetin alfa for the treatment of anemia in pts with LR-MDS (NCT07422480).

**Copyright Information:** (Online) ISSN: 2572-9241
©2026 The Author(s). HemaSphere is published by John Wiley & Sons Ltd on behalf of European Hematology Association. This is an open access Abstract Book distributed under the Attribution-NonCommercial-NoDerivs (CC BY-NC-ND), which allows third parties to download the articles and share them with others as long as they credit the author and the Abstract Book, but they cannot change the content in any way or use them commercially.
**Abstract Book Citations:** Authors, Title, HemaSphere, 2026;10:(S1):pages. The abstract book DOIs can be found on the "Author Information" page.
**Disclaimer:** Articles published in the journal HemaSphere exclusively reflect the opinions of the authors. The authors are responsible for all content in their abstracts including accuracy of the facts, statements, citing resources, etc.

### Methods

Eligible pts are ESA-naïve adults (≥18 years) with anemia due to LR-MDS (per Revised International Prognostic Scoring System) and TD (≥2–6 RBC units/8 weeks [wks]), <500 U/L serum erythropoietin (EPO) >2 weeks post-transfusion, <5% bone marrow blast count and <11.0 g/dL hemoglobin (Hgb). Key exclusion criteria include history of thromboembolic events <6 months prior to screening, clinically significant cardiovascular, hepatic or renal issues, BMI ≥40 kg/$m^2$ and prior therapy with ESAs, hypomethylating agents, luspatercept or immunomodulatory/suppressive agents or procedures (some exceptions applied). Following screening, eligible pts will be randomized 1:1 to receive subcutaneous injections of either elritercept 3.75 mg/kg once every 4 wks (with possible up-titration to 5 mg/kg after ≥8 wks) or epoetin alfa 450 IU/kg weekly (with possible up-titration to 1050 IU/kg after ≥8 wks, with a maximum total dose of 80,000 IU). Randomization will be stratified by ringed-sideroblast (RS) status (RS+ vs RS-), and baseline EPO levels (≤200 vs >200 U/L) and transfusion burden (low vs high [2-3 vs 4-6 units/8 wks]). RBC transfusions will be allowed at the investigator's discretion for low Hgb levels during the study. The study will enroll 210–300 pts across ~150 sites. Pts may be followed for up to 5 years after randomization (Figure 1).

### Results

The primary endpoint is the proportion of pts achieving TI ≥12 weeks from first dose through Week 24 with concurrent mean Hgb increase ≥1.5 g/dL from baseline. Key secondary and exploratory endpoints include additional efficacy measures (proportion of pts achieving TI and hematological improvement-erythroid at different time points), health-related QoL questionnaires (e.g. Functional Assessment of Chronic Illness Therapy-Fatigue score), safety assessment (frequency, severity and relationship of treatment-emergent adverse events as per the National Cancer Institute Common Terminology Criteria for Adverse Events v5.0), and pharmacokinetic and additional biomarker analyses. Transfusion verification and disease assessments will be performed periodically and as clinically indicated until the end of treatment.

### Summary/Conclusion

ELRISE is an actively recruiting phase 3 study evaluating elritercept for the treatment of anemia in pts with TD LR-MDS.

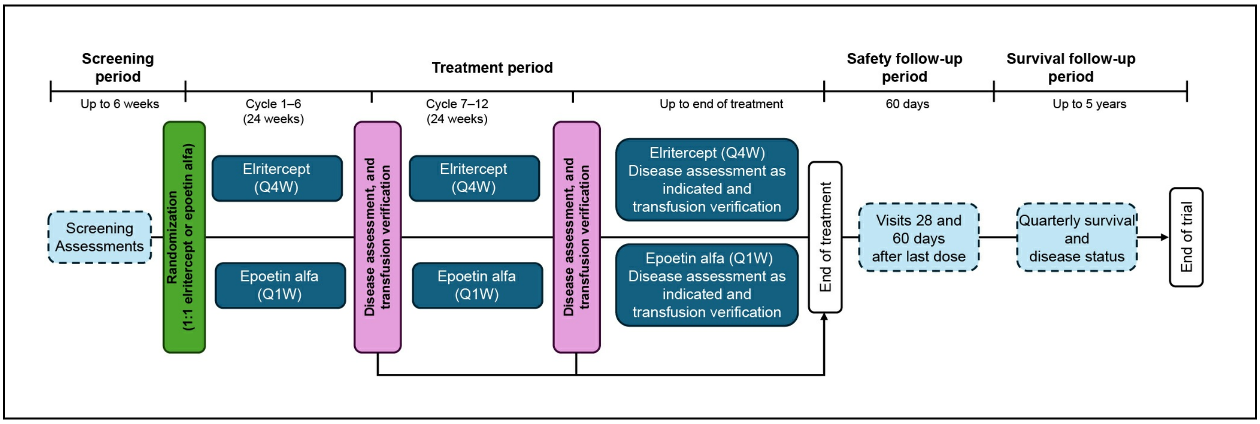

Figure 1. Schematic of the ELRISE study design

Copyright Information: (Online) ISSN: 2572-9241
©2026 The Author(s). HemaSphere is published by John Wiley & Sons Ltd on behalf of European Hematology Association. This is an open access Abstract Book distributed under the Attribution-NonCommercial-NoDerivs (CC BY-NC-ND), which allows third parties to download the articles and share them with others as long as they credit the author and the Abstract Book, but they cannot change the content in any way or use them commercially.
Abstract Book Citations: Authors, Title, HemaSphere, 2026;10:(S1):pages. The abstract book DOIs can be found on the "Author Information" page.
Disclaimer: Articles published in the journal HemaSphere exclusively reflect the opinions of the authors. The authors are responsible for all content in their abstracts including accuracy of the facts, statements, citing resources, etc.

### (PB3086) CLONAL HAEMATOPOIESIS OF INDETERMINATE POTENTIAL (CHIP) AS A BIOMARKER FOR RISK STRATIFICATION IN NON-SMALL-CELL LUNG CANCER (NSCLC)

**Topic:** 10. Myelodysplastic syndromes - Clinical

Harberth Fernandez Leyva*[1]

[1] *University Hospital Dorset, Haematology, Bournemouth, United Kingdom;*

### Background

Clonal haematopoiesis of indeterminate potential (CHIP) is defined by the age-related expansion of haematopoietic clones harbouring somatic mutations detectable in peripheral blood. These mutations may also be identified within tumour specimens as tumour-infiltrating clonal haematopoiesis (TI-CH), potentially influencing the tumour immune microenvironment and clinical outcomes. The role of CHIP in Non-Small-Cell Lung Cancer (NSCLC) remains incompletely understood.

### Aims

To determine the prevalence, molecular spectrum, and clinical significance of CHIP and TI-CH in patients with NSCLC receiving systemic therapy.

### Methods

We retrospectively analysed 103 patients with NSCLC evaluated between October 2023 and 2025 from a database of 305 lung cancer cases. All patients had received at least one line of systemic treatment; 61% were on active therapy at the time of clonal haematopoiesis assessment. Somatic mutations associated with CHIP were identified and categorised by functional pathway. Variant allele frequency (VAF) was recorded. Associations with clinicopathological features and survival outcomes were evaluated.

### Results

At least one CHIP-associated mutation was detected in 38/103 patients (36%), with prevalence increasing with age. CH-positive patients were older than CH-negative individuals. Epigenetic modifier genes (TET2, DNMT3A, ASXL1) were most frequently mutated (12%; median VAF 6.5%). DNA damage-response genes (PPM1D, TP53, CHEK2, ATM) were identified in 5% (median VAF 4%), and cohesin-complex genes (SMC3, SMC1A, RAD21, STAG2) in 3% (median VAF 5%). TET2 was the most prevalent single mutation and the strongest genetic predictor of TI-CH, associated with enhanced monocyte recruitment and a myeloid-enriched tumour microenvironment. TI-CH correlated with increased risk of disease recurrence or death and accelerated tumour-organoid growth. However, no statistically significant difference in overall survival was observed between CH-positive and CH-negative patients (log-rank $p = 0.218$).

### Summary/Conclusion

CHIP is common in NSCLC and increases with age. TI-CH appears to influence tumour immune architecture and disease progression, supporting its potential role as a biomarker for risk stratification. Nevertheless, its impact on overall survival remains uncertain in this cohort. Prospective studies are warranted to validate its prognostic and predictive utility.

**Copyright Information:** (Online) ISSN: 2572-9241
©2026 The Author(s). HemaSphere is published by John Wiley & Sons Ltd on behalf of European Hematology Association. This is an open access Abstract Book distributed under the Attribution-NonCommercial-NoDerivs (CC BY-NC-ND), which allows third parties to download the articles and share them with others as long as they credit the author and the Abstract Book, but they cannot change the content in any way or use them commercially.
**Abstract Book Citations:** Authors, Title, HemaSphere, 2026;10:(S1):pages. The abstract book DOIs can be found on the "Author Information" page.
**Disclaimer:** Articles published in the journal HemaSphere exclusively reflect the opinions of the authors. The authors are responsible for all content in their abstracts including accuracy of the facts, statements, citing resources, etc.

### (PB3088) ALLOGENEIC HEMATOPOIETIC STEM CELL TRANSPLANTATION FOR MYELODYSPLASTIC SYNDROMES:OUTCOMES AND RISK FACTORS

**Topic:** 10. Myelodysplastic syndromes - Clinical

Jie Zhao[1], Xiaolin Yu[2], Xiaochen Song[2], Qianqian Xiao[2], Jing Wang[2], Lei Deng[2], Fang Zhou*[2]

*[1]The 960th Hospital of The People's Liberation Army (PLA) Joint Logistics Support Force; Shandong First Medical University, Jinan, China; [2]The 960th Hospital of The People's Liberation Army (PLA) Joint Logistics Support Force, Jinan, China;*

### Background

Myelodysplastic syndromes (MDS) are heterogeneous clonal disorders of hematopoietic stem cells. Approximately 30% of patients progress to acute myeloid leukemia. Allogeneic hematopoietic stem cell transplantation (allo-HSCT) is the only curative option for high/very high-risk patients. However, pre-transplant risk factors increase post-transplant relapse and mortality. Identifying high-risk patients and mitigating these factors to improve survival remain critical clinical challenges.

### Aims

To evaluate outcomes of allo-HSCT in patients with MDS and to identify prognostic factors affecting transplant outcomes.

### Methods

We retrospectively analyzed the clinical data of 100 patients with MDS who underwent allo-HSCT between June 2013 and December 2024, aiming to evaluate overall survival (OS), disease-free survival (DFS), and non-relapse mortality (NRM), and to identify risk factors for transplant outcomes.

### Results

A total of 100 patients were included: 57 males and 43 females, with a median age of 46 years (IQR: 33–53). The median time to neutrophil engraftment was 13 days (range 8–28), and for platelets, 14 days (range 8–41). The median follow-up after transplantation was 11.2 months (range, 0.2–117.5). The 1-year OS and DFS rates were 66.5% (95% CI 55.8%–75.4%) and 61.3% (95% CI 51.3%–71.3%), respectively; the corresponding 2-year rates were 62.8% (95% CI 52.6%–73.0%) and 58.3% (95% CI 48.1%–68.9%). NRM was 30.6% (95% CI 21.6%–39.6%) at 1 year and 33.4% (95% CI 24.2%–42.6%) at 2 years. Kaplan-Meier univariate analysis identified the following factors as significantly associated with inferior OS: age ≥45 years (log-rank $\chi^2$=4.239, P=0.04), high/very high risk Revised International Prognostic Scoring System (IPSS-R) (log-rank $\chi^2$=9.096, P=0.011), grade III–IV acute graft-versus-host disease (aGVHD) (log-rank $\chi^2$=9.228, P=0.002), and absence of chronic graft-versus-host disease (cGVHD) (log-rank $\chi^2$=9.390, P=0.002). Similarly, these factors were significantly associated with inferior DFS: age ≥45 years (log-rank $\chi^2$=5.472, P=0.019), high/very high risk IPSS-R (log-rank $\chi^2$=11.253, P=0.004), grade III–IV aGVHD (log-rank $\chi^2$=6.247, P=0.012), and absence of cGVHD (log-rank $\chi^2$=9.330, P=0.002). Multivariable Cox regression identified age ≥45 years (OS: HR=2.378, 95% CI 1.143–4.949, P=0.020; DFS: HR=2.342, 95% CI 1.182–4.640, P=0.015) and grade III–IV aGVHD (OS: HR=3.259, 95% CI 1.539–6.900, P=0.002; DFS: HR=2.886, 95% CI 1.392–5.983, P=0.004) as independent adverse factors, while presence of cGVHD was protective for both OS (HR=0.298, 95% CI 0.142–0.623, P=0.001) and DFS (HR=0.345, 95% CI 0.177–0.672, P=0.002). IPSS-R risk stratification was significantly associated with OS (overall P=0.016) and DFS (overall P=0.011). Compared with the very low/low risk group, the high/very high risk group showed a trend toward increased risk for OS (HR=2.373, 95% CI 0.790–7.127, P=0.124) and DFS (HR=1.820, 95% CI 0.721–4.595, P=0.205), although neither reached statistical significance.

**Copyright Information:** (Online) ISSN: 2572-9241
©2026 The Author(s). HemaSphere is published by John Wiley & Sons Ltd on behalf of European Hematology Association. This is an open access Abstract Book distributed under the Attribution-NonCommercial-NoDerivs (CC BY-NC-ND), which allows third parties to download the articles and share them with others as long as they credit the author and the Abstract Book, but they cannot change the content in any way or use them commercially.
**Abstract Book Citations:** Authors, Title, HemaSphere, 2026;10:(S1):pages. The abstract book DOIs can be found on the “Author Information” page.
**Disclaimer:** Articles published in the journal HemaSphere exclusively reflect the opinions of the authors. The authors are responsible for all content in their abstracts including accuracy of the facts, statements, citing resources, etc.

**Summary/Conclusion**

Age ≥45 years, high/very high IPSS-R risk, and grade III–IV aGVHD were independent risk factors for worse OS and DFS, while cGVHD was a protective factor. Further studies with larger cohorts are needed to evaluate factors influencing the efficacy of allo-HSCT in MDS.

**Copyright Information:** (Online) ISSN: 2572-9241
©2026 The Author(s). HemaSphere is published by John Wiley & Sons Ltd on behalf of European Hematology Association. This is an open access Abstract Book distributed under the Attribution-NonCommercial-NoDerivs (CC BY-NC-ND), which allows third parties to download the articles and share them with others as long as they credit the author and the Abstract Book, but they cannot change the content in any way or use them commercially.
**Abstract Book Citations:** Authors, Title, HemaSphere, 2026;10:(S1):pages. The abstract book DOIs can be found on the "Author Information" page.
**Disclaimer:** Articles published in the journal HemaSphere exclusively reflect the opinions of the authors. The authors are responsible for all content in their abstracts including accuracy of the facts, statements, citing resources, etc.

### (PB3089) MORTALITY–DISABILITY DIVERGENCE IN MDS AND CLASSICAL MPNS: GBD 2023 NORTH AMERICA VERSUS WESTERN EUROPE

**Topic:** 10. Myelodysplastic syndromes - Clinical

Zeal Soni*[1], Tapan Giri[2]

*[1]Smt NHL Municipal Medical College, Ahmedabad, Gujarat, Ahmedabad, India;[2]University of Nevada, Reno, NV, Reno, United States of America;*

### Background

Myelodysplastic syndromes (MDS) and classical Philadelphia-negative myeloproliferative neoplasms (MPNs) including polycythemia vera, essential thrombocythemia, and primary myelofibrosis primarily affect older adults and represent related clonal myeloid neoplasms with overlapping age-driven disease burden. Major therapeutic and diagnostic advances may have altered population-level outcomes, yet regional differences in mortality and disability trends remain unclear.

### Aims

To compare long-term trends in incidence, prevalence, mortality, and disability burden of MDS and classical MPNs between high-income North America (HINA) and Western Europe (WE) using Global Burden of Disease (GBD) 2023 data.

### Methods

Age-standardized rates (ASR per 100,000) and absolute counts for adults aged ≥60 years were extracted from the GBD Results Tool (1990–2023) for incidence, prevalence, deaths, and disability-adjusted life years (DALYs). Estimated annual percent change (EAPC) quantified temporal trends. Decomposition analysis assessed the contribution of population aging to DALY changes.

### Results

In 2023, incidence ASR was higher in HINA than WE (8.63 vs 3.95). Prevalence ASR increased substantially in HINA (106.7 to 145.4) compared with WE (33.6 to 39.8). Mortality ASR rose more rapidly in WE (0.96 to 1.64; EAPC +1.63%) than in HINA (1.41 to 1.57; EAPC +0.32%). DALY ASR increased by 58% in WE (17.52 to 27.74; EAPC +1.40%) versus 8% in HINA (29.45 to 31.74; EAPC +0.23%). Among adults aged ≥60 years in 2023, deaths reached 18,994 in WE and 11,136 in HINA, while DALYs totaled 260,042 and 179,419, respectively. Post-2020 trends showed stabilization in HINA but continued DALY expansion in WE, largely driven by population aging. Directionally similar divergence patterns were observed across both disease groups.

### Summary/Conclusion

Despite lower incidence, Western Europe demonstrates accelerating mortality and disability burden from MDS and classical MPNs compared with high-income North America. Findings indicate widening outcome disparities in aging populations and support the need for earlier molecular diagnostics, equitable access to disease-modifying therapies, and integration of geriatric care. Linkage of registry and molecular datasets is required to clarify drivers of regional differences.

**Copyright Information:** (Online) ISSN: 2572-9241
©2026 The Author(s). HemaSphere is published by John Wiley & Sons Ltd on behalf of European Hematology Association. This is an open access Abstract Book distributed under the Attribution-NonCommercial-NoDerivs (CC BY-NC-ND), which allows third parties to download the articles and share them with others as long as they credit the author and the Abstract Book, but they cannot change the content in any way or use them commercially.
**Abstract Book Citations:** Authors, Title, HemaSphere, 2026;10:(S1):pages. The abstract book DOIs can be found on the “Author Information” page.
**Disclaimer:** Articles published in the journal HemaSphere exclusively reflect the opinions of the authors. The authors are responsible for all content in their abstracts including accuracy of the facts, statements, citing resources, etc.

### (PB3090) PROGNOSTIC IMPACT AND BIOLOGICAL BASIS OF BONE MARROW FIBROSIS IN MYELODYSPLASTIC NEOPLASMS: A COMPARATIVE ANALYSIS WITH PRIMARY MYELOFIBROSIS

**Topic:** 10. Myelodysplastic syndromes - Clinical

Wan-Hsuan Lee*[1], Chien-Chin Lin[2], Cheng-Hong Tsai[1], Feng-Ming Tien[1], Yen-Ling Peng[1], Mei-Hsuan Tseng[1], Yuan-Yeh Kuo[2], Shan-Chi Yu[1], Ming-Chih Liu[1], Chi-Yuan Yao[1], Chia-Lang Hsu[2], Chang-Tsu Yuan[1], Ming Yao[1], Bor-Sheng Ko[1], Hwei-Fang Tien[1], Hsin-An Hou[1], Wen-Chien Chou[1]

*[1]National Taiwan University Hospital, Internal medicine, Tapei, Taiwan; [2]National Taiwan University Hospital, Tapei, Taiwan;*

### Background

Bone marrow fibrosis (BMF) is a clinically relevant but incompletely characterized feature in myelodysplastic neoplasms (MDS). While evidence suggests inferior outcomes in MDS patients with fibrosis, its prognostic significance within contemporary molecular risk models (such as IPSS-M) and its biological distinction from primary myelofibrosis (PMF) remain unclear.

### Aims

We aimed to evaluate the prognostic impact of BMF in MDS, compare clinical and molecular features between MDS with fibrosis and PMF, and explore fibrosis-associated biological pathways using transcriptomic profiling.

### Methods

A total of 351 MDS patients (WHO 2016) and 162 PMF patients were included. Diagnostic BM samples underwent targeted next-generation sequencing (TruSight Myeloid Panel) and bulk RNA sequencing. Clinical characteristics, treatment modalities, and outcomes were systematically analyzed.

### Results

Among MDS patients, 20 (5.7%) had grade 2–3 BMF. Patients with MF 2–3 exhibited more severe anemia, higher bone marrow blast percentages, and were more frequently classified as "very high risk" by both IPSS-R and IPSS-M compared to those with MF 0–1. Regarding genetic and karyotypes differences, patients with grade 2-3 fibrosis had more tumor suppressor gene mutations, such as *TP53* and *CUX1*, but less *SF3B1* mutations and more complex karyotypes. Survival analyses demonstrated markedly inferior outcomes for MF 2–3 patients, with significantly shorter overall survival (OS) and higher leukemic transformation rates. Subgroup survival analysis in intermediate-risk IPSS-R and moderate low/moderate high-risk IPSS-M demonstrated significantly faster leukemic change for patients with MF 2–3. Additionally, there was a trend of shorter time to leukemic change and OS for patients with MF 2–3 in high or very high-risk IPSS-R/M. In multivariable analysis adjusting for age, sex, IPSS-M risk, and therapy-related status, MF grade 2–3 remained an independent predictor of inferior survival. Notably, the negative prognostic impact persisted even in patients without excess blasts.
Comparative analyses between patients with PMF and MDS (MF 2–3) revealed that proliferative PMF was characterized by significantly higher white blood cell counts and absolute neutrophil counts. Conversely, cytopenic PMF exhibited lower hemoglobin levels, similar to those observed in the MDS (MF 2–3) cohort. Regarding genetic landscapes, patients with MDS and cytopenic PMF demonstrated a higher prevalence of spliceosome complex mutations compared to those with proliferative PMF. Survival analysis further indicated that MDS patients experienced significantly higher rates of leukemic transformation and shorter overall survival than the PMF group.
Transcriptomic profiling in MDS with fibrosis identified up-regulation of genes involved in inflammatory signaling, cellular stress responses, and epigenetic regulation, showing enrichment of distinct fibrosis-associated pathways.

**Copyright Information:** (Online) ISSN: 2572-9241
©2026 The Author(s). HemaSphere is published by John Wiley & Sons Ltd on behalf of European Hematology Association. This is an open access Abstract Book distributed under the Attribution-NonCommercial-NoDerivs (CC BY-NC-ND), which allows third parties to download the articles and share them with others as long as they credit the author and the Abstract Book, but they cannot change the content in any way or use them commercially.
**Abstract Book Citations:** Authors, Title, HemaSphere, 2026;10:(S1):pages. The abstract book DOIs can be found on the "Author Information" page.
**Disclaimer:** Articles published in the journal HemaSphere exclusively reflect the opinions of the authors. The authors are responsible for all content in their abstracts including accuracy of the facts, statements, citing resources, etc.

### Summary/Conclusion

Advanced BMF identifies a biologically and clinically distinct high-risk subset of MDS characterized by adverse features and increased leukemic transformation independent of established prognostic models. Compared with PMF, fibrosis in MDS represents a more aggressive disease state driven by distinct molecular and microenvironmental mechanisms, supporting the need for fibrosis-adapted risk stratification and specific therapeutic strategies.

**Figure. Kaplan-Meier curves for proportion of leukemic change (a) and overall survival (b), stratified by fibrosis grade**

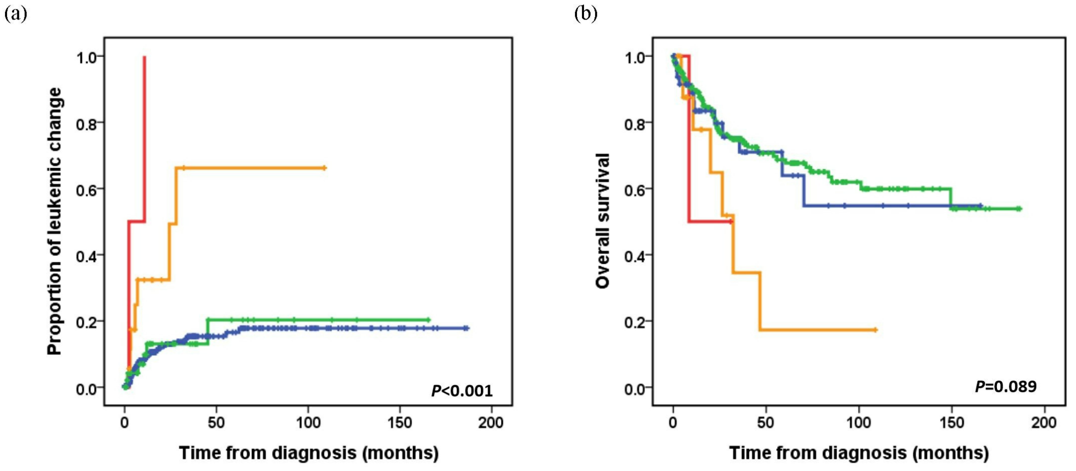

**MF 0 (n=280)**, Median time to leukemic change not reached
**MF 1 (n=51)**, Median time to leukemic change not reached
**MF 2 (n=18)**, Median time to leukemic change 28.1 months
**MF 3 (n=2)**, Median time to leukemic change 2.3 months

**MF 0 (n=280)**, Median OS not reached
**MF 1 (n=51)**, Median OS not reached
**MF 2 (n=18)**, Median OS 32.2 months
**MF 3 (n=2)**, Median t OS 8.4 months

**Copyright Information:** (Online) ISSN: 2572-9241
©2026 The Author(s). HemaSphere is published by John Wiley & Sons Ltd on behalf of European Hematology Association. This is an open access Abstract Book distributed under the Attribution-NonCommercial-NoDerivs (CC BY-NC-ND), which allows third parties to download the articles and share them with others as long as they credit the author and the Abstract Book, but they cannot change the content in any way or use them commercially.
**Abstract Book Citations:** Authors, Title, HemaSphere, 2026;10:(S1):pages. The abstract book DOIs can be found on the "Author Information" page.
**Disclaimer:** Articles published in the journal HemaSphere exclusively reflect the opinions of the authors. The authors are responsible for all content in their abstracts including accuracy of the facts, statements, citing resources, etc.

### (PB3091) CORRELATION OF NEUTROPHIL GRANULARITY INTENSITY (NEUT-GI) WITH NEUTROPHIL HYPOGRANULATION IN PERIPHERAL BLOOD SMEARS OF PATIENTS WITH MYELODYSPLASTIC SYNDROMES.

**Topic:** 10. Myelodysplastic syndromes - Clinical

Zuzanna Wierzchucka*[1], Iwona Kwiecień[1], Agata Raniszewska[1], Piotr Rzepecki[2], Elżbieta Rutkowska[1]

*[1]Military Institute of Medicine - National Research Institute, Laboratory of Hematology and Flow Cytometry, Department of Internal Medicine and Hematology, Warsaw, Poland; [2]Military Institute of Medicine - National Research Institute, Department of Internal Medicine and Hematology, Warsaw, Poland;*

### Background

Myelodysplastic syndromes (MDS) are a heterogeneous group of myeloid malignancies characterized by peripheral cytopenias and dysplastic hematopoiesis. Hypogranulated neutrophils observed in peripheral blood smears constitute an important morphological feature suggestive of dysplasia in the granulocytic lineage. However, peripheral blood smear is not always performed and subtle dysplastic changes may be overlooked. Automated hematology parameters such as Neutrophil Granularity Intensity (NEUT-GI) may serve as an objective indicator of neutrophil hypogranulation in suspicion of MDS.

### Aims

The aim of the study was to determine whether NEUT-GI reported in complete blood count (CBC) results may assist in early suspicion of MDS by reflecting neutrophil hypogranulation observed in peripheral blood smears.

### Methods

CBC analysis was performed using the Sysmex XN-1500 analyzer. NEUT-GI values were collected from 224 patients suspected of MDS. Peripheral blood smears were assessed for neutrophil hypogranulation. Based on the severity of hypogranulation observed in the smears, a semi-quantitative scale (0-5) was created, in which: 0 – no hypogranulation, 1 – single hypogranulated neutrophils, 2 – few hypogranulated neutrophils, 3 – hypogranulation affecting a subset of neutrophils, 4 – hypogranulation affecting the majority of neutrophils/subset of neutrophils being agranular, 5 – majority agranular neutrophils. Statistical analyses were performed using Jamovi (Spearman's correlation, Mann- Whitney, Kruskal-Wallis with post-hoc pairwise – Dunn's test with Bonferroni correction).

**Copyright Information:** (Online) ISSN: 2572-9241
©2026 The Author(s). HemaSphere is published by John Wiley & Sons Ltd on behalf of European Hematology Association. This is an open access Abstract Book distributed under the Attribution-NonCommercial-NoDerivs (CC BY-NC-ND), which allows third parties to download the articles and share them with others as long as they credit the author and the Abstract Book, but they cannot change the content in any way or use them commercially.
**Abstract Book Citations:** Authors, Title, HemaSphere, 2026;10:(S1):pages. The abstract book DOIs can be found on the "Author Information" page.
**Disclaimer:** Articles published in the journal HemaSphere exclusively reflect the opinions of the authors. The authors are responsible for all content in their abstracts including accuracy of the facts, statements, citing resources, etc.

### Results

NEUT-GI values differed significantly across hypogranulation severity scores with progressively lower values observed at higher scores (Table 1). A significant negative correlation between NEUT-GI and hypogranulation score was observed ($\rho$=-0.565; p<0.001). NEUT-GI values were significantly lower in patients with hypogranulation compared with those without (median 141, n=105; 153 n=119; p<0.001). Post-hoc pairwise comparisons showed significant differences in NEUT-GI values between grade 0 and grades 3-5 (adjusted p<0.001), as well as between grades 1 vs 4-5 and 2 vs 4-5 (adjusted p≤0.012).

| Hypogranulation score (peripheral blood smear) | N | NEUT- GI, mean ± SD | NEUT-GI, median (Q1-Q3) |
|---|---|---|---|
| 0 | 119 | 152 ± 7.49 | 153 (148-156) |
| 1 | 23 | 148 ± 6.22 | 148 (143-152) |
| 2 | 16 | 150 ± 7.65 | 149 (144-157) |
| 3 | 31 | 141 ± 9.56 | 139 (136-144) |
| 4 | 18 | 134 ± 11.29 | 131 (125-142) |
| 5 | 17 | 132 ± 10.28 | 132 (123-138) |

Table 1 NEUT-GI values according to neutrophil score in peripheral blood smears.

### Summary/Conclusion

NEUT-GI reflects neutrophil hypogranulation and may serve as an accessible, objective parameter supporting early suspicion of MDS. Routine reporting of NEUT-GI in CBC results may help in earlier diagnostic evaluation of MDS.

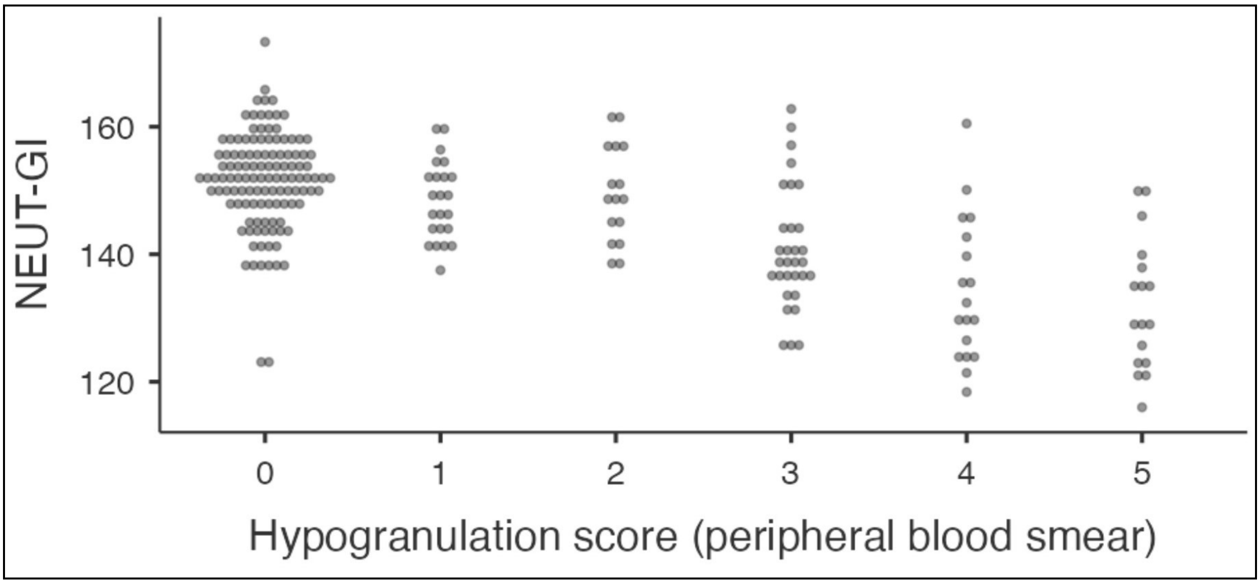

Figure 1 Distribution of NEUT-GI values according to neutrophil hypogranulation score in peripheral blood smears.

Copyright Information: (Online) ISSN: 2572-9241
©2026 The Author(s). HemaSphere is published by John Wiley & Sons Ltd on behalf of European Hematology Association. This is an open access Abstract Book distributed under the Attribution-NonCommercial-NoDerivs (CC BY-NC-ND), which allows third parties to download the articles and share them with others as long as they credit the author and the Abstract Book, but they cannot change the content in any way or use them commercially.
Abstract Book Citations: Authors, Title, HemaSphere, 2026;10:(S1):pages. The abstract book DOIs can be found on the "Author Information" page.
Disclaimer: Articles published in the journal HemaSphere exclusively reflect the opinions of the authors. The authors are responsible for all content in their abstracts including accuracy of the facts, statements, citing resources, etc.

### (PB3092) DYNAMICS OF SERUM INTERLEUKIN-1 AS A PREDICTIVE BIOMARKER OFRESPONSE TO AZACITIDINE IN PATIENTS WITH MDS-EB-2

**Topic:** 10. Myelodysplastic syndromes - Clinical

Halyna Starodub[1], Nadiya Goryainova[2], Nataliia Tretiak[1], Olga Basova[1], Bohdana Kuiavovych[1], Hanna Bortnik*[1]

*[1]STATE INSTITUTION «NATIONAL RESEARCH CENTER FOR RADIATION MEDICINE, HEMATOLOGY and ONCOLOGY», Department of Blood System Diseases,, Kyiv, Ukraine; [2]STATE INSTITUTION «NATIONAL RESEARCH CENTER FOR RADIATION MEDICINE, HEMATOLOGY and ONCOLOGY», STATE INSTITUTION «NATIONAL RESEARCH CENTER FOR RADIATION MEDICINE, HEMATOLOGY and ONCOLOGY», Kyiv, Ukraine;*

### Background

Myelodysplastic syndromes (MDS) are clonal hematopoietic disorders characterized by ineffective hematopoiesis and a high risk of progression to acute myeloid leukemia. Increasing evidence indicates that inflammatory dysregulation within the bone marrow microenvironment contributes to disease progression. Interleukin-1 (IL-1) is a key pro-inflammatory cytokine involved in suppression of normal hematopoiesis and expansion of malignant clones. However, the clinical value of IL-1 monitoring during hypomethylating therapy has not been clearly defined.

### Aims

To evaluate serum IL-1 dynamics and its association with clinical response in patients with MDS with excess blasts-2 (MDS-EB-2) treated with azacitidine.

### Methods

Thirty-three patients with MDS-EB-2 (18 men [54.5%], 15 women [45.5%]; age 49–78 years) were included. Diagnosis was established according to WHO 2008 criteria. Serum IL-1 levels were measured using enzyme-linked immunosorbent assay (ELISA) (Multiscan Ascent, LabSystems; Diaclone kits). Patients received azacitidine 75 mg/m²/day on days 1–7 every 28 days. Dose modifications were performed according to hematologic toxicity. Treatment response was assessed using standard hematologic criteria.

### Results

Complete or partial response was achieved in 5 patients (15.1%). Responders demonstrated a significant 1.5-fold decrease in serum IL-1 levels (23.0±2.1 to 14.5±0.5 pg/mL; $p \leq 0.05$), accompanied by a 1.9-fold reduction in bone marrow blasts (16.3±0.5% to 8.5±0.8%).
Seven patients (21.2%) showed disease progression or no response. In this group, blast percentage increased 1.9-fold (to 31.4±2.6%; $p \leq 0.05$), while IL-1 levels remained elevated (28.5±3.1 pg/mL), exceeding values observed in patients with stable disease (16.8±1.3 pg/mL) and responders.

**Copyright Information:** (Online) ISSN: 2572-9241
©2026 The Author(s). HemaSphere is published by John Wiley & Sons Ltd on behalf of European Hematology Association. This is an open access Abstract Book distributed under the Attribution-NonCommercial-NoDerivs (CC BY-NC-ND), which allows third parties to download the articles and share them with others as long as they credit the author and the Abstract Book, but they cannot change the content in any way or use them commercially.
**Abstract Book Citations:** Authors, Title, HemaSphere, 2026;10:(S1):pages. The abstract book DOIs can be found on the "Author Information" page.
**Disclaimer:** Articles published in the journal HemaSphere exclusively reflect the opinions of the authors. The authors are responsible for all content in their abstracts including accuracy of the facts, statements, citing resources, etc.

**Summary/Conclusion**

Reduction of serum IL-1 is associated with hematologic response to azacitidine, whereas persistently elevated IL-1 correlates with disease progression and increased blast burden. Monitoring IL-1 may serve as a clinically relevant biomarker for inflammatory activity and early prediction of treatment response in patients with high-risk MDS.

**Copyright Information:** (Online) ISSN: 2572-9241
©2026 The Author(s). HemaSphere is published by John Wiley & Sons Ltd on behalf of European Hematology Association. This is an open access Abstract Book distributed under the Attribution-NonCommercial-NoDerivs (CC BY-NC-ND), which allows third parties to download the articles and share them with others as long as they credit the author and the Abstract Book, but they cannot change the content in any way or use them commercially.
**Abstract Book Citations:** Authors, Title, HemaSphere, 2026;10:(S1):pages. The abstract book DOIs can be found on the "Author Information" page.
**Disclaimer:** Articles published in the journal HemaSphere exclusively reflect the opinions of the authors. The authors are responsible for all content in their abstracts including accuracy of the facts, statements, citing resources, etc.

### (PB3093) GENOMIC PROFILING REVEALS DISTINCT MUTATIONAL LANDSCAPES AND PROGRESSION-ASSOCIATED SIGNATURES IN ESSENTIAL THROMBOCYTHEMIA AND POST-ET MYELOFIBROSIS

**Topic:** 10. Myelodysplastic syndromes - Clinical

Xiaojing Bao[1], Jia Li[1], Xiudi Yang[1], Tian Zeng[1], Zhikang Zheng[1], Rumeng Li[1], Bingbing Wen[1], Qingmei Han[1], Jie Jin[1], Jian Huang*[1]

*[1]The First Affiliated Hospital, Zhejiang University School of Medicine, Hangzhou, China;*

### Background

Essential thrombocythemia (ET) is a clonal myeloproliferative neoplasm (MPN) characterized by thrombocytosis and a relatively indolent course, with median survival exceeding 20 years in low-risk patients. However, a subset of patients progresses to post-ET myelofibrosis (Post-ET MF), a complication associated with significantly impaired survival and therapeutic challenges. While driver mutations define disease subtypes and thrombosis risk, the genomic determinants of fibrotic transformation remain incompletely characterized. Understanding the comprehensive mutational profiles distinguishing ET from Post-ET MF may elucidate mechanisms of disease progression and inform risk stratification strategies beyond conventional clinical models.

### Aims

This study aims to compare the genomic landscapes, including driver and non-driver mutational profiles, between ET and Post-ET MF patients using next-generation sequencing (NGS), and to identify molecular signatures associated with fibrotic transformation.

### Methods

We performed targeted NGS on bone marrow specimens from 198 ET patients and 29 Post-ET MF patients using a 154-gene hematologic malignancy panel. Mutations with variant allele frequency (VAF) ≥2% were included. Driver mutations (*JAK2, CALR, MPL*) were analyzed, and non-driver mutations were categorized by functional pathways (include epigenetic regulation, spliceosome factor, DNA damage response, signaling). Mutational burden, gene frequencies, and pathway distributions were compared between groups (Figure A).

### Results

A total of 749 mutations across 121 genes were detected. ET patients harbored 614 mutations in 117 genes, while Post-ET MF patients had 135 mutations in 58 genes (Figure B). Post-ET MF patients exhibited significantly higher *CALR* mutation rates compared to ET (44.8% vs. 22.7%, P=0.01), with enrichment for type 2 variants, and lower triple-negative rates (6.9% vs. 27.3%, P=0.03). Simultaneously, Post-ET MF patients demonstrated significantly higher non-driver mutational burden (2 vs. 3 per patient, P=0.01) (Table 1). Distinct gene profiles emerged: ET patients showed frequent TET2 (20.2%), KMT2C (15.2%), and KMT2D (12.2%) mutations, whereas Post-ET MF patients were enriched for TET2 (31.0%), ASXL1 (24.1%), and TP53 (20.7%). ASXL1, TP53, and ZRSR2 mutations were significantly more prevalent in Post-ET MF (all P<0.05)(Figure C). Regarding functional pathways, both groups were dominated by epigenetic regulator and signaling pathway mutations. However, Post-ET MF showed significantly higher frequencies of DNA damage response and spliceosome factor mutations (P<0.05) (Figure D).

**Copyright Information:** (Online) ISSN: 2572-9241
©2026 The Author(s). HemaSphere is published by John Wiley & Sons Ltd on behalf of European Hematology Association. This is an open access Abstract Book distributed under the Attribution-NonCommercial-NoDerivs (CC BY-NC-ND), which allows third parties to download the articles and share them with others as long as they credit the author and the Abstract Book, but they cannot change the content in any way or use them commercially.
**Abstract Book Citations:** Authors, Title, HemaSphere, 2026;10:(S1):pages. The abstract book DOIs can be found on the "Author Information" page.
**Disclaimer:** Articles published in the journal HemaSphere exclusively reflect the opinions of the authors. The authors are responsible for all content in their abstracts including accuracy of the facts, statements, citing resources, etc.

### Summary/Conclusion

Comparative analysis of ET and post-ET MF samples reveals that fibrotic transformation is associated with distinct genomic features, including *CALR* driver mutations, accumulated non-driver mutations, and emergence of high-risk genes involving *ASXL1*, *TP53*, and *ZRSR2*. In contrast, stable ET is marked by triple-negative status and limited mutation burden. And DNA damage response and spliceosomal dysfunction may represent key mechanisms driving fibrotic transformation. These molecular signatures, integrated with established clinical risk factors, provide potential biomarkers for enhanced risk stratification and therapeutic targeting in ET patients.

This research was supported by the National Natural Science Foundation of China (No. 82570191) and the Zhejiang Provincial Health Highlevel Innovative Talent Project (2022-2026). *Correspondence to: Prof Jian Huang, E-mail: househuang@zju.edu.cn

(A)

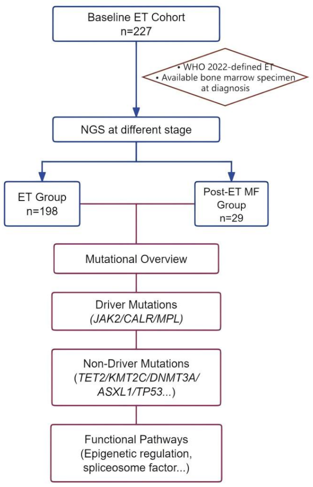

Table 1. Mutation profiles in ET vs. Post-ET MF patients

| Variables | Overall patients (n=227) | ET patients (n=198) | post-ET MF patients (n=29) | *P*-value |
|---|---|---|---|---|
| **Age (range)** | 45 (11-80) | 43 (11-80) | 62 (28-77) | **<0.001** |
| **Non-driver mutation count** | 2 (1-4) | 2 (1-3) | 3 (2-5) | **0.014** |
| **Driver mutation** | | | | |
| *JAK2* | 106 (46.7) | 92 (46.5) | 14 (48.3) | 0.885 |
| *CALR* | 58 (25.6) | 45 (22.7) | 13 (44.8) | **0.011** |
| *MPL* | 7 (3.1) | 7 (3.5) | 0 | 0.599 |
| Triple negative | 56 (24.7) | 54 (27.3) | 2 (6.9) | **0.032** |
| **Other gene mutation** | | | | |
| *TET2* | 49 (21.6) | 40 (20.2) | 9 (31.0) | 0.185 |
| *KMT2C* | 36 (15.9) | 30 (15.2) | 6 (20.7) | 0.446 |
| *KMT2D* | 26 (11.5) | 24 (12.1) | 2 (6.9) | 0.608 |
| *DNMT3A* | 23 (10.1) | 19 (9.6) | 4 (13.8) | 0.711 |
| *ASXL1* | 22 (9.7) | 15 (7.6) | 7 (24.1) | **0.005** |
| *KMT2A* | 17 (7.5) | 15 (7.6) | 2 (6.9) | 1.000 |
| *TP53* | 13 (5.7) | 7 (3.5) | 6 (20.7) | **<0.001** |
| *ZRSR2* | 10 (4.4) | 6 (3.0) | 4 (13.8) | **0.031** |
| *PPM1D* | 7 (3.1) | 4 (2.0) | 3 (10.3) | **0.046** |
| *SAMD9* | 5 (2.2) | 2 (1.0) | 3 (10.3) | **0.016** |
| *DDX41* | 3 (1.3) | 1 (0.5) | 2 (6.9) | **0.044** |

(B) (C) (D)

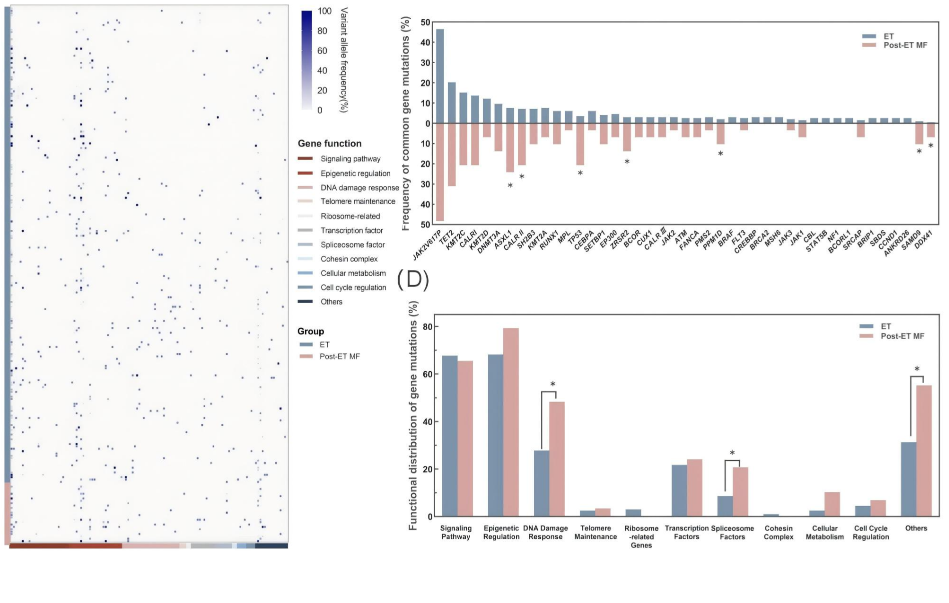

**Copyright Information:** (Online) ISSN: 2572-9241
©2026 The Author(s). HemaSphere is published by John Wiley & Sons Ltd on behalf of European Hematology Association. This is an open access Abstract Book distributed under the Attribution-NonCommercial-NoDerivs (CC BY-NC-ND), which allows third parties to download the articles and share them with others as long as they credit the author and the Abstract Book, but they cannot change the content in any way or use them commercially.
**Abstract Book Citations:** Authors, Title, HemaSphere, 2026;10:(S1):pages. The abstract book DOIs can be found on the “Author Information” page.
**Disclaimer:** Articles published in the journal HemaSphere exclusively reflect the opinions of the authors. The authors are responsible for all content in their abstracts including accuracy of the facts, statements, citing resources, etc.

### (PB3094) EARLY DISEASE PROGRESSION IN LOWER-RISK MYELODYSPLASTIC SYNDROMES: CLINICAL AND CYTOGENETIC PREDICTORS IN A REAL-WORLD COHORT

**Topic:** 10. Myelodysplastic syndromes - Clinical

Elif Naz Kabukcu[1], Furkan Kabukcu[1], Hakan Goker*[1], Haluk Demiroglu[1], Yahya Buyukasik[1], Elifcan Aladag[1]

*[1]Hacettepe, Department of Hematology, Altındağ, Türkiye;*

### Background

Lower-risk myelodysplastic syndromes (LR-MDS) are generally considered indolent; however, a subset of patients experiences early progression to higher-risk disease or acute myeloid leukemia (AML). Although certain cytogenetic abnormalities have traditionally been categorized as favorable, emerging data suggest substantial biological heterogeneity even within these groups.

### Aims

We aimed to identify clinical and cytogenetic predictors of early disease progression in a real-world LR-MDS cohort.

### Methods

We retrospectively analyzed 262 adult patients diagnosed with low- or intermediate-risk MDS (IPSS-R) between 2010 and 2024. Patients received supportive or conventional therapies according to institutional practice. Disease progression was defined as evolution to high/very high-risk MDS or AML. Time-to-progression (TTP) and overall survival (OS) were assessed using Kaplan–Meier methods. Logistic regression identified predictors of progression, and Cox proportional hazards models evaluated survival determinants.

### Results

The median age was 65 years (range 18–88), and 104 patients were female (39.7%) and 158 were male (60.3%). The median follow-up duration was 27.2 months (range 1–201). During follow-up, 23 patients (8.8%) developed disease progression. Median TTP was 18.6 months (range 1–202). Mortality among patients with progression was 82.6%, with a median OS of 24 months after progression. Baseline IPSS-R category was significantly associated with progression ($p=0.002$). Bone marrow blast percentage ≥5% independently predicted progression (OR 3.93; $p=0.007$). The presence of del(5q) was also independently associated with progression (OR 4.06; $p=0.031$). In unadjusted survival analysis, del(5q) was associated with inferior median OS (26.4 vs 47.3 months; $p=0.035$), but it did not retain independent prognostic significance in multivariable Cox analysis (HR 1.15; $p=0.634$).

### Summary/Conclusion

In this real-world LR-MDS cohort, disease progression occurred early in a subset of patients. Although del(5q) is traditionally considered favorable-risk, our findings suggest potential heterogeneity within this cytogenetic subgroup. Molecular profiling was not systematically available, and co-occurring genetic alterations could not be assessed; therefore, the observed association between del(5q) and progression should be interpreted with caution. Prospective studies incorporating comprehensive molecular data are warranted to refine risk stratification in LR-MDS

**Copyright Information:** (Online) ISSN: 2572-9241
©2026 The Author(s). HemaSphere is published by John Wiley & Sons Ltd on behalf of European Hematology Association. This is an open access Abstract Book distributed under the Attribution-NonCommercial-NoDerivs (CC BY-NC-ND), which allows third parties to download the articles and share them with others as long as they credit the author and the Abstract Book, but they cannot change the content in any way or use them commercially.
**Abstract Book Citations:** Authors, Title, HemaSphere, 2026;10:(S1):pages. The abstract book DOIs can be found on the "Author Information" page.
**Disclaimer:** Articles published in the journal HemaSphere exclusively reflect the opinions of the authors. The authors are responsible for all content in their abstracts including accuracy of the facts, statements, citing resources, etc.

### (PB3095) EVALUATING MLPA AS A COST-EFFECTIVE DIAGNOSTIC TOOL IN MYELODYSPLASTIC SYNDROMES: A RETROSPECTIVE COMPARISON WITH CYTOGENETICS AND FISH

**Topic:** 10. Myelodysplastic syndromes - Clinical

Anna Szilágyi*[1], László Rejtő[2], Zsófia Miltényi[3], Anikó Ujfalusi[1]

*[1]University of Debrecen, Faculty of Medicine, Department of Medical Genetics, Debrecen, Hungary;[2]Szabolcs-Szatmár-Bereg County Teaching Hospital, Jósa András Member Hospital, Department of Hematology, Nyíregyháza, Hungary; [3]University of Debrecen, Faculty of Medicine, Department of Hematology, Debrecen, Hungary;*

### Background

Myelodysplastic syndromes (MDS) are clonal myeloid neoplasms characterized by dysplastic and ineffective hematopoiesis, leading to peripheral cytopenias. Accurate diagnosis is often challenging due to heterogeneous clinical presentation and the complexity of underlying genomic alterations. Conventional karyotyping and fluorescence in situ hybridization (FISH) frequently fail to detect abnormalities, with more than half of cases yielding negative results. According to the 2020 ESMO guideline, optional FISH testing following negative or unsuccessful karyotyping includes 5q31, cen7, 7q31, cen8, *TP53*, 20q, and cenY. Given the number of required probes and the labor-intensive workflow, multiplex ligation-dependent probe amplification (MLPA) may offer a more efficient alternative for detecting copy number variations (CNVs).

### Aims

Our aim was to assess the diagnostic value of MLPA in detecting CNVs in MDS patients and to compare its performance with routine cytogenetics and FISH. MLPA enables simultaneous evaluation of multiple loci, including several incorporated into the IPSS-R for diagnostic and prognostic relevance.

### Methods

This retrospective study included 20 patients referred with MDS. Conventional chromosome analysis and FISH were performed on cultured bone marrow sample anticoagulated with sodium heparin. DNA was isolated from a separate bone marrow sample collected on the same day (anticoagulated with EDTA) and used for myeloid gene panel by next-generation sequencing (NGS). MLPA was performed using the SALSA MLPA Probemix P414 MDS kit (MRC Holland), which is specifically designed for detecting MDS-related CNVs as well as the *JAK2* V617F mutation.

### Results

Ten patients had MDS-related clonal chromosome abnormalities detected by karyotyping and/or FISH, while the remaining ten cases showed normal karyotype but carried small-scale oncogenic variants identified by NGS. MLPA confirmed cytogenetic abnormalities in 9/10 (90%) cytogenetically positive cases. One false negative result occurred in a sample with low-level (≤10%) del(5q) revealed by FISH. In two cases, MLPA refined cytogenetic findings by more precisely delineating the affected chromosomal regions. Among the ten cytogenetically negative cases, MLPA identified a clinically relevant CNV (*KMT2A* duplication) in 1/10 (10%) patients, providing additional prognostic information. MLPA detected *JAK2* V617F in 2/2 (100%) cases, concordant with NGS results, supporting its utility in distinguishing MDS from MDS/MPN overlap syndromes with a shorter turnaround time.

**Copyright Information:** (Online) ISSN: 2572-9241
©2026 The Author(s). HemaSphere is published by John Wiley & Sons Ltd on behalf of European Hematology Association. This is an open access Abstract Book distributed under the Attribution-NonCommercial-NoDerivs (CC BY-NC-ND), which allows third parties to download the articles and share them with others as long as they credit the author and the Abstract Book, but they cannot change the content in any way or use them commercially.
**Abstract Book Citations:** Authors, Title, HemaSphere, 2026;10:(S1):pages. The abstract book DOIs can be found on the “Author Information” page.
**Disclaimer:** Articles published in the journal HemaSphere exclusively reflect the opinions of the authors. The authors are responsible for all content in their abstracts including accuracy of the facts, statements, citing resources, etc.

**Summary/Conclusion**

MLPA showed strong concordance with conventional cytogenetics and provided added value through rapid processing, multilocus assessment, and finer resolution of selected abnormalities. It is cost-effective, culture-independent, though false-negative results may occur when the abnormal clone is present at low frequency. The study will be expanded to a larger cohort, including patients with unsuccessful cytogenetic analysis. With more robust data, revision of the diagnostic algorithm may be considered to support more efficient workflows in MDS diagnostics.

**Copyright Information:** (Online) ISSN: 2572-9241
©2026 The Author(s). HemaSphere is published by John Wiley & Sons Ltd on behalf of European Hematology Association. This is an open access Abstract Book distributed under the Attribution-NonCommercial-NoDerivs (CC BY-NC-ND), which allows third parties to download the articles and share them with others as long as they credit the author and the Abstract Book, but they cannot change the content in any way or use them commercially.
**Abstract Book Citations:** Authors, Title, HemaSphere, 2026;10:(S1):pages. The abstract book DOIs can be found on the "Author Information" page.
**Disclaimer:** Articles published in the journal HemaSphere exclusively reflect the opinions of the authors. The authors are responsible for all content in their abstracts including accuracy of the facts, statements, citing resources, etc.

### (PB3096) EXTENDED FOLLOW-UP OF LUSPATERCEPT TREATMENT IN LOWER-RISK, TRANSFUSION-DEPENDENT MYELODYSPLASTIC SYNDROMES WITH RING SIDEROBLASTS: HEMATOLOGIC RESPONSE AND MULTILINEAGE EFFECTS

**Topic:** 10. Myelodysplastic syndromes - Clinical

Mara Memoli*[1], Stefania Leone[2], Francesco Grimaldi[1], Simona Avilia[1], Novella Pugliese[1], Maria Di Perna[3], Catello Califano[3], Danilo De Novellis[4], Antonella Malandrini[5], Bianca Serio[4], Camilla Frieri[6], Giuseppe Gaeta[6], Chiara Masucci[6], Alessandro D'ambrosio[7], Paola Della Cioppa[8], Giuseppe Cerciello[9], Tiziana Izzo[2], Mario Annunziata[10], Annamaria Maddaloni[1], Cono Quagliano[1], Giorgia Battipaglia[1], Marco Picardi[1], Fabrizio Pane[1]

*[1]Department of clinical medicine and surgery, hematology unit, University of Naples Federico II, Naples, Italy;[2]Hematopoietic Stem Cell Transplantation Unit, AORN Cardarelli, Naples, Italy; [3]UOC Ematologia, Presidio Ospedaliero Andrea Tortora ASL Salerno, Pagani, Italy; [4]Hematology and Transplant Center, University Hospital San Giovanni di Dio e Ruggi d'Aragona, Department of Medicine, Surgery and Dentistry Scuola Medica Salernitana University of Salerno, Salerno, Italy; [5]Hematology and Transplant Center, University Hospital San Giovanni di Dio e Ruggi d'Aragona, Salerno, Italy; [6]Hematology and Bone Marrow Transplant Unit AORN S. Giuseppe Moscati, Avellino, Italy; [7]AORN S.Anna e S. Sebastiano, Caserta, Italy;[8]Hematology Unit, Azienda Sanitaria Locale Napoli 1 Centro, 80147, Naples, Italy; [9]San Pio Hospital, Hematology Unit, Benevento, Italy; [10]Division of Hematology, AORN Cardarelli, Naples, Italy;*

### Background

Luspatercept (Luspa) is a first-in-class erythroid maturation agent initially approved for transfusion-dependent anemia (RBC-TD) refractory/ineligible to erythropoiesis-stimulating agents (ESAs) in patients with lower risk Myelodysplastic Syndromes with Ring Sideroblasts (LR-MDS-RS). Despite evidence of Luspa efficacy in both clinical trials and real-world studies, long-term follow-up data in real-world settings are still lacking. Moreover, in registration studies, Luspa was effective in inducing a hematological improvement also on granulocytic and platelets lines.  Thus, Luspa long-term efficacy in real-life setting, as well as its impact on others cell lines is still an unexplored issue.

### Aims

This study aims to evaluate the long-term efficacy of Luspatercept on erythroid response in a real-world setting, as well as its impact on other haematopoietic cell lineages.

### Methods

We retrospectively collected data of 48 patients diagnosed with MDS with low blasts and *SF3B1* mutation (MDS-*SF3B1*) and/or ring sideroblasts, according to WHO 2022, and suffered from a RBC-TD anemia. Luspa treatment was administered in conformity with manufacturer instruction. Response was evaluated at 6 months, 1 and 2 year, applying IWG 2018 response criteria.  Data have been analyzed with GraphPad Prism 9.0 and R software version 4.3.2.

**Copyright Information:** (Online) ISSN: 2572-9241
©2026 The Author(s). HemaSphere is published by John Wiley & Sons Ltd on behalf of European Hematology Association. This is an open access Abstract Book distributed under the Attribution-NonCommercial-NoDerivs (CC BY-NC-ND), which allows third parties to download the articles and share them with others as long as they credit the author and the Abstract Book, but they cannot change the content in any way or use them commercially.
**Abstract Book Citations:** Authors, Title, HemaSphere, 2026;10:(S1):pages. The abstract book DOIs can be found on the "Author Information" page.
**Disclaimer:** Articles published in the journal HemaSphere exclusively reflect the opinions of the authors. The authors are responsible for all content in their abstracts including accuracy of the facts, statements, citing resources, etc.

### Results

Patient characteristics are summarized in Table 1. At 6 months, 23/42 (54.8%) and 15/42 (35.7%) patients reached a response and transfusion independence, respectively. At 1 year, 16/30 (53.3%) patients showed a response, including 12 patients who already responded at 6 months. At 2 years, 10/20 patients maintained a previous response and 2/20 patients were de novo responders. No patients had remarkable side effects, 21 (43.8%) patients discontinued Luspa principally due to lack of response. With a median follow-up of 29.8 months, Overall survival (OS) significantly differed in responders groups (p<0.05). Predictive factors of short-term response were IPSS-M (p<0.05), additional somatic mutations (p<0.05) and SF3B1 hotspot mutation (p<0.05), while monocyte count predicted 2 years response (p<0.05).
Considering the whole cohort, neutrophils at 1 year were significantly increased compared to pre-Luspa levels (p<0.05), while platelets did not showed any variation. Intriguing, monocytes were increased at all three time points compared to baseline (p<0.05). Specifically, long-term responders presented highter levels of neutrophils and monocytes, as well decreased level of LDH, compared to non-responders (p<0.05).

Table 1.

| **Patients characteristics** | **Total = 48** |
|---|---|
| **Baseline characteristics** | **Median (IQR)** |
| Age | 76.32 (67,23–80,94) |
| Male, n (%) | 25 (52,1) |
| Platelet count, × 10-9/L | 296 (151–360) |
| Neutrophil count, × 10-6/L | 2.21 (1,35-3,62) |
| Baseline transfusion-burden category | **n (%)** |
| - High Transfusion burden (≥4 units/8 weeks) | 28 (58,3) |
| - Low Transfusion burden (<4 units/8 weeks) | 20 (41,7) |
| Previous iron chelation therapy, n (%) | 21 (47,7) |
| Baseline genetics | **n (%)** |
| IPSS-R risk | |
| Very low | 6 (13,6) |
| Low | 31 (67,4) |
| Intermediate | 9 (19,6) |
| M-IPSS risk | |
| Very Low | 6 (14,3) |
| Low | 26 (61,9) |
| Moderately Low | 8 (19) |
| Moderately High | 1 (2,4) |
| High | 1 (2,4) |
| SF3B1 mutated | 44 (91,7) |
| SF3B1 hotspot | 19 (39,6) |
| Additional somatic mutations | 23 (52,3) |

### Summary/Conclusion

In our cohort, Luspa is capable of maintaining erythroid response in long-term follow-up. The possible capability of Luspa to induce a haematological improvement on granulocytic and monocyte lines needs further investigations.

**Copyright Information:** (Online) ISSN: 2572-9241
©2026 The Author(s). HemaSphere is published by John Wiley & Sons Ltd on behalf of European Hematology Association. This is an open access Abstract Book distributed under the Attribution-NonCommercial-NoDerivs (CC BY-NC-ND), which allows third parties to download the articles and share them with others as long as they credit the author and the Abstract Book, but they cannot change the content in any way or use them commercially.
**Abstract Book Citations:** Authors, Title, HemaSphere, 2026;10:(S1):pages. The abstract book DOIs can be found on the "Author Information" page.
**Disclaimer:** Articles published in the journal HemaSphere exclusively reflect the opinions of the authors. The authors are responsible for all content in their abstracts including accuracy of the facts, statements, citing resources, etc.

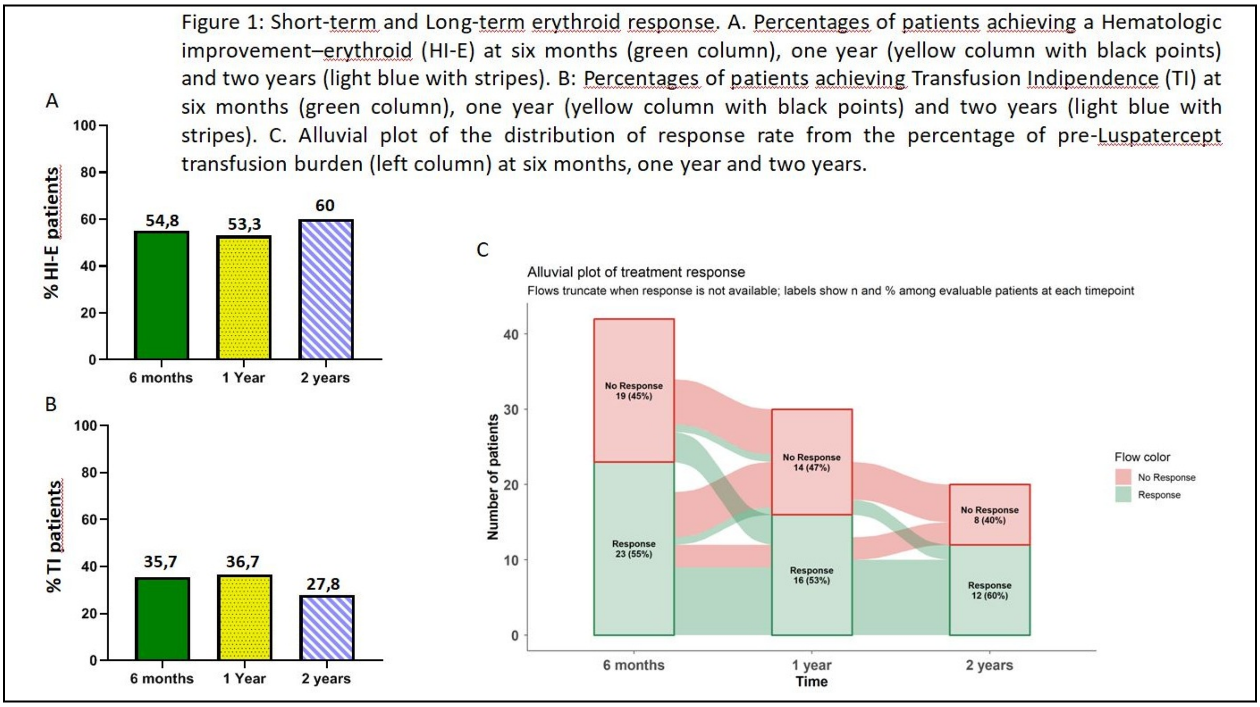

Figure 1: Short-term and Long-term erythroid response. A. Percentages of patients achieving a Hematologic improvement–erythroid (HI-E) at six months (green column), one year (yellow column with black points) and two years (light blue with stripes). B: Percentages of patients achieving Transfusion Indipendence (TI) at six months (green column), one year (yellow column with black points) and two years (light blue with stripes). C. Alluvial plot of the distribution of response rate from the percentage of pre-Luspatercept transfusion burden (left column) at six months, one year and two years.

**Copyright Information:** (Online) ISSN: 2572-9241
©2026 The Author(s). HemaSphere is published by John Wiley & Sons Ltd on behalf of European Hematology Association. This is an open access Abstract Book distributed under the Attribution-NonCommercial-NoDerivs (CC BY-NC-ND), which allows third parties to download the articles and share them with others as long as they credit the author and the Abstract Book, but they cannot change the content in any way or use them commercially.
**Abstract Book Citations:** Authors, Title, HemaSphere, 2026;10:(S1):pages. The abstract book DOIs can be found on the "Author Information" page.
**Disclaimer:** Articles published in the journal HemaSphere exclusively reflect the opinions of the authors. The authors are responsible for all content in their abstracts including accuracy of the facts, statements, citing resources, etc.

### (PB3097) IMMUNOPHENOTYPIC AND MOLECULAR GENETIC MARKERS IN MDS AS PREDICTORS OF DISEASE OUTCOME.

**Topic:** 10. Myelodysplastic syndromes - Clinical

Anna Shevchenko*[1], Ihar Iskrou[1, 2], Iryna Lendzina[2], Tatiana Lebedeva[2]

*[1]Minsk Scientific And Practical Center For Surgery, Transplantology and Hematology, depatment of hematology 2, Minsk, Belarus; [2]Minsk Scientific And Practical Center For Surgery, Transplantology and Hematology, Minsk, Belarus;*

### Background

The number of blasts in the bone marrow is a critical marker in the diagnosis and classification of MDS, and flow cytometry has become essential when morphological assessment is difficult or unavailable. In clinical practice, a comprehensive approach (cytomorphological examination and flow cytometry) allows for rapid evaluation and verification of MDS, especially in cases of low-cell count bone marrow.
The use of flow cytometry in patients with MDS is important not only for diagnosing the disease but also for predicting the risk of transformation to acute leukemia or death and assessing the effectiveness of treatment. Existing prognostic scales are based on cytomorphological and cytogenetic data, but an immunophenotypic approach to cell analysis is equally important for diagnostic purposes, not to mention for prognostic assessment and treatment response.

### Aims

The aim of the study is to improve treatment outcomes in patients with MDS by using immunophenotypic and molecular genetic markers to predict response.

### Methods

We conducted a multivariate analysis using the Cox-Markov model, specifically the three-state "disease-death" model, which reflects the probability of the presence of a specific parameter affecting the risk of death, as well as the risk of transformation to acute leukemia and subsequent risk of death. The multivariate analysis included variable immunophenotypic and molecular genetic markers characteristic of MDS. This statistical method allows us to determine the significance of various factors in the created model.

### Results

A prospective cohort study included 62 patients with newly diagnosed MDS. Based on the results obtained, reliable immunophenotypic and molecular genetic markers of unfavorable prognosis were identified: 1) the "diagnosis - death" model: CD 38<50% HR 3.7 (1.2-11.5 CI; p=0.022), CD 13>50% HR 8.7 (1.1-67.8 CI; p=0.04), complex aberrations >3 HR 5.8 (1.4-24.6 CI; p=0.015); 2) the "diagnosis - transformation" model: CD 71≥65% HR 4.1 (1.35-12.4 CI; p=0.013); CD 13>75% HR 2.8 (1.1-7.1 CI; p=0.034), complex aberrations >3 HR 2.8 (0.86-9.0 CI; p=0.087); 3) "transformation-death" model: CD 25 <5%HR 6.3 (1.4-28/4 CI; p=0.017), CD 33 <50% HR 6.6 (1.3-34.7 CI; p=0.026), trisomy 8 HR 7.9 (0.71-88 CI; p=0.093); complex aberrations >3 HR 0.22 (0.06-0.84 CI; p=0.027). Patients with two or more of the above-mentioned immunophenotypic and molecular genetic markers were assigned to unfavorable prognosis groups. When comparing two equally significant unfavorable prognosis groups before differentiated therapy (group 1) and after differentiated therapy (group 2), the following preliminary results were obtained (shown in the figure).
The figure shows that in group 1, partial remission and complete remission were achieved in 25% of cases over 6 months of observation, and in group 2, partial and complete remission were achieved in 81.25% of cases (Fisher test p=0.025).

**Copyright Information:** (Online) ISSN: 2572-9241
©2026 The Author(s). HemaSphere is published by John Wiley & Sons Ltd on behalf of European Hematology Association. This is an open access Abstract Book distributed under the Attribution-NonCommercial-NoDerivs (CC BY-NC-ND), which allows third parties to download the articles and share them with others as long as they credit the author and the Abstract Book, but they cannot change the content in any way or use them commercially.
**Abstract Book Citations:** Authors, Title, HemaSphere, 2026;10:(S1):pages. The abstract book DOIs can be found on the "Author Information" page.
**Disclaimer:** Articles published in the journal HemaSphere exclusively reflect the opinions of the authors. The authors are responsible for all content in their abstracts including accuracy of the facts, statements, citing resources, etc.

**Summary/Conclusion**

Immunophenotypic and molecular genetic markers of poor prognosis are predictors of disease outcome. By timely predicting the progression to acute leukemia and the expected life expectancy of patients with various MDS variants, even during diagnostic verification, clinicians can influence the achievement of remission and thereby improve the quality of life of patients with MDS.

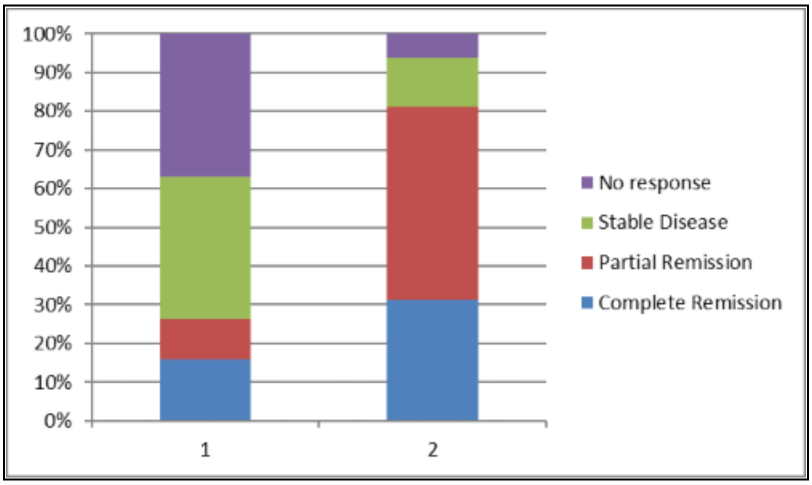

**Copyright Information:** (Online) ISSN: 2572-9241
©2026 The Author(s). HemaSphere is published by John Wiley & Sons Ltd on behalf of European Hematology Association. This is an open access Abstract Book distributed under the Attribution-NonCommercial-NoDerivs (CC BY-NC-ND), which allows third parties to download the articles and share them with others as long as they credit the author and the Abstract Book, but they cannot change the content in any way or use them commercially.
**Abstract Book Citations:** Authors, Title, HemaSphere, 2026;10:(S1):pages. The abstract book DOIs can be found on the "Author Information" page.
**Disclaimer:** Articles published in the journal HemaSphere exclusively reflect the opinions of the authors. The authors are responsible for all content in their abstracts including accuracy of the facts, statements, citing resources, etc.

### (PB3098) EFFICACY EVALUATION OF LUSPATERCEPT IN MYELODYSPLASTIC SYNDROMES (MDS) PATIENTS WITH SF3B1 MUTATIONS WHO HAVE FAILED HYPOMETHYLATING AGENT THERAPY

**Topic:** 10. Myelodysplastic syndromes - Clinical

Chengtao Qin[1], Ting Zhang[2], Yawen Zhang[1], Yuemin Gong[1], Guangsheng He*[1]

*[1]The First Affiliated Hospital with Nanjing Medical University, Nanjing, China;[2]Nanjing Jiangning Hospital, affiliated to Nanjing Medical University, Nanjing, China;*

### Background

Luspatercept is approved for treatment of anemia in MDS patients with lower IPSS-R risk. In China, medical insurance only covers HMAs in treatment of severe cytopenia in MDS, leading most transfusion-dependent MDS patients to have to choose HMAs. Outcome after HMAs failure is poor, especially in China, where there is a severe shortage of blood supply. Preceding luspatercept study did not enroll patients who are refractory to HMAs or relapse after HMAs.

### Aims

This study aimed to evaluate the efficacy of luspatercept in myelodysplastic syndromes (MDS) patients with SF3B1 mutations who have failed hypomethylating agent therapy in a prospective multicenter study (ChiCTR2300075883).

### Methods

Eligible patients were diagnosed with MDS with SF3B1 mutations, had failed prior HMA therapy, and required ≥2 units of red blood cells per 8 weeks. Patients received luspatercept subcutaneously every 3 weeks. The primary endpoint was red blood cell transfusion independence (RBC-TI) for ≥8 weeks during weeks 1-24. Secondary endpoints included RBC-TI for ≥12 and ≥16 weeks, erythroid hematological improvement (HI-E) , neutrophile hematological improvement (HI-N), platelet hematological improvement (HI-P) duration of transfusion independence, and safety.

### Results

From September 2023 to January 2026, 20 MDS patients with anemia were enrolled and treated with luspatercept for a median of 10 cycles.The median age was 68 years (range, 42–80 years), with an equal sex distribution (50% male and 50% female). Its baseline hematological parameters are shown in Table 1. Regarding disease risk stratification, the majority of patients were classified as lower risk according to IPSS-R (65% vs. 35% higher risk), whereas IPSS-M categorization showed a predominance of higher-risk disease (65% vs. 35% lower risk). Ring sideroblasts were present in 60% of patients (≥5% in 12 patients). With respect to prior treatment, 45% of patients had received erythropoiesis-stimulating agent (ESA) therapy without success, while 55% were ESA-naïve.(Table 1).Treatment efficacy was assessed using multiple clinically relevant endpoints. Six patients met the primary endpoint, which was the achievement of RBC-TI at 8 weeks. At the secondary endpoint, RBC-TI was achieved by 30% of patients at 12-week time points, with 20% maintaining TI for at least 16 weeks. Hematologic improvement was observed in 30% of patients for HI-E, 25% for HI-N, and 15% for HI-P. Among responders, the median duration of RBC transfusion independence was 146.5 days (range, 90–377 days). Notably, no Grade 3 adverse events were reported during the study period, indicating a favorable safety profile (Table 2).

### Summary/Conclusion

Luspatercept demonstrates clinical activity in transfusion-dependent MDS patients with SF3B1 mutations who have failed HMA therapy.

**Copyright Information:** (Online) ISSN: 2572-9241
©2026 The Author(s). HemaSphere is published by John Wiley & Sons Ltd on behalf of European Hematology Association. This is an open access Abstract Book distributed under the Attribution-NonCommercial-NoDerivs (CC BY-NC-ND), which allows third parties to download the articles and share them with others as long as they credit the author and the Abstract Book, but they cannot change the content in any way or use them commercially.
**Abstract Book Citations:** Authors, Title, HemaSphere, 2026;10:(S1):pages. The abstract book DOIs can be found on the “Author Information” page.
**Disclaimer:** Articles published in the journal HemaSphere exclusively reflect the opinions of the authors. The authors are responsible for all content in their abstracts including accuracy of the facts, statements, citing resources, etc.

Table 1 Baseline Patients Characteristics (Data are n (%) and median (range).)

| Characteristics | |
|---|---|
| **Age（yrs）** | 68[42-80] |
| **HB（g/L）** | 62.5[47.00-80.00] |
| **Neutrophil（×109/L）** | 1[0.19-13.40] |
| **Platelet（×109/L）** | 59[3.00-406.00] |
| **ErythropoieticprecursorsinBM（%）** | 52.4[4.50-79.80] |
| **RBC transfusion burden(unitsper8wks)** | 6.5[2-18] |
| **Gender** | |
| Male | 10(50%) |
| Female | 10(50%) |
| **Ring sideroblasts** | |
| ≥5% | 12(60%) |
| <5% | 8(40%) |
| **IPSS-R** | |
| lower risk | 13(65%) |
| higher risk | 7(35%) |
| **IPSS-M** | |
| lower risk | 7(35%) |
| higher risk | 13(65%) |
| **Erythropoiesis-stimulating agent therapy** | |
| failed | 9(45%) |
| naive | 11(55%) |

提取文字 更多

Table 2 Primary and Secondary Endp[illegible] median (range).)

| Endpoint | |
|---|---|
| **RBC- TI≥8w** | 6(30%) |
| **RBC- TI≥12w** | 6(30%) |
| **RBC- TI≥16w** | 4(20%) |
| **HI-E** | 6(30%) |
| **HI-N** | 5(25%) |
| **HI-P** | 3(15%) |
| **Duration of RBC-TI/d** | 146. 5[90-377] |
| **Grade 3 adverse event** | 0 |

**Copyright Information:** (Online) ISSN: 2572-9241
©2026 The Author(s). HemaSphere is published by John Wiley & Sons Ltd on behalf of European Hematology Association. This is an open access Abstract Book distributed under the Attribution-NonCommercial-NoDerivs (CC BY-NC-ND), which allows third parties to download the articles and share them with others as long as they credit the author and the Abstract Book, but they cannot change the content in any way or use them commercially.
**Abstract Book Citations:** Authors, Title, HemaSphere, 2026;10:(S1):pages. The abstract book DOIs can be found on the "Author Information" page.
**Disclaimer:** Articles published in the journal HemaSphere exclusively reflect the opinions of the authors. The authors are responsible for all content in their abstracts including accuracy of the facts, statements, citing resources, etc.

### (PB3099) THREE VENETOCLAX SCHEDULES AND CORRELATION WITH OVERALL SURVIVAL IN HIGH-RISK MYELODYSPLASTIC SYNDROME (MDS)

**Topic:** 10. Myelodysplastic syndromes - Clinical

Maher Salamoon*[1]

[1]*al bairouni university cancer center, hematology/oncology, Damascus, Syrian Arab Republic;*

### Background

MDS is a heterogenous disease from a morphologic and genetic perspective. It is considered a background for development of Acute Myelogenous Leukemia (AML) especially in patients with high-risk groups. A minority of patients are eligible for allogeneic bone marrow transplants. In this high-risk group, patients are treated the same way AML is treated especially with the emergence of new drugs such as anti-BCL-2 drugs in combination with hypomethylating agents (HMA)

### Aims

Is to evaluate the efficacy and safety of 3 Venetoclax doses schedule in combination with Azacitidine in newly diagnosed high-risk patients with MDS.

### Methods

Patients are given azacitidine 75mg/m² for 7 days in 28 days schedule in all the 30 recruited patients. Regarding Venetoclax, 10 patients received 400mg per day in parallel with Azacitidine (Arm 1), 10 patients received 200mg per day for 21 days (Arm 2) and the rest of 10 patients received 100mg per day in continuous (Arm 3). Bone marrow aspiration performed after each cycle with morphologic and flowcytometric studies. Toxicity profile was registered in each patient including adherence to treatment. Azole antifungal prophylaxis was not given to our patients.

### Results

We have included 30 newly diagnosed patients with high-risk AML with ages ranging between 48 and 77 years (63 years in median). A male to female ratio 2:1. Complete response was documented in 4 patients in arm 1 (40%), 5 patients in arm 2 (50%) and 4 patients in arm 3 (40%). Adherence to treatment was 90% in arm 1, 80% in arm 2 and 70% in arm 3. Neutropenia was documented in 100% of patients in arm 1, 80% in patients of arm 2 and 30% in patients with arm 3. In general, after the second cycle, all 30 patients could not adhere to the treatment plan as scheduled before. We have lost one patient in arm 1, two patients in arm 2 and one patient in arm 3 after the end of the first induction. The overall survival rate after 12 months of follow-up was 70% in arm 1, 80% in arm 2 and 80% in arm 3. Toxicity profile was worse in arm 1 compared with the other 2 arms. Tolerance and good adherence to treatment was correlated with better response in general irrespective of the studied arm and the dose taken p value 0.05.

### Summary/Conclusion

In high-risk MDS, treatment with Venetoclax and HMA demonstrates good response and survival irrespective of the dose of Venetoclax. However: low doses of Venetoclax confer good adherence and consequently good response. Bigger trials are warranted to reach the preferred dose of Venetoclax in high-risk MDS.

**Copyright Information:** (Online) ISSN: 2572-9241
©2026 The Author(s). HemaSphere is published by John Wiley & Sons Ltd on behalf of European Hematology Association. This is an open access Abstract Book distributed under the Attribution-NonCommercial-NoDerivs (CC BY-NC-ND), which allows third parties to download the articles and share them with others as long as they credit the author and the Abstract Book, but they cannot change the content in any way or use them commercially.
**Abstract Book Citations:** Authors, Title, HemaSphere, 2026;10:(S1):pages. The abstract book DOIs can be found on the “Author Information” page.
**Disclaimer:** Articles published in the journal HemaSphere exclusively reflect the opinions of the authors. The authors are responsible for all content in their abstracts including accuracy of the facts, statements, citing resources, etc.

### (PB3100) ANALYSIS OF THE DISTRIBUTION CHARACTERISTICS AND PROGNOSTIC IMPACT OF GENE MUTATIONS IN PATIENTS WITH MYELODYSPLASTIC SYNDROMES BASED ON NEXT-GENERATION SEQUENCING

**Topic:** 10. Myelodysplastic syndromes - Clinical

Rui Zhang*[1], Tao Wu[1]

[1] *The 940th Hospital of Joint Logistics Support force of Chinese People's Liberation Army, hematology, Gansu, China;*

### Background

Myelodysplastic syndrome (MDS) is a group of acquired clonal hematologic malignancies originating from hematopoietic stem cells, primarily characterized by ineffective hematopoiesis in the bone marrow and single or multilineage cytopenia in the peripheral blood caused by unilineage or multilineage dysplasia of myeloid hematopoietic stem and progenitor cells, with a high risk of transformation into acute myeloid leukemia (AML) in the advanced stage. The incidence of MDS has been on a yearly rise, with an increased prevalence in the aging population. Relevant studies have indicated that the global incidence of MDS is approximately 2 to 12 per 100,000 population, and the incidence in China is 0.23 to 1.51 per 100,000 population; over 80% of patients are diagnosed at the age of more than 60 years, with a slight male predominance. At present, the diagnosis of MDS is an exclusionary diagnosis that requires the elimination of other diseases with overlapping features such as reactive cytopenia or cellular dysplasia. Its diagnosis relies on a variety of laboratory examinations, centering on bone marrow morphocytology and cellular and molecular genetic detection techniques, supplemented by bone marrow histopathological examination and immunophenotyping via bone marrow flow cytometry. In recent years, with the continuous innovation of molecular detection technologies, next-generation sequencing (NGS) has been increasingly applied in the research, diagnosis and treatment of hematologic diseases due to its advantages of high accuracy (high sensitivity), comprehensiveness (high throughput) and low cost, leading to the gradual identification of an increasing number of mutated genes.

### Aims

To characterize the molecular genetic profile and identify prognostic factors in patients with myelodysplastic syndromes (MDS) using next-generation sequencing (NGS).

### Methods

Clinical data of newly diagnosed MDS patients admitted to the 940th Hospital of the Joint Logistics Support Force between January 2019 and December 2024 were retrospectively analyzed. NGS, Kaplan–Meier survival analysis, and Cox multivariate regression were used to assess risk factors for overall survival (OS). According to the Revised International Prognostic Scoring System (IPSS-R), 109 patients were divided into a lower-risk group (IPSS-R ≤ 3.5; n = 51) and a higher-risk group (IPSS-R > 3.5; n = 58).

### Results

The cohort comprised 109 patients (male-to-female ratio 1.18:1). Median age was 61 years (19–88) in the lower-risk group and 59 years (19–89) in the higher-risk group. All patients underwent NGS. Genes mutated in >10% of cases were ASXL1 (21.1 %), TET2 (17.4 %), U2AF1 (16.5 %), SF3B1 (11.9 %), TP53 (11.0 %), and DNMT3A (10.1 %). Univariate analysis showed that age group, IPSS-R category, number of mutations, and presence of ASXL1 or TP53 mutations significantly affected OS. Multivariate analysis identified age, ASXL1 mutation, and TP53 mutation as independent risk factors for OS.

**Copyright Information:** (Online) ISSN: 2572-9241
©2026 The Author(s). HemaSphere is published by John Wiley & Sons Ltd on behalf of European Hematology Association. This is an open access Abstract Book distributed under the Attribution-NonCommercial-NoDerivs (CC BY-NC-ND), which allows third parties to download the articles and share them with others as long as they credit the author and the Abstract Book, but they cannot change the content in any way or use them commercially.
**Abstract Book Citations:** Authors, Title, HemaSphere, 2026;10:(S1):pages. The abstract book DOIs can be found on the "Author Information" page.
**Disclaimer:** Articles published in the journal HemaSphere exclusively reflect the opinions of the authors. The authors are responsible for all content in their abstracts including accuracy of the facts, statements, citing resources, etc.

**Summary/Conclusion**

MDS prognosis is influenced by multiple factors. Mutation frequencies differ across age groups, and ASXL1 or TP53 mutations predict poor outcome.

**Copyright Information:** (Online) ISSN: 2572-9241
©2026 The Author(s). HemaSphere is published by John Wiley & Sons Ltd on behalf of European Hematology Association. This is an open access Abstract Book distributed under the Attribution-NonCommercial-NoDerivs (CC BY-NC-ND), which allows third parties to download the articles and share them with others as long as they credit the author and the Abstract Book, but they cannot change the content in any way or use them commercially.
**Abstract Book Citations:** Authors, Title, HemaSphere, 2026;10:(S1):pages. The abstract book DOIs can be found on the "Author Information" page.
**Disclaimer:** Articles published in the journal HemaSphere exclusively reflect the opinions of the authors. The authors are responsible for all content in their abstracts including accuracy of the facts, statements, citing resources, etc.

### (PB3101) CLINICAL AND PATHOLOGICAL CHARACTERISTICS AND TREATMENT OUTCOMES IN PATIENTS WITH MYELODYSPLASTIC SYNDROME AND OTHER RELATED MYELOID DISORDERS

**Topic:** 10. Myelodysplastic syndromes - Clinical

Angham Sahli*[1], Ahmed Alaskar[2], Hind Salama[1], Mohsen Alzahrani[3], Abdukrhman Al Raiza[1], Bader Alahmari[3], Husam Alsadi[1], Abdullah Alsaleh[3], Ayman Ibrahim[1], Mohammed Bakkar[1], Ayel Yahya[1], Khalid Aldosari[1], Sumayyah Altamimi[1], Ayman Almozaini[1], Rawan Al Mohamady[1], Duha Al Ahmadi[1], Lulu Bin Dayel[1], Ayman Hejazi[3], Mohammed Al Khayat[1]

*[1]Oncology Department, King Abdulaziz Medical City, Ministry of National Guards - Health Affairs, Riyadh, Saudi Arabia,, Riyadh, Saudi Arabia; [2]Oncology Department, King Abdulaziz Medical City, Ministry of National Guards - Health Affairs, Riyadh, Saudi Arabia, 2King Abdullah International Medical Research Center, Riyadh, Saudi Arabia, 3College of Medicine, King Saud bin Abdulaziz University for Health Sciences, Riyadh, Saudi Arabia, Riyadh, Saudi Arabia; [3]Oncology Department, King Abdulaziz Medical City, Ministry of National Guards - Health Affairs, Riyadh, Saudi Arabia, 2King Abdullah International Medical Research Center, Riyadh, Saudi Arabia, 3College of Medicine, King Saud bin Abdulaziz University for Health Sciences, Riyadh, Saudi A, Riyadh, Saudi Arabia;*

### Background

Myelodysplastic syndrome (MDS) are myeloid neoplasms characterized by clonal proliferation of hematopoietic stem cells, recurrent genetic abnormalities, myelodysplasia, ineffective hematopoiesis, peripheral blood cytopenia, and a high risk of evolution to acute myeloid leukemia (AML).Other myeloid disorders related to MDS are chronic myelomonocytic leukemia (CMML) and myelodysplastic syndrome/myeloproliferative neoplasm (MDS/MPN) overlap syndromes. Local data related to MDS are limited.

### Aims

The study amid to describe the clinical and pathological presentation, including cytogenetic and molecular landscape for patient with MDS, CMML and MDS/MPN overlap syndromes. It also described the management approach and treatment outcomes of the patient with myelodysplastic syndrome treated in our center. We also identified the risk factors for treatment outcomes and survival data.

### Methods

This study was a retrospective, descriptive, study that included all MDS, CMML, and MDS/MPN overlap syndromes patients who were diagnostic from Jan2010 to April  2025 this study was carried out by using the electronic chart review, it was conducted in hematology and oncology center of king Abdulaziz Medical City in Riyadh. Kaplan-Meier overall survival (OS) was used to estimate the survival function and the log-rank test was used to compare curves. Microsoft Excel was used for data entry and Jamovi software was used for statistical analysis.

**Copyright Information:** (Online) ISSN: 2572-9241
©2026 The Author(s). HemaSphere is published by John Wiley & Sons Ltd on behalf of European Hematology Association. This is an open access Abstract Book distributed under the Attribution-NonCommercial-NoDerivs (CC BY-NC-ND), which allows third parties to download the articles and share them with others as long as they credit the author and the Abstract Book, but they cannot change the content in any way or use them commercially.
**Abstract Book Citations:** Authors, Title, HemaSphere, 2026;10:(S1):pages. The abstract book DOIs can be found on the “Author Information” page.
**Disclaimer:** Articles published in the journal HemaSphere exclusively reflect the opinions of the authors. The authors are responsible for all content in their abstracts including accuracy of the facts, statements, citing resources, etc.

**Results**

The result showed the total number of cases (N= 102 ), 71.6% are classified as Myelodysplastic Syndromes (MDS), while 28.4% show an overlap between Myelodysplastic Syndromes and Myeloproliferative Neoplasms (MDS/MPN). The mean age of the total population is 64.1± 19.3 years. Among the total population, 64.7% are male, while 35.3% are female. Among the total population, 13.7% underwent Allo-HCT. In the MDS group, 12% underwent Allo-HCT, while in the MDS/MPN overlap group, 18.5% underwent Allo-HCT. Other patients' characteristics are available in table 1. After median follow up of 16 months, the median overall survival for all patients (MDS and Overlap) is 30.8 months. Median OS for MDS and overlap syndrome is 30.8 and 29 months, respectively with p-value of 0.905 not significant. 3-years cumulative incidence of AML transformation is 30.5 %. The median OS according to IPSS-R risk stratification as follow: very low not reached, low 48.7 months, intermediate 22 months, high 18 months, and very high 12.4 months, (p-value 0.0007), which is consistent with the expected prediction in published data. Patients who progressed to AML has worst outcome with median OS of 21.8 months vs. 37.4 months for MDS patients without AML transformation, p-value 0.011.

**Summary/Conclusion**

We showed that IPSS-R risk stratification system is applicable to our MDS patient population and was able to predict outcome similar to published data in the literatures. AML transformation is associated with poor outcome. Further analysis for predictors of AML transformation and impact of transplant are needed

**Copyright Information:** (Online) ISSN: 2572-9241
©2026 The Author(s). HemaSphere is published by John Wiley & Sons Ltd on behalf of European Hematology Association. This is an open access Abstract Book distributed under the Attribution-NonCommercial-NoDerivs (CC BY-NC-ND), which allows third parties to download the articles and share them with others as long as they credit the author and the Abstract Book, but they cannot change the content in any way or use them commercially.
**Abstract Book Citations:** Authors, Title, HemaSphere, 2026;10:(S1):pages. The abstract book DOIs can be found on the "Author Information" page.
**Disclaimer:** Articles published in the journal HemaSphere exclusively reflect the opinions of the authors. The authors are responsible for all content in their abstracts including accuracy of the facts, statements, citing resources, etc.

**Table 1**

| Parameter | Total N (%) N=102 | MDS n (%) n=75 | MDS/MPN Overlap n (%) n=27 |
|---|---|---|---|
| **Age at diagnosis (years)** | | | |
| Median (range) | 68 (17–97) | 68 (17–97) | 63 (31–88) |
| **AML Transformation** | | | |
| • Yes | 27 (26.5) | 20 (26.7) | 7 (25.9) |
| • No | 75 (73.5) | 55 (73.3) | 20 (74.1) |
| **IPSS-R Category** | | | |
| • Very low ≤ 1.5 | 18 (17.6) | 13 (17.3) | 5 (18.5) |
| • Low 1.5 – 3.0 | 28 (27.5) | 21 (28.0) | 7 (25.9) |
| • Intermediate 3.0 – 4.5 | 9 (8.8) | 6 (8.0) | 3 (11.1) |
| • High 4.5 – 6 | 20 (19.6) | 16 (21.3) | 4 (14.8) |
| • Very high > 6.5 | 19 (18.6) | 16 (21.3) | 3 (11.1) |
| • Not evaluable | 8 (7.8) | 3 (4.0) | 5 (18.5) |
| **Patient status** | | | |
| • Alive | 44 (43.1) | 33 (44) | 11 (40.7%) |
| • Dead | 58 (56.9) | 42 (56.0) | 16 (59.3) |
| **Constitutional symptoms** | | | |
| • Yes | 10 (9.8) | 4 (5.3) | 6 (22.2) |
| • No | 73 (71.6) | 55 (73.3) | 18 (66.7) |
| • unknown | 19 (18.6) | 16 (21.3) | 3 (11.1) |
| **initial management** | | | |
| • first-line systemic therapy | 47 (46.1) | 32 (42.7) | 15 (55.6) |
| • supportive care (ESA or GCSF) | 41 (40.2) | 33 (44.0) | 8 (29.6) |
| • observation | 12 (11.8) | 9 (12.0) | 3 (11.1) |
| • unknown | 2 (2.0) | 1 (1.3) | 1 (3.7) |
| • N/A | 0 (0.0) | 0 (0.0) | 0 (0.0) |
| **First Line of systemic therapy** | | | |
| • AZA | 29 (28.4) | 24 (32.0) | 5 (18.5) |
| • LEN | 13 (12.7) | 10 (13.3) | 3 (11.1) |
| • HU | 7 (6.9) | 0 (0.0) | 7 (25.9) |
| • Allo-SCT | 4 (3.9) | 2 (2.7) | 2 (7.4) |
| • Intensive CTX | 2 (2.0) | 1 (1.3) | 1 (3.7) |
| • N/A | 34 (33.3) | 28 (37.3) | 6 (22.2) |
| • Unknown | 1 (1.0) | 0 (0.0) | 1 (3.7) |
| • Other | 12 (11.8) | 10 (13.3) | 2 (7.4) |
| **BM-blast % (aspirate)** | | | |
| median and range | 3 (0–60) | 2 (0–60) | 3 (0–13) |
| **Monosomy 5 or 7** | | | |
| • Yes | 17 (16.7) | 15 (20.0) | 2 (7.4) |
| • No | 85 (83.3) | 60 (80.0) | 25 (92.6) |
| **Chromosome 17 abnormalities** | | | |
| • Yes | 7 (6.9) | 5 (6.7) | 2 (7.4) |
| • No | 95 (93.1) | 70 (93.3) | 25 (92.6) |
| **Best Response to first line** | | | |
| • NR/SD | 55 (53.9) | 41 (54.7) | 14 (51.9) |
| • Progress to AML | 19 (18.6) | 14 (18.7) | 5 (18.5) |
| • CR | 10 (9.8) | 7 (9.3) | 3 (11.1) |
| • HI | 8 (7.8) | 5 (6.7) | 3 (11.1) |
| • N/A | 10 (9.8) | 8 (10.7) | 2 (7.4) |

**Survival Plot**

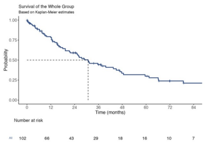

Cox Table- IPSS_R

| Explanatory | Levels | all | HR (Univariable) |
|---|---|---|---|
| IPSS_R | Very Low | 18 (19.1) | - |
| | Low | 28 (29.8) | 1.71 (0.67-4.36, p=0.258) |
| | Intermediate | 9 (9.6) | 3.16 (1.05-9.48, p=0.040) |
| | High | 20 (21.3) | 4.61 (1.68-12.69, p=0.003) |
| | Very High | 19 (20.2) | 8.29 (2.99-23.01, p<0.001) |

**Cumulative incedence of AML transformation**

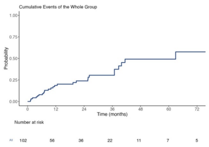

**Survival Plot - AML transformation**

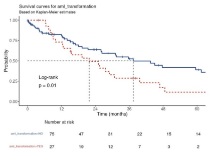

**Copyright Information:** (Online) ISSN: 2572-9241
©2026 The Author(s). HemaSphere is published by John Wiley & Sons Ltd on behalf of European Hematology Association. This is an open access Abstract Book distributed under the Attribution-NonCommercial-NoDerivs (CC BY-NC-ND), which allows third parties to download the articles and share them with others as long as they credit the author and the Abstract Book, but they cannot change the content in any way or use them commercially.
**Abstract Book Citations:** Authors, Title, HemaSphere, 2026;10:(S1):pages. The abstract book DOIs can be found on the "Author Information" page.
**Disclaimer:** Articles published in the journal HemaSphere exclusively reflect the opinions of the authors. The authors are responsible for all content in their abstracts including accuracy of the facts, statements, citing resources, etc.

### (PB3102) EFFICACY AND SAFETY OF SELINEXOR IN COMBINATION WITH AZACITIDINE FOR THE TREATMENT OF HIGHER-RISK MYELODYSPLASTIC SYNDROMES

**Topic:** 10. Myelodysplastic syndromes - Clinical

Jing-Dong Zhou[1, 2, 3], Yan Lin[4], Xu-Zhang Lu[5], Yuan Feng[1, 2, 3], Lei Yang[1, 2, 3], Wei Zhao[1, 2, 3], Jun Qian*[1, 2, 3]

*[1]Department of Hematology, Affiliated People's Hospital of Jiangsu University, zhenjiang, China;[2]Institute of Hematology, Jiangsu University, zhenjiang, China; [3]Zhenjiang Clinical Research Center of Hematology, zhenjiang, China; [4]The First People's Hospital of Changzhou, changzhou, China; [5]The Second People's Hospital of Changzhou, the Third Affi1iated Hospital of Nanjing Medical University, changzhou, China;*

### Background

Myelodysplastic syndromes (MDS) comprise a heterogeneous group of clonal myeloid disorders originating from hematopoietic stem cells. For patients with higher-risk MDS, the current therapeutic landscape is primarily centered on allogeneic hematopoietic stem cell transplantation (allo-HSCT) and hypomethylating agents (HMAs). However, due to the typically advanced age of this patient population and the high incidence of treatment-related complications, only about one-third of these high-risk patients are eligible for transplant. Meanwhile, the overall response rate to HMAs is limited, at approximately 50-60%, leaving nearly half of patients with primary resistance. Furthermore, even among responders, the duration of response is often brief, with a median of less than 18 months. Consequently, optimizing treatment strategies to improve survival in higher-risk MDS remains a significant unmet clinical need. In this context, combination regimens based on HMAs have emerged as a critical direction in clinical research.

### Aims

This study investigates the efficacy and safety of selinexor in combination with azacitidine for the treatment of higher-risk myelodysplastic syndromes.

### Methods

This study is a prospective, single-arm, multicenter clinical trial. Newly diagnosed patients with higher-risk MDS are screened, and those eligible for enrollment receive treatment with selinexor in combination with azacitidine according to the following regimen: azacitidine 75 mg/m² on days 1–7, and selinexor 60 mg on days 1, 8, and 15 of each 4-week cycle. Treatment is administered for a total of 6 cycles. The primary study endpoint is the assessment of the overall response rate (CR + PR + HI). Secondary endpoints include time to first response, duration of response, event-free survival, overall survival, adverse events and safety, and the duration of grade 3–4 cytopenias.

### Results

As of February 2026, a total of 6 participants have been enrolled in the study. The median age was 76 years (range: 67–82 years), consisting of 5 males and 1 female. Among them, 3 were classified as high-risk and 3 as very high-risk. Abnormal karyotypes were observed in 5 out of 6 cases (5/6). Commonly identified gene mutations included *ASXL1*, *TET2*, *STAG2*, and *RUNX1*. 2 participants completed all 6 cycles of treatment and achieved a CR . 1 participant died likely of sudden cardiac death before completing the full treatment course with a history of two coronary artery stenting procedures. 1 participant experienced disease progression during treatment. The remaining 2 participants are still undergoing treatment, their first assessment indicated SD. AEs were mild and resolved with supportive care.

**Copyright Information:** (Online) ISSN: 2572-9241
©2026 The Author(s). HemaSphere is published by John Wiley & Sons Ltd on behalf of European Hematology Association. This is an open access Abstract Book distributed under the Attribution-NonCommercial-NoDerivs (CC BY-NC-ND), which allows third parties to download the articles and share them with others as long as they credit the author and the Abstract Book, but they cannot change the content in any way or use them commercially.
**Abstract Book Citations:** Authors, Title, HemaSphere, 2026;10:(S1):pages. The abstract book DOIs can be found on the "Author Information" page.
**Disclaimer:** Articles published in the journal HemaSphere exclusively reflect the opinions of the authors. The authors are responsible for all content in their abstracts including accuracy of the facts, statements, citing resources, etc.

**Summary/Conclusion**

Selinexor in combination with azacitidine has demonstrated encouraging efficacy and a manageable safety profile in the treatment of higher-risk myelodysplastic syndromes. The study is ongoing, with the aim of providing further guidance for clinical practice.

**Copyright Information:** (Online) ISSN: 2572-9241
©2026 The Author(s). HemaSphere is published by John Wiley & Sons Ltd on behalf of European Hematology Association. This is an open access Abstract Book distributed under the Attribution-NonCommercial-NoDerivs (CC BY-NC-ND), which allows third parties to download the articles and share them with others as long as they credit the author and the Abstract Book, but they cannot change the content in any way or use them commercially.
**Abstract Book Citations:** Authors, Title, HemaSphere, 2026;10:(S1):pages. The abstract book DOIs can be found on the "Author Information" page.
**Disclaimer:** Articles published in the journal HemaSphere exclusively reflect the opinions of the authors. The authors are responsible for all content in their abstracts including accuracy of the facts, statements, citing resources, etc.

### (PB3103) TREATMENT OF HIGHER RISK MYELODYSPLASTIC SYNDROME HR-MDS WITH HIGH-DOSE CHEMOTHERAPY FOLLOWED BY LENALIDOMIDE AND AZACITIDINE: A THREE YEARS SURVIVAL UPDATE

**Topic:** 10. Myelodysplastic syndromes - Clinical

Maher Salamoon*[1]

*[1]al bairouni university cancer center, hematology/oncology, Damascus, Syrian Arab Republic;*

### Background

Higher-risk myelodysplastic syndromes (MDS) are defined by patients who fall into higher-risk group categories in the original or revised International Prognostic Scoring System. Survival for these patients is dismal, and treatment should be initiated rapidly. Standard therapies include the hypomethylating agents azacitidine and decitabine. Patients in relapses are refered to clinical trials.

### Aims

Studying new protocol in low-income countries where drug shortage is the rule and employing a new drug is challenging

### Methods

We have included 20 patients diagnosed with very high risk MDS according to international prognostic scoring system (IPSS). most of them demonstrated a blast percentage between 12-20%, chromosomal abnormalities and generalized cytopenia with a histology oscillating between RAEB- 1 and RAEB- 2. We have offered our patients an induction chemotherapy including Cytarabine 3 grams every week for 8 consecutive weeks followed by azacitadine 75mg/m2 per day for 5 consecutive days of 28-day cycle concomitant with lenalidomide 10 mg per day continuously. Treatment to be continued if there is a response with an acceptable toxicity. Bone marrow aspiration performed at the end of induction and every 3 months thereafter.

### Results

A complete response was documented in 5 out of 20 patients (25%) at the end of induction and 15 patients (75%) partial responders. 3 months after the start of the second block, another 3 patients joined the complete responders (40%). Progression free survival at 3 years was 55% (95% CI: 49.2-59.4) and an overall survival at y3 ears was 74% (95% CI: 70.2-79.5). the percentage of admission to intensive care unit following neutropenia was 8%. Neutropenia of grades 3 and 4 documented in 15% of patients mainly in the induction setting. After induction, patients were living a good quality of life.

### Summary/Conclusion

The combination of induction chemotherapy with high-dose Cytarabine  followed by hypomethylating agents plus immunomodulators seems to be a good choice in low income countries in treatment of higher risk MDS patients. this combination has demonstrated good tolerability and good safety profile

**Copyright Information:** (Online) ISSN: 2572-9241
©2026 The Author(s). HemaSphere is published by John Wiley & Sons Ltd on behalf of European Hematology Association. This is an open access Abstract Book distributed under the Attribution-NonCommercial-NoDerivs (CC BY-NC-ND), which allows third parties to download the articles and share them with others as long as they credit the author and the Abstract Book, but they cannot change the content in any way or use them commercially.
**Abstract Book Citations:** Authors, Title, HemaSphere, 2026;10:(S1):pages. The abstract book DOIs can be found on the “Author Information” page.
**Disclaimer:** Articles published in the journal HemaSphere exclusively reflect the opinions of the authors. The authors are responsible for all content in their abstracts including accuracy of the facts, statements, citing resources, etc.

### (PB3104) REAL-WORLD OUTCOMES OF THYMOGLOBULIN-BASED IMMUNOSUPPRESSION FOR ADULT APLASTIC ANEMIA IN COLOMBIA: A 12-YEAR SINGLE-CENTER COHORT

**Topic:** 10. Myelodysplastic syndromes - Clinical

Fabian Ahumada*[1], Elizabeth Arrieta[1], Katherine Arias[1], Oriana Arias[1, 2], Andrés Dominguez[1], Luisa Betancourth[1], Joaquin Rosales[1]

*[1]Fundación Valle del Lili, Valle del Cauca, Cali, Colombia;[2]Universidad Icesi, Facultad de medicina, Cali, Colombia;*

### Background

Real-world evidence on adult aplastic anemia (AA) outcomes in Latin America remains limited, particularly in settings where access to horse antithymocyte globulin (hATG) may be constrained and immunosuppression is predominantly thymoglobulin-based.

### Aims

To describe baseline severity, treatment patterns, and early response outcomes at 3 and 6 months among adults with AA treated between 2011 and 2023.

### Methods

We conducted a retrospective cohort study including adults (≥18 years) with AA who met predefined eligibility criteria and received first-line immunosuppressive therapy (IST). Patients were selected for first-line IST because they lacked an HLA-identical donor and/or were considered frail at diagnosis, precluding upfront transplantation. The primary outcome was overall response rate (ORR; complete response [CR] + partial response [PR]) at 3 months- follow up. Secondary outcomes included response at 6 months, time to second immunosuppression, time-to-first response.

### Results

Of 60 screened patients, 41 were included (mean age 50.4 years [SD 16.3]. At diagnosis, 88% were classified as severe/very severe AA. The most common first-line regimen was rabbit ATG + cyclosporine + eltrombopag (71%) (Table 1). ORR was 24% at 3 months (CR 2.4%, PR 22%) and 30% at 6 months (CR 9.8%, PR 20%) (Fig. 1). Twelve patients (29.3%) received second-line IST, with an ORR of 33% (CR 25%, PR 8.3%). The median time from initiation of first-line IST to the start of second-line IST was 131.5 [IQR 112, 319] days. The overall median time to response was 121 days [IQR 23, 217].

**Copyright Information:** (Online) ISSN: 2572-9241
©2026 The Author(s). HemaSphere is published by John Wiley & Sons Ltd on behalf of European Hematology Association. This is an open access Abstract Book distributed under the Attribution-NonCommercial-NoDerivs (CC BY-NC-ND), which allows third parties to download the articles and share them with others as long as they credit the author and the Abstract Book, but they cannot change the content in any way or use them commercially.
**Abstract Book Citations:** Authors, Title, HemaSphere, 2026;10:(S1):pages. The abstract book DOIs can be found on the "Author Information" page.
**Disclaimer:** Articles published in the journal HemaSphere exclusively reflect the opinions of the authors. The authors are responsible for all content in their abstracts including accuracy of the facts, statements, citing resources, etc.

| **Characteristics** | **N = 41** *1* |
|---|---|
| **Age** | 50.4 (16.3) |
| **Sex** | |
| Female | 17 (41%) |
| Male | 24 (59%) |
| **Comorbidities** | |
| Hypertension | 9 (22%) |
| Diabetes mellitus | 3 (7.3%) |
| PNH | 2 (4.9%) |
| Other malignancy | 3 (7.3%) |
| Other | 14 (34%) |
| None | 16 (39%) |
| **PNH clone at diagnosis** | |
| No | 30 (73%) |
| Yes | 11 (27%) |
| **Bone marrow cellularity** | 5 (5, 10) |
| **Severity of the disease at diagnosis** | |
| Severe | 24 (59%) |
| Very severe | 12 (29%) |
| Non severe | 5 (12%) |
| **Thymoglobulin dose** | 225 (200, 250) |
| **Number of thymoglobulin cycles received** | |
| 1 cycle | 25 (61%) |
| 2 cycles | 12 (29%) |
| **Inital immunosuppressive therapy regimen** | |
| rATG + CsA + EPAG | 29 (71%) |
| hATG + CsA | 1 (2.4%) |
| rATG + CsA | 8 (20%) |
| hATG + CsA + EPAG | 3 (7.3%) |

*1* n (%); Median (Q1, Q3)

### Summary/Conclusion

In this real-world cohort with predominantly severe and very severe AA, thymoglobulin-based IST was associated with modest early response rates, and outcomes after second-line non-transplant salvage remained limited. These findings underscore the need to optimize delivery of contemporary AA care and strengthen access to effective first line and rescue strategies such as alternative donors, like haploidentical family donors or unrelated donor searches.

**Copyright Information:** (Online) ISSN: 2572-9241
©2026 The Author(s). HemaSphere is published by John Wiley & Sons Ltd on behalf of European Hematology Association. This is an open access Abstract Book distributed under the Attribution-NonCommercial-NoDerivs (CC BY-NC-ND), which allows third parties to download the articles and share them with others as long as they credit the author and the Abstract Book, but they cannot change the content in any way or use them commercially.
**Abstract Book Citations:** Authors, Title, HemaSphere, 2026;10:(S1):pages. The abstract book DOIs can be found on the "Author Information" page.
**Disclaimer:** Articles published in the journal HemaSphere exclusively reflect the opinions of the authors. The authors are responsible for all content in their abstracts including accuracy of the facts, statements, citing resources, etc.

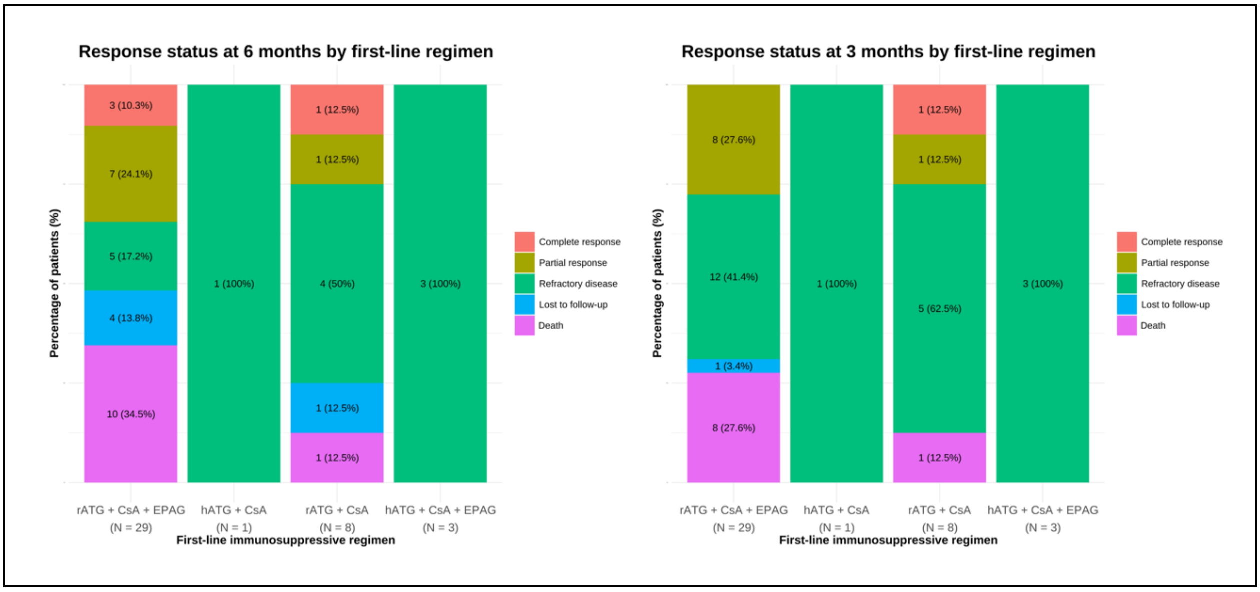

**Copyright Information:** (Online) ISSN: 2572-9241
©2026 The Author(s). HemaSphere is published by John Wiley & Sons Ltd on behalf of European Hematology Association. This is an open access Abstract Book distributed under the Attribution-NonCommercial-NoDerivs (CC BY-NC-ND), which allows third parties to download the articles and share them with others as long as they credit the author and the Abstract Book, but they cannot change the content in any way or use them commercially.
**Abstract Book Citations:** Authors, Title, HemaSphere, 2026;10:(S1):pages. The abstract book DOIs can be found on the "Author Information" page.
**Disclaimer:** Articles published in the journal HemaSphere exclusively reflect the opinions of the authors. The authors are responsible for all content in their abstracts including accuracy of the facts, statements, citing resources, etc.

### (PB3105) CSNK1A1 AND DDX41 MUTATIONS IN MYELODYSPLASTIC NEOPLASMS WITH DELETION OF LONG ARM OF CHROMOSOME 5

**Topic:** 10. Myelodysplastic syndromes - Clinical

Valeriya Okhota*[1], Alina Kokhno[1], Bella Biderman[1], Andrey Sudarikov[1], Valentina Dvirnik[1], Tatyana Obukhova[1], Elena Parovichnikova[1]

[1]*National Medical Research Center for Hematology, Moscow, Russia;*

### Background

*CSNK1A1* mutation is one of the most common mutations in myelodysplastic neoplasms with deletion of long arm chromosome 5 (MDS-5q), occurs in 5-10% cases. *CSNK1A1* haploinsufficiency ensures survival of cells with del(5q) by inhibiting p53 activity. *CSNK1A1* mutation may increase p53 inhibition and lead to lenalidomide resistance. *DDX41* mutation occurs in 4-5% of MDS-5q. Limited data suggest a positive impact of *DDX41* mutations on the clinical outcomes and sensitivity to lenalidomide in patients (pts) with MDS-5q.

### Aims

To investigate the frequency of *CSNK1A1* and *DDX41* mutations in a Russian cohort of MDS with 5q and to determine their impact on clinical course, response to lenalidomide and prognosis.

### Methods

From 2005 to 2025, 48 pts with available genetic material were followed at the National Medical Research Center for Hematology, Moscow. Forty-three (90%) were women. The median (Me) age was 64 (range, 19-79) years. The diagnoses were: MDS with isolated del(5q) – 38 (79%), MDS-EB-1–7 (15%), MDS-multi lineage dysplasia – 2 (4%) and MDS/MPN – 1 (2%). Based on IPSS-R risk category 17 (35%) pts were low risk, 30 (63%) were intermediate, and 1 (2%) were high. The del(5q) abnormality was identified by FISH in 9 (19%) pts. Additional cytogenetic alterations included trisomy 8, 20, t (7;12). All pts presented with anemia with Me Hb 66 (24-103) g/l, 38 (79%) pts were RBC transfusion-depended. The Me absolute neutrophil count was 1,77 (0,8 – 7,0) x$10^9$/l, Me platelets count 311 (42-888) x$10^9$/l.  Thirty-six (75%) pts received lenalidomide in a dose of 10 mg for 21 days, 12 (25%) in alternative schedules. Mutations in the genes CSNK1A1 (3-4 exons) and DDX41 (1-2,5-6,14-15 exons) were studied with high-throughput sequencing.

### Results

The Me overall survival (OS) in our cohort was 38,9 (4,1-128,9) months. Hematological response (HR) was reached in 25 (52%) pts, 19 (52%) became transfusion-independent. The Me time to HR was 8,0 (2,9–35,0) weeks. The Me duration of lenalidomide therapy was 8 (1 - 60) cycles. *CSNK1A1* mutation was identified in 4 (8,3%) pts, 3 pts with MDS-EB-1 and 1 with MDS-5q. One patient had *CSNK1A1* mutation in 2 hotspots – D140A and E98G with VAF 12,9% and 8,0%. In other 3 cases mutation was in D140V, D140Y and E98K points with VAF 6,6%, 14,0% and 28,4% respectively. Co-occurrence mutations were SF3B1 and ASXL1 in 2 cases. Deleted regions were q15q33 and q13q33.  Three pts (75%) had HR. The Me duration of HR was 46 (33,6–58,6) weeks. One patient achieved a partial cytogenetic response, but lost it in 16,6 weeks. The presence of the mutation did not significantly affect the achievement of HR ($p$ = 0,60), and OS ($p$ = 0,67). *DDX41* mutation was identified in 1 patient in point E542K with VAF 39,13%. This patient reached HR after 5 cycles of lenalidomide treatment. Duration of HR was 117 weeks.

**Copyright Information:** (Online) ISSN: 2572-9241
©2026 The Author(s). HemaSphere is published by John Wiley & Sons Ltd on behalf of European Hematology Association. This is an open access Abstract Book distributed under the Attribution-NonCommercial-NoDerivs (CC BY-NC-ND), which allows third parties to download the articles and share them with others as long as they credit the author and the Abstract Book, but they cannot change the content in any way or use them commercially.
**Abstract Book Citations:** Authors, Title, HemaSphere, 2026;10:(S1):pages. The abstract book DOIs can be found on the "Author Information" page.
**Disclaimer:** Articles published in the journal HemaSphere exclusively reflect the opinions of the authors. The authors are responsible for all content in their abstracts including accuracy of the facts, statements, citing resources, etc.

**Summary/Conclusion**

The frequency of *CSNK1A1* and *DDX41* mutations in pts with MDS and del(5q) was assessed. The frequency of *CSNK1A1* mutation consistent with data from international studies. *DDX41* mutation was identified in 1 patient. No statistically significant effect of *CSNK1A1* mutation on the rate of HR or OS was observed. The only patient harboring a *DDX41* mutation demonstrated a prolonged HR to lenalidomide therapy, which correlates with the hypothesis regarding the favorable prognostic value of this mutation. A limitation of our study was the small sample of pts. Further accumulation of cases is required to draw definitive conclusions.

**Copyright Information:** (Online) ISSN: 2572-9241
©2026 The Author(s). HemaSphere is published by John Wiley & Sons Ltd on behalf of European Hematology Association. This is an open access Abstract Book distributed under the Attribution-NonCommercial-NoDerivs (CC BY-NC-ND), which allows third parties to download the articles and share them with others as long as they credit the author and the Abstract Book, but they cannot change the content in any way or use them commercially.
**Abstract Book Citations:** Authors, Title, HemaSphere, 2026;10:(S1):pages. The abstract book DOIs can be found on the "Author Information" page.
**Disclaimer:** Articles published in the journal HemaSphere exclusively reflect the opinions of the authors. The authors are responsible for all content in their abstracts including accuracy of the facts, statements, citing resources, etc.

### (PB3106) OBSERVATIONAL AND GENETIC ASSOCIATION OF MYELODYSPLASTIC SYNDROMES (MDS) AND AUTOIMMUNE DISEASES IN ADULTS

**Topic:** 10. Myelodysplastic syndromes - Clinical

Qizhao Li[1], Shuqian Xu*[1]

[1]*Qilu Hospital of Shandong University, Jinan, China;*

### Background

About 25% of patients with myelodysplastic syndrome (MDS) also have combined autoimmune diseases (AIDs). However, the relationship between MDS and autoimmune disease, especially a causal relationship and the underlying pathophysiological mechanisms remains largely unknown.

### Aims

We aimed to evaluate the association between MDS and AIDs using a multicenter retrospective study, Mendelian randomization (MR), and bioinformatics analysis.

### Methods

A multicenter retrospective study was conducted to analyze the impact of autoimmune diseases on progression (conversion to acute myeloid leukemia) and survival in MDS patients. Bidirectional mendelian randomization (MR) was used to dissect the direct relationships between MDS and 9 autoimmune diseases, and we explored the mediation of abnormal immune cells through conditional analysis. Bioinformatic analysis was conducted to identify the potential diagnostic biomarkers of MDS with SLE.

### Results

About 26.6% of patients with MDS from all centers presented with AIDs. Compared to MDS patients without AIDs, MDS with AIDs was less likely to progress to acute myeloid leukemia (AML) (6.6% vs. 15.1%, p=0.037), and the pre-existing AIDs could be used as an independent protective factor of survival (HR: 0.504, p=0.048). Bidirectional MR results showed that MDS could cause the risk of systemic lupus erythematosus (SLE, OR: 1.09, p =0.015), although with no significant causal relationship in other AIDs. The effect of MDS on SLE may be partially mediated by naïve CD4+ T-cells (median proportion 6.9%) and CD45RA-CD4+ T memory cells (median proportion 9.0%). Furthermore, two hub genes (IFI27 and VSIG4) were identified by machine learning and curve analysis as potential diagnostic markers for MDS with SLE.

### Summary/Conclusion

In our study, the pre-existing AIDs can slow the progression of MDS to AML and the diagnosis time of AIDs also affected the survival of MDS. Genetically, there is a positive causal relationship between MDS and SLE, and naïve T and T memory cells play an important role as mediators. We also demonstrated that MDS and SLE share common diagnostic markers. These results suggest that the impact and mechanisms of AIDs in MDS need to be taken seriously, which could provide more accurate treatment guidance.

**Copyright Information:** (Online) ISSN: 2572-9241
©2026 The Author(s). HemaSphere is published by John Wiley & Sons Ltd on behalf of European Hematology Association. This is an open access Abstract Book distributed under the Attribution-NonCommercial-NoDerivs (CC BY-NC-ND), which allows third parties to download the articles and share them with others as long as they credit the author and the Abstract Book, but they cannot change the content in any way or use them commercially.
**Abstract Book Citations:** Authors, Title, HemaSphere, 2026;10:(S1):pages. The abstract book DOIs can be found on the “Author Information” page.
**Disclaimer:** Articles published in the journal HemaSphere exclusively reflect the opinions of the authors. The authors are responsible for all content in their abstracts including accuracy of the facts, statements, citing resources, etc.

### (PB3107) FROM MYELODYSPLASTIC SYNDROME TO ACUTE MYELOID LEUKEMIA: DIAGNOSTIC PATHWAYS AND REAL-WORLD DISEASE EVOLUTION

**Topic:** 10. Myelodysplastic syndromes - Clinical

Bianca-Andreea Cristian*[1, 2], Maria Tomosoiu[1, 2], Mihaela Popescu[1], Lidia Felicia Mihai[1], Florina Oana Gianina Pătrînoiu[1], Meilin Omer[1], Geanina Ofiteru[1], Oana-Diana Dragoi-Cozmaciuc[1], Viola Maria Popov[1], Mihaela Andreescu[1, 2]

*[1]Colentina Clinical Hospital, Hematology, Bucharest, Romania;[2]Titu Maiorescu University, Faculty of Medicine, Bucharest, Romania;*

### Background

Acute myeloid leukemia (AML) evolving from myelodysplastic syndromes (MDS) is biologically heterogeneous. While transformation risk in established MDS cohorts is well described, less is known about real-world diagnostic pathways, particularly cases in which persistent cytopenias may be overlooked and MDS is recognized only at a late stage or at AML presentation.

### Aims

To characterize real-world progression patterns from MDS to AML and to explore whether patients with retrospectively inferred (suspected) antecedent MDS demonstrate distinct temporal or biological features at transformation compared with those with previously confirmed MDS.

### Methods

We performed a single-center retrospective analysis of consecutive patients diagnosed between 2017 and 2025, initially identifying 25 individuals with AML evolving from MDS or with retrospectively inferred antecedent disease. Six patients were excluded from comparative analysis due to insufficient historical data. The remaining 19 patients were divided into two predefined groups: (1) confirmed MDS prior to AML (n=16) and (2) suspected antecedent MDS (n=3), defined retrospectively based on documented persistent, unexplained cytopenias prior to AML diagnosis without prior bone marrow confirmation. During the MDS phase, patients received treatment according to standard clinical practice, including hypomethylating agents (HMA), intensive chemotherapy, or supportive care. Given the small subgroup sizes and non-normal data distribution, nonparametric methods were used. Continuous variables were compared using the Mann–Whitney U test and categorical variables using Fisher's exact test. Statistical significance was defined as $p<0.05$.

### Results

Median age at AML diagnosis was 71 years (range 24–87). At transformation, median hemoglobin was 9.0 g/dL, median bone marrow blasts 18%, and median platelet count $102\times10^9$/L.
Among patients with confirmed MDS (n=16), time from MDS diagnosis to AML transformation differed significantly compared to the suspected antecedent subgroup (n=3) (Mann–Whitney p=0.045), indicating distinct temporal trajectories. Given the very small size of the suspected subgroup, between-group comparisons are interpreted cautiously.
Adverse cytogenetic risk at AML diagnosis was observed in 66.7% (2/3) of patients with suspected antecedent MDS versus 12.5% (2/16) of those with confirmed MDS. Given the minimal subgroup size, this finding is presented as a descriptive observation rather than a formal statistical inference (Fisher p=0.097). No meaningful differences were observed in hemoglobin, platelet count, or blast percentage at transformation.

**Copyright Information:** (Online) ISSN: 2572-9241
©2026 The Author(s). HemaSphere is published by John Wiley & Sons Ltd on behalf of European Hematology Association. This is an open access Abstract Book distributed under the Attribution-NonCommercial-NoDerivs (CC BY-NC-ND), which allows third parties to download the articles and share them with others as long as they credit the author and the Abstract Book, but they cannot change the content in any way or use them commercially.
**Abstract Book Citations:** Authors, Title, HemaSphere, 2026;10:(S1):pages. The abstract book DOIs can be found on the "Author Information" page.
**Disclaimer:** Articles published in the journal HemaSphere exclusively reflect the opinions of the authors. The authors are responsible for all content in their abstracts including accuracy of the facts, statements, citing resources, etc.

**Summary/Conclusion**

In this real-world cohort, AML evolution from MDS followed heterogeneous diagnostic and temporal pathways. A small subset of patients presented with AML despite prior unexplained cytopenias suggestive of unrecognized antecedent disease. While limited by small subgroup size and retrospective inference, the observed differences in transformation timing and cytogenetic distribution generate the hypothesis that delayed recognition of persistent cytopenias may influence biological presentation at AML diagnosis. Prospective validation in larger cohorts is warranted.

**Copyright Information:** (Online) ISSN: 2572-9241
©2026 The Author(s). HemaSphere is published by John Wiley & Sons Ltd on behalf of European Hematology Association. This is an open access Abstract Book distributed under the Attribution-NonCommercial-NoDerivs (CC BY-NC-ND), which allows third parties to download the articles and share them with others as long as they credit the author and the Abstract Book, but they cannot change the content in any way or use them commercially.
**Abstract Book Citations:** Authors, Title, HemaSphere, 2026;10:(S1):pages. The abstract book DOIs can be found on the "Author Information" page.
**Disclaimer:** Articles published in the journal HemaSphere exclusively reflect the opinions of the authors. The authors are responsible for all content in their abstracts including accuracy of the facts, statements, citing resources, etc.

### (PB3108) CLINICAL OUTCOMES OF HYPOMETHYLATING AGENTS IN THERAPY-RELATED MYELODYSPLASTIC SYNDROME: A SYSTEMATIC REVIEW OF PUBLISHED CASE SERIES AND REPORTS

**Topic:** 10. Myelodysplastic syndromes - Clinical

Joed Ivan Mata*[1], Lynn Bonifacio[1]

[1]*National Kidney and Transplant Institute, Division of Internal Medicine, Quezon City, Philippines;*

### Background

Therapy-related myelodysplastic syndrome (t-MDS) is a high-risk secondary myeloid neoplasm that develops after prior chemotherapy and/or radiotherapy. Compared with de novo MDS, it is more frequently associated with complex cytogenetics, chromosome 5/7 abnormalities, and inferior survival. Despite its aggressive biology, patients with therapy-related disease were largely excluded from pivotal hypomethylating agent (HMA) trials. As a result, treatment decisions rely primarily on retrospective data.

### Aims

To systematically review published evidence describing clinical outcomes of patients with therapy-related MDS treated with hypomethylating agents.

### Methods

A systematic PubMed search was performed through February 2026 using combinations of “therapy-related myelodysplastic syndrome,” “t-MDS,” “azacitidine,” “decitabine,” and “hypomethylating agents.” Case reports and case series reporting treatment outcomes in adult patients with t-MDS were included. Studies exclusively involving therapy-related acute myeloid leukemia were excluded. Extracted data included cytogenetic risk, treatment exposure, response according to IWG 2006 criteria, transfusion independence, and overall survival (OS). Owing to study heterogeneity, findings were synthesized descriptively.

### Results

Seven studies met inclusion criteria, encompassing approximately 190 therapy-related myeloid neoplasms, the majority consistent with WHO-defined t-MDS. Median patient age ranged from 65–70 years. Adverse cytogenetics were reported in up to 71% of patients, and complex karyotypes in approximately 40%, reflecting the high-risk nature of this population.

Azacitidine was the predominant agent and was generally administered using standard 75 mg/m² per 7-day schedule. Median treatment exposure ranged from 4 to 9 cycles. Overall response rates ranged from 39% to 71%, with complete remission observed in 11% to 43% of patients. Approximately 45–50% of evaluable patients achieved transfusion independence. Responses typically emerged after 3–4 cycles.

Median OS ranged from 10 to 14.5 months. In cohorts that included patients with concomitant active malignancy, outcomes with HMA therapy were comparable to those without active cancer, whereas patients treated with best supportive care alone experienced markedly inferior survival. Selected responders were successfully bridged to allogeneic transplantation. Isolated case reports also described cytogenetic remission following HMA exposure.

**Copyright Information:** (Online) ISSN: 2572-9241
©2026 The Author(s). HemaSphere is published by John Wiley & Sons Ltd on behalf of European Hematology Association. This is an open access Abstract Book distributed under the Attribution-NonCommercial-NoDerivs (CC BY-NC-ND), which allows third parties to download the articles and share them with others as long as they credit the author and the Abstract Book, but they cannot change the content in any way or use them commercially.
**Abstract Book Citations:** Authors, Title, HemaSphere, 2026;10:(S1):pages. The abstract book DOIs can be found on the “Author Information” page.
**Disclaimer:** Articles published in the journal HemaSphere exclusively reflect the opinions of the authors. The authors are responsible for all content in their abstracts including accuracy of the facts, statements, citing resources, etc.

**Summary/Conclusion**

Hypomethylating agents demonstrate meaningful hematologic activity in therapy-related MDS despite adverse biological features. Although long-term survival remains limited, response rates are comparable to those observed in high-risk de novo MDS. These findings support HMAs as a rational frontline approach for patients ineligible for intensive therapy and underscore the need for prospective studies dedicated to therapy-related disease.

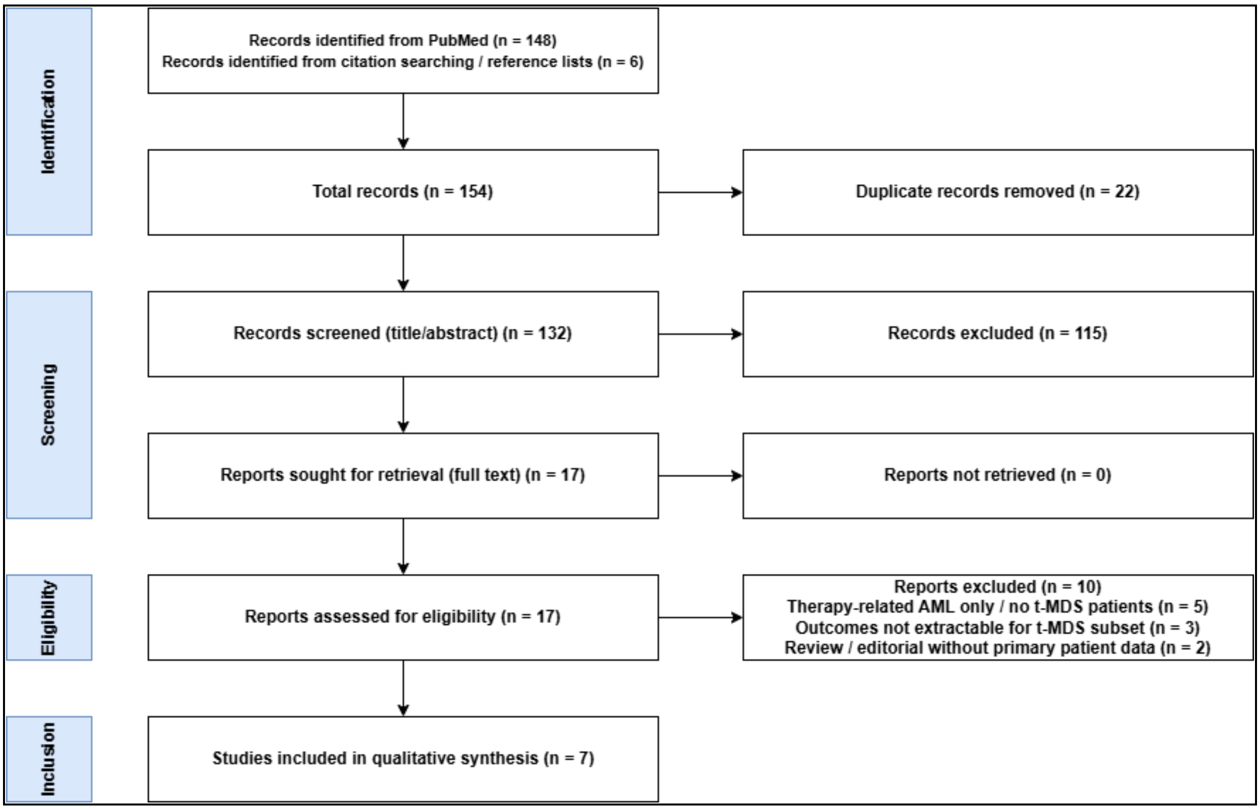

Figure 1: PRISMA Flow Diagram

**Copyright Information:** (Online) ISSN: 2572-9241
©2026 The Author(s). HemaSphere is published by John Wiley & Sons Ltd on behalf of European Hematology Association. This is an open access Abstract Book distributed under the Attribution-NonCommercial-NoDerivs (CC BY-NC-ND), which allows third parties to download the articles and share them with others as long as they credit the author and the Abstract Book, but they cannot change the content in any way or use them commercially.
**Abstract Book Citations:** Authors, Title, HemaSphere, 2026;10:(S1):pages. The abstract book DOIs can be found on the "Author Information" page.
**Disclaimer:** Articles published in the journal HemaSphere exclusively reflect the opinions of the authors. The authors are responsible for all content in their abstracts including accuracy of the facts, statements, citing resources, etc.

### (PB3109) HOTSPOT GENE MUTATIONS AND TREATMENT RESPONSE IN MYELODYSPLASTIC SYNDROMES (MDS): PREDICTIVE BIOMARKERS AND TARGETED STRATEGIES

**Topic:** 10. Myelodysplastic syndromes - Clinical

Rui Zhang*[1], Tao Wu[1]

[1] *The 940th Hospital of Joint Logistics Support force of Chinese People's Liberation Army, hematology, Gansu, China;*

### Background

MDS response to HMAs/chemotherapy is unpredictable; genomic biomarkers are missing

### Aims

Identify hotspot mutations that predict response to HMAs, chemotherapy or targeted agents and translate them into precision choices.

### Methods

Systematic review of 2020-2025 literature; 1,600+ MDS cases; response defined by 2018 IWG criteria; multivariate Cox for survival.

### Results

HMAs:ASXL1mut → HMA resistance (ORR 32 %), but VEN+HMA rescue (ORR 87 %);TET2mut/ASXL1wt → best HMA response (ORR 62 %); dual TET2-ASXL1 loses benefit (19 %);DNMT3A-R882 decitabine hypersensitivity via hemi-methylated enhancer trapping; median PFS 20.3 vs 50 mo WT;Chemotherapy:RUNX1, BCOR, EZH2 mutations independently cut response to cytarabine/anthracycline (ORR ≤19 %);Targeted therapyTP53 multi-hit: eprenetapopt + AZA ORR 73 %, median OS not reached at 12 mo; IDH1/2 mut: ivosidenib 83 % ORR, mOS 35.7 mo; enasidenib 53 % ORR, mOS 16.9 mo.

### Summary/Conclusion

ASXL1, TET2, DNMT3A, RUNX1, BCOR, EZH2, TP53, IDH1/2 mutations are actionable predictors; genotyping should guide front-line therapy and trial selection.

**Copyright Information:** (Online) ISSN: 2572-9241
©2026 The Author(s). HemaSphere is published by John Wiley & Sons Ltd on behalf of European Hematology Association. This is an open access Abstract Book distributed under the Attribution-NonCommercial-NoDerivs (CC BY-NC-ND), which allows third parties to download the articles and share them with others as long as they credit the author and the Abstract Book, but they cannot change the content in any way or use them commercially.
**Abstract Book Citations:** Authors, Title, HemaSphere, 2026;10:(S1):pages. The abstract book DOIs can be found on the "Author Information" page.
**Disclaimer:** Articles published in the journal HemaSphere exclusively reflect the opinions of the authors. The authors are responsible for all content in their abstracts including accuracy of the facts, statements, citing resources, etc.

### (PB3110) PATTERNS OF AZACITIDINE RESISTANCE IN HIGH-RISK MDS: REAL-WORLD DATA FROM A SINGLE CENTER.

**Topic:** 10. Myelodysplastic syndromes - Clinical

Pablo Prieto-Martinez*[1], Tzu Hua Chen Liang[1], Carlos Bravo Pérez[1], Maria Luz Amigo[1], Maria Lozano[1]

[1]*Hospital Universitario Morales Meseguer, Hematology, Murcia, Spain;*

### Background

Azacitidine is the standard first-line therapy for higher-risk myelodysplastic syndromes (HR-MDS), but real-world outcomes and resistance patterns are often inferior to pivotal trials, with heterogeneous failures and poor post-failure survival.

### Aims

To describe azacitidine exposure, response, and failure patterns in HR-MDS patients treated at a single Spanish center and to estimate overall survival azacitidine initiation and from treatment failure. To explore clonal evolution in a subset with serial NGS.

### Methods

We retrospectively included adults with intermediate-2/high-risk MDS (IPSS/IPSS-R >3.5) who started azacitidine between 2015–2025. Baseline characteristics, treatment details, and responses were collected from electronic records. Responses were classified using IWG-2023 criteria when marrow and blood parameters were available; otherwise, documented clinical improvement or progression was used. Azacitidine failure was defined as absence of response (primary refractoriness) or loss of response due to progression (secondary refractoriness). Failures were categorized as early (<6 cycles) or late (≥6 cycles). Overall response rate (ORR) included complete response (CR) and hematologic improvement (HI); response duration was measured from first documented response until failure in responders. Overall survival (OS) was calculated from azacitidine start to death.

### Results

Seventeen patients were analysed (median age 67.5 years; 53% males). A marrow-based response assessment was available in 15/17 (88%). Patients received a median of eight azacitidine cycles (range 3–81). By cycle six, best response was CR/HI in 47% (8/17), stable disease in 12% (2/17), and primary failure in 41% (7/17). ORR was 70.6%, and median response duration was 193.5 days. Median OS from azacitidine start was nearly 11.7 months, shorter than pivotal trial results. Median OS after azacitidine failure was 87 days. Only four patients had paired NGS; *ASXL1* mutations were the most frequent, followed by *NRAS* and *SRSF2*, with mainly rising VAFs at progression and one emergent *JAK2* mutation.
Four patients had early failure and thirteen failed after ≥6 cycles. Early failures were enriched for del(5q), including one case with additional high-risk abnormalities (del(7q) and TP53 deletion) and one patient with normal karyotype who had transient hematologic improvement. Later failures had a median response duration of 329 days and frequently showed poor-risk cytogenetics, including complex monosomal karyotypes and monosomy 7, but also trisomy 8 and normal or very good-risk karyotypes.
OS did not differ significantly between early and late failure groups (log-rank p=0.81), likely reflecting limited sample size.

**Copyright Information:** (Online) ISSN: 2572-9241
©2026 The Author(s). HemaSphere is published by John Wiley & Sons Ltd on behalf of European Hematology Association. This is an open access Abstract Book distributed under the Attribution-NonCommercial-NoDerivs (CC BY-NC-ND), which allows third parties to download the articles and share them with others as long as they credit the author and the Abstract Book, but they cannot change the content in any way or use them commercially.
**Abstract Book Citations:** Authors, Title, HemaSphere, 2026;10:(S1):pages. The abstract book DOIs can be found on the “Author Information” page.
**Disclaimer:** Articles published in the journal HemaSphere exclusively reflect the opinions of the authors. The authors are responsible for all content in their abstracts including accuracy of the facts, statements, citing resources, etc.

**Summary/Conclusion**

In this single-center HR-MDS cohort, azacitidine obtained an ORR of around 70% and a median OS of about 12 months, in line with other real-world data but clearly inferior to pivotal trials. The high failure rate and short post-failure survival emphasize the need for earlier consideration of transplantation and clinical trials, as well as improved biomarkers and therapies after hypomethylating agent failure. Notably, patients with poorer-risk cytogenetics at diagnosis often had more prolonged benefit than early progressors, whereas early failure clustered in del(5q), including TP53-altered cases, suggesting that specific genetic lesions may drive early resistance beyond baseline cytogenetic risk alone and supporting routine cytogenetic and molecular assessment at diagnosis and progression.

**Copyright Information:** (Online) ISSN: 2572-9241
©2026 The Author(s). HemaSphere is published by John Wiley & Sons Ltd on behalf of European Hematology Association. This is an open access Abstract Book distributed under the Attribution-NonCommercial-NoDerivs (CC BY-NC-ND), which allows third parties to download the articles and share them with others as long as they credit the author and the Abstract Book, but they cannot change the content in any way or use them commercially.
**Abstract Book Citations:** Authors, Title, HemaSphere, 2026;10:(S1):pages. The abstract book DOIs can be found on the "Author Information" page.
**Disclaimer:** Articles published in the journal HemaSphere exclusively reflect the opinions of the authors. The authors are responsible for all content in their abstracts including accuracy of the facts, statements, citing resources, etc.

### (PB3111) EFFECTIVE TREATMENT 3 CASES OF MDS BI-TP53/MDS-F WITH LOW-DOSE DECITABINE COMBINED WITH LOW-DOSE SELINEXOR

**Topic:** 10. Myelodysplastic syndromes - Clinical

Gaixiang Xu*[1], Jingjing Zhu[1], Hongyan Tong[2]

*[1]the First Affiliated Hospital,Zhejiang University School of Medicine, Hangzhou, China;[2]The First Affiliated Hospital, Zhejiang University School of Medicine, Hangzhou, China;*

### Background

According to the 2022 WHO classification, MDS-BiTP53 and MDS-f are listed as independent subtypes based on defined genetic abnormalities and morphologically, respectively. But these two subtypes often overlap clinically, with poor response to drug therapy and poor prognosis. A 10-day regimen of DAC showed very good efficacy in patients with TP53-mutated MDS or AML, leading to transient clearance of mutant cells.But some But some patients may not be able to tolerate 10 days of continuous low-dose decitabine�especially when the blood count is significantly abnormal at the onset in elderly patients. Here we report two cases of MDS BiTP53/MDS-f patients who received low-dose decitabine combined with selinexor and achieved rapid remission, including hematological and bone marrow responses.

### Aims

To evaluate the efficacy and safety of low-dose decitabine combined with selinexor in MDS-BiTP53/MDS-f patients

### Methods

Decitabine 25mg is administered intravenously once a week;selinexor 40mg is taken orally once a week, for continuous use with a 28 day cycle.

### Results

Both patients underwent next-generation sequencing (NGS), which identified a BiTP53 mutation.Bone marrow biopsy with reticulin staining demonstrated myelofibrosis-Grade 3 and Collagen fibrosis- Grade 1.Both was diagnosed MDS-BiTP53/MDS-f according to the 2022 WHO MDS classification and achieved CR within 3 months according to the 2023 IWG MDS response criteria.

Patient1:Female,68 years old.The chromosome karyotype changed from complex phenotype (45~47,XX,del(2)(q35),del(5)(q21q34),-7,del(9)(q34),add(11)(p15),add(12)(p13),-18,-21,+1~2mar[cp10]) to 46,XX,del(9)(q13q31)[1]/46,XX[10] .The bone marrow also showed the fibrosis changed for grade-3 to grade-1.
Patient2:Male,72years old.The chromosome karyotype changed from complex phenotype (42,XY,?del(2)(q13),-5,-7,add(7_)(?p15),-11,add(12)(q24.2),-14,-16,-17,-19,+3mar,1ace[6]/43, idem,+mar[1]/ 46,XY[4] ) to 46,XY[2]. The bone marrow also showed the fibrosis changed for grade-3 to grade-1.

Due to economic reasons, the patient did not undergo NGS again until the data collected.

**Copyright Information:** (Online) ISSN: 2572-9241
©2026 The Author(s). HemaSphere is published by John Wiley & Sons Ltd on behalf of European Hematology Association. This is an open access Abstract Book distributed under the Attribution-NonCommercial-NoDerivs (CC BY-NC-ND), which allows third parties to download the articles and share them with others as long as they credit the author and the Abstract Book, but they cannot change the content in any way or use them commercially.
**Abstract Book Citations:** Authors, Title, HemaSphere, 2026;10:(S1):pages. The abstract book DOIs can be found on the "Author Information" page.
**Disclaimer:** Articles published in the journal HemaSphere exclusively reflect the opinions of the authors. The authors are responsible for all content in their abstracts including accuracy of the facts, statements, citing resources, etc.

**Summary/Conclusion**

Low-dose decitabine combined with low-dose selenidazole once a week can quickly and effectively achieve hematological and bone marrow remission, but the sample size is small.We plan to conduct a prospective phase II clinical study in the future to further observe the effectiveness and safety of this regimen.Low-dose decitabine combined with selinexor and achieved rapid remission, including hematological and bone marrow responses.

**Copyright Information:** (Online) ISSN: 2572-9241
©2026 The Author(s). HemaSphere is published by John Wiley & Sons Ltd on behalf of European Hematology Association. This is an open access Abstract Book distributed under the Attribution-NonCommercial-NoDerivs (CC BY-NC-ND), which allows third parties to download the articles and share them with others as long as they credit the author and the Abstract Book, but they cannot change the content in any way or use them commercially.
**Abstract Book Citations:** Authors, Title, HemaSphere, 2026;10:(S1):pages. The abstract book DOIs can be found on the "Author Information" page.
**Disclaimer:** Articles published in the journal HemaSphere exclusively reflect the opinions of the authors. The authors are responsible for all content in their abstracts including accuracy of the facts, statements, citing resources, etc.

### (PB3112) A SCOPING ANALYSIS OF MATERNAL, OBSTETRIC, AND NEONATAL RESULTS IN PREGNANT WOMEN WITH MYELODYSPLASTIC SYNDROMES

**Topic:** 10. Myelodysplastic syndromes - Clinical

Shehab Fareed*[1], Fadel Alqatati[2], Marrita Rabadi[3], Maria Benkhadra[4], Angham Tartour[5], Ayman Dalol[3], Maymouna Hekmat[3], Ruba Aweer[3], Mohammed Mohammed[1], Abdulrahman F. Al-Mashdali[1], Naseema Gangat[6]

*[1]National Center for Cancer Care and Research (NCCCR), Hamad Medical Corporation, Department of Hematology, Doha, Qatar; [2]Hamad Medical Corporation, Department of Medicine, Doha, Qatar;[3]Qatar University, College of Medicine, Doha, Qatar; [4]National Center for Cancer Care and Research (NCCCR), Hamad Medical Corporation, Department of Pharmacy, Doha, Qatar; [5]Qatar University, Department of Population Medicine, College of Medicine, Doha, Qatar;[6]Mayo Clinic, Rochester, Minnesota, United States of America;*

**Background**

Clonal haematopoietic stem cell abnormalities known as myelodysplastic syndromes (MDS) are characterised by cytopenias, inefficient haematopoiesis, and an increased likelihood of developing acute myeloid leukaemia (AML). Because they primarily affect elderly persons, pregnancy-associated MDS is uncommon. Case reports and short series are the only sources of evidence. Cases may rise due to postponed childbearing and increased rate of survival from cancer. The literature on maternal, fetal, and disease-associated outcomes is compiled in this scoping review.

**Aims**

Analysing maternal and newborn outcomes according to the time of diagnosis, mapping available data on pregnancy outcomes in women with MDS, and identifying information shortages to guide future studies and clinical management.

**Methods**

The PRISMA-ScR standards were followed for carrying out a scoping review. We searched PubMed, EMBASE, and Google Scholar up until August 24, 2025. Included were case reports, case series, and observational studies with MDS pregnant patients. Demographics, disease classification (IPSS, FAB), methods of therapy, maternal outcomes, pregnancy outcomes, neonatal outcomes, and MDS development were all documented during data extraction. Stata 19.5 was used to conduct descriptive analysis.

**Results**

There were 21 articles totalling 69 pregnancies in 64 patients (14 case reports and 7 case series). 30 was the median age of mothers. 33 people (47.8%) received a diagnosis during pregnancy, whereas 36 patients (52.2%) had a diagnosis before becoming pregnant. IPSS and FAB categorisation reports was variable.

Women diagnosed prior to pregnancy had a higher incidence of stable illness (94.4% vs. 54.5%). Those who were diagnosed during pregnancy were more likely to experience progression of the disease (21.2%) and AML transformation (21.2%) . The group that died during pregnancy had a greater maternal death rate (21.2% vs. 5.6%). In total, 45.5% recovered, however 14% passed very soon after giving birth or within a few years(As shown in Figure1).

**Copyright Information:** (Online) ISSN: 2572-9241
©2026 The Author(s). HemaSphere is published by John Wiley & Sons Ltd on behalf of European Hematology Association. This is an open access Abstract Book distributed under the Attribution-NonCommercial-NoDerivs (CC BY-NC-ND), which allows third parties to download the articles and share them with others as long as they credit the author and the Abstract Book, but they cannot change the content in any way or use them commercially.
**Abstract Book Citations:** Authors, Title, HemaSphere, 2026;10:(S1):pages. The abstract book DOIs can be found on the “Author Information” page.
**Disclaimer:** Articles published in the journal HemaSphere exclusively reflect the opinions of the authors. The authors are responsible for all content in their abstracts including accuracy of the facts, statements, citing resources, etc.

**Summary/Conclusion**

Pregnancy in MDS is rare and current evidence is limited to case reports/series, precluding firm guideline-level recommendations. Available experience suggests that selected patients with low-risk, stable disease may achieve favorable maternal and neonatal outcomes, particularly when cytopenias are mild; however, severe thrombocytopenia or transfusion-refractory cytopenias increase maternal risk and may be associated with adverse fetal outcomes, including pregnancy loss. Management should be individualized with early recognition of complications, close surveillance, and multidisciplinary care, and future registries are needed to better define risk stratification and optimal supportive strategies.

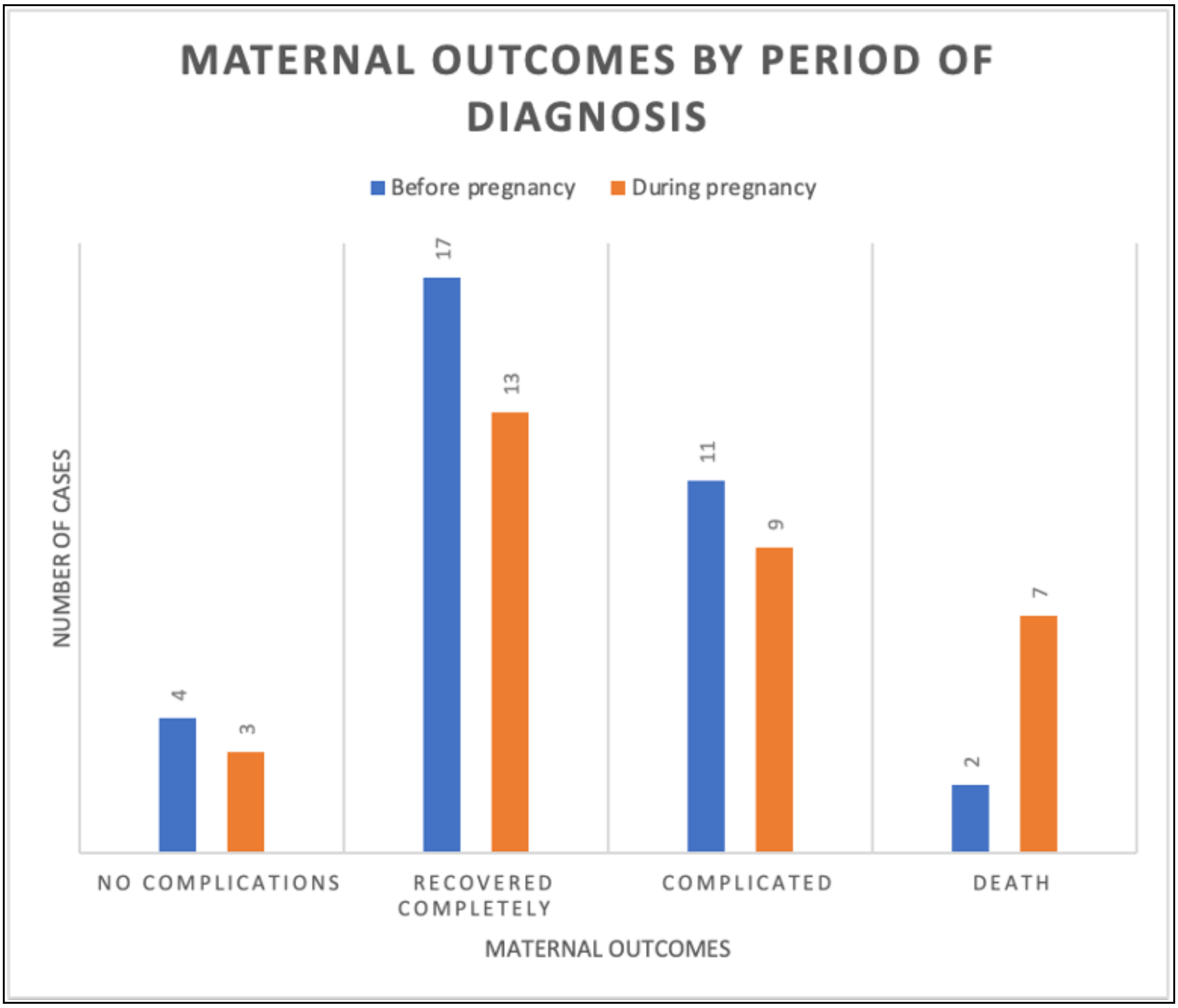

Figure 1. The elevated maternal risk associated with gestational diagnosis was highlighted by the considerably greater maternal death rate (21.2% vs. 5.6%) and comparable rates of recovery when MDS was identified during pregnancy.

**Copyright Information:** (Online) ISSN: 2572-9241
©2026 The Author(s). HemaSphere is published by John Wiley & Sons Ltd on behalf of European Hematology Association. This is an open access Abstract Book distributed under the Attribution-NonCommercial-NoDerivs (CC BY-NC-ND), which allows third parties to download the articles and share them with others as long as they credit the author and the Abstract Book, but they cannot change the content in any way or use them commercially.
**Abstract Book Citations:** Authors, Title, HemaSphere, 2026;10:(S1):pages. The abstract book DOIs can be found on the "Author Information" page.
**Disclaimer:** Articles published in the journal HemaSphere exclusively reflect the opinions of the authors. The authors are responsible for all content in their abstracts including accuracy of the facts, statements, citing resources, etc.

### (PB3113) THE INFLUENCE OF INTESTINAL FLORA AND ITS METABOLITE BUTYRIC ACID ON SEVERE APLASTIC ANEMIA

**Topic:** 11. Bone marrow failure syndromes incl. PNH - Biology & translational research

Xiaorui Meng[1], Rong Fu*[1]

[1] *Tianjin Medical University General Hospital, Tianjin, China;*

### Background

Aplastic anemia (AA) is an autoimmune disease mediated by T-lymphocyte hyperfunction and targeting the hematopoietic system. Recent studies have shown that gut flora metabolism has an important role in the human immune system, which in turn affects a number of immune system diseases.

### Aims

This study aims to explore the impact of gut microbiota and their metabolites on the pathogenesis and progression of SAA, and to conduct a preliminary investigation into the therapeutic potential of butyrate, a significantly altered metabolite, in the treatment of SAA.

### Methods

An immunomediated method was employed to establish a mouse model of severe aplastic anemia (SAA). Metagenomic sequencing was used to detect the fecal microbial abundance in mice, followed by analysis of metabolites associated with the differential bacterial communities identified.
Fecal microbiota transplantation (FMT) was conducted by transplanting SAA mouse microbiota into normal mice, and conversely, transplanting normal mouse microbiota into SAA mice. Subsequently, metagenomic sequencing was used to assess changes in gut microbiota abundance; routine blood tests were performed to analyze hematological parameters; flow cytometry was employed to evaluate changes in immune cell proportions; bone marrow HE staining was used to analyze changes in the bone marrow hematopoietic area; and intestinal mucosal HE staining along with immunohistochemical (IHC) staining for the intestinal goblet cell-specific protein MUC2 was performed to assess changes in the intestinal system.
Finally, SAA mice were treated with butyrate, a metabolite derived from the differential bacterial communities identified. Routine blood tests were used to analyze hematological changes; flow cytometry was employed to evaluate changes in immune cell proportions; and bone marrow HE staining was used to analyze changes in bone marrow hematopoietic function.

### Results

The abundance of Lachnospiraceae was significantly reduced in the gut microbiota of SAA mice, accompanied by pathological alterations in the intestinal system, including thinning of the intestinal mucosa and a decrease in the number of goblet cells.
After transplantation of SAA mouse microbiota into normal mice, the recipient mice exhibited reduced abundances of Lachnospiraceae and Oscillibacter, along with an increased abundance of Bacteroides, and the intestinal tissue underwent pathological changes.
Following transplantation of normal mouse microbiota into SAA mice, the recipients showed an increased abundance of Muribaculum, partial recovery of intestinal homeostasis, expanded bone marrow hematopoietic area, a decreased proportion of CD8+ T cells, and elevated peripheral blood counts.
Treatment of SAA mice with butyrate resulted in increased peripheral blood counts, expanded bone marrow hematopoietic area, elevated proportions of CD4+ T cells, NK cells, and Treg cells, and a reduced proportion of CD8+ T cells. Both CD8+ T cells and NK cells exhibited a downward trend in killing and secretory functions.

**Copyright Information:** (Online) ISSN: 2572-9241
©2026 The Author(s). HemaSphere is published by John Wiley & Sons Ltd on behalf of European Hematology Association. This is an open access Abstract Book distributed under the Attribution-NonCommercial-NoDerivs (CC BY-NC-ND), which allows third parties to download the articles and share them with others as long as they credit the author and the Abstract Book, but they cannot change the content in any way or use them commercially.
**Abstract Book Citations:** Authors, Title, HemaSphere, 2026;10:(S1):pages. The abstract book DOIs can be found on the “Author Information” page.
**Disclaimer:** Articles published in the journal HemaSphere exclusively reflect the opinions of the authors. The authors are responsible for all content in their abstracts including accuracy of the facts, statements, citing resources, etc.

**Summary/Conclusion**

Significant alterations in gut microbiota composition and intestinal homeostasis were observed in SAA mice. The SAA-derived microbiota exhibited pathogenic potential, while transplantation of normal microbiota or treatment with the microbial metabolite butyrate exerted therapeutic effects against SAA, the specific mechanisms of which warrant further investigation.

**Copyright Information:** (Online) ISSN: 2572-9241
©2026 The Author(s). HemaSphere is published by John Wiley & Sons Ltd on behalf of European Hematology Association. This is an open access Abstract Book distributed under the Attribution-NonCommercial-NoDerivs (CC BY-NC-ND), which allows third parties to download the articles and share them with others as long as they credit the author and the Abstract Book, but they cannot change the content in any way or use them commercially.
**Abstract Book Citations:** Authors, Title, HemaSphere, 2026;10:(S1):pages. The abstract book DOIs can be found on the "Author Information" page.
**Disclaimer:** Articles published in the journal HemaSphere exclusively reflect the opinions of the authors. The authors are responsible for all content in their abstracts including accuracy of the facts, statements, citing resources, etc.

### (PB3114) TWO TYPES OF STROMAL PRECURSORS FROM THE BONE MARROW OF PATIENTS WITH PAROXYSMAL NOCTURNAL HEMOGLOBINURIA REVEAL SIMILAR CHANGES IN GENE EXPRESSION, INDICATING ALTERATIONS IN MESENCHYMAL STEM CELLS

**Topic:** 11. Bone marrow failure syndromes incl. PNH - Biology & translational research

Irina Shipounova*[1], Alena Dorofeeva[1], Maria Finyakina[1], Vitaliy Latyshev[1], Elena Lukina[1], Elena Parovichnikova[1]

[1]*National Medical Research Center for Hematology, Moscow, Russia;*

### Background

Paroxysmal nocturnal hemoglobinuria (PNH) is a rare acquired nonmalignant clonal hematopoietic disorder. PNH is driven by the complete or partial absence of GPI-anchored proteins due to somatic mutations in PIGA gene in hematopoietic stem cells. PNH is accompanied by intra- and extravascular hemolysis, bone marrow (BM) failure, and a high risk of thrombosis. PNH pathogenesis is still unknown in details. Although BM insufficiency is revealed in most PNH patients, the information on BM stromal precursors is extremely rare.

### Aims

To analyze proliferation and expression level of genes regulating stromal and hematopoietic cells in two known types of BM stromal precursors, multipotent mesenchymal stromal cells (MMSCs) and colony-forming units of fibroblasts (CFU-Fs), from PNH patients and healthy donors.

### Methods

The study included 26 patients with PNH (11 m, 15 f, 40±3 y.o. (median 38.5), PNH-clone 75±6% (median 88)) and 31 healthy donors (15 m, 16 f, 35±2 y.o.(median 34)) as controls. BM samples were provided by diagnostic punctures (patients) or planned exfusions (donors). An informed consent was signed in all cases. MMSCs were isolated standardly and cultured up to passage 3. Time to passage 0 (P0), population doubling time (PDT) and total cell production were estimated. RNA was extracted from MMSCs on P1. CFU-F concentration was calculated microscopically after 2-week cultivation of 10^6 BM cells in aMEM+20% fetal bovine serum. Then, RNA from all colonies was extracted. Gene expression was analyzed with TaqMan real-time PCR. Data are presented as mean ± standard error of the mean. For normal distribution, the significance of differences was determined with Student t-test; otherwise, Mann-Whitney test was applied. Differences were considered significant at $p<0.05$.

### Results

As we had previously shown, MMSCs' proliferative abilities did not differ between donors and PNH patients. There were no changes in the CFU-F concentration either. However, the gene expression of eight genes was significantly higher both in MMSCs and in CFU-Fs from the BM of PNH patients, compared to control ones. These genes included some immunity factors (CFH, PDL1), growth factors and receptors (TGBF1, FGF2, FGFR1, PDGFRA, PDGFRB), and one regulating hematopoietic microenvironment (CXCL12) (Table). The expression levels of all these genes were 1.5-2.9 times higher in PNH patients' cells.
Table. Proliferative properties and genes' expression in MMSCs and CFU-Fs from the BM of healthy donors and PNH patients

**Copyright Information:** (Online) ISSN: 2572-9241
©2026 The Author(s). HemaSphere is published by John Wiley & Sons Ltd on behalf of European Hematology Association. This is an open access Abstract Book distributed under the Attribution-NonCommercial-NoDerivs (CC BY-NC-ND), which allows third parties to download the articles and share them with others as long as they credit the author and the Abstract Book, but they cannot change the content in any way or use them commercially.
**Abstract Book Citations:** Authors, Title, HemaSphere, 2026;10:(S1):pages. The abstract book DOIs can be found on the "Author Information" page.
**Disclaimer:** Articles published in the journal HemaSphere exclusively reflect the opinions of the authors. The authors are responsible for all content in their abstracts including accuracy of the facts, statements, citing resources, etc.

| | | MMSC | | CFU-F | |
|---|---|---|---|---|---|
| | | donors (N=14) | PNH patients (N=26) | donors (N=14) | PNH patients (N=26) |
| genes with significantly changed expression | **CFH** | 0.25±0.06 | 0.72±0.11* | 0.13±0.03 | 0.22±0.03* |
| | **FGF2** | 0.21±0.05 | 0.38±0.03* | 0.09±0.02 | 0.24±0.03* |
| | **PDGFRA** | 0.98±0.14 | 2.22±0.18* | 0.51±0.10 | 0.98±0.14* |
| | **PDGFRB** | 2.15±0.34 | 4.26±0.28* | 1.39±0.24 | 2.41±0.28* |
| | **FGFR1** | 1.09±0.15 | 1.75±0.09* | 0.75±0.06 | 1.80±0.28* |
| | **SDF1** | 0.50±0.06 | 1.10±0.11* | 1.10±0.17 | 1.86±0.23* |
| | **TGFb1** | 0.43±0.05 | 0.90±0.05* | 0.71±0.11 | 1.15±0.10* |
| | **PD-L1** | 0.11±0.02 | 0.21±0.03* | 0.17±0.03 | 0.26±0.04 |
| proliferative properties of BM stromal precursors | **Time to P0, days** | 14.55±0.63 | 15.54±0.56 | NA | NA |
| | **PDT, days** | 6.03±2.26 | 4.49±0.57 | NA | NA |
| | **cumulative MMSC production, 10^6** | 108.07±30.68 | 109.77±36.93 | NA | NA |
| | **CFU-F concentration, per 10^6 BM cells** | NA | NA | 36.01±8.42 | 50.93±9.00 |

* - indicates the significant difference ($p<0.05$), NA – not applicable

### Summary/Conclusion

There seem to be the changes in common mesenchymal precursor of MMSCs and CFU-F (possibly, true mesenchymal stem cells) from BM of PNH-patients. This phenomenon is unique and suggest some novel perspective in PNH pathogenesis research.

**Copyright Information:** (Online) ISSN: 2572-9241
©2026 The Author(s). HemaSphere is published by John Wiley & Sons Ltd on behalf of European Hematology Association. This is an open access Abstract Book distributed under the Attribution-NonCommercial-NoDerivs (CC BY-NC-ND), which allows third parties to download the articles and share them with others as long as they credit the author and the Abstract Book, but they cannot change the content in any way or use them commercially.
**Abstract Book Citations:** Authors, Title, HemaSphere, 2026;10:(S1):pages. The abstract book DOIs can be found on the "Author Information" page.
**Disclaimer:** Articles published in the journal HemaSphere exclusively reflect the opinions of the authors. The authors are responsible for all content in their abstracts including accuracy of the facts, statements, citing resources, etc.

### (PB3115) DECIPHERING BONE DISEASE IN GAUCHER DISEASE: INTEGRATED ENDOCRINE AND MOLECULAR SIGNATURES

**Topic:** 11. Bone marrow failure syndromes incl. PNH - Biology & translational research

Sonia Roca Esteve*[1, 2], Irene Serrano-Gonzalo[1, 2], Isidro Arévalo-Vargas[1, 2], Pilar Giraldo[1, 3, 4]

*[1]Fundación Española para el Estudio y Terapéutica de la Enfermedad de Gaucher y otras lisosomales (FEETEG), Zaragoza, Spain; [2]University of Zaragoza, Zaragoza, Spain;[3]QuironSalud Hospital, Hematology department, Zaragoza, Spain;[4]Spanish Group for Lysosomal Storage Diseases of the SEHH (GEEDL), Zaragoza, Spain;*

### Background

Skeletal involvement remains one of the most prevalent and disabling complications of Gaucher disease (GD), often insufficiently captured by classical disease biomarkers. Bone pathology in GD reflects a complex interplay between substrate accumulation, marrow infiltration, remodeling imbalance, and systemic regulators. Although disturbances in parathyroid hormone (PTH) and 25-hydroxyvitamin D (25-OH-D) have been reported, their integration with molecular regulators of bone homeostasis remains incompletely understood. MicroRNAs (miRNAs) are key post-transcriptional modulators of bone homeostasis. In a previous machine learning–driven study, we identified three bone-associated miRNAs linked to reduced bone mineral density (BMD). The relationship between these miRNAs and endocrine and bone parameters in GD has not yet been explored.

### Aims

Therefore, our main objective is to analyze these associations and to elucidate their potential role in the pathophysiology of skeletal involvement and alterations in PTH and 25-OH-D levels.

### Methods

We analyzed 34 GD patients who were evaluated at diagnosis for bone marrow involvement by MRI (Spanish S-MRI score) and skeletal involvement by DXA. Serum PTH, 25-OH-D, and classical GD biomarkers (chitotriosidase activity, CCL18/PARC, and glucosylsphingosine) were measured at diagnosis. The expression levels of the three bone-related miRNAs were obtained from circulating exosomes and were quantified using next-generation sequencing (NGS). All of these molecules were correlated with endocrine markers, skeletal parameters, and disease biomarkers.

### Results

The median age was 32.0 years (IQR 23.0–53.0). BMD loss was observed in 30% of patients, osteonecrosis in 20%, and bone crises in 17%. The median S-MRI score was 7.0 (IQR 1.5–10.0). Classical GD biomarkers showed no significant correlation with PTH levels or miRNA expression. In contrast, both PTH and miRNA profiles varied according to skeletal involvement. Although no direct association was observed between PTH and miRNA levels, each independently correlated with bone status, suggesting complementary and partially independent biological pathways contributing to skeletal damage.

**Copyright Information:** (Online) ISSN: 2572-9241
©2026 The Author(s). HemaSphere is published by John Wiley & Sons Ltd on behalf of European Hematology Association. This is an open access Abstract Book distributed under the Attribution-NonCommercial-NoDerivs (CC BY-NC-ND), which allows third parties to download the articles and share them with others as long as they credit the author and the Abstract Book, but they cannot change the content in any way or use them commercially.
**Abstract Book Citations:** Authors, Title, HemaSphere, 2026;10:(S1):pages. The abstract book DOIs can be found on the “Author Information” page.
**Disclaimer:** Articles published in the journal HemaSphere exclusively reflect the opinions of the authors. The authors are responsible for all content in their abstracts including accuracy of the facts, statements, citing resources, etc.

**Summary/Conclusion**

Skeletal disease in GD is highly prevalent and biologically multidimensional. Classical biomarkers inadequately reflect bone pathology, whereas endocrine (PTH) and molecular (bone-associated miRNAs) signals provide independent and complementary information. These findings support a translational framework integrating imaging, endocrine regulators, and circulating molecular signatures to improve risk stratification and personalized monitoring of bone disease in GD. Ongoing validation in larger cohorts aims to refine this multimodal predictive model and inform precision-based skeletal management strategies.

**Copyright Information:** (Online) ISSN: 2572-9241
©2026 The Author(s). HemaSphere is published by John Wiley & Sons Ltd on behalf of European Hematology Association. This is an open access Abstract Book distributed under the Attribution-NonCommercial-NoDerivs (CC BY-NC-ND), which allows third parties to download the articles and share them with others as long as they credit the author and the Abstract Book, but they cannot change the content in any way or use them commercially.
**Abstract Book Citations:** Authors, Title, HemaSphere, 2026;10:(S1):pages. The abstract book DOIs can be found on the "Author Information" page.
**Disclaimer:** Articles published in the journal HemaSphere exclusively reflect the opinions of the authors. The authors are responsible for all content in their abstracts including accuracy of the facts, statements, citing resources, etc.

### (PB3116) BONE MARROW FAILURE IS ASSOCIATED WITH CHANGES IN GENE EXPRESSION IN BOTH TYPES OF STROMAL PRECURSORS FROM THE BONE MARROW OF PATIENTS WITH PAROXYSMAL NOCTURNAL HEMOGLOBINURIA

**Topic:** 11. Bone marrow failure syndromes incl. PNH - Biology & translational research

Irina Shipounova*[1], Alena Dorofeeva[1], Maria Finyakina[1], Vitaliy Latyshev[1], Elena Lukina[1], Elena Parovichnikova[1]

[1]*National Medical Research Center for Hematology, Moscow, Russia;*

### Background

Paroxysmal nocturnal hemoglobinuria (PNH) is a rare, acquired disease of hematopoietic stem cells that belongs to the group of bone marrow (BM) failure syndromes. The disease is characterized by intra- and extravascular hemolysis, thrombophilia and a variable degree of cytopenia. PNH develops due to the complete or partial absence of GPI-anchored proteins caused by somatic mutations in PIGA gene in hematopoietic stem cells. There is very little data on the BM stromal precursors of PNH patients so the pathogenesis of BM failure (BMF) is not understood fully.

### Aims

To analyze proliferation and expression level of genes regulating stromal and hematopoietic cells in two known types of BM stromal precursors, multipotent mesenchymal stromal cells (MMSCs) and colony-forming units of fibroblasts (CFU-Fs), from PNH patients with and without BMF and healthy donors.

### Methods

The study included 26 patients with PNH (11 m, 15 f, 40±3 y.o. (median 38.5), PNH-clone 75±6% (median 88)) and 31 healthy donors (15 m, 16 f, 35±2 y.o.(median 34)) as controls. BMF was diagnosed in 22 PNH patients. BM samples were provided by diagnostic punctures (patients) or planned exfusions (donors). An informed consent was signed in all cases. MMSCs were isolated standardly and cultured up to passage 3. Population doubling time (PDT) and total cell production were estimated. RNA was extracted from MMSCs on P1. CFU-F concentration was calculated microscopically after 2-week cultivation of 10^6 BM cells in aMEM+20% fetal bovine serum. Then, RNA from all colonies was extracted. Gene expression was analyzed with TaqMan real-time PCR. Data are presented as mean ± standard error of the mean. For normal distribution, the significance of differences was determined with Student t-test; otherwise, Mann-Whitney test was applied. Differences were considered significant at $p<0.05$.

### Results

MMSCs' proliferative abilities did not differ neither between donors and PNH patients nor between PNH patients with or without BMF. The only exception was shortened PDT in MMSCs from patients without BMF (2.8±0.4 days vs 4.5±0.6 for donors' and 4.8±0.6 for PNH patients with BMF). There was dramatic decrease in the expression level of IL10 in MMSCs of the PNH patients without BMF (0.5±0.2 vs 3.3±0.6 for donors' and 2.2±0.7 for PNH patients with BMF). CFU-F concentration did not differ in those groups. The only difference in gene expression in CFU-Fs of the PNH patients without BMF was dramatic decrease in HLA-DR expression level (1.8±0.4 vs 4.5±1.0 for donors' and 4.1±0.7 for PNH patients with BMF). There were a few genes whose expression levels were significantly upregulated in stromal precursors from the BM of PNH patient with BMF compared to donors or PNH patients without BMF (Figure). These genes are involved in positive regulation of response to external stimuli, of cell migration or of MAPK activity.

**Copyright Information:** (Online) ISSN: 2572-9241
©2026 The Author(s). HemaSphere is published by John Wiley & Sons Ltd on behalf of European Hematology Association. This is an open access Abstract Book distributed under the Attribution-NonCommercial-NoDerivs (CC BY-NC-ND), which allows third parties to download the articles and share them with others as long as they credit the author and the Abstract Book, but they cannot change the content in any way or use them commercially.
**Abstract Book Citations:** Authors, Title, HemaSphere, 2026;10:(S1):pages. The abstract book DOIs can be found on the "Author Information" page.
**Disclaimer:** Articles published in the journal HemaSphere exclusively reflect the opinions of the authors. The authors are responsible for all content in their abstracts including accuracy of the facts, statements, citing resources, etc.

**Summary/Conclusion**

The state of stromal precursors from BM of PNH patients depends on whether they had BMFor not. Surprisingly, in stromal precursors from the BM of patients without BMF we have found down-regulated genes in comparison with healthy donors and PNH patients with BMF. The latter group revealed a few upregulated genes. It could suggest differences in pathogenesis of various forms of PNH.

| | | Donors (N=14) | PNH + BMF (N=22) | PNH – BMF (N=4) |
|---|---|---|---|---|
| MMSC | CFH | 0.25±0.06 | 0.77±0.11* | 0.45±0.30 |
| | IL1R1 | 0.36±0.07 | 0.52±0.04** | 0.31±0.05 |
| | ANGPT1 | 0.63±0.13 | 1.02±0.12* | 0.59±0.15 |
| | FGF2 | 0.21±0.05 | 0.40±0.03* | 0.25±0.07 |
| | CXCL12 | 0.50±0.06 | 1.15±0.12* | 0.81±0.15 |
| CFU-F | IDO1 | 0.002±0.001 | 0.003±0.001** | 0.001±0.000 |
| | IL1B | 0.19±0.04 | 0.26±0.05** | 0.12±0.03 |
| | NES | 0.43±0.12 | 0.78±0.16** | 0.31±0.07 |
| | FGFR1 | 0.75±0.06 | 1.89±0.30* | 1.29±0.13* |
| | FGF2 | 0.09±0.02 | 0.26±0.032* | 0.12±0.079 |

* indicates p<0.05 when compared to donors;
** indicates p<0.05 when compared to PNH-BMF

Figure. Genes, significantly upregulated in stromal precursors from the BM of PNH patients with BMF

**Copyright Information:** (Online) ISSN: 2572-9241
©2026 The Author(s). HemaSphere is published by John Wiley & Sons Ltd on behalf of European Hematology Association. This is an open access Abstract Book distributed under the Attribution-NonCommercial-NoDerivs (CC BY-NC-ND), which allows third parties to download the articles and share them with others as long as they credit the author and the Abstract Book, but they cannot change the content in any way or use them commercially.
**Abstract Book Citations:** Authors, Title, HemaSphere, 2026;10:(S1):pages. The abstract book DOIs can be found on the "Author Information" page.
**Disclaimer:** Articles published in the journal HemaSphere exclusively reflect the opinions of the authors. The authors are responsible for all content in their abstracts including accuracy of the facts, statements, citing resources, etc.

### (PB3117) TERT INTRON 4 RETENTION AS A NOVEL MECHANISM OF TELOMERE BIOLOGY DISORDERS

**Topic:** 11. Bone marrow failure syndromes incl. PNH - Biology & translational research

Fernanda Gutierrez-Rodrigues*[1], Mari Tokita[2], Magdalena Walkiewicz[2], Sarah Bannon[2], Sachiko Kajigaya[1], Justin Lack[3], Neelam Redekar[3], Tania MacHado[1], Jennifer Lotter[1], Nicholas Lee[1], Ludmila Prokunina-Olsson[4], Oscar Florez-Vargas[4], Logan Zeigler[4, 5], Sharon A. Savage[4], Neal Young[1], Bhavisha Patel[1], Emma Groarke[1]

*[1]Hematology Branch, National Heart, Lung and Blood Institute, National Institutes of Health, bethesda, United States of America; [2]Centralized Sequencing Program, Division of Intramural Research, National Institute of Allergy and Infectious Diseases, National Institutes of Health, bethesda, United States of America; [3]Integrated Data Sciences Section, Research Technologies Branch, National Institute of Allergy and Infectious Diseases, National Institutes of Health., bethesda, United States of America; [4]Division of Cancer Epidemiology and Genetics, National Cancer Institute, National Institutes of Health, bethesda, United States of America; [5]Cancer Genomics Research Laboratory, Frederick National Laboratory for Cancer Research, bethesda, United States of America;*

### Background

Intron 4 of *TERT*, encoding the telomerase reverse transcriptase, harbors single-nucleotide polymorphisms (SNPs) identified by GWAS as multi-cancer susceptibility loci. The rs10069690-T allele, found in 20-60% of the global population, results in increased alternative splicing of a catalytically inactive TERT-INS1b isoform due to intron 4 retention, thus reducing the ratio of full-length TERT, and accelerating age-dependent telomere length (TL) shortening in the general population. Alternative *TERT* splicing caused by rare intronic *TERT* variants has not been associated with telomere biology disorders (TBD), a group of Mendelian diseases caused by pathogenic germline variants in telomere-related genes, including *TERT*.

### Aims

Here, we report a novel molecular mechanism of TBD associated with *TERT* intron 4 retention due to a putatively pathogenic deep intronic mutation.

### Methods

A novel intronic variant 12-bp upstream of rs10069690 in *TERT* intron 4 was identified by whole-genome sequencing (WGS) in a family with autosomal recessive TBD participating in an IRB-approved study NCT05012111. TL was measured by flow-FISH in lymphocytes. *TERT* isoforms in activated peripheral blood lymphocytes were characterized by PacBio long-read RNA sequencing. *TERT* intron 4 retention was validated by PCR and Sanger sequencing.

**Copyright Information:** (Online) ISSN: 2572-9241
©2026 The Author(s). HemaSphere is published by John Wiley & Sons Ltd on behalf of European Hematology Association. This is an open access Abstract Book distributed under the Attribution-NonCommercial-NoDerivs (CC BY-NC-ND), which allows third parties to download the articles and share them with others as long as they credit the author and the Abstract Book, but they cannot change the content in any way or use them commercially.
**Abstract Book Citations:** Authors, Title, HemaSphere, 2026;10:(S1):pages. The abstract book DOIs can be found on the "Author Information" page.
**Disclaimer:** Articles published in the journal HemaSphere exclusively reflect the opinions of the authors. The authors are responsible for all content in their abstracts including accuracy of the facts, statements, citing resources, etc.

**Results**

The proband is an 18-yr-old female with the dyskeratosis congenita mucocutaneous triad, bone marrow failure, and TL <1$^{st}$ percentile (2.6kb). Parents (47 and 40yr) are consanguineous and asymptomatic with TL <10$^{th}$ percentile (4.4kb and 5kb, respectively). Two sisters (16 and 13yr) are asymptomatic and one sister (3yr) has mucocutaneous features; all siblings have TL <1$^{st}$ percentile (4.8, 6.5, and 4kb, respectively). WGS identified a novel homozygous *TERT* intron 4 variant chr5:g.1279687C>A, predicted to affect splicing, in the proband and in the 3yo sibling. Both parents and the 16yr sister are heterozygous for the same intronic *TERT* variant; the 13yr sister is negative for the variant. Given the proximity of the novel intronic *TER*T variant to the reported rs10069690-T allele, known to alter *TERT* splicing, we evaluated alternative *TERT* splicing in the family. Long-read RNA sequencing revealed a novel *TERT* isoform retaining intron 4 in both homozygous patients, which was not observed in the healthy control sample. This isoform likely generates a telomerase-inactive TERT isoform, similar to TERT-INS1b. Both full-length and intron 4 retention isoforms were detected in heterozygous carriers of the chr5:g.1279687C>A. Interestingly, the sister who tested negative for chr5:g.1279687C>A also had very short TL (a phenomenon seen in TBD families due to inheritance of short telomeres). Notably, she and her father harbor the rs10069690-T allele, which may have contributed to their TL shortening independently of chr5:g.1279687C>A.

**Summary/Conclusion**

In accordance with previous population-based studies, our data confirms *TERT* intron 4 as a locus associated with *TERT* alternative splicing and modulation of ratios of non-functional and full-length TERT isoforms. The combination of common and rare variants in non-coding regions of *TERT* may contribute to the heterogeneity in clinical presentation and TL among TBD-TERT families. Additionally, our findings highlight the utility of WGS for identifying pathogenic non-coding variants in Mendelian disorders.

**Copyright Information:** (Online) ISSN: 2572-9241
©2026 The Author(s). HemaSphere is published by John Wiley & Sons Ltd on behalf of European Hematology Association. This is an open access Abstract Book distributed under the Attribution-NonCommercial-NoDerivs (CC BY-NC-ND), which allows third parties to download the articles and share them with others as long as they credit the author and the Abstract Book, but they cannot change the content in any way or use them commercially.
**Abstract Book Citations:** Authors, Title, HemaSphere, 2026;10:(S1):pages. The abstract book DOIs can be found on the "Author Information" page.
**Disclaimer:** Articles published in the journal HemaSphere exclusively reflect the opinions of the authors. The authors are responsible for all content in their abstracts including accuracy of the facts, statements, citing resources, etc.

### (PB3118) THE INVOLVEMENT OF T-REGULATORY CELLS IN THE PATHOGENESIS OF CHRONIC IDIOPATHIC NEUTROPENIA

**Topic:** 11. Bone marrow failure syndromes incl. PNH - Biology & translational research

Nikoleta Bizymi*[1], Nikoletta Aresti[1], Athina Damianaki[1], Irene Mavroudi[1], Helen Papadaki[1]

*[1]Hemopoiesis Research Laboratory, School of Medicine, University of Crete, and Department of Hematology, University Hospital of Heraklion, Heraklion, Greece;*

**Background**

Chronic idiopathic neutropenia (CIN) is characterized by reduced counts of peripheral blood (PB) neutrophils below the lower limit of the normal distribution for a given ethnic population, of unknown cause. The pathophysiology of the condition, including immune dysregulation and an inflammatory bone marrow (BM) microenvironment with activated oligoclonal T-lymphocytes, has still not been described in detail.

**Aims**

In this study, we aimed to investigate the involvement of T-regulatory cells (Tregs) in the pathogenesis of CIN, which has not been studied yet.

**Methods**

A number of 41 adult CIN patients and 31 age- and sex-matched healthy controls were enrolled, fulfilling the previously described diagnostic criteria for CIN. Tregs were measured in the PB of all patients and controls and in the BM of seven patients, and were defined as $CD4^{+}CD25^{high}FoxP3^{+}$ cells, in the PB mononuclear cell (PBMC) and BMMC fraction, respectively. The acquisition was performed in a Cytomics FC500 flow cytometer (Beckman Coulter, Brea, CA, USA) and data were analyzed with the Kaluza software. Statistical analysis was performed with the appropriate statistical tests based on the distribution of the data and by using the GraphPad Prism Statistical PC software (La Jolla, CA, USA).

**Results**

Statistically significantly increased counts of Tregs were found (Mann–Whitney U test, P = 0.0214) in the PBMC fraction of the patients (median 1.24% of PBMCs, interquartile range 0.71% – 1.52%) compared to healthy controls (median 0.72% of PBMCs, interquartile range 0.51% – 1.09%). Tregs were shown to be statistically significantly elevated (Wilcoxon signed ranks test for paired samples, P = 0.0096) in the PBMC (median 1.04% of PBMCs, interquartile range 0.72% – 1.28%) compared to the BMMC (median 0.50% of BMMCs, interquartile range 0.31% – 0.68%) fraction in patients with CIN, probably explained by the induction of these cells peripherally rather than centrally.

**Summary/Conclusion**

The enhanced induction of Tregs in CIN may represent an attempt of the immune system to suppress the activated oligoclonal T-lymphocytes in the PB. However, functional and mechanistic studies are needed to better characterize the functionality as well as the involvement of these cells in the complex pathophysiology of the condition.

**Copyright Information:** (Online) ISSN: 2572-9241
©2026 The Author(s). HemaSphere is published by John Wiley & Sons Ltd on behalf of European Hematology Association. This is an open access Abstract Book distributed under the Attribution-NonCommercial-NoDerivs (CC BY-NC-ND), which allows third parties to download the articles and share them with others as long as they credit the author and the Abstract Book, but they cannot change the content in any way or use them commercially.
**Abstract Book Citations:** Authors, Title, HemaSphere, 2026;10:(S1):pages. The abstract book DOIs can be found on the “Author Information” page.
**Disclaimer:** Articles published in the journal HemaSphere exclusively reflect the opinions of the authors. The authors are responsible for all content in their abstracts including accuracy of the facts, statements, citing resources, etc.

### (PB3119) MOLECULAR CHARACTERIZATION OF PAROXYSMAL NOCTURNAL HEMOGLOBINURIA: SPECTRUM OF PIGA MUTATIONS AND ASSOCIATED SOMATIC ALTERATIONS

**Topic:** 11. Bone marrow failure syndromes incl. PNH - Biology & translational research

Patrycja Mensah-Glanowska*[1, 2], Magdalena Zawada[1, 2], Sylwia Czekalska[2], Agnieszka Balana-Nowak[1, 2], Daria Zawirska[2], Tomasz Sacha[1, 2]

*[1]Jagiellonian University Medical College, Department of Haematology, Krakow, Poland;[2]University Hospital in Krakow, Department of Haematology, Krakow, Poland;*

### Background

Paroxysmal nocturnal hemoglobinuria (PNH) is a rare hematologic disorder caused by somatic mutations in the *PIGA* gene, leading to deficiency of glycosylphosphatidylinositol (GPI)-anchored proteins, including the complement regulators CD55 and CD59. PNH is associated with an increased risk of clonal evolution to myelodysplastic neoplasms (MDS) and acute myeloid leukemia (AML). The clinical significance of additional somatic mutations in PNH remains incompletely understood.

### Aims

The aim of this study was to characterize the spectrum and number of *PIGA* mutations in patients with PNH and to identify co-occurring mutations associated with an increased risk of progression to MDS and/or AML.

### Methods

Twenty-three patients with PNH clone were analyzed using targeted next-generation sequencing (Pillar OncoReveal Myeloid Panel) at the Molecular Diagnostics Laboratory, University Hospital in Krakow. The cohort included patients with classical PNH, PNH associated with severe or moderate aplastic anemia (AA), PNH with bone marrow failure and SAA evolving to classical PNH. Disease duration ranged from 1 to 22 years. Twenty patients received complement pathway inhibitors, including both distal and proximal agents.

### Results

*PIGA* mutations were detected in all patients. A total of 32 variants were identified, including 29 coding and 3 intronic mutations (Figure 1). Recurrent variants were rare and limited to two sites (Arg77GlufsTer18 and Leu243Pro, each n=2). Thirteen patients harbored a single *PIGA*
mutation, while ten had multiple mutations (range 2 - 6). Additional somatic mutations were identified in 11 patients. The most frequently affected genes were *DNMT3A* (5 mutations in 4 patients), *TET2* (3 mutations in 3 patients), *U2AF1* (2 mutations in 2 patients), *ASXL1* (2 mutations in 2 patients), and *BCOR* (2 mutations in 1 patient). Mutations in *PHF6*, *CEBPA*, and *CALR* were detected in single cases. Three patients carried two additional somatic mutations besides *PIGA*.

### Summary/Conclusion

The mutational profile observed in this cohort is consistent with previously published data.While mutations in *PIGA*, *BCOR*, and *BCORL1* are generally associated with favorable prognosis, mutations in genes such as *DNMT3A* and *ASXL1* may indicate a higher risk of clonal progression.
In the era of effective complement inhibition and improved survival in PNH, next-generation sequencing may help identify patients at increased risk of evolution to MDS/AML and support risk-adapted monitoring strategies.

**Copyright Information:** (Online) ISSN: 2572-9241
©2026 The Author(s). HemaSphere is published by John Wiley & Sons Ltd on behalf of European Hematology Association. This is an open access Abstract Book distributed under the Attribution-NonCommercial-NoDerivs (CC BY-NC-ND), which allows third parties to download the articles and share them with others as long as they credit the author and the Abstract Book, but they cannot change the content in any way or use them commercially.
**Abstract Book Citations:** Authors, Title, HemaSphere, 2026;10:(S1):pages. The abstract book DOIs can be found on the "Author Information" page.
**Disclaimer:** Articles published in the journal HemaSphere exclusively reflect the opinions of the authors. The authors are responsible for all content in their abstracts including accuracy of the facts, statements, citing resources, etc.

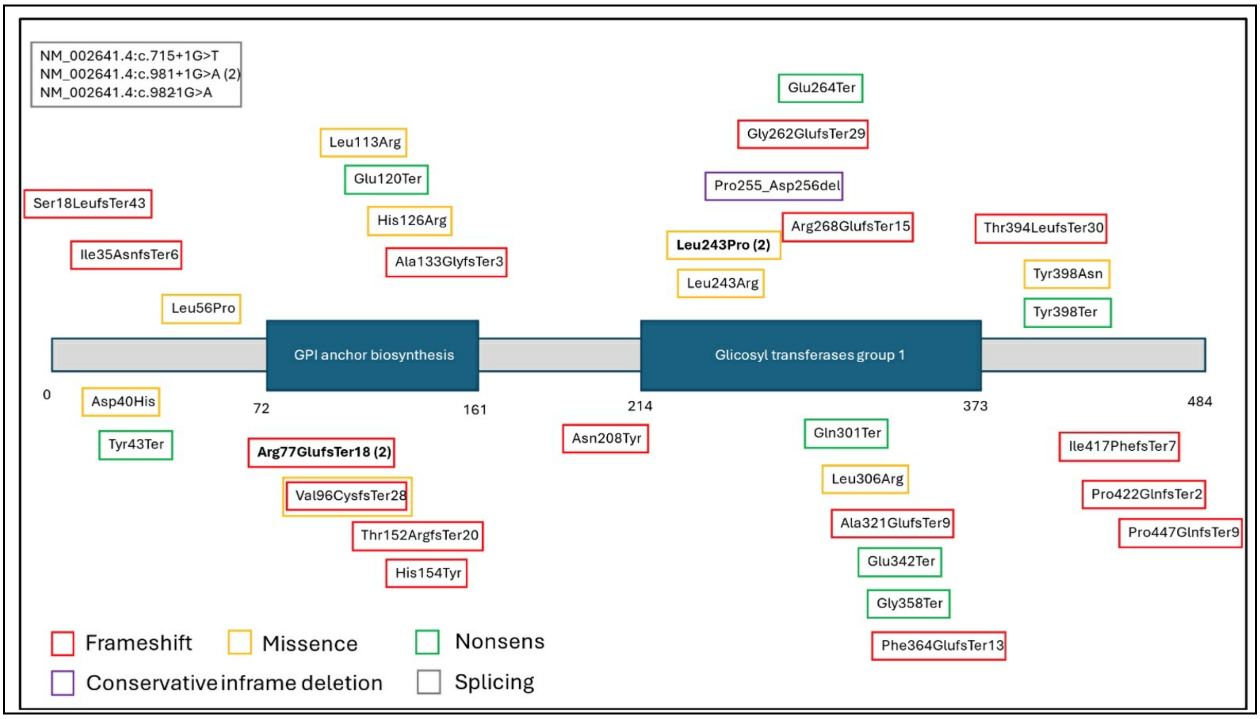

Figure 1. Distribution of PIGA in the cohort of 20 PNH patients. The type of detected mutation is highlighted with colored boxes. Frameshift (red boxes): These mutations cause a shift in the reading frame, potentially leading to the production of truncated or nonfunctional proteins. Missense (yellow boxes): These are single-nucleotide mutations that change one amino acid in the protein sequence. Nonsense (green boxes): These mutations create a premature stop codon, leading to a truncated protein. Conservative Inframe Deletion (purple box): These deletion do not affect the reading frame but can still impact the protein's function. Splicing (grey boxes): Mutations affecting the splicing process of the RNA, po tentially leading to incorrect protein synthesis.

**Copyright Information:** (Online) ISSN: 2572-9241
©2026 The Author(s). HemaSphere is published by John Wiley & Sons Ltd on behalf of European Hematology Association. This is an open access Abstract Book distributed under the Attribution-NonCommercial-NoDerivs (CC BY-NC-ND), which allows third parties to download the articles and share them with others as long as they credit the author and the Abstract Book, but they cannot change the content in any way or use them commercially.
**Abstract Book Citations:** Authors, Title, HemaSphere, 2026;10:(S1):pages. The abstract book DOIs can be found on the "Author Information" page.
**Disclaimer:** Articles published in the journal HemaSphere exclusively reflect the opinions of the authors. The authors are responsible for all content in their abstracts including accuracy of the facts, statements, citing resources, etc.

### (PB3120) DEVELOPING MRNA-BASED THERAPEUTICS FOR THE TREATMENT OF DIAMOND BLACKFAN ANEMIA SYNDROME

**Topic:** 11. Bone marrow failure syndromes incl. PNH - Biology & translational research

Olivia Delfino*[1], Susannah Brown[2], Nicole Thomsen[2], Jinshu He[3], Koula Diamand[3], Thomas Preiss[1], Eduardo Eyras[1], Ross D. Hannan[1], Amee J. George[1, 2, 3]

*[1]The Australian National University, The John Curtin School of Medical Research, Division of Genome Sciences and Cancer, Canberra, Australia; [2]The Australian National University, The John Curtin School of Medical Research, The National Therapeutic mRNA Platform, Canberra, Australia; [3]The Australian National University, The John Curtin School of Medical Research, The ANU Centre for Therapeutic Discovery, Canberra, Australia;*

### Background

Diamond-Blackfan Anemia Syndrome (DBAS) is a rare inherited bone marrow failure disorder presenting in early childhood with severe chronic anemia, congenital abnormalities, and increased cancer risk, most commonly caused by ribosomal protein haploinsufficiency.

Current therapies are limited: corticosteroids and chronic red cell transfusions carry substantial toxicities, while hematopoietic stem cell transplantation (HSCT), the only 'curative' option, is associated with significant morbidity due to conditioning regimens and graft-versus-host disease. Emerging autologous gene therapy reduces immune complications but still requires toxic marrow conditioning, poses mutagenesis risks, and faces challenges of *ex vivo* hematopoietic stem and progenitor cell (HSPC) manipulation.

mRNA-based therapeutics offer a non-genomic strategy to restore deficient protein expression. Previous studies have demonstrated proof-of-concept in Fanconi anemia models, and CD117-targeted lipid nanoparticle (LNP) delivery has enabled *in vivo* mRNA targeting of HSPCs, supporting exploration in hematopoietic disorders.

Recent studies identified biallelic loss-of-function mutations in *HEATR3*, a factor essential for 60S ribosomal maturation and 5S-ribonucleoprotein particle biogenesis, as the primary cause of autosomal recessive DBAS. Unlike most DBAS-associated genes, *HEATR3*-deficient cells remain viable, permitting controlled knockout models for quantitative evaluation of mRNA-mediated protein and functional rescue.

### Aims

To develop *HEATR3* mRNA therapeutics with enhanced stability, translation efficiency, and reduced immunogenicity, and to evaluate their capacity to restore HEATR3 expression and rescue defects in ribosome biogenesis, cellular proliferation, and erythropoiesis resulting from HEATR3 deficiency, establishing proof-of-concept for mRNA therapy in DBAS.

**Copyright Information:** (Online) ISSN: 2572-9241
©2026 The Author(s). HemaSphere is published by John Wiley & Sons Ltd on behalf of European Hematology Association. This is an open access Abstract Book distributed under the Attribution-NonCommercial-NoDerivs (CC BY-NC-ND), which allows third parties to download the articles and share them with others as long as they credit the author and the Abstract Book, but they cannot change the content in any way or use them commercially.
**Abstract Book Citations:** Authors, Title, HemaSphere, 2026;10:(S1):pages. The abstract book DOIs can be found on the "Author Information" page.
**Disclaimer:** Articles published in the journal HemaSphere exclusively reflect the opinions of the authors. The authors are responsible for all content in their abstracts including accuracy of the facts, statements, citing resources, etc.

### Methods

*HEATR3* mRNA candidates incorporating computational sequence optimization and chemical modifications were designed and synthesized. Lead candidates are identified by quantifying real-time translation kinetics and protein output using a NanoBiT luciferase system in K562 and *HEATR3*-null and wild-type A549 models.

Functional rescue is assessed by measuring mRNA-mediated rescue of ribosome biogenesis and proliferation defects in *HEATR3*-null A549 cells using polysome profiling and live-cell imaging. *HEATR3*-null and patient variant iPSCs are differentiated towards HSPCs to evaluate erythropoietic rescue via flow cytometry and erythroid culture assays. CD117-targeted LNPs are developed for *in vivo* bone marrow delivery, with therapeutic efficacy evaluated in *Heatr3*-deficient mouse models using hematopoietic endpoints.

### Results

*HEATR3* mRNA candidates have been synthesized, purified to quality control standards, and transfection conditions established across cellular models. *HEATR3*-null A549 cells reproducibly exhibit reduced proliferative capacity and impaired ribosome biogenesis, demonstrated by decreased 60S:40S ratios and reduced 80S ribosome abundance compared to wild-type controls. Quantitative evaluation of translation kinetics, protein expression, and rescue of HEATR3 function is ongoing across cellular systems.

### Summary/Conclusion

This work establishes a platform for mRNA-based protein replacement therapy targeting bone marrow failure in DBAS using *HEATR3* as a model system. These studies provide foundational evidence supporting the development of safer, non-genomic therapies aimed at achieving transfusion independence and reducing reliance on HSCT.

**Copyright Information:** (Online) ISSN: 2572-9241
©2026 The Author(s). HemaSphere is published by John Wiley & Sons Ltd on behalf of European Hematology Association. This is an open access Abstract Book distributed under the Attribution-NonCommercial-NoDerivs (CC BY-NC-ND), which allows third parties to download the articles and share them with others as long as they credit the author and the Abstract Book, but they cannot change the content in any way or use them commercially.
**Abstract Book Citations:** Authors, Title, HemaSphere, 2026;10:(S1):pages. The abstract book DOIs can be found on the "Author Information" page.
**Disclaimer:** Articles published in the journal HemaSphere exclusively reflect the opinions of the authors. The authors are responsible for all content in their abstracts including accuracy of the facts, statements, citing resources, etc.

### (PB3121) SHARED T-CELL RECEPTOR CLONOTYPES IN FAMILIAL APLASTIC ANEMIA ASSOCIATED WITH HLA-A*02:06 LOSS DUE TO 6P LOSS OF HETEROZYGOSITY

**Topic:** 11. Bone marrow failure syndromes incl. PNH - Biology & translational research

Tatsuya Imi*[1]

[1]*Kanazawa university, Kanazawa-shi, Japan;*

### Background

We previously investigated the pathophysiology of aplastic anemia (AA) in six pairs of relatives without a family history of hematologic disorders or congenital AA. In one mother–daughter pair, flow cytometric analysis revealed leukocytes lacking HLA-A2 expression. Both individuals shared the HLA-A*02:06-B*55:02-C*01:02-DRB1*09:01 haplotype, and their leukocytes exhibited loss of this haplotype due to loss of heterozygosity on chromosome 6p (6pLOH).

### Aims

We hypothesized that hematopoietic cells lacking HLA-A*02:06 had escaped cytotoxic T lymphocyte–mediated autoimmunity, and that shared T-cell receptor (TCR) clonotypes potentially recognizing a common autoantigen might be detectable in activated T cells from these patients.

### Methods

CD8⁺ T cells were isolated from peripheral blood samples obtained during hematologic remission after treatment from the mother–daughter pair with AA harboring HLA-A*02:06 loss due to 6pLOH,. TCR α- and β-chain repertoires were sequenced to identify shared and dominant T-cell clonotypes.

### Results

Distinct TCR repertoire patterns were identified in CD8⁺ T cells from all four blood or bone marrow samples of two patients. Marked clonal expansion was observed in the mother-daughter pair. The mother showed high clonality in the TCR α chain, predominantly TRAV19/TRAJ47, while the daughter exhibited two dominant TCR α clonotypes: TRAV38-2/TRAJ23 and TRAV21/TRAJ50.
Analysis of the TCR β chain revealed a striking dominance of the TRBV20-1/TRBJ2-1 clonotype in the mother (89.8% of total clones), which was also the most frequent clonotype in the daughter (17.7%).
In silico prediction of peptides complementary to the CDR3 regions of the TCR α- and β-chain repertoires identified several candidate peptides, including shared sequences. One predicted peptide showed similarity to several epitopes derived from Epstein Barr virus, although this peptide did not match known candidate autoantigens.

### Summary/Conclusion

In a familial AA setting, shared expansion of a dominant TCRβ clonotype associated with HLA-A*02:06 loss suggests antigen-driven T-cell selection and immune escape via 6pLOH. Integrated analysis of HLA genetics and TCR repertoires may provide a useful framework for identifying disease-relevant immune targets in AA. Further studies are required to validate the antigen specificity and pathogenic relevance of these findings.

**Copyright Information:** (Online) ISSN: 2572-9241
©2026 The Author(s). HemaSphere is published by John Wiley & Sons Ltd on behalf of European Hematology Association. This is an open access Abstract Book distributed under the Attribution-NonCommercial-NoDerivs (CC BY-NC-ND), which allows third parties to download the articles and share them with others as long as they credit the author and the Abstract Book, but they cannot change the content in any way or use them commercially.
**Abstract Book Citations:** Authors, Title, HemaSphere, 2026;10:(S1):pages. The abstract book DOIs can be found on the "Author Information" page.
**Disclaimer:** Articles published in the journal HemaSphere exclusively reflect the opinions of the authors. The authors are responsible for all content in their abstracts including accuracy of the facts, statements, citing resources, etc.

### (PB3122) PHENOTYPIC PROFILE OF PAROXYSMAL NOCTURNAL HEMOGLOBINURIA CLONES (≥ 5%) IN PATIENTS WITH ACQUIRED APLASTIC ANEMIA : A MONOCENTRIC STUDY

**Topic:** 11. Bone marrow failure syndromes incl. PNH - Biology & translational research

Salima Oukid*[1], Nabila Rekab[1], Fatima Lamraoui[1], Bouchakor Moussa Yamina[1], Khadoudj Mounira Benlabiod[1], Hamida Brahimi[1], Souad Taoussi[1], Ferdi Zineb[2], Guezlane Sherifa[1], Mohamed Mezroud[2], Ahmed Amine Ziani[1], Amina Rebouh[1], Bradai Mohamed[1]

*[1]Faculty of Medicine, University of Blida 1. Research Laboratory on Malignant Hemopathies and Hemoglobinopathies, Faculty of Medicine., Department of Hematology, EHS ELCC., Blida, Algeria; [2]Department of Hematology, EHS ELCC, Blida, Algeria;*

### Background

Screening for a Paroxysmal Nocturnal Hemoglobinuria (PNH) clone by flow cytometry (FCM) is currently recommended in all cases of acquired Aplastic Anemia (AA). Previous studies have reported that approximately 20–25% of AA patients harbor a detectable PNH clone.

### Aims

We report 229 cases of AA in which a PNH clone was investigated and monitored by FCM.

### Methods

This retrospective study was conducted from 2009 to January 2026. Screening for a PNH clone was systematically performed by FCM at the time of diagnosis of AA. The degree of glycosy-lphosphatidylinositol (GPI) - anchor deficiency was assessed using CD59a on red blood cells (RBCs), CD24, CD157 and FLAER on neutrophils, and CD14, CD157 and FLAER on monocytes. We included cases with a PNH clone defined as ≥ 5% GPI-deficient cells in at least two lineages. FCM monitoring was performed in cases with no detectable deficiency, moderate deficiency, or involvement of a single lineage. A total of 229 patients with AA were analyzed, including 112 women and 117 men, with a mean age of 34.3 years (15–79). AA was classified as moderate in 125 cases (54.8%) and severe in 104 cases (45.2%).

### Results

Among the 229 patients analyzed, no PNH clone ≥ 5% was detected by flow cytometry in 181 cases (79%), whereas 48 patients (21%) had a PNH clone ≥ 5%. These 48 patients included 21 women and 27 men, with a mean age of 36.4 years (17–70). AA was classified as moderate in 31 cases (64.6%) and severe in 17 cases (35.4%). Regarding the type of GPI deficiency, 3 cases (6.2%) were classified as partial (type II), 36 cases (75%) as total (type III), and 9 cases (18.8%) as mixed (types II/III). The mean clone size according to lineage was as follows: **RBC** (n : 44): CD59 :15.4% (0.1-82), **Neutrophils** (n : 48): mean 43.2% (5-99), **Monocytes** (n : 37): mean 31.6% (0.2-97). In 47 patients, deficiency was detected in only one lineage, indicating the need for regular monitoring by FCM immunophenotyping every six month, among them 13 patients developed a detectable clone during follow-up, highlighting the importance of continuous surveillance

**Copyright Information:** (Online) ISSN: 2572-9241
©2026 The Author(s). HemaSphere is published by John Wiley & Sons Ltd on behalf of European Hematology Association. This is an open access Abstract Book distributed under the Attribution-NonCommercial-NoDerivs (CC BY-NC-ND), which allows third parties to download the articles and share them with others as long as they credit the author and the Abstract Book, but they cannot change the content in any way or use them commercially.
**Abstract Book Citations:** Authors, Title, HemaSphere, 2026;10:(S1):pages. The abstract book DOIs can be found on the “Author Information” page.
**Disclaimer:** Articles published in the journal HemaSphere exclusively reflect the opinions of the authors. The authors are responsible for all content in their abstracts including accuracy of the facts, statements, citing resources, etc.

**Summary/Conclusion**

**Discussion**

Flow cytometry proved to be essential for diagnosing aplastic PNH, with a detection rate of 21%, consistent with previous reports (20-25%). In patients with moderate deficiency or initially absent clones, monitoring clone size is necessary. This finding is consistent with B. Höchsmann's study, which followed 155 patients and reported significant clone expansion in 28% of cases and the emergence of a new clone in 9%. During disease progression in our cohort, we observed clone size increase in 13 patients, emphasizing the clinical relevance of regular monitoring to detect clonal evolution and guide management strategies.

**Conclusion**

Flow cytometry is essential for the diagnosis and monitoring of PNH clones, particularly in AA. Early detection and monitoring of clones, especially those ≥5%, allow for adapted therapeutic management and a better understanding of disease progression. Regular surveillance is therefore crucial, even in cases with an initially negative result, to detect the emergence or expansion of clones.

**Copyright Information:** (Online) ISSN: 2572-9241
©2026 The Author(s). HemaSphere is published by John Wiley & Sons Ltd on behalf of European Hematology Association. This is an open access Abstract Book distributed under the Attribution-NonCommercial-NoDerivs (CC BY-NC-ND), which allows third parties to download the articles and share them with others as long as they credit the author and the Abstract Book, but they cannot change the content in any way or use them commercially.
**Abstract Book Citations:** Authors, Title, HemaSphere, 2026;10:(S1):pages. The abstract book DOIs can be found on the "Author Information" page.
**Disclaimer:** Articles published in the journal HemaSphere exclusively reflect the opinions of the authors. The authors are responsible for all content in their abstracts including accuracy of the facts, statements, citing resources, etc.

### (PB3123) RESEARCH ON THE MECHANISM OF DENDRITIC CELLS INVOLVEMENT IN THE IMMUNE ESCAPE OF PNH CLONES IN PATIENTS WITH CLASSICAL PAROXYSMAL NOCTURNAL HEMOGLOBINURIA

**Topic:** 11. Bone marrow failure syndromes incl. PNH - Biology & translational research

Yan Yang[1], Chaomeng Wang[2], Mengting Che[1], Hui Liu[1], Rong Fu*[2]

*[1]Tianjin Medical University General Hospital, Hematology, Tianjin, China;[2]Tianjin Medical University General Hospital, Hematology, tianjin, China;*

### Background

Paroxysmal nocturnal hemoglobinuria (PNH) is a rare acquired clonal disorder of hematopoietic stem cells. Its pathological mechanism is the abnormal synthesis of glycosyl phosphatidylinositol (GPI), which results in the deficiency of a cluster of GPI-anchored proteins (CD55, CD59, etc.) on the erythrocyte membrane. Clinically, it is characterized by intravascular hemolysis, bone marrow failure and a high risk of complicated thrombosis. Current studies have revealed that immune evasion, anti-apoptotic mechanisms and secondary gene mutations are all involved in the clonal proliferation of PNH, and immune abnormalities exert a pivotal role in the pathogenesis and progression of PNH.

### Aims

To analyze the changes in the immune microenvironment of patients with classical PNH via single-cell sequencing technology, and to investigate the alterations in the number, subpopulation distribution and biological functions of dendritic cells (DCs) in depth. Meanwhile, this study explores the expression and regulatory changes of the CCL5 gene and tumor necrosis factor (TNF) signaling pathway in PNH patients. The present study aims to further elucidate the underlying mechanism of immune evasion in classical PNH, thereby providing a theoretical basis for the development of novel therapeutic strategies.

### Summary/Conclusion

This study employed single-cell sequencing technology to conduct an in-depth investigation into the dynamic alterations in the immune microenvironment in cPNH. The findings revealed that, compared with HCs, the number of DCs was markedly increased in cPNH patients and predominantly composed of PNH clone, accompanied by a significant decrease in the proportion of $CD59^{+}$ DCs.
Further subtype stratification of DCs demonstrated an increased proportion of cDCs among total DCs in the $CD59^{-}$ DC subset of cPNH patients, while the proportion of pDCs was conversely reduced. These observations suggest that the altered number of DCs and the aberrant distribution of their subsets may contribute to the imbalance of the hematopoietic microenvironment in PNH.
In vitro flow cytometry (FCM) assays revealed that the proportion of DCs in the peripheral blood of patients with cPNH was elevated, accompanied by enhanced functional activity of the cells. Moreover, the enhanced function of DCs was closely associated with T lymphocytes, whereas no significant alterations were observed in the intrinsic functions of iDCs themselves. This finding suggests that DCs may drive the enhancement of T cell functional activity, which leads to hyperactivation of T lymphocytes and thereby contributes to the mechanisms underlying immune evasion in PNH.
CCL5 gene may regulate the functional activity of DCs through the TNF signaling pathway in PNH. This finding provides a theoretical basis for an in-depth investigation into the immunopathogenic mechanisms underlying PNH.

**Copyright Information:** (Online) ISSN: 2572-9241
©2026 The Author(s). HemaSphere is published by John Wiley & Sons Ltd on behalf of European Hematology Association. This is an open access Abstract Book distributed under the Attribution-NonCommercial-NoDerivs (CC BY-NC-ND), which allows third parties to download the articles and share them with others as long as they credit the author and the Abstract Book, but they cannot change the content in any way or use them commercially.
**Abstract Book Citations:** Authors, Title, HemaSphere, 2026;10:(S1):pages. The abstract book DOIs can be found on the "Author Information" page.
**Disclaimer:** Articles published in the journal HemaSphere exclusively reflect the opinions of the authors. The authors are responsible for all content in their abstracts including accuracy of the facts, statements, citing resources, etc.

### (PB3124) OSTEOGENIC POTENTIAL OF BONE MARROW DERIVED MULTIPOTENT MESENCHYMAL STROMAL CELLS FROM APLASTIC ANEMIA PATIENTS WITH ASEPTIC NECROSIS OF THE FEMORAL HEAD

**Topic:** 11. Bone marrow failure syndromes incl. PNH - Biology & translational research

Alena Dorofeeva*[1], Irina Shipounova[1], Ardavazd Vartikyan[1], Anastasia Abramova[1], Anton Luchkin[1], Zalina Fidarova[1], Vasily Mamonov[1], Elena Mikhailova[1]

[1]*National Medical Research Center for Hematology, Moscow, Russia;*

### Background

Aseptic osteonecrosis (AON) is a severe disease of the musculoskeletal system that develops in 8% of patients with aplastic anemia (AA). AON of the bone epiphyses can cause deformation of the articular surface, which leads to secondary degenerative changes in the joint, patient disability and may require surgical treatment. Multipotent mesenchymal stromal cells (MMSCs) play an important role in reparative regeneration and bone remodeling, including in cases of osteonecrosis. The MMSCs' osteogenic potential of AA patients with AON has not been studied

### Aims

To isolate bone marrow (BM) derived MMSCs from affected (femur metaphysis (FM)) and unaffected (iliac crest (IC)) areas of AA patients with AON and characterize their osteogenic potential

### Methods

The study included 12 AA patients with aseptic necrosis of the femoral head (ANFH) (7m, 5f, 20-72 y.o., median 36.5; 2 at the onset and 10 after immunosupressive therapy (IST)). Control group consisted of 15 AA patients without diagnosed ANFH (6m, 9f, 19-59 y.o., median 27; 2 at the onset and 13 after IST)

Patients' BM samples were obtained during diagnostic puncture and during planned hip arthroplasty, which was performed due to ANFH after informed consents had been signed. BM samples was obtained from both affected (FM) and unaffected (IC) areas in all cases of patients with ANFH and from IC in case of control group

MMSCs were isolated and cultured using standard method up to passage 3. The intensity of osteogenic differentiation was assessed as described previously (Dorofeeva et all, HemaSphere. 2025;9(S1):2796-2797). The Alizarin Red concentration corresponds to the intensity of MMSCs osteogenic differentiation

The data are presented as median and interquartile range. Significance of the differences was determined by the Mann-Whitney U-test or Wilcoxon signed-rank test. Differences were considered significant at $p < 0.05$

**Copyright Information:** (Online) ISSN: 2572-9241
©2026 The Author(s). HemaSphere is published by John Wiley & Sons Ltd on behalf of European Hematology Association. This is an open access Abstract Book distributed under the Attribution-NonCommercial-NoDerivs (CC BY-NC-ND), which allows third parties to download the articles and share them with others as long as they credit the author and the Abstract Book, but they cannot change the content in any way or use them commercially.
**Abstract Book Citations:** Authors, Title, HemaSphere, 2026;10:(S1):pages. The abstract book DOIs can be found on the "Author Information" page.
**Disclaimer:** Articles published in the journal HemaSphere exclusively reflect the opinions of the authors. The authors are responsible for all content in their abstracts including accuracy of the facts, statements, citing resources, etc.

**Results**

MMSCs from both affected and unaffected areas were successfully isolated from all patients with ANFH included in this study and their osteogenic potential had been analyzed

Surprisingly the Alizarin Red concentration was significantly higher in the group of IC derived MMSCs from AA patients with ANFH compared to that of control group (4390 (3667-4654) and 1791 (524-3500) µM, respectively; Figure). Therefore IC derived MMSCs from patients with ANFH differ from that of patients without ANFH by an increased ability to respond to osteogenic induction

The concentration of Alizarin Red was lower in the group of MMSC derived from the affected area compared to unaffected area of patients with ANFH (3237 (1041-4442) and 4390 (3667-4654) µM, respectively) however the differences were not significant. Out of 12 patients with ANFH 4 are characterized by decreased osteogenic capacity of MMSCs from affected compared to unaffected region and 8 had no well-marked differences (7 of which have high intensity of osteogenic differentiation in both areas and 1 have low). This may indicate different mechanisms of AON pathogenesis in AA and require additional studies

**Summary/Conclusion**

Difference in the ability of IC derived MMSCs to respond to osteogenic induction between patients with or without ANFH indicates systemic disturbance in osteogenesis in AA patients with AON

Functional MMSCs with high osteogenic potential are retained both in affected and unaffected bone regions in the most cases of AA patients with AON

The study was supported by a grant from Russian Science Foundation № 25-25-00648,
https://rscf.ru/en/project/25-25-00648/

**Copyright Information:** (Online) ISSN: 2572-9241
©2026 The Author(s). HemaSphere is published by John Wiley & Sons Ltd on behalf of European Hematology Association. This is an open access Abstract Book distributed under the Attribution-NonCommercial-NoDerivs (CC BY-NC-ND), which allows third parties to download the articles and share them with others as long as they credit the author and the Abstract Book, but they cannot change the content in any way or use them commercially.
**Abstract Book Citations:** Authors, Title, HemaSphere, 2026;10:(S1):pages. The abstract book DOIs can be found on the “Author Information” page.
**Disclaimer:** Articles published in the journal HemaSphere exclusively reflect the opinions of the authors. The authors are responsible for all content in their abstracts including accuracy of the facts, statements, citing resources, etc.

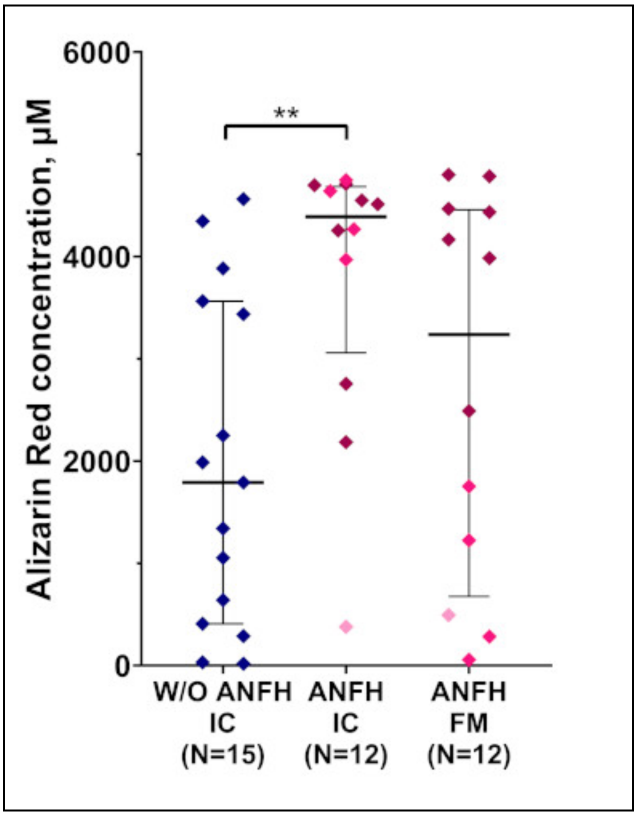

The intensity of MMSCs osteogenic differentiation in the groups of AA patients with or without ANFH. Each symbol corresponds to the value of Alizarin Red concentration detected for each MMSC sample. Patients with ANFH characterized by decreased osteogenic capacity in the affected region are indicated in bright pink, by high and low osteogenic capacity in both regions, dark pink and light pink, respectively. Median and interquartile range are indicated. ** - significant differences (p<0.01) ANFH ¬ aseptic necrosis of the femoral head, IC ¬ iliac crest, FM ¬femur metaphysis

**Copyright Information:** (Online) ISSN: 2572-9241
©2026 The Author(s). HemaSphere is published by John Wiley & Sons Ltd on behalf of European Hematology Association. This is an open access Abstract Book distributed under the Attribution-NonCommercial-NoDerivs (CC BY-NC-ND), which allows third parties to download the articles and share them with others as long as they credit the author and the Abstract Book, but they cannot change the content in any way or use them commercially.
**Abstract Book Citations:** Authors, Title, HemaSphere, 2026;10:(S1):pages. The abstract book DOIs can be found on the "Author Information" page.
**Disclaimer:** Articles published in the journal HemaSphere exclusively reflect the opinions of the authors. The authors are responsible for all content in their abstracts including accuracy of the facts, statements, citing resources, etc.

### (PB3125) GENDER-DEPENDENT ALTERATIONS IN THE ABILITY OF MULTIPOTENT MESENCHYMAL STROMAL CELLS TO RESPOND TO OSTEOGENIC INDUCTION IN APLASTIC ANEMIA

**Topic:** 11. Bone marrow failure syndromes incl. PNH - Biology & translational research

Alena Dorofeeva*[1], Irina Shipounova[1], Anastasia Abramova[1], Anton Luchkin[1], Zalina Fidarova[1], Elena Mikhailova[1]

[1]*National Medical Research Center for Hematology, Moscow, Russia;*

### Background

The hematopoietic stem cell (HSC) functions in close interaction with the stromal microenvironment formed by the mesenchymal stem cells and their differentiated descendants (osteoblasts and adipocytes). In aplastic anemia (AA), an autoimmune attack causes a decrease in the number of HSCs and, consequently, disrupts this interaction. This leads to alterations in the properties of stromal precursors, which may be dependent on the patient's gender

### Aims

To analyze the properties of bone marrow (BM) derived stromal precursors multipotent mesenchymal stromal cells (MMSCs) and colony forming units of fibroblasts (CFU-f) of AA patients depending on gender

### Methods

The study included 21 AA patients (11 m (18-50 y.o., median 22), 10 f (18-39 y.o., median 26)) and 16 healthy donors for allogenic HSC transplantation (8 m (18-58 y.o., median 29), 8 f (23-53 y.o., median 31)). Patients' BM samples were obtained during diagnostic punctures and donors' ones, during planned exfusions after informed consents had been signed.
MMSCs were isolated and cultured using standard method up to passage 3. Cumulative cell production and population doubling time were analyzed. The intensity of osteogenic and adipogenic differentiation was assessed as described previously (Dorofeeva et all, HemaSphere. 2025;9(S1):2796-2797). The resulted concentration of Alizarin Red and Oil Red O corresponds to the intensity of MMSCs differentiation in the osteogenic or adipogenic direction, respectively.
The CFU-f concentration was determined using the method described previously (Dorofeeva et all, Bull Exp Biol Med. 2023 Feb;174(4):538-543).
The data are presented as median and interquartile range. To determine the significance of differences between groups the Student's t-test or Mann-Whitney U-test were applied according to the normality of distribution. Differences were considered significant at $p < 0.05$

### Results

No gender-dependent differences were found in the AA patients' MMSCs population doubling time, cumulative cell production, ability to respond to adipogenic induction, and CFU-f concentration compared to donors.
Analysis of the ability of AA patients' MMSCs to respond to osteogenic induction revealed gender-dependent alterations oppositely directed compared to donors (Figure). The Alizarin Red concentration was lower in female AA patients (543 (79.5–780) µM) and higher in male AA patients (2961 (1336-3540) µM) compared to the gender-matched groups of donors (2784 (1412-4191) and 430 (56.6-1094) µM, respectively). Moreover, the differences were significant both between genders in donors' or patients' groups and between groups in each gender

**Copyright Information:** (Online) ISSN: 2572-9241
©2026 The Author(s). HemaSphere is published by John Wiley & Sons Ltd on behalf of European Hematology Association. This is an open access Abstract Book distributed under the Attribution-NonCommercial-NoDerivs (CC BY-NC-ND), which allows third parties to download the articles and share them with others as long as they credit the author and the Abstract Book, but they cannot change the content in any way or use them commercially.
**Abstract Book Citations:** Authors, Title, HemaSphere, 2026;10:(S1):pages. The abstract book DOIs can be found on the "Author Information" page.
**Disclaimer:** Articles published in the journal HemaSphere exclusively reflect the opinions of the authors. The authors are responsible for all content in their abstracts including accuracy of the facts, statements, citing resources, etc.

**Summary/Conclusion**

Oppositely directed alterations in the intensity of AA patients' MMSCs osteogenic differentiation were detected depending on gender. These alterations indicate a disturbance in osteogenesis in AA patients that differ between men and women.

The study was supported by a grant from Russian Science Foundation № 25-25-00648, https://rscf.ru/en/project/25-25-00648/

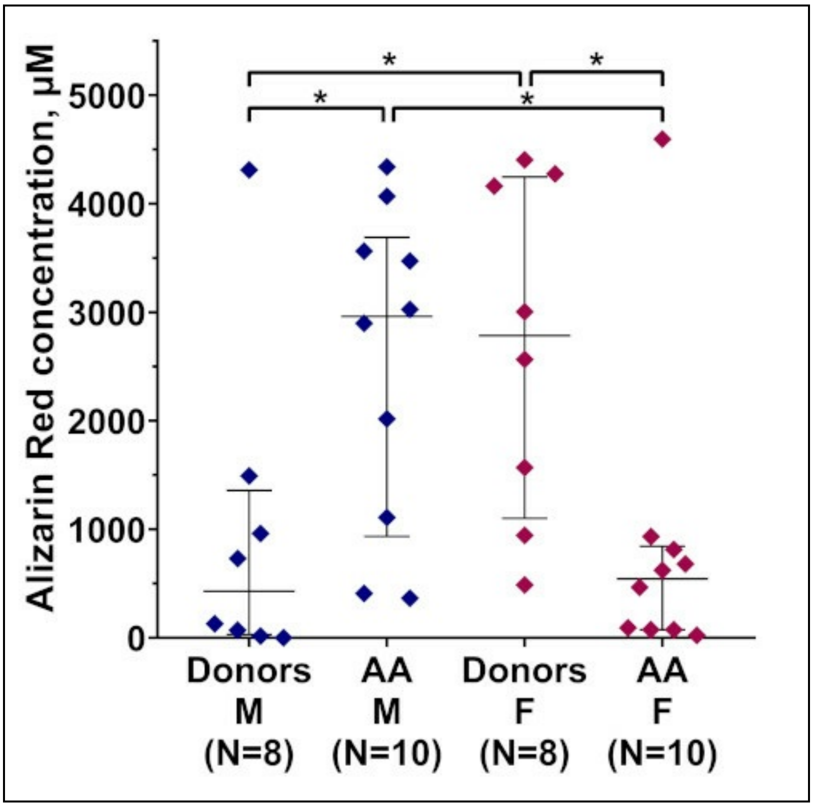

Figure. The intensity of MMSCs osteogenic differentiation in AA patients and donors groups depending on gender. Each symbol corresponds to the value of each donor or AA patient. Median and interquartile range are indicated. * - significant differences (p<0.05) M ¬ male, F ¬ female, AA ¬ aplastic anemia

**Copyright Information:** (Online) ISSN: 2572-9241
©2026 The Author(s). HemaSphere is published by John Wiley & Sons Ltd on behalf of European Hematology Association. This is an open access Abstract Book distributed under the Attribution-NonCommercial-NoDerivs (CC BY-NC-ND), which allows third parties to download the articles and share them with others as long as they credit the author and the Abstract Book, but they cannot change the content in any way or use them commercially.
**Abstract Book Citations:** Authors, Title, HemaSphere, 2026;10:(S1):pages. The abstract book DOIs can be found on the "Author Information" page.
**Disclaimer:** Articles published in the journal HemaSphere exclusively reflect the opinions of the authors. The authors are responsible for all content in their abstracts including accuracy of the facts, statements, citing resources, etc.

### (PB3126) IDENTIFICATION OF A NOVEL TERC VARIANT IN THE H/ACA BOX WITH REVERTANT SOMATIC MOSAICISM IN DYSKERATOSIS CONGENITA

**Topic:** 11. Bone marrow failure syndromes incl. PNH - Biology & translational research

René Mulder*[1], Dennis Bos[1], Arjan Buijs[1]

[1]*University Medical Center Groningen, Genetics, Groningen, The Netherlands;*

### Background

Dyskeratosis congenita and related biology disorders (DC/TBC) is a rare multisystem disorders caused by impaired telomere maintenance resulting in (very) short telomeres. Its clinical presentation is highly variable. Classic DC is characterized by a triad of dysplastic nails, lacy reticular pigmentation of the upper chest and/or neck, and oral leukoplakia, although this may not be present in all individuals. People with DC/TBD are at increased risk for progressive bone marrow failure (BMF), myelodysplastic syndrome or acute myelogenous leukemia, solid tumors (usually squamous cell carcinoma of the head/neck or anogenital cancer), and pulmonary fibrosis (Genereviews).
Heterozygous variants in *TERC* located at 3q26.2 are known to cause autosomal dominant dyskeratosis congenita (DKCA1; OMIM #127550).

### Aims

We describe a 53-year-old female with persistent microcytic anemia, pulmonary and hepatic fibrosis, and recurrent pancreatitis, who was referred for genetic evaluation of a suspected telomere disorder. Her mother died at the age of 52 from presumed liver disease.

### Results

Whole-exome sequencing (WES) using a BMF gene panel did not identify a pathogenic variant. Subsequent Sanger sequencing of the noncoding *TERC* gene revealed a novel variant, NR_001566.1:n.372A>G, located within the H/ACA box. Notably, the wild-type allele was overrepresented (~75%) in peripheral blood. SNP array analysis demonstrated three independent regions of acquired isodisomy involving chromosome 3q, including the *TERC* locus at 3q26.2, together accounting for approximately 75% of cells. These findings are consistent with somatic reversion events due to mitotic recombination, as previously reported in *TERC*-associated DC (PMID 22341970).
Flow-FISH analysis showed a marked reduction in telomere length compared with age-matched controls in all 6 tested cell lines, eg. telomere length was below or equal to 1st percentile. Based on the significantly shortened telomeres, published functional data from in vitro studies of a comparable A372U *TERC* mutation demonstrating severely impaired ribonucleoprotein formation and defective assembly with H/ACA proteins (PMID 19095616), absence of the variant from a population database (gnomAD v4), and a concordant clinical phenotype, the variant was classified as likely pathogenic.

### Summary/Conclusion

*In conclusion*, we report a novel *TERC* variant within the H/ACA box causing DKCA1, classified as likely pathogenic and accompanied by somatic reversion mosaicism.
This finding explains the patient's clinical phenotype and highlights the importance of recognizing that such variants may be associated with loss of heterozygosity of chromosome 3q.

**Copyright Information:** (Online) ISSN: 2572-9241
©2026 The Author(s). HemaSphere is published by John Wiley & Sons Ltd on behalf of European Hematology Association. This is an open access Abstract Book distributed under the Attribution-NonCommercial-NoDerivs (CC BY-NC-ND), which allows third parties to download the articles and share them with others as long as they credit the author and the Abstract Book, but they cannot change the content in any way or use them commercially.
**Abstract Book Citations:** Authors, Title, HemaSphere, 2026;10:(S1):pages. The abstract book DOIs can be found on the "Author Information" page.
**Disclaimer:** Articles published in the journal HemaSphere exclusively reflect the opinions of the authors. The authors are responsible for all content in their abstracts including accuracy of the facts, statements, citing resources, etc.

### (PB3127) GPI ANCHOR EXPRESSION AT EARLY STAGES OF MYELOID MATURATION IN NON-NEOPLASTIC PAEDIATRIC BONE MARROW.

**Topic:** 11. Bone marrow failure syndromes incl. PNH - Biology & translational research

Anna Stelmaszczyk-Emmel*[1], Dominika Zalewska[2], Katarzyna Popko[2], Urszula Demkow[2]

*[1]Medical University of Warsaw, Department of Laboratory Diagnostics and Clinical Immunology of Developmental Age, Warsaw, Poland; [2]Medical University of Warsaw, Student's Scientific Group at the Department of Laboratory Diagnostics and Clinical Immunology of Developmental Age, Warsaw, Poland;*

### Background

Paroxysmal nocturnal hemoglobinuria (PNH) diagnosis relies, in part, on the flow cytometric assessment of glycosylphosphatidylinositol (GPI) anchor expression on neutrophils. Many patients with suspected PNH exhibit a 'left shift' in their peripheral blood, characterized by the presence of immature myeloid precursors. Therefore, it is essential to determine the physiological expression of GPI anchors during the earlier stages of granulopoiesis in children and to evaluate whether excluding these cells from analysis impacts the final results.

### Aims

Evaluation of GPI anchor expression on immature myeloid precursors ($CD117^+$ and/or $CD34^+$ and/or $HLA\text{-}DR^+$) in the bone marrow of paediatric patients.

### Methods

GPI-anchor expression was assessed in 10 bone marrow samples from pediatric patients (mean age 10 ± 5.8 years) without neoplastic disease, who underwent routine immunophenotyping. Analysis was performed using 8-color flow cytometry (BD FACSLyric; FACSSuite software), with FLAER (Alexa Fluor 488, FL2S-C, Cedarlane) used for GPI-anchor detection.

### Results

Immature myeloid cells: myeloblasts (i.a. $CD34^+CD117^+HLA\text{-}DR^+$) and promyelocytes (i.a. $CD34^-CD117^+HLA\text{-}DR^-$) exhibited significantly lower GPI-anchor surface expression compared to mature bone marrow granulocytes (i.a. $CD34^-CD117^-CD64^-$). The mean fluorescence intensity (MFI) for GPI-anchor expression was 1973 ± 562.6 for myeloblasts and 2198 ± 2364.5 for promyelocytes, compared to 9295 ± 2922.2 for mature granulocytes ($p<0.05$ for both comparisons).

### Summary/Conclusion

Among myeloid cells, the expression of the GPI anchor molecule varies according to the stage of cell maturation. Due to the lower expression of GPI anchor on immature granulocytes, it is important to follow ICCS/ESCCA recommendations and use antibodies against CD15, CD24, or CD157 when analyzing blood samples. Expression should be assessed only on fully mature cells (neutrophils) using appropriate antigens and accurate gating, especially in children with suspected myelodysplastic syndrome, given the potential presence of immature cells in the peripheral blood.

**Copyright Information:** (Online) ISSN: 2572-9241
©2026 The Author(s). HemaSphere is published by John Wiley & Sons Ltd on behalf of European Hematology Association. This is an open access Abstract Book distributed under the Attribution-NonCommercial-NoDerivs (CC BY-NC-ND), which allows third parties to download the articles and share them with others as long as they credit the author and the Abstract Book, but they cannot change the content in any way or use them commercially.
**Abstract Book Citations:** Authors, Title, HemaSphere, 2026;10:(S1):pages. The abstract book DOIs can be found on the "Author Information" page.
**Disclaimer:** Articles published in the journal HemaSphere exclusively reflect the opinions of the authors. The authors are responsible for all content in their abstracts including accuracy of the facts, statements, citing resources, etc.

### (PB2535) STATISTICAL ANALYSIS OF EVH IN PNH PATIENTS TREATED WITH C5 INHIBITORS: A REAL-WORLD STUDY

**Topic:** 12. Bone marrow failure syndromes incl. PNH - Clinical

Xinyu Chen[1, 2], Hui Liu[1], Liyan Li[1], Mengting Che[1], Yan Yang[1, 2], Rong Fu*[1]

*[1]Tianjin Medical University General Hospital, Tianjin, China;[2]Tianjin Medical University, Tianjin, China;*

### Background

C5 inhibitors exert their therapeutic effect in Paroxysmal Nocturnal Hemoglobinuria (PNH) by blocking the terminal complement pathway, however, the upstream cascade remains active. Prolonged administration of these agents leads to substantial deposition of complement C3d on the surface of erythrocytes. These red blood cells are subsequently recognized and phagocytosed by hepatic macrophages upon reaching the liver, thereby inducing extravascular hemolysis (EVH). Nowadays, EVH remains clinical concerns. Real-world data on the incidence and characteristics of EVH in PNH patients receiving C5 inhibitors are needed.

### Aims

Analysis of the occurrence and clinical outcomes of EVH in PNH patients treated with C5 inhibitors in real-world clinical practice.

### Methods

Between March 1, 2021, and January 31, 2026, we prospectively followed 45 patients with PNH who were treated with C5 inhibitors at Tianjin Medical University General Hospital. The cohort included 31 cases of classical PNH and 14 cases of PNH with bone marrow failure. Patients received crovalimab (n=27), eculizumab (n=16), or ravulizumab (n=2). A decrease in hemoglobin (HB) by 10g/L, accompanied by an increase in reticulocyte percentage (RET%) and indirect bilirubin (IBIL), along with a positive Coombs test for C3d, is considered indicative of EVH occurrence. Statistical analyses included baseline data, efficacy evaluations at 24 weeks (HB, lactate dehydrogenase [LDH], Ret%, IBIL], EVH incidence, time of occurrence, and clinical outcomes.

### Results

C5 inhibitors showed significant efficacy in reducing LDH and increasing HB from baseline to 24 weeks ($p<0.05$). Among the 45 patients, 29 (64.4%) tested positive on the Coombs test, and 7 (15.6%) developed EVH. The median time from initiation of C5 inhibitor therapy to the first occurrence of EVH was 42 months (IQR 25, 53), and to the first positive Coombs test was 25 months (IQR 13, 45). The median interval from a positive Coombs test to the first episode of EVH was 0 months (IQR 0, 1.5). Following the onset of EVH, management strategies varied: 4 patients (57.1%) continued the current treatment regimen without specific intervention; 1 patient (14.3%) received blood transfusion; 1 patient (14.3%) had corticosteroids added to the regimen; and 1 patient (14.3%) was switched to a factor B inhibitor (iptacopan).

### Summary/Conclusion

In this real-world study, the occurrence of EVH in 15.6% of C5 inhibitor users demonstrates the favorable safety profile of these agents as a key therapeutic option for PNH. Theoretically, long-term use of C5 inhibitors inevitably leads to the accumulation of C3d on the surface of red blood cells, which increases the risk of EVH and may affect the continued use of C5 inhibitors. In this study, 42.9% of patients who developed EVH underwent adjustments to their treatment regimen. Thus, the occurrence of EVH poses a challenge to the long-term application of C5 inhibitors.

**Copyright Information:** (Online) ISSN: 2572-9241
©2026 The Author(s). HemaSphere is published by John Wiley & Sons Ltd on behalf of European Hematology Association. This is an open access Abstract Book distributed under the Attribution-NonCommercial-NoDerivs (CC BY-NC-ND), which allows third parties to download the articles and share them with others as long as they credit the author and the Abstract Book, but they cannot change the content in any way or use them commercially.
**Abstract Book Citations:** Authors, Title, HemaSphere, 2026;10:(S1):pages. The abstract book DOIs can be found on the "Author Information" page.
**Disclaimer:** Articles published in the journal HemaSphere exclusively reflect the opinions of the authors. The authors are responsible for all content in their abstracts including accuracy of the facts, statements, citing resources, etc.

Table.1 Baseline demographic and clinical characteristics

| Category | Total | Classic PNH | PNH with Bone Marrow Failure |
|---|---|---|---|
| *N* | 45 | 31 | 14 |
| *Gender* | | | |
| *Male* | 15 | 7 | 8 |
| *Female* | 30 | 24 | 6 |
| *Median age (years)* | 25 | 25 | 25.5 |
| *Median disease duration (years)* | 10 | 10 | 11.5 |
| *Median duration of complement inhibitor therapy (months)* | 55 | 55 | 39.5 |
| *Median time from medication to Coombs positivity (months)* | 38 | 38 | 35.5 |
| *Thrombosis incidence* | 17.78% (8/45) | 16.13% (5/31) | 21.43% (3/14) |
| *WBC ($x10^9/L$)* | 3.34 (2.74, 4.365) | 3.49 (2.68, 4.37) | 3.24 (2.755, 4.388) |
| *RBC ($x10^{12}/L$)* | 2.3 (1.925, 2.785) | 2.14 (1.93, 2.85) | 2.37 (1.848, 2.728) |
| *HB (g/L)* | 67.42 ± 14.1 | 65.35 ± 13.09 | 72 ± 15.63 |
| *PLT ($x10^9/L$)* | 131 (72.5, 176) | 153 (83, 204) | 85.5 (49, 140.8) |
| *RET (%)* | 9.23 (6.58, 12.13) | 9.585 (8.01, 12.26) | 7.43 (5.433, 9.895) |
| *LDH (U/L)* | 1662 (1138, 2609) | 1838 (1304, 2790) | 1144 (917.3, 2584) |
| *IBIL (μmol/L)* | 25.95 (14.7, 39.53) | 26.3 (14.2, 38.7) | 20.6 (15.55, 52.85) |
| *Cr (μmol/L)* | 49 (40, 59) | 45 (38, 53) | 57.5 (49.75, 69.75) |
| *D-Dimer (ng/ml)* | 472 (285.5, 684.8) | 466.5 (234, 642.5) | 585 (347, 1343) |
| *PNH Clone* | | | |
| *RBC (%)* | 46.12 (28.78, 74.68) | 44.67 (29.8, 74.98) | 48.81 (24.64, 76.05) |
| *Type II (%)* | 9.2 (4.22, 26) | 9.075 (4.053, 26.32) | 14.07 (5.085, 27.01) |
| *Type III (%)* | 27.14 (17.71, 46.57) | 25.92 (19.42, 47.22) | 29.89 (13.31, 48.89) |
| *WBC - Granulocytes (%)* | 90.19 (80.81, 95.57) | 90.97 (79.28, 95.62) | 89.62 (82, 94.93) |
| *FLARE* | | | |
| *CD14 (%)* | 90.3 (86.06, 97.13) | 90.47 (87.39, 97.33) | 90.12 (51.81, 96.97) |
| *CD24 (%)* | 96.83 (86.97, 98.69) | 95.8 (80.71, 98.73) | 97.97 (89.03, 98.16) |

Data presented as median (IQR) or mean ± SD as applicable. PNH: Paroxysmal Nocturnal Hemoglobinuria; WBC: White Blood Cell count; RBC: Red Blood Cell count; HB: Hemoglobin; PLT: Platelet count; RET: Reticulocyte percentage; LDH: Lactate Dehydrogenase; IBIL: Indirect Bilirubin; Cr: Creatinine.

Data presented as median (IQR) or mean ± SD as applicable. PNH: Paroxysmal Nocturnal Hemoglobinuria; WBC: White Blood Cell count; RBC: Red Blood Cell count; HB: Hemoglobin; PLT: Platelet count; RET: Reticulocyte percentage; LDH: Lactate Dehydrogenase; IBIL: Indirect Bilirubin; Cr: Creatinine.

**Copyright Information:** (Online) ISSN: 2572-9241
©2026 The Author(s). HemaSphere is published by John Wiley & Sons Ltd on behalf of European Hematology Association. This is an open access Abstract Book distributed under the Attribution-NonCommercial-NoDerivs (CC BY-NC-ND), which allows third parties to download the articles and share them with others as long as they credit the author and the Abstract Book, but they cannot change the content in any way or use them commercially.
**Abstract Book Citations:** Authors, Title, HemaSphere, 2026;10:(S1):pages. The abstract book DOIs can be found on the "Author Information" page.
**Disclaimer:** Articles published in the journal HemaSphere exclusively reflect the opinions of the authors. The authors are responsible for all content in their abstracts including accuracy of the facts, statements, citing resources, etc.

### (PB3128) CLINICAL BURDEN OF PNH PATIENTS WITH HEMOGLOBIN BETWEEN ≥10 AND

**Topic:** 12. Bone marrow failure syndromes incl. PNH - Clinical

Jörg Schubert*[1], Harish Negi[2], Aurelio Pio Nardozza[3], Nima Melzer[4], Sam Williamson[5], Yasmin Taylor[5], Bruno Fattizzo[6]

*[1]Klinik für Innere Medizin II - Elblandklinikum Riesa, Haematology, Oncology and Palliative Medicine, Weinbergstraße, Germany; [2]Novartis Healthcare Private Ltd, Hyderabad, India; [3]Novartis Farma S.p.A, Milan, Italy; [4]Novartis Pharma GmbH, Nürnberg, Germany; [5]Adelphi Real World, Rare Diseases, Bollington, United Kingdom; [6]Oncology Università degli Studi di Milano, Department of Oncology and Hematology, Milan, Italy;*

### Background

Paroxysmal nocturnal hemoglobinuria (PNH) is an ultra-rare clonal hematopoietic stem cell disorder which renders red blood cells sensitive to complement-mediated destruction. PNH is characterized by hemolytic anemia, bone marrow failure, and thrombosis. Common manifestations include fatigue, dyspnea, and chest/abdominal pain. Disease modifying treatments include terminal complement inhibitor therapies (Ci) which target complement 5 (C5i) and proximal Cis which target complement 3 (C3i), factor B or D. Improvements in hemoglobin (Hb) above 10 g/dL are viewed as beneficial, yet patients with Hb levels between ≥10 and <12 g/dl still face meaningful burden on their daily living.

### Aims

To understand the burdens of PNH in patients with Hb levels between ≥10 and <12 g/dl.

### Methods

Data were drawn from the Adelphi PNH II Disease Specific Programme™, a real-world cross–sectional survey of physicians and their PNH patients, conducted across Canada, France, Germany, Italy, Japan, Spain and United Kingdom from November 2023–January 2025. Physicians provided data on demographics, clinical symptoms, treatment, and PNH-related hospitalizations. Patients voluntarily provided self-reported data on symptoms, Functional Assessment of Chronic Illness Therapy (FACIT)-fatigue and work productivity and activity impairment (WPAI) questionnaires. Data were analyzed descriptively; missing data were not imputed.

### Results

Eighty physicians provided data for 234 PNH patients with Hb levels between ≥10 and <12 g/dl.Patients had a median (interquartile range; IQR) age of 48.0 (37.0–60.0) years, 56.4% were male and 59.9% were in full/part time employment. The median (IQR) time since PNH diagnosis was 2.2 (0.9–4.5) years. Of patients prescribed treatment at the time of survey (n=217), the majority were prescribed Ci (85.7%), with 79.3% and 6.5% prescribed C5 and C3 inhibitor treatment, respectively. Patients (n=181) had been prescribed Ci for a median (IQR) duration of 1.6 (0.7–2.8) years.
Among patients diagnosed since at least a year (n=160), 16.9% had at least one PNH-related hospitalization in the 12 months prior to survey, mostly due to infection (44.4%), followed by treatment of PNH-related complications (22.2%), and thrombotic event (14.8%). Additionally, 48.1% were admitted via the emergency room and 92.6% had to stay in hospital overnight for a median (IQR) 5.0 (4.0–7.0) nights.
Sixty-three patients provided self-reported data. The most common patient-reported symptom was tiredness (79.4%), followed by shortness of breath (47.6%), and lack of focus/brain fog (44.4%). Tiredness was also considered the most bothersome symptom by 61.2% of patients. The mean (standard deviation; SD) of FACIT-fatigue score was 38.5 (7.7). Via the WPAI, patients experienced a mean (SD) 25.1 (23.7) percentage overall work impairment (n=24), and mean (SD) 33.7 (25.2) percentage activity impairment (n=62).

**Copyright Information:** (Online) ISSN: 2572-9241
©2026 The Author(s). HemaSphere is published by John Wiley & Sons Ltd on behalf of European Hematology Association. This is an open access Abstract Book distributed under the Attribution-NonCommercial-NoDerivs (CC BY-NC-ND), which allows third parties to download the articles and share them with others as long as they credit the author and the Abstract Book, but they cannot change the content in any way or use them commercially.
**Abstract Book Citations:** Authors, Title, HemaSphere, 2026;10:(S1):pages. The abstract book DOIs can be found on the "Author Information" page.
**Disclaimer:** Articles published in the journal HemaSphere exclusively reflect the opinions of the authors. The authors are responsible for all content in their abstracts including accuracy of the facts, statements, citing resources, etc.

**Summary/Conclusion**

Overall, these findings highlight that even PNH patients with mild anemia (Hb levels between ≥10 and <12 g/dL) experience a significant burden on their daily living and working activities. A notable proportion also depend heavily on healthcare resources, reflecting the ongoing challenges associated with managing their condition. Addressing this unmet need is essential to closing gaps in care and ultimately improving patient outcomes.

**Copyright Information:** (Online) ISSN: 2572-9241
©2026 The Author(s). HemaSphere is published by John Wiley & Sons Ltd on behalf of European Hematology Association. This is an open access Abstract Book distributed under the Attribution-NonCommercial-NoDerivs (CC BY-NC-ND), which allows third parties to download the articles and share them with others as long as they credit the author and the Abstract Book, but they cannot change the content in any way or use them commercially.
**Abstract Book Citations:** Authors, Title, HemaSphere, 2026;10:(S1):pages. The abstract book DOIs can be found on the "Author Information" page.
**Disclaimer:** Articles published in the journal HemaSphere exclusively reflect the opinions of the authors. The authors are responsible for all content in their abstracts including accuracy of the facts, statements, citing resources, etc.

### (PB3129) EFFICACY AND SAFETY OF RAVULIZUMAB VERSUS ECULIZUMAB IN PAROXYSMAL NOCTURNAL HEMOGLOBINURIA: SYSTEMATIC REVIEW AND META-ANALYSIS

**Topic:** 12. Bone marrow failure syndromes incl. PNH - Clinical

Mehak Dang*[1], Sahithi Mani Chandana Paramkusam[2], Hosana Candreva[3], Abhinav Vardhan Paramkusam[4], Ankur Dhangar[5], Abdrabo Gamal Motawea[6], Lavanya Airen[7], Aashray Raj[8], Fahad Alabbas[9]

*[1]JSS Medical College, Mysore, India;[2]Katuri Medical College and Hospital, Guntur, India;[3]Department of Medicine, Rio de Janeiro State University, Department of Medicine, Rio de Janeiro, Brazil; [4]Jonelta Foundation School of Medicine - UPHSD, Las Pinas, Philippines; [5]Independent Researcher, Gurgaon, India; [6]Faculty of Medicine, Menoufia University, menoufia, Egypt; [7]Lady Hardinge Medical College, New Delhi, India; [8]Government Medical College, Bhavnagar, India;[9]Prince Sultan Military Medical City, Riyadh, Saudi Arabia;*

### Background

Paroxysmal nocturnal hemoglobinuria (PNH) is a rare clonal hematopoietic stem cell disorder caused by acquired mutations in the PIGA gene, leading to deficiency of the complement regulatory proteins CD55 and CD59. This results in uncontrolled terminal complement activation, membrane attack complex formation, chronic intravascular hemolysis, and increased thrombotic risk. Terminal complement inhibition with C5 inhibitors, including eculizumab and long-acting ravulizumab, is the standard of care, yet comparative efficacy and safety remain clinically relevant.

### Aims

To compare the efficacy and safety of ravulizumab versus eculizumab in adults with PNH and to evaluate outcomes by prior complement inhibitor exposure.

### Methods

This systematic review and meta-analysis followed the Cochrane Handbook and PRISMA guidelines and was registered with PROSPERO (CRD420261307071). Searches were conducted in Embase, PubMed, and Cochrane from inception to January 2026. Included studies were randomized controlled trials (RCTs), observational studies, and conference abstracts comparing Ravulizumab with Eculizumab in PNH. Primary outcomes were transfusion avoidance and breakthrough hemolysis. Secondary outcomes included hemoglobin stabilization, LDH normalization, transfusion burden reduction, and adverse events. Subgroup analyses were performed to compare outcomes in complement inhibitor-naïve patients and those previously exposed. Analyses were performed using R (version 4.5.2). Pooled effect estimates were calculated using a random-effects model with 95% Confidence Interval (CI). Heterogeneity was assessed using $I^2$ statistic.

**Copyright Information:** (Online) ISSN: 2572-9241
©2026 The Author(s). HemaSphere is published by John Wiley & Sons Ltd on behalf of European Hematology Association. This is an open access Abstract Book distributed under the Attribution-NonCommercial-NoDerivs (CC BY-NC-ND), which allows third parties to download the articles and share them with others as long as they credit the author and the Abstract Book, but they cannot change the content in any way or use them commercially.
**Abstract Book Citations:** Authors, Title, HemaSphere, 2026;10:(S1):pages. The abstract book DOIs can be found on the "Author Information" page.
**Disclaimer:** Articles published in the journal HemaSphere exclusively reflect the opinions of the authors. The authors are responsible for all content in their abstracts including accuracy of the facts, statements, citing resources, etc.

### Results

Nine studies comprising 1328 patients with PNH were included: two phase III RCTs and seven observational studies. Baseline characteristics were as follows (Ravulizumab and Eculizumab, respectively): Median age 46.06 (ranged from 40.5-67.5) and 45.6 years (ranged from 40.9-55.6); male sex 43.2% and 43.9%. Pooled analysis demonstrated statistically significant improvement in the transfusion avoidance (RR 1.21; 95% CI 1.07-1.36; $I^2$ = 56.3%) and reduction in the breakthrough hemolysis in the patients receiving ravulizumab compared to eculizumab (RR 0.52; 95% CI 0.30-0.90; $I^2$ =37.1%). LDH normalization significantly favoured Ravulizumab (RR 1.15; 95% CI 1.03-1.28; $I^2$=0%). Hemoglobin stabilization was comparable between the two drugs (RR 1.05; 95%CI 0.94-1.17; $I^2$=0%). Safety outcomes (mortality, thrombosis, incidence of headache, and serious infections) were not significantly different between the patients receiving Ravulizumab compared to Eculizumab.
Subgroup analyses showed improved transfusion avoidance among complement inhibitor-exposed patients (RR 1.17; 95% CI 1.01-1.35; $I^2$=52.9%), while the difference was not statistically significant among complement inhibitor–naïve patients (RR 1.20; 95% CI 0.94-1.51; $I^2$ = 66.5%). Breakthrough hemolysis was significantly reduced with ravulizumab in both complement inhibitor-naïve (RR 0.37; 95% CI 0.15-0.95; $I^2$=0%) and complement inhibitor-experienced patients (RR 0.17; 95% CI 0.03-0.87; $I^2$ = 0%).

### Summary/Conclusion

Our findings support that ravulizumab improves transfusion avoidance and reduces the incidence of breakthrough hemolysis, particularly among complement-experienced patients. The safety profiles of both agents are similar. Owing to its extended 8-week dosing interval, ravulizumab may offer meaningful reductions in treatment burden while maintaining sustained clinical efficacy.

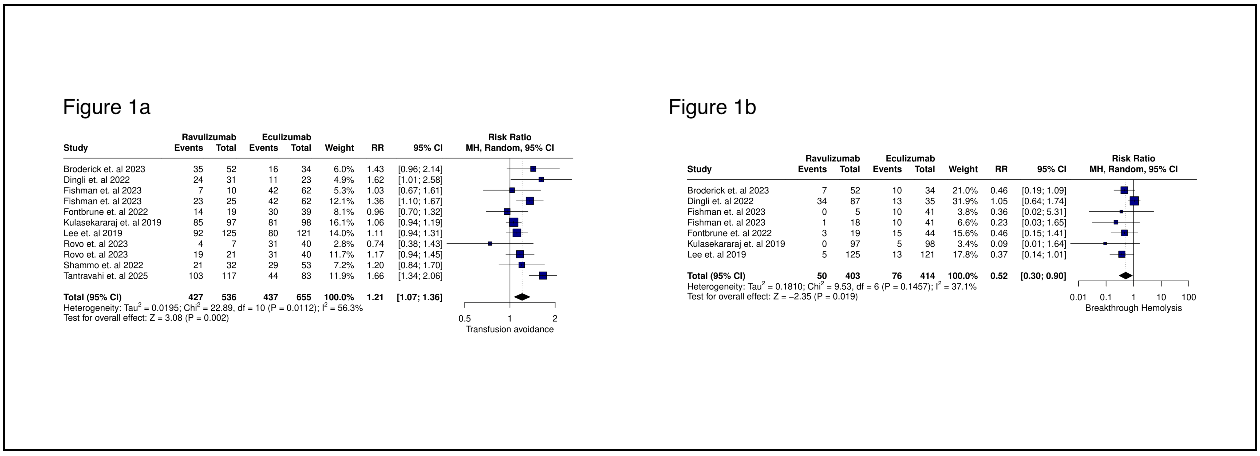

Figure 1a. demonstrates improvement in the transfusion avoidance in patients receiving ravulizumab compared to eculizumab. Figure 1b. demonstrates reduction in the breakthrough hemolysis in the patients receiving ravulizumab compared to eculizumab.

**Copyright Information:** (Online) ISSN: 2572-9241
©2026 The Author(s). HemaSphere is published by John Wiley & Sons Ltd on behalf of European Hematology Association. This is an open access Abstract Book distributed under the Attribution-NonCommercial-NoDerivs (CC BY-NC-ND), which allows third parties to download the articles and share them with others as long as they credit the author and the Abstract Book, but they cannot change the content in any way or use them commercially.
**Abstract Book Citations:** Authors, Title, HemaSphere, 2026;10:(S1):pages. The abstract book DOIs can be found on the "Author Information" page.
**Disclaimer:** Articles published in the journal HemaSphere exclusively reflect the opinions of the authors. The authors are responsible for all content in their abstracts including accuracy of the facts, statements, citing resources, etc.

### (PB3130) COMPARATIONS OF EFFICACY OF IMMUNOSUPPRESSIVE THERAPY AND ALLOGENEIC HEMATOPOIETIC STEM CELL TRANSPLANTATION IN SEVERE APLASTIC ANEMIA : A SINGLE-CENTER RETROSPECTIVE ANALYSIS

**Topic:** 12. Bone marrow failure syndromes incl. PNH - Clinical

Yatong Lv*[1], Yi Wang[1]

[1]*Shaanxi Provincial People's Hospital, Department of Hematology, Xi'an, China;*

### Background

Aplastic anemia (AA) is an immune-mediated haemotopoietic stem-cell (HSC) disorder, characterized by hypocellular bone marrow and pancytopenia, and subclassified into non-severe aplastic anemia (NSAA) and severe aplastic anemia (SAA) based on the bone marrow cellularity and cytopenia of peripheral blood (PB). While SAA with an absolute neutrophil count below $0.2\times10^9$/L, defined as very severe aplastic anemia (VSAA). Most of the patients with SAA will be died with infections within 6 months when lack of effective therapy. Recently, the mainly effective therapy of SAA include immunosuppressive therapy (IST) and allogeneic haematopoietic stem cell transplantation (allo-HSCT), this study retrospectively analysis the efficacy of these two therapy in SAA.

### Aims

To compare the clinical efficacy and outcome between various allo-HSCT and IST in SAA.

### Methods

This retrospective analysis included data from the SAA patients that treated with allo-HSCT or IST at our center between January 2014 and October 2025. The primary endpoint was to compare the overall survival (OS) and event-free survival (EFS) between allo-HSCT group and IST group. The secondary endpoints included early response rates, haematopoietic engraftment times, and graft-versus-host disease (GVHD) incidence. Follow-up ended on October 31, 2025, or at death. Categorical variables were compared using chi-square tests, quantitative variables with independent t-tests or non-parametric tests, survival analyzed via Kaplan-Meier analysis and log-rank tests for differences, and multivariate analyses using Cox proportional hazards regression. And p-value $< 0.05$ were considered significant.

**Copyright Information:** (Online) ISSN: 2572-9241
©2026 The Author(s). HemaSphere is published by John Wiley & Sons Ltd on behalf of European Hematology Association. This is an open access Abstract Book distributed under the Attribution-NonCommercial-NoDerivs (CC BY-NC-ND), which allows third parties to download the articles and share them with others as long as they credit the author and the Abstract Book, but they cannot change the content in any way or use them commercially.
**Abstract Book Citations:** Authors, Title, HemaSphere, 2026;10:(S1):pages. The abstract book DOIs can be found on the “Author Information” page.
**Disclaimer:** Articles published in the journal HemaSphere exclusively reflect the opinions of the authors. The authors are responsible for all content in their abstracts including accuracy of the facts, statements, citing resources, etc.

**Results**

1) Patient Characteristics
A cohort of 63 patients, 37 SAA and 26 VSAA were include. In which 26 of the patients received allo-HSCT (9 matched sibling donor HSCT [MSD-HSCT] and 17 were haploidentical HSCT [HID-HSCT]), while 37 of the patients received IST (porcine antilymphocyte globulin combined with cyclosporine A).
2) Adverse Events
In the allo-HSCT group, adverse events occurred in 31% (8/26) of the patients, with 15.3% (4/26) mortality, that 75% (3/4) of the patients developed grade III-IV acute GVHD within 3 months post-transplant, and 25% (1/4) developed post-transplant lymphoproliferative disorder at 4 months. In the IST group, severe adverse events occurred in 27% (10/37) of the patients, with 21.6% (8/37) mortality, that 87.5% (7/8) of the patients were infection-related, while 5.4% (2/37) of the patients developed paroxysmal nocturnal hemoglobinuria (PNH) transformation.
3) Efficacy Evaluation
Treatment responses were assessed at 3 and 6 months in all 63 patients. The overall response rate (ORR) in the allo-HSCT group was 92.3% at 3 months and 88.5% at 6 months, significantly higher than in the IST group (both was 56.8%; $p < 0.05$). However, 5-year OS (allo-HSCT 82.1% vs. IST 77.9%; $p = 0.499$) and EFS (allo-HSCT67.0% vs. IST 59.3%; $p = 0.568$) showed no significant differences. Subgroup analysis of MSD-HSCT (n=9) and HID-HSCT (n=17) revealed no significant  differences in 5-year OS (88.9% vs. 78.4%; $p = 0.667$) or EFS (88.9% vs. 61.8%; $p = 0.222$). While in VSAA patients, 5-year OS in the allo-HSCT (n=10) versus IST (n=16) groups was 90.0% vs. 68.2% ($p = 0.206$), but 5-year EFS was significantly higher in the allo-HSCT group (90.0% vs. 37.9%; $p = 0.019$).

**Summary/Conclusion**

Allo-HSCT was more suitable for VSAA, especially younger patients, while IST was more appropriate for older patients that with non-VSAA.

**Copyright Information:** (Online) ISSN: 2572-9241
©2026 The Author(s). HemaSphere is published by John Wiley & Sons Ltd on behalf of European Hematology Association. This is an open access Abstract Book distributed under the Attribution-NonCommercial-NoDerivs (CC BY-NC-ND), which allows third parties to download the articles and share them with others as long as they credit the author and the Abstract Book, but they cannot change the content in any way or use them commercially.
**Abstract Book Citations:** Authors, Title, HemaSphere, 2026;10:(S1):pages. The abstract book DOIs can be found on the “Author Information” page.
**Disclaimer:** Articles published in the journal HemaSphere exclusively reflect the opinions of the authors. The authors are responsible for all content in their abstracts including accuracy of the facts, statements, citing resources, etc.

### (PB3131) EFFICACY AND SAFETY OF THROMBOPOIETIN RECEPTOR AGONISTS COMBINED WITH INTENSIVE IMMUNOSUPPRESSIVE THERAPY PLUS UMBILICAL CORD BLOOD TRANSFUSION FOR SEVERE APLASTIC ANEMIA

**Topic:** 12. Bone marrow failure syndromes incl. PNH - Clinical

Fang Zhou*[1], Guokai Zhang[1], Jijia Li[1]

*[1]The 960th Hospital of The People's Liberation Army (PLA) Joint Logistics Support Force, Jinan, Shandong, China;*

### Background

Severe aplastic anemia (SAA) is a life-threatening bone marrow failure disease. Thrombopoietin receptor agonists (TPO-RAs) have emerged as promising therapeutic agents for SAA, but their efficacy and safety profile in conjunction with intensive immunosuppressive therapy combined with umbilical cord blood transfusions (IIST-UCB) remain uncertain.

### Aims

A retrospective study was conducted to evaluate the efficacy and safety of IIST-UCB+TPO-RA therapy and IIST-UCB of severe aplastic anemia.

### Methods

A retrospective analysis was conducted on the clinical data and outcomes of 36 patients with severe aplastic anemia who received different treatment regimens between 2018 and 2024. Among them,15 received TPO-RA within 30 days post-UCB infusion (IIST-UCB+TPO-RA group), and 21 received IIST-UCB alone (IIST-UCB group). The primary objectives were to compare overall survival and time to hematopoietic cell engraftment between SAA patients receiving the two different treatment regimens. Secondary endpoints included clinical indicators such as the incidence of infection, treatment-related mortality, and failure-free survival.

### Results

Results showed that the IIST-UCB+TPO-RA group had numerically shorter time for absolute neutrophil count (ANC) recovery to $0.5\times10^9$/L (13 vs. 25 d, $p = 0.126$) and significantly shorter time for hemoglobin (Hb) recovery to 60 g/L (37 vs. 51 d,$p$=0.044). The time for platelet count recovery to $50\times10^9$/L was also numerically shorter in the combined group (44.5 vs. 57.5 d, $p = 0.126$). A lower incidence of major complications (such as pulmonary infection) was observed in the IIST-UCB+TPO-RA group (61.5% vs. 65.0%), and the two-year event-free survival rate was improved (88.9% vs. 85.0%), though without statistical significance. Stratified analysis and multivariable regression confirmed that the combined regimen still promoted hematopoietic recovery regardless of baseline differences in TNC count, ABO matching, age, and disease severity.

### Summary/Conclusion

In this single-center retrospective study, IIST-UCB combined with TPO-RA showed favorable efficacy and safety in SAA treatment. The addition of TPO-RA enhanced hematopoietic recovery, accelerated hematological responses, and did not increase adverse events. For SAA patients without suitable donors, especially those with suboptimal UCB quality, IIST-UCB+TPO-RA is a viable first-line treatment option. Further prospective research is needed to comprehensively assess its efficacy.

**Copyright Information:** (Online) ISSN: 2572-9241
©2026 The Author(s). HemaSphere is published by John Wiley & Sons Ltd on behalf of European Hematology Association. This is an open access Abstract Book distributed under the Attribution-NonCommercial-NoDerivs (CC BY-NC-ND), which allows third parties to download the articles and share them with others as long as they credit the author and the Abstract Book, but they cannot change the content in any way or use them commercially.
**Abstract Book Citations:** Authors, Title, HemaSphere, 2026;10:(S1):pages. The abstract book DOIs can be found on the "Author Information" page.
**Disclaimer:** Articles published in the journal HemaSphere exclusively reflect the opinions of the authors. The authors are responsible for all content in their abstracts including accuracy of the facts, statements, citing resources, etc.

### (PB3132) REAL-WORLD PHYSICIANS AND PATIENTS PERSPECTIVES ON ADHERENCE, SATISFACTION WITH PEGCETACOPLAN, AND HEALTH RELATED QUALITY OF LIFE ACROSS EUROPE, THE UNITED STATES AND CANADA: A CROSS-SECTIONAL STUDY

**Topic:** 12. Bone marrow failure syndromes incl. PNH - Clinical

Jens Panse*[1, 2], Koo Wilson[3], Zalmai Hakimi[3], Barbara Czech[3], Brianne Kerr[4], Sam Williamson[4], Yasmin Taylor[4], Susanna Libby[4], Marc Bienz[5, 6]

*[1]Center for Integrated Oncology Aachen Bonn Cologne Düsseldorf (CIO ABCD), Aachen, Germany; [2]University Hospital RWTH Aachen, Department of Hematology, Oncology, Hemostaseology and Stem Cell Transplantation, Aachen, Germany; [3]Sobi, Stockholm, Sweden; [4]Adelphi Real World, Rare Diseases, Bollington, United Kingdom; [5]Jewish General Hospital, Division of Hematology, Montreal, Canada; [6]McGill University, Department of Medicine, Montreal, Canada;*

### Background

Paroxysmal nocturnal hemoglobinuria (PNH) is a rare disorder whereby red blood cells lack protection from physiologic complement activation. This leads to anemia, fatigue and decreased health-related quality of life (HRQoL). Current standard of care includes terminal complement inhibitors (Ci) that target C5, and proximal Ci that target C3, factor B or D (in combination with C5i). Pegcetacoplan (first C3/C3b inhibitor) controls intravascular and extravascular hemolysis and has demonstrated efficacy and safety in PNH patients.

### Aims

To describe physician and patient perspectives on treatment adherence, satisfaction, and HRQoL of PNH patients prescribed pegcetacoplan.

### Methods

Data were drawn from the Adelphi PNH Disease Specific Programme™, a cross-sectional survey of hematologists and their consulting PNH patients. Data were collected in Canada, France, Germany, Italy, Spain, the UK, and the US (November 2023–January 2025). Physicians provided data on patient demographics, clinical characteristics, treatment satisfaction, and adherence based on their own clinical judgement. Patients optionally reported data on symptomatology, treatment satisfaction, and HRQoL via EQ-5D-5L (German tariff) and EQ-visual analogue scale (VAS), Functional Assessment of Chronic Illness Therapy (FACIT)-Fatigue, and the Work Productivity and Activity Impairment (WPAI) questionnaire. Patients prescribed pegcetacoplan for ≥3 months at time of survey were included in this analysis. Analyses were descriptive, reported as mean (standard deviation; SD) or median (interquartile range; IQR); missing data were not imputed.

**Copyright Information:** (Online) ISSN: 2572-9241
©2026 The Author(s). HemaSphere is published by John Wiley & Sons Ltd on behalf of European Hematology Association. This is an open access Abstract Book distributed under the Attribution-NonCommercial-NoDerivs (CC BY-NC-ND), which allows third parties to download the articles and share them with others as long as they credit the author and the Abstract Book, but they cannot change the content in any way or use them commercially.
**Abstract Book Citations:** Authors, Title, HemaSphere, 2026;10:(S1):pages. The abstract book DOIs can be found on the "Author Information" page.
**Disclaimer:** Articles published in the journal HemaSphere exclusively reflect the opinions of the authors. The authors are responsible for all content in their abstracts including accuracy of the facts, statements, citing resources, etc.

**Results**

Overall, 54 physicians reported data on 77 PNH patients prescribed pegcetacoplan. Median (IQR) age was 45.0 (33.5–55.0) years, 64% were male and 58% were employed (full/part time). Median (IQR) time since initiation of pegcetacoplan was 0.6 (0.4–1.0) years.
At time of survey, median (IQR) hemoglobin (n=76) and lactate dehydrogenase (n=73) levels were 11.0 (10.2–12.0) g/dL and 232.0 (192.5–367.5) U/L, respectively. Complete adherence to treatment was reported for 87% of patients. Physicians perceived 25% of patients to have a 'good', 'very good' or 'excellent' quality of life at treatment initiation, increasing to 91% at time of survey.
Twenty-five patients self-reported data. Of those, 88% experienced symptoms with 24% reporting moderate or severe tiredness at time of survey.
Of a matched patient-physician sample (n=21), physicians were 'satisfied' or 'completely satisfied' with treatment overall for 95% of patients, whilst this was reported by 86% of patients. Physicians were 'satisfied' or 'completely satisfied' with treatment in relation to fatigue for 86% of patients, the same level of satisfaction was reported by 81% of patients.
Patients reported a mean (SD); EQ-VAS score of 71.2 (17.6), EQ-5D Utility score of 0.88 (0.15) and FACIT-Fatigue score (n=24) of 39.2 (10.7). From the WPAI (n=12), mean (SD) percentage absenteeism was 6.6 (14.8), presenteeism was 25.0 (13.8), and overall work impairment was 30.1 (17.3).

**Summary/Conclusion**

Results indicate that although there was some discordance in satisfaction level between physicians and patients, most were satisfied or completely satisfied with treatment and reported favorable HRQoL measures. These findings demonstrate that the perceived benefit of pegcetacoplan treatment in PNH patients in real-world practice is consistent with results from clinical trials and other real-world evidence.

**Copyright Information:** (Online) ISSN: 2572-9241
©2026 The Author(s). HemaSphere is published by John Wiley & Sons Ltd on behalf of European Hematology Association. This is an open access Abstract Book distributed under the Attribution-NonCommercial-NoDerivs (CC BY-NC-ND), which allows third parties to download the articles and share them with others as long as they credit the author and the Abstract Book, but they cannot change the content in any way or use them commercially.
**Abstract Book Citations:** Authors, Title, HemaSphere, 2026;10:(S1):pages. The abstract book DOIs can be found on the "Author Information" page.
**Disclaimer:** Articles published in the journal HemaSphere exclusively reflect the opinions of the authors. The authors are responsible for all content in their abstracts including accuracy of the facts, statements, citing resources, etc.

### (PB3133) SAFETY, PHARMACOKINETICS, AND PHARMACODYNAMICS OF MY008211A, AN ORAL COMPLEMENT FACTOR B INHIBITOR, IN SINGLE- AND MULTIPLE-ASCENDING-DOSE STUDIES IN HEALTHY PARTICIPANTS

**Topic:** 12. Bone marrow failure syndromes incl. PNH - Clinical

Xin Li*[1], Ping Zhang[1], Yuan Li[1], Wei Wang[2], Juan Du[2], Chunlan Tang[2], Li Liu[2], Rui Xia[2], Wilson Chunlin Wang[2], Lian MA[2]

*[1]The Third Hospital of Changsha, Changsha, China;[2]Wuhan Createrna Science and Technology Co., Ltd., Wuhan, China;*

### Background

Paroxysmal nocturnal hemoglobinuria (PNH) is a life-threatening disease characterized by red blood cell destruction, impaired bone marrow function, vasoconstriction, and platelet activation. MY008211A—an orally administered, selective complement factor B inhibitor that blocks activation of the alternative complement pathway (AP) upstream of C3 cleavage—is being developed as a treatment for PNH.

### Aims

To evaluate the safety, tolerability, pharmacokinetics (PK), and pharmacodynamics (PD) of MY008211A in first-in-human single-ascending-dose (SAD) and multiple-ascending-dose (MAD) studies (NCT05642585 and NCT05828472) in healthy volunteers.

### Methods

The SAD and MAD studies both used randomized, double-blind, placebo (PBO)-controlled designs and were conducted in China. In the SAD study, participants were randomized to receive single doses of MY008211A 50, 800, 1200, or 1600 mg (6:2 ratio vs PBO) or 100, 200, or 400 mg (8:2 ratio vs PBO). In the MAD study, participants were randomized (8:2 in each dose group) to receive 200 mg BID, 400 mg BID, 600 mg QD, and 600 mg BID of MY008211A or PBO for 7 consecutive days. Both studies evaluated adverse events (AEs), PK, and PD (including AP activity and C3 level). All participants provided informed consent.

### Results

In the SAD study, there were 35 men and 27 women; mean ages ranged from 22–30 years. The median $T_{max}$ ranged from 0.50–2.50 h, and $T_{½}$ was 8.65–14.87 h. Exposure increased with dose but was less than dose-proportional at higher dose levels. Complement AP activity decreased rapidly within 1–3 h after MY008211A administration, with near-maximal inhibition observed at peak concentrations. Among the 40 participants included in the MAD study (8 participants in each dose group and PBO group), there were 28 men and 12 women; mean age was 27 years. Median $T_{max}$ ranged from 0.63–2.50 h; steady-state was reached before the last dose, and the geometric mean $t_{½}$ of the last dose was 8.74–14.46 h. $C_{max}$ was dose proportional, and AUC was approximately dose proportional from 200–600 mg BID. AP activity was decreased in a dose-dependent manner vs PBO. Twice-daily dosing achieved sustained suppression over the dosing interval, with trough inhibition approaching 70% at the highest BID dose. The C3 level slightly decreased from baseline and subsequently increased to or above baseline, without significant dose dependence. Adverse events were predominantly mild and resolved without intervention, and no serious AEs or discontinuations due to AEs occurred during either study.

**Copyright Information:** (Online) ISSN: 2572-9241
©2026 The Author(s). HemaSphere is published by John Wiley & Sons Ltd on behalf of European Hematology Association. This is an open access Abstract Book distributed under the Attribution-NonCommercial-NoDerivs (CC BY-NC-ND), which allows third parties to download the articles and share them with others as long as they credit the author and the Abstract Book, but they cannot change the content in any way or use them commercially.
**Abstract Book Citations:** Authors, Title, HemaSphere, 2026;10:(S1):pages. The abstract book DOIs can be found on the “Author Information” page.
**Disclaimer:** Articles published in the journal HemaSphere exclusively reflect the opinions of the authors. The authors are responsible for all content in their abstracts including accuracy of the facts, statements, citing resources, etc.

**Summary/Conclusion**

In these Phase I studies, MY008211A showed a favorable overall safety profile and was well tolerated following single doses of 50-1600 mg and multiple doses of 200–600 mg BID. Steady-state concentration was achieved within 1 week of dosing, and AP activity was rapidly and dose-dependently decreased, with sustained suppression observed with BID regimens. These data support continued clinical development of MY008211A for the treatment of PNH.

**Copyright Information:** (Online) ISSN: 2572-9241
©2026 The Author(s). HemaSphere is published by John Wiley & Sons Ltd on behalf of European Hematology Association. This is an open access Abstract Book distributed under the Attribution-NonCommercial-NoDerivs (CC BY-NC-ND), which allows third parties to download the articles and share them with others as long as they credit the author and the Abstract Book, but they cannot change the content in any way or use them commercially.
**Abstract Book Citations:** Authors, Title, HemaSphere, 2026;10:(S1):pages. The abstract book DOIs can be found on the "Author Information" page.
**Disclaimer:** Articles published in the journal HemaSphere exclusively reflect the opinions of the authors. The authors are responsible for all content in their abstracts including accuracy of the facts, statements, citing resources, etc.

### (PB3134) IMMUNOSUPPRESSIVE TREATMENT IN ELDERLY PATIENTS (>60 YEARS) WITH ACQUIRED APLASTIC ANEMIA: A RETROSPECTIVE SINGLE-CENTER ANALYSIS

**Topic:** 12. Bone marrow failure syndromes incl. PNH - Clinical

Beatrice De Marco[1], Elisa Cerqui[1], Erika Borlenghi[1], Chiara Cattaneo[1], Margherita Oberti[1], Samuele Bagnasco[1], Eugenia Accorsi Buttini[1], Lorenzo Masina[1], Gabriele Magliano[2], Alessandra Tucci*[1]

*[1]Hematology, ASST Spedali Civili, ERN-EuroBloodNet, Brescia, Italy; [2]Unit of Blood Diseases and Bone Marrow Transplant, ASST Spedali Civili, Brescia, Italy;*

### Background

Patients aged >60 years with acquired aplastic anemia (AAA) present therapeutic challenges due to comorbidities and reduced tolerance to intensive treatments. Standard therapy consists of immunosuppressive treatment (IST) with anti-thymocyte globulin plus cyclosporine (ATG+CSA), with or without eltrombopag (ELT), or hematopoietic stem cell transplantation (HSCT) in selected cases.

### Aims

The study aimed to evaluate the feasibility and safety of ATG ± CSA in elderly patients with AAA.

### Methods

We retrospectively analyzed 40 consecutive patients aged >60 years diagnosed with AAA according to Camitta criteria and treated at our center between January 1989 and January 2025. Horse ATG was administered in 37 patients, whereas rabbit ATG was used in 3 patients.

**Copyright Information:** (Online) ISSN: 2572-9241
©2026 The Author(s). HemaSphere is published by John Wiley & Sons Ltd on behalf of European Hematology Association. This is an open access Abstract Book distributed under the Attribution-NonCommercial-NoDerivs (CC BY-NC-ND), which allows third parties to download the articles and share them with others as long as they credit the author and the Abstract Book, but they cannot change the content in any way or use them commercially.
**Abstract Book Citations:** Authors, Title, HemaSphere, 2026;10:(S1):pages. The abstract book DOIs can be found on the “Author Information” page.
**Disclaimer:** Articles published in the journal HemaSphere exclusively reflect the opinions of the authors. The authors are responsible for all content in their abstracts including accuracy of the facts, statements, citing resources, etc.

**Results**

Starting in 2018, ELT (up to 150 mg/day) was introduced after 3 months in non-responders. Treatment responses were assessed according to EBMT criteria: complete response (CR; hemoglobin >10 g/dL, absolute neutrophil count [ANC] >1.5 × $10^9$/L, platelets >100 × $10^9$/L); partial response (PR; transfusion independence); and no response (NR; persistent transfusion dependence).
The median age was 71 years (range, 60–84). Two patients (5%) had non-severe transfusion-dependent AA, 22 (55%) had severe AA, and 16 (40%) had very severe AA. A small PNH clone was detected in 9 patients (22%). The ATG dose was reduced by 50% in 11 of 40 patients (27%).
At 3 months, the overall response rate of the whole cohort (ORR; CR + PR) was 37% (15/40).
In the pre-eltrombopag era (before 2018), 23 patients received ATG alone or ATG combined with CSA. Among evaluable patients (n=19; 4 not evaluable for early death), the ORR at +12 weeks was 48%, including 8 CRs and 3 PRs.
In the post-eltrombopag era, 17 patients received ATG plus CSA. Response was not evaluable in 5 patients for early death. In the evaluable cohort, the ORR at +12 weeks was 33%, with no CRs and 4 PRs, while 8 patients (67%) were non-responders.
Among the 8 non-responders, 6 subsequently received ELT. After an additional 4 months, the ORR increased to 75% (9/12), with all initial responders maintaining their response. Five of 6 ELT-treated patients achieved a response (2 CRs and 3 PRs overall).
Thirty-three of the 40 patients (83%) developed treatment-related complications including infectious events (53%), cardiac events (20%), cerebrovascular events (16%) and fever of unknown origin (11%).
In 11 of 40 patients, a fatal event occured, including aspergillus pneumonia (n=2), acute myocardial infarction (n=1), sepsis (n=6), cerebral hemorrhage (n=1), and hepatorenal syndrome (n=1).
Overall, after a median follow-up of 2 years (range, 2–12), 62% (25/40) of patients died.
Median overall survival was 5.9 years, and the estimated 30-year overall survival was 25% (Figure 1).

**Summary/Conclusion**

Despite achieving hematologic responses, ATG-based IST was associated with substantial early mortality (27%) and significant morbidity in elderly patients. The addition of eltrombopag improved response rates with acceptable toxicity. Prospective studies are warranted to investigate CSA plus eltrombopag, without ATG, as a potentially safer first-line therapeutic strategy in elderly patients with AAA.

Fig. 1

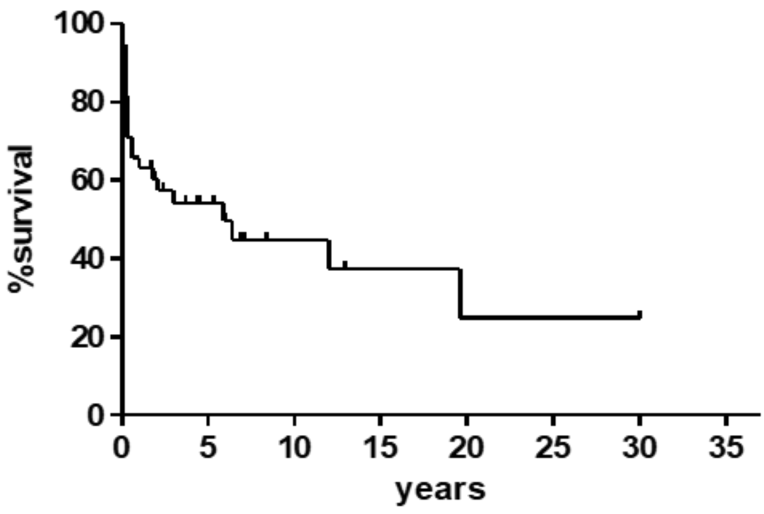

**Copyright Information:** (Online) ISSN: 2572-9241
©2026 The Author(s). HemaSphere is published by John Wiley & Sons Ltd on behalf of European Hematology Association. This is an open access Abstract Book distributed under the Attribution-NonCommercial-NoDerivs (CC BY-NC-ND), which allows third parties to download the articles and share them with others as long as they credit the author and the Abstract Book, but they cannot change the content in any way or use them commercially.
**Abstract Book Citations:** Authors, Title, HemaSphere, 2026;10:(S1):pages. The abstract book DOIs can be found on the "Author Information" page.
**Disclaimer:** Articles published in the journal HemaSphere exclusively reflect the opinions of the authors. The authors are responsible for all content in their abstracts including accuracy of the facts, statements, citing resources, etc.

### (PB3135) OPTIMIZING ORAL FRONT-LINE THERAPY IN APLASTIC ANEMIA: REAL-WORLD EFFICACY OF ELTROMBOPAG AND CYCLOSPORINE IN AN OUTPATIENT SETTING FROM LOW AND MIDDLE-INCOME COUNTRY

**Topic:** 12. Bone marrow failure syndromes incl. PNH - Clinical

Gaurav Ojha*[1]

[1]*Mahatma Gandhi Medical College and Hospital, Clinical Hematology, Jaipur, India;*

### Background

Acquired aplastic anemia (AA) stems from an immune-mediated depletion of hematopoietic stem cells, traditionally requiring intensive, hospital-based treatments. However, logistical and financial barriers often necessitate the use of oral-only regimens. Eltrombopag (EPAG), a thrombopoietin receptor agonist has revolutionized management by directly stimulating progenitor cells. Evaluating the effectiveness of combining oral EPAG with cyclosporine (CSA) as a primary treatment is essential for developing accessible, outpatient-based protocols.

### Aims

To investigate the clinical outcomes, safety profile, and transfusion-free intervals in a cohort of 40 patients with AA treated exclusively with an oral combination of EPAG and CSA in a real-world clinical environment.

### Methods

We analyzed data from 40 patients diagnosed with AA (including non-severe and severe variants). All participants received a front-line oral regimen consisting of CSA (initial dose 3-5 mg/kg/day) and EPAG (median starting dose 50 mg/day), with some receiving adjunctive Danazol. The study excluded patients receiving parenteral biologics or cellular therapies. Primary endpoints included hematologic response rates (Overall Response [OR] and Complete Response [CR]) at 3, 6, and 12 months, and the achievement of transfusion independence.

### Results

The preliminary analysis of the cohort (median age 35 years) demonstrated that oral dual therapy successfully induced hematologic recovery. At the 6-month landmark, the OR rate was 45%, with notable improvements in absolute neutrophil counts and platelet levels. Transfusion independence was reached by 48% of the cohort within the first 90 days of treatment. In patients with non-severe AA, response rates were even more robust, with 75% reaching a clinical response by one year. The safety profile was manageable; the most frequent adverse events were transient elevations in liver enzymes and mild gastrointestinal distress, which generally did not require treatment discontinuation. Clonal monitoring revealed the presence of PNH clones in 35% of patients, emphasizing the need for long-term surveillance.

### Summary/Conclusion

This study confirms that the oral combination of EPAG and CSA is an effective and safe front-line strategy for AA management. By eliminating the need for inpatient infusion-based therapies, this regimen offers a viable alternative for patients prioritized for outpatient care or those in resource-constrained settings. These findings suggest that oral dual therapy provides significant clinical benefit and can serve as a primary therapeutic pillar in modern AA management.

**Copyright Information:** (Online) ISSN: 2572-9241
©2026 The Author(s). HemaSphere is published by John Wiley & Sons Ltd on behalf of European Hematology Association. This is an open access Abstract Book distributed under the Attribution-NonCommercial-NoDerivs (CC BY-NC-ND), which allows third parties to download the articles and share them with others as long as they credit the author and the Abstract Book, but they cannot change the content in any way or use them commercially.
**Abstract Book Citations:** Authors, Title, HemaSphere, 2026;10:(S1):pages. The abstract book DOIs can be found on the "Author Information" page.
**Disclaimer:** Articles published in the journal HemaSphere exclusively reflect the opinions of the authors. The authors are responsible for all content in their abstracts including accuracy of the facts, statements, citing resources, etc.

### (PB3136) COMPARATIVE EFFICACY OF NOVEL COMPLEMENT INHIBITORS VERSUS ECULIZUMAB IN ADULT PATIENTS WITH PAROXYSMAL NOCTURNAL HEMOGLOBINURIA: A META-ANALYSIS OF RANDOMIZED TRIALS

**Topic:** 12. Bone marrow failure syndromes incl. PNH - Clinical

Luiza Veloso*[1], Isadora Mamede[2], Yasmim Nunes[3], Yasmin Faller[4], Luisa Medeiros Visentini[5], Ancy Jenil-Franco[6]

*[1]Federal University of the State of Rio de Janeiro, Rio de Janeiro, Brazil;[2]Federal University of São João del Rei, Divinópolis, Brazil; [3]Afya Faculty of Medical Sciences of Palmas, Palmas, Brazil;[4]University of Buenos Aires - UBA, Buenos Aires, Argentina; [5]University of Western Sao Paulo, São Paulo, Brazil; [6]MBBS, Internal Medicine Department, McLaren Bay Region, Bay City, United States of America;*

### Background

Paroxysmal Nocturnal Hemoglobinuria (PNH) is a rare, acquired, life-threatening clonal hematopoietic stem cell disorder characterized by complement-mediated intravascular hemolysis, resulting in anemia and an increased risk of thrombosis. The introduction of complement inhibitors transformed the disease management, modifying disease course by reducing the risk of thrombosis and transfusion dependency, while improving quality of life. Eculizumab, which was the first C5 inhibitor approved by the FDA for PNH, remains the standard treatment. However, persistent hemolysis remains a significant challenge and continues to be observed in the majority of patients. Recently, novel complement inhibitors, including longer-acting C5 and proximal complement inhibitors, were evaluated in randomized controlled trials (RCTs) against standard therapy.

### Aims

This systematic pairwise meta-analysis of Phase III RCTs aimed to compare the efficacy of novel complement inhibitors versus eculizumab in adults with PNH, with transfusion independence as primary endpoint.

### Methods

This meta-analysis was conducted in accordance with the PRISMA guidelines. Eligible RCTs were identified through a systematic search of PubMed, Embase, and Cochrane up to February 2026. The primary outcome was transfusion independence. Secondary outcomes included hemoglobin response and lactate dehydrogenase (LDH) normalization. Pooled risk ratios (RRs) with 95% confidence intervals (CIs) were calculated using a random-effects model with restricted maximum likelihood (REML) estimation and Hartung-Knapp-Sidik-Jonkman adjustment, given the small number of studies and potential heterogeneity.

### Results

A total of five RCTs comprising 800 patients (436 assigned to novel complement inhibitors and 364 to eculizumab) were included. Novel complement inhibitors did not reduce the need for transfusion compared to eculizumab (RR 1.34, 95% CI 0.58-3.10; $I^2$= 80%). Subgroup analysis showed consistent results among treatment-naive patients (RR 1.05, 95% CI 0.43-2.58; $I^2$= 13.9%), while substantial heterogeneity was observed in previously treated patients ($I^2$= 89.4%). Hemoglobin stabilization showed no difference (RR 1.02 95% CI 0.84-1.23; $I^2$= 42.1%), with consistent results in naïve patients (RR 1.05 95% CI 0.97-1.13; $I^2$= 0%). LDH normalization also did not differ significantly (RR 1.22, 95% CI 0.57-2.61; $I^2$= 82.8%). The PEGASUS trial was the main driver of heterogeneity in all the outcomes, likely due to being the only trial evaluating a C3 inhibitor; however, results remained consistent after its exclusion.

**Copyright Information:** (Online) ISSN: 2572-9241
©2026 The Author(s). HemaSphere is published by John Wiley & Sons Ltd on behalf of European Hematology Association. This is an open access Abstract Book distributed under the Attribution-NonCommercial-NoDerivs (CC BY-NC-ND), which allows third parties to download the articles and share them with others as long as they credit the author and the Abstract Book, but they cannot change the content in any way or use them commercially.
**Abstract Book Citations:** Authors, Title, HemaSphere, 2026;10:(S1):pages. The abstract book DOIs can be found on the “Author Information” page.
**Disclaimer:** Articles published in the journal HemaSphere exclusively reflect the opinions of the authors. The authors are responsible for all content in their abstracts including accuracy of the facts, statements, citing resources, etc.

**Summary/Conclusion**

In this meta-analysis, novel complement inhibitors provided comparable efficacy to eculizumab regarding transfusion avoidance and LDH normalization. Heterogeneity was primarily driven by the inclusion of proximal inhibitors in patients with suboptimal C5 response. Excluding these cases yielded neutral estimates. While a definitive class-wide superiority was not established, the data supports the non-inferiority of these novel agents. Their clinical value may also reside in improved administration routes (subcutaneous) and extended dosing intervals. Future mechanism-specific trials are required to refine patient selection for proximal versus terminal inhibition.

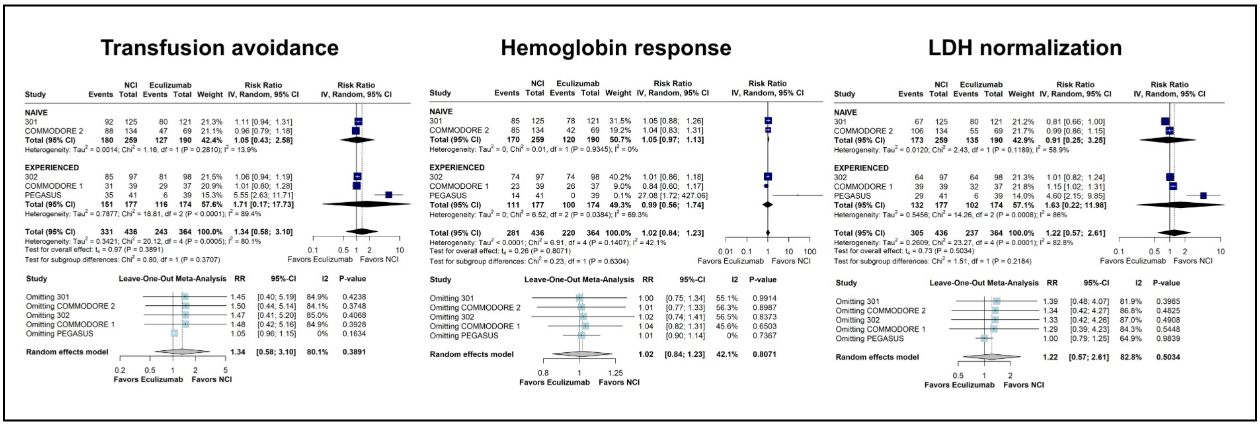

NCI, novel complement inhibitors; RR, risk ratio; CI, confidence interval; LDH, lactate dehydrogenase. Random-effects models were estimated using REML with Hartung–Knapp–Sidik–Jonkman adjustment. Leave-one-out analyses were performed to assess the influence of individual studies.

**Copyright Information:** (Online) ISSN: 2572-9241
©2026 The Author(s). HemaSphere is published by John Wiley & Sons Ltd on behalf of European Hematology Association. This is an open access Abstract Book distributed under the Attribution-NonCommercial-NoDerivs (CC BY-NC-ND), which allows third parties to download the articles and share them with others as long as they credit the author and the Abstract Book, but they cannot change the content in any way or use them commercially.
**Abstract Book Citations:** Authors, Title, HemaSphere, 2026;10:(S1):pages. The abstract book DOIs can be found on the "Author Information" page.
**Disclaimer:** Articles published in the journal HemaSphere exclusively reflect the opinions of the authors. The authors are responsible for all content in their abstracts including accuracy of the facts, statements, citing resources, etc.

### (PB3137) CARDIAC STRUCTURAL ABNORMALITIES, IRON OVERLOAD, AND PROGNOSTIC IMPLICATIONS IN FANCONI ANEMIA: A SINGLE-CENTER COHORT STUDY

**Topic:** 12. Bone marrow failure syndromes incl. PNH - Clinical

Beril Kiracı Şen*[1], Şule Ünal Cangül[1]

[1]*Hacettepe University Ihsan Dogramaci Children's Hospital, Pediatric Hematology Department, Ankara, Türkiye;*

### Background

**Background:**
Fanconi Anemia (FA) is a rare inherited bone marrow failure syndrome characterized by congenital anomalies, progressive cytopenias, and an increased risk of malignancy. Multisystem involvement is common, and congenital heart defects have been reported in approximately 5–7% of patients in previous series. The most frequently described abnormalities include septal defects such as atrial and ventricular septal defects, whereas complex malformations are uncommon. In addition to developmental structural anomalies, transfusion-dependent patients may develop secondary iron overload, which can result in myocardial dysfunction. Despite these observations, the full spectrum and clinical implications of cardiovascular involvement in FA remain insufficiently characterized. Given the role of the FANC pathway in embryogenesis and genomic stability, both developmental and acquired cardiac abnormalities may contribute to overall disease burden.

### Aims

**Objective:**
To evaluate the frequency and spectrum of cardiac abnormalities, assess transfusion-related myocardial iron overload, and determine whether cardiac involvement differs between prognostic risk groups in patients.

### Methods

This retrospective cohort study included patients diagnosed with FA and followed between 1975 and 2025 at a single tertiary referral center specialized in inherited bone marrow failure syndromes. Clinical, genetic, echocardiographic, and transfusion data were reviewed. Structural cardiac anomalies were classified by defect type. Iron overload was assessed using serum ferritin levels and, when available, T2* cardiac MRI parameters. Patients were stratified into good and poor prognosis groups based on predefined hematologic severity and clinical outcome criteria. The frequency of cardiac abnormalities between groups was compared.

**Copyright Information:** (Online) ISSN: 2572-9241
©2026 The Author(s). HemaSphere is published by John Wiley & Sons Ltd on behalf of European Hematology Association. This is an open access Abstract Book distributed under the Attribution-NonCommercial-NoDerivs (CC BY-NC-ND), which allows third parties to download the articles and share them with others as long as they credit the author and the Abstract Book, but they cannot change the content in any way or use them commercially.
**Abstract Book Citations:** Authors, Title, HemaSphere, 2026;10:(S1):pages. The abstract book DOIs can be found on the "Author Information" page.
**Disclaimer:** Articles published in the journal HemaSphere exclusively reflect the opinions of the authors. The authors are responsible for all content in their abstracts including accuracy of the facts, statements, citing resources, etc.

**Results**

At least one structural cardiac anomaly was identified in 52 of the total cohort of 267 patients. The most common defects were atrial septal defect (ASD) and patent foramen ovale (PFO), observed in 24 patients (46.2%). Ventricular septal defect (VSD) was present in 10 patients (19.2%), and isolated mitral valve prolapse (MVP) in 9 patients (17.3%). Less frequent anomalies included patent ductus arteriosus (7.7%), bicuspid aortic valve (3.8%), persistent left superior vena cava (1.9%), major aortopulmonary collateral arteries (1.9%), and one complex congenital malformation consisting of Tetralogy of Fallot with associated minor defects (1.9%).
Following the implementation of T2* magnetic resonance imaging (MRI) in 2007, 32 regularly followed patients who had received more than 20 transfusions were evaluated for iron overload. Among them, 14 underwent cardiac and/or hepatic T2* MRI. Cardiac iron accumulation was detected in 3 patients(21.4%). T2* MRI findings were normal in 9 of the 14 evaluated individuals. In 18 patients, T2* MRI could not be performed due to age of the patient.
When stratified by prognostic groups, structural cardiac anomalies were present in 18.4% of patients in the good prognosis group and 20.0% in the poor prognosis group, with no statistically significant difference (p=0.869). The distribution of other major congenital anomalies also did not differ significantly between prognostic categories.

**Summary/Conclusion**

Recognition of both congenital defects and transfusion-related myocardial iron overload is essential for comprehensive phenotypic characterization in specialized bone marrow failure centers.

**Copyright Information:** (Online) ISSN: 2572-9241
©2026 The Author(s). HemaSphere is published by John Wiley & Sons Ltd on behalf of European Hematology Association. This is an open access Abstract Book distributed under the Attribution-NonCommercial-NoDerivs (CC BY-NC-ND), which allows third parties to download the articles and share them with others as long as they credit the author and the Abstract Book, but they cannot change the content in any way or use them commercially.
**Abstract Book Citations:** Authors, Title, HemaSphere, 2026;10:(S1):pages. The abstract book DOIs can be found on the “Author Information” page.
**Disclaimer:** Articles published in the journal HemaSphere exclusively reflect the opinions of the authors. The authors are responsible for all content in their abstracts including accuracy of the facts, statements, citing resources, etc.

### (PB3138) SINGLE CENTRE REVIEW OF APLASTIC ANAEMIA: FANCONI, TELOMERE AND INHERITED BONE MARROW FAILURE TESTING

**Topic:** 12. Bone marrow failure syndromes incl. PNH - Clinical

Emily Groom-White[1], Malinthi Adikarama[1], Rebekah Gullett[1], Richard J. Kelly[1], Alexandra Pike[1], Talha Munir[1], Briony Robinson[1], Louise Arnold[1], Isla Storey[1], Catherine Barnfield[1], Nora Youngs[1], Matthew Holt[1], Petra Muus[1], Morag Griffin*[1]

[1]*St James Hospital, Haematology, Leeds, United Kingdom;*

### Background

Aplastic anaemia (AA) is a rare bone marrow failure disorder (BMF); current guidelines advise testing all patients <50 for Fanconi anaemia (FA) and for inherited bone marrow failure (IBMF) if phenotypic concerns/no response to ATG. With IBMF testing in adulthood,identification of mutations may increase,however interpretation and patient discussion can be complex. We report a tertiary centre service review of AA

### Methods

All adult patients with a diagnosis of BMF (historical or current) currently under active follow-up were reviewed. Patients identified by database search term: AA or BMF. Data collected: patient demographics,FA breakage testing,telomeres and IBMF testing. For those with identified abnormalities,clinical phenotype was assessed.

### Results

106 patients included, mean age at referral 44.4 years (range 4 months-90 years). 4 transferred from paediatrics. 63 were <50 years,45 (71%) had documented FA breakage testing with 1 confirmed FA (Hb 99g/l,neut 1x10*9/l,plt 70x10*9/l). Of those with no documented FA test,5/63 transferred after primary diagnosis/treatment,8/63 had a historical AA (pre-2010),4/63 didn't meet testing indications & 1 had an IBMF panel sent only. Telomere testing was undertaken in 46/63 (73%):26 (56%) >10$^{th}$ percentile (normal),8 (17%) <10 percentile & 5 (10%) <1 percentile; no results/awaited 6 (13%);8 with short telomeres had no identified IBMF. IBMF screens were sent for 39/63 (62%),8 (20%) positive results (see table; no results 3)

**Copyright Information:** (Online) ISSN: 2572-9241
©2026 The Author(s). HemaSphere is published by John Wiley & Sons Ltd on behalf of European Hematology Association. This is an open access Abstract Book distributed under the Attribution-NonCommercial-NoDerivs (CC BY-NC-ND), which allows third parties to download the articles and share them with others as long as they credit the author and the Abstract Book, but they cannot change the content in any way or use them commercially.
**Abstract Book Citations:** Authors, Title, HemaSphere, 2026;10:(S1):pages. The abstract book DOIs can be found on the "Author Information" page.
**Disclaimer:** Articles published in the journal HemaSphere exclusively reflect the opinions of the authors. The authors are responsible for all content in their abstracts including accuracy of the facts, statements, citing resources, etc.

| Patient | Age at presentation (years) | Telomere | IBMF | Clinical signs/symptoms | FBC result |
|---|---|---|---|---|---|
| 1 | 28 | Normal | Homozygous G6PC3 missense variant | Infections | Hb 150g/l<br>N 0.5x10*9/l<br>P 298 x10*9/l |
| 2 | <1 | Not available | Diamond Blackfan Anaemia (heterozygous RPS19) | Thumb & toe abnormalities | Hb 86g/l<br>N 3.8x10*9/l<br>P 213 x10*9/l |
| 3 | <1 | NT | FANCB variant c.1894T>G (VUS) | Septic arthritis,hearing issues,ovarian failure | Hb 134g/l<br>N 2.5x10*9/l<br>P 16x10*9/l |
| 4 | 25 | Short | Negative | No | Hb 78 g/L<br>N 3.5x10*9/l<br>P 37 x 10*9/l |
| 5 | 25 | Very short | Negative | No | Not available |
| 6 | 12 | Short | Negative | No | Hb 50g/L<br>N 0.2x10*9/L<br>P 5 x 10*9/l |
| 7 | 27 | Very short | Heterozygous pathogenic TERT mutation | Early greying | Hb 77 g/l<br>N 1.1x10*9/l<br>P 63x10*9/l |
| 8 | 28 | Very short | Heterozygous pathogenic TERT missense variant | No | Hb 108g/l<br>N 1.2x10*9/l<br>P 72 x10*9/l |
| 9 | 29 | Normal | Heterozygous TERT mutation c.1234C>T heterozygote (VUS) | No | Hb 97 g/l<br>N 1x10*9/l<br>P 49x10*9/l |
| 10 | 30 | Short | WRAP53 missense variant | Liver fibrosis,early grey | Hb 106 g/l<br>N 2x10*9/l<br>P 49x10*9/l |
| 12 | 39 | Very short | Negative | No | Hb 83 g/l<br>N 2.1x10*9/l<br>P 109x10*9/l |
| 13 | 40 | Short | Negative | No | Hb 91g/l<br>N 0.62x10*9/l<br>P 29x10*9/l |
| 14 | 41 | Normal | Dyskeratosis Congenita TERT mutation | Short stature,fatty liver | Hb 89xg/l<br>N 0.7x10*9/l<br>Plt 19x10*9/l |
| 15 | 43 | Very short | Negative | No | Hb 108g/l<br>N 0.89x10*9/l<br>P 33x10*9/l |
| 16 | 43 | Short | Negative | No | Hb 72g/l<br>N 0.3x10*9/l<br>P 27x10*9/l |
| 17 | 45 | Short | No results | Low BMI | Hb 69g/l<br>N 0.6x10*9/l<br>P 80x10*9/l |
| 18 | 49 | Very short | Heterozygous TERT mutation Chr5 g.1255513C>T (VUS) | Lung fibrosis,early grey, finger clubbing | Hb 101g/l<br>N 1.91x10*9/L<br>P 86x10*9/l |
| 19 | 49 | Short | Negative | No | Hb 39g/l<br>N 1.1x10*9/l<br>P 8x10*9/l |

NT=not tested

Hb=haemoglobin,N=neutrophils,P=platelets

Telomeres:Normal =>10$^{th}$ percentile,Short =<10$^{th}$percentile,very short =<1$^{st}$percentile

**Copyright Information:** (Online) ISSN: 2572-9241
©2026 The Author(s). HemaSphere is published by John Wiley & Sons Ltd on behalf of European Hematology Association. This is an open access Abstract Book distributed under the Attribution-NonCommercial-NoDerivs (CC BY-NC-ND), which allows third parties to download the articles and share them with others as long as they credit the author and the Abstract Book, but they cannot change the content in any way or use them commercially.
**Abstract Book Citations:** Authors, Title, HemaSphere, 2026;10:(S1):pages. The abstract book DOIs can be found on the "Author Information" page.
**Disclaimer:** Articles published in the journal HemaSphere exclusively reflect the opinions of the authors. The authors are responsible for all content in their abstracts including accuracy of the facts, statements, citing resources, etc.

**Summary/Conclusion**

We report a tertiary referral centre review of BMF patients. As expected in an adult population,the majority did not have IBMF. Three patients with a VUS on IBMF testing had clinical disease phenotype which may affect future management and have family screening implications. Eight patients had reduced telomere length with no current identified IBMF, however repeat future testing is advised if new variants are added to panels

**Copyright Information:** (Online) ISSN: 2572-9241
©2026 The Author(s). HemaSphere is published by John Wiley & Sons Ltd on behalf of European Hematology Association. This is an open access Abstract Book distributed under the Attribution-NonCommercial-NoDerivs (CC BY-NC-ND), which allows third parties to download the articles and share them with others as long as they credit the author and the Abstract Book, but they cannot change the content in any way or use them commercially.
**Abstract Book Citations:** Authors, Title, HemaSphere, 2026;10:(S1):pages. The abstract book DOIs can be found on the “Author Information” page.
**Disclaimer:** Articles published in the journal HemaSphere exclusively reflect the opinions of the authors. The authors are responsible for all content in their abstracts including accuracy of the facts, statements, citing resources, etc.

### (PB3139) INCIDENCE RATES OF SERIOUS INFECTIONS IN PATIENTS WITH PAROXYSMAL NOCTURNAL HEMOGLOBINURIA: A REAL-WORLD COMPARISON USING DATA FROM TRINETX ACROSS THE US, EUROPE, AND OTHER REGIONS

**Topic:** 12. Bone marrow failure syndromes incl. PNH - Clinical

Régis Peffault de Latour[1], Håkan Malmström*[2], Michael O'malley[2], Sara Bruce Wirta[2], Shihua Sun[2], Jonas Eriksson[2], Luis López Lázaro[2]

*[1]French Reference Center for Aplastic Anemia and Paroxysmal Nocturnal Hemoglobinuria; Université Paris Cité, Paris, France; [2]Swedish Orphan Biovitrum AB, Stockholm, Sweden;*

### Background

Robust estimates of serious infection (SI) incidence in patients with paroxysmal nocturnal hemoglobinuria (PNH) are needed to contextualize SI rates reported in single-arm pegcetacoplan studies. Pegcetacoplan is a proximal complement inhibitor used to treat PNH. Real-world data sources such as TriNetX LIVE™, which contain millions of contemporaneous, de-identified patient records coded using harmonized standards, provide an opportunity to generate such estimates and to characterize patient clinical profiles *(Ludwig et.al, Front. Pharmacol 2025; Usman et al. Blood 2024*).

### Aims

Here we aim to develop and apply proxy definitions of SI to estimate SI incidence rates in patients with PNH across the United States, Europe, and other regions.

### Methods

A retrospective cohort study was conducted using TriNetX LIVE™ (data access: January 2025), comprising de-identified electronic health records (EHR) from healthcare organizations. Prevalent PNH patients were identified using ICD-10-CM code D59.5. Patients receiving pegcetacoplan were excluded to estimate SI rates among those on other PNH treatments for later comparison with pegcetacoplan studies. SI were defined as inpatient or emergency department visits with either 1) overall infections: ICD-10-CM codes A00–B99; or 2) specified infections including bacterial, viral, fungal, protozoal, and mycobacterial infections identified by ICD-10-CM codes. Incidence rates for overall and specified SI were derived for 2022-2023 [per 100 patient years (95%CI)]. Prevalent patients per year were defined as those diagnosed with PNH before that year, and with at least one healthcare interaction during that year. Each prevalent patient contributed one patient-year to the denominator. Only the first SI event per patient and year was included.

**Copyright Information:** (Online) ISSN: 2572-9241
©2026 The Author(s). HemaSphere is published by John Wiley & Sons Ltd on behalf of European Hematology Association. This is an open access Abstract Book distributed under the Attribution-NonCommercial-NoDerivs (CC BY-NC-ND), which allows third parties to download the articles and share them with others as long as they credit the author and the Abstract Book, but they cannot change the content in any way or use them commercially.
**Abstract Book Citations:** Authors, Title, HemaSphere, 2026;10:(S1):pages. The abstract book DOIs can be found on the “Author Information” page.
**Disclaimer:** Articles published in the journal HemaSphere exclusively reflect the opinions of the authors. The authors are responsible for all content in their abstracts including accuracy of the facts, statements, citing resources, etc.

**Results**

Prevalent PNH patient counts per year are shown in Table 1. Overall SI incidence rates ranged from 10.2 (8.8-11.7) in 2023 to 10.5 (9.1-12.0) in 2022 in the US. The corresponding non-US rates ranged from 7.0 (5.0-9.0) in 2023 to 9.8 (7.0-13.0) in 2022 during the same period. Overall SI rates were similar in Europe for the year 2022 (11.1 (6.8-17.2)) and 2023 (10.5 (9.1-12.0)).
For specified SIs, incidence rates were 8.6 (7.4-10.0) in 2022 and 8.7 (7.4-10.1) in 2023 in the US. Corresponding European rates were slightly lower: (5.6 (2.7-10.2) in 2022; 5.3 (2.5-9.7) in 2023). Rates were numerically similar across regions, with overlapping confidence intervals.

**Table 1 Number of prevalent patients by year**

| Year/Region | US | Ex-US* | Europe* |
|---|---|---|---|
| 2022 | 2063 | 410 | 180 |
| 2023 | 1984 | 430 | 190 |

*Number rounded to the nearest 10

**Summary/Conclusion**

SI incidence rates in patients with PNH derived from structured and harmonized EHR data in TriNetX LIVE™ were consistent and comparable across geographies and years using two SI definitions. Overall SI rates were similar to previous US claims data (Patel et al., EHA 2021), with TriNetX LIVE™ representing a larger and more contemporary population. Although proxy definitions differ from those used in clinical studies, this approach provides a valid and practical method to contextualize SI rates in single-arm studies.

**Copyright Information:** (Online) ISSN: 2572-9241
©2026 The Author(s). HemaSphere is published by John Wiley & Sons Ltd on behalf of European Hematology Association. This is an open access Abstract Book distributed under the Attribution-NonCommercial-NoDerivs (CC BY-NC-ND), which allows third parties to download the articles and share them with others as long as they credit the author and the Abstract Book, but they cannot change the content in any way or use them commercially.
**Abstract Book Citations:** Authors, Title, HemaSphere, 2026;10:(S1):pages. The abstract book DOIs can be found on the "Author Information" page.
**Disclaimer:** Articles published in the journal HemaSphere exclusively reflect the opinions of the authors. The authors are responsible for all content in their abstracts including accuracy of the facts, statements, citing resources, etc.

### (PB3140) EARLY ACCESS PROGRAM FOR DANICOPAN (ALXN2040) AS ADD-ON TREATMENT TO ECULIZUMAB OR RAVULIZUMAB IN PATIENTS WITH PAROXYSMAL NOCTURNAL HEMOGLOBINURIA (PNH): THE ITALIAN REAL-WORLD.

**Topic:** 12. Bone marrow failure syndromes incl. PNH - Clinical

Elisabetta Metafuni[1], Antonio De Vivo[2], Andrea Patriarca[3], Marta Venturi[4], Simona Raso[5], Amalia Figuera[6], Antonella Sau[7], Bruna Greve[8], Milena Mirabile[9], Olga Mulas[10], Alessandro Costa[10], Eloise Beggiato[11], Antonell Carbone[12], Martina Salvatori[13], Anna Paola Iori[13], Simona Sica[14], Wilma Barcellini[15], Bruno Fattizzo*[15]

*[1]Fondazione Policlinico Universitario Agostino Gemelli, Scienze di Laboratorio e Ematologiche, Roma, Italy;[2]Policlinico S.orsola-Malpighi Arco Storico su Via Albetoni, Hematology Unit, Department of Experimental, Diagnostic and Specialty Medicine (DIMES), Bologna, Italy; [3]Scuola di Medicina - Università del Piemonte Orientale, Novara, Italy; [4]Policlinico S.orsola-Malpighi Arco Storico su Via Albetoni, Bologna, Italy; [5]Ospedali Riuniti Villa Sofia, Palermo, Italy; [6]AOU Policlinico "G.Rodolico-San Marco", Catania, Italy; [7]Ospedale Civile dello Spirito Santo, Pescara, Italy; [8]Grande Ospedale Metropolitano 'Bianchi Melacrino Morelli', Reggio di Calabria, Italy; [9]Ospedale di Civitanova Marche, Civitanova Marche, Italy; [10]Ospedale Oncologico Businco, Cagliari, Italy; [11]Azienda Ospedaliero Universitaria Città della Salute e della Scienza di Torino, Torino, Italy;[12]Ospedale Santa Scolastica, Cassino, Italy; [13]Istituto Ematologia Policlinico Umberto I, Roma, Italy; [14]Fondazione Policlinico Universitario Agostino Gemelli, Roma, Italy; [15]Ospedale Maggiore Ca' Granda Università Statale, Milano, Italy;*

### Background

Paroxysmal nocturnal hemoglobinuria (PNH) is a rare hematopoietic disease characterized by complement-mediated intravascular hemolysis. Although C5 inhibitors (C5i) secure effective control of intravascular hemolysis (IVH), a variable degree of consequent extravascular hemolysis (EVH) might be responsible for persistent anemia. Danicopan (ALXN2040) is an oral proximal complement inhibitor of alternative pathway factor D, conceived to control both EVH and IVH.

### Aims

We report the Italian collaborative real-world evidence on danicopan added to a C5i in patients with PNH. Fifteen patients were enrolled in the Alexion® early access program (EAP) from September 2023 to December 2025.

### Methods

Data collection included patient age and gender, PNH characteristics, PNH response to treatments, clinically significant adverse events, breakthrough hemolysis (BTH) episodes, transfusion support, and laboratory data at different time points (before danicopan start, after 1 month (M), 3M, 6M and 12M of danicopan).

**Copyright Information:** (Online) ISSN: 2572-9241
©2026 The Author(s). HemaSphere is published by John Wiley & Sons Ltd on behalf of European Hematology Association. This is an open access Abstract Book distributed under the Attribution-NonCommercial-NoDerivs (CC BY-NC-ND), which allows third parties to download the articles and share them with others as long as they credit the author and the Abstract Book, but they cannot change the content in any way or use them commercially.
**Abstract Book Citations:** Authors, Title, HemaSphere, 2026;10:(S1):pages. The abstract book DOIs can be found on the “Author Information” page.
**Disclaimer:** Articles published in the journal HemaSphere exclusively reflect the opinions of the authors. The authors are responsible for all content in their abstracts including accuracy of the facts, statements, citing resources, etc.

### Results

Data from 12 patients treated with danicopan+C5i were available (5 females, median age 36.6 ys, and 7 males, median age 58.1 ys). Patients mainly suffered from classical PNH (75%), followed by PNH/aplastic anemia (PNH/AA, 25%). At the time of enrollment in the danicopan EAP, ongoing C5i was eculizumab in 4 patients and ravulizumab in 8 patients. Seven patients (58.3%) had received at least one red blood cell (RBC) transfusion (median number per patient 4, range 1-6). Four patients experienced at least one BTH (median Hgb 6.7 g/dl), managed with RBC transfusions in all cases, and with an extra dose of C5i in two cases. The reasons to add danicopan were fatigue with persistent anemia due to EVH (n=6), and inadequate response (n=6). Laboratory results before starting danicopan, after 1M, 3M, 6M, and 12M of C5i+danicopan are reported in the table.

| Laboratory results | C5i alone (n=12) | 1M C5i+Danicopan (n=12) | 3M C5i+Danicopan (n=11) | 6M C5i+Danicopan (n=10) | 12M C5i+Danicopan (n=7) |
|---|---|---|---|---|---|
| Median Hemoglobin g/dl (range) | 9<br>(6.5-10.8) | 10.7<br>(7.4-13.2) | 11.3<br>(7.8-12.8) | 11.6<br>(6.7-13.8) | 11.1<br>(9.9-14) |
| Median reticulocytes x10^9/L (range) | 208<br>(12-248) | 139<br>(12-229) | 136<br>(19-190) | 117<br>(11-278) | 141<br>(110-277) |
| Median LDH xULN (range) | 1.1<br>(0.8-2.1) | 1<br>(0.6-3.4) | 0.95<br>(0.8-2.7) | 1.1<br>(0.8-1.8) | 1.2<br>(0.8-1.7) |
| Response: | | | | | |
| None n (%) | 1 (8.3) | 1 (8.3) | - | 1 (10) | - |
| Minor n (%) | 1 (8.3) | 1 (8.3) | 2 (18.1) | - | - |
| Partial n (%) | 7 (58.3) | 3 (25) | - | 3 (30) | 1 (14.3) |
| Good n (%) | 3 (25) | 4 (33.3) | 5 (45.5%) | 1 (10) | 4 (57.1) |
| Major n (%) | - | 1 (8.3) | 1 (9.1%) | 1 (10) | 1 (14.3) |
| Complete n (%) | - | 2 (16.7) | 3 (27.3%) | 4 (40) | 1 (14.3) |

Considering laboratory values, median Hgb value gradually increased: 10.7 g/dl (range 7.4-13.2) at 1M (+1.7 g/dl from baseline, p=0.02), 11.3 g/dl (range 7.8-12.8) at 3M (+2.3 g/dl from baseline, p=0.01), 11.6 g/dl (range 6.7-13.8) at 6M (+2.6 g/dl, p=0.05), and 11.1 g/dl (range 9.9-14) at 12M (+2.1 g/dl, p=0.003). To date, two patients stopped danicopan because of myalgia and tremors in one case, and liver toxicity in the other. Mild side effects were reported by 2 patients (20%): mild abdominal pain and diarrhea. The total number of RBC transfusions recorded in the cohort was 3, each corresponding to an episode of BTH. A total of 3 BTH were reported in two patients (25%) (median Hgb 7.9 g/dl). To date, the median duration of danicopan+C5i was 13.2 months (range 3-21).

### Summary/Conclusion

Danicopan added to C5i appeared to be a promising combination therapy for patients with PNH and clinically relevant residual EVH.

**Copyright Information:** (Online) ISSN: 2572-9241
©2026 The Author(s). HemaSphere is published by John Wiley & Sons Ltd on behalf of European Hematology Association. This is an open access Abstract Book distributed under the Attribution-NonCommercial-NoDerivs (CC BY-NC-ND), which allows third parties to download the articles and share them with others as long as they credit the author and the Abstract Book, but they cannot change the content in any way or use them commercially.
**Abstract Book Citations:** Authors, Title, HemaSphere, 2026;10:(S1):pages. The abstract book DOIs can be found on the "Author Information" page.
**Disclaimer:** Articles published in the journal HemaSphere exclusively reflect the opinions of the authors. The authors are responsible for all content in their abstracts including accuracy of the facts, statements, citing resources, etc.

### (PB3141) PAROXYSMAL NOCTURNAL HEMOGLOBINURIA (PNH), AN OVERVIEW IN BRAZIL

**Topic:** 12. Bone marrow failure syndromes incl. PNH - Clinical

Janaína Gioseffi[1], Catherine Moura*[1], Nina Melo[1]

*[1]Abrale - Brazilian Association of Blood Cancer, São Paulo, Brazil;*

### Background

Paroxysmal Nocturnal Hemoglobinuria (PNH) is a rare acquired hematologic disorder characterized by chronic hemolytic anemia, stemming from a mutation in the PIG-A gene. This mutation leads to a deficiency of GPI-anchored proteins (CD55 and CD59), rendering red blood cells vulnerable to complement-mediated destruction. Clinical manifestations include fatigue, dark urine, and a high risk of life-threatening thrombosis. Due to its rarity—with an estimated incidence of 1.3 cases per million in Brazil—diagnosis remains a significant challenge.

### Aims

The aim of the study was to perform a sociodemographic descriptive analysis of people with PNH within the scope of the Brazilian Unified Health System, regarding their outpatient procedures, hospitalizations, and geographic distribution.

### Methods

This cross-sectional, quantitative study analyzed public data from the Department of Informatics of the Unified Health System (2018–2022) regarding hospitalizations and outpatient production for ICD D59.5.

### Results

The analysis of outpatient care revealed a total of 14,659 procedures performed for an estimated 464 patients, resulting in an average of 32 procedures per individual. A major shift in the therapeutic landscape occurred in 2023 following the inclusion of Eculizumab in public protocols, which caused a sharp increase in both the volume of procedures and financial expenditure. Total outpatient costs reached approximately R$ 57 million, with an average cost of R$ 11,319.12 per procedure, driven primarily by the high cost of Eculizumab, which is capped at R$ 25,251.09 for government procurement. Regarding hospitalizations, 444 occurrences were recorded for 256 estimated patients, averaging two stays per person with a mean duration of one week. Critical care was required for 5% of these patients, and the in-hospital mortality rate was 4%. Total spending on hospitalizations exceeded R$ 670,000, averaging R$ 1,516.25 per stay.
The sociodemographic profile indicated that São Paulo led the country in both outpatient procedures (25.7%) and hospitalizations (22.7%), followed by Paraná and Rio Grande do Sul. A critical finding was that 50% of patients had to travel to a different municipality to access treatment. The patient population is predominantly female (53%) and self-identified as white (55.4%) or mixed-race (34.7%). Most affected individuals are in the 30–39 age group (24%), emphasizing the disease's burden during peak productive years. Qualitative insights from patients highlighted that late diagnoses are often linked to severe complications, and the treatment journey significantly disrupts daily and professional routines. Despite frustrations with long wait times and travel distances, there is a prevailing sense of hope regarding future gene therapies and simplified treatments.

**Copyright Information:** (Online) ISSN: 2572-9241
©2026 The Author(s). HemaSphere is published by John Wiley & Sons Ltd on behalf of European Hematology Association. This is an open access Abstract Book distributed under the Attribution-NonCommercial-NoDerivs (CC BY-NC-ND), which allows third parties to download the articles and share them with others as long as they credit the author and the Abstract Book, but they cannot change the content in any way or use them commercially.
**Abstract Book Citations:** Authors, Title, HemaSphere, 2026;10:(S1):pages. The abstract book DOIs can be found on the "Author Information" page.
**Disclaimer:** Articles published in the journal HemaSphere exclusively reflect the opinions of the authors. The authors are responsible for all content in their abstracts including accuracy of the facts, statements, citing resources, etc.

**Summary/Conclusion**

PNH imposes a profound physical and emotional burden on patients. While Eculizumab has revolutionized clinical outcomes, it has significantly increased public healthcare expenditures. The data underscores the urgent need for decentralized care to reduce patient displacement and faster diagnostic protocols to mitigate severe complications.

**Copyright Information:** (Online) ISSN: 2572-9241
©2026 The Author(s). HemaSphere is published by John Wiley & Sons Ltd on behalf of European Hematology Association. This is an open access Abstract Book distributed under the Attribution-NonCommercial-NoDerivs (CC BY-NC-ND), which allows third parties to download the articles and share them with others as long as they credit the author and the Abstract Book, but they cannot change the content in any way or use them commercially.
**Abstract Book Citations:** Authors, Title, HemaSphere, 2026;10:(S1):pages. The abstract book DOIs can be found on the "Author Information" page.
**Disclaimer:** Articles published in the journal HemaSphere exclusively reflect the opinions of the authors. The authors are responsible for all content in their abstracts including accuracy of the facts, statements, citing resources, etc.

### (PB3142) ALLOGENEIC HEMATOPOIETIC STEM CELL TRANSPLANTATION FOR CONGENITAL AMEGAKARYOCYTIC THROMBOCYTOPENIA: A 20-YEAR SINGLE-CENTER EXPERIENCE

**Topic:** 12. Bone marrow failure syndromes incl. PNH - Clinical

Belkis Dekhili[1], Farih Mehdid[1], Baazizi Mounira[1], Nadia Rahmoune[1], Ait Ouali Dina[1, 1], Hanane Bouarab[1], Sarah Zerkout[1], Fouzia Louar[1], Amira Fahem[1], Farida Harieche[1], Rose Marie Hamladji[1], Redhouane Ahmed Nacer[1], Malek Benakli*[1]

[1]*Hematology and Bone Marrow Transplantation department, Pierre and Marie Curie Center, Algiers, Algeria;*

### Background

Congenital Amegakaryocytic Thrombocytopenia (CAMT) is a rare cause of central thrombocytopenia characterized by a reduction or absence of megakaryocytes in the bone marrow. It results from mutations in the *MPL* gene, which encodes the thrombopoietin (TPO) receptor. While the initial presentation may mimic immune thrombocytopenic purpura (ITP), the disease rapidly progresses toward aplastic anemia. Allogeneic hematopoietic stem cell transplantation (HSCT) remains the only curative treatment once pancytopenia develops.

### Methods

Over a 20-year period (January 2005 to June 2025), 7 patients (pts) with CAMT underwent HSCT with sibling donors at our center. The median age was 6 years (range: 4–13), with a male-to-female ratio of 2.5. At diagnosis, 4 pts (57%) presented with isolated hemorrhagic syndrome, 2 pts (28%) with isolated anemia, and one (14%) with both. All pts had severe thrombocytopenia, and 6 pts (85%) had already progressed to pancytopenia. Bone marrow examination confirmed amegakaryocytosis in all cases, with hypocellular marrow in 42% and normo/hypercellular marrow in 57%. Chromosomal fragility testing was performed in all pts to rule out Fanconi anemia. Pre-transplant complications included frontotemporal hematoma and post-transfusion iron overload. Conditioning regimens were myeloablative (Busulfan-based) in 5 pts and reduced-intensity (Cyclophosphamide +/- ATG) in two. Graft sources included bone marrow (n=3) and peripheral blood stem cells (n=4). GVHD prophylaxis consisted of Cyclosporine and Methotrexate.

### Results

Two patients developed severe sepsis during the aplastic phase, one of which was fatal. No early graft rejection or CMV reactivation was observed. Acute GVHD occurred in 2 pts, including one grade IV steroid-resistant case. One patient developed extensive chronic GVHD with a favorable outcome. Late graft failure occurred in 4 pts (including both who received reduced-intensity conditioning); two of these were successfully rescued with a stem cell boost. As of June 2025, after a median follow-up of 84 months (range: 17–207), 4 pts are alive (three with full donor chimerism). Three pts died due to severe sepsis, acute GVHD, and late graft failure.

### Summary/Conclusion

Allogeneic HSCT is the definitive therapy for CAMT. It should be performed as early as possible, prior to the onset of severe infectious or hemorrhagic complications, while minimizing pre-transplant transfusions to reduce the risks of alloimmunization and iron overload. In our experience, superior outcomes were achieved using myeloablative conditioning regimens and bone marrow as the graft source.

**Copyright Information:** (Online) ISSN: 2572-9241
©2026 The Author(s). HemaSphere is published by John Wiley & Sons Ltd on behalf of European Hematology Association. This is an open access Abstract Book distributed under the Attribution-NonCommercial-NoDerivs (CC BY-NC-ND), which allows third parties to download the articles and share them with others as long as they credit the author and the Abstract Book, but they cannot change the content in any way or use them commercially.
**Abstract Book Citations:** Authors, Title, HemaSphere, 2026;10:(S1):pages. The abstract book DOIs can be found on the “Author Information” page.
**Disclaimer:** Articles published in the journal HemaSphere exclusively reflect the opinions of the authors. The authors are responsible for all content in their abstracts including accuracy of the facts, statements, citing resources, etc.

### (PB3143) SUPERIOR EFFICACY OF CSA PLUS ATG OVER CSA MONOTHERAPY IN PEDIATRIC TRANSFUSION-INDEPENDENT MODERATE APLASTIC ANEMIA

**Topic:** 12. Bone marrow failure syndromes incl. PNH - Clinical

Hongmin Li*[1], Lingling Fu[1], Bixi Yang[1], Ruixin Wang[1], Hui Chen[1], Jie MA[1]

[1]*Beijing Children's Hospital, Capital Medical University, National Center for Children's Health, Hematology, Beijing, China;*

### Background

Data on the management and outcomes of transfusion-independent moderate aplastic anemia (MAA) in pediatric patients remain scarce.

### Aims

This study aimed to compare the efficacy and safety of two immunosuppressive therapy (IST) regimens: cyclosporine (CsA) monotherapy versus CsA combined with anti-thymocyte globulin (ATG) in children with transfusion-independent MAA.

### Methods

We conducted a prospective randomized controlled trial involving 29 pediatric patients diagnosed with transfusion-independent MAA. Patients were randomly assigned to either the CsA monotherapy group (n=14) or the CsA+ATG combination therapy group (n=15).

### Results

Baseline characteristics were well-balanced between the two groups. The CsA+ATG group exhibited a higher overall response rate (ORR: 80% *vs.* 50%) and a significantly higher complete response rate (CRR: 60% *vs.* 7.1%, *P*=0.005) compared with the CsA monotherapy group. At 12 months post-treatment, the combination group demonstrated better hematologic recovery. The 36-month event-free survival (EFS) rate was higher in the combination group than in the monotherapy group (86.7% ± 8.8% *vs.* 32.5% ± 18.5%), though this difference did not attain statistical significance (P > 0.05), likely due to the small sample size. Shorter pre-treatment disease duration correlated with better response (*P*=0.045). Adverse events in both groups were mild to moderate and clinically manageable.

### Summary/Conclusion

In conclusion, CsA combined with ATG is superior to CsA monotherapy for pediatric patients with transfusion-independent MAA, with a favorable safety profile. Early initiation of IST (within 3 months of diagnosis) may correlate with better response.

**Copyright Information:** (Online) ISSN: 2572-9241
©2026 The Author(s). HemaSphere is published by John Wiley & Sons Ltd on behalf of European Hematology Association. This is an open access Abstract Book distributed under the Attribution-NonCommercial-NoDerivs (CC BY-NC-ND), which allows third parties to download the articles and share them with others as long as they credit the author and the Abstract Book, but they cannot change the content in any way or use them commercially.
**Abstract Book Citations:** Authors, Title, HemaSphere, 2026;10:(S1):pages. The abstract book DOIs can be found on the "Author Information" page.
**Disclaimer:** Articles published in the journal HemaSphere exclusively reflect the opinions of the authors. The authors are responsible for all content in their abstracts including accuracy of the facts, statements, citing resources, etc.

### (PB3144) FIRST REPORT OF REAL-WORLD EXPERIENCE WITH IPTACOPAN AS INITIAL ORAL MONOTHERAPY IN PATIENTS WITH PAROXYSMAL NOCTURNAL HEMOGLOBINURIA IN MEXICO

**Topic:** 12. Bone marrow failure syndromes incl. PNH - Clinical

Andrea Mirelly Lopez Flores*[1], Gregorio Campos Cabrera[2], Carlos Hernandez[3], Estefanía García Servín[2], Alva Nora Zaragoza Vazquez[4]

*[1]Instituto Mexicano del Seguro Social, Centro Médico Nacional La Raza, Ciudad de México, Mexico;[2]Laboratorios Fatima de Michoacán, Morelia, Mexico; [3]Instituto Mexicano Del Seguro Social, Centro Médico Nacional Siglo XXI, Ciudad de Mexico, Mexico; [4]Instituto Mexicano Del Seguro Social, Hospital General Regional No. 1, Chihuahua, Mexico;*

### Background

Paroxysmal nocturnal hemoglobinuria (PNH) is a non-malignant clonal disorder of hematopoietic stem cells characterized by PIGA mutations and GPI-anchor deficiency, that leads to the absence of CD55 and CD59, resulting in complement-mediated hemolysis and thrombosis.
C5 inhibitors have been the gold standard for 19 years, improving transfusion independence (TI) from 24.8% to 73.6%, however only 53.9% of patients maintain TI at 5 years, often due to residual extravascular hemolysis. Proximal complement inhibition has emerged as a novel therapeutic approach to address these unmet needs. Initial results among patients switched from C5 inhibition, 85% achieved a ≥2 g/dL Hb increase, and 70% reached Hb ≥12 g/dL without transfusions. In treatment-naïve patients, 94% achieved a ≥2 g/dL Hb increase.

### Aims

To evaluate the real-world efficacy of first-line iptacopan monotherapy in treatment-naïve PNH patients, focusing on hematologic recovery, hemolysis control, and quality of life (QoL).

### Methods

We analyzed the data of 16 treatment-naïve patients receiving oral iptacopan (200 mg BID) through a managed access program across three medical centers. Primary aims were changes in hemoglobin (Hb) and hemolysis markers (LDH, indirect bilirubin). Secondary aims included leukocytes and platelet counts, TI, breakthrough hemolysis (BTH), QoL via the FACIT-Fatigue scale (n=8) and adverse effects. Statistical significance was assessed using the Student's paired t-test.

### Results

Sixteen patients were enrolled. Mean Hb increased from 8.65 g/dL to 11.59 g/dL, representing a mean gain of 2.95 g/dL and 34% from baseline (p<0.001). Mean LDH showed a 74.5% reduction, decreasing from 1693.5 U/L at baseline to 430.3 U/L post-treatment (p<0.001). Mean indirect bilirubin declined from 1.75 to 0.77 mg/dL (p<0.001). Mean reticulocyte percentage decreased from 5.75% to 2.61%, reflecting a diminished hemolytic drive. Leukocyte counts remained stable (Δ+0.17) (p=0.7012), and platelet counts showed a modest increase (Δ+21x$10^9$/L) (p= 0.2527). Remarkably, 100% of the cohort achieved TI. In a subgroup analysis (n=8) mean FACIT-Fatigue scores increased from 23.4 to 32 points. Two patients (12.5%) experienced pharmacokinetic BTH, triggered by choledocholithiasis and thrombosed hemorrhoids, respectively. Regarding safety, 80% of patients reported mild headache during the first three days of use. One patient presented nummular eczema that did not require treatment discontinuation.

**Copyright Information:** (Online) ISSN: 2572-9241
©2026 The Author(s). HemaSphere is published by John Wiley & Sons Ltd on behalf of European Hematology Association. This is an open access Abstract Book distributed under the Attribution-NonCommercial-NoDerivs (CC BY-NC-ND), which allows third parties to download the articles and share them with others as long as they credit the author and the Abstract Book, but they cannot change the content in any way or use them commercially.
**Abstract Book Citations:** Authors, Title, HemaSphere, 2026;10:(S1):pages. The abstract book DOIs can be found on the "Author Information" page.
**Disclaimer:** Articles published in the journal HemaSphere exclusively reflect the opinions of the authors. The authors are responsible for all content in their abstracts including accuracy of the facts, statements, citing resources, etc.

### Summary/Conclusion

Iptacopan as first-line of treatment was associated with statistically significant suppression of hemolysis and clinically meaningful hemoglobin improvement. Beyond the biochemical response, the 8.6-point gain in the FACIT-Fatigue scale, which more than doubles the 3-point Minimal Clinically Important Difference, reflects a profound improvement in quality of life and a substantial reduction in disease-related burden. These findings reinforce the effectiveness of proximal complement inhibition as a robust frontline strategy and provide real-world evidence for the management of PNH in the Latin American setting.

| Characteristic | Total Cohort (N=16) |
|---|---|
| **Age, years,** mean (range) | 38.7 (19–67) |
| **Sex (Female/Male),** n | 8/8 |
| **Time since diagnosis, years,** mean (range) | 4.5 (0.2–17) |
| **PNH Subclassification,** n (%) | |
| Classic | 6 (37.5%) |
| In the setting of Bone Marrow Failure | 10 (62.5%) |
| **PNH Clone Size (Type II + III),** mean % (range) | |
| Granulocytes | 70.7 (4–100) |
| Monocytes | 73.4 (4–100) |
| **Prior History of Thrombosis,** n (%) | 4 (25.0%) |
| **Exposure to Iptacopan, weeks,** mean (range) | 32 (4–56) |

**Copyright Information:** (Online) ISSN: 2572-9241
©2026 The Author(s). HemaSphere is published by John Wiley & Sons Ltd on behalf of European Hematology Association. This is an open access Abstract Book distributed under the Attribution-NonCommercial-NoDerivs (CC BY-NC-ND), which allows third parties to download the articles and share them with others as long as they credit the author and the Abstract Book, but they cannot change the content in any way or use them commercially.
**Abstract Book Citations:** Authors, Title, HemaSphere, 2026;10:(S1):pages. The abstract book DOIs can be found on the “Author Information” page.
**Disclaimer:** Articles published in the journal HemaSphere exclusively reflect the opinions of the authors. The authors are responsible for all content in their abstracts including accuracy of the facts, statements, citing resources, etc.

### (PB3145) OUTCOMES OF DIRECT ORAL ANTICOAGULANT USE IN PATIENTS WITH BONE MARROW FAILURE SYNDROMES: A SYSTEMATIC REVIEW

**Topic:** 12. Bone marrow failure syndromes incl. PNH - Clinical

Stephanie Quon*[1], Katherine Zheng[1], Sarah Miller[1]

[1]*University of British Columbia, Vancouver, Canada;*

### Background

Patients with bone marrow failure syndromes face competing risks of thrombosis and bleeding due to cytopenias, transfusion dependence, and underlying inflammatory or clonal processes. Use of direct oral anticoagulants (DOACs) in this population is increasing, yet evidence guiding their safety and effectiveness remains limited and is largely extrapolated from related hematologic populations.

### Aims

To synthesize available evidence on thrombotic, bleeding, and mortality outcomes associated with DOAC use in patients with bone marrow failure syndromes and closely related conditions.

### Methods

We searched MEDLINE, Embase, and Cochrane CENTRAL from inception through June 2025 for studies reporting outcomes of DOAC therapy in adults with acquired or inherited bone marrow failure syndromes, including aplastic anemia and myelodysplastic syndromes. Outcomes included venous or arterial thrombosis, major bleeding, clinically relevant non-major bleeding, and all-cause mortality. Two reviewers independently screened studies, extracted data, and assessed risk of bias using ROBINS-I. Due to substantial heterogeneity, results were summarized descriptively.

### Results

Thirteen studies involving 2,146 patients were included, predominantly retrospective cohort studies. Indications for anticoagulation included atrial fibrillation, venous thromboembolism, and catheter-associated thrombosis. Annual rates of recurrent thromboembolism among patients treated with DOACs ranged from 2.8% to 6.5%. Major bleeding rates varied widely (3.5%–12.1% per year), with consistently higher bleeding risk observed in patients with severe thrombocytopenia or transfusion dependence. Comparative data were limited. In a cohort of patients with hematologic malignancies and venous thromboembolism, DOAC use was associated with a non-significant increase in a composite outcome of recurrent thrombosis or bleeding compared with low-molecular-weight heparin (adjusted HR 1.51, 95% CI 0.70–3.30). For major bleeding alone, the hazard ratio was 4.85 (95% CI 0.64–36.56), with wide confidence intervals. No studies reported comparative effect estimates specific to aplastic anemia or myelodysplastic syndromes.

### Summary/Conclusion

Evidence supporting DOAC use in bone marrow failure syndromes is limited and indirect. Available data suggest acceptable thrombotic control in selected patients but substantial bleeding risk in those with advanced cytopenias. Prospective, disease-specific studies are needed to inform patient selection and anticoagulation strategies in this high-risk population.

**Copyright Information:** (Online) ISSN: 2572-9241
©2026 The Author(s). HemaSphere is published by John Wiley & Sons Ltd on behalf of European Hematology Association. This is an open access Abstract Book distributed under the Attribution-NonCommercial-NoDerivs (CC BY-NC-ND), which allows third parties to download the articles and share them with others as long as they credit the author and the Abstract Book, but they cannot change the content in any way or use them commercially.
**Abstract Book Citations:** Authors, Title, HemaSphere, 2026;10:(S1):pages. The abstract book DOIs can be found on the "Author Information" page.
**Disclaimer:** Articles published in the journal HemaSphere exclusively reflect the opinions of the authors. The authors are responsible for all content in their abstracts including accuracy of the facts, statements, citing resources, etc.

### (PB3146) SEVERE IDIOPATHIC NEUTROPENIA: IMMUNOSUPPRESSIVE THERAPY IN CHILDREN REFRACTORY TO GRANULOCYTE COLONY-STIMULATING FACTOR (G-CSF).

**Topic:** 12. Bone marrow failure syndromes incl. PNH - Clinical

Maria Florencia Mininni*[1], Ana Clara Prieto[1], Estefania Rosetti[1], Florencia Novello[1], Andrea Carolina Candas[1], Lilian Diaz[1], Jaqueline Vanesa Avalos Gomez[1], Belen Bianco[1], Daniel Buamscha[1], Carolina Pepe[1], Carolina Cervio[1], Gabriela Sciuccati[1]

[1]*Hospital Garrahan, Hematology, Buenos Aires, Argentina;*

### Background

Severe idiopathic neutropenia (SIN) is a heterogeneous granulopoietic disorder that can result in a life-threatening condition. While immune dysregulation is hypothesized as a primary driver, its precise etiology is not fully understood and evidence for immunosuppressive therapy (IST) in G-CSF-refractory patients remains scarce.

### Aims

To describe the clinical characteristics and outcomes of pediatric cohort with SIN refractory to G-CSF treated with IST.

### Methods

Retrospective cohort study at a single tertiary care center in Argentina. Inclusion criteria: patients(pts) <18 years old with SIN (absolute neutrophil count (ANC) <0.2×10⁹/L), bone marrow myeloid hypoplasia/aplasia and G-CSF refractoriness diagnosed from May/2014 to Dec/2025. Pts with identified related causes (genetic, inflammatory, autoimmune, neoplastic or drug-induced) were excluded. IST included rabbit antithymocyte globulin(rATG) and/or cyclosporine(CsA). Clinical and laboratory outcomes were analyzed. Results are reported as median(m) and interquartile range [IQR].

### Results

Thirty-three pts were included: median age 6.2 years [IQR: 4.4–9.4], 60.6% male. Sixteen pts (48.5%)had a prior history of transient neutropenia: 3/16 achieved spontaneous remission while 13/16 responded to G-CSF. Genetic testing in 14 pts (42%) showed no *ELANE* variants. Patients´ baseline characteristics: Table 1.

**Table 1.** Clinical and Laboratory Characteristics at Diagnosis

| | n=33 |
|---|---|
| **Clinical Characteristics** | **n(%)** |
| Previous Neutropenia | 16(48.5) |
| Fever | 33(100) |
| Lymphadenopathy | 9(27) |

**Copyright Information:** (Online) ISSN: 2572-9241
©2026 The Author(s). HemaSphere is published by John Wiley & Sons Ltd on behalf of European Hematology Association. This is an open access Abstract Book distributed under the Attribution-NonCommercial-NoDerivs (CC BY-NC-ND), which allows third parties to download the articles and share them with others as long as they credit the author and the Abstract Book, but they cannot change the content in any way or use them commercially.
**Abstract Book Citations:** Authors, Title, HemaSphere, 2026;10:(S1):pages. The abstract book DOIs can be found on the "Author Information" page.
**Disclaimer:** Articles published in the journal HemaSphere exclusively reflect the opinions of the authors. The authors are responsible for all content in their abstracts including accuracy of the facts, statements, citing resources, etc.

| | |
|---|---|
| Splenomegaly | 8(24) |
| Oral Ulcers | 14(42) |
| Enteritis | 10(35) |
| **Concomitant Infections** | **n(%)** |
| Fungal | 21(63) |
| Bacterial | 16(48) |
| Viral | 14(42) |
| Parasitic | 1(3) |
| **Laboratory Findings** | **Median [IQR]** |
| ANC (x$10^9$/L) | 0[0-0.2] |
| Hemoglobin (g/dL) | 8.5[7.7-10] |
| Platelet Count (x$10^9$/L) | 365[217-557] |
| **Bone Marrow** | **n(%)** |
| Cellularity <50% | 25(78) |
| Myeloid Lineage <10% | 29(88) |
| Increased Plasma Cells | 28(84) |
| Moderate/Severe Fibrosis | 13(39) |
| Normal Karyotype | 33(100) |

IST was administered to 30 pts (90.9%). Overall response rate (ANC>1.5 ×$10^9$/L): 96.6% (29/30), mostly within the first week after rATG. Among the 29 responders, 6pts required intermittent G-CSF support and 3pts relapsed. Of these, 2/3 responded to a second

**Copyright Information:** (Online) ISSN: 2572-9241
©2026 The Author(s). HemaSphere is published by John Wiley & Sons Ltd on behalf of European Hematology Association. This is an open access Abstract Book distributed under the Attribution-NonCommercial-NoDerivs (CC BY-NC-ND), which allows third parties to download the articles and share them with others as long as they credit the author and the Abstract Book, but they cannot change the content in any way or use them commercially.
**Abstract Book Citations:** Authors, Title, HemaSphere, 2026;10:(S1):pages. The abstract book DOIs can be found on the "Author Information" page.
**Disclaimer:** Articles published in the journal HemaSphere exclusively reflect the opinions of the authors. The authors are responsible for all content in their abstracts including accuracy of the facts, statements, citing resources, etc.

IST cycle and 1/3 underwent hematopoietic stem cell transplantation. Five pts died due to infectious complications: 2 before IST and 3 after IST (including 2 with normal ANC). No severe IST-related adverse events were observed. Median follow-up: 1.7 years. The 2-year overall survival(OS) was 82%. IST is shown in Table 2.

| **IST** | **n=30** |
|---|---|
| Time to IST initiation (days), median [IQR] | 34[24-43] |
| rATG + CsA, n (%) | 25(83) |
| rATG alone, n (%) | 5(17) |
| rATG Dose (mg/kg/day), median [IQR] x 5days | 3.4[3.2-3.6] |
| Duration of IST (months), median [IQR] | 12.6[11.8-16.6] |
| **Response and Clinical Outcomes** | |
| Time to ANC recovery > 0.5 x$10^9$/L (days), median [IQR] | 9[7-12] |
| Length of Hospital Stay (days), median [IQR] | 38[30-81] |
| ICU Admission, n (%) | 16(53) |
| Relapse after IST, n (%) | 3(10) |
| Mortality, n (%) | 3(10) |

### Summary/Conclusion

In this pediatric cohort, rATG and CsA-based IST was highly effective and safe, yielding a 96.6% response rate. Prompteutrophil count recovery was crucial in the managing of life-threatening infections. This robust response strongly suggests that T-cell-mediated immune mechanisms drive granulopoiesis failure in these patients, making this IST a good choice for children with refractory SIN.

**Copyright Information:** (Online) ISSN: 2572-9241
©2026 The Author(s). HemaSphere is published by John Wiley & Sons Ltd on behalf of European Hematology Association. This is an open access Abstract Book distributed under the Attribution-NonCommercial-NoDerivs (CC BY-NC-ND), which allows third parties to download the articles and share them with others as long as they credit the author and the Abstract Book, but they cannot change the content in any way or use them commercially.
**Abstract Book Citations:** Authors, Title, HemaSphere, 2026;10:(S1):pages. The abstract book DOIs can be found on the "Author Information" page.
**Disclaimer:** Articles published in the journal HemaSphere exclusively reflect the opinions of the authors. The authors are responsible for all content in their abstracts including accuracy of the facts, statements, citing resources, etc.

### (PB3147) SEVERE APLASTIC ANEMIA IN A MOROCCAN CENTER: REAL-WORLD TREATMENT OUTCOMES

**Topic:** 12. Bone marrow failure syndromes incl. PNH - Clinical

Zineb Guessous*[1], Maryame Ahnach[1]

[1]*University Mohammed VI of Health Sciences, Casablanca, Morocco;*

### Background

Severe aplastic anemia is a rare and life-threatening hematologic disorder, for which allogeneic hematopoietic stem cell transplantation remains the only curative treatment. In emerging countries, implementing this therapeutic strategy poses major challenges. In real-world clinical practice, a substantial proportion of patients are managed with immunosuppressive therapy due to the lack of a compatible donor and organizational or socio-economic constraints. Real-world data analysis allows evaluation of the impact of these factors on management strategies and patient outcomes

### Methods

This is a retrospective descriptive study including a series of 34 adult patients with severe aplastic anemia followed at the Hôpital International Cheikh Khalifa in Casablanca between January 2017 and March 2025. Inclusion criteria comprised adults over 18 years diagnosed with SAA who received either allogeneic hematopoietic stem cell transplantation or immunosuppressive therapy consisting of antithymocyte globulin and cyclosporine, with or without eltrombopag. For immunosuppressed patients, ATG type, eltrombopag use, tolerability, and hematologic response were analyzed. For transplanted patients, conditioning, graft source, time to transplant, engraftment, complications, relapses, and overall survival were collected

### Results

Our cohort included 34 patients with severe aplastic anemia, of whom 10 underwent HLA-matched allogeneic hematopoietic stem cell transplantation. The remaining 24 patients, due to lack of a compatible donor or transplant contraindications, were treated with immunosuppressive therapy consisting of antithymocyte globulin ( ATG), glucocorticoids, and cyclosporine. Among the patients receiving ATG, the median age was 34 years, with a female predominance, and 75% had idiopathic disease. Rabbit ATG was used in 15 patients, while horse ATG could only be administered to 9 patients due to cost, temporary use authorization, and lack of financial coverage. Eltrombopag was introduced in 21 patients. Thirteen patients experienced significant adverse events. Complete hematologic response was achieved in 7 patients, partial response in 1 patient, and treatment failure in 2 cases. Six deaths were reported, and eight patients were lost to follow-up.
Among the 10 transplanted patients, the median age was 26.3 years. Nine patients received a conditioning regimen with cyclophosphamide and ATG, while one patient with a constitutional form received FB4 conditioning. The median time from diagnosis to transplant was 11 months (range, 6–28 months). The graft source was bone marrow in 60% of cases and peripheral blood stem cells in 40%. Engraftment was achieved in all patients. One case of moderate chronic cutaneous graft-versus-host disease and one cytomegalovirus reactivation occurred, both with favorable outcomes. One-year overall survival was estimated at 80%, with two early deaths due to septic shock and posterior reversible encephalopathy syndrome. To date, two late relapses have been observed.

**Copyright Information:** (Online) ISSN: 2572-9241
©2026 The Author(s). HemaSphere is published by John Wiley & Sons Ltd on behalf of European Hematology Association. This is an open access Abstract Book distributed under the Attribution-NonCommercial-NoDerivs (CC BY-NC-ND), which allows third parties to download the articles and share them with others as long as they credit the author and the Abstract Book, but they cannot change the content in any way or use them commercially.
**Abstract Book Citations:** Authors, Title, HemaSphere, 2026;10:(S1):pages. The abstract book DOIs can be found on the "Author Information" page.
**Disclaimer:** Articles published in the journal HemaSphere exclusively reflect the opinions of the authors. The authors are responsible for all content in their abstracts including accuracy of the facts, statements, citing resources, etc.

**Summary/Conclusion**

This real-world study demonstrates that management of severe aplastic anemia still relies predominantly on immunosuppressive therapy due to limited access to allogeneic transplantation. HLA-matched allogeneic stem cell transplantation, when feasible, provides satisfactory clinical outcomes. These findings highlight the impact of organizational and socio-economic constraints on therapeutic decisions and emphasize the need to optimize access to transplantation in resource-limited settings

**Copyright Information:** (Online) ISSN: 2572-9241
©2026 The Author(s). HemaSphere is published by John Wiley & Sons Ltd on behalf of European Hematology Association. This is an open access Abstract Book distributed under the Attribution-NonCommercial-NoDerivs (CC BY-NC-ND), which allows third parties to download the articles and share them with others as long as they credit the author and the Abstract Book, but they cannot change the content in any way or use them commercially.
**Abstract Book Citations:** Authors, Title, HemaSphere, 2026;10:(S1):pages. The abstract book DOIs can be found on the "Author Information" page.
**Disclaimer:** Articles published in the journal HemaSphere exclusively reflect the opinions of the authors. The authors are responsible for all content in their abstracts including accuracy of the facts, statements, citing resources, etc.

### (PB3148) EFFICACY OF ADDING PEGFILGRASTIM TO IMMUNOSUPPRESSIVE THERAPY IN APLASTIC ANEMIA PATIENTS- AN OPEN LABELED RANDOMIZED PILOT STUDY FROM SOUTH INDIA.

**Topic:** 12. Bone marrow failure syndromes incl. PNH - Clinical

Deepak Amalnath*[1], Aparna Sudhakaran[2]

*[1]Jawaharlal Institute of Postgraduate Medical Education & Research, Medicine, Puducherry, India;[2]Jawaharlal Institute of Postgraduate Medical Education & Research, medicine, puducherry, India;*

### Background

Aplastic anemia is a hematological disorder with pancytopenia and marrow hypocellularity. Immunosuppressive therapy (IST) with Anti Thymocyte Globulin and cyclosporine is recommended as the first-line therapy for patients who lack a matched sibling donor and for patients aged >35-50 years. Daily filgrastim has been recommended to reduce the incidence of infections following IST. Hence we sought to study the efficacy of single dose of Pegfilgrastim in reducing infections post IST.

### Aims

1. to study the efficacy of adding pegfilrastim in reducing infections following IST in aplastic anemia.
2. does pegfilgrastim improve response rate following IST

### Methods

This randomized open label active controlled trial recruited 65 newly diagnosed patients of AA> 13 years of age. The study was conducted from January 2024 to July 2025, at a tertiary referral centre in south India. Institute ehtical approval was obtained. Patients were randomised to peg filgrastim + IST arm and IST alone arm. The baseline clinical profile, treatment details and the hematological response of the patient were collected and recorded in the data sheet. Pegfilgrastim 6 mg single subcutaneous injection was administered subcutaneously once to experimental group on Day 1 of administration of ATG. After discharge, they were followed up monthly for 6 months for development of febrile neutropenia, response to treatment, survival status.

### Results

The intervention arm enrolled 33 patients as compared to the control arm (32 patients).The incidence of infection at 2 and 4 weeks following ATG was lower in the Pegfilgrastim group, though not statistically significant. Overall infection rates were 24% versus 28% at 2 weeks and 32% versus 44% at 4 weeks in the pegfilgrastim and control groups, respectively, with an absolute risk reduction of 12% at 4 weeks (NNT 8.33). The greatest reduction in infections was observed in patients with very severe aplastic anemia receiving pegfilgrastim.
Hematologic response, assessed by ANC recovery, was similar between groups at 3 and 6 months, with 56% achieving recovery at 6 months. Six-month mortality was comparable (36% vs 32%), and infections were the leading cause of death. Adverse events were similar between groups, with no pegfilgrastim-related toxicity observed.

### Summary/Conclusion

In this study, the use of pegfilgrastim in combination with IST did not reduce the overall incidence of infections or better response rates /mortality benefit.

**Copyright Information:** (Online) ISSN: 2572-9241
©2026 The Author(s). HemaSphere is published by John Wiley & Sons Ltd on behalf of European Hematology Association. This is an open access Abstract Book distributed under the Attribution-NonCommercial-NoDerivs (CC BY-NC-ND), which allows third parties to download the articles and share them with others as long as they credit the author and the Abstract Book, but they cannot change the content in any way or use them commercially.
**Abstract Book Citations:** Authors, Title, HemaSphere, 2026;10:(S1):pages. The abstract book DOIs can be found on the "Author Information" page.
**Disclaimer:** Articles published in the journal HemaSphere exclusively reflect the opinions of the authors. The authors are responsible for all content in their abstracts including accuracy of the facts, statements, citing resources, etc.

### (PB3149) A NOVEL DE NOVO RPS19 MUTATION CONFIRMS ATYPICAL DIAMOND-BLACKFAN ANEMIA IN AN ADULT PATIENT: A CASE REPORT

**Topic:** 12. Bone marrow failure syndromes incl. PNH - Clinical

Lijuan Zhang*[1], Yujiao Jia[1], Jiawei Zhao[1], Li Qin[1], Ruixue Zhang[1], Bingqing Huang[1], Jiao Chang[1, 1], Haoxu Wang[1]

[1]*Institute of Hematology, CAMS, Tian Jin, China;*

### Background

Diamond-Blackfan anemia (DBA) is a congenital bone marrow failure syndrome typically diagnosed in infancy or early childhood, with approximately 25% of cases caused by mutations in the ribosomal protein gene RPS19. Cases diagnosed in adulthood are uncommon and often exhibit nonspecific hematologic features. Peripheral blood findings may include isolated anemia with reduced erythroid parameters, while bone marrow examination typically shows a selective decrease in erythroid precursors with preservation of granulocytic and megakaryocytic lineages. These features closely mimic those of acquired pure red cell aplasia (PRCA), making clinical differentiation particularly challenging. In such complex cases diagnosed during adulthood, conventional diagnostic approaches often fail to identify the underlying etiology. Molecular testing for DBA-associated genes, including RPS19, has therefore become essential for establishing a definitive diagnosis.

### Aims

To investigate the clinicopathological characteristics and molecular diagnostic approaches for atypical DBA in adults, emphasizing the essential role of NGS in the accurate diagnosis of complex inherited bone marrow failure syndromes, and to provide insights for clinical practice.

### Methods

We conducted a retrospective analysis of a 34-year-old male with a history of anemia since six months of age and congenital polydactyly. Laboratory evaluations included complete blood count, bone marrow aspiration and biopsy. Targeted NGS for genetic anemia-related genes was performed, followed by Sanger sequencing for family segregation analysis.

### Results

A 34-year-old male patient was diagnosed with "pure red cell aplasia" at 6 months of age and was followed up long-term, with worsening anemia over the past 4 years. He had congenital polydactyly since childhood, which was surgically removed 29 years ago. Laboratory examinations at the current visit revealed: WBC $4.09\times10^9$/L, HGB 65g/L, PLT $408\times10^9$/L, showing isolated erythroid lineage reduction. Bone marrow pathology revealed normocellular marrow with trilineage (granulocytic, erythroid, megakaryocytic) hyperplasia (Figure 1a). Bone marrow smear demonstrated trilineage hyperplasia (Figure 1b), making it difficult to distinguish from acquired pure red cell aplasia.

Concurrently, targeted NGS was performed and identified a heterozygous variant in the RPS19 gene: c.173C>T (p.A58V). Sanger sequencing confirmed the presence of this mutation in the patient (Figure 1c). The dbscSNV database predicts a potential impact on mRNA splicing. This variant has not been reported in existing databases. Family Sanger sequencing verification showed that neither parent carried this variant (Figures 1d-e), confirming it as a de novo mutation. Based on the patient's history of anemia since infancy (6 months of age), congenital polydactyly, and the de novo mutation in a classic DBA-associated gene, and according to current DBA diagnostic criteria, the patient was definitively diagnosed with atypical DBA.

**Copyright Information:** (Online) ISSN: 2572-9241
©2026 The Author(s). HemaSphere is published by John Wiley & Sons Ltd on behalf of European Hematology Association. This is an open access Abstract Book distributed under the Attribution-NonCommercial-NoDerivs (CC BY-NC-ND), which allows third parties to download the articles and share them with others as long as they credit the author and the Abstract Book, but they cannot change the content in any way or use them commercially.
**Abstract Book Citations:** Authors, Title, HemaSphere, 2026;10:(S1):pages. The abstract book DOIs can be found on the "Author Information" page.
**Disclaimer:** Articles published in the journal HemaSphere exclusively reflect the opinions of the authors. The authors are responsible for all content in their abstracts including accuracy of the facts, statements, citing resources, etc.

### Summary/Conclusion

Adult-onset atypical DBA closely resembles acquired PRCA in both peripheral blood and bone marrow morphology, making diagnosis based solely on clinical presentation unreliable. For suspected atypical DBA cases, NGS-based molecular testing is essential for definitive diagnosis. The novel de novo mutation identified in this study expands the mutational spectrum of DBA and provides important insights for genetic counseling and clinical management of rare inherited bone marrow failure syndromes.

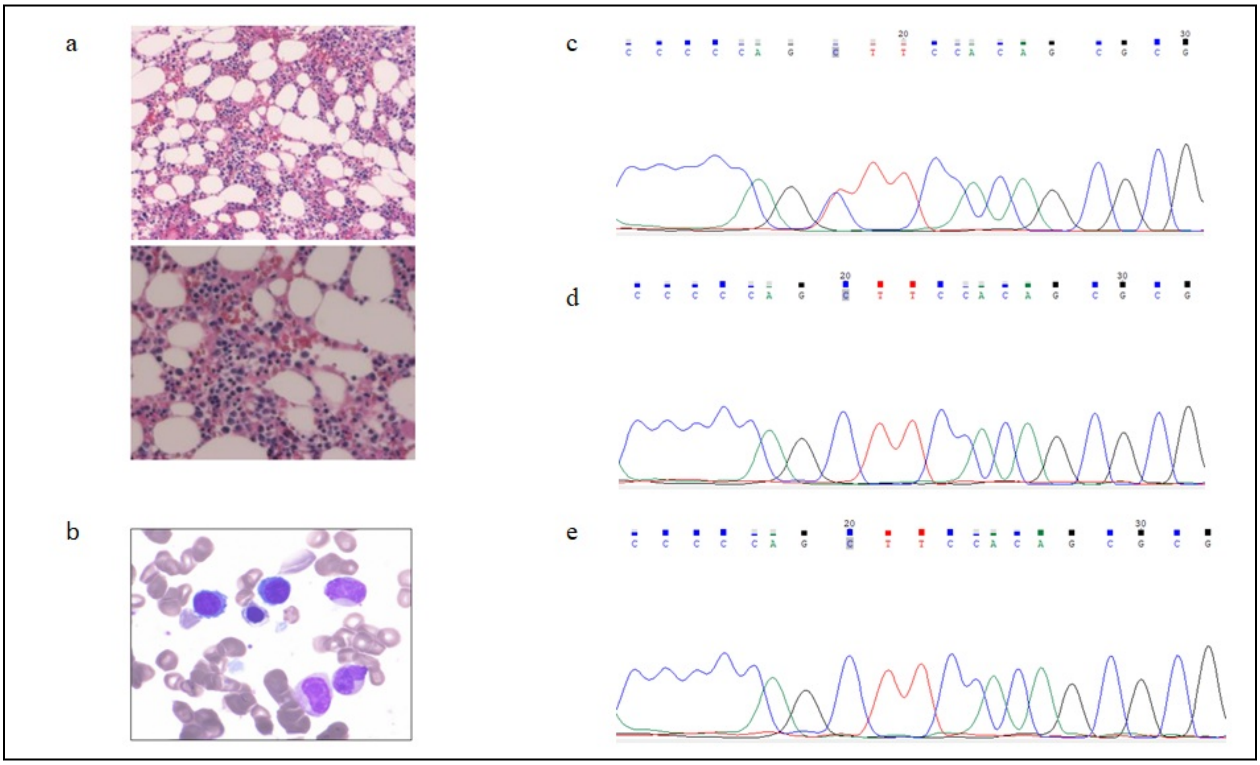

Figure 1. (a) Bone marrow pathology showing normocellular marrow with trilineage hyperplasia. (b) Bone marrow smear demonstrating trilineage hyperplasia. (c) Sanger sequencing confirmation of the RPS19 c.173C>T mutation in the patient. (d-e) Sanger sequencing showing absence of the mutation in the patient's father and mother.

**Copyright Information:** (Online) ISSN: 2572-9241
©2026 The Author(s). HemaSphere is published by John Wiley & Sons Ltd on behalf of European Hematology Association. This is an open access Abstract Book distributed under the Attribution-NonCommercial-NoDerivs (CC BY-NC-ND), which allows third parties to download the articles and share them with others as long as they credit the author and the Abstract Book, but they cannot change the content in any way or use them commercially.
**Abstract Book Citations:** Authors, Title, HemaSphere, 2026;10:(S1):pages. The abstract book DOIs can be found on the "Author Information" page.
**Disclaimer:** Articles published in the journal HemaSphere exclusively reflect the opinions of the authors. The authors are responsible for all content in their abstracts including accuracy of the facts, statements, citing resources, etc.

### (PB3150) CLINICAL SPECTRUM OF HOMOZYGOUS PATHOGENIC FANCD2 MUTATIONS: A CASE SERIES OF FOUR PEDIATRIC PATIENTS

**Topic:** 12. Bone marrow failure syndromes incl. PNH - Clinical

Çağrı Coşkun*[1], Ceren Damla Durmaz Özdinç[2], Arda Çetinkaya[2], Beril Kiracı Şen[3], Sule Unal Cangul[1]

*[1]Hacettepe University Faculty of Medicine, Department of Pediatric Hematology, Ankara, Türkiye;[2]Hacettepe University Faculty of Medicine, Department of Medical Genetics, Ankara, Türkiye; [3]Hacettepe University Faculty of Medicine, Department of Pediatrics, Ankara, Türkiye;*

### Background

Fanconi anemia (FA) is a rare and frequently autosomal recessive hereditary disease caused by mutations in genes involved in DNA damage response or repair. It is characterized by congenital malformations including growth retardation, renal anomalies, radius and thumb anomalies, progressive bone marrow failure, irregular skin pigmentation patterns, and cancer susceptibility. *FANCD2* is an FA gene that plays an important role in DNA double-strand break responses. It undergoes monoubiquitination in response to DNA damage and coordinates homologous recombination repair. Malformations are common in FA D2 patients, and hematological manifestations appear earlier and progress more rapidly compared to other patients. Homozygous pathogenic variants in the *FANCD2* gene are rare, and published data are limited to isolated case reports or small series with heterogeneous phenotypes.

### Aims

This abstract aims to describe the clinical, hematological, and genetic features of four pediatric patients with homozygous pathogenic *FANCD2* mutations and to broaden the phenotypic spectrum associated with *FANCD2* deficiency.

### Results

A total of 4 patients (3 males, 1 female) were included (median age at diagnosis: 44.5 months [range: 7–46 months]). Parental consanguinity was present in three cases, and two had a history of intrauterine growth restriction (IUGR). All patients exhibited microcephaly, microphthalmia, thumb anomalies, growth retardation, and short stature. Cardiac defects (VSD, ASD, PFO) were identified in three patients (Table 1).
Bicytopenia was observed at diagnosis in three patients, while macrocytosis was present in all. Elevated HbF levels were detected in three patients. Bone marrow cytogenetics were normal in all cases. All patients carried homozygous pathogenic *FANCD2* mutations. The median age of the patients at their last follow-up was 57.5 months (range: 21–89 months). Severe bone marrow failure developed in one patient who underwent HSCT, while one patient died at 7 years of age before transplantation due to sepsis. Annual follow-up with bone marrow aspiration examination was planned for the remaining two patients (Table 1).

### Summary/Conclusion

Homozygous pathogenic *FANCD2* mutations are associated with a severe and multisystemic phenotype characterized by congenital anomalies, growth failure, and early-onset hematologic abnormalities. Early genetic diagnosis is critical for surveillance, avoidance of DNA-damaging exposures, and timely HSCT planning.

**Copyright Information:** (Online) ISSN: 2572-9241
©2026 The Author(s). HemaSphere is published by John Wiley & Sons Ltd on behalf of European Hematology Association. This is an open access Abstract Book distributed under the Attribution-NonCommercial-NoDerivs (CC BY-NC-ND), which allows third parties to download the articles and share them with others as long as they credit the author and the Abstract Book, but they cannot change the content in any way or use them commercially.
**Abstract Book Citations:** Authors, Title, HemaSphere, 2026;10:(S1):pages. The abstract book DOIs can be found on the "Author Information" page.
**Disclaimer:** Articles published in the journal HemaSphere exclusively reflect the opinions of the authors. The authors are responsible for all content in their abstracts including accuracy of the facts, statements, citing resources, etc.

**Table 1.** Clinical and Genetic Characteristics of Patients with FANCD2 Homozygous Pathogenic FANCD2 Mutations

| Cases | Age (mo)/sex | Phenotype | Clonality/MDS-AML | Family history of cancer | Hematopoietic stem cell transplantation | Outcome |
|---|---|---|---|---|---|---|
| Case 1 | 7/M | Short stature<br>Microcephaly<br>Hypopigmentation | No | No | No | Alive |
| Case 2 | 46/M | Short stature<br>Microcephaly<br>Microphthalmia<br>Clinodactyly<br>Café au lait spots | No | No | No | Died |
| Case 3 | 46/M | Short stature<br>Microcephaly<br>Microphthalmia<br>Syndactyly<br>Polydactyly<br>hypertelorism | No | Yes | No | Alive |
| Case 4 | 43/F | Short stature<br>Microcephaly<br>Microphthalmia<br>Strabismus<br>Café au lait spots | No | Yes | Yes | Alive |

**Copyright Information:** (Online) ISSN: 2572-9241
©2026 The Author(s). HemaSphere is published by John Wiley & Sons Ltd on behalf of European Hematology Association. This is an open access Abstract Book distributed under the Attribution-NonCommercial-NoDerivs (CC BY-NC-ND), which allows third parties to download the articles and share them with others as long as they credit the author and the Abstract Book, but they cannot change the content in any way or use them commercially.
**Abstract Book Citations:** Authors, Title, HemaSphere, 2026;10:(S1):pages. The abstract book DOIs can be found on the "Author Information" page.
**Disclaimer:** Articles published in the journal HemaSphere exclusively reflect the opinions of the authors. The authors are responsible for all content in their abstracts including accuracy of the facts, statements, citing resources, etc.

### (PB3151) FIRST-IN-HUMAN, FIRST-IN-PATIENT STUDY OF ALN-CFB, AN INVESTIGATIONAL SIRNA TARGETING HEPATIC COMPLEMENT FACTOR B: TREATMENT OF PERSISTENT ANEMIA IN PATIENTS WITH PAROXYSMAL NOCTURNAL HEMOGLOBINURIA

**Topic:** 12. Bone marrow failure syndromes incl. PNH - Clinical

Morag Griffin[1], Jun Ho Jang[2], Austin G. Kulasekararaj[3], Nikhil Singh*[4], Arjun Gadhiya[4], Kishor Devalaraja-Narashimha[4], Karoline Meagher[4], Zhentao Tong[4], Lori Morton[4], Eric Bachman[4], Lorah Perlee[4], Umesh Chaudhari[4], Christopher Patriquin[5]

*[1]St James's University Hospital, Leeds, United Kingdom;[2]Samsung Medical Center, Seoul, South Korea;[3]King's College Hospital NHS Foundation Trust, London, United Kingdom;[4]Regeneron Pharmaceuticals, Inc., Tarrytown, United States of America;[5]University Health Network, Toronto General Hospital, Toronto, Canada;*

### Background

Paroxysmal nocturnal hemoglobinuria (PNH) is a rare, life-threatening disease characterized by complement-mediated hemolysis. Despite treatment with C5 inhibitors, many people with PNH remain anemic due to extravascular hemolysis (EVH), caused by deposition of proximal alternative pathway components and phagocytic removal of red blood cells. Complement factor B (CFB) is a critical component of the alternative pathway. ALN-CFB is a novel GalNAc-conjugated small interfering RNA targeting hepatic production of CFB; by cleaving *CFB* messenger RNA, single subcutaneous (SC) doses of ALN-CFB may deeply and durably reduce circulating CFB protein, abrogate EVH, and possibly control hemolysis in PNH.

### Aims

We describe the study design of a phase 1/2 trial that will evaluate the safety, tolerability, pharmacokinetics (PK), pharmacodynamics (PD) and preliminary efficacy of ALN-CFB in people with PNH and persistent anemia despite receiving a stable dose of an approved C5 inhibitor.

### Methods

This is an ongoing, first-in-human (FIH), first-in-patient, 2-part, randomized, double-blind, placebo-controlled study (NCT07187401). In Part A, up to 24 participants will be randomized to single-ascending SC doses of ALN-CFB or placebo on a background of their approved C5-inhibitor therapy. Interim safety, PK, and PD data from Part A will then be used to identify the dose and dosing regimen for Part B, the multiple-dosing safety and efficacy expansion part of the study. Key inclusion and exclusion criteria for Part A are shown in the Table.

Part A consists of a ~28-day treatment period with a post-treatment follow-up period lasting up to Day 365. The overall study duration will be approximately 57 weeks, including the screening period. The primary endpoint will be the occurrence and severity of treatment-emergent adverse events (AEs) during treatment until the end of the study. Secondary endpoints will include absolute and percentage change from baseline in CFB concentration, and concentrations of combined ALN-CFB and major metabolites in plasma (throughout the study) and urine (24 hours following ALN-CFB administration). Throughout the study, safety assessments will include vital signs, body weight, physical examination, electrocardiograms, laboratory tests, and monitoring of anti-drug antibodies and AEs.

### Results

Part A of the study is actively recruiting in the UK, Canada, and South Korea.

**Copyright Information:** (Online) ISSN: 2572-9241
©2026 The Author(s). HemaSphere is published by John Wiley & Sons Ltd on behalf of European Hematology Association. This is an open access Abstract Book distributed under the Attribution-NonCommercial-NoDerivs (CC BY-NC-ND), which allows third parties to download the articles and share them with others as long as they credit the author and the Abstract Book, but they cannot change the content in any way or use them commercially.
**Abstract Book Citations:** Authors, Title, HemaSphere, 2026;10:(S1):pages. The abstract book DOIs can be found on the "Author Information" page.
**Disclaimer:** Articles published in the journal HemaSphere exclusively reflect the opinions of the authors. The authors are responsible for all content in their abstracts including accuracy of the facts, statements, citing resources, etc.

### Summary/Conclusion

This FIH study will provide the first clinical evaluation of ALN-CFB in participants with PNH and persistent anemia on C5 inhibitors, generating safety, PK, PD and preliminary efficacy data and informing dose selection for future studies.

| Key inclusion criteria | Key exclusion criteria |
| --- | --- |
| • Adults (aged ≥18 years) with PNH<br>• Persistent anemia, defined as hemoglobin ≤10 g/dL (Canada) or ≤10.5 g/dL (UK and South Korea)<br>• Reticulocyte count of ≥100 x $10^9$/L at screening Visit 1<br>• ≥24 weeks on stable C5 inhibitor therapy (eg, eculizumab or approved biosimilar, ravulizumab or crovalimab) or on expanded access program | • Recipient of an organ/bone marrow transplant<br>• History of meningococcal infection or similar recurrent infections by other encapsulated bacterial organisms<br>• Any active, ongoing or recent infection requiring ongoing systemic treatment with antibiotics, antivirals or antifungals within 2 weeks of screening or during screening<br>• Laboratory evidence of bone marrow failure |

Copyright Information: (Online) ISSN: 2572-9241
©2026 The Author(s). HemaSphere is published by John Wiley & Sons Ltd on behalf of European Hematology Association. This is an open access Abstract Book distributed under the Attribution-NonCommercial-NoDerivs (CC BY-NC-ND), which allows third parties to download the articles and share them with others as long as they credit the author and the Abstract Book, but they cannot change the content in any way or use them commercially.
Abstract Book Citations: Authors, Title, HemaSphere, 2026;10:(S1):pages. The abstract book DOIs can be found on the "Author Information" page.
Disclaimer: Articles published in the journal HemaSphere exclusively reflect the opinions of the authors. The authors are responsible for all content in their abstracts including accuracy of the facts, statements, citing resources, etc.

### (PB3152) EFFICACY AND SAFETY OF IPTACOPAN IN PATIENTS WITH PAROXYSMAL NOCTURNAL HEMOGLOBINURIA: A MULTI-CENTER RETROSPECTIVE STUDY IN SOUTHERN CHINA

**Topic:** 12. Bone marrow failure syndromes incl. PNH - Clinical

Gaixiang Xu*[1], Lan Yao[2], Ruiqing Zhou[3], Wei Zhou[3], Tao Huang[4], Weibo Wu[5], Xiaolan Lai[6], Xiaolan Lian[7], Zifeng Wang[8], Jing Liu[9], Xiuqing Liu[9], Jingxiu Yan[9], Jieyu Wang[10], Meiqi Ding[11], Yanli Yang[12, 12], Yanjuan Lin[7], Fei Li[13], Shunqing Wang[14], Hongyan Tong[15]

*[1]the First Affiliated Hospital,Zhejiang University School of Medicine, Hangzhou, China;[2]the First Affiliated Hospital,Zhejiang University School of Medicine, Department of Hematology, Hangzhou, China; [3]Guangzhou First People's Hospital, Guangzhou, China; [4]The First Hospital of Quanzhou City, fujian, China;[5]Longyan First Affiliated Hospital of Fujian Medical University, Quanzhou, China; [6]Ningde Municipal hospital, Ningde, China; [7]Fujian Medical University Union Hospital, Fujian, China; [8]Nanchang University Affiliated Shangrao Hospital, Shangrao, China;[9]Nanchang University Affiliated Shangrao HospitalNanchang University Affiliated Shangrao Hospital, Shangrao, China; [10]Jiangxi Clinical Research Center For Hematologic Disease, Jiangxi Provincial Key Laboratory of Hematologica Disease, The First Affliated Hospital, Jiangxi Medical College, Nanchang, China., Nanchang, China; [11]Bozhou People's Hospital, Bozhou, China; [12]The first affiliated hospital of bengbu medical college, Bengbu, China; [13]The First Affiliated Hospital, Jiangxi Medical College, Nanchang University, Nanchang, China; [14]Guangzhou First People's Hospital, uangzhou, China; [15]The First Affiliated Hospital, Zhejiang University School of Medicine, Hangzhou, China;*

### Background

Paroxysmal nocturnal hemoglobinuria (PNH) is a rare acquired clonal disorder of hematopoietic stem cells (HSCs), characterized by complement mediated intravascular hemolysis, thrombosis, and bone marrow failure.Iptacopan, an oral factor B inhibitor, has demonstrated superior efficacy and safety in Phase III trials compared to C5 inhibitors, achieving greater increases in hemoglobin levels and reduced transfusion needs. But Iptacopan has been approved in China over one year, there were still fewer reports demonstrated the real-world efficacy and adverse events in  Chinese PNH patients. This multicenter, retrospective cohort study followed the efficacy and adverse safety of iptacopan in PNH  in real-world settings.

### Aims

To evaluate the efficacy and safety of Iptacopan in the treatment of PNH patients in the real world in China

### Methods

A retrospective study was conducted in 36 PNH patients,treated with full-dose Iptacopan for at least 3 months from Jun 2024 to July 2025 and were followed up for ≥1 month at 15 centers in Southern China. Laboratory and clinical parameters were collected at baseline and at 1, 2, 3, and 6 months post-treatment.Efficacy endpoints—including control of hemolysis and improvement in transfusion dependence—as well as the incidence of breakthrough hemolysis (BTH) and adverse events were evaluated.

**Copyright Information:** (Online) ISSN: 2572-9241
©2026 The Author(s). HemaSphere is published by John Wiley & Sons Ltd on behalf of European Hematology Association. This is an open access Abstract Book distributed under the Attribution-NonCommercial-NoDerivs (CC BY-NC-ND), which allows third parties to download the articles and share them with others as long as they credit the author and the Abstract Book, but they cannot change the content in any way or use them commercially.
**Abstract Book Citations:** Authors, Title, HemaSphere, 2026;10:(S1):pages. The abstract book DOIs can be found on the "Author Information" page.
**Disclaimer:** Articles published in the journal HemaSphere exclusively reflect the opinions of the authors. The authors are responsible for all content in their abstracts including accuracy of the facts, statements, citing resources, etc.

### Results

From December 2024 to July 2025, 40 patients diagnosed with PNH detected by FLAER flow cytometry and used iptacopan ≥1months were enrolled in our study. There were 15 females and 21 males, with median age 41.5 years at iptacopan start, ranging from 20 to 76 years. The median treatment duration was 5.44 months. Iptacopan demonstrated significant and rapid efficacy. Hemoglobin levels increased substantially from a baseline of 65.85 g/L to mean levels of 94.8 g/L at 3 weeks and 116.4 g/L at 5 months. Overall, 86% of patients achieved a hemoglobin increase of ≥20 g/L, and 42% reached an Hb level ≥120 g/L at the end of 24weeks.Concurrently, lactate dehydrogenase (LDH) levels normalized rapidly, decreasing from 1668 U/L to means near or below 400 U/L within months, indicating control of intravascular hemolysis. Among the 16 patients who were transfusion-dependent at baseline, 14 (87.5%) achieved transfusion independence. Hemoglobinuria episodes resolved in all but one patient. No new thrombotic events occurred during follow-up. A subgroup analysis of 9 patients previously treated with eculizumab showed a particularly robust response, with 100% achieving an Hb increase >20 g/L and 66.7% reaching ≥120 g/L. Two breakthrough hemolysis (BTH) events were reported; one due to unauthorized drug withdrawal and another triggered by infection. Both resolved with re-initiation of therapy/supportive care.Platelet and white blood cell counts remained stable throughout follow-up, and no clinically meaningful deterioration in renal function was observed. No  patients discontinued iptacopan due to severe treatment-related adverse events.

### Summary/Conclusion

This real-world data rom the Southern China PNH Collaborative Network shows that oral iptacopan induces hematologic responses in PNH, with most patients achieving meaningful Hb rises and transfusion independence by 3 months. The rate of BTH in this follow-up period was comparable to that reported in clinical trials. Modest increases in total , with the mean degree of rise consistent with trial data. No thrombotic events occurred. These findings support the effectiveness of iptacopan in the treatment of PNH in China.

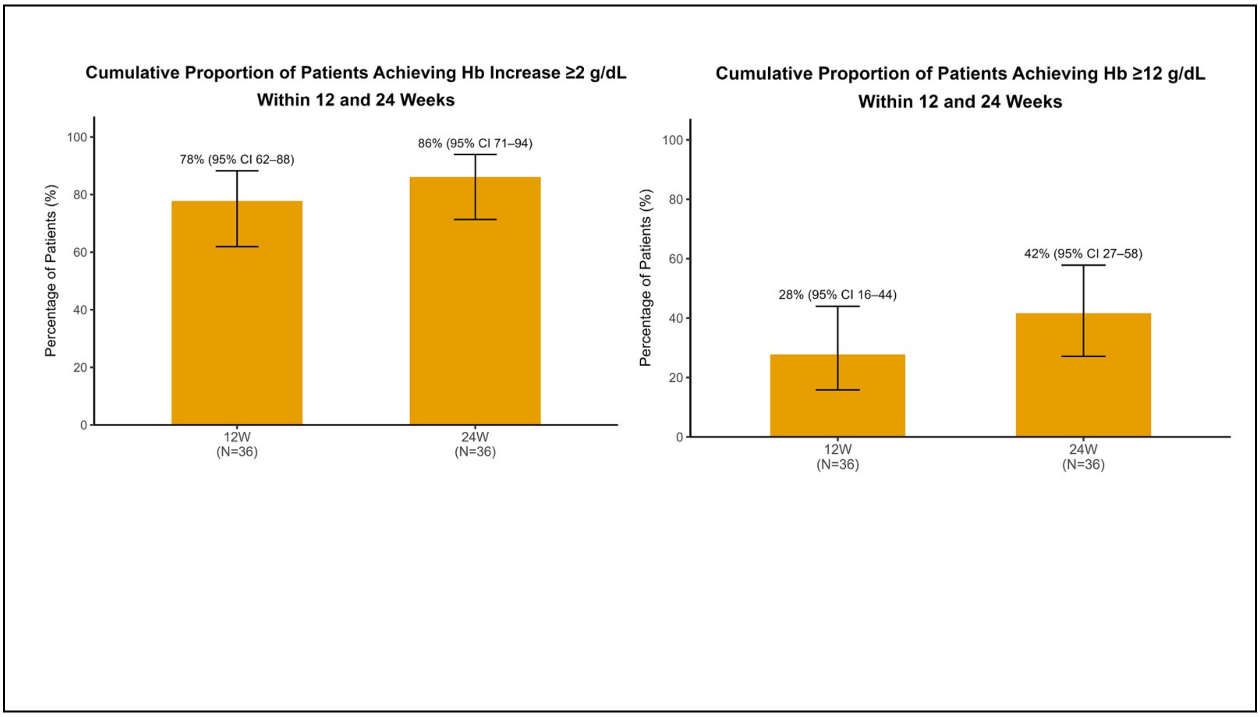

**Copyright Information:** (Online) ISSN: 2572-9241
©2026 The Author(s). HemaSphere is published by John Wiley & Sons Ltd on behalf of European Hematology Association. This is an open access Abstract Book distributed under the Attribution-NonCommercial-NoDerivs (CC BY-NC-ND), which allows third parties to download the articles and share them with others as long as they credit the author and the Abstract Book, but they cannot change the content in any way or use them commercially.
**Abstract Book Citations:** Authors, Title, HemaSphere, 2026;10:(S1):pages. The abstract book DOIs can be found on the "Author Information" page.
**Disclaimer:** Articles published in the journal HemaSphere exclusively reflect the opinions of the authors. The authors are responsible for all content in their abstracts including accuracy of the facts, statements, citing resources, etc.

### (PB3153) THE IMPROVEMENT OF RENAL FUNCTION BY COMPLEMENT INHIBITORS IN PATIENTS WITH PAROXYSMAL NOCTURNAL HEMOGLOBINURIA

**Topic:** 12. Bone marrow failure syndromes incl. PNH - Clinical

Chengtao Qin[1], Ting Zhang[2], Yawen Zhang[1], Yuemin Gong[1], Guangsheng He*[1]

*[1]The First Affiliated Hospital with Nanjing Medical University, Nanjing, China;[2]Nanjing Jiangning Hospital, affiliated to Nanjing Medical University, Nanjing, China;*

### Background

Patients with paroxysmal nocturnal hemoglobinuria (PNH) experience chronic intravascular hemolysis due to the deficiency of complement regulatory proteins. The released free hemoglobin induces kidney injury through hemosiderin deposition, inflammation, and nitric oxide (NO) depletion, which inducing renal failure being one of the leading causes of mortality. Some researchers have reported that eculizumab can effectively improve renal function in patients with PNH. However, there have been no reports on the impact of iptacopan on renal function in patients with PNH.

### Aims

This study presents the real world's comparative analysis of iptacopan and eculizumab in improving renal function in PNH patients (ChiCTR2500112964).

### Methods

A prospective observational registry including 18 PNH patients treated with iptacopan and 43 patients treated with eculizumab was coducted from September 2024 to January 2026. The staging of chronic kidney disease (CKD) is used to assess the severity of a patient's renal insufficiency or impairment.

### Results

Among the 61 PNH patients we followed up, renal impairment at baseline was observed in 14 out of 18 (77.8%) patients treated with iptacopan (including 6 patients with CKD stage 2–3 and 8 with CKD stage 1) and in 20 out of 43 (46.5%) patients receiving eculizumab (including 2 with CKD stage 4, 5 with CKD stage 3, 5 with CKD stage 2, and 8 with CKD stage 1).

After a median treatment duration of 9 months (range: 2–16 months) with iptacopan, renal function improved in 10 patients. Notably, 5 out of 6 (83.3%) patients with CKD stage 2–3 showed improvement to CKD stage 1 (median eGFR increase: 9.15 mL/min/1.73 m²), while 1 patient with CKD stage 1 achieved normal renal function. Similarly, following a median treatment duration of 6 months (range: 1–12 months) with eculizumab, 4 patients showed renal function improvement (median eGFR increase: 24.5 mL/min/1.73 m²), whereas 2 experienced deterioration (median eGFR decrease: 17.25 mL/min/1.73 m²).

Kaplan-Meier curve analysis was performed on patients with renal impairment at baseline to assess cumulative response rates. After 8 months of treatment, the cumulative response rate was 29.7% in the iptacopan group compared to 27.5% in the eculizumab group. By months 12, the cumulative response rate had risen to 66.2% for iptacopan-treated patients versus 63.7% for those receiving eculizumab (Figure 1).

**Copyright Information:** (Online) ISSN: 2572-9241
©2026 The Author(s). HemaSphere is published by John Wiley & Sons Ltd on behalf of European Hematology Association. This is an open access Abstract Book distributed under the Attribution-NonCommercial-NoDerivs (CC BY-NC-ND), which allows third parties to download the articles and share them with others as long as they credit the author and the Abstract Book, but they cannot change the content in any way or use them commercially.
**Abstract Book Citations:** Authors, Title, HemaSphere, 2026;10:(S1):pages. The abstract book DOIs can be found on the "Author Information" page.
**Disclaimer:** Articles published in the journal HemaSphere exclusively reflect the opinions of the authors. The authors are responsible for all content in their abstracts including accuracy of the facts, statements, citing resources, etc.

### Summary/Conclusion

Both eculizumab and iptacopan have been shown to improve renal function in patients with PNH.

Figure 1 K-M curve of the cumulative remission rate in patients with baseline renal impairment

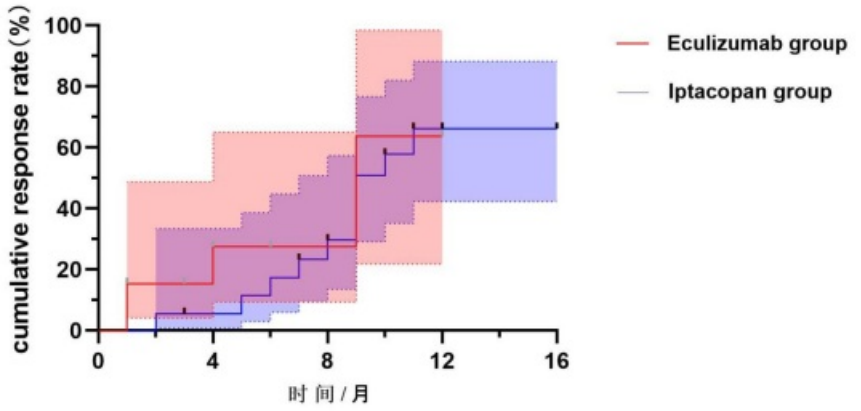

**Copyright Information:** (Online) ISSN: 2572-9241
©2026 The Author(s). HemaSphere is published by John Wiley & Sons Ltd on behalf of European Hematology Association. This is an open access Abstract Book distributed under the Attribution-NonCommercial-NoDerivs (CC BY-NC-ND), which allows third parties to download the articles and share them with others as long as they credit the author and the Abstract Book, but they cannot change the content in any way or use them commercially.
**Abstract Book Citations:** Authors, Title, HemaSphere, 2026;10:(S1):pages. The abstract book DOIs can be found on the "Author Information" page.
**Disclaimer:** Articles published in the journal HemaSphere exclusively reflect the opinions of the authors. The authors are responsible for all content in their abstracts including accuracy of the facts, statements, citing resources, etc.

### (PB3154) ACQUIRED APLASTIC ANAEMIA: CLINICO-HAEMATOLOGICAL FEATURES, MANAGEMENT AND LONG TERM OUTCOMES IN PATIENT INELIGIBLE FOR ALLOGENEIC TRANSPLANT

**Topic:** 12. Bone marrow failure syndromes incl. PNH - Clinical

Nabila Rekab*[1], Salima Oukid[1], Fatima Lamraoui[1], Hamida Brahimi[1], Khadoudj Mounira Benlabiod[1], Souad Taoussi[1], Yamina Bouchakour Moussa[1], Zineb Ferdi[1], Guezlane Sherifa[1], Mohamed Mezroud[1], Bradai Mohamed[1]

[1]*Université Saad Dahlab de Blida, Hematology, Ouled Yaich, Algeria;*

### Background

Idiopathic acquired aplastic anemia (AAA) is a rare bone marrow failure disorder.In patients who are not eligible for allogeneic hematopoietic stem cell transplantation (HSCT), immunosuppressive therapy (IST) with anti-thymocyte globulin (ATG) combined with cyclosporine A (CSA) remains the standard first-line treatment.Although survival and response rates have improved significantly over recent decades,long-term complications—including relapse,clonal evolution,and late treatment-related adverse events—continue to represent major clinical challenges.

### Aims

This study aimed to describe the clinical characteristicstreatment patterns,and outcomes of adult patients with AAA who were ineligible for HSCT (aged >40 years or lacking an HLA-matched donor) and treated with ATG plus CSA or CSA alone.

### Methods

We conducted a retrospective study including patients diagnosed with AAA between January 2013 and December 2024.The diagnosis was confirmed by bone marrow biopsy. All patients received supportive care,including blood product transfusions and antibiotic therapy,in addition to specific treatment.
Treatment strategy was determined according to disease severity, based on Camitta criteria.In our setting, immunosuppressive therapy (IST) consisted of oral CSA at 5 mg/kg/day from day 1 for a minimum duration of 12 months,administered either alone or in combination with rATG at 3.5 mg/kg/day for 5 consecutive days.CSA dosing was adjusted to maintain trough whole-blood levels between 150 and 250 ng/mL.
Hematologic response was assessed at 3, 6, 9,and 12 months,and at the end of treatment.Follow-up evaluations included complete blood counts,bone marrow examination, and cytogenetic analysis to detect potential clonal evolution, particularly progression to myelodysplastic syndrome (MDS).

**Copyright Information:** (Online) ISSN: 2572-9241
©2026 The Author(s). HemaSphere is published by John Wiley & Sons Ltd on behalf of European Hematology Association. This is an open access Abstract Book distributed under the Attribution-NonCommercial-NoDerivs (CC BY-NC-ND), which allows third parties to download the articles and share them with others as long as they credit the author and the Abstract Book, but they cannot change the content in any way or use them commercially.
**Abstract Book Citations:** Authors, Title, HemaSphere, 2026;10:(S1):pages. The abstract book DOIs can be found on the “Author Information” page.
**Disclaimer:** Articles published in the journal HemaSphere exclusively reflect the opinions of the authors. The authors are responsible for all content in their abstracts including accuracy of the facts, statements, citing resources, etc.

**Results**

A total of 45 patients were included, with a median age of 36 years (range,16–70) and a male-to-female ratio of 1.36. Anemia was the most common presenting feature, observed in 86% of patients. According to Camitta criteria, 16 patients (35%) had moderate AAA, while 29 (64%) had severe or very severe disease.
AA was idiopathic in 40 patients (88%),and associated with paroxysmal nocturnal hemoglobinuria (PNH) in 5 cases (11%).Five patients received rATG plus CSA,while 40 patients (88.8%) were treated with CSA alone.
The median follow-up duration was: 690 days (range 23- 3540)
In rATG plus CSA group, partial response (PR) was achieved in 2/5 patients at 3, 6,and 12 months. In the CSA alone group, at 3 months, responses were documented in 11 of 37 evaluable patients including 9 PR and 2 complete responses [CR]. At 6 months, 20 of 34 evaluable patients achieved a response (17 PR and 3 CR). At 12 months,18 of 31 evaluable patients responded, comprising 11CR (35%) and 7PR (22.5%),corresponding to an overall response rate of 58%.
During the follow up, relapse occurred in six patients, and nine patients developed a PNH clone.Five patients subsequently underwent allogeneic transplantation due to relapse (n=1), emergence of monosomy 7 (n=1), progression to MDS (n=2) or transformation to acute leukemia (n=1).The 2 year OS rate was 70.8%.

**Summary/Conclusion**

Immunosuppressive therapy significantly improves survival in idiopathic aplastic anemia; however, long-term complications, including relapse and clonal evolution, remain important challenges.Careful long-term monitoring is essential to optimize management and improve patient outcomes.The add of thrombopoietin receptor agonists in treatment recently will improve our results.

**Copyright Information:** (Online) ISSN: 2572-9241
©2026 The Author(s). HemaSphere is published by John Wiley & Sons Ltd on behalf of European Hematology Association. This is an open access Abstract Book distributed under the Attribution-NonCommercial-NoDerivs (CC BY-NC-ND), which allows third parties to download the articles and share them with others as long as they credit the author and the Abstract Book, but they cannot change the content in any way or use them commercially.
**Abstract Book Citations:** Authors, Title, HemaSphere, 2026;10:(S1):pages. The abstract book DOIs can be found on the “Author Information” page.
**Disclaimer:** Articles published in the journal HemaSphere exclusively reflect the opinions of the authors. The authors are responsible for all content in their abstracts including accuracy of the facts, statements, citing resources, etc.

### (PB3155) SHORT- AND LONG-TERM OUTCOMES OF EQUINE ANTITHYMOCYTE GLOBULIN-BASED DOUBLE AND TRIPLE IMMUNOSUPPRESSIVE THERAPY IN APLASTIC ANEMIA: A SYSTEMATIC REVIEW

**Topic:** 12. Bone marrow failure syndromes incl. PNH - Clinical

Austin G. Kulasekararaj*[1], Raj Gokani[2], Mark Latymer[2], Kevin Kallmes[3], Stephanie MacLeod[4], Frank Ernst[2], Régis Peffault de Latour[5]

*[1]King's College Hospital, National Institute for Health Research and Wellcome King's Clinical Research Facility and King's College London, London, UK, London, United Kingdom; [2]Pfizer Inc., New York, NY, United States of America;[3]Nested Knowledge, St Paul, MN, United States of America; [4]Value & Access, RTI Health Solutions, Durham, United States of America; [5]Assistance Publique- Hôpitaux de Paris, Saint-Louis Hospital, Paris, France;*

### Background

Acquired aplastic anemia (AA) is a rare, life-threatening bone marrow failure syndrome treated with immunosuppressive therapy (IST) or hematopoietic stem-cell transplantation (HSCT). Patients ineligible for HSCT are treated with IST, equine antithymocyte globulin (eATG) and cyclosporine. Eltrombopag was initially introduced as salvage therapy in refractory AA and was evaluated in combination with eATG-based IST, leading to its incorporation in upfront triple therapy (3T) in recent studies. While short-term outcomes have been reported, long-term outcome data remain limited and inconsistent.

### Aims

To systematically review short- and long-term efficacy outcomes of eATG-based double therapy (2T) and 3T in AA, focusing on overall survival (OS) and overall response rate (ORR) across age and disease-severity subgroups.

### Methods

An artificial intelligence (AI)-assisted SLR with validated human-in-the-loop tools was performed: search was conducted in Embase and PubMed/Medline (January 1980–November 2025). Randomized controlled trials (RCTs), non-interventional, and observational studies of 2T or 3T with equine or unspecified ATG were evaluated, with expert-adjudicated dual screening and human oversight in extraction of outcomes by therapy and subgroup. Key outcomes were OS and ORR, with subgroup sub-analyses by age, disease severity and forthcoming assessments on relapse and safety.

### Results

Of 2,360 unique records screened, 89 studies (11 RCTs [n=1,300], 11 non-interventional [n=118], 16 prospective cohort [n=1,822], 55 other observational [n=8,964], and 4 case series [n=57) were included. Results for OS and ORR at <5-yr and 5-yr timepoints were as follows:

**Table: OS and ORR by Therapy, Severity, and Age**

**Copyright Information:** (Online) ISSN: 2572-9241
©2026 The Author(s). HemaSphere is published by John Wiley & Sons Ltd on behalf of European Hematology Association. This is an open access Abstract Book distributed under the Attribution-NonCommercial-NoDerivs (CC BY-NC-ND), which allows third parties to download the articles and share them with others as long as they credit the author and the Abstract Book, but they cannot change the content in any way or use them commercially.
**Abstract Book Citations:** Authors, Title, HemaSphere, 2026;10:(S1):pages. The abstract book DOIs can be found on the "Author Information" page.
**Disclaimer:** Articles published in the journal HemaSphere exclusively reflect the opinions of the authors. The authors are responsible for all content in their abstracts including accuracy of the facts, statements, citing resources, etc.

| | Short-term <5-yrs | Long-term ≥5-yrs | Short-term <5-yrs |
|---|---|---|---|
| | **OS** | | **ORR** |
| **2T** | 2649/2966, 89.3% | 3290/3989, 82.5% | 4029/6318, 63.8% |
| Non-severe (NSAA) | 82/92, 89.1% | 253/269, 94.1% | 124/176, 70.5% |
| Severe (SAA) | 688/773, 89.0% | 869/1017, 85.4% | 1374/2098, 65.5% |
| SAA or very Severe (vSAA) | 1055/1170, 90.2% | 1068/1243, 85.9% | 596/1988, 30.0% |
| Pediatric | 1196/1300, 92.0% | 1725/1901, 90.7% | 1621/2581, 62.8% |
| Adults | 1453/1666, 87.2% | 1565/2088, 75.0% | 2408/3737, 64.4% |
| **3T** | 526/582, 90.4% | -- | 608/821, 74.1% |
| SAA/vSAA | 290/322, 90.1% | -- | 267/371, 72.0% |
| Pediatric | 90/98, 91.8% | -- | 83/112, 74.1% |
| Adults | 436/484, 90.1% | -- | 525/709, 74.0% |

OS was generally reported at one or fewer timepoints, and heterogenous, retrospective designs further limited both OS and ORR reporting. A substantial number of studies had unclear grouping of SAA vs. vSAA patients. There were no significant variations in outcomes by study type or for results of studies after 2017 (earliest published 3T) versus pre-2017.
ORR was higher with 3T than 2T, though across variable timepoints and study designs. Responses were lowest in patients with SAA and especially SAA/VSAA treated with 2T. Outcomes did not differ by study design or publication period.

### Summary/Conclusion

Both 2T and 3T IST were associated with high OS, with a trend toward higher ORR for 3T. Children demonstrated higher OS than adults, particularly those aged ≥5 yrs, while ORR was comparable across age groups. ORR was lower in SAA and VSAA treated with 2T. Inconsistent long-term outcome reporting, with many studies providing only one timepoint, limits longitudinal assessment and adds heterogeneity. More complete, longitudinal reporting by therapy and clinically relevant subgroups is needed to enable robust comparative assessment and inform optimal treatment strategies in AA.

**Copyright Information:** (Online) ISSN: 2572-9241
©2026 The Author(s). HemaSphere is published by John Wiley & Sons Ltd on behalf of European Hematology Association. This is an open access Abstract Book distributed under the Attribution-NonCommercial-NoDerivs (CC BY-NC-ND), which allows third parties to download the articles and share them with others as long as they credit the author and the Abstract Book, but they cannot change the content in any way or use them commercially.
**Abstract Book Citations:** Authors, Title, HemaSphere, 2026;10:(S1):pages. The abstract book DOIs can be found on the “Author Information” page.
**Disclaimer:** Articles published in the journal HemaSphere exclusively reflect the opinions of the authors. The authors are responsible for all content in their abstracts including accuracy of the facts, statements, citing resources, etc.

### (PB3156) A PIVOTAL PHASE 3 STUDY TO INVESTIGATE EFFICACY, SAFETY, AND TOLERABILITY OF MAVORIXAFOR IN PARTICIPANTS WITH PRIMARY CHRONIC NEUTROPENIA – TRIAL UPDATE

**Topic:** 12. Bone marrow failure syndromes incl. PNH - Clinical

Deb Steiner*[1], Katrina Adell[1], Susan Dubuc[1], Shelby Abel[1], Xiaoxi Li[1]

[1]*X4 Pharmaceuticals, Boston, United States of America;*

### Background

Primary chronic neutropenia (CN) is a rare immunodeficiency disorder characterized by absolute neutrophil count (ANC) <1500 cells/μL persisting for ≥3 months and associated with increased susceptibility to infections, which can be life threatening. The current standard of care for severe CN (ANC <500 cells/μL), injectable granulocyte colony-stimulating factor (G-CSF), is associated with side effects including bone pain, splenomegaly, thrombocytopenia, glomerulonephritis, vasculitis, and osteoporosis and at high doses is correlated with increased risks of myelodysplastic syndromes and leukemia in patients with congenital neutropenia.[1,2]
Treatment options for primary CN that provide the opportunity to limit or eliminate G-CSF use represent an unmet medical need. Mavorixafor, an oral C-X-C chemokine receptor 4 (CXCR4) antagonist that can increase circulating neutrophils and has the potential to reduce infection frequency, severity, and duration in patients with CN, is approved in the United States for the treatment of WHIM (warts, hypogammaglobulinemia, infections, and myelokathexis) syndrome. The current study aims to evaluate the efficacy, safety, and tolerability of mavorixafor in participants with primary CN experiencing recurrent and/or serious infections.

**Study Design and Methods**

The 52-week, Phase 3, randomized, double-blind, placebo-controlled, multicenter, global study will enroll approximately 176 participants aged ≥12 years with congenital or acquired primary autoimmune or idiopathic CN and ANC <1000 cells/μL at baseline. Participants must have had ≥2 recurrent and/or serious infections, considered to be likely related to their CN disorder, in the last 12 months.
Patients with 1 of 3 primary CN types (congenital, acquired idiopathic [CIN], and acquired autoimmune [AIN]) are being enrolled. Congenital neutropenia participants eligible for participation include, but are not limited to, those with: ELANE, HAX1, G6PC3, CSF3R, SRP54, CXCR2, WAS genetic mutations and Barth syndrome, Cohen syndrome, Glycogen storage disease type 1b, and Shwachman-Diamond syndrome. Excluded diagnoses include secondary neutropenia, aplastic anemia, WHIM syndrome, CN with cyclic presentation, and CN associated with immune dysregulation (e.g., CVID, ALPS, GATA2) or bone marrow failure (e.g., Fanconi anemia, Diamond-Blackfan anemia).
Participants are eligible regardless of current background CN therapy regimen. Approximately 40% of participants are expected to be receiving chronic G-CSF and must be willing to maintain a stable therapy regimen throughout the study.
Participants will be randomized 1:1 to receive placebo or mavorixafor orally once daily for 52 weeks, stratified by CN type (congenital or AIN/CIN) and use of G-CSF as background CN therapy. The co-primary endpoints are annualized infection rate and proportion of ANC responders (increase >500 cells/μL from baseline). Key secondary endpoints include incidence of oral ulcers and evaluation of quality of life using the PROMIS SF Fatigue Questionnaire. Participants can be offered the option of home health or telemedicine study visits. This pivotal study (EU CT Number: 2023-508482-32-00; ClinicalTrials.gov ID: NCT06056297), initiated in 2024, has >100 study sites activated globally and continues to enroll participants, with many sites accepting patient referrals.

### References

1. Fioredda, et al. *Hemasphere*. Apr 2025;9(4):e70113.
2. Parisi, et al. *J Clin Pathol*. Aug 16 2024;77(9):586-604.

**Copyright Information:** (Online) ISSN: 2572-9241
©2026 The Author(s). HemaSphere is published by John Wiley & Sons Ltd on behalf of European Hematology Association. This is an open access Abstract Book distributed under the Attribution-NonCommercial-NoDerivs (CC BY-NC-ND), which allows third parties to download the articles and share them with others as long as they credit the author and the Abstract Book, but they cannot change the content in any way or use them commercially.
**Abstract Book Citations:** Authors, Title, HemaSphere, 2026;10:(S1):pages. The abstract book DOIs can be found on the "Author Information" page.
**Disclaimer:** Articles published in the journal HemaSphere exclusively reflect the opinions of the authors. The authors are responsible for all content in their abstracts including accuracy of the facts, statements, citing resources, etc.

### (PB3157) PERSISTENT ANEMIA FOLLOWING ECULIZUMAB IN PATIENTS WITH PAROXYSMAL NOCTURNAL HEMOGLOBINURIA

**Topic:** 12. Bone marrow failure syndromes incl. PNH - Clinical

Ting Zhang[1], Chengtao Qin[2], Yuemin Gong[3], Yawen Zhang[2], Jinning Shi[1], Guangsheng He*[2]

*[1]Nanjing Jiangning Hospital, affiliated to Nanjing Medical University, Nanjing, China; [2]The First Affiliated Hospital of Nanjing Medical University, Jiangsu Province Hospital, Nanjing, Jiangsu, China, Nanjing, China; [3]The First Affiliated Hospital of Nanjing Medical University, Jiangsu Province Hospital, Nanjing, Jiangsu, China, Jiangsu Province Engineering Research Center of Cell and Gene Therapy for hematologic and lymphoid Diseases, Nanjing, China;*

### Background

Paroxysmal Nocturnal Hemoglobinuria (PNH) is a rare hemolytic disease. The use of complement C5 inhibitor has improved clinical symptoms and enhanced the long-term survival rate. However, nearly 40% of the patients are still anemic and require red blood cell transfusion.

### Aims

To investigate the factors contributing to persistent anemia in Chinese patients with PNH with high disease-activity (HDA) undergoing eculizumab therapy.

### Methods

A prospective registry-based study was conducted on 52 PNH patients with HDA treated for at least 6 months from December 2023 to March 2025, aiming to analyze the reasons for persistent anemia in patients despite eculizumab treatment.

### Results

Among 52 patients, 3 patient achieved hemoglobin (Hb) ≥ 120 g/L, 15 patients maintained Hb >100 g/L, and 34 patients exhibited Hb <100 g/L. After 6 months of eculizumab treatment, Hb >100 g/L was achieved in 27% (4/15) of elevated LDH patients vs 71% (15/21) of normal LDH patients (27% vs 71%, p=0.02) (Figure 1a). An evaluation of PNH clone size was conducted on 35 patients. Among them, 28 had a difference of more than 10% in PNH clones between red blood cells and neutrophils, and 7 had a difference of less than 10%. Only 43% of these 28 patients had Hb levels above 100 g/L, while 7 patients all had Hb levels above 100 g/L (43% vs 100%, p=0.02) (Figure 1b).

Among the 27 patients with concomitant AA (52%), the proportion of individuals with hemoglobin levels below 100 g/L was lower than that in patients with PNH alone (28% vs 65% p=0.02). After additional treatment with cyclosporine, hemoglobin levels improved in 15 cases (56%). Anti-C3d positivity via direct antiglobulin test (DAT) was detected in 19 patients (19/52, 37%), with median onset time of 20 weeks (2-40). Nine patients demonstrated C3d titers ≥ 1:64, All patients with C3d titer positive 1:64 were switched to iptacopan therapy, with Hb levels increasing to above 100 g/L.

Three patients experienced breakthrough hemolysis (BTH), all occurring after 32 (15-91) days of delayed eculizumab administration.

### Summary/Conclusion

Persistent anemia after eculizumab treatment in PNH is closely related to the presence of residual intravascular hemolysis, concomitant aplastic anemia, extravascular hemolysis, and breakthrough hemolysis.

**Copyright Information:** (Online) ISSN: 2572-9241
©2026 The Author(s). HemaSphere is published by John Wiley & Sons Ltd on behalf of European Hematology Association. This is an open access Abstract Book distributed under the Attribution-NonCommercial-NoDerivs (CC BY-NC-ND), which allows third parties to download the articles and share them with others as long as they credit the author and the Abstract Book, but they cannot change the content in any way or use them commercially.
**Abstract Book Citations:** Authors, Title, HemaSphere, 2026;10:(S1):pages. The abstract book DOIs can be found on the “Author Information” page.
**Disclaimer:** Articles published in the journal HemaSphere exclusively reflect the opinions of the authors. The authors are responsible for all content in their abstracts including accuracy of the facts, statements, citing resources, etc.

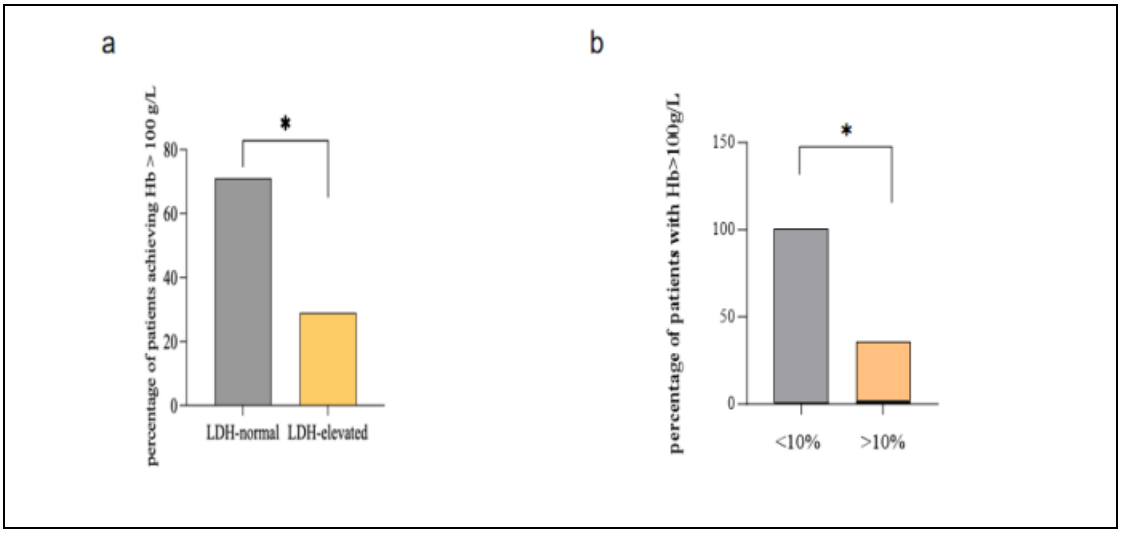

Figure1 Hb >100 g/L was achieved in 27%(4/15) of elevated LDH patients vs 71% (15/21) of normal LDH patients (27% vs 71%, p=0.02) (Figure 1a). With a difference of more than 10% in PNH clones size between red blood cells and neutrophils, 43% of 28 patients had Hb levels above 100 g/L, wihle all of 7 patients who with a difference of less than 10%, had Hb levels above 100 g/L (43% vs 100%, p=0.02) (Figure 1b).

**Copyright Information:** (Online) ISSN: 2572-9241
©2026 The Author(s). HemaSphere is published by John Wiley & Sons Ltd on behalf of European Hematology Association. This is an open access Abstract Book distributed under the Attribution-NonCommercial-NoDerivs (CC BY-NC-ND), which allows third parties to download the articles and share them with others as long as they credit the author and the Abstract Book, but they cannot change the content in any way or use them commercially.
**Abstract Book Citations:** Authors, Title, HemaSphere, 2026;10:(S1):pages. The abstract book DOIs can be found on the “Author Information” page.
**Disclaimer:** Articles published in the journal HemaSphere exclusively reflect the opinions of the authors. The authors are responsible for all content in their abstracts including accuracy of the facts, statements, citing resources, etc.

### (PB3158) ACQUIRED AMEGAKARYOCYTIC THROMBOCYTOPENIA AS A PRECURSOR TO MARROW FAILURE AND CLONAL DISEASE: A SCOPING REVIEW OF PROGRESSION CASES

**Topic:** 12. Bone marrow failure syndromes incl. PNH - Clinical

Areej Saad[1], Mohammed W H[1], Deena Mudawi[2], Motaz Almahmood[3], Duha Mohmmad[4], Bidoor Alabbas[5], Esra Sharief[6], Shahd Bota[1], Marrita Rabadi[7], Abdulrahman F. Al-Mashdali[2], Mohammed Mohammed[2], Samah Kohla[8], Shehab Fareed*[2]

*[1]College of medicine – Sudan International University, Khartoum, Sudan;[2]Hamad Medical Corporation, Hematology Department, NCCCR, Doha, Qatar; [3]Tower Health Phoenixville Hospital, Internal Medicine, Phoenixville, United States of America;[4]College of medicine – Sudan International University, Khartoum, South Sudan; [5]Saudi Commission for health specialities, Riyadh, Saudi Arabia; [6]College of medicine – Sudan International University, Khartoum, Sudan, Khartoum, Sudan;[7]College of Medicine - Qatar University, Doha, Qatar; [8]Hamad Medical Corporation, Department of laboratory Medicine and pathology, Hematology Division, Doha, Qatar;*

### Background

Acquired amegakaryocytic thrombocytopenia (AAMT) is a rare marrow disorder marked by severe thrombocytopenia with absent or markedly reduced megakaryocytes. Early presentations may resemble immune thrombocytopenia, which can delay recognition of evolving marrow failure or clonal disease.

### Aims

To summarize published cases of acquired AAMT that subsequently progressed to aplastic anemia (AA), pure red cell aplasia (PRCA), myelodysplastic syndrome (MDS), acute myeloid leukemia (AML), or large granular lymphocytic (LGL) leukemia, and to describe diagnostic features, treatment patterns, and outcomes

### Methods

A scoping review was conducted in accordance with PRISMA-ScR guidance. PubMed, Google Scholar, the Cochrane Library, and Qatar National Library resources were searched for English-language human reports published from 1950 through December 2025. Eligible studies were case reports or case series describing acquired AAMT followed by progression to AA, PRCA, MDS, AML, or LGL leukemia. Two reviewers independently screened records and extracted study data. Treatment responses were categorized as complete response, partial response, or no response.

### Results

Twenty-three reports comprising 24 patients published between 1978 and 2023 met inclusion criteria. Median age was 49 years (range, 16-82), and the sex distribution was balanced. Bleeding manifestations were reported in 14/24 patients. After exclusion of extreme outliers, the median presenting platelet count was 5.0 x10^9/L. The most frequent progression phenotypes were AA (13/24) and PRCA (9/24), whereas MDS (3/24), AML (2/24), and LGL leukemia (2/24) were less common. Cytogenetic data were reported in 12/24 cases, with one abnormal result (del(5q)). Molecular testing was available in 9/24 cases and identified clonal or abnormal findings in 3. Initial management often followed immune thrombocytopenia practice, most commonly corticosteroids (15/24) and IVIG (8/24), but responses were limited (complete response, n=3; partial response, n=3; no response, n=17 among 23 evaluable patients). Cyclosporine was the most frequently used subsequent therapy, and hematopoietic stem cell transplantation was performed in 3 patients. At last follow-up, 14 patients were alive and 8 had died.

**Copyright Information:** (Online) ISSN: 2572-9241
©2026 The Author(s). HemaSphere is published by John Wiley & Sons Ltd on behalf of European Hematology Association. This is an open access Abstract Book distributed under the Attribution-NonCommercial-NoDerivs (CC BY-NC-ND), which allows third parties to download the articles and share them with others as long as they credit the author and the Abstract Book, but they cannot change the content in any way or use them commercially.
**Abstract Book Citations:** Authors, Title, HemaSphere, 2026;10:(S1):pages. The abstract book DOIs can be found on the "Author Information" page.
**Disclaimer:** Articles published in the journal HemaSphere exclusively reflect the opinions of the authors. The authors are responsible for all content in their abstracts including accuracy of the facts, statements, citing resources, etc.

**Summary/Conclusion**

In published progression cases, acquired AAMT most often evolved into immune marrow-failure phenotypes, particularly AA and PRCA, while clonal myeloid transformation was uncommon but documented. Severe thrombocytopenia that is refractory to immune thrombocytopenia-directed therapy should prompt early bone marrow evaluation and close longitudinal surveillance for multilineage cytopenias and clonal evolution. Prospective studies with standardized cytogenetic and molecular assessment are needed to improve risk stratification and treatment selection.

**Copyright Information:** (Online) ISSN: 2572-9241
©2026 The Author(s). HemaSphere is published by John Wiley & Sons Ltd on behalf of European Hematology Association. This is an open access Abstract Book distributed under the Attribution-NonCommercial-NoDerivs (CC BY-NC-ND), which allows third parties to download the articles and share them with others as long as they credit the author and the Abstract Book, but they cannot change the content in any way or use them commercially.
**Abstract Book Citations:** Authors, Title, HemaSphere, 2026;10:(S1):pages. The abstract book DOIs can be found on the "Author Information" page.
**Disclaimer:** Articles published in the journal HemaSphere exclusively reflect the opinions of the authors. The authors are responsible for all content in their abstracts including accuracy of the facts, statements, citing resources, etc.

### (PB3159) DISSEMINATED KAPOSI SARCOMA COMPLICATING UNTREATED FANCONI ANEMIA–ASSOCIATED VERY SEVERE APLASTIC ANEMIA

**Topic:** 12. Bone marrow failure syndromes incl. PNH - Clinical

Rihab Ben Sghaier*[1], Firas Cherif[1], Nour Gaaloul[1], Monia Guermazi[1], Yasmine Ben Romdhane[2], Monia Zaier[1], Kmira Zahra[1], Haifa Regaieg[1], Badreddine Sriha[2], Bouteraa Walid[1], Yosra Ben Youssef[1]

*[1]Farhat Hached University Hospital, Clinical Hematology Department, Sousse, Tunisia;[2]Pathology department ,Farhat Hached University Hospital, Pathology, Sousse, Tunisia;*

### Background

Fanconi anemia (FA) is an inherited bone marrow failure syndrome characterized by chromosomal instability and high cancer susceptibility. Kaposi sarcoma (KS), an HHV-8–driven vascular tumor, is exceptionally rare in non-HIV, non-transplant patients with untreated severe aplastic anemia (SAA). The coexistence of both conditions is extremely rare.

### Methods

We report a case of very severe aplastic anemia secondary to Fanconi anemia complicated by disseminated Kaposi sarcoma, highlighting the diagnostic complexity, therapeutic limitations, and aggressive clinical course associated with this unusual combination.

### Results

A 25-year-old male was admitted with fever and severe hemorrhagic manifestations. Initial laboratory evaluation revealed profound pancytopenia (ANC <200/µL, platelets 16,000/µL, hemoglobin 3.7 g/dL). Bone marrow biopsy demonstrated marked hypocellularity (<25%) without dysplasia, excess blasts, fibrosis, or infiltrative disease. These findings fulfilled the criteria for very severe aplastic anemia. An etiological workup was subsequently performed: Cytogenetic analysis showed a normal karyotype, flow cytometry did not detect a PNH clone, viral serologies were negative, chromosomal breakage testing established the diagnosis of Fanconi anemia, despite absence of phenotypic abnormalities. HLA typing identified a matched sibling donor.
During hospitalization, the patient developed multiple violaceous cutaneous nodules. Skin biopsy demonstrated Kaposi sarcoma with CD34 and HHV-8 immunohistochemical positivity (Figure 1).
HHV-8 serology was negative. Thoracic imaging revealed multiple bilateral pulmonary nodules consistent with disseminated KS. Immunosuppressive therapy was contraindicated due to FA. Hematopoietic stem cell transplantation, the only curative option for FA-related SAA, was precluded by active disseminated KS. Systemic chemotherapy for KS was not feasible because of profound marrow aplasia.
Management was limited to supportive transfusion therapy. The clinical course was complicated by massive gastrointestinal bleeding leading to fatal hemorrhagic shock.

### Summary/Conclusion

This case illustrates a unique therapeutic impasse in which inherited marrow failure and aggressive HHV8-driven malignancy mutually exclude curative options. To our knowledge, this is the first reported case of disseminated KS complicating untreated FA-related very severe aplastic anemia. It underscores the vulnerability of FA patients to oncogenic viral proliferation even prior to therapeutic immunosuppression.

**Copyright Information:** (Online) ISSN: 2572-9241
©2026 The Author(s). HemaSphere is published by John Wiley & Sons Ltd on behalf of European Hematology Association. This is an open access Abstract Book distributed under the Attribution-NonCommercial-NoDerivs (CC BY-NC-ND), which allows third parties to download the articles and share them with others as long as they credit the author and the Abstract Book, but they cannot change the content in any way or use them commercially.
**Abstract Book Citations:** Authors, Title, HemaSphere, 2026;10:(S1):pages. The abstract book DOIs can be found on the “Author Information” page.
**Disclaimer:** Articles published in the journal HemaSphere exclusively reflect the opinions of the authors. The authors are responsible for all content in their abstracts including accuracy of the facts, statements, citing resources, etc.

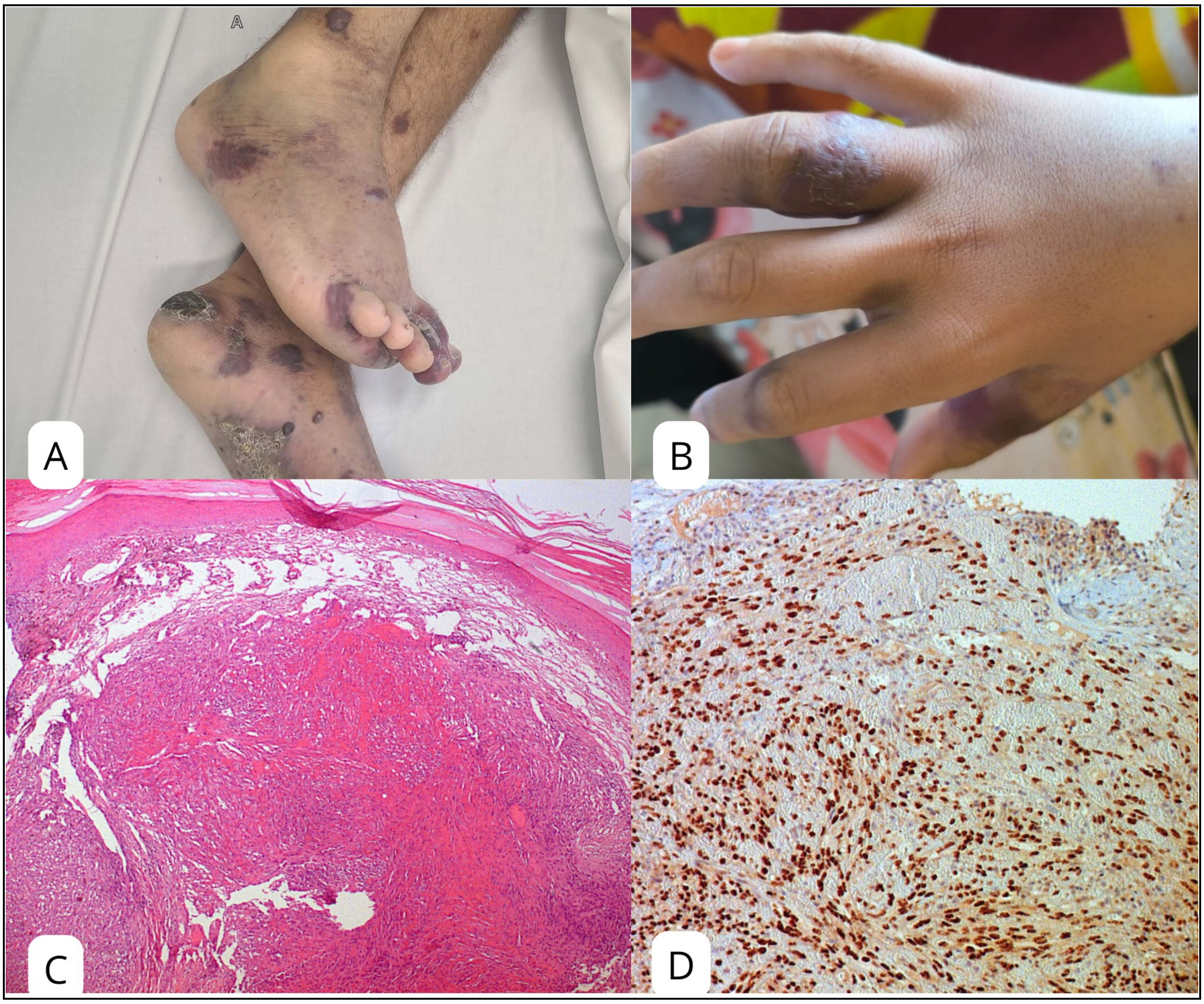

Figure 1. Clinicopathologic features of Kaposi sarcoma. (A, B) Multiple violaceous cutaneous nodules involving the hands and feet. (C) Low-power hematoxylin and eosin photomicrograph showing a dermal-based spindle-cell proliferation with intersecting fascicles and slit-like vascular spaces. (D) HHV-8 immunohistochemistry demonstrating diffuse nuclear positivity in tumor cells, confirming Kaposi Sarcoma.

**Copyright Information:** (Online) ISSN: 2572-9241
©2026 The Author(s). HemaSphere is published by John Wiley & Sons Ltd on behalf of European Hematology Association. This is an open access Abstract Book distributed under the Attribution-NonCommercial-NoDerivs (CC BY-NC-ND), which allows third parties to download the articles and share them with others as long as they credit the author and the Abstract Book, but they cannot change the content in any way or use them commercially.
**Abstract Book Citations:** Authors, Title, HemaSphere, 2026;10:(S1):pages. The abstract book DOIs can be found on the “Author Information” page.
**Disclaimer:** Articles published in the journal HemaSphere exclusively reflect the opinions of the authors. The authors are responsible for all content in their abstracts including accuracy of the facts, statements, citing resources, etc.

### (PB3160) NFKB1 INSUFFICIENCY PRESENTING AS CONGENITAL CYTOPENIAS

**Topic:** 12. Bone marrow failure syndromes incl. PNH - Clinical

Krishna Gundabolu*[1], Jeffrey Cannatella[2], Vijaya Bhatt[1]

[1]*University of Nebraska Medical Center, Internal Medicine - Division of Hematology Oncology, Omaha, United States of America;*
[2]*University of Nebraska Medical Center, Pathology, Microbiology, and Immunology, Omaha, United States of America;*

### Background

Cytopenias is a common reason for a hematology consultation. Around 70-80% of adults and 20% children with immune thrombocytopenia (ITP) have chronic ITP. Registry data show around 40-50% of chronic neutropenia and immune hemolytic anemia are autoimmune in nature (AIN & AIHA). A positive family history associated with chronic cytopenias often raise a concern for inherited bone marrow failure syndromes(IBFS), but other rare differentials remain a possibility.

### Aims

Outline a pedigree of family with chronic autoimmune cytopenias and diagnosed with NFKB1 associated common variable immunodeficiency (CVID).

### Methods

We identified a family with a strong history of thrombocytopenia and leucopenia. A 51-year-old woman with long standing history of cirrhosis and chronic ITP with multiple acute recurrences was referred for evaluation of pancytopenia. Quantitative immunoglobulin levels are normal. Workup was consistent with warm AIHA, AIN with severe neutropenia. Bone marrow biopsy showed normocellular marrow with left shifted myeloid precursors and unremarkable erythroid and megakaryocyte precursors without dyspoiesis. Karyotype was normal. Microarray analysis did not show copy number abnormalities or loss of heterozygosity. Based on family history, DNA extracted from buccal mucosa was sent for targeted exome sequencing analysis.

### Results

Next generation sequencing revealed autosomal dominant, pathogenic heterozygous frameshift variant NFKB1: c.376_377del; p.(V126Nfs*26). A similar variant was seen in multiple maternal relatives across generations. Heterozygous loss of function variants is described in Nuclear Factor-Kappa B and Activator Protein-1, a ubiquitous transcription factor shown to regulate inflammatory cytokines and adhesion molecules. Heterozygous loss of function variants has been associated with high frequency of autoimmunity, inflammation, lymphoproliferation, noninfectious enteritis, liver disease and malignancies.

### Summary/Conclusion

In the context of a strong family history with cytopenias and autoimmune disease, hereditary NFKB1 mutations should be ruled out. NFKB1 haploinsufficiency leads to a highly variable phenotype of primary immunodeficiency often presenting with multiple cytopenias. Congenital bone marrow failure syndromes often have hypoplastic bone marrow without the phenotype of CVID and autoimmune diseases, helping to distinguish between the diagnosis of IBFS and other rare possibility of hereditary NFKB1 mutation associated cytopenias.

**Copyright Information:** (Online) ISSN: 2572-9241
©2026 The Author(s). HemaSphere is published by John Wiley & Sons Ltd on behalf of European Hematology Association. This is an open access Abstract Book distributed under the Attribution-NonCommercial-NoDerivs (CC BY-NC-ND), which allows third parties to download the articles and share them with others as long as they credit the author and the Abstract Book, but they cannot change the content in any way or use them commercially.
**Abstract Book Citations:** Authors, Title, HemaSphere, 2026;10:(S1):pages. The abstract book DOIs can be found on the "Author Information" page.
**Disclaimer:** Articles published in the journal HemaSphere exclusively reflect the opinions of the authors. The authors are responsible for all content in their abstracts including accuracy of the facts, statements, citing resources, etc.

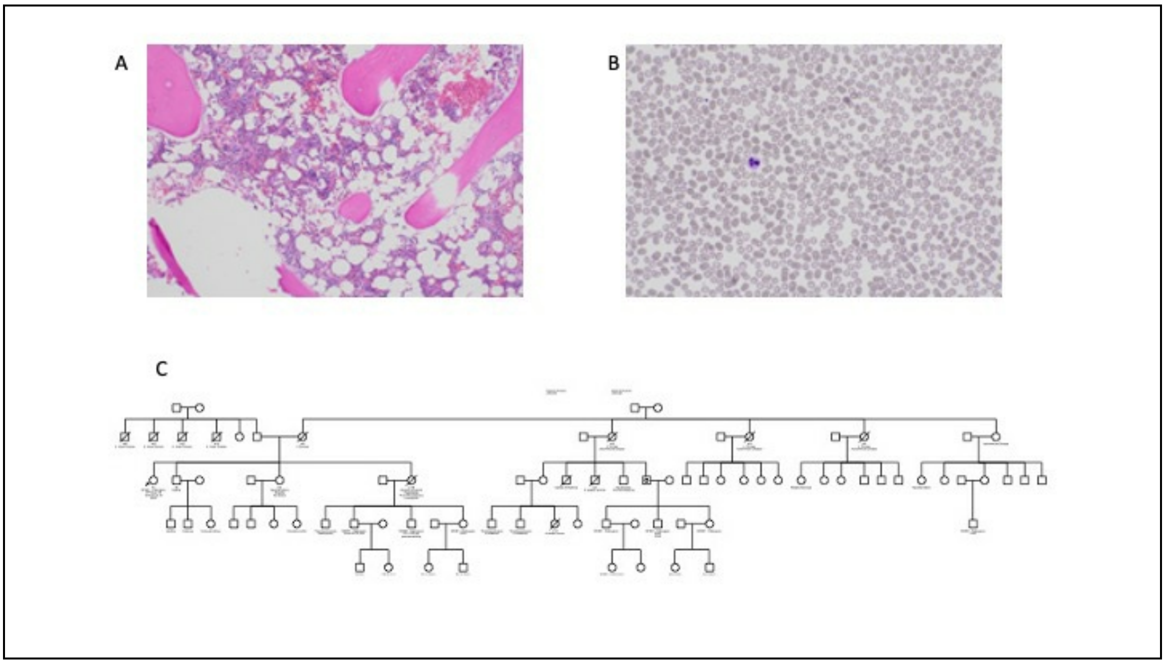

A)Bone marrow core biopsy (H&E stain, 100x magnification) showing normocellular marrow. No significant morphologic abnormality is seen. B)Peripheral blood smear (Wright stain, 400x magnification) showing neutropenia and thrombocytopenia without significant morphologic abnormality. C) Family Pedigree of multiple relatives with cytopenias and NFKB1 mutation

**Copyright Information:** (Online) ISSN: 2572-9241
©2026 The Author(s). HemaSphere is published by John Wiley & Sons Ltd on behalf of European Hematology Association. This is an open access Abstract Book distributed under the Attribution-NonCommercial-NoDerivs (CC BY-NC-ND), which allows third parties to download the articles and share them with others as long as they credit the author and the Abstract Book, but they cannot change the content in any way or use them commercially.
**Abstract Book Citations:** Authors, Title, HemaSphere, 2026;10:(S1):pages. The abstract book DOIs can be found on the "Author Information" page.
**Disclaimer:** Articles published in the journal HemaSphere exclusively reflect the opinions of the authors. The authors are responsible for all content in their abstracts including accuracy of the facts, statements, citing resources, etc.

### (PB3161) THE FIRST REPORTED CASE OF REVESZ SYNDROME IN SAUDI ARABIA: CLINICAL AND MOLECULAR CHARACTERIZATION

**Topic:** 12. Bone marrow failure syndromes incl. PNH - Clinical

Abrar Aljunaid*[1, 1], Rawan Harun[2], Gada Jadkarim[2], Muawiyah Qasem[2]

*[1]International Medical Center, Jeddah, Saudi Arabia;[2]international medical center, jeddah, Saudi Arabia;*

### Background

Revesz syndrome (RS) is a rare and severe variant of dyskeratosis congenita within the spectrum of telomere biology disorders. It is characterized by early-onset bone marrow failure, intracranial calcifications, exudative retinopathy, and markedly shortened telomeres. RS is most commonly associated with pathogenic mutations in *TINF2*, particularly in exon 6. Fewer than 20 genetically confirmed cases have been reported worldwide

### Aims

To describe a genetically confirmed case of RS and emphasize the importance of early genomic testing in infants presenting with pancytopenia and neurological abnormalities.

### Methods

Clinical data were obtained from multidisciplinary records. Laboratory evaluation included complete blood counts and bone marrow examination. Neuroimaging and ophthalmologic assessments were performed. Telomere length was measured using age-adjusted percentile comparison. Whole exome sequencing was conducted to identify pathogenic variants.

### Results

A male infant born preterm at 32 weeks' gestation presented in early infancy with progressive pancytopenia, severe neutropenia (absolute neutrophil count <200/μL), thrombocytopenia ($13 \times 10^9$/L), anemia (hemoglobin 7.5 g/dL), developmental delay, and failure to thrive. Bone marrow aspiration resulted in a dry tap. Neuroimaging showed bilateral thalamic and subcortical calcifications with obstructive hydrocephalus,(figure1).

Ophthalmologic evaluation revealed bilateral exudative retinopathy requiring laser photocoagulation and intravitreal bevacizumab. Telomere length was below the 1st percentile for age. Whole exome sequencing identified a heterozygous pathogenic *TINF2* variant, c.850A>C (p.Thr284Pro), in exon 6, confirming RS.

Despite transfusion support, ventricular tapping, endoscopic third ventriculostomy, and intensive care, the patient remained transfusion-dependent and developed Gram-negative sepsis with septic shock and disseminated intravascular coagulation, resulting in death.

### Summary/Conclusion

This case broadens the geographic spectrum of *TINF2*-associated RS and supports the link between exon 6 mutations and severe phenotype. Early genomic testing should be considered in infants with pancytopenia, intracranial calcifications, and exudative retinopathy to enable timely diagnosis and coordinated multidisciplinary management.

**Copyright Information:** (Online) ISSN: 2572-9241
©2026 The Author(s). HemaSphere is published by John Wiley & Sons Ltd on behalf of European Hematology Association. This is an open access Abstract Book distributed under the Attribution-NonCommercial-NoDerivs (CC BY-NC-ND), which allows third parties to download the articles and share them with others as long as they credit the author and the Abstract Book, but they cannot change the content in any way or use them commercially.
**Abstract Book Citations:** Authors, Title, HemaSphere, 2026;10:(S1):pages. The abstract book DOIs can be found on the "Author Information" page.
**Disclaimer:** Articles published in the journal HemaSphere exclusively reflect the opinions of the authors. The authors are responsible for all content in their abstracts including accuracy of the facts, statements, citing resources, etc.

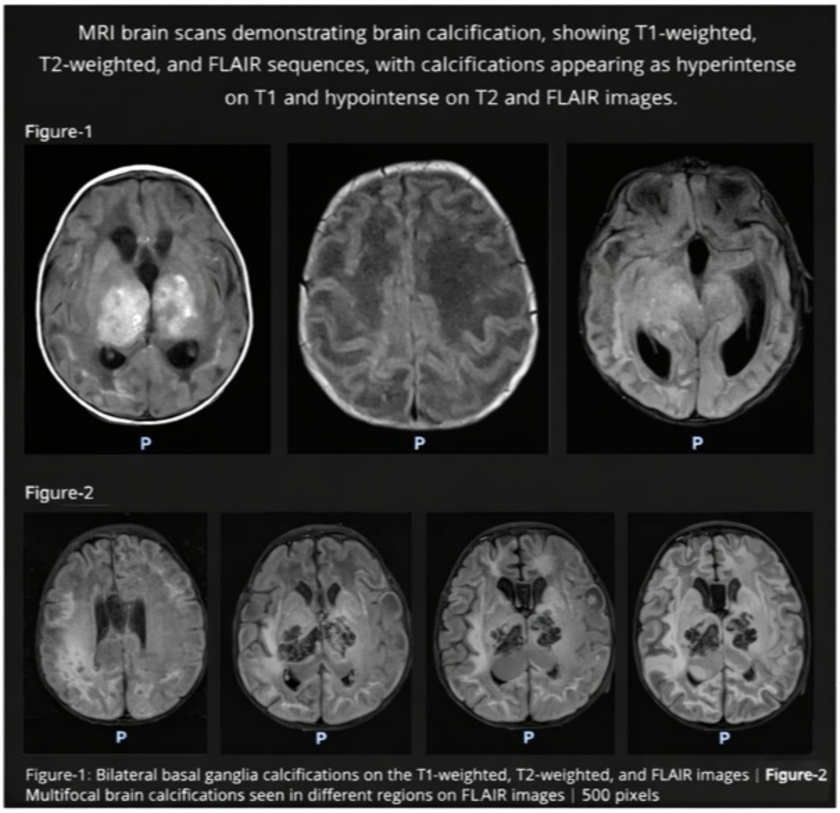

figure(1)

**Copyright Information:** (Online) ISSN: 2572-9241
©2026 The Author(s). HemaSphere is published by John Wiley & Sons Ltd on behalf of European Hematology Association. This is an open access Abstract Book distributed under the Attribution-NonCommercial-NoDerivs (CC BY-NC-ND), which allows third parties to download the articles and share them with others as long as they credit the author and the Abstract Book, but they cannot change the content in any way or use them commercially.
**Abstract Book Citations:** Authors, Title, HemaSphere, 2026;10:(S1):pages. The abstract book DOIs can be found on the "Author Information" page.
**Disclaimer:** Articles published in the journal HemaSphere exclusively reflect the opinions of the authors. The authors are responsible for all content in their abstracts including accuracy of the facts, statements, citing resources, etc.

### (PB3162) A SYSTEMATIC REVIEW OF REAL-WORLD EVIDENCE ON THERAPIES FOR MODERATE APLASTIC ANEMIA

**Topic:** 12. Bone marrow failure syndromes incl. PNH - Clinical

Britta Höchsmann*[1, 2], Marc Bierings[3], Frank Ernst[4], Raj Gokani[4], Morag Griffin[5], Kevin Kallmes[6], Stephanie MacLeod[7], Barbara Możejko-Pastewka[4], Sujith Samarasinghe[8], Carlo Dufour[9]

*[1]Institute of Transfusion Medicine, University of Ulm, Ulm, Ulm, Germany;[2]Institute of Clinical Transfusion Medicine and Immunogenetics, German Red Cross Blood Transfusion Service and University Hospital Ulm, Ulm, Germany; [3]Princess Maxima Center for Pediatric Oncology, Utrecht, The Netherlands; [4]Pfizer Inc., New York, NY, United States of America;[5]St James Hospital, Leeds Teaching Hospitals NHS Trust, Leeds, United Kingdom; [6]Nested Knowledge, St Paul, MN, United States of America; [7]Value & Access, RTI Health Solutions, Durham, United States of America;[8]Department of Haematology, Great Ormond Street Hospital, London, United Kingdom; [9]Hematology Unit, IRCCS G. Gaslini Children's Hospital Genova, Italy, Genova, Italy;*

### Background

Moderate aplastic anemia (mAA) is clinically heterogeneous with substantial variation in its definitions, and no universally accepted guidelines or therapies. Treatment choices include supportive care, monotherapy (cyclosporine [CSA], tacrolimus, daclizumab, thrombopoietin receptor agonists [TPO-RA], androgens), double or triple therapy, (combining horse or rabbit anti-thymocyte globulin [hATG or rATG], CSA/tacrolimus, and eltrombopag [EPAG]/TPO-RA or androgens), and/or hematopoietic stem-cell transplantation (HSCT). Comprehensive documentation of the treatment landscape and impact is limited.

### Aims

To inform evidence-based decision-making, we conducted a systematic literature review (SLR) of therapeutic outcomes in mAA.

### Methods

This artificial intelligence (AI)-assisted review followed a prespecified protocol. Study searches for any mAA therapy (adults and/or children) were conducted in Embase and PubMed/Medline (January 1980–November 2025). This SLR was assisted by validated human-in-the-loop AI review tools, with expert oversight. All searches, screening, extraction, and quality-control workflows were performed using AI and experts as independently adjudicated dual screeners and expert-curated extractions. Eligible study designs included randomized controlled trials (RCTs), observational, and non-randomized interventional studies; case reports were excluded. Key outcomes were overall survival (OS) and response rate (ORR).

**Copyright Information:** (Online) ISSN: 2572-9241
©2026 The Author(s). HemaSphere is published by John Wiley & Sons Ltd on behalf of European Hematology Association. This is an open access Abstract Book distributed under the Attribution-NonCommercial-NoDerivs (CC BY-NC-ND), which allows third parties to download the articles and share them with others as long as they credit the author and the Abstract Book, but they cannot change the content in any way or use them commercially.
**Abstract Book Citations:** Authors, Title, HemaSphere, 2026;10:(S1):pages. The abstract book DOIs can be found on the "Author Information" page.
**Disclaimer:** Articles published in the journal HemaSphere exclusively reflect the opinions of the authors. The authors are responsible for all content in their abstracts including accuracy of the facts, statements, citing resources, etc.

### Results

Of 2,539 unique records screened, 65 studies (5 RCT [n=374], 5 non-RCT single-arm [n=1,100], 14 prospective cohort [n=974], and 41 other observational [n=2,679]) reporting on patients with mAA were included. Studies spanned diverse eras (most reported in 2020-2025 [n=32]) and geographies (most reported in Asia [n=47]) with substantial heterogeneity in populations treated and outcomes reported. 'Non-severe' AA was reported in 43 and 'moderate' in 22 studies; Camitta criteria were used in four studies, and Seattle criteria in one. Eleven studies closely matched the Camitta definition and 14 the Seattle definition; 13 studies provided no definition.

OS across the therapies was generally consistent but ORR seemed to vary widely (**Table**).

**Table. Aggregated OS and ORR by Intervention Category for mAA**

| Intervention Category | OS, n/N (%) | ORR, n/N (%) | Follow-up period (months) |
|---|---|---|---|
| **Supportive Care** | 78/93 (83.9%) | -- | 3-278 |
| **Monotherapy** | | | |
| CSA | 166/197 (84.3%) | 242/499 (48.5%) | 1-278 |
| Tacrolimus | 99/101 (98.0%) | 39/101 (38.6%) | 14-36 |
| Daclizumab | 57/60 (95.0%) | 26/61 (42.6%) | 1-278 |
| EPAG | 43/46 (93.5%) | 113/213 (53.1%) | 1-278 |
| Androgens | 40/55 (72.7%) | 26/61 (42.6%) | 1-174 |
| **Double therapy** | | | |
| hATG+CSA | 216/229 (94.3%) | 86/143 (60.1%) | 1-221 |
| rATG+CSA | 86/100 (86.0%) | 64/99 (64.6%) | 1-166 |
| Unspecified ATG+CSA | 140/150 (93.3%) | 171/266 (64.3%) | 1-278 |
| CSA/Tacrolimus+TPO-RA (including EPAG) | 51/54 (94.4%) | 109/157 (69.4%) | 6-278 |
| CSA+androgens | 419/448 (93.5%) | 10/24 (41.7%) | 9-132 |
| **Triple therapy** | | | |
| ATG+CSA+EPAG | *Not reported* | 5/5 (100.0%) | 14-104 |
| **HSCT** | 49/60 (81.7%) | 23/29 (79.3%) | 1-166 |

### Summary/Conclusion

Across heterogenous studies, OS and ORR have been documented for monotherapies, double, and triple therapies; HSCT was only reported in small cohorts. Due to varied timepoints, missing data, and cohort selection, there was insufficient homogenous data for quantitative analysis. This SLR demonstrates a clear need for uniform definition of mAA and for comparative, prospective studies to enable the comparison of therapeutic effectiveness and to develop sufficient treatment algorithms.

**Copyright Information:** (Online) ISSN: 2572-9241
©2026 The Author(s). HemaSphere is published by John Wiley & Sons Ltd on behalf of European Hematology Association. This is an open access Abstract Book distributed under the Attribution-NonCommercial-NoDerivs (CC BY-NC-ND), which allows third parties to download the articles and share them with others as long as they credit the author and the Abstract Book, but they cannot change the content in any way or use them commercially.
**Abstract Book Citations:** Authors, Title, HemaSphere, 2026;10:(S1):pages. The abstract book DOIs can be found on the "Author Information" page.
**Disclaimer:** Articles published in the journal HemaSphere exclusively reflect the opinions of the authors. The authors are responsible for all content in their abstracts including accuracy of the facts, statements, citing resources, etc.

### (PB3163) ATLAS: A RANDOMIZED, DOUBLE-BLIND, PLACEBO-CONTROLLED, ADAPTIVE SEAMLESS PHASE 2/3 STUDY TO ASSESS THE SAFETY AND EFFICACY OF MOMELOTINIB IN PATIENTS WITH VEXAS SYNDROME

**Topic:** 12. Bone marrow failure syndromes incl. PNH - Clinical

David Beck*[1, 2], Mael Heiblig[3], Katja Sockel[4, 5], Martin Krusche[6], Hideaki Nakajima[7], Sinisa Savic[8, 9], Matteo Giovanni Della Porta[10], Jasmine Sahni[11], Catherine Ellis[11], Antara Majumdar[11], Meenakshi Srinivasan[12], Dwaipayan Patnaik[13], Mrinal Patnaik[14]

*[1]Center for Human Genetics and Genomics, New York University School of Medicine, New York, United States of America; [2]Division of Rheumatology, Department of Medicine, New York University School of Medicine, New York, United States of America; [3]Service d'Hématologie Clinique, Hospices Civils de Lyon, Hôpital Lyon Sud, Pierre Bénite, France;[4]Medical Clinic and Policlinic I, University Hospital Dresden, TU Dresden, Dresden, Germany; [5]German Cancer Consortium (DKTK), CHOICE Consortium, Partner Sites, Munich/Dresden, Germany; [6]Division of Rheumatology and Systemic Inflammatory Diseases III. Department of Medicine, University Medical Center Hamburg-Eppendorf, Hamburg, Germany; [7]Department of Stem Cell and Immune Regulation, Yokohama City University Graduate School of Medicine, Yokohama, Japan; [8]Leeds Institute of Rheumatic and Musculoskeletal Medicine, University of Leeds, Leeds, United Kingdom; [9]National Institute for Health and Care Research (NIHR)-Leeds Biomedical Research Centre, Leeds, United Kingdom; [10]Humanitas Clinical and Research Center-IRCCS, Milan, Italy; Department of Biomedical Sciences, Humanitas University, Milan, Italy; [11]GSK, Collegeville, PA, United States of America; [12]GSK, Waltham, MA, United States of America; [13]GSK, Baar, Switzerland; [14]Division of Hematology, Department of Internal Medicine, Mayo Clinic, Rochester, MN, United States of America;*

### Background

VEXAS (vacuoles, E1 enzyme, X-linked, autoinflammatory, somatic) syndrome is a rare disease caused by somatic *UBA1* mutations in hematopoietic progenitor cells, resulting in severe multisystemic inflammation and progressive bone marrow failure. Affected patients display rheumatologic, dermatologic, and hematologic involvement, often with debilitating constitutional symptoms. There are no approved therapies for VEXAS, and current non-glucocorticoid (GC) therapies do little to limit the adverse trajectory of the disease.
Momelotinib is a Janus kinase (JAK) 1/JAK2/activin A receptor type 1 inhibitor approved for treating myelofibrosis in adults with anemia. Momelotinib's broad kinase profile targeting multiple inflammatory pathways, along with a promising 2-person VEXAS case study, supports further exploration of momelotinib for treating VEXAS.

### Aims

To assess the efficacy, safety, and pharmacokinetics (PK) of momelotinib in patients with VEXAS syndrome.

**Copyright Information:** (Online) ISSN: 2572-9241
©2026 The Author(s). HemaSphere is published by John Wiley & Sons Ltd on behalf of European Hematology Association. This is an open access Abstract Book distributed under the Attribution-NonCommercial-NoDerivs (CC BY-NC-ND), which allows third parties to download the articles and share them with others as long as they credit the author and the Abstract Book, but they cannot change the content in any way or use them commercially.
**Abstract Book Citations:** Authors, Title, HemaSphere, 2026;10:(S1):pages. The abstract book DOIs can be found on the "Author Information" page.
**Disclaimer:** Articles published in the journal HemaSphere exclusively reflect the opinions of the authors. The authors are responsible for all content in their abstracts including accuracy of the facts, statements, citing resources, etc.

### Methods

ATLAS is a multicenter, double-blind, adaptive, inferentially seamless phase 2/3 study to assess the efficacy of momelotinib for VEXAS. Eligible patients have a *UBA1* mutation, inflammatory manifestations, and ongoing GC treatment. Exclusion criteria include platelet counts <50×$10^9$/L or absolute neutrophil counts <1×$10^9$/L, a history of severe corticosteroid toxicity, and high-/very high–risk myelodysplastic syndromes. Patients with >1 prior VEXAS-directed therapy or prior treatment with JAK2 inhibitors or hypomethylating agents are not eligible.
This phase 2/3 study will enroll ≈150 patients. Phase 2 will randomize patients 1:1:1 to receive 1 of 2 doses of momelotinib or placebo for 26 weeks, with randomization stratified by baseline GC dose. A protocol-defined GC taper regimen during blinded treatment is required, and GC rescue therapy for VEXAS flares will be permitted.
Phase 3 will have a randomized or open-label design, depending on prespecified interim analysis (IA) results. If superiority/futility for momelotinib vs placebo is not declared at the IA, an additional prespecified number of patients will be enrolled and randomized 1:1 to receive the momelotinib recommended phase 3 dose (RP3D) or placebo; all patients enrolled in phase 2 who have not met the disease progression definition will remain blinded until patients enrolled during phase 3 finish 26 weeks of blinded treatment. If superiority at either/both momelotinib doses is declared at the IA, phase 3 will be modified to an open-label, single-arm to enroll additional patients at the momelotinib RP3D to collect further safety and efficacy data.

### Results

In both phases, the primary endpoint will be overall response rate at week 26, defined as achieving complete/partial response as best overall response based on a composite of factors, including absence of flares and reduction of GC daily dose. Secondary endpoints will include RP3D identification (phase 2), safety, patient-reported outcomes, hematologic improvement, PK, and overall survival. Exploratory endpoints will include transfusion rates, GC dose changes, and clonal burden.

### Summary/Conclusion

There is a high medical need for novel therapies that address the rheumatologic, hematologic, and inflammatory manifestations and lower GC dose dependency in VEXAS. The seamless phase 2/3 study design of ATLAS will prospectively evaluate momelotinib vs placebo in combination with GCs as an option to address the critical needs of patients with VEXAS syndrome.
Funding: GSK

**Copyright Information:** (Online) ISSN: 2572-9241
©2026 The Author(s). HemaSphere is published by John Wiley & Sons Ltd on behalf of European Hematology Association. This is an open access Abstract Book distributed under the Attribution-NonCommercial-NoDerivs (CC BY-NC-ND), which allows third parties to download the articles and share them with others as long as they credit the author and the Abstract Book, but they cannot change the content in any way or use them commercially.
**Abstract Book Citations:** Authors, Title, HemaSphere, 2026;10:(S1):pages. The abstract book DOIs can be found on the "Author Information" page.
**Disclaimer:** Articles published in the journal HemaSphere exclusively reflect the opinions of the authors. The authors are responsible for all content in their abstracts including accuracy of the facts, statements, citing resources, etc.

### (PB3164) SUCCESSFUL USE OF NSAIDS IN GHOSAL SYNDROME THERAPY: A CLINICAL CASE REPORT

**Topic:** 12. Bone marrow failure syndromes incl. PNH - Clinical

Svetlana Lebedeva*[1], Sofya Lushchevich[1], Maria Sadovskaya[1], Maria Kurnikova[1], Alexey Pshonkin[1]

[1]*Dmitry Rogachev National Medical Research Center of Pediatric Hematology, Oncology and Immunology, Moscow, Russia;*

### Background

Ghosal syndrome (GS) is a rare autosomal recessive disorder characterized by diaphyseal cortical hyperostosis of long bones, bone marrow fibrosis, and hematopoietic impairment. To date, approximately 40 cases of GS have been reported in the literature, with the majority described in patients of Middle Eastern and Mediterranean descent. Initial manifestations typically appear in early childhood and progress over time. GS results from biallelic mutations in the TBXAS1 gene, which encodes thromboxane-A-synthase 1. Reduced TBXAS1 activity leads to decreased thromboxane A2 (TXA2) levels and subsequent elevation of PGE2. Increased PGE2 levels, in turn, suppress proliferation and differentiation of erythroid precursors in the bone marrow. Furthermore, impaired TBXAS1 function and elevated PGE2 levels indirectly lead to osteoclast insufficiency and disruption of bone remodeling processes, resulting in abnormal thickening of the cortical layer of long bones. The main drugs used for GS treatment are glucocorticoids and NSAIDs. Both drug groups inhibit arachidonic acid metabolism, leading to further blockade and consequent reduction of PGE2 levels.

### Aims

To describe a case of successful long-term NSAID therapy in a 3.5-year-old patient with Ghosal syndrome.

### Methods

A 3-year-old female patient was admitted to the National Medical Research Center for Pediatric Hematology, Oncology and Immunology for comprehensive evaluation due to anemia, thrombocytopenia, and bone marrow fibrosis confirmed by trephine biopsy. Anemic syndrome was first detected at 1 year of age, while thrombocytopenia was identified at 2 years of age. Prior to admission, the patient received iron supplementation therapy and red blood cell transfusion support at her local medical facility.

Comprehensive evaluation excluded hematological malignancies, myelodysplastic syndrome, hemoglobinopathies, Fanconi anemia, hemolytic anemia, and iron deficiency anemia. Patient samples were sent for whole-exome sequencing. While awaiting genetic results, therapy with subcutaneous romiplostim was initiated, during which the patient remained transfusion-dependent (2 red blood cell transfusions over 7 months).

### Results

Molecular genetic testing revealed mutations in exon 2 (p.Ser38Ter, nonsense mutation) and exon 14 (p.Asn571MetfsTer29, frameshift mutation) of the TBXAS1 gene. These mutations were subsequently identified in heterozygous state in both parents. Radiographic examination of long bones demonstrated cortical thickening of both femoral bones in central regions up to 7 mm.

Based on these findings, the diagnosis of GS was established. NSAID therapy with ibuprofen (10 mg/kg/day) combined with gastroprotective therapy was initiated. During follow-up, normalization of hemoglobin concentration and platelet count was observed. Control radiography of femoral bones after 2 years of NSAID therapy showed normalization of cortical thickness. No adverse effectswere reported throughout the NSAID treatment period. To date, the patient has maintained complete hematological response for 2.5 years while on NSAID therapy.

**Copyright Information:** (Online) ISSN: 2572-9241
©2026 The Author(s). HemaSphere is published by John Wiley & Sons Ltd on behalf of European Hematology Association. This is an open access Abstract Book distributed under the Attribution-NonCommercial-NoDerivs (CC BY-NC-ND), which allows third parties to download the articles and share them with others as long as they credit the author and the Abstract Book, but they cannot change the content in any way or use them commercially.
**Abstract Book Citations:** Authors, Title, HemaSphere, 2026;10:(S1):pages. The abstract book DOIs can be found on the "Author Information" page.
**Disclaimer:** Articles published in the journal HemaSphere exclusively reflect the opinions of the authors. The authors are responsible for all content in their abstracts including accuracy of the facts, statements, citing resources, etc.

**Summary/Conclusion**

NSAID therapy demonstrated efficacy and good tolerability in this pediatric patient with GS, suggesting its potential as a viable treatment option for this rare condition.

**Copyright Information:** (Online) ISSN: 2572-9241
©2026 The Author(s). HemaSphere is published by John Wiley & Sons Ltd on behalf of European Hematology Association. This is an open access Abstract Book distributed under the Attribution-NonCommercial-NoDerivs (CC BY-NC-ND), which allows third parties to download the articles and share them with others as long as they credit the author and the Abstract Book, but they cannot change the content in any way or use them commercially.
**Abstract Book Citations:** Authors, Title, HemaSphere, 2026;10:(S1):pages. The abstract book DOIs can be found on the "Author Information" page.
**Disclaimer:** Articles published in the journal HemaSphere exclusively reflect the opinions of the authors. The authors are responsible for all content in their abstracts including accuracy of the facts, statements, citing resources, etc.

### (PB3165) HAEMOPHAGOCYTIC LYMPHOHISTIOCYTOSIS IN A DISTRICT GENERAL HOSPITAL: A SIX MONTH CASE SERIES HIGHLIGHTING DIAGNOSTIC CHALLENGES AND THE VALUE OF EARLY MULTIDISCIPLINARY MANAGEMENT

**Topic:** 12. Bone marrow failure syndromes incl. PNH - Clinical

Benjamin Lau*[1], Manjula Sinnakirouchenan[1], Manahal Mateen[1], Akila Danga[1], Ruby Haji[1]

[1]*Hillingdon Hospital, London, United Kingdom;*

### Background

Haemophagocytic lymphohistiocytosis (HLH) is a rare but life-threatening hyperinflammatory syndrome in which delayed recognition contributes significantly to mortality. Although frequently associated with tertiary centres, HLH may present in district general hospitals (DGHs), where competing diagnoses such as sepsis or malignancy are more commonly considered. We describe four cases presenting to our DGH within six months to highlight the importance of maintaining a high index of suspicion across emergency, medical, intensive care, and haematology teams, the limitations of relying solely on H-scores to exclude the diagnosis, and the value of multidisciplinary investigations and management.

### Methods

Four adult patients diagnosed with HLH at our DGH over a six-month period were reviewed retrospectively. Clinical features, laboratory findings, H-scores, bone marrow results, treatments administered, and overall outcomes were analysed.

### Results

All four patients presented with non-specific systemic illness and were initially admitted under general medical teams. Ages ranged from 19 to 82 years. Presenting features included fever, dyspnoea, weakness and, in one case, pleuritic chest pain with pleural effusion. HLH was not suspected early in any admission and was only considered once markedly elevated ferritin levels were identified.

Clinical severity varied across the cohort. The first patient presented with pancytopenia and progressive functional decline without an identifiable underlying driver. The second presented with pneumonia complicated by pulmonary embolism, with HLH suspected following a rising ferritin trend. The third patient deteriorated rapidly with reduced consciousness requiring intensive care admission, with investigations suggesting a possible T-cell lymphoma as the underlying trigger. The fourth presented with persistent fever and cellulitis, complicated by severe acute kidney injury requiring renal replacement therapy.

H-scores ranged from intermediate to high probability in all cases. Diagnostic uncertainty persisted despite laboratory and clinical findings, and early bone marrow examination proved valuable in supporting the diagnosis. Soluble CD25 levels were only sent in two out of the four patients, likely as a reflection of the test not being run in-house, and both levels were elevated. Management required coordinated multidisciplinary input involving intensive care, renal, and rheumatology teams in addition to haematology. Treatment included corticosteroids, intravenous immunoglobulin, and anakinra in most patients, with rituximab or etoposide used selectively. Despite treatment, morbidity and mortality remained significant, with two out of the four patients dying and one remaining in intensive care, reflecting the aggressive clinical course of HLH and the current UK mortality data.

**Copyright Information:** (Online) ISSN: 2572-9241
©2026 The Author(s). HemaSphere is published by John Wiley & Sons Ltd on behalf of European Hematology Association. This is an open access Abstract Book distributed under the Attribution-NonCommercial-NoDerivs (CC BY-NC-ND), which allows third parties to download the articles and share them with others as long as they credit the author and the Abstract Book, but they cannot change the content in any way or use them commercially.
**Abstract Book Citations:** Authors, Title, HemaSphere, 2026;10:(S1):pages. The abstract book DOIs can be found on the "Author Information" page.
**Disclaimer:** Articles published in the journal HemaSphere exclusively reflect the opinions of the authors. The authors are responsible for all content in their abstracts including accuracy of the facts, statements, citing resources, etc.

### Summary/Conclusion

HLH can present in clusters within DGHs and is likely under-recognised. Ferritins should be checked early in non-specific presentations with fever, which may prompt consideration of the diagnosis in these centres where there is less experience of the disease and fewer cases are seen. Patients with intermediate H-scores should be considered for multidisciplinary work up, and early bone marrow examination can support the diagnosis if clinical suspicion is high. Increasing clinician awareness of HLH outside tertiary centres may reduce diagnostic delay and improve outcomes, and ongoing education with appropriate local clinical guidelines is essential.

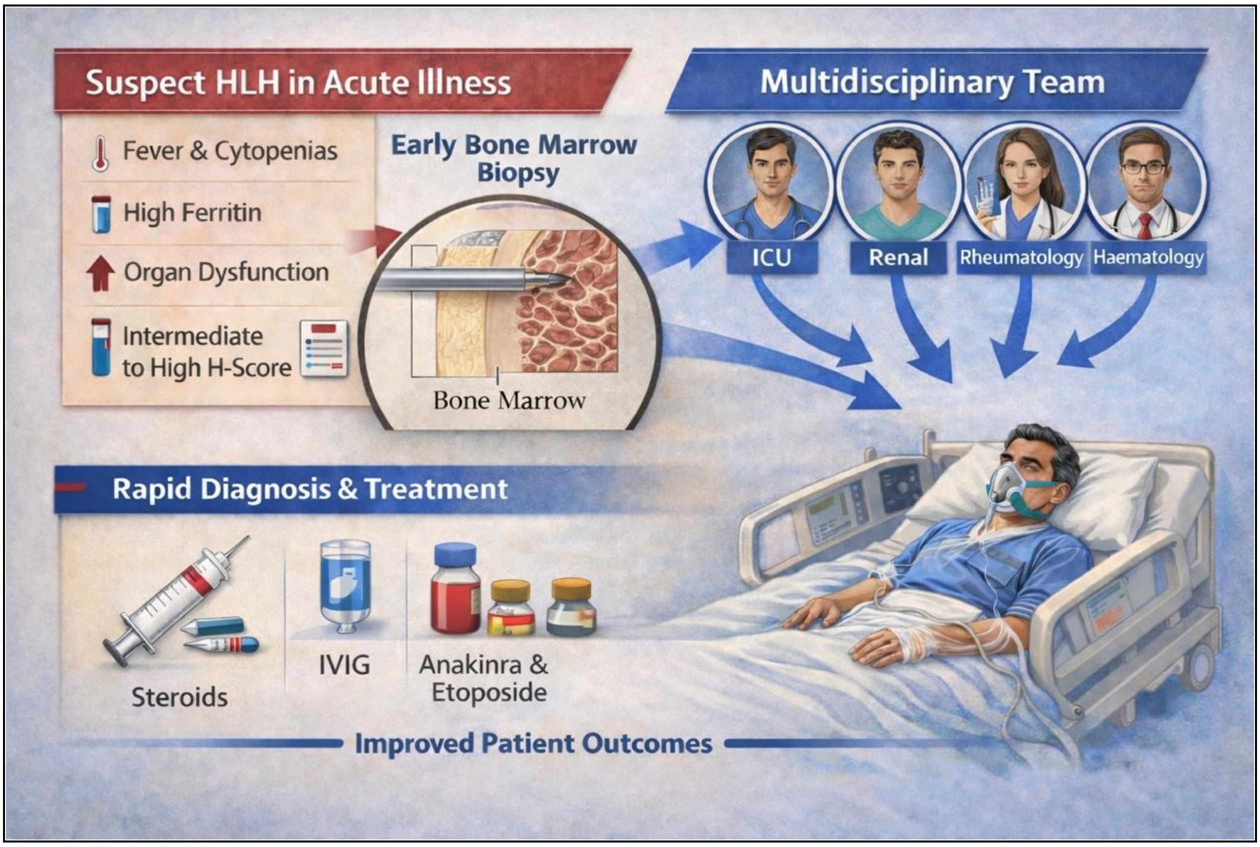

Infographic aimed at improving awareness of HLH, especially in non-tertiary settings

**Copyright Information:** (Online) ISSN: 2572-9241
©2026 The Author(s). HemaSphere is published by John Wiley & Sons Ltd on behalf of European Hematology Association. This is an open access Abstract Book distributed under the Attribution-NonCommercial-NoDerivs (CC BY-NC-ND), which allows third parties to download the articles and share them with others as long as they credit the author and the Abstract Book, but they cannot change the content in any way or use them commercially.
**Abstract Book Citations:** Authors, Title, HemaSphere, 2026;10:(S1):pages. The abstract book DOIs can be found on the "Author Information" page.
**Disclaimer:** Articles published in the journal HemaSphere exclusively reflect the opinions of the authors. The authors are responsible for all content in their abstracts including accuracy of the facts, statements, citing resources, etc.

### (PB3166) AUTOIMMUNE CYTOPENIAS AS THE INITIAL PRESENTATION OF COMMON VARIABLE IMMUNODEFICIENCY

**Topic:** 12. Bone marrow failure syndromes incl. PNH - Clinical

Juliana Castañeda-Fernandez[1], Ana Maria Bielsa[1], Stefania Gonzalez-Lopez[1], Pablo Martinez-Forga[1], Leire Etxebarria[1], Laura Villarroya Martínez[1], Rafael Monleon[1], Fernando Cadenas[1], Roberto Palacios[1], David Lozada[1], Angeles Montañes[1], María del Valle Recasens Flores*[1]

[1]*Hospital Universitario Miguel Servet, Hematology, Zaragoza, Spain;*

### Background

Common variable immunodeficiency (CVID) is the most frequent symptomatic primary immunodeficiency and may present at any age, with similar incidence in both genders. Diagnosis is based on reduced serum IgG levels in combination with low IgA and/or IgM, recurrent infections, impaired vaccine responses, and exclusion of other causes of hypogammaglobulinemia. Autoimmune cytopenias are common non-infectious manifestations, including immune thrombocytopenia (ITP), autoimmune hemolytic anemia (AIHA), Evans syndrome (AIHA-ITP) and neutropenia, and may precede or accompany the diagnosis of CVID.

### Aims

To describe autoimmune cytopenias as the initial manifestation of CVID and to highlight the importance of early immunological assessment in hematological practice.

### Methods

Observational, longitudinal, retrospective study of 5 patients with autoimmune cytopenias and CVID followed at a tertiary care Hematology Department. Clinical and analytical data related to diagnosis and treatment were collected from electronic medical records.

### Results

In the five cases analyzed, CVID was diagnosed at different ages, despite being a congenital defect. Recurrent infections were the most frequent symptom (80%), mainly respiratory and gastrointestinal, while 20% presented autoimmune cytopenia without previous infections. It should be noted that one patient initially diagnosed with ITP and normal levels of immunoglobulins developed recurrent infections and panhypogammaglobulinemia years later, allowing for a late diagnosis of CVID, thus highlighting the clinical heterogeneity of this disease.
Regarding hematological manifestations, ITP occurred in 40% of cases, AIHA in 20% and 20% presented with Evans syndrome. 60% of patients presented with splenomegaly.
Initial treatment was intravenous immunoglobulins (IVIG), with subsequent transition to subcutaneous therapy (SCIG) in 3 patients, available in our setting since 2018 with good tolerance and greater practicality. Only one mild adverse effect (AE) was reported with IVIG (pruritus, resolved with antihistamines), with no AEs with SCIG. During the follow-up, no infectious recurrences or autoimmune cytopenias were observed.

### Summary/Conclusion

CVID should be considered in patients presenting with autoimmune cytopenias, especially in cases of atypical course or poor treatment response. Assessment of immunoglobulin levels allows diagnosis and treatment. In our series, immunoglobulin replacement therapy was safe and effective in patients with CVID.

**Copyright Information:** (Online) ISSN: 2572-9241
©2026 The Author(s). HemaSphere is published by John Wiley & Sons Ltd on behalf of European Hematology Association. This is an open access Abstract Book distributed under the Attribution-NonCommercial-NoDerivs (CC BY-NC-ND), which allows third parties to download the articles and share them with others as long as they credit the author and the Abstract Book, but they cannot change the content in any way or use them commercially.
**Abstract Book Citations:** Authors, Title, HemaSphere, 2026;10:(S1):pages. The abstract book DOIs can be found on the "Author Information" page.
**Disclaimer:** Articles published in the journal HemaSphere exclusively reflect the opinions of the authors. The authors are responsible for all content in their abstracts including accuracy of the facts, statements, citing resources, etc.

### (PB3167) EFFICACY AND SAFETY OF ECULIZUMAB IN 47 PATIENTS WITH PAROXYSMAL NOCTURNAL HEMOGLOBINURIA IN CHINA:A MULTI-CENTER REAL-WORLD STUDY

**Topic:** 12. Bone marrow failure syndromes incl. PNH - Clinical

Gaixiang Xu*[1], Ying Xu[2], Ruiqing Zhou[2, 2], Wei Zhou[2, 2], Mingyue Zeng[3], Yumiao Li[4], Fang Zheng[5], Jishi Wang[6], Yanli Yang[7], Shunqing Wang[8], Hongyan Tong[9]

*[1]the First Affiliated Hospital,Zhejiang University School of Medicine, Hangzhou, China;[2]Guangzhou First People's Hospital, Department of Hematology, Guangzhou, China; [3]Guizhou Provincial People's Hospital, Department of Hematology, Guiyang, China; [4]Guangzhou First People's Hospital, Guangzhou, China;[5]The second people's hospital of Guiyang,Guiyang 550023,China, Guiyang, China; [6]The Affiliated Hospital of Guizhou Medical University, Guiyang, China;[7]The first affiliated hospital of bengbu medical college, Bengbu, China; [8]Guangzhou First People's Hospital, Department of Hematology, Guangzhou, China; [9]The First Affiliated Hospital, Zhejiang University School of Medicine, Hangzhou, China;*

### Background

Eculizumab specifically blocks the cleavage of terminal complement C5 and the formation of membrane attack complexes, thereby effectively inhibiting intravascular hemolysis and significantly reducing the risk of complications such as thrombosis and renal impairment. However, Eculizumab was not officially launched in mainland China until the end of 2022. Compared to the abundant international clinical data, real-world data on Eculizumab treatment for PNH in China remains relatively scarce.This study conducted a retrospective analysis of data from PNH patients treated with standard-dose Eculizumab registered in the multicenter Southern China PNH Collaborative Group, aiming to further evaluate the real-world efficacy and safety of Eculizumab in Chinese PNH patients.

### Aims

To evaluate the real-world efficacy and safety of standard-dose eculizumab in the treatment of patients with hemolytic paroxysmal nocturnal hemoglobinuria (PNH) in China.

### Methods

A retrospective analysis was conducted on the clinical data of 47 patients with PNH registered in the multicenter Southern China PNH Working Group from January 2023 to January 2025. Patients who received eculizumab treatment for  6 months were included. Clinical and laboratory parameters were collected at baseline and at 1, 2, 3, and 6 months post-treatment. Efficacy endpoints—including control of hemolysis and improvement in transfusion dependence—as well as the incidence of breakthrough hemolysis (BTH), extravascular hemolysis (EVH), and adverse events were evaluated.

**Copyright Information:** (Online) ISSN: 2572-9241
©2026 The Author(s). HemaSphere is published by John Wiley & Sons Ltd on behalf of European Hematology Association. This is an open access Abstract Book distributed under the Attribution-NonCommercial-NoDerivs (CC BY-NC-ND), which allows third parties to download the articles and share them with others as long as they credit the author and the Abstract Book, but they cannot change the content in any way or use them commercially.
**Abstract Book Citations:** Authors, Title, HemaSphere, 2026;10:(S1):pages. The abstract book DOIs can be found on the "Author Information" page.
**Disclaimer:** Articles published in the journal HemaSphere exclusively reflect the opinions of the authors. The authors are responsible for all content in their abstracts including accuracy of the facts, statements, citing resources, etc.

**Results**

A total of 47 patients were enrolled, comprising 20 males and 27 females with a median age of 35 years (range: 18–72). The cohort included 43 patients (91.5%) with classical PNH and 4 patients (8.5%) with PNH associated with bone marrow failure (PNH/BMF). The median duration of eculizumab treatment was 12 months (range: 6-24). In terms of efficacy: Compared with baseline, the frequency of paroxysmal hemoglobinuria was significantly reduced after treatment [0 (0-1) vs 3 (1-17) episodes/month]. At 6 months of treatment, 38 patients (80.9%) achieved lactate dehydrogenase (LDH) levels <1.5×ULN. Hemoglobin (HGB) levels increased from baseline in 39 patients (83.0%), with 25 patients (53.2%) achieving an increase of >20 g/L. Among the 19 patients who were transfusion-dependent at baseline,17(89.5%) achieved transfusion independence. No new thromboembolic events occurred during the follow-up period. In terms of safety: BTH occurred in 5 patients (10.6%), primarily associated with infections. EVH was observed in 6 patients (12.8%). Common adverse events included mild headache and upper respiratory tract infection. No serious adverse events (SAEs) or deaths were reported.

**Summary/Conclusion**

Standard-dose eculizumab provides rapid and sustained control of intravascular hemolysis in PNH patients in Southern China. It significantly improves anemia and transfusion independence with a favorable safety profile.

**Copyright Information:** (Online) ISSN: 2572-9241
©2026 The Author(s). HemaSphere is published by John Wiley & Sons Ltd on behalf of European Hematology Association. This is an open access Abstract Book distributed under the Attribution-NonCommercial-NoDerivs (CC BY-NC-ND), which allows third parties to download the articles and share them with others as long as they credit the author and the Abstract Book, but they cannot change the content in any way or use them commercially.
**Abstract Book Citations:** Authors, Title, HemaSphere, 2026;10:(S1):pages. The abstract book DOIs can be found on the “Author Information” page.
**Disclaimer:** Articles published in the journal HemaSphere exclusively reflect the opinions of the authors. The authors are responsible for all content in their abstracts including accuracy of the facts, statements, citing resources, etc.

### (PB3168) REAL-WORLD EXPERIENCE WITH IPTACOPAN IN PAROXYSMAL NOCTURNAL HEMOGLOBINURIA: EFFICACY IN TREATMENT-NAÏVE AND SWITCH PATIENTS

**Topic:** 12. Bone marrow failure syndromes incl. PNH - Clinical

Ting Wu*[1], Wei Wang[1], Xiaoqin Wang[1]

[1]*Huashan Hospital, Fudan University, Department of Hematology, Shanghai, China;*

### Background

- Iptacopan is the first oral proximal complement factor B inhibitor approved for paroxysmal nocturnal hemoglobinuria (PNH).
- Clinical trials have demonstrated its efficacy, but real-world data, particularly in Asian populations and in patients switching from C5 inhibitors, remain limited.
- This study reports the real-world experience of iptacopan in 16 PNH patients from a single center in China.

### Aims

To evaluate the real-world efficacy and safety of iptacopan in:

- Patients with PNH switching from C5 inhibitors (eculizumab, ravulizumab, crovalimab)
- Treatment-naïve patients receiving iptacopan as first-line therapy

### Methods

- **Study design:**Retrospective, single-center analysis
- **Setting:**Huashan Hospital, Fudan University, Shanghai, China
- **Patients:**16 patients with PNH treated with iptacopan
- **Data collected:** Demographics, baseline HGB, clone size, prior C5i therapy, switch reasons, response to iptacopan, safety

**Copyright Information:** (Online) ISSN: 2572-9241
©2026 The Author(s). HemaSphere is published by John Wiley & Sons Ltd on behalf of European Hematology Association. This is an open access Abstract Book distributed under the Attribution-NonCommercial-NoDerivs (CC BY-NC-ND), which allows third parties to download the articles and share them with others as long as they credit the author and the Abstract Book, but they cannot change the content in any way or use them commercially.
**Abstract Book Citations:** Authors, Title, HemaSphere, 2026;10:(S1):pages. The abstract book DOIs can be found on the “Author Information” page.
**Disclaimer:** Articles published in the journal HemaSphere exclusively reflect the opinions of the authors. The authors are responsible for all content in their abstracts including accuracy of the facts, statements, citing resources, etc.

**Results**

**Patient Characteristics**
A total of 16 PNH patients from a single center (Huashan Hospital, Fudan University) were included. The mean age at diagnosis was 40.8 years (range 18–73), with 10 males and 6 females. Thirteen patients (81%) had classic PNH, and 3 (19%) had AA-PNH. The mean hemoglobin at diagnosis was 70.1 g/L (range 51–100), and the mean granulocyte clone size was 91.7% (range 75.9–99.9). The reasons for initiating complement inhibition were hemolysis alone (13/16, 81%), hemolysis with thrombosis (2/16, 13%), and hemolysis with pregnancy (1/16, 6%).
**Prior C5 Inhibitor Therapy (n=11)**
Among the 11 patients who received prior C5 inhibitor therapy, 9 received eculizumab, 1 received ravulizumab, and 1 received crovalimab.
Response to prior C5i was as follows:

- **8 classic PNH patients**achieved good response
- **1 classic PNH patient**achieved partial response
- **1 AA-PNH patient (Patient 1)**achieved minor response with intermittent transfusion requirement
- **1 AA-PNH patient (Patient 2)**achieved partial response

The reasons for switching to iptacopan were:

- Breakthrough hemolysis (BTH) with extravascular hemolysis (EVH): 3/11
- EVH alone: 6/11
- Clinical trial termination: 2/11

**Efficacy of Iptacopan – Switch Group (n=11)**
The median time to response after switching to iptacopan was **0.7 months** (range 0.3–1.4).
Among the **9 classic PNH patients**, all achieved complete response, with a median time to complete response of**0.8 months** (range 0.3–1.3).
Among the **2 AA-PNH patients:**

- **Patient 1**achieved good response and became transfusion-independent
- **Patient 2**achieved only minor response due to transformation to MDS and required intermittent transfusions

**Efficacy of Iptacopan – Treatment-Naïve Group (n=5)**
In the 5 treatment-naïve patients, the mean hemoglobin at diagnosis was **76.6 g/L** (range 61–100).

- **3 classic PNH patients**all achieved complete response, with a median time to complete response of **8 months** (range 4.2–4.9)
- **1 AA-PNH patient (Patient 15)**achieved good response after **8 months** of treatment
- **1 classic PNH patient (Patient 12)**achieved good response after only **4 months**, though longer follow-up is needed

**Safety**
No meningococcal infections, thrombotic events, or treatment discontinuations due to adverse events.

**Copyright Information:** (Online) ISSN: 2572-9241
©2026 The Author(s). HemaSphere is published by John Wiley & Sons Ltd on behalf of European Hematology Association. This is an open access Abstract Book distributed under the Attribution-NonCommercial-NoDerivs (CC BY-NC-ND), which allows third parties to download the articles and share them with others as long as they credit the author and the Abstract Book, but they cannot change the content in any way or use them commercially.
**Abstract Book Citations:** Authors, Title, HemaSphere, 2026;10:(S1):pages. The abstract book DOIs can be found on the "Author Information" page.
**Disclaimer:** Articles published in the journal HemaSphere exclusively reflect the opinions of the authors. The authors are responsible for all content in their abstracts including accuracy of the facts, statements, citing resources, etc.

### Summary/Conclusion

- Iptacopan showed rapid and sustained HGB improvement in switch and treatment-naïve PNH patients.
- Classic PNH patients achieved rapid complete response after switching.
- AA-PNH patients showed variable response; one became transfusion-free.
- Treatment-naïve patients achieved good to complete responses.
- Well-tolerated with no safety concerns.
- Supports iptacopan as an effective oral option for PNH in real-world settings.

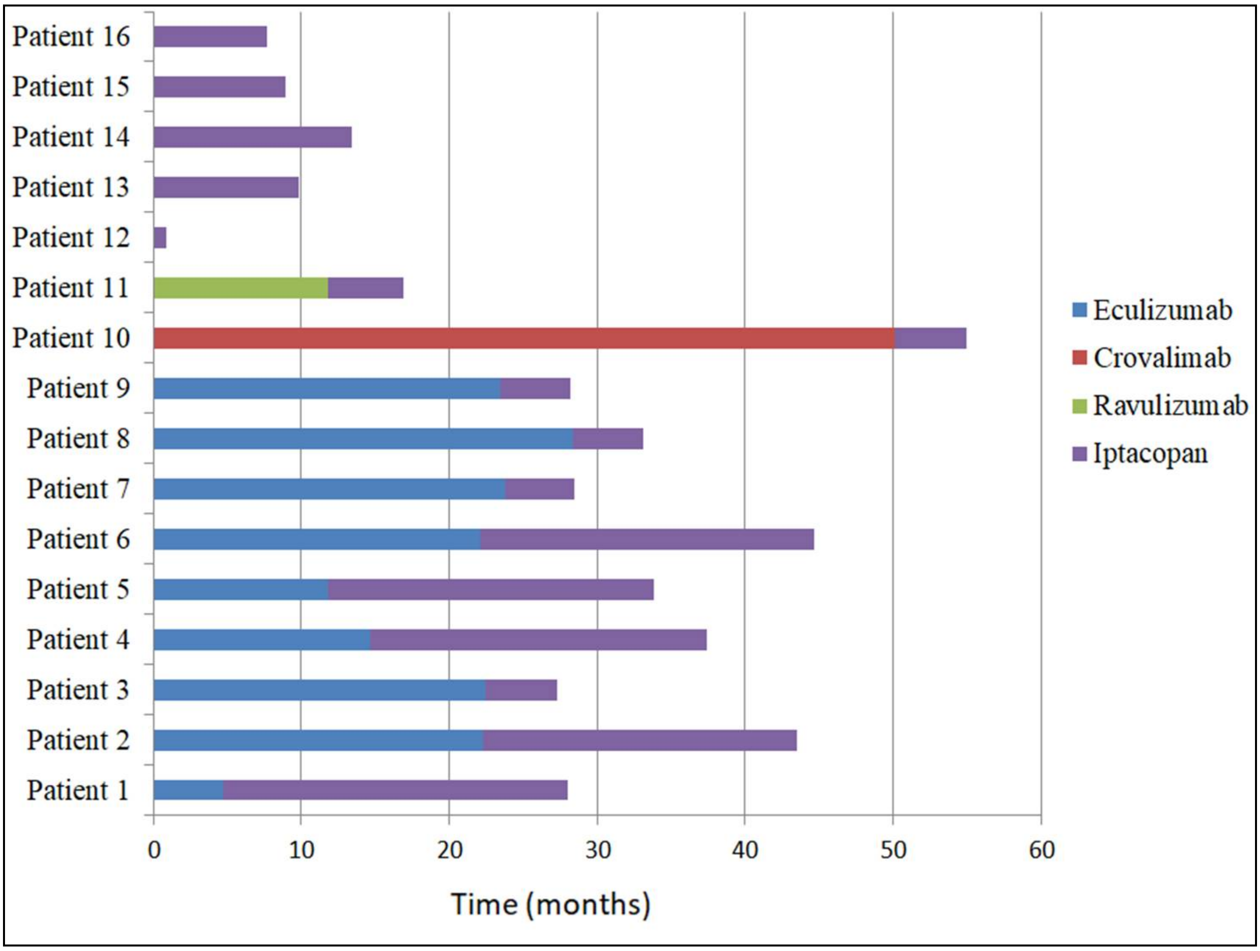

Patient treatment timeline

Copyright Information: (Online) ISSN: 2572-9241
©2026 The Author(s). HemaSphere is published by John Wiley & Sons Ltd on behalf of European Hematology Association. This is an open access Abstract Book distributed under the Attribution-NonCommercial-NoDerivs (CC BY-NC-ND), which allows third parties to download the articles and share them with others as long as they credit the author and the Abstract Book, but they cannot change the content in any way or use them commercially.
**Abstract Book Citations:** Authors, Title, HemaSphere, 2026;10:(S1):pages. The abstract book DOIs can be found on the "Author Information" page.
**Disclaimer:** Articles published in the journal HemaSphere exclusively reflect the opinions of the authors. The authors are responsible for all content in their abstracts including accuracy of the facts, statements, citing resources, etc.

### (PB3169) FLOW CYTOMETRY IN PAROXYSMAL NOCTURNAL HEMOGLOBINURIA: RELEVANCE OF THE MONITORIZATION OF THE ERYTHROID CLONE SIZE IN PATIENTS WITH COMPLEMENT INHIBITORS THERAPY.

**Topic:** 12. Bone marrow failure syndromes incl. PNH - Clinical

María Dolores Linares Latorre*[1], Beatriz Martín-Herreros[2], Lourdes Cordón Gallego[3], Paula Nuñez Lorenzo[3], Elvira Mora[1], Carlos Carretero Marquez[1], Rafael Andreu Lapiedra[1], Santiago Bonanad Boix[1], Maria Desamparados Sempere Talens[1]

*[1]Hospital La Fe, Hematology department, Valencia, Spain; [2]Instituto de Investigación Sanitaria La Fe, Hematology Research Group, Valencia, Spain; [3]Instituto de Investigación Sanitaria La Fe, Flow Cytometry Laboratory, Valencia, Spain;*

### Background

Paroxysmal nocturnal hemoglobinuria (PNH) is an ultrarare and non-neoplastic clonal acquired hematological disease characterized by complement-mediated hemolysis, aplasia, and thrombosis. The complement inhibitor (Ci) therapy has changed the natural history of PNH, by controlling intravascular hemolysis (IVH). Ci drugs available for proximal or terminal complement blockade require specific clinical and laboratory monitoring. Patients with terminal Ci therapy are at higher risk of extravascular hemolysis (EVH). High-sensitivity flow cytometry (HSFC) is essential for diagnosis and clinical follow-up. Evaluation of erythroid clone size is relevant in patients treated with Ci especially with persistent hemolysis, providing an objective biomarker of treatment efficacy.

### Aims

To evaluate the utility of monitoring the PNH erythroid clone by HSFC as an objective biomarker of response to Ci treatment and to assess its added value when combined with hemolysis biomarkers, especially in patients receiving terminal Ci.

### Methods

We retrospectively analyzed 10 patients (9 adults, 1 pediatric) treated with different Ci (eculizumab, ravulizumab, pegcetacoplan, and danicopan) over a 26-years period. Hemolysis biomarkers were collected at different time points during clinical follow-up: diagnosis, before and after treatment initiation, and therapy change. Detection of PNH clones was evaluated with HSFC, according to International Guidelines[1]. EVH was defined as persistent anemia with positive hemolysis biomarkers and direct Coombs test, with LDH <1.5 x UNL (upper normal levels).

### Results

Median age at diagnosis was 29 years (range 5-69), most were male (60%). The median time from diagnosis to first treatment was 64 months (range 7-398). Median erythroid clone size was 31.15% (range 16.02-84.24) before treatment with a median leucocyte (neutrophils or monocytes) clone size of 76% (range 38-97.2). All patients had hemolysis biomarkers of IVH. Nine patients started treatment with eculizumab and one patient with ravulizumab. During a median follow-up of 49.5 months, 3/10 patients required therapy modification due to persistent anemia compatible with EVH. Two patients changed to pegcetacoplan and 1 added danicopan to ravulizumab. At the time of treatment change, median erythroid clone size was 54.91%, and increased to 99.74% after the switch to proximal Ci, paralleling leukocyte clone size (98.04%) and the improvement of hemolysis biomarkers. The remaining patients (3) continued with eculizumab and 4 changed to ravulizumab to ease the treatment administration. All patients who maintained terminal Ci remained stable without IVH evidence. Two patients had long-term follow-up (16 years) under terminal Ci. All patients are alive but one dead unrelated to PNH.

**Copyright Information:** (Online) ISSN: 2572-9241
©2026 The Author(s). HemaSphere is published by John Wiley & Sons Ltd on behalf of European Hematology Association. This is an open access Abstract Book distributed under the Attribution-NonCommercial-NoDerivs (CC BY-NC-ND), which allows third parties to download the articles and share them with others as long as they credit the author and the Abstract Book, but they cannot change the content in any way or use them commercially.
**Abstract Book Citations:** Authors, Title, HemaSphere, 2026;10:(S1):pages. The abstract book DOIs can be found on the "Author Information" page.
**Disclaimer:** Articles published in the journal HemaSphere exclusively reflect the opinions of the authors. The authors are responsible for all content in their abstracts including accuracy of the facts, statements, citing resources, etc.

**Summary/Conclusion**

The assessment of the efficacy of inhibitor therapies should be a pivotal goal in the follow-up of patients with PNH. In real-world practice, combined assessment of erythroid clone size by HSFC and hemolysis biomarkers provides an objective measure of Ci efficacy. Especially in patients receiving C5 inhibitors, the PNH clone size discordance between leukocytes and erythrocytes may represent a surrogate marker of EVH and it is advised to be investigated. HSFC is a dynamic methodology for treatment monitoring in PNH with implications in the decision-making process.

1.Illingworth, A. J., Marinov, I., & Sutherland, D. R. (2019). *International Journal of Laboratory Hematology*, *41*, 73-81.

**Copyright Information:** (Online) ISSN: 2572-9241
©2026 The Author(s). HemaSphere is published by John Wiley & Sons Ltd on behalf of European Hematology Association. This is an open access Abstract Book distributed under the Attribution-NonCommercial-NoDerivs (CC BY-NC-ND), which allows third parties to download the articles and share them with others as long as they credit the author and the Abstract Book, but they cannot change the content in any way or use them commercially.
**Abstract Book Citations:** Authors, Title, HemaSphere, 2026;10:(S1):pages. The abstract book DOIs can be found on the "Author Information" page.
**Disclaimer:** Articles published in the journal HemaSphere exclusively reflect the opinions of the authors. The authors are responsible for all content in their abstracts including accuracy of the facts, statements, citing resources, etc.

### (PB3170) SARCOIDOSIS-ASSOCIATED HEMOPHAGOCYTIC LYMPHOHISTIOCYTOSIS: A RARE CATASTROPHIC OVERLAP WITH MULTIORGAN FAILURE

**Topic:** 12. Bone marrow failure syndromes incl. PNH - Clinical

Mai Abouzeid*[1], Mohamed Ghonim[2], Mudireddy Mythri[1]

*[1]North Mississippi Medical Centre, Internal Medicine, Tupelo, United States of America;[2]St Jude research Hospital, Immunology, Memphis, United States of America;*

### Background

Sarcoidosis is a systemic inflammatory condition defined by non-caseating granulomas affecting multiple organs. Hemophagocytic lymphohistiocytosis (HLH) is a severe syndrome marked by excessive cytokine release and dysregulated macrophage activity, often leading to rapid mortality. This case highlights the rare coexistence of sarcoidosis-associated HLH complicated by embolic events and multiorgan failure, emphasizing the importance of early recognition and multidisciplinary care.

### Aims

To describe a rare case of sarcoidosis-associated HLH complicated by embolic phenomena and multiorgan failure.

### Methods

A 59-year-old female with a past medical history of heart failure with preserved ejection fraction, previous aortic valve replacement, multisystem sarcoidosis affecting the liver and bone marrow, type II diabetes mellitus, and chronic pain syndrome presented with acute epigastric pain. Preliminary laboratory investigations indicated the presence of pancytopenia alongside elevated levels of liver enzymes, ferritin, LDH, D-dimer, and triglycerides, in conjunction with reduced fibrinogen and acute kidney injury. The combination of splenomegaly, cytopenias, hyperferritinemia, elevated LDH, D-dimer, triglycerides, and hypofibrinogenemia met criteria for secondary HLH (HScore >200). CD25 and NK cell activity were confirmed. High-dose intravenous methylprednisolone was initiated.

### Results

During hospitalization, she developed new paroxysmal atrial fibrillation and acute neurological changes. Brain MRI revealed multiple small bilateral subacute infarcts, while CTA abdomen/pelvis demonstrated splenic and right renal artery occlusions, consistent with embolic phenomena, so anticoagulation was initiated. Despite multidisciplinary management, her condition was complicated by pulmonary edema, acute hypoxemic respiratory failure, and undifferentiated shock. She required intubation and experienced pulseless electrical activity and cardiac arrest with a return to spontaneous circulation. Ultimately, her condition progressed to multiorgan failure, leading to a transition of care to comfort measures.

**Copyright Information:** (Online) ISSN: 2572-9241
©2026 The Author(s). HemaSphere is published by John Wiley & Sons Ltd on behalf of European Hematology Association. This is an open access Abstract Book distributed under the Attribution-NonCommercial-NoDerivs (CC BY-NC-ND), which allows third parties to download the articles and share them with others as long as they credit the author and the Abstract Book, but they cannot change the content in any way or use them commercially.
**Abstract Book Citations:** Authors, Title, HemaSphere, 2026;10:(S1):pages. The abstract book DOIs can be found on the "Author Information" page.
**Disclaimer:** Articles published in the journal HemaSphere exclusively reflect the opinions of the authors. The authors are responsible for all content in their abstracts including accuracy of the facts, statements, citing resources, etc.

**Summary/Conclusion**

Sarcoidosis-associated hemophagocytic lymphohistiocytosis, also termed macrophage activation syndrome-HLH (MAS-HLH), is an exceptionally rare but devastating overlap syndrome. Sarcoidosis provides a granulomatous trigger for macrophage hyperactivation, while HLH represents uncontrolled T-cell and macrophage activity leading to cytokine storm and multiorgan failure (1-3). Diagnosis is challenging because MAS-HLH mimics sepsis, malignancy, and autoimmune flares. In this case, the presence of cytopenias, hyperferritinemia, hypofibrinogenemia, and elevated triglycerides met the criteria for HLH, along with confirmatory suppression of NK cell activity and elevated soluble CD25. CD25 reflects T-cell activation and is a key biomarker in the HLH-2004 guidelines, correlating with disease severity (4). High-dose glucocorticoids are still the first-line therapy, while cyclosporine, anakinra, or etoposide may be required for refractory disease. Despite early immunosuppression, our patient's course was complicated by embolic events, atrial fibrillation, and multiorgan failure, highlighting the high mortality associated with adult HLH (1)(3)(5-6). This case highlights the serious implications of sarcoidosis-associated MAS-HLH and underscores the necessity for prompt diagnosis, urgent immunosuppression, and collaborative care to enhance outcomes in this uncommon yet impactful overlap syndrome.

**Copyright Information:** (Online) ISSN: 2572-9241
©2026 The Author(s). HemaSphere is published by John Wiley & Sons Ltd on behalf of European Hematology Association. This is an open access Abstract Book distributed under the Attribution-NonCommercial-NoDerivs (CC BY-NC-ND), which allows third parties to download the articles and share them with others as long as they credit the author and the Abstract Book, but they cannot change the content in any way or use them commercially.
**Abstract Book Citations:** Authors, Title, HemaSphere, 2026;10:(S1):pages. The abstract book DOIs can be found on the "Author Information" page.
**Disclaimer:** Articles published in the journal HemaSphere exclusively reflect the opinions of the authors. The authors are responsible for all content in their abstracts including accuracy of the facts, statements, citing resources, etc.

### (PB3171) CLINICAL EFFICACY OF HIGH-DOSE INTRAVENOUS IMMUNOGLOBULIN PULSE THERAPY IN CHILDREN WITH STAGE IV HIGH-RISK NEUROBLASTOMA COMPLICATED BY SECONDARY BONE MARROW FAILURE

**Topic:** 12. Bone marrow failure syndromes incl. PNH - Clinical

Jianming Fang*[1], Ran Zheng[1], Han Gong[1], Yingli Zhang[1], Wei Zhou[1], Jinyue Xiong[1], Hongyan Liu[1]

[1]*Beijing Jingdu Children's Hospital, Beijing, China;*

### Background

Myelodysplastic diseases are mainly divided into congenital and acquired types, among which acquired myelodysplasia is further classified into primary and secondary subtypes. Secondary myelodysplasia mainly includes the following categories: (1) Hematopoietic cell tumors, such as hairy cell leukemia (HCL), T-cell large granular lymphocytic leukemia (T-LGLL), multiple myeloma (MM), etc.; (2) Bone marrow infiltration by tumors of other systems; (3) Myelofibrosis; (4) Severe nutritional anemia; (5) Acute hematopoietic arrest (AHA); (6) Myelosuppression induced by radiotherapy and chemotherapy in neoplastic diseases, etc. Neuroblastoma (NB) is the most common extracranial solid tumor in children, with significant heterogeneity in its clinical manifestations and prognosis. The disease has an insidious onset, and most children have distant metastases at the time of diagnosis. For children with stage IV high-risk NB, combined treatment including chemotherapy, radiotherapy, surgery, and hematopoietic stem cell transplantation is required. However, children often develop secondary myelodysplasia after combined treatment; fortunately, clinical practice has shown that high-dose intravenous immunoglobulin (IVIG) pulse therapy can effectively improve the state of myelodysplasia in children to a certain extent. Recently, 6 children with stage IV high-risk NB were admitted to our center, all of whom developed secondary myelodysplasia after combined treatment and received high-dose IVIG pulse therapy after admission. The details of these cases are reported as follows:

### Aims

To investigate the improving effect of high-dose intravenous immunoglobulin (IVIG) pulse therapy on secondary bone marrow failure in children with stage IV high-risk neuroblastoma.

### Methods

We retrospectively analyzed the clinical data of 6 children with stage IV high-risk neuroblastoma complicated withsecondary bone marrow failure who received high-dose IVIG pulse therapy at Beijing Jingdu Children's Hospital between September 2024 and March 2025. Relevant literature was retrieved and reviewed from CNKI, Wanfang Medical Network, and PubMed databases.

### Results

Six children with stage IV high-risk neuroblastoma developed Secondary bone marrow failure after long-term combined treatment including chemotherapy, surgery, and radiotherapy. Following high-dose IVIG pulse therapy, bone marrow cellularity was significantly improved. The mean recovery time of neutrophils was 9.14 days, and the mean recovery time of platelets was 13.21 days. Transfusion requirements were significantly reduced.

### Summary/Conclusion

High-dose intravenous immunoglobulin pulse therapy can significantly ameliorate secondary bone marrow failure in children with stage IV high-risk neuroblastoma.

**Copyright Information:** (Online) ISSN: 2572-9241
©2026 The Author(s). HemaSphere is published by John Wiley & Sons Ltd on behalf of European Hematology Association. This is an open access Abstract Book distributed under the Attribution-NonCommercial-NoDerivs (CC BY-NC-ND), which allows third parties to download the articles and share them with others as long as they credit the author and the Abstract Book, but they cannot change the content in any way or use them commercially.
**Abstract Book Citations:** Authors, Title, HemaSphere, 2026;10:(S1):pages. The abstract book DOIs can be found on the "Author Information" page.
**Disclaimer:** Articles published in the journal HemaSphere exclusively reflect the opinions of the authors. The authors are responsible for all content in their abstracts including accuracy of the facts, statements, citing resources, etc.

### (PB3172) DIAGNOSTIC CHALLENGES OF SECONDARY HEMOPHAGOCYTIC LYMPHOHISTIOCYTOSIS MIMICKING AUTOIMMUNE CYTOPENIA IN A RESOURCE-LIMITED SETTING.

**Topic:** 12. Bone marrow failure syndromes incl. PNH - Clinical

Brandi Afontin Gabriel*[1, 2]

*[1]Universidad central del Este, Clinical and research., San pedro de Macoris, Dominican Republic;[2]Hospital General y De Especialidades Nuestra Señora de la Altagracia., Intern Medicine, La Altagracia, Dominican Republic;*

### Background

Hemophagocytic Lymphohistiocytosis (HLH) is a rare, life-threatening hyperinflammatory syndrome caused by uncontrolled immune activation . its clinical presentation is often nonspecific, frequently leading to misdiagnosis and delayed treatment. Thrombocytopenia is a common finding and may be mistaken for immune thrombocytopenic purpura (ITP), representing an important diagnostic pitfall with potentially fatal consequences.

### Aims

To highlight diagnostic challenges of secondary HLH initially misinterpreted as immune thrombocytopenia and to illustrate the impact of laboratory resources on Clinical outcome.

### Methods

We performed a retrospective Clinical, laboratory, and histopathological evaluation of a patient admitted with presumed autoimmune thrombocytopenia who subsequently fulfilled morphological criteria for HLH based on bone marrow examination.

### Results

A 40-year-old female with prior diagnosis of immune thrombocytopenia presented with progressive asthenia, mucosal bleeding, intermittent fever, and pancytopenia affecting all three hematopoietic lineages. Laboratory findings included neutropenia, relative lymphocytosis, mild transaminase elevation, and preserved triglycerides levels. Ferritin and soluble CD25 were unavailable. Urinalysis showed leukocyturia and abundant bacteria, raising suspicion of infectious triggers. Initial management targeted autoimmune cytopenia.
Due to delayed access to bone marrow biopsy result, HLH-directed therapy was not initiated promptly. Histopathology ultimately demonstrated hemophagocytosis consistent with secondary HLH. The patient deteriorated and died from cardiorespiratory arrest.

### Summary/Conclusion

Secondary HLH may masquerade as immune cytopenia, particularly in environments lacking access to diagnosis inflammatory biomarkers. Normal triglyceride levels do not exclude the diagnosis. Delayed recognition in resource-constrained settings may lead to fatal for improving survival.

**Copyright Information:** (Online) ISSN: 2572-9241
©2026 The Author(s). HemaSphere is published by John Wiley & Sons Ltd on behalf of European Hematology Association. This is an open access Abstract Book distributed under the Attribution-NonCommercial-NoDerivs (CC BY-NC-ND), which allows third parties to download the articles and share them with others as long as they credit the author and the Abstract Book, but they cannot change the content in any way or use them commercially.
**Abstract Book Citations:** Authors, Title, HemaSphere, 2026;10:(S1):pages. The abstract book DOIs can be found on the “Author Information” page.
**Disclaimer:** Articles published in the journal HemaSphere exclusively reflect the opinions of the authors. The authors are responsible for all content in their abstracts including accuracy of the facts, statements, citing resources, etc.

### (PB3173) THE MECHANISM OF STAT3 INHIBITORS INDUCING DNA DAMAGE IN MULTIPLE MYELOMA CELLS AND ENHANCING THE ANTI-TUMOR EFFECTS OF NK CELLS

**Topic:** 13. Myeloma and other monoclonal gammopathies - Biology & translational research

Xianghong Zhao[1], Zhaoyun Liu[1], Rong Fu*[1]

[1] *Tianjin Medical University General Hospital, Tianjin, China;*

### Background

Multiple myeloma (MM) is an incurable disease. DNA damage response (DDR) is a potential therapeutic target to induce the apoptosis of myeloma cells and expression of major histocompatibility complex class I polypeptide-related sequence A and B (MICA/B) to activate NK cell. Inhibiting the activity of signal transducers and activators of transcription 3 (STAT3) could induce the DDR of MM cells. Inhibition of Poly-ADP-ribose-polymerase 1(PARP1) can induce DDR in myeloma however no effect on the viability of tumor cells.

### Aims

To investigate the anti-tumour cellular effects and mechanisms of DNA damage-induced activation of NK cells by STAT3 inhibitors in MM cells.

### Methods

Using data of MMRF-CoMMpass studies to identify the prognostic value of STAT3 and PARP1. Using CCK-8 to explore the Half-maximal inhibitory concentration (IC50) of STAT3 inhibitor C188-9 in MM cell lines U266 and RPMI-8266. Using flow cytometry (FCM) to evaluated the apoptosis and the expression of MICA/B in MM cells. Using western-blot to measure the level of STAT3, H2AX and ATM. KEGG analysis was used to identify enriched pathways. We co-cultured MM cells with NK cells and measure the activation of NK cells and the level of apoptosis of MM cells by using FCM.

### Results

By using the data of MMRF-CoMMpass, we found that patients with higher expression was associated worse prognosis. The IC50 of C188-9 in U266 was 23.07μM while in RPMI-8226 was 44.16μM. The C188-9 can significantly induce the level of apoptosis and expression MICA/B of MM cells. Western blot should that inhibition of STAT3 could elevated the levels of H2AX and ATM. For PARP1 inhibitor BYK204165, it had no effect on the apoptosis and the level of MICA/B. However, when it combined with C188-9, it could induce higher levels of apoptosis and expression of MICA/B of MM cells. Western blot also verified higher level of H2AX and ATM when C188-9 combined with BYK204165.  The KEGG showed the nucleotide excision repair pathway upregulated in the treatment group. With the cotreatment of C188-9 and BYK204165, we found upregulation of NKG2D, perforin and granzyme B and heightened cytotoxicity in NK cells in Coculture of NK cells with the MM cells.

### Summary/Conclusion

We found STAT3 inhibitor induces apoptosis and DNA damage in MM cells and STAT3 inhibitor combined with PARP1 inhibitor induced apoptosis and DNA damage in MM cells and mediated the high expression of MICA/B in MM cells. What's more, STAT3 inhibitor combined with PARP1 inhibitor induces DNA damage in MM cells, mediates high expression of MICA/B in MM cells, and activates the anti-tumour effect of NK cells.

**Copyright Information:** (Online) ISSN: 2572-9241
©2026 The Author(s). HemaSphere is published by John Wiley & Sons Ltd on behalf of European Hematology Association. This is an open access Abstract Book distributed under the Attribution-NonCommercial-NoDerivs (CC BY-NC-ND), which allows third parties to download the articles and share them with others as long as they credit the author and the Abstract Book, but they cannot change the content in any way or use them commercially.
**Abstract Book Citations:** Authors, Title, HemaSphere, 2026;10:(S1):pages. The abstract book DOIs can be found on the "Author Information" page.
**Disclaimer:** Articles published in the journal HemaSphere exclusively reflect the opinions of the authors. The authors are responsible for all content in their abstracts including accuracy of the facts, statements, citing resources, etc.

### (PB3174) THE EPIGENETIC ROLE OF CYCLIN D1 IN T(11;14) MULTIPLE MYELOMA

**Topic:** 13. Myeloma and other monoclonal gammopathies - Biology & translational research

Katrina Fordwor-Hepburn*[1], Jason Taslim[1], Sophie Ball[1], Qizhen Jia[1], Yiming Huang[1], Wang Guo[1], Alexia Katsarou[1], Aristeidis Chaidos[1], Pierangela Sabbattini[1], Anastasios Karadimitris[1], Nicholas T Crump[1]

*[1]Hugh and Josseline Langmuir Centre for Myeloma Research, Centre for Haematology, Department of Immunology and Inflammation, Imperial College London, London, United Kingdom;*

### Background

The plasma cell malignancy multiple myeloma (MM) is the second most common hematological malignancy, with a worldwide incidence of 4.5-6 per 100000 per year. MM is characterized by myeloma initiating events (MIE) of either hyperdiploidy or chromosomal translocation. Oncogenic enhancer activity is a hallmark of MM, with these cancer specific distal regulatory elements driving the expression of key myeloma genes. The t(11;14) translocation is found in approximately 15-20% of MM cases, juxtaposing *CCND1* with the highly active *IgH* enhancer. This results in the overexpression of cyclin D1, a key regulator of the G1 to S phase transition of the cell cycle. Previous work on other malignancies has shown that cyclin D1 can also act as a transcriptional co-factor to promote a specific transcriptional program. Furthermore, examination of chromatin accessibility in patient-derived malignant plasma cells shows that t(11;14) MM patients have differential accessibility compared to other sub-types.

### Aims

To explore a role for cyclin D1 in driving a sub-type specific transcriptional program in t(11;14) MM that may contribute to myelomagenesis.

### Methods

We used tagmentation, a small cell-number chromatin immunoprecipitation (ChIP)- based approach, to generate a genome-wide profile of histone modifications including H3K27 acetylation (H3K27ac) in malignant plasma cells from the bone marrows of three t(11;14) and two t(4;14) patients. In one t(11;14) patient, we used the high-resolution 3D genome technique Micro-Capture-C (MCC) to measure enhancer-promoter interactions at key MM genes. In conjunction, we generated cyclin D1 ChIP-sequencing (ChIP-seq) data to characterize cyclin D1 chromatin binding in the t(11;14) MM cell line U266. By integrating these data sets, we identified enhancers and promoters associated with cyclin D1 binding that regulate key genes in t(11;14) MM. We compared Multiple Myeloma Research Foundation (MMRF) CoMMpass RNA-sequencing data from t(11;14) and t(4;14) patients to assess the link to transcriptional differences.

### Results

Analysis of H3K27ac of t(11;14) and t(4;14) patients revealed differential histone acetylation, indicating subtype-specific gene regulation. Those genes found to be differentially acetylated, overlapped markedly with those that differentially expressed, and higher expression levels correlated with higher enhancer and promoter acetylation in t(11;14) patients. Genes differentially expressed were also associated with cyclin D1 chromatin binding at both promoters and enhancers.

Our analysis also identified cyclin D1 chromatin binding at sites of differential acetylation. Using MCC we identified differentially acetylated, cyclin D1 bound enhancers that directly regulate key MM genes, including CXCR4, MYC and CD74.

**Copyright Information:** (Online) ISSN: 2572-9241
©2026 The Author(s). HemaSphere is published by John Wiley & Sons Ltd on behalf of European Hematology Association. This is an open access Abstract Book distributed under the Attribution-NonCommercial-NoDerivs (CC BY-NC-ND), which allows third parties to download the articles and share them with others as long as they credit the author and the Abstract Book, but they cannot change the content in any way or use them commercially.
**Abstract Book Citations:** Authors, Title, HemaSphere, 2026;10:(S1):pages. The abstract book DOIs can be found on the "Author Information" page.
**Disclaimer:** Articles published in the journal HemaSphere exclusively reflect the opinions of the authors. The authors are responsible for all content in their abstracts including accuracy of the facts, statements, citing resources, etc.

**Summary/Conclusion**

This work highlights the epigenetic role of cyclin D1 in t(11;14) MM. We find that cyclin D1 predominantly binds to chromatin at both enhancers and promoters, including at loci specifically acetylated in t(11;14) patients, arguing that cyclin D1 may contribute to their activity. This differential acetylation is also reflected at the gene expression level. By developing a better understanding of cyclin D1's transcriptional role in t(11;14) MM we aim to identify new targets to directly inhibit its epigenetic effect as a novel therapeutic strategy.

**Copyright Information:** (Online) ISSN: 2572-9241
©2026 The Author(s). HemaSphere is published by John Wiley & Sons Ltd on behalf of European Hematology Association. This is an open access Abstract Book distributed under the Attribution-NonCommercial-NoDerivs (CC BY-NC-ND), which allows third parties to download the articles and share them with others as long as they credit the author and the Abstract Book, but they cannot change the content in any way or use them commercially.
**Abstract Book Citations:** Authors, Title, HemaSphere, 2026;10:(S1):pages. The abstract book DOIs can be found on the "Author Information" page.
**Disclaimer:** Articles published in the journal HemaSphere exclusively reflect the opinions of the authors. The authors are responsible for all content in their abstracts including accuracy of the facts, statements, citing resources, etc.

### (PB3175) TARGETING DORMANT MULTIPLE MYELOMA CELLS BY INDUCING OXIDATIVE STRESS

**Topic:** 13. Myeloma and other monoclonal gammopathies - Biology & translational research

Chloe Harris*[1], Haider Al-Janabi[1], Sherif El-Khamisy[1, 2], Alanna Green[1]

*[1]University of Sheffield, Sheffield, United Kingdom;[2]University of Bradford, Bradford, United Kingdom;*

**Background**

Multiple myeloma is the 2$^{nd}$ most common blood cancer in Europe and remains incurable despite the armamentarium of current multi-drug treatment strategies. The presence of dormant myeloma cells, which exit the cell cycle to evade treatment, persists through minimal residual disease and later reactivate causing relapse. There are currently no clinically approved 'dormalytic' therapies targeting dormant myeloma cells, highlighting a major unmet need in myeloma therapy. We have identified elevated levels of reactive oxygen species (ROS) in dormant myeloma cells compared to proliferating myeloma and healthy cells, suggesting a possible dormant cell specific vulnerability. We hypothesise that ROS induction presents a novel therapeutic strategy in dormant myeloma and is the mechanism of our novel dormalytic, which can kill dormant cells in 3 myeloma mouse models (5TGM1, JJN3, U266), delaying relapse and increasing survival beyond standard-of-care.

**Aims**

To determine whether oxidative stress from ROS induction can effectively eliminate dormant myeloma cells and prevent disease relapse.

**Methods**

ROS inducing compound auranofin or a novel 'dormalytic' were used to induce ROS, ± antioxidant N-acetyl cysteine (NAC). Viability in proliferating cells was assessed in resazurin dose-response assays. The lipophilic membrane dye Vybrant™ DiD was used to identify dormant myeloma cells through dye-retention. DiD-stained myeloma cells were monocultured or co-cultured with osteoblast-lineage cells (MC3T3) to support dormancy and apoptosis (Annexin V), ROS (CellROX™) and viability were assessed by flow cytometry. Oxidative damage (8-oxo-G, 4HNE, nitrotyrosine) was measured by immunofluorescence microscopy, and functional assays were performed in dormant and proliferative myeloma cells treated with both compounds.

**Results**

Auranofin and the novel dormalytic reduced viability of myeloma cell lines (U266, JJN3, 5TGM1) at nanomolar concentrations, which was rescued by the antioxidant NAC (4mM, both drugs in U266 $p<1e-15$). Oxidative stress markers were increased, and apoptosis was induced in both proliferative and dormant (DiD+++) cells by both compounds, with largest NAC rescue seen in dormant populations (dormalytic $p=0.0028$, auranofin $p<0.0001$). Myeloma cells, particularly dormant cells, were less sensitive to both therapies when co-cultured with osteoblasts than in monoculture, indicating osteoblasts provide some protection from oxidative stress within the myeloma bone microenvironment. This highlights that dormant myeloma cells are vulnerable to ROS induction and oxidative stress and provides evidence of microenvironment-driven resistance.

**Summary/Conclusion**

Oxidative stress induction presents a possible novel therapeutic strategy to eliminate dormant myeloma cells, prevent relapse and progress towards better outcomes for myeloma blood cancer patients.

**Copyright Information:** (Online) ISSN: 2572-9241
©2026 The Author(s). HemaSphere is published by John Wiley & Sons Ltd on behalf of European Hematology Association. This is an open access Abstract Book distributed under the Attribution-NonCommercial-NoDerivs (CC BY-NC-ND), which allows third parties to download the articles and share them with others as long as they credit the author and the Abstract Book, but they cannot change the content in any way or use them commercially.
**Abstract Book Citations:** Authors, Title, HemaSphere, 2026;10:(S1):pages. The abstract book DOIs can be found on the "Author Information" page.
**Disclaimer:** Articles published in the journal HemaSphere exclusively reflect the opinions of the authors. The authors are responsible for all content in their abstracts including accuracy of the facts, statements, citing resources, etc.

### (PB3176) GENETIC ABERRATIONS IN MULTIPLE MYELOMA REVEALED BY OPTICAL GENOME MAPPING

**Topic:** 13. Myeloma and other monoclonal gammopathies - Biology & translational research

Balázs Kardos*[1], Anna Bekő[1], Zsófia Urbán[1], Fruzsina Szél[2], István Ádám Wiedemann[2], Gábor Barna[1], Gergő Papp[1], Dóra Kapczár[1], Ildikó Istenes[3], Beáta Deák[4], András Masszi[4], Zsuzsanna Szemlaky[5], Péter Rajnics[6], Márk Plander[7], Masszi Tamás Pál[2], András Matolcsy[1], Gabor Mikala[2], Gergely Varga[2], Csaba Bödör[1]

*[1]Semmelweis University, Department of Pathology and Experimental Cancer Research, Budapest, Hungary;[2]Semmelweis University, Department of Internal Medicine and Hematology, Budapest, Hungary; [3]Semmelweis University, Department of Internal Medicine and Oncology, Budapest, Hungary; [4]National Institute of Oncology, Budapest, Hungary;[5]South Pest Central Hospital National Hematology and Infectology Institute, Budapest, Hungary; [6]Kaposi Mór University Teaching Hospital of County Somogy, Kaposvár, Hungary; [7]Teaching Hospital Markusovszky, Szombathely, Hungary;*

### Background

Optical genome mapping (OGM) is a high-throughput cytogenetic analysis method enabling accurate and reliable detection of structural variants (SVs) and copy number alterations (CNAs) from 500 base pairs to the genome-wide range. In multiple myeloma (MM), OGM enhances traditional cytogenetic approaches by identifying recurrent genomic abnormalities and delivering high-resolution structural insights.

### Aims

We aimed to evaluate the applicability of OGM in multiple myeloma and to systematically define the landscape of structural and copy number alterations, including previously undescribed events and genomic complexity patterns.

### Methods

Diagnostic bone marrow aspirates (BMAs) from 80 patients with MM were collected at the time of diagnosis and during relapse (median age 70 years, range 44–91). All 80 patients were analyzed by OGM using the Saphyr system (Bionano Genomics, San Diego, USA). The raw data was analyzed using the Bionano Access software, detecting SVs and CNAs aligned to the reference genome. The resulting genome maps were used to evaluate the genomic profile of the samples and to identify biologically or clinically relevant alterations.

### Results

OGM was successful in all 80 investigations. Translocations involving the immunoglobulin heavy chain (IGH) region at Chr14q32.33 were detected in 38 cases (47.5%). Additionally, novel translocations affecting the IGH region were identified in 7 patients (8.75%). These translocations (t(1;14), t(2;14), t(9;14), t(14;19), and t(14;22)) are not recognized as recurrent or clinically significant IGH translocations in multiple myeloma in the medical literature. Simultaneous IGH translocations involving different partner chromosomes were identified in 9 cases (11.25%). The most frequent combinations included t(4;14) with t(8;14), followed by t(8;14) co-occurring with t(11;14) and other rare pairings, such as t(11;14) with t(2;14). These findings indicate the presence of complex IGH rearrangement patterns within individual genomes. The presence of 17p deletion (del(17p)) was demonstrated in 20 patients (25%). Deletions involving chromosome 1p (1p12, 1p21, 1p22, and 1p32) were identified in 26 cases (32.5%) and frequently affected more than one subregion within the same genome. Gain of 1q21 was observed in 30 cases (37.5%), with 8 patients demonstrating high-level amplification. Chromothripsis and chromoplexy were identified in 12 and 5 patients, respectively, highlighting the complex genomic landscape of the disease.

**Copyright Information:** (Online) ISSN: 2572-9241
©2026 The Author(s). HemaSphere is published by John Wiley & Sons Ltd on behalf of European Hematology Association. This is an open access Abstract Book distributed under the Attribution-NonCommercial-NoDerivs (CC BY-NC-ND), which allows third parties to download the articles and share them with others as long as they credit the author and the Abstract Book, but they cannot change the content in any way or use them commercially.
**Abstract Book Citations:** Authors, Title, HemaSphere, 2026;10:(S1):pages. The abstract book DOIs can be found on the "Author Information" page.
**Disclaimer:** Articles published in the journal HemaSphere exclusively reflect the opinions of the authors. The authors are responsible for all content in their abstracts including accuracy of the facts, statements, citing resources, etc.

**Summary/Conclusion**

Application of OGM provided a high-resolution characterization of the genomic landscape in multiple myeloma, revealing novel, previously unreported IGH translocations, concurrent IGH rearrangements, and regionally complex 1p deletions, while accurately identifying 1q21 gain and amplification. By resolving patterns of co-occurrence and structural complexity, OGM expands the spectrum of detectable abnormalities beyond conventional cytogenetics and may refine genomic risk assessment in MM.

**Funding**
LP2024-3, K21_137948, H2020-739593, TKP2021-EGA-24, TKP2021-NVA-15, EKÖP-2025-559, ELIXIR Hungary

**Copyright Information:** (Online) ISSN: 2572-9241
©2026 The Author(s). HemaSphere is published by John Wiley & Sons Ltd on behalf of European Hematology Association. This is an open access Abstract Book distributed under the Attribution-NonCommercial-NoDerivs (CC BY-NC-ND), which allows third parties to download the articles and share them with others as long as they credit the author and the Abstract Book, but they cannot change the content in any way or use them commercially.
**Abstract Book Citations:** Authors, Title, HemaSphere, 2026;10:(S1):pages. The abstract book DOIs can be found on the "Author Information" page.
**Disclaimer:** Articles published in the journal HemaSphere exclusively reflect the opinions of the authors. The authors are responsible for all content in their abstracts including accuracy of the facts, statements, citing resources, etc.

### (PB3177) MELFLUFEN TREATMENT MAY BE PARTICULARLY EFFECTIVE IN IMMUNOCOMPROMISED PATIENTS WITH MULTIPLE MYELOMA

**Topic:** 13. Myeloma and other monoclonal gammopathies - Biology & translational research

Philipp Sergeev*[1], Juho Miettinen[1], Maiju-Emilia Huppunen[1], Minna Suvela[1], Stefan Svensson Gelius[2], Caroline Heckman[1]

*[1]University of Helsinki, Institute for Molecular Medicine Finland (FIMM), Helsinki, Finland;[2]Oncopeptides AB, Stockholm, Sweden;*

### Background

Melflufen is an alkylating peptide-drug conjugate approved for relapsed/refractory multiple myeloma (MM). Upon hydrolysis by peptidases, it preferentially delivers alkylating metabolites within tumor cells. Despite its clinical relevance, the relationship between melflufen efficacy and the tumor microenvironment (TME) remains poorly understood. Here, we investigate the interplay between immune and malignant cells in the context of melflufen sensitivity, using single-cell RNA sequencing (scRNAseq) and flow cytometry-based drug sensitivity and resistance testing (DSRT) of bone marrow mononuclear cells (BM-MNCs) from MM patients ex vivo.

### Aims

To identify biomarkers of melflufen sensitivity and provide insights into patient stratification and treatment strategy.

### Methods

Frozen BM-MNCs from two MM patient cohorts were used for DSRT and scRNAseq after written informed consent, per Declaration of Helsinki. The first cohort (n=26, 0.5–5,000 nM, 72h) was assessed for melflufen sensitivity using antibody panels identifying T, B, NK, progenitor, and malignant plasma cells (PCs). Samples were stratified into high (HS, Q3) and low sensitivity (LS, Q1) by PC-specific drug sensitivity score (DSS); PC-negative samples were excluded (n=11). The second cohort (n=24, 0.1–100,000 nM, 72h) was evaluated by DSRT and scRNAseq, with samples classified as high sensitivity (HS: DSS >40), intermediate (IS: DSS 31–40), or low sensitivity (LS: DSS <31). Differential gene expression (DGE) analyses between HS and LS samples identified genes associated with sensitivity. Exhaustion score was computed as the average expression of relevant genes minus the aggregated expression of randomly sampled, expression-matched control features for each cell.

### Results

When assessing the impact of the tumor immune microenvironment on melflufen sensitivity, we observed higher melflufen sensitivity in MM cells negatively correlated with the abundance of CD8 effector cells, cytotoxic immune cells that normally drive anti-tumor responses, in both cohorts (Fig. 1A, B).
Gene expression analysis revealed higher exhaustion score, driven by elevated expression of *TOX*, *HAVCR2* (TIM-3), and *TIGIT* genes markers in HS samples, suggesting that samples with less functional immune cells tend to be more sensitive to melflufen (Fig. 1C). Additionally, we identified five peptidase enzymes upregulated in effector cells and MM PCs, including *ERAP2*, previously linked to melflufen sensitivity (Miettinen et al., 2021). Two further candidates, *PSMA3* and *USP16*, are involved in DNA damage response pathways, potentially explaining the previously observed enrichment of DNA repair gene signatures in melflufen-sensitive MM cells (Acs et al., 2025).

**Copyright Information:** (Online) ISSN: 2572-9241
©2026 The Author(s). HemaSphere is published by John Wiley & Sons Ltd on behalf of European Hematology Association. This is an open access Abstract Book distributed under the Attribution-NonCommercial-NoDerivs (CC BY-NC-ND), which allows third parties to download the articles and share them with others as long as they credit the author and the Abstract Book, but they cannot change the content in any way or use them commercially.
**Abstract Book Citations:** Authors, Title, HemaSphere, 2026;10:(S1):pages. The abstract book DOIs can be found on the "Author Information" page.
**Disclaimer:** Articles published in the journal HemaSphere exclusively reflect the opinions of the authors. The authors are responsible for all content in their abstracts including accuracy of the facts, statements, citing resources, etc.

**Summary/Conclusion**

Our findings suggest that melflufen works best when the immune system is depleted or exhausted, which is commonly seen in heavily pretreated MM patients. This includes patients with therapy-induced lymphopenia or those who have relapsed after immunomodulatory drugs that progressively impair immune function, or after CAR-T cell therapy or bispecific antibodies, where T cell exhaustion and depletion are frequent consequences. Together, these patient groups represent a clinically relevant population in whom melflufen may retain or even enhance its efficacy. Furthermore, ERAP2, PSMA3, and USP16 represent promising candidate biomarkers for predicting melflufen sensitivity, warranting further functional validation.

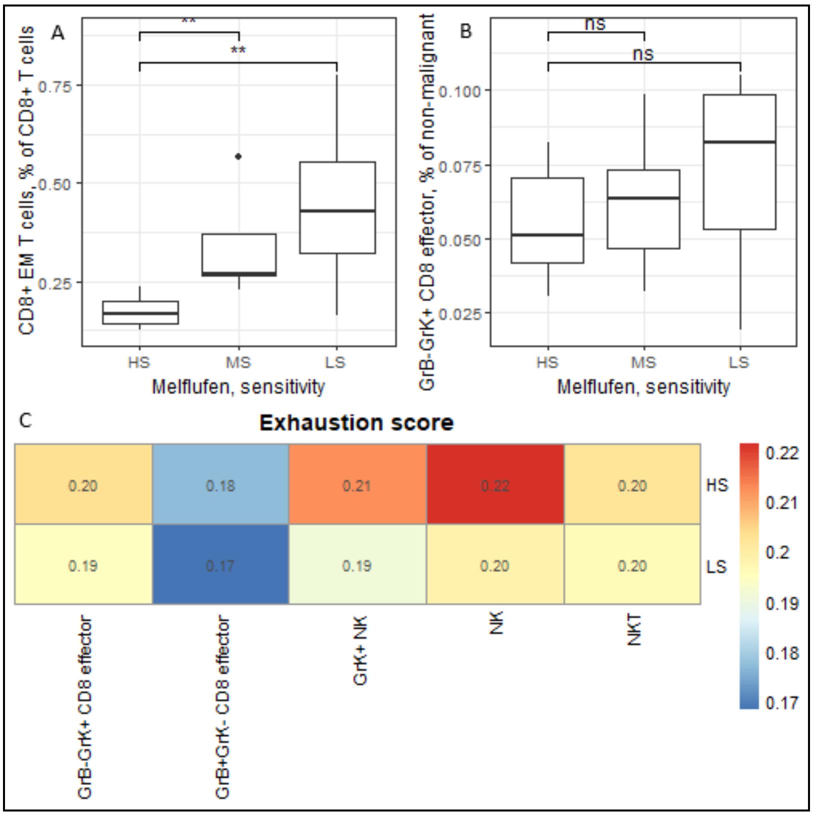

Figure 1. Melflufen HS samples demonstrates lower abundance of CD8+ effector cells in flow (A) and scRNAseq (B) cohorts and higher exhaustion for HS group (C).

**Copyright Information:** (Online) ISSN: 2572-9241
©2026 The Author(s). HemaSphere is published by John Wiley & Sons Ltd on behalf of European Hematology Association. This is an open access Abstract Book distributed under the Attribution-NonCommercial-NoDerivs (CC BY-NC-ND), which allows third parties to download the articles and share them with others as long as they credit the author and the Abstract Book, but they cannot change the content in any way or use them commercially.
**Abstract Book Citations:** Authors, Title, HemaSphere, 2026;10:(S1):pages. The abstract book DOIs can be found on the "Author Information" page.
**Disclaimer:** Articles published in the journal HemaSphere exclusively reflect the opinions of the authors. The authors are responsible for all content in their abstracts including accuracy of the facts, statements, citing resources, etc.

### (PB3178) DEVELOPMENTAL TOXICITY AND NEUROBEHAVIORAL EFFECTS OF POMALIDOMIDE ON ZEBRAFISH (DANIO RERIO) EMBRYOS: IMPLICATIONS FOR THE SAFETY PROFILE OF IMMUNOMODULATORY DRUGS

**Topic:** 13. Myeloma and other monoclonal gammopathies - Biology & translational research

Dimitrios Leonardos*[1], Maximos Leonardos[2], Panteleimon Takis[3], Lampros Lakkas[2], Eleftheria Hatzimichael[1], Constantine Stalikas[3]

*[1]University Hospital of Ioannina, Hematology, Ioannina, Greece; [2]University of Ioannina, Physiology, Ioannina, Greece; [3]University of Ioannina, Chemistry, Ioannina, Greece;*

### Background

Pomalidomide is a third-generation immunomodulatory drug (IMiD) used for relapsed/refractory multiple myeloma. Despite clinical efficacy, it belongs to a drug class historically associated with developmental toxicity, as exemplified by the thalidomide embryopathy. Pomalidomide acts through cereblon (CRBN)-mediated degradation of IKZF1/IKZF3 and demonstrates high blood-brain barrier penetration (30–40%), substantially exceeding that of lenalidomide (~5%). Given CRBN's critical role in neural stem cell survival and embryonic neurogenesis, evaluation of pomalidomide's developmental and neurobehavioral effects is warranted.

### Aims

To evaluate the developmental toxicity, neurobehavioral alterations, and cardiac effects of pomalidomide on zebrafish (*Danio rerio*) embryos, a vertebrate model sharing ~70% genetic homology with humans.

### Methods

Wild-type (AB) zebrafish embryos (N=780) were exposed to pomalidomide at 0 (control, n=300), 10 (n=180), 20 (n=180), and 40 µg/mL (n=120) from 24 hours post-fertilization. Developmental endpoints (body length, eye surface, heart rate, pericardial edema, malformations) were assessed at 24, 48, 72, and 96 hours. Behavioral analysis included locomotor activity under alternating dark/light conditions, thigmotaxis, and vibrational startle response (VSR) at two intensities, recorded using EthoVision XT (Noldus). Data were analyzed using GLM with Tukey's HSD post-hoc tests (p<0.05).

### Results

No mortality was observed up to 120 hours. Body length was significantly affected by pomalidomide concentration (p<0.001), exposure time (p<0.001), and their interaction (p=0.042), with reductions at 20 and 40 µg/mL at later timepoints (Fig. 1). Eye surface area was significantly affected by concentration (p<0.001), with progressively smaller areas at 40 µg/mL after 48 hours. Heart rate was influenced by concentration (p<0.001) and exposure time (p<0.001); 10–20 µg/mL induced tachycardia peaking at 72 hours, while 40 µg/mL showed a divergent decrease at 96 hours. Transient pericardial edema (<5%) was noted at 20–40 µg/mL at 72 hours, resolving by 96 hours.
Behavioral analysis revealed dose-dependent hyperlocomotion during dark phases at 20–40 µg/mL (p<0.001), with a significant concentration x light/dark interaction (p<0.001) (Fig. 2). Three behavioral subsets emerged: non-exposed/10 µg/mL (low activity), 20 µg/mL (intermediate), and 40 µg/mL (highest). Thigmotaxis showed a non-monotonic pattern: 10 µg/mL induced anxiety-like responses, whereas 20–40 µg/mL produced behavioral disinhibition with frequent inner-zone entries (p<0.001). The VSR was dose-dependently modulated, suggesting altered sensorimotor gating at higher concentrations.

**Copyright Information:** (Online) ISSN: 2572-9241
©2026 The Author(s). HemaSphere is published by John Wiley & Sons Ltd on behalf of European Hematology Association. This is an open access Abstract Book distributed under the Attribution-NonCommercial-NoDerivs (CC BY-NC-ND), which allows third parties to download the articles and share them with others as long as they credit the author and the Abstract Book, but they cannot change the content in any way or use them commercially.
**Abstract Book Citations:** Authors, Title, HemaSphere, 2026;10:(S1):pages. The abstract book DOIs can be found on the "Author Information" page.
**Disclaimer:** Articles published in the journal HemaSphere exclusively reflect the opinions of the authors. The authors are responsible for all content in their abstracts including accuracy of the facts, statements, citing resources, etc.

### Summary/Conclusion

Pomalidomide induces significant dose-dependent developmental toxicity and complex neurobehavioral alterations in zebrafish embryos, including impaired somatic growth, cardiac dysfunction, hyperlocomotion, and disrupted sensorimotor integration. These findings highlight the retained teratogenic potential of third-generation IMiDs. Further metabolomic assessment is essential to elucidate the underlying biochemical pathways and identify early biomarkers of neurotoxicity. The high CNS penetration and CRBN-mediated mechanism of pomalidomide warrant investigation regarding potential neurodevelopmental implications.

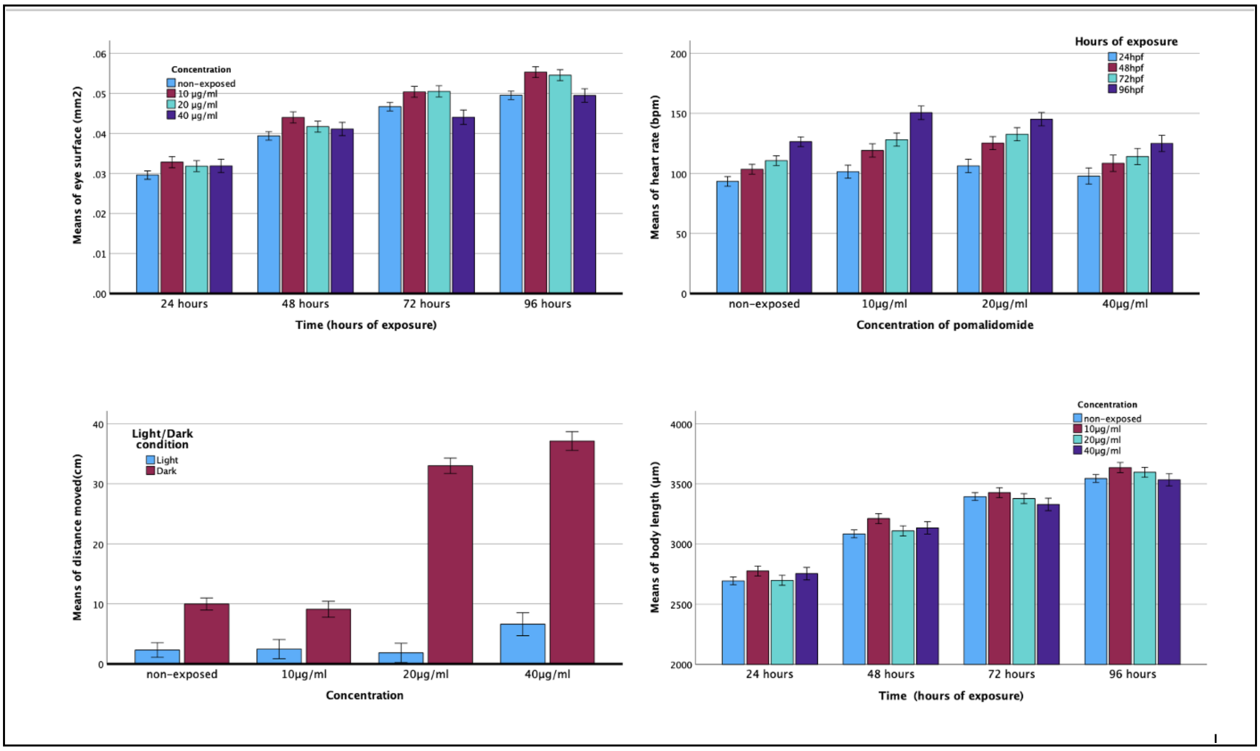

Figure 1. Developmental and behavioral effects of pomalidomide on zebrafish (Danio rerio) embryos. (A) Mean eye surface area (mm²) by concentration and exposure time. Specimens exposed to 40 µg/mL showed progressively smaller eye areas after 48 hours ($p<0.001$). (B) Mean heart rate (bpm) by concentration and exposure time. Tachycardia was observed at 10–20 µg/mL peaking at 72 hours, with a divergent decrease at 40 µg/mL by 96 hours ($p<0.001$). (C) Mean distance moved (cm) under light/dark conditions. Dose-dependent hyperlocomotion was observed during dark phases at 20–40 µg/mL ($p<0.001$). (D) Mean body length (µm) across exposure timepoints. Reductions were observed at 20 and 40 µg/mL at 72–96 hours ($p<0.001$). Error bars represent SEM.

**Copyright Information:** (Online) ISSN: 2572-9241
©2026 The Author(s). HemaSphere is published by John Wiley & Sons Ltd on behalf of European Hematology Association. This is an open access Abstract Book distributed under the Attribution-NonCommercial-NoDerivs (CC BY-NC-ND), which allows third parties to download the articles and share them with others as long as they credit the author and the Abstract Book, but they cannot change the content in any way or use them commercially.
**Abstract Book Citations:** Authors, Title, HemaSphere, 2026;10:(S1):pages. The abstract book DOIs can be found on the "Author Information" page.
**Disclaimer:** Articles published in the journal HemaSphere exclusively reflect the opinions of the authors. The authors are responsible for all content in their abstracts including accuracy of the facts, statements, citing resources, etc.

### (PB3179) PROGNOSTIC SIGNIFICANCE OF DYNAMIC CHANGES IN BONE MARROW B-CELLS, PLASMA CELLS AND T-CELLS IN WALDENSTRÖM MACROGLOBULINEMIA PATIENTS.

**Topic:** 13. Myeloma and other monoclonal gammopathies - Biology & translational research

Sarah Dobin*[1], Amine Moslemi[1], Lydia Montes[1], Laurette Lefeuvre[2], Daniela Robu[3], Stephanie Petit[3], Henri Sevestre[1], Jean Pierre Marolleau[1, 2], Hussein Ghamlouch[1, 2], Pierre Morel[1, 2]

*[1]CHU Amiens Picardie, Amiens, France;[2]Centre Universitaire de Recherche en Santé, Amiens, France;[3]Centre Hospitalier de Lens, Lens, France;*

### Background

In Waldenström Macroglobulinemia (WM), variable degrees of plasma cell (PC) differentiation are observed with the bone marrow (BM) B-cell infiltration, consistently with the identification of two biological WM subtypes, memory B-cell–like and plasma cell–like disease, the latter displaying higher CD38 expression. The role of BM microenvironment in tumor cell survival and treatment response is increasingly recognized. Response criteria rely mainly on serum IgM levels, but guidelines recommended a post-treatment BM evaluation using flow cytometry or immunohistochemistry (IHC) on BM biopsy (BMB). Indeed, a substantial B-cell depletion may occur despite persisting paraproteinemia. However, it remains unclear whether post-treatment bone marrow IHC findings provide prognostic information beyond conventional IgM-based response criteria.

### Aims

To evaluate the prognostic impact of dynamic changes in BM B-cells, PCs, T-cells, and stromal features (fibrosis) following therapy in WM patients.

### Methods

We reviewed BMB of 39 WM patients treated at the University Hospital of Amiens-Picardie and at the Hospital of Lens and who had paired BMB performed 0 to 128 days before treatment (median 35) and after therapy (median time from treatment initiation : 8.5 months, 4 to 16). MYD88 was mutated and del6q present in 95% and 43% of the evaluable patients, respectively. Chemoimmunotherapy was delivered in 32 patients and chemotherapy alone in 7 patients. It was first-line therapy in 26 patients, later line in 13. Independent pathological review of IHC checked the amount and the pattern of infiltration using the following monoclonal antibodies: anti-CD20, CD79a, CD138, CD3. Myelofibrosis was assessed with reticulin staining (MF0 vs. MF1 or 2). Additional markers (CD117, CD163, CD68 and CD5) were assessed in 12 patients. All patients gave their informed consent. Main endpoints were subsequent (from the time of post treatment BM biopsy) time to next treatment (STNT) and survival (SS).

### Results

At baseline, no IHC marker showed a prognostic impact. As expected, CD20+ and CD79a+ B-cell infiltrates decreased after therapy in 75% of patients. Changes in other cellular compartments were heterogeneous. For example, the amount of T-cells was unchanged in 55% of patients, the amount of CD138+ cells, increased, decreased or was unchanged in 44%; 36 % and 20% respectively. After treatment, greater CD20+ infiltration (>12%), CD79a+ infiltration (>20%) were significantly associated with shorter STNT (hazard ratio [HR]=2.5, p=0.01, and HR=3.7, p=0.0003, respectively). Greater CD138+ infiltration (>10%) and CD3+ infiltration (>10%) were associated with shorter SS (HR=3.1, p=0,05 and HR=4.0, p=0.04, respectively), The adverse prognostic value of high CD3+ cell count was also found in first-line patients (p=0,03). Finally, worsening reticulin fibrosis was associated with shorter STNT (HR=2.58, p=0.05).

**Copyright Information:** (Online) ISSN: 2572-9241
©2026 The Author(s). HemaSphere is published by John Wiley & Sons Ltd on behalf of European Hematology Association. This is an open access Abstract Book distributed under the Attribution-NonCommercial-NoDerivs (CC BY-NC-ND), which allows third parties to download the articles and share them with others as long as they credit the author and the Abstract Book, but they cannot change the content in any way or use them commercially.
**Abstract Book Citations:** Authors, Title, HemaSphere, 2026;10:(S1):pages. The abstract book DOIs can be found on the "Author Information" page.
**Disclaimer:** Articles published in the journal HemaSphere exclusively reflect the opinions of the authors. The authors are responsible for all content in their abstracts including accuracy of the facts, statements, citing resources, etc.

**Summary/Conclusion**

Post-treatment BM IHC evaluation provides prognostic information in WM: Persistent B-cell infiltration predicts earlier need for retreatment, while increased PC and T-cell infiltration is associated with inferior survival. These findings highlight the clinical relevance of bone marrow immune and tumor remodeling after therapy. Further studies are warranted to better decipher the prognostic impact of B-cell depletion, the role of PCs and T-cell subtypes on the clonal evolution of WM.

**Copyright Information:** (Online) ISSN: 2572-9241
©2026 The Author(s). HemaSphere is published by John Wiley & Sons Ltd on behalf of European Hematology Association. This is an open access Abstract Book distributed under the Attribution-NonCommercial-NoDerivs (CC BY-NC-ND), which allows third parties to download the articles and share them with others as long as they credit the author and the Abstract Book, but they cannot change the content in any way or use them commercially.
**Abstract Book Citations:** Authors, Title, HemaSphere, 2026;10:(S1):pages. The abstract book DOIs can be found on the "Author Information" page.
**Disclaimer:** Articles published in the journal HemaSphere exclusively reflect the opinions of the authors. The authors are responsible for all content in their abstracts including accuracy of the facts, statements, citing resources, etc.

### (PB3180) URINE PCR/ACR DISCORDANCE DEFINES A RENAL PROTEIN PHENOTYPE AND INDEPENDENTLY ASSOCIATES WITH RENAL IMPAIRMENT IN MULTIPLE MYELOMA

**Topic:** 13. Myeloma and other monoclonal gammopathies - Biology & translational research

Mamta Garg*[1], Chandhini Suresh[2]

*[1]University Hospitals Leicester, Haematology, Leicester, United Kingdom;[2]United Lincolnshire Hospitals, Lincoln, United Kingdom;*

### Background

Multiple myeloma (MM) is frequently complicated by renal impairment, often driven by monoclonal free light chains causing renal tubular injury and cast nephropathy. Renal recovery is a major determinant of prognosis, and delayed diagnosis remains common. Urine protein–creatinine ratio (PCR) is widely used to detect proteinuria, while albumin–creatinine ratio (ACR) specifically quantifies albumin excretion. In glomerular disease, PCR and ACR correlate closely. In contrast, in light-chain–mediated disease, PCR may be disproportionately elevated relative to ACR because light chains are non-albumin proteins.

Disproportionate elevation of PCR relative to ACR may indicate excess non-albumin protein, particularly light chains. Discordant urine PCR/ACR may therefore be a useful biomarker in making an earlier diagnosis of myeloma and predicting renal risk.

### Aims

We hypothesised that PCR/ACR discordance represents an early biomarker of light-chain nephropathy Specifically, we aimed to determine whether discordance:

1. Identifies BJ proteinuria
2. Independently predicts renal impairment
3. Reflects tumour burden or aggressive biology
4. Precedes clinical diagnosis

### Methods

We conducted a retrospective cohort study of 538 patients diagnosed with MM. PCR/ACR ratio was calculated to quantify discordance. Outcomes included Bence Jones (BJ) proteinuria, renal impairment at diagnosis (eGFR <60 mL/min/1.73m²), tumour burden markers, cytogenetic risk, and lead time between pre-diagnosis urine testing and formal diagnosis. Multivariate logistic regression incorporated log10-transformed variables and adjusted for age and PCR.

**Copyright Information:** (Online) ISSN: 2572-9241
©2026 The Author(s). HemaSphere is published by John Wiley & Sons Ltd on behalf of European Hematology Association. This is an open access Abstract Book distributed under the Attribution-NonCommercial-NoDerivs (CC BY-NC-ND), which allows third parties to download the articles and share them with others as long as they credit the author and the Abstract Book, but they cannot change the content in any way or use them commercially.
**Abstract Book Citations:** Authors, Title, HemaSphere, 2026;10:(S1):pages. The abstract book DOIs can be found on the "Author Information" page.
**Disclaimer:** Articles published in the journal HemaSphere exclusively reflect the opinions of the authors. The authors are responsible for all content in their abstracts including accuracy of the facts, statements, citing resources, etc.

**Results**

PCR exceeded twice ACR in 95.9% of the 538 patients.
Pre-diagnosis discordance (PCR >30 mg/mmol and PCR/ACR >2) was identified in 96 patients and preceded diagnosis by a median of 406 days (IQR 208–818). PCR increased significantly between pre-diagnostic and diagnostic sampling (paired Wilcoxon $p=4.48\times10^{-10}$).
At diagnosis, each 10-fold increase in PCR/ACR ratio was associated with a 2.05-fold increase in odds of renal impairment (95% CI 1.31–3.24). After adjustment for PCR and age, discordance remained independently associated (OR 1.65, 95% CI 1.03–2.64), whereas serum free light chains were not independently predictive of renal function.
For BJ positivity (n=297), PCR demonstrated superior discrimination (AUC 0.834) compared with PCR/ACR ratio (AUC 0.622; $p=3.4\times10^{-6}$).
Pre-diagnostic discordance was associated with increased odds of renal impairment at diagnosis (OR 2.45, 95% CI 1.50–3.91).

**Summary/Conclusion**

PCR/ACR discordance represents a renal protein phenotype associated with light-chain nephropathy in MM. Discordance independently predicts renal impairment and frequently precedes formal diagnosis by approximately one year. However, discordance shows only weak association with tumour burden markers and does not outperform PCR for BJ detection. Recognition of disproportionate non-albumin proteinuria may support earlier identification of patients at risk of renal injury.
In addition, discordant PCR and ACR can serve as a prompt to undertake a MM screen (serum protein electrophoresis and serum freelight assay) in patients with no symptoms and aid an earlier diagnosis of MM.

**Copyright Information:** (Online) ISSN: 2572-9241
©2026 The Author(s). HemaSphere is published by John Wiley & Sons Ltd on behalf of European Hematology Association. This is an open access Abstract Book distributed under the Attribution-NonCommercial-NoDerivs (CC BY-NC-ND), which allows third parties to download the articles and share them with others as long as they credit the author and the Abstract Book, but they cannot change the content in any way or use them commercially.
**Abstract Book Citations:** Authors, Title, HemaSphere, 2026;10:(S1):pages. The abstract book DOIs can be found on the "Author Information" page.
**Disclaimer:** Articles published in the journal HemaSphere exclusively reflect the opinions of the authors. The authors are responsible for all content in their abstracts including accuracy of the facts, statements, citing resources, etc.

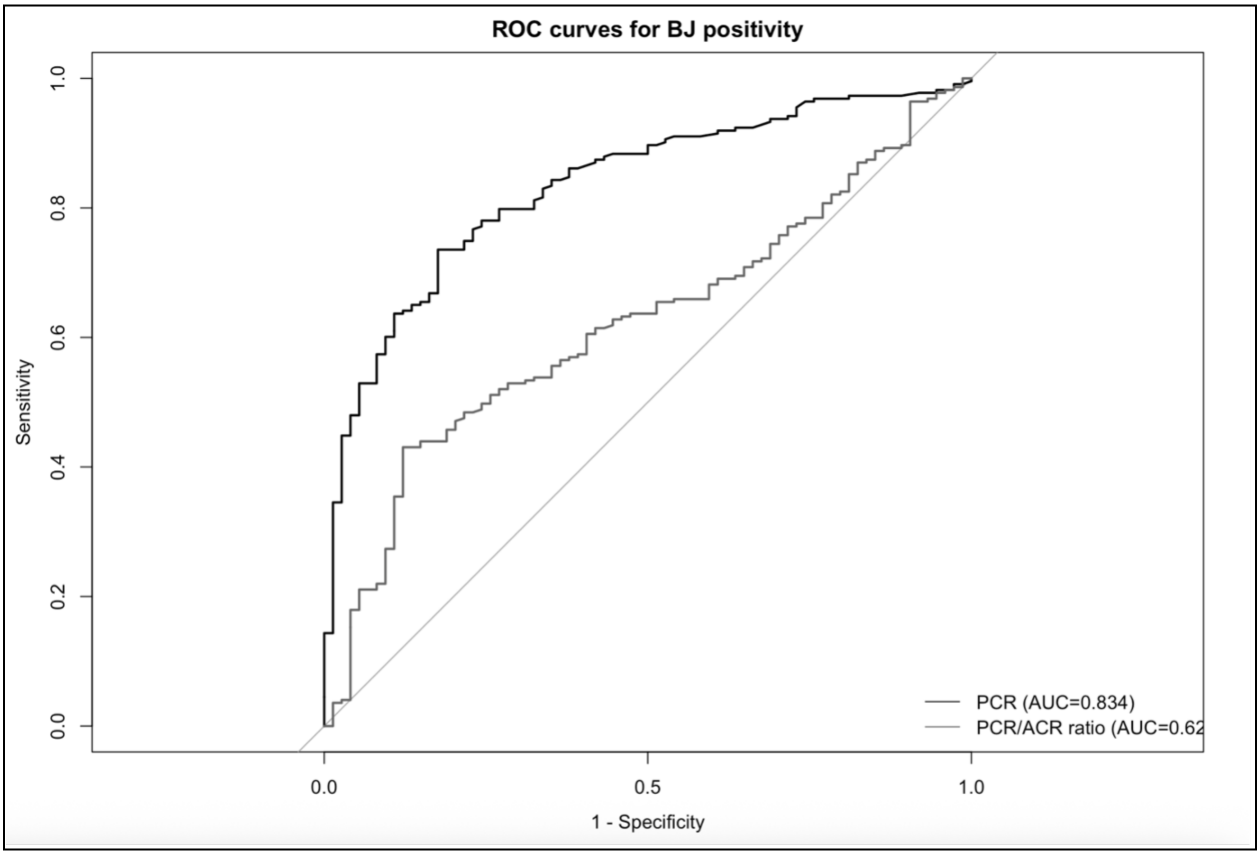

ROC curves for prediction of Bence Jones positivity. Receiver operating characteristic curves comparing urine PCR and PCR/ACR ratio for discrimination of Bence Jones proteinuria.

**Copyright Information:** (Online) ISSN: 2572-9241
©2026 The Author(s). HemaSphere is published by John Wiley & Sons Ltd on behalf of European Hematology Association. This is an open access Abstract Book distributed under the Attribution-NonCommercial-NoDerivs (CC BY-NC-ND), which allows third parties to download the articles and share them with others as long as they credit the author and the Abstract Book, but they cannot change the content in any way or use them commercially.
**Abstract Book Citations:** Authors, Title, HemaSphere, 2026;10:(S1):pages. The abstract book DOIs can be found on the "Author Information" page.
**Disclaimer:** Articles published in the journal HemaSphere exclusively reflect the opinions of the authors. The authors are responsible for all content in their abstracts including accuracy of the facts, statements, citing resources, etc.

### (PB3181) THE NONO INTERACTOME IN MULTIPLE MYELOMA: MAPPING PROTEIN PARTNERS AND REGULATORY PATHWAYS

**Topic:** 13. Myeloma and other monoclonal gammopathies - Biology & translational research

Domenica Ronchetti[1], Ilaria Silvestris[1], Valentina Traini[1], Giuseppina Fabbiano[2], Giulia Biancon[2], Marta Lionetti[1], Francesca Lazzaroni[2], Francesco Passamonti[2], Niccolò Bolli[2], Elisa Taiana*[2]

*[1]University of Milan, Department of Oncology and Hemato-Oncology, Milan, Italy;[2]IRCCS Fondazione Ca' Granda Ospedale Maggiore Policlinico, Hematology, Milan, Italy;*

### Background

Multiple myeloma (MM) is an incurable malignancy characterized by the uncontrolled proliferation of bone marrow plasma cells. The lncRNA NEAT1, the structural scaffold of paraspeckles (PSs), promotes MM progression by regulating DNA repair pathways and enhancing cell survival under stress conditions. NONO, a multifunctional RNA- and DNA-binding protein that stabilizes NEAT1 and is essential for PS biogenesis and structural integrity, is upregulated in MM and correlates with poor patient survival. Beyond its canonical role within PSs, NONO has been implicated in several additional cellular processes, indicating that it may exert broader regulatory functions and potentially contribute to MM pathobiology through mechanisms that remain to be fully elucidated.

### Aims

To comprehensively define the NONO interactome in MM cells, with the aim of uncovering its molecular partners and delineating the regulatory networks through which it contributes to MM pathobiology.

### Methods

Total protein extracts from MM cell lines (AMO-1, NCI-H929, and LP1) were immunoprecipitated using an anti-NONO antibody. A negative control was performed using the secondary antibody alone to assess non-specific binding. Immunoprecipitated samples were analysed by mass spectrometry at the University of Trento to characterize the NONO interactome.

### Results

The results demonstrated that NONO interacts with over one hundred distinct proteins across all three cell lines, based on a fold change threshold of $\log_2(FC)>2$ and an adjusted p-value < 0.05. Specifically, 167, 531, and 132 NONO-interacting proteins were identified in AMO-1, NCI-H929, and LP1 cells, respectively, highlighting both shared and cell line-specific interaction patterns. Notably, 38 proteins were consistently detected in all samples, defining a conserved NONO core interactome. Pathway enrichment analysis of these common interactors using the EnrichR tool revealed significant enrichment ($p<0.05$) in biological processes related to cell cycle control and proliferative signalling, supporting a role for NONO in sustaining malignant cell growth.
Strong associations were also observed with RNA metabolism, including processing and splicing, consistent with NONO's established function as an RNA-binding protein.
In addition, enriched pathways included cellular stress response mechanisms and DNA damage repair, indicating a potential contribution to genome surveillance.
Further links emerged with transcriptional regulation, chromatin remodelling, telomere maintenance and nucleosome organization, underscoring a broader involvement in chromatin dynamics and genome stability.
Finally, the identification of proteins involved in signal transduction, cell-cell communication, and mitochondrial dynamics suggests that NONO integrated nuclear and cytoplasmic regulatory networks, contributing to the maintenance of cellular homeostasis.

**Copyright Information:** (Online) ISSN: 2572-9241
©2026 The Author(s). HemaSphere is published by John Wiley & Sons Ltd on behalf of European Hematology Association. This is an open access Abstract Book distributed under the Attribution-NonCommercial-NoDerivs (CC BY-NC-ND), which allows third parties to download the articles and share them with others as long as they credit the author and the Abstract Book, but they cannot change the content in any way or use them commercially.
**Abstract Book Citations:** Authors, Title, HemaSphere, 2026;10:(S1):pages. The abstract book DOIs can be found on the "Author Information" page.
**Disclaimer:** Articles published in the journal HemaSphere exclusively reflect the opinions of the authors. The authors are responsible for all content in their abstracts including accuracy of the facts, statements, citing resources, etc.

**Summary/Conclusion**

This study confirms the involvement of NONO in pathways already described in the literature, further strengthening current knowledge of its biological functions in MM cells. Importantly, our analysis also uncovered additional pathways associations that, particularly in the context of MM, appear to be previously unreported and warrant further investigation. Elucidating these novel roles of NONO may provide deeper insights into the molecular mechanisms underlying MM pathogenesis, and potentially identify new biomarkers or therapeutic targets relevant to disease progression.

**Copyright Information:** (Online) ISSN: 2572-9241
©2026 The Author(s). HemaSphere is published by John Wiley & Sons Ltd on behalf of European Hematology Association. This is an open access Abstract Book distributed under the Attribution-NonCommercial-NoDerivs (CC BY-NC-ND), which allows third parties to download the articles and share them with others as long as they credit the author and the Abstract Book, but they cannot change the content in any way or use them commercially.
**Abstract Book Citations:** Authors, Title, HemaSphere, 2026;10:(S1):pages. The abstract book DOIs can be found on the “Author Information” page.
**Disclaimer:** Articles published in the journal HemaSphere exclusively reflect the opinions of the authors. The authors are responsible for all content in their abstracts including accuracy of the facts, statements, citing resources, etc.

### (PB3182) BONE MARROW STROMAL PROTEOMICS IDENTIFIES DETERMINANTS OF TREATMENT RESPONSE IN MULTIPLE MYELOMA.

**Topic:** 13. Myeloma and other monoclonal gammopathies - Biology & translational research

Aishwarya Dash*[1], Man Updesh Singh Sachdeva[1], Pankaj Malhotra[2], Manni Luthra Guptasarma[3], Sreejesh Sreedharanunni[1]

*[1]Postgraduate Institute of Medical Education and Research,Chandigarh,India, Hematology, Chandigarh, India;[2]Postgraduate Institute of Medical Education and Research,Chandigarh, Clinical Hematology and Medical Oncology, Chandigarh, India; [3]Postgraduate Institute of Medical Education and Research, Chandigarh, Immunopathology, Chandigarh, India;*

### Background

The bone marrow microenvironment particularly bone marrow mesenchymal stromal cells (BM-MSCs) is a critical regulator of multiple myeloma (MM) pathogenesis, and possibly the therapeutic response. BMMSCs govern malignant plasma-cell homing, survival, proliferation and through direct cell contact, soluble mediators, and extracellular vesicles. While the genetic and epigenetic landscape of MM plasma cells has been extensively characterized, the stromal proteome and its dynamic remodelling remain underexplored as determinants of treatment response and resistance.

### Aims

This study aims to decode proteomic alterations within the stromal niche that underpin therapeutic failure.

### Methods

BMMSCs were isolated and culture-expanded from newly diagnosed MM (NDMM) patients (n=10), and their purity confirmed by flow cytometry. High-resolution LC-MS–based proteomics was performed on patient-matched BMMSCs. Patients were stratified as good responders (GR; n=4) and included those who achieved stringent complete response (sCR) following bortezomib-based induction therapy, and the rest were categorised as poor responders (PR; n=4). Differential protein expression and pathway enrichment analyses were conducted. Selected candidate proteins were validated by Western blotting and confocal microscopy in an independent NDMM cohort (n=37). Statistical significance was assessed using the Mann–Whitney U test.

### Results

BMMSC proteomic profiles revealed a clear segregation between the PR and GR groups. PR-derived stromal cells exhibited pronounced enrichment of cholesterol biosynthesis, steroid & lipid metabolism, and rRNA-processing pathways, alongside suppression of glycosaminoglycan and glycosphingolipid metabolism. Among PR-specific differentially expressed proteins, **SEPTIN6** emerged as a key cytoskeletal regulator, displaying both overexpression and aberrant nuclear localization – indicating a possibility of stress-induced structural remodelling. LC-MS analysis further revealed significant upregulation of **TGF-β1** in PR stromal cells, reflecting a cytokine-driven activated state. Collectively, the SEPTIN6–TGF-β axis defines a mechanically stiff, immunosuppressive, and pro-adhesive stromal phenotype that is permissive to myeloma persistence. Validation in the independent cohort confirmed significantly higher SEPTIN6 expression in PR patients ($p < 0.05$).

**Copyright Information:** (Online) ISSN: 2572-9241
©2026 The Author(s). HemaSphere is published by John Wiley & Sons Ltd on behalf of European Hematology Association. This is an open access Abstract Book distributed under the Attribution-NonCommercial-NoDerivs (CC BY-NC-ND), which allows third parties to download the articles and share them with others as long as they credit the author and the Abstract Book, but they cannot change the content in any way or use them commercially.
**Abstract Book Citations:** Authors, Title, HemaSphere, 2026;10:(S1):pages. The abstract book DOIs can be found on the “Author Information” page.
**Disclaimer:** Articles published in the journal HemaSphere exclusively reflect the opinions of the authors. The authors are responsible for all content in their abstracts including accuracy of the facts, statements, citing resources, etc.

**Summary/Conclusion**

This study identifies a distinct proteomic signature of BMMSC remodeling associated with therapeutic resistance in MM. Aberrant overexpression and nuclear localization of SEPTIN6, coupled with enhanced TGF-β signaling, characterize a resistant stromal state. These findings position SEPTIN6 as a potential biomarker of poor response and highlight the stromal–cytoskeletal–cytokine axis as a promising target for overcoming microenvironment-mediated drug resistance.

**Copyright Information:** (Online) ISSN: 2572-9241
©2026 The Author(s). HemaSphere is published by John Wiley & Sons Ltd on behalf of European Hematology Association. This is an open access Abstract Book distributed under the Attribution-NonCommercial-NoDerivs (CC BY-NC-ND), which allows third parties to download the articles and share them with others as long as they credit the author and the Abstract Book, but they cannot change the content in any way or use them commercially.
**Abstract Book Citations:** Authors, Title, HemaSphere, 2026;10:(S1):pages. The abstract book DOIs can be found on the “Author Information” page.
**Disclaimer:** Articles published in the journal HemaSphere exclusively reflect the opinions of the authors. The authors are responsible for all content in their abstracts including accuracy of the facts, statements, citing resources, etc.

### (PB3183) DNA DAMAGE REPAIR (DDR) RELATED PROGNOSTIC RISK MODEL IN MULTIPLE MYELOMA BASED ON SINGLE-CELL AND BULK SEQUENCING

**Topic:** 13. Myeloma and other monoclonal gammopathies - Biology & translational research

Hongxiu Liu*[1], Yanping MA[1]

[1]*second hospital of shanxi medical university, Department of Hematology, Taiyuan, China;*

**Background**

Genomic rearrangements and instability are key pathological features of multiple myeloma (MM). However, the impact of DNA damage repair (DDR) on disease progression, the immune microenvironment, and genomic alterations in MM remains incompletely understood.

**Aims**

This study aimed to systematically evaluate the prognostic significance of DDR-related genes in multiple myeloma and to develop a DDR-based risk model integrating single-cell and bulk RNA sequencing data.

**Methods**

We screened DDR-related genes from single-cell and bulk RNA-seq datasets using differential expression analysis and weighted gene co-expression network analysis (WGCNA). Univariate Cox regression and LASSO analyses were performed to identify prognostic genes from the overlapping candidates. A prognostic signature was constructed based on gene expression levels and corresponding regression coefficients. Immune infiltration and mutation landscape analyses were conducted to characterize biological differences between risk groups. Functional enrichment analyses were performed to explore underlying molecular mechanisms. Finally, quantitative real-time PCR (qRT-PCR) was performed to validate the expression of key prognostic genes.

**Results**

Eight overlapping genes were identified at the intersection of WGCNA-derived hub genes and differentially expressed genes from scRNA-seq analysis. Univariate Cox regression and LASSO analyses identified three prognostic genes (PARP1, PCNA, and RAD23A), which were used to construct a DDR-related risk signature. Patients were stratified into high- and low-risk groups based on the median risk score. High-risk patients exhibited significantly poorer overall survival in both training and validation cohorts. Immune infiltration analysis revealed increased activated memory $CD4^+$ T cells, activated NK cells, and resting mast cells in the high-risk group. Mutation analysis showed higher frequencies of IGHV2-70 and IGLV3-1 alterations in high-risk patients, while KRAS and NRAS mutation rates were comparable between groups. Functional enrichment analyses suggested that the three key genes were associated with pathways related to protein processing, glycosylation, ribosome function, and oxidative stress. All three genes were upregulated in MM samples compared with controls, with PARP1 and RAD23A showing significant overexpression.

**Summary/Conclusion**

Altogether, the DDR-related prognostic model in MM may facilitate risk stratification and inform treatment decisions, with key prognostic genes potentially serving as biomarkers and therapeutic targets.

**Copyright Information:** (Online) ISSN: 2572-9241
©2026 The Author(s). HemaSphere is published by John Wiley & Sons Ltd on behalf of European Hematology Association. This is an open access Abstract Book distributed under the Attribution-NonCommercial-NoDerivs (CC BY-NC-ND), which allows third parties to download the articles and share them with others as long as they credit the author and the Abstract Book, but they cannot change the content in any way or use them commercially.
**Abstract Book Citations:** Authors, Title, HemaSphere, 2026;10:(S1):pages. The abstract book DOIs can be found on the “Author Information” page.
**Disclaimer:** Articles published in the journal HemaSphere exclusively reflect the opinions of the authors. The authors are responsible for all content in their abstracts including accuracy of the facts, statements, citing resources, etc.

### (PB3184) GUT LACTIC ACID BACTERIA PROMOTE MULTIPLE MYELOMA PROGRESSION BY UPREGULATING PRDX2 TO INHIBIT FERROPTOSIS AND REMODEL THE IMMUNE MICROENVIRONMENT

**Topic:** 13. Myeloma and other monoclonal gammopathies - Biology & translational research

Yue Chen[1, 2], Minjing Liao[1], Liansheng Zhang*[1, 2]

*[1]The Second Clinical Medical College, Lanzhou University, Lanzhou, China;[2]The Second Hospital & Clinical Medical College, Lanzhou University, Lanzhou, China;*

### Aims

Grounded in the increasingly recognized "gut microbiota-tumor immunity" axis, this study aimed to profile the expression patterns and prognostic value of the peroxiredoxin (PRDX) family in multiple myeloma (MM). Furthermore, we sought to elucidate the biological roles and immunological functions of PRDX2, alongside its regulation by the gut microbiota.

### Methods

Leveraging bioinformatics analyses and clinical sample evaluations, we compared the expression levels of PRDX family members across normal plasma cells, as well as newly diagnosed (NDMM) and relapsed MM cells. Survival analyses were conducted to assess the prognostic significance of PRDXs. Functional assays were performed to investigate the impact of PRDX2 on MM cell proliferation, ferroptosis resistance, and immune infiltration. Additionally, 16S rRNA gene sequencing was utilized to characterize disparities in the gut microbiota between healthy donors (HDs) and NDMM patients, followed by experimental validation of the regulatory effects exerted by these differentially enriched microbes on PRDX2.

### Results

Compared to normal plasma cells, PRDX2/4/5/6 expression levels were significantly elevated in MM cells. In relapsed MM, the expression of PRDX1/2/3/6 was further upregulated relative to baseline levels. Notably, elevated PRDX2 expression strongly correlated with adverse clinicopathological features and disease progression in MM. Enrichment analyses revealed that PRDX2 is implicated in diverse biological functions, influencing metabolic networks and modulating both ferroptosis and immune-related signaling pathways in MM cells. *In vitro* functional assays demonstrated that PRDX2 silencing significantly suppressed MM cell proliferation, sensitized cells to ferroptosis, and potentially modulated the MM immune microenvironment. Microbiome analyses identified a significantly higher abundance of lactic acid bacteria (LAB) in NDMM patients, which may drive the malignant progression of MM via a PRDX2-dependent mechanism.

### Summary/Conclusion

This study highlights the critical association between PRDX2, ferroptosis, and the tumor immune microenvironment, elucidating the pathogenic role of the "gut microbiota-PRDX2" axis in MM. These findings provide a theoretical foundation for the development of precision immunotherapies and comprehensive intervention strategies targeting this axis in MM.

**Copyright Information:** (Online) ISSN: 2572-9241
©2026 The Author(s). HemaSphere is published by John Wiley & Sons Ltd on behalf of European Hematology Association. This is an open access Abstract Book distributed under the Attribution-NonCommercial-NoDerivs (CC BY-NC-ND), which allows third parties to download the articles and share them with others as long as they credit the author and the Abstract Book, but they cannot change the content in any way or use them commercially.
**Abstract Book Citations:** Authors, Title, HemaSphere, 2026;10:(S1):pages. The abstract book DOIs can be found on the "Author Information" page.
**Disclaimer:** Articles published in the journal HemaSphere exclusively reflect the opinions of the authors. The authors are responsible for all content in their abstracts including accuracy of the facts, statements, citing resources, etc.

### (PB3185) TARGETING CD38 BEYOND DARATUMUMAB: A REAL-WORLD MULTICENTRE EXPERIENCE WITH ISATUXIMAB COMBINATIONS IN DARATUMUMAB-EXPOSED MULTIPLE MYELOMA.

**Topic:** 13. Myeloma and other monoclonal gammopathies - Biology & translational research

Paula Gili Herreros*[1], Xabier Gutierrez Lopez De Ocariz[2], Leticia Cañas De Dios[3], Patricia García Ramírez[1], Marta Callejas[1], Julio García Suárez[1], Juan Jose Gil Fernandez[1]

*[1]Hospital Universitario Príncipe de Asturias, Hematology, Alcalá de Henares, Spain;[2]Hospital Universitario Araba, Hematology, Vitoria-Gasteiz, Spain; [3]General Hospital Villarobledo, Hematology, Villarrobledo, Spain;*

### Background

Daratumumab-based regimens have improved outcomes in multiple myeloma (MM); however, patients who become refractory have a poor prognosis and no established sequential strategy. Despite targeting CD38, mechanistic differences between daratumumab and isatuximab may support the use of isatuximab as salvage therapy without a mandatory washout period.

### Aims

To evaluate the response to salvage therapy with isatuximab plus carfilzomib–dexamethasone (Isa-Kd) without a washout period in patients with daratumumab-refractory MM.

### Methods

This retrospective multicenter real-world study included MM patients treated with isatuximab after prior daratumumab exposure (June 2022–February 2026). Demographic, clinical, treatment, and response data were collected.

### Results

Twenty daratumumab-refractory MM patients were included (median age 74 years [IQR 61.75–75]); 60% had high-risk cytogenetics and median R-ISS was 2. Baseline characteristics and outcomes are shown in Table 1.
Eighty-five percent of patients received early salvage therapy with isatuximab (washout period <3 months). The median time between the last daratumumab dose and the first isatuximab dose was 37.5 days (IQR 31.5–49.5). A rapid objective response was observed in 80% (16/20) of patients. Among responders, strict complete response (sCR) was achieved in 10%, complete response (CR) in 5%, very good partial response (VGPR) in 30%, partial response (PR) in 25%, and stable disease (SD) in 10%. Among the four non-responders, 50% experienced an initial objective response before progression (after cycle 2 at 2 months and after cycle 8 at 8 months, respectively) (Figure 1).

***Table 1. Demographic characteristics, laboratory parameters, and salvage treatment with isatuximab.***

| | **Patients n = 20 (100%)** |
|---|---|
| **Age (years (IQR))** | 74 (61.75-75) |

**Copyright Information:** (Online) ISSN: 2572-9241
©2026 The Author(s). HemaSphere is published by John Wiley & Sons Ltd on behalf of European Hematology Association. This is an open access Abstract Book distributed under the Attribution-NonCommercial-NoDerivs (CC BY-NC-ND), which allows third parties to download the articles and share them with others as long as they credit the author and the Abstract Book, but they cannot change the content in any way or use them commercially.
**Abstract Book Citations:** Authors, Title, HemaSphere, 2026;10:(S1):pages. The abstract book DOIs can be found on the "Author Information" page.
**Disclaimer:** Articles published in the journal HemaSphere exclusively reflect the opinions of the authors. The authors are responsible for all content in their abstracts including accuracy of the facts, statements, citing resources, etc.

| | | | |
|---|---|---|---|
| Sex | Male | | 12 (66%) |
| | Female | | 8 (34%) |
| Diagnosis | MM subtype | IgG kappa | 6 (30%) |
| | | IgG lambda | 3 (15%) |
| | | LC lambda | 4 (20%) |
| | | LC kappa | 3 (15%) |
| | | IgA kappa | 3 (15%) |
| | | Plasma cell leukemia | 1 (5%) |
| | Cytogenetic | High risk | 12 (60%) |
| | R-ISS | 2-3 | 17 (85%) |
| Prior therapies | Dara-VRD | | 9 (45%) |
| | Dara-VCD | | 4 (20%) |
| | Dara-LenaD | | 5 (25%) |
| | Dara-VTD | | 3 (15%) |
| | ASCT | | 2 (10%) |
| | Line of treatment | 2 | 2 (10%) |
| | | 3 | 5 (25%) |
| | | 4 | 6 (30%) |

**Copyright Information:** (Online) ISSN: 2572-9241
©2026 The Author(s). HemaSphere is published by John Wiley & Sons Ltd on behalf of European Hematology Association. This is an open access Abstract Book distributed under the Attribution-NonCommercial-NoDerivs (CC BY-NC-ND), which allows third parties to download the articles and share them with others as long as they credit the author and the Abstract Book, but they cannot change the content in any way or use them commercially.
**Abstract Book Citations:** Authors, Title, HemaSphere, 2026;10:(S1):pages. The abstract book DOIs can be found on the "Author Information" page.
**Disclaimer:** Articles published in the journal HemaSphere exclusively reflect the opinions of the authors. The authors are responsible for all content in their abstracts including accuracy of the facts, statements, citing resources, etc.

| | | | |
|---|---|---|---|
| Isatuximab | | 5 | 4 (20%) |
| | | 6 | 3 (15%) |
| | Treatment regimen | Isa-KD | 11 (55%) |
| | | Isa-PomaD | 9 (45%) |
| | Number of cycles (median (IQR)) | | 7 (2-12.5) |
| | Time since last daratumumab dose (median days (IQR)) | | 37.5 (31.5-49.5) |
| Response | sCR | | 2 (10%) |
| | CR | | 1 (5%) |
| | VGPR | | 6 (30%) |
| | PR | | 5 (25%) |
| | SD | | 2 (10%) |
| | No response | | 4 (20%) |

*MM: multiple myeloma.ISS: International Staging System.R-ISS: Revised International Staging System. FLC: free light chains. K: kappa. L: lambda. MC: monoclonal component. Del: deletion. Dara-VRd: Daratumumab, Bortezomib, Lenalidomide, and Dexamethasone. Dara-VCD: Daratumumab, Bortezomib, Cyclophosphamide, and Dexamethasone. Dara-CyBorD: Daratumumab, Cyclophosphamide, Bortezomib, and Dexamethasone. Dara-Lena-D: Daratumumab, Lenalidomide, and Dexamethasone. Dara-VTD: Daratumumab, Bortezomib, Thalidomide, and Dexamethasone. ASCT: Autologous Stem Cell Transplantation. IQR: Interquartile Range. Isa-KD: Isatuximab, Carfilzomib, and Dexamethasone. Isa-PomaD: Isatuximab, Pomalidomide, and Dexamethasone. sCR: stringent complete response. CR: complete response.VGPR: very good partial response. PR: partial response. SD: stable disease.*

### Summary/Conclusion

Most patients achieved rapid and deep responses following isatuximab without a washout period, suggesting its potential as a sequential therapeutic strategy in daratumumab-refractory MM. Further real-world studies are warranted to validate this approach and optimize management in this clinical setting.

**Copyright Information:** (Online) ISSN: 2572-9241
©2026 The Author(s). HemaSphere is published by John Wiley & Sons Ltd on behalf of European Hematology Association. This is an open access Abstract Book distributed under the Attribution-NonCommercial-NoDerivs (CC BY-NC-ND), which allows third parties to download the articles and share them with others as long as they credit the author and the Abstract Book, but they cannot change the content in any way or use them commercially.
**Abstract Book Citations:** Authors, Title, HemaSphere, 2026;10:(S1):pages. The abstract book DOIs can be found on the “Author Information” page.
**Disclaimer:** Articles published in the journal HemaSphere exclusively reflect the opinions of the authors. The authors are responsible for all content in their abstracts including accuracy of the facts, statements, citing resources, etc.

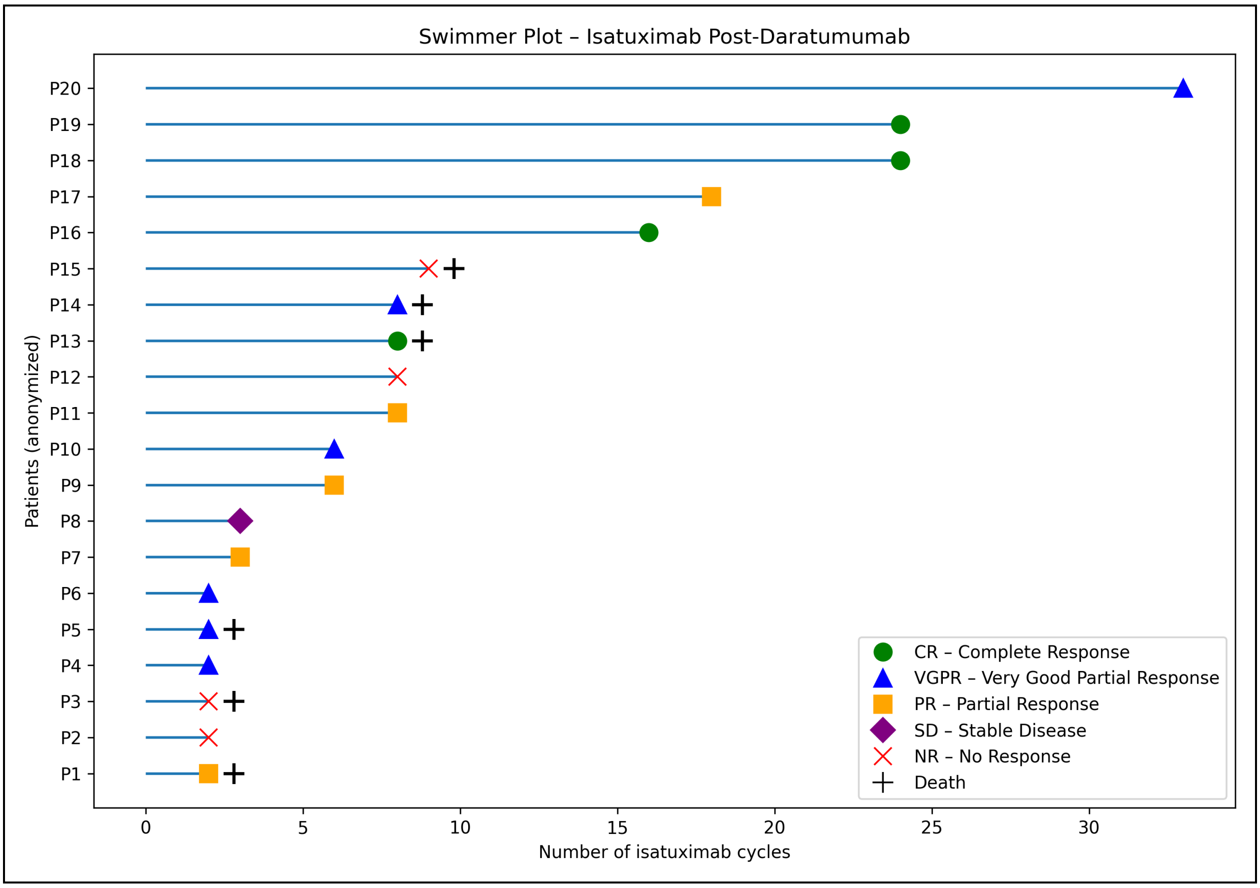

**Copyright Information:** (Online) ISSN: 2572-9241
©2026 The Author(s). HemaSphere is published by John Wiley & Sons Ltd on behalf of European Hematology Association. This is an open access Abstract Book distributed under the Attribution-NonCommercial-NoDerivs (CC BY-NC-ND), which allows third parties to download the articles and share them with others as long as they credit the author and the Abstract Book, but they cannot change the content in any way or use them commercially.
**Abstract Book Citations:** Authors, Title, HemaSphere, 2026;10:(S1):pages. The abstract book DOIs can be found on the "Author Information" page.
**Disclaimer:** Articles published in the journal HemaSphere exclusively reflect the opinions of the authors. The authors are responsible for all content in their abstracts including accuracy of the facts, statements, citing resources, etc.

### (PB3186) REAL-WORLD EFFECTIVENESS AND SAFETY OF BELANTAMAB MAFODOTIN–BASED CONTAINING REGIMEN IN RELAPSED/REFRACTORY MULTIPLE MYELOMA: A EMN LAZIO WORKING GROUP EXPERIENCE.

**Topic:** 13. Myeloma and other monoclonal gammopathies - Biology & translational research

Marco Zomparelli*[1], Angela Rago[2], Elena Rossi[3], Francesca Di Landro[3], Luca Franceschini[1], Manuela Rizzo[1], Federica Sisti[4], Luca Cupelli[5], Silvia Ferraro[5], Marcello Capriata[6], Monica Di Cecca[7], Martina Tomassi[8], Gianluca Maiorana[9], Alfonso Piciocchi[10], Tommaso Caravita Di Toritto[2]

*[1]Hematology, University Tor Vergata, UOC Patologie Linfoproliferative, Roma, Italy; [2]Ospedale Santo Spirito in Saxia, Roma, Italy; [3]Università' Cattolica Sacro Cuore Fondazione Policlinico A.Gemelli, IRCCS, Scienze di laboratorio ed Ematologiche, Roma, Italy; [4]Ospedale Santa Maria Goretti, Università Sapienza Polo Pontino, Medicina Traslazionale e di Precisione, Latina, Italy;[5]Ospedale Sant'Eugenio, Roma, Italy; [6]Azienda Ospedaliera Policlinico Umberto I, Roma, Italy;[7]Fondazione Policlinico Universitario Campus Bio Medico, UOC Ematologia Trapianto di cellule staminali, Roma, Italy; [8]Istituto Nazionale Tumori Regina Elena, UOC Oncoematologia clinica sperimentale, Roma, Italy; [9]AOU Sant'Andrea, Università La Sapienza, Medicina Clinica e Molecolare, Roma, Italy; [10]Gruppo Italiano Malattie Ematologiche dell'Adulto (GIMEMA) Foundation, Roma, Italy;*

### Background

Belantamab mafodotin–containing regimen with bortezomib- dexamethasone (BVd) or pomalidomide-dexamethasone (BPd) represent a promising therapeutic strategy for relapsed/refractory multiple myeloma (RRMM). However, real-world evidence on their effectiveness and safety in routine clinical practice remains limited.

### Aims

To evaluate the effectiveness and safety of BVd or BPd in a real-world multicenter cohort.

### Methods

We retrospectively and prospectively reviewed data of thirty-two pts who received belantamab-containing regimen for RRMM in 9 centers of EMN Lazio (Italy) between 2025 and 2026. Clinical characteristics, treatment exposure, responses according to International Myeloma Working Group (IMWG) criteria, and adverse events were collected. Overall survival (OS) was evaluated descriptively. Continuous variables were summarized using median and range and categorical variables as frequencies and percentages. Median follow-up was estimated from treatment initiation.

### Results

Thirty-two heavily pretreated patients were analyzed, 18/32 (56%) treated with BPd and 14/32 (43%) with BVd. 11/32 (34%) received treatment in second or third line. The median age was 70 ys (range 51-78). The median number of prior lines of therapy was 4 (range 2-5). The overall response rate (≥PR) was 81%, with very good partial response or better achieved in 47% of patients. Median time to best response was 2 months (range 1-9 months). Ocular adverse events were observed in approximately 22% of patients, mainly consisting of keratopathy and visual disturbances, and were manageable with dose modifications, without permanent treatment discontinuations. Hematologic toxicity occurred in 19% of patients. These 32 patients were followed for a median duration of 4 months [1-12+ months]. exploratory analyses suggested longer survival among patients achieving deeper responses (Figure 1).

**Copyright Information:** (Online) ISSN: 2572-9241
©2026 The Author(s). HemaSphere is published by John Wiley & Sons Ltd on behalf of European Hematology Association. This is an open access Abstract Book distributed under the Attribution-NonCommercial-NoDerivs (CC BY-NC-ND), which allows third parties to download the articles and share them with others as long as they credit the author and the Abstract Book, but they cannot change the content in any way or use them commercially.
**Abstract Book Citations:** Authors, Title, HemaSphere, 2026;10:(S1):pages. The abstract book DOIs can be found on the "Author Information" page.
**Disclaimer:** Articles published in the journal HemaSphere exclusively reflect the opinions of the authors. The authors are responsible for all content in their abstracts including accuracy of the facts, statements, citing resources, etc.

### Summary/Conclusion

In our real-word experience, the Bela cointaining regimen demonstrated high response rates and a manageable safety profile in heavily pre-treated RRMM pts. The adoption of these regimens in earlier treatment lines within a regional network supports (such as ocular specialist) further evaluation of their role earlier in the treatment pathway. Finally, further studies and registries with long follow-up are warranted.

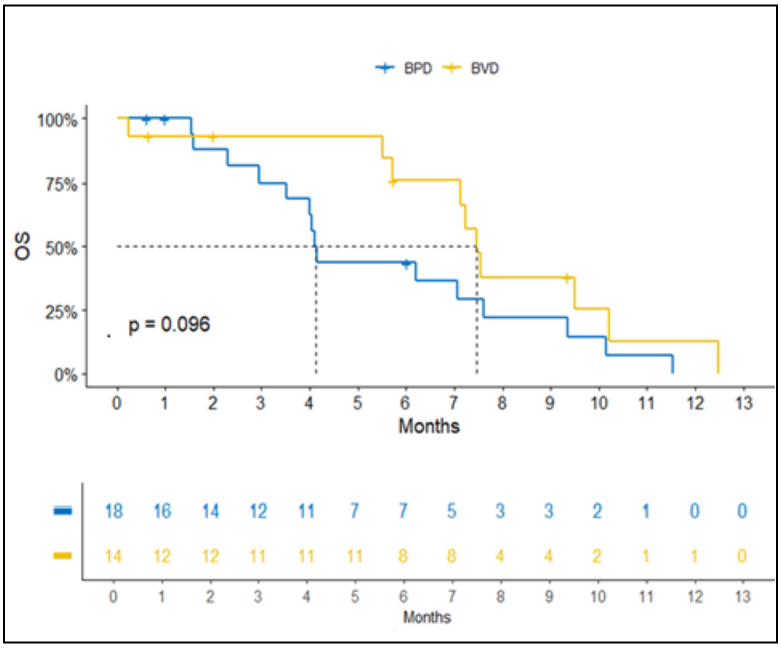

Figure: Overall survival according to depth of response (CR/VGPR vs other responses). Exploratory analysis suggests improved outcomes among patients achieving deeper responses.

**Copyright Information:** (Online) ISSN: 2572-9241
©2026 The Author(s). HemaSphere is published by John Wiley & Sons Ltd on behalf of European Hematology Association. This is an open access Abstract Book distributed under the Attribution-NonCommercial-NoDerivs (CC BY-NC-ND), which allows third parties to download the articles and share them with others as long as they credit the author and the Abstract Book, but they cannot change the content in any way or use them commercially.
**Abstract Book Citations:** Authors, Title, HemaSphere, 2026;10:(S1):pages. The abstract book DOIs can be found on the "Author Information" page.
**Disclaimer:** Articles published in the journal HemaSphere exclusively reflect the opinions of the authors. The authors are responsible for all content in their abstracts including accuracy of the facts, statements, citing resources, etc.

### (PB3187) TUMOR-SPECIFIC CELL-FREE DNA FOR MONITORING DISEASE ACTIVITY IN MULTIPLE MYELOMA WITH PARASKELETAL AND EXTRAMEDULLARY PLASMACYTOMAS

**Topic:** 13. Myeloma and other monoclonal gammopathies - Biology & translational research

Nela Sendlerova[1], Martin Stork*[1], Jana Kotaskova[1, 2], Andrea Mareckova[1], Johana Mayerova[1], Jakub Pawel Porc[2, 3], Tomas Reigl[2, 3], Klara Mensikova[1], Ivanna Biochuk[1], Jakub Cieślar[1], Michaela Bilcikova[1], Zdenek Adam[1], Marta Krejčí[1], Viera Sandecká[1], Zdenka Knechtova[1], Tomas Rohan[4], Zuzana Jelinkova[5], Milan Krticka[6], Vladimir Nekuda[6], Eva Ondrouskova[1], Viera Hrabcakova[1], Lenka Janska-Vojtkova[1], Marek Borsky[1], Marie Jarosova[1, 2, 3], Zdeněk Řehák[7], Ludek Pour[1]

*[1]Department of Internal Medicine, Hematology and Oncology, University Hospital Brno and Faculty of Medicine, Masaryk University, Brno, Czechia; [2]Institute of Medical Genetics and Genomics, University Hospital Brno and Faculty of Medicine, Masaryk University, Brno, Czechia; [3]Central European Institute of Technology, Masaryk University, Brno, Czechia;[4]Department of Radiology and Nuclear Medicine, University Hospital Brno and Faculty of Medicine, Masaryk University, Brno, Czechia; [5]Department of Burns and Plastic Surgery, Faculty of Medicine, Masaryk University, Brno, Czechia;[6]Trauma Surgery Department, University Hospital Brno and Faculty of Medicine, Masaryk University, Brno, Czechia; [7]Department of Nuclear Medicine, Masaryk Memorial Cancer Institute, Brno, Czechia;*

### Background

Treatment response in multiple myeloma (MM) is routinely assessed using M-protein/free light chains and bone marrow minimal residual disease (MRD) assessment. These approaches may underestimate disease activity in non-secretory or extraosseous MM (EMM), including PS (paraskeletal) and EMD (true extramedullary) plasmacytomas, where imaging methods (FDG-PET/CT) are often required. Circulating cell free DNA (cfDNA) measurement represents a minimally invasive method independent of secretory phenotype.

### Aims

We evaluated tumor-specific cfDNA (TS-cfDNA) as an experimental marker of EMM activity compared to International Myeloma Working Group response criteria, imaging methods and MRD assessment.

### Methods

Eleven patients with EMM treated at University Hospital Brno (2022-2025) were included, comprising 4 newly diagnosed patients (NDMM) and 7 relapsed/refractory patients (RRMM). All patients provided written informed consent.
The EMM tissue samples were obtained by surgical sampling or CT-guided biopsy and analyzed using in-house targeted NGS panel. An EMM-specific single-nucleotide variant was selected for longitudinal monitoring and subsequently quantified in serial peripheral blood (PB) samples using digital PCR. PB was prospectively collected at predefined time points according to treatment phase and response status.
PB was processed using standardized protocols, and cfDNA was subsequently isolated and measured. Digital PCR measurements were performed in triplicate.

**Copyright Information:** (Online) ISSN: 2572-9241
©2026 The Author(s). HemaSphere is published by John Wiley & Sons Ltd on behalf of European Hematology Association. This is an open access Abstract Book distributed under the Attribution-NonCommercial-NoDerivs (CC BY-NC-ND), which allows third parties to download the articles and share them with others as long as they credit the author and the Abstract Book, but they cannot change the content in any way or use them commercially.
**Abstract Book Citations:** Authors, Title, HemaSphere, 2026;10:(S1):pages. The abstract book DOIs can be found on the “Author Information” page.
**Disclaimer:** Articles published in the journal HemaSphere exclusively reflect the opinions of the authors. The authors are responsible for all content in their abstracts including accuracy of the facts, statements, citing resources, etc.

**Results**

In the NDMM cohort, 3 patients presented with PS and one with EMD. *KRAS* variants (p.Gly12Val, p.Gly12Asp, p.Gly13Asp, p.Gln61His) were selected for longitudinal monitoring. All patients achieved biochemical response with concordant FDG-PET/CT findings and MRD assessment. In all cases, TS-cfDNA declined to undetectable levels during treatment.
In the RRMM cohort, three patients presented with PS and four with EMD. Tracked variants included *BRAF*(p.Val600Glu, p.Glu695Lys), *NRAS* (p.Gln61Lys), and *KRAS* (p.Gly13Asp, p.Gly12Cys). Two patients with high-burden cutaneous EMD progressed shortly after treatment initiation, with persistently elevated TS-cfDNA levels. Another two EMD patients progressed after initial response, accompanied by a marked increase in tumor-specific cfDNA. Two patients with PS plasmacytomas progressing shortly after treatment initiation demonstrated persistently low and stable TS-cfDNA levels that did not reflect their clinical course. In contrast, only one long-term responding RRMM patient with PS plasmacytoma demonstrated a sustained decrease in TS-cfDNA levels, concordant with imaging findings (FDG-PET/CT).

**Summary/Conclusion**

Overall, the pilot results indicate that monitoring TS-cfDNA may represent a useful complementary marker of MM activity. The strongest performance was observed in EMD patients with high tumor burden, whereas the utility in PS plasmacytomas appeared limited, especially in cases with lower tumor volume.
Taken together, TS-cfDNA monitoring may provide additional dynamic information beyond standard response criteria and imaging, potentially supporting earlier detection of progression in selected patients.
Computational resources were provided by the e-INFRA CZ project (ID:90254), supported by MEYS CZ. Supported by Conceptual development of research organization (FNBr 65269705).

**Copyright Information:** (Online) ISSN: 2572-9241
©2026 The Author(s). HemaSphere is published by John Wiley & Sons Ltd on behalf of European Hematology Association. This is an open access Abstract Book distributed under the Attribution-NonCommercial-NoDerivs (CC BY-NC-ND), which allows third parties to download the articles and share them with others as long as they credit the author and the Abstract Book, but they cannot change the content in any way or use them commercially.
**Abstract Book Citations:** Authors, Title, HemaSphere, 2026;10:(S1):pages. The abstract book DOIs can be found on the "Author Information" page.
**Disclaimer:** Articles published in the journal HemaSphere exclusively reflect the opinions of the authors. The authors are responsible for all content in their abstracts including accuracy of the facts, statements, citing resources, etc.

### (PB3188) HETEROGENEITY OF THE PLASMA CELL CLONE AT THE ONSET OF PLASMA CELL NEOPLASMS: A COMPARATIVE ANALYSIS

**Topic:** 13. Myeloma and other monoclonal gammopathies - Biology & translational research

Veronika Pinchuk*[1], Ihar Iskrou[1], Iryna Lendzina[1], Viktirya Smolnikova[1]

[1]*State Institution "Minsk Scientific and Practical Center for Surgery, Transplantology and Hematology", Minsk, Belarus;*

**Background**

Plasma cell neoplasms (PCNs) exhibit clinical and biological heterogeneity, complicating early diagnosis. Characterizing clone biology at onset may reveal specific diagnostic patterns.

**Aims**

To comparatively analyze key biological characteristics of plasma cell clones in newly diagnosed PCNs to identify onset-specific patterns underlying their heterogeneity.

**Methods**

This retrospective single-center study included 68 treatment-naïve patients (36 women, 32 men; median age 64 years) with newly diagnosed PCNs: systemic AL-amyloidosis (n=22), MM (n=28), and other PCNs including MGUS, POEMS syndrome, plasmacytoma (n=18). All patients underwent comprehensive laboratory evaluation: complete blood count, biochemistry, serum/urine immunofixation, free light chain (FLC) assay, $\beta_2$-microglobulin, and multiparameter flow cytometry of bone marrow aspirates using a panel including CD138, CD38, CD56, CD117, CD27, CD28, CD81, CD20, and cytoplasmic κ/λ light chains. Amyloidosis was histologically confirmed by Congo red staining with apple-green birefringence. Statistical analysis used Mann–Whitney U and $\chi^2$ tests ($p \leq 0.05$); data presented as median (Q25–Q75).

**Results**

Amyloidosis patients showed significantly lower clonal plasma cell (PC) burden versus MM: median 2.5% (1.2–3.8%) vs. 20.6% (12.1–30.5%; p<0.001); PC range 0.8–9.7% in amyloidosis vs. 9.2–52.4% in MM. λ-light chain restriction predominated in amyloidosis (75%) versus κ-restriction in MM (74%; $\chi^2$=28.4, p<0.001). Amyloidosis was characterized by hypoproteinemia (total protein 55.0 g/L vs. 76.0 g/L in other PCNs; p<0.001), low-normal IgG (median 6.70 g/L vs. 8.42 g/L; p<0.05), and renal-related $\beta_2$-microglobulin elevation (median 120.0 mg/L, range 2.3–3345.8 vs. 3.88 mg/L; p<0.001). FLC analysis revealed λ-dominance in amyloidosis (median λ-FLC 89.5 mg/dL) versus κ-dominance in other PCNs (median κ-FLC 320.6 mg/dL; p<0.05); FLC-ratio (λ/κ) effectively discriminated groups. Immunofixation detected monoclonal protein in 90.9% of amyloidosis cases (predominantly IgG/λ, FLC-λ) and 71.4% of other PCNs (mainly IgG/κ, FLC-κ); one amyloidosis patient showed biclonal IgG/λ+IgG/κ. Immunophenotypically, amyloidosis clones retained expression of maturation markers CD27 (85%), CD56 (85%), CD117 (75%); other PCNs showed higher CD19 expression (47% vs. 10–11% in amyloidosis/MM; p<0.05) and absent CD81 (0%), suggesting distinct differentiation pathways. IgA/IgM levels showed no significant difference (p=0.18/p=0.82), except IgM elevation (>19 g/L) in 23.1% of other PCNs.

**Copyright Information:** (Online) ISSN: 2572-9241
©2026 The Author(s). HemaSphere is published by John Wiley & Sons Ltd on behalf of European Hematology Association. This is an open access Abstract Book distributed under the Attribution-NonCommercial-NoDerivs (CC BY-NC-ND), which allows third parties to download the articles and share them with others as long as they credit the author and the Abstract Book, but they cannot change the content in any way or use them commercially.
**Abstract Book Citations:** Authors, Title, HemaSphere, 2026;10:(S1):pages. The abstract book DOIs can be found on the "Author Information" page.
**Disclaimer:** Articles published in the journal HemaSphere exclusively reflect the opinions of the authors. The authors are responsible for all content in their abstracts including accuracy of the facts, statements, citing resources, etc.

**Summary/Conclusion**

Distinct biological patterns differentiate PCNs at onset. AL-amyloidosis features low clonal burden, λ-restriction, preserved maturation markers, and λ-FLC predominance. MM exhibits high clonal load and κ-restriction. Other PCNs show heterogeneous profiles and immature markers. Integrated assessment of light chain isotype, FLC-ratio, immunophenotype, and immunofixation enhances diagnostic accuracy.

**Copyright Information:** (Online) ISSN: 2572-9241
©2026 The Author(s). HemaSphere is published by John Wiley & Sons Ltd on behalf of European Hematology Association. This is an open access Abstract Book distributed under the Attribution-NonCommercial-NoDerivs (CC BY-NC-ND), which allows third parties to download the articles and share them with others as long as they credit the author and the Abstract Book, but they cannot change the content in any way or use them commercially.
**Abstract Book Citations:** Authors, Title, HemaSphere, 2026;10:(S1):pages. The abstract book DOIs can be found on the "Author Information" page.
**Disclaimer:** Articles published in the journal HemaSphere exclusively reflect the opinions of the authors. The authors are responsible for all content in their abstracts including accuracy of the facts, statements, citing resources, etc.

### (PB3189) EARLY DETECTION OF MULTIPLE MYELOMA AND MGUS PROGRESSION RISK USING ARTIFICIAL INTELLIGENCE AND ROUTINE LABORATORY DATA

**Topic:** 13. Myeloma and other monoclonal gammopathies - Biology & translational research

Ciro Rinaldi*[1], Syed Muhammad Ali Shah[2], Khaled Abdalgader Mohamed Omar Balawafi[3], Karim Tout[2], Atheer A Matroud[2]

*[1]United Lincolnshire Teaching Hospital and University of Lincoln, Haematology, Lincoln, United Kingdom;[2]De Montfort University-Dubai, Dubai, United Arab Emirates; [3]American University of Ras Al Khaimah, Ras Al Khaimah, United Arab Emirates;*

### Background

Multiple myeloma (MM) is a malignant plasma cell disorder that often presents with non-specific symptoms, resulting in delayed diagnosis and poorer outcomes. While artificial intelligence (AI) models using routine laboratory data have shown promise for MM detection, their ability to distinguish monoclonal gammopathy of undetermined significance (MGUS) from early MM and to identify patients at risk of progression remains limited.

### Aims

To generate an AI based tool able to predict Myeloma trasformation in patients with MGUS

### Methods

We conducted a retrospective study using routinely collected clinical and laboratory data from 89 patients evaluated at the Lincolnshire Hospital Group between 2003 and 2025. The cohort included confirmed MM cases, MGUS patients, and non-myeloma controls. Predictive features included hemoglobin, serum creatinine, calcium, albumin, immunoglobulin levels, paraprotein concentration, and derived laboratory indices. After rigorous data cleaning and preprocessing, eight supervised machine learning models were trained using a training–testing split. These included six machine learning algorithms (Lasso, gradient boosting, random forest, elastic-net, ridge regression, and support vector) and two baseline algorithms (Naïve (Mean) and Seasonal-Naïve). The training set included 69 patients (82%), and the test set included 20 patients (18%). Model performance was evaluated using precision, recall, F1 score, and regression evaluation metrics such as $R^2$ (R-squared) and MAE (Mean Absolute Error), with a specific focus on MGUS–MM differentiation.

### Results

The developed AI models demonstrated reliable performance in distinguishing MM from non-myeloma controls and showed improved discriminatory ability between MGUS and MM compared with standard laboratory threshold-based assessment. Specific laboratory feature patterns were associated with progression-consistent phenotypes, enabling risk stratification within the MGUS population.
The best-performing model, Lasso Regression, achieved 0.01 MAE (months) and a nearly perfect score of 1.00 for $R^2$. This meant the best prediction done by the model had a 0.47 months error, i.e. 14 days difference from the actual transformation date. Gradient Boosting and Random Forest achieved 1.04 and 1.15 MAE, and 0.90 and 0.95 $R^2$, respectively. The results were implemented as a prototype clinical decision-support tool capable of generating rapid risk predictions using routinely available laboratory data.

**Copyright Information:** (Online) ISSN: 2572-9241
©2026 The Author(s). HemaSphere is published by John Wiley & Sons Ltd on behalf of European Hematology Association. This is an open access Abstract Book distributed under the Attribution-NonCommercial-NoDerivs (CC BY-NC-ND), which allows third parties to download the articles and share them with others as long as they credit the author and the Abstract Book, but they cannot change the content in any way or use them commercially.
**Abstract Book Citations:** Authors, Title, HemaSphere, 2026;10:(S1):pages. The abstract book DOIs can be found on the "Author Information" page.
**Disclaimer:** Articles published in the journal HemaSphere exclusively reflect the opinions of the authors. The authors are responsible for all content in their abstracts including accuracy of the facts, statements, citing resources, etc.

**Summary/Conclusion**

This study advances existing AI-based diagnostic approaches by addressing the clinically important challenge of early MM detection and MGUS risk stratification. AI-driven analysis of routine laboratory data may support earlier identification of patients at risk of progression, enabling improved monitoring, timely referral, and enhanced clinical decision-making.

**Copyright Information:** (Online) ISSN: 2572-9241
©2026 The Author(s). HemaSphere is published by John Wiley & Sons Ltd on behalf of European Hematology Association. This is an open access Abstract Book distributed under the Attribution-NonCommercial-NoDerivs (CC BY-NC-ND), which allows third parties to download the articles and share them with others as long as they credit the author and the Abstract Book, but they cannot change the content in any way or use them commercially.
**Abstract Book Citations:** Authors, Title, HemaSphere, 2026;10:(S1):pages. The abstract book DOIs can be found on the "Author Information" page.
**Disclaimer:** Articles published in the journal HemaSphere exclusively reflect the opinions of the authors. The authors are responsible for all content in their abstracts including accuracy of the facts, statements, citing resources, etc.

### (PB3190) PROTEASOME INHIBITION TRIGGERS RIBOSOME COLLISIONS AND TRANSLATIONAL REPROGRAMMING IN MULTIPLE MYELOMA CELLS

**Topic:** 13. Myeloma and other monoclonal gammopathies - Biology & translational research

Jinglin Zhou[1], Nicholas T Crump[1], Leonie Rieger[2], Florian Bassermann[2], Lenka Besse[3, 4], Christoph Driessen[5], Holger Auner[1, 6, 7], Monica Roman-Trufero*[1, 6]

*[1]Imperial College London, Department of Immunology and Inflammation, London, United Kingdom;[2]TUM University Hospital, Department of Internal Medicine III, Munich, Germany; [3]Masaryk University, Department of Biology, Faculty of Medicine, Brno, Czechia; [4]Kantonsspital St. Gallen, Department of Hematology and Oncology, St. Gallen, Switzerland;[5]University of Zurich, Faculty of Medicine, Zurich, Switzerland; [6]Lausanne University Hospital, Division of Haematology and Central Haematology Laboratory, Lausanne, Switzerland; [7]University of Lausanne, Faculty of Biology and Medicine, Lausanne, Switzerland;*

### Background

Multiple myeloma (MM) cells are highly dependent on the proteostasis network, a vulnerability exploited therapeutically by proteasome inhibitors (PIs). While PI-based therapies have substantially improved patient outcomes, resistance remains a clinical problem. The molecular mechanisms of PI-induced proteostatic stress and of PI resistance remain incompletely defined. Proteasomal protein degradation and protein synthesis are tightly coordinated to maintain proteostasis. During translation, ribosome collisions (RCs) arise when elongating ribosomes stall, causing trailing ribosomes to collide on the mRNA and initiate a translational stress signalling cascade. Whether PIs trigger RCs in MM and how collision-associated stress is sensed and resolved has not been explored.

### Aims

To investigate whether PIs induce RCs in MM and how translational stress responses contribute to recovery from or resistance to PIs.

### Methods

MM cell lines were treated with carfilzomib (Cfz) for 1h to reproduce clinical pharmacokinetics and monitored for six days. RNA-seq and ribosome profiling (ribo-seq) were performed at 4h (acute stress), 24-48h (early recovery) and 6 days (late recovery) post-treatment. Intracellular amino acid levels were measured by metabolomics and global protein synthesis by puromycinylation. Gene and protein expression and phosphorylation were analysed by qRT-PCR and immunoblotting. Heterotopic and orthotopic cell line-derived xenograft (CDX) tumours were treated with Cfz and samples collected 1 or 6 days after treatment. Primary patient-derived MM cells were exposed *ex vivo* to a 1h pulse of Cfz and harvested 6h later.

**Copyright Information:** (Online) ISSN: 2572-9241
©2026 The Author(s). HemaSphere is published by John Wiley & Sons Ltd on behalf of European Hematology Association. This is an open access Abstract Book distributed under the Attribution-NonCommercial-NoDerivs (CC BY-NC-ND), which allows third parties to download the articles and share them with others as long as they credit the author and the Abstract Book, but they cannot change the content in any way or use them commercially.
**Abstract Book Citations:** Authors, Title, HemaSphere, 2026;10:(S1):pages. The abstract book DOIs can be found on the "Author Information" page.
**Disclaimer:** Articles published in the journal HemaSphere exclusively reflect the opinions of the authors. The authors are responsible for all content in their abstracts including accuracy of the facts, statements, citing resources, etc.

**Results**

Cfz treatment rapidly triggered RCs, activation of the ribotoxic stress response (RSR), engagement of the integrated stress response (ISR), depletion of intracellular amino acids and marked reduction in global protein synthesis. Despite translational repression, ribo-seq showed preferential translation of stress response genes and proteasomal subunits, consistent with a "proteasome bounce-back". In Cfz-treated CDX tumours ribosome quality control genes were upregulated and P38 phosphorylation and RPS10 ubiquitination increased, consistent with RSR activation *in vivo.* Moreover, activation of the RSR was observed in primary patient-derived MM cells following short-term Cfz exposure. Between 24h and 48h post Cfz, RCs were resolved and RSR and ISR signalling attenuated, while global protein synthesis recovered, transiently surpassing baseline levels. Integrated ribo- and RNA-seq revealed broad translatome reprogramming, characterised by enhanced translation of ribosomal subunits and translation factors. During late recovery, proliferating MM cells no longer showed evidence of RCs, or RSR and ISR signalling. However, Cfz-resistant MM cells retained a distinct translational programme with increased translation of components of the protein synthesis machinery. Pharmacological inhibition of the RSR or suppression of translational reactivation impaired MM cell recovery from proteasome inhibition, indicating that early RSR signalling and subsequent translational reactivation are required to overcome PI-induced stress.

**Summary/Conclusion**

Our findings establish RCs as an early consequence of proteasome inhibition in MM. RCs are accompanied by activation of the RSR, the ISR and repression of protein synthesis. Moreover, resolution of RCs and translational reactivation are critical for recovery from PI-induced stress and may be therapeutic targets to increase PIs efficacy.

**Copyright Information:** (Online) ISSN: 2572-9241
©2026 The Author(s). HemaSphere is published by John Wiley & Sons Ltd on behalf of European Hematology Association. This is an open access Abstract Book distributed under the Attribution-NonCommercial-NoDerivs (CC BY-NC-ND), which allows third parties to download the articles and share them with others as long as they credit the author and the Abstract Book, but they cannot change the content in any way or use them commercially.
**Abstract Book Citations:** Authors, Title, HemaSphere, 2026;10:(S1):pages. The abstract book DOIs can be found on the "Author Information" page.
**Disclaimer:** Articles published in the journal HemaSphere exclusively reflect the opinions of the authors. The authors are responsible for all content in their abstracts including accuracy of the facts, statements, citing resources, etc.

### (PB3191) ELTROMBOPAG TARGETS PNPO TO SUPPRESS WNT/Β-CATENIN SIGNALING AND SYNERGIZE WITH CYCLOPHOSPHAMIDE–PREDNISONE IN MULTIPLE MYELOMA

**Topic:** 13. Myeloma and other monoclonal gammopathies - Biology & translational research

Fan Zhou*[1], Wei Wei[1], Dongjiao Wang[1], Haimin Chen[1], Nian Zhou[1], Lixia Wu[1], Haotian Shi[1], Xiaoling Chen[1], Ying Zhang[1]

[1]*Zhabei Central Hospital in Shanghai Jing'an District, shanghai, China;*

### Background

Multiple myeloma (MM) is a plasma cell malignancy characterized by complex interactions with the bone marrow microenvironment and remains largely incurable despite therapeutic advances. Dysregulated Wnt/β-catenin signaling contributes to MM progression, drug resistance, and bone destruction. Pyridoxine-5′-phosphate oxidase (PNPO), a key enzyme in vitamin B6 metabolism, has recently emerged as a regulator of oncogenic signaling. Eltrombopag (ELT), an oral thrombopoietin receptor agonist approved for thrombocytopenia, was recently identified as a potent PNPO inhibitor capable of suppressing Wnt/β-catenin signaling. However, the therapeutic potential of ELT and its combination with standard chemotherapy in MM remains undefined.

### Aims

To investigate the role of PNPO in MM pathogenesis, evaluate the antimyeloma activity of ELT, and determine whether ELT synergizes with cyclophosphamide plus prednisone (CP) chemotherapy through modulation of Wnt/β-catenin signaling.

### Methods

PNPO expression was analyzed in MM patient samples and correlated with clinical outcomes. Antimyeloma activity of ELT was evaluated in vitro using RPMI-8226 and U266 MM cell lines, in vivo using murine xenograft models, and in patient-derived xenograft (PDX) models. Drug synergy between ELT and CP was assessed by CCK-8 assays and quantified using the coefficient of drug interaction (CDI) model. Wnt pathway activity was examined by Western blot analysis of phosphorylated GSK-3β, total GSK-3β, and β-catenin. Mechanistic studies investigated PNPO-mediated regulation of Wnt signaling via DVL3 oxidation. Preliminary retrospective clinical analyses were performed to explore associations between ELT exposure and treatment outcomes.

### Results

PNPO was significantly overexpressed in MM samples and associated with inferior survival and increased bone destruction. Mechanistically, PNPO promoted MM cell proliferation and osteoclast differentiation by activating Wnt/β-catenin signaling through DVL3 oxidation. ELT directly bound PNPO, inhibited Wnt pathway activation, and suppressed tumor growth and bone damage in vitro, in vivo, and in PDX models. Combination treatment with ELT and CP demonstrated strong synergistic cytotoxicity in both MM cell lines (CDI < 1), accompanied by enhanced suppression of phosphorylated GSK-3β and β-catenin levels. Preliminary clinical analyses suggested improved response rates and survival outcomes in patients exposed to ELT-containing regimens.

### Summary/Conclusion

PNPO is a critical driver of MM progression and bone pathology through activation of Wnt/β-catenin signaling. Eltrombopag exerts potent antimyeloma effects by targeting PNPO and synergizes with cyclophosphamide–prednisone chemotherapy. These findings support a novel, mechanism-based therapeutic strategy and provide strong rationale for clinical evaluation of ELT-based combination regimens in MM.

**Copyright Information:** (Online) ISSN: 2572-9241
©2026 The Author(s). HemaSphere is published by John Wiley & Sons Ltd on behalf of European Hematology Association. This is an open access Abstract Book distributed under the Attribution-NonCommercial-NoDerivs (CC BY-NC-ND), which allows third parties to download the articles and share them with others as long as they credit the author and the Abstract Book, but they cannot change the content in any way or use them commercially.
**Abstract Book Citations:** Authors, Title, HemaSphere, 2026;10:(S1):pages. The abstract book DOIs can be found on the "Author Information" page.
**Disclaimer:** Articles published in the journal HemaSphere exclusively reflect the opinions of the authors. The authors are responsible for all content in their abstracts including accuracy of the facts, statements, citing resources, etc.

### (PB3192) MAF CONVERTS METABOLIC STATE INTO SUPERENHANCER ARCHITECTURE AND DEPENDENCY IN MYELOMA

**Topic:** 13. Myeloma and other monoclonal gammopathies - Biology & translational research

Phyllis Sy Chong*[1], Sze Lynn, Julia Lim[2], Wee Joo Chng[2]

[1]*CSI Singapore/NUS Medicine, Singapore, Singapore;* [2]*CSI Singapore, Singapore, Singapore;*

### Background

Lineage-defining transcription factors establish superenhancers (SEs) that drive high transcriptional output, yet how metabolic rewiring sustains these regulatory hubs remains unclear. Multiple myeloma (MM) is a plasma cell malignancy characterized by recurrent cytogenetic drivers. Among these, the t(14;16) translocation represents a high-risk subtype, in which this rearrangement drives the pathological overexpression of the MAF transcription factor. Here, we present data linking MAF to epigenetic remodeling arising from its altered metabolic state.

### Aims

To examine how MAF coordinates metabolic and epigenomic programs to regulate histone acetylation, SE architecture and oncogenic transcriptional dependencies in MM.

### Methods

Integrated metabolomics, RNA-seq, chromatin profiling and chromatin architecture analyses were performed alongside genetic and pharmacologic perturbations targeting key metabolic enzymes. Loss-of-function approaches included CRISPR/Cas9 targeting of citrate transport and acetyl-CoA–generating pathways. Functional assays assessed mitochondrial function, nutrient dependencies and validation with diet interventions in vivo.

### Results

MAF induced a metabolically-driven hyperacetylated chromatin state characterized by elevated H3K27ac and SE activity through increased acetyl-CoA availability derived from glutamine-dependent anaplerosis into the tricarboxylic acid (TCA) cycle. Metabolic channeling of acetylation donors was facilitated by nucleus-localizing citrate synthase, while disruption of mitochondrial citrate export via SLC25A1 abrogated H3K27ac deposition. Epigenomic profiling revealed broad H3K27ac enrichment and chromatin accessibility at MAF-bound regions, confirming a MAF-specific SE landscape. Through integrative analyses, we prioritized ZC3H3 as a SE-driven dependency gene that is sensitive to glutamine modulation and essential for myeloma viability and tumorigenesis.

### Summary/Conclusion

These findings establish MAF as a metabolic gatekeeper linking glutamine-driven acetyl-CoA production to SE-dependent chromatin acetylation and oncogenic transcriptional programs. The dependency of MAF-high myeloma on metabolically sustained SEs creates conditional vulnerabilities exploitable through metabolic intervention or epigenetic targeting, providing a conceptual framework for therapeutic strategies in t(14;16) myeloma.

**Copyright Information:** (Online) ISSN: 2572-9241
©2026 The Author(s). HemaSphere is published by John Wiley & Sons Ltd on behalf of European Hematology Association. This is an open access Abstract Book distributed under the Attribution-NonCommercial-NoDerivs (CC BY-NC-ND), which allows third parties to download the articles and share them with others as long as they credit the author and the Abstract Book, but they cannot change the content in any way or use them commercially.
**Abstract Book Citations:** Authors, Title, HemaSphere, 2026;10:(S1):pages. The abstract book DOIs can be found on the "Author Information" page.
**Disclaimer:** Articles published in the journal HemaSphere exclusively reflect the opinions of the authors. The authors are responsible for all content in their abstracts including accuracy of the facts, statements, citing resources, etc.

### (PB3193) EPIGENETIC MODIFIER GSK-J4 ENHANCES SENSITIVITY TO VENETOCLAX IN MULTIPLE MYELOMA

**Topic:** 13. Myeloma and other monoclonal gammopathies - Biology & translational research

Stephanie Gilmore*[1], Lyndsey Flanagan[1, 1], Aisling Coughlan[1, 1], Ludovica Di Martino[1], Matthew Hiller[1], Siobhan Glavey[2], Triona Ni Chonghaile[1]

*[1]Royal College of Surgeons, Department of Physiology and Medical Physics, Dublin 2, Ireland;[2]Royal College of Surgeons, Department of Pathology, Dublin, Ireland;*

### Background

**Introduction:** Multiple myeloma (MM) is an incurable haematological cancer with increasing prevalence. MM cells are heterogeneous and rely on diverse anti-apoptotic BCL-2 family proteins (BCL-2, MCL-1, and BCL-XL), which enhance their survival. Venetoclax, an FDA-approved BCL-2 inhibitor, has shown promise in a subset of MM patients that are BCL-2-dependent t(11;14) cells. However, approximately only 20% of MM patients rely on BCL-2 for survival.

### Aims

**Aims:** To expand the therapeutic potential of venetoclax, we performed an epigenetic modifier screen and identified that GSK-J4 enhanced the sensitivity to venetoclax.

### Methods

**Methods:** Non-t(11;14), MCL-1–dependent MM cell lines (JJN3 and H929) and patient samples were treated with a combination of GSK-J4 and venetoclax. Cell death was assessed using Annexin V/PI.  mRNA and protein expression were accessed by RNA sequencing, qRT-PCR, and western blotting, following GSK-J4 treatment. BCMA (mature MM surface marker) were assessed after GSK-J4 treatment by flow cytometry. The SeahorseXFe96 assay was used to assess oxygen consumption rate, after GSK-J4 treatment.

### Results

**Results:** First, we confirmed that the combination of GSK-J4 and venetoclax enhanced cell killing in the MCL1-dependent, non-t (11;14) cell lines and in primary MM patient samples in comparison to either agent alone. Next, we assessed the transcriptional changes induced by GSK-J4 using RNA sequencing. GSK-J4 treatment induced an upregulation of BCL-2 mRNA and downregulation of MCL-1 mRNA, which was confirmed by qRT-PCR and Western blotting. Pathway analysis of the RNA sequencing revealed a downregulation of genes involved in OXPHOS. Next, we confirmed that GSK-J4 altered OXPHOS metabolism using the Seahorse assay. Indeed, we also identified an altered mitochondrial network using confocal microscopy and TMRE staining. Lastly, GSK-J4 decreased the expression of B-cell maturation antigen (BCMA) on the surface of MM cells, suggesting that the epigenetic modifier may be inducing a more immature phenotype, however this would need to be confirmed.

### Summary/Conclusion

**Conclusion:** Our findings suggest that the epigenetic modifier GSK-J4 is driving mature non-t(11;14) MM cells to a more immature phenotype. Thereby inducing a reliance on BCL-2 and an altered metabolic phenotype. This combination could expand the use of venetoclax into both t(11;14) and non-t(11;14) MM patients.

**Copyright Information:** (Online) ISSN: 2572-9241
©2026 The Author(s). HemaSphere is published by John Wiley & Sons Ltd on behalf of European Hematology Association. This is an open access Abstract Book distributed under the Attribution-NonCommercial-NoDerivs (CC BY-NC-ND), which allows third parties to download the articles and share them with others as long as they credit the author and the Abstract Book, but they cannot change the content in any way or use them commercially.
**Abstract Book Citations:** Authors, Title, HemaSphere, 2026;10:(S1):pages. The abstract book DOIs can be found on the "Author Information" page.
**Disclaimer:** Articles published in the journal HemaSphere exclusively reflect the opinions of the authors. The authors are responsible for all content in their abstracts including accuracy of the facts, statements, citing resources, etc.

### (PB3194) A NOVEL DIAGNOSTIC AND PROGNOSTIC BIOMARKER PANEL FOR MULTIPLE MYELOMA

**Topic:** 13. Myeloma and other monoclonal gammopathies - Biology & translational research

Zhang Liangjun*[1]

[1] *zigong first people's hospital, zigong, China;*

**Background**

Multiple myeloma (MM) is characterized by profound heterogeneity and a dysfunctional immune microenvironment. Current diagnostic systems, reliant on conventional parameters, lack granular insight into the immune landscape that dictates progression and therapy resistance.

**Aims**

This study aimed to develop an integrated diagnostic and prognostic model by combining routine clinical indices with deep immunophenotyping.

**Methods**

We conducted a comparative analysis of 53 newly diagnosed MM patients and 44 healthy controls (HCs). Comprehensive profiling included immunophenotyping of T-cell subsets (e.g., Th1, Th17, Tregs, Tfh), myeloid-derived suppressor cells (p-MDSCs), inflammatory cytokines (IL-6, IL-10, IL-21), and standard hematological/biochemical parameters. Machine learning approaches, including LASSO and Bayesian logistic regression, were used for biomarker selection and diagnostic model construction. A clinical nomogram was built for risk prediction. Prognostic utility was assessed using Cox regression and Kaplan-Meier survival analysis.

**Results**

MM patients exhibited significant immune dysregulation versus HCs, including severe T-cell lymphopenia, expansion of p-MDSCs (14.94% vs. 3.58%, p=0.002), a skewed Th profile (elevated Th17, Tregs; reduced Th1), and elevated pro-tumor cytokines (IL-6, IL-10, IL-21, all p<0.01). LASSO regression identified a 7-predictor panel, refined via Bayesian analysis to a core 3-marker diagnostic signature: hematocrit (HCT), albumin (ALB), and Tfh17 cell percentage. This signature formed the basis of a high-performance nomogram ($\chi^2 = 88.24$, $p < 0.0001$). For prognosis, multivariate Cox regression identified three independent factors: elevated %p-MDSC/MDSC (HR = 1.164, p=0.041) as a risk factor, and higher %Th2/Th (HR = 0.888, p=0.003) and ALB (HR = 0.903, p=0.044) as protective factors. Kaplan-Meier analysis confirmed strong survival stratification by these biomarkers (e.g., high p-MDSC group median survival 305 days vs. not reached in low group, p=0.002).

**Summary/Conclusion**

This study delineates a comprehensive landscape of immune dysfunction in MM and validates a novel, parsimonious diagnostic model integrating a key immune subset (Tfh17) with routine parameters (HCT, ALB). Furthermore, we identify a potent immune-centric prognostic signature (%p-MDSC/MDSC and %Th2/Th alongside ALB). Our findings underscore the critical role of the immune microenvironment and advocate for its integration into clinical practice for refined risk stratification and personalized therapy.

**Copyright Information:** (Online) ISSN: 2572-9241
©2026 The Author(s). HemaSphere is published by John Wiley & Sons Ltd on behalf of European Hematology Association. This is an open access Abstract Book distributed under the Attribution-NonCommercial-NoDerivs (CC BY-NC-ND), which allows third parties to download the articles and share them with others as long as they credit the author and the Abstract Book, but they cannot change the content in any way or use them commercially.
**Abstract Book Citations:** Authors, Title, HemaSphere, 2026;10:(S1):pages. The abstract book DOIs can be found on the "Author Information" page.
**Disclaimer:** Articles published in the journal HemaSphere exclusively reflect the opinions of the authors. The authors are responsible for all content in their abstracts including accuracy of the facts, statements, citing resources, etc.

### (PB3196) INTEGRATION OF DEMOGRAPHIC VARIABLES AND DISEASE BURDEN INDICATORS WITH SPECIFIC BONE BIOMARKERS MAY PREDICT THE PRESENCE OF IMAGING-DETECTED LYTIC LESIONS

**Topic:** 13. Myeloma and other monoclonal gammopathies - Biology & translational research

Patricia García Ramírez*[1], María Menor Gómez[1], María Jesús Ruíz-Álvarez[2], Nuria Rosón[1], Elena Rozas Timón[1], Marta Rosillo Coronado[3], Argeme Rodríguez Torres[3], Isabel Saiz Vergaz[1], Begoña Rodríguez Izquierdo[1], Julio García Suárez[1], Juan Jose Gil Fernandez[1]

*[1]Hospital Universitario Príncipe de Asturias, Hematology, Madrid, Spain;[2]Hospital Universitario Príncipe de Asturias, Clinical Chemistry, Madrid, Spain; [3]Hospital Universitario Ramón y Cajal, Clinical Chemistry, Madrid, Spain;*

### Background

Skeletal complications remain a major cause of morbidity in patients with multiple myeloma (MM), even in the era of immunochemotherapy. Approximately one in four patients with active MM presents with skeletal complications (bone pain requiring radiotherapy, pathological fractures and/or spinal cord compression) or develops them at first clinical progression after first-line treatment.

Plasma markers of bone resorption and formation, the C-terminal telopeptide of type I collagen (CTX-1) and the Procollagen type I N-terminal propeptide (P1NP), have been recommended by the International Osteoporosis Foundation and the International Federation of Clinical Chemistry as preferred markers for osteoporosis monitoring. Although data on their usefulness in MM are limited, they may be useful for treatment monitoring or response prediction.

### Aims

The primary objective of this study was to evaluate the diagnostic performance of bone biomarkers for detecting bone involvement in patients with plasma cell dyscrasias.

### Methods

Between November 2023 and December 2024, bone biomarkers were prospectively assessed in 49 patients with symptomatic multiple myeloma (sMM) (n=11), smouldering multiple myeloma (SMM) (n=14), or high-risk monoclonal gammopathy of undetermined significance (high-risk MGUS) (n=24), according to International Myeloma Working Group (IMWG) criteria.

Bone biomarkers analysed were CTX-1 and P1NP. In routine practice, all patients underwent whole-body low-dose computed tomography (CT) with bone window and/or whole-body magnetic resonance imaging (MRI) to evaluate lytic lesions.

A non-parametric analysis using the Kruskal–Wallis test evaluated differences in biomarker levels among groups, followed by Dunn's test for pairwise comparisons. Diagnostic performance was assessed using receiver operating characteristic (ROC) curve analysis to determine optimal cut-off values.

The predictive value of biochemical and clinical variables for lytic lesions detected by CT or MRI was evaluated using multivariable logistic regression.

**Copyright Information:** (Online) ISSN: 2572-9241
©2026 The Author(s). HemaSphere is published by John Wiley & Sons Ltd on behalf of European Hematology Association. This is an open access Abstract Book distributed under the Attribution-NonCommercial-NoDerivs (CC BY-NC-ND), which allows third parties to download the articles and share them with others as long as they credit the author and the Abstract Book, but they cannot change the content in any way or use them commercially.
**Abstract Book Citations:** Authors, Title, HemaSphere, 2026;10:(S1):pages. The abstract book DOIs can be found on the "Author Information" page.
**Disclaimer:** Articles published in the journal HemaSphere exclusively reflect the opinions of the authors. The authors are responsible for all content in their abstracts including accuracy of the facts, statements, citing resources, etc.

### Results

Statistically significant differences were observed in CTX-1 ($p = 0.0036$) and P1NP ($p = 0.0138$) levels, with a progressive increase from high-risk MGUS to symptomatic MM. CTX-1 proved to be the most robust indicator of structural bone damage.
ROC curve analysis identified an optimal CTX-1 cut-off value >0.335 ng/mL, providing a sensitivity of 78.6% and specificity of 76.9% for detecting lytic lesions. A moderate positive correlation between bone resorption and formation markers was confirmed ($p < 0.001$), suggesting preserved but insufficient coupling of bone remodelling.
Model 1, combining bone remodelling parameters with systemic markers (CTX-1, P1NP, age and β2-microglobulin), showed the best diagnostic performance with an area under the curve (AUC) of 0.865 (95% CI: 0.782–0.939), outperforming Model 2 based on bone biomarkers alone (AUC: 0.812). The Hosmer–Lemeshow test confirmed adequate goodness-of-fit ($p > 0.05$).

### Summary/Conclusion

The results of this study highlight the value of bone remodelling biomarkers, particularly CTX-1, as biochemical markers for identifying patients at high risk of bone disease. The superior diagnostic performance of Model 1 (AUC = 0.865) suggests that the integration of demographic variables and disease burden indicators (such as β2-microglobulin and age) with specific bone biomarkers may predict the presence of imaging-detected lytic lesions.

| MODEL | VARIABLES INCLUDED | AUC (BOOTSTRAP) | CI 95% |
|---|---|---|---|
| **MODEL 1** | CTX-1, P1NP, age and B2m | 0.865 | [0.782 – 0.939] |
| **MODEL 2** | CTX-1, P1NP, Calcium | 0.812 | [0.714 – 0.901] |

**TABLE 1. COMPARISON OF DIAGNOSTIC MODELS FOR DETECTION OF LYTIC LESIONS.** AUC: Area under the curve; CI: Confidence Interval; CTX-1: C-terminal telopeptide of type I collagen; P1NP: Procollagen type I N-terminal propeptide; β2m: beta-2 microglobulin.

TABLE 1. COMPARISON OF DIAGNOSTIC MODELS FOR DETECTION OF LYTIC LESIONS

**Copyright Information:** (Online) ISSN: 2572-9241
©2026 The Author(s). HemaSphere is published by John Wiley & Sons Ltd on behalf of European Hematology Association. This is an open access Abstract Book distributed under the Attribution-NonCommercial-NoDerivs (CC BY-NC-ND), which allows third parties to download the articles and share them with others as long as they credit the author and the Abstract Book, but they cannot change the content in any way or use them commercially.
**Abstract Book Citations:** Authors, Title, HemaSphere, 2026;10:(S1):pages. The abstract book DOIs can be found on the “Author Information” page.
**Disclaimer:** Articles published in the journal HemaSphere exclusively reflect the opinions of the authors. The authors are responsible for all content in their abstracts including accuracy of the facts, statements, citing resources, etc.

### (PB3197) ROLE AND MECHANISM OF MULTIPLE MYELOMA CELLS IN REGULATING MACROPHAGE POLARIZATION AND THE ANTITUMOR EFFECT VIA ENO1

**Topic:** 13. Myeloma and other monoclonal gammopathies - Biology & translational research

Zhaoyun Liu*[1], Zhiwei Wang[1], Rong Fu[1]

[1] *Tianjin Medical University General Hospital, Tianjin, China;*

### Background

Multiple myeloma (MM) is an incurable hematologic malignancy. Abnormalities in tumor cell energy metabolism and immune environment play important roles in MM development.

### Aims

The aim of this study was to investigate the role and mechanism of alpha-enolase (ENO1) in the regulation of cell proliferation and energy metabolism in MM, and to analyze the role and mechanism of ENO1 in the regulation of macrophage polarization and the anti-tumor effect.

### Methods

Bioinformatics combined with clinical samples to analyze the expression of ENO1 in MM cells and the correlation between ENO1 and the clinical prognosis of patients, flow cytometry and zymography to detect the role of ENO1 on the proliferation and energy metabolism of MM cells; Constructing a co-culture system of MM cells and macrophages, flow-cytometry to detect the polarization and killing function of macrophages, and western-blot to detect the expression of macrophage polarization-related proteins; MM nude mouse subcutaneous tumor model to verify the effect of ENO1 on macrophage proliferation and the anti-tumor effects.

### Results

Tumor cells from MM patients highly expressed ENO1 and correlated with poor prognosis of MM patients; after inhibiting the ENO1, the proliferation level of MM cells was reduced, and the apoptosis level was increased, and the cell cycle was blocked; then we found that after inhibiting the ENO1, p-Akt level was reduced and restored by supplementation of ENO1. M2-type macrophage was increased, and killing and phagocytosis of macrophage was decreased after co-culture of MM cells and macrophages, which was improved after ENO1 inhibition; exogenous L-lactic acid and microvesicles derived from MM cells with high expression of ENO1 similarly promoted macrophage M2 polarization and decreased macrophage function; after supplementation of ENO1, macrophages showed increased levels of p-AKT, p-GSK3-β, β-catenin, and PGC1α protein, increased levels of cellular OCR, and decreased levels of ECAR. In the MM nude mouse model, the volume of tumor mound was significantly reduced and the number of M1-type macrophages was increased in the group of infused macrophages combined with the ENO1 inhibitor.

### Summary/Conclusion

High expression of ENO1 may promote MM cell proliferation through PI3K/AKT pathway, ENO1 inhibitor could inhibit MM cell tumorigenesis while promoting macrophage polarization to M1 and increasing the killing ability of MM cells, targeting ENO1 is a novel and effective potential target for the treatment of MM.

**Copyright Information:** (Online) ISSN: 2572-9241
©2026 The Author(s). HemaSphere is published by John Wiley & Sons Ltd on behalf of European Hematology Association. This is an open access Abstract Book distributed under the Attribution-NonCommercial-NoDerivs (CC BY-NC-ND), which allows third parties to download the articles and share them with others as long as they credit the author and the Abstract Book, but they cannot change the content in any way or use them commercially.
**Abstract Book Citations:** Authors, Title, HemaSphere, 2026;10:(S1):pages. The abstract book DOIs can be found on the “Author Information” page.
**Disclaimer:** Articles published in the journal HemaSphere exclusively reflect the opinions of the authors. The authors are responsible for all content in their abstracts including accuracy of the facts, statements, citing resources, etc.

### (PB3198) GEOGRAPHIC VARIABILITY IN HEALTHCARE BURDEN OF MULTIPLE MYELOMA IN SPAIN: TRENDS IN HOSPITALIZATION AND MORTALITY (2016-2023).

**Topic:** 13. Myeloma and other monoclonal gammopathies - Biology & translational research

Ane Brieva Lopez*[1], Maria Ordoñez Garcia[1], Rafael Garcia Carretero[2], Mariana Cerrato Salas[1], Pavel Manuel Acevedo Pérez[1], Juan Sastre Ortega[1], Esther Jaro Arias[1], Alicia Lorenzo Jambrina[1], Maria Angeles Andreu Costa[1]

*[1]Hospital Universitario de Móstoles, Madrid, Spain;[2]Hospital Universitario Severo Ochoa, Madrid, Spain;*

### Background

The incidence of multiple myeloma (MM) shows marked geographical variability worldwide, with higher rates reported in North America, Australia and Western Europe compared to Asia and Africa, highlighting the influence of genetic, environmental and healthcare-related factors on its distribution. In Spain, accurate population-based data describing regional differences in healthcare burden of MM remain limited.

### Aims

The primary objective of this study is to characterize the geographic variability in healthcare burden of MM across Spanish regions between 2016 and 2023, and to analyze trends in hospitalization rates and in-hospital mortality per Spanish region.

### Methods

Nationwide retrospective study using the Spanish Minimum Basic Data Set for Hospitalization. All hospital admissions with a diagnosis of MM between 2016 and 2023 were included. Hospital admissions and in-hospital mortality were analyzed per region. We calculated hospitalization rates per 100,000 population, and in-hospital mortality per 100 admissions.

**Copyright Information:** (Online) ISSN: 2572-9241
©2026 The Author(s). HemaSphere is published by John Wiley & Sons Ltd on behalf of European Hematology Association. This is an open access Abstract Book distributed under the Attribution-NonCommercial-NoDerivs (CC BY-NC-ND), which allows third parties to download the articles and share them with others as long as they credit the author and the Abstract Book, but they cannot change the content in any way or use them commercially.
**Abstract Book Citations:** Authors, Title, HemaSphere, 2026;10:(S1):pages. The abstract book DOIs can be found on the “Author Information” page.
**Disclaimer:** Articles published in the journal HemaSphere exclusively reflect the opinions of the authors. The authors are responsible for all content in their abstracts including accuracy of the facts, statements, citing resources, etc.

### Results

Hospitalization rates varied substantially across Spanish regions (Table 1). The highest mean hospitalization rates were observed in Castilla y Leon (36.57), Galicia (35.07), and Madrid (33.94), whereas the lowest rates were recorded in Andalucia (19.82) and the Balearic Islands (20.71). In-hospital mortality rates also varied geographically. Northern and northwestern regions, such as Asturias, Galicia, and La Rioja exhibited higher in-hospital mortality rates compared with central and southern regions (Figure 1).

**Table 1.** Hospital Admissions and In-Hospital Mortality by Spanish Region (2016–2023).

| Region | **Hospital admissions** (rate per 100,000 population) | **In-hospital mortality** (rate per 100 admissions) |
|---|---|---|
| **Andalucía** | 13366 (19.82) | 1718 (2.55) |
| **Aragón** | 3451 (32.7) | 419 (3.97) |
| **Asturias** | 2666 (32.58) | 306 (3.74) |
| **Illes Balears** | 1904 (20.71) | 170 (1.85) |
| **Canarias** | 4325 (25.17) | 586 (3.41) |
| **Cantabria** | 1568 (33.65) | 114 (2.45) |
| **Castilla y León** | 7032 (36.57) | 815 (4.24) |
| **Castilla - La Mancha** | 4261 (26.1) | 465 (2.85) |
| **Catalunya** | 19459 (31.68) | 1584 (2.58) |
| **Comunitat Valenciana** | 11975 (29.86) | 1271 (3.17) |
| **Extremadura** | 2398 (28.04) | 283 (3.31) |
| **Galicia** | 7582 (35.07) | 943 (4.36) |
| **Madrid** | 18071 (33.94) | 1595 (2.99) |
| **Murcia** | 3945 (32.95) | 342 (2.86) |
| **Navarra** | 2207 (42.19) | 155 (2.96) |
| **Euskadi** | 6760 (38.29) | 627 (3.55) |
| **La Rioja** | 845 (33.23) | 118 (4.64) |
| **Ceuta** | 43 (6.38) | 10 (1.48) |
| **Melilla** | 98 (14.18) | 9 (1.3) |
| **Total** | 111956 (29.74) | 11530 (3.06) |

### Summary/Conclusion

The nationwide study reveals substantial geographic disparities in both hospitalization rates and in-hospital mortality for MM across Spain. Several factors may contribute to these differences. Regional variations in population age structures are likely relevant, as regions with older population would be expected to show higher MM-related hospital burden. Differences in healthcare access and diagnostic capacity may also play a role, particularly in regions hosting referral hematology centers for MM. Additionally, heterogeneity in data capture and coding practices within administrative hospital databases may account for the observed variability. The particularity low hospitalization rates observed in Ceuta and Melilla may reflect their small population size and referral of patients to mainland hospitals. Overall, these findings underscore the need for harmonized diagnostic and care pathways across Spain. Further investigation into the demographic, clinical, and healthcare system factors underlying regional MM burden should be warranted.

**Copyright Information:** (Online) ISSN: 2572-9241
©2026 The Author(s). HemaSphere is published by John Wiley & Sons Ltd on behalf of European Hematology Association. This is an open access Abstract Book distributed under the Attribution-NonCommercial-NoDerivs (CC BY-NC-ND), which allows third parties to download the articles and share them with others as long as they credit the author and the Abstract Book, but they cannot change the content in any way or use them commercially.
**Abstract Book Citations:** Authors, Title, HemaSphere, 2026;10:(S1):pages. The abstract book DOIs can be found on the “Author Information” page.
**Disclaimer:** Articles published in the journal HemaSphere exclusively reflect the opinions of the authors. The authors are responsible for all content in their abstracts including accuracy of the facts, statements, citing resources, etc.

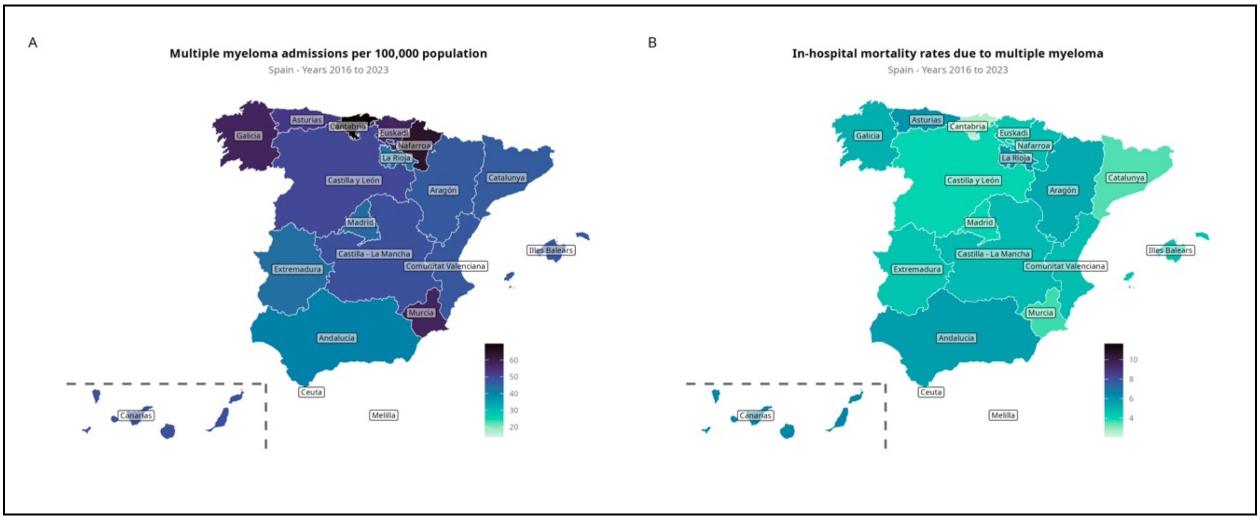

Figure 1. Geographical distribution fo hospitalization rate per 100,000 population (A) and distribution of in- hospital mortality rate per Spanish region (B).

**Copyright Information:** (Online) ISSN: 2572-9241
©2026 The Author(s). HemaSphere is published by John Wiley & Sons Ltd on behalf of European Hematology Association. This is an open access Abstract Book distributed under the Attribution-NonCommercial-NoDerivs (CC BY-NC-ND), which allows third parties to download the articles and share them with others as long as they credit the author and the Abstract Book, but they cannot change the content in any way or use them commercially.
**Abstract Book Citations:** Authors, Title, HemaSphere, 2026;10:(S1):pages. The abstract book DOIs can be found on the "Author Information" page.
**Disclaimer:** Articles published in the journal HemaSphere exclusively reflect the opinions of the authors. The authors are responsible for all content in their abstracts including accuracy of the facts, statements, citing resources, etc.

### (PB3199) GUT DERIVED METABOLIC PROFILING OF MGUS AND MYELOMA PATIENTS

**Topic:** 13. Myeloma and other monoclonal gammopathies - Biology & translational research

Catherine Chinnery*[1, 2], Ravindu De Silva[2], Anastasios Bampalis[1], Trey Koev[1], Dominic Fowler-Shorten[1], Alyssa Polski-Delve[1], Matthew Markham[1], George Bell[1], Rosie Taylor[1], Maddie Bristow[1], Aaron White[1], Kristian Bowles[1, 2], Naiara Beraza[3], Stuart Rushworth[1], Charlotte Hellmich[1, 2]

*[1]University of East Anglia, Norwich, United Kingdom;[2]Norfolk and Norwich University Hospital, Norwich, United Kingdom; [3]Quadram Institute, Norwich, United Kingdom;*

### Background

In the UK, approximately 6200 people are diagnosed with multiple myeloma (MM) every year. Whilst there are multiple lines of therapy for the disease, a cure is yet to be identified. MM is always preceded by the pre-malignant condition of monoclonal gammopathy of uncertain significance (MGUS), however there is limited knowledge as to the exact cause of progression. One previously identified risk factor for progression is obesity. This has led us to explore the role of the microbiome and changes in metabolism in this progression.

### Aims

The aim of this study is to identify changes in gut metabolites and metabolic pathways in patients with MGUS compared to MM. This will allow us to identify possible targets for prognostication or treatment of the disease.

### Methods

Serum and stool samples were collected from 43 patients with MGUS, MM, or smouldering myeloma (SM) following informed consent and under existing ethical approval. Metabolomic analysis was undertaken using nuclear magnetic resonance (NMR) to identify concentrations of 94 metabolites. Using MetaboAnalyst 6.0, differential metabolites were then identified using one-way ANOVA followed by Benjamini–Hochberg false discovery rate correction. Metabolites with adjusted p-values ($q < 0.05$) were considered significant.

### Results

Of the 43 patients, 9 had a diagnosis of MM, 25 of MGUS, and 9 of SM. Diagnosis was made in line with British Society of Haematology guidelines, using histological analysis of a bone marrow biopsy combined with serum blood test results and clinical history. All tissue samples were taken pre-treatment for their condition (if indicated).
Mean age of the MM patient group was 68.3 years old (SD 6.2), MGUS group 68.6 years old (SD 12.7), and SM group 71.6 years old (SD 7.8). Mean creatinine clearance was similar across all groups calculated using the Cockcroft-Gault equation, with CrCl of 83.91mL/min (SD 27.66) in MM, 81.71mL/min (SD 31.58) in MGUS, and 85.75mL/min (SD 46.45) in SM. Creatinine clearance is an important variable to consider as metabolite concentrations within the stool or serum may inversely correlate with renal clearance depending upon how they are excreted from the body.
Metabolomic analysis revealed changes in two key metabolites: phenylalanine and taurine. Phenylalanine is an essential amino acid and metabolised by gut bacteria. Phenylalanine was significantly increased within the stool of patients with MGUS and MM, however not in SM. Taurine, a metabolite derived largely from cysteine metabolism, is released into the gut following bile acid deconjugation by bacteria. Taurine was significantly increased in the stool of the SM patient group.

**Copyright Information:** (Online) ISSN: 2572-9241
©2026 The Author(s). HemaSphere is published by John Wiley & Sons Ltd on behalf of European Hematology Association. This is an open access Abstract Book distributed under the Attribution-NonCommercial-NoDerivs (CC BY-NC-ND), which allows third parties to download the articles and share them with others as long as they credit the author and the Abstract Book, but they cannot change the content in any way or use them commercially.
**Abstract Book Citations:** Authors, Title, HemaSphere, 2026;10:(S1):pages. The abstract book DOIs can be found on the "Author Information" page.
**Disclaimer:** Articles published in the journal HemaSphere exclusively reflect the opinions of the authors. The authors are responsible for all content in their abstracts including accuracy of the facts, statements, citing resources, etc.

**Summary/Conclusion**

Both phenylalanine and taurine represent differing microbial activity within the gut in patients with plasma cell dyscrasias. Previous studies have reported upon differences in concentrations of taurine and phenylalanine in the serum and bone marrow of patients with bone marrow malignancy. However, understanding how the concentration of such metabolites differs throughout the plasma cell dyscrasia disease course may give rise to an understanding of the metabolic pathways that may be driving disease progression. Following on from stool metabolomics as previously described, the study plans to perform metabolomics on paired serum samples from the same patients, therefore allowing a deeper understanding of the metabolic pathways affected in the development of plasma cell dyscrasias.

**Copyright Information:** (Online) ISSN: 2572-9241
©2026 The Author(s). HemaSphere is published by John Wiley & Sons Ltd on behalf of European Hematology Association. This is an open access Abstract Book distributed under the Attribution-NonCommercial-NoDerivs (CC BY-NC-ND), which allows third parties to download the articles and share them with others as long as they credit the author and the Abstract Book, but they cannot change the content in any way or use them commercially.
**Abstract Book Citations:** Authors, Title, HemaSphere, 2026;10:(S1):pages. The abstract book DOIs can be found on the "Author Information" page.
**Disclaimer:** Articles published in the journal HemaSphere exclusively reflect the opinions of the authors. The authors are responsible for all content in their abstracts including accuracy of the facts, statements, citing resources, etc.

### (PB3200) GENE-DRIVEN MONOCLONAL GAMMOPATHY/LYMPHOMA EVOLUTION IN SCHNITZLER SYNDROME: EVIDENCE FOR AN ALTERNATIVE AUTOINFLAMMATORY–ONCOGENIC AXIS

**Topic:** 13. Myeloma and other monoclonal gammopathies - Biology & translational research

Ferenc Sipos*[1], Györgyi Műzes[1]

[1]*Semmelweis University, Department of Hematology and Internal Medicine, Budapest, Hungary;*

### Background

Schnitzler syndrome (SchS) is a unique autoinflammatory condition characterized by chronic urticarial rash, monoclonal IgM gammopathy, systemic inflammation, and osteosclerosis. Despite the recognition of its potential lymphoproliferative transformation, the underlying molecular drivers remain poorly understood.

### Aims

To highlight the potential pathogenetic contribution of autoinflammatory- and lymphoma-associated gene variants in a patient with SchS who subsequently developed marginal zone lymphoma (MZL), thereby providing mechanistic insight beyond a conventional case report.

### Methods

Comprehensive clinical, laboratory, radiological, histopathological, and immunogenetic evaluation was performed. Targeted next-generation sequencing using an autoinflammatory disease panel assessed variants in genes including NLRP3, MYD88, MEFV, F2, ITGB2, and C2. Bone marrow histology and PET-CT were conducted at diagnosis and at relapse. Therapeutic response to IL-1β blockade and anti-CD20 therapy was monitored clinically and biochemically.

### Results

A 54-year-old woman fulfilled both Lipsker and Strasbourg criteria for SchS, presenting with recurrent non-pruritic urticaria, periodic fever, diffuse arthralgia, tibial osteosclerosis, lymphadenopathy, elevated inflammatory markers (ESR 101 mm/h; CRP 64 mg/L), and IgM κ monoclonal gammopathy (14.5 g/L). Autoimmune and infectious causes were excluded. Bone marrow examination initially showed no malignancy. Genetic analysis revealed two pathogenic/probably pathogenic variants in the MEFV gene (c.2084A>G, p.Lys695Arg, rs104895094), a 3′UTR F2 variant (c.*97G>A, rs1799963), and variants of uncertain significance in ITGB2 (c.1570G>A, p.Val524Ile) and C2 (c.841_868del). No clinically relevant alterations were detected in NLRP3 or MYD88. Complete clinical remission was achieved with the anti-IL-1β monoclonal antibody canakinumab. After two years, despite stable IgM levels, the patient developed B symptoms and bone pain. Repeat marrow biopsy confirmed marginal zone lymphoma. PET-CT excluded high-grade transformation. Addition of rituximab (375 mg/m² ×4) induced remission. Notably, the MEFV c.2084A>G variant has been described in MYD88 mutation–positive indolent B-cell lymphomas with CNS involvement, and F2 variants have co-occurred in selected cases. Although specific data on ITGB2 c.1570G>A are limited, ITGB2 (CD18) plays a key role in B-cell adhesion, tumor–microenvironment interaction, immune infiltration, and CAR-T responsiveness. Complement pathway alterations, including C2 variants, have been implicated in non-Hodgkin lymphoma susceptibility.

**Copyright Information:** (Online) ISSN: 2572-9241
©2026 The Author(s). HemaSphere is published by John Wiley & Sons Ltd on behalf of European Hematology Association. This is an open access Abstract Book distributed under the Attribution-NonCommercial-NoDerivs (CC BY-NC-ND), which allows third parties to download the articles and share them with others as long as they credit the author and the Abstract Book, but they cannot change the content in any way or use them commercially.
**Abstract Book Citations:** Authors, Title, HemaSphere, 2026;10:(S1):pages. The abstract book DOIs can be found on the “Author Information” page.
**Disclaimer:** Articles published in the journal HemaSphere exclusively reflect the opinions of the authors. The authors are responsible for all content in their abstracts including accuracy of the facts, statements, citing resources, etc.

### Summary/Conclusion

This rare case suggests that MEFV, F2, ITGB2, and C2 variants may create a proinflammatory and microenvironmentally permissive milieu predisposing to lymphomagenesis in SchS. The absence of MYD88 and NLRP3 mutations underscores an alternative genetic route linking autoinflammation and B-cell transformation. Our findings support a gene-centered model of SchS-associated lymphoma evolution and justify systematic genomic profiling in similar patients to identify biomarkers of malignant progression.

**Copyright Information:** (Online) ISSN: 2572-9241
©2026 The Author(s). HemaSphere is published by John Wiley & Sons Ltd on behalf of European Hematology Association. This is an open access Abstract Book distributed under the Attribution-NonCommercial-NoDerivs (CC BY-NC-ND), which allows third parties to download the articles and share them with others as long as they credit the author and the Abstract Book, but they cannot change the content in any way or use them commercially.
**Abstract Book Citations:** Authors, Title, HemaSphere, 2026;10:(S1):pages. The abstract book DOIs can be found on the "Author Information" page.
**Disclaimer:** Articles published in the journal HemaSphere exclusively reflect the opinions of the authors. The authors are responsible for all content in their abstracts including accuracy of the facts, statements, citing resources, etc.

### (PB3201) NOVEL ALTERNATIVE SPLICE VARIANTS OF GENES ENCODING LYSOSOME-ASSOCIATED MEMBRANE PROTEINS 1 AND 2 (LAMP1 AND LAMP2) IN MULTIPLE MYELOMA, REVEALED BY COMBINING NEXT-GENERATION AND NANOPORE SEQUENCING

**Topic:** 13. Myeloma and other monoclonal gammopathies - Biology & translational research

Christos Kontos[1], Konstantina Kalogerini[1], Konstantina Athanasopoulou[1], Ioannis Ntanasis-Stathopoulos[2], Andreas Scorilas[1], Ioannis Trougakos[3], Evangelos Terpos*[2]

*[1]National and Kapodistrian University of Athens, Faculty of Biology, Department of Biochemistry and Molecular Biology, Athens, Greece; [2]National and Kapodistrian University of Athens, School of Medicine, Department of Clinical Therapeutics, Athens, Greece; [3]National and Kapodistrian University of Athens, Faculty of Biology, Department of Cell Biology and Biophysics, Athens, Greece;*

### Background

Alternative splicing is a pivotal post-transcriptional mechanism that enhances gene expression complexity by generating multiple splice variants from a single gene, thereby greatly expanding the diversity and functional versatility of the transcriptome. Within the lysosome-associated membrane protein (LAMP) gene family, alternative splicing produces splice variants being translated into protein isoforms with distinct structural and functional properties that influence lysosomal dynamics, membrane trafficking, and cellular homeostasis. Dysregulation of expression of *LAMP* family members has been associated with cancer progression; however, their splicing pattern in multiple myeloma (MM) remains largely unexplored.

### Aims

We sought to elucidate the impact of *LAMP* isoforms on MM progression, we sought firstly to identify alternative splice variants generated by *LAMP* family precursor messenger RNAs (pre-mRNAs).

### Methods

We followed a combinatorial approach based on both next-generation sequencing (NGS) and nanopore sequencing. Ten established MM cell lines (AMO-1, H929, KMS-12-BM, L-363, LP-1, MM.1S, OPM-2, RPMI 8226, SK-MM-2, and U-266) were subcultured based on ATCC instructions, and total RNA was extracted and enriched for poly(A)+ RNAs. RNA integrity was verified by capillary electrophoresis prior to sequencing library preparation. 100-bp paired-end RNA sequencing was performed on the DNBSEQ-G400 platform, and high-quality reads were aligned to the GRCh38 human reference genome using HISAT2. Next, we focused on the alternative splicing of *LAMP1* and *LAMP2* transcripts, by targeted nanopore sequencing, as these two members were consistently and highly expressed across all MM cell lines. Thus, after first-strand cDNA synthesis using an oligo-dT adaptor primer, we performed nested 3′-rapid amplification of cDNA ends (3′-RACE), using gene-specific primers annealing on each distinct first exon of the currently annotated *LAMP1* and *LAMP2* transcripts. For sequencing data analysis, we used our *in-house*–built PERL algorithm called Alternative Splicing Detection Tool (ASDT).

**Copyright Information:** (Online) ISSN: 2572-9241
©2026 The Author(s). HemaSphere is published by John Wiley & Sons Ltd on behalf of European Hematology Association. This is an open access Abstract Book distributed under the Attribution-NonCommercial-NoDerivs (CC BY-NC-ND), which allows third parties to download the articles and share them with others as long as they credit the author and the Abstract Book, but they cannot change the content in any way or use them commercially.
**Abstract Book Citations:** Authors, Title, HemaSphere, 2026;10:(S1):pages. The abstract book DOIs can be found on the "Author Information" page.
**Disclaimer:** Articles published in the journal HemaSphere exclusively reflect the opinions of the authors. The authors are responsible for all content in their abstracts including accuracy of the facts, statements, citing resources, etc.

**Results**

NGS-based expression analysis revealed that *LAMP1* and *LAMP2* were consistently and highly expressed across all MM cell lines, whereas *LAMP3* and *LAMP5* exhibited minimal or undetectable expression; *CD68* displayed moderate levels. Focusing on *LAMP1* and *LAMP2*, we identified both annotated and novel splice junctions, including exon-skipping events, novel exons, and different 3′-UTRs, resulting in >20 splice variants produced by these two genes. *In silico* prediction of open reading frames (ORFs) using publicly available bioinformatic tools revealed that several of the novel *LAMP1* and *LAMP2* transcripts are predicted to encode new protein isoforms of these two genes, accordingly. Moreover, the landscape of miRNA-binding sites in the splice variants comprising distinct 3′-UTRs is different, compared to the currently annotated transcripts, thus affecting post-transcriptional / translational regulation by microRNAs (miRNAs) and other regulatory small non-coding RNAs. The expression patterns of novel splice variants varied significantly among the 10 MM cell lines.

**Summary/Conclusion**

Alternative splicing of *LAMP1* and *LAMP2* pre-mRNAs in MM produces several novel transcripts, most of which are highly likely to encode for new protein isoforms that may influence lysosomal function in MM. Moreover, *LAMP1* and *LAMP2* splice variants with shorter 3′-UTRs are differentially regulated by miRNAs and other regulatory small non-coding RNAs.

**Copyright Information:** (Online) ISSN: 2572-9241
©2026 The Author(s). HemaSphere is published by John Wiley & Sons Ltd on behalf of European Hematology Association. This is an open access Abstract Book distributed under the Attribution-NonCommercial-NoDerivs (CC BY-NC-ND), which allows third parties to download the articles and share them with others as long as they credit the author and the Abstract Book, but they cannot change the content in any way or use them commercially.
**Abstract Book Citations:** Authors, Title, HemaSphere, 2026;10:(S1):pages. The abstract book DOIs can be found on the “Author Information” page.
**Disclaimer:** Articles published in the journal HemaSphere exclusively reflect the opinions of the authors. The authors are responsible for all content in their abstracts including accuracy of the facts, statements, citing resources, etc.

### (PB3202) COMPARATIVE TRANSCRIPTOMIC PROFILING OF IGG AND IGA-PRODUCING HUMAN MULTIPLE MYELOMA CELL LINES REVEALS ISOTYPE-SPECIFIC GENE EXPRESSION SIGNATURES

**Topic:** 13. Myeloma and other monoclonal gammopathies - Biology & translational research

Konstantina Athanasopoulou[1], Panagiotis Adamopoulos[1], Ioannis Ntanasis-Stathopoulos[2], Christos Kontos[1], Evangelos Terpos*[2]

*[1]National and Kapodistrian University of Athens, Faculty of Biology, Department of Biochemistry and Molecular Biology, Athens, Greece; [2]National and Kapodistrian University of Athens, School of Medicine, Department of Clinical Therapeutics, Athens, Greece;*

### Background

Multiple myeloma (MM) is a hematological malignancy characterized by profound clinical and biological heterogeneity. The immunoglobulin A (IgA) subtype, comprising ~20% of cases, is frequently associated with a more aggressive clinical course, therapy resistance, and an increased propensity for extramedullary disease compared to the common IgG subtype. While clinical differences between IgG and IgA-producing myeloma subtypes are well-documented, the molecular and transcriptomic variations between these subsets remain incompletely understood.

### Aims

The present study aims to unveil differentially expressed genes between IgA and IgG isotypes as well as to identify pathogenic mechanisms, signaling alterations and potential therapeutic targets specific to the aggressive IgA isotype.

### Methods

We used 6 established human MM cell lines, grouped either as IgG-producing (L-363, LP-1, and RPMI 8226) or IgA-producing (AMO-1, MM.1S, and H929). Cells were cultured under standard conditions, followed by total RNA extraction and poly(A)+ RNA enrichment. Quality control was assessed using capillary electrophoresis prior to library preparation. Paired-end RNA sequencing was performed on the MGI DNBSEQ platform. Following stringent computational quality control, raw FASTQ reads were aligned to the GRCh38 human reference genome. Transcript quantification was performed using the StringTie algorithm, followed by differential expression modeling utilizing the DESeq2 framework. Downstream functional annotation, biological network construction, and pathway mapping were conducted using the ShinyGO platform. This included the incorporation of Gene Ontology (GO) terms, KEGG pathways, and the MSigDB hallmark gene sets to condense redundant pathways and identify distinct cancer-associated biological states.

### Results

Differential expression analysis revealed a highly distinct transcriptomic landscape. Notably, *NOTCH2*, a key receptor in cellular signaling and differentiation, was significantly underexpressed in the IgA-producing group, suggesting an attenuation of Notch-driven survival mechanisms. Conversely, a specific signature of 26 genes was upregulated in the IgA-producing group. Functional analysis demonstrated that these upregulated genes drive specific molecular pathways. Biological process analysis highlighted the regulation of cell cycle G1/S phase transition, inflammatory responses and extensive apoptotic processes. Molecular function revealed significant enrichment in phosphorylation-dependent protein binding and kinase activator activity. KEGG pathway analysis implicated the p53 signaling pathway alongside various stress-resistance profiles. Furthermore, MSigDB Hallmark analysis provided a clear picture of the IgA-associated cellular state, showing significant enrichment in hypoxia, mTORC1 signaling, TNFA signaling through NFKB and the p53 pathway.

**Copyright Information:** (Online) ISSN: 2572-9241
©2026 The Author(s). HemaSphere is published by John Wiley & Sons Ltd on behalf of European Hematology Association. This is an open access Abstract Book distributed under the Attribution-NonCommercial-NoDerivs (CC BY-NC-ND), which allows third parties to download the articles and share them with others as long as they credit the author and the Abstract Book, but they cannot change the content in any way or use them commercially.
**Abstract Book Citations:** Authors, Title, HemaSphere, 2026;10:(S1):pages. The abstract book DOIs can be found on the "Author Information" page.
**Disclaimer:** Articles published in the journal HemaSphere exclusively reflect the opinions of the authors. The authors are responsible for all content in their abstracts including accuracy of the facts, statements, citing resources, etc.

**Summary/Conclusion**

RNA-seq analysis highlights transcriptomic divergence between IgG and IgA-producing MM cell lines. The marked underexpression of *NOTCH2* and the upregulation of a specific 26-gene panel in the IgA-producing group suggest the presence of distinct regulatory networks. Functional enrichment identifies that IgA-producing cell lines exhibit increased p53 pathway activity, active mTORC1 signaling and hypoxia responses. Ultimately, these findings provide an initial step for understanding the biological heterogeneity of MM, revealing isotype-specific molecular dependencies and potential therapeutic targets.

**Copyright Information:** (Online) ISSN: 2572-9241
©2026 The Author(s). HemaSphere is published by John Wiley & Sons Ltd on behalf of European Hematology Association. This is an open access Abstract Book distributed under the Attribution-NonCommercial-NoDerivs (CC BY-NC-ND), which allows third parties to download the articles and share them with others as long as they credit the author and the Abstract Book, but they cannot change the content in any way or use them commercially.
**Abstract Book Citations:** Authors, Title, HemaSphere, 2026;10:(S1):pages. The abstract book DOIs can be found on the “Author Information” page.
**Disclaimer:** Articles published in the journal HemaSphere exclusively reflect the opinions of the authors. The authors are responsible for all content in their abstracts including accuracy of the facts, statements, citing resources, etc.

### (PB3203) GLYCOSYLATION SINGLE-CELL TRANSCRIPTOMIC PROFILING DECODES DRIVER MECHANISMS OF CIRCULATING PLASMA CELLS IN MULTIPLE MYELOMA

**Topic:** 13. Myeloma and other monoclonal gammopathies - Biology & translational research

Dian Tong[1], Xiaoyan Qu*[1]

[1] *first affiliated hospital with Nanjing medical university, nanjing, China;*

### Background

Circulating plasma cells (CPCs), identified by the presence of clonal plasma cells in peripheral blood (PB), are associated with dissemination and poor prognosis in multiple myeloma (MM). However, the gene expression characteristics of CPCs and their formation mechanisms remain unclear.

### Aims

Our study aimed to define malignant PC states associated with CPC emergence and to determine how glycosylation-associated programs within the tumor microenvironment contribute to dissemination in MM.

### Methods

We performed integrated single-cell transcriptomic and surface glycosylation profiling of paired bone marrow (BM) and PB samples from four newly diagnosed multiple myeloma (NDMM) patients. Spatial relationships were evaluated by multiplex immunohistochemistry (mIHC), and the functional impact of monocytes on malignant PC migratory behavior was assessed using in vitro assays, with external transcriptomic datasets used for validation.

### Results

We identified a distinctive cancer cell subcluster in both BM and PB that highly associated with a gene module and exhibited specific characteristics, suggesting its potential role as a CPC-initiating clone. In parallel, we characterized FOS-activated monocytes with elevated glycosylation and metabolic features, and evaluated their spatial relationship with CCNB1$^{+}$ tumor cells by mIHC as well as their functional effects on malignant PC migration in vitro.

### Summary/Conclusion

Our results demonstrated that the circulation of MM was driven by specific CPC-initiating cells with unique genetic profiles and interaction with TME cells.

**Copyright Information:** (Online) ISSN: 2572-9241
©2026 The Author(s). HemaSphere is published by John Wiley & Sons Ltd on behalf of European Hematology Association. This is an open access Abstract Book distributed under the Attribution-NonCommercial-NoDerivs (CC BY-NC-ND), which allows third parties to download the articles and share them with others as long as they credit the author and the Abstract Book, but they cannot change the content in any way or use them commercially.
**Abstract Book Citations:** Authors, Title, HemaSphere, 2026;10:(S1):pages. The abstract book DOIs can be found on the “Author Information” page.
**Disclaimer:** Articles published in the journal HemaSphere exclusively reflect the opinions of the authors. The authors are responsible for all content in their abstracts including accuracy of the facts, statements, citing resources, etc.

### (PB3204) SINGLE-CENTER RETROSPECTIVE STUDY OF CYTOGENETIC ABNORMALITIES IN PATIENTS WITH MONOCLONAL GAMMOPATHY OF UNDETERMINED SIGNIFICANCE

**Topic:** 13. Myeloma and other monoclonal gammopathies - Biology & translational research

Ernesto Manuel Talegón de la Fuente*[1], Aida Carril Barcia[1], Lara Eritzpokhoff[1], Gayane Avetisyan[2], Neus Torres Hernández[2], Cristian Garcia-Ruiz[2], Alejandro Berenguer-Rubio[2], Montiel Marco Soriano[1], Francisco Arellano Bruno[1], Eva Lora Pérez[1], Micaela Córcoles Molina[1], Irene Luna[1], Ana Isabel Vicente Sánchez[1], Marta Santiago[1], Maria Leonor Senent Peris[1], María Dolores Linares Latorre[1], Rafael Andreu Lapiedra[1], Maria Desamparados Sempere Talens[1], Mario Arnao[1], Esperanza Such[1], Javier De La Rubia Comos[1], David Martínez-Campuzano[2]

*[1]Hospital Universitari i Politècnic La Fe, Hematology and hemotherapy, Valencia, Spain;[2]Instituto de Investigación Sanitaria La Fe (IISLAFE), Valencia, Spain;*

### Background

Monoclonal gammopathy of undetermined significance (MGUS) is a premalignant plasma cell disorder with an estimated 1% annual risk of progression to smoldering or active multiple myeloma (SMM/MM), usually stratified by the Mayo Clinic model. Cytogenetic abnormalities (CA) are not included in this score but are often assessed at diagnosis. Fluorescent in situ hybridization (FISH) in MGUS can be technically challenging due to low plasma cell burden, and its clinical value remains unclear.

### Aims

To evaluate CA in CD138+ plasma cells from MGUS patients and their association with progression to SMM/MM.

### Methods

We conducted a retrospective single-center study including MGUS patients with available cytogenetic testing between December 2011 and November 2025. Bone marrow samples were enriched for CD138+ plasma cells, with a median purity of 35% (range, 22.7–60%), as assessed by microscopy or flow cytometry. FISH included probes for *IGH* rearrangements (IGHR), *TP53* deletion, and 1p32.3(CDKN2C)/1q21(CKS1B), but none of them assessed triploidy. Patients were stratified using Mayo Clinic criteria. Data were analyzed in R software using Chi-square and Fisher's exact tests.

### Results

Sixty-one patients were included (median age, 72 years; range, 41–89 years) with a median follow-up of 36.2 months (range, 0.2–170.4 months). FISH was evaluable in 92% (n = 56); 21% (n = 12) had only IGHR status available.
CA were detected in 27/56 patients. IGHR was detected in 32% (n = 18) of cases, but a partner gene was identified in only two cases (*MAF* and *CCND1*); del(17p) in 5% (n = 3); and 1q gain/amplification in 20% (n = 12). High-risk CA (HRCA) were identified in eight patients (14%); two of them progressed to MM (Mayo scores 0 and 1, progression at 14.2 and 2 years since the MGUS diagnosis, respectively). In contrast, the other six patients (75%) did not progress, with a median follow-up of 71.4 months (range, 11.1–170.4) and a median Mayo score of 0 (range, 0–2).
In the total cohort, eight patients (13%) progressed to MM. HRCA were not associated with progression (p = 1), and no differences were observed in progression-free survival according to cytogenetic risk (log-rank p = 1). Clinical and biological characteristics of progressing patients are shown in Table 1.

**Copyright Information:** (Online) ISSN: 2572-9241
©2026 The Author(s). HemaSphere is published by John Wiley & Sons Ltd on behalf of European Hematology Association. This is an open access Abstract Book distributed under the Attribution-NonCommercial-NoDerivs (CC BY-NC-ND), which allows third parties to download the articles and share them with others as long as they credit the author and the Abstract Book, but they cannot change the content in any way or use them commercially.
**Abstract Book Citations:** Authors, Title, HemaSphere, 2026;10:(S1):pages. The abstract book DOIs can be found on the "Author Information" page.
**Disclaimer:** Articles published in the journal HemaSphere exclusively reflect the opinions of the authors. The authors are responsible for all content in their abstracts including accuracy of the facts, statements, citing resources, etc.

### Summary/Conclusion

Most patients with MGUS and HRCA did not progress during follow-up, suggesting that cytogenetic testing showed limited prognostic value in this setting and may be restricted to selected cases. Low FISH efficiency and limited sample size were the main limitations.

**Table 1. Baseline characteristics at the time of MGUS diagnosis and at progression to SMM/MM**

| | MGUS diagnosis | | | | | | Progression to SMM/MM | | | | |
|---|---|---|---|---|---|---|---|---|---|---|---|
| **Patient** | Age (years) | Type of measurable disease | Mayo risk score | M-protein (mg/dL) | CA (% positive cells) | CD138+ purity (%) | Progression event | Time until progression (years) | Plasma cells in bone marrow, % | M-protein (mg/dL) | CA (% positive cells) |
| **1** | 65 | IgG lambda | 2 | 3.1 | Negative | 43 | MM | 8.6 | 46 | 3.89 | Negative |
| **2** | 65 | IgA lambda | 2 | 0.8 | Negative | 94 | MM | 12.2 | 10 | 0.9 | 1q+ (26%), +17 (47%) |
| **3** | 71 | IgA lambda +IgM kappa | 3 | 1.8+0.3 | 1q+ (18%) | 26 | SMM | 0.9 | 13 | 3.4 | 1q+ (51%) |
| **4** | 50 | IgG kappa | 0 | 0.9 | Negative | 32 | SMM | 4.7 | 12 | 0.6 | Negative |
| **5** | 51 | IgG kappa | 1 | 0,4 | *IGH-r** (42%) | 58 | SMM | 14.2 | 16 | 2.4 | 1q+ (31%); t(14,20) (by optical mapping) |
| **6** | 71 | IgG kappa | 0 | 0.9 | Negative | 92 | SMM | 8.9 | 30 | 3.1 | Negative |
| **7** | 74 | IgG kappa | 1 | 0.7 | *IGH-r** (42%), del17p (21%) | 38 | SMM> MM | 2 | 10 | 2.5 | *IGH*-r* (52 %), del17p (55%) |
| **8** | 69 | IgG kappa | 1 | 1.65 | Del14q (25%) | 22 | SMM> MM | 3.9 | 14 | 2.9 | 1q+ (51%), del14q (20%) |
| | | | | | | | | | | | |
| **Median (range)** | 67 (50-74) | | 1 (0-3) | 0.9 (0.8-3.1) | | 41 (22-94) | | 6.6 (0.9-12.2) | 13.5 (10-46) | 2.7 (0.6-3.89) | |

*IGH-r: IGH break-apart FISH positive; partner not identified.

Copyright Information: (Online) ISSN: 2572-9241
©2026 The Author(s). HemaSphere is published by John Wiley & Sons Ltd on behalf of European Hematology Association. This is an open access Abstract Book distributed under the Attribution-NonCommercial-NoDerivs (CC BY-NC-ND), which allows third parties to download the articles and share them with others as long as they credit the author and the Abstract Book, but they cannot change the content in any way or use them commercially.
Abstract Book Citations: Authors, Title, HemaSphere, 2026;10:(S1):pages. The abstract book DOIs can be found on the "Author Information" page.
Disclaimer: Articles published in the journal HemaSphere exclusively reflect the opinions of the authors. The authors are responsible for all content in their abstracts including accuracy of the facts, statements, citing resources, etc.

### (PB3205) PLATELET AGGREGATION AND THROMBIN GENERATION IN MULTIPLE MYELOMA: A PROSPECTIVE STUDY

**Topic:** 13. Myeloma and other monoclonal gammopathies - Biology & translational research

Mario Biglietto*[1], Rosaria Mormile[1], Alfonso Piciocchi[1], Francesca Fazio[1], Marcello Capriata[1], Silvia Sorella[1], Bianca Maria Mandelli[1], Deborah Kasmi[1], Erminia Baldacci[1], Antonio Chistolini[1], Maria Teresa Petrucci[1], Cristina Santoro[1]

[1]*Sapienza, University of Rome, Department of Translational and Precision Medicine, Rome, Italy;*

### Background

Multiple Myeloma (MM) is a plasma cell dyscrasia characterized by the clonal proliferation of plasma cells, which is associated with a significantly increased risk of both thrombotic and hemorrhagic complications.

### Aims

This ongoing prospective observational study aims to characterize platelet function and thrombin generation (TGA) in patients with newly diagnosed MM and to assess the impact of first-line therapy on these parameters.

### Methods

To date, eighteen patients with newly diagnosed MM have been enrolled. Platelet function was evaluated using closure time (PFA200) and light transmission aggregometry (Born method) with four agonists: ADP (5 µM), Epinephrine (Epi) (10 µM), Collagen (Coll) (2 µg/ml), and Arachidonic Acid (AA) (1 mM). Thrombin generation (TGA) was assessed using the Calibrated Automated Thrombogram (CAT) assay. Baseline results were compared with historical healthy control cohorts. Intra-patient analysis was performed on 11 patients after four cycles of induction therapy (D-VTd or DRd). Statistical analysis was performed using R software (version 4.4). Comparisons between patients and controls were performed using the Wilcoxon rank-sum test and Pearson's Chi-square test. For paired data the Wilcoxon signed-rank test was employed. Statistical significance was set at $p < 0.05$. All enrolled subjects provided informed consent according to the principles of the Declaration of Helsinki.

### Results

In this interim analysis, at diagnosis, MM patients exhibited significantly lower platelet aggregation compared to controls for ADP (p=0.008), Epinephrine (p=0.004), and Arachidonic Acid (p=0.026). Procoagulant markers were significantly elevated: vWF:Ag (p=0.002) and vWF:RCo (p=0.009), while Factor VIII (p=0.065) showed a trend toward elevation. TGA analysis revealed significantly prolonged Lag Time (p=0.021) and significantly higher ETP (p=0.009), Peak (p=0.003), and Velocity Index (p=0.014) in MM patients (Table 1). Following therapy, most parameters remained stable, showing persistence of high thrombin generation, elevated vWF/FVIII levels, and impaired platelet function. Only Mean Platelet Volume (MPV) showed a significant decrease (7.85 fl vs. 7.30 fl, p=0.024).

### Summary/Conclusion

This study highlights the coexistence of hypercoagulability and platelet dysfunction in MM. Based on these preliminary results, the reduction of tumor burden and the treatment don't seem to modify the evaluated parameters.

**Copyright Information:** (Online) ISSN: 2572-9241
©2026 The Author(s). HemaSphere is published by John Wiley & Sons Ltd on behalf of European Hematology Association. This is an open access Abstract Book distributed under the Attribution-NonCommercial-NoDerivs (CC BY-NC-ND), which allows third parties to download the articles and share them with others as long as they credit the author and the Abstract Book, but they cannot change the content in any way or use them commercially.
**Abstract Book Citations:** Authors, Title, HemaSphere, 2026;10:(S1):pages. The abstract book DOIs can be found on the "Author Information" page.
**Disclaimer:** Articles published in the journal HemaSphere exclusively reflect the opinions of the authors. The authors are responsible for all content in their abstracts including accuracy of the facts, statements, citing resources, etc.

| Comparison between MM patients and controls in terms of Platelet Aggregation (Born Method) | | | |
|---|---|---|---|
| | **Patients** N = 18[1] | **Controls** N = 14[1] | **p-value**[2] |
| **Sex (M, F)** | | | **0.004** |
| M | 13 (72%) | 3 (21%) | |
| F | 5 (28%) | 11 (79%) | |
| **Age (years)** | 62.2 [48.8; 82.8] | 48.5 [18.8; 93] | **0.034** |
| **Hb (g/dl)** | 12.1 [7.8; 14.6] | 13.7 [12; 15.5] | **0.023** |
| **Hct (%)** | 34.7 [24.9; 46] | 41.7 [35.8; 45.4] | **0.004** |
| **MCV (fl)** | 92.1 [63.5; 104.7] | 89.7 [76.1; 112.1] | 0.2 |
| **WBC/mmc** | 5360 [1660; 23980] | 7055 [4190; 9970] | 0.11 |
| **N/mmc** | 2765 [920; 22040] | 4095 [2090; 6890] | 0.2 |
| **L/mmc** | 1155 [480.00; 3020] | 2150 [1220; 2630] | **0.005** |
| **Plts/mmc** | 196500 [65000; 429000] | 224000 [35400; 425000] | 0.4 |
| **MPV (fl)** | 7.85 [5.90; 9.40] | 7.45 [5.90; 9.90] | 0.5 |
| **NLR** | 2.07 [0.93; 32.90] | 2.15 [0.99; 3.94] | 0.8 |
| **PLR** | 173.49 [60.6; 372.92] | 117.06 [13.46; 242.86] | 0.084 |
| **PT ratio** | 0.97 [0.84; 1.26] | 0.97 [0.88; 1.18] | > 0.9 |
| **PTT ratio** | 0.95 [0.79; 1.23] | 1 [0.9; 1.15] | 0.2 |
| **Fibrinogen (mg/dl)** | 363.5 [187; 682] | 306.5 [241; 495] | 0.5 |
| **ATIII (%)** | 102 [74; 126] | 103 [92; 114] | 0.9 |
| **FVIII (%)** | 182.55 [107; 580] | 149 [112; 178] | 0.065 |
| **vWF:Ag (%)** | 156.85 [57.2; 379] | 105.25 [66; 151] | **0.002** |
| **vWF:RCo (%)** | 151 [44; 645] | 108.5 [73; 147] | **0.009** |
| **vWF ratio** | 0.96 [0.74; 1.70] | 1.01 [0.91; 1.24] | 0.3 |
| **Coll/ADP (sec)** | 93.5 [70; 154] | 93 [77; 121] | > 0.9 |
| **Coll/Epi (sec)** | 137 [66; 209] | 124 [109; 160] | 0.4 |
| **ADP (%)** | 63 [19; 82] | 75 [61; 95] | **0.008** |
| **Collagen (%)** | 74 [36; 87] | 80 [71; 94] | 0.080 |
| **Epinefrine (%)** | 70 [4; 92] | 78 [65; 93] | **0.004** |
| **Arachidonic Acid (%)** | 65 [50; 87] | 75.5 [65; 87] | **0.026** |
| **Comparison between MM patients and controls in terms of TGA parameters** | | | |
| | **Patients** N = 18[1] | **Controls** N = 11[1] | **p-value**[2] |
| **Lag Time (min)** | 3.78 [2.06; 10.58] | 3.07 [2.57; 4.07] | **0.021** |
| **ETP (nM x min)** | 1405.84 [747.31; 2257.37] | 1067.34 [910.16; 1321.25] | **0.009** |
| **Peak (nM)** | 238.01 [45.92; 363.58] | 142.55 [110.23; 206.29] | **0.003** |
| **ttPeak (min)** | 7.41 [4.40; 14.42] | 7.24 [5.41; 8.91] | 0.8 |
| **Start Tail (min)** | 24.17 [20.77; 42.31] | 25.10 [19.26; 29.28] | 0.7 |
| **Velocity Index (nM/min)** | 63.47 [13.74; 130.52] | 36.37 [20.99; 73] | **0.014** |

[1] n (%); Median [range]
[2] Pearson's $\chi^2$; Wilcoxon's rank-sum test; Wilcoxon's exact test

**Copyright Information:** (Online) ISSN: 2572-9241
©2026 The Author(s). HemaSphere is published by John Wiley & Sons Ltd on behalf of European Hematology Association. This is an open access Abstract Book distributed under the Attribution-NonCommercial-NoDerivs (CC BY-NC-ND), which allows third parties to download the articles and share them with others as long as they credit the author and the Abstract Book, but they cannot change the content in any way or use them commercially.
**Abstract Book Citations:** Authors, Title, HemaSphere, 2026;10:(S1):pages. The abstract book DOIs can be found on the "Author Information" page.
**Disclaimer:** Articles published in the journal HemaSphere exclusively reflect the opinions of the authors. The authors are responsible for all content in their abstracts including accuracy of the facts, statements, citing resources, etc.

### (PB3206) SERUM PENTRAXIN-3 LEVELS AS A POTENTIAL BIOMARKER FOR THROMBOSIS RISK AND TREATMENT RESPONSE IN NEWLY DIAGNOSED MULTIPLE MYELOMA

**Topic:** 13. Myeloma and other monoclonal gammopathies - Biology & translational research

Ayşe Çİçek[1], Seda Yilmaz*[2], Ömer Özbek[3], Çiğdem Denİz[4], Meryem Eren Karanİs[5]

*[1]University of Health Sciences, Hamidiye Faculty of Medicine, Konya City Hospital, Internal Medicine, Konya, Türkiye;[2]University of Health Sciences, Hamidiye Faculty of Medicine, Konya City Hospital, Internal Medicine and Hematology, Konya, Türkiye; [3]University of Health Sciences, Hamidiye Faculty of Medicine, Konya City Hospital, Radiology, Konya, Türkiye;[4]University of Health Sciences, Hamidiye Faculty of Medicine, Konya City Hospital, Medical Biochemistry, Konya, Türkiye; [5]University of Health Sciences, Hamidiye Faculty of Medicine, Konya City Hospital, Medical Pathology, Konya, Türkiye;*

### Background

Thromboembolic events (TE) remains a major cause of morbidity in multiple myeloma (MM). Current risk models do not fully capture endothelial injury and inflammatory activation. Pentraxin-3 (PTX3) reflects vascular inflammation and may provide additional prognostic information.

### Aims

To investigate the relationship between PTX3 levels in MM and TE and treatment response, and to evaluate the usefulness of PTX3 as a complementary biomarker in clinical decision-making processes.

### Methods

Prospective single-center cohort of 33 newly diagnosed MM patients and 26 healthy controls. PTX3 was measured by ELISA at diagnosis and month 4. Doppler ultrasonography screening was performed. Correlations and logistic regression analyses were conducted.

### Results

The incidence of TE was 12.1% (n=4), and the events showed a heterogeneous course (jugular vein thrombosis, proximal deep vein thrombosis, superficial thrombophlebitis, cerebellar ischemic event). PTX3 levels at diagnosis showed a gradual increase trend from the control group towards cases with thrombosis and mortality (p=0.059).  PTX3 at 4 months showed a strong positive  correlation with M-protein (rho=0.428 at diagnosis; p=0.021 / rho=0.417 at the 2nd cycle; p=0.024 / rho=0.529 at the 4th cycle; p=0.003); additionally, a positive correlation was found with total protein-IgG-uric acid at diagnosis and HDL at the 4th month. The insufficient decrease in Delta PTX3 showed a significant positive correlation with M-protein levels at the end of the 4th cycle (rho=0.593; p=0.001). A significant decrease in PTX3 levels was detected in patients who started antiaggregant prophylaxis (p=0.025). In regression analyses, PTX3 was significant in distinguishing patients from healthy controls and showed an independent association with mortality even after adjustment for lactate dehydrogenase/total protein. During follow-up, mortality was observed only in the group without TE  (n=4); and causal interpretation was avoided due to the small sample size and time constraints.

**Copyright Information:** (Online) ISSN: 2572-9241
©2026 The Author(s). HemaSphere is published by John Wiley & Sons Ltd on behalf of European Hematology Association. This is an open access Abstract Book distributed under the Attribution-NonCommercial-NoDerivs (CC BY-NC-ND), which allows third parties to download the articles and share them with others as long as they credit the author and the Abstract Book, but they cannot change the content in any way or use them commercially.
**Abstract Book Citations:** Authors, Title, HemaSphere, 2026;10:(S1):pages. The abstract book DOIs can be found on the "Author Information" page.
**Disclaimer:** Articles published in the journal HemaSphere exclusively reflect the opinions of the authors. The authors are responsible for all content in their abstracts including accuracy of the facts, statements, citing resources, etc.

**Summary/Conclusion**

PTX3 stands out as a complementary marker associated with thrombotic tendency and residual disease/inflammation in MM.The use of PTX3 in conjunction with existing clinical scores (IMPEDE/SAVED/Khorana) and contextual triggers (central venous catheter, steroid load, etc.) may have clinical value for risk-adapted thromboprophylaxis and monitoring of treatment response. In conclusion, the apparent association of PTX3 levels with disease activity and thrombosis risk suggests that these biomarkers could be used as early risk markers in MM.

Table 1. Baseline PTX3 Levels by Group

| Group | Median PTX3 (pg/mL) |
|---|---|
| Healthy Controls | 52.38 |
| MM without TE | 60.11 |
| MM with TE | 86.92 |
| Exitus | 139.64 |

**Copyright Information:** (Online) ISSN: 2572-9241
©2026 The Author(s). HemaSphere is published by John Wiley & Sons Ltd on behalf of European Hematology Association. This is an open access Abstract Book distributed under the Attribution-NonCommercial-NoDerivs (CC BY-NC-ND), which allows third parties to download the articles and share them with others as long as they credit the author and the Abstract Book, but they cannot change the content in any way or use them commercially.
**Abstract Book Citations:** Authors, Title, HemaSphere, 2026;10:(S1):pages. The abstract book DOIs can be found on the "Author Information" page.
**Disclaimer:** Articles published in the journal HemaSphere exclusively reflect the opinions of the authors. The authors are responsible for all content in their abstracts including accuracy of the facts, statements, citing resources, etc.

### (PB3207) THE ABUNDANCE AND THERAPEUTIC POTENTIAL OF LIPID DROPLETS IN MULTIPLE MYELOMA

**Topic:** 13. Myeloma and other monoclonal gammopathies - Biology & translational research

Stefania Iliana Tziola*[1], Christine Dyrager[2], Berta Garrido-Zabala[1], Isabelle Holm[1], Torbjörn Karlsson[3], Fredrik Öberg[4], Andreas Lennartsson[5], Antonia Kalushkova[1], Patrick Nylund[1], Helena Jernberg Wiklund[1]

*[1]Uppsala University, Department of Immunology, Genetics and Pathology, Uppsala, Sweden;[2]Uppsala University, Department of Chemistry for Life Sciences, Uppsala, Sweden; [3]Uppsala University, Department of Medical Sciences, Division of Haematology, Uppsala, Sweden; [4]Medivir AB, Huddinge, Stockholm, Sweden;[5]Karolinska Institutet, Department of Biosciences and Nutrition, Stockholm, Sweden;*

### Background

Multiple myeloma (MM) is a heterogeneous haematological malignancy originating from the bone marrow. One of the main challenges facing patients diagnosed with MM, is that despite the recent advances in treatment and disease management, many patients will eventually relapse and develop treatment resistance. Interestingly, even though extensive research has been conducted into the heterogenetic nature of MM genotypes, no single genetic alteration has been established as principal pathogenic event highlighting the role of the intertwined dysregulation of several molecular mechanisms, including metabolic reprogramming, in MM development. Notably, malignant plasma cells dynamically alter key metabolic pathways to support their elevated energy demands resulting in disease progression. Previous studies have suggested that cancer cells meet their increased energetic demands needed to maintain a high proliferative rate by enhancing lipid synthesis and storage within LDs. Moreover, elevated LD abundance has been linked to treatment resistance. However, the dependency of LDs in MM has not been investigated yet.

### Aims

This research aims to evaluate the effect of *de novo* lipogenesis inhibition on LDs in human and murine MM cells. Furthermore, we aim to combine bortezomib, the standard-of-care for newly diagnosed MM patients, with *de novo* lipogenesis inhibition to overcome treatment resistance in non-responsive MM cells.

### Methods

The LD-BTD1 compound was used to image LDs by confocal microscopy. Moreover, to assess the clinical importance of the*de novo* lipogenesis proteins, we utilized expression and overall survival data from newly diagnosed MM patients within the MMRF cohort. For *de novo* lipogenesis inhibition, human MM cell lines as well as the 5T33U murine MM cell line were treated with BMS-303141 or fatostatin. The effect of the treatments on the metabolic activity and viability of the cells was evaluated using the AlamarBlue and apoptosis assays, respectively.

### Results

We identified increased abundance of LDs in MM cells by LD-BTD1 staining. By utilizing RNA-seq data from newly diagnosed MM patients within the MMRF cohort, we demonstrated that key gene transcripts of the *de novo* lipogenesis pathway (ACLY and SREBP1) where overexpressed in MM compared to healthy individuals and associated with worse prognosis. Interestingly, inhibition of ACLY (BMS-303141) and SREBPs (fatostatin) significantly reduced cell viability and induced apoptosis in human and murine MM cells.

**Copyright Information:** (Online) ISSN: 2572-9241
©2026 The Author(s). HemaSphere is published by John Wiley & Sons Ltd on behalf of European Hematology Association. This is an open access Abstract Book distributed under the Attribution-NonCommercial-NoDerivs (CC BY-NC-ND), which allows third parties to download the articles and share them with others as long as they credit the author and the Abstract Book, but they cannot change the content in any way or use them commercially.
**Abstract Book Citations:** Authors, Title, HemaSphere, 2026;10:(S1):pages. The abstract book DOIs can be found on the "Author Information" page.
**Disclaimer:** Articles published in the journal HemaSphere exclusively reflect the opinions of the authors. The authors are responsible for all content in their abstracts including accuracy of the facts, statements, citing resources, etc.

**Summary/Conclusion**

Taken together, our preliminary data suggest that MM cells rely on lipid droplets for their survival and proliferation. Moreover, we demonstrated that both human and murine MM cell lines are sensitive to *de novo* lipogenesis inhibition suggesting a novel therapeutic avenue for the treatment of MM.

**Copyright Information:** (Online) ISSN: 2572-9241
©2026 The Author(s). HemaSphere is published by John Wiley & Sons Ltd on behalf of European Hematology Association. This is an open access Abstract Book distributed under the Attribution-NonCommercial-NoDerivs (CC BY-NC-ND), which allows third parties to download the articles and share them with others as long as they credit the author and the Abstract Book, but they cannot change the content in any way or use them commercially.
**Abstract Book Citations:** Authors, Title, HemaSphere, 2026;10:(S1):pages. The abstract book DOIs can be found on the "Author Information" page.
**Disclaimer:** Articles published in the journal HemaSphere exclusively reflect the opinions of the authors. The authors are responsible for all content in their abstracts including accuracy of the facts, statements, citing resources, etc.

### (PB3209) INTRATUMORAL MICROBIOTA IN B-CELL LYMPHOMA AND MULTIPLE MYELOMA: AN EXPLORATORY STUDY

**Topic:** 13. Myeloma and other monoclonal gammopathies - Biology & translational research

Alba Rodríguez-García[1], Andrés Arroyo[1], Roberto Garcia-Vicente[1], Paula Sanchez-Llorca[1], Enrique Revilla[2], María Linares*[1, 3], Joaquín Martinez-Lopez[1]

*[1]Hematological Malignancies Clinical Research CRIS Unit H120-CNIO, Department of Hematology, Hospital Universitario 12 de Octubre, imas12, Universidad Complutense de Madrid, Madrid, Spain; [2]Hospital 12 de Octubre, Pathology Anatomy, Madrid, Spain; [3]Pharmacy School, Universidad Complutense de Madrid, Department of Biochemistry and Molecular Biology, Madrid, Spain;*

**Background**

The microbiota has emerged as a key modulator of tumor immunity and response to anticancer therapies. While most studies have focused on the gut microbiota, recent evidence supports the presence of intratumoral microbial communities with potential biological and clinical relevance. In hematologic malignancies, the role of the intratumoral microbiota remains poorly characterized.

**Aims**

The aim of this study was to characterize and compare the intratumoral microbiota of lymphoma-involved lymph nodes and multiple myeloma bone marrow samples with healthy tissues using 16S rRNA gene sequencing, in order to identify disease-associated microbial patterns.

**Methods**

Tumor samples from patients with diffuse large B-cell lymphoma (n=5) and multiple myeloma (n=5) were analyzed, each compared with tissue-matched healthy controls (lymph nodes, n=5; bone marrow, n=5). DNA was extracted from lymph nodes using the DNeasy PowerLyzer PowerSoil Kit and from bone marrow using the DNeasy Blood & Tissue Kit (Qiagen), following standardized protocols. The V3–V4 region of the 16S rRNA gene was amplified and sequenced on the Illumina MiSeq platform (2×300 bp). Raw reads were preprocessed by removing human DNA using Bowtie2, followed by quality filtering and ASV inference with DADA2. Potential contaminants were identified using Decontam, and non-informative sequences (singletons, mitochondrial and chloroplast reads) were removed prior to downstream analyses. Taxonomic assignment was conducted against the SILVA v138 database. Group comparisons of relative abundance were performed using the Wilcoxon rank-sum test, with p-values adjusted for multiple testing using the Benjamini–Hochberg method).

**Results**

In both lymphoma and multiple myeloma cohorts, a large proportion of ASVs were present in only a single sample or detected in control samples, indicating substantial technical noise. After prevalence-based filtering (retaining ASVs present in ≥2 clinical samples), the datasets were reduced to a small number of ASVs. In lymphoma, genera detected exclusively in controls included *Lactobacillus, Haemophilus, Enterococcus, Corynebacterium*, and *Achromobacter*, while *Allorhizobium–Neorhizobium–Pararhizobium* and *Escherichia–Shigella* were observed only in lymphoma samples (Fig.1A). *Bifidobacterium* (p=0.52), *Cutibacterium* (p=0.83), and *Streptococcus* (p=0.65) were present in both groups, with trends suggesting higher *Bifidobacterium* and *Streptococcus* in controls and higher *Cutibacterium* in lymphoma, but none of these differences were statistically significant (Fig.1B). In multiple myeloma, no genera was exclusive to cases or controls, and *Cutibacterium* (p=0.55) which was present in both groups, presented a trend toward higher abundance in controls, although no significant differences were observed (Fig.1C). Given the low number of retained ASVs, diversity and differential abundance analyses were considered unreliable and were not performed.

**Copyright Information:** (Online) ISSN: 2572-9241
©2026 The Author(s). HemaSphere is published by John Wiley & Sons Ltd on behalf of European Hematology Association. This is an open access Abstract Book distributed under the Attribution-NonCommercial-NoDerivs (CC BY-NC-ND), which allows third parties to download the articles and share them with others as long as they credit the author and the Abstract Book, but they cannot change the content in any way or use them commercially.
**Abstract Book Citations:** Authors, Title, HemaSphere, 2026;10:(S1):pages. The abstract book DOIs can be found on the "Author Information" page.
**Disclaimer:** Articles published in the journal HemaSphere exclusively reflect the opinions of the authors. The authors are responsible for all content in their abstracts including accuracy of the facts, statements, citing resources, etc.

### Summary/Conclusion

In this exploratory study, no disease-specific microbial signatures were detected. While some taxa showed relative abundance trends in certain groups, none was statistically significant, and we cannot exclude the possibility that these represent residual contamination. These findings highlight the challenges of studying low-biomass tumor-associated microbiota in hematological tumors and underscore the need for larger cohorts and rigorous contamination control in future investigations.

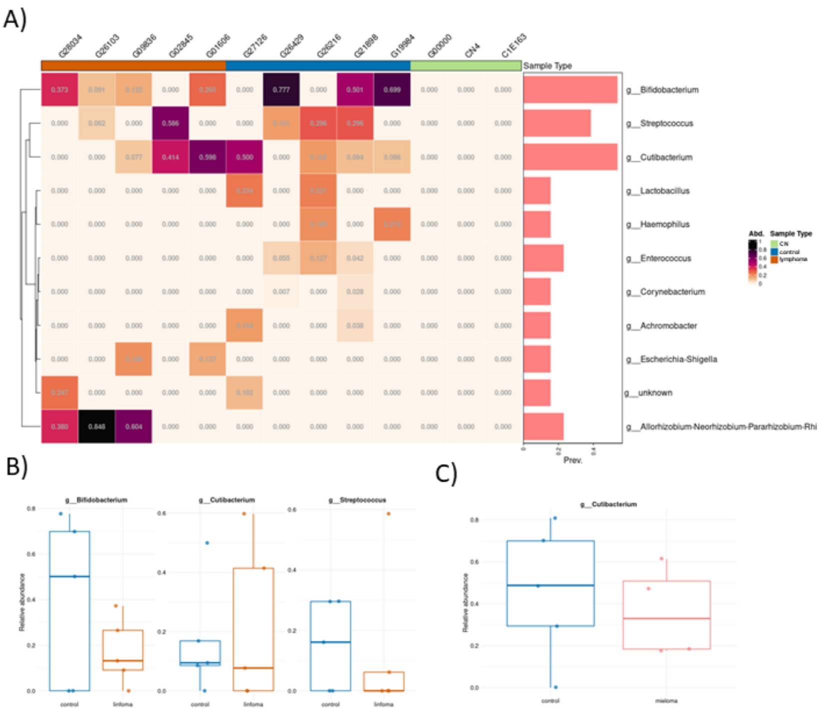

**Figure 1. A)** Heatmap of relative genus-level abundance across negative controls, healthy lymph node controls, and lymphoma samples following contaminant identification and preprocessing. Rows correspond to bacterial genera and columns to individual samples. Color intensity reflects normalized relative abundance. **B)** Box-and-whisker plots showing the relative abundance of *Bifidobacterium*, *Cutibacterium*, and *Streptococcus* in controls and lymphoma samples. Boxes represent the range, the central line indicates the median, and whiskers denote the minimum and maximum values. **C)** Box-and-whisker plot showing the relative abundance of *Cutibacterium*, in controls and myeloma samples. Boxes represent the range, the central line indicates the median, and whiskers denote the minimum and maximum values

**Copyright Information:** (Online) ISSN: 2572-9241
©2026 The Author(s). HemaSphere is published by John Wiley & Sons Ltd on behalf of European Hematology Association. This is an open access Abstract Book distributed under the Attribution-NonCommercial-NoDerivs (CC BY-NC-ND), which allows third parties to download the articles and share them with others as long as they credit the author and the Abstract Book, but they cannot change the content in any way or use them commercially.
**Abstract Book Citations:** Authors, Title, HemaSphere, 2026;10:(S1):pages. The abstract book DOIs can be found on the "Author Information" page.
**Disclaimer:** Articles published in the journal HemaSphere exclusively reflect the opinions of the authors. The authors are responsible for all content in their abstracts including accuracy of the facts, statements, citing resources, etc.

### (PB3210) SURVIVAL OF MULTIPLE MYELOMA AND PLASMA CELL NEOPLASMS: A POPULATION-BASED STUDY IN THE PROVINCE OF GIRONA, SPAIN (1994-2020)

**Topic:** 13. Myeloma and other monoclonal gammopathies - Biology & translational research

Kleber Garrochamba*[1, 2], Alicia Villavicencio[1, 3], Alberto Ameijide[4], Jan Trallero[1, 5], Aina Romaguera[1, 5], Arantza Sanvisens[1, 5], Marc Saez[6, 7], Rafael Marcos-Gragera[1, 5, 6, 7, 8]

*[1]Epidemiology Unit and Girona Cancer Registry, Catalan Institute of Oncology, Girona Biomedical Research Institute Dr. Josep Trueta (IDIBGI-CERCA), Girona, Spain; [2]Departamento de Ciencias de la Salud, Universidad Técnica Particular de Loja, Loja, Ecuador; [3]Facultad de la Salud Humana, Universidad Nacional de Loja, Loja, Ecuador;[4]Southern Catalonia Biomedical Research Institute - IRBCatSud, Sant Joan de Reus University Hospital, Reus, Spain; [5]Josep Carreras Leukaemia Research Institute, Badalona, Spain; [6]Research Group on Statistics, Econometrics and Health (GRECS), University of Girona, Girona, Spain; [7]CIBER of Epidemiology and Public Health (CIBERESP), Madrid, Spain;[8]Department of Medical Sciences, University of Girona, Girona, Spain;*

### Background

Multiple myeloma (MM) is a major cause of mortality among lymphoid malignancies (LMs). The observed survival (OS) and net survival (NS) of MM have improved significantly over the last two decades, particularly in Europe and North America . Assessing MM survival in relation to the latest advances is essential in the population for a better prognosis and treatment of MM from the perspective of hematology and oncology.

### Aims

To evaluate the survival of MM and plasma cell neoplasms (PCNs) according to sex, age, and period of diagnosis during 1994-2020 in the province of Girona, Spain.

### Methods

Cases of MM and PCNs recorded in the population-based cancer registry of Girona province between 1994 and 2020 were coded based on the International Classification of Diseases for Oncology (ICD-O-3), 3rd edition. Patient follow-up was until December 31, 2021. Kaplan-Meier was used for the OS analysis, and the log-rank method was used for determine the statistical significant differences by sex, age (31-69, 70-94 years), and period of diagnosis (1994-2001, 2002-2009, and 2010-2016). NS was determined using the Pohar-Perme method.

### Results

A total of 783 cases of MM and PCNs were recorded between 1994 and 2020, with 425 (54.3%) and 358 (45.7%) diagnosed in men and women, respectively. The median age was 72 [IQR:(63-79)] years. The 5-year OS was 36.0%[95% CI: 31.7-40.9] and 37.4% [95% CI: 32.6-42.8] in men and women, respectively. For the age groups 31-69 and 70-94 years, the 5-year OS was 54.3%[95% CI: 49.1-60.1] and 24.3%[95% CI: 20.7-28.6], respectively. For the periods 1994-2001 and 2010-2016, the 5-year OS was 27.9%[95% CI: 21.5-36.0] and 41.5%[95% CI: 36.4-47.3], respectively, with a significant increase in median survival from 1.86 to 3.65 years (p=0.02). The 5-year NS was slightly higher in women, 41.4% [95%CI: 36.1-47.5], compared to men, 41.0% [95%CI: 35.9-46.9]. The 5-year NS was higher for the 31-69 age group (56.5% [95% CI: 51.1-62.5]) than for the 70-94 age group (30.6% [95% CI: 25.9-36.1]). Finally, the 5-year NS was higher for the diagnosis period between 1994-2001 compared to the period 2010-2016 (p=0.03), 31.9% [95% CI: 24.6-41.3] and 46.5% [95% CI: 40.6-53.3], respectively.

**Copyright Information:** (Online) ISSN: 2572-9241
©2026 The Author(s). HemaSphere is published by John Wiley & Sons Ltd on behalf of European Hematology Association. This is an open access Abstract Book distributed under the Attribution-NonCommercial-NoDerivs (CC BY-NC-ND), which allows third parties to download the articles and share them with others as long as they credit the author and the Abstract Book, but they cannot change the content in any way or use them commercially.
**Abstract Book Citations:** Authors, Title, HemaSphere, 2026;10:(S1):pages. The abstract book DOIs can be found on the "Author Information" page.
**Disclaimer:** Articles published in the journal HemaSphere exclusively reflect the opinions of the authors. The authors are responsible for all content in their abstracts including accuracy of the facts, statements, citing resources, etc.

**Summary/Conclusion**

Survival rates for MM and PCNs in the province of Girona, Spain, are similar to those observed in other countries in Europe and North America. Prognosis for MM and PCNs have improved substantially since 2000. The results of treatments with T-cell therapy (CAR-T cells) (cilta-cel and ide-cel), bispecific T-cell engaging antibodies (BiTE), and CAR-NK cells in patients with relapsed/refractory MM are promising and will be reflected in the coming years.

**Copyright Information:** (Online) ISSN: 2572-9241
©2026 The Author(s). HemaSphere is published by John Wiley & Sons Ltd on behalf of European Hematology Association. This is an open access Abstract Book distributed under the Attribution-NonCommercial-NoDerivs (CC BY-NC-ND), which allows third parties to download the articles and share them with others as long as they credit the author and the Abstract Book, but they cannot change the content in any way or use them commercially.
**Abstract Book Citations:** Authors, Title, HemaSphere, 2026;10:(S1):pages. The abstract book DOIs can be found on the “Author Information” page.
**Disclaimer:** Articles published in the journal HemaSphere exclusively reflect the opinions of the authors. The authors are responsible for all content in their abstracts including accuracy of the facts, statements, citing resources, etc.

### (PB3211) SEX INFLUENCES THE PROGNOSTIC IMPACT OF TUMOR PATHWAYS IN MULTIPLE MYELOMA

**Topic:** 13. Myeloma and other monoclonal gammopathies - Biology & translational research

Mobina Shrestha*[1]

[1]*Sentara Albemarle Medical Center, Elizabeth City, United States of America;*

### Background

Men and women with multiple myeloma (MM) have small but consistent differences in outcomes. It is unclear whether tumor biology carries different clinical meaning across genders. We examined whether key transcriptional pathways have different prognostic effects in men and women.

### Aims

To investigate whether sex influences the prognostic significance of tumor pathway activity in newly diagnosed MM, with respect to early progression and survival outcomes.

### Methods

We analyzed gene expression data from 559 newly diagnosed MM patients (337 men, 222 women) from the GSE24080 cohort. Pathway activity was measured using single-sample gene set enrichment analysis of MSigDB Hallmark gene sets. We evaluated associations with 24-month event-free survival (EFS) and overall survival (OS) using age-adjusted logistic regression models that included sex-pathway interaction terms. For clinical interpretation, pathway scores were grouped into tertiles and high versus low activity was compared within each sex.

### Results

At 24 months, 21% of patients had progressed and 14% had died. A significant interaction between sex and the Inflammatory Response pathway was observed for EFS (interaction p=0.040). In men, high inflammatory pathway activity was associated with a significantly lower risk of early progression (OR 0.21, 95% CI 0.10-0.45). This protective effect was not seen in women (OR 0.69, 95% CI 0.30-1.63). For OS, a significant sex-modified effect was observed for the Apical Junction pathway (interaction p=0.037), suggesting differences in the prognostic role of cell adhesion and microenvironment-related signaling between men and women.

### Summary/Conclusion

These findings suggest that key tumor pathways may have different clinical implications in men and women with MM. Considering sex as a biological modifier may improve risk assessment and guide future studies exploring personalized treatment strategies.

**Copyright Information:** (Online) ISSN: 2572-9241
©2026 The Author(s). HemaSphere is published by John Wiley & Sons Ltd on behalf of European Hematology Association. This is an open access Abstract Book distributed under the Attribution-NonCommercial-NoDerivs (CC BY-NC-ND), which allows third parties to download the articles and share them with others as long as they credit the author and the Abstract Book, but they cannot change the content in any way or use them commercially.
**Abstract Book Citations:** Authors, Title, HemaSphere, 2026;10:(S1):pages. The abstract book DOIs can be found on the "Author Information" page.
**Disclaimer:** Articles published in the journal HemaSphere exclusively reflect the opinions of the authors. The authors are responsible for all content in their abstracts including accuracy of the facts, statements, citing resources, etc.

### (PB3212) EXPORTIN-1 INHIBITION MODULATES MONOCYTIC DIFFERENTIATION AND TUMOR-ASSOCIATED MACROPHAGE FUNCTION IN THE MULTIPLE MYELOMA TUMOR MICROENVIRONMENT

**Topic:** 13. Myeloma and other monoclonal gammopathies - Biology & translational research

Kereshmeh Tasbihi*[1], Katrin Bitterer[1], Christopher Lischer[1], Vivien Reinecke[1], Johannes Berges[2], Gina Nusser[1], Matthias Rex[1], Andreas MacKensen[1], Heiko Bruns[1]

*[1]University Hospital Erlangen, Department of Medicine 5, Erlangen, Germany;[2]Eberhard Karls University, Department of Dermatology, Tübingen, Germany;*

### Background

Despite major therapeutic advances, the persistence of malignant plasma cells in multiple myeloma is critically shaped by a supportive and highly adaptive immunologic tumor microenvironment (TME). Increasing evidence highlights a pivotal role of monocytes and tumor-associated macrophages (TAMs), in orchestrating immunoregulatory niches that influence recurrence and therapy resistance. Central to these processes is the dynamic regulation of nucleocytoplasmic transport, which precisely controls immune cell differentiation, signaling, and effector function. Exportin-1 (XPO1) is a key mediator of nuclear export and a central regulator of transcriptional and post-transcriptional cellular homeostasis.

### Aims

While XPO1 inhibition has demonstrated antineoplastic activity, its impact on the molecular-biological programming and functional plasticity of human monocytes and macrophages remains poorly understood. In this context, the present *in vitro* study was designed to elucidate the impact of XPO1 inhibition on the phenotypical differentiation and effector functions of monocytes and macrophages.

### Methods

Primary human monocytes and macrophages were generated from selectively isolated peripheral blood mononuclear cells of healthy donors. In addition, TAMs were isolated from human bone marrow samples. Cellular differentiation and phenotypic development were analyzed by microscopy, flow cytometry, and western blotting. Transcriptional and molecular profiles were assessed using RT-qPCR and Nanopore sequencing. Functional analyses included assays for viability, apoptosis, transmigration, and cytokine secretion, as well as phagocytosis. XPO1 inhibition-induced alterations in nucleocytoplasmic distribution of transcripts and proteins following biochemical cell fractionation were evaluated as well.

### Results

XPO1 inhibition interfered with macrophage colony-stimulating factor receptor-dependent differentiation programs of monocytes and macrophages at both transcriptional and translational levels. Transcriptomic profiling revealed differential regulation of gene clusters involved in immunoregulatory pathways, intracellular signaling, cytoskeletal organization, cellular motility, and inflammatory response modulation. While cell viability and apoptosis rates remained largely unaffected, XPO1-inhibited monocytes and macrophages exhibited significantly reduced migratory capacity in response to chemo-attractive stimuli. Furthermore, secretion of multiple pro-inflammatory cytokines and chemokines was markedly diminished. Biochemical fractionation of monocytes followed by RT-qPCR and Nanopore sequencing revealed XPO1 inhibition-dependent alterations in the nuclear and cytoplasmic distribution of distinct regulatory transcripts.

**Copyright Information:** (Online) ISSN: 2572-9241
©2026 The Author(s). HemaSphere is published by John Wiley & Sons Ltd on behalf of European Hematology Association. This is an open access Abstract Book distributed under the Attribution-NonCommercial-NoDerivs (CC BY-NC-ND), which allows third parties to download the articles and share them with others as long as they credit the author and the Abstract Book, but they cannot change the content in any way or use them commercially.
**Abstract Book Citations:** Authors, Title, HemaSphere, 2026;10:(S1):pages. The abstract book DOIs can be found on the "Author Information" page.
**Disclaimer:** Articles published in the journal HemaSphere exclusively reflect the opinions of the authors. The authors are responsible for all content in their abstracts including accuracy of the facts, statements, citing resources, etc.

**Summary/Conclusion**

This study demonstrates that XPO1-dependent nucleocytoplasmic transport critically governs differentiation trajectories and effector functions of human monocytes and macrophages. These findings provide mechanistic insight into how modulation of nuclear export reshapes myeloid cell behavior and immunoregulatory networks within the complex cellular TME. Finally, these insights establish a molecular rationale for exploiting nucleocytoplasmic transport pathways to therapeutically modulate myeloid-driven immune dysregulation and treatment resistance in disease-associated myeloma niches.

**Copyright Information:** (Online) ISSN: 2572-9241
©2026 The Author(s). HemaSphere is published by John Wiley & Sons Ltd on behalf of European Hematology Association. This is an open access Abstract Book distributed under the Attribution-NonCommercial-NoDerivs (CC BY-NC-ND), which allows third parties to download the articles and share them with others as long as they credit the author and the Abstract Book, but they cannot change the content in any way or use them commercially.
**Abstract Book Citations:** Authors, Title, HemaSphere, 2026;10:(S1):pages. The abstract book DOIs can be found on the "Author Information" page.
**Disclaimer:** Articles published in the journal HemaSphere exclusively reflect the opinions of the authors. The authors are responsible for all content in their abstracts including accuracy of the facts, statements, citing resources, etc.

### (PB3213) DECIPHERING THE CROSSTALK BETWEEN DNA DAMAGE RESPONSE NETWORK AND IMMUNE SURVEILLANCE IN MULTIPLE MYELOMA

**Topic:** 13. Myeloma and other monoclonal gammopathies - Biology & translational research

Elisavet Deligianni*[1], Panagiotis Malamos[1], Christina Papanikolaou[1], Dimitra Mavroeidi[1], Maria Gavriatopoulou[2], Evangelos Terpos[2], Vassilis Souliotis[1]

*[1]Institute of Chemical Biology, National Hellenic Research Foundation, 11635 Athens, Greece;[2]Department of Clinical Therapeutics, School of Medicine, National and Kapodistrian University of Athens, Alexandra General Hospital, Athens, Greece;*

### Background

Despite major therapeutic advances, Multiple Myeloma (MM) remains an incurable hematologic malignancy, largely due to the emergence of treatment resistance and recurrent relapse. Growing evidence underscores a critical interplay between the DNA Damage Response (DDR) network and the immune system. Recent findings show that DNA-damaging therapies can boost tumor immunogenicity and elicit antitumor defense. Exploiting this interaction may pave the way for designing combinatorial therapeutic strategies.

### Aims

This research investigates how chemotherapy-induced DNA damage reshapes the immunogenic phenotype of MM cells and modulates immune recognition pathways.

### Methods

Myeloma cell lines and a B lymphoblastoid cell line as a healthy control were analyzed at baseline and following exposure to specific genotoxic agents (Bortezomib, Carfilzomib, Melphalan and Oxaliplatin). DNA damage was assessed by quantification of apurinic/apyrimidinic (AP) sites, oxidative stress levels, interstrand crosslink repair capacity (ICL/R) and γH2AX accumulation. Flow cytometry was employed to monitor modifications in the Programmed Death-Ligand 1 (PD-L1) and Major Histocompatibility Complex Class I (MHC-I) expression, as well as hallmarks of immunogenic cell death (ICD) on MM cells. Cytoplasmic double-stranded DNA (dsDNA) accumulation and the subsequent induction of the cGAS-STING pathway were determined via confocal microscopy and western blotting, respectively.

### Results

Genotoxic drugs significantly elevated levels of DNA damage, AP-site formation, and oxidative stress across MM cell lines. Notably, chemotherapy successfully triggered ICD, marked by the translocation of calreticulin and HSP90 to the cell surface, alongside an upregulation of MHC-I expression to enhance antigen presentation. Interestingly, we observed an elevation in PD-L1 expression, suggesting a potential mechanism of adaptive immune resistance. Also, despite detectable cytosolic dsDNA at baseline, treatment appeared to suppress cGAS-STING pathway activity within the MM cell lines.

**Copyright Information:** (Online) ISSN: 2572-9241
©2026 The Author(s). HemaSphere is published by John Wiley & Sons Ltd on behalf of European Hematology Association. This is an open access Abstract Book distributed under the Attribution-NonCommercial-NoDerivs (CC BY-NC-ND), which allows third parties to download the articles and share them with others as long as they credit the author and the Abstract Book, but they cannot change the content in any way or use them commercially.
**Abstract Book Citations:** Authors, Title, HemaSphere, 2026;10:(S1):pages. The abstract book DOIs can be found on the “Author Information” page.
**Disclaimer:** Articles published in the journal HemaSphere exclusively reflect the opinions of the authors. The authors are responsible for all content in their abstracts including accuracy of the facts, statements, citing resources, etc.

**Summary/Conclusion**

These findings demonstrate that the DDR network is strongly linked to the immunostimulatory potential of specific chemotherapies. Inducing direct cytotoxicity while simultaneously priming the immune system provides a critical strategic advantage for the development of more effective therapeutic approaches in MM.

**Copyright Information:** (Online) ISSN: 2572-9241
©2026 The Author(s). HemaSphere is published by John Wiley & Sons Ltd on behalf of European Hematology Association. This is an open access Abstract Book distributed under the Attribution-NonCommercial-NoDerivs (CC BY-NC-ND), which allows third parties to download the articles and share them with others as long as they credit the author and the Abstract Book, but they cannot change the content in any way or use them commercially.
**Abstract Book Citations:** Authors, Title, HemaSphere, 2026;10:(S1):pages. The abstract book DOIs can be found on the "Author Information" page.
**Disclaimer:** Articles published in the journal HemaSphere exclusively reflect the opinions of the authors. The authors are responsible for all content in their abstracts including accuracy of the facts, statements, citing resources, etc.

### (PB3214) IMMUNOPHENOTYPIC FACTORS INFLUENCING THE CLINICAL COURSE OF MULTIPLE MYELOMA

**Topic:** 13. Myeloma and other monoclonal gammopathies - Biology & translational research

Azat Karabekov*[1], Vadim Kemaikin[1], Zhandos Burkitbayev[1], Gulnur Zhakhina[2], Dinara Zharlyganova[3], Inna Berger[4], Ulbolsyn Orymbayeva[5], Zhuldyz Iskakova[1], Rose Ibragimova[1], Nazym Temir[1], Gulnur Mamyr[1], Olga Kolesnikova[1], Fariza Shokubayeva[1], Akbota Tursyn[1], Meiramgul Yussupova[1], Aidana Shalkarbekova[1]

*[1]National Research Oncology Center LLP, oncohematology and bone marrow transplantation, Astana, Kazakhstan; [2]Nazarbayev University School of Medicine, Department of Medicine, Astana, Kazakhstan; [3]National Research Oncology Center LLP, science, Astana, Kazakhstan; [4]Republican Specialized Scientific and Practical Center of Hematology, oncohematology and bone marrow transplantation, Tashkent, Uzbekistan; [5]University Medical Center CF, Astana, Kazakhstan;*

### Background

CD56 expression has been proposed as a prognostic and predictive biomarker in multiple myeloma. However, its clinical relevance in the context of modern induction therapy and autologous stem cell transplantation (ASCT) remains controversial.

### Aims

Our goal was to evaluate the level of CD56 expression on pathological plasma cells and their effect on the frequency of objective response to induction therapy and OS rates in patients with newly diagnosed MM.

### Methods

The researchers studied individuals with newly diagnosed multiple myeloma. CD56 expression was assessed by flow cytometry at diagnosis. Assessment was performed to determine patients' responses to induction therapy and to measure their progression-free and overall survival rates.

### Results

The study included 88 participants, of whom 68 (77%) were CD56-positive, and 20 (23%) were CD56-negative. The study results showed that CD56(-) patients developed plasmacytomas at a 70% rate, while CD56(+) patients had a 46% rate (p=0.055). A similar pattern was observed for extramodular lesions (p=0.006). The induction response rate was lower in CD56-negative patients than in CD56-positive patients (65% vs 85%, p=0.043). Patients who did not experience relapse received more $CD34^{+}$ cells (11.9±5.71 vs 9.29±2.57 $\times 10^{6}$/kg, p=0.012) and had higher post-transplant response rates (91% vs 63%, p=0.002). The patients who received induction treatment before their disease showed better survival outcomes than patients who did not respond to treatment (90.2% vs 87.1%, p=0.029) and patients who did not experience relapse (92.7% vs 85.0%, p=0.022). CD56 status did not affect survival outcomes.

### Summary/Conclusion

CD56 expression is associated with disease burden and response to induction therapy in multiple myeloma, supporting its role as an early disease-modifying factor. However, its prognostic value appears limited in patients receiving high-dose chemotherapy with ASCT, suggesting that intensive treatment may mitigate the adverse impact of CD56 negativity.

Table 1. Baseline clinical and laboratory characteristics of study participants stratified by CD56 expression

**Copyright Information:** (Online) ISSN: 2572-9241
©2026 The Author(s). HemaSphere is published by John Wiley & Sons Ltd on behalf of European Hematology Association. This is an open access Abstract Book distributed under the Attribution-NonCommercial-NoDerivs (CC BY-NC-ND), which allows third parties to download the articles and share them with others as long as they credit the author and the Abstract Book, but they cannot change the content in any way or use them commercially.
**Abstract Book Citations:** Authors, Title, HemaSphere, 2026;10:(S1):pages. The abstract book DOIs can be found on the “Author Information” page.
**Disclaimer:** Articles published in the journal HemaSphere exclusively reflect the opinions of the authors. The authors are responsible for all content in their abstracts including accuracy of the facts, statements, citing resources, etc.

| | Total (n = 88) | CD56 | | |
|---|---|---|---|---|
| | | Negative (n = 20; 23%) | Positive (n = 68; 77%) | p-value |
| Age, years (median [IQR]) | 56 [49-61] | 51 [42-61] | 57 [51-62] | 0.114<br>0.211 |
| Gender, n (%) Female<br>Male | 46 (52)<br>42 (48) | 8 (40)<br>12 (60) | 38 (56)<br>30 (44) | |
| Blood paraprotein, n (%) IgA<br>IgG<br>IgM<br>Light-chain | 10 (11)<br>65 (74)<br>1 (1)<br>12 (14) | 2 (10)<br>13 (65)<br>0<br>5 (25) | 8 (12)<br>52 (76)<br>1 (2)<br>7 (10) | 0.395 |
| Bence–Jones protein, n (%)<br>Absent<br>Present | 36 (41)<br>52 (59) | 9 (45)<br>11 (55) | 27 (40)<br>41 (60) | 0.672 |
| Lymphocytes, $\times 10^9$/L (median [IQR])<br>Monocytes, $\times 10^9$/L (median [IQR])<br>IgA, g/L (median [IQR])<br>IgG, g/L (median [IQR])<br>IgM, g/L (median [IQR]) | 1.50 [1.12-2.07]<br>0.40 [0.25-0.60]<br>0.27 [0.14-0.70]<br>13.5 [5.57-44.9]<br>0.25 [0.12-0.41] | 1.70 [1.24-1.98]<br>0.36 [0.25-0.59]<br>0.18 [0.10-0.68]<br>11.12 [4.98-18.3]<br>0.13 [0.04-0.41] | 1.50 [1.10-2.09]<br>0.41 [0.25-0.60]<br>0.30 [0.19-0.89]<br>15.5 [5.91-53.2]<br>15.5 [5.91-53.2] | 0.819<br>0.679<br>0.313<br>0.353<br>0.201 |
| Bone marrow plasma cells by flow cytometry (%), (median [IQR])<br>Bone marrow plasma cells (morphology, %), (median [IQR]) | 15 [10-24]<br>24.6 [9.1-40.2] | 26 [16.5-35.5]<br>30.9 [7.8-41.8] | 13 [7.8-18]<br>23.9 [10.5-40] | <0.001<br>0.673 |
| Plasmocytoma, n (%) Absent<br>Present<br>Extramodular lesions, n (%)<br>Absent<br>Present | 43 (49)<br>45 (51)<br>81 (92)<br>7 (8) | 6 (30)<br>14 (70)<br>15 (75)<br>5 (25) | 37 (54)<br>31 (46)<br>66 (97)<br>2 (3) | 0.055<br><br>0.006 |

**Copyright Information:** (Online) ISSN: 2572-9241
©2026 The Author(s). HemaSphere is published by John Wiley & Sons Ltd on behalf of European Hematology Association. This is an open access Abstract Book distributed under the Attribution-NonCommercial-NoDerivs (CC BY-NC-ND), which allows third parties to download the articles and share them with others as long as they credit the author and the Abstract Book, but they cannot change the content in any way or use them commercially.
**Abstract Book Citations:** Authors, Title, HemaSphere, 2026;10:(S1):pages. The abstract book DOIs can be found on the "Author Information" page.
**Disclaimer:** Articles published in the journal HemaSphere exclusively reflect the opinions of the authors. The authors are responsible for all content in their abstracts including accuracy of the facts, statements, citing resources, etc.

| | | | | |
|---|---|---|---|---|
| Renal failure Yes<br>No | 20 (23)<br>68 (77) | 4 (20)<br>16 (80) | 16 (24)<br>52 (76) | 0.502 |
| Induction response, n (%)<br>Response<br>No response | 71 (81)<br>17 (19) | 13 (65)<br>7 (35) | 58 (85)<br>10 (15) | 0.043 |

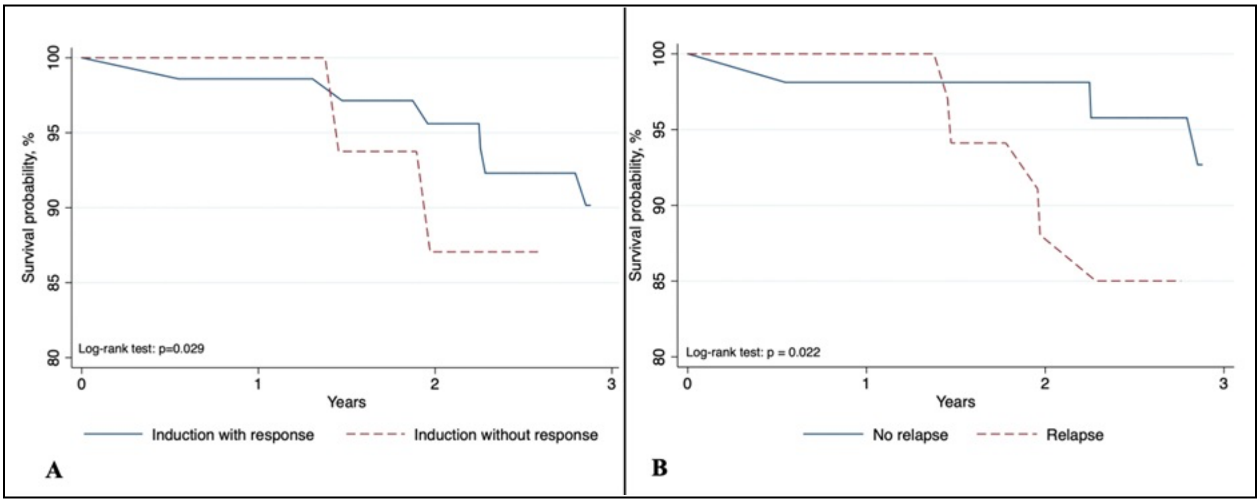

Figure 1. Overall survival according to induction response (A) and relapse status (B)

Copyright Information: (Online) ISSN: 2572-9241
©2026 The Author(s). HemaSphere is published by John Wiley & Sons Ltd on behalf of European Hematology Association. This is an open access Abstract Book distributed under the Attribution-NonCommercial-NoDerivs (CC BY-NC-ND), which allows third parties to download the articles and share them with others as long as they credit the author and the Abstract Book, but they cannot change the content in any way or use them commercially.
Abstract Book Citations: Authors, Title, HemaSphere, 2026;10:(S1):pages. The abstract book DOIs can be found on the "Author Information" page.
Disclaimer: Articles published in the journal HemaSphere exclusively reflect the opinions of the authors. The authors are responsible for all content in their abstracts including accuracy of the facts, statements, citing resources, etc.

### (PB3215) EVOLUTION OF PHOSPHATAEMIA AND SIGNALLING OF THE HORMONE FGF23 IN PATIENTS WITH NEWLY DIAGNOSED MULTIPLE MYELOMA: A POST-HOC STUDY

**Topic:** 13. Myeloma and other monoclonal gammopathies - Biology & translational research

Guillaume Courbon[1], Manon Sapet*[2], Stéphanie Mundweiler[1], Sebastien Charles[1], Amélie Cifuentes[1], Laurie Lugnier[1], Lionel Karlin[3], Tiphany Neel[4], Hubert Marotte[5], Emilie Chalayer[1, 2]

*[1]SAINBIOSE- INSERM U1059, Jean Monnet University, Ecole des Mines,, Saint-Etienne, France;[2]Division of Clinical Haematology, University Hospital, Saint Etienne, France; [3]Division of Clinical Haematology, HCL, Lyon, France;[4]Division of Rheumatology, University Hospital, Saint-Etienne, France; [5]Jean Monnet University, Division of Rheumatology, University Hospital, Ecole des Mines, SAINBIOSE, INSERM U1059, Saint-Etienne, France;*

### Background

Hypophosphatemia is described as a common abnormality in patients with multiple myeloma (MM, Cancarevic 2023). It can lead to defective bone mineralisation (osteomalacia) as well as probable cellular effects, as phosphate is an essential component of membranes, enzymes, and ATP. In its intact form, fibroblast growth factor 23 (iFGF23) is the phosphaturic hormone. iFGF23 is produced by bone and, in the presence of heparans, binds to FGFR1 and α-Klotho on renal membranes to inhibit phosphate reabsorption. Heparanases remove heparans from cellular membranes and matrices, and the presence of heparans is often correlated with low heparanase activity.

### Aims

To explore the relationship between phosphatemia, FGF23 and heparanase activity before and during treatment in patients with newly diagnosed MM (NDMM).

### Methods

This is an ancillary study of a prospective multicenter observational study that included NDMM patients without renal failure, assessed before and after the first treatment cycle. Plasma was obtained by two sequential centrifugations of citrated blood at 2,500×g for 15 min at room temperature. Inorganic phosphate was measured by UV method (BioLabo), iFGF23 by enzyme immunoassay (Quidel Ortho), and heparanase activity by enzymatic assay of heparan degradation (Takara Bio). Results are presented as mean±SEM.

### Results

Sixteen patients were included with a median age of 61 years (52-76). Patients had low serum phosphate levels, with an initial phosphate concentration of 1.04±0.07 mM decreasing to 0.82±0.05 mM (p=0.03); 60% of patients could be considered hypophosphataemic. Concentrations in iFGF23 were initially elevated at 63±11 pg/mL and decreased after one treatment cycle to 34±3 pg/mL (p=0.01), which was “abnormally normal” given the low phosphate levels. Levels of iFGF23 and phosphate were tightly correlated (r=0.75, p=0.0003). No significant creatinine variation was noted. Finally, baseline heparanase activity was 0.95±0.08 U/mL and decreased to 0.81±0.04 U/mL after one treatment cycle (p=0.04).

**Copyright Information:** (Online) ISSN: 2572-9241
©2026 The Author(s). HemaSphere is published by John Wiley & Sons Ltd on behalf of European Hematology Association. This is an open access Abstract Book distributed under the Attribution-NonCommercial-NoDerivs (CC BY-NC-ND), which allows third parties to download the articles and share them with others as long as they credit the author and the Abstract Book, but they cannot change the content in any way or use them commercially.
**Abstract Book Citations:** Authors, Title, HemaSphere, 2026;10:(S1):pages. The abstract book DOIs can be found on the “Author Information” page.
**Disclaimer:** Articles published in the journal HemaSphere exclusively reflect the opinions of the authors. The authors are responsible for all content in their abstracts including accuracy of the facts, statements, citing resources, etc.

### Summary/Conclusion

In this pilot study in patients with NDMM, we observed low circulating phosphate levels that worsened over time and would require corrective intervention in 60% of participants. We identified two potential mechanisms that may explain hypophosphataemia. First, inappropriate concentrations of the phosphaturic hormone iFGF23, which do not adapt to low phosphate levels as expected. Second, a reduction in heparanase activity, which may indicate persistence of heparans on renal cell membranes, thereby potentiating iFGF23 signalling. These preliminary results support further investigation of phosphate metabolism in larger cohorts of patients with NDMM and MM, as well as exploration of the underlying pathophysiological mechanisms.

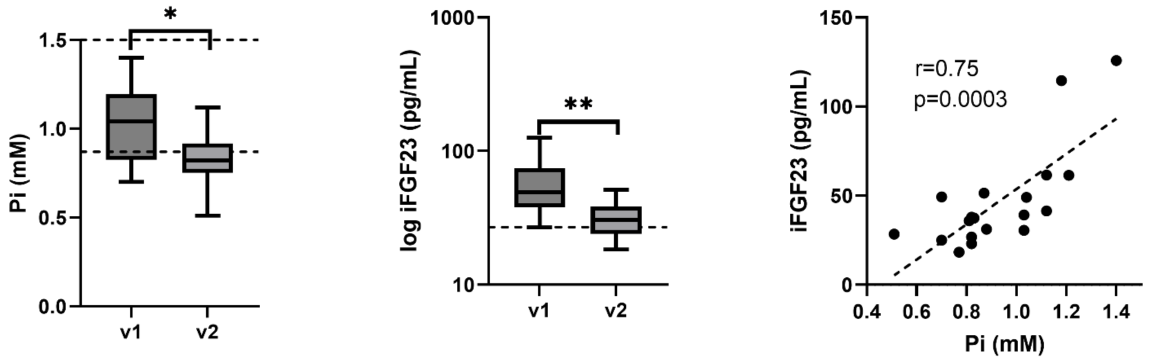

Figure: Evolution of phosphatemia (Pi), intact FGF23 (iFGF23) and correlation between Pi and iFGF23.

**Copyright Information:** (Online) ISSN: 2572-9241
©2026 The Author(s). HemaSphere is published by John Wiley & Sons Ltd on behalf of European Hematology Association. This is an open access Abstract Book distributed under the Attribution-NonCommercial-NoDerivs (CC BY-NC-ND), which allows third parties to download the articles and share them with others as long as they credit the author and the Abstract Book, but they cannot change the content in any way or use them commercially.
**Abstract Book Citations:** Authors, Title, HemaSphere, 2026;10:(S1):pages. The abstract book DOIs can be found on the "Author Information" page.
**Disclaimer:** Articles published in the journal HemaSphere exclusively reflect the opinions of the authors. The authors are responsible for all content in their abstracts including accuracy of the facts, statements, citing resources, etc.

### (PB3216) AGE-RELATED LOSS OF THE Y CHROMOSOME DRIVES MALE-BIASED CANCER BURDEN AND ADVERSE OUTCOMES

**Topic:** 13. Myeloma and other monoclonal gammopathies - Biology & translational research

Zuxi Feng[1], Minjing Liao[2], Liansheng Zhang*[1]

*[1]The Second Hospital of Lanzhou University, The Second Hospital of Lanzhou University, Lanzhou, China;[2]Lanzhou University, Second Clinical Medical College, China;*

### Background

Men exhibit higher incidence and mortality for most non-reproductive cancers, including multiple myeloma (MM). Age-related loss of the Y chromosome (LOY) has been implicated in male cancer progression, but its contribution to sex-biased differences and underlying mechanisms remains unclear.

### Aims

To investigate whether LOY drives male-biased cancer burden and to elucidate its biological and clinical consequences.

### Methods

Global Burden of Disease data and sex-stratified pan-cancer transcriptomic datasets were analyzed to assess male-biased incidence and mortality. A Y chromosome gene expression score (Chr.Y.Score) quantified Y-linked transcription in male tumors. Multi-omics analyses explored associations between LOY and tumor stemness, genomic instability, immune microenvironment, and clinical outcomes. In MM, bulk and single-cell RNA sequencing and FISH assays were performed on malignant and immune cells to evaluate LOY prevalence and its clinical correlations.

### Results

Men displayed higher incidence and mortality across most non-reproductive cancers, with disparities increasing with age. Reduced Y-linked gene expression correlated with older age at diagnosis, poorer survival, higher tumor stemness, genomic instability, and altered immune microenvironment. In MM, LOY was associated with increased tumor mutational burden, enhanced immunosuppressive signaling, and shorter overall survival. LOY was detected in malignant plasma cells and multiple immune cell populations, with malignant cell LOY predicting reduced CAR-T therapy response. FISH analyses identified a male MM subgroup with LOY, characterized by advanced age, higher tumor burden, and selective immune alterations.

### Summary/Conclusion

Age-related LOY is a male-specific factor driving tumor progression, shaping the immune microenvironment, impairing therapy response, and contributing to sex differences in cancer outcomes. These findings highlight the importance of incorporating sex-specific biology in cancer research and clinical management.

**Copyright Information:** (Online) ISSN: 2572-9241
©2026 The Author(s). HemaSphere is published by John Wiley & Sons Ltd on behalf of European Hematology Association. This is an open access Abstract Book distributed under the Attribution-NonCommercial-NoDerivs (CC BY-NC-ND), which allows third parties to download the articles and share them with others as long as they credit the author and the Abstract Book, but they cannot change the content in any way or use them commercially.
**Abstract Book Citations:** Authors, Title, HemaSphere, 2026;10:(S1):pages. The abstract book DOIs can be found on the “Author Information” page.
**Disclaimer:** Articles published in the journal HemaSphere exclusively reflect the opinions of the authors. The authors are responsible for all content in their abstracts including accuracy of the facts, statements, citing resources, etc.

### (PB3217) BASELINE PROLIFERATION ACTIVITY IDENTIFIES PATIENTS AT HIGHER RISK OF EARLY RELAPSE IN NEWLY DIAGNOSED MULTIPLE MYELOMA

**Topic:** 13. Myeloma and other monoclonal gammopathies - Biology & translational research

Mobina Shrestha*[1]

[1]*Sentara Albemarle Medical Center, Elizabeth City, United States of America;*

### Background

Even though treatments for multiple myeloma have improved, some patients relapse within the first two years. Early relapses are usually a sign of more aggressive disease and worse long-term outcomes. We examined whether tumor proliferation activity at diagnosis could help identify patients at higher risk of early progression.

### Aims

To evaluate whether a proliferation-based transcriptional index at diagnosis can identify patients with newly diagnosed multiple myeloma who are at increased risk of relapse or death within the first 24 months.

### Methods

We analyzed gene expression data from 559 newly diagnosed multiple myeloma patients using GSE24080 dataset. A Proliferation Index was created using the average activity of E2F and MYC gene sets, which reflect cell cycle and growth signals. We tested whether this index was associated with relapse or death within 24 months, adjusting for age and sex. Patients were divided into low, intermediate, and high proliferation groups.

### Results

Within 24 months, 21% of patients experienced disease progression. Each increase in the Proliferation Index was associated with about a two-fold higher risk of early relapses (Odds Ratio (OR): 2.08, 95% CI: 1.61-2.68, p<0.001). Patients in the highest proliferation group had a 35% early relapse rate, compared with 15% in the lowest group (OR: 3.15, p<0.001). Higher proliferation activity was also associated with increased early mortality (OR: 1.84, p<0.001). The model showed moderate predictive ability with AUC score of 0.67.

### Summary/Conclusion

Higher tumor proliferation activity at diagnosis is strongly associated with early relapse and death in multiple myeloma. A simple proliferation-based score may help identify patients who are at higher risk and could benefit from closer monitoring or more intensive upfront treatment strategies.

**Copyright Information:** (Online) ISSN: 2572-9241
©2026 The Author(s). HemaSphere is published by John Wiley & Sons Ltd on behalf of European Hematology Association. This is an open access Abstract Book distributed under the Attribution-NonCommercial-NoDerivs (CC BY-NC-ND), which allows third parties to download the articles and share them with others as long as they credit the author and the Abstract Book, but they cannot change the content in any way or use them commercially.
**Abstract Book Citations:** Authors, Title, HemaSphere, 2026;10:(S1):pages. The abstract book DOIs can be found on the “Author Information” page.
**Disclaimer:** Articles published in the journal HemaSphere exclusively reflect the opinions of the authors. The authors are responsible for all content in their abstracts including accuracy of the facts, statements, citing resources, etc.

### (PB3218) PREDICTING NON-RESPONSE TO VMP USING SIMPLE BASELINE PARAMETERS IN ELDERLY MULTIPLE MYELOMA PATIENTS : A REAL-WORLD STUDY

**Topic:** 13. Myeloma and other monoclonal gammopathies - Biology & translational research

Ahlem Slama*[1], Inaam Bizid[1], Nour Gaaloul[1], Zaineb Mlayah[1], Wiem Boufrikha[1], Nader Slama[1], Med.Adnene Laatiri[1], Sarra Boukhriss[1]

*[1]Fattouma Bourguiba University Hospital, Clinical Hematology Department, Monastir, Tunisia;*

### Background

Multiple myeloma (MM) in elderly patients is associated with heterogeneous clinical presentations and variable responses to standard therapies. The VMP regimen (bortezomib, melphalan, and prednisone) is widely used in transplant-ineligible patients, but a significant proportion fail to achieve an optimal response. Identifying baseline clinical, biological, and disease-related factors predictive of non-response is crucial to guide treatment decisions, personalize therapy, and improve outcomes, particularly in resource-limited settings where alternative regimens may be constrained.

### Aims

To identify baseline clinical, biological, and disease-related factors predicting failure to achieve an optimal response to VMP therapy in elderly patients with newly diagnosed MM.

### Methods

This retrospective study included newly diagnosed MM treated with VMP at the Haematology department of Monastir, Tunisia between 2019 and 2025.
Baseline clinical, laboratory, and disease-related parameters including age, sex, anemia, renal function, bone involvement, hypercalcemia, and ISS stage were collected.
Treatment response was evaluated according to IMWG criteria.
Univariate analyses were performed to identify factors associated with failure to achieve at least a partial response (PR). The statistical significance threshold P was set at 0,05.
Survival outcomes, including progression-free survival (PFS) and overall survival (OS), were estimated using Kaplan-Meier curves.

**Copyright Information:** (Online) ISSN: 2572-9241
©2026 The Author(s). HemaSphere is published by John Wiley & Sons Ltd on behalf of European Hematology Association. This is an open access Abstract Book distributed under the Attribution-NonCommercial-NoDerivs (CC BY-NC-ND), which allows third parties to download the articles and share them with others as long as they credit the author and the Abstract Book, but they cannot change the content in any way or use them commercially.
**Abstract Book Citations:** Authors, Title, HemaSphere, 2026;10:(S1):pages. The abstract book DOIs can be found on the "Author Information" page.
**Disclaimer:** Articles published in the journal HemaSphere exclusively reflect the opinions of the authors. The authors are responsible for all content in their abstracts including accuracy of the facts, statements, citing resources, etc.

### Results

A total of 53 elderly patients with newly diagnosed multiple myeloma were included. The median age was 72 years (range: 58–92). Sex ratio was 1. At baseline, 25 patients (47%) had elevated Red Cell Distribution Width (RDW > 14%), 31 patients (58%) presented with anemia (Haemoglobin ≤10 g/dL), 11 patients (21%) had renal impairment (creatinine clearance < 40 mL/min), two patients (4%) had hypercalcemia (> 2.73 mmol/L), and 46 (87%) had bone lesions. Twenty-one patients (40%) had International Staging System (ISS) stage ≥ 2.
Following VMP therapy, the overall response rate (ORR) was 54%, with 41% achieving very good partial response (VGPR). Patients with elevated RDW had lower ORR compared to those with normal RDW (44% vs 60%, P = 0.21), and a lower proportion achieved ≥ VGPR (35% vs 44%, P = 0.58). Renal impairment (P = 0,007) failure to achieve ≥ VGPR at interim evaluation (P = 0,001). Anemia (P = 0.37), baseline bone involvement (P = 0.14), hypercalcemia (P = 0.19), and ISS stage ≥ 2 (P = 0.18) were also associated with reduced response rates, although these did not reach statistical significance individually.
With a median follow-up of 56 months, patients with high RDW had shorter progression-free survival (PFS: 27% vs 91%, log-rank P = 0.22) and overall survival (OS: 33% vs 44%, log-rank P = 0.05) compared to patients with normal RDW. In univariate analysis, anemia (P = 0.045) and high RDW (P = 0.05) were associated with inferior OS.

### Summary/Conclusion

These findings highlight the value of simple, low-cost clinical and biological markers for early risk stratification in resource-limited settings and support the need for adapted therapeutic strategies and closer monitoring in patients with newly diagnosed MM at high risk of treatment failure.

**Copyright Information:** (Online) ISSN: 2572-9241
©2026 The Author(s). HemaSphere is published by John Wiley & Sons Ltd on behalf of European Hematology Association. This is an open access Abstract Book distributed under the Attribution-NonCommercial-NoDerivs (CC BY-NC-ND), which allows third parties to download the articles and share them with others as long as they credit the author and the Abstract Book, but they cannot change the content in any way or use them commercially.
**Abstract Book Citations:** Authors, Title, HemaSphere, 2026;10:(S1):pages. The abstract book DOIs can be found on the “Author Information” page.
**Disclaimer:** Articles published in the journal HemaSphere exclusively reflect the opinions of the authors. The authors are responsible for all content in their abstracts including accuracy of the facts, statements, citing resources, etc.

### (PB3219) REDOX STATUS AND DNA DAMAGE RESPONSE NETWORK IN THE TRANSFORMATION PROCESS OF MYELOMAGENESIS

**Topic:** 13. Myeloma and other monoclonal gammopathies - Biology & translational research

Panagiotis Malamos*[1], Elisavet Deligianni[1], Maria Gavriatopoulou[2], Evangelos Terpos[2], Vassilis Souliotis[1]

*[1]National Hellenic Research Foundation, Institute of Chemical Biology, Athens, Greece;[2]National and Kapodistrian University of Athens, Department of Clinical Therapeutics, athens, Greece;*

### Background

Multiple myeloma is a plasma cell malignancy characterized by clonal expansion within the bone marrow and progressive accumulation of genetic alterations. Persistent oxidative stress has emerged as a critical contributor to genomic instability, promoting DNA lesions and mutational burden. Cellular adaptation to DNA damage is mediated through the DNA damage response (DDR), a highly regulated, evolutionarily conserved, and complex network of signaling pathways. This network functions to maintain genome integrity by sensing DNA damage, signaling downstream effectors, and orchestrating repair processes.

### Aims

This study aims to elucidate the contribution of oxidative stress and DDR pathways to the malignant progression of multiple myeloma.

### Methods

Redox status, assessed by the GSH/GSSG ratio, apurinic/apyrimidinic (AP) sites, and DDR-related parameters; baseline DNA damage using alkaline comet assay, immunofluorescence based γH2Ax foci counting, chromatin condensation, DNA repair capacity through nucleotide excision repair (NER), interstrand crosslink repair (ICL/R), and double-strand break repair (DSB/R), as well as apoptosis rates were evaluated in bone marrow plasma cells (BMPCs) from 16 patients with MGUS, 18 with SMM, and 15 with MM.

### Results

Significant alterations in DDR-associated parameters were observed during myelomagenesis. Specifically, baseline DNA damage (single- and double-strand breaks), AP lesions, chromatin relaxation as well as NER, ICL/R and DSB/R capacities progressively increased in the order MGUS < SMM < MM. In parallel, a gradual decrease in the GSH/GSSG ratio and apoptosis rates was detected across disease stages.

### Summary/Conclusion

In conclusion, the transition from MGUS to SMM and ultimately to active MM is characterized by progressive dysregulation of redox status and DDR network, resulting in cumulative genomic instability that is subsequently exploited by malignant plasma cells to enhance survival, therapy resistance, and aggressive disease progression. Recognizing these mechanisms may help identify high-risk patients early and guide the development of targeted therapies.

**Copyright Information:** (Online) ISSN: 2572-9241
©2026 The Author(s). HemaSphere is published by John Wiley & Sons Ltd on behalf of European Hematology Association. This is an open access Abstract Book distributed under the Attribution-NonCommercial-NoDerivs (CC BY-NC-ND), which allows third parties to download the articles and share them with others as long as they credit the author and the Abstract Book, but they cannot change the content in any way or use them commercially.
**Abstract Book Citations:** Authors, Title, HemaSphere, 2026;10:(S1):pages. The abstract book DOIs can be found on the "Author Information" page.
**Disclaimer:** Articles published in the journal HemaSphere exclusively reflect the opinions of the authors. The authors are responsible for all content in their abstracts including accuracy of the facts, statements, citing resources, etc.

### (PB3220) DIAGNOSTIC PRACTICES AND BARRIERS OF HIGH-RISK MULTIPLE MYELOMA IN ENGLAND

**Topic:** 13. Myeloma and other monoclonal gammopathies - Biology & translational research

Hannah McNally*[1], Emma Soopramanien[2], Andrew Wilhelmsen[3], Joseph Smith[4]

*[1]Myeloma UK, Edinburgh, United Kingdom; [2]Costello Medical, London, United Kingdom; [3]Costello Medical, Manchester, United Kingdom; [4]Costello Medical, Cambridge, United Kingdom;*

### Background

Across England, there is no consistent definition of high-risk multiple myeloma (HRMM), an absence of a risk-stratified treatment pathway, and variation in the availability and use of diagnostic testing for HRMM *(Galley, 2025)*.

### Aims

This research explores the current diagnostic testing landscape for HRMM andidentifies key barriers to implementing risk-stratified testing and treatment in England. Findings will inform policy recommendations to standardise myeloma diagnostic and treatment pathways, supporting equitable access and clinically appropriate risk-stratification for all myeloma patients. Here, we present preliminary findings from a pilot survey.

### Methods

A targeted survey was developed to assess current diagnostic testing practices, barriers, and challenges affecting consistent implementation of risk-stratification testing in England. To pilot test the survey, it was distributed among consultant haematologists practicing in NHS Trusts in England. Following roll-out of the final survey, focus groups with clinicians and patients will be subsequently conducted to contextualise and enhance understanding of the survey findings.

### Results

Eight practicing consultant haematologists employed by NHS Trusts in England responded to the pilot survey. They reported 6–≥30 years' experience managing and diagnosing myeloma across university/teaching and district/general hospitals. All consultants (n=8) reported using fluorescent in-situ hybridisation (FISH) to diagnose HRMM; 7 each reported using Serum Free Light Chain (SFLC) and beta-2 microglobulin testing.  All consultants performed risk-stratification for HRMM, although one did so occasionally rather than routinely. The primary reasons for conducting risk-stratification were to inform treatment decisions (n=4) and to assess prognosis (n=3). The most frequently cited barrier to implementing risk-stratification was funding/reimbursement (n=4), followed by the absence of clear protocols or guidance (n=3).

Most consultants perceived their genetic testing practices to be consistent (n=7). The primary reasons for ordering genetic tests were to inform prognosis and to identify suitable patients for clinical trials or experimental therapies (both n=7). Key challenges affecting the use of genetic testing included inappropriate samples being collected (n=5) and delays in sample processing (n=4).

**Copyright Information:** (Online) ISSN: 2572-9241
©2026 The Author(s). HemaSphere is published by John Wiley & Sons Ltd on behalf of European Hematology Association. This is an open access Abstract Book distributed under the Attribution-NonCommercial-NoDerivs (CC BY-NC-ND), which allows third parties to download the articles and share them with others as long as they credit the author and the Abstract Book, but they cannot change the content in any way or use them commercially.
**Abstract Book Citations:** Authors, Title, HemaSphere, 2026;10:(S1):pages. The abstract book DOIs can be found on the "Author Information" page.
**Disclaimer:** Articles published in the journal HemaSphere exclusively reflect the opinions of the authors. The authors are responsible for all content in their abstracts including accuracy of the facts, statements, citing resources, etc.

**Summary/Conclusion**

Pilot findings suggest variation in risk-stratification practices across NHS Trusts in England. While all survey respondents agreed that risk-stratification is important, there was no clear consensus on HRMM diagnostic testing practices or what results can be utilised for. As the pilot survey was circulated to a small cohort, the current data may reflect some geographical limitations. The final survey will be distributed more widely across all NHS Trusts in England to assess regional differences in risk-stratification testing, supported by focus groups to explore clinical and patient perspectives.

**Copyright Information:** (Online) ISSN: 2572-9241
©2026 The Author(s). HemaSphere is published by John Wiley & Sons Ltd on behalf of European Hematology Association. This is an open access Abstract Book distributed under the Attribution-NonCommercial-NoDerivs (CC BY-NC-ND), which allows third parties to download the articles and share them with others as long as they credit the author and the Abstract Book, but they cannot change the content in any way or use them commercially.
**Abstract Book Citations:** Authors, Title, HemaSphere, 2026;10:(S1):pages. The abstract book DOIs can be found on the "Author Information" page.
**Disclaimer:** Articles published in the journal HemaSphere exclusively reflect the opinions of the authors. The authors are responsible for all content in their abstracts including accuracy of the facts, statements, citing resources, etc.

### (PB3221) FTO DRIVES MULTIPLE MYELOMA PROGRESSION VIA CHRM3-MEDIATED DYSREGULATION OF CALCIUM SIGNALING

**Topic:** 13. Myeloma and other monoclonal gammopathies - Biology & translational research

Hengyu Liu*[1], Bo Lu[2]

*[1]The Seventh Affiliated Hospital,Sun Yat-Sen University, Haematology, Shenzhen, China;[2]The Seventh Affiliated Hospital, Sun Yat-sen University, Shenzhen, Hematopathology, Shenzhen, Guangdong, China;*

### Background

Fat Mass and Obesity-Associated (FTO) is linked to multiple myeloma (MM) progression, but its downstream mechanisms are poorly understood.

### Aims

This study comprehensively elucidated the mechanistic roles of FTO and its downstream pathway in the progression of the disease, providing new perspectives for understanding the pathogenesis of MM.

### Methods

**Design/Participants**
The training set (GSE118985, MMRF-COMMPASS), validation set (GSE24870, GSE24080), and scRNA-seq dataset (GSE124310) of this study were derived from the GEO database and the UCSC Xena platform.
**Main Measures**
Machine learning and correlation analysis identified genes with consistent expression patterns as FTO downstream targets. Additionally, the expression levels of FTO and its downstream target genes in MM tissues were validated through immunohistochemistry. Single-cell RNA sequencing (scRNA-seq) elucidated cellular expression patterns.

### Results

FTO was found to be significantly upregulated in MM and associated with poor prognosis. Four potential downstream target genes (CHRM3, GINS3, RRM2, SHCBP1) were identified. Correlation and immunohistochemical analyses further focused the FTO-CHRM3 axis. Functional enrichment analysis unveiled that FTO and CHRM3 were involved in “DNA replication” and “cell cycle”. ScRNA-seq highlighted the monocyte-specific expression of SLC8A1 in the calcium signaling pathway in MM.

### Summary/Conclusion

FTO may drive the progression of MM by regulating CHRM3, thereby disrupting the calcium signaling pathway. The potential mechanisms of the FTO-CHRM3 axis in MM were explored, providing insights for targeted interventions.

**Copyright Information:** (Online) ISSN: 2572-9241
©2026 The Author(s). HemaSphere is published by John Wiley & Sons Ltd on behalf of European Hematology Association. This is an open access Abstract Book distributed under the Attribution-NonCommercial-NoDerivs (CC BY-NC-ND), which allows third parties to download the articles and share them with others as long as they credit the author and the Abstract Book, but they cannot change the content in any way or use them commercially.
**Abstract Book Citations:** Authors, Title, HemaSphere, 2026;10:(S1):pages. The abstract book DOIs can be found on the “Author Information” page.
**Disclaimer:** Articles published in the journal HemaSphere exclusively reflect the opinions of the authors. The authors are responsible for all content in their abstracts including accuracy of the facts, statements, citing resources, etc.

### (PB3222) BASELINE RDW AS A PROGNOSTIC MARKER IN ELDERLY MULTIPLE MYELOMA: A REAL-WORLD STUDY

**Topic:** 13. Myeloma and other monoclonal gammopathies - Biology & translational research

Ahlem Slama*[1], Inaam Bizid[1], Zaineb Mlayah[1], Nour Gaaloul[1], Wiem Boufrikha[1], Nader Slama[1], Med.Adnene Laatiri[1], Sarra Boukhriss[1]

[1]*Fattouma Bourguiba University Hospital, Clinical Hematology Department, Monastir, Tunisia;*

### Background

Multiple myeloma (MM) predominantly affects elderly patients. Current prognostic scores such as the International Staging System (ISS) do not fully capture biological vulnerability in this population. Red cell distribution width (RDW), a routinely available hematological parameter, reflecting inflammation, nutritional status and bone marrow dysfunction, has emerged as a potential low-cost prognostic biomarker. However, its prognostic value in elderly patients with MM remains insufficiently explored.

### Aims

To evaluate the impact of baseline RDW on treatment outcomes and survival in elderly patients with newly diagnosed MM.

### Methods

A retrospective analysis was carried out on elderly MM patients diagnosed between 2019 to 2025 and treated by the VMP regimen in the Hematology department of Monastir, Tunisia. Baseline RDW was assessed at diagnosis and patients were classified into normal or high RDW groups (>14 %). Treatment response and survival outcomes were analysed. Overall survival (OS) and progression-free survival (PFS) were estimated using the Kaplan–Meier method and compared by log-rank test. Cox regression analysis was performed to identify prognostic factors.

**Copyright Information:** (Online) ISSN: 2572-9241
©2026 The Author(s). HemaSphere is published by John Wiley & Sons Ltd on behalf of European Hematology Association. This is an open access Abstract Book distributed under the Attribution-NonCommercial-NoDerivs (CC BY-NC-ND), which allows third parties to download the articles and share them with others as long as they credit the author and the Abstract Book, but they cannot change the content in any way or use them commercially.
**Abstract Book Citations:** Authors, Title, HemaSphere, 2026;10:(S1):pages. The abstract book DOIs can be found on the "Author Information" page.
**Disclaimer:** Articles published in the journal HemaSphere exclusively reflect the opinions of the authors. The authors are responsible for all content in their abstracts including accuracy of the facts, statements, citing resources, etc.

### Results

A total of 53 elderly patients with newly diagnosed MM were included. The median baseline RDW level was 16,1% (range: 12,9 % - 21,9%). Among these, 25 patients (47%) presented with a higher pre-treatment RDW level. The mean RDW values of the normal-RDW group and high-RDW group were 14% (range, 12,9–14,6%) and 17% (range, 14.7–21,9%), respectively.
At baseline, patients with high RDW presented with significantly more adverse clinical and biological features. Renal impairment at diagnosis was more common in the high RDW group (32% vs 4%, P = 0.013), as was advanced disease stage, with a significantly higher proportion of ISS stage II patients (40% vs 18%, P = 0.006).
Regarding treatment outcomes, patients with elevated RDW showed inferior response rates. The overall response rate (ORR) was lower in the high RDW group (44% vs 60,7%, P = 0,2), with a reduced proportion of patients achieving ≥VGPR (35% vs 44%, P = 0.58). Early treatment-related complications, particularly infectious events within the first three months, were more frequently observed in patients with high RDW (21,4% vs 4%, P = 0.06), and these patients experienced a higher rate of treatment interruption or dose reduction.
After a median follow-up of 56 months, survival analysis showed a significant impact of RDW on outcomes. The 5-year OS rate was significantly lower in the high-RDW group (33% vs 92%, log-rank P = 0.05). The 5-year PFS rate was reduced in patients with elevated RDW (27% vs 91%, log-rank P = 0.22).
In univariate analysis, anemia (P = 0.045) and high RDW (P = 0.05) were significantly associated with inferior OS. In multivariate Cox regression analysis including ISS stage, anemia, renal dysfunction and baseline RDW did not remain an independent prognostic factor.

### Summary/Conclusion

Baseline RDW is associated with adverse clinical features and inferior survival outcomes in elderly patients with MM. While RDW did not retain independent prognostic significance in multivariate analysis, it appears to capture biological vulnerability not fully reflected by conventional staging systems. Future prospective studies should evaluate the integration of RDW into low-cost prognostic scores, and assess its potential role in guiding treatment.

**Copyright Information:** (Online) ISSN: 2572-9241
©2026 The Author(s). HemaSphere is published by John Wiley & Sons Ltd on behalf of European Hematology Association. This is an open access Abstract Book distributed under the Attribution-NonCommercial-NoDerivs (CC BY-NC-ND), which allows third parties to download the articles and share them with others as long as they credit the author and the Abstract Book, but they cannot change the content in any way or use them commercially.
**Abstract Book Citations:** Authors, Title, HemaSphere, 2026;10:(S1):pages. The abstract book DOIs can be found on the "Author Information" page.
**Disclaimer:** Articles published in the journal HemaSphere exclusively reflect the opinions of the authors. The authors are responsible for all content in their abstracts including accuracy of the facts, statements, citing resources, etc.

### (PB3223) PHASE 3 MAGNETISMM-6: ELRANATAMAB, DARATUMUMAB, AND LENALIDOMIDE VS DARATUMUMAB, BORTEZOMIB, LENALIDOMIDE, AND DEXAMETHASONE IN TRANSPLANT-INELIGIBLE PATIENTS WITH NEWLY DIAGNOSED MULTIPLE MYELOMA

**Topic:** 14. Myeloma and other monoclonal gammopathies - Clinical

Meletios A. Dimopoulos*[1], Hang Quach[2], Alexander Lesokhin[3], Karin Gogat-Marchant[4], Alexander Neuhof[5], Domenico Cieri[6], Ja Min Byun[7]

*[1]National and Kapodistrian University of Athens, Department of Clinical Therapeutics, Athens, Greece;[2]St. Vincent's Hospital, University of Melbourne, Melbourne, VIC, Australia;[3]Memorial Sloan Kettering Cancer Center, New York, NY, United States of America;[4]Pfizer Inc, Paris, France;[5]Pfizer Pharma GmbH, Berlin, Germany;[6]Pfizer SRL, Milan, Italy;[7]Seoul National University Hospital, Department of Internal Medicine, Seoul, South Korea;*

### Background

Elranatamab (ELRA), a B-cell maturation antigen-CD3 bispecific antibody, induced deep and durable responses with a manageable safety profile in patients with relapsed/refractory multiple myeloma (RRMM). MagnetisMM-6 (NCT05623020) is an open-label, 2-arm, randomized, phase 3 study to evaluate the efficacy and safety of ELRA in combination with daratumumab and lenalidomide (EDR) versus daratumumab, bortezomib, lenalidomide and dexamethasone (DVRd) in patients with transplant-ineligible (TI) newly diagnosed MM (NDMM). Evaluation of T-cell redirection therapies in early lines of therapy is supported by deep responses in patients with relapsed or refractory disease, and the potential benefit of an intact immune system earlier in the course of disease. The phase 3 dose of EDR used in MagnetisMM-6 Part 2 was informed by Part 1 of the study, in which EDR demonstrated early and promising efficacy and a manageable safety profile in patients with TI NDMM.

### Aims

To describe the design of MagnetisMM-6 Part 2 which will evaluate whether EDR provides superior clinical benefit compared with DVRd in patients with TI NDMM.

**Copyright Information:** (Online) ISSN: 2572-9241
©2026 The Author(s). HemaSphere is published by John Wiley & Sons Ltd on behalf of European Hematology Association. This is an open access Abstract Book distributed under the Attribution-NonCommercial-NoDerivs (CC BY-NC-ND), which allows third parties to download the articles and share them with others as long as they credit the author and the Abstract Book, but they cannot change the content in any way or use them commercially.
**Abstract Book Citations:** Authors, Title, HemaSphere, 2026;10:(S1):pages. The abstract book DOIs can be found on the "Author Information" page.
**Disclaimer:** Articles published in the journal HemaSphere exclusively reflect the opinions of the authors. The authors are responsible for all content in their abstracts including accuracy of the facts, statements, citing resources, etc.

### Methods

In Part 2, eligible patients are aged ≥18 years with TI (defined by age and/or comorbidities impacting transplant possibility) NDMM per IMWG criteria; ECOG performance status ≤2; and adequate hepatic, renal, and marrow function (Figure).
Approximately 870 patients will be enrolled in Part 2 and randomized 1:1 to receive EDR or DVRd. Randomization will be stratified by IMS/IMWG risk (high vs standard), region (North America vs Europe vs rest of the world), and age (<75 yrs vs ≥75 yrs). All patients will receive treatment until disease progression, withdrawal of consent, lost to follow-up, unacceptable toxicity, or study termination.
In the EDR arm (cycle [C] ≥1, 28 days), patients will receive subcutaneous (SC) ELRA as a step-up regimen of 12 mg day (D) 1, 32 mg D4, and 76 mg D8, followed by ELRA 76 mg SC every 4 weeks (Q4W); daratumumab 1800 mg SC weekly (QW) (D1, D8, D15, D22 in C1-C2), every 2 weeks (Q2W) (D1, D15 in C3-C6), and Q4W (D1 in C≥7); and oral lenalidomide 25 mg daily on D1-D21.
In the DVRd arm (C1-8, 21 days; C≥9, 28 days), patients will receive SC daratumumab QW in C1-C2, every 3 weeks in C3-C8, and Q4W in C≥9; SC bortezomib 1.3 mg/m$^2$ on D1, D4, D8, D11 in C1-C8; oral lenalidomide 25 mg daily D1-D14 in C1-C8, D1-D21 in C≥9; plus oral dexamethasone 20 mg on D1, D2, D4, D5, D8, D9, D11, D12 in C1-C8, and 40 mg on D1, D8, D15, D22 in C≥9 (20 mg on D1, D4, D8, D11 in C1-C8, and QW in C≥9 if age >75 years or BMI <18.5).

Dual primary endpoints are minimal residual disease (MRD) negative complete response (CR) rate ($10^{-5}$) at 12 months after randomization and progression-free survival (PFS) per IMWG criteria. The key secondary endpoint is overall survival; other secondary endpoints include overall and sustained MRD negative CR rate, duration of MRD negativity, objective and complete response rates, time to response, duration of response and complete response, PFS and PFS2 (time to second objective disease progression) by investigator, safety, pharmacokinetics, immunogenicity, and health-related quality of life measures. This study is funded by Pfizer.

### Summary/Conclusion

**Figure. MagnetisMM-6 Part 2 study design**

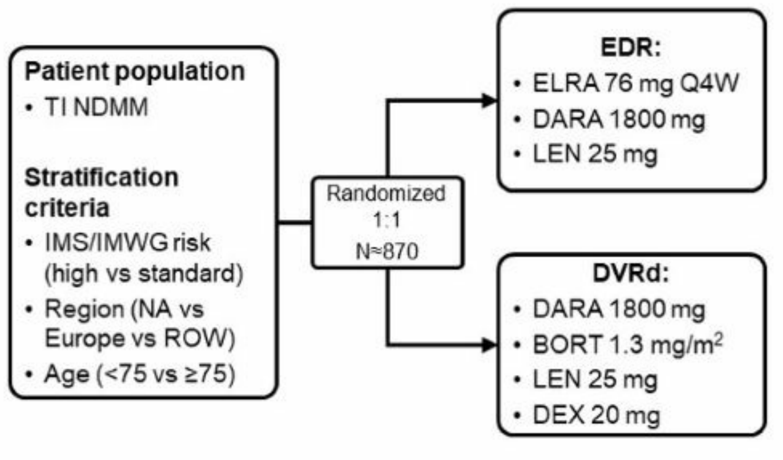

BORT=bortezomib; DARA=daratumumab; DEX=dexamethasone; ELRA=elranatamab; IMWG=International Myeloma Working Group; IMS=International Myeloma Society; LEN=lenalidomide; NA=North America; NDMM=newly diagnosed multiple myeloma; Q4W=every 4 weeks; ROW-rest of world; TI=transplant ineligible.

**Copyright Information:** (Online) ISSN: 2572-9241
©2026 The Author(s). HemaSphere is published by John Wiley & Sons Ltd on behalf of European Hematology Association. This is an open access Abstract Book distributed under the Attribution-NonCommercial-NoDerivs (CC BY-NC-ND), which allows third parties to download the articles and share them with others as long as they credit the author and the Abstract Book, but they cannot change the content in any way or use them commercially.
**Abstract Book Citations:** Authors, Title, HemaSphere, 2026;10:(S1):pages. The abstract book DOIs can be found on the "Author Information" page.
**Disclaimer:** Articles published in the journal HemaSphere exclusively reflect the opinions of the authors. The authors are responsible for all content in their abstracts including accuracy of the facts, statements, citing resources, etc.

### (PB3224) TRIAL IN PROGRESS: A PHASE 3 STUDY COMPARING AZD0120, A BCMA/CD19 DUAL-TARGETING CAR-T CELL THERAPY, VERSUS STANDARD REGIMENS IN PATIENTS WITH RELAPSED/REFRACTORY MULTIPLE MYELOMA (DURGA-4)

**Topic:** 14. Myeloma and other monoclonal gammopathies - Clinical

Hang Quach*[1], Shebli Atrash[2], Paula Rodríguez-Otero[3], Hans C. Lee[4], Elena Zamagni[5], Leyla Shune[6], Katja C. Weisel[7], Omar Nadeem[8], Juan Du[9], Marianne de Borja[10], Kamal Chamoun[11], Wenjuan Gu[12], Patricia Cheung[13], Grace Kao[11], Nina Shah[13], Natalie Callander[14]

*[1]University of Melbourne, St. Vincent's Hospital, Melbourne, VIC, Australia;[2]Levine Cancer Institute, Charlotte, NC, United States of America; [3]Clínica Universidad de Navarra, Pamplona, Spain;[4]Sarah Cannon Research Institute, Nashville, TN, United States of America; [5]University of Bologna, Bologna, Italy; [6]University of Kansas Medical Center, Kansas City, KS, United States of America; [7]University Medical Center of Hamburg-Eppendorf, Hamburg, Germany;[8]Dana-Farber Cancer Institute, Boston, MA, United States of America; [9]Renji Hospital, Shanghai Jiao Tong University School of Medicine, Department of Hematology, Shanghai, China; [10]AstraZeneca, Mississauga, ON, Canada; [11]AstraZeneca, Waltham, MA, United States of America; [12]AstraZeneca, Gaithersburg, MD, United States of America; [13]AstraZeneca, South San Francisco, CA, United States of America; [14]University of Wisconsin Carbone Cancer Center, Madison, WI, United States of America;*

### Background

Multiple myeloma (MM) remains a disease with substantial clinical burden, with patients requiring multiple lines of therapy (LOT). While advances such as BCMA-directed CAR-T cell therapy have improved outcomes, challenges persist, including disease relapse, treatment toxicities (cytokine release syndrome, immune effector cell-associated neurotoxicity syndrome [ICANS], and non-ICANS neurotoxicities), and limited access. AZD0120 is a first-in-class autologous BCMA/CD19 dual-targeting CAR-T cell therapy using rapid manufacturing for enhanced T cell fitness and faster availability to patients. Phase 1 trials have demonstrated deep, durable responses and favorable safety following a single AZD0120 infusion in newly diagnosed and relapsed/refractory MM (RRMM) (Du J, et al. ASH 2025, and Richard S, et al. ASH 2025).

### Aims

We present the study design of DURGA-4 (NCT07391657), a Phase 3 open-label, randomized, multicenter, global study comparing AZD0120 versus standard regimens in patients with RRMM.

### Methods

The study population will consist of patients aged ≥18 years with RRMM who have received 1–3 prior LOT including an immunomodulatory drug, and either a proteasome inhibitor or an anti-CD38 antibody. Additional inclusion criteria include documented diagnosis of MM and evidence of progressive disease per International Myeloma Working Group criteria, measurable disease at screening, and Eastern Cooperative Oncology Group performance status 0 or 1. Patients who have previously received any prior BCMA-targeted treatment, or CAR-T, CAR-NK or T cell engager therapy directed at any target will be excluded from study participation. Eligible patients will be randomized to receive either a single infusion of AZD0120 or standard therapy including DKd (daratumumab, carfilzomib, and dexamethasone), DPd (daratumumab, pomalidomide, and dexamethasone), PVd (pomalidomide, bortezomib, and dexamethasone), or Kd (carfilzomib and dexamethasone). Primary endpoints are progression-free survival (PFS) and minimal residual disease (MRD) negative complete response (CR) rate at 9 months. Key secondary endpoints include overall survival, CR rate, and objective response rate. After completing the initial study duration, patients in the AZD0120 arm will be followed up to 15 years post infusion as part of a long-term follow-up study.

**Copyright Information:** (Online) ISSN: 2572-9241
©2026 The Author(s). HemaSphere is published by John Wiley & Sons Ltd on behalf of European Hematology Association. This is an open access Abstract Book distributed under the Attribution-NonCommercial-NoDerivs (CC BY-NC-ND), which allows third parties to download the articles and share them with others as long as they credit the author and the Abstract Book, but they cannot change the content in any way or use them commercially.
**Abstract Book Citations:** Authors, Title, HemaSphere, 2026;10:(S1):pages. The abstract book DOIs can be found on the "Author Information" page.
**Disclaimer:** Articles published in the journal HemaSphere exclusively reflect the opinions of the authors. The authors are responsible for all content in their abstracts including accuracy of the facts, statements, citing resources, etc.

**Results**

This is a trial in progress and is expected to enroll ~500 patients in ~17 countries, with first patient enrollment planned in Q1 2026.

**Summary/Conclusion**

We hypothesize that in this Phase 3 study AZD0120 will demonstrate superior PFS and MRD negativity versus standard regimens in patients with RRMM.

**Copyright Information:** (Online) ISSN: 2572-9241
©2026 The Author(s). HemaSphere is published by John Wiley & Sons Ltd on behalf of European Hematology Association. This is an open access Abstract Book distributed under the Attribution-NonCommercial-NoDerivs (CC BY-NC-ND), which allows third parties to download the articles and share them with others as long as they credit the author and the Abstract Book, but they cannot change the content in any way or use them commercially.
**Abstract Book Citations:** Authors, Title, HemaSphere, 2026;10:(S1):pages. The abstract book DOIs can be found on the "Author Information" page.
**Disclaimer:** Articles published in the journal HemaSphere exclusively reflect the opinions of the authors. The authors are responsible for all content in their abstracts including accuracy of the facts, statements, citing resources, etc.

### (PB3225) DREAMM-15: A STUDY ASSESSING THE EFFICACY AND SAFETY OF EXTENDED DOSING OF BELANTAMAB MAFODOTIN IN COMBINATION WITH STANDARD OF CARE THERAPIES IN PATIENTS WITH RELAPSED/REFRACTORY MULTIPLE MYELOMA

**Topic:** 14. Myeloma and other monoclonal gammopathies - Clinical

Douglas Sborov*[1], Hans C. Lee[2], Yu Hu[3], Zhen Cai[4], Shinsuke Iida[5], Kihyun Kim[6], Alexis Talbot[7], Moritz Bewarder[8], Laura Abril Sabater[9], Feng Zhou[10], Kelly Brock[11], Sutapa Mukhopadhyay[12], Christos Nikolaidis[13], Jodie Wilkes[14], Omar Ladhani[15], Jacqueline Nielsen[15], Meletios A. Dimopoulos[16, 17], Evangelos Terpos[18]

*[1]Huntsman Cancer Institute at the University of Utah, Salt Lake City, UT, United States of America;[2]Sarah Cannon Research Institute, Nashville, TN, United States of America; [3]Union Hospital Tongji Medical College Huazhong University of Science and Technology, Wuhan, China; [4]Zhejiang University School of Medicine, Hangzhou, Zhejiang, China;[5]Nagoya City University Hospital, Nagoya-shi, Aichi-Ken, Japan; [6]Sungkyunkwan University Samsung Medical Center, Seoul, South Korea; [7]Hôpital Saint-Louis, Immuno-Haematology Department, Université Paris Cité, Paris, France; [8]Centrum für Hämatologie und Onkologie Bethanien, Frankfurt am Main, Hessen, Germany; [9]ICO Badalona - Hospital Universitari Germans Trias i Pujol, Badalona, Barcelona, Spain; [10]GSK, Mississauga, ON, Canada; [11]GSK, Collegeville, United States of America; [12]GSK, Collegeville, PA, United States of America; [13]GSK, Baar, Switzerland; [14]GSK, Stevenage, United Kingdom; [15]GSK, Chicago, IL, United States of America; [16]Department of Clinical Therapeutics, School of Medicine, National and Kapodistrian University of Athens, Athens, Greece; [17]Department of Medicine, Korea University, Seoul, South Korea; [18]Department of Clinical Therapeutics, National and Kapodistrian University of Athens, School of Medicine, Athens, Greece;*

### Background

Targeting B-cell maturation antigen (BCMA) has demonstrated transformational survival benefits for patients (pts) with relapsed/refractory multiple myeloma (RRMM) from first relapse. There is an unmet need for accessible anti-BCMA agents at the earliest opportunity across all sites of care.
Belantamab mafodotin (belamaf) is an antibody-drug conjugate representing a unique BCMA-targeting modality that provides clinical efficacy with minimal immunosuppression and no evidence of inducing T-cell exhaustion. Belamaf triplet combinations (DREAMM-7/8) have demonstrated superior progression-free survival, improved response rates, and higher minimal residual disease (MRD) negativity vs standard-of-care therapies, including in difficult-to-treat pt populations. Belamaf, bortezomib, and dexamethasone (DREAMM-7) has also demonstrated statistically significant improvement in overall survival vs an anti-CD38–containing triplet, daratumumab, bortezomib, and dexamethasone. Ocular events with belamaf treatment were managed through dose and schedule modifications, leading to median belamaf-free intervals of 8-12 weeks while preserving efficacy.
These observations, plus additional dose-finding studies and simulations, led to the design of DREAMM-15, which is evaluating a proactively extended dosing schedule of belamaf (2.5 mg/kg every 8 weeks [Q8W] in cycle 1, then 1.9 mg/kg Q8W for 2 additional cycles [cycles 2-3], and Q12W thereafter). With a reduced treatment regimen of only 4 doses of belamaf per year after the first 6 months, DREAMM-15 aims to achieve a favorable benefit/risk profile while maintaining efficacy and preserving pt quality of life.

### Aims

Evaluate the efficacy and safety of proactive extended dosing of belamaf in combination with pomalidomide and dexamethasone (BPd), bortezomib and dexamethasone (BVd), or carfilzomib and dexamethasone (BKd) in pts with RRMM.

**Copyright Information:** (Online) ISSN: 2572-9241
©2026 The Author(s). HemaSphere is published by John Wiley & Sons Ltd on behalf of European Hematology Association. This is an open access Abstract Book distributed under the Attribution-NonCommercial-NoDerivs (CC BY-NC-ND), which allows third parties to download the articles and share them with others as long as they credit the author and the Abstract Book, but they cannot change the content in any way or use them commercially.
**Abstract Book Citations:** Authors, Title, HemaSphere, 2026;10:(S1):pages. The abstract book DOIs can be found on the "Author Information" page.
**Disclaimer:** Articles published in the journal HemaSphere exclusively reflect the opinions of the authors. The authors are responsible for all content in their abstracts including accuracy of the facts, statements, citing resources, etc.

### Methods

DREAMM-15 (NCT07227311) is a phase 2, open label, nonrandomized, multicenter study. Pts aged ≥18 years with confirmed RRMM, 1-2 prior lines of treatment, measurable disease, and Eastern Cooperative Oncology Group performance status of 0-2 are eligible. Notable exclusion criteria include prior exposure to BCMA-targeted therapy and being intolerant/refractory to pomalidomide, bortezomib, or carfilzomib depending on group allocation. Approximately 200 pts will be allocated at a 2:2:1 ratio to the BPd, BVd, or BKd arm. Treatment in each group will continue until progressive disease, death, unacceptable toxicity, or withdrawal of consent, whichever occurs first. Dosing and timing of treatments can be found in the figure. This study will be open in 9 countries: USA, Germany, France, Spain, Netherlands, Greece, China, Japan, and Republic of Korea.
The primary endpoint is overall response rate (percent of pts with ≥confirmed partial response); key secondary endpoints include complete response rate, MRD status, duration of response, and safety. Findings from this study will inform a harmonized extended dosing regimen for belamaf across important RRMM regimens, further supporting a pt-centric treatment journey that is accessible across diverse sites of care.
Sponsored by GSK. Editorial support by Nucleus Global, an Inizio Company (GSK funded, study ID 224317). Drug linker technology licensed from Seagen Inc.; monoclonal antibody produced using POTELLIGENT Technology licensed from BioWa.

### Summary/Conclusion

**Figure: Study Design (Global n=200; US n=50)***
**Phase 2, Multicenter, Open-Label, Non-Randomized Study**

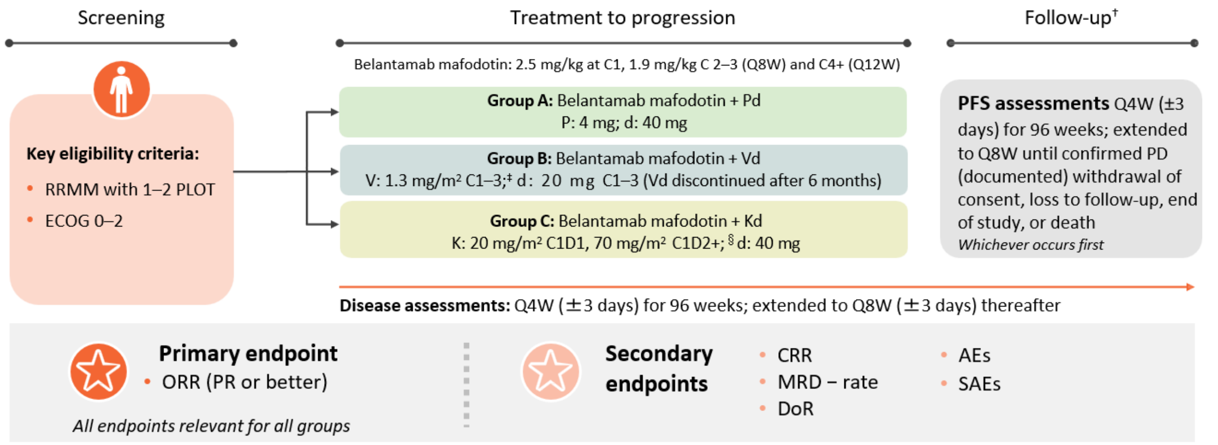

*Target enrolment number; †Participants who discontinue treatment for reasons other than PD; ‡Or six 28-day cycles, with bortezomib administered on Day 1, 8, 15, and 22; §Each carfilzomib cycle is 28 days; carfilzomib will be administered at 20 mg/m2 on Day 1 and then at 70 mg/m2 on Day 8, and Day 15 of the first carfilzomib cycle. Carfilzomib will be administered at 70 mg/m2 on Day 1, 8, 15 of each 28-day carfilzomib cycle thereafter. C, cycle; CRR, complete response rate; d, dexamethasone; DoR, duration of response; ECOG, Eastern Cooperative Oncology Group; K, carfilzomib; MRD, minimal residual disease; ORR, overall response rate; P, pomalidomide; PD, progressive disease; PLOT, prior lines of therapy; PR, partial response; Q#W, every # weeks; RRMM, relapsed/refractory multiple myeloma; (S)AE, (serious) adverse event; V, bortezomib.

**Copyright Information:** (Online) ISSN: 2572-9241
©2026 The Author(s). HemaSphere is published by John Wiley & Sons Ltd on behalf of European Hematology Association. This is an open access Abstract Book distributed under the Attribution-NonCommercial-NoDerivs (CC BY-NC-ND), which allows third parties to download the articles and share them with others as long as they credit the author and the Abstract Book, but they cannot change the content in any way or use them commercially.
**Abstract Book Citations:** Authors, Title, HemaSphere, 2026;10:(S1):pages. The abstract book DOIs can be found on the “Author Information” page.
**Disclaimer:** Articles published in the journal HemaSphere exclusively reflect the opinions of the authors. The authors are responsible for all content in their abstracts including accuracy of the facts, statements, citing resources, etc.

### (PB3226) CEVOLUTION: A RANDOMIZED PHASE III TRIAL COMPARING THE EFFICACY AND SAFETY OF CEVOSTAMAB PLUS POMALIDOMIDE AND DEXAMETHASONE VERSUS STANDARD OF CARE IN RELAPSED/REFRACTORY MULTIPLE MYELOMA

**Topic:** 14. Myeloma and other monoclonal gammopathies - Clinical

Rakesh Popat*[1], Adam D Cohen[2], Amrita Krishnan[3], Elena Zamagni[4], Hira Mian[5], Joshua Richter[6], Sammy Chebon[7], Diyva Samineni[8], Kerstin Trunzer[7], Tulika Tyagi[8], Leanne Richardson[9], Paresh Sewpaul[9], Tina Nielsen[7], Binod Dhakal[10]

*[1]University College London Hospitals NHS Foundation Trust, London, United Kingdom;[2]Abramson Cancer Center and University of Pennsylvania, Philadelphia, United States of America; [3]City of Hope Cancer Center, Irvine, United States of America;[4]IRCCS Azienda Ospedaliero-Universitaria di Bologna, Istituto di Ematologia "Seràgnoli", Bologna, Italy; [5]McMaster University, Hamilton, ON, Canada; [6]Tisch Cancer Institute, Icahn School of Medicine at Mount Sinai, New York, United States of America;[7]F. Hoffmann-La Roche Ltd, Basel, Switzerland; [8]Genentech, Inc., South San Francisco, United States of America;[9]Roche Products Ltd, Welwyn, United Kingdom; [10]Medical College of Wisconsin, Milwaukee, United States of America;*

### Background

Anti-CD38 antibodies (Abs), such as daratumumab (dara) and isatuximab, and immunomodulatory drugs (IMiDs), such as lenalidomide (len), are frequently used in the early lines of multiple myeloma (MM) therapy. New treatments that are efficacious in patients (pts) with prior anti-CD38 Ab and len exposure are needed to improve outcomes. Fc receptor-homolog 5 (FcRH5) is a type I membrane protein that is ubiquitously expressed on malignant plasma cells and represents a novel therapeutic target for MM. Cevostamab, a FcRH5xCD3 T-cell-engaging bispecific Ab, has shown promising activity and manageable safety as monotherapy in late-line relapsed/refractory MM (RRMM; Richter et al. ASH 2024). Combining cevostamab with MM therapies that augment T-cell activity could enhance efficacy. Pomalidomide (pom) is an IMiD that exhibits potent T-cell co-stimulatory effects and is clinically active when given alone or with dexamethasone (dex) in RRMM. In the Arm B randomized dose-expansion of the Phase Ib CAMMA 1 study, cevostamab plus pom-dex had manageable safety and induced deep and durable responses in pts with RRMM, the majority of whom were triple-class exposed and refractory to their last prior therapy (Harrison et al. ASH 2025).

### Aims

We describe for the first time the design of the open-label, multicenter, randomized Phase III CEVOLUTION study that aims to compare the efficacy and safety of cevostamab plus pom-dex vs standard of care (SOC) in pts with RRMM who have received an anti-CD38 Ab and len as part of 1–3 prior lines of therapy.

**Copyright Information:** (Online) ISSN: 2572-9241
©2026 The Author(s). HemaSphere is published by John Wiley & Sons Ltd on behalf of European Hematology Association. This is an open access Abstract Book distributed under the Attribution-NonCommercial-NoDerivs (CC BY-NC-ND), which allows third parties to download the articles and share them with others as long as they credit the author and the Abstract Book, but they cannot change the content in any way or use them commercially.
**Abstract Book Citations:** Authors, Title, HemaSphere, 2026;10:(S1):pages. The abstract book DOIs can be found on the "Author Information" page.
**Disclaimer:** Articles published in the journal HemaSphere exclusively reflect the opinions of the authors. The authors are responsible for all content in their abstracts including accuracy of the facts, statements, citing resources, etc.

### Methods

Additional inclusion criteria include measurable disease, an ECOG PS of 0 or 1 and informed consent. Prior exposure to pom and B-cell maturation antigen (BCMA), G protein-coupled receptor class C group 5 member D (GPRC5D) and/or FcRH5-targeted agents is not allowed. Pts will be randomized 1:1 to cevostamab plus pom-dex (Arm A) or investigator's choice of SOC (Arm B), stratified by number of prior lines (1 vs 2 vs 3), ISS stage (1 vs >1) and choice of SOC. In Arm A, step-up doses of cevostamab will be given on Cycle (C) 1 Day (D) 1, C1D2 (or D3 or D4) and C1D8. Target doses (70 mg) will be given on C1D9 (or D10 or D11), D1 and D15 of C2–7 (Q2W) and D1 of C8+ (Q4W). Pom (D1–21) will be given from C2+. In Arm B, pts will receive investigator's choice of either dara plus pom-dex, elotuzumab plus pom-dex or carfilzomib plus dex, with the choice informed by the pts prior treatment exposure and refractoriness to individual classes of agents. In both arms, treatment will continue until disease progression or unacceptable toxicity. The primary objective is to compare the efficacy of cevostamab plus pom-dex vs SOC based on dual primary endpoints of rate of minimal residual disease-negative complete response at the 10-5 level at 9 months and progression-free survival. Key secondary endpoints are the rate of very good partial response or better, overall survival, change from baseline in the disease symptom scale of the EORTC QLQ-MY20 at 9 months and change from baseline in the global health status/quality of life scale of the EORTC QLQ-C30 at 9 months. Safety, pharmacokinetics, immunogenicity and pharmacodynamics will also be assessed.

### Results

CEVOLUTION will enroll ~380 pts across ~140 global sites. First patient enrollment is expected in Q2 2026.

### Summary/Conclusion

CEVOLUTION is a pivotal Phase III trial that is expected to inform the clinical efficacy and safety of cevostamab plus pom-dex in pts with RRMM.

**Copyright Information:** (Online) ISSN: 2572-9241
©2026 The Author(s). HemaSphere is published by John Wiley & Sons Ltd on behalf of European Hematology Association. This is an open access Abstract Book distributed under the Attribution-NonCommercial-NoDerivs (CC BY-NC-ND), which allows third parties to download the articles and share them with others as long as they credit the author and the Abstract Book, but they cannot change the content in any way or use them commercially.
**Abstract Book Citations:** Authors, Title, HemaSphere, 2026;10:(S1):pages. The abstract book DOIs can be found on the "Author Information" page.
**Disclaimer:** Articles published in the journal HemaSphere exclusively reflect the opinions of the authors. The authors are responsible for all content in their abstracts including accuracy of the facts, statements, citing resources, etc.

### (PB3227) PHASE 3 EMN39/LUMYNA LINKER-MM6 STUDY DESIGN: DARATUMUMAB-LENALIDOMIDE-DEXAMETHASONE (DRD) INDUCTION FOLLOWED BY LINVOSELTAMAB VS. DRD IN TRANSPLANT-INELIGIBLE PATIENTS WITH NEWLY DIAGNOSED MYELOMA

**Topic:** 14. Myeloma and other monoclonal gammopathies - Clinical

Roberto Mina*[1, 2], Fredrik Schjesvold[3, 4], Laura Rosiñol Dachs[5], Meral Beksaç[6, 7], Shinsuke Iida[8], Evangelos Terpos[9], Sandra Bašić Kinda[10], Janusz Krawczyk[11], Elias K. Mai[12], Giuseppe Bertuglia[13], Kate Knorr[14], Gwen Ouellette[14], Emelie Asselbergs[15], Silvia Villa[1], Glenn S. Kroog[14], Serena Merante[1], Frida Bugge Askeland[3], Ka Lung Wu[16], Martin Schreder[17], Tomas Jelínek[18, 19], Christoph Driessen[20], Andrew Spencer[21], Hermann Einsele[22], Pieter Sonneveld[15, 23], Mario Boccadoro[1], Claudia Stege[15, 23]

*[1]European Myeloma Network, EMN, Italy; [2]Department of Hematology and Medical Oncology, Winship Cancer Institute, Emory University, Atlanta, GA, United States of America; [3]Oslo Myeloma Center, Department of Hematology, Oslo University Hospital, Oslo, Norway; [4]KG Jebsen Centre for B cell malignancies, Institute of Clinical Medicine, University of Oslo, Oslo, Norway; [5]Amyloidosis and Myeloma Unit, Department of Hematology, Hospital Clínic de Barcelona, IDIBAPS and PETHEMA/GEM, Barcelona and Spain, Spain; [6]Department of Hematology, Ankara University Faculty of Medicine, Ankara, Türkiye;[7]Department of Hematology, Ankara Liv Hospital, Istinye University, Ankara, Türkiye; [8]Department of Hematology and Oncology, Nagoya City University Graduate School of Medical Sciences, Nagoya, Japan; [9]Department of Clinical Therapeutics, National and Kapodistrian University of Athens, School of Medicine, Athens, Greece; [10]Division of Hematology, Department of Internal Medicine, University Hospital Center Zagreb, Zagreb, Croatia; [11]Department of Haematology, University Hospital Galway, Galway, Ireland; [12]Heidelberg Myeloma Center, Department of Internal Medicine V, Heidelberg University Hospital and Medical Faculty Heidelberg, Heidelberg University, Heidelberg, Germany; [13]Division of Hematology, AOU Città della Salute e della Scienza di Torino, University of Torino and Department of Molecular Biotechnology and Health Sciences, University of Torino, Torino, Italy; [14]Regeneron Pharmaceuticals, Inc, Tarrytown, NY, United States of America; [15]European Myeloma Network, EMN, The Netherlands; [16]ZAS Cadix, Antwerp, Belgium; [17]Wilhelminen Cancer Research Institute, c/o Department of Medicine I, Clinic Ottakring, Vienna, Austria; [18]Department of Haematooncology, University Hospital Ostrava, Ostrava, Czechia; [19]Department of Haematooncology, Faculty of Medicine, University of Ostrava, Ostrava, Czechia; [20]Department of Medical Oncology and Hematology, Cantonal Hospital St. Gallen, St. Gallen, Switzerland; [21]Malignant Haematology and Stem Cell Transplantation Service, Alfred Health-Monash University, Melbourne, Australia; [22]Department of Internal Medicine II, University Hospital Würzburg, Würzburg, Germany; [23]Department of Hematology, Erasmus MC Cancer Institute, Rotterdam, The Netherlands;*

### Background

Anti-CD38 monoclonal antibodies, lenalidomide and/or proteasome inhibitor combinations have improved the survival of transplant-ineligible (TIE), newly diagnosed multiple myeloma (NDMM) patients (pts). Progression-free survival (PFS) ranges between 5–8 years in TIE pts. Nonetheless, despite triplet/quadruplet regimens, a substantial proportion of TIE pts (40–70%) does not achieve measurable residual disease (MRD) negativity (Facon et al. Lancet Oncol 2025; Usmani et al. Nat Med 2025). Furthermore, multi-drug regimens are burdened by increased toxicity, especially in older pts. Hence, newer strategies to improve efficacy and tolerability in TIE-NDMM pts are needed. Linvoseltamab (LINVO), a BCMA-targeting bispecific antibody (BsAb) approved for relapsed/refractory (RRMM) pts, has demonstrated high efficacy and a favorable safety profile in heavily pre-treated pts, including older and frail ones. In the LINKER-MM1 study (Lee et al. Clin Lymph Myel Leuk 2026), LINVO resulted in overall (ORR) and complete response (CR) rates of 71% and 52%, respectively, and a 24-month PFS of 56%. The LINKER-MM4 study (Orlowski et al. ASH 2025), in which NDMM pts received single-agent LINVO, ORR and CR rates reached up to 86% and 75%, respectively. Studies have shown better efficacy and safety profiles with BsAbs in pts with lower tumor burden and soluble BCMA levels. In the IMMUNOPLANT study (Kazandjian et al. ASH 2025), 4–6 cycles of LINVO led to 100% MRD negativity ($10^{-6}$) in NDMM pts with residual disease following induction therapy. Based on this rationale, we designed the LUMYNA study comparing LINVO monotherapy, preceded by a debulking phase with daratumumab-lenalidomide-dexamethasone (DRd), to the DRd standard-of-care regimen as initial treatment for TIE-NDMM pts.

**Copyright Information:** (Online) ISSN: 2572-9241
©2026 The Author(s). HemaSphere is published by John Wiley & Sons Ltd on behalf of European Hematology Association. This is an open access Abstract Book distributed under the Attribution-NonCommercial-NoDerivs (CC BY-NC-ND), which allows third parties to download the articles and share them with others as long as they credit the author and the Abstract Book, but they cannot change the content in any way or use them commercially.
**Abstract Book Citations:** Authors, Title, HemaSphere, 2026;10:(S1):pages. The abstract book DOIs can be found on the "Author Information" page.
**Disclaimer:** Articles published in the journal HemaSphere exclusively reflect the opinions of the authors. The authors are responsible for all content in their abstracts including accuracy of the facts, statements, citing resources, etc.

**Methods**

EMN39/LUMYNA (LINKER-MM6; NCT06932562) is a randomized, open-label, phase 3 multicenter study enrolling NDMM pts transplant ineligible due to age (≥70 years) or comorbidities that are likely to negatively impact the tolerability of high-dose chemotherapy and ASCT, regardless of age. Key inclusion criteria include serum and urine measurable disease according to IMWG and a creatinine clearance ≥30 ml/min. Pts will be randomized 1:1 to receive DRd induction followed by LINVO monotherapy or continued DRd. Randomization will be stratified by FISH (standard- vs. high-risk) and frailty status.
In the experimental arm, pts will receive 4 28-day cycles of DRd induction to reduce tumor burden and soluble BCMA levels. LINVO intravenous administration will be initiated in cycle 5, with 2 step-up doses followed by full-dose LINVO, a weekly administration in cycles 6–7 and Q2W from cycle 8, in 28-day cycles. Pts achieving CR will switch to Q4W LINVO as early as cycle 9. In both treatment arms, daratumumab will be administered subcutaneously at the approved dose and schedule, lenalidomide orally at 25 mg on days 1–21 and dexamethasone orally at 40 mg (20 mg in pts aged ≥76 years) on days 1, 8, 15 and 22 of every 28-day cycle. Pts will be treated until disease progression, death, unacceptable toxicity or consent withdrawal. The primary endpoints are CR-MRD negativity rate (NGS, $10^{-5}$) and PFS. Overall survival is a key secondary endpoint. Additional endpoints include safety and health-related quality of life. The study opened for enrollment in September 2025.

**Copyright Information:** (Online) ISSN: 2572-9241
©2026 The Author(s). HemaSphere is published by John Wiley & Sons Ltd on behalf of European Hematology Association. This is an open access Abstract Book distributed under the Attribution-NonCommercial-NoDerivs (CC BY-NC-ND), which allows third parties to download the articles and share them with others as long as they credit the author and the Abstract Book, but they cannot change the content in any way or use them commercially.
**Abstract Book Citations:** Authors, Title, HemaSphere, 2026;10:(S1):pages. The abstract book DOIs can be found on the “Author Information” page.
**Disclaimer:** Articles published in the journal HemaSphere exclusively reflect the opinions of the authors. The authors are responsible for all content in their abstracts including accuracy of the facts, statements, citing resources, etc.

### (PB3229) TRIAL IN PROGRESS: QUINTESSENTIAL-2—A PHASE 3 STUDY OF ARLOCABTAGENE AUTOLEUCEL VERSUS STANDARD OF CARE IN ADULT PATIENTS WITH RELAPSED AND REFRACTORY MULTIPLE MYELOMA (RRMM) EXPOSED TO LENALIDOMIDE

**Topic:** 14. Myeloma and other monoclonal gammopathies - Clinical

Eyal Lebel*[1], Ahmed Abdulgawad[2], Stefania Bramanti[3], Gonzalo Garate[4], P. Joy Ho[5], Mikko Keränen[6], Yael Cohen[7], Maja Ølholm Vase[8], Amit Khot[9], Ida Kristensen[10], Richard Leblanc[11], Cindy Lee[12], Wojciech Legieć[13], Hila Magen[14], María-Victoria Mateos[15], Danielle Oh[16], Ludek Pour[17], Natalia Schutz[18], Alexandros Spyridonidis[19], Katja C. Weisel[20], Jan MacIej Zaucha[21], Sarah M. Larson[22], Asya Nina Varshavsky-Yanovsky[23], Kimberly Grazioli[24], Debashree Basudhar[24], Hongxiang Hu[24], Ya Meng[24], Lamis Eldjerou[24], Susan Bal[25], Simon Harrison[9], Rakesh Popat[26], Marc-Steffen Raab[27], Paula Rodríguez-Otero[28]

*[1]Hadassah Medical Center, Jerusalem, Israel;[2]Christie NHS Foundation Trust, Manchester, United Kingdom;[3]IRCCS Istituto Clinico Humanitas, Rozzano, Italy; [4]Hospital Aleman, Buenos Aires, Argentina; [5]Royal Prince Alfred Hospital and University of Sydney, Camperdown, NSW, Australia; [6]Helsinki University Hospital, Helsinki, Finland; [7]Tel Aviv University, Tel Aviv Sourasky Medical Center and Faculty of Medical and Health Sciences,, Tel Aviv, Israel; [8]Aarhus Universitetshospital, Aarhus, Midtjylland, Denmark; [9]Peter MacCallum Cancer Centre, Royal Melbourne Hospital and University of Melbourne, Melbourne, Victoria, Australia; [10]Odense Universitetshospital, Odense, Syddanmark, Denmark; [11]Hematology-Oncology and Cell Therapy University Institute, Hôpital Maisonneuve-Rosemont Research Center, Université de Montréal, Montreal, Quebec, Canada; [12]Royal Adelaide Hospital, Adelaide, South Australia, Australia; [13]St. John of Dukla Oncology Center of Lublin Land, Lublin, Poland;[14]Chaim Sheba Medical Center, Ramat-Gan, Faculty of Medicine, Tel Aviv University, Department of Hematology, Tel-Aviv, Israel; [15]Hospital Universitario de Salamanca, Salamanca, Spain; [16]Monash Health and Monash University, Clayton, Victoria, Australia;[17]Masaryk University and University Hospital Brno, Brno, Czechia; [18]Hospital Italiano de Buenos Aires, Buenos Aires, Argentina;[19]University Hospital of Patras, Rio-Patras, Greece; [20]University Medical Center Hamburg-Eppendorf, Hamburg, Germany; [21]Medical University of Gdańsk and University Clinical Center, Gdansk, Poland; [22]UCLA Medical Center, Los Angeles, California, United States of America; [23]Fox Chase Cancer Center Temple Health, Philadelphia, Pennsylvania, United States of America;[24]Bristol Myers Squibb, Princeton, New Jersey, United States of America; [25]University of Alabama at Birmingham, Birmingham, Alabama, United States of America; [26]University College London Hospital, London, United Kingdom; [27]Universitaetsklinikum Heidelberg, Heidelberg, Germany; [28]Clínica Universidad de Navarra, Pamplona, Spain;*

### Background

Despite advances in multiple myeloma (MM) treatment, most patients (pts) will relapse, highlighting the need for new drug classes in RRMM. Further, with the extensive use of lenalidomide (LEN), an immunomodulatory drug (IMiD), in frontline and maintenance therapies, LEN-refractoriness has become increasingly common and poses an additional challenge as the disease is less likely to respond to subsequent treatment. G protein-coupled receptor class C group 5 member D (GPRC5D) is a promising therapeutic target for MM as the receptor is highly expressed on malignant plasma cells; it has little to no expression on non-plasma cell immune populations and limited expression on other tissues. Arlocabtagene autoleucel (arlo-cel) is a GPRC5D-directed autologous CAR T cell therapy that has demonstrated safety and efficacy in pts with RRMM in a first-in-human phase 1 study. ORR was 94% and 91% in pts with 1-3 and ≥3 prior LOT (pLOT), respectively, after a single arlo-cel infusion ($150\times10^6$ CAR T-cells); ORR was 92% in pts with ≥3 pLOT following a $75\times10^6$ CAR T cell infusion.

### Aims

The objective is to describe the study design of the ongoing phase 3 QUINTESSENTIAL-2 study.

**Copyright Information:** (Online) ISSN: 2572-9241
©2026 The Author(s). HemaSphere is published by John Wiley & Sons Ltd on behalf of European Hematology Association. This is an open access Abstract Book distributed under the Attribution-NonCommercial-NoDerivs (CC BY-NC-ND), which allows third parties to download the articles and share them with others as long as they credit the author and the Abstract Book, but they cannot change the content in any way or use them commercially.
**Abstract Book Citations:** Authors, Title, HemaSphere, 2026;10:(S1):pages. The abstract book DOIs can be found on the "Author Information" page.
**Disclaimer:** Articles published in the journal HemaSphere exclusively reflect the opinions of the authors. The authors are responsible for all content in their abstracts including accuracy of the facts, statements, citing resources, etc.

### Methods

QUINTESSENTIAL-2 (NCT06615479) is a randomized, open-label, multicenter, phase 3 confirmatory study comparing the efficacy and safety of arlo-cel vs SOC in adults with RRMM and prior LEN. Key inclusion criteria were age ≥18 years, confirmed diagnosis of MM per IMWG criteria, 1-3 pLOT (may include a proteasome inhibitor, IMiD, anti-CD38 antibody, and BCMA-targeted therapy), be exposed to LEN (≥2 consecutive cycles, unless PD was the best response to LEN-containing treatment or if there was LEN intolerance or unacceptable toxicity), have measurable disease, and ECOG PS of 0 or 1. Pts who received prior GPRC5D-targeted therapy are excluded. Eligible pts will be randomized 1:1 across 2 arms. Arm A includes leukapheresis within 3-4 days of randomization, mandatory bridging therapy ≤6 days of randomization with DPd (daratumumab, pomalidomide, dexamethasone) or Kd (carfilzomib, dexamethasone) per Investigator choice and lymphodepleting chemotherapy prior to a single arlo-cel infusion. Arm B includes SOC of DPd or Kd per investigator choice, dosed per labeling, until PD. Primary endpoints are PFS and minimal residual disease (MRD) negativity in CR. Key secondary endpoints include OS and ORR. Other endpoints include safety, MRD-negative status, CR rate, time to response, duration of response, pharmacokinetics, and pt-reported outcomes. Pts will be followed for ≤5 years after the last pt is randomized, and with a long-term follow-up study (≤15 years post-infusion) for pts receiving arlo-cel. Anticipated enrollment is 440 pts at ~125 sites globally. The first pt was enrolled in March 2025.

©American Society of Hematology (2025). Reused with permission.

**Copyright Information:** (Online) ISSN: 2572-9241
©2026 The Author(s). HemaSphere is published by John Wiley & Sons Ltd on behalf of European Hematology Association. This is an open access Abstract Book distributed under the Attribution-NonCommercial-NoDerivs (CC BY-NC-ND), which allows third parties to download the articles and share them with others as long as they credit the author and the Abstract Book, but they cannot change the content in any way or use them commercially.
**Abstract Book Citations:** Authors, Title, HemaSphere, 2026;10:(S1):pages. The abstract book DOIs can be found on the “Author Information” page.
**Disclaimer:** Articles published in the journal HemaSphere exclusively reflect the opinions of the authors. The authors are responsible for all content in their abstracts including accuracy of the facts, statements, citing resources, etc.

### (PB3230) ASSOCIATION BETWEEN NON-CLASSICAL MUTATIONS IN THE KRAS, NRAS GENES AND EXTRAMEDULLARY DISEASE IN MULTIPLE MYELOMA

**Topic:** 14. Myeloma and other monoclonal gammopathies - Clinical

Maiia Soloveva*[1], Maksim Soloviev[1], Igor Yakutik[1], Bella Biderman[1], Elena Nikulina[1], Natalya Risinskaya[1], Tatiana Obukhova[1], Chabaeva Julia[1], Sergey Kulikov[1], Andrey Sudarikov[1], Larisa Mendeleeva[1]

[1]*National Medical Research Center for Hematology, Moscow, Russia;*

### Background

Extramedullary involvement in multiple myeloma is a rare and aggressive phenomenon. The role of liquid biopsy, based on plasma circulating tumor DNA (ctDNA) analysis, in MM continues to be explored. Mutations in the *KRAS* and *NRAS* genes typically affected codons 12, 13, and 61. Mutations in the *KRAS* and *NRAS* genes outside of these codons are rare and poorly understood.

### Aims

To compare clinical and laboratory parameters of MM patients with classical and non-classical mutations in the *KRAS* and *NRAS* genes and patients without mutations in tumor foci of various localizations (CD138+ bone marrow cells, ctDNA, plasmacytoma).

### Methods

A single-center prospective study from September 2021 to May 2025 included 161 patients (77 men and 84 women): 154 with newly diagnosed MM and 7 with plasma cell leukemia, aged 29 to 83 years (median 55 years). The diagnosis was established in accordance with the IMWG-2014 criteria. Positive immunomagnetic selection of CD138+ bone marrow cells was performed using a monoclonal antibody to CD138 according to the manufacturer's protocol. Cases with t(4;14), t(14;16), del17p13, del1p32 and 1q21 amplifications were classified as a high cytogenetic risk group.Mutations in the *KRAS* and *NRAS* genes were evaluated using high-throughput sequencing. The source of DNA for mutation analysis were CD138+ cells isolated from bone marrow samples; in 82 patients, ctDNA samples were examined in parallel. In addition, 9 plasmacytomas were also analyzed, using DNA isolated from material of tumor biopsies. Contingency table analysis was used to test hypotheses regarding differences in the distributions of categorical characteristics between comparison groups.

### Results

The frequency of the *KRAS* and *NRAS* gene mutations found in any of the tumor substrate in the entire group was43.5% (70 out of 161). Classic *KRAS* and *NRAS* gene mutations (affecting codons 12, 13, and 61) were detected in 58 patients, while non-classical mutations were detected in 12 patients (A59G, V29A, L19F, K88E, G60D (G179A), T50P (A148C) in the *KRAS* gene; L95P, Y64N, A83V, S87I (G260T), T50I in the *NRAS* gene). Rare mutations in the *KRAS* and *NRAS* genes were significantly more common in ctDNA compared with the bone marrow substrate (50% vs. 9%, $p = 0.01$). We observed an association between non-classical *KRAS/NRAS* gene mutations and extramedullary disease when comparing various clinical and laboratory parameters in 3 groups of patients (Table 1). Thus, extramedullary plasmacytomas were more common in patients with non-classical mutations (25%) compared to patients with classical mutations (6.9%) and patients without mutations (3%), p = 0.0327. The ability of tumor plasma cells to circulate in the bloodstream can also be considered as extramedullary involvement. In the group of patients with non-classical mutations, plasma cells were detected in the bloodstream twice as often (22%) compared to patients with classical mutations and without mutations. Moreover, plasma cell leukemia was detected more often in the group of patients with rare mutations compared to patients with classical mutations or without them, p = 0.0582. The identified association of non-classical *KRAS* and *NRAS* gene mutations  and extramedullary disease may indicate the specific activation of the RAS-ERK cascade in this category of patients.

**Copyright Information:** (Online) ISSN: 2572-9241
©2026 The Author(s). HemaSphere is published by John Wiley & Sons Ltd on behalf of European Hematology Association. This is an open access Abstract Book distributed under the Attribution-NonCommercial-NoDerivs (CC BY-NC-ND), which allows third parties to download the articles and share them with others as long as they credit the author and the Abstract Book, but they cannot change the content in any way or use them commercially.
**Abstract Book Citations:** Authors, Title, HemaSphere, 2026;10:(S1):pages. The abstract book DOIs can be found on the "Author Information" page.
**Disclaimer:** Articles published in the journal HemaSphere exclusively reflect the opinions of the authors. The authors are responsible for all content in their abstracts including accuracy of the facts, statements, citing resources, etc.

### Summary/Conclusion

Liquid biopsy can be used to identify new factors associated with an unfavorable prognosis in MM.This study was supported by the Russian Science Foundation (RSF), project No. 25-25-20201.

Table 1. Comparison of clinical and laboratory parameters in 3 groups of MM patients

| Parameters | Patients with MM depending on the presence and type of mutations in the KRAS/NRAS genes (n=161) | | | p |
|---|---|---|---|---|
| | classical mutation n =58 | non-classical mutation n=12 | without mutations n=91 | |
| Cytogenetic aberrations | | | | 0.1654 |
| high-risk | 26 из 57 (46%) | 8 из 12 (67%) | 53 из 88 (60%) | |
| standard | 31 ( 54%) | 4 (33%) | 35 (40%) | |
| ISS stage | | | | 0.5982 |
| III | 13 из 46 (28%) | 2 из 9 (22%) | 13 из 64 (20%) | |
| I or II | 33(72%) | 7 (78%) | 51(80%) | |
| Plasma cells in the blood | | | | 0.5345 |
| Yes | 5 из 47 (11%) | 2 из 9 (22%) | 10 из 83 (12%) | |
| No | 42 (89%) | 7 (78%) | 73 (88%) | |
| Plasma cell leukemia (≥5% plasma cells in the blood) | | | | **0.0582** |
| Yes | 1 из 47 (2%) | 2 из 9 (22%) | 4 из 83 (4.8%) | |
| No | 46 (98%) | 7 (78%) | 79 (95.2%) | |
| Type of paraprotein secretion | | | | 0.4586 |
| D | 3 из 58 (5%) | 1 из 12 (8%) | 3 из 91 (3%) | |
| G /A/ B-J | 55 (95%) | 11 (92%) | 88 (97%) | |
| Bone plasmocytomas | | | | 0.2391 |
| Yes | 32 (55%) | 9 (75%) | 45 (49.5%) | |
| No | 26 (45%) | 3 (25%) | 46 (50.5%) | |
| Extramedullary plasmacytomas | | | | **0.0327** |
| Yes | 4 (6.9%) | 3 (25%) | 3 (3%) | |
| No | 54 (93.1%) | 9(75%) | 88 (97%) | |
| Refractory course of the disease | | | | 0.3016 |
| Yes | 5 из 56 (9%) | 1 из 11 (9%) | 3 из 87 (3.5%) | |
| No | 51 (91%) | 10 (91%) | 84 (96.5%) | |

**Copyright Information:** (Online) ISSN: 2572-9241
©2026 The Author(s). HemaSphere is published by John Wiley & Sons Ltd on behalf of European Hematology Association. This is an open access Abstract Book distributed under the Attribution-NonCommercial-NoDerivs (CC BY-NC-ND), which allows third parties to download the articles and share them with others as long as they credit the author and the Abstract Book, but they cannot change the content in any way or use them commercially.
**Abstract Book Citations:** Authors, Title, HemaSphere, 2026;10:(S1):pages. The abstract book DOIs can be found on the “Author Information” page.
**Disclaimer:** Articles published in the journal HemaSphere exclusively reflect the opinions of the authors. The authors are responsible for all content in their abstracts including accuracy of the facts, statements, citing resources, etc.

### (PB3231) DARATUMUMAB-CVD THERAPY IN IGM-ASSOCIATED LIGHT CHAIN AMYLOIDOSIS: CLINICAL CHARACTERISTICS AND OUTCOMES

**Topic:** 14. Myeloma and other monoclonal gammopathies - Clinical

Suraj Vasudevan*[1, 2], Jahanzaib Khwaja[1, 2], Sriram Ravichandran[1, 2], Marianna Fontana[2], Carol Whelan[2], Helen Lachmann[2], Julian Gillmore[2], Ashutosh Wechalekar[1, 2]

*[1]University College London Hospitals NHS Foundation Trust, Amyloidosis, London, United Kingdom;[2]National Amyloidosis Centre, Amyloidosis, London, United Kingdom;*

### Background

Systemic light chain amyloidosis (AL) results from a clonal plasma cell or lymphoplasmacytic disorder producing misfolded immunoglobulin light chains that deposit as amyloid fibrils in target organs. IgM-associated AL (IgM AL) accounts for approximately 5–7% of AL and represents a biologically distinct subtype, characterised by a higher prevalence of neuropathy and nodal involvement and historically inferior treatment responses. Prior series reported haematological response rates of ~57% between 1990–2012, with modest improvement using rituximab-bendamustine–based regimens. Data on daratumumab-based therapy in this rare population remain limited.

### Aims

To characterise the clinical profile of patients with IgM AL treated with first-line daratumumab-based regimens and to evaluate haematological and survival outcomes.

### Methods

We identified 22 consecutive patients with IgM AL treated first-line with daratumumab-based therapy from the UK National Amyloidosis Centre database. Twenty-one patients received daratumumab-bortezomib-cyclophosphamide-dexamethasone (DVCD) and one received daratumumab-bortezomib-thalidomide-dexamethasone (DVTD). Haematological responses were assessed using ISA criteria. Outcomes included overall survival (OS), progression-free survival (PFS), and time to next treatment (TTNT). Patients with baseline difference between involved and uninvolved free light chains (dFLC) <50 mg/L were evaluated using low-dFLC response criteria (post-treatment dFLC <10 mg/L).

### Results

Median age was 73.5 years (range 43–82). Underlying lymphoplasmacytic lymphoma was present in 82%, with pure plasma cell dyscrasia in 18%. Lambda light chain predominated (77%). Median cardiac Mayo stage (2015 European modification) was stage 2; five patients had stage 3B disease. Organ involvement included renal (64%), cardiac (59%), liver (14%), gastrointestinal (9%), soft tissue (9%), and autonomic neuropathy (9%). Median baseline paraprotein was 10.5 g/L, dFLC 89.7 mg/L, NT-proBNP 3511.5 ng/L, troponin T 47 ng/L, and creatinine 100.5 µmol/L.
Haematological responses were observed in 71.4% (data unavailable for one patient): complete response in 2/21, very good partial response in 7/21, and partial response in 6/21. dFLC <20 mg/L was seen in 38.1%. Median overall survival was not reached with patients in >=VGRP having significantly better outcomes. Sixteen patients remain alive; seven are continuing or have completed daratumumab-based therapy, while nine proceeded to second-line treatment. Six patients died during daratumumab-based therapy, predominantly those with advanced cardiac involvement and higher baseline biomarkers.

**Copyright Information:** (Online) ISSN: 2572-9241
©2026 The Author(s). HemaSphere is published by John Wiley & Sons Ltd on behalf of European Hematology Association. This is an open access Abstract Book distributed under the Attribution-NonCommercial-NoDerivs (CC BY-NC-ND), which allows third parties to download the articles and share them with others as long as they credit the author and the Abstract Book, but they cannot change the content in any way or use them commercially.
**Abstract Book Citations:** Authors, Title, HemaSphere, 2026;10:(S1):pages. The abstract book DOIs can be found on the “Author Information” page.
**Disclaimer:** Articles published in the journal HemaSphere exclusively reflect the opinions of the authors. The authors are responsible for all content in their abstracts including accuracy of the facts, statements, citing resources, etc.

### Summary/Conclusion

First-line daratumumab-CVD in IgM AL amyloidosis is associated with encouraging haematological response rates and acceptable tolerability. These responses are comparable to those achieved with R-Bendamustine. Dara-VCD offers a new viable treatment alternative for patients with IgM related AL amyloidosis.

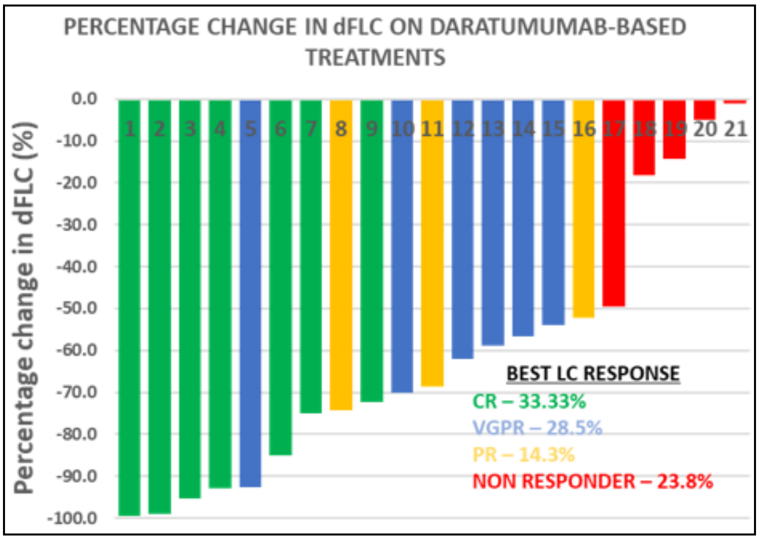

Percentage change in dFLC with Daratumumab-based therapy in IgM AL Amyloidosis

**Copyright Information:** (Online) ISSN: 2572-9241
©2026 The Author(s). HemaSphere is published by John Wiley & Sons Ltd on behalf of European Hematology Association. This is an open access Abstract Book distributed under the Attribution-NonCommercial-NoDerivs (CC BY-NC-ND), which allows third parties to download the articles and share them with others as long as they credit the author and the Abstract Book, but they cannot change the content in any way or use them commercially.
**Abstract Book Citations:** Authors, Title, HemaSphere, 2026;10:(S1):pages. The abstract book DOIs can be found on the "Author Information" page.
**Disclaimer:** Articles published in the journal HemaSphere exclusively reflect the opinions of the authors. The authors are responsible for all content in their abstracts including accuracy of the facts, statements, citing resources, etc.

### (PB3232) IMPACT OF BITE BRIDGING ON CRS KINETICS AFTER BCMA CAR-T: A MACHINE LEARNING SURVIVAL FRAMEWORK

**Topic:** 14. Myeloma and other monoclonal gammopathies - Clinical

Anushri Soni*[1], Kimberly Seymour[2, 2], Santiago Beltran[3], Emma Cordover[4], Lauren Shapiro[2], Noah Kornblum[2], Aditi Shastri[2], David Levitz[2], Mendel Goldfinger[2, 2], Kira Gritsman[2], Ioannis Mantzaris[2], Marina Konopleva[2], Dennis Cooper[2], Eric Feldman[2], Amit Verma[2], Alejandro Sica[2], Ridhi Gupta[2]

*[1]Jacobi Medical Center/Albert Einstein College of Medicine, New York City, United States of America;[2]Montefiore Einstein Comprehensive Cancer Center, New York City, United States of America; [3]Montefiore Medical Center, New York City, United States of America; [4]Albert Einstein College of Medicine, New York City, United States of America;*

### Background

Cytokine release syndrome(CRS) occurs in >80% of patients(pts) post BCMA CAR-T therapy in multiple myeloma(MM) with median onset 3–9 days depending on the product. While emerging data suggest bridging therapy prior to CAR-T infusion may reduce tumor burden and mitigate late toxicities, its role in CRS timing is poorly defined. Bispecific T cell engagers(BiTE) are often used as bridging and may alter CRS dynamics. As CAR-T treatment shifts to the outpatient setting, understanding this is essential for effective monitoring.

### Aims

To assess the impact of bridging therapy on CRS timing and dynamics after BCMA CAR-T in MM using a machine learning (ML)-based survival modeling.

### Methods

We retrospectively analyzed data from 51 MM pts who received BCMA CAR-T at our institute (Sep 2023-May 2025). Bridging therapy was categorized as BiTE (teclistamab n=3, talquetamab n=14, both n=1; total n=18); non-BiTE n=14, no bridging n=19. Median follow up was 13 months. Primary endpoint was time to first CRS event post CAR-T; pts without CRS were censored at last follow up. Pre-infusion variables were used as predictors, with Cox models adjusted for M spike prior to CAR-T, transplant, high-risk features, MRD positivity, comorbidities, ECOG status, and bridging therapy. Time-to-event associations were modeled using Cox proportional hazards regression. To reduce overfitting and account for correlated predictors, we applied penalized Cox regression (elastic net). A random survival forest, a tree-based ensemble method capturing nonlinear effects was used as a complementary approach. Model discrimination was assessed using cross-validated, time-dependent area under the curve (AUC). To evaluate early CRS risk, we used penalized logistic regression at fixed landmarks (days 3, 7, 14, and 17) and ridge-penalized piecewise Cox models across prespecified intervals (0-3, 3-7, and 7-17d), with bootstrap-derived 95% confidence intervals.

**Copyright Information:** (Online) ISSN: 2572-9241
©2026 The Author(s). HemaSphere is published by John Wiley & Sons Ltd on behalf of European Hematology Association. This is an open access Abstract Book distributed under the Attribution-NonCommercial-NoDerivs (CC BY-NC-ND), which allows third parties to download the articles and share them with others as long as they credit the author and the Abstract Book, but they cannot change the content in any way or use them commercially.
**Abstract Book Citations:** Authors, Title, HemaSphere, 2026;10:(S1):pages. The abstract book DOIs can be found on the “Author Information” page.
**Disclaimer:** Articles published in the journal HemaSphere exclusively reflect the opinions of the authors. The authors are responsible for all content in their abstracts including accuracy of the facts, statements, citing resources, etc.

### Results

Overall rate of CRS was 73% (Table). Most CRS events were grade 1, with one grade 2 event per group. All CRS events occurred within 17d (mean 6.1d, median 6.5d). BiTE bridging was associated with earlier CRS onset (median 5d vs 7d) and a higher late CRS free plateau, suggesting lower cumulative incidence. In piecewise Cox models, BiTE bridging increased CRS hazard on days 0-3 (HR 1.68, 95% CI 1.06-2.38), which attenuated on days 3-7 (HR 0.88, 95% CI 0.52-1.57) and reversed on days 7-17 (HR 0.52, 95% CI 0.38-0.78). ML models showed modest discrimination (Cox time dependent AUC 0.49±0.13 (D7), 0.42±0.05 (D14), 0.45±0.08 (D17); RSF AUC ≈0.21), reflecting early clustering of CRS events and limited baseline heterogeneity. Overall, the main signal captured was a timing-dependent shift in onset rather than strong patient-level risk stratification.

| Bridging | BiTE n=18 | Non-BiTe n=14 | None n=19 |
|---|---|---|---|
| CRS % | 61(n=11) | 86(n=12) | 79(n=15) |
| Median time to onset(days) | 5 | 7 | 7 |

### Summary/Conclusion

BiTE based bridging before CAR-T infusion is associated with earlier CRS onset by average of 2 days, without increased subsequent risk. The confined early hazard followed by reduced later risk supports front loaded CRS biology rather than sustained toxicity and is independent of patient/disease characteristics. These findings suggest intensified early monitoring would be important in BiTE bridged pts especially as treatment moves outpatient. ML-based survival methods illustrate how time dependent modeling refines toxicity characterization beyond crude incidence and may inform risk-adapted care pathways as datasets expand.

**Copyright Information:** (Online) ISSN: 2572-9241
©2026 The Author(s). HemaSphere is published by John Wiley & Sons Ltd on behalf of European Hematology Association. This is an open access Abstract Book distributed under the Attribution-NonCommercial-NoDerivs (CC BY-NC-ND), which allows third parties to download the articles and share them with others as long as they credit the author and the Abstract Book, but they cannot change the content in any way or use them commercially.
**Abstract Book Citations:** Authors, Title, HemaSphere, 2026;10:(S1):pages. The abstract book DOIs can be found on the "Author Information" page.
**Disclaimer:** Articles published in the journal HemaSphere exclusively reflect the opinions of the authors. The authors are responsible for all content in their abstracts including accuracy of the facts, statements, citing resources, etc.

### (PB3233) EARLY MORTALITY IN NEWLY DIAGNOSED MULTIPLE MYELOMA PATIENTS: BIOMARKERS AND PREDICTORS IN THE REAL-WORLD SETTINGS

**Topic:** 14. Myeloma and other monoclonal gammopathies - Clinical

Marko Mitrovic*[1], Aleksandra Sretenovic[1, 2], Igor Tanovic[1], Natalija Kecman[1], Zoran Bukumiric[3], Jelica Jovanović[4], Jelena Bila[1, 2]

*[1]Clinic of Hematology, University Clinical Center of Serbia, Ward for Multiple Myeloma, Belgrade, Serbia;[2]Medical Faculty, University of Belgrade, Belgrade, Serbia; [3]Medical Faculty, University of Belgrade, Institute of Statistics and Bioinformatics, Belgrade, Serbia; [4]Clinic of Hematology, University Clinical Center of Serbia, Belgrade, Serbia;*

### Background

Despite major therapeutic advances, recognizing biomarkers and predictors of the early mortality remains a significant challenge in multiple myeloma (MM), particularly bringing up treatment decision in the real-world settings.

### Aims

The Aim of study was to analyze in the real-world settings biomarkers and possible predictors of the early mortality within first 12 months in newly diagnosed MM (NDMM) patients (pts).

### Methods

Analyzed group consisted of 373 NDMM pts diagnosed and treated during period 2018-2025, at the Ward for multiple myeloma, Clinic of Hematology, University Clinical Center of Serbia (median age 63yrs, range 36-88yrs, males 185pts, 49.6%, female 188pts, 50.4%). IgG MM was present in 229pts (61.4%), IgA in 82pts (21.9%), Bence-Jones in 61pts (16.4%), IgD in 1pts (0.3%). Clinical stage (Salmon/Durie, CS) III had 290pts (77.8%), II 56pts (15%), I 27pts (7.2%). Renal impairment was present in 96pts (25.7%). ISS 1/2/3 score was observed in 103pts (27.6%), 107pts (28.7%), and 163pts (43.7%) respectively. R2-ISS 1/2/3/4 score was present in 69pts (18.5%); 89pts (23.9%); 177pts (47.5%), and 38pts (10.2%), respectively. According to the IMWG frailty score, 102pts (27.3%) were fit, 180pts (48.3%) intermediate-fit, and 91pts (24.4%) frail. Treatment with Bortezomib-based triplets was applied in 286pts (76.7%). A total of 241pts (64.6%) were transplant-eligible, and 131/241pts (54.4%) underwent autologous stem cell transplantation.

### Results

The early mortality (EM) outcome occurred in 63/373pts (16.9%). According to the timing of the EM outcome, the distribution was as follows: 13/63pts (20.6%) died within first 3 months, 18/63pts (28.6%) between 3–6 months, and 32/63pts (50.8%) between 6–12 months. The most common EM causes were: Infection in 26/62pts (41.9%), progressive disease in 21/62pts (33.9%), and multi-organ impairement in 15/62pts (24.2%). Patients with EM were characterized with: age ≥65yrs (mean 65.9 vs. 62.4 yrs, p=0.01), renal impairment (25/63pts, 39.7% vs. 71/310pts, 22.9%; p=0.005), cardiac-insufficiency (10/63pts, 15.9% vs. 23/310pts, 7.4%; p=0.03), IMWG frail status (28/63pts, 44.4% vs. 63/310pts, 20.3%, p<0.001); ECOG ≥2 (p<0.001), Charlson Comorbidity Index ≥3 (CCI, p=0.002), and transplant-ineligibilty (33/63pts, 52.4% vs. 99/310pts, 31.9%; p=0.002). The ISS3 score was strongly associated with EM (39/63pts, 61.9% vs. 124/310pts, 40.0%, p<0.001), as well as R2-ISS scores 3-4 (49/63pts, 77.8% vs. 166/310pts, 53.5%, p<0.001). In multivariable analysis, only frailty remained independently associated with EM (HR 2.47, 95% CI, range 1.47–4.15, p=0.001). The absence of treatment response emerged as the strongest EM predictor (HR 0.047, 95% CI, range 0.023–0.098, p<0.001).

**Copyright Information:** (Online) ISSN: 2572-9241
©2026 The Author(s). HemaSphere is published by John Wiley & Sons Ltd on behalf of European Hematology Association. This is an open access Abstract Book distributed under the Attribution-NonCommercial-NoDerivs (CC BY-NC-ND), which allows third parties to download the articles and share them with others as long as they credit the author and the Abstract Book, but they cannot change the content in any way or use them commercially.
**Abstract Book Citations:** Authors, Title, HemaSphere, 2026;10:(S1):pages. The abstract book DOIs can be found on the "Author Information" page.
**Disclaimer:** Articles published in the journal HemaSphere exclusively reflect the opinions of the authors. The authors are responsible for all content in their abstracts including accuracy of the facts, statements, citing resources, etc.

**Summary/Conclusion**

Specific patient characteristics as: age ≥65yrs, renal impairment, cardiac-insufficiency, frailty, ECOG ≥2, CCI ≥3, and transplant-ineligibilty, combined with disease aggressiveness biomarkers as ISS 3, R2-ISS 3-4 scores, infection as the the absence of the treatment response, represents the strong EM predictive factors, highlighting the need for early risk-adapted treatment and supportive strategies.

**Copyright Information:** (Online) ISSN: 2572-9241
©2026 The Author(s). HemaSphere is published by John Wiley & Sons Ltd on behalf of European Hematology Association. This is an open access Abstract Book distributed under the Attribution-NonCommercial-NoDerivs (CC BY-NC-ND), which allows third parties to download the articles and share them with others as long as they credit the author and the Abstract Book, but they cannot change the content in any way or use them commercially.
**Abstract Book Citations:** Authors, Title, HemaSphere, 2026;10:(S1):pages. The abstract book DOIs can be found on the "Author Information" page.
**Disclaimer:** Articles published in the journal HemaSphere exclusively reflect the opinions of the authors. The authors are responsible for all content in their abstracts including accuracy of the facts, statements, citing resources, etc.

### (PB3234) A PROSPECTIVE COMPARISON OF APONERMIN-BASED REGIMENS WEEKLY VERSUS MONTHLY IN PATIENTS(PTS) WITH RELAPSED/REFRACTORY MULTIPLE MYELOMA

**Topic:** 14. Myeloma and other monoclonal gammopathies - Clinical

Lefu Huang[1], Ying Tian[1], Yanru Zhang[1], Menghan Liu[1], Aijun Liu*[1]

*[1]Beijing Chaoyang Hospital, affiliated to Capital Medical University, Beijing, China;*

### Background

Multiple myeloma (MM) remains incurable, and therapeutic options for patients(pts) with relapsed/refractory MM (RRMM) are still limited. Aponermin (Apo), the first approved DR4/DR5 agonist, triggers the extrinsic apoptotic pathway through caspase cascade activation, presenting high anti-myeloma activity with low toxicity. The 2-month-ORR of the Apo-per two-week regimen in RRMM with EMD is much better than the phase III clinical trial of Apo-monthly (86.6% vs 30.4%). Due to its short half-life, weekly scheduled administration may be more effective than monthly administration. Here, we report the efficacy and safety of Apo-weekly regimens in patients with RRMM.

### Aims

This study was to evaluate the efficacy and safety of Apo-weekly regimens in patients with RRMM.

### Methods

We analyzed the Apo-weekly* data of three prospective, single-arm study(ChiCTR2500100365, ChiCTR2500106279, NCT05013190) in our center, with matched Apo-monthly data (matched for age and sex, Apo-based# regimens in the real world) as control. Patients with RRMM who had received ≥2 prior lines of therapy were enrolled in the weekly regimen cohort between July 2024 and December 2025. The primary endpoint was overall response rate (ORR). Secondary endpoints included very good partial response (VGPR) rate, progression-free survival (PFS), and safety.

**Copyright Information:** (Online) ISSN: 2572-9241
©2026 The Author(s). HemaSphere is published by John Wiley & Sons Ltd on behalf of European Hematology Association. This is an open access Abstract Book distributed under the Attribution-NonCommercial-NoDerivs (CC BY-NC-ND), which allows third parties to download the articles and share them with others as long as they credit the author and the Abstract Book, but they cannot change the content in any way or use them commercially.
**Abstract Book Citations:** Authors, Title, HemaSphere, 2026;10:(S1):pages. The abstract book DOIs can be found on the "Author Information" page.
**Disclaimer:** Articles published in the journal HemaSphere exclusively reflect the opinions of the authors. The authors are responsible for all content in their abstracts including accuracy of the facts, statements, citing resources, etc.

### Results

As of December 31, 2025, 52 of RRMM were enrolled, 26 of Apo-weekly* pts(in those trials) and matched Apo-monthly pts (Apo-based# regemin). The median age was 61 years vs 62years (range 38-79 vs 43-79), and the median number of prior lines of therapy both was 4 (range 2-12 vs 2-11), with the exception of high-risk cytogenetics(73.1%, 19/26 vs 57.7%,15/26), TP53 deletion was 34.6% (9/26) vs 7.7% (2/26), extramedullary disease (EMD) was 69.2%(18/26) vs 53.8%(14/26), including big EMD(diameter ≥5cm) 33.3%(6/18) vs 21.4%(3/14). Triple- class-resistance and BCMA-CART exposure were higher in the weekly group, 80.8% (21/26) vs 73.1%(19/26),3 vs 1, respectively.
Efficacy (Weekly vs. Monthly):
The weekly group demonstrated superior 2-month-ORR and ≥VGPR rate of 2 months compared to the monthly group 91.7%, (22/24) vs16%(4/25), 54.2%(13/24 ) vs 0%; including a complete response (CR) rate of the weekly group was 37.5%. Among pts with EMD in the weekly group, 2-month-ORR was higher,94.4%(17/18) vs 21.4%(3/14). Pts with TP53 deletion achieved a higher VGPR rate comparable to pts without TP53 deletion,57.2%(4/7, except one without tolerance to BsAbs, one died during treatment)vs 52.9%(9/17). 4 of pts with plasmablastic myeloma achieved optimal efficacy of CR, and 2 of pts with severe thrombocytopenia achieved optimal efficacy of CR in one month. After a median follow-up of 6 months (range 2-16), median PFS was not reached in the weekly group, 6-month-PFS rate was 94.1%, and 12-month PFS rate was 59.9%, respectively.
Safety (Weekly vs Monthly):
The most common treatment-related adverse events (TRAEs) in the weekly cohort vs monthly were leukopenia,77.0% vs 69.2%; anemia, 77.0% vs 80.8%; thrombocytopenia, 61.5% vs 65.4%; infections, 50.0%vs 46.2%. Grade ≥3 TRAEs included cytopenias 61.5% vs 53.8% and infections 26.9% vs19.2%.

### Summary/Conclusion

The weekly Aponermin-based regimen demonstrates promising efficacy in heavily pretreated RRMM patients, including those with EMD, TP53 deletion, plasmablastic myeloma and severe cytopenias. The safety profile was manageable. These findings support further investigation of weekly Aponermin schedules in RRMM.

**Copyright Information:** (Online) ISSN: 2572-9241
©2026 The Author(s). HemaSphere is published by John Wiley & Sons Ltd on behalf of European Hematology Association. This is an open access Abstract Book distributed under the Attribution-NonCommercial-NoDerivs (CC BY-NC-ND), which allows third parties to download the articles and share them with others as long as they credit the author and the Abstract Book, but they cannot change the content in any way or use them commercially.
**Abstract Book Citations:** Authors, Title, HemaSphere, 2026;10:(S1):pages. The abstract book DOIs can be found on the "Author Information" page.
**Disclaimer:** Articles published in the journal HemaSphere exclusively reflect the opinions of the authors. The authors are responsible for all content in their abstracts including accuracy of the facts, statements, citing resources, etc.

### (PB3235) A PRELIMINARY CLINICAL STUDY ON REDUCED-DOSE TALQUETAMAB COMBINED WITH PACE REGIMEN BRIDGE BCMA CAR-T THERAPY FOR RELAPSED/REFRACTORY MULTIPLE MYELOMA WITH MULTIPLE EXTRAMEDULLARY LESIONS

**Topic:** 14. Myeloma and other monoclonal gammopathies - Clinical

Yanli Feng*[1], Yi Wang[1]

[1] *Shaanxi Provincial People's Hospital, Department of Hematology, Xi'an, China;*

### Background

BCMA CAR-T therapy shows limited efficacy in patients with relapsed/refractory multiple myeloma (RRMM) accompanied by multiple extramedullary lesions (EMLs), and effective therapy remains scarce. Talquetamab, a bispecific antibody targeting GPRC5D�a myeloma-specific antigen has demonstrated promising clinical activity against MM with EMLs in earlier studies. However, its monotherapy is costly. The PACE regimen (cisplatin, pirarubicin, cyclophosphamide, and etoposide) also offers good responses in RRMM with EMLs, though its effects are short-lived. To improve cost-effectiveness, this study investigated reduced-dose Talquetamab combined with PACE as bridging therapy, followed by sequential BCMA CAR-T therapy (with or without autologous stem cell transplantation [ASCT]), to evaluate the safety and efficacy in these high-risk patients. It aims to provide new insights for managing high-risk RRMM with EMLs in the future.

### Aims

To assess the safety and clinical efficacy of reduced-dose Talquetamab combined with PACE as bridging therapy, followed by BCMA CAR-T therapy, in RRMM patients with multiple EMLs.

### Methods

This retrospective analysis included patients treated at our center from October 2025 to January 2026 who had RRMM with non-plasmacytoma EMLs, high-risk cytogenetic abnormalities, resistance after 3–5 prior lines of therapy. All patients expressed GPRC5D and BCMA, and without GPRC5D- or BCMA-targeted immunotherapy before, received reduced-dose Talquetamab combined with standard-dose PACE for one cycle to rapidly reduce tumor burden. And then received BCMA CAR-T therapy alone or autologous stem cell transplantation (ASCT) followed. Adverse events, clinical responses, and follow-up data were evaluated until February 25, 2026.

**Copyright Information:** (Online) ISSN: 2572-9241
©2026 The Author(s). HemaSphere is published by John Wiley & Sons Ltd on behalf of European Hematology Association. This is an open access Abstract Book distributed under the Attribution-NonCommercial-NoDerivs (CC BY-NC-ND), which allows third parties to download the articles and share them with others as long as they credit the author and the Abstract Book, but they cannot change the content in any way or use them commercially.
**Abstract Book Citations:** Authors, Title, HemaSphere, 2026;10:(S1):pages. The abstract book DOIs can be found on the "Author Information" page.
**Disclaimer:** Articles published in the journal HemaSphere exclusively reflect the opinions of the authors. The authors are responsible for all content in their abstracts including accuracy of the facts, statements, citing resources, etc.

**Results**

A cohort of 7 patients were enrolled. The therapy consisted 3 phases: (1) Bridging: reduced-dose Talquetamab (1.5 mg on days 1–2, 3 mg on day 4, and 3 mg on days 6–7; total 12 mg per cycle) combined with standard PACE (cisplatin 5 mg/m², pirarubicin 5 mg/m² , and cyclophosphamide 200 mg/m² continuous infusion over 96 hours on days 1–4 and 8–11; etoposide 20 mg/m²  days 8–11). (2) Stem cell mobilization/collection: post-bridging evaluation. (3) Consolidation: Three patients received ASCT followed by BCMA CAR-T (4.63–6.32 × $10^6$/kg) on days +3 to +7 post-transplant; other four patients received fludarabine-cyclophosphamide lymphodepletion followed by the same dose of BCMA CAR-T.
Severe myelosuppression occurred in all of the patients, pulmonary infection was 85.7% (6/7), oral ulcers was 42.8% (3/7), nail shedding was 14.3% (1/7), and skin desquamation was 42.8% (3/7). After CAR-T infusion, all patients experienced cytokine release syndrome (CRS): 85.7% (6/7) were grade 1–2 and 14.3% (1/7) were grade 3; no immune effector cell-associated neurotoxicity syndrome (ICANS) occurred. Infection after CAR-T infusion was 42.8% (3/7), all of them were grade 2.
Efficacy evaluation showed that all of the 7 patients were improved after one cycle bridging therapy: very good partial response (VGPR) in 42.8% (3/7) and partial response (PR) in 57.1% (4/7) of the patients. All patients recieved CAR-T (with or without ASCT) within one month, achieved 100% overall response rate (ORR) : complete response (CR) in 28.6% (2/7) and VGPR in 71.4% (5/7). All patients remain under follow-up.

**Summary/Conclusion**

The reduced-dose Talquetamab bridging BCMA CAR-T effectively improves the outcome of RRMM patients with multiple high-risk features, especially those who with multiple EMLs.

**Copyright Information:** (Online) ISSN: 2572-9241
©2026 The Author(s). HemaSphere is published by John Wiley & Sons Ltd on behalf of European Hematology Association. This is an open access Abstract Book distributed under the Attribution-NonCommercial-NoDerivs (CC BY-NC-ND), which allows third parties to download the articles and share them with others as long as they credit the author and the Abstract Book, but they cannot change the content in any way or use them commercially.
**Abstract Book Citations:** Authors, Title, HemaSphere, 2026;10:(S1):pages. The abstract book DOIs can be found on the "Author Information" page.
**Disclaimer:** Articles published in the journal HemaSphere exclusively reflect the opinions of the authors. The authors are responsible for all content in their abstracts including accuracy of the facts, statements, citing resources, etc.

### (PB3236) A REAL-WORLD STUDY ON THE EFFICACY AND SAFETY OF POMALIDOMIDE AS MAINTENANCE THERAPY FOR PATIENTS WITH NEWLY DIAGNOSED MULTIPLE MYELOMA

**Topic:** 14. Myeloma and other monoclonal gammopathies - Clinical

Zhao Yuqing[1], Chong Wang[2, 2], Jinxiao Yao[3], Hailong Yuan[4], Shuhao Mei[5], Ping Wang[6], Baijun Fang[7], Chao Li[8], Zheng Xu[9], Li Li[2, 10], Wenming Chen[11], Ling Qin*[1]

*[1]The First Affiliated Hospital, and College of Clinical Medicine of Henan University of Science and Technology, Luoyang, China; [2]The First Affiliated Hospital Of Zhengzhou University, Zhengzhou, China; [3]Nanyang Second General Hospital, Nanyang, China; [4]Hematology Center, The First Affiliated Hospital of Xinjiang Medical University, Xinjiang Institute of Hematology, Urumqi, China; [5]Xuchang Central Hospital, Xuchang, China; [6]Zhumadian Central Hospital, Zhumadian, China; [7]Henan Cancer Hospital, Zhengzhou, China; [8]Nanyang Central Hospital, Nanyang, China; [9]Xinyang Central Hospital, Xinyang, China; [10]The First Affiliated Hospital Of ZHENGZHOU University, Zhengzhou, China; [11]Beijing Chao-Yang Hospital, Beijing, China;*

### Background

Multiple myeloma (MM) is the second most common malignancy in the hematological system, and it remains a disease that cannot be cured at present. Maintenance therapy has the potential to significantly extend the duration of response and remission period in patients with MM, thereby ultimately improving their progression-free survival (PFS) and overall survival (OS). As a third-generation immunomodulatory drug, pomalidomide demonstrates a stronger anti-myeloma effect than lenalidomide. Furthermore, when compared to lenalidomide, pomalidomide exhibits fewer non-hematological side effects and is better tolerated.

### Aims

We aim to evaluate the clinical efficacy and safety of pomalidomide as a long-term maintenance treatment for patients with newly diagnosed MM (NDMM).

### Methods

Eligible patients with NDMM (aged ≥18 years) who had completed both induction and consolidation therapies and achieved at least a partial response (PR) were enrolled into the maintenance therapy phase. Pomalidomide was administered orally at a dosage of 4mg/day from day 1 to day 21 of a 28-day cycle. The primary end point was PFS, with key secondary end points being OS and safety.

**Copyright Information:** (Online) ISSN: 2572-9241
©2026 The Author(s). HemaSphere is published by John Wiley & Sons Ltd on behalf of European Hematology Association. This is an open access Abstract Book distributed under the Attribution-NonCommercial-NoDerivs (CC BY-NC-ND), which allows third parties to download the articles and share them with others as long as they credit the author and the Abstract Book, but they cannot change the content in any way or use them commercially.
**Abstract Book Citations:** Authors, Title, HemaSphere, 2026;10:(S1):pages. The abstract book DOIs can be found on the "Author Information" page.
**Disclaimer:** Articles published in the journal HemaSphere exclusively reflect the opinions of the authors. The authors are responsible for all content in their abstracts including accuracy of the facts, statements, citing resources, etc.

**Results**

As of December 31, 2025, a total of 73 patients with NDMM were enrolled in this study. There were 42 males (57.5%) and 31 females (42.5%), with a median age of 59 years (range 41-79 years). The median follow-up duration was 16.4 months (range 4.3-61.8 months). Neither the median PFS nor the OS had yet been reached. Among the 73 patients, the 2-year PFS rate was 65.1% , and the 2-year OS rate was 98.6%. For patients with standard-risk disease, the 2-year PFS rate was 69.0%, and the 2-year OS rate was 97.4%. For patients with high-risk disease, the 2-year PFS rate was 60.9%, and no deaths had occurred in this group as of the follow-up date.
After four cycles of pomalidomide maintenance therapy, the minimal residual disease (MRD) negativity rate increased from 47.9% to 56.2% compared to baseline; however, this difference was not statistically significant (P = 0.320). In contrast, the rate of deep response (CR/sCR) showed a significant improvement, rising from 63.0% to 83.6% (P = 0.0034). At the final follow-up, the MRD negativity rate had further increased to 68.5%, representing a significant improvement over baseline (P = 0.012). The deep response (CR/sCR) rate was 65.8%. Notably, the overall response distribution at this time point differed significantly from both baseline and the four-cycle assessment (P < 0.0001), primarily due to disease progression (PD) observed in 26.0% of patients.
During treatment, adverse events (AEs) occurred in 39.7% (29/73) of patients. Hematologic AEs were mild to moderate. Neutropenia (28.8%, n=21) and anemia (17.8%, n=13) were grade 1-2; thrombocytopenia (13.6%, n=10) included grade 3 (2.7%, n=2) and grade 4 (1.4%, n=1). Non-hematologic AEs were infrequent (grade 1-2): nausea (2.7%) and hand numbness (5.4%). One grade 5 AE (surgery-related sepsis) occurred. No patient discontinued due to drug-related AEs.

**Summary/Conclusion**

Pomalidomide maintenance therapy significantly enhances the depth of response in patients with newly diagnosed multiple myeloma (NDMM) and demonstrates good safety. This therapy may provide survival benefits to patients with NDMM.

**Copyright Information:** (Online) ISSN: 2572-9241
©2026 The Author(s). HemaSphere is published by John Wiley & Sons Ltd on behalf of European Hematology Association. This is an open access Abstract Book distributed under the Attribution-NonCommercial-NoDerivs (CC BY-NC-ND), which allows third parties to download the articles and share them with others as long as they credit the author and the Abstract Book, but they cannot change the content in any way or use them commercially.
**Abstract Book Citations:** Authors, Title, HemaSphere, 2026;10:(S1):pages. The abstract book DOIs can be found on the "Author Information" page.
**Disclaimer:** Articles published in the journal HemaSphere exclusively reflect the opinions of the authors. The authors are responsible for all content in their abstracts including accuracy of the facts, statements, citing resources, etc.

### (PB3237) STEADY-STATE MOBILIZATION WITH ON-DEMAND PLERIXAFOR AFTER CD38-ANTIBODY-BASED INDUCTION IN MULTIPLE MYELOMA PATIENTS

**Topic:** 14. Myeloma and other monoclonal gammopathies - Clinical

Maximilian Roehnert*[1], Anna Seifert[1], Karolin Trautmann[1], Christoph Röllig[1], Kristin Zimmer[1], Katharina Egger-Heidrich[1], Marius Bill[1], Lisa Heberling[1], Freya Schulze[1], Matthias Blechschmidt[1], Malte von Bonin[1], Martin Bornhauser[1, 2], Kristina Hölig[1], Raphael Teipel[1]

*[1]Department of Internal Medicine I, Faculty of Medicine and University Hospital Carl Gustav Carus TUD Dresden, University of Technology, Dresden, Germany; [2]National Center for Tumor Disease (NCT), Dresden, Germany;*

### Background

High-dose chemotherapy followed by autologous stem cell transplantation (ASCT) remains standard for fit patients with newly diagnosed multiple myeloma (MM). CD38 antibody–based quadruplet induction (e.g. Dara-VTd) improves depth of response but has been associated with impaired stem cell mobilization. Real-world data on steady-state mobilization with structured on-demand plerixafor use are limited.

### Aims

To compare mobilization kinetics, collection outcomes, and long-term graft utilization after Dara-VTd versus VCd induction using steady-state mobilization with on-demand plerixafor in a real-world setting.

### Methods

This retrospective single-center analysis included 153 ASCT-eligible MM patients treated between July 2017 and October 2023 (Dara-VTd n=85, VCd n=68). All patients underwent steady-state mobilization with G-CSF (10 µg/kg). Plerixafor was administered pre-emptively or as rescue based on peripheral blood $CD34^+$ counts. To capture intrinsic mobilization capacity independent of plerixafor and mobilization day, adjusted $CD34^+$ (adj$CD34^+$) was defined as the $CD34^+$ count measured before any plerixafor; in patients without plerixafor, this corresponded to the $CD34^+$ count immediately before first apheresis (LPH1). Primary endpoints were adj$CD34^+$, cumulative $CD34^+$ yield, and achievement of target collection (≥$4\times10^6$ $CD34^+$/kg).

### Results

Baseline characteristics were balanced. Dara-VTd induced deeper responses (≥VGPR 81% vs. 42%, p<0.01). adj$CD34^+$ prior to LPH1 was significantly lower after Dara-VTd (16 vs. 50/µL, p<0.01), indicating impaired intrinsic mobilization, and plerixafor use was higher (64% vs. 15%, p<0.01). Nevertheless, cumulative $CD34^+$ yields were comparable (6.4 vs. $6.0\times10^6$/kg, p=0.15), and target collection was achieved in 90% (Dara-VTd) versus 94% (VCd) of patients proceeding to apheresis. In multivariate analysis, Dara-VTd induction, prior radiation, and high tumor burden independently predicted impaired mobilization capacity, while no factor adversely affected $CD34^+$ yield after LPH1. Chemotherapy-based remobilization was required in 5% of patients and resulted in adequate grafts in all cases. After a median follow-up of 4.6 years, 45% of collected grafts remained unused, suggesting potential overcollection.

**Copyright Information:** (Online) ISSN: 2572-9241
©2026 The Author(s). HemaSphere is published by John Wiley & Sons Ltd on behalf of European Hematology Association. This is an open access Abstract Book distributed under the Attribution-NonCommercial-NoDerivs (CC BY-NC-ND), which allows third parties to download the articles and share them with others as long as they credit the author and the Abstract Book, but they cannot change the content in any way or use them commercially.
**Abstract Book Citations:** Authors, Title, HemaSphere, 2026;10:(S1):pages. The abstract book DOIs can be found on the "Author Information" page.
**Disclaimer:** Articles published in the journal HemaSphere exclusively reflect the opinions of the authors. The authors are responsible for all content in their abstracts including accuracy of the facts, statements, citing resources, etc.

### Summary/Conclusion

This large real-world cohort shows that steady-state mobilization with structured on-demand plerixafor compensates for impaired intrinsic mobilization after CD38 antibody–based induction, enabling successful graft collection in over 90% of patients. The high proportion of unused grafts may support re-evaluation of current collection targets in the era of highly effective induction and evolving salvage strategies in multiple myeloma.

| | Reference | **adjCD34+ pre-LPH1** (>=20 vs <20/µl) OR (95% CI; p) | **CD34+ yield post-LPH1** (>=4 vs <4x10^6/kg) OR (95% CI; p) |
|---|---|---|---|
| **Induction** | VCd | **0.1 (0.0-0.2; <0.01)** | 1.0 (0.2-3.5; 0.90) |
| **Sex** | Male | 1.5 (0.6-3.8; 0.40) | 1.3 (0.4-4.1; 0.70) |
| **Age** | >=60 y | 0.6 (0.2-1.5; 0.30) | 1.7 (0.6-5.3; 0.40) |
| **Radiation** | No | **0.2 (0.0-0.6; <0.01)** | 0.4 (0.1-1.6; 0.20) |
| **BM plasma cells** | <60% | **0.3 (0.1-0.8; <0.05)** | 1.1 (0.4-3.5; 0.90) |
| **ISS stage** | I-II | 0.8 (0.3-2.6; 0.70) | 0.6 (0.2-1.8; 0.30) |
| **Cytogenetics** | Std-risk | 1.5 (0.5-4.1; 0.50) | 0.9 (0.3-3.0; 0.90) |
| **Induction cycles** | <4 | 1.1 (0.4-3.0; 0.90) | 0.4 (0.1-1.4; 0.20) |
| **Response** | <VGPR | 2.2 (0.7-8.2; 0.20) | 0.6 (0.1-2.3; 0.40) |

Table: Multivariable logistic regression model for the mobilization capacity and apheresis yield

BM bone marrow plasma cell infiltration, ISS International Staging System stage, VGPR very good partial remission

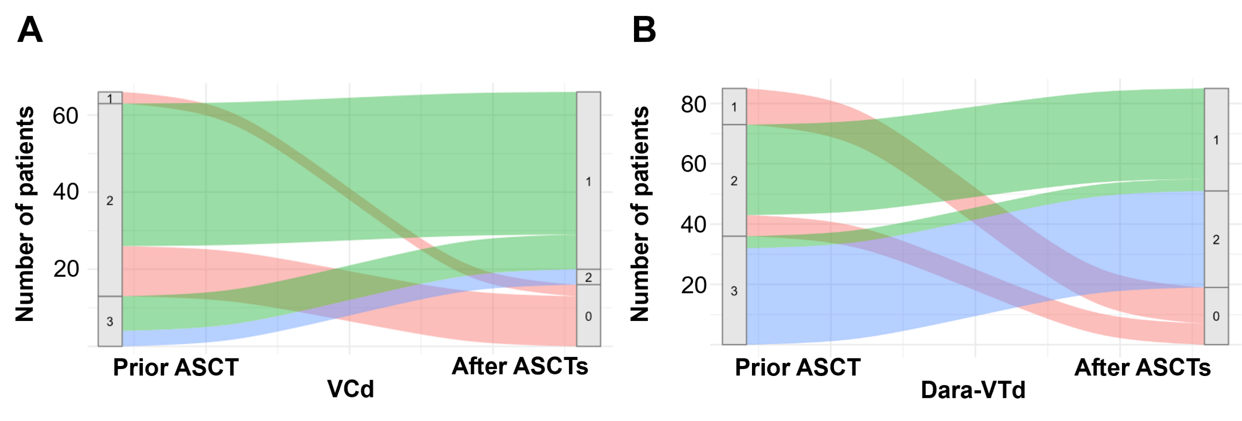

Figure. Sankey plots of available stem cell grafts. The plots show the number of collected grafts per patient prior to ASCT (grey bar) and their utilization after the last ASCT until the end of follow-up (median 4.6 years), including the proportion of grafts not yet infused. A: VCd; B: Dara-VTd.

**Copyright Information:** (Online) ISSN: 2572-9241
©2026 The Author(s). HemaSphere is published by John Wiley & Sons Ltd on behalf of European Hematology Association. This is an open access Abstract Book distributed under the Attribution-NonCommercial-NoDerivs (CC BY-NC-ND), which allows third parties to download the articles and share them with others as long as they credit the author and the Abstract Book, but they cannot change the content in any way or use them commercially.
**Abstract Book Citations:** Authors, Title, HemaSphere, 2026;10:(S1):pages. The abstract book DOIs can be found on the "Author Information" page.
**Disclaimer:** Articles published in the journal HemaSphere exclusively reflect the opinions of the authors. The authors are responsible for all content in their abstracts including accuracy of the facts, statements, citing resources, etc.

### (PB3238) CLINICAL SPECTRUM AND PATHOGENETIC MECHANISMS OF LIVER DYSFUNCTION IN IDIOPATHIC MULTICENTRIC CASTLEMAN DISEASE: A MULTICENTER STUDY AND NOVEL MURINE MODELS

**Topic:** 14. Myeloma and other monoclonal gammopathies - Clinical

Yi Liu*[1], David C. Uhlfelder[2], Yu-Han Gao[3], Danping Yu[4], Li Zhu[1], Jiaying Ge[1], Xuejiao Yin[1], Haitao Meng[1], Min Yang[1], Shengnan Ding[1], Qiumei Yao[1], Man Su[5], Jian Li[3], Hongyan Tong[1], Fang Yu[1], Lu Zhang[3], Ilgiz A. Mufazalov[2], Liangshun You[1]

*[1]The First Affiliated Hospital, College of Medicine, Zhejiang University, Hematology, Hangzhou, China;[2]Institute for Molecular Medicine, University Medical Center of the Johannes Gutenberg University, Mainz, Germany, Molecular Medicine, Mainz, Germany; [3]Department of Hematology, Peking Union Medical College Hospital, Chinese Academy of Medical Sciences and Peking Union Medical College, Beijing, China, Hematology, Beijing, China; [4]Department of Radiology, The First Affiliated Hospital, College of Medicine, Zhejiang University,, Radiology, Hangzhou, China; [5]Department of Oncology and Hematology, Beilun District People's Hospital, Ningbo, China., Ningbo, China;*

### Background

Idiopathic multicentric Castleman disease (iMCD) is a systemic hyperinflammatory disorder driven by IL-6 overproduction. While hepatic involvement is common, the longitudinal progression toward irreversible structural damage, such as liver cirrhosis, remains poorly characterized. This study aims to delineate the clinical spectrum of iMCD-associated liver dysfunction and elucidate the underlying molecular pathways.

### Methods

We conducted a multicenter retrospective study of 679 patients with HHV-8-negative iMCD from the First Affiliated Hospital of Zhejiang University and Peking Union Medical College Hospital. Clinical trajectories of 12 patients with significant liver dysfunction (6 with cirrhosis, 6 without) were analyzed. Histopathological evaluations were performed on human liver biopsies. To model the pathogenesis, we developed two novel mouse models: a Foxp3-Cre-dependent IL-6 overexpressing model (IL-6Fp3-OE, representing MCD) and an immunoglobulin-deficient version (IgHμγ1/μγ1 IL-6Fp3-OE, representing IgMi-MCD).

### Results

Patients with liver cirrhosis (LC) exhibited significantly longer diagnostic delays compared to the non-cirrhosis (NLC) group (median 39.3 vs 1.2 months) and poorer treatment responses. Liver dysfunction predominantly manifested as intrahepatic cholestasis (elevated ALP/γ-GT). Histology revealed nodular regenerative hyperplasia (NRH) and perivascular fibrosis prior to cirrhosis. In murine models, both groups exhibited marked chronic inflammatory infiltration (B and T cells) and perivascular fibrosis. Multiplex IHC showed robust upregulation of VEGFR2 and perivascular IL-6 signaling in the MCD model, whereas the plasma cell-deficient IgMi-MCD model exhibited only moderate VEGFR elevation without significant local IL-6 overexpression in the hepatic parenchyma.

### Summary/Conclusion

Our study establishes a strong correlation between prolonged uncontrolled iMCD activity and the development of irreversible liver cirrhosis. The distinct phenotypes observed between the MCD and IgMi-MCD models highlight the pivotal role of vascular endothelial cells, which appear to be integral to the amplification of inflammatory signaling and the progression of hepatic pathology. These findings underscore that early diagnosis and timely initiation of effective therapy are crucial to prevent end-stage hepatic complications in iMCD.

**Copyright Information:** (Online) ISSN: 2572-9241
©2026 The Author(s). HemaSphere is published by John Wiley & Sons Ltd on behalf of European Hematology Association. This is an open access Abstract Book distributed under the Attribution-NonCommercial-NoDerivs (CC BY-NC-ND), which allows third parties to download the articles and share them with others as long as they credit the author and the Abstract Book, but they cannot change the content in any way or use them commercially.
**Abstract Book Citations:** Authors, Title, HemaSphere, 2026;10:(S1):pages. The abstract book DOIs can be found on the "Author Information" page.
**Disclaimer:** Articles published in the journal HemaSphere exclusively reflect the opinions of the authors. The authors are responsible for all content in their abstracts including accuracy of the facts, statements, citing resources, etc.

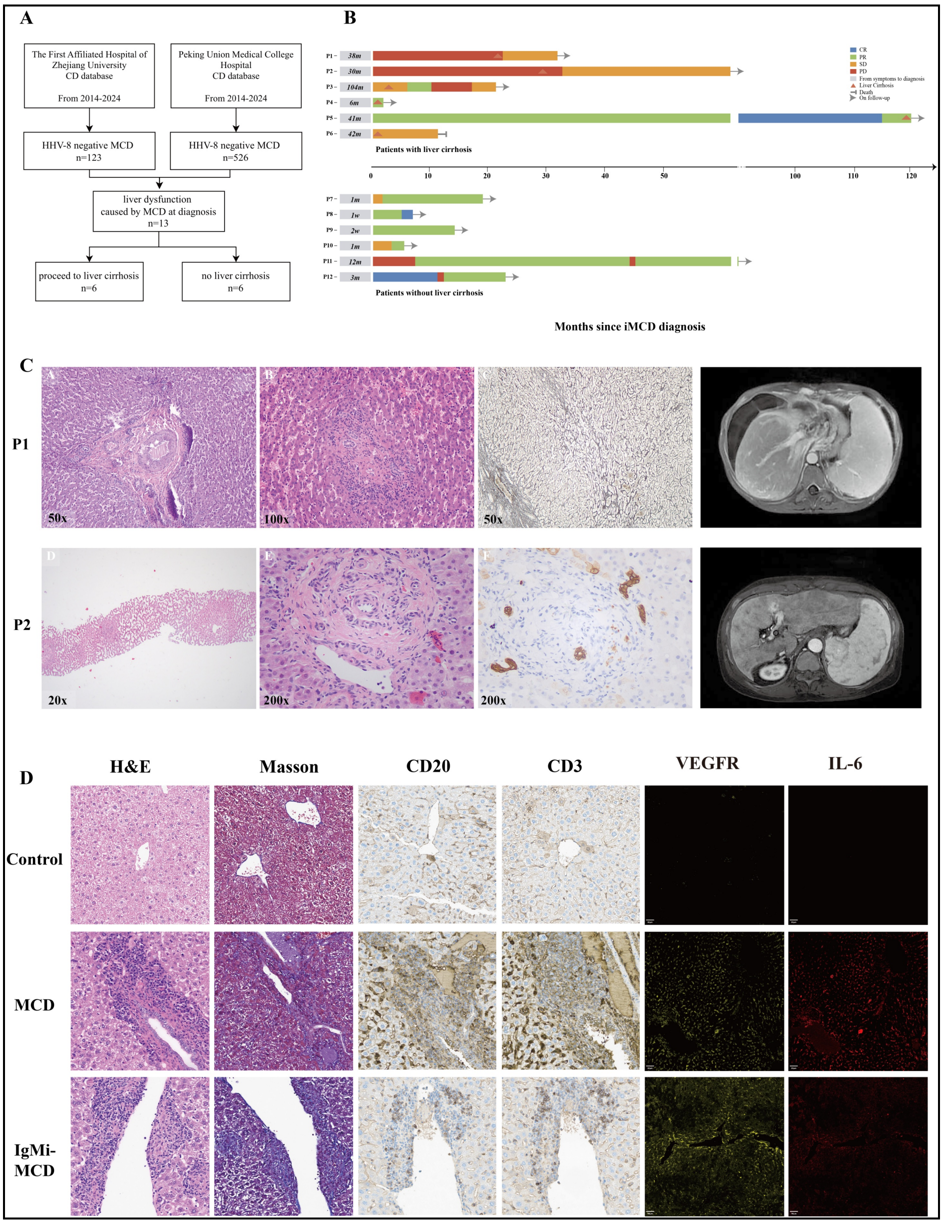

Figure 1. Clinical Spectrum and Pathogenetic Mechanisms of Liver Dysfunction in iMCD. (A) Study Design and Patient Enrollment (B) Clinical Trajectories and Treatment Response (C) Representative Histopathological and Radiological Findings (D) Comparative Analysis of Novel Murine Models

**Copyright Information:** (Online) ISSN: 2572-9241
©2026 The Author(s). HemaSphere is published by John Wiley & Sons Ltd on behalf of European Hematology Association. This is an open access Abstract Book distributed under the Attribution-NonCommercial-NoDerivs (CC BY-NC-ND), which allows third parties to download the articles and share them with others as long as they credit the author and the Abstract Book, but they cannot change the content in any way or use them commercially.
**Abstract Book Citations:** Authors, Title, HemaSphere, 2026;10:(S1):pages. The abstract book DOIs can be found on the "Author Information" page.
**Disclaimer:** Articles published in the journal HemaSphere exclusively reflect the opinions of the authors. The authors are responsible for all content in their abstracts including accuracy of the facts, statements, citing resources, etc.

### (PB3239) A PILOT STUDY OF ROMOSOZUMAB IN POSTMENOPAUSAL WOMEN WITH MYELOMA-RELATED BONE DISEASE AND OSTEOPOROSIS

**Topic:** 14. Myeloma and other monoclonal gammopathies - Clinical

Carlyn Rose Tan*[1], Andriy Derkach[2], David Nemirovsky[2], Malin Hultcrantz[1], Hani Hassoun[1], Sham Mailankody[1], Urvi A. Shah[1], Sridevi Rajeeve[1], Kylee MacLachlan[1], Hamza Hashmi[1], Maximilian Merz[1], Francesco Maura[1], Ross Firestone[1], Gunjan Shah[3], Michael Scordo[3], Heather Landau[3], Sergio Giralt[3], Daniel Lafontaine[4], Burcin Agridag Ucpinar[5], Heiko Schoder[5], Alexander Lesokhin[1], Neha Korde[1], Saad Z Usmani[1], Azeez Farooki[6]

*[1]Memorial Sloan Kettering Cancer Center, Myeloma Service, Department of Medicine, New York, United States of America; [2]Memorial Sloan Kettering Cancer Center, Department of Epidemiology and Biostatistics, New York, United States of America; [3]Memorial Sloan Kettering Cancer Center, Adult Bone Marrow Transplant Service, Department of Medicine, New York, United States of America; [4]Memorial Sloan Kettering Cancer Center, Departments of Medical Physics, New York, United States of America; [5]Memorial Sloan Kettering Cancer Center, Departments of Radiology, New York, United States of America; [6]Memorial Sloan Kettering Cancer Center, Endocrinology Service, Department of Medicine, New York, United States of America;*

### Background

Myeloma-related bone disease (MBD) affects up to 80% of patients with newly diagnosed multiple myeloma (NDMM). Although antiresorptive agents for MM inhibit bone resorption, they do not exert an anabolic effect to increase bone formation. Sclerostin is an osteocyte product that inhibits osteoblast maturation, impairs bone mineralization, and induces osteoblast apoptosis. Elevated sclerostin levels have been associated with advanced disease, presence of fractures, and abnormal bone remodeling in MM. Romosozumab (romo) is an anti-sclerostin monoclonal antibody approved in osteoporosis that has resulted in a rapid and marked increase in bone formation and moderate but sustained decrease in bone resorption, reflected in Procollagen Type 1 N-telopeptide (P1NP) and Type 1 collagen C-telopeptide (ß-CTX) levels, respectively.

### Aims

We report results from a 10-patient pilot study evaluating the efficacy (based on bone turnover marker levels; BTM) and safety of romosozumab in MM patients with MBD.

### Methods

Postmenopausal women with MM and evidence of MBD and osteoporosis were treated with romo in conjunction with standard MM therapy at Memorial Sloan Kettering. Patients received romo at 210mg SC monthly for 12 months after a prespecified washout period for prior antiresorptive agents. The primary endpoint was percent change in P1NP level after 1 month of romo compared to baseline. Key secondary endpoints include percent change in other BTMs (osteocalcin, bone alkaline phosphatase, ß-CTX), evidence of bone remineralization on imaging at 6 and 12 months, and change in bone mineral density (BMD) at 12 and 24 months.

**Copyright Information:** (Online) ISSN: 2572-9241
©2026 The Author(s). HemaSphere is published by John Wiley & Sons Ltd on behalf of European Hematology Association. This is an open access Abstract Book distributed under the Attribution-NonCommercial-NoDerivs (CC BY-NC-ND), which allows third parties to download the articles and share them with others as long as they credit the author and the Abstract Book, but they cannot change the content in any way or use them commercially.
**Abstract Book Citations:** Authors, Title, HemaSphere, 2026;10:(S1):pages. The abstract book DOIs can be found on the “Author Information” page.
**Disclaimer:** Articles published in the journal HemaSphere exclusively reflect the opinions of the authors. The authors are responsible for all content in their abstracts including accuracy of the facts, statements, citing resources, etc.

### Results

Ten patients completed 12 months of romo: 6 with NDMM and 4 with RRMM. Median age was 69 (interquartile range [IQR], 64-74) with 50% having high-risk cytogenetics per the IMS/IMWG high-risk definition. During romo therapy, 4 were on maintenance therapy, 5 on daratumumab-based regimens with 2 transitioning to CART, and 1 on observation. While on romo, 9 patients maintained or had deepening of their responses (7 ≥CR, 2 VGPR); 1 patient had stable disease and then developed progressive disease. All patients had received prior zoledronic acid but had been off for ≥3 months. Median baseline P1NP level of 27 mcg/L (IQR 24-56) increased to 116 (IQR 67-144) after 1 month of romo with a median percent change of 153% (IQR 104%-304%) (P=0.002). The median osteocalcin level was 13 ng/mL (IQR 11-23) at baseline and 46 ng/mL (23-58) at month 1 with median percent change of 149% (IQR 115%-183%) (P=0.002). Median bone alkaline phosphatase at baseline was 10.1 mcg/L (IQR 8-12) and increased to 19.5 mcg/L (116-25) at month 1 with median percent change of 96% (69%-107%) (P=0.002). There was no significant change between median ß-CTX baseline (202 pg/mL [IQR, 132-378]) and month 1 (201 pg/mL [134-402]) levels with median percent change of 2% (8%-35%) (P=0.8). No remineralization of target lytic lesions was seen on whole body PET/CT imaging. At 12 months, mean percent changes in BMD at the lumbar spine and total hip was +6.73% and +4.46%, respectively. The safety profile was consistent with known romo adverse events with no major adverse cardiac events reported.

### Summary/Conclusion

In this pilot study, romo has been well-tolerated and resulted in significant increases in bone formation markers, P1NP, osteocalcin, and bone alkaline phosphatase compared to baseline. Our findings suggest that romo has positive anabolic effects in MM patients without negatively impacting anti-MM therapy.

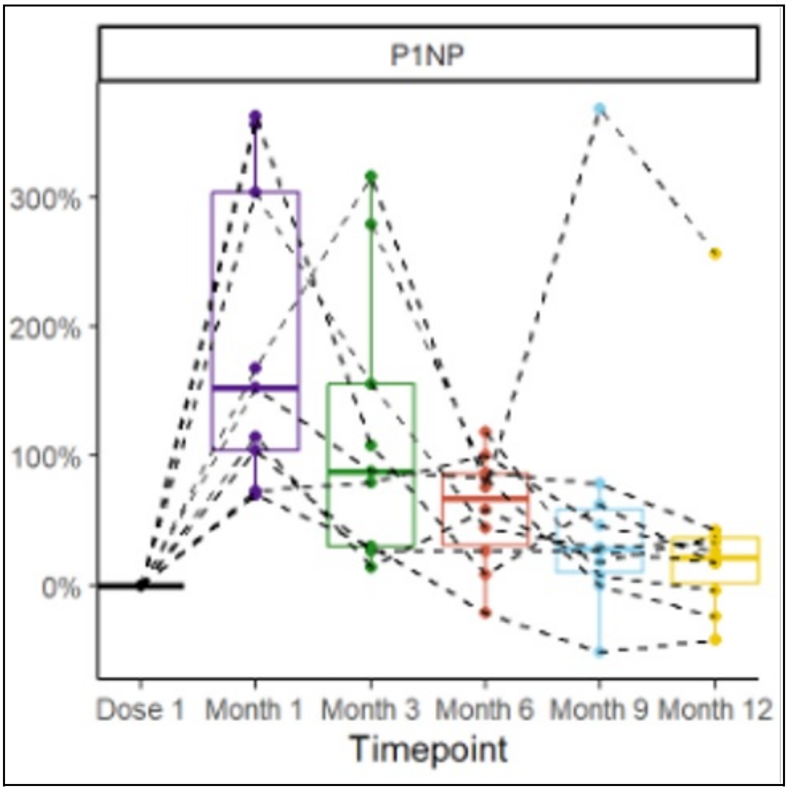

**Copyright Information:** (Online) ISSN: 2572-9241
©2026 The Author(s). HemaSphere is published by John Wiley & Sons Ltd on behalf of European Hematology Association. This is an open access Abstract Book distributed under the Attribution-NonCommercial-NoDerivs (CC BY-NC-ND), which allows third parties to download the articles and share them with others as long as they credit the author and the Abstract Book, but they cannot change the content in any way or use them commercially.
**Abstract Book Citations:** Authors, Title, HemaSphere, 2026;10:(S1):pages. The abstract book DOIs can be found on the "Author Information" page.
**Disclaimer:** Articles published in the journal HemaSphere exclusively reflect the opinions of the authors. The authors are responsible for all content in their abstracts including accuracy of the facts, statements, citing resources, etc.

### (PB3240) PERFORMANCE VALIDATION OF MASS SPECTROMETRY FOR URINARY BENCE JONES PROTEIN DETECTION AND ITS CLINICAL APPLICATION IN MULTIPLE MYELOMA AND AMYLOIDOSIS

**Topic:** 14. Myeloma and other monoclonal gammopathies - Clinical

Yu Chen[1, 2], Haitao Meng[1, 3], Min Yang*[1, 3]

*[1]Department of Hematology, The First Affiliated Hospital, College of Medicine, Zhejiang University, Hangzhou, Zhejiang, China; [2]Zhejiang Province Key Laboratory of Hematology Oncology Diagnosis and Treatment, Hangzhou, Zhejiang, China;[3]Zhejiang Provincial Clinical Research Center for Hematologic Diseases, Hangzhou, Zhejiang, China;*

### Background

The clinical necessity of urine immunofixation electrophoresis (uIFE), in the management of multiple myeloma (MM) and light chain amyloidosis (AL), is increasingly debated. This is attributable to its cumbersome procedure (requiring 24-hour collection), limited sensitivity, and subjective interpretation. These limitations become particularly critical in patients with renal impairment, where the accumulation of polyclonal free light chains can cause pseudo-normalization of the serum free light chain (sFLC) ratio, thereby creating a diagnostic "blind spot" in disease monitoring. Consequently, there is an urgent clinical need for a simpler, more objective urine-based test to circumvent this "serum ratio trap."

### Aims

This study aimed to evaluate a novel MALDI-TOF mass spectrometry (MS) assay for urinary Bence Jones protein detection as a potential replacement for uIFE. We directly compared its diagnostic performance with uIFE and assessed its clinical utility, particularly in patients with renal dysfunction.

### Methods

To address this practical clinical problem, we compared the diagnostic performance of traditional uIFE (in two modes: uIFE and uIFE-FLC) and MS (in two modes: IMS-IP Assay-urine and IMS-FLC Assay-urine) using urine samples from 273 patients with MM/AL. Based on their uIFE results, these patients were stratified into a negative cohort (n=160) and a positive cohort (n=113), and all samples were tested using both modes of the two methods. We specifically evaluated the correlation between the results obtained by the new methods and serum evidence (sIFE and sFLC), as well as their clinical utility in challenging scenarios such as renal impairment.

### Results

IMS-IP Assay-urine showed excellent overall agreement with uIFE (258/273, 94.51%; $\kappa$=0.889). In the negative cohort, IMS-IP Assay-urine detected 14 positive cases. In the positive cohort, it detected 112 positive cases, including 4 cases with biclonal proteins (identified as monoclonal by uIFE), 1 case with daratumumab interference (indistinguishable by uIFE), and 11 cases with intact M-proteins (identified as light-chain only by uIFE).IMS-IP Assay-urine provided N-glycosylation information for 14 patients.
IMS-FLC Assay-urine showed good overall agreement with uIFE-FLC (229/273, 83.88%; $\kappa$=0.678). In the negative cohort, uIFE-FLC detected 3 positive cases, whereas iMS-FLC Assay-urine detected 25. In the positive cohort, uIFE-FLC detected 90 positive cases, while IMS-FLC Assay-urine detected 112. IMS-FLC Assay-urine provided N-glycosylation information for 11 patients.
Critically, in 6 patients with renal impairment (median eGFR 50.1 mL/min/1.73m²)who were serum-negative (negative sIFE and normal sFLC ratio), uIFE-FLC detected 3 positive cases, whereas IMS-FLC Assay-urine detected all 6. With a limit of detection as low as 0.001 mg/mL, this method successfully identified disease activity missed by serum testing.

**Copyright Information:** (Online) ISSN: 2572-9241
©2026 The Author(s). HemaSphere is published by John Wiley & Sons Ltd on behalf of European Hematology Association. This is an open access Abstract Book distributed under the Attribution-NonCommercial-NoDerivs (CC BY-NC-ND), which allows third parties to download the articles and share them with others as long as they credit the author and the Abstract Book, but they cannot change the content in any way or use them commercially.
**Abstract Book Citations:** Authors, Title, HemaSphere, 2026;10:(S1):pages. The abstract book DOIs can be found on the "Author Information" page.
**Disclaimer:** Articles published in the journal HemaSphere exclusively reflect the opinions of the authors. The authors are responsible for all content in their abstracts including accuracy of the facts, statements, citing resources, etc.

**Summary/Conclusion**

This study confirms that mass spectrometry-based urinary Bence Jones protein detection is more sensitive, rapid, and objective than uIFE. This novel approach not only detects more monoclonal proteins but also distinguishes therapeutic drug interference and circumvents the impact of renal function on serum free light chain ratios. It provides critical complementary information for the diagnosis of multiple myeloma and related disorders, demonstrating particular value in complex scenarios such as renal impairment and seronegative disease.Consequently, it holds promise as a replacement for routine uIFE.

**Copyright Information:** (Online) ISSN: 2572-9241
©2026 The Author(s). HemaSphere is published by John Wiley & Sons Ltd on behalf of European Hematology Association. This is an open access Abstract Book distributed under the Attribution-NonCommercial-NoDerivs (CC BY-NC-ND), which allows third parties to download the articles and share them with others as long as they credit the author and the Abstract Book, but they cannot change the content in any way or use them commercially.
**Abstract Book Citations:** Authors, Title, HemaSphere, 2026;10:(S1):pages. The abstract book DOIs can be found on the "Author Information" page.
**Disclaimer:** Articles published in the journal HemaSphere exclusively reflect the opinions of the authors. The authors are responsible for all content in their abstracts including accuracy of the facts, statements, citing resources, etc.

### (PB3241) IMPACT OF PROTON PUMP INHIBITORS ON CLINICAL OUTCOMES IN NEWLY DIAGNOSED TRANSPLANT-INELIGIBLE MULTIPLE MYELOMA: A POST HOC ANALYSIS OF THE BENEFIT STUDY

**Topic:** 14. Myeloma and other monoclonal gammopathies - Clinical

Ilhame Bounouara[1], Manon Sapet*[1], Théo Maynand[1], Elisabeth Daguenet[1], Arthur Bobin[2], Murielle Roussel[3], Laurent Frenzel[4], Stephanie Harel[5], Olivier Decaux[6], Karim Belhadj Merzoug[7], Cyrille Touzeau[8], Margaret MacRo[9], Mohamad Mohty[10], Philippe Moreau[8], Jill Corre[11], Hervé Avet Loiseau[11], Lionel Karlin[12], Thierry Facon[13], Aurore Perrot[11], Titouan Cazaubiel[14], Salomon Manier[13], Xavier Leleu[2], Emilie Chalayer[1]

*[1]CHU Saint-Etienne, Hematology, Saint-Etienne, France;[2]CHU de Poitiers, Poitiers, France;[3]CHU Limoges, Limoges, France; [4]Hôpital Necker, Paris, France;[5]Hôpital Saint-Louis, Paris, France;[6]Chu Rennes, Rennes, France; [7]Hôpital Henri Mondor- APHP, Créteil, France; [8]CHU Nantes, Nantes, France; [9]CHU Caen, Caen, France; [10]Hôpital Saint-Antoine, Paris, France; [11]IUCT Oncopole, Toulouse, France; [12]Hôpital Lyon Sud-HCL, Saint-Genis-Laval, France; [13]Hôpital Claude Huriez- CHU de Lille, Lille, France; [14]CHU Bordeaux, Bordeaux, France;*

### Background

Proton pump inhibitors (PPIs) are frequently prescribed in newly diagnosed multiple myeloma (NDMM), particularly in elderly, transplant-ineligible (TI) patients receiving antithrombotic prophylaxis and corticosteroids. IPPs prevent bleeding from a gastric ulcer. However, they may increase susceptibility to infections, alter gut microbiota, and impact the immune response. Their influence on clinically relevant endpoints in NDMM remains unclear (Almansour, 2024).

### Aims

We performed a post hoc analysis of the BENEFIT trial to evaluate the impact of PPI exposure on bleeding events, infectious complications, minimal residual disease (MRD), progression-free survival (PFS), and overall survival (OS).

### Methods

We conducted a post hoc analysis of TI NDMM patients enrolled in the phase 3 BENEFIT trial. Patients were categorized according to PPI exposure during treatment (≥4 weeks) (Kiecka, 2023). Clinical endpoints were assessed at 18 months. The primary objective was to evaluate the association between bleeding events (all events) and PPI use. Secondary objectives were to evaluate the association between PPI use and MRD negativity (sensitivity $10^{\wedge 5}$), PFS, OS, and infections (pneumopathy and *Clostridium difficile* infection [CDI]). Associations were evaluated using the chi-squared or Fisher's exact test, as appropriate, and logistic regression models were used to estimate the odds ratio (OR) with a 95% confidence interval (CI).

### Results

Patients characteristics are described in the original study (Leleu, 2024). Among evaluable patients (n=270), PPI exposure (n=159, 59%) was balanced between treatment arms. Bleeding events at 18 months were observed in 4% of patients receiving PPIs vs. 7% in the group without PPIs (OR=0.6; 95% CI 0.2–1.7; p=0.32). Although not statistically significant, a decrease was noted with PPI exposure. No gastric bleeding event occurred in either group. Regarding pulmonary infections, no significant association was observed between groups (OR=0.9; 95% CI 0.5–1.6; p=0.71). Only one case of CDI was reported in a patient not receiving a PPI. Regarding prognostic outcomes, at 18 months, MRD negativity was not significantly different with respect to PPI exposure (OR=1.0; 95% CI 0.6–1.7; p=1.0), nor was PFS or OS.

**Copyright Information:** (Online) ISSN: 2572-9241
©2026 The Author(s). HemaSphere is published by John Wiley & Sons Ltd on behalf of European Hematology Association. This is an open access Abstract Book distributed under the Attribution-NonCommercial-NoDerivs (CC BY-NC-ND), which allows third parties to download the articles and share them with others as long as they credit the author and the Abstract Book, but they cannot change the content in any way or use them commercially.
**Abstract Book Citations:** Authors, Title, HemaSphere, 2026;10:(S1):pages. The abstract book DOIs can be found on the "Author Information" page.
**Disclaimer:** Articles published in the journal HemaSphere exclusively reflect the opinions of the authors. The authors are responsible for all content in their abstracts including accuracy of the facts, statements, citing resources, etc.

**Summary/Conclusion**

In this post-hoc analysis of the BENEFIT study, the rate of bleeding events was lower at 18 months in the PPI group, but this difference was not statistically significant. PPI use was not associated with an increased risk of pulmonary infections or CDI, nor with a poorer 18-month prognosis in TI NDMM patients. Although this study has some limitations related to its post-hoc design, these data do not suggest a major impact of PPIs on short-term clinical outcomes in this population. Larger studies with longer follow-up are needed to clarify their potential influence on bleeding risk and long-term survival.

**Copyright Information:** (Online) ISSN: 2572-9241
©2026 The Author(s). HemaSphere is published by John Wiley & Sons Ltd on behalf of European Hematology Association. This is an open access Abstract Book distributed under the Attribution-NonCommercial-NoDerivs (CC BY-NC-ND), which allows third parties to download the articles and share them with others as long as they credit the author and the Abstract Book, but they cannot change the content in any way or use them commercially.
**Abstract Book Citations:** Authors, Title, HemaSphere, 2026;10:(S1):pages. The abstract book DOIs can be found on the "Author Information" page.
**Disclaimer:** Articles published in the journal HemaSphere exclusively reflect the opinions of the authors. The authors are responsible for all content in their abstracts including accuracy of the facts, statements, citing resources, etc.

### (PB3242) DISTINCT CLINICAL FEATURES OF MULTIPLE MYELOMA WITH T(11;14): A POST HOC ANALYSIS OF PROSPECTIVE OBSERVATIONAL STUDY FOR MULTIPLE MYELOMA IN JAPAN (JSH-MM-15)

**Topic:** 14. Myeloma and other monoclonal gammopathies - Clinical

Taku Tsukamoto*[1], Yuju Shimura[1], Shinsuke Mizutani[1], Takahiro Fujino[1], Haruya Okamoto[1], Mitsuhiro Itagaki[2], Hiroshi Handa[3], Takayuki Shimizu[4], Morio Sawamura[5], Tadao Ishida[6], Satoru Kosugi[7], Shuichi Ota[8], Makoto Yoshimitsu[9], Yutaka Shimazu[10], Isamu Sugiura[11], Hirohiko Shibayama[12], Shin-Ichi Fuchida[13], Hiroyuki Takamatsu[14], Akiko M. Saito[15], Hitoshi Kiyoi[16], Akashi Koichi[17], Itaru Matsumura[18], Akifumi Takaori[10], Junya Kuroda[1], Shinsuke Iida[19]

*[1]Kyoto Prefectural University of Medicine, Kyoto, Japan;[2]Hiroshima Red Cross Hospital & Atomic-bomb Survivors Hospital, Hiroshima, Japan; [3]Gunma University Graduate School of Medicine, Gunma, Japan;[4]National Hospital Organization Tokyo Medical Center, Tokyo, Japan; [5]National Hospital Organization Shibukawa Medical Center, Shibukawa, Japan;[6]Japanese Red Cross Medical Center, Tokyo, Japan; [7]Toyonaka Municipal Hospital, Toyonaka, Japan; [8]Sapporo Hokuyu Hospital, Sapporo, Japan; [9]Kagoshima University, Kagoshima, Japan; [10]Kyoto University, Kyoto, Japan; [11]Toyohashi Municipal Hospital, Toyohashi, Japan; [12]National Hospital Organization Osaka National Hospital, Osaka, Japan; [13]Japan Community Health Care Organization Kyoto Kuramaguchi Medical Center, Kyoto, Japan; [14]Kanazawa University, Kanazawa, Japan; [15]NHO Nagoya Medical Center, Nagoya, Japan; [16]Nagoya University Graduate School of Medicine, Nagoya, Japan; [17]Kyushu University Graduate School of Medicine, Fukuoka, Japan; [18]Kindai University Faculty Of Medicine, Osaka, Japan; [19]Nagoya City University Graduate School of Medical Sciences, Nagoya, Japan;*

### Background

The chromosomal translocation t(11;14)(q13;q32) is detected in approximately 15–25% of patients with multiple myeloma (MM) and has traditionally been considered a standard-risk cytogenetic abnormality. However, recent studies suggest that t(11;14)-positive MM represents a biologically heterogeneous entity with clinical behavior distinct from other standard-risk subgroups. The prognostic relevance of conventional risk factors, depth of response, and optimal frontline treatment strategies in this population remain incompletely defined.

### Aims

To clarify the clinical characteristics, prognostic factors, and treatment outcomes of newly diagnosed t(11;14)-positive MM in the era of novel agents.

### Methods

We performed a post hoc analysis of the JSH-MM-15 study, a nationwide, multicenter prospective observational cohort enrolling Japanese patients with newly diagnosed MM between 2016 and 2018. Among 1,385 patients, 482 underwent fluorescence in situ hybridization testing for t(11;14); 131 (27%) were positive. Patients without t(11;14) were classified as standard-risk or high-risk based on the presence of t(4;14), t(14;16), or del(17p). Overall survival (OS), progression-free survival (PFS), treatment responses, and the prognostic impact of clinical variables and immunophenotypic markers (CD20 and CD56) were evaluated.

**Copyright Information:** (Online) ISSN: 2572-9241
©2026 The Author(s). HemaSphere is published by John Wiley & Sons Ltd on behalf of European Hematology Association. This is an open access Abstract Book distributed under the Attribution-NonCommercial-NoDerivs (CC BY-NC-ND), which allows third parties to download the articles and share them with others as long as they credit the author and the Abstract Book, but they cannot change the content in any way or use them commercially.
**Abstract Book Citations:** Authors, Title, HemaSphere, 2026;10:(S1):pages. The abstract book DOIs can be found on the "Author Information" page.
**Disclaimer:** Articles published in the journal HemaSphere exclusively reflect the opinions of the authors. The authors are responsible for all content in their abstracts including accuracy of the facts, statements, citing resources, etc.

**Results**

Patients with t(11;14)-positive MM showed OS intermediate between standard- and high-risk cytogenetic groups, while PFS was comparable to that of high-risk MM. Rates of deep responses, including ≥ complete response (CR)/stringent CR and ≥ very good partial response (VGPR), were significantly lower in t(11;14)-positive patients than in other risk groups. Notably, depth of response was not associated with OS or PFS in t(11;14)-positive MM, in contrast to t(11;14)-negative cases. Conventional prognostic factors such as ISS stage, del(17p), and peripheral blood plasma cell involvement showed limited prognostic impact in t(11;14)-positive patients.
Immunophenotypic analysis revealed a higher frequency of CD20 expression and a lower frequency of CD56 expression in t(11;14)-positive MM. Although CD20 expression alone did not affect survival, CD20-positive patients demonstrated improved OS and PFS when treated with proteasome inhibitor–containing regimens, whereas this benefit was not observed in CD20-negative patients. Combined CD20/CD56 expression identified subgroups with discordant response depth and survival outcomes.

**Summary/Conclusion**

t(11;14)-positive MM is a biologically and clinically distinct subgroup characterized by modest response depth, attenuated prognostic value of conventional risk factors, and treatment-dependent outcomes influenced by immunophenotypic features. These findings underscore the need for refined risk stratification and individualized therapeutic strategies for patients with t(11;14)-positive MM.

**Copyright Information:** (Online) ISSN: 2572-9241
©2026 The Author(s). HemaSphere is published by John Wiley & Sons Ltd on behalf of European Hematology Association. This is an open access Abstract Book distributed under the Attribution-NonCommercial-NoDerivs (CC BY-NC-ND), which allows third parties to download the articles and share them with others as long as they credit the author and the Abstract Book, but they cannot change the content in any way or use them commercially.
**Abstract Book Citations:** Authors, Title, HemaSphere, 2026;10:(S1):pages. The abstract book DOIs can be found on the "Author Information" page.
**Disclaimer:** Articles published in the journal HemaSphere exclusively reflect the opinions of the authors. The authors are responsible for all content in their abstracts including accuracy of the facts, statements, citing resources, etc.

### (PB3243) A PHASE 1/2, OPEN-LABEL, PLATFORM STUDY TO EVALUATE THE SAFETY AND EFFICACY OF ETENTAMIG MONOTHERAPY OR COMBINATION THERAPY IN PATIENTS WITH MULTIPLE MYELOMA

**Topic:** 14. Myeloma and other monoclonal gammopathies - Clinical

Karthik Nath*[1], Hila Magen[2], Peter Forsberg[3], Ariel Grajales-Cruz[4], John McKay[5], Darren Pan[6], Matthew J. Pianko[7], Fredrik Schjesvold[8], Faisal Rahman[9], Jovian Yu[9], Kojo Osei-Bonsu[9], Matthew Bentley[9], Sunny He[9], Hillary Peltier[9], Ly Pham[9], Jordan Morrissey[9], Eben Lichtman[10]

*[1]Icon Cancer Centre, Brisbane, Queensland, Australia;[2]Hematology Chaim Sheba Medical Center, Ramat-Gan, Faculty of Medicine, Tel Aviv University, Tel Aviv, Israel; [3]Colorado Blood Cancer Institute, Denver, CO, United States of America; [4]Malignant Hematology, Myeloma Section, H. Lee Moffitt Cancer Center and Research Institute, Tampa, FL, United States of America; [5]Atrium Health Wake Forest Baptist Comprehensive Cancer Center, Wake Forest University School of Medicine, Winston-Salem, NC, United States of America; [6]Department of Medicine, University of California, San Francisco, San Francisco, CA, United States of America; [7]Department of Internal Medicine, Division of Hematology/Oncology, Rogel Cancer Center, University of Michigan Medical School, Ann Arbor, MI, United States of America; [8]Oslo Myeloma Center, Department of Hematology, Oslo University Hospital, Oslo, Norway; [9]AbbVie Inc., North Chicago, IL, United States of America; [10]UNC Lineberger Comprehensive Cancer Center, Chapel Hill, NC, United States of America;*

### Background

Despite recent treatment advances, leading to substantial improvements in long-term outcomes, many patients (pts) with multiple myeloma (MM) still face considerable challenges and experience disease relapse, leading to multi-agent refractory disease. This highlights persistent unmet need for innovative treatments or novel combinations that provide robust efficacy with a manageable safety profile. B-cell maturation antigen (BCMA) is emerging as a key target in MM due to its highly selective expression in malignant plasma cells.
Etentamig (etenta) is being developed as a patient-centric, next-generation differentiated BCMA x CD3 bispecific T-cell engager composed of a bivalent BCMA-binding domain with high-avidity, low-affinity CD3-binding domain potentially reducing cytokine release syndrome (CRS), and a silenced Fc tail for extended half-life enabling convenient monthly (Q4W) dosing from the start. In a Phase 1 study, etenta monotherapy demonstrated encouraging efficacy and was well tolerated in pts with relapsed/refractory (RR) MM, with ≥3 prior lines of therapy.

### Aims

This platform study will determine the recommended dose of etenta—as a monotherapy or in combination with standard of care (SoC) or investigational agents—evaluating safety, tolerability, preliminary anti-MM activity, pharmacokinetics (PK), and immunogenicity.

**Copyright Information:** (Online) ISSN: 2572-9241
©2026 The Author(s). HemaSphere is published by John Wiley & Sons Ltd on behalf of European Hematology Association. This is an open access Abstract Book distributed under the Attribution-NonCommercial-NoDerivs (CC BY-NC-ND), which allows third parties to download the articles and share them with others as long as they credit the author and the Abstract Book, but they cannot change the content in any way or use them commercially.
**Abstract Book Citations:** Authors, Title, HemaSphere, 2026;10:(S1):pages. The abstract book DOIs can be found on the "Author Information" page.
**Disclaimer:** Articles published in the journal HemaSphere exclusively reflect the opinions of the authors. The authors are responsible for all content in their abstracts including accuracy of the facts, statements, citing resources, etc.

**Methods**

This is a Phase 1/2, open-label, multicenter, dose selection and optimization platform study (NCT06892522) utilizing a master protocol to evaluate etenta as monotherapy or in combination with other agents in pts with MM (**Figure**). Different etenta regimens will be evaluated in separate substudies (currently 4). Each substudy may include a dose-escalation phase (Phase 1) and/or a dose-expansion and selection phase (Phase 2). Eligible pts will be ≥18 years of age with an ECOG-PS of ≤1, with either newly diagnosed (ND) or RRMM, depending on the substudy. Different substudies will enroll pts with transplant-ineligible NDMM (TI NDMM), transplant-eligible NDMM (TE NDMM) post autologous stem cell transplant, or pts with RRMM, who received 1–3 prior lines of therapy.

All treatments are administered based on a 28-day cycle (C). Etenta will be administered at the cohort-designated dose starting from C1 Day (D) 4 onwards after one single 2 mg step-up dose on C1D1. If combined with daratumumab, etenta will be initiated as from C1D8, while lenalidomide and carfilzomib will be initiated from C2D1. Etenta may be administered in an outpatient setting if permitted per institutional guidelines; otherwise, pts are hospitalized 24 hours to monitor for cytokine release syndrome after receipt of etenta on C1D1 and C1D4.

The primary endpoint is to determine the dose-limiting toxicities (DLTs) of etenta monotherapy or in combinations. Secondary endpoints include treatment response as assessed by IMWG 2016 criteria. Additional endpoints include safety, PK, and immunogenicity.

**Results**

The platform design of this study enables evaluation of etenta dosing, safety, and efficacy as monotherapy or in combination with current SoC across multiple pt populations to inform future research. Dose level 1 has been cleared for the first 3 substudies.

**Summary/Conclusion**

This trial in progress highlights ongoing efforts to develop novel therapies for MM to improve pt outcomes.

**Copyright Information:** (Online) ISSN: 2572-9241
©2026 The Author(s). HemaSphere is published by John Wiley & Sons Ltd on behalf of European Hematology Association. This is an open access Abstract Book distributed under the Attribution-NonCommercial-NoDerivs (CC BY-NC-ND), which allows third parties to download the articles and share them with others as long as they credit the author and the Abstract Book, but they cannot change the content in any way or use them commercially.
**Abstract Book Citations:** Authors, Title, HemaSphere, 2026;10:(S1):pages. The abstract book DOIs can be found on the “Author Information” page.
**Disclaimer:** Articles published in the journal HemaSphere exclusively reflect the opinions of the authors. The authors are responsible for all content in their abstracts including accuracy of the facts, statements, citing resources, etc.

**Figure.** Study schema and key eligibility criteria

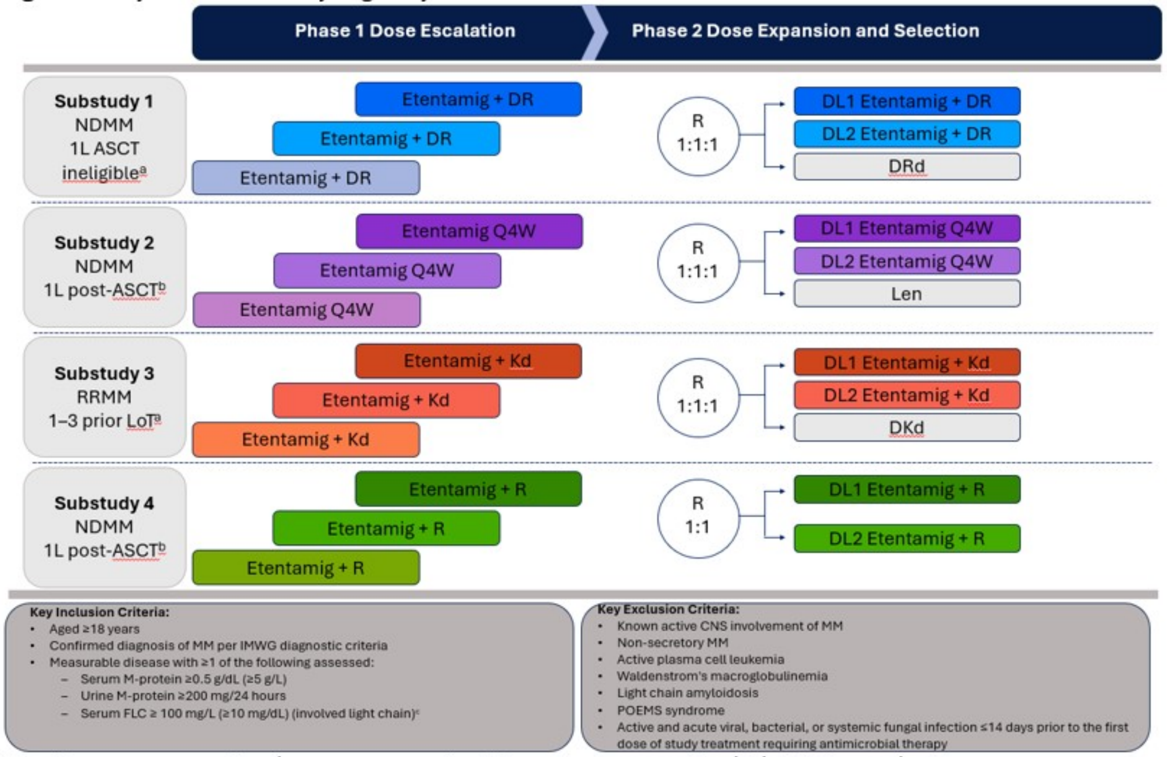

[a]Must have measurable diseases per central laboratory assessed within 28 days prior to enrollment.
[b]Must have measurable disease at the time of diagnosis.
[c]Only for patients without measurable serum or urine M-protein.
For each substudy that has a Phase 1 portion, each cohort will enroll 3–15 patients guided by BOIN design for dose escalation. The phase 2 portion will enroll up to 25 patients per cohort in each substudy.
ASCT, autologous stem cell transplant; CNS, central nervous system; DKd, daratumumab plus carfilzomib plus dexamethasone; DL, dose level; DR, daratumumab plus lenalidomide; DRd, daratumumab plus lenalidomide and dexamethasone; FLC, free light chain; IMWG, International Myeloma Working Group; Kd, carfilzomib plus dexamethasone; LoT, line(s) of therapy; NDMM, newly diagnosed multiple myeloma; POEMS, polyneuropathy, organomegaly, endocrinopathy, monoclonal protein, and skin changes; Q4W, every 4 weeks; R, lenalidomide; RRMM, relapsed/refractory multiple myeloma; SCT, stem cell transplant.

Copyright Information: (Online) ISSN: 2572-9241
©2026 The Author(s). HemaSphere is published by John Wiley & Sons Ltd on behalf of European Hematology Association. This is an open access Abstract Book distributed under the Attribution-NonCommercial-NoDerivs (CC BY-NC-ND), which allows third parties to download the articles and share them with others as long as they credit the author and the Abstract Book, but they cannot change the content in any way or use them commercially.
Abstract Book Citations: Authors, Title, HemaSphere, 2026;10:(S1):pages. The abstract book DOIs can be found on the "Author Information" page.
Disclaimer: Articles published in the journal HemaSphere exclusively reflect the opinions of the authors. The authors are responsible for all content in their abstracts including accuracy of the facts, statements, citing resources, etc.

### (PB3244) LONG-TERM EFFICACY OF TALQUETAMAB IN CHINESE PATIENTS WITH RELAPSED/REFRACTORY MULTIPLE MYELOMA FROM THE PHASE 2 MONUMENTAL-1 STUDY ANALYSIS OF 2-YEAR FOLLOW-UP

**Topic:** 14. Myeloma and other monoclonal gammopathies - Clinical

Gang An*[1], Jie Jin[2], Zhen Cai[2], Hongmei Jing[3], Chengcheng Fu[4], Pengcheng He[5], Zhongjun Xia[6], Qianqian Xu[7], Hongye Chen[8], Xue Gai[8], Dian Zhu[9], Xinchao Luo[9], Paula Barreyro[10], Lugui Qiu[1]

*[1]Institute of Hematology and Blood Diseases Hospital, Chinese Academy of Medical Sciences and Peking Union Medical College, Tianjin, China, Tianjin, China; [2]First Affiliated Hospital of Zhejiang University Medical College, Hangzhou, China, Hangzhou, China; [3]Peking University Third Hospital, Beijing, China, Beijing, China; [4]The First Affiliated Hospital of Soochow University, Suzhou, China, Suzhou, China; [5]First Affiliated Hospital of Xi'an Jiaotong University School of Medicine, Xi'an, China, Xi'an, China; [6]Sun Yat-Sen University Cancer Center, Guangzhou, China, Guangzhou, China; [7]Johnson & Johnson, Beijing, China, Medical Affairs, Beijing, China; [8]Johnson & Johnson, Beijing, China; [9]Johnson & Johnson, Shanghai, China; [10]Johnson & Johnson, Toronto, Canada;*

### Background

Talquetamab (tal) is the first and only approved BsAb targeting novel antigen, GPRC5D. Tal has shown overall response rates of >70% in patients(pts) with relapsed/refractory multiple myeloma (RRMM) in the MonumenTAL-1 study. Pts from China were not included in MonumenTAL-1 global cohort. Previously, China cohort had shown consistent efficacy and safety data with global cohort in a short follow-up time (mFU 9-16 mo).

### Aims

Here we report long-term survival and safety from a long-term follow-up time (mFU 27-34 mo).

### Methods

MonumenTAL-1 is a phase 1/2, open-label, multicenter study of tal monotherapy in RRMM pts. Phase 1 identified 2 RP2Ds, 0.4 mg/kg QW and 0.8 mg/kg Q2W, which were further assessed in phase 2. Chinese pts were enrolled in phase 2. CRS and ICANS were graded by ASTCT criteria; all other AEs were graded by CTCAE v4.03. Response was assessed per IMWG 2016 criteria.

### Results

29 pts were enrolled in 0.4mg/kg QW cohort and 12 pts were enrolled in 0.8mg/kg Q2W cohort. As of Sep 2025, median follow-up (mFU) was 34.4 mo and 27.2 mo respectively. Across cohorts, overall response rate (ORR, 66.7-69.0%) was unchanged vs previous results. Median duration of response (mDOR) and median progression-free-survival (mPFS) continued to show clinical benefit (mDOR: QW 16.6mo, Q2W 20.1mo; mPFS: QW 8.3mo, Q2W 10.7mo). In patients who achieved CR or better, mDOR was not reached in QW cohort (95%CI 16.6-NE) and 20.1mo in Q2W cohort, The median overall survival (OS) was not reached in both cohorts, and 24-mo OS rate was 59.8% and 63.6% respectively. As previously reported, the most common adverse events (AE) were hematologic AEs and CRS, and most common grade 3/4 AE was lymphopenia. With approximately additional 18- month follow-up, there were no new safety signals in both cohorts. No new TEAEs leading to treatment discontinuation were observed, including GPRC5D-associated (oral and dermatologic) AEs and weight loss.

**Copyright Information:** (Online) ISSN: 2572-9241
©2026 The Author(s). HemaSphere is published by John Wiley & Sons Ltd on behalf of European Hematology Association. This is an open access Abstract Book distributed under the Attribution-NonCommercial-NoDerivs (CC BY-NC-ND), which allows third parties to download the articles and share them with others as long as they credit the author and the Abstract Book, but they cannot change the content in any way or use them commercially.
**Abstract Book Citations:** Authors, Title, HemaSphere, 2026;10:(S1):pages. The abstract book DOIs can be found on the "Author Information" page.
**Disclaimer:** Articles published in the journal HemaSphere exclusively reflect the opinions of the authors. The authors are responsible for all content in their abstracts including accuracy of the facts, statements, citing resources, etc.

**Summary/Conclusion**

At an extended long-term follow-up, high ORR elicited by Tal in Chinese heavily pretreated RRMM patients were durable, especially in patients who achieved CR or better, and more than 60% patients stayed alive after 2 years.

Figure--Long-term Efficacy in MonumenTAL-1 China cohort

| Outcome | 0.4mg/kg QW (N=29) | 0.8mg/kg Q2W (N=12) |
|---|---|---|
| mFU, mo | 34.43 | 27.20 |
| mDOR, mo (95% CI) | 16.6 (5.8, NE) | 20.1 (2.8, NE) |
| mDOR in patients with ≥CR, , mo (95% CI) | NE (16.6, NE) | 20.1 (9.2, NE) |
| mPFS, mo (95% CI) | 8.3 (6.3, NE) | 10.7 (2.3, NE) |
| mPFS in patients with ≥CR, , mo (95% CI) | NE (17.9, NE) | 21.2 (10.6, NE) |
| mOS, mo (95% CI) | NE (15.7, NE) | NE (9.7, NE) |
| 24-mo OS rate, % (95% CI) | 59.8% (39.2,75.4) | 63.6% (29.7,84.5) |

**Copyright Information:** (Online) ISSN: 2572-9241
©2026 The Author(s). HemaSphere is published by John Wiley & Sons Ltd on behalf of European Hematology Association. This is an open access Abstract Book distributed under the Attribution-NonCommercial-NoDerivs (CC BY-NC-ND), which allows third parties to download the articles and share them with others as long as they credit the author and the Abstract Book, but they cannot change the content in any way or use them commercially.
**Abstract Book Citations:** Authors, Title, HemaSphere, 2026;10:(S1):pages. The abstract book DOIs can be found on the "Author Information" page.
**Disclaimer:** Articles published in the journal HemaSphere exclusively reflect the opinions of the authors. The authors are responsible for all content in their abstracts including accuracy of the facts, statements, citing resources, etc.

### (PB3246) CLINICAL OBSERVATION OF DV/RBD REGIMEN IN THE TREATMENT OF FIRST-RELAPSED MULTIPLE MYELOMA

**Topic:** 14. Myeloma and other monoclonal gammopathies - Clinical

Min Yang[1, 2], Lingwei Kong[1, 2], Liangshun You[1, 2], Haitao Meng*[1, 2]

[1]*Department of Hematology, The First Affiliated Hospital, College of Medicine, Zhejiang University, Hangzhou, Zhejiang, China;* [2]*Zhejiang Provincial Clinical Research Center for Hematologic Diseases, Hangzhou, Zhejiang, China;*

### Background

Multiple myeloma (MM), a clonal plasma cell malignancy, presents a therapeutic challenge, particularly in selecting therapy for patients with relapsed/refractory MM (RRMM), especially those with EMD. In this population, identifying the optimal regimen is critical. This study investigated the efficacy and safety of the DV/RBD regimen in patients with first-relapsed multiple myeloma espeically for EMD patients. It was designed as a single-center, prospective, exploratory clinical study.

### Aims

The objective for the phase 1 study is to assess the efficacy, safety,of DV/RBD regimen in first-relapsed and refractory MM patients espeically for EMD patients.

### Methods

Eighteen patients with first-relapsed multiple myeloma received chemotherapy with the DV/RBD regimen. Response criteria for multiple myeloma (MM) followed the 2016 International Myeloma Working Group (IMWG) response criteria. Common adverse events were graded according to the WHO "Common Acute and Subacute Toxicity Grading Criteria," and the severity of adverse events (AEs) during clinical treatment was assessed and recorded.

### Results

Among the 18 patients with first-relapsed multiple myeloma treated with the DV/RBD regimen, 15 (83.3%) achieved response, including: 1 (5.5%) sCR, 3 (16.7%) CR, 6 (33.3%) VGPR, 3 (16.7%) PR, 2 (11.1%) MR, and 2 (11.1%) SD, with 1 (5.5%) PD. Among the 5 patients with primary extramedullary multiple myeloma, the response rate reached 80%, including 1 (20%) CR, 2 (40%) PR, 1 (20%) MR, and 1 (20%) PD. Fatigue was the most common non-hematological adverse event, while neutropenia was the most common hematological adverse event. The median PFS was 16 months, and OS was 24 months. For primary EMM, the response rate after DV/RBD treatment was 80%, with a median PFS of 6.7 months and OS of 17.5 months. Conclusion: The DV/RBD regimen demonstrates high efficacy, good tolerability, and acceptable economic cost, and can be considered as an effective treatment option for first-relapsed multiple myeloma, particularly in cases with extramedullary involvement.

### Summary/Conclusion

Treatment with DV/RBD regimen was well tolerated with efficiant anti-myeloma activity.This regimen is expected to improve the prognosis of patients with RRMM especially for first relapse and refractory multiple myeloma.

**Copyright Information:** (Online) ISSN: 2572-9241
©2026 The Author(s). HemaSphere is published by John Wiley & Sons Ltd on behalf of European Hematology Association. This is an open access Abstract Book distributed under the Attribution-NonCommercial-NoDerivs (CC BY-NC-ND), which allows third parties to download the articles and share them with others as long as they credit the author and the Abstract Book, but they cannot change the content in any way or use them commercially.
**Abstract Book Citations:** Authors, Title, HemaSphere, 2026;10:(S1):pages. The abstract book DOIs can be found on the "Author Information" page.
**Disclaimer:** Articles published in the journal HemaSphere exclusively reflect the opinions of the authors. The authors are responsible for all content in their abstracts including accuracy of the facts, statements, citing resources, etc.

### (PB3248) REAL WORLD USE OF MELPHALAN FLUFENAMIDE (MELFLUFEN) IN RELAPSED/REFRACTORY MULTIPLE MYELOMA: PRELIMINARY RESULTS IN 60 PATIENTS FROM THE SPANISH REGISTRY

**Topic:** 14. Myeloma and other monoclonal gammopathies - Clinical

Adrian Alegre Amor[1], David Martínez-Campuzano*[2], Blanca Vegas[1], Brais Lamas[3], Clara Sopeña Pell-Llderton[3], Ana Gálvez Ruiz[3], María José Moreno[4], Valentín Cabañas Perianes[4], Dunia De Miguel Llorente[5], Maria Sanchez[6], Felipe de Arriba de la Fuente[6], Maria Paula Chavez Collazos[7], Xabier Gutierrez Lopez De Ocariz[8], Clara Espinosa Arnandis[9], Adriana Gascón[9], Juan Jose Gil Fernandez[10], Silvina Ríos[11], Pascual Fernandez[11], Maria Esther Clavero Sanchez[12], Alessandra Comai[13], Albert Pérez Montaña[14], Aránzazu Alonso Alonso[15], Ana Lopez De La Guia[16], Pablo Lorente Alegre[17], Aida Calo Pérez[18], Mario Montagud[19], Julio Dávila-Valls[20], Paola Beneit[21], Ricarda Garcia Sanchez[22], Isabel García Cabrera[23], Lucia Núñez[24], Maria Casanova[25], Maria Concepcion Alaez Uson[26], Ana Maria Dios Loureiro[27], Gladys Virginia Ibarra Fernandez[28], Marcos Lorenzo Perez[29], Maria Jesus Blanchard Rodriguez[30], Alfonso García de Coca[31], Natalia Tovar[32], Yolanda Gonzalez Montes[33], Maribel Orts[34], Miguel Paricio[35], Amelia Martinez Marin[36], Beatriz Aguado Bueno[1], Gonzalo Benzo Callejo[1], Mario Arnao[3], Javier De La Rubia Comos[3]

*[1]Hospital de La Princesa, Hematology, Madrid, Spain;[2]Instituto de Investigación Sanitaria La Fe, Valencia, Spain;[3]Hospital Universitario y Politécnico La Fe, Instituto de Investigación Sanitaria La Fe, Valencia, Spain; [4]Hospital Clínico Universitario Virgen de la Arricaxa, Hematology, Murcia, Spain; [5]Hospital Universitario de Guadalajara, Hematology, Guadalajara, Spain; [6]Hospital General Universitario Morales Meseguer, Hematology, Murcia, Spain; [7]Hosp. Universitario De Cabueñes, Hematology, Gijón, Spain; [8]Hospital de Txagorritxu, Hematology, Vitoria, Spain;[9]Hospital General Universitario de Castellón, Hematology, Castelló de la Plana, Spain; [10]Hospital Universitario Príncipe de Asturias, Hematology, Madrid, Spain; [11]Hospital General Universitario Dr. Balmis, Hematology, Alicante, Spain; [12]Hospital Virgen de las Nieves, Hematology, Granada, Spain;[13]Institut Català d'Oncologia (ICO) Hospital Duran i Reynals, Hematology, L´Hospitalet de Llobregat, Spain; [14]Hospital Son Espases, Hematology, Palma de Mallorca, Spain; [15]Hospital Quirón Madrid Juan Bravo, Hematology, Madrid, Spain;[16]Hospital Universitario La Paz, Hematology, Madrid, Spain; [17]Hospital Arnau de Vilanova de Valencia, hematology, Valencia, Spain;[18]Hospital del Henares, Hematology, Madrid, Spain; [19]Hospital de Vinaròs, Castellón, Spain;[20]Hospital Nuestra Señora de Sonsoles, Hematology, Ávila, Spain; [21]H.C.U. Sant Joan d'Alacant, Alicante, Spain;[22]Hospital Clínico de la Victoria Málaga, Hematology, Málaga, Spain;[23]Hospital Universitario Clínico San Cecilio, Hematology, Granada, Spain; [24]Hospital General Universitario de Ciudad Real, Hematology, Ciudad Real, Spain; [25]Hospital Costa del Sol, Hematology, Málaga, Spain;[26]Hospital HLA Moncloa., Hematology, Madrid, Spain; [27]Complejo Hospitalario Universitario de Pontevedra, Hematology, Pontevedra, Spain;[28]Ico Badalona, Hematology, Badalona, Spain; [29]Complexo Hospitalario Universitario de Vigo, Hematology, Vigo, Spain;[30]Hospital Universitario Ramón y Cajal, Hematology, Madrid, Spain; [31]Hospital Clínico de Valladolid, Hematology, Valladolid, Spain;[32]Hospital Clínic de Barcelona, Hematology, Barcelona, Spain; [33]Ico Girona, Hematology, Girona, Spain;[34]Hospital de Sagunto, Hematology, Valencia, Spain; [35]Hospital Universitario Lozano Blesa, Hematology, Zaragoza, Spain;[36]Hospital Universitario Vega Baja, Orihuela, Spain;*

### Background

Melphalan fluflenamide (Melflufen) is a peptide-conjugated drug that combined with dexamethasone (Melf-dex) is approved for patients with relapsed/refractory multiple myeloma (RRMM) after ≥3 prior lines of therapy. However, real-world evidence remains limited to small cohorts and data on patients pre-treated with novel immunotherapies are lacking.

### Aims

To evaluate the outcomes of Melf-dex use in patients with RRMM in the real-world setting from the Spanish Registry.

**Copyright Information:** (Online) ISSN: 2572-9241
©2026 The Author(s). HemaSphere is published by John Wiley & Sons Ltd on behalf of European Hematology Association. This is an open access Abstract Book distributed under the Attribution-NonCommercial-NoDerivs (CC BY-NC-ND), which allows third parties to download the articles and share them with others as long as they credit the author and the Abstract Book, but they cannot change the content in any way or use them commercially.
**Abstract Book Citations:** Authors, Title, HemaSphere, 2026;10:(S1):pages. The abstract book DOIs can be found on the “Author Information” page.
**Disclaimer:** Articles published in the journal HemaSphere exclusively reflect the opinions of the authors. The authors are responsible for all content in their abstracts including accuracy of the facts, statements, citing resources, etc.

### Methods

We included patients with RRMM from 35 centers in Spain treated with at least one cycle of Melf-dex in the real-world setting between 2021-2025. Medical records were reviewed using the REDCap platform. The study was approved by the CEIC from the coordinating center.

### Results

Sixty patients were included in the analysis. Thirty-nine out of 60 (65%) were females and the median age was 71 years (range, 60-95). The median time from diagnosis to treatment with Melf-dex was 68 months (12-249) and patients had received a median of 4 prior lines of therapy (range, 4-13). Fifty-seven (95%) and 34 (57%) were triple and penta-class refractory, respectively. Twenty-two (37%) patients presented with extramedullary disease and 21 (35%) had high-risk cytogenetics, including seven with ≥2 high-risk abnormalities. Thirty patients had previously received at least one anti-BCMA therapy and 14 (23%) anti-GPRC5D therapy, including 9 cases of dual exposure. A total of five patients (8%) had received CAR T-cell therapy. Median cycles of Melf-dex were 3 (range, 1-10) Overall response rate was 40% (24/60) in the total cohort and 45% (15/33) in the immunotherapy-exposed subgroup. A CR was achieved in two patients, including one receiving prior immunotherapy. In the immunotherapy-exposed vs non-exposed groups, the VGPR rate was 7% vs 6%, PR 30% vs 36%, and stable disease 28% vs 21%, respectively. With a median follow-up of 8.97 months (95% CI, 6.41-9.99), the median PFS of 4.27 months (95% CI, 2.73-7.36) and the median OS of 12.35 months (95% CI, 8.77-NR). Two patients received Melf-dex as a bridging therapy before CAR T-cell therapy, achieving PR. The most common adverse events observed were hematologic: thrombocytopenia (62%), neutropenia (47%), and anemia (42%). Supportive care included G-CSF in 27 patients (45%), EPO in 18 (30%), TPO agonists in 9 (15%), and platelet and red blood cell transfusions in 11 (18%) and 13 (22%), respectively. Infections were reported in 23 (38%), including 10 ≥ grade 3 cases. A total of 16 (27%) patients remain alive and continue treatment after a median of 6 months (range, 1-10), 11 of them with PR or better. Twenty-three patients (38%) died during follow-up, mainly due to disease progression.

### Summary/Conclusion

This is the largest report to date on the use of melflufen in patients with RRMM in the real-world setting. Although the retrospective nature of the study limits the strength of the findings, our data suggest that this regimen is a safe and effective option for these challenging patients, including those previously exposed to immunotherapies.

**Copyright Information:** (Online) ISSN: 2572-9241
©2026 The Author(s). HemaSphere is published by John Wiley & Sons Ltd on behalf of European Hematology Association. This is an open access Abstract Book distributed under the Attribution-NonCommercial-NoDerivs (CC BY-NC-ND), which allows third parties to download the articles and share them with others as long as they credit the author and the Abstract Book, but they cannot change the content in any way or use them commercially.
**Abstract Book Citations:** Authors, Title, HemaSphere, 2026;10:(S1):pages. The abstract book DOIs can be found on the “Author Information” page.
**Disclaimer:** Articles published in the journal HemaSphere exclusively reflect the opinions of the authors. The authors are responsible for all content in their abstracts including accuracy of the facts, statements, citing resources, etc.

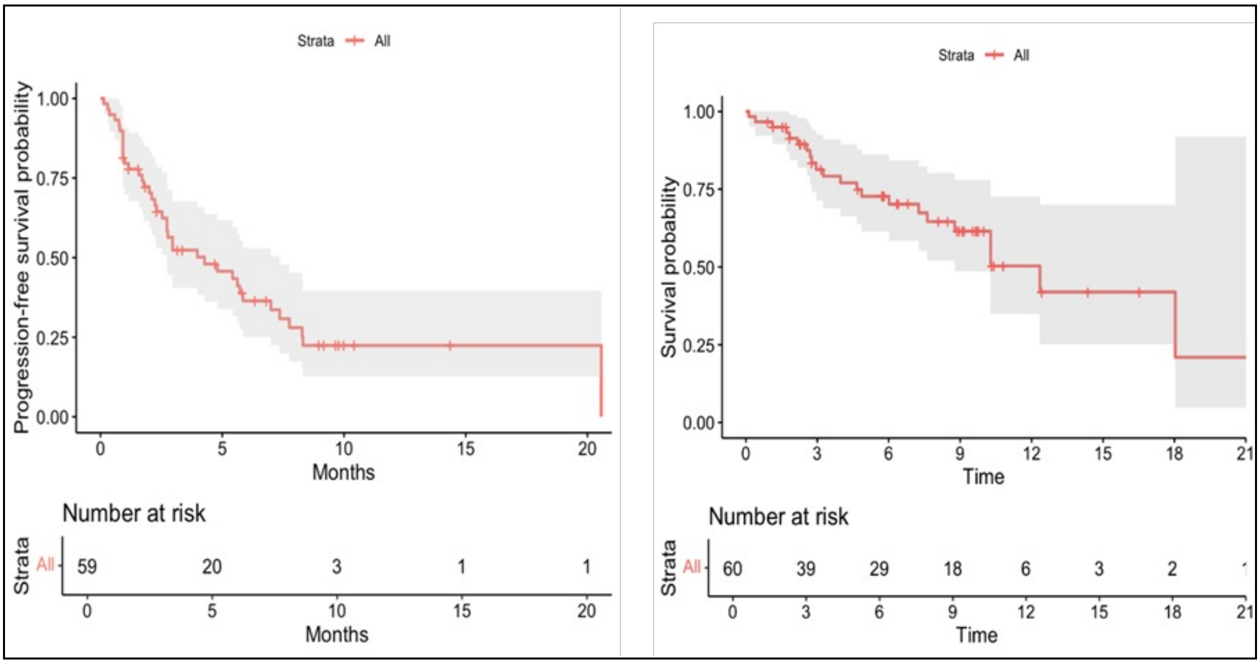

Figure 1. Progression-free survival (left) and overall free-survival (right) in the total cohort

**Copyright Information:** (Online) ISSN: 2572-9241
©2026 The Author(s). HemaSphere is published by John Wiley & Sons Ltd on behalf of European Hematology Association. This is an open access Abstract Book distributed under the Attribution-NonCommercial-NoDerivs (CC BY-NC-ND), which allows third parties to download the articles and share them with others as long as they credit the author and the Abstract Book, but they cannot change the content in any way or use them commercially.
**Abstract Book Citations:** Authors, Title, HemaSphere, 2026;10:(S1):pages. The abstract book DOIs can be found on the "Author Information" page.
**Disclaimer:** Articles published in the journal HemaSphere exclusively reflect the opinions of the authors. The authors are responsible for all content in their abstracts including accuracy of the facts, statements, citing resources, etc.

### (PB3249) REGIONAL COMPARISONS OF REAL-WORLD OUTCOMES FOLLOWING LENALIDOMIDE AND ANTI-CD38 EXPOSURE IN RELAPSED/REFRACTORY MULTIPLE MYELOMA: A PREAMBLE REGISTRY ANALYSIS IN THE US AND EUROPE

**Topic:** 14. Myeloma and other monoclonal gammopathies - Clinical

Karthik Ramasamy*[1], Claudio Cerchione[2], Kamaraj Karunanithi[3], Amol Dhamane[4], Amir Samuel[5], Jessica Katz[4], Teofilia Acheampong[4], Amanda Crosbie[4], Katie Gravagna[4], David J. Kuter[6], Ravi Vij[7]

*[1]Oxford University Hospitals NHS Trust, Oxford, United Kingdom;[2]Istituto Romagnolo per lo Studio dei Tumori "Dino Amadori" - IRST IRCCS, Meldola, Italy; [3]University Hospitals of North Midlands NHS Trust, Stoke-on-Trent, United Kingdom;[4]Bristol Myers Squibb, Princeton, United States of America; [5]Celgene International Sàrl, a Bristol-Myers Squibb Company, Boudry, Switzerland; [6]Massachusetts General Hospital, Harvard Medical School, Boston, United States of America;[7]Washington University School of Medicine, St. Louis, United States of America;*

### Background

Patients (pts) with relapsed/refractory multiple myeloma (RRMM) with prior lenalidomide (LEN) and anti-CD38 antibody exposure have poor outcomes. PREAMBLE (NCT01838512) is a multinational real-world (RW) registry capturing treatment patterns and clinical outcomes in pts with MM across US, Canada, and Europe (France, Germany, Italy, UK). Comparing RW data between regions may help to understand RRMM clinical trial population differences globally and heterogeneity in therapy access.

### Aims

To compare demographics, clinical characteristics, treatment patterns, progression-free survival (PFS), and overall survival (OS) between pts from US and Europe with RRMM in PREAMBLE who received ≥1 post-LEN/anti-CD38 therapy.

### Methods

Adults (≥18 yrs) with RRMM, prior LEN and anti-CD38 exposure, and ≥1 subsequent line (2016-2025) were included; initiation of subsequent line defined index date. Baseline characteristics and regimens were summarized by region. PFS/OS were estimated from index using Kaplan–Meier methods. Regional differences were assessed using multivariable Cox models adjusted for baseline covariates.

### Results

Among 346 pts (US n=122; Europe n=224), median age was 70 years, 58% were male, 23% had high-risk cytogenetics, and 49% had prior autologous transplant. Baseline characteristics were similar across regions. Index regimens were heterogenous overall; proteasome inhibitors (e.g. carfilzomib ± dexamethasone [dex], 9.8%) and immunomodulatory agents (e.g. pomalidomide + elotuzumab ± dex, 6.1%) were most frequently observed. Most pts (89%) were triple-class exposed before index. Median PFS was 5.4 months overall, with no difference between US and Europe (5.1 vs 5.8 months; p=0.87); median OS was also comparable (26.1 vs 25.9 months; p=0.75) between regions. In adjusted Cox models (**Table**), region was not associated with PFS (HR 1.00, 95% CI 0.76–1.31; p=0.99) or OS (HR 1.12, 95% CI 0.78–1.61; p=0.53). Multivariable analyses yielded consistent results over a more recent 2020–2025 period.

**Copyright Information:** (Online) ISSN: 2572-9241
©2026 The Author(s). HemaSphere is published by John Wiley & Sons Ltd on behalf of European Hematology Association. This is an open access Abstract Book distributed under the Attribution-NonCommercial-NoDerivs (CC BY-NC-ND), which allows third parties to download the articles and share them with others as long as they credit the author and the Abstract Book, but they cannot change the content in any way or use them commercially.
**Abstract Book Citations:** Authors, Title, HemaSphere, 2026;10:(S1):pages. The abstract book DOIs can be found on the "Author Information" page.
**Disclaimer:** Articles published in the journal HemaSphere exclusively reflect the opinions of the authors. The authors are responsible for all content in their abstracts including accuracy of the facts, statements, citing resources, etc.

**Summary/Conclusion**

In this RW RRMM cohort with prior LEN/anti-CD38 exposure, outcomes remain poor, highlighting the ongoing unmet need for more effective post-LEN/anti-CD38 treatment options. No clinically meaningful differences in PFS/OS were observed between US and European pts after adjustment for baseline characteristics.

**Table: Adjusted HRs for PFS and OS from Multivariable Cox Models (2016-2025)[a]**

| Variable | Category | PFS HR (95% CI) | PFS P-value[c] | OS HR (95% CI) | OS P-value[c] |
|---|---|---|---|---|---|
| Region (ref=US) | Europe | 1.00 (0.76-1.31) | 0.9908 | 1.12 (0.78-1.61) | 0.5286 |
| Age at index (ref=≤70 yrs) | >70 yrs | 0.85 (0.65-1.10) | 0.2172 | 1.12 (0.79-1.59) | 0.5175 |
| Sex (ref=Male) | Female | 0.95 (0.74-1.22) | 0.6716 | 1.02 (0.72-1.43) | 0.9311 |
| International Stage System stage (ref=Stage I) | II | 1.02 (0.65-1.59) | 0.8611 | 1.71 (0.90-3.27) | 0.4271 |
| | III | 1.17 (0.78-1.76) | | 1.45 (0.78-2.71) | |
| | Unknown | 1.08 (0.75-1.56) | | 1.51 (0.85-2.70) | |
| Cytogenetic high risk (ref=No)[b] | Yes | 1.25 (0.94-1.67) | 0.1239 | 1.30 (0.88-1.91) | 0.1907 |
| Triple-class exposed (ref=No) | Yes | 1.23 (0.74-2.05) | 0.4207 | 2.32 (0.91-5.88) | 0.0775 |
| Number of prior lines (ref=1) | 2 | 0.94 (0.53-1.67) | 0.5736 | 1.85 (0.66-5.19) | 0.5789 |
| | 3 | 1.14 (0.63-2.05) | | 2.08 (0.73-5.92) | |
| | 4+ | 1.20 (0.67-2.14) | | 2.01 (0.71-5.71) | |
| | Unknown | 1.35 (0.66-2.76) | | 2.69 (0.81-8.93) | |

[a]Separate multivariable Cox models were fit for PFS and OS; each model included all baseline covariates listed. [b]Defined as any of Revised-International Staging System III, t(4;14), t(14;16), t(14;20), del(17p), TP53 abnormality. [c]p-values ≤0.05 were deemed significant. CI, confidence interval; HR, hazard ratio; OS, overall survival; PFS, progression-free survival; ref, reference.

Copyright Information: (Online) ISSN: 2572-9241
©2026 The Author(s). HemaSphere is published by John Wiley & Sons Ltd on behalf of European Hematology Association. This is an open access Abstract Book distributed under the Attribution-NonCommercial-NoDerivs (CC BY-NC-ND), which allows third parties to download the articles and share them with others as long as they credit the author and the Abstract Book, but they cannot change the content in any way or use them commercially.
Abstract Book Citations: Authors, Title, HemaSphere, 2026;10:(S1):pages. The abstract book DOIs can be found on the "Author Information" page.
Disclaimer: Articles published in the journal HemaSphere exclusively reflect the opinions of the authors. The authors are responsible for all content in their abstracts including accuracy of the facts, statements, citing resources, etc.

### (PB3250) OCULAR TOXICITIES OF BELANTAMAB MAFODOTIN MONOTHERAPY IN RELAPSED/REFRACTORY MULTIPLE MYELOMA: A SYSTEMATIC REVIEW AND META-ANALYSIS

**Topic:** 14. Myeloma and other monoclonal gammopathies - Clinical

Mehak Dang*[1], Harkirat Kaur Sandhu[2], Hosana Candreva[3], Abdrabo Gamal Motawea[4], Ayodeji Arobo[5], Fernando Gibran-Nunes[6], Fahad Alabbas[7]

*[1]JSS Medical College, Mysore, India;[2]Punjab institute of medical science, Jalandhar, India; [3]Department of Medicine, Rio de Janeiro State University, Department of Medicine, Rio de Janeiro, Brazil; [4]Faculty of Medicine, Menoufia University, menoufia, Egypt; [5]Faculty of Dentistry, Obafemi Awolowo University, Ile-Ife, Nigeria;[6]Hematology, BMT and Cell Therapy Department-Hospital Sírio-Libanês, São Paulo, Brazil; [7]Prince Sultan Military Medical City, Riyadh, Saudi Arabia;*

### Background

Belantamab mafodotin, a BCMA-targeting antibody–drug conjugate, has demonstrated clinically meaningful responses in heavily pretreated relapsed/refractory multiple myeloma (RRMM). However, corneal adverse events have emerged as a dose-limiting toxicity and a major barrier to sustained treatment exposure. A comprehensive pooled evaluation of efficacy and ocular safety across dosing strategies remains lacking.

### Aims

To quantify pooled efficacy outcomes and characterize the incidence, severity, and dose-dependence of ocular toxicities associated with belantamab mafodotin monotherapy in RRMM.

### Methods

We conducted this systematic review and meta-analysis according to the Cochrane Handbook for Systematic Reviews of Interventions and PRISMA guidelines and prospectively registered the protocol in PROSPERO (CRD420261304280). A systematic literature search was performed across PubMed, Embase, and Cochrane Library from inception to January 2026. We included randomized controlled trials and observational studies that evaluated belantamab mafodotin monotherapy in adults with relapsed/refractory multiple myeloma. The outcomes assessed were Overall Response Rate (ORR), Complete Response (CR), Very Good Partial Response(VGPR), Partial Response (PR), Stable Disease (SD), and ocular toxicity. We pooled proportions using random-effects models with logit transformation and calculated 95% confidence intervals. We quantified heterogeneity using the $I^2$ statistic. Pre-specified subgroup analyses were conducted to evaluate the efficacy of belantamab mafodotin in triple- and penta-refractory disease and to assess dose-dependent differences in efficacy and ocular toxicity between 2.5 mg/kg and 3.4 mg/kg.

**Copyright Information:** (Online) ISSN: 2572-9241
©2026 The Author(s). HemaSphere is published by John Wiley & Sons Ltd on behalf of European Hematology Association. This is an open access Abstract Book distributed under the Attribution-NonCommercial-NoDerivs (CC BY-NC-ND), which allows third parties to download the articles and share them with others as long as they credit the author and the Abstract Book, but they cannot change the content in any way or use them commercially.
**Abstract Book Citations:** Authors, Title, HemaSphere, 2026;10:(S1):pages. The abstract book DOIs can be found on the "Author Information" page.
**Disclaimer:** Articles published in the journal HemaSphere exclusively reflect the opinions of the authors. The authors are responsible for all content in their abstracts including accuracy of the facts, statements, citing resources, etc.

### Results

Seventeen studies, including 1,621 patients, were analyzed (3 RCTs and 14 observational studies). The pooled overall response rate (ORR) was 43.5% (95% CI 38.8–48.3; $I^2 = 58.2\%$). Complete response was 10.19% (95% CI 7.93–13.0; $I^2 = 37.7\%$), very good partial response 18.54% (95% CI 14.31–23.69; $I^2 = 71.5\%$), and stable disease 24.37% (95% CI 21.16–27.90; $I^2 = 9.2\%$).
The pooled incidence of any-grade ocular toxicity was 64.70% (95% CI 53.02–74.86; $I^2 = 81.6\%$), and ≥grade 3 ocular events occurred in 18.21% (95% CI 8.10–35.99; $I^2 = 91.8\%$). Keratopathy was the most frequent manifestation (54.31%), followed by blurred vision (29.65%). Ocular toxicity led to dose delays in 47.04%, dose reductions in 33.08%, and permanent discontinuation in 16.32% (95% CI 8.37–29.41; $I^2 = 93.6\%$).

ORR was similar in triple-refractory 43.11% (95% CI 35.08-51.52; $I^2$=74.5%) and penta-refractory disease 44.14%(95% CI 23.49-67.03; $I^2$=83.8%), with no statistically significant difference between the subgroups (P = 0.93). Dose-dependent analysis showed numerically higher ORR at 3.4 mg/kg (46.03%) versus 2.5 mg/kg (42.65%), with increased ocular toxicity at the higher dose.

### Summary/Conclusion

Belantamab mafodotin demonstrates consistent antimyeloma activity in heavily pretreated RRMM, including triple- and penta-refractory populations. However, ocular toxicity is frequent and clinically impactful, with substantial treatment modification rates. The high heterogeneity and predominance of non-randomized data warrant cautious interpretation. Future prospective studies should refine dose optimization strategies and clarify the positioning of belantamab among emerging BCMA-targeted therapies.

### Pooled Outcomes of Belantamab Mafodotin for Multiple Myeloma

**Table 1a. Incidence of Ocular Adverse Events by Belantamab Mafodotin Dose**

| Ocular Adverse Event | Any Grade | | ≥ Grade 3 | |
|---|---|---|---|---|
| | 2.5 mg/kg | 3.4 mg/kg | 2.5 mg/kg | 3.4 mg/kg |
| Any Ocular Toxicity | **58.14%** (N = 952) | **82.95%** (N = 127) | **23.79%** (N = 586) | NA |
| Keratopathy | **52.88%** (N = 1,133) | **73.39%** (N = 162) | **18.40%** (N = 1,021) | **24.05%** (N= 127) |
| Decreased Visual Acuity | **11.72%** (N = 763) | **4.31%** (N = 162) | **4.92%** (N = 375) | **5.16%** (N = 39) |
| Blurred Vision | **30.86%** (N = 708) | **40.77%** (N = 162) | **5.18%** (N = 555) | **5.16%** (N = 39) |

**Table 1b. Efficacy Outcomes by Belantmab Mafodotin Dose**

| Outcome | 2.5 mg/kg | 3.4 mg/kg |
|---|---|---|
| Overall Response Rate | **42.65%** (95% CI, 37.32-48.15, N = 1,115) | **46.03%** (95% CI, 31.98-60.74, N = 163) |
| Very Good Partial Response | **17.24%** (95% CI, 12.40-23.46, N = 1,115) | **25.61%** (95% CI, 15.57-39.14, N = 163) |
| Stable Disease | **26.00%** (95% CI, 22.36-30.01, N = 574) | **20.78%** (95% CI, 14.60-28.71, N = 128) |

**NA:** Not Available; **N:** Number of Patients Pooled for the Outcome

Table 1- Pooled Outcomes of Belantamab Mafodotin for Multiple Myeloma stratified by dosage

**Copyright Information:** (Online) ISSN: 2572-9241
©2026 The Author(s). HemaSphere is published by John Wiley & Sons Ltd on behalf of European Hematology Association. This is an open access Abstract Book distributed under the Attribution-NonCommercial-NoDerivs (CC BY-NC-ND), which allows third parties to download the articles and share them with others as long as they credit the author and the Abstract Book, but they cannot change the content in any way or use them commercially.
**Abstract Book Citations:** Authors, Title, HemaSphere, 2026;10:(S1):pages. The abstract book DOIs can be found on the "Author Information" page.
**Disclaimer:** Articles published in the journal HemaSphere exclusively reflect the opinions of the authors. The authors are responsible for all content in their abstracts including accuracy of the facts, statements, citing resources, etc.

### (PB3251) ELRANATAMAB (ELRA) SAFETY DURING STEP-UP DOSING (SUD) BY CARE SETTING IN RELAPSED AND REFRACTORY MULTIPLE MYELOMA (RRMM)

**Topic:** 14. Myeloma and other monoclonal gammopathies - Clinical

Aaron Rosenberg*[1], Rahul Banerjee[2], Chai Kim[3], David Hughes[4], Guido Nador[5], Rickard Sandin[6], Jenna Collins[7], Trong Le[7], Amy Pierre[7], Ayush Kris[7], Yong Chen[3]

*[1]UC Davis Comprehensive Cancer Center, Sacramento, United States of America;[2]Fred Hutchinson Cancer Center, Seattle, United States of America; [3]Pfizer Inc, New York, United States of America;[4]Pfizer Inc, Cambridge, United States of America; [5]Pfizer Ltd, Surrey, United Kingdom;[6]Pfizer AB, Stockholm, Sweden;[7]Flatiron Health, New York, United States of America;*

### Background

ELRA is initiated with SUD to help mitigate immune-mediated adverse events, including cytokine release syndrome (CRS) and neurologic toxicity (NT), including immune effector cell–associated neurotoxicity syndrome (ICANS). Limited real-world (RW) data have compared SUD safety across inpatient versus outpatient care settings.

### Aims

To describe CRS and NT safety outcomes during ELRA SUD overall and between inpatient and outpatient settings.

### Methods

This retrospective, observational analysis used data from the Flatiron Health Database. Adult US patients (≥18 years) with RRMM and triple-class refractory initiating ELRA between 08/2023 and 07/2025 were included. Safety was evaluated across two dose periods —SUD 1 and SUD 2—and patients were categorized by care setting for each period.
Demographic, clinical characteristics, and safety outcomes, including CRS and NT, which includes ICANS, were summarized descriptively by incidence, grade, time to onset, resolution, and treatment. CRS and NT incidence reflects events during the index ELRA, stratified by—but not specific to—SUD period (Table).

**Copyright Information:** (Online) ISSN: 2572-9241
©2026 The Author(s). HemaSphere is published by John Wiley & Sons Ltd on behalf of European Hematology Association. This is an open access Abstract Book distributed under the Attribution-NonCommercial-NoDerivs (CC BY-NC-ND), which allows third parties to download the articles and share them with others as long as they credit the author and the Abstract Book, but they cannot change the content in any way or use them commercially.
**Abstract Book Citations:** Authors, Title, HemaSphere, 2026;10:(S1):pages. The abstract book DOIs can be found on the “Author Information” page.
**Disclaimer:** Articles published in the journal HemaSphere exclusively reflect the opinions of the authors. The authors are responsible for all content in their abstracts including accuracy of the facts, statements, citing resources, etc.

### Results

Among N=115 ELRA treated patients, 45% were male, median age was 71 years (37% ≥75 years), and median time from diagnosis to ELRA initiation was 7.2 years (IQR, 4.3–10.3), with median follow-up of 11.5 months (IQR, 6.4–15.6). Patients were treated in academic (35%), community (33%), or mixed academic–community (32%) settings.The most common sequence was inpatient SUD1–SUD2 (N=79, 69%), followed by fully outpatient SUD (N=26, 23%).
CRS and NT occurred in 35 (30%) and 15 (13%) of patients, respectively, with comparable incidence across care settings (Table). Events were predominately Grade 1-2 (CRS, N=33/115 [29%]; NT, N=11/115 [10%]). Grade ≥3 events were uncommon, with one Grade 3 CRS (outpatient), and two Grade 3–4 NT (inpatients). One CRS and two NT cases had missing grade. No Grade ≥3 CRS occurred in inpatients or NT in outpatients.
CRS and NT occurred early after ELRA initiation (median time to onset: 2.0 and 4.0 days, respectively) with comparable timing across care settings (Table). Resolution was documented for most CRS and NT events (N=34, 97% and N=12, 80%, respectively); two NT cases had ongoing resolution at last follow-up, and one NT case had no documented resolution status. Median time to resolution was short (CRS, 2.0 days [IQR, 1–5]; NT, 2.5 days [IQR, 1–11]).
Most patients received treatment for CRS (N=31, 89%) and NT (N=13, 87%), most commonly dexamethasone (CRS, 49%; NT, 80%), with tocilizumab used in 34% and 27%, respectively. No treatment CRS or NT -related discontinuations or deaths were documented.

**Table**. CRS and NT Incidence and Time to Onset

| | Inpatient SUD 1 (N=83) | Inpatient SUD 2 (N=80) | Outpatient SUD 1 (N=30) | Outpatient SUD 2 (N=26) | Overall (N=115)* |
|---|---|---|---|---|---|
| CRS, n (%) | 26 (31) | 26 (33) | 9 (30) | 7 (27) | 35 (30) |
| Time to CRS, median (IQR), days | 2.5 (2-6) | 2 (1-6) | 2 (1-2) | 2 (1-2) | 2 (1-4) |
| NT, n (%) | 11 (13) | 10 (13) | 4 (13) | 4 (15) | 15 (13) |
| Time to NT, median (IQR), days | 4 (2-6) | 3 (2-6) | 2.5 (1-18) | 2.5 (1-18) | 4 (1-6) |

*Note: N=2 patients with “not documented” care setting

### Summary/Conclusion

ELRA safety outcomes following SUD were comparable across inpatient and outpatient settings, with onset and resolution in line with MM-3; CRS incidence was numerically lower, while NT reflected broader real-world capture of neurologic toxicity and ICANS, supporting a practice shift toward outpatient ELRA SUD as a safe and feasible approach.

**Copyright Information:** (Online) ISSN: 2572-9241
©2026 The Author(s). HemaSphere is published by John Wiley & Sons Ltd on behalf of European Hematology Association. This is an open access Abstract Book distributed under the Attribution-NonCommercial-NoDerivs (CC BY-NC-ND), which allows third parties to download the articles and share them with others as long as they credit the author and the Abstract Book, but they cannot change the content in any way or use them commercially.
**Abstract Book Citations:** Authors, Title, HemaSphere, 2026;10:(S1):pages. The abstract book DOIs can be found on the “Author Information” page.
**Disclaimer:** Articles published in the journal HemaSphere exclusively reflect the opinions of the authors. The authors are responsible for all content in their abstracts including accuracy of the facts, statements, citing resources, etc.

### (PB3252) ANITOCABTAGENE AUTOLEUCEL CLINICAL TRIAL MANUFACTURING EXPERIENCE IN PATIENTS WITH RELAPSED/REFRACTORY OR NEWLY DIAGNOSED MULTIPLE MYELOMA

**Topic:** 14. Myeloma and other monoclonal gammopathies - Clinical

Krina Patel[1], Clare Spooner[2], Monique Giordana[2], Taryn Shuler[2], David Myers[2], María-Victoria Mateos*[3]

*[1]The University of Texas MD Anderson Cancer Center, Houston, TX, United States of America;[2]Kite, a Gilead Company, Santa Monica, CA, United States of America; [3]University Hospital of Salamanca/IBSAL/CIC/CIBERONC, Salamanca, Spain;*

### Background

Anitocabtagene autoleucel (anito-cel) is an autologous D-Domain–based anti-BCMA chimeric antigen receptor (CAR) T-cell therapy that has demonstrated deep and durable efficacy and manageable safety in a Phase 1 study and a Phase 2 registrational study (iMMagine-1) in 4L+ relapsed/refractory multiple myeloma (RRMM; Bishop, et al. ASH 2024; Patel, et al. ASH 2025). The D-Domain has a fast off-rate that facilitates high transduction efficiency and CAR expression, decreases risk of tonic signaling, and has potential for enhanced manufacturing efficiency and optimal tumor cytotoxicity. Anito-cel is also being investigated in patients with RRMM with 1-3 prior therapies and in patients with newly diagnosed multiple myeloma (NDMM). The anito-cel CAR T-cell therapy manufacturing process has been optimized by leveraging the learnings from the robust development process of another CAR T-cell therapy, axicabtagene ciloleucel (axi-cel). Axi-cel, an autologous anti-CD19 CAR T-cell therapy approved to treat patients with 2L large B-cell lymphoma based on the ZUMA-7 trial, has demonstrated a high manufacturing success rate (MSR) in both clinical trial and commercial real-world settings (100% and 99%, respectively; Alquist, et al. TCT 2024).

### Aims

To report initial anito-cel manufacturing experience from 2 clinical trials in patients with RRMM or NDMM.

### Methods

Pooled manufacturing outcomes from adults with RRMM or NDMM enrolled in iMMagine-3 or GEM-AnitoFIRST who underwent leukapheresis from 09/2024 to 10/2025 were included. First pass (FP)-MSR was defined as anito-cel lots manufactured on first attempt and within specification per total first attempt lots dispositioned plus lots not dispositioned due to termination in the period (excluding those terminated for patient withdrawal). The median turnaround time (mTAT) was defined as the time from leukapheresis to the quality release of final anito-cel product.

### Results

There was a total of 104 patients who underwent leukapheresis and whose lots were dispositioned as of October 31, 2025; anito-cel was successfully manufactured for 100% of these patients. The FP-MSR was 99.0% with 1/102 lots rejected on disposition (Table). The global mTAT was 18 days (interquartile range [IQR], 17-20 days) for the 101 lots released. Updated data to be presented.

**Copyright Information:** (Online) ISSN: 2572-9241
©2026 The Author(s). HemaSphere is published by John Wiley & Sons Ltd on behalf of European Hematology Association. This is an open access Abstract Book distributed under the Attribution-NonCommercial-NoDerivs (CC BY-NC-ND), which allows third parties to download the articles and share them with others as long as they credit the author and the Abstract Book, but they cannot change the content in any way or use them commercially.
**Abstract Book Citations:** Authors, Title, HemaSphere, 2026;10:(S1):pages. The abstract book DOIs can be found on the “Author Information” page.
**Disclaimer:** Articles published in the journal HemaSphere exclusively reflect the opinions of the authors. The authors are responsible for all content in their abstracts including accuracy of the facts, statements, citing resources, etc.

**Summary/Conclusion**

Our results demonstrate rapid and reliable anito-cel manufacturing for MM clinical trials globally, with high manufacturing success rates and consistent turnaround times. These results are consistent with axi-cel manufacturing outcomes and highlight the importance of leveraging axi-cel manufacturing experience in the development of anito-cel.

© 2026 American Society of Clinical Oncology, Inc. Reused with permission. This abstract was accepted and previously presented at the 2026 ASCO Annual Meeting. All rights reserved.

**Table**

| | |
|---|---|
| **FP-MSR, n/N (%)** | 101/102 (99.0) |
| **Global mTAT, days (IQR)** | 18 (17-20) |
| **Lots released, n** | 101 |

**Copyright Information:** (Online) ISSN: 2572-9241
©2026 The Author(s). HemaSphere is published by John Wiley & Sons Ltd on behalf of European Hematology Association. This is an open access Abstract Book distributed under the Attribution-NonCommercial-NoDerivs (CC BY-NC-ND), which allows third parties to download the articles and share them with others as long as they credit the author and the Abstract Book, but they cannot change the content in any way or use them commercially.
**Abstract Book Citations:** Authors, Title, HemaSphere, 2026;10:(S1):pages. The abstract book DOIs can be found on the "Author Information" page.
**Disclaimer:** Articles published in the journal HemaSphere exclusively reflect the opinions of the authors. The authors are responsible for all content in their abstracts including accuracy of the facts, statements, citing resources, etc.

### (PB3253) PATIENT CHARACTERISTICS AND INITIAL REAL-WORLD DOSING EXPERIENCE WITH BELANTAMAB MAFODOTIN-BASED COMBINATIONS FOR RELAPSED/REFRACTORY MULTIPLE MYELOMA

**Topic:** 14. Myeloma and other monoclonal gammopathies - Clinical

Elena Zamagni*[1], Vania Hungria[2], John Hartman[3], Omar Ladhani[3], Sandhya Sapra[3], Giancarlo Lorenzini[4], Philip Woods[5], François Bergey[6], Madaline Kinlay[6], Fabio Lawson[7], Mamta Garg[8]

*[1]University of Bologna, Bologna, Italy; [2]Clinica São Germano, São Paulo, Brazil; [3]GSK, Upper Providence, PA, United States of America; [4]GSK, Verona, Italy; [5]GSK, London, United Kingdom; [6]myTomorrows, Amsterdam, The Netherlands; [7]GSK, São Paulo, Brazil; [8]Leicester Royal Infirmary, Leicester, United Kingdom;*

### Background

Patients (pts) with multiple myeloma (MM) are often initially treated with triplet/quadruplet combination regimens comprising proteasome inhibitors, immunomodulators, and anti-CD38 therapies. However, most pts will experience progression after initial treatment. For pts with relapsed/refractory MM (RRMM), B-cell maturation antigen (BCMA)-directed therapies have been shown to deliver deep and durable responses with significant efficacy benefits compared to standards of care. The DREAMM-7[1] and DREAMM-8[2] clinical trials demonstrated robust efficacy of the anti-BCMA antibody-drug conjugate belantamab mafodotin (belamaf) plus bortezomib and dexamethasone (BVd) and belamaf plus pomalidomide and dexamethasone (BPd) vs standard-of-care triplet regimens for pts with RRMM who had received at least one prior line of therapy. A global managed access program was initiated to facilitate pt access to these treatments, and belamaf was subsequently granted regulatory approval in the UK in April 2025 and the European Union in July 2025.

### Aims

To examine pt characteristics and early dosing patterns in pts with RRMM who were treated with belamaf combinations outside of a clinical trial environment.

### Methods

A retrospective, multi-country chart review was initiated to examine pts treated with BVd or BPd in Brazil, Italy, and the UK, as part of the global managed access program. Due to belamaf's accelerated approval, this study also includes pts who received care upon market authorization in the UK. Data were captured on pt demographics, disease characteristics, clinical characteristics, and treatment history. The treatment period with belamaf was from Jan 2025 to Jan 2026.

**Copyright Information:** (Online) ISSN: 2572-9241
©2026 The Author(s). HemaSphere is published by John Wiley & Sons Ltd on behalf of European Hematology Association. This is an open access Abstract Book distributed under the Attribution-NonCommercial-NoDerivs (CC BY-NC-ND), which allows third parties to download the articles and share them with others as long as they credit the author and the Abstract Book, but they cannot change the content in any way or use them commercially.
**Abstract Book Citations:** Authors, Title, HemaSphere, 2026;10:(S1):pages. The abstract book DOIs can be found on the "Author Information" page.
**Disclaimer:** Articles published in the journal HemaSphere exclusively reflect the opinions of the authors. The authors are responsible for all content in their abstracts including accuracy of the facts, statements, citing resources, etc.

**Results**

In total, 62 pts were included in the study, of whom 52 (84%) were treated with BVd and 10 (16%) with BPd. Median treatment period was 144 (interquartile range [IQR] 93–208) days, and 49 (79%) pts remained on treatment at data cutoff. The median age was 72 (IQR 64–79) years and 9 of 52 (17%) pts with data available had high cytogenetic risk. Before starting treatment with belamaf-based combinations, pts received a median of 1 (IQR 1–2) prior therapies, with 29 (47%) pts being refractory to anti-CD38 agents. At data cutoff, pts had received a mean of 4.8 (standard deviation, 3) doses of belamaf.
Most (60/62 [97%]) pts started belamaf on a 2.5 mg/kg dose at planned schedules of Q3W (BVd) or Q4W (BPd, with planned reduction to 1.9 mg/kg Q4W from dose 2), and 2 (3%) pts started belamaf at 1.9 mg/kg. Dose reductions (45% of pts) and delays (63% of pts) were frequently utilized, but discontinuations were infrequent (Figure); 2 discontinuations occurred due to disease progression. For pts treated with BVd, the median/mean time between doses increased from 3.0/3.7 weeks during the first 3 months of treatment to 6.1/8.9 weeks for months 6–9. Thirty-nine of the 40 (98%) pts who received ≥4 doses were still on treatment at data cutoff.

**Summary/Conclusion**

Similar to the pivotal DREAMM-7 and DREAMM-8 clinical trials, physicians utilized extended dosing to enable pts to live well while on treatment. Real-world belamaf use in earlier lines of therapy signifies the unmet need for highly efficacious therapies at first relapse.

**References:**

1. Hungria et al. NEJM 2024
2. Dimopoulos et al. NEJM 2024

**Funding:** GSK (Study 224207). Drug linker technology licensed from Seagen Inc.; monoclonal antibody produced using POTELLIGENT Technology licensed from BioWa.

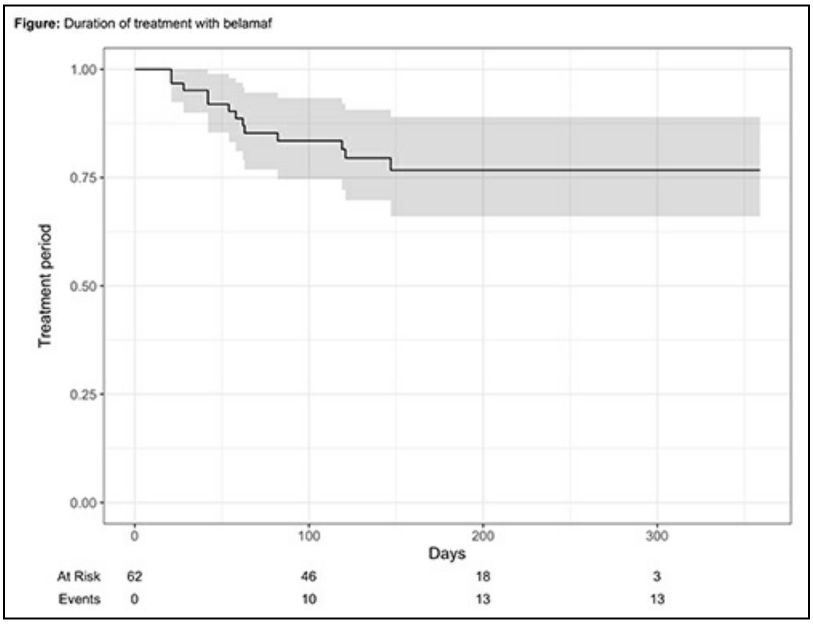

Duration of treatment with belamaf

**Copyright Information:** (Online) ISSN: 2572-9241
©2026 The Author(s). HemaSphere is published by John Wiley & Sons Ltd on behalf of European Hematology Association. This is an open access Abstract Book distributed under the Attribution-NonCommercial-NoDerivs (CC BY-NC-ND), which allows third parties to download the articles and share them with others as long as they credit the author and the Abstract Book, but they cannot change the content in any way or use them commercially.
**Abstract Book Citations:** Authors, Title, HemaSphere, 2026;10:(S1):pages. The abstract book DOIs can be found on the "Author Information" page.
**Disclaimer:** Articles published in the journal HemaSphere exclusively reflect the opinions of the authors. The authors are responsible for all content in their abstracts including accuracy of the facts, statements, citing resources, etc.

### (PB3254) EARLY ADMINISTRATION OF TOCILIZUMAB FOR GRADE 1 CYTOKINE RELEASE SYNDROME AT FIRST FEVER SIGNIFICANTLY REDUCES THE RISK OF PROGRESSING TO A HIGHER GRADE IN PATIENTS RECEIVING CEVOSTAMAB MONOTHERAPY

**Topic:** 14. Myeloma and other monoclonal gammopathies - Clinical

Paula Rodríguez-Otero*[1], Alexander Lesokhin[2], Suzanne Trudel[3], Amrita Krishnan[4], Chu-Fang Lin[5], Peter Kuebler[5], Tulika Tyagi[5], Elizabeth Germino[5], Anjali Vaze[5], Voleak Choeurng[5], Adam D Cohen[6]

*[1]Clínica Universidad de Navarra, Pamplona, Spain;[2]Memorial Sloan Kettering Cancer Center, New York, United States of America; [3]Princess Margaret Cancer Centre and University of Toronto, Toronto, Canada;[4]City of Hope Cancer Center, Irvine, United States of America; [5]Genentech, Inc., South San Francisco, United States of America;[6]Abramson Cancer Center and University of Pennsylvania, Philadelphia, United States of America;*

### Background

Recommendations for tocilizumab (TCZ) use in mitigating cytokine release syndrome (CRS) are reflected in American Society of Clinical Oncology (ASCO) and International Myeloma Working Group (IMWG) guidelines. However, institutional practice for early TCZ use varies. We sought to quantify the reduction in risk of progression to higher grades of CRS when TCZ was administered early at Grade (Gr) 1 CRS ('pre-emptive TCZ') in patients with relapsed/refractory multiple myeloma (RRMM) receiving cevostamab, a FcRH5xCD3 T-cell-engaging bispecific antibody, as monotherapy.

### Aims

To estimate the reduction in risk of progressing to Gr 2+ CRS associated with early TCZ administration in patients with fever indicative of Gr 1 CRS.

### Methods

We conducted a retrospective analysis of CRS data from Cycle 1 of the Phase I cevostamab monotherapy study in patients with RRMM (GO39775; NCT03275103; Richter et al. ASH 2024). Pre-emptive treatment was defined as TCZ administration after the onset of fever, but prior to hypotension or hypoxia. Entropy balancing weighting was used to address data imbalances. A survey-weighted generalized linear model (GLM) (svyglm function; R survey package) was used to derive the odds ratio (OR) and relative risk (RR), with corticosteroid (CS) use and baseline patient characteristics included as covariates. The 95% confidence intervals (CIs) for RR were calculated with a 1000-replicate bootstrap approach. A weighted multilevel mixed-effects model (R WeMix package) was used to ensure the treatment effect remained consistent when controlling for individual patient baseline variability. The frequency of Gr 3+ neutropenia was calculated as % of patients with absolute neutrophil counts (ANC) <1000/mm$^3$.

**Copyright Information:** (Online) ISSN: 2572-9241
©2026 The Author(s). HemaSphere is published by John Wiley & Sons Ltd on behalf of European Hematology Association. This is an open access Abstract Book distributed under the Attribution-NonCommercial-NoDerivs (CC BY-NC-ND), which allows third parties to download the articles and share them with others as long as they credit the author and the Abstract Book, but they cannot change the content in any way or use them commercially.
**Abstract Book Citations:** Authors, Title, HemaSphere, 2026;10:(S1):pages. The abstract book DOIs can be found on the "Author Information" page.
**Disclaimer:** Articles published in the journal HemaSphere exclusively reflect the opinions of the authors. The authors are responsible for all content in their abstracts including accuracy of the facts, statements, citing resources, etc.

### Results

A total of 324 patients were included in this post-hoc analysis. Pre-emptive TCZ reduced the risk of progression to Gr 2+ CRS with an OR of 0.18 (95% CI: 0.04, 0.73; **Figure**), RR of 0.21 (p=0.02) and bootstrap 95% CI of (0, 0.46). The protective effect of pre-emptive TCZ was corroborated in a secondary subject-specific analysis that yielded an OR of 0.15 (95% CI: 0.04, 0.56; p=0.0045), demonstrating that the reduction in CRS progression risk is consistent at both the population and individual patient level. CS administration preceding TCZ was adjusted for in the multivariable analysis, which showed a non-significant trend toward risk reduction (OR: 0.52; 95% CI: 0.16, 1.67; p=0.270), but did not alter the magnitude or significance of the primary pre-emptive TCZ effect.

Neutropenia is a recognized potential risk associated with TCZ. The frequency of Gr 3+ neutropenia in patients receiving TCZ for Gr 1 CRS (17.2%) was similar to, but numerically lower than, that observed in patients treated with TCZ for Gr 2+ CRS (22.2%).

### Summary/Conclusion

In this retrospective analysis, pre-emptive TCZ administration at Gr 1 CRS was associated with a statistically significant and clinically meaningful reduction in the risk of progression to Gr 2+ CRS. Incidence of Gr 3+ neutropenia associated with pre-emptive TCZ was comparable to, but numerically less frequent than, that observed with TCZ for Gr 2+ CRS. Taken together, the reduction in risk of high-grade CRS and manageable neutropenia profile with pre-emptive TCZ may improve the overall management of CRS. These data support IMWG guidelines for use of early TCZ treatment and the potential for clinical benefit in other diseases treated with T-cell-engaging bispecifc antibodies.

**Figure:** Generalized linear model results.

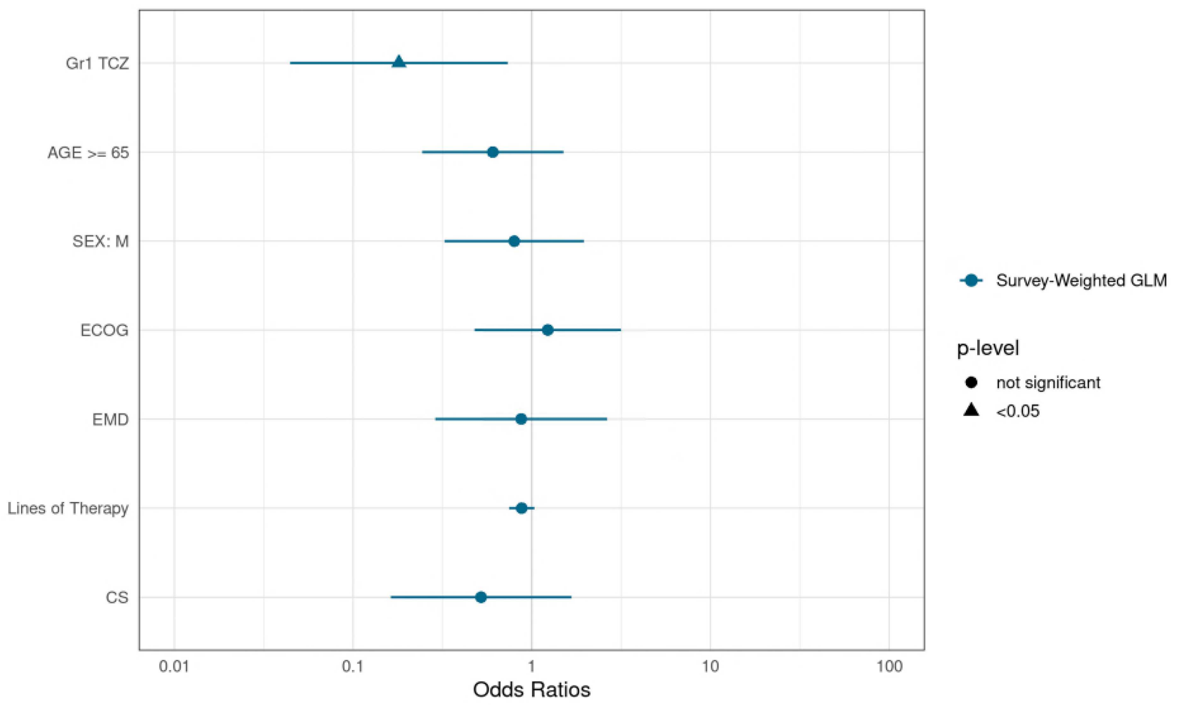

**Copyright Information:** (Online) ISSN: 2572-9241
©2026 The Author(s). HemaSphere is published by John Wiley & Sons Ltd on behalf of European Hematology Association. This is an open access Abstract Book distributed under the Attribution-NonCommercial-NoDerivs (CC BY-NC-ND), which allows third parties to download the articles and share them with others as long as they credit the author and the Abstract Book, but they cannot change the content in any way or use them commercially.
**Abstract Book Citations:** Authors, Title, HemaSphere, 2026;10:(S1):pages. The abstract book DOIs can be found on the "Author Information" page.
**Disclaimer:** Articles published in the journal HemaSphere exclusively reflect the opinions of the authors. The authors are responsible for all content in their abstracts including accuracy of the facts, statements, citing resources, etc.

### (PB3255) REAL-WORLD OUTCOMES OF DARATUMUMAB-LENALIDOMIDE-DEXAMETHASONE IN TRANSPLANT-INELIGIBLE NEWLY DIAGNOSED MULTIPLE MYELOMA: EXPERIENCE FROM THE RTM (REGIONAL TUSCAN MYELOMA NETWORK)

**Topic:** 14. Myeloma and other monoclonal gammopathies - Clinical

Irene Attucci*[1], Elisabetta Antonioli[1], Gabriele Buda[2], Maria Livia Del Giudice[2], Caterina Biagiotti[3], Veronica Candi[4], Sara Grammatico[5], Alessandro Gozzetti[6], Fabrizio Bonanni[7], Giulia Fontanelli[8], Laura Notarfranchi[9], Sofia Pilerci[10], Francesca Andreazzoli[10], Maria Messeri[1], Monica Bocchia[6], Sara Galimberti[2], Alessandro Maria Vannucchi[1]

*[1]Haematology Unit, Careggi University Hospital, Florence, Italy; [2]Department of Clinical and Experimental medicine, University of Pisa, Haematology Unit, Pisa, Italy; [3]S.O.S. Oncohematology, San Jacopo Hospital, Pistoia, Italy; [4]U.O.S Hematology, San Donato Hospital, Arezzo, Italy; [5]S.O.S. Oncohematology, Santo Stefano Hospital, Prato, Italy; [6]Department of Medical Science, Surgery and Neuroscience, Hematology, University of Siena, Siena, Italy; [7]S.O.S. Oncohematology and Clinical Hematology, Ospedale Nuovo San Giovanni di Dio, Florence, Italy; [8]SOS Oncohematology, San Giuseppe Hospital, Empoli, Florence, Italy; [9]UOC Ematologia Aziendale, Azienda USL Toscana Nordovest, Ospedale Nuovo Apuane, Massa Carrara, Italy; [10]U.O.S. Ematologia–Ospedale Versilia, Lido di Camaiore, Viareggio, Italy;*

### Background

In the pivotal MAIA trial, daratumumab-lenalidomide-dexamethasone (D-Rd) significantly improved progression-free survival (PFS) and overall survival (OS) in transplant-ineligible newly diagnosed multiple myeloma (NDMM). Based on International Myeloma Working Group 2021 guidelines, D-Rd was a preferred frontline regimen. However, real-world data, particularly in elderly and frail patients, remain limited.

### Aims

We aimed to assess the effectiveness and safety of D-Rd in a large, unselected cohort of transplant-ineligible NDMM patients treated in a real-world setting within the Regional Tuscan Myeloma Network.

### Methods

This multicentre retro-prospective observational study included 357 NDMM patients treated across 10 haematology centres in Tuscany (Italy) between January 2021 and December 2025. The study was conducted according to the Declaration of Helsinki and approved by local ethics committees; all patients provided written informed consent. Transplant ineligibility was defined as age ≥70 years and/or relevant comorbidities. Patients receiving ≥1 dose of each drug were included. Responses were assessed per IMWG criteria; adverse events (AEs) were graded per CTCAE v5.0.

**Copyright Information:** (Online) ISSN: 2572-9241
©2026 The Author(s). HemaSphere is published by John Wiley & Sons Ltd on behalf of European Hematology Association. This is an open access Abstract Book distributed under the Attribution-NonCommercial-NoDerivs (CC BY-NC-ND), which allows third parties to download the articles and share them with others as long as they credit the author and the Abstract Book, but they cannot change the content in any way or use them commercially.
**Abstract Book Citations:** Authors, Title, HemaSphere, 2026;10:(S1):pages. The abstract book DOIs can be found on the "Author Information" page.
**Disclaimer:** Articles published in the journal HemaSphere exclusively reflect the opinions of the authors. The authors are responsible for all content in their abstracts including accuracy of the facts, statements, citing resources, etc.

### Results

Median age was 75 years (range 61–86); 17% were ≥80 years and 10% <70 years with comorbidities; M/F ratio was 176/181. An ECOG performance status (PS) ≥ 2 at diagnosis was observed in 55 (15%) patients. ISS stage II and III were reported in 41% and 32%, respectively. FISH data were available in 157 patients (44%); among them, high-risk cytogenetic features were identified in 46 (29%): del(17p) (*n*=10), t(4;14) (*n*=22), t(14;16) (*n*=6), and +1q (*n*=44); 9 patients had double-hit abnormalities. Renal impairment (eGFR <60 mL/min) was present in 25%, and 4% had concomitant amyloidosis. After a median follow-up of 23 months, patients received a median of 18 D-Rd cycles (range 1-65) with a median treatment duration of 18 months. Overall response rate was 94%, with 70% achieving ≥VGPR. Median PFS was not reached (NR), 72% of patients remained progression-free at 40 months. Those achieving ≥VGPR, showed longer PFS (median NR vs 23 months; P<0.001). Age >80 years old or PS ECOG ≥ 2, did not significantly impact PFS. Median OS was NR, with an estimated 4-year OS of 81%. Treatment was discontinued in 91 patients (25%), mainly due to progression (53%), AEs (18%), or refractory disease (7%). Thirty-four deaths occurred, primarily due to disease progression (20%); five were treatment-related (mainly sepsis). The most common AEs (all grades) were neutropenia (44%), anaemia (19%), and thrombocytopenia (10%). Infections occurred in 31%, gastrointestinal toxicity in 13%, and renal toxicity in 7%. Grade ≥3 AEs included neutropenia (30%) and infections (10%). Hospitalization was required in 8% (n=29), mainly due to sepsis (n=17) and cardiovascular events (n=6). Lenalidomide dose reductions and discontinuations occurred in 41% and 29%, respectively; dexamethasone was reduced in 17% and discontinued in 17%. Fifty-five patients received second-line therapy, most commonly PVD (n=19), KD (n=8), or SVD (n=8); 11 received a bortezomib-based doublet.

### Summary/Conclusion

In this large real-world cohort of transplant-ineligible NDMM patients, including elderly and clinically frail individuals, D-Rd demonstrated high response rates, durable disease control, and a manageable safety profile. These findings support the effectiveness and feasibility of D-Rd in routine clinical practice.

**Copyright Information:** (Online) ISSN: 2572-9241
©2026 The Author(s). HemaSphere is published by John Wiley & Sons Ltd on behalf of European Hematology Association. This is an open access Abstract Book distributed under the Attribution-NonCommercial-NoDerivs (CC BY-NC-ND), which allows third parties to download the articles and share them with others as long as they credit the author and the Abstract Book, but they cannot change the content in any way or use them commercially.
**Abstract Book Citations:** Authors, Title, HemaSphere, 2026;10:(S1):pages. The abstract book DOIs can be found on the "Author Information" page.
**Disclaimer:** Articles published in the journal HemaSphere exclusively reflect the opinions of the authors. The authors are responsible for all content in their abstracts including accuracy of the facts, statements, citing resources, etc.

### (PB3256) REAL-WORLD DATA INDICATE THAT IXAZOMIB-LENALIDOMIDE-DEXAMETHASONE MAY NOT BE INFERIOR TO DARATUMUMAB-LENALIDOMIDE-DEXAMETHASONE IN ELDERLY RELAPSED/REFRACTORY MYELOMA PATIENTS

**Topic:** 14. Myeloma and other monoclonal gammopathies - Clinical

Hermina Sánta*[1], Laura Júlia Regáli[2], László Váróczy[3], Virág Réka Szita[4], István Wiedemann[4], Lóránt Varju[5], Laszlo Rejto[5], Norbert Sándor Bartha[5], Dorottya Máté[6], András Masszi[6], Márk Plander[7], Szabolcs Kosztolanyi[8], Hussain Alizadeh[8], Piroska Pettendi[9], Ildiko Kalmanne Istenes[10], Árpád Szomor[1], Peter Pal Remenyi[2], Masszi Tamás Pál[4], Gergely Varga[4], Gabor Mikala[4]

*[1]Fejér County University Hospital, Hematology, Székesfehérvár, Hungary;[2]South-Pest Central Hospital, Budapest, Hungary; [3]Faculty of Medicine, University of Debrecen, Debrecen, Hungary;[4]Semmelweis University, Department of Internal Medicine and Hematology, Budapest, Hungary; [5]Szabolcs-Szatmár-Bereg County Hospital, Nyíregyháza, Hungary; [6]National Institute of Oncology,, Budapest, Hungary; [7]Vas Vármegyei Markusovszky Egyetemi Oktató Kórház, Szombathely, Hungary; [8]University of Pécs-Clinical Centre, Pécs, Hungary; [9]Jász-Nagykun-Szolnok County Géza Hetényi Hospital, Szolnok, Hungary; [10]Semmelweis University, Department of Internal Medicine and Oncology, Budapest, Hungary;*

### Background

Despite significant advances in the treatment of relapsed/refractory multiple myeloma (RRMM), therapeutic decision-making remains challenging. Key unresolved issues include the optimal timing of treatment initiation in patients with biochemical versus clinical progression and the personalization of therapy. In situations where prior treatment efficacy, frailty, comorbidities, mobility limitations, social factors, drug availability, or patient preference preclude the use of newer therapies, the all-oral ixazomib–lenalidomide–dexamethasone (IRd) regimen represents a practical alternative.

### Aims

We aimed to provide real-world data to inform the choice between IRd and daratumumab–lenalidomide–dexamethasone (DRd) in the second-line setting, using recently published Hungarian real-world data on DRd for comparison.

### Methods

**Methods:** We conducted a multicenter retrospective analysis of 152 RRMM patients treated with IRd in Hungary between 1 January 2020 and 30 June 2025.

### Results

The median age at treatment initiation was 73.7 years. Treatment was initiated due to biochemical progression in 53.3% of patients, clinical progression in 38.2%, and intolerance or toxicity of prior therapy in 8.6%. Median progression-free survival (PFS) and overall survival (OS) were 18.7 and 34.7 months, respectively. Initiation of IRd during biochemical progression was associated with significantly longer PFS than treatment at clinical progression (24.3 vs. 15.6 months; $p = 0.004$), with an additional benefit observed in patients treated due to intolerance or toxicity of prior therapy (p = 0.04). In the second-line setting, median PFS was 24.5 months, while median OS was not reached. Adverse events occurred in 68.3% of patients; dose reductions were required in 18.4%, and treatment discontinuation due to intolerance or toxicity occurred in 21.6%. The most frequent toxicities were neutropenia (32.9%), thrombocytopenia (27.6%), diarrhoea (25.0%), peripheral neuropathy (25.3%), and infections (22.4%).

**Copyright Information:** (Online) ISSN: 2572-9241
©2026 The Author(s). HemaSphere is published by John Wiley & Sons Ltd on behalf of European Hematology Association. This is an open access Abstract Book distributed under the Attribution-NonCommercial-NoDerivs (CC BY-NC-ND), which allows third parties to download the articles and share them with others as long as they credit the author and the Abstract Book, but they cannot change the content in any way or use them commercially.
**Abstract Book Citations:** Authors, Title, HemaSphere, 2026;10:(S1):pages. The abstract book DOIs can be found on the "Author Information" page.
**Disclaimer:** Articles published in the journal HemaSphere exclusively reflect the opinions of the authors. The authors are responsible for all content in their abstracts including accuracy of the facts, statements, citing resources, etc.

### Summary/Conclusion

When compared with second-line DRd, outcomes with IRd were not significantly inferior (36-month OS from second-line initiation: 65.5% for DRd vs. 60.0% for IRd; p = 0.56) despite a difference in the median age at treatment initiation ( median 65 years for DRd vs median 73.7 years for IRd). Initiation of IRd during biochemical progression was associated with superior PFS compared with treatment at clinical progression, and an in-class transition of proteasome inhibitors showed the best results. These real-world data support IRd as a practical, convenient, all-oral treatment option for appropriately selected RRMM patients.

**Copyright Information:** (Online) ISSN: 2572-9241
©2026 The Author(s). HemaSphere is published by John Wiley & Sons Ltd on behalf of European Hematology Association. This is an open access Abstract Book distributed under the Attribution-NonCommercial-NoDerivs (CC BY-NC-ND), which allows third parties to download the articles and share them with others as long as they credit the author and the Abstract Book, but they cannot change the content in any way or use them commercially.
**Abstract Book Citations:** Authors, Title, HemaSphere, 2026;10:(S1):pages. The abstract book DOIs can be found on the "Author Information" page.
**Disclaimer:** Articles published in the journal HemaSphere exclusively reflect the opinions of the authors. The authors are responsible for all content in their abstracts including accuracy of the facts, statements, citing resources, etc.

### (PB3257) RURALITY AND ITS EFFECT ON TREATMENT AND SURVIVAL OF MULTIPLE MYELOMA IN AUSTRALIA:A STUDY FROM THE AUSTRALIA AND NEW ZEALAND MYELOMA AND RELATED DISEASES REGISTRY (ANZ MRDR)

**Topic:** 14. Myeloma and other monoclonal gammopathies - Clinical

Benjamin Reardon*[1, 2], Louise Imlay-Gillespie[3], Cameron Wellard[4], Elizabeth Moore[4], Ramya Kommineni[2], Erica Wood[4], Jillian De Malmanche[2], Bradley Augustson[5], Jessica Heenan[6], Wojt Janowski[2], Peter Mollee[7], Andrew Spencer[8]

*[1]University of Newcastle, School of Medicine and Public Health, Newcastle, Australia;[2]Calvary Mater Newcastle, Haematology, Newcastle, Australia; [3]Lismore Hospital, Haematology Department, Lismore, Australia;[4]Monash University, School of Public Health and Preventative Medicine, Melbourne, Australia; [5]Sir Charles Gairdner Hospital, Haematology Department, Nedlands, WA, Australia; [6]Launceston General Hospital, Haematology Department, Launceston, Australia;[7]Princess Alexandra Hospital, Brisbane, Australia; [8]The Alfred Hospital and Monash University, Department of Haematology, Melbourne, Australia;*

### Background

Multiple myeloma (MM) is the second most common haematological malignancy in Australia, accounting for 1.6% of all new cancer diagnoses, but 2.2% of deaths from cancer in Australia in 2022 (AIHW Cancer Data Australia 2025). 27% of the Australian population, or approximately 7 million people, live in rural and remote areas and face unique challenges to cancer care due to their geographic location (ABS Remoteness Areas 2026). Multiple myeloma is a complex malignancy requiring specialist care, yet patients in rural areas may face barriers to treatment access.

### Aims

The objective of our study was to assess differences in demographics including socio-economic status, clinical presentation, treatment practices and outcomes for MM patients living in non-major city areas versus major cities across Australia, using the Australia and New Zealand Myeloma and Related Diseases Registry (ANZ MRDR) data.

### Methods

We conducted a prospective cohort analysis of 4,353 patients with MM enrolled at Australian treatment centres through the ANZ MRDR between January 2013 and August 2025. Patients were classified as residing in major cities or non-major city areas at diagnosis.

### Results

A total of 4,353 patients receiving first-line therapy were included, of whom 1,434 (33.0%) resided in non-major city areas. Of all 45 treating sites 33 were from major cities (77.8%) and 12 from non-major city areas (22.2%). Baseline disease characteristics were similar across groups with median age of 67.9 and 68.6, respectively, though socioeconomic disadvantage was greater outside major cities. First-line treatment patterns were broadly comparable, with slightly shorter time to therapy initiation in non-major city patients (median 16 vs 19 days; $p = 0.005$). Clinical trial participation at second line was lower outside major cities (13.3% vs 17.2%;$p = 0.066$). Overall survival was significantly reduced for non-major city patients (median 78.5 vs 88.3 months; $p = 0.013$), while progression-free survival did not differ. However, the overall survival difference attenuated after adjustment for age, cytogenetic risk and socio-economic status with a HR of 1.01 (0.91 – 1.12, p=0.8).

**Copyright Information:** (Online) ISSN: 2572-9241
©2026 The Author(s). HemaSphere is published by John Wiley & Sons Ltd on behalf of European Hematology Association. This is an open access Abstract Book distributed under the Attribution-NonCommercial-NoDerivs (CC BY-NC-ND), which allows third parties to download the articles and share them with others as long as they credit the author and the Abstract Book, but they cannot change the content in any way or use them commercially.
**Abstract Book Citations:** Authors, Title, HemaSphere, 2026;10:(S1):pages. The abstract book DOIs can be found on the "Author Information" page.
**Disclaimer:** Articles published in the journal HemaSphere exclusively reflect the opinions of the authors. The authors are responsible for all content in their abstracts including accuracy of the facts, statements, citing resources, etc.

### Summary/Conclusion

Geographic disparities in multiple myeloma care persist in Australia, with shorter overall survival among patients outside major cities despite similar early treatment. Survival differences appear to arise later in the disease trajectory, with patients outside major cities less likely to receive subsequent lines of therapy following relapse. Strategies to improve access to later-line therapies and continuity of care are needed to address these inequities.

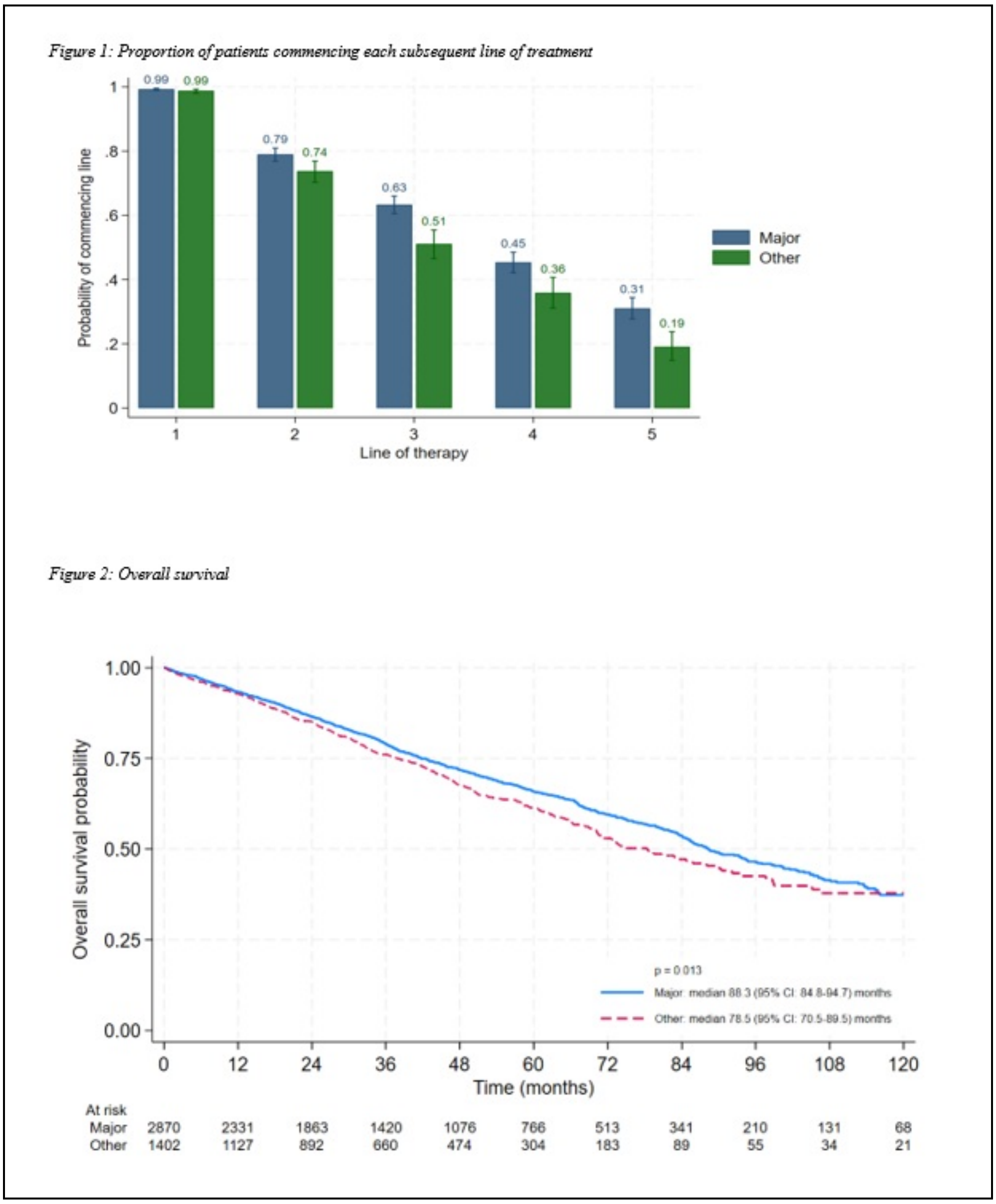

Figure 1: Proportion of patients commencing each subsequent line of treatment. Figure 2: Overall survival

**Copyright Information:** (Online) ISSN: 2572-9241
©2026 The Author(s). HemaSphere is published by John Wiley & Sons Ltd on behalf of European Hematology Association. This is an open access Abstract Book distributed under the Attribution-NonCommercial-NoDerivs (CC BY-NC-ND), which allows third parties to download the articles and share them with others as long as they credit the author and the Abstract Book, but they cannot change the content in any way or use them commercially.
**Abstract Book Citations:** Authors, Title, HemaSphere, 2026;10:(S1):pages. The abstract book DOIs can be found on the "Author Information" page.
**Disclaimer:** Articles published in the journal HemaSphere exclusively reflect the opinions of the authors. The authors are responsible for all content in their abstracts including accuracy of the facts, statements, citing resources, etc.

### (PB3258) DETERMINANTS OF OUTCOMES IN MULTIPLE MYELOMA: A COMPARATIVE ANALYSIS OF SUPER-RESPONDERS VS. EARLY RELAPSERS

**Topic:** 14. Myeloma and other monoclonal gammopathies - Clinical

Magaly Valeria Escobedo Cruz*[1], Karla Mariana Castro Bórquez[1], Martha Paola Anchondo Commesse[1], Ana Echenique Alcázar[1], Jennifer Ahlstrom[1], Miranda de la Luz Franco[1], Samuel Bennion[1], Jay R. Hydren[1], Jorge Arturo Hurtado Martinez[1]

*[1]HealthTree Foundation, South Jordan, United States of America;*

### Background

Quadruplet induction and immunotherapeutic approaches have revolutionized the treatment of Multiple Myeloma (MM), yet patient outcomes remain highly variable. A subset of patients achieves extraordinary treatment durability with their first-line of therapy (LoT1) lasting over 5 years (Super-Responders, SR), while others, unfortunately, relapse early, within 18 months (Early Relapsers, ER). Defining the unique clinical and genetic features that confer better outcomes is critical for advancing risk-adapted treatment strategies. This identification would facilitate treatment de-escalation for SR patients and enable the front-loading of more intensive therapies for the high-risk ER group.

### Aims

To describe the clinical, biochemical, and cytogenetic characteristics to identify commonalities among MM Super-Responders compared to Early Relapsers using real-world data.

### Methods

This retrospective descriptive study utilized validated real-world data (vRWD) from the HealthTree registry. The study population included patients with a confirmed diagnosis of Multiple Myeloma (MM) and at least one of the following cohort characteristics on verified medical data from our HealthTree registry: Group A Super-Responders (SR), defined by a continuous first-line therapy (LoT1) with a duration of ≥60 months; and Group B Early-Relapsers (ER), defined as LoT1 duration of ≤18 months. The analysis included primary variables (diagnosis, LoT1 duration, last followup date) were analyzed alongside secondary risk profiles, including High-Risk Cytogenetics (del(17p), 1q gain/amp, t(4;14), t(14;16), t(14;20)) and other markers like t(11;14) and trisomies 3 and 5. Clinical markers (R-ISS, B2M, subtype) and treatment factors (LoT1 agent count, maintenance, best response, TTNT) further defined the cohort. Group comparisons used standard univariable tests as appropriate. Continuous variables were reported with medians (Q1,Q3).

### Results

A total of 551 patients were analyzed. The SR group comprised 252 patients, while the ER, 299 patients. The SR patients' median age was 59 (52, 64), while the ER patients' was 60 (53, 67) ($p<0.001$). At diagnosis, SR patients presented with significantly lower median Beta-2-microglobulin 2.9 (2.2, 4.3) vs 3.3 (2.3, 5.3) mg/L ($p<0.05$). R-ISS I was more prevalent in SR (27 vs 16%, $p<0.05$), while Stage III patients were more prevalent in ER (8.3 vs 14, $p<0.05$). Trisomies 3 (SR 78% vs ER 87%, $p=0.7$) and 5 (SR 83% vs ER 66%, $p=0.2$) were similar, with t(11;14) being less prevalent in SR (44% vs 56%). ER patients had higher high-risk features: del(17p) (28% vs 20%), 1q Gain/Amp (61% vs 58%), and t(14;20) (6.7% vs 9.5%). SR utilized more transplant/HDT (56.4% vs 13.3%), The best response of SR patients during their LoT1 was more likely to be Complete Response (43% vs. 9.4%) a or sCR(29% vs 2.3%), in contrast ER patients were more likely to have had a Partial Response (39% vs 5.2%) or Progressive Disease (17% vs 3.2%), resulting in a median TTNT of 2538 days (2132, 3436) vs. 163 days (98, 292) for ER.

**Copyright Information:** (Online) ISSN: 2572-9241
©2026 The Author(s). HemaSphere is published by John Wiley & Sons Ltd on behalf of European Hematology Association. This is an open access Abstract Book distributed under the Attribution-NonCommercial-NoDerivs (CC BY-NC-ND), which allows third parties to download the articles and share them with others as long as they credit the author and the Abstract Book, but they cannot change the content in any way or use them commercially.
**Abstract Book Citations:** Authors, Title, HemaSphere, 2026;10:(S1):pages. The abstract book DOIs can be found on the “Author Information” page.
**Disclaimer:** Articles published in the journal HemaSphere exclusively reflect the opinions of the authors. The authors are responsible for all content in their abstracts including accuracy of the facts, statements, citing resources, etc.

### Summary/Conclusion

Despite the similar prevalence of high-risk features in the ER and SR group, the primary differentiator for long-term success versus early failure was the strong association with intensity and efficacy of first-line therapy (LoT1), with the SR cohort characterized by a highly effective treatment pathway. These findings support follow-up analyses adjusting for treatment selection and baseline risk to better disentangle prognostic biology from treatment effects.

**Table 1. Clinical Characteristics, Cytogenetics, and Treatment Patterns of Multiple Myeloma Super-Responders vs. Early Relapsers**

| | N | Super-Responder (≥60 mo) N = 252[1] | Early Relapser (≤18 mo) N = 299[1] | Overall N = 551[1] | p-value[2] |
|---|---|---|---|---|---|
| **Age at diagnosis** | 551 | 59 (52, 64) | 61 (53, 67) | 60 (53, 66) | <0.001 |
| **Gender** | 551 | | | | 0.9 |
| Female | | 131 (52%) | 153 (51%) | 284 (52%) | |
| Male | | 121 (48%) | 146 (49%) | 267 (48%) | |
| **Race** | 551 | | | | 0.11 |
| White | | 224 (89%) | 246 (82%) | 470 (85%) | |
| Black or African American | | 14 (5.6%) | 23 (7.7%) | 37 (6.7%) | |
| Other Race, White | | 3 (1.2%) | 10 (3.3%) | 13 (2.4%) | |
| Other Race | | 8 (3.2%) | 14 (4.6%) | 22 (4%) | |
| Asian | | 3 (1.2%) | 6 (2.0%) | 9 (1.6%) | |
| **Beta-2 Microglobulin** | 315 | 2.9 (2.2, 4.3) | 3.3 (2.3, 5.3) | 3.1 (2.2, 4.8) | 0.025 |
| Unknown | | 128 | 108 | 236 | |
| **ISS-Stage** | 312 | | | | 0.021 |
| ISS Stage 1 | | 48 (40%) | 47 (25%) | 95 (30%) | |
| ISS Stage 2 | | 51 (42%) | 99 (52%) | 150 (48%) | |
| ISS Stage 3 | | 22 (18%) | 45 (24%) | 67 (21%) | |
| Unknown | | 131 | 108 | 239 | |
| **R-ISS Stage** | 312 | | | | 0.041 |
| R-ISS Stage 1 | | 33 (27%) | 31 (16%) | 64 (21%) | |
| R-ISS Stage 2 | | 78 (64%) | 134 (70%) | 212 (68%) | |
| R-ISS Stage 3 | | 10 (8.3%) | 26 (14%) | 36 (12%) | |
| Unknown | | 131 | 108 | 239 | |
| **Time to next treatment (days)** | 551 | 2,538 (2,132, 3,436) | 163 (98, 292) | 451 (153, 2,477) | <0.001 |
| **Deletion 17p** | 253 | | | | 0.2 |
| Positive | | 21 (20%) | 41 (28%) | 62 (25%) | |
| Negative | | 83 (80%) | 108 (72%) | 191 (75%) | |
| Unknown | | 148 | 150 | 298 | |
| **gain/amp 1q** | 240 | | | | 0.6 |
| Positive | | 58 (58%) | 86 (61%) | 144 (60%) | |
| Negative | | 42 (42%) | 54 (39%) | 96 (40%) | |
| Unknown | | 152 | 159 | 311 | |
| **t 4;14** | 160 | | | | |
| Positive | | 20 (32%) | 25 (26%) | 45 (28%) | |
| Negative | | 43 (68%) | 72 (74%) | 115 (72%) | |
| Unknown | | 189 | 202 | 391 | |
| **t 14;16** | 135 | | | | 0.7 |
| Positive | | 8 (18%) | 21 (23%) | 29 (21%) | |
| Negative | | 36 (82%) | 70 (77%) | 106 (79%) | |
| Unknown | | 208 | 208 | 416 | |
| **t 14;20** | 66 | | | | 0.6 |
| Positive | | 2 (9.5%) | 3 (6.7%) | 5 (7.6%) | |
| Negative | | 19 (90%) | 42 (93%) | 61 (92%) | |
| Unknown | | 231 | 254 | 485 | |
| **t 11;14** | 217 | | | | 0.067 |
| Positive | | 36 (44%) | 79 (58%) | 115 (53%) | |
| Negative | | 45 (56%) | 57 (42%) | 102 (47%) | |
| Unknown | | 171 | 163 | 334 | |
| **Trisomy 3** | 38 | | | | 0.7 |
| Positive | | 18 (78%) | 13 (87%) | 31 (82%) | |
| Negative | | 5 (22%) | 2 (13%) | 7 (18%) | |
| Unknown | | 229 | 284 | 513 | |
| **Trisomy 5** | 67 | | | | 0.2 |
| Positive | | 29 (83%) | 21 (66%) | 50 (75%) | |
| Negative | | 6 (17%) | 11 (34%) | 17 (25%) | |
| Unknown | | 217 | 267 | 484 | |
| **Induction intensity** | 551 | | | | 0.8 |
| Mono (1 class) | | 16 (6.3%) | 19 (6.4%) | 35 (6.4%) | |
| Doublet (2 classes) | | 158 (63%) | 196 (66%) | 354 (64%) | |
| Triplet (3 classes) | | 64 (25%) | 71 (24%) | 135 (25%) | |
| Quadruplet (≥4 classes) | | 10 (4.0%) | 7 (2.3%) | 17 (3.1%) | |
| Other/Unknown | | 4 (1.6%) | 6 (2.0%) | 10 (1.8%) | |
| **Regimen Class** | 551 | | | | |
| PI+IMiD | | 43 (17%) | 126 (42%) | 169 (31%) | |
| PI+IMiD+Transplant/High-dose | | 103 (41%) | 24 (8.0%) | 127 (23%) | |
| CD38+PI+IMiD | | 9 (3.6%) | 42 (14%) | 51 (9.3%) | |
| PI+Alkylator | | 2 (0.8%) | 30 (10%) | 32 (5.8%) | |
| CD38+PI+IMiD+Transplant/High-dose | | 17 (6.7%) | 13 (4.3%) | 30 (5.4%) | |
| PI+IMiD+Alkylator+Transplant/High-dose | | 22 (8.7%) | 3 (1.0%) | 25 (4.5%) | |
| PI+IMiD+Alkylator | | 16 (6.3%) | 8 (2.7%) | 24 (4.4%) | |
| IMiD | | 8 (3.2%) | 11 (3.7%) | 19 (3.4%) | |
| Other/Unknown | | 32 (13%) | 42 (14%) | 74 (13%) | |
| **First Line of Therapy response** | 550 | | | | |
| Complete Response | | 108 (43%) | 28 (9.4%) | 136 (25%) | |
| Partial Response | | 13 (5.2%) | 116 (39%) | 129 (23%) | |
| Very Good Partial Response | | 45 (18%) | 59 (20%) | 104 (19%) | |
| Stringent Complete Response | | 74 (29%) | 7 (2.3%) | 81 (15%) | |
| Progressive Disease | | 8 (3.2%) | 50 (17%) | 58 (11%) | |
| Stable Disease | | 4 (1.6%) | 38 (13%) | 42 (7.6%) | |
| Unknown | | 0 | 1 | 1 | |

1 Median (Q1, Q3); n (%)

2 Wilcoxon rank sum test; Fisher's exact test; NA

Table 1. Clinical Characteristics, Cytogenetics, and Treatment Patterns of Multiple Myeloma Super-Responders vs. Early Relapsers A total of 551 patients were analyzed. They were subdivided in two cohorts, Super Responders were defined as those patients that remained more than 5 years on their first line of therapy, and Early relapsers as those the relapsed within 18 months of starting their first line of therapy.

**Copyright Information:** (Online) ISSN: 2572-9241
©2026 The Author(s). HemaSphere is published by John Wiley & Sons Ltd on behalf of European Hematology Association. This is an open access Abstract Book distributed under the Attribution-NonCommercial-NoDerivs (CC BY-NC-ND), which allows third parties to download the articles and share them with others as long as they credit the author and the Abstract Book, but they cannot change the content in any way or use them commercially.
**Abstract Book Citations:** Authors, Title, HemaSphere, 2026;10:(S1):pages. The abstract book DOIs can be found on the "Author Information" page.
**Disclaimer:** Articles published in the journal HemaSphere exclusively reflect the opinions of the authors. The authors are responsible for all content in their abstracts including accuracy of the facts, statements, citing resources, etc.

### (PB3259) CYTOGENETIC TESTING IN AL AMYLOIDOSIS IN ROUTINE CLINICAL PRACTICE, A POPULATION-BASED STUDY

**Topic:** 14. Myeloma and other monoclonal gammopathies - Clinical

Flores Weverling*[1, 2], Mirian Brink[2], Anne-Marie van der Kevie-Kersemaekers[3], Monique Minnema[1]

*[1]University Medical Center Utrecht, Department of Hematology, Utrecht, The Netherlands;[2]Netherlands Comperhensive Cancer Organisation (IKNL), Department of Research and Development, Utrecht, The Netherlands; [3]University Medical Center Utrecht, Department of Clinical Genetics, Utrecht, The Netherlands;*

### Background

Immunoglobulin light chain (AL) amyloidosis is a monoclonal plasma cell disease in which toxic light chains form amyloid fibrils resulting in organ damage. Over the past decade, focus on clarifying the prognostic relevance of cytogenetic abnormalities in AL amyloidosis has not led to clear guidance on treatment decisions. In addition, it is unclear how frequently cytogenetic testing is performed in routine clinical practice.

### Aims

In this population-based analysis we evaluated the use of cytogenetic testing in patients with AL amyloidosis in the Netherlands. Specifically, we assess whether the use of testing has increased over time and explore which characteristics are associated with the likelihood of undergoing cytogenetic testing.

### Methods

Patients ≥18 years diagnosed with AL amyloidosis between 2017-2024 were identified in the Netherlands cancer registry, which captures all cases based on nationwide pathology data. Cytogenetic testing was defined as assessment of one or more of the following abnormalities: chromosome 14 translocations (t(14q32), t(11;14), t(4;14), t(14;16)), chromosome 1 abnormalities (gain(1q), del(1p)), del17p, del13q14 and hyperdiploidy, regardless of whether results were positive or negative. Fluorescence in situ hybridization is routinely performed by the academic hospitals, complemented by single nucleotide polymorphism (SNP) array analysis.
We performed an univariable logistic regression to assess the annual increase of cytogenetic testing. Furthermore, we assessed for chromosome 14 if detailed characterization increased over the years. Subsequently, we performed a multivariable logistic regression to assess characteristics interacting with the likelihood to receive cytogenetic testing.

### Results

Among 1131 patients diagnosed between 2017–2024, 771 (68%) underwent cytogenetic testing. Testing frequency increased from 57% in 2017 to 75% in 2024 (OR per year of diagnosis, 1.08; 95% CI, 1.02–1.14). Of all tested patients, 96% were assessed for chromosome 14 (n = 741) and gain1q (n = 741), 97% for del17p (n = 746) and 84% for hyperdiploidy (n = 644). The proportion of fully characterized chromosome 14 abnormalities increased from 33% in 2017 to 75% in 2024 (OR per year of diagnosis, 1.27 (95% CI, 1.04-1.27).
Characteristics contributing to the likelihood of getting cytogenetic testing were a younger age (OR 1.06 [95% CI 1.04-1.07]), diagnosed and/or treated in an academic hospital (OR 1.44 [95% CI 1.02-2.06]), receiving treatment (OR 2.31 [95% CI 1.52-3.53]) and year of diagnosis (OR 1.13 [95% CI 1.06-1.21]).

**Copyright Information:** (Online) ISSN: 2572-9241
©2026 The Author(s). HemaSphere is published by John Wiley & Sons Ltd on behalf of European Hematology Association. This is an open access Abstract Book distributed under the Attribution-NonCommercial-NoDerivs (CC BY-NC-ND), which allows third parties to download the articles and share them with others as long as they credit the author and the Abstract Book, but they cannot change the content in any way or use them commercially.
**Abstract Book Citations:** Authors, Title, HemaSphere, 2026;10:(S1):pages. The abstract book DOIs can be found on the "Author Information" page.
**Disclaimer:** Articles published in the journal HemaSphere exclusively reflect the opinions of the authors. The authors are responsible for all content in their abstracts including accuracy of the facts, statements, citing resources, etc.

**Summary/Conclusion**

Cytogenetic testing in patients with AL amyloidosis has increased over the past seven years to 75%. Testing was more frequently performed in younger patients, patients receiving systemic therapy, and those diagnosed at academic hospitals. Furthermore, detailed characterization of chromosome 14 abnormalities increased substantially over time, reflecting a shift in daily practice from reporting generic t(14q32) rearrangements to identifying specific partner translocations such as t(11;14).
With this increased testing, further analysis on prognostic relevance of cytogenetic abnormalities in the current treatment landscape can be performed on a nationwide level.

**Copyright Information:** (Online) ISSN: 2572-9241
©2026 The Author(s). HemaSphere is published by John Wiley & Sons Ltd on behalf of European Hematology Association. This is an open access Abstract Book distributed under the Attribution-NonCommercial-NoDerivs (CC BY-NC-ND), which allows third parties to download the articles and share them with others as long as they credit the author and the Abstract Book, but they cannot change the content in any way or use them commercially.
**Abstract Book Citations:** Authors, Title, HemaSphere, 2026;10:(S1):pages. The abstract book DOIs can be found on the “Author Information” page.
**Disclaimer:** Articles published in the journal HemaSphere exclusively reflect the opinions of the authors. The authors are responsible for all content in their abstracts including accuracy of the facts, statements, citing resources, etc.

### (PB3260) DELAYED AND ATYPICAL NEUROTOXICITY FOLLOWING B-CELL MATURATION ANTIGEN-DIRECTED CHIMERIC ANTIGEN RECEPTOR-T CELL THERAPY: A SYSTEMATIC REVIEW AND META-ANALYSIS

**Topic:** 14. Myeloma and other monoclonal gammopathies - Clinical

Derek Tai*[1], Shreya Kansal[2], Kareem Latif[1], Karina Basmajian[3], Justin Nguyen[1], Daniel Park[4], Mojtaba Akhtari[1]

*[1]Loma Linda University Medical Center, Internal Medicine, Loma Linda, United States of America;[2]Touro University Nevada College of Osteopathic Medicine, Henderson, United States of America; [3]Arrowhead Regional Medical Center, Colton, United States of America; [4]Harbor UCLA Medical Center, Torrance, United States of America;*

### Background

Immune effector cell-associated neurotoxicity syndrome (ICANS) is a recognized complication of chimeric antigen receptor (CAR)-T cell therapy. However, emerging data from B-cell maturation antigen (BCMA)-directed CAR-T cell trials, particularly ciltacabtagene autoleucel (cilta-cel), suggest a distinct pattern of delayed atypical neurological toxicities not meeting ICANS criteria, including parkinsonism, cranial nerve palsy, and peripheral neuropathy. The incidence and reproducibility of these atypical neurological adverse reactions remain incompletely defined.

### Aims

We quantified the pooled incidence of ICANS and delayed non-ICANS neurological toxicities following BCMA-directed CAR-T cell therapy in five prospective clinical trials.

### Methods

We conducted a systematic review and meta-analysis of phase I-III prospective trials evaluating idecabtagene vicleucel (ide-cel) and cilta-cel in relapsed or refractory multiple myeloma. Random-effects generalized linear mixed models with logit transformation were used to estimate pooled incidence proportions. Heterogeneity was assessed using the $I^2$ statistic.

### Results

Five prospective trials met inclusion criteria: KarMMa, KarMMa-3, CARTITUDE-1, CARTITUDE-4, and CRB-401 encompassing over 700 treated patients. The pooled incidence of any-grade ICANS was 16% following ide-cel (95% CI, 13-20%, $p$=0.72, $I^2$=0%) and 9% following cilta-cel (95% CI, 3-21%, $p$<0.05, $I^2$=89.8%). Severe ICANS (grade ≥3) occurred in approximately 3% of ide-cel recipients (95% CI, 2-6%, $p$=0.99, $I^2$=0%).

The median onset of delayed neurotoxicity was 27 days. Delayed non-ICANS neurological toxicity was observed exclusively with cilta-cel and occurred in 14% of treated patients (95% CI, 10–18%, $p$=0.49, $I^2$=0%). Subtype-specific pooled incidences were 2% for parkinsonism (95% CI, 0-11%, $p$=0.02, $I^2$=80.2%), 4% for cranial nerve palsy (95% CI, 1-17%, $p$=0.03, $I^2$=78.9%), and 3% for peripheral neuropathy (95% CI, 1-6%, $p$=0.91, $I^2$=0%). Parkinsonism and cranial nerve palsy were significant and heterogeneity was minimal for overall non-ICANS events and peripheral neuropathy but substantial for parkinsonism and cranial nerve palsy.

**Copyright Information:** (Online) ISSN: 2572-9241
©2026 The Author(s). HemaSphere is published by John Wiley & Sons Ltd on behalf of European Hematology Association. This is an open access Abstract Book distributed under the Attribution-NonCommercial-NoDerivs (CC BY-NC-ND), which allows third parties to download the articles and share them with others as long as they credit the author and the Abstract Book, but they cannot change the content in any way or use them commercially.
**Abstract Book Citations:** Authors, Title, HemaSphere, 2026;10:(S1):pages. The abstract book DOIs can be found on the “Author Information” page.
**Disclaimer:** Articles published in the journal HemaSphere exclusively reflect the opinions of the authors. The authors are responsible for all content in their abstracts including accuracy of the facts, statements, citing resources, etc.

**Summary/Conclusion**

Cilta-cel is associated with a reproducible delayed neurological toxicity phenotype distinct from classical ICANS, affecting up to 14% of patients. This meta-analysis has identified parkinsonism and cranial nerve palsy as the most significant atypical neurotoxicites in a pooled analysis of cilta-cel. Practicing oncologists, neurologists, and pharmacists need not only to be aware of these potential toxicities, but also to establish strategies for extended neurological monitoring beyond the traditional ICANS window and highlight the need for standardized classification and mechanistic investigation of atypical neurological adverse events associated with CAR-T therapy.

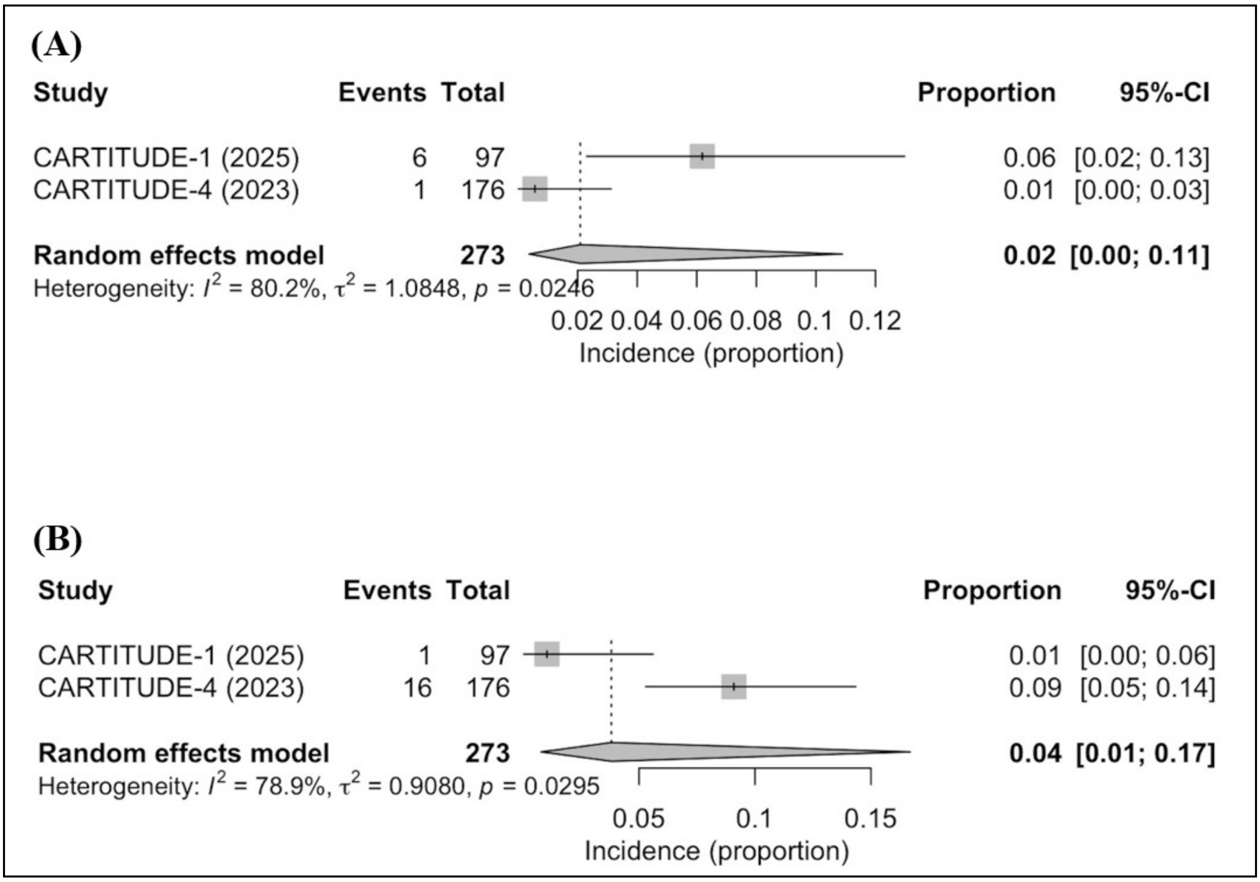

Forest Plot describing delayed atypical neurological toxicity following cilta-cel from CARTITUDE-1 and CARTITUDE-4 trials. (A) Parkinsonism delayed neurological toxicity after cilta-cel showing 2% incidence (95% confidence interval 0-11%, p=0.02, heterogeneity I^2=80.2%). (B) Cranial nerve palsy delayed neurological toxicity after cilta-cel showing 4% incidence (95% confidence interval, 1-17%, p=0.03, heterogeneity I^2=78.9%).

**Copyright Information:** (Online) ISSN: 2572-9241
©2026 The Author(s). HemaSphere is published by John Wiley & Sons Ltd on behalf of European Hematology Association. This is an open access Abstract Book distributed under the Attribution-NonCommercial-NoDerivs (CC BY-NC-ND), which allows third parties to download the articles and share them with others as long as they credit the author and the Abstract Book, but they cannot change the content in any way or use them commercially.
**Abstract Book Citations:** Authors, Title, HemaSphere, 2026;10:(S1):pages. The abstract book DOIs can be found on the "Author Information" page.
**Disclaimer:** Articles published in the journal HemaSphere exclusively reflect the opinions of the authors. The authors are responsible for all content in their abstracts including accuracy of the facts, statements, citing resources, etc.

### (PB3261) THE EVOLVING OUTCOMES OF NEWLY DIAGNOSED (NDMM) HIGH-RISK CYTOGENETIC MULTIPLE MYELOMA IN CHINESE PATIENTS: A 15-YEAR REAL-WORLD ANALYSIS WITH US BENCHMARKING

**Topic:** 14. Myeloma and other monoclonal gammopathies - Clinical

Jingyu Xu[1], Meng Shu*[2], Hsingwen Chung[3], Yuntong Liu[1], Wenqiang Yan[1], Qirui Bai[1], Ning Dai[1], Lingna Li[1], Jian Cui[1], Jieqiong Zhou[1], Yating Li[1], Chenxing Du[1], Shuhui Deng[1], Weiwei Sui[1], Yan Xu[1], Hong Qiu[3], Lugui Qiu[1], Gang An[1]

*[1]State Key Laboratory of Experimental Hematology, National Clinical Research Center for Blood Diseases, Haihe Laboratory of Cell Ecosystem, Institute of Hematology & Blood Diseases Hospital, Chinese Academy of Medical Sciences & Peking Union Medical College, Tianjin, China; [2]Johnson & Johnson, Shanghai, China; [3]Johnson & Johnson, Raritan, United States of America;*

### Background

High-risk cytogenetic abnormalities in multiple myeloma (MM) remain associated with poor prognosis despite global therapeutic advancements. Real-world evidence from these Chinese populations is limited, and cross-regional benchmark is scarce.

### Aims

To examine 15-year trends in survival outcomes among newly diagnosed multiple myeloma (NDMM) patients with high-risk cytogenetics in China, identify independent predictors of progression-free survival (PFS) and overall survival (OS), and benchmark outcomes against a contemporaneous US real-world cohort.

### Methods

A retrospective cohort study was conducted in adults (≥18 years) with NDMM and high-risk cytogenetics treated at a major Chinese myeloma center between January 2008 and June 2023. Multivariable stratified Cox regression, stratified by age group and sex, was used to identify independent predictors of PFS and OS. The proportional hazards assumption was assessed using log-minus log survival plots. Outcomes were benchmarked against high-risk cytogenetics treated MM patients from the Flatiron Health US database.

### Results

A total of 341 Chinese patients were included (median age 57 years; 12.0% aged >65 years; 48.11% ISS stage III). Median follow-up was 33.1 months. Median PFS improved substantially across treatment eras: 17.9 months (2008–2012), 29.9 months (2013–2017), and 42.3 months (2018–2023). Median OS increased from 42.1 months (2008-2012) to 66.9 months (2013-2017) and was not reached (2018-2023).

In multivariable analysis, autologous stem cell transplantation (ASCT) and CD38 antibody–based induction were independently associated with improved PFS. ISS stage III predicted worse OS, while ASCT, CD38 antibody–based induction, and PI+IMiD–based induction predicted improved OS.

Benchmarking against 1,545 US patients (median age 68 years; similar ISS III distribution) showed parallel improvements in PFS across eras (29.3, 34.4, and 42.6 months) and relatively stable OS around 54 months, and consistent prognostic associations for ISS stage, ASCT, and CD38 antibody–based therapy.

**Copyright Information:** (Online) ISSN: 2572-9241
©2026 The Author(s). HemaSphere is published by John Wiley & Sons Ltd on behalf of European Hematology Association. This is an open access Abstract Book distributed under the Attribution-NonCommercial-NoDerivs (CC BY-NC-ND), which allows third parties to download the articles and share them with others as long as they credit the author and the Abstract Book, but they cannot change the content in any way or use them commercially.
**Abstract Book Citations:** Authors, Title, HemaSphere, 2026;10:(S1):pages. The abstract book DOIs can be found on the "Author Information" page.
**Disclaimer:** Articles published in the journal HemaSphere exclusively reflect the opinions of the authors. The authors are responsible for all content in their abstracts including accuracy of the facts, statements, citing resources, etc.

**Summary/Conclusion**

Survival outcomes for high-risk cytogenetic MM patients in China have significantly improved over the past 15 years, largely driven by increased use of ASCT and novel induction regimens. Advanced ISS stage remains an adverse prognostic factor. Alignment with US real-world patterns highlights shared global drivers of treatment optimization for high-risk MM.

**Copyright Information:** (Online) ISSN: 2572-9241
©2026 The Author(s). HemaSphere is published by John Wiley & Sons Ltd on behalf of European Hematology Association. This is an open access Abstract Book distributed under the Attribution-NonCommercial-NoDerivs (CC BY-NC-ND), which allows third parties to download the articles and share them with others as long as they credit the author and the Abstract Book, but they cannot change the content in any way or use them commercially.
**Abstract Book Citations:** Authors, Title, HemaSphere, 2026;10:(S1):pages. The abstract book DOIs can be found on the "Author Information" page.
**Disclaimer:** Articles published in the journal HemaSphere exclusively reflect the opinions of the authors. The authors are responsible for all content in their abstracts including accuracy of the facts, statements, citing resources, etc.

### (PB3262) DESIGN OF THE PHASE 2 ALANIS STUDY: BELANTAMAB MAFODOTIN IN COMBINATION WITH BORTEZOMIB, CYCLOPHOSPHAMIDE, AND DEXAMETHASONE (VCD) IN PATIENTS WITH NEWLY DIAGNOSED AMYLOID LIGHT CHAIN AMYLOIDOSIS

**Topic:** 14. Myeloma and other monoclonal gammopathies - Clinical

Shaji Kumar[1], Ashutosh Wechalekar[2], Vaishali Sanchorawala[3], Giovanni Palladini[4, 5], M. Teresa Cibeira[6], Matthew J. Rees[7], Hang Quach[7], Zeyad Khalaf[8], Jorge Mouro[9], Lydia Eccersley[8], Martin Winiger[9], Julia Branicka[10], Sutapa Mukhopadhyay[11], Veena Vincent[12], Efstathios Kastritis*[13]

*[1]Mayo Clinic, Rochester, MN, United States of America;[2]University College London, London, United Kingdom; [3]Boston Medical Center, Boston, MA, United States of America; [4]Department of Molecular Medicine, University of Pavia, Pavia, Italy; [5]Amyloidosis Research and Treatment Center, Foundation IRCCS Policlinico Sal Matteo, Pavia, Italy; [6]Hospital Clinic of Barcelona, IDIBAPS, Barcelona, Spain; [7]St Vincent's Hospital Melbourne, University of Melbourne, Melbourne, VIC, Australia;[8]GSK, London, United Kingdom; [9]GSK, Baar, Switzerland; [10]ICON Clinical Research, Warsaw, Poland; [11]GSK, Upper Providence, PA, United States of America; [12]GSK, Bengaluru, India; [13]National and Kapodistrian University of Athens, Athens, Greece;*

### Background

Amyloid light chain (AL) amyloidosis is a plasma cell disorder with an unmet need for new treatment options that produce deep responses. B-cell maturation antigen (BCMA)-targeting therapies have shown encouraging preliminary results. Belantamab mafodotin (belamaf) is a BCMA-targeting antibody-drug conjugate that has efficacy as monotherapy in relapsed/refractory AL amyloidosis (Kastritis *Blood*. 2024).

### Aims

Here we present the design of the ALANIS study of belamaf in combination with VCd in newly diagnosed (ND) patients with AL amyloidosis.

**Copyright Information:** (Online) ISSN: 2572-9241
©2026 The Author(s). HemaSphere is published by John Wiley & Sons Ltd on behalf of European Hematology Association. This is an open access Abstract Book distributed under the Attribution-NonCommercial-NoDerivs (CC BY-NC-ND), which allows third parties to download the articles and share them with others as long as they credit the author and the Abstract Book, but they cannot change the content in any way or use them commercially.
**Abstract Book Citations:** Authors, Title, HemaSphere, 2026;10:(S1):pages. The abstract book DOIs can be found on the "Author Information" page.
**Disclaimer:** Articles published in the journal HemaSphere exclusively reflect the opinions of the authors. The authors are responsible for all content in their abstracts including accuracy of the facts, statements, citing resources, etc.

### Methods

ALANIS (NCT07224672) is a phase 2, open-label, multicenter, international, randomized, dose optimization study. Eligible patients are aged ≥18 years with histologically confirmed ND AL amyloidosis, measurable clonal disease, an Eastern Cooperative Group Performance Status of 0–2, and adequate organ function, who are not eligible for high-dose chemotherapy with autologous stem cell transplant. Patients will be excluded if they have other plasma cell dyscrasias, significant non-AL amyloidosis-related cardiovascular conditions, Mayo stage 3B disease, or corneal epithelial disease other than mild punctate keratopathy.
Patients (N=40) will be randomized (1:1) to receive 2 different dose levels of belamaf in combination with VCd weekly for 24 weeks in the dose optimization phase. Randomization will be stratified by cardiac stage (I/II vs IIIa). From 24 weeks, patients will receive belamaf monotherapy at a longer planned cycle length for 18 months. Planned interim analysis will determine the optimal belamaf dose from the dose optimization phase. The dose expansion phase will enroll 20 additional patients to receive the selected dose of belamaf (plus VCd for 24 weeks, followed by monotherapy for 18 months). In both phases, treatment will continue up to 24 months or until progressive disease, death, start of a new anti-AL amyloidosis therapy, unacceptable toxicity, withdrawal of consent, or early study termination, whichever occurs first. Dose modifications (delays/interruptions or reductions) are permitted to mitigate adverse events. The primary endpoint is efficacy defined by complete hematologic response (CHR) rate. Key secondary endpoints are safety (adverse events, serious adverse events, ocular findings on ophthalmic examination), organ response rates for kidney, heart, and liver, duration of CHR, and pharmacokinetics. Exploratory endpoints include major organ deterioration progression-free survival (MOD-PFS; a composite endpoint including time to death and clinical manifestation of cardiac/renal failure [whichever comes first]), hematologic progressive disease, and overall survival. No formal hypothesis will be tested on CHR rate. This study is planned to start enrollment in March 2026.

### Summary/Conclusion

**Funding:** GSK. Drug linker technology licensed from Seagen Inc.; monoclonal antibody produced using POTELLIGENT Technology licensed from BioWa.

**Copyright Information:** (Online) ISSN: 2572-9241
©2026 The Author(s). HemaSphere is published by John Wiley & Sons Ltd on behalf of European Hematology Association. This is an open access Abstract Book distributed under the Attribution-NonCommercial-NoDerivs (CC BY-NC-ND), which allows third parties to download the articles and share them with others as long as they credit the author and the Abstract Book, but they cannot change the content in any way or use them commercially.
**Abstract Book Citations:** Authors, Title, HemaSphere, 2026;10:(S1):pages. The abstract book DOIs can be found on the “Author Information” page.
**Disclaimer:** Articles published in the journal HemaSphere exclusively reflect the opinions of the authors. The authors are responsible for all content in their abstracts including accuracy of the facts, statements, citing resources, etc.

### (PB3263) PROGNOSTIC IMPACT OF BASELINE CHARACTERISTICS IN EXTRAMEDULLARY MYELOMA: A RETROSPECTIVE MULTICENTRIC ANALYSIS

**Topic:** 14. Myeloma and other monoclonal gammopathies - Clinical

Maria Beatrice Ferrari*[1], Francesco Pierpaoli[1], Anna Mochi[1], Elena Agostani[1], Francesca Guidotti[2], Anna Maria Cafro[3], Stefano Crippa[1], Mauro Turrini[2], Alessandra Perego[1], Sara Pezzatti[1], Carlo Gambacorti-Passerini[1]

*[1]University of Milan Bicocca, Hematology, Milan, Italy; [2]Valduce Hospital, Hematology, Como, Italy; [3]ASST Niguarda Hospital, Milan, Italy;*

### Background

Extramedullary myeloma (EMM) represents an aggressive clinical phenotype of plasma cell neoplasia characterized by clonal proliferation outside the bone marrow microenvironment. Escape from stromal dependence is associated with genomic instability, treatment resistance, and inferior survival. Current staging systems incompletely capture this biological heterogeneity, and real-world data describing baseline features and outcome determinants at diagnosis remain scarce.

### Aims

To characterize the clinical, pathologic, and cytogenetic features of newly diagnosed EMM and to identify independent predictors of survival in a multicenter cohort.

### Methods

We performed a retrospective study across four Italian hematology centers including 87 patients diagnosed with EMM between 2010 and 2024. Disease phenotype was categorized as paraskeletal (PSD) or bone-independent EMM according to imaging and histologic criteria. Baseline assessment included PET/CT metabolic activity and number of extramedullary sites, LDH, β2-microglobulin, hemoglobin, creatinine, calcium, bone marrow plasma cell infiltration, and FISH cytogenetics [del(17p), del(13q), del(1p), 1q gain/amplification, t(11;14), t(4;14), t(14;16), t(14;20)]. Overall survival (OS) was the primary endpoint and progression-free survival (PFS) secondary. Survival curves were estimated by Kaplan–Meier analysis and prognostic variables tested using Cox proportional hazards models. Multiple imputation and cross-validated feature selection were applied to improve model robustness.

### Results

Median age at diagnosis was 65 years (range 39–82); 53% were female. PSD accounted for 75% of cases and bone-independent EMM for 25%. Multiple extramedullary sites were present in 41% of patients and were more frequent in bone-independent disease. High-risk cytogenetic abnormalities were common, including 1q gain/amplification (18%), del(17p) (14%), del(13q) (13%), del(1p) (9%), and high-risk IgH translocations (12%). Median OS for the entire cohort was 48 months (95% CI 43–108) and median PFS 23 months (95% CI 18–36). Patients with bone-independent EMM showed significantly inferior OS compared with PSD, with earlier relapse and reduced post-progression survival, consistent with a more treatment-resistant phenotype. PSD cases demonstrated relatively prolonged disease control, suggesting partial persistence of bone marrow microenvironment dependence. On univariate analysis, anemia, renal impairment, del(1p), EMM phenotype, age, LDH, and marrow plasma cell burden were associated with reduced OS. Multivariable modeling confirmed bone-independent phenotype (HR 2.8, p=0.004), age (HR 1.08/year, p=0.0003), LDH (HR 1.16 per 50 U/L, p=0.0001), and marrow plasma cell percentage (HR 1.02 per point, p=0.006) as independent predictors. First-line treatment was heterogeneous and included PI/IMiD triplets, daratumumab-based combinations, intensive chemotherapy ± radiotherapy, and autologous transplantation, reflecting the absence of standardized algorithms

**Copyright Information:** (Online) ISSN: 2572-9241
©2026 The Author(s). HemaSphere is published by John Wiley & Sons Ltd on behalf of European Hematology Association. This is an open access Abstract Book distributed under the Attribution-NonCommercial-NoDerivs (CC BY-NC-ND), which allows third parties to download the articles and share them with others as long as they credit the author and the Abstract Book, but they cannot change the content in any way or use them commercially.
**Abstract Book Citations:** Authors, Title, HemaSphere, 2026;10:(S1):pages. The abstract book DOIs can be found on the "Author Information" page.
**Disclaimer:** Articles published in the journal HemaSphere exclusively reflect the opinions of the authors. The authors are responsible for all content in their abstracts including accuracy of the facts, statements, citing resources, etc.

### Summary/Conclusion

Bone-independent EMM identifies a biologically distinct subgroup with markedly inferior survival compared with paraskeletal disease, supporting the concept of stromal escape as a driver of aggressive behavior. Elevated LDH, age, and marrow tumor burden further refine risk stratification. Despite contemporary therapy, outcomes remain poor, underscoring the need for EMM-specific prognostic models biology-driven therapeutic strategies

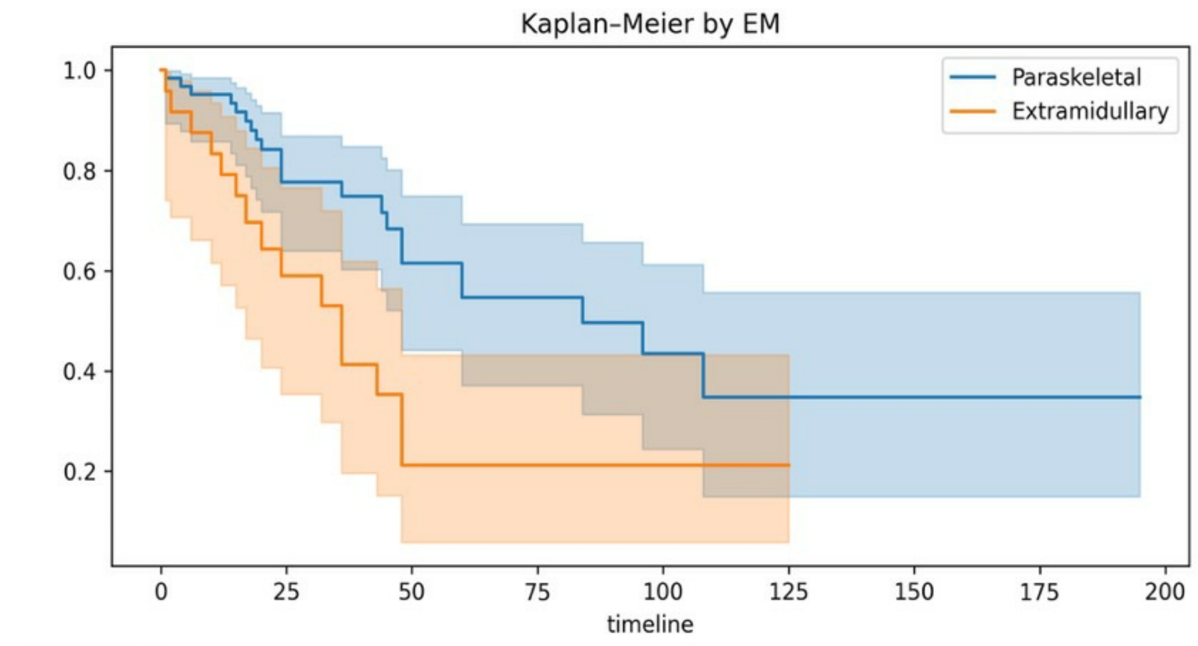

| | 0 | 25 | 50 | 75 | 100 | 125 | 150 | 175 | 200 |
|---|---|---|---|---|---|---|---|---|---|
| **Paraskeletal** | | | | | | | | | |
| At risk | 63 | 33 | 18 | 11 | 5 | 3 | 2 | 2 | 0 |
| Censored | 0 | 18 | 28 | 33 | 37 | 38 | 39 | 39 | 41 |
| Events | 0 | 12 | 17 | 19 | 21 | 22 | 22 | 22 | 22 |
| **Extramidullary** | | | | | | | | | |
| At risk | 24 | 11 | 3 | 2 | 2 | 0 | 0 | 0 | 0 |
| Censored | 0 | 4 | 6 | 7 | 7 | 9 | 9 | 9 | 9 |
| Events | 0 | 9 | 15 | 15 | 15 | 15 | 15 | 15 | 15 |

Overall Survival in pure EMD and Paraskeletal EMD.EMD OS was 36 months while Paraskeletal OS was 84 months.

**Copyright Information:** (Online) ISSN: 2572-9241
©2026 The Author(s). HemaSphere is published by John Wiley & Sons Ltd on behalf of European Hematology Association. This is an open access Abstract Book distributed under the Attribution-NonCommercial-NoDerivs (CC BY-NC-ND), which allows third parties to download the articles and share them with others as long as they credit the author and the Abstract Book, but they cannot change the content in any way or use them commercially.
**Abstract Book Citations:** Authors, Title, HemaSphere, 2026;10:(S1):pages. The abstract book DOIs can be found on the "Author Information" page.
**Disclaimer:** Articles published in the journal HemaSphere exclusively reflect the opinions of the authors. The authors are responsible for all content in their abstracts including accuracy of the facts, statements, citing resources, etc.

### (PB3264) SELINEXOR, BORTEZOMIB AND DEXAMETHASONE SHOWS EFFICACY IN HEPTA-REFRACTORY MULTIPLE MYELOMA - A MULTICENTER ANALYSIS

**Topic:** 14. Myeloma and other monoclonal gammopathies - Clinical

Maximilian Al-Bazaz*[1], Winfried Alsdorf[1], Lisa Leypoldt[1, 2], Piet Sonnemann[1], Christoph Schaefers[1], Jule Artzenroth[1], Leandra Bartke[1], Jan Vorwerk[3], Theo Leitner[3], Markus Maulhardt[4], Kerstin Brinkert[5], Annamaria Brioli[5], Tim Richardson[6], Udo Holtick[6], Stefan Hillmann[7], Hans Juergen Dr. Salwender[7], Cyrus Khandanpour[8, 9], Carsten Bokemeyer[1], Katja C. Weisel[1], Ricardo Kosch[1]

*[1]University Medical Center Hamburg-Eppendorf, Department of Oncology, Hematology and Bone Marrow Transplantation with Division of Pneumology, Hamburg, Germany; [2]University Medical Center Hamburg-Eppendorf, Mildred Scheel Early Career Center, Hamburg, Germany; [3]University Medical Center Schleswig-Holstein, Campus Lübeck, Department of Hematology, Oncology, Lübeck, Germany; [4]University Medical Center Göttingen, Department of Hematology and Medical Oncology, Göttingen, Germany; [5]Hannover Medical School, Clinic for Hematology, Hemostaseology, Oncology and Stem Cell Transplantation, Hannover, Germany; [6]University Hospital of Cologne, Department I of Internal Medicine, Cologne, Germany; [7]Asklepios Klinik Altona and St. Georg, Asklepios Tumorzentrum Hamburg, Hamburg, Germany; [8]Klinikum Oldenburg, Department of Hematology and Oncology, Oldenburg, Germany; [9]Carl von Ossietzky University, University Medicine Oldenburg, Oldenburg, Germany;*

### Background

Outcomes in multiple myeloma (MM) have been substantially improved and transformed with immunotherapies targeting BCMA and GPRC5D. However, patients frequently relapse, and the emergence of patients who are not only penta-class refractory (resistance to two proteasome inhibitors, two immunomodulatory drugs, and an anti-CD38 antibody) but also refractory to BCMA- and GPRC5D-directed therapies (*hepta-refractory*) represents an urgent and distinct clinical challenge. Selinexor, a selective inhibitor of nuclear export targeting XPO1, forces the nuclear retention of tumor suppressors and inhibits oncoprotein translation. This antigen-independent mechanism offers a strong rationale for overcoming cross-resistance to T-cell therapies. While the combination of selinexor, bortezomib, and dexamethasone (SVd) is established, its efficacy in the post-immunotherapy setting remains undefined.

### Aims

We aimed to evaluate the efficacy and safety of the SVd regimen in a hepta-refractory patient population.

### Methods

We conducted a multicenter retrospective analysis of patients with relapsed or refractory MM treated at six German tertiary centers from 12/2023 to 10/2025. Eligible patients had penta-refractory disease and documented progression following BCMA- and GPRC5D-directed therapies. The cohort comprised 18 patients who received selinexor (60 to 100 mg weekly), bortezomib (1.3 $mg/m^2$ weekly), and dexamethasone (20 to 40 mg weekly). Primary endpoints were overall response rate (ORR) and progression-free survival rate (PFS), responses were assessed per IMWG criteria.

**Copyright Information:** (Online) ISSN: 2572-9241
©2026 The Author(s). HemaSphere is published by John Wiley & Sons Ltd on behalf of European Hematology Association. This is an open access Abstract Book distributed under the Attribution-NonCommercial-NoDerivs (CC BY-NC-ND), which allows third parties to download the articles and share them with others as long as they credit the author and the Abstract Book, but they cannot change the content in any way or use them commercially.
**Abstract Book Citations:** Authors, Title, HemaSphere, 2026;10:(S1):pages. The abstract book DOIs can be found on the "Author Information" page.
**Disclaimer:** Articles published in the journal HemaSphere exclusively reflect the opinions of the authors. The authors are responsible for all content in their abstracts including accuracy of the facts, statements, citing resources, etc.

**Results**

Patients were exceptionally heavily pretreated, with a median of seven prior lines of therapy (range, 5–11) over a median of 9.5 years. The median age was 63 years (range, 40–76), and all had undergone prior autologous stem cell transplantation. High-risk features included active extramedullary disease in 50% (9/18 patients) of the cohort and del(17p) in 33% (6/18 patients). SVd demonstrated substantial clinical activity, achieving a 61% ORR (11/18 patients) and a 78% disease control rate (14/18 patients). The median time to first response was 0.9 months. Median PFS was 4.3 months, and median overall survival rate reached 17.1 months. Among the nine patients presenting with extramedullary disease, 44% (4/9 patients) achieved complete or near-complete radiographic resolution. The regimen provided meaningful disease control in 4/6 patients with del(17p) and successfully bridged three patients to subsequent CAR T apheresis and therapy. The safety profile was manageable and consistent with known toxicities. Hematologic adverse events were most frequent, including grade 3 to 4 thrombocytopenia in 63%, anemia in 53%, and neutropenia in 25%. Non-hematologic adverse events, such as fatigue and nausea, were primarily limited to Grades 1 and 2. Importantly, no severe infections or treatment-related deaths occurred. Selinexor dose reduction was required in five patients and discontinued in one patient, while bortezomib was interrupted or discontinued in four patients.

**Summary/Conclusion**

In conclusion, SVd demonstrates significant efficacy and in *hepta-refractory* MM (penta-refractory MM with progression following BCMA- and GPRC5D-directed therapies). The regimen's ability to induce rapid responses, control extramedullary disease, and serve as a disease controlling therapy to subsequent cellular therapies positions it as a vital tool in the management of late-line MM in the "post-immunotherapy" setting.

**Copyright Information:** (Online) ISSN: 2572-9241
©2026 The Author(s). HemaSphere is published by John Wiley & Sons Ltd on behalf of European Hematology Association. This is an open access Abstract Book distributed under the Attribution-NonCommercial-NoDerivs (CC BY-NC-ND), which allows third parties to download the articles and share them with others as long as they credit the author and the Abstract Book, but they cannot change the content in any way or use them commercially.
**Abstract Book Citations:** Authors, Title, HemaSphere, 2026;10:(S1):pages. The abstract book DOIs can be found on the "Author Information" page.
**Disclaimer:** Articles published in the journal HemaSphere exclusively reflect the opinions of the authors. The authors are responsible for all content in their abstracts including accuracy of the facts, statements, citing resources, etc.

### (PB3265) THE IMPACT OF FIRST-LINE DARATUMUMAB ON LIGHT-CHAIN AMYLOIDOSIS SURVIVAL: A REAL-WORLD ANALYSIS OF 70 PATIENTS

**Topic:** 14. Myeloma and other monoclonal gammopathies - Clinical

Fruzsina Szél*[1], István Wiedemann[1], Szabolcs Svorenj[1], Mate Huszar[1], Virag Reka Szita[1], Zoltán Pozsonyi[1], Gabor Mikala[1], Masszi Tamás Pál[1], Gergely Varga[1]

*[1]Semmelweis University, Department of Internal Medicine and Hematology, Budapest, Hungary;*

### Background

Daratumumab-based induction therapy has established itself as the gold standard for light-chain (AL) amyloidosis following the landmark ANDROMEDA trial (Kastritis et al. N Engl J Med 2021). However, several clinical questions remain, including the optimal combination partners, the ideal number of treatment cycles, and the appropriate dosing of adjunct therapies such as dexamethasone. Furthermore, strategies for managing suboptimal responses are still subjects of debate. Real-world evidence is essential to address these gaps and refine clinical practice.

### Aims

To evaluate the real-world impact of daratumumab-based frontline therapy on survival and organ responses in patients with AL amyloidosis treated at our institution.

### Methods

We performed a retrospective analysis of 70 patients with AL amyloidosis treated between 2013 and 2025 at our institution. Daratumumab became available in 2022 and its use was partially restricted by reimbursement limitations. Clinical characteristics, treatment regimens, survival outcomes, and organ responses were analyzed. Survival was evaluated using overall survival (OS) and major organ deterioration–progression-free survival (MOD-PFS).

### Results

The annual incidence of diagnosed cases increased significantly over time, with two-thirds of patients diagnosed in the latter half of the study period, likely reflecting increased clinical awareness and more streamlined referral pathways.
Within the cohort, 24 patients received frontline daratumumab-based therapy, predominantly in combination with bortezomib and dexamethasone (D-VCd-like). Lenalidomide was substituted for bortezomib in three cases due to severe baseline neuropathy. The median duration of daratumumab treatment was 8 cycles, while cyclophosphamide was generally omitted. Dexamethasone was administered at a reduced dose of 8 mg weekly in all but one patient.
The 6-year OS was 61% in the daratumumab group compared to 39% in the non-daratumumab group (p=0.12). Among the 17 patients meeting ANDROMEDA inclusion criteria, the 6-year OS reached 73%, consistent with long-term outcomes reported by Yohannan et al. (Blood Neoplasia, 2025), with no deaths beyond 24 months. Similarly, the 24-month MOD-PFS was comparable to ANDROMEDA results. Daratumumab-based induction resulted in superior organ response rates compared to other regimens (cardiac response: 73.4% vs. 46.5%, p=0.04; renal response: 55.6% vs. 23.3%, p=0.08).

### Summary/Conclusion

These real-world data support the frontline use of daratumumab in AL amyloidosis. De-escalated dexamethasone dosing and a limited number of treatment cycles may maintain favorable clinical outcomes without jeopardizing efficacy.

**Copyright Information:** (Online) ISSN: 2572-9241
©2026 The Author(s). HemaSphere is published by John Wiley & Sons Ltd on behalf of European Hematology Association. This is an open access Abstract Book distributed under the Attribution-NonCommercial-NoDerivs (CC BY-NC-ND), which allows third parties to download the articles and share them with others as long as they credit the author and the Abstract Book, but they cannot change the content in any way or use them commercially.
**Abstract Book Citations:** Authors, Title, HemaSphere, 2026;10:(S1):pages. The abstract book DOIs can be found on the “Author Information” page.
**Disclaimer:** Articles published in the journal HemaSphere exclusively reflect the opinions of the authors. The authors are responsible for all content in their abstracts including accuracy of the facts, statements, citing resources, etc.

### (PB3266) OUTCOMES OF PATIENTS WITH INADEQUATE EARLY RESPONSE TO DARATUMUMAB UPFRONT THERAPY

**Topic:** 14. Myeloma and other monoclonal gammopathies - Clinical

Foteini Theodorakakou*[1], Despina Fotiou[1], Eirini Solia[1], Nikolaos Kanellias[1], Vassiliki Spiliopoulou[1], Magdalini Migkou[1], Evangelos Eleutherakis-Papaiakovou[1], Ioannis Ntanasis-Stathopoulos[1], Panagiotis Malandrakis[1], Asimina Papanikolaou[2], Maria Gavriatopoulou[1], Evangelos Terpos[1], Meletios A. Dimopoulos[1], Efstathios Kastritis[1]

*[1]National and Kapodistrian University of Athens, Department of Clinical Therapeutics, Athens, Greece;[2]Evangelismos Hospital, Athens, Greece;*

### Background

The introduction of daratumumab substantially improved rates of rapid and deep hematologic and organ responses; however, there is a group of patients (pts) with inadequate hematologic response to frontline daratumumab.

### Aims

To evaluate the outcomes of pts with AL amyloidosis who did not achieve ≥hemVGPR after 1 month of daratumumab-based therapy.

### Methods

We identified 50 newly diagnosed pts treated with daratumumab-based regimens and had not achieved ≥hemVGPR after 1 month of treatment, of which in 21 pts treatment was modified and 29 did not change therapy.

**Copyright Information:** (Online) ISSN: 2572-9241
©2026 The Author(s). HemaSphere is published by John Wiley & Sons Ltd on behalf of European Hematology Association. This is an open access Abstract Book distributed under the Attribution-NonCommercial-NoDerivs (CC BY-NC-ND), which allows third parties to download the articles and share them with others as long as they credit the author and the Abstract Book, but they cannot change the content in any way or use them commercially.
**Abstract Book Citations:** Authors, Title, HemaSphere, 2026;10:(S1):pages. The abstract book DOIs can be found on the “Author Information” page.
**Disclaimer:** Articles published in the journal HemaSphere exclusively reflect the opinions of the authors. The authors are responsible for all content in their abstracts including accuracy of the facts, statements, citing resources, etc.

**Results**

Median age was 67 years; 53% were females; 92% had cardiac and 49% renal involvement. Per Mayo stage 8%/33%/18%/41% were stage 1/2/3a/3b and 49% harbored t(11;14). Frontline therapy was daratumumab with bortezomib in 53% and daratumumab monotherapy in 47%.
More pts in the early change group were Mayo stage 3b (50% vs 34%), had significantly higher levels of hsTnT (median 115 vs 56.5 ng/L, p=0.046), lower BMPC (median 10% vs 20%, p=0.009) and a trend toward more impaired GLS (median -11.8% vs -14.5%, p=0.074). Also, they were more likely to have received daratumumab monotherapy, as they had more severe cardiac disease (82% vs 21%, p<0.001). The modification of therapy included addition of bortezomib in 15 pts, of venetoclax in 6 and of pomalidomide in 1.
At 1-month landmark, hematologic response was hemPR in 93% and NR in 7% for those who did not change therapy vs PR in 62% and NR in 38% for those who did (p=0.005). At 3-months, hematologic response rates were 50% CR/VGPR and 50% PR for those who did not change therapy vs 35% CR/VGPR, 45% PR and 20% NR (p=0.066); 10/24 pts among those who did not change therapy, improved their response vs 10/20 among those who modified their treatment. At 6-month landmark hematologic response rates were 74% CR/VGPR and 26% PR for those who did not change therapy vs 47% CR/VGPR, 29% PR and 12% NR (p=0.148).
According to dFLC at 6 months, 61% of pts who did not change therapy had dFLC < 20mg/L vs 29% (p=0.266).
Numerically more pts in the group of those who did not change therapy achieved organ responses: at 3-month 42% vs 30% had cardiac and 17% vs 15% renal response, at 6-month 39% vs 35% and 22% vs 12%, and at 12-month 67% vs 43% and 14% vs 25%; regarding graded cardiac responses more patients in the group that remained on initial treatment, achieved carCR/VGPR (18% vs 12% at 6 months and 34% vs 25% at 12 months) but the differences were not statistically significant.
Median follow up for the whole cohort was 31 months; 7 pts died in the group that did not change therapy and 11 in the group that received early salvage. Pts who received early salvage had significantly inferior survival (median OS 12.2 months vs NR, p=0.047). Among pts who achieved CR/VGPR at 3 months those who received salvage therapy had significantly inferior survival (median OS of 27.7 months vs NR, p=0.012) and similarly at 6 months (Fig1B&C). After adjustment for Mayo stage 3b and hem response at 3 months having received early salvage therapy remained an independent predictor of poor survival (HR 8.179, 95% CI 1.584-42.235, p=0.012).

**Summary/Conclusion**

A proportion of pts with AL amyloidosis with less severe cardiac disease who fail to achieve ≥hemVGPR after 1 month of daratumumab-based therapy can improve their hematologic response and achieve meaningful cardiac responses. Patients with advanced cardiac AL who fail to achieve rapid deep hematologic response can be partly salvaged but still their outcome is poor.

**Copyright Information:** (Online) ISSN: 2572-9241
©2026 The Author(s). HemaSphere is published by John Wiley & Sons Ltd on behalf of European Hematology Association. This is an open access Abstract Book distributed under the Attribution-NonCommercial-NoDerivs (CC BY-NC-ND), which allows third parties to download the articles and share them with others as long as they credit the author and the Abstract Book, but they cannot change the content in any way or use them commercially.
**Abstract Book Citations:** Authors, Title, HemaSphere, 2026;10:(S1):pages. The abstract book DOIs can be found on the "Author Information" page.
**Disclaimer:** Articles published in the journal HemaSphere exclusively reflect the opinions of the authors. The authors are responsible for all content in their abstracts including accuracy of the facts, statements, citing resources, etc.

### (PB3268) ANTI-CD38 QUADRUPLET VS TRIPLET INDUCTION IN TRANSPLANT-ELIGIBLE NDMM: A BAYESIAN NETWORK META-ANALYSIS COMPARING D-VRD AND ISA-RVD

**Topic:** 14. Myeloma and other monoclonal gammopathies - Clinical

Shankar Biswas*[1], Yashasvi Srivastava[1], Urooj Iftekhar[2]

*[1]Ivano-Frankivsk National Medical University, Internal Medicine, Ivano-Frankivsk, Ukraine; [2]Tbilisi State Medical University, Internal Medicine, Tbilisi, Georgia;*

### Background

Anti-CD38 monoclonal antibody-based quadruplet regimens have become the preferred induction for transplant-eligible newly diagnosed multiple myeloma (TE-NDMM), with D-VRd (daratumumab, bortezomib, lenalidomide, dexamethasone) and Isa-RVd (isatuximab, bortezomib, lenalidomide, dexamethasone) both demonstrating superiority over VRd triplet therapy. However, no head-to-head trial has compared these two quadruplets, and the relative efficacy of daratumumab- versus isatuximab-based induction remains uncertain.

### Aims

To perform a Bayesian network meta-analysis (NMA) indirectly comparing the efficacy of D-VRd, Isa-RVd, and VRd for induction therapy in TE-NDMM, using progression-free survival (PFS) and MRD negativity ($10^{-5}$) as co-primary outcomes.

### Methods

We conducted a systematic review (PRISMA/Cochrane; PROSPERO registered) searching PubMed, Embase, CENTRAL, and conference proceedings through January 2026. Eligible studies were RCTs comparing anti-CD38 quadruplets (D-VRd or Isa-RVd) to VRd in TE-NDMM. Three trials were included: PERSEUS (D-VRd vs VRd; N=709), GRIFFIN (D-VRd vs VRd; N=207), and GMMG-HD7 (Isa-RVd vs VRd; N=660), totaling 1,576 patients connected through the VRd common comparator. Bayesian fixed-effect NMA was performed using the gemtc package with contrast-based models (4 chains, 50,000 iterations, 10,000 burn-in). Treatment rankings were assessed via SUCRA. Risk of bias was evaluated using Cochrane RoB 2. Sensitivity analyses included random-effects modeling, exclusion of the phase II GRIFFIN trial, and use of a weighted HR for GMMG-HD7.

### Results

In pairwise class-effect meta-analysis, anti-CD38 quadruplets significantly improved PFS versus VRd (HR 0.55; 95% CI 0.44–0.68) and MRD negativity (OR 2.80; 95% CI 2.26–3.45). The Bayesian NMA ($I^2$=0.5%; DIC=3.99) demonstrated that both quadruplets significantly improved PFS versus VRd, with D-VRd showing a greater magnitude of benefit: D-VRd vs VRd HR 0.42 (95% CrI 0.31–0.58), Isa-RVd vs VRd HR 0.70 (95% CrI 0.52–0.95). The indirect comparison favored D-VRd over Isa-RVd (HR 0.61; 95% CrI 0.39–0.93). For MRD negativity at $10^{-5}$, D-VRd vs VRd OR 3.50 (95% CrI 2.63–4.66) and Isa-RVd vs VRd OR 2.13 (95% CrI 1.56–2.92). SUCRA rankings consistently favored D-VRd (PFS: 99.4%; MRD: 99.4%) over Isa-RVd (PFS: 50.0%; MRD: 50.6%) and VRd (PFS: 0.5%; MRD: 0.0%). Results were robust across all sensitivity analyses. Subgroup analysis revealed a significant interaction for cytogenetic risk (p=0.042), with greater quadruplet benefit in standard-risk disease (HR 0.48) versus high-risk (HR 0.78).

**Copyright Information:** (Online) ISSN: 2572-9241
©2026 The Author(s). HemaSphere is published by John Wiley & Sons Ltd on behalf of European Hematology Association. This is an open access Abstract Book distributed under the Attribution-NonCommercial-NoDerivs (CC BY-NC-ND), which allows third parties to download the articles and share them with others as long as they credit the author and the Abstract Book, but they cannot change the content in any way or use them commercially.
**Abstract Book Citations:** Authors, Title, HemaSphere, 2026;10:(S1):pages. The abstract book DOIs can be found on the "Author Information" page.
**Disclaimer:** Articles published in the journal HemaSphere exclusively reflect the opinions of the authors. The authors are responsible for all content in their abstracts including accuracy of the facts, statements, citing resources, etc.

### Summary/Conclusion

This first NMA comparing anti-CD38 quadruplets in TE-NDMM demonstrates that both D-VRd and Isa-RVd significantly improve PFS and MRD negativity over VRd. The indirect comparison suggests D-VRd may confer superior PFS benefit versus Isa-RVd. These findings should be interpreted cautiously given cross-trial heterogeneity, differing follow-up durations, and the absence of direct comparative data. Head-to-head trials are warranted to confirm these findings.

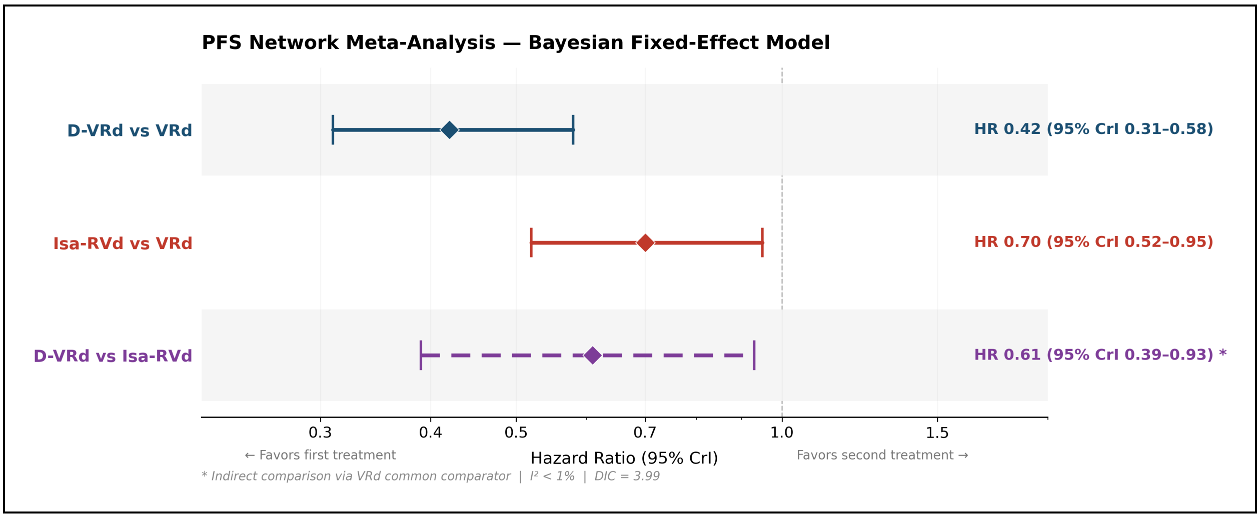

Abbreviations: CrI, credible interval; D-VRd, daratumumab-bortezomib-lenalidomide-dexamethasone; DIC, deviance information criterion; HR, hazard ratio; Isa-RVd, isatuximab-bortezomib-lenalidomide-dexamethasone; PFS, progression-free survival; VRd, bortezomib-lenalidomide-dexamethasone. The D-VRd vs Isa-RVd comparison (dashed line) represents an indirect estimate via the VRd common comparator; $I^2$ <1% indicates negligible heterogeneity; DIC=3.99 supports fixed-effect model fit. Asterisk () denotes statistical significance (95% CrI excludes 1.0).*

**Copyright Information:** (Online) ISSN: 2572-9241
©2026 The Author(s). HemaSphere is published by John Wiley & Sons Ltd on behalf of European Hematology Association. This is an open access Abstract Book distributed under the Attribution-NonCommercial-NoDerivs (CC BY-NC-ND), which allows third parties to download the articles and share them with others as long as they credit the author and the Abstract Book, but they cannot change the content in any way or use them commercially.
**Abstract Book Citations:** Authors, Title, HemaSphere, 2026;10:(S1):pages. The abstract book DOIs can be found on the "Author Information" page.
**Disclaimer:** Articles published in the journal HemaSphere exclusively reflect the opinions of the authors. The authors are responsible for all content in their abstracts including accuracy of the facts, statements, citing resources, etc.

### (PB3269) IINFECTIONS FOLLOWING COMBINED THERAPY WITH A GPRC5DXCD3 BISPECIFIC (TALQUETAMAB) OR BCMA BISPECIFIC ANTIBODIES (ELRANATAMAB/TECLISTAMAB) AND TOCILIZUMAB, IN MULTIPLE MYELOMA PATIENTS

**Topic:** 14. Myeloma and other monoclonal gammopathies - Clinical

Bojana Mandic*[1], Lanzhu Yue[1], Olga Tesarova[1], Maria Ahmad[1], Heidi Cho[1], Jaime Smith[1], Nancy Lunney[1], Ehtesham Iqbal[1]

[1]*Parexel, Global Medical Services, Raleigh, United States of America;*

### Background

The treatment landscape for multiple myeloma has evolved with the FDA approval of talquetamab, a GPRC5D×CD3 bispecific antibody (BsAb), and the BCMA×CD3 BsAbs elranatamab and teclistamab. These agents demonstrate high response rates but are associated with cytokine release syndrome (CRS), immune effector cell–associated neurotoxicity syndrome (ICANS), and infections. Tocilizumab is routinely used for CRS treatment and prophylaxis. However, combined immune effects including BsAb-mediated plasma cell depletion and IL-6 blockade from tocilizumab may contribute to increased infection risk. Only the talquetamab + tocilizumab study has specifically analyzed infection rates, while elranatamab and teclistamab publications include prophylaxis cohorts where infections are reported but not consistently evaluated.

### Aims

Given the complex interplay between BsAb immunomodulation and IL-6 inhibition, this study assesses infection rates in real-world multiple myeloma patients receiving BsAbs with tocilizumab and evaluates whether BsAb target (GPRC5D vs BCMA) influences infection risk.

### Methods

A retrospective real-world data analysis was conducted using the TriNetX database on November 7, 2025, across 83 healthcare organizations. Patients with multiple myeloma were identified and stratified into two cohorts: (1) talquetamab recipients who also received an interleukin-6 inhibitor (IL6i), excluding elranatamab/teclistamab; and (2) elranatamab or teclistamab recipients who also received an IL6i. Propensity score matching (1:1) was performed to balance baseline characteristics, including age and prior immunoglobulin use. The index date was the first BsAb administration. Primary outcomes included incidence of infections specifically sepsis, respiratory infections, and cytomegalovirus (CMV) and all-cause mortality. Risk ratios (RR), hazard ratios (HR), and 95% confidence intervals (CI) were calculated, and Kaplan–Meier analysis with log-rank testing compared survival.

### Results

A total of 204 patients were identified: 51 in the talquetamab + IL6i cohort and 153 in the elranatamab/teclistamab + IL6i cohort. After matching, 51 patients remained in each group. No statistically significant difference in all-cause mortality was observed (19.61% talquetamab vs 23.53% elranatamab/teclistamab; HR 0.771, 95% CI 0.339–1.751; p=0.534). Infection rates were also similar between cohorts. Sepsis occurred in 15.69% vs 17.65% (RR 0.889, 95% CI 0.369–2.144; p=0.792), respiratory infections in 41.18% vs 37.25% (RR 1.105, 95% CI 0.672–1.817; p=0.686), and CMV in 0% vs 3.92% (p=0.153). Overall infection incidence was comparable (62.75% vs 60.78%; RR 1.032, 95% CI 0.760–1.402; p=0.838).

**Copyright Information:** (Online) ISSN: 2572-9241
©2026 The Author(s). HemaSphere is published by John Wiley & Sons Ltd on behalf of European Hematology Association. This is an open access Abstract Book distributed under the Attribution-NonCommercial-NoDerivs (CC BY-NC-ND), which allows third parties to download the articles and share them with others as long as they credit the author and the Abstract Book, but they cannot change the content in any way or use them commercially.
**Abstract Book Citations:** Authors, Title, HemaSphere, 2026;10:(S1):pages. The abstract book DOIs can be found on the "Author Information" page.
**Disclaimer:** Articles published in the journal HemaSphere exclusively reflect the opinions of the authors. The authors are responsible for all content in their abstracts including accuracy of the facts, statements, citing resources, etc.

**Summary/Conclusion**

Real-world data show elevated but not statistically significant differences in infection risk or all-cause mortality between patients treated with talquetamab + tocilizumab and those treated with BCMA-directed BsAbs + tocilizumab. Given the essential role of tocilizumab in CRS management, understanding infection risk in this population is critical. Larger studies are needed to clarify infection patterns and guide prevention strategies in patients receiving BsAbs with IL-6 inhibition.

**Copyright Information:** (Online) ISSN: 2572-9241
©2026 The Author(s). HemaSphere is published by John Wiley & Sons Ltd on behalf of European Hematology Association. This is an open access Abstract Book distributed under the Attribution-NonCommercial-NoDerivs (CC BY-NC-ND), which allows third parties to download the articles and share them with others as long as they credit the author and the Abstract Book, but they cannot change the content in any way or use them commercially.
**Abstract Book Citations:** Authors, Title, HemaSphere, 2026;10:(S1):pages. The abstract book DOIs can be found on the "Author Information" page.
**Disclaimer:** Articles published in the journal HemaSphere exclusively reflect the opinions of the authors. The authors are responsible for all content in their abstracts including accuracy of the facts, statements, citing resources, etc.

### (PB3270) REAL-WORLD EVALUATION OF INTRAVENOUS IMMUNOGLOBULIN USE IN COMBINATION WITH BISPECIFIC ANTIBODIES IN PATIENTS WITH MULTIPLE MYELOMA

**Topic:** 14. Myeloma and other monoclonal gammopathies - Clinical

Vincent Rajkumar*[1], Meletios Dimopoulos[2], Mike Gart[3], Jing-Zhou Hou[4], Rushir Choksi[4], Taral Patel[5], Simon Blanc[6], Jeffrey Scott[3], Anna Rui[3], Don Parris[3], Cecelia Wall[3], Brandon Wai Hua Wang[3], Prateesh Varughese[3], María-Victoria Mateos[7]

*[1]Mayo Clinic, Rochester, United States of America; [2]University of Athens School of Medicine, Athens, Greece; [3]IntegraConnect, West Palm Beach, United States of America; [4]University of Pittsburgh Medical Center, Pittsburgh, United States of America; [5]Zangmeister Cancer Center, Columbus, United States of America; [6]Broward Health, Fort Lauderdale, United States of America; [7]University Hospital of Salamanca, Salamanca, Spain;*

### Background

Bispecific antibodies (BsAbs) have improved outcomes for patients (pts) with relapsed/ refractory multiple myeloma (MM), offering deep, durable responses. However, BsAb therapy carries increased infection risk, likely due to hypogammaglobulinemia and immune dysregulation. Intravenous immunoglobulin (IVIG) mitigates infection risk and manage hypogammaglobulinemia, but real-world (RW) evidence describing IVIG utilization, timing, and outcomes in pts receiving BsAbs remains limited. We describe IVIG use with BsAbs for MM and evaluate clinical outcomes in US community oncology practices.

### Aims

To characterize RW patterns and timing of IVIG use and evaluate treatment duration and outcomes among pts with MM receiving BsABs in US Oncology practices.

### Methods

Adult pts with MM initiating BsAb therapy on or after 01-Jan-2022 were identified in the Integra PrecisionQ database of deidentified electronic health records. The Index date was BsAb start. Eligible pts had ≥90 days follow-up. Discontinuation was defined as no treatment in prior 60 days. BsAb therapies included teclistamab, elranatamab, and talquetamab. IVIG exposure was categorized as administration ≤30 days of BsAb initiation (early-IVIG), >30 days after initiation (delayed-IVIG), or no IVIG. All pts required ≥30 days of therapy. Time to BsAb discontinuation (TTD) and time to next treatment or death (TTNT/D) were assessed, with group comparisons by log-rank test.

**Copyright Information:** (Online) ISSN: 2572-9241
©2026 The Author(s). HemaSphere is published by John Wiley & Sons Ltd on behalf of European Hematology Association. This is an open access Abstract Book distributed under the Attribution-NonCommercial-NoDerivs (CC BY-NC-ND), which allows third parties to download the articles and share them with others as long as they credit the author and the Abstract Book, but they cannot change the content in any way or use them commercially.
**Abstract Book Citations:** Authors, Title, HemaSphere, 2026;10:(S1):pages. The abstract book DOIs can be found on the “Author Information” page.
**Disclaimer:** Articles published in the journal HemaSphere exclusively reflect the opinions of the authors. The authors are responsible for all content in their abstracts including accuracy of the facts, statements, citing resources, etc.

### Results

492 pts initiated BsAb therapy. Median age was 72 years (IQR 66–78). Among pts with known ECOG performance status, 84% (n=354) had ECOG . 293 pts (59.6%) received IVIG during BsAb therapy; 162 (33%) as early IVIG, and 131 (27%) as delayed IVIG. Among pts with IgG labs available +/-30 days of treatment, pts with IgG level <400 comprised of 31.6% (80/253) for pts who received IVIG and 22.1% (32/145) for pts who did not receive IVIG. Median TTD was 13.5 months for pts who received IVIG versus 4.4 months for pts who did not receive IVIG (P<0.001)(Table 1). Median TTD was similar for early IVIG versus delayed IVIG, 12.1 months and 14.8 months, respectively. Median TTNT/D was 28.8 months for pts who received IVIG versus 16.8 months for pts who did not receive IVIG (P<0.001)(Table 1). Median TTNT/D was 31.5 and 27.4 months for delayed-and early-IVIG pts, both significantly longer than 16.8 months for pts who did not receive IVIG, P<0.05 for each comparison.

**Table 1: Outcomes by IVIG Administration Status**

| **Treatment Groups** | **No IVIG (n=199)** | **IVIG (n=293)** | **P value** |
|---|---|---|---|
| **Treatment Discontinuation** | | | |
| Median Duration (mo) | 4.4 | 13.5 | <0.001 |
| Median Follow-up (mo) | 17.4 | 15.1 | |
| | | | |
| **Time to Next Treatment or Death** | | | |
| Median TTNT or Death (mo) | 16.8 | 28.8 | <0.001 |
| Median Follow-up (mo) | 17.4 | 15.1 | |

### Summary/Conclusion

In this RW cohort of MM pts treated s, 40% with BsAb did not receive IVIG. IVIG use was associated with substantially longer treatment duration and delayed time to next treatment or death versus no IVIG. We did not observe differences in outcomes between early and late administration of IVIG, which may reflect baseline cohort differences. Clinical trial data suggest benefit with routine IVIG use in pts receiving BsAb therapy, and our findings support this. Further studies are warranted to better define the role and optimal timing of IVIG in this setting.

**Copyright Information:** (Online) ISSN: 2572-9241
©2026 The Author(s). HemaSphere is published by John Wiley & Sons Ltd on behalf of European Hematology Association. This is an open access Abstract Book distributed under the Attribution-NonCommercial-NoDerivs (CC BY-NC-ND), which allows third parties to download the articles and share them with others as long as they credit the author and the Abstract Book, but they cannot change the content in any way or use them commercially.
**Abstract Book Citations:** Authors, Title, HemaSphere, 2026;10:(S1):pages. The abstract book DOIs can be found on the "Author Information" page.
**Disclaimer:** Articles published in the journal HemaSphere exclusively reflect the opinions of the authors. The authors are responsible for all content in their abstracts including accuracy of the facts, statements, citing resources, etc.

### (PB3271) UPDATED RESULTS FROM THE PHASE 2 IZALCO STUDY: EFFICACY AND SAFETY OF ISATUXIMAB SUBCUTANEOUS PLUS CARFILZOMIB AND DEXAMETHASONE IN RELAPSED/REFRACTORY MULTIPLE MYELOMA

**Topic:** 14. Myeloma and other monoclonal gammopathies - Clinical

Marcelo Capra*[1], Gurdeep Parmar[2], Fernanda Seguro[3], Vania Hungria[4], Meletios A. Dimopoulos[5, 6], Sosana Delimpasi[7], Junichiro Yuda[8], Ivan Špička[9], Roman Hájek[10], Christine Soufflet[11], Disa Yu[12], Victorine Koch[13], Florence Suzan[11], Jiri Minarik[14], Hang Quach[15]

*[1]Centro Integrado de Hematologia e Oncologia, Hospital Mãe de Deus, Porto Alegre, Porto Alegre, Brazil;[2]Illawarra Cancer Care Centre, Wollongong, NSW, Australia; [3]Hematologia e Hemoterapia do Hospital das Clinicas – FMUSP, São Paulo, Brazil; [4]Clinica São Germano, São Paulo, Brazil; [5]Department of Clinical Therapeutics, School of Medicine, National and Kapodistrian University of Athens, Athens, Greece; [6]Department of Medicine, Korea University, Seoul, South Korea; [7]Department of Hematology and Bone Marrow Transplantation Unit, Evangelismos General Hospital, Athens, Greece; [8]Department of Hematology and Experimental Therapeutics, National Cancer Center Hospital East, Kashiwa, Japan; [9]Departments of Medicine and Hematology, First Faculty of Medicine, Charles University and General Hospital, Prague, Czechia; [10]Department of Haemato-Oncology, University Hospital Ostrava and Faculty of Medicine, University of Ostrava, Ostrava, Czechia; [11]Sanofi R&D, Vitry-sur-Seine, France; [12]Sanofi, Cambridge, MA, United States of America; [13]Excelya on behalf of Sanofi Research & Development, Vitry-sur-Seine, France; [14]Department of Hemato-Oncology, Faculty of Medicine and Dentistry, Palacký University and University Hospital Olomouc, Olomouc, Czechia; [15]Clinical Haematology Service, St Vincent's Hospital, University of Melbourne, Melbourne, VIC, Australia;*

### Background

In combination with conventional doublet and triplet therapy, isatuximab (Isa) provides significant benefit to patients across the multiple myeloma (MM) spectrum. Subcutaneous (SC) administration of Isa could be a more convenient option for patients and caregivers. The Phase 3 IRAKLIA trial in relapsed/refractory MM (RRMM) patients demonstrated non-inferiority in efficacy and pharmacokinetics of Isa SC delivered via a wearable on-body injector (OBI) in combination with pomalidomide and dexamethasone (Pd) vs Isa IV Pd. The Phase 2 IZALCO study (NCT05704049) also showed efficacy and safety of Isa SC plus carfilzomib and dexamethasone (Kd), either by manual or OBI injections, in RRMM patients. Following exposure to both methods, patients showed higher preference for OBI vs manual SC injection.

### Aims

To report updated efficacy and safety results from the IZALCO study.

### Methods

Isa SC 1400 mg was given weekly in Cycle (C)1 then biweekly. In Part 1, patients received Isa injected SC manually. In Part 2, patients were randomized to Isa administered SC via OBI (Isa SC OBI; C1-C3) followed by manual SC injection (C4-C6), or to manual SC injection (C1-C3) followed by Isa SC OBI (C4-C6). Starting at C7, patients could choose either treatment method. All patients were treated with carfilzomib (20 mg/m$^2$ on Day [D]1-2 then 56 mg/m$^2$ on D8-9, 15-16 at C1, then D1-2, 8-9, and 15-16) and dexamethasone (20 mg on D1-2, 8-9, 15-16, and 22-23). All patients provided informed consent.

**Copyright Information:** (Online) ISSN: 2572-9241
©2026 The Author(s). HemaSphere is published by John Wiley & Sons Ltd on behalf of European Hematology Association. This is an open access Abstract Book distributed under the Attribution-NonCommercial-NoDerivs (CC BY-NC-ND), which allows third parties to download the articles and share them with others as long as they credit the author and the Abstract Book, but they cannot change the content in any way or use them commercially.
**Abstract Book Citations:** Authors, Title, HemaSphere, 2026;10:(S1):pages. The abstract book DOIs can be found on the "Author Information" page.
**Disclaimer:** Articles published in the journal HemaSphere exclusively reflect the opinions of the authors. The authors are responsible for all content in their abstracts including accuracy of the facts, statements, citing resources, etc.

**Results**

A total of 74 RRMM patients were enrolled (8 in Part 1; 66 in the randomized cohort, Part 2). At the data cut-off of November 10, 2025, 59 patients remained on study. At study entry, patients had median age of 65 (44-85) years, median weight of 75.9 (40.0-129.0) kg, and median of 1 prior line of therapy (1-5), and 56.8% had ISS stage I. The median duration of Isa SC OBI administration was 12 mins and manual SC injection was 6 mins. None of the Isa SC injections, manual or OBI, were interrupted. Median time to best response was 5.3 months (95% CI: 3.25-6.24). At median follow-up of 21.9 months, median progression free survival (PFS) was not reached (NR) (95% CI: 16.23-NR). PFS at 18 months was 61.5% (95% CI: 48.7–72.0). Overall survival (OS) at 18 months was 80.8% (95% CI: 69.8-88.2); median OS was not reached (95% CI: NR-NR). Overall, grade (G) ≥3 TEAEs occurred in 66.2%, and serious TEAEs in 51.4% patients. Infusion reaction (IR) on C1D1 occurred in 2 patients (2.7%) with manual SC injections; no IRs occurred with OBI. Six (8.1%) patients had 13 (0.54%) injection-site reactions (ISR), all G1, in 2425 manual or OBI injections. Only 1 ISR was deemed related to OBI. Patient satisfaction with injection method remained consistently high with Isa SC OBI up to C25D15.

**Summary/Conclusion**

Updated results of the IZALCO study continue to show the efficacy and safety of Isa SC plus Kd delivered either by manual injection or Isa SC OBI, while demonstrating high patient satisfaction and preference for Isa SC OBI. These findings are consistent with those reported in the Phase 3 IKEMA study (Isa IV plus Kd).

© 2026 American Society of Clinical Oncology, Inc. Reused with permission. This abstract was accepted and previously presented at the 2026 ASCO Annual Meeting. All rights reserved.

**Copyright Information:** (Online) ISSN: 2572-9241
©2026 The Author(s). HemaSphere is published by John Wiley & Sons Ltd on behalf of European Hematology Association. This is an open access Abstract Book distributed under the Attribution-NonCommercial-NoDerivs (CC BY-NC-ND), which allows third parties to download the articles and share them with others as long as they credit the author and the Abstract Book, but they cannot change the content in any way or use them commercially.
**Abstract Book Citations:** Authors, Title, HemaSphere, 2026;10:(S1):pages. The abstract book DOIs can be found on the “Author Information” page.
**Disclaimer:** Articles published in the journal HemaSphere exclusively reflect the opinions of the authors. The authors are responsible for all content in their abstracts including accuracy of the facts, statements, citing resources, etc.

### (PB3272) REAL-WORLD CHARACTERISTICS, TREATMENT PATTERNS AND OUTCOMES OF PATIENTS WITH NEWLY DIAGNOSED MULTIPLE MYELOMA IN CHINA TREATED WITH FIRST-LINE DARATUMUMAB-BASED REGIMENS DVRD AND DRD

**Topic:** 14. Myeloma and other monoclonal gammopathies - Clinical

Yating Li*[1], Yuntong Liu[1], Canchan Cui[2], Haoyan Cai[2], Bijie Xun[2], Chohan Lee[3], Chi-Yin Liao[3], Cathy Kwang-Wei Wu[4], David Bin-Chia Wu[5, 6, 7], Shimin Bi[8], Jia Zhong[8], Xiaochen Zhang[8], Gang An[1]

*[1]Institute of Hematology and Blood Diseases Hospital, Tianjin, China;[2]Johnson & Johnson, China Medical Affairs, Beijing, China; [3]Johnson & Johnson Innovative Medicine, Global Real-world Evidence, Raritan, United States of America; [4]Johnson& Johnson, Singapore, Singapore; [5]Johnson & Johnson Innovative Medicine, Asia Pacific Market Access, Singapore, Singapore;[6]Saw Swee Hock School of Public Health, National University of Singapore, Singapore, Singapore; [7]School of Pharmacy, Faculty of Health & Medical Sciences, Taylor's University Malaysia, Subang Jaya, Malaysia; [8]Analysis Group, Boston, United States of America;*

### Background

Daratumumab, an anti-CD38 monoclonal antibody, has demonstrated efficacy in newly diagnosed multiple myeloma (NDMM). In China, frontline treatment patterns have evolved following approval of daratumumab for transplant ineligible patients in 2021, national reimbursement in 2022, and approval for transplant eligible patients in 2026. However, real world evidence describing patient characteristics, treatment patterns, and outcomes of daratumumab-based regimens, particularly DVRd and DRd, remains limited.

### Aims

To describe real-world characteristics, treatment patterns, and clinical outcomes of NDMM patients receiving first-line (1L) DVRd with or without stem cell transplantation (SCT), or DRd without SCT, in routine clinical practice in China (D:daratumumab; V:bortezomib; R:lenalidomide; d:dexamethasone).

### Methods

This retrospective observational cohort study used data from the National Longitudinal Cohort of Hematological Diseases, a nationwide Chinese registry with prospective follow-up (~56,000 patients across 13 hematologic disorders). Adults (≥18 years) with NDMM who received 1L DVRd (±SCT) or DRd (no SCT) after 5 July 2019 and had ≥2 months of follow-up were included.

### Results

Among 252 NDMM patients, 184 received DVRd (83 with SCT; 101 without SCT) and 68 received DRd. Median follow-up was 30.6 months for DVRd (33.1 with SCT; 25.7 without SCT) and 21.4 months for DRd. DVRd patients were younger than those receiving DRd (median age 57 vs 69 years). ISS stage III disease occurred in 38% of DVRd and 46% of DRd; ECOG ≥2 was observed in 6% of DVRd and 12% of DRd. Among evaluable patients, real-world ORR was 89% with DVRd (91% with SCT; 88% without SCT) and 84% with DRd; ≥CR rates were 55% (68% with SCT; 41% without SCT) and 45%, respectively. Median real-world PFS (rwPFS) and real-world OS (rwOS) were not reached; 1/2/3-year rwPFS/rwOS were 95.1/87.9/87.0% and 97.3/94.0/93.1% for DVRd, and 86.1/73.0/69.0% and 96.9/92.6/89.1% for DRd. Within DVRd, 2-year rwPFS/rwOS was 97.6/97.6% with SCT versus 78.6/90.5% without SCT.

### Summary/Conclusion

**Copyright Information:** (Online) ISSN: 2572-9241
©2026 The Author(s). HemaSphere is published by John Wiley & Sons Ltd on behalf of European Hematology Association. This is an open access Abstract Book distributed under the Attribution-NonCommercial-NoDerivs (CC BY-NC-ND), which allows third parties to download the articles and share them with others as long as they credit the author and the Abstract Book, but they cannot change the content in any way or use them commercially.
**Abstract Book Citations:** Authors, Title, HemaSphere, 2026;10:(S1):pages. The abstract book DOIs can be found on the "Author Information" page.
**Disclaimer:** Articles published in the journal HemaSphere exclusively reflect the opinions of the authors. The authors are responsible for all content in their abstracts including accuracy of the facts, statements, citing resources, etc.

Frontline treatment selection in real-world practice reflects patient fitness, with DVRd more commonly used in younger, fitter patients and DRd in older, frailer populations. Real-world outcomes were consistent with clinical trial data, supporting the effectiveness of both regimens in NDMM. DVRd demonstrated deep and durable responses across SCT and non-SCT settings, while DRd provided meaningful benefit in patients with less favorable baseline characteristics.

**Table:**

| N (%) unless otherwise specified | DVRd | | | DRd without SCT N=68 |
|---|---|---|---|---|
| | Overall N=184 | With SCT N=83 | Without SCT N=101 | |
| Age at 1L initiation (yrs), median (range) | 57(26,83) | 53(32,73) | 61(26,83) | 69(38,83) |
| Male | 100(54) | 41(49) | 59(58) | 42(62) |
| ECOG | | | | |
| 0-1 | 168(91) | 81(98) | 87(86) | 56(82) |
| ≥2 | 11(6) | 2(2) | 9(9) | 8(12) |
| Unknown | 5(3) | 0(0) | 5(5) | 4(6) |
| ISS | | | | |
| Stage I | 55(30) | 30(36) | 25(25) | 13(19.1) |
| Stage II | 49(27) | 26(31) | 23(23) | 21(31) |
| Stage III | 70(38) | 25(30) | 45(45) | 31(46) |
| Unknown | 10(5) | 2(2) | 8(8) | 3(4) |
| rwORR* | 134/150(89) | 70/77(91) | 64/73(88) | 43/51(84) |
| rw≥CR* | 82(55) | 52(68) | 30(41) | 23(45) |
| rwPFS rates, % | | | | |

**Copyright Information:** (Online) ISSN: 2572-9241
©2026 The Author(s). HemaSphere is published by John Wiley & Sons Ltd on behalf of European Hematology Association. This is an open access Abstract Book distributed under the Attribution-NonCommercial-NoDerivs (CC BY-NC-ND), which allows third parties to download the articles and share them with others as long as they credit the author and the Abstract Book, but they cannot change the content in any way or use them commercially.
**Abstract Book Citations:** Authors, Title, HemaSphere, 2026;10:(S1):pages. The abstract book DOIs can be found on the "Author Information" page.
**Disclaimer:** Articles published in the journal HemaSphere exclusively reflect the opinions of the authors. The authors are responsible for all content in their abstracts including accuracy of the facts, statements, citing resources, etc.

| | | | | |
|---|---|---|---|---|
| **1-yr** | 95.1 | 98.8 | 92.0 | 86.1 |
| **2-yr** | 87.9 | 97.6 | 78.6 | 73.0 |
| **3-yr** | 87.0 | 97.6 | 76.7 | 69.0 |
| **rwOS rates, %** | | | | |
| **1-yr** | 97.3 | 98.8 | 96.0 | 96.9 |
| **2-yr** | 94.0 | 97.6 | 90.5 | 92.6 |
| **3-yr** | 93.1 | 97.6 | 88.7 | 89.1 |

*Estimated among evaluable patients

**Copyright Information:** (Online) ISSN: 2572-9241
©2026 The Author(s). HemaSphere is published by John Wiley & Sons Ltd on behalf of European Hematology Association. This is an open access Abstract Book distributed under the Attribution-NonCommercial-NoDerivs (CC BY-NC-ND), which allows third parties to download the articles and share them with others as long as they credit the author and the Abstract Book, but they cannot change the content in any way or use them commercially.
**Abstract Book Citations:** Authors, Title, HemaSphere, 2026;10:(S1):pages. The abstract book DOIs can be found on the "Author Information" page.
**Disclaimer:** Articles published in the journal HemaSphere exclusively reflect the opinions of the authors. The authors are responsible for all content in their abstracts including accuracy of the facts, statements, citing resources, etc.

### (PB3273) MAINTAINED RENAL FUNCTION WITH MELFLUFEN THERAPY IN RRMM: RESULTS FROM ANALYSIS OF POOLED CLINICAL DATA

**Topic:** 14. Myeloma and other monoclonal gammopathies - Clinical

Fredrik Schjesvold*[1], Sara Bringhen[2], Meletios A. Dimopoulos[3, 4], Ludek Pour[5], María-Victoria Mateos[6], Albert Oriol[7], Alessandra Larocca[8], Maria Theresa Krauth[9], Marcus Thuresson[10], Stefan Norin[10], Pieter Sonneveld[11], Paul Richardson[12]

*[1]Oslo Myeloma Center, Department of Hematology, Oslo University Hospital, Oslo, Norway;[2]Azienda Ospedaliera Santa Croce e Carle, Department of Hematology, Cuneo, Italy; [3]Department of Clinical Therapeutics, School of Medicine, National and Kapodistrian University of Athens, Athens, Greece; [4]Department of Medicine, Korea University, Seoul, South Korea;[5]Department of Internal Medicine, Hematology, and Oncology, University Hospital Brno, Brno, Czechia; [6]University Hospital of Salamanca/IBSAL/CIC/CIBERONC, Salamanca, Spain; [7]Institut Català d'Oncologia and Institut Josep Carreras, Badalona, Barcelona, Spain; [8]Department of Molecular Biotechnology and Health Sciences, Division of Hematology University of Torino, Torino, Italy; [9]Department of Medicine I, Division of Hematology and Hemostaseology, Medical University of Vienna, Vienna, Austria; [10]Oncopeptides AB, Stockholm, Sweden; [11]Department of Hematology, Erasmus MC Cancer Institute, Rotterdam, Netherlands., Rotterdam, The Netherlands; [12]Department of Medical Oncology, Dana-Farber Cancer Institute, Jerome Lipper Center for Multiple Myeloma Research, Harvard Medical School, Boston, United States of America;*

**Background**

Melphalan flufenamide (also known as melflufen) is a lipophilic peptide–drug conjugate that improves intracellular delivery of alkylating agents and activation within malignant cells. Melflufen is approved by EMA, in combination with dexamethasone, for the treatment of adult patients with multiple myeloma who have received at least three prior lines of therapies and whose disease is triple-class refractory. For patients with a prior autologous stem cell transplantation, the time to progression should be at least 3 years from transplantation.

**Aims**

The purpose was to investigate changes in renal function during treatment with melflufen in a pooled patient population from three clinical studies and to compare renal function over time in patients treated with melflufen vs pomalidomide.

**Methods**

Patients with relapsed/refractory multiple myeloma (RRMM) from the single-arm studies O-12-M1 and OP-106 HORIZON, and the randomized Phase 3 study OP-103 OCEAN were included. Patients received either intravenous melflufen 40 mg on day 1 or oral pomalidomide 4 mg daily on days 1-21 of each 28-day cycle. In O-12-M1, 28 patients received melflufen in 21-day cycles. All patients also received 40 mg dexamethasone weekly, or 20 mg if ≥75 years of age. All studies required an estimated creatinine clearance ≥ 45 ml/min at screening.

Renal function was assessed from baseline to day 1 in cycle 7 (C7) and from baseline to end of treatment (EOT). The analysis comprised a pooled cohort of melflufen-treated patients and a separate comparison between melflufen and pomalidomide in patients from the OCEAN study. In addition, outcomes were assessed in a subgroup of melflufen treated patients with baseline eGFR <60 mL/min/1.73 m². Safety assessment focused on renal function.

**Copyright Information:** (Online) ISSN: 2572-9241
©2026 The Author(s). HemaSphere is published by John Wiley & Sons Ltd on behalf of European Hematology Association. This is an open access Abstract Book distributed under the Attribution-NonCommercial-NoDerivs (CC BY-NC-ND), which allows third parties to download the articles and share them with others as long as they credit the author and the Abstract Book, but they cannot change the content in any way or use them commercially.
**Abstract Book Citations:** Authors, Title, HemaSphere, 2026;10:(S1):pages. The abstract book DOIs can be found on the "Author Information" page.
**Disclaimer:** Articles published in the journal HemaSphere exclusively reflect the opinions of the authors. The authors are responsible for all content in their abstracts including accuracy of the facts, statements, citing resources, etc.

**Results**

The pooled analysis included 430 patients treated with melflufen. The median eGFR at baseline was 81.2 mL/min/1.73m², 74 patients (17%) had eGFR <60. Reason for EOT was predominantly due to progressive disease (54.0%) or adverse events (20.7%).

The result from the pooled melflufen group showed that renal function remained stable throughout the course of treatment (figure 1). Mean eGFR showed no clinical meaningful change from baseline to C7, or from baseline to EOT. Importantly, patients with baseline eGFR <60 maintained a stable eGFR with no meaningful decline observed during their treatment with melflufen.

Adverse events were reported in 99.5% of patients in the pooled melflufen group, most commonly thrombocytopenia and neutropenia. Renal or urinary adverse events, as defined by the MedDRA System Organ Class, were infrequent (10.7%), with low rates of acute kidney injury (2.3%) and renal failure (1.4%). Serious adverse events of renal and urinary disorders were reported in 3%.

The comparative analysis between melflufen and pomalidomide included 228 patients receiving melflufen and 246 patients receiving pomalidomide from the OCEAN trial. Baseline characteristics were similar between the groups including eGFR (median 81.4 mL/min/1.73m² for melflufen vs. 79.6 for pomalidomide). No differences in renal function over time were observed between treatments (figure 1).

**Summary/Conclusion**

Renal function remained stable during treatment with melflufen, including in patients with moderate renal impairment, and no signal of increased toxicity was observed. These results indicate that melflufen can be administered in patients with RRMM without negatively affecting renal function or safety outcomes.

**Copyright Information:** (Online) ISSN: 2572-9241
©2026 The Author(s). HemaSphere is published by John Wiley & Sons Ltd on behalf of European Hematology Association. This is an open access Abstract Book distributed under the Attribution-NonCommercial-NoDerivs (CC BY-NC-ND), which allows third parties to download the articles and share them with others as long as they credit the author and the Abstract Book, but they cannot change the content in any way or use them commercially.
**Abstract Book Citations:** Authors, Title, HemaSphere, 2026;10:(S1):pages. The abstract book DOIs can be found on the "Author Information" page.
**Disclaimer:** Articles published in the journal HemaSphere exclusively reflect the opinions of the authors. The authors are responsible for all content in their abstracts including accuracy of the facts, statements, citing resources, etc.

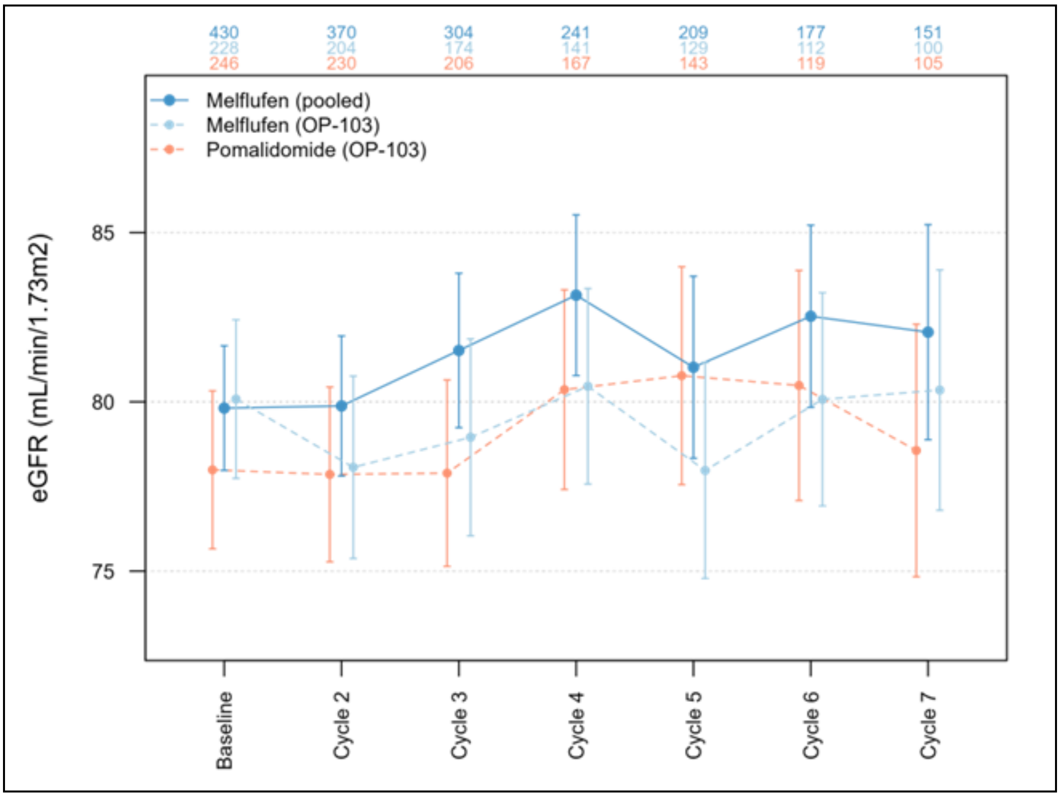

Figure 1. eGFR values from baseline to Day 1 in Cycle 7 in patients treated with melflufen + dexamethasone or pomalidomide + dexamethasone. Mean and 95% confidence intervals.

**Copyright Information:** (Online) ISSN: 2572-9241
©2026 The Author(s). HemaSphere is published by John Wiley & Sons Ltd on behalf of European Hematology Association. This is an open access Abstract Book distributed under the Attribution-NonCommercial-NoDerivs (CC BY-NC-ND), which allows third parties to download the articles and share them with others as long as they credit the author and the Abstract Book, but they cannot change the content in any way or use them commercially.
**Abstract Book Citations:** Authors, Title, HemaSphere, 2026;10:(S1):pages. The abstract book DOIs can be found on the "Author Information" page.
**Disclaimer:** Articles published in the journal HemaSphere exclusively reflect the opinions of the authors. The authors are responsible for all content in their abstracts including accuracy of the facts, statements, citing resources, etc.

### (PB3274) INCIDENCE AND IMPACT OF FRAILTY ON TREATMENT TOLERABILITY AND QUALITY OF LIFE IN NEWLY DIAGNOSED MULTIPLE MYELOMA: A MULTICENTER PROSPECTIVE STUDY FROM THE CATALAN MYELOMA GROUP

**Topic:** 14. Myeloma and other monoclonal gammopathies - Clinical

Carles Tolosa Ridao*[1], Noemí Llober[2], Laura Rodriguez[2], Itziar Carro Arostegui[3], Montserrat Garcia Garcia[1], Iago Arribas Castro[3], Cristina Baca Cano[4], Rahma Ben Azaiz Ben Lahsen[4], Maria Elena Cabezudo Perez[5], Sheila Sanchez Perez[5], Laura Rosiñol Dachs[2, 6], Josep Maria Marti Tutusaus[1], Carlos Fernández de Larrea[2, 6], Maria Queralt Salas[2, 6]

*[1]Hospital Universitari Mútua Terrassa, Hematology Department, Terrassa, Spain;[2]Hospital Clínic de Barcelona, Hematology Department, Barcelona, Spain; [3]ICO Hospitalet, Hematology Department, Hospitalet de Llobregat, Spain;[4]Hospital del Mar, Hematology Department, Barcelona, Spain; [5]Hospital Moisés Broggi, Sant Joan Despí, Spain;[6]Institut d'Investigacions Biomèdiques August Pi i Sunyer (IDIBAPS), Barcelona, Spain;*

### Background

Frailty syndrome is increasingly recognized as a key factor in the evaluation of newly diagnosed multiple myeloma (NDMM). Although its assessment is recommended, there is no consensus on standardized methodologies for its evaluation in clinical practice.

### Aims

This pilot analysis evaluates frailty in NDMM and investigates its impact on treatment tolerability.

### Methods

Observational prospective study of 78 consecutive NDMM patients from 5 centers of the Catalan MM Group. Frailty was assessed at diagnosis using the Hematopoietic Cell Transplant Frailty Scale, which integrates eight variables and classifies patients as fit, pre-frail, or frail. Social status (Gijón scale), cognitive function (Mini-Cog), and quality of life (EQ-5D-3L) were evaluated. Due to differences in treatment strategies, the impact of frailty on outcomes was evaluated separately in patients ≤70 and >70 years (Fig1).

**Copyright Information:** (Online) ISSN: 2572-9241
©2026 The Author(s). HemaSphere is published by John Wiley & Sons Ltd on behalf of European Hematology Association. This is an open access Abstract Book distributed under the Attribution-NonCommercial-NoDerivs (CC BY-NC-ND), which allows third parties to download the articles and share them with others as long as they credit the author and the Abstract Book, but they cannot change the content in any way or use them commercially.
**Abstract Book Citations:** Authors, Title, HemaSphere, 2026;10:(S1):pages. The abstract book DOIs can be found on the "Author Information" page.
**Disclaimer:** Articles published in the journal HemaSphere exclusively reflect the opinions of the authors. The authors are responsible for all content in their abstracts including accuracy of the facts, statements, citing resources, etc.

### Results

At diagnosis, 12 (15%) patients were classified as fit, 35 (45%) as pre-frail and 31 (40%) as frail. As expected, frail patients tended to be older (p=0.06) and associated with advanced ISS stage (p=0.003), KPS <90% (p<0.01), and CRAB symptoms at presentation (p<0.01) (Tab1). Social impairment was also more prevalent in frail patients (p=0.05), whereas cognitive assessment did not differ between groups (p=0.14). QoL progressively worsened across fit, pre-frail and frail groups, with greater impairment in mobility, self-care and daily activities observed in frail patients (p<0.05).
Among patients >70 years (n=34), 50% were frail, 41.2% pre-frail and 8.8% fit. Frail patients had a higher proportion of ISS stage III (p<0.01), worse KPS (p<0.01), and poorer social status than pre-frail and fit ones (47% vs 33% and 7%, p=0.04). Frailty was associated with worse QoL in self-care (p=0.02) and daily activities (p<0.01). Frail patients showed a trend toward higher hospitalizations rates (33% vs. 0% and 0%, p=0.13), dose reductions (77.8% vs. 30% and 50%, p=0.09), treatment discontinuations (23% vs. 21% and 33%, p=0.82), and lower complete response rates (18% vs 67% and 30%, p=0.2).
Among patients ≤70 years (n=44), 32% patients were frail, 48% prefrail and 20% fit. Frail patients are more frequently presented with CRAB symptoms (p=0.01) and worse QoL, particularly in mobility (p<0.01), self-care (p=0.02), daily-activities (p<0.01) and pain (p=0.02). Although not significant, frail patients had higher hospitalizations (30% vs 15% vs 0%, p=0.54), dose reductions (40% vs 38.5% vs 0%, p=0.28) and treatment discontinuations (40% vs 7% vs 0%, p=0.09) than pre-frail and fit ones.

| | Fit N= 12 (15.4) | Pre-Frail N= 35 (44.9) | Frail N= 31 (39.7) | P-value |
|---|---|---|---|---|
| **Age** | | | | |
| Continuous (range) | 61 (37-81) | 67.8 (27-88) | 75 (52-89) | 0.06 |
| Age >70 years | 3 (25) | 14 (40) | 17 (54.8) | 0.17 |
| **ISS** | | | | |
| III | 0 | 4 (11.4) | 13 (41.9) | **0.003** |
| **Diagnostic criteria** | | | | |
| CRAB symptoms | 8 (66.7) | 35 (100) | 29 (92.3) | **<0.01** |
| **First line therapy** | | | | |
| Anti-CD38+VRd | 7 (58.3) | 12 (34.3) | 9 (29) | 0.38 |
| DRd | 1 (8.3) | 9 (25.7) | 12 (38.7) | |
| Clinical trial | 3 (25) | 11 (31.4) | 6 (19.4) | |
| Other | 1 (8.3) | 3 (8.6) | 4 (12.9) | |
| **KPS** | | | | |
| <90% | 2 (16.7) | 15 (42.9) | 26 (83.9) | **<0.01** |
| **Abnormal Gijón >10** | 2 (16.7) | 4 (11.4) | 11 (35.5) | 0.05 |

### Summary/Conclusion

Frailty was prevalent in NDMM and associated with greater disease burden, impaired functional and social status, and poorer QoL. Notably, frailty was linked to increased treatment discontinuation and hospitalization, particularly in patients >70 years, supporting its routine evaluation in clinical practice and the potential implementation of corrective measures to improve tolerability and outcomes.

**Copyright Information:** (Online) ISSN: 2572-9241
©2026 The Author(s). HemaSphere is published by John Wiley & Sons Ltd on behalf of European Hematology Association. This is an open access Abstract Book distributed under the Attribution-NonCommercial-NoDerivs (CC BY-NC-ND), which allows third parties to download the articles and share them with others as long as they credit the author and the Abstract Book, but they cannot change the content in any way or use them commercially.
**Abstract Book Citations:** Authors, Title, HemaSphere, 2026;10:(S1):pages. The abstract book DOIs can be found on the “Author Information” page.
**Disclaimer:** Articles published in the journal HemaSphere exclusively reflect the opinions of the authors. The authors are responsible for all content in their abstracts including accuracy of the facts, statements, citing resources, etc.

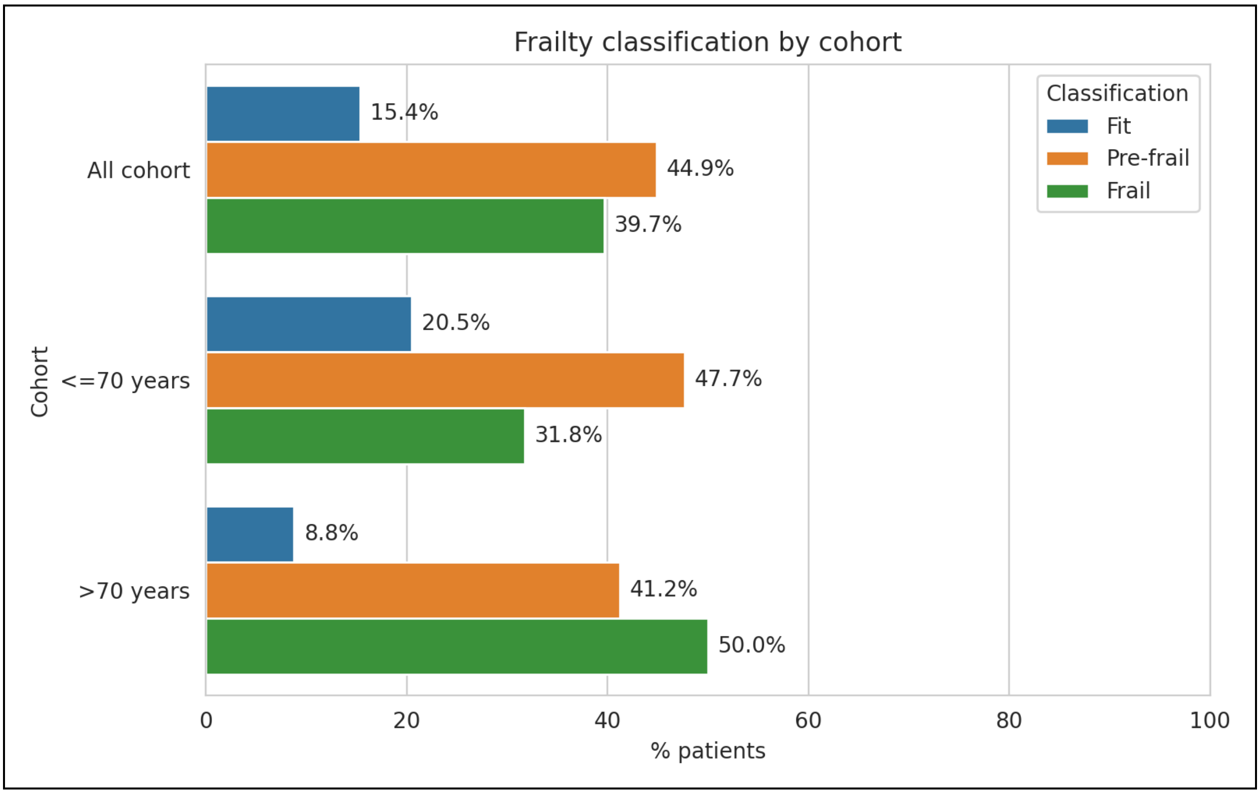

Figure 1. Frailty classification in all cohort and subgroups

**Copyright Information:** (Online) ISSN: 2572-9241
©2026 The Author(s). HemaSphere is published by John Wiley & Sons Ltd on behalf of European Hematology Association. This is an open access Abstract Book distributed under the Attribution-NonCommercial-NoDerivs (CC BY-NC-ND), which allows third parties to download the articles and share them with others as long as they credit the author and the Abstract Book, but they cannot change the content in any way or use them commercially.
**Abstract Book Citations:** Authors, Title, HemaSphere, 2026;10:(S1):pages. The abstract book DOIs can be found on the "Author Information" page.
**Disclaimer:** Articles published in the journal HemaSphere exclusively reflect the opinions of the authors. The authors are responsible for all content in their abstracts including accuracy of the facts, statements, citing resources, etc.

### (PB3275) APONERMIN+SD REGIMEN: AN EFFECTIVE AND SAFE PREFERRED BRIDGING STRATEGY BEFORE BCMA CAR-T THERAPY IN RELAPSED/REFRACTORY MULTIPLE MYELOMA PATIENTS

**Topic:** 14. Myeloma and other monoclonal gammopathies - Clinical

Meilan Chen[1], Juan Li*[1]

[1] *The First Affiliated Hospital of Sun Yat-sen University, Guangzhou, China;*

### Background

Bridging therapy is crucial for patients with relapsed/refractory multiple myeloma (R/R MM) prior to BCMA CAR-T cell infusion, aiming to rapidly reduce tumor burden, control disease progression, and improve the efficacy and safety of subsequent CAR-T therapy. The Aponermin + SD (Selinexor+dexamethasone) regimen is a commonly used chemotherapy combination, and its value as a preferred bridging strategy before CAR-T therapy needs to be further validated.

### Aims

The study aimed to explore the effective and safe of Aponermin+SD regimen as bridging strategy before BCMA CAR-T therapy in R/RMM.

### Methods

This research included 6 R/RMM patients who received the Apo+SD regimen as bridging therapy before BCMA CAR-T cell infusion. We analyzed the effective and safe of Aponermin+SD regimen as bridging strategy before BCMA CAR-T therapy.

### Results

A total of 6 patients with R/R MM were enrolled, with a median age of 61.0 years (range, 48-72). The cohort included 4 males and 2 females. High-risk cytogenetic abnormalities were common, including t(4;14) (33.3%), t(14;16) (16.7%), t(14;20) (16.7%), and gain/amplification of 1q21 (66.7%). Additionally, 66.7% of patients had penta-drug resistance, and 50.0% had double-hit myeloma.Efficacy Outcomes: After Apo+SD regimen, 4 of 6 patients (66.7%) achieved a very good partial response (VGPR), 2 of 6 patients (33.3%) achieved partial response (PR),and resulting in an overall response rate (ORR) of 100%.It has no obvious toxic and is well-tolerated by patients.All 6 patients (100%) achieved MRD-negative status at 1 month post-CAR-T infusion, and this negative status was sustained in subsequent follow-up evaluations.

### Summary/Conclusion

The Apo +SD regimen, as a bridging therapy before BCMA CAR-T infusion, is an effective, safe strategy .

**Copyright Information:** (Online) ISSN: 2572-9241
©2026 The Author(s). HemaSphere is published by John Wiley & Sons Ltd on behalf of European Hematology Association. This is an open access Abstract Book distributed under the Attribution-NonCommercial-NoDerivs (CC BY-NC-ND), which allows third parties to download the articles and share them with others as long as they credit the author and the Abstract Book, but they cannot change the content in any way or use them commercially.
**Abstract Book Citations:** Authors, Title, HemaSphere, 2026;10:(S1):pages. The abstract book DOIs can be found on the “Author Information” page.
**Disclaimer:** Articles published in the journal HemaSphere exclusively reflect the opinions of the authors. The authors are responsible for all content in their abstracts including accuracy of the facts, statements, citing resources, etc.

### (PB3277) A REAL-WORLD COMPARISON OF DARATUMUMAB PLUS DCEP VERSUS OTHER TARGETED REGIMENS IN RELAPSED/REFRACTORY MULTIPLE MYELOMA WITH EXTRAMEDULLARY DISEASE

**Topic:** 14. Myeloma and other monoclonal gammopathies - Clinical

Xuzhao Zhang*[1], Luyu Yang[1], Qian Luo[1], Huawei Jiang[1], Zhaoxing Wu[1], Xi Qiu[1], Yongsheng Xie[1], Yun Liang[1], Wenbin Qian[1], Qunyi Guo[1], Yang Xu[2]

*[1]Taizhou Hospital of Zhejiang Province, Hematology, Taizhou, China;[2]The Second Affiliated Hospital of Zhejiang University School of Medicine, Hangzhou, China;*

### Background

Relapsed/refractory multiple myeloma (RRMM) patients with extramedullary disease (EMD) face an extremely poor prognosis and limited treatment options. The combination of daratumumab with DCEP (dexamethasone, cyclophosphamide, etoposide, cisplatin; Dara-DCEP) has demonstrated encouraging efficacy, but comparative real-world evidence (RWE) supporting its use remains limited.

### Aims

This retrospective study aims to compare the efficacy and safety of DARA-DCEP with other targeted therapies in this high-risk patient group.

### Methods

In this retrospective study, we included 40 patients with relapsed/refractory multiple myeloma and extramedullary disease (RRMM with EMD) treated between 2019 and 2024: 16 received daratumumab plus DCEP (Dara-DCEP), and 24 received other targeted regimens (including daratumumab-, carfilzomib-, or pomalidomide-based combinations). Outcomes assessed included overall response rate (ORR), ≥very good partial response (≥VGPR), complete response (CR; with stringent CR [sCR] specified when applicable), progression-free survival (PFS), and safety profiles.

### Results

Baseline characteristics were balanced between the two groups. Treatment with daratumumab plus DCEP (Dara-DCEP) resulted in significantly higher rates of overall response (ORR: 93.8% vs. 54.2%; p=0.012) and ≥very good partial response (≥VGPR: 62.5% vs. 16.7%; p=0.025) compared to other targeted regimens. Although the median progression-free survival (PFS) was longer with Dara-DCEP (12.0 vs. 6.0 months), this difference did not reach statistical significance (HR 0.60, 95% CI 0.28-1.30; p=0.194). Subgroup analysis demonstrated that achieving deep responses (CR or ≥VGPR) was associated with prolonged PFS in the overall cohort; however, this association reached statistical significance only in patients receiving other targeted therapies (p<0.01), but not in the Dara-DCEP group (p=0.38). Safety profiles were comparable between arms, with predominantly manageable hematologic toxicities (grade 3-4 neutropenia/thrombocytopenia) observed in both groups.

### Summary/Conclusion

Dara-DCEP demonstrates significantly higher response rates in RRMM patients with EMD, with manageable hematologic toxicities. These findings support further exploration of Dara-DCEP as a potential salvage immunochemotherapy option for this high-risk population.

**Copyright Information:** (Online) ISSN: 2572-9241
©2026 The Author(s). HemaSphere is published by John Wiley & Sons Ltd on behalf of European Hematology Association. This is an open access Abstract Book distributed under the Attribution-NonCommercial-NoDerivs (CC BY-NC-ND), which allows third parties to download the articles and share them with others as long as they credit the author and the Abstract Book, but they cannot change the content in any way or use them commercially.
**Abstract Book Citations:** Authors, Title, HemaSphere, 2026;10:(S1):pages. The abstract book DOIs can be found on the "Author Information" page.
**Disclaimer:** Articles published in the journal HemaSphere exclusively reflect the opinions of the authors. The authors are responsible for all content in their abstracts including accuracy of the facts, statements, citing resources, etc.

### (PB3278) BEYOND FUNCTIONAL HIGH-RISK: ULTRA-EARLY RELAPSE AFTER AUTOLOGOUS STEM CELL TRANSPLANTATION IDENTIFIES AN EVEN HIGHER-RISK SUBGROUP IN MULTIPLE MYELOMA

**Topic:** 14. Myeloma and other monoclonal gammopathies - Clinical

Ziqiao Lv[1], Xiaozhe Li[1], Juan Li*[1]

[1] *The First Affiliated Hospital of Sun Yat-sen University, Department of Hematology, Guangzhou, China;*

### Background

With the development of novel agents and the increasing application of transplantation strategies, the outcomes of patients with multiple myeloma (MM) have significantly improved. However, a considerable proportion of patients still fail to benefit from these advances, and marked heterogeneity remains in treatment response and survival outcomes. Identifying high-risk MM patients has therefore become increasingly important. Although the MM risk stratification system has undergone multiple revisions, and the concept of functional high-risk disease has been proposed in recent years—significantly improving prognostic accuracy—we continue to observe a subset of patients who relapse even earlier and experience inferior survival. This study aims to characterize these ultra-early relapse patients and explore potential therapeutic strategies to improve their survival outcomes.

### Aims

To identify patients with ultra-early relapse (within 6 months after transplantation) among those with multiple myeloma.

### Methods

A total of 468 newly diagnosed MM patients who underwent ASCT at the Department of Hematology, First Affiliated Hospital of Sun Yat-sen University between 2003 and 2025 were retrospectively analyzed. Using a novel risk stratification approach, patients with ultra-early relapse were identified. Baseline characteristics were summarized, and potential treatment strategies to improve survival were explored.

### Results

After a median follow-up of 57 months, 60 patients (12.8%) were classified as having functional high-risk MM (disease progression or relapse within 18 months of induction therapy initiation or within 12 months after ASCT). These patients had significantly worse outcomes compared with non-functional high-risk patients (median OS 35.6 months vs. 120.4 months, $P < 0.001$). Among the functional high-risk group, a subset of patients relapsed even earlier and demonstrated poorer survival outcomes. We defined patients who experienced disease progression or relapse within 6 months after ASCT despite receiving standard therapy as having ultra-early relapse. Twenty-nine patients (6.2%) had ultra-early relapse (within 6 months post-transplant), 31 patients (6.6%) had early relapse (6–12 months post-transplant), and 408 patients (87.2%) had late relapse. Compared with other patients, those with ultra-early relapse were more likely to present with elevated LDH levels (198 U/L vs. 169 U/L, $P = 0.001$), extramedullary disease (20.7% vs. 7.7%, $P < 0.001$), high-risk cytogenetics (51.7% vs. 29.2%, $P = 0.021$), and poorer response to induction therapy. Overall survival was significantly shorter in the ultra-early relapse group compared with the early and late relapse groups (25.5 months vs. 40.5 months vs. 120.4 months). Furthermore, among patients with ultra-early relapse, those who received CD38 monoclonal antibody therapy had better outcomes than those who did not receive immunotherapy (median OS not reached vs. 20.2 months, $P = 0.048$).

**Copyright Information:** (Online) ISSN: 2572-9241
©2026 The Author(s). HemaSphere is published by John Wiley & Sons Ltd on behalf of European Hematology Association. This is an open access Abstract Book distributed under the Attribution-NonCommercial-NoDerivs (CC BY-NC-ND), which allows third parties to download the articles and share them with others as long as they credit the author and the Abstract Book, but they cannot change the content in any way or use them commercially.
**Abstract Book Citations:** Authors, Title, HemaSphere, 2026;10:(S1):pages. The abstract book DOIs can be found on the "Author Information" page.
**Disclaimer:** Articles published in the journal HemaSphere exclusively reflect the opinions of the authors. The authors are responsible for all content in their abstracts including accuracy of the facts, statements, citing resources, etc.

### Summary/Conclusion

Even among patients classified as functional high-risk MM, a subset experiences earlier disease progression or relapse and has markedly inferior survival outcomes. More precise predictive models are needed to identify ultra-early relapse patients. For this subgroup, earlier initiation of intensified treatment strategies, such as CAR-T therapy or bispecific antibody therapy, may provide improved clinical benefit.

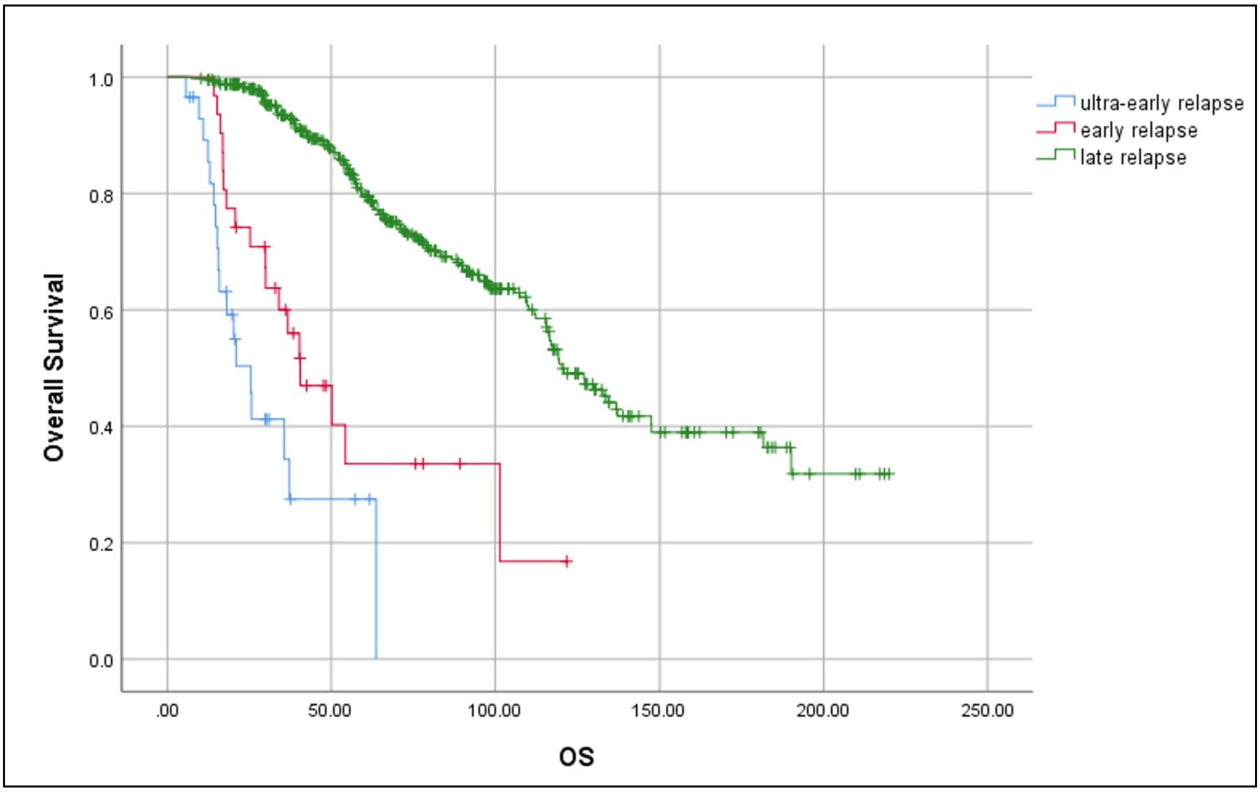

**Copyright Information:** (Online) ISSN: 2572-9241
©2026 The Author(s). HemaSphere is published by John Wiley & Sons Ltd on behalf of European Hematology Association. This is an open access Abstract Book distributed under the Attribution-NonCommercial-NoDerivs (CC BY-NC-ND), which allows third parties to download the articles and share them with others as long as they credit the author and the Abstract Book, but they cannot change the content in any way or use them commercially.
**Abstract Book Citations:** Authors, Title, HemaSphere, 2026;10:(S1):pages. The abstract book DOIs can be found on the "Author Information" page.
**Disclaimer:** Articles published in the journal HemaSphere exclusively reflect the opinions of the authors. The authors are responsible for all content in their abstracts including accuracy of the facts, statements, citing resources, etc.

### (PB3279) BASELINE AND EARLY DYNAMIC CHANGES OF AFR AND RAR: NOVEL AND ACCESSIBLE PROGNOSTIC BIOMARKERS IN MULTIPLE MYELOMA

**Topic:** 14. Myeloma and other monoclonal gammopathies - Clinical

Shuxia Zhu[1], Xinyi Zhang[1], Luning Cui[1], Dong Zheng[1], Huiying Mao[1], Mengxiong Shi[1], Yibo Xia[2], Songfu Jiang[1], Yongyong MA*[1]

*[1]The First Affiliated Hospital of Wenzhou Medical University, Wenzhou, China;[2]Wenzhou Medical University, Wenzhou, China;*

### Background

Multiple myeloma (MM) is characterized by significant clinical heterogeneity. Current risk stratification models, while essential, often fall short in real-world practice due to complexity and cost. There is an urgent need for simple, reproducible, and cost-effective biomarkers derived from routine blood tests to refine prognostication and guide personalized treatment strategies. The albumin-to-fibrinogen ratio (AFR) and red cell distribution width-to-albumin ratio (RAR), reflecting nutritional and inflammatory status, have emerged as promising prognostic indicators in solid tumors but remain largely unexplored in MM.

### Aims

In this study, we aimed to investigate the predictive ability of baseline values as well as dynamic changes in AFR and RAR on the prognosis of patients with MM.

### Methods

We conducted a retrospective analysis of 200 consecutive patients with newly diagnosed MM who received at least three cycles of induction therapy. AFR (albumin/fibrinogen) and RAR (RDW/albumin) were measured at baseline and after three cycles of treatment. Optimal cut-off values were determined using maximally selected log-rank statistics. We assessed the association of these biomarkers with response (≥VGPR, CR) and survival outcomes (PFS, OS) in the overall population and in an ASCT subgroup. Furthermore, patients with serial measurements (n=151) were stratified into four dynamic categories based on baseline levels and their evolution (increase/decrease) post-induction to model real-time risk.

### Results

With a median age of 65 years, the optimal cut-offs were 11.72 for AFR and 0.49 for RAR.

Predicting Response: High baseline AFR predicted superior response (≥VGPR: 67.6% vs. 44.2%, p=0.002), while low baseline RAR predicted higher CR rates (30.4% vs. 12.0%, p=0.005).

Predicting Survival: In univariate analysis, low AFR and high RAR were significantly associated with inferior PFS (p=0.025; p<0.001) and OS (p=0.015; p=0.002). Multivariate analysis confirmed both as independent predictors for OS (p=0.027; p=0.009), while RAR remained an independent predictor for PFS (p=0.017).

Clinical Utility in High-Risk Patients: Notably, in the ASCT subgroup, the adverse prognostic impact of low AFR or high RAR was abrogated, suggesting ASCT may overcome the poor risk associated with these baseline features.

Value of Early Dynamic Assessment: Crucially, patients with persistently high RAR (high at both baseline and post-induction) experienced significantly worse PFS and OS compared to those with persistently low RAR (p<0.001 for both). This identifies a "RAR-high" failure group with ultra-high risk immediately after induction.

**Copyright Information:** (Online) ISSN: 2572-9241
©2026 The Author(s). HemaSphere is published by John Wiley & Sons Ltd on behalf of European Hematology Association. This is an open access Abstract Book distributed under the Attribution-NonCommercial-NoDerivs (CC BY-NC-ND), which allows third parties to download the articles and share them with others as long as they credit the author and the Abstract Book, but they cannot change the content in any way or use them commercially.
**Abstract Book Citations:** Authors, Title, HemaSphere, 2026;10:(S1):pages. The abstract book DOIs can be found on the "Author Information" page.
**Disclaimer:** Articles published in the journal HemaSphere exclusively reflect the opinions of the authors. The authors are responsible for all content in their abstracts including accuracy of the facts, statements, citing resources, etc.

### Summary/Conclusion

Baseline AFR and RAR are powerful, independent predictors of response and survival in MM, offering a cost-effective method to refine initial risk stratification. Most importantly, the early dynamic monitoring of RAR provides a novel and practical tool for real-time prognostic refinement. Patients with persistently elevated RAR represent a distinct subgroup with a dismal prognosis who may be candidates for alternative or intensified therapeutic strategies. These easily accessible biomarkers warrant further prospective validation to integrate them into routine clinical decision-making.

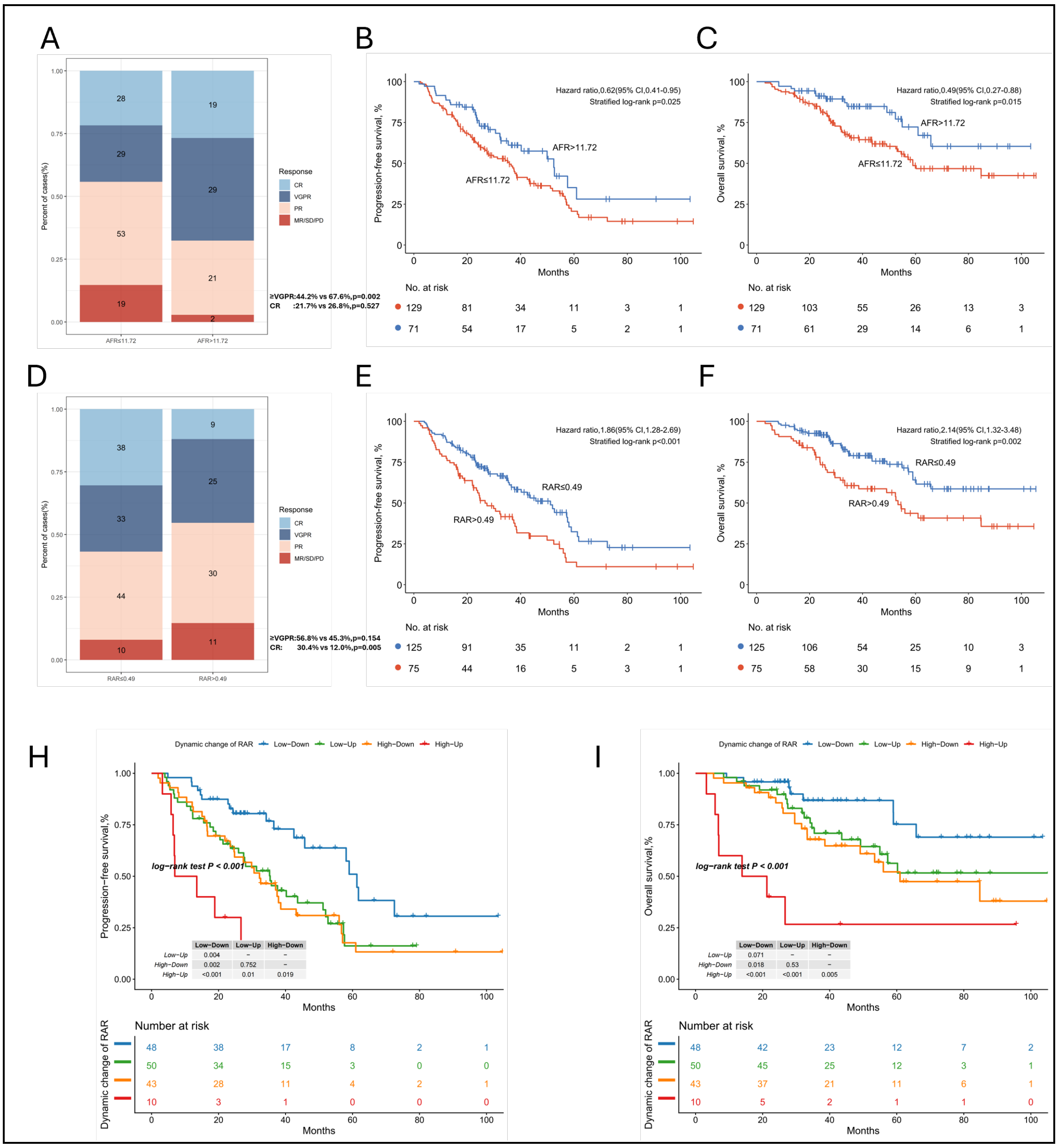

**Copyright Information:** (Online) ISSN: 2572-9241
©2026 The Author(s). HemaSphere is published by John Wiley & Sons Ltd on behalf of European Hematology Association. This is an open access Abstract Book distributed under the Attribution-NonCommercial-NoDerivs (CC BY-NC-ND), which allows third parties to download the articles and share them with others as long as they credit the author and the Abstract Book, but they cannot change the content in any way or use them commercially.
**Abstract Book Citations:** Authors, Title, HemaSphere, 2026;10:(S1):pages. The abstract book DOIs can be found on the "Author Information" page.
**Disclaimer:** Articles published in the journal HemaSphere exclusively reflect the opinions of the authors. The authors are responsible for all content in their abstracts including accuracy of the facts, statements, citing resources, etc.

### (PB3280) DUAL-ANTIGEN-TARGETING T-CELL IMMUNOTHERAPIES FOR RELAPSE/REFRACTORY MULTIPLE MYELOMA: A SYSTEMATIC REVIEW AND META-ANALYSIS

**Topic:** 14. Myeloma and other monoclonal gammopathies - Clinical

Minyue Zhang*[1], Xiaoqing Zhu[2], Ming Zhou[2], Juan Du[1]

*[1]Ren Ji Hospital, Shanghai Jiao Tong University School of Medicine, Department of Hematology, Shanghai, China;[2]Ningbo Hangzhou Bay Hospital, Department of Hematology, Ningbo, China;*

### Background

Relapsed/refractory multiple myeloma (RRMM) remains challenging despite major advances in BCMA-directed therapies. Dual-targeted chimeric antigen receptor T-cell (CAR-T) treatment and T-cell–engaging (TCE) immunotherapies, including trispecific antibodies (TsAbs) or bispecific antibodies (BsAbs) combined with anti-CD38 monoclonal antibodies (CD38 mAbs), show promising efficacy, yet comparative efficacy and safety are unclear.

### Aims

We aim to perform systematic review and meta-analysis to evalute the efficacy and safety of dual-targeted CAR-T versus TCE-based therapies in RRMM.

### Methods

We conducted comprehensive literature search of PubMed, EMBASE, Cochrane Library, clinical trial registries, and international conference abstracts (through Dec 31, 2025) for studies investigating dual-targeted CAR-T or TCE-based therapies (TsAbs, dual BsAbs or BsAbs plus CD38 mAbs) as third-line or later-line treatments in RRMM. Outcomes included pooled overall response rate (ORR)complete response (CR) rate, minimal residual disease negative (MRD-neg) rate and the incidence of adverse events, including severe cytokine release syndrome (CRS), neurotoxicity, infection, and hematologic toxicities. MRD-neg was defined according to each included study's assessment method and sensitivity threshold, which varied from $10^{-4}$ to $10^{-6}$.

### Results

A total of 28 publications involving 964 patients were included. Of these, 17 studies evaluated dual-target CAR-T (10 BCMA/CD19, 2 BCMA/GPRC5D, 1 BCMA/CS1, and 4 BCMA/CD38), and 11 studies evaluated TCE-based therapies (4 TsAbs targeting GPRC5D/BCMA/CD3, 1 TsAbs targeting BCMA/CD38/CD3, 2 dual BsAbs, and 4 BsAbs plus CD38 mAbs). Single-arm meta-analysis demonstrated higher pooled ORR with dual-targeting CAR-T versus TCE-based therapies (93.11% vs. 79.75%; $p<0.0001$). In contrast, there were no significant differences in deep responses, including pooled CR (60.76% vs. 44.44%; $p=0.136$) and MRD-neg rates (85.58% vs. 72.88%; $p=0.5757$). Regarding safety, CAR-T therapy was associated with higher rates of grade ≥3 CRS (8.05% vs. 1.09%; $p=0.0005$) and grade ≥3 neutropenia (71.94% vs. 47.26%; $p=0.0174$). Conversely, TCE-based therapies showed higher incidence of grade ≥3 infection (30.29% vs. 15.61%; $p=0.0238$). However, no significant differences were observed in the incidence of grade ≥3 neurotoxicity, thrombocytopenia or anemia between the two modalities.

### Summary/Conclusion

In RRMM, dual-targeted CAR-T therapy provides superior ORR compared with TCE-based regimens, while achieving comparable deep responses. CAR-T treatment presents higher risks of severe CRS and neutropenia, whereas TCE-based regimens carry greater risk of severe infection, highlighting the need for individualized treatment.

**Copyright Information:** (Online) ISSN: 2572-9241
©2026 The Author(s). HemaSphere is published by John Wiley & Sons Ltd on behalf of European Hematology Association. This is an open access Abstract Book distributed under the Attribution-NonCommercial-NoDerivs (CC BY-NC-ND), which allows third parties to download the articles and share them with others as long as they credit the author and the Abstract Book, but they cannot change the content in any way or use them commercially.
**Abstract Book Citations:** Authors, Title, HemaSphere, 2026;10:(S1):pages. The abstract book DOIs can be found on the "Author Information" page.
**Disclaimer:** Articles published in the journal HemaSphere exclusively reflect the opinions of the authors. The authors are responsible for all content in their abstracts including accuracy of the facts, statements, citing resources, etc.

### (PB3281) REAL-WORLD COMPARISON OF UPFRONT PLERIXAFOR PLUS G-CSF VERSUS CYCLOPHOSPHAMIDE-BASED MOBILIZATION IN MULTIPLE MYELOMA: A SINGLE-CENTER STUDY FROM CHINA

**Topic:** 14. Myeloma and other monoclonal gammopathies - Clinical

Xiaoyan Han*[1], Xi Huang[1], Yaqin Xiong[2], Zhen Cai[1]

*[1]Zhejiang University School of Medicine First Affiliated Hospital, Bone Marrow Transplantation Center, Hangzhou, China;*
*[2]Zhongshan Hospital, Fudan University, Department of Hematology, Shanghai, China;*

### Background

Autologous hematopoietic stem cell transplantation (ASCT) remains a frontline therapy for transplant-eligible multiple myeloma (MM) patients. While Plerixafor plus G-CSF (PLE+G-CSF) is effective, its high cost in China necessitates a more resource-efficient approach.

### Aims

To evaluate the efficacy, safety, and cost-effectiveness of an innovative "one-vial" plerixafor mobilization strategy compared to high-dose cyclophosphamide (CY+G-CSF) in the Chinese clinical setting.

### Methods

We retrospectively analyzed 220 MM patients (PLE+G-CSF, n=98; CY+G-CSF, n=122) undergoing mobilization at our center (2016–2020). The PLE group utilized a unique "one-vial" protocol: a standard dose (0.24 mg/kg) was injected on Day 4, and any residual volume in the vial was used for a second harvest if the first-day yield was <$2\times10^6$ CD34+ cells/kg. Primary endpoints included the successful collection rate (≥$2\times10^6$/kg) and total mobilization costs.

### Results

Mobilization Efficiency: The PLE group achieved a significantly higher success rate than the CY group (85.7% vs. 74.6%, p=0.042). The median number of CD34+ cells collected on the first day was significantly higher in the PLE group (3.3 vs. $1.6\times10^6$/kg, p<0.001).
Impact of Prior Therapy: Notably, the PLE strategy maintained superior mobilization efficacy even in patients with >4 cycles of lenalidomide exposure or those progressing after daratumumab-containing induction, who are typically "hard-to-mobilize."
Kinetics: 69.4% of PLE patients achieved the collection target in a single apheresis, compared to only 41.0% in the CY group (p<0.001).
Safety: The CY group experienced a much higher incidence of febrile neutropenia (42.6% vs. 0%, p<0.001) and required more intravenous antibiotics (45.9% vs. 29.6%; P = 0.014) .
Economics: Although the drug cost of plerixafor was higher, the PLE strategy significantly reduced hospitalization duration and ancillary medical costs. Multivariate analysis identified absolute platelet count ≥$204\times10^9$/L and day -1 PB CD34+ count ≥11/μL as independent predictors for ideal collection (>$4\times10^6$/kg).
Post-Transplant: No significant differences were found in neutrophil/platelet engraftment time or 2-year PFS/OS between the two cohorts.

**Copyright Information:** (Online) ISSN: 2572-9241
©2026 The Author(s). HemaSphere is published by John Wiley & Sons Ltd on behalf of European Hematology Association. This is an open access Abstract Book distributed under the Attribution-NonCommercial-NoDerivs (CC BY-NC-ND), which allows third parties to download the articles and share them with others as long as they credit the author and the Abstract Book, but they cannot change the content in any way or use them commercially.
**Abstract Book Citations:** Authors, Title, HemaSphere, 2026;10:(S1):pages. The abstract book DOIs can be found on the "Author Information" page.
**Disclaimer:** Articles published in the journal HemaSphere exclusively reflect the opinions of the authors. The authors are responsible for all content in their abstracts including accuracy of the facts, statements, citing resources, etc.

**Summary/Conclusion**

The "one-vial" plerixafor strategy provides a superior balance of efficacy, safety, and economy for MM patients in China. It significantly improves mobilization success and reduces infection-related complications compared to high-dose CY. This model offers a sustainable "Chinese experience" for optimizing stem cell mobilization in resource-limited or cost-sensitive healthcare systems.

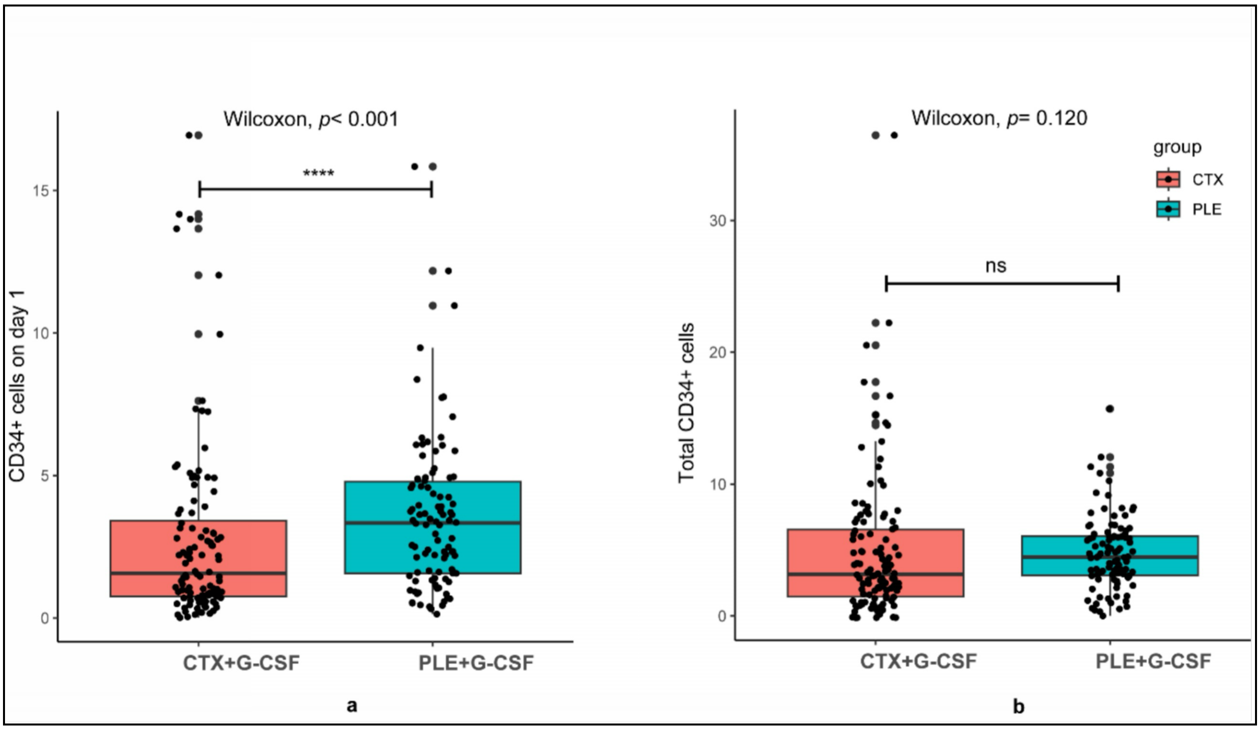

**Copyright Information:** (Online) ISSN: 2572-9241
©2026 The Author(s). HemaSphere is published by John Wiley & Sons Ltd on behalf of European Hematology Association. This is an open access Abstract Book distributed under the Attribution-NonCommercial-NoDerivs (CC BY-NC-ND), which allows third parties to download the articles and share them with others as long as they credit the author and the Abstract Book, but they cannot change the content in any way or use them commercially.
**Abstract Book Citations:** Authors, Title, HemaSphere, 2026;10:(S1):pages. The abstract book DOIs can be found on the "Author Information" page.
**Disclaimer:** Articles published in the journal HemaSphere exclusively reflect the opinions of the authors. The authors are responsible for all content in their abstracts including accuracy of the facts, statements, citing resources, etc.

### (PB3282) REAL WORLD OUTCOMES OF YOUNG MULTIPLE MYELOMA PATIENTS AND FACTORS INFLUENCING OUTCOMES: A SINGLE CENTRE EXPERIENCE IN INDIA

**Topic:** 14. Myeloma and other monoclonal gammopathies - Clinical

Souvik Saha*[1], Harshal Mamlekar[1], Sanjeev Sanjeev[1], Manish Singh[1], Dinesh Chandra[1], Khaliqur Rahman[1], Ruchi Gupta[1], Rajesh Kashyap[1]

[1]*Sanjay Gandhi Postgraduate Institute of Medical Sciences, Department of Hematology, Lucknow, India;*

### Background

Multiple myeloma is primarily a disease of elderly population with a median age of 65-70 years.However, various Indian studies have shown a median age of 54-58 years, which is a decade younger compared to Western population.Although there are Western studies documenting the outcomes of young myeloma patients, there is a scarcity of data in Indian literature.

### Aims

1. To document the baseline characteristics of young myeloma patients
2. Assessment of Induction outcomes, PFS and OS and the factors influencing survival

### Methods

In this retrospective observational study, newly diagnosed multiple myeloma patients <=50 years of age, presenting at our institute between 2020 till 2025 were included.The clinical and laboratory data were retrieved from Hospital records.Induction outcomes were noted.PFS was defined as the duration between initiation of therapy and disease progression.OS was defined as duration between date of diagnosis and death, or censored at the date of last follow up.

**Copyright Information:** (Online) ISSN: 2572-9241
©2026 The Author(s). HemaSphere is published by John Wiley & Sons Ltd on behalf of European Hematology Association. This is an open access Abstract Book distributed under the Attribution-NonCommercial-NoDerivs (CC BY-NC-ND), which allows third parties to download the articles and share them with others as long as they credit the author and the Abstract Book, but they cannot change the content in any way or use them commercially.
**Abstract Book Citations:** Authors, Title, HemaSphere, 2026;10:(S1):pages. The abstract book DOIs can be found on the "Author Information" page.
**Disclaimer:** Articles published in the journal HemaSphere exclusively reflect the opinions of the authors. The authors are responsible for all content in their abstracts including accuracy of the facts, statements, citing resources, etc.

**Results**

A total of 104 patients were included in the study.3 patients died before the initiation of therapy.The median follow up period was 20.5 months.At the end of Induction, VGPR was attained in 56.4% patients, PR in 30.8%, SD in 4.3% and 8.5% patients had PD.We did not find any significant association of baseline factors with Induction outcomes.The 3 year PFS was 82.9% and 3 year OS was 85.8%.Patients presenting with plasma cell leukemia (p=0.009) had significantly poorer PFS.On immunofixation, light chain myeloma had significantly better PFS as compared to non light chain disease (p=0.037).R ISS Stage I had significantly better PFS, but there was no difference between R ISS II and III.Patients who attained VGPR post Induction had significant improvement in PFS unlike patients who failed to attain VGPR.Also,PFS was significantly poorer in functional high risk patients.On evaluation of factors affecting OS, plasma cell leukemia, R ISS Stage III and FHR disease were significantly associated with poorer outcomes.There was no significant improvement in PFS or OS in patients receiving Daratumumab and those undergoing autologous HSCT.This was possibly due to very few patients receiving Daratumumab (n=6) and HSCT (n=9) due to resource constraints.We did not find any association of anemia, hypercalcemia, kidney injury, extramedullary disease and cytogenetics with survival outcomes.

| | n=104 |
|---|---|
| Median age | 45 years |
| Sex | |
| · Male | 71 (68.3%) |
| · Female | 33 (31.7%) |
| Anemia | 86 (82.7%) |
| Renal injury | 54 (51.9%) |
| Hypercalcemia | 25 (24%) |
| Lytic lesion | 65 (62.5%) |
| Extramedullary disease | 13 (12.5%) |
| Plasma cell leukemia | 7 (6.7%) |
| Immunofixation | |
| · IgG kappa | 39 (37.5%) |
| · IgG lambda | 17 (16.3%) |
| · IgA kappa | 6 (5.8%) |
| · IgA lambda | 7 (6.7%) |
| · Kappa LC | 24 (23.1%) |
| · Lambda LC | 11 (10.6%) |
| Cytogenetics | |
| · High risk | 32 (30.8%) |
| · Standard risk | 59 (56.7%) |
| R ISS staging | |
| · Stage I | 6 (5.8%) |
| · Stage II | 51 (49%) |
| · Stage III | 35 (33.7%) |
| Functional high risk | 15/79 (18.9%) |

**Copyright Information:** (Online) ISSN: 2572-9241
©2026 The Author(s). HemaSphere is published by John Wiley & Sons Ltd on behalf of European Hematology Association. This is an open access Abstract Book distributed under the Attribution-NonCommercial-NoDerivs (CC BY-NC-ND), which allows third parties to download the articles and share them with others as long as they credit the author and the Abstract Book, but they cannot change the content in any way or use them commercially.
**Abstract Book Citations:** Authors, Title, HemaSphere, 2026;10:(S1):pages. The abstract book DOIs can be found on the "Author Information" page.
**Disclaimer:** Articles published in the journal HemaSphere exclusively reflect the opinions of the authors. The authors are responsible for all content in their abstracts including accuracy of the facts, statements, citing resources, etc.

**Summary/Conclusion**

Our study reports one of the largest cohorts of young myeloma patients in India.We found that plasma cell leukemia, R ISS staging and FHR disease were consistently associated with both PFS and OS.Although light chain disease and VGPR post Induction were associated with better PFS, they did not convert to OS benefit.The major drawbacks of our study were that very few patients underwent HSCT and we did not have a comparator arm of patients >50 years.Hence, further Indian studies to address these limitations are required.

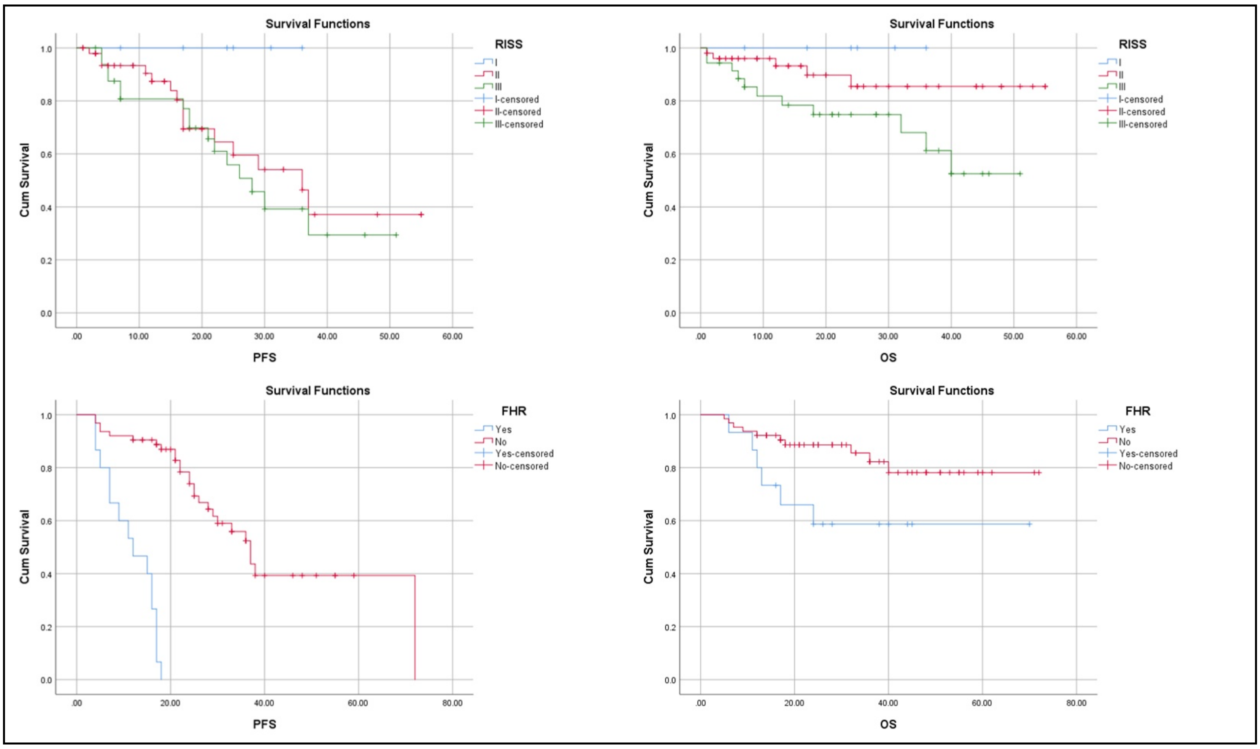

**Copyright Information:** (Online) ISSN: 2572-9241
©2026 The Author(s). HemaSphere is published by John Wiley & Sons Ltd on behalf of European Hematology Association. This is an open access Abstract Book distributed under the Attribution-NonCommercial-NoDerivs (CC BY-NC-ND), which allows third parties to download the articles and share them with others as long as they credit the author and the Abstract Book, but they cannot change the content in any way or use them commercially.
**Abstract Book Citations:** Authors, Title, HemaSphere, 2026;10:(S1):pages. The abstract book DOIs can be found on the "Author Information" page.
**Disclaimer:** Articles published in the journal HemaSphere exclusively reflect the opinions of the authors. The authors are responsible for all content in their abstracts including accuracy of the facts, statements, citing resources, etc.

### (PB3283) BONE MARROW SUVMAX ON 18F-FDG PET / CT REFLECTS DISEASE BURDEN AND IMMUNOSUPPRESSIVE MICROENVIRONMENT IN NEWLY DIAGNOSED MULTIPLE MYELOMA

**Topic:** 14. Myeloma and other monoclonal gammopathies - Clinical

Hao Xiong[1], Lijuan Li[1], Liansheng Zhang*[1]

[1] *The Second Hospital of Lanzhou University, Department of Hematolog, Lanzhou, GanSu, China;*

### Background

Multiple myeloma (MM) progression is associated with both increasing tumor burden and remodeling of the immune microenvironment. The maximum standardized uptake value (SUVmax) derived from ^18F-FDG PET/CT reflects metabolic activity, but its relationship with immunological parameters in MM remains insufficiently characterized.

### Aims

To investigate the association between bone marrow SUVmax and disease burden indicators, circulating cytokines, and immune cell subsets in newly diagnosed MM patients.

### Methods

This retrospective single-center study included 49 newly diagnosed MM patients (August 2021–December 2024). All patients underwent ^18F-FDG PET/CT using a Discovery 690 scanner. Bone marrow SUVmax was recorded, and patients were stratified into low (n=32) and high (n=17) SUVmax groups. Peripheral blood lymphocyte subsets were analyzed by flow cytometry (BD Canto II), and serum cytokines were quantified using a flow cytometry–based capture assay. Clinical characteristics, laboratory parameters, and immune-related variables were compared between groups. Correlation analyses were performed between SUVmax and measured parameters.

### Results

Median age was 60 years; 69.4% were male. Most patients presented with stage II or higher disease, and 87.8% had bone involvement at diagnosis. High SUVmax was associated with a greater proportion of R-ISS stage III disease, severe myeloma bone disease, pathological fractures, elevated serum calcium levels, and increased bone marrow plasma cell infiltration. Immunologically, the high SUVmax group exhibited lower proportions of CD4+ T cells and higher proportions of regulatory T cells (Treg), together with increased serum IL-4 and IL-10 levels. SUVmax positively correlated with serum calcium (total and corrected), bone marrow plasma cell percentage, IL-4, IL-6, IL-10, IL-12p70, TNF-α, and Treg proportion, and negatively correlated with white blood cell count and CD4+ T cell proportion.

### Summary/Conclusion

Bone marrow SUVmax is closely associated with disease burden and immunosuppressive immune alterations in MM. SUVmax may serve as an imaging biomarker reflecting both tumor load and immune microenvironment status.

**Copyright Information:** (Online) ISSN: 2572-9241
©2026 The Author(s). HemaSphere is published by John Wiley & Sons Ltd on behalf of European Hematology Association. This is an open access Abstract Book distributed under the Attribution-NonCommercial-NoDerivs (CC BY-NC-ND), which allows third parties to download the articles and share them with others as long as they credit the author and the Abstract Book, but they cannot change the content in any way or use them commercially.
**Abstract Book Citations:** Authors, Title, HemaSphere, 2026;10:(S1):pages. The abstract book DOIs can be found on the "Author Information" page.
**Disclaimer:** Articles published in the journal HemaSphere exclusively reflect the opinions of the authors. The authors are responsible for all content in their abstracts including accuracy of the facts, statements, citing resources, etc.

### (PB3284) ASSOCIATION BETWEEN EARLY RESPONSE TO TREATMENT AND LONG-TERM SURVIVAL OUTCOMES AMONG REAL-WORLD PATIENTS WITH RELAPSED/REFRACTORY (R/R) MULTIPLE MYELOMA

**Topic:** 14. Myeloma and other monoclonal gammopathies - Clinical

Patricia Prince[1], Nileesa Gautam[1], Guido Nador[2], Soraya Djema[3], Alexander Russell*[2]

*[1]Aetion, a Datavant Company, New York, United States of America;[2]Pfizer R&D UK Ltd, Surrey, United Kingdom;[3]Pfizer, Algiers, Algeria;*

### Background

In multiple myeloma (MM), early treatment response endpoints, such as overall response rate (ORR), are commonly used in accelerated approvals as surrogate endpoints. Limited information is available on the prognostic value of early response endpoints outside clinical trials.

### Aims

This study aimed to assess the association between depth of treatment response and later-term outcomes such as real-world progression-free survival (rwPFS) and overall survival (rwOS).

### Methods

This non-interventional, retrospective cohort study assessed the association between early response to treatment and longer-term survival outcomes using an electronic health record (EHR) database (COTA Real World Evidence MM database). Adult patients (≥ 18 years old) were included in this study if they had a diagnosis of MM and were treated with second-line (2L) systemic anti-cancer treatment between 1/1/2015 and 30/9/2023 (index date). Response to treatment was assigned using International Myeloma Working Group (IMWG) Uniform Response Criteria for MM, based on available laboratory data at baseline and during follow-up. Patients were categorized based on their best outcome as real-world stringent complete response (rwsCR), complete response (rwCR), very good partial response (rwVGPR), partial response (rwPR), stable disease (rwSD), progressive disease (rwPD), or missing response data. Patients with and without a positive response to treatment were compared in terms of rwPFS and rwOS using Cox proportional hazards regression, including hazard ratios (HRs) and 95% confidence intervals (CIs). Two definitions of positive response were used: rwVGPR or better (rwCR, rwsCR, rwVGPR) and rwPR or better (rwCR, rwsCR, rwVGPR, rwPR).

### Results

In the database, 2,670 patients with r/r MM were treated with 2L systemic anti-cancer therapy and included in this study. Median age was 68 years (IQR: 60, 75), 55.0% of patients were male, and 68.3% patients were White. Combination treatment with a proteasome inhibitor (PIs) and an immunomodulatory drug (IMiD) was the most common 2L treatment (40.5%). 1,214 (45.5%) patients did not have the available lab data to determine response to treatment and were excluded from the association analyses. In 2L, 87 (3.3%) patients had a response of rwVGPR or better, and 952 (35.7%) patients had a response of rwPR or better. Patients with a rwVGPR or better had a median rwOS of 69.7 months compared to 53.2 months among those without a positive response (HR: 0.52 [95% CI: 0.34, 0.79]); patients with a rwPR or better had a median rwOS of 63.5 months compared to 40.0 months without a positive response (HR: 0.64 [95% CI: 0.54, 0.76]). Patients with a response of rwVGPR or better had a median rwPFS of 64.3 months compared to 24.5 months among those without a positive response (HR: 0.39 [95% CI: 0.27, 0.57]); patients with a response of rwPR or better had a median rwPFS of 43.4 months compared to 5.3 months without a positive response (HR: 0.28 [95% CI: 0.25, 0.33]).

**Copyright Information:** (Online) ISSN: 2572-9241
©2026 The Author(s). HemaSphere is published by John Wiley & Sons Ltd on behalf of European Hematology Association. This is an open access Abstract Book distributed under the Attribution-NonCommercial-NoDerivs (CC BY-NC-ND), which allows third parties to download the articles and share them with others as long as they credit the author and the Abstract Book, but they cannot change the content in any way or use them commercially.
**Abstract Book Citations:** Authors, Title, HemaSphere, 2026;10:(S1):pages. The abstract book DOIs can be found on the "Author Information" page.
**Disclaimer:** Articles published in the journal HemaSphere exclusively reflect the opinions of the authors. The authors are responsible for all content in their abstracts including accuracy of the facts, statements, citing resources, etc.

**Summary/Conclusion**

This real-world study demonstrated that patients with an early-term positive prognostic outcome (rwCR/rwsCR or rwPR/rwVGPR) experienced prolonged survival. Although these real-world study results do not establish full surrogacy, these favorable findings contribute to the growing body of evidence from diverse study designs supporting early-term positive outcomes as a surrogate.

**Copyright Information:** (Online) ISSN: 2572-9241
©2026 The Author(s). HemaSphere is published by John Wiley & Sons Ltd on behalf of European Hematology Association. This is an open access Abstract Book distributed under the Attribution-NonCommercial-NoDerivs (CC BY-NC-ND), which allows third parties to download the articles and share them with others as long as they credit the author and the Abstract Book, but they cannot change the content in any way or use them commercially.
**Abstract Book Citations:** Authors, Title, HemaSphere, 2026;10:(S1):pages. The abstract book DOIs can be found on the "Author Information" page.
**Disclaimer:** Articles published in the journal HemaSphere exclusively reflect the opinions of the authors. The authors are responsible for all content in their abstracts including accuracy of the facts, statements, citing resources, etc.

### (PB3285) EXPERT CONSENSUS ON DEFINING PROGNOSTIC FACTORS AND TREATMENT-EFFECT MODIFIERS IN MULTIPLE MYELOMA

**Topic:** 14. Myeloma and other monoclonal gammopathies - Clinical

Karthik Ramasamy*[1], James Farrell[2], Gordon Cook[3], Reuben Benjamin[4], Dean Smith[5], Martin Kaiser[6], Emma Searle[7], Neil Rabin[8], Jaimal Kothari[9], Sebastian Theurich[10], K Martin Kortum[11], Christof Scheid[12], Hermann Einsele[13], Hans Juergen Dr. Salwender[14], Sunil Lakhwani[15], Rafael Alberto Alonso Fernandez[16], Maria Ester Clavero[17], Esther González García[18], Mario Arnao[19], Mercedes Gironella Mesa[20], Xabier Gutierrez Lopez De Ocariz[21], Venancio Conesa Garcia[22], Anthony Levy[23], Lionel Karlin[24], Benjamin Hebraud[25], Shanti Neff-Baro[26], Atara Laor[27], Syed Raza[28], Joseph Cappelleri[29], Ashraf Chaudhary[30], Graham Jackson[31]

*[1]Department of Haematology, Oxford University Hospitals NHS Foundation Trust, Oxford, United Kingdom;[2]Pfizer Ltd, Dublin, Ireland; [3]Leeds Institute of Cancer & Pathology, University of Leeds, Leeds, United Kingdom;[4]School of Cancer & Pharmaceutical Sciences, King's College London, London, United Kingdom; [5]Department of Haematology, City Hospital, Nottingham, United Kingdom; [6]The Royal Marsden Hospital and The Institute of Cancer Research, Department of Haemato-oncology, London, United Kingdom; [7]The Christie Hospital, The University of Manchester, Manchester, United Kingdom; [8]Department of Haematology, University College London Hospitals, London, United Kingdom;[9]Department of Clinical Haematology, Oxford University Hospitals NHS Foundation Trust, Oxford, United Kingdom; [10]Department of Medicine III, LMU University Hospital, Munich, Germany; German Cancer Consortium (DKTK), Munich Site, and German Cancer Research Center, Heidelberg, Germany; Comprehensive Cancer Center Munich, LMU University Munich, Munich, Germany; Bavarian Cancer Research Center (BZKF), LMU University Hospital,, Munich, Germany; [11]Würzburg University Hospital, Wurzburg, Germany; [12]Cologne University Hospital, Cologne, Germany; [13]Medizinische Klinik und Poliklinik II, Universitätsklinikum Würzburg, Würzburg, Germany; [14]Asklepios Hospital Hamburg, Altona, Hematology, Oncology and Palliative Care, 22763, Hamburg, Germany; [15]Hospital Universitario de Canarias, Universidad de La Laguna, Tenerife, Spain;[16]Department of Hematology, Hospital Universitario 12 de Octubre, Universidad Complutense de Madrid, Departamento de Medicina, H12O CNIO Clinical Research Hematology Unit; CIBERONC, Madrid, Spain; [17]Hematology Department, Virgen de las Nieves University Hospital, 18012 Granada, Spain; [18]Hospital Universitario de Cabueñes, Gijón, Spain;[19]Servicio de Hematología y Hemoterapia, Hospital Universitario y Politécnico La Fe, Valencia, Spain; [20]Hospital Universitari Vall d'Hebron, Barcelona, Spain;[21]Hematology Department, Hospital Universitario de Álava, Vitoria, Spain; [22]Hospital General de Elche, 03203 Alicante, Spain;[23]Service d'Hematologie et Therapie Cellulaire, and Inserm CIC U1402, 86000 Poitiers, France; [24]Hôpital Lyon Sud, Hospices Civils de Lyon, Pierre-Benite, France; [25]Hematology Department, Institut Universitaire du Cancer de Toulouse-Oncopole, Toulouse, France; [26]Amaris Ltd, Paris, France; [27]Amaris Ltd, Montreal, Canada; [28]Amaris Ltd, Toronto, Canada; [29]Pfizer Inc, New York, United States of America; [30]Pfizer Inc, Collegeville, United States of America; [31]Northern Institute for Cancer Research, Newcastle University, Newcastle upon Tyne, United Kingdom;*

### Background

Multiple myeloma (MM) is a relapsing-remitting bone cancer with variable outcomes depending on prognostic factors.
Prognostic factors are measurable variables that predict clinical outcomes in MM patients and inform risk stratification and treatment decisions by distinguishing subgroups with different prognoses.
Treatment effect modifiers are variables that influence how the effect of a treatment varies across different subgroups of a population. The treatment landscape has significantly changed over the last 2 years and historical publications lack relevance. Given this and the complexity of MM, identifying and adjusting for prognostic factors and treatment effect modifiers in clinical and real-world evidence studies is critical to reliably evaluate treatment benefit and provide accurate assessments in indirect treatment comparisons.

**Copyright Information:** (Online) ISSN: 2572-9241
©2026 The Author(s). HemaSphere is published by John Wiley & Sons Ltd on behalf of European Hematology Association. This is an open access Abstract Book distributed under the Attribution-NonCommercial-NoDerivs (CC BY-NC-ND), which allows third parties to download the articles and share them with others as long as they credit the author and the Abstract Book, but they cannot change the content in any way or use them commercially.
**Abstract Book Citations:** Authors, Title, HemaSphere, 2026;10:(S1):pages. The abstract book DOIs can be found on the “Author Information” page.
**Disclaimer:** Articles published in the journal HemaSphere exclusively reflect the opinions of the authors. The authors are responsible for all content in their abstracts including accuracy of the facts, statements, citing resources, etc.

### Aims

To achieve consensus on key prognostic factors and treatment effect modifiers in 2L+ MM.

### Methods

The Delphi technique is an established approach for eliciting and synthesizing expert. It is a structured, iterative process that enables the convergence of views toward consensus. This is achieved through multiple rounds of questionnaires, with feedback provided between rounds to allow participants to reconsider their views considering the group response (**Figure1**).

In this study, the Delphi process consisted of three rounds of electronic surveys completed by a panel of 25 experts in MM. All responses were anonymized to minimize peer influence. Panel members represented a multinational cohort from Germany, the UK, France, and Spain.

In each round, participants were asked to indicate their level of agreement regarding whether specific clinical variables (from previous systematic literature reviews) were prognostic or modified treatment effect. Consensus was defined a priori as ≥70% agreement among respondents. In the first round, experts were also invited to propose further relevant variables not included in the initial questionnaire.

During the second round, all items that did not achieve consensus in round 1 were re-presented along with aggregated feedback showing the distribution of agreement from the previous round. Experts were asked whether they wished to revise their initial responses. Newly suggested variables in round 1 were also introduced for evaluation.

The third and final round followed the same procedure: variables still lacking consensus were re-presented with summary feedback and panellists were given a final opportunity to reconsider their responses. This iterative process concluded with the identification of variables meeting the predefined consensus threshold.

### Results

The 25 experts reached consensus on 20 variables; age, median time since diagnosis, International Staging System (ISS) disease stage, high-risk cytogenetics, extramedullary disease, number of prior lines of therapy, ECOG PS, CD38 refractory status, lenalidomide refractory status, creatinine clearance, MRD status, frailty, comorbidities, prior BCMA exposure, triple class exposed status, triple class refractory status, LDH levels, gene expression profiling, circulating plasma cells and cytopenia.

### Summary/Conclusion

This Delphi panel builds on previous publications which rely on systematic literature reviews or face-to-face expert meetings with limited numbers of clinicians which can be subject to bias.

The large sample of MM experts provides the most robust framework for identifying prognostic factors and treatment effect modifiers.

**Copyright Information:** (Online) ISSN: 2572-9241
©2026 The Author(s). HemaSphere is published by John Wiley & Sons Ltd on behalf of European Hematology Association. This is an open access Abstract Book distributed under the Attribution-NonCommercial-NoDerivs (CC BY-NC-ND), which allows third parties to download the articles and share them with others as long as they credit the author and the Abstract Book, but they cannot change the content in any way or use them commercially.
**Abstract Book Citations:** Authors, Title, HemaSphere, 2026;10:(S1):pages. The abstract book DOIs can be found on the “Author Information” page.
**Disclaimer:** Articles published in the journal HemaSphere exclusively reflect the opinions of the authors. The authors are responsible for all content in their abstracts including accuracy of the facts, statements, citing resources, etc.

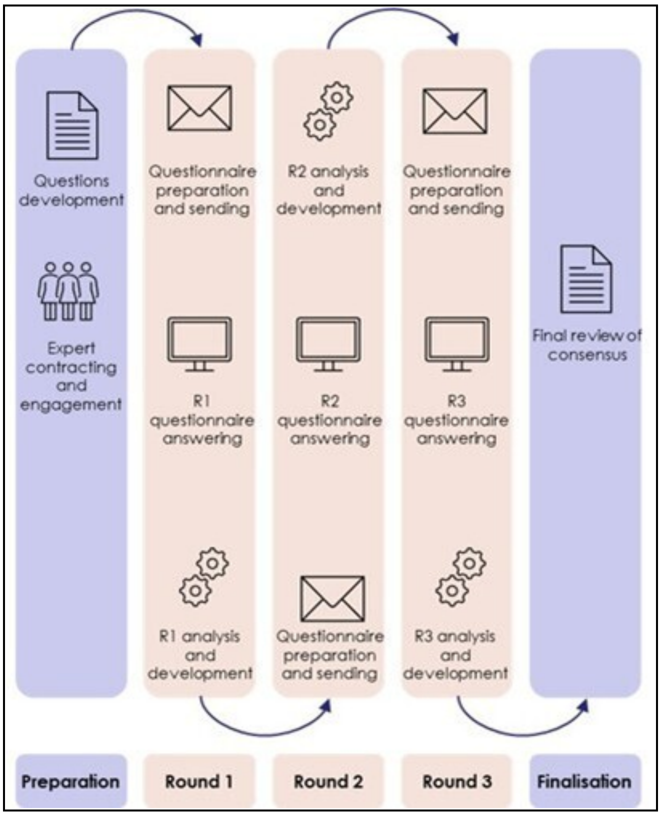

**Copyright Information:** (Online) ISSN: 2572-9241
©2026 The Author(s). HemaSphere is published by John Wiley & Sons Ltd on behalf of European Hematology Association. This is an open access Abstract Book distributed under the Attribution-NonCommercial-NoDerivs (CC BY-NC-ND), which allows third parties to download the articles and share them with others as long as they credit the author and the Abstract Book, but they cannot change the content in any way or use them commercially.
**Abstract Book Citations:** Authors, Title, HemaSphere, 2026;10:(S1):pages. The abstract book DOIs can be found on the "Author Information" page.
**Disclaimer:** Articles published in the journal HemaSphere exclusively reflect the opinions of the authors. The authors are responsible for all content in their abstracts including accuracy of the facts, statements, citing resources, etc.

### (PB3286) REAL-WORLD OVERALL SURVIVAL IN RELAPSED OR REFRACTORY MULTIPLE MYELOMA (MM) IN TAIWAN TREATED WITH AND WITHOUT DARATUMUMAB-BASED THERAPY

**Topic:** 14. Myeloma and other monoclonal gammopathies - Clinical

Shih-Pei Shen*[1, 2], David Bin-Chia Wu[3, 4, 5], Chao-Hsiun Tang[2], Cathy Kwang-Wei Wu[3], Lee Anne Rothwell[6], Hsin-An Hou[1]

*[1]Division of Hematology, Department of Internal Medicine, National Taiwan University Hospital, Taipei, Taiwan;[2]Taipei Medical University, Taipei, Taiwan; [3]Johnson& Johnson, Singapore, Singapore; [4]Saw Swee Hock School of Public Health, National University of Singapore, Singapore, Singapore; [5]School of Pharmacy, Faculty of Health & Medical Sciences, Taylor's University Malaysia, Subang Jaya, Malaysia; [6]Johnson & Johnson (Janssen-Cilag Pty Ltd), North Ryde, Australia;*

### Background

Daratumumab (dara), an anti-CD38 monoclonal antibody, was approved for relapsed or refractory multiple myeloma (MM) in Taiwan in 2017 and reimbursed in 2020 with a cap of 22 infusions. Frontline dara became reimbursable in 2025 but is limited to transplant-eligible patients, resulting in delayed use of novel agents in routine practice.

### Aims

This analysis aimed to evaluate the survival benefit associated with dara use in Taiwanese patients with MM.

### Methods

This population-based study utilized the Taiwan National Health Insurance Research Database (NHIRD) and the linked Registry of Catastrophic Illness (RCI). The study included adults (≥18 years) with newly diagnosed MM (ICD-10 C90.0/C90.0X) recorded in the RCI from 2013–2022 and excluded those with pre-existing primary malignancies. Patients were followed until death or the end of the study period (Dec. 31, 2023), whichever occurred first. Baseline characteristics selected through hematologists' expert opinions and literature review were adjusted using inverse probability of treatment weighting (IPTW). Real-world overall survival (rwOS) was estimated from the initiation of second-line (2L) therapy to minimize the immortal time bias by Kaplan–Meier, and HRs were calculated using Cox regression models adjusted for age, sex, comorbidities, and diagnosis period.

### Results

Of 2479 patients included, 461 (18.6%) patients received dara and 2018 (81.4%) patients never received dara by the end of the study period. Among dara-treated patients, 285 (61.8%) initiated therapy in 2L and 176 (38.2%) in third-line (3L) or later. Before IPTW, substantial imbalance was observed: diagnosis period was highly skewed toward 2018–2022 in the dara-treated group (2018–2022: 88% vs 43%, SMD=1.08), 1L regimens differed markedly (VTd/VTCd/VRd: 83% vs 59% SMD=0.54), and time from 1L to 2L therapy was longer among dara group (≤180 days 24% vs 46%, SMD=0.47; ≥361 days: 54% vs 30%, SMD=0.50). Geographic distribution showed mild imbalance, with fewer dara-treated patients in Northern Taiwan (43% vs 54%, SMD=0.24) and more in Central (26% vs 18%, SMD=0.20) and Southern Taiwan (16% vs 11%, SMD=0.14). After IPTW adjustment, covariate balance improved substantially (Table 1). Only two covariates showed minimal residual imbalance and were not statistically significant: lower wage-based socioeconomic status (SMD=0.12, overall p=0.58) and Charlson Comorbidity Index ≥2 (SMD=0.14, overall p=0.47). Patients treated with dara had significantly longer rwOS from initiation of 2L than those never treated with dara (Figure 1. Median rwOS [95% CI] not reached with dara vs 2.54 [2.31–2.75] years without dara; adjusted HR [95% CI] 0.54[0.46–0.63], p<0.0001).

**Copyright Information:** (Online) ISSN: 2572-9241
©2026 The Author(s). HemaSphere is published by John Wiley & Sons Ltd on behalf of European Hematology Association. This is an open access Abstract Book distributed under the Attribution-NonCommercial-NoDerivs (CC BY-NC-ND), which allows third parties to download the articles and share them with others as long as they credit the author and the Abstract Book, but they cannot change the content in any way or use them commercially.
**Abstract Book Citations:** Authors, Title, HemaSphere, 2026;10:(S1):pages. The abstract book DOIs can be found on the "Author Information" page.
**Disclaimer:** Articles published in the journal HemaSphere exclusively reflect the opinions of the authors. The authors are responsible for all content in their abstracts including accuracy of the facts, statements, citing resources, etc.

### Summary/Conclusion

In real-world practice, treatment sequencing is often influenced by reimbursement and access constraints, leading to delayed use of novel agents. Despite 2L reimbursement approval in Taiwan, most patients did not receive dara and many initiated it only after 3L. Patients treated with dara demonstrated longer OS compared with those never receiving it at the end of the study period. Given the rapid progression and high mortality of relapsed/refractory MM, delaying novel therapy may leave patients without the opportunity to ever receive it, thereby forfeiting a potential survival benefit. These findings support the importance of administering the most effective therapy as early as feasible.

**Table 1. Baseline Characteristics**

| | Before IPTW | | | After IPTW | | |
|---|---|---|---|---|---|---|
| | With Dara % | Without Dara % | SMD | With Dara % | Without Dara % | SMD |
| **Diagnosis period** | | | | | | |
| 2013–2017 | 12 | 57 | 1.08 | 47 | 49 | 0.03 |
| 2018–2022 | 88 | 43 | 1.08 | 53 | 51 | 0.03 |
| **Male** | 55 | 53 | 0.04 | 49 | 53 | 0.09 |
| **Age** | | | | | | |
| <50 | 3 | 4 | 0.08 | 2 | 4 | 0.09 |
| 50-69 | 45 | 45 | 0.01 | 46 | 45 | 0.02 |
| ≥70 | 52 | 51 | 0.02 | 52 | 51 | 0.01 |
| **SES** | | | | | | |
| Higher wage employees | 9 | 7 | 0.07 | 6 | 8 | 0.05 |
| Medium wage employees | 16 | 18 | 0.06 | 16 | 18 | 0.05 |
| Lower wage employees | 9 | 8 | 0.02 | 5 | 8 | 0.12 |
| Self-employees or professional union member | 40 | 41 | 0.03 | 43 | 41 | 0.03 |
| Non-fixed income | 26 | 25 | 0.03 | 29 | 25 | 0.10 |
| **CCI** | | | | | | |
| 0 | 31 | 31 | 0.00 | 34 | 31 | 0.05 |
| 1 | 24 | 23 | 0.02 | 27 | 23 | 0.10 |
| ≥2 | 45 | 46 | 0.01 | 39 | 46 | 0.14 |
| **MM associated comorbidities** | 57 | 59 | 0.04 | 59 | 59 | - |
| **Medical center hospital type** | 62 | 63 | 0.03 | 58 | 63 | 0.10 |
| **Geographical areas** | | | | | | |
| Taipei/ Northern | 43 | 54 | 0.24 | 56 | 52 | 0.08 |
| Central | 26 | 18 | 0.20 | 19 | 20 | 0.01 |
| Southern | 16 | 11 | 0.14 | 9 | 12 | 0.09 |
| Kaohsiung & Pingtung/ Eastern | 15 | 17 | 0.03 | 15 | 16 | 0.03 |
| **1L VTd/VTCd/VRd** | 83 | 59 | 0.54 | 59 | 64 | 0.09 |
| **Time from 1L to 2L (days)** | | | | | | |
| ≤180 | 24 | 46 | 0.47 | 41 | 42 | 0.01 |
| 181-360 | 23 | 25 | 0.05 | 22 | 24 | 0.05 |
| ≥361 | 54 | 30 | 0.50 | 37 | 34 | 0.06 |

Abbreviations: IPTW, inverse probability of treatment weighting; Dara, daratumumab; SMD, standardized mean difference; SES, social economic status; CCI, Charlson Comorbidity Index; MM, multiple myeloma; 1L, first-line; 2L, second-line; V, bortezomib; T, thalidomide; d, dexamethasone; C, cyclophosphamide; R, lenalidomide

**Figure 1. Adjusted Kaplan–Meier rwOS From Initiation of 2L Therapy**

| | Median rwOS [95%CI] | Adjusted HR [95%CI] | Crude HR [95%CI] |
|---|---|---|---|
| With dara | Not reached | 0.54 [0.46–0.63], p<.0001 | 0.55 [0.47–0.68], p<.0001 |
| Without dara | 2.54 [2.31–2.75] years | Ref. | Ref. |

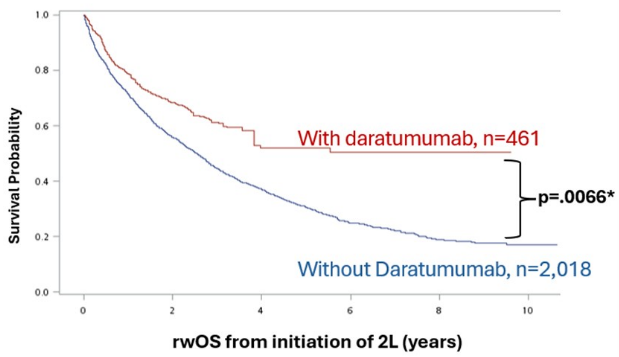

Abbreviations: rwOS, real-world overall survival; HR: hazard ratio
Study period: From the initiation of 2L to the occurrence of death or the end of the study (Dec. 31, 2023)

**Copyright Information:** (Online) ISSN: 2572-9241
©2026 The Author(s). HemaSphere is published by John Wiley & Sons Ltd on behalf of European Hematology Association. This is an open access Abstract Book distributed under the Attribution-NonCommercial-NoDerivs (CC BY-NC-ND), which allows third parties to download the articles and share them with others as long as they credit the author and the Abstract Book, but they cannot change the content in any way or use them commercially.
**Abstract Book Citations:** Authors, Title, HemaSphere, 2026;10:(S1):pages. The abstract book DOIs can be found on the "Author Information" page.
**Disclaimer:** Articles published in the journal HemaSphere exclusively reflect the opinions of the authors. The authors are responsible for all content in their abstracts including accuracy of the facts, statements, citing resources, etc.

### (PB3287) LAGOON (OP-115): A NON-INTERVENTIONAL STUDY OF MELPHALAN FLUFENAMIDE (MELFLUFEN) PLUS DEXAMETHASONE IN PATIENTS WITH RELAPSED AND/OR REFRACTORY MULTIPLE MYELOMA IN SPAIN ACCORDING TO APPROVED LABEL

**Topic:** 14. Myeloma and other monoclonal gammopathies - Clinical

Enrique M. Ocio*[1], Esther González García[2], Albert Pérez Montaña[3], Xabier Gutierrez Lopez De Ocariz[4], Valentín Cabañas Perianes[5], Estrella Cruz Carrillo[6], Pernilla Omberg[7], Stefan Norin[7], María-Victoria Mateos[8]

*[1]Hospital Universitario Marqués de Valdecilla (IDIVAL) Universidad de Cantabria, Hematology Department, Santander, Spain; [2]Hospital Universitario de Cabueñes, Department of Hematology, Gijón, Spain;[3]Hospital Universitario Son Espases, Department of Hematology, Palma, Spain; [4]Hospital Universitario De Álava, Department of Hematology, Vitoria-Gasteiz, Spain;[5]Hospital Universitario Virgen de La Arrixaca, Department of Hematology, Murcia, Spain; [6]Hospital Universitario Virgen del Rocío, Instituto de Biomedicina de Sevilla (IBIS)/CSIC/Universidad de Sevilla, Department of Hematology, Sevilla, Spain; [7]Oncopeptides AB, Stockholm, Sweden; [8]University Hospital of Salamanca and Cancer Research Center (IBMCC-CSICUSAL), Hematology Department, Salamanca, Spain;*

### Background

Melphalan flufenamide (also known as melflufen) is a lipophilic alkylating peptide-drug conjugate that utilizes increased peptidase and esterase expression to rapidly release hydrophilic cytotoxic alkylating agents inside tumor cells. Melflufen is EMA-approved, in combination with dexamethasone, for adult patients with RRMM who have received at least 3 prior lines of therapy and who are refractory to a proteasome Inhibitor (PI), an immunomodulatory drug (IMiD) and an anti-CD38 monoclonal antibody. For patients with prior autologous stem cell transplantation, the time to progression should be at least 36 months from transplantation. After the completion of the large melflufen studies (pivotal phase 2 study HORIZON and confirmatory phase 3 study OCEAN), other new drugs have become available and updated guidelines have been adopted. Therefore, it is important to study the effectiveness and safety of melflufen in the current treatment landscape.

### Aims

LAGOON (OP-115) is a non-interventional study that aims to observe the effectiveness of melflufen plus dexamethasone, used according to approved label, for treating RRMM in routine clinical practice. The patients in this observational study will receive the same treatment and undergo the same diagnostic procedures as they would have if they were not included in the study.

### Methods

LAGOON is a prospective observational study in Spain enrolling 50 adults treated who receive melflufen plus dexamethasone as 4th or later-line treatment per the approved label, by their treating physician prior to enrollment. Retrospective inclusion within 4 weeks is allowed if no efficacy assessment occurred. Patients will receive melflufen 40 mg intravenously on day 1 and dexamethasone 40 mg on days 1, 8, 15, and 22 of each 28-day cycle until disease progression, unacceptable toxicity, or physician determines it is not in patient's best interest to continue treatment.

### Results

The primary endpoint is overall response rate (ORR) per the International Myeloma Working Group Uniform Response Criteria. Secondary endpoints include best response, duration of response (DOR), clinical benefit rate (CBR), time to next treatment (TTNT), time to progression (TTP), progression-free survival (PFS), overall survival (OS), incidence and severity of adverse events, and quality of life (EORTC QLQ-C30). The study is ongoing and has enrolled 6 patients to date.

**Copyright Information:** (Online) ISSN: 2572-9241
©2026 The Author(s). HemaSphere is published by John Wiley & Sons Ltd on behalf of European Hematology Association. This is an open access Abstract Book distributed under the Attribution-NonCommercial-NoDerivs (CC BY-NC-ND), which allows third parties to download the articles and share them with others as long as they credit the author and the Abstract Book, but they cannot change the content in any way or use them commercially.
**Abstract Book Citations:** Authors, Title, HemaSphere, 2026;10:(S1):pages. The abstract book DOIs can be found on the "Author Information" page.
**Disclaimer:** Articles published in the journal HemaSphere exclusively reflect the opinions of the authors. The authors are responsible for all content in their abstracts including accuracy of the facts, statements, citing resources, etc.

**Summary/Conclusion**

This study will provide real-world data on the effectiveness and safety of melflufen plus dexamethasone in RRMM. As patients in clinical trials are often younger and have fewer comorbidities than the general MM population, real-world evidence is essential to understand treatment outcomes in routine practice. The study is funded by Oncopeptides AB.

**Copyright Information:** (Online) ISSN: 2572-9241
©2026 The Author(s). HemaSphere is published by John Wiley & Sons Ltd on behalf of European Hematology Association. This is an open access Abstract Book distributed under the Attribution-NonCommercial-NoDerivs (CC BY-NC-ND), which allows third parties to download the articles and share them with others as long as they credit the author and the Abstract Book, but they cannot change the content in any way or use them commercially.
**Abstract Book Citations:** Authors, Title, HemaSphere, 2026;10:(S1):pages. The abstract book DOIs can be found on the "Author Information" page.
**Disclaimer:** Articles published in the journal HemaSphere exclusively reflect the opinions of the authors. The authors are responsible for all content in their abstracts including accuracy of the facts, statements, citing resources, etc.

### (PB3289) REAL-WORLD SAFETY AND EFFICACY OF APONERMIN AND DARATUMUMAB-BASED REGIMENS IN PATIENTS WITH MULTIPLE MYELOMA

**Topic:** 14. Myeloma and other monoclonal gammopathies - Clinical

Da Gao*[1], Liping Su[2], Han Donghai[1], Peiling Zhang[3], Shengjin Fan[4], Dong Lu[5], Yan Chen[6], Baijun Fang[7], Xiaoqi Qin[8], Yan Yi[9], Zhao Yu[9], Aijun Liu[10], Wenming Chen[11]

*[1]The Affiliated Hospital of Inner Mongolia Medical University, Hohhot, China;[2]Shanxi Province Cancer Hospital/ Shanxi Hospital Affiliated to Cancer Hospital, shanxi, China; [3]Tongji Hospital, Tongji Medical College, Huazhong University of Science and Technology, Wuhan, China, wuhan, China; [4]THE FIRST AFFILIATED HOSPITAL OF HARBIN MEDICAL UNIVERSITY, Harbin, China; [5]The Third Xiangya Hospital of Central South University, changsha, China;[6]The Eighth Affiliated Hospital, Sun Yat-sen University, guangzhou, China; [7]Henan Institute of Hematology, The Affiliated Cancer Hospital of Zhengzhou University & Henan Cancer Hospital, zhengzhou, China; [8]The Second Hospital of Shanxi Medical university, taiyuan, China;[9]Southern Medical University Third Affiliated Hospital, guangzhou, China; [10]Beijing Chao-yang Hospital, Capital Medical University, beijin, China; [11]Beijing Chao-yang Hospital, Capital Medical University, beijing, China;*

### Background

Multiple myeloma, characterized by clonal proliferation of malignant plasma cells, is prone to progress to relapsed and refractory multiple myeloma (RRMM). Aponermin is a recombinant circularly permuted tumor necrosis factor-related apoptosis-inducing ligand (TRAIL). Its fundamental significance lies in its unique mechanism of action. It is the world's first and only approved death receptor 4/death receptor 5 (DR4/DR5) agonist. Non-clinical evidence had shown a synergistic effect between TRAIL agonists and CD38 monoclonal antibodies�suggesting that the combination of these two agents warrants further clinical exploration.

### Methods

We retrospectively analyzed the clinical data of 19 patients with RRMM who were treated with aponermin combined with daratumumab-based regimens for at least 2 cycles. The primary endpoint was overall response rate (ORR). The secondary endpoints included duration of response (DOR), and adverse events.

### Results

The median age was 65 years (range 51-86), with 57.89% male patients. Among all patients, 26.32% were R-ISS stage II and 31.58% stage III. Notably, 42.10% of patients presented with extramedullary disease, all with paraosseous involvement. High-risk cytogenetic abnormalities were present in 50% of the patients, with 1q21 gain/amplification in 36.84%, t(4;14) in 21.1%, and t (14;16) in 5.26%. Double-hit myeloma was present in 36.84% of cases. The number of prior lines of therapy was 2 (range 1-6), with 63.15% previously triple-class exposed to proteasome inhibitors, immunomodulatory drugs, and CD38 monoclonal antibodies. Treatment regimens consisted of aponermin combined with daratumumab and various backbone therapies: dexamethasone (31.58%), Proteasome Inhibitor (PI,including carfizomib and bortezomib)-dexamethasone (31.58%), Immunomodulatory Drugs(pomalidomide)-dexamethasone (36.84%). Patients received a median of 4 treatment cycles (range 2-10). Efficacy analysis demonstrated an overall response rate of 78.94%, with complete response in 21.05%, very good partial response in 10.52%, and partial response in 47.36% of patients. For Apo-Dd regimen, the ORR was 66.6%, for Apo-Dd-PI regimen, the ORR was 83.3%, and for Apo-Dd-imids, the ORR was 87.5%. The DOR was 3.7 months (range 2.13-9.3). The safety profile was favorable, with manageable adverse events including anemia (5.26%) and thrombocytopenia (5.26%).

**Copyright Information:** (Online) ISSN: 2572-9241
©2026 The Author(s). HemaSphere is published by John Wiley & Sons Ltd on behalf of European Hematology Association. This is an open access Abstract Book distributed under the Attribution-NonCommercial-NoDerivs (CC BY-NC-ND), which allows third parties to download the articles and share them with others as long as they credit the author and the Abstract Book, but they cannot change the content in any way or use them commercially.
**Abstract Book Citations:** Authors, Title, HemaSphere, 2026;10:(S1):pages. The abstract book DOIs can be found on the "Author Information" page.
**Disclaimer:** Articles published in the journal HemaSphere exclusively reflect the opinions of the authors. The authors are responsible for all content in their abstracts including accuracy of the facts, statements, citing resources, etc.

**Summary/Conclusion**

Real-world data demonstrate that aponermin and daratumumab-based regimens exhibit promising efficacy and favorable safety profiles, offering a potential new treatment option for this challenging population.

**Copyright Information:** (Online) ISSN: 2572-9241
©2026 The Author(s). HemaSphere is published by John Wiley & Sons Ltd on behalf of European Hematology Association. This is an open access Abstract Book distributed under the Attribution-NonCommercial-NoDerivs (CC BY-NC-ND), which allows third parties to download the articles and share them with others as long as they credit the author and the Abstract Book, but they cannot change the content in any way or use them commercially.
**Abstract Book Citations:** Authors, Title, HemaSphere, 2026;10:(S1):pages. The abstract book DOIs can be found on the "Author Information" page.
**Disclaimer:** Articles published in the journal HemaSphere exclusively reflect the opinions of the authors. The authors are responsible for all content in their abstracts including accuracy of the facts, statements, citing resources, etc.

### (PB3290) AUTOLOGOUS STEM CELL TRANSPLANTATION WITHOUT CRYOPRESERVATION IN MULTIPLE MYELOMA: FEASIBILITY AND LONG-TERM OUTCOMES IN A MOROCCAN RESOURCE-LIMITED SETTING

**Topic:** 14. Myeloma and other monoclonal gammopathies - Clinical

Ikram Sadki*[1], Habiba Bennesser Alaoui[1], Abdellah Seghiri[1], Soumaya Barkhoukh[1], Zahida Aqodad[1], Samia Sabri[1], Siham Hamaz[1], Houda Bachir[1], Khalid Serraj Andaloussi[1]

*[1]Immuno-Hematology and Cellular Therapy Laboratory. Faculty of Medicine and Pharmacy of Oujda (FMPO ). University of Mohamed First of Oujda. Morocco, Department of Internal Medicine, Mohammed VI University Hospital of Oujda, Oujda, Morocco;*

### Background

Autologous stem cell transplantation (ASCT) remains the standard of care for transplant-eligible multiple myeloma (MM). However, limited access to cryopreservation facilities may restrict its implementation in low- and middle-income countries. Data on outcomes using fresh, non-cryopreserved grafts are limited

### Aims

To evaluate feasibility, toxicity, depth of response, and durability of disease control following ASCT using fresh grafts in a Moroccan tertiary center.

### Methods

We retrospectively analyzed 26 consecutive MM patients (<65 years) who underwent ASCT between July 2018 and July 2025 at Mohammed VI University Hospital, Oujda. Mobilization was performed using G-CSF, with collection initiated at $CD34^+$ >10/µL and targeting $\geq 2\times10^6$ $CD34^+$/kg. Fresh grafts stored for <48 hours without cryopreservation were infused after melphalan 200 mg/m² conditioning. Responses were assessed according to IMWG criteria. Consolidation and maintenance were administered per institutional practice

### Results

All patients successfully mobilized and achieved $\geq 2\times10^6$ $CD34^+$/kg (mean infused dose $4.12\times10^6$/kg; 57.7% achieved $\geq 4\times10^6$/kg). Median neutropenia onset was 4.8 days, with a mean duration of 8.3 days and recovery at day +12.6. Febrile neutropenia occurred in 73%, with documented infection in 15%. No major hemorrhagic events were observed. Grade ≥2 mucositis was rare (3.8%).
At 2–3 months post-ASCT, ≥VGPR was achieved in 73.9% of evaluable patients (CR 26%). After consolidation, ≥VGPR increased to 90.9% among 22 evaluable patients (CR 27%).
After a median follow-up of 37 months, relapse occurred in 21.7% of patients, with a mean time to relapse of 31.6 months. Overall mortality was 13%, and early transplant-related mortality was 3.8%.

### Summary/Conclusion

ASCT using fresh, non-cryopreserved grafts is feasible, safe, and associated with deep and durable responses, with a relapse-free interval exceeding 30 months. Despite limited infrastructure, outcomes appear comparable to international standards. This strategy represents a pragmatic and scalable model to expand access to high-quality MM care in resource-constrained settings.

**Copyright Information:** (Online) ISSN: 2572-9241
©2026 The Author(s). HemaSphere is published by John Wiley & Sons Ltd on behalf of European Hematology Association. This is an open access Abstract Book distributed under the Attribution-NonCommercial-NoDerivs (CC BY-NC-ND), which allows third parties to download the articles and share them with others as long as they credit the author and the Abstract Book, but they cannot change the content in any way or use them commercially.
**Abstract Book Citations:** Authors, Title, HemaSphere, 2026;10:(S1):pages. The abstract book DOIs can be found on the "Author Information" page.
**Disclaimer:** Articles published in the journal HemaSphere exclusively reflect the opinions of the authors. The authors are responsible for all content in their abstracts including accuracy of the facts, statements, citing resources, etc.

### (PB3291) REAL-WORLD OUTCOMES OF ELRANATAMAB IN CANADA: A CANADIAN MYELOMA RESEARCH GROUP DATABASE STUDY

**Topic:** 14. Myeloma and other monoclonal gammopathies - Clinical

Guido Lancman*[1], Donna Reece[1], Arleigh McCurdy[2], Engin Gul[3], Smriti Sharma[3], Jiandong Su[3], Michael Sebag[4], Hira Mian[5], Kevin Song[6], Steven Shih[7], Christopher Venner[8], Darrell White[9], Julie Stakiw[10], Anthony Reiman[11], Rami Kotb[12], Martha Louzada[13], Muhammad Aslam[14], Debra Bergstrom[15], Rayan Kaedbey[16], Jesse Shustik[17], Ashley Freeman[17], Eve St-Hilaire[18], Shyam Ravisankar[19], Richard Leblanc[20]

*[1]Princess Margaret Cancer Centre, Toronto, Canada;[2]The Ottawa Hospital, Ottawa, Canada;[3]Canadian Myeloma Research Group, Toronto, Canada; [4]Department of Medicine, McGill University, Montreal, Canada;[5]Juravinski Cancer Center, Hamilton, Canada; [6]Vancouver General Hospital, Vancouver, Canada;[7]Cross Cancer Institute, Edmonton, Canada;[8]BC Cancer Agency, Vancouver, Canada; [9]Queen Elizabeth II Health Sciences Centre, Dalhousie University, Halifax, Canada; [10]Saskatoon Cancer Centre, Saskatoon, Canada; [11]Department of Oncology, Saint John Regional Hospital, Saint John, Canada;[12]Cancer Care Manitoba, Winnipeg, Canada; [13]London Regional Cancer Center, London, Canada; [14]Allan Blair Cancer Centre, Regina, Canada; [15]Newfoundland and Labrador Health Services, St John's, Canada;[16]Jewish General Hospital, Montreal, Canada;[17]BC Cancer Agency, Victoria, Canada; [18]Dr. Georges-L.-Dumont University Hospital Centre, Moncton, Canada;[19]William Osler Health System, Brampton, Canada; [20]Hematology-Oncology and Cell Therapy University Institute, Hôpital Maisonneuve-Rosemont Research Center, Université de Montréal, Montreal, Canada;*

### Background

Elranatamab is approved in Canada for adult patients with relapsed or refractory multiple myeloma and at least 3 prior lines of therapy, including an immunomodulatory drug, proteasome inhibitor, and CD38 monoclonal antibody, and who have demonstrated disease progression on the last therapy, based on the results of the MagnetisMM-3 trial; however real-world data are needed on efficacy and safety.

### Aims

To describe the efficacy and safety of elranatamab, administered through a compassionate access program, in patients across Canada.

### Methods

We conducted a retrospective study through the Canadian Myeloma Research Group database, encompassing 21 sites and over 10,000 myeloma patients, to evaluate all patients treated with elranatamab outside of clinical trials. Patients receiving at least one full dose of elranatamab were included, while patients receiving combination treatments in addition to elranatamab were excluded. Results are presented descriptively. All survival analyses are estimated with the Kaplan-Meier method, with log-rank test used to compares survival curves. Chi-square test was used to compare dichotomous variables between groups. $p<0.05$ was considered statistically significant.

**Copyright Information:** (Online) ISSN: 2572-9241
©2026 The Author(s). HemaSphere is published by John Wiley & Sons Ltd on behalf of European Hematology Association. This is an open access Abstract Book distributed under the Attribution-NonCommercial-NoDerivs (CC BY-NC-ND), which allows third parties to download the articles and share them with others as long as they credit the author and the Abstract Book, but they cannot change the content in any way or use them commercially.
**Abstract Book Citations:** Authors, Title, HemaSphere, 2026;10:(S1):pages. The abstract book DOIs can be found on the “Author Information” page.
**Disclaimer:** Articles published in the journal HemaSphere exclusively reflect the opinions of the authors. The authors are responsible for all content in their abstracts including accuracy of the facts, statements, citing resources, etc.

### Results

A total of 39 patients were treated with elranatamab monotherapy. Patients were 46% female, median age of 66, and had received a median of 4 lines of therapy (range, 3-8) over a median of 6.3 years since diagnosis. 85% were triple-class refractory and 15% had received a prior BCMA therapy. 32% were high risk with either t (4;14), t (14;16), or del17p.
The overall response rate (ORR) in response-evaluable patients was 72% (23/32),with 59% achieving at least VGPR. ORR was 74% without prior BCMA and 60% with prior BCMA therapy. There was no significant difference in ORR by prior lines of therapy. At a median follow up of 10.8 months, the median PFS was 4.2 months and estimated 12-month PFS was 30%. Among patients achieving at least VGPR, the median PFS was 12.3 months. The median duration of response was 6.0 months for all responders and 11.6 months for patients achieving at least VGPR. Median overall survival was 8.9 months (Table 1).
67% had CRS (49% gr 1, 18% gr 2) with 26% requiring tocilizumab, while 18% had ICANS (gr 1: 8 %, gr 2: 10%; an additional 3 patients experienced confusion without CRS and with multiple confounding factors and were not counted as ICANS). 41% of patients had at least one gr 3 or higher infection, with a serious infection incidence of 0.8 per patient-year. Prophylactic IVIG or SCIG was used in 62%; these patients had significantly lower rates of gr 3+ infections (29% vs 64%, p=0.03), and a significantly prolonged infection-free survival (median NE vs 0.6 months, 12-month infection-free survival 61% vs 31%; HR 0.32 [0.12-0.88], p=0.02) (Figure 1).

Table 1 Overall PFS, OS and by sub-groups

| Groups | N | PFS (95% CI) in months | OS (95% CI) in months |
|---|---|---|---|
| Overall | 39 | 4.2 (1.8, 14.6) | 8.9 (5.3, NR) |
| Prior BCMA | 6 | 4.7 (0.6, NR) | 5.3 (0.6, 7.6) |
| No prior BCMA | 33 | 4.2 (1.8, NR) | 10.6 (3.1, NR) |
| ≥VGPR | 19 | 12.3 (11.0, NR) | NRY (8.9, NR) |
| <VGPR | 13 | 1.8 (0.9, NR) | 5.0 (2.2, 10.6) |
| ≥PR | 23 | 11.0 (6.6, NR) | NRY (7.6, NR) |
| <PR | 9 | 0.9 (0.3, NR) | 3.4 (0.4, 7.3) |

### Summary/Conclusion

In this real-world Canadian cohort, elranatamab demonstrated similar response rates to the MagnetisMM-3trial but with numerically lower DOR and PFS, which may reflect trial selection for fitter patients and less functionally aggressive disease. Prophylactic IVIG/SCIG use significantly decreased the risk of grade 3 or higher infections, consistent with prior reports.

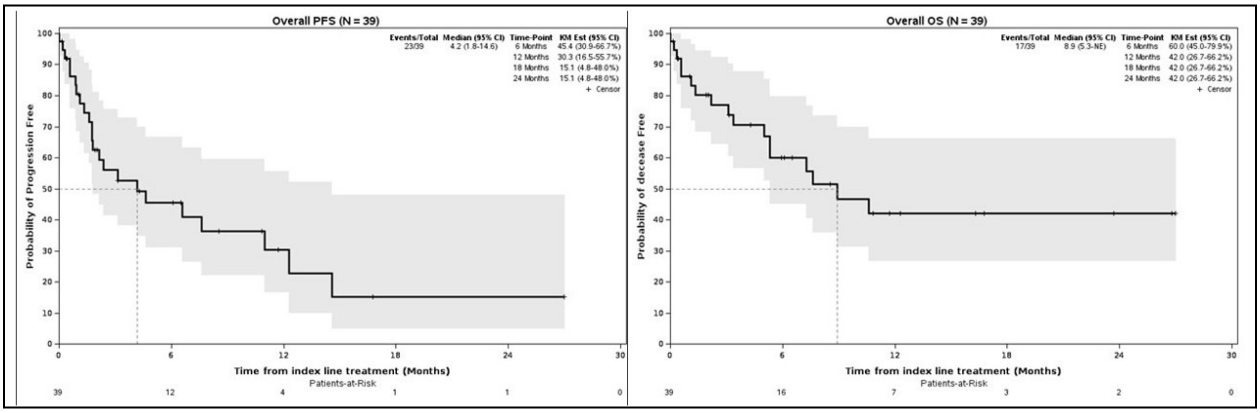

Figure 1: Progression Free Survival (PFS) and Overall Survival (OS)

**Copyright Information:** (Online) ISSN: 2572-9241
©2026 The Author(s). HemaSphere is published by John Wiley & Sons Ltd on behalf of European Hematology Association. This is an open access Abstract Book distributed under the Attribution-NonCommercial-NoDerivs (CC BY-NC-ND), which allows third parties to download the articles and share them with others as long as they credit the author and the Abstract Book, but they cannot change the content in any way or use them commercially.
**Abstract Book Citations:** Authors, Title, HemaSphere, 2026;10:(S1):pages. The abstract book DOIs can be found on the "Author Information" page.
**Disclaimer:** Articles published in the journal HemaSphere exclusively reflect the opinions of the authors. The authors are responsible for all content in their abstracts including accuracy of the facts, statements, citing resources, etc.

### (PB3292) USING MINIMAL RESIDUAL DISEASE AS A SURVIVAL PREDICTOR FOR PATIENTS WITH NEWLY DIAGNOSED MULTIPLE MYELOMA

**Topic:** 14. Myeloma and other monoclonal gammopathies - Clinical

Aashal Shah*[1], Fayaz Ahmed Mubarak Ali[2]

*[1]GMERS Medical College and Civil Hospital, Pharmacology, Valsad, India;[2]Guwahati Medical College and Hospital, Pathology, Guwahati, India;*

### Background

There has been a huge shift in the therapy of multiple myeloma (MM) with the introduction of immunomodulatory drugs, monoclonal antibodies and proteasome inhibitors. Treatment advancement has prolonged survival in MM patients. With improvement in therapeutic outcomes, the need for sensitive prognostic markers have increased to accurately predict the long-term survival prognosis.

### Aims

Minimal Residual Disease (MRD) is defined as the small number of malignant plasma cells remaining in patient's body even after achieving complete remission. This study was conducted with an aim to evaluate the prognostic significance of MRD in newly diagnosed MM patients.

### Methods

This was a prospective and observational study conducted at a tertiary care hospital for a duration of three years. Newly diagnosed patients of MM, being treated with standard quadruplet induction regimen containing daratumumab, bortezomib, lenalidomide and dexamethasone followed by autologous stem cell transplantation were included in the study. Next-generation flow cytometry (NGF) was used to assess MRD at the end of induction regimen. Patients were followed up for a period of one year. The outcomes evaluated included overall survival (OS), progression-free survival (PFS) and depth of response. Kaplan-Meier survival estimates were used for analysis. $P<0.05$ was considered as statistically significant.

### Results

Total 103 patients were included in the data analysis. Following the quadruplet induction therapy, 64.38% patients achieved MRD negativity. MRD-negative patients had significantly higher two-year PFS as compared to MRD-positive patients (87.91% vs 61.77%, $P < 0.01$). MRD-negative patients had significanly higher two-year OS (92.86% vs 75.52%, $P < 0.01$). 28.41% patients had high-risk cytogenetics and they had significantly lower MRD negativity rates (41.08% vs 69.87%, $P < 0.05$). Adverse events due to drugs occurred in 18.04% patients. There was no reported mortality during the follow-up period.

### Summary/Conclusion

Thus, MRD-negative newly diagnosed MM patients, post induction therapy had higher overall chances of survival. Integrating MRD assessment in routine practice will help guide response-based therapy and also improve the disease prognosis. Further studies using larger sample size and multiple centres care required to establish the clinical role of MRD in the prognosis of MM patients.

**Copyright Information:** (Online) ISSN: 2572-9241
©2026 The Author(s). HemaSphere is published by John Wiley & Sons Ltd on behalf of European Hematology Association. This is an open access Abstract Book distributed under the Attribution-NonCommercial-NoDerivs (CC BY-NC-ND), which allows third parties to download the articles and share them with others as long as they credit the author and the Abstract Book, but they cannot change the content in any way or use them commercially.
**Abstract Book Citations:** Authors, Title, HemaSphere, 2026;10:(S1):pages. The abstract book DOIs can be found on the "Author Information" page.
**Disclaimer:** Articles published in the journal HemaSphere exclusively reflect the opinions of the authors. The authors are responsible for all content in their abstracts including accuracy of the facts, statements, citing resources, etc.

### (PB3293) MULTIPLE MYELOMA IN A RESTRICTED-ACCESS HEALTHCARE SETTING: SURVIVAL OUTCOMES AND PROGNOSTIC RISK FACTORS

**Topic:** 14. Myeloma and other monoclonal gammopathies - Clinical

Francisca Ramirez Aspiazu*[1], Manuel Granja[2], Jhoana Ramirez[3], Maria Pacheco[4], Melissa Joan Ortega Espinosa[5], Lorena Loaiza[6], Monica Barzallo[7], Siboney Granja[2], Jairo Quinonez[8]

*[1]Solca Guayaquil, Onco-Hematology, Guayaquil, Ecuador;[2]Carlos Andrade Marin Hospital, Onco-Hematology, Quito, Ecuador; [3]Teodoro Maldonado Carbo Hospital, Onco-Hematology, Guayaquil, Ecuador; [4]Solca Cuenca, Onco-Hematology, Cuenca, Ecuador; [5]Manual Ignacio Monteros Hospital, Onco-Hematology, Loja, Ecuador;[6]Solca Loja Hospital, Onco-Hematology, Loja, Ecuador; [7]Solca Manabi, Onco-Hematology, Manabi, Ecuador; [8]IRHED specialties medical center, Internal Medicine, Guayaquil, Ecuador;*

### Background

Multiple myeloma (MM) represents approximately 10% of hematologic malignancies worldwide, with increasing incidence across both high-income and low- and middle-income countries. Despite therapeutic advances, outcomes in resource-limited settings remain suboptimal. In Latin America, access to cytogenetics, novel agents, and autologous stem-cell transplantation varies widely. This study provides the first national overview of MM in Ecuador and highlights system-level gaps.

### Aims

Multiple myeloma (MM) represents approximately 10% of hematologic malignancies worldwide, with increasing incidence across both high-income and low- and middle-income countries. Despite therapeutic advances, outcomes in resource-limited settings remain suboptimal. In Latin America, access to cytogenetics, novel agents, and autologous stem-cell transplantation varies widely. This study provides the first national overview of MM in Ecuador and highlights system-level gaps.

### Methods

This retrospective cross-sectional study included adults diagnosed with multiple myeloma between January 2015 and December 2024 across 10 Ecuadorian hematology reference centers.
Eligible patients were ≥18 years, had confirmed MM according to International Myeloma Working Group (IMWG) criteria, and available medical records. Plasma cell leukemia, solitary plasmacytoma, and cases with missing key diagnostic data were excluded. Baseline data included demographic characteristics, clinical features at diagnosis (anemia, hypercalcemia, renal dysfunction, bone disease), laboratory parameters, and ISS stage. Cytogenetic abnormalities were recorded when available.
Treatment variables included first-line regimens, access to novel agents, Autologous Stem Cell Transplantation (ASCT), and maintenance therapy. Responses were assessed using IMWG criteria. Overall and progression-free survival were analyzed using Kaplan–Meier methods, with group comparisons performed using appropriate statistical tests.

**Copyright Information:** (Online) ISSN: 2572-9241
©2026 The Author(s). HemaSphere is published by John Wiley & Sons Ltd on behalf of European Hematology Association. This is an open access Abstract Book distributed under the Attribution-NonCommercial-NoDerivs (CC BY-NC-ND), which allows third parties to download the articles and share them with others as long as they credit the author and the Abstract Book, but they cannot change the content in any way or use them commercially.
**Abstract Book Citations:** Authors, Title, HemaSphere, 2026;10:(S1):pages. The abstract book DOIs can be found on the “Author Information” page.
**Disclaimer:** Articles published in the journal HemaSphere exclusively reflect the opinions of the authors. The authors are responsible for all content in their abstracts including accuracy of the facts, statements, citing resources, etc.

### Results

Among 648 patients, 59.3% were ≤65 years. Median age was 63 years; females comprised 58.5%. IgG was the predominant isotype (56%), with age-related differences in heavy-chain distribution. ISS stage III was frequent in both groups (~50%). Older patients more often had high-risk cytogenetics, renal failure, and lower bortezomib use, while younger patients more frequently received bortezomib-based regimens (p<0.001).
Response was evaluable in 490 patients: stringent complete response (sCR) 13.9%, complete response (CR) 21.0%, very good partial response (VGPR) 8.3%, and partial response (PR) 14.0%. Bortezomib-based regimens achieved higher sCR/CR rates than non-bortezomib therapies (p<0.001), particularly in patients ≤65 years (p=0.028). Median OS for the cohort was 54 months, with a 5-year OS of 45.6%. Improved OS was associated with bortezomib use, age ≤65 years, and absence of hypercalcemia, renal failure, or anemia (all p<0.01).

### Summary/Conclusion

Multiple myeloma outcomes in Ecuador reflect restricted healthcare access. Bortezomib-based therapy and younger age were associated with deeper responses and improved survival, highlighting the need to expand access to effective treatments and diagnostic resources.

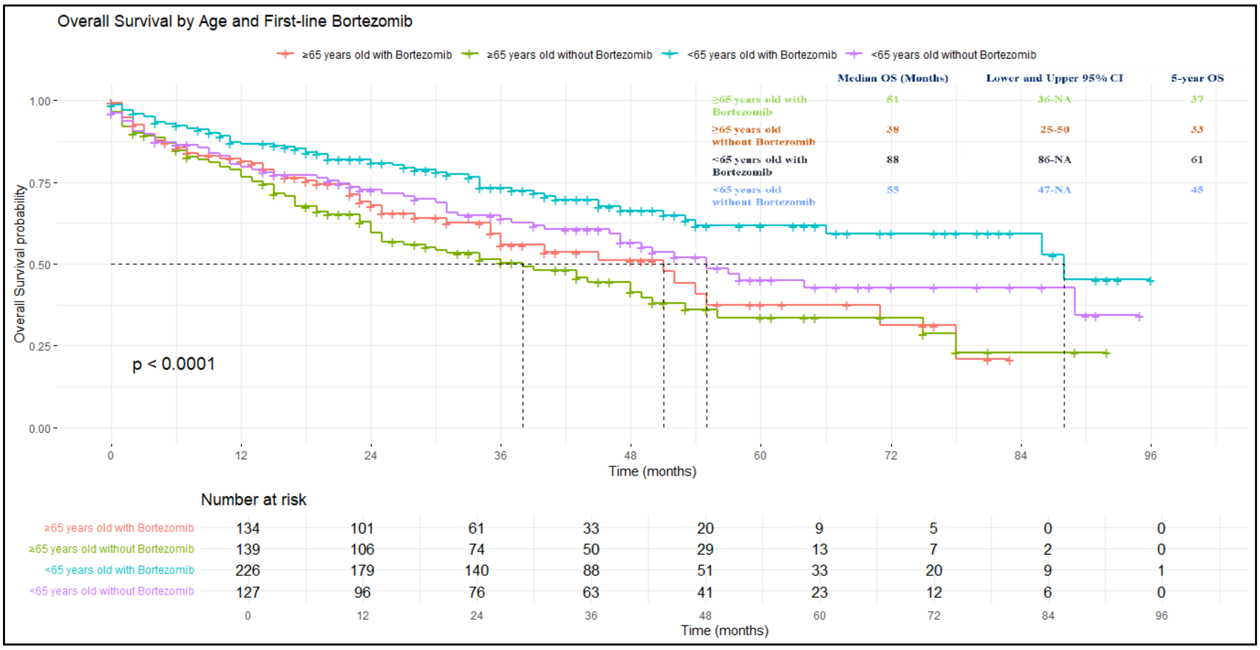

**Copyright Information:** (Online) ISSN: 2572-9241
©2026 The Author(s). HemaSphere is published by John Wiley & Sons Ltd on behalf of European Hematology Association. This is an open access Abstract Book distributed under the Attribution-NonCommercial-NoDerivs (CC BY-NC-ND), which allows third parties to download the articles and share them with others as long as they credit the author and the Abstract Book, but they cannot change the content in any way or use them commercially.
**Abstract Book Citations:** Authors, Title, HemaSphere, 2026;10:(S1):pages. The abstract book DOIs can be found on the "Author Information" page.
**Disclaimer:** Articles published in the journal HemaSphere exclusively reflect the opinions of the authors. The authors are responsible for all content in their abstracts including accuracy of the facts, statements, citing resources, etc.

### (PB3294) PREDICTIVE VALUE OF HIGH-THROUGHPUT IN VITRO DRUG SENSITIVITY TESTING FOR THERAPEUTIC EFFICACY IN RELAPSED/REFRACTORY MULTIPLE MYELOMA

**Topic:** 14. Myeloma and other monoclonal gammopathies - Clinical

Hongyun Liu[1], Yunjing Zeng[1], Jiali Li[1], Dezhi Huang[1], Li Gao[1], Xi Zhang*[1]

[1]*Xinqiao Hospital of Army Medical University, CHONGQING, China;*

### Aims

To evaluate the predictive value of high-throughput in vitro drug sensitivity testing for therapeutic efficacy in patients with relapsed/refractory multiple myeloma (RRMM).

### Methods

A prospective observational study was conducted, enrolling 141 patients with multiple myeloma (MM) according to standardized inclusion and exclusion criteria. Baseline characteristics, post-relapse treatment regimens, clinical efficacy, and results from high-throughput in vitro drug sensitivity assays were collected. The degree of drug sensitivity among patients with different treatment responses and across various drugs/regimens was analyzed. The concordance between in vitro drug sensitivity results and actual clinical efficacy was evaluated.

### Results

The majority of patients had previously received three to four lines of therapy. Most patients with cytogenetic abnormalities exhibited at least two chromosomal aberrations. The most common post-relapse treatment regimen was VRD (bortezomib, lenalidomide, dexamethasone). After one treatment cycle, the majority of patients achieved stable disease (SD), while only 10 cases achieved complete response (CR). Moderate sensitivity predominated across all response groups. The distribution of drug sensitivity levels differed significantly among the four clinical response groups ($P < 0.05$), and drug sensitivity also varied significantly between different agents and treatment regimens ($P < 0.05$). The overall concordance rate between drug sensitivity results and clinical outcomes was 65.25% (92/141), with a positive concordance rate of 58.16% (82/141) and a negative concordance rate of 7.09% (10/141).

### Summary/Conclusion

High-throughput in vitro drug sensitivity testing has predictive value for post-relapse treatment efficacy in MM patients. It may serve as a clinical decision-making tool to optimize treatment regimens for RRMM, enabling more rational, personalized, and effective therapeutic strategies.

**Copyright Information:** (Online) ISSN: 2572-9241
©2026 The Author(s). HemaSphere is published by John Wiley & Sons Ltd on behalf of European Hematology Association. This is an open access Abstract Book distributed under the Attribution-NonCommercial-NoDerivs (CC BY-NC-ND), which allows third parties to download the articles and share them with others as long as they credit the author and the Abstract Book, but they cannot change the content in any way or use them commercially.
**Abstract Book Citations:** Authors, Title, HemaSphere, 2026;10:(S1):pages. The abstract book DOIs can be found on the "Author Information" page.
**Disclaimer:** Articles published in the journal HemaSphere exclusively reflect the opinions of the authors. The authors are responsible for all content in their abstracts including accuracy of the facts, statements, citing resources, etc.

### (PB3295) THE ROLE OF KINETICS OF HEMATOLOGIC RESPONSE IN MULTIPLE MYELOMA WITH OBVIOUS RENAL IMPAIRMENT

**Topic:** 14. Myeloma and other monoclonal gammopathies - Clinical

Ying Tian[1], Lefu Huang[1], Yanru Zhang[1], Menghan Liu[1], Aijun Liu*[1]

[1]*Beijing Chaoyang Hospital, affiliated to Capital Medical University, Beijing, China;*

### Background

Rapid and deep reduction of involved serum free light chain(iFLC) has been linked to renal recovery in myeloma, yet the value of early percent reduction at distinct timepoints in real-world practice remains uncertain.

### Aims

This study aimed to evaluate the relationship between hematologic kinetics and renal recovery in newly diagnosed multiple myeloma (NDMM) and relapsed/refractory multiple myeloma (RRMM) with obvious renal impairment.

### Methods

We retrospectively analyzed 47 patients (pts) of NDMM with obvious renal impairment according to IMWG criteria (ChiCTR2200058235). Patients received induction therapy at least 4-6 cycles, 2-week-iFLC, 4-week-iFLC, and M protein, creatinine/CCr/ eGFR were collected. Primary endpoint: renal recovery of 3 months, key endpoints: renal recovery of 6 months, progression-free survival (PFS).

### Results

At data cutoff, a total of 49 patients(pts) were enrolled, with a median age of 63 years (range:47 -79), males 65.31%, ISS Stage Ⅰ/Ⅱ/Ⅲ: 4.08%/10.2%/81.63%, DS stage Ⅰ/Ⅱ/Ⅲ: 2.04% /6.12%/77.55%. IgG/IgA/IgD/light chain/non-secretory types: 22.45%/18.37%/20.41%/38.78%/2.04%. Among light chain subtypes, λ/κ: 61.22%/36.73%. NDMM/RRMM:61.22%/38.78%. According to the degree of renal impairment, pts with stage 5/4/3: 42.86%/28.57%/28.57%, 18.37% of pts received dialysis, and 12.24% developed oliguria during treatment. The median (interquartile range) baseline iFLC level was 4314.00 (746.33–11274.59) mg/L. The baseline median (interquartile range) serum creatinine was 287.90 (216.00–488.00) μmol/L, and the baseline median (interquartile range) estimated glomerular filtration rate(eGFR) was 17.47 (9.50–26.17) ML/min/1.73m².
Hematologic response:
One-month- overall response rate(ORR) rate was 61.2%(30/49), One-month-Complete remission(CR) rate was 26.5%(13/49). 87.8% of pts involved FLC(iFLC) decreased 50%, 77.6% of pts iFLC decreased 80%, 55.1% of pts iFLC decreased 90% at one month.
renal response:
Three-month- MR rate 55.0%(27/49), Three-month-PR and CR rate 20.4%(10/49); Six-month- MR rate 62.2%(28/45), Six-month-PR and CR rate 31.1%(14/45). One-month- CR of hematologic response related to renal response in three months (p=0.019, OR=19.7) and six months (p=0.029, OR=17.9).
The 0–6-month creatinine slope showed minimal correlation with 2-week/4-week iFLC reduction (ρ = −0.037, P = 0.803; ρ = −0.054, P = 0.718). In the multivariable model, neither 2-week nor 4-week iFLC reduction (per 10% decrease) showed significant independent association with 1-month renal efficacy: 1-month Δcreatinine: β=0.044 (-0.172 ~ 0.261,95%), p=0.688; β=-0.033 (-0.295 ~ 0.229,95%), p=0.808).

Copyright Information: (Online) ISSN: 2572-9241
©2026 The Author(s). HemaSphere is published by John Wiley & Sons Ltd on behalf of European Hematology Association. This is an open access Abstract Book distributed under the Attribution-NonCommercial-NoDerivs (CC BY-NC-ND), which allows third parties to download the articles and share them with others as long as they credit the author and the Abstract Book, but they cannot change the content in any way or use them commercially.
**Abstract Book Citations:** Authors, Title, HemaSphere, 2026;10:(S1):pages. The abstract book DOIs can be found on the "Author Information" page.
**Disclaimer:** Articles published in the journal HemaSphere exclusively reflect the opinions of the authors. The authors are responsible for all content in their abstracts including accuracy of the facts, statements, citing resources, etc.

**Summary/Conclusion**

This study suggest obvious renal remission at 3 and 6 months can be predicted by the deep reduction of iFLC at 4 weeks with deep hematologic response, such as CR.

**Copyright Information:** (Online) ISSN: 2572-9241
©2026 The Author(s). HemaSphere is published by John Wiley & Sons Ltd on behalf of European Hematology Association. This is an open access Abstract Book distributed under the Attribution-NonCommercial-NoDerivs (CC BY-NC-ND), which allows third parties to download the articles and share them with others as long as they credit the author and the Abstract Book, but they cannot change the content in any way or use them commercially.
**Abstract Book Citations:** Authors, Title, HemaSphere, 2026;10:(S1):pages. The abstract book DOIs can be found on the “Author Information” page.
**Disclaimer:** Articles published in the journal HemaSphere exclusively reflect the opinions of the authors. The authors are responsible for all content in their abstracts including accuracy of the facts, statements, citing resources, etc.

### (PB3296) EVOLVING OUTCOMES IN MULTIPLE MYELOMA: A 30-YEAR SINGLE CENTER REAL-WORLD EXPERIENCE.

**Topic:** 14. Myeloma and other monoclonal gammopathies - Clinical

Claudia Bellofiore*[1], Giuliana Giunta[2], Elvira Scalisi[2], Vittorio Del Fabro[3], Federica Elia[1, 2], Bruno Garibaldi[1], Chiara Finocchiaro[2], Francesco Di Raimondo[1, 2], Alessandra Romano[1, 2], Concetta Conticello[1]

*[1]Division of Hematology, Azienda Policlinico-S.Marco, University of Catania – Catania IT, Catania, Italy; [2]Postgraduate school of Hematology, University of Catania - Catania IT, Catania, Italy; [3]Faculty of Medicine and Surgery, "Kore" University of Enna – Enna, enna, Italy;*

### Background

In the last two decades therapeutic armamentarium for treatment of MM is increased both with the introduction of novel drugs and combinations and by introducing consolidation and maintenance therapy. Life expectancy and outcomes of MM patients has remarkably improved. Particularly, maintenance therapy, either such as continuous upfront therapy in transplant ineligible patients or after an induction intensified therapy, has demonstrated to have a pivotal role in improving patient survival either upfront or at relapse. Moreover, the efficacy of new therapies induced a decrease in relapse and refractory (RR) patients

### Aims

To evaluate changes in patient characteristics, treatment patterns, survival outcomes, and time to next treatment (TTNT) across different therapeutic eras in a large single-center real-world MM cohort.

### Methods

We retrospectively analyzed 1052 consecutive MM patients diagnosed and treated at our Institution between January 2005 and September 2025. Most patients were managed outside clinical trials. Patients were stratified according to year of diagnosis into two cohorts: Group A (2005–2015) and Group B (2016–2025). Overall survival (OS) was calculated from diagnosis to death or last follow-up. TTNT was defined as the interval between initiation of one therapy line and the start of the subsequent line.

### Results

A total of 1052 patients were included: 446 in group A and 546 in group B. Baseline characteristics are reported in table 1. Median age at diagnosis was 65 years, with 52% of patients aged >65; 54% were male. Cytogenetic data by FISH were available for 497 patients, with greater availability in the later era. Patients received a median of two lines of therapy (range 1–3); approximately 25% were triple-exposed and 5% penta-exposed. Overall, 22% of patients were enrolled in clinical trials. After a median follow-up of 107 months (95% CI: 98–243), median OS for the entire cohort was 65 months (95% CI: 58–69). Considering patients <65 years, median OS was superior in group B compared to group A (not reached vs 75 months, p<0.001). Autologous stem cell transplantation (ASCT) was performed in 338 patients, 43% of whom received tandem ASCT. ASCT was associated with superior OS in both eras (Group A: 95 vs 49 months, p<0.001; Group B: not reached vs 41 months, p<0.001). Participation in clinical trials also conferred a significant survival advantage (Group A: 80 vs 50 months, p<0.001; Group B: 107 vs 63 months, p<0.001). In RRMM, TTNT progressively shortened with advancing lines of therapy, reaching a median of 4 months after third-line treatment. Beyond the eighth line, TTNT appeared to stabilize, likely reflecting the impact of novel drugs. Sixteen patients received bispecific antibodies across multiple lines (second to tenth); in this subgroup, median progression-free survival was not reached. In the ninth line, TTNT increased from 4 months in the overall population to 11 months in bispecific-treated patients, suggesting a meaningful clinical benefit despite small sample size and limited statistical power.

**Copyright Information:** (Online) ISSN: 2572-9241
©2026 The Author(s). HemaSphere is published by John Wiley & Sons Ltd on behalf of European Hematology Association. This is an open access Abstract Book distributed under the Attribution-NonCommercial-NoDerivs (CC BY-NC-ND), which allows third parties to download the articles and share them with others as long as they credit the author and the Abstract Book, but they cannot change the content in any way or use them commercially.
**Abstract Book Citations:** Authors, Title, HemaSphere, 2026;10:(S1):pages. The abstract book DOIs can be found on the "Author Information" page.
**Disclaimer:** Articles published in the journal HemaSphere exclusively reflect the opinions of the authors. The authors are responsible for all content in their abstracts including accuracy of the facts, statements, citing resources, etc.

**Summary/Conclusion**

This 30-year single-center real-world analysis highlights the continuous improvement in MM outcomes driven by therapeutic innovation. The sequential integration of IMiDs, proteasome inhibitors, monoclonal antibodies, cellular therapies, and bispecific antibodies has reshaped treatment strategies and survival expectations in routine clinical practice.

**Table 1.** Patients' characteristics.

| | Entire cohort (N=1052) Median (IQR) N (%) | Patients diagnosed between 2005-2015 (N=446) Median (IQR) N (%) | Patients diagnosed between 2015-2025 (N=546) Median (IQR) N (%) | P |
|---|---|---|---|---|
| Age, years | 65 (64 – 66) | 65 (64 – 66) | 66 (65 – 67) | **.016** |
| Male sex | 576 (54) | 238 (53) | 305 (72) | .432 |
| Age<65 years | 513 (48) | 228 (51) | 239 (43) | **.021** |
| HR Cytogenetics | 119 (34) | 23 (25) | 94 (37) | **.028** |
| Extramedullary | 29 (2) | 7 (1) | 20 (4) | **.043** |
| AL amyloidosis | 71 (7) | 21 (5) | 45 (8) | **.025** |
| N. of therapies | 2 (1 – 3) | 2 (1 – 3) | 1 (1 – 2) | **<.001** |
| Triple exposed | 237 (22) | 53 (12) | 180 (33) | **<.001** |
| Penta exposed | 55 (5) | 20 (4) | 33 (6) | .281 |
| Trial enrolled | 212 (20) | 169 (37) | 36 (6) | **<.001** |

**Copyright Information:** (Online) ISSN: 2572-9241
©2026 The Author(s). HemaSphere is published by John Wiley & Sons Ltd on behalf of European Hematology Association. This is an open access Abstract Book distributed under the Attribution-NonCommercial-NoDerivs (CC BY-NC-ND), which allows third parties to download the articles and share them with others as long as they credit the author and the Abstract Book, but they cannot change the content in any way or use them commercially.
**Abstract Book Citations:** Authors, Title, HemaSphere, 2026;10:(S1):pages. The abstract book DOIs can be found on the “Author Information” page.
**Disclaimer:** Articles published in the journal HemaSphere exclusively reflect the opinions of the authors. The authors are responsible for all content in their abstracts including accuracy of the facts, statements, citing resources, etc.

### (PB3297) EVALUATING THE CLINICAL BENEFIT OF ANTI-CD38 THERAPY IN MULTIPLE MYELOMA USING ESMO-MCBS:H AND ASCO-VF

**Topic:** 14. Myeloma and other monoclonal gammopathies - Clinical

Bushra Salman*[1], Amna Mohamed[2], Mahmood Al-Abri[1], Murtadha Khabori[3]

*[1]National Hematology and Bone Marrow Transplant Center, University Medical City, Pharmacy, Muscat, Oman; [2]University of Technology and Applied Sciences, Pharmacy, Muscat, Oman; [3]National Hematology and Bone Marrow Transplant Center, University Medical City, Muscat, Oman;*

### Background

The European Society for Medical Oncology Magnitude of Clinical Benefit Scale for Hematologic Malignancies (ESMO-MCBS:H) and the American Society of Clinical Oncology Value Framework (ASCO-VF) are widely used to assess the clinical benefit of cancer therapies. However, their applicability and alignment in evaluating anti-CD38 therapies for multiple myeloma (MM) remain unclear.

### Aims

This study aimed to compare these frameworks and identify discrepancies in their assessments.

### Methods

Twelve pivotal randomized controlled trials (RCTs) supporting FDA approvals of daratumumab and isatuximab were evaluated using ESMO-MCBS:H v1.1 and ASCO-VF v2.0. Data on efficacy, toxicity, and quality of life (QoL) were extracted, and scores were calculated for each trial. Spearman's rho was used to assess correlations between ESMO-MCBS:H, ASCO-VF, and predicted ESMO-MCBS grades.

### Results

Significant discrepancies were observed between the frameworks. ESMO-MCBS:H tended to apply more conservative grading, particularly in trials like MAIA and IKEMA, where ASCO-VF captured long-term survival benefits more comprehensively. Conversely, trials such as POLLUX and CASTOR showed inflated ESMO-MCBS:H scores due to reliance on the lower limit of the 95% confidence interval (CI). Statistical analysis revealed weak correlations between ESMO-MCBS:H and ASCO-VF ($|\rho| < 0.3$, $p > 0.05$), indicating fundamentally different methodologies. Key limitations included ESMO-MCBS:H's rigid toxicity criteria and ASCO-VF's reliance on patient-reported outcomes, which overlooked laboratory-based toxicities. In addition, premature evaluations based on immature data in trials like CASSIOPEIA and PERSEUS compromised scoring accuracy.

### Summary/Conclusion

This study highlights significant discrepancies between ESMO-MCBS:H and ASCO-VF in assessing anti-CD38 therapies for MM, underscoring the need for further refinement of both frameworks. Future research should focus on validating ESMO-MCBS:H in MM, integrating real-world evidence, and incorporating patient-centered outcomes to enhance their clinical applicability and accuracy. These improvements will support evidence-based treatment decisions and sustainable formulary management in MM and other hematologic malignancies.

**Copyright Information:** (Online) ISSN: 2572-9241
©2026 The Author(s). HemaSphere is published by John Wiley & Sons Ltd on behalf of European Hematology Association. This is an open access Abstract Book distributed under the Attribution-NonCommercial-NoDerivs (CC BY-NC-ND), which allows third parties to download the articles and share them with others as long as they credit the author and the Abstract Book, but they cannot change the content in any way or use them commercially.
**Abstract Book Citations:** Authors, Title, HemaSphere, 2026;10:(S1):pages. The abstract book DOIs can be found on the "Author Information" page.
**Disclaimer:** Articles published in the journal HemaSphere exclusively reflect the opinions of the authors. The authors are responsible for all content in their abstracts including accuracy of the facts, statements, citing resources, etc.

### (PB3298) LISAFTOCLAX COMBINED WITH IXAZOMIB AND DEXAMETHASONE AFTER CART FOR MAINTENANCE THERAPY IN TRANSPLANT-INELIGIBLE RELAPSED/REFRACTORY HIGH-RISK MULTIPLE MYELOMA : TWO CASE REPORTS AND LITERATURE REVIEW

**Topic:** 14. Myeloma and other monoclonal gammopathies - Clinical

Weige Xu[1, 2], Xue Qiao[1], Linlin Zhang[1], Lina Xing[1], Jingnan Zhang[1], Xiaonan Guo[1], Shukai Qiao*[1]

*[1]Second Hospital of Hebei Medical University, Department of Hematology, Shijiazhuang , Hebei, China;[2]The Second Affiliated Hospital of Xingtai Medical College, Department of Hematology, Xingtai, China;*

### Background

Multiple myeloma (MM) is an incurable malignancy. The treatment mainly includes induction therapy, MRD clearance therapy and maintenance therapy. For high-risk/ultro-high-risk or relapsed/refractory MM , optimizing the eradication of minimal residual disease (MRD) and sustaining long-term suppression of MRD at low levels continues is a formidable challenge.

### Methods

Herein, we report two patients with relapsed/refractory MM, however, when ixazomib, lisaftoclax and dexamethasone were added to the treatment regimen after CART, the patients achieved a very good maintenance. Treatment Regimen All drugs was administered orally. Ixazomib 4mg d1,8,15; Lisaftoclax 400mg d1-14;dexamethasone 20mg d1,8,15. One treatment cycle is defined as 28 days. Treatment will be discontinued in the event of uncontrollable active infection or organ injury.

### Results

Case 1 involves a patient with multiple ultro - high risk characteristics, including a complex karyotype, unfavorable genetic mutations, recurrent EMI, and who has undergone multiple lines of therapy. The case 2 involves a patient under long-term follow-up. At initial diagnosis, the patient presented with few adverse prognostic factors and demonstrated sensitivity to the VRD triple-drug combination regimen. However, the disease progressed recurrently, with a growing number of osteolytic lesions and fracture sites, persistently elevated M-protein levels and plasma cell counts, and the emergence of a complex chromosomal karyotype, resulting in failure to reduce tumor burden. In our management, we administered ILD maintenance therapy subsequent to CAR-T treatment, and minimal residual disease (MRD) has remained at low levels to date. Notably, the entire ILD treatment course for both patients was conducted on an outpatient basis, featuring convenient oral administration and favorable safety profiles.

### Summary/Conclusion

These cases demonstrate that the ILD combination therapy can achieve optimizing the eradication of MRD and sustaining long-term suppression of MRD at low levels continues, offering a promising therapeutic option for patients with limited treatment choices. Formal evaluations of this regimen in patients with high-risk/ultro-high-risk or relapsed/refractory MM may be meaningful.

**Copyright Information:** (Online) ISSN: 2572-9241
©2026 The Author(s). HemaSphere is published by John Wiley & Sons Ltd on behalf of European Hematology Association. This is an open access Abstract Book distributed under the Attribution-NonCommercial-NoDerivs (CC BY-NC-ND), which allows third parties to download the articles and share them with others as long as they credit the author and the Abstract Book, but they cannot change the content in any way or use them commercially.
**Abstract Book Citations:** Authors, Title, HemaSphere, 2026;10:(S1):pages. The abstract book DOIs can be found on the “Author Information” page.
**Disclaimer:** Articles published in the journal HemaSphere exclusively reflect the opinions of the authors. The authors are responsible for all content in their abstracts including accuracy of the facts, statements, citing resources, etc.

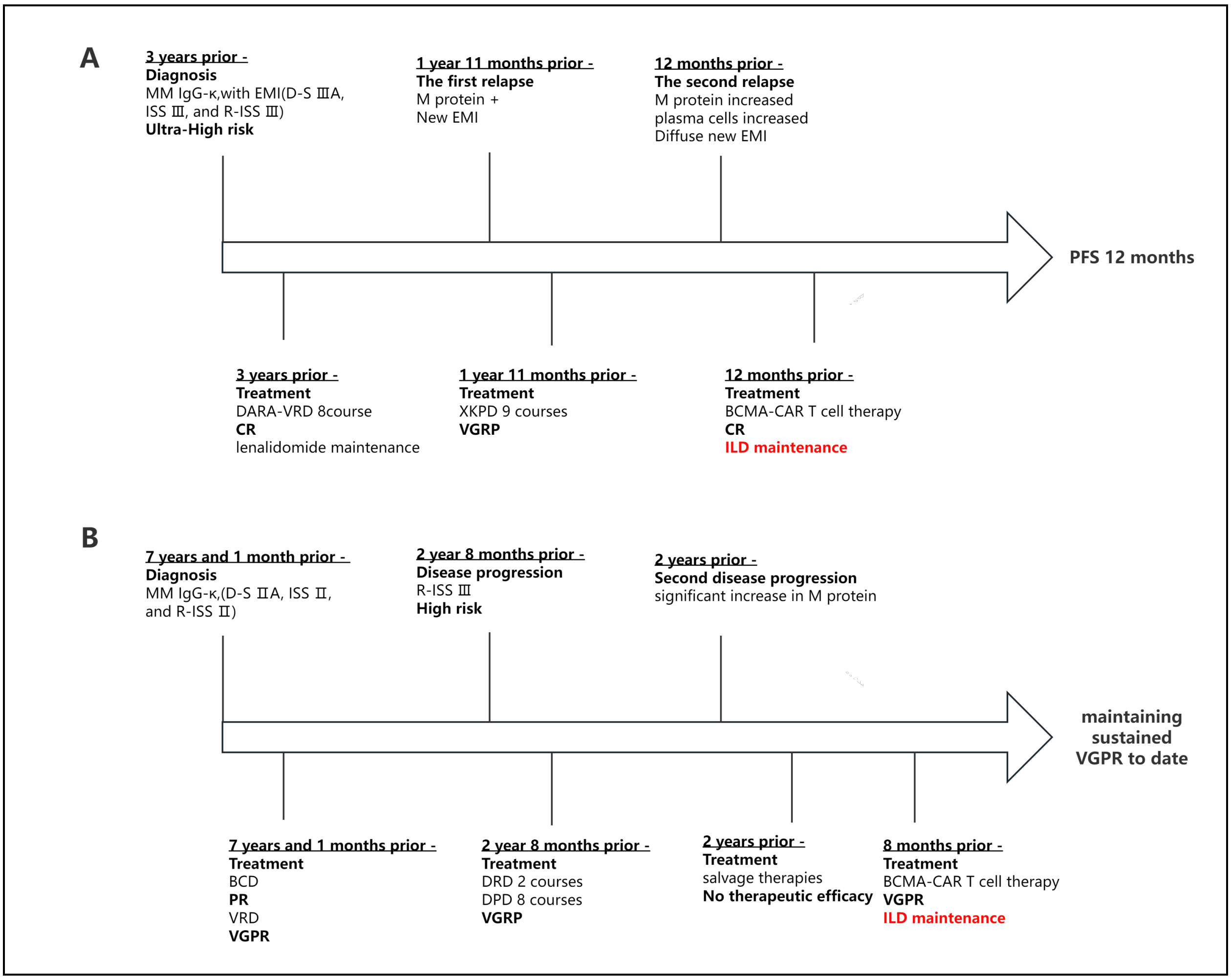

**Copyright Information:** (Online) ISSN: 2572-9241
©2026 The Author(s). HemaSphere is published by John Wiley & Sons Ltd on behalf of European Hematology Association. This is an open access Abstract Book distributed under the Attribution-NonCommercial-NoDerivs (CC BY-NC-ND), which allows third parties to download the articles and share them with others as long as they credit the author and the Abstract Book, but they cannot change the content in any way or use them commercially.
**Abstract Book Citations:** Authors, Title, HemaSphere, 2026;10:(S1):pages. The abstract book DOIs can be found on the "Author Information" page.
**Disclaimer:** Articles published in the journal HemaSphere exclusively reflect the opinions of the authors. The authors are responsible for all content in their abstracts including accuracy of the facts, statements, citing resources, etc.

### (PB3299) METABOLIC TUMOR BURDEN ON $^{18}$F-FDG PET/CT AS AN INDEPENDENT PROGNOSTIC MARKER IN MULTIPLE MYELOMA

**Topic:** 14. Myeloma and other monoclonal gammopathies - Clinical

Edibe Eker*[1], Istemi Serin[1], Melis Mammacıoğlu[1], Gizem Şirin[2], Kübranur Toplutaş[3], Halil Kapucu[3], Mesut Ayer[1]

*[1]Başakşehir Çam and Sakura City Hospital, Hematology, Istanbul, Türkiye;[2]Başakşehir Çam and Sakura City Hospital, Internal Medicine, Istanbul, Türkiye; [3]Başakşehir Çam and Sakura City Hospital, Nuclear Medicine, Istanbul, Türkiye;*

### Background

Multiple myeloma (MM) is a malignant hematologic disease characterized by clonal plasma cell proliferation in the bone marrow and marked biological and clinical heterogeneity. Despite major improvements in survival with novel therapeutic agents, considerable variability in clinical course and prognosis persists. This underscores the need for reliable prognostic biomarkers that better reflect tumor burden and disease biology.Conventional staging systems, including Durie–Salmon, ISS, and Revised ISS (R-ISS), do not adequately capture disease distribution, focal lesion burden, or metabolic activity. Consequently, functional imaging modalities have gained increasing importance.18F-FDG PET/CT enables integrated metabolic and morphologic assessment and is recommended by IMWG and EANM guidelines for staging, treatment response evaluation, and detection of residual active disease in MM. Given the limitations of SUVmax, volumetric metabolic parameters such as metabolic tumor volume (MTV) and total lesion glycolysis (TLG) have emerged as promising prognostic markers.

### Aims

This study aimed to evaluate the prognostic significance of PET/CT-derived metabolic and morphologic parameters in patients with MM.

### Results

A total of 148 patients with MM were included (mean age 66.1±10.8 years), of whom 53.6% were classified as R-ISS stage III. On PET/CT, measurable tMTV and TLG were detected in 82.4% of patients, while SUVmax was observed in 84.5%. Focal bone lesions were present in 60.8% of cases, most commonly involving the vertebrae. Paramedullary disease was identified in 37.8% and extramedullary disease in 11.5% of patients. Median tMTV, TLG, and SUVmax values were 92.55, 272, and 5.2, respectively. β2-microglobulin levels showed weak but significant positive correlations with both tMTV and TLG ($p<0.05$). Patients who died during follow-up had significantly higher tMTV, TLG, SUVmax, and Deauville scores compared with survivors ($p<0.05$). No significant association was observed between PET metabolic parameters and disease progression. In univariate Cox regression analysis, tMTV, SUVmax, and the number of osteolytic lesions were significantly associated with overall survival. In multivariate analysis, only tMTV remained an independent prognostic factor for overall survival (HR=1.0007; p=0.01).

**Copyright Information:** (Online) ISSN: 2572-9241
©2026 The Author(s). HemaSphere is published by John Wiley & Sons Ltd on behalf of European Hematology Association. This is an open access Abstract Book distributed under the Attribution-NonCommercial-NoDerivs (CC BY-NC-ND), which allows third parties to download the articles and share them with others as long as they credit the author and the Abstract Book, but they cannot change the content in any way or use them commercially.
**Abstract Book Citations:** Authors, Title, HemaSphere, 2026;10:(S1):pages. The abstract book DOIs can be found on the "Author Information" page.
**Disclaimer:** Articles published in the journal HemaSphere exclusively reflect the opinions of the authors. The authors are responsible for all content in their abstracts including accuracy of the facts, statements, citing resources, etc.

**Summary/Conclusion**

This study demonstrates that volumetric metabolic parameters derived from 18F-FDG PET/CT, particularly total metabolic tumor volume, provide strong and independent prognostic information in multiple myeloma. While SUVmax reflects focal metabolic activity, it fails to capture the overall tumor burden and loses prognostic significance in multivariate analysis. Similarly, TLG did not retain independent prognostic value, possibly due to its susceptibility to wide variability. Our findings support the concept that tMTV represents a comprehensive marker of disease burden, integrating spatial extent and metabolic activity, and may better reflect the aggressive biological behavior of MM. The strong association between higher tMTV and mortality further highlights its potential clinical relevance. In conclusion, PET/CT-based assessment of metabolic tumor burden, particularly tMTV, may provide incremental prognostic value beyond established clinical risk models and contribute to more accurate prognostic evaluation and personalized treatment strategies in patients with multiple myeloma.

**Copyright Information:** (Online) ISSN: 2572-9241
©2026 The Author(s). HemaSphere is published by John Wiley & Sons Ltd on behalf of European Hematology Association. This is an open access Abstract Book distributed under the Attribution-NonCommercial-NoDerivs (CC BY-NC-ND), which allows third parties to download the articles and share them with others as long as they credit the author and the Abstract Book, but they cannot change the content in any way or use them commercially.
**Abstract Book Citations:** Authors, Title, HemaSphere, 2026;10:(S1):pages. The abstract book DOIs can be found on the "Author Information" page.
**Disclaimer:** Articles published in the journal HemaSphere exclusively reflect the opinions of the authors. The authors are responsible for all content in their abstracts including accuracy of the facts, statements, citing resources, etc.

Table 1: Evaluation of the Effect of 18F-FDG PET/CT Values on Overall Survival Using Univariate Cox Regression Analysis

| **18F-FDG PET/CT Değerleri** | **Hazard Ratio** | **%95 Confidence Interval** | **p-value** |
|---|---|---|---|
| tMTV | 1.0008 | 1.0004-1.001 | **0.0003** |
| TLG | 1.000 | 0.999-1.000 | 0.40 |
| SUVmax | 1.07 | 1.01-1.13 | **0.02** |
| PET Focal Lesion Deauville Score | 1.19 | 0.99-1.43 | 0.07 |
| Number of osteolytic lesions on CT | 1.32 | 1.01-1.71 | **0.04** |
| Bone Marrow Deauville Score | 1.12 | 0.86-1.44 | 0.40 |

**Copyright Information:** (Online) ISSN: 2572-9241
©2026 The Author(s). HemaSphere is published by John Wiley & Sons Ltd on behalf of European Hematology Association. This is an open access Abstract Book distributed under the Attribution-NonCommercial-NoDerivs (CC BY-NC-ND), which allows third parties to download the articles and share them with others as long as they credit the author and the Abstract Book, but they cannot change the content in any way or use them commercially.
**Abstract Book Citations:** Authors, Title, HemaSphere, 2026;10:(S1):pages. The abstract book DOIs can be found on the "Author Information" page.
**Disclaimer:** Articles published in the journal HemaSphere exclusively reflect the opinions of the authors. The authors are responsible for all content in their abstracts including accuracy of the facts, statements, citing resources, etc.

### (PB3300) DEPRESSION AND ANXIETY FOLLOWING MULTIPLE MYELOMA: A DANISH NATIONWIDE REGISTER-BASED COHORT STUDY

**Topic:** 14. Myeloma and other monoclonal gammopathies - Clinical

Klara Frederikke Koue Rydahl*[1, 2, 3, 4], Andreas Kiesbye Øvlisen[5], Trine Allerslev Horsbøl[6], Sören Möller[7], Lene Kongsgaard Nielsen[1, 2, 4, 8], Søren Dinesen Østergaard[3, 4], Tarec Christoffer El-Galaly[4, 9, 10, 11], Christopher Garne Rohde[3, 4]

*[1]Quality of Life Research Center, Department of Haematology, Odense University Hospital, Odense, Denmark;[2]Department of Clinical Research, University of Southern Denmark, Odense, Denmark; [3]Department of Affective Disorders, Aarhus University Hospital, Aarhus, Denmark; [4]Department of Clinical Medicine, Aarhus University, Aarhus, Denmark; [5]Department of Haematology, Aalborg University Hospital, Aalborg, Denmark; [6]National Institute of Public Health, University of Southern Denmark, Copenhagen, Denmark; [7]Department of Public Health, Epidemiology, Biostatistics, and Biodemography, University of Southern Denmark, Odense, Denmark; [8]Department of Haematology, Gødstrup Hospital, Herning, Denmark;[9]Department of Haematology, Aarhus University Hospital, Aarhus, Denmark; [10]Department of Clinical Epidemiology, Aarhus University Hospital, Aarhus, Denmark; [11]Department of Molecular Medicine, Aarhus University, Aarhus, Denmark;*

### Background

Patients with multiple myeloma (MM) may be at elevated risk of developing depression and anxiety due to high disease burden and treatment-related complications. However, the incidence of depression and anxiety following MM diagnosis is largely unknown. Mental health issues are associated with decreased treatment adherence, impaired quality of life, and increased mortality. Therefore, knowledge of the incidence and risk factors for development of depression and anxiety among MM patients is needed.

### Aims

The aim of this study was to investigate the risk of developing incident depression and/or anxiety among patients with MM compared to sex- and age-matched comparators from the general Danish population.

### Methods

This register-based cohort study included patients diagnosed with MM between January 2017 and June 2024, who were matched with five age- and sex-matched comparators from the general Danish population. Both MM patients and matched comparators had no history of hematological malignancy and/or mental disorders 10 years preceding the index date (date of MM diagnosis). Depression and anxiety were defined as having a hospital record with a diagnosis of depression or anxiety or the use of either antidepressants, anxiolytics, or pregabalin with the indication of depression or anxiety. All individuals were followed up from the index date until the date of depression and/or anxiety, death, emigration, or June 1, 2024, whichever occurred first. The risk of depression and/or anxiety was investigated using an age- and sex-adjusted hazard ratio, as well as a fully adjusted hazard ratio for socioeconomics (cohabitation, education, and occupational status) and comorbidity (heart, pulmonary, renal, gastrointestinal, rheumatic disease, cancer, dementia, and diabetes mellitus).

**Copyright Information:** (Online) ISSN: 2572-9241
©2026 The Author(s). HemaSphere is published by John Wiley & Sons Ltd on behalf of European Hematology Association. This is an open access Abstract Book distributed under the Attribution-NonCommercial-NoDerivs (CC BY-NC-ND), which allows third parties to download the articles and share them with others as long as they credit the author and the Abstract Book, but they cannot change the content in any way or use them commercially.
**Abstract Book Citations:** Authors, Title, HemaSphere, 2026;10:(S1):pages. The abstract book DOIs can be found on the "Author Information" page.
**Disclaimer:** Articles published in the journal HemaSphere exclusively reflect the opinions of the authors. The authors are responsible for all content in their abstracts including accuracy of the facts, statements, citing resources, etc.

### Results

In total, 3,871 MM patients and 19,305 matched comparators were included. The median age was 73 years (interquartile range 65-79), and 58.9% were male. The number of MM patients with an outcome of depression and/or anxiety during follow-up was 315, and among matched comparators, the number was 660. The incidence rate was 31.20 per 1000 person-years (95% CI 27.94-34.84) among MM patients, and 10.37 per 1000 person-years (95% CI 9.60-11.19) among matched comparators.
MM was associated with increased risk of depression and/anxiety (HR: 2.97, 95% CI 2.59-3.39) compared with age- and sex matched comparators. When fully adjusted for socioeconomics and comorbidity, MM was still associated with increased risk of depression and/or anxiety (HR 2.56, 95% CI 2.23-2.94). The association was slightly stronger for men (HR 2.71, 95% CI 2.26-3.26), than women (HR 2.40, 95% CI 1.96-2.95) with overlapping confidence intervals. Across age categories, the association was strongest among patients aged 61-75 years (HR 3.13, 95% CI 2.52-3.88), and weaker among those ≤60 years (HR 2.28, 95% CI 1.49-3.48), and >75 years (HR 2.25, 95% CI 1.84-2.76). The cumulative incidence curves showed the highest risk during the initial period after MM diagnosis.

### Summary/Conclusion

Patients with MM are at elevated risk of developing depression and/or anxiety. These findings emphasize the need for focus on mental illness following MM. Furthermore, these results underline the need for future studies investigating whether screening for mental illness and/or preventive interventions may be beneficial in detecting and limiting psychopathology following MM.

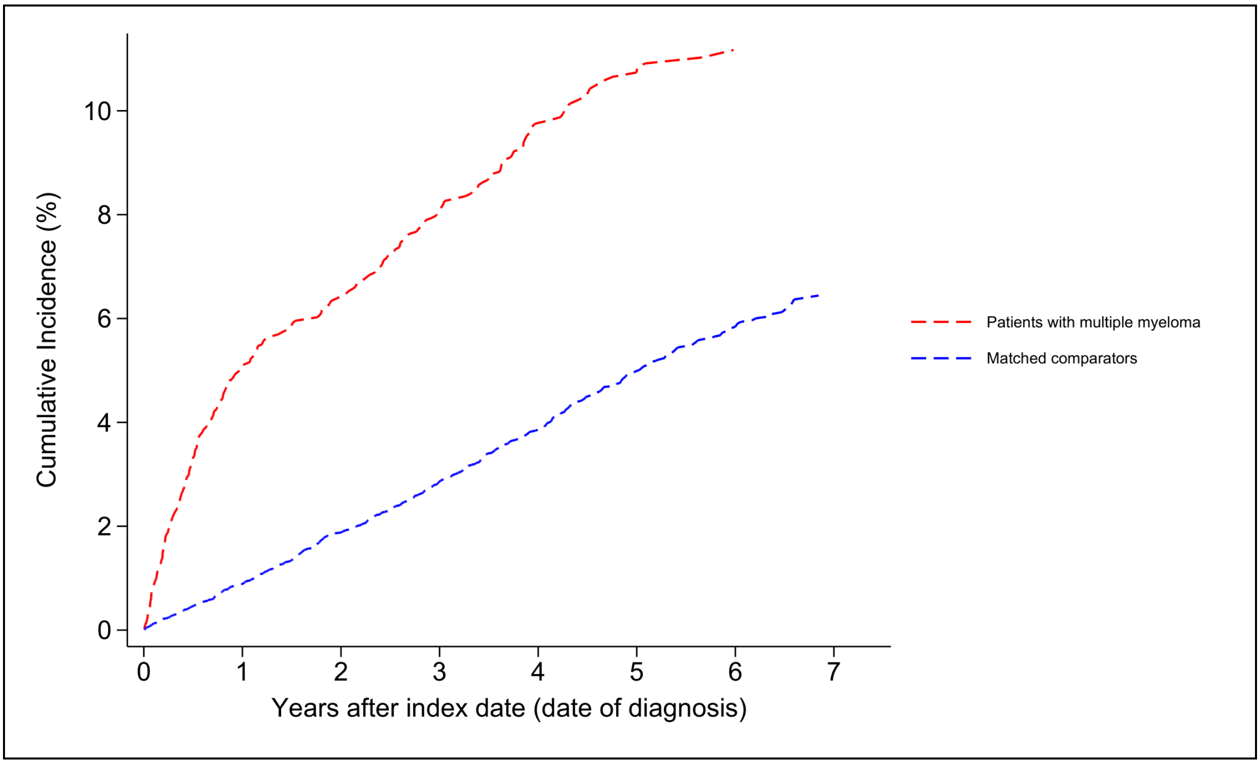

Cumulative incidence curves of time to depression and/or anxiety among patients with MM and matched comparators from the general Danish population

**Copyright Information:** (Online) ISSN: 2572-9241
©2026 The Author(s). HemaSphere is published by John Wiley & Sons Ltd on behalf of European Hematology Association. This is an open access Abstract Book distributed under the Attribution-NonCommercial-NoDerivs (CC BY-NC-ND), which allows third parties to download the articles and share them with others as long as they credit the author and the Abstract Book, but they cannot change the content in any way or use them commercially.
**Abstract Book Citations:** Authors, Title, HemaSphere, 2026;10:(S1):pages. The abstract book DOIs can be found on the "Author Information" page.
**Disclaimer:** Articles published in the journal HemaSphere exclusively reflect the opinions of the authors. The authors are responsible for all content in their abstracts including accuracy of the facts, statements, citing resources, etc.

### (PB3301) EFFICACY ANALYSIS OF SELINEXOR-BASED REGIMENS IN THE TREATMENT OF TRIPLE-CLASS REFRACTORY OR BEYOND MULTIPLE MYELOMA WITH EXTRAMEDULLARY DISEASE: A REAL-WORLD STUDY

**Topic:** 14. Myeloma and other monoclonal gammopathies - Clinical

Man Shen[1], Xin Li*[1]

[1]*Beijing Chaoyang Hospital, Capital Medical University, Beijing, China;*

### Background

Previous clinical studies have reported preliminary efficacy of selinexor-containing regimens in the treatment of high-risk multiple myeloma patients.

### Aims

To investigate the efficacy of selinexor-based regimens in multiple myeloma (MM) patients with triple-class refractory or beyond accompanied by extramedullary disease.

### Methods

A retrospective analysis was conducted on 53 patients with relapsed/refractory MM who received selinexor-based regimens at Beijing Chao-Yang Hospital, Capital Medical University, from August 2022 to June 2025. Among them, 36 patients presented with triple-class refractory or beyond disease complicated by extramedullary lesions. The treatment regimens included selinexor, bortezomib, and dexamethasone (SVD) in 3 patients; selinexor, carfilzomib, and dexamethasone (SKD) in 16 patients; selinexor, pomalidomide, and dexamethasone (SPD) in 8 patients; selinexor combined with aponermin (CPT) in 6 patients; and selinexor combined with other agents in 2 patients. The objective response rate (ORR) and progression-free survival (PFS) associated with selinexor-containing regimens were evaluated in this patient subset.

### Results

The 36 MM patients with triple-class refractory or beyond complicated by extramedullary disease received a median of 5 cycles (range, 1-14) of selinexor-containing therapy. The overall ORR was 41.7%, and the clinical benefit rate (CBR) was 52.8%. The median time to response was 2 months (range, 1-4 months). The estimated median PFS for the 36 patients was 6.62 months (95% CI: 4.94-8.29 months), and the estimated median overall survival (OS) following selinexor treatment was 20 months (95% CI: 3.50-36.40 months). Within this cohort, a comparison between pomalidomide-naive and pomalidomide-exposed/refractory subgroups revealed a significant difference in estimated median PFS: 8.07 months vs. 4.17 months (95% CI: 2.35-5.99 months), respectively ($P = 0.021$). Regarding OS post-selinexor treatment, the 1-year OS rates for the two subgroups were 82.1% and 32.7%, respectively, with estimated median OS not reached (NE) in the naive group vs. 9.1 months (95% CI: 5.14-13.06 months) in the exposed/refractory group ($P = 0.003$). Among all selinexor-containing regimens, the SKD-based regimen subgroup exhibited the longest estimated median PFS at 8.07 months (95% CI: 5.55-10.59 months), followed by the selinexor plus aponermin subgroup with a median PFS of 7.03 months (95% CI: 1.65-12.42 months).

### Summary/Conclusion

Selinexor-based regimens offer an effective treatment option for MM patients with triple-class refractory or beyond complicated by extramedullary disease. Patients without prior pomalidomide exposure derived significant survival benefits from selinexor-based therapies. Furthermore, the SKD-based combination regimen also demonstrated a PFS benefit in this patient population.

**Copyright Information:** (Online) ISSN: 2572-9241
©2026 The Author(s). HemaSphere is published by John Wiley & Sons Ltd on behalf of European Hematology Association. This is an open access Abstract Book distributed under the Attribution-NonCommercial-NoDerivs (CC BY-NC-ND), which allows third parties to download the articles and share them with others as long as they credit the author and the Abstract Book, but they cannot change the content in any way or use them commercially.
**Abstract Book Citations:** Authors, Title, HemaSphere, 2026;10:(S1):pages. The abstract book DOIs can be found on the “Author Information” page.
**Disclaimer:** Articles published in the journal HemaSphere exclusively reflect the opinions of the authors. The authors are responsible for all content in their abstracts including accuracy of the facts, statements, citing resources, etc.

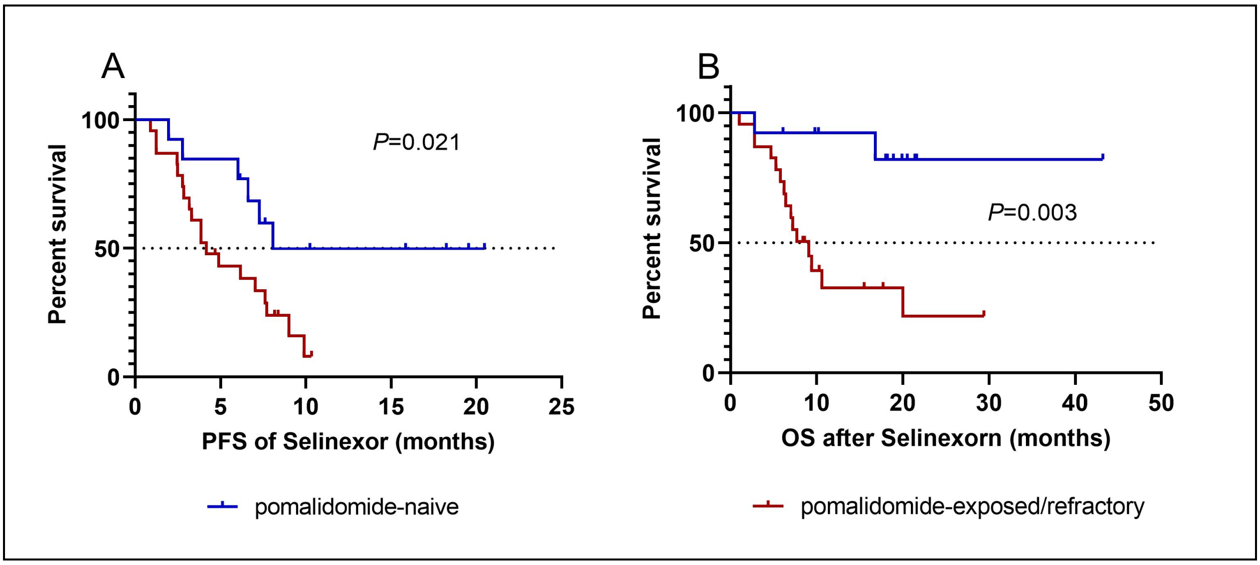

**Copyright Information:** (Online) ISSN: 2572-9241
©2026 The Author(s). HemaSphere is published by John Wiley & Sons Ltd on behalf of European Hematology Association. This is an open access Abstract Book distributed under the Attribution-NonCommercial-NoDerivs (CC BY-NC-ND), which allows third parties to download the articles and share them with others as long as they credit the author and the Abstract Book, but they cannot change the content in any way or use them commercially.
**Abstract Book Citations:** Authors, Title, HemaSphere, 2026;10:(S1):pages. The abstract book DOIs can be found on the "Author Information" page.
**Disclaimer:** Articles published in the journal HemaSphere exclusively reflect the opinions of the authors. The authors are responsible for all content in their abstracts including accuracy of the facts, statements, citing resources, etc.

### (PB3302) REAL-WORLD TREATMENT PATTERNS AND OUTCOMES OF RELAPSED/REFRACTORY MULTIPLE MYELOMA IN CHINA: INSIGHTS FROM THE NICHE-MM REGISTRY (2018–2025)

**Topic:** 14. Myeloma and other monoclonal gammopathies - Clinical

Yuntong Liu[1], Xiaoyan Yang[2], Yating Li[1], Yining Li[2], Zengyi Hao[2], Shimin Bi[3], Jia Zhong[3], Gang An*[1]

*[1]China Academy of Medical Science & Peking Union Medical College, Institute of Hematology & Blood Disease Hospital, Tianjin, China; [2]GlaxoSmithKline (China) Investment Co., Ltd., Shanghai, China;[3]Beijing Analysis International Consulting Co., Ltd., Beijing, China;*

### Background

The treatment landscape for relapsed/refractory multiple myeloma (RRMM) has evolved substantially in recent years with the introduction of novel agents and combination regimens. However, real-world data on treatment patterns and clinical outcomes among patients with RRMM in China remain limited. Understanding treatment utilization and patient outcomes in routine clinical practice, particularly following refractory to lenalidomide (len) and anti-CD38 monoclonal antibodies (anti-CD38), is critical to inform clinical decision-making and optimize patient outcomes.

### Aims

To describe real-world patient characteristics, treatment patterns, and clinical outcomes of patients with RRMM in China, including subgroups by len and anti-CD38 refractoriness.

### Methods

A non-interventional, retrospective study was conducted using real-world data from the National Longitudinal Cohort of Hematological Diseases in China. Adult patients with a confirmed diagnosis of RRMM between January 1, 2018 and September 15, 2025, and with ≥2 follow-up visits were included; a minimum of 6 months of follow-up was required for response and outcome analyses. Demographic, disease and clinical characteristics, treatment patterns, and clinical outcomes were summarized descriptively. Progression-free survival (PFS) and overall survival (OS) were estimated using the Kaplan-Meier method. Same analyses were conducted in subgroups.

### Results

A total of 539 patients were included (median age at RRMM diagnosis, 59.3 years [range: 29.9-83.5]; 54.2% male), with a median follow-up of 18.0 months. Most patients had an ECOG performance status of 0 or 1 (82.7%) and ISS stage II (34.9%) or III (42.5%), and comorbidities were reported in 73.8%. Treatment patterns were heterogeneous after RR. In 2L (n=461), IMiD-based (67.2%), PI-based (61.8%), and anti-CD38-based (46.9%) regimens were commonly used. Heterogeneity persisted in 3L+ (n=182), with continued use of PI-based (56.6%), IMiD-based (52.2%), and anti-CD38-based (45.6%) therapies; CAR-T therapy was administered in 22.5% of patients. Overall use of anti-CD38-based regimens increased over time, from 18.9% in 2019-2021 to 52.2% in 2023-2025. Among 2L patients with response assessments (n=233), the complete response or better (≥CR) rate was 27.0%. Median PFS from 2L initiation was 16.0 months (95% confidence interval [CI]: 13.5-19.8), and median PFS from 3L initiation was 11.9 months (95% CI: 8.8-18.1). Median OS from RRMM diagnosis to death was not reached [NR] (74.5-NR). In refractory subgroups, 233 patients (43.2%) were len-refractory. Median PFS from 2L initiation was 11.9 months (95% CI: 9.1-14.3), and from 3L initiation was 9.5 months (95% CI: 5.2-18.1). A total of 158 patients (29.3%) were anti-CD38-refractory, of whom 95 (60.1%) were also len-refractory. The ≥CR rate in anti-CD38-refractory patients was 23.8%. Median PFS from 2L initiation was 11.9 months (95% CI: 9.1-16.3), and from 3L initiation was 9.1 months (95% CI: 5.0-13.4).

**Copyright Information:** (Online) ISSN: 2572-9241
©2026 The Author(s). HemaSphere is published by John Wiley & Sons Ltd on behalf of European Hematology Association. This is an open access Abstract Book distributed under the Attribution-NonCommercial-NoDerivs (CC BY-NC-ND), which allows third parties to download the articles and share them with others as long as they credit the author and the Abstract Book, but they cannot change the content in any way or use them commercially.
**Abstract Book Citations:** Authors, Title, HemaSphere, 2026;10:(S1):pages. The abstract book DOIs can be found on the “Author Information” page.
**Disclaimer:** Articles published in the journal HemaSphere exclusively reflect the opinions of the authors. The authors are responsible for all content in their abstracts including accuracy of the facts, statements, citing resources, etc.

**Summary/Conclusion**

This study provides a comprehensive overview of treatment patterns and clinical outcomes among patients with RRMM in China. The heterogeneity of post-RR treatment and suboptimal PFS highlight a substantial unmet need for effective and durable therapeutic options, especially for patients refractory to len or anti-CD38. Analysis with extended follow-up data is warranted to characterize long-term outcomes.

Funding: GSK (Study ID: 224324)

**Copyright Information:** (Online) ISSN: 2572-9241
©2026 The Author(s). HemaSphere is published by John Wiley & Sons Ltd on behalf of European Hematology Association. This is an open access Abstract Book distributed under the Attribution-NonCommercial-NoDerivs (CC BY-NC-ND), which allows third parties to download the articles and share them with others as long as they credit the author and the Abstract Book, but they cannot change the content in any way or use them commercially.
**Abstract Book Citations:** Authors, Title, HemaSphere, 2026;10:(S1):pages. The abstract book DOIs can be found on the "Author Information" page.
**Disclaimer:** Articles published in the journal HemaSphere exclusively reflect the opinions of the authors. The authors are responsible for all content in their abstracts including accuracy of the facts, statements, citing resources, etc.

### (PB3304) PROGNOSTIC VALUE OF CIRCULATING PLASMA CELLS DETECTED BY FLOW CYTOMETRY VERSUS PERIPHERAL BLOOD SMEAR IN PATIENTS WITH MULTIPLE MYELOMA

**Topic:** 14. Myeloma and other monoclonal gammopathies - Clinical

Li Bao*[1], Bin Chu[1], Mengru Liu[1], Qi Yan[1], Na Ji[1, 1], Shan Gao[1], Lei Shi[1], Lijuan Fang[1], Mengzhen Wang[1], Yuan Chen[1], Minqiu Lu[1]

[1]*Beijing Jishuitan Hospital, Capital Medical University, Beijing, China;*

### Background

The presence of circulating plasma cells (CPCs) has been confirmed to be closely associated with poor prognosis in Multiple myeloma (MM) patients. Peripheral blood smear and flow cytometry are two common methods for detecting CPCs. The former is simple, low-cost, and widely available but has limited sensitivity. The latter is highly sensitive and capable of identifying clonality but requires specialized equipment and expertise, limiting its availability compared to smear. This study retrospectively analyzed clinical data from 294 newly diagnosed MM (NDMM) patients to systematically compare the detection efficacy of the two methods, their correlation with clinical characteristics and prognosis, and their value in predicting plasma cell leukemia-like ultra-high-risk patients, aiming to provide a basis for selecting more appropriate detection techniques in clinical practice.

### Aims

To compare the sensitivity, concordance, and prognostic predictive value of flow cytometry versus peripheral blood smear for detecting CPCs in NDMM patients and primary plasma cell leukemia.

### Methods

Clinical data of 294 NDMM patients were retrospectively analyzed. Using flow cytometry as the reference standard, the sensitivity and concordance of blood smear for detecting CPCs were calculated. The correlation of plasma cell levels detected by both methods with patient clinical characteristics and progression-free survival (PFS) and overall survival (OS) were compared.

### Results

Among the 294 patients, the median proportion of clonal plasma cells by flow cytometry was 0.04% (range 0-62.70%), with 173 patients (58.8%) being positive (≥0.01%). By smear, the median proportion was 0% (range 0-69%), with 67 patients (22.8%) being positive (≥1%). Using flow cytometry as the gold standard, blood smear detection showed a sensitivity of 60.0%, specificity of 93.1%, positive predictive value of 95.1%, negative predictive value of 51.3%, and a Kappa coefficient of 0.231 for concordance. Patients were grouped based on CPC proportion to compare survival differences. With a median follow-up of 31 (21.53-42.87) months, median PFS for patients with flow cytometry CPCs <0.05%, 0.05%-2%, and ≥2% was 55, 19, and 15 months, respectively (P=0.0003); median OS was not reached, 59, and 24 months, respectively (P=0.0001). For patients with smear CPCs <1%, 1-4%, and ≥5%, median PFS was 40, 19, and 12 months (P=0.0001), and median OS was not reached, 58, and 24 months (P=0.0006), respectively. To predict plasma cell leukemia-like ultra-high-risk patients, patients were re-categorized into three groups based on combined detection: Group 1 (flow cytometry <2% and smear <5%), Group 2 (flow cytometry ≥2% and smear <5%), and Group 3 (flow cytometry ≥2% and smear ≥5%). Their median PFS was 36, 18, and 8 months (P=0.0001), and median OS was not reached, 54, and 13 months (P=0.0001), respectively. Multivariate analysis identified flow cytometry CPCs ≥0.05% as independent predictors of poor prognosis in NDMM patients, indicating shorter PFS and OS.

**Copyright Information:** (Online) ISSN: 2572-9241
©2026 The Author(s). HemaSphere is published by John Wiley & Sons Ltd on behalf of European Hematology Association. This is an open access Abstract Book distributed under the Attribution-NonCommercial-NoDerivs (CC BY-NC-ND), which allows third parties to download the articles and share them with others as long as they credit the author and the Abstract Book, but they cannot change the content in any way or use them commercially.
**Abstract Book Citations:** Authors, Title, HemaSphere, 2026;10:(S1):pages. The abstract book DOIs can be found on the “Author Information” page.
**Disclaimer:** Articles published in the journal HemaSphere exclusively reflect the opinions of the authors. The authors are responsible for all content in their abstracts including accuracy of the facts, statements, citing resources, etc.

**Summary/Conclusion**

Both smear and flow cytometry detection of CPCs have prognostic predictive value, but flow cytometry demonstrates significantly superior sensitivity and specificity. For predicting plasma cell leukemia-like ultra-high-risk patients, the combined use of both techniques is more precise than using either alone.

**Copyright Information:** (Online) ISSN: 2572-9241
©2026 The Author(s). HemaSphere is published by John Wiley & Sons Ltd on behalf of European Hematology Association. This is an open access Abstract Book distributed under the Attribution-NonCommercial-NoDerivs (CC BY-NC-ND), which allows third parties to download the articles and share them with others as long as they credit the author and the Abstract Book, but they cannot change the content in any way or use them commercially.
**Abstract Book Citations:** Authors, Title, HemaSphere, 2026;10:(S1):pages. The abstract book DOIs can be found on the "Author Information" page.
**Disclaimer:** Articles published in the journal HemaSphere exclusively reflect the opinions of the authors. The authors are responsible for all content in their abstracts including accuracy of the facts, statements, citing resources, etc.

### (PB3305) ESA CO-ADMINISTRATION INDEPENDENTLY INCREASES THROMBOTIC RISK DURING LENALIDOMIDE THERAPY IN MULTIPLE MYELOMA: A REAL-WORLD COHORT STUDY

**Topic:** 14. Myeloma and other monoclonal gammopathies - Clinical

Anthony Naassan*[1], Raina Fatima[1], Matthew Naassan[1], Carly Chisholm[1]

*[1]Lakeridge Health, Durham Regional Cancer Centre, Oshawa, Canada;*

### Background

Lenalidomide is a cornerstone of myeloma therapy but carries a well-recognized thrombotic risk, with venous thromboembolism (VTE) rates of up to 26% reported in clinical trials. Erythropoiesis stimulating agents (ESAs) are also established risk factors for thrombosis and are co-administered to manage disease and treatment-related anemia. However, the incremental thrombotic risk associated with combining these two drug classes remains poorly characterized.

### Aims

We aim to quantify the independent association between ESA co-administration and thrombosis during lenalidomide therapy, and to evaluate the SAVED-VTE score as a risk stratification tool in a real-world Canadian cohort.

### Methods

We reviewed 229 multiple myeloma patients who began lenalidomide treatment between January 2013 and February 2025 at the Durham Regional Cancer Centre, with follow-up through January 2026. The primary outcome was venous thromboembolism (VTE) occurring during active lenalidomide therapy, identified by retrospective chart review. ESA co-administration was defined as concurrent use at any point during lenalidomide treatment. VTE risk was stratified using the SAVED-VTE score (High-risk defined as score >=2). Time-to-event analysis was performed using multivariable Cox proportional hazards regression, adjusting for SAVED high-risk category, baseline hemoglobin, creatinine clearance, sex, and prophylaxis type. Survival outcomes were compared via Kaplan-Meier methods and log-rank tests, with model discrimination assessed by AUC. Statistical analysis was performed using SPSS (v29.0) and Python (v3.11). AI-assisted coding tools were used to optimize Python script efficiency, with all outputs cross-validated to ensure analytical rigor.

### Results

Two hundred twenty-nine patients were included (median follow-up 11.2 months). Of these, 37 patients (16.2%) received ESA co-administration. A total of 21 VTE events occurred overall (9.2%); the VTE rate was significantly higher in the ESA group compared to the non-ESA group (21.9% vs. 6.8%, p=0.009). Seventy-six patients (33.2%) were classified as SAVED high risk. Primary thromboprophylaxis included ASA (n=173, 75.5%) or anticoagulation (NOAC, warfarin or LMWH; n=29, 12.7%). On multivariable Cox regression, ESA co-exposure was independently associated with an increased VTE risk (HR 6.08, 95% CI 1.84-20.01, p=0.003). SAVED high-risk category was the strongest predictor of VTE (HR 8.36, 95% CI 2.92-23.92, p<0.001), while anticoagulation showed a non-significant trend towards protection (HR 0.24, 95% CI 0.05-1.06, p=0.059). Baseline hemoglobin, creatinine clearance, and sex were not independently associated with VTE risk. Kaplan-Meier analysis confirmed significantly lower VTE-free survival in ESA-exposed patients (log-rank p=0.003) and in SAVED high-risk patients (log-rank p<0.001). The model demonstrated excellent discrimination (AUC=0.811).

**Copyright Information:** (Online) ISSN: 2572-9241
©2026 The Author(s). HemaSphere is published by John Wiley & Sons Ltd on behalf of European Hematology Association. This is an open access Abstract Book distributed under the Attribution-NonCommercial-NoDerivs (CC BY-NC-ND), which allows third parties to download the articles and share them with others as long as they credit the author and the Abstract Book, but they cannot change the content in any way or use them commercially.
**Abstract Book Citations:** Authors, Title, HemaSphere, 2026;10:(S1):pages. The abstract book DOIs can be found on the "Author Information" page.
**Disclaimer:** Articles published in the journal HemaSphere exclusively reflect the opinions of the authors. The authors are responsible for all content in their abstracts including accuracy of the facts, statements, citing resources, etc.

**Summary/Conclusion**

ESA co-administration independently increases VTE risk during lenalidomide therapy, with a six-fold hazard of thrombosis, even when adjusting for baseline risk scores and prophylaxis type. The SAVED-VTE score effectively stratified risk in this real-world cohort and the addition of an ESA further compounds the risk in a clinically significant manner. These findings support heightened thromboprophylaxis consideration in patients receiving concurrent ESA and lenalidomide and validate the SAVED score as a practical risk stratification tool in clinical practice.

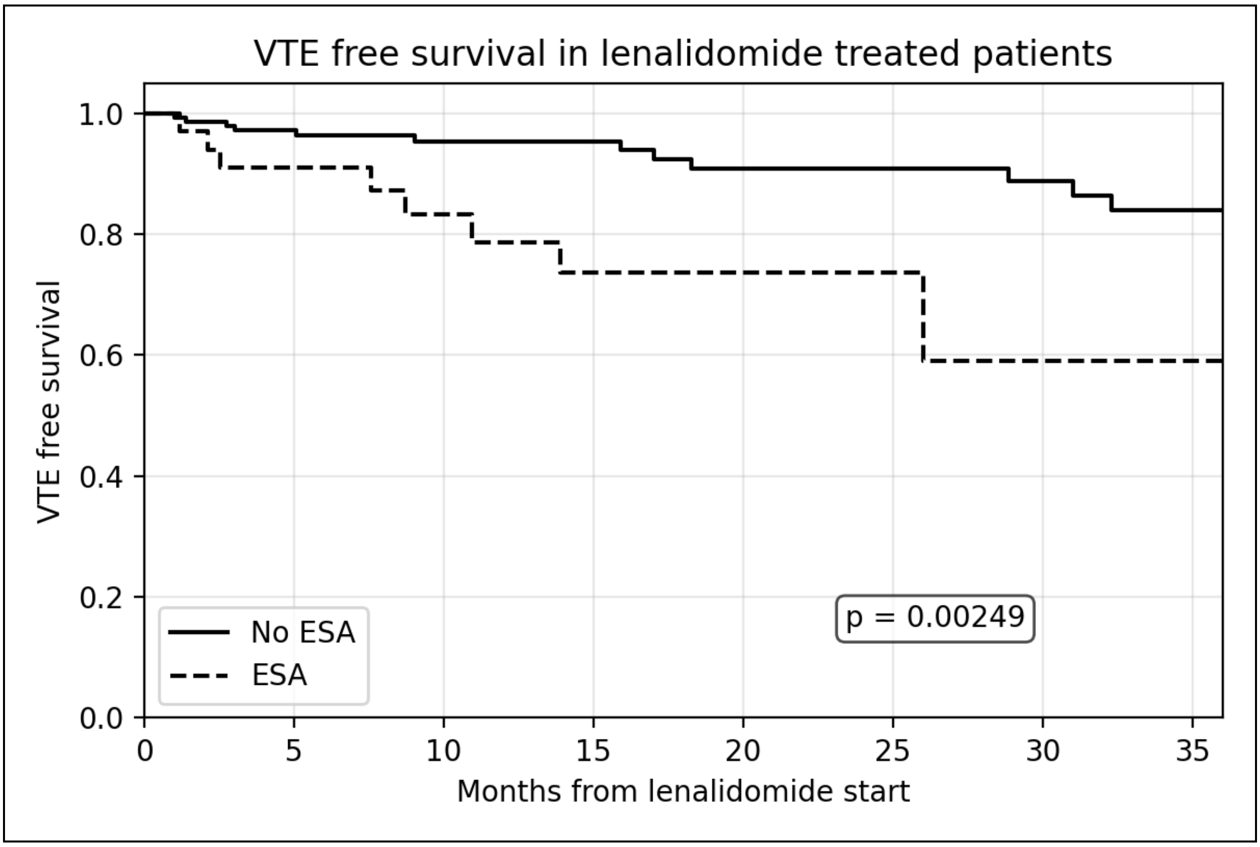

Figure 1. Kaplan-Meier estimates of VTE-free survival stratified by ESA co-administration during lenalidomide therapy.

**Copyright Information:** (Online) ISSN: 2572-9241
©2026 The Author(s). HemaSphere is published by John Wiley & Sons Ltd on behalf of European Hematology Association. This is an open access Abstract Book distributed under the Attribution-NonCommercial-NoDerivs (CC BY-NC-ND), which allows third parties to download the articles and share them with others as long as they credit the author and the Abstract Book, but they cannot change the content in any way or use them commercially.
**Abstract Book Citations:** Authors, Title, HemaSphere, 2026;10:(S1):pages. The abstract book DOIs can be found on the "Author Information" page.
**Disclaimer:** Articles published in the journal HemaSphere exclusively reflect the opinions of the authors. The authors are responsible for all content in their abstracts including accuracy of the facts, statements, citing resources, etc.

### (PB3306) CLINICAL CHARACTERISTICS AND ADJUSTED IN-HOSPITAL OUTCOMES OF ACUTE PANCREATITIS IN PATIENTS WITH MULTIPLE MYELOMA: A NATIONWIDE ANALYSIS

**Topic:** 14. Myeloma and other monoclonal gammopathies - Clinical

Hala Soomro*[1], Mohammad Ahsen Soomro[2], Amninder Singh[2]

*[1]Brown University of Health Sciences, Internal Medicine, Providence, United States of America;[2]The Wright Center for Graduate Medical Education, Cardiology, Scranton, United States of America;*

### Background

Acute pancreatitis (AP) is a common cause of hospitalization and may be influenced by underlying hematologic malignancies. Multiple myeloma (MM) is associated with systemic complications—including hypercalcemia, renal dysfunction, and coagulopathy—that may plausibly modify AP severity. However, population-level data evaluating AP outcomes in patients with MM are limited.

### Aims

To compare clinical characteristics, in-hospital mortality, and resource utilization of AP hospitalizations in patients with and without MM using a nationally representative dataset.

### Methods

We conducted a retrospective cohort study using the National Inpatient Sample (2016–2022). Hospitalizations with a principal diagnosis of AP (ICD-10-CM K85.x) were identified. The MM cohort was defined by a secondary diagnosis of multiple myeloma (C90.00, C90.02). Exclusions included MGUS/smoldering myeloma, plasma cell leukemia, chronic pancreatitis, and pancreatic cancer. Outcomes included in-hospital mortality, length of stay (LOS), and total hospital charges. Comorbidity burden was assessed using Elixhauser ICD-10 refined variables. Survey-weighted multivariable regression models adjusted for age, sex, and major comorbidities were performed.

### Results

A total of 312 AP hospitalizations with MM (weighted n≈1,560) and 301,912 without MM (weighted n≈1,509,559) were identified. MM patients were older (median 68 vs. 51 years) and had higher rates of congestive heart failure, renal failure, and coagulopathy. Hypercalcemia (7.1% vs. 1.0%) and acute kidney injury (29.5% vs. 12.4%) were more common in the MM cohort, while alcohol use disorder was less prevalent (6.7% vs. 29.8%).
In-hospital mortality was similar between groups (0.64% vs. 0.66%). After multivariable adjustment, MM was not independently associated with mortality (adjusted OR 0.38, 95% CI 0.09–1.53; p=0.17). However, MM was independently associated with longer hospitalization, corresponding to a 10.7% increase in LOS (95% CI 3.9–17.9%; p=0.0017). Total hospital charges were also higher in the MM cohort.

### Summary/Conclusion

In this nationally representative analysis, AP hospitalizations in patients with MM were characterized by older age, greater comorbidity burden, and increased resource utilization. Despite higher rates of hypercalcemia and renal dysfunction, MM was not independently associated with increased in-hospital mortality after adjustment. These findings suggest preserved short-term mortality outcomes but increased care complexity in MM patients presenting with AP, highlighting the importance of early recognition and management of MM-related metabolic and renal complications.

**Copyright Information:** (Online) ISSN: 2572-9241
©2026 The Author(s). HemaSphere is published by John Wiley & Sons Ltd on behalf of European Hematology Association. This is an open access Abstract Book distributed under the Attribution-NonCommercial-NoDerivs (CC BY-NC-ND), which allows third parties to download the articles and share them with others as long as they credit the author and the Abstract Book, but they cannot change the content in any way or use them commercially.
**Abstract Book Citations:** Authors, Title, HemaSphere, 2026;10:(S1):pages. The abstract book DOIs can be found on the "Author Information" page.
**Disclaimer:** Articles published in the journal HemaSphere exclusively reflect the opinions of the authors. The authors are responsible for all content in their abstracts including accuracy of the facts, statements, citing resources, etc.

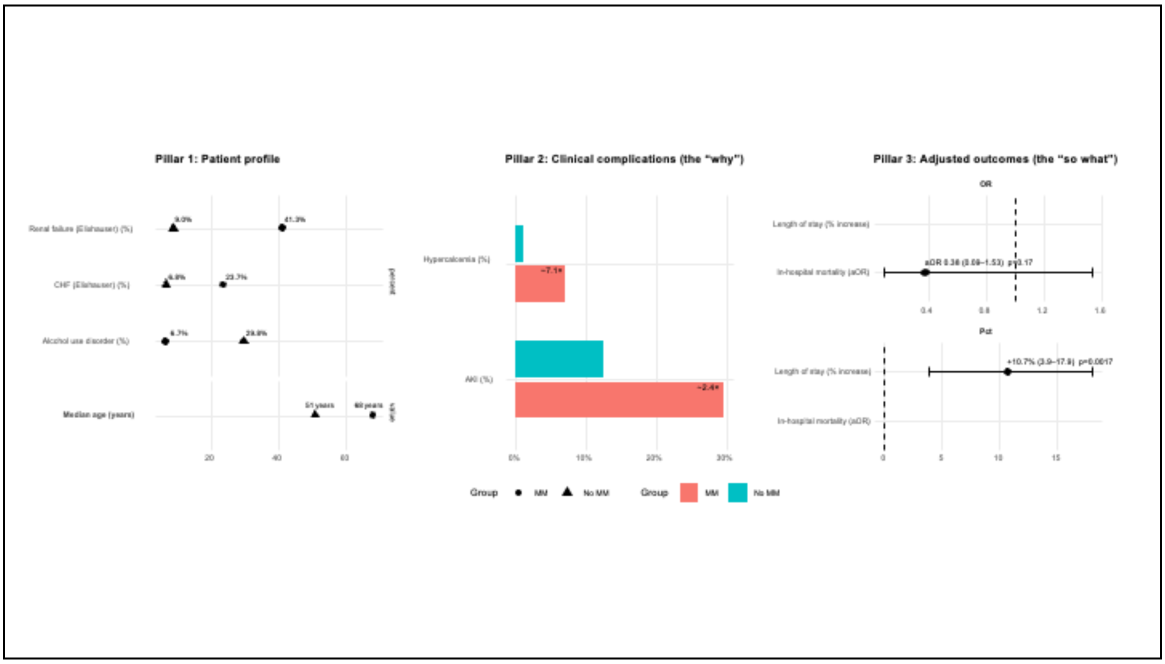

Figure 1. Three pillars of AP outcomes in multiple myeloma. Pillar 1: Baseline characteristics. Pillar 2: Fold-increase in prevalence of hypercalcemia and acute kidney injury (MM vs. no MM). Pillar 3: Adjusted outcomes from multivariable regression models (mortality aOR; LOS % increase). Models adjusted for age, sex, CHF, renal failure, and coagulopathy.

**Copyright Information:** (Online) ISSN: 2572-9241
©2026 The Author(s). HemaSphere is published by John Wiley & Sons Ltd on behalf of European Hematology Association. This is an open access Abstract Book distributed under the Attribution-NonCommercial-NoDerivs (CC BY-NC-ND), which allows third parties to download the articles and share them with others as long as they credit the author and the Abstract Book, but they cannot change the content in any way or use them commercially.
**Abstract Book Citations:** Authors, Title, HemaSphere, 2026;10:(S1):pages. The abstract book DOIs can be found on the "Author Information" page.
**Disclaimer:** Articles published in the journal HemaSphere exclusively reflect the opinions of the authors. The authors are responsible for all content in their abstracts including accuracy of the facts, statements, citing resources, etc.

### (PB3307) ULTRA-EARLY MORTALITY (≤90 DAYS) AND ITS PREDICTORS IN NEWLY DIAGNOSED MULTIPLE MYELOMA TREATED WITH BORTEZOMIB-BASED REGIMENS: AN AMBISPECTIVE REAL-WORLD STUDY FROM A RESOURCE-LIMITED LMIC SETTING

**Topic:** 14. Myeloma and other monoclonal gammopathies - Clinical

Rajat Goyal*[1], Gopal Bohra[1], Satyendra Khichar[1], Abhishek Purohit[1]

[1] *All India Institute of Medical Sciences, Jodhpur, General Medicine, Jodhpur, India;*

### Background

Predictors of early mortality in patients with newly diagnosed multiple myeloma treated with bortezomib-based regimens in low- and middle-income countries (LMICs) remain poorly characterised. Available data are limited, particularly from resource-constrained settings, where delayed presentations are common. We therefore aimed to identify baseline factors associated with mortality within 90 days of diagnosis in newly diagnosed multiple myeloma patients treated at a tertiary public hospital in western India.

### Aims

To determine the incidence and identify baseline predictors of ultra-early mortality (≤90 days) in newly diagnosed multiple myeloma patients treated with bortezomib-based regimens in a resource-limited public-sector setting in western India.

### Methods

We conducted an ambispective observational cohort study including 93 patients with newly diagnosed multiple myeloma, comprising 61 prospectively enrolled patients treated from July 2024 onwards and 32 retrospectively identified patients treated between July 2022 and June 2024, at a tertiary-care public hospital in western India.
The primary outcome was ultra-early mortality, defined as death occurring within 90 days of diagnosis. Baseline demographic, clinical, laboratory, and available cytogenetic parameters were recorded at the time of diagnosis.
Continuous variables were summarised as medians and compared between patients with and without ultra-early mortality using the Mann–Whitney U test. Categorical variables were compared using the chi-square test or Fisher's exact test, as appropriate. A two-sided *p-value* < 0.05 was considered statistically significant.

### Results

The study included 93 patients with newly diagnosed multiple myeloma (median age 62 years; 60% male). Twenty-one patients (22.6%) died within 3 months. Patients who died within 3 months had significantly worse renal function at diagnosis, with higher baseline creatinine (median 2.69 vs 1.64 mg/dL; p = 0.022) and urea levels (66 vs 39 mg/dL; p = 0.003). Haemoglobin levels at diagnosis were also significantly lower among patients who died within 3 months (median 7.0 vs 8.35 g/dL; p = 0.023). In the subset with available cytogenetic data (n = 60), high-risk cytogenetics was associated with ultra-early mortality (p = 0.041). Neither baseline creatinine nor haemoglobin levels differed significantly between high-risk and standard-risk cytogenetic groups (p = 0.65 and p = 0.699, respectively), suggesting that renal dysfunction, anemia, and disease biology may independently contribute to early mortality.

**Copyright Information:** (Online) ISSN: 2572-9241
©2026 The Author(s). HemaSphere is published by John Wiley & Sons Ltd on behalf of European Hematology Association. This is an open access Abstract Book distributed under the Attribution-NonCommercial-NoDerivs (CC BY-NC-ND), which allows third parties to download the articles and share them with others as long as they credit the author and the Abstract Book, but they cannot change the content in any way or use them commercially.
**Abstract Book Citations:** Authors, Title, HemaSphere, 2026;10:(S1):pages. The abstract book DOIs can be found on the "Author Information" page.
**Disclaimer:** Articles published in the journal HemaSphere exclusively reflect the opinions of the authors. The authors are responsible for all content in their abstracts including accuracy of the facts, statements, citing resources, etc.

**Summary/Conclusion**

This ambispective real-world study from a resource-limited public-sector setting in western India demonstrates that nearly one-quarter of patients with newly diagnosed multiple myeloma die within 90 days of diagnosis. Ultra-early mortality was associated with baseline renal dysfunction, anemia, and high-risk cytogenetics. Early identification of high-risk patients at diagnosis may facilitate timely referral to specialised centres and optimisation of therapeutic and supportive care strategies, including early initiation of renal replacement therapy where indicated and optimisation of anemia , as well as early consideration of transplant pathways where feasible. These findings provide a rationale for developing risk-adapted triaging models to reduce preventable early deaths in newly diagnosed multiple myeloma.

**Copyright Information:** (Online) ISSN: 2572-9241
©2026 The Author(s). HemaSphere is published by John Wiley & Sons Ltd on behalf of European Hematology Association. This is an open access Abstract Book distributed under the Attribution-NonCommercial-NoDerivs (CC BY-NC-ND), which allows third parties to download the articles and share them with others as long as they credit the author and the Abstract Book, but they cannot change the content in any way or use them commercially.
**Abstract Book Citations:** Authors, Title, HemaSphere, 2026;10:(S1):pages. The abstract book DOIs can be found on the "Author Information" page.
**Disclaimer:** Articles published in the journal HemaSphere exclusively reflect the opinions of the authors. The authors are responsible for all content in their abstracts including accuracy of the facts, statements, citing resources, etc.

### (PB3308) EVALUATION OF PROGNOSTIC FACTORS IN PATIENTS WITH MULTIPLE MYELOMA IN A RESOURCE-LIMITED SETTING : ABOUT A SENEGALESE COHORT OF 99-PATIENTS

**Topic:** 14. Myeloma and other monoclonal gammopathies - Clinical

Nata Dieng*[1], Mohamed Keita[2], Kevine Vanessa Mandom Youmssi[1], Mouhamadou Ndiaye[1], El Hadj Tafsir Niaw Gueye[1], Elimane Seydi Bousso[1], Mamadou Wague Gueye[1], Sara Boury Gning[1]

*[1]Hôpital Principal de Dakar, Medicine, Dakar, Senegal;[2]Hôpital Militaire Ouakam, Dakar, Senegal;*

### Background

Multiple myeloma (MM) is a plasma cell malignancy whose prognosis is determined by tumor-related cytogenetic features and clinical-biological parameters at diagnosis. In low-resource settings, delayed diagnosis and limited access to novel therapies may significantly influence survival outcomes.

### Aims

This study aimed to evaluate prognostic factors associated with overall survival among patients with MM treated in a tertiary referral center in Senegal.

### Methods

We conducted a retrospective monocentric study including 99 patients diagnosed with MM according to the International Myeloma Working Group (IMWG 2009/2014) criteria between April 2011 and May 2022 at the Hôpital Principal de Dakar. Demographic, clinical (CRAB features), laboratory (hemoglobin, serum creatinine, calcium, albumin, β2-microglobulin), immunological, radiological, and staging data (Salmon–Durie and ISS) were collected. Treatment regimens and outcomes were recorded. Overall survival (OS) was estimated using the Kaplan–Meier method. Bivariate analysis was performed to identify factors associated with mortality.Any difference less than 0,05 was considered statiscally significant.

### Results

The mean age at diagnosis was 62.4 ± 10.1 years, with a slight male predominance (54.5%). Most patients were diagnosed at advanced stage (68.7% Salmon–Durie stage III; 61.6% ISS stage II). Severe anemia (hemoglobin < 8.5 g/dL) was present in 51.5%, renal impairment in 57%, hypercalcemia in 50.5%, and hypoalbuminemia (< 35 g/L) in 71.4% of patients. Elevated β2-microglobulin (> 5.5 mg/L) was observed in 36.2% of tested patients. First-line therapy consisted mainly of melphalan–prednisone (53.5%) and melphalan–prednisone–thalidomide (26.3%), while bortezomib-based regimens were used in 11.1% of cases. After a median follow-up of 16 months, overall survival was 60.0% at 12 months and 36.4% at 24 months. Factors significantly associated with shorter survival included age ≥ 65 years, renal impairment, hypercalcemia, hypoalbuminemia, and Salmon–Durie stage III.

### Summary/Conclusion

In this resource-limited setting, multiple myeloma is predominantly diagnosed at advanced stages, with a high burden of adverse clinical and biological prognostic factors. Easily accessible parameters such as renal function, albumin level, calcium, and hemoglobin retain major prognostic value and may guide risk stratification where cytogenetic testing is limited. Improving early detection and access to novel therapeutic agents is crucial to enhance survival outcomes.

**Copyright Information:** (Online) ISSN: 2572-9241
©2026 The Author(s). HemaSphere is published by John Wiley & Sons Ltd on behalf of European Hematology Association. This is an open access Abstract Book distributed under the Attribution-NonCommercial-NoDerivs (CC BY-NC-ND), which allows third parties to download the articles and share them with others as long as they credit the author and the Abstract Book, but they cannot change the content in any way or use them commercially.
**Abstract Book Citations:** Authors, Title, HemaSphere, 2026;10:(S1):pages. The abstract book DOIs can be found on the "Author Information" page.
**Disclaimer:** Articles published in the journal HemaSphere exclusively reflect the opinions of the authors. The authors are responsible for all content in their abstracts including accuracy of the facts, statements, citing resources, etc.

### (PB3310) REAL WORLD DATA ON INITIATION AND DOSE RAMP-UP OF TECLISTAMAB USING TOCILUZIMAB PROPHYLAXIS AND MANAGEMENT OF TOXICITIES IN A DISTRICT GENERAL HOSPITAL SETTING WITH NO PREVIOUS BISPECIFIC EXPERIENCE

**Topic:** 14. Myeloma and other monoclonal gammopathies - Clinical

Eddy Ukponahunsi*[1], Margarida Rodrigues[1], Diana Rance[1], Deirdre Peters[1], Barry MacDonagh[1], Richard Hinton[1], Mary McCloy[1]

[1] *Our Lady of Lourdes Hospital, Haematology, Drogheda, Ireland;*

### Background

Teclistamab (TEC) targets BCMA /CD3 and in Majestec-1 demonstrated efficacy& safety with impressive responses. The first bispecific for relapsed refractory (RR) myeloma reimbursed in the Republic of Ireland, many centres will have no experience of implementing these therapies/mitigating toxicities.

### Aims strategy to initiate TEC, mitigate cytokine release syndrome (CRS), optimise anti-infectives , monitor response , extend frequency of TEC dosing in a district general hospital (DGH).

### Methods

Single centre retrospective analysis: triple class exposed,RR myeloma patients treated with TEC, prophylaxed against CRS(ie pre 1st doseTEC,tociluzimab 8mg/kg IV< 4 hours, dexamethasone/acetaminophen). All myeloma types defined,& Eastern Cooperative Oncology Group (ECOG)/International Staging System (ISS ) scores. Full blood count (FBC), lactate dehydrogenase (LDH), Beta 2 microglobulin, creatinine, calcium, serum free light chains (SFLCs), serum protein electrophoresis (SPEP) - to define toxicities/responses 2 /4 weekly.
All had bone marrow biopsies with FISH.

**Copyright Information:** (Online) ISSN: 2572-9241
©2026 The Author(s). HemaSphere is published by John Wiley & Sons Ltd on behalf of European Hematology Association. This is an open access Abstract Book distributed under the Attribution-NonCommercial-NoDerivs (CC BY-NC-ND), which allows third parties to download the articles and share them with others as long as they credit the author and the Abstract Book, but they cannot change the content in any way or use them commercially.
**Abstract Book Citations:** Authors, Title, HemaSphere, 2026;10:(S1):pages. The abstract book DOIs can be found on the "Author Information" page.
**Disclaimer:** Articles published in the journal HemaSphere exclusively reflect the opinions of the authors. The authors are responsible for all content in their abstracts including accuracy of the facts, statements, citing resources, etc.

**Results**

n=5 patients treated with TEC, all male, age range 52-79 years, median 57 years. All triple class exposed, RR disease; range of disease duration at initiation of TEC 2-9 years, median disease duration 3 years.
n=2/5 IgG Kappa , n=1/5 IgG Lambda, n=1/5 light chain disease,n=1/5 IgA lambda .
n=1/5 had plasmacytomatous disease.
ECOG score prior to treatment: range 0-2,median score 1.
n=5/5, ISS score =3.
LDH normal at treatment initiation:range 137-188 U/L, median 160 U/L.
Beta2 microglobulin level - range: 1.5-4.01 mg/L, median 1.93mg/L.
All serum creatinine levels, normal range at baseline, all normocalcaemic.
FISH at baseline, 17p deletion in n=4/5, and the following were also documented: t(4,14), tetraploidy, 1q+, 14q+, 15q+, 11q13, 4q32, 13qdel, 16qdel.
n=5/5 received prophylaxis for PJP, HSV/VZV & subcutaneous (SC ) or intravenous (IV) immunoglobulin (Ig) .
n=5/5 were admitted for initial dose ramping,receiving CRS prophylaxis with tociluzimab, dexamethasone & acetaminophen. n=4/5 had no CRS, n=1/5 had grade 1 CRS at 36 hours post 1st dose TEC, receiving 1 further dose tociluzimab . No further tociluzimab was required, no further CRS prophylaxis was given in the remainder of the dose ramp up phase. N=5 /5 had no evidence of immune effector cell-associated neurotoxicity syndrome (ICANS).
n=2/5 tumour flair
First treatment response seen: n=5/5 4 wks. Best response : n=3/5 4 wks, n=1/5 6 wks, n=1/5 6 months.
Serious infection :n=2/5 , both 4 months from initiation, with anti infectives/Ig.n=1/2 grade 3 febrile neutropenia, bacterial sepsis(staphlococcus aureus, streptococcus australis on blood culture (BC)), good response to antiinfectives & recovery; the other, n=1/2 no neutropenia, severev pharyngeal infection, negative BC. On recovery, n=2/2 ,previous best response to TEC at 4 weeks, restarted monthly TEC (no further infections)
Neutropenia : n=4/5. n=1/4, grade 3 + treatment interruption; n=1/4 grade 3 , had 2 weeks GCSF,resolving pre next TEC; n=1/4 grade 2 , 1 weeks duration (not treated, no TEC delay); n=1/4 grade1, 4 weeks duration.
TEC dosing schedule: n=3/5 weekly for 3 months, fortnightly 3 months ,then monthly.
n=2/5 :severe infections at 4 months , resumed monthly TEC .

**Summary/Conclusion**

DGH TEC initiation achievable, tociluzimab mitigates CRS, with no ICANS observed.
Requires anti infective prophylaxis/ Ig; n=2/5 severe infection - on recovery TEC reinitiated.
Responses ongoing, treatment well tolerated.

**Copyright Information:** (Online) ISSN: 2572-9241
©2026 The Author(s). HemaSphere is published by John Wiley & Sons Ltd on behalf of European Hematology Association. This is an open access Abstract Book distributed under the Attribution-NonCommercial-NoDerivs (CC BY-NC-ND), which allows third parties to download the articles and share them with others as long as they credit the author and the Abstract Book, but they cannot change the content in any way or use them commercially.
**Abstract Book Citations:** Authors, Title, HemaSphere, 2026;10:(S1):pages. The abstract book DOIs can be found on the "Author Information" page.
**Disclaimer:** Articles published in the journal HemaSphere exclusively reflect the opinions of the authors. The authors are responsible for all content in their abstracts including accuracy of the facts, statements, citing resources, etc.

### (PB3311) SAFETY AND RELIABILITY OF ISATUXIMAB SUBCUTANEOUS ON-BODY INJECTOR: RESULTS ACROSS THE PHASE 3 IRAKLIA, PHASE 2 IZALCO AND PHASE 1B TCD15484 TRIALS

**Topic:** 14. Myeloma and other monoclonal gammopathies - Clinical

Xavier Leleu*[1], Claudio Cerchione[2], Sikander Ailawadhi[3], Jiri Minarik[4], Ivan Špička[5], Albert Oriol[6], Enrique M. Ocio[7], Richard Leblanc[8], Gurdeep Parmar[9], Eduardo Yanez[10], Carmen Cao[11], Christine Rojas[12], Zhongjun Xia[13], Yafei Wang[14], Umer Khan[15], Maya Stefanova-Urena[15], Florence Suzan[16], Philippe Moreau[17], Hang Quach[18]

*[1]University Hospital, Poitiers, France; [2]Hematology Unit, Istituto Romagnolo per lo Sudio dei Tumori 'Dino Amadori'-IRST IRCCS, Meldola (FC), Italy; [3]Mayo Clinic, Jacksonville, United States of America; [4]Department of Hemato-Oncology, Faculty of Medicine and Dentistry, Palacky University and University Hospital, Olomouc, Olomouc, Czechia; [5] 1st Department of Medicine – Department of Hematology, First Faculty of Medicine, Charles University and General Hospital, Prague, Czechia; [6]Institut Català d'Oncologia and Josep Carreras Research Institute, Hospital Germans Trias i Pujol, Barcelona, Spain; [7]Hospital Universitario Marqués de Valdecilla (IDIVAL), Universidad de Cantabria, Santander, Spain; [8]Hematology-Oncology and Cell Therapy University Institute, Hôpital Maisonneuve-Rosemont Research Center, Université de Montréal, Montreal, QC, Canada; [9]Illawarra Cancer Care Centre, University of Wollongong, NSW, Australia; [10]Clinical Oncology, Universidad de la Frontera, Temuco, Chile; [11]Instituto Nacional del Cancer, Santiago, Chile;[12]Centro de Investigaciones Clínicas Viña del Mar, Viña del Mar, Chile;[13]State Key Laboratory of Oncology in South China, Guangdong Provincial Clinical Research Center for Cancer, Sun Yat-Sen University Cancer Center, Guangzhou, China; [14]Tianjin Medical University Cancer Institute and Hospital, Tianjin, China;[15]Sanofi, Morristown, United States of America; [16]Sanofi, R&D, Vitry-sur-Seine, France; [17]University Hospital Hôtel-Dieu, Nantes, France; [18]Clinical Hematology Service, St. Vincent's Hospital Melbourne and University of Melbourne, Fitzroy, Australia;*

### Background

Subcutaneous (SC) drug delivery options have become more widely available in multiple myeloma (MM). SC delivery of isatuximab (Isa), an anti-CD38 monoclonal antibody, has been investigated across clinical trials via a novel hands-free on-body injector (OBI). Isa SC OBI features a small, retractable needle, establishing the first device-enabled delivery of an oncologic treatment. The Phase 3 IRAKLIA, Phase 2 IZALCO, and Phase 1b (TCD15484) trials consistently demonstrated similar efficacy and safety of Isa SC OBI vs Isa IV in relapsed/refractory MM (RRMM) patients (pts) except a notable lower rate of systemic infusion reactions with Isa SC OBI.

### Aims

Here, we present a cross-trial analysis exploring the safety and reliability of Isa SC OBI in RRMM pts.

### Methods

Pts with RRMM in IRAKLIA (N=263) and the Phase 1b expansion cohort (N=22) received 1400 mg Isa SC OBI plus pomalidomide and dexamethasone (d). In IZALCO, RRMM pts (N=64) received 1400 mg Isa SC OBI + carfilzomib (K)-d. In all three trials, patients provided informed consent prior to enrollment. Pts were monitored for 4 hrs (IRAKLIA) or 1-2 hrs (IZALCO) following first administration and for 1 hr for the following cycle (C) 1 administrations to assess local tolerability. Pre-infusion medications included montelukast (C1), steroids, acetaminophen and H1 antihistamines. Pts without systemic infusion-related reactions (IRRs) after 4 consecutive administrations of Isa SC OBI had subsequent pre-medication reassessed at investigator's discretion. Safety and device performance explored in this cross-trial analysis of company sponsored studies were assessed in the Isa SC OBI cohorts by the duration of injection, IRRs, local injection-site reactions (ISRs), and injection/device success, which were defined similarly across all trials.

**Copyright Information:** (Online) ISSN: 2572-9241
©2026 The Author(s). HemaSphere is published by John Wiley & Sons Ltd on behalf of European Hematology Association. This is an open access Abstract Book distributed under the Attribution-NonCommercial-NoDerivs (CC BY-NC-ND), which allows third parties to download the articles and share them with others as long as they credit the author and the Abstract Book, but they cannot change the content in any way or use them commercially.
**Abstract Book Citations:** Authors, Title, HemaSphere, 2026;10:(S1):pages. The abstract book DOIs can be found on the "Author Information" page.
**Disclaimer:** Articles published in the journal HemaSphere exclusively reflect the opinions of the authors. The authors are responsible for all content in their abstracts including accuracy of the facts, statements, citing resources, etc.

**Results**

The median duration of Isa SC OBI injection was 13 mins in IRAKLIA, 12 mins in IZALCO, and 10 mins in the Phase 1b trial (6426 total injections, N=349 pts). IRRs across trials occurred in 6/6426 (0.09%) injections and 4/349 (1.15%) pts. IRRs were mostly grade (G)1 (3/6426, 0.05%) with 1 G3 (0.02%); none leading to discontinuation. In IRAKLIA, after C1, 80/263 (30.4%) pts did not require premedication (931/5145 injections, 18.1%); no IRRs occurred in this group. Across trials, ISRs occurred in 21/349 (6.02%) pts resulting in 35/6426 (0.54%) ISR events. Most ISRs were G1 (33, 0.51%), the remaining were G2 (2, 0.03%) and generally occurred on the day of injection. 99.9% (6421/6426) of injections in 98.6% (344/349) pts were successful (completed without interruption) across all trials.

**Summary/Conclusion**

These findings support overall low, self-limiting rates of IRRs and ISRs across trials, with >6000 (99.9%) Isa SC OBI injections successfully delivered. Isa SC OBI shows reproducible safety and reliability in IRAKLIA, IZALCO and Phase 1b trials, supporting it as a novel administration option for MM pts and its potential for future exploration in at-home administration.

© 2026 American Society of Clinical Oncology, Inc. Reused with permission. This abstract was accepted and previously presented at the 2026 ASCO Annual Meeting. All rights reserved.

**Copyright Information:** (Online) ISSN: 2572-9241
©2026 The Author(s). HemaSphere is published by John Wiley & Sons Ltd on behalf of European Hematology Association. This is an open access Abstract Book distributed under the Attribution-NonCommercial-NoDerivs (CC BY-NC-ND), which allows third parties to download the articles and share them with others as long as they credit the author and the Abstract Book, but they cannot change the content in any way or use them commercially.
**Abstract Book Citations:** Authors, Title, HemaSphere, 2026;10:(S1):pages. The abstract book DOIs can be found on the “Author Information” page.
**Disclaimer:** Articles published in the journal HemaSphere exclusively reflect the opinions of the authors. The authors are responsible for all content in their abstracts including accuracy of the facts, statements, citing resources, etc.

### (PB3312) "ANTI-CD38–VRD QUADRUPLET REGIMENS IN GERIATRIC PATIENTS WITH MULTIPLE MYELOMA INELIGIBLE FOR AUTOLOGOUS TRANSPLANTATION: A REAL-WORLD STUDY OF FEASIBILITY AND SAFETY."

**Topic:** 14. Myeloma and other monoclonal gammopathies - Clinical

Belkacem Mansour*[1], Xavier Leleu[2]

*[1]JACQUES COEUR HOSPITAL, HEMATOLOGY-ONCOLOGY, BOURGES, France; [2]CHU POITIERS, HEMATOLOGY AND CELLULAR THERAPY, POITIERS, France;*

### Background

Anti-CD38–VRd–based quadruplet therapy in geriatric patients with transplant-ineligible multiple myeloma: a real-world evaluation of feasibility and safety.

### Aims

Assessment of the impact of comprehensive geriatric oncology evaluation on therapeutic decision-making in older patients in routine clinical practice: available data remain scarce, particularly among the very elderly. We herein report the experience of our center in a geriatric population.

### Methods

This observational study included 21 patients aged 75 to 85 years, including 6 patients aged ≥80 years, treated for newly diagnosed multiple myeloma ineligible for autologous stem cell transplantation between January and December 2025. Patients received Isa-VRd (n=7) or Dara-VRd (n=14) (Table 1).

The evaluated endpoints included depth of response according to IMWG criteria, treatment tolerability, progression-free survival, and overall survival, estimated using the Kaplan–Meier method. A comprehensive geriatric assessment was performed on patients aged ≥80 years.

**Copyright Information:** (Online) ISSN: 2572-9241
©2026 The Author(s). HemaSphere is published by John Wiley & Sons Ltd on behalf of European Hematology Association. This is an open access Abstract Book distributed under the Attribution-NonCommercial-NoDerivs (CC BY-NC-ND), which allows third parties to download the articles and share them with others as long as they credit the author and the Abstract Book, but they cannot change the content in any way or use them commercially.
**Abstract Book Citations:** Authors, Title, HemaSphere, 2026;10:(S1):pages. The abstract book DOIs can be found on the "Author Information" page.
**Disclaimer:** Articles published in the journal HemaSphere exclusively reflect the opinions of the authors. The authors are responsible for all content in their abstracts including accuracy of the facts, statements, citing resources, etc.

### Results

The clinical and biological characteristics of the patients are presented in Table 1. After one cycle of treatment, 80% of patients treated with Isa-VRd and 71% of those treated with Dara-VRd had achieved at least a very good partial response (VGPR). After four cycles, all patients (100%) achieved a deep response, defined as VGPR or complete response (CR) (Figure 1).

Six patients aged over 80 years were included in the study; five received Dara-VRd and one received Isa-VRd. The results of the comprehensive geriatric assessment are detailed in Table 3. According to the Balducci decision-making algorithm, all patients aged over 80 years were considered eligible for standard oncologic treatment, confirming their fitness to receive a quadruplet regimen combining an anti-CD38 monoclonal antibody with VRd.

Progression-free survival (PFS), estimated using the Kaplan–Meier method, showed a 12-month PFS comparable to that reported in the academic BENEFIT and CEPHEUS studies, with the median PFS not reached at the time of analysis (80% for the Bourges Hospital cohort versus 85.2% in the BENEFIT study by Leleu et al.) (Figure 2). Similarly, overall survival (OS) analysis demonstrated a high 12-month OS consistent with published data, with the median OS not reached (85% for the Bourges Hospital cohort versus 91% in the BENEFIT study cohort [Leleu et al.]) (Figure 3).

Treatment tolerability is summarized in Table 2. Under Isa-VRd, the main adverse events observed were grade 2 anemia, grade 1 thrombocytopenia, grade 2 hypertension, and one case of esophageal abscess. Under Dara-VRd, grade 3 hematologic toxicity was reported in one patient, as well as one episode of arrhythmia. Overall tolerability was considered acceptable, including among patients aged over 80 years.

### Summary/Conclusion

In real-world settings, anti-CD38–VRd quadruplet regimens are effective and well tolerated in geriatric patients with transplant-ineligible multiple myeloma, including those aged over 80 years following an appropriate comprehensive geriatric assessment.

These findings further support the role of these regimens in the routine management of fit older patients.

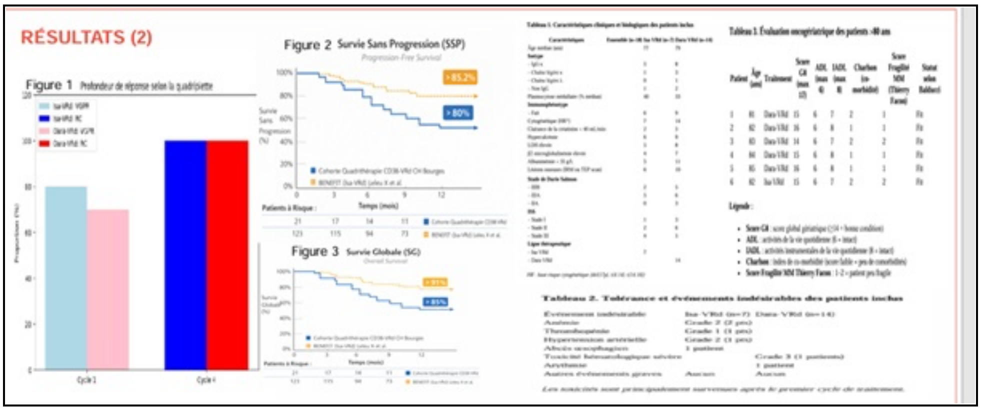

**Copyright Information:** (Online) ISSN: 2572-9241
©2026 The Author(s). HemaSphere is published by John Wiley & Sons Ltd on behalf of European Hematology Association. This is an open access Abstract Book distributed under the Attribution-NonCommercial-NoDerivs (CC BY-NC-ND), which allows third parties to download the articles and share them with others as long as they credit the author and the Abstract Book, but they cannot change the content in any way or use them commercially.
**Abstract Book Citations:** Authors, Title, HemaSphere, 2026;10:(S1):pages. The abstract book DOIs can be found on the "Author Information" page.
**Disclaimer:** Articles published in the journal HemaSphere exclusively reflect the opinions of the authors. The authors are responsible for all content in their abstracts including accuracy of the facts, statements, citing resources, etc.

### (PB3313) DARATUMUMAB-BASED THERAPY IN HIGH-RISK CYTOGENETIC MULTIPLE MYELOMA: A REAL-WORLD RETROSPECTIVE CYTOGENETIC SUBGROUP ANALYSIS

**Topic:** 14. Myeloma and other monoclonal gammopathies - Clinical

Hengyu Liu[1], Bo Lu*[1]

[1] *The Seventh Affiliated Hospital, Sun Yat-sen University, Shenzhen, Hematopathology, Shenzhen, Guangdong, China;*

### Background

High-risk cytogenetic abnormalities (HRCAs)—including del(17p), t(4;14), t(14;16), and 1q21 +(1q21 gain/amplification)—are associated with poor prognosis in multiple myeloma (MM). Although daratumumab-based regimens improve MM outcomes, their effectiveness across specific high-risk cytogenetic subgroups remains unclear.

### Aims

This multicenter retrospective study evaluated the real-world efficacy of daratumumab in high-risk MM (HRMM).

### Methods

Forty-one HRMM patients carrying ≥1 HRCA and treated with daratumumab-containing combinations were analyzed. Endpoints included progression-free survival (PFS), overall survival (OS), overall response rate (ORR), ≥very good partial response (VGPR), and progressive disease (PD). Subgroup analyses were performed based on cytogenetic abnormality and HRCA count.

### Results

Among 41 patients, ORR was 75.6%, with 63.4% achieving ≥VGPR and 17.1% experiencing PD. The median PFS was 18 months. OS was not reached at a median follow-up of 10 months. Outcomes varied markedly by cytogenetic subgroup. Patients with t(4;14) had significantly lower response rates (ORR 41.7% vs 89.7% for those without t(4,14), $P = 0.003$; ≥VGPR 33.3% vs 75.9%, $P = 0.015$) and shorter median PFS (8 vs 18 months, $P = 0.04$). In contrast, patients with 1q21 + showed higher ORR (82.4% vs 42.9%, $P = 0.047$) and longer PFS (20 vs 8 months, $P = 0.012$) than those without 1q21+.

### Summary/Conclusion

Daratumumab-based regimens show meaningful activity in HRMM but with substantial variation across cytogenetic subgroups. In particular, t(4;14) conferred inferior responses and shorter PFS. Conversely, 1q21+ myeloma was associated with higher response rates and extended PFS, suggesting a distinct benefit from daratumumab in that subgroup.

**Copyright Information:** (Online) ISSN: 2572-9241
©2026 The Author(s). HemaSphere is published by John Wiley & Sons Ltd on behalf of European Hematology Association. This is an open access Abstract Book distributed under the Attribution-NonCommercial-NoDerivs (CC BY-NC-ND), which allows third parties to download the articles and share them with others as long as they credit the author and the Abstract Book, but they cannot change the content in any way or use them commercially.
**Abstract Book Citations:** Authors, Title, HemaSphere, 2026;10:(S1):pages. The abstract book DOIs can be found on the “Author Information” page.
**Disclaimer:** Articles published in the journal HemaSphere exclusively reflect the opinions of the authors. The authors are responsible for all content in their abstracts including accuracy of the facts, statements, citing resources, etc.

### (PB3314) DARATUMUMAB-BASED THERAPY IN NEWLY DIAGNOSED MULTIPLE MYELOMA: A SINGLE-CENTER REAL-WORLD STUDY OF DYNAMIC TRANSPLANT ELIGIBILITY, DEEP RESPONSE, AND PROGNOSIS IN THE 2025 IMS-IMWG ERA

**Topic:** 14. Myeloma and other monoclonal gammopathies - Clinical

Lijing Shen*[1], Xiaofeng Han[1], Fei Xiao[1], Lu Zhong[1], Minyue Zhang[1], Shasha Lu[1], Yi Fang[1], Beiwen Ni[1], Jian Hou[1], Juan DU[1]

[1]*Ren Ji Hospital, Shanghai Jiao Tong University School of Medicine, Haematology, Shanghai, China;*

### Background

Multiple myeloma (MM) is an incurable plasma cell malignancy. In recent years, the therapeutic goal of induction therapy for newly diagnosed multiple myeloma (NDMM) has been defined as achieving sustained deep remission to reduce the risk of clonal evolution. As the first-in-class anti-CD38 monoclonal antibody, daratumumab (Dara) exerts potent multi-faceted anti-tumor effects and significantly enhances the depth of remission in patients. As early as 2018, our center pioneered the exploratory use of daratumumab-based induction regimens in clinical practice for NDMM patients with comorbidities, advanced age, high-risk cytogenetic abnormalities and other conditions, and established a long-term continuous follow-up dataset.

### Aims

This real-world study aimed to address key clinical questions: 1) Efficacy of daratumumab (Dara)-based regimens by transplant status; 2) Dynamic frailty assessment for transplant eligibility; 3) Correlation of pre-transplant remission depth with post-transplant outcomes; 4) Validation of 2025 IMS-IMWG high-risk criteria; 5) Safety of subcutaneous (SC) vs. intravenous (IV) Dara.

### Methods

A single-center retrospective analysis of 58 NDMM patients receiving Dara-based induction therapy (2018–2025) was done, stratified into TE (n=10) and TIE (n=48). Baseline features, treatment responses (2016 IMWG), MRD status (sensitivity $\geq 10^{-6}$), PFS, OS and AEs were collected and analyzed. Dynamic frailty was assessed via IMWG-FI at baseline and pre-transplant. Cox models identified independent PFS prognostic factors, validated against 2025 IMS-IMWG high-risk cytogenetics (17p deletion, t(4;14), gain 1q, triple-hit). Efficacy and safety endpoints were compared between TE/TIE cohorts and SC/IV Dara groups. Correlation analysis explored the link between pre-transplant remission depth and post-transplant clinical outcomes.

### Results

Median follow-up was 46.5 months (TE: 32.0 months, diagnosed 2022–2025; TIE: 52.0 months, diagnosed 2018–2019). Overall ORR was 96.6%, with 41.4% achieving MRD⁻ remission. TE patients had a 70.0% post-transplant MRD⁻ rate, mPFS and OS not reached (NR). TIE patients had an ORR of 95.8% and MRD⁻ rate of 37.5%, with mPFS 1753 days and OS NR. 30% of TE patients improved from intermediate-fit to fit post-Dara induction. Pre-transplant VGPR+ strongly correlated with post-transplant deep remission (r=0.83, P<0.001) and superior PFS (HR=0.15, P=0.024). Multivariate Cox analysis confirmed 2025 IMS-IMWG high-risk cytogenetics (HR=2.89, P=0.020), pre-transplant remission ≤PR (HR=4.12, P=0.013) and IMWG-FI ≥2 (HR=3.07, P=0.014) as independent adverse PFS prognostic factors. SC Dara had a markedly lower infusion reaction rate than IV Dara (10.3% vs. 28.6%, P=0.042).

**Copyright Information:** (Online) ISSN: 2572-9241
©2026 The Author(s). HemaSphere is published by John Wiley & Sons Ltd on behalf of European Hematology Association. This is an open access Abstract Book distributed under the Attribution-NonCommercial-NoDerivs (CC BY-NC-ND), which allows third parties to download the articles and share them with others as long as they credit the author and the Abstract Book, but they cannot change the content in any way or use them commercially.
**Abstract Book Citations:** Authors, Title, HemaSphere, 2026;10:(S1):pages. The abstract book DOIs can be found on the "Author Information" page.
**Disclaimer:** Articles published in the journal HemaSphere exclusively reflect the opinions of the authors. The authors are responsible for all content in their abstracts including accuracy of the facts, statements, citing resources, etc.

### Summary/Conclusion

In real-world practice, Dara-based therapy yields deep, durable remission in TE and TIE NDMM patients. Dynamic frailty assessment post-Dara induction optimizes transplant eligibility by improving frailty in intermediate-fit TE patients, and pre-transplant VGPR+ strongly predicts favorable post-transplant outcomes. The 2025 IMS-IMWG high-risk cytogenetic criteria reliably stratify prognosis in Dara-treated NDMM, with high-risk cytogenetics, pre-transplant remission ≤PR and IMWG-FI ≥2 as independent adverse prognostic factors. SC Dara cuts infusion reaction rates markedly, boosting treatment safety and feasibility, and is thus the preferred formulation for NDMM clinical use.

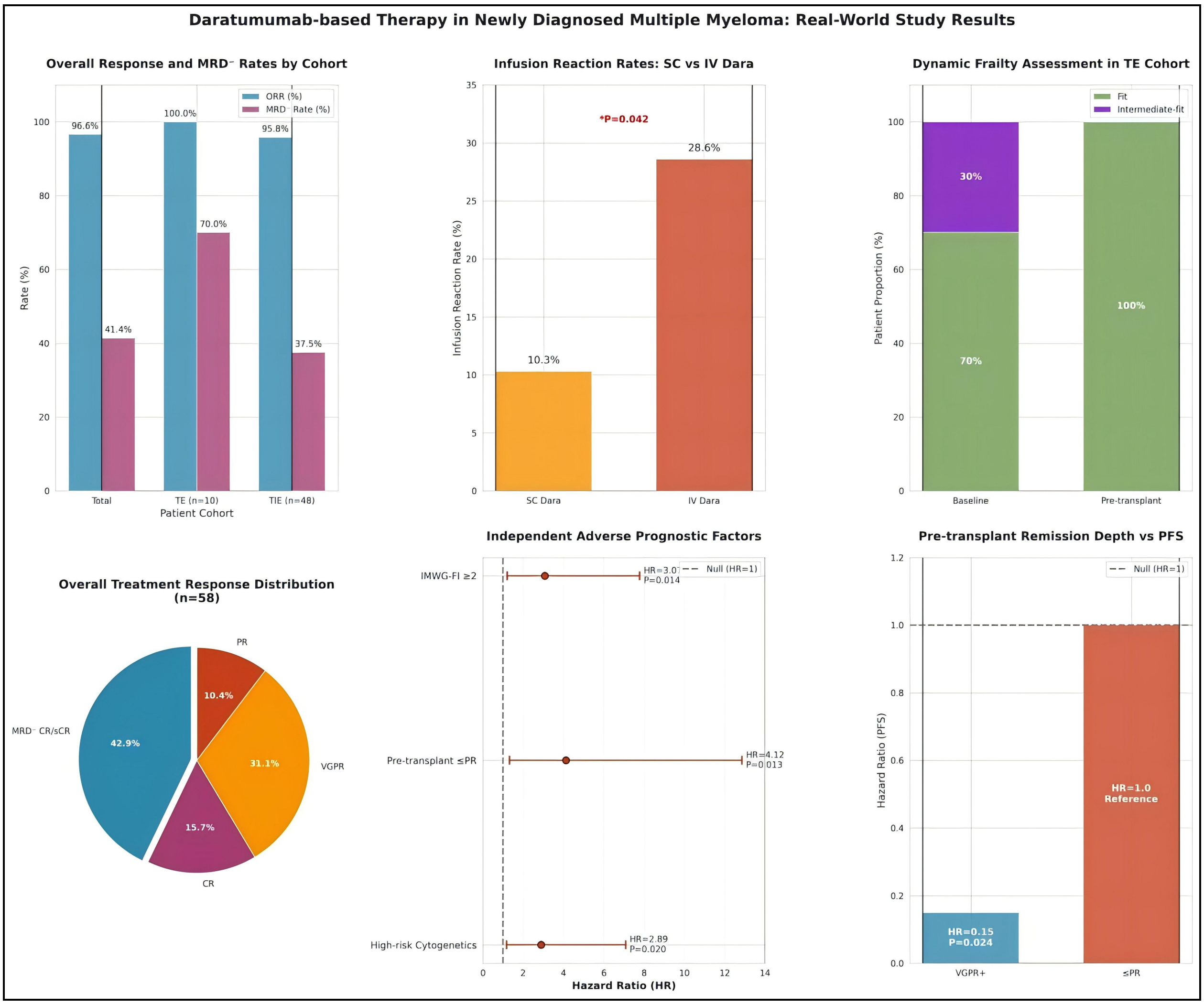

**Copyright Information:** (Online) ISSN: 2572-9241
©2026 The Author(s). HemaSphere is published by John Wiley & Sons Ltd on behalf of European Hematology Association. This is an open access Abstract Book distributed under the Attribution-NonCommercial-NoDerivs (CC BY-NC-ND), which allows third parties to download the articles and share them with others as long as they credit the author and the Abstract Book, but they cannot change the content in any way or use them commercially.
**Abstract Book Citations:** Authors, Title, HemaSphere, 2026;10:(S1):pages. The abstract book DOIs can be found on the "Author Information" page.
**Disclaimer:** Articles published in the journal HemaSphere exclusively reflect the opinions of the authors. The authors are responsible for all content in their abstracts including accuracy of the facts, statements, citing resources, etc.

### (PB3315) CLINICAL OBSERVATION OF POMALIDOMIDE COMBINED WITH DEXAMETHASONE IN THE TREATMENT OF POEMS SYNDROME UNSUITABLE FOR AUTOLOGOUS HEMATOPOIETIC STEM CELL TRANSPLANTATION

**Topic:** 14. Myeloma and other monoclonal gammopathies - Clinical

Xiaofang Wei[1], Qike Zhang*[1], Qiaolin Chen[1], Fei Liu[1], Yang Chen[1], Jinli Jian[1], Youfan Feng[1], Yuan Fu[1]

*[1]GANSU PROVINCIAL HOSPITAL, Lanzhou, China;*

### Background

POEMS syndrome is a clinical syndrome characterized by predominantly sensorimotor peripheral neuropathy, frequently accompanied by multisystem involvement and associated with plasma cell dyscrasia. Its name is derived from the first letters of its five major clinical manifestations: polyneuropathy (P), organomegaly (O), endocrinopathy (E), monoclonal gammopathy (M), and skin changes (S). POEMS syndrome has an incidence of less than 1 per 100,000 population and is thus a rare disease, characterized by multisystem involvement and high clinical heterogeneity. Pomalidomide, which is a third-generation analogue of thalidomide, exhibits greater anti-myeloma efficacy compared to lenalidomide.

### Aims

To explore the efficacy and safety of the pomalidomide plus dexamethasone regimen (Pd regimen) in the treatment of patients with POEMS syndrome who are unsuitable for autologous hematopoietic stem cell transplantation (auto-HSCT).

### Methods

We collected the clinical data of 9 patients with POEMS syndrome who were unsuitable for autologous hematopoietic stem cell transplantationand received Pd regimen at Gansu Provincial People's Hospital from January 2021 to August 2025. A retrospective analysis was performed to summarize their clinical characteristics and prognosis.

### Results

Among the 9 patients, 7 were male and 2 were female, with a median age of 63 (range 51-76) years.There was 1 patient in the low-risk group, 5 in the intermediate-risk group, and 3 in the high-risk group. All 9 patients received at least 6 cycles of pomalidomide plus dexamethasone. The median follow-up time was 26 (range 8-50) months.The 2-year progression-free survival (PFS) rate was 100%, and no patient experienced disease progression. The overall survival (OS) rate was 100%. The overall hematological response rate was 77.8% (7 patients), with a complete response rate of 55.6% (5 patients).All patients achieved neurological remission, and the median Overall Neurological Severity Score (ONLS) decreased from 5 (range 3-10) to 1 (range 0-4).The serum vascular endothelial growth factor (VEGF) response rate was 100%, and the serum VEGF level normalized in 77.8% (7 patients) of the patients.The pomalidomide plus dexamethasone regimen was well tolerated, with no treatment-related deaths and no grade 3 or 4 hematological or non-hematological toxicities.

### Summary/Conclusion

The pomalidomide plus dexamethasone regimen is safe and effective for the treatment of patients with POEMS syndrome who are unsuitable for autologous hematopoietic stem cell transplantation.

**Copyright Information:** (Online) ISSN: 2572-9241
©2026 The Author(s). HemaSphere is published by John Wiley & Sons Ltd on behalf of European Hematology Association. This is an open access Abstract Book distributed under the Attribution-NonCommercial-NoDerivs (CC BY-NC-ND), which allows third parties to download the articles and share them with others as long as they credit the author and the Abstract Book, but they cannot change the content in any way or use them commercially.
**Abstract Book Citations:** Authors, Title, HemaSphere, 2026;10:(S1):pages. The abstract book DOIs can be found on the "Author Information" page.
**Disclaimer:** Articles published in the journal HemaSphere exclusively reflect the opinions of the authors. The authors are responsible for all content in their abstracts including accuracy of the facts, statements, citing resources, etc.

### (PB3316) EVALUATION OF SAFETY AND EFFICACY OF DENOSUMAB IN PATIENTS WITH MULTIPLE MYELOMA IN REAL LIFE: A SINGLE-CENTER EXPERIENCE

**Topic:** 14. Myeloma and other monoclonal gammopathies - Clinical

Alessandra Romano*[1, 2], Mohammadreza Khosropoor[2], Bruno Garibaldi[2], Claudia Bellofiore[2], Concetta Conticello[2], Francesco Di Raimondo[1, 2]

*[1]Università degli Studi di Catania, Dipartimento di Chirurgia e Specialità Medico Chirurgiche, Catania, Italy;[2]AOU Policlinico Rodolico San Marco, UOC Ematologia, Catania, Italy;*

### Background

A key clinical feature of multiple myeloma (MM) is osteolytic bone disease, predisposing to skeletal-related events (SREs). Renal impairment (RI), a cardinal feature of MM, further complicates treatment strategies. A phase 3 trial showed that the bone-directed agent denosumab 120 mg given subcutaneous every 4 weeks was noninferior to zoledronate in delaying time to first SRE in patients with newly diagnosed multiple myeloma (NDMM).

### Aims

Since efficacy and safety in patients with moderate-to-severe RI is underexplored in relapsed-refractory patients in real-life settings we have designed a prospective study in a single center.

### Methods

We designed a prospective study to evaluate denosumab efficacy and safety in NDMM and RRMM patients with moderate-to-severe RI (eGFR based on CKD-EPI <45 ml/min/1.73m2).

### Results

This analysis included 70 MM patients: 50 (71.4%) newly-diagnosed (NDMM) and 20 (28.5%) relapsed/refractory (RRMM; median lines of prior treatment: 3); median age 72 years (range 48 – 90); 42 (60.0%) patients were males. All patients had bone disease at the time of denosumab initiation and 14/20 (70%) RRMM patients had been given zoledronic acid previously. Median eGFR was 36.75 (7.4-45.0) ml/min/1.73m2. Only 2 patients were on dialysis; concomitant hypercalcemia was present in 6 (8.5%) patients. Patients with hypercalcemia at baseline received one dose of zoledronic acid, dose-adjusted based on kidney function, due to restrictions in prescription by local regulatory agency AIFA. All patients received denosumab at a dose of 120 mg monthly, with normal baseline calcium levels.
Best response to MM treatment for NDMM patients treated with daratumumab-based regimens was as follows: 18 (36.0%) patients achieved ≥CR, 20 (40.0%) VGPR and 11 (22.0%) PR; median time to response was 40 (28-56) days. Response rates were lower in RRMM patients, as follows: 4 (20.0%) patients achieved ≥CR, 8 (40.0%) VGPR and 8 (40.0%) PR; median time to response was 60 (32-90) days.
After a median follow-up of 30.3 (2.3-38.2) months, 46 patients (65.7%) are still receiving denosumab, while 24 (34.2%) have discontinued, mainly due to dental infections (N=8, 11.4%), comorbidities (N=3, 4.2) and death (N=11, 15.7%), mostly attributed to disease progression (72.8%).
In line with previous reports, serious toxicities associated with denosumab include osteonecrosis of the jaw (N=6, 8.6%), hypocalcemia (N=2, 2.80%), and atypical femoral fracture events (N=3, 4.2%), with no significant differences between NDMM and RRMM patients.

**Copyright Information:** (Online) ISSN: 2572-9241
©2026 The Author(s). HemaSphere is published by John Wiley & Sons Ltd on behalf of European Hematology Association. This is an open access Abstract Book distributed under the Attribution-NonCommercial-NoDerivs (CC BY-NC-ND), which allows third parties to download the articles and share them with others as long as they credit the author and the Abstract Book, but they cannot change the content in any way or use them commercially.
**Abstract Book Citations:** Authors, Title, HemaSphere, 2026;10:(S1):pages. The abstract book DOIs can be found on the “Author Information” page.
**Disclaimer:** Articles published in the journal HemaSphere exclusively reflect the opinions of the authors. The authors are responsible for all content in their abstracts including accuracy of the facts, statements, citing resources, etc.

**Summary/Conclusion**

Despite the emergence of novel and effective antitumor therapies, there remains a strong rationale for the clinical utility of denosumab for SRE prevention in both NDMM and RRMM settings.

**Copyright Information:** (Online) ISSN: 2572-9241
©2026 The Author(s). HemaSphere is published by John Wiley & Sons Ltd on behalf of European Hematology Association. This is an open access Abstract Book distributed under the Attribution-NonCommercial-NoDerivs (CC BY-NC-ND), which allows third parties to download the articles and share them with others as long as they credit the author and the Abstract Book, but they cannot change the content in any way or use them commercially.
**Abstract Book Citations:** Authors, Title, HemaSphere, 2026;10:(S1):pages. The abstract book DOIs can be found on the "Author Information" page.
**Disclaimer:** Articles published in the journal HemaSphere exclusively reflect the opinions of the authors. The authors are responsible for all content in their abstracts including accuracy of the facts, statements, citing resources, etc.

### (PB3317) TRANSPLANT INELIGIBLE MULTIPLE MYELOMA A SYSTEMATIC REVIEW MEA-ANALYSIS COMPARING CYCLOPHOSPHAMIDE BORTEZOMIB DEXAMETHASONE AND DARATUMUMAB LENALIDOMIDE DEXAMETHASONE WITH FOCUS ON RENAL IMPAIRMENT

**Topic:** 14. Myeloma and other monoclonal gammopathies - Clinical

Mahmoud Meleha*[1], Yasmine Heremes[1], Islam Abdelkader[1]

[1]*Warrington Hospital, Haematology, Warrington, United Kingdom;*

### Background

Multiple myeloma is a common haematological malignancy, and a substantial proportion of newly diagnosed patients are not eligible for autologous stem cell transplantation due to age, frailty, or comorbidity. In transplant-ineligible newly diagnosed multiple myeloma, treatment selection must balance efficacy, toxicity, comorbidity profile, and resource availability. Cyclophosphamide, bortezomib, and dexamethasone remain widely used in routine clinical practice, while daratumumab, lenalidomide, and dexamethasone have demonstrated superior outcomes compared with lenalidomide and dexamethasone alone. However, no direct randomised comparison exists between these regimens. Renal impairment is frequent at diagnosis and represents a clinically relevant determinant of treatment choice.

### Aims

To systematically compare the efficacy and safety of cyclophosphamide bortezomib dexamethasone and daratumumab lenalidomide dexamethasone as frontline therapy in transplant ineligible newly diagnosed multiple myeloma, with specific focus on outcomes in patients with baseline renal impairment.

### Methods

We systematically searched PubMed/MEDLINE, ClinicalTrials.gov, and conference databases through December 2025 for studies evaluating CVD or DRd in MM patients. Primary outcomes included progression-free survival (PFS) and overall survival (OS). Secondary outcomes included overall response rate (ORR), complete response (CR) rate, and safety parameters. Quality assessment used the Cochrane Risk of Bias tool. Statistical analysis employed random effects models with hazard ratios (HR) and 95% confidence intervals (CI).

### Results

We identified 23 studies (CVD: 15 studies, n=1,847 patients; DRd: 8 studies, n=2,314 patients). DRd demonstrated superior efficacy in transplant-ineligible newly diagnosed MM (NDMM) with a median PFS not reached versus 9-15 months for CVD (HR 0.56, 95% CI 0.43-0.73, $p<0.001$). Five-year OS was 66.3% for DRd versus 45-55% for CVD (HR 0.68, 95% CI 0.53-0.86, $p=0.002$). ORR was significantly higher with DRd (92.9% vs 76.8%, OR 3.89, 95% CI 2.34-6.47, $p<0.001$). Grade ≥3 infections were more common with DRd (28.3% vs 17.2%, RR 1.64, 95% CI 1.32-2.04, $p<0.001$), while peripheral neuropathy favoured DRd (4.2% vs 19.6%, RR 0.21, 95% CI 0.14-0.32, $p<0.001$). In severe renal impairment (CrCl <30 mL/min), CVD showed preserved efficacy without dose adjustment

**Copyright Information:** (Online) ISSN: 2572-9241
©2026 The Author(s). HemaSphere is published by John Wiley & Sons Ltd on behalf of European Hematology Association. This is an open access Abstract Book distributed under the Attribution-NonCommercial-NoDerivs (CC BY-NC-ND), which allows third parties to download the articles and share them with others as long as they credit the author and the Abstract Book, but they cannot change the content in any way or use them commercially.
**Abstract Book Citations:** Authors, Title, HemaSphere, 2026;10:(S1):pages. The abstract book DOIs can be found on the "Author Information" page.
**Disclaimer:** Articles published in the journal HemaSphere exclusively reflect the opinions of the authors. The authors are responsible for all content in their abstracts including accuracy of the facts, statements, citing resources, etc.

**Summary/Conclusion**

DRd represents the superior treatment option for transplant-ineligible NDMM with adequate renal function, demonstrating significantly improved PFS, OS, and response rates despite higher infection risk. CVD remains the preferred regimen for patients with severe renal impairment or dialysis dependence due to its favourable pharmacokinetics and established safety profile. Treatment selection should be individualised based on renal function, comorbidities, and access to care.

**Copyright Information:** (Online) ISSN: 2572-9241
©2026 The Author(s). HemaSphere is published by John Wiley & Sons Ltd on behalf of European Hematology Association. This is an open access Abstract Book distributed under the Attribution-NonCommercial-NoDerivs (CC BY-NC-ND), which allows third parties to download the articles and share them with others as long as they credit the author and the Abstract Book, but they cannot change the content in any way or use them commercially.
**Abstract Book Citations:** Authors, Title, HemaSphere, 2026;10:(S1):pages. The abstract book DOIs can be found on the "Author Information" page.
**Disclaimer:** Articles published in the journal HemaSphere exclusively reflect the opinions of the authors. The authors are responsible for all content in their abstracts including accuracy of the facts, statements, citing resources, etc.

### (PB3318) EFFICACY AND SURVIVAL OUTCOMES OF IXAZOMIB-BASED REGIMENS IN A REAL-WORLD CHINESE COHORT RE-STRATIFIED BY IMS/IMWG 2024 CRITERIA

**Topic:** 14. Myeloma and other monoclonal gammopathies - Clinical

Ning An*[1], Miao Zheng[1], Peiling Zhang[1], Di Wang[1], Wei Huang[1], Donghua Zhang[1], Yi Tang[1], Zhenya Hong[1], Li Meng[1], Chunrui Li[1]

[1]*Department of Hematology, Tongji Hospital, Tongji Medical College, Huazhong University of Science and Technology, Wuhan, China;*

### Background

Ixazomib-based therapy is widely used in multiple myeloma (MM) and has shown clinical benefit in both trials and real-world practice, including in patients with adverse cytogenetics. With the IMS/IMWG 2024 update to high-risk disease definitions, we re-stratified a real-world cohort and reassessed outcomes with ixazomib-based regimens, focusing on patients classified as high-risk by the new criteria.

### Aims

To assess the impact of ixazomib-based therapy on treatment persistence, response depth, and overall survival in high-risk multiple myeloma patients, and to determine whether ixazomib can mitigate adverse high-risk biology under the updated IMS/IMWG 2024 definitions.

### Methods

We conducted a retrospective, single-center study of MM patients who received ≥1 cycle of ixazomib at Tongji Hospital, Tongji Medical College, Huazhong University of Science and Technology (February 2019–July 2025). Patients were reclassified using IMS/IMWG 2024 criteria. Treatment patterns, best response, and survival outcomes (PFS, OS) were collected retrospectively. The data cut-off was February 2026.

**Copyright Information:** (Online) ISSN: 2572-9241
©2026 The Author(s). HemaSphere is published by John Wiley & Sons Ltd on behalf of European Hematology Association. This is an open access Abstract Book distributed under the Attribution-NonCommercial-NoDerivs (CC BY-NC-ND), which allows third parties to download the articles and share them with others as long as they credit the author and the Abstract Book, but they cannot change the content in any way or use them commercially.
**Abstract Book Citations:** Authors, Title, HemaSphere, 2026;10:(S1):pages. The abstract book DOIs can be found on the “Author Information” page.
**Disclaimer:** Articles published in the journal HemaSphere exclusively reflect the opinions of the authors. The authors are responsible for all content in their abstracts including accuracy of the facts, statements, citing resources, etc.

**Results**

In total, 113 patients were included (median age 61 years [36–84]; 54.0% male; 41.6% ≥65 years; ISS stage III 39.8%). At ixazomib initiation, 98/113 (86.7%) had newly diagnosed MM and 15/113 (13.3%) had relapsed/refractory MM. Regimen composition was IRd in 56/113 (49.6%), Id in 14/113 (12.4%), IPd in 14/113 (12.4%), and ixazomib plus daratumumab-containing regimens in 29/113 (25.7%). Prior exposure rates were lenalidomide >1 cycle 67/113 (59.3%), bortezomib 78/113 (69.0%), and anti-CD38 25/113 (22.1%); effective anti-CD38 exposure (≥2 cycles) occurred in 21/113 (18.6%). By IMS/IMWG 2024, 63/113 (55.8%) were high-risk and 50/113 (44.2%) standard-risk; high-risk features included 1q21 abnormality in 50/113 (44.2%) and TP53 abnormality in 11/113 (9.7%). Median follow-up was 22 months (2–83). Among patients switching to ixazomib for oral convenience (n=47), 34/47 (72.3%) remained on treatment. Among those switching due to inadequate efficacy (n=49), 22/31 evaluable patients (71.0%) achieved deeper responses than pre-switch, with no progression observed by data cut-off. Among those switching due to intolerance (n=17), 10/17 (58.8%) continued therapy, with no discontinuations attributed to adverse events. Among 109 evaluable patients, best responses were ≥CR in 54/109 (49.5%), VGPR in 21/109 (19.3%), PR in 25/109 (22.9%), MR in 2/109 (1.8%), and PD in 7/109 (6.4%); there were nine deaths. Median PFS was 31 months (95% CI 19.4–42.6) and median OS was not reached. High-risk patients had a median PFS of 24 months (95% CI 18.5–29.5) and a median OS of 57 months (95% CI 33.0–81.0), whereas median PFS and OS were not reached in standard-risk patients. PFS did not differ significantly between risk groups (P=0.168), while OS differed significantly (P=0.013), suggesting ixazomib-based therapy may partially mitigate adverse high-risk biology under IMS/IMWG 2024 definitions.

**Summary/Conclusion**

In routine practice, ixazomib-based regimens were associated with strong treatment persistence, favorable tolerability, and durable and/or deepening responses. In patients classified as high-risk by IMS/IMWG 2024 criteria, the observed survival signal warrants confirmation in larger cohorts, supporting ixazomib as a practical option for continuous oral therapy.

**Copyright Information:** (Online) ISSN: 2572-9241
©2026 The Author(s). HemaSphere is published by John Wiley & Sons Ltd on behalf of European Hematology Association. This is an open access Abstract Book distributed under the Attribution-NonCommercial-NoDerivs (CC BY-NC-ND), which allows third parties to download the articles and share them with others as long as they credit the author and the Abstract Book, but they cannot change the content in any way or use them commercially.
**Abstract Book Citations:** Authors, Title, HemaSphere, 2026;10:(S1):pages. The abstract book DOIs can be found on the “Author Information” page.
**Disclaimer:** Articles published in the journal HemaSphere exclusively reflect the opinions of the authors. The authors are responsible for all content in their abstracts including accuracy of the facts, statements, citing resources, etc.

### (PB3319) STEADY-STATE HEMATOPOIETIC CELL MOBILIZATION FOR NEWLY DIAGNOSED MULTIPLE MYELOMA PATIENTS RECEIVING DARATUMUMAB-BASED VERSUS NON DARATUMUMAB-BASED INDUCTION THERAPY: A REAL-WORLD EXPERIENCE

**Topic:** 14. Myeloma and other monoclonal gammopathies - Clinical

Li Bao[1], Xin Zhao*[1]

*[1]Beijing Jishuitan Hospital, Capital Medical University, Beijing, China;*

### Background

Daratumumab-based induction therapy have recently become the standard of care for patients with newly diagnosed multiple myeloma who are candidates for autologous stem cell transplantation. Previous reports have shown that daratumumab might impair stem cell mobilization and collection, leading to an increased requirement for plerixafor use and impaired stem cell engraftment. In the context of Daratumumab, protocols for mobilization vary significantly.

### Aims

Therefore, we conducted a single-center retrospective analysis in order to evaluate steady-state stem cell mobilization and harvest in patients receiving daratumumab-based induction therapy versus non daratumumab-based induction therapy.

### Methods

From Jan 2023 till May 2025, we retrospectively reviewed patients receiving daratumumab-based induction therapy and non daratumumab-based induction therapy followed by stem cell mobilization in a steady-state protocol using 10 μg/kg granulocyte colony-stimulating factor (G-CSF) for 5 days. Propensity score 1:5 matching with replacement (ie, that each participant treated with daratumumab was matched to the participant with the closest propensity score treated with non-daratumumab therapy, even if they had already been matched) was used to handle confounding factors, with the balance of covariate distribution between daratumumab-based and non-daratumumab groups assessed using propensity score matched(PSM).

### Results

Overall,168 patients (daratumumab-based 21 patients, non- daratumumab-based 147 patients) were included in the analysis. After PSM,21 patients were included in daratumumab-based,105 patients were included in non-daratumumab based. Patient characteristics were well balanced between groups. There was no significant difference in median days of apheresis to reach the patient-specific $CD34^+$ goal and mean cumulative $CD34^+$ cell collection. Plerixafor was used in 71.4% (daratumumab-based) and 49.5% (non daratumumab-based) cases ($p = 0.06$).

### Summary/Conclusion

Steady-state hematopoietic cell mobilization in patients undergoing daratumumab-based induction therapy is feasible, although patients with daratumumab-based induction therapy more frequently requires plerixafor.

**Copyright Information:** (Online) ISSN: 2572-9241
©2026 The Author(s). HemaSphere is published by John Wiley & Sons Ltd on behalf of European Hematology Association. This is an open access Abstract Book distributed under the Attribution-NonCommercial-NoDerivs (CC BY-NC-ND), which allows third parties to download the articles and share them with others as long as they credit the author and the Abstract Book, but they cannot change the content in any way or use them commercially.
**Abstract Book Citations:** Authors, Title, HemaSphere, 2026;10:(S1):pages. The abstract book DOIs can be found on the "Author Information" page.
**Disclaimer:** Articles published in the journal HemaSphere exclusively reflect the opinions of the authors. The authors are responsible for all content in their abstracts including accuracy of the facts, statements, citing resources, etc.

**Table 1 Baseline characteristics of MM patients**

| Characteristic | Before propensity score matching | | | After propensity score matching | | |
|---|---|---|---|---|---|---|
| | Dara-based(n=21) | Non dara-based(n=143) | P value | Dara-based(n=21) | Non dara-based(n=105) | P value SMD |
| Age,yr,median(range) | 61(51,71) | 58(28,68) | 0.04 | 61(56,63) | 59(56,63) | 0.36 |
| Sex(Male/Female) | 15/6 | 76/71 | 0.09 | 15/6 | 61/44 | 0.25 |
| Subtype, n(%) | | | | | | |
| IgG | 7(33.3) | 58(39.5) | | 7(33.3) | 42(40.0) | |
| IgA | 4(19.0) | 31(21.1) | | 4(19.0) | 22(21.0) | |
| Kap | 5(23.8) | 28(19.0) | | 5(23.8) | 18(17.1) | |
| lam | 4(19.0) | 19(12.9) | | 4(19.0) | 16(15.2) | |
| Nonsecretory | 1(4.8) | 11(7.5) | 0.56 | 1(4.8) | 7(6.7) | 0.92 |
| ISS stage,n(%) | | | | | | |
| I | 4(19.0) | 70(48.0) | | 4(19.0) | 34(32.4) | |
| II | 7(33.3) | 38(26.0) | | 7(33.3) | 36(34.3) | |
| III | 10(47.6) | 38(26.0) | 0.01 | 10(47.6) | 35(33.3) | 0.36 |
| RISS stage,n(%) | | | | | | |
| I | 1(4.8) | 47(32.9) | | 1(4.8) | 15(14.3) | |
| II | 18(85.7) | 77(53.8) | | 18(85.7) | 73(69.5) | |
| III | 2(9.5) | 19(13.3) | 0.06 | 2(9.5) | 17(16.2) | 0.40 |
| Disease status prior to ASCT,n(%) | | | | | | |
| CR | 5(24.0) | 45(31.0) | | 5(24.0) | 5(24.0) | |
| VGPR | 11(52.0) | 74(50.0) | | 56(53.0) | 11(52.0) | |
| PR | 5(24.0) | 28(19.0) | 0.69 | 28(37.0) | 5(24.0) | 0.90 |
| MRD+/- | 9/12 | 75/72 | 0.48 | 9/12 | 56/49 | 0.38 |

**Table 2 PBSC mobilization and collection yields**

| Characteristic | Before propensity score matching | | | After propensity score matching | | |
|---|---|---|---|---|---|---|
| | Dara-based(n=21) | Non dara-based(n=147) | P value | Dara-based(n=21) | Non dara-based(n=105) | P value SMD |
| pB $CD34^+$ cells/uL on day of mobilization day 4,median(SD) | 12(15) | 17(14) | 0.12 | 12(15) | 16(15) | 0.27 |
| $CD34^+$ cells collected at first apheresis( × $10^6$cells/kg bw),median(SD) | 3.45(1.74) | 3.28(1.87) | 0.68 | 3.45(1.74) | 3.34(1.86) | 0.81 |
| Cumulative yield of $CD34^+$ cells collected( × $10^6$ cells/kg bw),median(SD) | 4.65(1.37) | 4.31(1.81) | 0.31 | 4.65(1.37) | 4.45(1.71) | 0.56 |
| Plerixafor use, n(%) | 15(71.4) | 56(38.1) | 0.004 | 15(71.4) | 52(49.5) | 0.06 |
| Days for stem cell collection,median(range) | 2(1,2) | 2(1,3) | 0.23 | 2(1,2) | 2(1,3) | 0.23 |

Copyright Information: (Online) ISSN: 2572-9241
©2026 The Author(s). HemaSphere is published by John Wiley & Sons Ltd on behalf of European Hematology Association. This is an open access Abstract Book distributed under the Attribution-NonCommercial-NoDerivs (CC BY-NC-ND), which allows third parties to download the articles and share them with others as long as they credit the author and the Abstract Book, but they cannot change the content in any way or use them commercially.
**Abstract Book Citations:** Authors, Title, HemaSphere, 2026;10:(S1):pages. The abstract book DOIs can be found on the "Author Information" page.
**Disclaimer:** Articles published in the journal HemaSphere exclusively reflect the opinions of the authors. The authors are responsible for all content in their abstracts including accuracy of the facts, statements, citing resources, etc.

### (PB3320) COMPARATIVE OVERALL SURVIVAL OF IMMUNE EFFECTORS AND NOVEL TRIPLETS IN RELAPSED/REFRACTORY MULTIPLE MYELOMA: A BAYESIAN NETWORK META-ANALYSIS

**Topic:** 14. Myeloma and other monoclonal gammopathies - Clinical

Shankar Biswas*[1], Yashasvi Srivastava[1], Urooj Iftekhar[2, 2]

*[1]Ivano-Frankivsk National Medical University, Internal Medicine, Ivano-Frankivsk, Ukraine; [2]Tbilisi State Medical University, Internal Medicine, Tbilisi, Georgia;*

### Background

The treatment landscape for relapsed/refractory multiple myeloma (RRMM) has expanded rapidly with BCMA-targeted therapies including the antibody-drug conjugate belantamab mafodotin (belamaf), CAR T-cell therapy ciltacabtagene autoleucel (cilta-cel), and anti-CD38-based triplets. While recent network meta-analyses (NMAs) have compared proteasome inhibitor-backbone regimens using progression-free survival, no NMA has comprehensively compared overall survival (OS) across both bortezomib-dexamethasone (Vd) and pomalidomide-dexamethasone (Pd) backbone regimens, incorporating immune effector therapies alongside conventional triplets.

### Aims

To compare OS of novel RRMM treatments spanning two structurally disconnected evidence networks using Bayesian NMA.

### Methods

A systematic review (MEDLINE, Embase, Cochrane, conference proceedings through January 2026) identified phase II/III RCTs of approved RRMM regimens reporting OS hazard ratios. Bayesian random-effects NMA was conducted using gemtc/JAGS (4 chains, 100,000 iterations, informative log-normal heterogeneity prior per Turner et al.). Network connectivity analysis revealed two disconnected clusters: Cluster A (Vd-backbone: Kd, PVd, BPd vs Vd; 3 RCTs, n=1,790) and Cluster B (Pd-backbone: Isa-Pd, DPd, EPd, cilta-cel vs Pd; 4 RCTs, n=1,147). Sensitivity analyses included vague priors, fixed-effects models, exclusion of the phase II ELOQUENT-3, and inclusion of KarMMa-3 (ide-cel). DREAMM-7 (BVd vs DVd) was reported as standalone direct evidence, being disconnected from either cluster without CASTOR bridging data. Frequentist NMA via netmeta validated Bayesian estimates. Risk of bias was assessed using RoB 2.

### Results

Eleven RCTs (N=5,783) were included; 7 contributed to the primary NMA. In Cluster A (vs Vd), point estimates favored all active treatments: BPd HR 0.72 (95%CrI 0.15-3.42), Kd HR 0.76 (0.27-2.41), PVd HR 0.94 (0.29-2.97); no comparison reached statistical significance ($I^2$=33%). In Cluster B (vs Pd), cilta-cel demonstrated the most favorable point estimate: HR 0.45 (0.06-2.84), followed by EPd HR 0.59 (0.15-2.29), Isa-Pd HR 0.78 (0.20-3.25), and DPd HR 0.82 (0.21-3.12); no comparison was statistically significant ($I^2$=24%). SUCRA rankings placed cilta-cel (85.4%) and EPd (68.2%) highest in Cluster B. In standalone analysis, DREAMM-7 yielded the strongest OS signal in the entire dataset: BVd vs DVd HR 0.58 (0.43-0.79). Sensitivity analyses including vague heterogeneity priors and KarMMa-3 inclusion were consistent with primary results.

**Copyright Information:** (Online) ISSN: 2572-9241
©2026 The Author(s). HemaSphere is published by John Wiley & Sons Ltd on behalf of European Hematology Association. This is an open access Abstract Book distributed under the Attribution-NonCommercial-NoDerivs (CC BY-NC-ND), which allows third parties to download the articles and share them with others as long as they credit the author and the Abstract Book, but they cannot change the content in any way or use them commercially.
**Abstract Book Citations:** Authors, Title, HemaSphere, 2026;10:(S1):pages. The abstract book DOIs can be found on the "Author Information" page.
**Disclaimer:** Articles published in the journal HemaSphere exclusively reflect the opinions of the authors. The authors are responsible for all content in their abstracts including accuracy of the facts, statements, citing resources, etc.

### Summary/Conclusion

This is the first Bayesian NMA comparing OS across both Vd- and Pd-backbone RRMM regimens incorporating immune effector therapies. While cilta-cel and belamaf-based regimens showed the most favorable OS point estimates, wide credible intervals reflect sparse per-cluster evidence. The structural disconnection between backbone networks precludes cross-cluster ranking without bridging trials. DREAMM-7 (BVd vs DVd) was the only comparison demonstrating statistically significant OS benefit. These findings highlight an urgent need for OS-powered trials and bridging studies to enable unified treatment ranking in RRMM.

**Copyright Information:** (Online) ISSN: 2572-9241
©2026 The Author(s). HemaSphere is published by John Wiley & Sons Ltd on behalf of European Hematology Association. This is an open access Abstract Book distributed under the Attribution-NonCommercial-NoDerivs (CC BY-NC-ND), which allows third parties to download the articles and share them with others as long as they credit the author and the Abstract Book, but they cannot change the content in any way or use them commercially.
**Abstract Book Citations:** Authors, Title, HemaSphere, 2026;10:(S1):pages. The abstract book DOIs can be found on the “Author Information” page.
**Disclaimer:** Articles published in the journal HemaSphere exclusively reflect the opinions of the authors. The authors are responsible for all content in their abstracts including accuracy of the facts, statements, citing resources, etc.

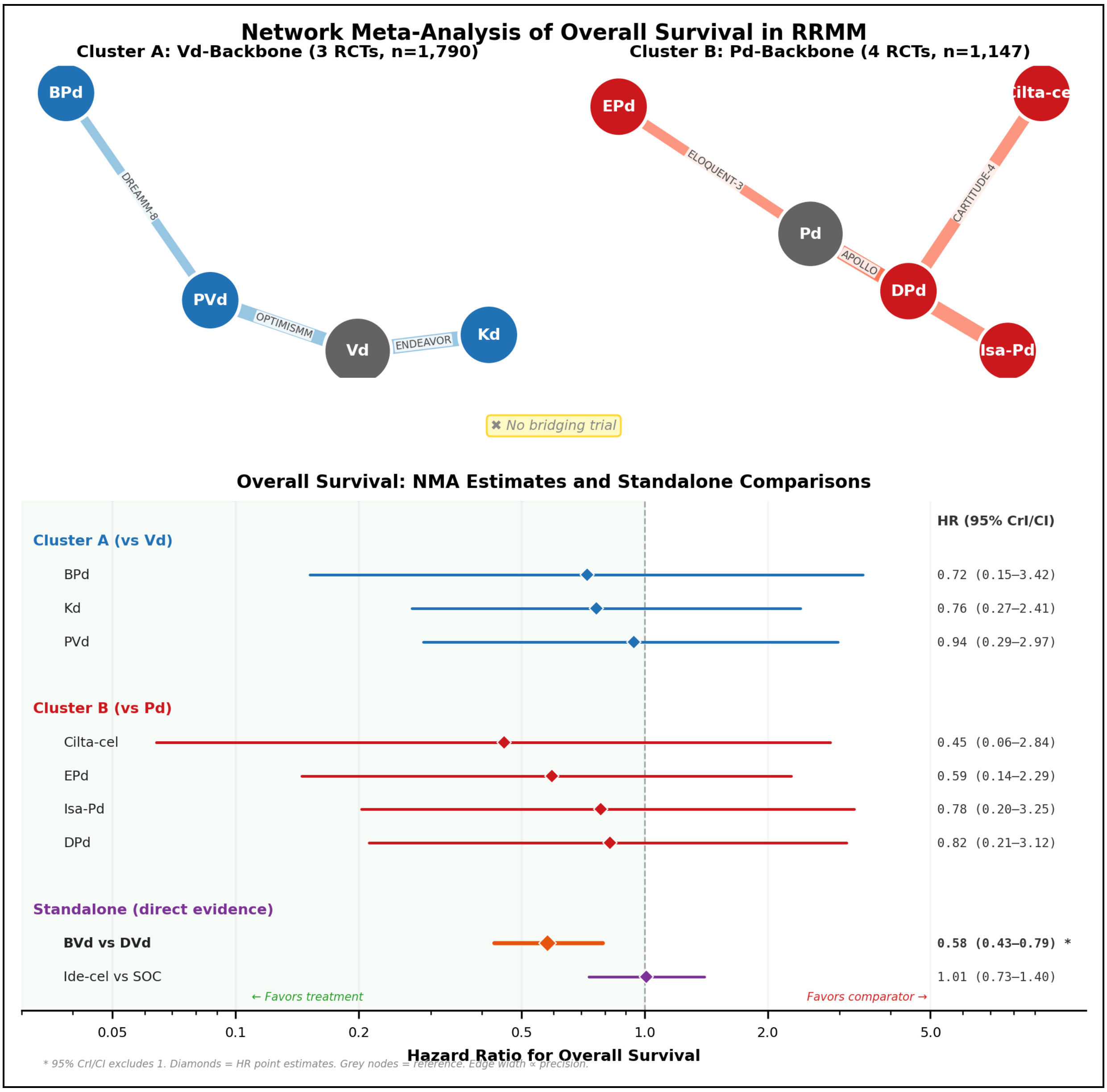

Abbreviations: BPd, belantamab mafodotin-pomalidomide-dexamethasone; BVd, bortezomib-based regimen; CI, confidence interval; Cilta-cel, ciltacabtagene autoleucel; CrI, credible interval; DPd, daratumumab-pomalidomide-dexamethasone; DVd, daratumumab-bortezomib-dexamethasone; EPd, elotuzumab-pomalidomide-dexamethasone; HR, hazard ratio; Ide-cel, idecabtagene vicleucel; Isa-Pd, isatuximab-pomalidomide-dexamethasone; Kd, carfilzomib-dexamethasone; NMA, network meta-analysis; Pd, pomalidomide-dexamethasone; PVd, pomalidomide-bortezomib-dexamethasone; RCT, randomized controlled trial; RRMM, relapsed/refractory multiple myeloma; SOC, standard of care; Vd, bortezomib-dexamethasone. Cluster A (Vd-backbone) and Cluster B (Pd-backbone) could not be connected by NMA due to absence of a bridging trial; estimates within each cluster are from Bayesian NMA (CrI), while standalone comparisons represent direct trial evidence (CI). Asterisk () denotes statistical significance where 95% CrI/CI excludes 1.0. Grey nodes = reference comparators; edge width reflects precision.*

**Copyright Information:** (Online) ISSN: 2572-9241
©2026 The Author(s). HemaSphere is published by John Wiley & Sons Ltd on behalf of European Hematology Association. This is an open access Abstract Book distributed under the Attribution-NonCommercial-NoDerivs (CC BY-NC-ND), which allows third parties to download the articles and share them with others as long as they credit the author and the Abstract Book, but they cannot change the content in any way or use them commercially.
**Abstract Book Citations:** Authors, Title, HemaSphere, 2026;10:(S1):pages. The abstract book DOIs can be found on the "Author Information" page.
**Disclaimer:** Articles published in the journal HemaSphere exclusively reflect the opinions of the authors. The authors are responsible for all content in their abstracts including accuracy of the facts, statements, citing resources, etc.

### (PB3321) BASELINE AND DYNAMIC EASIX SCORE AS AN INDEPENDENT PREDICTOR OF MORTALITY FOR MULTIPLE MYELOMA IN THE LATIN AMERICAN CONTEXT: A FIRTH-PENALIZED LOGISTIC REGRESSION ANALYSIS

**Topic:** 14. Myeloma and other monoclonal gammopathies - Clinical

Gledy Jimenez Tornero*[1], Susy Bazán Ruiz[2], Eduardo Bendezu[2], David Orccosupa Quispe[2], Romain Orccosupa Quispe[1], Sommer Porras[1], María Alejandra Vera Vasquez[1], Rosa Vengoa[1], Cesar Samanez[3], Viviana Olaya Colorado[4], Alejandro Munoz[4], Michael Amarillo[4], Diana Martinez[4], Catalina Zapata[4], Leidy Valencia[4], Marco Ruiz[5]

*[1]Hospital Alberto Sabogal, Hematology Oncology, Lima, Peru; [2]Hospital Nacional Edgardo Rebagliti, Hematology Oncology, Lima, Peru; [3]Oncosalud-AUNA, Lima, Peru; [4]Centro Oncologico Antioquia, Medellin, Colombia; [5]Miami Cancer Insttitute/Florida International University, Miami, United States of America;*

### Background

The Endothelial Activation and Stress Index (EASIX) is a composite biomarker that may capture endothelial dysfunction linked to adverse outcomes. We assessed whether baseline and dynamic EASIX scores, expressed as $\log_2$(EASIX) to yield "per-doubling" interpretability, is associated with mortality for Multiple Myeloma in the Latin American Context.

### Aims

To evaluate the EASIX score as an independent predictor of mortality in a Latinomaerican cohort of Myeloma Multiple patients and to identify the relationship between the dynamic EASIX score and therapeutic intervention.

### Methods

We analyzed 167 patients overall (3 centers in Lima, Peru, 1 center in Medellin, Colombia with data from 2019-2025) ; mortality occurred in 24/167 (14.4%). Sample consisted of 54 females (35.5%) and 98 (64.5%) males, respectively; 15 observations had missing sex. For multivariable modeling, complete-case analysis yielded 88 patients, with 14 deaths (15.9%). Given the rare event rate and potential small-sample bias, we fit a Firth-penalized logistic regression including age, body mass index (BMI), sex, and $\log_2$(EASIX_pre). Model discrimination was summarized by the c-statistic. We also compared pre- and post-EASIX on the $\log_2$ scale using paired t-tests in patients with both time points.

### Results

The Firth model converged and demonstrated acceptable discrimination (c=0.735). $\text{Log}_2$(EASIX_pre) was independently associated with mortality (β=0.464; *P*=0.0099), corresponding to an odds ratio (OR) of 1.59 (95% CI: 1.14, 2.30) per doubling of EASIX score. Age, BMI, and gender were not statistically significant covariates (*P*>0.05 for all). Baseline EASIX was right-skewed (median 0.77; IQR 0.45–1.81). Among paired observations (n=55), **$\log_2$(EASIX_post)** was lower than **$\log_2$(EASIX_pre)** (mean difference −0.56; 95% CI −0.89 to −0.22; *P*=0.0015), indicating a reduction in endothelial stress over time.

### Summary/Conclusion

In this cohort, higher baseline EASIX was independently associated with increased mortality; each **doubling** of EASIX conferred a **~59% increase** in the odds of death. The model's discrimination was acceptable despite a rare outcome and missingness, and paired analyses suggested that EASIX is dynamic and may decrease over time. These findings support EASIX as a clinically meaningful and interpretable biomarker of mortality risk in the Latin American setting.

**Copyright Information:** (Online) ISSN: 2572-9241
©2026 The Author(s). HemaSphere is published by John Wiley & Sons Ltd on behalf of European Hematology Association. This is an open access Abstract Book distributed under the Attribution-NonCommercial-NoDerivs (CC BY-NC-ND), which allows third parties to download the articles and share them with others as long as they credit the author and the Abstract Book, but they cannot change the content in any way or use them commercially.
**Abstract Book Citations:** Authors, Title, HemaSphere, 2026;10:(S1):pages. The abstract book DOIs can be found on the "Author Information" page.
**Disclaimer:** Articles published in the journal HemaSphere exclusively reflect the opinions of the authors. The authors are responsible for all content in their abstracts including accuracy of the facts, statements, citing resources, etc.

### (PB3322) IMPACT OF THERAPEUTIC ADVANCES ON ATTRITION RATES IN MULTIPLE MYELOMA: A 30-YEAR SINGLE CENTER REAL-WORLD EXPERIENCE

**Topic:** 14. Myeloma and other monoclonal gammopathies - Clinical

Claudia Bellofiore*[1], Giuliana Giunta[2], Elvira Scalisi[2], Federica Elia[1], Bruno Garibaldi[1], Chiara Finocchiaro[2], Francesco Di Raimondo[1, 2], Alessandra Romano[1, 2], Concetta Conticello[1]

*[1]Division of Hematology, Azienda Policlinico-S.Marco, University of Catania – Catania IT, Catania, Italy;[2]Postgraduate school of Hematology, University of Catania - Catania IT, Catania, Italy;*

### Background

The therapeutic landscape of multiple myeloma (MM) has profoundly changed with the introduction of immunomodulatory drugs, proteasome inhibitors, monoclonal antibodies, cellular therapies and bispecific antibodies. While these advances have improved survival, conventional endpoints may not fully reflect treatment feasibility in routine practice. However, traditional efficacy endpoints may not fully capture the real-world benefit of these advances. In this context, attrition rate (AR)—defined as the proportion of patients who die without receiving the subsequent line of therapy (LOT) after progression—represents a clinically meaningful indicator of treatment feasibility, disease aggressiveness, and patient vulnerability across the MM disease course.

### Aims

Aim of the present study is to assess changes in attrition rates over time and to correlate them with evolving treatment strategies in a real-world MM population.

### Methods

We retrospectively evaluated 1052 consecutive patients diagnosed and treated for MM at our Institution between January 2005 and September 2025. Most patients were managed outside clinical trials. Patients were stratified by therapeutic era: Group A (2005–2015; n=446) and Group B (2016–2025; n=546). AR was calculated after each LOT.

### Results

Patient characteristics are summarized in Table 1. After a median follow-up of 107 months (95% CI 98–243), median overall survival (OS) for the entire cohort was 65 months (95% CI 58–69). Treatment strategies evolved over time. Patients diagnosed up to 2015 received a median of 2 LOTs (range 1–10), compared with 1 LOT (range 1–12) in the later period. In Group A, bortezomib-based regimens were the predominant first-line treatment (52%). In Group B, anti-CD38–based combinations were more frequently used (36%), including combinations with bortezomib (21%) or lenalidomide (15%). Re-treatment with backbone agents across multiple lines was more common in Group A (bortezomib 29%, lenalidomide 13%) compared with Group B (8% and 5%, respectively). AR increased with successive LOTs in both eras but was consistently lower in the more recent cohort. In 2005–2015, AR was 27% after first line, 37% after second line, and 33% and 42% after third and fourth lines, respectively. In 2016–2025, AR decreased to 10%, 20%, 28%, and 31% at the corresponding time points. Overall, AR ranged from 27–42% in Group A and 10–31% in Group B. Overall response rates showed modest improvement in Group A (3% to 5%) but were lower in Group B (48% to 21%), likely reflecting earlier use of highly effective regimens in upfront settings.

**Copyright Information:** (Online) ISSN: 2572-9241
©2026 The Author(s). HemaSphere is published by John Wiley & Sons Ltd on behalf of European Hematology Association. This is an open access Abstract Book distributed under the Attribution-NonCommercial-NoDerivs (CC BY-NC-ND), which allows third parties to download the articles and share them with others as long as they credit the author and the Abstract Book, but they cannot change the content in any way or use them commercially.
**Abstract Book Citations:** Authors, Title, HemaSphere, 2026;10:(S1):pages. The abstract book DOIs can be found on the "Author Information" page.
**Disclaimer:** Articles published in the journal HemaSphere exclusively reflect the opinions of the authors. The authors are responsible for all content in their abstracts including accuracy of the facts, statements, citing resources, etc.

**Summary/Conclusion**

Despite substantial therapeutic advances and improvements in supportive care, treatment attrition remains a relevant challenge throughout the MM disease course. However, AR has significantly decreased in the modern treatment era, suggesting improved treatment feasibility. Further efforts to optimize sequencing strategies and maintain patients on active therapy are warranted to further reduce attrition in real-world practice.

**Table 1.** Patients' characteristics

| | Entire cohort (N=1052) Median (IQR) N (%) | Patients diagnosed between 2005-2015 (N=446) Median (IQR) N (%) | Patients diagnosed between 2015-2025 (N=546) Median (IQR) N (%) | P |
|---|---|---|---|---|
| Age, years | 65 (64 – 66) | 65 (64 – 66) | 66 (65 – 67) | **.016** |
| Male sex | 576 (54) | 238 (53) | 305 (72) | .432 |
| Age<65 years | 513 (48) | 228 (51) | 239 (43) | **.021** |
| HR Cytogenetics | 119 (34) | 23 (25) | 94 (37) | **.028** |
| Extramedullary | 29 (2) | 7 (1) | 20 (4) | **.043** |
| AL amyloidosis | 71 (7) | 21 (5) | 45 (8) | **.025** |
| N. of therapies | 2 (1 – 3) | 2 (1 – 3) | 1 (1 – 2) | **<.001** |
| Triple exposed | 237 (22) | 53 (12) | 180 (33) | **<.001** |
| Penta exposed | 55 (5) | 20 (4) | 33 (6) | .281 |
| Trial enrolled | 212 (20) | 169 (37) | 36 (6) | **<.001** |
| **Agents to which patients were exposed across different lines of treatment** | | | | |
| Bispecific antibodies | 13 (1) | 2 (0.4) | 11 (2) | **.031** |
| Lenalidomide | 658 (66) | 247 (55) | 411 (75) | **<001** |
| Pomalidomide | 159 (16) | 78 (17) | 81 (15) | .259 |
| Bortezomib | 761 (76) | 376 (84) | 385 (71) | **<001** |
| Kyprolis | 112 (11) | 41 (9) | 71 (13) | .059 |
| Ixazomib | 26 (2) | 8 (2) | 18 (3) | .145 |
| Bendamustine | 32 (3) | 28 (6) | 4 (1) | **<001** |
| Daratumumab | 356 (36) | 57 (13) | 299 (55) | **<001** |
| Elotuzumab | 24 (2) | 6 (1) | 18 (3) | **.047** |
| Isatuximab | 29 (3) | 4 (1) | 25 (5) | **<001** |
| DCEP | 57 (6) | 38 (9) | 19 (3) | **<001** |
| Belantamab | 20 (2) | 9 (2) | 11 (2) | .991 |
| Melflufen | 12 (1) | 2 (0.4) | 10 (2) | **.049** |
| Selinexor | 10 (1) | 1 (0.1) | 9 (3) | **.025** |
| CAR-T | 3 (0.3) | 0 | 3 (1) | .166 |
| Mezigodomide | 3 (0.3) | 2 (0.1) | 1 (0.1) | .515 |

**Copyright Information:** (Online) ISSN: 2572-9241
©2026 The Author(s). HemaSphere is published by John Wiley & Sons Ltd on behalf of European Hematology Association. This is an open access Abstract Book distributed under the Attribution-NonCommercial-NoDerivs (CC BY-NC-ND), which allows third parties to download the articles and share them with others as long as they credit the author and the Abstract Book, but they cannot change the content in any way or use them commercially.
**Abstract Book Citations:** Authors, Title, HemaSphere, 2026;10:(S1):pages. The abstract book DOIs can be found on the "Author Information" page.
**Disclaimer:** Articles published in the journal HemaSphere exclusively reflect the opinions of the authors. The authors are responsible for all content in their abstracts including accuracy of the facts, statements, citing resources, etc.

### (PB3323) CLINICAL CHARACTERISTICS, TREATMENT AND SURVIVAL OF PATIENTS WITH AL AMYLOIDOSIS IN MOSCOW

**Topic:** 14. Myeloma and other monoclonal gammopathies - Clinical

Ekaterina Zotina*[1, 2], Marat Mingalimov[1, 2], Alina Ahmadeeva[1], Dmitrij Polyakov[1], Maria Orlova[2], Marina Lysenko[1], Sergej Usatyuk[1], Nadiya Frolova[1], Oleg Kotenko[1], Elena Baryakh[1, 2], Elena Misyurina[1, 2]

*[1]Moscow Clinical Science and Research Center 52, Moscow, Russia;[2]I.M. Sechenov First Moscow State Medical University (Sechenovskiy University), Department of Hematology, Moscow, Russia;*

### Background

Amyloid light chain (AL) amyloidosis is characterized by the insoluble amyloid fibril's deposition from monoclonal immunoglobulin light chains, which leads to impaired structure and function of internal organs. There is severe lack of data on the clinical characteristics and treatment of patients with AL amyloidosis in Russia.

### Aims

To describe the clinical characteristics, treatments, and survival in patients with newly diagnosed AL amyloidosisin Moscow city hospitals.

### Methods

The retrospective study included patients with newly diagnosed AL amyloidosis who were observed and treated inMoscow city hospitals between 2018 and 2025. The clinical and laboratory characteristics of patients, treatment, and survival were analyzed.

### Results

The study included 205 patients, and 53% were male. The patients' ages ranged from 30 to 86 years, with a median age of 65 years. In 27% patients, AL amyloidosis was concomitant with multiple myeloma. The median time from the first symptoms to diagnosis was 22 months. Multiorgan involvement occurred in 48% of patients. At the time of diagnosis, 78%, 45%, and 21% of patients, respectively, had kidney, heart, and gastrointestinal tract involvement. In 74% of patients, the lambda light chain was involved in the pathological process. Overall, 67% of patients received anticancer therapy. As a first-line therapy bortezomib-based regimen was used in 57%, daratumumab-based – in 23%, lenalidomide-based – in 12% and melphalan-based therapy – in 8%. Only 4% of the patients received high-dose chemotherapy with autologous hematopoietic stem cell transplantation. In the general group hematological response (complete, very good partial or partial remission) was observed in 79% of patients, cardiac and renal responses at 6 months were 27% and 34%, respectively. Median overall survival was 49 months (95 CI: 38.2-57.6). Median progression-free survival was 13 months (95 CI: 9.1-15.4).

### Summary/Conclusion

This study provides important information on the clinical characteristics, treatment regimens, and survival of a large group of patients with AL amyloidosis in real-world clinical practice in Russia.

**Copyright Information:** (Online) ISSN: 2572-9241
©2026 The Author(s). HemaSphere is published by John Wiley & Sons Ltd on behalf of European Hematology Association. This is an open access Abstract Book distributed under the Attribution-NonCommercial-NoDerivs (CC BY-NC-ND), which allows third parties to download the articles and share them with others as long as they credit the author and the Abstract Book, but they cannot change the content in any way or use them commercially.
**Abstract Book Citations:** Authors, Title, HemaSphere, 2026;10:(S1):pages. The abstract book DOIs can be found on the "Author Information" page.
**Disclaimer:** Articles published in the journal HemaSphere exclusively reflect the opinions of the authors. The authors are responsible for all content in their abstracts including accuracy of the facts, statements, citing resources, etc.

### (PB3324) 25 YEARS OF MULTIPLE MYELOMA CARE IN COLOMBIA. REAL-WORLD DATA FROM A MIDDLE-INCOME COUNTRY. ACHO'S RENEHOC REGISTRY REPORT

**Topic:** 14. Myeloma and other monoclonal gammopathies - Clinical

Kenny Mauricio Galvez Cardenas*[1], Patricia Isabel Martinez Alvarez[2], Claudia Sossa-Melo[3, 4], Luis Salazar[3, 5], Henry Idrobo[6], Diana Cuervo[7], Gomez Rigoberto[8], Guillermo Enrique Quintero Vega[9], Isabel Munevar Lopez[10], Pedraza Morales Julian Eduardo[11], Jheremy Enrique Reyes Castellanos[12], Domingo Saavedra Ramirez[13], Juan Carlos Serrano[14], Lina Maria Gaviria Jaramillo[15], Mario Correa[16], Jose Lobaton[17], Monica Ozuna Perez[18], Carlos Bermudez Silva[19], Juan Manuel Herrera[20], Viviana Olaya Colorado[21], Carlos Roberto Varón[22], Carmen Rosales-Oliveros[23], Paola Guerrero[20], Mauricio Andres Alzate Arias[15], Claudia Del Pilar Agudelo Lopez[24], Virginia Abello-Polo[25]

*[1]Hospital Pablo Tobón Uribe, Medellín, Colombia;[2]Clínica PortoAzúl, Barranquilla, Colombia; [3]Fundacion Oftalmologica De Santander Heliport, Floridablanca, Colombia; [4]Universidad Autónoma de Bucaramanga, FOSUNAB, Bucaramanga, Colombia; [5]Universidad Autónoma de Bucaramanga, Bucaramanga, Colombia;[6]Centro Médico Julián Coronel, Cali, Colombia; [7]Hospital de San José, Bogotá, Colombia; [8]Hemato Oncólogos De Imbanaco, Cali, Colombia;[9]Fundación Santa Fe De Bogotá, Bogotá, Colombia; [10]Hemato oncólogos asociados, Bogota, Colombia; [11]Clínica Colsubsidio Calle 127, Bogotá, Colombia; [12]Clínica Los Nogales, Bogotá, Colombia; [13]Clinica Vida, Medellín, Colombia; [14]Clinica De Cancerologia Del Norte De Santander Ltda, Ragonvalia, Colombia; [15]Fundación Hospitalaria San Vicente de Paúl, Medellín, Colombia; [16]Clínica De Los Remedios, Cali, Colombia; [17]Oncomedica, Montería, Colombia; [18]Grun Los Cobos, Bogotá, Colombia; [19]Clinica San Diego, Bogotá, Colombia; [20]Imbanaco, Cali, Colombia; [21]Centro Oncológico de Antioquia Medellin, Medellin, Colombia;[22]Unidad Hematólogica Especialista, Cúcuta, Colombia; [23]Sociedad de Oncología y Hematología del Cesar (SOHEC), Valledupar, Colombia; [24]Fundación Santa Fe de Bogotá, Bogotá, Colombia;[25]Fundación Centro para el Tratamiento e Investigación sobre Cáncer (CTIC), Bogotá, D.C., Colombia;*

### Background

Multiple myeloma (MM) is the second most common hematologic malignancy and represents a substantial clinical and economic burden due to its chronic course, repeated relapses, and escalating treatment costs. During the last 25 years treatment paradigms have evolved notably resulting in better outcomes in terms of survival and quality of life for MM patients.
In low- and middle-income countries (LMICs) such as Colombia, structural, financial, and access-related barriers limit the optimal and timely implementation of new technologies for diagnosis, prognosis, and treatment; that lags behind countries with greater resources. Real-world data(RWD) from LMICs are essential for health care planing and policy development.

### Aims

RENEHOC (Registro Epidemiológico de Enfermedades Hematológicas en Colombia) is a multicenter clinical registry established in January 2018 to generate RWD on diagnosis, treatment patterns, and outcomes in hematologic malignancies. This study evaluated changes in the implementation of diagnostic, prognostic and therapeutic tools in a cohort of MM patients over the last 25 years.

### Methods

An ambispective registry of adult MM patients, treated in IRB approved centers, registered between 2018 and December 2024. Descriptive statistics were used for patient’s demographic, clinical characteristics, diagnosis and treatment patterns.

**Copyright Information:** (Online) ISSN: 2572-9241
©2026 The Author(s). HemaSphere is published by John Wiley & Sons Ltd on behalf of European Hematology Association. This is an open access Abstract Book distributed under the Attribution-NonCommercial-NoDerivs (CC BY-NC-ND), which allows third parties to download the articles and share them with others as long as they credit the author and the Abstract Book, but they cannot change the content in any way or use them commercially.
**Abstract Book Citations:** Authors, Title, HemaSphere, 2026;10:(S1):pages. The abstract book DOIs can be found on the “Author Information” page.
**Disclaimer:** Articles published in the journal HemaSphere exclusively reflect the opinions of the authors. The authors are responsible for all content in their abstracts including accuracy of the facts, statements, citing resources, etc.

### Results

A total of 2125 patients were included; the earliest recorded diagnosis dated back to 1996. 1090 (51.2%) were female, median age at diagnosis was 67 years(IQR 60-75), most patients were diagnosed in an advance stage Durie Salmon III (n=1415; 66%). A total of 1120 patients (57.4%) were insured under the contributory healthcare system. 1656 (77.9%) were diagnosed after 2012.
Access to advanced diagnostic evaluation has improved over the time but remains limited: FISH cytogenetic was consistently reported after 2014; with 453 (21.5%) of the total cohort having a report. However, this figure increased from 18% from beteween 2014-2017, to 27% between 2018-2021, reaching 45% after 2022.
Similarly, PET-CT, was incorporated routinely after 2014. Overall, 305 (14%) patients had a PET-CT at diagnosis. However widely availability remains limited across centers, its use increased from 10% between 2014-2017, to 21% between 2018-2021, and to 29% after 2022.
Treatments patterns also changed significantly, Bortezomib was approved in 2005, and 70% of patients received bortezomib-based triplet therapy as induction. Overall, 1260 (59.2%) received either CyBorD (n=696;33%), VTD (n=333;16%) or VRd (n=247;12%). Between 2005-2014 CyBorD and VTD were the predominant regimens. From 2015 onwards, there was a progressive increase in VRd induction, with marked rise during 2020-2024 (CyBorD 38%, VRd 35%9). By 2024, 49% of patients received VRd in first-line. Daratumumab was approved as first-line in 2024, only 19 patients received It in this setting overall.
611 (29%) patients underwent ASCT, without any significant changes over time.

### Summary/Conclusion

Over the past 25 years, management of MM in Colombia, has evolved substantially, mirroring global advances. Our data shows how adoption of new technologies is gradual, remains limited and heterogeneous across centers, reflecting implementation challenges and health systems constrains in LMIC.
These findings highlight the importance of RWD, generated through registries to better understand the changing landscape of disease management; as a foundation for pharmacoeconomic, epidemiologic, and outcomes research in LMIC.

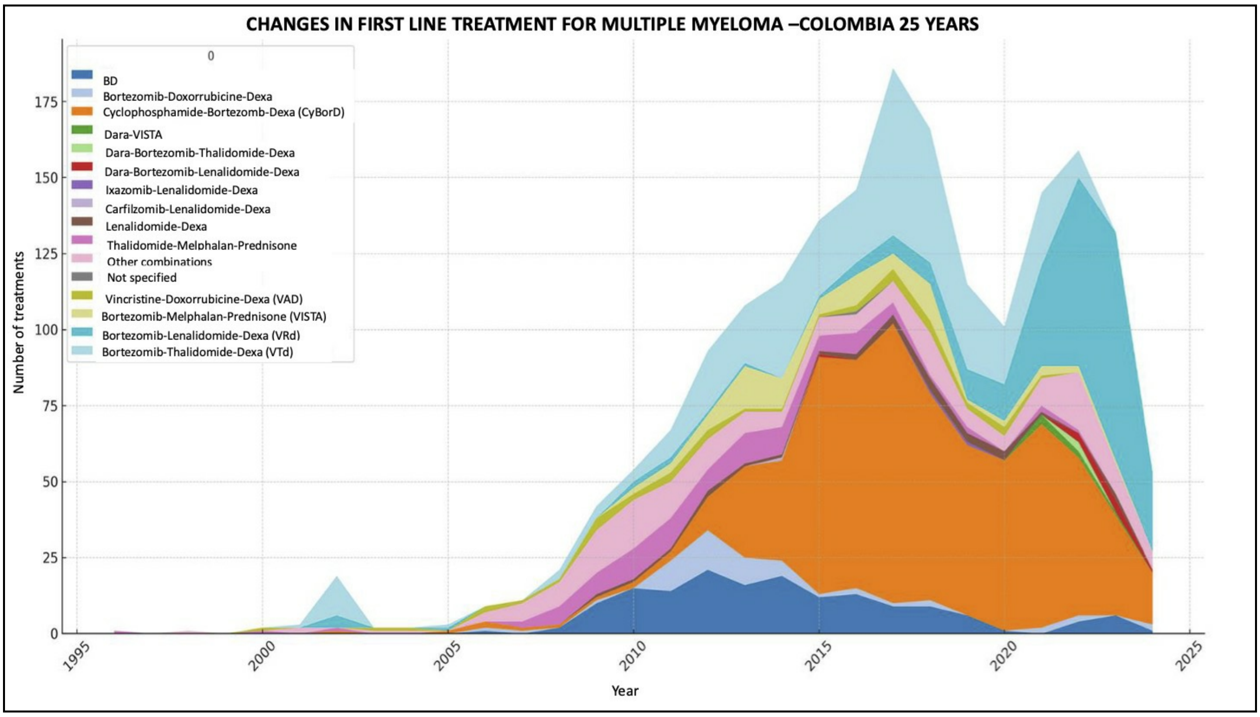

**Copyright Information:** (Online) ISSN: 2572-9241
©2026 The Author(s). HemaSphere is published by John Wiley & Sons Ltd on behalf of European Hematology Association. This is an open access Abstract Book distributed under the Attribution-NonCommercial-NoDerivs (CC BY-NC-ND), which allows third parties to download the articles and share them with others as long as they credit the author and the Abstract Book, but they cannot change the content in any way or use them commercially.
**Abstract Book Citations:** Authors, Title, HemaSphere, 2026;10:(S1):pages. The abstract book DOIs can be found on the "Author Information" page.
**Disclaimer:** Articles published in the journal HemaSphere exclusively reflect the opinions of the authors. The authors are responsible for all content in their abstracts including accuracy of the facts, statements, citing resources, etc.

### (PB3325) EFFICACY AND SAFETY OF APONERMIN COMBINED WITH POMALIDOMIDE-BASED REGIMENS IN RELAPSED/REFRACTORY MULTIPLE MYELOMA

**Topic:** 14. Myeloma and other monoclonal gammopathies - Clinical

Xin Li*[1], Yan Yi[2], Qian Cheng[1], Liping Su[3], Peiling Zhang[4], Fan Zhou[5], Wenyin Hou[6], Wen Gao[7], Jing Bao[8], Xiaoqi Qin[9], Wang Yaomei[10], Zhong Jinfa[11], Lan Lingqiong[12], Wenming Chen[7]

*[1]The Third Xiangya Hospital of Central South University, changsha, China;[2]Southern Medical University Third Affiliated Hospital, guangzhou, China; [3]Shanxi Province Cancer Hospital/ Shanxi Hospital Affiliated to Cancer Hospital, taiyuan, China;[4]Tongji Medical College, Huazhong University of Science and Technology, wuhan, China; [5]Zhabei Central Hospital of Jing'an District, Shanghai, shanghai, China; [6]THE FIRST AFFILIATED HOSPITAL OF HARBIN MEDICAL UNIVERSITY, harbin, China; [7]Beijing Chao-yang Hospital, Capital Medical University, beijing, China; [8]The First Affiliated Hospital of Anhui Medical University, hefei, China; [9]The Second Hospital of Shanxi Medical university, taiyuan, China;[10]Henan Institute of Hematology, The Affiliated Cancer Hospital of Zhengzhou University & Henan Cancer Hospital, zhengzhou, China; [11]Second Affiliated Hospital of Fujian Medical University, quanzhou, China; [12]Longyan Second Hospital, longyan, China;*

### Background

Multiple myeloma (MM) is a common hematologic malignancy. Patients with relapsed/refractory multiple myeloma (RRMM) are often faced with limited treatment options due to disease progression and drug tolerance after multiple lines of therapy. Aponermin (Apo) has been approved for the treatment of RRMM, but additional studies are needed.

### Aims

To evaluate the efficacy and safety of Apo combined with pomalidomide-based regimens in RRMM patients.

### Methods

This is a multicenter retrospective study including patients who received at least two cycles of Apo plus pomalidomide-based therapy between June 2024 and January 2026. The primary endpoint is overall response rate (ORR). The secondary endpoints included rate of very good partial response or better(≥VGPRR), and adverse events.

### Results

A total of 40 RRMM patients were included in this study The median patient age was 66 years (range: 43-86), with a slight male predominance (78.8%). Disease stages were predominantly advanced (Durie-Salmon stage II/III: 84.4%; ISS stage II/III: 68.8%; R-ISS stage II/III: 53.6%). Patients had received a median of 2 prior lines of therapy (range: 1-7), with 56.3% (18/32) being triple-class exposed (PI, IMiD, and anti-CD38). Extramedullary disease (EMD) was present in 33.3% (11/33) of patients, and 56.3% (18/32) harbored high-risk cytogenetic abnormalities (HRCA). Regimens administered included APO+PD (n=19), APO+DPD (n=8), and APO+KPD (n=5). The median number of treatment cycles was 4 (range: 2-11). The overall ORR was 56.3% (18/32), with a ≥VGPR rate of 21.9% (7/32). The ORR showed an increasing trend with prolonged treatment, reaching 70.0% (14/20) in patients who completed ≥4 cycles. Subgroup analyses revealed no significant differences in ORR between patients with and without HRCA (61.1% vs. 50.0%), with and without EMD (45.5% vs. 61.9%), or between those with and without triple-class exposure (55.6% vs. 53.8%). Among the different regimens, the APO+DPD combination yielded the highest ORR (75%), compared to 47.4% for APO+PD and 60% for APO+KPD. The most common treatment-emergent adverse events were nausea (5%), thrombocytopenia (5%), leukopenia (5%), lymphocytopenia (5%), manageable with supportive care.

**Copyright Information:** (Online) ISSN: 2572-9241
©2026 The Author(s). HemaSphere is published by John Wiley & Sons Ltd on behalf of European Hematology Association. This is an open access Abstract Book distributed under the Attribution-NonCommercial-NoDerivs (CC BY-NC-ND), which allows third parties to download the articles and share them with others as long as they credit the author and the Abstract Book, but they cannot change the content in any way or use them commercially.
**Abstract Book Citations:** Authors, Title, HemaSphere, 2026;10:(S1):pages. The abstract book DOIs can be found on the "Author Information" page.
**Disclaimer:** Articles published in the journal HemaSphere exclusively reflect the opinions of the authors. The authors are responsible for all content in their abstracts including accuracy of the facts, statements, citing resources, etc.

**Summary/Conclusion**

Aponermin combined with pomalidomide-based regimens demonstrate a favorable overall response rate in treating RRMM patients. This combination appears to provide clinical benefit even for patients with high-risk cytogenetic abnormalities, extramedullary disease, or those who have failed multiple prior lines of therapy.

**Copyright Information:** (Online) ISSN: 2572-9241
©2026 The Author(s). HemaSphere is published by John Wiley & Sons Ltd on behalf of European Hematology Association. This is an open access Abstract Book distributed under the Attribution-NonCommercial-NoDerivs (CC BY-NC-ND), which allows third parties to download the articles and share them with others as long as they credit the author and the Abstract Book, but they cannot change the content in any way or use them commercially.
**Abstract Book Citations:** Authors, Title, HemaSphere, 2026;10:(S1):pages. The abstract book DOIs can be found on the "Author Information" page.
**Disclaimer:** Articles published in the journal HemaSphere exclusively reflect the opinions of the authors. The authors are responsible for all content in their abstracts including accuracy of the facts, statements, citing resources, etc.

### (PB3326) EFFICACY AND SAFETY OF APONERMIN-SELINEXOR-DEXAMETHASONE (APO-SD) IN HEAVILY PRE-TREATED RELAPSED/REFRACTORY MULTIPLE MYELOMA

**Topic:** 14. Myeloma and other monoclonal gammopathies - Clinical

Chunrui Li*[1], Wenming Chen[2], Ning An[1], Di Wang[1], Xin Li[3], Peiling Zhang[1], Weijian Zhu[4], Meiling Li[5], Rujiao Dong[6], Yaping Liao[7], Wen Gao[2]

*[1]Tongji Medical College, Huazhong University of Science and Technology, wuhan, China;[2]Beijing Chao-yang Hospital, Capital Medical University, beijing, China; [3]The Third Xiangya Hospital of Central South University, changsha, China;[4]Zhuhai People's Hospital, Zhuhai, China; [5]Second Affiliated Hospital of Fujian Medical University, quanzhou, China;[6]Shenzhen Second People's Hospital, Shenzhen, China; [7]The Eighth Affiliated Hospital, Sun Yat-sen University, Shenzhen, China;*

### Background

Multiple myeloma (MM) is an incurable disease with frequent relapses, requiring continuous treatment. Recently, Aponermin(Apo) has been approved in China for the treatment of MM, with several clinical trials evaluating Aponermin-based therapies in relapsed/refractory MM (RRMM) patients. The results have shown significant improvements in survival rates. Aponermin is a recombinant cyclically modified human tumor necrosis factor-related apoptosis-inducing ligand (TRAIL) that activates death receptor 4 (DR4) and death receptor 5 (DR5) on the surface of tumor cells, triggering intracellular cascades to exert anti-tumor effects. Currently, this drug has been shown to synergize with proteasome inhibitors to enhance anti-myeloma activity. However, the efficacy and safety of combining Aponermin with Selinexor are still unclear.

### Aims

This study reports the efficacy and safety of the Aponermin-Selinexor-Dexamethasone (Apo-Sd) regimen in RRMM patients. The primary endpoints included overall response rate (ORR), duration of response (DOR), and adverse events.

### Methods

This was a multi-center retrospective study that included patients with relapsed/refractory MM who received Aponermin-Selinexor-Dexamethasone-based therapy. The main aim was to compare the efficacy and safety of the Aponermin-Selinexor+low-dose Dexamethasone regimen in RRMM.

### Results

As of February 10, 2026, 33 patients with relapsed/refractory multiple myeloma received the Aponermin-Selinexor-Dexamethasone-based regimen. The treatment regimens included ApoSd (n=24), ApoSK (Carfilzomib)d (n=3), and ApoSP (Pomalidomide)d (n=6). The median age of patients was 60 years, with 51.5% being male. 30.3% patients had extramedullary disease, and 54.5% had high-risk cytogenetic features(including 12.1% with p53 loss/mutation ). The median number of prior lines of treatment was 3, with 2 patients having received autologous stem cell transplants. 23 patients had been exposed to triple therapy. The overall response rate (ORR) was 51.2%, the complete response rate (CRR) was 15.2%. For patients with traceable disease progression (n=16), median time to first response is 1.5 months, and median progression-free survival (PFS) was 7.9 months (95% CI: 2.7 months to 13.0 months). The most common treatment-emergent adverse events were thrombocytopenia (12.1%), Anemia (9.1%), liver dysfunction(9.1%), manageable with supportive care.

**Copyright Information:** (Online) ISSN: 2572-9241
©2026 The Author(s). HemaSphere is published by John Wiley & Sons Ltd on behalf of European Hematology Association. This is an open access Abstract Book distributed under the Attribution-NonCommercial-NoDerivs (CC BY-NC-ND), which allows third parties to download the articles and share them with others as long as they credit the author and the Abstract Book, but they cannot change the content in any way or use them commercially.
**Abstract Book Citations:** Authors, Title, HemaSphere, 2026;10:(S1):pages. The abstract book DOIs can be found on the "Author Information" page.
**Disclaimer:** Articles published in the journal HemaSphere exclusively reflect the opinions of the authors. The authors are responsible for all content in their abstracts including accuracy of the facts, statements, citing resources, etc.

**Summary/Conclusion**

The Apo-Sd-based regimen shows promising efficacy in heavily pretreated RRMM patients, including those with high-risk prognostic factors and those who failed CAR-T therapy. Encouraging response rates were observed even in patients with multiple treatment failures and drug resistance. This highlights the unique mechanism of action of Aponermin and its synergistic effect with the Sd regimen. The safety of this combination is manageable. These findings support further evaluation of this novel combination regimen in larger clinical trials.

**Copyright Information:** (Online) ISSN: 2572-9241
©2026 The Author(s). HemaSphere is published by John Wiley & Sons Ltd on behalf of European Hematology Association. This is an open access Abstract Book distributed under the Attribution-NonCommercial-NoDerivs (CC BY-NC-ND), which allows third parties to download the articles and share them with others as long as they credit the author and the Abstract Book, but they cannot change the content in any way or use them commercially.
**Abstract Book Citations:** Authors, Title, HemaSphere, 2026;10:(S1):pages. The abstract book DOIs can be found on the “Author Information” page.
**Disclaimer:** Articles published in the journal HemaSphere exclusively reflect the opinions of the authors. The authors are responsible for all content in their abstracts including accuracy of the facts, statements, citing resources, etc.

### (PB3327) CLINICAL FEATURES AND PROGNOSTIC FACTORS OF CARDIAC LIGHT CHAIN AMYLOIDOSIS: A SINGLE-CENTER STUDY OF 37 PATIENTS

**Topic:** 14. Myeloma and other monoclonal gammopathies - Clinical

Huawei Jiang*[1], Xi Qiu[1], Qian Luo[1], Yang Xu[1]

[1]*Department of Hematology, The Second Affiliated Hospital of Zhejiang University School of Medicine, Hangzhou, China;*

### Background

Cardiac light chain (LC) amyloidosis is a rare and serious condition with a poor prognosis. It is often associated with plasma cell dyscrasias, with heart failure being the most common symptom. This study aims to examine the clinical characteristics, treatment response, and prognostic factors in patients with cardiac LC amyloidosis.

### Aims

To analyze the clinical characteristics and treatment outcomes of patients with biopsy-confirmed cardiac AL amyloidosis and to identify independent predictors of achieving a Very Good Partial Response (VGPR) or better in cardiac function.

### Methods

We retrospectively reviewed 37 patients diagnosed with cardiac AL amyloidosis via endomyocardial biopsy at a single center between November 2020 and November 2025, excluding patients with kidney involvement. Clinical data, including demographics, disease stage (Mayo 2004 classification), heart function (NYHA classification), treatment regimens, and survival outcomes, were collected and analyzed. Heart function response was defined as achieving at least a VGPR. A multi-factorial Firth logistic regression analysis was performed to identify independent predictors of heart function response.

### Results

The cohort comprised 21 males and 16 females, with a median age of 63 years (range: 30–70). λ light-chain type accounted for 83.8% (n=31). Arrhythmia was prevalent in 62.2% (n=23). At baseline, 83.8% (n=31) were in NYHA class II-III. According to Mayo 2004 staging: Stage I (n=2), II (n=9), IIIa (n=21), and IIIb (n=5). Risk stratification identified 29 intermediate-risk and 8 high-risk patients. Anti-plasma cell therapy was administered to 36 patients, 19 of whom received Daratumumab-based regimens. The median time to best hematologic response was 3 months (range: 0–29). The median overall survival (OS) was 21.5 months (range: 1–60). Firth logistic regression analysis showed that the Mayo 2004 stage and the latency period from symptom onset to diagnosis were independent predictors of heart function response. Higher Mayo stage was significantly associated with a lower heart function response rate (OR=0.06, P=0.002), while a latency period exceeding 19 months was associated with a higher likelihood of heart function improvement (OR=28.72, P=0.005). Long-term treatment showed a positive trend toward better outcomes (OR=2.73, P=0.250), though this was not statistically significant after multivariable adjustment.

### Summary/Conclusion

In this cohort of isolated cardiac AL amyloidosis, Mayo 2004 stage and diagnostic latency are independent predictors of cardiac response. The high prevalence of advanced Mayo stages (70.3% of patients in late stages) underscores the importance of early detection. The unexpected positive correlation between longer latency and better response suggests that longer latency may serve as a biological marker of a "slower disease progression" phenotype, with the possibility of survival bias, reflecting a more indolent pathological feature.

**Copyright Information:** (Online) ISSN: 2572-9241
©2026 The Author(s). HemaSphere is published by John Wiley & Sons Ltd on behalf of European Hematology Association. This is an open access Abstract Book distributed under the Attribution-NonCommercial-NoDerivs (CC BY-NC-ND), which allows third parties to download the articles and share them with others as long as they credit the author and the Abstract Book, but they cannot change the content in any way or use them commercially.
**Abstract Book Citations:** Authors, Title, HemaSphere, 2026;10:(S1):pages. The abstract book DOIs can be found on the "Author Information" page.
**Disclaimer:** Articles published in the journal HemaSphere exclusively reflect the opinions of the authors. The authors are responsible for all content in their abstracts including accuracy of the facts, statements, citing resources, etc.

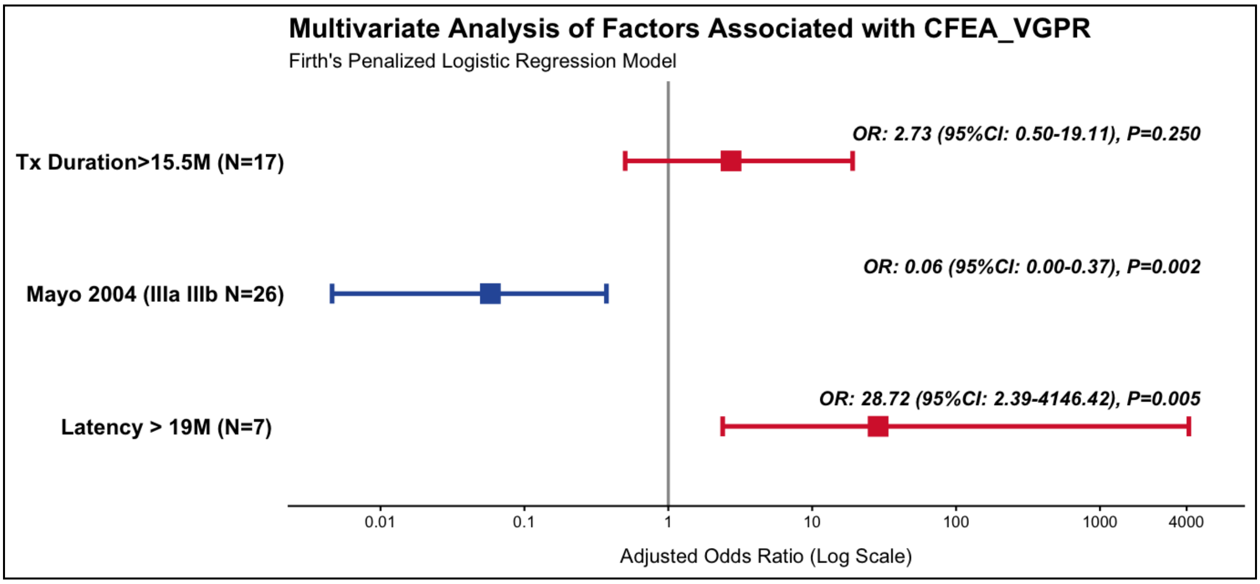

Figure 1: Multivariate analysis of factors associated with CFEA_VGPR using Firth's penalized logistic regression model. The adjusted odds ratios (ORs) with 95% confidence intervals (CIs) are shown for three factors: treatment duration (Tx Duration >15.5M), Mayo 2004 stage (IIIa/IIIb), and latency (>19M). The Mayo 2004 stage and latency were found to be significant predictors of VGPR achievement, while treatment duration did not show a significant association (p = 0.250).

**Copyright Information:** (Online) ISSN: 2572-9241
©2026 The Author(s). HemaSphere is published by John Wiley & Sons Ltd on behalf of European Hematology Association. This is an open access Abstract Book distributed under the Attribution-NonCommercial-NoDerivs (CC BY-NC-ND), which allows third parties to download the articles and share them with others as long as they credit the author and the Abstract Book, but they cannot change the content in any way or use them commercially.
**Abstract Book Citations:** Authors, Title, HemaSphere, 2026;10:(S1):pages. The abstract book DOIs can be found on the "Author Information" page.
**Disclaimer:** Articles published in the journal HemaSphere exclusively reflect the opinions of the authors. The authors are responsible for all content in their abstracts including accuracy of the facts, statements, citing resources, etc.

### (PB3328) UPDATED RESULTS OF ISATUXIMAB PLUS POMALIDOMIDE AND DEXAMETHASONE IN CHINESE PATIENTS WITH RELAPSED/REFRACTORY MULTIPLE MYELOMA: 2-YEAR FOLLOW-UP OF A REAL-WORLD PROSPECTIVE STUDY

**Topic:** 14. Myeloma and other monoclonal gammopathies - Clinical

Jianhua You*[1, 2], Wenting Chen[1, 2, 3, 4], Lie Lin[3], Xiaojing Yan[5], Xi Zhang[6], Weiwei Tian[7], Wenming Chen[8], Yafei Wang[9, 10], Yingxue Wang[11], Dan Gao[12], Lei Zhou[12], Zhini Wang[12], Liu Gang[13], Weili Zhao[1, 2]

*[1]Shanghai Institute of Hematology, State Key Laboratory of Medical Genomics, National Research Center for Translational Medicine at Shanghai, Ruijin Hospital, Shanghai Jiao Tong University School of Medicine, Shanghai, China; [2]Department of Hematology, Ruijin-Hainan Hospital, Shanghai Jiao Tong University School of Medicine, Boao, Hainan, China; [3]Department of Hematology, Hainan General Hospital (Hainan Affiliated Hospital of Hainan Medical University), Haikou, Hainan, China; [4]School of Biomedical Engineering, Hainan University, Haikou, Hainan, China; [5]Department of Hematology, The First Hospital of China Medical University, Shenyang, Liaoning, China; [6]Medical Center of Hematology, Xinqiao Hospital of Army Medical University, Chongqing, China; [7]Department of Hematology, Shanxi Bethune Hospital, Shanxi Academy of Medical Sciences, Tongji Shanxi Hospital, Third Hospital of Shanxi Medical University, Taiyuan, Shanxi, China; [8]Department of Hematology, Beijing Chao-Yang Hospital, Capital Medical University, Beijing, China; [9]Department of Hematology, Tianjin Medical University Cancer Institute and Hospital, Tianjin, China; [10]Department of Blood and Marrow Transplantation, Tianjin Cancer Hospital Airport Hospital, Tianjin, China; [11]Department of Rheumatology, The Second Hospital of Shandong University, Jinan, Shandong, China; [12]Research and Development, Sanofi, Beijing, China; [13]Oncology Medical Affairs, Sanofi, Shanghai, China;*

### Background

Multiple myeloma (MM) is an incurable hematologic malignancy, with increasing incidence and mortality in China. The combination of pomalidomide/dexamethasone with isatuximab (Isa-Pd) was approved in China based on the findings of the global ICARIA-MM phase 3 study and the prospective observational real-world IsaFiRsT study in Chinese patients. In the primary analysis of IsaFiRsT (median follow-up 8.41 months), Isa-Pd was effective in Chinese patients with relapsed/refractory multiple myeloma (RRMM) and had an acceptable safety profile, consistent with findings from ICARIA-MM. Here, we report the 2-year follow-up results of IsaFiRsT (ChiCTR2200062878).

### Aims

To provide longer follow-up evidence of the real-world effectiveness and safety of Isa-Pd in Chinese patients with RRMM.

### Methods

Patients aged ≥18 years with RRMM (≥2 prior therapies, including lenalidomide and a proteasome inhibitor) and measurable serum/urine M-protein who provided written informed consent were eligible. Isa-Pd initiation was determined independently by treating physicians (TP) prior to enrollment. The label-recommended dose of isatuximab (10 mg/kg intravenously weekly in Cycle 1, then every 2 weeks) plus pomalidomide (4 mg/day, Days 1–21 of repeated 28-day cycles) and dexamethasone (40 mg [20 mg if ≥75 years]) weekly on days 1, 8, 15, and 22 of each cycle. Treatment could be adjusted based on the TP's clinical judgment and was continued until disease progression or unacceptable toxicity. The primary endpoint was overall response rate (ORR); secondary endpoints included progression-free survival (PFS), overall survival (OS), duration of response (DOR), and safety.

**Copyright Information:** (Online) ISSN: 2572-9241
©2026 The Author(s). HemaSphere is published by John Wiley & Sons Ltd on behalf of European Hematology Association. This is an open access Abstract Book distributed under the Attribution-NonCommercial-NoDerivs (CC BY-NC-ND), which allows third parties to download the articles and share them with others as long as they credit the author and the Abstract Book, but they cannot change the content in any way or use them commercially.
**Abstract Book Citations:** Authors, Title, HemaSphere, 2026;10:(S1):pages. The abstract book DOIs can be found on the "Author Information" page.
**Disclaimer:** Articles published in the journal HemaSphere exclusively reflect the opinions of the authors. The authors are responsible for all content in their abstracts including accuracy of the facts, statements, citing resources, etc.

**Results**

A total of 24 patients were enrolled (median 62 years; 45.8% male; median 3 prior lines of therapy). At the March 12, 2025, data cut-off (median follow-up: 23.8 months), 12 patients remained on treatment; reasons for discontinuation among the remaining 12 included disease progression (n=8), adverse events (n=3), and patient withdrawal (n=1). The median (range) duration of exposure was 21.1 (0.23–24.9) months. ORR was 82.6% (19/23; 95% CI 61.2, 95.0), 60.9% achieved complete response or better, and 78.3% achieved very good partial response or better (Table). Median (range) time to first response was 1.18 (0.9–3.1) months. The median PFS and DOR were not reached (NR); The estimated PFS rate at 12 months was 82.6% (95% CI 60.1, 93.1). The estimated OS rate at 12-month was 87.0% (95% CI 64.8, 95.6) . All patients had at least 1 treatment-emergent adverse event (TEAE; any grade) and ≥1 Grade ≥3 TEAE; 22 patients (91.7%) had serious TEAEs. Most frequent (>15%) Grade ≥3 TEAEs were neutrophil count decreased (79.2%), white blood cell count decreased (58.3%), anemia (50.0%), pneumonia (45.8%), platelet count decreased (37.5%), and hypokalemia (25.0%). Ten patients (41.7%) experienced ≥1 infusion reaction (IR) with isatuximab. Only 1 patient (4.2%) reported Grade 3 IR; no Grade >3 IRs were reported. Eight patients died during the on- or post-treatment period, 6 due to progressive disease (all during post-treatment period), and 2 due to AE. No new safety signals were identified.

**Summary/Conclusion**

Consistent with the primary analysis, 2-year follow-up of IsaFiRsT further confirms that Isa-Pd provides a deep and rapid response and demonstrates sustained effectiveness in heavily pretreated Chinese patients with RRMM, with an acceptable long-term safety profile in a real-world setting.

**Table.** Summary of effectiveness

| | **Isa-Pd (n=23[b])** |
|---|---|
| Best overall response, n (%) | |
| sCR | 13 (56.5) |
| CR | 1 (4.3) |
| VGPR | 4 (17.4) |
| PR | 1 (4.3) |
| SD | 4 (17.4) |
| ORR, % (95% CI)[a] | 82.6 (61.2, 95.0) |
| ≥CR, n (%) | 14 (60.9) |
| ≥VGPR, n (%) | 18 (78.3) |
| Median DOR, months (95% CI) | NR (18.8, NE) |
| Median PFS, months (95% CI) | NR (14.5, NE) |
| PFS, % (95% CI) | |
| 12 months | 82.6 (60.1, 93.1) |
| 20 months | 59.0 (35.6, 76.3) |
| OS, % (95% CI) | |
| 12 months | 87.0 (64.8, 95.6) |
| 20 months | 82.6 (60.1,93.1) |

[a] Defined as an sCR, CR, VGPR, or PR. [b] 1 patient was not evaluable for response.
Isa-Pd, isatuximab with pomalidomide and dexamethasone; sCR, stringent complete response; CR, complete response; VGPR, very good partial response; PR, partial response; SD, stable disease; ORR, overall response rate; CI, confidence interval; DOR, duration of response; NE, not evaluable; NR, not reached; PFS, progression-free survival; OS, overall survival

**Copyright Information:** (Online) ISSN: 2572-9241
©2026 The Author(s). HemaSphere is published by John Wiley & Sons Ltd on behalf of European Hematology Association. This is an open access Abstract Book distributed under the Attribution-NonCommercial-NoDerivs (CC BY-NC-ND), which allows third parties to download the articles and share them with others as long as they credit the author and the Abstract Book, but they cannot change the content in any way or use them commercially.
**Abstract Book Citations:** Authors, Title, HemaSphere, 2026;10:(S1):pages. The abstract book DOIs can be found on the "Author Information" page.
**Disclaimer:** Articles published in the journal HemaSphere exclusively reflect the opinions of the authors. The authors are responsible for all content in their abstracts including accuracy of the facts, statements, citing resources, etc.

### (PB3329) THE VALIDATION OF A MULTIPLE MYELOMA MINIMAL RESIDUAL DISEASE FLOW CYTOMETRY ASSAY FOR USE IN MULTI-CENTRIC CLINICAL TRIALS.

**Topic:** 14. Myeloma and other monoclonal gammopathies - Clinical

Eirini Kamileri*[1], Christele Gonneau[1], Yoav Peretz[2]

*[1]Labcorp, Flow Cytometry, Geneva, Switzerland; [2]Labcorp, Flow Cytometry, Indianapolis, United States of America;*

### Background

Flow cytometry is a high-throughput and sensitive technique suitable for the detection of very rare cell populations amidst complex mixtures. Consequently, it has become a platform of choice for the detection of minimal residual disease (MRD) in various types of leukemia.

### Aims

For Multiple Myeloma (MM) patients undergoing treatment, the detection of MRD helps determine if any cancerous cells escape treatment. This biomarker can constitute an important prognostic measure and help predict the likelihood of disease relapse and/or progression. Therefore, there is considerable interest around the use of MRD as a potential surrogate endpoint in clinical trials.

### Methods

Labcorp has validated and adapted a highly sensitive next-generation flow cytometry assay to detect MRD in MM patients based on EuroFlow™ Consortium published recommendations (Kalina et.al., 2012) and the MM MRD assay developed by Waters Biosciences (formerly Becton Dickinson (BD) Biosciences)/Cytognos. Limit of Detection (LOD), Lower Limit of Quantification (LLOQ), precision and stability for key reported parameters of the assay, including abnormal plasma cells (PC) frequency, have been validated per published CLSI H62 guidelines and within CAP/CLIA compliance.

### Results

MM MRD assay validation experiments demonstrated high sensitivity (for abnormal PC, LLOQ was established at 23 events or 0.00039% (%Total Nucleated Cells, TNC)), acceptable assay performance (for abnormal PC intra-assay precision %CV was established at 7.67% (%TNC)) and confirmed published sample stability of 48 hours post-collection (Stetler-Stevenson et.al., 2016).

### Summary/Conclusion

Concluding that, MRD by flow cytometry offers a high-quality, sensitive, and robust approach to be used for multi-centric clinical trials and run in a CAP/CLIA clinical laboratory environment.

**Copyright Information:** (Online) ISSN: 2572-9241
©2026 The Author(s). HemaSphere is published by John Wiley & Sons Ltd on behalf of European Hematology Association. This is an open access Abstract Book distributed under the Attribution-NonCommercial-NoDerivs (CC BY-NC-ND), which allows third parties to download the articles and share them with others as long as they credit the author and the Abstract Book, but they cannot change the content in any way or use them commercially.
**Abstract Book Citations:** Authors, Title, HemaSphere, 2026;10:(S1):pages. The abstract book DOIs can be found on the “Author Information” page.
**Disclaimer:** Articles published in the journal HemaSphere exclusively reflect the opinions of the authors. The authors are responsible for all content in their abstracts including accuracy of the facts, statements, citing resources, etc.

### (PB3330) HEALTH CARE PROFESSIONALS (HCPS) PERSPECTIVES ON TREATMENT AND MANAGEMENT OF LIGHT CHAIN (AL) AMYLOIDOSIS: AN INTERNATIONAL CROSS-SECTIONAL SURVEY STUDY

**Topic:** 14. Myeloma and other monoclonal gammopathies - Clinical

Jamie Zigterman*[1], Kristen Hsu[1], Phaedra Johnson[1], Isabelle Lousada[1], Efstathios Kastritis[2], Paolo Milani[3], Angela Dispenzieri[4]

*[1]Amyloidosis Research Consortium, Research, Newton, United States of America;[2]National and Kapodistrian University of Athens, Athens, Greece;[3]University of Pavia, Department of Molecular Medicine, Pavia, Italy;[4]Mayo Clinic, Hematology, Rochester, United States of America;*

### Background

Light chain (AL) amyloidosis is a rare multi-systemic disease where abnormal proteins in plasma cells misfold and create amyloid fibrils that can deposit in organs and tissues, where they cause damage and increased risk of mortality. Cardiac involvement and depth of hematologic response, measured by difference between involved and uninvolved free light chains (dFLC), are the most important factors in determining AL amyloidosis prognosis however consensus criteria for hematologic progression may be outdated. In most countries, daratumumab (Dara) in combination with cyclophosphamide, bortezomib, and dexamethasone (CyBorD)is the preferred first-line therapy (FLT) for AL amyloidosis. This prospective study examined healthcare professional (HCP) perspectives on treatment and management of newly diagnosed and relapsed/refractory AL amyloidosis patients building on research by Milani, et al (2017).

### Aims

The goal of the study is to understand HCPs management of patients with newly diagnosed and relapsed/refractory AL amyloidosis, including their treatment preferences and goals for hematologic and organ response.

### Methods

The Amyloidosis Research Consortium (ARC) conducted an international cross-sectional online survey from April to July 2025. HCPs who treat AL amyloidosis identified in ARC's database were sent an email with individual links to complete the survey. Members of the International Society of Amyloidosis also received email notifications for study participation. Measures included HCP demographics, practice characteristics, FLT and SLT preferences, clinical trial perspectives, and use of clinical criteria including dFLC, relapsed/refractory definitions, and hematologic and organ progression. Target enrollment was 50 HCPs. Final enrollment was 54 HCPs with evaluable data from 48 respondents. Results were analyzed descriptively.

### Results

HCPs were primarily male (67%), hematologists or oncologists (86%, 10%), and based in the US (60%) or Europe (30%). Most treat patients in academic centers (94%) and have been treating patients with AL for at least 10 or more years (81%). HCPs prioritized rapid deep hematologic responses as shown by their practice patterns: 73% will switch therapy if there is < partial response (PR) within 2 treatment cycles; 65% will switch therapy if there is < very good partial response (VGPR) within 3 cycles; and 53% would switch therapy for less than a complete response (CR) by 6 months. The decision to re-initiate therapy is primarily determined by time between relapse/progression and discontinuation of last therapy (38%) and dFLC level at diagnosis (38%). Most were uncomfortable to very uncomfortable waiting for dFLC values to reach 50 mg/L (61%) to meet eligibility criteria for a trial of relapsed patients, especially for patients relapsing from CR. Only 21% would wait to satisfy consensus hematologic progression criteria to start therapy among patients relapsing from VGPR.

**Copyright Information:** (Online) ISSN: 2572-9241
©2026 The Author(s). HemaSphere is published by John Wiley & Sons Ltd on behalf of European Hematology Association. This is an open access Abstract Book distributed under the Attribution-NonCommercial-NoDerivs (CC BY-NC-ND), which allows third parties to download the articles and share them with others as long as they credit the author and the Abstract Book, but they cannot change the content in any way or use them commercially.
**Abstract Book Citations:** Authors, Title, HemaSphere, 2026;10:(S1):pages. The abstract book DOIs can be found on the "Author Information" page.
**Disclaimer:** Articles published in the journal HemaSphere exclusively reflect the opinions of the authors. The authors are responsible for all content in their abstracts including accuracy of the facts, statements, citing resources, etc.

**Summary/Conclusion**

Survey results provide contemporary insights into HCP preferences for FLT, criteria for determining when to reinitiate therapy, dFLC values for determining clinical trial eligibility, and definitions of hematologic and organ progression following the global approval of Dara in 2021. These findings also provide insights into the real-world management of more advanced AL patients and may help inform future hematologic consensus definitions and refine trial eligibility criteria.

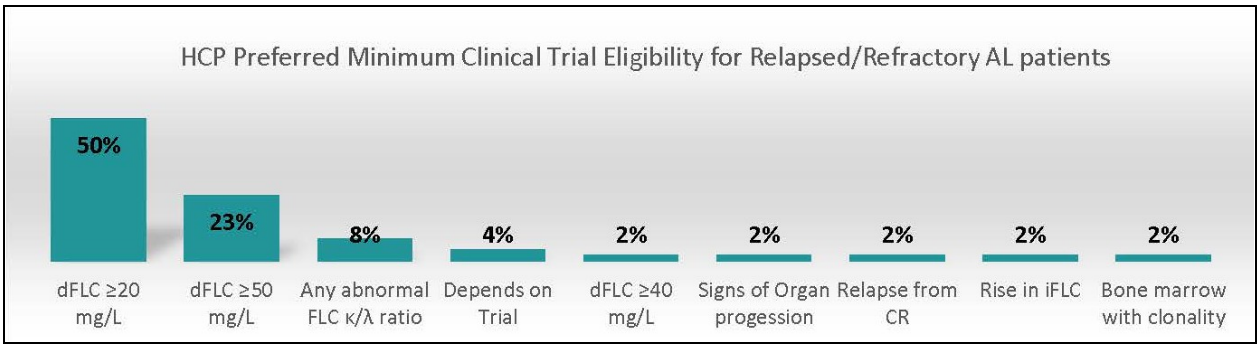

**Copyright Information:** (Online) ISSN: 2572-9241
©2026 The Author(s). HemaSphere is published by John Wiley & Sons Ltd on behalf of European Hematology Association. This is an open access Abstract Book distributed under the Attribution-NonCommercial-NoDerivs (CC BY-NC-ND), which allows third parties to download the articles and share them with others as long as they credit the author and the Abstract Book, but they cannot change the content in any way or use them commercially.
**Abstract Book Citations:** Authors, Title, HemaSphere, 2026;10:(S1):pages. The abstract book DOIs can be found on the "Author Information" page.
**Disclaimer:** Articles published in the journal HemaSphere exclusively reflect the opinions of the authors. The authors are responsible for all content in their abstracts including accuracy of the facts, statements, citing resources, etc.

### (PB3331) DISTRIBUTION OF IGG SUBCLASSES AND THEIR ASSOCIATION WITH RISK STRATIFICATION IN IGG-ASSOCIATED PLASMA CELL DISORDERS: ANALYSIS OF 491 PATIENTS

**Topic:** 14. Myeloma and other monoclonal gammopathies - Clinical

Aileen Schenk*[1], Britta Besemer[1], Claudia Lengerke[1], Andreas Peter[2], Reinhild Klein[1]

*[1]University Hospital Tübingen, Department of Hematology, Oncology, Clinical Immunology and Rheumatology, Tübingen, Germany; [2]University Hospital Tübingen, Institute for Clinical Chemistry and Pathobiochemistry, Department for Diagnostic Laboratory Medicine, Tübingen, Germany;*

### Background

IgG is the most common heavy chain isotype found in plasma cell disorders (PCD). However, the clinical relevance of IgG subclasses remains insufficiently characterized.

### Aims

This study aimed to analyze the distribution of IgG subclasses in IgG-associated PCD and to evaluate potential differences in established risk stratification systems across disease entities.

### Methods

A total of 491 patients with IgG-associated PCD were analyzed IgG subclasses were quantified by nephelometry. Subclass assignment was defined by an increased relative proportion of the respective IgG subclass (IgG1≥75%, IgG2≥25%, IgG3≥10%, IgG4≥8%).. Subclass distribution was assessed descriptively. Differences in risk stratification within each disease entity were evaluated using the chi-square test. For multiple myeloma (MM), risk was evaluated using the Revised International Staging System (R-ISS) and the presence of high-risk cytogenetics. For smoldering multiple myeloma (SMM), the 2/20/20 model ± cytogenetics was applied. For monoclonal gammopathy of undetermined significance (MGUS), risk stratification was performed according to the Rajkumar criteria.

### Results

IgG1 was the predominant subclass (76%, n=371), followed by IgG2 (14%, n=70), IgG4 (6%, n=31), and IgG3 (4%, n=19). The distribution of disease entities (MGUS, SMM, MM) was homogeneous across subclasses.
In MM, no statistically significant differences were observed between IgG subclasses regarding R-ISS stage or the prevalence of high-risk cytogenetics. Similarly, no significant differences were detected in SMM using the 2/20/20 model ± cytogenetics.
In MGUS, patients with the IgG3 subclass were significantly more frequently classified as high risk according to Rajkumar criteria ($p < 0.05$).

### Summary/Conclusion

IgG1 was the dominant subclass in IgG-associated plasma cell disorders in this cohort. While most analyses did not reveal any significant differences between subclasses, IgG3 appeared to be associated with adverse risk features,particularly in MM, where a higher proportion of patients showed an elevated R-ISS score and high-risk cytogenetics. However, these findings did not reach statistical significance, likely due to the small size of the IgG3 subgroup (n=19). Larger studies are required to clarify the prognostic relevance of IgG subclasses in plasma cell disorders.

**Copyright Information:** (Online) ISSN: 2572-9241
©2026 The Author(s). HemaSphere is published by John Wiley & Sons Ltd on behalf of European Hematology Association. This is an open access Abstract Book distributed under the Attribution-NonCommercial-NoDerivs (CC BY-NC-ND), which allows third parties to download the articles and share them with others as long as they credit the author and the Abstract Book, but they cannot change the content in any way or use them commercially.
**Abstract Book Citations:** Authors, Title, HemaSphere, 2026;10:(S1):pages. The abstract book DOIs can be found on the "Author Information" page.
**Disclaimer:** Articles published in the journal HemaSphere exclusively reflect the opinions of the authors. The authors are responsible for all content in their abstracts including accuracy of the facts, statements, citing resources, etc.

### (PB3332) ADDITION OF CARFILZOMIB TO DCEP (K-DCEP) IS A HIGHLY EFFECTIVE REGIMEN IN HEAVILY PRE-TREATED AND REFRACTORY MULTIPLE MYELOMA WITHOUT EXCESS TOXICITY

**Topic:** 14. Myeloma and other monoclonal gammopathies - Clinical

Shivem Shah*[1], Lia Jueng[1], Roger Pearse[1], Ruben Niesvizky[1], Adriana Rossi[2], Mateo Mejia Saldarriaga[1]

*[1]Weill Cornell, New York City, United States of America; [2]Icahn School of Medicine at Mount Sinai, New York City, United States of America;*

### Background

In patients with relapsed/refractory multiple myeloma (RRMM), dexamethasone, cyclophosphamide, etoposide, and cisplatin (DCEP) is often used as salvage therapy, frequently in combinations with thalidomide and bortezomib. Currently, most patients will have extensive exposure to IMiD's, PI's, and BCMA directed agents. Carfilzomib (K) is highly active even after previous PI use. In this heavily pre-treated population, the efficacy and toxicity of adding K to DCEP are not well characterized.

### Aims

We evaluate the efficacy and toxicity of K-DCEP as a salvage regimen in RRMM patients.

### Methods

We performed a retrospective analysis in all patients diagnosed with MM who last were treated at an academic center between 2020-2026 and who received K-DCEP. Each 4-day cycle included DCEP daily and K on day 1 (20 mg/m2) and day 4 (36 mg/m2). Patients with evaluable response data were included, with responses determined based on International Myeloma Working Group (IMWG) criteria. Grade ≥ 3 adverse events (per CTCAE v.5) were collected for 30 days post treatment. Due to addition of K, cardiac toxicities - heart failure, pulmonary edema, pHTN, arrhythmias, and cardiac arrest - were collected for 90 days post treatment.

### Results

14 patients with 1 patient receiving two independent cycles > 6 months apart (n = 15, median age 62 (range: 52-92) with 11/15 female) received K-DCEP.  7 patients also received daratumumab (Dara). 67% were had high risk cytogenetics (as defined by IMWG); median R-ISS score was 2. Median prior treatment lines was 8 with 86.7% triple-class (IMiD, PI, aCD38) and 60% penta-class (2 IMiD, 2 PI, aCD38) exposed. Previous treatment included autologous stem cell transplant (40%), any anti-BCMA therapy (46.7%) (including Blenrep (14.7%), BiTE therapy (42.85%), and CAR-T (42.85%)), chemotherapy (93.3%), K (86.7% of which 69.2% were refractory), and Dara (86.7% of which 69.2% were refractory). Overall response rate (ORR) was 80%; best responses included 1 CR, 3 VGPR, and 8 PR. Median free progression survival was 70 days with median overall survival of 257 days (**Fig. 1A & B**). After treatment, median time to discharge was 6 days, and median time to next treatment was 28.5 days. In terms of toxicities, median time to ANC nadir was 2 days with median time to recovery (>0.5 cells * 10^3/uL for two days) being 2.5 days, with 80% patients receiving prophylactic G-CSF. Median days to platelet recovery (>20 cells 10^3/uL for two days without transfusion) was 5 days, with median transfusion burden being 0 unit of platelets and 2 units of pRBCs. Additional toxicities were primarily dyspnea (20%), AKI (6.7%), AST/ALT elevation (6.7%), and 40% infection (n = 3 bacteremia, 2 viral pneumonia, and 1 with shingles). Importantly, there were no cardiac events in the 90 days following K-DCEP in this cohort.

**Copyright Information:** (Online) ISSN: 2572-9241
©2026 The Author(s). HemaSphere is published by John Wiley & Sons Ltd on behalf of European Hematology Association. This is an open access Abstract Book distributed under the Attribution-NonCommercial-NoDerivs (CC BY-NC-ND), which allows third parties to download the articles and share them with others as long as they credit the author and the Abstract Book, but they cannot change the content in any way or use them commercially.
**Abstract Book Citations:** Authors, Title, HemaSphere, 2026;10:(S1):pages. The abstract book DOIs can be found on the "Author Information" page.
**Disclaimer:** Articles published in the journal HemaSphere exclusively reflect the opinions of the authors. The authors are responsible for all content in their abstracts including accuracy of the facts, statements, citing resources, etc.

### Summary/Conclusion

K-DCEP can is a highly effective salvage regimen in heavily pretreated RRMM and should be considered even with previous K exposure. With most patients using G-CSF support, toxicities were similar to historical cohorts treated with DCEP, with no clear signal for increased hematological , transfusion support, hospital stays, or cardiac toxicities. The ORR observed here (80%) suggests this regimen can provide adequate cytoreduction and disease control, especially for those with previous T-cell engager or CAR-T exposure, or when access to these agents is not possible.

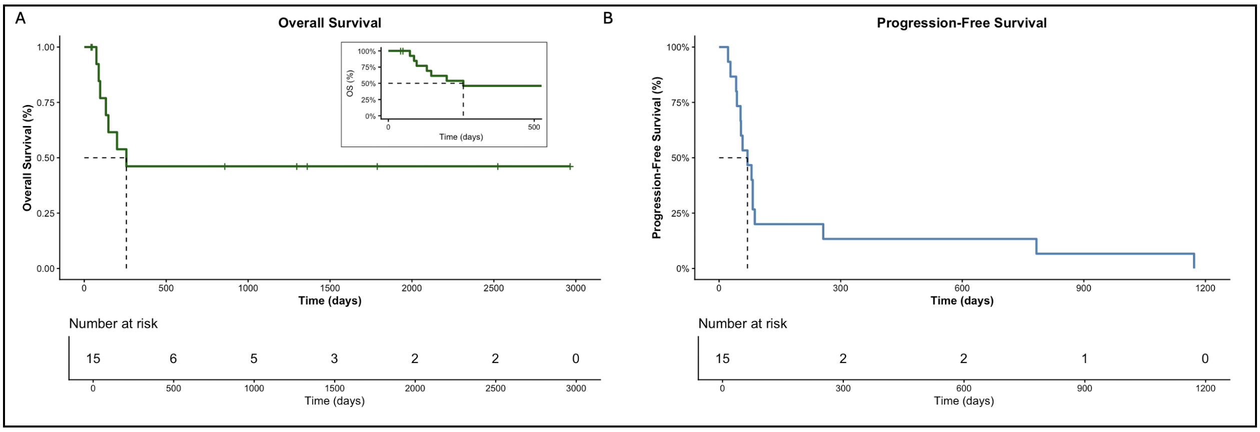

Figure 1. A) Kaplan-Meier overall survival curve 2.) Kaplan-Meier progression free survival

**Copyright Information:** (Online) ISSN: 2572-9241
©2026 The Author(s). HemaSphere is published by John Wiley & Sons Ltd on behalf of European Hematology Association. This is an open access Abstract Book distributed under the Attribution-NonCommercial-NoDerivs (CC BY-NC-ND), which allows third parties to download the articles and share them with others as long as they credit the author and the Abstract Book, but they cannot change the content in any way or use them commercially.
**Abstract Book Citations:** Authors, Title, HemaSphere, 2026;10:(S1):pages. The abstract book DOIs can be found on the "Author Information" page.
**Disclaimer:** Articles published in the journal HemaSphere exclusively reflect the opinions of the authors. The authors are responsible for all content in their abstracts including accuracy of the facts, statements, citing resources, etc.

### (PB3333) APONERMIN COMBINED WITH THALIDOMIDE-DEXAMETHASONE (TD) OR TD PLUS OTHER AGENTS (TD+) IN RELAPSED/REFRACTORY MULTIPLE MYELOMA

**Topic:** 14. Myeloma and other monoclonal gammopathies - Clinical

Chuanying Geng*[1], Huihan Wang[2], Guangzhong Yang[1], Weiwei Tian[3], Yanping MA[4], Yafei Guo[5], Lan Lingqiong[6], Jingrong Wang[7], Baijun Fang[8], Xiaoling Chen[9], Wenming Chen[1]

*[1]Beijing Chao-yang Hospital, Capital Medical University, beijing, China;[2]Shengjing Hospital of China Medical University, Shenyang, China; [3]Shanxi Bethune Hospital, Shanxi, China; [4]The Second Hospital of Shanxi Medical university, taiyuan, China; [5]Second Affiliated Hospital of Fujian Medical University, quanzhou, China;[6]Longyan Second Hospital, longyan, China;[7]Shanxi Province Cancer Hospital/ Shanxi Hospital Affiliated to Cancer Hospital, taiyuan, China; [8]Henan Institute of Hematology, The Affiliated Cancer Hospital of Zhengzhou University & Henan Cancer Hospital, zhengzhou, China; [9]Zhabei Central Hospital of Jing'an District, Shanghai, shanghai, China;*

### Background

Aponermin, a recombinant circularly permuted TRAIL, activates death receptors on tumor cells and has shown synergy with proteasome inhibitors and corticosteroids in multiple myeloma (MM). Thalidomide-dexamethasone (Td) remains a backbone regimen for relapsed/refractory MM (RRMM), but the benefit of adding aponermin to Td or Td-based combinations is unclear.

### Aims

To evaluate the efficacy and safety of aponermin combined with Td (Apo-Td) versus aponermin combined with Td plus other agents (Apo-Td+) in patients with RRMM. The primary endpoint was overall response rate (ORR) per IMWG criteria. Secondary endpoints included progression-free survival (PFS).

### Methods

This retrospective, multi-center study enrolled patients with RRMM who had received at least two prior lines of therapy. Patients were assigned to Apo-Td (thalidomide + dexamethasone) or Apo-Td+ (thalidomide + dexamethasone + additional drugs) based on physician's choice. Aponermin was administered at 10 mg/kg on days 1–5 of each 28-day cycle.

### Results

Between May 2024 and January 2026, 18 patients were enrolled (9 per group). Baseline characteristics: median age ≤65 years in 61.1% (11/18), male 66.7% (12/18), IgA type 61.1% (11/18), ISS stage III 53.3% (8/15 evaluable), high-risk cytogenetics 33.3% (3/9 evaluable), and extramedullary disease 47.1% (8/17 evaluable). Median prior lines of therapy were 2 (range 1–6) in the Apo-Td group and 4 (2–14) in the Apo-Td+ group (p=0.226) (Table 1). At a median follow-up of 2.5 months, ORR was 57.1% (4/7) in the Apo-Td group and 87.5% (7/8) in the Apo-Td+ group (p=0.412). The ≥VGPR rate was 14.3% vs 25.0%, respectively (Table 2). Median PFS was 5.4 months (Apo-Td) vs 4.9 months (Apo-Td+) (p=0.741).

### Summary/Conclusion

Aponermin-based regimens showed promising activity in heavily pretreated RRMM, with numerically higher response rates in the Apo-Td+ group, though not statistically significant. The combination was well-tolerated. Larger studies are warranted to identify optimal partners for aponermin in RRMM.

**Copyright Information:** (Online) ISSN: 2572-9241
©2026 The Author(s). HemaSphere is published by John Wiley & Sons Ltd on behalf of European Hematology Association. This is an open access Abstract Book distributed under the Attribution-NonCommercial-NoDerivs (CC BY-NC-ND), which allows third parties to download the articles and share them with others as long as they credit the author and the Abstract Book, but they cannot change the content in any way or use them commercially.
**Abstract Book Citations:** Authors, Title, HemaSphere, 2026;10:(S1):pages. The abstract book DOIs can be found on the "Author Information" page.
**Disclaimer:** Articles published in the journal HemaSphere exclusively reflect the opinions of the authors. The authors are responsible for all content in their abstracts including accuracy of the facts, statements, citing resources, etc.

| Table 1. Baseline Clinical and Biological Characteristics of MM Patients | | | | | |
|---|---|---|---|---|---|
| Characteristics | | all n=18 n (%) | Td n=9 n (%) | Td+ n=9 n (%) | p value |
| Sex | | | | | |
| | Male | 12 (66.7) | 7 (77.8) | 5 (55.6) | 0.317 |
| | Female | 6 (33.3) | 2 (22.2) | 4 (44.4) | |
| Age | | | | | |
| | ≤65 years | 11 (61.1) | 5 (55.6) | 6 (66.7) | 0.629 |
| | >65 years | 7 (38.9) | 4 (44.4) | 3 (33.3) | |
| Monoclonal protein type | | | | | |
| | IgG | 5 (27.8) | 4 (44.4) | 1 (11.1) | 0.270 |
| | IgA | 11 (61.1) | 4 (44.4) | 7 (77.8) | |
| | IgD | 2 (11.1) | 1 (11.1) | 1 (11.1) | |
| ISS stage | | | | | |
| | I | 4 (26.7) | 1 (14.3) | 3 (37.5) | 0.412 |
| | II | 3 (20.0) | 1 (14.3) | 2 (25.0) | |
| | III | 8 (53.3) | 5 (71.4) | 3 (37.5) | |
| Median no. of prior (range) | | 2(1-14) | 2(1-6) | 4(2-14) | 0.226 |
| Previous therapy | | | | | |
| | PIs/IMIDs/CD38 | 3 (27.3) | 1 (16.7) | 2 (18.2) | 0.387 |
| | ASCT | 4 (33.3) | 2 (28.6) | 2 (40.0) | 0.679 |
| Cytogenetic abnormalities by FISH | | | | | |
| | High risk | 3 (33.3) | 1 (33.3) | 2 (33.3) | 0.679 |
| | Standard risk | 6 (66.7) | 2 (66.7) | 4 (66.7) | |
| Extramedullary disease | | | | | |
| | Yes | 8 (47.1) | 3 (37.5) | 5 (55.6) | 0.457 |
| | No | 9 (52.9) | 5 (62.5) | 4 (44.4) | |

Abbreviations: Td: Thalidomide and dexamethasone; Td+: Thalidomide, dexamethasone and other drugs; ISS: International Staging System;Pis: Protease inhibitors; IMiD: immunomodulatory; ASCT: autologous stem cell transplant

**Table 2. Best response rate after induction therapy in all patients**

| | Overall n (%) | Td n (%) | Td+ n (%) |
|---|---|---|---|
| sCR/CR | 3 (20.0) | 1 (14.3) | 2 (25.0) |
| VGPR/PR | 8 (53.3) | 3 (42.9) | 5 (62.5) |
| < PR | 4 (26.7) | 3 (42.9) | 1 (12.5) |

Abbreviations: Td: Thalidomide and dexamethasone; Td+: Thalidomide, dexamethasone and other drugs; sCR: stringent complete response; CR: complete response; VGPR: very good partial response; PR: partial response

**Copyright Information:** (Online) ISSN: 2572-9241
©2026 The Author(s). HemaSphere is published by John Wiley & Sons Ltd on behalf of European Hematology Association. This is an open access Abstract Book distributed under the Attribution-NonCommercial-NoDerivs (CC BY-NC-ND), which allows third parties to download the articles and share them with others as long as they credit the author and the Abstract Book, but they cannot change the content in any way or use them commercially.
**Abstract Book Citations:** Authors, Title, HemaSphere, 2026;10:(S1):pages. The abstract book DOIs can be found on the "Author Information" page.
**Disclaimer:** Articles published in the journal HemaSphere exclusively reflect the opinions of the authors. The authors are responsible for all content in their abstracts including accuracy of the facts, statements, citing resources, etc.

### (PB3334) ALLG MM26-D1/AMN009 NOVEL COMBINATIONS FOR ORPHAN MYELOMA (NORM) PLATFORM STUDY

**Topic:** 14. Myeloma and other monoclonal gammopathies - Clinical

Georgia McCaughan*[1, 2, 3], John Reynolds[4], Tzu-Yang Wang[5], Angelina Yong[6, 7], Kihyun Kim[8], Jeffrey Huang[9], Christian Bryant[10, 11], Nicole Wong Doo[12, 13], Matthew Ku[14], Wee Joo Chng[15], Hang Quach[14, 16]

*[1]St Vincent's Hospital, Haematology, Sydney, Australia;[2]Garvan Institute of Medical Research, Sydney, Australia;[3]University of New South Wales, School of Clinical Medicine, Sydney, Australia; [4]Monash University, Australian Centre for Blood Diseases, Melbourne, Australia; [5]Princess Alexandra Hospital, Department of Haematology, Woolloongabba, Australia;[6]Central Adelaide Local Health Network, Department of Haematology, Adelaide, Australia; [7]Adelaide University, Adelaide Medical School, Adelaide, Australia; [8]Samsung Medical Centre, Haematology-Oncology, Seoul, South Korea;[9]National Taiwan University Hospital, Taipei, Taiwan; [10]Royal Prince Alfred Hospital, Department of Haematology, Camperdown, Australia;[11]University of Sydney, Sydney Medical School, Sydney, Australia; [12]Concord Hospital, Department of Haematology, Sydney, Australia; [13]University of Sydney, Concord Clinical School, Sydney, Australia;[14]St Vincent's Hospital, Department of Haematology, Melbourne, Australia; [15]National University of Singapore, Department of Haematolog-Oncology, Singapore, Singapore; [16]University of Melbourne, Faculty of Medicine, Melbourne, Australia;*

### Background

In multiple myeloma (MM), patients with non-measurable disease, renal impairment, extramedullary myeloma (EMD) or central nervous system (CNS) involvement are frequently excluded from clinical trials. These biologically and clinically high-risk groups represent significant unmet need, and their underrepresentation has resulted in limited evidence to guide management. To address this, we have established a multi-arm platform trial to explore novel agents in these underserved populations.

### Aims

The NORM platform recruits patients with relapsed/refractory MM (RRMM) meeting one of four strata: renal impairment (CrCl <30ml/min; stratum A); non-measurable disease (stratum B); EMD (stratum C) and CNS disease (stratum D). Agents may be evaluated in one or more strata (Figure 1).

The primary objective is to determine efficacy within each stratum, defined as the proportion of patients who achieve≥partial response (IMWG), at any time up to cycle 4. In stratum B, response assessment will incorporate PET-based criteria (Zagmani 2021).

In stratum D, primary CNS lymphoma response criteria will be utilised (Abrey 2005). Secondary endpoints include progression free, overall survival, safety and quality of life.

### Methods

In each stratum, efficacy will be investigated using a dual-criteria, Bayesian, Proof-of-Concept (PoC) approach with a minimally informative prior for the response rate.

The first treatment domain was selinexor, pomalidomide and dexamethasone (SPd) in patients who were refractory, or had a contraindication, to bortezomib and lenalidomide. Dosing was given in 28-day cycles: selinexor 60mg weekly, pomalidomide 4mg day 1-21, Dexamethasone 40mg weekly. Planned recruitment was 40 patients in Strata A-C and 20 in D. PoC for efficacy of SPd in a stratum will be claimed if 2 criteria are met: Stratum A or B, observed ORR ≥40% and posterior probability (PP) that the true ORR is ≥30%, given the data, is ≥0.90 and in stratum C or D, observed ORR ≥30% and PP(true ORR ≥20% | data) ≥ 0.90.

**Copyright Information:** (Online) ISSN: 2572-9241
©2026 The Author(s). HemaSphere is published by John Wiley & Sons Ltd on behalf of European Hematology Association. This is an open access Abstract Book distributed under the Attribution-NonCommercial-NoDerivs (CC BY-NC-ND), which allows third parties to download the articles and share them with others as long as they credit the author and the Abstract Book, but they cannot change the content in any way or use them commercially.
**Abstract Book Citations:** Authors, Title, HemaSphere, 2026;10:(S1):pages. The abstract book DOIs can be found on the "Author Information" page.
**Disclaimer:** Articles published in the journal HemaSphere exclusively reflect the opinions of the authors. The authors are responsible for all content in their abstracts including accuracy of the facts, statements, citing resources, etc.

### Results

The study closed early due to funding withdrawal. 13 patients were enrolled in Stratum C, 12 received SPd; median (range) age = 61 (50 – 83), median (range) number of prior lines = 4 (2 – 7). The protocol allowed monitoring of the primary endpoint to commence when $\geq$ 12 patients became evaluable in a stratum. In Stratum C, the ORR was 5/12 (41.7%), exceeding the threshold (30%) in the first of the dual PoC criteria, and the posterior probability that the true response rate is greater than equal to 20% is 0.9499 exceeding the level of proof (0.90). Hence PoC, can, per protocol, be claimed for Stratum C. 9 patients were recruited to Stratum A, 5 to Stratum B and 4 to Stratum D; not meeting the criteria for monitoring the primary endpoint.
All patients experienced an adverse event. The most common Grade 3/4 adverse events were haematological; neutropenia (53.3%), thrombocytopenia (40%), anaemia (36.7%). Grade 3/4 infections were reported in 16.7%. Any grade fatigue was seen in 40%, nausea in 33.3% and anorexia in 10%. One grade 5 adverse event was reported.

### Summary/Conclusion

Recruitment of patients typically excluded from conventional trials was feasible, supporting the practicality of this PoC platform for rapid evaluation of novel regimens in high-risk MM subgroups . SPd showed clinically meaningful activity in RRMM with EMD, meeting the protocol defined PoC. Toxicities were primarily haematological; nausea and anorexia were infrequent and generally manageable with supportive care.

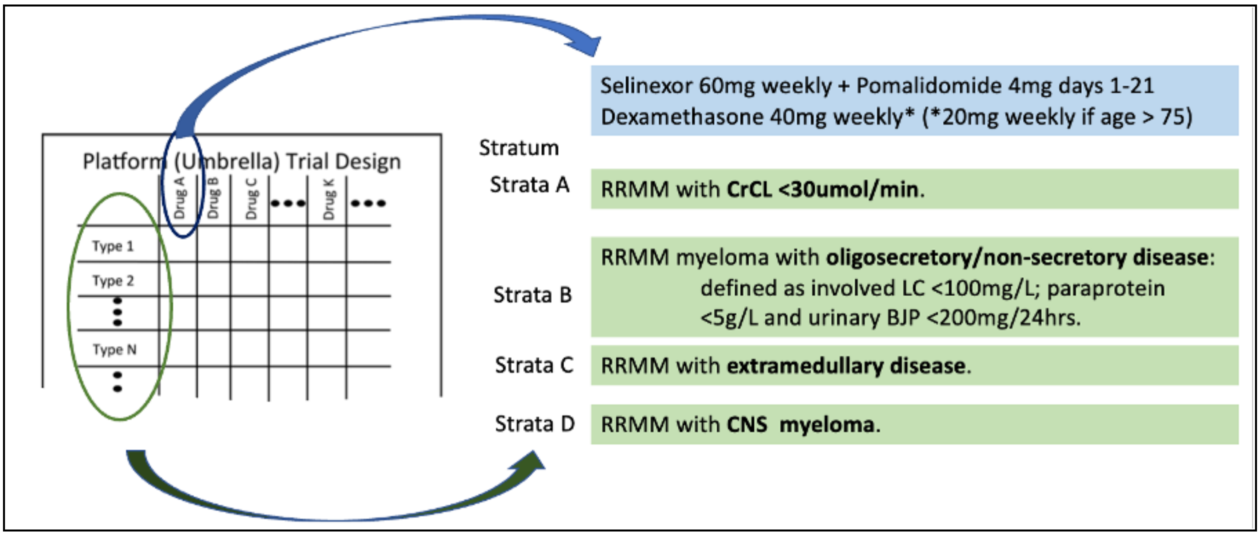

Figure 1: NORM Platform Study Design

Copyright Information: (Online) ISSN: 2572-9241
©2026 The Author(s). HemaSphere is published by John Wiley & Sons Ltd on behalf of European Hematology Association. This is an open access Abstract Book distributed under the Attribution-NonCommercial-NoDerivs (CC BY-NC-ND), which allows third parties to download the articles and share them with others as long as they credit the author and the Abstract Book, but they cannot change the content in any way or use them commercially.
Abstract Book Citations: Authors, Title, HemaSphere, 2026;10:(S1):pages. The abstract book DOIs can be found on the "Author Information" page.
Disclaimer: Articles published in the journal HemaSphere exclusively reflect the opinions of the authors. The authors are responsible for all content in their abstracts including accuracy of the facts, statements, citing resources, etc.

### (PB3335) RENAL RESPONSE OF PATIENTS WITH MULTIPLE MYELOMA WITH RENAL DYSFUNCTION TO DARATUMUMAB-BASED TREATMENT

**Topic:** 14. Myeloma and other monoclonal gammopathies - Clinical

Jiali Li*[1], Song Dong[1], Xixi Xiang[1], Yunjing Zeng[1], Li Gao[1], Xi Zhang[1]

[1]*Medical Center of Hematology. Institute of Science Innovation for Blood Ecology and Intelligent Cells. Xinqiao Hospital of Army Medical University, Chongqing, China;*

### Background

Kidney injury is the most common complications of multiple myeloma (MM). Up to 50% of MM patients have kidney injury at the time of diagnosis, and 2-4% of them need dialysis. Rapid reversal of renal injury is very important to improve the clinical efficacy. Daratumumab is an anti-CD 38 monoclonal antibody, which has significant clinical effect in MM. In order to evaluate the therapeutic effect of Daratumumab -based regimen on multiple myeloma complicated with kidney injury.

### Aims

To analyze the treatment, efficacy of Daratumumab in MM patients with kidney injury.

### Methods

Patients with diagnosed multiple myeloma. Inclusion criteria: adult patients with age ≥18 years, symptomatic NDMM or RRMM, RI (defined eGFR by MDRD [illegible]40ml/min). Daratumumab has been used for more than one cycle. The primary endpoint was renal ORR. Secondary endpoints included maior renal response (≥PR), hematologica ORR, progression-free survival (PFS), overall survival(OS).

### Results

Between Aug 22,2021 and Jun 30, 2025, 68 patients were enrolled. with a median age of 67 years (range: 49-84). Thirty-four patients (50%) used Daratumumab in a relapsed/refractory condition. Twenty-five patients used Daratumumab in the first-line treatment, accounting for 36.8%. The median time from diagnosis of myeloma to the start of Daratumumab was 2 months. The proportion of ISS Ill was 55.9%. The most common cytogenetics change is 1q21 apm. Median serum creatine level and eGFR were 418.9 umol/L (interquartile range (IQR): 151.2-1184) and 13 ml/min (IQR: 4-39), respectively. The primary endpoint with renal ORR of total patients at 1 months,2 months,3 months was 30.88%( including 22.06% renal PR and 8.82% renal CR), 30.88%( including 23.53% renal PR and 10.29% renal CR), 25%(including 11.76% renal PR and 13.24% renal CR),respectively. The renal ORR of NDMM with RI at 2 months,3 months was 40%(10% renal CR), 40%( 25% renal CR),respectively. The median follow-up time was 11.2 months[illegible]the estimated 12-month survival rate was 91.5%.

### Summary/Conclusion

This real-world study demonstrates that a Daratumumab-based regimen is highly effective in treating multiple myeloma (MM) patients complicated with severe kidney injury. The benefit was particularly pronounced in newly diagnosed multiple myeloma (NDMM) patients, where the renal ORR at three months was 40%. These findings support the early initiation of daratumumab to optimize renal outcomes in MM patients with renal impairment.

**Copyright Information:** (Online) ISSN: 2572-9241
©2026 The Author(s). HemaSphere is published by John Wiley & Sons Ltd on behalf of European Hematology Association. This is an open access Abstract Book distributed under the Attribution-NonCommercial-NoDerivs (CC BY-NC-ND), which allows third parties to download the articles and share them with others as long as they credit the author and the Abstract Book, but they cannot change the content in any way or use them commercially.
**Abstract Book Citations:** Authors, Title, HemaSphere, 2026;10:(S1):pages. The abstract book DOIs can be found on the "Author Information" page.
**Disclaimer:** Articles published in the journal HemaSphere exclusively reflect the opinions of the authors. The authors are responsible for all content in their abstracts including accuracy of the facts, statements, citing resources, etc.

### (PB3336) DOES PELVIC BONE INVOLVEMENT DEFINE A DISTINCT PROGNOSTIC PHENOTYPE IN MULTIPLE MYELOMA? A RETROSPECTIVE SURVIVAL ANALYSIS

**Topic:** 14. Myeloma and other monoclonal gammopathies - Clinical

Sengul Baran Yerlikaya[1], Ozlem Beyler*[1], Cengiz Demir[1]

[1]*Gazi Yasargil Training and Research Hospital, Diyarbakir, Türkiye;*

### Background

Skeletal involvement is a defining feature of multiple myeloma (MM) and represents both a diagnostic and prognostic hallmark of the disease. Osteolytic lesions occur in the majority of patients and are a major cause of morbidity through skeletal-related events. However, while the overall presence of bone disease is well established as a marker of symptomatic MM, the prognostic significance of specific anatomical skeletal localization remains insufficiently characterized. The pelvis represents a region of high marrow activity and substantial biomechanical load, and its involvement may reflect either biologically aggressive disease behavior or simply advanced skeletal dissemination.

### Aims

This study aimed to evaluate whether pelvic lytic bone involvement is associated with adverse clinicopathological features and inferior overall survival in patients with multiple myeloma.

### Methods

We retrospectively analyzed 91 consecutive MM patients followed at a tertiary center. Associations between pelvic involvement and clinical, laboratory, and cytogenetic variables were evaluated using chi-square and Fisher's exact tests. Overall survival (OS) was calculated from diagnosis to death or last follow-up using Kaplan–Meier methodology and compared by log-rank testing. Due to limited event numbers, a parsimonious multivariable Cox regression model including pelvic involvement, ISS stage, and cytogenetic risk was constructed.

**Copyright Information:** (Online) ISSN: 2572-9241
©2026 The Author(s). HemaSphere is published by John Wiley & Sons Ltd on behalf of European Hematology Association. This is an open access Abstract Book distributed under the Attribution-NonCommercial-NoDerivs (CC BY-NC-ND), which allows third parties to download the articles and share them with others as long as they credit the author and the Abstract Book, but they cannot change the content in any way or use them commercially.
**Abstract Book Citations:** Authors, Title, HemaSphere, 2026;10:(S1):pages. The abstract book DOIs can be found on the "Author Information" page.
**Disclaimer:** Articles published in the journal HemaSphere exclusively reflect the opinions of the authors. The authors are responsible for all content in their abstracts including accuracy of the facts, statements, citing resources, etc.

### Results

Pelvic involvement was significantly associated with younger age (≤65 years: 69.5% vs 46.9%, p=0.034), extensive skeletal disease (other lytic lesions: 98.3% vs 40.6%, p<0.001), and plasmacytoma presence (40.7% vs 12.5%, p=0.005). Patients with pelvic lesions were more frequently treated in advanced therapy lines (p=0.004), suggesting a greater disease burden. No statistically significant differences were observed in ISS, R-ISS distribution, gender, or cytogenetic risk between groups. Median follow-up was 27.9 months, during which seven deaths occurred. Kaplan–Meier analysis showed no significant difference in OS between patients with and without pelvic involvement (log-rank p=0.434). In multivariable Cox regression, pelvic involvement was not independently associated with mortality (HR 0.55, 95% CI 0.12–2.50, p=0.443), while increasing ISS stage demonstrated a trend toward higher risk (HR 2.48, 95% CI 0.82–7.45, p=0.107).

**Table 1. Comparison of Clinical and Demographic Characteristics According to Pelvic Lytic Bone Involvement**

| Variable | Pelvic (-) n=32 | Pelvic (+) n=59 | p-value |
|---|---|---|---|
| Age ≤65 years | 15 (46.9%) | 41 (69.5%) | 0.034 |
| Sex (Male) | 20 (62.5%) | 34 (57.6%) | 0.651 |
| ISS Stage III | 8 (25.0%) | 20 (33.9%) | 0.672 |
| R-ISS Stage III | 2 (6.3%) | 6 (10.3%) | 0.808 |
| Cytogenetic abnormality (+) | 6 (18.8%) | 9 (15.3%) | 0.668 |
| Any lytic lesion present | 10 (31.3%) | 49 (83.1%) | **<0.001** |
| Plasmacytoma present | 4 (12.5%) | 24 (40.7%) | **0.005** |
| Other bone lytic lesion present | 13 (40.6%) | 58 (98.3%) | **<0.001** |
| Advanced therapy lines (≥3rd line) | 11 (34.4%) | 50 (84.7%) | **0.004** |

**Copyright Information:** (Online) ISSN: 2572-9241
©2026 The Author(s). HemaSphere is published by John Wiley & Sons Ltd on behalf of European Hematology Association. This is an open access Abstract Book distributed under the Attribution-NonCommercial-NoDerivs (CC BY-NC-ND), which allows third parties to download the articles and share them with others as long as they credit the author and the Abstract Book, but they cannot change the content in any way or use them commercially.
**Abstract Book Citations:** Authors, Title, HemaSphere, 2026;10:(S1):pages. The abstract book DOIs can be found on the "Author Information" page.
**Disclaimer:** Articles published in the journal HemaSphere exclusively reflect the opinions of the authors. The authors are responsible for all content in their abstracts including accuracy of the facts, statements, citing resources, etc.

**Summary/Conclusion**

Pelvic lytic bone involvement in MM strongly correlates with disseminated skeletal disease and plasmacytoma formation, supporting its association with increased tumor burden. However, in this cohort, pelvic localization did not independently predict inferior overall survival. These findings suggest that pelvic involvement may reflect disease extent rather than intrinsic biological aggressiveness.

**Copyright Information:** (Online) ISSN: 2572-9241
©2026 The Author(s). HemaSphere is published by John Wiley & Sons Ltd on behalf of European Hematology Association. This is an open access Abstract Book distributed under the Attribution-NonCommercial-NoDerivs (CC BY-NC-ND), which allows third parties to download the articles and share them with others as long as they credit the author and the Abstract Book, but they cannot change the content in any way or use them commercially.
**Abstract Book Citations:** Authors, Title, HemaSphere, 2026;10:(S1):pages. The abstract book DOIs can be found on the "Author Information" page.
**Disclaimer:** Articles published in the journal HemaSphere exclusively reflect the opinions of the authors. The authors are responsible for all content in their abstracts including accuracy of the facts, statements, citing resources, etc.

### (PB3337) REAL-WORLD ADHERENCE TO ANTI-PNEUMOCOCCAL VACCINATION GUIDELINES IN NEWLY TREATED MULTIPLE MYELOMA PATIENTS IN FRANCE: A NATIONWIDE PMSI ANALYSIS

**Topic:** 14. Myeloma and other monoclonal gammopathies - Clinical

Manon Sapet*[1], Ilhame Bounouara[1], Margot Brossard[1], Tiphany Neel[1], Charlotte Doublet[1], Anthony Levy[2], Ugo Thevenet[3], Fabien Tinquaut[1], Emilie Chalayer[1]

*[1]CHU Saint-Etienne, Hematology, Saint-Etienne, France;[2]Centre Hospitalier Jacques Lacarin, Vichy, France; [3]Centre Hospitalier de Roanne, Roanne, France;*

### Background

Patients with multiple myeloma (MM) are at high risk of invasive pneumococcal infections due to disease-related and treatment-induced immunosuppression. International and national guidelines recommend sequential anti-pneumococcal vaccination with PCV13 followed by PPSV23 in all MM patients. However, real-world adherence to these recommendations remains poorly documented in France.

### Aims

We aimed to assess nationwide pneumococcal vaccination coverage in newly treated MM patients using medico-administrative databases.

### Methods

We conducted a nationwide retrospective study including newly treated MM patients receiving first chemotherapy during the study period between March 1, 2021 and March 1, 2023. We used the National Health Data System (SNDS). MM patients were identified from the French national hospital database (PMSI-MCO) using the following criteria: (i) primary diagnosis C90 associated with chemotherapy code Z51.1 as a related diagnosis, or (ii) primary diagnosis Z51.1 with related diagnosis C90. To restrict the cohort to newly treated patients, we excluded individuals previously identified with MM before the index period. The final cohort was cross-matched with outpatient healthcare consumption databases to identify pneumococcal vaccine dispensations using predefined UCD codes. PCV13 and PPSV23 were retrieved. Vaccination was considered regardless of timing within the study period.
The primary objective was to evaluate overall pneumococcal vaccination coverage. Secondary objectives included assessment of adherence to the recommended sequential vaccination schedule and identification of patients receiving incomplete vaccination.

### Results

Between March 2021 and March 2023, 12,423 newly treated MM patients received a first chemotherapy course in France (46% female, (mean [SD] age, 70.6 [11.3] years). Overall, 3.933 patients (31.7%) received at least one pneumococcal vaccine during the study period. Adherence to the recommended sequential schedule was observed in only 2,421 patients (19.5%). Indeed, 2,421 patients received both PCV13 and PPSV23 (optimal sequential schedule), 810 received PCV13 only, 702 received PPSV23 only and 8,490 received no pneumococcal vaccine. Thus, 68.3% of newly treated MM patients had no documented pneumococcal vaccination, and 12.2% received an incomplete vaccination strategy.

**Copyright Information:** (Online) ISSN: 2572-9241
©2026 The Author(s). HemaSphere is published by John Wiley & Sons Ltd on behalf of European Hematology Association. This is an open access Abstract Book distributed under the Attribution-NonCommercial-NoDerivs (CC BY-NC-ND), which allows third parties to download the articles and share them with others as long as they credit the author and the Abstract Book, but they cannot change the content in any way or use them commercially.
**Abstract Book Citations:** Authors, Title, HemaSphere, 2026;10:(S1):pages. The abstract book DOIs can be found on the "Author Information" page.
**Disclaimer:** Articles published in the journal HemaSphere exclusively reflect the opinions of the authors. The authors are responsible for all content in their abstracts including accuracy of the facts, statements, citing resources, etc.

**Summary/Conclusion**

In this large nationwide real-world analysis, pneumococcal vaccination coverage among newly treated MM patients in France remains suboptimal. Fewer than one in five patients received the recommended sequential PCV13–PPSV23 schedule. Although this study has some limitations due to the source of the data which does not allow the integration of hospital vaccination, and to provide the percentage of patients who received a prescription but did not carry out the vaccination compared to those who did not receive a vaccination prescription, these findings highlight a substantial gap between guideline recommendations and real-world practice and underscore the need for structured vaccination pathways integrated into MM care to improve infection prevention in this highly vulnerable population.

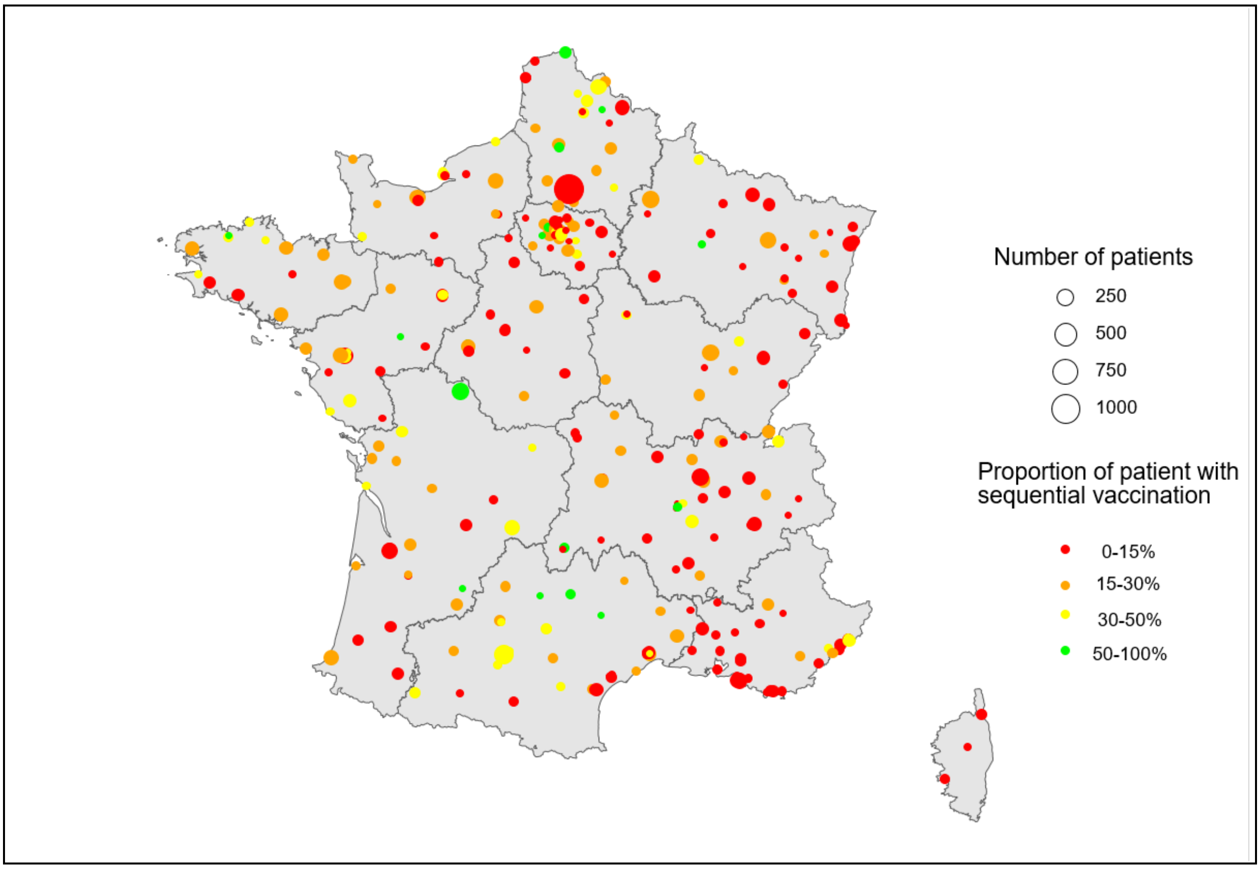

Regional disparities in vaccination adherence for the pneumococcal vaccines in France between 2021-2023

Copyright Information: (Online) ISSN: 2572-9241
©2026 The Author(s). HemaSphere is published by John Wiley & Sons Ltd on behalf of European Hematology Association. This is an open access Abstract Book distributed under the Attribution-NonCommercial-NoDerivs (CC BY-NC-ND), which allows third parties to download the articles and share them with others as long as they credit the author and the Abstract Book, but they cannot change the content in any way or use them commercially.
Abstract Book Citations: Authors, Title, HemaSphere, 2026;10:(S1):pages. The abstract book DOIs can be found on the "Author Information" page.
Disclaimer: Articles published in the journal HemaSphere exclusively reflect the opinions of the authors. The authors are responsible for all content in their abstracts including accuracy of the facts, statements, citing resources, etc.

### (PB3338) CLINICAL CHARACTERISTICS AND PROGNOSTIC ANALYSIS OF EXTRAMEDULLARY DISEASE IN MULTIPLE MYELOMA

**Topic:** 14. Myeloma and other monoclonal gammopathies - Clinical

Jingliang Zhao*[1], Qirui Bai[1], Qile Qiu[1], Jiaying Song[1], Siyu Kong[1], Kun Zhu[1], Yifan Zhang[1], Shengtao Li[1], Lin Zhang[1], Yanping MA[1], Xiaoqi Qin[1]

[1]*Second Hospital of Shanxi Medical University, Hematology, Taiyuan, China;*

### Background

Extramedullary disease (EMD) in multiple myeloma (MM) is associated with poor prognosis and can be classified into bone-related (bEMD) and soft-tissue (sEMD) subtypes. However, the clinical heterogeneity and prognostic factors between these subtypes remain poorly characterized, and a simple risk stratification tool for EMD patients is lacking.

### Aims

To compare the clinical characteristics and prognosis of bone-related extramedullary disease (bEMD) and soft-tissue extramedullary disease (sEMD) in patients with multiple myeloma (MM), and to construct a simple prognostic scoring system for risk stratification.

### Methods

The clinical data of 118 MM patients with EMD admitted to the Second Hospital of Shanxi Medical University from August 2011 to August 2025 were retrospectively analyzed. Among them, there were 72 cases in the bEMD group and 46 cases in the sEMD group. The clinical baseline data, laboratory indicators, cytogenetic abnormalities and survival data of the two groups of patients at the time of initial diagnosis were collected. The Kaplan-Meier method was used to analyze the progression-free survival (PFS) and overall survival (OS). The Cox proportional hazard regression model was used to screen independent prognostic factors, and a prognostic risk scoring system was constructed and verified based on key indicators.

### Results

The serum β2-microglobulin (β2-MG) level of patients in the sEMD group was significantly higher than that in the bEMD group (6.4 mg/L vs 4.5 mg/L, $p = 0.007$), and the proportion of relapsed and refractory patients in the sEMD group was higher (41.3% vs 15.3%, $p = 0.002$). Survival analysis showed that the median progression-free survival (PFS) and overall survival (OS) of the sEMD group were significantly shorter than those of the bEMD group (12.0 months vs 29.0 months, $p < 0.001$; 25.0 months vs 67.0 months, $p = 0.009$). Multivariate Cox regression analysis showed that thrombocytopenia, elevated serum β2-MG, multi-site extramedullary infiltration and P53 deletion were independent adverse prognostic factors for PFS and OS in MM patients with EMD (all $p < 0.05$). The risk scoring system based on β2-MG (<3.5 mg/L scored 0, 3.5–5.5 mg/L scored 1, >5.5 mg/L scored 2), thrombocytopenia (<100×$10^9$/L scored 1) and multi-site extramedullary infiltration (≥2 sites scored 1) divided the patients into low-risk group (0-2 points) and high-risk group (3-4 points). The difference between the two groups in PFS (27.0 months vs 10.0 months, $p < 0.001$) and OS (54.0 months vs 22.0 months, $p = 0.008$) was significant.

**Copyright Information:** (Online) ISSN: 2572-9241
©2026 The Author(s). HemaSphere is published by John Wiley & Sons Ltd on behalf of European Hematology Association. This is an open access Abstract Book distributed under the Attribution-NonCommercial-NoDerivs (CC BY-NC-ND), which allows third parties to download the articles and share them with others as long as they credit the author and the Abstract Book, but they cannot change the content in any way or use them commercially.
**Abstract Book Citations:** Authors, Title, HemaSphere, 2026;10:(S1):pages. The abstract book DOIs can be found on the "Author Information" page.
**Disclaimer:** Articles published in the journal HemaSphere exclusively reflect the opinions of the authors. The authors are responsible for all content in their abstracts including accuracy of the facts, statements, citing resources, etc.

**Summary/Conclusion**

sEMD is more clinically aggressive and has a worse prognosis than bEMD. Thrombocytopenia, high β2-MG level, multi-site extramedullary infiltration and P53 deletion are key adverse prognostic factors in MM patients with EMD. The simple risk scoring system constructed in this study has good risk stratification ability and provides reference for clinical individualized treatment strategy formulation.

**Copyright Information:** (Online) ISSN: 2572-9241
©2026 The Author(s). HemaSphere is published by John Wiley & Sons Ltd on behalf of European Hematology Association. This is an open access Abstract Book distributed under the Attribution-NonCommercial-NoDerivs (CC BY-NC-ND), which allows third parties to download the articles and share them with others as long as they credit the author and the Abstract Book, but they cannot change the content in any way or use them commercially.
**Abstract Book Citations:** Authors, Title, HemaSphere, 2026;10:(S1):pages. The abstract book DOIs can be found on the "Author Information" page.
**Disclaimer:** Articles published in the journal HemaSphere exclusively reflect the opinions of the authors. The authors are responsible for all content in their abstracts including accuracy of the facts, statements, citing resources, etc.

### (PB3339) EXAMINING THE ROLE OF NURSES IN MULTIPLE MYELOMA (MM) CARE: INSIGHTS FROM A EUROPEAN SURVEY

**Topic:** 14. Myeloma and other monoclonal gammopathies - Clinical

Maria Teresa San Miguel-Arregui*[1], Yvonne Barnes[2], Fiona Pereira[3], Stefanie Teloniatis[4]

*[1]Cancer Centre Clinica Universidad De Navarra, Pamplona, Spain;[2]Sanofi, Cambridge, United States of America;[3]IQVIA Ltd, London, United Kingdom;[4]IQVIA Ltd, Athens, Greece;*

### Background

Nurses in Europe play a central and multifaceted role in MM care, contributing to clinical management, patient education, and psychosocial support. However, their roles and responsibilities across the patient journey remain inconsistently defined and often underrecognized. A structured survey-based mapping of MM nursing roles across Europe offers a pragmatic way to clarify responsibilities and identify challenges to inform actionable strategies for optimal care delivery.

### Aims

To map the role of nurses in MM care across Europe, identify key challenges they face in clinical practice, and explore opportunities to strengthen their contribution to patient care.

### Methods

We conducted a 30-minute online survey between May and June 2025 among 60 nurses across hospitals and specialized clinics in France, Germany, Italy, Spain, and the UK (n=12/country). Eligible participants were MM nurses with >1 year of experience who spent ≥75% of their professional time in clinical practice and managed >10 patients with MM per year. The survey comprised closed and open-ended questions on nursing roles across the patient journey, involvement in multidisciplinary teams (MDTs), availability of MM-specific training and guidelines, key challenges in clinical practice, perceived impact on patient care, and aspirations for professional development. Data were analyzed using descriptive statistics. All participants provided written informed consent.

### Results

All 60 nurses completed the survey: advanced nurse practitioners (28%), oncology nurses (28%), hematology nurses (27%), and clinical nurse specialists (17%). The mean MM care experience was 9.5 (range 6–15) years. Most nurses identified themselves as key decision-makers across the MM patient journey (**Fig. 1A**). This involved counseling (72%), patient information and education (67%), and quality-of-life assessment (65%) at diagnosis, and medication preparation (72%), medication administration (70%), and patient education (65%) during treatment and follow-up. Further, most nurses (95%) participate in MDTs, with roles primarily focused on patient needs, such as home care (68%), symptom management (67%), and psychosocial support (56%). Over half (58%) were aware of guidelines defining the nursing role in MM, but 52% reported the guidelines to be nonspecific or locally developed. While 60% agreed that general nursing education covers some aspects of MM care, more than half reported insufficient MM-specific training for nurses in continuing medical education programs. Key challenges included nurse shortages (62%), healthcare and hospital resource/capacity pressures (45%), and lack of financial incentives for specialized nurses (38%) (**Fig. 1B**). Despite nearly all nurses (98%) believing their role in MM care should be better supported, owing to their proximity to patients (24%) and provision of holistic care (20%), existing challenges continue to limit their ability to provide optimal care in practice.

**Copyright Information:** (Online) ISSN: 2572-9241
©2026 The Author(s). HemaSphere is published by John Wiley & Sons Ltd on behalf of European Hematology Association. This is an open access Abstract Book distributed under the Attribution-NonCommercial-NoDerivs (CC BY-NC-ND), which allows third parties to download the articles and share them with others as long as they credit the author and the Abstract Book, but they cannot change the content in any way or use them commercially.
**Abstract Book Citations:** Authors, Title, HemaSphere, 2026;10:(S1):pages. The abstract book DOIs can be found on the "Author Information" page.
**Disclaimer:** Articles published in the journal HemaSphere exclusively reflect the opinions of the authors. The authors are responsible for all content in their abstracts including accuracy of the facts, statements, citing resources, etc.

**Summary/Conclusion**

Although nurses play a central role across the MM patient journey, their ability to fully consolidate this role and deliver optimal care is hindered by limited specialized training, lack of standardized guidelines, and broader systemic barriers. Addressing these gaps through clearer role definitions, improved MM-specific training, and stronger structural support could strengthen nursing practice, ultimately enhancing patient experience and outcomes in MM care.

**Figure 1. Key decision-making roles (A) and challenges (B) reported by nurses involved in MM care**

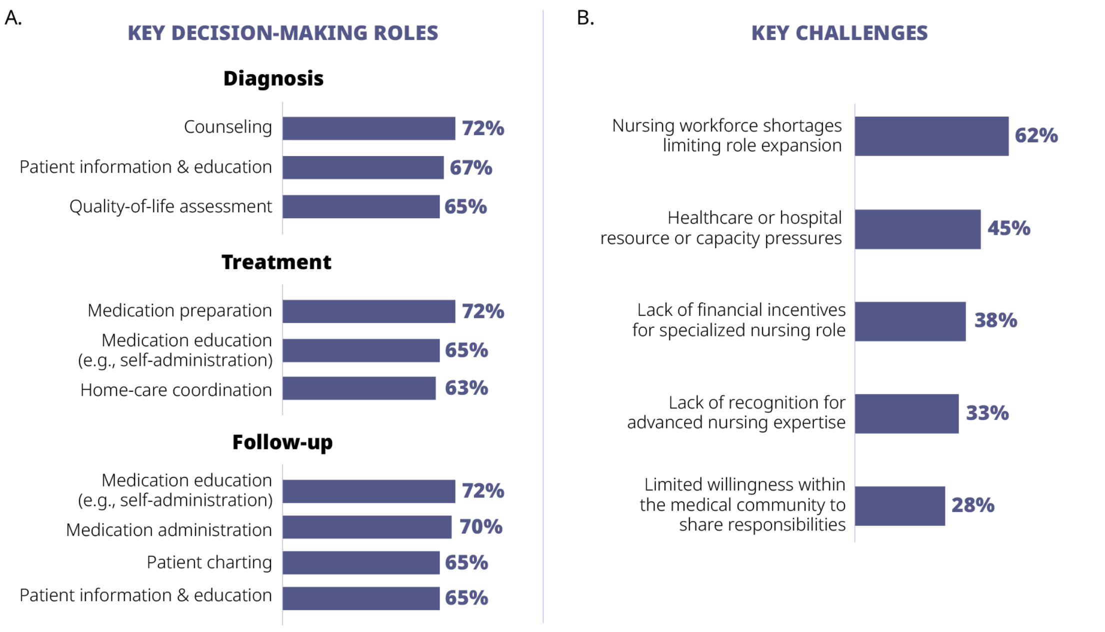

**Copyright Information:** (Online) ISSN: 2572-9241
©2026 The Author(s). HemaSphere is published by John Wiley & Sons Ltd on behalf of European Hematology Association. This is an open access Abstract Book distributed under the Attribution-NonCommercial-NoDerivs (CC BY-NC-ND), which allows third parties to download the articles and share them with others as long as they credit the author and the Abstract Book, but they cannot change the content in any way or use them commercially.
**Abstract Book Citations:** Authors, Title, HemaSphere, 2026;10:(S1):pages. The abstract book DOIs can be found on the "Author Information" page.
**Disclaimer:** Articles published in the journal HemaSphere exclusively reflect the opinions of the authors. The authors are responsible for all content in their abstracts including accuracy of the facts, statements, citing resources, etc.

### (PB3340) DETERMINANTS OF 10-YEAR OVERALL SURVIVAL IN MULTIPLE MYELOMA: A MULTICENTER RETROSPECTIVE CASE-CONTROL STUDY IN THE CHINESE POPULATION

**Topic:** 14. Myeloma and other monoclonal gammopathies - Clinical

Yinyi Zhang*[1], Yi Li[1], Donghua He[1], Yang Yang[1], Ying Lu[2], Xiaoyan Han[1], Guifang Ouyang[3], Minlei Zhao[4], Gaofeng Zheng[1], Lihong Shou[5], Junyu Zhang[4], He Jingsong[1], Zhen Cai[1]

*[1]The First Affiliated Hospital of Zhejiang University School of Medicine, Hangzhou, China;[2]Department of Hematology, The Affiliated People's Hospital of Ningbo University, Ningbo, China; [3]The First Affiliated Hospital of Ningbo University, Ningbo, China; [4]Lishui Central Hospital, Lishui, China;[5]HUZHOU CENTRAL HOSPITAL, HUZHOU, China;*

### Background

Multiple myeloma (MM) is a clonal hematologic malignancy of aberrant plasma cells. While novel therapies over the past two decades have markedly prolonged MM patients' overall survival (OS), data on clinical characteristics and prognostic factors of very long-term survivors (VLTS, OS ≥10 years)—especially large-scale multicenter data from Chinese populations remain scarce.

### Aims

This retrospective case-control study enrolled MM patients with OS ≥10 years as case group and contemporaneously treated patients without 10-year survival as controls, aiming to identify clinical features and independent prognostic factors of 10-year VLTS in Chinese MM patients and provide evidence-based guidance for clinical individualized treatment and prognostic stratification.

### Methods

Inclusion criteria (case group): 1) Symptomatic MM diagnosed per International Myeloma Working Group (IMWG) criteria; 2) Age ≥18 years at diagnosis; 3) OS ≥10 years from first-line therapy initiation; 4) Complete clinical/follow-up data.Exclusion criteria (all subjects): Missing key prognostic/survival data.The control group comprised contemporaneously treated MM patients meeting the above inclusion criteria except for OS <10 years.

Demographic, baseline clinical/laboratory, treatment, response and survival data were extracted from electronic medical records. Categorical variables were reported as percentages, continuous variables as medians (interquartile range, IQR). Intergroup comparisons, survival analysis, and prognostic factor identification were performed using Chi-square/Mann-Whitney U tests, Kaplan-Meier with log-rank test, and multivariable Cox/logistic regression models, respectively.

**Copyright Information:** (Online) ISSN: 2572-9241
©2026 The Author(s). HemaSphere is published by John Wiley & Sons Ltd on behalf of European Hematology Association. This is an open access Abstract Book distributed under the Attribution-NonCommercial-NoDerivs (CC BY-NC-ND), which allows third parties to download the articles and share them with others as long as they credit the author and the Abstract Book, but they cannot change the content in any way or use them commercially.
**Abstract Book Citations:** Authors, Title, HemaSphere, 2026;10:(S1):pages. The abstract book DOIs can be found on the "Author Information" page.
**Disclaimer:** Articles published in the journal HemaSphere exclusively reflect the opinions of the authors. The authors are responsible for all content in their abstracts including accuracy of the facts, statements, citing resources, etc.

**Results**

We enrolled 96 MM patients with OS ≥10 years (case group) and 140 contemporaneous non-10-year survivors (controls). vs controls, cases had significantly more favorable baseline prognoses: lower HRCAs rate (11.5% vs 39.3%, p<0.001), higher ISS stage I and R-ISS stage I proportions (all p<0.001), lower beta-2-microglobulin, and lower incidences of anemia, hypercalcemia, elevated LDH and renal impairment (all p<0.05).
For treatment, cases had significantly higher rates of ASCT (28.1% vs 8.6%), maintenance therapy (85.4% vs 35.7%), CR after 4 first-line cycles (47.4% vs 22.1%) and best overall response (65.3% vs 28.6%, all p<0.001). Median PFS was significantly longer in cases (82.1 vs 18.3 months, p<0.001), with no significant intergroup difference in relapse rates (86.5% vs 90.7%, p=0.398).
Multivariable Cox regression identified HRCAs (HR 1.82, 95%CI 1.26-2.64, p=0.001), elevated LDH (HR 1.56, 95%CI 1.01-2.41, p=0.043) and renal impairment (HR 1.80, 95%CI 1.14-2.84, p=0.012) as independent adverse OS prognostic factors; higher non-clonal bone marrow plasma cell proportion was an independent protective factor (HR 0.99, 95%CI 0.98-1.00, p=0.031).
Multivariable logistic regression confirmed HRCAs as the sole independent negative predictor of 10-year VLTS (OR 0.28, 95%CI 0.12-0.57, p<0.001).

**Summary/Conclusion**

HRCAs, elevated LDH and renal impairment are independent adverse OS prognostic factors in MM patients. Notably, HRCAs are the sole independent negative predictor of 10-year VLTS. Patients with deep first-line response, ASCT receipt and maintenance therapy are more likely to achieve ≥10-year VLTS. This study characterizes clinical features and independent determinants of 10-year VLTS in Chinese MM patients, providing real-world evidence to guide prognostic stratification and individualized treatment for newly diagnosed MM.

**Copyright Information:** (Online) ISSN: 2572-9241
©2026 The Author(s). HemaSphere is published by John Wiley & Sons Ltd on behalf of European Hematology Association. This is an open access Abstract Book distributed under the Attribution-NonCommercial-NoDerivs (CC BY-NC-ND), which allows third parties to download the articles and share them with others as long as they credit the author and the Abstract Book, but they cannot change the content in any way or use them commercially.
**Abstract Book Citations:** Authors, Title, HemaSphere, 2026;10:(S1):pages. The abstract book DOIs can be found on the "Author Information" page.
**Disclaimer:** Articles published in the journal HemaSphere exclusively reflect the opinions of the authors. The authors are responsible for all content in their abstracts including accuracy of the facts, statements, citing resources, etc.

### (PB3341) DYNAMIC INFLAMMATORY HEMATOLOGIC MARKERS DURING INDUCTION THERAPY AND CLINICAL OUTCOMES IN NEWLY DIAGNOSED MULTIPLE MYELOMA

**Topic:** 14. Myeloma and other monoclonal gammopathies - Clinical

Alexandra-Ştefania Stroe-Ionescu*[1, 2], Ana Maria Patrascu[1, 2], Janina Georgiana Goanta[1, 2], Alina Maria Ilie[2], Mihail-Virgil Boldeanu[1, 2], Ionela Rotaru[1, 2]

[1]*University of Medicine and Pharmacy of Craiova, Craiova, Romania;*[2]*Filantropia Clinical Hospital, Craiova, Romania;*

### Background

Systemic inflammation plays a central role in multiple myeloma (MM) biology, influencing tumor progression and treatment response. Inflammation-based hematologic indices derived from the complete blood count are inexpensive and widely available biomarkers, but their longitudinal prognostic value during induction therapy remains insufficiently defined.

### Aims

To evaluate changes in inflammation-based hematologic indices from diagnosis to after four cycles of induction therapy in newly diagnosed MM and to assess their association with progression-free survival (PFS) and overall survival (OS).

### Methods

We conducted a retrospective single-center cohort study including 122 consecutive patients with newly diagnosed MM. Neutrophil-to-lymphocyte ratio (NLR), platelet-to-lymphocyte ratio (PLR), monocyte-to-lymphocyte ratio (MLR), systemic immune-inflammation index (SII), pan-immune-inflammation value (PIV), and red cell distribution width (RDW) were assessed at diagnosis and after four induction cycles. Paired comparisons were performed using non-parametric tests. Survival analyses were performed using Kaplan–Meier estimates and Cox regression models. A landmark approach was applied for post–four-cycle analyses. ROC curves were used to determine outcome-oriented cut-off values.

### Results

Median age was 68 years (IQR 62–75), and 51.6% had ISS stage III disease. During a median follow-up of 11.3 months (IQR 4.5–26.6), 52 patients (42.6%) experienced progression or relapse and 57 (46.7%) died. All evaluated inflammatory indices increased significantly after induction therapy (all $p < 0.05$). At diagnosis, baseline inflammatory indices showed limited prognostic value; however, RDW ≥14.8% was associated with inferior PFS (HR 1.58, 95% CI 1.03–2.43; $p = 0.037$). After four cycles, RDW demonstrated improved discrimination for OS (AUC 0.651; cut-off 15.8%) and PFS (AUC 0.636; cut-off 16.8%). In landmark analyses, elevated post-treatment RDW was associated with inferior OS (HR 2.21, 95% CI 1.28–3.82; $p = 0.004$) and PFS (HR 2.04, 95% CI 1.26–3.30; $p = 0.004$). Other inflammatory indices were not significantly associated with survival outcomes. In multivariable landmark models adjusting for age, ISS stage, renal function, LDH, and bone marrow plasmocyte percentage, RDW showed a consistent but attenuated association with outcomes, while LDH remained independently associated with OS.

### Summary/Conclusion

Among multiple inflammation-based hematologic indices, RDW showed the most consistent association with clinical outcomes, particularly when assessed after induction therapy. Early post-treatment RDW assessment may provide complementary prognostic information in routine clinical practice. Prospective validation in larger cohorts is warranted.

**Copyright Information:** (Online) ISSN: 2572-9241
©2026 The Author(s). HemaSphere is published by John Wiley & Sons Ltd on behalf of European Hematology Association. This is an open access Abstract Book distributed under the Attribution-NonCommercial-NoDerivs (CC BY-NC-ND), which allows third parties to download the articles and share them with others as long as they credit the author and the Abstract Book, but they cannot change the content in any way or use them commercially.
**Abstract Book Citations:** Authors, Title, HemaSphere, 2026;10:(S1):pages. The abstract book DOIs can be found on the “Author Information” page.
**Disclaimer:** Articles published in the journal HemaSphere exclusively reflect the opinions of the authors. The authors are responsible for all content in their abstracts including accuracy of the facts, statements, citing resources, etc.

### (PB3342) SUSTAINING CONTEMPORARY NDMM CARE DURING WARTIME: EARLY OUTCOMES OF AN INTEGRATED ALL-UNDER-ONE-ROOF PATHWAY

**Topic:** 14. Myeloma and other monoclonal gammopathies - Clinical

Anna Sergeieva*[1], Zinaida Stupakova[1], Kateryna Khurdepa[1], Bohdana Kuiavovych[1], Kateryna Davydova[1]

*[1]National Research Center for Radiation Medicine, Oncology and Hematology of NAMS of Ukraine, Blood pathology department, Kyiv, Ukraine;*

### Background

In leading centers, multiple myeloma (MM) care integrates cytogenetic risk assessment, systemic therapy, and autologous stem cell transplantation (ASCT) within a single program. In Ukraine, MM care has historically been fragmented, with limited access to cytogenetic testing and ASCT outside major centers. Since 2022, these limitations have persisted within a healthcare system operating under wartime conditions. In 2024, we launched an integrated all-under-one-roof (AUOR) pathway for newly diagnosed MM (NDMM), bringing diagnostics, induction, and ASCT into one coordinated program. We evaluated its feasibility and early outcomes.

### Aims

To evaluate outcomes of an integrated AUOR NDMM care pathway implemented during active wartime conditions.

### Methods

In this retrospective analysis, we compared consecutive NDMM patients treated within the AUOR pathway from January 2024 to December 2025 with a historical institutional cohort treated from January 2022 to December 2023. Primary endpoints were the rate of baseline cytogenetic risk assessment by FISH and the proportion of transplant-eligible patients proceeding to ASCT. Secondary endpoints included time from diagnosis to treatment initiation, depth of response after induction, time to ASCT, MRD assessment by multiparameter flow cytometry (sensitivity $10^{-5}$), early post-ASCT outcomes, and progression-free survival (PFS). PFS was defined as the time from treatment initiation to progression/relapse or death from any cause and was estimated using the Kaplan-Meier method.

### Results

Thirty-four consecutive NDMM patients were treated within the AUOR pathway (median age 65.5 years, range 41-87). Baseline characteristics were comparable with the historical cohort (n=40; median age 67 years, range 46-90), including ISS III disease (47% vs 55%), extramedullary disease (32% vs 25%), and circulating plasma cells (12% vs 8%). Cytogenetic assessment increased from 23% in the historical cohort to 74% with AUOR, identifying high-risk abnormalities in 40% of AUOR patients, including 15% with double-hit features. Median time from diagnosis to treatment initiation decreased from 18 to 8 days (IQR 4-16). Transplant eligibility was similar (71% vs 75%), but ASCT rates among transplant-eligible patients increased from 10% to 42%, and median time to ASCT decreased from 9.3 to 4.0 months. Induction was predominantly VRD-based (68% vs 65%), with similar ≥VGPR rates (62% vs 68%). MRD assessment increased from 73% to 97%. Among transplanted AUOR patients, post-ASCT MRD negativity reached 80%, with no day +100 relapse or non-relapse mortality. At a median follow-up of 10.3 months for the AUOR cohort and 11.6 months for the historical cohort, 1-year PFS was 73% (95% CI 53-86) versus 62% (95% CI 47-77), respectively. Median PFS was not reached in either cohort (9 vs 15 events).

**Copyright Information:** (Online) ISSN: 2572-9241
©2026 The Author(s). HemaSphere is published by John Wiley & Sons Ltd on behalf of European Hematology Association. This is an open access Abstract Book distributed under the Attribution-NonCommercial-NoDerivs (CC BY-NC-ND), which allows third parties to download the articles and share them with others as long as they credit the author and the Abstract Book, but they cannot change the content in any way or use them commercially.
**Abstract Book Citations:** Authors, Title, HemaSphere, 2026;10:(S1):pages. The abstract book DOIs can be found on the “Author Information” page.
**Disclaimer:** Articles published in the journal HemaSphere exclusively reflect the opinions of the authors. The authors are responsible for all content in their abstracts including accuracy of the facts, statements, citing resources, etc.

**Summary/Conclusion**

The AUOR NDMM care model enabled coordinated, risk-adapted management within a unified institutional framework. Implementation of this pathway substantially improved cytogenetic coverage, accelerated treatment initiation, and expanded access to ASCT while maintaining response depth and early transplant safety, despite advanced disease burden and wartime healthcare delivery. Together, these findings challenge the notion that comprehensive NDMM care is feasible only in well-resourced and stable healthcare environments and support its applicability in other resource-constrained or crisis-affected settings.

**Copyright Information:** (Online) ISSN: 2572-9241
©2026 The Author(s). HemaSphere is published by John Wiley & Sons Ltd on behalf of European Hematology Association. This is an open access Abstract Book distributed under the Attribution-NonCommercial-NoDerivs (CC BY-NC-ND), which allows third parties to download the articles and share them with others as long as they credit the author and the Abstract Book, but they cannot change the content in any way or use them commercially.
**Abstract Book Citations:** Authors, Title, HemaSphere, 2026;10:(S1):pages. The abstract book DOIs can be found on the "Author Information" page.
**Disclaimer:** Articles published in the journal HemaSphere exclusively reflect the opinions of the authors. The authors are responsible for all content in their abstracts including accuracy of the facts, statements, citing resources, etc.

### (PB3343) REAL-WORLD EFFICACY AND SAFETY OF CARFILZOMIB-BASED COMBINATION REGIMENS IN PATIENTS WITH MULTIPLE MYELOMA

**Topic:** 14. Myeloma and other monoclonal gammopathies - Clinical

Xiuli Sun[1], Wei Cai*[1], Xiushuai Dong[2], Jinghua Wang[2], Dewan Zhao[1]

*[1]the First Affiliated Hospital of Dalian Medical University, Dalian, China, Dalian, China;[2]the Second Affiliated Hospital, Harbin Medical University, Harbin, China, Harbin, China;*

### Background

Carfilzomib has been widely used in patients with multiple myeloma (MM), but its real-world efficacy and safety profiles in the Chinese MM population require further exploration.

### Aims

To characterize clinical outcomes in real-world MM patients, estimate the treatment efficacy and safety of carfilzomib-based regimens, and explore potential factors influencing overall response rate (ORR) and progression-free survival (PFS).

### Methods

The retrospective study enrolled MM patients treated with carfilzomib-based regimens at two centers in China (August 1, 2022 - December 30, 2025). Logistic regression was used for predictors of ORR. Survival analyzed with Kaplan-Meier and Cox regression method was implemented to evaluate PFS and OS.

### Results

A total of 120 patients were included. The median age was 60 years (range: 41–79) and 50.8% were male. 60.2% of patients were classified as stage Ⅲ, 19.0% had high cytogenetic risk, and 6.7% exhibited a “double-hit” profile. Prior exposure to bortezomib was observed in 99.1% of patients, 45.7% had received prior anti-CD38 monoclonal antibody (mAb), and 9.5% had undergone autologous stem cell transplantation (ASCT). The median number of prior treatment lines was 3 (range: 1-7). Carfilzomib-based doublet regimens were used in 5.8% of patients, triplets regimens in 65.8%, and quadruplets or regimens in 28.3%. The most common triplet regimens combined carfilzomib and dexamethasone (Kd) with lenalidomide or pomalidomide. Half of patients receiving quadruplets regimens treated with Kd plus anti-CD38 mAb-based therapy. Patients received a median of 5 treatment cycles (range: 1-7). Complete response (CR) was achieved in 17.1%, very good partial response (VGPR) in 16.2%, partial response (PR) in 30.5%, stable disease (SD) in 3.8%, and progressive disease (PD) in 32.4%, ORR was 63.8%. With a median follow-up of 9 months (range: 1–27), the median PFS was 11.6 months, and the median OS was 24.8 months. Elderly patients (≥65 years) demonstrated worse ORR than younger patients, with an ORR of 58.3% vs 72.6% ($p<0.05$). Patients who had no response to carfilzomib-based combination therapy or received more than 3 prior treatment lines, had shorter PFS ($p<0.05$), but there was no difference in OS. Overweight (BMI 25–29.9 kg/m$^2$) patients may have longer PFS vs normal or underweight patients (BMI ≤24.9 kg/m$^2$) , although the difference was not statistically significant. DS stage, ISS stage, hyperlipidemia, LDH, or combine anti-CD38 mAb regimen had no significant difference in PFS.
In terms of safety, 14.2% of patients developed cardiovascular adverse events (CVAEs), including heart failure, arrhythmia, elevated troponin I levels, and pericardial effusion. Three patients experienced grade ≥3 CVAEs, all of them relieved following symptomatic treatment. Hematologic toxicities were the most common adverse events (AEs), occurring in 65.1% of patients, 28.3% experiencing grade ≥3 AEs. The second most common AEs were infections (30.1%). Additional, complications observed included renal toxicity, diarrhea, pulmonary embolism, oral ulcers, allergic dermatitis, ankle joint swelling, and headache.

**Copyright Information:** (Online) ISSN: 2572-9241
©2026 The Author(s). HemaSphere is published by John Wiley & Sons Ltd on behalf of European Hematology Association. This is an open access Abstract Book distributed under the Attribution-NonCommercial-NoDerivs (CC BY-NC-ND), which allows third parties to download the articles and share them with others as long as they credit the author and the Abstract Book, but they cannot change the content in any way or use them commercially.
**Abstract Book Citations:** Authors, Title, HemaSphere, 2026;10:(S1):pages. The abstract book DOIs can be found on the “Author Information” page.
**Disclaimer:** Articles published in the journal HemaSphere exclusively reflect the opinions of the authors. The authors are responsible for all content in their abstracts including accuracy of the facts, statements, citing resources, etc.

### Summary/Conclusion

Carfilzomib-based combination regimens demonstrated promising efficacy and manageable safety profiles in MM patients. Elderly patients exhibited worse ORR, those who did not respond to Carfilzomib-based therapy or had received more than 3 prior treatments lines had shorter PFS, while overweight status may be associated with improved PFS.

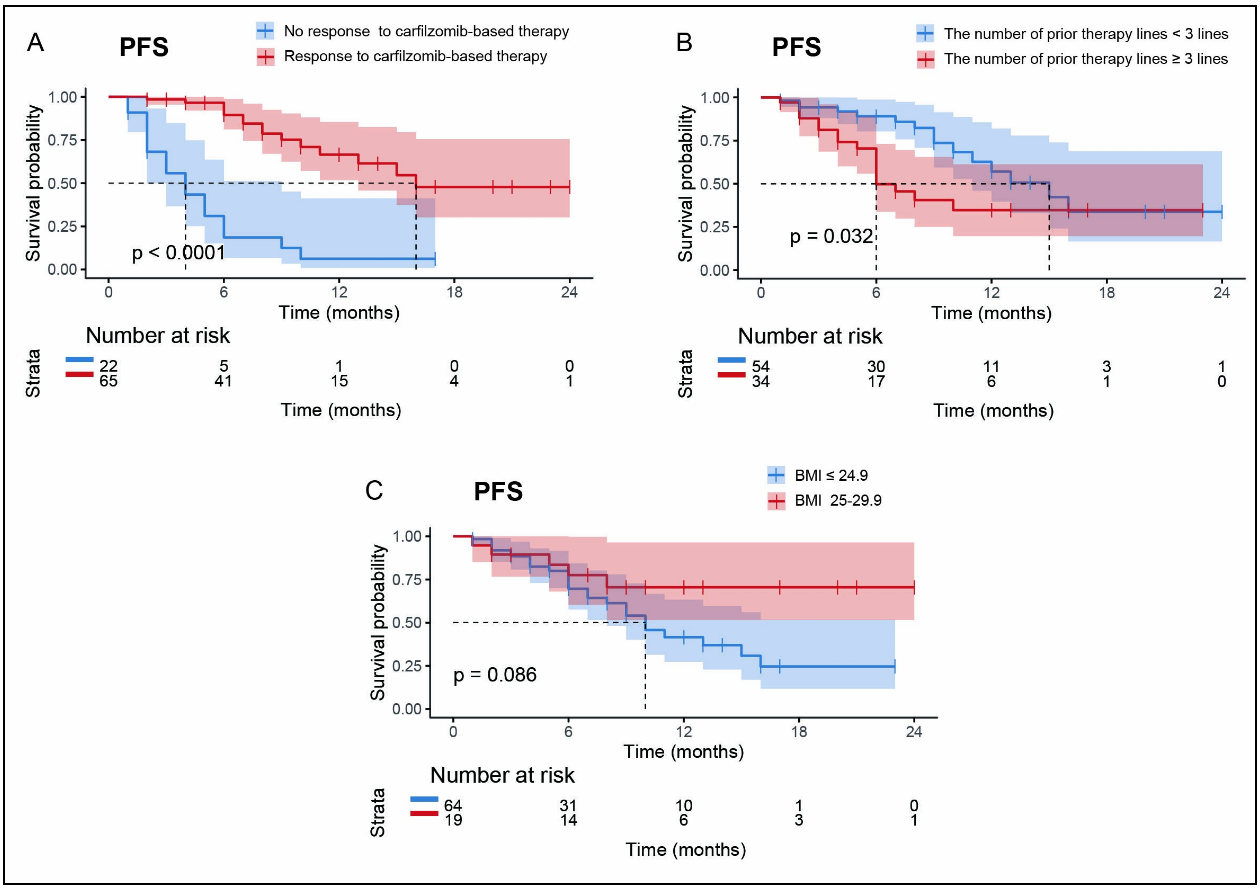

Figure 1. Kaplan-Meier PFS Curves A.PFS curves stratified by treatment response of carfilzomib-based therapy. B. PFS curves stratified by prior therapy lines. C. PFS curves stratified by BMI.

Copyright Information: (Online) ISSN: 2572-9241
©2026 The Author(s). HemaSphere is published by John Wiley & Sons Ltd on behalf of European Hematology Association. This is an open access Abstract Book distributed under the Attribution-NonCommercial-NoDerivs (CC BY-NC-ND), which allows third parties to download the articles and share them with others as long as they credit the author and the Abstract Book, but they cannot change the content in any way or use them commercially.
Abstract Book Citations: Authors, Title, HemaSphere, 2026;10:(S1):pages. The abstract book DOIs can be found on the "Author Information" page.
Disclaimer: Articles published in the journal HemaSphere exclusively reflect the opinions of the authors. The authors are responsible for all content in their abstracts including accuracy of the facts, statements, citing resources, etc.

### (PB3344) TECLISTAMAB INDUCED REMISSION IN REFRACTORY MONOCLONAL GAMMOPATHY ASSOCIATED COAGULATION DISORDERS

**Topic:** 14. Myeloma and other monoclonal gammopathies - Clinical

Johannes Thaler*[1], Karoline Gleixner[1], Lisbeth Eischer[1], Christian Sillaber[1], Peter Quehenberger[1], Maria Theresa Krauth[1], Cihan Ay[1], Hermine Agis[1]

*[1]Medizinische Universität Wien, Medicine I, Wien, Austria;*

### Background

Teclistamab is a bispecific monoclonal antibody that targets B-cell maturation antigen (BCMA) on plasma- and B-cells and CD3 on T-cells, thereby redirecting cytotoxic T-cells to eliminate BCMA-expressing cells.

### Aims

To investigate teclistamab in refractory monoclonal gammopathy associated coagulation disorders (MGCD).

### Methods

MGCD were defined as bleeding or thrombotic disorders that are caused by a monoclonal immunoglobulin or free light chain that directly binds to, inhibits, or accelerates the clearance of coagulation factors or regulatory proteins, thereby producing an acquired, paraprotein-driven coagulation disorder.

### Results

Four patients with refractory MGCD were treated with teclistamab (Figure 1). Standard therapies including IVIG, coagulation factor concentrates, or anticoagulation had failed in these patients. Two patients with acquired von Willebrand disease and one patient with acquired factor XI deficiency had severe bleeding complications. One patient had catastrophic antiphospholipid syndrome (APS) and thrombotic complications. Teclistamab treatment led to a rapid decline of pathogenic monoclonal antibodies in all four patients and normalization of coagulation parameters. In the three patients with bleeding complications, factor levels normalized after initiation of teclistamab and bleeding events stopped. Patient 2 developed severe diarrhea, which was not caused by infection and was probably due to autoimmune colitis, which strongly improved during the observation period. In the patient with catastrophic APS, anti-cardiolipin and β2-glykoprotein antibodies normalized and no further thromboembolic events were reported, leg ulcers due to livedoid vasculopathy persisted and sero-negative joint pain newly emerged, but improved over time.

### Summary/Conclusion

Teclistamab effectively eradicated autoreactive plasma cells responsible for MGCD and induced immunologic remissions.

**Copyright Information:** (Online) ISSN: 2572-9241
©2026 The Author(s). HemaSphere is published by John Wiley & Sons Ltd on behalf of European Hematology Association. This is an open access Abstract Book distributed under the Attribution-NonCommercial-NoDerivs (CC BY-NC-ND), which allows third parties to download the articles and share them with others as long as they credit the author and the Abstract Book, but they cannot change the content in any way or use them commercially.
**Abstract Book Citations:** Authors, Title, HemaSphere, 2026;10:(S1):pages. The abstract book DOIs can be found on the "Author Information" page.
**Disclaimer:** Articles published in the journal HemaSphere exclusively reflect the opinions of the authors. The authors are responsible for all content in their abstracts including accuracy of the facts, statements, citing resources, etc.

**A Patient characteristics**

| | Patient 1<br>Acquired factor XI deficiency | Patient 2<br>Acquired von Willebrand Syndrome | Patient 3<br>Acquired von Willebrand Syndrome | Patient 4<br>Catastrophic antiphospholipid syndrome |
|---|---|---|---|---|
| **Age - yr** | 64, m | 66, m | 71, m | 48, m |
| **Sex** | Male | Male | Male | Male |
| **Clinical presentation** | Spontaneous hematomas | Severe anemia due to gastrointestinal bleedings | Prolonged bleeding after minor skin injuries | Livedoid vasculopathy of the legs, recurrent deep vein thromboses, myocardial infarction, thalamic stroke |
| **Pre-treatment coagulation parameters** | Factor XI activity: <1 IU /dL | Von Willebdand factor activity: 4 IU/dL<br>Factor VIII activity: 8 IU/dL | Von Willebdand factor activity: <4 IU/dL<br>Factor VIII activity: 8 IU/dL | Anticardiolipin IgG: >120 (mg/mL)<br>ß2-Glycoprotein IgG: >100 (mg/mL) |
| **Paraprotein** | IgA-kappa | IgG-kappa | IgG-kappa | IgG-lambda |
| **Plasma cells in bone marrow** | Monotypic, 15% | Monotypic, 15% | Monotypic, 30% | Polyclonal, <5% |
| **Diagnosis** | Monoclonal antibody-mediated acquired factor XI deficiency | Monoclonal antibody-mediated acquired von Willebrand disease | Monoclonal antibody-mediated acquired von Willebrand disease | Monoclonal antibody-mediated antiphospholipid syndrome |
| **Response to teclistamab treatment** | Normalization of factor XI activity | Normalization of von Willebrand factor- and factor VIII activity | Normalization of von Willebrand factor- and factor VIII activity | Normalization of anticardiolipin- and ß2-Glycoprotein IgG |
| **Side effects of teclistamab** | None | Transient colitis with diarrhea | None | Transient sero-negative joint pain |
| **Outcome** | Cessation of bleeding tendency | Cessation of bleeding tendency | Cessation of bleeding tendency | No further arterial or venous thrombo-embolic events but persistence of leg ulcers |
| **Length of follow-up after first dose - wk** | 33 | 41 | 31 | 24 |

**B Schematic treatment protocol**

Day 1 Teclistamab 0.06 mg/kg → Day 3 Teclistamab 0.3 mg/kg → Day 5 Teclistamab 1.5 mg/kg → Maintenance Teclistamab 1.5 mg/kg

**C Response to teclistamab**

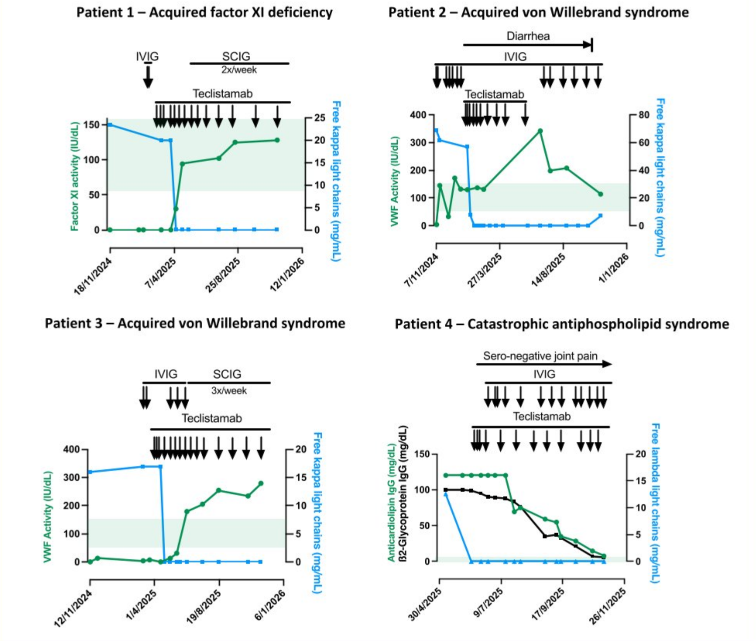

**Copyright Information:** (Online) ISSN: 2572-9241
©2026 The Author(s). HemaSphere is published by John Wiley & Sons Ltd on behalf of European Hematology Association. This is an open access Abstract Book distributed under the Attribution-NonCommercial-NoDerivs (CC BY-NC-ND), which allows third parties to download the articles and share them with others as long as they credit the author and the Abstract Book, but they cannot change the content in any way or use them commercially.
**Abstract Book Citations:** Authors, Title, HemaSphere, 2026;10:(S1):pages. The abstract book DOIs can be found on the “Author Information” page.
**Disclaimer:** Articles published in the journal HemaSphere exclusively reflect the opinions of the authors. The authors are responsible for all content in their abstracts including accuracy of the facts, statements, citing resources, etc.

### (PB3345) RISK ASSESSMENT IN NEWLY DIAGNOSED MYELOMA USING NGS, FISH, AND CONVENTIONAL CYTOGENETICS: A SINGLE-CENTER EXPERIENCE.

**Topic:** 14. Myeloma and other monoclonal gammopathies - Clinical

Işil Erdoğan Özünal*[1], Mesut Tiglioglu[1], Tayfun Elibol[1], Pınar Tığlıoğlu[1], Tahacan Aydin[1], Özkan Kaya[1], Erman Öztürk[1]

*[1]İstanbul Medeniyet University Göztepe Prof. Dr. Süleyman Yalçın City Hospital, Hematology, İstanbul, Türkiye;*

### Background

Multiple myeloma (MM) is a genetically heterogeneous plasma cell malignancy in which genetic abnormalities critically influence prognosis and treatment decisions. As recommended by the International Myeloma Study Group (IMWG), fluorescence in situ hybridization (FISH) forms the basis of genomic risk stratification. However, the reliability and cut-off values for FISH translocations are debatable in daily practice, as plasma cell sorting is not available in many centers. Nevertheless, conventional karyotyping and next-generation sequencing (NGS) can support FISH and thus detect subclonal mutations. However, there is still no clear consensus regarding the application of a standard NGS panel in MM patients. EMN specifically recommends NGS for detecting TP53 mutations in newly diagnosed MM.
Because it particularly affects the elderly population, myeloma can be associated with cytogenetic anomalies characteristic of myelodysplastic syndrome (MDS). Phenotypic anomalies associated with MDS can be observed in 1 out of every 10 myeloma patients, and this is associated with poor survival.Therefore, the presence of myeloid mutations remains important for the diagnosis of myeloma.

### Aims

The aim of this study is to compare mutations identified in the myeloid NGS panel from bone marrow samples of newly diagnosed myeloma patients with those obtained using other methods.

### Methods

Targeted NGS was performed as part of the routine diagnostic workup using an Illumina NextSeq platform and a 75-gene myeloid-oriented panel. TP53, KRAS, NRAS, BRAF, DNMT3A, TET2, SF3B1, and ASXL1 mutations are considered high-risk molecular signatures. Variants with allele frequencies≥5% were reported and categorized according to AMP/ASCO/CAP guidelines.
Conventional karyotyping classified metaphase chromosome numbers and structures as diploidy, complex karyotype, other anomalies, and normal findings.

### Results

During the data collection period, the findings of 220 newly diagnosed myeloma patients were evaluated. t(4;14) was found positive in 6 out of 133 patients (2.7%). t(11;14) was found positive in 14 out of 116 patients (6.4%). Del 17p was detected in 7 of 133 patients (3.2%), and del 13q in 9 of 117 patients (4.1%).
Conventional karyotyping was performed on 163 patients, with normal results in 139 patients (63.2%). Three patients had no metaphases that could be assessed. Y chromosome deletion (n=9) and hyperdiploidy (n=4) were other anomalies observed.
Of the 59 patients in whom NGS could be performed, 35 (15.9%) did not have any pathological variants. The most frequently observed anomalies were DNMT3A (n=5), TET2 (n=4), KRAS (n=4), NRAS (n=2), and Jak-2 (n=2) mutations.

**Copyright Information:** (Online) ISSN: 2572-9241
©2026 The Author(s). HemaSphere is published by John Wiley & Sons Ltd on behalf of European Hematology Association. This is an open access Abstract Book distributed under the Attribution-NonCommercial-NoDerivs (CC BY-NC-ND), which allows third parties to download the articles and share them with others as long as they credit the author and the Abstract Book, but they cannot change the content in any way or use them commercially.
**Abstract Book Citations:** Authors, Title, HemaSphere, 2026;10:(S1):pages. The abstract book DOIs can be found on the "Author Information" page.
**Disclaimer:** Articles published in the journal HemaSphere exclusively reflect the opinions of the authors. The authors are responsible for all content in their abstracts including accuracy of the facts, statements, citing resources, etc.

**Summary/Conclusion**

Since FISH can be challenging to interpret, particularly when plasma cell sorting is not performed, as in our center, NGS may detect genetic anomalies in myeloma patients that are missed by FISH and conventional karyotyping, and that may affect prognosis.

**Copyright Information:** (Online) ISSN: 2572-9241
©2026 The Author(s). HemaSphere is published by John Wiley & Sons Ltd on behalf of European Hematology Association. This is an open access Abstract Book distributed under the Attribution-NonCommercial-NoDerivs (CC BY-NC-ND), which allows third parties to download the articles and share them with others as long as they credit the author and the Abstract Book, but they cannot change the content in any way or use them commercially.
**Abstract Book Citations:** Authors, Title, HemaSphere, 2026;10:(S1):pages. The abstract book DOIs can be found on the "Author Information" page.
**Disclaimer:** Articles published in the journal HemaSphere exclusively reflect the opinions of the authors. The authors are responsible for all content in their abstracts including accuracy of the facts, statements, citing resources, etc.

### (PB3346) DECENTRALISING MYELOMA CARE; FEASIBILITY AND ADVANTAGES OF HOME-BASED BORTEZOMIB SELF-ADMINISTRATION FROM A REGIONAL PERSPECTIVE IN AUSTRALIA

**Topic:** 14. Myeloma and other monoclonal gammopathies - Clinical

Rohullah Sultani*[1], Amanda Ormerod[1], Tricia Wright[1]

*[1]Latrobe Regional Health, Victoria, Australia, Haematology, Traralgon, Australia;*

### Background

Home-based chemotherapy (HBC) benefits are well established. HBC is convenient, cost-effective, flexible with greater patient satisfaction. Health services (HS) benefit from improving efficiencies in ambulatory chemotherapy day unit (CDU) and reducing HS costs. Regional centres face barriers to the implementation of these important service improvement activities related to wide distances consumers live from HS and health service resources. Latrobe Regional Health (LRH) is the referral centre for Gippsland in Victoria Australia. Gippsland covers 41,500 Square Kilometres, roughly the size of Switzerland. The LRH haematology department has an established relationship with a metropolitan multiple myeloma (MM) service with a successful bortezomib at home (B@home) program.

### Aims

To evaluate the feasibility of implementing a home-based Bortezomib self-administration program in regional centre.

### Methods

LRH haematology service adapted the metropolitan B@home protocol and implemented an ethics endorsed single-centre retrospective cohort study. Adult patients (over 18 years), planned to or receiving a Bortezomib-based regimen, deemed competent by the treating haematologist and chemotherapy nurse (CN) for self-administration with access to telecommunication for clinical monitoring in the community were deemed eligible. Patients self-administered their first cycle of Bortezomib in the CDU under supervision and day one of each subsequent cycle. Patients self-administered all remaining doses at home. The CDU allocates 90 minutes for each Bortezomib scheduled dose. Patients were enrolled in the haematology symptom and urgent review service (SURS). Symptoms were monitored remotely for each dose delivered at home using the UKONS (United Kingdom Oncology Nursing Society) 24-Hour Triage Tool. Patients were reviewed by their treating haematologist at the end of each cycle. Patient-reported perspectives and symptoms reported through SURS were reviewed. CDU-related cost benefits were calculated in collaboration with LRH's finance office.

### Results

Thirty MM patients were screened for the program with 5 (17%) deemed eligible. Reasons for failure to recruit at screening included 15 (50%) individuals receiving daratumumab in combination with bortezomib; 5 (17%) enrolled in a clinical trial and 5(17%) too frail to self-inject. One out of five individuals recruited was transplant eligible. The average distance travelled to the CDU was 44.4 km. A total of 7.5 hours ($1500 AUD) of CDU chair availability was released per week. Patients reported reduced travel time and improved productivity at home. There were no reported adverse effects, logistical issues around delivery, storage, or administration and no infections requiring antimicrobial therapy.

**Copyright Information:** (Online) ISSN: 2572-9241
©2026 The Author(s). HemaSphere is published by John Wiley & Sons Ltd on behalf of European Hematology Association. This is an open access Abstract Book distributed under the Attribution-NonCommercial-NoDerivs (CC BY-NC-ND), which allows third parties to download the articles and share them with others as long as they credit the author and the Abstract Book, but they cannot change the content in any way or use them commercially.
**Abstract Book Citations:** Authors, Title, HemaSphere, 2026;10:(S1):pages. The abstract book DOIs can be found on the "Author Information" page.
**Disclaimer:** Articles published in the journal HemaSphere exclusively reflect the opinions of the authors. The authors are responsible for all content in their abstracts including accuracy of the facts, statements, citing resources, etc.

**Summary/Conclusion**

We describe a successful implementation of a B@home program.  Recruitment was restricted by external factors including enrolment in a clinical trial, availability of daratumumab on the pharmaceutical benefits scheme during the study which improved access to Bortezomib and daratumumab combination treatment requiring attendance in the CDU.  Importantly only a small proportion of individuals were excluded due to patient factors and no adverse safety signals were observed. This small cohort study has demonstrated the ability for a regional site to implement a consumer centred, safe, and cost-effective HBC.  The program is now being expanded to daratumumab and other suitable cancer therapies.

**Copyright Information:** (Online) ISSN: 2572-9241
©2026 The Author(s). HemaSphere is published by John Wiley & Sons Ltd on behalf of European Hematology Association. This is an open access Abstract Book distributed under the Attribution-NonCommercial-NoDerivs (CC BY-NC-ND), which allows third parties to download the articles and share them with others as long as they credit the author and the Abstract Book, but they cannot change the content in any way or use them commercially.
**Abstract Book Citations:** Authors, Title, HemaSphere, 2026;10:(S1):pages. The abstract book DOIs can be found on the “Author Information” page.
**Disclaimer:** Articles published in the journal HemaSphere exclusively reflect the opinions of the authors. The authors are responsible for all content in their abstracts including accuracy of the facts, statements, citing resources, etc.

### (PB3347) PHASE II STUDY TO EVALUATE THE EFFICACY AND SAFETY OF TECLISTAMAB COMBINED WITH DARATUMUMAB IN NEWLY DIAGNOSED MULTIPLE MYELOMA WITH CONCURRENT LIGHT CHAIN AMYLOIDOSIS, THE STAMINA TRIAL

**Topic:** 14. Myeloma and other monoclonal gammopathies - Clinical

Jing Li*[1], Chenqi Yu[1], Yang Yang[1], Yuhong Ren[1], Yian Zhang[1], Jiadai Xu[1], Peng Liu[1]

[1]*Zhongshan Hospital Fudan University, Shanghai, China;*

### Background

Up to 10–20% of biopsy-confirmed immunoglobulin light-chain amyloidosis (AL) coexists with multiple myeloma (MM), and patients with newly diagnosed MM and concurrent AL experience markedly worse outcomes. Previous reports from Zhongshan Hospital revealed that NDMM+AL patients had worse progression-free survival (PFS), overall survival (OS), and higher early mortality than those with isolated MM or AL, underscoring a critical unmet need. Teclistamab (Tec), a BCMA×CD3 bispecific antibody, and subcutaneous daratumumab (Dara), an anti-CD38 monoclonal antibody, have synergistic mechanisms that may deliver rapid, deep responses with a manageable safety profile when delivered in combination, providing a strong rationale for combination therapy in this population. The Regimen of Tec plus Dara has been previously evaluated in RRMM patients in the MajesTEC-3 study with unprecedented benefit. The safety-run in cohort from the MajesTEC-7 study also reported an acceptable safety profile for this combination in transplant ineligible or deferred patients.

### Aims

The primary aim is to evaluate the efficacy and safety of teclistamab plus daratumumab (Tec-Dara) in NDMM+AL patients. The exploratory aims include assessing depth of response by minimal residual disease (MRD) and correlating baseline RNA-sequencing features as well as risk factors with outcomes.

### Methods

This is a prospective, open-label, single-center, single-arm phase II trial at Fudan University Zhongshan Hospital, planning to enroll 30 patients meeting prespecified criteria. Eligible patients aged ≥18 years, with IMWG confirmed NDMM, measurable disease, and biopsy-confirmed AL will be included. Patients who were previously treated (including autologous stem cell transplant) for MM or AL are excluded.

Teclistamab will be administered subcutaneously with step-up dosing in Cycle 1 (0.06 mg/kg Day 2; 0.3 mg/kg Day 4; 1.5 mg/kg Days 8 and 15), then 3.0 mg/kg every 4 weeks in Cycles 2–24. Daratumumab 1800mg is administered subcutaneously weekly in Cycles 1–2, every 2 weeks in Cycles 3–6, thereafter every 4 weeks in Cycles 7–24. Treatment will continue until disease progression, unacceptable toxicity, or completion of the trial treatment of 24 cycles. Disease assessments will be performed following IMWG criteria every 28±7 days, and organ responses are evaluated using cardiac/renal biomarkers; safety is graded per CTCAE. Patients enter long-term follow-up after end-of-treatment.

The primary endpoint is PFS rate at 1 year. Key secondary endpoints include hematologic overall response rate (ORR), ≥VGPR, CR, organ response at planned timepoints, MRD negativity, time to first response, duration of response (DoR), median PFS, OS, time to next therapy (TTNT), and safety profile. Exploratory endpoints including evaluation of MRD-defined (NGF/NGS) depth of response and associations of baseline RNA-seq features with efficacy and survival.

**Copyright Information:** (Online) ISSN: 2572-9241
©2026 The Author(s). HemaSphere is published by John Wiley & Sons Ltd on behalf of European Hematology Association. This is an open access Abstract Book distributed under the Attribution-NonCommercial-NoDerivs (CC BY-NC-ND), which allows third parties to download the articles and share them with others as long as they credit the author and the Abstract Book, but they cannot change the content in any way or use them commercially.
**Abstract Book Citations:** Authors, Title, HemaSphere, 2026;10:(S1):pages. The abstract book DOIs can be found on the “Author Information” page.
**Disclaimer:** Articles published in the journal HemaSphere exclusively reflect the opinions of the authors. The authors are responsible for all content in their abstracts including accuracy of the facts, statements, citing resources, etc.

**Results**

This is an ongoing study in which first enrollment has been completed on Dec. 15$^{th}$, 2025. The planned primary endpoint readout will be in July 2028.

**Summary/Conclusion**

This phase 2 study is designed to assess the efficacy and safety of Teclistamab in combination with Daratumumab in newly diagnosed MM with concurrent AL populations whose survival outcomes were extremely poor under previous regimens.

**Copyright Information:** (Online) ISSN: 2572-9241
©2026 The Author(s). HemaSphere is published by John Wiley & Sons Ltd on behalf of European Hematology Association. This is an open access Abstract Book distributed under the Attribution-NonCommercial-NoDerivs (CC BY-NC-ND), which allows third parties to download the articles and share them with others as long as they credit the author and the Abstract Book, but they cannot change the content in any way or use them commercially.
**Abstract Book Citations:** Authors, Title, HemaSphere, 2026;10:(S1):pages. The abstract book DOIs can be found on the "Author Information" page.
**Disclaimer:** Articles published in the journal HemaSphere exclusively reflect the opinions of the authors. The authors are responsible for all content in their abstracts including accuracy of the facts, statements, citing resources, etc.

### (PB3348) SHOULD COGNITIVE IMPAIRMENT BE AN EXCLUSION CRITERIA FOR TREATING MULTIPLE MYELOMA WITH BISPECIFIC ANTIBODIES? A UK TERTIARY CENTRE EXPERIENCE OF NEUROTOXICITY COMPLICATING TREATMENT

**Topic:** 14. Myeloma and other monoclonal gammopathies - Clinical

Sally Keat*[1], Saarah Rajah[2], Yousef Ezaydi[1], Andrew Chantry[1], Elisa Roldan Galvan[1]

*[1]Sheffield Teaching Hospitals NHS Foundation Trust, Sheffield, United Kingdom;[2]University of Sheffield, School of Medicine and Population Health, Sheffield, United Kingdom;*

### Background

The NICE approved bispecific antibodies (bsAb) available for myeloma in the UK target either BCMA or GPRC5D. The risk of immune effector cell associated neurotoxicity syndrome (ICANS) is quoted as up to 15%, higher with those targeting GPRC5D than BCMA. This side effect is most commonly low grade (grade 1-2). Neurotoxicity other than headaches is rarely documented.
Much of the documented experience of neurotoxicity in T-cell redirecting therapies is from CAR-T. Parkinsonian features are a recognised side effect of BCMA CAR-T and other neurological features have been noted- mostly headache and peripheral neuropathy. Pre-existing cognitive impairment or neurological abnormalities have not been found to increase risk of ICANS in patients undergoing CAR-T therapy.
The experience of ICANS in bsAb at our centre is different; our recorded rate of ICANS at any grade is 39%. It has forced us to question some of our processes in patient selection and management.

### Aims

To present our real- world experience of neuro-toxicity in bsAb treatment of myeloma. Additionally, to explore why our rate of ICANS is higher and whether this can be attributed to a failure of effective patient screening or assessment.

### Methods

A retrospective analysis of those receiving their first cycle of a bsAb between January 2023 and February 2026 in a tertiary hospital in the UK. Data were collected from paper notes, discharge summaries and electronic patient records. Descriptive analysis was used to summarise the information.

### Results

28 patients with myeloma received a bsAb between Jan 2023 and Feb 2026: 12 women, 16 men. Median age was 66 years (range 37 to 84). Median prior lines of therapy was 4 (range 3 -10). ECOG ranged from 0-3 (26% ECOG 1, 39% ECOG 2 and 7% ECOG 3). 43 % (12) received elranatamab, 39% (11) received teclistamab and 17% (5) received talquetamab. 0 patients had a diagnosis of dementia prior to the bispecific exposure.
39% (11) experienced ICANS. 6 received elranatamab, 4 teclistamab and 1 talquetamab. The majority (7 of 11) were grade 1, but 2 patients had grade 2 and there were 2 documented grade 3 (one had a subarachnoid haemorrhage). 4 (36%) patients refused any further treatment. 2 of them were in CR. Detailed collateral histories after commencing treatment revealed 6 of the 11 patients likely had some baseline cognitive impairment and a higher degree of frailty than previously recognised.
5 patients developed psychotic features with paranoid ideation, 2 with auditory hallucinations. 3 were given antipsychotics to which they responded. 1 patient developed suicidal ideation. Headaches were a common feature. No patients on BCMA bsAb developed any parkinsonian symptoms.

**Copyright Information:** (Online) ISSN: 2572-9241
©2026 The Author(s). HemaSphere is published by John Wiley & Sons Ltd on behalf of European Hematology Association. This is an open access Abstract Book distributed under the Attribution-NonCommercial-NoDerivs (CC BY-NC-ND), which allows third parties to download the articles and share them with others as long as they credit the author and the Abstract Book, but they cannot change the content in any way or use them commercially.
**Abstract Book Citations:** Authors, Title, HemaSphere, 2026;10:(S1):pages. The abstract book DOIs can be found on the "Author Information" page.
**Disclaimer:** Articles published in the journal HemaSphere exclusively reflect the opinions of the authors. The authors are responsible for all content in their abstracts including accuracy of the facts, statements, citing resources, etc.

**Summary/Conclusion**

ICANS at even low grade is extremely distressing and may prompt patients to refuse further treatment. Psychotic features such as paranoid delusions and hallucinations may occur and steroids may exacerbate them. Detailed collateral histories revealed significant pre-existing frailty which hadn't been noted in clinic assessments. For patients with psychosis, involvement of psychiatry and the use of antipsychotics was effective.
The investigators are using these data to assess patient eligibility more thoughtfully, rather than using it as an exclusion. We have instituted the Addenbrookes Cognitive Examination to screen patients at the time of bsAb eligibility discussion to ensure they are given accurate information regarding their risk of neurotoxicity.

**Copyright Information:** (Online) ISSN: 2572-9241
©2026 The Author(s). HemaSphere is published by John Wiley & Sons Ltd on behalf of European Hematology Association. This is an open access Abstract Book distributed under the Attribution-NonCommercial-NoDerivs (CC BY-NC-ND), which allows third parties to download the articles and share them with others as long as they credit the author and the Abstract Book, but they cannot change the content in any way or use them commercially.
**Abstract Book Citations:** Authors, Title, HemaSphere, 2026;10:(S1):pages. The abstract book DOIs can be found on the "Author Information" page.
**Disclaimer:** Articles published in the journal HemaSphere exclusively reflect the opinions of the authors. The authors are responsible for all content in their abstracts including accuracy of the facts, statements, citing resources, etc.

### (PB3349) RISK STRATIFICATION, TREATMENT PATTERNS, AND SURVIVAL OUTCOMES IN NEWLY DIAGNOSED MULTIPLE MYELOMA PATIENTS: A REAL-WORLD ANALYSIS FROM THE CHINESE MYELOMA COMMITTEE

**Topic:** 14. Myeloma and other monoclonal gammopathies - Clinical

Xuelin Dou*[1], Aijun Liao[2], Jun Luo[3], Chunrui Li[4], Ling Qin[5], Yuping Zhong[6], Liru Wang[7], Jinlei Qi[8], Yang Liu[9], Nan Peng[9], Lei Wen[9], Sinan Gu[10], Lifen Kuang[11], Jia Song[12], Zhongqing Li[13], Maigeng Zhou[8], Rong Fu[12], Juan Li[11], Juan Du[10], Jin Lu[9]

*[1]Peking University People's Hospital, Beijing, China; [2]Shengjing Hospital of China Medical University, Shenyang, China; [3]The First Affiliated Hospital of Guangxi Medical University, NanNing GuangXi,530021,China, Nanning, China; [4]Department of Hematology, Tongji Hospital, Tongji Medical College, Huazhong University of Science and Technology, wuhan, China; [5]The First Affiliated Hospital, and College of Clinical Medicine of Henan University of Science and Technology, luoyang, China; [6]Department of Hematology, Qingdao Hospital, University of Health and Rehabilitation Science (Qingdao Municipal Hospital), qingdao, China; [7]Department of Hematology, Fu Xing Hospital, Capital Medical University, Beijing, China; [8]National Center for Chronic and Noncommunicable Disease Control and Prevention, Chinese Center for Disease Control and Prevention, Beijing, China; [9]Peking University People's Hospital, Peking University Institute of Hematology, National Clinical Research Center for Hematologic Disease, Beijing Key Laboratory of Hematopoietic Stem Cell Transplantation, Peking University, Beijing, Beijing, China; [10]Department of Hematology, The Myeloma & Lymphoma Center, The Second Affiliated Hospital of Naval Medical University (Shanghai Changzheng Hospital), Shanghai, China; [11]First Affiliated Hospital of Sun Yat-sen University, Guangzhou, China; [12]Tianjin Medical University General Hospital, Tianjin, China; [13]The First Affiliated Hospital of Guangxi Medical University, Nanning, China;*

### Background

Multiple myeloma (MM) remains a heterogeneous malignancy with rising prevalence. A decade ago, our multicenter analysis defined the distinct clinical profile of MM in China, characterized by a younger age of onset and high-risk presentatio. However, those findings reflected an era of limited bortezomib access and heterogeneous care. Following the National Volume-Based Procurement (NVBP) policy, proteasome inhibitor-based triplets (e.g., RVD) have become the standard of care, necessitating an updated benchmark.

### Aims

Building on our previous multicenter benchmark, we analyzed 1,384 newly diagnosed multiple myeloma patients from 10 representative centers of the Chinese Myeloma Committee to establish a reliable standard for the novel agent era.

### Methods

Survival outcomes were verified by the National Mortality Surveillance System to ensure data accuracy.

### Results

The median age was 62 years, confirming the persistent younger onset of Chinese patients compared to Western populations. Treatment patterns have evolved significantly: RVD became the dominant induction (51.8%), and upfront autologous stem cell transplantation (ASCT) rates in eligible patients rose to 40.0% (vs. 14.4% historically). During a median follow-up of 46.6 months, the progression-free survival reached 44.2 months, with a 4-year overall survival rate of 66.4%. Validating the 2025 IMS/IMWG Consensus Genomic Staging (CGS), we identified 26.2% of patients as high-risk. Notably, patients harboring ≥ two CGS high-risk factors (3.8%) exhibited significantly inferior prognosis.

**Copyright Information:** (Online) ISSN: 2572-9241
©2026 The Author(s). HemaSphere is published by John Wiley & Sons Ltd on behalf of European Hematology Association. This is an open access Abstract Book distributed under the Attribution-NonCommercial-NoDerivs (CC BY-NC-ND), which allows third parties to download the articles and share them with others as long as they credit the author and the Abstract Book, but they cannot change the content in any way or use them commercially.
**Abstract Book Citations:** Authors, Title, HemaSphere, 2026;10:(S1):pages. The abstract book DOIs can be found on the "Author Information" page.
**Disclaimer:** Articles published in the journal HemaSphere exclusively reflect the opinions of the authors. The authors are responsible for all content in their abstracts including accuracy of the facts, statements, citing resources, etc.

### Summary/Conclusion

This study validates the 2025 CGS in an Asian cohort and demonstrates that treatment standardization has significantly improved real-world survival, while highlighting the distinct poor outcomes in patients with multiple CGS risk factors.

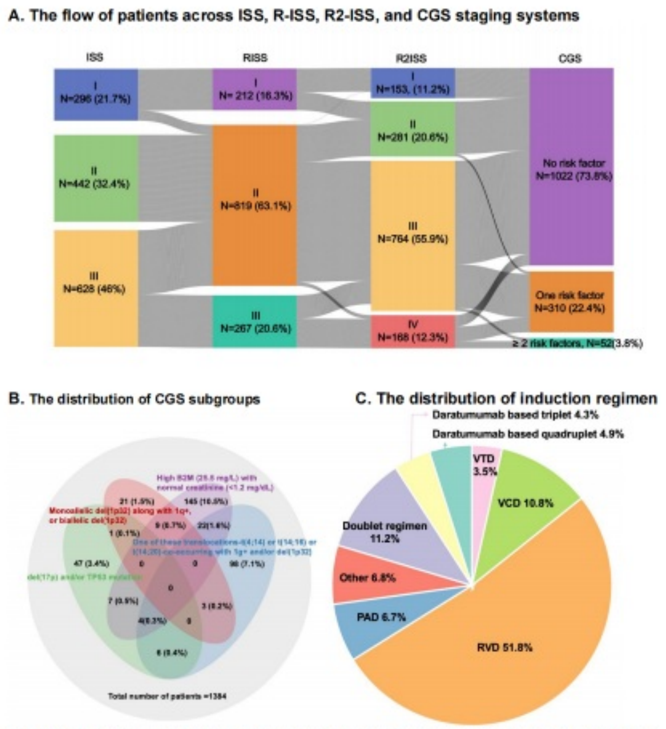

**Figure. The distribution of disease stagings and induction regimen of the multicenter cohort.** (A) Sankey plot illustrating the flow of patients across ISS, R-ISS, R2-ISS, and CGS staging systems. (B) Venn diagram detailing the distribution and overlap of the 2025 CGS high-risk subgroups. (C) Pie chart showing the distribution of first-line induction regimens.
Abbreviations: RVD: lenalidomide, bortezomib, and dexamethasone.
VCD: bortezomib, cyclophosphamide, and dexamethasone.
VTD: bortezomib, thalidomide, and dexamethasone.
PAD: bortezomib, doxorubicin or liposomal doxorubicin, and dexamethasone.
Doublet regimen: Two-drug combinations including bortezomib and dexamethasone (Vd), ixazomib and dexamethasone (Id), lenalidomide and dexamethasone (Rd), pomalidomide and dexamethasone (Pd), and daratumumab and dexamethasone (Dd).

**Copyright Information:** (Online) ISSN: 2572-9241
©2026 The Author(s). HemaSphere is published by John Wiley & Sons Ltd on behalf of European Hematology Association. This is an open access Abstract Book distributed under the Attribution-NonCommercial-NoDerivs (CC BY-NC-ND), which allows third parties to download the articles and share them with others as long as they credit the author and the Abstract Book, but they cannot change the content in any way or use them commercially.
**Abstract Book Citations:** Authors, Title, HemaSphere, 2026;10:(S1):pages. The abstract book DOIs can be found on the “Author Information” page.
**Disclaimer:** Articles published in the journal HemaSphere exclusively reflect the opinions of the authors. The authors are responsible for all content in their abstracts including accuracy of the facts, statements, citing resources, etc.

### (PB3350) INDUCTION STRATEGIES AND SURVIVAL IN TRANSPLANT-ELIGIBLE MULTIPLE MYELOMA: A TUNISIAN 20-YEAR EXPERIENCE

**Topic:** 14. Myeloma and other monoclonal gammopathies - Clinical

Faten Kallel*[1], Maha Charfi[1], Wafa Sakli[1], Rahma Mallek[1], Kharrat Khadija[1], Imen Frikha[1], Yousra Fakhfakh Derbel[1], Asma Koubaa[1], Imen Ben Amor[1], Aicha Ben Kahla[1], Charfi Hanen[1], Nour Siala[1], Moez Medhaffar[1], Moez Elloumi[1]

[1]*Clinical Hematology Department, Hedi Chaker, Sfax, Tunisia;*

### Background

The introduction of novel agents has profoundly transformed the prognosis of multiple myeloma. In Tunisia, access to these therapies has been gradual and resource-dependent. The transition from doublet to triplet induction regimens represents a major therapeutic advancement in settings with limited resources.

### Aims

This study aimed to evaluate the impact of evolving induction strategies on survival outcomes in young, transplant-eligible patients with multiple myeloma treated at the hematology department of Sfax, Tunisia, over a 20-year period.

### Methods

We conducted a retrospective study including all newly diagnosed patients aged ≤65 years between June 2003 and June 2023. Two treatment periods were compared: period 1 (2003–2016), during which patients received doublet induction with dexamethasone and thalidomide; and period 2 (2016–2023), during which patients received risk-adapted triplet induction. High-risk patients (defined by ISS stage III, unfavorable cytogenetics, or renal failure) were treated with VTD (bortezomib, thalidomide, and dexamethasone), while standard-risk patients received CTD (cyclophosphamide, thalidomide, and dexamethasone). All patients were scheduled for autologous stem cell transplantation. Primary endpoints were response rate according to IMWG criteria, overall survival (OS), and progression-free survival (PFS).

### Results

A total of 203 patients were included: 100 in period 1 and 103 in period 2, with a median age of 53 years. Triplet induction significantly improved pre-transplant response quality. Although overall response rates were similar between periods (73% vs. 67%), the rate of very good partial response or better (≥VGPR) increased from 11% to 35% ($p<0.001$), reaching 44% in the VTD subgroup. This improvement in response depth translated into enhanced survival outcomes: median OS increased from 42 months (95% CI: 35–49) to 60 months (95% CI: 52–68), with 5-year OS rising from 44.6% to 65.7%. Median PFS extended from 28 to 38 months. Transplant feasibility remained stable at approximately 60% across both periods, although the median time to transplantation increased from 6 to 9 months in the recent period.

### Summary/Conclusion

Alignment with international standards through triplet induction resulted in an 18-month gain in overall survival. The 5-year OS rate in our cohort (65.7%) is comparable to published data (65–70%); however, deep response rates (44% with VTD) remain below those reported in pivotal trials (>60%), a gap likely attributable to prolonged waiting times for autologous transplantation. Strategic priorities include the adoption of universal VTD induction to maximize minimal residual disease negativity and ensuring access to daratumumab at relapse to align with current standards of care.

**Copyright Information:** (Online) ISSN: 2572-9241
©2026 The Author(s). HemaSphere is published by John Wiley & Sons Ltd on behalf of European Hematology Association. This is an open access Abstract Book distributed under the Attribution-NonCommercial-NoDerivs (CC BY-NC-ND), which allows third parties to download the articles and share them with others as long as they credit the author and the Abstract Book, but they cannot change the content in any way or use them commercially.
**Abstract Book Citations:** Authors, Title, HemaSphere, 2026;10:(S1):pages. The abstract book DOIs can be found on the “Author Information” page.
**Disclaimer:** Articles published in the journal HemaSphere exclusively reflect the opinions of the authors. The authors are responsible for all content in their abstracts including accuracy of the facts, statements, citing resources, etc.

### (PB3351) PROGNOSTIC VALUE OF INTERPHASE FISH IN MULTIPLE MYELOMA: IMPLICATIONS FOR PRACTICE IN IRELAND BASED ON A SINGLE CENTER EXPERIENCE

**Topic:** 14. Myeloma and other monoclonal gammopathies - Clinical

Tushar Pramod*[1], Jimmy Abdelnour[1], Ahmed Bannaga[1], Senthil Kumar[1], Brian Hennessy[1], Ezzat El Hassadi[1]

[1]*University Hospital Waterford, Department of Haematology, Waterford, Ireland;*

### Background

Interphase fluorescence in situ hybridization (iFISH) identifies recurrent chromosomal abnormalities in multiple myeloma (MM) that have strong and reproducible associations with progression-free and overall survival. International groups (IMWG, EMN) recognise specific iFISH abnormalities as high-risk features that modify prognosis and (increasingly) treatment selection. Ireland has a stable and ageing MM population, improving survival overall, but variable availability of advanced diagnostics and novel therapies as has been the experience in our cancer centre[2]. This study provides real-world, population-specific insight into FISH-based MM biology within the Irish patients.

### Aims

To evaluate the frequency and prognostic impact of MM FISH abnormalities in our institution and compared with reported literature.

### Methods

We retrospectively analysed clinical data from 69 patients with MM diagnosed and treated at our centre; FISH analysis was successful in 67 cases. Ethical approval was obtained and patient consent waived. Demographic data, disease subtype, IPI score, and FISH abnormalities were collected from electronic medical records and laboratory reports. IMWG prognostic criteria were used for FISH-based risk stratification, and genomic subgroups were examined for prognostic impact. Treatment regimens and responses were assessed using IMWG response criteria. Survival analyses were performed using Prism v10.06, with statistical significance defined as $p<0.05$ using the log-rank (Mantel–Cox) test.

### Results

Median age at diagnosis was 68 years, with a male predominance. IgG myeloma was the most common subtype (65%). Cytogenetic abnormalities were identified in 54% of patients, most frequently gain of 1q21 (33.3%). High-risk cytogenetics according to IMS 2025 criteria were present in 26.1% of patients. 10.8% harboured multiple high-risk lesions. A very good partial response or better was achieved in 72.5% of patients, and 37.7% underwent autologous stem cell transplantation. Median OS was significantly inferior in patients with high-risk versus standard-risk cytogenetics (48 vs 88 months, $p=0.016$) and in patients aged >65 years versus <65 years (74 vs 127 months, $p=0.024$). PFS and OS trends, and the frequency of high-risk FISH abnormalities, were comparable to published international data[1].

**Copyright Information:** (Online) ISSN: 2572-9241
©2026 The Author(s). HemaSphere is published by John Wiley & Sons Ltd on behalf of European Hematology Association. This is an open access Abstract Book distributed under the Attribution-NonCommercial-NoDerivs (CC BY-NC-ND), which allows third parties to download the articles and share them with others as long as they credit the author and the Abstract Book, but they cannot change the content in any way or use them commercially.
**Abstract Book Citations:** Authors, Title, HemaSphere, 2026;10:(S1):pages. The abstract book DOIs can be found on the "Author Information" page.
**Disclaimer:** Articles published in the journal HemaSphere exclusively reflect the opinions of the authors. The authors are responsible for all content in their abstracts including accuracy of the facts, statements, citing resources, etc.

**Summary/Conclusion**

This study reports real-world outcomes of patients with MM treated at a regional Irish cancer centre, focusing on the cytogenetic landscape and its prognostic significance. Demographic characteristics and the distribution of genetic abnormalities were comparable to large international series. High-risk cytogenetic features were associated with inferior survival outcomes, consistent with established prognostic models. These findings confirm that the biological behaviour and clinical impact of cytogenetic risk in MM are reproducible in an Irish population, supporting the generalisability of international risk stratification frameworks and reinforcing the importance of cytogenetic profiling in routine clinical practice.

**References:**

1. **Risk Rewritten: The Biology-Driven 2025 International Myeloma Society/International Myeloma Working Group Criteria for High-Risk Multiple Myeloma** (2025) *The Hematologist*, 22(6), American Society of Hematology.
2. National Cancer Registry Ireland. Multiple Myeloma Factsheet 2018–2020. Cork, Ireland: NCRI; 2022.

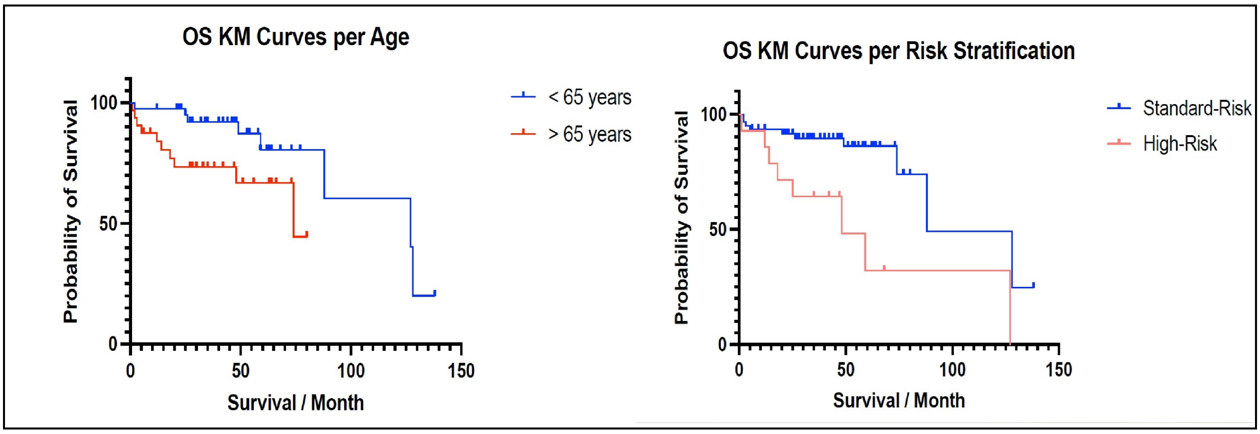

**Copyright Information:** (Online) ISSN: 2572-9241
©2026 The Author(s). HemaSphere is published by John Wiley & Sons Ltd on behalf of European Hematology Association. This is an open access Abstract Book distributed under the Attribution-NonCommercial-NoDerivs (CC BY-NC-ND), which allows third parties to download the articles and share them with others as long as they credit the author and the Abstract Book, but they cannot change the content in any way or use them commercially.
**Abstract Book Citations:** Authors, Title, HemaSphere, 2026;10:(S1):pages. The abstract book DOIs can be found on the "Author Information" page.
**Disclaimer:** Articles published in the journal HemaSphere exclusively reflect the opinions of the authors. The authors are responsible for all content in their abstracts including accuracy of the facts, statements, citing resources, etc.

### (PB3352) SAFETY AND EFFICACY OF ISATUXIMAB IN NEWLY DIAGNOSED FRAIL PATIENTS OF MULTIPLE MYELOMA PATIENTS

**Topic:** 14. Myeloma and other monoclonal gammopathies - Clinical

Maher Salamoon*[1]

[1]*al bairouni university cancer center, hematology/oncology, Damascus, Syrian Arab Republic;*

### Background

Multiple myeloma is the second most common hematological malignancy, with an age-standardized incidence of approximately 5 cases per 100,000 individuals. Despite improvements in survival following the introduction of immunomodulatory drugs (IMiDs) and proteasome inhibitors (PIs), multiple myeloma remains incurable .Therefore, the goal of treatment is to prolong survival and maintain quality of life. Treatment of frail patient is still a challenge even in the era of Anti-CD38 drugs

### Aims

Is to assess both efficacy and safety profile in newly diagnosed frail MM patients. Reporting the adverse events on rapid infusion over 30 minutes after the standard first cycle taking into consideration the comorbidities.

### Methods

Patients are given Isatuximab at a dose of 10mg/kg every 14 days till disease progression combined with weekly Bortezomib 3.5mg SC every week for a sum of 12 cycles in 10 patients and Lenalidomide at a dose of 10 mg per day in continuous in 5 patients. Dexamethasone given at a dose of 8 mg every 4 days. Treatment delivered as scheduled till disease progression or undesirable toxicity.

### Results

We have included 15 patients with NDMM with ages ranging between 68-82 (76 in median) with male to female ratio of 1.5:1. Patients presented with several comorbidities such as diabetes, hypertension, hypo and hyperthyroidism, prostatic hypertrophy and hyperlipidemia. 12 patients fell in the category intermediate-go while 3 in slow-go according to the frailty index score.
After the end of 3 months of treatment, complete response was observed in 9 patients manifested by complete clearance of plasma cells from the bone marrow and disappearance of gamma peak. 6 patients demonstrated objective response. Regarding safety profile, we started short infusion of Isatuximab beginning from the second cycle and it was tolerable in most of our patients. We documented several cases of neutropenia with different grades as well as thrombocytopenia in others while acute enal failure documented in 4 patients resolved after proper treatment. Isatuximab significantly improved PFS (HR = 0.69, 95% CI: 0.55–0.88, p = 0.002) and MRD negativity rates (RR = 1.30, 95% CI: 1.10–1.53, p < 0.003). the follow-up is 12 months so far and all patients are censored and documented alive by January 2026.

### Summary/Conclusion

Isatuximab-based regimens significantly improve PFS and depth of response with a manageable safety profile. It is considered to be a choice in NDMM patients with frail criteria due to its tolerance and low toxicity profile in combination with Lenalidomide, Bortezomib and Dexamethasone.

**Copyright Information:** (Online) ISSN: 2572-9241
©2026 The Author(s). HemaSphere is published by John Wiley & Sons Ltd on behalf of European Hematology Association. This is an open access Abstract Book distributed under the Attribution-NonCommercial-NoDerivs (CC BY-NC-ND), which allows third parties to download the articles and share them with others as long as they credit the author and the Abstract Book, but they cannot change the content in any way or use them commercially.
**Abstract Book Citations:** Authors, Title, HemaSphere, 2026;10:(S1):pages. The abstract book DOIs can be found on the “Author Information” page.
**Disclaimer:** Articles published in the journal HemaSphere exclusively reflect the opinions of the authors. The authors are responsible for all content in their abstracts including accuracy of the facts, statements, citing resources, etc.

### (PB3353) CARDIOVASCULAR ADVERSE EVENTS IN NEWLY DIAGNOSED MULTIPLE MYELOMA: INCIDENCE, RISK FACTORS, AND PROGNOSTIC IMPACT

**Topic:** 14. Myeloma and other monoclonal gammopathies - Clinical

Dewan Zhao*[1], Wangjie Chen[1], Boliang Li[2], Guirong Song[2], Xiuli Sun[1]

*[1]The First Affiliated Hospital of Dalian Medical University, Dalian, China;[2]Dalian Medical University, Dalian, China;*

### Background

Novel-agent frontline therapy has improved outcomes in multiple myeloma (MM), yet cardiovascular and thromboembolic events remain clinically relevant in modern care, reflecting both disease vulnerability and therapy exposure and potentially compromising progression-free survival (PFS). Real-world evidence on cardiovascular adverse events (CVAEs) in newly diagnosed MM remains limited, with heterogeneous definitions and grading and suboptimal generalizability of risk-prediction models.

### Aims

To characterize CVAEs during guideline-concordant frontline therapy (≥4 cycles) in newly diagnosed MM, identify independent risk factors, and explore associations with PFS.

### Methods

We retrospectively enrolled newly diagnosed MM patients treated at The First Affiliated Hospital of Dalian Medical University (January 1, 2011–February 1, 2025) who received ≥4 frontline cycles. We excluded cardiac amyloidosis, severe baseline cardiovascular disease (myocardial infarction/stroke within 6 months or New York Heart Association class III–IV heart failure), and concurrent active malignancy or prior cancer chemo-radiotherapy. CVAEs were defined per the 2022 European Society of Cardiology cardio-oncology guideline and graded by the Common Terminology Criteria for Adverse Events (CTCAE) v5.0 (grades 1–5). Baseline characteristics were compared using t/Mann–Whitney U and $\chi^2$/Fisher's exact tests. Time to first CVAE was analyzed using Cox regression; variables with univariable P<0.10 and clinically relevant covariates were entered into multivariable models with checks for collinearity and proportional hazards, and the final model was derived by backward stepwise likelihood ratio testing. PFS was evaluated by Kaplan–Meier and log-rank tests, with age-stratified sensitivity analyses (<65 vs ≥65 years).

### Results

A total of 164 patients were included (median follow-up 207.5 days [IQR 124–431.75]; mean age 64.36±9.09 years; 51.22% male). IgG MM accounted for 56.10%; Durie–Salmon stage III, 76.83%; ISS stage III, 44.51%; and 34.15% had prior hypertension. The most common frontline regimen was a proteasome inhibitor (PI) plus an immunomodulatory drug (IMiD) ± daratumumab (55.49%); 95.12% achieved ≥partial response (PR) after four cycles. CVAEs occurred in 37.20% (61/164), the most common categories were arrhythmia (32.79%), hypertension (27.87%), and heart failure (14.75%). Patients with CVAEs were older (P=0.005) and more frequently had prior hypertension (P<0.001), coronary artery disease (P=0.014), atrial fibrillation (AF; P=0.050), and ischemic stroke (P=0.027), while regimen distribution and ≥PR rates were similar (both P>0.05). Independent predictors of CVAEs were age (HR=1.045, 95% CI 1.009–1.081; P=0.013), WBC (per $1\times10^9$/L increase; HR=1.163, 95% CI 1.021–1.325; P=0.023), LDH (per 1 U/L increase; HR=1.003, 95% CI 1.001–1.005; P=0.007), prior atrial fibrillation (HR=13.229, 95% CI 3.778–46.318; P<0.001), and prior hypertension (HR=2.422, 95% CI 1.438–4.081; P<0.001). Age-stratified analyses suggested distinct risk profiles (<65 years: triglycerides and LDH; ≥65 years: high-sensitivity troponin I, WBC, and prior hypertension). PFS was inferior among patients with CVAEs (log-rank P=0.013).

**Copyright Information:** (Online) ISSN: 2572-9241
©2026 The Author(s). HemaSphere is published by John Wiley & Sons Ltd on behalf of European Hematology Association. This is an open access Abstract Book distributed under the Attribution-NonCommercial-NoDerivs (CC BY-NC-ND), which allows third parties to download the articles and share them with others as long as they credit the author and the Abstract Book, but they cannot change the content in any way or use them commercially.
**Abstract Book Citations:** Authors, Title, HemaSphere, 2026;10:(S1):pages. The abstract book DOIs can be found on the "Author Information" page.
**Disclaimer:** Articles published in the journal HemaSphere exclusively reflect the opinions of the authors. The authors are responsible for all content in their abstracts including accuracy of the facts, statements, citing resources, etc.

### Summary/Conclusion

CVAEs were frequent during frontline therapy in newly diagnosed MM and were independently predicted by age, WBC, LDH, and baseline atrial fibrillation and hypertension. CVAEs were associated with inferior PFS, supporting early risk stratification and proactive surveillance.

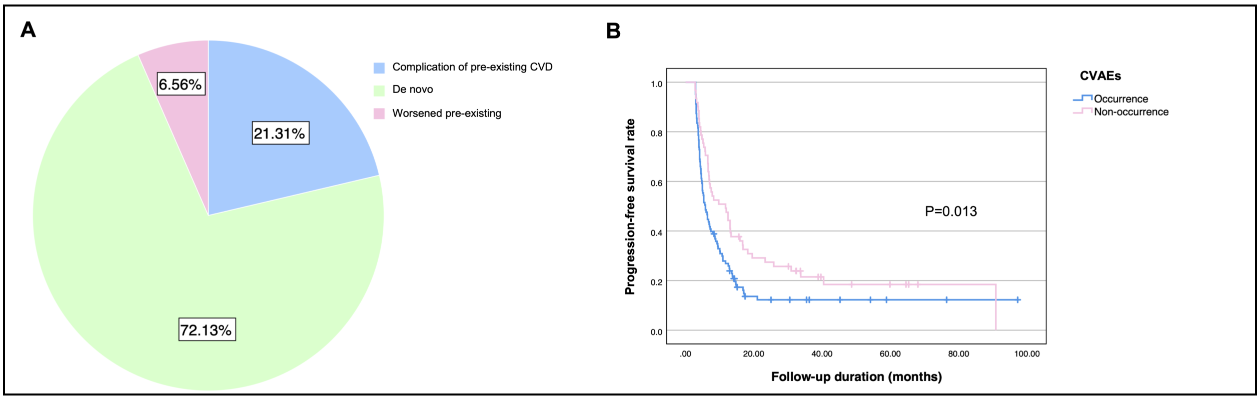

Figure 1. Patterns of cardiovascular adverse events (CVAEs) and progression-free survival (PFS) in newly diagnosed multiple myeloma. (A) Proportions of CVAE presentations among patients who developed CVAEs during follow-up (n=61): De novo (New-onset) (44/61, 72.13%), Worsened pre-existing (4/61, 6.56%), and Complication of pre-existing cardiovascular disease (CVD) (13/61, 21.31%). (B) Kaplan–Meier curves of PFS comparing patients with CVAEs (n=61) versus without CVAEs (n=103). PFS was significantly lower in the CVAE group (log-rank P=0.013).

**Copyright Information:** (Online) ISSN: 2572-9241
©2026 The Author(s). HemaSphere is published by John Wiley & Sons Ltd on behalf of European Hematology Association. This is an open access Abstract Book distributed under the Attribution-NonCommercial-NoDerivs (CC BY-NC-ND), which allows third parties to download the articles and share them with others as long as they credit the author and the Abstract Book, but they cannot change the content in any way or use them commercially.
**Abstract Book Citations:** Authors, Title, HemaSphere, 2026;10:(S1):pages. The abstract book DOIs can be found on the “Author Information” page.
**Disclaimer:** Articles published in the journal HemaSphere exclusively reflect the opinions of the authors. The authors are responsible for all content in their abstracts including accuracy of the facts, statements, citing resources, etc.

### (PB3354) ANALYSIS OF MULTIPLE MYELOMA TREATMENT COSTS IN THE BRAZILIAN PUBLIC HEALTH SYSTEM (SUS)

**Topic:** 14. Myeloma and other monoclonal gammopathies - Clinical

Janaína Gioseffi[1], Catherine Moura*[1], Nina Melo[1]

[1] *Abrale - Brazilian Association of Blood Cancer, São Paulo, Brazil;*

### Background

The Brazilian healthcare system is currently navigating a complex epidemiological transition characterized by the prevalence of non-communicable chronic diseases, with cancer emerging as a critical priority. Between 2023 and 2024, the Unified Health System (SUS) incorporated 67 new technologies, 16 of which were dedicated specifically to oncology. Financially, oncology treatment represents a substantial portion of the budget, accounting for approximately 14% of total health expenditures and reaching 32% within the specific sub-area of hospital and ambulatory assistance in 2024.

### Aims

The primary objective of this research, conducted by the *Oncology Observatory*, was to identify the direct costs of first-line treatment for Multiple Myeloma (C90) from the perspective of the public health system. The study sought to quantify the financial impact starting from a confirmed diagnosis.

### Methods

The methodology employed was a descriptive research approach using secondary budget data from the Management System of the Unified Health System (SUS) Table of Procedures, Medicines, and Orthotics, Prosthetics and Special Materials (OPM), and the Drug Market Regulation Chamber systems of the Brazilian Ministry of Health, incorporating an 18% Tax on the Circulation of Goods and Services. The analysis for this hematological neoplasm covered a one-year period and considered the patient's eligibility for Hematopoietic Stem Cell Transplantation (HSCT). To ensure clinical accuracy, the protocols were validated by specialists.

### Results

The results indicate that the initial cost related solely to the diagnosis of Multiple Myeloma is R$ 1,842.30. However, the subsequent treatment path creates a significant financial divide based on the patient's clinical profile. For patients eligible for HSCT, the total cost escalates to R$ 263,686.48 in one year, covering intensive phases such as stem cell mobilization, conditioning chemotherapy, the transplant procedure itself, and ongoing maintenance. In contrast, patients ineligible for the transplant follow a regimen based on Bortezomib, Melphalan, and Prednisone, resulting in a lower annual cost of R$ 108,953.70.

### Summary/Conclusion

In the discussion of these findings, the study notes that the high cost of transplantation in Brazil is somewhat mirrored in international literature; for instance, U.S. data shows post-transplant costs around US$ 60,000. Despite the high initial investment, HSCT is often considered the most cost-effective first-line option due to the gain in life years compared to the high costs associated with repeated hospitalizations in non-transplant scenarios. Finally, the study concludes that treatment costs increase significantly as the disease progresses or requires more interventions. This underscores the urgent need for strategic actions focused on prevention and early diagnosis to strengthen the sustainability of the public health system.

**Copyright Information:** (Online) ISSN: 2572-9241
©2026 The Author(s). HemaSphere is published by John Wiley & Sons Ltd on behalf of European Hematology Association. This is an open access Abstract Book distributed under the Attribution-NonCommercial-NoDerivs (CC BY-NC-ND), which allows third parties to download the articles and share them with others as long as they credit the author and the Abstract Book, but they cannot change the content in any way or use them commercially.
**Abstract Book Citations:** Authors, Title, HemaSphere, 2026;10:(S1):pages. The abstract book DOIs can be found on the "Author Information" page.
**Disclaimer:** Articles published in the journal HemaSphere exclusively reflect the opinions of the authors. The authors are responsible for all content in their abstracts including accuracy of the facts, statements, citing resources, etc.

### (PB3355) TRANSITION TO IXAZOMIB-BASED MAINTENANCE FOLLOWING DARATUMUMAB-BASED INDUCTION FOR CONTINUOUS THERAPY IN NEWLY DIAGNOSED MULTIPLE MYELOMA: A RETROSPECTIVE MULTICENTER STUDY

**Topic:** 14. Myeloma and other monoclonal gammopathies - Clinical

Yajuan Cui[1], Chanjuan Shen[2], Haimei Chen[2], Feiyue Zhu[3], Xiaoyan Liu[3], Ping Zhu[4], Limei Fu[4], Lijun Huang[5], Naping Tang[6], Jianwang Xu[7], Hongxia Xiang[7], Zeyu Luo[8], Min Liang[9], Dan Yi[10], Wen Cao[10], Donghua Zhang[11], Defang Jin[11], Kaibo Zhu[12], Zhongjun Huo[12], Hongling Peng[1], Ruijuan Li*[1]

*[1]The Second Xiangya Hospital, Central South University, Changsha, China;[2]Zhuzhou Hospital Affiliated to Xiangya School of Medicine, Central South University, Zhuzhou, China; [3]Central Hospital of Loudi, Loudi, China;[4]Chenzhou First People's Hospital, Department of Hematology, Chenzhou, China; [5]Hunan Cancer Hospital and the Affiliated Cancer Hospital of Xiangya School of Medicine, Central South University, Changsha, China; [6]Huaihua Central Hospital, Huaihua, China;[7]Yueyang People's Hospital, Yueyang, China; [8]The First Affiliated Hospital of South China University, Hengyang, China;[9]Xiangxi Tujia and Miao Autonomous Prefecture People's Hospital, Jishou, China; [10]The Second Affiliated Hospital of Hunan University of Chinese Medicine, Changsha, China; [11]Central Hospital of Shaoyang, Shaoyang, China; [12]Central Hospital of Xiangtan, Xiangtan, China;*

### Background

Daratumumab-based regimens have demonstrated favorable efficacy in newly diagnosed multiple myeloma (NDMM). However, prolonged administration poses challenges, including infection risks and limited treatment convenience. Ixazomib-based regimens offer a more convenient and tolerable maintenance strategy. Real-world evidence regarding the transition from daratumumab-based induction to to ixazomib-based maintenance remains limited.

### Aims

To address this clinical gap, we conducted a multicenter retrospective study using real-world data from 13 hospitals in Hunan Province, China, to evaluate treatment efficacy and health-related quality of life (HRQoL) outcomes following the switch from daratumumab-based induction to ixazomib-based maintenance.

### Methods

We enrolled 47 NDMM patients from 13 hospitals who received a median of 6 cycles (range 1–21) of daratumumab-based induction, achieved at least minimal response (MR), and subsequently switched to oral ixazomib-based maintenance. Maintenance regimens included ixazomib monotherapy (3–4 mg, days 1, 8, 15) or combination with lenalidomide/pomalidomide. Endpoints included response rates (IMWG criteria), progression-free survival (PFS), overall survival (OS), adverse events (CTCAE v5.0), and HRQoL.

**Copyright Information:** (Online) ISSN: 2572-9241
©2026 The Author(s). HemaSphere is published by John Wiley & Sons Ltd on behalf of European Hematology Association. This is an open access Abstract Book distributed under the Attribution-NonCommercial-NoDerivs (CC BY-NC-ND), which allows third parties to download the articles and share them with others as long as they credit the author and the Abstract Book, but they cannot change the content in any way or use them commercially.
**Abstract Book Citations:** Authors, Title, HemaSphere, 2026;10:(S1):pages. The abstract book DOIs can be found on the "Author Information" page.
**Disclaimer:** Articles published in the journal HemaSphere exclusively reflect the opinions of the authors. The authors are responsible for all content in their abstracts including accuracy of the facts, statements, citing resources, etc.

**Results**

A total of 47 patients met eligibility criteria and were included in the analysis. The cohort comprised 24 (51%) male patients with a median age of 63 years (range 40–76). High-risk cytogenetic abnormalities (del[17p], t[4;14], gain or amp[1q]) were present in 30 patients (63.8%), 22 (46.8%) had Revised International Staging System (R-ISS) stage III disease, and 30 (63.8%) were ineligible for autologous stem cell transplantation. At a median follow-up of 26 months (range 5–153), the median duration of maintenance therapy was 5 months (range 1–41). Median PFS was not reached; the 2-year PFS and OS rates were 97.1% and 100.0%, respectively. Among the 47 patients, a total 43 patients were evaluable for efficacy after the transition to ixazomib-based maintenance. Prior to transition, the rates of ≥VGPR and ≥CR were 65.1% (28/43) and 41.9% (18/43), respectively. Following ixazomib-based maintenance, these rates improved to 86.0% (37/43) for ≥VGPR and 69.8% (30/43) for ≥CR. The most common adverse events were hematologic toxicities (neutropenia: 11%, grade 3: 0%; thrombocytopenia: 4%, grade 3: 2%; anemia: 40%, grade 3: 0%), gastrointestinal events (diarrhea: 6.4%, grade 3: 2.1%; nausea: 2.1%, grade 3: 0%), rash (2.1%, grade 3), fatigue (2.1%, grade 2), upper respiratory tract infection (2.1%, grade 2), heart failure (2.1%, grade 2), and peripheral neuropathy (4.3%, grade 2). No treatment discontinuations due to adverse events were reported. HRQoL was assessed using the Treatment-Related Quality of Life Scale for Chinese Patients with Multiple Myeloma (total score: 144). Among patients receiving ixazomib-based maintenance, 29.7% scored 120–144, 59.5% scored 90–119, 8.1% scored 60–89, and 2.7% scored <60.

**Summary/Conclusion**

Transitioning to ixazomib-based maintenance following daratumumab-based induction is a feasible, safe, and highly effective strategy for NDMM. This approach sustains deep responses, ensures excellent survival outcomes, and preserves quality of life, supporting its utility as a sustainable long-term treatment option in real-world practice.

**Copyright Information:** (Online) ISSN: 2572-9241
©2026 The Author(s). HemaSphere is published by John Wiley & Sons Ltd on behalf of European Hematology Association. This is an open access Abstract Book distributed under the Attribution-NonCommercial-NoDerivs (CC BY-NC-ND), which allows third parties to download the articles and share them with others as long as they credit the author and the Abstract Book, but they cannot change the content in any way or use them commercially.
**Abstract Book Citations:** Authors, Title, HemaSphere, 2026;10:(S1):pages. The abstract book DOIs can be found on the “Author Information” page.
**Disclaimer:** Articles published in the journal HemaSphere exclusively reflect the opinions of the authors. The authors are responsible for all content in their abstracts including accuracy of the facts, statements, citing resources, etc.

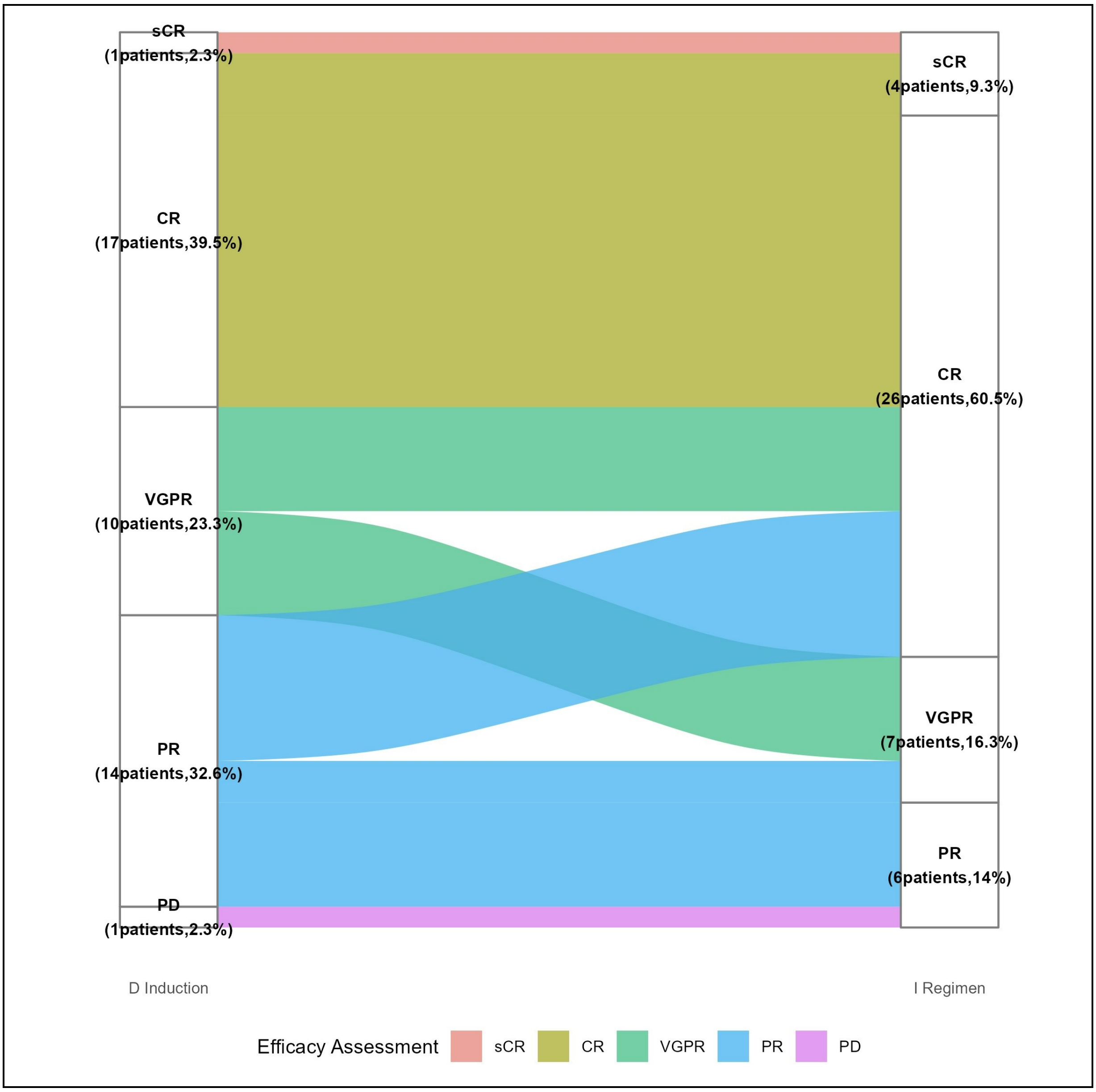

Fig. 1 Efficacy Assessment Changes in Multiple Myeloma Patients (Dara-based Induction switch to I-based maintenance)

**Copyright Information:** (Online) ISSN: 2572-9241
©2026 The Author(s). HemaSphere is published by John Wiley & Sons Ltd on behalf of European Hematology Association. This is an open access Abstract Book distributed under the Attribution-NonCommercial-NoDerivs (CC BY-NC-ND), which allows third parties to download the articles and share them with others as long as they credit the author and the Abstract Book, but they cannot change the content in any way or use them commercially.
**Abstract Book Citations:** Authors, Title, HemaSphere, 2026;10:(S1):pages. The abstract book DOIs can be found on the "Author Information" page.
**Disclaimer:** Articles published in the journal HemaSphere exclusively reflect the opinions of the authors. The authors are responsible for all content in their abstracts including accuracy of the facts, statements, citing resources, etc.

### (PB3356) ADVANCING PERSONALIZED MANAGEMENT IN MULTIPLE MYELOMA: THE UNTAPPED VALUE OF MINIMAL RESIDUAL DISEASE-GUIDED TREATMENT

**Topic:** 14. Myeloma and other monoclonal gammopathies - Clinical

Mihaela Musat[1], Jessicca Rege[1], Rozee Liu[1], Anna Forsythe*[1], Saro Sarkisian[2]

*[1]Oncoscope-AI, Miami, United States of America;[2]Frederick Health, Frederick, United States of America;*

### Background

Minimal residual disease (MRD)-driven treatment strategies have been integrated in guidelines and clinical practice in other hematologic cancers such as chronic and acute leukemias, while their usefulness in multiple myeloma (MM) is still unclear. MM guidelines recommend the assessment of MRD status for prognostication and while MRD negativity rate became an important trial endpoint, the role of MRD-guided treatment decisions in MM is still under investigation.

### Aims

We explored MRD-guided treatment strategies and associated outcomes in multiple myeloma.

### Methods

Utilizing REal-time AI-assisted Living systematic review (REAL-SLR) conducted in MM, according to PRISMA and Cochrane guidelines. PubMed and conference proceedings were reviewed to identify trials reporting outcomes of treatments for MM. For each included study, trial methodology, efficacy, safety and quality of life data was extracted.

### Results

As of February 25$^{th}$, 2026, 715 studies included in REAL-SLR database, 20 studies involved treatment decisions based on MRD status, most often during consolidation and maintenance phases (8 studies each). Four studies investigated the role of active therapy for persistent MRD positivity or MRD relapse.
MRD surveillance as alternative to indefinite maintenance and adjusted maintenance schedule in MRD negative patients are strategies that may benefit standard-risk patients. MASTER and GEM2014 trials showed a cumulative incidence of MRD relapse/progression of 0%-4% at 12 months after therapy cessation and 16%-33% at 5 years, respectively, among patients with standard risk who did not receive (MASTER) or stopped (GEM2014) maintenance upon achieving MRD negativity. Alternatively, lenalidomide maintenance did not show significant improvement in progression-free survival, overall survival or sustained MRD negativity compared to no maintenance in patients who achieved MRD negativity after autologous stem-cell transplant (ASCT).
Consolidation and ASCT benefited patients who were MRD positive post-induction, leading to deeper response and conversion to MRD negative status. However, MIDAS study showed that MRD outcomes did not significantly improve with ASCT compared to isatuximab, carfilzomib, lenalidomide, and dexamethasone consolidation in patients who achieved MRD negative status post-induction. Moreover, tandem ASCT did not confer better MRD outcomes compared to single ASCT among patients who were MRD positive post-induction.
Early intervention upon MRD relapse and treatment of persistent MRD with daratumumab significantly delayed progression, restored MRD negativity and resulted in high rates of conversion to MRD negative, as shown in PREDATOR-MRD trial and a study by Yan L et al.

**Copyright Information:** (Online) ISSN: 2572-9241
©2026 The Author(s). HemaSphere is published by John Wiley & Sons Ltd on behalf of European Hematology Association. This is an open access Abstract Book distributed under the Attribution-NonCommercial-NoDerivs (CC BY-NC-ND), which allows third parties to download the articles and share them with others as long as they credit the author and the Abstract Book, but they cannot change the content in any way or use them commercially.
**Abstract Book Citations:** Authors, Title, HemaSphere, 2026;10:(S1):pages. The abstract book DOIs can be found on the "Author Information" page.
**Disclaimer:** Articles published in the journal HemaSphere exclusively reflect the opinions of the authors. The authors are responsible for all content in their abstracts including accuracy of the facts, statements, citing resources, etc.

**Summary/Conclusion**

Use of MRD status to guide treatment in MM holds great potential for improving outcomes and reduce burden of treatment including risks of serious adverse events. MRD-guided strategies require further, more systematic investigations, including predefined studies, as they represent viable options for personalizing therapy while maintaining effectiveness and potentially minimizing side effects and overall costs.

**Copyright Information:** (Online) ISSN: 2572-9241
©2026 The Author(s). HemaSphere is published by John Wiley & Sons Ltd on behalf of European Hematology Association. This is an open access Abstract Book distributed under the Attribution-NonCommercial-NoDerivs (CC BY-NC-ND), which allows third parties to download the articles and share them with others as long as they credit the author and the Abstract Book, but they cannot change the content in any way or use them commercially.
**Abstract Book Citations:** Authors, Title, HemaSphere, 2026;10:(S1):pages. The abstract book DOIs can be found on the "Author Information" page.
**Disclaimer:** Articles published in the journal HemaSphere exclusively reflect the opinions of the authors. The authors are responsible for all content in their abstracts including accuracy of the facts, statements, citing resources, etc.

### (PB3357) SECOND MALIGNANCIES IN MULTIPLE MYELOMA: A SINGLE-CENTER COHORT

**Topic:** 14. Myeloma and other monoclonal gammopathies - Clinical

Onur Turkoz[1], Elifcan Aladag*[1], Yahya Buyukasik[1], Haluk Demiroglu[1], Hakan Goker[1]

[1]*Hacettepe, Department of Hematology, Altındağ, Türkiye;*

**Background**

Despite prolonged survival in the novel agent era, second malignancies remain an important long-term concern in patients with multiple myeloma (MM). The development of second cancers likely reflects complex interactions between disease-related immune dysregulation, host susceptibility, aging, and treatment exposure.

**Aims**

To evaluate the incidence, distribution, survival impact, and clinical associations of second malignancies in a single-center MM cohort

**Methods**

We retrospectively analyzed 495 MM patients (median age 64 years; 59.4% male) with a median follow-up of 50.7 months. Cumulative incidence of second malignancies was estimated accounting for death as a competing event. Multivariable logistic regression was performed to explore associations with clinical and treatment-related variables. Standardized incidence ratios (SIR) were calculated using age- and sex-adjusted expected cancer rates from the general population.

**Results**

Eighteen patients (3.6%) developed a second malignancy after MM diagnosis. Median time to development was 97.3 months. Second malignancies included thyroid (n=3), lung (n=2), colon (n=2), skin (n=2), acute myeloid leukemia (n=1), breast (n=1), gastric (n=1), ovarian (n=1), renal cell carcinoma (n=1), hepatocellular carcinoma (n=1), brain tumor (n=1), ampullary carcinoma (n=1), and sarcoma (n=1). Solid tumors predominated, and only one case of therapy-related acute myeloid leukemia was observed. The 5- and 10-year cumulative incidence rates were 1.6% and 5%, respectively. In exploratory multivariable analysis, daratumumab exposure was statistically associated with higher odds of second malignancy (OR 4.40, 95% CI 1.08–15.69), whereas cyclophosphamide exposure was associated with lower odds (OR 0.31, 95% CI 0.09–0.88). Lenalidomide exposure and autologous stem cell transplantation were not significantly associated. Given the limited number of events and retrospective design, these findings should not be interpreted as causal. The overall SIR was 0.75 (95% CI 0.53–1.07), indicating no statistically significant excess risk compared with the general population.

**Summary/Conclusion**

In this single-center cohort, the cumulative incidence of second malignancies was low and did not exceed population-based expectations. Events were predominantly solid tumors, and therapy-related myeloid neoplasms were rare. The development of second malignancies in MM appears multifactorial, likely involving disease biology, immune dysregulation, host factors, and treatment exposure. Larger prospective studies are warranted to better define these relationships.

**Copyright Information:** (Online) ISSN: 2572-9241
©2026 The Author(s). HemaSphere is published by John Wiley & Sons Ltd on behalf of European Hematology Association. This is an open access Abstract Book distributed under the Attribution-NonCommercial-NoDerivs (CC BY-NC-ND), which allows third parties to download the articles and share them with others as long as they credit the author and the Abstract Book, but they cannot change the content in any way or use them commercially.
**Abstract Book Citations:** Authors, Title, HemaSphere, 2026;10:(S1):pages. The abstract book DOIs can be found on the "Author Information" page.
**Disclaimer:** Articles published in the journal HemaSphere exclusively reflect the opinions of the authors. The authors are responsible for all content in their abstracts including accuracy of the facts, statements, citing resources, etc.

### (PB3358) EVALUATING VARIATION IN THE MANAGEMENT OF MONOCLONAL GAMMOPATHY OF UNDETERMINED SIGNIFICANCE (MGUS) AMONG CANADIAN HEMATOLOGISTS: A QUALITY ASSURANCE STUDY

**Topic:** 14. Myeloma and other monoclonal gammopathies - Clinical

Christina Tam*[1], Siraj Mithoowani[1], Mark Andrew[1]

[1]*McMaster University, Department of Medicine, Hamilton, Canada;*

### Background

Monoclonal gammopathy of undetermined significance (MGUS) is a premalignant plasma cell disorder affecting about 5% of adults over 50, carrying lifelong risk of progression to multiple myeloma or related conditions. In Canada, no national standard exists for MGUS monitoring, leading to wide variation in follow-up intervals, investigations, and thresholds for additional assessment. Such inconsistencies may delay recognition of disease progression in some patients while creating unnecessary clinical testing in others. Understanding everyday MGUS management is essential for identifying gaps, informing standardization, and guiding future Canadian recommendations.

### Aims

This quality-assurance study aimed to characterize how Canadian hematologists investigate and follow MGUS, quantify variation in responses to standardized case scenarios, and explore potential associations between management patterns and clinician demographics, experience, training, and guideline use.

### Methods

A descriptive, cross-sectional online survey was distributed through the Canadian Hematology Society and McMaster University email lists between December 22, 2025 and February 15, 2026. Participants were eligible to enter a $100 gift card raffle upon completion. Eligible participants included hematology residents, fellows, and independent practitioners. The self-administered survey consisted of demographics, clinician background, and ten MGUS case-based scenarios with structured response options. Case scenarios had no evidence of end-organ dysfunction or myeloma-defining events. Data were analyzed descriptively. This study was approved by the Hamilton Integrated Research Ethics Board #19296.

### Results

Forty hematologists completed the survey. Most respondents were 30–39 and 40–49 years (32.5% each), followed by 50–59 years (22.5%). Most respondents practiced in Ontario (65%). Years of independent practice varied widely: 32.5% had 11–20 years of experience, while 27.5% had fewer than five. Fewer than half (47.5%) had received education or training in plasma cell disorders in the past five years. Guideline use was diverse – 50% relied on IMWG recommendations, 27.5% used local or institutional guidance, and 25% reported using no specific guideline. Some respondents cited ASH, NCCN, or other guidelines.
Case-based responses showed substantial variability. Strong agreement occurred only in clearly low-risk or non-MGUS presentations. For example, in a case featuring beta–gamma bridging and no detectable M-protein, 90% recommended discharge from clinic. In contrast, in a case featuring an elderly woman with elevated IgM (4.7 g/L): 20.0% recommended discharge from clinic, 32.5% follow-up, and 47.5% additional investigations. Among the latter group, testing choices varied. 26.3% ordered bone marrow examination without skeletal imaging, 21.1% chose skeletal imaging without bone marrow examination, 36.8% opted for neither, and 15.8% included both.

**Copyright Information:** (Online) ISSN: 2572-9241
©2026 The Author(s). HemaSphere is published by John Wiley & Sons Ltd on behalf of European Hematology Association. This is an open access Abstract Book distributed under the Attribution-NonCommercial-NoDerivs (CC BY-NC-ND), which allows third parties to download the articles and share them with others as long as they credit the author and the Abstract Book, but they cannot change the content in any way or use them commercially.
**Abstract Book Citations:** Authors, Title, HemaSphere, 2026;10:(S1):pages. The abstract book DOIs can be found on the “Author Information” page.
**Disclaimer:** Articles published in the journal HemaSphere exclusively reflect the opinions of the authors. The authors are responsible for all content in their abstracts including accuracy of the facts, statements, citing resources, etc.

### Summary/Conclusion

This survey reveals large inconsistency in MGUS management among Canadian hematologists, likely influenced by differences in experience, training, and guideline adoption. While consensus emerged in low-risk or non-MGUS scenarios, management decisions diverged widely in intermediate or ambiguous cases. These findings highlight the need for standardized MGUS guidance to reduce practice variation. As fewer than half of respondents reported recent plasma-cell–specific training, expanding educational resources may also improve coherence in MGUS management across Canada.

**Copyright Information:** (Online) ISSN: 2572-9241
©2026 The Author(s). HemaSphere is published by John Wiley & Sons Ltd on behalf of European Hematology Association. This is an open access Abstract Book distributed under the Attribution-NonCommercial-NoDerivs (CC BY-NC-ND), which allows third parties to download the articles and share them with others as long as they credit the author and the Abstract Book, but they cannot change the content in any way or use them commercially.
**Abstract Book Citations:** Authors, Title, HemaSphere, 2026;10:(S1):pages. The abstract book DOIs can be found on the "Author Information" page.
**Disclaimer:** Articles published in the journal HemaSphere exclusively reflect the opinions of the authors. The authors are responsible for all content in their abstracts including accuracy of the facts, statements, citing resources, etc.

### (PB3359) DELAYED DIAGNOSIS AND ADVANCED ORGAN INVOLVEMENT IN LIGHT-CHAIN AMYLOIDOSIS: INSIGHTS FROM A TERTIARY CARE CENTRE IN NORTHERN INDIA

**Topic:** 14. Myeloma and other monoclonal gammopathies - Clinical

Nitin Gupta*[1], Naveenvairamoorthy Dakshinamoorthy[1], Naman Bansal[1], Navneet Mishra[1], Meena Verma[1]

*[1]GANGA RAM INSTITUTE OF POST MEDIAL EDUCATION AND RESEARCH, CLINICAL HEMATOLOGY AND BONE MARROW/STEM CELL TRANSPLANT, NEW DELHI, India;*

### Background

Despite significant progress and improving outcomes in the management of plasma cell disorders, AL amyloidosis remains diagnostically and therapeutically challenging. With the availability of serum-free light chain assay, more cases of AL amyloidosis are being diagnosed who were earlier missed as secondary amyloidosis. Novel agents like bortezomib are being used in almost all the patients. There is lack of published data from India about diagnosis and outcome of AL amyloidosis in current era.

### Aims

To study the clinical profile and treatment outcomes in AL amyloidosis.

### Methods

A retrospective analysis of AL amyloidosis patients who were diagnosed in our hospital from January 2016 to September 2025 was done. Demographic, laboratory and treatment outcome data were collected from the departmental database. The haematological response was assessed as per international consensus guidelines for AL amyloidosis.

### Results

Seventy-seven patients were diagnosed with AL amyloidosis during the study period. Of these, 59 (77%) were males and 18 (23%) were females, with a median age of 61 years (range 39-81 years). Underlying multiple myeloma was noted in 18/77 (23%) of cases. The median time from symptom onset to diagnosis was 8 months (range 1-36 months), highlighting significant diagnostic delay. In 77% (59/77) of cases, the amyloid light chain type was lambda, while in 23% (18/77) it was kappa. Only light chain involvement (with no heavy chain) was seen in 53% (41/77), among which 33 (80%) were lambda and 8 (20%) were kappa. The median dFLC at diagnosis was 177 mg/L (range 12-1887 mg/L). The most involved organs were the kidneys and heart (57% each, 44/77), followed by the gastrointestinal tract (21%, 16/77). Two or more organs were involved in 45% (35/77) of patients. All were treated with bortezomib-based protocols. Fifty-one patients (75%) who received treatment at our hospital were available for follow-up and were included in the survival analysis. Forty-one patients (80%) responded to first-line therapy: a complete hematological response was seen in 22/51 (43%), a very good partial response in 4/51 (8%), and a partial response in 14/51 (27%). Ten patients (20%) had no response or progressed while on first-line therapy. The median follow-up duration was 13 months (6 months to 76 months). Twenty-nine patients were alive, while 22 (43%) succumbed to amyloid-related complications. The median overall survival was 55 months, and the survival rates were 75%, 62%, and 40% at 1, 2, and 5 years, respectively.In multivariate analysis, cardiac involvement (HR 3.2, 95% CI 1.3-7.8, p=0.012), involvement of more than two organs (HR 2.8, 95% CI 1.2-6.5, p=0.02), and less than VGPR to first-line therapy (HR 4.1, 95% CI 1.4-12.3, p=0.012) remained independent predictors of poor survival. (Figure 1)

**Copyright Information:** (Online) ISSN: 2572-9241
©2026 The Author(s). HemaSphere is published by John Wiley & Sons Ltd on behalf of European Hematology Association. This is an open access Abstract Book distributed under the Attribution-NonCommercial-NoDerivs (CC BY-NC-ND), which allows third parties to download the articles and share them with others as long as they credit the author and the Abstract Book, but they cannot change the content in any way or use them commercially.
**Abstract Book Citations:** Authors, Title, HemaSphere, 2026;10:(S1):pages. The abstract book DOIs can be found on the "Author Information" page.
**Disclaimer:** Articles published in the journal HemaSphere exclusively reflect the opinions of the authors. The authors are responsible for all content in their abstracts including accuracy of the facts, statements, citing resources, etc.

**Summary/Conclusion**

Serum free light chain assay in appropriate clinical syndrome setting clinches the diagnosis in majority of patients. Fat pad and rectal biopsy demonstration of amyloid deposit helps in tissue demonstration of amyloid if affected tissue biopsy is not available. The overall prognosis of patients with multi-organ involvement and patients with reduced cardiac ejection fractions remains poor. Patients who achieve complete hematologic response experience improved organ function and survival. These findings emphasize the importance of early diagnosis and effective treatment strategies tailored to individual patient profiles

Figure 1 A Survival according to response to therapy

P=0.023 (1064 days vs. NR)

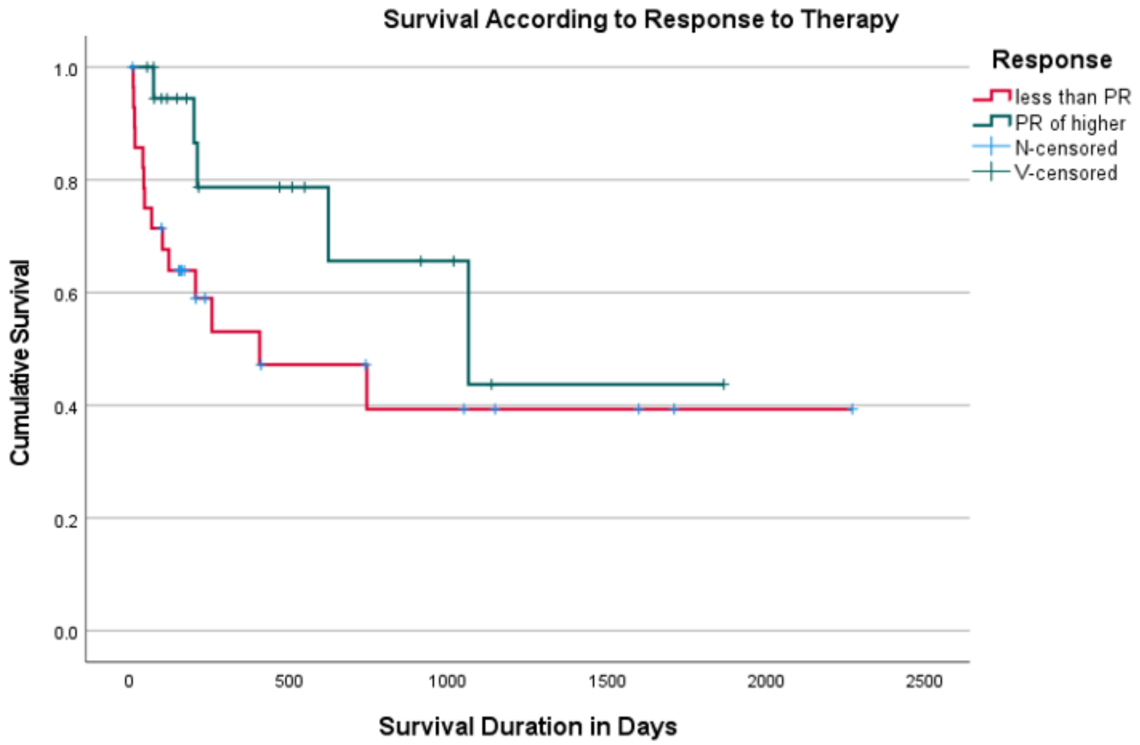

**Copyright Information:** (Online) ISSN: 2572-9241
©2026 The Author(s). HemaSphere is published by John Wiley & Sons Ltd on behalf of European Hematology Association. This is an open access Abstract Book distributed under the Attribution-NonCommercial-NoDerivs (CC BY-NC-ND), which allows third parties to download the articles and share them with others as long as they credit the author and the Abstract Book, but they cannot change the content in any way or use them commercially.
**Abstract Book Citations:** Authors, Title, HemaSphere, 2026;10:(S1):pages. The abstract book DOIs can be found on the "Author Information" page.
**Disclaimer:** Articles published in the journal HemaSphere exclusively reflect the opinions of the authors. The authors are responsible for all content in their abstracts including accuracy of the facts, statements, citing resources, etc.

### (PB3360) EXTRAMEDULLARY DISEASE VERSUS PARAMEDULLARY LESIONS IN NEWLY DIAGNOSED MULTIPLE MYELOMA: CLINICAL CHARACTERISTICS AND PROGNOSTIC IMPACT IN A SINGLE-CENTRE COHORT

**Topic:** 14. Myeloma and other monoclonal gammopathies - Clinical

Harberth Fernandez Leyva*[1]

[1]*NHS University hospital of Dorset, haematology, Bournemouth, United Kingdom;*

### Background

Extramedullary disease (EMD) represents an aggressive manifestation of multiple myeloma (MM) and remains associated with poor prognosis despite advances in novel therapies. The reported incidence of EMD ranges from 3–30% at diagnosis and increases in relapsed or refractory disease. Paramedullary lesions (PLs), arising from bone lesions with soft tissue extension, may differ biologically and prognostically from true EMD. However, their distinct clinical impact remains incompletely defined, and controversies persist regarding classification and optimal management of plasmacytomas in newly diagnosed multiple myeloma (NDMM).

### Aims

To evaluate the clinical characteristics and prognostic impact of EMD compared with PLs in patients with NDMM.

### Methods

We performed a retrospective single-centre analysis of 30 consecutive NDMM patients presenting with plasmacytomas between 2020 and 2025. Whole-body MRI and 18F-FDG PET/CT were performed according to clinical indication at diagnosis. Patients were classified as having EMD and/or PLs. Baseline characteristics, International Staging System (ISS) stage, progression-free survival (PFS), and overall survival (OS) were analyzed. Survival outcomes were assessed using hazard ratios (HR) with 95% confidence intervals (CI). Multivariate analysis was conducted to evaluate independent prognostic factors.

### Results

Among 30 patients, 22 presented with EMD and 19 with PLs; some patients exhibited both manifestations. Patients with EMD more frequently had advanced ISS stage (ISS II–III), suggesting more aggressive disease biology.
After a median follow-up of 24 months, PFS did not significantly differ between EMD and PL groups (HR 0.93; 95% CI 0.51–1.87). In contrast, OS was significantly inferior in patients with EMD compared with those with PLs (HR 3.76; 95% CI 1.81–7.92; $p = 0.017$). Multivariate analysis confirmed EMD at diagnosis as an independent adverse prognostic factor for OS ($p = 0.038$). Notably, patients with initial EMD demonstrated shorter survival after first progression, consistent with more aggressive plasma cell behavior.

### Summary/Conclusion

In this single-centre cohort of NDMM patients with plasmacytomas, EMD and PLs demonstrated distinct prognostic implications. While PFS was comparable, EMD at diagnosis was independently associated with significantly inferior OS. These findings support the concept that true extramedullary spread reflects biologically aggressive disease distinct from paramedullary extension. Despite the limited sample size, our data highlight the need for refined risk stratification and prospective studies to establish standardized diagnostic definitions and evidence-based treatment strategies for this high-risk subgroup in the era of novel agents.

**Copyright Information:** (Online) ISSN: 2572-9241
©2026 The Author(s). HemaSphere is published by John Wiley & Sons Ltd on behalf of European Hematology Association. This is an open access Abstract Book distributed under the Attribution-NonCommercial-NoDerivs (CC BY-NC-ND), which allows third parties to download the articles and share them with others as long as they credit the author and the Abstract Book, but they cannot change the content in any way or use them commercially.
**Abstract Book Citations:** Authors, Title, HemaSphere, 2026;10:(S1):pages. The abstract book DOIs can be found on the "Author Information" page.
**Disclaimer:** Articles published in the journal HemaSphere exclusively reflect the opinions of the authors. The authors are responsible for all content in their abstracts including accuracy of the facts, statements, citing resources, etc.

### (PB3361) EFFICACY OF IXAZOMIB, LENALIDOMIDE, AND DEXAMETHASONE (IRD) IN RELAPSED/REFRACTORY MULTIPLE MYELOMA AFTER ≥3 PRIOR LINES OF THERAPY: A REAL-WORLD SINGLE-CENTER EXPERIENCE

**Topic:** 14. Myeloma and other monoclonal gammopathies - Clinical

Oksana Karnabeda[1, 2], Tetiana Yehorova[2], Ulyana Melnyk[3, 4], Yuliia Makarets*[2]

*[1]National Research Center for Radiation Medicine of National Academy of Medical Sciences of Ukraine, Kyiv, Ukraine;[2]Arensia Exploratory Medicine, Kyiv, Ukraine; [3]Kyiv City Clinical Hospital № 9, Kyiv, Ukraine;[4]Kyiv City Clinical Hospital № 7, Kyiv, Ukraine;*

### Background

Ixazomib, lenalidomide, and dexamethasone (IRd) is a well-established triplet regimen for relapsed/refractory multiple myeloma (RRMM). However, real-world data on its activity in patients treated beyond the third line of therapy remain limited. Understanding IRd performance in heavily pretreated populations is important to guide therapeutic sequencing.

### Aims

To evaluate the objective response rate (ORR) and progression-free survival (PFS) in RRMM patients treated with IRd after ≥3 prior lines, and to assess the relationship between the number of previous therapies and clinical outcomes

### Methods

A retrospective single-center study was conducted at the National Cancer Institute (Kyiv, Ukraine) and included 24 RRMM patients treated with IRd between 2023 and 2025. Responses were assessed per IMWG criteria. Patients were stratified by the number of prior therapy lines: 3 lines, 4–6 lines, and ≥6 lines. Spearman correlation was used to analyze the relationship between prior therapy lines and ORR. Progression-free survival (PFS) was evaluated using the Kaplan–Meier method

### Results

Among the 24 patients (median age 63 years, range 42–78), the ORR (CR+PR) was: 33.3% for 3 prior lines, 28.6% for 4–6 prior lines, and 25.0% for ≥6 prior lines. A strong negative correlation was observed between the number of prior therapy lines and response rate ($\rho = -1.00$, $p < 0.001$). The median PFS for the entire cohort was 13.9 months, with a trend toward shorter PFS in patients with more prior treatments. Treatment was generally well tolerated, with no grade ≥3 toxicities leading to discontinuation.

### Summary/Conclusion

IRd remains an effective and well-tolerated regimen for RRMM patients even after multiple prior lines of therapy. Although response rates and PFS decline with increasing treatment exposure, meaningful clinical benefit is observed beyond the sixth line in real-world practice. These findings support the use of IRd as a viable late-line treatment option for RRMM.

**Copyright Information:** (Online) ISSN: 2572-9241
©2026 The Author(s). HemaSphere is published by John Wiley & Sons Ltd on behalf of European Hematology Association. This is an open access Abstract Book distributed under the Attribution-NonCommercial-NoDerivs (CC BY-NC-ND), which allows third parties to download the articles and share them with others as long as they credit the author and the Abstract Book, but they cannot change the content in any way or use them commercially.
**Abstract Book Citations:** Authors, Title, HemaSphere, 2026;10:(S1):pages. The abstract book DOIs can be found on the "Author Information" page.
**Disclaimer:** Articles published in the journal HemaSphere exclusively reflect the opinions of the authors. The authors are responsible for all content in their abstracts including accuracy of the facts, statements, citing resources, etc.

### (PB3362) AUTOLOGOUS STEM CELL TRANSPLANTATION AS BRIDGING THERAPY PRIOR TO IDECABTAGENE VICLEUCEL (IDE-CEL) IN MULTIPLE MYELOMA: A SINGLE-CENTRE EXPERIENCE

**Topic:** 14. Myeloma and other monoclonal gammopathies - Clinical

Jakub Cieślar[1], Ivanna Biochuk*[1], Klara Mensikova[1], Martin Stork[1], Nela Sendlerová[2], Viera Sandecká[1], Marta Krejčí[1], Zdenek Adam[1], Frantisek Folber[1], Ludek Pour[1]

*[1]University Hospital Brno and Faculty of Medicine, Department of Internal Medicine, Hematology and Oncology, Brno, Czechia;*
*[2]Faculty of Medicine, Masaryk University, Brno, Czechia;*

**Background**

Despite advances in novel therapeutic strategies, multiple myeloma (MM) remains incurable for the majority of patients. A major clinical challenge is refractory disease characterized by short survival.

CAR T-cell therapy represents an important treatment option for this population. However, manufacturing and logistical delays may lead to disease progression or death before infusion.

Bridging therapy is administered to maintain disease control during the interval between leukapheresis and CAR T-cell infusion; however, standardized treatment strategies have not yet been established.

**Aims**

To evaluate the safety and disease control of autologous stem cell transplantation (ASCT) used as bridging therapy prior to CAR-T (Ide-cel) in heavily pretreated MM patients, and to describe early efficacy and survival outcomes.

**Methods**

We conducted a retrospective single-centre analysis of 8 refractory MM patients who underwent ASCT as bridging therapy prior to CAR-T cell treatment. Conditioning consisted of high-dose melphalan. All patients received standard lymphodepletion with fludarabine and cyclophosphamide prior to CAR-T.

**Copyright Information:** (Online) ISSN: 2572-9241
©2026 The Author(s). HemaSphere is published by John Wiley & Sons Ltd on behalf of European Hematology Association. This is an open access Abstract Book distributed under the Attribution-NonCommercial-NoDerivs (CC BY-NC-ND), which allows third parties to download the articles and share them with others as long as they credit the author and the Abstract Book, but they cannot change the content in any way or use them commercially.
**Abstract Book Citations:** Authors, Title, HemaSphere, 2026;10:(S1):pages. The abstract book DOIs can be found on the "Author Information" page.
**Disclaimer:** Articles published in the journal HemaSphere exclusively reflect the opinions of the authors. The authors are responsible for all content in their abstracts including accuracy of the facts, statements, citing resources, etc.

**Results**

Eight heavily pretreated refractory MM patients were analyzed (median age 55 years, range 48–74; 4 males). Median of prior therapy lines was 4 (range 3–8), all patients were refractory to PI, IMiDs and anti CD38 antibody, 3 had received bispecific antibodies.

Leukapheresis was successful in all patients. The median length of hospitalization for ASCT was 17 days (range 15–18). Bridging ASCT resulted in disease control in the majority of evaluable patients and no patient progressed between leukapheresis and CAR-T infusion. Ide-cel was administered after a median of 40.5 days (range 37–80) from leukapheresis and 35.5 days (range 28–71) from ASCT. The median length of hospitalization for CAR-T administration was 16.5 days.

Cytokine release syndrome (CRS) occurred in five patients, including gr. 1 in 4 patients and 1 case of gr. 3; all cases were treated with tocilizumab. No cases of immune effector cell-associated neurotoxicity syndrome (ICANS) were observed. All patients developed pancytopenia (including gr. III neutropenia) and hypogammaglobulinemia; however, no infectious complications were documented in association with these cytopenias. One patient died due to infection occurring after disease progression following CAR-T cell therapy.

**Summary/Conclusion**

ASCT as bridging therapy to CAR-T was safe, with hospitalization duration and toxicity rates comparable to other CAR-T approaches. No patient experienced disease progression between leukapheresis and CAR-T infusion, supporting ASCT as a clinically effective strategy to maintain disease control during the manufacturing interval.

**Copyright Information:** (Online) ISSN: 2572-9241
©2026 The Author(s). HemaSphere is published by John Wiley & Sons Ltd on behalf of European Hematology Association. This is an open access Abstract Book distributed under the Attribution-NonCommercial-NoDerivs (CC BY-NC-ND), which allows third parties to download the articles and share them with others as long as they credit the author and the Abstract Book, but they cannot change the content in any way or use them commercially.
**Abstract Book Citations:** Authors, Title, HemaSphere, 2026;10:(S1):pages. The abstract book DOIs can be found on the “Author Information” page.
**Disclaimer:** Articles published in the journal HemaSphere exclusively reflect the opinions of the authors. The authors are responsible for all content in their abstracts including accuracy of the facts, statements, citing resources, etc.

### (PB3363) IMPACT OF AUTOLOGOUS STEM CELL TRANSPLANTATION ON OVERALL SURVIVAL IN PATIENTS WITH MULTIPLE MYELOMA

**Topic:** 14. Myeloma and other monoclonal gammopathies - Clinical

Sviatlana Haurylava*[1], Iryna Lendzina[1], Ihar Iskrou[2], Tatiana Lebedeva[1]

*[1]State Institution "Minsk Scientific and Practical Center for Surgery, Transplantology and Hematology", Minsk, Belarus;[2]Institute Of Advanced Training And Retraining Of Healthcare Personnel Of BSMU, Minsk, Belarus;*

### Background

In multiple myeloma (MM), cytogenetic alterations are widely recognized as the most robust indicators of adverse clinical outcomes. Currently, fluorescence in situ hybridization (FISH) continues to serve as the reference method for genetic risk assessment in routine clinical settings. Patients are classified as high-risk when harboring specific aberrations, including del(17p), translocations t(4;14), t(14;16), or t(14;20), amplification or gain of chromosome 1q (gain 1q), deletion of 1p, or mutations in the TP53 gene. When two or more of these high-risk features coexist, the disease is termed "double-hit myeloma"; the presence of three or more such abnormalities defines "triple-hit myeloma." Among patients who meet eligibility criteria, autologous hematopoietic stem cell transplantation (auto-HSCT) represents a fundamental component of consolidation strategies within the overall therapeutic approach for MM. However, whether auto-HSCT can offset the poor survival outcomes conferred by high-risk genetic abnormalities remains uncertain.

### Aims

To evaluate the effect of auto-HSCT on survival outcomes in MM patients with adverse cytogenetic profiles.

### Methods

A total of 107 patients with histologically confirmed MM, diagnosed between 2017 and 2025 and subjected to FISH-based cytogenetic profiling, were included in this study. High-risk status was assigned to individuals carrying del(17p), t(4;14), t(14;16), or co-occurrence of these abnormalities. Among the high-risk subgroup (n=17), auto-HSCT was performed in 11 patients, while 6 patients were managed without transplantation. Three patients underwent salvage tandem auto-HSCT following disease relapse or progression.

### Results

Cytogenetic aberrations were identified in 65.4% of the study population (n=70). Among these, 17 patients (24.3%) were stratified into the high-risk cytogenetic group, while 53 patients (75.7%) were classified as standard-risk. Within the high-risk cohort, the most frequent abnormality was t(4;14), observed in 10 patients (58.8%), followed by t(14;16) in 3 patients (17.6%) and TP53 mutations/deletions in 2 patients (11.8%). A double-hit profile—defined as the co-occurrence of t(4;14) and t(14;16)—was detected in 2 patients (11.8%), placing them in a very-high-risk category.
Survival analysis was performed in 82 patients with available follow-up data. The estimated 5-year overall survival (OS) was 78.9% in the standard-risk group (median OS not reached) compared to 57.1% in the high-risk group (median OS: 80 months); this difference did not reach statistical significance (p=0.09).
Auto-HSCT was performed in 11 patients within the high-risk subgroup. Among the remaining high-risk patients, auto-HSCT was not administered in 5 cases due to primary refractoriness to induction therapy, and 1 patient declined further disease-specific treatment. Two-year OS was significantly higher in high-risk patients who underwent auto-HSCT compared to those who did not (100% vs. 33%, respectively; p=0.045). In the standard-risk cohort, 2-year OS was 89.9% among patients who received auto-HSCT (n=29) and 92.3% among those managed without transplantation (n=40).

**Copyright Information:** (Online) ISSN: 2572-9241
©2026 The Author(s). HemaSphere is published by John Wiley & Sons Ltd on behalf of European Hematology Association. This is an open access Abstract Book distributed under the Attribution-NonCommercial-NoDerivs (CC BY-NC-ND), which allows third parties to download the articles and share them with others as long as they credit the author and the Abstract Book, but they cannot change the content in any way or use them commercially.
**Abstract Book Citations:** Authors, Title, HemaSphere, 2026;10:(S1):pages. The abstract book DOIs can be found on the "Author Information" page.
**Disclaimer:** Articles published in the journal HemaSphere exclusively reflect the opinions of the authors. The authors are responsible for all content in their abstracts including accuracy of the facts, statements, citing resources, etc.

**Summary/Conclusion**

Among patients with multiple myeloma, those classified as standard-risk demonstrated superior overall survival compared to their high-risk counterparts. Notably, autologous hematopoietic stem cell transplantation was associated with a statistically significant improvement in overall survival specifically within the high-risk patient cohort.

**Copyright Information:** (Online) ISSN: 2572-9241
©2026 The Author(s). HemaSphere is published by John Wiley & Sons Ltd on behalf of European Hematology Association. This is an open access Abstract Book distributed under the Attribution-NonCommercial-NoDerivs (CC BY-NC-ND), which allows third parties to download the articles and share them with others as long as they credit the author and the Abstract Book, but they cannot change the content in any way or use them commercially.
**Abstract Book Citations:** Authors, Title, HemaSphere, 2026;10:(S1):pages. The abstract book DOIs can be found on the "Author Information" page.
**Disclaimer:** Articles published in the journal HemaSphere exclusively reflect the opinions of the authors. The authors are responsible for all content in their abstracts including accuracy of the facts, statements, citing resources, etc.

### (PB3364) TRENDS IN FRONTLINE DARATUMUMAB USE OVER TIME AND DIFFERENCES IN UTILIZATION BY INSURANCE TYPE AMONG PATIENTS WITH MULTIPLE MYELOMA

**Topic:** 14. Myeloma and other monoclonal gammopathies - Clinical

Anthony Corsi*[1], Phyu Thin Naing[2], Kelvin Zheng[3], Divya Chukkalore[3], Richa Thakur[3], Salman Khan[4], David Chung[5], Jessica Caro[5]

*[1]Donald and Barbara Zucker School of Medicine of Hofstra/Northwell at NS/LIJ, Department of Internal Medicine, Manhasset, United States of America; [2]Vanderbilt University Medical Center, Department of Hematology/Oncology, Nashville, United States of America; [3]Donald and Barbara Zucker School of Medicine of Hofstra/Northwell at NS/LIJ, Department of Hemtology/Oncology, Manhasset, United States of America; [4]Donald and Barbara Zucker School of Medicine of Hofstra/Northwell Health at Staten Island, Department of Internal Medicine, New York, United States of America; [5]Northwell Health, Department of Hematology/Oncology, New Hyde Park, United States of America;*

### Background

Daratumumab (Dara), an anti-CD38 monoclonal antibody, has been incorporated into frontline regimens for multiple myeloma since 2019 for both transplant-ineligible (MAIA trial) and transplant-eligible (CASSIOPEIA trial) patients. Use of Dara as a frontline agent is varied among providers and may be influenced by insurance coverage.

### Aims

This study analyzed temporal trends in frontline Dara use and assessed the impact of insurance type on its use within Northwell Health, the largest healthcare system in New York.

### Methods

We conducted a retrospective cohort study of patients diagnosed with multiple myeloma between 2021 and 2024 at Northwell Health. Patients were identified using ICD-10 codes and confirmed by chart review. Those without a confirmed diagnosis or who were lost to follow-up were excluded. Data collection included demographics, Dara start date, time to treatment, practice setting, and insurance type. Frontline Dara use was defined as initiation within 90 days of diagnosis.

### Results

Among the eligible 278 patients, 128 (46.0%) patients were male and 150 (54.0%) were female, with a median age of 71 years at diagnosis. Racial distribution was White (41.0%), Black or African American (32.7%), Asian (5.4%), Native American (0.4%), Other (12.6%), and Unknown (7.9%). Overall, 136 (48.9%) patients received Dara as frontline therapy, with use increasing over time: 8/54 (14.81%) patients in 2021, 19/61 (31.5%) in 2022, 42/81 (51.85%) in 2023, and 67/82 (81.7%) in 2024. Among patients receiving frontline Dara, 175 (63.0%) were treated at academic practices and 103 (37.0%) were treated at community practices, with frontline Dara use increasing similarly at both sites over time: 3/31 (10%) vs 5/21 (24%) in 2021, 13/37 (35%) vs 6/24 (25%) in 2022, 26/51 (51%) vs 16/30 (53%) in 2023, and 44/54 (81%) vs 23/28 (82%) in 2024. No year-specific differences in frontline Dara use between treatment sites were significant ($p \geq 0.16$). Frontline Dara use did significantly differ by insurance type ($\chi^2 = 8.24$, $p=0.04$): 32.4% of patients with Medicaid, 50.4% with Medicare, 59.7% with private insurance, and 40.0% with unknown insurance.

**Copyright Information:** (Online) ISSN: 2572-9241
©2026 The Author(s). HemaSphere is published by John Wiley & Sons Ltd on behalf of European Hematology Association. This is an open access Abstract Book distributed under the Attribution-NonCommercial-NoDerivs (CC BY-NC-ND), which allows third parties to download the articles and share them with others as long as they credit the author and the Abstract Book, but they cannot change the content in any way or use them commercially.
**Abstract Book Citations:** Authors, Title, HemaSphere, 2026;10:(S1):pages. The abstract book DOIs can be found on the "Author Information" page.
**Disclaimer:** Articles published in the journal HemaSphere exclusively reflect the opinions of the authors. The authors are responsible for all content in their abstracts including accuracy of the facts, statements, citing resources, etc.

### Summary/Conclusion

Since its FDA approval, frontline Dara utilization has steadily increased across practice settings within Northwell Health. Academic and community sites both showed a similar increase in use of frontline Dara over time. Insurance type appears to significantly influence access, with lower frontline Dara use in patients with Medicaid compared to those with private insurance. These findings highlight the need for system-level strategies to promote equitable access to novel therapies and mitigate insurance-related barriers to care.

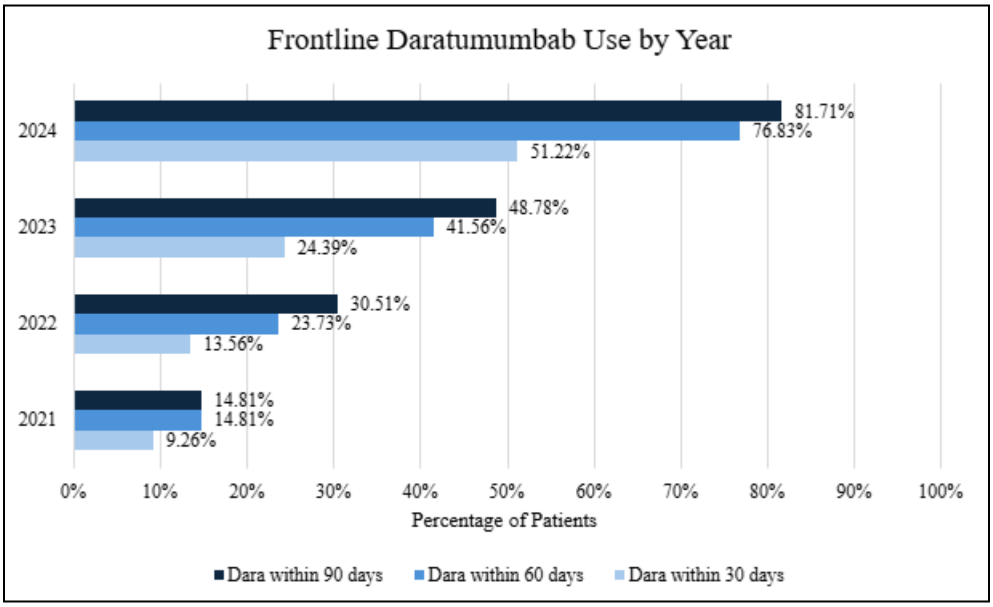

Figure 1. Trends in time to daratumumab initiation from time of diagnosis between 2021 and 2024.

**Copyright Information:** (Online) ISSN: 2572-9241
©2026 The Author(s). HemaSphere is published by John Wiley & Sons Ltd on behalf of European Hematology Association. This is an open access Abstract Book distributed under the Attribution-NonCommercial-NoDerivs (CC BY-NC-ND), which allows third parties to download the articles and share them with others as long as they credit the author and the Abstract Book, but they cannot change the content in any way or use them commercially.
**Abstract Book Citations:** Authors, Title, HemaSphere, 2026;10:(S1):pages. The abstract book DOIs can be found on the "Author Information" page.
**Disclaimer:** Articles published in the journal HemaSphere exclusively reflect the opinions of the authors. The authors are responsible for all content in their abstracts including accuracy of the facts, statements, citing resources, etc.

### (PB3365) THE EMERGING ‘TRANSPLANT DEFERRED’ POPULATION IN NEWLY DIAGNOSED MULTIPLE MYELOMA (NDMM) REPRESENTS A SUBSTANTIAL EVIDENCE GAP IN THE NOVEL-AGENT ERA

**Topic:** 14. Myeloma and other monoclonal gammopathies - Clinical

Shaji Kumar*[1], Molly Purser[2], Alex Bates[3], Zeyad Khalaf[3]

*[1]Mayo Clinic, Rochester, MN, United States of America;[2]GSK, Upper Providence, PA, United States of America;[3]GSK, London, United Kingdom;*

### Background

Patients with multiple myeloma who are potentially transplant eligible (TE) but do not proceed to upfront autologous stem cell transplantation (ASCT) are considered ‘transplant deferred’ (TD). TD remains inconsistently defined and TD-NDMM is often treated as equivalent to transplant-ineligible (TI) NDMM, complicating interpretation and evidence translation. Trials that include a TD population may be more in line with clinical practice, and as a result easier to generalize to the broader NDMM population.

### Aims

To systematically review the NDMM trial literature since 2021 to identify the emergence of TD as a distinct subgroup in trials, and to assess whether TD-specific outcomes are reported in these trials.

### Methods

A systematic literature review (SLR) was conducted to identify prospective, interventional NDMM trials published between January 2022 and January 2026 that evaluated pharmacological interventions for NDMM. Databases were searched using defined terms relating to NDMM, and included TE, TI, and TD populations in the scope. Trials were screened for inclusion, and reporting of TD-specific outcomes was extracted.

### Results

In total, 59 NDMM study publications were identified, but only 5 explicitly defined or operationalized a TD population. However, most trials that targeted a TI population did not explicitly exclude patients with TD-NDMM and therefore had the possibility to include unremarked TD patients. Although no study recruited specifically for a TD population, 5 distinguished between TD-NDMM and TI-NDMM as part of their eligibility criteria. These were CEPHEUS, MagnetisMM-6, CC-220-MM-001, US MM-6, and AFT-41. None of these studies reported outcomes for the TD population as a separate subgroup.
No studies targeting a TE population described including a TD population. Studies that described a *de facto* TD population (for example, testing early vs delayed ASCT in TE-NDMM) were excluded from the search as they did not test a pharmacological intervention.

### Summary/Conclusion

This SLR demonstrated that prospective evidence in a TD population remains fragmented and inadequately reported. Patients with TD-NDMM have generally been included in trials targeting a TI population, and some recent trials have explicitly highlighted that a TD population might exist within the study. Standardized definitions and prespecified reporting of TD-specific outcomes are needed to address an evidence gap regarding the generalizability of trials with and without TD-specific subpopulations.
**Funding:** GSK (Study 300291)

**Copyright Information:** (Online) ISSN: 2572-9241
©2026 The Author(s). HemaSphere is published by John Wiley & Sons Ltd on behalf of European Hematology Association. This is an open access Abstract Book distributed under the Attribution-NonCommercial-NoDerivs (CC BY-NC-ND), which allows third parties to download the articles and share them with others as long as they credit the author and the Abstract Book, but they cannot change the content in any way or use them commercially.
**Abstract Book Citations:** Authors, Title, HemaSphere, 2026;10:(S1):pages. The abstract book DOIs can be found on the “Author Information” page.
**Disclaimer:** Articles published in the journal HemaSphere exclusively reflect the opinions of the authors. The authors are responsible for all content in their abstracts including accuracy of the facts, statements, citing resources, etc.

### (PB3366) GAMMAGA: PROJECT FOR EDUCATION AND DIAGNOSIS OF GAUCHER DISEASE (GD) AND ACID SPHINGOMYELINASE DEFICIENCY (ASMD) IN PATIENTS WITH MONOCLONAL GAMMOPATHY OF UNDETERMINED SIGNIFICANCE OR MULTIPLE MYELOMA

**Topic:** 14. Myeloma and other monoclonal gammopathies - Clinical

Albert Pérez Montaña[1], Alejandro Contento Gonzalo*[2], Carola Diaz Aizpun[2], Xabier Gutierrez Lopez De Ocariz[3], Erik de Cabo[4], Dolores Gómez Toboso[5], Beatriz Cuevas Ruiz[6], Bolivar Luis Diaz Jordan[7], Yolanda Gonzalez Montes[8], Marta Gomez Nuñez[9], Amalia Domingo Gonzalez[10], Carmen Pérez Martínez[11], Alejandro Ponce Navarro[12], Antonio Cruz Díaz[13], Abelardo Bárez[14], Maria Carmen Albo Lopez[15], Maria Mar Meijon Ortigueira[16], Jose Angel Mendez Sanchez[17], Javier De La Rubia Comos[18], Andres Madinaveitia[19], Marta Morado[20]

*[1]Hospital Universitario Son Espases, Hematology, Palma de Mallorca, Spain; [2]Hospital Regional Universitario de Málaga, Hematology, Malaga, Spain; [3]Hospital Universitario de Alava-Txagorritxu, Hematology, Vitoria, Spain;[4]Hospital del Bierzo, Hematology, Ponferrada, Spain; [5]Hospital de Manises, Hematology, Valencia, Spain;[6]Hospital Universitario de Burgos, H, Burgos, Spain; [7]Hospital de Valdepeñas, Hematology, Ciudad Real, Spain;[8]Hospital Universitario Josep Trueta, Hematology, Girona, Spain; [9]Hospital Parc Tauli, Hematology, Sabadell, Spain; [10]Hospital Fundacion Jimenez Diaz, Hematology, Madrid, Spain; [11]Hospital Clinico Universitraio de Valladolid, Hematology, Valladolid, Spain;[12]Hospital Universitario de Torrecardenas, Almeria, Spain; [13]Hospital Virgen de las Nieves, Granada, Spain; [14]Complejo Asistencial de Avila, Avila, Spain; [15]Hospital Alvaro Cunqueiro, Vigo, Spain; [16]Hospital Ramon y Cajal, Madrid, Spain; [17]Complejo Hospitalario de Orense, Orense, Spain;[18]Hospital Universitario y Politecnico la Fé, Valencia, Spain; [19]Hospital de Alcañiz, Teruel, Spain;[20]Hospital La Paz, Madrid, Spain;*

### Background

Gaucher disease (GD) is a rare lysosomal disease (LD) caused by a deficiency of the enzyme glucocerebrosidase (GCase), leading to the accumulation of glucosylceramide (BGl-22) and glucosylsphingosine (Lyso-Gl1). Acid sphingomyelinase deficiency (ASMD), though less prevalent, causes sphingomyelin accumulation. These glycolipid deposits in macrophages and induce organ damage and a global inflammatory state that leads to continuous immune activation. A strong association has been demonstrated between GD and monoclonal gammopathies (MG) (monoclonal gammopathies of uncertain significance (MGUS) or multiple myeloma (MM)). Recent studies also show a high incidence of MGUS in ASMD. Both LD commonly present with splenomegaly and/or thrombocytopenia, but diagnosis requires high clinical suspicion. Given this association with MG, the presence of a monoclonal spike with splenomegaly and/or thrombocytopenia should prompt clinicians to rule out these LD. An Italian study found that 0.1% of MG patients have GD.

### Aims

The objective of this project is to analyze the prevalence of ASMD and GD in adult patients diagnosed with MGUS or MM who also present splenomegaly/splenectomy and/or thrombocytopenia.

### Methods

This project includes two programs: an educational program to increase awareness about the association between MG and GD/ASMD; and a prospective epidemiological program in which enzymatic quantification of glucosylceramide and sphingomyelin is performed using DBS samples from patients with MGUS or MM and splenomegaly, splenectomy and/or thrombocytopenia (not secondary to antineoplastic treatment). Samples with values below the normal range undergo confirmatory genetic testing.

**Copyright Information:** (Online) ISSN: 2572-9241
©2026 The Author(s). HemaSphere is published by John Wiley & Sons Ltd on behalf of European Hematology Association. This is an open access Abstract Book distributed under the Attribution-NonCommercial-NoDerivs (CC BY-NC-ND), which allows third parties to download the articles and share them with others as long as they credit the author and the Abstract Book, but they cannot change the content in any way or use them commercially.
**Abstract Book Citations:** Authors, Title, HemaSphere, 2026;10:(S1):pages. The abstract book DOIs can be found on the "Author Information" page.
**Disclaimer:** Articles published in the journal HemaSphere exclusively reflect the opinions of the authors. The authors are responsible for all content in their abstracts including accuracy of the facts, statements, citing resources, etc.

**Results**

From November 2023 to December 2025, 38 educational sessions were held in 19 hospitals across the country, with a total of 502 attendees, 67% of whom were hematologists.
Additionally, samples and clinical data from 80 patients diagnosed with MG and presenting splenomegaly/splenectomy and/or thrombocytopenia were collected.
59 patients were men (73.8%). Median age was 71 years (IQR 62.8–79). 35 patients (60.3%) had non-IgM MGUS, 13 (22.4%) had IgM MGUS, 10 (17.2%) had light-chain MGUS, and 22 (27.5%) had MM. Splenomegaly was observed in 50 patients (62.5%), and splenectomy in 4 (5%).
Up to date, 8 samples showed enzymatic activity below normal limits: 2 with low GCase and 8 with low acid sphingomyelinase.
No biallelic pathogenic variants were detected, but 2 samples remain pending of genetic confirmation. Completed results will be presented as soon as would be available.

**Summary/Conclusion**

The association of monoclonal gammopathies with GD and ASMD is significant, making it essential to consider these LD in patients with MG and thrombocytopenia/splenomegaly, even if prevalence is low.
Most haematologists participating in this program reported increased awareness and diagnostic capability regarding ASMD and GD.

**Copyright Information:** (Online) ISSN: 2572-9241
©2026 The Author(s). HemaSphere is published by John Wiley & Sons Ltd on behalf of European Hematology Association. This is an open access Abstract Book distributed under the Attribution-NonCommercial-NoDerivs (CC BY-NC-ND), which allows third parties to download the articles and share them with others as long as they credit the author and the Abstract Book, but they cannot change the content in any way or use them commercially.
**Abstract Book Citations:** Authors, Title, HemaSphere, 2026;10:(S1):pages. The abstract book DOIs can be found on the “Author Information” page.
**Disclaimer:** Articles published in the journal HemaSphere exclusively reflect the opinions of the authors. The authors are responsible for all content in their abstracts including accuracy of the facts, statements, citing resources, etc.

### (PB3367) IMPEDE-VTE RISK STRATIFICATION AND REAL-WORLD THROMBOTIC PROPHYLAXIS IN MULTIPLE MYELOMA, NORTHERN MOROCCO

**Topic:** 14. Myeloma and other monoclonal gammopathies - Clinical

Siham Belkadi*[1], Oumayma Hari[1], Imane El Boutahiri[1], Safae Regragui[1]

[1]*University Hospital Center Mohammed VI, Clinical Hematology Service, Tangier, Morocco;*

### Background

Thromboembolic complications, estimated at 6–13% in the international literature, represent the second leading cause of death in multiple myeloma patients. Yet, they remain poorly documented, particularly in North Africa where local data are lacking. Current guidelines recommend stratified prophylaxis according to the IMPEDE-VTE score: aspirin for low-risk patients; low-molecular-weight heparin (LMWH) for intermediate- and high-risk patients.

### Aims

This first study on a North Moroccan cohort aims to:

1. Analyze real-world prophylactic practices, marked by predominant aspirin use due to economic accessibility
2. Evaluate the applicability of the IMPEDE-VTE score in this context
3. Assess the incidence of thrombotic events

### Methods

We conducted a single-center retrospective study in the hematology department of Tangier University Hospital Center, including 126 patients with multiple myeloma diagnosed and followed between 2019 and 2025 (median follow-up, 17 months).
IMPEDE-VTE score was retrospectively calculated for 100 patients (79.4%), due to complete data availability. Patients were then classified as low, intermediate, or high risk according to the score's proposed classification (cumulative points).
We performed a cross-analysis between the IMPEDE-VTE score, type of prophylaxis used, and the occurrence of thromboembolic events (TEEs) to assess their concordance or discordance.
Predictive factors for TEEs were evaluated using univariate and multivariate tests.

### Results

Among 126 multiple myeloma patients (50.8% women [n=64], 49.2% men [n=62]), with median age 60.5 years, TEEs data were available for 105 patients (83.3%), allowing analysis of complications under universal prophylaxis (n=105): aspirin in 82 patients (78.1%) and LMWH in 23 patients (21.9%).
Thirteen TEEs were observed (overall incidence 12.4%), predominantly venous: 4 at diagnosis, 8 under active treatment (7.6%), with median onset of 17.5 days.
Cross-analysis revealed all events occurred under aspirin, with particularly high incidence (30.0%) in high-risk group under aspirin. No event occurred under LMWH regardless of risk level.
Major predictive factors included prolonged bedrest (OR=30.61; p=0.001), amyloidosis (p<0.001), hypercalcemia (p=0.014) and IMiD exposure (p=0.001).

**Copyright Information:** (Online) ISSN: 2572-9241
©2026 The Author(s). HemaSphere is published by John Wiley & Sons Ltd on behalf of European Hematology Association. This is an open access Abstract Book distributed under the Attribution-NonCommercial-NoDerivs (CC BY-NC-ND), which allows third parties to download the articles and share them with others as long as they credit the author and the Abstract Book, but they cannot change the content in any way or use them commercially.
**Abstract Book Citations:** Authors, Title, HemaSphere, 2026;10:(S1):pages. The abstract book DOIs can be found on the “Author Information” page.
**Disclaimer:** Articles published in the journal HemaSphere exclusively reflect the opinions of the authors. The authors are responsible for all content in their abstracts including accuracy of the facts, statements, citing resources, etc.

**Summary/Conclusion**

This first Moroccan study documents 7.6% thromboembolic complications, comparable to international literature (6-13%), despite universal prophylaxis dominated by aspirin in a resource-limited setting. We identified three major factors: prolonged bedrest, systemic amyloidosis, and hypercalcemia, which are not included in the IMPEDE-VTE score. These results question population specificity. Our study proposes an adapted local thromboprophylaxis algorithm and call for prospective multicenter validation to adapt Moroccan thromboprophylactic practices.

**Copyright Information:** (Online) ISSN: 2572-9241
©2026 The Author(s). HemaSphere is published by John Wiley & Sons Ltd on behalf of European Hematology Association. This is an open access Abstract Book distributed under the Attribution-NonCommercial-NoDerivs (CC BY-NC-ND), which allows third parties to download the articles and share them with others as long as they credit the author and the Abstract Book, but they cannot change the content in any way or use them commercially.
**Abstract Book Citations:** Authors, Title, HemaSphere, 2026;10:(S1):pages. The abstract book DOIs can be found on the "Author Information" page.
**Disclaimer:** Articles published in the journal HemaSphere exclusively reflect the opinions of the authors. The authors are responsible for all content in their abstracts including accuracy of the facts, statements, citing resources, etc.

### (PB3368) TREATMENT OF HIGH-RISK SMOLDERING MULTIPLE MYELOMA (HRSMM) WITH ISATUXIMAB, LENALIDOMIDE, ORAL CYCLOPHOSPHAMIDE AND DEXAMETHASONE: A SURVIVAL ADVANTAGE IN A CASE SERIES STUDY

**Topic:** 14. Myeloma and other monoclonal gammopathies - Clinical

Maher Salamoon*[1]

*[1]al bairouni university cancer center, hematology/oncology, Damascus, Syrian Arab Republic;*

### Background

High risk smoldering multiple myeloma was treated traditionally with observation only, however; transformation to multiple myeloma takes place within at least 2 years of diagnosis. Some patients do not take serious medical observations, making the transformation more aggressive at presentation. Several trials were conducted, shedding light on treatment smoldering multiple myeloma patients at higher risk to transform into more serious diseases and most studies investigated the role of anti-CD38 along with immune modulators such as Lenalidomide and Dexamethasone.

### Aims

In our study we are trying to show the importance of early treatment of HRSMM patients. Those patients are rarely diagnosed in our country because they are followed by physicians other than haemato-oncologists. Some are seen by nephrologists or orthopedists. In this study, we tried to demonstrate the impact of early treatment on progression free survival in those patients.

### Methods

We have included 12 patients diagnosed with HRSMM with ages ranging between 42 and 70 (62 in median) and a male to female ratio of 3:1. All patients diagnosed had 2 out of the following 3 criteria: serum M-protein more than 2 gr/dl, free light chain ratio more than 20 and/or bone marrow plasma cells more than 20%. Patients were treated on 28-day cycles with ISA 20 mg/kg IV on days 1, 8, 15, and 22 of cycle (C) 1, days 1 and 15 of C2-6, and day 1 of C7-30, together with LEN 10 mg PO in continuous, oral Cyclophosphamide 100mg PO in continuous and Dexamethasone 8mg every week. The primary endpoint was overall response rate (ORR) after the 6$^{th}$ cycle. Secondary endpoints were progression-free survival (PFS), defined as time from enrollment to development of symptomatic multiple myeloma, ORR after completion of 18 and 30 cycles, and overall survival (OS).

### Results

The overall response rate ORR was 91.6% after the 24$^{th}$ cycle improved from 66.6% after the 6$^{th}$ cycle to 83% after the 12 cycles. Only one patient progressed after the 17 cycles. Complete responses were documented in 7 patients (58%) while all others responded between partial response and very good partial response. Deep response documented to happen between cycle 15 and 21 (17 in median). With a median follow-up of 2 years the progression free survival was 91.6% (95% CI: 86.2-100). Hematologic side effects with grades between 1-2 were documented effects such as nausea, diarrhea, constipation fatigue and skin rashes ranged between 8-32% in general and they were tolerable.

**Copyright Information:** (Online) ISSN: 2572-9241
©2026 The Author(s). HemaSphere is published by John Wiley & Sons Ltd on behalf of European Hematology Association. This is an open access Abstract Book distributed under the Attribution-NonCommercial-NoDerivs (CC BY-NC-ND), which allows third parties to download the articles and share them with others as long as they credit the author and the Abstract Book, but they cannot change the content in any way or use them commercially.
**Abstract Book Citations:** Authors, Title, HemaSphere, 2026;10:(S1):pages. The abstract book DOIs can be found on the “Author Information” page.
**Disclaimer:** Articles published in the journal HemaSphere exclusively reflect the opinions of the authors. The authors are responsible for all content in their abstracts including accuracy of the facts, statements, citing resources, etc.

**Summary/Conclusion**

The ORR of our study shows that the combination of anti-CD38, immune-modulators, oral chemotherapy and Dexamethasone is a good tolerable combination in high risk smoldering multiple myeloma patients with a good safety profile. Though the number is modest, however; results are comparable with other trials

**Copyright Information:** (Online) ISSN: 2572-9241
©2026 The Author(s). HemaSphere is published by John Wiley & Sons Ltd on behalf of European Hematology Association. This is an open access Abstract Book distributed under the Attribution-NonCommercial-NoDerivs (CC BY-NC-ND), which allows third parties to download the articles and share them with others as long as they credit the author and the Abstract Book, but they cannot change the content in any way or use them commercially.
**Abstract Book Citations:** Authors, Title, HemaSphere, 2026;10:(S1):pages. The abstract book DOIs can be found on the “Author Information” page.
**Disclaimer:** Articles published in the journal HemaSphere exclusively reflect the opinions of the authors. The authors are responsible for all content in their abstracts including accuracy of the facts, statements, citing resources, etc.

### (PB3370) IXAZOMIB- VERSUS BORTEZOMIB-BASED PROTEASOME INHIBITOR TRIPLET REGIMENS IN ELDERLY, TRANSPLANT-INELIGIBLE NEWLY DIAGNOSED MULTIPLE MYELOMA: A SCOPING REVIEW OF REAL-WORLD EVIDENCE

**Topic:** 14. Myeloma and other monoclonal gammopathies - Clinical

Yan Wang[1], Sha Liu[2], Jian-qing Mi*[1]

*[1]Shanghai Institute of Hematology, State Key Laboratory of Medical Genomics, National Research Center for Translational Medicine at Shanghai, Ruijin Hospital, Shanghai Jiao Tong University School of Medicine, Shanghai, China; [2]Takeda (China) International Trading Co., Ltd, Shanghai, China., Shanghai, China;*

### Background

Frontline therapy for elderly patients with transplant-ineligible newly diagnosed multiple myeloma (TIE-NDMM) remains challenging in real-world clinical practice. Due to advanced age, frailty, and the presence of multiple comorbidities, a substantial proportion of these patients face barriers to healthcare access and exhibit limited tolerance to continuous therapy. Although bortezomib-based triplets are widely used, their long-term use is often constrained by cumulative toxicities, most notably peripheral neuropathy, and the burden of parenteral administration. The oral proteasome inhibitor, ixazomib, offers a potential alternative to address these challenges.

### Aims

This study aimed to compare the real-world effectiveness and safety of ixazomib- versus bortezomib-based triplet regimens as first-line treatment in elderly (≥65 years) patients with TIE-NDMM.

### Methods

Following PRISMA-ScR 2020, major international literature databases were searched from the inception of databases to April 18, 2025. Eligible studies included elderly (≥65 years) TIE-NDMM patients treated with ixazomib- or bortezomib-based (I-based or V-based) triplet regimens as first-line treatment. The primary outcomes were progression-free survival (PFS) and duration of treatment (DOT). Secondary outcomes included grade ≥3 adverse events (AEs), serious adverse events (SAEs), peripheral neuropathy (PN), and treatment discontinuation. These outcomes were all presented as median values derived from the included studies.

### Results

Nineteen studies comprising 12,905 patients were included. Notably, the treatment regimens evaluated in these studies did not involve monoclonal antibodies. The median PFS was 17.8 months (range, 13.9-21.6) for I-based treatment and 19.4 months (range, 11.9-26.5) for V-based treatment. The median DOT was 9.3 months (range, 5.6-13.1) for I-based treatment and 6.0 months (range, 3.7-11.2) for V-based treatment. I-based regimens were associated with lower toxicity-related discontinuation (5-12% vs. 10.6–30.0%), lower rates of ≥ grade 3 AEs (23.7-29.4% vs. 27.2-80.2%), SAEs (18.3%-25.0% vs. 45%-68.4%), and PN (0%-8.3% vs. 18.2%-46.8%). Notably, PFS estimates for I-based regimens were derived predominantly from studies conducted in frail elderly populations, whereas V-based estimates were generally derived from studies enrolling a mixed population of frail and non-frail elderly patients. I-based regimens maintained PFS comparable to that of bortezomib-based regimens and demonstrated better treatment tolerability.

**Copyright Information:** (Online) ISSN: 2572-9241
©2026 The Author(s). HemaSphere is published by John Wiley & Sons Ltd on behalf of European Hematology Association. This is an open access Abstract Book distributed under the Attribution-NonCommercial-NoDerivs (CC BY-NC-ND), which allows third parties to download the articles and share them with others as long as they credit the author and the Abstract Book, but they cannot change the content in any way or use them commercially.
**Abstract Book Citations:** Authors, Title, HemaSphere, 2026;10:(S1):pages. The abstract book DOIs can be found on the “Author Information” page.
**Disclaimer:** Articles published in the journal HemaSphere exclusively reflect the opinions of the authors. The authors are responsible for all content in their abstracts including accuracy of the facts, statements, citing resources, etc.

**Summary/Conclusion**

This scoping review suggests that ixazomib-based regimens are as effective as bortezomib-based therapies in older patients with transplant-ineligible NDMM in first-line treatment, while offering an improved safety and tolerability profile. These findings should be interpreted with consideration of potential heterogeneity across studies, yet still support ixazomib-based regimens as an effective and more tolerable alternative to bortezomib-based backbones for this population, particularly among frail patients in real-world practice.

**Copyright Information:** (Online) ISSN: 2572-9241
©2026 The Author(s). HemaSphere is published by John Wiley & Sons Ltd on behalf of European Hematology Association. This is an open access Abstract Book distributed under the Attribution-NonCommercial-NoDerivs (CC BY-NC-ND), which allows third parties to download the articles and share them with others as long as they credit the author and the Abstract Book, but they cannot change the content in any way or use them commercially.
**Abstract Book Citations:** Authors, Title, HemaSphere, 2026;10:(S1):pages. The abstract book DOIs can be found on the “Author Information” page.
**Disclaimer:** Articles published in the journal HemaSphere exclusively reflect the opinions of the authors. The authors are responsible for all content in their abstracts including accuracy of the facts, statements, citing resources, etc.

### (PB3371) CLINICAL AND EPIDEMIOLOGICAL CHARACTERIZATION OF PATIENTS WITH MULTIPLE MYELOMA IN EL SALVADOR: A REAL-WORLD RETROSPECTIVE STUDY

**Topic:** 14. Myeloma and other monoclonal gammopathies - Clinical

Karla Fernanda Rodriguez Estrada*[1], Luis Rivas[1], Maria de Lourdes Miranda Rivas[1]

[1]*Salvadoran Social Security Institute, San Salvador, El Salvador;*

### Background

Multiple myeloma (MM) is one of the most common hematologic malignancies worldwide; however, epidemiological and clinical data from Central America are scarce. The lack of local data limits the understanding of disease presentation, diagnostic delays, and risk stratification in this region.

### Aims

To describe the clinical and epidemiological characteristics of patients newly diagnosed with multiple myeloma in a national referral oncology center in El Salvador.

### Methods

A retrospective, observational, descriptive, cross-sectional study was conducted at the Oncology Hospital of the Salvadoran Social Security Institute. Patients with newly diagnosed MM between January 2022 and December 2024 were included. Sociodemographic variables, clinical presentation, time to diagnosis, and disease risk according to the International Staging System (ISS) were analyzed.

### Results

Seventy-nine patients were included. The mean age was 59 years (range 36–86), with male predominance (56%) and mainly urban residence (82%). Time to diagnosis was between 3–6 months in 39% of patients, while 34% experienced delays longer than 6 months. Bone pain was the most frequent presenting symptom (65%), followed by anemia (24%). Bone involvement at diagnosis was observed in 91% of cases. According to ISS, nearly half of the patients presented with high-risk disease (ISS stage III, 49%) (Table 1).

### Summary/Conclusion

This study represents the first real-world clinical and epidemiological characterization of multiple myeloma in El Salvador. Patients frequently present with diagnostic delays, extensive bone disease, and advanced-stage MM, patterns comparable to other Latin American reports. These findings provide a foundation for regional comparisons, support the need for earlier diagnostic strategies, and may serve as a starting point for future multicenter and prospective studies in Central America.

**Copyright Information:** (Online) ISSN: 2572-9241
©2026 The Author(s). HemaSphere is published by John Wiley & Sons Ltd on behalf of European Hematology Association. This is an open access Abstract Book distributed under the Attribution-NonCommercial-NoDerivs (CC BY-NC-ND), which allows third parties to download the articles and share them with others as long as they credit the author and the Abstract Book, but they cannot change the content in any way or use them commercially.
**Abstract Book Citations:** Authors, Title, HemaSphere, 2026;10:(S1):pages. The abstract book DOIs can be found on the "Author Information" page.
**Disclaimer:** Articles published in the journal HemaSphere exclusively reflect the opinions of the authors. The authors are responsible for all content in their abstracts including accuracy of the facts, statements, citing resources, etc.

| Table 1. Baseline clinical characteristics of newly diagnosed multiple myeloma patients (n = 79) | |
|---|---|
| **Characteristic** | **Value** |
| Age, years, mean (range) | 59 (36–86) |
| Male sex, n (%) | 44 (56) |
| Urban residence, n (%) | 65 (82) |
| Time to diagnosis, n (%) | |
| < 3 months | 21 (27) |
| 3–6 months | 31 (39) |
| > 6 months | 27 (34) |
| **Main presenting symptom, n (%)** | |
| Bone pain | 51 (65) |
| Anemia | 19 (24) |
| **Bone involvement at diagnosis, n (%)** | 72 (91) |
| **ISS stage, n (%)** | |
| Stage I (low risk) | 23 (29) |
| Stage II (intermediate risk) | 17 (22) |
| Stage III (high risk) | 39 (49) |

**Copyright Information:** (Online) ISSN: 2572-9241
©2026 The Author(s). HemaSphere is published by John Wiley & Sons Ltd on behalf of European Hematology Association. This is an open access Abstract Book distributed under the Attribution-NonCommercial-NoDerivs (CC BY-NC-ND), which allows third parties to download the articles and share them with others as long as they credit the author and the Abstract Book, but they cannot change the content in any way or use them commercially.
**Abstract Book Citations:** Authors, Title, HemaSphere, 2026;10:(S1):pages. The abstract book DOIs can be found on the "Author Information" page.
**Disclaimer:** Articles published in the journal HemaSphere exclusively reflect the opinions of the authors. The authors are responsible for all content in their abstracts including accuracy of the facts, statements, citing resources, etc.

### (PB3372) THERAPEUTIC TRANSITION IN NEWLY DIAGNOSED MULTIPLE MYELOMA: REAL-WORLD ANALYSIS OF INDUCTION OPTIMIZATION IN MEXICAN REFERRAL CENTERS.

**Topic:** 14. Myeloma and other monoclonal gammopathies - Clinical

Karla Sánchez Hernández*[1], Mauricio Servin Casio[2], Gilberto Israel Barranco Lampon[3], Christian Ramos[3], Carlos Martinez Murillo[3], Alonso Hernández Company[3], Emmanuel Martinez Moreno[3], Gerardo Santiago Jiménez[3], Edgar Cordero[3], Amaya Llorente-Chávez[1], Luis Felipe Rubalcava Lara[1], Irma Karen Pellon Tellez[1], Omar Eduardo Fernandez Vargas[1], Efreen Horacio Montaño-Figueroa[3], Humberto Baldemar Castellanos Sinco[3], Brenda Lizeth Acosta Maldonado[1], Etsel Camargo Romero[3], Juan Francisco Zazueta Pozos[3]

*[1]Incan Nueva Torre de Hospitalización, HEMATOLOGY, Ciudad de México, Mexico;[2]IMSS Hospital General Regional 1 Carlos MacGregor, Hematology, Ciudad de México, Mexico; [3]Hospital General de México Doctor Eduardo Liceaga, Hematology, Ciudad de México, Mexico;*

### Background

The therapeutic landscape of newly diagnosed multiple myeloma (NDMM) has evolved substantially over the past decade, driven by the incorporation of immunomodulatory drugs (IMiDs) and, more recently, anti-CD38 monoclonal antibodies into frontline regimens. While randomized clinical trials have demonstrated superior response depth and progression-free survival with lenalidomide-based and daratumumab-containing combinations compared to first-generation thalidomide-based regimens, real-world data from middle-income countries remain limited. Mexico represents a heterogeneous healthcare environment in which therapeutic availability varies across public academic referral centers.

### Aims

This study aims to compare survival outcomes and depth of response among patients with NDMM treated with thalidomide-based, lenalidomide-based, or daratumumab-containing frontline regimens across three major academic referral centers in Mexico.

### Methods

We conducted a multicenter, retrospective, observational cohort study involving patients with NDMM treated at three major academic referral centers in Mexico. Eligible patients were those aged ≥18 years with a confirmed diagnosis of symptomatic MM according to IMWG criteria, who initiated first-line induction therapy between 2020 to 2025. To ensure a homogeneous comparison of immunomodulatory backbones, we applied strict exclusion criteria. Patients with incomplete clinical records, those who received palliative care only, or those treated with non-IMiD/non-Anti-CD38 regimens were excluded from the primary efficacy analysis.

**Copyright Information:** (Online) ISSN: 2572-9241
©2026 The Author(s). HemaSphere is published by John Wiley & Sons Ltd on behalf of European Hematology Association. This is an open access Abstract Book distributed under the Attribution-NonCommercial-NoDerivs (CC BY-NC-ND), which allows third parties to download the articles and share them with others as long as they credit the author and the Abstract Book, but they cannot change the content in any way or use them commercially.
**Abstract Book Citations:** Authors, Title, HemaSphere, 2026;10:(S1):pages. The abstract book DOIs can be found on the “Author Information” page.
**Disclaimer:** Articles published in the journal HemaSphere exclusively reflect the opinions of the authors. The authors are responsible for all content in their abstracts including accuracy of the facts, statements, citing resources, etc.

**Results**

A total of 171 patients were screened. Of these, 19 were excluded (10 due to missing data, 9 due to non-standard regimens). The final evaluable population consisted of 152 patients: Thalidomide-based (n=86, 56.6%), lenalidomide-based (n=34, 22.4%), and anti-CD38-based (n=32, 21.0%). The median age at diagnosis was 58 years (range: 28–92), reflecting a younger demographic compared to other cohorts. Baseline characteristics were balanced across groups (Table 1). While Overall Response Rates (ORR) were comparable, the quality of response improved significantly with novel agents. The rate of deep response (≥VGPR) was 34.9% in the thalidomide cohort versus 58.8% and 53.1% in the lenalidomide and anti-CD38 cohorts, respectively. Survival analysis revealed a profound disparity in early attrition. The thalidomide cohort exhibited an early mortality rate of 29.1% (25 deaths), predominantly driven by infection and progression. In contrast, mortality was dramatically reduced in the lenalidomide (5.9%, 2 deaths) and anti-CD38 (3.1%, 1 death) cohorts. Kaplan-Meier analysis confirmed a significant survival advantage for the optimized cohorts (Log-Rank p=0.011, Figure 1).

**Summary/Conclusion**

In this multicenter real-world cohort of Mexican patients with NDMM, optimization of the induction backbone was associated with clinically meaningful improvements in depth of response and early survival. Although overall response rates were comparable, lenalidomide- and anti-CD38–based regimens significantly increased ≥VGPR rates and markedly reduced early mortality compared with thalidomide-based therapy. The high early attrition observed in the thalidomide cohort underscores the vulnerability of patients treated with first-generation regimens in resource-variable settings.

**Copyright Information:** (Online) ISSN: 2572-9241
©2026 The Author(s). HemaSphere is published by John Wiley & Sons Ltd on behalf of European Hematology Association. This is an open access Abstract Book distributed under the Attribution-NonCommercial-NoDerivs (CC BY-NC-ND), which allows third parties to download the articles and share them with others as long as they credit the author and the Abstract Book, but they cannot change the content in any way or use them commercially.
**Abstract Book Citations:** Authors, Title, HemaSphere, 2026;10:(S1):pages. The abstract book DOIs can be found on the "Author Information" page.
**Disclaimer:** Articles published in the journal HemaSphere exclusively reflect the opinions of the authors. The authors are responsible for all content in their abstracts including accuracy of the facts, statements, citing resources, etc.

### (PB3373) SPONTANEOUS RUPTURE OF SPLEEN IN SYSTEMIC LIGHT-CHAIN AMYLOIDOSIS

**Topic:** 14. Myeloma and other monoclonal gammopathies - Clinical

Yi Xu*[1, 2], Ai Guan[1, 2], Chengyang Xu[1, 2], Kaini Shen[1, 2], Jian Li[1, 2]

*[1]Peking Union Medical College Hospital, Hematology, Beijing, China;[2]Chinese Academy of Medical Sciences and Peking Union Medical College, Beijing, China;*

### Background

Spontaneous splenic rupture is a rare but severe complication in systemic immunoglobulin light-chain (AL) amyloidosis. However, comprehensive characterization of its clinical features, risk factors, and outcomes remains limited.

### Aims

To characterize the clinical presentation, organ involvement, coagulation abnormalities, splenic pathology, and treatment outcomes of patients with AL amyloidosis who developed spontaneous splenic rupture.

### Methods

We retrospectively reviewed all patients diagnosed with biopsy-proven AL amyloidosis at our center between 2015 and 2025. Patients who developed spontaneous splenic rupture were included. Clinical data were systematically collected and summarized, including baseline characteristics, organ involvement, splenic imaging and pathology findings, management strategies for rupture, and clinical outcomes.

### Results

A total of eight patients with biopsy-confirmed AL amyloidosis who experienced spontaneous splenic rupture were included. None had a history of liver cirrhosis, severe infection, or hypersplenism. The median age at diagnosis was 57 years (range, 39-66), and 5/8 (62.5%) were male. Lambda light-chain disease predominated (7/8, 87.5%). Most patients presented with advanced disease: four were stage IIIa and two were stage IIIb according to the modified Mayo 2004 staging system. All patients had cardiac involvement (100%), followed by renal involvement (87.5%), and hepatic involvement was present in up to 75% of patients. Three patients had Factor X (FX) activity <50%, including two with severe deficiency (<10%). The median bone marrow plasma cell percentage was 3.5% (range, 2-14%), and the median difference between involved and uninvolved free light chains was 275.3 mg/L.
Among patients with available pre-rupture imaging, all demonstrated splenomegaly. Splenic rupture occurred within approximately six months before or after the diagnosis of AL amyloidosis, with 7/8 events occurring prior to initiation of anti–plasma cell therapy. Management strategies varied: six patients underwent emergency splenectomy, one received transcatheter arterial embolization, and one was managed conservatively. The two patients who did not undergo splenectomy had markedly reduced FX activity with significant coagulopathy, and both experienced recurrent splenic rupture or bleeding during the disease course. Among the six patients with evaluable splenic pathology, four showed amyloid deposition with restricted light-chain expression. In one patient who did not undergo splenectomy, $^{18}$F-florbetapir PET-CT demonstrated amyloid accumulation within the spleen.
All patients subsequently received anti-plasma cell therapy, including daratumumab-based regimens (n=4), bortezomib-based regimens (n=3), and melphalan–thalidomide–dexamethasone (n=1). Six patients achieved a hematologic response of ≥very good partial response. During follow-up, four patients died; none of the deaths were directly attributable to splenic rupture.

**Copyright Information:** (Online) ISSN: 2572-9241
©2026 The Author(s). HemaSphere is published by John Wiley & Sons Ltd on behalf of European Hematology Association. This is an open access Abstract Book distributed under the Attribution-NonCommercial-NoDerivs (CC BY-NC-ND), which allows third parties to download the articles and share them with others as long as they credit the author and the Abstract Book, but they cannot change the content in any way or use them commercially.
**Abstract Book Citations:** Authors, Title, HemaSphere, 2026;10:(S1):pages. The abstract book DOIs can be found on the "Author Information" page.
**Disclaimer:** Articles published in the journal HemaSphere exclusively reflect the opinions of the authors. The authors are responsible for all content in their abstracts including accuracy of the facts, statements, citing resources, etc.

### Summary/Conclusion

Spontaneous splenic rupture is a rare but clinically significant manifestation of AL amyloidosis. In this cohort, most events occurred early in the disease course, often before initiation of therapy. Hepatic involvement was frequently observed, and patients often presented with advanced-stage disease. Most cases of splenic rupture were associated with splenic amyloid deposition, while severe coagulation factor deficiency posed significant therapeutic challenges.

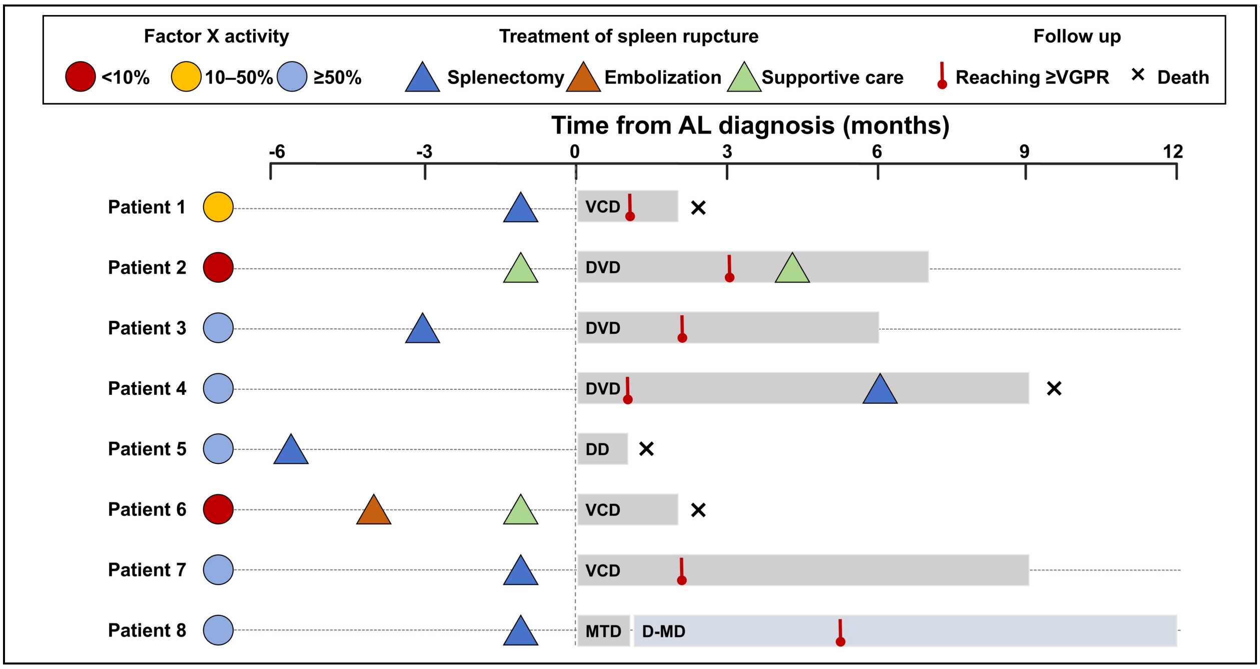

Figure 1. Patients swimmer plot depicting the timepoints of spontaneous splenic rupture, with corresponding Factor X activity at rupture.

Copyright Information: (Online) ISSN: 2572-9241
©2026 The Author(s). HemaSphere is published by John Wiley & Sons Ltd on behalf of European Hematology Association. This is an open access Abstract Book distributed under the Attribution-NonCommercial-NoDerivs (CC BY-NC-ND), which allows third parties to download the articles and share them with others as long as they credit the author and the Abstract Book, but they cannot change the content in any way or use them commercially.
Abstract Book Citations: Authors, Title, HemaSphere, 2026;10:(S1):pages. The abstract book DOIs can be found on the "Author Information" page.
Disclaimer: Articles published in the journal HemaSphere exclusively reflect the opinions of the authors. The authors are responsible for all content in their abstracts including accuracy of the facts, statements, citing resources, etc.

### (PB3374) PROGNOSTIC VALUE OF RED CELL DISTRIBUTION WIDTH IN MULTIPLE MYLOMA: A SINGLE-CENTER EXPERIENCE

**Topic:** 14. Myeloma and other monoclonal gammopathies - Clinical

Marta Diaz-Lopez*[1], Sara Delgado-Del Rosario[2], Tessa Casañas-Denboer[2], Lorena Rivero-Peña[2], Ana Jing Garcia-Perez-Febles[2], Alejandro Martin Martin[1], Sunil Lakhwani[1, 2]

[1]*Hospital Universitario de Canarias, Hematology and Hemotherapy, San Cristóbal de La Laguna, Spain;*[2]*University of La Laguna, Faculty of Medicine, San Cristobal de Laguna, Spain;*

### Background

The prognosis of Multiple Myeloma (MM) is heterogeneous, and widely used prognostic scores such as ISS and R-ISS do not always accurately identify high-risk patients. Red cell distribution width (RDW) at diagnosis has shown prognostic value in several hematologic malignancies. However, available data in MM are inconsistent and different RDW cut-offs have been proposed.

### Aims

To evaluate whether RDW at diagnosis is associated with progression-free survival (PFS), overall survival (OS), and response to first-line treatment.

### Methods

We conducted a retrospective study including newly diagnosed MM patients treated at the Department of Hematology and Hemotherapy of the University Hospital of the Canary Islands (Spain) between 2011 and 2015. Receiver operating characteristic (ROC) curve analysis was performed to determine the optimal RDW cut-off for predicting shorter PFS.

**Copyright Information:** (Online) ISSN: 2572-9241
©2026 The Author(s). HemaSphere is published by John Wiley & Sons Ltd on behalf of European Hematology Association. This is an open access Abstract Book distributed under the Attribution-NonCommercial-NoDerivs (CC BY-NC-ND), which allows third parties to download the articles and share them with others as long as they credit the author and the Abstract Book, but they cannot change the content in any way or use them commercially.
**Abstract Book Citations:** Authors, Title, HemaSphere, 2026;10:(S1):pages. The abstract book DOIs can be found on the "Author Information" page.
**Disclaimer:** Articles published in the journal HemaSphere exclusively reflect the opinions of the authors. The authors are responsible for all content in their abstracts including accuracy of the facts, statements, citing resources, etc.

### Results

A total of 83 patients were included; 50 (60.2%) were male and the mean age was 68.7 years. Initially, we evaluated whether a normal RDW (<15%) versus elevated RDW (≥15%) at diagnosis was associated with differences in PFS, but no statistically significant differences were observed (p=0.148).
ROC analysis identified 14% as the optimal cut-off for predicting shorter PFS (Figure 1A). Patients with RDW <14% had significantly longer PFS compared to those with RDW ≥14% (p=0.005; Figure 1B).
In multivariable analysis including variables significant in univariable analysis (age, β2-microglobulin, creatinine, ISS, and RDW), RDW remained an independent prognostic factor for PFS (p=0.006; HR 0.56; 95% CI 0.31–0.99; Table 1).
Regarding response to first-line treatment, RDW <14% was associated with higher complete response rates (50% vs. 22.8%; p=0.013) and higher rates of at least very good partial response (80.8% vs. 49.1%; p=0.007). No statistically significant differences in OS were observed (p=0.104).

Table 1 – Comparative of the univariant and multivariant analysis.

| VARIABLES | UNIVARIANT ANALYSIS | | | MULTIVARIANT ANALYSIS | | |
|---|---|---|---|---|---|---|
| | *pvalue* | *HR* | CI 95% | *pvalue* | *HR* | CI 95% |
| **Age** | **0,006** | **1,03** | **1,01 – 1,05** | **0,021** | **1,03** | **1,01 – 1.05** |
| Inmunoparesis | 0,170 | 0.37 | 0,09 – 1,53 | -- | -- | -- |
| Albúmina | 0,164 | 0,75 | 0,51 – 1,12 | -- | -- | -- |
| LDH | 0,268 | 1,42 | 0,76 – 2,64 | -- | -- | -- |
| **ß2-microglobuline** | **< 0,001** | **1,12** | **1,06 – 1,17** | 0,426 | 1,06 | 0,92 – 1,22 |
| **ISS** | **0,009** | **1,46** | **1,10 – 1,45** | 0,506 | 0,88 | 0,60 – 1,28 |
| %PC en BM | 0.240 | 1,01 | 0,99 – 1.35 | -- | -- | -- |
| **Creatinine** | **< 0,001** | **1,47** | **1,24 – 1,74** | **< 0,001** | **1,52** | **1,26 – 1,83** |
| **RDW 14%** | **0,006** | **0,48** | **0,29 – 0,81** | **0,048** | **0,56** | **0.31 – 0,99** |

*HR: Hazard ratio; CI: confidence interval; ISS: International Staging System; PC: plasmatic cells; BM: bone marrow. RDW: red cell distribution width.*

### Summary/Conclusion

RDW <14% at diagnosis was associated with longer PFS and better first-line response, but not OS. Further validation is needed.

**Copyright Information:** (Online) ISSN: 2572-9241
©2026 The Author(s). HemaSphere is published by John Wiley & Sons Ltd on behalf of European Hematology Association. This is an open access Abstract Book distributed under the Attribution-NonCommercial-NoDerivs (CC BY-NC-ND), which allows third parties to download the articles and share them with others as long as they credit the author and the Abstract Book, but they cannot change the content in any way or use them commercially.
**Abstract Book Citations:** Authors, Title, HemaSphere, 2026;10:(S1):pages. The abstract book DOIs can be found on the "Author Information" page.
**Disclaimer:** Articles published in the journal HemaSphere exclusively reflect the opinions of the authors. The authors are responsible for all content in their abstracts including accuracy of the facts, statements, citing resources, etc.

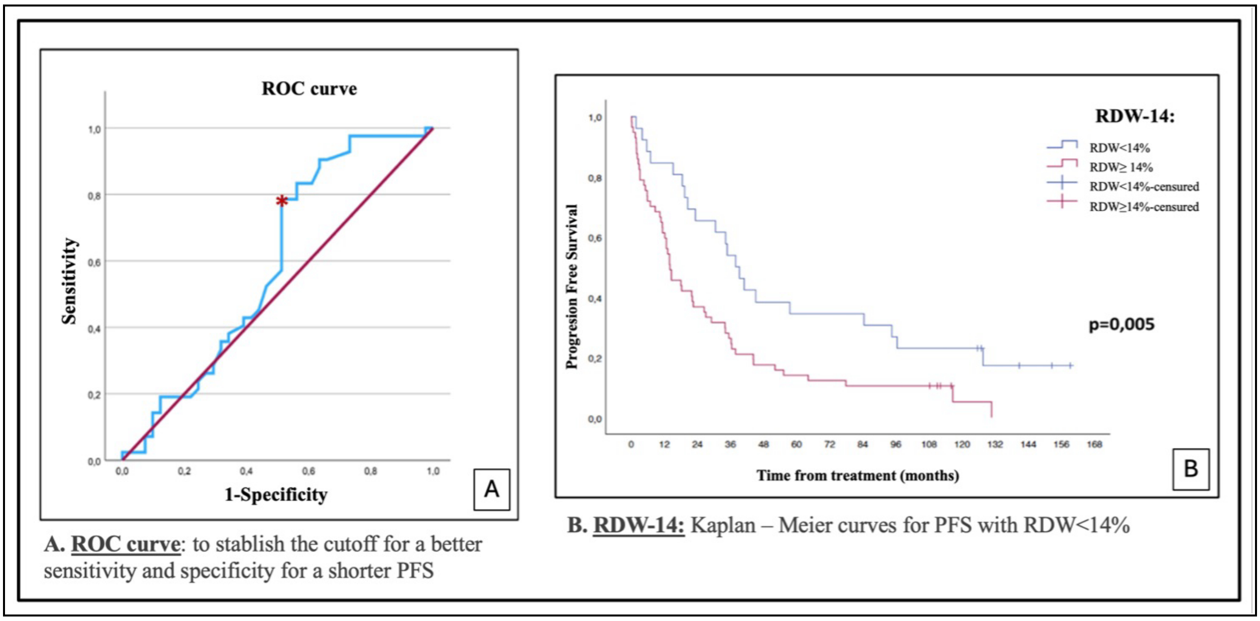

Figure 1: A) ROC Curve. B) RDW 14.

**Copyright Information:** (Online) ISSN: 2572-9241
©2026 The Author(s). HemaSphere is published by John Wiley & Sons Ltd on behalf of European Hematology Association. This is an open access Abstract Book distributed under the Attribution-NonCommercial-NoDerivs (CC BY-NC-ND), which allows third parties to download the articles and share them with others as long as they credit the author and the Abstract Book, but they cannot change the content in any way or use them commercially.
**Abstract Book Citations:** Authors, Title, HemaSphere, 2026;10:(S1):pages. The abstract book DOIs can be found on the "Author Information" page.
**Disclaimer:** Articles published in the journal HemaSphere exclusively reflect the opinions of the authors. The authors are responsible for all content in their abstracts including accuracy of the facts, statements, citing resources, etc.

### (PB3375) SERUM IL-6 AND VEGF AS BIOMARKERS OF TREATMENT RESPONSE IN NEWLY DIAGNOSED MULTIPLE MYELOMA: A PROSPECTIVE OBSERVATIONAL STUDY FROM WESTERN INDIA

**Topic:** 14. Myeloma and other monoclonal gammopathies - Clinical

Naman Lodha*[1], Gopal Bohra[1], Abhishek Purohit[1], Naresh Midha[1], Deepak Sharma[1]

[1]*All India Institute of Medical Sciences College, Internal Medicine, Jodhpur, India;*

### Background

Multiple myeloma (MM) progression is driven by complex interactions between malignant plasma cells and the bone marrow microenvironment. Cytokines such as interleukin-6 (IL-6) and vascular endothelial growth factor (VEGF) play key roles in plasma cell survival, angiogenesis, osteolysis, and drug resistance. While elevated cytokine levels have been associated with aggressive disease biology, prospective data evaluating their role in prognostication and treatment response in the Indian population remain limited.

### Aims

To evaluate the role of serum IL-6 and VEGF levels in (1) disease prognostication at diagnosis and (2) treatment response following induction therapy in newly diagnosed multiple myeloma patients.

### Methods

This prospective observational study enrolled 42 newly diagnosed MM patients between September 2022 and January 2024 at a tertiary care center in Western India. Baseline evaluation included complete blood counts, biochemical parameters, serum protein electrophoresis, immunofixation, serum free light chain assay, β2-microglobulin, bone marrow examination, and International Staging System (ISS) classification. Serum IL-6 and VEGF levels were measured at diagnosis. Treatment response was assessed after three cycles of induction therapy using International Myeloma Working Group (IMWG) criteria. Patients were categorized as responders (≥ partial response) or non-responders (stable or progressive disease). Statistical analysis was performed using SPSS v20. Non-parametric tests were applied due to skewed cytokine distribution. A p-value ≤ 0.05 was considered statistically significant.

### Results

The median age was 63.5 years (range 40–79), with a male-to-female ratio of 1.47:1. Median baseline IL-6 and VEGF levels were 15.8 pg/mL and 92.9 pg/mL, respectively. No significant association was observed between baseline cytokine levels and ISS stage. IL-6 demonstrated weak positive correlations with renal parameters, free light chain ratio, and β2-microglobulin. Among 23 patients who completed three cycles of induction therapy, all achieved at least partial response. A significant reduction in both IL-6 and VEGF levels was observed post-treatment ($p < 0.001$). Baseline cytokine levels did not significantly differ between patients completing therapy and those who did not.

### Summary/Conclusion

In this prospective cohort, baseline IL-6 and VEGF levels were not associated with ISS staging, suggesting limited prognostic utility at diagnosis. However, significant post-treatment reductions in cytokine levels among responders indicate that IL-6 and VEGF may serve as dynamic biomarkers of treatment response in multiple myeloma. Larger longitudinal studies incorporating survival endpoints are warranted to validate these findings.

**Copyright Information:** (Online) ISSN: 2572-9241
©2026 The Author(s). HemaSphere is published by John Wiley & Sons Ltd on behalf of European Hematology Association. This is an open access Abstract Book distributed under the Attribution-NonCommercial-NoDerivs (CC BY-NC-ND), which allows third parties to download the articles and share them with others as long as they credit the author and the Abstract Book, but they cannot change the content in any way or use them commercially.
**Abstract Book Citations:** Authors, Title, HemaSphere, 2026;10:(S1):pages. The abstract book DOIs can be found on the “Author Information” page.
**Disclaimer:** Articles published in the journal HemaSphere exclusively reflect the opinions of the authors. The authors are responsible for all content in their abstracts including accuracy of the facts, statements, citing resources, etc.

### (PB3376) SERUM Β2-MICROGLOBULIN AS AN INDEPENDENT PROGNOSTIC FACTOR IN JAPANESE PATIENTS AGED ≥80 YEARS WITH MULTIPLE MYELOMA

**Topic:** 14. Myeloma and other monoclonal gammopathies - Clinical

Tsutomu Kobayashi*[1], Harunori Suzuki[1], Koichi Nonaka[1], Ayako Muramatsu[1], Mio Sugitani[1], Yosuke Matsumoto[1], Toshiki Iwai[1], Hitoji Uchiyama[1]

[1]*Japanese Red Cross Kyoto Daiichi Hospital, Hematology, Kyoto, Japan;*

### Background

Multiple myeloma (MM) predominantly affects the elderly; however, limited data are available regarding outcomes in patients aged ≥80 years. Although triplet regimens such as daratumumab, lenalidomide, and dexamethasone are considered promising induction therapies for newly diagnosed MM, very elderly or frail patients may not tolerate such treatments due to comorbidities and treatment-related toxicities. Furthermore, it remains unclear whether prognostic factors in very elderly patients are similar to those observed in younger populations.

### Aims

This study aimed to clarify the current treatment patterns and prognostic factors in very elderly patients with MM. Although preliminary results from this cohort were previously reported, the present study provides a dedicated analysis of survival outcomes and prognostic factors in very elderly patients with MM.

### Methods

We retrospectively analyzed 45 consecutive patients aged ≥80 years with newly diagnosed MM treated at our institution between 2009 and 2023. Overall survival (OS) was estimated using the Kaplan–Meier method. Categorical variables were compared using Fisher's exact test, and continuous variables were analyzed using the Mann–Whitney U-test. Factors evaluated for their impact on OS included age, comorbidities, initial treatment strategy, use of anti-CD38 antibodies, and pretreatment serum albumin, LDH, β2-microglobulin (β2MG), and eGFR. Multivariable analysis was performed using the Cox proportional hazards model.

### Results

The median age was 83 years (range, 80–92), and 25 (56%) were male. Doublet regimens were administered as first-line therapy in 51% of patients, while best supportive care (BSC) was selected in 18%. With a median follow-up of 19.6 months, the median OS was 58 months. Twelve patients were transferred to other hospitals for end-of-life care and subsequently lost to follow-up. In a sensitivity analysis considering these cases as death events, the median OS was 20 months. Among patients who received anti-MM treatment, no significant difference in OS was observed between doublet and triplet regimens; however, the use of anti-CD38 antibodies showed a trend toward improved OS ($p=0.09$). Patients who received BSC were significantly older than those who received active treatment (median age; 87 vs. 82 years, $p<0.01$), although age itself was not associated with OS. In both univariable and multivariable analyses, elevated serum β2MG, according to the ISS stratification, was independently associated with inferior OS (HR 4.10, 95% CI 1.71–9.84; $p<0.01$).

**Copyright Information:** (Online) ISSN: 2572-9241
©2026 The Author(s). HemaSphere is published by John Wiley & Sons Ltd on behalf of European Hematology Association. This is an open access Abstract Book distributed under the Attribution-NonCommercial-NoDerivs (CC BY-NC-ND), which allows third parties to download the articles and share them with others as long as they credit the author and the Abstract Book, but they cannot change the content in any way or use them commercially.
**Abstract Book Citations:** Authors, Title, HemaSphere, 2026;10:(S1):pages. The abstract book DOIs can be found on the "Author Information" page.
**Disclaimer:** Articles published in the journal HemaSphere exclusively reflect the opinions of the authors. The authors are responsible for all content in their abstracts including accuracy of the facts, statements, citing resources, etc.

**Summary/Conclusion**

In very elderly patients with newly diagnosed MM, serum β2MG appears to be a key prognostic factor. Treatment intensity (doublet vs. triplet regimens) was not clearly associated with survival in this cohort. Given the retrospective design and limited sample size, larger studies are warranted to confirm these findings.

**Copyright Information:** (Online) ISSN: 2572-9241
©2026 The Author(s). HemaSphere is published by John Wiley & Sons Ltd on behalf of European Hematology Association. This is an open access Abstract Book distributed under the Attribution-NonCommercial-NoDerivs (CC BY-NC-ND), which allows third parties to download the articles and share them with others as long as they credit the author and the Abstract Book, but they cannot change the content in any way or use them commercially.
**Abstract Book Citations:** Authors, Title, HemaSphere, 2026;10:(S1):pages. The abstract book DOIs can be found on the "Author Information" page.
**Disclaimer:** Articles published in the journal HemaSphere exclusively reflect the opinions of the authors. The authors are responsible for all content in their abstracts including accuracy of the facts, statements, citing resources, etc.

### (PB3377) REAL-WORLD OUTCOMES OF SELINEXOR-BASED THERAPY IN RELAPSED/REFRACTORY MULTIPLE MYELOMA: A MULTICENTER STUDY FROM TÜRKIYE

**Topic:** 14. Myeloma and other monoclonal gammopathies - Clinical

Zehra Narlı Özdemir*[1], Berke Han Orhan[1], Sibel Kabukçu Hacıoğlu[2], Zehra Akşit Bozkına[3], Ayşe Hilal Eroğlu Küçükdiler[4], Gülsüm Akgün Çağlıyan[2], Serkan Guven[5], Zekeriya Aksöz[6], Ayşe Uysal[6], Ufuk Demirci[7], Osman Can Öztürk[8], Fatos Dilan Koseoglu[9], Ünal Ataş[10], Celal Acar[11], Oktay Bilgir[1], İnci Alacacıoğlu[8], Irfan Yavasoglu[4], Seçkin Çağırgan[11], Güray Saydam[3], Güner Hayri Özsan[12]

*[1]İzmir City Hospital, Hematology, İzmir, Türkiye;[2]Pamukkale University Faculty of Medicine, Hematology, Denizli, Türkiye;[3]Ege University Faculty of Medicine, Department of Hematology, İzmir, Türkiye; [4]Adnan Menderes University Faculty of Medicine, Department of Hematology, Aydın, Türkiye; [5]Çanakkale Mehmet Akif Ersoy State Hospital, Department of Hematology, Çanakkale, Türkiye; [6]Fırat University Faculty of Medicine, Department of Hematology, Elazığ, Türkiye;[7]Manisa Celal Bayar University Faculty of Medicine, Department of Hematology, Manisa, Türkiye; [8]Dokuz Eylül University Faculty of Medicine, Department of Hematology, İzmir, Türkiye; [9]Bakırçay University Çiğli Training and Research Hospital, Department of Hematology, İzmir, Türkiye; [10]Akdeniz University Faculty of Medicine, Department of Hematology, Antalya, Türkiye;[11]İzmir Medical Point Hospital, Department of Hematology, İzmir, Türkiye; [12]İzmir Tınaztepe University Faculty of Medicine, Department of Hematology, İzmir, Türkiye;*

### Aims

This multicenter, real-world (RW) study was conducted in Turkiye to report on the effectiveness, safety, and patterns of use of selinexor in patients (pts) with relapsed/refractory multiple myeloma (RRMM).

### Methods

This retrospective study collected patient records from hospital data after obtaining ethics committee approval. The research focused on patients aged 18 or older with a confirmed diagnosis of relapsed/refractory multiple myeloma (RRMM) who received selinexor. The main goal was to describe the patients' demographic and clinical features. Secondary goals included characterizing adverse events (AEs) and assessing survival and response outcomes.

**Copyright Information:** (Online) ISSN: 2572-9241
©2026 The Author(s). HemaSphere is published by John Wiley & Sons Ltd on behalf of European Hematology Association. This is an open access Abstract Book distributed under the Attribution-NonCommercial-NoDerivs (CC BY-NC-ND), which allows third parties to download the articles and share them with others as long as they credit the author and the Abstract Book, but they cannot change the content in any way or use them commercially.
**Abstract Book Citations:** Authors, Title, HemaSphere, 2026;10:(S1):pages. The abstract book DOIs can be found on the "Author Information" page.
**Disclaimer:** Articles published in the journal HemaSphere exclusively reflect the opinions of the authors. The authors are responsible for all content in their abstracts including accuracy of the facts, statements, citing resources, etc.

**Results**

Data on the real-world use of selinexor in Turkiye was collected from **12** sites, covering **68** patients. Treatment initiation occurred between January 9, 2023, and February 16, 2026. Patient demographics and treatment outcomes are summarized in the table. Patients had advanced disease, with a median time of **66** months from Multiple Myeloma (MM) diagnosis to selinexor initiation (based on 54 patients). They had received a median of **4** prior lines of therapy, distributed as follows:

- 1-3 prior lines: 28 patients
- 4-5 prior lines: 22 patients
- 6-9 prior lines: 18 patients

All patients completed at least one cycle of selinexor:

- 1 cycle: 17 patients
- 2 cycles: 16 patients
- 3 cycles: 14 patients
- 4-6 cycles: 10 patients
- 7-10 cycles: 8 patients

The real-world use of Selinexor in Turkiye involved a variety of combination therapies and dosing schedules, primarily in 67 patients (pts) across different regimens:

| **Combination Regimen** | **Selinexor Dose and Frequency** | **Number of Patients (n)** |
|---|---|---|
| **SVd** (Selinexor, Bortezomib, Dexamethasone) | 100 mg once weekly (w)<br>80 mg once weekly (w)<br>60 mg once weekly (w)<br>40 mg once weekly (w)<br>Dose unknown | 26<br>5<br>5<br>5<br>1 |
| **Sd** (Selinexor, Dexamethasone) | 80 mg twice weekly (bw)<br>60 mg twice weekly (bw)<br>60 mg once weekly (w)<br>40 mg once weekly (w) | 10<br>2<br>2<br>1 |
| **SPd** (Selinexor, Pomalidomide, Dexamethasone) | 100 mg once weekly (w)<br>60 mg once weekly (w)<br>40 mg once weekly (w) | 1<br>1<br>3 |
| **SV** (Selinexor, Bortezomib) | 100 mg once weekly (w) | 2 |
| **SKd** (Selinexor, Carfilzomib, Dexamethasone) | 80 mg once weekly (w)<br>40 mg once weekly (w) | 1<br>1 |
| **SDd** (Selinexor, Daratumumab, Dexamethasone) | Dose unknown | 1 |

Patient characteristics and treatment outcomes are presented in Table 1. Dose reduction of Selinexor was necessary for 35 patients, while 30 patients did not require a dose reduction. The most common Grade 3 or 4 adverse events were hematological: thrombocytopenia (38%), leukopenia (19%), and anemia (18%). Non-hematological Grade 3/4 toxicities included nausea (16%), fatigue (13%), infections (10%), and diarrhea (4%). Treatment was discontinued due to disease progression in 15 patients, intolerance in 10 patients, and death (exitus) in 4 patients.

**Copyright Information:** (Online) ISSN: 2572-9241
©2026 The Author(s). HemaSphere is published by John Wiley & Sons Ltd on behalf of European Hematology Association. This is an open access Abstract Book distributed under the Attribution-NonCommercial-NoDerivs (CC BY-NC-ND), which allows third parties to download the articles and share them with others as long as they credit the author and the Abstract Book, but they cannot change the content in any way or use them commercially.
**Abstract Book Citations:** Authors, Title, HemaSphere, 2026;10:(S1):pages. The abstract book DOIs can be found on the "Author Information" page.
**Disclaimer:** Articles published in the journal HemaSphere exclusively reflect the opinions of the authors. The authors are responsible for all content in their abstracts including accuracy of the facts, statements, citing resources, etc.

### Summary/Conclusion

Our real-world experience with selinexor in Turkiye involved heavily pretreated patients who received various regimens over a short follow-up period. While patient characteristics, effectiveness, and safety outcomes generally align with other real-world studies, some data are missing due to the retrospective nature of collection. The observed response rates in this patient group are encouraging. However, there is a clear necessity to standardize treatment management practices to ensure optimal patient outcomes.

**Table 1: Patient characteristics and treatment outcomes**

| | |
|---|---|
| Age (median, min-max) | 65,5 (46-78) |
| Gender (female, male) | 36 F / 32 M |
| ECOG PS | 0 (27), 1 (31), 2 (9), 3 (1) |
| ISS | 1 (21), 2 (22), 3 (17) |
| R-ISS | 1 (12), 2 (15), 3 (15) |
| Prior therapies | n (%) |
| PI (bortezomib, carfilzomib, ixazomib) | 68 (100%), 36 (53%), 11 (16%) |
| IMID (lenalidomide, pomalidomide, thalidomide) | 63 (93%), 36 (53%), 9 (13%) |
| Anti-CD38 mAb (daratumumab) | 46 (68%) |
| Triple-class treated (PI, IMID, Anti-CD38 mAb) | 44 (65%) |
| Penta-treated (=2PIs, =2IMIDs,1 Anti-CD38 mAb) | 30 (44%) |
| ASCT (Autologous Stem Cell Transplant) | 51 (75%) |
| Best overall response, n (%) | N= 62 (17 pts had 1 cycle of selinexor) |
| sCR | 1 |
| CR | 5 |
| VGPR | 9 |
| PR | 11 |
| MR | 2 |
| SD | 13 |
| PD | 19 |

Table 1: Patient characteristics and treatment outcomes

Copyright Information: (Online) ISSN: 2572-9241
©2026 The Author(s). HemaSphere is published by John Wiley & Sons Ltd on behalf of European Hematology Association. This is an open access Abstract Book distributed under the Attribution-NonCommercial-NoDerivs (CC BY-NC-ND), which allows third parties to download the articles and share them with others as long as they credit the author and the Abstract Book, but they cannot change the content in any way or use them commercially.
Abstract Book Citations: Authors, Title, HemaSphere, 2026;10:(S1):pages. The abstract book DOIs can be found on the "Author Information" page.
Disclaimer: Articles published in the journal HemaSphere exclusively reflect the opinions of the authors. The authors are responsible for all content in their abstracts including accuracy of the facts, statements, citing resources, etc.

### (PB3378) HEMATOLOGY-ONCOLOGISTS' PERSPECTIVES ON AN ON-BODY INJECTOR FOR ADMINISTRATION OF LARGE-VOLUME SUBCUTANEOUS DRUGS

**Topic:** 14. Myeloma and other monoclonal gammopathies - Clinical

Carlyn Rose Tan*[1], Muhamed Baljević[2], Sikander Ailawadhi[3], Erin Comerford[4], Karnav Dalal[4], Aidee Ayala Camargo[4]

*[1]Memorial Sloan Kettering Cancer Center, New York, NY, United States of America;[2]Department of Hematology-Oncology, Vanderbilt University Medical Center, Nashville, TN, United States of America; [3]Mayo Clinic, Jacksonville, FL, United States of America; [4]Sanofi, Cambridge, MA, United States of America;*

### Background

Isatuximab (Isa), an anti-CD38 monoclonal antibody, has been investigated as a subcutaneous (SC) formulation administered through an innovative on-body injector (OBI) in relapsed/refractory multiple myeloma. The Isa SC OBI provides hands-free administration through a small retractable needle and was preferred by both patients and healthcare providers (HCPs) over manual SC administration in the IZALCO trial. Data generated on physician preferences regarding the Isa SC OBI were limited to 3 physicians, with no other data on their perceptions available.

### Aims

Here we assess physicians' perspectives on the value and usability of the OBI and preferences for the OBI vs previous experience with large-volume SC manual syringe injections.

### Methods

This was a qualitative semi-structured cross-sectional double-blinded survey of 50 US hematology-oncologists (heme-oncs), who provided informed consent for participation. Participants were shipped an unbranded device with a vial of surrogate solution that mimicked the fill volume and viscosity of Isa SC, guided to conduct a self-demonstration of the preparation and administration, and then interviewed remotely one-on-one. Physicians who had previous experience with the OBI were excluded.

### Results

Heme-oncs were from private practices (n=16), academic cancer centers (n=12), teaching hospitals affiliated with (n=8) or not affiliated with an academic center (n=7), and community hospitals (n=7). Median percentage time in direct patient care was 90% (range 50–100%). Most (92%; 46/50) reported a positive perception of the OBI, and most (90%; 45/50) thought the OBI was valuable for their practice. Unaided mentions of value were driven mainly by the simple, intuitive workflow (easy to learn, easy to prepare; n=29) and the hidden needle/patient comfort (n=28). Potential disadvantages mentioned included device reliability (n=5) and cost/reimbursement (n=3). Among 7 features, those ranked in the top 3 were (1) simple one-step vial-to-device preparation, (2) potential to reduce preparation time in the pharmacy compared to large-volume (≥10 mL) SC manual injections, (3) hands-free administration. All heme-oncs agreed they would feel confident with treatment by an HCP using the OBI in the clinic. Most (94%; 46/49) agreed they would feel confident with a pharmacist preparing/loading the OBI outside the sterile hood, and similarly most (92%; 45/49) agreed they would feel confident with a nurse preparing/loading the OBI by the patient's chairside. Vast majority (96%; 48/50) thought the OBI required less resources and effort (eg, pharmacist/nurse time) for preparation and administration than large-volume SC manual injections, and most (92%; 46/50) would choose the OBI over large-volume SC manual injections, assuming no differences in efficacy, safety, and/or cost and that both methods were suitable for the patient.

**Copyright Information:** (Online) ISSN: 2572-9241
©2026 The Author(s). HemaSphere is published by John Wiley & Sons Ltd on behalf of European Hematology Association. This is an open access Abstract Book distributed under the Attribution-NonCommercial-NoDerivs (CC BY-NC-ND), which allows third parties to download the articles and share them with others as long as they credit the author and the Abstract Book, but they cannot change the content in any way or use them commercially.
**Abstract Book Citations:** Authors, Title, HemaSphere, 2026;10:(S1):pages. The abstract book DOIs can be found on the "Author Information" page.
**Disclaimer:** Articles published in the journal HemaSphere exclusively reflect the opinions of the authors. The authors are responsible for all content in their abstracts including accuracy of the facts, statements, citing resources, etc.

**Summary/Conclusion**

Heme-oncs who completed hands-on demonstrations with the OBI device recognized significant clinical value, driven primarily by streamlined preparation workflows and hands-free administration capability. Most identified the OBI as requiring fewer clinical resources and less procedural effort compared to current practice, with a clear preference over large-volume SC manual injections. Most expressed confidence in clinic staff's ability to prepare the OBI both outside sterile hood environments or by patient chairside, demonstrating flexibility for integration into clinical workflows.

**Copyright Information:** (Online) ISSN: 2572-9241
©2026 The Author(s). HemaSphere is published by John Wiley & Sons Ltd on behalf of European Hematology Association. This is an open access Abstract Book distributed under the Attribution-NonCommercial-NoDerivs (CC BY-NC-ND), which allows third parties to download the articles and share them with others as long as they credit the author and the Abstract Book, but they cannot change the content in any way or use them commercially.
**Abstract Book Citations:** Authors, Title, HemaSphere, 2026;10:(S1):pages. The abstract book DOIs can be found on the "Author Information" page.
**Disclaimer:** Articles published in the journal HemaSphere exclusively reflect the opinions of the authors. The authors are responsible for all content in their abstracts including accuracy of the facts, statements, citing resources, etc.

### (PB3379) DARATUMUMAB ALONGSIDE CYCLOPHOSPHAMIDE AND DEXAMETHASONE FOR ELDERLY TRANSPLANT-INELIGIBLE MYELOMA

**Topic:** 14. Myeloma and other monoclonal gammopathies - Clinical

Jiao Lu*[1], Miao Sun[1], Jiawei Geng[1], Qiaoyan Han[1], Chunhua Liu[1], Haoyue Chen[1], Lu Chen[1], Xin Jiang[1], Tong Lin[1], Bing Lu[1], Xiaorui Li[2]

*[1]Jingjiang People's Hospital Affiliated to Yangzhou University, Taizhou, China;[2]Jingjiang Maternal and Child Health Care Hospital, Taizhou, China;*

### Background

For patients diagnosed with multiple myeloma who are not suitable for transplantation, the combination of daratumumab with standard therapy leads to improved outcomes.

### Aims

We aimed to evaluate the overall response to the DCd (daratumumab alongside cyclophosphamide and dexamethasone) regimen in patients with multiple myeloma who were not eligible for transplantation.

### Methods

In this study, we have reviewed the clinical data of 23 patients diagnosed with multiple myeloma who were treated with the daratumumab alongside cyclophosphamide and dexamethasone (DCd regimen) at our center. This group included seven patients with newly diagnosed MM, 12 patients with relapsed/refractory MM, and four patients who were in remission after prior therapy. The median age was 75 years, with most patients being elderly and transplant-ineligible individuals.

### Results

Over half of the patients (52%) had four treatment cycles or more. The total number of cycles ranged from 0.5 to 15 (median five cycles), with three patients receiving fewer than one cycle and one patient switching regimens after completing one cycle. Eighteen patients were evaluable for response. The overall response rate (ORR) was 83.3%, with a ≥VGPR rate of 61.1%. The most common chemotherapy-related adverse event was infection, primarily pulmonary. But no patients required permanent discontinuation or regimen change due to severe infection.

### Summary/Conclusion

This study demonstrates that the DCd regimen is generally well tolerated and yields high response rates, even in patients with relapsed or refractory multiple myeloma. It represents a particularly suitable treatment option for elderly patients who are not candidates for transplantation.

**Copyright Information:** (Online) ISSN: 2572-9241
©2026 The Author(s). HemaSphere is published by John Wiley & Sons Ltd on behalf of European Hematology Association. This is an open access Abstract Book distributed under the Attribution-NonCommercial-NoDerivs (CC BY-NC-ND), which allows third parties to download the articles and share them with others as long as they credit the author and the Abstract Book, but they cannot change the content in any way or use them commercially.
**Abstract Book Citations:** Authors, Title, HemaSphere, 2026;10:(S1):pages. The abstract book DOIs can be found on the “Author Information” page.
**Disclaimer:** Articles published in the journal HemaSphere exclusively reflect the opinions of the authors. The authors are responsible for all content in their abstracts including accuracy of the facts, statements, citing resources, etc.

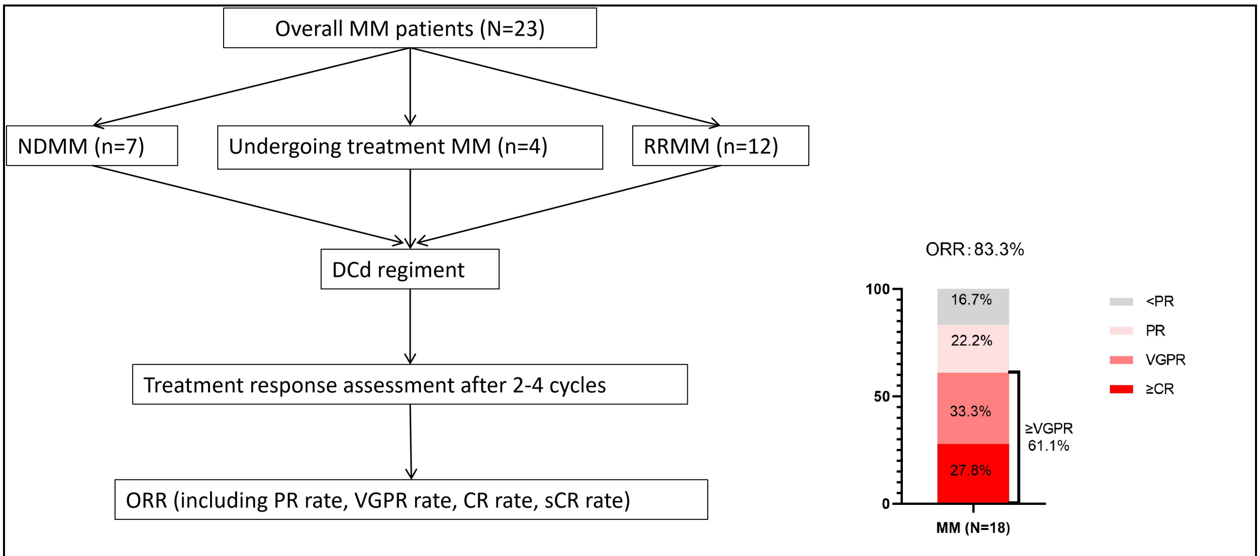

**A. Research flowchart.** Patient Distribution and Management Process

**B.** ORR of DCd therapy in MM patients

Figure A. Research flowchart. Patient Distribution and Management Process. A total of 23 MM patients were enrolled in the study, including 7 cases with newly diagnosed MM (NDMM), 4 with previously treated MM, and 12 with relapsed/refractory MM (RRMM). All patients had received the DCd therapy. The primary endpoint was overall response rate (ORR), encompassing partial response (PR), very good partial response (VGPR), complete response (CR), and strict complete response (sCR). Figure B. ORR of DCd therapy in MM patients. Among the 18 MM patients with evaluable treatment responses, more than half were relapsed/refractory multiple myeloma (RRMM). The overall response rate (ORR) to DCd therapy was 83.3%, with a probability of very good partial response (VGPR) or better at 61.1% and a complete response (CR) rate of 27.8%.

**Copyright Information:** (Online) ISSN: 2572-9241
©2026 The Author(s). HemaSphere is published by John Wiley & Sons Ltd on behalf of European Hematology Association. This is an open access Abstract Book distributed under the Attribution-NonCommercial-NoDerivs (CC BY-NC-ND), which allows third parties to download the articles and share them with others as long as they credit the author and the Abstract Book, but they cannot change the content in any way or use them commercially.
**Abstract Book Citations:** Authors, Title, HemaSphere, 2026;10:(S1):pages. The abstract book DOIs can be found on the “Author Information” page.
**Disclaimer:** Articles published in the journal HemaSphere exclusively reflect the opinions of the authors. The authors are responsible for all content in their abstracts including accuracy of the facts, statements, citing resources, etc.

### (PB3380) SURVIVAL IMPROVEMENT IN PRIMARY PLASMA CELL LEUKEMIA: A RETROSPECTIVE ANALYSIS OF NOVEL AGENT-BASED REGIMENS AND STEM CELL TRANSPLANTATION

**Topic:** 14. Myeloma and other monoclonal gammopathies - Clinical

Hua Bai*[1], Yong Xu[1], Bing Chen[1]

*[1]Nanjing Drum Tower Hospital, Affiliated Hospital of Medical School, Nanjing University, Department of Hematology, Nanjing, China;*

### Background

Primary plasma cell leukemia (pPCL) is a rare and aggressive malignancy. In 2021, the IMWG revised the diagnostic threshold for pPCL from ≥20% to ≥5% circulating plasma cells (CPCs). Despite novel agent-based therapies, outcomes remain poor with median overall survival (OS) <12 months. This study characterizes clinical profiles and treatment outcomes in pPCL patients diagnosed by updated criteria.

### Aims

To analyze clinical characteristics, prognostic factors, and efficacy of novel agent-based induction regimens in pPCL patients, with focus on achieving deep responses and long-term survival.

### Methods

We retrospectively analyzed 50 pPCL patients (per 2021 IMWG criteria) treated at Nanjing Drum Tower Hospital from March 2014 to January 2026. All received proteasome inhibitor (PI) and/or immunomodulatory drug (IMiD)-based induction. Clinical/laboratory features, cytogenetics (FISH), treatment responses (IMWG criteria), OS, and time to next therapy (TTNT) were evaluated. Multivariate Cox regression identified independent prognostic factors.

### Results

Median age: 63 years (range 43-81). Advanced-stage disease predominated. Common cytogenetic abnormalities: 1q21+, del(13q14), t(11;14). Median follow-up: 23 months. Median OS: 15 months; median TTNT: 9 months. Achieving ≥VGPR after first-line therapy independently predicted superior OS (HR=0.093, 95%CI 0.021-0.451, p=0.003) and longer TTNT (HR=0.081, 95%CI 0.021-0.411, p<0.001). Regimens incorporating venetoclax (Ven) or autologous stem cell transplantation (ASCT) achieved higher ≥VGPR rates (p<0.05) and significantly prolonged OS (p<0.001). Ven-treated patients achieved ≥VGPR regardless of t(11;14) status, though remissions were transient in non-t(11;14) cases. Post-hoc analysis confirmed comparable outcomes between patients with 5-19% versus ≥20% CPCs, validating the updated diagnostic criteria.

### Summary/Conclusion pPCL maintains poor prognosis despite novel agents. Achieving ≥VGPR with first-line therapy is the critical determinant of survival. PI/IMiD combinations intensified with Ven (guided by t(11;14) status) and consolidated by ASCT may optimize response depth and long-term outcomes. These findings support the 2021 IMWG diagnostic revision and highlight the need for prospective evaluation of quadruplet regimens incorporating anti-CD38 antibodies.

**Copyright Information:** (Online) ISSN: 2572-9241
©2026 The Author(s). HemaSphere is published by John Wiley & Sons Ltd on behalf of European Hematology Association. This is an open access Abstract Book distributed under the Attribution-NonCommercial-NoDerivs (CC BY-NC-ND), which allows third parties to download the articles and share them with others as long as they credit the author and the Abstract Book, but they cannot change the content in any way or use them commercially.
**Abstract Book Citations:** Authors, Title, HemaSphere, 2026;10:(S1):pages. The abstract book DOIs can be found on the "Author Information" page.
**Disclaimer:** Articles published in the journal HemaSphere exclusively reflect the opinions of the authors. The authors are responsible for all content in their abstracts including accuracy of the facts, statements, citing resources, etc.

### (PB3381) EFFICACY AND SAFETY OF CARFILZOMIB IN 128 PATIENTS WITH RELAPSED OR REFRACTORY MULTIPLE MYELOMA: REAL-WORLD EXPERIENCE FROM A SINGLE CENTER

**Topic:** 14. Myeloma and other monoclonal gammopathies - Clinical

Guezlane Sherifa*[1, 1], Hamida Brahimi[1], Souad Taoussi[1, 1], Salima Oukid[1], Bradai Mohamed[1]

*[1]EHS ELCC, Blida, Algeria, Hematology department, Blida, Algeria;*

### Background

Multiple myeloma remains incurable and is characterized by repeated relapses requiring successive lines of therapy. Carfilzomib (Cfz) is a second-generation proteasome inhibitor with superior efficacy over bortezomib (Btz) and a significantly improved safety profile, notably a markedly lower incidence of peripheral neuropathy, making it a valuable option in relapsed/refractory settings. We report our real-world experience with carfilzomib-based regimens in relapsed/refractory multiple myeloma (RRMM).

### Aims

To describe the efficacy and safety of carfilzomib in adult patients with multiple myeloma treated in second line and beyond (≥L2).

### Methods

Retrospective monocentric study, 128 RRMM patients, September 2023–December 2025, 1–3 prior lines. Median age 60.5 years; ECOG ≥2: 53.9%; ISS III: 49.5%; Btz-refractory: 80.5%; Len-refractory: 27.3%. Prior therapy: VTd (62%), VCd (35%), VRd (3%); 28.1% had previously undergone autologous stem cell transplantation (ASCT). Regimens: KRd (58.6%), KCd (21.1%; Len-refractory or renal impairment), Kd (20.3%; Len-refractory).. Cfz 56 mg/m² (82%) or 70 mg/m² (18%), days 1, 8, 15 of 28-day cycles. Response per IMWG criteria. TRM defined as death due to toxicity or infection without progression; pre-progression TRM (TRMb) occurred before any progression. Intercurrent deaths were censored.

### Results

The overall response rate (ORR) was 50% (64/128), including complete response (CR) in 20.3%, very good partial response (VGPR) in 23.4%, and partial response (PR) in 6.2%. High-quality responses (CR+VGPR) reached 43.8%. A numerically higher ORR was observed with KRd vs other combinations. Clinically meaningful responses were achieved in Btz-refractory patients. Median time to response was 4 months (2–9); median duration of response 6 months (2–21); median number of cycles 6 (1–18).

Median overall survival (OS) was 25.7 months. Among 44 deaths (34.4%), 13 (10.2%) were treatment-related (TRM), including 7 pre-progression TRM (TRMb, 5.5%), predominantly infectious. Four intercurrent deaths were censored. OS at 12 months: 66.3% (95% CI: 57.0–75.6); at 24 months: 52.1% (95% CI: 40.0–64.2).

Median Progression-Free Survival (PFS) was 12.6 months (55 events, 73 censored). PFS at 12 months: 50.1% (95% CI: 39.5–60.8); at 24 months: 28.0% (95% CI: 10.5–45.6). PFS was significantly better with KRd vs KCd and Kd; Better outcomes were observed in patients with only one prior line of therapy.

The safety profile was favorable. One patient (0.8%) discontinued due to cardiac toxicity. Hematologic toxicities: thrombocytopenia 46.9% (grade 3: 14.8%, grade 4: 7.8%), anemia 38.3%, neutropenia 27.3% (grade 3–4: 7.8%), febrile neutropenia 1.6%. Non-hematologic toxicities: infections 13.3% (pulmonary 11.7%), heart failure 8.6%, thromboembolic events 3.9%, hypertension 3.1%, renal failure 0.8%. No peripheral neuropathy, dyspnea, or infusion-related reactions were observed, confirming the favorable neurological safety profile of Cfz compared with Btz.

**Copyright Information:** (Online) ISSN: 2572-9241
©2026 The Author(s). HemaSphere is published by John Wiley & Sons Ltd on behalf of European Hematology Association. This is an open access Abstract Book distributed under the Attribution-NonCommercial-NoDerivs (CC BY-NC-ND), which allows third parties to download the articles and share them with others as long as they credit the author and the Abstract Book, but they cannot change the content in any way or use them commercially.
**Abstract Book Citations:** Authors, Title, HemaSphere, 2026;10:(S1):pages. The abstract book DOIs can be found on the “Author Information” page.
**Disclaimer:** Articles published in the journal HemaSphere exclusively reflect the opinions of the authors. The authors are responsible for all content in their abstracts including accuracy of the facts, statements, citing resources, etc.

### Summary/Conclusion

This real-world study confirms the efficacy and good tolerability of carfilzomib-based regimens in high-risk RRMM, including patients largely refractory to bortezomib and with impaired performance status. An ORR of 50%, high-quality responses of 43.8%, median PFS of 12.6 months, and median OS of 25.7 months represent clinically meaningful outcomes in this challenging population. The near absence of peripheral neuropathy and favorable hematologic tolerability are major advantages supporting improved quality of life.

**Copyright Information:** (Online) ISSN: 2572-9241
©2026 The Author(s). HemaSphere is published by John Wiley & Sons Ltd on behalf of European Hematology Association. This is an open access Abstract Book distributed under the Attribution-NonCommercial-NoDerivs (CC BY-NC-ND), which allows third parties to download the articles and share them with others as long as they credit the author and the Abstract Book, but they cannot change the content in any way or use them commercially.
**Abstract Book Citations:** Authors, Title, HemaSphere, 2026;10:(S1):pages. The abstract book DOIs can be found on the "Author Information" page.
**Disclaimer:** Articles published in the journal HemaSphere exclusively reflect the opinions of the authors. The authors are responsible for all content in their abstracts including accuracy of the facts, statements, citing resources, etc.

### (PB3382) IMPLICATION OF DIABETES MELLITUS IN THE DEVELOPMENT AND PROGNOSIS OF MULTIPLE MYELOMA: A SINGLE-CENTER EXPERIENCE.

**Topic:** 14. Myeloma and other monoclonal gammopathies - Clinical

Marta Diaz-Lopez*[1], Ana Jing Garcia-Perez-Febles[2], Lorena Rivero-Peña[2], Tessa Casañas-Denboer[2], Sara Delgado del Rosario[2], Alejandro Martin Martin[1], Sunil Lakhwani[1, 2]

*[1]Hospital Universitario de Canarias, Hematology and Hemotherapy, San Cristóbal de La Laguna, Spain;[2]University of La Laguna, Faculty of Medicine, San Cristobal de Laguna, Spain;*

### Background

Diabetes mellitus (DM) has been associated with an increased risk of several malignancies and adverse outcomes in cancer. However, its role in multiple myeloma (MM) remains unclear.

### Aims

To assess the prevalence of DM in MM patients compared with the general population of the Canary Islands and to evaluate its prognostic impact on survival and response to first-line treatment.

### Methods

We performed a retrospective analysis of patients diagnosed and treated for MM at the Department of Hematology, Complejo Hospitalario Universitario de Canarias, between 2011 and 2015. DM prevalence in the general population was obtained from data published by the Canary Islands Health Observatory (population-based survey, 2015). Overall survival (OS), progression-free survival (PFS), and first-line response rates were analyzed according to DM status (pre-existing DM, DM developed during follow-up, and no DM).

### Results

Eighty-three patients were included; 50 (60.2%) were male and the mean age at diagnosis was 68.7 years. Pre-existing DM was present in 27.7% (23/83) of patients, and 13.3% (11/83) developed DM during MM evolution.
Among patients aged 45–64 years, DM prevalence was significantly higher in MM patients than in the general population (24% vs 11.7%; p=0.033), whereas no significant difference was observed in those aged ≥65 years (29.1% vs 25.3%; p=0.410).
No significant differences were observed in OS or PFS according to DM status (p=0.870 and p=0.715, respectively). However, response to first-line therapy was inferior in patients with pre-existing DM (**Table 1**); the rate of very good partial response or better was 39.1% compared with 66.7% in patients without DM at diagnosis (p=0.022).
Metformin or insulin use and body mass index ≥30 kg/m² were not associated with OS, PFS, or response rates.
**Table 1:** Comparison of best response rates to first-line treatment in patients with and without diabetes mellitus.

| RESPONSE | Pre-existing DM | DM developed during follow-up | No-DM |
|---|---|---|---|
| CR/sCR | 6 (26,1%) | 3 (27,3%) | 17 (34,7%) |
| VGPR | 3 (13%) | 4 (36,4%) | 16 (32.7%) |
| RP | 13 (56,5%) | 4 (36,4%) | 15 (30,6%) |
| SD | 1 (4,3%) | 0 (0%) | 2 (2,4%) |

*DM: diabetes mellitus; CR: complete response; sCR: stringent complete response; VGPR: very good partial response; PR: partial response; SD: stable disease.*

**Copyright Information:** (Online) ISSN: 2572-9241
©2026 The Author(s). HemaSphere is published by John Wiley & Sons Ltd on behalf of European Hematology Association. This is an open access Abstract Book distributed under the Attribution-NonCommercial-NoDerivs (CC BY-NC-ND), which allows third parties to download the articles and share them with others as long as they credit the author and the Abstract Book, but they cannot change the content in any way or use them commercially.
**Abstract Book Citations:** Authors, Title, HemaSphere, 2026;10:(S1):pages. The abstract book DOIs can be found on the "Author Information" page.
**Disclaimer:** Articles published in the journal HemaSphere exclusively reflect the opinions of the authors. The authors are responsible for all content in their abstracts including accuracy of the facts, statements, citing resources, etc.

**Summary/Conclusion**

In our cohort, MM patients younger than 65 years showed a higher prevalence of DM compared with the general population. Although DM was not associated with OS or PFS, pre-existing DM was associated with inferior response to first-line treatment.

**Copyright Information:** (Online) ISSN: 2572-9241
©2026 The Author(s). HemaSphere is published by John Wiley & Sons Ltd on behalf of European Hematology Association. This is an open access Abstract Book distributed under the Attribution-NonCommercial-NoDerivs (CC BY-NC-ND), which allows third parties to download the articles and share them with others as long as they credit the author and the Abstract Book, but they cannot change the content in any way or use them commercially.
**Abstract Book Citations:** Authors, Title, HemaSphere, 2026;10:(S1):pages. The abstract book DOIs can be found on the "Author Information" page.
**Disclaimer:** Articles published in the journal HemaSphere exclusively reflect the opinions of the authors. The authors are responsible for all content in their abstracts including accuracy of the facts, statements, citing resources, etc.

### (PB3383) FRONTLINE BORTEZOMIB–MELPHALAN–PREDNISONE IN TRANSPLANT-INELIGIBLE ELDERLY MULTIPLE MYELOMA PATIENTS: A SINGLE-CENTER REAL-WORLD STUDY

**Topic:** 14. Myeloma and other monoclonal gammopathies - Clinical

Ahlem Slama*[1], Inaam Bizid[1], Daami Mayssa[1], Zaineb Mlayah[1], Wiem Boufrikha[1], Nader Slama[1], Med.Adnene Laatiri[1], Sarra Boukhriss[1]

*[1]Fattouma Bourguiba University Hospital, Clinical Hematology Department, Monastir, Tunisia;*

### Background

Multiple myeloma (MM) predominantly affects elderly patients, in whom therapeutic management is often challenged by comorbidities and frailty. The bortezomib–melphalan–prednisone (VMP) regimen remains a widely used frontline option. However, real-world data on clinical characteristics, treatment tolerance, and long-term outcomes in this vulnerable population are limited, particularly in resource-constrained settings.

### Aims

To describe the clinical and biological features of elderly patients with MM treated with VMP and to evaluate treatment responses, toxicity, and survival outcomes.

### Methods

A retrospective descriptive and analytic study was carried out on elderly MM patients (age > 65 years) diagnosed between January 2019 and December 2025 and who were treated by the VMP regimen in the Hematology Department of Monastir, Tunisia.
We used SPSS to analyse the collected data.

**Copyright Information:** (Online) ISSN: 2572-9241
©2026 The Author(s). HemaSphere is published by John Wiley & Sons Ltd on behalf of European Hematology Association. This is an open access Abstract Book distributed under the Attribution-NonCommercial-NoDerivs (CC BY-NC-ND), which allows third parties to download the articles and share them with others as long as they credit the author and the Abstract Book, but they cannot change the content in any way or use them commercially.
**Abstract Book Citations:** Authors, Title, HemaSphere, 2026;10:(S1):pages. The abstract book DOIs can be found on the “Author Information” page.
**Disclaimer:** Articles published in the journal HemaSphere exclusively reflect the opinions of the authors. The authors are responsible for all content in their abstracts including accuracy of the facts, statements, citing resources, etc.

### Results

A total of 53 patients were included. Sex ratio was 1. The median age was 72 years (range: 58–92). Bone pain was the most frequent presenting symptom (68%). Comorbidities were present in 87%, mainly hypertension (45%) and diabetes (32%). The ECOG performance status was ≥2 in 60% of cases.
Radiological abnormalities were observed in 46 patients (87%). Low-dose CT scans, performed in 40% of cases, revealed lytic bone lesions (81%) and pathological fractures (19%). Spinal MRI was performed in 72% of patients, showing bone marrow replacement (71%), epidural involvement (10%), and extramedullary plasmacytomas (10%).
Anemia, renal impairment and hypercalcemia was present in 58%, 25%, 4% of patients respectively. Complete immunoglobulin MM was observed in 85 % of cases (IgG: 68%, IgA: 32%), while 15 % had light-chain MM (κ: 75%, λ: 25%).
A bone marrow infiltration exceeding 10% by dystrophic plasma cells was observed in all cases, and associated myelodysplastic features were identified in 6%.
The International Staging System (ISS), was assessable in 56% (stage I: 15%, stage II: 29%, stage III: 12%)
Based on the Revised Myeloma Comorbidity Index (R -MCI), patients were classified as fit (28%), intermediate-fit (64%), or frail (8%).
Most patients (79%) received at least six cycles of VMP.
Following VMP treatment, 32% achieved very good partial response, 28% partial response, 6% had stable disease and 25% experienced disease progression.
Patients with progression, received lenalidomide–dexamethasone (96%) or botezomib-lenalidomide-dexamethasone (4%) as second line therapy.
Supportive care included bisphosphonates in all patients (zoledronic acid: 76%, ibandronate: 24%) and systematic antiviral prophylaxis. Analgesics were required in 43%, and 9% received palliative radiotherapy. Initial transfusion support was needed in 28%, with 75% achieving transfusion independence.
Treatment-related toxicities were hematologic in 9% and non-hematologic in 13%, mainly neuropathy (71%) and infections (43%).
Overall survival was 86% at 2 years and 65% at 5 years. Progression-free survival was 86% at 2 years and 36% at 5 years.

### Summary/Conclusion

In this real-world cohort of transplant-ineligible elderly MM patients, frontline VMP achieved meaningful response rates and durable survival despite a high burden of comorbidities. Toxicity was manageable, supporting VMP as an effective and feasible frontline option, particularly in resource-constrained settings.

**Copyright Information:** (Online) ISSN: 2572-9241
©2026 The Author(s). HemaSphere is published by John Wiley & Sons Ltd on behalf of European Hematology Association. This is an open access Abstract Book distributed under the Attribution-NonCommercial-NoDerivs (CC BY-NC-ND), which allows third parties to download the articles and share them with others as long as they credit the author and the Abstract Book, but they cannot change the content in any way or use them commercially.
**Abstract Book Citations:** Authors, Title, HemaSphere, 2026;10:(S1):pages. The abstract book DOIs can be found on the "Author Information" page.
**Disclaimer:** Articles published in the journal HemaSphere exclusively reflect the opinions of the authors. The authors are responsible for all content in their abstracts including accuracy of the facts, statements, citing resources, etc.

### (PB3384) EXPERIENCE WITH THE DVD REGIMEN IN TRANSPLANT-INELIGIBLE MULTIPLE MYELOMA PATIENTS: A SINGLE-CENTER RETROSPECTIVE ANALYSIS

**Topic:** 14. Myeloma and other monoclonal gammopathies - Clinical

Carmen Gil Barroso[1], Ana Felicidad de Las Nieves Egea*[1], Ana Rosa Carranco Falcón[1], Juan Francisco Domínguez Rodríguez[1]

*[1]Hospital Juan Ramón Jiménez, Huelva, Spain;*

### Background

The management of multiple myeloma (MM) in patients ineligible for autologous stem cell transplantation remains a clinical challenge, particularly in the setting of relapse or refractory disease. The combination of daratumumab, bortezomib, and dexamethasone (DVd) has demonstrated significant improvements in progression-free survival (PFS) and overall survival (OS). However, real-world evidence, especially in elderly patients, those with a high comorbidity burden, or a higher proportion of lenalidomide-refractory disease, remains limited.

### Aims

To describe the efficacy and safety of the DVd regimen in transplant-ineligible MM patients treated at our center.

### Methods

We conducted a single-center retrospective study including 23 MM patients treated with DVd between 2022 and 2025. All patients had an indication for treatment and had received between 0 and 4 prior lines of therapy. Baseline characteristics, treatment efficacy (response and survival outcomes), and adverse events (AEs) were analyzed. Responses were assessed according to IMWG criteria.

### Results

The median age at DVd initiation was 67 years (range 45–87); 56.5% were male. ISS stage III was present in 52.2% (n=12) and R-ISS stage III in 39.1% (n=9). At diagnosis, more than 60% (n=14) had anemia, renal impairment, or hypercalcemia; baseline laboratory data were unavailable in 13% (n=3). Bone involvement was observed in 65.2% (n=15) and extramedullary disease in 34.8% (n=8). The clinical characteristics at diagnosis can be seen in Table 1.
Prior lines of therapy were distributed as follows: 0 (26.1%), 1 (39.1%), 2 (17.4%), 3 (13%), and 4 (4.3%). Disease progression on prior therapy occurred in 52.2% (n=12), and 33.3% (n=7) were lenalidomide-refractory. The median time from diagnosis to DVd initiation was 8 months.
Twelve patients (52.2%) experienced AEs, none grade >2. The most frequent AEs were peripheral neuropathy (n=7) and infections (n=2). Seven patients (30.4%) discontinued DVd due to toxicity (n=4) or intercurrent comorbidities (n=3). Only 30.4% (n=7) received daratumumab maintenance, and 26.1% (n=6) required subsequent therapy.
The overall response rate (ORR) was 56.5% (n=13), including complete response (CR) in 13% (n=3), stringent CR in 8.7% (n=2), partial response (PR) in 21.7% (n=5), and very good partial response (VGPR) in 13% (n=3). Three patients (13%) were not evaluable.
After a median follow-up of 29 months (range 0–234), nine patients had died due to renal failure (n=2), infections (n=3), disease progression (n=2), or other causes (n=2). The estimated 60-month OS was 63 ± 12% (Figure 1). PFS from DVd initiation was 38.1 ± 10% at 30 months and 19 ± 11% at 47 months (Figure 2). The median time to event from DVd initiation was 11 months.

**Copyright Information:** (Online) ISSN: 2572-9241
©2026 The Author(s). HemaSphere is published by John Wiley & Sons Ltd on behalf of European Hematology Association. This is an open access Abstract Book distributed under the Attribution-NonCommercial-NoDerivs (CC BY-NC-ND), which allows third parties to download the articles and share them with others as long as they credit the author and the Abstract Book, but they cannot change the content in any way or use them commercially.
**Abstract Book Citations:** Authors, Title, HemaSphere, 2026;10:(S1):pages. The abstract book DOIs can be found on the "Author Information" page.
**Disclaimer:** Articles published in the journal HemaSphere exclusively reflect the opinions of the authors. The authors are responsible for all content in their abstracts including accuracy of the facts, statements, citing resources, etc.

### Summary/Conclusion

In our experience, the ORR and median time to event with DVd were lower than those reported in pivotal studies, likely reflecting a higher proportion of patients with advanced disease (ISS III) and lenalidomide-refractory status. Nevertheless, the estimated OS was clinically meaningful, and the safety profile was favorable. Larger real-world studies with longer follow-up are warranted to draw more robust conclusions.

| TABLE 1: CLINICAL CHARACTERISTICS AT DIAGNOSIS | n (%) |
|---|---|
| SEX | |
| - Male | 13 (56.5%) |
| - Female | 10 (43.5%) |
| MEAN AGE (Range) | 67 (45-87) |
| TYPE OF MYELOMA | |
| - Kappa light Chain | 9 (39.1%) |
| - Lambdalight chain | 1 (4.3%) |
| - IgG Kappa | 6 (26.1%) |
| - IgA Lambda | 2 (8.7%) |
| - IgA Kappa | 3 (13%) |
| - Monoclonal gammopathy of renal significance | 2 (8.7%) |
| ISS | |
| - Stage I | 4 (17.4%) |
| - Stage II | 4 (17.4%) |
| - Stage III | 12 (52.2%) |
| - Baseline laboratory data unavailable | 3 (13%) |
| R-ISS | |
| - Stage I | 2 (8.7%) |
| - Stage II | 6 (26.1%) |
| - Stage III | 9 (39.1%) |
| - Baseline laboratory data unavailable | 6 (26.1%) |
| ANEMIA (Hb < 10 g/dL) | |
| - YES | 14 (60.9%) |
| - NO | 6 (26.1%) |
| - Baseline laboratory data unavailable | 3 (13%) |
| RENAL FAILURE (Cr > 1 mg/dL) | |
| - YES | 15 (65.2%) |
| - NO | 5 (21.7%) |
| - Baseline laboratory data unavailable | 3 (13%) |
| HYPERCALCEMIA (Ca > 9 mg/dL) | |
| - YES | 14 (60.9%) |
| - NO | 6 (26.1%) |
| - Baseline laboratory data unavailable | 3(13%) |
| BONE INVOLVEMENT | |
| - YES | 15 (65.2%) |
| - NO | 8 (34.8%) |
| EXTRAMEDULLARY DISEASE | |
| - YES | 8 (34.8%) |
| - NO | 15 (65.2%) |
| CYTOGENETICS | |
| - Standard risk | 11 (47,8%) |
| - High risk | 9 (39.1%) |
| - Cytogenetic study not performed | 3 (13%) |
| REASON FOR DVd INITIATON | |
| - Biochemical relapse | 10 (43,5%) |
| - Metabolic relapse | 2 (8,7%) |
| - Newly diagnosed disease | 7 (30,4%) |
| - Prior treatment-related toxicity | 4 (17,4%) |

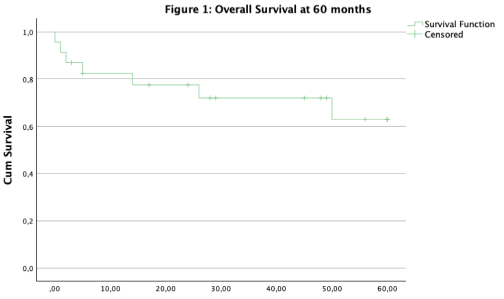

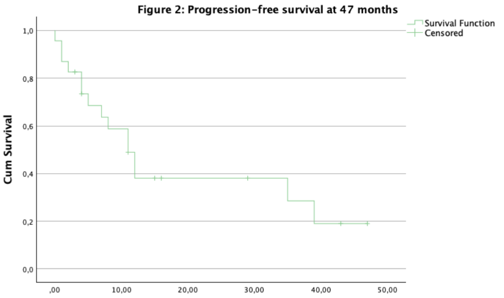

Clinical characteristics at diagnosis, OS and PFS.

**Copyright Information:** (Online) ISSN: 2572-9241
©2026 The Author(s). HemaSphere is published by John Wiley & Sons Ltd on behalf of European Hematology Association. This is an open access Abstract Book distributed under the Attribution-NonCommercial-NoDerivs (CC BY-NC-ND), which allows third parties to download the articles and share them with others as long as they credit the author and the Abstract Book, but they cannot change the content in any way or use them commercially.
**Abstract Book Citations:** Authors, Title, HemaSphere, 2026;10:(S1):pages. The abstract book DOIs can be found on the "Author Information" page.
**Disclaimer:** Articles published in the journal HemaSphere exclusively reflect the opinions of the authors. The authors are responsible for all content in their abstracts including accuracy of the facts, statements, citing resources, etc.

### (PB3385) ALTERNATIVE CHEMOMOBILIZATION REGIMENS FOR STEM CELL COLLECTION IN MULTIPLE MYELOMA: REAL-WORLD INDUCTION PRACTICE

**Topic:** 14. Myeloma and other monoclonal gammopathies - Clinical

Anastasia Semenova*[1], Stanislav Kamyshanov[2], Gayane Tumyan[1], Galina Petrova[1], Olga Baranova[1, 2], Arman Arakelyan[1], Nikita Zemlyakov[2], Alena Cherentsova[1]

*[1]The Blokhin Russian Cancer Research Center, Moscow, Russia;[2]Pirogov Russian National Research Medical University, Moscow, Russia;*

### Background

ASCT remains a key component of first-line therapy for transplant-eligible MM. Anti-CD38 induction (e.g., Dara-VRd) may reduce mobilization, and limited plerixafor access makes alternative strategies important. We evaluated real-world stem cell collection (SCC) outcomes after VRd vs Dara-VRd using alternative mobilization regimens in a single center.

### Aims

To assess SCC efficiency after VRd vs Dara-VRd induction by comparing CD34+ cell yield and the number of apheresis across chemomobilization regimens, and to evaluate the impact of induction cycles, depth of response, and chemomobilization regimen on these outcomes.

### Methods

At the Blokhin National Medical Research Center of Oncology, 95 transplant-eligible patients with newly diagnosed MM undergoing planned ASCT were included between 2021 and 2026. Induction therapy was VRd in 81/95 patients and Dara-VRd in 14/95. Median age was 51(44–61), and 45/95(47.4%) were male.

SCC was planned after 3 cycles. Successful collection was defined as $\geq 2\times 10^6$ CD34+ cells/kg. Mobilization consisted of etoposide 375 mg/m² on days 1–2 / gemcitabine 1250 mg/$m^2$ day 1, followed by G-CSF for ≥8 days (median 9, range 8–13). Two patients required remobilization with plerixafor. Treatment response was assessed per IMWG 2016.

To address potential confounding between the induction and the number of induction cycles in the association of chemomobilization with SCC outcomes, generalized linear models were fitted separately for each endpoint (Poisson regression for count outcomes and gamma regression with a log link for continuous right-skewed outcomes).

**Copyright Information:** (Online) ISSN: 2572-9241
©2026 The Author(s). HemaSphere is published by John Wiley & Sons Ltd on behalf of European Hematology Association. This is an open access Abstract Book distributed under the Attribution-NonCommercial-NoDerivs (CC BY-NC-ND), which allows third parties to download the articles and share them with others as long as they credit the author and the Abstract Book, but they cannot change the content in any way or use them commercially.
**Abstract Book Citations:** Authors, Title, HemaSphere, 2026;10:(S1):pages. The abstract book DOIs can be found on the “Author Information” page.
**Disclaimer:** Articles published in the journal HemaSphere exclusively reflect the opinions of the authors. The authors are responsible for all content in their abstracts including accuracy of the facts, statements, citing resources, etc.

**Results**

By February 2026, all patients (n=95) accomplished induction cycles (median 3 (3–4)). Best responses were ≥CR 40 (42.1%), VGPR 28 (29.5%), PR 13 (13.7%), SD 2 (2.1%), and PD 9 (9.5%); 3 patients (3.1%) died during induction. ORR was 85.3%.
SCC was successful in 84/95 (88.4%). 11/95 did not proceed due to death (n=4), refusal (n=4), progression (n=2), or insufficient mobilization (n=1). Median days from the last induction course to apheresis – 30 (5–58). 82 patients collected a sufficient dose for 2 ASCT procedures and 2 patients for 1.
Etoposide-based mobilization (n=58) yielded a higher CD34+ collection than gemcitabine-based (n=26): 10.66 (6.9–12.85) vs 6.65 (5.46–8.23)×10^6/kg, with fewer apheresis sessions: 1 (1–2) vs 3 (3–4).
Etoposide-based chemomobilization was associated with a higher number of collected CD34+ compared with gemcitabine-based (10.66 and 6.65, respectively; p<0.001). The differences were statistically significant across both parametric and non-parametric tests.
Patients receiving gemcitabine as chemomobilization required a significantly higher number of apheresis (median 3) compared with the etoposide group (median 1). This difference was statistically significant (p<0.001).
Induction regimen, number of induction cycles, and depth of response did not demonstrate a significant confounding effect on the association between the type of chemomobilization and outcomes (number of apheresis and collected stem cell yield) (p>0.05).
Chemomobilization regimens demonstrated an acceptable toxicity profile, predominantly hematologic. The number of secondary malignancies was n=1.
Patients successfully underwent ASCT (n=63).

**Summary/Conclusion**

Etoposide-based regimen demonstrated higher collection efficiency without signals of clinically meaningful toxicity. The addition of daratumumab to VRd induction did not result in a statistically significant deterioration of mobilization parameters.

**Copyright Information:** (Online) ISSN: 2572-9241
©2026 The Author(s). HemaSphere is published by John Wiley & Sons Ltd on behalf of European Hematology Association. This is an open access Abstract Book distributed under the Attribution-NonCommercial-NoDerivs (CC BY-NC-ND), which allows third parties to download the articles and share them with others as long as they credit the author and the Abstract Book, but they cannot change the content in any way or use them commercially.
**Abstract Book Citations:** Authors, Title, HemaSphere, 2026;10:(S1):pages. The abstract book DOIs can be found on the "Author Information" page.
**Disclaimer:** Articles published in the journal HemaSphere exclusively reflect the opinions of the authors. The authors are responsible for all content in their abstracts including accuracy of the facts, statements, citing resources, etc.

### (PB3387) MOMENTUM: A PHASE 2, OPEN-LABEL, SINGLE-ARM, MULTICENTER STUDY TO EVALUATE THE EFFICACY OF CEMSIDOMIDE + DEXAMETHASONE IN SUBJECTS WITH RELAPSED/REFRACTORY MULTIPLE MYELOMA

**Topic:** 14. Myeloma and other monoclonal gammopathies - Clinical

Binod Dhakal*[1], Karthik Ramasamy[2], Amro Ali[3], Leah Leahy[3], Uma Chandrasekaran[3], Riadh Lobbardi[3], Eunju Hurh[3], Leonard Reyno[3], Martin Kaiser[4]

*[1]Medical College of Wisconsin, Milwaukee, United States of America;[2]Oxford University Hospital NHS Trust, Oxford, United Kingdom; [3]C4 Therapeutics, Watertown, United States of America; [4]The Royal Marsden Hospital and The Institute of Cancer Research, London, United Kingdom;*

### Background

Cemsidomide is a novel, highly potent, cereblon-based, IKZF1/3 MonoDAC® degrader, having a similar mechanism of action as BMS's IMiD® or CELMoD® degraders for MM. Cemsidomide displays catalytic activity enabling rapid and deep target degradation with high binding affinity to overcome resistance due to low cereblon levels. Cemsidomide binds to cereblon to facilitate the recruitment and ubiquitination of IKZF1/3 leading to the proteasomal degradation of both proteins. IKZF1/3 degradation induces multiple myeloma cell death, activation of fully differentiated T-cells which prevents T-cell exhaustion and promotes secretion of key immune stimulating cytokines. In a Phase 1 study, cemsidomide, given orally QD over 14 days with a 14 day “off” period (14/14) in combination with weekly dexamethasone in a heavily pre-treated patient population (7 median prior lines), was well tolerated and demonstrated durable anti-myeloma activity at increasing dose levels. A 53% ORR was observed as the highest dose of 100 µg QD 14/14, with a 36% ORR observed across all dose levels (Dhakal IMS 2025).

### Aims

MOMENTUM (NCT07284758) is a Phase 2 multi-center, open-label, single-arm, global study to further evaluate the anti-myeloma activity as well as safety, tolerability, and PK/PD of cemsidomide.

### Methods

The study will enroll approximately 100 patients. The patient population eligible for this study include those who have received at least 3 prior antimyeloma regimens, which must have included an IKZF1/3 degrader other than cemsidomide, a proteasome inhibitor, an anti-CD38 antibody, and a TCE or CAR-T therapy (unless not indicated). Prior TCE or CAR-T therapy may be one or more targets including but not limited to: BCMA, GPRC5D, FcRH5, and CD38. Patients must have an ECOG score of ≤2, and adequate organ function prior to dosing. Patients will receive cemsidomide 100 µg QD 14/14 and dexamethasone in a 28-day cycle until progressive disease, AE requiring treatment discontinuation, or withdrawal of consent. The primary endpoint of this study is ORR per IMWG response Criteria (IMS 2025) assessed by an Independent Review Committee (IRC). Secondary endpoints include assessment of DOR, PFS, and OS. Exploratory objectives include antimyeloma activity assessed by investigator, degradation of IKF1/3, quantification of protein expression, and patient QoL assessments. Exposure-response relationships between cemsidomide and relevant markers of safety and efficacy will also be analyzed.  A safety review committee and an IRC will be established to oversee safety and efficacy throughout the trial.

### Results

The study is open to enrollment as of December 2025 and is currently recruiting.

**Copyright Information:** (Online) ISSN: 2572-9241
©2026 The Author(s). HemaSphere is published by John Wiley & Sons Ltd on behalf of European Hematology Association. This is an open access Abstract Book distributed under the Attribution-NonCommercial-NoDerivs (CC BY-NC-ND), which allows third parties to download the articles and share them with others as long as they credit the author and the Abstract Book, but they cannot change the content in any way or use them commercially.
**Abstract Book Citations:** Authors, Title, HemaSphere, 2026;10:(S1):pages. The abstract book DOIs can be found on the “Author Information” page.
**Disclaimer:** Articles published in the journal HemaSphere exclusively reflect the opinions of the authors. The authors are responsible for all content in their abstracts including accuracy of the facts, statements, citing resources, etc.

### (PB3388) PHASE II STUDY OF POST-TRANSPLANT MAINTENANCE THERAPY WITH ISATUXIMAB PLUS LENALIDOMIDE FOR HIGH-RISK MULTIPLE MYELOMA (HRMM)

**Topic:** 14. Myeloma and other monoclonal gammopathies - Clinical

Samer A. Srour*[1], Roland Bassett[2], Qaiser Bashir[1], Jing Christine Ye[3], George Chen[1], Neeraj Saini[1, 3], Yosra Aljawai[1], Jin Im[1], Paul Lin[1], Yago Nieto[1], Partow Kebriaei[1], Uday R. Popat[1], Mahmoud R. Gaballa[3], Oren Pasvolsky[3], Sheeba K. Thomas[3], Krina Patel[3], Robert Z Orlowski[3], Richard E. Champlin[1], Elizabeth J. Shpall[1], Muzaffar H. Qazilbash[1]

*[1]The University of Texas MD Anderson Cancer Center, Department of Stem Cell Transplantation and Cellular Therapy, Houston, United States of America; [2]The University of Texas MD Anderson Cancer Center, Department of Biostatics, Houston, United States of America; [3]The University of Texas MD Anderson Cancer Center, Department of Lymphoma/Myeloma, Houston, United States of America;*

### Background

Despite the remarkable advances in novel therapeutics, patients with HRMM continue to have poor prognosis, with relapse being the main cause of treatment failure. Lenalidomide (Len) with or without proteasome inhibitors is considered standard maintenance after autologous stem cell transplantation (ASCT) in HRMM. Incorporation of anti-CD38 monoclonal antibodies in maintenance therapy hypothesized to improve HRMM outcomes. Isatuximab (Isa), an anti-CD38 monoclonal antibody, showed promising results in heavily pretreated patients and as in the frontline setting. In this phase 2 clinical trial our aim is to determine the safety and efficacy of Isa + Len as maintenance therapy for HRMM after ASCT. We present here an interim safety and efficacy analysis.

### Methods

Adult patients with newly diagnosed HRMM, up to age 78 years, were eligible. HRMM was defined as: R-SS stage II/III or ISS stage III; presence of at least 2 of the following high-risk genetic abnormalities by fluorescence in situ hybridization (FISH): t(4:14), t(14:16), t(14:20), deletion (del) 17/17p or gain (amp) 1q21; del(17p) by conventional cytogenetic analysis regardless of ISS/R-ISS stage. Conditioning regimen used was at the discretion of treating physician (melphalan alone +/- busulfan). Maintenance started 60-180 days after ASCT. Treatment consisted of IV Isa at 10 mg/kg on days 1, 8, 15 and 22 of cycle 1, days 1 and 15 of cycles 2-3, and day 1 from cycles 4-39; and oral Len 10 mg daily on days 1-21 of cycles 1-3, and if tolerated could be increased to days 1-28 in subsequent cycles. The primary endpoint is progression-free survival (PFS) at 3 years post-ASCT. Selected secondary objectives: overall survival (OS), overall best response rate (VGPR + CR/sCR per the IMWG criteria), and measurable residual disease (MRD) at $10^{-5}$ by next generation flow cytometry (NGF) after 3 cycles, and at 12 and 24 months after starting maintenance therapy.

### Results

Twenty-five patients enrolled between 10/23 and 01/2025, of which 23 with a median age of 59 (range, 29-77) years were evaluable for safety and efficacy at the data cutoff for this analysis. All patients had HRMM, and 52% had ≥2 HR cytogenetic abnormalities. Fourteen (61%) received melphalan as conditioning and 9 (39%) received busulfan + melphalan. At the time of ASCT, 87% had achieved a ≥VGPR (CR/sCR: 9%) and 41% were MRD -ve by NGF. At 3 months after transplant, 96% achieved ≥VGPR (CR/sCR: 65%) and 68% were MRD negative. After 3 cycles of Isa + Len (n=22), 96% achieved a ≥VGPR (CR/sCR: 77%) and 77% were MRD negative. After a median follow-up of 12.4 months, median PFS and OS were not reached, none of the patients died during the study period and 2 patients progressed (figure). No unexpected serious adverse effects attributable to Isa + Len were noted.

**Copyright Information:** (Online) ISSN: 2572-9241
©2026 The Author(s). HemaSphere is published by John Wiley & Sons Ltd on behalf of European Hematology Association. This is an open access Abstract Book distributed under the Attribution-NonCommercial-NoDerivs (CC BY-NC-ND), which allows third parties to download the articles and share them with others as long as they credit the author and the Abstract Book, but they cannot change the content in any way or use them commercially.
**Abstract Book Citations:** Authors, Title, HemaSphere, 2026;10:(S1):pages. The abstract book DOIs can be found on the "Author Information" page.
**Disclaimer:** Articles published in the journal HemaSphere exclusively reflect the opinions of the authors. The authors are responsible for all content in their abstracts including accuracy of the facts, statements, citing resources, etc.

**Summary/Conclusion**

Isatuximab plus lenalidomide maintenance therapy after ASCT is safe and feasible in patients HRMM, with encouraging survival and low rates of progression. Longer follow up is needed. The study is ongoing (NCT05776979).

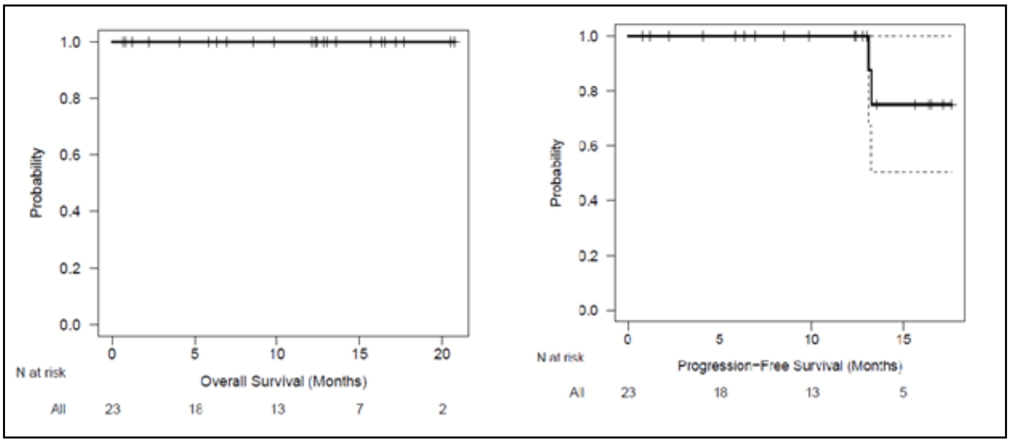

**Copyright Information:** (Online) ISSN: 2572-9241
©2026 The Author(s). HemaSphere is published by John Wiley & Sons Ltd on behalf of European Hematology Association. This is an open access Abstract Book distributed under the Attribution-NonCommercial-NoDerivs (CC BY-NC-ND), which allows third parties to download the articles and share them with others as long as they credit the author and the Abstract Book, but they cannot change the content in any way or use them commercially.
**Abstract Book Citations:** Authors, Title, HemaSphere, 2026;10:(S1):pages. The abstract book DOIs can be found on the "Author Information" page.
**Disclaimer:** Articles published in the journal HemaSphere exclusively reflect the opinions of the authors. The authors are responsible for all content in their abstracts including accuracy of the facts, statements, citing resources, etc.

### (PB3389) STROMAL PROTECTION OF JAK2V617F-POSITIVE CELLS AGAINST JAK2 INHIBITORS INVOLVES BOTH PARACRINE AND CONTACT-DEPENDENT MECHANISMS

**Topic:** 15. Myeloproliferative neoplasms - Biology & translational research

Maya Koren-Michowitz*[1, 2], Odit Gutwein[1, 2], Katrin Herzog Tzarfati[1], Arie Apel[1], Ori Perlmuter[1], Ariela Berkowitz[1], Zehavit Rogov[2]

*[1]Shamir Medical Center, Hematology, Beer Yakov, Israel; [2]Gray Faculty of Medicine, Tel Aviv University, Tel-Aviv, Israel;*

### Background

Bone marrow stromal cells support malignant cell survival and contribute to therapy resistance in myeloproliferative neoplasms (MPN). Stromal co-culture has been reported to attenuate JAK2 inhibitor responses in JAK2V617F-positive models, but the relative contribution of soluble versus contact-dependent mechanisms and the key stromal regulators remain unclear.

### Aims

This study aimed to characterize the nature of stromal-mediated protection (paracrine vs. contact-dependent) and investigate the role of differentially expressed stromal genes in resistance to the JAK2 inhibitors.

### Methods

RNA-seq analysis was conducted on HS-5 stromal cells co-cultured with peripheral blood CD34+ cells from myelofibrosis (MF) patients or normal cord blood (CB) CD34+ cells, with and without Fedratinib. The differential expression of candidate genes was validated by qPCR. The role of ATP1B2 in stromal-mediated protection was assessed by MISSION® esiRNA knockdown in stroma, followed by viability assays under drug treatment.
To study the mechanism of stromal protection further, JAK2V617F-positive SET2 and UKE1 cells were co-cultured with the HS-5 stromal line in monoculture, Transwell (TW), and direct contact (CO) configurations. Cells were treated with 1.5 µM Fedratinib or 0.5 µM Pacritinib for 48 hours. Cell viability was quantified by flow cytometry.

### Results

RNA-seq analysis of stromal cells co-cultured with CD34+ cells either with or without JAK2 inhibition  revealed 49 candidate genes (10 upregulated, 39 downregulated) in co-culture with MF cells, and 24 candidate genes (6 upregulated, 18 downregulated) in co-culture with  CB CD34+ cells, with 9 genes shared between both conditions. Gene expression changes identified by RNA-seq were further validated by real-time qPCR and ATP1B2 was selected for further studies.
The relative contribution of contact and paracrine dependent mechanisms of stromal protection, was studied in co-culture with the JAK2 positive SET2 and UKE1 cells. Both Fedratinib and Pacritinib markedly reduced monoculture SET2 and UKE1 cells viability. HS-5 co-culture increased cell survival, with stronger stromal protection in direct contact (CO) than Transwell (TW). Under Fedratinib, SET2 and UKE1 cells viability increased 3.5-fold in TW and 5.4 (SET2) to 7.9-fold (UKE1) in CO. Similarly, under Pacritinib, HS-5 increased UKE1 viability 7.0-fold in TW and 19.8-fold in CO relative to conditions without HS-5. Stromal ATP1B2 silencing further enhanced viability under drug treatment, increasing viability 1.25-fold in Fedratinib-treated SET2 ($p < 0.05$) and 1.34-fold in Fedratinib-treated UKE1 ($p < 0.01$) compared to control siRNA, and by 1.30-fold in Pacritinib-treated UKE1. Notably, while ATP1B2 was upregulated in HS-5 cells RNA-seq during co-culture, its knockdown further increased stromal protection of the JAK2 positive cells.

**Copyright Information:** (Online) ISSN: 2572-9241
©2026 The Author(s). HemaSphere is published by John Wiley & Sons Ltd on behalf of European Hematology Association. This is an open access Abstract Book distributed under the Attribution-NonCommercial-NoDerivs (CC BY-NC-ND), which allows third parties to download the articles and share them with others as long as they credit the author and the Abstract Book, but they cannot change the content in any way or use them commercially.
**Abstract Book Citations:** Authors, Title, HemaSphere, 2026;10:(S1):pages. The abstract book DOIs can be found on the “Author Information” page.
**Disclaimer:** Articles published in the journal HemaSphere exclusively reflect the opinions of the authors. The authors are responsible for all content in their abstracts including accuracy of the facts, statements, citing resources, etc.

**Summary/Conclusion**

Stromal cells protect JAK2V617F-positive cells through both paracrine and contact-dependent mechanisms, with stronger protection in direct contact than Transwell conditions. Stromal ATP1B2 depletion increases JAK2V617F cell viability under JAK2 inhibitor treatment in co-culture models, suggesting ATP1B2 is a functional component of stromal-mediated drug response. These findings underscore the complexity of stromal regulation under JAK2 inhibition and support further mechanistic studies of ATP1B2-associated pathways as potential targets to modulate stromal protection and drug resistance.

**Copyright Information:** (Online) ISSN: 2572-9241
©2026 The Author(s). HemaSphere is published by John Wiley & Sons Ltd on behalf of European Hematology Association. This is an open access Abstract Book distributed under the Attribution-NonCommercial-NoDerivs (CC BY-NC-ND), which allows third parties to download the articles and share them with others as long as they credit the author and the Abstract Book, but they cannot change the content in any way or use them commercially.
**Abstract Book Citations:** Authors, Title, HemaSphere, 2026;10:(S1):pages. The abstract book DOIs can be found on the “Author Information” page.
**Disclaimer:** Articles published in the journal HemaSphere exclusively reflect the opinions of the authors. The authors are responsible for all content in their abstracts including accuracy of the facts, statements, citing resources, etc.

### (PB3390) SH2B3 P242S GERMLINE VARIANT ACTIVATES THE JAK2/STAT5 PATHWAY TO MEDIATE ABNORMAL ERYTHROCYTE PROLIFERATION

**Topic:** 15. Myeloproliferative neoplasms - Biology & translational research

Yifan Duan*[1], Yizhen Wang[1], Guangshuai Teng[1], Ke Shang[1], Xiaojing Wei[1], Yuhui Zhang[1], Chenxiao Du[1], Yan Wang[1], Yanqi Li[1], Huiqin Zhang[1], Lan Peng[1], Yanjie Lan[1], Zonghong Shao[1], Yuan Zhou[2], Weiping Yuan[1, 2], Jie Bai[1]

*[1]The Second Hospital of Tianjin Medical University, Department of Hematology, Tianjin, China;[2]Institute of Hematology & Blood Diseases Hospital, Chinese Academy of Medical Sciences & Peking Union Medical College, State Key Laboratory of Experimental Hematology, National Clinical Research Center for Blood Diseases, Tianjin, China;*

### Background

Idiopathic erythrocytosis (IE) is a heterogeneous disease characterized by abnormal erythroid hyperplasia after excluding polycythemia vera (PV) and other definite etiologies. Its molecular pathogenesis remains unclear, and most patients have germline genetic abnormalities.

### Aims

This study aimed to analyze the clinical characteristics and genetic variant spectrum of IE patients, explore the regulatory mechanism of the key variant *SH2B3* P242S on erythrocyte proliferation, and provide a molecular basis for IE pathogenesis.

### Methods

A total of 54 IE patients admitted to the Department of Hematology, The Second Hospital of Tianjin Medical University from September 2019 to July 2025 were retrospectively enrolled, with 131 PV patients as controls. Clinical and laboratory characteristics were compared between the two groups. Whole-Exome Sequencing (WES) detected erythroid-related pathogenic/susceptibility gene variants in IE patients, with germline verification via hair follicle samples. SWISS-MODEL predicted the impact of *SH2B3* P242S on protein conformation. K562 cell lines were constructed: empty vector control (VEC), *SH2B3* wild-type (*SH2B3* WT), and *SH2B3* P242S mutant groups. Cell proliferation, erythroid antigen expression, and morphological changes were detected. Differentially expressed genes and enriched signaling pathways were screened, and JAK2/STAT5 pathway activation was verified.

**Copyright Information:** (Online) ISSN: 2572-9241
©2026 The Author(s). HemaSphere is published by John Wiley & Sons Ltd on behalf of European Hematology Association. This is an open access Abstract Book distributed under the Attribution-NonCommercial-NoDerivs (CC BY-NC-ND), which allows third parties to download the articles and share them with others as long as they credit the author and the Abstract Book, but they cannot change the content in any way or use them commercially.
**Abstract Book Citations:** Authors, Title, HemaSphere, 2026;10:(S1):pages. The abstract book DOIs can be found on the "Author Information" page.
**Disclaimer:** Articles published in the journal HemaSphere exclusively reflect the opinions of the authors. The authors are responsible for all content in their abstracts including accuracy of the facts, statements, citing resources, etc.

### Results

① Compared with PV patients, IE patients had a higher male proportion, younger median onset age, lower median WBC, PLT, and HCT levels, lower incidences of splenomegaly, chromosomal abnormalities, EEC positivity, thrombosis, pruritus, and EPO reduction, and significantly longer thrombus-free survival. ② IE patients carried 22 erythroid-related germline variant genes, among which *SH2B3* (52%) and *ZFPM1* (10%) had the highest variant frequencies. Variants were mostly distributed on chromosomes 12 and 16, mainly as heterozygous variants, and there was a strong correlation between *ZFP36* and *INPP5D* gene variants. Sixteen IE patients carried the *SH2B3 P242S* germline variant, including 2 homozygous variants and 14 heterozygous variants. ③ Protein conformation prediction showed that the *SH2B3 P242S* variant could disrupt the protein turn structure maintained by proline, leading to loose local conformation and reduced protein stability. ④ In cell function experiments, the proliferation rate of K562 cells in the *SH2B3* P242S group was significantly higher than that in the VEC group, with significantly increased expression levels of CD235a and CD71. RNA-seq analysis indicated that the expression levels of erythroid proliferation, differentiation, and anti-apoptosis-related genes such as *IGF1, IRS1, KIT, GLRX2, BCL2* , and *BACH2* were significantly upregulated in the *SH2B3 P242S* group, and differentially expressed genes were enriched in signaling pathways such as JAK/STAT and PI3K/Akt. Western blot confirmed that the phosphorylation levels of JAK2 and STAT5 were significantly higher in the *SH2B3* P242S group than in the VEC group, whereas the *SH2B3* WT group showed no significant effect on pathway activation.

### Summary/Conclusion

*SH2B3* is a high-frequency germline variant in IE patients. The *SH2B3* P242S germline variant disrupts protein structural stability, activates the JAK2/STAT5 signaling pathway, upregulates erythroid-related gene expression, and promotes abnormal erythrocyte proliferation and differentiation, which may be a key molecular mechanism of Idiopathic erythrocytosis.

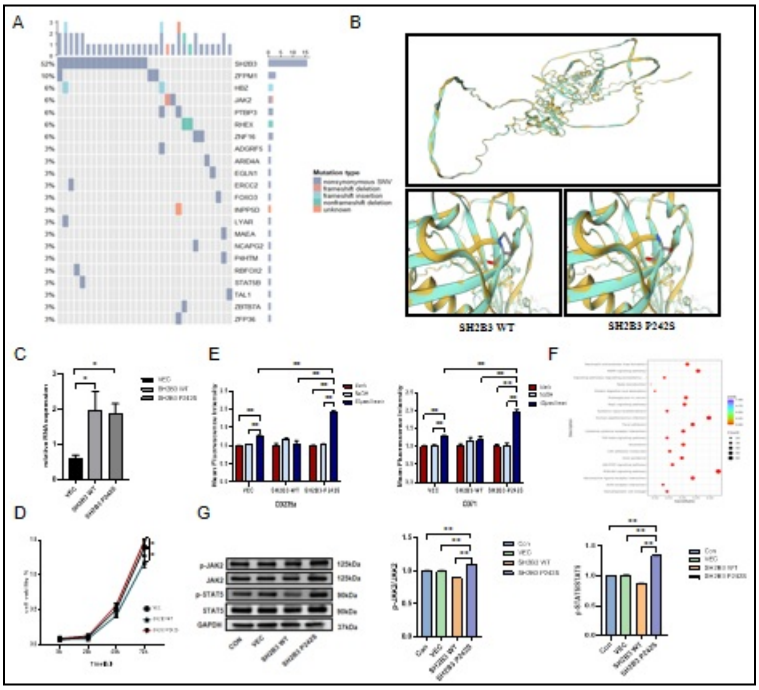

Figure. SH2B3 P242S germline variant activates the JAK2/STAT5 pathway to drive abnormal erythroid proliferation (A)Distribution of germline variants in erythroid-related genes of IE patients. (B) SWissModel prediction of protein conformational changes. (C) RT-PCR detection of SH2B3 expression in each group. (D) CCK-8 assay for cell proliferation.(E) Flow cytometry analysis of erythroid antigen expression in each group.(F) KEGG enrichment analysis. (G) Western blot detection of JAK2/STAT5 pathway-related proteins.

**Copyright Information:** (Online) ISSN: 2572-9241
©2026 The Author(s). HemaSphere is published by John Wiley & Sons Ltd on behalf of European Hematology Association. This is an open access Abstract Book distributed under the Attribution-NonCommercial-NoDerivs (CC BY-NC-ND), which allows third parties to download the articles and share them with others as long as they credit the author and the Abstract Book, but they cannot change the content in any way or use them commercially.
**Abstract Book Citations:** Authors, Title, HemaSphere, 2026;10:(S1):pages. The abstract book DOIs can be found on the "Author Information" page.
**Disclaimer:** Articles published in the journal HemaSphere exclusively reflect the opinions of the authors. The authors are responsible for all content in their abstracts including accuracy of the facts, statements, citing resources, etc.

### (PB3391) AUTOIMMUNE PHENOTYPES AND MUTATIONAL PATTERNS IN MYELOID NEOPLASMS: AGE-DEPENDENT ASSOCIATIONS AND PROGNOSTIC IMPACT OF CLONAL HEMATOPOIESIS.

**Topic:** 15. Myeloproliferative neoplasms - Biology & translational research

Sara Galimberti*[1], Amedeo Votto[2], Ledjia Sijoni[1, 2], Ines Rina[1], Giovanni Fulvio[1], Antonello Di Paolo[1], Guido Bocci[1], Ilaria Puxeddu[1], Marta Mosca[1], Chiara Baldini[1]

*[1]DEPARTMENT OF CLINICAL AND EXPERIMENTAL MEDICINE, UNIVERSITY OF PISA, PISA, Italy; [2]department of clinical and experimental medicine, University of Pisa, Pisa, Italy;*

### Background

Autoimmune diseases (AD) are frequently observed in patients with myeloid neoplasms, but their biological and prognostic implications remain incompletely understood. The interaction between immune dysregulation and somatic mutational profiles may contribute to disease heterogeneity and clinical outcomes.

### Aims

To investigate the distribution of ADs across myeloid diagnostic categories, characterize their associated mutational patterns, and explore their prognostic impact.

### Methods

We analyzed 107 patients with hematologic diagnoses categorized as acute leukemia (AL), myelofibrosis (MF), myeloproliferative neoplasms (MPN), and myelodysplastic syndromes (MDS). Patients were stratified by autoimmune status into no AD (AD1), rheumatologic AD (AD2), and non-rheumatologic AD (AD3). Mutational profiles were assessed and explored using hierarchical clustering heatmaps to evaluate associations with diagnosis and AD category. Survival analyses were performed using Kaplan–Meier estimates and multivariable Cox regression models adjusted for age, diagnosis, and selected mutations.

### Results

Overall, 75.7% of patients had no AD, 6.5% had rheumatologic AD, and 17.8% had non-rheumatologic AD. The distribution of AD differed across diagnostic groups, with rheumatologic AD more frequent in MF (18.5%) compared to MPN (4.2%), AL (2.8%), and MDS (0%). In patients ≥65 years, rheumatologic AD was markedly enriched in MF (50%), whereas this association was not observed in younger individuals. Mutational analysis revealed distinct patterns according to AD phenotype. Rheumatologic ADs were associated with a prominent epigenetic signature, particularly TET2 (86%) and ASXL1 (57%), defining an "epigenetic cluster," especially within MF. In contrast, non-rheumatologic ADs showed higher prevalence of spliceosome mutations, including U2AF1 (21%) and ZRSR2 (11%), and enrichment of SRSF2 in MDS. Patients without AD displayed no specific mutational enrichment. Hierarchical clustering confirmed the presence of distinct molecular subgroups shaped by the combination of autoimmune phenotype and hematologic diagnosis. Kaplan–Meier curves suggested qualitative survival differences among AD groups, but limited sample size precluded definitive conclusions. In multivariable Cox analysis, SRSF2 mutation emerged as an independent predictor of worse overall survival (OS: HR 5.50, 95% CI 1.24–24.38, p=0.025) and progression-free survival (PFS: HR 3.13, 95% CI 1.03–9.57, p=0.045). ASXL1 showed a trend toward adverse OS (HR 2.03, p=0.092), while KRAS was not independently associated with survival. Acute leukemia was associated with significantly worse PFS compared to MDS (HR 4.20, p=0.026)

**Copyright Information:** (Online) ISSN: 2572-9241
©2026 The Author(s). HemaSphere is published by John Wiley & Sons Ltd on behalf of European Hematology Association. This is an open access Abstract Book distributed under the Attribution-NonCommercial-NoDerivs (CC BY-NC-ND), which allows third parties to download the articles and share them with others as long as they credit the author and the Abstract Book, but they cannot change the content in any way or use them commercially.
**Abstract Book Citations:** Authors, Title, HemaSphere, 2026;10:(S1):pages. The abstract book DOIs can be found on the "Author Information" page.
**Disclaimer:** Articles published in the journal HemaSphere exclusively reflect the opinions of the authors. The authors are responsible for all content in their abstracts including accuracy of the facts, statements, citing resources, etc.

**Summary/Conclusion**

Autoimmune phenotypes in myeloid neoplasms are associated with distinct, non-random mutational patterns. Rheumatologic AD correlates with an epigenetic mutational signature, particularly in MF and in older patients, whereas non-rheumatologic AD is more frequently linked to spliceosome mutations. Although survival differences by AD phenotype remain exploratory, SRSF2 mutation independently predicts adverse outcome. These findings support a biologically meaningful interaction between immune dysregulation and the molecular architecture of myeloid neoplasms.

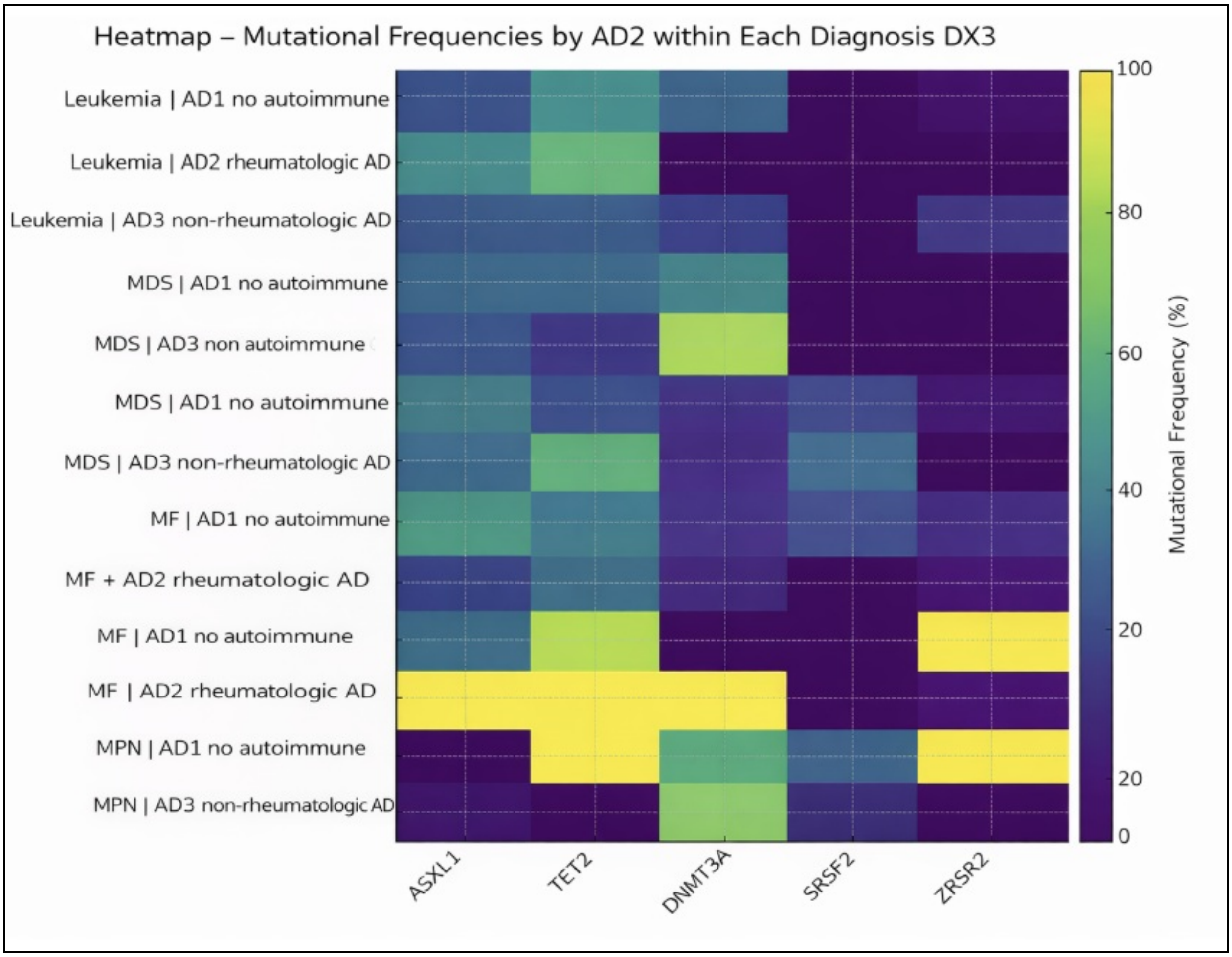

Figure 1. Heat map

**Copyright Information:** (Online) ISSN: 2572-9241
©2026 The Author(s). HemaSphere is published by John Wiley & Sons Ltd on behalf of European Hematology Association. This is an open access Abstract Book distributed under the Attribution-NonCommercial-NoDerivs (CC BY-NC-ND), which allows third parties to download the articles and share them with others as long as they credit the author and the Abstract Book, but they cannot change the content in any way or use them commercially.
**Abstract Book Citations:** Authors, Title, HemaSphere, 2026;10:(S1):pages. The abstract book DOIs can be found on the “Author Information” page.
**Disclaimer:** Articles published in the journal HemaSphere exclusively reflect the opinions of the authors. The authors are responsible for all content in their abstracts including accuracy of the facts, statements, citing resources, etc.

### (PB3392) GENOME-WIDE CRISPR-CAS9 SCREENING IDENTIFIES NOVEL MEDIATORS OF RUXOLITINIB RESISTANCE IN MYELOPROLIFERATIVE NEOPLASMS

**Topic:** 15. Myeloproliferative neoplasms - Biology & translational research

Qingmei Han[1], Rumeng Li[1], Bingbing Wen[2], Xiaoyan MA[1], Jia Li[1], Tian Zeng[1], Zhikang Zheng[3], Xiaojing Bao[3], Jian Huang*[1]

*[1]The First Affiliated Hospital, Zhejiang University School of Medicine, Hangzhou, China;[2]The second hospital of shenzhen, Shenzhen, China; [3]The Fourth Affiliated Hospital, Zhejiang University School of Medicine, Hangzhou, China;*

### Background

Philadelphia-negative myeloproliferative neoplasms (MPNs) are a group of diseases, including primary myelofibrosis (PMF), essential thrombocythemia (ET), and polycythemia vera (PV). Ruxolitinib, as a JAK1/2 inhibitor, can reduce splenomegaly and overproduction of blood cells. However, prolonged ruxolitinib treatment often leads to loss of therapeutic efficacy or intolerable side effects, ultimately resulting in treatment discontinuation in many patients. Genome-scale CRISPR-Cas9 screen has emerged as a robust, unbiased tool for discovering novel cancer vulnerabilities and therapeutic targets.

### Aims

In this study, we performed CRISPR-Cas9 knockout screening in vitro to identify genes whose loss was involved in resistance to ruxolitinib.

### Methods

We employed the genome-scale CRISPR-Cas9 knockout library and utilized the SET-2 cell line as a myeloproliferative neoplasm (MPN) model. The screening process involved several key steps. The first was GFP control virus infection and drug concentration tests,and next was the generation of stable Cas9-expressing SET-2 cells. After that, the gRNA library was introduced into stable Cas9-expressing SET-2 cells, followed by puromycin selection to enrich successfully infected cells, named as Human GeCKO SET-2. Human GeCKO SET-2 cells were treated with 150 nM ruxolitinib for twenty-one days. Finally, genomic DNA was extracted from surviving cells, and the gRNA sequences were amplified and analyzed via next-generation sequencing (NGS). Bioinformatic tools were used to identify enriched or depleted gRNAs, linking gene knockouts to phenotypic changes.

### Results

The screen identified 114 candidate genes with significantly enriched gRNAs (FDR < 0.25), implicating them in ruxolitinib resistance. Pathway enrichment analysis revealed strong associations with Gene Ontology terms related to translation, including cytoplasmic and mitochondrial translation. KEGG analysis further highlighted aminoacyl-tRNA biosynthesis, the proteasome, and RNA polymerase, suggesting a systemic dependence of resistant cells on gene expression and protein homeostasis. Functional characterization of the enriched genes uncovered a multi-layered adaptive network . Among the top candidates, IGF2BP3 (an RNA stability regulator), CAMTA1 (a transcription factor), and CDC34 (a ubiquitin-conjugating enzyme) pointed to enhanced transcriptional output, translational control, and protein stability as adaptive mechanisms. Enrichment of GSTM3 indicated involvement of metabolic detoxification and oxidative stress defense. Several neuron-associated genes, such as FAM71C, GPRIN2, and ABHD12B, were also strongly enriched, potentially reflecting activation of neurodevelopmental programs. Notably, these resistance-associated alterations appeared to operate independently of the canonical JAK-STAT pathway, suggesting a bypass mechanism driven by alternative survival pathways.

**Copyright Information:** (Online) ISSN: 2572-9241
©2026 The Author(s). HemaSphere is published by John Wiley & Sons Ltd on behalf of European Hematology Association. This is an open access Abstract Book distributed under the Attribution-NonCommercial-NoDerivs (CC BY-NC-ND), which allows third parties to download the articles and share them with others as long as they credit the author and the Abstract Book, but they cannot change the content in any way or use them commercially.
**Abstract Book Citations:** Authors, Title, HemaSphere, 2026;10:(S1):pages. The abstract book DOIs can be found on the "Author Information" page.
**Disclaimer:** Articles published in the journal HemaSphere exclusively reflect the opinions of the authors. The authors are responsible for all content in their abstracts including accuracy of the facts, statements, citing resources, etc.

**Summary/Conclusion**

This genome-wide CRISPR screen provides a comprehensive genetic landscape of ruxolitinib resistance in MPN cells and reveals a complex functional network underlying the resistant phenotype. These findings expand the understanding of resistance mechanisms and identify potential targets for combination therapeutic strategies in MPN.

This research was supported by the National Natural Science Foundation of China (No. 82570191) and the Zhejiang Provincial Health High level Innovative Talent Project (2022-2026).

*Correspondence to: Prof Jian Huang, E-mail: househuang@zju.edu.cn.

**Copyright Information:** (Online) ISSN: 2572-9241
©2026 The Author(s). HemaSphere is published by John Wiley & Sons Ltd on behalf of European Hematology Association. This is an open access Abstract Book distributed under the Attribution-NonCommercial-NoDerivs (CC BY-NC-ND), which allows third parties to download the articles and share them with others as long as they credit the author and the Abstract Book, but they cannot change the content in any way or use them commercially.
**Abstract Book Citations:** Authors, Title, HemaSphere, 2026;10:(S1):pages. The abstract book DOIs can be found on the "Author Information" page.
**Disclaimer:** Articles published in the journal HemaSphere exclusively reflect the opinions of the authors. The authors are responsible for all content in their abstracts including accuracy of the facts, statements, citing resources, etc.

### (PB3393) ELUCIDATING THE IMPACTS OF TYROSINE KINASE INHIBITORS ON KIT SIGNALLING IN A SYSTEMIC MASTOCYTOSIS CELL LINE

**Topic:** 15. Myeloproliferative neoplasms - Biology & translational research

Ben Weekes*[1], Darrin McKenzie[1], Graeme Greenfield[2], Bobby Graham[1], Ciaren Graham[1]

*[1]Queens University Belfast, School of Biological Sciences, Belfast, United Kingdom;[2]Queens University Belfast, Patrick G. Johnston Centre for Cancer Research, Belfast, United Kingdom;*

### Background

Mastocytosis is a rare clonal haematological malignancy characterised by the over proliferation of neoplastic mast cells throughout the body. The disease is classified by the World Health Organisation (WHO) into cutaneous mastocytosis (CM) and systemic mastocytosis (SM), with the latter further sub-divided into indolent (ISM) or advanced systemic mastocytosis (AdvSM).

A gain of function *KIT D816V* mutation is observed in ~80-90% of SM patients, resulting in constitutive activation of KIT signalling. Despite similar allele variant frequencies of *KIT D816V* in ISM and AdvSM, these subtypes differ markedly in prognosis and life expectancy, with ISM patients having a normal overall survival rate whilst AdvSM patients survive 2-4 years from time of diagnosis, despite advancements in treatment through the introduction of targeted tyrosine kinase inhibitors (TKI) such as Avapritinib. This suggests an unmet need for deeper understanding of KIT signalling and how it influences mastocytosis aetiology as well as new potential therapeutic options.

### Aims

This study aims to address the gap in knowledge of KIT signalling in mastocytosis, with hopes that a deeper understanding of molecular mechanisms involved will allow for more effective treatment options for patients.

### Methods

To address this gap, this study employed a quantitative proteomics and molecular biology-based approach to investigate KIT-driven signalling  making use of the SM mast cell lines HMC1.1 and HMC1.2, the latter of which harbours a *KIT D816V* mutation. Cells were treated with Avapritinib (1µM ,24 hours), followed by sodium deoxycholate (SDC) based cell lysis, automated protein aggregation capture (PAC) on bead digestion via an OpenTrons OT-2, a technique never before implemented in mastocytosis research.  Samples were analysed using DIA-PASEF on a timsTOF-HT mass spectrometer coupled to an Evosep ONE system. Data were processed using Spectronaut (v20) with a 1% FDR, and differentially expressed proteins defined by $p \leq 0.05$ and log2FC ≥ 0.58.

### Results

Proteomic profiling revealed over 8000 proteins identified in both cell lines, with a greater degree of signalling dysregulation in*KIT D816V* mutant cells with 14% of the HMC1.2 proteome significantly altered compared to 7% in HMC1.1 cells. Notably, dysregulated proteins in HMC1.2 cells included cyclin dependent kinases and histone modifying proteins, implicating epigenetic regulation as a potential driver of neoplastic mast cell proliferation. Functional validation supported a role for histone modifying proteins in driving neoplastic mast cell proliferation. Furthermore, treatment of both cell lines with a histone demethylase (KDM) PROTAC followed by  MTS assay analysis revealed sensitivity of *KIT D816V*-mutant HMC1.2cells, with an $IC_{50}$ of 2.42µM following 72 hour treatment, highlighting epigenetic regulation as a potential therapeutic option. Ongoing work will explore whether combined targeting of KIT signalling and histone modifying proteins enhances therapeutic efficacy in neoplastic mast cells.

**Copyright Information:** (Online) ISSN: 2572-9241
©2026 The Author(s). HemaSphere is published by John Wiley & Sons Ltd on behalf of European Hematology Association. This is an open access Abstract Book distributed under the Attribution-NonCommercial-NoDerivs (CC BY-NC-ND), which allows third parties to download the articles and share them with others as long as they credit the author and the Abstract Book, but they cannot change the content in any way or use them commercially.
**Abstract Book Citations:** Authors, Title, HemaSphere, 2026;10:(S1):pages. The abstract book DOIs can be found on the “Author Information” page.
**Disclaimer:** Articles published in the journal HemaSphere exclusively reflect the opinions of the authors. The authors are responsible for all content in their abstracts including accuracy of the facts, statements, citing resources, etc.

**Summary/Conclusion**

Collectively, these findings highlight novel signalling and epigenetic pathways associated with *KIT D816V*-driven mastocytosis, highlighting histone modifying proteins as potential therapeutic targets. This work supports the use of proteomics to uncover non-genetic drivers of disease progression and provides a foundation for the development of new treatment strategies for systemic mastocytosis.

**Copyright Information:** (Online) ISSN: 2572-9241
©2026 The Author(s). HemaSphere is published by John Wiley & Sons Ltd on behalf of European Hematology Association. This is an open access Abstract Book distributed under the Attribution-NonCommercial-NoDerivs (CC BY-NC-ND), which allows third parties to download the articles and share them with others as long as they credit the author and the Abstract Book, but they cannot change the content in any way or use them commercially.
**Abstract Book Citations:** Authors, Title, HemaSphere, 2026;10:(S1):pages. The abstract book DOIs can be found on the "Author Information" page.
**Disclaimer:** Articles published in the journal HemaSphere exclusively reflect the opinions of the authors. The authors are responsible for all content in their abstracts including accuracy of the facts, statements, citing resources, etc.

### (PB3394) OPTICAL GENOME MAPPING IN TRIPLE-NEGATIVE ESSENTIAL THROMBOCYTHEMIA

**Topic:** 15. Myeloproliferative neoplasms - Biology & translational research

Irene Botella Pérez*[1, 2], Marta Salido[3], Lierni Fernández Ibarrondo[1], Maria Alicia Senin Magan[1, 4], Patricia Velez Tenza[1, 4], María Rodríguez-Rivera[3], Barbara Ymaine[1, 3], Laura Camacho[1, 3], Concepción Fernández-Rodriguez[1, 3], Raquel Longarón[1, 3], Daniel Martinez[3], Andrea Sala Pages[1], Ramon Diez-Feijoo Varela[1, 2, 4], Anna Puiggros[3], Carlos Besses[1], Blanca Espinet[3], Beatriz Bellosillo[1, 2, 3]

*[1]Hospital del Mar Research Institute, Group of Applied Clinical Research in Hematology, Barcelona, Spain;[2]Pompeu Fabra University, Barcelona, Spain; [3]Hospital del Mar, Department of Pathology, Barcelona, Spain;[4]Hospital del Mar, Department of Hematology, Barcelona, Spain;*

**Background**

Myeloproliferative neoplasms (MPNs) are characterized by recurrent driver mutations (*JAK2*, *CALR*, or *MPL*). However, around 10-15% of patients with essential thrombocythemia (ET) lack driver mutations and are classified as triple-negative ET (TN-ET). These cases represent a diagnostic challenge and are generally associated with a lower thrombotic risk. Optical genome mapping (OGM) is a recently implemented cytogenetic technology based on the analysis of ultra–high molecular weight DNA (>250 kb) that allows genome-wide detection of structural variants with high resolution and sensitivity. By overcoming several limitations of conventional cytogenetic techniques, OGM has demonstrated its utility in myelofibrosis where conventional cytogenetics has a scarce diagnostic yield, and might be a promising approach to improve the genomic characterization of TN-ET.

**Aims**

To evaluate the applicability of OGM in patients with TN-ET to identify cytogenetic alterations including structural variants.

**Methods**

Peripheral blood (PB) samples of 19 patients diagnosed with TN-ET were collected. Samples were processed for OGM according to the manufacturer's protocol (Bionano, San Diego, USA), achieving an average genome coverage of approximately 300x. Structural variants (5kb) and regions of homozygosity (≥20Mb) were analyzed with Bionano Access software and filtering using Rare Variant Analysis v1.8 and *De Novo* Assembly pipelines. Moreover, variants were reviewed in Variant Intelligence Application (VIA) (Bionano Genomics). In parallel, molecular characterization was performed in all patients using next-generation sequencing (NGS). Libraries were prepared using custom QIAseq DNA panels (QIAGEN) including myeloid-associated genes and sequenced on Illumina technology.

**Results**

The median age was 52 years, and 78.9% of patients were females. The cytogenetic study using OGM was informative in 18/19 cases. A normal cytogenetic profile, with no clinically relevant structural variants, was observed in the whole cohort. Analysis by NGS showed somatic gene mutations in 7/19 patients (36.8%), with a total of 12 variants identified, involving epigenetic regulation (*TET2* and *ASXL1*), cell signaling (*CBL* and *SH2B3*) and DNA damage repair (*PPM1D* and *TP53*). During the disease follow-up, progression to acute myeloid leukemia (AML) was observed in the patient who carried a *TP53* mutation (Figure 1).

**Copyright Information:** (Online) ISSN: 2572-9241
©2026 The Author(s). HemaSphere is published by John Wiley & Sons Ltd on behalf of European Hematology Association. This is an open access Abstract Book distributed under the Attribution-NonCommercial-NoDerivs (CC BY-NC-ND), which allows third parties to download the articles and share them with others as long as they credit the author and the Abstract Book, but they cannot change the content in any way or use them commercially.
**Abstract Book Citations:** Authors, Title, HemaSphere, 2026;10:(S1):pages. The abstract book DOIs can be found on the "Author Information" page.
**Disclaimer:** Articles published in the journal HemaSphere exclusively reflect the opinions of the authors. The authors are responsible for all content in their abstracts including accuracy of the facts, statements, citing resources, etc.

### Summary/Conclusion

This study provides a preliminary assessment of OGM in a cohort of TN-ET patients. Despite the high resolution and demonstrated utility of OGM in other myeloid disorders, including myelofibrosis, no additional clinically relevant alterations were identified that could improve risk stratification or patient management in this cohort. Molecular characterization demonstrated clonal findings in more than one third of the cases. These results highlight the marked biological heterogeneity of this entity and the current limitations of available diagnostic tools, underscoring the need for novel approaches to improve disease characterization and clinical management.

**Acknowledgments.** Instituto de Salud Carlos III (ISCIII), PI19/0005, PI23/00626, co-funded by the European Union; 2021SGR628, PT20/00023, and Xarxa de Banc de Tumors de Catalunya.

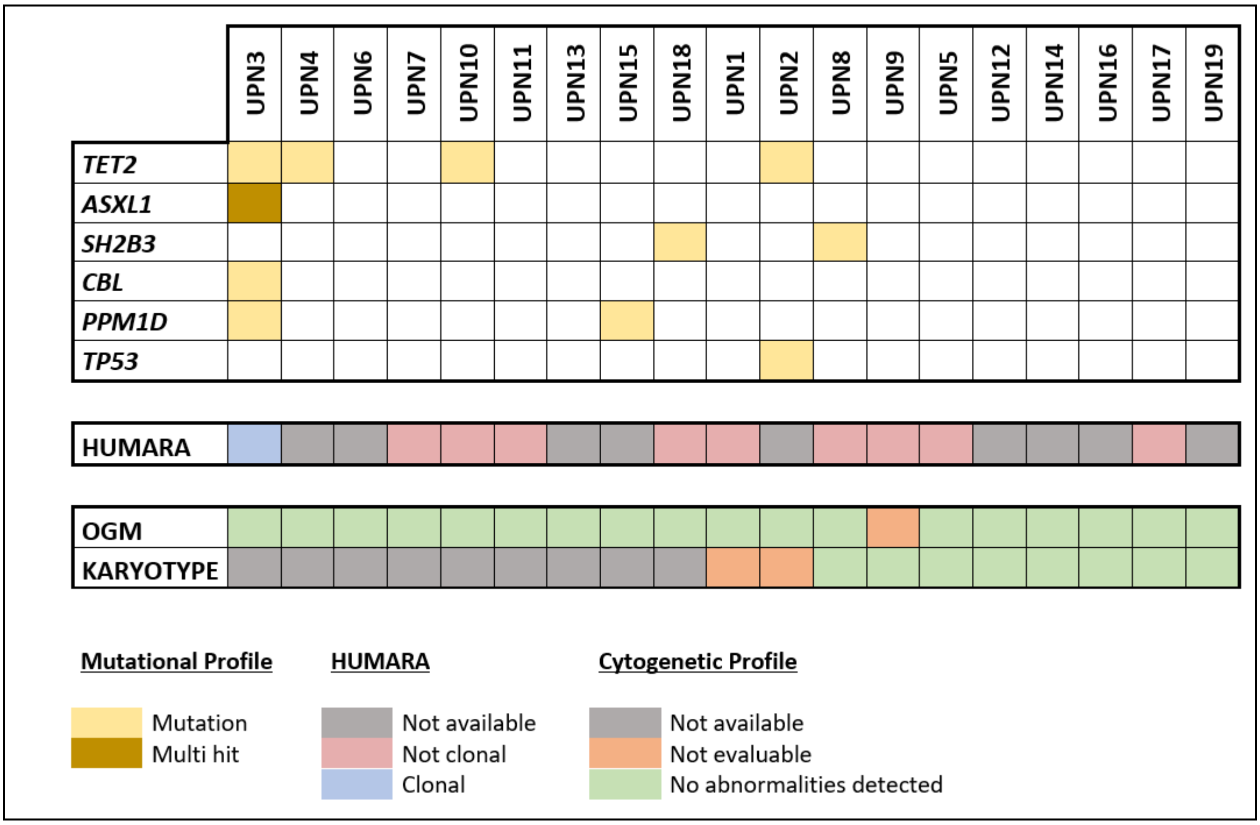

Figure 1. Integrated NGS and cytogenetic profiling of 19 patients with TN-ET.

**Copyright Information:** (Online) ISSN: 2572-9241
©2026 The Author(s). HemaSphere is published by John Wiley & Sons Ltd on behalf of European Hematology Association. This is an open access Abstract Book distributed under the Attribution-NonCommercial-NoDerivs (CC BY-NC-ND), which allows third parties to download the articles and share them with others as long as they credit the author and the Abstract Book, but they cannot change the content in any way or use them commercially.
**Abstract Book Citations:** Authors, Title, HemaSphere, 2026;10:(S1):pages. The abstract book DOIs can be found on the "Author Information" page.
**Disclaimer:** Articles published in the journal HemaSphere exclusively reflect the opinions of the authors. The authors are responsible for all content in their abstracts including accuracy of the facts, statements, citing resources, etc.

### (PB3395) EXOSOMAL MIR-146A, MIR-155 AND MIR-451A DYNAMICS DURING INTERFERON-ALPHA THERAPY IN PHILADELPHIA-NEGATIVE MYELOPROLIFERATIVE NEOPLASMS

**Topic:** 15. Myeloproliferative neoplasms - Biology & translational research

Rodolfo Ortiz-Flores*[1], Julio Torres González[1], Alejandro Escamilla Sanchez[1, 2], Regina García Delgado[1, 3]

*[1]Instituto de Investigaciones Biomédicas de Málaga (IBIMA-Plataforma Bionand) - Hospital Universitario Virgen de la Victoria, IBIMA-BE21: Hematología e Inmunoterapia, Málaga, Spain; [2]Universidad de Málaga, Departamento de Fisiología Humana, Histología Humana, Anatomía Patológica y Educación Físico-Deportiva, Facultad de Medicina, Malaga, Spain; [3]Hospital Universitario Virgen de la Victoria, Departamento de Hematología y Hemoterapia, Malaga, Spain;*

### Background

Interferon-alpha (IFN-α) is a disease-modifying therapy in Philadelphia-negative myeloproliferative neoplasms (MPN), exerting immunomodulatory and potential anticlonal effects. However, circulating molecular correlates of IFN exposure remain insufficiently characterized. Exosomal microRNAs (miRNAs) regulate inflammatory and hematopoietic signaling pathways and may reflect treatment-associated immune remodeling.

### Aims

To evaluate whether IFN-α exposure duration in patients with MPN is associated with dynamic changes in exosomal inflammation-related miRNAs.

### Methods

Plasma samples from 24 MPN patients treated with IFN-α were stratified by treatment duration (<6 months, n=10; 6–24 months, n=5; >24 months, n=9). Exosomal miRNAs were isolated using standardized protocols and quantified by double-normalized RT-qPCR. A predefined panel including miR-146a, miR-155, miR-451a, miR-223, miR-150, miR-29a, miR-210, miR-34a, miR-126 and miR-221 was analyzed. Comparisons across exposure strata were exploratory and non-parametric.

### Results

All selected exosomal miRNAs were consistently detectable across treatment groups. Progressive modulation of miR-146a and miR-155 expression was observed according to IFN exposure duration, with a concordant directional pattern in miR-451a. Patients treated for >24 months displayed distinct expression profiles compared with early exposure (<6 months), suggesting treatment-duration–dependent immune adaptation. In contrast, miR-223 showed an early decrease between the <6-month and 6–24-month groups followed by stabilization in long-term exposure, supporting a biphasic treatment-associated pattern.

### Summary/Conclusion

Exosomal inflammation-related miRNAs demonstrate dynamic modulation during IFN-α therapy in MPN. The coordinated patterns observed for miR-146a, miR-155 and miR-451a suggest progressive immune remodeling with sustained treatment, while miR-223 may reflect early adaptation. These preliminary translational findings warrant validation in longitudinal and mechanistic studies.

**Copyright Information:** (Online) ISSN: 2572-9241
©2026 The Author(s). HemaSphere is published by John Wiley & Sons Ltd on behalf of European Hematology Association. This is an open access Abstract Book distributed under the Attribution-NonCommercial-NoDerivs (CC BY-NC-ND), which allows third parties to download the articles and share them with others as long as they credit the author and the Abstract Book, but they cannot change the content in any way or use them commercially.
**Abstract Book Citations:** Authors, Title, HemaSphere, 2026;10:(S1):pages. The abstract book DOIs can be found on the “Author Information” page.
**Disclaimer:** Articles published in the journal HemaSphere exclusively reflect the opinions of the authors. The authors are responsible for all content in their abstracts including accuracy of the facts, statements, citing resources, etc.

### (PB3396) THE SOMATIC MUTATIONAL LANDSCAPE OF MYELOFIBROSIS WITH OR WITHOUT CYTOPENIA

**Topic:** 15. Myeloproliferative neoplasms - Biology & translational research

Fengling Liu[1], Leyu Wang[1], Qi Wang[1], Bing Han*[1]

[1]*Peking Union Medical College Hospital, Chinese Academy of Medical Sciences., Beijing, China;*

**Background**

Cytopenia represents a clinically challenging phenotype in myelofibrosis (MF), associated with inferior survival and therapeutic resistance. However, the somatic and immunogenetic features underlying cytopenia remain poorly defined.

**Aims**

This study aimed to investigate the associations between somatic mutational profiles, HLA diversity, and cytopenic severity in MF.

**Methods**

Whole-exome sequencing was performed in MF patients. High-resolution HLA typing was inferred using the HLA-HD algorithm. Patients were stratified into non-cytopenic and cytopenic groups, with the latter further subdivided into Group 1 (isolated anemia) and Group 2 (non-isolated anemia).

**Results**

The non-cytopenic group was primarily characterized by enrichment of cell cycle-related pathways, with lower involvement of hematopoietic regulatory, oncogenic signaling, and inflammatory processes compared with the cytopenic group. Group 1 was predominantly defined by epigenetic regulatory and showed limited features of genomic instability with immune dysregulation relative to Group 2. The non-cytopenic group demonstrated a lower *JAK2* V617F variant allele frequency and absence of MPL mutations. It was further characterized by reduced involvement of alterations affecting RNA splicing, DNA methylation, chromatin modification, and signaling pathways compared with cytopenic group. Group 1 showed higher *JAK2* clonal burden and enrichment of chromatin modifier alterations, while demonstrating limited involvement of signaling pathway and DNA methylation-related mutations relative to Group 2. The non-cytopenic group presented a significantly lower frequency of *HLA-A**03:01 than the cytopenic group, whereas Group 1 showed lower frequencies of *HLA-C**06:02 and *HLA-DPB1**04:02 compared with Group 2. The non-cytopenic group lacked significant co-occurrence between *HLA-A**03:01 and *SF3B1* and between *HLA-A**30:01 and *ZRSR2*, which were observed in the cytopenic group and in Group 2.

**Summary/Conclusion**

Cytopenia in MF defines a biologically distinct state characterized by altered mutational landscape, inflammatory and oncogenic pathway dysregulation, and specific HLA susceptibility alleles. The co-occurrence between HLA risk alleles and somatic mutations suggests a potential interaction between immunogenetic background and clonal evolution.

**Copyright Information:** (Online) ISSN: 2572-9241
©2026 The Author(s). HemaSphere is published by John Wiley & Sons Ltd on behalf of European Hematology Association. This is an open access Abstract Book distributed under the Attribution-NonCommercial-NoDerivs (CC BY-NC-ND), which allows third parties to download the articles and share them with others as long as they credit the author and the Abstract Book, but they cannot change the content in any way or use them commercially.
**Abstract Book Citations:** Authors, Title, HemaSphere, 2026;10:(S1):pages. The abstract book DOIs can be found on the “Author Information” page.
**Disclaimer:** Articles published in the journal HemaSphere exclusively reflect the opinions of the authors. The authors are responsible for all content in their abstracts including accuracy of the facts, statements, citing resources, etc.

### (PB3397) EXPOSURE TO AIR POLLUTANTS CORRELATES WITH AN INCREASED INCIDENCE OF MYELOPROLIFERATIVE NEOPLASMS: A POPULATION-BASED STUDY

**Topic:** 15. Myeloproliferative neoplasms - Biology & translational research

Alessandro Bosi*[1], Emanuela Carloni[2], Ilaria Cozzi[2], Matilde Zijno[2], Enrica Santelli[2], Massimo Stafoggia[2], Paola Michelozzi[2], Daniela D'ippoliti[2], Pier Mannuccio Mannucci[1, 3], Francesco Passamonti[1, 3]

*[1]Fondazione IRCCS Ca' Granda Ospedale Maggiore Policlinico, Hematology Unit, Milan, Italy; [2]Department of Epidemiology, Lazio Region Health Service / ASL Roma 1, Rome, Italy; [3]University of Milan, Milan, Italy;*

### Background

Exposure to air pollutants is associated with an increased risk of any-cause and lung cancer mortality. Association with hematological malignancies (HM) is less clear. Although incidence of multiple myeloma and non-Hodgkin's lymphomas seems unrelated, a recent study potentially linked exposure to $PM_{2.5}$, $NO_3$ or black carbon (BC) to incidence or severity of myelodysplastic neoplasms (MDS). Furthermore, when analyzing subtypes more granularly, incidence of mantle cell lymphoma, Hodgkin's lymphoma and marginal zone lymphoma may emerge to correlate as well. The association with other malignancies, including myeloproliferative neoplasms (MPN), remains under investigated.

### Aims

This study aims to investigate the association between both conventional and emerging air pollutants with incidence of HM using data from the Lazio Cancer Registry. This study was funded by Fondazione Umberto Veronesi.

### Methods

All residents aged ≥30 years in the municipality of Rome (as by census) were evaluated for new diagnosis of an HM (ICD-10: C81-C86, C88-C90-C92, C94-C96) between 2013 and 2019, as by the Lazio Cancer Registry. All prevalent cases of any tumor diagnosis, except for non-melanoma skin cancer, were excluded due to potentially higher risk of HM after chemo/radiotherapy.
The annual concentrations of particular matter ($PM_{2.5}$), nitrogen dioxide ($NO_2$), BC and ultra fine particles (UFP) were obtained through land use regression models and were geocoded at baseline individuals addresses. Hazard ratios (HR) were calculated for each pollutant interquartile range (IQR) using Cox regression model, adjusting for individual and area-level covariates.

**Copyright Information:** (Online) ISSN: 2572-9241
©2026 The Author(s). HemaSphere is published by John Wiley & Sons Ltd on behalf of European Hematology Association. This is an open access Abstract Book distributed under the Attribution-NonCommercial-NoDerivs (CC BY-NC-ND), which allows third parties to download the articles and share them with others as long as they credit the author and the Abstract Book, but they cannot change the content in any way or use them commercially.
**Abstract Book Citations:** Authors, Title, HemaSphere, 2026;10:(S1):pages. The abstract book DOIs can be found on the "Author Information" page.
**Disclaimer:** Articles published in the journal HemaSphere exclusively reflect the opinions of the authors. The authors are responsible for all content in their abstracts including accuracy of the facts, statements, citing resources, etc.

### Results

The cohort consisted of 1,629,126 subjects, 7,780 (0.48%) of which developed a new HM in the seven-year observation time, resulting in a incidence rate (i) of 477.6 new cases/100.000 persons. We identified 1799 leukemias (i 110.4): 92 were chronic myeloid leukemia (CML, i 5.6), 1,007 were acute leukemias (i 61.8), and 390 were chronic lymphocytic leukemia (i 23.9). We also identified 1392 multiple myelomas (i 85.4), 377 MDSs (i 23.1), 595 MPNs (i 36.5) and 3,531 lymphomas (i 216.7), 336 of which were Hodgkin's (i 20.6). The map of estimated exposure to $PM_{2.5}$ is shown in Figure 1 (left-hand side)

After adjusting for covariates, a significantly higher hazard ratio (HR) for developing MPNs emerged for higher exposure to ultra fine particles (UFP; HR 1.13, $CI_{95\%}$ 1.01-1.26, for IQR increment of 2673.7 #/$cm^3$) and the PM components Nickel (HR 1.15, $CI_{95\%}$ 1.02 -1.29, for IQR increment of 0.5 ng/$m^3$), Sulfur (HR 1.20, $CI_{95\%}$ 1.05 -1.36, for IQR increment of 88.3 ng/$m^3$) and Zinc (HR 1.17, $CI_{95\%}$ 1.05 -1.31, for IQR increment of 5.4 ng/$m^3$). Moreover, for a 0.7 ng/$m^3$ IQR increment of vanadium, we estimated a statistically significant HR of 1.30 ($CI_{95\%}$ 1.03 - 1.64) to develop CML. No other significant associations were found between the evaluated pollutants and HM. HRs for developing MPN by increasing exposure to pollutants are shown in fFigure 1 (right-hand side).

### Summary/Conclusion

Increasing exposure to traffic-related air pollutants seems to correlate with an increased incidence of MPNs in adult popolation. Further studies are needed to confirm this finding and explore its pathogenetic underpinnings.

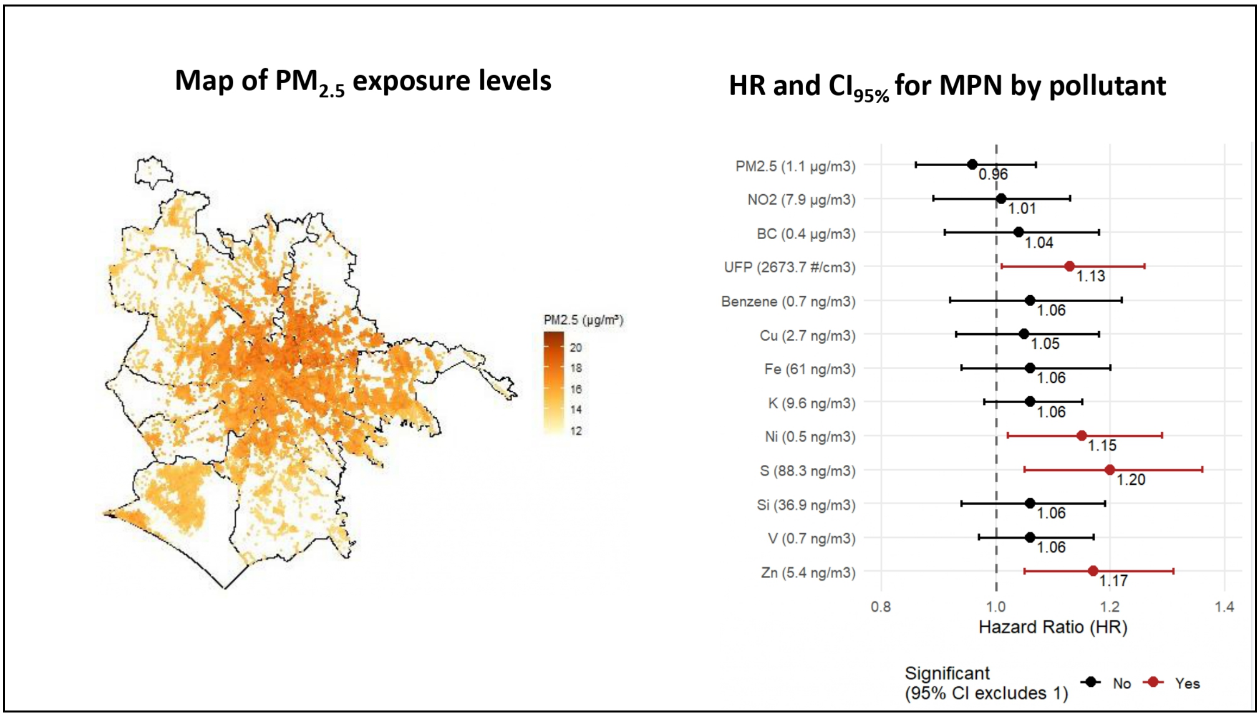

Figure 1: map of exposure to PM2.5 (on the left) and HRs for developing MPN by pollutant (on the right)

Copyright Information: (Online) ISSN: 2572-9241
©2026 The Author(s). HemaSphere is published by John Wiley & Sons Ltd on behalf of European Hematology Association. This is an open access Abstract Book distributed under the Attribution-NonCommercial-NoDerivs (CC BY-NC-ND), which allows third parties to download the articles and share them with others as long as they credit the author and the Abstract Book, but they cannot change the content in any way or use them commercially.
Abstract Book Citations: Authors, Title, HemaSphere, 2026;10:(S1):pages. The abstract book DOIs can be found on the "Author Information" page.
Disclaimer: Articles published in the journal HemaSphere exclusively reflect the opinions of the authors. The authors are responsible for all content in their abstracts including accuracy of the facts, statements, citing resources, etc.

### (PB3398) A MODERN THROMBOSIS RISK MODEL FOR MYELOPROLIFERATIVE NEOPLASMS: INTEGRATING INFLAMMATION, MOLECULAR MARKERS, AND SPLENIC BIOLOGY IN SINGLE CENTER REAL-WORLD COHORT.

**Topic:** 15. Myeloproliferative neoplasms - Biology & translational research

Noha Eisa[1, 2], Nouran Alhashimi*[1], Mohammed Almakadi[3], X Abdulaziz Khiyami[4], Dalal Aldhafeeri[5], Ali Alahmadi[3], Naif Aljohani[1]

*[1]King Faisal Specialist Hospital and research center, Jeddah, Saudi Arabia, Department of Oncology, Jeddah, Saudi Arabia; [2]Oncology Center-Mansoura University, Internal medicine, Mansoura, Egypt; [3]King Faisal Specialist Hospital and research center, Madinah, Department of Oncology, Madinah, Saudi Arabia; [4]King Faisal Specialist Hospital & Research Centre, Radiology department, Jeddah, Saudi Arabia; [5]King Faisal Specialist Hospital and research center, Madinah, Madinah, Saudi Arabia;*

### Background

Thrombosis remains the leading cause of morbidity and mortality in patients with myeloproliferative neoplasms (MPNs), yet current risk models such as r-IPSET-T rely mainly on age, prior thrombosis, and JAK2 status without incorporating inflammatory, splenic, or broader molecular features.

### Aims

To evaluate the clinical, inflammatory, molecular, and splenic predictors of thrombosis in a real-world MPN cohort and develop a modern thrombosis risk framework for identifying high-risk patients

### Methods

In this retrospective real-world cohort of 83 patients with ET, PV, MF (including pre- and post-PV MF), or MPN-unclassifiable, we evaluated clinical, laboratory, molecular, and splenic predictors of thrombosis and explored a modernized risk framework.

### Results

Thrombosis occurred in 29% (24/83). On multivariable analysis, splenomegaly emerged as the strongest independent predictor (OR 6.4; 95% CI 1.5–26.6; p=0.013), while higher platelet count showed an inverse association (OR 0.71 per $100\times10^9$/L; p=0.023); age, sex, and JAK2 positivity were not significant. IPSS-PMF and DIPSS-Plus were not associated with thrombotic risk. In ET, all thrombotic events occurred in the high r-IPSET-T category, whereas in PV, MIPSS-PV demonstrated a stepwise gradient (0% low, 33% intermediate, 83% high). A conceptual composite model integrating splenomegaly, leukocytosis, JAK2 positivity, smoking, and ≥2 cardiovascular comorbidities generated distinct estimated risk strata (38% very low, 66% intermediate, 89% very high).

### Summary/Conclusion

Splenic involvement is a major independent predictor of thrombosis in this unselected MPN cohort. r-IPSET-T and MIPSS-PV aligned with thrombotic gradients, whereas MF survival scores did not. A simple biology-integrated framework incorporating splenomegaly, leukocytosis, and clinical factors may enhance thrombosis risk discrimination beyond traditional tools. Prospective validation is needed.

**Copyright Information:** (Online) ISSN: 2572-9241
©2026 The Author(s). HemaSphere is published by John Wiley & Sons Ltd on behalf of European Hematology Association. This is an open access Abstract Book distributed under the Attribution-NonCommercial-NoDerivs (CC BY-NC-ND), which allows third parties to download the articles and share them with others as long as they credit the author and the Abstract Book, but they cannot change the content in any way or use them commercially.
**Abstract Book Citations:** Authors, Title, HemaSphere, 2026;10:(S1):pages. The abstract book DOIs can be found on the "Author Information" page.
**Disclaimer:** Articles published in the journal HemaSphere exclusively reflect the opinions of the authors. The authors are responsible for all content in their abstracts including accuracy of the facts, statements, citing resources, etc.

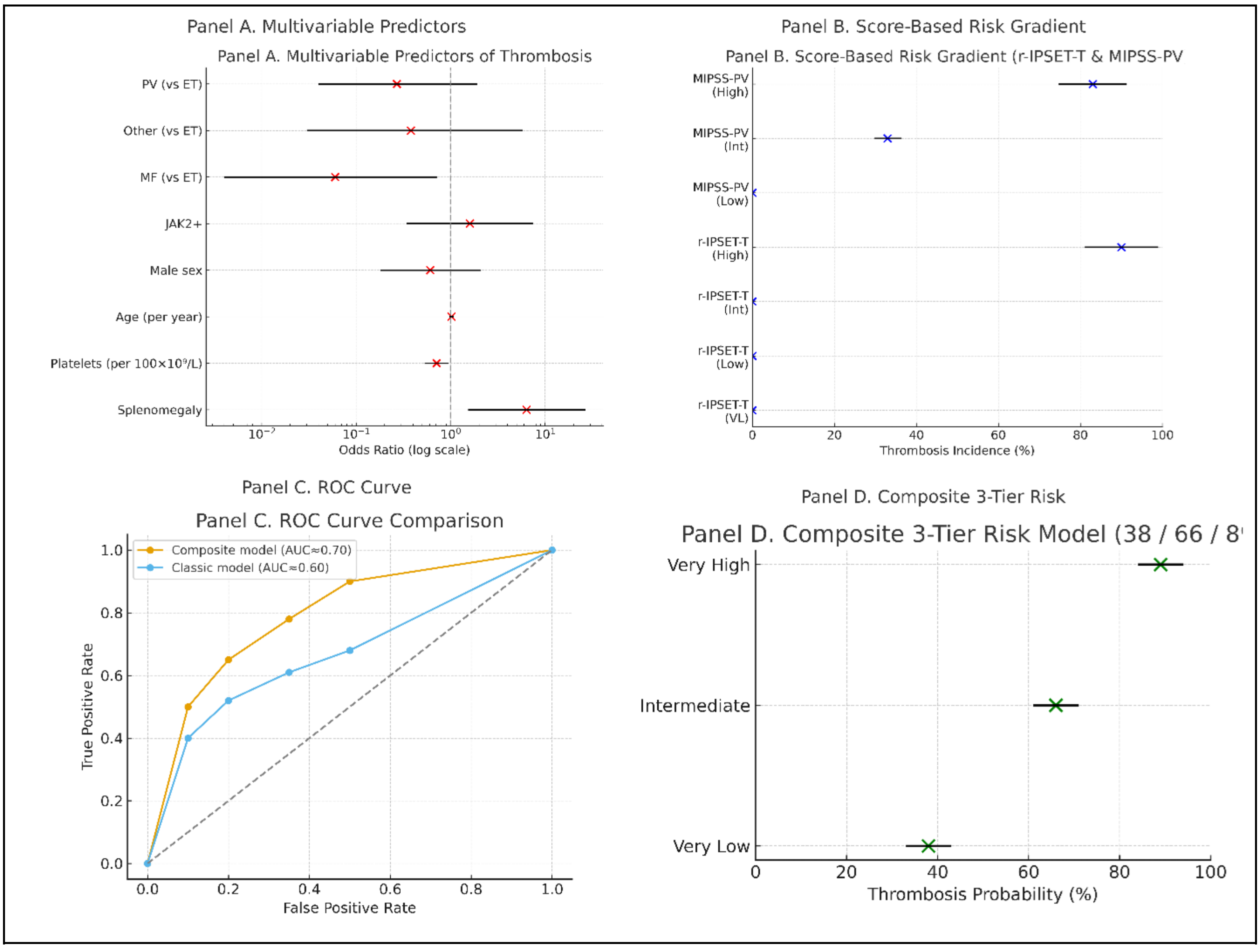

Panel A: Multivariable logistic regression showing splenomegaly and lower platelet count as independent predictors of thrombosis Panel B: Score-based gradients demonstrate sharp separation with r-IPSET-T and MIPSS-PV Panel C: ROC curves comparing composite vs. classic prediction models Panel D: Three-tier composite model showing stepwise thrombosis risk (38%, 66%, and 89%)

**Copyright Information:** (Online) ISSN: 2572-9241
©2026 The Author(s). HemaSphere is published by John Wiley & Sons Ltd on behalf of European Hematology Association. This is an open access Abstract Book distributed under the Attribution-NonCommercial-NoDerivs (CC BY-NC-ND), which allows third parties to download the articles and share them with others as long as they credit the author and the Abstract Book, but they cannot change the content in any way or use them commercially.
**Abstract Book Citations:** Authors, Title, HemaSphere, 2026;10:(S1):pages. The abstract book DOIs can be found on the "Author Information" page.
**Disclaimer:** Articles published in the journal HemaSphere exclusively reflect the opinions of the authors. The authors are responsible for all content in their abstracts including accuracy of the facts, statements, citing resources, etc.

### (PB3399) JAK2 V617F ALLELE BURDEN MATTERS FOR RISK OF VENOUS THROMBOSIS AND OVERALL SURVIVAL IN ESSENTIAL THROMBOCYTHEMIA

**Topic:** 15. Myeloproliferative neoplasms - Biology & translational research

Lurdes Zamora Plana[1], Anna Angona Figueras[2], Beatriz Cuevas[3], Manuel Pérez Encinas[4], Gonzalo Caballero Navarro[5], Francisca Ferrer Marin[6], Irene Pastor[7], Maria Alicia Senin Magan[8], Maria Teresa Gomez Casares[9], Gonzalo Carreño Gómez-Tarragona[10], Elena Magro Mazo[11], Raúl Pérez López[12], Eduardo Arellano Rodrigo[13], Juan Manuel Alonso Dominguez[14], Lucía Guerrero Fernández[15], Maria Isabel Mata Vazquez[16], Maria Laura Fox[17], Valentin Garcia Gutierrez[18], Maria Carmen Garcia Hernandez[19], Juan Carlos Hernandez Boluda[7], Blanca Xicoy Cirici[1], Alberto Alvarez Larran*[13, 20]

*[1]ICO-Hospital Universitari Germans Trias i Pujol - Josep Carreras Leukemia Research Institute, Badalona, Spain;[2]ICO Girona. IDIBGI. Josep Carreras Leukemia Research Institute, Girona, Spain; [3]Hospital Universitario de Burgos, Burgos, Spain;[4]Hospital Clínico Universitario de Santiago, Universidad de Santiago de Compostela., Santiago de Compostela, Spain; [5]Hospital Universitario Miguel Servet, Zaragoza, Spain; [6]Hospital Morales Messeguer. IIS-Fundación Jimenez Díaz. UCAM. CIBERER, Murcia, Spain; [7]Hospital Clínico de Valencia, Valencia, Spain; [8]Hospital del Mar-Barcelona, Barcelona, Spain;[9]Hospital Universitario de Gran Canaria Dr Negrín. Instituto de Investigacion Sanitaria de Canarias (IISC)., Las Palmas de Gran Canaria, Spain; [10]Hospital Universitario 12 de Octubre, Madrid, Spain; [11]Hospital Universitario Príncipe de Asturias, Alcalá de Henares, Spain; [12]Hospital Clínico Universitario Virgen de la Arrixaca, Murcia, Spain; [13]Hospital Clínic de Barcelona, Barcelona, Spain; [14]Hospital Universitario Fundación Jiménez Díaz, IIS-FJD, UAM, Madrid, Spain; [15]Complejo Asistencial de Palencia, Palencia, Spain; [16]Hospital Universitario Costa del Sol, Málaga, Spain; [17]Hospital Universitario Vall d'Hebron, Barcelona, Spain; [18]Hospital Ramón y Cajal. Instituto Ramón y Cajal de Investigación Sanitaria. Universidad de Alcalá, Madrid, Spain; [19]Hospital General de Alicante, Alicante, Spain; [20]On behalf of the MPN Spanish Group (GEMFIN)., Spain, Spain;*

### Background

Essential thrombocythemia (ET) is a myeloproliferative neoplasm (MPN) characterized by elevated platelet count, megakaryocytic hyperproliferation, and increased risk of thrombo-haemorrhagic complications. Risk stratification according to IPSET-thrombosis includes the presence of the *JAK2* V617F mutation without considering its variant allele frequency (VAF). Recent studies have identified a *JAK2* V617F VAF≥50% as an independent risk factor for venous thrombosis in MPN but this value is uncommon in ET.

### Aims

To study the impact of *JAK2* V617F allele burden on thrombotic risk and overall survival in *JAK2* V617F ET patients.

### Methods

By December 2024, 3074 patients with ET diagnosed after year 2000 were included in the Spanish Registry (*JAK2* V617F-mutated n=1963). ET diagnosis was performed according to WHO/ICC prevailing classification. A total of 905 patients with *JAK2* V617F VAF measured within the first year of diagnosis were selected for the present study. All participants provided written informed consent for inclusion in the registry. *JAK2* V617F was analysed in whole peripheral blood using Q-PCR with TaqMan probes. Allele burden was calculated as the percentage of mutant allele relative to total (mutant + wild type) *JAK2*. For centres where study was done in granulocytes, *JAK2* ratio was corrected by their percentage. To assess the impact of *JAK2* V617F allele burden, a 10% cut-off was established.

### Results

Main laboratory and clinical variables are summarized in **Table 1**. A total of 287 patients (32%) had *JAK2* V617F VAF <10%. Low

**Copyright Information:** (Online) ISSN: 2572-9241
©2026 The Author(s). HemaSphere is published by John Wiley & Sons Ltd on behalf of European Hematology Association. This is an open access Abstract Book distributed under the Attribution-NonCommercial-NoDerivs (CC BY-NC-ND), which allows third parties to download the articles and share them with others as long as they credit the author and the Abstract Book, but they cannot change the content in any way or use them commercially.
**Abstract Book Citations:** Authors, Title, HemaSphere, 2026;10:(S1):pages. The abstract book DOIs can be found on the "Author Information" page.
**Disclaimer:** Articles published in the journal HemaSphere exclusively reflect the opinions of the authors. The authors are responsible for all content in their abstracts including accuracy of the facts, statements, citing resources, etc.

*JAK2* V617F VAF (<10%) was associated with significant better overall survival (OS) compared with a high *JAK2* V617F VAF (≥10%) (**Figure 1A**). In multivariate analysis adjusted for sex, age, cardiovascular risk factors, and history of thrombosis, *JAK2* V617F ≥10% remained an independent predictor of OS (HR 2.63, 95%CI: 1.57-4.39, p<0.001). *JAK2* V617F ≥10% was significantly associated with a higher probability of venous thrombosis (**Figure 1B**). In patients without history of previous thrombosis (n=851), *JAK2* V617F <10% was associated with lower rate of venous thrombosis (**Figure 1C**). When *JAK2 V617F* is modelled as a continuous variable in patients without history of prior thrombosis, higher VAF was associated with increased risk of venous thrombosis adjusted by age: for every 1% increase in *JAK2* V617F VAF, the risk of venous thrombosis increased by approximately 2.1% (HR 1.021, 95% CI 1.003–1.04, p=0.02). There were no significant differences in the probability of arterial thrombosis according to *JAK2* V617F VAF.

**Table 1**. Main laboratory and clinical variables from *JAK2* V617F ET patients (n=905)

| **Variable** | **Value** |
|---|---|
| Age, years | 66.2 (52.5–75.6) |
| Female sex, n (%) | 580 (64.1) |
| CVRF, n (%)<br>Smoking, n (%)<br>Hypertension, n (%)<br>Diabetes mellitus, n (%) | 530 (63.5)<br>141 (16.6)<br>423 (49.8)<br>135 (15.8) |
| Haemoglobin (g/L) | 145.0 (137.0–154.0) |
| Haematocrit (%) | 44.0 (41.0–46.2) |
| Leukocytes (×10⁹/L) | 8.7 (7.3–10.5) |
| Platelets (×10⁹/L) | 635.5 (541.0–763.8) |
| Arterial thrombosis history (pre/at diagnosis), n (%) | 146 (16.1) |
| Venous thrombosis history (pre/at diagnosis), n (%) | 54 (6.0) |
| Arterial and/or venous thrombosis history (pre/at diagnosis), n (%) | 196 (21.7) |

**Copyright Information:** (Online) ISSN: 2572-9241
©2026 The Author(s). HemaSphere is published by John Wiley & Sons Ltd on behalf of European Hematology Association. This is an open access Abstract Book distributed under the Attribution-NonCommercial-NoDerivs (CC BY-NC-ND), which allows third parties to download the articles and share them with others as long as they credit the author and the Abstract Book, but they cannot change the content in any way or use them commercially.
**Abstract Book Citations:** Authors, Title, HemaSphere, 2026;10:(S1):pages. The abstract book DOIs can be found on the "Author Information" page.
**Disclaimer:** Articles published in the journal HemaSphere exclusively reflect the opinions of the authors. The authors are responsible for all content in their abstracts including accuracy of the facts, statements, citing resources, etc.

| | |
|---|---|
| Classical stratification<br>Low risk<br>High risk | <br>280 (30.9)<br>625 (69.1) |
| IPSET-t<br>Low<br>Intermediate<br>High | <br>—<br>153 (17.3)<br>729 (82.7) |
| IPSET-t revised<br>Very low<br>Low<br>Intermediate<br>High | <br>—<br>280 (30.9)<br>—<br>625 (69.1) |

Median follow-up: 3.85 years (0.1–22.8)

### Summary/Conclusion

*JAK2* V617F VAF <10% measured within the first year of diagnosis is associated with better outcome in patients with ET.

**Figure 1**. Survival (a), probability of venous thrombosis (b) and probability of venous thrombosis without previous venous thrombosis (c) in ET patients according to the *JAK2*V617F mutant allele burden

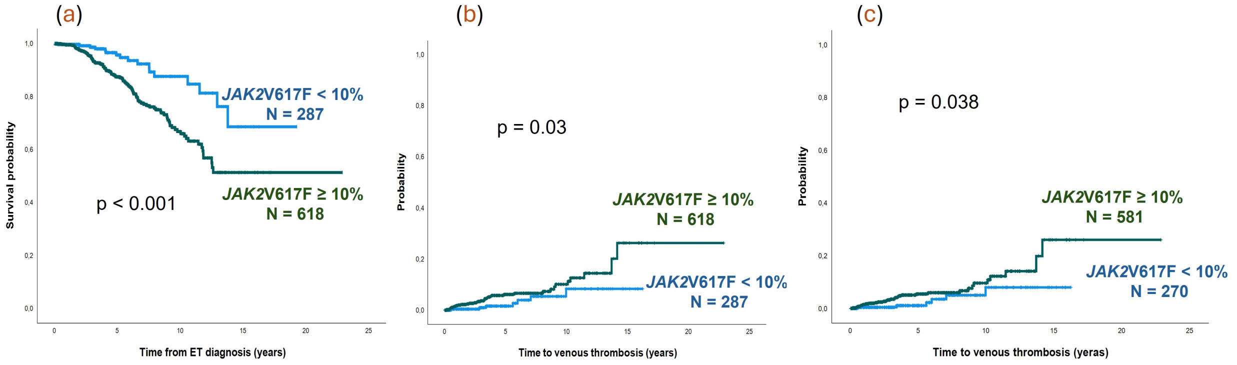

**Copyright Information:** (Online) ISSN: 2572-9241
©2026 The Author(s). HemaSphere is published by John Wiley & Sons Ltd on behalf of European Hematology Association. This is an open access Abstract Book distributed under the Attribution-NonCommercial-NoDerivs (CC BY-NC-ND), which allows third parties to download the articles and share them with others as long as they credit the author and the Abstract Book, but they cannot change the content in any way or use them commercially.
**Abstract Book Citations:** Authors, Title, HemaSphere, 2026;10:(S1):pages. The abstract book DOIs can be found on the "Author Information" page.
**Disclaimer:** Articles published in the journal HemaSphere exclusively reflect the opinions of the authors. The authors are responsible for all content in their abstracts including accuracy of the facts, statements, citing resources, etc.

### (PB3400) EARLY AND LONGITUDINAL CHANGES IN LYMPHOCYTE SUBPOPULATIONS IN MYELOFIBROSIS PATIENTS TREATED WITH MOMELOTINIB

**Topic:** 15. Myeloproliferative neoplasms - Biology & translational research

Novella Pugliese*[1], Vincenza Cerbone[2], Assunta Lombardi[1], Roberta Spisso[1], Annamaria Maddaloni[1], Fabrizio Pane[1]

*[1]University of Naples Federico II, Napoli, Italy;[2]CEINGE Biotwcnologie Avanzate, Napoli, Italy;*

### Background

Momelotinib, a JAK1/2 inhibitor, is used in myelofibrosis to control splenomegaly and anemia. Its impact on immune recovery remains poorly characterized.

### Aims

To assess immune reconstitution in myelofibrosis patients receiving momelotinib by evaluating longitudinal changes in lymphocyte subpopulations up to 3 months after treatment initiation.

### Methods

We conducted a prospective study in patients receiving momelotinib. Peripheral blood lymphocyte subpopulations were measured at baseline and at months 3, 6, 9, and 12 using flow cytometry. The following populations were quantified: total lymphocytes, $CD3^+$ T cells, $CD4^+$ T helper cells, $CD8^+$ cytotoxic T cells, $CD19^+$ B cells, $CD25^+CD4^+$ regulatory T cells (Tregs), $CD56^+$ NK cells, $CD3^+CD56^+$ NKT cells, HLA-$DR^+$ activated T cells, $CD3^+DR^+$ activated T cells, naïve ($CD45RA^+$) and memory ($CD45RO^+$) T subsets, including $CD3^+CD4^+CD45RA^+/CD45RO^+$ T helper and $CD3^+CD8^+CD45RA^+/CD45RO^+$ cytotoxic T cells, $CD57^+$ and $CD16^+$ cytotoxic cells, $CD11b^+$ and $CD11c^+$ myeloid-like populations, and additional subsets including $CD3^+CD29^+$, $CD29^+$, $CD7^+$, $CD2^+$, and $CD4^+/CD8^+$ ratios. Longitudinal changes were analyzed using linear mixed models (LMM - subject-specific effects) and generalized estimating equations (GEE - population-averaged effects) in patients with complete paired samples at baseline, month 3, and month 6. β coefficients and p-values were reported.

### Results

Data from 26 patients were analyzed (median age 66.6 years, 53.9% male; 2 received momelotinib as first-line therapy, 15 as second-line, and 9 as third-line). At month 3, significant increases were observed in $CD3^+CD29^+$ ($\beta = +399.8$, LMM $p = 0.0252$), $CD3^+CD56^+$ ($\beta = +34.99$, GEE $p = 0.0089$), and $CD29^+$ ($\beta = +506.3$, GEE $p = 0.0512$). By month 6, early markers $CD3^+CD29^+$and $CD29^+$ stabilized, whereas $CD3^+CD56^+$ ($\beta = +34.99$, LMM $p = 0.099$; GEE $p = 0.0089$), $CD3^+DR^+$ ($\beta = +39.56$, GEE $p = 0.0292$), HLA-$DR^+$ ($\beta = +35.59$, GEE $p = 0.00226$), and $CD3^+CD8^+CD45RA^+$ naïve cytotoxic T cells ($\beta = +56.54$, GEE $p = 0.0294$) showed significant increases. No significant longitudinal changes were observed in other lymphocyte subsets that remained relatively stable.

**Copyright Information:** (Online) ISSN: 2572-9241
©2026 The Author(s). HemaSphere is published by John Wiley & Sons Ltd on behalf of European Hematology Association. This is an open access Abstract Book distributed under the Attribution-NonCommercial-NoDerivs (CC BY-NC-ND), which allows third parties to download the articles and share them with others as long as they credit the author and the Abstract Book, but they cannot change the content in any way or use them commercially.
**Abstract Book Citations:** Authors, Title, HemaSphere, 2026;10:(S1):pages. The abstract book DOIs can be found on the "Author Information" page.
**Disclaimer:** Articles published in the journal HemaSphere exclusively reflect the opinions of the authors. The authors are responsible for all content in their abstracts including accuracy of the facts, statements, citing resources, etc.

### Summary/Conclusion

Momelotinib induces a biphasic immune modulation in myelofibrosis patients, with an early expansion of NKT-like and effector T cells followed by selective activation of naïve and activated cytotoxic T cells by month 6. These changes can be reliably monitored in peripheral blood and reflect targeted immune restoration without global immunosuppression. Population-averaged analyses (GEE) highlight consistent trends across patients despite inter-individual variability. Early modulation of NK-T cells, activated T cells, and naïve cytotoxic T cells may serve as biomarkers of immune recovery and guide patient monitoring. While these immunological changes suggest potential improvement in immune competence, their impact on infection risk requires evaluation during extended follow-up and dedicated clinical studies.

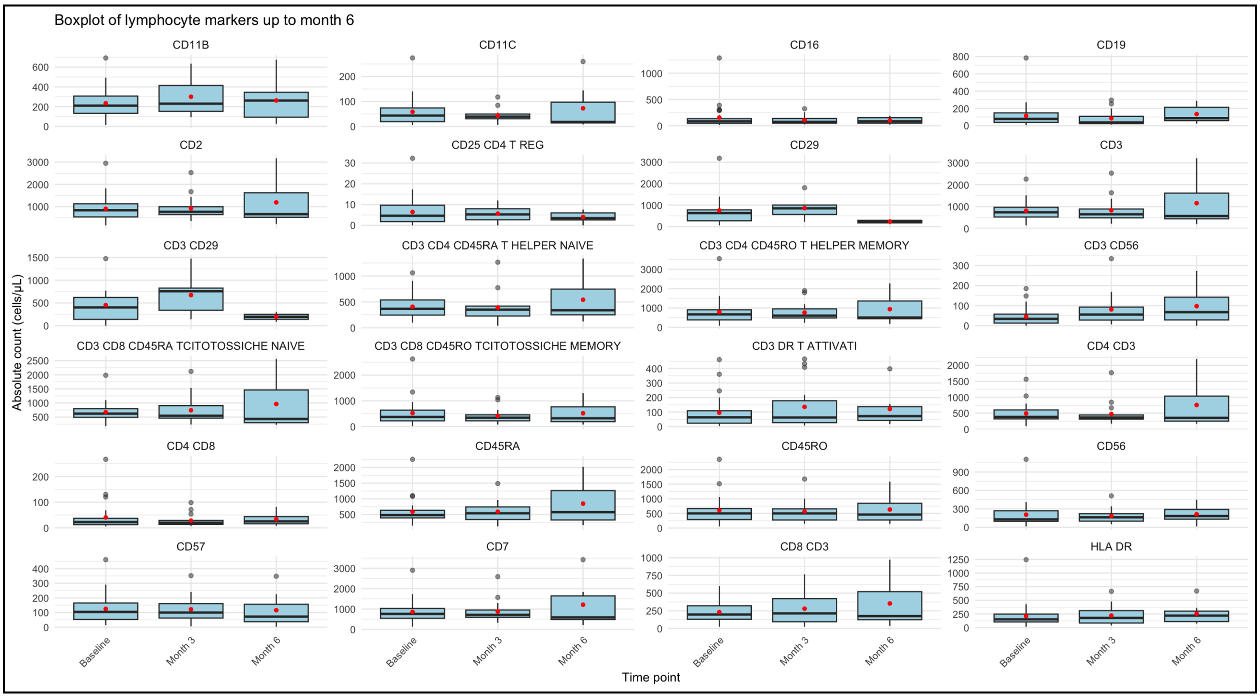

**Copyright Information:** (Online) ISSN: 2572-9241
©2026 The Author(s). HemaSphere is published by John Wiley & Sons Ltd on behalf of European Hematology Association. This is an open access Abstract Book distributed under the Attribution-NonCommercial-NoDerivs (CC BY-NC-ND), which allows third parties to download the articles and share them with others as long as they credit the author and the Abstract Book, but they cannot change the content in any way or use them commercially.
**Abstract Book Citations:** Authors, Title, HemaSphere, 2026;10:(S1):pages. The abstract book DOIs can be found on the "Author Information" page.
**Disclaimer:** Articles published in the journal HemaSphere exclusively reflect the opinions of the authors. The authors are responsible for all content in their abstracts including accuracy of the facts, statements, citing resources, etc.

### (PB3402) PLASMA PROTEOMIC SIGNATURES AT DIAGNOSIS IN MYELOPROLIFERATIVE NEOPLASMS FOR NOVEL DIAGNOSTIC BIOMARKERS

**Topic:** 15. Myeloproliferative neoplasms - Biology & translational research

Xiangfu Zhong*[1], Ingrid Lundahl[2], Axel Rosell[2], Kajsa Ax[2], Roza Chaireti[2], Johanna Ungerstedt[1]

*[1]Linköping University, Division of Surgery Orthopedics and Oncology, Department of Biomedical and Clinical Sciences, Linköping, Sweden; [2]Karolinska Institutet, Stockholm, Sweden;*

### Background

While thrombosis is frequent in Philadelphia-negative myeloproliferative neoplasms (MPN), biomarkers for predicting thrombosis, as well as biomarkers specific for MPN and MPN subtypes, e.g. discriminating pre-fibrotic myelofibrosis from ET, or triple negative ET from reactive thrombocytosis, remain an unmet medical need. To date, large-scale proteome analyses to bridge these gaps are lacking.

### Methods

| Variable | MPN (N = 55) | non MPN (N = 30) | p-value |
|---|---|---|---|
| AGE | 67 ± 13 | 56 ± 15 | 0.002 |
| GENDER | | | 0.011 |
| FEMALE | 36 (65%) | 11 (37%) | |
| MALE | 19 (35%) | 19 (63%) | |
| THROMBOSIS | 16 (29%) | 7 (23%) | 0.6 |
| MUTATIONS | | | |
| CALR | 7 (13%) | 1 (3.3%) | |
| JAK2 | 43 (78%) | 3 (10%) | |
| MPL | 3 (5.5%) | 0 (0%) | |
| TRIP.NEG. | 2 (3.6%) | 26 (86.7%) | |
| HEMOGLOBIN AT DISGNOSIS | 144 ± 16 | 157 ± 18 | <0.001 |
| PLATELET AT DISGNOSIS | 640 ± 228 | 352 ± 129 | < 0.001 |
| LEUKOCYTE AT DISGNOSIS | 8.94 ± 2.94 | 8.19 ± 2.50 | 0.2 |
| ANTICOAGULANT TREATMENT | 22 (40%) | 11 (37%) | 0.8 |

To address this, we collected plasma samples from patients referred to the Hematology clinic at Karolinska University Hospital in Stockholm, Sweden due to suspected MPN. Peripheral blood samples were collected in conjunction with the first outpatient clinic visit. In total, plasma samples from 55 newly diagnosed MPN patients, and 30 controls were analyzed by proximity extension assay using Olink Explore 384 Inflammation I and Cardiometabolic I panels, comprising a total of 734 unique proteins. Patient characteristics and blood counts are shown in Table 1. Female patients were over-represented in the MPN group due to a large proportion of ET patients. Of note, the frequency of history of thrombosis was similar in the groups. However, hemoglobin levels were significantly (p-value < 0.001) higher in the non MPN group (Table 1).

**Copyright Information:** (Online) ISSN: 2572-9241
©2026 The Author(s). HemaSphere is published by John Wiley & Sons Ltd on behalf of European Hematology Association. This is an open access Abstract Book distributed under the Attribution-NonCommercial-NoDerivs (CC BY-NC-ND), which allows third parties to download the articles and share them with others as long as they credit the author and the Abstract Book, but they cannot change the content in any way or use them commercially.
**Abstract Book Citations:** Authors, Title, HemaSphere, 2026;10:(S1):pages. The abstract book DOIs can be found on the "Author Information" page.
**Disclaimer:** Articles published in the journal HemaSphere exclusively reflect the opinions of the authors. The authors are responsible for all content in their abstracts including accuracy of the facts, statements, citing resources, etc.

### Results

Differential expression analysis identified 205 significantly altered plasma proteins, of which 189 were elevated in MPN compared to controls (Figure 1 A). The most strongly upregulated proteins in MPN included MMP1 and GP1BA, the latter previously linked to MPN risk. Functional enrichment analysis demonstrated a significant overrepresentation of Hemostasis and Platelet Activation pathways, highlighting dysregulated platelet biology in MPN.
Cox LASSO regression identified GP1BA and SORT1 as the strongest predictors of MPN disease (Figure 1 B). Increased levels of soluble transferrin receptor (TFRC, also known as CD71) and decreased levels of EPO strongly predicted PV and may be of pathobiological relevance as TFRC levels higher than those in PV are only found in iron deficiency anemia.

### Summary/Conclusion

In conclusion, proteomic patterns identified significantly altered proteins enriched in Hemostasis and Platelet Activation pathways. Elevated levels of GP1BA and SORT1 were strong predictors of MPN, and TFRC and EPO were strong predictors of PV, suggesting a potential as novel disease biomarkers.

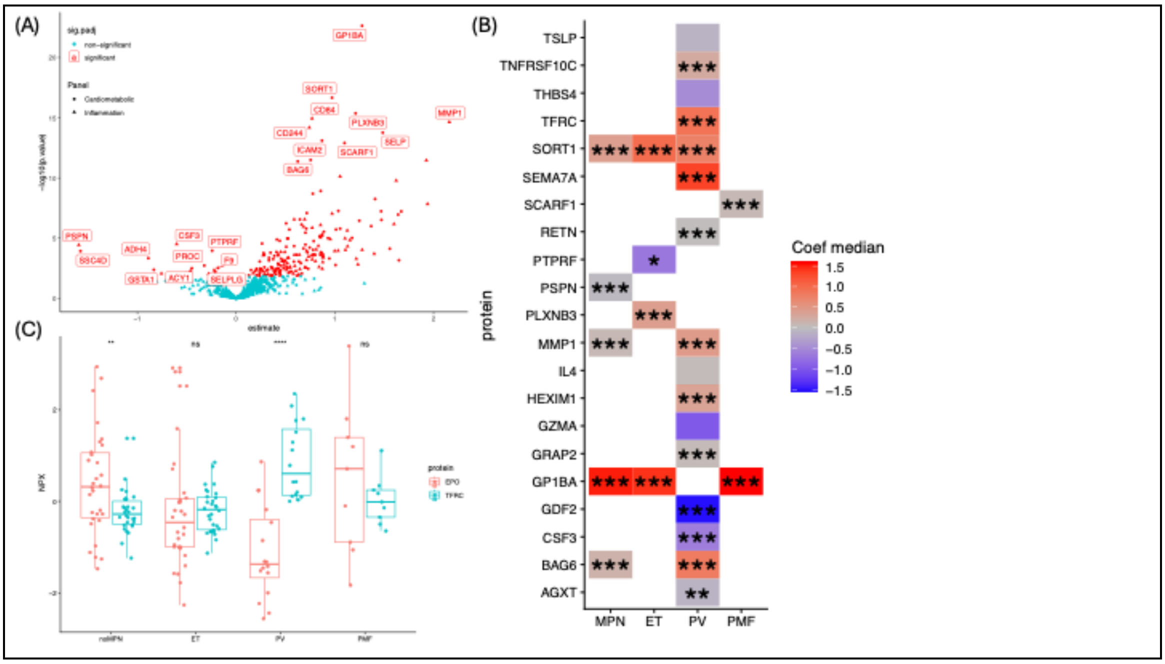

Figure 1. (A) Volcano plot protein differences between MPN and non-MPN. (B) Cox LASSO regression demonstrating plasma protein biomarkers specific for MPN subtypes ET, PV and PMF. (C) TFRC is an excellent marker of PV and in combination TFRC and EPO are excellent biomarkers.

**Copyright Information:** (Online) ISSN: 2572-9241
©2026 The Author(s). HemaSphere is published by John Wiley & Sons Ltd on behalf of European Hematology Association. This is an open access Abstract Book distributed under the Attribution-NonCommercial-NoDerivs (CC BY-NC-ND), which allows third parties to download the articles and share them with others as long as they credit the author and the Abstract Book, but they cannot change the content in any way or use them commercially.
**Abstract Book Citations:** Authors, Title, HemaSphere, 2026;10:(S1):pages. The abstract book DOIs can be found on the “Author Information” page.
**Disclaimer:** Articles published in the journal HemaSphere exclusively reflect the opinions of the authors. The authors are responsible for all content in their abstracts including accuracy of the facts, statements, citing resources, etc.

### (PB3403) COEXISTENCE OF BCR::ABL1 AND JAK2 MUTATIONS IN MYELOPROLIFERATIVE NEOPLASMS: A CASE SERIES HIGHLIGHTING DUAL CLONAL HEMATOPOIESIS

**Topic:** 15. Myeloproliferative neoplasms - Biology & translational research

Rene Amadore*[1], Rowel David Yap[1], Anne Kristine Quero-Taggaoa[1]

[1]*University of the Philippines - Philippine General Hospital, Division of Hematology, Department of Medicine, Manila, Philippines;*

### Background

BCR::ABL1-positive chronic myeloid leukemia (CML) and BCR::ABL1-negative myeloproliferative neoplasms (MPNs), including polycythemia vera (PV) and essential thrombocythemia (ET), are traditionally regarded as mutually exclusive due to distinct molecular drivers. However, rare cases demonstrating coexistence of BCR::ABL1 rearrangements and JAK2 mutations suggest the presence of dual clonal hematopoiesis. Data on this entity remain limited, particularly in Southeast Asia.

### Aims

To describe the clinical characteristics, diagnostic challenges, and management strategies of patients harboring concurrent BCR::ABL1 and JAK2 mutations, and to explore implications for clonal evolution in MPNs.

### Methods

We retrospectively reviewed three male patients (age range: 25–62 years) initially diagnosed with CML and managed at a tertiary referral center in the Philippines. All patients received tyrosine kinase inhibitor (TKI) therapy and were monitored with serial complete blood counts, bone marrow examinations, cytogenetic studies, and molecular assays. JAK2 mutation testing was pursued following persistent or newly emergent hematologic abnormalities despite achievement of molecular response to TKI therapy.

### Results

All patients achieved major or deep molecular response to TKI therapy targeting BCR::ABL1. Subsequent molecular evaluation demonstrated JAK2 mutations in all three cases. Two patients developed overt PV 8–9 years after initial CML diagnosis, presenting with erythrocytosis and recurrent splenomegaly despite sustained molecular remission. One patient demonstrated persistent thrombocytosis despite deep molecular response and was reclassified as having concomitant ET following repeat JAK2 testing. Bone marrow findings supported BCR::ABL1-negative MPN morphology, including increased megakaryocytes with clustering and hyperlobation. Management consisted of continued TKI therapy combined with individualized cytoreductive strategies, including hydroxyurea, phlebotomy, and anagrelide. Adequate hematologic control was achieved in all cases.

Table 1. Clinical and Molecular Characteristics of Patients with Concurrent BCR::ABL1 and JAK2 Mutations

| Case | Age / Sex | Initial Diagnosis | Years to JAK2 Detection | Final MPN Diagnosis | Molecular Status at JAK2 Detection | Management |
|---|---|---|---|---|---|---|
| 1 | 62 / Male | CML, chronic phase | 9 years | Polycythemia vera | Sustained major molecular response (BCR::ABL1 0.059%) | Continued TKI + hydroxyurea + intermittent phlebotomy |
| 2 | 51 / Male | CML, chronic phase | 4 years | Essential thrombocythemia | Deep molecular response (BCR::ABL1 0.00015%) | Continued TKI + hydroxyurea + anagrelide |
| 3 | 25 / Male | CML, chronic phase | 8 years | Polycythemia vera | Major molecular response (BCR::ABL1 0.021%) | Continued TKI + hydroxyurea + aspirin + intermittent phlebotomy |

**Copyright Information:** (Online) ISSN: 2572-9241
©2026 The Author(s). HemaSphere is published by John Wiley & Sons Ltd on behalf of European Hematology Association. This is an open access Abstract Book distributed under the Attribution-NonCommercial-NoDerivs (CC BY-NC-ND), which allows third parties to download the articles and share them with others as long as they credit the author and the Abstract Book, but they cannot change the content in any way or use them commercially.
**Abstract Book Citations:** Authors, Title, HemaSphere, 2026;10:(S1):pages. The abstract book DOIs can be found on the “Author Information” page.
**Disclaimer:** Articles published in the journal HemaSphere exclusively reflect the opinions of the authors. The authors are responsible for all content in their abstracts including accuracy of the facts, statements, citing resources, etc.

**Summary/Conclusion**

This first Philippine case series supports the concept of dual clonal MPNs and highlights that suppression of the BCR::ABL1 clone may permit expansion of a pre-existing or concomitant JAK2-mutated clone. Awareness of this entity is essential to prevent misinterpretation as TKI resistance or CML relapse. In CML patients with unexplained erythrocytosis, thrombocytosis, or splenomegaly despite molecular remission, evaluation for coexisting BCR::ABL1-negative MPNs should be considered. Larger studies and international registries are needed to define optimal diagnostic and therapeutic strategies for this rare but clinically relevant condition.

**Copyright Information:** (Online) ISSN: 2572-9241
©2026 The Author(s). HemaSphere is published by John Wiley & Sons Ltd on behalf of European Hematology Association. This is an open access Abstract Book distributed under the Attribution-NonCommercial-NoDerivs (CC BY-NC-ND), which allows third parties to download the articles and share them with others as long as they credit the author and the Abstract Book, but they cannot change the content in any way or use them commercially.
**Abstract Book Citations:** Authors, Title, HemaSphere, 2026;10:(S1):pages. The abstract book DOIs can be found on the "Author Information" page.
**Disclaimer:** Articles published in the journal HemaSphere exclusively reflect the opinions of the authors. The authors are responsible for all content in their abstracts including accuracy of the facts, statements, citing resources, etc.

### (PB3404) ALTERED LEVELS OF CYTOKINES INVOLVED IN FIBROCYTE DIFFERENTIATION IN PATIENTS WITH LYMPHOID MALIGNANCIES COMPLICATED BY SECONDARY MYELOFIBROSIS

**Topic:** 15. Myeloproliferative neoplasms - Biology & translational research

Noriaki Tachi[1], Takaaki Maekawa*[1, 2], Nobuyuki Mukai[1], Yurika Yanaki[1], Emi Kemmoku[1], Takuto Shonai[1], Kota Kawasaki[1], Takehiro Sone[1], Kohei Takada[1], Hiraku Ogata[1], Keita Saito[1], Toshikuni Kawamura[1], Shoichiro Kato[1], Yukiko Osawa[1], Shinichi Kobayashi[1]

*[1]National Defense Medical College, Division of Hematology, Department of Internal Medicine, Tokorozawa, Japan;[2]National Defense Medical College Hospital, Division of Palliative Care, Tokorozawa, Japan;*

### Background

The pathogenesis of myelofibrosis (MF), a myeloproliferative neoplasm, involves the thrombopoietin signaling pathway in fibrocytes, spindle-shaped fibroblast-like cells derived from monocytes. Lymphoid malignancies, such as multiple myeloma and lymphoma, can also induce secondary MF, which is associated with poor outcomes. However, the underlying mechanism remains unknown, and effective therapeutic strategies are needed. We previously reported increased *CCL22* and *TIMP1* mRNA expression in IM-9 cells, a fibrocyte-inducing cell line, as well as increased CCL22 and TIMP1 levels in IM-9 culture supernatants, and elevated serum CCL22 and TIMP1 levels in patients with MF secondary to lymphoid malignancies. We therefore analyzed changes in cytokines involved in fibrocyte differentiation to understand the underlying mechanisms.

### Aims

To elucidate the mechanisms of MF in lymphoid malignancies and identify therapeutic targets

### Methods

In culture supernatants of five lymphoid malignancy cell lines (IM-9, KMS-12-BM, KMS-11, KMM-1, and LICR-LON HMy2) and in serum samples of 80 patients with lymphoid malignancies whose MF grade (MF- 0, 1, 2) in the bone marrow was evaluated, we analyzed the levels of cytokines reportedly involved in regulating fibrocyte differentiation and validated in our experimental paradigms, using LEGENDplex® (BioLegend) and Quantikine® enzyme-linked immunosorbent assay kits (R&D Systems). The following cytokines were evaluated: IL-4, IL-13, IFN-γ, IL-12p70, IL-18, TNF-α, CCL2, IL-1Ra, CCL4, CXCL1, IL-6, CCL22, IL-7, M-CSF, and CHI3L1.

### Results

CCL22, CCL2, IL-7, and M-CSF levels were significantly higher in IM-9 supernatants than in the supernatants of the other cell lines. In patients with lymphoid malignancies, IL-18, CCL4, and CHI3L1 levels were higher in tumors with a higher fibrocyte precursor cell (FPC) ratio than in those with a lower FPC ratio. Tumors with a higher FPC ratio tended to have higher levels of CCL22, TNF-α, IL-6, and IL-1Ra and lower IL-7 levels. IL-18, CCL4, TNF-α, IL-6, and CHI3L1 levels were significantly higher; IL-7 levels were significantly lower; and IL-1Ra levels tended to be higher in patients with splenomegaly than in those without splenomegaly. IL-18 and CHI3L1 levels were significantly higher; IL-7 and IFN-γ levels were significantly lower, and IL-4 levels tended to be higher in patients with MF-1 or MF-2 than in those with MF-0.

**Copyright Information:** (Online) ISSN: 2572-9241
©2026 The Author(s). HemaSphere is published by John Wiley & Sons Ltd on behalf of European Hematology Association. This is an open access Abstract Book distributed under the Attribution-NonCommercial-NoDerivs (CC BY-NC-ND), which allows third parties to download the articles and share them with others as long as they credit the author and the Abstract Book, but they cannot change the content in any way or use them commercially.
**Abstract Book Citations:** Authors, Title, HemaSphere, 2026;10:(S1):pages. The abstract book DOIs can be found on the "Author Information" page.
**Disclaimer:** Articles published in the journal HemaSphere exclusively reflect the opinions of the authors. The authors are responsible for all content in their abstracts including accuracy of the facts, statements, citing resources, etc.

### Summary/Conclusion

Differences in the levels of eight cytokines involved in fibrocyte differentiation between patients with higher and lower FPC ratios suggest a role for these cytokines in fibrocyte-mediated secondary MF associated with lymphoid tumors. IL-18 can promote the differentiation of circulating fibrocytes in aging individuals. Moreover, organ fibrosis associated with CHI3L1 is believed to primarily involve macrophages; however, our study suggests its involvement in a fibrocyte-mediated mechanism that induces MF (manuscript under review). We are investigating the hypothesis that elevated serum IL-18 promotes M2 macrophage differentiation at local sites (such as the bone marrow and spleen), thereby enhancing monocyte-to-fibrocyte differentiation through the secretion of CCL22 and related factors. In summary, serum levels of multiple cytokines involved in fibrocyte differentiation, including IL-18 and CHI3L1, were significantly altered in patients with lymphoid malignancies presenting with secondary MF, splenomegaly, and an increased FPC ratio.

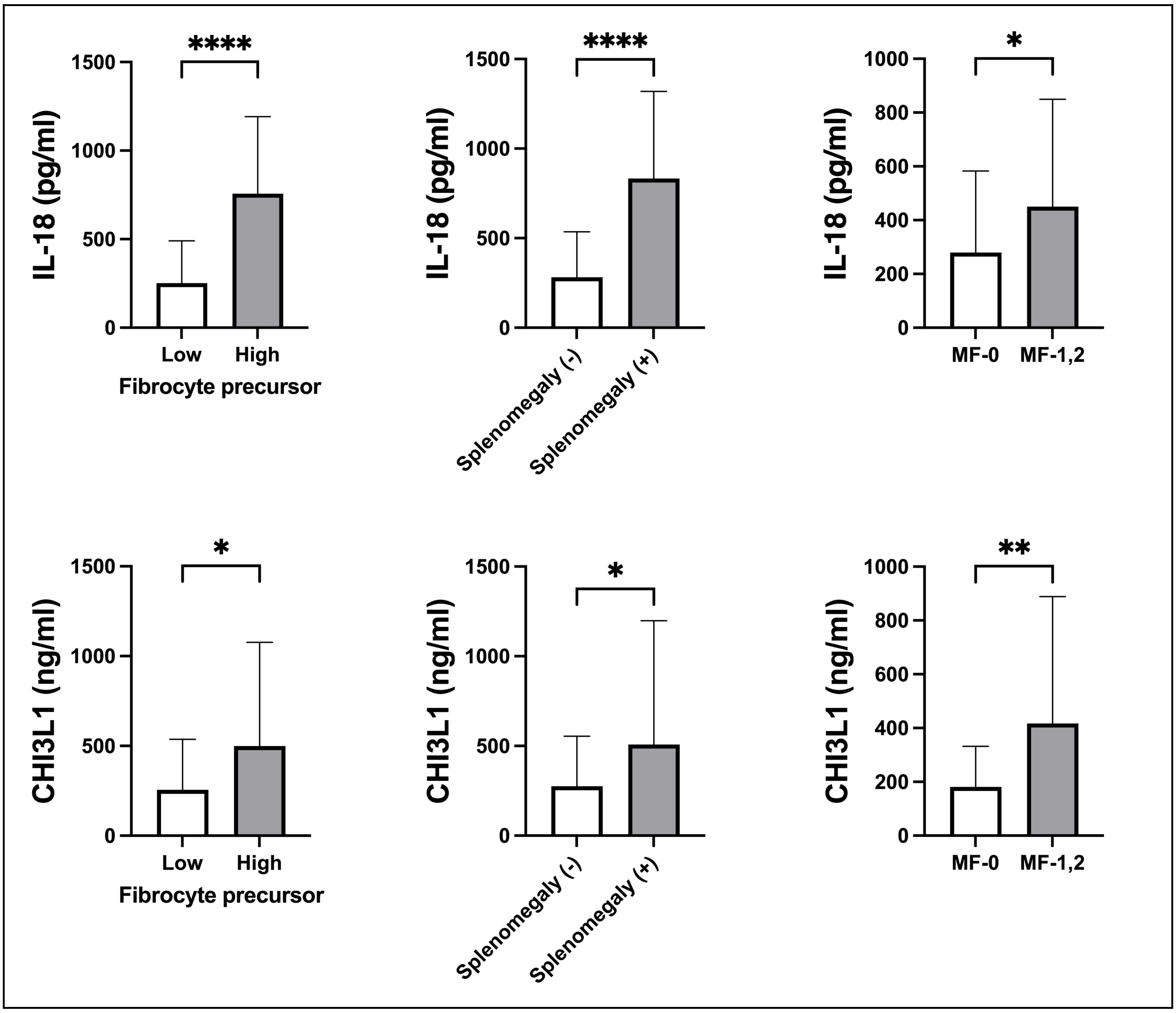

**Copyright Information:** (Online) ISSN: 2572-9241
©2026 The Author(s). HemaSphere is published by John Wiley & Sons Ltd on behalf of European Hematology Association. This is an open access Abstract Book distributed under the Attribution-NonCommercial-NoDerivs (CC BY-NC-ND), which allows third parties to download the articles and share them with others as long as they credit the author and the Abstract Book, but they cannot change the content in any way or use them commercially.
**Abstract Book Citations:** Authors, Title, HemaSphere, 2026;10:(S1):pages. The abstract book DOIs can be found on the "Author Information" page.
**Disclaimer:** Articles published in the journal HemaSphere exclusively reflect the opinions of the authors. The authors are responsible for all content in their abstracts including accuracy of the facts, statements, citing resources, etc.

### (PB3405) PRIMARY MYELOFIBROSIS: FROM DISEASE PHENOTYPE AND AGE TO THE MOLECULAR ABERRATION PROFILE

**Topic:** 15. Myeloproliferative neoplasms - Biology & translational research

Krzysztof Lewandowski*[1], Zuzanna Kanduła[1, 1], Małgorzata Marcinkowska-Swojak[2], Magdalena Rakoczy[2], Weronika Litowczenko[2], Edyta Paczkowska[3, 3], Marta Sobas[4], Luiza Handschuh[2]

*[1]University of Medical Sciences, Hematology and Bone Marrow Transplantation, Poznań, Poland;[2]Institute of Bioorganic Chemistry Polish Academy of Sciences, Poznań, Poland; [3]Pomeranian Medical University, General Pathology, Szczecin, Poland; [4]Bydgoszcz University, Hematology, Bydgoszcz, Poland;*

### Background

Primary myelofibrosis (PMF) is a neoplastic disorder, diagnosed in most cases at >50 years of age and manifested by two main clinically different phenotypes: myeloproliferative or cytopenic.

### Aims

The aim of the study was to analyze the molecular profile of PMF patients (pts, n=74) depending on the age at the time of diagnosis [<50y (n= 20) and ≥50y (n=54)], disease phenotype (myeloproliferative n=18, cytopenic n=32), and the driver mutation status.

### Methods

Whole-genome sequencing (WGS) was performed on a NovaSeq 6000 (Illumina) system using the S4 Reagent Kit v1.5 (with 300-cycle chemistry, cat. No. 20028312), achieving an average coverage of 30x per sample. Variants were excluded if they had an eVAI pathogenicity score (PS) below 2, sample coverage below 6, alternate variant coverage below 3, and sample quality below 30. Additionally, multi-allelic and intron variants, except splice-site variants and those classified as pathogenic or likely pathogenic, were removed.

### Results

The mean number of DNA sequence variants detected. depending on the driver mutation type. was 26.4, 26.4, 21.8 and 21.3 per patient in *JAK2*, *MPL*, *CALR* mutation positive patients, and triple negative cases, respectively. Similar analysis performed dependently from the blast cells percentage in PB/BM samples showed an increase of the mean variant number with an increase of overage blast cell content [in B0 (0-5%), B1 (5-10%), B2 (11-19%) and B2 (≥20%) group up to 23.3, 21.9, 29.5 and 28.0 variants/patient, respectively]. The following mutations were detected with more than 5% frequency in the whole studied patient group (n=74) - *ACADS, ASXL1, BTD, CALR, CFHR5, COL9A2, COQ2, EZH2, FMN2, JAK2, LAMB3, MPL, PLEKHG2, REN, RPGR, RYR3, SLC45A1, SLCO1B3, SNORD118, ZMYM3.* The analysis of *JAK2*V617F, *CALR,* and *MPL* driver mutation frequency in patients with the myeloproliferative and cytopenic PMF phenotype revealed their presence in 50.0% vs 49.6%, 33.3% vs 15.6% and 0% vs 12% of pts, respectively. The higher frequency of *PLEKHG2* mutations in the myeloproliferative pts group (16.7% vs 0%; p=0.04) was confirmed. The frequency of other mutations detected with at least 5% frequency did not show statistically significant differences between pts with different PMF phenotypes. A similar analysis performed in patients <50 and $^{3}$50 years revealed *MPL* mutation positivity in only 4 patients from the $^{3}$50 years group. No difference in driver *JAK2*V617F and *CALR* mutations frequency between the studied groups (45% vs 48.1% and 20% vs 20.4%, respectively) was noted. However, a significantly higher frequency of *ASXL1* mutations in patients $^{3}$50 years old (2% vs 23%, respectively, p=0.01) was found. The frequency of other mutations occurring with $^{3}$5% frequency in the studied pts cohort showed no difference between the studied pts age groups.

**Copyright Information:** (Online) ISSN: 2572-9241
©2026 The Author(s). HemaSphere is published by John Wiley & Sons Ltd on behalf of European Hematology Association. This is an open access Abstract Book distributed under the Attribution-NonCommercial-NoDerivs (CC BY-NC-ND), which allows third parties to download the articles and share them with others as long as they credit the author and the Abstract Book, but they cannot change the content in any way or use them commercially.
**Abstract Book Citations:** Authors, Title, HemaSphere, 2026;10:(S1):pages. The abstract book DOIs can be found on the “Author Information” page.
**Disclaimer:** Articles published in the journal HemaSphere exclusively reflect the opinions of the authors. The authors are responsible for all content in their abstracts including accuracy of the facts, statements, citing resources, etc.

**Summary/Conclusion**

The obtained results revealed only subtle differences in the profile of molecular changes in DNA in PMF patients depending on age and phenotypic manifestation of the disease. However, a larger study group would be necessary to investigate this further.

**Copyright Information:** (Online) ISSN: 2572-9241
©2026 The Author(s). HemaSphere is published by John Wiley & Sons Ltd on behalf of European Hematology Association. This is an open access Abstract Book distributed under the Attribution-NonCommercial-NoDerivs (CC BY-NC-ND), which allows third parties to download the articles and share them with others as long as they credit the author and the Abstract Book, but they cannot change the content in any way or use them commercially.
**Abstract Book Citations:** Authors, Title, HemaSphere, 2026;10:(S1):pages. The abstract book DOIs can be found on the "Author Information" page.
**Disclaimer:** Articles published in the journal HemaSphere exclusively reflect the opinions of the authors. The authors are responsible for all content in their abstracts including accuracy of the facts, statements, citing resources, etc.

### (PB3406) IS THERE ANY RELATIONSHIP BETWEEN STROMAL MODIFICATIONS IN MYELOFIBROSIS AND THE MOLECULAR SIGNATURE OF THE HEMATOPOIETIC PROGENITOR?

**Topic:** 15. Myeloproliferative neoplasms - Biology & translational research

María Isabel Montero Cuadrado[1], Juan José Borrero[1], Elena Soria Sardise[1], Eduardo Rodríguez-Arbolí*[1], Estrella Cruz Carrillo[1]

*[1]Hospital Universitario Virgen del Rocío, Sevilla, Spain;*

### Background

The abnormal communication between stromal cell types and hematopoietic progenitors in the bone marrow of patients with myelofibrosis leads to inflammation and fibrosis of the medullary niche conditioning the phenotype of the disease. The three mutations driving myeloproliferative syndromes and the addition of new molecular changes in the progenitor cell could play a role in the different bone marrow microenvironments observed in myelofibrosis.

### Aims

From a clinical database of 207 patients of a single center with myelofibrosis diagnosis , we analysed medical histories of 81 patients with myelofibrosis (MF) regarding epidemiological data, molecular status and histological examination of bone marrow.

### Methods

Diagnosis of MF was made according to the WHO 2016. Molecular tests for detection for JAK2, MPL and CALR mutations were performed in all patients and next-generation sequencing in elegible transplant patients. Non driver mutations were classified as follows: signaling pathways (LNK1, CBL, NRAS/KRAS, PTPN1), epigenetics (TET2, DNMT3A, IDH1/2, ASXL1), splicing(SF3B1, SRSF2, U2AF1, ZRSR2) and DNA damage repair genes (TP53). The histological study of the bone marrow considered the appearance and arrangement of megakaryocytes, cellularity, the presence of collagenous fibrosis , reticulin fibrosis or both, the degree of fibrosis and the presence of osteosclerosis..Survival analysis was performed by means of Kaplan-Meier curves and differences between KM curves were evaluated using the Log-rank test. *P* values less than 0.05 were considered satistically significant . Analyses were performed with SPSS software v.15.

### Results

Median age was 64 years (range 28–84); 61 % patients were females. *JAK2*V617F was present in 65% ; *CALR* mutations in 20,5% and *MPL*W515K/L in 13,3% patients. Next generation sequencing was made in 48 patients (59%). 60,4% had additional mutations: signaling pathways (18,7%), epigenetics(46,9%), splicing (32,6%) and TP53(14,5%). Bone marrow cellularity was high in 57% of patients. normal in 10,6% and low in 32% of them; collagen fibrosis was observed in 10,8% patients, reticulin fibrosis in 68,9% and both in 20,2%, with grade 1,2 and 3 fibrosis in 23,6%, 36,8% and 39,4% respectively; osteosclerosis was detected in 15,7% of them and associated with presence of collagen fibrosis ($p$<0,05%).Most of *JAK2*V617F and *MPL*W515K/L patients had reticulin fibrosis only (75%) whilst collagen fibrosis was observed in 50% of *CALR* mutated patients ($p$<0,05%). Osteosclerosis was higher in *MPL*W515K/L patients (33%). Additional mutations were correlated with collagen fibrosis (44% vs 23%) .

**Copyright Information:** (Online) ISSN: 2572-9241
©2026 The Author(s). HemaSphere is published by John Wiley & Sons Ltd on behalf of European Hematology Association. This is an open access Abstract Book distributed under the Attribution-NonCommercial-NoDerivs (CC BY-NC-ND), which allows third parties to download the articles and share them with others as long as they credit the author and the Abstract Book, but they cannot change the content in any way or use them commercially.
**Abstract Book Citations:** Authors, Title, HemaSphere, 2026;10:(S1):pages. The abstract book DOIs can be found on the “Author Information” page.
**Disclaimer:** Articles published in the journal HemaSphere exclusively reflect the opinions of the authors. The authors are responsible for all content in their abstracts including accuracy of the facts, statements, citing resources, etc.

**Summary/Conclusion**

1.- Patients with calreticulin mutations had a higher proportion of collagen fibrosis; given that fibroblasts and osteoblasts, in which calreticulin plays an important role, are involved in type I collagen synthesis, this result could be related to paracrine secretion of mutated calreticulin into the stroma. 2.-Osteosclerosis was more frequent in *MPL*W515K/L patients which could indicate greater interaction between pathological megakaryocytes and osteoblasts to be taken into account in patients who are candidates for transplantation due to the greater need for niche remodelling in engraftment.3.- Additional mutations occur more frequently in patients with collagen and reticulin fibrosis, although they do not appear to have a significant influence on bone marrow niche alterations.

**Copyright Information:** (Online) ISSN: 2572-9241
©2026 The Author(s). HemaSphere is published by John Wiley & Sons Ltd on behalf of European Hematology Association. This is an open access Abstract Book distributed under the Attribution-NonCommercial-NoDerivs (CC BY-NC-ND), which allows third parties to download the articles and share them with others as long as they credit the author and the Abstract Book, but they cannot change the content in any way or use them commercially.
**Abstract Book Citations:** Authors, Title, HemaSphere, 2026;10:(S1):pages. The abstract book DOIs can be found on the "Author Information" page.
**Disclaimer:** Articles published in the journal HemaSphere exclusively reflect the opinions of the authors. The authors are responsible for all content in their abstracts including accuracy of the facts, statements, citing resources, etc.

### (PB3407) PREVALENCE OF THE JAK2 V617F MUTATION IN THE PERIPHERAL BLOOD OF INDIVIDUALS PRESENTING WITH ACUTE MYOCARDIAL INFARCTION

**Topic:** 15. Myeloproliferative neoplasms - Biology & translational research

David Ross*[1], Romana Panagopoulos[2], Momina Allahwala[2], Rosanna Tavella[2], Nur Hezrin Shahrin[1], Naomi Wattchow[2], Rajesh Pathy[2], Christina Bursill[2], Chung Kok[1], John Beltrame[2], Susan Branford[1], Peter Psaltis[3]

*[1]SA Pathology, Adelaide, Australia; [2]Adelaide University, Adelaide, Australia; [3]South Australian Health and Medical Research Institute, Adelaide, Australia;*

### Background

*JAK2* V617F is the most common mutation in myeloproliferative neoplasms and is associated with an increased risk of arterial and venous thrombosis. Studies of *JAK2* clonal haematopoiesis (CH) in the adult population have also reported an association with arterial thrombosis typically based on a variant allele frequency (VAF) ≥2%, but the significance of mutations at a lower VAF is unclear.

### Aims

To assess the prevalence and clinical associations of low-level *JAK2* V617F in individuals presenting with acute myocardial infarction (AMI).

### Methods

Peripheral blood samples were available from two ongoing cardiovascular studies in Adelaide, South Australia. VISION-CAD (n=120) is an observational study of AMI; CADOSA (n=62) is a registry enrolling individuals undergoing coronary angiography for any reason. DNA was extracted from leukocyte pellets or Buffy coats. Digital droplet PCR (BioRad) for *JAK2* V617F was performed in triplicate according to the manufacturer's instructions with an average limit of detection around 0.003%. A targeted hybridization capture panel comprising the 18 genes most frequently involved in CH was sequenced using Illumina technology. Variants were called using oncogenicity criteria and a minimum VAF of 0.2%. Non-parametric statistics were used to compare groups.

**Copyright Information:** (Online) ISSN: 2572-9241
©2026 The Author(s). HemaSphere is published by John Wiley & Sons Ltd on behalf of European Hematology Association. This is an open access Abstract Book distributed under the Attribution-NonCommercial-NoDerivs (CC BY-NC-ND), which allows third parties to download the articles and share them with others as long as they credit the author and the Abstract Book, but they cannot change the content in any way or use them commercially.
**Abstract Book Citations:** Authors, Title, HemaSphere, 2026;10:(S1):pages. The abstract book DOIs can be found on the "Author Information" page.
**Disclaimer:** Articles published in the journal HemaSphere exclusively reflect the opinions of the authors. The authors are responsible for all content in their abstracts including accuracy of the facts, statements, citing resources, etc.

### Results

The average age of the cohort was 64 years and 72% were male. *JAK2* V617F was detected in 22/182 individuals (12.1%) with a median VAF of 0.006% (range 0.001%-50.7%). One patient had a diagnosis of polycythaemia vera. The highest VAF in the remaining patients was 0.5%. Comparing individuals with and without *JAK2* V617F there was no significant difference in age, sex, or prevalent cardiovascular risk factors (smoking, diabetes mellitus, hyperlipidaemia, hypertension). Individuals with *JAK2* V617F had higher leukocyte counts and a higher proportion of patients having CH in multiple genes (other than *JAK2*) or CH with VAF ≥2% (see Table). The genes most frequently mutated were *DNMT3A*, *TET2*, *ASXL1*, and *TP53*. The median VAF of additional CH variants in patients with *JAK2* V617F was 1.9% compared with 0.7% in the remainder of the cohort.

| Table | *JAK2* V617F | | P-value |
|---|---|---|---|
| | No (n=160) | Yes (n=22) | |
| Female sex | 28% | 27% | NS |
| Ever smoked | 67% | 68% | NS |
| Haemoglobin (g/L) | 139 | 146 | NS |
| Platelets (x$10^9$/L) | 234 | 255 | NS |
| **White cells (x$10^9$/L)** | **8.5** | **10.7** | **0.015** |
| Neutrophils | 5.5 | 6.2 | NS |
| Any CH | 58% | 68% | NS |
| **Multiple CH** | **28%** | **59%** | **0.003** |
| **CHIP (≥2% VAF)** | **15%** | **36%** | **0.014** |

NS = P-value >0.10

### Summary/Conclusion

*JAK2* V617F CH at low VAF (<2%) is frequent in individuals presenting with ischaemic cardiovascular disease without other features of MPN, and commonly occurs in the presence of additional CH variants. The relative contribution of the different variants to arterial thrombotic risk is unclear, but may provide incremental information in addition to conventional cardiovascular risk factors alone. Mechanistic studies are required to understand the biological significance of the *JAK2* V617F mutation when present in a minor fraction of cells.

**Copyright Information:** (Online) ISSN: 2572-9241
©2026 The Author(s). HemaSphere is published by John Wiley & Sons Ltd on behalf of European Hematology Association. This is an open access Abstract Book distributed under the Attribution-NonCommercial-NoDerivs (CC BY-NC-ND), which allows third parties to download the articles and share them with others as long as they credit the author and the Abstract Book, but they cannot change the content in any way or use them commercially.
**Abstract Book Citations:** Authors, Title, HemaSphere, 2026;10:(S1):pages. The abstract book DOIs can be found on the “Author Information” page.
**Disclaimer:** Articles published in the journal HemaSphere exclusively reflect the opinions of the authors. The authors are responsible for all content in their abstracts including accuracy of the facts, statements, citing resources, etc.

### (PB3408) TPSAB1 COPY NUMBER ANALYSIS BY PLATE-BASED DIGITAL PCR IN HEREDITARY ALPHA TRYPTASEMIA (HαT): A SINGLE-CENTER EXPERIENCE

**Topic:** 15. Myeloproliferative neoplasms - Biology & translational research

Salvatore Piras*[1], Giovanna Piras[1], Margherita Deidda[2], Giusi Taurisano[2], Maria Luisa Di Martino[2], Stefano Del Giacco[2], Maria Pina Barca[2], Angelo Domenico Palmas[1]

*[1]"San Francesco" Hospital, Hematology Unit, CTMO and Specialized Laboratory, NUORO, Italy; [2]AOU Cagliari, Unit of Internal Medicine, Allergy and Clinical Immunology, Cagliari, Italy;*

### Background

Hereditary alpha tryptasemia (HαT) is an autosomal dominant genetic trait caused by increased copy number of the *TPSAB1* gene, which encodes α-tryptase, and has an estimated prevalence of 4–7% in the general population. It is the most common inherited cause of elevated basal serum tryptase (BST). Elevated BST also constitutes a minor diagnostic criterion for systemic mastocytosis, a clonal myeloid neoplasm most often driven by the *KIT* D816V variant. Because HαT and systemic mastocytosis can both present with increased BST and mediator-related symptoms, precise molecular discrimination is essential to prevent diagnostic misclassification and ensure appropriate clinical management.

### Aims

To validate a plate-based digital PCR assay for detecting *TPSAB1* copy number variations and to determine the prevalence of HαT in a cohort of patients evaluated for suspected mast cell disorders. We also assessed the correlations between *TPSAB1* copy number, basal serum tryptase (BST) levels, and *KIT* D816V mutation status.

### Methods

Genomic DNA from 115 patients (111 peripheral blood and 4 bone marrow samples) referred for suspected HαT and/or mast cell–related disorders was analyzed. An additional set of 50 healthy controls was included. *TPSAB1* copy number and *KIT* D816V mutation status were assessed using a plate-based digital PCR (QuantStudio Absolute Q, Thermo Fisher Scientific). Basal serum tryptase (BST) levels were collected when available. Continuous variables were summarized as median (IQR) and compared using non-parametric tests, while categorical variables were reported as frequencies with 95% confidence intervals.

### Results

HαT-associated genotypes were identified in 15 of 115 patients (13.0%). The most frequent genotype overall was α/β,β/β (42.6%), followed by β/β,β/β (28.7%) and α/β,α/β (15.7%). BST values were available for 96 of 115 subjects (83.5%), of whom 52 (54.2%) had BST >11.4 ng/mL. Elevated BST was observed in 13 of 15 HαT carriers (86.7%). Median BST levels were higher in HαT-positive compared with HαT-negative individuals (14.4 vs 10.6 ng/mL, p=0.13). *KIT* D816V (VAF range 0.03%–25%) was detected in 25 of 115 patients (21.7%), exclusively among non-HαT subjects. Among healthy controls, *TPSAB1* genotypes were distributed as follows: 56% α/β,β/β; 24% β/β,β/β; and 20% α/β,α/β.

**Copyright Information:** (Online) ISSN: 2572-9241
©2026 The Author(s). HemaSphere is published by John Wiley & Sons Ltd on behalf of European Hematology Association. This is an open access Abstract Book distributed under the Attribution-NonCommercial-NoDerivs (CC BY-NC-ND), which allows third parties to download the articles and share them with others as long as they credit the author and the Abstract Book, but they cannot change the content in any way or use them commercially.
**Abstract Book Citations:** Authors, Title, HemaSphere, 2026;10:(S1):pages. The abstract book DOIs can be found on the "Author Information" page.
**Disclaimer:** Articles published in the journal HemaSphere exclusively reflect the opinions of the authors. The authors are responsible for all content in their abstracts including accuracy of the facts, statements, citing resources, etc.

**Summary/Conclusion**

Assessment of *TPSAB1* copy number remains technically challenging. Plate-based digital PCR provides a robust and accurate method for detecting copy number variations, ensuring high diagnostic precision. HαT was identified in 13% of cases and was associated with higher BST levels. The absence of *KIT* D816V mutations among HαT carriers reinforces the concept that HαT and clonal mast cell disease represent biologically distinct entities requiring different diagnostic and clinical management pathways. Ongoing studies aim to characterize the distribution of *TPSAB1* genotypes within the Sardinian population, a genetically isolated and distinctive background.

**Copyright Information:** (Online) ISSN: 2572-9241
©2026 The Author(s). HemaSphere is published by John Wiley & Sons Ltd on behalf of European Hematology Association. This is an open access Abstract Book distributed under the Attribution-NonCommercial-NoDerivs (CC BY-NC-ND), which allows third parties to download the articles and share them with others as long as they credit the author and the Abstract Book, but they cannot change the content in any way or use them commercially.
**Abstract Book Citations:** Authors, Title, HemaSphere, 2026;10:(S1):pages. The abstract book DOIs can be found on the “Author Information” page.
**Disclaimer:** Articles published in the journal HemaSphere exclusively reflect the opinions of the authors. The authors are responsible for all content in their abstracts including accuracy of the facts, statements, citing resources, etc.

### (PB3409) DYNAMIC ROLES FOR ILC2S IN THE PATHOGENESIS OF BONE MARROW FIBROSIS

**Topic:** 15. Myeloproliferative neoplasms - Biology & translational research

Maria Angeles Pinero Garasa*[1], Egon Urgard[2], Jonathan Coquet[2], Johanna Ungerstedt[3], Itziar Martinez Gonzalez[1], Sergio Martinez Høyer[1]

*[1]Karolinska Institutet, Stockholm, Sweden; [2]University of Copenhagen, Copenhagen, Denmark; [3]Linköping University, Linköping, Sweden;*

### Background

Primary myelofibrosis (PMF) is a subtype of myeloproliferative neoplasm (MPN) that leads to progressive and irreversible bone marrow fibrosis. Recent evidence from mouse models of PMF show that type 2 inflammation contributes to disease pathogenesis, as blockades of IL-4 and IL-13 signaling delayed fibrotic onset. However, the cellular players originating such signals remain poorly defined. Group 2 innate lymphoid cells (ILC2) are innate lymphocytes present throughout the organism that do not react against specific antigens but instead are highly responsive to alarmins released upon damage, such as IL-33. Upon activation, ILC2s produce large amounts of type 2 cytokines, including IL-13 and IL-4, and therefore are considered key initiators and orchestrators of type 2 immune responses. Remarkably, ILC2-derived IL-13 has been shown to be involved in fibrotic responses in different tissues.

### Aims

Our work aims to understand the contribution of ILC2s to the inflammatory response that promotes the progression of PMF.

### Methods

We transplanted primary bone marrow cKIT+ cells transduced with a retrovirus expressing MPL W515L (or MPL WT as control) into sublethally irradiated recipients. Peripheral blood, spleen and bone marrow were harvested at different time points after transplantation to study immune cell populations using high dimensional flow cytometry.

### Results

At early disease stages, we observed a significant increase in mature KLRG1+ ILC2s in peripheral blood. Concomitantly, KLRG1+ ILC2s were reduced in the bone marrow suggesting local activation and egress into circulation. As disease progressed and transplanted animals developed splenomegaly, we observed accumulation of CD25+ ILC2s in the spleen. CD25 encodes for the receptor of IL-2, and therefore splenic ILC2s may be in an activated state. At late stages, when fibrosis is present, ILC2s exhibited markedly elevated expression of the inhibitory receptor PD-1, both in the bone marrow and spleen. In contrast, although splenic T cell subsets also expressed PD-1, they did not display a comparable accumulation.
Furthermore, analysis of peripheral blood mononuclear cells from patients in early stages of the disease showed a shift towards an ILC2 phenotype within the ILC compartment, suggesting that the disease development promotes the circulation of ILC2s. In addition, functional analyses of purified ILC2s from MPN patients showed their competence to produce the type 2 cytokines

### Summary/Conclusion

Together, our findings suggest a stage specific involvement of ILC2s in the pathogenesis of MPN, switching from an activated state in pre-fibrosis towards an exhausted phenotype at fibrosis onset. Our study may uncover previously unrecognized features of the lymphoid microenvironment in MPN progression, emphasizing the potential contribution of ILC2s to the development of bone marrow fibrosis in myeloid malignancies.

**Copyright Information:** (Online) ISSN: 2572-9241
©2026 The Author(s). HemaSphere is published by John Wiley & Sons Ltd on behalf of European Hematology Association. This is an open access Abstract Book distributed under the Attribution-NonCommercial-NoDerivs (CC BY-NC-ND), which allows third parties to download the articles and share them with others as long as they credit the author and the Abstract Book, but they cannot change the content in any way or use them commercially.
**Abstract Book Citations:** Authors, Title, HemaSphere, 2026;10:(S1):pages. The abstract book DOIs can be found on the “Author Information” page.
**Disclaimer:** Articles published in the journal HemaSphere exclusively reflect the opinions of the authors. The authors are responsible for all content in their abstracts including accuracy of the facts, statements, citing resources, etc.

### (PB3410) EPIGENETIC LINEAGE TRACING OF CIRCULATING CELL-FREE DNA REVEALS DIFFERENTIAL CLONAL OUTPUT IN HEMATOLOGICAL MALIGNANCIES

**Topic:** 15. Myeloproliferative neoplasms - Biology & translational research

Selin Toprak Yalçın*[1, 2], Esra Nur Demirtas[1, 2], Selçuk Sözer Tokdemir[1]

[1]*Istanbul University, Department of Genetics, Aziz Sancar Institute of Experimental Medicine, İstanbul, Türkiye,*[2]*Istanbul University, Institute of Graduate Studies in Health Sciences, İstanbul, Türkiye;*

### Background

Hematological malignancies arise from clonal transformations within the hematopoietic hierarchy. However, the relative contribution of specific lineages (e.g., erythroid, megakaryocytic) to circulating cell-free DNA (cfDNA) is still not fully determined. While molecular mutation profiling quantifies clonal burden, it is insufficient for determining lineage origin. Since DNA methylation patterns are lineage-specific and epigenetically stable, cfDNA methylation signatures can be used as a non-invasive tracer of the cellular origin of circulating DNA.

### Aims

To determine whether lineage-specific methylation patterns in cfDNA can discriminate differential hematopoietic lineage contributions in hematological malignancies.

### Methods cfDNA was isolated from conditioned media of cell lines with QIAamp Circulating Nucleic Acid kit. Bisulfite-converted DNA underwent methylation-specific PCR (MSP) targeting the erythroid-associated FECH promoter CpG region and the megakaryocyte-associated MK-1 locus. GAPDH served as a normalization control. Lineage specificity was validated in reference cell lines: HEL (erythroid), MEG01 (megakaryocytic), and HUVEC (non-hematopoietic control). Methylation percentages were calculated using the 2^-(ΔCt) method, where ΔCt = Ct(GAPDH) − Ct(target): %Methylation = 2^-(ΔCt)_methylated / [2^-(ΔCt)_methylated + 2^-(ΔCt)_unmethylated] × 100 (Livak and Schmittgen, 2001; Fackler et al., 2004).

### Results

Reference validation demonstrated clear lineage discrimination. FECH was markedly hypomethylated in HEL cells (unmethylated ΔCt = 3.27, corresponding to 97% unmethylated fraction), while MK-1 was hypomethylated in MEG01 cells (unmethylated ΔCt = 4.2, 99% unmethylated fraction). HUVEC controls showed near-equal methylated and unmethylated fractions, consistent with their non-hematopoietic origin.

### Summary/Conclusion

Lineage-specific methylation profiling of cfDNA enables epigenetic monitoring of hematopoietic cell origin signals in hematological malignancies. Unlike mutation-based assays that measure clonal burden, cfDNA methylation signatures provide information on lineage allocation and differential cellular contribution to circulating DNA. This cell line validation provides proof-of-concept that cfDNA methylation can serve as a dynamic biomarker for monitoring disease biology, lineage shifts, and treatment-related clonal remodeling.

**Copyright Information:** (Online) ISSN: 2572-9241
©2026 The Author(s). HemaSphere is published by John Wiley & Sons Ltd on behalf of European Hematology Association. This is an open access Abstract Book distributed under the Attribution-NonCommercial-NoDerivs (CC BY-NC-ND), which allows third parties to download the articles and share them with others as long as they credit the author and the Abstract Book, but they cannot change the content in any way or use them commercially.
**Abstract Book Citations:** Authors, Title, HemaSphere, 2026;10:(S1):pages. The abstract book DOIs can be found on the "Author Information" page.
**Disclaimer:** Articles published in the journal HemaSphere exclusively reflect the opinions of the authors. The authors are responsible for all content in their abstracts including accuracy of the facts, statements, citing resources, etc.

### (PB3411) MITOCHONDRIAL DNA DAMAGE CONTRIBUTES TO THE CYTOTOXIC EFFECT OF THE PEPTIDE–DRUG CONJUGATE MELFLUFEN

**Topic:** 15. Myeloproliferative neoplasms - Biology & translational research

Ulrica Westermark*[1], Carina Norström[1], Kristina Witt Mulder[1], Komal Shahzad[1], Karl-Johan Fasth[1], Karthik Selvaraj[2], Pádraig D'arcy[2]

*[1]Oncopeptides AB, Stockholm, Sweden; [2]Linköpings Universitet, Department of Biomedical and Clinical Sciences, Linköping, Sweden;*

### Background

Peptide–Drug Conjugates (PDCs) rapidly cross cell membranes due to their high lipophilicity, bypassing transporter-mediated resistance. Once inside cancer cells, they are hydrolyzed by upregulated peptidases and esterases, generating intracellular accumulation of the hydrophilic payload and thereby inducing DNA damage. The PDC compound melflufen is approved for relapsed/refractory multiple myeloma (MM). Mitochondrial dysfunction is common in cancer cells and is an emerging therapeutic target. Given the hydrophobic nature of the PDC compounds we investigated whether mitochondrial involvement contributes to the cytotoxic effect of melflufen.

### Aims

To determine whether mitochondrial DNA damage contributes to melflufen-induced cytotoxicity in cancer cells.

### Methods

Cellular and mitochondrial localization of melflufen and its metabolites were evaluated in MM cells and isolated mitochondria. DNA damage to nuclear and mitochondrial DNA was measured by long-range PCR. Effects on mitochondrial membrane potential and reactive oxygen species (ROS) were assessed by live cell imaging. Metabolic consequences were analyzed using Seahorse assays. Correlation between mitochondrial gene expression and melflufen sensitivity was evaluated using bioinformatic approaches.

### Results

Melflufen undergoes intracellular hydrolysis resulting in accumulation of the payload melphalan both in whole cells and in the mitochondria. In addition, melflufen can not only be hydrolyzed in the cytoplasm of the cell but also inside the mitochondria. Treatment with melflufen trigger rapid mitochondrial membrane hyperpolarization followed by depolarization and cell death. Melflufen induced damage to both nuclear and mitochondrial DNA, a feature not observed with other alkylators, such as melphalan and cyclophosphamide. The induction of cell death by other alkylators is not preceded by changes in mitochondrial membrane potential. Bioinformatic analyses revealed correlations between mitochondrial gene-expression profiles and cellular sensitivity to melflufen across diverse mitochondrial pathways.

### Summary/Conclusion

Melflufen targets mitochondria and induces mitochondrial DNA damage that contributes to its cytotoxic mechanism. Dual targeting of nuclear and mitochondrial DNA may allow melflufen to overcome resistance mediated by cell cycle regulation and DNA repair pathways. These findings highlight mitochondrial involvement as an important component of the activity against myeloma by melflufen and support further exploration of PDC compounds as modulators of mitochondrial vulnerability in cancer.

**Copyright Information:** (Online) ISSN: 2572-9241
©2026 The Author(s). HemaSphere is published by John Wiley & Sons Ltd on behalf of European Hematology Association. This is an open access Abstract Book distributed under the Attribution-NonCommercial-NoDerivs (CC BY-NC-ND), which allows third parties to download the articles and share them with others as long as they credit the author and the Abstract Book, but they cannot change the content in any way or use them commercially.
**Abstract Book Citations:** Authors, Title, HemaSphere, 2026;10:(S1):pages. The abstract book DOIs can be found on the "Author Information" page.
**Disclaimer:** Articles published in the journal HemaSphere exclusively reflect the opinions of the authors. The authors are responsible for all content in their abstracts including accuracy of the facts, statements, citing resources, etc.

### (PB3412) CO-OCCURRENCE OF DUAL DRIVER MUTATIONS IN MYELOPROLIFERATIVE NEOPLASMS: FREQUENCY AND PHENOTYPIC IMPLICATIONS IN AN ASIAN COHORT

**Topic:** 15. Myeloproliferative neoplasms - Biology & translational research

Chih-Cheng Chen**[1, 2], Ying-Hsuan Wang[1, 2]

[1]*National Yang Ming Chiao Tung University, School of Medicine, Faculty of Medicine, Taipei, Taiwan;*[2]*Taipei Veterans General Hospital, Department of Oncology, Taipei, Taiwan;*

### Background

Canonical driver mutations in myeloproliferative neoplasms (MPNs)—*JAK2*V617F, *JAK2* exon 12, *CALR*, and *MPL*—all activate the JAK–STAT signaling axis and have historically been regarded as mutually exclusive events. Emerging evidence, however, increasingly challenges this paradigm, with concurrent driver mutations being documented in a subset of MPN patients. Data specific to Asian populations remain scarce.

### Aims

This study sought to establish the prevalence of double driver mutations in an Asian MPN cohort and to delineate the associated clinical and hematologic characteristics.

### Methods

A retrospective analysis was conducted on 142 consecutively enrolled MPN patients carrying heterozygous *JAK2*V617F (n = 106), *CALR* (n = 26), *MPL* (n = 7), or *JAK2* exon 12 (n = 3) mutations. All subjects underwent comprehensive molecular screening for co-existing canonical driver mutations. *JAK2*V617F status and mutant allele burden (AB) were quantified by quantitative competitive allele-specific TaqMan PCR (qCAST-PCR), while *CALR*, *MPL*, and *JAK2* exon 12 mutations were assessed by conventional PCR and Sanger sequencing.

### Results

Concurrent driver mutations were detected in 5 out of 142 patients (3.5%). All five harbored co-existing *JAK2*V617F, with two cases showing *JAK2*/*CALR* co-mutation, two with *JAK2*/*MPL*, and one with simultaneous *JAK2*V617F and *JAK2* exon 12 mutations. Notably, the co-occurrence rate was substantially elevated among patients with low *JAK2* AB (0.1–10%), at 11.1%, versus 1.4% in those with AB >10%. For further phenotypic analysis, the four cases with bona fide double drivers (excluding the *JAK2*V617F/exon 12 patient due to potential biological overlap) were examined. All four had low *JAK2* AB (range: 0.37–2.7%). Compared with single-driver patients carrying similarly low *JAK2* AB, double-driver patients exhibited distinct hematologic features, with notably higher platelet counts at the time of diagnosis. Conversely, their hematologic profiles were broadly similar to those of patients with *CALR*- or *MPL*-mutated MPNs. This phenotypic pattern suggests that when *JAK2* AB is low, the non-*JAK2* driver may be the dominant determinant of disease phenotype.

**Copyright Information:** (Online) ISSN: 2572-9241
©2026 The Author(s). HemaSphere is published by John Wiley & Sons Ltd on behalf of European Hematology Association. This is an open access Abstract Book distributed under the Attribution-NonCommercial-NoDerivs (CC BY-NC-ND), which allows third parties to download the articles and share them with others as long as they credit the author and the Abstract Book, but they cannot change the content in any way or use them commercially.
**Abstract Book Citations:** Authors, Title, HemaSphere, 2026;10:(S1):pages. The abstract book DOIs can be found on the "Author Information" page.
**Disclaimer:** Articles published in the journal HemaSphere exclusively reflect the opinions of the authors. The authors are responsible for all content in their abstracts including accuracy of the facts, statements, citing resources, etc.

### Summary/Conclusion

Although uncommon, co-occurrence of two MPN driver mutations is not negligible in Asian patients, with a pronounced enrichment in cases carrying low *JAK2* AB. The phenotypic resemblance of dual-driver cases to *CALR*- and *MPL*-mutated MPNs, together with the allele burden–dependent enrichment, substantiates the concept of hierarchical driver dominance and clonal complexity within this disease group. These observations underscore the clinical utility of systematic screening for all canonical driver mutations at MPN diagnosis, which may enhance disease classification accuracy and guide more tailored patient management.

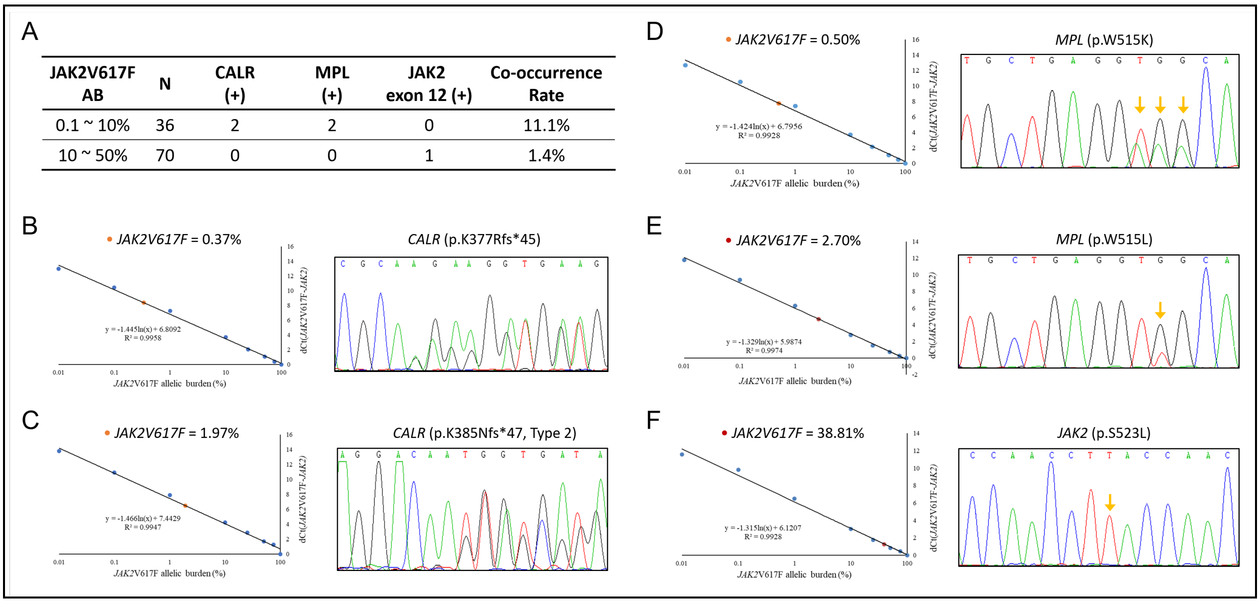

**Copyright Information:** (Online) ISSN: 2572-9241
©2026 The Author(s). HemaSphere is published by John Wiley & Sons Ltd on behalf of European Hematology Association. This is an open access Abstract Book distributed under the Attribution-NonCommercial-NoDerivs (CC BY-NC-ND), which allows third parties to download the articles and share them with others as long as they credit the author and the Abstract Book, but they cannot change the content in any way or use them commercially.
**Abstract Book Citations:** Authors, Title, HemaSphere, 2026;10:(S1):pages. The abstract book DOIs can be found on the "Author Information" page.
**Disclaimer:** Articles published in the journal HemaSphere exclusively reflect the opinions of the authors. The authors are responsible for all content in their abstracts including accuracy of the facts, statements, citing resources, etc.

### (PB3413) STAGE-DEPENDENT IMMUNE PROFILING OF T AND NK CELLS IN MULTIPLE MYELOMA PATIENTS USING FLOW CYTOMETRY

**Topic:** 15. Myeloproliferative neoplasms - Biology & translational research

Myung-Won Lee*[1], Sang-Woo Lee[2], Thi Thuy Duong Pham[2], Ik-Chan Song[1], Jeong Suk Koh[1], Wonhyoung Seo[1], Jung-Hyun Park[3], Deog-Yeon Jo[1]

*[1]Department of Internal Medicine, College of Medicine, Chungnam National University, Daejeon, South Korea;[2]Department of Medical Science, College of Medicine, Chungnam National University, Department of Internal Medicine, Daejeon, South Korea; [3]Translational Immunology Institute, College of Medicine, Chungnam National University, Daejeon, South Korea;*

### Background

Despite therapeutic advances, Multiple Myeloma (MM) remains an incurable disease, largely because the bone marrow immune microenvironment deteriorates over time. However, the precise phenotypic changes of T and NK cells—specifically the link between terminal differentiation and exhaustion—remain unclear.

### Aims

This study aimed to characterize the unique immune landscape of relapsed bone marrow in a Korean cohort, focusing on surface markers associated with the paradoxical expansion of terminally differentiated CD8+ T cells and concurrent NK cell dysfunction

### Methods

We analyzed bone marrow mononuclear cells from 18 individuals across four distinct disease stages (Normal, MGUS, NDMM, and RRMM) using multiparametric spectral flow cytometry (AURORA, CYTEK). By utilizing a comprehensive high-dimensional panel comprising 17 markers targeting differentiation, exhaustion, and senescence, and employing UMAP analysis, we were able to visualize the global restructuring of the immune architecture.

### Results

Our analysis revealed a dramatic restructuring of the immune landscape in Relapsed/Refractory MM (RRMM). Crucially, while we observed a numerical expansion of CD8+ T cells, this was driven by an accumulation of terminally differentiated effector memory (EMRA) cells rather than functional effectors. These cells were characterized by the elevated expression of inhibitory receptors (PD-1, TIGIT, TIM-3) and senescence markers (KLRG1) alongside the death receptor FAS. Significantly, NK cells also underwent a similar shift, acquiring T cell-like exhaustion features and, along with CD8+ T cells, showing a striking upregulation of FAS. This indicates a high susceptibility to apoptosis, which was further compounded by a significant expansion of regulatory T cells (Tregs).

### Summary/Conclusion

Ultimately, the relapsed marrow is fundamentally restructured into a "senescent-exhausted" state dominated by functionally inert and apoptosis-prone effector cells. These findings imply that to achieve durable disease control, future therapies must go beyond simple checkpoint blockade and actively target immune senescence to restore effector cell longevity

**Copyright Information:** (Online) ISSN: 2572-9241
©2026 The Author(s). HemaSphere is published by John Wiley & Sons Ltd on behalf of European Hematology Association. This is an open access Abstract Book distributed under the Attribution-NonCommercial-NoDerivs (CC BY-NC-ND), which allows third parties to download the articles and share them with others as long as they credit the author and the Abstract Book, but they cannot change the content in any way or use them commercially.
**Abstract Book Citations:** Authors, Title, HemaSphere, 2026;10:(S1):pages. The abstract book DOIs can be found on the “Author Information” page.
**Disclaimer:** Articles published in the journal HemaSphere exclusively reflect the opinions of the authors. The authors are responsible for all content in their abstracts including accuracy of the facts, statements, citing resources, etc.

### (PB3414) DEVELOPMENT AND VALIDATION OF A NOVEL PERIPHERAL BLOOD-BASED BIOMARKER PANEL FOR THE NON-INVASIVE DIAGNOSIS OF MULTIPLE MYELOMA AND PRECURSOR STATES

**Topic:** 15. Myeloproliferative neoplasms - Biology & translational research

Irit Avivi Mazza*[1], Yael Michaeli[2], Miriam Neaman[3], Lynne Itelson[2], Yuval Ebenstein[4], Tamir Shragai[1], Irit Segalovich[1], Shahar Zikrin[2], Efstathios Kastritis[5]

*[1]Hematology division, Tel Aviv Sourasky Medical center, Tel Aviv, Israel;[2]JaxBio Technologies Ltd., Netanya, Israel; [3]Department of Hematology, Tel Aviv Sourasky Medical Center, Tel Aviv, Israel , Department of Chemistry, Raymond and Beverly Sackler Faculty of Exact Sciences, Tel Aviv University, Tel Aviv, Israel; [4]Department of Biomedical Engineering, Faculty of Engineering, Tel Aviv Univesity, Tel Aviv, Israel , Department of Chemistry, Raymond and Beverly Sackler Faculty of Exact Sciences, Tel Aviv University, Tel Aviv, Israel, Tel Aviv, Israel; [5]Department of Clinical Therapeutics, National and Kapodistrian University of Athens, School of Medicine, Athens, Greece, Athens, Greece;*

### Background

Early detection and accurate risk stratification of plasma cell dyscrasias (PCD) remain significant clinical challenges, often requiring invasive bone marrow biopsies and intensive longitudinal monitoring. Identifying the precise transition from precursor states—Monoclonal Gammopathy of Undetermined Significance (MGUS) and Smoldering Multiple Myeloma (SMM)—to active Multiple Myeloma (MM) is critical for timely intervention. The prospective, multicenter SANGUINE study (NCT05735704) aims to establish a novel epigenetic liquid biopsy for the non-invasive diagnosis of hematological malignancies. Building on our initial discovery stage (ICML 2025, Abstract #420), which established unique methylome signatures for PCD, we developed the HemaChip platform. This targeted, methylation-based microarray is designed for sensitive, scalable, and cost-effective testing to distinguish healthy individuals from patients across the PCD spectrum.

### Aims

To validate our epigenetic HemaChip in diagnosing different PCD subtypes.

### Methods

DNA was extracted from blood samples obtained from patients with MM, SMM, MGUS, and healthy controls. Epigenetic profiling was first conducted using a high-content commercial methylation discovery array. An in-house deep neural network (DNN) analytical pipeline identified methylome targets associated with disease presence and progression stage. These selected biomarkers were integrated into the HemaChip platform. Utilizing a proprietary workflow, Genomic DNA (analyzing full buffy coat without separation) was fluorescently labeled, hybridized to the array, and processed. Finally, discovery-derived classification models were applied to the HemaChip data to evaluate diagnostic discrimination and analytical consistency.

**Copyright Information:** (Online) ISSN: 2572-9241
©2026 The Author(s). HemaSphere is published by John Wiley & Sons Ltd on behalf of European Hematology Association. This is an open access Abstract Book distributed under the Attribution-NonCommercial-NoDerivs (CC BY-NC-ND), which allows third parties to download the articles and share them with others as long as they credit the author and the Abstract Book, but they cannot change the content in any way or use them commercially.
**Abstract Book Citations:** Authors, Title, HemaSphere, 2026;10:(S1):pages. The abstract book DOIs can be found on the "Author Information" page.
**Disclaimer:** Articles published in the journal HemaSphere exclusively reflect the opinions of the authors. The authors are responsible for all content in their abstracts including accuracy of the facts, statements, citing resources, etc.

### Results

During the discovery phase, high-content methylation profiling was performed on a large cohort (N=425) comprising nine hematological conditions and age-matched controls. From ~1 million methylation sites, the DNN pipeline identified ~1,000 highly predictive targets able to differentiate between these entities, providing the foundational targets for the HemaChip. The HemaChip assay was subsequently clinically validated in a cohort of 119 participants: MM (n=32), MGUS/SMM (n=23), and age- and sex-matched healthy controls (n=64). Against healthy controls, the assay demonstrated high diagnostic performance for active MM, achieving 88% sensitivity, 92% specificity, and an Area Under the Curve (AUC) of 0.90 using a 130-biomarker signature (Fig 1A). Similarly, the platform effectively distinguished precursor states (MGUS/SMM) from healthy individuals with 87% sensitivity, 81% specificity and an AUC of 0.90 utilizing a 125-biomarker panel (Fig 1B). Furthermore, a refined 40-biomarker .
panel successfully discriminated against active MM from asymptomatic precursor states, correctly classifying 91% of MM cases and 81% of MGUS/SMM cases. (Fig 1C).

### Summary/Conclusion

The HemaChip epigenetic liquid biopsy provides a highly accurate, cost-effective, and minimally invasive peripheral blood alternative to bone marrow biopsies across all plasma cell dyscrasias. Crucially, its robust differentiation of MGUS/SMM from active MM addresses a major unmet clinical need. These continuously validated findings support its future use for scalable longitudinal monitoring, empowering clinicians to accurately detect disease progression and initiate timely therapy. Expanded validation data will be presented at EHA

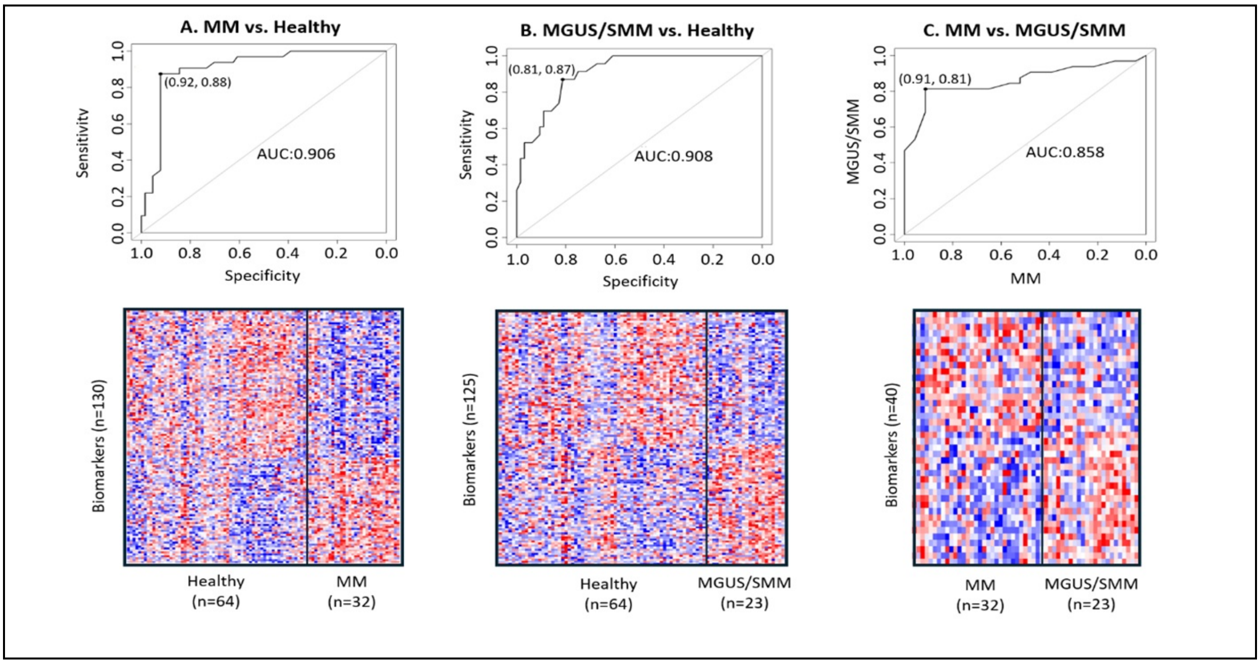

**Copyright Information:** (Online) ISSN: 2572-9241
©2026 The Author(s). HemaSphere is published by John Wiley & Sons Ltd on behalf of European Hematology Association. This is an open access Abstract Book distributed under the Attribution-NonCommercial-NoDerivs (CC BY-NC-ND), which allows third parties to download the articles and share them with others as long as they credit the author and the Abstract Book, but they cannot change the content in any way or use them commercially.
**Abstract Book Citations:** Authors, Title, HemaSphere, 2026;10:(S1):pages. The abstract book DOIs can be found on the “Author Information” page.
**Disclaimer:** Articles published in the journal HemaSphere exclusively reflect the opinions of the authors. The authors are responsible for all content in their abstracts including accuracy of the facts, statements, citing resources, etc.

### (PB3415) REAL-WORLD TREATMENT PATTERNS AND OUTCOMES IN PATIENTS WITH MYELOFIBROSIS TREATED FIRST-LINE WITH PACRITINIB OR RUXOLITINIB

**Topic:** 16. Myeloproliferative neoplasms - Clinical

James Rossetti*[1], Douglas Tremblay[2], Abiola Oladapo[3], Michael Marrone[3], Caleb Paydar[4], Djibril Liassou[4], Sarah Buckley[3], Purvi Suthar[3], Adina Geprifti[4], Michael Vredenburg[3], Bruce Feinberg[4], Naveen Pemmaraju[5]

*[1]University of Pittsburgh Medical Center, Pittsburgh, United States of America;[2]Icahn School of Medicine at Mount Sinai, New York, United States of America; [3]Sobi, Inc., Waltham, United States of America;[4]Cardinal Health, Dublin, United States of America; [5]University of Texas MD Anderson Cancer Center, Houston, United States of America;*

### Background

In patients (pts) with myelofibrosis (MF), cytopenias are a poor prognostic factor associated with poor response to therapy and shorter survival. JAK inhibitors such as ruxolitinib (RUX) are often used in the first-line (1L) setting to address splenomegaly and symptoms. As the utility of RUX is limited in cytopenic pts, pacritinib (PAC) is often selected as 1L therapy. Real-world (RW) evidence is limited in describing pt characteristics, treatment patterns and outcomes in pts receiving RUX or PAC in the 1L.

### Aims

To describe RW treatment patterns and outcomes of pts with MF in the United States treated with PAC or RUX in the 1L setting.

### Methods

This was a multisite chart review of pts with MF treated with RUX or PAC for ≥1 month between 06/2022 and 07/2024, with ≥6 months of follow-up through 01/2025 or death, whichever is earlier. Pts were followed from initiation of RUX or PAC (index) until the earliest of end of the study, the end of data availability for the pt, or death. Evaluated data included pt characteristics, treatment patterns, palpable spleen lenght based on ultrasound (total lenght minus 10cm), MF-related symptoms, and overall survival through Day 180. Data were summarized descriptively; the Kaplan Meier method was used to estimate survival probabilities.

### Results

A total of 103 pts were treated with 1L RUX and 104 pts received 1L PAC. Compared to pts receiving PAC, those who received RUX were less cytopenic at index (median platelet [PLT] count 144 vs 45 x$10^9$/L and median hemoglobin [Hb] 9.8 vs 9 g/dL) and had less advanced disease (peripheral blasts ≥2% in 35.3% vs 54.5% and percentage of pts with fibrosis grade 3 in 16.5% vs 22.8%). However, baseline spleen size category and presence of MF symptoms were comparable (**Table 1**).
Most pts initiated 1L treatment with RUX or PAC at the full therapeutic dose, with 91% of RUX pts receiving ≥30 mg daily and 100% of PAC pts receiving 400 mg total daily. A higher proportion had ≥1 dose reduction on RUX (19.4%; 20/103) than PAC (14.4%; 15/104). After follow-up 88.4% remained on RUX (median 11 months follow-up) and 73.1% of pts remained on PAC (median 10.3 months follow-up).
Among evaluable pts with spleen ultrasound measurements at index and Day 180, median spleen length reduction (SLR) was 41% (n=24) for RUX and 45% (n=23) for PAC. The percentage of evaluable pts achieving SLR ≥50% on RUX and PAC were 33.3% [8/24] and 43.5% [10/23], respectively. A majority in both groups experienced a decrease in the number of MF symptoms from index to Day 180 (81.6% [n=102] and 80.8% [n=100]). Similarly, the number of symptoms reported decreased by a median of 80% (RUX) and 67% (PAC) from index to Day 180. At the end of the observation period, 92.2% (95/103) of RUX pts and 89.4% (93/104) of PAC pts were still alive. The 6-month probability of survival was 97.1% (95% CI: 91.2-99.1) and 96.2% (95% CI: 90.1-98.5%) for RUX and PAC, respectively.

**Copyright Information:** (Online) ISSN: 2572-9241
©2026 The Author(s). HemaSphere is published by John Wiley & Sons Ltd on behalf of European Hematology Association. This is an open access Abstract Book distributed under the Attribution-NonCommercial-NoDerivs (CC BY-NC-ND), which allows third parties to download the articles and share them with others as long as they credit the author and the Abstract Book, but they cannot change the content in any way or use them commercially.
**Abstract Book Citations:** Authors, Title, HemaSphere, 2026;10:(S1):pages. The abstract book DOIs can be found on the "Author Information" page.
**Disclaimer:** Articles published in the journal HemaSphere exclusively reflect the opinions of the authors. The authors are responsible for all content in their abstracts including accuracy of the facts, statements, citing resources, etc.

**Summary/Conclusion**

These RW data demonstrate that 1L PAC is used in more advanced cytopenic MF pts with lower PLT counts, whereas RUX is used in pts with higher blood counts. Despite these population differences and the worse prognosis typically observed for cytopenic pts, PAC and RUX pts appeared to have similar 6-month survival probabilities, and both achieved clinically meaningful spleen and symptom responses in the 1L setting. The activity of PAC observed in a higher risk, higher disease burden population warrants investigation of its use in more favorable pts.

**Table 1** Patient Characteristics and Treatment Outcomes by 1L Treatment

| | Ruxolitinib (n=103) | Pacritinib (n=104) |
|---|---|---|
| **Patient Characteristics at Index** | | |
| Age at MF diagnosis, median (IQR), years | 70 (65.0 – 75.0) | 72 (65.5 – 77.0) |
| Male, n (%) | 53 (51.5) | 57 (54.8) |
| White, n (%) | 76 (73.8) | 70 (67.3) |
| Bone marrow fibrosis grade 3, n/N (%) | 16/97 (16.5) | 21/92 (22.8) |
| Platelet count (x $10^9$/L), median (IQR) | 144 (111 – 202) | 45 (40 – 49) |
| Hemoglobin (g/dL), median (IQR) | 9.8 (8.9 – 11.0) | 9.0 (8.2 – 10.1) |
| Peripheral blast category (evaluable), n | 51 | 66 |
| 0- <2 | 33 (64.7) | 30 (45.5) |
| 2- <5 | 18 (35.3) | 34 (51.5) |
| >5 | 0 | 2 (3.0) |
| Palpable spleen length based on ultrasound, median (IQR) | 9 cm (7 – 10) (n=36) | 7 cm (6 – 9) (n=35) |
| Baseline spleen category (evaluable), n | 62 | 63 |
| Not palpable, n% | 4 (6.4) | 4 (6.4) |
| Mild enlargement (5-10 cm palpable), n% | 28 (45.2) | 30 (47.6) |
| Moderate enlargement (11-20 cm palpable), n% | 29 (46.8) | 25 (39.7) |
| Severe enlargement (>20 cm palpable), n% | 1 (1.6) | 4 (6.4) |
| Any MF symptom present | 99.0% | 96.2% |
| **Treatment outcomes from Index to Day 180** | | |
| SLR*, % (median, IQR) | 41% (IQR: 22.5-50.0) (n=24) | 45% (IQR: 33.3-66.7) (n=23) |
| % of patients with SLR ≥50%* at Day 180 | 33.3% (8/24) | 43.5% (10/23) |
| % of patients with reduction in MF-symptoms reported | 81.6% (n=102) | 80.8% (n=100) |
| # of symptoms decreased, % (median, IQR) | 80% (50-100) | 67% (50-100) |

*Spleen length measured by ultrasound.
1L, first-line; IQR, interquartile range; MF, myelofibrosis; SLR, spleen length reduction.

**Copyright Information:** (Online) ISSN: 2572-9241
©2026 The Author(s). HemaSphere is published by John Wiley & Sons Ltd on behalf of European Hematology Association. This is an open access Abstract Book distributed under the Attribution-NonCommercial-NoDerivs (CC BY-NC-ND), which allows third parties to download the articles and share them with others as long as they credit the author and the Abstract Book, but they cannot change the content in any way or use them commercially.
**Abstract Book Citations:** Authors, Title, HemaSphere, 2026;10:(S1):pages. The abstract book DOIs can be found on the “Author Information” page.
**Disclaimer:** Articles published in the journal HemaSphere exclusively reflect the opinions of the authors. The authors are responsible for all content in their abstracts including accuracy of the facts, statements, citing resources, etc.

### (PB3416) LONG-TERM CLINICAL BENEFIT WITH AVAPRITINIB IN INDOLENT SYSTEMIC MASTOCYTOSIS: PIONEER LONG-TERM RESULTS WITH FOLLOW-UP THROUGH 4 YEARS

**Topic:** 16. Myeloproliferative neoplasms - Clinical

Jens Panse*[1, 2], Cem Akin[3], Tsewang Tashi[4], Deepti H. Radia[5], Ingunn Dybedal[6], Andreas Reiter[7], Celalettin Ustun[8], Prithviraj Bose[9], Daniel J. Deangelo[10], Lindsay Rein[11], Pankit Vachhani[12], Jason Gotlib[13], Sigurd Broesby-Olsen[14], Frank Siebenhaar[15, 16], Karin Hartmann[17, 18, 19], Vishnu Sundaresh[20], Thanai Pongdee[21], Vito Sabato[22], Cristina Bulai Livideanu[23], Massimo Triggiani[24], Tracy I. George[2, 25], Hui-Min Lin[26], Ilda Bidollari[26], Kate Newberry[26], Guang Yang[26], Ashley Doyle[26], Janet Hong[26], Benjamin Lampson[26], Mariana Castells[27], Ivan Alvarez-Twose[28]

*[1]Department of Oncology, Hematology, Hemostaseology, and Stem Cell Transplantation,University Hospital Aachen, Medical Faculty, RWTH Aachen University, Aachen, Germany; [2]Center for Integrated Oncology (CIO), Aachen, Bonn, Cologne, Düsseldorf (ABCD), Aachen, Germany; [3]University of Michigan, Ann Arbor, MI, United States of America; [4]Huntsman Cancer Institute, University of Utah, Salt Lake City, UT, United States of America; [5]Guy's & St Thomas' NHS Foundation Trust, London, United Kingdom; [6]Department of Hematology, Oslo University Hospital, Oslo, Norway; [7]Department of Hematology and Oncology, University Hospital Mannheim, Heidelberg University, Mannheim, Germany; [8]Department of Internal Medicine, Division of Hematology, Oncology and Cell Therapy, Section of Bone Marrow Transplantation and Cellular Therapy, Rush Medical College, Chicago, IL, United States of America; [9]Department of Leukemia, Division of Cancer Medicine, The University of Texas MD Anderson Cancer Center, Houston, TX, United States of America; [10]Dana-Farber Cancer Institute, Boston, MA, United States of America; [11]Department of Medicine, Duke University School of Medicine, Durham, NC, United States of America; [12]Department of Medicine, University of Alabama at Birmingham, Birmingham, AL, United States of America;[13]Stanford Cancer Institute/Stanford University School of Medicine, Stanford, CA, United States of America; [14]Department of Dermatology and Allergy Centre, Odense University Hospital, Odense, Denmark; [15]Institute of Allergology, Charité–Universitätsmedizin Berlin, Corporate member of Freie Universität Berlin and Humboldt-Universität zu Berlin, Berlin, Germany; [16]Fraunhofer Institute for Translational Medicine and Pharmacology ITMP, Immunology and Allergology, Berlin, Germany; [17]Division of Allergy, Department of Dermatology, University Hospital Basel and University of Basel, Basel, Switzerland; [18]Department of Clinical Research, University Hospital Basel and University of Basel, Basel, Switzerland; [19]Department of Biomedicine, University Hospital Basel and University of Basel, Basel, Switzerland; [20]Division of Endocrinology, University of Utah School of Medicine, Salt Lake City, UT, United States of America; [21]Division of Allergic Diseases, Mayo Clinic, Rochester, MN, United States of America; [22]Department of Immunology, Allergology and Rheumatology, University of Antwerp, and Antwerp University Hospital, Antwerp, Belgium; [23]Department of Dermatology, Expert Center of Mastocytosis (CEREMAST), Toulouse University Hospital, Toulouse, France; [24]Division of Allergy and Clinical Immunology, University of Salerno, Salerno, Italy;[25]ARUP Laboratories, Department of Pathology, University of Utah School of Medicine, Salt Lake City, UT, United States of America; [26]Blueprint Medicines Corporation, Cambridge, MA, United States of America; [27]Brigham and Women's Hospital, Harvard Medical School, Boston, MA, United States of America; [28]Institute of Mastocytosis Studies of Castilla-La Mancha, Virgen del Valle Hospital, Toledo, Spain;*

### Background

Indolent systemic mastocytosis (ISM), a clonal mast cell disease primarily driven by the *KIT* D816V mutation, can cause long-term debilitating symptoms and impact bone health. Avapritinib, a potent, selective KIT D816V inhibitor, is approved in patients with moderate-to-severe ISM in the EU and in adults with ISM in the USA.

### Aims

To report the long-term efficacy and safety of avapritinib for the treatment of ISM.

**Copyright Information:** (Online) ISSN: 2572-9241
©2026 The Author(s). HemaSphere is published by John Wiley & Sons Ltd on behalf of European Hematology Association. This is an open access Abstract Book distributed under the Attribution-NonCommercial-NoDerivs (CC BY-NC-ND), which allows third parties to download the articles and share them with others as long as they credit the author and the Abstract Book, but they cannot change the content in any way or use them commercially.
**Abstract Book Citations:** Authors, Title, HemaSphere, 2026;10:(S1):pages. The abstract book DOIs can be found on the "Author Information" page.
**Disclaimer:** Articles published in the journal HemaSphere exclusively reflect the opinions of the authors. The authors are responsible for all content in their abstracts including accuracy of the facts, statements, citing resources, etc.

### Methods

PIONEER (NCT03731260) enrolled patients with moderate-to-severe ISM symptoms. Patients initiated avapritinib 25 mg once daily (QD) + best supportive care across Parts 1, 2, or 3. In Part 3, dose increase to 50 mg was permitted based on investigator discretion and disease burden. Long-term efficacy was assessed by ISM-Symptom Assessment Form (ISM-SAF; ©2018 Blueprint Medicines Corporation) total symptom score (TSS) and optional dual-energy X-ray absorptiometry (DXA) scans. Safety was assessed.

### Results

As of September 17, 2025, 226 patients initiated avapritinib 25 mg QD with a median (range) treatment duration of 46.5 (0.7–67.2) months. Avapritinib maintained durable improvement through ~4 years in TSS (**Figure 1**) and across all symptom domains (skin, gastrointestinal, and neurocognitive). Avapritinib-treated patients also showed improved bone mineral density (BMD) over time (mean change [standard deviation] from baseline in BMD in the lumbar spine was 1.60% [5.53, n=60] at 1 year and 2.73% [9.30, n=26] at 4 years). Seventy-four patients increased to 50 mg QD with a median (range) treatment duration after dose increase of 16.5 (0.2–33.6) months with improvements in TSS at 6- and 12-months post-escalation. Avapritinib was well tolerated at doses of 25 mg QD long-term and 50 mg QD, with low rates of treatment-related discontinuations and no new safety concerns identified at either dose.

### Summary/Conclusion

Avapritinib is an effective chronic treatment for ISM, as demonstrated by the long-term symptom improvement and well-tolerated safety profile during ~4 years of treatment. By targeting the underlying KIT D816V driver mutation, benefits with avapritinib may extend beyond symptom improvements and address other health consequences of ISM as suggested by improved BMD findings.

**Figure 1: Mean change in ISM-SAF TSS at 1 year and 4 years**

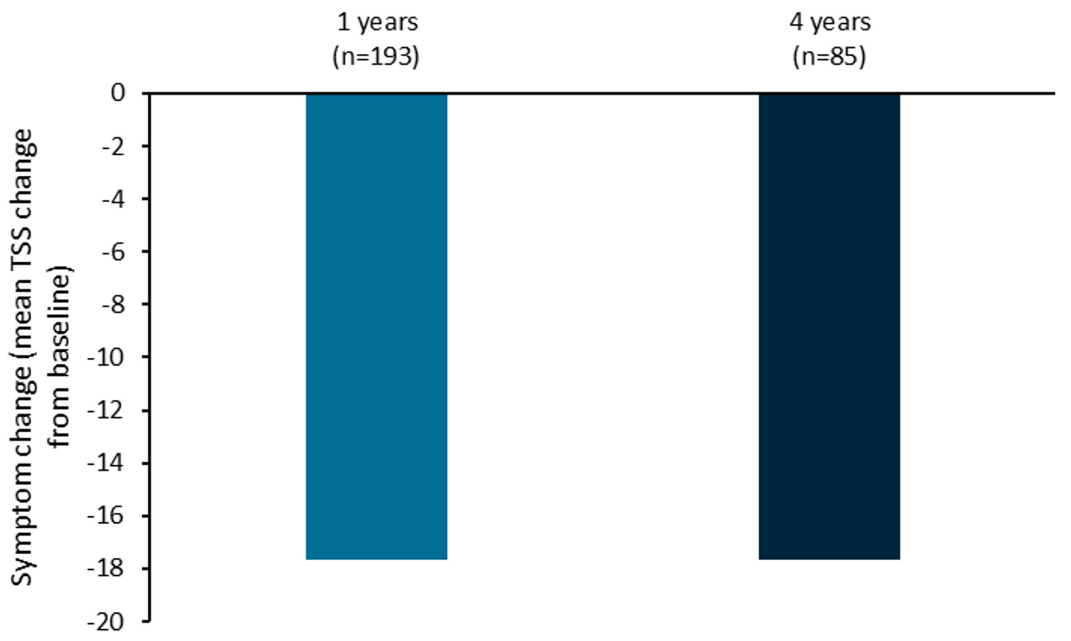

ISM-SAF, Indolent Systemic Mastocytosis - Symptom Assessment Form; TSS, total symptom score.

**Copyright Information:** (Online) ISSN: 2572-9241
©2026 The Author(s). HemaSphere is published by John Wiley & Sons Ltd on behalf of European Hematology Association. This is an open access Abstract Book distributed under the Attribution-NonCommercial-NoDerivs (CC BY-NC-ND), which allows third parties to download the articles and share them with others as long as they credit the author and the Abstract Book, but they cannot change the content in any way or use them commercially.
**Abstract Book Citations:** Authors, Title, HemaSphere, 2026;10:(S1):pages. The abstract book DOIs can be found on the "Author Information" page.
**Disclaimer:** Articles published in the journal HemaSphere exclusively reflect the opinions of the authors. The authors are responsible for all content in their abstracts including accuracy of the facts, statements, citing resources, etc.

### (PB3417) THE COMBINATION OF RUXOLITINIB AND THE CDK4/6 INHIBITOR ABEMACICLIB DEMONSTRATES SAFETY AND EFFICACY IN PREVIOUSLY TREATED MYELOFIBROSIS PATIENTS: RESULTS OF A PHASE I STUDY

**Topic:** 16. Myeloproliferative neoplasms - Clinical

Jan Bewersdorf[1], Andriy Derkach[2], Amer M. Zeidan[1], Emily Burton[2], Katherine Kan[2], Marissa Guiliani[2], Jeetayu Biswas[2], Michael Mauro[2], Brian Chernak[2], Tamanna Haque[2], Nikolai Podoltsev[1], Prithviraj Bose[3], Ross Levine[2], Raajit K Rampal*[2]

*[1]Yale University School of Medicine, Department of Internal Medicine, New Haven, United States of America;[2]Memorial Sloan Kettering Cancer Center, New York, United States of America; [3]MD Anderson Cancer Center, Houston, United States of America;*

### Background

Janus kinase (JAK) inhibitors such as ruxolitinib (RUX) improve disease-related symptoms and splenomegaly in patients (pts) with myelofibrosis (MF) but do not change the underlying disease biology. JAK-STAT activating mutations are a hallmark of MF and promote G1/S-phase cell cycle transition via increased expression of CDC25A, Cyclin D and CDK6. Preclinical studies testing the combination of JAK inhibitors and CDK4/6 inhibitors have demonstrated disease-directed biologic effects beyond those seen with JAK inhibition alone.

### Aims

We conducted a phase I dose-escalation trial (NCT05714072) to evaluate the safety of combination therapy with RUX and the CDK4/6 inhibitor abemaciclib (ABE) in pts with MF with inadequate response to RUX.

### Methods

This phase I dose escalation trial enrolled pts with MF who were treated with RUX for ≥12 weeks and had an inadequate response to RUX, defined as either persistent radiographic splenomegaly (≥450 $cm^3$) or MPN-SAF Total Symptom Score (TSS ≥5). Pts with platelets ≥75 × $10^9$/L and < 10% circulating or bone marrow blasts were eligible to enroll. Three escalating dose levels (DL) of ABE (50 mg, 100 mg, and 150 mg all BID) were combined with fixed doses of 10mg BID or 15mg BID of RUX using a “3+3” design. The primary endpoint was safety and identification of the recommended phase II dose of the combination. CTCAE v5.0 was used to grade adverse events (AEs). Dose-limiting toxicities (DLTs) were defined as grade ≥3 AEs during the first 28 days of combination therapy if related to either study drug. Secondary endpoints included spleen volume reduction by 25% (SVR25) and 35% (SVR35) and absolute MPN-SAF TSS change.

**Copyright Information:** (Online) ISSN: 2572-9241
©2026 The Author(s). HemaSphere is published by John Wiley & Sons Ltd on behalf of European Hematology Association. This is an open access Abstract Book distributed under the Attribution-NonCommercial-NoDerivs (CC BY-NC-ND), which allows third parties to download the articles and share them with others as long as they credit the author and the Abstract Book, but they cannot change the content in any way or use them commercially.
**Abstract Book Citations:** Authors, Title, HemaSphere, 2026;10:(S1):pages. The abstract book DOIs can be found on the “Author Information” page.
**Disclaimer:** Articles published in the journal HemaSphere exclusively reflect the opinions of the authors. The authors are responsible for all content in their abstracts including accuracy of the facts, statements, citing resources, etc.

### Results

As of 11/30/2025, enrollment is complete, and 11 pts were enrolled (3 pts each at DL0 and DL1, 5 at DL2). Median age at enrollment was 66 years [range: 54-76], 8 pts had a *JAK2*$^{V617F}$ and 3 pts a *CALR* mutation. All pts had DIPSS intermediate/high risk disease. Baseline disease characteristics included (medians, with range): spleen volume 1991cm$^3$ (531-3587cm$^3$), MPN-SAF TSS 33 (14-47), WBC 26.4/μL (5-70/μL), peripheral blood blasts 2% (0-7%), LDH 943 U/L (202-2915 U/L). Median time on study was 5 cycles (range: 1 -20) with 7 pts still on treatment at time of data cut-off. No DLTs or AE-related treatment discontinuations were observed. All AEs except for grade 3 anemia (n=2) and neutropenia (n=1), were grade 1/2 with diarrhea (62.5%), anemia (25%), and neutropenia (25%) being the most common treatment-emergent AEs. At data cut-off, 9 of 11 treated pts were evaluable for spleen volume and symptom response. 7 of 9 evaluable patients (78%) achieved SVR25 and 4 of 9 patients (44%) achieved SVR35, with median SVR of -31% (-55.3% - +19.6%; **Figure**). 6 out of 9 pts had TSS reduction with median change in absolute TSS of -4 (-13 - +12). Leukocytosis resolved in 7 out of 9 evaluable patients by cycle 4 (median baseline and C4 WBC 26.5/μL (13.5-73.3/μL) and 6.8/μL (3.9-22.5/μL), respectively). Data on bone marrow fibrosis, cytokines and molecular responses will be presented at the meeting.

### Summary/Conclusion

The combination of RUX and the CDK 4/6 inhibitor ABE among pts with advanced, previously treated MF is safe and has encouraging efficacy. A phase II trial of RUX + ABE at a dose of 100mg BID in previously treated MF pts is ongoing.

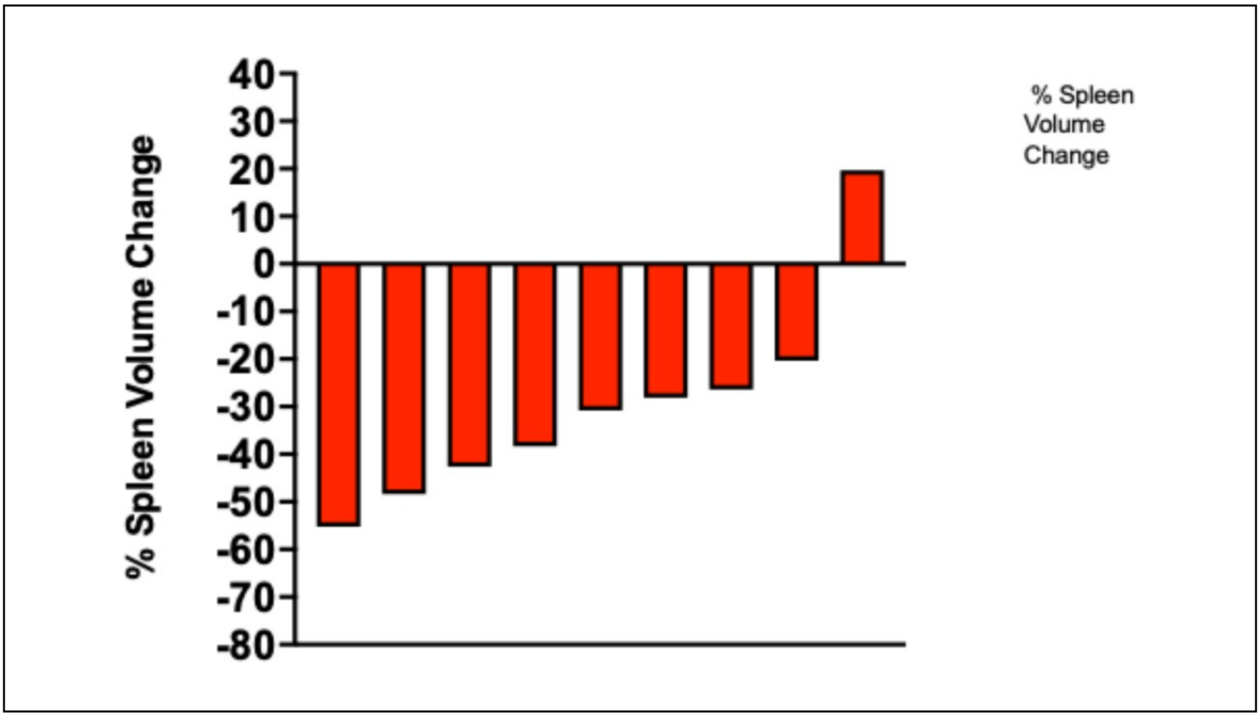

Figure: Maximum spleen volume reduction from baseline

**Copyright Information:** (Online) ISSN: 2572-9241
©2026 The Author(s). HemaSphere is published by John Wiley & Sons Ltd on behalf of European Hematology Association. This is an open access Abstract Book distributed under the Attribution-NonCommercial-NoDerivs (CC BY-NC-ND), which allows third parties to download the articles and share them with others as long as they credit the author and the Abstract Book, but they cannot change the content in any way or use them commercially.
**Abstract Book Citations:** Authors, Title, HemaSphere, 2026;10:(S1):pages. The abstract book DOIs can be found on the "Author Information" page.
**Disclaimer:** Articles published in the journal HemaSphere exclusively reflect the opinions of the authors. The authors are responsible for all content in their abstracts including accuracy of the facts, statements, citing resources, etc.

### (PB3418) FROM IMMUNOSUPPRESSION TO IMMUNE REBALANCING: EARLY IMMUNOLOGICAL EFFECTS OF MOMELOTINIB IN MYELOFIBROSIS

**Topic:** 16. Myeloproliferative neoplasms - Clinical

Andrea Duminuco*[1], Nunziatina Laura Parrinello[1], Sara Marino[1], Annalisa Santisi[1], Arianna Sbriglione[1], Paolo Antonio Cavaleri[1], Cesarina Giallongo[2], Francesco Di Raimondo[1], Giuseppe Alberto Maria Palumbo[1]

*[1]AOU Policlinico "G.Rodolico-San Marco", Catania, Italy; [2]Department of Biomedical and Biotechnological Sciences, Division of Medical Biochemistry, Catania, Italy;*

### Background

Myelofibrosis (MF) is a clonal myeloproliferative neoplasm characterized by bone marrow fibrosis, cytopenias, splenomegaly, and constitutional symptoms. Ruxolitinib revolutionized treatment, improving symptom control and reducing splenomegaly, but it induces significant immunosuppression. Momelotinib (MMB), a JAK1/JAK2/ACVR1 inhibitor, provides anemia benefit via ACVR1, hepcidin modulation. While its effects on inflammatory cytokines (particularly IL-6 and IL-1β) are documented, the specific impact on lymphocyte subpopulations and their cytokine secretory profile remains poorly characterized.

### Aims

To longitudinally characterize peripheral immune cell subsets in MF patients on MMB, focusing on T-lymphocyte differentiation/trafficking markers, NK cell subsets, and monocyte inflammatory/immunosuppressive phenotypes.

### Methods

This study enrolled MF patients receiving MMB. Peripheral blood was collected at baseline, week 4, and week 24 for multiparameter flow cytometry of T-cell subsets, NK cells, and monocytes, and for intracellular cytokine staining after stimulation. Serum cytokines were measured by multiplex assay, and immunological changes were correlated with standard clinical response endpoints.

**Copyright Information:** (Online) ISSN: 2572-9241
©2026 The Author(s). HemaSphere is published by John Wiley & Sons Ltd on behalf of European Hematology Association. This is an open access Abstract Book distributed under the Attribution-NonCommercial-NoDerivs (CC BY-NC-ND), which allows third parties to download the articles and share them with others as long as they credit the author and the Abstract Book, but they cannot change the content in any way or use them commercially.
**Abstract Book Citations:** Authors, Title, HemaSphere, 2026;10:(S1):pages. The abstract book DOIs can be found on the “Author Information” page.
**Disclaimer:** Articles published in the journal HemaSphere exclusively reflect the opinions of the authors. The authors are responsible for all content in their abstracts including accuracy of the facts, statements, citing resources, etc.

**Results**

We enrolled 28 MF patients (median age 72 years). At week 4 post-MMB initiation, significant immunological remodeling was observed.
Despite a modest absolute increase (+0.08 points, from 3.50% to 3.58%), Treg showed marked relative expansion (+42.7%), suggesting preserved immunoregulatory capacity during treatment.
Central memory CD4+CD197+ cells increased by 7.2 points (58.5%→65.8%), while CD8+CD197+ expanded +1.3 points (+29.6% relative). Effector memory CD8+CD197- decreased by 6.5 points (81.4%→74.9%), reflecting redistribution toward less-activated phenotypes. L-selectin+ populations (CD4+CD62L+, CD8+CD62L+) showed modest increases (+3.7 and +1.8 points).
Immunosuppressive monocytes (CD14+HLADR-LOW) underwent reduction (-7.2 points: 14.2%→7.0%, -43.8% relative), while inflammatory monocytes (CD14+CD16+) increased (+5.2 points: 16.4%→21.6%, +17.1%), indicating a favorable rebalancing of innate immunity.
T-helper cells showed a modest reduction (-4.1 points), T-cytotoxic remained stable (+1.3 points), while activated CD3-/CD8+/CD16+ subsets expanded (+0.7 points, +23.1% relative).
As part of an exploratory analysis with a reduced number of patients, marked increases in intracellular cytokine production were detected. CD4+ T cells producing IFN-γ doubled from 11.9% to 23.9% (+12.0 points, +21.7% relative change), while CD3+ IFN-γ+ cells increased from 17.7% to 31.4% (+13.7 points). IL-2-producing cells expanded across CD3+ (+10.8 points, from 60.9% to 71.7%) and CD4+ subsets (+7.9 points, from 72.2% to 80.1%). TNF-α production increased in CD4+ (+14.1 points, from 66.3% to 80.4%) and CD8+ compartments (+5.8 points, from 63.5% to 69.3%), indicating enhanced effector function.

**Summary/Conclusion**

Nine parameters showed absolute changes >5 points (six increases, three decreases), with the most pronounced shifts in cytokine production, where T cells showed marked functional enhancement, regulatory T cells exhibited relative expansion, and immunosuppressive monocytes were dramatically depleted. These findings suggest a favorable rebalancing of innate/adaptive immunity, potentially contributing to clinical responses while preserving immune competence. Expanded follow-up is ongoing.

**Copyright Information:** (Online) ISSN: 2572-9241
©2026 The Author(s). HemaSphere is published by John Wiley & Sons Ltd on behalf of European Hematology Association. This is an open access Abstract Book distributed under the Attribution-NonCommercial-NoDerivs (CC BY-NC-ND), which allows third parties to download the articles and share them with others as long as they credit the author and the Abstract Book, but they cannot change the content in any way or use them commercially.
**Abstract Book Citations:** Authors, Title, HemaSphere, 2026;10:(S1):pages. The abstract book DOIs can be found on the “Author Information” page.
**Disclaimer:** Articles published in the journal HemaSphere exclusively reflect the opinions of the authors. The authors are responsible for all content in their abstracts including accuracy of the facts, statements, citing resources, etc.

### (PB3419) UPDATED ANALYSIS OF OVERALL SURVIVAL (OS) WITH IMETELSTAT IN PATIENTS WITH RELAPSED OR REFRACTORY (R/R) MYELOFIBROSIS (MF) FROM IMBARK VS REAL-WORLD (RW) DATA, AND ASSESSMENT OF RW TREATMENT PATTERNS

**Topic:** 16. Myeloproliferative neoplasms - Clinical

Andrew Kuykendall*[1], Hana Qasim[1], Ghada Araji[1], Libo Sun[2], Qi Xia[2], Michelle Mudge-Riley[2], Joseph Eid[2], Rami S. Komrokji[1]

*[1]Moffitt Cancer Center, Tampa, United States of America; [2]Geron Corporation, Foster City, United States of America;*

### Background

Imetelstat showed significantly longer median OS (mOS) in patients with Janus kinase inhibitor (JAKi) R/R intermediate-2–risk or high-risk MF in the Phase 2 IMbark trial (NCT02426086) versus closely matched RW patients treated with best available therapy (BAT) after ruxolitinib (RUX; hazard ratio [HR], 0.35; *P*=.0019). The MF treatment landscape changed considerably in recent years, potentially impacting OS.

### Aims

To present updated OS analysis of IMbark patients versus a larger RW pt cohort after extended follow-up.

### Methods

The updated RW dataset included patients who discontinued RUX and were subsequently treated with BAT at Moffitt Cancer Center between 2010 and 2025. To assess changes in RW treatment patterns, baseline/disease characteristics and OS of patients diagnosed before/after 2016 and 2019 were compared. To update the IMbark versus RW OS analysis, a closely matched RW cohort was identified using IMbark eligibility criteria, including patients diagnosed before 2016 who received RUX but were R/R. The mOS was measured from time of JAKi discontinuation to death or censored at last follow-up. Propensity score approaches using average treatment effect for stabilized inverse probability treatment weighting (sIPTW) and overlap population (ATO) were implemented to adjust for baseline covariates/prognostic factors that may impact outcomes.

### Results

The RW treatment pattern assessment included 96 RW patients. Time from diagnosis to start of RUX and duration of RUX treatment were significantly shorter for patients diagnosed after 2016 versus before (**Table**). Similar changes were observed after 2019 versus before. The mOS in patients diagnosed after 2016 and 2019 was significantly longer versus before. In patients who had no other JAKi as first treatment after RUX (n=72), mOS was also longer for patients diagnosed after 2016 and 2019 vs before. These differences in OS benefit remained when patients were censored at the time of transplant.
The updated mOS analysis included 59 IMbark patients who had provided informed consent at the time of the clinical trial, and 54 closely matched RW patients. With a median follow-up of 46.2 mo for IMbark patients and 48.2 mo for RW patients, mOS (95% CI) was 30.7 mo (25.5-36.9) with imetelstat in IMbark vs 15.4 mo (13.1-30.5) with BAT in RW (HR, 0.512; *P*=.003) per the unweighted analysis. Propensity-weighted analyses (sIPTW, ATO) had similar results.

**Copyright Information:** (Online) ISSN: 2572-9241
©2026 The Author(s). HemaSphere is published by John Wiley & Sons Ltd on behalf of European Hematology Association. This is an open access Abstract Book distributed under the Attribution-NonCommercial-NoDerivs (CC BY-NC-ND), which allows third parties to download the articles and share them with others as long as they credit the author and the Abstract Book, but they cannot change the content in any way or use them commercially.
**Abstract Book Citations:** Authors, Title, HemaSphere, 2026;10:(S1):pages. The abstract book DOIs can be found on the "Author Information" page.
**Disclaimer:** Articles published in the journal HemaSphere exclusively reflect the opinions of the authors. The authors are responsible for all content in their abstracts including accuracy of the facts, statements, citing resources, etc.

### Summary/Conclusion

This updated post hoc analysis confirms a significantly more favorable OS benefit with imetelstat versus BAT in patients with R/R MF and poor prognosis consistent with the previous report (Kuykendall AT, et al. *Ann Hematol.* 2022;101(1):139-146). A significant improvement in OS in RW patients over the last decade was also demonstrated, which was independent of transplant status, and potentially due to shorter time from diagnosis to start of RUX and earlier switch to next-line JAKi or clinical trial. These results were also observed in patients treated with no other JAKi therapy after RUX. Understanding evolving treatment patterns are key considerations for interpreting future clinical trial outcomes.

**Table. Updated RW Data by Time of Diagnosis**

| | Before 2016 | After 2016 | Before 2019 | After 2019 |
|---|---|---|---|---|
| **Total population, n** | 54 | 42 | 69 | 27 |
| **Time from diagnosis to RUX start (mean), mo** | 46.7 | 10.5 | 39.7 | 8.2 |
| | *P*=.002 | | *P*=.015 | |
| **RUX duration (mean), mo** | 30.4 | 13.5 | 26.1 | 15.0 |
| | *P*=.015 | | *P*=.151 | |
| **Transplant, n (%)** | 3 (5.6) | 4 (9.5) | 5 (7.2) | 2 (7.4) |
| mOS (95% CI), mo | 15.4 (13.1-30.5) | 34.2 (24.2-NE) | 16.6 (14.2-30.0) | 39.8 (34.2-NE) |
| | HR=0.492; *P*=.008 | | HR=0.462; *P*=.018 | |
| mOS censored at time of transplant (95% CI), mo | 16.4 (12.0-30.7) | 30.0 (10.7-NE) | 16.6 (13.1-29.5) | 39.8 (34.2-NE) |
| | HR=0.565; *P*=.037 | | HR=0.429; *P*=.010 | |
| **Patients who had no other JAKi as first tx after RUX, n** | 42 | 30 | 55 | 17 |
| mOS (95% CI), mo | 15.4 (13.1-30.5) | 30.0 (14.2-NE) | 16.4 (14.2-30.0) | 49.6 (14.1-NE) |
| | HR=0.514; *P*=.028 | | HR=0.482; *P*=.055 | |
| mOS censored at time of transplant (95% CI), mo | 16.4 (12.0-30.5) | 25.1 (14.1-NE) | 16.4 (13.1-29.5) | 49.6 (14.1-NE) |
| | HR=0.600; *P*=.090 | | HR=0.425; *P*=.026 | |

HR, hazard ratio; JAKi, Janus kinase inhibitor; mOS, median overall survival; NE, not estimable; RUX, ruxolitinib; RW, real world; tx, treatment.

**Copyright Information:** (Online) ISSN: 2572-9241
©2026 The Author(s). HemaSphere is published by John Wiley & Sons Ltd on behalf of European Hematology Association. This is an open access Abstract Book distributed under the Attribution-NonCommercial-NoDerivs (CC BY-NC-ND), which allows third parties to download the articles and share them with others as long as they credit the author and the Abstract Book, but they cannot change the content in any way or use them commercially.
**Abstract Book Citations:** Authors, Title, HemaSphere, 2026;10:(S1):pages. The abstract book DOIs can be found on the "Author Information" page.
**Disclaimer:** Articles published in the journal HemaSphere exclusively reflect the opinions of the authors. The authors are responsible for all content in their abstracts including accuracy of the facts, statements, citing resources, etc.

### (PB3420) NEUTROPHIL EXTRACELLULAR TRAP FORMATION IS INCREASED IN PATIENTS WITH MYELOPROLIFERATIVE NEOPLASMS AND DECREASES WITH CYTOREDUCTIVE TREATMENT

**Topic:** 16. Myeloproliferative neoplasms - Clinical

Ingrid Lundahl*[1, 2], Johanna Ungerstedt[3, 4, 5], Axel Rosell[2, 4, 6], Roza Chaireti[1, 2, 7]

*[1]Karolinska Institutet, Department of Molecular Medicine and Surgery, Stockholm, Sweden; [2]Karolinska University Hospital, Department of Hematology, Stockholm, Sweden; [3]Linköping University, Department of Biomedical and Clinical Sciences, Linköping, Sweden; [4]Karolinska Institutet, HERM, Department of Medicine, Stockholm, Sweden; [5]Linköping University Hospital, Hematology Clinic, Linköping, Sweden; [6]Danderyd Hospital, Karolinska Institutet, Department of Clinical Sciences, Stockholm, Sweden; [7]Karolinska Institutet, Department of Medicine, Solna, Stockholm, Sweden;*

### Background

Neutrophil extracellular trap (NET) formation has been implicated in the pathogenesis of thrombosis in myeloproliferative neoplasms (MPN), although their exact role and the effect of cytoreductive therapy on NETs levels remain unclear.

### Aims

We assessed NETs levels measured as citrullinated histone H3 (H3Cit)-DNA in MPN patients at diagnosis and following cytoreductive treatment, compared with patients with secondary erythrocytosis, thrombocytosis and/or leukocytosis. We further investigated whether cytoreductive treatment lowers H3Cit-DNA in MPN and whether H3Cit-DNA levels are influenced by MPN type, molecular status, *JAK2* allelic burden, anticoagulant and antiplatelet treatment and previous history of thrombosis.

### Methods

We assessed NET formation, measured as citrullinated histone H3 (H3Cit)-DNA, in MPN patients at diagnosis and following cytoreductive treatment, compared with non-MPN patients with secondary erythrocytosis, thrombocytosis and/or leukocytosis. We further investigated whether cytoreductive treatment lowers H3Cit-DNA in MPN and whether H3Cit-DNA levels are influenced by MPN type, molecular status, *JAK2* allelic burden, antithrombotic treatment and prior thrombosis.

**Copyright Information:** (Online) ISSN: 2572-9241
©2026 The Author(s). HemaSphere is published by John Wiley & Sons Ltd on behalf of European Hematology Association. This is an open access Abstract Book distributed under the Attribution-NonCommercial-NoDerivs (CC BY-NC-ND), which allows third parties to download the articles and share them with others as long as they credit the author and the Abstract Book, but they cannot change the content in any way or use them commercially.
**Abstract Book Citations:** Authors, Title, HemaSphere, 2026;10:(S1):pages. The abstract book DOIs can be found on the "Author Information" page.
**Disclaimer:** Articles published in the journal HemaSphere exclusively reflect the opinions of the authors. The authors are responsible for all content in their abstracts including accuracy of the facts, statements, citing resources, etc.

### Results

Patients with MPN were predominantly female and older than non-MPN patients, although the prevalence of previous thrombosis was similar between the two groups. H3Cit-DNA levels were higher in MPN patients compared to non-MPN patients (p=0.029) but did not differ according to MPN subtype, mutational status or previous thrombosis. H3Cit-DNA levels decreased after cytoreductive treatment (p<0.0001) but remained unchanged in untreated patients. A similar reduction was observed in patients receiving both cytoreductive and antithrombotic therapy (p=0.0008), but not with antithrombotic therapy alone.

| | In total | MPN | No MPN | p-value |
|---|---|---|---|---|
| **N (%)** | 87 (100) | 56 (64.4) | 31 (35.6) | |
| **Age in years, median (range)** | 66 (20–86) | 70 (34–86) | 55 (20–83) | 0.008 |
| **Sex, n (%)** | | | | |
| Female | 47 (54.0) | 37 (66.1) | 10 (32.3) | 0.002 |
| Male | 40 (46.0) | 19 (33.9) | 21 (67.7) | |
| **Previous thrombosis, n (%)** | | | | |
| Yes | 22 (25.3) | 15 (26.8) | 7 (22.6) | 0.666 |
| No | 65 (74.7) | 41 (73.2) | 24 (77.4) | |
| **Previous thrombosis, type, n (%)** | | | | |
| Arterial | 12 (54.6) | 8 (53.3) | 4 (57.1) | |
| Venous | 6 (27.3) | 4 (26.7) | 2 (28.6) | |
| Both | 3 (13.6) | 2 (13.3) | 1 (14.3) | 0.921 |
| Unknown | 1 (4.6) | 1 (6.7) | 0 (0) | |
| **MPN subtype, n (%)** | | | | |
| PV | | 16 (28.6) | | |
| ET | | 30 (53.6) | | |
| PMF | | 8 (14.3) | | |
| MPN-U | | 1 (1.8) | | |
| MDS/MPN | | 1 (1.8) | | |
| **Molecular status, n (%)** | | | | |
| *JAK2* | 47 (54.0) | 44 (78.6) | 3 (9.7) | |
| *CALR* | 8 (9.2) | 7 (12.5) | 1 (3.2) | <0.001 |
| *MPL* | 3 (3.5) | 3 (5.4) | 0 (0) | |
| Triple-negative | 29 (33.3) | 2 (3.6) | 27 (87.1) | |

Table showing patient characteristics of the overall cohort (n=87), patients with MPN (n=56), and non-MPN patients (n=31). The JAK2 and CALR positivity in the non-MPN control group was considered as clonal hematopoiesis.
Abbreviations: MPN = myeloproliferative neoplasm, PV = polycythemia vera, ET = essential thrombocythemia, PMF = primary myelofibrosis, MPN-U = MPN unclassifiable, MDS = myelodysplastic syndrome

### Summary/Conclusion

We demonstrated higher H3Cit-DNA levels in MPN patients compared to non-MPN patients with elevated blood counts not fulfilling MPN criteria, and that cytoreductive treatment reduced the levels of H3Cit-DNA in serial samples. Taken together, these findings indicate that excessive NET formation is a characteristic of MPN which is targetable with cytoreductive therapy. Future studies combining NET and thromboinflammatory markers may lead to improved thrombotic risk stratification and further elucidate the thrombotic mechanisms in MPN.

**Copyright Information:** (Online) ISSN: 2572-9241
©2026 The Author(s). HemaSphere is published by John Wiley & Sons Ltd on behalf of European Hematology Association. This is an open access Abstract Book distributed under the Attribution-NonCommercial-NoDerivs (CC BY-NC-ND), which allows third parties to download the articles and share them with others as long as they credit the author and the Abstract Book, but they cannot change the content in any way or use them commercially.
**Abstract Book Citations:** Authors, Title, HemaSphere, 2026;10:(S1):pages. The abstract book DOIs can be found on the "Author Information" page.
**Disclaimer:** Articles published in the journal HemaSphere exclusively reflect the opinions of the authors. The authors are responsible for all content in their abstracts including accuracy of the facts, statements, citing resources, etc.

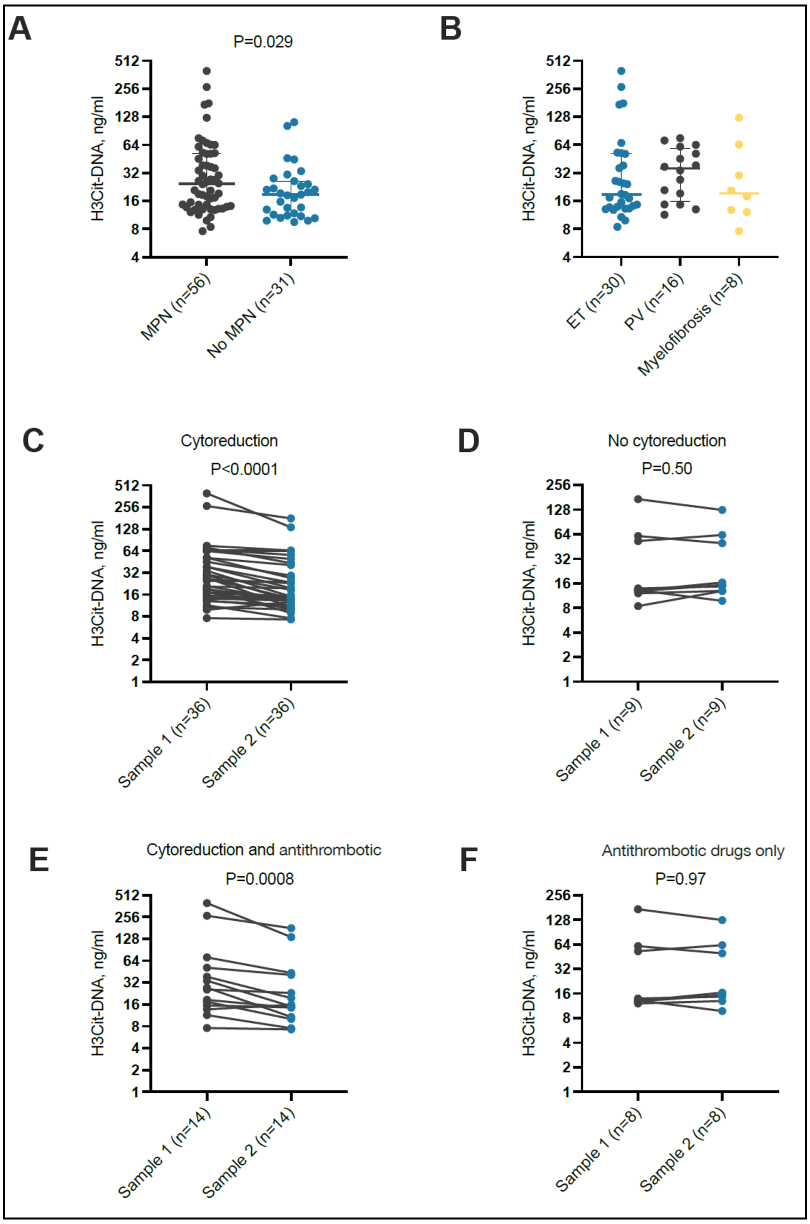

**Copyright Information:** (Online) ISSN: 2572-9241
©2026 The Author(s). HemaSphere is published by John Wiley & Sons Ltd on behalf of European Hematology Association. This is an open access Abstract Book distributed under the Attribution-NonCommercial-NoDerivs (CC BY-NC-ND), which allows third parties to download the articles and share them with others as long as they credit the author and the Abstract Book, but they cannot change the content in any way or use them commercially.
**Abstract Book Citations:** Authors, Title, HemaSphere, 2026;10:(S1):pages. The abstract book DOIs can be found on the "Author Information" page.
**Disclaimer:** Articles published in the journal HemaSphere exclusively reflect the opinions of the authors. The authors are responsible for all content in their abstracts including accuracy of the facts, statements, citing resources, etc.

### (PB3421) COMPARATIVE PREDICTIVE PERFORMANCE OF QRISK3, SCORE2, AND PREVENT IN 568 PATIENTS WITH BCR::ABL1-NEGATIVE MYELOPROLIFERATIVE NEOPLASMS

**Topic:** 16. Myeloproliferative neoplasms - Clinical

Alessandro Costa*[1, 2], Olga Mulas[1, 2], Federica Pilo[2], Martina Pettinau[2], Eleonora Atzeni[1], Daniela Mantovani[2], Giovanni Caocci[1, 2]

*[1]Department of Medical Sciences and Public Health, University of Cagliari, 09121 Cagliari, Italy, Caglari, Italy;[2]Hematology and BMT Unit, "A. Businco" Hospital, ARNAS Brotzu, Cagliari, Cagliari, Italy;*

### Background

Patients with *BCR::ABL1*-negative myeloproliferative neoplasms (MPN) have a persistent prothrombotic phenotype. Whether general-population cardiovascular risk scores improve thrombotic risk stratification in MPN remains uncertain.

### Aims

To evaluate QRISK3, ESC SCORE2/OP and PREVENT for prediction of incident thrombotic events and overall survival (OS) in MPN.

### Methods

Cardiovascular scores were calculated at baseline. The primary endpoint was time to first thrombotic event (myocardial infarction, ischemic stroke, arterial thrombosis or venous thromboembolism). Cause-specific Cox models and Fine–Gray competing-risk analyses (death as competing event) were performed. Discrimination was assessed using Harrell's C-index. OS was analyzed with Cox models (continuous and categorical forms).

**Copyright Information:** (Online) ISSN: 2572-9241
©2026 The Author(s). HemaSphere is published by John Wiley & Sons Ltd on behalf of European Hematology Association. This is an open access Abstract Book distributed under the Attribution-NonCommercial-NoDerivs (CC BY-NC-ND), which allows third parties to download the articles and share them with others as long as they credit the author and the Abstract Book, but they cannot change the content in any way or use them commercially.
**Abstract Book Citations:** Authors, Title, HemaSphere, 2026;10:(S1):pages. The abstract book DOIs can be found on the "Author Information" page.
**Disclaimer:** Articles published in the journal HemaSphere exclusively reflect the opinions of the authors. The authors are responsible for all content in their abstracts including accuracy of the facts, statements, citing resources, etc.

### Results

We retrospectively analyzed 568 consecutive patients with MPN. Median age was 64.5 years (IQR 51.6–74.4) and 49.3% were male. Median follow-up was 5.2 years (IQR 2.50–10.48). ESC SCORE2/OP categories were distributed as: low risk, *n*=174 (33.3%); intermediate risk, *n*=199 (38.0%); high risk, *n*=150 (28.7%). Median QRISK3 was 11.8% (IQR 4.0–22.2); 46.3% were <10%, 24.3% 10–19.9%, and 29.4% ≥20%. Median PREVENT was 8.4% (IQR 3.2–16.6); 46.0% were <7.5%, 37.9% 7.5–19.9%, and 16.1% ≥20%.
ESC categories were not associated with overall thrombotic risk (HR 1.21, 95% CI 0.79–1.85; *p*=0.39; C-index 0.56), confirmed in competing-risk analysis (*p*=0.30). Continuous QRISK3 was associated with thrombotic risk (HR 1.03 per 1% increase, 95% CI 1.00–1.05; *p*=0.034; C-index 0.61). When analyzed categorically, only the ≥20% group showed increased risk compared with <10% (HR 2.30, 95% CI 1.14–4.63; *p*=0.0198). In competing-risk analysis, QRISK3 ≥20% remained significantly associated with ATE+VTE (sHR 2.28, 95% CI 1.15–4.49; *p*=0.0177; Gray's test *p*=0.0145). PREVENT was not significantly associated with thrombotic risk when modeled continuously (HR 1.04; *p*=0.072; C-index 0.57). However, PREVENT ≥20% identified a high-risk subgroup compared with <7.5% (HR 2.80, 95% CI 1.27–6.21; *p*=0.0110). Competing-risk analysis confirmed the association for PREVENT ≥20% (sHR 2.75, 95% CI 1.26–6.00; *p*=0.0110; Gray's test *p*=0.0028). For OS, continuous QRISK3 showed the highest discrimination (C-index 0.83; HR 1.09; *p*<0.001), followed by continuous PREVENT (C-index 0.812; HR 1.15; *p*<0.001), whereas ESC showed lower discrimination (C-index 0.716). Importantly, both QRISK3 and PREVENT remained significantly associated with OS when analyzed categorically. For QRISK3, the 10–19.9% group showed HR 6.19 (*p*<0.001) and the ≥20% group HR 24.0 (*p*<0.001). For PREVENT, 7.5–19.9% group showed HR 4.80 (*p*<0.001) and the ≥20% group HR 26.1 (*p*<0.001). ESC categories were also associated with OS, but with lower discriminatory capacity. In low-risk subgroup (i.e. age ≤65 y, no thrombotic history; *n*=154; 9 events), categorical analyses were not statistically estimable due to sparse event distribution. When modeled continuously, neither QRISK3 (sHR 0.94; *p*=0.64) nor PREVENT (sHR 0.83; *p*=0.35) was significantly associated with thrombotic risk.

### Summary/Conclusion

ESC SCORE2/OP does not discriminate thrombotic risk in MPN. In contrast, very-high-risk thresholds (≥20%) of QRISK3 and PREVENT identify subgroups with significantly increased arterial and venous thrombosis. Both scores show strong and consistent association with OS. Extreme cardiovascular risk categories may therefore complement MPN-specific models for integrated risk assessment.

**Copyright Information:** (Online) ISSN: 2572-9241
©2026 The Author(s). HemaSphere is published by John Wiley & Sons Ltd on behalf of European Hematology Association. This is an open access Abstract Book distributed under the Attribution-NonCommercial-NoDerivs (CC BY-NC-ND), which allows third parties to download the articles and share them with others as long as they credit the author and the Abstract Book, but they cannot change the content in any way or use them commercially.
**Abstract Book Citations:** Authors, Title, HemaSphere, 2026;10:(S1):pages. The abstract book DOIs can be found on the "Author Information" page.
**Disclaimer:** Articles published in the journal HemaSphere exclusively reflect the opinions of the authors. The authors are responsible for all content in their abstracts including accuracy of the facts, statements, citing resources, etc.

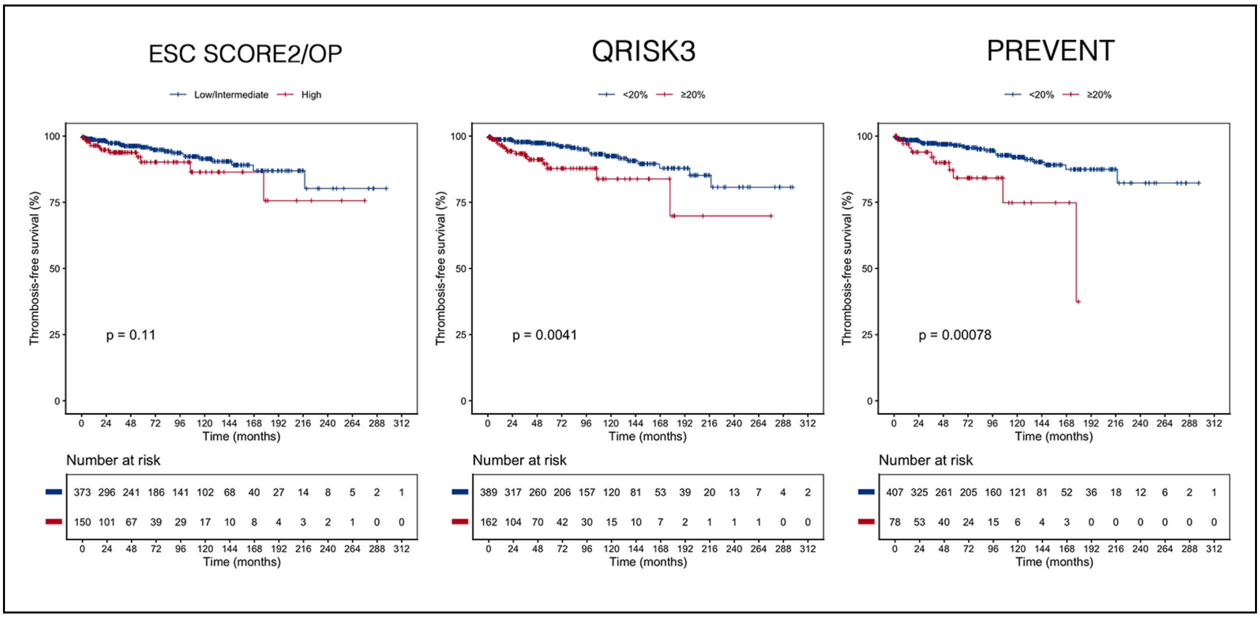

Figure. Thrombosis-free survival according to cardiovascular risk categories in patients with BCR::ABL1-negative myeloproliferative neoplasms Kaplan–Meier curves showing thrombosis-free survival stratified by (A) ESC SCORE2/OP categories (low/intermediate vs high risk), (B) QRISK3 categories (<20%, ≥20%), and (C) PREVENT categories (<20%, ≥20%).

**Copyright Information:** (Online) ISSN: 2572-9241
©2026 The Author(s). HemaSphere is published by John Wiley & Sons Ltd on behalf of European Hematology Association. This is an open access Abstract Book distributed under the Attribution-NonCommercial-NoDerivs (CC BY-NC-ND), which allows third parties to download the articles and share them with others as long as they credit the author and the Abstract Book, but they cannot change the content in any way or use them commercially.
**Abstract Book Citations:** Authors, Title, HemaSphere, 2026;10:(S1):pages. The abstract book DOIs can be found on the "Author Information" page.
**Disclaimer:** Articles published in the journal HemaSphere exclusively reflect the opinions of the authors. The authors are responsible for all content in their abstracts including accuracy of the facts, statements, citing resources, etc.

### (PB3422) TRANSITORY NATURE OF SGLT-2 INHIBITOR ASSOCIATED ERYTHROCYTOSIS

**Topic:** 16. Myeloproliferative neoplasms - Clinical

Marko Lucijanic*[1], Irzal Hadzibegovic[1], Marin Pavlov[1], Ivana Jurin[1]

[1]*University Hospital Dubrava, Hematology department, Zagreb, Croatia;*

### Background

Sodium-glucose cotransporter 2 inhibitors (SGLT2i), initially registered for diabetes mellitus and subsequently gaining a central role in the treatment of heart failure and chronic kidney disease, are widely used medications with the peculiar side effect of promoting erythrocytosis of unknown significance. Since substantial proportion of patients exposed to SGLT2i surpasses the threshold for polycythemia vera (PV) evaluation, and concerns about unrecognized myeloproliferative neoplasm (MPN) and potential thrombotic risk exist, further characterization of this phenomenon is needed.

### Aims to evaluate the extent and longitudinal trends of SGLT2i-associated erythrocytosis.

### Methods

We evaluated the occurrence of erythrocytosis among prospectively enrolled heart failure patients exposed to SGLT2is at our institution between May 2021 and May 2023. Hemoglobin levels were assessed at baseline, and at 6 and 12 months of SGLT2i use. Erythrocytosis was defined according to the WHO 2022 PV criteria: hemoglobin ≥160 g/L for females and ≥165 g/L for males. None of the patients had a known prior MPN, nor were any diagnosed with MPN upon further evaluation.

### Results

A total of 848 SGLT2i-exposed patients with heart failure were evaluated, of whom 278 (32.6%) were female. The median age was 69 years.
The median baseline hemoglobin concentration was 136 g/L, with 3.7% of patients qualifying as having polycythemia at baseline. At 6 and 12 months of SGLT2i treatment, the median hemoglobin concentration was 140 g/L and 141 g/L, respectively, with 5.2% and 3.5% of patients qualifying as having polycythemia, respectively. Hemoglobin concentration at both time points was significantly higher in comparison to baseline ($P<0.05$ for both comparisons).
Although the overall proportion of patients with erythrocytosis did not significantly differ between the baseline, 6-, and 12-month time points, the structure of patients with erythrocytosis shifted over time (Figure). Only approximately 1% of patients had persistent erythrocytosis throughout the baseline, 6, and 12 month periods. However, during the first 6 months, 4% of patients developed de novo erythrocytosis, while 2% experienced resolution compared to baseline. In the subsequent 6-month period, 3% of non-polycythemic patients developed erythrocytosis, and 3% of polycythemic patients experienced resolution. No significant increase in MACE was observed among consistently polycythemic patients.

### Summary/Conclusion

Although SGLT2i-treated heart failure patients experience an average increase in hemoglobin levels, MPN-range erythrocytosis is rare, developing in up to 5% of patients. The phenomenon of erythrocytosis is transient in many cases, with a continuous exchange of patients developing or resolving erythrocytosis over time. Only a small fraction (1%) of patients experiences persistent erythrocytosis; these individuals likely represent candidates for further hematologic follow-up.

**Copyright Information:** (Online) ISSN: 2572-9241
©2026 The Author(s). HemaSphere is published by John Wiley & Sons Ltd on behalf of European Hematology Association. This is an open access Abstract Book distributed under the Attribution-NonCommercial-NoDerivs (CC BY-NC-ND), which allows third parties to download the articles and share them with others as long as they credit the author and the Abstract Book, but they cannot change the content in any way or use them commercially.
**Abstract Book Citations:** Authors, Title, HemaSphere, 2026;10:(S1):pages. The abstract book DOIs can be found on the "Author Information" page.
**Disclaimer:** Articles published in the journal HemaSphere exclusively reflect the opinions of the authors. The authors are responsible for all content in their abstracts including accuracy of the facts, statements, citing resources, etc.

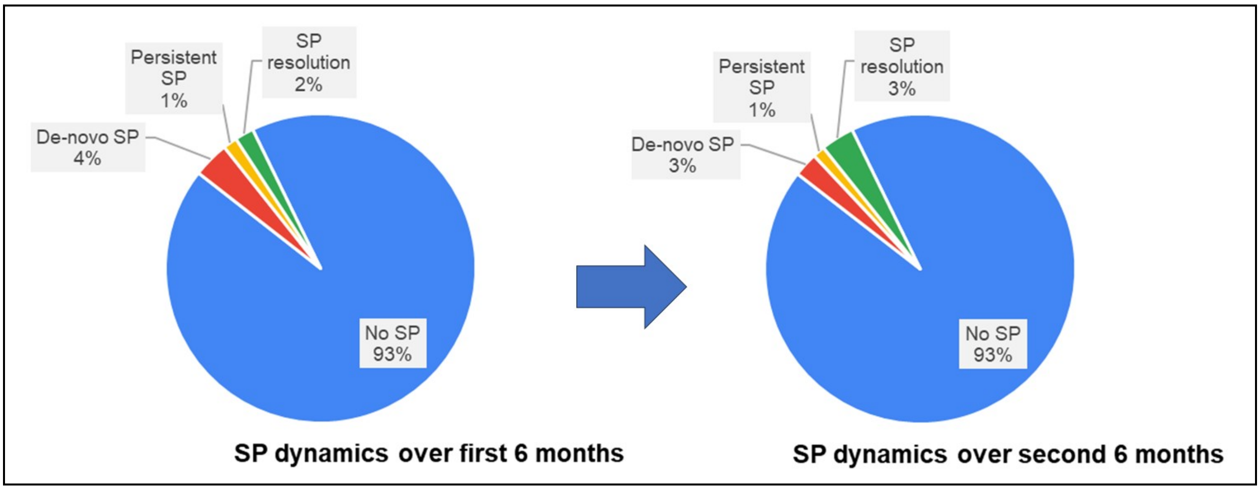

**Copyright Information:** (Online) ISSN: 2572-9241
©2026 The Author(s). HemaSphere is published by John Wiley & Sons Ltd on behalf of European Hematology Association. This is an open access Abstract Book distributed under the Attribution-NonCommercial-NoDerivs (CC BY-NC-ND), which allows third parties to download the articles and share them with others as long as they credit the author and the Abstract Book, but they cannot change the content in any way or use them commercially.
**Abstract Book Citations:** Authors, Title, HemaSphere, 2026;10:(S1):pages. The abstract book DOIs can be found on the "Author Information" page.
**Disclaimer:** Articles published in the journal HemaSphere exclusively reflect the opinions of the authors. The authors are responsible for all content in their abstracts including accuracy of the facts, statements, citing resources, etc.

### (PB3423) EFFECT OF TREATMENT WITH SODIUM-GLUCOSE CO-TRANSPORTER 2 INHIBITORS AMONG PATIENTS ON OUTCOMES AMONG PATIENTS WITH POLYCYTHEMIA VERA AND TYPE 2 DIABETES MELLITUS

**Topic:** 16. Myeloproliferative neoplasms - Clinical

Orly Leiva[1], Gabriela Hobbs*[2], Rushad Patell[3], Katherine Feder[4]

*[1]University of Kentucky Gill Heart & Vascular Institute, Lexington, Kentucky, United States of America;[2]Massachusetts General Hospital, Boston, Massachusetts, United States of America; [3]Beth Israel Deaconess Medical Center, Boston, Massachusetts, United States of America; [4]Dana Farber Cancer Institute, Boston, Massachusetts, United States of America;*

### Background

Polycythemia vera (PV) is a Philadelphia-chromosome negative myeloproliferative neoplasm (MPN) characterized by clonal erythrocytosis. The leading cause of death in PV is secondary to cardiovascular (CV) complications, including arterial and venous thrombosis and heart failure (HF). Sodium-glucose co-transporter-2 inhibitors (SGLT2-I) are increasingly common medications shown to have mortality and cardiovascular benefits in cardiometabolic disorders including HF, chronic kidney disease (CKD), and type 2 diabetes mellitus (T2DM). However, SGLT2-I have been shown to increase hematocrit levels which is a potential concern for their use in patients with PV.

### Aims

To investigate the effect of SGLT2-I on outcomes in patients with PV and T2DM compared to PV patients receiving other medications for T2DM.

### Methods

We leveraged a large, international, retrospective database (TriNetX) to conduct a target trial emulation to investigate the effect of SGLT2-I use on outcomes in patients with PV with T2DM from 2014 to 2025. Patients with PV and T2DM who were treated with SGLT2-I (SGTL2-I group) were compared with patients treated with dipeptidyl peptidase IV inhibitor (DPP4-I) or sulfonylurea (DPP4/sulfonylurea group). Index date was defined as time from first prescription of either SGLT2-I or DPP4-I/sulfonylurea. Outcomes of interest included death, thrombosis, heart failure, first instance of uncontrolled hematocrit (HCT) of > 45%, and hematologic progression at five years from index date. Propensity score matching was performed using multivariable logistic regression with demographic, CV and non-CV comorbidities, and PV characteristics (hematocrit at index, cytoreduction) as co-variables. Cox proportional hazards regression was utilized to evaluate effect of SGLT2-I on outcomes.

### Results

A total of 433 patients treated with SGLT2-I were matched with 433 DPP4/sulfonylurea patients. After matching, the mean follow-up time in SGLT2-I group was 669 days (SD 549) and 1037 days in DPP4/sulfonylurea group (SD 674). After matching, variables were balanced between groups including age (66.1 vs 65.9 years), prior HF (31.2% vs 31.9%), CKD (34.9% vs 34.6%), aspirin use (64.7% vs 61.2%), hydroxyurea (42.0% vs 38.3%), and hematocrit (mean 46.3% vs 45.7%). Treatment with SGLT2-I was associated with decreased risk of death (8.1% vs 18.2%; HR 0.59, 95% CI 0.40 – 0.89). Patients with SGLT2-I had numerically number lower rates of HF but no association on time-to-event analysis were observed (8.3% vs 14.5%, p = 0.020; HR 0.72, 95% CI 0.44 – 1.20) compared with DPP4/sulfonylurea. Treatment with SGLT2-I was associated with faster time to first hematocrit of >45% after index (HR 1.32, 95% CI 1.12 – 1.57). However, there was no association between SGLT2-I and risk of thrombosis (25.2% vs 27.5%; HR 1.12, 95% CI 0.86 – 1.45).

**Copyright Information:** (Online) ISSN: 2572-9241
©2026 The Author(s). HemaSphere is published by John Wiley & Sons Ltd on behalf of European Hematology Association. This is an open access Abstract Book distributed under the Attribution-NonCommercial-NoDerivs (CC BY-NC-ND), which allows third parties to download the articles and share them with others as long as they credit the author and the Abstract Book, but they cannot change the content in any way or use them commercially.
**Abstract Book Citations:** Authors, Title, HemaSphere, 2026;10:(S1):pages. The abstract book DOIs can be found on the "Author Information" page.
**Disclaimer:** Articles published in the journal HemaSphere exclusively reflect the opinions of the authors. The authors are responsible for all content in their abstracts including accuracy of the facts, statements, citing resources, etc.

### Summary/Conclusion

In this observational, international cohort study of patients with PV and T2DM, SGLT2-I therapy was associated with reduced risk of death and numerically lower rates of HF compared with DPP4/sulfonylurea, Figure. Moreover, although SGLT2-I treated patients experienced HCT elevations, this did not translate to worse PV related outcomes. Our study results suggest that SGLT2-I therapy is safe and efficacious in patients with PV and T2DM and should be used according to current guidelines.

**Figure**

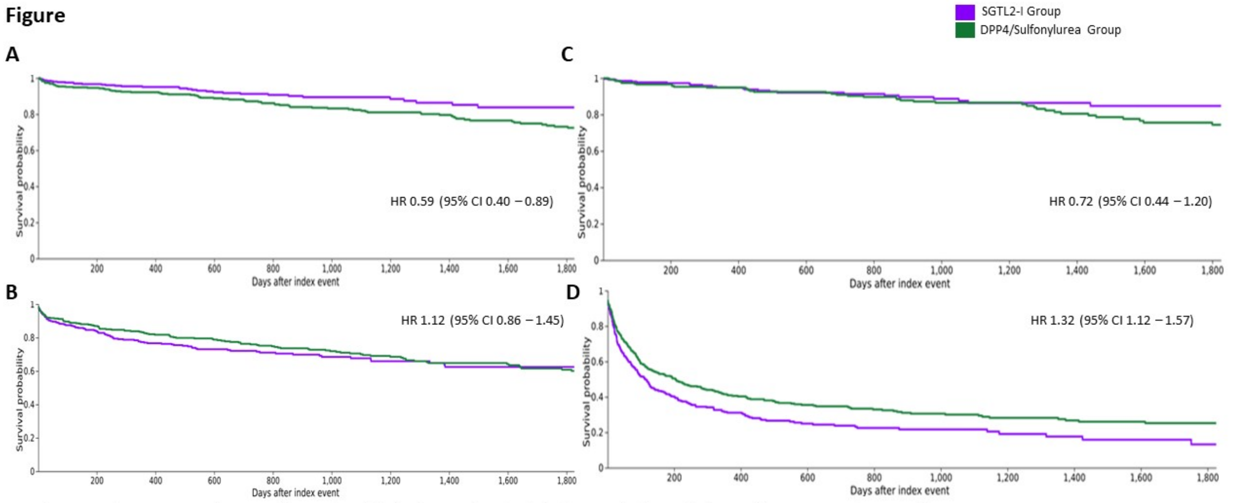

Kaplan-Meier curve of overall survival (A), thrombosis (B), heart failure (C), and hematocrit > 45%

**Copyright Information:** (Online) ISSN: 2572-9241
©2026 The Author(s). HemaSphere is published by John Wiley & Sons Ltd on behalf of European Hematology Association. This is an open access Abstract Book distributed under the Attribution-NonCommercial-NoDerivs (CC BY-NC-ND), which allows third parties to download the articles and share them with others as long as they credit the author and the Abstract Book, but they cannot change the content in any way or use them commercially.
**Abstract Book Citations:** Authors, Title, HemaSphere, 2026;10:(S1):pages. The abstract book DOIs can be found on the "Author Information" page.
**Disclaimer:** Articles published in the journal HemaSphere exclusively reflect the opinions of the authors. The authors are responsible for all content in their abstracts including accuracy of the facts, statements, citing resources, etc.

### (PB3424) PHILADELPHIA-NEGATIVE MYELOPROLIFERATIVE NEOPLASMS AND ACQUIRED VON WILLEBRAND SYNDROME: ONLY PERTINENT AT DIAGNOSIS ?

**Topic:** 16. Myeloproliferative neoplasms - Clinical

Fanny Lemarié*[1], Jérémy Picard[2], Hubert Galinat[3], Brigitte Pan-Petesch[1, 4], Christophe Nicole[5], Laura Herbreteau[1], Lanig Civi[1], Laëtitia Rio[1], Wayne-Corentin Lambert[3], Vincent Olivier[3], Simon Jaouen[3], Eric Lippert[3, 6], Jean Christophe Ianotto[1, 4, 6]

*[1]Service d'Hématologie et d'Hémostase clinique, Institut de Cancéro-Hématologie, CHU de Brest, Brest, France;[2]Service d'Épidémiologie et de Prévention du Risque Infectieux, CHU de Brest, Brest, France; [3]Laboratoire d'Hématologie, CHU de Brest, Brest, France; [4]Université de Bretagne Occidentale, Inserm, UMR 1034, GETBO, Brest, France;[5]Service d'Onco-Hématologie, Centre Hospitalier des Pays de Morlaix, Morlaix, France; [6]FIM, France Intergroupe des syndromes myéloprolifératifs, Paris, France;*

### Background

Philadelphia-negative myeloproliferative neoplasms (MPN) are associated with both thrombotic and bleeding complications. Acquired von Willebrand syndrome (AVWS), particularly reported in Essential Thrombocythemia (ET), affects 10% to 50% of patients, although its role in bleeding risk remains uncertain.

### Aims

To characterize patients with AVWS at the time of MPN diagnosis and evaluate the association with subsequent clinical events.

### Methods

In our ambispective single-center cohort study, 178 patients diagnosed with polycythemia vera (PV), ET, or prefibrotic myelofibrosis (pre-PMF) were included. AVWS was assessed at diagnosis and was defined by a VWF:GPIbM/VWF:Ag ratio <0.65. All patient provided informed consent according to local ethics requirements.

### Results

Prevalence of AVWS was 38% in the whole cohort (46.3% in PV, 33% in ET and 36.4% in pre-PMF cases). Patients with AVWS showed a more proliferative profile with elevated platelets, leukocytes, hematocrit and LDH levels. The incidence of AVWS increased with the number of elevated blood cell counts, particularly in patients with three myeloid lineages implications (OR= 6.14, 95% CI: 2.22–18.9, p<0.001). In multivariable analysis, female sex (OR=2.52, 95% CI:1.15-5.78, p=0.02), thrombocytosis > 700 $\times 10^9$/L (OR=10, 95% CI:4.48–24.3, p<0.001) and polycythemia (OR=3.75, 95% CI:1.54-9.61, p=0.004) remained independently associated with AVWS. History of bleeding was significantly higher in AVWS patients (17.65% vs. 5.5%, p=0.018). Hemorrhage-free survival and thrombosis-free survival were comparable between the two groups (p=0.45 and p=0.61, respectively).

### Summary/Conclusion

AVWS affects over one-third of newly diagnosed MPN patients, particularly women and those with thrombocytosis or polycythemia. These results suggest AVWS is not solely driven by extreme thrombocytosis but also by overall myeloproliferative burden.

**Copyright Information:** (Online) ISSN: 2572-9241
©2026 The Author(s). HemaSphere is published by John Wiley & Sons Ltd on behalf of European Hematology Association. This is an open access Abstract Book distributed under the Attribution-NonCommercial-NoDerivs (CC BY-NC-ND), which allows third parties to download the articles and share them with others as long as they credit the author and the Abstract Book, but they cannot change the content in any way or use them commercially.
**Abstract Book Citations:** Authors, Title, HemaSphere, 2026;10:(S1):pages. The abstract book DOIs can be found on the "Author Information" page.
**Disclaimer:** Articles published in the journal HemaSphere exclusively reflect the opinions of the authors. The authors are responsible for all content in their abstracts including accuracy of the facts, statements, citing resources, etc.

**Figure 1. Distribution of patients according to platelet count.**

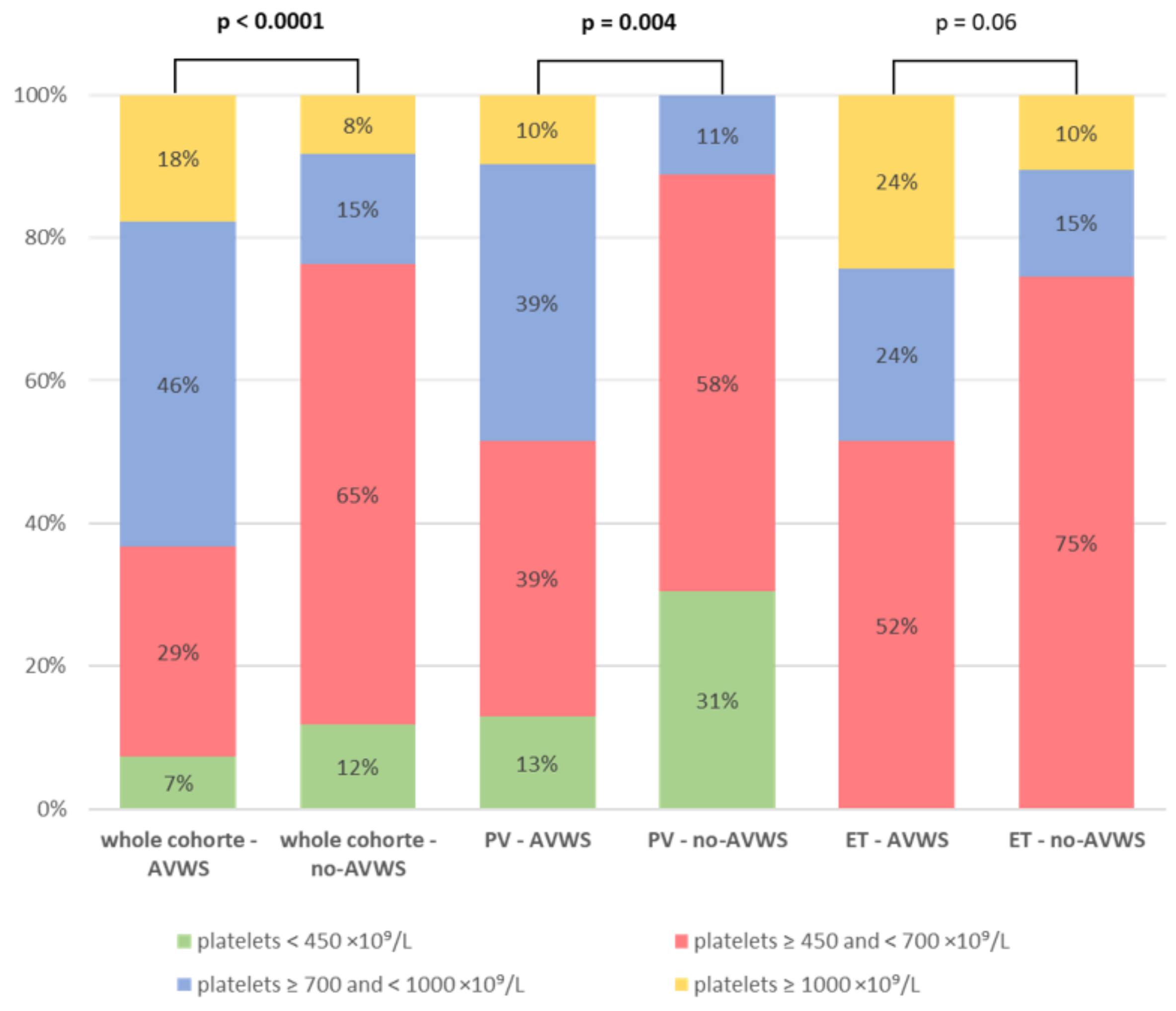

**Copyright Information:** (Online) ISSN: 2572-9241
©2026 The Author(s). HemaSphere is published by John Wiley & Sons Ltd on behalf of European Hematology Association. This is an open access Abstract Book distributed under the Attribution-NonCommercial-NoDerivs (CC BY-NC-ND), which allows third parties to download the articles and share them with others as long as they credit the author and the Abstract Book, but they cannot change the content in any way or use them commercially.
**Abstract Book Citations:** Authors, Title, HemaSphere, 2026;10:(S1):pages. The abstract book DOIs can be found on the "Author Information" page.
**Disclaimer:** Articles published in the journal HemaSphere exclusively reflect the opinions of the authors. The authors are responsible for all content in their abstracts including accuracy of the facts, statements, citing resources, etc.

### (PB3425) AGE-SPECIFIC RISK OF HEMATOLOGIC MALIGNANCIES AFTER ACUTE MYOCARDIAL INFARCTION: A NATIONWIDE COHORT STUDY

**Topic:** 16. Myeloproliferative neoplasms - Clinical

Kyoung Ha Kim*[1], Seug Yun Yoon[1], Jong-Ho Won[1]

[1]*Soonchunhyang University Seoul Hospital, Division of Hematology & Medical Oncology, Department of Internal Medicine, Seoul, South Korea;*

### Background

Survival after acute myocardial infarction (AMI) has improved, particularly among younger patients. As early cardiovascular mortality declines, non-cardiac outcomes are increasingly recognized. Hematologic malignancies share pathogenic mechanisms with atherosclerosis, including chronic inflammation and immune dysregulation. In addition, clonal hematopoiesis of indeterminate potential has emerged as a biological link between premature atherosclerotic cardiovascular disease and myeloid neoplasms. Several population-based studies, including our prior nationwide cohort analysis, have reported an increased incidence of hematologic malignancies following AMI. However, whether this association varies according to age has not been well characterized. We hypothesized that if shared clonal or inflammatory mechanisms contribute to both conditions, the relative association would be more pronounced in younger patients with premature AMI.

### Aims

To evaluate age-stratified risks of incident hematologic malignancies after AMI, with particular focus on patients younger than 50 years.

### Methods

We conducted a nationwide retrospective cohort study using the Korean National Health Insurance Service database. Patients newly diagnosed with AMI between 2003 and 2016 were identified using strict criteria requiring hospitalization, coronary angiography, and serial troponin testing. Individuals with prior hematologic malignancies were excluded. Propensity-score matching (1:2) based on age and sex was used to select controls. Participants were stratified into four age groups (<50, 50–60, 60–70, and ≥70 years). The primary outcome was incident hematologic malignancies. Incidence rates were calculated per 1,000 person-years. Hazard ratios were estimated using Fine–Gray competing-risk regression with death as a competing event, adjusting for socioeconomic status and major comorbidities. Standardized incidence ratios were calculated using national cancer statistics.

### Results

After matching, 294,398 patients with AMI and 558,084 controls were analyzed. During follow-up, 2,886 hematologic malignancies occurred in the AMI group and 7,297 in controls. Incidence was higher in the AMI group (1.23 vs. 0.91 per 1,000 person-years). AMI was independently associated with increased risk of hematologic malignancies (adjusted hazard ratio 1.42, 95% confidence interval 1.34–1.52), with significant interaction by age. Among individuals younger than 50 years, incidence rates were 0.77 versus 0.39 per 1,000 person-years, corresponding to more than twofold increased risk (hazard ratio 2.01, 95% confidence interval 1.62–2.51). Relative risk attenuated with age (1.57 for 50–60 years, 1.37 for 60–70 years, and 1.32 for ≥70 years) (Figure). Subtype analyses showed particularly elevated risks for myeloproliferative neoplasms and multiple myeloma, with more pronounced excess risk in younger age groups.

**Copyright Information:** (Online) ISSN: 2572-9241
©2026 The Author(s). HemaSphere is published by John Wiley & Sons Ltd on behalf of European Hematology Association. This is an open access Abstract Book distributed under the Attribution-NonCommercial-NoDerivs (CC BY-NC-ND), which allows third parties to download the articles and share them with others as long as they credit the author and the Abstract Book, but they cannot change the content in any way or use them commercially.
**Abstract Book Citations:** Authors, Title, HemaSphere, 2026;10:(S1):pages. The abstract book DOIs can be found on the "Author Information" page.
**Disclaimer:** Articles published in the journal HemaSphere exclusively reflect the opinions of the authors. The authors are responsible for all content in their abstracts including accuracy of the facts, statements, citing resources, etc.

**Summary/Conclusion**

AMI is associated with increased risk of hematologic malignancies, with a disproportionately stronger relative effect in patients younger than 50 years. These findings support the hypothesis that shared inflammatory or clonal hematopoietic mechanisms may link premature cardiovascular disease and malignant transformation. Further translational studies integrating genomic profiling are warranted to clarify age-dependent mechanisms and refine risk assessment in younger AMI survivors.

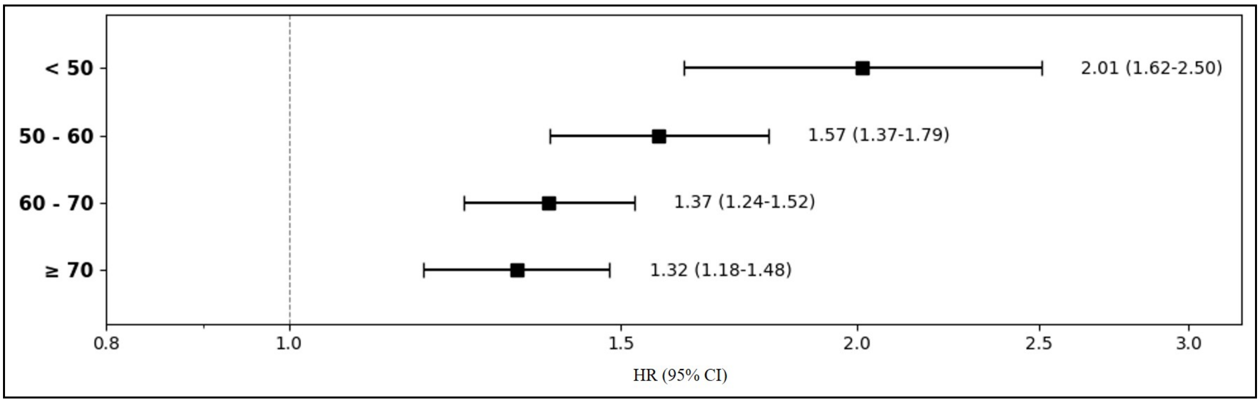

**Copyright Information:** (Online) ISSN: 2572-9241
©2026 The Author(s). HemaSphere is published by John Wiley & Sons Ltd on behalf of European Hematology Association. This is an open access Abstract Book distributed under the Attribution-NonCommercial-NoDerivs (CC BY-NC-ND), which allows third parties to download the articles and share them with others as long as they credit the author and the Abstract Book, but they cannot change the content in any way or use them commercially.
**Abstract Book Citations:** Authors, Title, HemaSphere, 2026;10:(S1):pages. The abstract book DOIs can be found on the "Author Information" page.
**Disclaimer:** Articles published in the journal HemaSphere exclusively reflect the opinions of the authors. The authors are responsible for all content in their abstracts including accuracy of the facts, statements, citing resources, etc.

### (PB3426) AI-BASED SCORE VERSUS CONVENTIONAL PROGNOSTIC SCORES IN MYELOFIBROSIS: A SINGLE-CENTER VALIDATION STUDY

**Topic:** 16. Myeloproliferative neoplasms - Clinical

Marta Ricci*[1, 2], Ludovica Lo Turco[2], Maria Helena Parrinello[1, 2], Francesca Lunghi[1], Fabio Ciceri[1, 2], Annalisa Ruggeri[1], Daniele Sannipoli[1]

*[1]IRCCS San Raffaele, Hematology and Bone Marrow Transplantation Unit, Milan, Italy; [2]Università Vita-Salute San Raffaele, Milan, Italy;*

### Background

Accurate estimation of overall survival (OS) in myelofibrosis (MF) is essential for optimal management given the marked clinical heterogeneity and the lack of curative options other than allogeneic hematopoietic stem cell transplantation (allo-HCT). Several prognostic models have been developed. More recently, machine learning–based tools have been proposed to enable individualized risk assessment, including the AIPSS-MF (Artificial Intelligence Prognostic Scoring System for Myelofibrosis) and the AlloRisk stratifier for patients who undergo allo-HCT.

### Methods

We retrospectively validated these models in a cohort of 95 patients diagnosed with MF according to WHO 2016/2022 criteria and followed at IRCCS San Raffaele, Milan. AlloRisk was evaluated in the 35 patients undergoing allo-HCT. Scores were calculated according to original publications. To minimize transplant-related confounding, comparative analyses between AIPSS and conventional models focused on the non-transplanted cohort.

### Results

Among 95 patients, median age at diagnosis was 59 years and 51 were male. Driver mutations were JAK2V617F 64%, CALR 27%, and MPL 8%. Among 49 patients with available NGS data, ASXL1 mutations were detected in 55%, and ≥2 high molecular risk mutations in 32 patients. After a median follow-up of 51.5 months, 32 patients had died. According to IPSS, 23% were low risk, 28% intermediate-1, 32% intermediate-2, and 19% high risk. MIPSS70 classified evaluable patients as low (n=7), intermediate (n=38), and high risk (n=26). Thirty-five patients underwent allo-HCT and were distributed across AlloRisk categories as follows: low (n=7), intermediate-1 (n=3), intermediate-2 (n=16), and high risk (n=9).
In non-transplanted patients (n=60), AIPSS showed slightly higher discriminative ability for OS than IPSS (C-index 0.73 vs 0.71). In this cohort, AIPSS provided higher OS estimates than IPSS in 45/60 cases (75%), with a median difference of +2.7 years. The upward shift was most pronounced in IPSS low risk (median Δ +5.4 years) and remained clinically relevant in intermediate-1 (+2.1), intermediate-2 (+2.3), and high-risk groups (+2.6). Among 36 patients with molecular data, AIPSS predicted longer survival than MIPSS70 in 29 (81%) (median Δ +2.6 years), particularly in the high-risk category, suggesting heterogeneity within this group. Using a 5-year OS threshold, 10/60 patients (17%) showed discordant classification, with AIPSS predicting >5-year survival despite <5-year estimates by conventional models; observed median OS was 8.0 years with 5-year OS of 80%. Among transplanted patients, 25/35 (71%) had AIPSS-predicted OS >5 years at diagnosis. Patients with favorable AIPSS but high AlloRisk appeared to experience non-negligible early transplant mortality.

**Copyright Information:** (Online) ISSN: 2572-9241
©2026 The Author(s). HemaSphere is published by John Wiley & Sons Ltd on behalf of European Hematology Association. This is an open access Abstract Book distributed under the Attribution-NonCommercial-NoDerivs (CC BY-NC-ND), which allows third parties to download the articles and share them with others as long as they credit the author and the Abstract Book, but they cannot change the content in any way or use them commercially.
**Abstract Book Citations:** Authors, Title, HemaSphere, 2026;10:(S1):pages. The abstract book DOIs can be found on the "Author Information" page.
**Disclaimer:** Articles published in the journal HemaSphere exclusively reflect the opinions of the authors. The authors are responsible for all content in their abstracts including accuracy of the facts, statements, citing resources, etc.

### Summary/Conclusion

In this real-world MF cohort, the machine learning–based AIPSS provided individualized and frequently more favorable survival estimates compared with conventional prognostic scores. Clinically meaningful discordance emerged in a subset of patients, particularly around the 5-year threshold commonly used to guide transplant decisions. These findings support a potential complementary role for AIPSS in refining risk stratification and optimizing timing of allo-HCT. Prospective validation in larger cohorts is warranted.

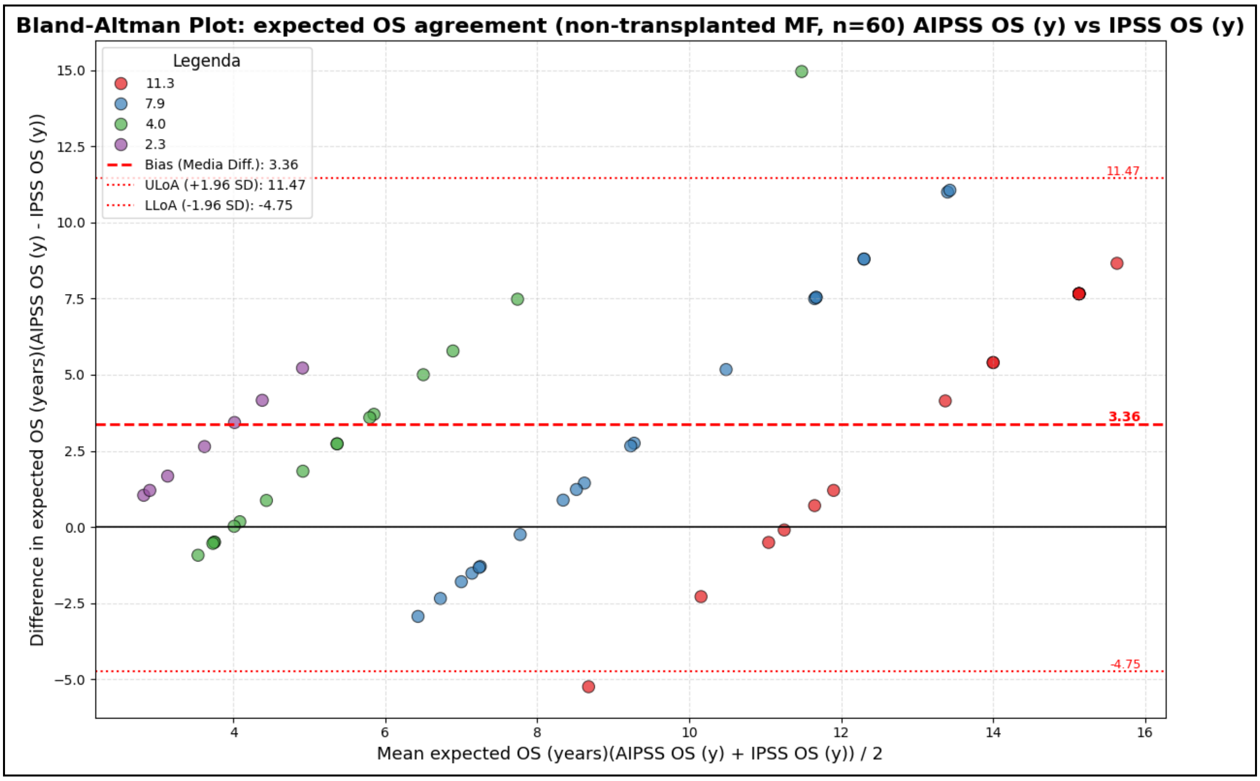

Figure 1. Bland–Altman analysis showed a positive systematic bias of AIPSS versus IPSS (mean difference +3.37 years) with wide limits of agreement (−4.73 to +11.47 years), indicating non-interchangeability of the two models at the individual level. The diagonal banding reflects the discrete nature of IPSS estimates and the continuous stratification provided by AIPSS.

**Copyright Information:** (Online) ISSN: 2572-9241
©2026 The Author(s). HemaSphere is published by John Wiley & Sons Ltd on behalf of European Hematology Association. This is an open access Abstract Book distributed under the Attribution-NonCommercial-NoDerivs (CC BY-NC-ND), which allows third parties to download the articles and share them with others as long as they credit the author and the Abstract Book, but they cannot change the content in any way or use them commercially.
**Abstract Book Citations:** Authors, Title, HemaSphere, 2026;10:(S1):pages. The abstract book DOIs can be found on the "Author Information" page.
**Disclaimer:** Articles published in the journal HemaSphere exclusively reflect the opinions of the authors. The authors are responsible for all content in their abstracts including accuracy of the facts, statements, citing resources, etc.

### (PB3427) BASELINE PREDICTORS OF SURVIVAL AND SPLEEN RESPONSE IN RUXOLITINIB-TREATED MYELOFIBROSIS: DEVELOPMENT OF THE R-PSS (RUXOLITINIB-PROGNOSTIC SCORING SYSTEM) IN A REAL-WORLD COHORT

**Topic:** 16. Myeloproliferative neoplasms - Clinical

Alessandro Laganà*[1], Emilia Scalzulli[1], Marialaura Bisegna[1], Ida Carmosino[1], Deborah Kasmi[1], Martina Gherardini[1], Anna Laura Faccini[1], Maurizio Martelli[1], Massimo Breccia[1]

[1]*Sapienza Università di Roma, Hematology, Department of Translational and Precision Medicine, Roma, Italy;*

### Background

In Myelofibrosis (MF), conventional prognostic scores are primarily designed for risk stratification at diagnosis. Currently, there is a lack of baseline tools specifically tailored to predict outcomes upon JAK-inhibitor initiation, as the RR6 score provides post-6-month-treatment guidance.

### Aims

To evaluate the rate of IWG-MRT defined-spleen response (SR) and overall survival (OS) of a real-life RUX-treated MF cohort, to identify baseline clinical factors as predictors of SR and OS, and to develop a model easily and broadly applicable in routine clinical practice at RUX-Start.

### Methods

We analyzed a single-center cohort of 235 JAKi naïve RUX-treated MF patients. Eligibility was based on national indications: PMF or SMF; DIPSS ≥int-1; palpable spleen ≥5 cm below LCM; PLTs count ≥$50\times10^9$/L. SR was assessed by physical examination as per clinical practice. For survival analysis patients undergoing allo-HSCT were censored at transplant date. Multivariate Cox proportional hazard was used to identify independent predictors. Model performance was assessed using C-index, AIC, and reclassification metrics (NRI/IDI).

**Copyright Information:** (Online) ISSN: 2572-9241
©2026 The Author(s). HemaSphere is published by John Wiley & Sons Ltd on behalf of European Hematology Association. This is an open access Abstract Book distributed under the Attribution-NonCommercial-NoDerivs (CC BY-NC-ND), which allows third parties to download the articles and share them with others as long as they credit the author and the Abstract Book, but they cannot change the content in any way or use them commercially.
**Abstract Book Citations:** Authors, Title, HemaSphere, 2026;10:(S1):pages. The abstract book DOIs can be found on the "Author Information" page.
**Disclaimer:** Articles published in the journal HemaSphere exclusively reflect the opinions of the authors. The authors are responsible for all content in their abstracts including accuracy of the facts, statements, citing resources, etc.

### Results

Median age at RUX-start was 65.9 years (IQR, 56.3–73.6), with 124 males (52.8%). PMF accounted for 109 (46.4%) cases, SMF for 126 (53.6%), with 37 (15.7%) cases of early-MF. After a median follow-up of 66.5 months, the overall SR rate was 49.8%, with 93 deaths (39.6%) and an estimated median OS of 82.5 months of the whole cohort.
Multivariate analysis, that included all regressors with a p<0.1 in univariate analysis, identified the following baseline factors as independent predictors of shortened OS: age ≥68 years (HR 2.94; 95% CI 1.76–4.91), BM Fibrosis Grade 3 (HR 2.41; 95% CI 1.48–3.94), anemia (M<11 g/dL, F<10 g/dL) (HR 2.41; 95% CI 1.46–3.96), PLTs < $150\times10^9$/L (HR 2.27; 95% CI 1.39–3.73), PB Blasts 1–2% (HR 2.23; 95% CI 1.35–3.69) and ≥3% (HR 4.05; 95% CI 2.17–7.58). The R-PSS was developed by assigning weighted points to the identified predictors, and patients were classified into four distinct risk categories: Low (0-2), Int-1 (3-5) HR 6.77 (95% CI 2.51–18.26, p<0.001), Int-2 (6-7) HR 15.42 (95% CI 5.66–41.97, p<0.001) and High (≥8) HR 61.89 (95% CI 22.45–170.57, p<0.001). Internal validation via bootstrapping (1000 samples) confirmed the robustness of the model, with p=0.001 for all risk tiers. The R-PSS demonstrated a significantly higher C-index (0.807) compared to both baseline DIPSS (0.737; p=0.004) and RR6 score (0.735; p=0.004) and achieved the lowest AIC (696.1 vs 737.0 for RR6 and 758.7 for DIPSS). Moreover, at 60 months, the R-PSS significantly improved the accuracy of risk assignment compared to DIPSS by 50.3% (NRI 0.503, P=0.003) and compared to RR6 by 39.9% (NRI 0.399, P=0.011), as well as model's overall predictive power by 17.2% over DIPSS (IDI 0.172, p<0.001) and by 20.0% over RR6 (IDI 0.200, p<0.001).
Interestingly, R-PSS showed a high accuracy in predicting the absence of overall SR, with an AUC of 0.772, superior to the DIPSS (AUC 0.732) and statistically non-inferior to the RR6 score (AUC 0.817%, p=0.09), despite the latter being a model that incorporates spleen size reduction over time itself.

### Summary/Conclusion

RUX remains an effective standard of care for MF. In this context, the clinically-based tool R-PSS may help in identifying patients at high risk of treatment failure, helping clinicians in early clinical decision-making, allowing optimal patient selection for novel therapeutic strategies and/or allo-HSCT referral soon after RUX-start.

**Copyright Information:** (Online) ISSN: 2572-9241
©2026 The Author(s). HemaSphere is published by John Wiley & Sons Ltd on behalf of European Hematology Association. This is an open access Abstract Book distributed under the Attribution-NonCommercial-NoDerivs (CC BY-NC-ND), which allows third parties to download the articles and share them with others as long as they credit the author and the Abstract Book, but they cannot change the content in any way or use them commercially.
**Abstract Book Citations:** Authors, Title, HemaSphere, 2026;10:(S1):pages. The abstract book DOIs can be found on the "Author Information" page.
**Disclaimer:** Articles published in the journal HemaSphere exclusively reflect the opinions of the authors. The authors are responsible for all content in their abstracts including accuracy of the facts, statements, citing resources, etc.

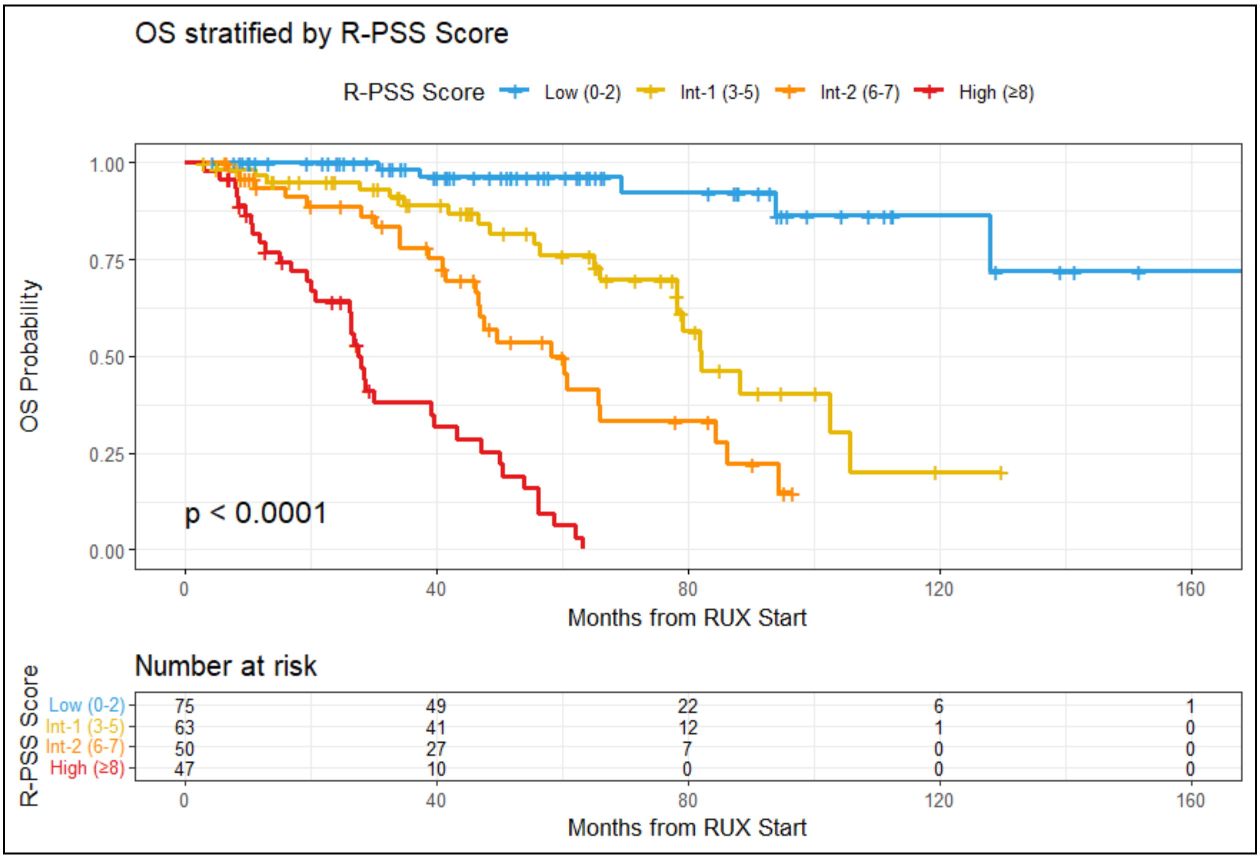

Kaplan-Meier cumulative overall survival (OS) curves for the 235 patients with myelofibrosis (MF) treated with Ruxolitinib (RUX) stratified by R-PSS (Ruxolitinib-Prognostic Scoring System). The R-PSS weighted score point allocation is as follows: Bone Marrow Fibrosis (grade 3 vs 2-1) = 2 pts; PLTs < 150x10^9/L = 2 pts; Hb sex-dependent anemia = 2 pts; Peripheral blood Blasts 1-2% = 2 pts, over 3% = 4 pts; and Age 68 years or more = 3 pts.

**Copyright Information:** (Online) ISSN: 2572-9241
©2026 The Author(s). HemaSphere is published by John Wiley & Sons Ltd on behalf of European Hematology Association. This is an open access Abstract Book distributed under the Attribution-NonCommercial-NoDerivs (CC BY-NC-ND), which allows third parties to download the articles and share them with others as long as they credit the author and the Abstract Book, but they cannot change the content in any way or use them commercially.
**Abstract Book Citations:** Authors, Title, HemaSphere, 2026;10:(S1):pages. The abstract book DOIs can be found on the "Author Information" page.
**Disclaimer:** Articles published in the journal HemaSphere exclusively reflect the opinions of the authors. The authors are responsible for all content in their abstracts including accuracy of the facts, statements, citing resources, etc.

### (PB3428) FATHERHOOD IN MYELOPROLIFERATIVE NEOPLASMS – A POPULATION BASED MATCHED COHORT STUDY OF 2 056 MEN

**Topic:** 16. Myeloproliferative neoplasms - Clinical

Anna Ravn Landtblom*[1, 2], Lovisa Vennström[3], Ivette Raices Cruz[4], Malin Hultcrantz[5, 6], Therese Andersson[7]

*[1]Center for Hematology and Regenerative Medicine, Department of Medicine, Huddinge, Karolinska Institutet, Stockholm, Sweden; [2]Department of Hematology, Karolinska University Hospital, Stockholm, Sweden;[3]Department of Hematology, Sahlgrenska University Hospital, Gothenburg, Sweden; [4]Division of Biostatistics, Institute of Environmental Medicine, Karolinska Institutet, Stockholm, Sweden; [5]Department of Medicine, Solna, Karolinska Institutet, Stockholm, Sweden;[6]Myeloma Service, Department of Medicine, Memorial Sloan Kettering Cancer Center, New York, United States of America; [7]Department of Medical Epidemiology and Biostatistics, Karolinska Institutet, Stockholm, Sweden;*

### Background

Although a majority of patients with myeloproliferative neoplasms (MPN) are diagnosed in older ages, 15-20% are younger than 45 at diagnosis. Common cytoreductive treatments may interfere with male fertility, and somatic and psychologic effects of MPN may also affect the possibility and choice to father children. Fatherhood in MPN is less studied than pregnancy and no previous study assess childbirth rates in men with MPN.

### Aims

The aim of the study was to estimate rates of childbirth in male MPN patients, and compare to matched population controls.

### Methods

Men diagnosed with MPN in Sweden 1973-2021, aged 15-50 at diagnosis, were identified from the Swedish Cancer Register and the Patient Register. Ten controls were matched to each by age and sex, from the Register of Total population. Biological children to the included patients and controls were identified from the Multi-Generation Register. Information of prescribed drugs were retrieved from the Prescribed Drug Register.
Hazard ratios (HR) with 95% confidence intervals (CI) were calculated using flexible parametric models. Separate analyses were performed by MPN subtype and age at diagnosis.
We also performed a nested case-control design where MPN patients with childbirth were age-matched to MPN patients without childbirth, and compared dispensations of cytoreductive treatments during the last 2 years prior to the childbirth.

**Copyright Information:** (Online) ISSN: 2572-9241
©2026 The Author(s). HemaSphere is published by John Wiley & Sons Ltd on behalf of European Hematology Association. This is an open access Abstract Book distributed under the Attribution-NonCommercial-NoDerivs (CC BY-NC-ND), which allows third parties to download the articles and share them with others as long as they credit the author and the Abstract Book, but they cannot change the content in any way or use them commercially.
**Abstract Book Citations:** Authors, Title, HemaSphere, 2026;10:(S1):pages. The abstract book DOIs can be found on the "Author Information" page.
**Disclaimer:** Articles published in the journal HemaSphere exclusively reflect the opinions of the authors. The authors are responsible for all content in their abstracts including accuracy of the facts, statements, citing resources, etc.

### Results

We identified 2,056 men with MPN and 20,466 matched comparators. Forty-three percent had polycythemia vera, 37% had essential thrombocythemia, 8% primary myelofibrosis, and 12% has MPN-unclassifiable. At diagnosis, the median age was 43 years, 8.5% were aged 15-25, 16.8% 26-35, 33.1% 36-44, and 41.6% 45-50.

The overall HR of childbirth in men with MPN in relation to controls was 0.89 (0.79-1.01), based on 270 childbirths in men with MPN and 3,389 in controls. HRs were similar across MPN subtypes, but significantly reduced in men diagnosed with MPN at age 15-25, HR 0.70 (0.54-0.91), but not in older age categories. Estimated birth rates over time after diagnose with 95% CIs are shown in Figure 1. As a sensitivity analysis, patients identified from the Cancer Register and Patient Register were analysed separately, with similar HRs.
Mean number of children was lower at diagnosis in MPN compared to controls, 1.40 compared to 1.49 ( p=0.006). A significantly lower proportion of men with MPN had at least one child at diagnosis, 63.7% compared to 66.9%, (p=0.003).
In the nested case control study, of men with MPN who had a child, 75% had no dispensation of any cytoreductive treatment during the last 2 years prior to childbirth, 6% had hydroxyurea, 15.6% interferon, 0% anagrelide, and 3.4% had received more than one cytoreductive treatment. Among their MPN controls, the corresponding numbers were 65%, 12.1%, 13.8%, 3.4% and 5.2%.

### Summary/Conclusion

This large and population-based study of men with MPN is the first to systematically estimate childbirth rates, and compare to matched controls. The childbirth rates were lower in the youngest age group, the overall childbirth rate did not differ significantly from matched non-MPN controls. Pattern of cytoreductive treatment differed between men with childbirths and without during the 2 years preceding childbirth, whether it was intentional and/or a causal effect of the treatment or not is not known. The results of this study are reassuring, and important information for men diagnosed with MPN who plans to start a family.

**Copyright Information:** (Online) ISSN: 2572-9241
©2026 The Author(s). HemaSphere is published by John Wiley & Sons Ltd on behalf of European Hematology Association. This is an open access Abstract Book distributed under the Attribution-NonCommercial-NoDerivs (CC BY-NC-ND), which allows third parties to download the articles and share them with others as long as they credit the author and the Abstract Book, but they cannot change the content in any way or use them commercially.
**Abstract Book Citations:** Authors, Title, HemaSphere, 2026;10:(S1):pages. The abstract book DOIs can be found on the “Author Information” page.
**Disclaimer:** Articles published in the journal HemaSphere exclusively reflect the opinions of the authors. The authors are responsible for all content in their abstracts including accuracy of the facts, statements, citing resources, etc.

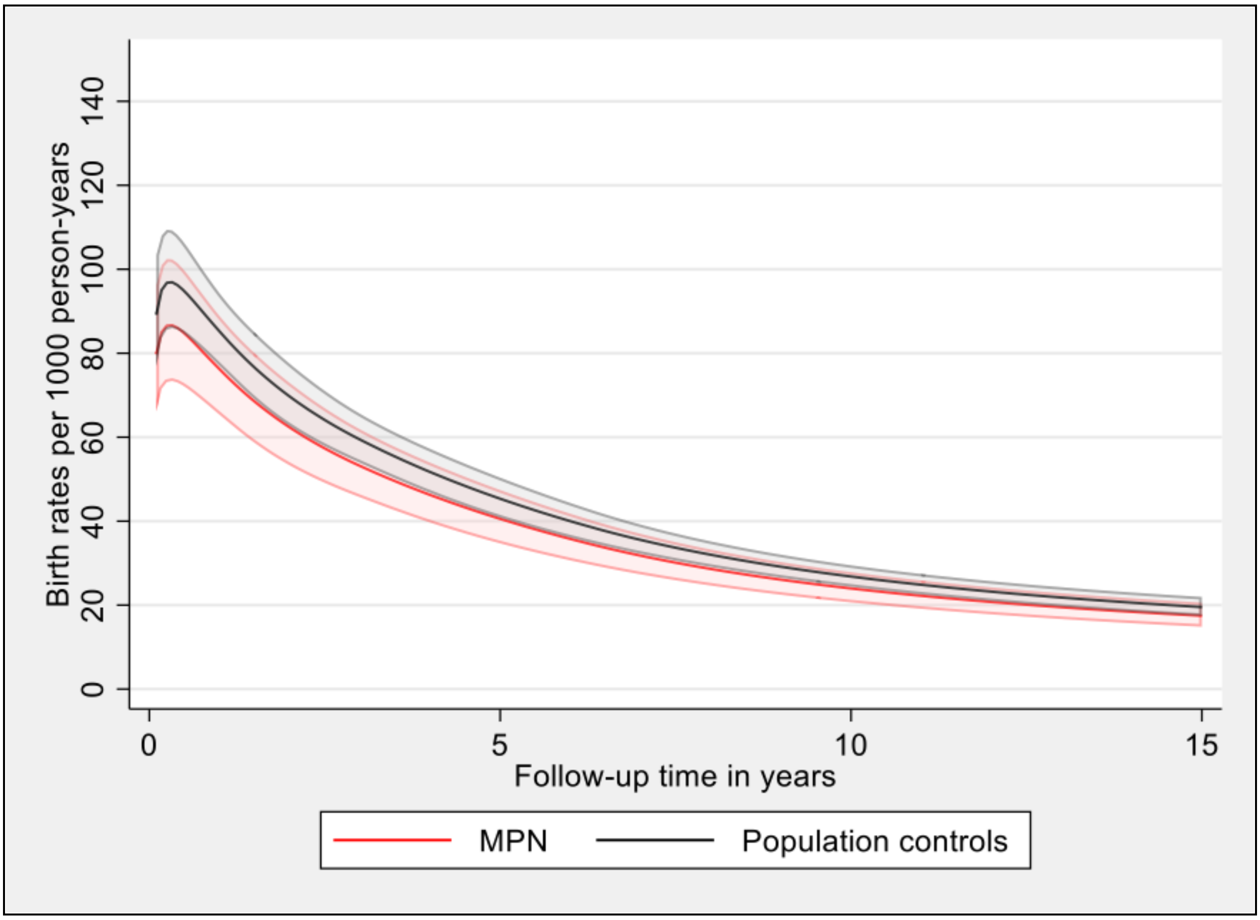

Figure 1. Estimated birth rates per 1000 person-years over time after diagnose with 95% CIs, for MPN patients and population controls.

Copyright Information: (Online) ISSN: 2572-9241
©2026 The Author(s). HemaSphere is published by John Wiley & Sons Ltd on behalf of European Hematology Association. This is an open access Abstract Book distributed under the Attribution-NonCommercial-NoDerivs (CC BY-NC-ND), which allows third parties to download the articles and share them with others as long as they credit the author and the Abstract Book, but they cannot change the content in any way or use them commercially.
Abstract Book Citations: Authors, Title, HemaSphere, 2026;10:(S1):pages. The abstract book DOIs can be found on the "Author Information" page.
Disclaimer: Articles published in the journal HemaSphere exclusively reflect the opinions of the authors. The authors are responsible for all content in their abstracts including accuracy of the facts, statements, citing resources, etc.

### (PB3429) SYSTEMIC INFLAMMATION IMPROVES RISK PREDICTION IN MYELOFIBROSIS BEYOND DIPSS: DEVELOPMENT OF THE DIMOL SCORE

**Topic:** 16. Myeloproliferative neoplasms - Clinical

Amedeo Votto*[1], Andrea Duminuco[2], Giulio Lavenia[3], Matilde Maggiorelli[1], Angela Brunetti[1], Giuseppe Palumbo[2], Sara Galimberti[1]

*[1]University of Pisa, Hematology Unit, Department of Clinical and Experimental Medicine, Pisa, Italy;[2]Università degli Studi di Catania, Dipartimento di Scienze Mediche, Chirurgiche e Tecnologie avanzate "G.F. Ingrassia", Catania, Italy; [3]Università di Catania, Scuola di Specializzazione di Ematologia, Catania, Italy;*

### Background

Chronic inflammation is a key driver of myelofibrosis (MF) pathogenesis, contributing to clonal evolution, bone marrow fibrosis, constitutional symptoms, and cardiovascular complications. However, widely used prognostic models such as DIPSS and MYSEC-PM do not incorporate inflammatory status. Emerging evidence suggests that systemic inflammatory indices—including neutrophil-to-lymphocyte ratio (NLR), monocyte-to-lymphocyte ratio (MLR), and systemic inflammation response index (SIRI)—may predict survival in MF.

### Aims

To evaluate the prognostic impact of inflammatory indices in MF patients treated with ruxolitinib and to develop an inflammation-integrated prognostic model.

### Methods

We retrospectively analyzed 166 consecutive MF patients (98 primary MF; 68 secondary MF) treated with ruxolitinib at the Hematology Units of Pisa and Catania (Italy) between 2013 and 2023. Diagnosis was established according to WHO 2008/2016 criteria. Inflammatory indices (NLR, MLR, PLR, WLR, SII, SIRI) were calculated at diagnosis. Overall survival (OS) was estimated using Kaplan–Meier analysis. Variables significant in univariate Cox regression were included in multivariate models. Based on independent predictors, a novel prognostic score combining DIPSS and MLR (DiMoL score) was developed.

### Results

Median age was 68 years (M:F=1.2). Driver mutations were distributed as follows: JAK2V617F 76%, CALR 16%, MPL 1%. According to DIPSS, 11% were low-risk, 36% intermediate-1, 40% intermediate-2, and 13% high-risk. Median OS was 84 months; 6-year OS was 54%. In univariate analysis, OS was significantly associated with DIPSS category, age >65 years, and symptom burden, but not with gender, splenomegaly, or driver mutation. Among inflammatory markers, elevated MLR ($p<0.01$) and SIRI ($p<0.008$) were associated with inferior OS. In multivariate analysis, only MLR retained independent prognostic significance (HR 1.80, $p=0.03$). Combining DIPSS with MLR dichotomized at the cohort median (0.55), assigning 1 point for MLR ≥ 0.55 and 0 for MLR < 0.55; the DiMoL score (DIPSS + MLR) stratified patients into three risk groups with 6-year OS of 90% (low), 52% (intermediate), and 22% (high), improving discrimination compared with DIPSS alone.

**Copyright Information:** (Online) ISSN: 2572-9241
©2026 The Author(s). HemaSphere is published by John Wiley & Sons Ltd on behalf of European Hematology Association. This is an open access Abstract Book distributed under the Attribution-NonCommercial-NoDerivs (CC BY-NC-ND), which allows third parties to download the articles and share them with others as long as they credit the author and the Abstract Book, but they cannot change the content in any way or use them commercially.
**Abstract Book Citations:** Authors, Title, HemaSphere, 2026;10:(S1):pages. The abstract book DOIs can be found on the "Author Information" page.
**Disclaimer:** Articles published in the journal HemaSphere exclusively reflect the opinions of the authors. The authors are responsible for all content in their abstracts including accuracy of the facts, statements, citing resources, etc.

**Summary/Conclusion**

Systemic inflammation, as reflected by MLR, independently predicts survival in MF patients treated with ruxolitinib. Integration of MLR into the DiMoL score refines risk stratification and identifies more homogeneous prognostic subgroups than DIPSS alone. These findings support the incorporation of simple inflammatory biomarkers into clinical practice and provide a rationale for prospective studies evaluating inflammation-targeted strategies in MF.

**Copyright Information:** (Online) ISSN: 2572-9241
©2026 The Author(s). HemaSphere is published by John Wiley & Sons Ltd on behalf of European Hematology Association. This is an open access Abstract Book distributed under the Attribution-NonCommercial-NoDerivs (CC BY-NC-ND), which allows third parties to download the articles and share them with others as long as they credit the author and the Abstract Book, but they cannot change the content in any way or use them commercially.
**Abstract Book Citations:** Authors, Title, HemaSphere, 2026;10:(S1):pages. The abstract book DOIs can be found on the “Author Information” page.
**Disclaimer:** Articles published in the journal HemaSphere exclusively reflect the opinions of the authors. The authors are responsible for all content in their abstracts including accuracy of the facts, statements, citing resources, etc.

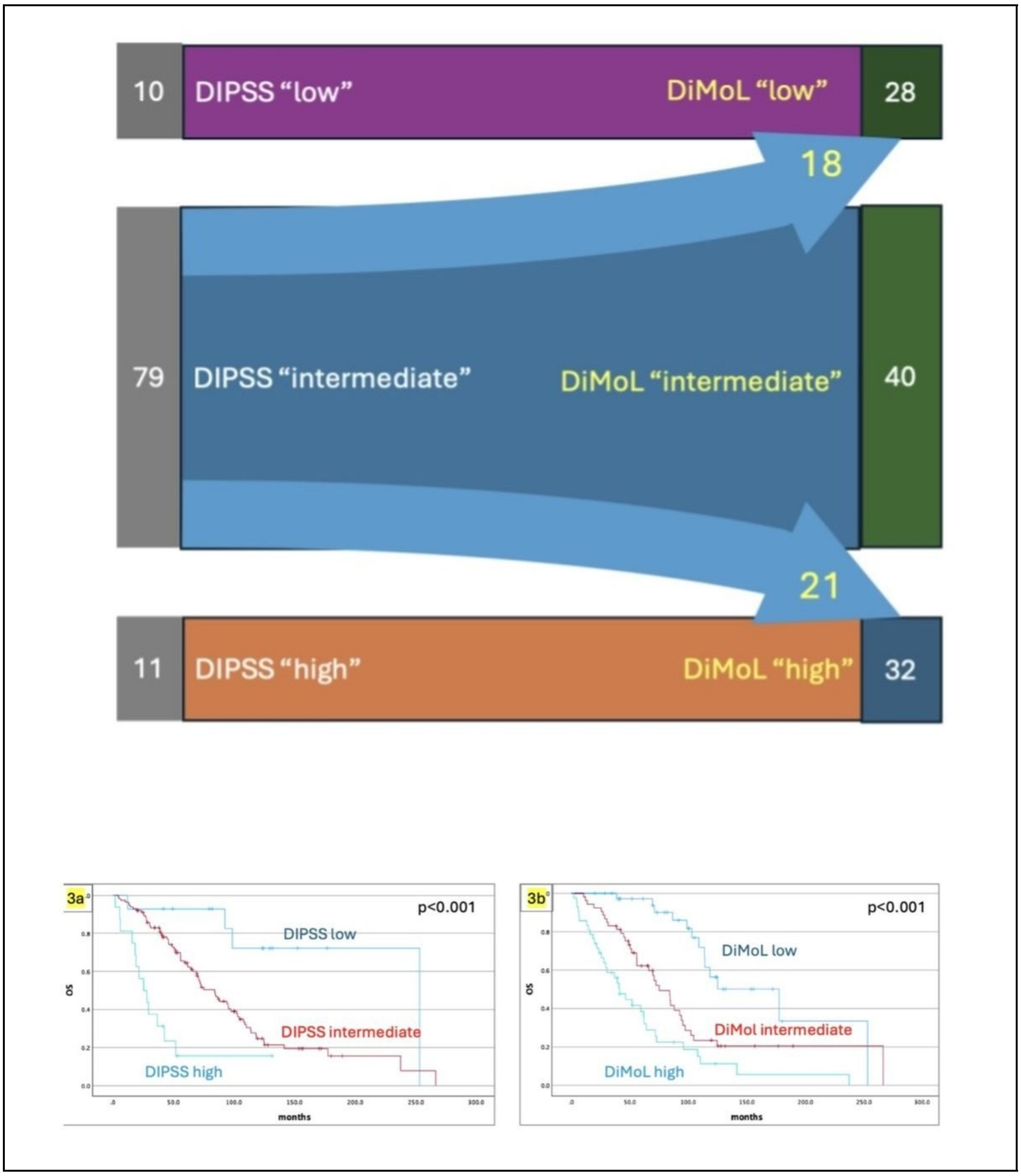

The upper panel is a flow-style diagram showing how patients classified according to DIPSS (low, intermediate, and high risk) are redistributed when reclassified using the DiMoL score. The numbers displayed within each category represent the number of patients in each risk group. Arrows indicate the transitions between corresponding DIPSS and DiMoL categories, highlighting reclassification patterns—particularly shifts within the intermediate group. The lower panel presents two Kaplan–Meier overall survival (OS) curves. Panel 3a shows OS stratified by DIPSS risk categories (low, intermediate, high), while panel 3b shows OS stratified by DiMoL categories. In both analyses, survival significantly differs among risk groups ($p < 0.001$). The curves demonstrate progressive worsening survival from low- to high-risk categories, supporting the prognostic value of both scoring systems and allowing visual comparison of their discriminative capacity.

**Copyright Information:** (Online) ISSN: 2572-9241
©2026 The Author(s). HemaSphere is published by John Wiley & Sons Ltd on behalf of European Hematology Association. This is an open access Abstract Book distributed under the Attribution-NonCommercial-NoDerivs (CC BY-NC-ND), which allows third parties to download the articles and share them with others as long as they credit the author and the Abstract Book, but they cannot change the content in any way or use them commercially.
**Abstract Book Citations:** Authors, Title, HemaSphere, 2026;10:(S1):pages. The abstract book DOIs can be found on the “Author Information” page.
**Disclaimer:** Articles published in the journal HemaSphere exclusively reflect the opinions of the authors. The authors are responsible for all content in their abstracts including accuracy of the facts, statements, citing resources, etc.

### (PB3430) BASELINE CHARACTERISTICS BY PRE-SPECIFIED CYTOREDUCTIVE THERAPY IN HIGH-RISK POLYCYTHEMIA VERA IN THE PHASE III MITHRIDATE TRIAL WITH DESCRIPTIVE COMPARISON TO MAJIC-PV

**Topic:** 16. Myeloproliferative neoplasms - Clinical

Anastasia Kosmidou*[1], Rebecca Boucher[2], Natalia Morelli[2], Mamta Garg[3], Ali Mahdi[4], Mary Frances McMullin[5], Frances Wadelin[6], Joanne Ewing[7], Anna Godfrey[8], Jean Christophe Ianotto[9], Stéphane Courby[10], Marc Bernard[11], Viviane Dubruille[12], Dana Ranta[13], Aimee Jackson[2], Claire Harrison[14], Jean-Jacques Kiladjian[15], Adam Mead[1, 16]

*[1]MRC Weatherall Institute of Molecular Medicine (Oxford University & John Radcliffe Hospital), Oxford, United Kingdom;[2]Cancer Research UK Clinical Trials Unit, University of Birmingham, Birmingham, United Kingdom; [3]University Hospitals Leicester, Leicester, United Kingdom; [4]Aneurin Bevan University Health Board, Newport, United Kingdom; [5]Queen's University, Belfast, Belfast, United Kingdom; [6]Nottingham University Hosptial, Nottingham, United Kingdom; [7]University Hospitals Birmingham, Birmingham, United Kingdom; [8]Cambridge University Hospitals NHS Foundation Trust, Cambridge, United Kingdom; [9]Chu Brest - Ici - Hematologie Clinique, Brest, France; [10]Centre Hospitalier Universitaire de Grenoble, Grenoble, France; [11]CHU Rennes - Pontchaillou Hospital, Rennes, France; [12]Hopital Hotel Dieu Et Hme-umac, Nantes, France; [13]University Hospital of Nancy (CHU Nancy), Nancy, France; [14]Guy's and St Thomas' NHS Foundation Trust, London, United Kingdom; [15]Institut De Recherche Saint Louis - Université De Paris, Inserm, Paris, France; [16]Churchill Hospital, Oxford, United Kingdom;*

### Background

Management of high-risk polycythemia vera (PV) requires cytoreduction to mitigate thromboembolic risk and disease transformation. While hydroxycarbamide (HC) and interferon (IFN) are established first-line therapies, and ruxolitinib is a proven second-line option (MAJIC-PV), whether ruxolitinib treatment might improve outcomes as a first line treatment remains uncertain. MITHRIDATE (NCT04116502) is a landmark Phase III trial across UK and France, evaluating ruxolitinib as first-line therapy, directly comparing it against investigator pre-specified choice of HC or IFN. Because treatment selection in PV is often subjective, a detailed analysis of baseline characteristics is essential to ensure the trial is recruiting a clinically relevant and generalizable population that accurately reflects the global high-risk PV landscape.

### Aims

To describe baseline characteristics by pre-specified HC versus IFN in MITHRIDATE and compare these descriptively with MAJIC-PV baseline data.

### Methods

This exploratory analysis included patients enrolled in the MITHRIDATE trial for whom HC or IFN were specified as BAT prior to randomization. All patients provided written informed consent. Baseline demographic, clinical, and laboratory characteristics were summarized descriptively. MITHRIDATE represents the largest prospective randomized trial in PV to date, designed to provide definitive evidence with potential to change global standard-of-care.

**Copyright Information:** (Online) ISSN: 2572-9241
©2026 The Author(s). HemaSphere is published by John Wiley & Sons Ltd on behalf of European Hematology Association. This is an open access Abstract Book distributed under the Attribution-NonCommercial-NoDerivs (CC BY-NC-ND), which allows third parties to download the articles and share them with others as long as they credit the author and the Abstract Book, but they cannot change the content in any way or use them commercially.
**Abstract Book Citations:** Authors, Title, HemaSphere, 2026;10:(S1):pages. The abstract book DOIs can be found on the “Author Information” page.
**Disclaimer:** Articles published in the journal HemaSphere exclusively reflect the opinions of the authors. The authors are responsible for all content in their abstracts including accuracy of the facts, statements, citing resources, etc.

### Results

Of 285 MITHRIDATE patients in the study to date, HC was pre-specified in 208 (73%) and IFN in 77 (27%). Two thirds of patients enrolled were treatment naïve. HC-selected patients were older than IFN-selected patients (median 67 vs 61 years) and had higher predicted cardiovascular risk (QRisk3 16.9 vs 11.2), while prior thrombosis rates were similar (27% vs 26%). IFN-selected patients were more often employed (56% vs 29%) and had higher symptom burden. Venesection within 3 months pre-randomization was common (59% overall; HC 61%, IFN 53%). Palpable splenomegaly was present in 15% overall and was more frequent with IFN than HC selection (23% vs 12%); when present, median spleen size below the costal margin was larger in HC than IFN (10 (2-15.9) vs 4 (2–8.8) cm). IFN-selected patients had higher RBC count (6.17 vs 5.71 ×10^12/L) and platelets (539 vs 444 ×10^9/L) with lower MCV (78.7 vs 82.7 fL) than HC. Compared with MAJIC-PV, MITHRIDATE patients were older at diagnosis (64 vs 57 years) but similar in age at randomization (65 vs 66) and venesection was more frequent (59% vs 26%). Prior thrombosis was less frequent in MITHRIDATE versus MAJIC-PV (27% vs 36%), as was palpable splenomegaly (15% vs 25%), consistent with consistent with a true front-line, earlier-stage population in MITHRIDATE. The pattern of older age with HC selection versus IFN was consistent across trials.

### Summary/Conclusion

With 285 patients randomized, MITHRIDATE is set to become the largest prospective trial in the PV landscape. These baseline data confirm the enrolment of a clinically representative high-risk population and reflect the real-world differences in patients selected for treatment with HC versus IFN in first and second line setting. By providing the first large-scale, head-to-head evidence for ruxolitinib against specific BAT in the front-line setting, MITHRIDATE is uniquely positioned to redefine the global standard of care for high-risk PV. The study remains open to recruitment in the UK and France.

**Table 1:** Selected baseline characteristics for MITHRIDATE by pre-specified best available therapy

| Characteristic | **HC (n=208)** | **IFN (n=77)** | **Overall (n=285)** |
|---|---|---|---|
| Age, years | 67 (61–74) | 61 (55–65) | 65 (60–72) |
| QRisk3 | 16.9 (9.9–26.1) | 11.2 (6–16.7) | 14.6 (9.3–23.7) |
| Prior thrombosis, n (%) | 56 (27) | 20 (26) | 76 (27) |
| Venesection $\leq$3 months, n (%) | 126 (61) | 41 (53) | 167 (59) |
| Palpable splenomegaly, n (%) | 24 (12) | 18 (23) | 42 (15) |
| Spleen size if palpable (cm) | 10 (2–15.9) | 4 (2–8.8) | 5 (2–13.75) |
| RBC count ($\times 10^{12}$/L) | 5.71 (4.87–6.45) | 6.17 (5.44–6.84) | 5.82 (5–6.56) |
| Platelet count ($\times 10^{9}$/L) | 444 (314.5–621) | 539 (345–815) | 465 (324–687) |
| MCV (fL) | 82.7 (73.2–93.35) | 78.7 (70–87) | 81.2 (71.7–91.5) |

Abbreviations: HC, hydroxycarbamide; IFN, interferon; IQR, interquartile range; QRisk3, QResearch Cardiovascular Risk 3; RBC, red blood cell; MCV, mean corpuscular volume. Continuous variables are median (IQR) unless stated.

**Copyright Information:** (Online) ISSN: 2572-9241
©2026 The Author(s). HemaSphere is published by John Wiley & Sons Ltd on behalf of European Hematology Association. This is an open access Abstract Book distributed under the Attribution-NonCommercial-NoDerivs (CC BY-NC-ND), which allows third parties to download the articles and share them with others as long as they credit the author and the Abstract Book, but they cannot change the content in any way or use them commercially.
**Abstract Book Citations:** Authors, Title, HemaSphere, 2026;10:(S1):pages. The abstract book DOIs can be found on the “Author Information” page.
**Disclaimer:** Articles published in the journal HemaSphere exclusively reflect the opinions of the authors. The authors are responsible for all content in their abstracts including accuracy of the facts, statements, citing resources, etc.

### (PB3431) MUTATIONAL LANDSCAPE AND OUTCOME OF CALRETICULIN MUTATED MYELOFIBROSIS

**Topic:** 16. Myeloproliferative neoplasms - Clinical

Lucia Masarova*[1], Prithviraj Bose[1], Lingsha Zhou[1], Mahesh Swaminathan[1], Keyur Patel[1], Guillermo Garcia-Manero[1], Naveen Pemmaraju[1]

[1]*MD Anderson Cancer Center, Leukemia, Houston, United States of America;*

### Background

Calreticulin (CALR) mutation is the second most common driver mutation detected in patients with myelofibrosis (MF); both post essential thrombocythemia MF (PETMF) and primary (PMF). Type 1/ like-1 CALR (52-bp deletion of the exon 9) is the most frequent (vs type 2: 5-bp insertion and others) and possess favorable overall survival (OS). Presence of additional mutations (MUT), such as high molecular risk mutations (HMR: *ASXL1, SRSF2, U2AF1Q157, EZH2, IDH1/2*) have negative impact on OS of PMF. More specific data for CALR mutated patients and their genomics as it correlates to outcomes is needed.

### Aims

We aimed to investigate characteristics and outcome of MF CALR patients who underwent 81- gene next generation sequencing (NGS) at our institution. We focused on disease phenotypes, CALR types (1: type 1 & like-1; 2: type 2 & like-2; others) and additional MUT.

### Methods

We analyzed data from patient records using descriptive statistics (significant findings: p-value < 0.05). OS analysis (Kaplan Meier method with log rank test) was conducted from the time of NGS.

**Copyright Information:** (Online) ISSN: 2572-9241
©2026 The Author(s). HemaSphere is published by John Wiley & Sons Ltd on behalf of European Hematology Association. This is an open access Abstract Book distributed under the Attribution-NonCommercial-NoDerivs (CC BY-NC-ND), which allows third parties to download the articles and share them with others as long as they credit the author and the Abstract Book, but they cannot change the content in any way or use them commercially.
**Abstract Book Citations:** Authors, Title, HemaSphere, 2026;10:(S1):pages. The abstract book DOIs can be found on the "Author Information" page.
**Disclaimer:** Articles published in the journal HemaSphere exclusively reflect the opinions of the authors. The authors are responsible for all content in their abstracts including accuracy of the facts, statements, citing resources, etc.

**Results**

The study incudes 212 patients: 133 PMF and 79 PETMF. 47 PMF and 25 PETMF patients were sequenced later during their disease, of which 92% and 98%, respectively, occurred < 10 years from MF diagnosis. PMF (vs PETMF) had higher type 1 (71% vs 57%) and lower type 2 CALR (20% vs 35%, respectively). Median age of *CALR*-mutated (type 1 and 2) PMF (57-56 years) was lower vs PETMF (62-63 years), except for PMF CALR-others (median 69 years). The rate of anemia (hemoglobin < 10 g/dL) was highest in PETMF (all types; ~ 43%) followed by PMF *CALR* type 1 (34% vs 22% *CALR* type 2). More PETMF patients (all types; ~ 48%) had higher DIPSS scores (vs 30-35% for PMF).
Overall, 52% of *CALR*-mutated PMF (n:82) vs 78% of *CALR*-mutated PETMF (n:57) had additional mutations (MUT): 57% of PETMF type 2 [new vs later: 53%-64%), 61% of PMF type 1 [53%-74%], 74% of PMF type 2 [61%-NA] and 80% of PETMF type 2 [79%-84%]. HMR among MUT was higher in *CALR*-mutated PMF (63% vs 51%). 2+ MUT were seen in 26-29% of PMF *CALR* types 1-2 vs 40-54% of PETMF *CALR* types 1-2. The most common MUT in PMF included *ASXL1* (n: 35, 43%), *TET2* (n: 21, 26%), *RAS* (n: 11, 14%), *TP53* and *EZH2* (each in 10, 12%) and *IDH1l2* (n: 9, 11%) and in PETMF: *ASXL1* (n: 25, 45%), *TET2* (n: 15, 27%), *SF3B1* (n: 7, 12.5%), *RAS* and *EZH2* (each in 6, 11%). In addition, 85% of PMF and 81% of PETMF, had variants of unknown significance or germline variants; > 10% of patients had these in *TET2, EZH2, BCOR, KDM6A, TERT, CBL, PIGA.*
Overall, 19% PMF and 22% PETMF patients received allogeneic stem cell transplantation. One PMF and 3 PETMF patients progressed to acute leukemia. CALR type 1 had superior OS to CALR type 2 only in those sequenced at diagnosis (e.g., excluding later sequencing). There was no difference in OS between CALR 1 PMF : PETMF and CALR 2 PMF : PETMF patients. In PMF (both CALR types), 2+ MUT, even without the presence of HMR, had significantly inferior OS to 1 MUT (even if 1 HMR); while OS of 1 MUT was comparable with and without HMR (Figure). In PETMF, OS was inferior with presence of HMR, and 1 MUT had similar OS as 2 MUT.

**Summary/Conclusion**

In our large dataset, we provide further details on mutational profiles and outcomes of CALR mutated PMF and PETMF patients. Our findings underscore the prognostic importance of testing for NGS with emphasis of additional mutational analysis beyond the driver mutations in all patients with CALR-mutated MF.

**Copyright Information:** (Online) ISSN: 2572-9241
©2026 The Author(s). HemaSphere is published by John Wiley & Sons Ltd on behalf of European Hematology Association. This is an open access Abstract Book distributed under the Attribution-NonCommercial-NoDerivs (CC BY-NC-ND), which allows third parties to download the articles and share them with others as long as they credit the author and the Abstract Book, but they cannot change the content in any way or use them commercially.
**Abstract Book Citations:** Authors, Title, HemaSphere, 2026;10:(S1):pages. The abstract book DOIs can be found on the "Author Information" page.
**Disclaimer:** Articles published in the journal HemaSphere exclusively reflect the opinions of the authors. The authors are responsible for all content in their abstracts including accuracy of the facts, statements, citing resources, etc.

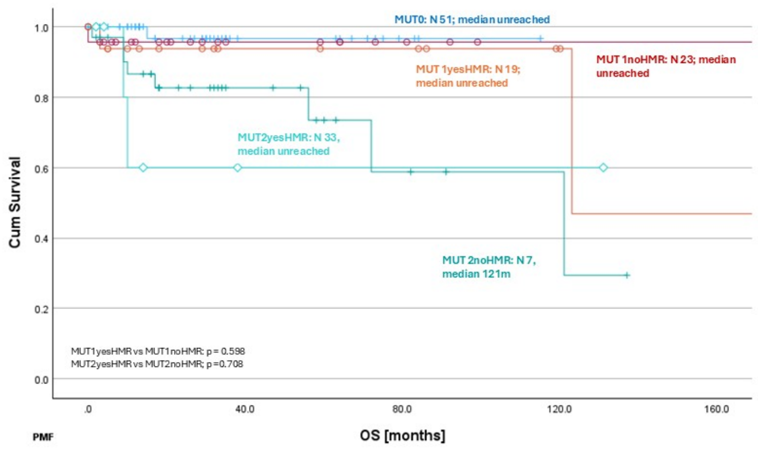

**Copyright Information:** (Online) ISSN: 2572-9241
©2026 The Author(s). HemaSphere is published by John Wiley & Sons Ltd on behalf of European Hematology Association. This is an open access Abstract Book distributed under the Attribution-NonCommercial-NoDerivs (CC BY-NC-ND), which allows third parties to download the articles and share them with others as long as they credit the author and the Abstract Book, but they cannot change the content in any way or use them commercially.
**Abstract Book Citations:** Authors, Title, HemaSphere, 2026;10:(S1):pages. The abstract book DOIs can be found on the "Author Information" page.
**Disclaimer:** Articles published in the journal HemaSphere exclusively reflect the opinions of the authors. The authors are responsible for all content in their abstracts including accuracy of the facts, statements, citing resources, etc.

### (PB3432) RETROMOMELO: REAL-WORLD MOMELOTINIB USE AND OUTCOMES AFTER SWITCH BACK TO ANOTHER JAK INHIBITOR IN MYELOFIBROSIS - THE FRENCH EXPERIENCE FROM THE FIM GROUP

**Topic:** 16. Myeloproliferative neoplasms - Clinical

Mathilde Chanut*[1], Imane Belhadi[1], Jose Miguel Torregrosa Diaz[2], Emmanuel Raffoux[1], Juliette Soret-Dulphy[1], Clemence Mediavilla[3], Geoffroy Venton[4], Vincent Alcazer[5], Lenaig Le Clech[6], Jean Christophe Ianotto[7], Alberto Santagostino[8], Mathieu Meunier[9], Mathilde Hunault[10], Ugo Thevenet[11], Elsa Lestang[12], Antoine Bonnet[13], Rafael Daltro de Oliveira[1], Ahmad Kanj Al Jijakli[14], Florence Sabirou[15], Jean-Jacques Kiladjian[1], Lina Benajiba[1]

*[1]Hôpital Saint Louis, Paris, France;[2]Centre hospitalier universitaire de Poitiers, Poitiers, France;[3]CHU de Bordeaux, Hôpital Haut-Lévèque, Bordeaux, France; [4]University hopsital Marseille Conception, Marseille, France; [5]Hospices Civils de Lyon, Lyon, France; [6]Centre Hospitalier de Cornouaille, Quimper, France; [7]University Hospital of Brest, Brest, France; [8]Hospital of Troyes, Troyes, France; [9]University Hospital of Grenoble, Grenoble, France; [10]University Hospital of Angers, Angers, France; [11]Roanne Hospital, Roanne, France; [12]Saint Nazaire Hospital, Saint Nazaire, France; [13]Bretagne Atlantique Hospital, Vannes, France; [14]Argenteuil Hospital, Argenteuil, France; [15]Libourne Hospital, Libourne, France;*

### Background

Momelotinib (MMB) is a novel therapy for myelofibrosis that inhibits JAK1, JAK2, and activin A receptor type 1 (ACVR1), a key regulator of iron metabolism through hepcidin modulation. Despite recent regulatory approval, real-world data on MMB remain limited, particularly regarding the feasibility and effectiveness of switching back to another JAK inhibitor (JAKi) in case of intolerance, toxicity or of lost response to MMB. The French Intergroup on Myeloproliferative Neoplasms (FIM) therefore initiated the RETROMOMELO study to evaluate the effectiveness and safety of momelotinib in routine clinical practice, as well as to characterize treatment strategies involving switching back to ruxolitinib (RUX) or fedratinib (FEDRA).

### Methods

RETROMOMELO is a retrospective multicenter study of MF patients treated with MMB under compassionate use between February 2023 and May 2025 across 16 French centers. Baseline characteristics, prior therapies, transfusion status, treatment duration, response, adverse events, and subsequent JAKi transitions were collected. Continuous and categorical variables are reported as medians (IQR) and proportions respectively.

**Copyright Information:** (Online) ISSN: 2572-9241
©2026 The Author(s). HemaSphere is published by John Wiley & Sons Ltd on behalf of European Hematology Association. This is an open access Abstract Book distributed under the Attribution-NonCommercial-NoDerivs (CC BY-NC-ND), which allows third parties to download the articles and share them with others as long as they credit the author and the Abstract Book, but they cannot change the content in any way or use them commercially.
**Abstract Book Citations:** Authors, Title, HemaSphere, 2026;10:(S1):pages. The abstract book DOIs can be found on the "Author Information" page.
**Disclaimer:** Articles published in the journal HemaSphere exclusively reflect the opinions of the authors. The authors are responsible for all content in their abstracts including accuracy of the facts, statements, citing resources, etc.

**Results**

Fifty patients were included (54% male), median age 79 years (IQR 75-82). Diagnoses were primary MF (60%), post-ET MF (26%), and post-PV MF (14%). JAK2 V617F was present in 68%, CALR type 1 in 10%, and MPL in 10%. Most patients (91%) had prior RUX exposure; 60% were transfusion-dependent (TD) at MMB initiation. According to DIPSS+, 44% were high-risk and 40% intermediate-2.
MMB was given as second-line therapy in 42% and third-line in 30%. Prior JAK inhibitors were discontinued abruptly in 86%, and with overlap in 8%; corticosteroids were used as bridging therapy in 8%.
Under MMB, median spleen reduction before and after MMB treatment was 1.0 cm (IQR 0–4; p<0.001) and hemoglobin (Hb) increased by 2.65 g/dL (IQR 1.12–3.80; p<0.001). Fatigue decreased from 72% to 53% (p=0.07). MMB was discontinued in 46% after a median of 6.9 months (IQR 3.0–10.9). Main reasons were intolerance (n=10), refractory/progressive disease (n=8), transplantation (n=2), and leukemic transformation (n=1). The most frequent non-hematologic adverse events were diarrhea (n=10), abdominal pain (n=8), nausea (n=7), and dizziness (n=12); grade ≥3 events were limited to diarrhea (n=2).
Among the 12 patients who rechallenged another JAKi, 2 switched to FEDRA and 10 to RUX after a median of 14 months of MMB exposure (IQR 10.9-19.3 months). Reasons for treatment change included intolerance (*n*=6), response loss (systemic symptoms *n*=2; splenomegaly *n*=1; transfusion benefit *n* = 1), primary treatment failure with stable or increased transfusion requirements (*n*=1) and leukemic transformation (n=1).
At the time of switch back, patients demonstrated excellent tolerance to the other JAKi. Splenomegaly improved in 50% of patients, and constitutional symptoms improved in three-quarters of patients. TD patients at the time of MMB initiation maintained their hemoglobin response after discontinuation and JAKi switch and did not become TD again after a median follow-up of 5.4 months (IQR 3.0–13.6).

**Summary/Conclusion**

In this real-world cohort, MMB showed expected hematologic benefit and manageable toxicity. Importantly, switching back to RUX or FEDRA was feasible and associated with good tolerability and preserved clinical responses, supporting JAKi treatment sequencing strategies in MF.

**Copyright Information:** (Online) ISSN: 2572-9241
©2026 The Author(s). HemaSphere is published by John Wiley & Sons Ltd on behalf of European Hematology Association. This is an open access Abstract Book distributed under the Attribution-NonCommercial-NoDerivs (CC BY-NC-ND), which allows third parties to download the articles and share them with others as long as they credit the author and the Abstract Book, but they cannot change the content in any way or use them commercially.
**Abstract Book Citations:** Authors, Title, HemaSphere, 2026;10:(S1):pages. The abstract book DOIs can be found on the "Author Information" page.
**Disclaimer:** Articles published in the journal HemaSphere exclusively reflect the opinions of the authors. The authors are responsible for all content in their abstracts including accuracy of the facts, statements, citing resources, etc.

### (PB3433) EVALUATION OF GECACITINIB IN PATIENTS WITH MYELOFIBROSIS REAL-WORLD EVIDENCE FROM 52 CONSECUTIVE PATIENTS

**Topic:** 16. Myeloproliferative neoplasms - Clinical

Qiang Wang*[1], Xuan Zhou[1], Yan Zhang[1], Bingyu Lin[1], Jiayi Li[1], Li Xuan[1], Qifa Liu[1], Xiaoli Liu[1], Na Xu[1]

[1]*Nanfang Hospital, Southern Medical University CN, Hematology, Guangzhou, China;*

### Background

Primary and post-polycythemia vera/essential thrombocythemia myelofibrosis (MF) are myeloproliferative neoplasms (MPN) characterized by splenomegaly, constitutional symptoms, haematological abnormalities, and risks of vascular complications and blast transformation. Ruxolitinib alleviates MF-related splenomegaly and symptoms but exhibits suboptimal haematological regulatory efficacy in cytopenic patients and fails to achieve durable splenic reduction. Gecacitinib, a JAK/ACVR1 inhibitor, exhibits anti-MF activity. However, real-world evidence regarding its use in both ruxolitinib-naïve and ruxolitinib-exposed patients remains scarce. This retrospective real-world study evaluated the clinical performance of gecacitinib in MF patients.

### Aims

To evaluate the efficacy and safety of gecacitinib in both ruxolitinib-naïve and ruxolitinib-exposed patients with MF in a real-world setting.

### Methods

A total of 52 patients with MF (12 ruxolitinib-naïve, 40 ruxolitinib-exposed) treated with gecacitinib from June 2025 to February 2026 were enrolled and followed up for 24 weeks. Oral dosage was individually adjusted based on baseline haematological parameters. Efficacy endpoints were splenic response evaluated per the 2022 Ultrasound Specification. Safety endpoints encompassed the incidence of any-grade and grade ≥ 3 treatment-related adverse events (TRAEs), graded by CTCAE v5.0, and causes of treatment discontinuation.

### Results

The ruxolitinib-naïve cohort (n=12) was aged 36 to 69 years (median age 61 years; 7 males and 5 females). 92% had primary myelofibrosis (PMF), 58% had moderate to severe anaemia (HGB < 80 g/L) and 50% had thrombocytopenia (PLT < 125 × $10^9$/L). Among the 12 patients, the overall splenic response rate was 90%, with 40% achieving ≥ 30% splenic volume reduction. The ruxolitinib-exposed cohort (n=40) was aged 38 to 78 years (median age 65 years; 22 males and 18 females). Similarly, 46% had moderate to severe anaemia and 46% had thrombocytopenia, with leukocytosis in 40% of patients and neutrophilia in 32% of patients. The overall splenic volume reduction response rate was 74%, with 16% achieving ≥ 30% splenic volume reduction. Haematological response: In naïve patients (baseline HGB < 80 g/L, n=8), mean HGB levels increased from 66 g/L to 149 g/L (126% improvement). The overall naïve cohort had HGB elevation from 83 g/L to 149 g/L. In exposed patients (baseline HGB < 80 g/L, n=18), median HGB rose from 63 g/L to 82 g/L (30% improvement), and the overall exposed cohort had HGB improvement from 81 g/L to 97 g/L. Subgroups with baseline HGB > 80 g/L maintained stable levels in both cohorts. Gecacitinib induced bidirectional normalization of PLT, WBC, and neutrophil counts without adverse effects. All non-haematological AEs in the 52 patients were grade 1 - 2. In the naïve cohort, 67% had any AEs, with fever, oedema, and infection each occurring in 17%. In the exposed cohort, 58% had any AEs, including diarrhoea (13%) and dizziness/peripheral neuralgia (8%). The overall treatment discontinuation rate was 15% (hematopoietic stem cell transplantation (n=2), leukemic transformation (n=1), infection (n=1)), of which 2.5% were attributed to TRAEs (mild intolerance).

**Copyright Information:** (Online) ISSN: 2572-9241
©2026 The Author(s). HemaSphere is published by John Wiley & Sons Ltd on behalf of European Hematology Association. This is an open access Abstract Book distributed under the Attribution-NonCommercial-NoDerivs (CC BY-NC-ND), which allows third parties to download the articles and share them with others as long as they credit the author and the Abstract Book, but they cannot change the content in any way or use them commercially.
**Abstract Book Citations:** Authors, Title, HemaSphere, 2026;10:(S1):pages. The abstract book DOIs can be found on the "Author Information" page.
**Disclaimer:** Articles published in the journal HemaSphere exclusively reflect the opinions of the authors. The authors are responsible for all content in their abstracts including accuracy of the facts, statements, citing resources, etc.

**Summary/Conclusion**

Our data demonstrates that gecacitinib exhibits a superior efficacy and a favorable safety profile for MF patients, particularly in those with anaemia. Larger multicentre long-term real-world studies are needed to confirm durable efficacy and safety.

**Copyright Information:** (Online) ISSN: 2572-9241
©2026 The Author(s). HemaSphere is published by John Wiley & Sons Ltd on behalf of European Hematology Association. This is an open access Abstract Book distributed under the Attribution-NonCommercial-NoDerivs (CC BY-NC-ND), which allows third parties to download the articles and share them with others as long as they credit the author and the Abstract Book, but they cannot change the content in any way or use them commercially.
**Abstract Book Citations:** Authors, Title, HemaSphere, 2026;10:(S1):pages. The abstract book DOIs can be found on the "Author Information" page.
**Disclaimer:** Articles published in the journal HemaSphere exclusively reflect the opinions of the authors. The authors are responsible for all content in their abstracts including accuracy of the facts, statements, citing resources, etc.

### (PB3434) EUROPEAN REAL WORLD EVIDENCE STUDY OF JAK INHIBITOR COMBINATION WITH INTERFERON FOR MYELOPROLIFERATIVE NEOPLASMS - EXPLORING INDICATION, TOLERABILITY, RESPONSE AND OUTCOME.

**Topic:** 16. Myeloproliferative neoplasms - Clinical

Vincent Olivier[1, 2], Wayne-Corentin Lambert[1, 2], Olga Chyrko[3], Vincent Alcazer[4], Mathilde Chanut[5], Jean Christophe Ianotto[2, 6], Claire Harrison[7], Alesia Khan[8], Eric Lippert*[1, 2], Marta Sobas[3]

*[1]Chu Brest, Laboratory of Hematology, Brest, France;[2]FIM, France Intergroupe des syndromes Myéloprolifératifs, Paris, France; [3]Collegium Medicum in Bydgoszcz, Nicolaus Copernicus University in Toruń, Department of Hematology, Bydgoszcz, Poland; [4]Centre Hospitalier Lyon Sud, Department of Hematology, Pierre-Bénite, France; [5]CHU St-Louis, Department of Haematology, Paris, France; [6]CHU Brest, Department of Haematology, Brest, France;[7]Guy's and St Thomas' NHS Foundation Trust, Department of Hematology, London, United Kingdom; [8]Leeds Teaching Hospitals NHS Trust, Department of Hematology, Leeds, United Kingdom;*

### Background

Treatment of Myeloproliferative Neoplasms (MPNs) has focused on spleen and symptom control, however this is shifting towards disease modification. In polycythaemia vera (PV), PROUD/CONTI-PV studies of ropeginterferon alfa-2b (ropegIFN) showed significantly improved event-free (EFS) and overall survival (OS) compared with best available therapy (BAT). The MAJIC-PV study similarly demonstrated improved molecular response (MR) and EFS with JAK inhibitor (JAKi) ruxolitinib.
As both agents ruxolitinib and ropegIFN are effective as monotherapy, their combination is appealing. The COMBI I/II and RUXOPEG trials indicate that JAKi plus IFN therapy may be effective in PV and myelofibrosis (MF), achieving haematologic responses, symptom improvement, MR, EFS and OS. FEDORA trial evaluated a similar approach using JAKi fedratinib with ropeg shows encouraging early results.

### Methods

We have initiated a Europe- and UK-wide retrospective real-world evidence study collecting data on patients treated with any combination of a JAKi (ruxolitinib, momelotinib, or fedratinib) and interferon (IFN): ropegIFN or pegylatedIFN alfa-2a. Patients enrolled in trials are excluded.
Collected variables include demographic, clinical characteristics, disease subtype, year of diagnosis, driver mutation allele burden, and extended molecular sequencing data.
Matched controls will be identified for each patient based on age, sex, disease subtype, and year of diagnosis. Equivalent clinical and molecular data will be captured to enable comparative statistical analyses between groups.

**Copyright Information:** (Online) ISSN: 2572-9241
©2026 The Author(s). HemaSphere is published by John Wiley & Sons Ltd on behalf of European Hematology Association. This is an open access Abstract Book distributed under the Attribution-NonCommercial-NoDerivs (CC BY-NC-ND), which allows third parties to download the articles and share them with others as long as they credit the author and the Abstract Book, but they cannot change the content in any way or use them commercially.
**Abstract Book Citations:** Authors, Title, HemaSphere, 2026;10:(S1):pages. The abstract book DOIs can be found on the "Author Information" page.
**Disclaimer:** Articles published in the journal HemaSphere exclusively reflect the opinions of the authors. The authors are responsible for all content in their abstracts including accuracy of the facts, statements, citing resources, etc.

### Results

Clinicians from France, Poland, Germany, Spain, and the UK have committed to data collection of over 100 patients. So far, of 25 recorded patients (12PV, 6ET, 7MF), JAKi (ruxolitinib) was added to IFN in most cases (16: 9PV, 4ET, 3MF). Indication for the combination was insufficient response to monotherapy in 80% (67% in PV, 83% in ET, 100% in MF), followed by pruritus (using IFN), and need to decrease the dose of either drug in monotherapy (10% each). The combination was prescribed as 3$^{rd}$ line (median, range:1-6). After a median follow-up of 12.5 months, 72% were still receiving the combination therapy (92% of PV, 50% of ET, 57% of MF), 3 discontinued for lack of response (one PV, ET and MF each), other causes included stem cell transplantation (2), blastic transformation (1) or solid tumor (1). Adverse effects included cytopenias (6), abdominal pain/discomfort (3) and infections (1). ELN response was stable in 6 patients (1 PV, 2 ET, 3 MF), improved in 8 (4 PV, 1 ET, 3 MF), achievement of complete response in 8 (5 PV, 3 ET, 0 MF), but degraded in 2 (1 ET, 1 MF)(Figure). Median time to best response was (92d: 86 for PV, 150 for ET, 175 for MF).

### Summary/Conclusion

Preliminary results indicate good tolerability and promising response, especially in PV. Full cohort data collection, with matched controls, will evaluate safety and tolerability of the combination in a real-world setting, helping to determine whether it represents a viable treatment option. We aim to understand which populations are most likely to benefit from combination therapy.
These findings will help inform design of future large-scale trials and may support reimbursement considerations. We welcome collaboration from clinicians using this combination therapy, inviting them to contact us if interested in participating in the study.

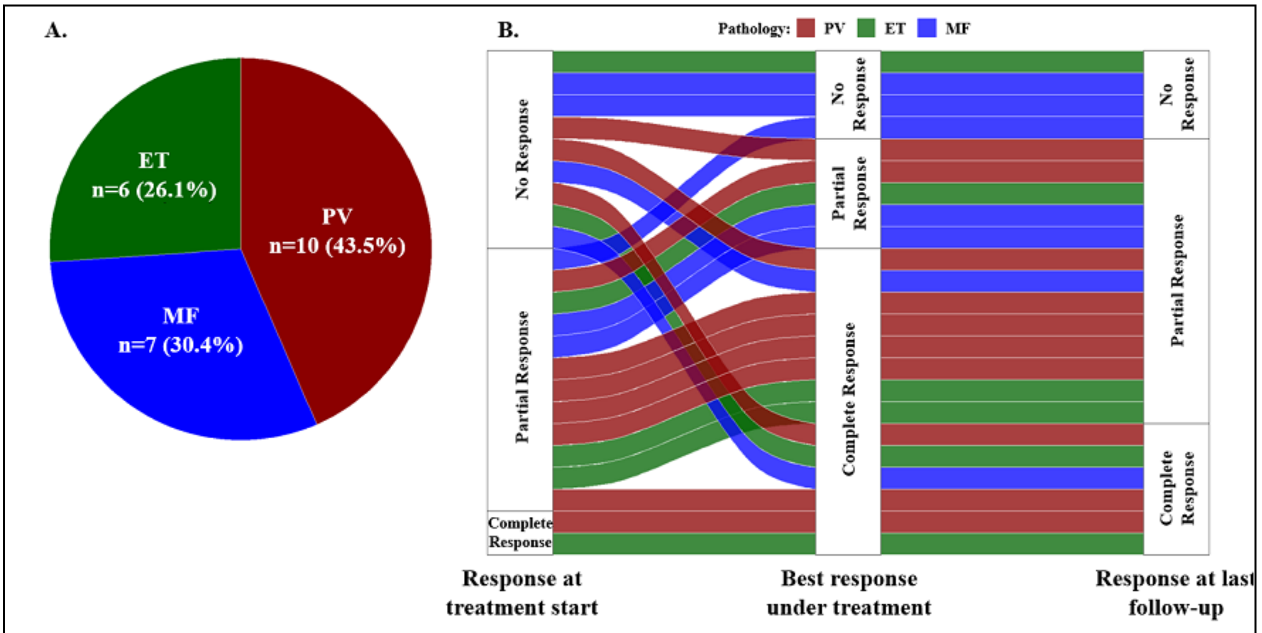

**Evaluation of ELN response in patients treated with a combination of JAK2 inhibitor and interferon.** *(A) Pie chart showing the distribution of MPN subgroups at the start of dual therapy. (B) Sankey diagram illustrating the longitudinal evolution of ELN response from treatment initiation to best response and last follow-up.*

**Copyright Information:** (Online) ISSN: 2572-9241
©2026 The Author(s). HemaSphere is published by John Wiley & Sons Ltd on behalf of European Hematology Association. This is an open access Abstract Book distributed under the Attribution-NonCommercial-NoDerivs (CC BY-NC-ND), which allows third parties to download the articles and share them with others as long as they credit the author and the Abstract Book, but they cannot change the content in any way or use them commercially.
**Abstract Book Citations:** Authors, Title, HemaSphere, 2026;10:(S1):pages. The abstract book DOIs can be found on the "Author Information" page.
**Disclaimer:** Articles published in the journal HemaSphere exclusively reflect the opinions of the authors. The authors are responsible for all content in their abstracts including accuracy of the facts, statements, citing resources, etc.

### (PB3435) PATIENT-REPORTED OUTCOME MEASURES IN PATIENTS WITH POLYCYTHEMIA VERA AND ESSENTIAL THROMBOCYTHEMIA TREATED WITH RUXOLITINIB OR BEST AVAILABLE THERAPY IN THE RANDOMIZED PHASE 2B RUXOBEAT CLINICAL TRIAL

**Topic:** 16. Myeloproliferative neoplasms - Clinical

Susanne Isfort[1], Florian Heidel[1], Carl Crodel[2], Philipp Jost[3, 4], Philippe Schafhausen[5], Lino Lars Teichmann[6], Martin Griesshammer[7], Heiko Becker[8], Denise Walther[9], Katja Sockel[10], Joachim Goethert[11], Daniel Sasca[12], Andreas Reiter[13], Konstanze Döhner[14], Mathias Hänel[15], Haifa Kathrin Al-Ali[16], Aristoteles Giagounidis[17], Nikolaschristiancornelius Bubnoff[18], Martine Klausmann[19], Marinela Augustin[20], Markus Schaich[21], Andreas Hochhaus[2], Frank Stegelmann[14], Michelle Zentis[22, 23], Maike Kortmann[22, 23], Julia Frank[24], Anna Hagemeier[24], Martin Hellmich[24, 25], Tim Brümmendorf[22, 23], Steffen Koschmieder*[22, 23]

*[1]Hannover Medical School (MHH), Hannover, Germany; [2]Jena University Hospital, Jena, Germany; [3]Medical University of Graz, Graz, Austria; [4]Technical University of Munich, Munich, Germany; [5]University Medical Center Hamburg-Eppendorf, Hamburg, Germany; [6]University Hospital Bonn, Bonn, Germany; [7]Johannes Wesling Medical Centre Minden, University of Bochum, Minden, Germany; [8]University of Freiburg and German Cancer Consortium (DKTK), partner site Freiburg, Freiburg, Germany; [9]Otto-von-Guericke University Medical Center Magdeburg, Magdeburg, Germany; [10]University Hospital Dresden, TU Dresden, Dresden, Germany; [11]University Hospital Essen, Essen, Germany; [12]University Medical Center Mainz, and DKTK, partner site Mainz, Mainz, Germany; [13]University Hospital Mannheim, Heidelberg University, Mannheim, Germany; [14]University Hospital of Ulm, Ulm, Germany; [15]Klinikum Chemnitz, Chemnitz, Germany; [16]University Hospital Halle (Saale), Halle (Saale), Germany; [17]Marienhospital Düsseldorf, Düsseldorf, Germany; [18]University of Lübeck, Lübeck, Germany; [19]Gemeinschaftspraxis, Aschaffenburg, Aschaffenburg, Germany; [20]Paracelsus Medizinische Privatuniversität, Nürnberg, Germany; [21]Rems-Murr-Klinikum, Winnenden, Germany; [22]RWTH Aachen University, Faculty of Medicine, Department of Hematology, Oncology, Hemostaseology, and Stem Cell Transplantation, Aachen, Germany; [23]Center for Integrated Oncology Aachen, Bonn, Cologne, Düsseldorf (CIO ABCD), Aachen, Germany; [24]University of Cologne, Cologne, Germany; [25]University Medical Center Göttingen, Göttingen, Germany;*

### Background

In the ongoing phase 2b RuxoBEAT clinical trial (NCT02577926), high-risk patients with polycythemia vera (PV) or essential thrombocythemia (ET) were randomized 1:1 to receive ruxolitinib (RUX) or best available therapy (BAT). Preliminary analyses have shown that both treatments were able to control blood counts, but RUX appeared to be more effective in alleviating symptoms.

### Aims

Here, we focused on detailed patient-reported outcome measures (PROMs) as a measurement of symptoms, both at baseline (BL) and at 6 months of therapy (M6).

### Methods

Patients in the RUX arm received a starting dose of 10 mg twice daily (BID) and were allowed to dose-increase to 20 mg BID (PV) or 25 mg BID (ET). PROM differences between RUX and BAT at BL and M6 and between BL and M6 for each treatment and disease subtype were calculated using Fisher´s exact test/McNemar Test orMann-Whitney-*U* test/ Wilcoxon Signed-Rank Test, without correction for multiple testing.

**Copyright Information:** (Online) ISSN: 2572-9241
©2026 The Author(s). HemaSphere is published by John Wiley & Sons Ltd on behalf of European Hematology Association. This is an open access Abstract Book distributed under the Attribution-NonCommercial-NoDerivs (CC BY-NC-ND), which allows third parties to download the articles and share them with others as long as they credit the author and the Abstract Book, but they cannot change the content in any way or use them commercially.
**Abstract Book Citations:** Authors, Title, HemaSphere, 2026;10:(S1):pages. The abstract book DOIs can be found on the "Author Information" page.
**Disclaimer:** Articles published in the journal HemaSphere exclusively reflect the opinions of the authors. The authors are responsible for all content in their abstracts including accuracy of the facts, statements, citing resources, etc.

### Results

For this exploratory analysis, a total of 171 patients (77 with PV and 94 with ET) were randomized to RUX (n=86) or BAT (n=85). BL characteristics did not reveal relevant significant differences in median age, sex, blood counts, spleen length, or PROMs before onset of therapy. At M6, PROMs for early satiety (p=0.038), abdominal discomfort (p=0.021), fatigue (p<0.001), headache (p=0.011), concentrations problems (p=0.033), night sweats (p=0.036), and pruritus (p=0.001), but not dizziness, numbness, bone pain, fever, weight loss, or overall quality of life (QOL), were lower with RUX than with BAT. Also, from BL to M6, RUX but not BAT significantly improved mean scores for early satiety (p=0.027), night sweats (p<0.001), and pruritus (p=0.006). BL scores for pruritus were higher in PV vs. ET (p<0.001), in keeping with pruritus as a characteristic symptom for PV. At M6, this pruritus difference between disease subtypes was still present (p=0.006).
We then analyzed correlations between the individual PROMs as well as between PROMs and other clinical parameters. At BL, significant correlations for PROMs (all p<0.05) were found between overall QOL and fatigue (r=0.71) or concentration problems (r=0.60), as well as between fatigue and concentration problems (r=0.65) or dizziness (r=0.60). At M6, the mot pronounced correlations between PROMs (all p<0.05) were found for overall QOL and fatigue (r=0.68), concentration problems (r=0.68), and dizziness (r=0.62), and, in addition, between fatigue and concentration problems (r=0.71) and concentration problems and dizziness (r=0.70). Age, WBC, PLT, HCT, and spleen length showed only weak correlations at BL, both among each other and with PROMs. Finally, the following intraindividual PROMs at BL showed considerable correlations (r=0.6 or higher) (all p<0.05) with those at M6: fatigue, headache, concentration problems, dizziness, pruritus, bone pain, and overall QOL, even when the data were not stratified for disease subtype and treatment, suggesting consistency of these symptoms over time.

### Summary/Conclusion

First-line treatment with RUX in high-risk patients with PV or ET was superior to BAT in improving early satiety, abdominal discomfort, fatigue, headache, concentration problems, night sweats, and pruritus at 6 months of therapy. Pruritus at baseline was more characteristic for PV than ET. Our results further suggest that PV and ET patients may particularly benefit from improvement of fatigue and concentration problems.

**Copyright Information:** (Online) ISSN: 2572-9241
©2026 The Author(s). HemaSphere is published by John Wiley & Sons Ltd on behalf of European Hematology Association. This is an open access Abstract Book distributed under the Attribution-NonCommercial-NoDerivs (CC BY-NC-ND), which allows third parties to download the articles and share them with others as long as they credit the author and the Abstract Book, but they cannot change the content in any way or use them commercially.
**Abstract Book Citations:** Authors, Title, HemaSphere, 2026;10:(S1):pages. The abstract book DOIs can be found on the "Author Information" page.
**Disclaimer:** Articles published in the journal HemaSphere exclusively reflect the opinions of the authors. The authors are responsible for all content in their abstracts including accuracy of the facts, statements, citing resources, etc.

### (PB3436) IMPACT OF RAPID VERSUS SLOW TITRATION OF ROPEGINTERFERON ALFA-2B ON JAK2 V617F ALLELE BURDEN IN JAPANESE PATIENTS WITH POLYCYTHEMIA VERA

**Topic:** 16. Myeloproliferative neoplasms - Clinical

Toshiaki Sato*[1], Rieko Sagara[1], Tomoyuki Tanaka[1], Albert Qin[2], Norio Komatsu[1, 3, 4]

*[1]PharmaEssentia Japan KK, Tokyo, Japan; [2]PharmaEssentia Corporation, Taipei, Taiwan; [3]Juntendo University Graduate School of Medicine, Department of Hematology, Tokyo, Japan; [4]Juntendo University Graduate School of Medicine, Department of Advanced Hematology, Tokyo, Japan;*

### Background

Ropeginterferon alfa-2b (ropegIFN) was approved in Japan for polycythemia vera (PV) patients in February 2023, with a slow titration regimen starting at 100 µg (50 µg in patients receiving other cytoreductive agents) and increasing in 50 µg increments every two weeks to a maximum dose of 500 µg. In February 2026, a rapid titration regimen (250–350–500 µg every two weeks) was additionally approved.

### Aims

Given the difference in pharmacologic exposure between these regimens, we explored their association with changes in *JAK2* V617F allele burden (AB) and evaluated the relationship between cumulative drug exposure and molecular response.

### Methods

Data from two phase 3 studies in Japanese PV patients were analyzed: A19-201 study (slow titration) and A23-301 study (rapid titration). The primary analysis assessed mean change in *JAK2* V617F AB from baseline to Week 24 within each study. Descriptive comparisons were drawn between cumulative exposure and magnitude of *JAK2* V617F AB reduction. Molecular response was assessed through individual-level analyses.

### Results

Baseline characteristics were generally comparable between the A23-301 study (n = 21) and the A19-201 study (n = 29), including median age (56.0 vs 54.0 years) and proportion of male patients (52.4% vs 44.8%). The proportion of patients achieving a complete hematologic response (CHR) at Week 24 was 57.1% in A23-301 and 55.2% in A19-201. At Week 24, the mean reduction in *JAK2* V617F AB was greater in the rapid-titration A23-301 study than in the slow-titration A19-201 study (−12.66% vs. −7.50%). These findings suggest a numerically greater reduction with rapid titration. Individual *JAK2* V617F AB trajectories for 21 patients in A23-301 (Figure A) demonstrated an overall decreasing trend with inter-patient variability. Among the 12 patients who maintained 500 µg through Week 24 (planned cumulative dose through Week 24:6100 µg), five achieved a partial response. In contrast, among eight patients whose Week 24 dose was reduced to 350 µg or 250 µg rather than maintained at 500 µg, only one patient (#1) achieved a partial molecular response (Figure B). The patient maintained 500 µg until Week 22 (skipped at Week 24 due to an adverse event) and received 91% of the planned cumulative dose. The actual cumulative doses for the eight reduced-dose patients ranged from 51% to 91% of the planned amount (mean 73.1%; 4459 µg), which was comparable to the cumulative dose in the A19-201 study (4700 µg). In A19-201, patients whose Week 24 dose remained ≤350 µg received relatively lower cumulative doses and showed modest reductions in *JAK2* V617F AB.

**Copyright Information:** (Online) ISSN: 2572-9241
©2026 The Author(s). HemaSphere is published by John Wiley & Sons Ltd on behalf of European Hematology Association. This is an open access Abstract Book distributed under the Attribution-NonCommercial-NoDerivs (CC BY-NC-ND), which allows third parties to download the articles and share them with others as long as they credit the author and the Abstract Book, but they cannot change the content in any way or use them commercially.
**Abstract Book Citations:** Authors, Title, HemaSphere, 2026;10:(S1):pages. The abstract book DOIs can be found on the “Author Information” page.
**Disclaimer:** Articles published in the journal HemaSphere exclusively reflect the opinions of the authors. The authors are responsible for all content in their abstracts including accuracy of the facts, statements, citing resources, etc.

### Summary/Conclusion

These exploratory findings suggest cumulative exposure to ropegIFN may be associated with greater reduction in *JAK2* V617F AB. A rapid titration regimen enabling earlier attainment of higher exposure may facilitate deeper molecular responses. Further controlled analyses are warranted to confirm these observations.

Figure
Individual changes in *JAK2* V617F AB in patients with dose reduction in the A23-301 Study

A

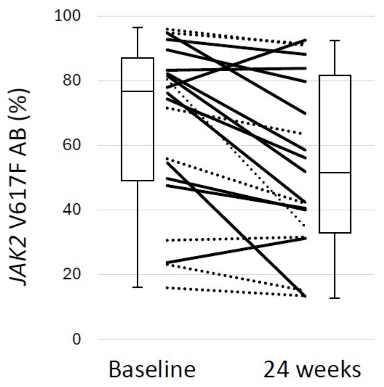

Solid line: 500μg at EoT
Dashed line: dose reduction at EoT

B

| Patient number | #1 | #2 | #3 | #4 | #5 | #6 | #7 | #8 |
|---|---|---|---|---|---|---|---|---|
| Dose at EoT*(μg) | 250 | 350 | 350 | 250 | 250 | 350 | 350 | 250 |
| AB at baseline (%) | 80.4 | 94.8 | 15.9 | 55.8 | 95.9 | 30.6 | 23.1 | 71.5 |
| AB at Week24 (%) | 34.7 | 91.4 | 13.4 | 42.0 | 90.9 | 31.6 | 15.0 | 63.4 |
| ΔAB(%) | -56.8 | -3.6 | -15.7 | -24.7 | -5.2 | 3.3 | -35.1 | -11.3 |
| Actual cumulative dose (μg) | 5550 | 5150 | 5050 | 3350 | 3700 | 4700 | 5050 | 3100 |
| Dose achievement rate** | 91.0 | 84.4 | 82.8 | 54.9 | 60.7 | 77.0 | 82.8 | 50.8 |

*: End of Treatment
**: Actual to planned dose ratio (based on 6100 μg)

**Copyright Information:** (Online) ISSN: 2572-9241
©2026 The Author(s). HemaSphere is published by John Wiley & Sons Ltd on behalf of European Hematology Association. This is an open access Abstract Book distributed under the Attribution-NonCommercial-NoDerivs (CC BY-NC-ND), which allows third parties to download the articles and share them with others as long as they credit the author and the Abstract Book, but they cannot change the content in any way or use them commercially.
**Abstract Book Citations:** Authors, Title, HemaSphere, 2026;10:(S1):pages. The abstract book DOIs can be found on the "Author Information" page.
**Disclaimer:** Articles published in the journal HemaSphere exclusively reflect the opinions of the authors. The authors are responsible for all content in their abstracts including accuracy of the facts, statements, citing resources, etc.

### (PB3437) INVOLVEMENT OF THE RESPIRATORY TRACT AND ITS DIAGNOSTIC AND THERAPEUTIC CONSEQUENCES IN PATIENTS WITH IDIOPATHIC HYPEREOSINOPHILIC SYNDROME

**Topic:** 16. Myeloproliferative neoplasms - Clinical

Vito Dangelo*[1], Juliana Schwaab[1], Nicole Naumann[1], Anna-Sophie Schübler[2], Leonard Krenz[1], Alice Fabarius[1], Wolf-Karsten Hofmann[1], Georgia Metzgeroth[1], Andreas Reiter[1], Johannes Lübke[1]

*[1]University Hospital Mannheim, Department of Hematology and Oncology, Mannheim, Germany;[2]University Hospital Mannheim, Department of Nephrology, Immunology and Rheumatology, Mannheim, Germany;*

### Background

Idiopathic hypereosinophilic syndrome (iHES) is defined by persistent eosinophilia (≥1.5x10$^{9}$/L) and heterogeneous eosinophilic organ involvement with or without dysfunction. In patients (pts) with multi-organ involvement, particularly when affecting the respiratory tract (RT), distinguishing iHES from its main differential diagnosis, eosinophilic granulomatosis with polyangiitis (EGPA), remains diagnostically challenging, particularly in the absence of vasculitis and myeloperoxidase (MPO)-specific antineutrophil cytoplasmic antibodies (ANCA).

### Aims

To characterize the involvement of the RT (IRT) and its diagnostic and therapeutic consequences in iHES.

### Methods

Using data from the German Registry on Disorders of Eosinophils and Mast Cells (GREM), we sought to investigate IRT in 172 iHES pts by respiratory function test (RFT, 76/172, 44%), imaging (99/172, 58%), rhinoscopy (86/172, 50%) and cytology/histology (74/172, 43%). Cardiac involvement was assessed by cardiac magnetic resonance imaging (MRI; 102/172, 59%), echocardiography (83/172, 48%) and cardiac biomarkers (133/172, 77%) with cardiomyopathy being defined as left-ventricular ejection fraction <50%. Clonal eosinophilia (e.g., tyrosine kinase fusion genes, somatic point mutations) was excluded by genetic analyses and bone marrow histology. MPO-ANCA and histologic evidence of vasculitis were absent in all pts. An EGPA-like phenotype (EGPA-PT) was defined using MIRRA trial criteria (NCT02020889).

### Results

IRT was observed in 125/172 (73%) pts. Sinonasal abnormalities were present in 86/172 pts (50%), with sinusitis in 76/86 (88%) and nasal polyps in 34/86 (40%). All pts with polyposis had sinusitis, whereas only 34/86 (40%) with sinusitis showed polyposis. A history of asthma was reported in 73/172 (42%) pts. Thoracic imaging (available in 103/172, 60%) revealed parenchymal opacities (49/103, 48%; ground-glass, 8/49, 16%; consolidations, 41/49, 84%), airway abnormalities (22/103, 21%; bronchial wall thickening, 4/22, 18%; bronchiectasis, 8/22, 36%; other signs of bronchitis, 10/22, 46%), pleural effusion (12/103, 12%), nodules (14/103, 14%), emphysema (4/103, 4%) or interlobular septal thickening (4/103, 4%). Tissue eosinophilia was identified in 49/172 (28%) pts (bronchoalveolar lavage, 22/49, 45%; lung biopsy, 32/49, 65%). An EGPA-PT was identified in 35/172 (20%) pts. These pts exhibited a higher number of involved organ systems as compared to non-EGPA-PT pts (median 5 vs 3, P<0.001). EGPA-PT pts more frequently presented with asthma (100% vs 28%; P<0.001), sinonasal abnormalities (96% vs 38%; P<0.001), pulmonary involvement beyond asthma (89% vs 42%; P<0.001), as well as cardiac involvement (77% vs 54%, P=0.013) and cardiomyopathy (26% vs 7%, P=0.003). Treatment data were available for 159/172 pts (92%): systemic glucocorticosteroids (146/159, 92%), mepolizumab (70/159 pts, 44%), benralizumab (11/159, 7%), and immunosuppressive agents (59/159, 37%).

**Copyright Information:** (Online) ISSN: 2572-9241
©2026 The Author(s). HemaSphere is published by John Wiley & Sons Ltd on behalf of European Hematology Association. This is an open access Abstract Book distributed under the Attribution-NonCommercial-NoDerivs (CC BY-NC-ND), which allows third parties to download the articles and share them with others as long as they credit the author and the Abstract Book, but they cannot change the content in any way or use them commercially.
**Abstract Book Citations:** Authors, Title, HemaSphere, 2026;10:(S1):pages. The abstract book DOIs can be found on the "Author Information" page.
**Disclaimer:** Articles published in the journal HemaSphere exclusively reflect the opinions of the authors. The authors are responsible for all content in their abstracts including accuracy of the facts, statements, citing resources, etc.

### Summary/Conclusion

In iHES, IRT and cardiac involvement are frequently co-occurring. The differentiation between iHES with or without an EGPA-PT and ANCA-negative EGPA remains elusive and may reflect a shared disease spectrum rather than distinct entities. In clinical practice, however, treatment options largely overlap including glucocorticosteroids, IL-5/IL-5 receptor antibodies (e.g., mepolizumab, benralizumab) and immunosuppressive agents (e.g., methotrexate, cyclophosphamide).

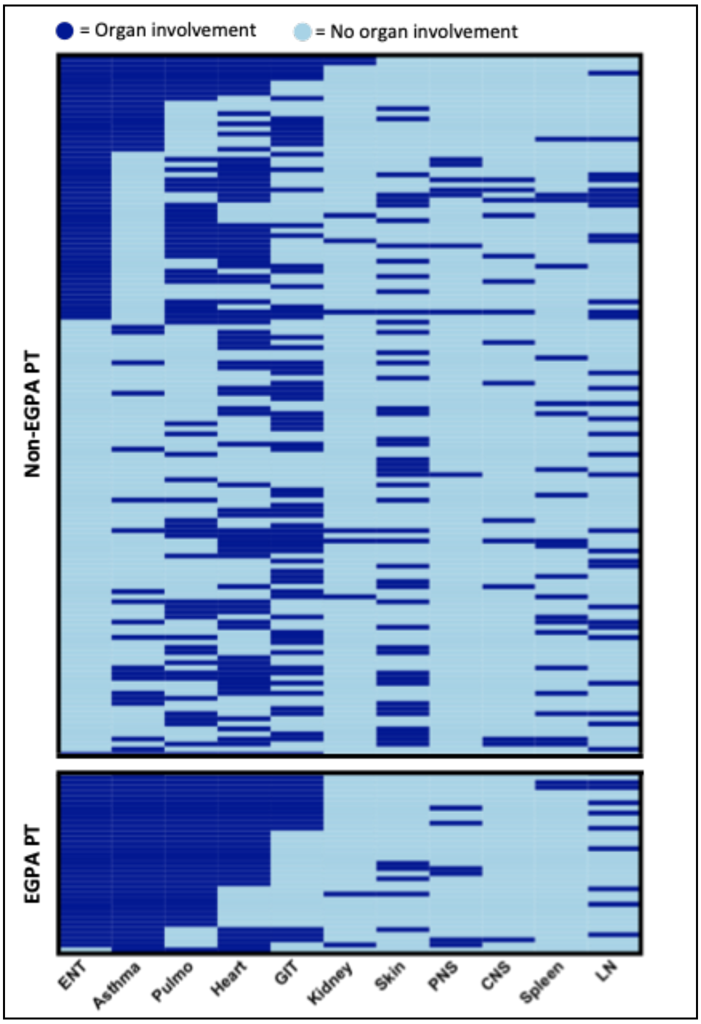

Organ involvement in patients with EGPA-like phenotype (meeting MIRRA criteria) in comparison to patients without EGPA-like phenotype. Abbreviations: CNS, central nervous system; EGPA, eosinophilic granulomatosis with polyangiitis; ENT, ear-nose-throat; GIT, gastrointestinal tract (including liver); LN, lymph node; PNS, peripheral nervous system; PT, phenotype; Pulmo, pulmonary involvement beyond asthma.

**Copyright Information:** (Online) ISSN: 2572-9241
©2026 The Author(s). HemaSphere is published by John Wiley & Sons Ltd on behalf of European Hematology Association. This is an open access Abstract Book distributed under the Attribution-NonCommercial-NoDerivs (CC BY-NC-ND), which allows third parties to download the articles and share them with others as long as they credit the author and the Abstract Book, but they cannot change the content in any way or use them commercially.
**Abstract Book Citations:** Authors, Title, HemaSphere, 2026;10:(S1):pages. The abstract book DOIs can be found on the "Author Information" page.
**Disclaimer:** Articles published in the journal HemaSphere exclusively reflect the opinions of the authors. The authors are responsible for all content in their abstracts including accuracy of the facts, statements, citing resources, etc.

### (PB3438) CLINICAL PHENOTYPE, GENETIC LANDSCAPE, AND OUTCOME IN PATIENTS WITH CO-OCCURRING MUTATIONS IN SF3B1 AND JAK2, CALR, OR MPL

**Topic:** 16. Myeloproliferative neoplasms - Clinical

Johannes Lübke*[1], Juliana Schwaab[1], Manja Meggendorfer[2], Parvis Sadjadian[3], Katja Sockel[4], Joachim Goethert[5], Nicole Naumann[1], Vito Dangelo[1], Alexander Streuer[1], Alice Fabarius[1], Georgia Metzgeroth[1], Peter Herhaus[6], Torsten Haferlach[2], Wolf-Karsten Hofmann[1], Nicholas Cross[7], Andreas Hochhaus[8], Katharina Götze[6], Susanne Isfort[9], Martin Griesshammer[3], Carl Crodel[8], Andreas Reiter[1]

*[1]University Hospital Mannheim, Department of Hematology and Oncology, Manheim, Germany; [2]MLL Munich Leukemia Laboratory, Munich, Germany; [3]Johannes Wesling Medical Center Minden, University of Bochum, University Clinic for Hematology, Oncology, Haemostaseology and Palliative Care, Bochum, Germany; [4]University Hospital Carl Gustav Carus, Faculty of Medicine Carl Gustav Carus, Department of Internal Medicine I, TU Dresden, Germany; [5]University Hospital Essen, University of Duisburg-Essen, Department of Hematology and Stem Cell Transplantation, Essen, Germany; [6]Technical University of Munich (TUM) School of Medicine, Department of Medicine III, Munich, Germany; [7]University of Southampton, Faculty of Medicine, Southampton, Germany; [8]University Hospital Jena, Department of Hematology and Oncology, Jena, Germany; [9]Hannover Medical School (MHH), Department of Hematology, Hemostasis, Oncology and Stem Cell Transplantation, Hannover, Germany;*

### Background

In chronic myeloid neoplasms, canonical driver mutations in *JAK2*, *CALR*, or *MPL* and spliceosome mutations in *SF3B1* are associated with distinct morphologic phenotypes. In myelodysplastic/myeloproliferative neoplasms with *SF3B1* mutation and thrombocytosis (MDS/MPN-*SF3B1*-T), mutations in *JAK2*, *CALR*, or *MPL* frequently co-occur with *SF3B1*. However, this co-occurrence is not diagnostic of MDS/MPN-*SF3B1*-T, but data on its incidence across other myeloid neoplasms is limited.

### Aims

To characterize the clinical phenotype, genetic landscape and outcome of patients (pts) harboring concurrent *SF3B1* and *JAK2*, *CALR*, or *MPL* mutations.

### Methods

In this multicenter retrospective study (four Germany centers), 54 pts with co-occurring mutations in *SF3B1* and in *JAK2*/*CALR*/*MPL* were identified. Diagnoses were assigned per 2022 WHO Classification of Haematolymphoid Tumours. Targeted next-generation sequencing was performed using a 63-gene (36/54, 68%) or a 22-gene panel (18/54, 32%).

**Copyright Information:** (Online) ISSN: 2572-9241
©2026 The Author(s). HemaSphere is published by John Wiley & Sons Ltd on behalf of European Hematology Association. This is an open access Abstract Book distributed under the Attribution-NonCommercial-NoDerivs (CC BY-NC-ND), which allows third parties to download the articles and share them with others as long as they credit the author and the Abstract Book, but they cannot change the content in any way or use them commercially.
**Abstract Book Citations:** Authors, Title, HemaSphere, 2026;10:(S1):pages. The abstract book DOIs can be found on the "Author Information" page.
**Disclaimer:** Articles published in the journal HemaSphere exclusively reflect the opinions of the authors. The authors are responsible for all content in their abstracts including accuracy of the facts, statements, citing resources, etc.

### Results

Median age at diagnosis was 64 years (range 21-94); 34/54 (63%) pts were male. Primary diagnoses included MDS/MPN-*SF3B1*-T in 13/54 (24%), classical myeloproliferative neoplasms in 35/54 (65%) pts (polycythemia vera, 8/54, 15%; essential thrombocythemia, 11/54, 20%; prefibrotic and overt primary myelofibrosis, 16/54, 30%), and other myeloid neoplasms (6/54, 11%; **Figure**). The main reasons for not meeting criteria for an MDS/MPN-*SF3B1*-T diagnosis included a platelet count <450 x$10^9$/L in 17/41 pts (41%) and/or a hemoglobin level >12/13 g/dL (females/males) in 10/41 pts (24%). The median leukocyte count was 12.0 x$10^9$/L (range 3.2–103.8), with leukoerythroblastosis present in 31/54 (57%) and blasts ≥5% in 4/54 (7%) pts. Splenomegaly was observed in 38/54 (70%) pts. Bone marrow was hypercellular in 42/47 (89%) pts. Dysplasia of megakaryopoiesis, erythropoiesis, and granulopoiesis was reported in 29/48 (60%), 23/48 (48%), and 15/48 (31%) pts, respectively. Myelofibrosis grade 2 or 3 was reported in 24/48 (50%) pts. Ring sideroblasts >15% were present in 8/44 (18%) pts. The median *SF3B1* variant allele frequency (VAF) was 33% (range 2–49). All *SF3B1* variants were missense mutations in the HEAT-repeat region, most commonly K700E (19/54, 35%) and K666N (18/54, 33%). The concurrent driver mutations were identified in *JAK2* (40/54, 74%; median VAF 36%, range 2–96), *CALR* (8/54, 15%; median VAF 38%, range 25–64), and *MPL* (8/54, 15%; median VAF 17%, range 2–75). Two pts harbored concomitant *JAK2* and *MPL* mutations. The median number of additional somatic mutations in 54 pts was 1 (range 0–5), most commonly involving *TET2* (33%), *ASXL1* (17%), *SRSF2* (7%), and *CUX1* (7%). A median of 2 treatment lines were administered (range 0–4) including JAK inhibitors in 32/54 (59%) and luspatercept in 8/54 (15%) pts. After a median follow-up of 5.2, 11.2, and 8.2 years from diagnosis, 6 pts experienced a thromboembolic event, 10 pts progressed to secondary myelofibrosis, and 7 pts to MPN blast phase / secondary acute myeloid leukemia. The median overall survival (mOS) for the entire cohort was 19.8 years. *CALR* mutated pts had superior mOS compared with non-*CALR* mutated pts (not reached vs. 18.6 years; P=0.016).

### Summary/Conclusion

In this multicenter cohort selected for pts with concurrent *SF3B1* and *JAK2/CALR/MPL* mutations, the predominant clinico-morphologic phenotype was consistent with classical MPNs, most frequently including the different subtypes of myelofibrosis, rather than with the phenotype of an MDS/MPN-*SF3B1*-T.

**Copyright Information:** (Online) ISSN: 2572-9241
©2026 The Author(s). HemaSphere is published by John Wiley & Sons Ltd on behalf of European Hematology Association. This is an open access Abstract Book distributed under the Attribution-NonCommercial-NoDerivs (CC BY-NC-ND), which allows third parties to download the articles and share them with others as long as they credit the author and the Abstract Book, but they cannot change the content in any way or use them commercially.
**Abstract Book Citations:** Authors, Title, HemaSphere, 2026;10:(S1):pages. The abstract book DOIs can be found on the "Author Information" page.
**Disclaimer:** Articles published in the journal HemaSphere exclusively reflect the opinions of the authors. The authors are responsible for all content in their abstracts including accuracy of the facts, statements, citing resources, etc.

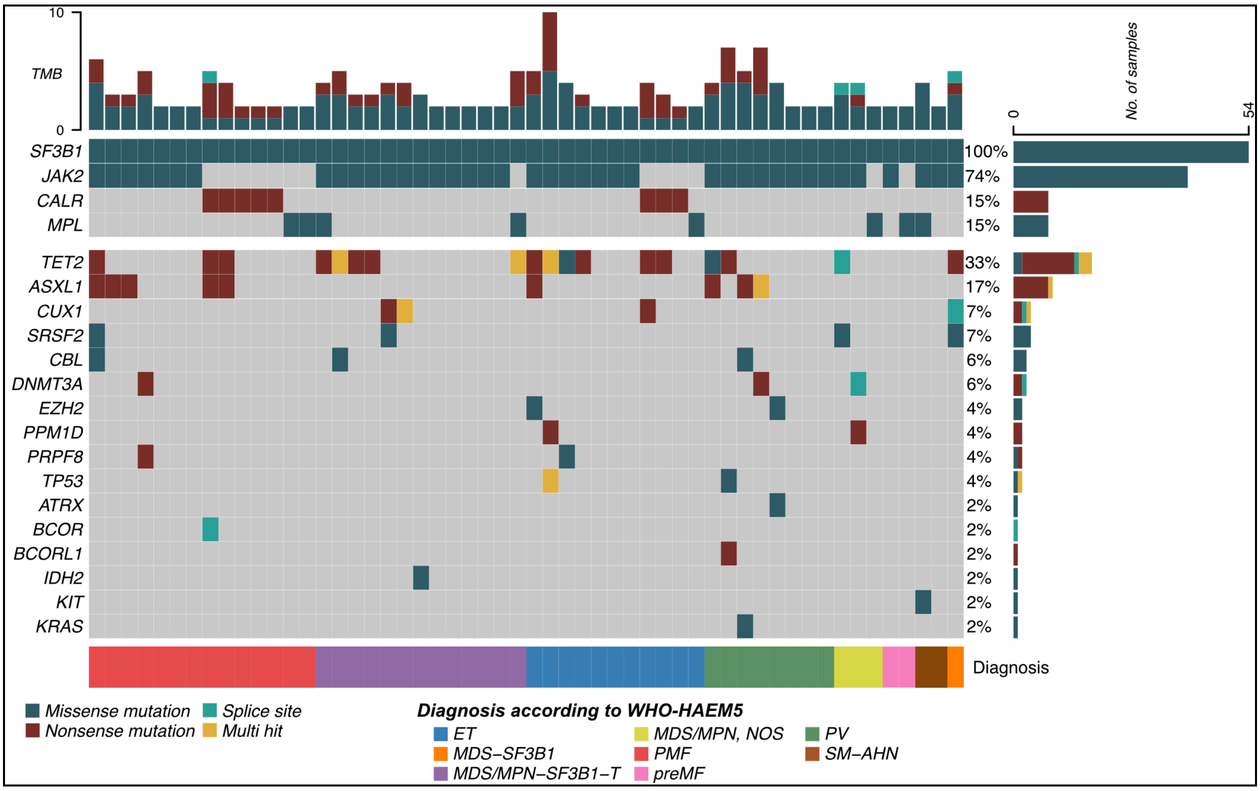

Figure: Genetic landscape of SF3B1 and JAK2, CALR, or MPL co-mutated patients. Abbreviations: ET, essential thrombocythemia; MDS-SF3B1, myelodysplastic neoplasm with SF3B1 mutation; MDS/MPN-SF3B1-T, myelodysplastic/myeloproliferative neoplasm with SF3B1 mutation and thrombocytosis; MDS/MPN, NOS, myelodysplastic/myeloproliferative neoplasm, not otherwise specified; No, number; PMF, primary myelofibrosis; preMF, prefibrotic myelofibrosis; PV, polycythaemia vera; SM-AHN, systemic mastocytosis with an associated hematologic neoplasm; TMB, tumor mutational burden; WHO-HAEM5, World Health Organization Classification of Haematolymphoid Tumours, 5th edition

**Copyright Information:** (Online) ISSN: 2572-9241
©2026 The Author(s). HemaSphere is published by John Wiley & Sons Ltd on behalf of European Hematology Association. This is an open access Abstract Book distributed under the Attribution-NonCommercial-NoDerivs (CC BY-NC-ND), which allows third parties to download the articles and share them with others as long as they credit the author and the Abstract Book, but they cannot change the content in any way or use them commercially.
**Abstract Book Citations:** Authors, Title, HemaSphere, 2026;10:(S1):pages. The abstract book DOIs can be found on the "Author Information" page.
**Disclaimer:** Articles published in the journal HemaSphere exclusively reflect the opinions of the authors. The authors are responsible for all content in their abstracts including accuracy of the facts, statements, citing resources, etc.

### (PB3439) OB756, A NOVEL SELECTIVE JAK2 INHIBITOR, IN PATIENTS WITH MYELOFIBROSIS AND BASELINE THROMBOCYTOPENIA (<100×10⁹/L), ANEMIA (<100 G/L), AND MASSIVE SPLENOMEGALY: AN EXPLORATORY ANALYSIS

**Topic:** 16. Myeloproliferative neoplasms - Clinical

Jian Huang*[1], Rumeng Li[1], Lianlian Fan[2], Jishi Wang[3, 3], Zhimei Wu[3], Sujun Gao[4], Jin Zhang[5], Zhongxing Jiang[6], Xiaojing Yan[7], Hu Zhou[8], Jie Jin[1]

*[1]The First Affiliated Hospital, Zhejiang University School of Medicine, Hangzhou, China;[2]Department of Hematology, The First Affiliated Hospital of Guizhou Medical University, Guiyang, Guizhou, China, Guizhou, China; [3]Department of Hematology, The Affiliated Hospital of Guilin Medical University, Guilin, Guangxi, China, Guilin, China; [4]Department of Hematology, The First Hospital of Jilin University, Changchun, Jilin, China, Changchun, China; [5]Department of Hematology, Sir Run Run Shaw Hospital, Zhejiang University School of Medicine, Hangzhou, Zhejiang, China, Hangzhou, China; [6]Department of Hematology, The First Affiliated Hospital of Zhengzhou University, Zhengzhou, Henan, China, Zhengzhou, China; [7]Department of Hematology, The First Affiliated Hospital of China Medical University, Shenyang, Liaoning, China, Shenyang, China; [8]Department of Hematology, Henan Cancer Hospital, Zhengzhou, Henan, China, Zhengzhou,, China;*

### Background

Myelofibrosis (MF) patients frequently develop thrombocytopenia during disease diagnosis or treatment, with approximately 25% of MF patients presenting with moderate to severe thrombocytopenia at diagnosis. For patients with poor baseline characteristics such as severe cytopenia (thrombocytopenia, anemia), high-risk mutations, or massive splenomegaly, treatment options are extremely limited and clinical prognosis is poor. Existing JAK inhibitors generally require platelet counts ≥100×10⁹/L, failing to meet the clinical needs of this population.

### Aims

This study aims to evaluate the efficacy and safety of novel JAK2 inhibitor OB756 in myelofibrosis patients with severe baseline cytopenia and massive splenomegaly who are ineligible for existing JAK inhibitors due to platelet count restrictions.

### Methods

This study aimed to evaluate the safety and efficacy of OB756 in intermediate- to high-risk MF patients with platelet counts <100×10⁹/L, hemoglobin <100 g/L, or spleen volume >1500 cm³. This project integrated CTR20211396 and CTR2500105524, enrolling patients with primary MF (PMF), post-polycythemia vera MF (Post-PV-MF), or post-essential thrombocythemia MF (Post-ET-MF). Inclusion criteria included: Dynamic International Prognostic Scoring System (DIPSS) intermediate-2 or high risk, spleen volume >1500 cm³, platelet count <100×10⁹/L, and hemoglobin <100 g/L. The primary endpoint was the proportion of patients achieving ≥35% reduction in spleen volume from baseline at Week 24 (SVR35, assessed by Independent Review Committee [IRC]). Key secondary endpoints included: proportion of patients achieving ≥50% reduction in Myeloproliferative Neoplasm Symptom Assessment Form Total Symptom Score (MPN-SAF TSS) from baseline; safety.

**Copyright Information:** (Online) ISSN: 2572-9241
©2026 The Author(s). HemaSphere is published by John Wiley & Sons Ltd on behalf of European Hematology Association. This is an open access Abstract Book distributed under the Attribution-NonCommercial-NoDerivs (CC BY-NC-ND), which allows third parties to download the articles and share them with others as long as they credit the author and the Abstract Book, but they cannot change the content in any way or use them commercially.
**Abstract Book Citations:** Authors, Title, HemaSphere, 2026;10:(S1):pages. The abstract book DOIs can be found on the "Author Information" page.
**Disclaimer:** Articles published in the journal HemaSphere exclusively reflect the opinions of the authors. The authors are responsible for all content in their abstracts including accuracy of the facts, statements, citing resources, etc.

### Results

A total of 12 patients receiving OB756 20 mg twice daily (BID) were enrolled across the two trials. Baseline characteristics were as follows: median age 58 years (range 51-74), 8 females (66.7%); ECOG performance status score 1 in 2 patients (16.7%), 2 in 8 patients (66.7%), and 3 in 2 patients (16.7%). Among enrolled patients, anemia was Grade 2 in 7 patients, Grade 3 in 3 patients, and Grade 4 in 1 patient; thrombocytopenia was Grade 1 in 9 patients and Grade 2 in 3 patients. Efficacy results showed: IRC-assessed SVR35 rate at Week 24 was 66.7% (8/12). Hematologic monitoring demonstrated relatively stable platelet counts, hemoglobin, and white blood cell counts during treatment without significant further decline; additionally, 4 patients achieved normalized platelet counts at Week 24. Regarding symptom improvement, 58.3% (7/12) of patients achieved ≥50% reduction in MPN-SAF TSS at Week 24.Safety analysis showed no obvious worsening of hematologic toxicity beyond the existing baseline thrombocytopenia and anemia. For non-hematologic toxicities, Grade 3 or higher infection occurred in 8.3% (1/12).

### Summary/Conclusion

**Conclusion:** In summary, OB756 demonstrated favorable tolerability and significant clinical efficacy in intermediate- to high-risk MF patients with severe cytopenia and massive splenomegaly, particularly showing outstanding efficacy in rapidly reducing spleen volume, with low incidence of non-hematologic toxicities. This study provides a new treatment option for MF patients with poor baseline characteristics.

This research was supported by the National Natural Science Foundation of China (No. 82570191) and the Zhejiang Provincial Health High level Innovative Talent Project (2022-2026).* Correspondence to:Jian Huang, email: househuang@zju.edu.cn

**Copyright Information:** (Online) ISSN: 2572-9241
©2026 The Author(s). HemaSphere is published by John Wiley & Sons Ltd on behalf of European Hematology Association. This is an open access Abstract Book distributed under the Attribution-NonCommercial-NoDerivs (CC BY-NC-ND), which allows third parties to download the articles and share them with others as long as they credit the author and the Abstract Book, but they cannot change the content in any way or use them commercially.
**Abstract Book Citations:** Authors, Title, HemaSphere, 2026;10:(S1):pages. The abstract book DOIs can be found on the “Author Information” page.
**Disclaimer:** Articles published in the journal HemaSphere exclusively reflect the opinions of the authors. The authors are responsible for all content in their abstracts including accuracy of the facts, statements, citing resources, etc.

### (PB3440) CLINICAL, GENOMIC, AND TREATMENT-RELATED OUTCOMES OF PATIENTS WITH HISTIOCYTIC SARCOMA TREATED BETWEEN 2000 TO 2025

**Topic:** 16. Myeloproliferative neoplasms - Clinical

Jenna Mahns*[1], Katherine Chapis[1], Daniel Sullivan[1], Nathan Roberts[2], Mark Sorial[1], Eric D. Jacobsen[1]

[1]*Dana-Farber Cancer Institute, Boston, United States of America;* [2]*University of Virginia, Charlottesville, VA, United States of America;*

### Background

Histiocytic sarcoma (HS) is a rare malignant histiocytic neoplasm that has varied presentation and may present concurrent with other malignancies. There is no standardized therapy, and management is guided by extent of disease with surgery, radiotherapy, combined modality therapy (Surg/RT), and chemotherapy (CC) as options. Relevant prognostic clinical or genomic factors are unknown, and data to guide systemic therapy are limited.

### Aims

To describe prognostic clinical, treatment, and genomic factors among patients with HS.

### Methods

This was a retrospective single arm cohort study of adult patients with pathologically diagnosed HS managed at a tertiary medical center from 2000-2025. Endpoints included progression-free (PFS) and overall survival (OS). Patients treated with multiple therapies could contribute survival time for each unique line of therapy (cross-over design). PFS was measured from each therapy start to progression, next line of therapy, or death. Surg/RT was not considered a censoring or PFS event. Estimates were adjusted for the line of therapy for each regimen. OS was measured from diagnosis to death. Genomic details were characterized based on cellular pathway. Kaplan-Meier and Cox regression methods were used.

### Results

A total of 47 patients were included. Median age was 53 (range 18-86) yr with 30 (63.8%) being male. 16 (34%) had concurrent malignancies (13 [27.7%] hematologic, 3 [6.4%] solid, and 1 [2.1%] both). HS affected ≥2 sites in 20 (42.6%) and 1 site in 27 (57.4%). 30 (65.2%) had nodal involvement. Of the 47 patients, 45 (95.7%) received treatment with a median 2 (range 1-8) lines of systemic therapy. Definitive surgery, RT, and Surg/RT were used in 9 (20%), 5 (11.1%), and 9 (20%), respectively, and was the only modality in 16 (35.6%). A total of 15 (33.3%) and 10 (22.2) received CHOP and ifosfamide-based CC, respectively. Others included antimetabolite CC (9 [20%; e.g. cladribine]), pembrolizumab (7 [15.6%]), MEK inhibitors (6 [13.3%]), and IMIDs (6 [13.3%]). 5 patients underwent stem cell transplant (11.1%; 4 [8.9%] allogeneic and 1 [2.2%] autologous). With a median follow up of 64.2 (95%CI 36.2-92.2) mo, median (95%CI) OS was 81.2 (33.2-129.2) mo overall, and 25.6 (8.5-42.7) vs 82.3 (0-200.1) mo in patients with ≥2 sites vs 1 site (p=0.034). Median (95%CI) PFS was 82.3 (54.5-110.1) mo with Surg/RT alone, 3.2 (1.7-4.7) mo for CC regimens (p<0.001 vs Surg/RT), and 10.1 (1.3-18.9) mo for nonCC therapies (p<0.001 vs Surg/RT; p=0.116 vs CC; Figure 1). Among patients who received systemic therapy, median (95%CI) OS was not reached (NR-NR) vs 24.7 (17-32.4) mo (p=0.023) in those with and without a concurrent malignancy, respectively. Genomic details were available for 8 (17%) patients, all with identified variants with median of 6 (range 1-13) variants. 7 (87.5%) patients had alterations in epigenetic dysregulation and survival signaling pathways. All variants were unique across patients with the exception of FAT1, IKZF1, MED12, and RPTOR, which were identified in 2 patients each.

**Copyright Information:** (Online) ISSN: 2572-9241
©2026 The Author(s). HemaSphere is published by John Wiley & Sons Ltd on behalf of European Hematology Association. This is an open access Abstract Book distributed under the Attribution-NonCommercial-NoDerivs (CC BY-NC-ND), which allows third parties to download the articles and share them with others as long as they credit the author and the Abstract Book, but they cannot change the content in any way or use them commercially.
**Abstract Book Citations:** Authors, Title, HemaSphere, 2026;10:(S1):pages. The abstract book DOIs can be found on the "Author Information" page.
**Disclaimer:** Articles published in the journal HemaSphere exclusively reflect the opinions of the authors. The authors are responsible for all content in their abstracts including accuracy of the facts, statements, citing resources, etc.

### Summary/Conclusion

Patients with HS with 1 site of disease or had concurrent malignancies had significantly better OS. The latter finding may be explained by overestimation of the number of sites affected by HS due to confounding by the presence of another malignancy. Surg/RT was associated with significantly better PFS in patients with 1 site of disease, whereas nonCC resulted in improved PFS in those with ≥2 sites. Genomic alterations may inform future management strategies.

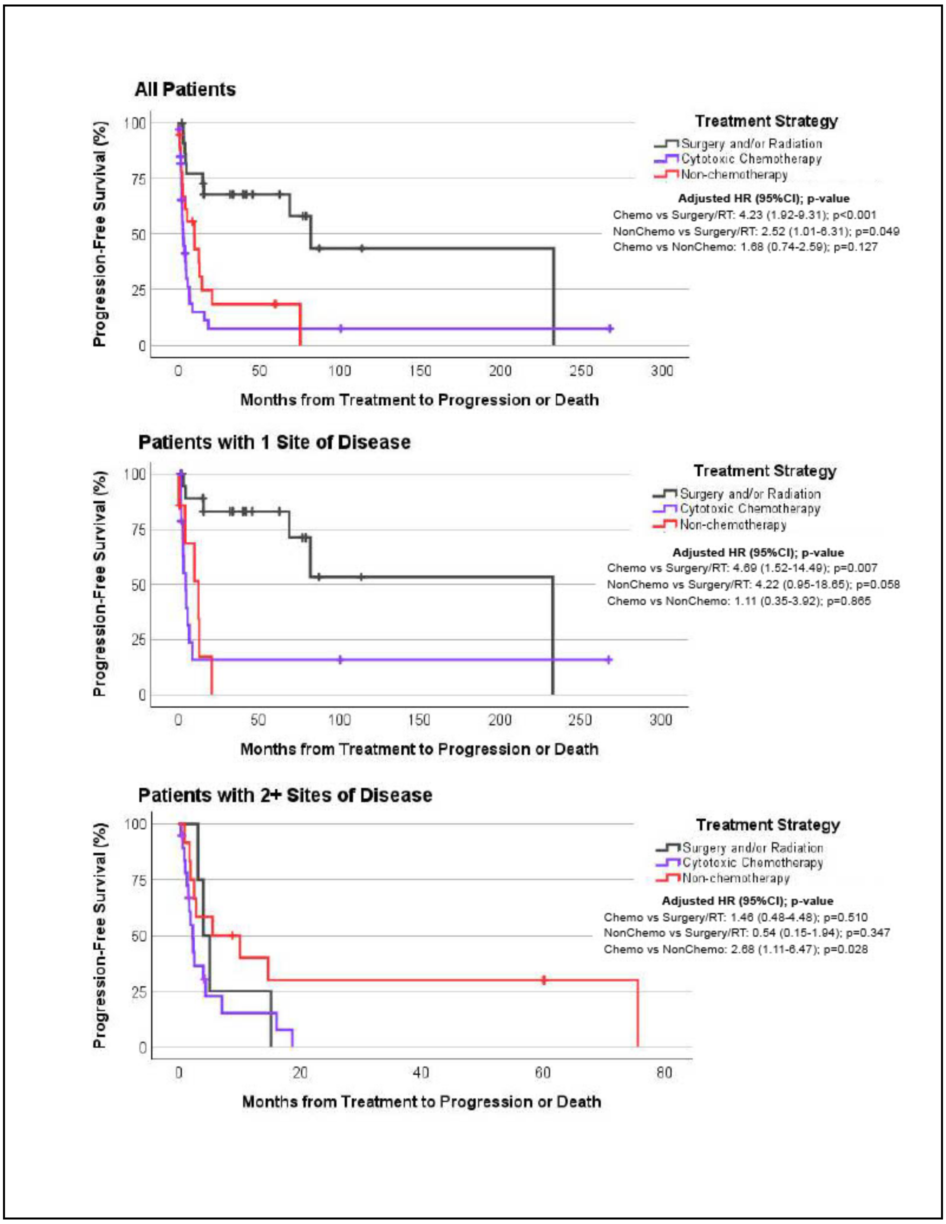

Figure 1: Progression-Free Survival Based on Therapeutic Strategies in patients with Histiocytic Sarcoma

**Copyright Information:** (Online) ISSN: 2572-9241
©2026 The Author(s). HemaSphere is published by John Wiley & Sons Ltd on behalf of European Hematology Association. This is an open access Abstract Book distributed under the Attribution-NonCommercial-NoDerivs (CC BY-NC-ND), which allows third parties to download the articles and share them with others as long as they credit the author and the Abstract Book, but they cannot change the content in any way or use them commercially.
**Abstract Book Citations:** Authors, Title, HemaSphere, 2026;10:(S1):pages. The abstract book DOIs can be found on the “Author Information” page.
**Disclaimer:** Articles published in the journal HemaSphere exclusively reflect the opinions of the authors. The authors are responsible for all content in their abstracts including accuracy of the facts, statements, citing resources, etc.

### (PB3441) EVALUATION OF THE FREQUENCY, RISK FACTORS, AND OUTCOMES OF SPLANCHNIC VEIN THROMBOSIS IN MYELOPROLIFERATIVE NEOPLASMS: A RETROSPECTIVE COHORT STUDY

**Topic:** 16. Myeloproliferative neoplasms - Clinical

Ufuk Demirci*[1], Mehmet Baysal[2], Elif Aksoy[3], Metban Mastanzade[4], Denis Bozer[5], Duygu Gül[6], Emine Gulturk[3], Fazıl Çağrı Hunutlu[7], Kubra Oral[8], Ayfer Gedük[9], Reyhan Diz Kucukkaya[10], Burak Canbaz[11], Deniz Ozmen[12], İbrahim Beyhan[13], Ayse Kaya[14], Ahmet Yigitbasi[15], Mehmet Yilmaz[16], Rafiye Ciftciler[17], İlknur Kara[18], Ali Turunç[19], Sevil Sadri[20], Süreyya Yiğit Kaya[21], Samet Yaman[22], Ayse Hilal Eroglu Kucukdiler[23], Fatos Dilan Koseoglu[24], Serkan Guven[25], Ali Doğan[26], Mehmet Can Ugur[27], Serhat Çelik[28], Aysun Şentürk Yikilmaz[29], Tarik Onur Tiryaki[30], Taha Ulutan Kars[31], Melda Comert[6], Zekeriya Aksöz[8], Abdulkerim Yildiz[22], İpek Yönal Hindilerden[4], Fehmi Hindilerden[3], Guray Saydam[5], Vildan Özkocaman[7], Ayşe Uysal[8], Meltem Olga Akay[32], Ahmet Muzaffer Demir[15], Emel Gürkan[19], Leylagul Kaynar[21], Seval Akpınar[2], Fahir Özkalemkaş[7], Elif Gülsüm Ümit[15], Ahmet Emre Eşkazan[12]

*[1]Celal Bayar University, Medical Faculty, Hematology, Manisa, Türkiye;[2]Namık Kemal University, Medical Faculty, Hematology, Tekirdağ, Türkiye; [3]University of Health Sciences Türkiye, Bakırköy Dr. Sadi Konuk Training and Research Hospital, Hematology, Istanbul, Türkiye; [4]Istanbul University, Istanbul Medical Faculty, Hematology, Istanbul, Türkiye;[5]Ege University, Medical Faculty, Hematology, Izmir, Türkiye; [6]University of Health Sciences Türkiye, Gülhane Training and Research Hospital, Hematology, Ankara, Türkiye; [7]Bursa Uludağ University, Medical Faculty, Hematology, Bursa, Türkiye;[8]Fırat University, Medical Faculty, Hematology, Elazığ, Türkiye; [9]Kocaeli University, Medical Faculty, Hematology, Kocaeli, Türkiye; [10]Istanbul Hemostasis and Thrombosis Institute, Hematology, Istanbul, Türkiye; [11]Istanbul University-Cerrahpaşa, Cerrahpaşa Faculty of Medicine, Internal Medicine, Istanbul, Türkiye; [12]Istanbul University-Cerrahpaşa, Cerrahpaşa Faculty of Medicine, Hematology, Istanbul, Türkiye; [13]Koç University, Medical Faculty, Internal Medicine, Istanbul, Türkiye; [14]University of Health Sciences Türkiye, Van Training and Research Hospital, Hematology, Van, Türkiye; [15]Trakya University, Medical Faculty, Hematology, Edirne, Türkiye; [16]Sanko University, Medical Faculty, Hematology, Gaziantep, Türkiye; [17]Selçuk University, Medical Faculty, Hematology, Konya, Türkiye; [18]Namık Kemal University, Medical Faculty, Internal Medicine, Tekirdağ, Türkiye;[19]Çukurova University, Medical Faculty, Hematology, Adana, Türkiye; [20]Bursa City Hospital, Medical Faculty, Hematology, Bursa, Türkiye;[21]Medipol University, Medical Faculty, Hematology, Istanbul, Türkiye; [22]Hitit University, Medical Faculty, Hematology, Çorum, Türkiye;[23]Adnan Menderes University, Medical Faculty, Hematology, Aydın, Türkiye; [24]Bakırçay University, Medical Faculty, Hematology, Izmır, Türkiye; [25]Çanakkale Mehmet Akif Ersoy State Hospital, Hematology, Çanakkale, Türkiye;[26]Yüzüncü Yıl University, Medical Faculty, Hematology, Van, Türkiye; [27]Tepecik Training and Research Hospital, Department of Internal Medicine, Division of,, Türkiye, Hematology, Izmir, Türkiye; [28]Yenimahalle State Hospital, Hematology, Ankara, Türkiye; [29]Denizli State Hospital, Hematology, Denizli, Türkiye; [30]University of Health Sciences Türkiye, Hamidiye Etfal Training and Research Hospital, Hematology, Istanbul, Türkiye; [31]Konya City Hospital, Medical Faculty, Hematology, Konya, Türkiye;[32]Koç University, Medical Faculty, Hematology, Istanbul, Türkiye;*

### Background

Philadelphia chromosome–negative myeloproliferative neoplasms (MPNs), including polycythemia vera (PV), essential thrombocythemia (ET), and myelofibrosis (MF), are clonal stem cell disorders associated with an increased thrombotic risk. Splanchnic vein thrombosis (SVT) is a distinct and clinically significant manifestation that contributes substantially to morbidity and mortality.

### Aims

To evaluate the clinical characteristics, molecular profile, management, and outcomes of SVT in patients with MPNs.

**Copyright Information:** (Online) ISSN: 2572-9241
©2026 The Author(s). HemaSphere is published by John Wiley & Sons Ltd on behalf of European Hematology Association. This is an open access Abstract Book distributed under the Attribution-NonCommercial-NoDerivs (CC BY-NC-ND), which allows third parties to download the articles and share them with others as long as they credit the author and the Abstract Book, but they cannot change the content in any way or use them commercially.
**Abstract Book Citations:** Authors, Title, HemaSphere, 2026;10:(S1):pages. The abstract book DOIs can be found on the "Author Information" page.
**Disclaimer:** Articles published in the journal HemaSphere exclusively reflect the opinions of the authors. The authors are responsible for all content in their abstracts including accuracy of the facts, statements, citing resources, etc.

**Methods**

This multicenter retrospective study was conducted by the Turkish Society of Hematology. Patients were identified according to the 2008 and 2016 WHO diagnostic criteria. Clinical, molecular, treatment, and outcome data were retrospectively analyzed.

**Results**

A total of 289 patients with MPN-associated SVT were included (PV: 101, ET: 119, MF: 69). The median age at diagnosis was 49 years, and 74% of patients were younger than 60 years. Prior thrombosis outside the splanchnic circulation was present in 14.9% of patients. The *JAK2 V617F* mutation was the most frequent driver mutation (85.5%), followed by CALR (2.1%) and MPL (0.3%), while the remaining patients were triple-negative. Next-generation sequencing (n=15) identified additional mutations, including *ASXL1, EZH2, SETBP1, ARID1A, FLT3, NF1, KCNA4,* and *SUZ12.*

SVT was the presenting event or occurred prior to MPN diagnosis in 67.5% of patients. Twenty-nine patients had received cytoreductive therapy prior to SVT, including hydroxycarbamide (n=16), ruxolitinib (n=5), anagrelide (n=4), and pegylated interferon (n=4). The mean overall survival was 310.8 months and was significantly higher in patients younger than 60 years ($p<0.01$). During follow-up, 11 patients developed post-PV MF and 19 developed post-ET MF. Leukemic transformation occurred in two patients (one PV and one MF).

Bleeding complications were observed in 27% of patients receiving anticoagulant therapy. Moderate-to-severe bleeding occurred in 24 patients, and severe bleeding occurred in 5 patients. Among patients with Grade III bleeding, 23 had gastrointestinal bleeding and 6 had esophageal variceal bleeding (5 receiving low-molecular-weight heparin, 13 vitamin K antagonists [VKA], 5 rivaroxaban, and 1 apixaban). All Grade IV bleeding events were upper gastrointestinal bleeding (4 receiving VKA and 1 apixaban). No bleeding-related deaths were observed. Anticoagulant therapy was discontinued due to bleeding in 17 patients.

Anticoagulant therapy was discontinued due to recanalization in 30 patients, with a mean treatment duration of 23.6 months in this subgroup. Among 82 patients who achieved early recanalization within 3 months, only 21 discontinued anticoagulant therapy shortly thereafter, whereas the majority continued treatment for a longer duration.

**Summary/Conclusion**

MPN-associated SVT predominantly affects younger patients and may occur in those harboring non-*JAK2* driver mutations. Cytoreductive therapy alone does not fully prevent SVT. Since most clinically significant bleeding events originated from the gastrointestinal tract—particularly esophageal varices—gastric protection and close surveillance of esophageal varices are essential in these patients. Direct oral anticoagulants appear to be a safe treatment option in patients with MPN-associated SVT.

**Copyright Information:** (Online) ISSN: 2572-9241
©2026 The Author(s). HemaSphere is published by John Wiley & Sons Ltd on behalf of European Hematology Association. This is an open access Abstract Book distributed under the Attribution-NonCommercial-NoDerivs (CC BY-NC-ND), which allows third parties to download the articles and share them with others as long as they credit the author and the Abstract Book, but they cannot change the content in any way or use them commercially.
**Abstract Book Citations:** Authors, Title, HemaSphere, 2026;10:(S1):pages. The abstract book DOIs can be found on the "Author Information" page.
**Disclaimer:** Articles published in the journal HemaSphere exclusively reflect the opinions of the authors. The authors are responsible for all content in their abstracts including accuracy of the facts, statements, citing resources, etc.

**Table 1.** Distribution of splanchnic vein thrombosis characteristics according to diagnosis.

| | | PV (n=101) | ET (n=119) | MF (n=69) | *p value* |
|---|---|---|---|---|---|
| **Gender, n (%)** | **Female**<br>**Male** | 43 (42.6)<br>58 (57.4) | 74 (62.2)<br>45 (37.8) | 28 (40.6)<br>41 (59.4) | [a]**0.003** |
| **Median age, years** | **(Min-Max)** | 49 (17-89) | 50 (22-98) | 53 (24-79) | [b]0.365 |
| **Age category, years** | **≤60**<br>**>60** | 82 (81.2)<br>19 (18.8) | 89 (74.8)<br>30 (25.2) | 49 (71.0)<br>20 (29.0) | |
| **Comorbidity, n (%)** | **Yes** | 33 (32.7) | 49 (41.2) | 24 (34.8) | [a]0.398 |
| **Follow-up** | **Median (Min-Max)** | 87.9 (0.4-439.9) | 95.5 (1-340) | 75.2 (2.5-290.2) | [c]0.196 |
| **Survival status, n (%)** | **Dead** | 6 (5.9) | 17 (14.3) | 17 (24.6) | [a]**0.002** |
| **Thrombosis history, n (%)** | **Yes** | 20 (19.8) | 15 (12.6) | 8 (11.6) | [a]0.222 |
| **Card Risk Factor ≥1, n (%)** | **Yes** | 34 (33.7) | 51 (42.9) | 23 (33.8) | [a]0.289 |
| **Portal hypertension, n (%)** | **No**<br>**Yes** | 25 (26.6)<br>69 (73.4) | 32 (30.8)<br>72 (69.2) | 11 (17.7)<br>51 (82.3) | [a]0.180 |
| **Recanalization in 3 months, n (%)** | **No**<br>**Yes**<br>**Minimal** | 50 (65.8)<br>25 (32.9)<br>1 (1.3) | 47 (56)<br>36 (42.9)<br>1 (1.2) | 36 (65.5)<br>18 (32.7)<br>1 (1.8) | [d]0.639 |
| **Bleeding, n (%)** | **Yes** | 25 (24.8) | 28 (23.5) | 25 (36.2) | [a]0.137 |
| **Bleeding grade, n (%)** | **No**<br>**Minimal**<br>**Moderate**<br>**Moderately severe**<br>**Severe** | 76 (75.2)<br>7 (6.9)<br>10 (9.9)<br>7 (6.9)<br>1 (1) | 91 (76.5)<br>2 (1.7)<br>14 (11.8)<br>12 (10.1)<br>0 (0) | 44 (63.8)<br>3 (4.3)<br>13 (18.8)<br>5 (7.2)<br>4 (5.8) | [d]**0.044** |

[a]Pearson's Chi Square Test
[b]One Way Anova Test&Bonferroni Test
[c]Kruskal Wallis Test
[d]Fisher Freeman Halton Test
**Abbreviations:** PV: Polycythemia vera, ET: Essential thrombocythemia, MF: Myelofibrosis

**Copyright Information:** (Online) ISSN: 2572-9241
©2026 The Author(s). HemaSphere is published by John Wiley & Sons Ltd on behalf of European Hematology Association. This is an open access Abstract Book distributed under the Attribution-NonCommercial-NoDerivs (CC BY-NC-ND), which allows third parties to download the articles and share them with others as long as they credit the author and the Abstract Book, but they cannot change the content in any way or use them commercially.
**Abstract Book Citations:** Authors, Title, HemaSphere, 2026;10:(S1):pages. The abstract book DOIs can be found on the "Author Information" page.
**Disclaimer:** Articles published in the journal HemaSphere exclusively reflect the opinions of the authors. The authors are responsible for all content in their abstracts including accuracy of the facts, statements, citing resources, etc.

### (PB3442) LONGITUDINAL GENOMIC SHIFTS ASSOCIATED WITH DISEASE TRANSFORMATION IN PATIENTS WITH POLYCYTHEMIA VERA (PV) ENROLLED IN REVEAL

**Topic:** 16. Myeloproliferative neoplasms - Clinical

Stephen Oh*[1], Michael Grunwald[2], Taehyung (Simon) Kim[3], Erin Crowgey[3], Jennifer Hamer-Maansson[3], Valkal Bhatt[3], Jeffrey Zwicker[4, 5]

*[1]Washington University School of Medicine, St. Louis, MO, United States of America;[2]Atrium Health Levine Cancer Institute and Wake Forest University School of Medicine, Charlotte, NC, United States of America; [3]Incyte Corporation, Wilmington, DE, United States of America; [4]Memorial Sloan Kettering Cancer Center, New York, NY, United States of America;[5]Weill Cornell Medical College, Department of Medicine, New York, NY, United States of America;*

### Background

Molecular markers associated with PV progression to myelofibrosis (MF) or acute myeloid leukemia remain limited. The Prospective Observational Study of Patients With PV in US Clinical Practices Trial (REVEAL; NCT02252159) enrolled patients (pts) between 2014 and 2019 for a median follow-up of 4 years. Optional blood and saliva biospecimens were collected at usual care visits. Of 2510 pts enrolled in REVEAL, 114 had MF transformation (Oh S. *Blood.* 2024).

### Aims

To investigate longitudinal molecular characteristics associated with PV transformation to MF via whole exome sequencing (WES) of pt samples from REVEAL.

### Methods

WES was performed on biospecimens from PV pts who transformed to MF during the study, determined using modified WHO criteria (Grunwald MR. *Blood* 2023). Pts with an enrollment sample and ≥1 longitudinal sample (collected close to/after transformation) were included. Biospecimens were sequenced (average coverage depth, 300×); processed using Genome Analysis Toolkit best practices; variants were called via Mutect2. Enrollment and longitudinal samples were collected on average 23 months (range 1.8, 50.5) before transformation and 2.3 months (range –2.7, 18.0) after transformation, respectively. Where multiple longitudinal samples were available, that collected nearest to transformation was analyzed.

**Copyright Information:** (Online) ISSN: 2572-9241
©2026 The Author(s). HemaSphere is published by John Wiley & Sons Ltd on behalf of European Hematology Association. This is an open access Abstract Book distributed under the Attribution-NonCommercial-NoDerivs (CC BY-NC-ND), which allows third parties to download the articles and share them with others as long as they credit the author and the Abstract Book, but they cannot change the content in any way or use them commercially.
**Abstract Book Citations:** Authors, Title, HemaSphere, 2026;10:(S1):pages. The abstract book DOIs can be found on the “Author Information” page.
**Disclaimer:** Articles published in the journal HemaSphere exclusively reflect the opinions of the authors. The authors are responsible for all content in their abstracts including accuracy of the facts, statements, citing resources, etc.

### Results

Of 2510 pts enrolled in REVEAL, 41 had available enrollment and longitudinal samples.All 41 pts were *JAK2*V617F-positive at enrollment. Mean *JAK2*V617F variant allele frequency (VAF) was not significantly different between enrollment and transformation time points (0.768 [range 0.056, 0.958] vs 0.796 [0.009, 0.963]; *P*=0.708). However, pts could be grouped individually into 3 patterns at transformation based on an increase (>5%; n=17), decrease (>5%; n=11), or stable (≤5% increase/decrease; n=13) *JAK2*V617F VAF at transformation vs enrollment. Among the 17 pts with increased *JAK2*V617F VAF at transformation, 12 (71%) carried copy-neutral loss of heterozygosity (CN-LOH) at chr9p at enrollment; the remaining 5 pts acquired CN-LOH at chr9p at transformation. Of the 11 pts with >5% decrease in *JAK2*V617F VAF at transformation, 3 (27%) had acquired/expanded variants in genes including *KRAS* and *STAG2*; 2 had consistent *IDH2* and *TET2* variants. Five of the 13 pts (38%) with stable *JAK2*V617F VAF acquired/expanded an additional variant(s) in genes at transformation including *CBL*, *RUNX1*, *DNMT3A*, or *GNAS*; 1 had a consistent *TP53* variant (VAF range 0.289, 0.302). Additional variants in myeloid malignancy-associated genes were identified in 18/41 (44%) pts.

Changes in *JAK2*V617F VAF were driven by CN-LOH at chr9p (linear regression; [F(1,39)]=409.9; *P*<0.001). CN-LOH at chr9p was detected in 35/41 (85%) pts at enrollment and 38/41 (93%) pts at transformation. Loss of CN-LOH at chr9p was observed in 2/35 (6%) pts at transformation; both carried somatic mutations in myeloid malignancy-associated genes (*IDH2* and *KRAS*). 5/6 (83%) pts without CN-LOH at chr9p at enrollment acquired this during transformation, which was accompanied by increased *JAK2*V617F VAF.

### Summary/Conclusion

These data demonstrate multiple patterns of clonal progression in pts enrolled in REVEAL and suggest a general pattern of increasing molecular burden post-transformation, which supports the clinical need for a comprehensive genomic model beyond driver mutation VAF. These findings warrant further genomic analyses to facilitate the monitoring and treatment of pts with PV at high-risk of transformation.

**Copyright Information:** (Online) ISSN: 2572-9241
©2026 The Author(s). HemaSphere is published by John Wiley & Sons Ltd on behalf of European Hematology Association. This is an open access Abstract Book distributed under the Attribution-NonCommercial-NoDerivs (CC BY-NC-ND), which allows third parties to download the articles and share them with others as long as they credit the author and the Abstract Book, but they cannot change the content in any way or use them commercially.
**Abstract Book Citations:** Authors, Title, HemaSphere, 2026;10:(S1):pages. The abstract book DOIs can be found on the “Author Information” page.
**Disclaimer:** Articles published in the journal HemaSphere exclusively reflect the opinions of the authors. The authors are responsible for all content in their abstracts including accuracy of the facts, statements, citing resources, etc.

### (PB3443) GECACITINIB TENDS TO RESTORE IMMUNE FUNCTION IN RUXOLITINIB EXPOSED MYELOFIBROSIS PATIENTS

**Topic:** 16. Myeloproliferative neoplasms - Clinical

Yunfan Yang*[1], Huanling Zhu[1], Yu Wu[1], Yan Li[1], Ling Pan[1]

[1]*Department of Hematology, Institute of Hematology, West China Hospital, Sichuan University, Chengdu, China;*

**Background**

Gecacitinib (GCA, also known as jaktinib) is a novel dual inhibitor targeting JAK (JAK1, JAK2, JAK3, TYK2) and ACVR1. It has been shown to reduce inflammation and splenomegaly, increase hemoglobin levels, thereby significantly reducing anemia and transfusion dependency in myelofibrosis (MF) patients. Ruxolitinib (RUX) is associated with significant immune suppression and increased risks of opportunistic infections and skin cancer partly due to reduced cellular immunity, The effects of Gecacitinib on immune effector cell populations have not been previously described.

**Aims**

To evaluate GCA efficacy on immune function in patients with myelofibrosis(MF) after transitioning from RUX.

**Methods**

Patients were monitored for 24 weeks after starting GCA. The study planned to enroll 40 myelofibrosis patients converted to GCA who were intolerant or refractory to RUX. At baseline whilst on RUX, 4 weeks, 12 weeks, and 24 weeks, the patients were tested for peripheral blood mononuclear cells, analyzed the changes of immune cell subsets and cytokines. 10 healthy subjects were used as controls (HC).

**Results**

As of February 24, 2026, 14 MF patients starting GCA at either 200 mg (n=11) or 100mg (n=3) daily, following prior RUX resistance/intolerance (median time on RUX=1.6 years), were included (primary MF n=10, secondary MF n=4). The median age was 64 years (females n=3, males n=11).The number of patients evaluable for immune function was 14, 14, 11, and 9 at baseline, 4, 12, and 24 weeks, respectively.
**Immune cell recovery:**
Patients showed significantly reduced frequencies of T cells, B cells, NK cells, and DC cells, significantly increased levels of the cytokine IL-2 at baseline whilst on RUX compared to HC ($p < 0.05$). Following the switch to GCA, absolute counts of T lymphocytes, Treg cells, B cells, NK cells, and DC cells tended to increase compared to baseline. The median CD3+CD4+ T lymphocytes counts were 169, 419, 391 and 484 cells/μL at baseline, 4, 12, and 24 weeks, respectively. B cells counts for the same time points were 56, 83, 82 and 90 cells/μL, respectively. Treg cells counts were 21, 42, 42 and 38 cells/μL, respectively. NK cells counts were 89, 119, 142 and 153 cells/μL, respectively. DC cells counts were 0.61, 3.57, 1.34 and 1.86 cells/μL, respectively. MDSCs counts showed a decreasing trend.

**Changes in Cytokine Levels:**
Inflammatory cytokine levels generally decreased after switching to GCA. The median values for IL-2 were 1.13, 1.00, 0.74, and 0.74 pg/mL at baseline, 4, 12, and 24 weeks, respectively; for IL-4: 1.87, 2.79, 1.21, and 1.21 pg/mL; for IL-6: 4.10, 6.70, 1.25, and 1.69 pg/mL; and for IL-10: 3.12, 1.38, 0, and 1.69 pg/mL.

**Copyright Information:** (Online) ISSN: 2572-9241
©2026 The Author(s). HemaSphere is published by John Wiley & Sons Ltd on behalf of European Hematology Association. This is an open access Abstract Book distributed under the Attribution-NonCommercial-NoDerivs (CC BY-NC-ND), which allows third parties to download the articles and share them with others as long as they credit the author and the Abstract Book, but they cannot change the content in any way or use them commercially.
**Abstract Book Citations:** Authors, Title, HemaSphere, 2026;10:(S1):pages. The abstract book DOIs can be found on the “Author Information” page.
**Disclaimer:** Articles published in the journal HemaSphere exclusively reflect the opinions of the authors. The authors are responsible for all content in their abstracts including accuracy of the facts, statements, citing resources, etc.

### Summary/Conclusion

We observed a trend toward rapid recovery of immune cell frequencies after starting GCA therapy, including recovery of total lymphocytes and MDSCs cells showed a downward trend. Concurrently, inflammation-related cytokines tended to decrease. These findings suggest a potential positive impact on immune function in MF patients transitioning from RUX to GCA. However, as the sample size collected to date remains relatively small, individual data may introduce bias. Further data from more patients will be disclosed to confirm these preliminary observations.

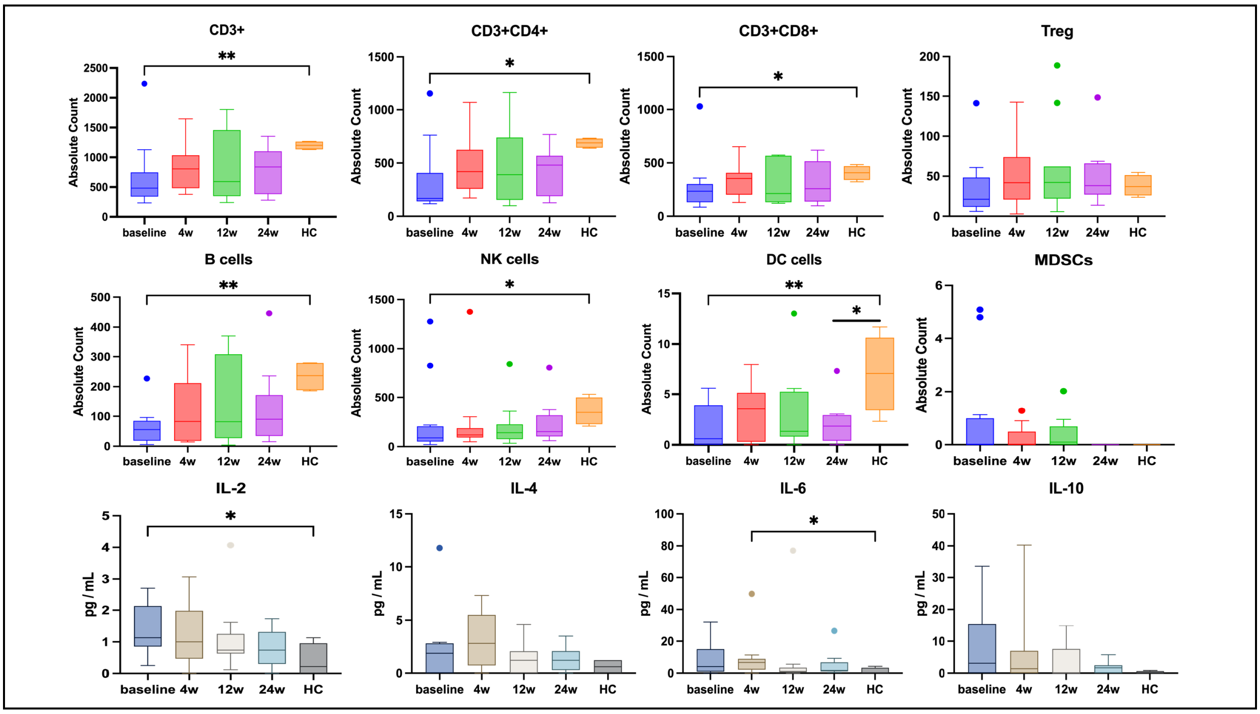

Figure1.Immune cell frequencies and cytokines at baseline,4 weeks, 12 weeks and 24 weeks in GCA-treated patients vs healthy controls.

Copyright Information: (Online) ISSN: 2572-9241
©2026 The Author(s). HemaSphere is published by John Wiley & Sons Ltd on behalf of European Hematology Association. This is an open access Abstract Book distributed under the Attribution-NonCommercial-NoDerivs (CC BY-NC-ND), which allows third parties to download the articles and share them with others as long as they credit the author and the Abstract Book, but they cannot change the content in any way or use them commercially.
Abstract Book Citations: Authors, Title, HemaSphere, 2026;10:(S1):pages. The abstract book DOIs can be found on the "Author Information" page.
Disclaimer: Articles published in the journal HemaSphere exclusively reflect the opinions of the authors. The authors are responsible for all content in their abstracts including accuracy of the facts, statements, citing resources, etc.

### (PB3445) TREATMENT STRATEGY AND THROMBOTIC STATUS SHAPE BLEEDING RISK IN 15,495 PATIENTS WITH MYELOPROLIFERATIVE NEOPLASMS

**Topic:** 16. Myeloproliferative neoplasms - Clinical

Stefanie Antonilli*[1], Anna Ravn Landtblom[2, 3], Malin Hultcrantz[4], Nurgul Batyrbekova[1], Ingrid Glimelius[5], Matthew Maurer[6], Therese Andersson[7], Caroline Dietrich[1]

*[1]Karolinska Institutet, Department of Medicine Solna, Stockholm, Sweden; [2]Karolinska Institutet, Department of Medicine Huddinge, Stockholm, Sweden; [3]Karolinska University Hospital, Department of Hematology, Stockholm, Sweden; [4]Memorial Sloan Kettering Cancer Center, Myeloma Service, New York, United States of America; [5]Uppsala University, Department of Immunology, Genetics and Pathology, Uppsala, Sweden; [6]Mayo Clinic, Department of Quantitative Health Sciences, Rochester, United States of America; [7]Karolinska Institutet, Department of Medical Epidemiology and Biostatistics, Stockholm, Sweden;*

### Background

Patients with myeloproliferative neoplasms (MPNs) have an elevated risk of both thrombosis and bleeding. Management often includes anticoagulants, antiplatelet agents, and cytoreductive therapy, which aim to reduce thrombotic risk but may increase the risk of bleeding. How treatment strategies and thrombotic events jointly influence bleeding risk remains underexplored in large, real-world cohorts.

### Aims

To characterise how bleeding rate varies by treatment strategies in patients living with MPN and how thrombotic events influence this rate.

### Methods

We identified 15,495 patients diagnosed with MPN between 2006 and 2021, aged 18-90, using Swedish nationwide health registers. Treatment strategies included anticoagulant monotherapy (e.g., warfarin, direct oral anticoagulants [DOACs]), antiplatelet monotherapy (e.g., aspirin, clopidogrel), cytoreductive monotherapy (e.g., hydroxyurea, interferon), and combination therapy (≥2 agents). Follow-up began at MPN diagnosis and ended at first bleeding, death, or administrative censoring. Patients entered follow-up as untreated or on therapy and were classified as post-thrombotic at baseline if they had experienced a thrombotic event within one year before MPN diagnosis. During follow-up, treatment and thrombosis status were updated in response to changes in treatment strategy or incident thrombotic events. A Cox proportional hazards model adjusted for age, sex, year of diagnosis, and MPN subtype was used to estimate hazard ratios (HRs) and 95% confidence intervals (CIs) for bleeding, with treatment and thrombosis as time-varying exposures.

**Copyright Information:** (Online) ISSN: 2572-9241
©2026 The Author(s). HemaSphere is published by John Wiley & Sons Ltd on behalf of European Hematology Association. This is an open access Abstract Book distributed under the Attribution-NonCommercial-NoDerivs (CC BY-NC-ND), which allows third parties to download the articles and share them with others as long as they credit the author and the Abstract Book, but they cannot change the content in any way or use them commercially.
**Abstract Book Citations:** Authors, Title, HemaSphere, 2026;10:(S1):pages. The abstract book DOIs can be found on the “Author Information” page.
**Disclaimer:** Articles published in the journal HemaSphere exclusively reflect the opinions of the authors. The authors are responsible for all content in their abstracts including accuracy of the facts, statements, citing resources, etc.

### Results

The cohort was 52% female, with a median age of 71 years. The most common MPN subtypes were polycythaemia vera (39%) and essential thrombocythemia (38%), followed by MPN-unclassifiable (16%) and primary myelofibrosis (7%). Among the 10,505 patients who at some point received combination therapy, antiplatelet was the most common agent (79%). The median follow-up time was 2.0 years (interquartile range: 0.2-5.4).

Before or in the absence of thrombosis, anticoagulant- and cytoreductive monotherapy both significantly increased the bleeding rate compared with untreated patients; HR=1.59 (95% CI: 1.33-1.90) and 1.48 (95% CI: 1.19-1.84), respectively (Figure 1). No differences were observed among patients receiving antiplatelet monotherapy or combination therapy.

After a thrombotic event, bleeding rates were increased across all treatments compared to untreated patients without thrombosis; relative rates ranged from 1.50 (95% CI: 1.23-1.83) for antiplatelets to 3.90 (95% CI: 1.93-7.87) for cytoreductive therapy. When using patients with a thrombosis and treated with anticoagulant monotherapy as the reference, antiplatelets and combination therapy were associated with reduced bleeding rates (HR=0.70, 95% CI: 0.54-0.92, and HR=0.76, 95% CI: 0.61-0.95).

### Summary/Conclusion

This nationwide study suggests that treatment strategy and thrombosis jointly influence bleeding in patients with MPN. Antiplatelet-containing strategies – both monotherapy and combination therapy – were consistently associated with no increased bleeding rates. Modelling treatment and thrombosis as time-varying exposures provides a more accurate reflection of real-world treatment dynamics. These findings highlight a potential protective role of antiplatelet therapy and may support individualized treatment decisions and bleeding risk assessment in MPN.

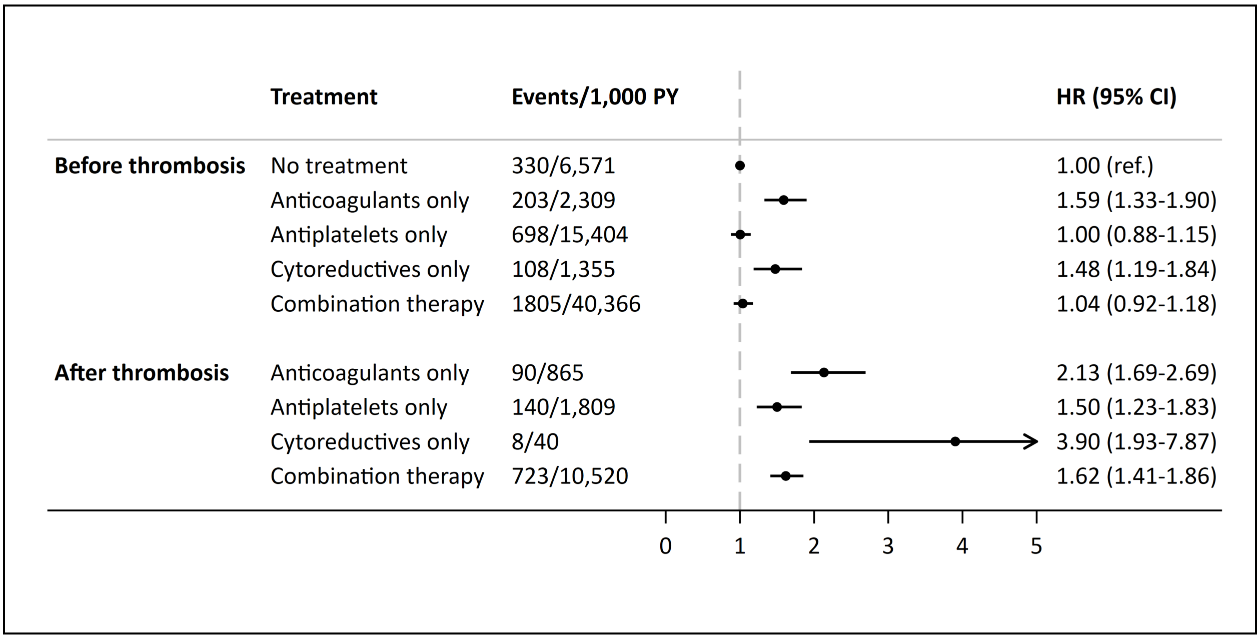

Figure 1. HRs (95% CIs) for bleeding from a Cox model with treatment category and thrombotic status as time-varying exposure. Adjusted for age, sex, year, and MPN subtype. PY = person-years.

**Copyright Information:** (Online) ISSN: 2572-9241
©2026 The Author(s). HemaSphere is published by John Wiley & Sons Ltd on behalf of European Hematology Association. This is an open access Abstract Book distributed under the Attribution-NonCommercial-NoDerivs (CC BY-NC-ND), which allows third parties to download the articles and share them with others as long as they credit the author and the Abstract Book, but they cannot change the content in any way or use them commercially.
**Abstract Book Citations:** Authors, Title, HemaSphere, 2026;10:(S1):pages. The abstract book DOIs can be found on the "Author Information" page.
**Disclaimer:** Articles published in the journal HemaSphere exclusively reflect the opinions of the authors. The authors are responsible for all content in their abstracts including accuracy of the facts, statements, citing resources, etc.

### (PB3446) PREDICTORS OF ANEMIA RESPONSE TO MOMELOTINIB IN MYELOFIBROSIS: EVIDENCE FROM A LARGE REAL-WORLD COHORT

**Topic:** 16. Myeloproliferative neoplasms - Clinical

Lucía Pérez Lamas*[1, 2, 3], Adrian Segura Diaz[4], Juan Carlos Hernandez Boluda[5], Alberto Alvarez Larran[6], Maria Isabel Mata Vazquez[7], Regina García Delgado[8], Sara Garrido Paniagua[9], Alberto Marín[10], Elvira Mora[11], Maria Alicia Senin Magan[12], Natalia de Las Heras[13], Paula Gómez Fernández[14], Teresa Cobo Rodríguez[15], Maria Carmen Albo Lopez[16], Santiago Ossorio Prendes[17], Gemma Azaceta[18], Manuel Mateo Perez Encinas[19], Raul Perez Lopez[20], Sonia Garces Piquer[21], Angela Gil[22], M Angeles Fernández Rodríguez[23], Blanca Xicoy Cirici[24], Armando Luaña Galan[25], Adriana Hernando Megido[26], Federico Herrera[27], Esther Herrera[28], Isabel Diez Campos[29], Raisa Peralta[30], Aitor Abuín Blanco[31], Ylenia Morales Ruiz[32], Sofía Martín[33], María Pérez Sala[34], Reyes Jimenez-Bárcenas[35], Diana Sanchez Argüello[36], Eloi Cañamero Giro[37], Francisco Pérez Leal[38], Santiago Redondo[39], Luiz Andre Aguilar[40], Mercedes Gasior Kabat[41], Luis Miguel Juarez-Salcedo[42], Julio Dávila-Valls[43], Marta Gomez Nuñez[44], Begoña Navas Elorza[45], Aurelia Tejedor[46], Williana Torres Jiménez[47], Olga Pérez López[48], Joan Alfons Gonzálvez Fernández[49], Alvaro Lorenzo Vizcaya[50], Teresa Hernandez Santamaria[51], Inmaculada Castillo Valero[52], Sonia González de Villambrosia Pellón[53], Paola Beneit[54], Daniel Naya[55], Carlos Aguilar[56], Fernando Marco de Lucas[57], Ana Royuela[1, 2], Valentin Garcia Gutierrez[58]

*[1]Hospital Universitario Puerta de Hierro, Hematology, Madrid, Spain;[2]Instituto Investigación Sanitaria Puerta de Hierro, Madrid, Spain; [3]Universidad de Alcalá, Madrid, Spain; [4]Hospital Universitario Doctor Negrín, Las Palmas de Gran Canaria, Spain; [5]Hospital Clinico Universitario de Valencia, Valencia, Spain; [6]Hospital Clínic de Barcelona, Barcelona, Spain;[7]Hospital Universitario Costa del Sol, Málaga, Spain; [8]Hospital Clínico Universitario Virgen de la Victoria, Málaga, Spain;[9]Hospital Universitari Vall d'Hebron, Barcelona, Spain; [10]Hospital General Universitario de Albacete, Albacete, Spain;[11]Hospital Universitario y Politécnico de La Fe, Valencia, Spain; [12]Hospital Duran i Reynals — ICO L'Hospitalet, Barcelona, Spain; [13]Hospital Universitario de León, León, Spain;[14]Hospital Universitario Son Espases, Palma de Mallorca, Spain; [15]Hospital Universitario del Sureste, Madrid, Spain; [16]Complexo Hospitalario Universitario de Vigo - IISGS, Vigo, Spain;[17]Hospital Universitario Gregorio Marañon, Madrid, Spain; [18]Hospital Clínico Universitario Lozano Blesa, Zaragoza, Spain; [19]Hospital Clínico Universitario de Santiago, Santiago de Compostela, Spain; [20]Hospital Virgen de la Arrixaca, IMIB, Murcia, Spain;[21]Hospital General de Valencia, Valencia, Spain; [22]Hospital Universitario de Guadalajara, Guadalajara, Spain;[23]Hospital Universitario Central de Asturias, Asturias, Spain; [24]Hospital Germans Trias i Pujol, Badalona, Spain;[25]Hospital Arnau de Vilanova, Lleida, Spain; [26]Hospital de Cabueñes, Asturias, Spain;[27]Hospital de Getafe, Madrid, Spain; [28]Hospital Lluis Alcanyís de Xàtiva, Valencia, Spain; [29]Hospital Universitario de Girona Doctor Josep Trueta (ICO Girona), Girona, Spain;[30]Centro San Miguel, Navarra, Spain; [31]Complejo Hospitalario Universitario de Lugo, Lugo, Spain;[32]Clínica Universitaria Materno-Insular, Gran Canaria, Spain; [33]Hospital de Alcañiz, Teruel, Spain;[34]Hospital de Elda, Alicante, Spain;[35]Hospital de la Serranía de Ronda, Málaga, Spain; [36]Hospital de Laredo, Cantabria, Spain;[37]Hospital de Mataró, Barcelona, Spain;[38]Hospital Don Benito-Villanueva, Badajoz, Spain; [39]Hospital HM Puerta del Sur, Móstoles. Instituto de Investigación Sanitaria HM Hospitales, Madrid, Spain; [40]Hospital Joan XXIII de Tarragona, Tarragona, Spain;[41]Hospital La Paz, Madrid, Spain; [42]Hospital La Princesa, Madrid, Spain; [43]Hospital Nuestra Señora de Sonsoles, Ávila, Spain;[44]Hospital Universitario Parc Taulí de Sabadell, Sabadell, Spain; [45]Hospital Clínico San Carlos, Madrid, Spain;[46]Hospital General Universitario Santa Lucía, Cartagena, Spain;[47]Hospital Virgen de la Luz, Cuenca, Spain; [48]Hospital Universitario de Jerez, Jerez de la Frontera, Spain;[49]Hospital General Universitario de Elche, Elche, Spain; [50]Hospital Universitario Lucus Augusti, Lugo, Spain;[51]Hospital Universitario Donostia, Donostia, Spain; [52]Hospital Universitario La Plana, Castellón, Spain; [53]Hospital Universitario Marqués de Valdecilla, Santander, Spain;[54]Hospital Universitario San Juan, Alicante, Spain; [55]Hospital Universitario Infanta Elena, Madrid, Spain; [56]Hospital Universitario Santa Bárbara, Soria, Spain; [57]Hospital Universitario de Basurto, Basurtu-Zorrotza, Spain;[58]Hospital Universitario Ramón y Cajal — IRYCIS; Universidad de Alcalá, Madrid, Spain;*

**Copyright Information:** (Online) ISSN: 2572-9241
©2026 The Author(s). HemaSphere is published by John Wiley & Sons Ltd on behalf of European Hematology Association. This is an open access Abstract Book distributed under the Attribution-NonCommercial-NoDerivs (CC BY-NC-ND), which allows third parties to download the articles and share them with others as long as they credit the author and the Abstract Book, but they cannot change the content in any way or use them commercially.
**Abstract Book Citations:** Authors, Title, HemaSphere, 2026;10:(S1):pages. The abstract book DOIs can be found on the "Author Information" page.
**Disclaimer:** Articles published in the journal HemaSphere exclusively reflect the opinions of the authors. The authors are responsible for all content in their abstracts including accuracy of the facts, statements, citing resources, etc.

### Background

Momelotinib (MMB) has shown anemia benefit in trials; however, real-world data with extended follow-up remain limited, and variables that might serve as predictors of response remain poorly characterized.

### Aims

Assess MMB effectiveness for anemia response in a large real-world cohort and identify baseline predictors of response.

### Methods

Retrospective, multicenter study of adult MF patients (pts) with disease-related anemia treated with MMB in Spain. Pts were JAK inhibitor (JAKi)–naïve or previously exposed. Anemia response was assessed per revised 2024 IWG-ELN criteria.

### Results

238 adult MF pts from 79 centers were included. Median follow-up was 10 months (mos) (1–57). At last follow-up, 158 (66.4%) remained on MMB. Reason for permanent discontinuation were: toxicity 14%, lack of efficacy 16%, progression 17%, AML transformation 7%, allo-transplant 11%, death 32%, other 3%. Median age was 74 years, 59.7% males. MF type was primary in 130 and secondary in 106. Driver mutations were JAK2 65%, CALR 19%, MPL 8%, triple-negative 8%. Pre-MMB karyotype: normal/single abnormalities 62%, unfavorable 8%, very high risk 3%, not available 27%.
61 pts (25.6%) were JAKi–naïve, while 177 (74.3%) had prior JAKi exposure (99% received ruxolitinib; median exposure 16.2 mos). 70% prior-JAKi pts started MMB immediately or within 1 month of ruxolitinib stop; later starters median interval was 3.5 mos (1–54). Previous therapies included erythropoietin stimulating agents (74%) and danazol (13%). Baseline median hemoglobin (Hb) was 8.3 g/dL; 66% were transfusion-dependent.
Among 209 pts evaluable for anemia response, 149 (71%) achieved a hematologic response (50% major, 21% minor) during follow-up(Fig.1a). Prior JAKi-treated pts showed a trend toward better response rates (major 55%, minor 18%, no response 27%) than JAKi-naive pts (39%/28%/33%). Median time to anemia response was 2 mos (IQR 2.4). Accounting for competing risks, median time to response was 7 mos(Fig.1b). Median response duration not reached. In univariate analysis of anemia response within 6 mos, only an unfavorable karyotype was significantly associated with poorer response(tab1). Baseline Hb, blast count, reticulocyte%, and splenomegaly showed non-significant trends.

**Copyright Information:** (Online) ISSN: 2572-9241
©2026 The Author(s). HemaSphere is published by John Wiley & Sons Ltd on behalf of European Hematology Association. This is an open access Abstract Book distributed under the Attribution-NonCommercial-NoDerivs (CC BY-NC-ND), which allows third parties to download the articles and share them with others as long as they credit the author and the Abstract Book, but they cannot change the content in any way or use them commercially.
**Abstract Book Citations:** Authors, Title, HemaSphere, 2026;10:(S1):pages. The abstract book DOIs can be found on the “Author Information” page.
**Disclaimer:** Articles published in the journal HemaSphere exclusively reflect the opinions of the authors. The authors are responsible for all content in their abstracts including accuracy of the facts, statements, citing resources, etc.

| Variable (value prior MMB start) | | observations | OR(95% CI) | p |
|---|---|---|---|---|
| Age | | 175 | 1.0(0.9-1.0) | 0.33 |
| Sex | | 182 | 1.2(0.6-2.2) | 0.64 |
| Time from diagnosis to MMB | | 182 | 1.0(0.9-1.1) | 0.96 |
| Hb | | 182 | 1.3(1.0-1.7) | **0.10** |
| Platelet | | 179 | 1.0(0.9-1.0) | 0.31 |
| WBC | | 177 | 1.0(0.9-1.0) | 0.32 |
| Reticulocyte% | | 45 | 1.3(0.7-2.0) | **0.21** |
| Blast% | | 161 | 0.9(0.7-1.0) | **0.12** |
| Ferritin | | 78 | 1.0(0.9-1.0) | 0.08 |
| eGFR | | 97 | 1.0(0.9-1.0) | 0.88 |
| Time under transfusion support | | 48 | 1.0(0.9-1.1) | 0.34 |
| Transfusion requirement | | 179 | 0.8(0.4-1.5) | 0.49 |
| nº RBC units/mo | | 113 | 0.9(0.8-1.1) | 0.40 |
| Median time on prior ruxolitinib | | 132 | 1.0(0.9-1.0) | 0.58 |
| Constitutional symptoms | | 182 | 1.0(0.5-1.8) | 0.99 |
| Spleen size | | 163 | 0.7(0.3-1.2) | **0.20** |
| Prior ESA use | | 182 | 1.0(0.5-2.0) | 0.94 |
| Prior JAKi | | 182 | 0.9(0.5-1.8) | 0.82 |
| Prior ruxolitinib dose | | 123 | 0.6(0.3-1.5) | 0.32 |
| MF type | 1º vs post-ET | 180 | 1.1(0.5-2.1) | 0.86 |
| | 1º vs post-PV | 180 | 1.7(0.6-4.6) | 0.33 |
| Karyotype | unfavorable vs normal | 131 | 0.3(0.1-0.8) | **0.02** |
| | high risk vs normal | 131 | 0.5(0.1-3.6) | 0.48 |
| DIPSS | intermediate-2 vs intermediate-1 | 172 | 2.2(0.7-7.0) | 0.26 |
| | high risk vs intermediate-1 | 172 | 0.9(0.3-3.0) | 0.78 |
| Reason for ruxolitinib stop | resistance vs intolerance | 122 | 0.8(0.3-1.7) | 0.50 |

**Table1.**Univariate logistic regression assessing predictors of anemia response

### Summary/Conclusion

In this large real-world cohort, MMB induced rapid, clinically meaningful anemia responses; unfavorable karyotype predicted poorer response, while other baseline variables showed no significant effect.

**Copyright Information:** (Online) ISSN: 2572-9241
©2026 The Author(s). HemaSphere is published by John Wiley & Sons Ltd on behalf of European Hematology Association. This is an open access Abstract Book distributed under the Attribution-NonCommercial-NoDerivs (CC BY-NC-ND), which allows third parties to download the articles and share them with others as long as they credit the author and the Abstract Book, but they cannot change the content in any way or use them commercially.
**Abstract Book Citations:** Authors, Title, HemaSphere, 2026;10:(S1):pages. The abstract book DOIs can be found on the "Author Information" page.
**Disclaimer:** Articles published in the journal HemaSphere exclusively reflect the opinions of the authors. The authors are responsible for all content in their abstracts including accuracy of the facts, statements, citing resources, etc.

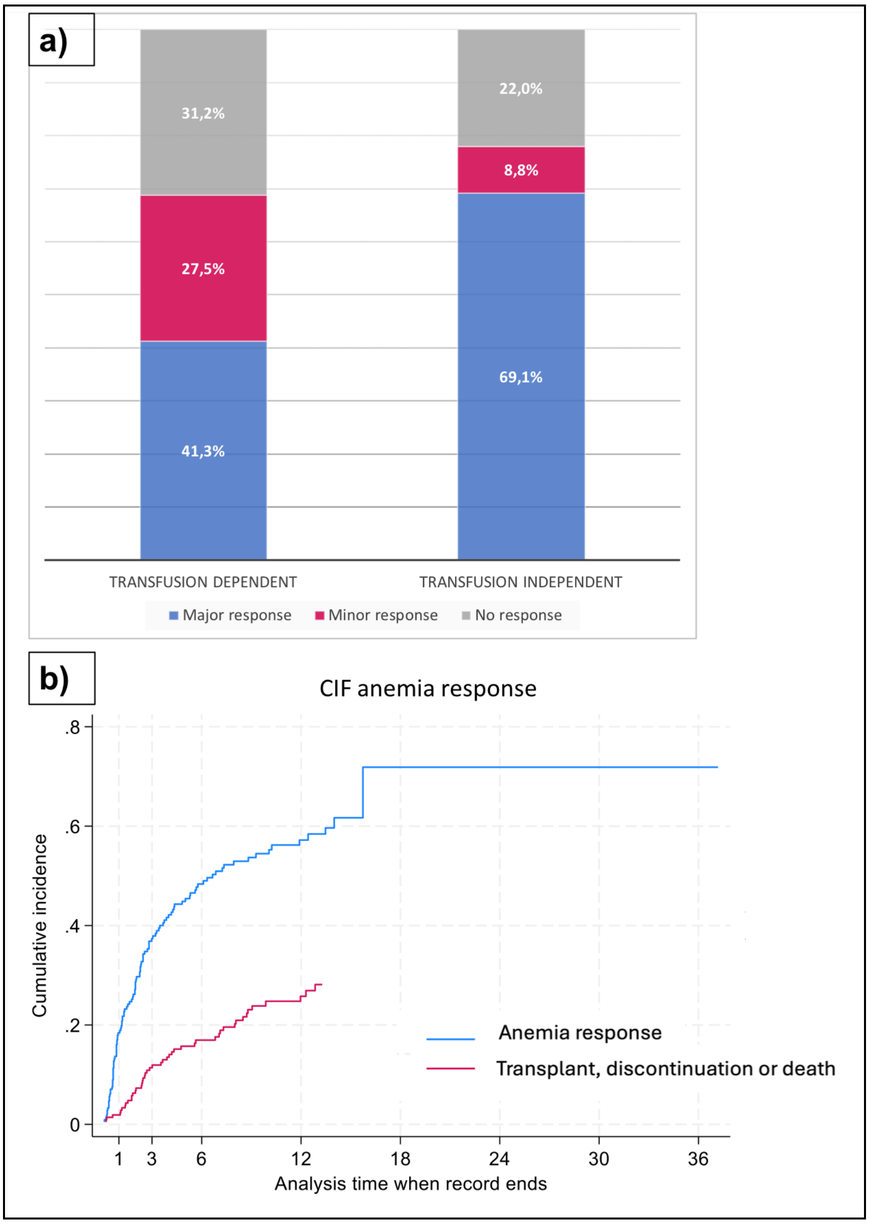

Figure 1. a) Anemia response divided by transfusion dependent/non dependent. b) Cumulative incidence function of anemia response within the first 6 months of momelotinib treatment. The curve represents the probability of achieving anemia response over time, considering allogeneic transplant, treatment discontinuation, and death as competing events.

**Copyright Information:** (Online) ISSN: 2572-9241
©2026 The Author(s). HemaSphere is published by John Wiley & Sons Ltd on behalf of European Hematology Association. This is an open access Abstract Book distributed under the Attribution-NonCommercial-NoDerivs (CC BY-NC-ND), which allows third parties to download the articles and share them with others as long as they credit the author and the Abstract Book, but they cannot change the content in any way or use them commercially.
**Abstract Book Citations:** Authors, Title, HemaSphere, 2026;10:(S1):pages. The abstract book DOIs can be found on the "Author Information" page.
**Disclaimer:** Articles published in the journal HemaSphere exclusively reflect the opinions of the authors. The authors are responsible for all content in their abstracts including accuracy of the facts, statements, citing resources, etc.

### (PB3447) PROGNOSTIC ROLE OF INFLAMMATION IN MPN:EVALUATING NLR, RAR, AND COMORBIDITY BURDEN

**Topic:** 16. Myeloproliferative neoplasms - Clinical

Isidora Arsenovic*[1], Andrijana Kojic[1], Andjela Nikolic[1], Nikola Lemajić[1], Mihailo Smiljanic[1], Natasa Čolović[1, 2], Andrija Bogdanovic[1, 2], Danijela Lekovic[1, 2]

*[1]Clinic of Hematology, University Clinical Center Serbia, Belgrade,Serbia, Belgrade, Serbia;[2]Faculty of Medicine, University of Belgrade, Belgrade, Serbia;*

### Background

Chronic inflammation contributes to thrombosis and mortality in MPN. Simple inflammatory biomarkers such as NLR and red cell distribution width–to–albumin ratio (RAR) may reflect this pro-inflammatory state, while the Charlson Comorbidity Index (CCI) captures comorbidity burden.

### Aims

To evaluated the association of NLR, RAR and CCI with thrombosis and overall survival (OS) in MPN patients.

### Methods

We retrospectively analyzed 437 consecutive MPN patients, 205 (47%) PV, 58 (13%) ET, and 174 (40%) PMF patients, diagnosed per current WHO criteria at single university center. Clinical-laboratory, thrombotic and survival data were collected. NLR and RAR were analyzed as continuous variables and dichotomized according to ROC analysis (cut-offs: NLR 4.5; RAR 3.0). CCI was analyzed in 46% of patients.

### Results

In our cohort 259 (59.3%) were females with median age of 66y (range 18-87). Mean follow-up was 24m (3-228).
During follow-up 25 patients (5.7%) (15 PV; 2 ET; 8 PMF) had thrombosis as follows: 14 pts (3.2%) arterial (CVI (n=7); AMI (n=4), TIA (n=2), splenic infarct (n=1)) and 11 pts venous (2.5%) (DVT (n=9) and PE (n=2)).
Mean RAR was 3.91 ± 0.87, with subtype values of 3.91 ± 0.82 in PV, 3.47 ± 0.65 in ET, and 4.05 ± 0.93 in PMF without difference between PV and PMF (p=0.109), but significant differences between PV and ET (p=0.001) as well as ET and PMF (p<0.001). Mean NLR didn't differ significantly across MPN subtypes (PV 4.3 ± 3.2; ET 3.9 ± 3.1; PMF 3.8 ± 2.1). Distribution of CCI groups was: no comorbidities 22 pts (10.9%), mild 84 pts (41.8%), moderate 63 (31.3%), and severe 32 pts (15.9%), with no significant differences across MPN subtypes.
RAR was higher in pts who developed thrombosis during f-up compared to those without (4.40 vs 3.88; p=0.004). The difference was significant in development of arterial events (4.47 vs 3.89; p=0.013) but not venous. NLR ≥4.5 and severity of CCI were also associated with thrombotic events (p=0.003 and p=0.005, respectively). In multivariate Cox regression analysis for thrombosis, only CCI score and NLR ≥4.5 remained independent predictors (HR 2.22, p=0.002 and HR 2.69, p=0.032, respectively).
Estimated mean OS was 155m with mortality rate in whole group 10.5% distributed as PV 34.8%, ET 4.3%, and PMF 60.9%. Mean RAR was significantly higher in patients who died compared to alive patients (4.58±1.00 vs 3.83±0.82; p<0.001). By ROC analyses RAR ≥3.0 showed 100% sensitivity and 88.3% specificity as adverse survival prognostic parameter (p=0.02) (Figure 1). Mean survival in NLR ≥4.5 group was shorter 78m while in NLR <4.5 group was 180m (p<0.001). Kaplan–Meier demonstrated significantly worse survival for patients with severe CCI (78m) compared with mild-moderate group (103m vs 104m, respectively; p=0.005) and was not reached in MPN patients without comorbidities. In multivariate survival COX regression model, CCI remained most important predictor of mortality (HR 2.35; p<0.001) among all tested parameters including RAR and NLR.

**Copyright Information:** (Online) ISSN: 2572-9241
©2026 The Author(s). HemaSphere is published by John Wiley & Sons Ltd on behalf of European Hematology Association. This is an open access Abstract Book distributed under the Attribution-NonCommercial-NoDerivs (CC BY-NC-ND), which allows third parties to download the articles and share them with others as long as they credit the author and the Abstract Book, but they cannot change the content in any way or use them commercially.
**Abstract Book Citations:** Authors, Title, HemaSphere, 2026;10:(S1):pages. The abstract book DOIs can be found on the "Author Information" page.
**Disclaimer:** Articles published in the journal HemaSphere exclusively reflect the opinions of the authors. The authors are responsible for all content in their abstracts including accuracy of the facts, statements, citing resources, etc.

### Summary/Conclusion

By regression model independent predictors of thrombosis are NLR ≥4.5 and severe CCI while only severity of CCI has an independent effect on survival. RAR is also associated with higher risk of both thrombosis and mortality meaning that NLR and RAR serve collectively as indicators of systemic inflammation in MPNs cytokine-mediated burden with the additional inflammatory contribution arising from comorbidities analyzed by CCI.

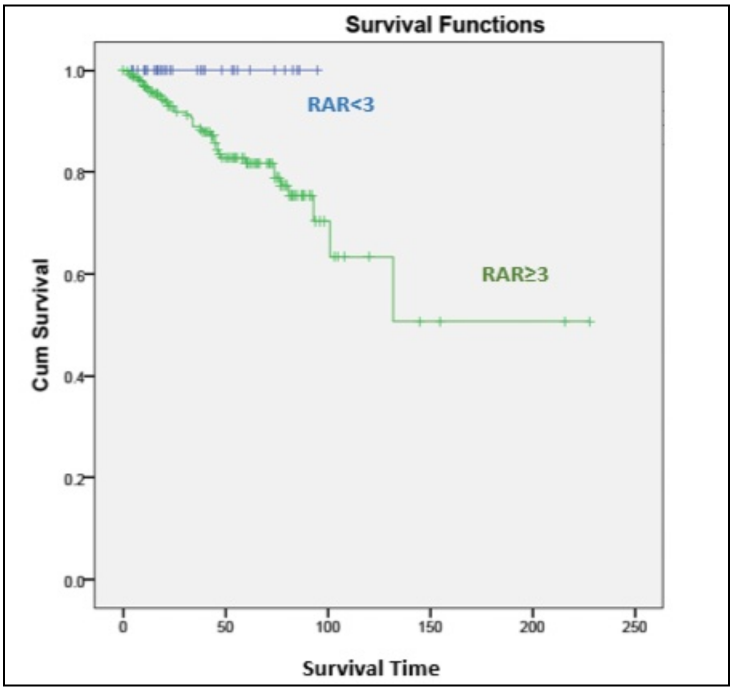

Figure 1. Kaplan-Meier Survival by RAR Cut-off (3.0) in MPN Patients

Copyright Information: (Online) ISSN: 2572-9241
©2026 The Author(s). HemaSphere is published by John Wiley & Sons Ltd on behalf of European Hematology Association. This is an open access Abstract Book distributed under the Attribution-NonCommercial-NoDerivs (CC BY-NC-ND), which allows third parties to download the articles and share them with others as long as they credit the author and the Abstract Book, but they cannot change the content in any way or use them commercially.
Abstract Book Citations: Authors, Title, HemaSphere, 2026;10:(S1):pages. The abstract book DOIs can be found on the "Author Information" page.
Disclaimer: Articles published in the journal HemaSphere exclusively reflect the opinions of the authors. The authors are responsible for all content in their abstracts including accuracy of the facts, statements, citing resources, etc.

### (PB3448) ANEMIA RECOVERY IDENTIFIES PROGNOSTIC HETEROGENEITY IN CYTOPENIC MYELOFIBROSIS: A POPULATION BASED REAL-WORLD ANALYSIS

**Topic:** 16. Myeloproliferative neoplasms - Clinical

Regina García Delgado*[1, 2, 3], Julio Torres González[1], Alejandro Escamilla Sanchez[1, 2], Alberto Esteban Medina[4], Ana Lopez Díaz[5], Carlos Loucera[4, 6], Laura Garcia Sanchis[7], Carlos Yubero[7], Joaquín Dopazo[4, 6]

*[1]BE21 Hematology and immunotherapy, Biomedical Research Institute and Nanomedicine Platform of Málaga (IBIMA Plataforma BIONAND), Málaga, Spain; [2]University of Málaga, Faculty of Medicine, Málaga, Spain;[3]Virgen de la Victoria Hospital, Hematology Service, Málaga, Spain; [4]Platform of Computational Medicine, Fundación Progreso y Salud (FPS), Hospital Virgen del Rocio, Sevilla, Spain; [5]Telomera SLU, Madrid, Spain;[6]Institute of Biomedicine of Seville, IBiS, University Hospital Virgen del Rocío/CSIC/University of Sevilla, Sevilla, Spain; [7]GlaxoSmithKline, Madrid, Spain;*

### Background

Cytopenic overt myelofibrosis (MF) is recognized as a high-risk clinical phenotype, yet prognostic assessment in real-world settings is often limited by heterogeneous data capture and restricted access to cytogenetic and molecular profiling. Most current real-world prognostic information derives from observational studies and specific registries, which have been instrumental in refining risk stratification. However, these data sources are inherently limited by selection bias, over-representation of large academic centers, manual data entry, and heterogeneity in the timing of baseline variable capture. Unlike disease registries, population-based cohorts minimize referral bias and better reflect real-world disease biology and outcomes. This approach is particularly relevant in MF, a heterogeneous entity in which extreme biological phenotypes, such as cytopenic presentations, may encompass clinically and prognostically distinct subgroups. While baseline cytopenias have been demonstrated to have a prognostic impact in patients with MF, whether simple hematologic dynamics—particularly anemia recovery—can refine risk stratification within this adverse phenotype, remains unclear.

### Aims

To explore the prognostic value of dynamic changes in hemoglobin levels in real-world MF patients.

### Methods

Using a population-based cohort from the Andalusian Public Health System (2017–2023), we identified all adults diagnosed with overt MF and classified them as cytopenic or proliferative based on hemoglobin and platelet counts at diagnosis. The index date corresponded to the MF diagnosis. A predefined subcohort of anemic patients (Hb <10 g/dL) was evaluated for hemoglobin recovery, defined as two consecutive Hb ≥10 g/dL measurements ≥15 days apart. Survival outcomes included event-free survival (EFS), overall survival (OS), and time to acute myeloid leukemia (AML) transformation. Competing risk analyses were performed using Aalen–Johansen estimators, and longitudinal hemoglobin trajectories were modeled with linear mixed effects models. Multivariable logistic regression assessed factors associated with adverse events.

**Copyright Information:** (Online) ISSN: 2572-9241
©2026 The Author(s). HemaSphere is published by John Wiley & Sons Ltd on behalf of European Hematology Association. This is an open access Abstract Book distributed under the Attribution-NonCommercial-NoDerivs (CC BY-NC-ND), which allows third parties to download the articles and share them with others as long as they credit the author and the Abstract Book, but they cannot change the content in any way or use them commercially.
**Abstract Book Citations:** Authors, Title, HemaSphere, 2026;10:(S1):pages. The abstract book DOIs can be found on the “Author Information” page.
**Disclaimer:** Articles published in the journal HemaSphere exclusively reflect the opinions of the authors. The authors are responsible for all content in their abstracts including accuracy of the facts, statements, citing resources, etc.

**Results**

1629 patients were analyzed with a median follow-up of 2,6 [1,5-4,5] years. Cytopenic patients (n=472) (defined as Hb<10 g/dL and/or platelet counts <100.00/uL) showed consistently inferior OS (HR=3,41 95% CI [2,86-4,05]), and AML free survival (HR=2,61 95% CI [1,78-3,83]) compared with the proliferative phenotype (n=1157), with a graded worsening in patients presenting with two cytopenias. Of the 370 anemic patients, 330 had post-index hemoglobin data, and 172 (52%) achieved recovery. Recovery was associated with markedly improved EFS, OS, and a lower cumulative incidence of AML transformation. In contrast, non-recovered individuals exhibited high early event rates and excess mortality. Multivariable analysis confirmed anemia recovery as an independent protective factor, reducing the risk of AML transformation or death ≈66% (OR=0,34, 95% CI [0,18-0,65], p=0.001).

**Summary/Conclusion**

In this unselected, population-based cohort, cytopenic overt MF at diagnosis was validated as a high-risk phenotype. Importantly, anemia recovery emerged as a powerful dynamic prognostic marker, identifying a subgroup with significantly improved survival despite cytopenic presentation. These findings highlight the clinical value of simple hematologic monitoring and support incorporating anemia recovery into routine prognostic assessment and therapeutic decision-making in MF.
Funded by GSK (study 219691).

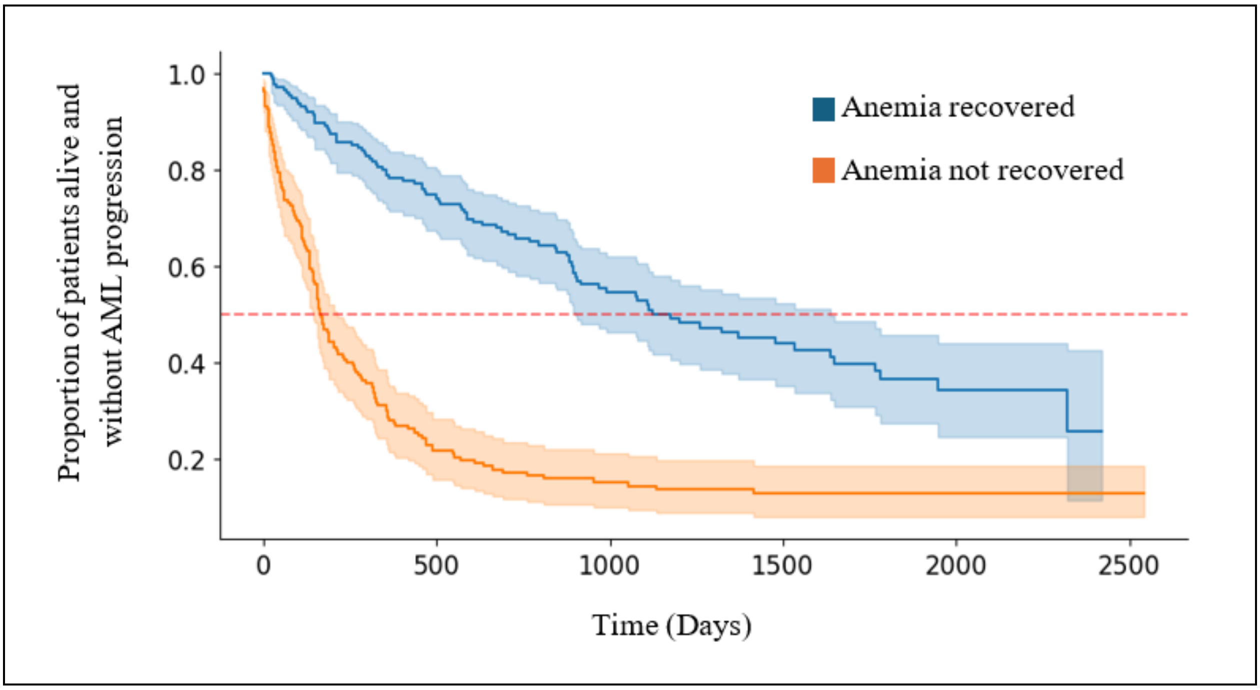

Figure 1: Event-free survival according to anemia-recovered and not recovered patients. Kaplan–Meier estimates illustrate the event-free survival of patients classified as recovered (blue) versus not recovered (orange). The x-axis indicates follow-up duration (in days), while the y-axis represents the corresponding event-free survival probability on a 0–1 scale. Shaded regions denote the 95% confidence intervals for each survival curve. Differences between groups were evaluated using the log-rank test (p < 0.001).

**Copyright Information:** (Online) ISSN: 2572-9241
©2026 The Author(s). HemaSphere is published by John Wiley & Sons Ltd on behalf of European Hematology Association. This is an open access Abstract Book distributed under the Attribution-NonCommercial-NoDerivs (CC BY-NC-ND), which allows third parties to download the articles and share them with others as long as they credit the author and the Abstract Book, but they cannot change the content in any way or use them commercially.
**Abstract Book Citations:** Authors, Title, HemaSphere, 2026;10:(S1):pages. The abstract book DOIs can be found on the “Author Information” page.
**Disclaimer:** Articles published in the journal HemaSphere exclusively reflect the opinions of the authors. The authors are responsible for all content in their abstracts including accuracy of the facts, statements, citing resources, etc.

### (PB3449) ESTIMATED PREVALENCE OF POLYCYTHEMIA VERA IN EUROPE: 2025-2030

**Topic:** 16. Myeloproliferative neoplasms - Clinical

Haifa Kathrin Al-Ali*[1], Samantha Martel[2], Caleigh Sawicki[2], Meng Yang[2], Cathy Lally[3], Chloe Barrera[3], W. Dana Flanders[3], Paola Guglielmelli[4]

*[1]University Hospital Halle, Halle (Saale), Germany; [2]Takeda Development Center Americas, Inc. (TDCA), Cambridge, United States of America; [3]Epidemiologic Research & Methods, Emory University, Chapel Hill, United States of America;[4]University of Florence, Florence, Italy;*

### Background

Polycythemia vera (PV) is a myeloproliferative neoplasm characterized by overproduction of red blood cells, leading to increased blood viscosity and elevated hematocrit, with a higher risk of cardiovascular and thrombotic events, and progression to myelofibrosis and acute myeloid leukemia. Determining PV prevalence is essential for characterizing disease burden; however, it is methodologically challenging, and country-level estimates are limited, outdated and reported inconsistently.

### Aims

This study aims to generate contemporary representative estimates of PV prevalence in the European Union (EU) and United Kingdom (UK).

### Methods

A modified life table approach was used to estimate PV prevalence based on age-specific incidence rate (IR) and age-of-onset-specific relative survival. Country-specific, age-specific IRs from national data sources were used for UK (Haematological Malignancy Research Network from 2010-2019), Belgium (Belgium Cancer Registry from 2018), Finland (Finnish Cancer Registry from 2019-2023) and Sweden (Swedish Cancer Registry from 2023). For the remaining countries that did not have country-specific representative incidence, EU-wide age-specific PV IRs were derived from combining available data sources from UK, Belgium, Finland and Sweden to inform each country-specific prevalence modeling. Survival estimates from the published limited EUROCARE-6 analysis with broader age groups and shorter follow-up were comparable to those from the US Surveillance, Epidemiology, and End Results (SEER) program. To obtain granular age groups and extended follow-up, relative survival was then derived from 20-year observed survival for PV from SEER 21, long-term (21- 40 year) observed survival from published data, and expected survival in each country-specific general population. Age-specific and country-specific population estimates from the United Nations were used to estimate 2025-2030 prevalence.

### Results

For countries with available age-specific incidence rates, the overall age-adjusted IRs in UK, Belgium, Finland and Sweden were 1.9, 2.0, 2.4 and 1.8 per 100,000 person-years, respectively. For the remaining countries, an overall average age-adjusted IR of 2.1 per 100,000 person-year was estimated. Correspondingly, the estimated incident cases in the EU (+UK) in 2025 is 10,723. The median survival ranged from 107 to 142 months across countries. The country-specific prevalence in 2025 ranged from 17.7 to 30.9 per 100,000 persons (Figure 1). By 2030, the estimated country-specific prevalence increased to 19.4 to 32.9 per 100,000 persons. This estimated prevalence corresponds to a total of 126,957 patients living with PV across all countries in 2025 and 135,040 patients by 2030.

**Copyright Information:** (Online) ISSN: 2572-9241
©2026 The Author(s). HemaSphere is published by John Wiley & Sons Ltd on behalf of European Hematology Association. This is an open access Abstract Book distributed under the Attribution-NonCommercial-NoDerivs (CC BY-NC-ND), which allows third parties to download the articles and share them with others as long as they credit the author and the Abstract Book, but they cannot change the content in any way or use them commercially.
**Abstract Book Citations:** Authors, Title, HemaSphere, 2026;10:(S1):pages. The abstract book DOIs can be found on the "Author Information" page.
**Disclaimer:** Articles published in the journal HemaSphere exclusively reflect the opinions of the authors. The authors are responsible for all content in their abstracts including accuracy of the facts, statements, citing resources, etc.

**Summary/Conclusion**

Using nationally representative data sources, we generated contemporary estimates of PV prevalence in Europe for 2025-2030, leveraging age-specific incidence rates and life-table model. These estimates will allow for a clearer, more robust characterization of the disease burden among individuals living with PV in Europe and help inform healthcare resource utilization and support more effective disease management.

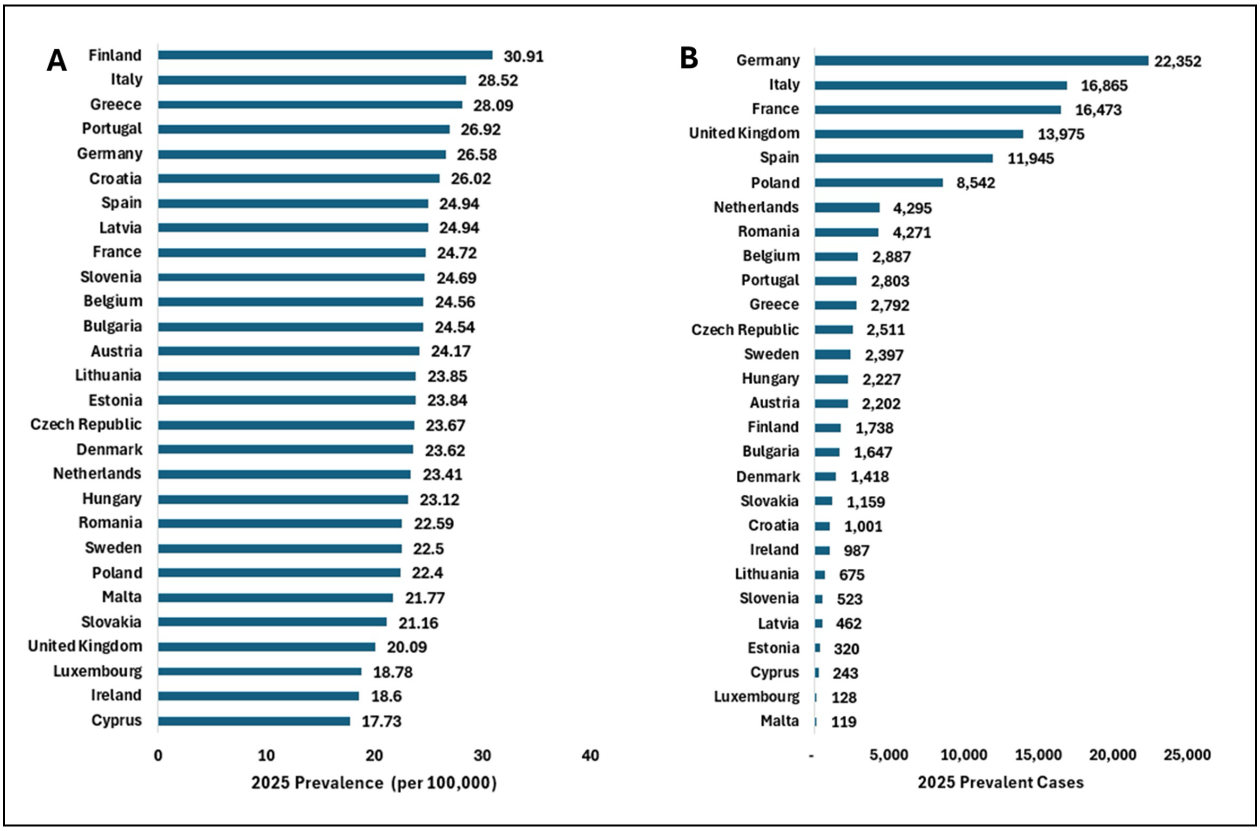

Figure 1. A) 2025 Prevalence per 100,000 and B) 2025 Prevalent Cases of PV in European Union and United Kingdom

**Copyright Information:** (Online) ISSN: 2572-9241
©2026 The Author(s). HemaSphere is published by John Wiley & Sons Ltd on behalf of European Hematology Association. This is an open access Abstract Book distributed under the Attribution-NonCommercial-NoDerivs (CC BY-NC-ND), which allows third parties to download the articles and share them with others as long as they credit the author and the Abstract Book, but they cannot change the content in any way or use them commercially.
**Abstract Book Citations:** Authors, Title, HemaSphere, 2026;10:(S1):pages. The abstract book DOIs can be found on the "Author Information" page.
**Disclaimer:** Articles published in the journal HemaSphere exclusively reflect the opinions of the authors. The authors are responsible for all content in their abstracts including accuracy of the facts, statements, citing resources, etc.

### (PB3450) CLINICAL CHARACTERISTICS AND PROGNOSTIC ANALYSIS FOR OTHER MALIGNANCIES AMONG PATIENTS WITH MYELOPROLIFERATIVE NEOPLASMS

**Topic:** 16. Myeloproliferative neoplasms - Clinical

Xiaojing Wei*[1], Guangshuai Teng[1], Dailian Zhang[1], Yuhui Zhang[1], Chenxiao Du[1], Yifan Duan[1], Yan Wang[1], Huiqin Zhang[1], Lan Peng[1], Jie Bai[1]

[1] *The Second Hospital of Tianjin Medical University, Department of Hematology, Tianjin, China;*

### Background

Myeloproliferative neoplasms (MPNs) are a group of clonal proliferation of hematopoietic stem and progenitor cells in the bone marrow, mainly including polycythemia vera (PV), essential thrombocythemia (ET), primary myelofibrosis (PMF), and pre-primary myelofibrosis (pre-PMF). In recent years, with the prolonged survival of MPN patients and the advancement of diagnosis and treatment, the reported cases of MPN complicated with other malignancies (including MPN secondary to other malignancies and MPN with secondary malignancies) have been increasing gradually. The mechanisms and prognostic factors of these patients have become research hotspots in clinical practice.

### Aims

To investigate the clinical features, risk factors, and prognostic factors of myeloproliferative neoplasm (MPN) patients with other cancers.

### Methods

A retrospective analysis was performed on the clinical data of 476 patients with myeloproliferative neoplasms (MPN) admitted to the Second Hospital of Tianjin Medical University from April 18, 2018 to March 21, 2024. Among them, 42 patients were complicated with other cancers (cancers-combined group), and 168 MPN patients without other cancers were selected as the control group (non-cancers-combined group) by 1:4 matching according to gender and age. The clinical characteristics, cytokine levels, gene mutation characteristics, etc. were compared between the two groups. The Kaplan-Meier method was used to analyze the factors influencing the occurrence of secondary cancers in MPN patients and the prognosis of MPN patients with other cancers, and the Cox regression model was used to analyze the risk factors for the occurrence of secondary cancers in MPN patients and prognostic factors.

### Results

Among the other cancers, solid tumors accounted for 78.6%, and hematological tumors accounted for 21.4%. The hemoglobin level in the cancer-combined group was significantly lower than that in the MPN group [140 (111.5, 177.8) g/L vs 164 (124.3, 187.8) g/L, *P*=0.049]. In the polycythemia vera (PV) subtype, the thrombosis rate was lower in the cancer-combined group (15.8% vs 50.5%, *P*=0.022)⊠Table 1⊠. Mutation frequencies of *DDX41* and *TET1* were also significantly increased (*P*<0.05)(Figure 1),and the levels of IL-6 and IL-22 in the tumor-combined group were significantly higher than those in the non-cancer group (*P*⊠0.05, respectively) (Figure 2). Cox regression indicated that age≥60 years (*HR*=3.485, *95%CI*⊠1.512-8.031, *P*=0.003), myelofibrosis (MF) grade≥2 (*HR*=4.185, *95%CI*: 1.586-11.041, *P*=0.004), and *JAK2*$^{V617F}$ mutation burden≥50% (*HR*=2.611, *95%CI*: 1.129-6.039, *P*=0.025) were independent risk factors for secondary cancer)(Figure 3). Patients with MPN secondary to prior cancers had poorer prognosis than those with secondary cancers after MPN (*P*<0.01) )(Figure 4). Age≥60 years, male, PMF subtype, and history of thrombosis were risk factors for poor prognosis in cancer-combined patients (all *P*<0.05) (Figure 5).

**Copyright Information:** (Online) ISSN: 2572-9241
©2026 The Author(s). HemaSphere is published by John Wiley & Sons Ltd on behalf of European Hematology Association. This is an open access Abstract Book distributed under the Attribution-NonCommercial-NoDerivs (CC BY-NC-ND), which allows third parties to download the articles and share them with others as long as they credit the author and the Abstract Book, but they cannot change the content in any way or use them commercially.
**Abstract Book Citations:** Authors, Title, HemaSphere, 2026;10:(S1):pages. The abstract book DOIs can be found on the "Author Information" page.
**Disclaimer:** Articles published in the journal HemaSphere exclusively reflect the opinions of the authors. The authors are responsible for all content in their abstracts including accuracy of the facts, statements, citing resources, etc.

Summary/Conclusion

MPN patients have a relatively high incidence of secondary cancers. Age≥60 years, MF grade≥2, and high *JAK2*$^{V617F}$ mutation burden are high-risk factors for secondary cancers.

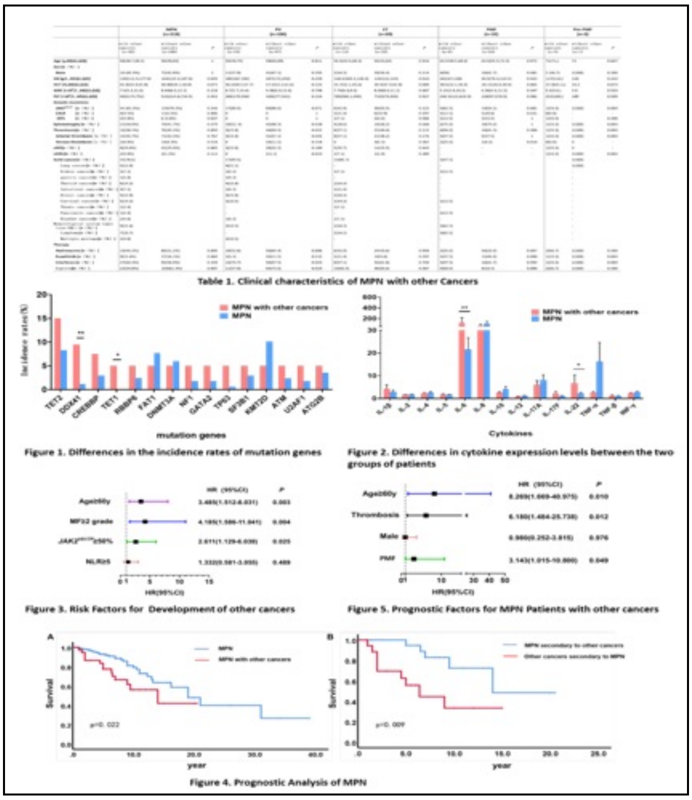

Copyright Information: (Online) ISSN: 2572-9241
©2026 The Author(s). HemaSphere is published by John Wiley & Sons Ltd on behalf of European Hematology Association. This is an open access Abstract Book distributed under the Attribution-NonCommercial-NoDerivs (CC BY-NC-ND), which allows third parties to download the articles and share them with others as long as they credit the author and the Abstract Book, but they cannot change the content in any way or use them commercially.
Abstract Book Citations: Authors, Title, HemaSphere, 2026;10:(S1):pages. The abstract book DOIs can be found on the "Author Information" page.
Disclaimer: Articles published in the journal HemaSphere exclusively reflect the opinions of the authors. The authors are responsible for all content in their abstracts including accuracy of the facts, statements, citing resources, etc.

### (PB3451) LONG TERM LOW RISK PEDIATRIC LANGERHANS CELL HISTIOCYTOSIS (LCH). DOES CENTRAL NERVOUS SYSTEM CNS + RISK SITE BONES AFFECT THE OUTCOME? A 15-YEAR OF EXPERIENCE.

**Topic:** 16. Myeloproliferative neoplasms - Clinical

Mohamed Sedky*[1], Seham Gohar[1], Nashwa Ezz Eldeen[1], Hani Abdel Rahman Sayed[1], Hala Taha[2], Iman Zaky[3], Saida Zakaria[4], Salma Abdelkarim[5], Ahmed Elghoneimy[6], Mona Atteya[7], Alaa Elhaddad[1]

*[1]CCHE 57357, Pediatric Oncology, Cairo, Egypt; [2]CCHE 57357, Pathology, Cairo, Egypt; [3]CCHE 57357, Radiology, Cairo, Egypt; [4]CCHE 57357, Nursing, Cairo, Egypt; [5]CCHE 57357, clinical research, Cairo, Egypt; [6]CCHE 57357, Orthopedics, Cairo, Egypt; [7]CCHE 57357, Endocrinology, Cairo, Egypt;*

### Background

Low risk organ Langerhans Cell Histiocytosis (LCH RO-) usually carries good prognosis. However, central nervous system risk sites (CNS+) craniofacial bones can cause morbidity.

### Aims

LCH RO- outcome according to presence of CNS+.

### Methods

Single center retrospective study of 395 LCH RO- with CNS+ or CNS- associated to multi (MS) or single system (SS), through LCHIII/IV protocols.

### Results

CNS+ group n= 164 (42%) CNS- group n= 231(58%). Of them MS CNS+ n= 56, SS CNS+ n=108, MS CNS - n= 44, SS CNS- n= 187. At diagnosis, CNS + was associated with age less than 2 years, weight and height retardation < minus (-) 2 SD Z score-, more cutaneous and diabetes insipidus (DI) while less appendicular bone involvement; all with statistically significant results. Similarly, more single and multiple reactivations (REA). 5-year event free survival (EFS) in MS CNS+ 60.7 % [95% CI 0.49-0.75], MS CNS- 62.6 % [95% CI 0.50, 0.79], SS CNS+70% [95% CI 0.62-0.79], SS CNS- 82 % [95% CI 0.77-0.88] p 0.001. EFS with long 12-week/short 6-week induction: MS CNS+ 63% / 50 %, MS CNS- 68% / 55 %, SS CNS+ 68 % / 79 %, SS CNS- 70 % / 79.5 % respectively, local treatment SS CNS+ 67 % and SSCNS-91% p < 0.001. Reactivation free survival (RFS) Long > 6 months / short ≤ 6 months. maintenance: SS CNS+ 71% / 76 % SS CNS- 78 % / 74 % local treatment SS CNS-90 %, p 0.03. At last follow up, REA by DI in 11% of CNS+ vs 5% of CNS- p 0.012, all seven Neuro Degenerative Disorder (NDD) patients in CNS+, and weight and height retardation in 6.7% of CNS+ vs 2.6% of CNS- p 0.018.

### Summary/Conclusion

On long term follow up, CNS+ risk bone involvement was associated with lower EFS as well as with a triad of NDD, DI and growth retardation. Long induction improved MS but not SS EFS. Contrarily, long maintenance improved SS CNS- but not SS CNS+ RFS.

**Copyright Information:** (Online) ISSN: 2572-9241
©2026 The Author(s). HemaSphere is published by John Wiley & Sons Ltd on behalf of European Hematology Association. This is an open access Abstract Book distributed under the Attribution-NonCommercial-NoDerivs (CC BY-NC-ND), which allows third parties to download the articles and share them with others as long as they credit the author and the Abstract Book, but they cannot change the content in any way or use them commercially.
**Abstract Book Citations:** Authors, Title, HemaSphere, 2026;10:(S1):pages. The abstract book DOIs can be found on the "Author Information" page.
**Disclaimer:** Articles published in the journal HemaSphere exclusively reflect the opinions of the authors. The authors are responsible for all content in their abstracts including accuracy of the facts, statements, citing resources, etc.

### (PB3452) ICC/WHO-DEFINED AND NGS-ANNOTATED IDIOPATHIC HYPEREOSINOPHILIA: MUTATIONS, CLINICAL AND TREATMENT DETAILS, AND PREDICTORS OF SURVIVAL

**Topic:** 16. Myeloproliferative neoplasms - Clinical

Shraddha Patel*[1], Yousuf Muhammad[1], Rania Abdelaziz[1], Priyansh Faldu[1], Shivani Udyawar[1], Aysel Kaplan Baryraktar[1], Abiola Bolarinwa[1], Cinthya Zepeda Mendoza[1], Kaaren Reichard[2], David Viswanatha[2], Rong He[2], Naseema Gangat[1], Animesh Pardanani[1], Ayalew Tefferi[1]

*[1]Mayo Clinic, Division of Hematology, Rochester, United States of America;[2]Mayo Clinic, Division of Pathology and Laboratory Medicine, Rochester, United States of America;*

### Background

Idiopathic hypereosinophilia (iHE) is characterized by persistent peripheral blood hypereosinophilia (absolute eosinophil count [AEC] ≥1.5×$10^9$/L and ≥10% circulating eosinophils), with (iHES) or without associated organ damage, and requires exclusion of other reactive or clonal conditions, including myeloid/lymphoid neoplasms with eosinophilia and tyrosine kinase gene fusions (M/LN-eo-TK), lymphocyte variant HES (LV-HES), and chronic eosinophilic leukemia, not otherwise specified (CEL-NOS). We retrospectively reviewed next-generation sequencing (NGS)-annotated cases of iHE/iHES.

### Methods

Diagnosis of cases with iHE/iHES, and distinction from M/LN-eo-TK, LV-HES and CEL-NOS, were according to the 2023 International Consensus Classification (*AJH 98: 1286-1306*). CLIA-approved NGS testing was used to curate mutational data. Organ involvement was determined by imaging, biopsy, and clinical presentation. Conventional statistical methods were utilized.

### Results

A total of 48 NGS-annotated patients with ICC/WHO-defined iHE/iHES (median age 56 years; 52% males) were considered. All study patients were symptomatic at presentation: gastrointestinal (n=12), pulmonary (n=12), cutaneous (n=9), constitutional (n=6), cardiac (n=6) and neurologic (n=3); thrombotic complications after diagnosis were reported in 5 patients. Pathogenic mutations were documented in 6 (12%) patients: *DNMT3A* (VAF 4%), *ASXL1 + DNMT3A (36% and 11%), TET2* (41%), *SF3B1* (47%), *CBL* (2%), and *CHEK2* (48%) and were significantly associated with age ≥65 years (83% vs. 24%; *p*<0.01) and lower likelihood of organ involvement (83% vs. 100%; *p*=0.04). Clonal T-cell receptor gene rearrangement (TCR), without associated abnormal T cell immunophenotype, was present in 3 patients and correlated with a higher rate of skin involvement (100% vs. 29%; *p*<0.01). Mepolizumab was administered to 35 (73%) patients and enabled corticosteroid discontinuation in 20 (59%); median AECs at treatment initiation and 6, and 12 months post-treatment were 2.6, 0.1, and 0.07 (×$10^9$/L), respectively. At a post-treatment median follow-up of 14 months, mepolizumab was discontinued in 13 (37%) patients, with continued treatment more likely in the presence of allergy history (63% vs. 16%; p<0.01). Other medications on record included: corticosteroids (n=46), hydroxyurea (n=8), dupilumab (n=7), benralizumab (n=6), imatinib (n=6), ruxolitinib (n=3) and alemtuzumab (n=2). One patient underwent hematopoietic cell transplantation with symptomatic improvement but persistent hypereosinophilia; another patient underwent cardiac transplantation with moderate symptomatic benefit.
At a median follow-up of 29 months, two deaths were documented (one related to iHES), with median overall survival (OS) exceeding the last follow-up. OS was comparable in patients with or without pathogenic mutations (p=0.56) or clonal TCR (p=0.69). In univariate analysis, cardiac involvement (2-year OS: 55% vs. 87%; p=0.01) and absolute lymphocyte count <1.0 ×$10^9$/L (59% vs. 100%; p<0.01) predicted inferior survival; the latter remained significant in multivariable analysis (p=0.02).

**Copyright Information:** (Online) ISSN: 2572-9241
©2026 The Author(s). HemaSphere is published by John Wiley & Sons Ltd on behalf of European Hematology Association. This is an open access Abstract Book distributed under the Attribution-NonCommercial-NoDerivs (CC BY-NC-ND), which allows third parties to download the articles and share them with others as long as they credit the author and the Abstract Book, but they cannot change the content in any way or use them commercially.
**Abstract Book Citations:** Authors, Title, HemaSphere, 2026;10:(S1):pages. The abstract book DOIs can be found on the "Author Information" page.
**Disclaimer:** Articles published in the journal HemaSphere exclusively reflect the opinions of the authors. The authors are responsible for all content in their abstracts including accuracy of the facts, statements, citing resources, etc.

**Summary/Conclusion**

The current study reveals enrichment of CHIP-associated mutations in older patients with iHE/iHES that were prognostically neutral, underscoring caution in their interpretation. The study also confirms previous observations regarding the adverse survival impact of cardiac involvement and lymphocytopenia and real-world efficacy of mepolizumab as a steroid sparing agent.

**Copyright Information:** (Online) ISSN: 2572-9241
©2026 The Author(s). HemaSphere is published by John Wiley & Sons Ltd on behalf of European Hematology Association. This is an open access Abstract Book distributed under the Attribution-NonCommercial-NoDerivs (CC BY-NC-ND), which allows third parties to download the articles and share them with others as long as they credit the author and the Abstract Book, but they cannot change the content in any way or use them commercially.
**Abstract Book Citations:** Authors, Title, HemaSphere, 2026;10:(S1):pages. The abstract book DOIs can be found on the "Author Information" page.
**Disclaimer:** Articles published in the journal HemaSphere exclusively reflect the opinions of the authors. The authors are responsible for all content in their abstracts including accuracy of the facts, statements, citing resources, etc.

### (PB3453) PATHOLOGIC FRACTURES INDEPENDENTLY INCREASE MORTALITY AND HEALTHCARE UTILIZATION IN HOSPITALIZED PATIENTS WITH MULTIPLE MYELOMA: A CONTEMPORARY NATIONAL ANALYSIS

**Topic:** 16. Myeloproliferative neoplasms - Clinical

Lydia Francis*[1], Benjamin Easow[1], Sandhra Jiby[2], Krishna Basarakodu[1]

*[1]Southeast Health, Internal Medicine, Dothan, United States of America;[2]Dr. Somervell Memorial CSI Medical College Hospital, Trivandrum, India;*

### Background

Skeletal complications remain a major source of morbidity in multiple myeloma (MM). Although pathologic fractures are common in MM, contemporary national estimates of their impact on inpatient mortality and healthcare utilization are limited.

### Aims

To determine whether pathologic fractures are independently associated with increased inpatient mortality, prolonged length of stay, and higher hospital charges among adults hospitalized with multiple myeloma in the United States.

### Methods

We performed a cross-sectional analysis of adult MM hospitalizations in the 2022 National Inpatient Sample (NIS). MM was identified using ICD-10-CM codes (C90.00–C90.02). Pathologic fractures were defined using ICD-10-CM codes M84.4x and M84.5x. Survey weights were applied to generate national estimates. Primary outcome was inpatient mortality. Secondary outcomes included length of stay (LOS) and total hospital charges. Weighted multivariable logistic regression evaluated mortality, and weighted log-linear regression models evaluated LOS and charges. Models adjusted for age, sex, race, primary payer, income quartile, hospital region, teaching status, bed size, and APR-DRG severity. Age-stratified analyses (<65 vs ≥65 years) assessed effect heterogeneity.

### Results

Among 23,245 unweighted hospitalizations representing 116,225 weighted adult multiple myeloma admissions in 2022, 7.25% had a pathologic fracture. In unadjusted weighted analyses, hospitalizations with pathologic fracture had longer length of stay (9.97 vs 7.68 days) and higher total charges ($146,746 vs $114,647), while crude mortality was similar (5.22% vs 5.62%). In multivariable weighted analyses adjusting for demographics, payer, hospital characteristics, and APR-DRG severity, pathologic fracture was independently associated with increased inpatient mortality (adjusted OR 1.13, 95% CI 1.01–1.27), 21% longer length of stay (multiplier 1.21, 95% CI 1.16–1.27), and 18% higher hospital charges (multiplier 1.18, 95% CI 1.12–1.23). In age-stratified adjusted models, the association between fracture and mortality did not significantly differ by age group (<65 years: OR 1.19, 95% CI 0.99–1.43; ≥65 years: OR 1.05, 95% CI 0.79–1.41; interaction p=0.30).

### Summary/Conclusion

In this contemporary national analysis, pathologic fractures among hospitalized adults with multiple myeloma were independently associated with higher inpatient mortality and substantially greater healthcare utilization. These findings highlight the persistent clinical and economic burden of skeletal complications in MM and emphasizes the importance of preventive and early therapeutic strategies to mitigate fracture risk.

**Copyright Information:** (Online) ISSN: 2572-9241
©2026 The Author(s). HemaSphere is published by John Wiley & Sons Ltd on behalf of European Hematology Association. This is an open access Abstract Book distributed under the Attribution-NonCommercial-NoDerivs (CC BY-NC-ND), which allows third parties to download the articles and share them with others as long as they credit the author and the Abstract Book, but they cannot change the content in any way or use them commercially.
**Abstract Book Citations:** Authors, Title, HemaSphere, 2026;10:(S1):pages. The abstract book DOIs can be found on the "Author Information" page.
**Disclaimer:** Articles published in the journal HemaSphere exclusively reflect the opinions of the authors. The authors are responsible for all content in their abstracts including accuracy of the facts, statements, citing resources, etc.

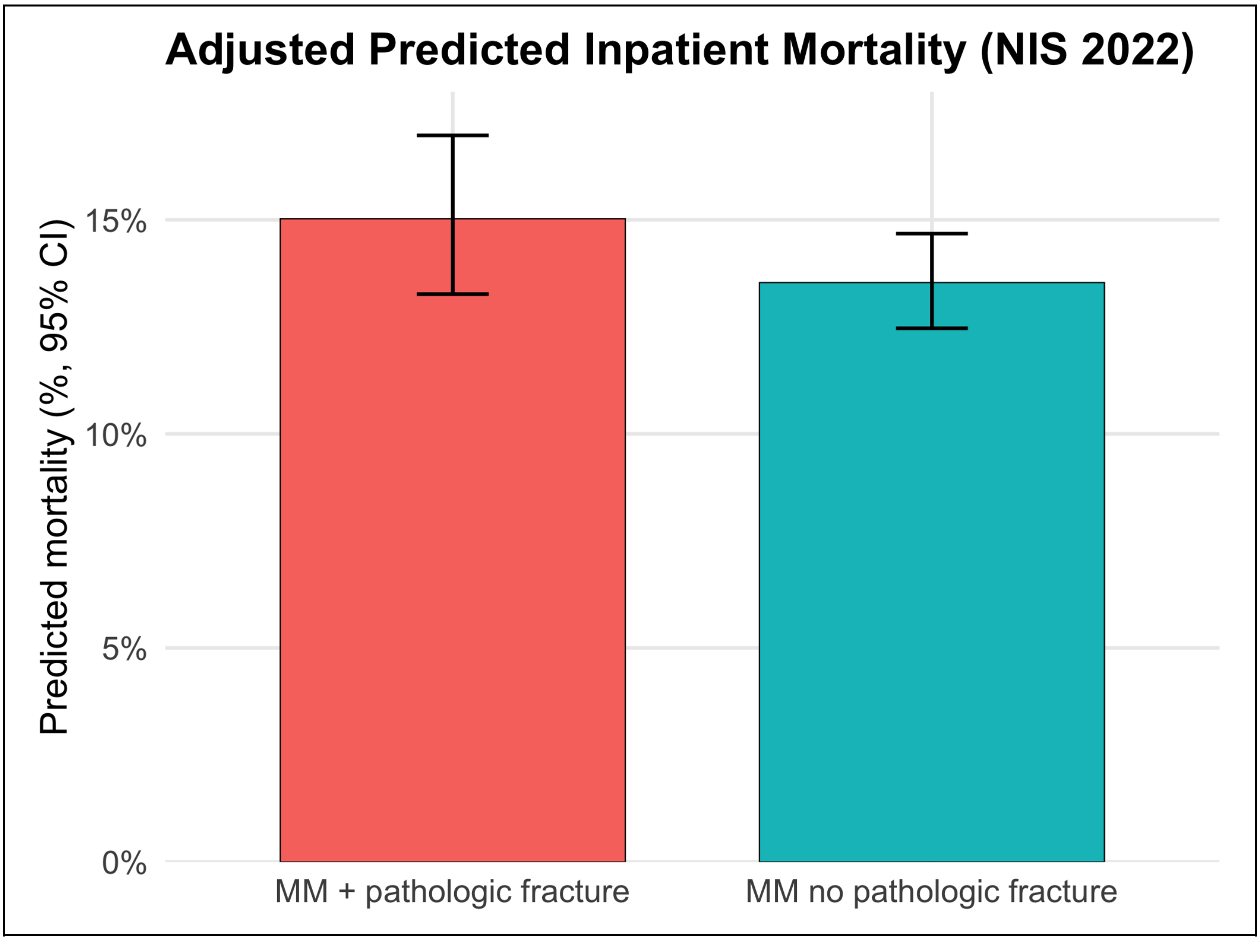

NIS = National Inpatient Sample. Estimates are weighted to generate nationally representative results. Adjusted mortality derived from multivariable logistic regression adjusting for age, sex, race, primary payer, income quartile, hospital region, teaching status, bed size, and APR-DRG severity of illness. Error bars represent 95% confidence intervals.

**Copyright Information:** (Online) ISSN: 2572-9241
©2026 The Author(s). HemaSphere is published by John Wiley & Sons Ltd on behalf of European Hematology Association. This is an open access Abstract Book distributed under the Attribution-NonCommercial-NoDerivs (CC BY-NC-ND), which allows third parties to download the articles and share them with others as long as they credit the author and the Abstract Book, but they cannot change the content in any way or use them commercially.
**Abstract Book Citations:** Authors, Title, HemaSphere, 2026;10:(S1):pages. The abstract book DOIs can be found on the "Author Information" page.
**Disclaimer:** Articles published in the journal HemaSphere exclusively reflect the opinions of the authors. The authors are responsible for all content in their abstracts including accuracy of the facts, statements, citing resources, etc.

### (PB3454) LONG-TERM FOLLOW-UP OUTCOMES AND RISK FACTORS FOR DISEASE PROGRESSION IN 2064 PATIENTS WITH ESSENTIAL THROMBOCYTHEMIA: A REAL-WORLD MULTICENTER STUDY FROM ZHEJIANG PROVINCE, CHINA

**Topic:** 16. Myeloproliferative neoplasms - Clinical

Xiaojing Bao[1], Jia Li[1], Xiudi Yang[1], Tian Zeng[1], Zhikang Zheng[1], Rumeng Li[1], Bingbing Wen[1], Qingmei Han[1], Jie Jin[1], Jian Huang*[1]

[1] *The First Affiliated Hospital, Zhejiang University School of Medicine, Hangzhou, China;*

### Background

Essential thrombocythemia (ET), the most prevalent myeloproliferative neoplasm (MPN), traditionally presents with a favorable prognosis. However, emerging evidence highlights a significant risk of disease progression to myelofibrosis (MF) and acute myeloid leukemia (AML) during long-term follow-up, particularly in the second decade after diagnosis. Large-scale real-world data from China remain limited.

### Aims

This study aims to investigate the clinical characteristics, long-term survival outcomes, and risk factors for disease progression in a large cohort of ET patients from China.

### Methods

This multicenter retrospective real-world study conducted across 35 hematology centers in Zhejiang Province, China, enrolled 2064 patients diagnosed with ET according to WHO criteria between January 2001 and December 2025. Data collection included demographic characteristics, laboratory parameters , bone marrow pathology, driver mutation status. Survival outcomes including overall survival (OS), myelofibrosis-free survival (MFS), and leukemia-free survival (LFS). Cox proportional hazards regression was performed to identify independent risk factors for progression to post-ET myelofibrosis (post-ET MF) (Figure A).

### Results

The study cohort comprised 2064 ET patients with a median diagnostic age of 59 years (range, 4-93 years), 45.6% were male. Prior thrombosis history was present in 13.2%, bleeding history in 3.7%, and smoking history in 15.2%. Constitutional symptoms were present in 32.9% and palpable splenomegaly in 17.3% at diagnosis. Driver mutations were detected in approximately 80% of patients: *JAK2* in 64.9%, *CALR* in 16.0%, *MPL* in 1.1%, and triple-negative status in 18.0% (Figure B). At diagnosis, the median PLT was 750×10⁹/L (IQR, 585-975), median WBC 8.9×10⁹/L (7.0-11.5), median Hb 134 g/L (122-147), median RDW 13.9% (13.1-15.3), median LDH 243 U/L (202-304), and 22.2% of bone marrow biopsies showed mild reticulin fibrosis (MF-1). With a median follow-up of 47 months (18-79), the 5-year, 10-year, and 15-year OS rates were 98.3%, 95.5%, and 91.9%, respectively (Figure D). The 5-year, 10-year, and 15-year MFS rates were 95.9%, 82.1%, and 64.5%, respectively (Figure E). Notably, no significant differences in MFS were observed among different driver mutation subgroups (*JAK2* vs. *CALR* vs. triple-negative, P>0.05). The 5-year, 10-year, and 15-year LFS rates were 99.5%, 99.1%, and 95.7%, respectively (Figure F). Multivariate analysis identified five independent risk factors for progression to post-ET MF: age at diagnosis >60 years (HR 2.3, 95%CI 1.5-3.7), smoking history (2.7, 1.7-4.4), splenomegaly (5.6, 3.4-9.1), MF-1 grade fibrosis at diagnosis (1.8, 1.2-2.8), and RDW >15% (3.0, 1.8-5.0) (Figure C).

**Copyright Information:** (Online) ISSN: 2572-9241
©2026 The Author(s). HemaSphere is published by John Wiley & Sons Ltd on behalf of European Hematology Association. This is an open access Abstract Book distributed under the Attribution-NonCommercial-NoDerivs (CC BY-NC-ND), which allows third parties to download the articles and share them with others as long as they credit the author and the Abstract Book, but they cannot change the content in any way or use them commercially.
**Abstract Book Citations:** Authors, Title, HemaSphere, 2026;10:(S1):pages. The abstract book DOIs can be found on the "Author Information" page.
**Disclaimer:** Articles published in the journal HemaSphere exclusively reflect the opinions of the authors. The authors are responsible for all content in their abstracts including accuracy of the facts, statements, citing resources, etc.

### Summary/Conclusion

ET patients in this large Chinese cohort demonstrate excellent long-term survival. However, the risk of myelofibrosis transformation accumulates significantly during the 10-15 year disease course, affecting approximately one-third of long-term survivors. Age at diagnosis >60 , smoking history , splenomegaly, MF-1 grade fibrosis at diagnosis, and RDW >15% are independent predictors of fibrotic progression. These findings underscore the importance of dynamic risk assessment and enhanced monitoring strategies for long-term ET survivors.

This research was supported by the National Natural Science Foundation of China (No. 82570191) and the Zhejiang Provincial Health Highlevel Innovative Talent Project (2022-2026). *Correspondence to: Prof Jian Huang, E-mail: househuang@zju.edu.cn

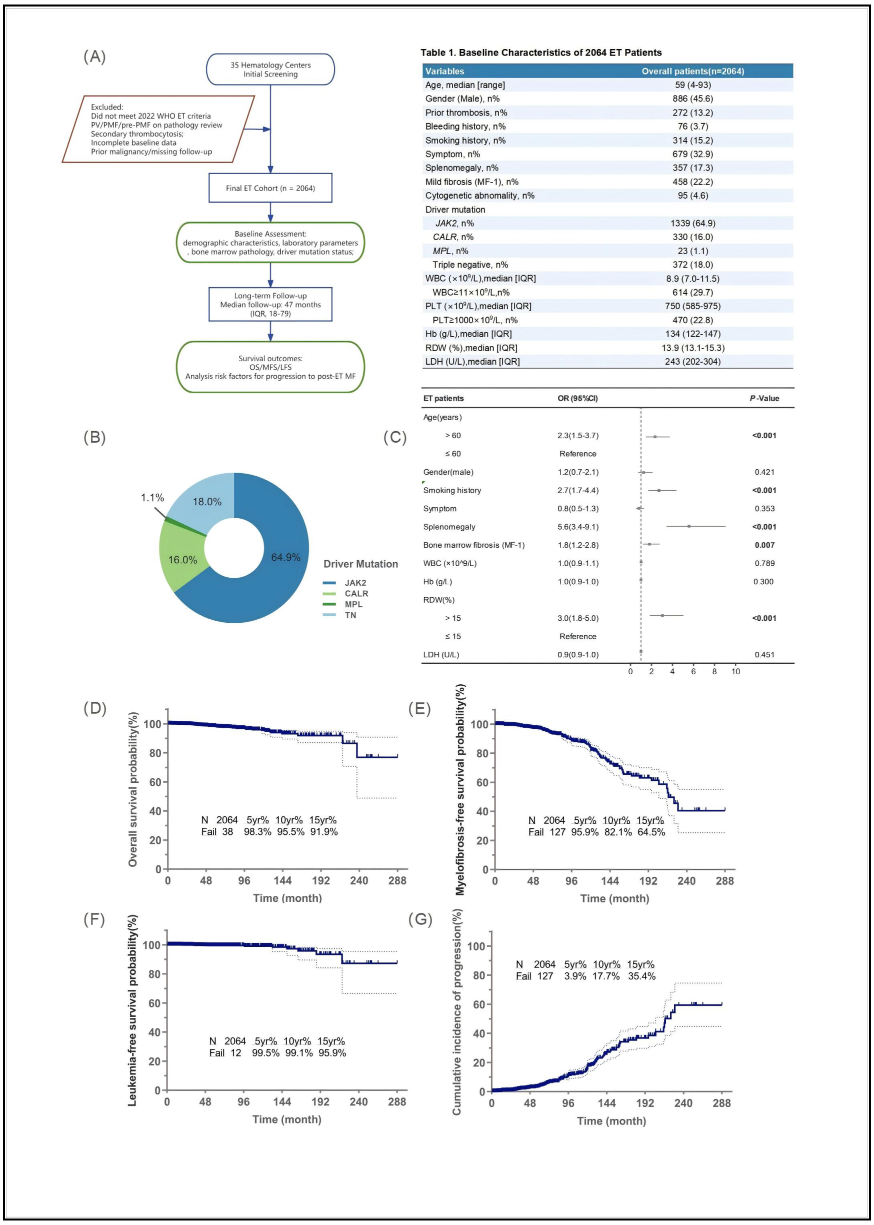

**Table 1. Baseline Characteristics of 2064 ET Patients**

| Variables | Overall patients(n=2064) |
|---|---|
| Age, median [range] | 59 (4-93) |
| Gender (Male), n% | 886 (45.6) |
| Prior thrombosis, n% | 272 (13.2) |
| Bleeding history, n% | 76 (3.7) |
| Smoking history, n% | 314 (15.2) |
| Symptom, n% | 679 (32.9) |
| Splenomegaly, n% | 357 (17.3) |
| Mild fibrosis (MF-1), n% | 458 (22.2) |
| Cytogenetic abnomality, n% | 95 (4.6) |
| Driver mutation | |
| *JAK2*, n% | 1339 (64.9) |
| *CALR*, n% | 330 (16.0) |
| *MPL*, n% | 23 (1.1) |
| Triple negative, n% | 372 (18.0) |
| WBC ($\times10^9$/L),median [IQR] | 8.9 (7.0-11.5) |
| WBC≥11×$10^9$/L,n% | 614 (29.7) |
| PLT ($\times10^9$/L),median [IQR] | 750 (585-975) |
| PLT≥1000×$10^9$/L, n% | 470 (22.8) |
| Hb (g/L),median [IQR] | 134 (122-147) |
| RDW (%),median [IQR] | 13.9 (13.1-15.3) |
| LDH (U/L),median [IQR] | 243 (202-304) |

| ET patients | OR (95%CI) | *P*-Value |
|---|---|---|
| Age(years) | | |
| > 60 | 2.3(1.5-3.7) | **<0.001** |
| ≤ 60 | Reference | |
| Gender(male) | 1.2(0.7-2.1) | 0.421 |
| Smoking history | 2.7(1.7-4.4) | **<0.001** |
| Symptom | 0.8(0.5-1.3) | 0.353 |
| Splenomegaly | 5.6(3.4-9.1) | **<0.001** |
| Bone marrow fibrosis (MF-1) | 1.8(1.2-2.8) | **0.007** |
| WBC (×10^9/L) | 1.0(0.9-1.1) | 0.789 |
| Hb (g/L) | 1.0(0.9-1.0) | 0.300 |
| RDW(%) | | |
| > 15 | 3.0(1.8-5.0) | **<0.001** |
| ≤ 15 | Reference | |
| LDH (U/L) | 0.9(0.9-1.0) | 0.451 |

**Copyright Information:** (Online) ISSN: 2572-9241
©2026 The Author(s). HemaSphere is published by John Wiley & Sons Ltd on behalf of European Hematology Association. This is an open access Abstract Book distributed under the Attribution-NonCommercial-NoDerivs (CC BY-NC-ND), which allows third parties to download the articles and share them with others as long as they credit the author and the Abstract Book, but they cannot change the content in any way or use them commercially.
**Abstract Book Citations:** Authors, Title, HemaSphere, 2026;10:(S1):pages. The abstract book DOIs can be found on the “Author Information” page.
**Disclaimer:** Articles published in the journal HemaSphere exclusively reflect the opinions of the authors. The authors are responsible for all content in their abstracts including accuracy of the facts, statements, citing resources, etc.

### (PB3455) REAL-WORLD HEMATOLOGIC OUTCOMES WITH MOMELOTINIB IN PATIENTS WITH MYELOFIBROSIS AND ANEMIA: A GERMAN RETROSPECTIVE CHART REVIEW

**Topic:** 16. Myeloproliferative neoplasms - Clinical

Haifa Kathrin Al-Ali*[1], Christine Schumacher[2], Martin Krammer[2], Fulya Sen Nikitas[3], Bella Vo[4], Sandra Joksaite[3], Julia Moore[4], Shiyuan Zhang[4], Yinong Liang[5], Josephine Bryar[5], Gwyneth Wei[5], Aida York[5], Wendy Cheng[5]

*[1]University Hospital Halle, Halle, Germany; [2]GSK, Munich, Germany; [3]GSK, London, United Kingdom; [4]GSK, Collegeville, PA, United States of America; [5]Berkeley Research Group, Boston, MA, United States of America;*

### Background

Myelofibrosis (MF) is a rare but debilitating myeloproliferative neoplasm, with a reported 2021 incidence of 1.2-1.8 cases per 100,000 persons in Germany. In Jan 2024, the European Commission approved the JAK1/JAK2/ACVR1 inhibitor momelotinib to treat disease-related splenomegaly or symptoms in adult patients (pts) with primary or secondary MF and moderate to severe anemia, including those who are JAK inhibitor (JAKi) naive or experienced with ruxolitinib. Early real-world evidence on momelotinib use in Germany included primarily JAKi-experienced pts treated for a short duration in academic centers.

### Aims

To conduct a broader real-world, retrospective chart review of pts treated with momelotinib in Germany using a representative, post–marketing authorization MF population.

### Methods

Retrospective data on momelotinib-treated pts with primary or secondary MF and hemoglobin (Hb) <10 g/dL were extracted by German academic and community physicians. The index date was the first prescription of momelotinib between Feb 15, 2024 (commercial availability) and Jun 30, 2025. The baseline period was ≥12 wk prior to and including the index date; the treatment period was ≥12 wk after and not including the index date until the end of data availability or death. Outcomes in this interim analysis included descriptive changes in Hb levels, platelet (PLT) counts, and red blood cell transfusion status since baseline (**Figure**); assessment time points were wk 12 and 24 post index, using the most recent laboratory value to each time point based on physician judgment (2-wk window).

**Copyright Information:** (Online) ISSN: 2572-9241
©2026 The Author(s). HemaSphere is published by John Wiley & Sons Ltd on behalf of European Hematology Association. This is an open access Abstract Book distributed under the Attribution-NonCommercial-NoDerivs (CC BY-NC-ND), which allows third parties to download the articles and share them with others as long as they credit the author and the Abstract Book, but they cannot change the content in any way or use them commercially.
**Abstract Book Citations:** Authors, Title, HemaSphere, 2026;10:(S1):pages. The abstract book DOIs can be found on the “Author Information” page.
**Disclaimer:** Articles published in the journal HemaSphere exclusively reflect the opinions of the authors. The authors are responsible for all content in their abstracts including accuracy of the facts, statements, citing resources, etc.

### Results

Charts were extracted from a total of 77 pts, including 39 JAKi naive and 38 JAKi experienced (35 [92%] prior ruxolitinib). Overall median Hb increased from 8.7 g/dL at baseline to 10.1 g/dL at wk 24. Median (IQR) Hb change from baseline to wk 24 was 1.3 (1.0-2.1) g/dL overall, 1.6 (1.1-2.1) g/dL in JAKi-naive pts, and 1.0 (0.6-2.1) g/dL in JAKi-experienced pts; 50% of evaluable JAKi-naive pts had an Hb increase of ≥2 g/dL. Results were consistent in sensitivity analyses that extended the wk 24 Hb assessment window from 2 to 6 wk to capture additional pts (n=25), as relatively few pts (n=13) had Hb values recorded within 2 wk of wk 24 due to incomplete data entry.

Overall median PLTs increased from $166.5\times10^9$/L at baseline to $191\times10^9$/L at wk 24; 41% of evaluable pts had baseline PLTs $\geq200\times10^9$/L, increasing to 49% at wk 24 (including increases from 46% to 57% of JAKi-naive and 36% to 39% of JAKi-experienced pts).

The proportion of transfusion-independent (TI) pts, defined as having no transfusions and no Hb <8 g/dL in the 12 wk prior to and including the time point of interest, also increased. While only 43 pts (56%) were TI at baseline, 71% (40/56 evaluable pts) were TI at wk 24, including 77% (24/31 evaluable pts) who were TI at baseline and maintained this status at wk 24 and 64% (16/25 evaluable pts) with baseline transfusion needs who became TI at wk 24. The proportion of TI pts increased from baseline to wk 24 in both JAKi-naive (62% to 86%) and -experienced (50% to 57%) pts.

### Summary/Conclusion

In a representative population of JAKi-naive and -experienced pts with MF in Germany, momelotinib was associated with early and sustained improvements in hematologic parameters and reduced transfusion burden, including maintenance of TI status when used earlier in the JAKi-naive setting, supporting line-agnostic use in pts with MF and anemia.

**Funding:** GSK (study #300285).

**Copyright Information:** (Online) ISSN: 2572-9241
©2026 The Author(s). HemaSphere is published by John Wiley & Sons Ltd on behalf of European Hematology Association. This is an open access Abstract Book distributed under the Attribution-NonCommercial-NoDerivs (CC BY-NC-ND), which allows third parties to download the articles and share them with others as long as they credit the author and the Abstract Book, but they cannot change the content in any way or use them commercially.
**Abstract Book Citations:** Authors, Title, HemaSphere, 2026;10:(S1):pages. The abstract book DOIs can be found on the “Author Information” page.
**Disclaimer:** Articles published in the journal HemaSphere exclusively reflect the opinions of the authors. The authors are responsible for all content in their abstracts including accuracy of the facts, statements, citing resources, etc.

**Figure.** Hematologic Parameters at Key Assessment Time Points

| **Hb level, median (IQR), g/dL** | **Baseline** | **Week 12[a]** | **Week 24[a]** |
|---|---|---|---|
| Overall (N=77) | n=74<br>8.7 (7.5-9.1) | n=69<br>9.6 (8.7-10.0) | 2 weeks: n=13<br>10.1 (9.9-10.8)<br>6 weeks: n=25<br>10.2 (9.9-11.0) |
| Naive (n=39) | n=38<br>9.0 (7.2-9.2) | n=33<br>9.7 (9.1-10.0) | 2 weeks: n=8<br>10.3 (10.0-10.6)<br>6 weeks: n=15<br>10.6 (10.1-11.2) |
| Experienced (n=38) | n=36<br>8.3 (7.8-9.0) | n=36<br>9.0 (8.4-9.9) | 2 weeks: n=5<br>10.0 (9.9-11.0)<br>6 weeks: n=10<br>9.7 (9.1-10.1) |
| **PLT count, median (IQR), $\times 10^9$/L** | **Baseline** | **Week 12[b]** | **Week 24[b]** |
| Overall (N=77) | n=66<br>166.5 (100-301) | n=59<br>170 (130-257.5) | n=39<br>191 (120-293) |
| Naive (n=39) | n=33<br>178 (120-450) | n=31<br>180 (131-267) | n=21<br>220 (117-310) |
| Experienced (n=38) | n=33<br>150 (95-250) | n=28<br>155 (118-250.5) | n=18<br>175 (125-283.5) |
| **TI, n (%)** | **Baseline** | **Week 12[b]** | **Week 24[b]** |
| Overall (N=77) | N=77<br>43 (55.8) | N=77<br>57 (74.0) | n=56<br>40 (71.4) |
| Naive (n=39) | n=39<br>24 (61.5) | n=39<br>27 (69.2) | n=28<br>24 (85.7) |
| Experienced (n=38) | n=38<br>19 (50.0) | n=38<br>30 (78.9) | n=28<br>16 (57.1) |

Sample size at each time point is based on the number of evaluable patients with data available at that time point.
Hb, hemoglobin; PLT, platelet; TI, transfusion independent.
[a] Hb analyses based on both 2-week and 6-week windows are shown at week 24, with the 6-week window increasing the number of evaluable patients. The 6-week window did not substantially increase patient numbers at week 12; therefore, data are not shown. The lower number of Hb-evaluable patients at week 24 reflects loss to follow-up, missing assessments, or incomplete data entry by physicians at the time of this interim analysis. [b] PLT values were assessed at fixed time points using the 2-week window and transfusion status was determined at each exact time point based on the prior 12 weeks; thus, sensitivity analyses based on a 6-week window were not applied to these data.

Figure. Hematologic Parameters at Key Assessment Time Points. Sample size at each time point is based on the number of evaluable patients with data available at that time point. Hb, hemoglobin; PLT, platelet; TI, transfusion independent. a Hb analyses based on both 2-week and 6-week windows are shown at week 24, with the 6-week window increasing the number of evaluable patients; the 6-week window did not substantially increase patient numbers at week 12; therefore, data are not shown. The lower number of Hb-evaluable patients at week 24 reflects loss to follow-up, missing assessments, or incomplete data entry by physicians at the time of this interim analysis. b PLT values were assessed at fixed time points using the 2-week window and transfusion status was determined at each exact time point based on the prior 12 weeks; thus, sensitivity analyses based on a 6-week window were not applied to these data.

**Copyright Information:** (Online) ISSN: 2572-9241
©2026 The Author(s). HemaSphere is published by John Wiley & Sons Ltd on behalf of European Hematology Association. This is an open access Abstract Book distributed under the Attribution-NonCommercial-NoDerivs (CC BY-NC-ND), which allows third parties to download the articles and share them with others as long as they credit the author and the Abstract Book, but they cannot change the content in any way or use them commercially.
**Abstract Book Citations:** Authors, Title, HemaSphere, 2026;10:(S1):pages. The abstract book DOIs can be found on the "Author Information" page.
**Disclaimer:** Articles published in the journal HemaSphere exclusively reflect the opinions of the authors. The authors are responsible for all content in their abstracts including accuracy of the facts, statements, citing resources, etc.

### (PB3456) LOW-DOSE HYPOMETHYLATING AGENTS IN THE TREATMENT OF CYTOPENIC CHRONIC MYELOPROLIFERATIVE NEOPLASMS: A RETROSPECTIVE ANALYSIS FROM A SINGLE CENTER

**Topic:** 16. Myeloproliferative neoplasms - Clinical

Tingting Cui*[1], Minghui Duan[2]

*[1]Beijing No.6 Hospital, Hematology, Beijing, China; [2]Peking Union Medical College Hospital, Beijing, China;*

### Background

Chronic myeloproliferative neoplasms (MPNs) including myelofibrosis (MF) and related disorders, are frequently complicated by significant cytopenias (anemia and/or thrombocytopenia) during their disease course. These cytopenias often limit the use of conventional therapies like JAK inhibitors or immunomodulatory drugs, leading to a poor prognosis and representing a major unmet clinical need. Hypomethylating agents (HMAs) have shown efficacy in higher-risk myeloid neoplasms, but their role and optimal dosing in cytopenic MPNs are not well defined.

### Aims

To evaluate the efficacy and safety of low-dose HMAs (Azacitidine or Decitabine) in patients with chronic MPNs presenting with cytopenias, and to analyze the impact of genetic mutations on treatment response.

### Methods

We conducted a retrospective analysis of patients diagnosed with chronic MPNs at Peking Union Medical College Hospital between July 2015 and July 2025. Inclusion criteria were: 1) Confirmed diagnosis of MPN, and 2) Cytopenia defined as hemoglobin (Hb) <100 g/L and/or platelet count <100×10⁹/L. Patients received low-dose HMA therapy: Azacitidine at a starting dose of 200 mg/m²/d for 7 days per 28-day cycle (dose adjusted based on tolerance, max 700 mg/m²/cycle), or fixed-dose Decitabine (50 mg once weekly). The overall response rate (ORR) was defined as achieving either hematological improvement (HI) according to IWG-2018 criteria, or a survival benefit (stable disease, SD, lasting >14 months). Genetic profiling for driver and non-driver mutations was performed.

### Results

A total of 39 patients were included (61.6% male; median age 57 years [range, 22-77]). Among them, 32 patients (82.1%) had a prior history of ruxolitinib treatment, and 29 patients (74.4%) received combination therapy containing thalidomide.Driver mutation distribution: JAK2V617F (56.4%), CALR (20.4%), MPL (5.0%), and triple-negative (17.9%). According to MIPSS-70+ Version 2.0, the cohort was predominantly high-risk (64.7%) or very high-risk (30.0%). DIPSS-Plus scoring classified 61.5% as Int-2 and 35.8% as high-risk.The ORR was **66.7% (26/39)** . Among responders:13 patients (50.0%) achieved platelet response.1 patient (3.9%) achieved erythroid response (who also had a survival benefit).13 patients (50.0%) achieved a survival benefit (SD >14 months).5 patients (19.2%) achieved both platelet response and survival benefit.Next-generation sequencing revealed that 84.6% of patients harbored at least one non-driver mutation. The most frequently mutated genes were ASXL1 (51.3%) , SRSF2 (25.6%), and TET2 (20.5%).

**Copyright Information:** (Online) ISSN: 2572-9241
©2026 The Author(s). HemaSphere is published by John Wiley & Sons Ltd on behalf of European Hematology Association. This is an open access Abstract Book distributed under the Attribution-NonCommercial-NoDerivs (CC BY-NC-ND), which allows third parties to download the articles and share them with others as long as they credit the author and the Abstract Book, but they cannot change the content in any way or use them commercially.
**Abstract Book Citations:** Authors, Title, HemaSphere, 2026;10:(S1):pages. The abstract book DOIs can be found on the "Author Information" page.
**Disclaimer:** Articles published in the journal HemaSphere exclusively reflect the opinions of the authors. The authors are responsible for all content in their abstracts including accuracy of the facts, statements, citing resources, etc.

**Summary/Conclusion**

Low-dose hypomethylating agents demonstrate promising efficacy in the difficult-to-treat population of cytopenic MPN patients, achieving an overall response rate of 66.7%. Responses were primarily driven by platelet improvement and disease stabilization. This regimen offers a valuable treatment option, particularly in this high-risk cohort with high prevalence of adverse mutations like ASXL1. Further prospective studies are warranted to validate these findings and optimize patient selection.

**Copyright Information:** (Online) ISSN: 2572-9241
©2026 The Author(s). HemaSphere is published by John Wiley & Sons Ltd on behalf of European Hematology Association. This is an open access Abstract Book distributed under the Attribution-NonCommercial-NoDerivs (CC BY-NC-ND), which allows third parties to download the articles and share them with others as long as they credit the author and the Abstract Book, but they cannot change the content in any way or use them commercially.
**Abstract Book Citations:** Authors, Title, HemaSphere, 2026;10:(S1):pages. The abstract book DOIs can be found on the “Author Information” page.
**Disclaimer:** Articles published in the journal HemaSphere exclusively reflect the opinions of the authors. The authors are responsible for all content in their abstracts including accuracy of the facts, statements, citing resources, etc.

### (PB3457) NEUTROPHIL-TO-LYMPHOCYTE (NLR) AND PLATELET-TO-LYMPHOCYTE RATIOS (PLR) DO NOT CORRELATE WITH SYMPTOM BURDEN IN PATIENTS WITH MYELOPROLIFERATIVE NEOPLASMS

**Topic:** 16. Myeloproliferative neoplasms - Clinical

Ivan Krecak*[1, 2], Marko Lucijanic[3, 4], Hrvoje Holik[5]

*[1]General Hospital of Sibenik-Knin County, Sibenik, Croatia;[2]Faculty of medicine Rijeka, Rijeka, Croatia;[3]University Hospital Dubrava, Zagreb, Croatia; [4]School of medicine Zagreb, Zagreb, Croatia;[5]Dr. Josip Bencevic Hospital, Slavonski Brod, Croatia;*

### Background

Patients with BCR::ABL1-negative myeloproliferative neoplasms (MPNs), essential thrombocythemia (ET), polycythemia vera (PV) and myelofibrosis (MF), suffer from debilitating disease-related symptoms. High neutrophil-to-lymphocyte ratio (NLR) and platelet-to-lymphocyte ratios (PLR) have been associated with inferior outcomes in MPNs. Moreover, normalization of NLR during interferon treatment correlated with a decrease in *JAK2* variant allele fraction as well as with improved clinical outcomes.

### Aims

To analyze whether NLR and PLR may also be associated with MPN-related symptoms.

### Methods

This prospective study was conducted at two hematology centers in Croatia and included patients with MPNs whose diagnosis was reassessed according to 2022 WHO criteria in the period between 2021-2023. After providing consent, participants fulfilled the Myeloproliferative Neoplasm Symptom Assessment Form (MPN-SAF) and had their blood samples taken at the time of study entry. Statistics were performed with MedCalc Statistical Software (v. 23.4.8). Continuous variables between two groups were compared using the Mann-Whitney U test and Spearman correlation was used to analyze associations between two continuous variables. Multiple regression was used to assess associations between different variables and the total symptom score (TSS).

### Results

A total of 107 MPN patients (ET=44, PV=35, MF=28) were included; median age was 68 years (35-90), 54.2% were females, median TSS was 16 (range 0-50), median NLR 3.4 (range 1.1-14.7), median PLR 270 (range 40-994), 81 patients used cytoreduction (hydroxyurea=73) and 79 (73.8%) had at least one cardiovascular risk factor (CVRF). There were no significant differences in NLR, PLR and TSS among different MPN subtypes (p>0.050 for all analyses).
As a continuous variable, NLR did not correlate with any of the symptoms assessed with the MPN-SAF in the overall cohort, nor when stratified according to the use of cytoreduction (p>0.050 for all analyses). On the other hand, higher PLR weakly correlated with higher scores of concentration problems (rho=0.271, p=0.013) and night sweats (rho=0.277, p=0.011) in the overall cohort, as well as with concentration problems (rho=0.288, p=0.022) and bone pains (rho=0.297, p=0.011) in the cytoreductive treatment group, and moderately with fatigue (rho=0.505, p=0.023) in the untreated group. Specific patient symptoms were then additionally stratified according to median NLR (3.4) and PLR (270) value. As shown in Figure 1, there were no statistically significant associations of higher NLR and PLR with any of the disease symptoms as measured with the MPN-SAF (p>0.050 for all analyses). Finally, in the multiple regression analysis, neither higher NLR (p=0.198) nor PLR (p=0.855) were associated with higher TSS when being adjusted for female sex (t=2.84, p=0.005), cytoreductive treatment (t =-1.91, p=0.050), advanced age (p=0.137), MPN subtype (p=0.832), or the presence of at least one CVRF (p=0.130).

**Copyright Information:** (Online) ISSN: 2572-9241
©2026 The Author(s). HemaSphere is published by John Wiley & Sons Ltd on behalf of European Hematology Association. This is an open access Abstract Book distributed under the Attribution-NonCommercial-NoDerivs (CC BY-NC-ND), which allows third parties to download the articles and share them with others as long as they credit the author and the Abstract Book, but they cannot change the content in any way or use them commercially.
**Abstract Book Citations:** Authors, Title, HemaSphere, 2026;10:(S1):pages. The abstract book DOIs can be found on the “Author Information” page.
**Disclaimer:** Articles published in the journal HemaSphere exclusively reflect the opinions of the authors. The authors are responsible for all content in their abstracts including accuracy of the facts, statements, citing resources, etc.

**Summary/Conclusion**

Despite the fact that reduction of NLR has been shown to correlate with improved clinical outcomes in MPNs, this study signals that it may not be accompanied by amelioration of symptom burden. This observation may be relevant, especially when considering that NLR is being increasingly proposed as a surrogate biomarker of treatment efficacy. Prospective studies are needed to assess whether longitudinal NLR/PLR changes during treatment with different cytoreductive medications are associated with symptom burden in MPNs.

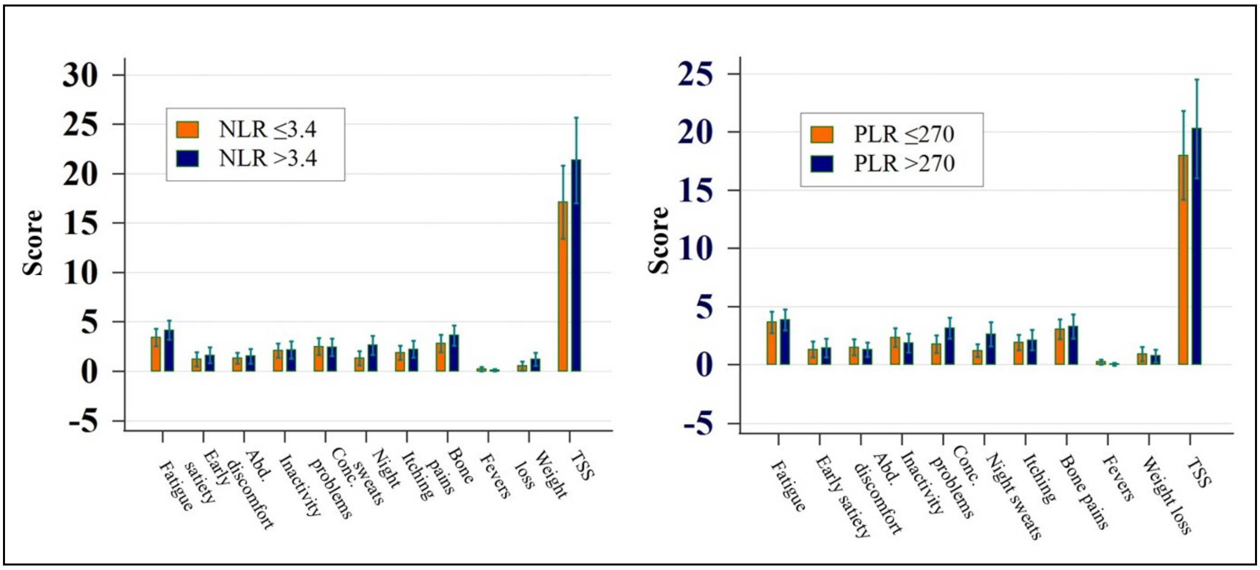

**Copyright Information:** (Online) ISSN: 2572-9241
©2026 The Author(s). HemaSphere is published by John Wiley & Sons Ltd on behalf of European Hematology Association. This is an open access Abstract Book distributed under the Attribution-NonCommercial-NoDerivs (CC BY-NC-ND), which allows third parties to download the articles and share them with others as long as they credit the author and the Abstract Book, but they cannot change the content in any way or use them commercially.
**Abstract Book Citations:** Authors, Title, HemaSphere, 2026;10:(S1):pages. The abstract book DOIs can be found on the "Author Information" page.
**Disclaimer:** Articles published in the journal HemaSphere exclusively reflect the opinions of the authors. The authors are responsible for all content in their abstracts including accuracy of the facts, statements, citing resources, etc.

### (PB3458) CLINICAL CHARACTERISTICS, TREATMENT OUTCOMES, AND GENETIC PROFILING OF LANGERHANS CELL HISTIOCYTOSIS: A SINGLE-CENTER RETROSPECTIVE STUDY OF 202 PEDIATRIC PATIENTS

**Topic:** 16. Myeloproliferative neoplasms - Clinical

Qihong Lin*[1], Shilin Liu[1]

[1] *Shenzhen Children's Hospital, SHEN ZHEN, China;*

**Background**

Langerhans cell histiocytosis (LCH) is a rare clonal myeloid disorder characterized by heterogeneous clinical manifestations and variable prognosis in children. Comprehensive studies detailing its clinical spectrum, therapeutic efficacy, and genetic correlates, especially in high-risk multi-organ disease, are still needed.

**Aims**

To evaluate the clinical characteristics, treatment outcomes, and genetic profiles of pediatric LCH patients managed between January 2019 and October 2025.

**Methods**

A retrospective review was conducted of 202 pediatric LCH patients (male: n=137, female: n=65). Data included demographics, genetic mutations (BRAF V600E, MAP2K1, others), disease extent (classified per SS/MS-RO± and CNS-risk criteria), treatment modalities (chemotherapy and/or targeted therapy), response (assessed as AD-B/AD-S/AD-I/AD-W or NAD), and long-term outcomes.

**Results**

Median age at diagnosis was 3.1 years (range: 1 month–13 years). Genetic analysis in 168 patients identified BRAF V600E in 78 (46.4%), MAP2K1 in 32 (19.0%), and other mutations in 28 (16.7%). Multi-system disease with risk-organ involvement (MS-RO+) was noted in 84 patients (41.6%). First-line vincristine/prednisone-based therapy in 178 patients yielded complete/partial response (AD-B/AD-S) in 134 (75.3%). Targeted therapy (dabrafenib/trametinib) was administered to 56 patients, primarily those with BRAF V600E or MAP2K1 mutations or refractory disease. Disease progression or suboptimal response prompted second-line therapy (cytarabine, cladribine, clofarabine, or FCN-159) in 64 patients. Relapse occurred in 42 patients (20.8%) at a median of 2.1 years. At last follow-up, no one died and 146 patients (72.3%) remained in sustained remission (NAD).

**Summary/Conclusion**

This large pediatric LCH cohort demonstrates diverse clinical presentation and underscores the prognostic and therapeutic relevance of genetic profiling. Risk-stratified and mutation-directed treatment strategies enable favorable long-term remission in most patients, supporting the integration of early molecular testing into routine clinical management to optimize outcomes.

**Copyright Information:** (Online) ISSN: 2572-9241
©2026 The Author(s). HemaSphere is published by John Wiley & Sons Ltd on behalf of European Hematology Association. This is an open access Abstract Book distributed under the Attribution-NonCommercial-NoDerivs (CC BY-NC-ND), which allows third parties to download the articles and share them with others as long as they credit the author and the Abstract Book, but they cannot change the content in any way or use them commercially.
**Abstract Book Citations:** Authors, Title, HemaSphere, 2026;10:(S1):pages. The abstract book DOIs can be found on the "Author Information" page.
**Disclaimer:** Articles published in the journal HemaSphere exclusively reflect the opinions of the authors. The authors are responsible for all content in their abstracts including accuracy of the facts, statements, citing resources, etc.

### (PB3459) BCR::ABL1 NEGATIVE MYELOPROLIFERATIVE NEOPLASMS REPRESENT A DISTINCT ENTITY IN THE PAEDIATRIC AND YOUNG ADULT POPULATION – A MULTI-CENTRE RETROSPECTIVE STUDY

**Topic:** 16. Myeloproliferative neoplasms - Clinical

Abbie Wilson*[1, 2], Katherine Stark[3], Victoria Campbell[4], Anthony Bench[4], Claire McKeeve[5], Gillian Horne[1, 2]

*[1]Beatson West of Scotland Cancer Centre, Glasgow, United Kingdom;[2]Paul O'Gorman Leukaemia Research Centre, Glasgow, United Kingdom; [3]Royal Infirmary of Edinburgh, Edinburgh, United Kingdom;[4]Western General Hospital, Edinburgh, United Kingdom; [5]West of Scotland Genomics Centre, Glasgow, United Kingdom;*

### Background

*BCR::ABL1* negative myeloproliferative neoplasms (MPNs) are traditionally regarded as disorders of older adults. However, up to 27% of cases occur in children and young people. Initial studies have suggested that MPNs may be under-diagnosed in younger people due to increased incidence of 'triple-negative' disease compared to adults. Small studies indicate potential biological and clinical differences in this population, but robust evidence remains limited.

### Aims

To characterise the molecular landscape, clinical phenotype and treatment indications of MPNs in the paediatric and young adult population.

### Methods

We conducted a mult-centre retrospective study of patients aged 0-30 years undergoing *JAK2*, *MPL*, or *CALR* testing between 2019-2024 at two of the largest genomics testing centres in Scotland. Clinical records were screened to identify those with diagnoses of confirmed MPN. Details collected included basic demographics, genetic testing, pathology reports and access to specialist services. All data were anonymised and analysed centrally.

### Results

Forty-four children and young adults were included in our study, of which 79.5% had Essential Thrombocythaemia (ET). Median age was 24 years (range 10-30 years). Half of patients were JAK2 positive and around 30% were 'triple negative'. Of those recorded, 28% of patients had splenomegaly at presentation. Next-generation sequencing was utilised in 8 patients (18.2%) however none were reported to have clinically significant variants. 21.4% of patients required cytoreductive treatment, most commonly with Pegylated-interferon. Although only around 10% of patients experienced an arterial or venous thrombosis, those reported were clinically significant in nature. Long-term aspirin monotherapy represented the predominant thromboprophylaxis strategy.

### Summary/Conclusion

These findings support growing evidence that MPNs in children and young people represent a distinct clinical and molecular subgroup characterised by higher rates of triple-negative disease and unique management considerations. Ongoing expansion of this cohort to include patients aged 30-40 years will enable further evaluation of age-related molecular variants, including *ASXL1*, and assessment of disease implications in more women of child-bearing age. The expanded cohort (>120 patients aged less than 40 years) will represent one of the largest Scottish datasets examining young adults with MPNs.

**Copyright Information:** (Online) ISSN: 2572-9241
©2026 The Author(s). HemaSphere is published by John Wiley & Sons Ltd on behalf of European Hematology Association. This is an open access Abstract Book distributed under the Attribution-NonCommercial-NoDerivs (CC BY-NC-ND), which allows third parties to download the articles and share them with others as long as they credit the author and the Abstract Book, but they cannot change the content in any way or use them commercially.
**Abstract Book Citations:** Authors, Title, HemaSphere, 2026;10:(S1):pages. The abstract book DOIs can be found on the "Author Information" page.
**Disclaimer:** Articles published in the journal HemaSphere exclusively reflect the opinions of the authors. The authors are responsible for all content in their abstracts including accuracy of the facts, statements, citing resources, etc.

### (PB3460) UNDERSTANDING DISEASE BURDEN AND LIVED EXPERIENCES IN PATIENTS WITH POLYCYTHAEMIA VERA BY CONDUCTING A REAL-WORLD SOCIAL MEDIA LISTENING STUDY ACROSS EUROPE

**Topic:** 16. Myeloproliferative neoplasms - Clinical

Alesia Khan*[1], Susanne Isfort[2], Darren Callanan[3], William Jones[3], Claudia Serra Monraba[4], Maricruz Linares[5], Peter Loffelhardt[6], Jonathan Mathias[7]

*[1]Leeds Teaching Hospitals NHS Trust, Haematological Malignancy Diagnostic Service, Leeds, United Kingdom;[2]Hannover Medical School, 2Haematology, Haemostasis, Oncology and Stem Cell Transplantation Comprehensive Cancer Centre Lorraine (CCC-N), Hannover, Germany; [3]Partners4Access, London, United Kingdom; [4]AOP Orphan Pharmaceuticals, Vienna, Austria; [5]Odd Blood Club and Collaborator MPN Global, Madrid, Spain;[6]Global MPN Scientific Foundation, Barcelona, Spain;[7]MPN Voice, London, United Kingdom;*

### Background

Polycythaemia vera (PV) is a rare, chronic myeloproliferative neoplasm that carries a substantial physical and emotional burden, significantly impacting patients' quality of life. Despite available therapies used to control the haematological parameters and reduce complications of the disease, the literature highlights that patients still face a high disease burden, often driven by the symptoms of the disease. A novel social listening method was used to capture real-world perspectives on the burden associated with PV.

### Aims

To capture real-world perspectives on the burden associated with PV by conducting a social listening study across Europe.

### Methods

Publicly available posts mentioning PV were retrospectively collected from over 100 million social media channels across 14 European countries between January 2019 and June 2025. Mentions were filtered by relevance and source, and categorised via a natural language model.

### Results

In total, 48,128 relevant mentions were identified, filtered from 3.6 million PV mentions. Using natural language model classification, mentions were categorised into three groups: disease burden (52.0%), therapy mention (38.2%), and treatment burden (9.7%). Among posts categorised as discussing disease burden, fatigue and sleep-related burdens were among the most frequently cited (18.6%), followed by skin (13.1%), pain and musculoskeletal (11.5%), and psychological (11.1%) symptoms (Figure 1a) Among the skin symptom burden category, pruritus was the most frequently mentioned, accounting for 51.4% (n=170) of all mentions associated with “skin” . Bruising and dryness were the next most frequently reported concerns, together representing over 20% (n=71) of mentions. Mentions of skin cancer accounted for 4.5% (n=15) of all skin-related discussions (Figure 1b).
In our investigation, PV appears to show a ~2.6-fold increase in relevant social media conversations compared with metastatic breast cancer. This disparity may occur due to PV being rare and with low awareness, which may contribute to the observed increase in patient engagement with online communities for support.
High negative disease burden reporting (34.1% vs 7.4% positive) and psychological distress may be related to a disconnect between patients and healthcare professionals (HCPs). Patients report inadequate control of fatigue, pruritus, and other symptoms, while studies show HCPs often underestimate symptom severity. Indicating a degree of misalignment between some patients and clinicians in treatment priorities.

**Copyright Information:** (Online) ISSN: 2572-9241
©2026 The Author(s). HemaSphere is published by John Wiley & Sons Ltd on behalf of European Hematology Association. This is an open access Abstract Book distributed under the Attribution-NonCommercial-NoDerivs (CC BY-NC-ND), which allows third parties to download the articles and share them with others as long as they credit the author and the Abstract Book, but they cannot change the content in any way or use them commercially.
**Abstract Book Citations:** Authors, Title, HemaSphere, 2026;10:(S1):pages. The abstract book DOIs can be found on the “Author Information” page.
**Disclaimer:** Articles published in the journal HemaSphere exclusively reflect the opinions of the authors. The authors are responsible for all content in their abstracts including accuracy of the facts, statements, citing resources, etc.

### Summary/Conclusion

This study, with novel real-world methodology, highlights areas of unmet need where current disease management strategies may inadequately address the burden experienced by patients. Narratives depict an important disconnect between the clinicians' priorities in treating the underlying disease pathophysiology and the burden experienced by patients.
Strategies to address these challenges span clinical development, market access and clinical practice. Key strategies include mandating the inclusion of the patient voice in clinical trial design and health technology appraisals, facilitating the expansion of treatment options, and increasing the resources (e.g., educational material) available to clinicians treating patients with PV.

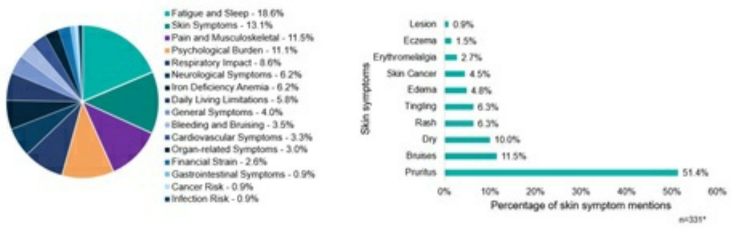

Figure 1a and b

**Copyright Information:** (Online) ISSN: 2572-9241
©2026 The Author(s). HemaSphere is published by John Wiley & Sons Ltd on behalf of European Hematology Association. This is an open access Abstract Book distributed under the Attribution-NonCommercial-NoDerivs (CC BY-NC-ND), which allows third parties to download the articles and share them with others as long as they credit the author and the Abstract Book, but they cannot change the content in any way or use them commercially.
**Abstract Book Citations:** Authors, Title, HemaSphere, 2026;10:(S1):pages. The abstract book DOIs can be found on the "Author Information" page.
**Disclaimer:** Articles published in the journal HemaSphere exclusively reflect the opinions of the authors. The authors are responsible for all content in their abstracts including accuracy of the facts, statements, citing resources, etc.

### (PB3461) THROMBOTIC RISK AND PREDICTORS OF PROGRESSION TO OVERT MYELOFIBROSIS IN PRE-FIBROTIC MYELOFIBROSIS: A MULTICENTER PROSPECTIVE COHORT STUDY

**Topic:** 16. Myeloproliferative neoplasms - Clinical

Novella Pugliese*[1], Davide Pio Abagnale[1], Maria Di Perna[2], Laura De Fazio[3], Francesco Mendicino[4], Fabrizio Pane[1]

*[1]University of Naples Federico II, Napoli, Italy;[2]Hospital "Andrea Tortora", Pagani, Italy; [3]Azienda Universitaria Ospedaliera Renato Dulbecco, Catanzaro, Italy; [4]Azienda Ospedaliera "Mariano Santo" di Cosenza, Cosenza, Italy;*

### Background

Pre-fibrotic myelofibrosis (pre-MF) is a heterogeneous myeloproliferative neoplasm characterized by variable thrombotic risk and potential progression to overt myelofibrosis (MF). Risk stratification tools are largely derived from overt MF or essential thrombocythemia settings and may not fully capture disease-specific prognostic features in pre-MF.

### Aims

To evaluate thrombotic risk, identify predictors of fibrotic progression, and assess the impact of therapeutic strategies in a prospective cohort of patients with primary and secondary pre-MF.

### Methods

We conducted a multicenter prospective cohort study including 80 adult patients diagnosed with primary or secondary (post-PV/post-ET) pre-fibrotic MF according to 2022 WHO criteria across four Italian hematology centers. Clinical, hematologic, and molecular data were collected at diagnosis and during follow-up (up to 60 months). Outcomes included overall survival (OS), thrombosis-free survival (TFS), myelofibrosis-free survival (MFS), thrombosis incidence (TI), and MF incidence (MI). Survival analyses were performed using the Kaplan–Meier method and Cox regression models.

### Results

Median age at diagnosis was 62 years; 66% harbored JAK2V617F mutation. Median follow-up was 3.36 years. Five-year OS was 96.8%.
Fourteen patients (18%) had a history of thrombosis at baseline, and three new thrombotic events were observed during follow-up. The 5-year TFS was 80.9%, reflecting both prior and incident events. The IPSET score significantly stratified TFS (p=0.02), with 5-year TFS rates of 100% in very low/low-risk, 90% in intermediate-risk, and 72% in high-risk patients. In multivariate analysis, spleen size independently predicted worse TFS (HR 1.2, 95% CI 1.03–1.39, p=0.017). A spleen size ≥14.5 cm identified patients with significantly inferior 5-year TFS (62% vs 87%, p=0.008).
Eight patients (10%) progressed to overt MF. Five-year MFS was 85.8%. IPSS did not significantly predict progression. In univariate analysis, higher hemoglobin levels were associated with improved MFS (HR 0.62, p=0.033). A hemoglobin cut-off of 11.2 g/dL effectively stratified risk, with 5-year MFS of 54% in patients below this threshold versus 97% in those ≥11.2 g/dL (p=0.00034).
Treatment (hydroxyurea, peg-interferon, or ruxolitinib) did not significantly impact TFS or MFS. Ruxolitinib was mainly used in patients with more advanced phenotypic features and was well tolerated.

**Copyright Information:** (Online) ISSN: 2572-9241
©2026 The Author(s). HemaSphere is published by John Wiley & Sons Ltd on behalf of European Hematology Association. This is an open access Abstract Book distributed under the Attribution-NonCommercial-NoDerivs (CC BY-NC-ND), which allows third parties to download the articles and share them with others as long as they credit the author and the Abstract Book, but they cannot change the content in any way or use them commercially.
**Abstract Book Citations:** Authors, Title, HemaSphere, 2026;10:(S1):pages. The abstract book DOIs can be found on the "Author Information" page.
**Disclaimer:** Articles published in the journal HemaSphere exclusively reflect the opinions of the authors. The authors are responsible for all content in their abstracts including accuracy of the facts, statements, citing resources, etc.

**Summary/Conclusion**

Pre-fibrotic MF shows favorable overall survival but non-negligible thrombotic and progression risks. IPSET remains a valid tool for thrombotic risk stratification, while spleen size emerges as an independent predictor of thrombosis. Hemoglobin ≥11.2 g/dL identifies patients with lower risk of fibrotic evolution, suggesting the need for recalibration of prognostic thresholds in pre-PMF. A disease-specific prognostic model integrating clinical and molecular features may improve patient stratification and guide earlier therapeutic intervention.

**Copyright Information:** (Online) ISSN: 2572-9241
©2026 The Author(s). HemaSphere is published by John Wiley & Sons Ltd on behalf of European Hematology Association. This is an open access Abstract Book distributed under the Attribution-NonCommercial-NoDerivs (CC BY-NC-ND), which allows third parties to download the articles and share them with others as long as they credit the author and the Abstract Book, but they cannot change the content in any way or use them commercially.
**Abstract Book Citations:** Authors, Title, HemaSphere, 2026;10:(S1):pages. The abstract book DOIs can be found on the “Author Information” page.
**Disclaimer:** Articles published in the journal HemaSphere exclusively reflect the opinions of the authors. The authors are responsible for all content in their abstracts including accuracy of the facts, statements, citing resources, etc.

### (PB3462) HAPLOIDENTICAL HEMATOPOIETIC STEM CELL TRANSPLANTATION FOR PATIENTS WITH MYELOFIBROSIS: A CHINESE MULTICENTER RETROSPECTIVE STUDY

**Topic:** 16. Myeloproliferative neoplasms - Clinical

Yuqian Sun*[1], Jun Zhu[2], Jia Chen[3], Erlie Jiang[4], Borui Tang[1], Xinchuan Chen[5], Yanmin Zhao[6], Wei Shi[7], Xi Zhang[8], Fang Zhou[9], Ming-Feng Zhao[10], Jinsong Yan[11], Yehui Tan[12], Shuangnian Xu[13], Guanchen Bai[14], Cao Weijie[15], Yang Cao[16], Xinhong Fei[17], Jian Zhou[18], Sanbin Wang[19], Peng Zhao[20], Kourong Miao[21], Ying Lu[22], Li Ding[23], Zhiling Yan[24], Liping Dou[25], Mei Lan[26], Shunqing Wang[27], Zhiguo Wang[28], Hai Yi[29, 29], Ming Jiang[30], Xiaosheng Fang[31], Hao Zhang[32], Xiao-Jun Huang[1]

*[1]Institute of Hematology, Peking University, Beijing, China;[2]Shanghai Zhaxin Hospital, Shanghai, China;[3]The First Affiliated Hospital of Soochow University, Suzhou, China; [4]Institute of Hematology & Blood Diseases Hospital, Chinese Academy of Medical Sciences, Tianjin, China; [5]West China Hospital, Sichuan University, chengdu, China;[6]the First Affiliated Hospital, Zhejiang University, Hangzhou, China; [7]Union Hospital, Tongji Medical College, Huazhong University of Science and Technology, wuhan, China; [8]Chongqing Xinqiao Hospital, chongqing, China; [9]The 960th Hospital of The People's Liberation Army (PLA) Joint Logistics Support Force, jinan, China; [10]Tianjin First Central Hospital, tianjin, China; [11]the Second Hospital of Dalian Medical University, dalian, China; [12]Department of Hematology,The First Hospital of Jilin University, changchun, China;[13]Southwest Hospital, Third Military Medical University (Army Medical University), chongqing, China; [14]Tai'an Central Hospital, tai'an, China; [15]The First Affiliated Hospital of Zhengzhou University, zhengzhou, China;[16]Tongji Hospital, Tongji Medical College, Huazhong University of Science and Technology, wuhan, China; [17]Aerospace Center Hospital, beijing, China; [18]The Affiliated Cancer Hospital of Zhengzhou University & Henan Cancer Hospital, zhengzhou, China; [19]920th Hospital of Joint Logistics Support Force of People's Liberation Army of China, Kunming, China; [20]Affiliated Hospital of Guizhou Medical University, guiyang, China; [21]Jiangsu Province Hospital, nanjing, China;[22]Ningbo University Affiliated People's Hospital, ningbo, China;[23]Air Force Medical Center, beijing, China; [24]The Affiliated Hospital of Xuzhou Medical University, xuzhou, China;[25]Department of Hematology, the First Medical Center of Chinese PLA General Hospital, beijing, China; [26]The People's Hospital of Guangxi Zhuang Autonomous Region,, nanning, China; [27]Guangzhou First People's Hospital, guangzhou, China;[28]Institute of Hematology, Harbin Medical University, harbin, China; [29]The General Hospital of Western Theater Command, chengdu, China;[30]The First Affiliated Hospital of Xinjiang Medical University, Urumchi, China; [31]Shandong Provincial Hospital, jinan, China; [32]Affiliated Hospital of Jining Medical College, jining, China;*

### Background

Despite the emergence of Janus kinase (JAK) inhibitors, allogeneic hematopoietic stem cell transplantation (allo-HSCT) remains the only curative treatment for myelofibrosis (MF). Haploidentical HSCT (Haplo-HSCT) expands access to transplantation for patients lacking an HLA-matched sibling donor or matched unrelated donor. However, data on Haplo-HSCT for MF are currently scarce.

### Aims

This study aimed to evaluate the real-world outcomes and risk factors of Haplo-HSCT in the treatment of MF in China.

**Copyright Information:** (Online) ISSN: 2572-9241
©2026 The Author(s). HemaSphere is published by John Wiley & Sons Ltd on behalf of European Hematology Association. This is an open access Abstract Book distributed under the Attribution-NonCommercial-NoDerivs (CC BY-NC-ND), which allows third parties to download the articles and share them with others as long as they credit the author and the Abstract Book, but they cannot change the content in any way or use them commercially.
**Abstract Book Citations:** Authors, Title, HemaSphere, 2026;10:(S1):pages. The abstract book DOIs can be found on the “Author Information” page.
**Disclaimer:** Articles published in the journal HemaSphere exclusively reflect the opinions of the authors. The authors are responsible for all content in their abstracts including accuracy of the facts, statements, citing resources, etc.

### Methods

This study retrospectively analyzed clinical data of 127 MF patients who underwent haplo-HSCT at 32 hematology centers in China between December 2008 and December 2024. Kaplan-Meier survival analysis and competing risk models were used to calculate the 1-year and 3-year overall survival (OS), disease-free survival (DFS), cumulative incidence of relapse (CIR), and non-relapse mortality (NRM) rates post-transplantation. Competing risk models were also applied to estimate the incidence of graft failure (GF) and engraftment. Competing risk regression analysis was performed to identify factors associated with GF. The adjusted probabilities of OS and DFS were estimated using the Cox proportional hazards model, with adjustment for other significant clinical variables included in the final multivariate models.

### Results

Among the 127 patients, 83 were diagnosed with primary myelofibrosis (PMF), 16 with post-essential thrombocythemia/polycythemia vera MF (post-ET/PV MF), and 28 with MF transformed into acute myeloid leukemia (MF-AML). The last follow-up date was December 31, 2024. The neutrophil engraftment rate at day 28 was 92.9% and the platelet engraftment rate at day 100 was 62.5%. The 60-day good graft function (GGF) rate was 62.5%. Graft failure occurred in 17 patients (14 primary, 3 secondary), corresponding to an incidence of 13.2%. Splenomegaly negatively affected GGF rates (non-massive splenomegaly: 71.4%, massive splenomegaly: 44.1%; P = 0.008) and increased the incidence of GF (non-massive splenomegaly: 8.8%, massive splenomegaly: 21.8%, P = 0.038). The incidences of Epstein-Barr virus (EBV) infection and cytomegalovirus (CMV) infection were 25.2% and 20.5%, respectively. The incidences of acute graft-versus-host disease (GVHD, grades II–IV) and chronic GVHD (moderate to severe) were 44% and 25%, respectively.
With a median follow-up duration of 830 days, the 3-year OS and DFS rates were 60.3% and 56.7%, respectively, with a CIR of 8.45% and an NRM of 34.3%.  In univariate analysis, only massive splenomegaly was associated with OS (hazard ratio [HR] = 2.924; 95% confidence interval [CI], 0.993–8.605; P = 0.051) and DFS (hazard ratio [HR] = 2.431; 95% confidence interval [CI], 0.893–6.617; P = 0.082). Pre-transplant white blood cell count, hemoglobin level, platelet count, MF grading, Hematopoietic Cell Transplantation-Comorbidity Index (HCT-CI), donor-specific antibodies, graft mononuclear cell count, and CD34+ cell count were not associated with survival, engraftment, GVHD, or infection.

### Summary/Conclusion

Haploidentical HSCT (Haplo-HSCT) is a feasible curative option for patients with MF. However, the relatively high incidences of NRM and GF compromise the efficacy of Haplo-HSCT, particularly in patients with massive splenomegaly. These findings highlight the importance of pre-transplantation management.

**Copyright Information:** (Online) ISSN: 2572-9241
©2026 The Author(s). HemaSphere is published by John Wiley & Sons Ltd on behalf of European Hematology Association. This is an open access Abstract Book distributed under the Attribution-NonCommercial-NoDerivs (CC BY-NC-ND), which allows third parties to download the articles and share them with others as long as they credit the author and the Abstract Book, but they cannot change the content in any way or use them commercially.
**Abstract Book Citations:** Authors, Title, HemaSphere, 2026;10:(S1):pages. The abstract book DOIs can be found on the “Author Information” page.
**Disclaimer:** Articles published in the journal HemaSphere exclusively reflect the opinions of the authors. The authors are responsible for all content in their abstracts including accuracy of the facts, statements, citing resources, etc.

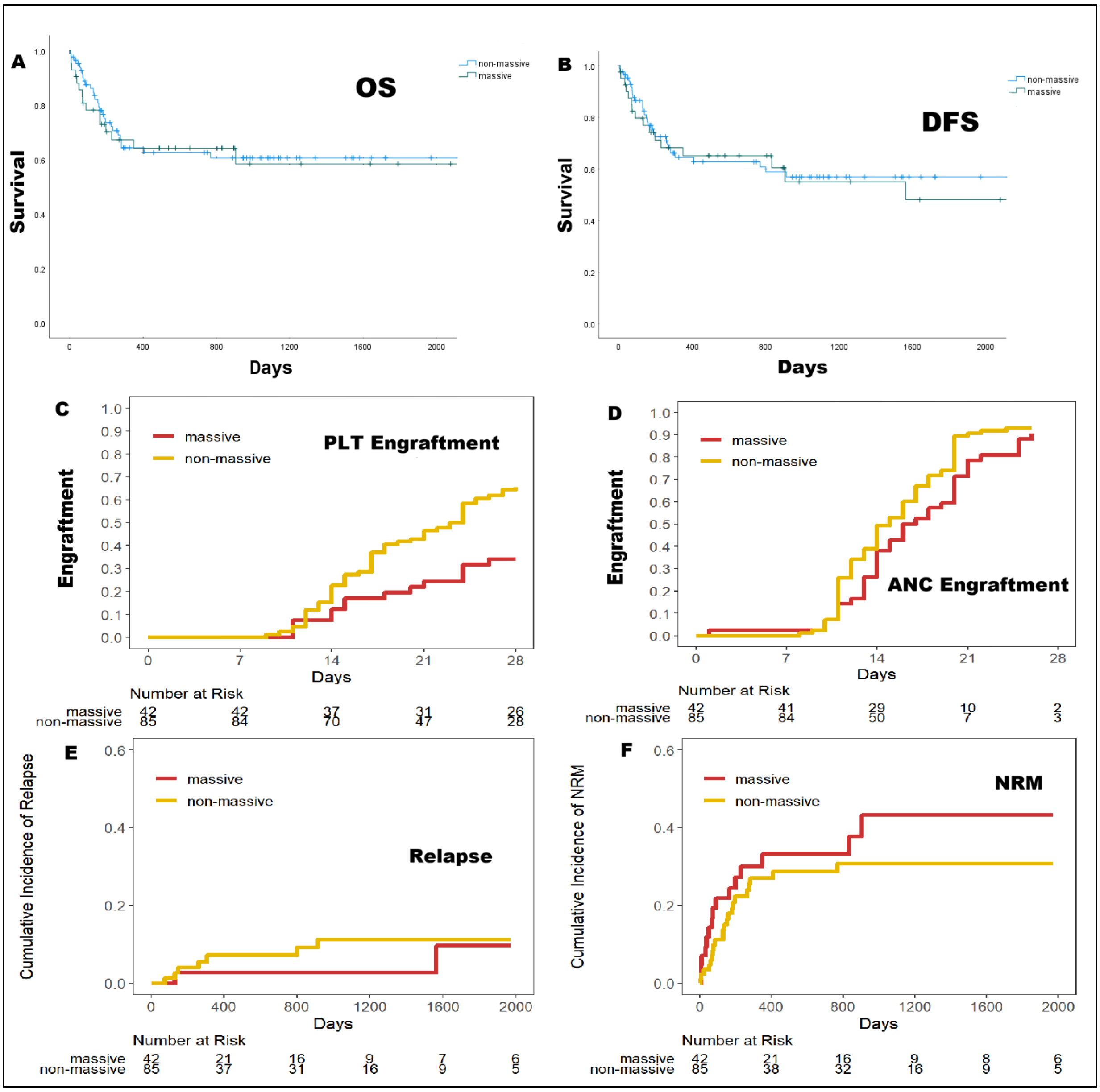

Figure 1. (A) Overall survival based on pretransplant splenomegaly. (B) Disease-free survival based on pretransplant splenomegaly. (C) PLT engraftment based on pretransplant splenomegaly. (D) ANC engraftment based on pretransplant splenomegaly.(E) Relapse based on pretransplant splenomegaly. (F) Non-relapse mortality based on pretransplant splenomegaly.

Copyright Information: (Online) ISSN: 2572-9241
©2026 The Author(s). HemaSphere is published by John Wiley & Sons Ltd on behalf of European Hematology Association. This is an open access Abstract Book distributed under the Attribution-NonCommercial-NoDerivs (CC BY-NC-ND), which allows third parties to download the articles and share them with others as long as they credit the author and the Abstract Book, but they cannot change the content in any way or use them commercially.
Abstract Book Citations: Authors, Title, HemaSphere, 2026;10:(S1):pages. The abstract book DOIs can be found on the "Author Information" page.
Disclaimer: Articles published in the journal HemaSphere exclusively reflect the opinions of the authors. The authors are responsible for all content in their abstracts including accuracy of the facts, statements, citing resources, etc.

### (PB3463) ROPEGINTERFERON ALFA-2B IS EFFECTIVE AND SAFE IN ESSENTIAL THROMBOCYTHEMIA PATIENTS LACKING CYTOREDUCTIVE TREATMENT OPTIONS INDEPENDENT OF TREATMENT HISTORY AND RISK GROUP

**Topic:** 16. Myeloproliferative neoplasms - Clinical

Francesca Palandri*[1, 2], Jiří Mayer[3, 4], Ciprian Tomuleasa[5, 6], Haifa Kathrin Al-Ali[7], Florian Heidel[8], Valentin Garcia Gutierrez[9, 10], Andreas Reiter[11], Eloise Beggiato[12], Joanna Gora-Tybor[13, 14], Mihaela Lazaroiu[15], Sebastian Grosicki[16], Alberto Alvarez Larran[17], Francisca Ferrer Marin[18], Tomasz Sacha[19], Valerio De Stefano[20], Konstanze Döhner[21], Massimo Breccia[22], Paola Guglielmelli[23], Arpád Illés[24], Chloé James[25, 26], Ana Manuela Crisan[27], Steffen Koschmieder[28, 29], Evangelos Terpos[30], Veronika Buxhofer-Ausch[31, 32], Stefan Schmidt[33], Albert Wölfler[34], Zsolt Nagy[35], Stefanie Schlager[36], Sandra Zehentmeier[36], Victoria Empson[36], Martin Unger[36], Kurt Krejcy[36], Christoph Klade[36], Jean-Jacques Kiladjian[37]

*[1]IRCCS Azienda Ospedaliero-Universitaria di Bologna, Bologna, Italy;[2]Istituto di Ematologia Seràgnoli, Bologna, Italy; [3]University Hospital Brno, Department of Internal Medicine, Hematology and Oncology, Brno, Czechia; [4]Masaryk University Brno, Brno, Czechia; [5]Ion Chiricuta Institute of Oncology, Iuliu Hatieganu University of Medicine and Pharmacy, Hematology Department, Cluj-Napoca, Romania; [6]Medfuture Research Center for Advanced Medicine, Cluj-Napoca, Romania;[7]University Hospital Halle (Saale), Krukenberg Cancer Center Halle, Halle, Germany; [8]Hannover Medical School (MHH), Clinic for Hematology, Hemostasis, Oncology and Stem Cell Transplantation, Hannover, Germany; [9]Hospital Universitario Ramón y Cajal, Madrid (IRYCIS), Madrid, Spain; [10]Universidad de Alcalá, Madrid, Spain; [11]University Hospital Mannheim, Medical Clinic III, Hematology and Internistic Oncology, Mannheim, Germany; [12]University Hospital City of Health and Science of Turin - Hospital Molinette, Complex Structure of Hematology, Torino, Italy; [13]Copernicus Memorial Hospital, Department of Hematooncology, Lodz, Poland; [14]Medical University of Lodz, Department of Hematology, Lodz, Poland;[15]Policlinica de Diagnostic Rapid Brasov, Department of Hematology, Brasov, Romania; [16]Medical University of Silesia, Katowice, Poland; [17]Hospital Clínic of Barcelona, Department of Hematology, Barcelona, Spain; [18]Morales Meseguer University General Hospital, Regional Center of Blood Donation. CIBERER. UCAM. IMIB-Murcia, Murcia, Spain; [19]Jagiellonian University Hospital, Department of Hematology, Kraków, Poland; [20]University Polyclinic Foundation "Agostino Gemelli" - IRCCS, Service of Hematology, Rome, Italy;[21]University Hospital Ulm, Department of Internal Medicine III, Ulm, Germany; [22]Sapienza University of Rome, Department of Translational and Precision Medicine, Rome, Italy; [23]Careggi University Hospital, Department of Hematology, Florence, Italy;[24]University of Debrecen, Faculty of Medicine, Department of Internal Medicine, Division of Hematology, Debrecen, Hungary; [25]University Bordeaux, INSERM, BMC, U1034, F-33600 Pessac, Bordeaux, France; [26]Bordeaux University Hospital, Laboratory of Hematology, Bordeaux, France; [27]Fundeni Clinical Institute, Center for Hematology and Bone Marrow Transplantation, București, Romania; [28]RWTH Aachen University, Faculty of Medicine, Department of Hematology, Oncology, Hemostaseology, and Stem Cell Transplantation (Medical Clinic IV), Aachen, Germany; [29]Center for Integrated Oncology (CIO-ABCD), Aachen, Germany; [30]National and Kapodistrian University of Athens, Department of Clinical Therapeutics, School of Medicine, Athens, Greece; [31]Ordensklinikum Linz Elisabethinen, Department of Internal Medicine I for Hematology with Stem Cell Transplantation, Hemostaseology and Medical Oncology, Linz, Austria; [32]Medical Faculty, Johannes Kepler University Linz, Linz, Austria; [33]Medical University Innsbruck, Department of Internal Medicine V (Hematology and Oncology), Innsbruck, Austria; [34]Medical University Graz, Department of Internal Medicine, Clinical Divison of Hematology, Graz, Austria; [35]Semmelweis University, Department of Internal Medicine and Hematology, Division of Hematology, Budapest, Hungary; [36]AOP Health, Vienna, Austria; [37]Hôpital Saint-Louis, Centre d'Investigations Cliniques CIC 1427, Inserm, Université Paris Cité, Paris, France;*

### Background

Many patients with essential thrombocythemia (ET) require cytoreduction but cannot receive available therapies such as hydroxyurea (HU) or anagrelide (ANA) due to resistance, intolerance or ineligibility. The ongoing ROP-ET trial (NCT06514807) evaluates ropeginterferon alfa-2b in this heterogeneous, under-served population. The study met its primary endpoint (durable response per modified ELN criteria, ASH 2025); however, the magnitude of benefit across patient subgroups is unknown.

Copyright Information: (Online) ISSN: 2572-9241
©2026 The Author(s). HemaSphere is published by John Wiley & Sons Ltd on behalf of European Hematology Association. This is an open access Abstract Book distributed under the Attribution-NonCommercial-NoDerivs (CC BY-NC-ND), which allows third parties to download the articles and share them with others as long as they credit the author and the Abstract Book, but they cannot change the content in any way or use them commercially.
**Abstract Book Citations:** Authors, Title, HemaSphere, 2026;10:(S1):pages. The abstract book DOIs can be found on the "Author Information" page.
**Disclaimer:** Articles published in the journal HemaSphere exclusively reflect the opinions of the authors. The authors are responsible for all content in their abstracts including accuracy of the facts, statements, citing resources, etc.

### Aims

To assess efficacy and safety of ropeginterferon alfa-2b across subgroups defined by: 1) thrombotic risk category according to the revised IPSET-thrombosis score; 2) type of driver mutation; 3) prior cytoreductive history.

### Methods

Prospective, single arm, multicenter, phase III study enrolling ET patients requiring cytoreduction but who are intolerant, refractory, and/or ineligible to receive approved therapies. Month-12 clinical response was assessed as composite of blood count remission, absence of thrombotic/hemorrhagic events and disease progression, absence/non-progression of disease-related signs, and symptom improvement/non-progression and was analyzed in patient subgroups.

### Results

A total of 132 patients were enrolled. IPSET-thrombosis scores were available for 130 patients: 57 patients (43.8%) were high-risk and 73 (56.2%) were non–high-risk, including 26 very-low-risk patients (20.0%), of whom 22 (84.6%) had previously received cytoreductive therapy. Month-12 response rates were comparable across risk categories, indicating that the efficacy of ropeginterferon alfa-2b was independent of thrombotic-risk level (high-risk: 56.4% [95% CI 42.3–69.7]; non-high-risk: 63.2% [95% CI 50.7–74.6]). Single driver mutations in *JAK2* or *CALR* were detected by sequencing in 71/130 (54.6%) and 35/130 (26.9%) patients, respectively (n=2 not available). *MPL* mutations were uncommon (1.5%), as were multiple driver mutations (*JAK2* plus *CALR* or *MPL*, 2.3%). Overall, 14.6% of patients were triple-negative. Response rates at Month 12 were comparable in patients carrying *JAK2* or *CALR* mutations (68.7% [95% CI: 56.2-79.4] vs. 63.6% [95% CI: 45.1-79.6]).
Treatment-naïve patients ineligible for alternative therapies comprised 16.7% of the study population (22/132). Compared with pretreated patients, these patients had a lower median age (51.5 [range: 32-73] vs. 58 [22-87] years), shorter time since diagnosis (median: 0.9 [range: 0-5] vs. 4.4 [0-25] years) and higher median baseline platelet count (717 [range: 214-1022] vs. 570 [201-1958] x$10^9$/L). A higher Month-12 response rate was seen in treatment-naïve patients versus pretreated patients (85.7% [95% CI: 63.7; 97.0] vs. 54.8% [95% CI: 44.7; 64.6]).
Ropeginterferon alfa-2b treatment up to Month 12 was well tolerated and no notable differences in safety were observed across patient subgroups. Three (2.3%) patients discontinued due to treatment-related adverse events. While 7/132 (5.3%) patients overall had adverse drug reactions grade ≥ 3, none occurred in cytoreduction-naïve patients.

### Summary/Conclusion

Ropeginterferon alfa-2b was effective and well tolerated in ET patients requiring cytoreduction but unable to receive approved treatments, regardless of thrombotic risk category, and driver mutation (*JAK2* vs. *CALR).* A remarkably high response rate and good tolerability among a small group of cytoreduction-naïve patients warrants further investigation of ropeginterferon alfa-2b in the first-line setting.

**Copyright Information:** (Online) ISSN: 2572-9241
©2026 The Author(s). HemaSphere is published by John Wiley & Sons Ltd on behalf of European Hematology Association. This is an open access Abstract Book distributed under the Attribution-NonCommercial-NoDerivs (CC BY-NC-ND), which allows third parties to download the articles and share them with others as long as they credit the author and the Abstract Book, but they cannot change the content in any way or use them commercially.
**Abstract Book Citations:** Authors, Title, HemaSphere, 2026;10:(S1):pages. The abstract book DOIs can be found on the "Author Information" page.
**Disclaimer:** Articles published in the journal HemaSphere exclusively reflect the opinions of the authors. The authors are responsible for all content in their abstracts including accuracy of the facts, statements, citing resources, etc.

### (PB3464) SEX INFLUENCES PROGNOSIS OF ADOLESCENTS AND YOUNG ADULTS WITH CLASSICAL BCR::ABL1 NEGATIVE MYELOPROLIFERATIVE NEOPLASMS

**Topic:** 16. Myeloproliferative neoplasms - Clinical

Yanan Cai*[1], Yuebo Wang[2], Ping Zhang[3]

*[1]Henan Provincial People's Hospital, Zhengzhou University, China, Department of Hematology, Zhengzhou, China;[2]Henan Provincial People's Hospital, Zhengzhou University, China, Clinical Research Service Center, Zhengzhou, China; [3]Henan Provincial People's Hospital, Zhengzhou University, China, Department of Hematology, Zhengzhou, China;*

### Background

Classical *BCR::ABL1* negative myeloproliferative neoplasms (MPNs) mainly includes polycythemia vera (PV), essential thrombocythemia (ET) and primary myelofibrosis (PMF). Adolescents and young adults (AYA), aged 16-39 years, make up a largely underrepresented subset of MPNs. It has been reported that sex was associated with survival prognosis in MPNs. However, large-scale data are still lacking in AYA-MPNs.

### Aims

We conducted a retrospective study utilizing the SEER database to address the prognostic value of sex in MPNs.

### Methods

We assembled a retrospective cohort of patients with PV, ET and PMF diagnosed between 2000 and 2021 in SEER-2017 Registry. Follow up ended at death, last follow-up, or December 31, 2021, whichever occurred earliest. We excluded the patients from further analysis if they: (1) aged<16 or ≥40 years old at diagnosis, (2) with missing data on age, race, survival status or follow-up information, (3) survival months<3 months, (4) reported from autopsy or death certificate only. Overall survival (OS) was defined as the time from diagnosis to the last follow-up or death from any cause. The Kaplan-Meier and Cox proportional hazards regression analysis were performed to calculate the impact of sex on survival outcomes. Variables significant in univariate analysis were included in multivariate analysis. Propensity score matching (PSM) was performed with ratio of 1:1 using nearest-neighbor matching (caliper = 0.1) of the standard deviation of the propensity score logit. Statistical significance was set at $P<0.05$ for two-sided tests.

**Copyright Information:** (Online) ISSN: 2572-9241
©2026 The Author(s). HemaSphere is published by John Wiley & Sons Ltd on behalf of European Hematology Association. This is an open access Abstract Book distributed under the Attribution-NonCommercial-NoDerivs (CC BY-NC-ND), which allows third parties to download the articles and share them with others as long as they credit the author and the Abstract Book, but they cannot change the content in any way or use them commercially.
**Abstract Book Citations:** Authors, Title, HemaSphere, 2026;10:(S1):pages. The abstract book DOIs can be found on the "Author Information" page.
**Disclaimer:** Articles published in the journal HemaSphere exclusively reflect the opinions of the authors. The authors are responsible for all content in their abstracts including accuracy of the facts, statements, citing resources, etc.

### Results

Of 3593 patients diagnosed with MPNs, 1907 patients (53.1%) were female. In entire MPNs, male patients presented with older age of diagnosis (median age : 33.0 years versus 32.0 years, $P$<0.001) and higher incidence of PV (54.6% versus 21.1%; $P$<0.001), while female patients with higher incidence of ET and PMF (73.9% versus 42.2% and 4.9% versus 3.3%, respectively, $P$<0.001). In subset analysis, an older age of onset in males versus females was only restricted to ET (median age : 33.0 years versus 31.0 years, $P$<0.001). The age by sex in PV and PMF were similar ($P$=0.947 and 0.693).

The median follow-up of the entire MPNs was 88.0 months [interquartile range (IQR), 41.0-160.0 months] with 109.0 months (IQR, 50.0-177.5 months), 78.0 months (IQR, 37.0-149.0 months) and 78.0 months (IQR, 29.0-146.0 months) in PV, ET and PMF. 242 death (6.7%) were recorded overall including: 116 patients with PV (8.8%), 95 with ET (4.5%) and 31 with PMF (20.8%).

Kaplan-Meier analysis revealed that males suffered shorter OS against females in entire MPNs (median survival not reached; $P$<0.001). Shortened OS in male against female was only restricted in ET (median survival not reached, $P$<0.001) whereas similar OS by sex in PV and PMF (median survival not reached for both, $P$=0.56 and 0.71). In multivariate analysis, the HR was 1.54 (95% CI 1.16-2.04; $P$=0.003) for male versus female in entire MPNs. In PV, age group, annual income and marital status but race retained significance on multivariable analysis. In ET, male still remained as a predictor of decreased OS (HR 2.61, 95% CI 1.73-3.92, $P$<0.001). Multivariate analysis was not conducted in PMF because only race was significant in univariate analysis. After PSM, the impact of sex on OS was consistent in matched patients with MPNs, as well as across PV, ET and PMF.

### Summary/Conclusion

Sex should be considered in prognosis stratification of AYA patients with ET.

**Copyright Information:** (Online) ISSN: 2572-9241
©2026 The Author(s). HemaSphere is published by John Wiley & Sons Ltd on behalf of European Hematology Association. This is an open access Abstract Book distributed under the Attribution-NonCommercial-NoDerivs (CC BY-NC-ND), which allows third parties to download the articles and share them with others as long as they credit the author and the Abstract Book, but they cannot change the content in any way or use them commercially.
**Abstract Book Citations:** Authors, Title, HemaSphere, 2026;10:(S1):pages. The abstract book DOIs can be found on the "Author Information" page.
**Disclaimer:** Articles published in the journal HemaSphere exclusively reflect the opinions of the authors. The authors are responsible for all content in their abstracts including accuracy of the facts, statements, citing resources, etc.

A

**Table 1. Univariate and multivariate Cox regression analysis for overall survival in entire MPNs**

| | | Univariate Analysis | | Multivariate Analysis | |
|---|---|---|---|---|---|
| | Number (%) | HR (95%CI) | *P* Value | HR (95%CI) | *P* Value |
| Sex | | | | | |
| Female | 1907 (53.1) | Ref | | Ref | |
| Male | 1686 (46.9) | 1.69 (1.30, 2.19) | <0.001 | 1.54 (1.16, 2.04) | 0.003 |
| Age group, years | | | | | |
| 16-20 | 259 (7.2) | Ref | | Ref | |
| 21-25 | 426 (11.9) | 1.54 (0.70, 3.36) | 0.279 | 1.70 (0.78, 3.71) | 0.185 |
| 26-30 | 702 (19.5) | 1.57 (0.75, 3.26) | 0.231 | 1.97 (0.94, 4.12) | 0.071 |
| 31-35 | 1025 (28.5) | 2.19 (1.10, 4.38) | 0.026 | 2.87 (1.42, 5.79) | 0.003 |
| 36-39 | 1181 (32.9) | 2.67 (1.35, 5.26) | 0.005 | 3.41 (1.71, 6.81) | 0.001 |
| Race | | | | | |
| Black | 540 (15.0) | Ref | | Ref | |
| White | 2661 (74.1) | 0.50 (0.37, 0.68) | <0.001 | 0.55 (0.40, 0.75) | <0.001 |
| Other# | 392 (10.9) | 0.47 (0.28, 0.78) | 0.003 | 0.60 (0.36, 1.01) | 0.053 |
| Marital status | | | | | |
| married | 1465 (40.8) | Ref | | Ref | |
| single | 1740 (48.4) | 2.05 (1.55, 2.71) | <0.001 | 2.26 (1.68, 3.04) | <0.001 |
| unknown | 388 (10.8) | 0.94 (0.54, 1.64) | 0.833 | 0.91 (0.52, 1.60) | 0.746 |
| Year of Diagnosis | | | | | |
| 2000-2004 | 475 (13.2) | 1.85 (1.10, 3.10) | 0.02 | 1.68 (0.99, 2.83) | 0.052 |
| 2005-2009 | 797 (22.2) | 1.49 (0.90, 2.46) | 0.123 | 1.42 (0.86, 2.36) | 0.172 |
| 2010-2014 | 791 (22.0) | 1.89 (1.15, 3.11) | 0.013 | 1.71 (1.03, 2.82) | 0.036 |
| > 2014 | 1530 (42.6) | Ref | | Ref | |
| Annual income | | | | | |
| <$70000 | 1125 (31.3) | Ref | | Ref | |
| ≥$70000 | 2466 (68.6) | 0.81 (0.62, 1.05) | 0.109 | 0.85 (0.65, 1.12) | 0.252 |
| missing | 2 (0.1) | 0.00 (0.00, Inf) | 0.993 | 0.00 (0.00, Inf) | 0.993 |
| Diagnosis | | | | | |
| PV | 1323 (36.8) | Ref | | Ref | |
| ET | 2121 (59.0) | 0.64 (0.49, 0.84) | 0.001 | 0.79 (0.59, 1.06) | 0.12 |
| PMF | 149 (4.1) | 2.88 (1.93, 4.27) | <0.001 | 3.08 (2.04, 4.67) | <0.001 |

HR, hazard ratio; CI, confidence interval; Ref: reference. PV, polycythemia vera; ET, essential thrombocythemia; PMF, primary myelofibrosis.

### Other included American Indian/Alaska Native and Asian/Pacific Islander.

B

**Table 2. Univariate and multivariate Cox regression analysis for overall survival in polycythemia vera**

| | | Univariate Analysis | | Multivariate Analysis | |
|---|---|---|---|---|---|
| | Number (%) | HR (95%CI) | *P* Value | HR (95%CI) | *P* Value |
| Sex | | | | | |
| Female | 403 (30.5) | Ref | | | |
| Male | 920 (69.5) | 1.13 (0.75, 1.72) | 0.56 | | |
| Age group, years | | | | | |
| 16-20 | 87 (6.6) | Ref | | Ref | |
| 21-25 | 151 (11.4) | 2.84 (0.81, 9.96) | 0.103 | 3.28 (0.93, 11.52) | 0.064 |
| 26-30 | 237 (17.9) | 2.46 (0.73, 8.37) | 0.148 | 3.06 (0.90, 10.43) | 0.073 |
| 31-35 | 383 (28.9) | 2.57 (0.79, 8.43) | 0.119 | 3.81 (1.15, 12.57) | 0.028 |
| 36-39 | 465 (35.1) | 3.69 (1.15, 11.82) | 0.028 | 5.39 (1.67, 17.41) | 0.005 |
| Race | | | | | |
| Black | 134 (10.1) | Ref | | Ref | |
| White | 1069 (80.8) | 0.57 (0.33, 0.97) | 0.039 | 0.64 (0.38, 1.10) | 0.11 |
| Other# | 120 (9.1) | 0.67 (0.30, 1.48) | 0.323 | 0.92 (0.41, 2.05) | 0.842 |
| Marital status | | | | | |
| married | 513 (38.8) | Ref | | Ref | |
| single | 675 (51.0) | 2.07 (1.36, 3.16) | 0.001 | 2.43 (1.57, 3.75) | <0.001 |
| unknown | 135 (10.2) | 1.21 (0.55, 2.63) | 0.636 | 1.21 (0.55, 2.64) | 0.64 |
| Year of Diagnosis | | | | | |
| 2000-2004 | 239 (18.1) | 1.64 (0.72, 3.71) | 0.239 | | |
| 2005-2009 | 341 (25.8) | 1.35 (0.60, 3.04) | 0.465 | | |
| 2010-2014 | 302 (22.8) | 1.65 (0.73, 3.75) | 0.233 | | |
| > 2014 | 441 (33.3) | Ref | | | |
| Annual income | | | | | |
| <$70000 | 476 (36.0) | Ref | | Ref | |
| ≥$70000 | 847 (64.0) | 0.69 (0.48, 1.00) | 0.047 | 0.66 (0.45, 0.96) | 0.028 |

HR, hazard ratio; CI, confidence interval; Ref: reference.

### Other included American Indian/Alaska Native and Asian/Pacific Islander.

C

**Table 3. Univariate and multivariate Cox regression analysis for overall survival in essential thrombocythemia**

| | | Univariate Analysis | | Multivariate Analysis | |
|---|---|---|---|---|---|
| | Number (%) | HR (95%CI) | *P* Value | HR (95%CI) | *P* Value |
| Sex | | | | | |
| Female | 1410 (66.5) | Ref | | Ref | |
| Male | 711 (33.5) | 2.67 (1.78, 4.02) | <0.001 | 2.61 (1.73, 3.92) | <0.001 |
| Age group, years | | | | | |
| 16-20 | 168 (7.9) | Ref | | | |
| 21-25 | 266 (12.5) | 0.77 (0.26, 2.30) | 0.646 | | |
| 26-30 | 442 (20.8) | 0.96 (0.37, 2.51) | 0.938 | | |
| 31-35 | 593 (28.0) | 1.60 (0.67, 3.82) | 0.292 | | |
| 36-39 | 652 (30.7) | 1.58 (0.67, 3.76) | 0.298 | | |
| Race | | | | | |
| Black | 379 (17.9) | Ref | | Ref | |
| White | 1488 (70.2) | 0.43 (0.27, 0.67) | <0.001 | 0.49 (0.31, 0.77) | 0.002 |
| Other# | 254 (12.0) | 0.31 (0.14, 0.71) | 0.005 | 0.40 (0.17, 0.92) | 0.03 |
| Marital status | | | | | |
| married | 883 (41.6) | Ref | | Ref | |
| single | 1003 (47.3) | 2.14 (1.37, 3.33) | 0.001 | 1.82 (1.15, 2.87) | 0.01 |
| unknown | 235 (11.1) | 0.76 (0.29, 1.96) | 0.571 | 0.73 (0.28, 1.88) | 0.509 |
| Year of Diagnosis | | | | | |
| 2000-2004 | 209 (9.9) | 1.72 (0.78, 3.77) | 0.177 | | |
| 2005-2009 | 429 (20.2) | 1.34 (0.64, 2.80) | 0.44 | | |
| 2010-2014 | 454 (21.4) | 1.88 (0.92, 3.83) | 0.084 | | |
| > 2014 | 1029 (48.5) | Ref | | | |
| Annual income | | | | | |
| <$70000 | 601 (28.3) | Ref | | | |
| ≥$70000 | 1518 (71.6) | 1.15 (0.72, 1.84) | 0.566 | | |
| missing | 2 (0.1) | 0.00 (0.00, Inf) | 0.994 | | |

HR, hazard ratio; CI, confidence interval; Ref: reference.

### Other included American Indian/Alaska Native and Asian/Pacific Islander.

D

**Table 4. Univariate and multivariate Cox regression analysis for overall survival in primary myelofibrosis**

| | | Univariate Analysis | | Multivariate Analysis | |
|---|---|---|---|---|---|
| PMF | Number (%) | HR (95%CI) | *P* Value | HR (95%CI) | *P* Value |
| Sex | | | | | |
| Female | 94 (63.1) | Ref | | | |
| Male | 55 (36.9) | 0.87 (0.42, 1.82) | 0.714 | | |
| Age group, years | | | | | |
| 16-20 | 4 (2.7) | Ref | | | |
| 21-25 | 9 (6.0) | (0.00, Inf) | 0.998 | | |
| 26-30 | 23 (15.4) | (0.00, Inf) | 0.998 | | |
| 31-35 | 49 (32.9) | (0.00, Inf) | 0.998 | | |
| 36-39 | 64 (43.0) | (0.00, Inf) | 0.998 | | |
| Race | | | | | |
| Black | 27 (18.1) | Ref | | Ref | |
| White | 104 (69.8) | 0.42 (0.18, 0.94) | 0.034 | 0.42 (0.18, 0.94) | 0.034 |
| Other# | 18 (12.1) | 0.52 (0.16, 1.70) | 0.28 | 0.52 (0.16, 1.70) | 0.28 |
| Marital status | | | | | |
| married | 69 (46.3) | Ref | | | |
| single | 62 (41.6) | 2.04 (0.97, 4.30) | 0.059 | | |
| unknown | 18 (12.1) | 0.77 (0.17, 3.43) | 0.727 | | |
| Year of Diagnosis | | | | | |
| 2000-2004 | 27 (18.1) | 1.33 (0.31, 5.66) | 0.696 | | |
| 2005-2009 | 27 (18.1) | 2.08 (0.52, 8.29) | 0.301 | | |
| 2010-2014 | 35 (23.5) | 1.91 (0.48, 7.60) | 0.357 | | |
| > 2014 | 60 (40.3) | Ref | | | |
| Annual income | | | | | |
| <$70000 | 48 (32.2) | Ref | | | |
| ≥$70000 | 101 (67.8) | 0.87 (0.42, 1.83) | 0.722 | | |

HR, hazard ratio; CI, confidence interval; Ref: reference; Inf, infinite.

### Other included American Indian/Alaska Native and Asian/Pacific Islander.

Univariate and multivariate Cox regression analysis for overall survival in MPNs

**Copyright Information:** (Online) ISSN: 2572-9241
©2026 The Author(s). HemaSphere is published by John Wiley & Sons Ltd on behalf of European Hematology Association. This is an open access Abstract Book distributed under the Attribution-NonCommercial-NoDerivs (CC BY-NC-ND), which allows third parties to download the articles and share them with others as long as they credit the author and the Abstract Book, but they cannot change the content in any way or use them commercially.
**Abstract Book Citations:** Authors, Title, HemaSphere, 2026;10:(S1):pages. The abstract book DOIs can be found on the "Author Information" page.
**Disclaimer:** Articles published in the journal HemaSphere exclusively reflect the opinions of the authors. The authors are responsible for all content in their abstracts including accuracy of the facts, statements, citing resources, etc.

### (PB3465) SOCIOECONOMIC DEPRIVATION IS ASSOCIATED WITH INCREASED MORTALITY RISK AMONG PATIENTS WITH MYELOPROLIFERATIVE NEOPLASMS

**Topic:** 16. Myeloproliferative neoplasms - Clinical

Nauman Butt*[1], Temitope Erinfolami[2], Samah Alimam[3], Mamta Garg[4], Gillian Thomas[5], Tobe Aleksander[5], Brogan Johnston[1], Toyyib Abdulkareem[2], Nurunnahar Akter[2], Indrani Karpha[1, 2], Yeong Lim[1, 2], Mark Bishton[6], Nagesh Kalakonda[1, 2], Andrew Pettitt[1, 2], Catrin Tudur Smith[2], Patrick Harrington[7]

*[1]The Clatterbridge Cancer Centre NHS Foundation Trust, Liverpool, United Kingdom;[2]University Of Liverpool, Liverpool, United Kingdom; [3]University College London Hospitals NHS Foundation Trust, London, United Kingdom;[4]University Hospitals of Leicester NHS Trust, Leicester, United Kingdom; [5]MPN Voice, London, United Kingdom; [6]University of Nottingham & Nottingham University Hospitals NHS Trust, Nottingham, United Kingdom; [7]Guy's and St Thomas NHS Foundation Trust, London, United Kingdom;*

### Background

UNCOVER is a national blood cancer initiative integrating NHS data curated by National Cancer Registration and Analysis Service (NCRAS) with linked datasets offering a comprehensive view of MPN patient characteristics and outcomes.

### Aims

To describe the epidemiology and survival of patients diagnosed with essential thrombocythaemia (ET), polycythaemia vera (PV), and primary myelofibrosis (PMF) in England between 2014-2022.

### Methods

NCRAS datasets identified newly diagnosed ET, PV, and PMF patients using International Classification of Diseases codes with linked sources providing socio-demographics, index of multiple deprivation (IMD), Charlson comorbidity index, and survival outcomes.

**Copyright Information:** (Online) ISSN: 2572-9241
©2026 The Author(s). HemaSphere is published by John Wiley & Sons Ltd on behalf of European Hematology Association. This is an open access Abstract Book distributed under the Attribution-NonCommercial-NoDerivs (CC BY-NC-ND), which allows third parties to download the articles and share them with others as long as they credit the author and the Abstract Book, but they cannot change the content in any way or use them commercially.
**Abstract Book Citations:** Authors, Title, HemaSphere, 2026;10:(S1):pages. The abstract book DOIs can be found on the "Author Information" page.
**Disclaimer:** Articles published in the journal HemaSphere exclusively reflect the opinions of the authors. The authors are responsible for all content in their abstracts including accuracy of the facts, statements, citing resources, etc.

**Results**

A total of 19,223 MPN patients were captured (ET n=12,878; PV n=6,746; PMF n=2,602). Incidence increased significantly with age. In ET, 59% were female and 82% White; in PV, 57% were male and 86% White; and in PMF, 65% were male and 86% White.

Socioeconomic deprivation significantly influenced incidence with higher incidence rate ratios (IRR) observed in the most deprived quintile for ET (IRR 1.09, 95% CI 1.02-1.15), PV (IRR 1.16, 95% CI 1.07-1.26, p=0.001), and PMF (IRR 1.10, 95% CI 1.05-1.15, p=0.001).
Five-year overall survival (OS) was highest in ET at 75% (95% CI 75–76%) and PV at 73% (95% CI 72–74%). PMF survival was markedly poorer, falling from 83% (95% CI 82–84%) at one year to 43% (95% CI 41–45%) at five years.
In ET, OS and cause-specific survival (CSS) were poorer in men (OSHR 1.32, 95% CI 1.24–1.41; CSHR 1.56, 95% CI 1.35–1.80) and declined with age and comorbidity. In PV, HRs were similarly higher in men (OSHR 1.22, 95% CI 1.12–1.33; CSHR 1.39, 95% CI 1.13–1.70), with worse outcomes in older or more comorbid patients. In PMF, both HRs were also increased in men (OSHR 1.26, 95% CI 1.13–1.40; CSHR 1.17, 95% CI 1.01–1.35).

Deprivation significantly worsened outcomes across all MPN subtypes (Table 1). The most deprived IMD quintile showed inferior OS and CSS in ET (OSHR 1.43, 95% CI 1.28-1.59; CSHR 1.47, 95% CI 1.15-1.88) and poorer OS in PV (OSHR 1.29, 95% CI 1.11-1.49), with a trend toward lower OSHR in PMF (p=0.085). Greater deprivation was also associated with higher cardiac and vascular mortality across all MPN subtypes (ET CSHR 1.33, 95%CI 1.04-1.70; PV CSHR 1.69, 95%CI 1.26-2.27; PMF CSHR 2.01, 95%CI 1.23-3.27)

**Summary/Conclusion**

UNCOVER provides a unique overview of UK MPN incidence and outcomes, revealing for the first time that greater socioeconomic deprivation is associated with poorer survival and highlighting the need to address potential health inequalities in MPN care.

**Copyright Information:** (Online) ISSN: 2572-9241
©2026 The Author(s). HemaSphere is published by John Wiley & Sons Ltd on behalf of European Hematology Association. This is an open access Abstract Book distributed under the Attribution-NonCommercial-NoDerivs (CC BY-NC-ND), which allows third parties to download the articles and share them with others as long as they credit the author and the Abstract Book, but they cannot change the content in any way or use them commercially.
**Abstract Book Citations:** Authors, Title, HemaSphere, 2026;10:(S1):pages. The abstract book DOIs can be found on the "Author Information" page.
**Disclaimer:** Articles published in the journal HemaSphere exclusively reflect the opinions of the authors. The authors are responsible for all content in their abstracts including accuracy of the facts, statements, citing resources, etc.

| MPN | IMD Quintiles | OS HR | OS 95% CI | OS global p-value | CSS HR | CSS 95% CI | CSS global p-value |
|---|---|---|---|---|---|---|---|
| ET | | | | <0.001 | | | 0.018 |
| | 5 - least deprived | — | — | | — | — | |
| | 4 | 1.06 | 0.96, 1.17 | | 1.06 | 0.85, 1.31 | |
| | 3 | 1.19 | 1.07, 1.31 | | 1.25 | 1.00, 1.55 | |
| | 2 | 1.25 | 1.12, 1.39 | | 1.22 | 0.96, 1.55 | |
| | 1 - most deprived | 1.43 | 1.28, 1.59 | | 1.47 | 1.15, 1.88 | |
| PV | | | | <0.001 | | | >0.9 |
| | 5 - least deprived | — | — | | — | — | |
| | 4 | 0.95 | 0.83, 1.08 | | 0.87 | 0.66, 1.16 | |
| | 3 | 1.1 | 0.97, 1.25 | | 0.94 | 0.70, 1.25 | |
| | 2 | 1.22 | 1.07, 1.40 | | 0.98 | 0.72, 1.34 | |
| | 1 - most deprived | 1.29 | 1.11, 1.49 | | 0.95 | 0.67, 1.34 | |
| MF | | | | 0.085 | | | 0.5 |
| | 5 (least deprived) | — | — | | — | — | |
| | 4 | 1.04 | 0.90, 1.21 | | 1.08 | 0.89, 1.30 | |
| | 3 | 1.02 | 0.87, 1.19 | | 0.91 | 0.75, 1.12 | |
| | 2 | 1.12 | 0.96, 1.32 | | 1.06 | 0.86, 1.31 | |
| | 1 (most deprived) | 1.25 | 1.05, 1.48 | | 1.08 | 0.86, 1.36 | |

Table 1. Effect of Index of Multiple Deprivation (IMD) on Overall Survival (OS) and Cause-Specific Survival (CSS) in MPN

**Copyright Information:** (Online) ISSN: 2572-9241
©2026 The Author(s). HemaSphere is published by John Wiley & Sons Ltd on behalf of European Hematology Association. This is an open access Abstract Book distributed under the Attribution-NonCommercial-NoDerivs (CC BY-NC-ND), which allows third parties to download the articles and share them with others as long as they credit the author and the Abstract Book, but they cannot change the content in any way or use them commercially.
**Abstract Book Citations:** Authors, Title, HemaSphere, 2026;10:(S1):pages. The abstract book DOIs can be found on the "Author Information" page.
**Disclaimer:** Articles published in the journal HemaSphere exclusively reflect the opinions of the authors. The authors are responsible for all content in their abstracts including accuracy of the facts, statements, citing resources, etc.

### (PB3466) DYNAMIC CHANGES IN BONE MARROW FIBROSIS DURING TREATMENT INDEPENDENTLY PREDICT OUTCOMES IN MPN-ASSOCIATED MYELOFIBROSIS

**Topic:** 16. Myeloproliferative neoplasms - Clinical

Ziyue Mi[1], Yuhuan Zheng[1], Ling Pan[1], Huanling Zhu[1], Yu Wu[1], Yunfan Yang*[1]

[1]*Department of Hematology, Institute of Hematology, West China Hospital, Sichuan University, Chengdu, China;*

### Background

Myeloproliferative neoplasm (MPN)–associated myelofibrosis includes primary myelofibrosis (PMF) and secondary myelofibrosis (SMF) evolving from polycythemia vera or essential thrombocythemia. Bone marrow fibrosis grade reflects disease severity and has established prognostic value at diagnosis. Recently, reversal or stabilization of fibrosis has become an important therapeutic objective and a common secondary endpoint in trials of disease-modifying therapies. However, the clinical and prognostic significance of dynamic changes in fibrosis grade over time remains unclear. This study evaluated the clinical impact of changes in bone marrow fibrosis grade during treatment in patients with MPN-associated myelofibrosis.

### Aims

This study evaluated the clinical impact of changes in bone marrow fibrosis grade during treatment in patients with MPN-associated myelofibrosis.

### Methods

This retrospective cohort study included 142 patients with PMF or SMF who underwent ≥2 bone marrow biopsies with fibrosis grading. Patients were classified according to fibrosis progression status during follow-up. Clinical data and outcomes were collected, including survival endpoints, clinical events, and hematologic deterioration. Progression-free survival (PFS) was defined according to the IWG-MRT/ELN (2013) consensus criteria as the time from PMF or SMF diagnosis to disease progression or death. Event-free survival (EFS) was defined as the time from PMF or SMF diagnosis to the first occurrence of major bleeding, thrombotic events, leukemic transformation, or death. Kaplan–Meier analysis was performed to estimate survival and CBC stability probability, with comparisons using the log-rank test. Cox proportional hazards regression was used to evaluate the impact of fibrosis progression on time-to-event outcomes. Hazard ratios (HRs) with 95% confidence intervals (CIs) were calculated. Median follow-up was 74 months.

### Results

Kaplan–Meier analysis showed no significant difference in overall survival (OS) between fibrosis progression and non-progression groups (log-rank P = 0.5587). However, fibrosis progression was associated with significantly inferior PFS (log-rank P = 0.0153) and significantly reduced CBC stability probability (log-rank P = 0.0003), while differences in leukemia-free survival (LFS) (P = 0.1204) and EFS (P = 0.3621) were not statistically significant. In Cox analysis, fibrosis progression independently predicted inferior OS (HR 2.39, P = 0.04) and PFS (HR 3.01, P < 0.001), higher risks of leukemic transformation (HR 3.29, P = 0.002) and CBC deterioration (HR 4.49, P < 0.001), and a nonsignificant trend toward thrombotic or bleeding events (HR 1.95, P = 0.066) ; these associations remained consistent after excluding patients who underwent allogeneic hematopoietic stem cell transplantation.

**Copyright Information:** (Online) ISSN: 2572-9241
©2026 The Author(s). HemaSphere is published by John Wiley & Sons Ltd on behalf of European Hematology Association. This is an open access Abstract Book distributed under the Attribution-NonCommercial-NoDerivs (CC BY-NC-ND), which allows third parties to download the articles and share them with others as long as they credit the author and the Abstract Book, but they cannot change the content in any way or use them commercially.
**Abstract Book Citations:** Authors, Title, HemaSphere, 2026;10:(S1):pages. The abstract book DOIs can be found on the “Author Information” page.
**Disclaimer:** Articles published in the journal HemaSphere exclusively reflect the opinions of the authors. The authors are responsible for all content in their abstracts including accuracy of the facts, statements, citing resources, etc.

### Summary/Conclusion

Bone marrow fibrosis grade progression during treatment is an adverse prognostic factor in patients with MPN-associated myelofibrosis. It is independently associated with inferior OS and PFS, increased risk of leukemic transformation, and hematologic deterioration. A trend toward increased thrombotic and bleeding events was observed. Dynamic assessment of bone marrow fibrosis provides prognostic information beyond baseline evaluation and may aid disease monitoring and risk stratification.

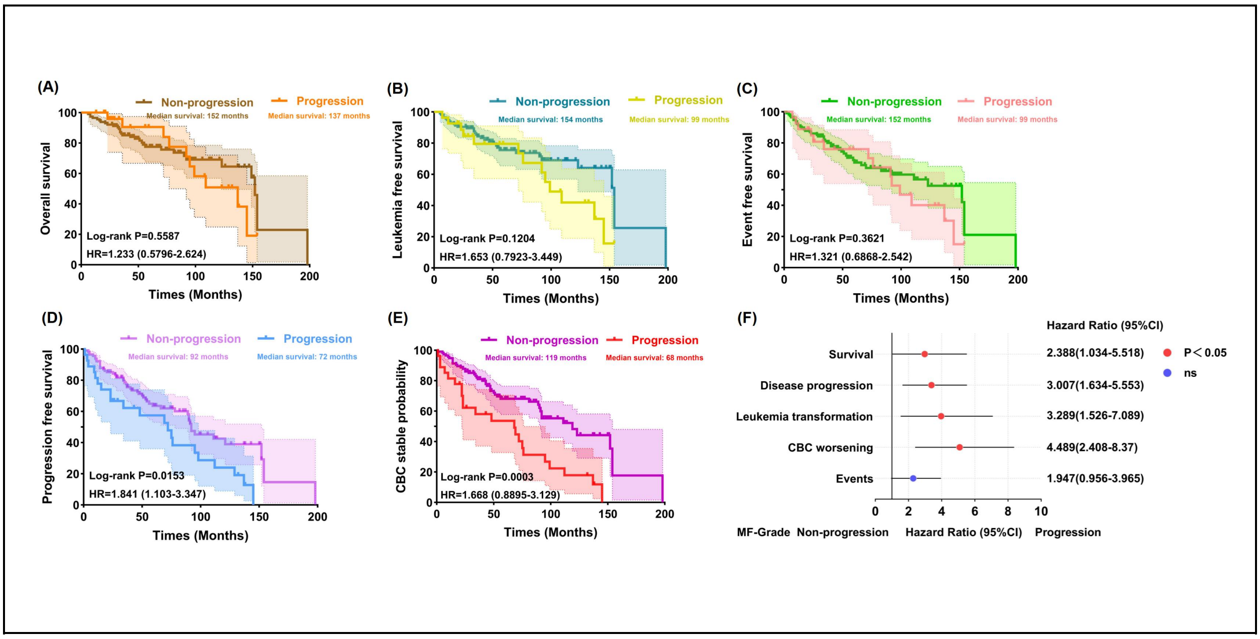

Kaplan–Meier analysis for OS(A), LFS(B), EFS(C), PFS(D) and CBC stable probability(E) between progression and non-progression group. Cox proportional hazards regression analysis(F) for survival, disease progression, leukemia transformation, CBC deterioration and thrombotic or bleeding events between progression and non-progression group.

**Copyright Information:** (Online) ISSN: 2572-9241
©2026 The Author(s). HemaSphere is published by John Wiley & Sons Ltd on behalf of European Hematology Association. This is an open access Abstract Book distributed under the Attribution-NonCommercial-NoDerivs (CC BY-NC-ND), which allows third parties to download the articles and share them with others as long as they credit the author and the Abstract Book, but they cannot change the content in any way or use them commercially.
**Abstract Book Citations:** Authors, Title, HemaSphere, 2026;10:(S1):pages. The abstract book DOIs can be found on the "Author Information" page.
**Disclaimer:** Articles published in the journal HemaSphere exclusively reflect the opinions of the authors. The authors are responsible for all content in their abstracts including accuracy of the facts, statements, citing resources, etc.

### (PB3467) EVALUATING THROMBOTIC RISK AND HEMATOCRIT EFFECT OF INTRAVENOUS IRON REPLETION IN CYTOREDUCED PATIENTS WITH POLYCYTHEMIA VERA

**Topic:** 16. Myeloproliferative neoplasms - Clinical

Katherine Feder*[1, 2], Jessica Freydman[2], Andrew Webb[3], Chi-Joan How[4], Michelle Lee[2], Layla Van Doren[5], Gabriela Hobbs[2]

*[1]Dana-Farber Cancer Institute, Boston, United States of America; [2]Mass General Brigham Cancer Institute, Massachusetts General Hospital, Boston, United States of America; [3]Massachusetts General Hospital, Boston, United States of America; [4]Brigham And Women's Hospital, Boston, United States of America; [5]Yale School of Medicine, New Haven, United States of America;*

### Background

Polycythemia vera (PV) is characterized by erythrocyte hyperproliferation and resultant iron deficiency. Iron stimulates erythropoiesis and is generally avoided in PV. Iron deficiency negatively impacts quality of life (QoL) and increases thrombotic risk. Guidelines recommend balancing severity of iron deficiency with risk of erythrocytosis. Yet, the role and safety of iron replacement is not well studied in PV patients controlled on cytoreduction.

### Aims

Evaluate hematocrit (HCT) control and rate of thrombosis after administration of intravenous (IV) iron.

### Methods

This is a retrospective analysis of patients with PV at Mass General Brigham (MGB) receiving cytoreduction (ruxolitinib, hydroxyurea, ropeginterferon alfa, or peginterferon alfa-2a). Demographics, comorbidities, iron studies, complete blood counts, erythropoietin stimulating agents, and IV iron administration were extracted from the MGB electronic database from 2015-2026 using Structured Query Language with venous thromboembolic events confirmed via manual chart review. HCT was evaluated 4-8 weeks after IV iron and thrombosis within 6 months. An IV iron course was defined as iron administration within 30 days or sequential doses without a gap ≥ 14 days.

### Results

710 PV patients on cytoreduction were identified; 98 (13.8%) received IV iron. Of the 98, 48% were male, 90% white, 29% had heart failure, 36% chronic kidney disease, and 27% gastrointestinal bleeding. Prior to iron repletion, median transferrin saturation was 7% (2-35%), ferritin 27ng/mL (2-1237ng/mL), and HCT 33.5% (21.6-59.1%); 92% of courses had a pre-infusion HCT <45%. Median courses of IV iron was 1 (1-15 courses) per patient with 58% receiving one course. Median dose per course was 510mg (100-1800mg) and median total iron per patient was 1000mg (100-8785mg). Of 67 patients with available pre/post infusion HCT, 15 (22%) had a HCT rise from <45% to ≥45% (median post HCT 47.1%, range 45.0-59.1%). This increase occurred after 18 of 115 (15.6%) iron courses.
Of 81 patients with ≥ 6 months of follow-up after the first iron dose, 2 (2.5%) had a thrombotic event within 6 months of iron due to provoking factors. Both had a history of venous thrombosis. HCT at the time of thrombosis were 27.3% and 27.6%.

**Copyright Information:** (Online) ISSN: 2572-9241
©2026 The Author(s). HemaSphere is published by John Wiley & Sons Ltd on behalf of European Hematology Association. This is an open access Abstract Book distributed under the Attribution-NonCommercial-NoDerivs (CC BY-NC-ND), which allows third parties to download the articles and share them with others as long as they credit the author and the Abstract Book, but they cannot change the content in any way or use them commercially.
**Abstract Book Citations:** Authors, Title, HemaSphere, 2026;10:(S1):pages. The abstract book DOIs can be found on the "Author Information" page.
**Disclaimer:** Articles published in the journal HemaSphere exclusively reflect the opinions of the authors. The authors are responsible for all content in their abstracts including accuracy of the facts, statements, citing resources, etc.

**Summary/Conclusion**

In this cohort of PV patients receiving cytoreductive therapy, IV iron was not associated with increased venous thrombotic events within 6 months compared to limited historic event rates (Barbui et al 2023, Griesshammer et al 2019). Although HCT rose above 45% in 16% of IV iron courses, the rise was modest, suggesting limited clinical impact. Thrombotic events occurred exclusively in patients with a history of thrombosis with provoking factors. The low median number of iron courses per patient may reflect clinician hesitancy and absence of clear guidance regarding IV iron use in PV. Study limitations include retrospective design, lack of patient reported outcomes, limited ability to assess the duration of HCT elevation, potential changes in cytoreduction, and exclusion of arterial thrombosis. This analysis provides reassurance for short-term safety of IV iron in patients with PV on cytoreduction and highlights the need for prospective studies to define patient selection, monitoring strategies, symptom benefit, and longer-term thrombotic risk. Given the high prevalence of iron deficiency in PV and its impact on QoL, establishing an evidence-based framework for iron repletion in cytoreduced patients is important and clinically meaningful.

**Copyright Information:** (Online) ISSN: 2572-9241
©2026 The Author(s). HemaSphere is published by John Wiley & Sons Ltd on behalf of European Hematology Association. This is an open access Abstract Book distributed under the Attribution-NonCommercial-NoDerivs (CC BY-NC-ND), which allows third parties to download the articles and share them with others as long as they credit the author and the Abstract Book, but they cannot change the content in any way or use them commercially.
**Abstract Book Citations:** Authors, Title, HemaSphere, 2026;10:(S1):pages. The abstract book DOIs can be found on the “Author Information” page.
**Disclaimer:** Articles published in the journal HemaSphere exclusively reflect the opinions of the authors. The authors are responsible for all content in their abstracts including accuracy of the facts, statements, citing resources, etc.

### (PB3468) THE DISCRIMINATIVE VALUE OF FLOW CYTOMETRY-BASED ANALYSIS OF CIRCULATING MYELOBLASTS IN ESSENTIAL THROMBOCYTHEMIA AND PRIMARY MYELOFIBROSIS

**Topic:** 16. Myeloproliferative neoplasms - Clinical

Gréta Garami*[1], Yasaman Farhini[1], Malak Abdelmaksoud[1], Laszlo Szerafin[2], Zsuzsanna Hevessy[3], Judit Bedekovics[4], Gábor Méhes[4], Arpád Illés[1], Zsofia Simon[1], Ferenc Magyari[1], Laszlo Imre Pinczes[1]

*[1]University of Debrecen, Faculty of Medicine, Division of Hematology, Department of Internal Medicine, Debrecen, Hungary; [2]Szabolcs-Szatmár-Bereg County Teaching Hospital, Department of Hematology, Nyíregyháza, Hungary;[3]University of Debrecen, Faculty of Medicine, Department of Laboratory Medicine, Debrecen, Hungary; [4]University of Debrecen, Faculty of Medicine, Department of Pathology, Debrecen, Hungary;*

### Background

Essential thrombocythemia (ET) and primary myelofibrosis (PMF) are chronic myeloproliferative neoplasms (MPN) with overlapping driver mutations and clinical symptoms. Distinguishing between them is crucial, as both PMF subtypes, namely prefibrotic (PPMF) and overt (POMF), carry a higher risk of leukemic transformation and death compared to ET. Currently, an invasive bone marrow biopsy is required to differentiate ET from PMF subtypes, as no non-invasive method reliably distinguishes ET from PPMF and POMF.

### Aims

Our aim was to assess the discriminative value of circulating myeloblasts determined by flow cytometry (CMB-FC) in newly diagnosed ET and PMF patients.

### Methods

We quantified CMB-FCs with an eight-color multiparametric flow cytometry panel (CD117+/CD34+/CD45dim) performed on peripheral blood samples as absolute counts (CMB-FC#) in newly diagnosed patients with ET and PMF. To assess the value of CMB-FC# as indicators of disease activity at diagnosis, we examined their associations with well-established patient- and disease-associated prognostic markers of ET and PMF by Pearson correlation. CMB-FC# levels among ET, PPMF, and POMF were compared using the Kruskal-Wallis test (K-W). Diagnostic ability was determined by multinominal logistic regression and receiver operating characteristic (ROC) analysis.

### Results

Altogether, 74 patients had sufficient data for analysis (ET=27, PPMF=26, POMF=21). The biological plausibility of CMB-FC# as a potential biomarker was confirmed by its significant correlations with disease activity markers in ET and PMF. CMB-FC# proved to be a reliable marker for separating ET from PPMF and POMF as its distributions differed between diagnostic categories (K-W $P<0.001$). Post-hoc pairwise comparisons demonstrated significant differences between all pairs (ET vs. PPMF $P=0.009$, ET vs. POMF $P<0.001$, PPMF vs. POMF $P=0.007$). In multinomial logistic regression analysis, increasing CMB-FC# levels were associated with both PPMF and POMF compared to ET ($P=0.004$ and $P<0.001$, respectively). Predicted probability analysis demonstrated a progressive shift in diagnostic likelihood with increasing CMB-FC# levels (Figure 1). Evaluation of the diagnostic performance through ROC analysis demonstrated that CMB-FC# counts yielded an excellent discrimination between ET and PPMF (AUC=0.81), ET and POMF (AUC=0.91) and PPMF and POMF (AUC=0.85).

**Copyright Information:** (Online) ISSN: 2572-9241
©2026 The Author(s). HemaSphere is published by John Wiley & Sons Ltd on behalf of European Hematology Association. This is an open access Abstract Book distributed under the Attribution-NonCommercial-NoDerivs (CC BY-NC-ND), which allows third parties to download the articles and share them with others as long as they credit the author and the Abstract Book, but they cannot change the content in any way or use them commercially.
**Abstract Book Citations:** Authors, Title, HemaSphere, 2026;10:(S1):pages. The abstract book DOIs can be found on the "Author Information" page.
**Disclaimer:** Articles published in the journal HemaSphere exclusively reflect the opinions of the authors. The authors are responsible for all content in their abstracts including accuracy of the facts, statements, citing resources, etc.

**Summary/Conclusion**

Our results provide evidence that CMB-FC# enable us to differentiate between ET, PPMF and POMF with a non-invasive blood test. CMB-FC# not only positively correlates with markers of disease severity but also demonstrates a progressive shift in diagnostic likelihood between ET, PPMF and POMF with increasing levels.

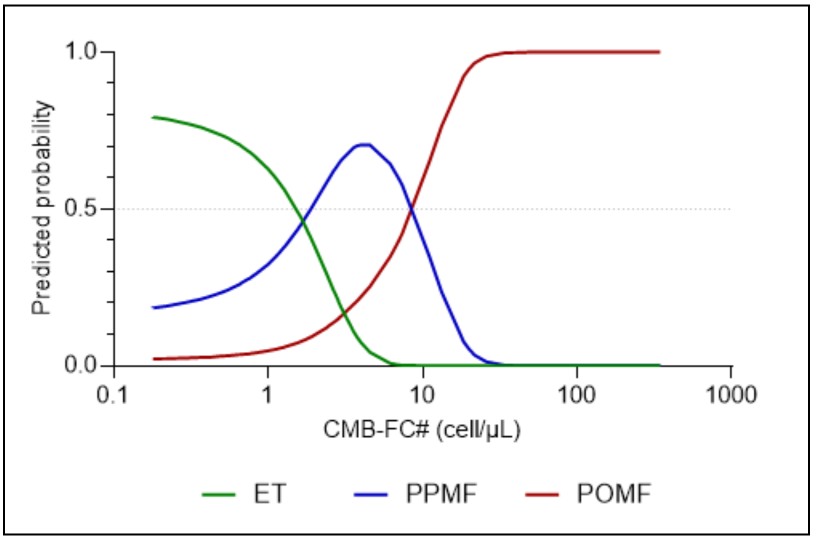

**Copyright Information:** (Online) ISSN: 2572-9241
©2026 The Author(s). HemaSphere is published by John Wiley & Sons Ltd on behalf of European Hematology Association. This is an open access Abstract Book distributed under the Attribution-NonCommercial-NoDerivs (CC BY-NC-ND), which allows third parties to download the articles and share them with others as long as they credit the author and the Abstract Book, but they cannot change the content in any way or use them commercially.
**Abstract Book Citations:** Authors, Title, HemaSphere, 2026;10:(S1):pages. The abstract book DOIs can be found on the "Author Information" page.
**Disclaimer:** Articles published in the journal HemaSphere exclusively reflect the opinions of the authors. The authors are responsible for all content in their abstracts including accuracy of the facts, statements, citing resources, etc.

### (PB3469) STUDY ON VASCULAR ENDOTHELIAL FUNCTION IN PATIENTS WITH POLYCYTHEMIA VERA: A SINGLE-CENTER ANALYSIS

**Topic:** 16. Myeloproliferative neoplasms - Clinical

Weronika Lebowa*[1, 2], Brygida Marczyk[3], Marzena Frołow[3], Alicja Podolak[3], Stefan Chłopicki[3], Tomasz Sacha[1]

*[1]Department of Hematology and Internal Medicine, Jagiellonian University Hospital, Kraków, Poland; [2]Jagiellonian University Medical College, Doctoral School of Medical and Health Sciences, Kraków, Poland; [3]Jagiellonian University, Jagiellonian Center for Experimental Therapeutics (JCET), Kraków, Poland;*

### Background

Polycythemia vera (PV) is characterized by an increased risk of thrombotic events. Vascular endothelial dysfunction is one of the factors promoting development of thrombotic processes. Cytoreductive drugs are expected to reduce the risk of vascular complications in patients with PV modifying both the prothrombotic phenotype of the endothelium and its functional status.

### Aims

To evaluate the vascular endothelium function in PV treated with cytoreductive drugs, using panel of functional tests devoted to assess endothelial function in conduit vessels and microcirculation.

### Methods

82 PV patients were included in this prospective single-center study: 69 patients treated with cytoreductive therapy and 13 PV controls matched for gender and age without cytoreduction. The laboratory panel included peripheral blood count, basic biochemical tests, and coagulation and fibrinolysis tests. The panel of functional tests of the endothelium included flow-mediated dilation (FMD), reactive hyperemia peripheral arterial tonometry (RH-PAT), and flow-mediated skin fluorescence (FMSF). The study was approved by the local ethics committee. Statistical analysis was performed using IBM SPSS Statistics version 29.0.0.0 software.

### Results

The mean age of the PV patients was 45 years (range 20-60), 58.5% were women. The incidence of predominantly venous thrombosis in this group was 19.5% and was correlated with lower protein C activity, higher von Willebrand factor (vWF) concentrations and longer aPTT.
The most frequent cytoreductive treatment was ropeginterferon alfa-2b (66.7%), followed by peginterferon alfa-2a (11.6%), hydroxyurea (10.1%), ruxolitinib (8.7%), and combined therapy with ropeginterferon alfa-2b and ruxolitinib (2.9%).
Significantly lower FMD was observed in PV patients receiving cytoreductive therapy (regardless of the type) for 6 to 24 months compared to the control group and patients treated for less than 6 months or longer than 24 months (median FMD 3.1% vs 4.3%; p=0.032). Patients treated for 6 to 24 months had higher concentrations of vWF and factor VIII than in the control group. Similar trends in FMD were observed in patients treated with ropeginterferon alfa-2b, with the lowest FMD observed in patients treated for 9 to 24 months (median FMD 2%, while the median FMD in patients treated for longer than 24 months was 4.4%).
Patients treated with ruxolitinib had lower factor VIII concentrations (104.2% vs. 139.9%, p=0.043) and higher protein S activity (118.2% vs. 100.1%, p=0.002) compared to the control group.
In the linear regression model (r2=0.691, p=0.011) in patients treated for 6 to 24 months, FMD [%] was dependent on the level of protein S (p=0.035) – with a 1% increase in the level of protein S, FMD [%] increases by 0.111%; the concentration of vWF (p=0.037) – with a 1% increase in the concentration of vWF, FMD [%] decreases by 0.048% and the aPTT (p=0.028) – with a prolongation of aPTT by 1 second, FMD [%] decreases by 0.45%.

**Copyright Information:** (Online) ISSN: 2572-9241
©2026 The Author(s). HemaSphere is published by John Wiley & Sons Ltd on behalf of European Hematology Association. This is an open access Abstract Book distributed under the Attribution-NonCommercial-NoDerivs (CC BY-NC-ND), which allows third parties to download the articles and share them with others as long as they credit the author and the Abstract Book, but they cannot change the content in any way or use them commercially.
**Abstract Book Citations:** Authors, Title, HemaSphere, 2026;10:(S1):pages. The abstract book DOIs can be found on the "Author Information" page.
**Disclaimer:** Articles published in the journal HemaSphere exclusively reflect the opinions of the authors. The authors are responsible for all content in their abstracts including accuracy of the facts, statements, citing resources, etc.

**Summary/Conclusion**

This analysis demonstrates the impairment of endothelial function in conduit vessels measured as FMD [%] was present in PV patients and seemed to correlate with low protein S activity and high vWF concentrations. Ruxolitinib but not other drugs tended to increase protein S activity suggesting a beneficial effect on the prothrombotic phenotype of the endothelium. Assessment of endothelial function by FMD in patients with PV may be helpful to better predict thrombotic complications, but further studies are needed to substantiate this findings.

**Copyright Information:** (Online) ISSN: 2572-9241
©2026 The Author(s). HemaSphere is published by John Wiley & Sons Ltd on behalf of European Hematology Association. This is an open access Abstract Book distributed under the Attribution-NonCommercial-NoDerivs (CC BY-NC-ND), which allows third parties to download the articles and share them with others as long as they credit the author and the Abstract Book, but they cannot change the content in any way or use them commercially.
**Abstract Book Citations:** Authors, Title, HemaSphere, 2026;10:(S1):pages. The abstract book DOIs can be found on the "Author Information" page.
**Disclaimer:** Articles published in the journal HemaSphere exclusively reflect the opinions of the authors. The authors are responsible for all content in their abstracts including accuracy of the facts, statements, citing resources, etc.

### (PB3470) BELOW THE THRESHOLD, ABOVE THE RISK: THROMBOTIC IMPLICATIONS OF JAK2 VAF

**Topic:** 16. Myeloproliferative neoplasms - Clinical

Laura De Fazio*[1], Claudia Simio[1], Giulio Caridà[1], Luciano Levato[1], Matteo Molica[1], Marco Rossi[1]

[1]*Azienda Ospedaliero Universitaria Renato Dulbecco, Department of Hematology-Oncology, Catanzaro, Italy;*

### Background

Highly sensitive molecular assays increasingly detect very low-level JAK2 V617F mutations in individuals not fulfilling diagnostic criteria for myeloproliferative neoplasms (MPNs). The clinical significance of detectable but subthreshold JAK2 V617F mutation burden, particularly regarding thrombotic risk, remains unclear. While overt MPNs are strongly associated with thrombosis, the risk profile of patients with very low allele burden is not well established definitely.

### Aims

To evaluate thrombotic risk and clinical characteristics in patients harboring detectable JAK2 V617F mutation with variant allele frequency (VAF) <1%.

### Methods

We conducted a retrospective single-center study including 169 patients screened for MPN in our Institution from April 2024 to February 2026 with detectable JAK2 V617F mutation and VAF <1% through high-sensitivity molecular testing. Patients with MPN or CML diagnosis at baseline were excluded. Clinical data, laboratory parameters, cardiovascular and metabolic risk factors, and antithrombotic treatments (i.e. antiplatelet therapy and anticoagulation) were collected. Thrombotic events were defined according to international criteria.

### Results

Overall, 169 patients (122 M; 47 F) aged 56.6±16.8 years, were screened for JAK2 mutation through quantitative PCR; mean VAF of JAK2 was 0,023% ± 0,011%; mean EPO 9,32 ± 4,97 mUI/mL. In the overall cohort, prevalence was: thrombotic events 26%, chronic renal insufficiency 14.2%, obstructive sleep apnea 20.1%, prior myocardial infarction 15.4%, hypertension 68%, atrial fibrillation 4.1%, diabetes 19.5%, dyslipidemia 40.1%, COPD 10.7%, smoking 31.5%, and obesity 31.4%. No differences regarding Thrombotic events, IRC, IMA, AI, FA, DM, dyslipidemia, smoking, or obesity emerged according to sex. Non-cardiological comorbidities (79% vs 57%; $\chi 2$=7.130; *df =1*; p=0.008) were more frequent among women. Conversely, men exhibited higher rates of OSAS (25% vs 6%; $\chi 2$= 7.643; p=0.006) and COPD (14% vs 2%; $\chi 2$=4.970; p=0.026).Thrombotic events were more frequent among those with older age, dyslipidemia and antiplatelet therapy at first evaluation. In the multivariable model, JAK2 VAF emerged as the strongest independent predictor of thrombotic events (OR = $2.95 \times 10^{18}$, 95% CI $2.32 \times 10^{3}$–$3.74 \times 10^{33}$, $p = 0.017$), indicating a very large effect size. Age also remained significantly associated (OR = 1.081, 95% CI 1.046–1.117, $p < 0.001$), but with a comparatively modest magnitude.

### Summary/Conclusion

JAK2 VAF values <1% were found to be associated with an increased thrombotic risk in a small cohort. These findings highlight the need for further investigation into the relationship between subthreshold JAK2 positivity in patients with erythrocytosis and additional cardiovascular risk factors.

**Copyright Information:** (Online) ISSN: 2572-9241
©2026 The Author(s). HemaSphere is published by John Wiley & Sons Ltd on behalf of European Hematology Association. This is an open access Abstract Book distributed under the Attribution-NonCommercial-NoDerivs (CC BY-NC-ND), which allows third parties to download the articles and share them with others as long as they credit the author and the Abstract Book, but they cannot change the content in any way or use them commercially.
**Abstract Book Citations:** Authors, Title, HemaSphere, 2026;10:(S1):pages. The abstract book DOIs can be found on the “Author Information” page.
**Disclaimer:** Articles published in the journal HemaSphere exclusively reflect the opinions of the authors. The authors are responsible for all content in their abstracts including accuracy of the facts, statements, citing resources, etc.

### (PB3471) IMMATURE PLATELET FRACTION REFLECTS DYSREGULATED THROMBOPOIESIS IN PHILADELPHIA-NEGATIVE MPNS

**Topic:** 16. Myeloproliferative neoplasms - Clinical

Ivan Zekanovic*[1], Tina Marketin[1], Martina Moric Peric[1], Ante Vulić[2], Marija Milos[3, 4], Anamarija Bogic[3], Marta Marčinković[1], Margareta Radic Antolic[3], Josip Knezevic[3], Ljiljana Jurlina[1], Ana Boban[2, 5]

*[1]General Hospital Zadar, Department of Internal Medicine, Zadar, Croatia;[2]University Hospital Centre Zagreb, Department of Hematology, Zagreb, Croatia; [3]University Hospital Centre Zagreb, Department of Laboratory Diagnostics, Zagreb, Croatia; [4]University of Mostar, Faculty of Pharmacy, Mostar, Bosnia and Herzegovina;[5]University of Zagreb, School of Medicine, Zagreb, Croatia;*

### Background

Thrombotic events represent the leading cause of morbidity and mortality in patients with Philadelphia chromosome-negative myeloproliferative neoplasms (Ph⁻ MPN), particularly in those aged >60 years. Despite extensive research, the precise mechanisms driving the prothrombotic state in MPN remain incompletely understood. Immature (reticulated) platelets are newly released from megakaryocytes, are larger, contain more dense granules and residual mRNA, and exhibit greater thrombotic potential than mature platelets. The immature platelet fraction (IPF) reflects the proportion of newly released, reticulated, highly reactive platelets and has emerged as a marker of thrombopoietic activity in various prothrombotic conditions.

### Aims

We conducted a prospective study to characterize IPF in newly diagnosed Ph⁻ MPN patients, compare them with non-MPN controls, and explore its variation according to subtype, age, and JAK2V617F status in order to evaluate its potential relevance as a marker of hypercoagulability.

### Methods

This prospective observational study was performed at General Hospital Zadar and University Hospital Centre Zagreb, Croatia, between March 2024 and October 2025. All adult patients undergoing diagnostic bone marrow examination for suspected Ph⁻ MPN were eligible. Peripheral blood samples were collected at the time of diagnostic bone marrow biopsy. Descriptive statistics were used for the summary presentation of patient characteristics. Continuous variables were compared with the Mann–Whitney U test, and continuous variables across groups using the Kruskal–Wallis test.

**Copyright Information:** (Online) ISSN: 2572-9241
©2026 The Author(s). HemaSphere is published by John Wiley & Sons Ltd on behalf of European Hematology Association. This is an open access Abstract Book distributed under the Attribution-NonCommercial-NoDerivs (CC BY-NC-ND), which allows third parties to download the articles and share them with others as long as they credit the author and the Abstract Book, but they cannot change the content in any way or use them commercially.
**Abstract Book Citations:** Authors, Title, HemaSphere, 2026;10:(S1):pages. The abstract book DOIs can be found on the "Author Information" page.
**Disclaimer:** Articles published in the journal HemaSphere exclusively reflect the opinions of the authors. The authors are responsible for all content in their abstracts including accuracy of the facts, statements, citing resources, etc.

### Results

Forty-five patients with newly diagnosed Ph⁻ MPN and 27 controls were enrolled. Among patients with MPN, 24 (53%) patients were diagnosed as having essential thrombocythemia, 13 (28%) patients with polycythemia vera, 5 (11%) with unclassified MPN, and 3 (6%) with primary myelofibrosis. There were 19 (42%) females in the Ph-MPN group and 8 (29%) in the control group. Patients with Ph⁻ MPN were significantly older than controls (median 65 vs. 50 years) and had higher platelet counts (median 572 × $10^9$/L vs. 238 × $10^9$/L, $p < 0.0001$) and LDH (median 226 vs. 178 U/L, $p < 0.001$). JAK2V617F was positive in 75% of MPN patients overall (92% of PV, 70% of ET, 66% of PMF, 80% of unclassified MPN). The IPF values were higher in Ph-MPN patients (median 27, range 6-126) in comparison to the control group (median 10.9, range 3.6-41, p< 0.0001). No differences were observed in IPF values between JAK2-positive and JAK2-negative MPN patients (median 26 vs.40.7, p= 0.25). However, higher values of hematocrit were found among JAK2-positive patients (median 0.47 vs. 0.41, p= 0.032). When comparing the Ph-MPN patients in the age group older than 60 years we found values of IPF (median 38.8, range 10.2-126) which were higher compared to Ph-MPN who were younger than 60 years (median 23.5, range 6-50, p=0.021), but we did not found any difference between the platelet count between these two age groups (median 572 vs. 604, p=0.84).

### Summary/Conclusion

IPF is significantly elevated in Philadelphia chromosome-negative myeloproliferative neoplasms and shows subtype- and age-specific patterns. These findings could highlight IPF as a readily available marker of dysregulated thrombopoiesis that may complement conventional hematological parameters. Further studies are warranted to determine whether IPF can improve thrombotic risk stratification in Ph⁻ MPNs.

**Copyright Information:** (Online) ISSN: 2572-9241
©2026 The Author(s). HemaSphere is published by John Wiley & Sons Ltd on behalf of European Hematology Association. This is an open access Abstract Book distributed under the Attribution-NonCommercial-NoDerivs (CC BY-NC-ND), which allows third parties to download the articles and share them with others as long as they credit the author and the Abstract Book, but they cannot change the content in any way or use them commercially.
**Abstract Book Citations:** Authors, Title, HemaSphere, 2026;10:(S1):pages. The abstract book DOIs can be found on the "Author Information" page.
**Disclaimer:** Articles published in the journal HemaSphere exclusively reflect the opinions of the authors. The authors are responsible for all content in their abstracts including accuracy of the facts, statements, citing resources, etc.

### (PB3472) SYMPTOMATIC HIP AND KNEE OSTEOARTHRITIS IS ASSOCIATED WITH HIGHER SYMPTOM BURDEN AND HIGHER THROMBOTIC RISK IN PATIENTS WITH MYELOPROLIFERATIVE NEOPLASMS

**Topic:** 16. Myeloproliferative neoplasms - Clinical

Hrvoje Holik*[1], Ivan Krecak[2, 3, 4], Marko Lucijanic[5, 6, 7], Ivan Samardžić[8], Danijel Pilipac[9], Ivana Ljubicic Vucinic[1], Bozena Coha[1], Blaženka Miškić[10, 11], Silva Zupančić Šalek[12]

*[1]General Hospital dr. Josip Benčević, Hematology and Oncology, Slavonski Brod, Croatia;[2]General Hospital of Sibenik-Knin County, Department of Internal Medicine, Šibenik, Croatia; [3]University of Rijeka, Faculty of Medicine, Rijeka, Croatia;[4]University of Applied Sciences Šibenik, Šibenik, Croatia; [5]University Hospital Dubrava, Hematology Department,, Zagreb, Croatia; [6]University Hospital Dubrava, Scientific Research and Translational Medicine Department, Zagreb, Croatia;[7]University of Zagreb, School of Medicine, Zagreb, Croatia; [8]General Hospital dr. Josip Benčević, Department of Orthopaedic Surgery, Slavonski Brod, Croatia; [9]General Hospital of Sibenik-Knin County, Department of Orthopaedic Surgery, Šibenik, Croatia; [10]General Hospital dr. Josip Benčević, Department of Internal Medicine, Slavonski Brod, Croatia;[11]University of Osijek Josip Juraj Strossmayer, Faculty of Dental Medicine, Osijek, Croatia; [12]Josip Juraj Strossmayer University of Osijek, Faculty of Medicine, Osijek, Croatia;*

### Aims

Patients with BCR::ABL1-negative myeloproliferative neoplasms (MPNs), essential thrombocythemia (ET), polycythemia vera (PV), and myelofibrosis (MF), have a high prevalence of symptomatic osteoarthritis (OA; Holik et al., *Life,* 2023) and are burdened with cardiovascular complications. This secondary analysis from that particular study additionally evaluated the potential impact of OA on MPN-related symptoms and thrombotic risk.

### Methods

This was a prospective two-center study conducted in Croatia. Consecutive MPN patients were included in the period from 2021-2023. Participants fulfilled the Myeloproliferative Symptom Assessment Form (MPN-SAF) at the time of study entry and were evaluated by orthopedic surgeon; those with suspected symptomatic hip and knee OA (joint symptoms) additionally underwent X-ray imaging of the symptomatic joint(s) with O being defined as grade≥1 according to the radiological Kellgreen-Lawrence classification. The chi-square and the Mann-Whitney tests were used to analyze differences in categorical and continuous variables, respectively. Multiple regression analysis was performed to analyze the impact of OA on TSS. Time-to-thrombosis (TTT; death being a censoring event) was measured from the time of diagnosis until first arterial or venous event. Survival analyses were performed using the Kaplan-Meier method, the log-rank test, and the Cox regression analysis.

**Copyright Information:** (Online) ISSN: 2572-9241
©2026 The Author(s). HemaSphere is published by John Wiley & Sons Ltd on behalf of European Hematology Association. This is an open access Abstract Book distributed under the Attribution-NonCommercial-NoDerivs (CC BY-NC-ND), which allows third parties to download the articles and share them with others as long as they credit the author and the Abstract Book, but they cannot change the content in any way or use them commercially.
**Abstract Book Citations:** Authors, Title, HemaSphere, 2026;10:(S1):pages. The abstract book DOIs can be found on the “Author Information” page.
**Disclaimer:** Articles published in the journal HemaSphere exclusively reflect the opinions of the authors. The authors are responsible for all content in their abstracts including accuracy of the facts, statements, citing resources, etc.

**Results**

107 MPN patients (ET=44, PV=35, MF=28) were included; median age was 68 years (range 35-90) and 54.2% were females. Symptomatic OA of any localisation was detected in 64 (59.8%) patients and was associated with older age (p<0.001), *JAK2* mutation (p=0.018), prior thrombosis (p=0.018), higher body weight (p<0.001), higher body mass index (p<0.001), higher uric acid (p=0.008), arterial hypertension (p=0.008), hyperlipidemia (p<0.001), analgetics (p=0.015) and opioid use (p=0.026). Median TSS was 16 (range 0-50) and was significantly higher in patients with symptomatic OA (p<0.001), as were the majority of MPN-SAF items (Figure 1A). In the multivariate linear regression, presence of OA (p<0.001) and female sex (p=0.003) were independently of each other associated with higher TSS score when additionally adjusted for age, MF phenotype, cytoreductive treatment and cardiovascular risk factors (p>0.050 for all analyses).
The median follow-up was 44 months (3-72); 10 (9.3%) thrombotic events occurred (arterial=8, venous=2) during this time. Patients with symptomatic OA had statistically significantly inferior TTT (HR 3.61, p=0.046) as presented in Figure 1B. Other variables associated with an inferior TTT were age>60 years, prior thrombosis, arterial hypertension, non-aspirin use and male sex (p<0.050 for all analyses); there was no difference in TTT among different MPN disease phenotypes (p=0.386). Symptomatic OA was independently (HR 5.22, p=0.002) of male sex (HR 4.30, p=0.042) and non-aspirin use (HR 5.42, p=0.019) associated with an inferior TTT when additionally adjusted for advanced age, prior thrombosis, arterial hypertension, MPN phenotype and cytoreductive treatment (p>0.050 for all analyses); the results remained consistent for hip vs. knee OA (p<0.050 for both analyses).

**Summary/Conclusion**

This study suggests that symptomatic OA may be independently associated with higher symptom burden and higher thrombotic risk in MPNs emphasizing the importance of early OA recognition in order to provide appropriate and timely patient counselling.

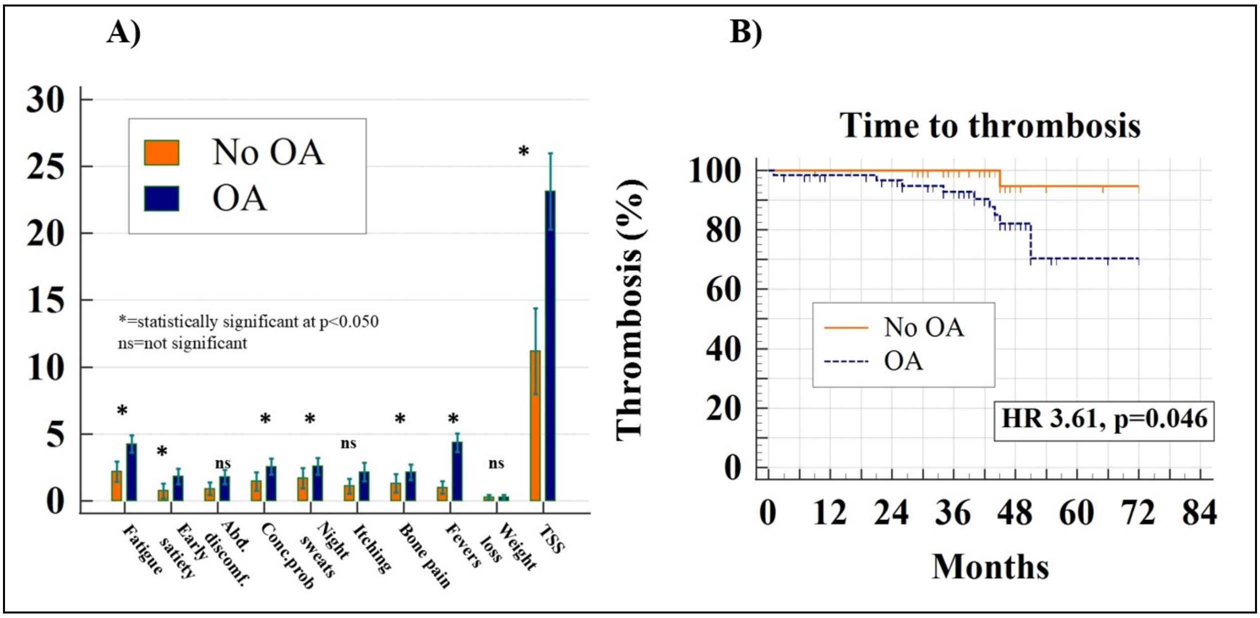

**Copyright Information:** (Online) ISSN: 2572-9241
©2026 The Author(s). HemaSphere is published by John Wiley & Sons Ltd on behalf of European Hematology Association. This is an open access Abstract Book distributed under the Attribution-NonCommercial-NoDerivs (CC BY-NC-ND), which allows third parties to download the articles and share them with others as long as they credit the author and the Abstract Book, but they cannot change the content in any way or use them commercially.
**Abstract Book Citations:** Authors, Title, HemaSphere, 2026;10:(S1):pages. The abstract book DOIs can be found on the "Author Information" page.
**Disclaimer:** Articles published in the journal HemaSphere exclusively reflect the opinions of the authors. The authors are responsible for all content in their abstracts including accuracy of the facts, statements, citing resources, etc.

### (PB3473) ASSOCIATION OF CLINICAL AND MOLECULAR RISK FACTORS WITH SEVERITY OF ANEMIA ASSOCIATED WITH MYELOFIBROSIS

**Topic:** 16. Myeloproliferative neoplasms - Clinical

Verna Cheung*[1], Eshetu Atenafu[1], Andrea Arruda[1], Jaime Claudio[1], Taylor Nye[1], Amulya Bhagirath[1], Ann George[1], Jessica Danquah[1], Steven M Chan[1], Marta Davidson[1], Aniket Bankar[1], Hassan, Sibai[1], Dawn Maze[1], Vikas Gupta[1]

[1]*Princess Margaret Cancer Centre, Department of Biostatistics, Toronto, Canada;*

### Background

Anemia with hemoglobin under 100g/L is recognized as a poor prognostic factor in patients with myelofibrosis (MF). Close to 50% of patients have transfusion requiring anemia within 1-year of diagnosis. MF-related anemia can be challenging to manage as it is often multifactorial including: related to underlying disease or associated complications, inflammation, abnormal iron metabolism, treatment, and comorbidities. As part of standard of care, a detailed anemia work-up (DAW) is completed according to the consensus expert recommendations for MF patients with anemia (Koshminder et al., 2024).

### Methods

To better understand anemia associated with MF, we conducted a retrospective chart review of MF patients presenting with anemia, at the Princess Margaret Cancer Centre between August 2024 to February 2026. The data set contains patient baseline characteristics such as sex, fibrosis grade, EPO level, DIPSS, MIPSS70 2.0 at index date (date of anemia work-up), prior bleeding history, molecular mutations (*JAK2, CALR, MPL, ASXL1, SF3B1, SRSF2, U2AF1, ZRSR2, IDH1, IDH2, EZH2, and TP53*), and clinical parameters such as complete blood counts, iron studies, and CRP. Anemia was defined by both the sex-specific range proposed in 2024 by the International-Working Group-European LeukemiaNet (Tefferi et al, 2024), and the recent consensus recommendations (Koshminder et al, 2024).

### Results

A total of 129 patients were included in the analysis; 46 (36%) female, and 83 (64%) male. The median age was 72 (range 31-87) years. Through our DAW other cause of anemia were found, i.e. bleeding (9%), hemoglobinopathy (4%), concurrent malignancies (5%). More male vs female patients had moderate (N=49 [61.2%] vs N=31 [38.8%]) and severe anemia (N=8 [53.3%] vs N=7 [46.7%], p=0.19). MPN driver mutations (*JAK2, CALR and MPL*) did not have a significant impact to severity of anemia (p=0.18). Interestingly 32% of patients had a spliceosome mutation. Amongst the spliceosome mutations, *SF3B1, SRSF2, U2AF1, ZRSR2,* only *U2AF1* were associated with severity of anemia (p= 0.003). Clinical parameters such as platelet level (p=0.0006), albumin (p=0.006), reticulocyte count (p=0.003), ferritin (p<0.0001), and EPO level <250 (p=0.0154) were associated with severity of anemia, even when adjusted for sex-specific hemoglobin. Additionally, when adjusted for sex-specific severity of anemia, the presence of *SF3B1* had an impact in severe anemia (N=9 [25%] p=0.002), as well as *U2AF1* (N=10 [27%] p=0.0035) for both sexes. Sankey plot shows (Fig 1) progressive worsening of anemia in majority of patients and higher number of deaths in patients with moderate and severe anemia in patients with 6 months follow up.

### Summary/Conclusion

This study highlighting the importance of DAW for anemia assessment in MF as a significant number of patients have anemia in MF due to causes other than underlying disease or its treatment. MPN specific treatment options unlikely to be effective in these patients. Male sex and spliceosome mutations such as SF3B1 and U2AF1 have significant associations with severity of anemia.

**Copyright Information:** (Online) ISSN: 2572-9241
©2026 The Author(s). HemaSphere is published by John Wiley & Sons Ltd on behalf of European Hematology Association. This is an open access Abstract Book distributed under the Attribution-NonCommercial-NoDerivs (CC BY-NC-ND), which allows third parties to download the articles and share them with others as long as they credit the author and the Abstract Book, but they cannot change the content in any way or use them commercially.
**Abstract Book Citations:** Authors, Title, HemaSphere, 2026;10:(S1):pages. The abstract book DOIs can be found on the "Author Information" page.
**Disclaimer:** Articles published in the journal HemaSphere exclusively reflect the opinions of the authors. The authors are responsible for all content in their abstracts including accuracy of the facts, statements, citing resources, etc.

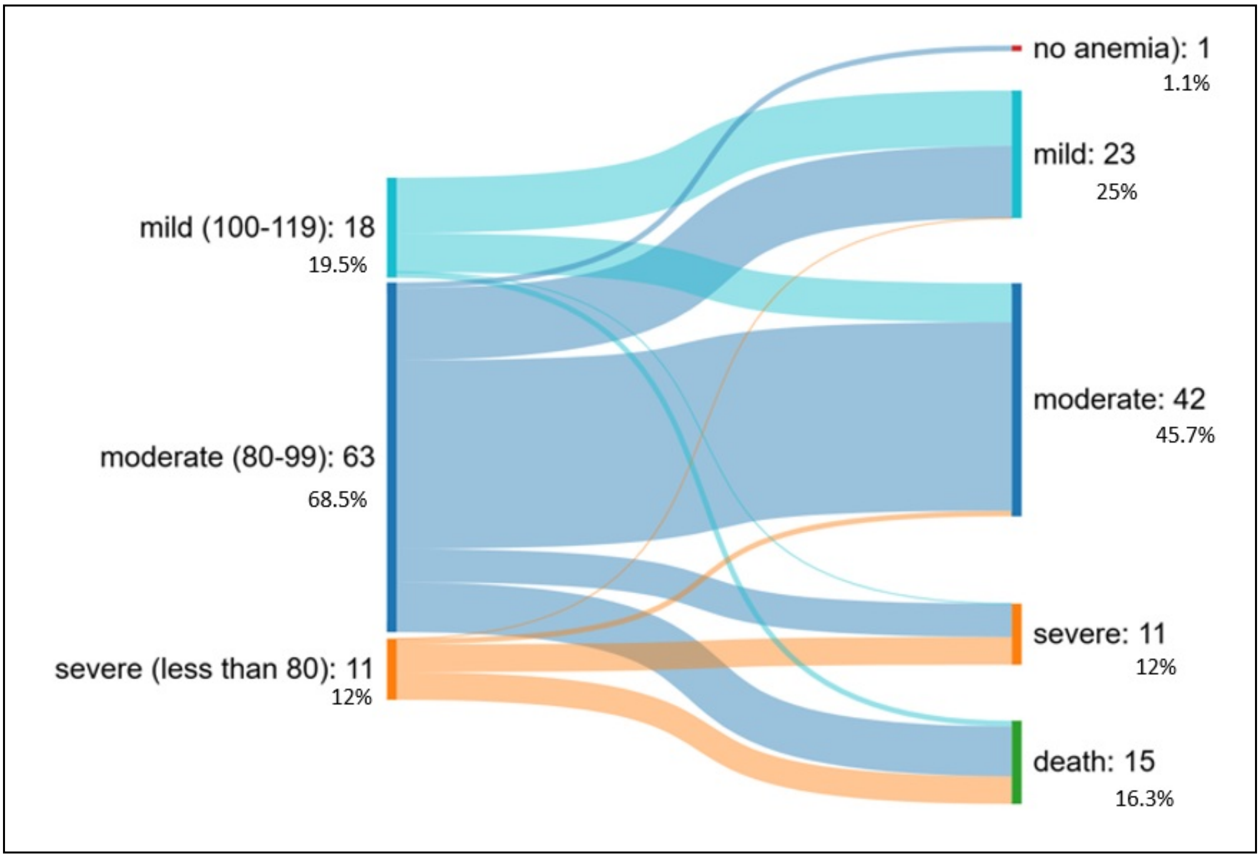

Figure 1 change of anemia over 6 months follow-up

**Copyright Information:** (Online) ISSN: 2572-9241
©2026 The Author(s). HemaSphere is published by John Wiley & Sons Ltd on behalf of European Hematology Association. This is an open access Abstract Book distributed under the Attribution-NonCommercial-NoDerivs (CC BY-NC-ND), which allows third parties to download the articles and share them with others as long as they credit the author and the Abstract Book, but they cannot change the content in any way or use them commercially.
**Abstract Book Citations:** Authors, Title, HemaSphere, 2026;10:(S1):pages. The abstract book DOIs can be found on the "Author Information" page.
**Disclaimer:** Articles published in the journal HemaSphere exclusively reflect the opinions of the authors. The authors are responsible for all content in their abstracts including accuracy of the facts, statements, citing resources, etc.

### (PB3474) REAL WORLD PRACTICE REVEALS HEMATOLOGIC INDICES AS EFFICACY PREDICTORS FOR MYELOFIBROSIS PATIENTS SWITCHING FROM RUXOLITINIB TO GECACITINIB

**Topic:** 16. Myeloproliferative neoplasms - Clinical

Xinjie Chen*[1], Wenfang Wang[1], Yuanfei Mao[2], Xiaodong Gao[1], Sujiang Zhang[1], Wen Wu[1]

*[1]Shanghai Institute of Hematology, State Key Laboratory of Medical Genomics, National Research Center for Translational Medicine at Shanghai, Ruijin Hospital Shanghai Jiao Tong University School of Medicine, Shanghai, China; [2]Clinical Hematology Center , Shanghai Municipal Hospital of Traditional Chinese Medicine, Shanghai University of Traditional Chinese Medicine, Shanghai, China;*

### Background

Gecacitinib (GCA), a dual JAK/ACVR1 inhibitor, has shown promising efficacy in improving anemia and reducing transfusion dependence in myelofibrosis (MF) patients who are intolerant or refractory to ruxolitinib (Ruxo). In real-world clinical practice, MF patients switching from Ruxo to GCA often present with poor clinical conditions and an urgent need for early efficacy assessment, highlighting the critical importance of reliable early predictors of treatment response. However, significant heterogeneity in clinical response exists among these patients, and the lack of validated early efficacy predictors hinders the implementation of individualized treatment strategies and timely therapeutic adjustments.

### Aims

This study aimed to explore the predictive value of baseline and dynamic hematologic indices for clinical efficacy in MF patients switching from Ruxo to GCA, and to identify the optimal early time point for predicting anemia response.

### Methods

A total of 37 MF patients who switched from Ruxo to GCA in real-world practice were enrolled. Serial hemoglobin (HGB) levels were measured at baseline and at Weeks 2, 4, 6, 8, and 12. Anemia response was defined as a ≥10% increase in HGB level at Week 12 compared with baseline.

### Results

The clinical characteristics of the 37 enrolled patients are summarized in Table 1. The median duration of prior Ruxo treatment was 12 months (range, 2–96 months), and 37.8% of patients had a high baseline white blood cell (WBC>$10\times10^9$/L).
At Week 12 after GCA initiation, the overall anemia response rate was 48.6%. A high baseline WBC count was negatively associated with anemia response, as evidenced by a higher HGB elevation rate in patients with a baseline WBC count ≤$10\times10^9$/L (65.2%) compared with those with a count >$10\times10^9$/L (28.6%). Anemia responders with baseline WBC ≤$10\times10^9$/L also experienced a greater absolute HGB increase after GCA treatment (median ΔHGB: +30 g/L vs. +12 g/L in responders with high baseline WBC, P = 0.032; Figure A).
Dynamic HGB changes from Week 2 to Week 8 were significantly associated with Week 12 anemia response, with Week 6 identified as the optimal predictive time point (OR = 12.73, P = 0.006). Anemia responders had a significantly greater median HGB elevation at Week 6 (+18.5 g/L) compared with non-responders (-1 g/L, P<0.001, Figure B). Notably, anemia response was not associated with baseline anemia severity, platelet counts, or disease risk scores (DIPSS/MIPSS).

**Copyright Information:** (Online) ISSN: 2572-9241
©2026 The Author(s). HemaSphere is published by John Wiley & Sons Ltd on behalf of European Hematology Association. This is an open access Abstract Book distributed under the Attribution-NonCommercial-NoDerivs (CC BY-NC-ND), which allows third parties to download the articles and share them with others as long as they credit the author and the Abstract Book, but they cannot change the content in any way or use them commercially.
**Abstract Book Citations:** Authors, Title, HemaSphere, 2026;10:(S1):pages. The abstract book DOIs can be found on the "Author Information" page.
**Disclaimer:** Articles published in the journal HemaSphere exclusively reflect the opinions of the authors. The authors are responsible for all content in their abstracts including accuracy of the facts, statements, citing resources, etc.

### Summary/Conclusion

In real-world practice for MF patients switching from Ruxo to GCA, HGB elevation at week 6 serves as a simple and effective early predictor of anemia response. A baseline WBC count >10×10⁹/L is associated with inferior anemia response, while severe baseline anemia and DIPSS, or MIPSS scores do not impact the HGB-improving efficacy of GCA. Dynamic monitoring of HGB changes at early timepoint after GCA initiation can guide individualized treatment decisions for Ruxo-exposed MF patients in real-world clinical settings. Given that GCA is often combined with other erythropoietic agents in real-world practice, larger sample sizes and prolonged following are warranted for further exploration.

**Table 1. Baseline characteristics and therapy response**

| | |
|---|---|
| **Age in years, median (range)** | 69 (37-81) |
| **Age > 65 years, n** | 21 (56.8%) |
| **Gender, n** | |
| Male | 19 (34.5%) |
| Female | 18 (32.7%) |
| **Type of MF, n (%)** | |
| Primary MF | 29 (78.4%) |
| Post-PV/ET MF | 8 (21.6%) |
| **WBC (*10^9/L), median (range)** | 6.3 (1.4-111.27) |
| **Hb (g/L), median (range)** | 73 (42-119) |
| **PLT (*10^9/L), median (range)** | 153 (2-1053) |
| **DIPSS (n,%)** | |
| Intermediate-1 | 4 (10.8%) |
| Intermediate-2 | 28 (75.7%) |
| High | 5 (13.5%) |
| **MIPSS70 (n,%)** | |
| Low | 2 (5.4%) |
| Intermediate | 18 (48.6%) |
| High | 17 (45.9%) |
| ***Spleen length below costal margin, median (range), cm** | 8 (0-24) |
| **Driver mutations, n** | |
| *JAK2$^{V617F}$* | 20 (54.0%) |
| *CALR* | 7 (18.9%) |
| *MPL* | 3 (8.1%) |
| Triple-negative | 7 (18.9%) |
| **Time since MF diagnosis, months (range)** | 12 (2-132) |
| **Ruxolitinib duration before Gecacitinib switch,months (range)** | 12 (2-96) |
| **Treatment Regimen, n (%)** | |
| Gecacitinib monotherapy | 19 (51.4%) |
| Gecacitinib + erythropoietic agents | 18 (48.6%) |

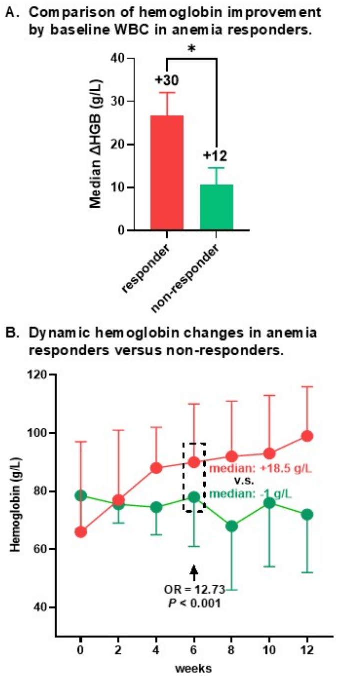

**Copyright Information:** (Online) ISSN: 2572-9241
©2026 The Author(s). HemaSphere is published by John Wiley & Sons Ltd on behalf of European Hematology Association. This is an open access Abstract Book distributed under the Attribution-NonCommercial-NoDerivs (CC BY-NC-ND), which allows third parties to download the articles and share them with others as long as they credit the author and the Abstract Book, but they cannot change the content in any way or use them commercially.
**Abstract Book Citations:** Authors, Title, HemaSphere, 2026;10:(S1):pages. The abstract book DOIs can be found on the "Author Information" page.
**Disclaimer:** Articles published in the journal HemaSphere exclusively reflect the opinions of the authors. The authors are responsible for all content in their abstracts including accuracy of the facts, statements, citing resources, etc.

### (PB3475) COMPARISON OF ROTEM IN PATIENTS WITH MYELOPROLIFERATIVE NEOPLASMS AND CONTROLS WITH NON-CLONAL POLYCYTHEMIA AND/OR THROMBOLEUKOCYTOSIS AND FOLLOWING INITIATION OF CYTOREDUCTION

**Topic:** 16. Myeloproliferative neoplasms - Clinical

Alsu Gatina*[1], Ingrid Lundahl[1], Samuel Wiqvist[2], Jovan Antovic[1], Roza Chaireti[1]

*[1]Karolinska Institutet, Molecular Medicine and Surgery, Stockholm, Sweden; [2]Karolinska Institutet, Learning, Informatics, Management and Ethics, Stockholm, Sweden;*

### Background

Haemostatic profiles in patients with myeloproliferative neoplasms (MPN) using rotational thromboelastometry (ROTEM) remain insufficiently characterized, limiting its role as a diagnostic and follow-up method.

### Aims

To compare ROTEM parameters between patients with and without MPN and to evaluate changes following cytoreduction.

### Methods

This prospective cohort study included 92 consecutive adults evaluated for suspected MPN, of whom 59 had MPN and 33 were used as controls. Blood samples were obtained at baseline and at 3 and 6 months after cytoreduction.
Whole-blood haemostasis was assessed using ROTEM. Clinical variables included MPN diagnosis (polycythaemia vera, essential thrombocythaemia, myelofibrosis), mutational status (JAK2, CALR), haemoglobin, platelet count, and leucocyte count.
The median age was 64.5 years (IQR 53.0-75.3) overall, 70.0 years (56.0-77.0) in patients with MPN and 55.0 years (IQR 46.0-70.0) in controls. Among controls, 12 were female and 21 male, whereas the MPN group comprised 40 females and 19 males. Within MPN, 16 had polycythaemia vera, 32 essential thrombocythaemia, and 9 myelofibrosis. JAK2 and CALR mutations were present in 47 and 7 patients, respectively.
Between-group differences were analysed using Mann–Whitney U or Kruskal–Wallis tests, correlations using Spearman's rank test, and longitudinal within-patient changes using the Friedman test. Two-sided p values $< 0.05$ were considered statistically significant.

### Results

MPN differed from controls in clot firmness and fibrinolysis (INTEM CFT, a-angle, A5, MCF, EXTEM/FIBTEM ML, $p < 0.05$). Haemoglobin correlated inversely with clot firmness, whereas platelet count correlated positively with propagation and firmness. ROTEM profiles were comparable between CALR- and JAK2-mutated patients. Clot firmness varied across diagnostic subtypes, being highest in essential thrombocythaemia. Following initiation of cytoreduction and over time, firmness parameters decreased.

**Copyright Information:** (Online) ISSN: 2572-9241
©2026 The Author(s). HemaSphere is published by John Wiley & Sons Ltd on behalf of European Hematology Association. This is an open access Abstract Book distributed under the Attribution-NonCommercial-NoDerivs (CC BY-NC-ND), which allows third parties to download the articles and share them with others as long as they credit the author and the Abstract Book, but they cannot change the content in any way or use them commercially.
**Abstract Book Citations:** Authors, Title, HemaSphere, 2026;10:(S1):pages. The abstract book DOIs can be found on the "Author Information" page.
**Disclaimer:** Articles published in the journal HemaSphere exclusively reflect the opinions of the authors. The authors are responsible for all content in their abstracts including accuracy of the facts, statements, citing resources, etc.

**Summary/Conclusion**

MPN is associated with altered clot firmness and fibrinolysis compared to controls, related mainly to blood cell counts and diagnosis rather than mutational status, with reduced clot strength following cytoreduction. ROTEM reflects therefore part of the hypercoagulability of MPN and could potentially be used as a diagnostic marker as well as a measure of the effect of cytoreduction.

**Table 1. Baseline characteristics**

| Characteristic | All (n=92) | MPN (n=59) | Controls (n=33) |
|---|---|---|---|
| Age, median (IQR), years | 64.5 (53.0–75.3) | 70.0 (56.0–77.0) | 55.0 (46.0–70.0) |
| Female, n (%) | 52 (57%) | 40 (68%) | 12 (36%) |
| Male, n (%) | 40 (43%) | 19 (32%) | 21 (64%) |
| Previous thrombosis, n (%) | 23 (25%) | 16 (27%) | 7 (21%) |
| Polycythaemia vera (MPN) | | 16 (27%) | |
| Essential thrombocythaemia (MPN) | | 32 (54%) | |
| Myelofibrosis (MPN) | | 9 (15%) | |
| JAK2 mutation (MPN) | | 47 (80%) | |
| CALR mutation (MPN) | | 7 (12%) | |

Values are median (IQR) or n (%).

**Copyright Information:** (Online) ISSN: 2572-9241
©2026 The Author(s). HemaSphere is published by John Wiley & Sons Ltd on behalf of European Hematology Association. This is an open access Abstract Book distributed under the Attribution-NonCommercial-NoDerivs (CC BY-NC-ND), which allows third parties to download the articles and share them with others as long as they credit the author and the Abstract Book, but they cannot change the content in any way or use them commercially.
**Abstract Book Citations:** Authors, Title, HemaSphere, 2026;10:(S1):pages. The abstract book DOIs can be found on the "Author Information" page.
**Disclaimer:** Articles published in the journal HemaSphere exclusively reflect the opinions of the authors. The authors are responsible for all content in their abstracts including accuracy of the facts, statements, citing resources, etc.

### (PB3477) BEYOND SIX MONTHS: EXTENDED REAL-WORLD VALIDATION OF THE RR6 MODEL FOR EARLY RISK STRATIFICATION IN RUXOLITINIB-TREATED MYELOFIBROSIS

**Topic:** 16. Myeloproliferative neoplasms - Clinical

Erika Morsia*[1], Dorela Lame[1], Michelangelo Pianelli[1], Riccardo Ceccarelli[1], Sonia Morè[1], Antonella Poloni[1]

[1] *Department of Clinical and Molecular Sciences, Università Politecnica delle Marche, Ancon, Italy;*

### Background

Ruxolitinib represents a standard therapy for patients with primary and secondary myelofibrosis (MF), leading to symptom control and spleen reduction. Nevertheless, treatment failure, resistance, or intolerance frequently emerge and are associated with reduced overall survival (OS). Traditional prognostic scores such as DIPSS and MYSEC-PM were developed at diagnosis and do not adequately capture prognosis during ruxolitinib treatment. The RR6 model was recently proposed to identify early suboptimal responders after six months of therapy.

### Aims

To externally validate the RR6 prognostic model in a real-world cohort of MF patients treated with ruxolitinib and to investigate its prognostic applicability at both 6 and 12 months of treatment.

### Methods

We conducted a retrospective single-center analysis including patients with primary MF or secondary MF (post–polycythemia vera or post–essential thrombocythemia) diagnosed according to WHO/ICC 2022 criteria and treated with ruxolitinib for at least six months at the Hematology Clinic of Ancona between 2011 and 2024. Clinical, laboratory, molecular, and cytogenetic variables were collected at baseline, treatment initiation, and at 3, 6, and 12 months. RR6 scores were calculated using the validated online calculator (), integrating ruxolitinib dose (<20 mg twice daily), spleen response (≤30% reduction), and transfusion dependence. Prognostic performance was assessed against DIPSS (PMF) and MYSEC-PM (SMF) using Kaplan–Meier survival analysis, Cox proportional hazards models, and concordance index (C-index).

### Results

A total of 128 patients were analyzed (52.3% male; median age 70 years, range 23–92), including 49.2% with PMF and 50.8% with SMF (25.8% post-PV and 25% post-ET). Driver mutations were distributed as follows: JAK2 V617F in 72.7%, CALR in 11.7%, MPL in 1.6%, and 14% triple-negative cases. High-molecular-risk mutations (including ASXL1, U2AF1, and IDH2) were identified in a minority of patients. The most frequent starting ruxolitinib dose was 20 mg twice daily (42.2%). Survival analyses included 109 patients with at least six months of follow-up (median follow-up 32.6 months). At 6 months, RR6 stratified patients into low- (16.5%), intermediate- (41.3%), and high-risk (50.5%) categories, with significantly inferior OS observed in the high-risk group ($p<0.01$). While RR6 correlated with conventional prognostic scores, it demonstrated superior capability in identifying early treatment failure. Importantly, RR6 maintained prognostic value at 12 months, improving risk discrimination particularly among intermediate-risk patients. Univariate Cox analysis showed that RR6 score, age >65 years, baseline anemia (Hb <10 g/dL), ongoing transfusion dependence, and absence of spleen response at 12 months were associated with poorer OS. The model showed strong predictive accuracy according to C-index analysis.

**Copyright Information:** (Online) ISSN: 2572-9241
©2026 The Author(s). HemaSphere is published by John Wiley & Sons Ltd on behalf of European Hematology Association. This is an open access Abstract Book distributed under the Attribution-NonCommercial-NoDerivs (CC BY-NC-ND), which allows third parties to download the articles and share them with others as long as they credit the author and the Abstract Book, but they cannot change the content in any way or use them commercially.
**Abstract Book Citations:** Authors, Title, HemaSphere, 2026;10:(S1):pages. The abstract book DOIs can be found on the “Author Information” page.
**Disclaimer:** Articles published in the journal HemaSphere exclusively reflect the opinions of the authors. The authors are responsible for all content in their abstracts including accuracy of the facts, statements, citing resources, etc.

**Summary/Conclusion**

In this real-world validation study, the RR6 model proved to be an effective dynamic prognostic tool for patients with MF receiving ruxolitinib. Its ability to identify high-risk patients early and to retain prognostic relevance at 12 months supports its use in guiding timely therapeutic decisions, including treatment modification or transplant evaluation, ultimately promoting a more personalized management approach in myelofibrosis.

**Copyright Information:** (Online) ISSN: 2572-9241
©2026 The Author(s). HemaSphere is published by John Wiley & Sons Ltd on behalf of European Hematology Association. This is an open access Abstract Book distributed under the Attribution-NonCommercial-NoDerivs (CC BY-NC-ND), which allows third parties to download the articles and share them with others as long as they credit the author and the Abstract Book, but they cannot change the content in any way or use them commercially.
**Abstract Book Citations:** Authors, Title, HemaSphere, 2026;10:(S1):pages. The abstract book DOIs can be found on the "Author Information" page.
**Disclaimer:** Articles published in the journal HemaSphere exclusively reflect the opinions of the authors. The authors are responsible for all content in their abstracts including accuracy of the facts, statements, citing resources, etc.

### (PB3478) DYNAMIC DNA METHYLATION CHANGES DURING DISEASE COURSE IN JUVENILE MYELOMONOCYTIC LEUKEMIA

**Topic:** 16. Myeloproliferative neoplasms - Clinical

Chenmeng Liu*[1], Zixi Yin[1], Xingchen Wang[1], Wenbin An[1], Yang Wan[1], Li Zhang[1], Yao Zou[1], Yumei Chen[1], Xiaojuan Chen[1], Ye Guo[1], Wenyu Yang[1], Xiaofan Zhu[1]

[1]*Institute of Hematology & Blood Diseases Hospital, Chinese Academy of Medical Sciences & Peking Union Medical College, tianjin, China;*

### Background

Juvenile myelomonocytic leukemia (JMML) is a rare myelodysplastic/myeloproliferative neoplasm that arises during infancy and early childhood. Aberrant DNA methylation has been identified as an independent prognostic factor in JMML. However, studies investigating the dynamic changes of DNA methylation throughout disease progression, including remission and relapse, remain limited.

### Aims

This study aimed to characterize longitudinal DNA methylation changes in JMML patients throughout disease progression, including remission and relapse, and to evaluate the concordance between bone marrow and peripheral blood methylation profiles.

### Methods

DNA methylation was assessed in 15 JMML patients. Two patients were profiled using the Illumina Infinium MethylationEPIC 850K BeadChip (EPIC array), while the remaining 11 patients were analyzed by targeted bisulfite sequencing of 59,855 JMML-related CpG sites using next-generation sequencing (NGS). Site-level gene set enrichment analysis (GSEA) was performed with Hallmark pathways, using CpG methylation changes as the ranking metric to evaluate pathway-level DNA methylation alterations across treatment stages. For the 13 patients with paired bone marrow (BM) and peripheral blood (PB) samples, Pearson correlation coefficients were calculated to assess concordance between BM and PB methylation profiles.

**Copyright Information:** (Online) ISSN: 2572-9241
©2026 The Author(s). HemaSphere is published by John Wiley & Sons Ltd on behalf of European Hematology Association. This is an open access Abstract Book distributed under the Attribution-NonCommercial-NoDerivs (CC BY-NC-ND), which allows third parties to download the articles and share them with others as long as they credit the author and the Abstract Book, but they cannot change the content in any way or use them commercially.
**Abstract Book Citations:** Authors, Title, HemaSphere, 2026;10:(S1):pages. The abstract book DOIs can be found on the “Author Information” page.
**Disclaimer:** Articles published in the journal HemaSphere exclusively reflect the opinions of the authors. The authors are responsible for all content in their abstracts including accuracy of the facts, statements, citing resources, etc.

**Results**

Pearson correlation coefficients between bone marrow (BM) and peripheral blood (PB) methylation profiles were >0.85 in all patients, with nine exceeding 0.90, indicating high concordance and supporting the use of PB as a surrogate for longitudinal methylation analysis. In three patients monitored from diagnosis to remission, genome-wide CpG methylation levels decreased significantly (median $\Delta\beta$ = -0.098, mean $\Delta\beta$ = -0.102, Wilcoxon $P < 2\times10^{-16}$), indicating a global hypomethylation trend during remission. Conversely, in three patients followed from remission to relapse, methylation levels increased significantly (median $\Delta\beta$ = 0.086, mean $\Delta\beta$ = 0.102, Wilcoxon $P < 2\times10^{-16}$), suggesting global remethylation at relapse.
Longitudinal 850K methylation analysis in two patients across diagnosis, remission, and relapse revealed that certain promoter CpG sites underwent demethylation during remission and re-methylation at relapse. The number of reversible promoter CpG sites was 13,260 for P12 (18.2% of total promoter CpGs) and 4,884 for P13 (6.7%), with 3,771 overlapping sites. Multi-timepoint NGS analysis of three patients showed stable genome-wide methylation during remission in two patients with sustained remission, whereas the patient who relapsed exhibited pronounced methylation fluctuations at relapse, followed by re-stabilization upon re-remission.
In the patient exhibiting methylation fluctuations at relapse, clustering of highly variable CpG sites identified two epigenetic subgroups (Cluster 2 and Cluster 6) with reversible, disease state–dependent methylation changes, characterized by relative hypermethylation at diagnosis and relapse and demethylation during remission. GSEA revealed enrichment of EMT, developmental, and KRAS-related pathways in Cluster 2, while Cluster 6 was enriched for EMT-associated gene sets.

**Summary/Conclusion**

Peripheral blood methylation closely mirrors bone marrow, supporting its use for longitudinal monitoring. In one patient with relapse-associated methylation fluctuations, reversible CpG sites in Clusters 2 and 6 may be linked to relapse and could serve as potential biomarkers for disease progression.

**Copyright Information:** (Online) ISSN: 2572-9241
©2026 The Author(s). HemaSphere is published by John Wiley & Sons Ltd on behalf of European Hematology Association. This is an open access Abstract Book distributed under the Attribution-NonCommercial-NoDerivs (CC BY-NC-ND), which allows third parties to download the articles and share them with others as long as they credit the author and the Abstract Book, but they cannot change the content in any way or use them commercially.
**Abstract Book Citations:** Authors, Title, HemaSphere, 2026;10:(S1):pages. The abstract book DOIs can be found on the "Author Information" page.
**Disclaimer:** Articles published in the journal HemaSphere exclusively reflect the opinions of the authors. The authors are responsible for all content in their abstracts including accuracy of the facts, statements, citing resources, etc.

### (PB3479) A PHASE 1 MULTICENTER STUDY OF INCB160058, A JAK2V617F MUTANT–SELECTIVE INHIBITOR, IN PATIENTS WITH MYELOFIBROSIS, POLYCYTHEMIA VERA, OR ESSENTIAL THROMBOCYTHEMIA: STUDY DESIGN AND RATIONALE

**Topic:** 16. Myeloproliferative neoplasms - Clinical

Jason Gotlib*[1], John Mascarenhas[2], Raajit K Rampal[3], Abdulraheem Yacoub[4], Ashwin Kishtagari[5], Elie Traer[6], Jamile Shammo[7], Achal Awasthi[8], Josu Santamaria[8], Molly Halloran[8], Naveen Pemmaraju[9]

*[1]Stanford Cancer Institute, Stanford, CA, United States of America; [2]Tisch Cancer Institute, Icahn School of Medicine at Mount Sinai, Division of Hematology/Oncology, New York, NY, United States of America; [3]Memorial Sloan Kettering Cancer Center, Department of Medicine, Leukemia Service, New York, NY, United States of America; [4]The University of Kansas Cancer Center, Division of Hematologic Malignancies and Cellular Therapeutics, Westwood, United States of America; [5]Vanderbilt Ingram Cancer Center, Nashville, United States of America; [6]Oregon Health and Science University, Portland, United States of America; [7]Northwestern Medical Group, Chicago, United States of America; [8]Incyte Corporation, Wilmington, United States of America; [9]The University of Texas MD Anderson Cancer Center, Department of Leukemia, Houston, United States of America;*

### Background

Current Janus kinase (JAK) inhibitors provide clinical but not curative benefit for patients (pts) with myeloproliferative neoplasms (MPNs). Unmet need exists for mutant-selective therapies that target JAK2V617F, a key driver of MPNs. The *JAK*2V617F mutation occurs in the pseudokinase domain and leads to cytokine-independent signaling and oncogenic cell proliferation. INCB160058 is a first-in-class small-molecule JAK2V617F mutant–selective inhibitor designed to uniquely bind the JAK2 pseudokinase domain, selectively inhibiting JAK2V617F signaling while preserving physiologic, cytokine-dependent signaling. Preclinically, INCB160058 inhibited aberrant signaling and proliferation in *JAK2*V617F+ cells with minimal impact on wild-type counterparts, offering potential eradication of malignant clones while minimizing adverse events due to broader JAK inhibition.

### Aims

This phase 1, global, open-label, dose-escalation/expansion study (NCT06313593) will investigate the safety, tolerability, pharmacokinetics (PK), pharmacodynamics, and preliminary clinical efficacy of INCB160058 in pts with *JAK2*V617F+ myelofibrosis (MF), polycythemia vera (PV), or essential thrombocythemia (ET).

**Copyright Information:** (Online) ISSN: 2572-9241
©2026 The Author(s). HemaSphere is published by John Wiley & Sons Ltd on behalf of European Hematology Association. This is an open access Abstract Book distributed under the Attribution-NonCommercial-NoDerivs (CC BY-NC-ND), which allows third parties to download the articles and share them with others as long as they credit the author and the Abstract Book, but they cannot change the content in any way or use them commercially.
**Abstract Book Citations:** Authors, Title, HemaSphere, 2026;10:(S1):pages. The abstract book DOIs can be found on the "Author Information" page.
**Disclaimer:** Articles published in the journal HemaSphere exclusively reflect the opinions of the authors. The authors are responsible for all content in their abstracts including accuracy of the facts, statements, citing resources, etc.

### Methods

Pts with MF, PV, or ET will be treated with INCB160058 monotherapy; pts with MF after suboptimal response to ruxolitinib (rux) will receive INCB160058 as an add-on to rux (MF SubOpt R). Pts with MF must have primary MF of intermediate-1 or higher risk per Dynamic International Prognostic Scoring System or secondary MF (post-ET or post-PV) of intermediate-1 or higher risk per Myelofibrosis Secondary to PV and ET Prognostic Model; have evaluable splenomegaly (≥450 mL per radiographic imaging or palpable ≥5 cm below left subcostal margin); be refractory, resistant, intolerant, or have lost response to ≥1 JAK inhibitor after ≥12 wks of treatment; and have a platelet count (PLT) ≥50×$10^9$/L. The MF SubOpt R cohort will include pts with the same criteria but requires rux treatment for ≥12 wks (stable dose past 8 wks) with no expected benefit from further rux monotherapy and PLT ≥75×$10^9$/L. Pts with PV and ET must be refractory, resistant, intolerant, or have lost response to ≥1 standard cytoreductive therapy. Pts with ET must be high risk (≥1 of the following: aged ≥60 y, history of thrombosis, history of ET-related major bleeding, or bleeding risk [ie, PLT >1000×$10^9$/L or history of PLT >1000×$10^9$/L and ongoing cytoreductive therapy]) with PLT >450×$10^9$/L. Select exclusion criteria include previous or planned hematopoietic stem cell transplant and MPN therapy within the washout period (exception for rux in MF SubOpt R). Dose escalation will enroll up to ~66 pts (MF, ~36; MF SubOptR, ~12; PV or ET, ~18). A statistical hybrid design with a 28-day dose-limiting toxicity window will be used to assess safety and identify the maximum tolerated dose and/or recommended dose for expansion (RDE); ≥3 pts will be enrolled at each dose level. Dose expansion will enroll ~15 pts per RDE and disease type. If >1 RDE is selected, pts will be randomized 1:1. The primary endpoint is safety/tolerability. Key secondary endpoints include standard PK parameters, overall response, ≥50% reduction in MPN Symptom Assessment Form Total Symptom Score at Wk 24, and for MF pts, spleen volume reduction at Wks 12 and 24 (**Figure**). Change in *JAK2*V617F variant allele frequency is an exploratory endpoint.

### Results

The study is currently recruiting.

### Summary/Conclusion

This study will explore the potential of this novel JAK2V617F-selective approach.

**Copyright Information:** (Online) ISSN: 2572-9241
©2026 The Author(s). HemaSphere is published by John Wiley & Sons Ltd on behalf of European Hematology Association. This is an open access Abstract Book distributed under the Attribution-NonCommercial-NoDerivs (CC BY-NC-ND), which allows third parties to download the articles and share them with others as long as they credit the author and the Abstract Book, but they cannot change the content in any way or use them commercially.
**Abstract Book Citations:** Authors, Title, HemaSphere, 2026;10:(S1):pages. The abstract book DOIs can be found on the “Author Information” page.
**Disclaimer:** Articles published in the journal HemaSphere exclusively reflect the opinions of the authors. The authors are responsible for all content in their abstracts including accuracy of the facts, statements, citing resources, etc.

**Figure. Study Design and Endpoints of an Open-Label, Phase 1, Dose Escalation/Expansion Study of INCB160058 in Patients With Previously Treated, *JAK2*V617F-Mutated MF, PV, or ET (NCT06313593)**

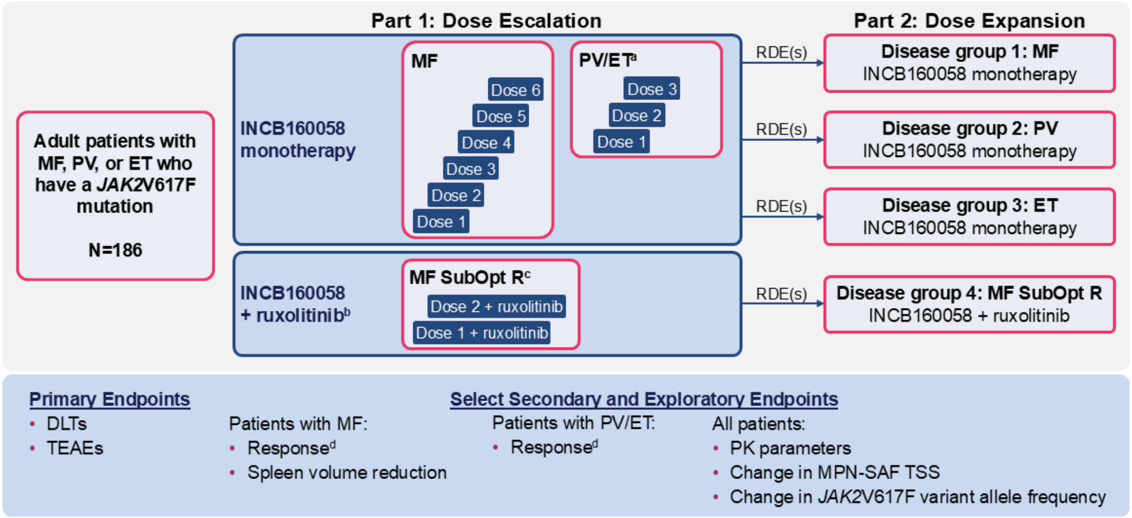

[a]Initiation for patients with PV or ET when ≥2 dose levels in MF have been declared tolerable. [b]Stable dose of ruxolitinib prior to enrollment to be continued. [c]Initiation for patients with MF SubOpt R at 1 dose level lower than the highest declared tolerable dose in MF. [d]Per revised International Working Group-Myeloproliferative Neoplasms Research and Treatment and European LeukemiaNet criteria (2013).

DLT, dose-limiting toxicity; ET, essential thrombocythemia; JAK, Janus kinase; MF, myelofibrosis; MPN-SAF, Myeloproliferative Neoplasm Symptom Assessment Form; PK, pharmacokinetic; PV, polycythemia vera; RDE, recommended dose for expansion; SubOpt R, suboptimal response to ruxolitinib; TEAE, treatment-emergent adverse event; TSS, total symptom score.

**Copyright Information:** (Online) ISSN: 2572-9241
©2026 The Author(s). HemaSphere is published by John Wiley & Sons Ltd on behalf of European Hematology Association. This is an open access Abstract Book distributed under the Attribution-NonCommercial-NoDerivs (CC BY-NC-ND), which allows third parties to download the articles and share them with others as long as they credit the author and the Abstract Book, but they cannot change the content in any way or use them commercially.
**Abstract Book Citations:** Authors, Title, HemaSphere, 2026;10:(S1):pages. The abstract book DOIs can be found on the "Author Information" page.
**Disclaimer:** Articles published in the journal HemaSphere exclusively reflect the opinions of the authors. The authors are responsible for all content in their abstracts including accuracy of the facts, statements, citing resources, etc.

### (PB3480) A REAL-WORLD STUDY OF GECACITINIB IN THE TREATMENT OF MYELOFIBROSIS FROM CHINA: A SINGLE-CENTER RETROSPECTIVE STUDY

**Topic:** 16. Myeloproliferative neoplasms - Clinical

Jin Mao*[1], Miao Miao[1], Depei Wu[1]

[1] *The First Affiliated Hospital of Soochow University, Suzhou, China;*

**Background**

Although ruxolitinib ameliorates splenomegaly and constitutional symptoms in patients with myelofibrosis (MF), 21% to 47% of patients need to discontinue ruxolitinib because of intolerance, drug resistance, or other reasons. Gecacitinib is a dual-target inhibitor of JAK and ACVR1. While clinical studies show gecacitinib effectively reduces spleen size, relieves symptoms, and improves anemia with limited side effects in MF, real-world data are scarce.

**Aims**

To analyze the efficacy and safety of gecacitinib in the treatment of MF in the real world, to guide clinical practice better.

**Methods**

A single-center retrospective study included adult patients with MF who received gecacitinib therapy at the First Affiliated Hospital of Soochow University was conducted. Patient demographics, efficacy, and safety data were collected from the hospital HIS and via follow-ups.

**Results**

Between Jul. 2024 and Feb. 2026, 37 patients with MF were enrolled, including 27 primary MF, 6 with post-PV-MF, and 4 with post-ET-MF. 22 patients were male and 15 were female, with a median age of 61 years (37–75). The median PLT was 151×10⁹/L, with 17 (45.9%) <100, median Hb was 90 g/L, with 23 (62.2%) <100, and 6 (16.2%) were RBC transfusion-dependent. 2 (5.4%), 11 (29.7%), and 24 (64.9%) patients were MF-1, MF-2, and MF-3 respectively. Mutation analysis showed JAK2V617F in 21 (60%), CALR in 8 (22.8%), and MPL in 3 (8.6%). Out of 35 NGS-tested patients, 17 had HMR (high-molecular-risk) mutations, including 14 with ASXL1 mutations. The median MPN-SAF-TSS score was 5. 19 (51.4%). By DIPSS risk stratification, 19 (51.4%), 13 (35.1%) and 5 (13.5%) patients were classified as intermediate-1, intermediate-2 and high risk. The median disease duration was 9m (1-120), and the median follow-up time after initiation treatment was 5.5m (0.5-19). 25 (67.6%) patients had prior ruxolitinib exposure, with 20 patients discontinuing due to cytopenias. Up to last follow-up, 31 patients remained on treatment. 12 patients have completed 24-week therapy. Initial dosing was 100 mg BID in 9, 50 mg BID in 25 (7 maintained, 14 escalated to 100 mg BID), and 50 mg QD in 3 patients. Among 12 patients who completed 24 weeks of treatment and had baseline splenomegaly, 9 (75%) achieved a reduction in spleen volume, including 5 (41.7%) who reached SVR35. At Week 24, 66.7% (8/12) patients achieved TSS50. 3/6 transfusion-dependent patients at baseline became independent. Among patients who were non-transfusion-dependent and had HGB≤100g/L at baseline, 42.1% (8/19) experienced an increase of at least 20g/L in HGB. In 17 patients with baseline PLT <100 ×10⁹/L, only 2 discontinued gecacitinib due to significant platelet decline. The overall adverse event (AE) rate was 65%, with grade≥3 AEs in 21.6%, and serious AE in 13.5%. Grade ≥3 treatment-emergent adverse events included infection, thrombocytopenia, elevated creatinine, and gastrointestinal hemorrhage. Non-hematologic TEAEs included bone pain, infection, fever, fatigue, rash, elevated creatinine, and gastrointestinal hemorrhage.

**Copyright Information:** (Online) ISSN: 2572-9241
©2026 The Author(s). HemaSphere is published by John Wiley & Sons Ltd on behalf of European Hematology Association. This is an open access Abstract Book distributed under the Attribution-NonCommercial-NoDerivs (CC BY-NC-ND), which allows third parties to download the articles and share them with others as long as they credit the author and the Abstract Book, but they cannot change the content in any way or use them commercially.
**Abstract Book Citations:** Authors, Title, HemaSphere, 2026;10:(S1):pages. The abstract book DOIs can be found on the "Author Information" page.
**Disclaimer:** Articles published in the journal HemaSphere exclusively reflect the opinions of the authors. The authors are responsible for all content in their abstracts including accuracy of the facts, statements, citing resources, etc.

**Summary/Conclusion**

Gecacitinib showed promising efficacy and a favorable safety profile in MF treatment. While previous trials recommended a starting dose of 100 mg bid, we found that a lower initial dose also provided significant efficacy, potentially with fewer AEs. Although prior studies suggested use only in patients with platelets≥100×$10^9$/L, our results indicate relative safety even in those with lower platelet counts. Longer follow-up is needed in future studies.

**Copyright Information:** (Online) ISSN: 2572-9241
©2026 The Author(s). HemaSphere is published by John Wiley & Sons Ltd on behalf of European Hematology Association. This is an open access Abstract Book distributed under the Attribution-NonCommercial-NoDerivs (CC BY-NC-ND), which allows third parties to download the articles and share them with others as long as they credit the author and the Abstract Book, but they cannot change the content in any way or use them commercially.
**Abstract Book Citations:** Authors, Title, HemaSphere, 2026;10:(S1):pages. The abstract book DOIs can be found on the "Author Information" page.
**Disclaimer:** Articles published in the journal HemaSphere exclusively reflect the opinions of the authors. The authors are responsible for all content in their abstracts including accuracy of the facts, statements, citing resources, etc.

### (PB3481) REAL-WORLD HEMOGLOBIN MODULATION PATTERNS WITH GECACITINIB IN MYELOFIBROSIS: IMPACT OF BASELINE ANEMIA SEVERITY

**Topic:** 16. Myeloproliferative neoplasms - Clinical

Tian Zeng[1], Xiudi Yang[1], Rumeng Li[1], Bingbing Wen[1], Qinmei Han[1], Zhikang Zheng[1], Xiaojing Bao[1], Jian Huang*[1]

[1] *The First Affiliated Hospital, Zhejiang University School of Medicine, Hangzhou, China;*

### Background

Ruxolitinib, the first approved JAK inhibitor, is frequently associated with treatment-emergent anemia, which may limit its long-term use. Gecacitinib, a dual JAK/ACVR1 inhibitor, has demonstrated clinically meaningful improvements in anemia in clinical trials. However, real-world data regarding hemoglobin (HGB) response across different baseline anemia severity remain limited.

### Aims

In this study, we aimed to evaluate real-world HGB modulation patterns in MF patients treated with Gecacitinib, and to assess the impact of baseline anemia severity on treatment response and safety outcomes.

### Methods

We retrospectively analyzed consecutive MF patients treated with Gecacitinib in routine clinical practice. All patients were followed for at least 3 months. Patients were stratified according to baseline HGB: Severe anemia: HGB <80 g/L and mild/moderate anemia: HGB ≥80 g/L. Anemia response was based on the 2024 IWG-ELN criteria.

### Results

A total of 34 patients were included. The median age was 63.0 years (IQR 59.0–71.8), and 22 patients (64.7%) were male. Regarding treatment initiation, 12 patients (35.3%) received a starting dose of 100 mg BID, while 18 patients (52.9%) were initiated at 50 mg BID. Ten patients (29.4%) were ruxolitinib-naïve. The median baseline HGB level was 76.0 g/L (IQR 63.25–101.0), with 17 patients (50.0%) presenting with HGB <80 g/L and 17 (50.0%) with HGB ≥80 g/L. As expected, median baseline HGB (63.0 g/L vs 101.0 g/L, $P < 0.001$) and PLT (median 92 vs 170 $\times 10^9$/L, $P = 0.014$) differed significantly between the two groups. Overall, HGB levels demonstrated a fluctuating but overall upward trend during treatment. Distinct response patterns were observed according to baseline anemia severity. Among patients with severe anemia, mean HGB increased markedly from 59.9 g/L at baseline to 77.6 g/L at Week 4. Although a transient decline was observed at Week 8 (69.4 g/L), levels recovered to 77.7 g/L by Week 12, representing a mean increase of 17.9 g/L at Week 12. In contrast, patients with mild/moderate anemia experienced an initial decrease from 111.0 g/L to 102.5 g/L at Week 2, followed by gradual recovery to 118.9 g/L at Week 12, with a mean increase of 7.9 g/L. Notably, a higher proportion of patients in the severe anemia group achieved a major anemia response (>15 g/L increase) compared to the mild/moderate group (47.1% vs 28.6%). Treatment was generally well tolerated. The most common adverse event was thrombocytopenia (6 patients, 17.6%), with a similar incidence in patients with baseline HGB >80 g/L and ≤80 g/L (3/17 in each group). Fever occurred in 4 patients (11.8%), all of whom had prior ruxolitinib exposure, highlighting the need for careful monitoring during JAK inhibitor transition in real-world practice. Grade ≥3 TRAEs occurred in 5 patients (14.7%). One patient (3.6%) permanently discontinued Gecacitinib due to a TRAE. Temporary treatment interruptions due to thrombocytopenia were reported in three patients.

**Copyright Information:** (Online) ISSN: 2572-9241
©2026 The Author(s). HemaSphere is published by John Wiley & Sons Ltd on behalf of European Hematology Association. This is an open access Abstract Book distributed under the Attribution-NonCommercial-NoDerivs (CC BY-NC-ND), which allows third parties to download the articles and share them with others as long as they credit the author and the Abstract Book, but they cannot change the content in any way or use them commercially.
**Abstract Book Citations:** Authors, Title, HemaSphere, 2026;10:(S1):pages. The abstract book DOIs can be found on the "Author Information" page.
**Disclaimer:** Articles published in the journal HemaSphere exclusively reflect the opinions of the authors. The authors are responsible for all content in their abstracts including accuracy of the facts, statements, citing resources, etc.

**Summary/Conclusion**

In this real-world study, Gecacitinib demonstrates clinically relevant HGB improvement even in patients with severe baseline anemia, supporting its potential role in anemia-directed management of MF. Moreover, the treatment demonstrated a manageable safety profile in routine clinical practice.

This research was supported by the National Natural Science Foundation of China (No. 82570191) and the Zhejiang Provincial Health High level Innovative Talent Project (2022-2026).

†Correspondence to: Prof Jian Huang, E-mail: househuang@zju.edu.cn

**Copyright Information:** (Online) ISSN: 2572-9241
©2026 The Author(s). HemaSphere is published by John Wiley & Sons Ltd on behalf of European Hematology Association. This is an open access Abstract Book distributed under the Attribution-NonCommercial-NoDerivs (CC BY-NC-ND), which allows third parties to download the articles and share them with others as long as they credit the author and the Abstract Book, but they cannot change the content in any way or use them commercially.
**Abstract Book Citations:** Authors, Title, HemaSphere, 2026;10:(S1):pages. The abstract book DOIs can be found on the "Author Information" page.
**Disclaimer:** Articles published in the journal HemaSphere exclusively reflect the opinions of the authors. The authors are responsible for all content in their abstracts including accuracy of the facts, statements, citing resources, etc.

### (PB3482) TREATMENT OF ADVANCED SYSTEMIC MASTOCYTOSIS WITH THE KIT INHIBITOR AVAPRITINIB: A RIMA (RETE ITALIANA MASTOCITOSI) REAL-LIFE DATASET COLLECTION

**Topic:** 16. Myeloproliferative neoplasms - Clinical

Chiara Elena*[1], Francesca Crupi[2], Marianna Criscuolo[3], Cristina Papayannidis[4], Alessandra Romano[5], Jacqueline Ferrari[1], Massimiliano Bonifacio[6], Michela Rondoni[7], Mariarita Sciume[8], Aniello Sottolano[9], Marco Frigeni[10], Sofia Sciabolacci[11], Valentina Giai[12], Monia Marchetti[13], Giulia Arrigo[14], Erika Morsia[15], Michelina Santopietro[16], Elena Crisà[17], Maria Bruna Greve[18], Carmen Fava[19], Umberto Gianelli[20], Raffaella Santi[21], Emanuela Boveri[1], Patrizia Bonadonna[22], Roberta Zanotti[23], Francesco Mannelli[2]

*[1]Fondazione IRCCS Policlinico San Matteo, Pavia, Division of Molecular Hematology and Precision Medicine, Pavia, Italy; [2]Centro Ricerca e Innovazione Malattie Mieloproliferative (CRIMM), AOU Careggi, Università di Firenze, SOD Ematologia, Firenze, Italy; [3]Fondazione Policlinico Universitario A. Gemelli IRCCS, Roma, Dipartimento di Scienze di Laboratorio ed Ematologiche, Roma, Italy; [4]IRCCS Azienda Ospedaliero-Universitaria di Bologna, Istituto di Ematologia "Seràgnoli", Bologna, Italy; [5]University of Catania, Catania, Italy; [6]University of Verona, Department of Engineering for Innovation Medicine, Section of Innovation Biomedicine, Verona, Italy; [7]Azienda Unità Sanitaria Locale della Romagna, U.O.C. di Ematologia, Ravenna, Italy; [8]Fondazione IRCCS Ca' Granda Ospedale Maggiore Policlinico Milano, S.C. Ematologia, Milano, Italy;[9]Università degli Studi di Salerno, Dipartimento di Medicina, Salerno, Italy; [10]ASST Papa Giovanni XXIII, Hematology and Bone Marrow Transplant Unit, Bergamo, Italy; [11]Università degli Studi di Perugia, Perugia, Italy; [12]Città della Salute e della Scienza, Divisione di Ematologia, Torino, Italy; [13]Azienda Ospedaliera Nazionale SS. Antonio e Biagio e Cesare Arrigo, Unit of Hematology, Alessandria, Italy;[14]AO Ospedali Riuniti Villa-Sofia Cervello, U.O.C Oncoematologia, Palermo, Italy; [15]AOU delle Marche, Hematology Unit, Ancona, Italy; [16]AO San Camillo-Forlanini, Haematology and Haematopoietic Stem Cells Transplant Unit, Roma, Italy; [17]Candiolo Cancer Institute, FPO-IRCCS, Hematology Division, Candiolo, Italy; [18]GOM Reggio Calabria, UOC Ematologia, Reggio Calabria, Italy; [19]AO Ordine Mauriziano di Torino, Università di Torino, Divisione di Ematologia, Dip di Scienze Cliniche e Biologiche,, Torino, Italy; [20]ASST Santi Paolo e Carlo, Milano, Italy;[21]Università degli Studi di Firenze, AOU Careggi, Firenze, Italy;[22]Ospedale San Bortolo, Allergology Unit, Vicenza, Italy; [23]IRCSS Ospedale Sacro Cuore Don Calabria, Negrar di Valpolicella, Italy;*

### Background

Avapritinib, a highly selective inhibitor of KIT D816V, has demonstrated substantial clinical activity in advanced systemic mastocytosis (AdvSM) in clinical trials. However, data on its use in real-world (RW) clinical practice remain limited.

### Aims

To evaluate the clinical efficacy, safety profile and management in a RW dataset of AdvSM patients treated with avapritinib in Italy.

### Methods

This is a multicenter, observational retrospective study conducted at Italian referral centers for systemic mastocytosis. Adult patients (pts) with AdvSM according to WHO/ICC treated with avapritinib from its approval were included. Response to treatment was evaluated by hematological, molecular, and histopathological assessment, according to clinical practice. Bone marrow (BM) trephine biopsies were collected and centrally reviewed.

**Copyright Information:** (Online) ISSN: 2572-9241
©2026 The Author(s). HemaSphere is published by John Wiley & Sons Ltd on behalf of European Hematology Association. This is an open access Abstract Book distributed under the Attribution-NonCommercial-NoDerivs (CC BY-NC-ND), which allows third parties to download the articles and share them with others as long as they credit the author and the Abstract Book, but they cannot change the content in any way or use them commercially.
**Abstract Book Citations:** Authors, Title, HemaSphere, 2026;10:(S1):pages. The abstract book DOIs can be found on the "Author Information" page.
**Disclaimer:** Articles published in the journal HemaSphere exclusively reflect the opinions of the authors. The authors are responsible for all content in their abstracts including accuracy of the facts, statements, citing resources, etc.

### Results

A total of 41 pts from 19 Italian centers were included (Table1). Median age was similar to that observed in the PATHFINDER trial (62,4 vs 68 yrs). SM subtypes were distributed as follows: 27 ASM (65,8%), 12 SM-AHN (29,3%), 2 MCL (4,9%). The proportion between ASM and AHN in our cohort was opposite compared to the PATHFINDER trial (20.0% and 66.0% respectively). Since the histopathological diagnosis was centrally reviewed, the main cause of this difference may lie in clinicians' therapeutic choice. Additional somatic mutations were present in 15/19 evaluable pts: the most frequently mutated gene was *TET2* (31,6%), followed by *DNMT3A, ASXL1, SRSF2.*

Table 1. Clinical and biological features at the time of avapritinib treatment.

| | |
|---|---|
| M/F | 19/22 |
| Age, median (range) | 62,4 (29,3-82,5) |
| WBC count, x$10^9$/L | 6,43 (1,7-58,0) |
| Hb level, g/dl | 12,0 (6,4-16,1) |
| PLT count, x$10^9$/L | 196 (46,0-485) |
| sTryptase, ng/ml | 149,5 (7,80-677) |
| c-Kit D816V-mut pts, n(%) | 34 (82,3) |
| Skin involvement, n(%) | 22 (53,6) |
| BM cellularity increased, n(%) | 19 (46,3) |
| BM fibrosis, n(%) | 22 (57,9) |
| MC phenotype: CD2/CD25/CD30+, n(%) | 12 (50)/25 (92.5)/3 (16,6) |
| Cytogenetic abnormalities, n(%) | 2/31 (6,4) |

Thirty-one pts (75,6%) received avapritinib as 2nd line (27 after 1st line midostaurin), whereas 10 pts as ≥3rd line. After a median follow-up of 41,63 months (range 7,42-159,25), 7 patients died (1 HSCT complications, 1 complications after subdural hematoma, 1 infection, 2 progressive disease, 2 unknown cause). Median duration of treatment was 39,2 (range 5,2-77,5) months. The overall response rate (ORR) was 75,6%, with a marked reduction in tryptase and C-KIT burden. The ORR was slightly higher than that reported in PATHFINDER (66% in previously treated pts): this observation could be explained with the lower percentage of heavily (>2 lines) pretreated patients in our cohort. Sequential histological evaluation was available in 12 pts: an overall reduction in cellularity and a >50% reduction in MC infiltrate were observed in 11 and 12 pts, respectively, with a ≥1-grade improvement in fibrosis in 8.
The most frequently reported adverse events were edema (58,5%), cognitive events (21,9%), cytopenias and gastrointestinal toxicity (7,3% each), mainly grade 1–2, and were generally manageable through dose modification or temporary treatment interruption.

### Summary/Conclusion

In an Italian RW setting, avapritinib demonstrated high effectiveness, also assessed by histopathological response, and an acceptable safety profile in patients with AdvSM, with outcomes consistent with those reported in clinical trials. These findings support the role of avapritinib as an effective and safe therapeutic option for AdvSM in routine clinical practice.

**Copyright Information:** (Online) ISSN: 2572-9241
©2026 The Author(s). HemaSphere is published by John Wiley & Sons Ltd on behalf of European Hematology Association. This is an open access Abstract Book distributed under the Attribution-NonCommercial-NoDerivs (CC BY-NC-ND), which allows third parties to download the articles and share them with others as long as they credit the author and the Abstract Book, but they cannot change the content in any way or use them commercially.
**Abstract Book Citations:** Authors, Title, HemaSphere, 2026;10:(S1):pages. The abstract book DOIs can be found on the "Author Information" page.
**Disclaimer:** Articles published in the journal HemaSphere exclusively reflect the opinions of the authors. The authors are responsible for all content in their abstracts including accuracy of the facts, statements, citing resources, etc.

### (PB3483) INCIDENCE AND PREVALENCE OF MYELOPROLIFERATIVE NEOPLASMS – A POPULATION-BASED STUDY FROM SWEDEN

**Topic:** 16. Myeloproliferative neoplasms - Clinical

Anna Ravn Landtblom*[1, 2], Yuliya Leontyeva[3], Francesca Coutinho[4], Xiaoqin Yang[4], Therese Andersson[3]

*[1]Center for Hematology and Regenerative Medicine, Department of Medicine, Huddinge, Karolinska Institutet, Stockholm, Sweden; [2]Department of Hematology, Karolinska University Hospital, Stockholm, Sweden; [3]Department of Medical Epidemiology and Biostatistics, Karolinska Institutet, Stockholm, Sweden; [4]Merck Sharp and Dohme LLC, Rahway, United States of America;*

### Background

Incidence of myeloproliferative neoplasms (MPNs) vary across studies, commonly described to be 2-5 per 100,000 person years (PY), with large variation depending on methodology and geographical region, while prevalence is less well characterized.

### Aims

Accurate epidemiologic information is important for health care planning and resource utilization. The aim of this study was to assess incidence and prevalence of MPNs over time in a population-based setting, using national health care registers.

### Methods

Adult patients in Sweden with MPN were identified from either the Cancer Register (from 1973), Blood Cancer Quality Register (from 2008) or the Patient Register (from 1987 for inpatient and 2001 for outpatient care). Incidence per 100,000 PY, both crude and age-standardized to the year 2000 was calculated. Yearly point prevalence per 100,000 based on MPN patients diagnosed during or prior to the study period, and alive on January 1st each year, for years 2008-2020 was calculated. Information on cytoreductive drugs was retrieved from the Prescribed Drug Register, 2008-2020.

**Copyright Information:** (Online) ISSN: 2572-9241
©2026 The Author(s). HemaSphere is published by John Wiley & Sons Ltd on behalf of European Hematology Association. This is an open access Abstract Book distributed under the Attribution-NonCommercial-NoDerivs (CC BY-NC-ND), which allows third parties to download the articles and share them with others as long as they credit the author and the Abstract Book, but they cannot change the content in any way or use them commercially.
**Abstract Book Citations:** Authors, Title, HemaSphere, 2026;10:(S1):pages. The abstract book DOIs can be found on the "Author Information" page.
**Disclaimer:** Articles published in the journal HemaSphere exclusively reflect the opinions of the authors. The authors are responsible for all content in their abstracts including accuracy of the facts, statements, citing resources, etc.

**Results**

For incidence, the identified study population consisted of 13,023 individuals, 54.7% were identified from multiple registers, 45.1% from Patient Register alone, and 0.2% from Cancer Register alone. The prevalence analysis was based on 33,979 individuals. The crude incidence rate per 100,000 PY in 2020 for MPN overall was 13.0, for essential thrombocythemia (ET) 5.7, for polycythemia vera (PV) 4.8, and for primary myelofibrosis (PMF) 1.0 (Table 1). The prevalence per 100,000 on January 1st for MPN overall was 138.7 in 2020, for ET 59.1, PV 54.3, and PMF 8.2 (Table 1). During the study period, ET surpassed PV in both incidence and prevalence. Temporal trends are demonstrated in Figure 1.

| | Crude incidence | Age standardized incidence* | Prevalence Jan 1st |
|---|---|---|---|
| MPN | 13.0 (12.3 ; 13.8) | 12.2 (11.5 ; 13.0) | 138.7 |
| ET | 5.7 (5.2 ; 6.2) | 5.4 (4.9 ; 5.9) | 59.1 |
| PV | 4.8 (4.3 ; 5.3) | 4.5 (4.0 ; 5.0) | 54.3 |
| PMF | 1.0 (0.8 ; 1.3) | 0.9 (0.7 ; 1.2) | 8.2 |
| MPN-U | 1.5 (1.3 ; 1.8) | 1.4 (1.2 ; 1.7) | 17.0 |

Table 1. Crude and age-standardized incidence, point prevalence in 2020 per 100,000 person-years and 95% confidence intervals.
*Age-standardization to the general population in Sweden older than 18 years in 2000
To further evaluate MPN diagnoses captured from the Patient Register, we reviewed the proportions of MPN patients ever treated with cytoreductive therapy among patients only identified in the Patient Register to those in the Cancer Register (using the incidence study population 2008-2020). The proportions were 47% and 83% respectively. Hence, patients from both registers were included.

**Summary/Conclusion**

In this population-based study, the incidence and prevalence of MPN is higher than previously demonstrated. As degree of certainty and validation of the different registers vary, there is a risk of overestimation, but when taking into account our case ascertainment, the results of this study clearly demonstrate a higher incidence than previously described. The increasing prevalence over time is likely mainly due to earlier diagnosis related to molecular markers and updated diagnostic criteria. The results of this study fill a key evidence gap and can inform healthcare planning, resource allocation, and future studies on temporal trends, diagnostic pathways and subtype-specific outcomes in MPN.

**Copyright Information:** (Online) ISSN: 2572-9241
©2026 The Author(s). HemaSphere is published by John Wiley & Sons Ltd on behalf of European Hematology Association. This is an open access Abstract Book distributed under the Attribution-NonCommercial-NoDerivs (CC BY-NC-ND), which allows third parties to download the articles and share them with others as long as they credit the author and the Abstract Book, but they cannot change the content in any way or use them commercially.
**Abstract Book Citations:** Authors, Title, HemaSphere, 2026;10:(S1):pages. The abstract book DOIs can be found on the "Author Information" page.
**Disclaimer:** Articles published in the journal HemaSphere exclusively reflect the opinions of the authors. The authors are responsible for all content in their abstracts including accuracy of the facts, statements, citing resources, etc.

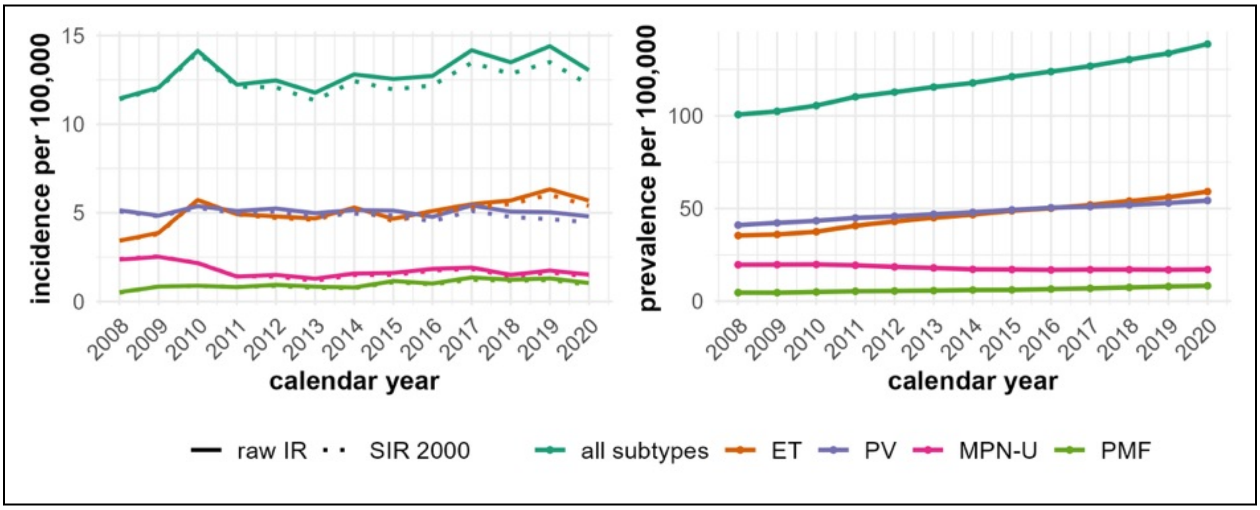

Figure 1. Temporal trends in incidence and prevalence of Myeloproliferative Neoplasms in Sweden

**Copyright Information:** (Online) ISSN: 2572-9241
©2026 The Author(s). HemaSphere is published by John Wiley & Sons Ltd on behalf of European Hematology Association. This is an open access Abstract Book distributed under the Attribution-NonCommercial-NoDerivs (CC BY-NC-ND), which allows third parties to download the articles and share them with others as long as they credit the author and the Abstract Book, but they cannot change the content in any way or use them commercially.
**Abstract Book Citations:** Authors, Title, HemaSphere, 2026;10:(S1):pages. The abstract book DOIs can be found on the "Author Information" page.
**Disclaimer:** Articles published in the journal HemaSphere exclusively reflect the opinions of the authors. The authors are responsible for all content in their abstracts including accuracy of the facts, statements, citing resources, etc.

### (PB3484) CLONAL HEMATOPOIESIS IS A ROBUST PREDICTOR OF RESPONSE TO INTERFERON-Α IN ERDHEIM-CHESTER DISEASE AND MYELOPROLIFERATIVE NEOPLASMS.

**Topic:** 16. Myeloproliferative neoplasms - Clinical

Jerome Razanamahery*[1], Francesco Pegoraro[2], Dana Bossert[3], Matthias Papo[4], Felicien Triboulet[4], Ludwig Serge Aho[1], Jean-Francois Emile[5], Francesco Poeta[2], Bernard Bonnotte[1], Louis Terriou[6], Tanguy Le Scornet[7], Noemie Gensous[8], Etienne Riviere[9], Adrien Guilloteau[1], Marc Maynadie[1], Zahir Amoura[4], Fleur Cohen-Aubart[4], Eli Diamond[3], Augusto Vaglio[2], Julien Haroche[4]

*[1]Dijon university hospital, Dijon, France; [2]University of Florence, florence, Italy; [3]Memorial Sloan Kettering cancer, New york, United States of America; [4]Pitié Salpetriere hospital, Paris, France; [5]Ambroise Paré Hospital, Boulogne, France; [6]Lille University Hospital, Lille, France; [7]Nantes University Hospital, nantes, France; [8]Bordeaux University Hospital, Bordeaux, France; [9]Bordeaux university hospital, PESSAC, France;*

### Background

Erdheim–Chester disease (ECD) is a rare clonal histiocytosis driven by MAPK-pathway mutations. While kinase inhibitors are effective, their use is often limited by access and long-term tolerability. Interferon-alpha (IFN-α) remains a frontline therapy for ECD patients and other myeloid neoplasms (MPN), yet clinical and molecular predictors of response are not well- defined.

### Aims

The aim is to evaluate IFN-α in ECD and MPN and identify predictors of response.

### Methods

We conducted a multicenter retrospective study of 377 patients (ECD, n=300; MPN, n=77) treated with IFN-α in France, Italy and the USA. The primary endpoint was the **clinical benefit rate (CBR)**, encompassing complete response, partial response, or stable disease (SD) leading to interferon continuation. Predictors were identified using multivariable logistic regression and machine-learning models.

**Copyright Information:** (Online) ISSN: 2572-9241
©2026 The Author(s). HemaSphere is published by John Wiley & Sons Ltd on behalf of European Hematology Association. This is an open access Abstract Book distributed under the Attribution-NonCommercial-NoDerivs (CC BY-NC-ND), which allows third parties to download the articles and share them with others as long as they credit the author and the Abstract Book, but they cannot change the content in any way or use them commercially.
**Abstract Book Citations:** Authors, Title, HemaSphere, 2026;10:(S1):pages. The abstract book DOIs can be found on the “Author Information” page.
**Disclaimer:** Articles published in the journal HemaSphere exclusively reflect the opinions of the authors. The authors are responsible for all content in their abstracts including accuracy of the facts, statements, citing resources, etc.

### Results

In the ECD cohort (n=300; median age 59 years; 87% male; 67% BRAF$^{V600E}$), the overall CBR was 83% (n=249), with majority of patients with stable disease (n=222). Responders were older at diagnosis (median 59 vs 54 years; p=0.0189), at symptom onset (56 vs 51 years; p=0.0231), and at IFN-α initiation (59 vs 54 years; p=0.0175). They were frequently men (89% vs 81%; p=0.0262) with longer disease duration (median 58 vs 10.8 months; p<0.0001). Multivariable analysis identified **ENT involvement** (OR 3.99; 95% CI 1.42–11.19; p=0.0089) and **clonal hematopoiesis (CH)** (OR 2.81; 95% CI 1.13–6.98; p=0.0270) as independent positive predictors. Conversely, **female sex** (OR 0.36; 95% CI 0.14–0.95; p=0.0406) and **digestive tract involvement** (OR 0.20; 95% CI 0.06–0.68; p=0.0109) were negative predictors.
Among patients receiving IFN-α as first line therapy (n=255; CBR:88%), responders were older (59 vs 54 years; p=0.05), exhibited higher baseline CRP (83% vs 36%; p=0.0448), and carried more myeloid driver mutations on NGS (median 1 vs 0; p=0.0013). In this subgroup, CH was the sole independent predictor of response (OR 21.9; 95% CI 4.39–140). In twelve *TET2*- mutated responders, IFN-α was associated with a significant CRP decrease (p=0.0007) despite stable variant allele frequencies. Machine-learning models consistently ranked CH as the most powerful predictor of efficacy.
In the MPN cohort (n=77), the CBR was 79%. First-line IFN-α treatment was more frequent among responders (72% vs 43%; p=0.0416) and predicted response in univariate analysis (OR 3.33; 95% CI 1.08–10.73; p=0.0368). In the pooled cohort (n=377), both CH (OR 1.94; 95% CI 1.01–3.73) and first-line therapy (OR 1.90; 95% CI 1.01–3.58) remained independent predictors of response.

### Summary/Conclusion

In this multicenter retrospective study, clonal hematopoiesis is a robust, independent predictor of IFN-α efficacy in ECD. Machine-learning analyses reinforce CH's predictive value and support integrating CH testing into molecular stratification to guide IFN-α use in ECD.

**Copyright Information:** (Online) ISSN: 2572-9241
©2026 The Author(s). HemaSphere is published by John Wiley & Sons Ltd on behalf of European Hematology Association. This is an open access Abstract Book distributed under the Attribution-NonCommercial-NoDerivs (CC BY-NC-ND), which allows third parties to download the articles and share them with others as long as they credit the author and the Abstract Book, but they cannot change the content in any way or use them commercially.
**Abstract Book Citations:** Authors, Title, HemaSphere, 2026;10:(S1):pages. The abstract book DOIs can be found on the "Author Information" page.
**Disclaimer:** Articles published in the journal HemaSphere exclusively reflect the opinions of the authors. The authors are responsible for all content in their abstracts including accuracy of the facts, statements, citing resources, etc.

### (PB3485) A DEEP SAMPLING AND SINGLE MOLECULE DIGITAL COUNTING ASSAY FOR RARE MOLECULE DETECTION

**Topic:** 16. Myeloproliferative neoplasms - Clinical

William Overman[1], Jung Won Keum[2], Cindy Wang[3], Jesse Soliman[2], Emily Blair[2], Eleen Shum*[2], Tricia Zwiefelhofer[3]

*[1]LabCorp, Research Triangle Park, United States of America;[2]Countable Labs, Palo Alto, United States of America;[3]LabCorp, San Diego, United States of America;*

### Background

Rare variant detection in liquid biopsy and hematologic malignancies remains analytically challenging. Improving sensitivity requires increased DNA input; however, conventional digital PCR (dPCR) platforms are constrained by limited partition numbers and input capacity, restricting analytical range and precision at very low mutant allele frequencies (MAFs). We evaluated Countable PCR, a next-generation partition-based PCR platform that generates ~30 million micro-reactions per 50 µL assay and supports DNA inputs exceeding 1 µg, enabling deep sampling within a single well.

### Methods

A multiplexed assay targeting the clinically relevant KIT (D816V) mutation associated with systemic mastocytosis (SM) was analytically validated across 15 blood and bone marrow specimens at multiple testing sites. Performance was compared with a reference dPCR method. Linearity, background signal, input tolerance, and inter-site concordance were assessed using clinical samples, synthetic controls, and contrived mixtures across a range of variant allele frequencies.

### Results

Standard dPCR achieved a 0.03% MAF limit of detection only by pooling three wells (78,000 partitions, 100 ng input), as higher inputs resulted in image saturation and reduced precision. In contrast, Countable PCR accurately quantified variants across 150–1,452 ng DNA in a single well, maintaining linearity and low background. MAF values demonstrated near-perfect concordance with the orthogonal method (Pearson $r = 0.9997$; $p = 1.03 \times 10^{-22}$). Synthetic controls showed 99.95% detection accuracy (5 variant-positive vs. 10,026 wild-type partitions). Contrived samples at 300 ng and 1 µg inputs demonstrated 100% concordance across expected allele frequencies.

### Summary/Conclusion

Countable PCR enables high-input, single-molecule quantification with exceptional sensitivity and reproducibility in a streamlined workflow. Deep sampling within a single reaction supports robust rare variant detection while reducing well pooling and associated costs, making this approach well suited for precision molecular diagnostics in clinical laboratory settings.

**Copyright Information:** (Online) ISSN: 2572-9241
©2026 The Author(s). HemaSphere is published by John Wiley & Sons Ltd on behalf of European Hematology Association. This is an open access Abstract Book distributed under the Attribution-NonCommercial-NoDerivs (CC BY-NC-ND), which allows third parties to download the articles and share them with others as long as they credit the author and the Abstract Book, but they cannot change the content in any way or use them commercially.
**Abstract Book Citations:** Authors, Title, HemaSphere, 2026;10:(S1):pages. The abstract book DOIs can be found on the "Author Information" page.
**Disclaimer:** Articles published in the journal HemaSphere exclusively reflect the opinions of the authors. The authors are responsible for all content in their abstracts including accuracy of the facts, statements, citing resources, etc.

### (PB3486) INTESTINAL INFLAMMATION IN PATIENTS WITH MYELOPROLIFERATIVE NEOPLASMS

**Topic:** 16. Myeloproliferative neoplasms - Clinical

Ivan Krecak*[1, 2], Iva Sesar Mrkela[3], Zvonimir Marko Skugor[2], Josipa Budimir[2], Goran Hauser[1, 4], Zinaida Perić[1, 5], Marko Skelin[1, 2]

*[1]Faculty of medicine Rijeka, Rijeka, Croatia;[2]General Hospital of Sibenik-Knin County, Sibenik, Croatia;[3]Rijeka Pharmacy, Rijeka, Croatia; [4]University Hospital Center Rijeka, Division of Gastroenterology, Rijeka, Croatia;[5]University Hospital Rijeka, Division of Hematology, Rijeka, Croatia;*

### Background

Patients with BCR::ABL1 negative myeloproliferative neoplasms (MPNs), essential thrombocythemia (ET), polycythemia vera (PV), and myelofibrosis (MF), suffer from different constitutional symptoms. Calprotectin is an acute phase reactant released from activated leukocytes with an important role in inflammation-linked tissue remodelling. In everyday practice, fecal calprotectin (fCAL) measurements are used for diagnosis of inflammatory bowel disease (IBD) and for monitoring IBD activity, assessment of treatment responses, and prediction of relapse.

### Aims

The aim of this study was to investigate fCAL in MPNs and its clinical associations.

### Methods

This is an ongoing prospective two-center study in Croatia enrolling consecutive MPN patients diagnosed according to World Health Organization 2022 criteria. Exclusion criteria were pregnancy, other solid cancers, acute intestinal infection and diverticulitis, known IBD, and the use of immunosupresants, non-steroidal anti-inflammatory drugs and proton pump inhibitors. All patients had their blood and stool samples taken at the time of study entry. Myeloproliferative Symptom Assessment Form was also fulfilled at study entry. The Bristol Stool Chart was used to classify feces into seven types (obstipation 1-2; normal stool 3-4; diarrhoea 5-7). fCAL (reference range <20 µg/g) was assessed by particle turbidimetric immunoassay (Abbot Allinity I analyzer). All patients with fCAL levels >200 µg/g were advised to perform colonoscopy. In cases with fCAL levels in the range of 50-200 µg/g stool samples were repeated once again; in persistently elevated levels, colonoscopy was advised. Statistics were performed with MedCalc Software. The chi-square and the Mann-Whitney U tests were used to analyze differences in categorical and continuous variables between different patient groups, respectively.

**Copyright Information:** (Online) ISSN: 2572-9241
©2026 The Author(s). HemaSphere is published by John Wiley & Sons Ltd on behalf of European Hematology Association. This is an open access Abstract Book distributed under the Attribution-NonCommercial-NoDerivs (CC BY-NC-ND), which allows third parties to download the articles and share them with others as long as they credit the author and the Abstract Book, but they cannot change the content in any way or use them commercially.
**Abstract Book Citations:** Authors, Title, HemaSphere, 2026;10:(S1):pages. The abstract book DOIs can be found on the "Author Information" page.
**Disclaimer:** Articles published in the journal HemaSphere exclusively reflect the opinions of the authors. The authors are responsible for all content in their abstracts including accuracy of the facts, statements, citing resources, etc.

**Results**

Seventy patients (ET=14, PV=44, MF=11, and MPN-u=2) were included; median age was 62 years (range 20-83), 35 (50%) were females, 58 (82.9%) were JAK2 positive and 52 (74.3%) were treated with cytoreduction (mostly hydroxyurea, n=32, and interferons, n=9).

A total of 24 (34.3%) patients had elevated (>20 μg/g) fCAL with a median value of 107.5 μg/g (range 21-282) and 19 (27.1%) of patients had values >50 μg/g which is statistically significantly higher than in the general population of similar age (16.7% from the TREND study; p=0.019). There was a trend for an increase in the proportion of patients with elevated fCAL levels from ET (15.4%) to PV (34.1%) and MF (54.5%; p=0.043, Figure 1). Patients with elevated fCAL had higher leukocyte count (p=0.040), higher C-reactive protein (CRP; p=0.009), lower hemoglobin (p=0.043), more frequently had prior venous thrombosis (p=0.029), presented with diarrhoea (p=0.028) and with higher symptom scores of abdominal discomfort (p=0.048), inactivity (p=0.045) and fatigue (p=0.060). There were no associations of fCAL with age, sex, disease duration, JAK2 mutation, splenomegaly, cardiovascular comorbidities, cytoreductive, antiplatelet or anticoagulant treatment (p>0.050 for all analyses). Fourteen patients underwent colonoscopy and none of them were diagnosed with IBD.

**Summary/Conclusion**

A significant proportion of MPN patients has elevated fCAL levels indicating its limited role as a tool for IBD screening in this patient population. Correlations of higher fCAL levels with higher leukocytes, higher CRP, diarrhoea and abdominal discomfort may suggest its pathophysiological role in intestinal inflammation in MPNs.

**Copyright Information:** (Online) ISSN: 2572-9241
©2026 The Author(s). HemaSphere is published by John Wiley & Sons Ltd on behalf of European Hematology Association. This is an open access Abstract Book distributed under the Attribution-NonCommercial-NoDerivs (CC BY-NC-ND), which allows third parties to download the articles and share them with others as long as they credit the author and the Abstract Book, but they cannot change the content in any way or use them commercially.
**Abstract Book Citations:** Authors, Title, HemaSphere, 2026;10:(S1):pages. The abstract book DOIs can be found on the "Author Information" page.
**Disclaimer:** Articles published in the journal HemaSphere exclusively reflect the opinions of the authors. The authors are responsible for all content in their abstracts including accuracy of the facts, statements, citing resources, etc.

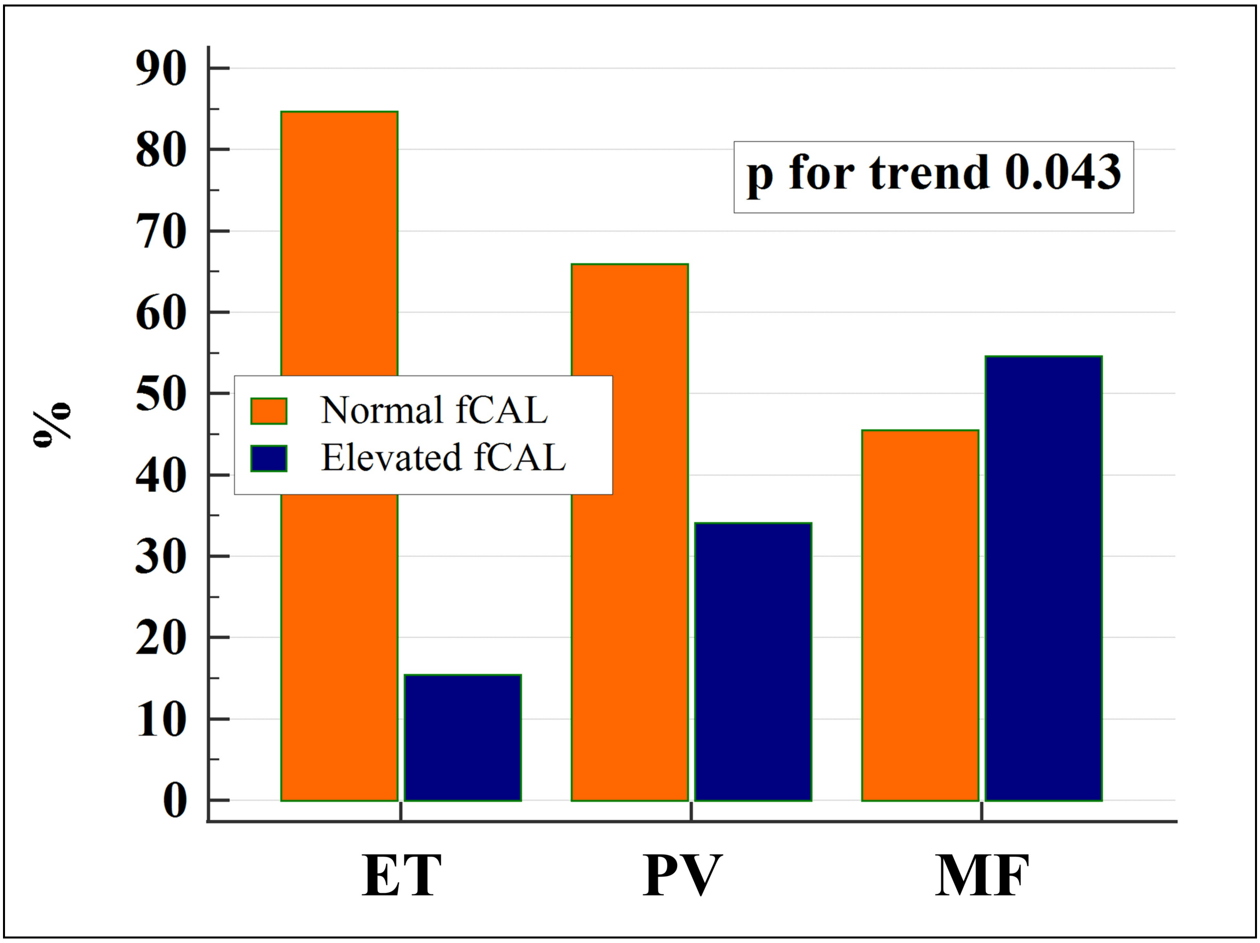

Copyright Information: (Online) ISSN: 2572-9241
©2026 The Author(s). HemaSphere is published by John Wiley & Sons Ltd on behalf of European Hematology Association. This is an open access Abstract Book distributed under the Attribution-NonCommercial-NoDerivs (CC BY-NC-ND), which allows third parties to download the articles and share them with others as long as they credit the author and the Abstract Book, but they cannot change the content in any way or use them commercially.
Abstract Book Citations: Authors, Title, HemaSphere, 2026;10:(S1):pages. The abstract book DOIs can be found on the "Author Information" page.
Disclaimer: Articles published in the journal HemaSphere exclusively reflect the opinions of the authors. The authors are responsible for all content in their abstracts including accuracy of the facts, statements, citing resources, etc.

### (PB3487) COMPARATIVE VALUE OF NOVEL DIPSS-R VERSUS DIPSS BASED PROGNOSTICATION IN PATIENTS WITH MYELOFIBROSIS

**Topic:** 16. Myeloproliferative neoplasms - Clinical

Marko Lucijanic*[1], Davor Galusic[2], Vlatka Periša[3], Ivan Zekanovic[4], Martina Morić Perić[4], Hrvoje Holik[5], Ena Soric[1], Rajko Kušec[1], Danijela Lekovic[6], Ivan Krecak[7]

*[1]University Hospital Dubrava, Hematology department, Zagreb, Croatia; [2]University Hospital Center Split, Hematology department, Split, Croatia; [3]University Hospital Center Osijek, Hematology department, Osijek, Croatia; [4]General Hospital Zadar, Internal medicine department, Zadar, Croatia; [5]Genral Hospital Josip Bencevic Slavonski Brod, Internal medicine department, Slavonski Brod, Croatia; [6]University Clinical Center Srbija, Hematology department, Belgrade, Serbia; [7]General Hospital of Šibenik-Knin County, Internal medicine department, Šibenik, Croatia;*

### Background

The recently developed revised Dynamic International Prognostic Scoring System (DIPSS-R) by Tefferi et al. aims to improve the widely applicable DIPSS-based prognostication in patients with myelofibrosis. The score exclusively includes disease-related factors (two degrees of anemia and leukocytosis, respectively; circulatory blasts; absolute monocyte count; and platelet count) and deliberately excludes age and constitutional symptoms as host-related and unreliable, respectively. The authors developed and validated the score in two independent patient cohorts.

### Aims

We aimed to further validate and explore the prognostic utility of DIPSS-R as compared to DIPSS in patients with myelofibrosis.

### Methods

This was a retrospective, multinational, multicentric study of newly diagnosed patients with primary or secondary myelofibrosis from 6 Croatian and 1 Serbian hematologic center.Patients were treated during the period from 6/2004 to 2/2026, and their diagnoses were reassessed according to the 2022 WHO criteria. The DIPSS and DIPSS-R were calculated according to the original criteria. All statistical analyses were performed using MedCalc statistical software version 23.4.5 (MedCalc Software Ltd., Ostend, Belgium).

**Copyright Information:** (Online) ISSN: 2572-9241
©2026 The Author(s). HemaSphere is published by John Wiley & Sons Ltd on behalf of European Hematology Association. This is an open access Abstract Book distributed under the Attribution-NonCommercial-NoDerivs (CC BY-NC-ND), which allows third parties to download the articles and share them with others as long as they credit the author and the Abstract Book, but they cannot change the content in any way or use them commercially.
**Abstract Book Citations:** Authors, Title, HemaSphere, 2026;10:(S1):pages. The abstract book DOIs can be found on the “Author Information” page.
**Disclaimer:** Articles published in the journal HemaSphere exclusively reflect the opinions of the authors. The authors are responsible for all content in their abstracts including accuracy of the facts, statements, citing resources, etc.

### Results

A total of 285 patients with myelofibrosis were analyzed (90 pre-fibrotic, 106 overt-fibrotic, 46 post-PV, and 43 post-ET MF). The median age was 68 years. A total of 175 (61.4%) patients were male, 198 (72.8%) were JAK2-mutated, 23 (10.4%) CALR-mutated, and 6 (2.7%) MPL-mutated. The median overall survival (OS) was 66.1 months, and the median follow-up was 90 months.
Both DIPSS-R (Figure panel A) and DIPSS adequately risk-stratified OS and achieved moderate to good, but fairly similar, prognostic accuracy in the overall cohort (Harrell's C-index: 0.691 vs. 0.698) and in both overt PMF (0.640 vs. 0.646) and pre-PMF (0.772 vs. 0.783) patients.
A total of 52 (19.1%) and 44 (15.4%) patients were stratified into low-risk, 58 (21.3%) and 112 (39.3%) into intermediate-1-risk, 108 (39.7%) and 106 (37.2%) into intermediate-2-risk, and 54 (19.9%) and 23 (8.1%) into high-risk categories by DIPSS-R and DIPSS, respectively. Both scores provided concordant results in 99 (36.5%) and 115 (42.4%) patients belonging to lower- and higher-risk categories, respectively. However, discordant risk stratification was observed in 57 (21%) patients, with DIPSS-R recognizing an additional 46 (17%) DIPSS low/lower-risk patients as higher risk, and DIPSS recognizing an additional 11 (4.1%) DIPSS-R low/lower-risk patients as higher risk. Discordantly stratified patients had a similar clinical course regardless of the score used, with intermediate prognosis compared to low- and high-risk patients. However, in the subgroup of patients aged ≤65 years (who are standardly considered SCT candidates and cannot obtain age-related adverse points in the DIPSS), only DIPSS-R identified an additional subgroup of 26 (22.4%) discordantly risk-stratified patients (panel B), providing important additional prognostic information.

### Summary/Conclusion

DIPSS-R and DIPSS provide similar overall prognostic accuracy in patients with myelofibrosis. Nevertheless, DIPSS-R recognizes a higher proportion of patients with adverse disease features and unfavorable clinical course who would otherwise be classified as low/lower-risk. This may be especially important among younger patients, where earlier initiation of specific treatments and a more potent therapeutic approaches can yield better outcomes.

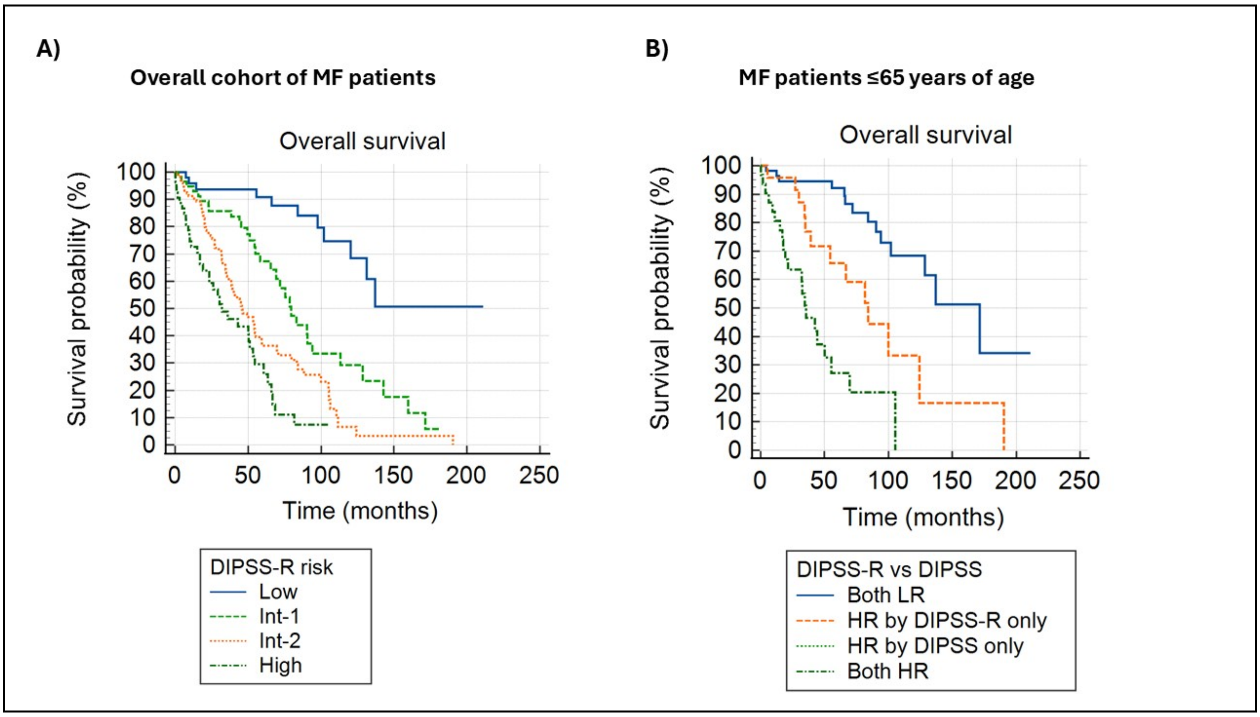

**Copyright Information:** (Online) ISSN: 2572-9241
©2026 The Author(s). HemaSphere is published by John Wiley & Sons Ltd on behalf of European Hematology Association. This is an open access Abstract Book distributed under the Attribution-NonCommercial-NoDerivs (CC BY-NC-ND), which allows third parties to download the articles and share them with others as long as they credit the author and the Abstract Book, but they cannot change the content in any way or use them commercially.
**Abstract Book Citations:** Authors, Title, HemaSphere, 2026;10:(S1):pages. The abstract book DOIs can be found on the "Author Information" page.
**Disclaimer:** Articles published in the journal HemaSphere exclusively reflect the opinions of the authors. The authors are responsible for all content in their abstracts including accuracy of the facts, statements, citing resources, etc.

### (PB3488) REAL-WORLD APPLICATION OF 2024 IWG-ELN CRITERIA FOR ANEMIA RESPONSE IN MOMELOTINIB-TREATED MYELOFIBROSIS

**Topic:** 16. Myeloproliferative neoplasms - Clinical

Francesca Duca*[1, 2], Fabrizio Cavalca[2], Elsa Masedu[1, 2], Carlo Gambacorti-Passerini[1, 2], Elena Elli[2]

[1]*University of Milano Bicocca, Monza, Italy;* [2]*IRCCS - Ospedale San Gerardo, Monza, Italy;*

### Background

Treatment for myelofibrosis (MF) relies on JAK inhibitors (JAKi), though their potential is often limited by cytopenias. Momelotinib (MMB) targets JAK1/JAK2 and ACVR1, reducing hepcidin to improve erythropoiesis. The 2024 IWG-ELN criteria provide standardized anemia response (AR) assessment, but real-world data applying these criteria to MMB-treated patients (pts) remain limited.

### Aims

Primary endpoint was to evaluate AR in a real-world cohort of MF pts (JAK-naive or JAK-pretreated) using the recently proposed 2024 IWG-ELN criteria. The secondary endpoint was to describe the safety profile of MMB treatment.

### Methods

We conducted a real-world, single-center analysis of MF pts treated with MMB (August 2023-February 2026). All pts were anemic at baseline and two subgroups were identified based on transfusion dependence (TD) status. AR was assessed using 2024 IWG-ELN criteria at 3, 6, and 12 months. Adverse events were graded according to CTCAE v5.0. Data were analyzed using standard statistical software.

**Copyright Information:** (Online) ISSN: 2572-9241
©2026 The Author(s). HemaSphere is published by John Wiley & Sons Ltd on behalf of European Hematology Association. This is an open access Abstract Book distributed under the Attribution-NonCommercial-NoDerivs (CC BY-NC-ND), which allows third parties to download the articles and share them with others as long as they credit the author and the Abstract Book, but they cannot change the content in any way or use them commercially.
**Abstract Book Citations:** Authors, Title, HemaSphere, 2026;10:(S1):pages. The abstract book DOIs can be found on the “Author Information” page.
**Disclaimer:** Articles published in the journal HemaSphere exclusively reflect the opinions of the authors. The authors are responsible for all content in their abstracts including accuracy of the facts, statements, citing resources, etc.

### Results

We enrolled 29 pts (median age 76 years, 14 males) with primary (n=15) or secondary MF (n=14). Most pts harbored JAK2 mutation (62%). DIPSS score at MMB initiation was intermediate-2 in 83% and high risk in 14%. Twenty pts (69%) had prior JAKi exposure, predominantly Ruxolitinib (Rux, 95%), while 9 pts (31%) were JAKi-naive. Among pretreated pts, 68% received Rux ≤10 mg BID before discontinuation (median duration 12.3 months, range 3.5-92), and RR6 score indicated intermediate/high risk in 88%. Most pts (79%) shifted to MMB within 7 days of JAKi discontinuation. At baseline, 69% were TD and 24% had thrombocytopenia (PLT <100×10⁹/L, including one pt with PLT ≤50×10⁹/L). All pts started MMB at 200 mg/day.
In TD pts (Fig.1), overall response rate (ORR; major + minor) increased from 56% at 3 months to 87% at 6 months, primarily driven by minor responses (19% to 56%), while major responses remained stable (38% vs 31%); nonetheless, this pattern reflects the predominance of partial responses providing meaningful transfusion burden reduction. All non-TD pts at baseline maintained transfusion independence throughout follow-up: the median Hb increased from 9.8 to 12.6 g/dL at 3 months and remained at 12.7 g/dL at 6 months.
AR rates were comparable between JAKi-naive and JAKi-pretreated pts: ORR 60% vs 55% at 3 months and 100% vs 88% at 6 months.
Overall, transfusion independence rates were 55%, 44%, and 40% at 3, 6, and 12 months.
The median MMB treatment duration was 6.7 months (range 0.4-54) with median follow-up of 10.7 months (range 0.7-54). At data cut-off of February 20, 2026, 21 (72%) pts were still on treatment.
Safety profile was favorable: only 7% of pts required dose reduction and 4 (14%) pts permanently discontinued due to toxicity. Most common hematological adverse events were thrombocytopenia (n=1, grade 3) and paradoxical erythrocytosis (n=2, grade 4) requiring phlebotomy. Extra-hematological adverse events (grade 2-4) included infections (n=2), hepatotoxicity (n=1), rash (n=1), dizziness (n=1), and hyperamylasemia (n=1).

### Summary/Conclusion

This real-world application of 2024 IWG-ELN criteria shows progressive ORR increase in TD pts and maintained TI in non-TD pts, with outcomes comparable to clinical trials and optimal safety in both JAKi-naive and pretreated pts. Low discontinuation rate supports MMB as effective in real-world practice. Predictive factors for AR durability warrant further investigation.

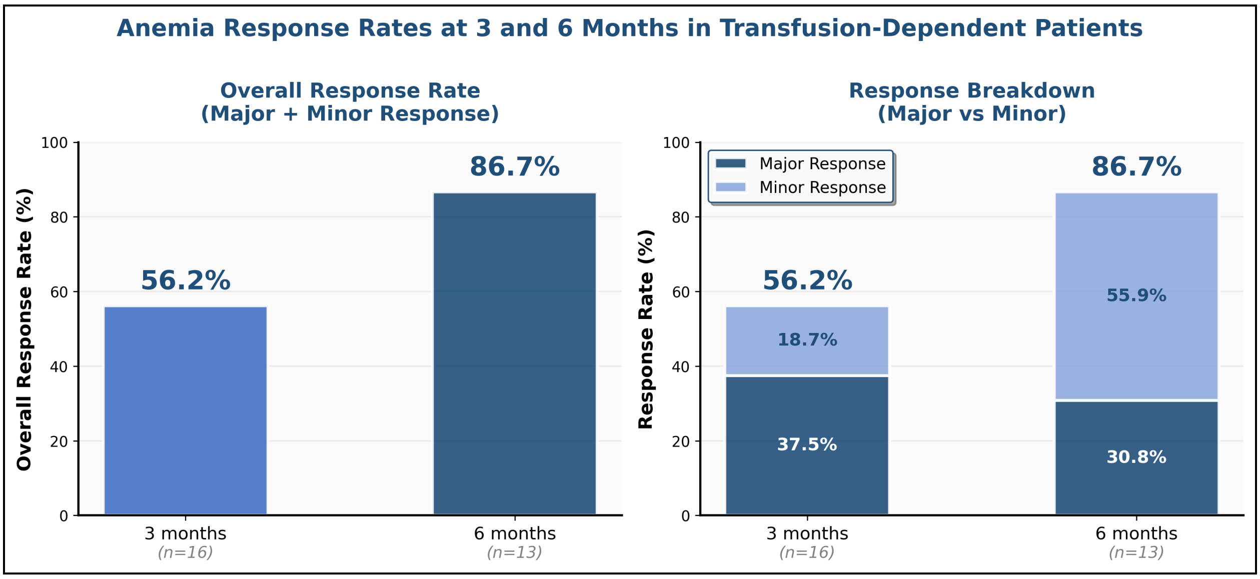

Figure 1. Anemia response rates at 3 and 6 months in TD patients showing ORR increase from 56% to 87%, primarily driven by minor responses.

**Copyright Information:** (Online) ISSN: 2572-9241
©2026 The Author(s). HemaSphere is published by John Wiley & Sons Ltd on behalf of European Hematology Association. This is an open access Abstract Book distributed under the Attribution-NonCommercial-NoDerivs (CC BY-NC-ND), which allows third parties to download the articles and share them with others as long as they credit the author and the Abstract Book, but they cannot change the content in any way or use them commercially.
**Abstract Book Citations:** Authors, Title, HemaSphere, 2026;10:(S1):pages. The abstract book DOIs can be found on the “Author Information” page.
**Disclaimer:** Articles published in the journal HemaSphere exclusively reflect the opinions of the authors. The authors are responsible for all content in their abstracts including accuracy of the facts, statements, citing resources, etc.

### (PB3489) EPIDEMIOLOGY, TREATMENT PATTERNS AND OUTCOMES IN MYELOFIBROSIS IN GERMANY: A HEALTH CLAIMS-BASED ANALYSIS

**Topic:** 16. Myeloproliferative neoplasms - Clinical

Julia Lovera[1], Vukašin Višković[2], Marc Feuerbach[3], Rainer Ehneß[3], Pamela Lenti[3], Isabel Barnstorf[3], Ariane Höer[4], Haifa Kathrin Al-Ali*[5]

*[1]IGES GmbH, Berlin, Germany; [2]InGef, Berlin, Germany; [3]Novartis Pharma GmbH, Nuremberg, Germany; [4]IGES Institut, Berlin, Germany; [5]University Hospital Halle, Medical Faculty of the Martin-Luther University Halle-Wittenberg, Halle (Saale), Germany;*

### Background

Myelofibrosis (MF) is a rare, life-threatening myeloproliferative neoplasm associated with substantial morbidity and mortality. In Germany, data on MF epidemiology and real-world treatment patterns remain limited. Treatment primarily aims to alleviate disease-related splenomegaly and symptoms and improve quality of life. According to German guideline recommendations, ruxolitinib (RUX) is the standard therapy for patients with symptomatic MF, while hydroxyurea (HU), which was considered standard therapy before the approval of RUX, is still used for cytoreduction in clinical practice.

### Aims

This study aimed to estimate the incidence and prevalence of MF in Germany and describe real-world treatment patterns and treatment outcomes of patients receiving RUX and HU.

### Methods

A retrospective German claims data analysis was conducted. Prevalent and incident patient populations were identified for each reference year from 2018 to 2023. Adults were included if they were continuously insured for ≥2 years prior to and during the respective reference year and had either ≥2 confirmed outpatient diagnoses or 1 inpatient diagnosis of MF (ICD-10-GM D47.4). Additionally, patients were classified as incident if no MF diagnosis was recorded during a 24-month diagnosis-free period prior to 2018. A longitudinal cohort was derived from the prevalent patient populations and stratified by initial therapy (RUX or HU). These patients were followed longitudinally until death, loss to follow-up, treatment gap, treatment switch, or the end of the observation period. Time to proxy-defined HU failure and time to anemia following initiation of RUX were estimated using Kaplan–Meier analyses.

**Copyright Information:** (Online) ISSN: 2572-9241
©2026 The Author(s). HemaSphere is published by John Wiley & Sons Ltd on behalf of European Hematology Association. This is an open access Abstract Book distributed under the Attribution-NonCommercial-NoDerivs (CC BY-NC-ND), which allows third parties to download the articles and share them with others as long as they credit the author and the Abstract Book, but they cannot change the content in any way or use them commercially.
**Abstract Book Citations:** Authors, Title, HemaSphere, 2026;10:(S1):pages. The abstract book DOIs can be found on the "Author Information" page.
**Disclaimer:** Articles published in the journal HemaSphere exclusively reflect the opinions of the authors. The authors are responsible for all content in their abstracts including accuracy of the facts, statements, citing resources, etc.

### Results

The study estimated a prevalence of MF of 8.2 per 100,000 insured individuals in 2023, with comparable rates among women (8.1) and men (8.3). The MF incidence rate in 2023 was 2.7 per 100,000 individuals, with consistently higher incidence observed among men, particularly in age groups aged 60 years and older.
Among the 380 patients included in the prevalent cohort, 47.9% (n = 182) initiated RUX and 26.1% (n = 99) initiated HU. Across both treatment cohorts, the most frequent reasons for censoring were treatment gaps, followed by treatment switches. Patients initiating HU were censored substantially earlier than those receiving RUX, with a median time to censoring of 8 months vs. 16 months, respectively.
Among RUX patients, the median dose at observation start was 20 mg/day, while the median time-averaged daily dose was 23 mg. Treatment duration differed by dose level, with a shorter median time to censoring observed in patients receiving an average RUX dose of ≤20 mg/day compared with >20 mg/day (12.7 vs. 19.6 months).
During follow-up, anemia-related ICD-10 diagnoses were observed in 20.4% of patients treated with RUX who did not have anemia at baseline. The median time to anemia onset in these patients was 4.9 months. Among patients receiving HU, 43.4% exhibited HU failure, with a median time to first occurrence of 11.6 months.

### Summary/Conclusion

Despite evolving evidence, gaps may exist between MF treatment guidelines and real-world practice in Germany. About 3 out of 10 MF patients started on HU, with ~40% failing therapy within a year. Findings suggest that RUX may have been administered at lower doses in a subset of patients. This highlights the need for guideline-aligned treatment, timely therapy adjustments and improved disease management in routine care.

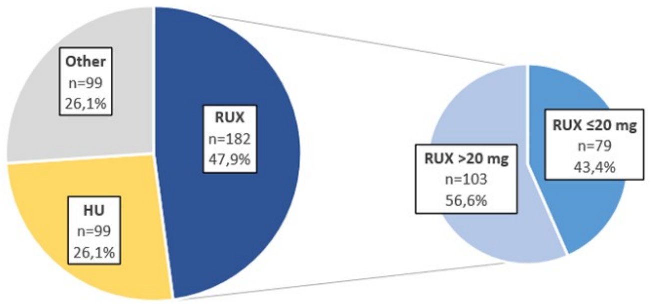

Among patients with MF, fewer than half received RUX, with 43% treated at doses ≤20 mg/day. Abbreviations: HU – Hydroxyurea, MF – Myelofibrosis, mg – milligram, n – number, RUX – Ruxolitinib. Note: The category “Other” includes patients receiving treatments other than those shown, as well as patients not receiving any treatment.

**Copyright Information:** (Online) ISSN: 2572-9241
©2026 The Author(s). HemaSphere is published by John Wiley & Sons Ltd on behalf of European Hematology Association. This is an open access Abstract Book distributed under the Attribution-NonCommercial-NoDerivs (CC BY-NC-ND), which allows third parties to download the articles and share them with others as long as they credit the author and the Abstract Book, but they cannot change the content in any way or use them commercially.
**Abstract Book Citations:** Authors, Title, HemaSphere, 2026;10:(S1):pages. The abstract book DOIs can be found on the “Author Information” page.
**Disclaimer:** Articles published in the journal HemaSphere exclusively reflect the opinions of the authors. The authors are responsible for all content in their abstracts including accuracy of the facts, statements, citing resources, etc.

### (PB3490) FOXP1 MUTATIONS IN PATIENTS WITH MYELOPROLIFERATIVE NEOPLASMS TRANSFORMING TO MYELOFIBROSIS

**Topic:** 16. Myeloproliferative neoplasms - Clinical

Diego Elrio[1, 2], Emmanuelle Verger[1, 2], Ying-Jung Huang[3], Nathalie Parquet[4], Nabih Maslah[1, 2], Stephane Giraudier[1, 2], Yasuhito Nannya[5], Jean-Jacques Kiladjian[2], Lee-Yung Shih[6], Bruno Cassinat*[2, 7]

*[1]Hopital Saint-Louis, AP-HP, Biologie Cellulaire, Paris, France;[2]INSERM UMR 1342, IRSL, Hopital Saint-Louis, Paris, France; [3]Division of Hematology-Oncology, Chang Gung Memorial Hospital, Taoyuan, Taiwan;[4]AP-HP hopital Saint-Louis, Apherese therapeutique, Paris, France; [5]Division of Hematopoietic Disease Control, The Institute of Medical Science, The University of Tokyo, Tokyo, Japan; [6]Division of Hematology-Oncology, Chang Gung Memorial Hospital at Linkou, and School of Medicine, Chang Gung University, Taoyuan, Taiwan; [7]Hopital Saint-Louis, AP-HP, Paris, France;*

### Background

Myeloproliferative neoplasms (MPNs) are clonal hematopoietic disorders driven by mutations acquired in JAK2, MPL or CALR genes, and comprise three different entities: essential thrombocythemia (ET), polycythemia vera (PV) and primary myelofibrosis (PMF). ET and PV are characterized by a chronic evolution inconstantly followed by a transformation to secondary myelofibrosis (sMF), a more aggressive disorder with severe symptoms and reduced life expectancy. In addition to driver mutations, MPN patients may acquire several additional mutations either in the driver clone or in independent clones, making MPNs oligoclonal diseases. Several genes have been shown mutated in MPNs, some of which are associated to an adverse outcome and included in prognostic scores such as the MIPSS70 and MIPSS70 plus.

### Aims

Recently, Takamori et al. (Leukemia 2025) showed for the first time the presence of mutations in the FOXP1 transcription factor in a subgroup of patients with MPN that transformed to sMF. To better define the role of these mutations in the progression of MPN, we sequenced the FOXP1 gene in a cohort of patients who evolved from a chronic phase of MPN to sMF.

### Methods

A cohort of 27 MPN patients (17 ET and 10 PV) was analyzed on whole blood DNA taken before (mean delay: 81 months) and after (mean delay: 15 months) evolution to sMF. MPN diagnosis was based on WHO criteria. The FOXP1 sequencing was performed on a MiSeq i100 plus platform using the TruSeq Nano library preparation kit (Illumina). Also, allele-specific ddPCR was performed on a QX200 instrument (BioRad) using allele-specific assays.

**Copyright Information:** (Online) ISSN: 2572-9241
©2026 The Author(s). HemaSphere is published by John Wiley & Sons Ltd on behalf of European Hematology Association. This is an open access Abstract Book distributed under the Attribution-NonCommercial-NoDerivs (CC BY-NC-ND), which allows third parties to download the articles and share them with others as long as they credit the author and the Abstract Book, but they cannot change the content in any way or use them commercially.
**Abstract Book Citations:** Authors, Title, HemaSphere, 2026;10:(S1):pages. The abstract book DOIs can be found on the “Author Information” page.
**Disclaimer:** Articles published in the journal HemaSphere exclusively reflect the opinions of the authors. The authors are responsible for all content in their abstracts including accuracy of the facts, statements, citing resources, etc.

**Results**

We identified 3 patients (1 type 1 CALR-mutated ET and 2 JAK2V617F-mutated PV) with 3 different FOXP1 truncating mutations: p.(W534*), p.(L573F fs*7) and p.(T426N fs*35). In each case the variant allelic frequency (VAF) was higher after the transformation to sMF (28% to 45%; 31% to 45%; 1% to 43%). Sequential samples covering several years of follow-up were available for all 3 patients. They were analysed by NGS and FOXP1-targeted ddPCR. In all cases VAFs of sequential analyses allowed to determine that both the driver and FOXP1 mutations were present in the same clone. In the CALR-mutated patient, FOXP1 was the only additional mutation. In the JAK2-mutated patients we found additional mutations in DNMT3A and IDH2 (1 case) or ASXL1 (1 case). During the chronic phase, JAK2-mutated patients received Interferon alpha (IFNa) while the CALR-mutated patient received hydroxyurea. All these treatments failed to induce any molecular response. Interestingly, at the time of sMF diagnosis, one JAK2-mutated patient became resistant to IFNa (135µg) which was later combined with ruxolitinib (25mg x2). This combined treatment induced a complete molecular response (JAK2, ASXL1 and FOXP1 mutations) and bone marrow fibrosis decreased from grade 2 to grade 0 after 4 years. None of these patients with FOXP1 mutations evolved to MDS or acute leukemia.

**Summary/Conclusion**

This study confirms the presence of FOXP1 mutations in a subset (11% in our study) of patients with MPN who evolve to sMF. These mutations may be present in the JAK2-mutant clone, which seems to be efficiently targeted by the combination of IFNa with ruxolitinib. This study also shows that the mutational profile evolves during the course of MPN with clear prognostic implications, reinforcing the role of molecular monitoring made possible by the implementation of NGS technologies in most laboratories.

**Copyright Information:** (Online) ISSN: 2572-9241
©2026 The Author(s). HemaSphere is published by John Wiley & Sons Ltd on behalf of European Hematology Association. This is an open access Abstract Book distributed under the Attribution-NonCommercial-NoDerivs (CC BY-NC-ND), which allows third parties to download the articles and share them with others as long as they credit the author and the Abstract Book, but they cannot change the content in any way or use them commercially.
**Abstract Book Citations:** Authors, Title, HemaSphere, 2026;10:(S1):pages. The abstract book DOIs can be found on the "Author Information" page.
**Disclaimer:** Articles published in the journal HemaSphere exclusively reflect the opinions of the authors. The authors are responsible for all content in their abstracts including accuracy of the facts, statements, citing resources, etc.

### (PB3491) IMPACT OF ADDITIONAL MUTATIONS ON FIRST-LINE TREATMENT RESPONSE AND DISEASE PROGRESSION IN ESSENTIAL THROMBOCYTHEMIA

**Topic:** 16. Myeloproliferative neoplasms - Clinical

Novella Pugliese*[1], Daniela D'angelo[1], Annamaria Vincenzi[1], Maria Di Perna[2], Roberta Spisso[1], Assunta Lombardi[1], Barbara Izzo[3], Fabrizio Pane[1]

*[1]University of Naples Federico II, Napoli, Italy;[2]Hospital “Andrea Tortora”, Pagani, Italy; [3]CEINGE Biotwcnologie Avanzate, Napoli, Italy;*

### Background

Patients with essential thrombocythemia (ET) have a chronic disease course with risk of hematologic transformation. While driver mutations (JAK2, CALR, MPL) are key for diagnosis and prognosis, additional mutations may influence treatment response and disease evolution. Early identification of patients less likely to respond to first-line therapy is crucial to optimize outcomes.

### Aims

We aimed to assess the impact of additional mutations on response to first-line treatment and evolution to myelofibrosis (MF).

### Methods

In this retrospective study, 147 ET patients received hydroxyurea (HU, n = 115) or pegylated interferon (peg-IFN, n = 32). Molecular profiling was performed using a 30-gene myeloid panel by next-generation sequencing. Response was defined according to ELN criteria as complete (CR), partial (PR), or no response (NR). Kaplan–Meier, multistate models, and Cox regression were used to evaluate time to CR and progression to MF.

### Results

CR rates were similar between hydroxyurea (HU) and pegylated interferon (peg-IFN) (77.4% vs 78.1%). Median time to CR was slightly shorter with peg-IFN (2 vs 2.6 months). Discontinuation for intolerance tended to be higher with peg-IFN (18.7% vs 7.8%), whereas resistance-related discontinuation was higher with HU (14.8% vs 9.4%). Overall, response distribution was comparable between treatments.
JAK2V617F mutations were associated with higher CR, CALR type 1 with more PR, and CALR type 2 with more NR, irrespective of treatment. The most frequently observed additional mutations involved TET2 (61 patients, 41.5%), SETBP1 (48 patients, 32.6%), EZH2 (24 patients, 16.3%), and ASXL1 (23 patients, 15.6%). Cox analysis showed that ≥1 additional mutation at diagnosis significantly reduced the likelihood of achieving CR over time (HR 0.46; $P < .05$) and delayed responses, despite no significant difference in final response rates ($p = 0.15$).
Analysis of myelofibrotic transformation showed no significant difference between HU and peg-IFN, with only 10 events observed, limiting statistical power. There was no difference in progression risk between patients achieving CR or PR. Notably, none of the patients without additional mutations evolved, whereas those with ≥2 additional mutations showed a trend toward reduced myelofibrosis-free survival, suggesting increased clonal instability.

**Copyright Information:** (Online) ISSN: 2572-9241
©2026 The Author(s). HemaSphere is published by John Wiley & Sons Ltd on behalf of European Hematology Association. This is an open access Abstract Book distributed under the Attribution-NonCommercial-NoDerivs (CC BY-NC-ND), which allows third parties to download the articles and share them with others as long as they credit the author and the Abstract Book, but they cannot change the content in any way or use them commercially.
**Abstract Book Citations:** Authors, Title, HemaSphere, 2026;10:(S1):pages. The abstract book DOIs can be found on the “Author Information” page.
**Disclaimer:** Articles published in the journal HemaSphere exclusively reflect the opinions of the authors. The authors are responsible for all content in their abstracts including accuracy of the facts, statements, citing resources, etc.

**Summary/Conclusion**

In ET, the number of additional mutations significantly affects time to CR independently of driver mutation status and treatment. While progression to MF remains infrequent, additional mutations may indicate underlying clonal instability. These findings support the integration of comprehensive molecular profiling into clinical decision-making to guide personalized therapy and anticipate disease evolution.

**Copyright Information:** (Online) ISSN: 2572-9241
©2026 The Author(s). HemaSphere is published by John Wiley & Sons Ltd on behalf of European Hematology Association. This is an open access Abstract Book distributed under the Attribution-NonCommercial-NoDerivs (CC BY-NC-ND), which allows third parties to download the articles and share them with others as long as they credit the author and the Abstract Book, but they cannot change the content in any way or use them commercially.
**Abstract Book Citations:** Authors, Title, HemaSphere, 2026;10:(S1):pages. The abstract book DOIs can be found on the "Author Information" page.
**Disclaimer:** Articles published in the journal HemaSphere exclusively reflect the opinions of the authors. The authors are responsible for all content in their abstracts including accuracy of the facts, statements, citing resources, etc.

### (PB3492) SHEAR-WAVE ELASTOGRAPHY IDENTIFIES A DYNAMIC SURROGATE IMAGING ENDPOINT OF MOMELOTINIB ACTIVITY IN A REAL-WORLD MYELOFIBROSIS COHORT

**Topic:** 16. Myeloproliferative neoplasms - Clinical

Amedeo Votto*[1], Maria Costanza Caparello[1], Angela Brunetti[1], Matilde Maggiorelli[1], Sara Galimberti[1], Edoardo Benedetti[1]

[1]*University of Pisa, Hematology Unit, Department of Clinical and Experimental Medicine, Pisa, Italy;*

### Background

In pivotal momelotinib (MMB) trials, spleen response is defined by MRI/CT-based spleen volume reduction (SVR35). While volumetric imaging represents the regulatory standard, it is not universally accessible and does not capture treatment-induced biomechanical remodeling of splenic tissue. In myelofibrosis (MF), splenic enlargement reflects complex changes in extramedullary hematopoiesis, vascular congestion, and stromal remodeling. Shear-wave elastography (SWE) enables quantitative assessment of splenic stiffness and may dynamically reflect microenvironmental modifications induced by JAK inhibition beyond mere dimensional changes.

### Aims

To evaluate spleen response at 6 months in MF patients treated with momelotinib and to determine whether SWE-derived stiffness reduction correlates with anatomical spleen response, potentially serving as a dynamic surrogate imaging endpoint of treatment activity.

### Methods

Twenty-three consecutive MF patients treated with momelotinib in a real-world setting were retrospectively analyzed. Spleen longitudinal diameter (cm) and stiffness (kPa) were assessed at baseline and 6 months using standardized high-resolution ultrasound with SWE performed by experienced operators. Percentage changes were calculated for both parameters. Spleen response was defined as ≥35% reduction in longitudinal diameter, consistent with SVR35 conceptual thresholds. Correlation analyses (Spearman), linear regression modeling, and exploratory predictor assessments were performed.

### Results

Median age at MMB initiation was 67 years (range 52–82). Driver mutations included JAK2V617F (62%), CALR (25%), and triple-negative status (13%). At 6 months, 4/23 patients (17%) achieved ≥35% spleen reduction, while the majority demonstrated measurable splenic shrinkage, supporting overall treatment activity. Percentage spleen reduction strongly correlated with stiffness decline (Spearman $\rho$=0.71, 95% CI 0.42–0.87; p=0.0001), indicating a robust monotonic association between anatomical and biomechanical changes. Linear regression confirmed that 38% of stiffness variability was explained by spleen reduction ($R^2$=0.38). Responders exhibited significantly greater stiffness reduction compared with non-responders (p=0.044), reinforcing the biological coherence of elastographic changes with clinical response. Notably, baseline spleen size and baseline stiffness were not predictive of response, suggesting that dynamic stiffness modulation — rather than static baseline measurements — reflects treatment sensitivity. Hemoglobin improved in 18/23 patients (78%), and 12/17 (70%) transfusion-dependent patients experienced reduced transfusion requirements.

**Copyright Information:** (Online) ISSN: 2572-9241
©2026 The Author(s). HemaSphere is published by John Wiley & Sons Ltd on behalf of European Hematology Association. This is an open access Abstract Book distributed under the Attribution-NonCommercial-NoDerivs (CC BY-NC-ND), which allows third parties to download the articles and share them with others as long as they credit the author and the Abstract Book, but they cannot change the content in any way or use them commercially.
**Abstract Book Citations:** Authors, Title, HemaSphere, 2026;10:(S1):pages. The abstract book DOIs can be found on the “Author Information” page.
**Disclaimer:** Articles published in the journal HemaSphere exclusively reflect the opinions of the authors. The authors are responsible for all content in their abstracts including accuracy of the facts, statements, citing resources, etc.

### Summary/Conclusion

SWE captures biologically meaningful, treatment-induced alterations in splenic biomechanics and closely parallels anatomical spleen reduction in MF patients receiving momelotinib. These findings support stiffness decline as a dynamic surrogate imaging endpoint that may complement conventional MRI-defined SVR35 assessment. Prospective multicenter validation is warranted to determine whether elastography-based parameters can enhance response monitoring and serve as pragmatic imaging endpoints in both clinical trials and routine practice.

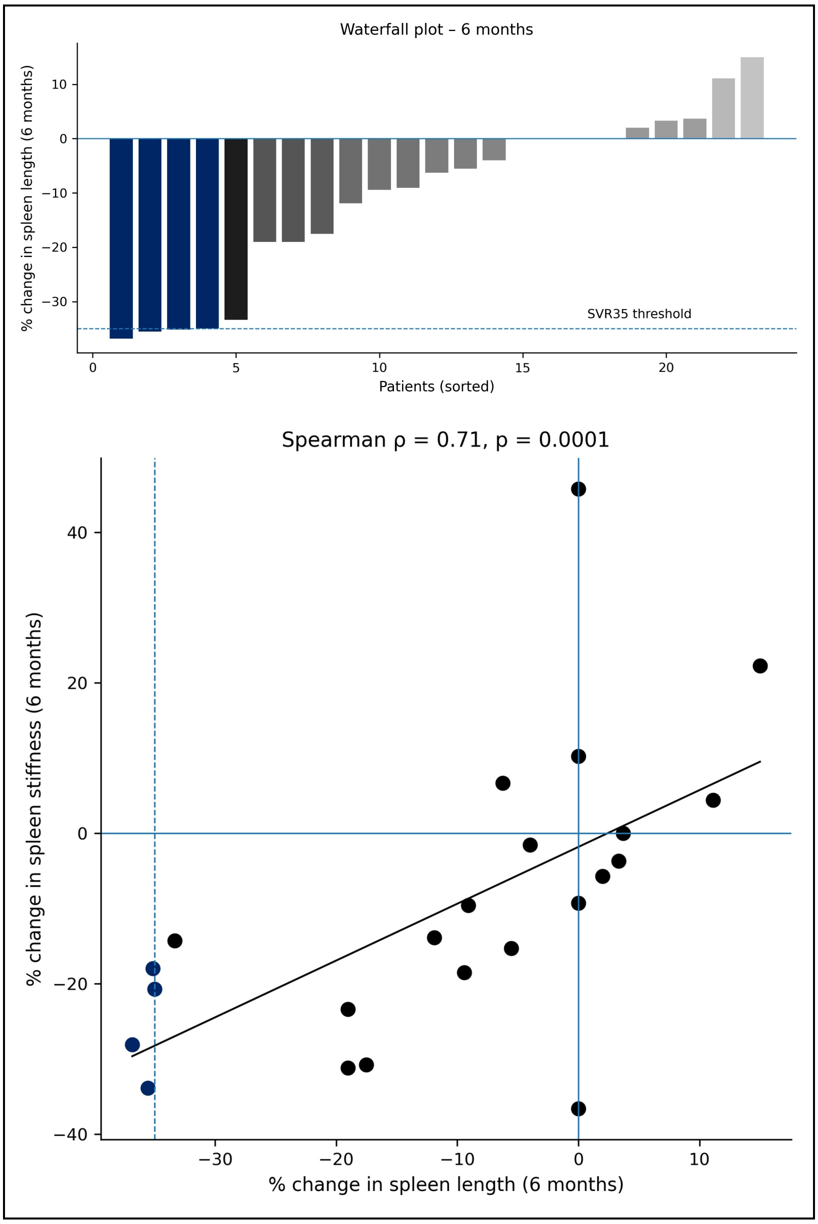

The upper panel shows a waterfall plot indicating the percentage change in spleen length for individual patients, highlighting those who achieved the SVR35 threshold. The lower panel presents a scatter plot showing a significant positive correlation between changes in spleen length and spleen stiffness (Spearman ρ = 0.71, p = 0.0001), indicating that reductions in spleen size are strongly associated with improvements in spleen stiffness.

**Copyright Information:** (Online) ISSN: 2572-9241
©2026 The Author(s). HemaSphere is published by John Wiley & Sons Ltd on behalf of European Hematology Association. This is an open access Abstract Book distributed under the Attribution-NonCommercial-NoDerivs (CC BY-NC-ND), which allows third parties to download the articles and share them with others as long as they credit the author and the Abstract Book, but they cannot change the content in any way or use them commercially.
**Abstract Book Citations:** Authors, Title, HemaSphere, 2026;10:(S1):pages. The abstract book DOIs can be found on the “Author Information” page.
**Disclaimer:** Articles published in the journal HemaSphere exclusively reflect the opinions of the authors. The authors are responsible for all content in their abstracts including accuracy of the facts, statements, citing resources, etc.

### (PB3494) THE DIAGNOSTIC VALUE OF NEXT-GENERATION-SEQUENCING IN PATIENTS WITH SUSTAINED ELEVATED BLOOD COUNTS

**Topic:** 16. Myeloproliferative neoplasms - Clinical

Jessica Smeds*[1], Philipp Kaufmann[1], Stefan Balabanov[1], Jeroen Simon Goede[2], Tobias Silzle[3], Rudolf Andreas Benz[4], Adrian Schmidt[5], Alexandre Theocharides[1, 6]

*[1]University Hospital Zurich, Department of Medical Oncology and Hematology, Zurich, Switzerland; [2]Kantonsspital Winterthur, Winterthur, Switzerland; [3]Kantonsspital Graubünden, Chur, Switzerland; [4]Spital Thurgau AG, Münsterlingen, Switzerland; [5]Stadtspital Zürich, Zürich, Switzerland; [6]Comprehensive Cancer Center Zurich, Zurich, Switzerland;*

### Background

Sustained elevation of red blood cells, platelets, or leukocytes may be indicative of myeloproliferative neoplasms (MPN).  While, the exclusion of secondary causes represents a diagnostic challenge, the detection of somatic mutations in driver and non-driver genes strongly suggests the presence of an underlying myeloid neoplasm. Next-generation-sequencing (NGS) analysis has transformed the diagnostic approach to patients with suspected MPN and led to the integration of the mutational profile into WHO criteria and prognostic scoring systems.

### Aims

In order to evaluate the role of NGS as an initial diagnostic step in individuals with sustained elevated blood counts, we retrospectively analyzed the results of >400 NGS analyses performed at five Swiss centers in individuals without a known MPN diagnosis and integrated clinical as well as hematological parameters. Based on the analyzed data we aimed to develop a scoring system to assess the likelihood of detecting MPN driver and non-driver mutations by NGS in selected individuals with sustained elevated blood counts.

### Methods

Descriptive statistics were performed to analyze the characteristics of the study cohort (n=404) including indications to perform NGS with a MPN gene panel (*ASXL1, CALR, CSF3R, EZH2, IDH1, IDH2, JAK2, MPL, SETBP1, SF3B1, SRSF2, TET2, U2AF1*). Using an univariate model, we screened for clinical and hematological parameters associated with the detection of mutations typically found in MPN and selected explanatory variables for a pre-analytical score using randomForestRSC variance importance. To form the weight-adjusted items we determined binary variable thresholds and variable weights using hyperopt to maximize ROC area under the curve in a xGBoost classifier. In addition to the integrated cross-validation we tested the score on an independent validation cohort (n=110).

**Copyright Information:** (Online) ISSN: 2572-9241
©2026 The Author(s). HemaSphere is published by John Wiley & Sons Ltd on behalf of European Hematology Association. This is an open access Abstract Book distributed under the Attribution-NonCommercial-NoDerivs (CC BY-NC-ND), which allows third parties to download the articles and share them with others as long as they credit the author and the Abstract Book, but they cannot change the content in any way or use them commercially.
**Abstract Book Citations:** Authors, Title, HemaSphere, 2026;10:(S1):pages. The abstract book DOIs can be found on the "Author Information" page.
**Disclaimer:** Articles published in the journal HemaSphere exclusively reflect the opinions of the authors. The authors are responsible for all content in their abstracts including accuracy of the facts, statements, citing resources, etc.

### Results

The most frequent indications that led to NGS analysis were erythrocytosis (30%) followed by thrombocytosis (25%) and cardiovascular events (19%). NGS detected MPN associated mutations in 100 individuals (25%) with *JAK2*-V617F being the most frequent mutation. The diagnostic work-up resulted in the diagnosis of 84 MPN according to WHO 2022 (21%) and 161 secondary causes (40%), while no underlying cause for blood count alterations were identified in 150 cases (37%). The multivariate analysis revealed a significant and independent association with the NGS result in seven of the collected clinical and laboratory parameters. We then developed a six-parameter score based on basophil count, platelet count, red blood cell distribution width (RDW), age, presence of myeloid precursors in the peripheral blood and hematocrit (Table 1). The score predicted the detection of MPN associated mutations with a sensitivity of 92% and specificity of 64% in the study cohort. The validation in the independent cohort confirmed the score accuracy (sensitivity 88%, specificity 67%).

### Summary/Conclusion

The distinction between primary and secondary etiologies in patients with sustained elevated blood counts remains a diagnostic challenge. We developed a scoring system based on clinical and laboratory parameters to support decision-making regarding whether to proceed with NGS analysis. While prospective trials are required to determine the role of the approach, this newly developed score may be integrated in diagnostic algorithms for individuals with sustained elevated blood counts.

| Parameters: | Points: |
|---|---|
| Basophils > 0.29 G/l | 5 |
| Platelets ≥ 410 G/l | 4 |
| RDW ≥ 14 % | 3 |
| Age ≥ 68 y | 1 |
| Presence of myeloid precursors | 1 |
| Hematocrite ≥ 0.49 l/l | 1 |
| Total score ≥ 4 supports NGS analysis | |
| Total score < 4 does not support NGS analysis | |

Table 1: Pre-analytical scoring system supporting the decision-making in the diagnostic path of patients with sustained elevated blood counts. Parameters of the score need to repeatedly match the criteria.

**Copyright Information:** (Online) ISSN: 2572-9241
©2026 The Author(s). HemaSphere is published by John Wiley & Sons Ltd on behalf of European Hematology Association. This is an open access Abstract Book distributed under the Attribution-NonCommercial-NoDerivs (CC BY-NC-ND), which allows third parties to download the articles and share them with others as long as they credit the author and the Abstract Book, but they cannot change the content in any way or use them commercially.
**Abstract Book Citations:** Authors, Title, HemaSphere, 2026;10:(S1):pages. The abstract book DOIs can be found on the "Author Information" page.
**Disclaimer:** Articles published in the journal HemaSphere exclusively reflect the opinions of the authors. The authors are responsible for all content in their abstracts including accuracy of the facts, statements, citing resources, etc.

### (PB3495) REAL-WORLD APPLICATION OF CLINICAL AND MOLECULAR-ENHANCED PROGNOSTIC MODELS IN PRIMARY AND SECONDARY MYELOFIBROSIS IN A MULTIETHNIC SOUTHEAST ASIAN COHORT

**Topic:** 16. Myeloproliferative neoplasms - Clinical

Than Hein[1], David Wong*[2], Charles Chuah[1], Gee Fung How[1], Jeffrey Quek[1], Si Jie Khoo[1], Jing Yuan Tan[1], Christopher Tham[1]

*[1]Singapore General Hospital, Haematology, Singapore, Singapore;[2]Duke-NUS Medical School, Singapore, Singapore;*

### Background

Prognostic models for myelofibrosis (MF), such as DIPSS, MIPSS70, MIPSS70-Plus v2.0, and MYSEC-PM, were largely developed in Western populations. Their applicability in Southeast Asian cohorts remains underexplored. Molecular-enhanced systems incorporate cytogenetic and next-generation sequencing (NGS) for mutation profiling. However, such molecular profiling has not been routinely assessed in real-world patients in Asia until recent years.

### Aims

This study evaluates the performance of established clinical and molecular-enhanced prognostic models in primary (PMF) and secondary MF (SMF) within a multiethnic Singaporean cohort, and assesses the incremental value of molecular data in a real-world setting.

### Methods

A retrospective review of the patient registry identified 146 adult Singaporean MF patients (PMF n=73; SMF n=73). Clinical, laboratory, cytogenetic, and molecular data were collected, and prognostic scores were calculated. Overall survival (OS) was analyzed using Kaplan–Meier estimates and Firth-penalized Cox regression to mitigate small-sample bias. Discrimination was assessed using Harrell's C-index, Uno's C-index, and time-dependent AUC. The study was approved by the local institutional review board.

### Results

Median follow-up was 75.9 months for PMF and 24.4 months for SMF. The cohortincluded Chinese patients (PMF 73%, SMF 71%), Malays (PMF 23%, SMF 18%), Indians (3%), and other minor Asian ethnicities. Driver mutations were predominantly JAK2 (PMF 66%; SMF 74%). More than one high-molecular-risk (HMR) mutation was detected in 18% of PMF and 25% of SMF among those tested.

Risk stratification using Kaplan–Meier analysis was significant for DIPSS and DIPSS-Plus in both PMF and SMF ($p < 0.001$). MIPSS70-Plus v2.0 demonstrated significant separation in PMF (p=0.011), although the analysis was limited by a smaller sample size of molecularly characterized patients.

In PMF, MIPSS70-Plus v2.0 showed the highest Harrell's C-index (0.831), with time-dependent AUC ranging from 0.835 to 0.892. In SMF, DIPSS-Plus achieved the highest discrimination (Harrell's C 0.813), particularly at earlier time points, whereas molecular-enhanced models demonstrated comparable or improved discrimination at later intervals.

Across both cohorts, age ≥65 years, anaemia, thrombocytopenia, and transfusion dependence were consistently associated with inferior OS. In SMF, circulating blasts ≥2%, unfavourable/complex karyotype, and TP53 mutation were additional adverse prognostic factors.

Copyright Information: (Online) ISSN: 2572-9241
©2026 The Author(s). HemaSphere is published by John Wiley & Sons Ltd on behalf of European Hematology Association. This is an open access Abstract Book distributed under the Attribution-NonCommercial-NoDerivs (CC BY-NC-ND), which allows third parties to download the articles and share them with others as long as they credit the author and the Abstract Book, but they cannot change the content in any way or use them commercially.
**Abstract Book Citations:** Authors, Title, HemaSphere, 2026;10:(S1):pages. The abstract book DOIs can be found on the "Author Information" page.
**Disclaimer:** Articles published in the journal HemaSphere exclusively reflect the opinions of the authors. The authors are responsible for all content in their abstracts including accuracy of the facts, statements, citing resources, etc.

**Summary/Conclusion**

Established MF prognostic models demonstrate good discrimination in a Southeast Asian multiethnic cohort. Molecular-enhanced scores show promising discriminatory capacity; however, their statistical significance is limited by a small sample size of this real-world dataset. Conventional clinical models remain robust and clinically applicable where molecular profiling is limited. Our study highlights the importance of incorporating NGS-based molecular profiling into routine clinical practice in our region.

**Copyright Information:** (Online) ISSN: 2572-9241
©2026 The Author(s). HemaSphere is published by John Wiley & Sons Ltd on behalf of European Hematology Association. This is an open access Abstract Book distributed under the Attribution-NonCommercial-NoDerivs (CC BY-NC-ND), which allows third parties to download the articles and share them with others as long as they credit the author and the Abstract Book, but they cannot change the content in any way or use them commercially.
**Abstract Book Citations:** Authors, Title, HemaSphere, 2026;10:(S1):pages. The abstract book DOIs can be found on the "Author Information" page.
**Disclaimer:** Articles published in the journal HemaSphere exclusively reflect the opinions of the authors. The authors are responsible for all content in their abstracts including accuracy of the facts, statements, citing resources, etc.

### (PB3496) DIGITAL PCR–BASED QUANTIFICATION OF KIT D816V IDENTIFIES DISEASE BURDEN ACROSS MASTOCYTOSIS SUBTYPES

**Topic:** 16. Myeloproliferative neoplasms - Clinical

Simge Erdem*[1], Hazel Taş[2], Aynur Dağlar Aday[3], Ayşe Gül Bayrak Tokaç[3], Zeynep Yegin Katran[4], Ali Yılmaz Altay[5], Şükrü Öztürk[3]

*[1]Istanbul University Istanbul Faculty of Medicine, Department of Internal Medicine, Division of Hematology, Istanbul, Türkiye; [2]Istanbul University Istanbul Faculty of Medicine, Department of Internal Medicine, Istanbul, Türkiye;[3]Istanbul University Istanbul Faculty of Medicine, Department of Medical Genetics, Istanbul, Türkiye; [4]University of Health Sciences, Süreyyapaşa Training and Research Hospital, Department of Allergy and Immunology, Istanbul, Türkiye; [5]Istanbul University Istanbul Faculty of Medicine, Department of Medical Pathology, Istanbul, Türkiye;*

### Background

Mastocytosis is a clonal hematologic disorder characterized by abnormal mast cell accumulation, and the KIT D816V mutation represents a key diagnostic and prognostic biomarker. Accurate detection and quantification of low variant allele fractions (VAF) remain challenging with conventional real-time PCR (rtPCR). Digital PCR (dPCR) enables highly sensitive and absolute quantification and may better reflect biological disease burden.

### Aims

To determine the frequency and quantitative allele burden of KIT D816V using dPCR and to evaluate its clinical relevance across the mastocytosis spectrum.

### Methods

We retrospectively analyzed 128 adult patients diagnosed according to the 2022 WHO criteria and followed between 2020–2025, including cutaneous mastocytosis (CM), systemic mastocytosis (SM) subtypes, and mast cell activation syndrome. Peripheral blood DNA samples were analyzed using QuantStudio Absolute Q dPCR. Clinical and laboratory data were collected from medical records. Patients were harmonized into CM, non-advanced SM (ISM/SSM), and advanced SM (ASM/SM-AHN/MCL). Concordance with routine rtPCR was assessed. Continuous variables were compared using non-parametric tests. A p-value $<0.05$ was considered statistically significant. The study was conducted in accordance with the Declaration of Helsinki and approved by the local ethics committee.

**Copyright Information:** (Online) ISSN: 2572-9241
©2026 The Author(s). HemaSphere is published by John Wiley & Sons Ltd on behalf of European Hematology Association. This is an open access Abstract Book distributed under the Attribution-NonCommercial-NoDerivs (CC BY-NC-ND), which allows third parties to download the articles and share them with others as long as they credit the author and the Abstract Book, but they cannot change the content in any way or use them commercially.
**Abstract Book Citations:** Authors, Title, HemaSphere, 2026;10:(S1):pages. The abstract book DOIs can be found on the "Author Information" page.
**Disclaimer:** Articles published in the journal HemaSphere exclusively reflect the opinions of the authors. The authors are responsible for all content in their abstracts including accuracy of the facts, statements, citing resources, etc.

### Results dPCR detected KIT D816V in 84.8% (106/125 evaluable cases), with a median VAF of 0.19% (range 0–31.44). Concordance between rtPCR and dPCR was moderate ($\kappa=0.502$, $p<0.001$), with no significant discordance by McNemar test ($p=0.332$). Using rtPCR as reference, dPCR showed 93.9% sensitivity and 52% specificity, identifying additional low-level mutations.

As summarized in Table 1, allele burden increased significantly across harmonized disease categories (Kruskal–Wallis $p=0.002$). Median dPCR VAF values were 0.012% in CM, 0.173% in non-advanced SM, and 0.760% in advanced SM. VAF correlated positively with serum tryptase levels ($r=0.485$, $p=0.035$). Patients with VAF ≥2% exhibited lower hemoglobin ($p=0.014$), higher alkaline phosphatase ($p=0.012$), and elevated β2-microglobulin levels ($p=0.001$). Advanced disease was significantly associated with B symptoms and hepatosplenomegaly (all $p<0.001$).

**Table-1: Molecular and laboratory characteristics across harmonized mastocytosis groups**

| VARIABLE | CM | NON-ADVANCED SM | ADVANCED SM | p value |
|---|---|---|---|---|
| **KIT D816V VAF (qPCR, %)** | 0.012 (0.00–4,428) | 0, 0173 (0,00-27,474) | 0.76 (0.00–34,640) | 0.002 |
| **KIT D816V VAF (ddPCR, %)** | 0.025 (0.00–5,721) | 0.207 (0.00–27,334 | 0,862 (0.00–31,440) | <0,001 |
| **Serum tryptase (μg/L)** | 7.56 (1,9–126,0) | 26 (2,8-220) | 86,5 (6,5–486) | <0,001 |
| **Hemoglobin (g/dL)** | 14.00 (10,6–16,4) | 13,9 (9,3–17,3) | 11 (6,00–15,4) | <0.001 |
| **Alkaline phosphatase (U/L)** | 61.50 (38-212) | 75 (33-336) | 152,5 (47–1090) | <0.001 |
| **ß-2 microglobulin (mg/L)** | 1,8 (1,4-2,5) | 1,9 (1,3-3,4) | 2,8 (1,4-6,3) | 0.001 |

*Continuous variables are presented as median (interquartile range, IQR). P values were calculated using the Kruskal–Wallis test. CM: cutaneous mastocytosis; ddPCR: droplet digital polymerase chain reaction; qPCR: quantitative polymerase chain reaction; SM: systemic mastocytosis; VAF: variant allele fraction*

### Summary/Conclusion

Quantitative KIT D816V assessment by dPCR reflects biological disease burden across the mastocytosis spectrum. Increasing allele burden correlates with advanced phenotype, organ involvement, and treatment intensity. dPCR-based VAF measurement may enhance molecularly guided risk stratification and support precision medicine approaches in mastocytosis.

**Copyright Information:** (Online) ISSN: 2572-9241
©2026 The Author(s). HemaSphere is published by John Wiley & Sons Ltd on behalf of European Hematology Association. This is an open access Abstract Book distributed under the Attribution-NonCommercial-NoDerivs (CC BY-NC-ND), which allows third parties to download the articles and share them with others as long as they credit the author and the Abstract Book, but they cannot change the content in any way or use them commercially.
**Abstract Book Citations:** Authors, Title, HemaSphere, 2026;10:(S1):pages. The abstract book DOIs can be found on the "Author Information" page.
**Disclaimer:** Articles published in the journal HemaSphere exclusively reflect the opinions of the authors. The authors are responsible for all content in their abstracts including accuracy of the facts, statements, citing resources, etc.

### (PB3497) SOLID SECOND PRIMARY MALIGNANCIES (SPMS) IN PATIENTS WITH POLYCYTHEMIA VERA (PV): A GLOBAL SYSTEMATIC LITERATURE REVIEW

**Topic:** 16. Myeloproliferative neoplasms - Clinical

Francesca Palandri*[1], Andreas Reiter[2], Dasha Cherepanov[3], Samantha Martel[3], Meng Yang[3], Nicholas Parr[4], Eva Gonzalez Viana[5], Sarah Ronnebaum[6], Joseph Shatzel[7]

*[1]IRCCS Azienda Ospedaliero-Universitaria di Bologna, Bologna, Italy;[2]University Hospital Mannheim, Heidelberg University, Mannheim, Germany; [3]Takeda Development Center Americas, Inc. (TDCA), Cambridge, United States of America;[4]Thermo Fisher Scientific, Inc, Waltham, MA, United States of America; [5]Evidence Synthesis, PPD™ Evidera™ Health Economics & Market Access, Thermo Fisher Scientific, London, United Kingdom; [6]Previous: Evidera; Current: Precision AQ, Washington DC, United States of America; [7]Oregon Health & Science University, Portland, United States of America;*

### Background

PV is a chronic myeloproliferative neoplasm (MPN) characterized by an increased red blood cell production and risk of transformation to secondary myelofibrosis or secondary acute myeloid leukemia. Although inconsistent, studies have suggested a higher risk of developing SPMs in PV patients.

### Aims

To identify and synthesize published literature on the occurrence of solid SPMs in PV patients.

### Methods

Literature was searched (July 2025) in EMBASE, MEDLINE, PsycINFO, and Cochrane Database of Systematic Reviews (limits: observational studies, English language, 2000-onwards, conference abstracts 2023-onwards). Studies in MPN patients not reporting PV-specific data were excluded. At title/abstract screening, a machine learning model (iteratively calibrated with citations from prior reviews) estimated inclusion probabilities to prioritize records for human review. Abstracts and full-text records were then independently screened by two reviewers per prespecified eligibility criteria. Data were extracted by one reviewer, validated by a second.

### Results

1,506 database citations and 198 hand-searched abstracts were reviewed, of which 39 studies reporting on solid SPMs in PV were identified. Most studies were retrospective and from the US or Europe. Data collection began as early as 1957; in most studies, median follow-up ranged from 3 to 7 years after diagnosis, study or treatment start. Sample sizes ranged from 24 to 6,636 patients. Most patient cohorts were primarily white with median/mean age ≥50 years (range 32–74). Melanoma and non-melanoma skin cancer (NMSC) data had the highest evidence strength due to many available studies (9 and 12, respectively) with generally consistent data. NMSC ranged between 1.1% and 18.5%, with most studies reporting (MSR) between 2.0% and 4.2% (Table; Figure). The next-highest published estimates in MSR ranges were for prostate (0.3-2.9% in males; 0.7-2.4% overall), breast (1.5-2.5% in females; 1.1-6.9% overall) and lung (0.3-2.4%) cancers. In four US and European studies comparing cancer risk in PV patients to the general population, PV patients had significantly higher incidence of melanoma, NMSC, kidney and lung cancer (most standardized incidence ratios: 1.7–1.9).

### Summary/Conclusion

**Copyright Information:** (Online) ISSN: 2572-9241
©2026 The Author(s). HemaSphere is published by John Wiley & Sons Ltd on behalf of European Hematology Association. This is an open access Abstract Book distributed under the Attribution-NonCommercial-NoDerivs (CC BY-NC-ND), which allows third parties to download the articles and share them with others as long as they credit the author and the Abstract Book, but they cannot change the content in any way or use them commercially.
**Abstract Book Citations:** Authors, Title, HemaSphere, 2026;10:(S1):pages. The abstract book DOIs can be found on the "Author Information" page.
**Disclaimer:** Articles published in the journal HemaSphere exclusively reflect the opinions of the authors. The authors are responsible for all content in their abstracts including accuracy of the facts, statements, citing resources, etc.

Published evidence indicates patients with PV experience a broad range of solid SPMs and increased incidence of certain cancers, including NMSC, compared with the general population.

**Table. SPM Occurrence[a] Evidence Summary**

| Cancer | Reported Estimates,<br>% range (No. of studies) | Evidence Strength[b] |
|---|---|---|
| NMSC | 1.1–18.5 (12),<br>MSR 2.0–4.2 (7) | High; 12 studies, LH |
| Melanoma | 0.4–6.5 (9),<br>MSR 0.4–0.7 (8) | High; 9 studies, LH |
| Prostate | Males only: 0.3–5.4 (8),<br>MSR 0.3–2.9 (6);<br>All pts.: 0.7–2.4 (4) | Medium; 12 studies, MH |
| Breast | Females only: 1.5–19.2 (6),<br>MSR 1.5–2.5 (5);<br>All pts.: 1.1–11.1 (6),<br>MSR 1.1–6.9 (5) | Medium; 12 studies, MH |
| Kidney | 0.2–2.4 (10),<br>MSR 0.2–0.7 (8) | Medium; 10 studies, MH |
| Colon/Colorectal | 0.3–19.8 (9),<br>MSR 0.3–1.3 (8) | Medium; 9 studies, MH |
| Lung | 0.3–10.2 (14),<br>MSR 0.3–2.4 (11) | Medium; 14 studies, MH |
| Thyroid | 0.03–0.36 (4) | Low; 4 studies, MH |
| Parathyroid | 0.3 (1) | Insufficient; 1 study |

LH: limited heterogeneity (H); MH: moderate H; MSR: most studies reported; No: number; Pts: patients.

**Copyright Information:** (Online) ISSN: 2572-9241
©2026 The Author(s). HemaSphere is published by John Wiley & Sons Ltd on behalf of European Hematology Association. This is an open access Abstract Book distributed under the Attribution-NonCommercial-NoDerivs (CC BY-NC-ND), which allows third parties to download the articles and share them with others as long as they credit the author and the Abstract Book, but they cannot change the content in any way or use them commercially.
**Abstract Book Citations:** Authors, Title, HemaSphere, 2026;10:(S1):pages. The abstract book DOIs can be found on the "Author Information" page.
**Disclaimer:** Articles published in the journal HemaSphere exclusively reflect the opinions of the authors. The authors are responsible for all content in their abstracts including accuracy of the facts, statements, citing resources, etc.

[a]We use “occurrence” (i.e., % of PV pts. that developed an SPM during follow-up) because use of prevalence/incidence terms varied by study.

[b]Assessed per Agency for Healthcare Research and Quality framework.

**Figure. NMSC Occurrence, %**

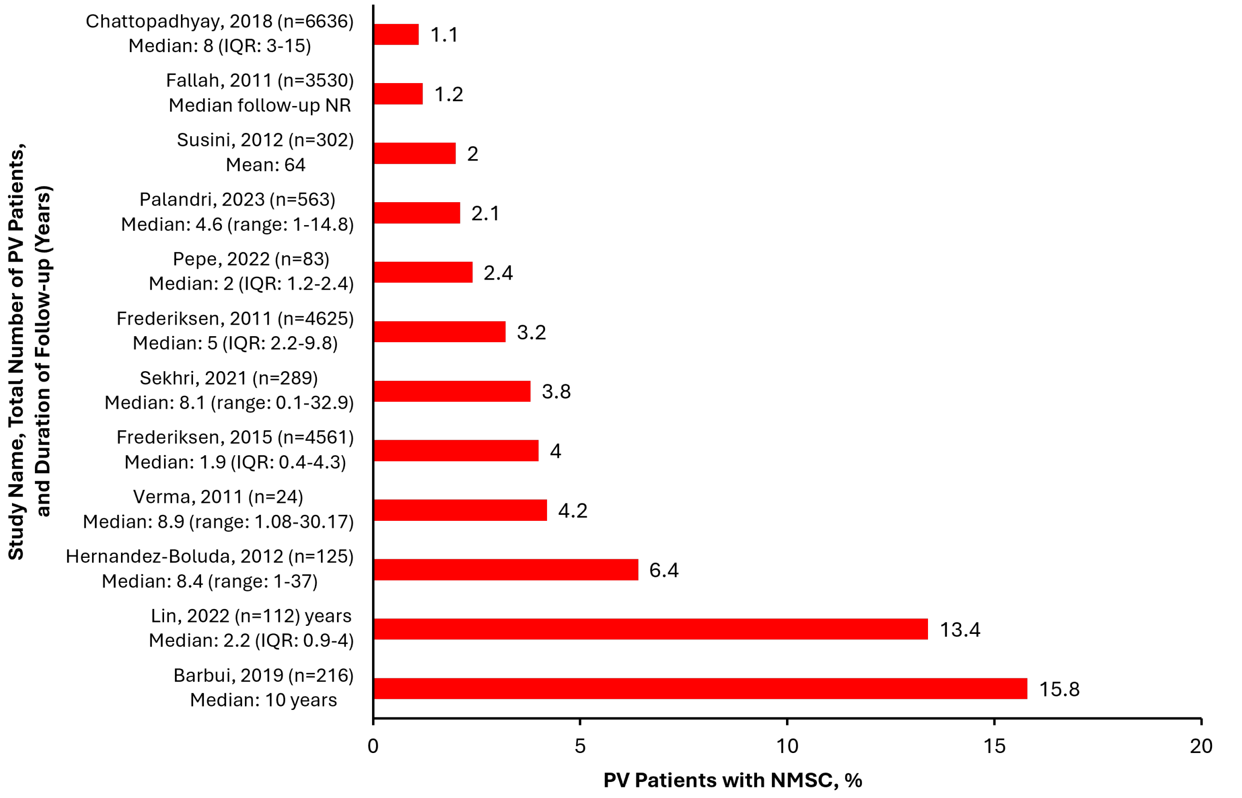

**Copyright Information:** (Online) ISSN: 2572-9241
©2026 The Author(s). HemaSphere is published by John Wiley & Sons Ltd on behalf of European Hematology Association. This is an open access Abstract Book distributed under the Attribution-NonCommercial-NoDerivs (CC BY-NC-ND), which allows third parties to download the articles and share them with others as long as they credit the author and the Abstract Book, but they cannot change the content in any way or use them commercially.
**Abstract Book Citations:** Authors, Title, HemaSphere, 2026;10:(S1):pages. The abstract book DOIs can be found on the “Author Information” page.
**Disclaimer:** Articles published in the journal HemaSphere exclusively reflect the opinions of the authors. The authors are responsible for all content in their abstracts including accuracy of the facts, statements, citing resources, etc.

### (PB3498) BURDENSOME, INCONVENIENT AND PAINFUL: REAL-WORLD EXPERIENCES OF PHLEBOTOMY REPORTED BY PATIENTS WITH POLYCYTHEMIA VERA IN THE UNITED STATES.

**Topic:** 16. Myeloproliferative neoplasms - Clinical

Abdulraheem Yacoub*[1], Kristen Pettit[2], Chloe Hammond[3], Alicia Cerretani[4], Amber Simpson[3], Katie Lewis[3], James Shaw[5], Luis Hernandez[4], Angela Fan[4]

*[1]University of Kansas Medical Center, Kansas City, United States of America;[2]University of Michigan, Ann Arbor, United States of America; [3]Adelphi Real World, Bollington, United Kingdom; [4]Takeda Pharmaceuticals America, Inc., Cambridge, United States of America; [5]Takeda Development Center Americas, Inc., Cambridge, United States of America;*

### Background

Polycythemia vera (PV) is a myeloproliferative neoplasm, for which recurrent phlebotomy is a common management strategy. However, in addition to inconsistent hematocrit (HCT) control, evidence suggests that phlebotomy is often poorly tolerated, may worsen quality of life (QoL), and exacerbate disease-associated symptoms, thus indicating a need for less burdensome alternatives.

### Aims

This study aimed to characterize the overall burden of phlebotomy to patients (pts) and explore QoL in pts with PV who have received phlebotomy in routine clinical practice.

### Methods

Data were collected using the Adelphi PV Disease Specific Programme™, a United States cross-sectional survey (August 2025-February 2026) with retrospective chart review. Physicians reported data for the next five pts with PV who met inclusion criteria. The same pts voluntarily reported their experiences of phlebotomy and QoL via patient-reported outcome measures (PROMs), including the Patient-Reported Outcomes Measurement Information System (PROMIS) Fatigue Short-Form and Work Productivity and Activity Impairment Questionnaire (WPAI).

**Copyright Information:** (Online) ISSN: 2572-9241
©2026 The Author(s). HemaSphere is published by John Wiley & Sons Ltd on behalf of European Hematology Association. This is an open access Abstract Book distributed under the Attribution-NonCommercial-NoDerivs (CC BY-NC-ND), which allows third parties to download the articles and share them with others as long as they credit the author and the Abstract Book, but they cannot change the content in any way or use them commercially.
**Abstract Book Citations:** Authors, Title, HemaSphere, 2026;10:(S1):pages. The abstract book DOIs can be found on the “Author Information” page.
**Disclaimer:** Articles published in the journal HemaSphere exclusively reflect the opinions of the authors. The authors are responsible for all content in their abstracts including accuracy of the facts, statements, citing resources, etc.

**Results**

Nine physicians provided data for 44 pts who reported their experience with PV and QoL. Pts had a median (interquartile range, IQR) age of 58.5 (51.2, 67.8) years and 50% were male.
Of pts who had at least one phlebotomy in the 12 months prior to survey (n=31), 90% had uncontrolled HCT levels (any HCT level was ≥45%), and had a mean (SD) of 3.0 (2.9) phlebotomies. For pts who had ever received phlebotomy since diagnosis (n=35), 77% reported that phlebotomy frequency was burdensome to some degree, with the majority reporting phlebotomy to be inconvenient (81%), time-consuming (69%), painful (60%) and stressful (58%) (**Figure**). Pts also frequently reported light-headedness (63%), bruising (51%), dehydration (37%) and needle site reactions (37%) during or following phlebotomy.
Pts who had received at least one phlebotomy in the 12 months prior to survey had a median (IQR) PROMIS T-score of 56.6 (48.1, 61.1) (n=28 where all PROMIS items were completed). On individual PROMIS items, 84% of pts (n=26) reported feeling fatigued to some extent in the 7 days prior to survey, with 84% reporting feeling "bothered" by their fatigue. Through the WPAI, pts (n=29 where known) reported median (IQR) 30.0% (20.0, 65.0) activity impairment due to their PV.
On average, pts (n=23 where known) spent median (IQR) of 2.0 (1.0, 3.0) hours in the clinic/hospital receiving phlebotomy, and 0.3 (0.2, 1.0) hours traveling to and from their phlebotomy. For pts who worked (n=21), they missed a median (IQR) of 4.0 (2.0, 6.0) hours from work for each phlebotomy procedure, while those who did not work (n=10) missed a median (IQR) of 2.0 (1.8, 3.0) hours out of their day. Annually, pts (n=17 where known) reported paying mean (SD) $218.8 (483.71) out-of-pocket towards their phlebotomy.

**Summary/Conclusion**

In this real-world study, phlebotomy was shown to be substantially burdensome to pts across physical, emotional, financial and social factors. Phlebotomy did not consistently control HCT, leaving pts at risk for thrombotic events, and was frequently rated as inconvenient and time-consuming, compounded by the time that pts missed from work to receive phlebotomy. Despite receiving phlebotomy management, most patients reported fatigue and notable impairment to daily activities. These findings underscore a critical need for newer, less burdensome alternatives to phlebotomy that control symptoms, HCT levels and improve QoL.

**Copyright Information:** (Online) ISSN: 2572-9241
©2026 The Author(s). HemaSphere is published by John Wiley & Sons Ltd on behalf of European Hematology Association. This is an open access Abstract Book distributed under the Attribution-NonCommercial-NoDerivs (CC BY-NC-ND), which allows third parties to download the articles and share them with others as long as they credit the author and the Abstract Book, but they cannot change the content in any way or use them commercially.
**Abstract Book Citations:** Authors, Title, HemaSphere, 2026;10:(S1):pages. The abstract book DOIs can be found on the "Author Information" page.
**Disclaimer:** Articles published in the journal HemaSphere exclusively reflect the opinions of the authors. The authors are responsible for all content in their abstracts including accuracy of the facts, statements, citing resources, etc.

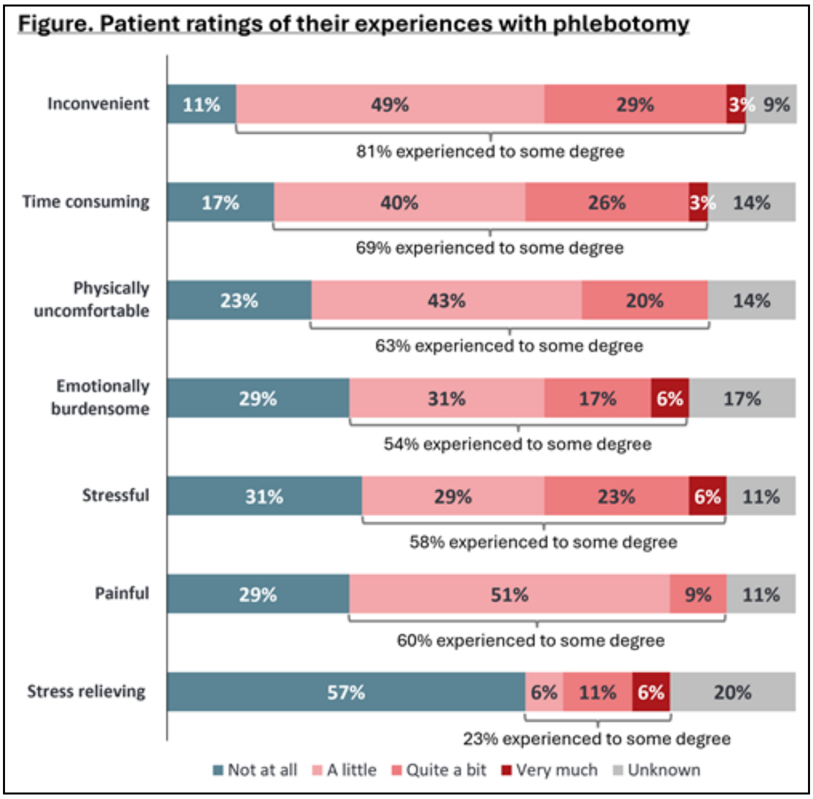

**Copyright Information:** (Online) ISSN: 2572-9241
©2026 The Author(s). HemaSphere is published by John Wiley & Sons Ltd on behalf of European Hematology Association. This is an open access Abstract Book distributed under the Attribution-NonCommercial-NoDerivs (CC BY-NC-ND), which allows third parties to download the articles and share them with others as long as they credit the author and the Abstract Book, but they cannot change the content in any way or use them commercially.
**Abstract Book Citations:** Authors, Title, HemaSphere, 2026;10:(S1):pages. The abstract book DOIs can be found on the "Author Information" page.
**Disclaimer:** Articles published in the journal HemaSphere exclusively reflect the opinions of the authors. The authors are responsible for all content in their abstracts including accuracy of the facts, statements, citing resources, etc.

### (PB3499) AGE-RELATED DISTRIBUTION OF GENE MUTATION PROFILES AND ITS PROGNOSTIC SIGNIFICANCE IN ADULT PATIENTS WITH POLYCYTHEMIA VERA: A MULTICENTER STUDY

**Topic:** 16. Myeloproliferative neoplasms - Clinical

Jia Li[1], Yanxia Han[2], Honglan Qian[3], Xiao Yan[4], Jie Jin[1], Hongyan Tong[1], Jian Huang*[1]

*[1]The First Affiliated Hospital, Zhejiang University School of Medicine, Hangzhou, China;[2]The Second Hospital of Jiaxing, Jiaxing, China; [3]The First Affiliated Hospital of Wenzhou Medical University, Wenzhou, China;[4]Department of Hematology, The First Affiliated Hospital of Ningbo University, Ningbo, China;*

### Background

Polycythemia vera (PV) predominantly affects middle-aged and elderly individuals, with young patients accounting for a small proportion. Previous studies have demonstrated differences in clinical characteristics and prognosis among PV patients of different ages; however, the underlying molecular basis remains unclear, and systematic analyses regarding the age-specific distribution of gene mutation profiles and their prognostic significance are insufficient.

### Aims

To investigate the spectrum of hematologic malignancy-related gene mutations in adult patients with PV across different age groups and to analyze their impact on various prognostic outcomes.

### Methods

Clinical data from 773 adult patients with PV across 25 centers in Zhejiang Province were retrospectively analyzed. Patients were stratified into three groups based on age at diagnosis: young (<40 years), middle-aged (40-59 years), and elderly (≥60 years). Bone marrow samples from 142 patients were subjected to next-generation sequencing (NGS) targeting 154 hematologic malignancy-related genes. Mutation profiles and functional classifications were analyzed, and their associations with thrombosis-free survival (TFS), myelofibrosis-free survival (MFS), and progression-free survival (PFS) were evaluated.

### Results

NGS analysis revealed that epigenetic regulator genes were the most frequently mutated category among non-driver genes (64.08%). The mutational spectrum exhibited heterogeneity across age groups: *ASXL1* was the most common mutation in the young group (18.75%); *TET2* (20.25%) and *KMT2C* (17.72%) predominated in the middle-aged group; and *TET2* (21.28%) and *KMT2C* (14.89%) were most frequent in the elderly group. Prognostic analysis demonstrated that *ASXL1* mutation was associated with shorter TFS in the overall cohort (HR=4.60, *P*=0.022), with this effect being particularly pronounced in the elderly group (HR=15.20, *P*<0.001). *IDH1/2* mutations were associated with shorter MFS (HR=7.37, *P*=0.008) and PFS (HR=7.37, *P*=0.008) in the middle-aged group. NF1 mutation correlated with shorter MFS (HR=3.47, *P*=0.035) and PFS (HR=3.22, *P*=0.048) in the overall cohort, with its impact on MFS being most prominent in the elderly group (HR=17.28, *P*=0.001).

**Copyright Information:** (Online) ISSN: 2572-9241
©2026 The Author(s). HemaSphere is published by John Wiley & Sons Ltd on behalf of European Hematology Association. This is an open access Abstract Book distributed under the Attribution-NonCommercial-NoDerivs (CC BY-NC-ND), which allows third parties to download the articles and share them with others as long as they credit the author and the Abstract Book, but they cannot change the content in any way or use them commercially.
**Abstract Book Citations:** Authors, Title, HemaSphere, 2026;10:(S1):pages. The abstract book DOIs can be found on the “Author Information” page.
**Disclaimer:** Articles published in the journal HemaSphere exclusively reflect the opinions of the authors. The authors are responsible for all content in their abstracts including accuracy of the facts, statements, citing resources, etc.

### Summary/Conclusion

PV patients of different ages exhibit distinct gene mutation profiles. *ASXL1*, *IDH1/2*, and *NF1* mutations serve as key molecular markers influencing prognosis, with their risk effects demonstrating age dependency. These findings provide a rationale for age- and molecular feature-based precision risk stratification and management in PV.

This research was supported by the National Natural Science Foundation of China (No. 82570191) and the Zhejiang Provincial Health High level Innovative Talent Project (2022-2026).

Correspondence to: Prof Jian Huang, E-mail: househuang@zju.edu.cn.

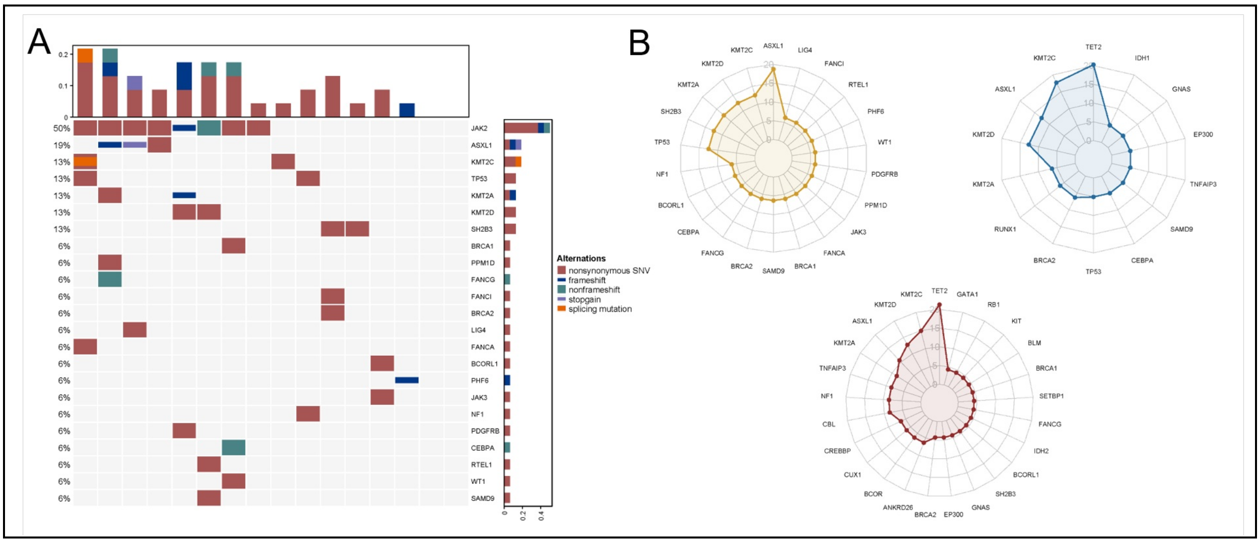

Figure 1. (A) Distribution of gene mutation types in PV patients aged <40 years. (B) Proportions of blood tumor genes in PV patients across different age groups, in the order of age <40 years, age 40–59 years, and age ≥60 years (Note: In figure B, mutations with a positive rate of ≥4% in each group, excluding the JAK2 gene, are described).

**Copyright Information:** (Online) ISSN: 2572-9241
©2026 The Author(s). HemaSphere is published by John Wiley & Sons Ltd on behalf of European Hematology Association. This is an open access Abstract Book distributed under the Attribution-NonCommercial-NoDerivs (CC BY-NC-ND), which allows third parties to download the articles and share them with others as long as they credit the author and the Abstract Book, but they cannot change the content in any way or use them commercially.
**Abstract Book Citations:** Authors, Title, HemaSphere, 2026;10:(S1):pages. The abstract book DOIs can be found on the "Author Information" page.
**Disclaimer:** Articles published in the journal HemaSphere exclusively reflect the opinions of the authors. The authors are responsible for all content in their abstracts including accuracy of the facts, statements, citing resources, etc.

### (PB3500) CLINICAL SIGNIFICANCE OF LOW JAK2V617F VARIANT ALLELE FREQUENCY (≤1%) IN CHRONIC MYELOPROLIFERATIVE NEOPLASMS AND THE DIAGNOSTIC VALUE OF LONGITUDINAL MOLECULAR MONITORING

**Topic:** 16. Myeloproliferative neoplasms - Clinical

Eren Arslan Davulcu*[1], Elif Aksoy[1], Fehmi Hindilerden[1], İpek Yönal Hindilerden[2], Emine Gültürk[1], Metban Mastanzade[2], Öykü Arslan[1], Aynur Dağlar Aday[3], Mustafa Nuri Yenerel[2], Sevgi Besisik[2]

*[1]Bakırköy Dr Sadi Konuk Treaining and Research Hospital, Hematology Clinic, İSTANBUL, Türkiye;[2]Istanbul Universıty Istanbul Medical Faculty, Internal Medicine, Hematology Department, İSTANBUL, Türkiye; [3]Istanbul Universıty Istanbul Medical Faculty, Medical Genetics, İSTANBUL, Türkiye;*

### Background

Highly sensitive molecular techniques enable detection of very low JAK2V617F variant allele frequencies (VAF ≤1%) in patients evaluated for suspected myeloproliferative neoplasms (MPNs). However, low-level JAK2V617F mutations may also occur in non-malignant clonal hematopoiesis, creating diagnostic uncertainty. The clinical significance of low VAF and the value of longitudinal molecular monitoring remain poorly defined.

### Aims

This study aims to assess the diagnostic significance and clinical implications of low-level JAK2V617F (VAF ≤1%) and to evaluate the added value of next-generation sequencing (NGS) and longitudinal molecular monitoring in patients with suspected MPN.

### Methods

We conducted a multicenter study of 72 adult patients with peripheral blood JAK2V617F VAF ≤1% who underwent bone marrow biopsy. Clinical, laboratory, histopathologic, and molecular data were collected. Patients were classified according to WHO 2022 criteria. JAK2V617F was quantified by real-time PCR, and targeted NGS was performed using a 75-gene myeloid panel. Longitudinal VAF changes were assessed in patients with follow-up samples.

### Results

Seventy-two patients (82% male) with a median age of 52 years were included. Indications for testing were erythrocytosis (67%), thrombocytosis (13%), leukocytosis (13%), splenomegaly (4%), and thrombosis (3%). Median JAK2V617F VAF was 0.0619%. Based on integrated clinical and histopathologic evaluation, 21 patients (30%) were diagnosed with MPN, including polycythemia vera (PV) (18%), myelofibrosis (MF) (7%), and essential thrombocythemia (ET) (4%). Idiopathic or secondary erythrocytosis was diagnosed in 49%, and 22% had non-hematologic causes. NGS data were available for 38 patients. NGS detected additional somatic mutations in 15.7% of patients, including CALR, SH2B3, TET2, NRAS, DNMT3A, and ZRSR2. Notably, all ET cases (n=3) harbored additional mutations, underscoring the diagnostic value of extended molecular profiling. Molecular monitoring of JAK2V617F VAF was performed in 28 patients with a median follow-up of 12 months (range, 3–35). The median VAF at follow-up was 0.0422% (range 0–1.4295%). Over time, VAF increased in 46% (n=13), decreased in 46% (n=13), and remained stable in 7% (n=2). VAF exceeded 1% in three patients (1.4295%, 1.1139%, and 1.54%). The first patient had a prior diagnosis of PV, whereas the remaining patients—evaluated for splenomegaly or idiopathic erythrocytosis—showed no clinical or laboratory progression. The splenomegaly subgroup exhibited the highest VAF (erythrocytosis 0.149%, thrombocytosis 0.114%, splenomegaly 0.485%, other 0.099%; $p=0.039$). Among patients with definitive diagnoses, mean VAFs were similar across groups (PV 0.176%, ET 0.196%, MF 0.268%, idiopathic erythrocytosis 0.137%, other 0.115%; $p=0.677$). JAK2V617F allele frequency was higher in MPN (PV, ET, MF) than non-MPN patients (0.201 vs 0.130), although the difference did not reach statistical significance ($p=0.093$).

**Copyright Information:** (Online) ISSN: 2572-9241
©2026 The Author(s). HemaSphere is published by John Wiley & Sons Ltd on behalf of European Hematology Association. This is an open access Abstract Book distributed under the Attribution-NonCommercial-NoDerivs (CC BY-NC-ND), which allows third parties to download the articles and share them with others as long as they credit the author and the Abstract Book, but they cannot change the content in any way or use them commercially.
**Abstract Book Citations:** Authors, Title, HemaSphere, 2026;10:(S1):pages. The abstract book DOIs can be found on the “Author Information” page.
**Disclaimer:** Articles published in the journal HemaSphere exclusively reflect the opinions of the authors. The authors are responsible for all content in their abstracts including accuracy of the facts, statements, citing resources, etc.

**Summary/Conclusion**

Integrated assessment of bone marrow histopathology and molecular profiling is crucial for accurate diagnosis in patients with low-level JAK2V617F VAF. In our cohort, NGS confirmed all ET cases, while its impact was less in other MPN subtypes. Longitudinal VAF monitoring showed no immediate clinical changes, but ongoing evaluation may enable early detection of clonal evolution and timely recognition of overt myeloid neoplasia.

**Copyright Information:** (Online) ISSN: 2572-9241
©2026 The Author(s). HemaSphere is published by John Wiley & Sons Ltd on behalf of European Hematology Association. This is an open access Abstract Book distributed under the Attribution-NonCommercial-NoDerivs (CC BY-NC-ND), which allows third parties to download the articles and share them with others as long as they credit the author and the Abstract Book, but they cannot change the content in any way or use them commercially.
**Abstract Book Citations:** Authors, Title, HemaSphere, 2026;10:(S1):pages. The abstract book DOIs can be found on the "Author Information" page.
**Disclaimer:** Articles published in the journal HemaSphere exclusively reflect the opinions of the authors. The authors are responsible for all content in their abstracts including accuracy of the facts, statements, citing resources, etc.

### (PB3501) SPLENECTOMY PROMOTES POST-TRANSPLANT HEMATOPOIETIC STEM CELL HOMING IN PATIENTS WITH MYELOFIBROSIS BY RESTORING CXCL12 CONCENTRATION GRADIENT

**Topic:** 16. Myeloproliferative neoplasms - Clinical

Yi Liao*[1], Danping Zhu[1], Borui Tang[1], Rui MA[1], Xueyi Luo[1], Xiao-Jun Huang[1], Yuqian Sun[1]

*[1]Peking University People's Hospital, Peking University Institute of Hematology, National Clinical Research Center for Treatment of Hematological Disease, Beijing, China, National Clinical Research Center for Treatment of Hematological Disease, Beijing, China;*

### Background

Allogeneic hematopoietic stem cell transplantation (allo-HSCT) is the only curative therapy for myelofibrosis (MF). Patients with massive splenomegaly have a higher risk of poor graft function after allo-HSCT. Splenectomy improves hematopoietic recovery post-transplant, but the mechanism remains unclear. We hypothesize that splenectomy may (i)reduce splenic pooling of hematopoietic stem cells(HSCs), enhance their homing; (ii)relieve hematopoiesis inhibition caused by inflammatory factors released by spleen.

### Aims

The aim of this study was to investigate the possible mechanisms by which splenectomy promotes post-transplant hematologic recovery in patients with MF.

### Methods

We prospectively collected 6 samples obtained from 6 patients with MF (4, splenectomy before HSCT; 2, splenectomy after HSCT); 3 samples (termed "normal controls") were from 1 patient who had received splenectomy due to traumatic splenic rupture, and 2 samples of public database (GSM7898197/198) for single-cell sequencing analysis.

Serum cytokines in peripheral blood(PB) and spleen were detected in MF patients received splenectomy. Immunohistochemical stains were performed in spleen of MF patients.

We retrospectively analyzed clinical data of 82 MF patients undergoing HSCT at Peking University People's Hospital from Dec. 2006 to Oct. 2025, to evaluate the impact of splenectomy.

**Copyright Information:** (Online) ISSN: 2572-9241
©2026 The Author(s). HemaSphere is published by John Wiley & Sons Ltd on behalf of European Hematology Association. This is an open access Abstract Book distributed under the Attribution-NonCommercial-NoDerivs (CC BY-NC-ND), which allows third parties to download the articles and share them with others as long as they credit the author and the Abstract Book, but they cannot change the content in any way or use them commercially.
**Abstract Book Citations:** Authors, Title, HemaSphere, 2026;10:(S1):pages. The abstract book DOIs can be found on the “Author Information” page.
**Disclaimer:** Articles published in the journal HemaSphere exclusively reflect the opinions of the authors. The authors are responsible for all content in their abstracts including accuracy of the facts, statements, citing resources, etc.

**Results**

Among 6 patients, no perioperative nor postoperative complications were observed. In 2 patients received splenectomy after HSCT, increase in blood counts was observed postoperatively. In 4 patients received splenectomy before HSCT, all achieved fast engraftment after HSCT.
Serial plasma cytokine assays were performed in 3 MF patients before and after splenectomy. No significant changes in PB cytokines were seen postoperatively. However, splenic serum CXCL12 levels were significantly and persistently higher in MF patients than in their peripheral blood or normal controls (Figure 1A). Additionally, PB CXCL12 levels in MF patients decreased after splenectomy (Figure 1B). Compared to healthy spleens, immunohistochemistry of spleens from MF patients revealed significantly higher expression of P-selectin, VCAM-1(Figure 1D).

60,668 transcriptomes met QC (6741/sample, 3431-9906) revealing 10 clusters(Figure 1D). Compositional analysis identified a proportional relative depletion of B cell and cDC and enrichment of neutrophils in MF vs. normal(Figure 1E). Moreover, we found that CXCL12 is mainly produced by Macro/mono cluster (Figure 1C).
In the clinical cohort, patients were divided into 3 groups according to spleen management: splenectomy group, massive splenomegaly (spleen diameter exceeding 22cm or extending more than 15cm below the left costal margin) without splenectomy group, and normal group. Patients undergoing splenectomy demonstrated a significant higher incidence of platelet engraftment at day 180 after HSCT (Figure 1H).

**Summary/Conclusion**

In summary, our study suggests that: (i) splenectomy may be an effective treatment for hematopoietic recovery in patients with massive splenomegaly;(ii) MF splenic microenvironment is characterized by increased levels of CXCL12, VCAM-1 and P-selectin expression, which contributes to the localization of MF HSCs to the spleen and the establishment of extramedullary hematopoiesis; (iii)splenectomy may promote post-transplant hematopoietic stem cell homing in MF patients by restoring the CXCL12 concentration gradient, thereby promoting hematologic recovery in patients with MF (Figure 1I).

**Copyright Information:** (Online) ISSN: 2572-9241
©2026 The Author(s). HemaSphere is published by John Wiley & Sons Ltd on behalf of European Hematology Association. This is an open access Abstract Book distributed under the Attribution-NonCommercial-NoDerivs (CC BY-NC-ND), which allows third parties to download the articles and share them with others as long as they credit the author and the Abstract Book, but they cannot change the content in any way or use them commercially.
**Abstract Book Citations:** Authors, Title, HemaSphere, 2026;10:(S1):pages. The abstract book DOIs can be found on the "Author Information" page.
**Disclaimer:** Articles published in the journal HemaSphere exclusively reflect the opinions of the authors. The authors are responsible for all content in their abstracts including accuracy of the facts, statements, citing resources, etc.

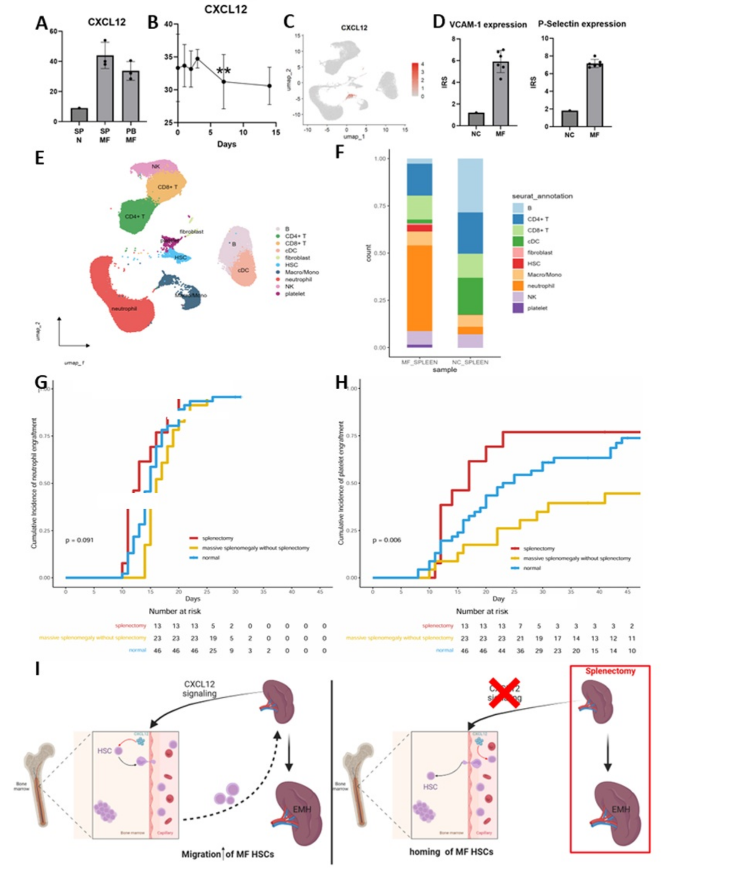

**Figure 1**: (A)CXCL12 in splenic and PB MF plasma of 3 patients and normal splenic plasma; (B) CXCL12 in PB MF plasma of 3 patients during the perioperative period. (D)Immunohistochemical rating score (IRS) for VCAM-1 and P-selectin expression in spleen of MF patients and healthy controls. The outcomes of single-cell RNA sequencing: (E) The integrated dataset comprised of 60668 single cells from three Normal and six MF samples; (F) Percentage of cells in each category; (C) UMAP plot of integrated dataset showing presence of CXCL12. The outcomes of allogeneic hematopoietic stem cell transplantation in MF patients: Cumulative incidence of (G) neutrophil engraftment (P=0.091), (H) platelet engraftment (P=0.006) in 180 days. (I) Inversion of the CXCL12 concentration gradient in spleen and bone marrow of patients with MF. CXCL12 produced by spleen encourages the transfer and engraftment of the HSCs to the spleen in MF patients. Splenectomy promotes post-transplant hematopoietic stem cell homing in patients with MF by restoring CXCL12 concentration gradient.

**Copyright Information:** (Online) ISSN: 2572-9241
©2026 The Author(s). HemaSphere is published by John Wiley & Sons Ltd on behalf of European Hematology Association. This is an open access Abstract Book distributed under the Attribution-NonCommercial-NoDerivs (CC BY-NC-ND), which allows third parties to download the articles and share them with others as long as they credit the author and the Abstract Book, but they cannot change the content in any way or use them commercially.
**Abstract Book Citations:** Authors, Title, HemaSphere, 2026;10:(S1):pages. The abstract book DOIs can be found on the "Author Information" page.
**Disclaimer:** Articles published in the journal HemaSphere exclusively reflect the opinions of the authors. The authors are responsible for all content in their abstracts including accuracy of the facts, statements, citing resources, etc.

### (PB3502) SEX-SPECIFIC DIFFERENCES IN RESPONSE TO RUXOLITINIB VERSUS BEST AVAILABLE THERAPY IN PATIENTS WITH POLYCYTHEMIA VERA TREATED IN THE RANDOMIZED PHASE 2B RUXO-BEAT CLINICAL TRIAL

**Topic:** 16. Myeloproliferative neoplasms - Clinical

Julia Stomper[1, 2], Susanne Isfort[1, 2, 3], Lino Lars Teichmann[4], Philippe Schafhausen[5], Florian Heidel[3, 6], Carl Crodel[6], Andreas Hochhaus[6], Martin Griesshammer[7], Katja Sockel[8], Philipp Jost[9, 10], Denise Walther[11], Konstanze Döhner[12], Joachim Goethert[13], Markus Radsak[14], Marinela Augustin[15], Heiko Becker[16], Aristoteles Giagounidis[17], Markus Schaich[18], Martine Klausmann[19], Nikolaschristiancornelius Bubnoff[20], Frank Stegelmann[12], Maike Kortmann[2, 21], Michelle Zentis[2, 21], Julia Frank[22], Anna Hagemeier[22], Martin Hellmich[22, 23], Tim Brümmendorf[1, 2], Steffen Koschmieder*[1, 2]

*[1]RWTH Aachen University Medical Center, Hematology, Oncology, Hemostaseology, and Stem Cell Transplantation, Aachen, Germany; [2]Center for Integrated Oncology Aachen Bonn Cologne Düsseldorf (CIO ABCD), Hematology, Oncology, Hemostaseology, and Stem Cell Transplantation, Aachen, Germany; [3]Hannover Medical School (MHH), Hematology, Hemostasis, Oncology and Stem Cell Transplantation, Hannover, Germany; [4]University Hospital Bonn, Medicine III, Bonn, Germany; [5]University Medical Center Hamburg-Eppendorf, Oncology, Hematology, and Bone Marrow Transplantation with Section of Pneumology, Hamburg, Germany; [6]Jena University Hospital, Medicine II, Jena, Germany; [7]Johannes Wesling Medical Centre Minden, University of Bochum, Haematology, Oncology, Haemostaseology and Palliative Care, Minden, Germany; [8]University Hospital Dresden, TU Dresden, Medical Clinic and Policlinic I, Dresden, Germany; [9]Technical University of Munich, Medical Department III, Hematology and Oncology, and Center for Translational Cancer Research, Translatum, Munich, Germany; [10]Medical University of Graz, Division of Clinical Oncology, Internal Medicine, Graz, Austria; [11]Otto-von-Guericke University Medical Center Magdeburg, Hematology/Oncology, Magdeburg, Germany; [12]University Hospital of Ulm, Internal Medicine III, Ulm, Germany; [13]West German Cancer Center, University Hospital Essen, Hematology and Stem Cell Transplantation, Essen, Germany; [14]University Medical Center Mainz, Hematology and Medical Oncology, Mainz, Germany; [15]Klinikum Nürnberg Nord, Paracelsus Medizinische Privatuniversität, Oncology/Hematology, Nürnberg, Germany; [16]Medical Center, Faculty of Medicine and German Cancer Consortium (DKTK), partner site Freiburg, Hematology, Oncology and Stem Cell Transplantation, Freiburg, Germany; [17]Marienhospital Düsseldorf, Oncology, Hematology and Palliative Care, Düsseldorf, Germany; [18]Rems-Murr-Klinikum, Hematology, Oncology and Palliative Medicine, Winnenden, Germany; [19]Gemeinschaftspraxis, Aschaffenburg, Germany; [20]University Medical Center Schleswig-Holstein and University Cancer Center Schleswig-Holstein (UCCSH), University of Lübeck, Hematology and Oncology, Lübeck, Germany; [21]RWTH Aachen University, Center for Translational & Clinical Research (CTC-A), Aachen, Germany; [22]Institute of Medical Statistics and Computational Biology, Faculty of Medicine and University Hospital Cologne, Cologne, Germany; [23]University Medical Center Göttingen, Medical Statistics, Göttingen, Germany;*

### Background

Polycythemia vera (PV) is more common in males than females. While males have a higher incidence of high-risk mutations and worse outcomes, females have more severe symptoms. Sex differences in response to therapy in patients with PV remain largely unknown.

### Aims

We aimed to characterize sex-specific differences in response to ruxolitinib (RUX) vs. best available therapy (BAT) in patients with high-risk PV treated within the Ruxo-BEAT trial.

**Copyright Information:** (Online) ISSN: 2572-9241
©2026 The Author(s). HemaSphere is published by John Wiley & Sons Ltd on behalf of European Hematology Association. This is an open access Abstract Book distributed under the Attribution-NonCommercial-NoDerivs (CC BY-NC-ND), which allows third parties to download the articles and share them with others as long as they credit the author and the Abstract Book, but they cannot change the content in any way or use them commercially.
**Abstract Book Citations:** Authors, Title, HemaSphere, 2026;10:(S1):pages. The abstract book DOIs can be found on the "Author Information" page.
**Disclaimer:** Articles published in the journal HemaSphere exclusively reflect the opinions of the authors. The authors are responsible for all content in their abstracts including accuracy of the facts, statements, citing resources, etc.

### Methods

High-risk patients with PV enrolled in the ongoing multicenter, open-label, phase-IIb Ruxo-BEAT trial (NCT02577926) were randomized 1:1 to RUX vs. BAT. Patients in the RUX arm received a starting dose of 10 mg BID and were allowed to increase their dose up to 20 mg BID, while BAT dose was administered as per investigator decision. Here, in an exploratory analysis, we evaluated the response to RUX and BAT at the end of month 6 of treatment among female and male participants, separately. Response assessment was based on the Barosi criteria (Barosi 2009). Complete response (CR) was defined as absence of PV-related symptoms including headache, itching, concentration problems, and dizziness (score 0), as well as normalization of blood counts (PLT $\leq$ 400 x $10^9$/L, WBC < 10 x $10^9$/L, HCT < 45% without phlebotomy) and splenomegaly (spleen size < 13 cm by ultrasound). Partial response (PR) was defined as at least an HCT < 45% without the necessity for phlebotomy in the past 3 months. Additional analyses were performed with a symptom threshold of < 3 and normalization of blood counts and splenomegaly defining CR. Fisher's Exact Test was used to compare the response between the two treatment arms among females and males. Wilcoxon rank-sum test was used to compare baseline characteristics between females and males.

### Results

A total of 29 female and 48 male patients with PV were randomly assigned to RUX treatment or BAT. At baseline, females had a significantly lower spleen length (p=0.007) and reported significantly more severe dizziness (p=0.013) and bone pain (p=0.001) compared to males. Blood counts at baseline were similar between females and males.
Responses at 6 months of treatment were numerically higher in females, with 24/29 (83%) achieving an overall response (CR or PR), as compared to 32/38 (67%) in males (p=0.187). Among females, the proportion of patients who achieved overall response at the end of month 6 tended to be higher in the RUX arm than in the BAT arm (17/18, 94.4% vs. 7/11, 63.6%, p=0.054). In contrast, among males, the proportion of patients who achieved overall response was similar between the two treatment arms (18/25, 72.0% in the RUX arm vs. 14/23, 60.9% in the BAT arm, p=0.543). When the same analysis was performed with CR defined as a symptom score of < 3, the response rates in the two treatment arms among females were identical to the ones based on a symptom threshold of 0. The response rates among males were comparable to the ones based on a cutoff of 0 (symptom threshold 3: overall response of 76.0% in the RUX arm and 69.6% in the BAT arm, p=0.749).

### Summary/Conclusion

Spleen length and the severity of dizziness and bone pain differed between females and males with PV at baseline. Women with PV tended to benefit more from RUX compared to BAT while men had similar outcomes with both treatment types, warranting further research on sex differences in response to therapy in patients with myeloproliferative neoplasms.

**Copyright Information:** (Online) ISSN: 2572-9241
©2026 The Author(s). HemaSphere is published by John Wiley & Sons Ltd on behalf of European Hematology Association. This is an open access Abstract Book distributed under the Attribution-NonCommercial-NoDerivs (CC BY-NC-ND), which allows third parties to download the articles and share them with others as long as they credit the author and the Abstract Book, but they cannot change the content in any way or use them commercially.
**Abstract Book Citations:** Authors, Title, HemaSphere, 2026;10:(S1):pages. The abstract book DOIs can be found on the "Author Information" page.
**Disclaimer:** Articles published in the journal HemaSphere exclusively reflect the opinions of the authors. The authors are responsible for all content in their abstracts including accuracy of the facts, statements, citing resources, etc.

### (PB3503) REAL-WORLD CHARACTERISTICS, TREATMENT PATTERNS, AND SURVIVAL IN PATIENTS WITH MYELOFIBROSIS AND THOSE USING RUXOLITINIB: A NATIONWIDE STUDY STRATIFIED BY BASELINE AND EARLY TRANSFUSION STATUS

**Topic:** 16. Myeloproliferative neoplasms - Clinical

Yi-Chen Chen[1], Thi Thuy Dung Nguyen[2], Tzu-Lan Huang[3], Audrey Yi Wen Chang[3], Sin-Syue Li*[4], Shiyuan Zhang[5], Zi-Yang Peng[2], Yi-Hsin Chang[2, 6], Huang Tz Ou[2, 6]

*[1]GSK, Singapore, Singapore; [2]National Cheng Kung University, Institute of Clinical Pharmacy and Pharmaceutical Sciences, College of Medicine, Tainan, Taiwan; [3]GSK, Taipei, Taiwan; [4]National Cheng Kung University, Tainan, Taiwan; [5]GSK, Pennsylvania, United States of America; [6]National Cheng Kung University, Department of Pharmacy, College of Medicine, Tainan, Taiwan;*

### Background

Although ruxolitinib (RUX), a widely used Janus Kinase inhibitor, improves myelofibrosis (MF)-related symptoms and splenomegaly in randomized trials, treatment-emergent cytopenia can occur, particularly early after initiation. Real-world evidence on how baseline characteristics and early changes in transfusion status related to RUX dosing patterns and overall survival (OS) in Asians is limited.

### Aims

We characterized baseline characteristics of patients with MF and those initiating RUX, stratified by baseline transfusion status. Among RUX-treated patients, we further evaluated the association of early changes in transfusion status with RUX dosing patterns and OS.

### Methods

This study used Taiwan's national claims data linked to the Taiwan Cancer Registry to identify patients diagnosed with MF from 01/06/2012 to 12/31/2019. Patients initiating RUX between 10/01/2016 and 12/31/2019 comprised the RUX-treated cohort. The index date was the first record of MF diagnosis (overall MF cohort), or the first RUX prescription (RUX-treated cohort), and followed them until death or 12/31/2022 (whichever occurred first). Baseline transfusion status in 12 weeks prior to the index date was classified as transfusion-independent (TI; no transfusion record), transfusion-dependent (TD; receiving ≥6 red blood cell units within 12 weeks or ≥4 units within 8 weeks), or transfusion-requiring (TR; not meeting TI or TD criteria). Among RUX-treated patients with baseline TI, early transfusion status changes to TR (TI->TR) or TD (TI->TD) within 12 weeks of initiation were evaluated as post-hoc analysis. Baseline characteristics were examined during the 6 months prior to or at the index date. Monthly RUX dosing changes were evaluated over 6 months from RUX initiation, stratified by baseline and early transfusion status. The OS of the RUX-treated cohort with early changes in transfusion status was assessed using the Kaplan–Meier method.

**Copyright Information:** (Online) ISSN: 2572-9241
©2026 The Author(s). HemaSphere is published by John Wiley & Sons Ltd on behalf of European Hematology Association. This is an open access Abstract Book distributed under the Attribution-NonCommercial-NoDerivs (CC BY-NC-ND), which allows third parties to download the articles and share them with others as long as they credit the author and the Abstract Book, but they cannot change the content in any way or use them commercially.
**Abstract Book Citations:** Authors, Title, HemaSphere, 2026;10:(S1):pages. The abstract book DOIs can be found on the "Author Information" page.
**Disclaimer:** Articles published in the journal HemaSphere exclusively reflect the opinions of the authors. The authors are responsible for all content in their abstracts including accuracy of the facts, statements, citing resources, etc.

**Results**

Overall, 406 patients with MF were identified (mean age 64.2 years; 63.8% male; median follow-up of 3.6 years), and 71.2/22.2/6.6% were TI/TR/TD at baseline, respectively. Compared with TI patients (mean age 62.9 years), those with TR (68.2 years) or TD (64.9 years) were older and had higher comorbidity burdens (i.e., mean Charlson Comorbidity Index scores: TI [1.09], TR [1.78], TD [1.96]). Among 167 patients initiating RUX (median follow-up: 3.8 years), 53.9% (n=90) were TI at baseline. Patients with baseline TR/TD started at lower mean daily doses than TI patients (17.2 vs 19.9 mg/day), and the mean daily dose remained at about 20 mg/day during follow-up. Within the baseline TI subgroup, 25 (27.8%) transitioned to TR, and 8 (8.9%) transitioned to TD during the first 12 weeks.Dose adjustments during the first 6 months were more frequent among patients transitioning to TD than those remaining TI or transitioning to TR. Median OS was 67.2 months for patients remaining TI, 51.4 months for TI→TR, and 14.6 months for TI→TD ($p < 0.0001$).

**Summary/Conclusion**

In this nationwide real-world study, baseline and early changes in transfusion status were associated with ruxolitinib dosing patterns and survival outcomes. Progression from TI to TR or TD within 3 months after ruxolitinib initiation identified a subgroup with substantially poorer survival, thus highlighting the importance of proactive management strategies and the development of novel therapies to reduce transfusion burden and improve survival outcomes in patients with myelofibrosis.
**Funding:** GSK (study ID 221307).

**Copyright Information:** (Online) ISSN: 2572-9241
©2026 The Author(s). HemaSphere is published by John Wiley & Sons Ltd on behalf of European Hematology Association. This is an open access Abstract Book distributed under the Attribution-NonCommercial-NoDerivs (CC BY-NC-ND), which allows third parties to download the articles and share them with others as long as they credit the author and the Abstract Book, but they cannot change the content in any way or use them commercially.
**Abstract Book Citations:** Authors, Title, HemaSphere, 2026;10:(S1):pages. The abstract book DOIs can be found on the "Author Information" page.
**Disclaimer:** Articles published in the journal HemaSphere exclusively reflect the opinions of the authors. The authors are responsible for all content in their abstracts including accuracy of the facts, statements, citing resources, etc.

**Figure. Overall survival among RUX-treated patients with TI at baseline stratified by early transfusion status changes (i.e., TI→TR, TI→TD, remaining TI)**

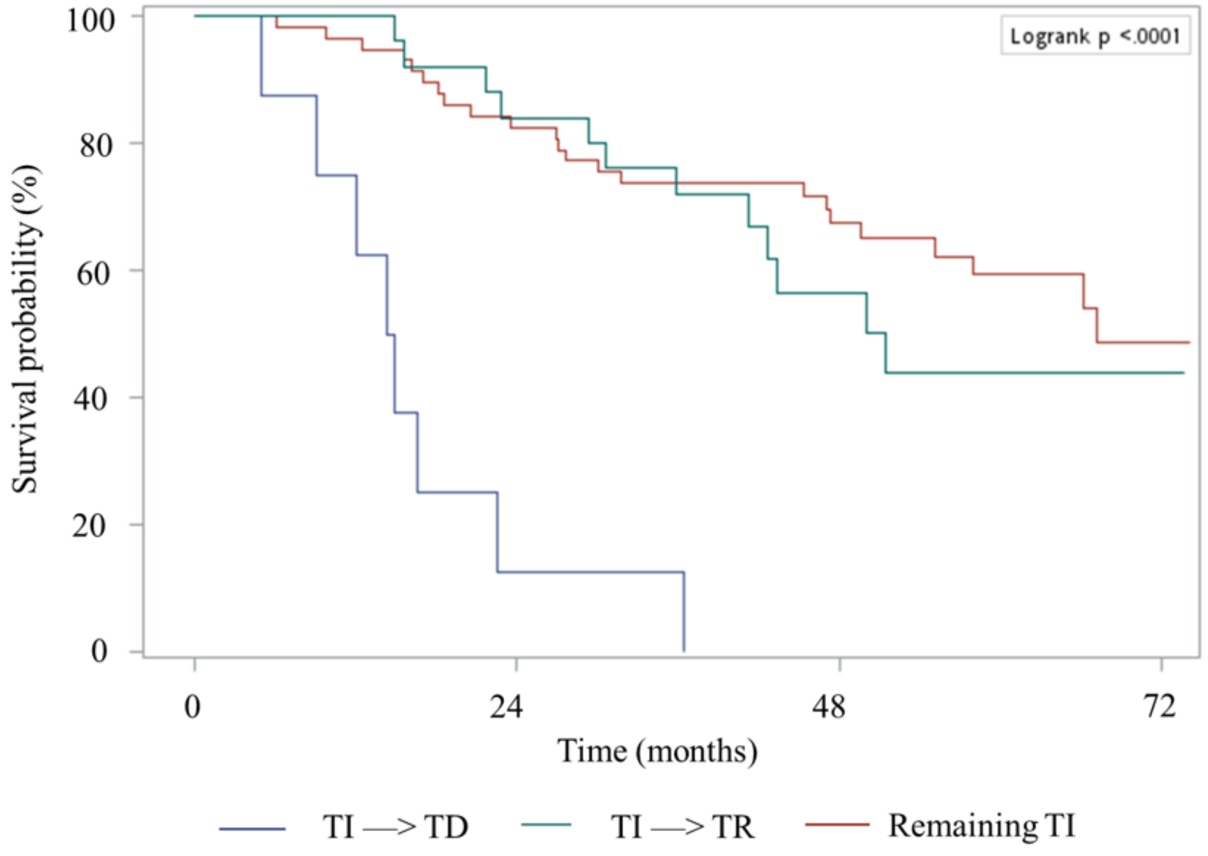

Abbreviations: RUX, ruxolitinib; TD, transfusion dependent; TI, transfusion independent; TR, transfusion requiring.

**Copyright Information:** (Online) ISSN: 2572-9241
©2026 The Author(s). HemaSphere is published by John Wiley & Sons Ltd on behalf of European Hematology Association. This is an open access Abstract Book distributed under the Attribution-NonCommercial-NoDerivs (CC BY-NC-ND), which allows third parties to download the articles and share them with others as long as they credit the author and the Abstract Book, but they cannot change the content in any way or use them commercially.
**Abstract Book Citations:** Authors, Title, HemaSphere, 2026;10:(S1):pages. The abstract book DOIs can be found on the "Author Information" page.
**Disclaimer:** Articles published in the journal HemaSphere exclusively reflect the opinions of the authors. The authors are responsible for all content in their abstracts including accuracy of the facts, statements, citing resources, etc.

### (PB3504) DIAGNOSIS, DISEASE BURDEN, AND TREATMENT IN ESSENTIAL THROMBOCYTHEMIA: A COMPARATIVE QUALITATIVE STUDY OF CLINICIAN AND PATIENT PERSPECTIVES IN THE UNITED STATES

**Topic:** 16. Myeloproliferative neoplasms - Clinical

Xiaoqin Yang[1], Mavis Obeng-Kusi[1], Francesca Coutinho[1], Jason Ritchie[2], Kimberly Kelly[2], Carolina Alonzo[2], Ana Buenfil[2], Michael Chladek[2], Martin Paczkowski*[2], Ruben Mesa[3]

*[1]Merck & Co., Inc, Rahway, United States of America;[2]IQVIA, New York, United States of America;[3]Atrium Health Wake Forest Baptist Comprehensive Cancer Center, Winston-Salem, United States of America;*

### Background

Essential thrombocythemia (ET) is a rare myeloproliferative neoplasm with variable clinical presentation. While some patients are asymptomatic, others experience persistent symptoms despite available therapies, contributing to differences between patient and clinician perceptions of disease burden.

### Aims

To explore diagnostic experiences, symptoms and treatment perspectives among patients with ET and clinicians who treat them.

### Methods

This U.S.-based qualitative study included semi-structured interviews conducted between May and December 2025 with a purposive sample of 20 hematologist-oncologists and 40 adults with ET who received cytoreductive therapy. Open-ended guides assessed diagnosis, symptoms, health related quality of life (HRQoL), and treatment. Transcripts were coded and thematically analyzed.

**Copyright Information:** (Online) ISSN: 2572-9241
©2026 The Author(s). HemaSphere is published by John Wiley & Sons Ltd on behalf of European Hematology Association. This is an open access Abstract Book distributed under the Attribution-NonCommercial-NoDerivs (CC BY-NC-ND), which allows third parties to download the articles and share them with others as long as they credit the author and the Abstract Book, but they cannot change the content in any way or use them commercially.
**Abstract Book Citations:** Authors, Title, HemaSphere, 2026;10:(S1):pages. The abstract book DOIs can be found on the "Author Information" page.
**Disclaimer:** Articles published in the journal HemaSphere exclusively reflect the opinions of the authors. The authors are responsible for all content in their abstracts including accuracy of the facts, statements, citing resources, etc.

### Results

**Demographics:** Specialists practiced in community(n=12) and academic (n=8) settings with 3–30 years of ET experience. At interview, patients were receiving hydroxyurea (HU)(n=21), peginterferon alfa 2a(n=9), ruxolitinib(n=5), anagrelide(n=2), or no treatment(n=3). Median age at interview was 64.5 years (38–82); 70% female.

**Diagnostic Journey:** Most specialists (83.3%) described a diagnostic process of ≤3 weeks after referral from a primary care physician (PCP). Delays to diagnosis, most often due to late referrals by PCPs for elevated platelet counts, were reported by 87.4%. When asked to describe the typical diagnostic process, specialists reported blood screening (95%), genetic testing (95%), ruling other potential diagnoses (75%); bone marrow biopsy was also mentioned by 70%, though 16.7% noted patient reluctance

Patients reported more challenging journeys: 22.5% consulted ≥3 providers before diagnosis; 17.9% were previously misdiagnosed, and many lacked confidence in provider ET knowledge (47.1%) or felt unheard (40.5%), prompting 55% to seek second opinions.

**Symptoms and HRQoL:** Specialists acknowledged some symptoms but often described patients as largely asymptomatic with minimal daily impact. Patients described broader and more persistent symptoms, especially fatigue (46.7%), night sweats (33.3%), itch (26.7%), and gastrointestinal issues (20%). Patients reported that symptoms affected leisure, emotional health, physical functioning, exercise, work, and sleep.

**Treatment Decision Making:** Most specialists (94.7%) used platelet reduction as the primary marker of effectiveness and were generally satisfied with available therapies, often minimizing ongoing symptom burden. Patients valued platelet control but were more dissatisfied with available therapies (76.2%) and reported a wide range of side effects (90%), which were a contributing factor among the 68.8% of patients who discontinued treatment. Specialists highlighted the need for more targeted, disease-modifying therapies (44.4%) and improved ET education among referring non-specialist physicians (50%). Patients emphasized the need for more effective (54.5%) treatments and cited cost and insurance coverage (36.4%), with affordability concerns primarily related to non-HU therapies.

### Summary/Conclusion

Clinicians and patients differed in their views of diagnosis, symptom burden, and treatment. Patients described prolonged diagnostic pathways and substantial HRQoL impacts. Both groups recognized the need for improved ET therapies. Findings highlight the need for better education and information resources, improved symptom recognition, and development of innovative therapies.

**Copyright Information:** (Online) ISSN: 2572-9241
©2026 The Author(s). HemaSphere is published by John Wiley & Sons Ltd on behalf of European Hematology Association. This is an open access Abstract Book distributed under the Attribution-NonCommercial-NoDerivs (CC BY-NC-ND), which allows third parties to download the articles and share them with others as long as they credit the author and the Abstract Book, but they cannot change the content in any way or use them commercially.
**Abstract Book Citations:** Authors, Title, HemaSphere, 2026;10:(S1):pages. The abstract book DOIs can be found on the “Author Information” page.
**Disclaimer:** Articles published in the journal HemaSphere exclusively reflect the opinions of the authors. The authors are responsible for all content in their abstracts including accuracy of the facts, statements, citing resources, etc.

### (PB3506) REAL WORLD HAEMATOLOGICAL OUTCOMES FOLLOWING TRANSITION TO ROPEGINTERFERON ALFA-2B DURING PEGYLATED INTERFERON SHORTAGE

**Topic:** 16. Myeloproliferative neoplasms - Clinical

Llywelyn Cadman-Davies*[1], Alesia Khan[2], Madeline Johns[3], Charlotte Moss[3], Claire Woodley[1], Hayder Hussain[4], Frances Wadelin[5], Rebecca Frewin[6], Mamta Garg[7], Catherine Loughran[7], Pete Dyer[8], Anna Godfrey[9], Luke Carter-Brzezinski[10], Sabia Rashid[11], Claire Harrison[1], Priya Sriskandarajah[1]

*[1]Guy's and St Thomas' NHS Foundation Trust, London, United Kingdom;[2]Leeds Teaching Hospital Trust, Leeds, United Kingdom; [3]King's College London, London, United Kingdom; [4]University Hospital Birmingham NHS Trust, Birmingham, United Kingdom; [5]Nottingham University Hospitals, Nottingham, United Kingdom; [6]Gloucestershire Royal Hospital, Gloucester, United Kingdom; [7]University Hospitals of Leicester, Leicester, United Kingdom;[8]Royal Stoke University Hospital of North Staffordshire, Stoke-on-Trent, United Kingdom; [9]Cambridge University Hospitals, Cambridge, United Kingdom; [10]University of Manchester, Manchester, United Kingdom; [11]Lewisham and Greenwich NHS Trust - Lewisham Hospital &Amp; Community, London, United Kingdom;*

### Background

Real-world data describing outcomes in patients with myeloproliferative neoplasms (MPN) transitioning between pegylated interferon alfa-2a (Pegasys®, pegIFN) and ropeginterferon alfa-2b (Besremi®, ropegIFN) are limited. We undertook a multicentre evaluation of ropegIFN use during the 2024 international pegIFN shortage, focusing on patients who transitioned from pegIFN.

### Aims

To evaluate haematological response, molecular outcomes and tolerability of ropegIFN following pegIFN transition during supply disruption across a national network.

### Methods

Retrospective data were collected from 23 UK sites using a central REDCap platform between August 2024 and 1 January 2026. Variables captured included baseline demographics, prior therapy exposure, European Leukemia Net (ELN)-defined haematological response, dosing, adverse events, and paired molecular assessments where available. Data were anonymised and collected under local governance and network ethics approval.

**Copyright Information:** (Online) ISSN: 2572-9241
©2026 The Author(s). HemaSphere is published by John Wiley & Sons Ltd on behalf of European Hematology Association. This is an open access Abstract Book distributed under the Attribution-NonCommercial-NoDerivs (CC BY-NC-ND), which allows third parties to download the articles and share them with others as long as they credit the author and the Abstract Book, but they cannot change the content in any way or use them commercially.
**Abstract Book Citations:** Authors, Title, HemaSphere, 2026;10:(S1):pages. The abstract book DOIs can be found on the “Author Information” page.
**Disclaimer:** Articles published in the journal HemaSphere exclusively reflect the opinions of the authors. The authors are responsible for all content in their abstracts including accuracy of the facts, statements, citing resources, etc.

**Results**

A total 206 patients received ropegIFN; 111 (54%) had prior pegIFN exposure (switch cohort) and 95 (46%) were ropegIFN-naïve. Within the switch cohort (n=111), mean age was 58.5 years (range 24–87) and 52% were female. Diagnoses were essential thrombocythaemia (ET, n=50), polycythaemia vera (PV, n=38), post-ET myelofibrosis (n=7), primary myelofibrosis (n=6), post-PV myelofibrosis (n=5), MPN not otherwise specified (n=4), and MPN/MDS overlap (n=1).
Best recorded haematological response at any timepoint included complete or partial response in 71/111 (64%), stable disease in 24/111 (22%) and progression in 3/111 (3%); 13/111 (12%) patients had no evaluable response due to incomplete follow-up. Median ropegIFN dose was 125 micrograms (range 50–500), administered at a 2-weekly interval (range 2–8 weeks).
Across the full cohort (n=206), adverse events occurred in 23% (n=48/206); in the switch cohort, adverse events occurred in 23% (n=26/111). Treatment discontinuation occurred in 13% overall (n=27/206) and 14% in switch patients (n=15/111), with toxicity-related discontinuation of 8% (n=17/206) and 8% (n=9/111), respectively. Adverse event–related discontinuations included hepatic dysfunction, neuropathy, migraine, ocular symptoms, and vascular events, each occurring in ≤ 2 patients. Non-toxicity-related discontinuations across the full cohort (n=10/206) reflected therapeutic strategy changes including switch to ruxolitinib (n=2), allogeneic stem cell transplantation (n=1), trial enrolment (n=1), and lack of efficacy (n=6).
Among *JAK2*-mutated patients in the switch group with paired molecular assessments (n=22), 12 (55%) achieved ≥25% reduction in allele burden, including 7 (32%) achieving ≥50% reduction and 1 (5%) achieving complete molecular response (VAF <1%) at 52 weeks. Two patients (9%) demonstrated ≥25% increase. A further 28 patients with paired molecular data are undergoing analysis.

**Summary/Conclusion**

RopegIFN was associated with achievement or ongoing haematological response in patients transitioning from pegIFN. Molecular responses were observed despite relatively short treatment exposure. Importantly, transition from pegIFN to ropegIFN was well tolerated with low discontinuation rates.
Limitations of this study include retrospective design, incomplete molecular data and limited follow-up. However, although the study design does not permit direct comparison between IFN formulations, our findings demonstrate ropegIFN as effective and support therapeutic substitution during supply disruption in MPN patients.

**Copyright Information:** (Online) ISSN: 2572-9241
©2026 The Author(s). HemaSphere is published by John Wiley & Sons Ltd on behalf of European Hematology Association. This is an open access Abstract Book distributed under the Attribution-NonCommercial-NoDerivs (CC BY-NC-ND), which allows third parties to download the articles and share them with others as long as they credit the author and the Abstract Book, but they cannot change the content in any way or use them commercially.
**Abstract Book Citations:** Authors, Title, HemaSphere, 2026;10:(S1):pages. The abstract book DOIs can be found on the “Author Information” page.
**Disclaimer:** Articles published in the journal HemaSphere exclusively reflect the opinions of the authors. The authors are responsible for all content in their abstracts including accuracy of the facts, statements, citing resources, etc.

### (PB3507) EHA GUIDELINE ON THE DIAGNOSIS AND MANAGEMENT OF MYELOPROLIFERATIVE NEOPLASMS IN PATIENTS AGED 25 YEARS OR YOUNGER: INTEGRATING BEST AVAILABLE EVIDENCE IN A RARE DISEASE SETTING

**Topic:** 16. Myeloproliferative neoplasms - Clinical

Julia Schroer*[1], Nicole Skoetz[1], Mario Csenar[1], Luise Stach von Goltzheim[1], Samah Alimam[2], Yvonne Lisa Behrens[3], Nicolas Boissel[4], Barbara Burroni[5], Maike Büttner-Herold[6], Holger Cario[7], Timothy Devos[8], Martin Griesshammer[9], Claire Harrison[10], Chloe James[11], Axel Karow[12], Charikleia Kelaidi[13], Jean-Jacques Kiladjian[14], Steffen Koschmieder[15], Nicole Elena Kucine[16], Danijela Lekovic[17, 18], Maaike Luesink[19], Mary Frances McMullin[20], Wolfgang Novak[21], Helen Oram[22], Isabelle Plo[23], Lee-Yung Shih[24], Radek C Skoda[25], Alessandro Maria Vannucchi[26], Jacek Wachowiak[27], Daniel Wiseman[28], Elisa De Vrieze[29], Madelyn Flickinger[30], Mónica Pérez-Carbonell[31], Alice Watson[32], Magdalena Roszkowska-Lwow[33], Hanna Simpson[28], Claire Woodley[10], Jean Christophe Ianotto[34], Marta Sobas[35]

*[1]Institute of Public Health, Faculty of Medicine and University Hospital Cologne, University of Cologne, Cologne, Germany; [2]Department of Haematology, University College London Hospitals NHS Foundation Trust, London, United Kingdom;[3]Institute of Medical Genetics, Carl von Ossietzky University Oldenburg, Oldenburg, Germany; [4]Hopital Saint-Louis, AP-HP, Paris, France; [5]Department of Pathology, GHU Paris Centre - Cochin, AP-HP, Université Paris Cité, INSERM 1138, Paris, France;[6]Department of Nephropathology, Institute of Pathology, Universitätsklinikum Erlangen, Friedrich-Alexander-Universität Erlangen-Nürnberg (FAU), Erlangen, Germany; [7]Department of Pediatrics and Adolescent Medicine, University Medical Centre, Ulm, Germany; [8]Department of Hematology, University Hospitals Leuven and Department of Microbiology and Immunology, Laboratory of Molecular Immunology (Rega Institute), Katholieke Universiteit Leuven, Leuven, Belgium; [9]University Clinic for Hematology, Oncology, Hemostaseology and Palliative Care, Johannes Wesling Medical Center Minden, University of Bochum, Minden, Germany; [10]Department of Hematology, Guy's and St Thomas' National Health Service Foundation Trust, London, United Kingdom; [11]Biology of Cardiovascular Diseases, University of Bordeaux, INSERM, UMR1034, Pessac, France; [12]Department of Pediatrics and Adolescent Medicine, University Hospital Erlangen, Friedrich-Alexander-Universität (FAU) Erlangen-Nürnberg, Erlangen, Germany; [13]Oncology Department "Panagiotis & Aglaia Kyriakou" Children's Hospital, Athens, Greece; [14]Assistance Publique - Hôpitaux de Paris, Hôpital Saint-Louis, Université Paris Cité, Inserm CIC1427, Paris, France; [15]Department of Hematology, Oncology, Hemostaseology, and Stem Cell Transplantation, RWTH Aachen University and Center for Integrated Oncology (CIO-ABCD), Aachen, Germany; [16]Department of Pediatrics, Weill Cornell Medicine, New York, United States of America; [17]Clinic of Hematology, University Clinical Center of Serbia, Belgrade, Serbia;[18]Faculty of Medicine, University of Belgrade, Belgrade, Serbia; [19]Princess Máxima Center for Paediatric Oncology, Utrecht, The Netherlands;[20]Haematology, Belfast City Hospital, Queen's University Belfast, Belfast, United Kingdom; [21]Department of Pediatrics and Medical University Vienna Children's Cancer Research Institute, St. Anna Children's Hospital, Vienna, Austria; [22]Leswisham and Greenwich NHS Trust, London, United Kingdom; [23]Université Paris-Saclay, Gustave Roussy, INSERM U1287, Cellules souches hématopoïétiques et développement d'hémopathies malignes, Villejuif, France; [24]Division of Hematology-Oncology, Chang Gung Memorial Hospital-Linkou and Chang Gung University, Taoyuan, Taiwan; [25]Baylor College of Medicine, Dan L. Duncan Comprehensive Cancer Center, Houston, United States of America; [26]CRIMM, Center for Research and Innovation of Myeloproliferative Neoplasms, Azienda Ospedaliero-Universitaria Careggi, Florence, Italy; [27]Department of Pediatric Oncology, Hematology and Transplantology, Medical University of Karol Marcinkowski Poznan, Poznan, Poland; [28]The Christie NHS Foundation Trust, Manchester, United Kingdom; [29]Patient Advocate, Ghent, Belgium; [30]Patient Advocate, George Mason University, Fairfax, United States of America; [31]Patient Advocate, MPN ESPAÑA association, Barcelona, Spain;[32]Patient Advocate, London, United Kingdom; [33]Wroclaw Medical University, Wroclaw, Poland; [34]Department of Hematology, Institute of Oncology and Hematology, University Hospital, Brest, France; [35]Department of Hematology, Blood Neoplasms and Bone Marrow Transplantation, Wroclaw Medical University, Wroclaw, Poland;*

**Copyright Information:** (Online) ISSN: 2572-9241
©2026 The Author(s). HemaSphere is published by John Wiley & Sons Ltd on behalf of European Hematology Association. This is an open access Abstract Book distributed under the Attribution-NonCommercial-NoDerivs (CC BY-NC-ND), which allows third parties to download the articles and share them with others as long as they credit the author and the Abstract Book, but they cannot change the content in any way or use them commercially.
**Abstract Book Citations:** Authors, Title, HemaSphere, 2026;10:(S1):pages. The abstract book DOIs can be found on the "Author Information" page.
**Disclaimer:** Articles published in the journal HemaSphere exclusively reflect the opinions of the authors. The authors are responsible for all content in their abstracts including accuracy of the facts, statements, citing resources, etc.

### Background

Approximately 10–20% of Philadelphia-negative chronic myeloproliferative neoplasms (MPNs), including essential thrombocythemia (ET), polycythemia vera (PV), and myelofibrosis (MF), are diagnosed before the age of 40 years. In children, adolescents, and young adults (CAYA), these diseases exhibit distinct biological and clinical characteristics compared with later-onset disease. Although age above 60 years is an established thrombotic risk factor in MPNs, younger patients frequently develop thrombotic events, particularly venous thromboses at atypical sites. Their long life expectancy confers a sustained risk of disease progression and long-term treatment-related complications. Managing young patients is further complicated by unique psychosocial challenges, underscoring the need for a clinical guideline dedicated to CAYA with MPNs, which is currently being developed by the European Hematology Association (EHA) MPN Specialized Working Group.

### Aims

To establish standards for the diagnosis and management of MPNs in patients aged 25 years or younger by integrating the best available evidence and expert consensus.

### Methods

Four working groups were established: 1. diagnosis, 2. assessment of thrombosis, hemorrhage, disease evolution, and prognosis risk, 3. therapy, and 4. quality of life. Each group comprises multidisciplinary clinical experts, guideline methodologists, and patient representatives. Collaboratively, questions were predefined in the Population, Intervention, Comparison, Outcome (PICO) format and classified as candidates for evidence-based versus consensus-only recommendations. Systematic work-up of the evidence, following the Grading of Recommendations, Assessment, Development and Evaluation (GRADE) approach, is currently ongoing. Within a structured evidence-to-recommendation framework, the guideline panel will determine the direction and strength of each recommendation. All recommendations will undergo formal consensus through a Delphi process. Consensus-only recommendations will be informed by population-specific scoping searches, including observational studies and case series. Given the underrepresentation of CAYA in randomized controlled trials (RCTs) of MPN, evidence-based recommendations will be informed by comparative population-specific observational studies and indirect evidence from RCTs in adults with MPNs and in similar, more prevalent indications, depending on the question. A systematic search for studies and cases in CAYA with MPNs in MEDLINE and EMBASE was conducted on 7 May 2025.

### Results

More than 30 PICOs were formulated, addressing diagnostic strategies, including bone marrow biopsy, testing for driver and germline mutations, and splenic ultrasound, as well as pharmacological management of MPNs in CAYA with or without prior thrombotic events. Beyond that, the guideline will address thrombophilia testing, risk stratification, and various supportive interventions to alleviate the psychosocial burden of the disease and improve clinical outcomes. The search for studies and cases of MPNs in CAYA yielded 3,559 records (Figure 1). After screening, 426 reports were eligible, 316 of which were case reports of less than five patients, some of which dating back several decades to a time preceding contemporary diagnostic criteria.

### Summary/Conclusion

This represents the first international guideline addressing the diagnosis and management of MPNs in CAYA, in which population-specific evidence is scarce.

**Copyright Information:** (Online) ISSN: 2572-9241
©2026 The Author(s). HemaSphere is published by John Wiley & Sons Ltd on behalf of European Hematology Association. This is an open access Abstract Book distributed under the Attribution-NonCommercial-NoDerivs (CC BY-NC-ND), which allows third parties to download the articles and share them with others as long as they credit the author and the Abstract Book, but they cannot change the content in any way or use them commercially.
**Abstract Book Citations:** Authors, Title, HemaSphere, 2026;10:(S1):pages. The abstract book DOIs can be found on the "Author Information" page.
**Disclaimer:** Articles published in the journal HemaSphere exclusively reflect the opinions of the authors. The authors are responsible for all content in their abstracts including accuracy of the facts, statements, citing resources, etc.

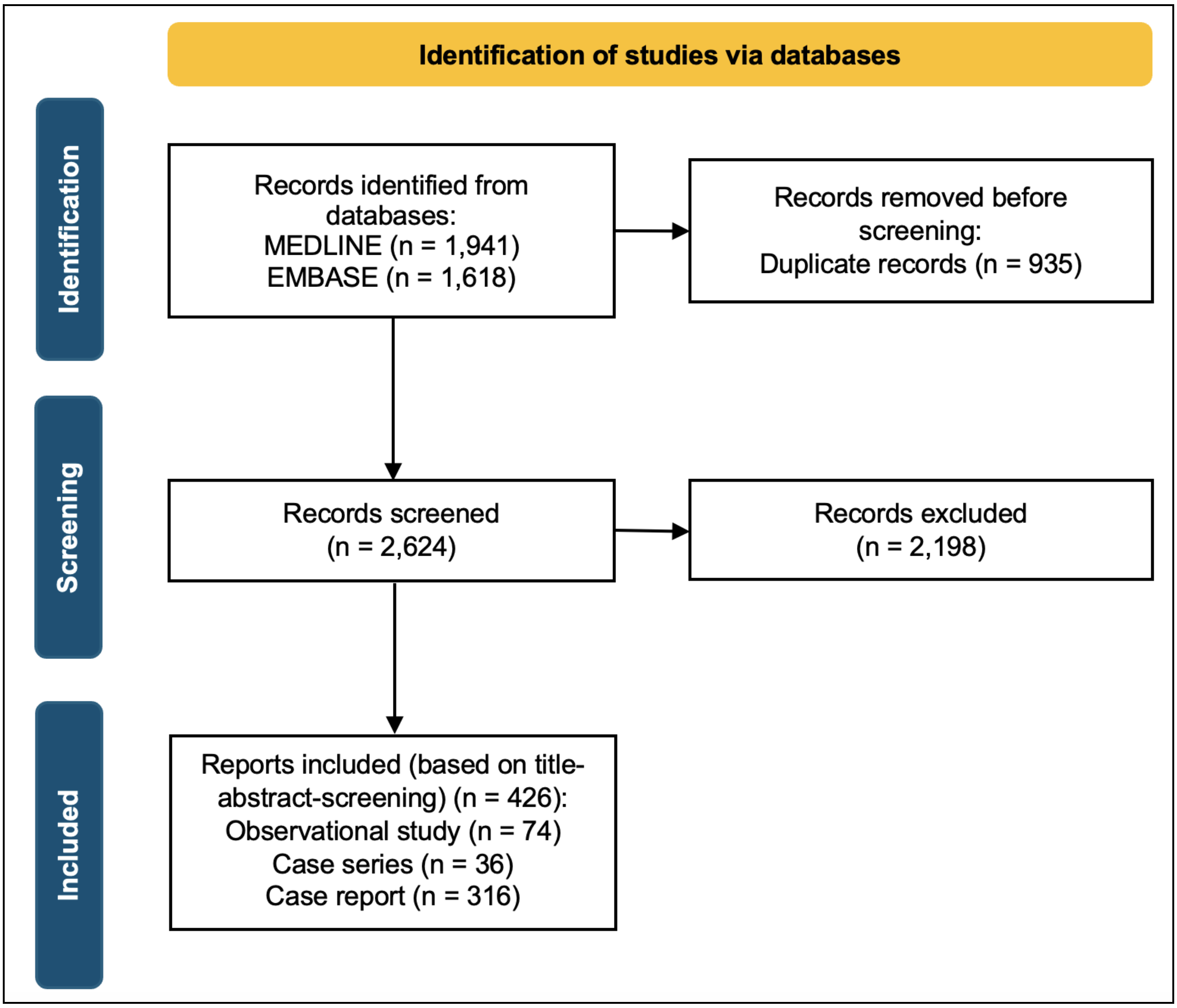

**Copyright Information:** (Online) ISSN: 2572-9241
©2026 The Author(s). HemaSphere is published by John Wiley & Sons Ltd on behalf of European Hematology Association. This is an open access Abstract Book distributed under the Attribution-NonCommercial-NoDerivs (CC BY-NC-ND), which allows third parties to download the articles and share them with others as long as they credit the author and the Abstract Book, but they cannot change the content in any way or use them commercially.
**Abstract Book Citations:** Authors, Title, HemaSphere, 2026;10:(S1):pages. The abstract book DOIs can be found on the "Author Information" page.
**Disclaimer:** Articles published in the journal HemaSphere exclusively reflect the opinions of the authors. The authors are responsible for all content in their abstracts including accuracy of the facts, statements, citing resources, etc.

### (PB3508) PHASE 2 STUDY TO ASSESS THE SAFETY AND EFFICACY OF BOMEDEMSTAT (IMG-7289) IN COMBINATION WITH MOMELOTINIB IN PATIENTS WITH MYELOFIBROSIS

**Topic:** 16. Myeloproliferative neoplasms - Clinical

Ciro Rinaldi*[1], Simon Archer[1], Sarah Fahy[1], Hannah Finch[1]

[1]*United Lincolnshire Teaching Hospital and University of Lincoln, Haematology, Lincoln, United Kingdom;*

### Background

Myelofibrosis (MF) is a chronic myeloproliferative neoplasm characterized by splenomegaly, cytopenias, inflammatory symptoms, and progressive marrow fibrosis. Momelotinib, a JAK1/JAK2 and ACVR1 inhibitor, improves splenomegaly and anemia; however, a substantial proportion of patients experience inadequate spleen, symptom, or hematologic responses. Bomedemstat (IMG-7289), an oral irreversible inhibitor of lysine-specific demethylase 1 (LSD1), has demonstrated disease-modifying activity in MF, including effects on megakaryopoiesis, inflammatory signaling, and fibrosis. Preclinical and clinical data support complementary mechanisms between JAK inhibition and LSD1 inhibition.

### Aims

This is an open-label, single-arm, multicenter phase 2 study enrolling approximately 40 adults with primary or secondary MF (post–polycythemia vera or post–essential thrombocythemia) with intermediate-2 or high-risk disease.

### Methods

This is an open-label, single-arm, multicenter phase 2 study enrolling approximately 50–60 adults with primary or secondary MF (post–polycythemia vera or post–essential thrombocythemia) with intermediate-2 or high-risk disease. All patients receive momelotinib 200 mg orally once daily. After 12 weeks of momelotinib monotherapy, patients with protocol-defined suboptimal response—based on spleen volume reduction, symptom improvement, hematologic parameters, or transfusion requirements—initiate combination therapy with bomedemstat 50 mg orally once daily, with dose titration per MF-specific guidelines, through week 24. Two cohorts are included: (1) patients previously treated with momelotinib who demonstrate suboptimal response after ≥12 weeks of therapy, and (2) patients with baseline cytopenias initiating momelotinib within the study who demonstrate suboptimal response at week 12.

### Results

This is a Trial In progress

**Copyright Information:** (Online) ISSN: 2572-9241
©2026 The Author(s). HemaSphere is published by John Wiley & Sons Ltd on behalf of European Hematology Association. This is an open access Abstract Book distributed under the Attribution-NonCommercial-NoDerivs (CC BY-NC-ND), which allows third parties to download the articles and share them with others as long as they credit the author and the Abstract Book, but they cannot change the content in any way or use them commercially.
**Abstract Book Citations:** Authors, Title, HemaSphere, 2026;10:(S1):pages. The abstract book DOIs can be found on the “Author Information” page.
**Disclaimer:** Articles published in the journal HemaSphere exclusively reflect the opinions of the authors. The authors are responsible for all content in their abstracts including accuracy of the facts, statements, citing resources, etc.

**Summary/Conclusion**

The primary endpoint is the proportion of patients achieving ≥35% spleen volume reduction at week 24 by MRI or CT. Secondary endpoints include ≥50% reduction in total symptom score, changes in symptom burden, transfusion independence, hematologic responses, overall response rate, duration of response, progression-free survival, overall survival, and safety and tolerability. Exploratory endpoints include molecular and biomarker analyses assessing changes in driver mutation allele burden, inflammatory cytokines, and markers of differentiation and fibrosis.

The study will run in 5 Countries (UK, France, Spain, Dubai, USA), for a total of 20 Sites. Enrollment expected to start in September 2026.

ISRCTN98283910

**Copyright Information:** (Online) ISSN: 2572-9241
©2026 The Author(s). HemaSphere is published by John Wiley & Sons Ltd on behalf of European Hematology Association. This is an open access Abstract Book distributed under the Attribution-NonCommercial-NoDerivs (CC BY-NC-ND), which allows third parties to download the articles and share them with others as long as they credit the author and the Abstract Book, but they cannot change the content in any way or use them commercially.
**Abstract Book Citations:** Authors, Title, HemaSphere, 2026;10:(S1):pages. The abstract book DOIs can be found on the “Author Information” page.
**Disclaimer:** Articles published in the journal HemaSphere exclusively reflect the opinions of the authors. The authors are responsible for all content in their abstracts including accuracy of the facts, statements, citing resources, etc.

### (PB3509) BASELINE CHARACTERISTICS AT DIAGNOSIS IN CHINESE MYELOFIBROSIS PATIENTS: A CROSS-SECTIONAL STUDY (CHAMPION-001)

**Topic:** 16. Myeloproliferative neoplasms - Clinical

Zefeng Xu*[1, 2], Jian Huang[1, 3], Bing Li[1, 2], Tiejun Qin[1, 2], Hu Zhou[1, 4], Miao Miao[1, 5], Na Xu[1, 6], Pengcheng He[1, 7], Liu Liu[1, 8], Zunmin Zhu[1, 9], Jianyu Weng[1, 10], Wen Wu[1, 11], Xin Du[1, 12], Jieyu Wang[1, 13], Qin Wen[1, 14], Xuliang Shen[1, 15], Yunfan Yang[1, 16], Yongzhong Su[1, 17], Qian Jiang[1, 18], Hongxia Shi[1, 18], Chunyan Chen[1, 19], Shuangnian Xu[20], Ruijuan Wang[1, 21], Ming Jiang[1, 22], Xiaoyu Zhu[1, 23], Guangxun Gao[1, 24], Zeping Zhou[1, 25], Yong Wu[1, 26], Min Zhang[1, 27], Zhenfang Liu[1, 28], Hai Lin[1, 29], Yuhua Li[1, 30], Zhenyu Yan[1, 31], Qingshu Zeng[1, 32], Lifang Huang[1, 33], Aijun Liao[1, 34], Wuhan Hui[1, 35], Zhiqiang Sun[1, 36], Zhenling Li[1, 37], Qing Leng[1, 38], Yajing Xu[1, 39], Weiwei Qin[1, 40], Yanping MA[1, 41], Zhijian Xiao[1, 2]

*[1]The China Hematology Alliance Myeloproliferative Neoplasms Investigation Organization, Tianjin, China;[2]Institute of Hematology and Blood Diseases Hospital, Chinese Academy of Medical Sciences & Peking Union Medical College, Tianjin, China; [3]The First Affiliated Hospital, Zhejiang University School of Medicine, Hangzhou, China; [4]The Affiliated Cancer Hospital of Zhengzhou University & Henan Cancer Hospital, Hemostasis and Thrombosis Diagnostic Engineering Research Center of Henan Province, Zhengzhou, China; [5]The First Affiliated Hospital of Soochow University, Suzhou, China;[6]Nanfang Hospital, Southern Medical University, Guangzhou, China; [7]The First Affiliated Hospital of Xi'an Jiaotong University, Xi'an, China;[8]The First Affiliated Hospital of Zhengzhou University, Zhengzhou, China; [9]Henan Provincial People's Hospital, Zhengzhou, China; [10]Guangdong Provincial People's Hospital, Guangzhou, China; [11]Ruijin Hospital, Shanghai Jiao Tong University School of Medicine, Shanghai, China; [12]Shenzhen Second People's Hospital, Shenzhen, China; [13]The First Affiliated Hospital of Nanchang University, Nanchang, China; [14]Xinqiao Hospital, Third Military Medical University, Chongqing, China;[15]Heping Hospital Affiliated to Changzhi Medical College, Changzhi, China; [16]West China Hospital, Sichuan University, Chengdu, China; [17]The First Affiliated Hospital of Shantou University Medical College, Shantou, China; [18]Peking University People's Hospital, Peking University Institute of Hematology, Beijing, China; [19]Qilu Hospital of Shandong University, Jinan, China;[20]The Southwest Hospital of Army Medical University, Chongqing, China; [21]Nanyang Central Hospital, Nanyang, China;[22]The First Affiliated Hospital of Xinjiang Medical University, Urumqi, China; [23]Anhui Provincial Hospital, Hefei, China;[24]Xijing Hospital of Air Force Military Medical University, Xi'an, China; [25]The Second Affiliated Hospital of Kunming Medical University, Kunming, China;[26]Fujian Institute of Hematology, Fujian Medical University Union Hospital, Fuzhou, China; [27]Union Hospital, Tongji Medical College, Huazhong University of Science and Technology, Wuhan, China; [28]The First Affiliated Hospital of Guangxi Medical University, Nanning, China;[29]The First Hospital of Jilin University, Changchun, China; [30]Zhujiang Hospital of Southern Medical University, Guangzhou, China;[31]North China University of Science and Technology Affiliated Hospital, Tangshan, China; [32]The First Affiliated Hospital of Anhui Medical University, Hefei, China; [33]Shanxi Bethune Hospital, Taiyuan, China; [34]Shengjing Hospital of China Medical University, Shenyang, China; [35]Xuanwu Hospital, Capital Medical University, Beijing, China;[36]Shenzhen Hospital of Southern Medical University, Shenzhen, China; [37]China-Japan Friendship Hospital, Beijing, China; [38]Anshan Center Hospital, Anshan, China; [39]Xiangya Hospital of Central South University, Changsha, China; [40]Tangdu Hospital, Fourth Military Medical University, Xi'an, China; [41]Second Hospital of Shanxi Medical University, Taiyuan, China;*

### Background

Myelofibrosis (MF) is a rare Philadelphia chromosome-negative myeloproliferative neoplasm with heterogeneous clinical features. While epidemiological and clinical data from Western populations are established, a comprehensive profile of baseline characteristics at diagnosis in Chinese MF patients is lacking.

**Copyright Information:** (Online) ISSN: 2572-9241
©2026 The Author(s). HemaSphere is published by John Wiley & Sons Ltd on behalf of European Hematology Association. This is an open access Abstract Book distributed under the Attribution-NonCommercial-NoDerivs (CC BY-NC-ND), which allows third parties to download the articles and share them with others as long as they credit the author and the Abstract Book, but they cannot change the content in any way or use them commercially.
**Abstract Book Citations:** Authors, Title, HemaSphere, 2026;10:(S1):pages. The abstract book DOIs can be found on the “Author Information” page.
**Disclaimer:** Articles published in the journal HemaSphere exclusively reflect the opinions of the authors. The authors are responsible for all content in their abstracts including accuracy of the facts, statements, citing resources, etc.

**Aims**

The China Hematology Alliance Myeloproliferative Neoplasms Investigation Organization (CHAMPION) conducted this cross-sectional CHAMPION-001 study to describe the demographic, clinical, laboratory, and molecular characteristics of Chinese MF patients at diagnosis.

**Methods**

This multicenter study enrolled consecutive patients diagnosed with primary MF (PMF) or secondary MF (post-PV/post-ET MF) across in China. Data collected at diagnosis included demographics, symptom burden (MPN-SAF TSS), physical examination, spleen size, laboratory results, molecular genetics. Cytogenetic analysis and bone marrow histopathology were based on local reports. Risk stratification was performed using the Dynamic International Prognostic Scoring System (DIPSS). DIPSS scores were determined using patient characteristics at baseline. Descriptive statistics were used.

**Results**

At data cutoff (October 28, 2025), a total of 2495 patients diagnosed between January 2003 and August 2025 were included (PMF: 71.2%; post-PV MF: 11.1%; post-ET MF: 17.7%). A refined Core Analysis Cohort (CAC, n=1463) meeting all predefined criteria – including availability of bone marrow fibrosis grade, spleen size assessment by palpation or abdominal ultrasound, key blood cell counts, and bone marrow aspiration results – was analyzed for comprehensive baseline characterization.
In the CAC, the median age at diagnosis was 62 years (IQR, 53.0-70.0), with a male-to-female ratio of 0.96:1. The median spleen was palpable 10 cm (IQR, 5.0-14.0) below the left costal margin. Anemia (<100 g/L) was present in 43.9%, thrombocytopenia (<100 × $10^9$/L) in 18.3%, and leukocytosis (>25 × $10^9$/L) in 10.4%. Elevated lactate dehydrogenase (above reference range) was observed in 84.1% (median: 416.0 [IQR, 285.0-631.8]), ferritin >1000 ng/mL in 10.6%, and erythropoietin >500 mIU/mL in 10.9%. Among 1392 patients tested, *JAK2*$^{V617F}$ was identified in 60.3% (median VAF: 49.0% [IQR, 28.0-80.9]), *CALR* in 16.8% (median VAF: 31.6% [IQR, 25.72-39.40]; type 1 in 44.9%, type 2 in 14.5%, other rare mutations in 16.7%, and unknown in 23.9%), and *MPL* in 6.3% (median VAF: 46.3% [IQR, 22.1-64.9]; approximately one-third were *W515* mutations). Triple-negative cases accounted for 9.6%. The most common non-driver mutations were ASXL1, TET2 and TP53. Chromosomal abnormalities were detected in 21.6% of 1132 patients tested, with trisomy 8 being the most frequent. Among patients with evaluable data, the DIPSS risk distribution was: low in 19.8%, intermediate-1 in 41.5%, intermediate-2 in 34.1%, and high in 4.5%. Among 334 patients with treatment data, JAK inhibitors were most common, with 79.0% receiving ruxolitinib and 14.1% gecacitinib (approved in China in May 2025). Other frequently used therapies included hydroxyurea (20.1%), thalidomide/lenalidomide (17.1%), interferon (16.5%), and androgens (13.8%).

**Summary/Conclusion**

This study, the largest cross-sectional analysis to date, provides a detailed profile of baseline characteristics in Chinese MF patients at diagnosis, addressing a significant gap in the literature. Further longitudinal studies are warranted to evaluate the prognostic impact of these features.

**Copyright Information:** (Online) ISSN: 2572-9241
©2026 The Author(s). HemaSphere is published by John Wiley & Sons Ltd on behalf of European Hematology Association. This is an open access Abstract Book distributed under the Attribution-NonCommercial-NoDerivs (CC BY-NC-ND), which allows third parties to download the articles and share them with others as long as they credit the author and the Abstract Book, but they cannot change the content in any way or use them commercially.
**Abstract Book Citations:** Authors, Title, HemaSphere, 2026;10:(S1):pages. The abstract book DOIs can be found on the "Author Information" page.
**Disclaimer:** Articles published in the journal HemaSphere exclusively reflect the opinions of the authors. The authors are responsible for all content in their abstracts including accuracy of the facts, statements, citing resources, etc.

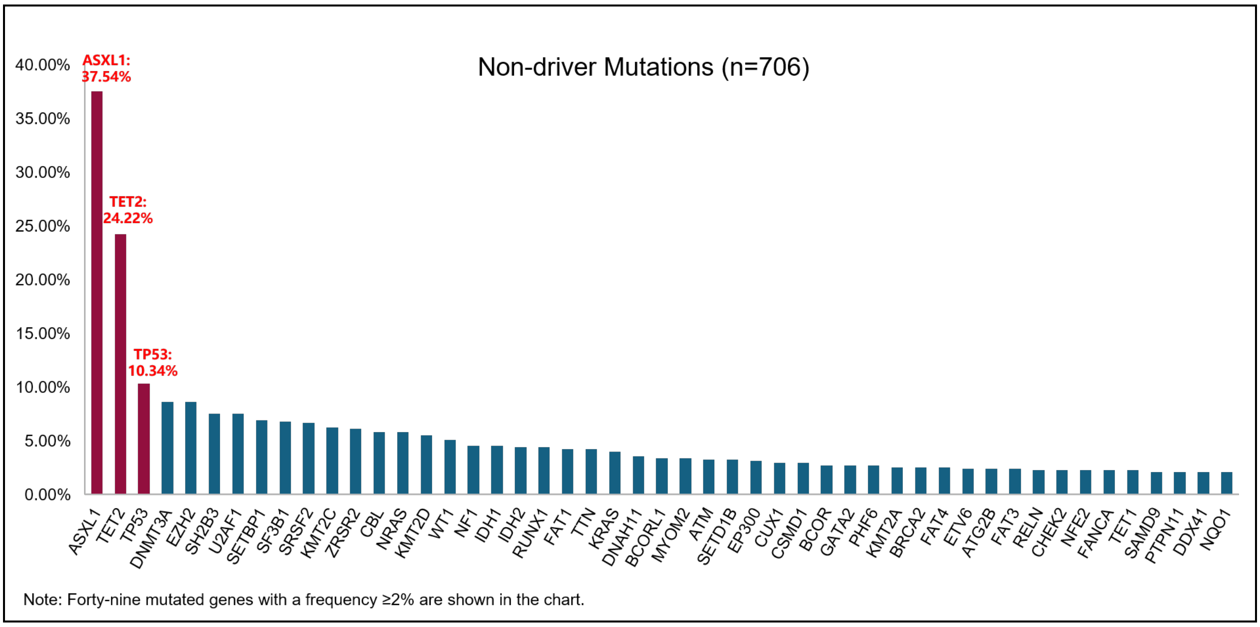

**Copyright Information:** (Online) ISSN: 2572-9241
©2026 The Author(s). HemaSphere is published by John Wiley & Sons Ltd on behalf of European Hematology Association. This is an open access Abstract Book distributed under the Attribution-NonCommercial-NoDerivs (CC BY-NC-ND), which allows third parties to download the articles and share them with others as long as they credit the author and the Abstract Book, but they cannot change the content in any way or use them commercially.
**Abstract Book Citations:** Authors, Title, HemaSphere, 2026;10:(S1):pages. The abstract book DOIs can be found on the "Author Information" page.
**Disclaimer:** Articles published in the journal HemaSphere exclusively reflect the opinions of the authors. The authors are responsible for all content in their abstracts including accuracy of the facts, statements, citing resources, etc.

### (PB3510) MULTI-CENTER REAL WORLD ANALYSIS OF EFFICACY AND SAFETY OF GECACITINIB IN THE TREATMENT OF MYELOFIBROSIS

**Topic:** 16. Myeloproliferative neoplasms - Clinical

Chen Cao*[1], Minran Zhou[1], Ran Wang[1], Sai MA[1], Changrong Wei[2], Qing Meng[2], Yanli Wang[3], Qian Zhou[3], Zhao Li[4], Shaoling Wu[5], Yan Liu[6], Yuqi Sang[7], Jianmin Guan[7], Sheng Yang[8], Chunyan Chen[1]

*[1]Qilu Hospital of Shandong University, Jinan, China;[2]Linyi People's Hospital, Linyi, China;[3]Linyi Central Hospital, Linyi, China;[4]Qilu Hospital of Shandong University (Qingdao), Qingdao, China;[5]The Affiliated Hospital of Qingdao University, Qingdao, China;[6]Zibo Central Hospital, Zibo, China;[7]Heze Municipal Hospital, Heze, China;[8]Binzhou People's Hospital,Binzhou, Binzhou, China;*

### Background

Gecacitinib (approved in China) inhibits both JAK and activin receptor 1,potentially improving anemia and reducing transfusion dependence.Clinical data on gecacitinib for MF remain limited.

### Aims

To evaluate the efficacy and safety of gecacitinib in MF patients in the multicenter study.

### Methods

Retrospective study of 44 MF patients receiving gecacitinib across 8 Chinese centers Baseline data included demographics,spleen length,MPN10 score,blood counts, fibrosis grade,karyotype,driver/non-driver mutations,transfusion dependence,and MF subtype.Risk stratified by IPSS/DIPSS.Adverse events graded per CTCAE v5.0.

**Copyright Information:** (Online) ISSN: 2572-9241
©2026 The Author(s). HemaSphere is published by John Wiley & Sons Ltd on behalf of European Hematology Association. This is an open access Abstract Book distributed under the Attribution-NonCommercial-NoDerivs (CC BY-NC-ND), which allows third parties to download the articles and share them with others as long as they credit the author and the Abstract Book, but they cannot change the content in any way or use them commercially.
**Abstract Book Citations:** Authors, Title, HemaSphere, 2026;10:(S1):pages. The abstract book DOIs can be found on the "Author Information" page.
**Disclaimer:** Articles published in the journal HemaSphere exclusively reflect the opinions of the authors. The authors are responsible for all content in their abstracts including accuracy of the facts, statements, citing resources, etc.

**Results**

4.1Baseline Characteristics
Median age 65 years (37–86).HMR mutations: 8 patients (ASXL1: 75%). Cytogenetics:+8 in 3,complex karyotype in 1. 39 patients had prior ruxolitinib.Switch reasons: disease progression (13) or worsening cytopenias/fatigue.By DIPSS:high-risk 47.7% vs IPSS 68.2%. 9 IPSS high-risk patients reclassified as intermediate-2 (66.7%) or intermediate-1 (33.3%).
4.2Efficacy
4.2.1Transfusion independence: Baseline transfusion history in 45.5% patients (20/44); 7/20 (35%) were RBC transfusion dependent.Within 24 weeks, 3/7 (42.9%) became transfusion independent.
4.2.2Anemia response:Among 21 evaluable patients, hemoglobin increased in 11(52.4%) after 2 weeks.In 21 patients with baseline HGB <100 g/L(non-transfusion dependent),7(33.3%) achieved HGB increase≥20 g/L within 24 weeks.
4.2.3Spleen response was observed in 83.3% of patients(25/30). According to DIPSS, response rates for ≥35% and ≥50% spleen reduction were 57.1% and 28.6% in high-risk patients (n=14),58.3% and 33.3% in inter-2 patients (n=12), and both 66.7% in inter-1 patients (n=3).By disease subtype, patients with PMF (n=25) had ≥35% and ≥50% reduction rates of 68% and 40%, compared to 20% and 0% in post-ET/PV patients (n=5).
4.2.4Symptom improvement: 56.3% (9/16) achieved TSS50 within 24 weeks.
4.2.5Survival and Safety
Median OS was 33.0 months.1 patient progressed to accelerated phase with new EZH2, CBL, RUNX1 mutations and del(11q).In terms of safety, grade≥3 hematologic adverse events included anemia (30.4%), thrombocytopenia(19.6%), and neutropenia (6.5%); grade≥3 non-hematologic adverse events included pneumonia (13.0%) and tuberculosis (2.2%). 3 deaths occurred (cerebral hemorrhage, respiratory failure, severe pneumonia).

**Summary/Conclusion**

Gecacitinib demonstrates promising efficacy in MF, with spleen reduction in 83.3%, transfusion independence in 42.9%, and symptom improvement in 56.3%.The safety profile was manageable.These real-world data support gecacitinib as an effective option for MF, including in ruxolitinib pretreated patients.

**Copyright Information:** (Online) ISSN: 2572-9241
©2026 The Author(s). HemaSphere is published by John Wiley & Sons Ltd on behalf of European Hematology Association. This is an open access Abstract Book distributed under the Attribution-NonCommercial-NoDerivs (CC BY-NC-ND), which allows third parties to download the articles and share them with others as long as they credit the author and the Abstract Book, but they cannot change the content in any way or use them commercially.
**Abstract Book Citations:** Authors, Title, HemaSphere, 2026;10:(S1):pages. The abstract book DOIs can be found on the “Author Information” page.
**Disclaimer:** Articles published in the journal HemaSphere exclusively reflect the opinions of the authors. The authors are responsible for all content in their abstracts including accuracy of the facts, statements, citing resources, etc.

### (PB3511) LONG-TERM FOLLOW-UP OF A SERIES OF 165 POLYCYTHEMIA VERA PATIENTS FROM A SINGLE CENTER

**Topic:** 16. Myeloproliferative neoplasms - Clinical

Evangelia-Faidra Triantafyllou*[1], Pantelis Tsirkinidis[1], Maria Efstathopoulou[2], Chrysovalantou Chatzidimitriou[3], Alexandros MacHairas[4], Iliana Konstantinou[4], Anastasia Kopsaftopoulou[4], Maria Dimitrakoudi[4], Olga Tsopra[4], Alexia Piperidou[4], Athanasios Liaskas[4], Aikaterini Kolotsiou[4], Georgios Petros Barakos[4], Konstantinos Angelopoulos[4], Panagiota Maria Arapaki[4], Ioannis Asimakopoulos[4], Marina Belia[4], Dimitrios Galopoulos[4], Aikaterini Bitsani[4], Panagiota Petsa[4], Aikaterini Benekou[4], Fotios Panitsas[4], Maria Dimopoulou[4], Helen Plata[4], Panagiotis Tsaftaridis[4], Marina Siakantaris[4], Panayiotis Panayiotidis[4], Maria Dimou[4], Gerassimos Pangalis[2], Theodoros Vassilakopoulos[4], Maria Angelopoulou[4]

*[1]401 General Military Hospital of Athens, Athens, Greece;[2]Medical Center Psychikon Branch, Athens, Greece;[3]Attikon University Hospital, Athens, Greece; [4]Laiko General Hospital, Athens, Greece;*

### Background

Polycythaemia vera (PV) is a JAK2-mutated myeloproliferative neoplasm (MPN) characterized by erythrocytosis, increased risk for thrombosis (venous and arterial), while progression into myelofibrosis (post-PV MF) and acute myeloid leukemia (AML) are relatively rare. Studying the risk for progression to MF and AML requires long-term follow-up, which is often missed in every day clinical practice.

### Aims

To evaluate clinical characteristics, long-term outcomes, prognostic factors and risk for progression to MF and AML of a series of PV patients from a single center.

### Methods

We analyzed 165 consecutive PV patients diagnosed and followed between 1995-2023 from a single center, with a median follow-up of 8,4 years (0,58-36,2).

**Copyright Information:** (Online) ISSN: 2572-9241
©2026 The Author(s). HemaSphere is published by John Wiley & Sons Ltd on behalf of European Hematology Association. This is an open access Abstract Book distributed under the Attribution-NonCommercial-NoDerivs (CC BY-NC-ND), which allows third parties to download the articles and share them with others as long as they credit the author and the Abstract Book, but they cannot change the content in any way or use them commercially.
**Abstract Book Citations:** Authors, Title, HemaSphere, 2026;10:(S1):pages. The abstract book DOIs can be found on the “Author Information” page.
**Disclaimer:** Articles published in the journal HemaSphere exclusively reflect the opinions of the authors. The authors are responsible for all content in their abstracts including accuracy of the facts, statements, citing resources, etc.

### Results

The median age of our patients was 66 years (17-89) and 93 were males. At diagnosis 26.3% were low-risk and 73.8% were high-risk for thrombosis according to ELN 2011. According to the 2013 IWG score (for survival in PV), 18.8%, 37.6% and 43.6% were low, intermediate and high risk respectively. 52% of patients had PV-related symptoms at diagnosis, the most common being pruritus(14.7%). Median values of WBC, ANC(absolute neutrophil counts), NLR(neutrophil to lymphocyte ratio), Hb, Ht, MCV, PLT were 9.9x$10^9$/L, 7.2x$10^9$/L, 4.1, 17.3 gr/dl, 53.2%, 81.8 fl, and 557x$10^9$/L. LDH levels were elevated in 58.2% and serum erythropoietin levels were reduced in 72.6%. JAK2V617F mutation was found in 114/122 patients (93.4%), while 3/122 were JAK2EXON12 mutated (2.5%) and 16% had an abnormal karyotype. Bone marrow biopsy revealed panmyelosis in 61.5%, fibrosis grade 1 in 27% and grade 2 in 1.6%. 17% of patients had a thrombotic episode. Factors associated with higher risk for thrombosis were a. ELN 2011 thrombotic risk (23.7% high risk patients had a thrombotic event vs 0% low-risk ones, p<0,0001) b. Ht>53% (p=0.05) and c. elevated LDH (p=0.05). First line treatment was hydroxyrea in 89.9%, while 8.6% and 1.4% were treated with phlebotomy only and interferon respectively. ANC>10x$10^9$/L (p=0.05), NLR>4 (p=0.05) and high risk 2013 IWG score (p=0.04) were associated with inferior response to first line treatment. Second line treatment included interferon (41.7%), ruxolitinib (20.8%), anagrelide (29.2%) and busulfan (8.4%). During follow-up, 10% experienced progression to MF, 2.3% to AML and 2.3% to MF followed by AML. WBC>13x$10^9$ /L (p=0.015) was a significant adverse predictive factor for progression, while ANC>10x$10^9$/L, Ht>52% and elevated LDH were marginally significant. At last follow-up, 3.5% were alive without treatment, 55.8% alive under cytoreductive treatment, 7.1% were alive with disease progression, 10.6% had died of disease, 20.4% died of other causes and 2.7% died from second malignancy. Median overal survival (OS) was 14.4 years (9.9-18.8), with OS at 10 and 15 years being 72% and 48%. Median disease specific survival (DSS) was 30.3 years and DSS at 10 and 15 years was 97% and 89%. Age >65years (p=0.002), disease progression (p=0.008), panmyelosis (p=0.05) and high risk 2013 IWG score (p=0.02) were found as significant adverse prognostic factors for DSS (figure).

### Summary/Conclusion

PV is associated with excellent long-term outcome. Approximately 15% of PV patients progress into MF and AML. Increased WBC, ANC, Ht and LDH predicted for progression of disease. Age >65years, disease progression, panmyelosis and high risk 2013 IWG score represent adverse prognostic factors for DSS. Elevated Ht and LDH are associated with increased risk for thrombosis.

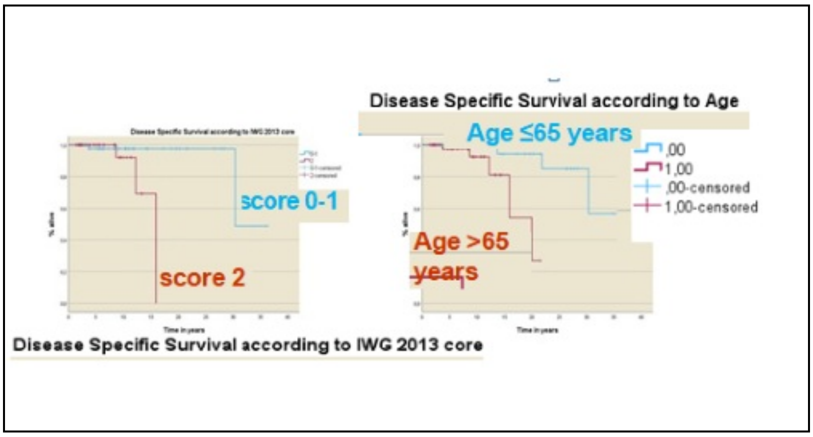

DISEASE SPECIFIC SURVIVAL ACCORDING TO AGE AND 2013 IWG SCORE

**Copyright Information:** (Online) ISSN: 2572-9241
©2026 The Author(s). HemaSphere is published by John Wiley & Sons Ltd on behalf of European Hematology Association. This is an open access Abstract Book distributed under the Attribution-NonCommercial-NoDerivs (CC BY-NC-ND), which allows third parties to download the articles and share them with others as long as they credit the author and the Abstract Book, but they cannot change the content in any way or use them commercially.
**Abstract Book Citations:** Authors, Title, HemaSphere, 2026;10:(S1):pages. The abstract book DOIs can be found on the "Author Information" page.
**Disclaimer:** Articles published in the journal HemaSphere exclusively reflect the opinions of the authors. The authors are responsible for all content in their abstracts including accuracy of the facts, statements, citing resources, etc.

### (PB3512) PRELIMINARY DATA ON SERUM OSTEOPROTEGERIN AND RANKL LEVELS IN PATIENTS WITH MYELOPROLIFERATIVE NEOPLASMS (MPNS)

**Topic:** 16. Myeloproliferative neoplasms - Clinical

Annita-Ioanna Gkioka*[1], George Oikonomou[1], Alexandros Alexandropoulos[1], Alexandros Gkiokas[1], Calliope Zerzi[1], Panagiotis Stoikos[1], Georgia Gryllaki[1], Vasilliki Bartzi[1], Panayiotis Panayiotidis[1], Marie Christine Kyrtsonis[1]

*[1]Hematology Section of First Department of Propedeutic Internal Medicine, National and Kapodistrian University of Athens, Greece ., Athens, Greece;*

### Background

The osteoprotegerin (OPG)/ Receptor activator of nuclear factor kappa-B (RANKL) axis plays a central role in bone metabolism and immune regulation and has been implicated in neoplasm microenvironment interactions. OPG acts as a decoy receptor for RANKL, inhibiting osteoclast activation and bone resorption. Given the recognized bone disease in MF and the inflammatory milieu characteristic of MPNs, we hypothesized that circulating OPG and RANKL levels may reflect disease-related microenvironmental dysregulation and potentially correlate with clinical outcomes. However, the clinical relevance of serum OPG and RANKL levels in MPNs remains insufficiently defined.

### Aims

To  assess serum soluble OPG and RANKL levels in patients with Ph chromosome- negative MPNs and explore their association with disease subtype and overall survival.

### Methods

Sixteen patients (median age 70 years, range 48–83) diagnosed with Ph chromosome negative (-) MPNs [polycythemia vera (PV) n=2, essential thrombocythemia (ET) n=7, MF n=5, and MDS/MPN overlap n=2] were included after informed consent. Frozen serum samples were analyzed for soluble OPG and RANKL using ELISA (DuoSet R&D Quantikine) according to the manufacturer's instructions. Fifteen healthy individuals (His) served as controls. Median values of patients were used as cut-offs; values above the median were defined as high. The OPG/RANKL ratio was calculated to assess pathway balance. Median overall survival (OS) of the cohort was 55 months (range 3–208). Statistical analysis was performed using SPSS v29.

### Results

Patients with MF exhibited  significantly higher OPG levels (median 2015.36 pg/mL; range 888.09–8517.18) compared with other MPN subtypes (median 1264.45 pg/mL; range 889–2194, p=0.014) and healthy individuals (median 857.18 pg/mL; range 60.5–2024.92, p=0.005). Median RANKL levels in MF were 277 pg/mL (range 0–1401.13), compared to undetectable levels (range undetectable levels –834.88) in other MPNs (p=0.001) and 16 pg/mL (range 0–945.14) in controls (p=0.0015). The median OPG/RANKL ratio was 790.89 (range 2–2015) in MF, (range 2–2114)  in other MPNs, and 54.61 (range 0–2024.32) in healthy individuals.  One patient with MF who progressed to acute myeloid leukemia (AML) 14 months after diagnosis had an especially increased OPG value 2519.91 pg/mL. In the overall cohort, high serum OPG levels were significantly associated with inferior OS ($p<0.05$).

**Copyright Information:** (Online) ISSN: 2572-9241
©2026 The Author(s). HemaSphere is published by John Wiley & Sons Ltd on behalf of European Hematology Association. This is an open access Abstract Book distributed under the Attribution-NonCommercial-NoDerivs (CC BY-NC-ND), which allows third parties to download the articles and share them with others as long as they credit the author and the Abstract Book, but they cannot change the content in any way or use them commercially.
**Abstract Book Citations:** Authors, Title, HemaSphere, 2026;10:(S1):pages. The abstract book DOIs can be found on the "Author Information" page.
**Disclaimer:** Articles published in the journal HemaSphere exclusively reflect the opinions of the authors. The authors are responsible for all content in their abstracts including accuracy of the facts, statements, citing resources, etc.

**Summary/Conclusion**

These preliminary findings describe the distribution of serum OPG and RANKL levels among Philadelphia chromosome–negative Myeloproliferative Neoplasms and demonstrate that patients with Primary Myelofibrosis (MF) exhibit higher levels compared with other subtypes. Elevated circulating OPG levels are associated with inferior overall survival in MPN patients, particularly in MF. Further studies are needed.

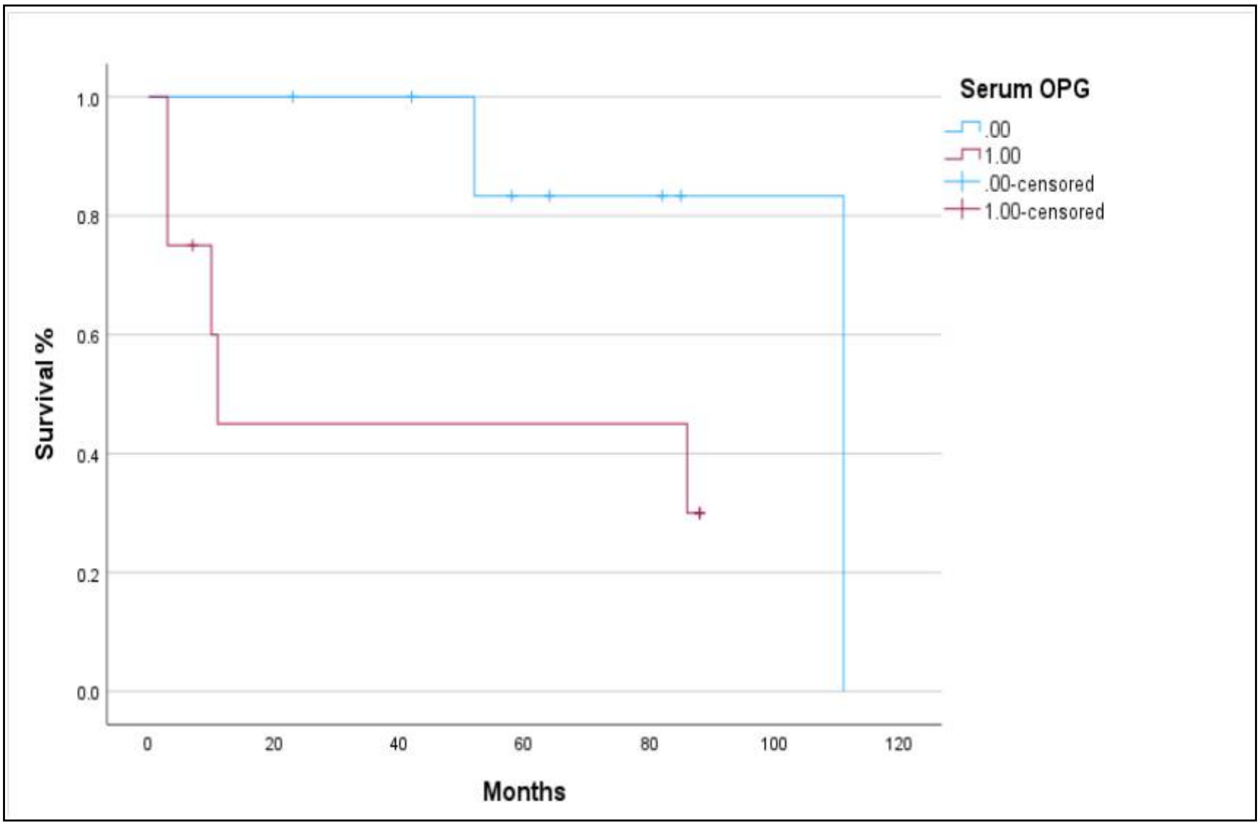

Overall survival according to OPG levels in patients with MPN

**Copyright Information:** (Online) ISSN: 2572-9241
©2026 The Author(s). HemaSphere is published by John Wiley & Sons Ltd on behalf of European Hematology Association. This is an open access Abstract Book distributed under the Attribution-NonCommercial-NoDerivs (CC BY-NC-ND), which allows third parties to download the articles and share them with others as long as they credit the author and the Abstract Book, but they cannot change the content in any way or use them commercially.
**Abstract Book Citations:** Authors, Title, HemaSphere, 2026;10:(S1):pages. The abstract book DOIs can be found on the "Author Information" page.
**Disclaimer:** Articles published in the journal HemaSphere exclusively reflect the opinions of the authors. The authors are responsible for all content in their abstracts including accuracy of the facts, statements, citing resources, etc.

### (PB3513) UTILITY OF NEXT-GENERATION SEQUENCING (NGS) IN PHILADELPHIA-NEGATIVE MYELOPROLIFERATIVE NEOPLASMS

**Topic:** 16. Myeloproliferative neoplasms - Clinical

Concepción Sánchez Chamizo*[1], Ruth Stuckey[1], Juan Francisco Lopez Rodriguez[1], Yanira Florido[1], Adrian Segura Diaz[1], Cristina Bilbao Sieyro[1], Guillermo Santana[1], Amagoia Ruiz Martin[1], Sara Medina[1], Raquel García-Talavera Hernández[1], Minia Grobas Sebio[1], Natalia Esteban[1], Carol Herrera Hernández[1], Monica Betancor[1], Jose Antonio Moreno[1], Guillermo Rodríguez Rodríguez[1], M Teresa Gómez Casares[1]

[1]*Hospital Universitario de Gran Canaria Doctor Negrín, Las Palmas de Gran Canaria, Spain;*

### Background

Classical myeloproliferative neoplasms (MPNs) are characterized by the presence of driver mutations in *JAK2*, *CALR*, or *MPL*, typically detected through targeted techniques such as PCR. However, a proportion of patients who are categorized as "triple negative" may harbour other variants that cannot be identified by conventional methods. Next-generation sequencing (NGS) enables the simultaneous analysis of multiple genes, including non-canonical variants in driver genes and mutations in other myeloid genes associated with clonality that may have diagnostic, prognostic, or familial relevance.

### Aims

To evaluate the diagnostic utility of NGS in patients with MPNs by identifying variants not previously detected by conventional PCR (standard techniques).

### Methods

A retrospective study of 289 patients with suspected or confirmed MPNs who underwent NGS testing with a myeloid panel at our center. Findings were classified into five groups according to diagnostic relevance: (1) classical driver mutations negative by PCR but positive by NGS, (2) double driver mutations, (3) presence of non-canonical variants in driver genes, (4) likely germline variants, and (5) somatic mutations associated with clonality in triple-negative patients.

**Copyright Information:** (Online) ISSN: 2572-9241
©2026 The Author(s). HemaSphere is published by John Wiley & Sons Ltd on behalf of European Hematology Association. This is an open access Abstract Book distributed under the Attribution-NonCommercial-NoDerivs (CC BY-NC-ND), which allows third parties to download the articles and share them with others as long as they credit the author and the Abstract Book, but they cannot change the content in any way or use them commercially.
**Abstract Book Citations:** Authors, Title, HemaSphere, 2026;10:(S1):pages. The abstract book DOIs can be found on the "Author Information" page.
**Disclaimer:** Articles published in the journal HemaSphere exclusively reflect the opinions of the authors. The authors are responsible for all content in their abstracts including accuracy of the facts, statements, citing resources, etc.

**Results**

Following a negative PCR, 8 patients (2.8%) presented driver mutations not previously detected by PCR (6 polycythemia vera, PV, and 2 essential thrombocythemia, ET).
An additional 3 patients (1.03%) presented non-canonical variants not previously detected. These findings suggest the presence of variants in non-hotspot regions and/or low–allele frequency mutations detectable only through NGS.
7 patients (2.4%) presented double molecular drivers; in 5/7 cases, the coexistence was only revealed after NGS testing, all involving the combination of *JAK2* and *MPL* mutations (in all cases, variant allele frequency (VAF) of JAK2 was significantly higher than VAF of MPL mutations (<5%).
3 patients (1.0%) carrying *JAK2 V617F* also presented non-canonical variants in the same gene, suggesting intraclonal heterogeneity or potential cooperative events within the JAK–STAT pathway.
Surprisingly 12 patients (5.9%) presented variants compatible with hereditary predisposition (genes involved included *SH2B3*, *DDX41*, *CSF3R*, *RUNX1* *JAK3* and *MPL)*. Among the latter two, previously reported mutations were identified in 2/4 *JAK2*-mutated cases and 1/2 *MPL*-mutated cases. Germline origin was confirmed in 3 cases (two *JAK2* variants confirmed in hair samples and one *MPL* variant confirmed in saliva). Confirmatory studies are ongoing in the remaining cases.
27 patients (9.3%) negative for classical driver mutations by PCR and confirmed by NGS presented somatic alterations in other genes associated with myeloid neoplasms: *TP53* (n=2), *STAG2* (n=2), *RUNX1* (n=1), *DNMT3A* (n=10), *TET2* (n=7), *ASXL1* (n=1), *WT1* (n=1), *CBL* (n=2), and *PHF6* (n=2). The identification of myeloid clonality supports the diagnosis of MPN in the absence of typical driver mutations.

**Summary/Conclusion**

NGS enables more comprehensive molecular characterization of MPNs, identifying drivers not detected by PCR, complex clonal architecture (double drivers), non-canonical variants, unsuspected germline predisposition, and somatic clonality in triple-negative patients. Its systematic incorporation improves diagnostic resolution and provides biologically relevant information on the clonal evolution of these neoplasms.

**Copyright Information:** (Online) ISSN: 2572-9241
©2026 The Author(s). HemaSphere is published by John Wiley & Sons Ltd on behalf of European Hematology Association. This is an open access Abstract Book distributed under the Attribution-NonCommercial-NoDerivs (CC BY-NC-ND), which allows third parties to download the articles and share them with others as long as they credit the author and the Abstract Book, but they cannot change the content in any way or use them commercially.
**Abstract Book Citations:** Authors, Title, HemaSphere, 2026;10:(S1):pages. The abstract book DOIs can be found on the "Author Information" page.
**Disclaimer:** Articles published in the journal HemaSphere exclusively reflect the opinions of the authors. The authors are responsible for all content in their abstracts including accuracy of the facts, statements, citing resources, etc.

### (PB3514) SYSTEM-WIDE VARIABILITY IN SECONDARY POLYCYTHEMIA MANAGEMENT: A NATIONWIDE ENTROPY-BASED ANALYSIS

**Topic:** 16. Myeloproliferative neoplasms - Clinical

Sinan Mersin*[1], Ahmet Emre Eşkazan[2]

[1]*Mersin University Faculty of Medicine, Mersin, Türkiye;*[2]*İstanbul University Cerrahpaşa Faculty of Medicine, İstanbul, Türkiye;*

### Background

Secondary polycythemia is commonly encountered in hematology practice; however, evidence-based recommendations remain limited, resulting in heterogeneous real-world management.

### Aims

To evaluate national diagnostic and therapeutic practices in secondary polycythemia and identify determinants of variability among hematologists.

### Methods

We conducted a nationwide electronic survey among hematologists in Türkiye assessing diagnostic strategies (EPO testing, *JAK2* analyses, bone marrow biopsy), therapeutic approaches (phlebotomy, antiplatelet therapy), and follow-up responsibility. Subgroup analyses were performed by experience, institution, and workload.

### Results

A total of 144 hematologists participated, representing approximately one-third of the national hematology workforce. For differentiation of primary versus secondary polycythemia, EPO testing was widely used (59.9% in all patients; 33.8% after initial evaluation), yet subnormal cut-offs varied (median 4.0 mU/mL, IQR 4.0–5.0). *JAK2 V617F* testing was universal in 45.1% and EPO-guided in 32.4%. Bone marrow biopsy was recommended in *JAK2*-positive patients by 41.7%, whereas 20.8% considered it unnecessary. Phlebotomy strategies varied markedly, including symptom-dependent and fixed hematocrit-based approaches. Antiplatelet therapy was guided by cardiovascular risk in 63.6% and considered unnecessary in 14.7%. Diagnostic and therapeutic strategies did not differ significantly by years of experience or institutional setting, indicating systemic rather than training-related variability. High outpatient workload was associated with greater use of cardiovascular risk–based antiplatelet strategies ($p = 0.015$), possibly reflecting increased exposure to comorbid populations and pragmatic decision-making in high-volume settings. Centers where phlebotomy was managed within hematology showed a trend toward more standardized hematocrit targets ($p = 0.055$), suggesting specialty-led management may promote more objective thresholds (Table 1). Despite laboratory variability, clinical history-taking was highly consistent, with >90% systematically evaluating smoking exposure, altitude, and erythromelalgia. Quantitative dispersion analysis using normalized Shannon entropy (a measure of distributional variability) demonstrated minimal dispersion in clinical history assessment, but marked heterogeneity across laboratory and procedural domains, with the highest entropy observed in phlebotomy target selection (Figure 1).
Table-1: Main Findings.

**Copyright Information:** (Online) ISSN: 2572-9241
©2026 The Author(s). HemaSphere is published by John Wiley & Sons Ltd on behalf of European Hematology Association. This is an open access Abstract Book distributed under the Attribution-NonCommercial-NoDerivs (CC BY-NC-ND), which allows third parties to download the articles and share them with others as long as they credit the author and the Abstract Book, but they cannot change the content in any way or use them commercially.
**Abstract Book Citations:** Authors, Title, HemaSphere, 2026;10:(S1):pages. The abstract book DOIs can be found on the "Author Information" page.
**Disclaimer:** Articles published in the journal HemaSphere exclusively reflect the opinions of the authors. The authors are responsible for all content in their abstracts including accuracy of the facts, statements, citing resources, etc.

| | Key Finding | n (%) |
|---|---|---|
| Participants | ≤10 years of experience | 77 (54.0) |
| | ≥4 suspected cases/week | 70 (49.0) |
| Clinical History Assessment | Smoking | 143 (99.3) |
| | High Altitude | 133 (92.4) |
| | Erythromelalgia symptoms | 133 (92.4) |
| Diagnostic Approaches | EPO was requested for all patients | 85 (59.9) |
| | *JAK2 V617F* is independent of EPO | 64 (45.1) |
| | *JAK2 V617F* guided by EPO | 46 (32.4) |
| | Bone marrow biopsy if *JAK2+* | 60 (41.7) |
| | Bone marrow biopsy is unnecessary | 30 (20.8) |
| Therapeutic Strategies | Symptom-based phlebotomy (HCT >50%) | 34 (23.6) |
| | Phlebotomy considered unnecessary | 31 (21.5) |
| | CV risk–based antiplatelet | 91 (63.6) |
| | Antiplatelet unnecessary | 21 (14.7) |
| Care Responsibility | Follow-up by cause specific specialist | 109 (75.7) |
| | Follow-up by a hematologist | 11 (7.6) |

### Summary/Conclusion

Secondary polycythemia management exhibits substantial nationwide heterogeneity that appears systemic rather than physician-driven, highlighting a need for consensus-based diagnostic algorithms and standardized therapeutic guidance.

**Copyright Information:** (Online) ISSN: 2572-9241
©2026 The Author(s). HemaSphere is published by John Wiley & Sons Ltd on behalf of European Hematology Association. This is an open access Abstract Book distributed under the Attribution-NonCommercial-NoDerivs (CC BY-NC-ND), which allows third parties to download the articles and share them with others as long as they credit the author and the Abstract Book, but they cannot change the content in any way or use them commercially.
**Abstract Book Citations:** Authors, Title, HemaSphere, 2026;10:(S1):pages. The abstract book DOIs can be found on the "Author Information" page.
**Disclaimer:** Articles published in the journal HemaSphere exclusively reflect the opinions of the authors. The authors are responsible for all content in their abstracts including accuracy of the facts, statements, citing resources, etc.

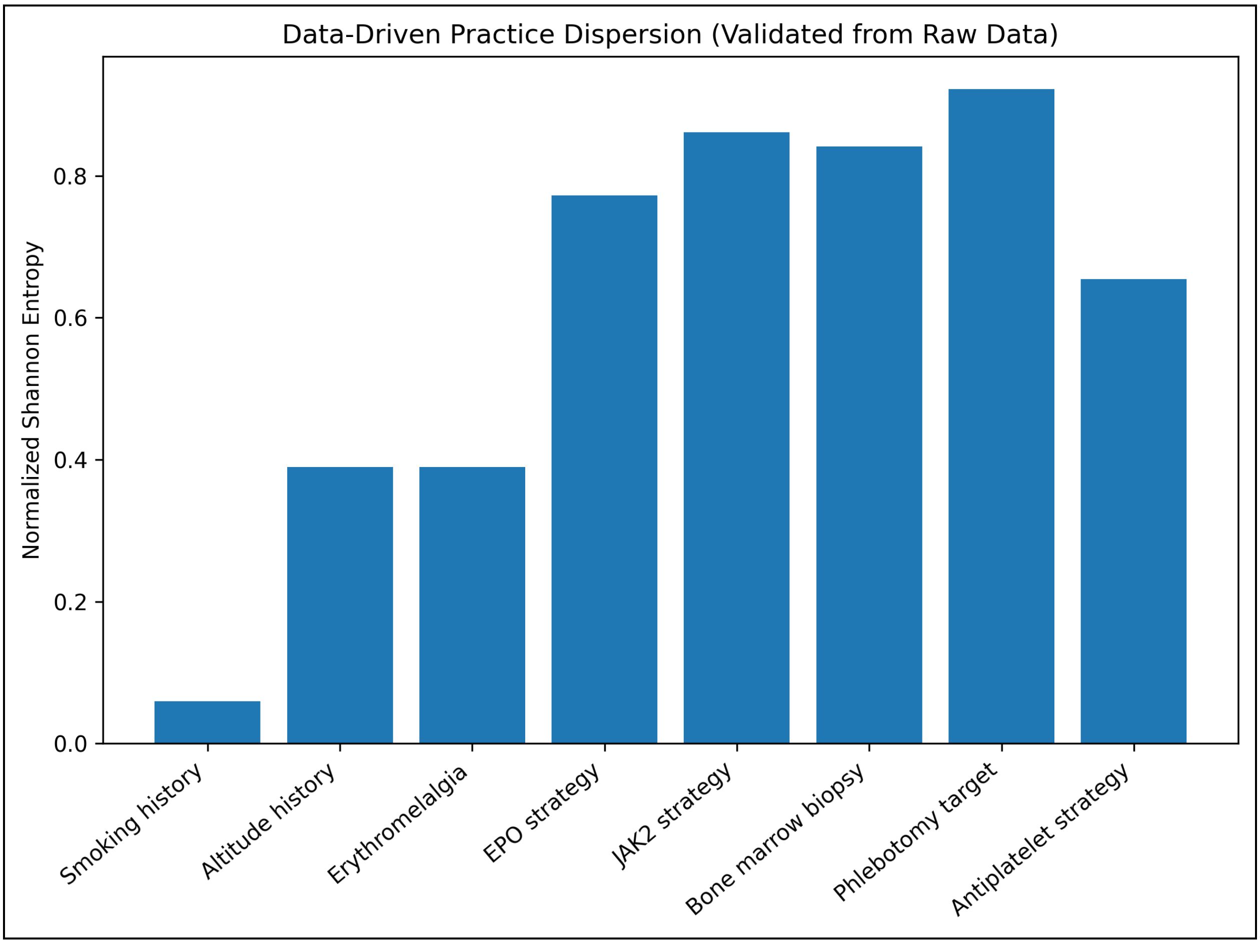

Figure 1. Quantitative Dispersion Analysis.

**Copyright Information:** (Online) ISSN: 2572-9241
©2026 The Author(s). HemaSphere is published by John Wiley & Sons Ltd on behalf of European Hematology Association. This is an open access Abstract Book distributed under the Attribution-NonCommercial-NoDerivs (CC BY-NC-ND), which allows third parties to download the articles and share them with others as long as they credit the author and the Abstract Book, but they cannot change the content in any way or use them commercially.
**Abstract Book Citations:** Authors, Title, HemaSphere, 2026;10:(S1):pages. The abstract book DOIs can be found on the "Author Information" page.
**Disclaimer:** Articles published in the journal HemaSphere exclusively reflect the opinions of the authors. The authors are responsible for all content in their abstracts including accuracy of the facts, statements, citing resources, etc.

### (PB3515) EXPLORATORY MACHINE LEARNING–BASED IDENTIFICATION OF PREDICTORS OF DISEASE PROGRESSION AND MORTALITY IN POLYCYTHEMIA VERA

**Topic:** 16. Myeloproliferative neoplasms - Clinical

Giulia Benevolo*[1], Jan Berger[2], Andrea Evangelista[2], Giuseppe Lanzarone[1], Andrea Cavallaro[3], Maria Antonella De Luca[3], Giacomo Ortone[3], Benedetto Bruno[1, 3], Eloise Beggiato[1]

*[1]Hematology U, Azienda Ospedaliero Universitaria Città della Salute e della Scienza, Turin, Italy;[2]Clinical Epidemiology U, Città della Salute e della Scienza and CPO Piemonte, Turin, Italy; [3]University of Turin, Department of Molecular Biotechnology and Health Science, Turin, Italy;*

### Background

Polycythemia vera (PV) is a chronic myeloproliferative neoplasm characterized by heterogeneous clinical trajectories, including progression to myelofibrosis (MF) and death. Identifying predictors of adverse outcomes remains challenging, particularly in real-world cohorts with limited event numbers. Machine learning (ML) approaches may help uncover clinically relevant patterns in an exploratory, hypothesis-generating setting.

### Aims

To explore clinical predictors of myelofibrosis transformation and overall survival (OS) in patients with PV using interpretable ML-based models.

### Methods

This single-center cohort study included PV patients diagnosed between 2008 and 2017 in Turin, Italy. Outcomes of interest were transformation to MF and OS. Random Survival Forest models were trained for each endpoint, with feature selection performed using the Boruta algorithm. Model discrimination was assessed using the concordance index (C-index) under repeated 5-fold cross-validation.

### Results

A total of 101 patients were included (median age at diagnosis 68 years; median follow-up 10.1 years). During follow-up, 7 MF transformations and 33 deaths were observed.
For MF transformation, spleen size at diagnosis emerged as the most influential predictor, followed by elevated lactate dehydrogenase and hematocrit levels, yielding good model discrimination (C-index 0.90).). Even presence of pre-diagnosis thrombosis, higher JAK2 allele burden and hemoglobin levels were consistently associated with higher risk of MF transformation (Figure 1).
For OS, age at diagnosis showed the strongest association, followed by leukocyte count and hematocrit, with good discrimination (C-index 0.82).

### Summary/Conclusion

In this exploratory ML-based analysis of PV patients, interpretable ensemble models identified clinically plausible predictors of MF transformation and mortality. Feature selection improved model performance across endpoints. Although limited by sample size and low event numbers, these findings support the use of explainable ML approaches to complement traditional risk stratification and to generate hypotheses for validation in larger cohorts.

**Copyright Information:** (Online) ISSN: 2572-9241
©2026 The Author(s). HemaSphere is published by John Wiley & Sons Ltd on behalf of European Hematology Association. This is an open access Abstract Book distributed under the Attribution-NonCommercial-NoDerivs (CC BY-NC-ND), which allows third parties to download the articles and share them with others as long as they credit the author and the Abstract Book, but they cannot change the content in any way or use them commercially.
**Abstract Book Citations:** Authors, Title, HemaSphere, 2026;10:(S1):pages. The abstract book DOIs can be found on the "Author Information" page.
**Disclaimer:** Articles published in the journal HemaSphere exclusively reflect the opinions of the authors. The authors are responsible for all content in their abstracts including accuracy of the facts, statements, citing resources, etc.

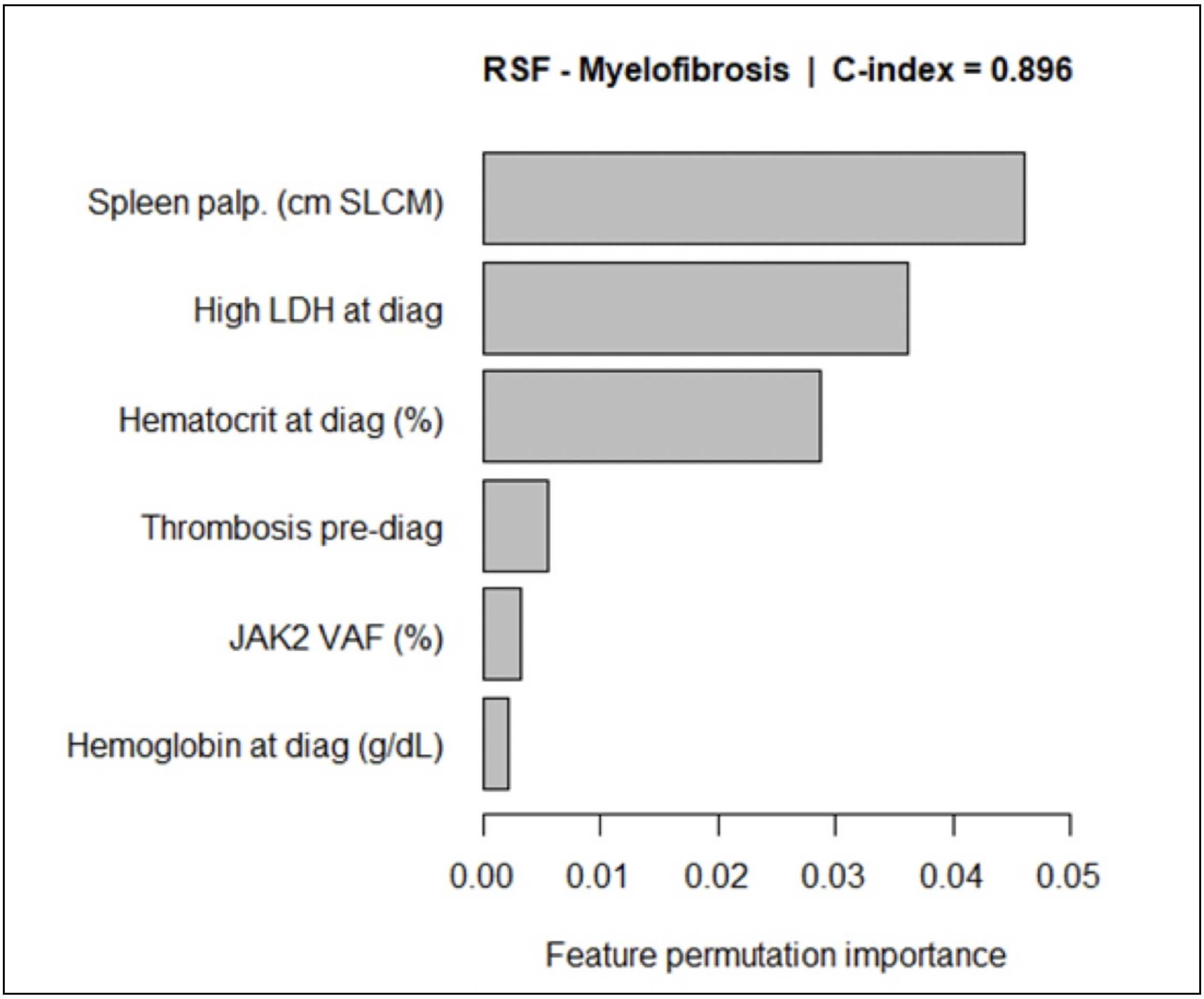

Predictors of MF transformation

**Copyright Information:** (Online) ISSN: 2572-9241
©2026 The Author(s). HemaSphere is published by John Wiley & Sons Ltd on behalf of European Hematology Association. This is an open access Abstract Book distributed under the Attribution-NonCommercial-NoDerivs (CC BY-NC-ND), which allows third parties to download the articles and share them with others as long as they credit the author and the Abstract Book, but they cannot change the content in any way or use them commercially.
**Abstract Book Citations:** Authors, Title, HemaSphere, 2026;10:(S1):pages. The abstract book DOIs can be found on the "Author Information" page.
**Disclaimer:** Articles published in the journal HemaSphere exclusively reflect the opinions of the authors. The authors are responsible for all content in their abstracts including accuracy of the facts, statements, citing resources, etc.

### (PB3516) ASSOCIATION BETWEEN JAK2V617F ALLELE BURDEN, CLINICAL FORMS, AND THROMBOEMBOLIC RISK GROUPS IN POLYCYTHEMIA VERA.

**Topic:** 16. Myeloproliferative neoplasms - Clinical

Fidan Khalilova*[1], Azer Kerimov[1], Gulnar Garayeva[1]

[1] *National Hematology and Blood Transfusion Center, Hematology, Baku, Azerbaijan;*

### Background

In some sources, the JAK2V617F mutation is considered as an independent risk factor for thrombosis during myeloproliferative neoplasms. However, the effect of the allele burden (AB) of JAK2V617F on the occurrence of thrombosis and the course and outcome of the polycythemia vera (PV) remains open.

### Aims

To investigate AB of the JAK2V617F gene mutation in overt PV (oPV) and masked PV (mPV) according to the thrombotic risk groups.

### Methods

We retrospectively analyzed 209 patients diagnosed with PV, registered between 2014 and 2020. The diagnosis was confirmed by the presence of the JAK2V617F gene and three-lineage hyperplasia of the bone marrow. An analysis of the patients general blood tests identified cases with hemoglobin (HGB) levels below the cutoff value specified in the 2008 WHO classification. As a result, we divided the patients into two clinical forms: classic and masked. Patients of each clinical form were stratified into 2 thromboembolic risk groups: low risk group (LRG) and high risk group (HRG). Comparisons between oPV and mPV patients across four AB categories (1–25%, 26–50%, 51–75%, 76–100%) were performed using two-way analysis of variance (ANOVA) with PV type and AB as factors. Distribution of JAK2 allele burden in oPV and mPV patients stratified by risk group was compared using Chi-square test of independence ($\chi^2$). Continuous data are presented as mean ± 95% confidence intervals (CI), and categorical data are reported as counts (percentages). All statistical tests were two-sided, and a p-value < 0.05 was considered statistically significant.

**Copyright Information:** (Online) ISSN: 2572-9241
©2026 The Author(s). HemaSphere is published by John Wiley & Sons Ltd on behalf of European Hematology Association. This is an open access Abstract Book distributed under the Attribution-NonCommercial-NoDerivs (CC BY-NC-ND), which allows third parties to download the articles and share them with others as long as they credit the author and the Abstract Book, but they cannot change the content in any way or use them commercially.
**Abstract Book Citations:** Authors, Title, HemaSphere, 2026;10:(S1):pages. The abstract book DOIs can be found on the "Author Information" page.
**Disclaimer:** Articles published in the journal HemaSphere exclusively reflect the opinions of the authors. The authors are responsible for all content in their abstracts including accuracy of the facts, statements, citing resources, etc.

### Results

209 PV  patients were divided into 2 clinical forms: overt and masked. oPV was detected in 136 patients (65.1%), mPV in 73 patients (34.9%). The AB of the JAK2V617F mutation was compared in 136 oPV and 73 mPV patients. The AB in oPV was (mean ± 95% CI) 53.4 ± 3.5, and in mPV- 34.9 ± 5.3 ($p < 0.001$). The AB of the JAK2V617F mutation found in patients in oPV and mPV was divided into groups by quartiles compared according to the thrombotic risk groups. The JAK2V617F AB differed significantly between overt oPV and mPV patients within the LRG ($p = 0.001$, $\chi^2$ test). In contrast, no significant differences were observed in the HRG across AB categories ( $p = 0.24$). Within LRG, higher ABs (26–100%) were more frequent in oPV than mPV, whereas lower burdens (1–25%) predominated in mPV. In HRG, the distribution of AB was similar between groups, with no statistically significant differences (Table 1).

**Table 3.** Distribution of PV patients by AB and thrombotic risk groups.

| Risk Group | Allele Burden (%) | oPV n=136 (100%) | mPV n=73(100%) | p-value ($\chi^2$) |
|---|---|---|---|---|
| LRG | 1–25 | 6 (4.4%) | 17 (23.3%) | 0.001 |
| | 26–50 | 25 (18.4%) | 16 (21.9%) | ___ |
| | 51–75 | 21 (15.4%) | 4 (5.5%) | ___ |
| | 76–100 | 10 (7.4%) | 1 (1.4%) | ___ |
| HRG | 1–25 | 4 (3%) | 8 (9.9%) | 0.24 |
| | 26–50 | 38 (27.9%) | 15 (20.5%) | ___ |
| | 51–75 | 19 (14%) | 8 (11%) | ___ |
| | 76–100 | 13 (9.5%) | 4 (5.5%) | ___ |

AB-allele burden. HRG-high risk group. LRG-low risk group. mPV-masked polycythemia vera. oPV-overt polycythemia vera.

### Summary/Conclusion oPV is associated with a significantly higher JAK2V617F AB compared with mPV. A distinct distribution pattern of AB was observed in the LRG, where higher AB were more common in oPV, while mPV was characterized by predominantly lower ABs. In contrast, no significant differences were detected within the HRG. These findings suggest that JAK2V617F AB may be associated with clinical heterogeneity in PV; however, further prospective studies are required to clarify its independent prognostic and clinical significance.

**Copyright Information:** (Online) ISSN: 2572-9241
©2026 The Author(s). HemaSphere is published by John Wiley & Sons Ltd on behalf of European Hematology Association. This is an open access Abstract Book distributed under the Attribution-NonCommercial-NoDerivs (CC BY-NC-ND), which allows third parties to download the articles and share them with others as long as they credit the author and the Abstract Book, but they cannot change the content in any way or use them commercially.
**Abstract Book Citations:** Authors, Title, HemaSphere, 2026;10:(S1):pages. The abstract book DOIs can be found on the "Author Information" page.
**Disclaimer:** Articles published in the journal HemaSphere exclusively reflect the opinions of the authors. The authors are responsible for all content in their abstracts including accuracy of the facts, statements, citing resources, etc.

### (PB3517) REAL LIFE OUTCOME OF MOMELOTINIB IN PATIENTS WITH PRIMARY OR SECONDARY MYELOFIBROSIS: PRELIMINARY RESULTS OF A RETROSPECTIVE EXPERIENCE OF TWO PUGLIA CENTERS

**Topic:** 16. Myeloproliferative neoplasms - Clinical

Domenico D'agostino[1], Silvia Sibilla[1], Teresa Maria Santeramo[2], Vera Carluccio[2], Stefania Citiso[1], Giuseppina Greco[1], Rosa De Francesco[1], Francesca Donatelli[1], Nicoletta Pizzileo[1], Francesco Aji[1], Guido Volpe[1], Francesca Anna Ciardo De Marti[1], Maria Rosaria Morciano[1], Antonella Orlando[1], Vincenzo Pavone[1], Giuseppe Tarantini[2], Anna Mele*[1]

*[1]Cardinale G.Panico Hospital, Hematology and Bone Marrow Transplant, TRICASE, Italy; [2]Mons. Dimiccoli Hospital, Hematology, Barletta, Italy;*

### Background

Momelotinib is JAK1/2 and ACVR1 pathway inhibitor. This molecule favours haematopoiesis, reducing hepcidin and increasing iron availability in such a way as to offer a specific advantage on anaemia beyond splenic and symptomatic control. SIMPLIFY 1/2 e MOMENTUM trial confirm transfusion independence (TI) in 30-80% of patients with greater results if momelotinib is used during moderate anaemia (Hb 8-10 g/dL). TI goal is linked with a better quality of life[4–6].

### Aims

In this retrospective analysis, the efficacy and safety of momelotinib as first or >= second line treatment was evaluated in a real word setting of patients with primary or secondary myelofibrosis (MF).

### Methods

This study included MF patient treated with momelotinib between From April 2025 to February 2026 at two Puglia centers. Baseline disease characteristics, prior therapies transfusion status, hematologic response and adverse events were collected. Blood tests, were analyzed at +3, +6 and +9 months after momelotinib therapy.

### Results

Thirty one MF patients with >= moderate anemia were enrolled. Diagnoses were primary MF (17- 55%), post essential thrombocythemia MF (11) and MF after MDS/MPN (3). Molecular profiling revealed JAK2 mutation in 14 patients (45%). Ten patients (32%) were triple negative. According to prognosis score, 8 (26%) patients had >= intermediate-2 DIPSS; 4 (13%) patients had >= Intermediate-2 MYSEC, and 4 (13%) had >= intermediate-2 MIPSS70. Median age was 76 years.
In our cohort, 9 (29%) patients received momelotinib in first line. Momelotinib was used as in >=2 second line in 22 (71%) cases, of whom 10 in third line. A majority of patients (85%) had received ruxolitinib as prior therapy. A total of 24 patients received exogenous erytropoietin. Hydroxyurea and deferasirox were added in case of leucocytosis or hyperferritinemia, respectively. Furthermore, 38% (12/31) of patients were transfusion dependent at baseline.
Following treatment initiation, the hemoglobin levels increased >=1,8g/dl in more than 40% of patients (13/31). In first line, 3 patients (33%) improved anemia earlier with a median of 3 months. In second line, 8 patients (66%) experienced anaemia improvement with a median of 6 months. In third line, improvement of anemia was observed in 2 (20%) patients with a median of 6 months.
Trasfusion independence (TI) was achieved in 75% of patients with median of 6 months. The JAKi-naïve patients achieved TI early at 3 months (5 of 9 patients).
Momelotinib was discontinued in 12% of patients after a median duration of 6,1 months. Among the 4 patients who discontinued, reasons included progression in leukemia (2), diverticulosis (1) and intolerance (1). One patients died for viral meningencefalite.

**Copyright Information:** (Online) ISSN: 2572-9241
©2026 The Author(s). HemaSphere is published by John Wiley & Sons Ltd on behalf of European Hematology Association. This is an open access Abstract Book distributed under the Attribution-NonCommercial-NoDerivs (CC BY-NC-ND), which allows third parties to download the articles and share them with others as long as they credit the author and the Abstract Book, but they cannot change the content in any way or use them commercially.
**Abstract Book Citations:** Authors, Title, HemaSphere, 2026;10:(S1):pages. The abstract book DOIs can be found on the "Author Information" page.
**Disclaimer:** Articles published in the journal HemaSphere exclusively reflect the opinions of the authors. The authors are responsible for all content in their abstracts including accuracy of the facts, statements, citing resources, etc.

**Summary/Conclusion**

This real world experience is limited by short follow-up duration. Nonetheless, our results support that momelotinib is an effective and well-tolerated treatment for MF patients in routine clinical practice, demonstrating efficacy in both JAK-naïve or JAK-exposed patients with >= moderate anemia.

**Copyright Information:** (Online) ISSN: 2572-9241
©2026 The Author(s). HemaSphere is published by John Wiley & Sons Ltd on behalf of European Hematology Association. This is an open access Abstract Book distributed under the Attribution-NonCommercial-NoDerivs (CC BY-NC-ND), which allows third parties to download the articles and share them with others as long as they credit the author and the Abstract Book, but they cannot change the content in any way or use them commercially.
**Abstract Book Citations:** Authors, Title, HemaSphere, 2026;10:(S1):pages. The abstract book DOIs can be found on the "Author Information" page.
**Disclaimer:** Articles published in the journal HemaSphere exclusively reflect the opinions of the authors. The authors are responsible for all content in their abstracts including accuracy of the facts, statements, citing resources, etc.

### (PB3520) EFFECTS OF LONG-TERM HYDROXYUREA THERAPY ON BONE MARROW MEGAKARYOCYTE MORPHOLOGY AND ITS CORRELATION WITH PROGNOSIS IN PATIENTS WITH ESSENTIAL THROMBOCYTHEMIA AND POLYCYTHEMIA VERA

**Topic:** 16. Myeloproliferative neoplasms - Clinical

Zhikang Zheng[1, 1], Tian Zeng[1], Rumeng Li[1], Qinmei Han[1], Jia Li[1], Xiaojing Bao[1], Bingbing Wen[1], Xiaoyan MA[1], Yingqing Xu[1], Jie Jin[1], Jian Huang*[1]

*[1]The First Affiliated Hospital, Zhejiang University School of Medicine, Hangzhou, China;*

### Background

Essential Thrombocythemia（ET）and Polycythemia Vera（PV） are hematological malignancies driven by abnormal bone marrow hematopoietic cell proliferation, with hydroxyurea(HU) as first-line therapies. However, their effects on megakaryocyte (MK) morphology and prognostic relevance remain unclear.

### Aims

To analyze MK morphology in patients with ET/PV, investigate the impact of long-term HU therapy on disease progression, and clarify its association with myelofibrosis (MF) and leukemic transformation, thereby providing evidence for clinical monitoring and treatment optimization.

### Methods

A retrospective analysis was conducted on bone marrow smears from 335 patients with ET (n=192) and PV (n=143) treated at the First Affiliated Hospital, Zhejiang University School of Medicine, and the Fourth Affiliated Hospital, Zhejiang University School of Medicine, between October 2014 and December 2025. A total of 16,965 MKs in ET patients and 9,675 MKs in PV patients were evaluated. MKs were classified into 12 morphological types according to WHO criteria. Patients in both groups were categorized based on HU therapy duration: no cytoreductive therapy (control group), HU therapy for <5 years, 5-10 years, and ≥10 years. The Mann-Whitney U test was used to compare median percentages of MK morphological types between groups.

### Results

In patients with ET, the proportions of normal MK and emperipolesis MK were decreased in the hydroxyurea group compared with the control group (P=0.0003; P=0.0058), while the proportions of naked nuclei, small, bulbous, separated nuclear, and non-lobated MK were increased (P=0.0396; P=0.0026; P<0.0001; P<0.0001; P=0.0118); compared with HU treatment for <5 years, the proportion of hyperchromatic MK increased from 1% to 3.5% in patients treated for 5–10 years (P=0.0135), and compared with treatment for 5–10 years, the proportion of naked nuclei MK increased from 4% to 7% in those treated for ≥10 years (P=0.0351). In patients with polycythemia vera (PV), the proportions of normal MK, emperipolesis MK, and small MK were decreased in the hydroxyurea group versus the control group (P=0.0018; P=0.0182; P=0.0171), whereas the proportions of naked nuclei, bulbous, and separated nuclear MK were increased (P=0.0106; P=0.0261; P<0.0001); HU was associated with reduced normal MK and emperipolesis MK and increased naked nuclei, bulbous, and separated nuclear MK, consistent with the findings in ET, and furthermore, prolonged HU treatment did not alter MK morphology in PV patients. Previous studies have reported that naked nuclei, bulbous, separated nuclear, and non-lobated MK are associated with MF and correlated with shorter progression-free survival (PFS), indicating a poor prognosis.

**Copyright Information:** (Online) ISSN: 2572-9241
©2026 The Author(s). HemaSphere is published by John Wiley & Sons Ltd on behalf of European Hematology Association. This is an open access Abstract Book distributed under the Attribution-NonCommercial-NoDerivs (CC BY-NC-ND), which allows third parties to download the articles and share them with others as long as they credit the author and the Abstract Book, but they cannot change the content in any way or use them commercially.
**Abstract Book Citations:** Authors, Title, HemaSphere, 2026;10:(S1):pages. The abstract book DOIs can be found on the “Author Information” page.
**Disclaimer:** Articles published in the journal HemaSphere exclusively reflect the opinions of the authors. The authors are responsible for all content in their abstracts including accuracy of the facts, statements, citing resources, etc.

**Summary/Conclusion**

In ET patients, HU therapy is associated with an increase in MKs with high-risk morphological features for MF, and the percentage of Naked nuclei MKs increases with prolonged treatment duration, particularly in patients treated for ≥10 years. Similar findings in PV patients corroborate the association between HU therapy and the emergence of naked nuclei, bulbous, and separated nuclear MK. Long-term hydroxyurea treatment can lead to an increase in these MKs, increasing the risk of PV/ET converting to MF.

1.This research was supported by the National Natural Science Foundation of China (No. 82570191) and the Zhejiang Provincial Health Highlevel Innovative Talent Project (2022-2026).
2.† Correspondence to: Prof Jian Huang, E-mail:househuang@zju.edu.cn

**Copyright Information:** (Online) ISSN: 2572-9241
©2026 The Author(s). HemaSphere is published by John Wiley & Sons Ltd on behalf of European Hematology Association. This is an open access Abstract Book distributed under the Attribution-NonCommercial-NoDerivs (CC BY-NC-ND), which allows third parties to download the articles and share them with others as long as they credit the author and the Abstract Book, but they cannot change the content in any way or use them commercially.
**Abstract Book Citations:** Authors, Title, HemaSphere, 2026;10:(S1):pages. The abstract book DOIs can be found on the "Author Information" page.
**Disclaimer:** Articles published in the journal HemaSphere exclusively reflect the opinions of the authors. The authors are responsible for all content in their abstracts including accuracy of the facts, statements, citing resources, etc.

### (PB3522) IMPACT OF BONE RESORPTIVE INHIBITORS ON DISEASE, SKELETAL, AND TREATMENT-RELATED OUTCOMES IN HISTIOCYTIC DISORDERS

**Topic:** 16. Myeloproliferative neoplasms - Clinical

Katherine Chapis*[1], Jenna Mahns[1], Daniel Sullivan[1], Mark Sorial[1], Eric D. Jacobsen[1]

[1]*Dana-Farber Cancer Institute, Boston, United States of America;*

### Background

Histiocytic disorders (HD) are rare diseases of myeloid derivation. Bone involvement, including lytic lesions in the jaw, skull, vertebrae, extremities and other bones is a significant cause of morbidity. Uniform treatment strategies have not been defined and can include surgery, radiotherapy, or systemic therapy depending on the size, location, and/or extent of lesions. Case series and small studies have suggested that bisphosphonates may improve bone-specific disease outcomes but the efficacy and optimal duration of therapy with bisphosphonates and denosumab remain to be defined.

### Aims

To describe the effects of bisphosphonates or denosumab on efficacy and safety-related outcomes in patients with histiocytic disorders.

### Methods

This was a retrospective cohort study of adult patients with HD affecting bone managed at a tertiary academic medical center and received denosumab or an intravenous bisphosphonate between 2015-2025. Patients with hemophagocytic lymphohistiocytosis and histiocytic sarcoma were excluded. Endpoints included radiologic disease response, progression-free (PFS) and overall survival (OS) from the start of bone modifier, and rates of skeletal fractures and osteonecrosis. Time was measured until death (OS), radiologic progression, or start of next HD-directed therapy (PFS). Chi-squared, Fisher's exact, and Kaplan-Meier with log-rank methods were used where applicable.

### Results

A total of 9 patients were identified and included in analyses. Median age was 44 (range 20-66) years, 6 (66.7%) were male, 8 (88.9%) had Langerhans histiocytosis (LCH), and 1 (11.1%) had Rosai Dorfman Disease. No patients had baseline osteoporosis or concurrent malignancies. Two (22.2%) patients received denosumab and 7 (77.8%) received zoledronic acid. Seven (77.8%) received bone modifying therapy as first line, 1 (11.1%) received it in second line, and 1 (11.1%) received it in fourth line. One (11.1%) patient received concurrent definitive surgery, and 2 (22.2%) received concurrent radiation therapy.The median (95%CI) duration of bone modifier therapy was 5.7 (4.8-6.5) months with a range of 1.9-35.3 months. Eight (88.9%) patients achieved an overall response (OR; complete [CR]+partial [PR] response). Six (66.7%) patients achieved a CR, of which 2 (22.2%) converted to a CR with continued bone modifier therapy (1 with PR and 1 with SD initially, Figure 1). One (11.1%) converted from an initial SD to PR. Median (95%CI) time to best CR and OR was 4.8 (2-7.7) and 3.8 (3.5-4.1) months, respectively. After a median (range) follow up of 15.4 (5.3-97) months, 1 (11.1%) patient had SD to zoledronic acid, and 2 (22.2%) patients (1 with denosumab and 1 with zoledronic acid) had recurrent disease (median PFS of 88.9 months). No patients died during follow up. There were no occurrences of fracture or osteonecrosis during or after therapy with a bone modifier.

**Copyright Information:** (Online) ISSN: 2572-9241
©2026 The Author(s). HemaSphere is published by John Wiley & Sons Ltd on behalf of European Hematology Association. This is an open access Abstract Book distributed under the Attribution-NonCommercial-NoDerivs (CC BY-NC-ND), which allows third parties to download the articles and share them with others as long as they credit the author and the Abstract Book, but they cannot change the content in any way or use them commercially.
**Abstract Book Citations:** Authors, Title, HemaSphere, 2026;10:(S1):pages. The abstract book DOIs can be found on the "Author Information" page.
**Disclaimer:** Articles published in the journal HemaSphere exclusively reflect the opinions of the authors. The authors are responsible for all content in their abstracts including accuracy of the facts, statements, citing resources, etc.

### Summary/Conclusion

Among this cohort of patients with histiocytic disorders involving the bone receiving denosumab or zoledronic acid, disease response rates, PFS, and OS were high with short courses of bone modifying therapy. No cases of osteonecrosis were reported, and no fractures were seen during follow up, including in patients with recurrent disease, representing potentially long-term protection against skeletal-related events. Bone resorptive inhibitors should be considered and more work is needed to define their full benefit across different bone modifying drugs or HD histological subtypes.

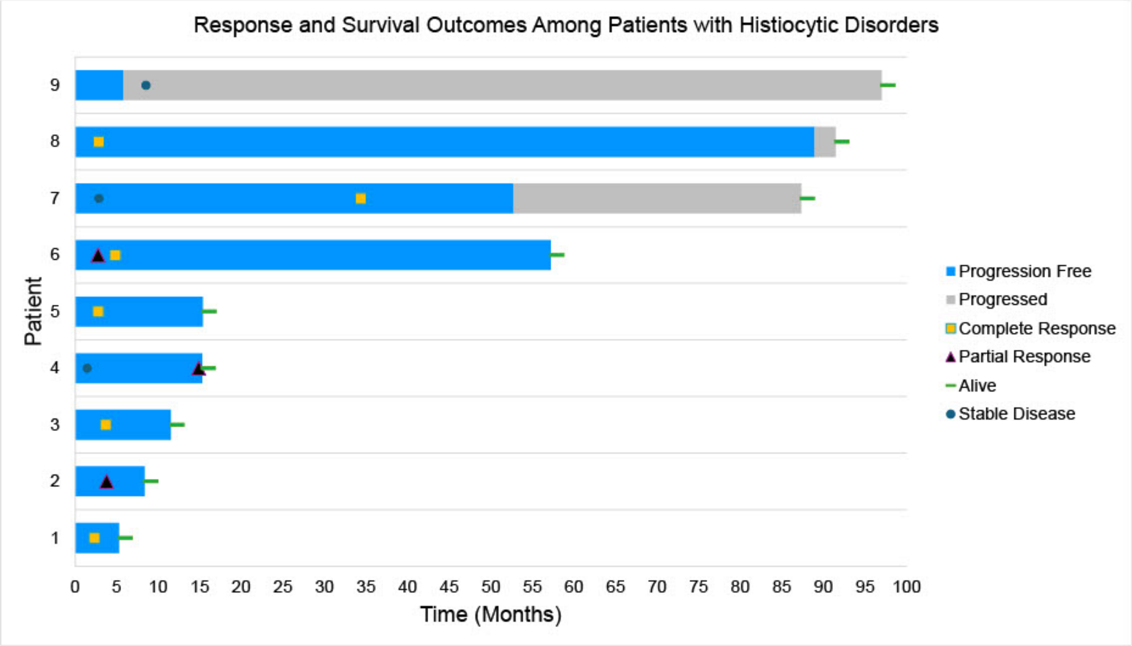

Response and Survival Outcomes Among Patients with Histiocytic Disorders

Copyright Information: (Online) ISSN: 2572-9241
©2026 The Author(s). HemaSphere is published by John Wiley & Sons Ltd on behalf of European Hematology Association. This is an open access Abstract Book distributed under the Attribution-NonCommercial-NoDerivs (CC BY-NC-ND), which allows third parties to download the articles and share them with others as long as they credit the author and the Abstract Book, but they cannot change the content in any way or use them commercially.
Abstract Book Citations: Authors, Title, HemaSphere, 2026;10:(S1):pages. The abstract book DOIs can be found on the "Author Information" page.
Disclaimer: Articles published in the journal HemaSphere exclusively reflect the opinions of the authors. The authors are responsible for all content in their abstracts including accuracy of the facts, statements, citing resources, etc.

### (PB3523) CLINICAL AND MOLECULAR CHARACTERISTICS AND PROGNOSTIC ANALYSIS OF ADULT POLYCYTHEMIA VERA: A MULTI-CENTER COHORT STUDY

**Topic:** 16. Myeloproliferative neoplasms - Clinical

Jia Li[1], Yanxia Han[2], Honglan Qian[3], Xiao Yan[4], Jie Jin[1], Hongyan Tong[1], Jian Huang*[1]

*[1]The First Affiliated Hospital, Zhejiang University School of Medicine, Hangzhou, China;[2]The Second Hospital of Jiaxing, Jiaxing, China; [3]The First Affiliated Hospital of Wenzhou Medical University, Wenzhou, China;[4]Department of Hematology, The First Affiliated Hospital of Ningbo University, Ningbo, China;*

### Background

Polycythemia vera (PV) is a myeloproliferative neoplasm characterized by erythroid proliferation, frequently accompanied by thrombotic events and the risk of transformation to myelofibrosis (MF) or acute leukemia. Systemic analyses based on large cohorts delineating the clinical and molecular characteristics and prognostic data of patients with PV are currently lacking in China.

### Aims

To investigate the clinical and molecular characteristics, prognosis, and prognostic factors in patients with PV.

### Methods

Clinical data were retrospectively collected from 773 adult patients diagnosed with PV across 25 medical centers in Zhejiang Province. Bone marrow samples from 142 patients were subjected to next-generation sequencing (NGS) targeting hematologic malignancy-related genes. The primary endpoints assessed were overall survival (OS), thrombosis-free survival (TFS), myelofibrosis-free survival (MFS), and progression-free survival (PFS).

### Results

The median age at diagnosis was 60 years, with males comprising 60.93% of the cohort. Splenomegaly was present in 44.1% of patients, and 22.78% exhibited MF-1 grade myelofibrosis. The mutational frequencies were 91.85% for *JAK2 V617F*, 1.68% for *JAK2* exon 12 mutations, while 6.47% were *JAK2* mutation-negative with low erythropoietin (EPO) levels. NGS analysis identified *TET2*, *KMT2C*, and *ASXL1* as the most prevalent non-driver gene mutations, with epigenetic regulator genes constituting the most frequently mutated category (64.08%). Patients harboring *JAK2* mutations demonstrated a significantly higher median number of gene mutations compared to JAK2 mutation-negative patients with low EPO (2.0 vs. 1.0, $P$=0.007). Over a median follow-up of 61 months, the overall mortality rate, post-diagnostic thrombosis incidence, and myelofibrosis transformation rate were 1.55%, 6.59%, and 9.31%, respectively. The 10-year OS, TFS, MFS, and PFS rates were 98.4%, 91.0%, 81.7%, and 79.9%, respectively. Multivariate analysis confirmed that a history of thrombosis (HR 4.93, 95% CI 1.39-17.46, $P$=0.014) and increasing age (HR 1.11, 95% CI 1.03-1.20, $P$=0.008) were independent risk factors for inferior OS.

**Copyright Information:** (Online) ISSN: 2572-9241
©2026 The Author(s). HemaSphere is published by John Wiley & Sons Ltd on behalf of European Hematology Association. This is an open access Abstract Book distributed under the Attribution-NonCommercial-NoDerivs (CC BY-NC-ND), which allows third parties to download the articles and share them with others as long as they credit the author and the Abstract Book, but they cannot change the content in any way or use them commercially.
**Abstract Book Citations:** Authors, Title, HemaSphere, 2026;10:(S1):pages. The abstract book DOIs can be found on the "Author Information" page.
**Disclaimer:** Articles published in the journal HemaSphere exclusively reflect the opinions of the authors. The authors are responsible for all content in their abstracts including accuracy of the facts, statements, citing resources, etc.

### Summary/Conclusion

This study systematically delineates the clinical and molecular landscape of PV. A history of thrombosis and advanced age are identified as independent adverse prognostic factors for overall survival. The high frequency of epigenetic regulator gene mutations, coupled with an increased mutational burden in *JAK2* mutation-positive patients, constitutes a significant basis for disease heterogeneity in PV.

This research was supported by the National Natural Science Foundation of China (No. 82570191) and the Zhejiang Provincial Health High level Innovative Talent Project (2022-2026).

Correspondence to: Prof Jian Huang, E-mail: househuang@zju.edu.cn.

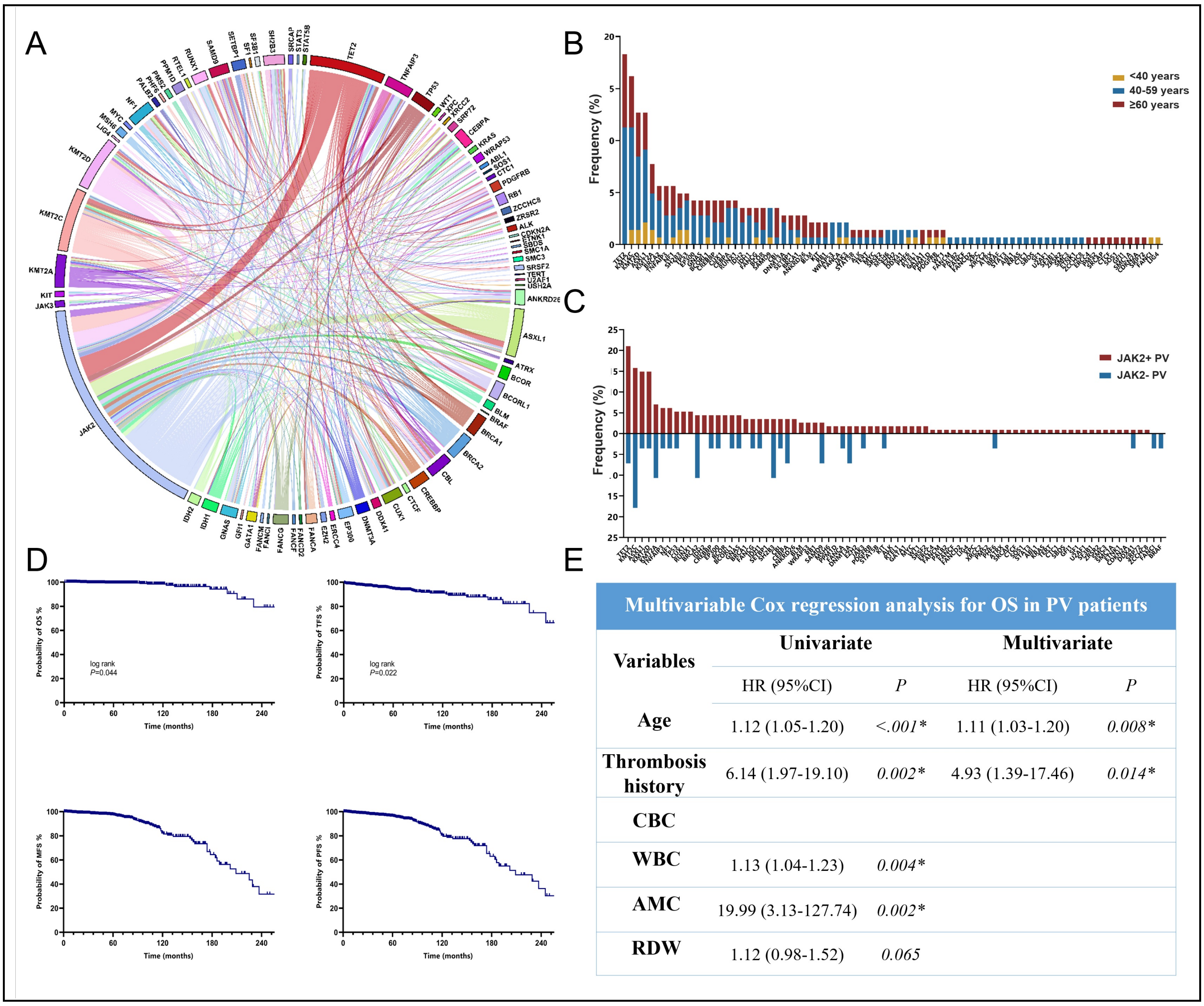

| Multivariable Cox regression analysis for OS in PV patients | | | | |
|---|---|---|---|---|
| Variables | Univariate | | Multivariate | |
| | HR (95%CI) | *P* | HR (95%CI) | *P* |
| Age | 1.12 (1.05-1.20) | *<.001** | 1.11 (1.03-1.20) | *0.008** |
| Thrombosis history | 6.14 (1.97-19.10) | *0.002** | 4.93 (1.39-17.46) | *0.014** |
| CBC | | | | |
| WBC | 1.13 (1.04-1.23) | *0.004** | | |
| AMC | 19.99 (3.13-127.74) | *0.002** | | |
| RDW | 1.12 (0.98-1.52) | *0.065* | | |

Figure 1. (A) Co-occurrence of 79 hematologic gene mutations in 142 patients with PV. (B) Landscape of hematologic gene mutations in 142 patients with PV. (C) Distribution of hematologic gene mutations in patients with PV stratified by JAK2 mutation status. (D) Prognostic outcomes of patients with PV. (E) Multivariable Cox regression analysis of factors influencing overall survival (OS) in patients with PV.

**Copyright Information:** (Online) ISSN: 2572-9241
©2026 The Author(s). HemaSphere is published by John Wiley & Sons Ltd on behalf of European Hematology Association. This is an open access Abstract Book distributed under the Attribution-NonCommercial-NoDerivs (CC BY-NC-ND), which allows third parties to download the articles and share them with others as long as they credit the author and the Abstract Book, but they cannot change the content in any way or use them commercially.
**Abstract Book Citations:** Authors, Title, HemaSphere, 2026;10:(S1):pages. The abstract book DOIs can be found on the "Author Information" page.
**Disclaimer:** Articles published in the journal HemaSphere exclusively reflect the opinions of the authors. The authors are responsible for all content in their abstracts including accuracy of the facts, statements, citing resources, etc.

### (PB3524) AGE AS A PROGNOSTIC FACTOR IN ADULT POLYCYTHEMIA VERA: A MULTICENTER REAL-WORLD STUDY

**Topic:** 16. Myeloproliferative neoplasms - Clinical

Jia Li[1], Yanxia Han[2], Honglan Qian[3], Xiao Yan[4], Jie Jin[1], Hongyan Tong[1], Jian Huang*[1]

[1]*The First Affiliated Hospital, Zhejiang University School of Medicine, Hangzhou, China;*[2]*The Second Hospital of Jiaxing, Jiaxing, China;* [3]*The First Affiliated Hospital of Wenzhou Medical University, Wenzhou, China;*[4]*Department of Hematology, The First Affiliated Hospital of Ningbo University, Ningbo, China;*

### Background

Polycythemia vera (PV) predominantly affects middle-aged and elderly individuals, with young patients accounting for less than 8% of cases. These patients exhibit distinct clinical characteristics and face long-term exposure to disease- and treatment-related complications. Data on age-specific characteristics of PV in Asian populations remain limited.

### Aims

To investigate the clinical and molecular characteristics, prognostic differences, and influencing factors in adult patients with PV across different age groups.

### Methods

Clinical data from 773 adult patients with PV across 25 centers were retrospectively collected and stratified into three groups: young (<40 years, n=57), middle-aged (40-59 years, n=316), and elderly (≥60 years, n=400). Baseline characteristics and outcomes were compared, and age-stratified prognostic factor analyses were performed using Cox models.

### Results

Compared to young patients, middle-aged and elderly patients had a higher proportion of constitutional symptoms. The elderly group exhibited significantly higher rates of WBC ≥$11\times10^9$/L, elevated ANC, and LDH levels compared to younger groups (all $P<0.05$), with RDW increasing progressively with age. JAK2 V617F mutation positivity (70.18% vs. 90.82% vs. 95.75%) and median VAF increased significantly with age. The 10-year OS rates for the three groups were 100%, 99.0%, and 97.6%, respectively ($P=0.044$); 10-year TFS rates were 96.7%, 93.5%, and 88.6%, respectively ($P=0.022$). No significant differences were observed in MFS or PFS among groups. In patients aged <60 years, thrombosis history (HR=7.81) and increasing age (HR=1.13) were independent risk factors for TFS, while splenomegaly was the primary factor affecting MFS (HR=3.23). In patients aged ≥60 years, thrombosis history was an independent risk factor for both OS (HR=10.42) and TFS (HR=2.34), while MF-1 grade was independently associated with inferior MFS (HR=6.05). For disease progression, independent risk factors were splenomegaly and abnormal karyotype in patients <60 years, versus increasing age and elevated RDW in patients ≥60 years.

**Copyright Information:** (Online) ISSN: 2572-9241
©2026 The Author(s). HemaSphere is published by John Wiley & Sons Ltd on behalf of European Hematology Association. This is an open access Abstract Book distributed under the Attribution-NonCommercial-NoDerivs (CC BY-NC-ND), which allows third parties to download the articles and share them with others as long as they credit the author and the Abstract Book, but they cannot change the content in any way or use them commercially.
**Abstract Book Citations:** Authors, Title, HemaSphere, 2026;10:(S1):pages. The abstract book DOIs can be found on the “Author Information” page.
**Disclaimer:** Articles published in the journal HemaSphere exclusively reflect the opinions of the authors. The authors are responsible for all content in their abstracts including accuracy of the facts, statements, citing resources, etc.

### Summary/Conclusion

PV exhibits significant age-related heterogeneity in clinical and molecular characteristics and prognosis. Thrombosis history is a central factor influencing survival, particularly in elderly patients. Splenomegaly serves as a key predictor of disease progression in younger patients. These findings support age- and risk-oriented individualized management.

This research was supported by the National Natural Science Foundation of China (No. 82570191) and the Zhejiang Provincial Health High level Innovative Talent Project (2022-2026).

Correspondence to: Prof Jian Huang, E-mail: househuang@zju.edu.cn.

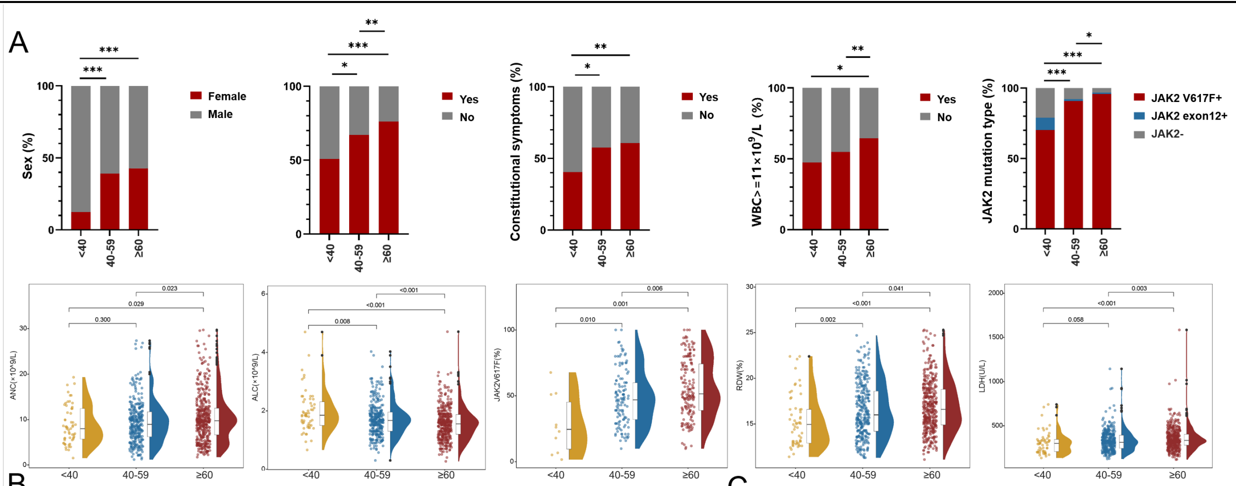

B

| Variables | TFS | | | MFS | | | PFS | | |
|---|---|---|---|---|---|---|---|---|---|
| | Univariate | Multivariate | | Univariate | Multivariate | | Univariate | Multivariate | |
| | P | HR (95%CI) | P | P | HR (95%CI) | P | P | HR (95%CI) | P |
| **Male** | 0.097 | 0.48 (0.18-1.30) | 0.148 | 0.456 | | | 0.638 | | |
| **Age** | 0.007* | 1.13 (1.01-1.27) | 0.042* | 0.044* | 1.04 (0.98-1.10) | 0.19 | 0.040* | 1.03 (0.98-1.09) | 0.269 |
| **Splenomegaly** | 0.995 | | | 0.002* | 3.23 (1.36-7.65) | 0.008* | 0.006* | 3.16 (1.25-7.97) | 0.015* |
| **Constitutional symptoms** | 0.505 | | | 0.099 | 1.50 (0.67-3.38) | 0.324 | 0.211 | | |
| **Thrombosis history** | <.001* | 7.81 (2.76-22.08) | <.001* | 0.601 | | | 0.688 | | |
| **CVR** | 0.942 | | | 0.105 | | | 0.176 | | |
| **MF-1 grade** | 0.165 | | | 0.163 | | | 0.246 | | |
| **Abnormal karyotype** | 0.338 | | | 0.158 | | | 0.024* | 4.44 (1.29-15.28) | 0.018* |
| **LDH** | 0.662 | | | 0.020* | 1.00 (1.00-1.00) | 0.391 | 0.015* | 1.00 (1.00-1.00) | 0.471 |
| **CBC** | | | | | | | | | |
| **WBC** | 0.608 | | | 0.249 | | | 0.154 | | |
| **AMC** | 0.573 | | | 0.169 | | | 0.204 | | |
| **HB** | 0.936 | | | 0.147 | | | 0.108 | | |
| **RDW** | 0.454 | | | 0.016* | 1.06 (0.93-1.20) | 0.415 | 0.033* | 1.00 (0.87-1.15) | 0.955 |
| **PLT** | 0.735 | | | 0.991 | | | 0.909 | | |

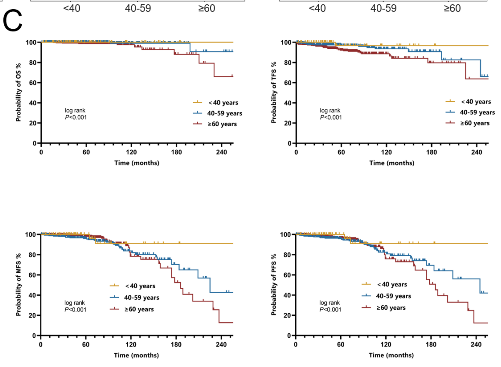

Figure 1. (A) Clinical, laboratory characteristics and driver gene differences in PV patients stratified by age. (B) Analysis of factors influencing different prognostic outcomes in patients aged <60 years (N=373). (C) Differences in prognostic outcomes among PV patients stratified by age.

**Copyright Information:** (Online) ISSN: 2572-9241
©2026 The Author(s). HemaSphere is published by John Wiley & Sons Ltd on behalf of European Hematology Association. This is an open access Abstract Book distributed under the Attribution-NonCommercial-NoDerivs (CC BY-NC-ND), which allows third parties to download the articles and share them with others as long as they credit the author and the Abstract Book, but they cannot change the content in any way or use them commercially.
**Abstract Book Citations:** Authors, Title, HemaSphere, 2026;10:(S1):pages. The abstract book DOIs can be found on the "Author Information" page.
**Disclaimer:** Articles published in the journal HemaSphere exclusively reflect the opinions of the authors. The authors are responsible for all content in their abstracts including accuracy of the facts, statements, citing resources, etc.

### (PB3525) REAL-WORLD (RW) CHART REVIEW OF CYTOREDUCTIVE THERAPY (CRT) PATTERNS, ADVERSE EVENTS AND TREATMENT MODIFICATIONS IN POLYCYTHEMIA VERA (PV) ACROSS PHYSICIAN PRACTICE SETTINGS IN THE US

**Topic:** 16. Myeloproliferative neoplasms - Clinical

Naveen Pemmaraju*[1], Angela Fan[2], Swapna Munnangi[3], Alicia Cerretani[2], Grace Gahlon[3], Shelby Corman[3], Luis Hernandez[2]

*[1]University of Texas MD Anderson Cancer Center, Houston, United States of America;[2]Takeda Pharmaceuticals America, Inc., Cambridge, United States of America; [3]Precision AQ, Bethesda, United States of America;*

### Background

PV is a chronic myeloproliferative neoplasm treated with phlebotomy (PHL), aspirin and CRT. However, RW evidence on how CRTs are initiated and modified is limited. Understanding RW CRT use, drivers of CRT selection and modification, and treatment-related adverse events (TRAEs), overall and by practice setting is key for improving PV care.

### Aims

To describe RW CRT use, sequencing, and TRAEs in PV, and to assess physician-reported drivers of CRT selection and treatment modification overall and by practice setting.

### Methods

A retrospective chart review abstracted de-identified medical records of adults (≥18 years) newly diagnosed with PV between 8/2020-7/2023 with ≥24 months of follow-up unless deceased. Hematologists/oncologists captured demographics, clinical characteristics, CRT sequencing, dose-titration patterns, treatment rationale, and practice setting (community [C], academic [A], mixed [M]) using structured electronic case report forms. Outcomes included CRT utilization; median time from diagnosis to CRT initiation; rationale for CRT selection, dose titration, and discontinuation, and TRAE frequency and management. Data were summarized for the overall cohort and by practice setting.

### Results

The study included 128 PV patients (median age at diagnosis 62 years; follow-up 2.3 years) across C (n=60), A (n=50), and M (n=18) settings. Overall, 71% (91/128) received PHL and 73% (93/128) received CRT (55% hydroxyurea [HU], 8% interferon [IFN], 20% ruxolitinib [RUX]). Median time from diagnosis to CRT initiation was 407 days. First-line CRT use varied by setting with HU more common in C than A (62% vs 48%), while RUX was used more often in A (22% vs 2%). A planned hematocrit (HCT) target < 45% was documented for 69% (64/93) of CRT initiators. CRT was most often initiated for hematologic abnormalities (63%, 59/93), which comprised inadequate HCT control (52%, 48/93), thrombocytosis (18%, 17/93), and leukocytosis (12%, 11/93). C physicians more often cited age ≥60 (50% vs 30%) and thrombocytosis (36% vs 5%) as drivers of CRT selection than A physicians. Among those who switched CRT (n=13), HU to RUX was the most common sequence (69%), followed by HU to IFN (15%), with similar distribution across settings. TRAEs were documented in 44% (41/93), most frequently fatigue (28%, 26/93) and anemia (12%, 11/93). TRAE management commonly included dose reduction (32%, 13/41), treatment pause (20%, 8/41), or discontinuation (12%, 5/41). CRT discontinuation occurred in 13% (12/93), typically due to inadequate HCT control (50%, 6/12). During CRT exposure, dose titrations (defined as dose increase or decrease) occurred in HU (44%, 31/71), IFN (55%, 6/11), and RUX (8%, 2/25). Among HU-treated patients (n=71), 37% required a dose increase and 7% a decrease. For HU-titrated patients (n=31), inadequate HCT control drove most dose changes (81%).

**Copyright Information:** (Online) ISSN: 2572-9241
©2026 The Author(s). HemaSphere is published by John Wiley & Sons Ltd on behalf of European Hematology Association. This is an open access Abstract Book distributed under the Attribution-NonCommercial-NoDerivs (CC BY-NC-ND), which allows third parties to download the articles and share them with others as long as they credit the author and the Abstract Book, but they cannot change the content in any way or use them commercially.
**Abstract Book Citations:** Authors, Title, HemaSphere, 2026;10:(S1):pages. The abstract book DOIs can be found on the "Author Information" page.
**Disclaimer:** Articles published in the journal HemaSphere exclusively reflect the opinions of the authors. The authors are responsible for all content in their abstracts including accuracy of the facts, statements, citing resources, etc.

### Summary/Conclusion

In PV, CRT is frequently modified through dose adjustments, pauses, or discontinuations, mainly due to hematologic abnormalities, particularly inadequate HCT control, and treatment tolerability. Treatment patterns and physician-reported drivers of CRT selection also differed by setting. Inadequate HCT control continues to drive treatment changes, highlighting persistent gaps in achieving consistent HCT control and the need for more effective, better-tolerated therapies to support reliable HCT control in routine practice.

**Copyright Information:** (Online) ISSN: 2572-9241
©2026 The Author(s). HemaSphere is published by John Wiley & Sons Ltd on behalf of European Hematology Association. This is an open access Abstract Book distributed under the Attribution-NonCommercial-NoDerivs (CC BY-NC-ND), which allows third parties to download the articles and share them with others as long as they credit the author and the Abstract Book, but they cannot change the content in any way or use them commercially.
**Abstract Book Citations:** Authors, Title, HemaSphere, 2026;10:(S1):pages. The abstract book DOIs can be found on the "Author Information" page.
**Disclaimer:** Articles published in the journal HemaSphere exclusively reflect the opinions of the authors. The authors are responsible for all content in their abstracts including accuracy of the facts, statements, citing resources, etc.

### (PB3526) WHOLE-EXOME SEQUENCING IN JAK2 UNMUTATED ERYTHROCYTOSIS: A STUDY OF A COHORT FROM A TERTIARY REFERENCE HOSPITAL

**Topic:** 16. Myeloproliferative neoplasms - Clinical

Ningning Liu*[1], Zhijian Xiao[2], Zefeng Xu[3], Bing Li[4], Tiejun Qin[1], Shiqiang Qu[1], Lijuan Pan[1], Meng Jiao[1], Qingyan Gao[1, 1]

[1]*Institute of Hematology and Blood Diseases Hospital, Chinese Academy of Medical Sciences & Peking Union Medical College, Tianjin, China;* [2]*Institute Of Hematology & Blood Diseases Hospital, Chinese Academy Of Medical Sciences, Tianjin, China;* [3]*Institute of Hematology and Blood Diseases Hospital, Chinese Academy of Medical Science & Peking Union Medical College, Tianjin, China;* [4]*Blood Diseases Hospital, CAMS, Tianjin, China;*

### Background

Erythrocytosis may be observed in polycythemia vera (PV) with *JAK2* mutations, or may remain unexplained (idiopathic erythrocytosis, IE) after excluding the primary and secondary causes.

### Aims

We investigated the clinical features and molecular profile of *JAK2* unmutated erythrocytosis in a cohort from a tertiary reference center.

### Methods

A total of 553 consecutive patients with erythrocytosis were tested for *JAK2* mutations; those who were negative underwent whole-exome sequencing (WES).

### Results

Clinical characteristics and outcomes were compared between IE and *JAK2* mutated PV. Among 553 patients, 456 (82.5%) were diagnosed with *JAK2* mutated PV, including 3 with non-canonical mutations detected only by WES. Of the 97 *JAK2* unmutated patients, 7 (1.3%) were diagnosed with *JAK2* unmutated PV, 2 (0.4%) with myeloproliferative neoplasm, unclassifiable (MPN-U), 9 (1.6%) with acquired erythrocytosis, 7 (1.3%) with congenital erythrocytosis (ECYT), and 72 (13.0%) with IE. IE patients were younger, predominantly male, and exhibited isolated erythrocytosis with higher erythropoietin and ferritin levels, along with significantly lower levels of interleukin (IL)-1β, IL-4, IL-6, IL-12, IL-17, interferon (IFN)-α, and IFN-γ compared to PV patients. Somatic mutations were detected in only 4 (6.7%) IE patients, including 3 with *DNMT3A* mutations and 1 with a *TET2* mutation, whereas germline variants across multiple pathways were frequently identified, such as *HFE*, *FANCA*, and *UGT1A1*.

### Summary/Conclusion

Overall, IE is heterogeneous, often with germline variants, and comprehensive molecular evaluation beyond *JAK2* testing is warranted.

**Copyright Information:** (Online) ISSN: 2572-9241
©2026 The Author(s). HemaSphere is published by John Wiley & Sons Ltd on behalf of European Hematology Association. This is an open access Abstract Book distributed under the Attribution-NonCommercial-NoDerivs (CC BY-NC-ND), which allows third parties to download the articles and share them with others as long as they credit the author and the Abstract Book, but they cannot change the content in any way or use them commercially.
**Abstract Book Citations:** Authors, Title, HemaSphere, 2026;10:(S1):pages. The abstract book DOIs can be found on the "Author Information" page.
**Disclaimer:** Articles published in the journal HemaSphere exclusively reflect the opinions of the authors. The authors are responsible for all content in their abstracts including accuracy of the facts, statements, citing resources, etc.

### (PB3527) NEUTROPHIL-TO-LYMPHOCYTE RATIO AS AN INFLAMMATORY MARKER IN POLYCYTHEMIA VERA PATIENTS TREATED WITH ROPEGINTERFERON ALFA-2B

**Topic:** 16. Myeloproliferative neoplasms - Clinical

Bianca Nistor*[1], Viola Maria Popov[2], Mirela Carmen Voicu[2], Mihaela Andreescu[3]

*[1]spitalul clinic colentina, hematology, Bucuresti, Romania;[2]spitalul clinic colentina, Bucuresti, Romania;[3]spitalul clinic colentina, titu maiorescu, Bucuresti, Romania;*

### Background

Polycythemia vera (PV) is a chronic myeloproliferative neoplasm characterized by persistent inflammation and myeloid activation. The neutrophil-to-leukocyte ratio (NLR) has emerged as a simple marker of systemic inflammatory status; however, data regarding its longitudinal dynamics during treatment with ropeginterferon alfa-2b in real-world settings remain limited.

### Aims

To evaluate longitudinal changes in NLR as an inflammatory marker in patients with PV treated with ropeginterferon alfa-2b in a real-world cohort.

### Methods

We performed a retrospective analysis of PV patients receiving ropeginterferon alfa-2b. Hematologic parameters were collected at baseline and during follow-up at 1, 3, 6, and 12 months. NLR was calculated as the ratio between absolute neutrophil and lymphocyte counts. Longitudinal trends and distribution patterns of NLR were assessed.

### Results

A total of 24 patients were included, with a median age of 57 years. Median baseline NLR was 3.67 (IQR 2.16–4.97; n=22), showing a slight decrease at 1 month (3.46, IQR 1.86–5.04; n=22) and comparable values at 3 months (3.76, IQR 2.06–4.64; n=19). At 6 months, median NLR decreased to 3.08 (IQR 1.71–5.83; n=11), suggesting a potential reduction in inflammatory burden during treatment. At 12 months, median NLR was 4.30 (IQR 1.65–4.44; n=5), with increased variability likely related to limited patient numbers at extended follow-up. Boxplot analysis demonstrated broader dispersion at baseline with a trend toward stabilization of NLR values over time under ropeginterferon alfa-2b therapy.

### Summary/Conclusion

In this real-world cohort, treatment with ropeginterferon alfa-2b was associated with dynamic changes in NLR, suggesting modulation of systemic inflammatory status in PV patients. NLR may represent an accessible biomarker for monitoring inflammatory dynamics during interferon therapy.

**Copyright Information:** (Online) ISSN: 2572-9241
©2026 The Author(s). HemaSphere is published by John Wiley & Sons Ltd on behalf of European Hematology Association. This is an open access Abstract Book distributed under the Attribution-NonCommercial-NoDerivs (CC BY-NC-ND), which allows third parties to download the articles and share them with others as long as they credit the author and the Abstract Book, but they cannot change the content in any way or use them commercially.
**Abstract Book Citations:** Authors, Title, HemaSphere, 2026;10:(S1):pages. The abstract book DOIs can be found on the "Author Information" page.
**Disclaimer:** Articles published in the journal HemaSphere exclusively reflect the opinions of the authors. The authors are responsible for all content in their abstracts including accuracy of the facts, statements, citing resources, etc.

### (PB3528) BEYOND DONATION: HARNESSING ROUTINE CBCS FOR EARLY DETECTION OF MYELOPROLIFERATIVE NEOPLASMS

**Topic:** 16. Myeloproliferative neoplasms - Clinical

Juan Pablo MacÍas Flores*[1], Gilberto Israel Barranco Lampon[1], Christian Ramos[1], Camila Terreros[2, 2], Abril Yañez Cervantes[1]

*[1]Hospital General de México Dr. Eduardo Liceaga, Hematology, Ciudad de México, Mexico,[2]Universidad Nacional Autónoma de México, Mexico City, Mexico;*

### Background

A substantial proportion of myeloproliferative neoplasms (MPNs) are diagnosed incidentally after routine laboratory abnormalities or disease-related complications, adversely affecting morbidity and survival. Blood donation centers routinely perform complete blood counts (CBCs) in apparently healthy individuals and may therefore offer an opportunity for early detection of hematologic alterations suggestive of MPNs.

### Aims

To identify CBC abnormalities among apparently healthy blood donors that may indicate underlying MPNs.

### Methods

Retrospective descriptive study of blood donors evaluated in 2024 at the Hospital General de México Dr. Eduardo Liceaga Blood Bank. Hematologic parameters and donor characteristics were extracted from clinical records. Abnormal CBCs were classified by affected cell line and by additional alterations suggestive of MPNs according to international criteria. Descriptive statistics summarized frequencies and patterns.

### Results

Among the 26,720 donors evaluated, the majority were male (63.4%), with a mean age of 37 years. Erythrocytosis was identified in 286 donors (1.1%), predominantly in men. Leukocyte abnormalities were more frequent: leukocytosis occurred in 884 donors (3.3%), neutrophilia in 1,167 (4.4%), and lymphocytosis in 1,719 (6.4%). Other findings included basophilia (n = 1,295; 4.8%), eosinophilia (n = 460; 1.7%), and monocytosis (n = 116; 0.4%). Thrombocytosis was observed in 175 donors (0.7%), occurring more frequently in women.

Within the erythrocytosis subgroup, 16 donors (5.6%) presented with concomitant leukocytosis (10.6–15.8 × $10^3$/µL), 57 (19.9%) with basophilia (0.2–3.1 × $10^3$/µL), 6 (2.1%) with eosinophilia (0.5–0.9 × $10^3$/µL), and 1 (0.3%) with thrombocytosis (493 × $10^3$/µL). Furthermore, 96 of these donors (33.6%) exhibited elevated systolic blood pressure (>130 mmHg), and 23 (8.0%) had a body mass index (BMI) >35 kg/m².

Among donors with leukocytosis, one case of a leukemoid reaction was identified (64.6 × $10^3$/µL). Subgroup analyses of specific leukocyte lines revealed the following: in the monocytosis group, 22 donors (19.0%) had absolute counts ≥1.0 × $10^3$/µL and ≥10% of total leukocytes; in the basophilia group, 22 donors (1.7%) had counts ≥0.5 × $10^3$/µL and ≥10% of total leukocytes; and in the eosinophilia group, 11 donors (2.4%) had counts ≥1.5 × $10^3$/µL and ≥10% of total leukocytes. Additionally, within the lymphocytosis group, 17 donors (1.0%) had absolute lymphocyte counts >5.0 × $10^3$/µL.

**Copyright Information:** (Online) ISSN: 2572-9241
©2026 The Author(s). HemaSphere is published by John Wiley & Sons Ltd on behalf of European Hematology Association. This is an open access Abstract Book distributed under the Attribution-NonCommercial-NoDerivs (CC BY-NC-ND), which allows third parties to download the articles and share them with others as long as they credit the author and the Abstract Book, but they cannot change the content in any way or use them commercially.
**Abstract Book Citations:** Authors, Title, HemaSphere, 2026;10:(S1):pages. The abstract book DOIs can be found on the "Author Information" page.
**Disclaimer:** Articles published in the journal HemaSphere exclusively reflect the opinions of the authors. The authors are responsible for all content in their abstracts including accuracy of the facts, statements, citing resources, etc.

### Summary/Conclusion

Routine CBC screening in apparently healthy blood donors reveals a clinically significant subset of individuals with hematological profiles suggestive of underlying MPNs. The identification of single-lineage abnormalities—such as erythrocytosis and thrombocytosis—alongside high-risk multi-lineage alterations (e.g., concurrent erythrocytosis with basophilia or leukocytosis) highlights the latent diagnostic value of blood donation centers. Establishing standardized referral protocols for donors presenting with these suspicious, incidental CBC patterns could facilitate the early diagnosis and management of MPNs, potentially mitigating disease-related complications and improving long-term survival.

***Table 1. Demographics and results***

| Variables | Women | Men |
|---|---|---|
| N (%) | 26,720 (100%) | |
| Sex (%) | 9768 (36.6%) | 16952 (63.4%) |
| Age (y), mean (SD) | 36.7 (±10.98) | 37.4 (±11.7) |
| Hb (g/dL), mean (SD) | 14.35 (±1.33) | 16.69 (±1.08) |
| Min, Max | 5.03-21.4 | 6.7-29.65 |
| Erythrocytosis (%) | 286 (1.1%) | |
| | 5 (1.7%) | 281 (98.3%) |
| Hct (%), mean (SD) | 43.08 (±3.56) | 49.3 (±3.09) |
| Min, Max | 18.29-64 | 24.5-77.8 |
| Leukocytes ($10^3$/uL), mean (SD) | 7.36 (±1.84) | 6.94 (±1.59) |
| Min, Max | 2.9-64.6 | 2.51- 18.9 |
| Leukocytosis (%) | 884 (3.3%) | |
| | 475 (53.7%) | 409 (46.3%) |
| Neutrophils ($10^3$/uL), mean (SD) | 4.25 (±1.44) | 3.88 (±1.23) |
| Min, Max | 0.60-51 | 0-13.8 |
| Neutrophilia (%) | 1167 (4.4%) | |
| | 594 (50.9%) | 573 (49.1%) |
| Lymphocytes ($10^3$/uL), mean (SD) | 2.4 (±0.60) | 2.30 (±0.59) |
| Min, Max | 0.67-6.44 | 0.46-6.35 |
| Lymphocytosis (%) | 1719 (6.4%) | |
| | 738 (43%) | 981 (57%) |
| Monocytes ($10^3$/uL), mean (SD) | 0.44 (±0.15) | 0.47 (±0.17) |
| Min, Max | 0.9-2.3 | 0.01-5 |
| Monocytosis (%) | 116 (0.4%) | |
| | 21 (18.1%) | 95 (81.9%) |
| Basophils ($10^3$/uL), mean (SD) | 0.08 (±0.17) | 0.1 (±0.22) |
| Min, Max | 0-6.4 | 0-3.39 |
| Basophilia (%) | 1295 (4.9%) | |
| | 271 (20.9%) | 1024 (79.1%) |
| Eosinophils ($10^3$/uL), mean (SD) | 0.16 (±0.13) | 0.17 (±0.11) |
| Min, Max | 0-6.5 | 0-1.8 |
| Eosinophilia (%) | 460 (1.7%) | |
| | 152 (33%) | 308 (67%) |
| Platelets ($10^3$/uL), mean( SD) | 289.93 (±65) | 248.36 (±53.61) |
| Min, Max | 12-655.78 | 4.6-601.04 |
| Thrombocytosis (%) | 175 (0.7%) | |
| | 153 (87.4%) | 22 (12.6%) |

**Copyright Information:** (Online) ISSN: 2572-9241
©2026 The Author(s). HemaSphere is published by John Wiley & Sons Ltd on behalf of European Hematology Association. This is an open access Abstract Book distributed under the Attribution-NonCommercial-NoDerivs (CC BY-NC-ND), which allows third parties to download the articles and share them with others as long as they credit the author and the Abstract Book, but they cannot change the content in any way or use them commercially.
**Abstract Book Citations:** Authors, Title, HemaSphere, 2026;10:(S1):pages. The abstract book DOIs can be found on the “Author Information” page.
**Disclaimer:** Articles published in the journal HemaSphere exclusively reflect the opinions of the authors. The authors are responsible for all content in their abstracts including accuracy of the facts, statements, citing resources, etc.

### (PB3529) DESIGN VERIFICATION TESTING OF A HIGHLY SENSITIVE MULTIPLEX QPCR ASSAY THAT DETECTS THE JAK2 V617F VARIANT COMMONLY PRESENT IN MYELOPROLIFERATIVE NEOPLASMS

**Topic:** 16. Myeloproliferative neoplasms - Clinical

Jonathan Turner*[1], Jamie Myers[1], Cale Heiniger[1], Mahesh Yarasi[1], Kevin Kelnar[1], John Milligan[1], Brian Haynes[1]

[1]*Bio-Techne, Austin, United States of America;*

**Background**

Approximately 95% of patients with polycythemia vera (PV) and 60% of patients with essential thrombocythemia or primary myelofibrosis (PMF) are positive for the *JAK2* V617F variant, the most common driver mutation in myeloproliferative neoplasms (MPNs). Testing for V617F is routine when diagnosing MPNs and interferon a-2b and pegylated interferon a-2a treatment in PV patients and to predict outcomes after allogenic transplantation in PMF patients.

**Aims**

To characterize assay sensitivity and accuracy when detecting the V617F variant in whole blood or bone marrow aspirate DNA samples using a multiplex design and a single qPCR reaction.

**Methods**

Study-specific sample panels were generated using 37 unique V617F-positive and -negative whole blood, bone marrow aspirate, and cell line samples. DNA was isolated from blood and bone marrow using the QIAamp DNA Blood Mini Kit (Qiagen). The Wizard® DNA Purification Kand System (Roche) were also used to isolate DNA from blood. DNA was quantified using a Nanodrop spectrophotometer. Linearity, DNA input range, kit freeze-thaws, sample type and isolation method, single-site precision, qPCR platform equivalency, and analytical sensitivity studies were performed on ABI 7500 Fast Dx, QuantStudio 5 Dx, and QuantStudio 7 Pro Dx instruments to characterize performance of the. Run data were exported as .txt or .sds files then uploaded and analyzed using the custom software analysis tool.

**Results**

Limits of blank (LOB), detection (LOD), and quantification (LOQ) were determined based on the classical nonparametric analysis methods described in CLSI document EP17-A2; 165 ng of sample DNA were used in all reactions across three qPCR platforms using two kit lots. V617F was undetected in 284/285 V617F-negative sample reactions; the resulting LOB was zero. LOD and LOQ were determined based on 360 reactions total consisting of four unique blood samples with target VAF values of 0.01%; the established assay LOD is < 0.1%. Results satisfied variability criteria at VAF values below LOD, indicating that assay LOQ is equal to LOD.

Two V617F-positive and one negative blood samples were tested to evaluate performance with DNA inputs ranging from 10 to 615 ng. V617F was detected, and VAF values passed precision criteria, in all four positive sample replicates at each input level. None of the eight negative sample replicates at each input level generated a VAF at or above LOD.

Three isolation methods were assessed for eight V617F-positive and eight negative blood samples tested in triplicate on all three qPCR platforms. Blood sample results were compared to those obtained for six V617F-positive and eight negative bone marrow DNA samples isolated using the QIAamp kit. All results passed percent positive and negative agreement and precision criteria, and regression analysis demonstrated linear performance across the VAF range, for all three isolation methods and both sample types.

**Copyright Information:** (Online) ISSN: 2572-9241
©2026 The Author(s). HemaSphere is published by John Wiley & Sons Ltd on behalf of European Hematology Association. This is an open access Abstract Book distributed under the Attribution-NonCommercial-NoDerivs (CC BY-NC-ND), which allows third parties to download the articles and share them with others as long as they credit the author and the Abstract Book, but they cannot change the content in any way or use them commercially.
**Abstract Book Citations:** Authors, Title, HemaSphere, 2026;10:(S1):pages. The abstract book DOIs can be found on the "Author Information" page.
**Disclaimer:** Articles published in the journal HemaSphere exclusively reflect the opinions of the authors. The authors are responsible for all content in their abstracts including accuracy of the facts, statements, citing resources, etc.

**Summary/Conclusion**

The assay quantifies the *JAK2* V617F variant in blood and bone marrow samples at allele frequencies < 0.1% in a single multiplex reaction with a streamlined workflow. With a total assay time of less than two hours from sample to result, this high sensitivity assay has the potential to aid in reliable identification of the V617F variant associated with myeloproliferative neoplasms.

**Copyright Information:** (Online) ISSN: 2572-9241
©2026 The Author(s). HemaSphere is published by John Wiley & Sons Ltd on behalf of European Hematology Association. This is an open access Abstract Book distributed under the Attribution-NonCommercial-NoDerivs (CC BY-NC-ND), which allows third parties to download the articles and share them with others as long as they credit the author and the Abstract Book, but they cannot change the content in any way or use them commercially.
**Abstract Book Citations:** Authors, Title, HemaSphere, 2026;10:(S1):pages. The abstract book DOIs can be found on the “Author Information” page.
**Disclaimer:** Articles published in the journal HemaSphere exclusively reflect the opinions of the authors. The authors are responsible for all content in their abstracts including accuracy of the facts, statements, citing resources, etc.

### (PB3530) REAL WORLD DATA ON SAFETY AND EFFICACY OF ROPEGINTERFERON ALPHA-2B FOR THE TREATMENT OF POLYCYTHEMIA VERA

**Topic:** 16. Myeloproliferative neoplasms - Clinical

Thomas Thomopoulos*[1], Anthi Bouchla[1], Christina Apostolopoulou[1], Pinelopi Vryttia[1], Artemis Zormpa[1], Ioulia Markaki[1], Georgia Karamanou[1], Papageorgiou Sotirios[1], Vasiliki Pappa[1]

*[1]Attikon University General Hospital, Hematology Unit, Second Department of Internal Medicine and Research Institute, ATHENS, Greece;*

### Background

Ropeginterferon alpha-2b (ropeg-IFN) is a mono-pegylated long-acting interferon that has demonstrated the ability to suppress the neoplastic clone in patients with polycythemia vera (PV), thereby decelerating disease progression to post-PV myelofibrosis (MF) or acute myeloid leukemia (AML). Following its regulatory approval by the EMA and FDA, it has been integrated into clinical practice for both frontline treatment of high-risk patients and as a rescue therapy following hydroxyurea (HU) failure. While the safety and efficacy results from major clinical trials have been remarkable, these findings are often confined to highly controlled settings. Consequently, a scarcity of real-world data (RWD) persists, which hampers the effective extrapolation of trial results into daily clinical practice.

### Aims

We evaluated the efficacy and safety of ropeg-IFN in a cohort of PV patients treated in the real-world setting of a tertiary hospital in Athens, Greece.

### Methods

Our cohort comprised PV patients requiring cytoreduction upfront or following HU failure. Patients received starting doses of 125 µg, followed by 250 µg from week 4 onwards. In cases where a sustained complete hematological response (CHR) was achieved at 12 months, dosing intervals were extended to assess the feasibility of treatment-free remission. JAK2 VAF was measured via NGS at baseline and 12 months. CHR was defined as per ELN criteria, and overall response was assessed using 2013 revised ELN criteria requiring bone marrow (BM) remission for complete response (CR). A provisional category of Complete Response/unconfirmed (CRu) was used for patients without 12-month BM assessment.

**Copyright Information:** (Online) ISSN: 2572-9241
©2026 The Author(s). HemaSphere is published by John Wiley & Sons Ltd on behalf of European Hematology Association. This is an open access Abstract Book distributed under the Attribution-NonCommercial-NoDerivs (CC BY-NC-ND), which allows third parties to download the articles and share them with others as long as they credit the author and the Abstract Book, but they cannot change the content in any way or use them commercially.
**Abstract Book Citations:** Authors, Title, HemaSphere, 2026;10:(S1):pages. The abstract book DOIs can be found on the "Author Information" page.
**Disclaimer:** Articles published in the journal HemaSphere exclusively reflect the opinions of the authors. The authors are responsible for all content in their abstracts including accuracy of the facts, statements, citing resources, etc.

### Results

By late January 2026, 42 cases of PV, including 8 JAK2-wildtype, were treated with ropeg-IFN. During a median follow-up of 26.5 months, discontinuation occurred in 38.1% of cases, primarily due to futility or toxicity (14.3%). Adverse events emerged in 54.8% of patients and were mostly low-grade. CHR was achieved by half of patients within 5.8 months. The 12-month and 24-month CHR estimates (50% and 86.7%) approximated trial data. The 12-month objective response rate reached 63.6%, including 3 CR cases. Partial molecular response (MR) was documented in 3 of 6 eligible patients. Prior HU exposure and resistance were inversely associated with CHR and ORR. Notably, lymphocyte-to-monocyte (LMR) and neutrophil-to-lymphocyte (NLR) ratios strongly correlated with JAK2 VAF% at both baseline and 12 months. Absolute change in NLR at 12 months was almost collinear to JAK2 VAF% shifts, rendering NLR a potential surrogate marker for molecular response.

**Table 1. Rates and reasons for ropeg-IFN discontinuation among 42 pateints having recieved at least on e dose of ropeg-IFN**

| | |
|---|---|
| Overall discontinuation rate, n (%) | 16 (38.1) |
| Reasons for ropeg-IFN discontinuation, % | |
| -No response/disease progression<br>*Development of post-PV MF*<br>*Transformation into AML* | 11.9<br>9.5<br>2.4 |
| -Drug-related adverse events<br>*Severe psychiatric symptoms of irritability, mood changes and, depression (n:3)*<br>*Clinically overt hypothyroidism (n:2)*<br>*Severe flu-like syndrome (n:1)* | 14.3 |
| -Deaths from unrelated causes | 4.8 |
| -Lost to follow-up | 7.1 |

### Summary/Conclusion

Real-world efficacy and safety of ropeg-IFN align with clinical trial results. The intriguing correlation of inexpensive markers such as LMR and NLR with JAK2 VAF and MR rates suggests they could serve as predictive biomarkers to guide treatment personalization.

**Copyright Information:** (Online) ISSN: 2572-9241
©2026 The Author(s). HemaSphere is published by John Wiley & Sons Ltd on behalf of European Hematology Association. This is an open access Abstract Book distributed under the Attribution-NonCommercial-NoDerivs (CC BY-NC-ND), which allows third parties to download the articles and share them with others as long as they credit the author and the Abstract Book, but they cannot change the content in any way or use them commercially.
**Abstract Book Citations:** Authors, Title, HemaSphere, 2026;10:(S1):pages. The abstract book DOIs can be found on the "Author Information" page.
**Disclaimer:** Articles published in the journal HemaSphere exclusively reflect the opinions of the authors. The authors are responsible for all content in their abstracts including accuracy of the facts, statements, citing resources, etc.

### (PB3531) CO-DESIGN OF A DIGITAL RESOURCE FOR PATIENTS WITH MYELOPROLIFERATIVE NEOPLASM ASSOCIATED SPLANCHNIC VEIN THROMBOSIS (MPN-SVT) AND CLINICIANS INVOLVED IN THEIR CARE

**Topic:** 16. Myeloproliferative neoplasms - Clinical

Mallika Sekhar*[1], Vicki Tsianakas[2, 2], Freya Brown[2], Jo Armes[3], Mandy Lomax[4, 4], Colin Mitchell[4], David Patch[1]

*[1]Royal Free London NHS Trust, Haematology, London, United Kingdom;[2]Kings College London, London, United Kingdom; [3]University of Surrey, London, United Kingdom;[4]Experts by Experience, London, United Kingdom;*

### Background

Myeloproliferative Neoplasms associated Splanchnic Vein Thrombosis (MPN-SVT) is a rare, lifelong condition in which blood clots form in the abdominal veins due to an underlying MPN. Despite the need for long-term, multidisciplinary management, care is often fragmented and poorly aligned with patient needs. Evidence shows that patients experience MPN and SVT as an inseparable condition shaped by uncertainty, limited understanding, and unmet support needs, highlighting a gap in care.

### Aims

Develop an intervention in collaboration with service-users and healthcare professionals (HCPs) to improve care experiences for people with MPN-SVT. Objectives were to: (1) reach consensus on priority areas for improvement, (2) identify feasible interventions, and (3) co-design the agreed intervention.

### Methods

This work formed part of a wider two-phase Experience-based Co-design study (EBCD). Phase 1 explored experiences of living with and delivering care to people with MPN-SVT. This abstract reports on Phase 2, the co-design study.
Participants were adults with MPN-SVT and HCPs working across four hospital sites in haematology, hepatology, gastroenterology, or primary care roles in England, recruited via re-contacting Phase 1 participants, professional networks, and a national patient charity. Thirty participants took part (13 patients; 17 HCPs), which comprised four stages: (1) priority-setting through separate online events for service users and HCPs, (2) a joint, hybrid co-design workshop, (3) development of iterative intervention through mixed co-design teams, (4) a final celebration and dissemination planning event. Descriptive summaries documented priorities, decisions, and co-designed outputs.

### Results

HCP and service users identified and prioritised 4 areas of unmet needs: journey to diagnosis, understanding the condition, communication within and between healthcare teams, and access to support. A resource to understand MPN-SVT was identified as top priority by both groups and was deemed achievable in budget and timeline. The outcome of this is a co-designed, publicly available digital resource providing clear condition information, care pathway guidance, tools to support clinical communication, and accessible signposting to practical and psychosocial support[1].

**Copyright Information:** (Online) ISSN: 2572-9241
©2026 The Author(s). HemaSphere is published by John Wiley & Sons Ltd on behalf of European Hematology Association. This is an open access Abstract Book distributed under the Attribution-NonCommercial-NoDerivs (CC BY-NC-ND), which allows third parties to download the articles and share them with others as long as they credit the author and the Abstract Book, but they cannot change the content in any way or use them commercially.
**Abstract Book Citations:** Authors, Title, HemaSphere, 2026;10:(S1):pages. The abstract book DOIs can be found on the "Author Information" page.
**Disclaimer:** Articles published in the journal HemaSphere exclusively reflect the opinions of the authors. The authors are responsible for all content in their abstracts including accuracy of the facts, statements, citing resources, etc.

### Summary/Conclusion

This study demonstrates the feasibility and value of using EBCD to address unmet care and support needs in people living with MPN-SVT. Grounded in shared patient and clinician experience, the resource addresses unmet needs with potential to enhance care experiences.

References
https://mpn-svt.er.kcl.ac.uk

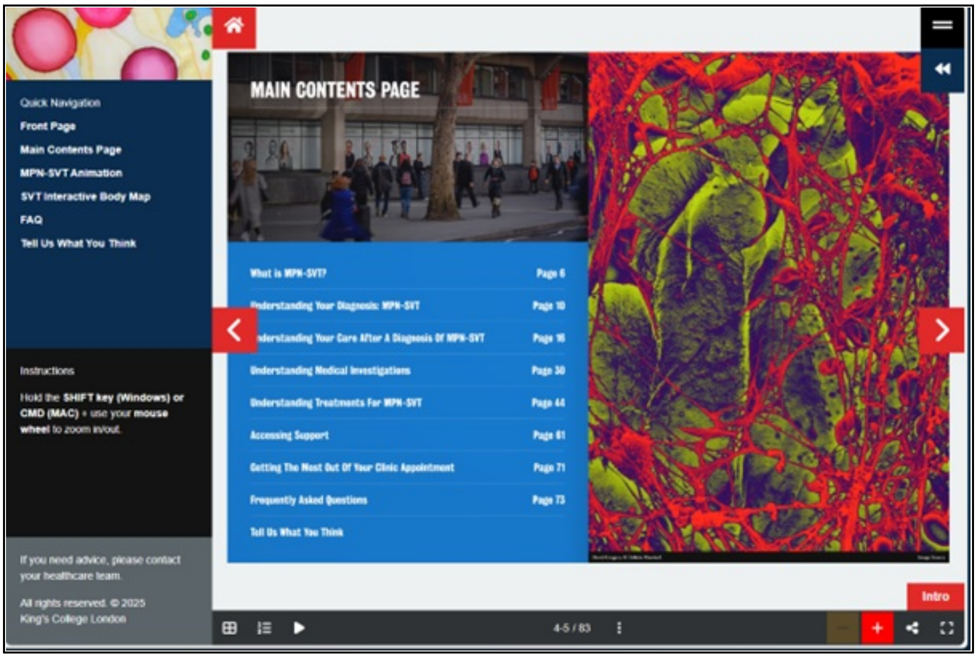

Front index of interactive resource

**Copyright Information:** (Online) ISSN: 2572-9241
©2026 The Author(s). HemaSphere is published by John Wiley & Sons Ltd on behalf of European Hematology Association. This is an open access Abstract Book distributed under the Attribution-NonCommercial-NoDerivs (CC BY-NC-ND), which allows third parties to download the articles and share them with others as long as they credit the author and the Abstract Book, but they cannot change the content in any way or use them commercially.
**Abstract Book Citations:** Authors, Title, HemaSphere, 2026;10:(S1):pages. The abstract book DOIs can be found on the "Author Information" page.
**Disclaimer:** Articles published in the journal HemaSphere exclusively reflect the opinions of the authors. The authors are responsible for all content in their abstracts including accuracy of the facts, statements, citing resources, etc.

### (PB3532) TREATMENT DISRUPTION DURING THE INTERNATIONAL PEGYLATED INTERFERON SHORTAGE: RESULTS OF AN INTERNATIONAL PATIENT SURVEY IN MYELOPROLIFERATIVE NEOPLASMS

**Topic:** 16. Myeloproliferative neoplasms - Clinical

Llywelyn Cadman-Davies*[1], Alesia Khan[2], Claire Harrison[1], Claire Woodley[1], Madeline Johns[3], Charlotte Moss[3], Priya Sriskandarajah[1]

*[1]Guy's and St Thomas' NHS Foundation Trust, London, United Kingdom;[2]Leeds Teaching Hospital Trust, Leeds, United Kingdom; [3]King's College London, London, United Kingdom;*

### Background

Pegylated interferon alfa 2a PEGASYS is a disease modifying therapy for essential thrombocythaemia ET and polycythaemia vera PV. Since 2023, international supply shortages have disrupted access, creating an unplanned real world test of treatment continuity and switching.

### Aims

To quantify the impact of PEGASYS shortage on dosing interruption and switching, and to assess patient reported outcomes following transition between interferon formulations.

### Methods

An international cross sectional survey was conducted using REDCap and disseminated via MPN Voice. Data included demographics, diagnosis, interferon exposure within 12 months, missed doses and discontinuation with reasons, MPN10 symptom burden, tolerability and treatment preference. Analyses were descriptive.

### Results

520 respondents completed the survey. Age data were available for 505; 39.0 percent were aged 45 to 59 years and 37.4 percent 60 to 74 years. Sixty percent were female. Diagnoses were ET in 48.5 percent and PV in 40.4 percent. Within 12 months, 53.5 percent received PEGASYS, 25.3 percent ropeginterferon alfa 2b BESREMi, and 13.5 percent both formulations.

Among PEGASYS treated respondents, 20.8 percent reported missed doses, predominantly due to supply constraints. Of those discontinuing PEGASYS, 58.1 percent switched to BESREMi. Access related missed dosing was lower with BESREMi at 8.0 percent.

Following switch from PEGASYS to BESREMi, most respondents reported acceptable tolerability and maintained adherence. MPN10 scores remained broadly stable without consistent signal of symptom deterioration. Among patients exposed to both agents, 82.1 percent preferred BESREMi and 52.6 percent were unwilling to switch back once stabilised.

### Summary/Conclusion

The PEGASYS shortage resulted in substantial real world treatment disruption in ET and PV, including missed dosing and enforced switching. Patient reported symptom control was generally maintained after transition to ropeginterferon. These findings demonstrate both the vulnerability of disease modifying therapy to supply instability and the clinical feasibility of alternative interferon strategies, with implications for service planning and drug procurement resilience.

**Copyright Information:** (Online) ISSN: 2572-9241
©2026 The Author(s). HemaSphere is published by John Wiley & Sons Ltd on behalf of European Hematology Association. This is an open access Abstract Book distributed under the Attribution-NonCommercial-NoDerivs (CC BY-NC-ND), which allows third parties to download the articles and share them with others as long as they credit the author and the Abstract Book, but they cannot change the content in any way or use them commercially.
**Abstract Book Citations:** Authors, Title, HemaSphere, 2026;10:(S1):pages. The abstract book DOIs can be found on the “Author Information” page.
**Disclaimer:** Articles published in the journal HemaSphere exclusively reflect the opinions of the authors. The authors are responsible for all content in their abstracts including accuracy of the facts, statements, citing resources, etc.

### (PB3533) A PHASE 2/3 STUDY OF ELENESTINIB IN ISM: A TRIAL-IN-PROGRESS UPDATE OF NOVEL ENDPOINTS AND BIOMARKERS AIMED AT EVALUATING DISEASE MODIFICATION (HARBOR)

**Topic:** 16. Myeloproliferative neoplasms - Clinical

Olivier Hermine[1], Tsewang Tashi[2], Jens Panse*[3], Tse-Chieh Teh[4], William Shomali[5], Renata Cabral[6], Robert Bird[7], Joseph Jurcic[8], Elizabeth A. Griffiths[9], Gandhi Laurent Damaj[10], Philippe Schafhausen[11], Chiara Elena[12], Juliana Schwaab[13], Ingunn Dybedal[14], Cristina Papayannidis[15], Hussein Abbas[16], Pankit Vachhani[17], Cristina Bulai Livideanu[18], Mariana Castells[19], Vito Sabato[20], Frank Siebenhaar[21, 22], Cem Akin[23], Clive Grattan[24], Thanai Pongdee[25], Pedro Giavina-Bianchi[26], Paul Van Daele[27], Marie Pierre Gourin[28], Lydia Zachariadou[29], Kevin He[29], Sam Tamulevich[29], Robyn Scherber[29], Deepti Radia[30], Tracy I. George[31], Ivan Alvarez-Twose[32]

*[1]Hopital Necker, APHP, CEREMAST, Imagine, Paris, France;[2]Huntsman Cancer Institute, University of Utah, Salt Lake City, UT, United States of America; [3]Department of Oncology, Hematology, Hemostaseology and Stem Cell Transplantation, Universitätsklinikum Aachen, Aachen, Germany; [4]Department of Clinical Haematology, The Alfred Hospital, Melbourne, Victoria, Australia; [5]Stanford University School of Medicine, Stanford, CA, United States of America; [6]Unidade Local de Saude de Santo António and ICBAS-School of Medicine and Biomedical Sciences, University of Porto, Porto, Portugal; [7]Princess Alexandra Hospital, Woolloongabba, Queensland, Australia; [8]Columbia University Medical Center, New York, NY, United States of America; [9]Roswell Park Comprehensive Cancer Center, Buffalo, NY, United States of America; [10]CHU de Caen, Caen, France; [11]Department of Oncology, Hematology, and Bone Marrow Transplantation with Section of Pneumology, University Medical Center Hamburg-Eppendorf, Hamburg, Germany; [12]Department of Molecular Medicine & Department of Hematology and Oncology, University of Pavia, Pavia, Italy; [13]University Hospital Mannheim, Heidelberg University, Mannheim, Germany; [14]Oslo Universitetssykehus HF Rikshospitalet, Oslo, Norway; [15]IRCCS, Azienda Ospedaliero-Universitaria di Bologna, Istituto di Ematologia Seràgnoli, Bologna, Italy; [16]The University of Texas MD Anderson Cancer Center, Houston, TX, United States of America; [17]University of Alabama at Birmingham: The Kirklin Clinic, Birmingham, AL, United States of America;[18]Département de dermatologie, CEREMAST CHU de Toulouse, Toulouse, France; [19]Brigham and Women's Mastocytosis Center, Division of Allergy and Clinical Immunology, Boston, MA, United States of America; [20]Allergology and Rheumatology, University of Antwerp and Antwerp University Hospital, Antwerp, Belgium; [21]Institute of Allergology, Charité-Universitätsmedizin Berlin, Corporate Member of Freie Universität Berlin and Humboldt-Universität zu Berlin, Berlin, Germany; [22]Fraunhofer Institute for Translational Medicine and Pharmacology ITMP, Immunology and Allergology, Berlin, Germany; [23]University of Michigan, Ann Arbor, MI, United States of America; [24]St John's Institute of Dermatology, Guy's and St Thomas' NHS Foundation Trust, London, United Kingdom; [25]Division of Allergic Diseases, Mayo Clinic, Rochester, MN, United States of America;[26]Clinical and Immunology Division, University of São Paulo Medical School, São Paulo, Brazil; [27]Erasmus Medical Centre, Rotterdam, The Netherlands; [28]Centre Hospitalier et Universitaire de Limoges, Limoges, France;[29]Blueprint Medicines Corporation, Cambridge, MA, United States of America; [30]Guy's and St Thomas' NHS Foundation Trust - Guy's Hospital, London, United Kingdom;[31]ARUP Laboratories, University of Utah, Salt Lake City, UT, United States of America; [32]Hospital Virgen del Valle, Toledo, Spain;*

### Background

Systemic mastocytosis (SM), which includes indolent SM (ISM), is a rare clonal mast cell disease driven by the *KIT* D816V mutation in ~95% of cases. ISM is characterized by mast cell (MC) accumulation and aberrant activation, resulting in chronic and debilitating manifestations such as anaphylaxis, skin lesions, gastrointestinal and neurocognitive symptoms, and osteoporosis and fractures, leading to diminished quality of life (QoL). Elenestinib is a next-generation inhibitor designed for potent and highly selective KIT D816V inhibition (half-maximal inhibitory concentration of KIT D816V, 3.1 nM; kinase selectivity score, 0.035) and limited central nervous system penetration. Elenestinib is currently being evaluated in the double-blind, placebo-controlled, global, phase 2/3 HARBOR randomized controlled trial (RCT; NCT04910685). Part 1 of HARBOR previously showed that elenestinib has a similar tolerability profile to placebo.

**Copyright Information:** (Online) ISSN: 2572-9241
©2026 The Author(s). HemaSphere is published by John Wiley & Sons Ltd on behalf of European Hematology Association. This is an open access Abstract Book distributed under the Attribution-NonCommercial-NoDerivs (CC BY-NC-ND), which allows third parties to download the articles and share them with others as long as they credit the author and the Abstract Book, but they cannot change the content in any way or use them commercially.
**Abstract Book Citations:** Authors, Title, HemaSphere, 2026;10:(S1):pages. The abstract book DOIs can be found on the "Author Information" page.
**Disclaimer:** Articles published in the journal HemaSphere exclusively reflect the opinions of the authors. The authors are responsible for all content in their abstracts including accuracy of the facts, statements, citing resources, etc.

### Aims

HARBOR is an ongoing study designed to evaluate the disease-modifying efficacy and safety of elenestinib in patients with ISM and smoldering SM (SSM). The safety, tolerability, and efficacy in Part 1 supports the Part 2 and Part 3 design.

### Methods

In HARBOR Part 2, ~350 patients will be randomized 2:1 to elenestinib 75 mg once daily (QD) + symptom-directed therapy (SDT) or placebo + SDT. After completing the double-blind Part 2 (Week 48), patients may roll over to 75 mg QD + SDT in Part 3 (open-label) with an option to increase to 100 mg elenestinib QD + SDT, for a total treatment duration of ~5 years. There will be two additional open-label cohorts: one evaluating the efficacy and tolerability of elenestinib 75 mg QD (with optional increase to 100 mg QD) in 40 patients previously treated with an approved selective KIT D816V inhibitor (Part K) and one evaluating the safety and efficacy of elenestinib 100 mg QD + SDT in ~20 patients with SSM (Part S). Key inclusion criteria for Part 2 include centrally confirmed ISM diagnosis and a moderate to severe total symptom score (TSS) per the ISM-Symptom Assessment Form (ISM-SAF; ©2018 Blueprint Medicines Corporation) based on a 14-day average. The primary endpoint is the mean change in ISM-SAF TSS from baseline to Week 48. Key secondary endpoints include the proportion of patients achieving serum tryptase normalization, undetectable or ≥50% reduction in *KIT* D816V variant allele fraction, symptom control measured by TSS, change in bone mineral density, change in the annualized rate of anaphylaxis, and change in QoL measures.

### Results

HARBOR is the largest RCT in ISM to date, Part 2 has initiated and is expanding globally, currently across 24 countries (**Figure**).

### Summary/Conclusion

Elenestinib is a highly selective and potent next-generation KIT D816V inhibitor that has limited central nervous system penetration. HARBOR Part 2 has been designed to assess the impact of a selective KIT inhibitor more robustly than in any other non-advanced SM trial. Novel and relevant endpoints, including bone health and anaphylaxis, will enable assessment of disease modification and the study timelines reflect the chronic nature of the disease. Dosing has been optimized to include treatment flexibility by evaluating multiple active dosing regimens.

**Copyright Information:** (Online) ISSN: 2572-9241
©2026 The Author(s). HemaSphere is published by John Wiley & Sons Ltd on behalf of European Hematology Association. This is an open access Abstract Book distributed under the Attribution-NonCommercial-NoDerivs (CC BY-NC-ND), which allows third parties to download the articles and share them with others as long as they credit the author and the Abstract Book, but they cannot change the content in any way or use them commercially.
**Abstract Book Citations:** Authors, Title, HemaSphere, 2026;10:(S1):pages. The abstract book DOIs can be found on the "Author Information" page.
**Disclaimer:** Articles published in the journal HemaSphere exclusively reflect the opinions of the authors. The authors are responsible for all content in their abstracts including accuracy of the facts, statements, citing resources, etc.

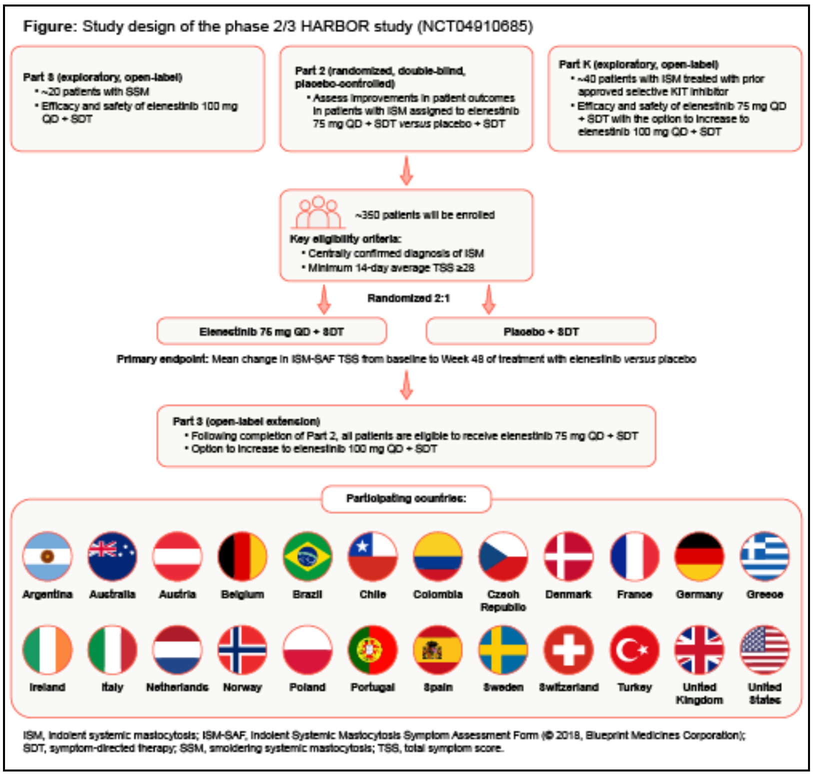

**Copyright Information:** (Online) ISSN: 2572-9241
©2026 The Author(s). HemaSphere is published by John Wiley & Sons Ltd on behalf of European Hematology Association. This is an open access Abstract Book distributed under the Attribution-NonCommercial-NoDerivs (CC BY-NC-ND), which allows third parties to download the articles and share them with others as long as they credit the author and the Abstract Book, but they cannot change the content in any way or use them commercially.
**Abstract Book Citations:** Authors, Title, HemaSphere, 2026;10:(S1):pages. The abstract book DOIs can be found on the “Author Information” page.
**Disclaimer:** Articles published in the journal HemaSphere exclusively reflect the opinions of the authors. The authors are responsible for all content in their abstracts including accuracy of the facts, statements, citing resources, etc.

### (PB3534) VALIDATION OF TRIPLE A SCORE IN POLYCITEMIA VERA IN A SINGLE PORTUGUESE CENTER

**Topic:** 16. Myeloproliferative neoplasms - Clinical

Maria Inês Pinto*[1], Bárbara Marques[2], Nelson Cardoso[1], Catarina Pinto[3], Ana Matos[3], Rosário Pinto Leite[3], Patrícia Ferraz[1], Manuel Cunha[1]

*[1]Unidade Local de Saúde de Trás-os-Montes e Alto Douro, Clinical Hematology, Vila Real, Portugal;[2]Unidade Local de Saúde de Coimbra, Clinical Hematology, Coimbra, Portugal; [3]Unidade Local de Saúde de Trás-os-Montes e Alto Douro, Genetic Laboratory, Vila Real, Portugal;*

### Background

Polycythemia vera (PV) is a JAK2-mutated myeloproliferative neoplasm (MPN) charaterized by erythrocytosis, splenomegaly and aquagenic pruritus. PV is associated with an increased risk of thrombotic events and it can adversely progress into secondary myelofibrosis (SMF) or acute myeloid leukemia (AML). Standard European Leukemia Network (ELN) risk stratification for thrombosis and overall survival (OS) in patients with PV is based on advanced age and history of thrombosis. Pathophysiologically, a pronounced clonal myeloproliferation and an host immune dysregulation may be predictive of adverse outcomes. In 2023, it was developed a novel prognostic score, the triple A score, which incorporates age, absolute neutrophil count (ANC) and absolute lymphocyte count (ALC) to discriminate OS and thrombotic risk in essential thrombocythemia. This score assigns: 4 points for age >70 years; 2 points for age 50–70 years; 1 point each for ANC> $8x10^9$/L and ALC <$1.7x10^9$/L. It classifies patients as low-risk (0–1 points), intermediate-1 risk (int-1)(3 points), intermediate-2 risk (int-2)(4 points), and high-risk (5–6 points). Regarding PV, very few studies validated its use and to our knowledge it was not evaluated in any Portuguese cohort.

### Aims

To investigate the role of triple A score predicting mortality and thrombosis risk in PV on a portuguese single center that covers an area of 360.000 inhabitants.

### Methods

Retrospectively, 58 patients were diagnosed with PV between 2020 and 2023, which corresponds to an incidence of 3.4 cases/100.000 inhabitants/year. All patients included presented: elevated hemoglobin concentration (> 16.5 g/dL in men; >16.0 g/dL in women) or elevated hematocrit (> 49% in men; >48% in women); *JAK2 V617F* mutation; a subnormal or low serum erythropoietin level or a bone marrow biopsy compatible with PV. All high ELN risk patients received cytoreductive therapy.

**Copyright Information:** (Online) ISSN: 2572-9241
©2026 The Author(s). HemaSphere is published by John Wiley & Sons Ltd on behalf of European Hematology Association. This is an open access Abstract Book distributed under the Attribution-NonCommercial-NoDerivs (CC BY-NC-ND), which allows third parties to download the articles and share them with others as long as they credit the author and the Abstract Book, but they cannot change the content in any way or use them commercially.
**Abstract Book Citations:** Authors, Title, HemaSphere, 2026;10:(S1):pages. The abstract book DOIs can be found on the "Author Information" page.
**Disclaimer:** Articles published in the journal HemaSphere exclusively reflect the opinions of the authors. The authors are responsible for all content in their abstracts including accuracy of the facts, statements, citing resources, etc.

### Results

Male patients were 26 (44,8%) and the median age at diagnosis was 75 (22-98) years; 13 patients had splenomegaly; median hemoglobin was 17,6 g/dL(15.4-21.2) and median hematocrit 53,2% (48.0-65.8); median platelets were 446 $x10^9$/L(139-937), ANC $7.2x10^9$/L(3.1-25.7), ALC 1.6 $x10^9$/L(0.56-5.1). There was a total of 19 (32,7%) thrombotic events, only 4 (6,9%) occurred after the diagnosis. High ELN risk disease was recorded in 53 patients (91,4%) and low risk in 5 (8,6%). Based on triple A model, a total of 1(1.7%), 22(37,9%), 9(15.5%) and 26(44.8%) were classified as low, int-1, int-2, and high risk, respectively. 18 high ELN risk patients were reclassified as int-1 and 9 patients as int-2. The median event-free survival (EFS) and OS were not reached. The 5-year EFS and OS rates were 55.6% and 59.1%. Only 1 patient progressed into SMF and none transformed to AML. 17 patients (29,3%) died. ELN score showed a positive but non-significant association with mortality risk (HR=4.91; 95%CI:0.16–155.69, p=0.367). Nevertheless, the triple A score demonstrated a significant association with increased mortality risk (HR=1.72; 95%CI:1.08–2.76, p=0.023).

### Summary/Conclusion

This study highlights the high incidence of PV in our center compared to the reported in the literature. Our results validate the superiority of triple A score predicting mortality compared to the traditional ELN. As it is easy applicable, this study favours its incorporation on prognosis stratification of PV. In the future, triple A score could represent a useful tool for guiding more precisely decisions regarding the use of cytoreductive therapy.

**Copyright Information:** (Online) ISSN: 2572-9241
©2026 The Author(s). HemaSphere is published by John Wiley & Sons Ltd on behalf of European Hematology Association. This is an open access Abstract Book distributed under the Attribution-NonCommercial-NoDerivs (CC BY-NC-ND), which allows third parties to download the articles and share them with others as long as they credit the author and the Abstract Book, but they cannot change the content in any way or use them commercially.
**Abstract Book Citations:** Authors, Title, HemaSphere, 2026;10:(S1):pages. The abstract book DOIs can be found on the "Author Information" page.
**Disclaimer:** Articles published in the journal HemaSphere exclusively reflect the opinions of the authors. The authors are responsible for all content in their abstracts including accuracy of the facts, statements, citing resources, etc.

### (PB3535) DOES HAPLOIDENTICAL TRANSPLANTATION IMPROVE MYELOFIBROSIS OUTCOMES IN REAL-WORLD? A SINGLE-CENTER EXPERIENCE FROM THE BRAZILIAN PUBLIC HEALTH CARE SYSTEM

**Topic:** 16. Myeloproliferative neoplasms - Clinical

Lucas Sales*[1], Caio Justino[1], Laura Vassalli[1], Amanda Chucre[1], Fernanda Santos Azevedo[1], Pedro Henrique Moraes[1], Eurides Rosa[1], Vinicius Molla[1], Celso Arrais-Rodrigues[1], Alex Sandes[1]

*[1]Paulista School of Medicine, Federal University of São Paulo (EPM–UNIFESP), São Paulo-SP, Brazil;*

### Background

Primary myelofibrosis (PMF) is a BCR::ABL1-negative myeloproliferative neoplasm characterized by highly heterogeneous clinical course. Risk stratification using validated tools such as DIPSS-Plus and the Myelofibrosis Transplant Scoring System (MTSS) is essential to guide therapeutic decision-making. Treatment options include supportive care, JAK inhibitors (JAKi), and allogeneic hematopoietic stem cell transplantation (allo- HSCT), the only potentially curative modality. Real-world data from Latin American public healthcare settings remain scarce.

### Aims

To evaluate overall survival (OS) in a cohort of PMF patients treated at a Brazilian public referral center, stratified by therapeutic approach: allo-HSCT versus best available therapy (BAT). Secondary objectives included analysis of MTSS-based outcomes and HSCT-specific endpoints (TRM and GVHD).

### Methods

Retrospective, observational cohort study conducted at Hospital São Paulo – UNIFESP between 2019 and 2026. Patients diagnosed with PMF per WHO criteria were included and assigned to two treatment arms: (A) allo-HSCT; (B) BAT (JAKi, hydroxyurea, immunomodulatory agents, EPO, transfusion support, and corticosteroids). Risk stratification was performed using DIPSS- Plus and MTSS. OS was estimated by Kaplan-Meier method and groups were compared by log-rank test ($p < 0.05$).

### Results

A total of 107 patients were included (56% male; median age: 64 years, range 36–91; median follow-up: 46 months). Driver mutations: JAK2 V617F 58.9%, triple-negative 26.2%, CALR 13.1%, MPL 1.9%. By DIPSS-Plus: 14.7% low-risk, 28.4% intermediate-1, 35.8% intermediate-2, and 19.3% high- risk. Overall survival at 5 years was 73.8% (median not reached; median follow- up 153 months). OS differed significantly according to DIPSS-Plus (low-risk: not reached vs. high-risk: 86 months; $p = 0.01$). Sixteen patients underwent allo-HSCT. Median age was 57 y (range 41–72); 68.8% were male. Donor distribution: 37% matched unrelated, 37% matched related, and 25% haploidentical. Reduced-intensity conditioning (RIC) was used in 62% of patients; peripheral blood was the stem cell source in 87%. Post-transplant cyclophosphamide (PTCy)-based GVHD prophylaxis was used in 75% of patients. Acute GVHD grade II–IV occurred in 43%; grade III–IV in 6%. The 2- year cumulative incidence of chronic GVHD was 50%, with only 6% requiring prolonged immunosuppression. NRM at day 100 was 18%, with infection as the leading cause of death (18.8%). Among intermediate-2 and high-risk patients, a borderline difference in OS was observed between the allo-HSCT and BAT groups (median 29 vs. 153 months; $p = 0.049$). However, no significant difference in OS was identified between patients undergoing allo-HSCT with low or intermediate MTSS risk ($n = 9$) and those treated with BAT (median 70 vs. 53 months; $p = 0.855$).

**Copyright Information:** (Online) ISSN: 2572-9241
©2026 The Author(s). HemaSphere is published by John Wiley & Sons Ltd on behalf of European Hematology Association. This is an open access Abstract Book distributed under the Attribution-NonCommercial-NoDerivs (CC BY-NC-ND), which allows third parties to download the articles and share them with others as long as they credit the author and the Abstract Book, but they cannot change the content in any way or use them commercially.
**Abstract Book Citations:** Authors, Title, HemaSphere, 2026;10:(S1):pages. The abstract book DOIs can be found on the "Author Information" page.
**Disclaimer:** Articles published in the journal HemaSphere exclusively reflect the opinions of the authors. The authors are responsible for all content in their abstracts including accuracy of the facts, statements, citing resources, etc.

### Summary/Conclusion

This cohort demonstrates that allo-HSCT is feasible within the Brazilian public healthcare system. PTCy and RIC were associated with acceptable GVHD and TRM rates. Haploidentical donors represented one quarter of transplants, expanding access where matched donors are limited. MTSS proved clinically relevant, with outcomes varying according to transplant risk stratification, supporting its integration into decision-making. Larger prospective studies are warranted to validate MTSS-guided strategies and define the optimal role of haploidentical HSCT in high-risk PMF within public healthcare systems.

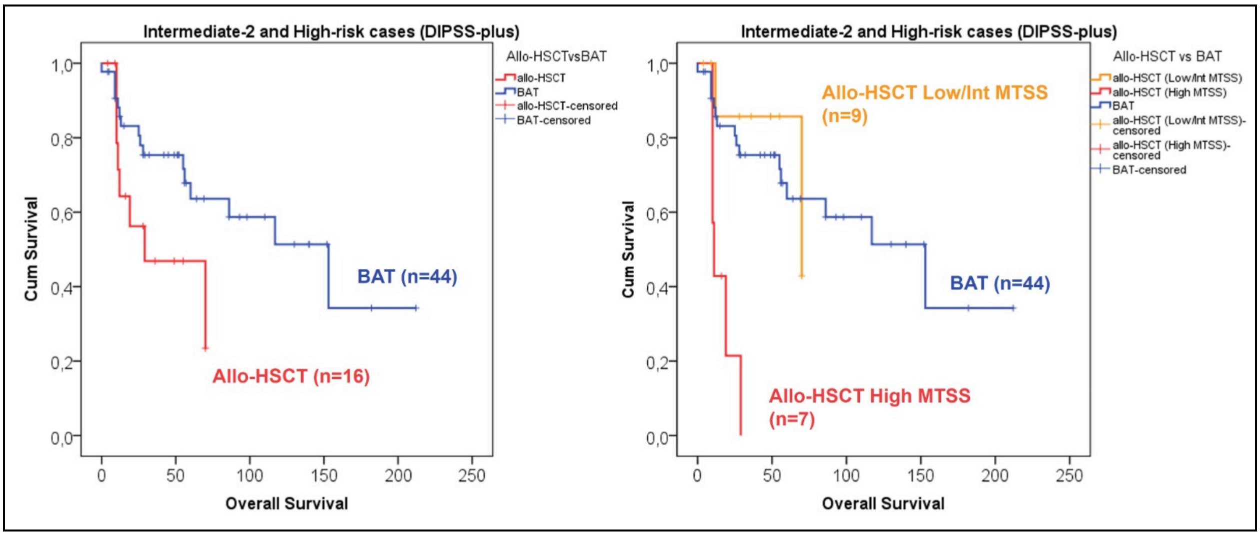

**Copyright Information:** (Online) ISSN: 2572-9241
©2026 The Author(s). HemaSphere is published by John Wiley & Sons Ltd on behalf of European Hematology Association. This is an open access Abstract Book distributed under the Attribution-NonCommercial-NoDerivs (CC BY-NC-ND), which allows third parties to download the articles and share them with others as long as they credit the author and the Abstract Book, but they cannot change the content in any way or use them commercially.
**Abstract Book Citations:** Authors, Title, HemaSphere, 2026;10:(S1):pages. The abstract book DOIs can be found on the "Author Information" page.
**Disclaimer:** Articles published in the journal HemaSphere exclusively reflect the opinions of the authors. The authors are responsible for all content in their abstracts including accuracy of the facts, statements, citing resources, etc.

### (PB3536) UNMASKING DIABETES MELLITUS IN PATIENTS WITH BCR::ABL1 NEGATIVE MYELOPROLIFERATIVE NEOPLASMS USING CONTINUOUS GLUCOSE MONITORING

**Topic:** 16. Myeloproliferative neoplasms - Clinical

Josipa Budimir*[1], Sanja Klobucar[2, 3], Marko Skelin[1, 2], Zvonimir Marko Skugor[1], Dijana Detel[2], Zinaida Perić[2, 3], Marko Lucijanic[4, 5], Ivan Krecak[1, 2]

*[1]General Hospital of Sibenik-Knin County, Sibenik, Croatia;[2]Faculty of medicine Rijeka, Rijeka, Croatia;[3]University Hospital Rijeka, Division of Hematology, Rijeka, Croatia; [4]University Hospital Dubrava, Zagreb, Croatia;[5]School of medicine Zagreb, Zagreb, Croatia;*

### Background

Patients with *BCR::ABL1* negative myeloproliferative neoplasms (MPNs), essential thrombocythemia (ET), polycythemia vera (PV), and myelofibrosis (MF) are characterized by strong myeloproliferation and high cardiovascular risk. Strict control of cardiovascular comorbidities such as intermediate hyperglycemia (IH) and diabetes mellitus (DM) in MPNs is imperative. According to literature data, the prevalence of DM in MPN is unusually low (<15%). Possible reasons are tumor-induced hypoglycemia and shorter erythrocyte half-life, which can potentially cause falsely lower fasting plasma glucose (FPG) and HbA1c values, as well as the potential effect of hydroxyurea on the promotion of HbF synthesis.

### Aims

The aim of this study was to analyze whether the use of continuous glucose monitoring (CGM) may help in identifying more MPN patients with dysglycemia than standard diagnostic work-up, as these devices measure interstitial glucose concentration and are not dependent on blood cell counts.

### Methods

This prospective multicenter study was conducted in three hematology centers in Croatia (General Hospital of Sibenik-Knin County, University Hospital Center Rijeka, and University Hospital Dubrava) during 2025-2026 and included patients with MPNs diagnosed according to 2022 World Health Organization criteria. Blood samples were taken at study entry. Classical diagnostic work-up for the detection of IH and DM (FPG, HbA1c and oral glucose tolerance test-OGTT) was performed in all patients expect in those already diagnosed with DM. The CGM device (Dexcom One®) was installed at the time of study entry and was kept on for 10 days. The diagnosis of IH and DM using classical criteria was made according to American Diabetes Association criteria; definition of these conditions according to CGM readings was defined as the time in tight range (TITR; reference range 3.9-7.8 mmol/L) of 70-89% for IH and <70% for DM. Statistics were performed with MedCalc Statistical Software (v. 23.4.8).

**Copyright Information:** (Online) ISSN: 2572-9241
©2026 The Author(s). HemaSphere is published by John Wiley & Sons Ltd on behalf of European Hematology Association. This is an open access Abstract Book distributed under the Attribution-NonCommercial-NoDerivs (CC BY-NC-ND), which allows third parties to download the articles and share them with others as long as they credit the author and the Abstract Book, but they cannot change the content in any way or use them commercially.
**Abstract Book Citations:** Authors, Title, HemaSphere, 2026;10:(S1):pages. The abstract book DOIs can be found on the "Author Information" page.
**Disclaimer:** Articles published in the journal HemaSphere exclusively reflect the opinions of the authors. The authors are responsible for all content in their abstracts including accuracy of the facts, statements, citing resources, etc.

### Results

A total of 34 patients were included (ET=10, PV=18, MF=6); median age was 70.5 years (36-85), 14 (41.2%) were female, and 24 (70.6%) were treated with cytoreduction (mostly hydroxyurea, 75%). At baseline, one (2.9%) and 8 (23.5%) patients had IH and DM recorded as such in the medical documentation, respectively. The median FPG, HbA1c and post-OGTT glucose were 5.7 mmol/L (range 4.5-12.2), 5.2% (range 4.1-7.3) and 6.3 mmol/L (range 3.6-11.2), respectively. The median time in range (TIR; reference range 3.9-10 mmol/L) and TITR were 97% (range 31-100) and 79% (range 2-98), respectively. There were no associations of IH/DM defined by the TITR with patient age, sex, disease phenotype, presence of splenomegaly nor any of the blood cell count components ($p>0.050$ for all analyses).
In MPN patients without a prior diagnosis of IH or DM (n=25), IH was diagnosed in 11 (44%) patients and DM in two (8%) patients by standard diagnostics. After CGM placement, IH and DM were diagnosed in 8 (32%) and 8 patients (32%), respectively. A patient with prior diagnosis of IH was diagnosed with DM (Figure 1).

### Summary/Conclusion

The total prevalence of IH (n=9, 26.4%) and DM (n=16, 47%) in this study was actually quite high in MPN patients. Therefore, traditional diagnostic methods for the detection of dysglycemia in MPNs seem to perform suboptimally. Considering the known detrimental impact of dysglycemia on cardiovascular outcomes, these results suggest that CGM may help in the diagnosis, monitoring and treatment of MPN patients with IH/DM.

| Subjects n=34 | Standard diagnostics using American Diabetes Association criteria (FPG, OGTT, HbA1c) | Continous glucose monitoring (IH definition as TITR$<$70-89%; DM as TITR$<$70%) |
|---|---|---|
| No prior IH and DM, n=25 | IH, n=11 (44%)<br>DM, n=2 (8%) | IH, n=8 (32%)<br>DM, n=8 (32%) |
| Prior IH, n=1 | IH, n=1 | Diagnosed as DM (TITR 58%) |

IH=intermediate hyperglycemia, DM=diabetes mellitus, TITR=time in tight range, FPD=fasting plasma glucose, OGTT=oral glucose tolerance test, HbA1c=glycated hemoglobin.

Copyright Information: (Online) ISSN: 2572-9241
©2026 The Author(s). HemaSphere is published by John Wiley & Sons Ltd on behalf of European Hematology Association. This is an open access Abstract Book distributed under the Attribution-NonCommercial-NoDerivs (CC BY-NC-ND), which allows third parties to download the articles and share them with others as long as they credit the author and the Abstract Book, but they cannot change the content in any way or use them commercially.
Abstract Book Citations: Authors, Title, HemaSphere, 2026;10:(S1):pages. The abstract book DOIs can be found on the "Author Information" page.
Disclaimer: Articles published in the journal HemaSphere exclusively reflect the opinions of the authors. The authors are responsible for all content in their abstracts including accuracy of the facts, statements, citing resources, etc.

### (PB3537) CLINICAL OUTCOMES OF MOMELOTINIB IN A REAL-WORLD COHORT OF PATIENTS WITH MYELOFIBROSIS

**Topic:** 16. Myeloproliferative neoplasms - Clinical

Esther Ortega Vida*[1], Francisco Jose Jimenez Najar[1], Vanesa Fernández Vilchez[1], Juan Antonio López[1]

[1]*Complejo Hospitalario de Jaén, Hematology, Jaén, Spain;*

**Background**

Momelotinib is a JAK1/JAK2 and ACVR1 inhibitor with demonstrated efficacy in anemia, splenomegaly and symptom control in patients with myelofibrosis. However, real-world evidence remains limited, particularly regarding treatment patterns and management of proliferative features emerging during therapy. Data on the need for concomitant cytoreductive treatment with hydroxyurea in patients receiving momelotinib are scarce.

**Aims**

To describe real-world clinical outcomes of momelotinib in patients with myelofibrosis treated at a tertiary referral center, focusing on hematologic response, symptom burden, splenic response, safety, and proliferative features requiring cytoreductive therapy.

**Methods**

We conducted a retrospective real-world study including patients with myelofibrosis treated with momelotinib at a tertiary hospital. Baseline demographic and clinical variables, mutational status and prior treatments were collected. Hemoglobin levels, transfusion requirements, symptom evolution and splenic response were assessed. Proliferative features were evaluated through serial leukocyte counts, defining clinically relevant leukocytosis as persistent values >20 x10$^9$/L without reactive causes. Hydroxyurea initiation after momelotinib was recorded as a proliferative endpoint. Outcomes were described using descriptive statistics.

**Results**

Ten patients were included, with a median age of 78.0 years (IQR 73.3–81.6), and 60% were female. Primary myelofibrosis was present in 40%, while 60% had secondary disease. JAK2 and triple-negative mutations were each observed in 40% of patients. Six patients (60%) had received prior ruxolitinib. Median baseline hemoglobin was 9.95 g/dL (IQR 8.80–10.78).
Following momelotinib initiation, median hemoglobin at last follow-up was 11.25 g/dL (IQR 10.30–11.62), with a median increase of +1.35 g/dL (IQR +0.48 to +2.50). A clinically meaningful hemoglobin response (≥1 g/dL increase) was achieved in 7 patients (70%). Among nine evaluable patients, two were transfusion-dependent at baseline, with a reduction in median red blood cell units from 33.0 to 24.5 during the first six months of treatment.
Symptom improvement was observed in 5 patients, with stability in the remaining 5. Splenic reassessment was available in four patients, showing size reduction in two cases. Momelotinib demonstrated a favorable safety profile with a low discontinuation rate.
Regarding proliferative features, median leukocyte count increased from 17.8 x10$^9$/L at baseline to a median peak of 34.9 x10$^9$/L during treatment. Persistent leukocytosis >20 x10$^9$/L occurred in 7 patients (70%), leading to initiation of hydroxyurea in the same proportion. Median time from momelotinib initiation to hydroxyurea introduction was 4.1 months. Hydroxyurea was initiated due to progressive disease-related leukocytosis in the absence of reactive causes, representing a new cytoreductive requirement emerging during momelotinib therapy.

**Copyright Information:** (Online) ISSN: 2572-9241
©2026 The Author(s). HemaSphere is published by John Wiley & Sons Ltd on behalf of European Hematology Association. This is an open access Abstract Book distributed under the Attribution-NonCommercial-NoDerivs (CC BY-NC-ND), which allows third parties to download the articles and share them with others as long as they credit the author and the Abstract Book, but they cannot change the content in any way or use them commercially.
**Abstract Book Citations:** Authors, Title, HemaSphere, 2026;10:(S1):pages. The abstract book DOIs can be found on the “Author Information” page.
**Disclaimer:** Articles published in the journal HemaSphere exclusively reflect the opinions of the authors. The authors are responsible for all content in their abstracts including accuracy of the facts, statements, citing resources, etc.

### Summary/Conclusion

In this real-world cohort from a tertiary center, momelotinib provided clinically meaningful improvement in anemia and symptom burden with acceptable tolerability. However, a subset of patients developed proliferative features characterized by progressive leukocytosis requiring hydroxyurea. These findings highlight the importance of close hematologic monitoring and suggest that combination treatment strategies may be required in routine practice. Larger studies are needed to better characterize proliferative patterns during momelotinib therapy.

**Figure 1. Baseline patient characteristics**

| | N=10 |
|---|---|
| Age, years (median/range) | 78 (73.3–81.6) |
| Sex (male/female) | 40%/60% |
| Type of driver mutation | |
| - JAK2 | 40% |
| - CALR | 10% |
| - MPL | 10% |
| - Triple-negative | 40% |
| Myelofibrosis type | |
| - Primary myelofibrosis | 40% |
| - Post-polycythemia vera myelofibrosis | 20% |
| - Post-essential thrombocythemia myelofibrosis | 40% |
| Prognostic score | |
| - Low risk | 20% |
| - Intermediate risk | 40% |
| - High risk | 40% |
| Line of therapy for momelotinib | |
| - First-line momelotinib | 40% |
| - Second-line momelotinib | 30% |
| - ≥3rd-line momelotinib | 30% |
| Prior JAK inhibitor exposure | 60% |
| Splenomegaly | 50% |
| Baseline symptoms | 70% |

**Figure 2. Hemoglobin evolution during first 6 months of momelotinib**

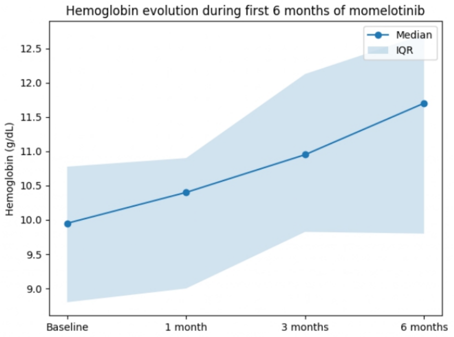

Copyright Information: (Online) ISSN: 2572-9241
©2026 The Author(s). HemaSphere is published by John Wiley & Sons Ltd on behalf of European Hematology Association. This is an open access Abstract Book distributed under the Attribution-NonCommercial-NoDerivs (CC BY-NC-ND), which allows third parties to download the articles and share them with others as long as they credit the author and the Abstract Book, but they cannot change the content in any way or use them commercially.
Abstract Book Citations: Authors, Title, HemaSphere, 2026;10:(S1):pages. The abstract book DOIs can be found on the "Author Information" page.
Disclaimer: Articles published in the journal HemaSphere exclusively reflect the opinions of the authors. The authors are responsible for all content in their abstracts including accuracy of the facts, statements, citing resources, etc.

### (PB3538) CORRELATION OF MEGAKARYOCYTE MORPHOLOGY WITH ADVERSE PROGNOSIS IN ESSENTIAL THROMBOCYTHEMIA

**Topic:** 16. Myeloproliferative neoplasms - Clinical

Zhikang Zheng[1], Tian Zeng[1], Rumeng Li[1], Qinmei Han[1], Jia Li[1], Xiaoyan MA[1], Bingbing Wen[1], Xiaojing Bao[1], Yingqing Xu[1], Jie Jin[1], Jian Huang*[1]

[1] *The First Affiliated Hospital, Zhejiang University School of Medicine, Hangzhou, China;*

### Background

Essential thrombocythemia (ET) carries a risk of progression to myelofibrosis (MF) or acute myeloid leukemia (AML). While megakaryocyte (MK) morphological abnormalities are hallmark features of ET, the prognostic significance of specific MK subtypes in predicting disease progression remains incompletely defined.

### Aims

Essential thrombocythemia (ET) carries a risk of progression to myelofibrosis (MF) or acute myeloid leukemia (AML). While megakaryocyte (MK) morphological abnormalities are hallmark features of ET, the prognostic significance of specific MK subtypes in predicting disease progression remains incompletely defined.

### Methods

A retrospective analysis was performed on bone marrow smears from 163 patients with ET at the First Affiliated Hospital and Fourth Affiliated Hospital of Zhejiang University School of Medicine between October 2014 and December 2025. A total of 14,727 MK were counted under microscopy, among whom 121 patients had available followup data and 12 patients developed progression to myelofibrosis or leukemia. MK morphology was classified into 12 categories according to the WHO criteria, including normal MK, emperipolesis MK, staghorn like MK, naked nuclei MK, cluster MK, large MK, small MK, hyperchromatic MK, abnormal N:C ratio MK, bulbous MK, separated nuclear MK, and nonlobated MK. After excluding the timedependent effects of cluster MK and abnormal N:C ratio MK on survival, Cox regression analysis was conducted using the median percentages of the remaining 10 MK morphological types.

### Results

Among the 121 ET patients, univariate analysis demonstrated that naked nuclei MK was positively associated with shorter progressionfree survival (PFS) (HR=1.14; 95%CI 1.05–1.22; P<0.001), and separated nuclear MK was also positively correlated with reduced PFS (HR=1.10; 95%CI 1.01–1.20; P=0.023). Further multivariate analysis identified naked nuclei MK as an independent risk factor for shorter PFS (HR=1.12; 95%CI 1.04–1.22; P=0.005), suggesting that an elevated percentage of naked nuclei MK may increase the risk of disease progression by 12%. No significant associations were observed between other MK morphological types and PFS.

**Copyright Information:** (Online) ISSN: 2572-9241
©2026 The Author(s). HemaSphere is published by John Wiley & Sons Ltd on behalf of European Hematology Association. This is an open access Abstract Book distributed under the Attribution-NonCommercial-NoDerivs (CC BY-NC-ND), which allows third parties to download the articles and share them with others as long as they credit the author and the Abstract Book, but they cannot change the content in any way or use them commercially.
**Abstract Book Citations:** Authors, Title, HemaSphere, 2026;10:(S1):pages. The abstract book DOIs can be found on the "Author Information" page.
**Disclaimer:** Articles published in the journal HemaSphere exclusively reflect the opinions of the authors. The authors are responsible for all content in their abstracts including accuracy of the facts, statements, citing resources, etc.

**Summary/Conclusion**

Naked nuclei and separated nuclear MK represent highrisk factors for progression of ET to MF and AML, among which naked nuclei MK serves as an independent risk factor for PFS.

1.This research was supported by the National Natural Science Foundation of China (No. 82570191) and the Zhejiang Provincial Health Highlevel Innovative Talent Project (2022-2026).
2.† Correspondence to: Prof Jian Huang, E-mail:househuang@zju.edu.cn

**Copyright Information:** (Online) ISSN: 2572-9241
©2026 The Author(s). HemaSphere is published by John Wiley & Sons Ltd on behalf of European Hematology Association. This is an open access Abstract Book distributed under the Attribution-NonCommercial-NoDerivs (CC BY-NC-ND), which allows third parties to download the articles and share them with others as long as they credit the author and the Abstract Book, but they cannot change the content in any way or use them commercially.
**Abstract Book Citations:** Authors, Title, HemaSphere, 2026;10:(S1):pages. The abstract book DOIs can be found on the "Author Information" page.
**Disclaimer:** Articles published in the journal HemaSphere exclusively reflect the opinions of the authors. The authors are responsible for all content in their abstracts including accuracy of the facts, statements, citing resources, etc.

### (PB3539) DIAGNOSTIC AND THERAPEUTIC INEQUITIES IN PHILADELPHIA-NEGATIVE MYELOPROLIFERATIVE NEOPLASMS: A MULTINATIONAL LATIN AMERICAN PHYSICIAN SURVEY

**Topic:** 16. Myeloproliferative neoplasms - Clinical

Carlos Murilo Barbosa Junior*[1], Mariana Guaraná[2], Cristiana Solza[1]

*[1]State University of Rio de Janeiro, Hematology, Rio de Janeiro, Brazil;[2]Gaffrée and Guinle University Hospital, Rio de Janeiro, Brazil;*

### Background

Optimal management of Philadelphia-negative myeloproliferative neoplasms (Ph- MPNs)—including polycythemia vera (PV), essential thrombocythemia (ET), and myelofibrosis (MF)—requires accurate molecular diagnosis and risk-adapted therapeutic strategies. Real-world data on clinical practice patterns in Latin America remain limited, particularly regarding access to diagnostic tools and novel therapies.

### Aims

To evaluate current diagnostic and therapeutic practices, as well as perceived barriers in the management of Ph- MPNs among Latin American hematologists.

### Methods

In 2025, a 31-item anonymous, web-based survey was distributed to hematologists practicing in Brazil. The survey assessed physician demographics, institutional characteristics, diagnostic resources, therapeutic decision-making, and perceived challenges in Ph- MPN management. In 2026, the survey was expanded to additional Latin American countries. Data were collected using the REDCap platform.

### Results

A total of 180 responses were obtained from Brazil, Mexico, Argentina, Guatemala, Paraguay, and the Dominican Republic. Among respondents, 41% had more than 15 years of clinical experience. The majority (84%) were based in Brazil, with 70% of Brazilian participants practicing in the Southeast region. Practice settings included public institutions (17%), private institutions (22%), and both sectors (61%).
Most respondents (69%) reported the absence of a dedicated Ph- MPN clinic at their institution, and 21% lacked access to a hematopathologist for bone marrow specimen evaluation. Although nearly all participants had access to JAK2 mutation testing, 25% reported no access to CALR and MPL mutation analysis, and 50% indicated that these tests were available exclusively in the private setting. Next-generation sequencing was unavailable to 56% of respondents.
Only 14% reported routinely using the MPN-SAF symptom assessment tool, with higher utilization among physicians managing more than 30 patients per month.
For MF treatment, JAK inhibitors were predominantly accessible in the private sector (71%). Despite the high regional incidence of tuberculosis, only 12% of respondents reported routine tuberculosis screening prior to initiating JAK inhibitor therapy. For patients with high-risk MF, 75% considered allogeneic hematopoietic stem cell transplantation a therapeutic option.
The most frequently reported barriers to optimal MPN care were limited access to molecular and genetic testing (83%) and restricted availability of novel therapies (71%).

**Copyright Information:** (Online) ISSN: 2572-9241
©2026 The Author(s). HemaSphere is published by John Wiley & Sons Ltd on behalf of European Hematology Association. This is an open access Abstract Book distributed under the Attribution-NonCommercial-NoDerivs (CC BY-NC-ND), which allows third parties to download the articles and share them with others as long as they credit the author and the Abstract Book, but they cannot change the content in any way or use them commercially.
**Abstract Book Citations:** Authors, Title, HemaSphere, 2026;10:(S1):pages. The abstract book DOIs can be found on the “Author Information” page.
**Disclaimer:** Articles published in the journal HemaSphere exclusively reflect the opinions of the authors. The authors are responsible for all content in their abstracts including accuracy of the facts, statements, citing resources, etc.

**Summary/Conclusion**

This study represents the first Brazilian survey evaluating real-world management of Ph- MPNs and is currently being expanded across Latin America. The findings demonstrate substantial disparities in access to diagnostic and therapeutic resources, particularly within public healthcare systems, and reveal underutilization of validated symptom assessment tools. Ongoing data collection will enable more comprehensive analyses. These results underscore the need for targeted strategies to improve equitable access to molecular diagnostics and innovative therapies across the region.

**Copyright Information:** (Online) ISSN: 2572-9241
©2026 The Author(s). HemaSphere is published by John Wiley & Sons Ltd on behalf of European Hematology Association. This is an open access Abstract Book distributed under the Attribution-NonCommercial-NoDerivs (CC BY-NC-ND), which allows third parties to download the articles and share them with others as long as they credit the author and the Abstract Book, but they cannot change the content in any way or use them commercially.
**Abstract Book Citations:** Authors, Title, HemaSphere, 2026;10:(S1):pages. The abstract book DOIs can be found on the "Author Information" page.
**Disclaimer:** Articles published in the journal HemaSphere exclusively reflect the opinions of the authors. The authors are responsible for all content in their abstracts including accuracy of the facts, statements, citing resources, etc.

### (PB3540) DEVELOPMENT OF EXPERT CONSENSUS ON THE MANAGEMENT OF MYELOFIBROSIS PATIENTS WITH ANAEMIA AND/OR THROMBOCYTOPENIA: A MODIFIED DELPHI PANEL

**Topic:** 16. Myeloproliferative neoplasms - Clinical

Gabriela Hobbs*[1], Donal McLornan[2], Kazuya Shimoda[3], Richard Perry[4], Charlotte Oswald[4], Lisa Perrett[4], Owain Morris[4]

*[1]Mass General Brigham, Boston, United States of America; [2]Department and Division of Haematology, University College Hospital London, London, United Kingdom; [3]Division of Hematology, Diabetes, and Endocrinology, Department of Medicine, University of Miyazaki, Miyazaki, Japan; [4]Adelphi Values PROVE, Bollington, United Kingdom;*

### Background

Myelofibrosis (MF) is a progressive bone marrow neoplasm characterized by splenomegaly, constitutional symptoms, and frequent cytopenias which impact prognosis and complicate therapeutic decisions. Evidence-based guidance on management of MF patients with anaemia and/or thrombocytopenia remains limited and clinicians often face challenging decisions.

### Aims

To develop consensus recommendations on the management of MF patients with anaemia and/or low platelets, exploring clinical practice and perceptions of JAK inhibitor (JAKi) use to optimise patient outcomes.

### Methods

A double-blinded modified Delphi panel is underway (**Fig 1**). The Delphi methodology allows iterative consensus generation.
A panel of 23 clinical experts actively managing MF patients with anaemia and/or thrombocytopenia from EU5 countries, Japan and the US was recruited to participate in the Delphi. A Steering Committee (SC) of three established MF experts was convened to review study materials, support study interpretation, and provide ongoing expert opinion. The panel and SC are mutually blinded, and both are blinded to the study sponsor. The study sponsor is blinded to the identities of both panelists and SC members.
The first-round survey mostly consisted of open-ended and multiple-choice questions to obtain exploratory insights, following input from SC review. First-round responses were analysed to generate statements for exploration in the second round using a 9-point Likert scale. Consensus will be defined as ≥80% agreement on a rating of 7–9 (1=strongly disagree, 9=strongly agree).
In line with the Delphi methodology, the second-round survey will include presentation of individual and group first-round results to guide exploration of topics and consensus statement elicitation. New topics will also be included. Statements that do not reach consensus will be taken forward to the consensus meeting for discussion of changes to enable consensus.

**Copyright Information:** (Online) ISSN: 2572-9241
©2026 The Author(s). HemaSphere is published by John Wiley & Sons Ltd on behalf of European Hematology Association. This is an open access Abstract Book distributed under the Attribution-NonCommercial-NoDerivs (CC BY-NC-ND), which allows third parties to download the articles and share them with others as long as they credit the author and the Abstract Book, but they cannot change the content in any way or use them commercially.
**Abstract Book Citations:** Authors, Title, HemaSphere, 2026;10:(S1):pages. The abstract book DOIs can be found on the "Author Information" page.
**Disclaimer:** Articles published in the journal HemaSphere exclusively reflect the opinions of the authors. The authors are responsible for all content in their abstracts including accuracy of the facts, statements, citing resources, etc.

### Results

This Delphi seeks to develop consensus recommendations from clinical experts around key themes that will be explored further during the second round and consensus meeting (**Table 1**).

**Table 1. Consensus themes and question topics**

| Themes | Question Topics (not exhaustive) |
|---|---|
| **1.** Current management of new onset or worsening anaemia with JAKi | > Approaches to the management of new/worsening anaemia |
| **2.** Current management of MF patients with anaemia and/or low platelets | > Considerations in choosing the most appropriate first-line JAKi<br>> Challenges and strategies to manage anaemia and/or thrombocytopenia |
| **3**. Dosing strategies in the management of JAKi-naïve or -experienced MF patients with anaemia and/or low platelets | > JAKi dose initiations in JAKi-naïve patients<br>> Adjustments following inadequate response to JAKi, such as dose modification, switching JAKis, or adding supportive care |
| **4.** Considerations regarding initiation and sequencing ('switching') of JAKis in JAKi-naïve or -experienced MF patients | > Dosing strategies and approaches for switching JAKis ensuring maintenance of therapeutic benefit |
| **5.** Considerations for JAKi discontinuation in MF | > Optimal duration of JAKi prior to discontinuation<br>> Factors that influence JAKi discontinuation and approaches to mitigate |

### Summary/Conclusion

This study will ultimately provide expert consensus to inform best practice for managing anaemia and/or thrombocytopenia in MF patients irrespective of JAKi status. Recommendations will address gaps in MF management and support clinicians in optimizing patient care.

**Copyright Information:** (Online) ISSN: 2572-9241
©2026 The Author(s). HemaSphere is published by John Wiley & Sons Ltd on behalf of European Hematology Association. This is an open access Abstract Book distributed under the Attribution-NonCommercial-NoDerivs (CC BY-NC-ND), which allows third parties to download the articles and share them with others as long as they credit the author and the Abstract Book, but they cannot change the content in any way or use them commercially.
**Abstract Book Citations:** Authors, Title, HemaSphere, 2026;10:(S1):pages. The abstract book DOIs can be found on the "Author Information" page.
**Disclaimer:** Articles published in the journal HemaSphere exclusively reflect the opinions of the authors. The authors are responsible for all content in their abstracts including accuracy of the facts, statements, citing resources, etc.

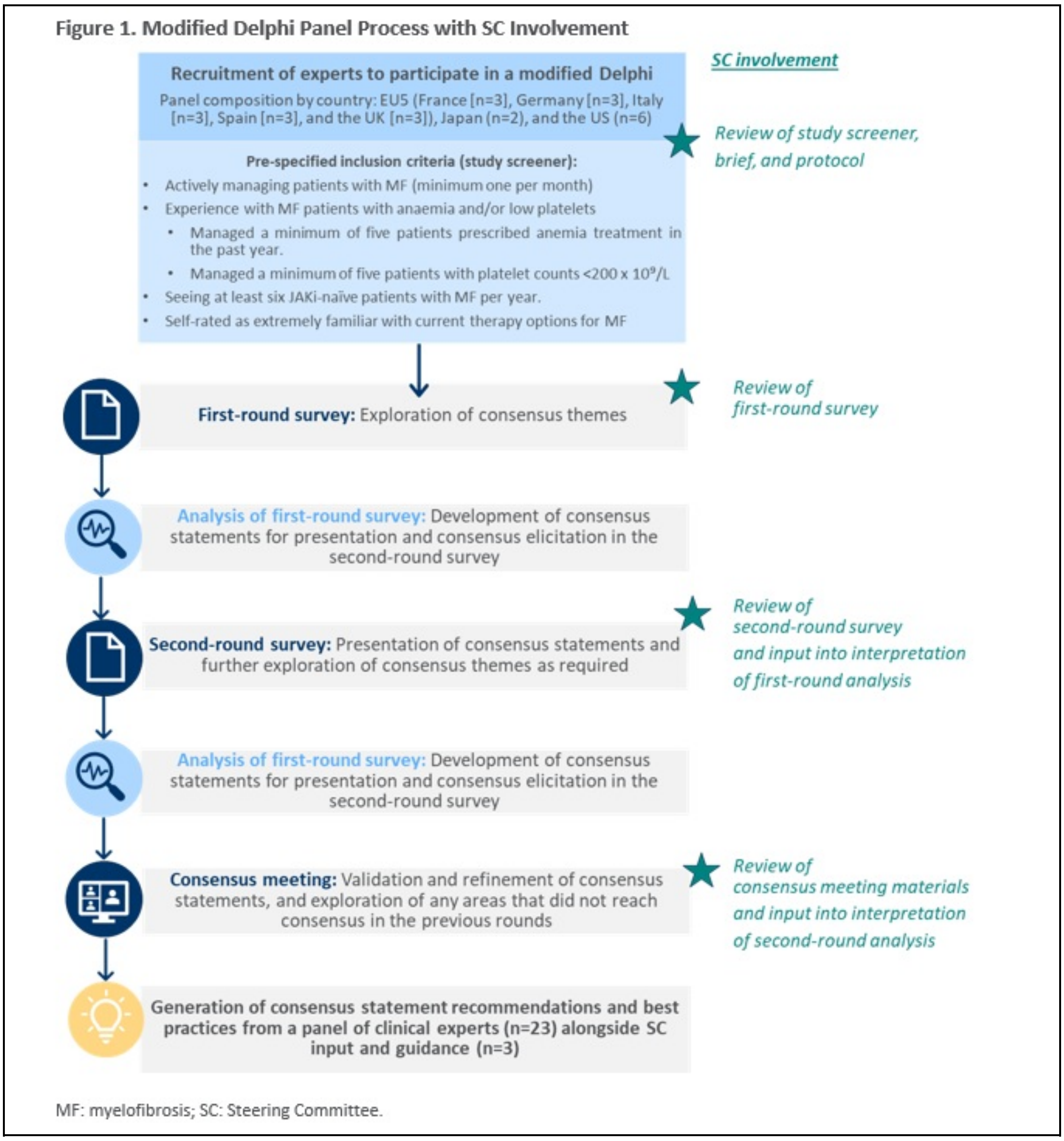

**Copyright Information:** (Online) ISSN: 2572-9241
©2026 The Author(s). HemaSphere is published by John Wiley & Sons Ltd on behalf of European Hematology Association. This is an open access Abstract Book distributed under the Attribution-NonCommercial-NoDerivs (CC BY-NC-ND), which allows third parties to download the articles and share them with others as long as they credit the author and the Abstract Book, but they cannot change the content in any way or use them commercially.
**Abstract Book Citations:** Authors, Title, HemaSphere, 2026;10:(S1):pages. The abstract book DOIs can be found on the “Author Information” page.
**Disclaimer:** Articles published in the journal HemaSphere exclusively reflect the opinions of the authors. The authors are responsible for all content in their abstracts including accuracy of the facts, statements, citing resources, etc.

### (PB3541) MORTALITY AND OUTCOMES AFTER CARDIOPULMONARY RESUSCITATION AND AFTER INTENSIVE CARE IN SWEDISH MYELOPROLIFERATIVE NEOPLASM PATIENTS

**Topic:** 16. Myeloproliferative neoplasms - Clinical

Helna Pettersson*[1, 2], Lena von Bahr[3]

*[1]NU-Hospital Group Vastra Gotaland Region, Hematology, Uddevalla, Sweden;[2]Sahlgrenska Akademi, Gothenburg, Sweden; [3]Sahlgrenska University Hospital, Hematology and Coagulation, Gothenburg, Sweden;*

### Background

Myeloproliferative Neoplasm (MPN) patients have an increased risk of thromboembolic, cardiovascular complications and infections. In addition to this, the chronic nature of MPN and increasing age of MPN patients implicates impending need for Cardiopulmonary Resuscitation (CPR) and/or Intensive Care (ICU) at some point in patient care. There are no existing data describing the outcome and mortality after CPR or ICU care among MPN patients in Sweden.

### Aims

Describe the 30-day mortality rate after CPR among MPN patients after cardiac arrest between 2008-2023.
Describe achievement of ROSC.
Describe the 30-day mortality rate after ICU admission between 2021-2023.

### Methods

This is a retrospective cohort registry study describing CPR mortality and ICU mortality among MPN patients diagnosed between 2008-2022. Data were collected from the Swedish MPN Quality Registry, Swedish Cardiopulmonary Resuscitation Registry, Swedish Intensive Care Registry and National Patient Registry. Descriptive and inferential statistics were performed.

### Results

There were 77 CPR events and 212 ICU admissions.
The 30-day mortality rate after CPR was 71% on 77 cardiac arrest (CA) episodes on 76 MPN patients who underwent CPR between 2008-2023, with follow-up time to June 2024. The median age of 75 (21-96), 47% were females. 4 of these 77 events were out of hospital cardiac arrests (OHCA). ROSC was achieved in 52% of patients but only 45% were alive at the end of CPR. 35% were alive at day 3 and 29% at day 30 after CPR.
The 30-day mortality rate after ICU admission was 32.9% with 212 ICU admissions on 170 Swedish MPN patients between the period of 2021-2023. The follow up time was until March 2025. 26 patients had more than one ICU admission and a maximum of 5 admissions in one patient. The ICU stay was at the average 3.2 days, median of 2 days and maximum of 27 days. The median age of the patients was 68 (21-88). The most common cause of ICU admission is septic chock. After multivariable analysis, high age >60, renal failure as primary ICU cause, MPN-U patient group and length of ICU stay were independently associated with increased 30-day mortality.

**Copyright Information:** (Online) ISSN: 2572-9241
©2026 The Author(s). HemaSphere is published by John Wiley & Sons Ltd on behalf of European Hematology Association. This is an open access Abstract Book distributed under the Attribution-NonCommercial-NoDerivs (CC BY-NC-ND), which allows third parties to download the articles and share them with others as long as they credit the author and the Abstract Book, but they cannot change the content in any way or use them commercially.
**Abstract Book Citations:** Authors, Title, HemaSphere, 2026;10:(S1):pages. The abstract book DOIs can be found on the "Author Information" page.
**Disclaimer:** Articles published in the journal HemaSphere exclusively reflect the opinions of the authors. The authors are responsible for all content in their abstracts including accuracy of the facts, statements, citing resources, etc.

**Summary/Conclusion**

Despite the achievement of ROSC in 52% of MPN patients, only 29% remain alive at day 30 after CPR. Whilst this result is comparable to previous results among the general population, the inability to independently sustain vital organ function after CPR still entails unnecessary suffering.
68% survival rate with ICU care is quite at par with previous studies on highly specialized ICU wards. Although recent evidence suggests better outcomes, the prognostic factors for survival remain inconsistent, making decision making around code status and ICU care still difficult.

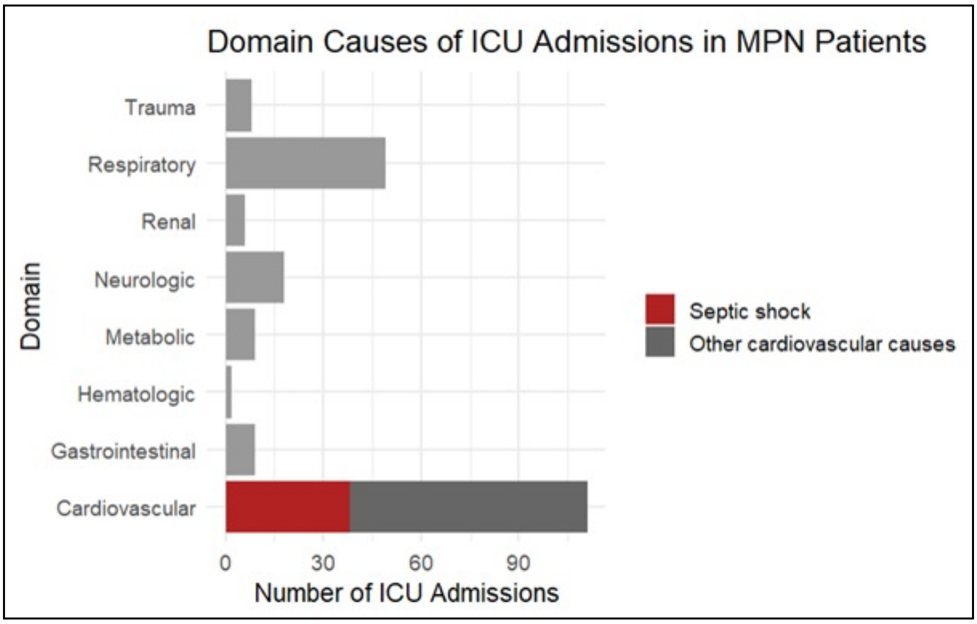

**Copyright Information:** (Online) ISSN: 2572-9241
©2026 The Author(s). HemaSphere is published by John Wiley & Sons Ltd on behalf of European Hematology Association. This is an open access Abstract Book distributed under the Attribution-NonCommercial-NoDerivs (CC BY-NC-ND), which allows third parties to download the articles and share them with others as long as they credit the author and the Abstract Book, but they cannot change the content in any way or use them commercially.
**Abstract Book Citations:** Authors, Title, HemaSphere, 2026;10:(S1):pages. The abstract book DOIs can be found on the "Author Information" page.
**Disclaimer:** Articles published in the journal HemaSphere exclusively reflect the opinions of the authors. The authors are responsible for all content in their abstracts including accuracy of the facts, statements, citing resources, etc.

### (PB3542) DISPARITIES IN TREATMENT MANAGEMENT AND OUTCOMES OF WHITE AND NON-WHITE ADOLESCENTS AND YOUNG ADULTS WITH MYELOPROLIFERATIVE NEOPLASMS.

**Topic:** 16. Myeloproliferative neoplasms - Clinical

Shruti Gajjar*[1], Sandro Pino-Ascencio[2], Julia Keating[3], Katherine Feder[4], Amir Fathi[5], Chi-Joan How[6], Michelle Lee[7], Gabriela Hobbs[8]

*[1]Massachusetts General Hospital, Mass General Brigham Cancer Institute, Boston, United States of America;[2]Massachusetts General Hospital, Leukemia, Boston, United States of America; [3]Dana-Farber Cancer Institute, Department of Data Science, Boston, United States of America; [4]Dana Farber Cancer Institute/Mass General Brigham, Hematology, Boston, United States of America; [5]Massachusetts General Hospital Cancer Center, Cancer Center. Hematology Oncology, Boston, United States of America; [6]Brigham And Women's Hospital, Hematology, Boston, United States of America;[7]Mass General Brigham Cancer Institute, Massachusetts General Hospital, Oncology, Boston, United States of America; [8]Massachusetts General Hospital, Leukemia Department, Boston, United States of America;*

### Background

Health disparities have been described across hematologic malignancies. However, there are limited data analyzing differences in access to care, treatments offered, and outcomes among white and non-white adolescents and young adults (AYA; ages 13–39) with myeloproliferative neoplasms (MPNs). We've collected data on AYA MPN patients at our tertiary academic center, where approximately 30% of patients identified as non-white. Thus, we sought to analyze differences in treatment and outcomes among white and non-white MPN AYA patients.

### Aims

We aim to describe the difference in incidence of thrombosis, cardiovascular conditions, treatments offered, and social determinants of health (SDOH) across both white and non-white MPN AYA patients.

### Methods

We conducted a retrospective study of AYA patients with MPN treated at Mass General Brigham between 2007–2025. Patients diagnosed at ages 15–39 and meeting 2022 WHO criteria for MPN were included. Data abstracted from the electronic medical record included demographics, diagnostics, cardiovascular (CV) risk factors, thrombotic and bleeding events, risk stratification, and management. Descriptive statistics were conducted with R version 4.3.2.

**Copyright Information:** (Online) ISSN: 2572-9241
©2026 The Author(s). HemaSphere is published by John Wiley & Sons Ltd on behalf of European Hematology Association. This is an open access Abstract Book distributed under the Attribution-NonCommercial-NoDerivs (CC BY-NC-ND), which allows third parties to download the articles and share them with others as long as they credit the author and the Abstract Book, but they cannot change the content in any way or use them commercially.
**Abstract Book Citations:** Authors, Title, HemaSphere, 2026;10:(S1):pages. The abstract book DOIs can be found on the "Author Information" page.
**Disclaimer:** Articles published in the journal HemaSphere exclusively reflect the opinions of the authors. The authors are responsible for all content in their abstracts including accuracy of the facts, statements, citing resources, etc.

**Results**

88 AYA patients with MPN were identified, of which 65 (73.9%) were white and 23 (26.1%) were non-white. Median age at diagnosis was 29 (16, 41) and 53 patients were female (60%). Age at diagnosis (p=0.4), gender (p=0.8), and MPN subtype (p=0.2) were similar across groups. Most patients (74%) were diagnosed incidentally with abnormal blood counts and 55 patients (62.5%) had at least one CV risk factor, e.g. HTN, HL, DM, BMI >25, tobacco use. Across both cohorts, 13 arterial/venous thrombotic events (56.5%) occurred within 1 year prior to diagnosis, and 10 arterial/venous thrombotic events (43.5%) occurred in the year after diagnosis, but the prevalence of cardiovascular risk factors and thrombotic events did not differ significantly by race. Among the SDOH variables, Area Deprivation Index and insurance status were also comparable with most patients (94%) using commercial insurance. In terms of cytoreduction received, number of treatments, and first line of treatment, there was no statistically significant difference. There was no statistically significant difference in the average months from first abnormal CBC to first hematology visit (p=0.9) for white patients (3, IQR 0–11) and non-white patients (2, IQR 0–22). Only non-white patients identified other languages besides English as their preferred language. Among the full cohort, median follow-up time is 3.9 years (95% CI: 2.9, 4.6 years).

**Summary/Conclusion**

In this analysis, we did not see statistically significant differences for patient characteristics, SDOH, nor treatment in white and non-white MPN AYA patients. However, we found a high percentage of thrombotic events in the year leading up todiagnosis and more variability, despite lacking statistical significance, in the time to first hematology visit for non-white patients, which should be explored in larger cohorts. Earlier referrals may play a role in preventing thrombotic complications in the year prior to diagnosis in this population. Lastly, the high incidence of thrombosis as well as comorbid cardiovascular conditions in this patient population is surprising and highlights the importance of developing treatment guidelines that are applicable to younger patients.

**Copyright Information:** (Online) ISSN: 2572-9241
©2026 The Author(s). HemaSphere is published by John Wiley & Sons Ltd on behalf of European Hematology Association. This is an open access Abstract Book distributed under the Attribution-NonCommercial-NoDerivs (CC BY-NC-ND), which allows third parties to download the articles and share them with others as long as they credit the author and the Abstract Book, but they cannot change the content in any way or use them commercially.
**Abstract Book Citations:** Authors, Title, HemaSphere, 2026;10:(S1):pages. The abstract book DOIs can be found on the “Author Information” page.
**Disclaimer:** Articles published in the journal HemaSphere exclusively reflect the opinions of the authors. The authors are responsible for all content in their abstracts including accuracy of the facts, statements, citing resources, etc.

### (PB3543) PRESENTATION AND MANAGEMENT OF PRIMARY MYELOFIBROSIS IN AZERBAIJAN: A RETROSPECTIVE NATIONAL CENTRE ANALYSIS

**Topic:** 16. Myeloproliferative neoplasms - Clinical

Elmir Guluyev*[1], Madad Abbasov[2], Gulnar Garayeva[1], Yeter Zuflugarova[1], Roberto Passera[3], Azer Kerimov[1], Semra Aydin[4]

*[1]National Hematology and Transfusiology Center, Baku, Azerbaijan;[2]Main Clinical Hospital of the Ministry of Defense of Azerbaijan, Baku, Azerbaijan;[3]University of Torino, Torino, Italy;[4]University Hospital Bonn, Department of Oncology, Hematology, Immuno-Oncology and Rheumatology, Bonn, Germany;*

### Background

The COMFORT trials have shown that ruxolitinib improves survival rates in patients with primary myelofibrosis (PMF) of intermediate-2 or high International Prognostic Score System (IPSS) risk compared to a placebo. Notwithstanding the principle of universal public healthcare, access to specific high-cost targeted drugs, such as ruxolitinib are constricted in Azerbaijan.

### Aims

The objective of the present study was to investigate the clinical and haematological parameters of PMF patients in Azerbaijan.

### Methods

A cohort of 241 consecutive, clinically well characterized, adult PMF patients, treated at the Azerbaijan National Center for Hematology and Transfusiology in Baku, were included in the analysis. Risk-stratification according to the IPSS was performed at diagnosis, including a total symptom scoring (TSS) assessment and repeated in the course of the disease. Overall survival (OS) was defined from time of diagnosis to death from any cause and was estimated using the Kaplan-Meier Method.

### Results

Median age at diagnosis was 59 years (range: 19-80); 22.7% of patients classified as pre-fibrotic PMF, while 77.3% showed overt myelofibrosis. A total of 27.2% stratified as intermediate-2 or high risk patients, while the majority were low/intermediate-1 (72.8%) patients. The median spleen size at diagnosis measured 20 cm (range 9–34 cm) and the median Total Symptom Score (TSS) marked 25 (range 0–79), indicating a highly symptomatic PMF cohort. Ruxolitinib treatment was given to 54 (22.4%) of patients, half of them bridged with short best available therapy (BAT), mainly hydroxyurea, while 187 (77.6%) received only BAT.

The median follow-up of the entire cohort was 3.3 years (interquartile range [IQR]: 1.7–5.7 years), and the median OS was 10.6 years. Age separated significantly with regard to OS. Patients ≥60 years had a shorter overall survival with 5.5 years (IQR: 2.9 – 10.4) compared to patients of <60 years with a median of 19 years (IQR: 8.4 – not reached), p<0.001. Stratifying the patients on the basis of IPSS showed a trend but failed important statistical significancy; low-/intermediate-1 risk patients had a median OS of 13.3. years (IQR: 3.1 – 19), while intermediate-2/high-risk patients had a median OS of 5.2 years (IQR: 3.8 – 9.1), p=0.048.

Interestingly, the bone marrow cellularity detected in 88% of bone marrow biopsies, associated highly with OS; 168 patients revealed a hypercellular bone marrow and had a median OS of 13.3 years (IQR: 6.6 – 19), while 44 patients showed an hypocellular marrow and had a median OS of 5.2 (2.2 – not reached) years, p=0.004. As expected, Ruxolitinib treatment improved survival significantly. Ruxolitinib treated patients had a longer median OS of 19 years (IQR: 10.4 – 19) than patients under BAT alone with 8.9 years (3,2 – not reached), p<0.001. Beside Ruxolitinib treatment, age, bone marrow cellularity and spleen size at diagnosis were confirmed in uni- and multivariate analysis.

**Copyright Information:** (Online) ISSN: 2572-9241
©2026 The Author(s). HemaSphere is published by John Wiley & Sons Ltd on behalf of European Hematology Association. This is an open access Abstract Book distributed under the Attribution-NonCommercial-NoDerivs (CC BY-NC-ND), which allows third parties to download the articles and share them with others as long as they credit the author and the Abstract Book, but they cannot change the content in any way or use them commercially.
**Abstract Book Citations:** Authors, Title, HemaSphere, 2026;10:(S1):pages. The abstract book DOIs can be found on the "Author Information" page.
**Disclaimer:** Articles published in the journal HemaSphere exclusively reflect the opinions of the authors. The authors are responsible for all content in their abstracts including accuracy of the facts, statements, citing resources, etc.

**Summary/Conclusion**

To the best of our knowledge, this study is the first analysis of outcome related factors in PMF patients in Azerbaijan, where limited targeted therapies are available. Ruxolitinib treatment, patients age, spleen size at diagnosis and bone marrow cellularity associated significantly with OS, possibly underlining the inflammatory nature of the disease.

**Copyright Information:** (Online) ISSN: 2572-9241
©2026 The Author(s). HemaSphere is published by John Wiley & Sons Ltd on behalf of European Hematology Association. This is an open access Abstract Book distributed under the Attribution-NonCommercial-NoDerivs (CC BY-NC-ND), which allows third parties to download the articles and share them with others as long as they credit the author and the Abstract Book, but they cannot change the content in any way or use them commercially.
**Abstract Book Citations:** Authors, Title, HemaSphere, 2026;10:(S1):pages. The abstract book DOIs can be found on the “Author Information” page.
**Disclaimer:** Articles published in the journal HemaSphere exclusively reflect the opinions of the authors. The authors are responsible for all content in their abstracts including accuracy of the facts, statements, citing resources, etc.

### (PB3544) LANGERHANS CELL HISTIOCYTOSIS AND OTHER HISTIOCYTOSIS: A SINGLE CENTRE EXPERIENCE

**Topic:** 16. Myeloproliferative neoplasms - Clinical

Marianna Criscuolo*[1], Martina Quattrone[2], Luana Fianchi[2], Antonio Giordano[1], Alessia Di Pilla[2], Sara Caterina Brunetti[1], Livio Pagano[2]

[1]*Fondazione Policlinico Universitario A. Gemelli IRCCS, Dipartimento di Scienze di Laboratorio ed Ematologiche, Rome, Italy;* [2]*Università Cattolica del Sacro Cuore. Fondazione Policlinico Universitario A. Gemelli IRCCS, Dipartimento di Scienze Radiologiche Ed Ematologiche. Dipartimento di Diagnostica Per Immagini, Radioterapia Oncologica Ed Ematologia, Rome, Italy;*

### Background

Histiocytosis represents a heterogeneous group of rare disorders characterized by a wide variety of clinical manifestations, depending on histological subtypes and extent of organ involvement. For patients with histiocytosis, the accurate identification of the subtype and disease spread is essential to establish appropriate therapeutic management and follow-up, as this is a potentially fatal disease.

### Aims

To evaluate the clinical and laboratory characteristics associated with a better prognosis.

### Methods

We retrospectively reviewed the medical records of 33 patients followed at our institution to evaluate clinical presentation, organ involvement, and therapeutic choice

**Copyright Information:** (Online) ISSN: 2572-9241
©2026 The Author(s). HemaSphere is published by John Wiley & Sons Ltd on behalf of European Hematology Association. This is an open access Abstract Book distributed under the Attribution-NonCommercial-NoDerivs (CC BY-NC-ND), which allows third parties to download the articles and share them with others as long as they credit the author and the Abstract Book, but they cannot change the content in any way or use them commercially.
**Abstract Book Citations:** Authors, Title, HemaSphere, 2026;10:(S1):pages. The abstract book DOIs can be found on the “Author Information” page.
**Disclaimer:** Articles published in the journal HemaSphere exclusively reflect the opinions of the authors. The authors are responsible for all content in their abstracts including accuracy of the facts, statements, citing resources, etc.

### Results

Male to female ratio was 0.94, with a median age at diagnosis of 50 yo (range 17-85). Histopathologic subtypes were as follows: 21 patients with Langerhans cell histiocytosis (LCH), 4 with Erdheim-Chester Disease (ECD), 3 with Rosai-Dorfman Disease (RDD) and 5 with Histiocytic sarcoma (HS).

Among the 21 patients with LCH, 9 had a unifocal unisystemic disease with bone involvement: 4 were treated with indomethacin with or without surgical removal, while 5 underwent surgery only. All 9 patients achieved disease remission. Two other LCH patients had unisystemic disease localized to the lungs and vocal folds, respectively: they were treated with a course of steroids, obtaining complete remission (CR). Ten patients were diagnosed with multisystemic disease or unisystemic disease involving visceral and/or at-risk organs. Five of them were treated with vinblastine plus steroids, all obtaining CR except one who underwent 6 courses of cladribine, for partial response. Among other 3 patients, one was previously treated with indomethacin with progression of disease, one patient with steroids only for prevalent bone involvement, and one patient was unable to underwent chemotherapy for concomitant late stage hepatic disease. Finally, 2 patients are still completing diagnostic work-up.

Among the 4 patients with ECD, 2 were treated with cladribine with no response and one of them eventually died. Two other patients were treated with vinblastine plus steroids for CNS involvement: one obtained a CR while the other started interferon-α maintenance, which is still ongoing.

Three patients with RDD were treated with surgery for isolate nodular lesion, interferon-α for lymph node involvement and elderly age, and vinblastine plus steroids for multisystemic and CNS involvement, respectively.

Five patients with HS were treated with chemotherapy, except one who underwent cladribine; all died from progressive disease.

Overall, 3 patients with LCH and 1 with ECD were diagnosed with another malignant neoplasm (breast, lung, melanoma, and thyroid), while 2 other patients with LCH and 1 with ECD were diagnosed with benign neoplasm (pilomatricoma, prolactinoma and parotid).

Table 1. Characteristics of involved sites

| | LCH (*21*) | | ECD (*4*) | RDD (*4*) | HS (*5*) |
|---|---|---|---|---|---|
| | unisystemic | multisystemic | | | |
| bone | 9 | 4 | 2 | 2 | |
| lung | 1 | 3 | | | |
| thyroid | 1 | | | 1 | |
| SNC | 1 | 3 | 3 | 1 | 1 |
| vocal folds | 1 | | | | |
| lymph nodes | 1 | 3 | 1 | 3 | 2 |
| skin | | 1 | | 1 | |
| liver | | 1 | | | |
| pararenal | | | 2 | | |
| cardiac | | | 2 | | |
| intra-abdominal | | | | | 2 |
| tot | 14 | 15 | 10 | 8 | 5 |

### Summary/Conclusion

Prognosis for patients with histiocytosis largely varies, depending mostly on histological subtypes and involved sites. These characteristics guide clinicians in the choice of the proper treatment. Regarding HS, prognosis is particularly poor and claims for an improvement in biological knowledge and targeted therapy. Concomitant surveillance for other neoplasms should be performed.

**Copyright Information:** (Online) ISSN: 2572-9241
©2026 The Author(s). HemaSphere is published by John Wiley & Sons Ltd on behalf of European Hematology Association. This is an open access Abstract Book distributed under the Attribution-NonCommercial-NoDerivs (CC BY-NC-ND), which allows third parties to download the articles and share them with others as long as they credit the author and the Abstract Book, but they cannot change the content in any way or use them commercially.
**Abstract Book Citations:** Authors, Title, HemaSphere, 2026;10:(S1):pages. The abstract book DOIs can be found on the “Author Information” page.
**Disclaimer:** Articles published in the journal HemaSphere exclusively reflect the opinions of the authors. The authors are responsible for all content in their abstracts including accuracy of the facts, statements, citing resources, etc.

### (PB3545) PREVALENCE OF THROMBOSIS AND CARDIOVASCULAR RISK FACTORS IN PATIENTS WITH POLYCYTHAEMIA VERA; A MULTI CENTRE DESCRIPTIVE ANALYTICAL STUDY IN THE WESTERN PROIVINCE OF SRI LANKA

**Topic:** 16. Myeloproliferative neoplasms - Clinical

Ahalyaa Sivashangar*[1], Sayanthan Sivakumaran[1], Indika Somaratne[2], Yenifa Samaraweera[3], Visaka Ratnamalala[4], Sanjeewa Pathirage[4], Yasintha Costa[5], A.A.N.I. Amarasinghe[5], Senani Williams[6], Lallindra Gooneratne[1]

*[1]Faculty of Medicine, University of Colombo,, Department of Pathology, Colombo, Sri Lanka;[2]General Sir John Kotelawala Defence University,, Department of Paraclinical Sciences, Ratmalana, Sri Lanka; [3]Colombo South Teaching Hospital, Department of Haematology, Dehiwala, Sri Lanka; [4]National Hospital of Sri Lanka,, Department of Haematology, Colombo, Sri Lanka; [5]Colombo North Teaching Hospital,, Department of Haematology, Ragama, Sri Lanka;[6]Faculty of Medicine, University of Kelaniya,, Department of Pathology, Ragama, Sri Lanka;*

### Background

Polycythaemia vera (PV) is a Philadelphia chromosome–negative myeloproliferative neoplasm characterized by clonal panmyelosis and a significant risk of developing thromboembolic events (TEs), both arterial and venous, compared to age and sex-matched controls. Advanced age (≥ 60 years) and previous history of thrombosis are well established and accepted predictors of future thrombosis. The presence of either one classifies a patient as high-risk. The literature suggests that coexistence of cardiovascular risk factors (CVRFs) may further increase the risk of TEs. Data  from South Asian populations is limited in this regard.

### Aims

To describe the prevalence of TEs and CVRFs in PV patients in Sri Lanka.

### Methods

A retrospective descriptive cross-sectional study conducted at six tertiary care centres in the Western Province of Sri Lanka. All adult patients with PV diagnosed as per WHO 2016 criteria were recruited. Clinical, demographic and laboratory data were extracted from medical records and patient interviews. Thrombotic events at diagnosis and follow-up were classified as arterial or venous. Correlations between TEs and CVRFs were analyzed using chi-square and non-parametric tests.

**Copyright Information:** (Online) ISSN: 2572-9241
©2026 The Author(s). HemaSphere is published by John Wiley & Sons Ltd on behalf of European Hematology Association. This is an open access Abstract Book distributed under the Attribution-NonCommercial-NoDerivs (CC BY-NC-ND), which allows third parties to download the articles and share them with others as long as they credit the author and the Abstract Book, but they cannot change the content in any way or use them commercially.
**Abstract Book Citations:** Authors, Title, HemaSphere, 2026;10:(S1):pages. The abstract book DOIs can be found on the "Author Information" page.
**Disclaimer:** Articles published in the journal HemaSphere exclusively reflect the opinions of the authors. The authors are responsible for all content in their abstracts including accuracy of the facts, statements, citing resources, etc.

**Results**

A total of 265 patients were recruited. Median age was 66 years with 61.1% males. 78.1% were high-risk. A mutation in JAK2 V617F was detected in 99.6% and exon 12 detected in 0.4% patient. A mean haemoglobin (Hb) of 183 g/L and mean haematocrit (HCT) of 57.4% were observed at presentation. 43.3% of patients had baseline total white blood cell count (WBC) of > 15.0 x$10^9$/L and 64.5 % had a baseline platelet count (PLT) of > 450 x $10^9$/L.

87 patients had TEs at presentation. Cumulative prevalence of 32.8%. Arterial events were predominant (90.8%). Cerebrovascular events and acute coronary syndrome were most common. Venous thrombosis (9.2%) mainly involved the splanchnic circulation. During follow-up 5.3% developed TEs.

Prevalence of traditional CVRFs for arterial thrombosis, diabetes mellitus (DM), hypertension (HTN), dyslipidemia (DL) and smoking were 30.6%, 50.2%, 35.5 % and 18.9 % respectively. Patients with DL showed a statistically significant correlation with arterial thrombosis (40 %; p= 0.022). None of the other CVRFs showed a statistical significance, independently. Coexistence of HTN + DL (42%; p=0.02) and HTN + DL + DM (46.9 %; p=0.038) showed a statistically significant correlation with arterial thrombosis. WBC, Hb, HCT and PLT did not show a significant association with an increased risk of arterial thrombosis.

**Summary/Conclusion**

Approximately a third of patients with PV in this Sri Lankan cohort experienced TEs. This is similar to the prevalence reported from studies in other countries. Over 90% of TEs were arterial. PV patients with DL had a higher proportion of arterial thrombosis compared to those without DL. DM, HTN and smoking were not independent risk factors for arterial thrombosis. Coexistence of HTN + DL and HTN + DL + DM with PV, showed a statistically significant correlation with developing arterial thrombosis. None of the haematological parameters showed a positive correlation with TEs. Therefore, these findings provide important regional data and underscore the need for sustained thrombosis prevention strategies and refined risk stratification tailored to this population.

**Copyright Information:** (Online) ISSN: 2572-9241
©2026 The Author(s). HemaSphere is published by John Wiley & Sons Ltd on behalf of European Hematology Association. This is an open access Abstract Book distributed under the Attribution-NonCommercial-NoDerivs (CC BY-NC-ND), which allows third parties to download the articles and share them with others as long as they credit the author and the Abstract Book, but they cannot change the content in any way or use them commercially.
**Abstract Book Citations:** Authors, Title, HemaSphere, 2026;10:(S1):pages. The abstract book DOIs can be found on the "Author Information" page.
**Disclaimer:** Articles published in the journal HemaSphere exclusively reflect the opinions of the authors. The authors are responsible for all content in their abstracts including accuracy of the facts, statements, citing resources, etc.

### (PB3546) SENTRY2—A PHASE II STUDY EVALUATING SELINEXOR MONOTHERAPY IN PATIENTS WITH JAKI-NAIVE MYELOFIBROSIS AND MODERATE THROMBOCYTOPENIA

**Topic:** 16. Myeloproliferative neoplasms - Clinical

Joseph M Scandura*[1], Aaron T. Gerds[2], Ellen Ritchie[1], Yi Chai[3], Mark Tomer[3], Raajit K Rampal[4]

*[1]Weill Cornell Medicine, New York, United States of America;[2]Cleveland Clinic, Taussig Cancer Institute, Cleveland, United States of America; [3]Karyopharm Therapeutics, Newton, United States of America;[4]Memorial Sloan Kettering Cancer Center, New York, United States of America;*

### Background

Myelofibrosis (MF) is a myeloproliferative neoplasm driven by dysregulated JAK/STAT activity and inflammatory cytokine dysregulation. The current standard of care, including JAK inhibitors (JAKi), can improve splenomegaly and symptoms but has limited frequency and depth of response. Furthermore, thrombocytopenia, which commonly occurs in MF and is associated with poor outcomes, is an on-target side effect of JAK/STAT inhibition; reduced dosing of JAKis to manage adverse effects further reduce their efficacy.

The Phase I portion of SENTRY (NCT04562389) showed that the combination of selinexor (SEL) and ruxolitinib (RUX) in JAKi-naïve patients with MF generally demonstrated good tolerability with manageable adverse events and provided preliminary evidence of rapid, deep, and sustained spleen and symptom responses. Exploratory analyses indicated that SEL may reduce NF-κB–mediated proinflammatory cytokine production, consistent with a potential mechanism of action where inhibition of XPO1 by SEL disrupts TPO-1 and NF-κB signaling, which are functionally overactive in MF. Additionally, evidence of bone marrow fibrosis improvement and normalization of erythroid and megakaryocytic progenitors was observed. The potential for SEL single-agent activity was evaluated in patients refractory/intolerant to JAKi in the ESSENTIAL trial (NCT03627403), which showed that SEL monotherapy was generally tolerable, with preliminary signs of efficacy. Coupled with the potential for SEL to restore bone marrow homeostasis and regulate proinflammatory cytokines, these results provide a rationale to explore SEL monotherapy in MF subpopulations with high unmet need, such as those with thrombocytopenia.

### Aims

To evaluate the efficacy and safety of SEL monotherapy in patients with JAKi-naïve MF and moderate thrombocytopenia.

### Methods

SENTRY2 (NCT05980806) is a two-arm, sequential, multicenter, open-label, Phase II study evaluating efficacy of SEL (40 mg or 60 mg QW; N=29 per arm) in patients with JAKi-naïve MF and thrombocytopenia (platelet count: ≥50x$10^9$/L). Optional RUX, pacritinib, or momelotinib treatment (based on platelet count) may be added for patients whose spleen volume reduction (SVR) from baseline is <10% at week 12 or <35% at week 24. Select inclusion criteria include age ≥18 years, spleen volume ≥450 $cm^3$, DIPSS intermediate-1 with symptoms or higher, platelet counts ≥50x$10^9$/L, and ineligibility for stem cell transplantation. Select exclusion criteria include >10% blasts in peripheral blood or bone marrow and previous treatment with JAKi for MF. Primary endpoint is SVR ≥35% at week 24. Key secondary endpoints are safety, ≥50% reduction in Total Symptom Score at week 24, anemia response at week 24, overall survival, and overall response rate. Exploratory endpoints include duration of spleen response, progression-free survival, changes in variant allele frequency, and bone marrow fibrosis. Written informed consent will be obtained from all patients before any study-related procedures are conducted.

**Copyright Information:** (Online) ISSN: 2572-9241
©2026 The Author(s). HemaSphere is published by John Wiley & Sons Ltd on behalf of European Hematology Association. This is an open access Abstract Book distributed under the Attribution-NonCommercial-NoDerivs (CC BY-NC-ND), which allows third parties to download the articles and share them with others as long as they credit the author and the Abstract Book, but they cannot change the content in any way or use them commercially.
**Abstract Book Citations:** Authors, Title, HemaSphere, 2026;10:(S1):pages. The abstract book DOIs can be found on the “Author Information” page.
**Disclaimer:** Articles published in the journal HemaSphere exclusively reflect the opinions of the authors. The authors are responsible for all content in their abstracts including accuracy of the facts, statements, citing resources, etc.

### (PB3547) DEMOGRAPHIC, CLINICAL CHARACTERISTICS OF BCR/ABL NEGATIVE MYELOPROLIFERATIVE NEOPLASMS PATIENTS IN OMAN, SINGLE CENTER EXPERIENCE

**Topic:** 16. Myeloproliferative neoplasms - Clinical

Zahra Alghammari*[1], Khalid Nasser Alhashmi[2]

*[1]Hematopathology Residency Training Program, Oman Medical Specialty Board, Muscat, Oman; [2]Hematology Department, Armed Forced Hospital, Muscat, Oman;*

### Background

MPNs are a heterogeneous group of clonal hematopoietic stem cell disorders, characterized by increased proliferation of one or more myeloid lineages in the bone marrow. Primarily affect adult to elderly patients and males predominant. The clinical- laboratory features of MPNs depend on the main lineage of differentiation and molecular landscape. JAK2V617F mutation was observed in 95% in PV, 50% in ET, and 50% in PMF. Other cytogenic and molecular markers: CALR, and MPL and Non driver mutations, such as ASXL1, DNMTA, IDH2, and SRSF2. MPNs patient at high risk of thrombotic and hemorrhagic complications, BM failure, and blast transformation.

### Aims

The primary aim of this study was to describe clinical and molecular characteristics of patients with classical BCR-ABL1-negative myeloproliferative neoplasms In Oman. The secondary objective was to describe demographic characteristics, clinical presentations, complications, and molecular mutations at diagnosis and to determine the genetic profile of JAK2V617F, CALR exon 9 and MPL genes among Omani patients and correlate these mutations with clinical and hematologic parameters in myeloproliferative neoplasm (MPN).

### Methods

This was a retrospective observational cohort study conducted in the Department of Hematology-Oncology at Royal Hospital from January 2015 to December 2020.

### Results

A total of 128 patients were assessed, 50 with Polycythemia Vera, 62 with Essential Thrombocythemia, and 11 with Primary Myelofibrosis. The mean age was 56 years (range = 14−95 years) with male predominance (56%). Of the overall MPN cohort, 56% were presented with symptoms including vascular events (22%). The mutational status analysis revealed that 98 (77%) harbored the JAK2V617F mutation, 27 (21%) CALR mutation, one (0.8%) had MPL, one (0.8%) had both JAK2V617F and CALR and one (0.8%) patient was negative. Among the PV patients, 49 (98%) were positive for the JAK2V617F mutation, one (2%) presented with CALR mutation. Fifteen patients were presented with thrombosis, including 11 patients with arterial thrombosis and others with venous thrombosis. For ET, there were more arterial thrombosis events seen in those with JAK2 V617F mutation compared to CALR mutation (18.2% vs 4.8%). The JAK2V617F mutation is associated with higher presenting Hemoglobin, Hematocrit and White cell counts, while the CALR mutation is associated with lower presenting HB and HCT but higher Platelets. The bleeding rate was only 4.5%. Disease progression was noted in three patients: two patients progressed to MF from ET and PV and one patient developed secondary acute myeloid leukemia post-myelofibrosis.

**Copyright Information:** (Online) ISSN: 2572-9241
©2026 The Author(s). HemaSphere is published by John Wiley & Sons Ltd on behalf of European Hematology Association. This is an open access Abstract Book distributed under the Attribution-NonCommercial-NoDerivs (CC BY-NC-ND), which allows third parties to download the articles and share them with others as long as they credit the author and the Abstract Book, but they cannot change the content in any way or use them commercially.
**Abstract Book Citations:** Authors, Title, HemaSphere, 2026;10:(S1):pages. The abstract book DOIs can be found on the "Author Information" page.
**Disclaimer:** Articles published in the journal HemaSphere exclusively reflect the opinions of the authors. The authors are responsible for all content in their abstracts including accuracy of the facts, statements, citing resources, etc.

**Summary/Conclusion**

The clinical and frequency of somatic mutations in our cohort, stratified according to the respective disease, was consistent with the literature data, despite some limitations. Further multi-center prospective studies with long-term follow-up should be designed to determine the link between the JAK2 gene and thrombosis in MPNs, disease progression and overall survival.

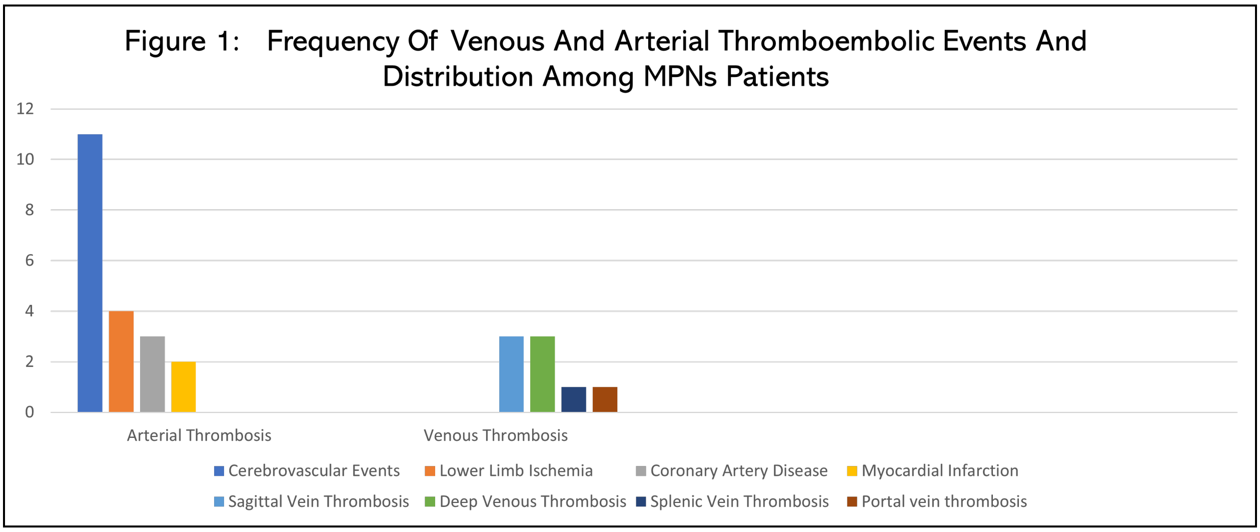

**Copyright Information:** (Online) ISSN: 2572-9241
©2026 The Author(s). HemaSphere is published by John Wiley & Sons Ltd on behalf of European Hematology Association. This is an open access Abstract Book distributed under the Attribution-NonCommercial-NoDerivs (CC BY-NC-ND), which allows third parties to download the articles and share them with others as long as they credit the author and the Abstract Book, but they cannot change the content in any way or use them commercially.
**Abstract Book Citations:** Authors, Title, HemaSphere, 2026;10:(S1):pages. The abstract book DOIs can be found on the "Author Information" page.
**Disclaimer:** Articles published in the journal HemaSphere exclusively reflect the opinions of the authors. The authors are responsible for all content in their abstracts including accuracy of the facts, statements, citing resources, etc.

### (PB3548) THE RATIONALE FOR THE ROUTINE USAGE OF NEXT GENERATION SEQUENCING (NGS) METHOD FOR DETECTION OF ADDITIONAL MUTATIONS IN ESSENTIAL THROMBOCYTHEMIA PATIENTS-SINGLE CENTRE EXPERIENCE

**Topic:** 16. Myeloproliferative neoplasms - Clinical

Marija Popova Labachevska*[1], Bojan Labachevski[2], Zlate Stojanoski[1], Marica Pavkovikj[1], Olivera Gjeorgjieva Janev[1], Aleksandra Pivkova Veljanovska[1], Dushko Dukovski[1], Sonja Genadieva Stavrikj[1]

*[1]University clinic for hematology, hematology, Skopje, Macedonia;[2]Institute of Preclinical and Clinical Pharmacology with Toxicology, University Cyril and Methodious, Skopje, Macedonia;*

### Background

Essential thrombocythemia (ET) is a BCR-ABL negative chronic myeloproliferative neoplasm (MPN) characterized by a sustained increase in platelet production due to clonal proliferation of megakaryocytes. Approximately 10–15% of ET patients lack the three canonical driver mutations . Although it is often indolent and compatible with near-normal life expectancy, the central clinical concern is the risk of thrombotic and hemorrhagic events and transformation to myelofibrosis or rarely in acute leukemia. Several risk stratification systems have been developed over the years. Currently, in clinical practice the Revised IPSET-Thrombosis (r-IPSET-T) is the preferred scoring system. Lately, more profound gene analysis led to the discovery of additional mutations  and following studies have proven their prognostic relevance in MPN in terms of disease evolution and progression. As a result, the Mutation-Enhanced International Prognostic System for Essential Thrombocythemia (MIPSS-ET) has been developed using specific mutations along with clinical factors for more refined risk stratification of ET patients.

### Aims

The aim of the study was to evaluate the importance of NGS analysis in ET patients.

### Methods

We analyzed the molecular landscape in 23 ET patients diagnosed, followed and treated at the University clinic of hematology in Skopje, R.N Macedonia. Median age of the patients was 51.4 years (range 30-70). Diagnose of ET was obtained using the European Leukemia Net (ELN) criteria. Detection of JAK2V617F, MPL and CALR mutations was performed using allele specific PCR assay with subsequent capillary electrophoresis on an automated genetic analyzer using peripheral blood. For identification of additional mutations, bone marrow was used and next generation sequencing (NGS) method was performed on the Ilumina platform covering 37 genes often present in myeloid neoplasms. The obtained data were statistically processed using the statistical program STATISTICA 8.0 and SPSS Statistics 23.0.

### Results

From 23 patients, 5 (21.7%) were “triple negative” cases, 11(47.8%) were JAK2V617F mutation positive, 5 (21.7%) CALR positive and 2 (8.7%) MPL positive cases. From total of 23 patients, 10 (44%) showed presence of ≥2 additional mutations, 7 (30%) presence of one additional mutation and 6 (26%) didn’t have additional mutations. Most prevalent mutations were the ASXL1, TET2 and DNMT3A mutations which were present in 5 (21.7%) patients, CEBPA mutation in two (8.7%) patients and SF3B1 and TP53 in one (4.3%) patient. The correlation between number of additional mutations and hematological parameters was not associated with statistically significant differences –hemoglobin ($p=0.52$), WBC ($p=0.77$), hematocrit ($p=0.22$) and platelet count (Plt) ($p=0.73$) in our cohort. NGS method confirmed the diagnosis of ET in four of five triple negative cases while in one patient clonality was excluded.

**Copyright Information:** (Online) ISSN: 2572-9241
©2026 The Author(s). HemaSphere is published by John Wiley & Sons Ltd on behalf of European Hematology Association. This is an open access Abstract Book distributed under the Attribution-NonCommercial-NoDerivs (CC BY-NC-ND), which allows third parties to download the articles and share them with others as long as they credit the author and the Abstract Book, but they cannot change the content in any way or use them commercially.
**Abstract Book Citations:** Authors, Title, HemaSphere, 2026;10:(S1):pages. The abstract book DOIs can be found on the “Author Information” page.
**Disclaimer:** Articles published in the journal HemaSphere exclusively reflect the opinions of the authors. The authors are responsible for all content in their abstracts including accuracy of the facts, statements, citing resources, etc.

**Summary/Conclusion**

Next-generation sequencing (NGS) is important in ET primarily for clonality determination in triple negative patients, but also for providing prognostic informations. However, in our study presence of clonal disease mutations didnt have effect on treatment desicion. Despite its benefits, its routine use in all ET patients remains limited by its high cost compared to traditional, single-gene testing.

**Copyright Information:** (Online) ISSN: 2572-9241
©2026 The Author(s). HemaSphere is published by John Wiley & Sons Ltd on behalf of European Hematology Association. This is an open access Abstract Book distributed under the Attribution-NonCommercial-NoDerivs (CC BY-NC-ND), which allows third parties to download the articles and share them with others as long as they credit the author and the Abstract Book, but they cannot change the content in any way or use them commercially.
**Abstract Book Citations:** Authors, Title, HemaSphere, 2026;10:(S1):pages. The abstract book DOIs can be found on the "Author Information" page.
**Disclaimer:** Articles published in the journal HemaSphere exclusively reflect the opinions of the authors. The authors are responsible for all content in their abstracts including accuracy of the facts, statements, citing resources, etc.

### (PB3549) MOLECULAR REMISSION IN THE TREATMENT OF POLYCYTHEMIA VERA: SHORT-TERM EFFECT OF ROPEGINTERFERON ALFA2-B

**Topic:** 16. Myeloproliferative neoplasms - Clinical

Veronica Vecchio*[1], Rossella Cacciola[1], Emma Cacciola[2]

*[1]University of Catania, Catania, Italy; [2]University of Catania, CATANIA, Italy;*

### Background

Polycythemia vera (PV) is a Philadelphia (Ph)-negative myeloproliferative neoplasm associated with the JAK2V617F mutation. The variant allele frequency (VAF) of JAK2V617F correlates with the neoplastic evolution in myelofibrosis and acute myeloid leukemia. A decrease of VAF signals the clearance of neoplastic cells haboring JAK2V617F and this is crucial in reducing the risk of disease progression. Ropeginterferone alfa-2b (ropeg) is a monopegylated interferon that selectively inhibits the mutation JAK2V617F leading to a decrease in the JAK2V617F VAF. Long-term studies suggest substantial reductions in JAK2V627F VAF at 18 and 24 months indicating disease-modifying potential effect.

### Aims

We studied 12 HU-intolerant patients with PV diagnosed according to WHO/ICC criteria enrolled from december 2024 to december 2025 on ropeg to evaluate JAK2V617F VAF from baseline to the end of follow-up.

### Methods

The mean age of patients was 64 years (57-70 years). All patients were positive for JAK2V617F with baseline mean VAFs of 27% and were on ropeg with a mean biweekly dose of 250±150 µg.

### Results

After 36 weeks, all patients exhibited a reduction in JAK2V617F VAF from baseline to less than 10% (9%). According to the 2009 European LeukemiaNet critera and using the 2013 European LeukemiaNet update, 14/15 patients had a partial molecular response (PMR) and 1/15 had a complete molecular response (CMR). All patients achieved a complete hematologic remission (CHR). Our data show a short-term reduction in JAK2V617F VAF and without progression of disease.

### Summary/Conclusion

The PMR observed to less than one year may be a predictor of early CMR. Interestingly, the observed CMR was associated with leukopenia that may reflect depletion of JAK2V617F-positive leukocytes, consistent with ropeg's anticlonal activity. Greater data collection is need to confirm this short-term molecular effect of ropeginterferon alfa-2b therapy in PV.

**Copyright Information:** (Online) ISSN: 2572-9241
©2026 The Author(s). HemaSphere is published by John Wiley & Sons Ltd on behalf of European Hematology Association. This is an open access Abstract Book distributed under the Attribution-NonCommercial-NoDerivs (CC BY-NC-ND), which allows third parties to download the articles and share them with others as long as they credit the author and the Abstract Book, but they cannot change the content in any way or use them commercially.
**Abstract Book Citations:** Authors, Title, HemaSphere, 2026;10:(S1):pages. The abstract book DOIs can be found on the "Author Information" page.
**Disclaimer:** Articles published in the journal HemaSphere exclusively reflect the opinions of the authors. The authors are responsible for all content in their abstracts including accuracy of the facts, statements, citing resources, etc.

### (PB3550) REAL-WORLD OUTCOMES OF MYELOFIBROSIS IN AN UNDERREPRESENTED LATIN AMERICAN POPULATION: EARLY EXPERIENCE FROM THE HOSPITAL GENERAL DE MÉXICO DR. EDUARDO LICEAGA

**Topic:** 16. Myeloproliferative neoplasms - Clinical

Gilberto Israel Barranco Lampon*[1], Christian Ramos[1], Carlos Martinez Murillo[1], Gerardo Santiago Jiménez[1], Alonso Hernández Company[1], Edgar Cordero[1], Emmanuel Martinez Moreno[1], Etsel Camargo[1], Juan Francisco Zazueta Pozos[1], Irma Olarte[2], Adolfo Martínez[2], Iveth Mendoza[2], Rafael Cerón[3], Adrian De la Cruz[4], Anel García[2]

*[1]Hospital General de México Dr. Eduardo Liceaga, Hematology, Mexico City, Mexico;[2]Hospital General de México Dr. Eduardo Liceaga, Molecular Biology Laboratory, Mexico City, Mexico; [3]Hospital General de México Dr. Eduardo Liceaga, Molecular Biology Department, Mexico City, Mexico; [4]Hospital General de México Dr. Eduardo Liceaga, Mexico City, Mexico;*

### Background

Myelofibrosis (MF) is a heterogeneous myeloproliferative neoplasm characterized by bone marrow fibrosis, splenomegaly, cytopenias, and risk of leukemic transformation. Most survival data derive from European and North American cohorts, while real-world evidence from Latin America remains limited.

### Aims

The aim of this study was to characterize clinical features, treatment exposure, and overall survival (OS) of MF patients treated at a large public tertiary referral center in Mexico

### Methods

We conducted a retrospective cohort study including adult patients diagnosed with primary or secondary MF and followed at a public tertiary referral center in Mexico. Clinical and treatment data were extracted from a structured institutional database and updated clinical records. Duplicate entries were consolidated by unique identifiers.
Treatment exposure was defined as any documented use of ruxolitinib or other MF-directed therapies during follow-up. OS was defined from diagnosis to death or last follow-up and estimated using the Kaplan–Meier method. Exploratory log-rank analyses evaluated OS according to transfusion requirement.

### Results

Eighteen unique MF patients were identified. Median age at diagnosis was 67 years (IQR 58–77). Fibrotic MF was documented in 55.6%, prefibrotic MF in 16.7%, and subtype was unavailable in 27.8%. Secondary MF following PV or ET was recorded in 5.6% of patients.
Transfusion requirement for red blood cell concentrates was documented in 61.1% of cases. At last evaluation, median hemoglobin, leukocyte count, platelet count, and spleen size were consistent with advanced symptomatic disease in a substantial proportion of patients.
Regarding treatment exposure, 50% of patients received ruxolitinib at any time during follow-up, 61% received hydroxyurea, 11% received interferon, and 17% received danazol. One patient underwent splenectomy. No allogeneic hematopoietic cell transplantation was documented in the available records.
Fifteen patients had complete dates available for survival analysis. With two deaths observed, KM-estimated OS at 2 years was 100%, and at 5 years 40%. Median OS was 3.09 years. However, survival estimates remain immature due to limited follow-up and substantial early censoring. In exploratory analysis, OS differed according to transfusion requirement (log-rank $p \approx 0.046$), though interpretation is limited by the small number of events.

**Copyright Information:** (Online) ISSN: 2572-9241
©2026 The Author(s). HemaSphere is published by John Wiley & Sons Ltd on behalf of European Hematology Association. This is an open access Abstract Book distributed under the Attribution-NonCommercial-NoDerivs (CC BY-NC-ND), which allows third parties to download the articles and share them with others as long as they credit the author and the Abstract Book, but they cannot change the content in any way or use them commercially.
**Abstract Book Citations:** Authors, Title, HemaSphere, 2026;10:(S1):pages. The abstract book DOIs can be found on the "Author Information" page.
**Disclaimer:** Articles published in the journal HemaSphere exclusively reflect the opinions of the authors. The authors are responsible for all content in their abstracts including accuracy of the facts, statements, citing resources, etc.

**Summary/Conclusion**

This real-world cohort from a large Mexican tertiary referral center provides early insight into MF characteristics and treatment patterns in an underrepresented Latin American population. A high proportion of patients required transfusional support and cytoreductive therapy, and half received ruxolitinib during follow-up.
Survival estimates are limited by small sample size and short follow-up; nevertheless, these findings highlight the importance of generating regional outcome data. Ongoing cohort expansion and longitudinal follow-up are underway to provide more mature survival analyses and evaluate the impact of treatment access on outcomes.

**Copyright Information:** (Online) ISSN: 2572-9241
©2026 The Author(s). HemaSphere is published by John Wiley & Sons Ltd on behalf of European Hematology Association. This is an open access Abstract Book distributed under the Attribution-NonCommercial-NoDerivs (CC BY-NC-ND), which allows third parties to download the articles and share them with others as long as they credit the author and the Abstract Book, but they cannot change the content in any way or use them commercially.
**Abstract Book Citations:** Authors, Title, HemaSphere, 2026;10:(S1):pages. The abstract book DOIs can be found on the “Author Information” page.
**Disclaimer:** Articles published in the journal HemaSphere exclusively reflect the opinions of the authors. The authors are responsible for all content in their abstracts including accuracy of the facts, statements, citing resources, etc.

### (PB3551) SAFETY AND EFFICACY OF RUXOLITINIB TREATMENT IN PATIENTS WITH MYELOFIBROSIS - A SINGLE-CENTER EXPERIENCE

**Topic:** 16. Myeloproliferative neoplasms - Clinical

Petra Belohlavkova*[1], Tomas Kupsa[1], Pavel Zak[1], Jakub Radocha[1]

*[1]Charles University Hospital, 4th Department of Internal Medicine – Hematology, Hradec Kralove, Czechia;*

### Background

Ruxolitinib has been the standard treatment for patients with myelofibrosis for many years.

### Aims

This study assesses the efficacy and safety of ruxolitinib (Rux) in patients with myelofibrosis under real-world clinical conditions. We evaluated the impact of DIPSS/DIPSS-plus, splenic response, baseline peripheral blood platelet and blast counts, and initial Rux dose on overall patient survival, including progression-free survival (PFS).

### Methods

A total of 54 patients diagnosed with myelofibrosis, with a median age at diagnosis of 65 years (range: 41–80 years), were evaluated. The median follow-up period following initiation of ruxolitinib The median duration of follow-up from the initiation of ruxolitinib was 29 months (M) (range 4 - 128 M) and the median initial Rux dose was 30 mg/dose/daily (range 10 - 40 mg/dose/daily).

### Results

An improvement in constitutional symptoms was observed in 52 patients (96.3%), while spleen size reduction was noted in 41 patients (75.9%). Treatment discontinuation occurred in 13 patients (24.1%) due to primary resistance and in 2 patients (3.7%) due to hematologic toxicity. Disease progression accounted for the majority of deaths, occurring in 17 patients (31.5%). The median duration of Rux treatment was 18 months (range: 6–128 months). The study identified an association between overall survival and factors including DIPSS or DIPSS-plus score and baseline platelet count ($\leq 150\times10^9$/L).
Our analysis confirmed the already published conclusions that achieving any splenic response correlates with a longer overall survival in patients (93% and 93% vs. 42%, respectively) ($p = 0.006$) and also correlates highly significantly with the risk of disease progression ($p < 0.0001$) (Figure).

### Summary/Conclusion

Our analysis confirmed that any reduction in spleen size is associated with better patient survival and lower incidence of progression. We further observed that if no splenic response is achieved at month 6 of treatment, a significant response does not occur in subsequent months. These patients represent the highest risk group for disease progression and shortest survival.

**Copyright Information:** (Online) ISSN: 2572-9241
©2026 The Author(s). HemaSphere is published by John Wiley & Sons Ltd on behalf of European Hematology Association. This is an open access Abstract Book distributed under the Attribution-NonCommercial-NoDerivs (CC BY-NC-ND), which allows third parties to download the articles and share them with others as long as they credit the author and the Abstract Book, but they cannot change the content in any way or use them commercially.
**Abstract Book Citations:** Authors, Title, HemaSphere, 2026;10:(S1):pages. The abstract book DOIs can be found on the “Author Information” page.
**Disclaimer:** Articles published in the journal HemaSphere exclusively reflect the opinions of the authors. The authors are responsible for all content in their abstracts including accuracy of the facts, statements, citing resources, etc.

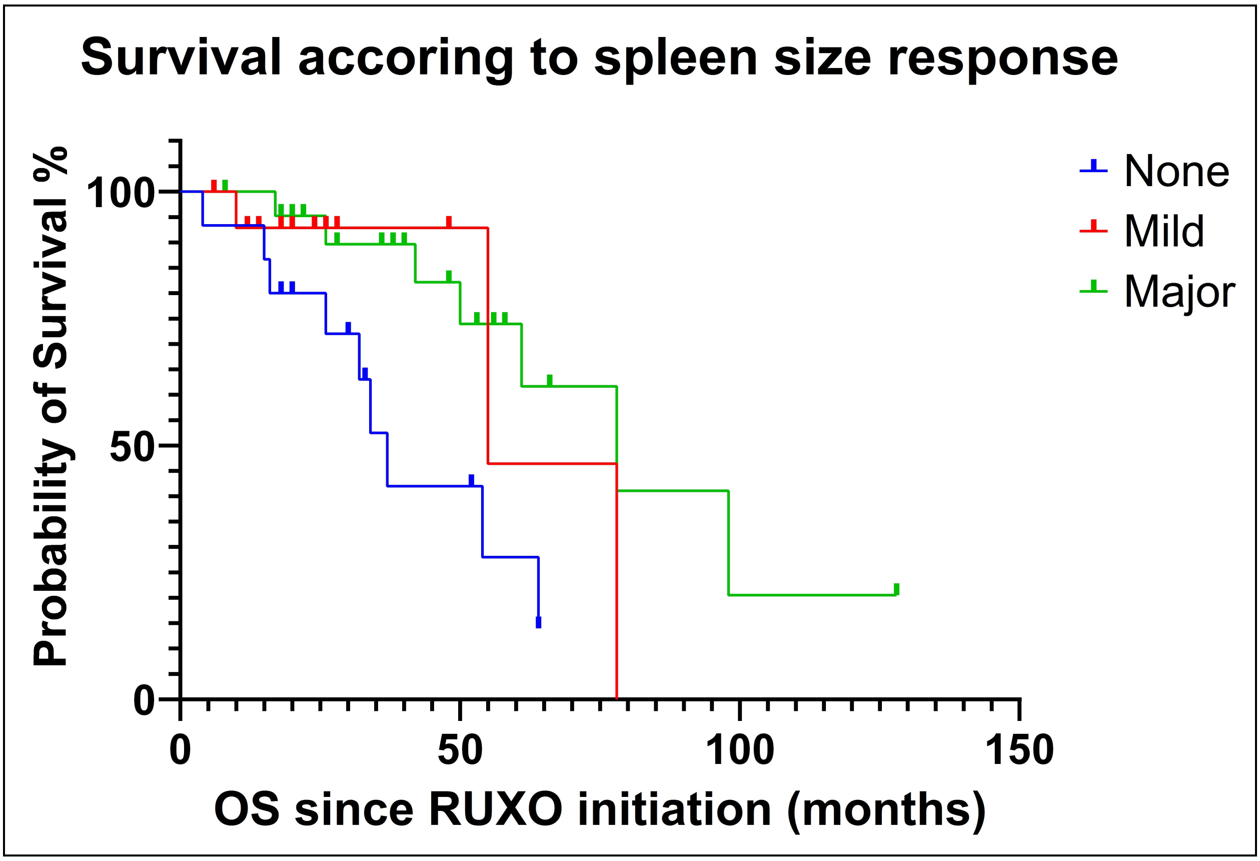

**Copyright Information:** (Online) ISSN: 2572-9241
©2026 The Author(s). HemaSphere is published by John Wiley & Sons Ltd on behalf of European Hematology Association. This is an open access Abstract Book distributed under the Attribution-NonCommercial-NoDerivs (CC BY-NC-ND), which allows third parties to download the articles and share them with others as long as they credit the author and the Abstract Book, but they cannot change the content in any way or use them commercially.
**Abstract Book Citations:** Authors, Title, HemaSphere, 2026;10:(S1):pages. The abstract book DOIs can be found on the "Author Information" page.
**Disclaimer:** Articles published in the journal HemaSphere exclusively reflect the opinions of the authors. The authors are responsible for all content in their abstracts including accuracy of the facts, statements, citing resources, etc.

### (PB3552) REAL WORLD OUTCOMES OF TREATMENT WITH RUXOLITINIB FOR POLYCYTHAEMIA VERA PATIENTS AT A SINGLE CENTRE DISTRICT GENERAL HOSPITAL

**Topic:** 16. Myeloproliferative neoplasms - Clinical

Tanveer Hamid*[1], Margarida Rodrigues[1], Diana Rance[1], Deirdre Peters[1], Vilma Hirsch[1], Richard Hinton[1], Mary McCloy[1]

*[1]Our Lady of Lourdes Hospital, Haematology, Drogheda, Ireland;*

### Background

Polycythaemia Vera (PV) is characterised by  Janus Kinase/signal transducer and activator of transcription (JAK/STAT) activation, thrombotic and haemorrhagic events, sytemic symptoms, and risk of disease transformation. In high-risk patients with PV , the JAK inhibitor ruxolitinib normalises blood counts and improves symptoms. Here, we report a retrospective study on a cohort of high-risk PV patients treated with ruxolitinib,who exhibited intolerance or resistance to hydroxycarbamide. Outcomes of interest included change in haematocrit levels and subsequent phlebotomy requirements, documented spleen size and symptom burden , with reference to the MAJIC-PV Study.

### Aims

To demonstrate replication of the findings of the MAJIC-PV study in a real world setting.

### Methods

This is a single centre retrospective analysis of outcomes of PV patients who were intolerant or resistant to hydroxycarbamide (HC) and were initiated on treatment with ruxolitinib.Clinical response was assessed by evaluating full blood count (FBC) and haematocrit (HCT) levels,documenting venesection frequency prior to initiation of ruxolitinib therapy and at designated time points thereafter, documenting spleen size and defining symptom burden using the Myeloproliferative Neoplasm Symptom Assessment Form (MPN-SAF).

### Results

A total of 12 patients with PV were followed in this analysis, all  JAK2V617F-positive, and identified as either resistant/intolerant to hydroxycarbamide. The age range was 48-81 years , with median age 72.5 years. There were 7 males and 5 females, and the duration of disease before initiation of ruxolitinib ranged from 2-216 months, median duration 77.5 months. Numbers of previous lines of therapy ranged from 1-2 , with a median of 1 previous therapy.n=4 (33%) were defined as HC intolerant, and n=8 (66%) defined as resistant. n=1 (8.33%) had a history of thrombosis,n=3 (25%) had a history of haemorrhage, and n=2 (16.6%) had diabetes, with n=7(58%) having hypertension.
Palpable spenomegaly was defined at baseline in n=4 (33%).Baseline FBC indices showed a median HCT of 0.473 (range 0.419-0.537). Looking at baseline venesection, 6months before initiation, n=9 , required venesection ranging from 1-7 episodes, with median 5 episodes. After 3 months of ruxolitinib, n=1 (1/12) required venesection - this was only 1 episode. At 6 months post initiation, n=5 had required a venesection, but the range was 1-2 episodes , with a median of 1 venesection in these 5 cases. Splenomegaly was no longer clinically apparent at the 3 month time point, and was subsequently maintained at T=6 months. MPN-SAF  scores at base line ranged from 0-11 with fatigue, pruritus, abdominal discomfort, night sweats and bone pain, being the most common symptoms. at T=3 months and at 6months, all symptoms had completely resolved except in n=2 patients with persisting fatigue. No deaths , thrombotic or haemorrhagic events, or infective issues/varicella zoster occurred. There were no discontinuations of ruxolitinib, and all n=12 remain on treatment

**Copyright Information:** (Online) ISSN: 2572-9241
©2026 The Author(s). HemaSphere is published by John Wiley & Sons Ltd on behalf of European Hematology Association. This is an open access Abstract Book distributed under the Attribution-NonCommercial-NoDerivs (CC BY-NC-ND), which allows third parties to download the articles and share them with others as long as they credit the author and the Abstract Book, but they cannot change the content in any way or use them commercially.
**Abstract Book Citations:** Authors, Title, HemaSphere, 2026;10:(S1):pages. The abstract book DOIs can be found on the “Author Information” page.
**Disclaimer:** Articles published in the journal HemaSphere exclusively reflect the opinions of the authors. The authors are responsible for all content in their abstracts including accuracy of the facts, statements, citing resources, etc.

**Summary/Conclusion**

Ruxolitinib is safe and well-tolerated in this cohort, with no discontinuations in therapy or infective complications. All patients with splenomegaly have had resolution on intitiation of ruxolitinib. There has been significant reduction in venesection requirements post initiation of therapy, and MPN-SAF scores demonstrate an improvement in well being with resolution of symptoms which has been maintained. This real world data reflects the findings in the MAJIC-PV Study

**Copyright Information:** (Online) ISSN: 2572-9241
©2026 The Author(s). HemaSphere is published by John Wiley & Sons Ltd on behalf of European Hematology Association. This is an open access Abstract Book distributed under the Attribution-NonCommercial-NoDerivs (CC BY-NC-ND), which allows third parties to download the articles and share them with others as long as they credit the author and the Abstract Book, but they cannot change the content in any way or use them commercially.
**Abstract Book Citations:** Authors, Title, HemaSphere, 2026;10:(S1):pages. The abstract book DOIs can be found on the "Author Information" page.
**Disclaimer:** Articles published in the journal HemaSphere exclusively reflect the opinions of the authors. The authors are responsible for all content in their abstracts including accuracy of the facts, statements, citing resources, etc.

### (PB3553) JAK2-POSITIVE HYPEREOSINOPHILIC SYNDROME PRESENTING WITH VASCULAR AND GI COMPLICATIONS

**Topic:** 16. Myeloproliferative neoplasms - Clinical

Rogermar Villanueva*[1]

[1]*University of the Philippines- Philippines General Hospital, Internal Medicine- Division of Hematology, Manila, Philippines;*

### Background

Hypereosinophilic syndrome (HES) is a rare disorder characterized by sustained eosinophilia and organ damage, which may arise from clonal myeloid neoplasms. JAK2- mutated HES is exceptionally uncommon and may overlap with myelofibrosis and atypical thrombosis.

### Aims

We present a diagnostically complex case of JAK2-positive HES with myelofibrosis in a woman with prior splenectomy, highlighting its rare gastrointestinal and vascular complications.

### Methods

This report describes a single patient, using data obtained retrospectively.

### Results

A 34-year-old woman presented with persistent abdominal pain and a tender left flank mass. She had a history of splenectomy following abdominal trauma. Cross-sectional imaging revealed an infarcted accessory spleen and left gonadal vein thrombosis. Laboratory investigations showed marked leukocytosis, sustained eosinophilia, and thrombocytosis. Further imaging and endoscopic evaluation identified non-cirrhotic portal hypertension, ascites, and colitis. Histopathological examination of colonic biopsies confirmed eosinophilic infiltration. Bone marrow biopsy demonstrated a hypercellular marrow with WHO grade 3 fibrosis. Molecular analysis detected a JAK2 V617F mutation, with no evidence of FIP1L1-PDGFRA fusion, CHIC2 deletion, PDGFRA or PDGFRB rearrangements, or BCR-ABL1. The patient was referred for surgical assessment of the 1infarcted accessory spleen; however, operative management was not indicated. She was initially treated with hydroxyurea, systemic corticosteroids, and anticoagulation. Due to ongoing symptoms and persistent laboratory abnormalities, ruxolitinib was subsequently initiated.

### Summary/Conclusion

This case illustrates a rare clonal eosinophilic syndrome complicated by splanchnic thrombosis, eosinophilic colitis, and portal hypertension. It underscores the importance of early molecular testing and a multidisciplinary approach in patients presenting with eosinophilia and multisystem involvement.

**Copyright Information:** (Online) ISSN: 2572-9241
©2026 The Author(s). HemaSphere is published by John Wiley & Sons Ltd on behalf of European Hematology Association. This is an open access Abstract Book distributed under the Attribution-NonCommercial-NoDerivs (CC BY-NC-ND), which allows third parties to download the articles and share them with others as long as they credit the author and the Abstract Book, but they cannot change the content in any way or use them commercially.
**Abstract Book Citations:** Authors, Title, HemaSphere, 2026;10:(S1):pages. The abstract book DOIs can be found on the "Author Information" page.
**Disclaimer:** Articles published in the journal HemaSphere exclusively reflect the opinions of the authors. The authors are responsible for all content in their abstracts including accuracy of the facts, statements, citing resources, etc.

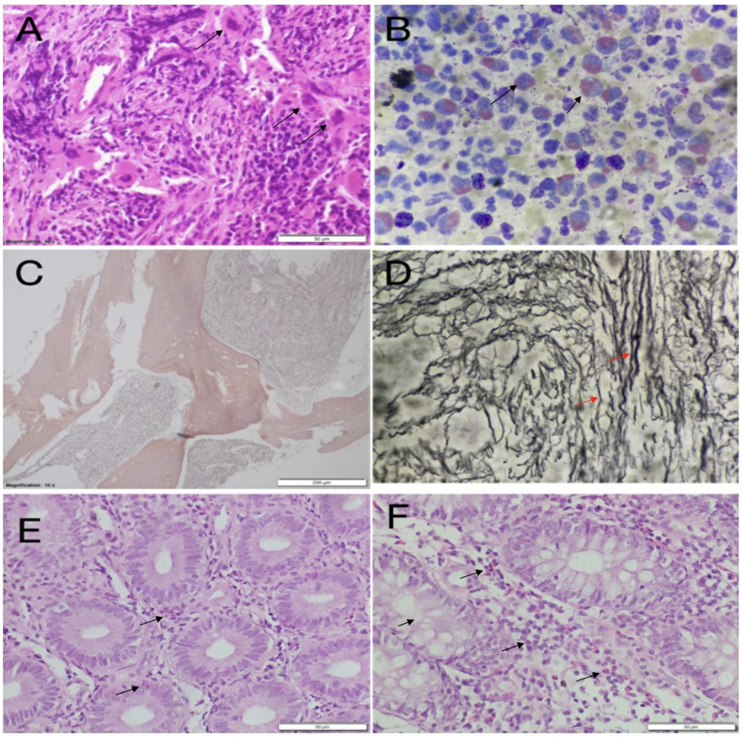

**Figure 2. Histopathologic findings demonstrating hematologic and gastrointestinal involvement in JAK2-positive HES with myelofibrosis**. **(A)** High-power field (H&E stain) of bone marrow core biopsy showing marked hypercellularity (90–95%) with trilineage hematopoiesis and loosely clustered megakaryocytes (black arrow). **(B)** Bone marrow aspirate smear with numerous eosinophils (black arrow) under high-power magnification. **(C–D)** Low-power (C) and high-power (D) views of reticulin-stained bone marrow core revealing dense reticulin deposition (red arrows), consistent with WHO Grade 3 myelofibrosis. **(E–F)** High-power fields of colonic mucosal biopsies showing focal mild to moderate chronic inflammation with dense eosinophilic infiltration (up to 20 eosinophils (black arrow) per high-power field), consistent with eosinophilic colitis.

**Copyright Information:** (Online) ISSN: 2572-9241
©2026 The Author(s). HemaSphere is published by John Wiley & Sons Ltd on behalf of European Hematology Association. This is an open access Abstract Book distributed under the Attribution-NonCommercial-NoDerivs (CC BY-NC-ND), which allows third parties to download the articles and share them with others as long as they credit the author and the Abstract Book, but they cannot change the content in any way or use them commercially.
**Abstract Book Citations:** Authors, Title, HemaSphere, 2026;10:(S1):pages. The abstract book DOIs can be found on the “Author Information” page.
**Disclaimer:** Articles published in the journal HemaSphere exclusively reflect the opinions of the authors. The authors are responsible for all content in their abstracts including accuracy of the facts, statements, citing resources, etc.

### (PB3554) ACUTE CORONARY SYNDROME IN ESSENTIAL THROMBOCYTHEMIA: A CASE SERIES AND CLINICAL INSIGHTS

**Topic:** 16. Myeloproliferative neoplasms - Clinical

Marta Hernández Durán*[1], Sara Rioja Abad[2], Alejandro Llopis Gimenez[1], Henar de Las Heras Sacedo[1], Ruth Arnau Teruel[1], Ignacio Hernández González[2], Natalia Carpizo Jiménez[1], Ana Campano García[1], Beatriz González Mena[1], Lucía Silvestre Cristóbal[1], Alberto Cantalapiedra[1]

*[1]Hospital Río Hortega, Hematology, Valladolid, Spain; [2]Hospital Río Hortega, Cardiology, Valladolid, Spain;*

### Background

Essential thrombocythemia (ET) is a chronic Philadelphia chromosome–negative myeloproliferative neoplasm characterized by excessive clonal platelet production and megakaryocytic hyperplasia in the bone marrow. Its incidence ranges from 0.2 to 2.5 cases per 100,000 per year, mostly affecting individuals aged 50–70. ET induces a hypercoagulable state due to hyperactive clonal platelets, chronic inflammation, and oxidative stress, which shifts the endothelium to a procoagulant state. Consequently, arterial events, including acute coronary syndrome (ACS), are frequent, with incidences ranging from 2% to 31%.

### Aims

To describe the clinical experience and outcomes of patients with ACS and ET at a tertiary hospital.

### Methods

A descriptive and retrospective case series was conducted, including patients diagnosed with ET and ACS (occurring either before or after the cardiovascular event) between 2022 and 2025. Data on demographics, cardiovascular risk factors (CVRF), ET and ACS characteristics, treatment, and clinical evolution were collected.

### Results

Four patients were identified between 2022 and 2025.

- **Case 1:** A 51-year-old female (without CVRF) presented with STEMI and a left anterior descending (LAD) artery lesion treated with a stent. Platelet count was 1,313,000/µL. JAK2 mutation was present (38% burden), and ET was confirmed post-MI. No recurrences occurred under hydroxyurea and aspirin

- **Case 2:** A 31-year-old male with ET (diagnosed 2020) presented with STEMI while on anagrelide. Angiography showed a right coronary artery lesion treated with a stent. Platelet count was 505,000/µL

**Copyright Information:** (Online) ISSN: 2572-9241
©2026 The Author(s). HemaSphere is published by John Wiley & Sons Ltd on behalf of European Hematology Association. This is an open access Abstract Book distributed under the Attribution-NonCommercial-NoDerivs (CC BY-NC-ND), which allows third parties to download the articles and share them with others as long as they credit the author and the Abstract Book, but they cannot change the content in any way or use them commercially.
**Abstract Book Citations:** Authors, Title, HemaSphere, 2026;10:(S1):pages. The abstract book DOIs can be found on the "Author Information" page.
**Disclaimer:** Articles published in the journal HemaSphere exclusively reflect the opinions of the authors. The authors are responsible for all content in their abstracts including accuracy of the facts, statements, citing resources, etc.

• **Case 3:** A 52-year-old female with multiple CVRF and ET (JAK2+, diagnosed 2012) presented with STEMI (LAD lesion) while on hydroxyurea. She was treated with a stent; platelet count was 433,000/µL

• **Case 4:** A 50-year-old female with CVRF and ET (diagnosed 2012, JAK2 negative) presented with MINOCA (myocardial infarction with non-obstructive coronary arteries) while on anagrelide. Vasospastic angina was diagnosed after ruling out other causes

Table 1. Hematological factors

| Case | Age/Gender | ET diagnosis pre-ACS/date | IPSET-Thrombosis risk | Platelets ($10^3$/µL) | JAK2 mutation | Treatment on the event/postevent |
|---|---|---|---|---|---|---|
| 1 | 51/Female | No, post-ACS (2024) | Low | 1313 | Yes (38%) | None / Hydroxyurea + ASA |
| 2 | 31/Male | Yes (2020) | Low | 505 | Unknown | Anagrelide + ASA / Anagrelide + ASA |
| 3 | 52/Female | Yes (2012) | High | 433 | Yes (28%) | Hydroxyurea + ASA / Hydroxyurea + ASA |
| 4 | 50/Female | Yes (2012) | Low | 263 | None | Anagrelide + ASA / Anagrelide + ASA |

Table 2. Cardiological factors

| Case | Age/Gender | CV risk factors | Presentation ACS/year | Coronary angiography | Recurrence |
|---|---|---|---|---|---|
| 1 | 51/Female | None | STEMI/2024 | LAD | None |
| 2 | 31/Male | None | STEMI/2022 | Right coronary | None |
| 3 | 52/Female | Yes | STEMI/2025 | LAD | None |
| 4 | 50/Female | Yes | MINOCA/2025 | Non-obstructive lesions | Chest pain episodes |

**Copyright Information:** (Online) ISSN: 2572-9241
©2026 The Author(s). HemaSphere is published by John Wiley & Sons Ltd on behalf of European Hematology Association. This is an open access Abstract Book distributed under the Attribution-NonCommercial-NoDerivs (CC BY-NC-ND), which allows third parties to download the articles and share them with others as long as they credit the author and the Abstract Book, but they cannot change the content in any way or use them commercially.
**Abstract Book Citations:** Authors, Title, HemaSphere, 2026;10:(S1):pages. The abstract book DOIs can be found on the "Author Information" page.
**Disclaimer:** Articles published in the journal HemaSphere exclusively reflect the opinions of the authors. The authors are responsible for all content in their abstracts including accuracy of the facts, statements, citing resources, etc.

**Summary/Conclusion**

ACS in ET often affects young adults, sometimes without classical CVRF. Notably, anagrelide is a rare but documented cause of coronary spasm (1-5%). Because recurrence after MI is frequent in ET patients, it is crucial to consider ET as an underlying cause of cardiovascular events to ensure early diagnosis and prompt treatment. Our findings suggest that thrombotic risk is determined mainly by qualitative platelet abnormalities rather than by the absolute platelet count, as events occurred across a wide range of platelet levels. In this series, timely management prevented recurrences. Futhermore, 75% of the patients were IPSET 'low-risk', suggesting current stratification tools underestimate acute arterial event potential.

**Copyright Information:** (Online) ISSN: 2572-9241
©2026 The Author(s). HemaSphere is published by John Wiley & Sons Ltd on behalf of European Hematology Association. This is an open access Abstract Book distributed under the Attribution-NonCommercial-NoDerivs (CC BY-NC-ND), which allows third parties to download the articles and share them with others as long as they credit the author and the Abstract Book, but they cannot change the content in any way or use them commercially.
**Abstract Book Citations:** Authors, Title, HemaSphere, 2026;10:(S1):pages. The abstract book DOIs can be found on the “Author Information” page.
**Disclaimer:** Articles published in the journal HemaSphere exclusively reflect the opinions of the authors. The authors are responsible for all content in their abstracts including accuracy of the facts, statements, citing resources, etc.

### (PB3556) DEEP LEARNING-ENHANCED BASELINE PET FOR PROGNOSTIC PREDICTION IN CLASSICAL HODGKIN LYMPHOMA: A RETROSPECTIVE STUDY

**Topic:** 17. Hodgkin lymphoma - Clinical

Haoan Zhang[1], Hang Yu[2], Xinyuan Chen[3], Yue Teng[4], Yimu Ji[2], Chongyang Ding[5], Shangdong Liu[2], Chong Jiang[6], Jingyan Xu*[1]

*[1]China Pharmaceutical University Nanjing Drum Tower Hospital, Nanjing, China; [2]Nanjing University of Posts and Telecommunications, School of Computer Science,, Nanjing, China; [3]Nanjing Drum Tower Hospital Clinical College of Nanjing University of Chinese Medicine, Nanjing, China; [4]Nanjing Drum Tower Hospital, Affiliated Hospital of Medical School, Nanjing University, Nanjing, China; [5]The First Affiliated Hospital of Nanjing Medical University, Jiangsu Provincial People's Hospital, Nanjing, China; [6]West China Hospital, Sichuan University, Chengdu, China;*

### Background

Classical Hodgkin lymphoma (cHL) is a prevalent lymphoid malignancy, primarily affecting adolescents and young adults. Although most patients are cured, 10–20% develop refractory or relapsed disease, typically within two years . Current prognostic scoring systems for cHL employ the European Organization for Research and Treatment of Cancer (EORTC), German Hodgkin Study Group (GHSG), and National Comprehensive Cancer Network (NCCN) criteria for limited-stage disease, and the International Prognostic Score (IPS) for advanced-stage disease. However, these prognostic scores have limited ability to identify patients with poor prognosis. Consequently, the development of a precision prognostic model is critical for making decisions in cHL

### Aims

This study aims to develop an automated deep learning-based method for tumor segmentation and derive a radiomics score (RadScore) from PET images. We further propose a multiparametric model incorporating RadScore to predict 2-year progression-free survival (2y-PFS) in cHL patients.

### Methods

A total of 192 patients with cHL from two hospitals were included in this retrospective study. The deep learning architectures named MedNeXt and Mambular were employed for automatic tumor segmentation and RadScore construction, respectively, using PET images. RadScore were used to predict 2-year progression-free survival (2y-PFS). Furthermore, the multiparametric model was developed based on the results of the Cox proportional hazards regression model, and the model was assessed using the concordance index (C-index), calibration curves, and time-dependent ROC curves.

### Results

MedNeXt achieved accurate tumor segmentation. RadScore demonstrated significant predictive ability (AUC:0.782 in validation) and prognostic relevance ($P<0.05$) for 2y-PFS. The multiparametric model demonstrated superior efficacy in predicting 2y-PFS (C-index: 0.712 in validation) compared to other models. Calibration curves demonstrated the good consistency, and time-dependent ROC curves confirmed that the multi-parameter model provided high predictive accuracy at multiple time points.

### Summary/Conclusion

RadScore demonstrated significant prognostic value in cHL patients and could serve as an independent prognostic factor. Moreover, the multiparametric model integrating RadScore effectively identified patients with poor prognosis and shows clinical translation potential.

**Copyright Information:** (Online) ISSN: 2572-9241
©2026 The Author(s). HemaSphere is published by John Wiley & Sons Ltd on behalf of European Hematology Association. This is an open access Abstract Book distributed under the Attribution-NonCommercial-NoDerivs (CC BY-NC-ND), which allows third parties to download the articles and share them with others as long as they credit the author and the Abstract Book, but they cannot change the content in any way or use them commercially.
**Abstract Book Citations:** Authors, Title, HemaSphere, 2026;10:(S1):pages. The abstract book DOIs can be found on the "Author Information" page.
**Disclaimer:** Articles published in the journal HemaSphere exclusively reflect the opinions of the authors. The authors are responsible for all content in their abstracts including accuracy of the facts, statements, citing resources, etc.

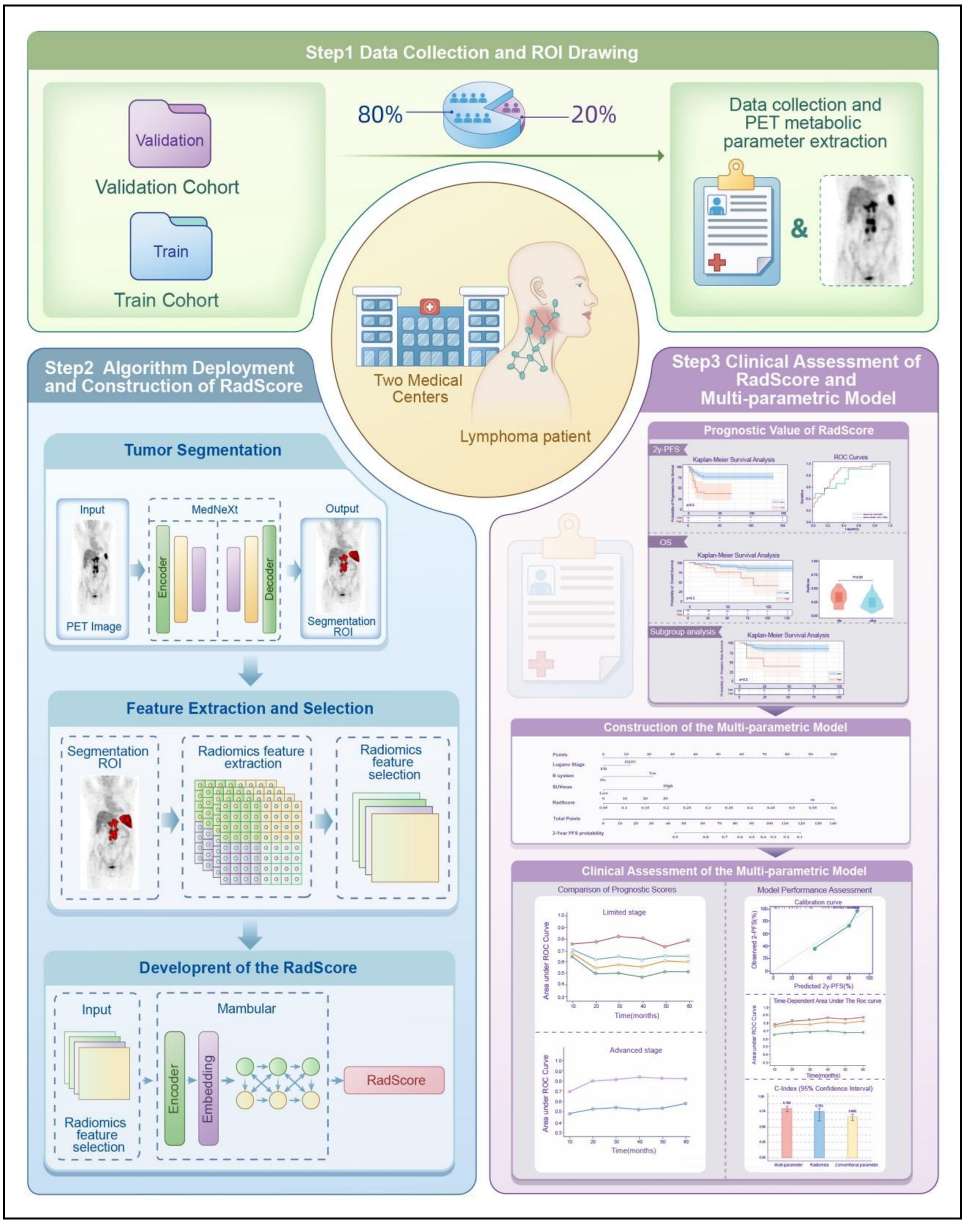

**Copyright Information:** (Online) ISSN: 2572-9241
©2026 The Author(s). HemaSphere is published by John Wiley & Sons Ltd on behalf of European Hematology Association. This is an open access Abstract Book distributed under the Attribution-NonCommercial-NoDerivs (CC BY-NC-ND), which allows third parties to download the articles and share them with others as long as they credit the author and the Abstract Book, but they cannot change the content in any way or use them commercially.
**Abstract Book Citations:** Authors, Title, HemaSphere, 2026;10:(S1):pages. The abstract book DOIs can be found on the "Author Information" page.
**Disclaimer:** Articles published in the journal HemaSphere exclusively reflect the opinions of the authors. The authors are responsible for all content in their abstracts including accuracy of the facts, statements, citing resources, etc.

### (PB3557) FROM LONG-TERM BENEFIT TO RISK-STRATIFIED MANAGEMENT: A 5-YEAR REAL-WORLD MULTICENTER STUDY OF PD-1 INHIBITORS IN CHINESE RELAPSED/REFRACTORY HODGKIN LYMPHOMA

**Topic:** 17. Hodgkin lymphoma - Clinical

Wenyan Yu*[1], Yi Song[1], Jian-qing Mi[1]

[1]*Shanghai Institute of Hematology, State Key Laboratory of Medical Genomics, National Research Center for Translational Medicine at Shanghai, Ruijin Hospital Affiliated to Shanghai Jiao Tong University School of Medicine, Shanghai, China;*

### Background

Despite the established efficacy of domestic PD-1 inhibitors (sintilimab, tislelizumab) in relapsed and refractory classical Hodgkin lymphoma (R/R cHL), their long-term clinical outcomes, adverse events and strategies for optimal risk-adapted management remain unclear.

### Aims

To provide extended real-world evidence and develop a novel prognostic model to guide personalized risk-adapted therapy.

### Methods

We conducted a retrospective multicenter study of 74 patients with R/R cHL (≥2 prior lines) initiating sintilimab or tislelizumab monotherapy between January 2019 and September 2021. The primary endpoint was progression-free survival at 24 months (PFS24). A novel risk score was derived from multivariate analysis. Targeted sequencing was additionally performed.

### Results

With a median follow-up of 67 months (range, 4-78), the 2-y and 5-y PFS rates were 47.3% and 35.0%, and OS rates were 90.5% and 88.1%, respectively. The median PFS was 23.5 months, while the median OS was not reached. PFS24 served as a critical prognostic benchmark. Smoothed annual hazard plot revealed a peak risk of progression within the initial 24 months, declining to <10% beyond 5 years.The patients who failed to reach PFS24 had a significant inferior OS (5-year OS 95.0% vs 79.2%, *p*=0.01). Key predictors of PFS24 failure included early non-CR; Ann Arbor IV and refractoriness to most recent prior therapy. A novel risk model stratified patients into low-, intermediate-, and high-risk groups with distinct 2-year PFS (76.2% vs 50.0% vs 20.6%, *p*<0.0001) and 5-year PFS (65.9% vs. 29.9% vs. 16.0%; *p* < 0.0001). Patients who discontinued therapy without progression, maintained a 2-year PFS of 75% post-cessation. Subgroup analysis revealed distinct exposure-dependent patterns: low-risk patients maintained prolonged remission (median PFS 49.5 months) even with <1 year of therapy, whereas intermediate-risk patients required ≥2 years of treatment for optimal outcomes (*p*=0.0052). Somatic mutations were observed in 44 out of 49 patients. TP53 (30.77% vs 8.7%, *p*=0.08) and B2M (38.5% vs 17.4%, *p*=0.125) mutations were enriched in non-PFS24 subgroup, while LRP1B mutations showed a negative correlation with early progression (*p*=0.086). Delayed irAEs occurred in 9.5% of patients, while chronic irAEs developed in 29.7%, primarily persistent endocrine and neurological toxicities.

**Copyright Information:** (Online) ISSN: 2572-9241
©2026 The Author(s). HemaSphere is published by John Wiley & Sons Ltd on behalf of European Hematology Association. This is an open access Abstract Book distributed under the Attribution-NonCommercial-NoDerivs (CC BY-NC-ND), which allows third parties to download the articles and share them with others as long as they credit the author and the Abstract Book, but they cannot change the content in any way or use them commercially.
**Abstract Book Citations:** Authors, Title, HemaSphere, 2026;10:(S1):pages. The abstract book DOIs can be found on the "Author Information" page.
**Disclaimer:** Articles published in the journal HemaSphere exclusively reflect the opinions of the authors. The authors are responsible for all content in their abstracts including accuracy of the facts, statements, citing resources, etc.

### Summary/Conclusion

This study provides the longest real-world evidence for domestic PD-1 inhibitors in R/R cHL, and establishes PFS24 as a robust prognostic benchmark. The proposed risk model stratifies patients into distinct outcome groups, supporting personalized risk-adapted management (e.g., de-escalation for low-risk, adequate exposure for intermediate-risk, and early intensification for high-risk patients). Future prospective validation in larger cohorts and the integration of molecular biomarkers with clinical and imaging data are warranted to refine the novel risk model for further precision immunotherapy in R/R HL, particularly in Asian clinical settings.

.

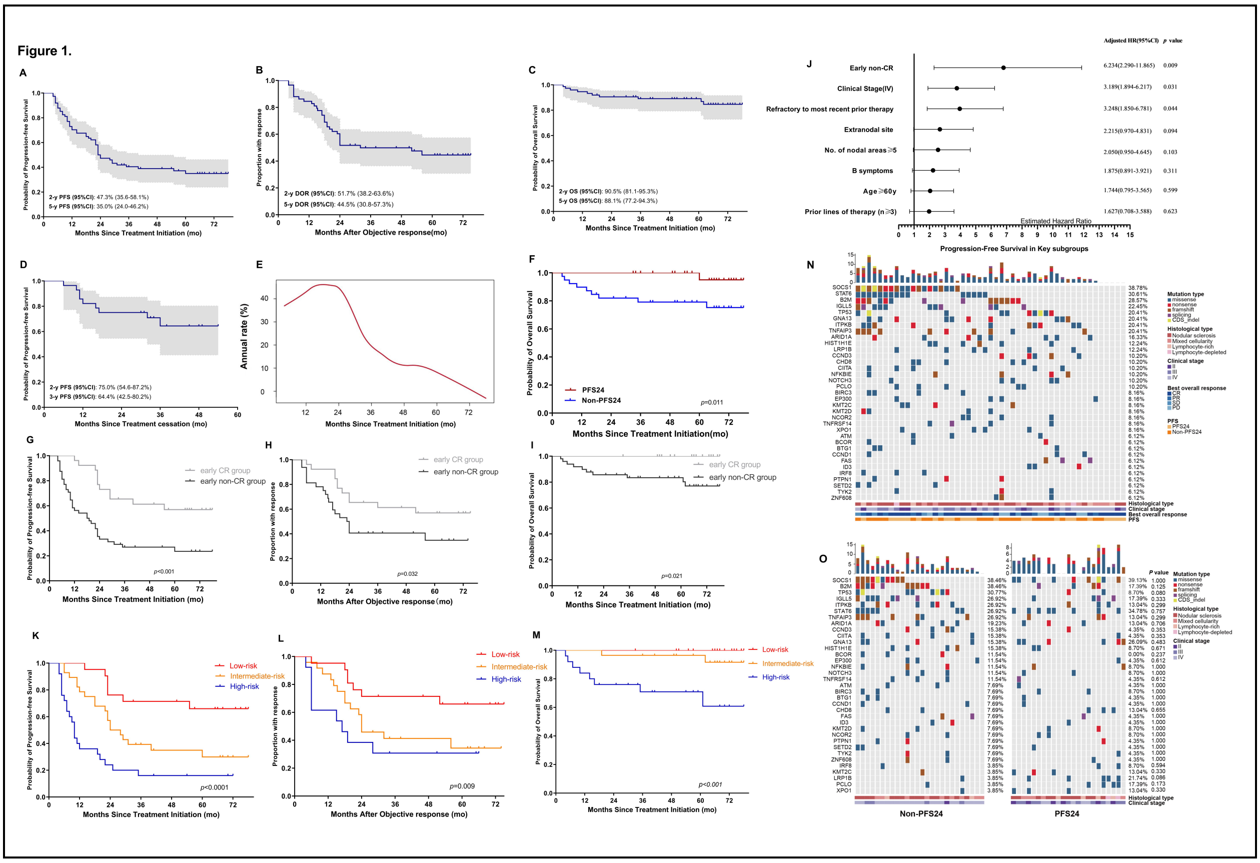

**Copyright Information:** (Online) ISSN: 2572-9241
©2026 The Author(s). HemaSphere is published by John Wiley & Sons Ltd on behalf of European Hematology Association. This is an open access Abstract Book distributed under the Attribution-NonCommercial-NoDerivs (CC BY-NC-ND), which allows third parties to download the articles and share them with others as long as they credit the author and the Abstract Book, but they cannot change the content in any way or use them commercially.
**Abstract Book Citations:** Authors, Title, HemaSphere, 2026;10:(S1):pages. The abstract book DOIs can be found on the "Author Information" page.
**Disclaimer:** Articles published in the journal HemaSphere exclusively reflect the opinions of the authors. The authors are responsible for all content in their abstracts including accuracy of the facts, statements, citing resources, etc.

### (PB3558) FRONTLINE THERAPY AND OUTCOMES OF CLASSICAL HODGKIN LYMPHOMA IN MALAYSIA: A NATIONWIDE MULTICENTRE REAL-WORLD COHORT

**Topic:** 17. Hodgkin lymphoma - Clinical

Yang Liang Boo*[1], Leong Tze Shin[2], Chee Yoong Wong[3], Jerome Tan Tsen Chuen[3], Lily Wong Lee Lee[4], Yin Man Lim[4], Nur Aini Syakirah Ahmad Shuyuti[5], Ai Sim Goh[6], Si Yuan Ng[7], Kamini Kirubamoorthy[8], Muhamad Izzat Abdul Rahman[9], Ahlam Naila Kori[9], Chee Chiat Liong[10], Yea Bing Tham[11], Lai Chee Chow[12], Xin Yee Tan[13], Kar Ying Yong[14], Kam Hoo Yip[15], Guang Yong Chong[16], Kim Wah Ho[17], Kian Meng Chang[17], Mohd Haris Fadzillah Abdul Rahman[18], Yong Khee Guan[19], Kim Yen Goh[20], Ching Soon Teoh[21], Tengku Ahmad Hidayat Tengku K Aziz[22], Frances Leow May Yan[22], Sen Mui Tan[3, 18], Lee Ping Chew[2], Soo Min Lim[1], Gin Gin Gan[10]

*[1]Sultanah Aminah Hospital, Johor Bahru, Malaysia;[2]Sarawak General Hospital, Kuching, Malaysia;[3]Ampang Hospital, Ampang, Malaysia; [4]Queen Elizabeth Hospital, Kota Kinabalu, Malaysia; [5]Raja Perempuan Zainab II Hospital, Kota Bahru, Malaysia; [6]Penang General Hospital, Pulau Pinang, Malaysia; [7]Melaka Hospital, Melaka, Malaysia; [8]Raja Permaisuri Bainun Hospital, Ipoh, Malaysia; [9]Tengku Ampuan Afzan Hospital, Kuantan, Malaysia; [10]Universiti Malaya Medical Centre, Kuala Lumpur, Malaysia; [11]Tuanku Ja'afar Hospital, Seremban, Malaysia; [12]Tengku Ampuan Rahimah Hospital, Klang, Malaysia; [13]Sibu Hospital, Sibu, Malaysia; [14]Miri Hospital, Miri, Malaysia; [15]Sultanah Nur Zahirah Hospital, Kuala Terengganu, Malaysia; [16]Hospital Canselor Tuanku Muhriz UKM, Kuala Lumpur, Malaysia; [17]Sunway Medical Centre, Bandar Sunway, Malaysia; [18]Subang Jaya Medical Centre, Subang Jaya, Malaysia; [19]Pantai Hospital Melaka, Melaka, Malaysia;[20]Ampang Puteri Specialist Hospital, Ampang, Malaysia; [21]Loh Guan Lye Specialist Centre, Pulau Pinang, Malaysia;[22]Beacon Hospital, Selangor, Malaysia;*

### Background

Classical Hodgkin lymphoma (cHL) is among the most curable hematologic malignancies, with outcomes improved by risk-adapted frontline strategies incorporating interim positron emission tomography (PET) and novel agents. However, major global disparities persist in access to PET imaging and newer therapies. In low- and middle-income countries, frontline treatment relies largely on conventional chemotherapy, with decisions often constrained by resource availability rather than biological risk. Although Malaysia is an upper-middle-income country, access to advanced diagnostics and treatments remains heterogeneous. Nationwide real-world data from such settings are scarce but essential to contextualize international recommendations and guide equitable cHL care.

### Aims

To describe the clinical characteristics, frontline treatment strategies, and outcomes of patients with cHL treated in routine clinical practice across Malaysia.

### Methods

This retrospective nationwide multicentre cohort study included patients aged >12 years diagnosed with cHL between January 2018 and December 2023 across 23 public and private centres. Baseline demographic, clinical, treatment, and outcome data were extracted from medical records. Disease staging was performed using the Ann Arbor system, and risk stratification was based on German Hodgkin Study Group (GHSG) criteria. Treatment response was assessed using the Lugano classification. Progression-free survival (PFS) and overall survival (OS) were estimated using the Kaplan–Meier method, and Cox proportional hazards regression was performed to identify prognostic factors.

**Copyright Information:** (Online) ISSN: 2572-9241
©2026 The Author(s). HemaSphere is published by John Wiley & Sons Ltd on behalf of European Hematology Association. This is an open access Abstract Book distributed under the Attribution-NonCommercial-NoDerivs (CC BY-NC-ND), which allows third parties to download the articles and share them with others as long as they credit the author and the Abstract Book, but they cannot change the content in any way or use them commercially.
**Abstract Book Citations:** Authors, Title, HemaSphere, 2026;10:(S1):pages. The abstract book DOIs can be found on the "Author Information" page.
**Disclaimer:** Articles published in the journal HemaSphere exclusively reflect the opinions of the authors. The authors are responsible for all content in their abstracts including accuracy of the facts, statements, citing resources, etc.

**Results**

Of 1,407 patients diagnosed with cHL, 1,262 treatment-naïve patients were included in final analysis. The median age at diagnosis was 29 years (range,12–81), and 53.4% were male. B symptoms were present in 67.0%, bulky disease in 43.7%, and extranodal involvement in 34.4%. According to GHSG criteria, 69.8% had advanced-stage disease. The most commonly used frontline regimen was ABVD (69.8%). Escalated BEACOPP was administered in 7.7%, while 18.5% received combinations of ABVD and BEACOPP within PET-adapted strategies. Overall, PET-adapted approaches were employed in 29.2% of patients, and consolidative radiotherapy in 22.3%. At completion of frontline therapy, 60.9% achieved complete response (CR), 21.9% partial response, 2.6% stable disease, and 14.6% progressive disease. Primary refractory disease occurred in 24.9%, and disease relapse after CR in 8.0%. With a median follow-up of 33 months, the estimated 3-year PFS and OS were 68.5% and 93.6%, respectively. On multivariate analysis, age >45 years (HR 4.70, p<0.001), extranodal disease (HR 2.99, p=0.001), and failure to achieve CR after frontline therapy (HR 19.89, p<0.001) were independently associated with inferior OS. Failure to achieve CR was also the strongest predictor of inferior PFS (HR 16.20, p<0.001).

**Summary/Conclusion**

In this nationwide real-world cohort, conventional chemotherapy remains the backbone of frontline treatment for cHL in Malaysia. Although OS is relatively high, PFS and rates of primary refractory disease reveal a clear outcome gap compared with high-income countries. These findings underscore the urgent need to expand access to risk-adapted strategies and novel therapies in resource-limited settings to reduce global disparities in cHL care.

*YLB and LTS contributed equally to this work.

**Copyright Information:** (Online) ISSN: 2572-9241
©2026 The Author(s). HemaSphere is published by John Wiley & Sons Ltd on behalf of European Hematology Association. This is an open access Abstract Book distributed under the Attribution-NonCommercial-NoDerivs (CC BY-NC-ND), which allows third parties to download the articles and share them with others as long as they credit the author and the Abstract Book, but they cannot change the content in any way or use them commercially.
**Abstract Book Citations:** Authors, Title, HemaSphere, 2026;10:(S1):pages. The abstract book DOIs can be found on the "Author Information" page.
**Disclaimer:** Articles published in the journal HemaSphere exclusively reflect the opinions of the authors. The authors are responsible for all content in their abstracts including accuracy of the facts, statements, citing resources, etc.

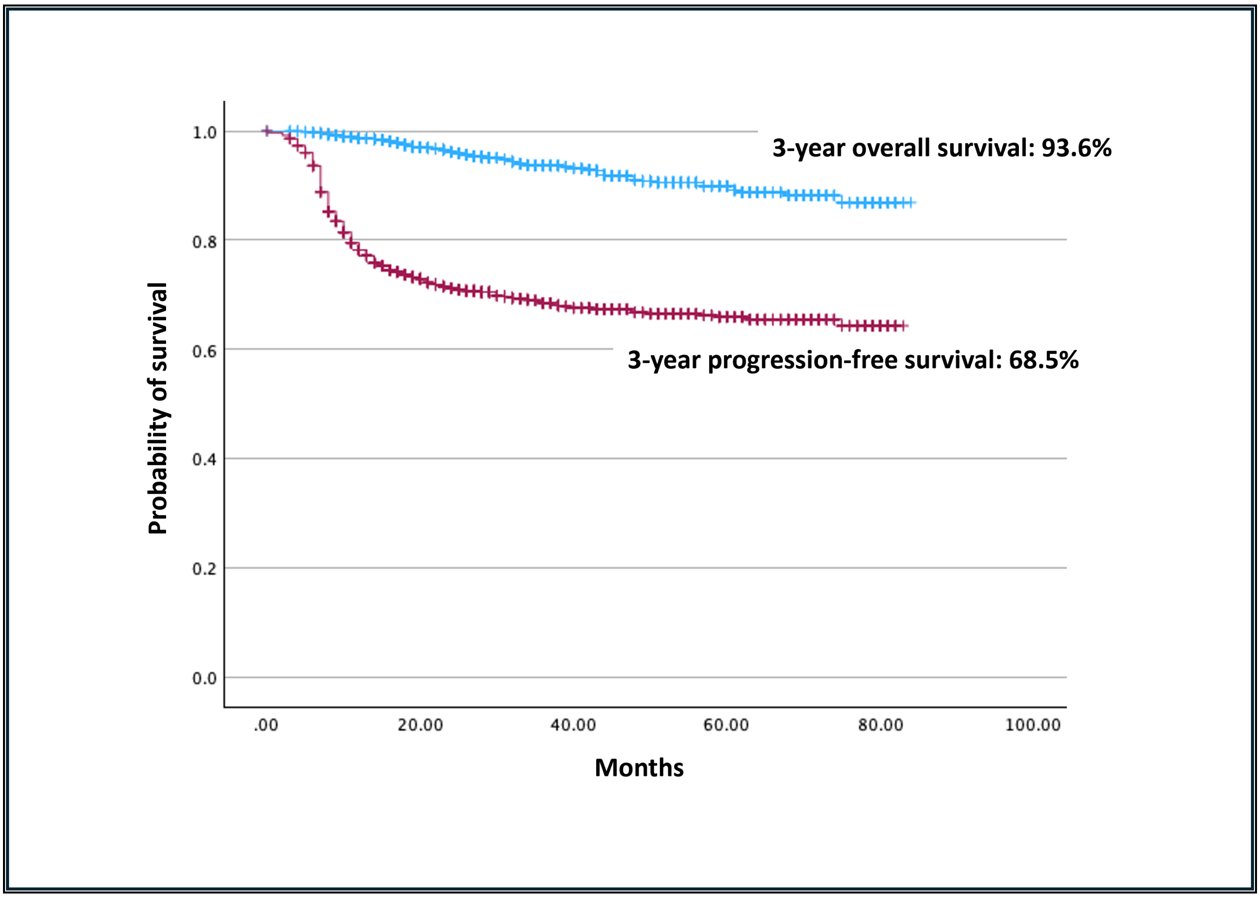

Kaplan-Meier survival curve for 3-year OS and PFS for the entire cohort of patients.

**Copyright Information:** (Online) ISSN: 2572-9241
©2026 The Author(s). HemaSphere is published by John Wiley & Sons Ltd on behalf of European Hematology Association. This is an open access Abstract Book distributed under the Attribution-NonCommercial-NoDerivs (CC BY-NC-ND), which allows third parties to download the articles and share them with others as long as they credit the author and the Abstract Book, but they cannot change the content in any way or use them commercially.
**Abstract Book Citations:** Authors, Title, HemaSphere, 2026;10:(S1):pages. The abstract book DOIs can be found on the "Author Information" page.
**Disclaimer:** Articles published in the journal HemaSphere exclusively reflect the opinions of the authors. The authors are responsible for all content in their abstracts including accuracy of the facts, statements, citing resources, etc.

### (PB3559) NODULAR LYMPHOCYTE PREDOMINANT HODGKIN'S LYMPHOMA WITH TRANSFORMATION TO DIFFUSE LARGE B-CELL LYMPHOMA

**Topic:** 17. Hodgkin lymphoma - Clinical

Elena Dorokhina*[1], Tatyana Moiseeva[1], Alla Kovrigina[1], Irina Shupletsova[1], Yulia Chabaeva[1], Lubov Al-Radi[1], Alina Kokhno[1]

[1]*National Research Center for Hematology, Moscow, Russia;*

### Background

Nodular lymphocyte predominant Hodgkin's lymphoma (NLPHL) is a rare indolent disease (1:1,000,000 population per year) with a relapsing course and the potential for transformation to DLBCL, according to literature data, ranging from 6 to 12%. Unfavorable prognostic value of a THRLBCL-like histopathologic growth pattern (HGP) and their predominance (>50% of the section area) in the tumor substrate for results of induction therapy has been statistically significantly demonstrated by our center, based on its own data analysis.  The possibility of histological transformation with a change in the HGP from favorable to unfavorable within a single nosological entity or tumor transformation (TT) from NLPHL to DLBCL has been demonstrated in NLPHL. Its frequency and prognostic significance have not been determined yet due to the rarity of this pathology.

### Aims to evaluate the frequency of transformation of NLPHL to DLBCL, its prognostic value, and the effectiveness of therapy

### Methods the results of therapy for 150 patients (pts) with NLPHL, treated from 2010 to 2023 in National Research Center for Hematology, were analyzed depending on the presence of THRLBCL-like histopathologic growth pattern of the tumor substrate. The group of patients without THRLBCL-like pattern consisted of 87 (58%) pts, with <50% of THRLBCL-like pattern - 31 (21%) pts, and with predominance of THRLBCL-like sites (> 50%) in the tumor substrate - in 32 (21%) pts.

### Results

Transformation to DLBCL was verified in 12/150 (8%) patients. All had THRLBCL-like histopathologic growth pattern in tumor substrate: male/female – 10/2, age range: 23-69 (47) years, stage II – 1/12, stage III – 1/12, stage IV – 10/12, bulky – 5/12 (42%).
In this group: 6/12 pts had TT in DLBCL at the moment of initial diagnosis, 5/12 pts within 1-4 years after NLPHL verification, and 1/12 after 31 year.
All patients received first-line therapy: 2/12 pts received R-ABVD, 5/12 pts - R-BEACOP-14, 4/12 pts - R-DA-EPOCH and R-CHOP, and 1/12 pts - NHLBFM90. Complete remission (CR) was achieved in 4/12 (30%) pts (3 after R-BEACOP-14 and 1 after NHLBFM90), disease progression (DP) was achieved in 7/12 pts, and partial remission - in 1/12 pts. Of the pts with treatment failure, 3/8 died from DP on first-line therapy; the remainder underwent second-line therapy: 2/5 achieved CR, and one underwent allo-HSCT; 1/5 pts died from DP, and 2/5 had a partial response and continue treatment. Thus, after a 4-year follow-up, 8 patients are alive.
In groups of pts with and without transformation to DLBCL, the 3-year overall survival (OS) was 50% and 97%, p <0.001 (A); the 3-year event-free survival (EFS) was 61% and 83%, p = 0.01 (B), respectively.
In patients with NLPHL with transformation to DLBCL, the 3-year OS significantly decreases to 50% compared to the groups of pts with NLPHL without THRLBCL-like HGP, with <50%, and with >50% THRLBCL-like HGP – 100%, 100%, and 68%, respectively (p < 0.001) (C). The 3-year EFS is 61%, compared to the groups of pts with NLPHL without THRLBCL-like HGP, with <50%, and with >50% THRLBCL-like HGP – 94%, 88%, and 43%, respectively, p = 0.006 (D)

**Copyright Information:** (Online) ISSN: 2572-9241
©2026 The Author(s). HemaSphere is published by John Wiley & Sons Ltd on behalf of European Hematology Association. This is an open access Abstract Book distributed under the Attribution-NonCommercial-NoDerivs (CC BY-NC-ND), which allows third parties to download the articles and share them with others as long as they credit the author and the Abstract Book, but they cannot change the content in any way or use them commercially.
**Abstract Book Citations:** Authors, Title, HemaSphere, 2026;10:(S1):pages. The abstract book DOIs can be found on the "Author Information" page.
**Disclaimer:** Articles published in the journal HemaSphere exclusively reflect the opinions of the authors. The authors are responsible for all content in their abstracts including accuracy of the facts, statements, citing resources, etc.

### Summary/Conclusion

Transformation to DLBCL is verified in 8% of patients with NLPHL. It is associated with THRLBCL-like HGP. It is an unfavorable prognostic factor – the complete response to standard first-line therapy is 30%, while the 3-year OS rate does not exceed 50%. Intensification of first-line therapy is necessary in this group of patients

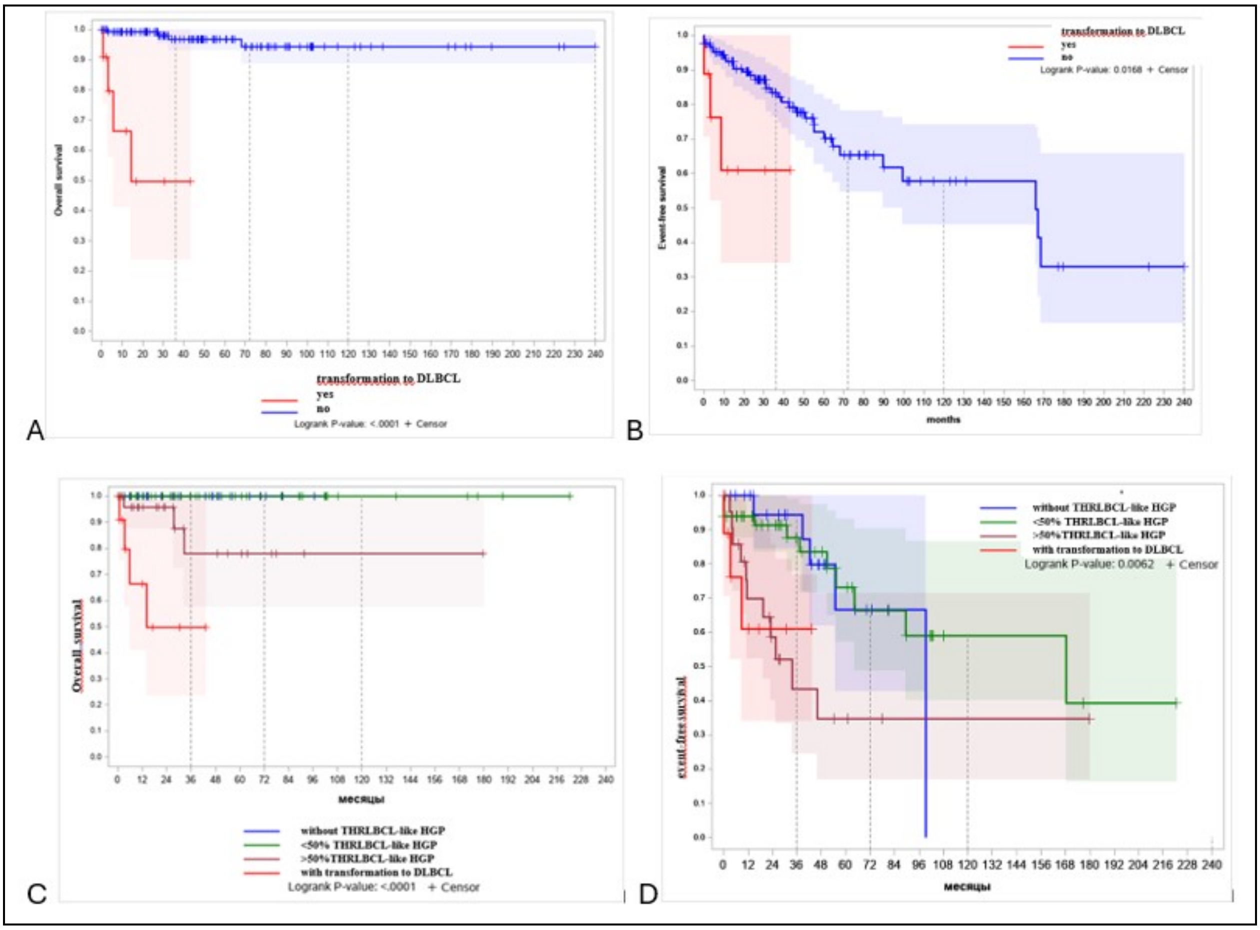

A,B -3-year overall survival and 3-year event-free survival pts with transformation to DLBCL, C,D -3-year overall survival and 3-year event-free survival pts with transformation to DLBCL, compared to the groups of pts with NLPHL without THRLBCL-like HGP, with <50%, and with >50% THRLBCL-like HGP

**Copyright Information:** (Online) ISSN: 2572-9241
©2026 The Author(s). HemaSphere is published by John Wiley & Sons Ltd on behalf of European Hematology Association. This is an open access Abstract Book distributed under the Attribution-NonCommercial-NoDerivs (CC BY-NC-ND), which allows third parties to download the articles and share them with others as long as they credit the author and the Abstract Book, but they cannot change the content in any way or use them commercially.
**Abstract Book Citations:** Authors, Title, HemaSphere, 2026;10:(S1):pages. The abstract book DOIs can be found on the "Author Information" page.
**Disclaimer:** Articles published in the journal HemaSphere exclusively reflect the opinions of the authors. The authors are responsible for all content in their abstracts including accuracy of the facts, statements, citing resources, etc.

### (PB3561) LUNG FUNCTION ASSESSMENT BY DIFFUSING CAPACITY OF THE LUNGS FOR CARBON MONOXIDE (DLCO) MEASUREMENT IN PATIENTS WITH CLASSICAL HODGKIN LYMPHOMA RECEIVING FIRST-LINE TREATMENT WITH NIVOLUMAB-AVD

**Topic:** 17. Hodgkin lymphoma - Clinical

Athanasios Liaskas*[1], Alexia Piperidou[1], Georgia Poultsaki[1], Maria Dimitrakoudi[1], Aikaterini Kolotsiou[1], Anastasia Kopsaftopoulou[1], Rodanthi Fioretzaki[1], Alexandros MacHairas[1], Marina Belia[1], Panagiota Maria Arapaki[1], Iliana Konstantinou[1], Ioannis Asimakopoulos[1], Fotios Panitsas[1], Aikaterini Benekou[1], Aikaterini Bitsani[1], Panagiotis Tsaftaridis[1], Helen Plata[1], Maria Dimou[1], Marina Siakantaris[1], Maria Angelopoulou[1], Theodoros Vassilakopoulos[1]

[1]*Department of Hematology and BMT of the National and Kapodistrian University of Athens, "Laikon" General Hospital of Athens, Athens, Greece;*

### Background

Nivolumab-AVD (N-AVD) has shown outstanding efficacy in patients with advanced-stage classical Hodgkin Lymphoma (cHL), establishing a new standard of care, while introducing a novel toxicity profile driven by immune-related adverse events (IRAEs). Pneumonitis is a well-recognized complication of bleomycin in patients previously treated with ABVD. Similarly, treatment with immune checkpoint inhibitors, including nivolumab, can also lead to pneumonitis through an immune-mediated mechanism, raising concerns about potential clinical or subclinical pulmonary impairment during therapy.

### Aims

To evaluate potential pulmonary impairment using standard pulmonary function testing and sequential changes in patients with cHL treated with N-AVD.

### Methods

The study included patients with stage III and IV cHL treated with N-AVD as a first-line therapy. Single-breath Diffusing Capacity of the Lungs for Carbon Monoxide corrected for Hemoglobin (DLCOc-SB) measurements were performed at predefined time points: prior to treatment initiation, after four cycles of N-AVD, and at treatment completion. In cases where significant abnormalities were detected, patients underwent serial pulmonary function testing after treatment at regular intervals to monitor progression or recovery. All patients underwent PET/CT after 2 cycles and chest CT after 3 cycles of N-AVD. Clinical evaluation and symptom assessment were constantly performed during treatment and afterwards to correlate functional changes with clinical findings.

**Copyright Information:** (Online) ISSN: 2572-9241
©2026 The Author(s). HemaSphere is published by John Wiley & Sons Ltd on behalf of European Hematology Association. This is an open access Abstract Book distributed under the Attribution-NonCommercial-NoDerivs (CC BY-NC-ND), which allows third parties to download the articles and share them with others as long as they credit the author and the Abstract Book, but they cannot change the content in any way or use them commercially.
**Abstract Book Citations:** Authors, Title, HemaSphere, 2026;10:(S1):pages. The abstract book DOIs can be found on the “Author Information” page.
**Disclaimer:** Articles published in the journal HemaSphere exclusively reflect the opinions of the authors. The authors are responsible for all content in their abstracts including accuracy of the facts, statements, citing resources, etc.

**Results**

Among 40 patients who had completed treatment with N-AVD up to the time of the present analysis, 19 had baseline and interim DLCOc-SB assessments and 10/19 also had at least 1 post-treatment assessment. The median age of the 19 patients was 28 years (range 16–79), with 11% aged ≥60 years; 42% were male and 53% had stage IV disease based on PET/CT. The median value of DLCOc-SB at baseline was 83.8% of the predicted value [interquartile range (IQR) 79.5-95.7%; range 55.8-120.8%], while after four cycles of N-AVD it was 80% (IQR 74.4-89.2%; range 56.3-117.2%). In fact 16/19 patients had a reduction in DLCOc-SB by a median of 4.5% (range 0.5-10.12%) and 3 had an increase of 0.3-3.7%. The difference was statistically significant ($p<0.001$ by the Wilcoxon signed rank test). In the 10 patients with available post-treatment assessment the median DLCOc-SB was 82.9% (IQR 65.2-93.6%; range 49.3-106.3%) versus 83.3% (IQR 71.7-96.6%; range 55.8-120.8%) at baseline ($p=0.33$) and 78.9% (IQR 67.7-91.2%; range 56.3-117.2%) at the interim assessment ($p=0.96$). Comparing the baseline and post-treatment evaluation, 7/10 patients had a reduction in SLCOc-SB by a median of 5.1% (range 0.1-30.2%) and 3 had an increase of 6.8-8.8%. Comparing the interim and post-treatment evaluation, 5/10 patients had a reduction in DLCOc-SB by a median of 10.0% (range 0.6-27.6%) and 5/10 had an increase of 1.4-12.4% (median 9.8%). Importantly, none of the patients developed clinical symptoms of pneumonitis even in the long-term and only 1/19 had a reduction in SLCOc-SB by >20%.

**Summary/Conclusion**

In our study, patients with cHL treated with N-AVD demonstrated a statistically significant decline of TLCOc SB during treatment. Some TLCOc-SB reductions did not recover early post-treatment but only a single patient had >20% reduction and none of the 19 patients had clinically or radiologically evident pneumonitis. These findings suggest that N-AVD has a favorable pulmonary safety profile.

**Copyright Information:** (Online) ISSN: 2572-9241
©2026 The Author(s). HemaSphere is published by John Wiley & Sons Ltd on behalf of European Hematology Association. This is an open access Abstract Book distributed under the Attribution-NonCommercial-NoDerivs (CC BY-NC-ND), which allows third parties to download the articles and share them with others as long as they credit the author and the Abstract Book, but they cannot change the content in any way or use them commercially.
**Abstract Book Citations:** Authors, Title, HemaSphere, 2026;10:(S1):pages. The abstract book DOIs can be found on the “Author Information” page.
**Disclaimer:** Articles published in the journal HemaSphere exclusively reflect the opinions of the authors. The authors are responsible for all content in their abstracts including accuracy of the facts, statements, citing resources, etc.

### (PB3562) GENOMIC INSTABILITY IN CLASSICAL HODGKIN LYMPHOMA: ASSOCIATION WITH METABOLIC TUMOR BURDEN AND CIRCULATING TUMOR DNA DETECTABILITY.

**Topic:** 17. Hodgkin lymphoma - Clinical

Anna Kravtsova*[1], Kostoeva Hava[1], Alexey Subbotin[2], Elena Nikulina[1], Natalya Risinskaya[1], Tatiana Nenasheva[1], Elena Rybkina[1], Darya Drokova[1], Ivan Bolshakov[1], Rasul Iusupov[3], Anna Sergeeva[1], Kira Lavrishinets[1], Erdni Arangaev[1], Olga Soboleva[1], Valentina Dvirnik[1], Yana Mangasarova[1, 4], Evgeniy Zvonkov[1, 4], Andrey Sudarikov[1]

*[1]National Medical Research Center for Hematology, Moscow, Russia;[2]N.N. Blokhin National Medical Research Center of Oncology, Moscow, Russia; [3]Lomonosov Moscow State University, Moscow, Russia;[4]The Russian University of Medicine, Moscow, Russia;*

**Background**

Classical Hodgkin lymphoma (cHL) is characterized by malignant Hodgkin and Reed–Sternberg cells (HRS cells) with a profoundly altered genomic landscape. Recurrent alterations of immune-regulatory loci, most notably 9p24.1, modulate checkpoint signaling and provide a biological basis for PD-1 blockade efficacy. However, whether the burden and pattern of genomic instability (GI) predict response to immune checkpoint inhibition or serve as prognostic markers in cHL remains unclear.

**Aims**

To characterize GI landscape in cHL using sorted tumor cells and to evaluate its association with systemic disease metrics, including PET-derived parameters and circulating tumor-derived DNA.

**Methods**

Tumor samples from 16 treatment-naïve cHL patients who attended National Medical Research Center for Hematology (Moscow, Russia) between October 2024 and November 2025 and were treated according to PET-adapted first-line protocol incorporating nivolumab (NCT07209059) were analyzed. For all patients, baseline and interim PET/CT data were collected and evaluated.
Tumor DNA was obtained from flow-sorted HRS cells. Microsatellite instability (MSI) and loss of heterozygosity (LOH) were assessed using a multiplex STR-based CORDIS Plus system, CORDIS MSI panel, and a custom microsatellite marker panel targeting immune-regulatory loci: PD-L1/PD-L2 (9p24.1), HLA (6p21.3), and CIITA (16p13.13). In selected patients with high MSI and/or high genomic instability burden, matched cell free DNA (cfDNA) samples were additionally evaluated.

**Copyright Information:** (Online) ISSN: 2572-9241
©2026 The Author(s). HemaSphere is published by John Wiley & Sons Ltd on behalf of European Hematology Association. This is an open access Abstract Book distributed under the Attribution-NonCommercial-NoDerivs (CC BY-NC-ND), which allows third parties to download the articles and share them with others as long as they credit the author and the Abstract Book, but they cannot change the content in any way or use them commercially.
**Abstract Book Citations:** Authors, Title, HemaSphere, 2026;10:(S1):pages. The abstract book DOIs can be found on the "Author Information" page.
**Disclaimer:** Articles published in the journal HemaSphere exclusively reflect the opinions of the authors. The authors are responsible for all content in their abstracts including accuracy of the facts, statements, citing resources, etc.

**Results**

MSI-H was identified in 5/16 patients (31%) using the CORDIS MSI panel. Allele imbalance (LOH and/or EMAST) in immune-regulatory regions was detected in 11/16 cases (69%), most frequently involving HLA and PD-L1/PD-L2, while CIITA alterations were observed in 3/16 patients (19%).
Importantly, Expanded CORDIS Plus profiling identified additional instability patterns not captured by CORDIS MSI panel. Multi-locus LOH (≥2 chromosomal regions) was observed in 6/16 patients (38%), and EMAST was detected in 4/16 cases (25%), including tumors without MSI-H. Overall, a high GI, defined by MSI-H and/or multi-locus LOH and/or EMAST, was present in 8/16 patients (50%).
Baseline PET/CT characteristics demonstrated heterogeneity in metabolic activity, with SUVmax ranging from 8.0 to 28.9. The highest parameters were predominantly observed in patients with high GI. Elevated MTV (up to 334) and TLG (up to 3665) were detected in this subgroup, suggesting an association between GI and increased metabolic tumor burden.
Despite higher baseline metabolic parameters, GI was not associated with inferior early response. After two treatment cycles, most patients achieved ≥80% reduction in SUVmax and Deauville score 3. In patients with MSI-H and/or pronounced EMAST, identical instability patterns were detectable in cfDNA despite a low tumor fraction of 0.5–1.5%, confirming the feasibility of highly sensitive non-invasive detection of tumor-specific genomic alterations.

**Summary/Conclusion**

GI is a frequent feature of classical Hodgkin lymphoma when assessed in purified HRS cells. A high GI is associated with increased MTV but not with impaired early metabolic response in patients treated with nivolumab–containing regimens, indicating preserved chemo- and immunosensitivity. The detection of identical instability patterns in low-fraction cfDNA supports the feasibility of non-invasive assessment and warrants further investigation of GI profiling for prognostic stratification and immunotherapy guidance in cHL.

**Copyright Information:** (Online) ISSN: 2572-9241
©2026 The Author(s). HemaSphere is published by John Wiley & Sons Ltd on behalf of European Hematology Association. This is an open access Abstract Book distributed under the Attribution-NonCommercial-NoDerivs (CC BY-NC-ND), which allows third parties to download the articles and share them with others as long as they credit the author and the Abstract Book, but they cannot change the content in any way or use them commercially.
**Abstract Book Citations:** Authors, Title, HemaSphere, 2026;10:(S1):pages. The abstract book DOIs can be found on the “Author Information” page.
**Disclaimer:** Articles published in the journal HemaSphere exclusively reflect the opinions of the authors. The authors are responsible for all content in their abstracts including accuracy of the facts, statements, citing resources, etc.

### (PB3563) LP-IPS–BASED RISK STRATIFICATION IN NODULAR LYMPHOCYTE-PREDOMINANT HODGKIN LYMPHOMA: A NINE-CENTRE TURKISH COHORT

**Topic:** 17. Hodgkin lymphoma - Clinical

Taner Tan*[1], Fatma Keklik Karadag[2], Ahmet Umur Topçu[1], Arda Bayar[3], Ebru Pekguc[4], Zafer Serenli[5], Sureyya Yigit Kaya[6], Serkan Guven[7], Hacer Berna Afacan Öztürk[8], Leylagül Kaynar[6], Fahir Özkalemkaş[9, 9], Guray Saydam[10], Meltem Olga Akay[11]

*[1]Koc University Hospital, Hematology, Istanbul, Türkiye; [2]Ege University, hematology, Izmir, Türkiye; [3]Marmara University, Hematology, Istanbul, Türkiye; [4]American Hospital, Hematology, Istanbul, Türkiye; [5]Doruk Hospital Nilufer, Hematology, Bursa, Türkiye; [6]Medipol Mega University Hospital, Hematology, Istanbul, Türkiye; [7]Canakkale State Hospital, hematology, Canakkale, Türkiye; [8]Etlik City Hospital, Hematology, Ankara, Türkiye; [9]Bursa Uludag Unirversity, Hematology, Bursa, Türkiye; [10]Ege University, Hematology, Izmir, Türkiye; [11]Koç University Hospital, hematology, istanbul, Türkiye;*

### Background

Nodular lymphocyte-predominant Hodgkin lymphoma (NLPHL) is a rare, indolent Hodgkin lymphoma subtype with B-cell antigen expression and generally favourable long-term outcomes, despite relapse risk. We retrospectively evaluated NLPHL patients and assessed prognosis using the Lymphocyte-Predominant International Prognostic Score (LP-IPS). (Binkley et al., J Clin Oncol 2024)

### Aims

In this study, we aimed to retrospectively evaluate patients with NLPHL and assess their prognosis using the recently defined Lymphocyte-Predominant International Prognostic Score (Binkley et al., J Clin Oncol 2024)

### Methods

We analysed 105 NLPHL patients diagnosed between 2008–2025 from nine centres in Türkiye. Clinical/laboratory features, stage, treatment, relapse, and survival were collected. LP-IPS was calculated using: age ≥45, stage III–IV, haemoglobin <10.5 g/dL, and splenic involvement; patients were grouped as low (0–1), intermediate (2), and high (3–4). Prognostic factors were assessed by univariate Cox regression. OS and PFS by LP-IPS were evaluated with Kaplan–Meier analysis.

### Results

Median age was 43 years (range 19–85) and 61.9% were male. Median follow-up was 3.5 years. Stage III–IV disease was present in 52.4%; B symptoms in 27.6%; extranodal involvement in 32.4%; splenic involvement in 27.6%; Hb <10.5 g/dL in 11.4%. Most common regimens were R-CHOP (41.0%) and ABVD (38.1%). Relapse occurred in 22.9% and 12.4% died. For PFS, B symptoms (HR 3.86, p=0.002) and Hb <10.5 g/dL (HR 4.40, p=0.005) were significant predictors. For OS, ≥2 B symptoms (HR 5.39, p=0.004), extranodal involvement (HR 4.76, p=0.011), splenic involvement (HR 4.07, p=0.017), and Hb <10.5 g/dL (HR 8.15, p<0.001) were significant. LP-IPS classified 61.0% low-, 22.9% intermediate-, and 16.2% high-risk. Kaplan–Meier analyses showed significant differences among LP-IPS groups for both PFS (p=0.022) and OS (p<0.001), with the high-risk group having the shortest survival.

**Copyright Information:** (Online) ISSN: 2572-9241
©2026 The Author(s). HemaSphere is published by John Wiley & Sons Ltd on behalf of European Hematology Association. This is an open access Abstract Book distributed under the Attribution-NonCommercial-NoDerivs (CC BY-NC-ND), which allows third parties to download the articles and share them with others as long as they credit the author and the Abstract Book, but they cannot change the content in any way or use them commercially.
**Abstract Book Citations:** Authors, Title, HemaSphere, 2026;10:(S1):pages. The abstract book DOIs can be found on the “Author Information” page.
**Disclaimer:** Articles published in the journal HemaSphere exclusively reflect the opinions of the authors. The authors are responsible for all content in their abstracts including accuracy of the facts, statements, citing resources, etc.

### Summary/Conclusion

In this multicentre study including 105 patients with NLPHL treated across nine centres in Türkiye, several clinical and laboratory parameters potentially influencing survival were evaluated. Although some factors were significant in univariate analyses, Kaplan–Meier survival analyses demonstrated that patients in the high-risk LP-IPS group had significantly shorter progression-free survival and overall survival compared to lower-risk groups. These findings support the prognostic value and clinical applicability of the LP-IPS in NLPHL, a rare subtype of Hodgkin lymphoma, while also highlighting the clinical heterogeneity of the disease. The LP-IPS may serve as a practical tool for identifying high-risk patients and could help guide risk-adapted treatment strategies, including potential treatment de-escalation in low-risk patients within the context of future prospective studies.

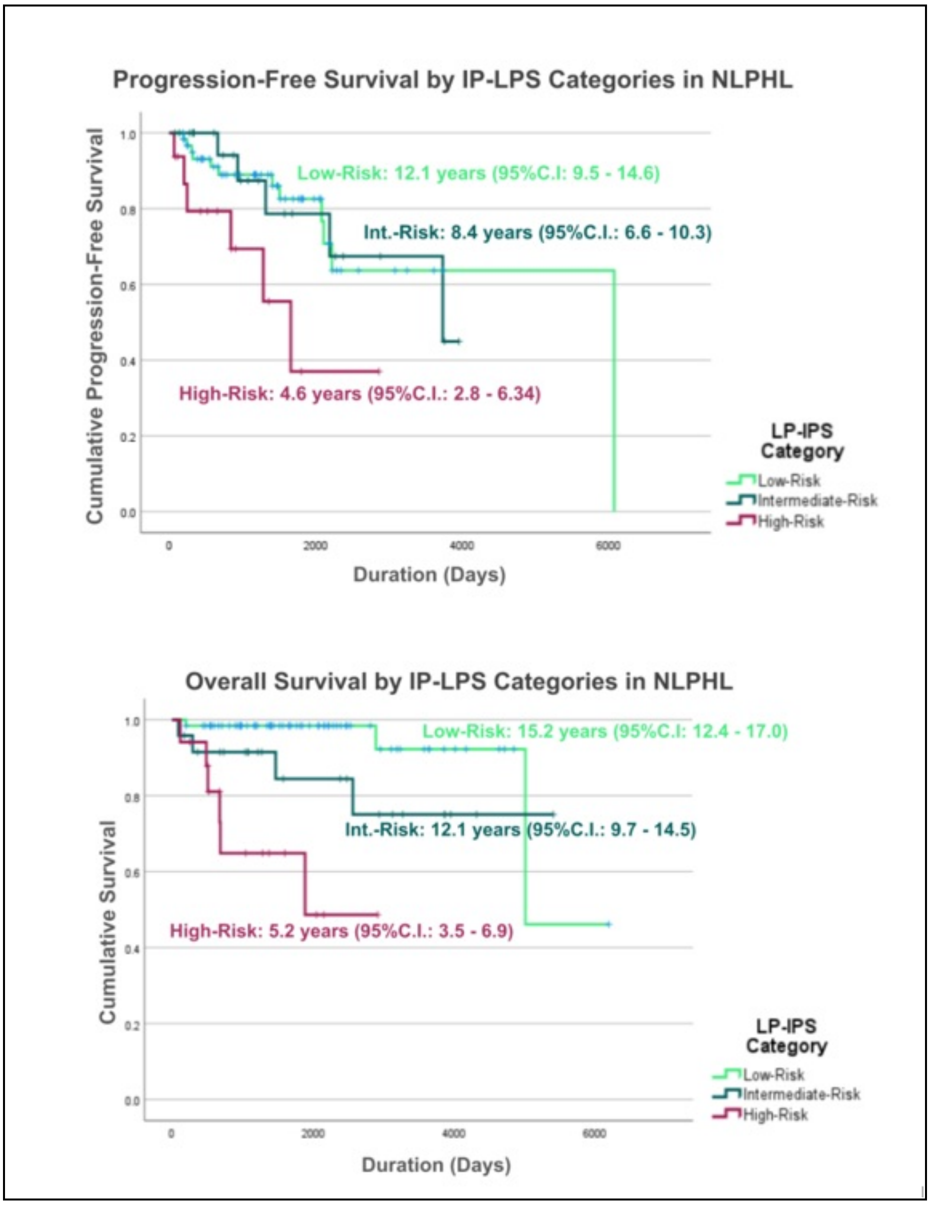

**Copyright Information:** (Online) ISSN: 2572-9241
©2026 The Author(s). HemaSphere is published by John Wiley & Sons Ltd on behalf of European Hematology Association. This is an open access Abstract Book distributed under the Attribution-NonCommercial-NoDerivs (CC BY-NC-ND), which allows third parties to download the articles and share them with others as long as they credit the author and the Abstract Book, but they cannot change the content in any way or use them commercially.
**Abstract Book Citations:** Authors, Title, HemaSphere, 2026;10:(S1):pages. The abstract book DOIs can be found on the "Author Information" page.
**Disclaimer:** Articles published in the journal HemaSphere exclusively reflect the opinions of the authors. The authors are responsible for all content in their abstracts including accuracy of the facts, statements, citing resources, etc.

### (PB3565) NIVOLUMAB IN FIRST-LINE THERAPY FOR THRLBCL-LIKE PATTERN OF NODULAR LYMPHOCYTE PREDOMINANT HODGKIN LYMPHOMA

**Topic:** 17. Hodgkin lymphoma - Clinical

Elena Dorokhina*[1], Tatyana Moiseeva[1], Alla Kovrigina[1], Irina Shupletsova[1, 1], Lubov Al-Radi[1], Alina Kokhno[1]

[1]*National Research Center for Hematology, Moscow, Russia;*

### Background

Nodular lymphocyte predominant Hodgkin lymphoma (NLPHL) is a rare, indolent disease characterized by morphological and immunohistochemical heterogeneity. According to the National research center for hematology, patients with a THRLBCL-like histopathologic growth pattern and their predominance (>50% of the section area) in the tumor substrate are rare (21% of cases). The disease course is characterized by a poor prognosis: the complete response to first-line therapy (R-BEACOPP-14, R-CHOP) does not exceed 37%, 5-year OS and EFS are 70% and 30%, respectively, and the early relapse rate reaches 60%. Increased PD-L1 expression by tumor cells and the microenvironment in the THRLBCL-like pattern has been demonstrated in our center, which justifies the use of nivolumab in first-line therapy to increase efficacy and prevent early relapses.

### Aims

To evaluate the efficacy of R-BEACOPP-14 in combination with nivolumab in the treatment of patients with THRLBCL-like pattern of NLPHL

### Methods

From 2022 to 2025, 11 patients with NHLBC with a THRLBCL-like pattern >50% in the tumor substrate were treated at the National research center for hematology with R-BEACOPP-14 plus nivolumab at a dose of 40 mg: men/women – 9/2, age 33-57 (42) years, IPS – 2-5, stage IV – 11/11 (100%), bone lesions – 8/11 (73%), liver – 6/11 (55%), bone marrow – 9/11 (82%), bulky – 9/11 (82%). All cases underwent preliminary immunohistochemistry and were PD-L1 positive.

### Results

Eight courses of nivo-R-BEACOPP-14 and maintenance therapy with nivolumab 40 mg every 2 weeks for 6 months were administered to 6/11 patients, all achieved complete remission. Six courses without maintenance therapy due to early complete responses after courses 2 and 4 received 4/11 patients. One patient was admitted in critical condition, with renal and hepatic failure, liver cirrhosis, and died of infection after 2 courses. Currently, 10/11 patients after treatment maintain a complete response with follow-up period of 6-36 (23) months, with no relapses.

### Summary/Conclusion

The combination of standard chemotherapy with immune checkpoint inhibitors in first-line treatment of NLPHL patients in the most unfavorable prognostic group with a THRLBCL-like histopathologic growth pattern allows to achieve a long-term complete remissions, to avoid early relapses, and to preserve hematopoietic reserves, given the recurrent nature of the disease. Further observation in a larger group of patients is necessary.

**Copyright Information:** (Online) ISSN: 2572-9241
©2026 The Author(s). HemaSphere is published by John Wiley & Sons Ltd on behalf of European Hematology Association. This is an open access Abstract Book distributed under the Attribution-NonCommercial-NoDerivs (CC BY-NC-ND), which allows third parties to download the articles and share them with others as long as they credit the author and the Abstract Book, but they cannot change the content in any way or use them commercially.
**Abstract Book Citations:** Authors, Title, HemaSphere, 2026;10:(S1):pages. The abstract book DOIs can be found on the “Author Information” page.
**Disclaimer:** Articles published in the journal HemaSphere exclusively reflect the opinions of the authors. The authors are responsible for all content in their abstracts including accuracy of the facts, statements, citing resources, etc.

### (PB3566) OUTCOMES OF PEDIATRIC HODGKIN LYMPHOMA PATIENTS TREATED WITH ABVD REGIMEN. A RETROSPECTIVE COHORT STUDY

**Topic:** 17. Hodgkin lymphoma - Clinical

Hani Abdel Rahman Sayed*[1, 2], Emad Moussa[3], Mohamed Sedky Sedky[3], Nesreen Ali[3], Walaa Elsayed[3], Maram Farouk[3], Sally Talaat[3, 3], Hanaa Rashad[3], Eman Khorshed[2], Mohamed Zaghlool[3], Esraa Roshdy[3], Madeha Elwakeel[2], Sarah Badwy[3]

*[1]Children's Cancer Hospital Egypt, Pediatric Oncology, Cairo, Egypt;[2]National Cancer Institute, Cairo 'University, Pediatric Oncology, Cairo, Egypt; [3]Children's Cancer Hospital Egypt, Pediatric, Cairo, Egypt;*

### Background

Hodgkin lymphoma (HL), a highly curable malignancy has a peak incidence in adolescent and young adults. Chemotherapy regimen ABVD and involved field radiotherapy (IFRT) is the gold-standard treatment for early and advanced stages of HL in adults. The favorable groups are stages IA-IIA without bulky disease, while unfavorable groups are stages IIIB and IV. Most current pediatric trials focus on strategies aimed at reducing late effects of therapy.

### Aims

To analyze data of patients who received first line chemotherapy ABVD and IFRT at the Children Cancer Hospital Egypt 57357 (CCHE) over 12 years period.

### Methods

A retrospective analysis including all patients < 18 years of age, diagnosed and treated as classic HL during the period from 2007-2019. Patients were stratified as low (stage IA/IB/IIA with ESR<30 and without bulky disease), intermediate (stage IEA/IEB/IIA/IIIA with ESR ≥ 30 mm/hour, or with bully disease), and high risk (stage IIBE/IIIAE/IIIB/IV) disease. Chemotherapy consisted of 4 cycles for low, and 6 for intermediate and high risk patients, followed by IFRT total dose 1,980 Gy. The median follows up period was 105 months (range 6- 217 months).

### Results

This study included 1378 patients. Their median age was 9.3 years. The majority (70%) were from 5-15 years of age. There was a strong male predominance (72%). The most common pathologic subtype was nodular sclerosis (54%), followed by mixed cellularity (38%). Stage 2A was he most common (31%), followed by IA (15%). Low risk patients were the most common (46%), followed by high risk (31%). Two hundred thirteen patients (15.4 %) had relapse (187, 13.5%) or refractory (26, 0.18%) disease. Following salvage chemotherapy, 123 (8.9%) patients received a hematopoietic stem cell transplant (HSCT), mainly autologous (98%). The 5 years overall survival (OS) for the entire cohort was 95.3% (95% CI 94.2%-96.4%), while the 5 years progression free survival (PFS) probability was 86.7% (95% CI 84.9% -88.5%). The 5 years PFS for low risk group was 94.2%, compared to 86.8% for intermediate risk and 75.6% for high risk patients (P<0.001). Second malignant neoplasm (SMN) was detected in 31 patients (0.2%) predominantly cancer thyroid (58%). One hundred patients died (7.2%), mainly from disease progression including 28 post HSCT and 6 post SMN.

**Copyright Information:** (Online) ISSN: 2572-9241
©2026 The Author(s). HemaSphere is published by John Wiley & Sons Ltd on behalf of European Hematology Association. This is an open access Abstract Book distributed under the Attribution-NonCommercial-NoDerivs (CC BY-NC-ND), which allows third parties to download the articles and share them with others as long as they credit the author and the Abstract Book, but they cannot change the content in any way or use them commercially.
**Abstract Book Citations:** Authors, Title, HemaSphere, 2026;10:(S1):pages. The abstract book DOIs can be found on the "Author Information" page.
**Disclaimer:** Articles published in the journal HemaSphere exclusively reflect the opinions of the authors. The authors are responsible for all content in their abstracts including accuracy of the facts, statements, citing resources, etc.

**Summary/Conclusion**

ABVD regimen at our institution has an excellent outcome. late effects of therapy, include an elevated risk for SMN, cardio toxicity, pulmonary toxicity, and endocrine dysfunction. Recently, new treatment strategies have been developed to balance the intensity of therapy needed to maintain disease free survival while preserving overall survival.

**Copyright Information:** (Online) ISSN: 2572-9241
©2026 The Author(s). HemaSphere is published by John Wiley & Sons Ltd on behalf of European Hematology Association. This is an open access Abstract Book distributed under the Attribution-NonCommercial-NoDerivs (CC BY-NC-ND), which allows third parties to download the articles and share them with others as long as they credit the author and the Abstract Book, but they cannot change the content in any way or use them commercially.
**Abstract Book Citations:** Authors, Title, HemaSphere, 2026;10:(S1):pages. The abstract book DOIs can be found on the "Author Information" page.
**Disclaimer:** Articles published in the journal HemaSphere exclusively reflect the opinions of the authors. The authors are responsible for all content in their abstracts including accuracy of the facts, statements, citing resources, etc.

### (PB3567) SPATIAL TRANSCRIPTOMICS OF THE HODGKIN LYMPHOMA MICROENVIRONMENT: TAM CLUSTERS AND IMMUNE CHECKPOINTS

**Topic:** 17. Hodgkin lymphoma - Clinical

Lily Filatova*[1], Egor Volchkov[1, 2, 3], Irina Larionova[4, 5], Gleb Shestopalov[6], Pavel Yamshikov[4, 5], Dmitry Abramov[2, 3], Ilya Sidorov[2, 3], Vlada Tuliavko[1], Alexandr Ikonnikov[1], Natalia Miakova[2]

*[1]Research Institute of Molecular and Cellular Medicine, Peoples' Friendship University of Russia named after Patrice Lumumba (RUDN University), Research Laboratory of Single-Cell Biology, Moscow, Russia; [2]National Medical Research Center for Pediatric Hematology, Oncology and Immunology, named by Dmitry Rogachev, Moscow, Russia; [3]Hospital named by Sergey Botkin, Moscow, Russia; [4]Laboratory of Cancer Progression Biology, Cancer Research Institute, Tomsk National Research Medical Center, Tomsk, Russia; [5]Laboratory of Translational Cellular and Molecular Biomedicine, National Research Tomsk State University, Tomsk, Russia; [6]Russian Children's Clinical Hospital, N. I. Pirogov Russian National Research Medical University, Moscow, Russia;*

### Background

A morphological feature of classical Hodgkin's lymphoma is the presence of a functionally active tumor microenvironment (TME). It is likely that the composition and spatial organization of immune and stromal cells critically shape immune evasion and even treatment response. Spatial transcriptomics enables simultaneous assessment of gene expression and tissue localization, providing insight into cellular interactions within the TME.

### Aims

To characterize spatially resolved transcriptional domains in HL and identify key tumor–immune interaction axes, with a focus on tumor-associated macrophages (TAMs) and T-cell exhaustion markers.

### Methods

Lymph node sections from four patients with morphologically confirmed HL were analyzed using the Visium Spatial Gene Expression platform (10x Genomics, USA) with H&E-guided imaging. Spot clustering, normalization, and differential gene expression analyses were performed. TAM-related markers (*CD68, CD163, APOE, APOC1, C1QA, C1QB, C1QC, CD14, SPP1*) and immune checkpoint/exhaustion markers (*HAVCR2/TIM3, CTLA4, PDCD1, LAG3, TIGIT, CD96, VSIR/VISTA*) were evaluated. Putative ligand–receptor interactions were inferred using curated databases and CellChat-based annotation.

### Results

A total of 11 different clusters were identified among the analyzed samples (an example of spatial arrangement in Figure 1). Reproducible macrophage-enriched clusters were identified across samples. These clusters showed strong expression of *CD68* and complement components *C1QA/B/C*, as well as a lipid-associated transcriptional program marked by *APOE* and *APOC1*. Distinct polarization patterns were observed: cluster 8 exhibited higher *CD163* and *CD14* expression compared to cluster 3, while SPP1 expression was focal. Spatially overlapping regions demonstrated elevated expression of inhibitory receptors, particularly in cluster 8 and partially in cluster 3, including *HAVCR2, CTLA4, PDCD1, LAG3, TIGIT/CD96*, and *VSIR*. Potential immunosuppressive ligand-receptor axes identified include *SELPLG–SELL* (identified by CellChat), *HAVCR2–LGALS9/HMGB1/CEACAM1, PDCD1–CD274/PDCD1LG2, CTLA4/CD28–CD80/CD86, TIGIT/CD96–PVR/NECTIN2, HLA-E/HLA-F–VSIR*, as well as TAM-associated pairs *SPP1–CD44* and *MMP9–CD44.*

**Copyright Information:** (Online) ISSN: 2572-9241
©2026 The Author(s). HemaSphere is published by John Wiley & Sons Ltd on behalf of European Hematology Association. This is an open access Abstract Book distributed under the Attribution-NonCommercial-NoDerivs (CC BY-NC-ND), which allows third parties to download the articles and share them with others as long as they credit the author and the Abstract Book, but they cannot change the content in any way or use them commercially.
**Abstract Book Citations:** Authors, Title, HemaSphere, 2026;10:(S1):pages. The abstract book DOIs can be found on the "Author Information" page.
**Disclaimer:** Articles published in the journal HemaSphere exclusively reflect the opinions of the authors. The authors are responsible for all content in their abstracts including accuracy of the facts, statements, citing resources, etc.

### Summary/Conclusion

Spatial transcriptomic profiling using Visium revealed heterogeneous TAM-rich domains associated with immune checkpoint upregulation and potential ligand–receptor interactions that support localized immunosuppression in HL. These findings refine our understanding of TME architecture and may help prioritize therapeutic targets for further validation.

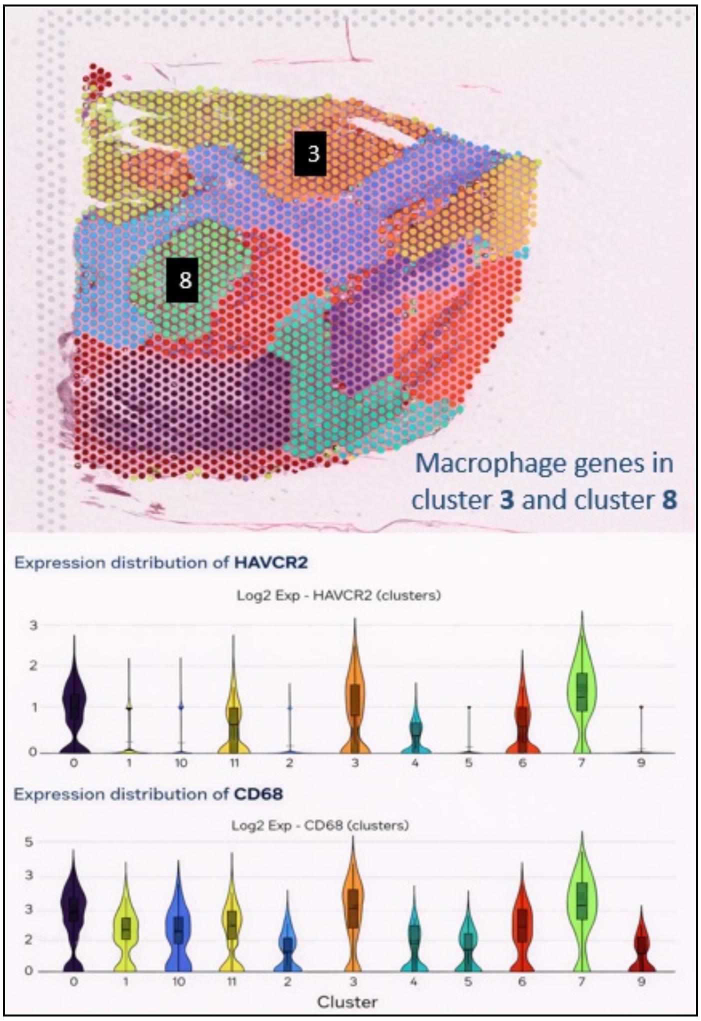

Figure 1. Spatial arrangement of clusters and expression level of the HAVCR2 and CD68 genes in the analyzed clusters in a Hodgkin's lymphoma sample

**Copyright Information:** (Online) ISSN: 2572-9241
©2026 The Author(s). HemaSphere is published by John Wiley & Sons Ltd on behalf of European Hematology Association. This is an open access Abstract Book distributed under the Attribution-NonCommercial-NoDerivs (CC BY-NC-ND), which allows third parties to download the articles and share them with others as long as they credit the author and the Abstract Book, but they cannot change the content in any way or use them commercially.
**Abstract Book Citations:** Authors, Title, HemaSphere, 2026;10:(S1):pages. The abstract book DOIs can be found on the "Author Information" page.
**Disclaimer:** Articles published in the journal HemaSphere exclusively reflect the opinions of the authors. The authors are responsible for all content in their abstracts including accuracy of the facts, statements, citing resources, etc.

### (PB3568) AHOD2131: A RANDOMIZED PHASE 3 INTERIM RESPONSE ADAPTED TRIAL COMPARING STANDARD THERAPY WITH IMMUNO-ONCOLOGY THERAPY FOR CHILDREN AND ADULTS WITH NEWLY DIAGNOSED STAGE I/II CLASSIC HODGKIN LYMPHOMA.

**Topic:** 17. Hodgkin lymphoma - Clinical

Boyu Hu*[1], Jennifer Seelisch[2], Lindsay Renfro[3], Adam Duvall[4], Yue Wu[5], Steve Cho[6], Bradford S. Hoppe[7], Sarah Milgrom[8], Raymond Mailhot[9], Andrea Lo[10], David Hodgson[11], Lisa Giulino-Roth[12], Natalie Grover[13], Ann Lacasce[14], Justine Kahn[15], Susan Parsons[16], Pamela Hinds[17], Pamela Allen[18], Andrew M. Evens[19], Heiko Schoder[20], Sharon Castellino[21], Kara Kelly[22]

*[1]Huntsman Cancer Institute at The University of Utah, Salt Lake City, UT, United States of America;[2]Children's Hospital London Health Sciences Centre, Western University, London, ON, Canada; [3]University of Southern California, Los Angeles, CA, United States of America; [4]University of Chicago Comprehensive Cancer Center, Chicago, IL, United States of America; [5]University of Florida, COG Data Center, Gainesville, FL, United States of America; [6]MD Anderson Cancer Center, Houston, TX, United States of America; [7]Mayo Clinic, Radiation Oncology, Jacksonville, FL, United States of America; [8]Children's Hospital of Colorado, Aurora, CO, United States of America; [9]University of Miami, Miami, FL, United States of America; [10]BC Cancer, Vancouver, BC, Canada; [11]University of Toronto, Toronto, ON, Canada; [12]Weill Cornell Medicine, New York, NY, United States of America; [13]University of North Carolina, Chapel Hill, NC, United States of America; [14]Dana-Farber/Harvard Cancer Center, Boston, MA, United States of America; [15]NYP/Columbia University Medical Irving Center/Herbert Irving Comprehensive Cancer Center, New York, NY, United States of America; [16]Tufts Medical Center, Boston, MA, United States of America; [17]Children's National Medical Center, Washington, DC, United States of America; [18]University Hospital/Winship Cancer Institute, Atlanta, GA, United States of America; [19]Rutgers Cancer Institute of New Jersey, New Brunswick, NJ, United States of America;[20]Memorial Sloan Kettering Cancer Center, New York, NY, United States of America; [21]Children's Healthcare of Atlanta, Atlanta, GA, United States of America; [22]Roswell Park Comprehensive Cancer Center, Buffalo, NY, United States of America;*

### Background

Chemotherapy with or without radiotherapy (RT) is currently the standard frontline treatment for early-stage (ES) classic Hodgkin lymphoma (cHL) and has achieved cure rates >90%. In recent years however, the development of novel treatment paradigms for ES cHL have aimed to maintain these historically high cure rates while minimizing the acute and long-term toxicities associated with chemotherapy and RT given that the disease overly affects adolescents and young adults. Incorporation of novel immunotherapy (IO) agents into the frontline setting offers new potential therapy options that could reduce patient exposure to chemotherapy and RT but still improve progression-free survival (PFS) while maintaining overall survival (OS).

### Aims

AHOD2131 (NCT05675410) is a randomized, phase 3 trial for patients ages 5 to 60 years with newly diagnosed stage I/II cHL, investigating the addition of combination brentuximab vedotin (BV) with nivolumab (BV/Nivo) compared to standard chemotherapy +/- RT. The primary objective of the study is to compare the PFS of patients treated through a response-adapted, superiority design with either standard or IO (i.e. BV/Nivo) therapies with the key secondary objective of demonstrating non-inferiority of 12-year OS. There are 11 additional secondary and exploratory study aims that focus on patient-reported outcomes (PROs), disease biology and imaging.

**Copyright Information:** (Online) ISSN: 2572-9241
©2026 The Author(s). HemaSphere is published by John Wiley & Sons Ltd on behalf of European Hematology Association. This is an open access Abstract Book distributed under the Attribution-NonCommercial-NoDerivs (CC BY-NC-ND), which allows third parties to download the articles and share them with others as long as they credit the author and the Abstract Book, but they cannot change the content in any way or use them commercially.
**Abstract Book Citations:** Authors, Title, HemaSphere, 2026;10:(S1):pages. The abstract book DOIs can be found on the "Author Information" page.
**Disclaimer:** Articles published in the journal HemaSphere exclusively reflect the opinions of the authors. The authors are responsible for all content in their abstracts including accuracy of the facts, statements, citing resources, etc.

**Methods**

AHOD2131 is a collaborative study developed between pediatric and adult oncology members of the National Cancer Institute's National Clinical Trial Network (NCTN), aiming to harmonize IO-based treatment approaches for ES cHL. Study champions from each North American (NA) cooperative group (Children's Oncology Group [COG], SWOG, ECOG-ACRIN, Alliance, NRG) and experts in imaging, radiation oncology, lymphoma biology and PROs were included in the development and execution of AHOD2131. AHOD2131 was activated in April 2023 with a target enrollment of 1875 patients over 5 years.
Patients are initially stratified based on the European Organisation for Research and Treatment of Cancer (EORTC) criteria for ES HL favorable or unfavorable risk with an additional criterion of mediastinal bulky disease ≥10 cm also considered unfavorable. All patients, regardless of risk assessment, receive 2 initial cycles of ABVD followed by an interim PET2 that is rapidly centrally reviewed. Those patients who are PET2 positive (slow early response [SER]) will undergo separate randomizations as compared to those who are PET2 negative (rapid early response [RER]). Patients with SER will receive involved site RT after completion of further systemic therapy while those with RER will receive systemic therapy only (Figure).

**Results**

As of 31 January 2026, 389 NA sites have activated the study and 737 participants have enrolled. Out of the patients enrolled, 49% are <18 years old and 66% are ES unfavorable risk.

**Summary/Conclusion**

This COG-led pan-NCTN trial will be the largest study for ES cHL in the history of the NA cooperative groups and the first to enroll both pediatric and adult patients. AHOD2131 strengthens the effort between NA cooperative groups to conduct collaborative clinical trials and aims to harmonize an improved standard of care for ES cHL across the age continuum.

We would like to acknowledge NCTN Operations Center Grant U10CA180886, NCTN Statistics & Data Center Grant U10CA180899, NCORP Grant UG1CA189955 & St. Baldrick's Foundation for their support of this clinical trial.

**Copyright Information:** (Online) ISSN: 2572-9241
©2026 The Author(s). HemaSphere is published by John Wiley & Sons Ltd on behalf of European Hematology Association. This is an open access Abstract Book distributed under the Attribution-NonCommercial-NoDerivs (CC BY-NC-ND), which allows third parties to download the articles and share them with others as long as they credit the author and the Abstract Book, but they cannot change the content in any way or use them commercially.
**Abstract Book Citations:** Authors, Title, HemaSphere, 2026;10:(S1):pages. The abstract book DOIs can be found on the "Author Information" page.
**Disclaimer:** Articles published in the journal HemaSphere exclusively reflect the opinions of the authors. The authors are responsible for all content in their abstracts including accuracy of the facts, statements, citing resources, etc.

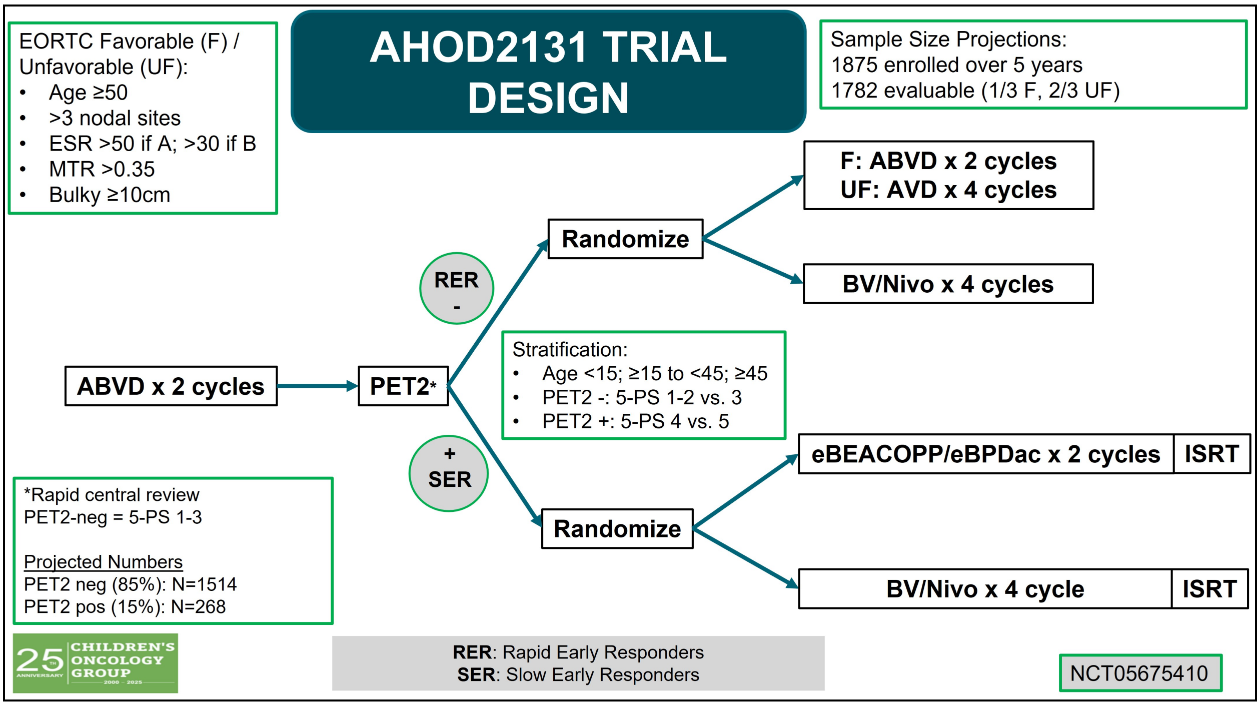

ISRT: Involved site radiotherapy

Copyright Information: (Online) ISSN: 2572-9241
©2026 The Author(s). HemaSphere is published by John Wiley & Sons Ltd on behalf of European Hematology Association. This is an open access Abstract Book distributed under the Attribution-NonCommercial-NoDerivs (CC BY-NC-ND), which allows third parties to download the articles and share them with others as long as they credit the author and the Abstract Book, but they cannot change the content in any way or use them commercially.
Abstract Book Citations: Authors, Title, HemaSphere, 2026;10:(S1):pages. The abstract book DOIs can be found on the "Author Information" page.
Disclaimer: Articles published in the journal HemaSphere exclusively reflect the opinions of the authors. The authors are responsible for all content in their abstracts including accuracy of the facts, statements, citing resources, etc.

### (PB3569) PROGNOSTIC IMPACT OF THE SIMPLIFIED GERIATRIC ASSESSMENT IN OLDER PATIENTS WITH CLASSICAL HODGKIN LYMPHOMA

**Topic:** 17. Hodgkin lymphoma - Clinical

Vittorio Ruggero Zilioli*[1], Silvia Ferrari[1], Silvia Bolis[2], Annalisa Arcari[3], Cristina Muzi[1], Emanuele Ravano[1], Elisa Bossi[2], Erika Meli[1], Erika Ravelli[1], Giovanni Rindone[2], Lorenzo Del Castello[4], Carlo Gambacorti-Passerini[2, 5], Davide Bernasconi[4, 6], Roberto Cairoli[1, 5]

*[1]Division of Hematology, ASST Grande Ospedale Metropolitano Niguarda, Milano, Italy; [2]Division of Hematology, IRCCS San Gerardo dei Tintori, Monza, Italy; [3]Division of Hematology, Ospedale Guglielmo da Saliceto, Piacenza, Italy;[4]Bicocca Bioinformatics Biostatistics and Bioimaging Centre - B4 School of Medicine and Surgery, University of Milano- Bicocca, Monza, Italy; [5]University of Milano-Bicocca, Milano, Italy; [6]Research Innovation and Technology Transfer, ASST Grande Ospedale Metropolitano Niguarda, Milano, Italy;*

### Background

Older adults (≥60 years) with classical Hodgkin lymphoma (cHL) have historically inferior outcomes compared with younger patients. This is multifactorial, reflecting more aggressive disease biology, comorbidity burden, frail status, lack of standardized treatment approaches, and treatment-related toxicity. Pretreatment evaluation of functional status and comorbidities through a simplified geriatric assessment (sGA) may help guide treatment selection in cHL, identifying fit patients suitable for curative-intent chemotherapy regimens and unfit or frail patients who may benefit from less intensive, adapted approaches.

### Aims

To assess the feasibility and the prognostic impact of the sGA in older patients newly diagnosed with cHL.

### Methods

We conducted a retrospective, observational, multicentre study including 103 consecutive patients aged ≥ 60 years with newly diagnosed cHL treated with ABVD/ABVD-like regimens between 2010 and 2023 at three haematology centres in Italy. For each patient, a retrospective sGA was performed, including Activities of Daily Living (ADL), Instrumental Activities of Daily Living (IADL), and the Cumulative Illness Rating Scale for Geriatrics (CIRS-G). Patients were stratified into three categories (FIT, UNFIT, FRAIL) according to sGA, and the prognostic impact of this stratification was analysed. Survival was estimated using the Kaplan–Meier method and Cox proportional hazards models.

### Results

Of 103 patients, 85.6% were classified as fit, 8.7% as unfit, and 5.8% as frail. Treatment intensity differed according to functional status, with reduced-dose regimens more frequently administered to unfit (55.6%) and frail (66.7%) patients compared with fit individuals (24.7%). No significant differences in complete remission (CR) rates were observed across sGA categories. However, sGA demonstrated a significant prognostic impact on survival outcomes. In univariate analysis, unfit status was associated with inferior progression-free survival (PFS) both in the whole cohort (HR 3.28, 95% CI 1.26–8.54, p=0.015), and within the full-dose ABVD subgroup (HR 3.85, 95% CI 1.16–12.81, p=0.028), while frail status showed a borderline association (HR 2.87, 95% CI 1.00–8.22, p=0.049). Frail status (HR 6.67, 95% CI 1.82–24.44, p=0.004) and impaired functional independence (ADL p<0.001; IADL p=0.033) strongly predicted inferior overall survival (OS).

**Copyright Information:** (Online) ISSN: 2572-9241
©2026 The Author(s). HemaSphere is published by John Wiley & Sons Ltd on behalf of European Hematology Association. This is an open access Abstract Book distributed under the Attribution-NonCommercial-NoDerivs (CC BY-NC-ND), which allows third parties to download the articles and share them with others as long as they credit the author and the Abstract Book, but they cannot change the content in any way or use them commercially.
**Abstract Book Citations:** Authors, Title, HemaSphere, 2026;10:(S1):pages. The abstract book DOIs can be found on the "Author Information" page.
**Disclaimer:** Articles published in the journal HemaSphere exclusively reflect the opinions of the authors. The authors are responsible for all content in their abstracts including accuracy of the facts, statements, citing resources, etc.

**Summary/Conclusion**

The sGA proved to be a feasible and clinically relevant tool to profile functional status in older patients with cHL, sGA-frail status and functional impairment emerged as strong determinants of survival, while not significantly affecting response rates. Prospective data are needed to better define the prognostic role of sGA in older cHL and its utility in guiding therapeutic decision-making.

**Copyright Information:** (Online) ISSN: 2572-9241
©2026 The Author(s). HemaSphere is published by John Wiley & Sons Ltd on behalf of European Hematology Association. This is an open access Abstract Book distributed under the Attribution-NonCommercial-NoDerivs (CC BY-NC-ND), which allows third parties to download the articles and share them with others as long as they credit the author and the Abstract Book, but they cannot change the content in any way or use them commercially.
**Abstract Book Citations:** Authors, Title, HemaSphere, 2026;10:(S1):pages. The abstract book DOIs can be found on the "Author Information" page.
**Disclaimer:** Articles published in the journal HemaSphere exclusively reflect the opinions of the authors. The authors are responsible for all content in their abstracts including accuracy of the facts, statements, citing resources, etc.

### (PB3570) OPTIMIZATION OF FIRST-LINE THERAPY IN PATIENTS WITH ADVANCED CLASSICAL HODGKIN LYMPHOMA: EFFICACY AND TOXICITY OF N-AVD COMPARED WITH EBEACOPP IN PROSPECTIVE CLINICAL TRIAL. REAL CLINICAL PRACTICE

**Topic:** 17. Hodgkin lymphoma - Clinical

Diana Ivanova*[1], Elena Baryakh[1], Elena Misyurina[1], Tatiana Tolstykh[1], Tatiana Chudnova[1], Julia Zhuravleva[1], Dmitrii Lebedev[2], Ekaterina Zotina[1], Marat Mingalimov[1], Olga Kochneva[1]

*[1]Первый Московский Государственный Медицинский Университет имени И. М. Сеченова, Hematology, Moscow, Russia;*
*[2]Городская клиническая больница N 52, Москва, Russia;*

### Background

Classical Hodgkin lymphoma (cHL) is one of the most common malignant tumors in young patients, with the peak incidence occurring between 15 and 35 years of age. Over the past decades, HL has become a highly curable disease not only in patients with localized but also with widespread stages. Thanks to the use of chemoradiation therapy, it was possible to improve the results of first-line therapy and cure more than 80% of patients. However, the use of intensive treatment regimens is associated with the risk of hematological toxicity and severe infectious complications. The use of immune checkpoint inhibitors in combination with doxorubicin, vinblastine and dacarbazine (AVD) in the first-line therapy of cHL is one of the possible strategies to increase the efficacy of therapy in both young and elderly patients.

### Aims

To evaluate the efficacy and toxicity of the N-AVD protocol compared with eBEACOPP in patients with newly cHL with advanced disease.

### Methods

The prospective study included 48 patients who received therapy using the N-AVD protocol, 32 - eBEACOPP. Patients received nivolumab dose of 3 mg/kg, days 1 and 15 of a 28-day cycle in combination chemotherapy AVD (doxorubicin 25 mg/m$^2$, vinblastine 6 mg/m$^2$, dacarbazine 375 mg/m$^2$) was then administered for 6 cycles followed by interim PET-CT (PET2). The median age in the AVD was 34 years (range, 18–60); there were 25 men (52%) and 23 women (48%), while in the eBEACOPP-36 years (range, 18–59); 14 men (44%) and 18 women (56%). Advanced disease was in 56% of cases (n=27) in the N-AVD, 81% (n=26) in the eBEACOPP. ECOG ≥2 in the N-AVD–25% (n=12) and 25% (n=8) in the eBEACOPP, IPS 4-7 - 37% (n=10) in the N-AVD, 19% (n=5) in the eBEACOPP, type of nodular sclerosis – 94% (n=45%) in the N-AVD, 97% (n=31) in the eBEACOPP.

**Copyright Information:** (Online) ISSN: 2572-9241
©2026 The Author(s). HemaSphere is published by John Wiley & Sons Ltd on behalf of European Hematology Association. This is an open access Abstract Book distributed under the Attribution-NonCommercial-NoDerivs (CC BY-NC-ND), which allows third parties to download the articles and share them with others as long as they credit the author and the Abstract Book, but they cannot change the content in any way or use them commercially.
**Abstract Book Citations:** Authors, Title, HemaSphere, 2026;10:(S1):pages. The abstract book DOIs can be found on the “Author Information” page.
**Disclaimer:** Articles published in the journal HemaSphere exclusively reflect the opinions of the authors. The authors are responsible for all content in their abstracts including accuracy of the facts, statements, citing resources, etc.

**Results**

A CR was achieved in 97% of patients (n = 30), a PR in 3% (n = 1) who received N-AVD, 23 patients (92%)–eBEACOPP, and disease progression-2 patients (8%). Hematological toxicity (grade III-IV neutropenia) developed in 14% of cases in the N-AVD, 100%-eBEACOPP (p<0.001). All patients who received eBEACOPP required G-CSF, 11 patients (23%) in the N-AVD. Anemia III-IV grade was recorded in 10% of cases in the eBEACOPP, III-IV grade thrombocytopenia was recorded in 9% of cases. No cases of III-IV grade anemia or thrombocytopenia were observed in the N-AVD (p<0.001). Febrile neutropenia was statistically significantly higher in the eBEACOPP compared to N-AVD: 19 episodes (12%) versus 1 (p=0.001). Pneumonia requiring antibacterial therapy was recorded in 2% of cases in the N-AVD (n=2), compared to 6%-eBEACOPP (n=7). There were 3 cases of severe infectious complications in the eBEACOPP (clostridial colitis, soft tissue infection), which is why the protocol was deescalated to ABVD. Among the irAE in the N-AVD, hypothyroidism II grade was observed in 5% (n=2), colitis in 2% (n=1), and toxic hepatitis grade II - 8% (n=4). Consolidation radiotherapy was administered to 12 patients (48%) - eBEACOPP, 5 patients (16%) - N-AVD. With a median follow-up of 12 months, 1-year PFS was 100% after N-AVD [HR95% CI: 100%, 100%] versus 86.8% - eBEACOPP [HR 95% CI 63.4% - 95.7%] (p = 0.0494)(*Fig.1)*, 1-year OS was 100% after N-AVD versus 96.7% - eBEACOPP (p=0.21)(*Fig.2)*

**Summary/Conclusion**

The obtained results of the study demonstrate high efficacy and acceptable toxicity of the N-AVD protocol in patients with newly diagnosed cHL, especially in extremely severe patients with generalized HL with a high prognostic index and poor somatic status.

**Copyright Information:** (Online) ISSN: 2572-9241
©2026 The Author(s). HemaSphere is published by John Wiley & Sons Ltd on behalf of European Hematology Association. This is an open access Abstract Book distributed under the Attribution-NonCommercial-NoDerivs (CC BY-NC-ND), which allows third parties to download the articles and share them with others as long as they credit the author and the Abstract Book, but they cannot change the content in any way or use them commercially.
**Abstract Book Citations:** Authors, Title, HemaSphere, 2026;10:(S1):pages. The abstract book DOIs can be found on the "Author Information" page.
**Disclaimer:** Articles published in the journal HemaSphere exclusively reflect the opinions of the authors. The authors are responsible for all content in their abstracts including accuracy of the facts, statements, citing resources, etc.

*Fig. 1.* 1-year progression-free survival

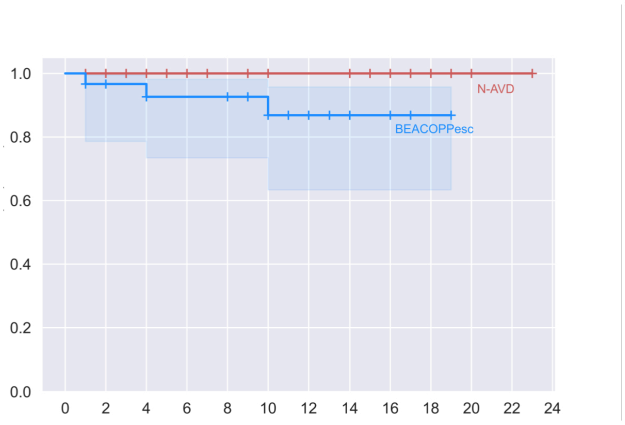

***p* = 0,049**

***Fig. 2.*** 1-year overall survival

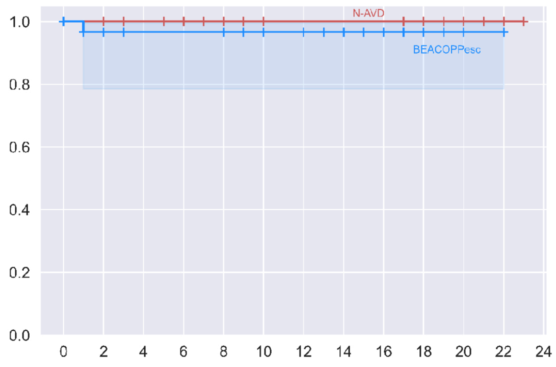

***p* = 0,21**

**Copyright Information:** (Online) ISSN: 2572-9241
©2026 The Author(s). HemaSphere is published by John Wiley & Sons Ltd on behalf of European Hematology Association. This is an open access Abstract Book distributed under the Attribution-NonCommercial-NoDerivs (CC BY-NC-ND), which allows third parties to download the articles and share them with others as long as they credit the author and the Abstract Book, but they cannot change the content in any way or use them commercially.
**Abstract Book Citations:** Authors, Title, HemaSphere, 2026;10:(S1):pages. The abstract book DOIs can be found on the "Author Information" page.
**Disclaimer:** Articles published in the journal HemaSphere exclusively reflect the opinions of the authors. The authors are responsible for all content in their abstracts including accuracy of the facts, statements, citing resources, etc.

### (PB3571) FIRST-LINE NIVOLUMAB-AVD FOR EARLY-STAGE UNFAVORABLE AND ADVANCED-STAGE CLASSICAL HODGKIN LYMPHOMA: A META-ANALYSIS

**Topic:** 17. Hodgkin lymphoma - Clinical

Priscille Neves Rupert Jones[1], Gabriel Lucas Ferreira Silva[1], Isabella Ferreira Michelon[2], Julia Marques Fernandes[3], Jasmina Veta Darkovski[4], Oleksandr Teteria[5], Fernando Gibran-Nunes*[6]

*[1]University of Pernambuco, Recife, Brazil; [2]University of Virginia Comprehensive Cancer Center, Division of Hematology and Oncology, Charlottesville, United States of America; [3]Albert Einstein Israeli Institute of Health Sciences, São Paulo, Brazil; [4]University Clinic for Gynecology and Obstetrics, Center for Gynecological and Breast Cancer, Ulm, Germany;[5]Institute of Hematology and Transfusion Medicine, Department of Hematological Diagnostics and Internal Medicine, Hematology Clinic, Warsaw, Poland; [6]Sírio-Libanês Hospital, Hematology, BMT and Cell Therapy Department, São Paulo, Brazil;*

### Background

Frontline therapy cures most patients with classical Hodgkin lymphoma (cHL), yet relapse remains clinically relevant in unfavorable early-stage and advanced-stage disease. ABVD has long been a standard frontline regimen but is limited by bleomycin-related pulmonary toxicity. Brentuximab vedotin–based approaches improved disease control in advanced-stage disease, although at the cost of treatment-related toxicities, particularly peripheral neuropathy. Given the frequent 9p24.1 alterations leading to PD-1 pathway overexpression in cHL, nivolumab plus AVD (N-AVD) represents a biologically rational frontline strategy; however, published studies have used different administration approaches, and pooled outcome estimates remain limited.

### Aims

To synthesize efficacy and safety outcomes of frontline N-AVD in adolescents and adults with unfavorable early-stage or advanced-stage cHL.

### Methods

We conducted a PRISMA-compliant systematic review and meta-analysis of studies evaluating frontline N-AVD in newly diagnosed cHL. We searched PubMed, Embase, and the Cochrane Library from inception to November 2025. The protocol was prospectively registered in PROSPERO (CRD420261279343). We used a random-effects model for binary endpoints and reconstructed individual patient data from Kaplan-Meier curves for time-to-event outcomes. Primary endpoints were overall survival (OS) and progression-free survival (PFS). Secondary endpoints included complete response (CR) rate, partial response (PR) rate disease progression (DP) and immune-related adverse events (IrAE).

**Copyright Information:** (Online) ISSN: 2572-9241
©2026 The Author(s). HemaSphere is published by John Wiley & Sons Ltd on behalf of European Hematology Association. This is an open access Abstract Book distributed under the Attribution-NonCommercial-NoDerivs (CC BY-NC-ND), which allows third parties to download the articles and share them with others as long as they credit the author and the Abstract Book, but they cannot change the content in any way or use them commercially.
**Abstract Book Citations:** Authors, Title, HemaSphere, 2026;10:(S1):pages. The abstract book DOIs can be found on the “Author Information” page.
**Disclaimer:** Articles published in the journal HemaSphere exclusively reflect the opinions of the authors. The authors are responsible for all content in their abstracts including accuracy of the facts, statements, citing resources, etc.

### Results

Three studies including 645 patients met the inclusion criteria. Endpoint-specific denominators varied according to outcome reporting. Median OS was not reached. Estimated OS was 98.1% at 12 and 24 months and 97.7% at 36 months. Median PFS was not reached (lower 95% CI 50.0 months). Estimated PFS was 96.2% at 12 months, 93.1% at 24 months, and 91.2% at 36 months. Pooled complete response rate was 98.6% (143/160). The pooled PR was 10.9% (16/152; 95% CI, 5.5–20.3).
DP occurred in 2.5% of evaluable patients (4/160; 95% CI 0.7–8.5). Immune-related adverse events occurred in 9.9% of patients (9/91; 95% CI 5.2–17.9%).

**Table 1: Progression-free survival**

| PFS | Estimate (95% CIs) |
|---|---|
| Median PFS, months | Not reached (95% CI, 50.0–NR) |
| PFS at 12 months, % | 96.2 (95% CI, 94.7–97.7) |
| PFS at 24 months, % | 93.1 (95% CI, 91.1–95.3) |
| PFS at 36 months, % | 91.2 (95% CI, 88.6–94.0) |

**Table 2: Overall survival**

| OS | Estimate (95% CIs) |
|---|---|
| Median OS (months) | Not reached |
| OS at 12 months (%) | 98.1 (95% CI 97.1–99.2) |
| OS at 24 months (%) | 98.1 (95% CI 97.1–99.2) |
| OS at 36 months (%) | 97.7 (95% CI 96.3–99.1) |

### Summary/Conclusion

Frontline N-AVD was associated with high complete response rates, durable progression-free survival, and sustained overall survival, with a manageable safety profile. These findings support N-AVD as a valid first-line therapeutic option for unfavorable early-stage and advanced-stage cHL.

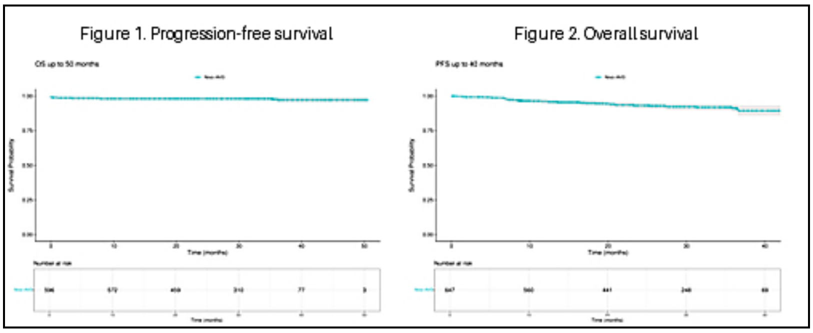

**Copyright Information:** (Online) ISSN: 2572-9241
©2026 The Author(s). HemaSphere is published by John Wiley & Sons Ltd on behalf of European Hematology Association. This is an open access Abstract Book distributed under the Attribution-NonCommercial-NoDerivs (CC BY-NC-ND), which allows third parties to download the articles and share them with others as long as they credit the author and the Abstract Book, but they cannot change the content in any way or use them commercially.
**Abstract Book Citations:** Authors, Title, HemaSphere, 2026;10:(S1):pages. The abstract book DOIs can be found on the “Author Information” page.
**Disclaimer:** Articles published in the journal HemaSphere exclusively reflect the opinions of the authors. The authors are responsible for all content in their abstracts including accuracy of the facts, statements, citing resources, etc.

### (PB3572) PEGYLATED LIPOSOMAL DOXORUBICIN IN HEAVILY PRETREATED ADULT PATIENTS WITH CLASSICAL HODGKIN LYMPHOMA: REAL-WORLD DATA FROM GUSTAVE ROUSSY COHORT

**Topic:** 17. Hodgkin lymphoma - Clinical

Cristina Garcia-Herce*[1, 2, 3], Thomas Hueso[3, 4], Jean-Marie Michot[3, 4], Laurentiu Agrigoroaie[5], Camille Bigenwald[4], David Ghez[4], Alina Danu[4], Peggy Dartigues[6], Cecilia Carpio[1, 2], Christophe Massard[3], Pau Abrisqueta[1, 2], Francisco Bosch Albareda[1, 2], Vincent Ribrag[3, 4]

*[1]Vall d'Hebron Hospital, Clinical Hematology department, Barcelona, Spain;[2]Vall d'Hebron insititute of Oncology, Clinical Trial department, Barcelona, Spain; [3]Gustave Roussy, Early Drug Development department, Villejuif, France; [4]Gustave Roussy, Clinical Hematology department, Villejuif, France; [5]Gustave Roussy, Nuclear Medicine department, Villejuif, France; [6]Gustave Roussy, Pathology department, Villejuif, France;*

### Background

Despite major advances with brentuximab vedotin (BV) and checkpoint inhibitors (CPI), a subset of patients with relapsed/refractory (R/R) classical Hodgkin lymphoma (cHL) remains with limited therapeutic options. Pegylated liposomal doxorubicin (PLD) allows safe anthracycline re-exposure and may enhance immunogenic cell death when combined with CPI.

### Aims

To evaluate the efficacy of PLD in R/R cHL.

### Methods

This retrospective study included adult patients treated in a real-world setting with PLD for R/R cHL from a Gustave Roussy cohort between 2009 and 2025, after prior anthracycline-based first-line therapy and with preserved baseline cardiac function. The primary endpoint was the overall response rate (ORR), and the secondary endpoint was event-free survival (EFS) defined as time from PLD initiation to progression, death, or treatment discontinuation due to toxicity. Patients were categorized into two groups: those receiving a CPI-based regimen with PLD (PLD+CPI) and those receiving a PLD combined with chemotherapy (PLD+CT). An exploratory analysis evaluated PLD + CPI in patients previously exposed to CPI. Responses were assessed according to Cheson or Lugano criteria. Response rates were compared using Fisher’s exact test.

**Copyright Information:** (Online) ISSN: 2572-9241
©2026 The Author(s). HemaSphere is published by John Wiley & Sons Ltd on behalf of European Hematology Association. This is an open access Abstract Book distributed under the Attribution-NonCommercial-NoDerivs (CC BY-NC-ND), which allows third parties to download the articles and share them with others as long as they credit the author and the Abstract Book, but they cannot change the content in any way or use them commercially.
**Abstract Book Citations:** Authors, Title, HemaSphere, 2026;10:(S1):pages. The abstract book DOIs can be found on the “Author Information” page.
**Disclaimer:** Articles published in the journal HemaSphere exclusively reflect the opinions of the authors. The authors are responsible for all content in their abstracts including accuracy of the facts, statements, citing resources, etc.

**Results**

Thirty-six patients treated with PLD were included. Median age at diagnosis was 30 (range 16–75) years and 38.9% were female. Most patients had stage III–IV disease at diagnosis (61.1%). First-line therapy was ABVD-based in 58.3% and BEACOPP-based in 30.6%; 52.9% were primary refractory to first-line treatment. Prior to PLD initiation, patients had received a median of 5 lines of therapy (range 2–11), with a median time from diagnosis to PLD of 3.35 (range 0.7–19) years. Previous exposure to CPI was observed in 16 patients (44.4%), of whom 9 (56.3%) were CPI non-responders. BV had been previously administered in 19 patients (52.7%), including 12 non-responders. Prior autologous stem cell transplantation (SCT) was performed in 18 cases (50.0%), and allogeneic SCT in 1 (2.8%).
Eleven patients received PLD+CPI regimens: PLD-pembrolizumab (n=6), PLD-pembrolizumab-vinblastine (n=1), or PLD-pembrolizumab-gemcitabine-vinblastine (n=4). Twenty-five patients received PLD+CT regimens: PLD-gemcitabine-vinblastine (n=15), PLD-vinblastine (n=6), PLD-gemcitabine (n=1), PLD-BV-gemcitabine-vinblastine (n=2), or PLD-BV (n=1). Treatment discontinuation due to PLD-related toxicities occurred in 3 patients, including two cases of grade 3 cardiac toxicity and one case of grade 3 cutaneous toxicity.
Best responses to PLD-based regimens are shown in *Figure 1.* The ORR was higher with PLD+CPI than with PLD+CT (81.8% vs. 60%), although the difference was not statistically significant (p=0.26). Median follow-up from PLD initiation was 12.0 years (95% CI: 4.85–not reached), with a median event-free survival of 12 months (95% CI: 6.9–18).
Ten of 11 patients treated with PLD+CPI had prior CPI exposure (9 CPI non-responders, 1 prior PR). Among these 10 patients, 8 achieved an objective response (CR, n=4; PR, n=4); 1 patient had stable disease and 1 was non-evaluable. In responders, response maintenance rates at 6 and 12 months were 71% and 43%, respectively; three responses were ongoing at last follow-up, including one patient consolidated with allogeneic stem cell transplantation.

**Summary/Conclusion**

These findings suggest that incorporating an anthracycline may represent a rational combination strategy with CPIs to restore CPI sensitivity in previously refractory patients.

**Copyright Information:** (Online) ISSN: 2572-9241
©2026 The Author(s). HemaSphere is published by John Wiley & Sons Ltd on behalf of European Hematology Association. This is an open access Abstract Book distributed under the Attribution-NonCommercial-NoDerivs (CC BY-NC-ND), which allows third parties to download the articles and share them with others as long as they credit the author and the Abstract Book, but they cannot change the content in any way or use them commercially.
**Abstract Book Citations:** Authors, Title, HemaSphere, 2026;10:(S1):pages. The abstract book DOIs can be found on the “Author Information” page.
**Disclaimer:** Articles published in the journal HemaSphere exclusively reflect the opinions of the authors. The authors are responsible for all content in their abstracts including accuracy of the facts, statements, citing resources, etc.

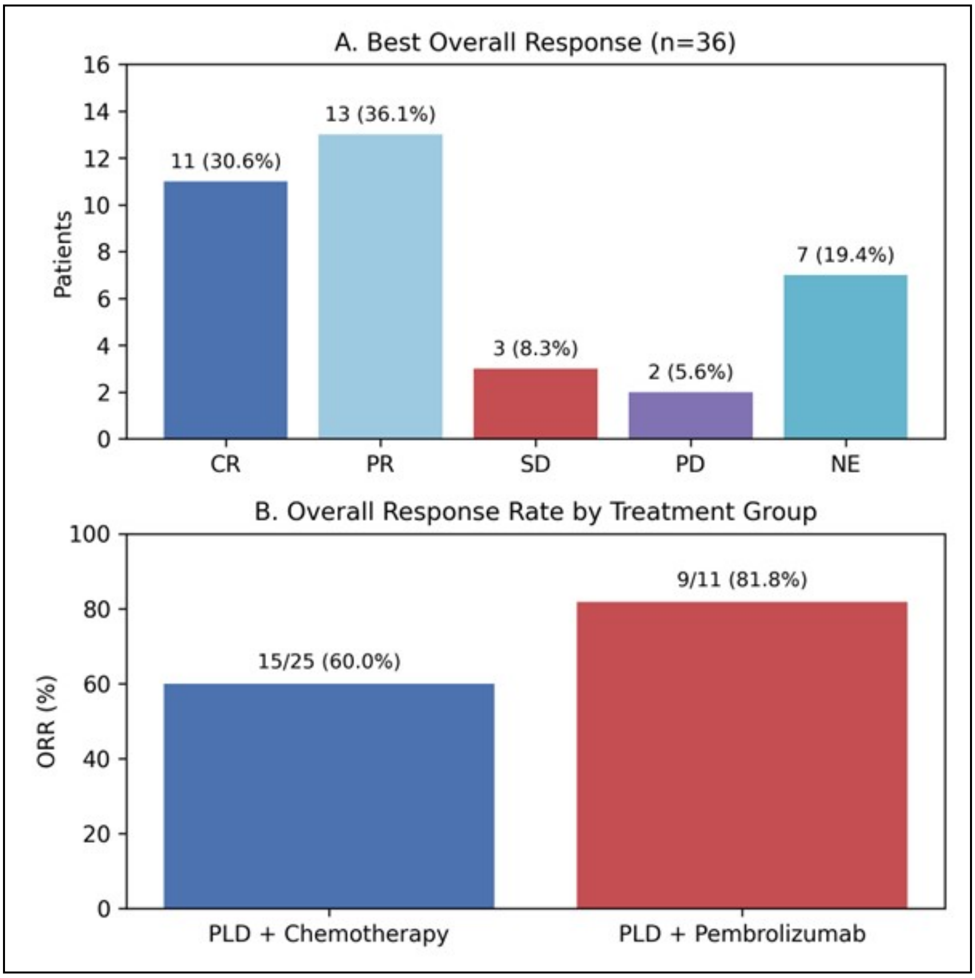

CR, complete response; PR, partial response; SD, stable disease; PD, progressive disease; NE, not evaluable; ORR, overall response rate.

**Copyright Information:** (Online) ISSN: 2572-9241
©2026 The Author(s). HemaSphere is published by John Wiley & Sons Ltd on behalf of European Hematology Association. This is an open access Abstract Book distributed under the Attribution-NonCommercial-NoDerivs (CC BY-NC-ND), which allows third parties to download the articles and share them with others as long as they credit the author and the Abstract Book, but they cannot change the content in any way or use them commercially.
**Abstract Book Citations:** Authors, Title, HemaSphere, 2026;10:(S1):pages. The abstract book DOIs can be found on the "Author Information" page.
**Disclaimer:** Articles published in the journal HemaSphere exclusively reflect the opinions of the authors. The authors are responsible for all content in their abstracts including accuracy of the facts, statements, citing resources, etc.

### (PB3573) SARCOPENIA IN OLDER PATIENTS WITH CLASSICAL HODGKIN LYMPHOMA: PROGNOSTIC AND TOXICITY-RELATED IMPACT IN A REAL-WORLD COHORT

**Topic:** 17. Hodgkin lymphoma - Clinical

Silvia Ferrari*[1], Vittorio Ruggero Zilioli[1], Maria Elena Pellegrino[2], Cristina Muzi[1], Emanuele Ravano[1], Erika Meli[1], Erika Ravelli[1], Lorenzo Del Castello[3], Davide Bernasconi[3, 4], Angelo Vanzulli[5, 6], Roberto Cairoli[1, 7], Domenico Albano[5, 8]

*[1]Division of Hematology, ASST Grande Ospedale Metropolitano Niguarda, Milano, Italy; [2]Department of Radiology, IRCCS Istituto Ortopedico Galeazzi, Milano, Italy; [3]Bicocca Bioinformatics Biostatistics and Bioimaging Centre - B4 School of Medicine and Surgery, University of Milano- Bicocca, Monza, Italy; [4]Research Innovation and Technology Transfer, ASST Grande Ospedale Metropolitano Niguarda, Milano, Italy; [5]Department of Radiology, ASST Grande Ospedale Metropolitano Niguarda, Milano, Italy; [6]Department of Oncology and Hemato-Oncology, Università degli Studi di Milano, Milano, Italy;[7]University of Milano-Bicocca, Milano, Italy; [8]Department of Biomedical, Surgical and Dental Sciences, Università degli Studi di Milano, Milano, Italy;*

### Background

Older adults with classical Hodgkin lymphoma (cHL) have historically experienced inferior outcomes compared with younger populations due to several factors, including aggressive disease biology, lack of standardized treatment approaches, frailty, comorbidities, and treatment-related toxicities. Sarcopenia is a clinical syndrome characterized by a progressive decline in skeletal muscle mass and strength, leading to disability, impaired quality of life, and mortality. In diffuse large B-cell lymphoma and solid tumours, sarcopenia has emerged as an independent adverse prognostic factor associated with increased treatment-related toxicity and worse survival. Evidence regarding the prognostic role of sarcopenia in cHL remains limited.

### Aims

To investigate the prognostic role and toxicity-related impact of sarcopenia in older cHL patients.

### Methods

We performed a retrospective analysis of 54 consecutive patients aged ≥ 60 years with newly diagnosed cHL, treated with either full-dose (FD) or reduced-dose (RD) ABVD/ABVD-like regimens between 2010 and 2023 at Niguarda Hospital (Milan, Italy). Sarcopenia was assessed on baseline computed tomography (CT) by calculating the skeletal muscle area (SMA) on a single axial slice at the level of the third lumbar vertebra (L3). The SMA was normalized for patient height to derive the skeletal muscle index (L3 SMI, $cm^2/m^2$). Image segmentation and quantitative analysis were performed automatically using CoreSlicer, open-source AI-based software. Sarcopenia was subsequently established according to the Lanic cut-off, which defines sarcopenia based on body mass index (BMI)-adjusted SMA, and the Nakamura cut-off, which applies sex-specific thresholds derived from a healthy reference cohort.

**Copyright Information:** (Online) ISSN: 2572-9241
©2026 The Author(s). HemaSphere is published by John Wiley & Sons Ltd on behalf of European Hematology Association. This is an open access Abstract Book distributed under the Attribution-NonCommercial-NoDerivs (CC BY-NC-ND), which allows third parties to download the articles and share them with others as long as they credit the author and the Abstract Book, but they cannot change the content in any way or use them commercially.
**Abstract Book Citations:** Authors, Title, HemaSphere, 2026;10:(S1):pages. The abstract book DOIs can be found on the “Author Information” page.
**Disclaimer:** Articles published in the journal HemaSphere exclusively reflect the opinions of the authors. The authors are responsible for all content in their abstracts including accuracy of the facts, statements, citing resources, etc.

**Results**

According to the Lanic criteria, 79.6% of patients were sarcopenic (100% among those receiving RD-ABVD and 70.3% in the FD-ABVD subgroup). Using the Nakamura cut-off, the prevalence was 48.1% (76.5% vs 35.1%, respectively). Sarcopenia, regardless of the definition applied, was not significantly associated with complete response (CR), progression-free survival (PFS), or overall survival (OS). Five-year OS was comparable between sarcopenic and non-sarcopenic patients using both cut-offs. A non-significant trend toward inferior OS was observed in patients treated with RD ABVD when sarcopenia was defined according to Nakamura (66.7% vs 100%, p=0.09). Toxicity patterns differed according to the cut-off used. While no significant differences were observed using the Lanic criteria, sarcopenia defined by the Nakamura cut-off was associated with a higher incidence of non–neutropenia-related infections (42% vs 14%, p=0.033) and pulmonary toxicity (16% vs 0%, p=0.029), particularly manifesting as reduced diffusing capacity of the lung for carbon monoxide (DLCO). In the subgroup of patients receiving FD ABVD, pulmonary toxicity remained more frequent among sarcopenic patients (31% vs 0%, p=0.011).

**Summary/Conclusion**

Sarcopenia is highly prevalent in older cHL patients. Although it did not significantly impact response rates, PFS, or OS, sarcopenia defined by the Nakamura cut-off identified patients at increased risk of infectious and pulmonary toxicity. Our data suggest that CT-based sarcopenia assessment, particularly using sex-specific thresholds, may improve toxicity risk stratification in older cHL patients treated with ABVD-like regimens. More data and prospective studies are needed in order to better define the real role of sarcopenia in cHL.

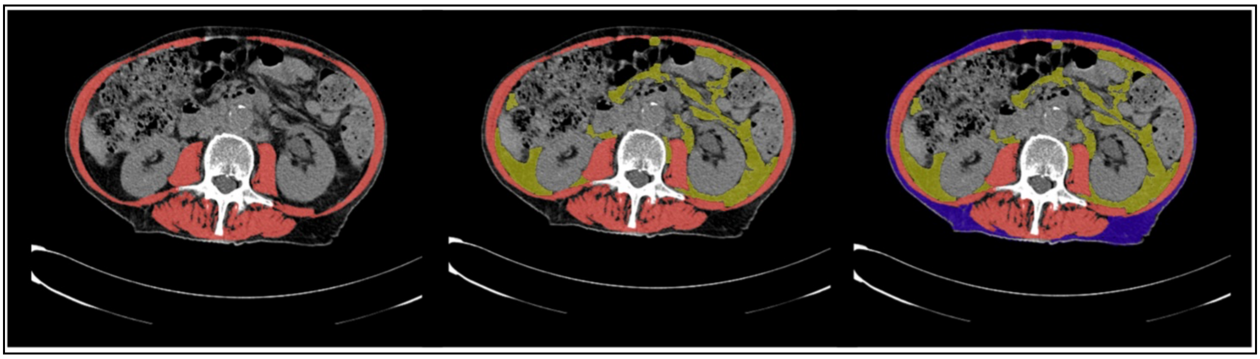

AI-assisted CT segmentation for sarcopenia assessment (skeletal muscle - red; visceral adipose tissue - yellow; subcutaneous adipose tissue - blue)

**Copyright Information:** (Online) ISSN: 2572-9241
©2026 The Author(s). HemaSphere is published by John Wiley & Sons Ltd on behalf of European Hematology Association. This is an open access Abstract Book distributed under the Attribution-NonCommercial-NoDerivs (CC BY-NC-ND), which allows third parties to download the articles and share them with others as long as they credit the author and the Abstract Book, but they cannot change the content in any way or use them commercially.
**Abstract Book Citations:** Authors, Title, HemaSphere, 2026;10:(S1):pages. The abstract book DOIs can be found on the "Author Information" page.
**Disclaimer:** Articles published in the journal HemaSphere exclusively reflect the opinions of the authors. The authors are responsible for all content in their abstracts including accuracy of the facts, statements, citing resources, etc.

### (PB3574) PROGNOSTIC UTILITY OF THE C-REACTIVE PROTEIN– ALBUMIN–LYMPHOCYTE (CALLY) INDEX IN HODGKIN LYMPHOMA

**Topic:** 17. Hodgkin lymphoma - Clinical

Fatma Keklik Karadag*[1], Denis Cetin[1], Didem Koca[1], Murat Aysin[2], Ajda Gunes[1], Fahri Sahin[1], Mahmut Töbü[1], Filiz Vural[1], Güray Saydam[1], Nur Soyer[1]

*[1]Ege University, Hematology, Izmir, Türkiye; [2]Balıkesir University, Public Health, Balıkesir, Türkiye;*

### Background

In order to evaluate and classify patients' inflammation, nutritional state, and immunity, the CALLY index provided points based on albumin level, lymphocyte count, and CRP. In a variety of solid tumors, including gastrointestinal tumors, renal cell carcinoma, malignant thyroid tumors, breast cancer, and lung cancer, it has shown effective in identifying malnutrition and linking its severity to adverse clinical outcomes. To date, the CALLY index has shown minimal utility in hematologic malignancies.

### Aims

Considering the inflammation/nutrition status prior to treatment is important for identifying high-risk groups with a poor prognosis in hematologic neoplasms, we conducted this study to utilize the CALLY index to evaluate the incidence of malnutrition among classical Hodgkin (HL) patients and to analyze its impact on clinical prognosis.

### Methods

This retrospective study examined the data of newly diagnosed HL patients treated with ABVD (adriamycin, bleomycin, vinblastine, and dacarbazine) regimen from January 2010 to December 2024 at Ege University Department of Hematology. The ideal cutoff value for the CALLY index was determined by receiver-operating characteristic (ROC) curve analysis using Youden's index. The Kaplan-Meier method was used to estimate the survival curves for each group, and the log-rank test was used to compare them. Overall survival (OS) and progression-free survival (PFS) analyses were performed according to the cut-off value, and Cox regression analyses were conducted to identify factors affecting prognosis.

### Results

A total of 291 patients were included with a median age of 39 years (range, 18-78). The most common pathologic subtype was nodular sclerosis (54.3%); moreover, 45.4% of patients had B symptoms, and 53.3% of the study group had advanced-stage disease. The optimal cut-off value for the CALLY index was determined to be 2.5 based on ROC analysis (AUC: 0.634, 95% GA: 0.520-0.747, P: 0.027) (Figure 1A). Patients were divided into two groups: CALLY ≥2.5, 18.6% of patients, and CALLY <2.5, 81.4% of patients. The 1-year and 5-year PFS rates were 97.9% and 92.3%, respectively in patients with CALLY ≥2.5, whereas 94.3% and 86.9% in patients with CALLY <2.5 (p=0.036) (Figure 1B). The 1- year and 5-year OS was significantly higher in patients with CALLY ≥2.5 (98% for both of them vs 92% and 89.6%, respectively p=0.035)(Figure 1C). Additionally, univariate regression analysis revealed that age, presence of any other comorbidity, and high IPS-7 score were related to lower OS in our study group (p values were <0.001, 0.004, and <0.001, respectively).

**Copyright Information:** (Online) ISSN: 2572-9241
©2026 The Author(s). HemaSphere is published by John Wiley & Sons Ltd on behalf of European Hematology Association. This is an open access Abstract Book distributed under the Attribution-NonCommercial-NoDerivs (CC BY-NC-ND), which allows third parties to download the articles and share them with others as long as they credit the author and the Abstract Book, but they cannot change the content in any way or use them commercially.
**Abstract Book Citations:** Authors, Title, HemaSphere, 2026;10:(S1):pages. The abstract book DOIs can be found on the "Author Information" page.
**Disclaimer:** Articles published in the journal HemaSphere exclusively reflect the opinions of the authors. The authors are responsible for all content in their abstracts including accuracy of the facts, statements, citing resources, etc.

### Summary/Conclusion

The CALLY Index serves as an independent and rapidly applicable prognostic indicator of PFS and OS in HL. It enhances conventional clinical models by adding indicators of nutritional status and systemic inflammation.

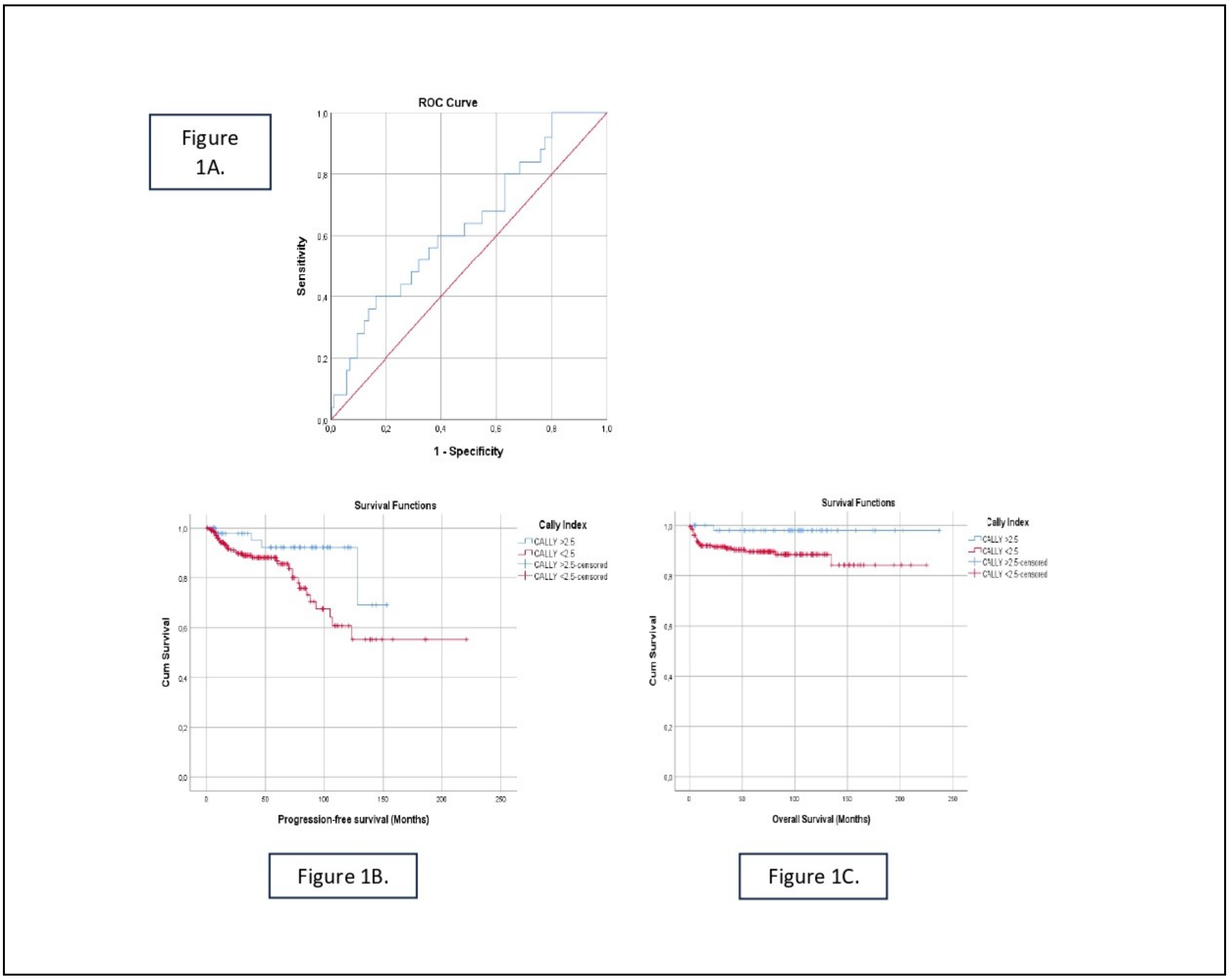

Figure 1A. ROC curve, Figure 1B. Progression Free Survival (PFS), Figure 1C. Overall Survival (OS)

**Copyright Information:** (Online) ISSN: 2572-9241
©2026 The Author(s). HemaSphere is published by John Wiley & Sons Ltd on behalf of European Hematology Association. This is an open access Abstract Book distributed under the Attribution-NonCommercial-NoDerivs (CC BY-NC-ND), which allows third parties to download the articles and share them with others as long as they credit the author and the Abstract Book, but they cannot change the content in any way or use them commercially.
**Abstract Book Citations:** Authors, Title, HemaSphere, 2026;10:(S1):pages. The abstract book DOIs can be found on the "Author Information" page.
**Disclaimer:** Articles published in the journal HemaSphere exclusively reflect the opinions of the authors. The authors are responsible for all content in their abstracts including accuracy of the facts, statements, citing resources, etc.

### (PB3575) IMPACT OF CLINICAL TRIAL ELIGIBILITY ON SURVIVAL IN PATIENTS WITH ADVANCED-STAGE HODGKIN LYMPHOMA

**Topic:** 17. Hodgkin lymphoma - Clinical

İlknur Kilic[1], Fatma Gecgel-Arikan[1], Asu Fergün Aydın[1], Isik Atagunduz[1], Tayfur Toptas*[1]

[1]*Marmara University Pendik Research And Education Hospital, Hematology, Pendik, Türkiye;*

### Background

There are universal laboratory and clinical parameters, which are used for the inclusion of patients in the randomized controlled clinical trials. A significant group of patients are not represented in clinical trials because of in eligibility for these criteria. This renders the generalizability of the data obtained from clinical trials questionable.

### Aims

The aim of this study is to reveal the course of disease in patients with advanced stage Hodgkin lymphoma (HL) who are not eligible for randomized controlled clinical trials and to determine the factors that may affect this course.

### Methods

Baseline study eligibility criteria of RATHL and ECHELON-1 trials, which included patients with advanced HL, were retrieved. A total of 236 patients with advanced stage HL were classified as "eligible and ineligible" using the eligibility criteria of these studies. Survival times of patients who were eligible and ineligible according to the inclusion criteria were calculated by the Kaplan-Meier method and compared with the log-rank test.

### Results

Among 236 patients with advanced HL, 31% (n=71) did not meet any of the study inclusion criteria. When the inclusion criteria of the RATHL and ECHELON-1 studies were used, it was observed that 20% (n=46) and 27% (n=63) of the patients were ineligible, respectively. Overall survival rates at five years for patients who were eligible, and ineligible were 94.3% and 86.4%, respectively (p= 0.04). Among the study eligibility criteria, only serum total bilirubin level was an independent predictor of overall survival (Hazard ratio: 2.33, 95% confidence interval: 2.33-29.1, p=0.001).

### Summary/Conclusion

Our study showed that one-third of patients did not meet the inclusion criteria and that these patients had a worse disease outcome in comparison to those who met the inclusion criteria. There is a unmet need for management and treatment strategies of these patients.

**Copyright Information:** (Online) ISSN: 2572-9241
©2026 The Author(s). HemaSphere is published by John Wiley & Sons Ltd on behalf of European Hematology Association. This is an open access Abstract Book distributed under the Attribution-NonCommercial-NoDerivs (CC BY-NC-ND), which allows third parties to download the articles and share them with others as long as they credit the author and the Abstract Book, but they cannot change the content in any way or use them commercially.
**Abstract Book Citations:** Authors, Title, HemaSphere, 2026;10:(S1):pages. The abstract book DOIs can be found on the “Author Information” page.
**Disclaimer:** Articles published in the journal HemaSphere exclusively reflect the opinions of the authors. The authors are responsible for all content in their abstracts including accuracy of the facts, statements, citing resources, etc.

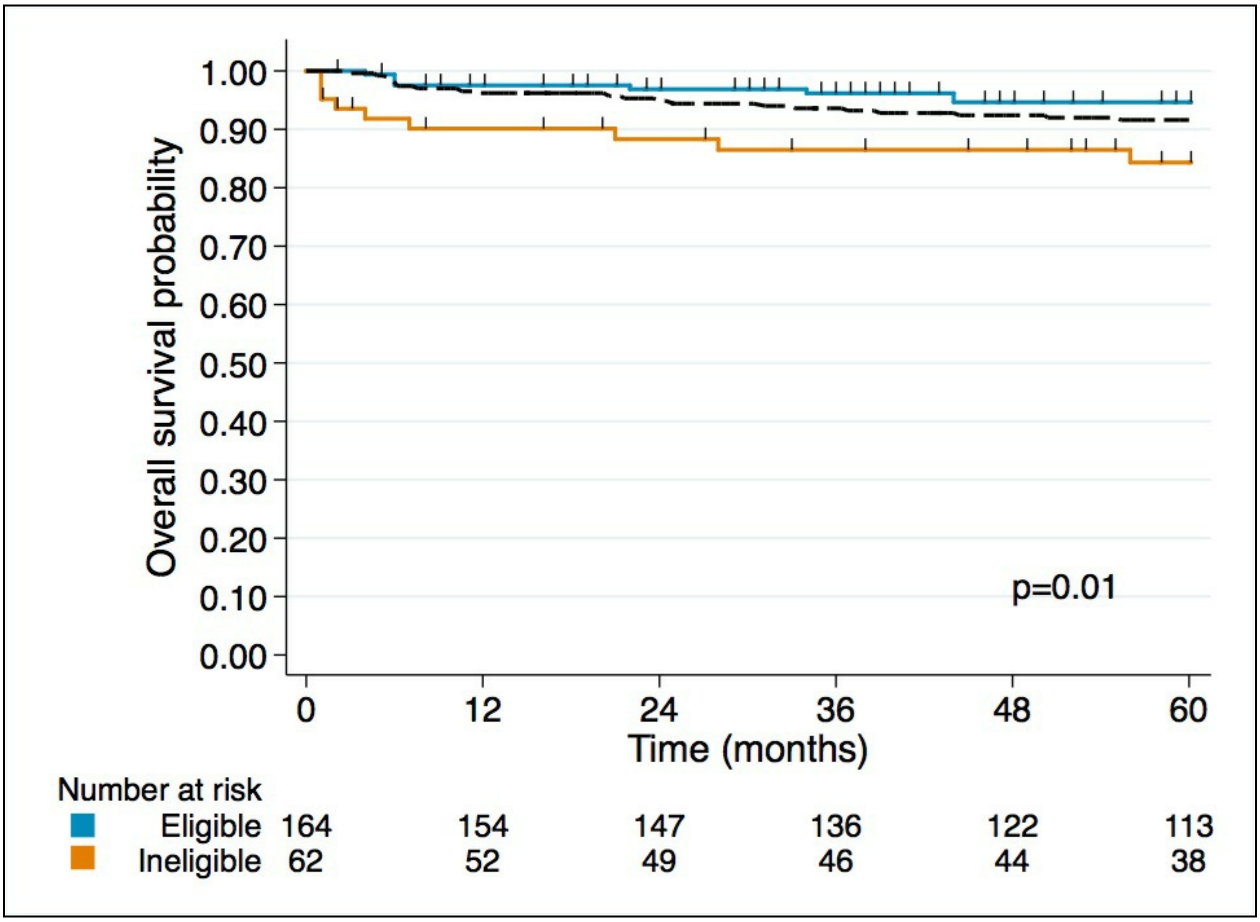

Overall survival for patients who are eligible and ineligible for clinical trials and comparison with BV-AVD arm in Echelon-1 study as plotted by dashed line.

**Copyright Information:** (Online) ISSN: 2572-9241
©2026 The Author(s). HemaSphere is published by John Wiley & Sons Ltd on behalf of European Hematology Association. This is an open access Abstract Book distributed under the Attribution-NonCommercial-NoDerivs (CC BY-NC-ND), which allows third parties to download the articles and share them with others as long as they credit the author and the Abstract Book, but they cannot change the content in any way or use them commercially.
**Abstract Book Citations:** Authors, Title, HemaSphere, 2026;10:(S1):pages. The abstract book DOIs can be found on the "Author Information" page.
**Disclaimer:** Articles published in the journal HemaSphere exclusively reflect the opinions of the authors. The authors are responsible for all content in their abstracts including accuracy of the facts, statements, citing resources, etc.

### (PB3576) CHARACTERISTICS OF MICROSATELLITE INSTABILITY IN NODULAR SCLEROSIS CLASSICAL HODGKIN LYMPHOMA

**Topic:** 17. Hodgkin lymphoma - Clinical

Dmitry Diakonov*[1], Vitaliy Rosin[1], Olga Zhizhina[1], Elena Vaneeva[1], Maria Berdnikova[1], Ekaterina Tregubova[1]

*[1]The Federal State-Financed Scientific Institution Kirov Research Institute of Hematology and Blood Transfusion under the Federal Medical Biological Agency, Kirov, Russia;*

### Background

Classical Hodgkin lymphoma (cHL) encompasses four pathohistological variants, the most common of which is nodular sclerosis. The disease is characterized by a variable clinical course and prognosis, fibrous changes in lymph nodes and other organs with the formation of nodule-like structures containing large mononuclear or multinuclear Hodgkin and/or Reed-Sternberg tumor cells. Microsatellite instability (MSI) is one of the factors determining the prognosis and treatment strategy for a number of oncological diseases (colorectal cancer, endometrial cancer, etc.). Her emergence is due to a malfunction of the gene repair system. Consequently, errors build up in DNA replication and recombination processes, resulting in a loss of genome stability and the appearance of numerous mutations, which act as triggers for carcinogenesis. The assessment of MSI status is performed using PCR and immunohistochemical methods (evaluating the expression of proteins encoded by 4 genes: MLH1, MSH2, MSH6, PMS2). Research on microsatellite instability in nodular sclerosis Hodgkin lymphoma is limited, with inconclusive findings.

### Aims

Assess the MSI status in tumor cells in cHL.

### Methods

79 patients with newly diagnosed cHL, nodular sclerosis subtype (median age - 36 years) were examined. The research was conducted using biopsies of tumor tissue and lymph nodes. The expression of MSI markers (MLH-1, MSH2, MSH6, PMS2) within Hodgkin and/or Reed-Sternberg cells was evaluated using immunohistochemistry and visualized with the Axio Scope A1 light microscope. Nuclear expression of all 4 proteins was defined as the norm (stable MSI status). Loss of expression of ≥1 marker was defined as a defect in the DNA repair system. Positive internal control was confirmed by nuclear staining in tumor microenvironment lymphocytes and fibroblasts.

### Results

Expression of all 4 MSI proteins was detected in 74 (93.7%) of 79 patients with nodular sclerosis cHL. The absence of at least one of the 4 markers was detected in 5 (6.3%) patients (unstable MSI status). In all cases, loss of MSH6 protein expression was observed. In 3 (60%) of 5 patients, the absence of positive reactions to MSH2 and PMS2 was additionally determined; in 2 (40%) cases, to MLH-1. Complete loss of marker expression was observed in 1 patient, accounting for 20% of all cases with detected unstable MSI status and 1.2% of all patients with nodular sclerosis cHL.

### Summary/Conclusion

Microsatellite instability was detected by immunohistochemistry in 6.3% of patients with nodular sclerosis cHL. Most often, its occurrence is associated with the loss of MSH6 protein expression. In subsequent studies, it is planned to correlate MSI status data with treatment response and patient survival.

**Copyright Information:** (Online) ISSN: 2572-9241
©2026 The Author(s). HemaSphere is published by John Wiley & Sons Ltd on behalf of European Hematology Association. This is an open access Abstract Book distributed under the Attribution-NonCommercial-NoDerivs (CC BY-NC-ND), which allows third parties to download the articles and share them with others as long as they credit the author and the Abstract Book, but they cannot change the content in any way or use them commercially.
**Abstract Book Citations:** Authors, Title, HemaSphere, 2026;10:(S1):pages. The abstract book DOIs can be found on the “Author Information” page.
**Disclaimer:** Articles published in the journal HemaSphere exclusively reflect the opinions of the authors. The authors are responsible for all content in their abstracts including accuracy of the facts, statements, citing resources, etc.

### (PB3577) INFLAMMATION-BASED SCORES AND THEIR DYNAMIC CHANGES AS PROGNOSTIC INDICATORS IN CLASSICAL HODGKIN LYMPHOMA

**Topic:** 17. Hodgkin lymphoma - Clinical

Hakki Onur Kirkizlar*[1], Tugcan Alp Kirkizlar[1], Guray Aygun[1], Ahmet Balci[1], Ahmet Muzaffer Demir[1]

[1]*Trakya University, Hematology, Edirne, Türkiye;*

### Background

Classical Hodgkin lymphoma (cHL) is characterized by an inflammatory tumor microenvironment. Although inflammation-based biomarkers such as the Glasgow Prognostic Score (GPS), modified GPS (mGPS), and C-reactive protein/albumin ratio (CAR) have been studied in various malignancies, their dynamic changes and prognostic value in cHL remain unclear.

### Aims

This study aimed to clarify the potential prognostic and response related value significance of baseline and interim inflammation-based parameters in cHL patients treated with ABVD chemotherapy.

### Methods

In this retrospective single-center cohort study, 112 newly diagnosed cHL patients treated with ABVD between 2012 and 2025 were analyzed. Laboratory parameters and CAR, GPS, and mGPS were assessed at diagnosis and interim evaluation. Overall survival (OS) and progression-free survival (PFS) were analyzed using Kaplan–Meier and Cox regression models.

### Results

The median age at diagnosis was 34.5y and 59.8% of patients were male. According to the Ann Arbor stage, 55.4% of pts had early-stage disease (stage I–II). Interim assessment showed complete response in 67.8% of patients (Table 1).
Interim assessment revealed significant reductions in LDH, CRP and CAR levels, accompanied by a significant increase in serum albumin ($p<0.001$). GPS and mGPS scores also decreased significantly over time ($p<0.001$, Table 2). The mean interim CAR was 0.26 ± 0.61, with a mean ΔCAR of −1.29 ± 2.14. Advanced-stage disease was associated with higher baseline inflammatory scores ($p<0.001$, Table 3). When stratified according to interim complete remission (CR) status, both GPS and mGPS declined significantly in the CR and non-CR groups ($p<0.001$); however, the extent of change did not differ significantly between groups (0.400, 0.500, respectively).
Median time to progression was 54months, with a median follow-up of 68.5 months. The mortality rate was 7.1%. The estimated mean OS for the entire cohort was 151.43 months (95% CI, 143.18–159.68). Stratified by baseline GPS, mean OS was 145.1 months for GPS 0, 157.5 months for GPS 1, and 136.8 months for GPS 2 (*p*: 0.184), indicating no statistically significant differences between groups. Similarly, for mGPS, mean OS was 146.1 months for mGPS 0, 157.3 months for mGPS 1, and 136.8 months for mGPS 2 (*p*: 0.196), showing no significant differences between the groups.
Baseline GPS and mGPS were not significantly associated with OS or PFS. In multivariate analysis, older age, shorter PFS, and higher CAR independently predicted worse OS.

**Copyright Information:** (Online) ISSN: 2572-9241
©2026 The Author(s). HemaSphere is published by John Wiley & Sons Ltd on behalf of European Hematology Association. This is an open access Abstract Book distributed under the Attribution-NonCommercial-NoDerivs (CC BY-NC-ND), which allows third parties to download the articles and share them with others as long as they credit the author and the Abstract Book, but they cannot change the content in any way or use them commercially.
**Abstract Book Citations:** Authors, Title, HemaSphere, 2026;10:(S1):pages. The abstract book DOIs can be found on the "Author Information" page.
**Disclaimer:** Articles published in the journal HemaSphere exclusively reflect the opinions of the authors. The authors are responsible for all content in their abstracts including accuracy of the facts, statements, citing resources, etc.

### Summary/Conclusion

Inflammation-based markers reflect disease burden in cHL and significantly improve during treatment. While GPS and mGPS did not independently predict survival in this cohort, CAR emerged as an independent prognostic factor for OS. These findings suggest that CAR may provide additional prognostic information beyond conventional clinical variables in patients with cHL.

**Table 1. Clinical characteristics and treatment outcomes of the study population**

| Parameters | Values |
|---|---|
| Age (years) | Median: 34,5 (18-72) |
| Sex (Female/Male) (%) | 40,2 / 59,8 |
| Histological Subtype (%) | NS: 42/ LR: 23,2/ LP: 2,7/ MC: 25-<br>Undetermined: 7,1 |
| Ann-Arbor stage (%) | I-II: 55,4 / III-IV: 44,6 |
| CAR @ diagnosis | Mean: 1,56 (± 2,21) |
| GPS @ diagnosis (%) | 0: 36,6/ 1: 47,3/ 2: 16,1 |
| mGPS @ diagnosis (%) | 0: 37,5/ 1: 46,4/ 2: 16,1 |
| Interim assessment (%) | CR: 67,8/ PR: 22,3/ SD: 7,2/ PD: 2,7 |
| CAR @ interim | Mean: 0,26 (± 0,61) |
| GPS @ interim (%) | 0: 76,8/ 1: 21,4/ 2: 1,8 |
| mGPS @ interim (%) | 0: 77,7/ 1: 20,5/ 2: 1,8 |
| ΔCAR | Mean -1,29 (± 2,14) |
| ΔCAR (%) | Mean: -23,84 (±144,18) |
| Follow-up (months) | Median: 68,5 (min.-max.: 6-164) |

**Table 2. Changes in laboratory and inflammatory parameters between diagnosis and interim assessment (n = 112)**

| Variable | Diagnosis (Mean ± SD / Median) | Interim (Mean ± SD / Median) | p-value |
|---|---|---|---|
| **CAR** | 1.56 ± 2.21 / 0.62 | 0.26 ± 0.61 / 0.08 | <0.001* |
| **GPS** | 0.79 ± 0.57 / 1.00 | 0.25 ± 0.45 / 0.00 | <0.001$^{\alpha}$ |
| **mGPS** | 0.79 ± 0.57 / 1.00 | 0.24 ± 0.44 / 0.00 | <0.001$^{\alpha}$ |

*: Wilcoxon signed-rank, $^{\alpha}$: Marginal Homogeneity

**Table 3. GPS, mGPS and CAR according to Ann-Arbor staging**

| Variable | Ann Arbor stage I-II (n: 62) | Ann Arbor stage III-IV (n: 50) | p-value |
|---|---|---|---|
| **GPS** | 0: 54,8%<br>1: 35,5%<br>2: 9,7% | 0: 14%<br>1: 62%<br>2: 24% | <0,001* |
| **mGPS** | 0: 56,5%<br>1: 33,9%<br>2: 9,7% | 0: 14%<br>1: 62%<br>2: 24% | <0,001* |
| **CAR** | Mean rank: 42,63<br>Median: 0,140 | Mean rank: 73,70<br>Median: 1,860 | <0,001 $^{\alpha}$ |

*: $X^2$ test, ; $^{\alpha}$: Mann-Whitney U test

Table 1 and 2 and 3

Copyright Information: (Online) ISSN: 2572-9241
©2026 The Author(s). HemaSphere is published by John Wiley & Sons Ltd on behalf of European Hematology Association. This is an open access Abstract Book distributed under the Attribution-NonCommercial-NoDerivs (CC BY-NC-ND), which allows third parties to download the articles and share them with others as long as they credit the author and the Abstract Book, but they cannot change the content in any way or use them commercially.
Abstract Book Citations: Authors, Title, HemaSphere, 2026;10:(S1):pages. The abstract book DOIs can be found on the “Author Information” page.
Disclaimer: Articles published in the journal HemaSphere exclusively reflect the opinions of the authors. The authors are responsible for all content in their abstracts including accuracy of the facts, statements, citing resources, etc.

### (PB3578) INDIVIDUALIZED MANAGEMENT OF RELAPSED OR REFRACTORY CLASSICAL HODGKIN LYMPHOMA IN A RESOURCE-LIMITED SETTING: A SINGLE-CENTER UKRAINIAN EXPERIENCE

**Topic:** 17. Hodgkin lymphoma - Clinical

Anisiia Luchkiv*[1], Oleksandr Istomin[1], Nataliia Bindiuzhenko[1], Hanna Brudna[1], Andrii Budzyn[1], Olha Karpenko[1], Oleksandr Lysytsia[1]

[1]*National Specialized Children's Hospital "Ohmatdyt", Kyiv, Ukraine;*

### Background

The Ukrainian national cancer registry reported 661 newly diagnosed cases of Hodgkin lymphoma and 139 deaths in 2023 reflecting a persistent mortality burden despite the high curability of this disease. Outcomes in relapsed or refractory classical Hodgkin lymphoma (r/r cHL) strongly depend on effective salvage therapy and timely transplant consolidation. In resource-limited healthcare systems, restricted access to novel agents may result in unfavorable results.In Ukraine, state-funded salvage therapy options are limited to platinum- or gemcitabine-containing regimens.

### Aims

To evaluate the outcomes of patients with r/r cHL managed using a risk-adapted salvage and transplant strategy at our center.

### Methods

We performed a retrospective single-center analysis of all patients with r/r classical Hodgkin lymphoma who underwent hematopoietic stem cell transplantation (HSCT) at National Specialized Children's Hospital «Ohmatdyt» between December 2023 and December 2025. At our center, we recognized the urgent need for innovative treatment approaches for this group of patients, given the lack of reimbursement for the novel drugs. Thanks to dedicated efforts in reallocating internal resources and the support of charitable organizations, we were able to offer salvage regimens, including brentuximab vedotin (BV) and PD-1 inhibitors. A risk-adapted approach was used, with chemotherapy-based salvage reserved for late relapse and targeted or immune-based therapy prioritized for higher-risk disease. From 2025, following updated treatment recommendations, PD-1 inhibitor-based regimens became the preferred salvage strategy in patients with primary refractory disease and early relapse. Treatment response was assessed by PET/CT. Overall response rate (ORR), complete metabolic response (CMR), and preliminary survival outcomes were evaluated.

**Copyright Information:** (Online) ISSN: 2572-9241
©2026 The Author(s). HemaSphere is published by John Wiley & Sons Ltd on behalf of European Hematology Association. This is an open access Abstract Book distributed under the Attribution-NonCommercial-NoDerivs (CC BY-NC-ND), which allows third parties to download the articles and share them with others as long as they credit the author and the Abstract Book, but they cannot change the content in any way or use them commercially.
**Abstract Book Citations:** Authors, Title, HemaSphere, 2026;10:(S1):pages. The abstract book DOIs can be found on the "Author Information" page.
**Disclaimer:** Articles published in the journal HemaSphere exclusively reflect the opinions of the authors. The authors are responsible for all content in their abstracts including accuracy of the facts, statements, citing resources, etc.

### Results

33 patients underwent 34 HSCT procedures, including three allogeneic transplants. One patient received allogeneic HSCT following relapse after prior autologous transplantation. Baseline characteristics are presented in Table 1. Following salvage therapy, ORR was 100%, with CMR achieved in 94% of patients. Among the cohort, 53% responded to BV–containing regimens, 38% to PD-1 inhibitor–based therapy, and 9% to chemotherapy-alone salvage. All patients successfully proceeded to HSCT consolidation. HSCT was allowed in patients with partial metabolic response only after prior PD-1 inhibitor exposure. Survival data are preliminary due to the median follow-up of 12 months; however, Kaplan–Meier estimated 12-month overall survival (OS) and event-free survival (EFS) were 94% and 84%, respectively (fig. 1). The overall mortality rate was 6% (n=2). Observed toxicities were consistent with expected transplant-related complications.

| **Patients, n** | **33** |
|---|---|
| Male, % | 45 |
| Median age at diagnosis, (range) | 26 (12-51) |
| B-symptoms, % | 81 |
| Advanced stage, % | 85 |
| Refractory, % | 48 |
| Previous radiotherapy, % | 39 |
| Median number of salvage lines, (range) | 2 (1-6) |

### Summary/Conclusion

These results demonstrate the effectiveness of a risk-adapted salvage and transplant approach for r/r cHL patients, even in a limited-resource setting. This experience may be particularly relevant for other healthcare systems facing similar resource constraints.

**Copyright Information:** (Online) ISSN: 2572-9241
©2026 The Author(s). HemaSphere is published by John Wiley & Sons Ltd on behalf of European Hematology Association. This is an open access Abstract Book distributed under the Attribution-NonCommercial-NoDerivs (CC BY-NC-ND), which allows third parties to download the articles and share them with others as long as they credit the author and the Abstract Book, but they cannot change the content in any way or use them commercially.
**Abstract Book Citations:** Authors, Title, HemaSphere, 2026;10:(S1):pages. The abstract book DOIs can be found on the "Author Information" page.
**Disclaimer:** Articles published in the journal HemaSphere exclusively reflect the opinions of the authors. The authors are responsible for all content in their abstracts including accuracy of the facts, statements, citing resources, etc.

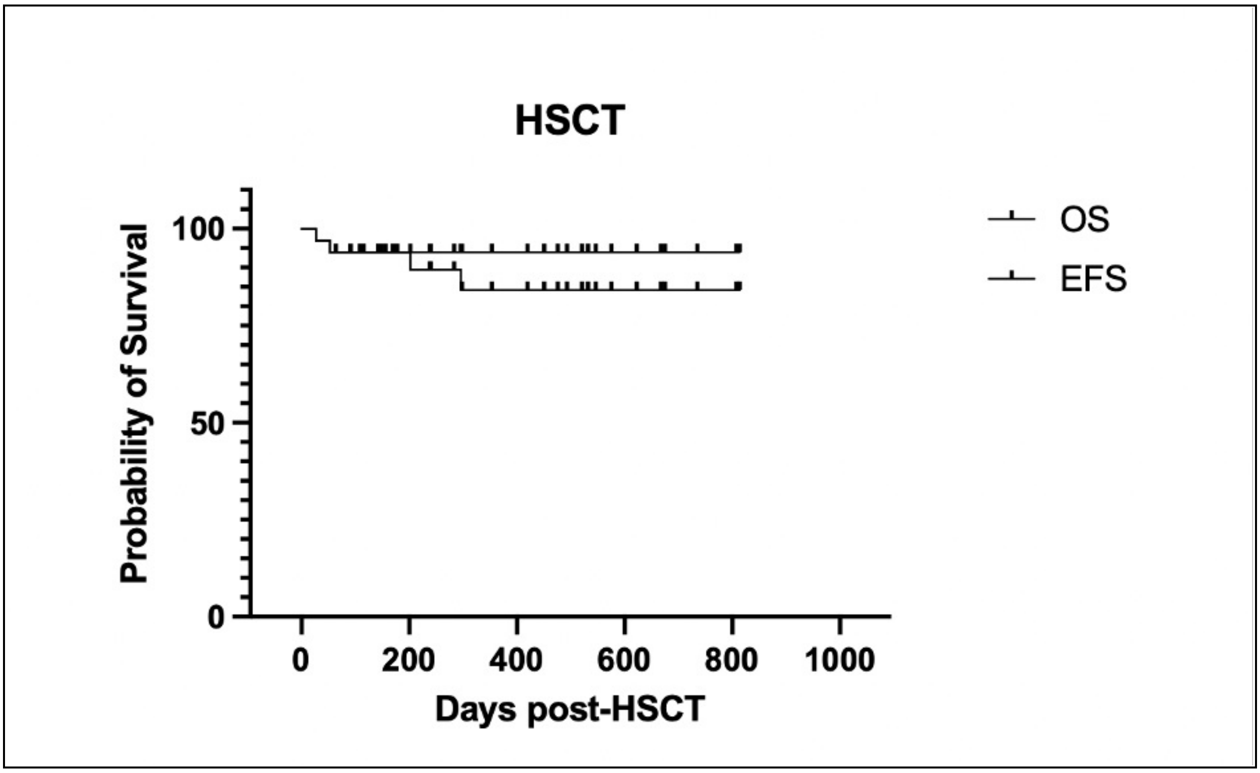

Figure 1

Copyright Information: (Online) ISSN: 2572-9241
©2026 The Author(s). HemaSphere is published by John Wiley & Sons Ltd on behalf of European Hematology Association. This is an open access Abstract Book distributed under the Attribution-NonCommercial-NoDerivs (CC BY-NC-ND), which allows third parties to download the articles and share them with others as long as they credit the author and the Abstract Book, but they cannot change the content in any way or use them commercially.
Abstract Book Citations: Authors, Title, HemaSphere, 2026;10:(S1):pages. The abstract book DOIs can be found on the "Author Information" page.
Disclaimer: Articles published in the journal HemaSphere exclusively reflect the opinions of the authors. The authors are responsible for all content in their abstracts including accuracy of the facts, statements, citing resources, etc.

### (PB3579) INTERIM AND END-OF-TREATMENT (EOT) PET/CT ASSESSMENT AFTER NIVOLUMAB, DOXORUBICIN, VINBLASTINE, AND DACARBAZINE (N-AVD) IN ADVANCED-STAGE CLASSICAL HODGKIN LYMPHOMA (CHL): A SINGLE-CENTER EXPERIENCE

**Topic:** 17. Hodgkin lymphoma - Clinical

Alexia Piperidou*[1], Athanasios Liaskas[2], Anastasia Kopsaftopoulou[2], Maria Dimitrakoudi[2], Iliana Konstantinou[2], Marina Belia[2], Maria Arapaki[2], Rodanthi Fioretzaki[2], Aikaterini Kolotsiou[2], Alexandros MacHairas[2], Georgia Poultsaki[2], Chrysovalantou Chatzidimitriou[2], Ioannis Asimakopoulos[2], Angeliki Georgopoulou[2], Calliope Zerzi[2], Panagiotis Tsaftaridis[2], Fotios Panitsas[2], Aikaterini Benekou[2], Panagiota Petsa[2], Helen Plata[2], Aikaterini Bitsani[2], Maria Dimou[2], Marina Siakantaris[2], Maria Angelopoulou[2], Theodoros Vassilakopoulos[2]

*[1]National and Kapodistrian University of Athens, Athens, Greece; [2]National and Kapodistrian University of Athens, Department of Hematology and Bone Marrow Transplantation, Athens, Greece;*

### Background

Based on the recent randomized SWOG S1826 trial, the N-AVD regimen is superior to BV-AVD in patients with stage III/IV cHL, with impressive results in terms of disease control, albeit with relatively short follow-up. We adopted N-AVD as first-line therapy for all patients with stage III/IV cHL starting in July 2023. PET/CT has been a cornerstone for response assessment in cHL -both for interim and EoT evaluation- with outstanding prognostic value after conventional chemotherapy regimens. However, limitations may exist due to false positives (FP) with evolving treatments and novel agents such as checkpoint inhibitors.

### Aims

To present our institutional experience with PET/CT assessment after N-AVD.

### Methods

We conducted a retrospective analysis of patients with cHL who received first-line treatment with N-AVD at our center. Interim PET (iPET) after 2 cycles and EoT-PET after N-AVD were assessed according to the Deauville Score (DS) and were considered positive in case of DS4-5.

**Copyright Information:** (Online) ISSN: 2572-9241
©2026 The Author(s). HemaSphere is published by John Wiley & Sons Ltd on behalf of European Hematology Association. This is an open access Abstract Book distributed under the Attribution-NonCommercial-NoDerivs (CC BY-NC-ND), which allows third parties to download the articles and share them with others as long as they credit the author and the Abstract Book, but they cannot change the content in any way or use them commercially.
**Abstract Book Citations:** Authors, Title, HemaSphere, 2026;10:(S1):pages. The abstract book DOIs can be found on the "Author Information" page.
**Disclaimer:** Articles published in the journal HemaSphere exclusively reflect the opinions of the authors. The authors are responsible for all content in their abstracts including accuracy of the facts, statements, citing resources, etc.

**Results**

A total of 40 patients had completed N-AVD up to the time of the present analysis. The median age was 29 years (range 16-79), with 13% aged ≥60 years; 51% were males,44% had B symptoms and 63% had the nodular sclerosing subtype. Overall, 64% had stage IV based on PET/CT. *iPET-positive patients:* Only 4/40 patients (10%) had a positive iPET, all with DS4: in 3/4 patients the EoT PET/CT was negative indicating potential FP results in the interim assessment, whereas only one patient experienced disease progression confirmed by subsequent PET/CT (DS5) having received BrECADD after N-AVDx3. *iPET-negative patients:* Despite a negative iPET in 36 patients, 7/36 (19%) had positive PET/CT (5 with DS4, 2 with DS5): in 3/5 patients with DS4 serial PET/CT evaluation (3mo post-EoT) was negative, while only 2/5 patients with DS4 and 2/2 with DS5 experienced actual treatment failure at EoT confirmed by subsequent PET/CT analysis and/or biopsy. Among these 4 patients, 2 proceeded to salvage therapy with BrESHAP and autoSCT due to systemic disease and one (with EoT PET/CT DS5) underwent radiotherapy due to localized disease at the initially involved sites at mediastinum. Further histological investigation of the second patient with EoT PET/CT DS5 revealed the presence of a metachronous lymphoid malignancy (DLBCL) necessitating chemotherapy initiation. An additional patient experienced early relapse (<12 months post EoT) despite both negative iPET and EoT-PET. *Total patient population:*Overall 6/40 patients have experienced treatment failure so far (15%). With a median follow-up of 20.7 months, the 2-year progression-free survival (PFS) rate was 82%.

**Summary/Conclusion**

In our cohort, N-AVD demonstrated high efficacy, with the vast majority of patients achieving complete metabolic response and only 6 treatment failures observed to date, including 1 metachronous DLBCL. The rate of iPET positivity was low (10%) with no DS5 cases. Interestingly 5/6 treatment failures occurred in patients with a negative iPET. However, interpretation of PET/CT findings in the context of N-AVD requires particular caution due to FP and potential overestimation of residual disease activity, as 3/6 EoT-positive but iPET-negative patients had FP EoT results. Therefore, PET/CT results should be evaluated within the broader clinical context, and isolated metabolic findings should not automatically prompt treatment modification without careful consideration.

**Copyright Information:** (Online) ISSN: 2572-9241
©2026 The Author(s). HemaSphere is published by John Wiley & Sons Ltd on behalf of European Hematology Association. This is an open access Abstract Book distributed under the Attribution-NonCommercial-NoDerivs (CC BY-NC-ND), which allows third parties to download the articles and share them with others as long as they credit the author and the Abstract Book, but they cannot change the content in any way or use them commercially.
**Abstract Book Citations:** Authors, Title, HemaSphere, 2026;10:(S1):pages. The abstract book DOIs can be found on the "Author Information" page.
**Disclaimer:** Articles published in the journal HemaSphere exclusively reflect the opinions of the authors. The authors are responsible for all content in their abstracts including accuracy of the facts, statements, citing resources, etc.

### (PB3581) DIMINISHED PROGNOSTIC VALUE OF INTERIM PET IN HODGKIN LYMPHOMA TREATED WITH BRENTUXIMAB VEDOTIN PLUS AVD: A RETROSPECTIVE ANALYSIS

**Topic:** 17. Hodgkin lymphoma - Clinical

Kareem Latif*[1], Isaiah Courtad[2], Avery Stuart[2], Faisal Ansari[1], Ravand Samaeekia[3], Mojtaba Akhtari[4]

*[1]Loma Linda University Medical Center, Internal Medicine, Loma Linda, United States of America;[2]Loma Linda University Medical School, Loma Linda, United States of America; [3]University of California, Irvine, Hematology/Oncology, Irvine, United States of America; [4]Loma Linda University Medical Center, Hematology/Oncology, Loma Linda, United States of America;*

### Background

Interim PET after two cycles of chemotherapy (PET2) has historically guided risk-adapted treatment in Hodgkin lymphoma (HL). However, the relevance of PET2 in the context of frontline brentuximab vedotin plus doxorubicin, vinblastine and dacarbazine (AVD) [BV+AVD] remains uncertain, as recent trials suggest improved outcomes may override early metabolic prognostication.

### Aims

To evaluate whether PET2 retains prognostic or clinical utility in patients receiving BV+AVD, particularly in predicting end-of-treatment (EOT) remission.

### Methods

We retrospectively reviewed 55 patients with newly diagnosed classical HL treated with frontline BV+AVD at our institution. All patients underwent interim PET2 (after 2 cycles) and EOT PET imaging. PET2-negative was defined as Deauville 1–3; PET2-positive as 4–5. The primary endpoint was the diagnostic performance of PET2 for prediction of EOT PET positivity.

### Results

Of 55 patients (median age 22 years [range 11-77]), 51 (92.7%) were PET2-negative and 4 (7.3%) were PET2-positive. EOT PET was negative in 48 (87.3%) and positive in 7 (12.7%). Among the 7 patients with EOT PET-positive disease, 3 were PET2-positive and 4 were PET2-negative. Among the 48 patients with EOT PET-negative disease, 47 were PET2-negative and 1 was PET2-positive. PET2 demonstrated a sensitivity of 42.9% (95% CI, 15.8%–75.0%), specificity of 97.9% (95% CI, 89.1%–99.6%), positive predictive value of 75.0% (95% CI, 30.1%–95.4%), and negative predictive value of 92.2% (95% CI, 81.5%–96.9%) for prediction of EOT PET positivity. The positive likelihood ratio was 20.57 (95% CI, 2.47–171.34), and the negative likelihood ratio was 0.584 (95% CI, 0.307–1.110).

### Summary/Conclusion

In patients with HL treated with BV+AVD, the majority of patients were PET2 negative, and PET2 appears to have limited clinical utility. A negative PET2 provides little additional reassurance beyond the low baseline risk of EOT PET positivity and may simply reflect the effectiveness of BV+AVD rather than meaningfully identify who is truly safe from an unfavorable EOT PET. Taken together, these findings call into question the value of routine interim PET for guiding treatment decisions in the BV+AVD era.

**Copyright Information:** (Online) ISSN: 2572-9241
©2026 The Author(s). HemaSphere is published by John Wiley & Sons Ltd on behalf of European Hematology Association. This is an open access Abstract Book distributed under the Attribution-NonCommercial-NoDerivs (CC BY-NC-ND), which allows third parties to download the articles and share them with others as long as they credit the author and the Abstract Book, but they cannot change the content in any way or use them commercially.
**Abstract Book Citations:** Authors, Title, HemaSphere, 2026;10:(S1):pages. The abstract book DOIs can be found on the "Author Information" page.
**Disclaimer:** Articles published in the journal HemaSphere exclusively reflect the opinions of the authors. The authors are responsible for all content in their abstracts including accuracy of the facts, statements, citing resources, etc.

### (PB3582) PET GUIDED TREATMENT FOR NEWLY DIAGNOSED ADVANCED HODGKIN LYMPHOMA

**Topic:** 17. Hodgkin lymphoma - Clinical

Fatima Lamraoui*[1], Nabila Rekab[1], Khadoudj Mounira Benlabiod[1], Hamida Brahimi[1], Zineb Ferdi[2], Souad Taoussi[1], Salima Oukid[1], Yamina Bouchakor Moussa[1], Mohamed Mezroud[2], Amina Rebouh[1], Amed Amine Ziani[1], Guezlane Sherifa[1], Bradai Mohamed[1]

*[1]Université Saad Dahlab de Blida, Medecine, Ouled Yaich, Algeria; [2]Hôpital Frantz Fanon, Hematology, Blida, Algeria;*

### Background

Over the last decades, the management of advanced-stage Hodgkin Lymphoma (HL) has considerably evolved. The efficacy of both ABVD and escalated BEACOPP regimens has been well demonstrated; however, this comes at the cost of significantly increased short-term and long-term toxic effects.
With the aim of reducing toxicity without compromising efficacy, the AHL 2011 study demonstrated that a positron emission tomography (PET) driven de-escalation strategy after two cycles of BEACOPP provides similar PFS and OS while minimizing early toxicity compared with a standard treatment

### Aims

Our aim is to assess the efficacy of a PET guided strategy after two cycles of esc BEACOPP in patients with advanced HL.

### Methods

This retrospective analysis includes patients with advanced HL (stage III, IV or IIB with mediastinum/thorax ratio>0.33 or extranodal involvement) who received a PET driven strategy after 2 BEACOP delivering 4 ABVD in PET 2 negative and 4 BEACOPP in PET2 positive patients.

### Results

Seventy one patients were enrolled between January 2017 and December 2024, with a median age of 27 (18-55) years, 59% were female, 53 (74.6%) had B symptoms, 26 (36.6%) had stage I or IIB with risk factors, 14 (19.7%) stage III and 31 (43.6%) stage IV, 48 (67.6%) had a bulk disease and 35 (49.2%) had an IPS of 3 or greater. All of patients had evaluable PET2 scans, 58 (81.6%) of them had a negative PET2 scan: 40 assigned to receive 4 cycles of ABVD and 18 received 2 cycles of ABVD + radiotherapy, while 13 (18.3%) had a positive PET 2 scan, 9 of them received 2 additional BEACOPP, the PET4 scan was negative in 5 patients; consolidative radiotherapy was administrated in six patients. A salvage therapy was indicated in 5 patients for primary refractory disease.
At the time of the analysis (01/02/2026), median follow up was 37 months (14 – 102), 4 patients relapsed and we regret two deaths, one due to disease progression and the other following a hypereosinophilic syndrome, no treatment related deaths were observed. 5 year progression free survival (PFS) was 82.6% and overall survival (OS) 96.3%; there was no significant difference in OS according to Bulky disease, IPS score or PET2 status; however the difference in PFS was significant according to PET2 status (87.8% vs 59.3% p 0.001).

**Copyright Information:** (Online) ISSN: 2572-9241
©2026 The Author(s). HemaSphere is published by John Wiley & Sons Ltd on behalf of European Hematology Association. This is an open access Abstract Book distributed under the Attribution-NonCommercial-NoDerivs (CC BY-NC-ND), which allows third parties to download the articles and share them with others as long as they credit the author and the Abstract Book, but they cannot change the content in any way or use them commercially.
**Abstract Book Citations:** Authors, Title, HemaSphere, 2026;10:(S1):pages. The abstract book DOIs can be found on the “Author Information” page.
**Disclaimer:** Articles published in the journal HemaSphere exclusively reflect the opinions of the authors. The authors are responsible for all content in their abstracts including accuracy of the facts, statements, citing resources, etc.

**Summary/Conclusion**

PET-guided strategies have transformed the management of advanced HL by enabling early adjustment of treatment intensity based on metabolic response, thereby allowing for a more personalised approach. They offer the possibility of reducing toxicity in good responders while optimizing disease control in high risk patients. Nevertheless, certains limitations remain, notably the risk of relapse after de-escalation.

**Copyright Information:** (Online) ISSN: 2572-9241
©2026 The Author(s). HemaSphere is published by John Wiley & Sons Ltd on behalf of European Hematology Association. This is an open access Abstract Book distributed under the Attribution-NonCommercial-NoDerivs (CC BY-NC-ND), which allows third parties to download the articles and share them with others as long as they credit the author and the Abstract Book, but they cannot change the content in any way or use them commercially.
**Abstract Book Citations:** Authors, Title, HemaSphere, 2026;10:(S1):pages. The abstract book DOIs can be found on the "Author Information" page.
**Disclaimer:** Articles published in the journal HemaSphere exclusively reflect the opinions of the authors. The authors are responsible for all content in their abstracts including accuracy of the facts, statements, citing resources, etc.

### (PB3583) A REAL-WORLD ANALYSIS OF FIRST LINE NIVOLUMAB COMBINED WITH DOXORUBICIN, VINBLASTINE, AND DACARBAZINE (N-AVD) IN CLASSICAL HODGKIN LYMPHOMA (CHL) – A SINGLE CENTER COHORT STUDY

**Topic:** 17. Hodgkin lymphoma - Clinical

Liat Shargian*[1, 2], Gabriel Zollmann[1], Meirav Kedmi[1], Elena Ribakovsky[1], Ronit Marcus[1], Maya Zlotnick[1], Irina Amitai[1], Ohad Benjamini[1], Yafit Segman[1], Natalia Orlova[1], Abraham Avigdor[1, 2]

*[1]Division of Hematology and Bone Marrow Transplantation ,Chaim Sheba Medical Center, Division of Hematology and Bone Marrow Transplantation, Ramat Gan, Israel; [2]Gray Faculty of Medical and Health Sciences, Tel-Aviv University, Tel-Aviv University, Tel-Aviv, Israel;*

### Background

Anti-PD-1 therapies are safe and effective in the setting of R/R cHL. The phase 3 SWOG S1826 trial, comparing first-line N-AVD with Brentuximab-AVD, demonstrated superior outcomes with 3-year PFS of 91% vs. 82%. However, data on adverse events and efficacy outside of a clinical trial remaine limited. Hence, we sought to evaluate safety profile and efficacy outcomes of patients (pts) treated with N-AVD in a real-world setting.

### Methods

This retrospective single-center study included cHL pts treated with ≥1 cycle of N-AVD. Baseline characteristics were descriptively summarized; response was assessed per Lugano criteria. PFS and OS were estimated using Kaplan–Meier methods with log-rank testing.

**Copyright Information:** (Online) ISSN: 2572-9241
©2026 The Author(s). HemaSphere is published by John Wiley & Sons Ltd on behalf of European Hematology Association. This is an open access Abstract Book distributed under the Attribution-NonCommercial-NoDerivs (CC BY-NC-ND), which allows third parties to download the articles and share them with others as long as they credit the author and the Abstract Book, but they cannot change the content in any way or use them commercially.
**Abstract Book Citations:** Authors, Title, HemaSphere, 2026;10:(S1):pages. The abstract book DOIs can be found on the “Author Information” page.
**Disclaimer:** Articles published in the journal HemaSphere exclusively reflect the opinions of the authors. The authors are responsible for all content in their abstracts including accuracy of the facts, statements, citing resources, etc.

**Results**

Between 10.2023 and 2.2026, 34 newly diagnosed cHL pts received N-AVD; 94% (32/34) had de novo disease and two were previously treated for follicular lymphoma. Median age was 37 years (18–76), 56% were female, 82% had stage III–IV disease and 18% stage IIB. Median IPS was 3 (0–4), 26% had bulky disease (>7.5 cm). EBV status (EBER ISH) was available in 25 patients, with 16% positive. No autoimmune history was noted, except for one patient with celiac disease.
All pts completed six treatment cycles. Treatment was modified in 12% (4/34): one patient switched from N-AVD to N-Gemzar-Vinblastine due to prior anthracycline exposure, one required vinblastine dose reduction for neuropathy, and nivolumab was modified in two pts due to immune-related adverse events (irAEs). One patient developed grade 2 hepatitis and pancreatitis after the first nivolumab dose, resolving with steroids after omission of a single dose. The second patient with pre-existing celiac disease developed colitis, treated with steroids and discontinuation of nivolumab at cycle 4b, with resolution. Any AEs and grade ≥3 AEs occurred in 94% and 67% of pts, respectively. Neutropenia occurred in 94% (67% ≥G3), with G-CSF given to 88%. Infections affected 25% (10% ≥G3), neuropathy 17% (3% ≥G3), and hospitalization occurred in 20% (9% febrile neutropenia), including one ICU admission. IrAEs occurred in 59% (9% ≥G3), commonly rash/pruritus (26%), hepatotoxicity (12% G1/2, 3% ≥G3), arthralgia/arthritis (12%/3%), hypothyroidism (9%), and colitis (6% total). Steroids were used in 21% and levothyroxine in 6%; all irAEs resolved.
All pts had an interim PET-CT after 2 cycles, with OR and CR of 100% and 94%, respectively. At end of treatment, OR and CR were 97% and 94%. After a median follow-up of 14 months (8–28), median PFS was not reached; estimated 1- and 2-year PFS were 94% (95% CI: 76–99%) and 89% (95% CI: 61–97%). Four pts required second-line therapy: one with residual FDG uptake at EOT PET/CT received radiation; among three with progression/relapse, one elderly patient received radiation only, and two (relapsing at 6 and 12 months) were salvaged with BV-ICE followed by auto-HCT. All pts remain alive in CR (CR1, n=30; CR2, n=4). All progressions occurred in stage IV (3/24) vs. none in stage IIB unfavorable/III (0/10). The small sample precluded statistical analysis or Cox regression.

**Summary/Conclusion**

The data from this single center cohort of pts show that survival outcomes of pts with advanced stage cHL treated with N-AVD in real world setting are comparable with the outcomes of pts treated in the pivotal SWOG S1826 trial. Most AEs were grade 1-2 and their incidence were similar to those reported in the SWOG trial. Our data supports the use of N-AVD for first line advanced stage cHL.

**Copyright Information:** (Online) ISSN: 2572-9241
©2026 The Author(s). HemaSphere is published by John Wiley & Sons Ltd on behalf of European Hematology Association. This is an open access Abstract Book distributed under the Attribution-NonCommercial-NoDerivs (CC BY-NC-ND), which allows third parties to download the articles and share them with others as long as they credit the author and the Abstract Book, but they cannot change the content in any way or use them commercially.
**Abstract Book Citations:** Authors, Title, HemaSphere, 2026;10:(S1):pages. The abstract book DOIs can be found on the “Author Information” page.
**Disclaimer:** Articles published in the journal HemaSphere exclusively reflect the opinions of the authors. The authors are responsible for all content in their abstracts including accuracy of the facts, statements, citing resources, etc.

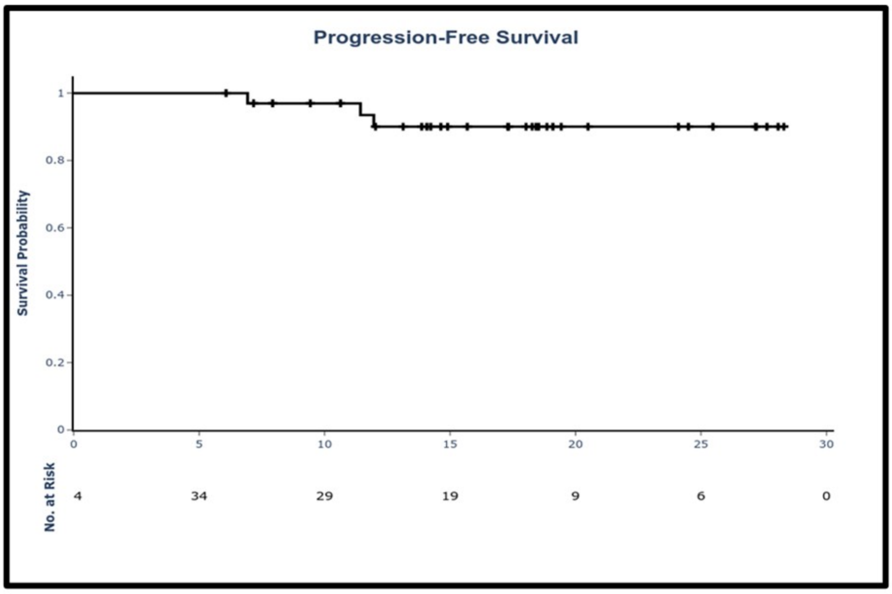

Kaplan–Meier curve: Progression-free survival of patients treated with first line N-AVD protocol

**Copyright Information:** (Online) ISSN: 2572-9241
©2026 The Author(s). HemaSphere is published by John Wiley & Sons Ltd on behalf of European Hematology Association. This is an open access Abstract Book distributed under the Attribution-NonCommercial-NoDerivs (CC BY-NC-ND), which allows third parties to download the articles and share them with others as long as they credit the author and the Abstract Book, but they cannot change the content in any way or use them commercially.
**Abstract Book Citations:** Authors, Title, HemaSphere, 2026;10:(S1):pages. The abstract book DOIs can be found on the "Author Information" page.
**Disclaimer:** Articles published in the journal HemaSphere exclusively reflect the opinions of the authors. The authors are responsible for all content in their abstracts including accuracy of the facts, statements, citing resources, etc.

### (PB3584) BRENTUXIMAB VEDOTIN-BASED THERAPY FOR RELAPSED/REFRACTORY HODGKIN LYMPHOMA IN CHINA: A REAL-WORLD, MULTICENTER ANALYSIS

**Topic:** 17. Hodgkin lymphoma - Clinical

Nan Zhong*[1], Jun Wang[2], Xiaojun Xu[3], Guowei Li[4], Zhiqiang Sun[5], Liang Shao[6], Wei Zhou[7], Xiaoqing Li[8], Huijing Wu[9], Dan Hao[5], Jingye Meng[10], Bo Lu[3], Yongqian Li[11], Peng Chen[12], Hao Zhou[13], Zhi Guo[1]

*[1]Affiliated Nanshan Hospital of Shenzhen University, Shenzhen, China;[2]Hongkong university Shenzhen Hospital, Shenzhen, China; [3]The Seventh Affiliated Hospital, Sun Yat-sen University, Shenzhen, China;[4]Huizhou Municipal Central Hospital, Huizhou, China; [5]Shenzhen Hospital of Southern Medical University, Shenzhen, China;[6]Zhongnan Hospital of Wuhan University, Wuhan, China; [7]Guangzhou First People's Hospital, Guangzhou, China;[8]The First Affiliated Hospital of Shenzhen University, Shenzhen, China; [9]Hubei Cancer Hospital, Wuhan, China; [10]Shenzhen Third People's Hospital, Shenzhen, China; [11]Shantou Central Hospital, Shantou, China; [12]The Fifth Medical Center of PLA General Hospital, Beijing, China;[13]Union Hospital, Tongji Medical College, Huazhong University of Science and Technology, Wuhan, China;*

**Background**

Despite advancements in therapy, managing relapsed/refractory Hodgkin lymphoma (R/R HL) remains challenging. Brentuximab vedotin (BV), an anti-CD30 antibody-drug conjugate, has demonstrated efficacy in R/R HL. This retrospective study aimed to evaluate the effectiveness and safety of BV-based regimens in real-world clinical practice across China.

**Methods**

This study retrospectively enrolled 65 patients with R/R HL who received BV-containing regimens across 13 centers in China between 2021 and 2025. Regimens included BV monotherapy, BV plus chemotherapy, BV plus PD-1 inhibitors, or BV combined with both PD-1 inhibitors and chemotherapy. Treatment continued until completion of 16 cycles of BV, disease progression, or unacceptable toxicity. The primary endpoint was progression-free survival (PFS). Secondary endpoints included tumor response, overall survival (OS), and safety.

**Copyright Information:** (Online) ISSN: 2572-9241
©2026 The Author(s). HemaSphere is published by John Wiley & Sons Ltd on behalf of European Hematology Association. This is an open access Abstract Book distributed under the Attribution-NonCommercial-NoDerivs (CC BY-NC-ND), which allows third parties to download the articles and share them with others as long as they credit the author and the Abstract Book, but they cannot change the content in any way or use them commercially.
**Abstract Book Citations:** Authors, Title, HemaSphere, 2026;10:(S1):pages. The abstract book DOIs can be found on the "Author Information" page.
**Disclaimer:** Articles published in the journal HemaSphere exclusively reflect the opinions of the authors. The authors are responsible for all content in their abstracts including accuracy of the facts, statements, citing resources, etc.

**Results**

The median age of the 65 patients was 36 years (range: 14-75), with 63.1% male. The most common histological subtype was nodular sclerosis (72.3%), 12.3% were EBV-positive, 73.8% had advanced-stage disease, and 56.9% had extranodal involvement. Patients had received a median of 2 prior treatment lines (range: 1-6), including 36.9% who had prior PD-1 inhibitor therapy, 9.2% with prior BV exposure, and 10.8% who underwent autologous stem cell transplantation (ASCT). The median treatment cycles in the current line were 7 (range: 1-17). Based on whether PD-1 inhibitors were combined, patients were further divided into BV ± chemotherapy (Group A, n=18) and BV + PD-1 ± chemotherapy (Group B, n=47). Baseline characteristics were generally well-balanced between the two groups, except for prior PD-1 treatment (11.1% vs. 45.8%, p=0.008) and the number of treatment cycles in the current line (median: 4 vs. 8, p=0.002).
With a median follow-up of 14.8 months (range: 0.3-50.5), 59 evaluable patients achieved an overall objective response rate (ORR) of 83.3% (complete response [CR]: 49.2%; partial response [PR]: 33.9%), with stable disease (SD) in 11.9% and progressive disease (PD) in 5.1%. The median PFS was not reached, and the estimated 2-year PFS rate was 74.1%.
Further analysis showed no significant differences in tumor response between Group A (n=15) and Group B (n=44) (ORR: 80.0% vs. 84.1%; CR: 60% vs. 45.5%, p=0.623). PFS also did not differ significantly between the two groups (p=0.525) and was not influenced by prior PD-1 inhibitor therapy.
Regarding safety, the main adverse events included myelosuppression, immune-mediated dermatitis, peripheral neuropathy, and liver function impairment, which were mostly grade 1–2 and generally well-tolerated.

**Summary/Conclusion**

This study confirms that BV-containing regimens are effective for treating R/R HL, with an estimated 2-year PFS rate of 74.1%. However, due to the relatively short follow-up period, these findings require further validation through longer-term follow-up and additional prospective studies.

**Copyright Information:** (Online) ISSN: 2572-9241
©2026 The Author(s). HemaSphere is published by John Wiley & Sons Ltd on behalf of European Hematology Association. This is an open access Abstract Book distributed under the Attribution-NonCommercial-NoDerivs (CC BY-NC-ND), which allows third parties to download the articles and share them with others as long as they credit the author and the Abstract Book, but they cannot change the content in any way or use them commercially.
**Abstract Book Citations:** Authors, Title, HemaSphere, 2026;10:(S1):pages. The abstract book DOIs can be found on the "Author Information" page.
**Disclaimer:** Articles published in the journal HemaSphere exclusively reflect the opinions of the authors. The authors are responsible for all content in their abstracts including accuracy of the facts, statements, citing resources, etc.

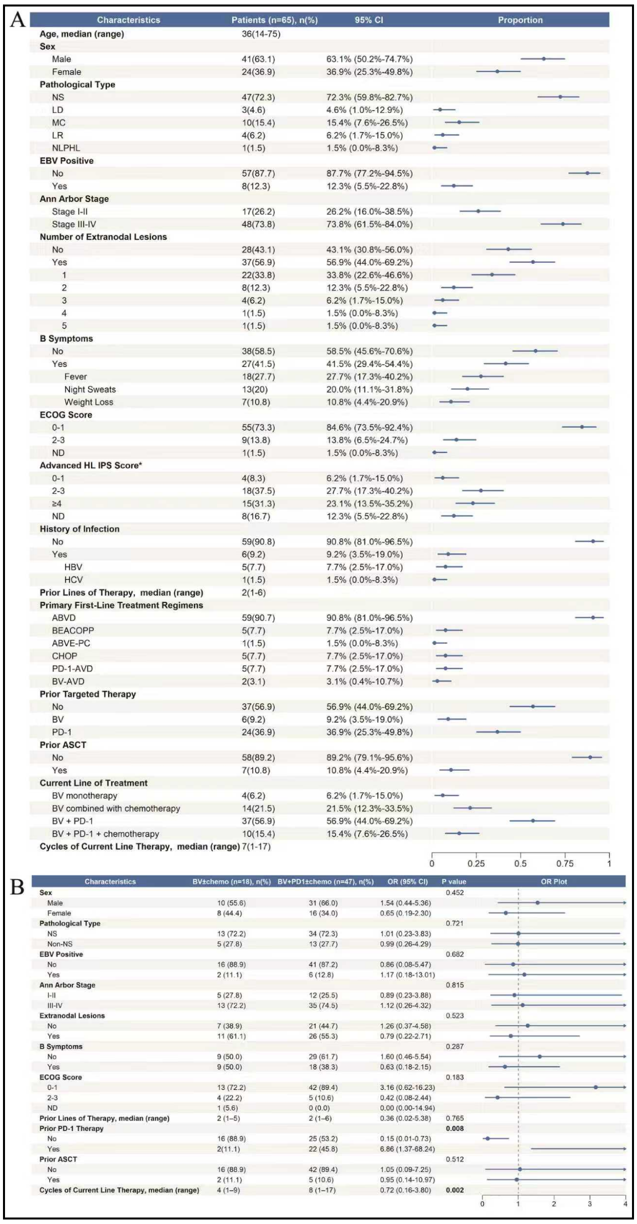

A

| Characteristics | Patients (n=65), n(%) | 95% CI |
|---|---|---|
| **Age, median (range)** | 36(14-75) | |
| **Sex** | | |
| Male | 41(63.1) | 63.1% (50.2%-74.7%) |
| Female | 24(36.9) | 36.9% (25.3%-49.8%) |
| **Pathological Type** | | |
| NS | 47(72.3) | 72.3% (59.8%-82.7%) |
| LD | 3(4.6) | 4.6% (1.0%-12.9%) |
| MC | 10(15.4) | 15.4% (7.6%-26.5%) |
| LR | 4(6.2) | 6.2% (1.7%-15.0%) |
| NLPHL | 1(1.5) | 1.5% (0.0%-8.3%) |
| **EBV Positive** | | |
| No | 57(87.7) | 87.7% (77.2%-94.5%) |
| Yes | 8(12.3) | 12.3% (5.5%-22.8%) |
| **Ann Arbor Stage** | | |
| Stage I-II | 17(26.2) | 26.2% (16.0%-38.5%) |
| Stage III-IV | 48(73.8) | 73.8% (61.5%-84.0%) |
| **Number of Extranodal Lesions** | | |
| No | 28(43.1) | 43.1% (30.8%-56.0%) |
| Yes | 37(56.9) | 56.9% (44.0%-69.2%) |
| 1 | 22(33.8) | 33.8% (22.6%-46.6%) |
| 2 | 8(12.3) | 12.3% (5.5%-22.8%) |
| 3 | 4(6.2) | 6.2% (1.7%-15.0%) |
| 4 | 1(1.5) | 1.5% (0.0%-8.3%) |
| 5 | 1(1.5) | 1.5% (0.0%-8.3%) |
| **B Symptoms** | | |
| No | 38(58.5) | 58.5% (45.6%-70.6%) |
| Yes | 27(41.5) | 41.5% (29.4%-54.4%) |
| Fever | 18(27.7) | 27.7% (17.3%-40.2%) |
| Night Sweats | 13(20) | 20.0% (11.1%-31.8%) |
| Weight Loss | 7(10.8) | 10.8% (4.4%-20.9%) |
| **ECOG Score** | | |
| 0-1 | 55(73.3) | 84.6% (73.5%-92.4%) |
| 2-3 | 9(13.8) | 13.8% (6.5%-24.7%) |
| ND | 1(1.5) | 1.5% (0.0%-8.3%) |
| **Advanced HL IPS Score*** | | |
| 0-1 | 4(8.3) | 6.2% (1.7%-15.0%) |
| 2-3 | 18(37.5) | 27.7% (17.3%-40.2%) |
| ≥4 | 15(31.3) | 23.1% (13.5%-35.2%) |
| ND | 8(16.7) | 12.3% (5.5%-22.8%) |
| **History of Infection** | | |
| No | 59(90.8) | 90.8% (81.0%-96.5%) |
| Yes | 6(9.2) | 9.2% (3.5%-19.0%) |
| HBV | 5(7.7) | 7.7% (2.5%-17.0%) |
| HCV | 1(1.5) | 1.5% (0.0%-8.3%) |
| **Prior Lines of Therapy, median (range)** | 2(1-6) | |
| **Primary First-Line Treatment Regimens** | | |
| ABVD | 59(90.7) | 90.8% (81.0%-96.5%) |
| BEACOPP | 5(7.7) | 7.7% (2.5%-17.0%) |
| ABVE-PC | 1(1.5) | 1.5% (0.0%-8.3%) |
| CHOP | 5(7.7) | 7.7% (2.5%-17.0%) |
| PD-1-AVD | 5(7.7) | 7.7% (2.5%-17.0%) |
| BV-AVD | 2(3.1) | 3.1% (0.4%-10.7%) |
| **Prior Targeted Therapy** | | |
| No | 37(56.9) | 56.9% (44.0%-69.2%) |
| BV | 6(9.2) | 9.2% (3.5%-19.0%) |
| PD-1 | 24(36.9) | 36.9% (25.3%-49.8%) |
| **Prior ASCT** | | |
| No | 58(89.2) | 89.2% (79.1%-95.6%) |
| Yes | 7(10.8) | 10.8% (4.4%-20.9%) |
| **Current Line of Treatment** | | |
| BV monotherapy | 4(6.2) | 6.2% (1.7%-15.0%) |
| BV combined with chemotherapy | 14(21.5) | 21.5% (12.3%-33.5%) |
| BV + PD-1 | 37(56.9) | 56.9% (44.0%-69.2%) |
| BV + PD-1 + chemotherapy | 10(15.4) | 15.4% (7.6%-26.5%) |
| **Cycles of Current Line Therapy, median (range)** | 7(1-17) | |

B

| Characteristics | BV±chemo (n=18), n(%) | BV+PD1±chemo (n=47), n(%) | OR (95% CI) | P value |
|---|---|---|---|---|
| **Sex** | | | | 0.452 |
| Male | 10 (55.6) | 31 (66.0) | 1.54 (0.44-5.36) | |
| Female | 8 (44.4) | 16 (34.0) | 0.65 (0.19-2.30) | |
| **Pathological Type** | | | | 0.721 |
| NS | 13 (72.2) | 34 (72.3) | 1.01 (0.23-3.83) | |
| Non-NS | 5 (27.8) | 13 (27.7) | 0.99 (0.26-4.29) | |
| **EBV Positive** | | | | 0.682 |
| No | 16 (88.9) | 41 (87.2) | 0.86 (0.08-5.47) | |
| Yes | 2 (11.1) | 6 (12.8) | 1.17 (0.18-13.01) | |
| **Ann Arbor Stage** | | | | 0.815 |
| I-II | 5 (27.8) | 12 (25.5) | 0.89 (0.23-3.88) | |
| III-IV | 13 (72.2) | 35 (74.5) | 1.12 (0.26-4.32) | |
| **Extranodal Lesions** | | | | 0.523 |
| No | 7 (38.9) | 21 (44.7) | 1.26 (0.37-4.58) | |
| Yes | 11 (61.1) | 26 (55.3) | 0.79 (0.22-2.71) | |
| **B Symptoms** | | | | 0.287 |
| No | 9 (50.0) | 29 (61.7) | 1.60 (0.46-5.54) | |
| Yes | 9 (50.0) | 18 (38.3) | 0.63 (0.18-2.15) | |
| **ECOG Score** | | | | 0.183 |
| 0-1 | 13 (72.2) | 42 (89.4) | 3.16 (0.62-16.23) | |
| 2-3 | 4 (22.2) | 5 (10.6) | 0.42 (0.08-2.44) | |
| ND | 1 (5.6) | 0 (0.0) | 0.00 (0.00-14.94) | |
| **Prior Lines of Therapy, median (range)** | 2 (1–5) | 2 (1–6) | 0.36 (0.02-5.38) | 0.765 |
| **Prior PD-1 Therapy** | | | | **0.008** |
| No | 16 (88.9) | 25 (53.2) | 0.15 (0.01-0.73) | |
| Yes | 2(11.1) | 22 (45.8) | 6.86 (1.37-68.24) | |
| **Prior ASCT** | | | | 0.512 |
| No | 16 (88.9) | 42 (89.4) | 1.05 (0.09-7.25) | |
| Yes | 2 (11.1) | 5 (10.6) | 0.95 (0.14-10.97) | |
| **Cycles of Current Line Therapy, median (range)** | 4 (1–9) | 8 (1–17) | 0.72 (0.16-3.80) | **0.002** |

A. Baseline characteristics of the intention-to-treat population. B. A comparative analysis of baseline characteristics between two treatment cohorts: BV±chemotherapy and BV+PD1±chemotherapy.

**Copyright Information:** (Online) ISSN: 2572-9241
©2026 The Author(s). HemaSphere is published by John Wiley & Sons Ltd on behalf of European Hematology Association. This is an open access Abstract Book distributed under the Attribution-NonCommercial-NoDerivs (CC BY-NC-ND), which allows third parties to download the articles and share them with others as long as they credit the author and the Abstract Book, but they cannot change the content in any way or use them commercially.
**Abstract Book Citations:** Authors, Title, HemaSphere, 2026;10:(S1):pages. The abstract book DOIs can be found on the "Author Information" page.
**Disclaimer:** Articles published in the journal HemaSphere exclusively reflect the opinions of the authors. The authors are responsible for all content in their abstracts including accuracy of the facts, statements, citing resources, etc.

### (PB3585) REAL-WORLD CLINICAL CHARACTERISTICS, TREATMENT PATTERNS AND OUTCOMES OF CLASSICAL HODGKIN LYMPHOMA IN A MULTI-ETHNIC ASIAN POPULATION: A 15-YEAR SINGLE-CENTRE EXPERIENCE

**Topic:** 17. Hodgkin lymphoma - Clinical

Roni Wang*[1], Esther Hian Li Chan[1], Victor Ling[1], William Chan[1], Sanjay de Mel[1], Gui Yi Ho[1], Edwin Thong[1], Yen Lin Chee[1], Michelle Limei Poon[1]

[1]*National University Hospital, Singapore, Singapore;*

### Background

The therapeutic landscape of classical Hodgkin lymphoma (cHL) has shifted remarkably over the past two decades, with breakthrough advances including brentuximab vedotin (BV), checkpoint inhibitors, and PET-adapted treatment strategies.

### Aims

We examined clinical characteristics, treatment patterns, and outcomes of cHL in a multi-ethnic Asian population to determine the real-world need for novel therapeutics.

### Methods

We conducted a retrospective cohort analysis of cHL patients (N=177) treated at the National University Cancer Institute Singapore from 2009–2025 with first-line chemotherapy. Survival analyses were performed using the Kaplan-Meier method and multivariate Cox proportional hazards modelling. Advanced stage and elderly (>60 years) patients were analysed as pre-specified subgroups, and temporal trends were assessed across three diagnostic eras: before 2015 (n=44), 2015–2020 (n=72), and after 2020 (n=61).

### Results

The cohort comprised 100 male and 77 female patients with a median age of 29 years (range 12–83). GHSG stage was early favourable (EF) in 10%, early unfavourable (EU) in 27%, and advanced (Adv) in 63%; bulky disease and B symptoms were present in 30% and 40% respectively. At a median follow-up of 49 months, 5-year PFS was 74% and OS 94%, with PFS differing significantly by GHSG stage (EF 100%, EU 88%, Adv 63%; p<0.001) but no difference in OS (p=0.167). Elderly patients (10% of cohort) were more likely to have ECOG PS $\geq$1, higher LDH, B symptoms and Adv disease. They had comparable PFS to younger patients (61.5% vs 74.9%, p=0.479) but markedly worse 5-year OS (68.1% vs 96.0%, p<0.001), reflecting excess non-lymphoma mortality and higher comorbidity burden.
Within the Adv cohort, majority (41%) were treated with 6-ABVD (41%) or RATHL approach (32%) as first line therapy, and 16% received BV-based induction. Interim PET post cycle 2 (iPET2) was performed in 90% of patients and was negative (Deauville 1–3) in 70%. On multivariate analysis, iPET2 positivity was the strongest predictor of inferior PFS (HR 4.88, p=0.002), while BV use was independently associated with improved PFS (HR 0.11, p=0.033).
Treatment patterns evolved significantly across eras, with radiotherapy use declining significantly from 36% to 12% (p=0.004) and BV use rising from 0% to 34% (p<0.001). Despite a trend towards higher-risk (Elevated LDH 14% to 43%, more advanced stage disease 60.5% to 75.0%) and more comorbid patients (ECOG>1 0 to 14%) in the modern era, 5-year PFS and OS remained comparable (p=0.304 and p=0.477 respectively).

**Copyright Information:** (Online) ISSN: 2572-9241
©2026 The Author(s). HemaSphere is published by John Wiley & Sons Ltd on behalf of European Hematology Association. This is an open access Abstract Book distributed under the Attribution-NonCommercial-NoDerivs (CC BY-NC-ND), which allows third parties to download the articles and share them with others as long as they credit the author and the Abstract Book, but they cannot change the content in any way or use them commercially.
**Abstract Book Citations:** Authors, Title, HemaSphere, 2026;10:(S1):pages. The abstract book DOIs can be found on the "Author Information" page.
**Disclaimer:** Articles published in the journal HemaSphere exclusively reflect the opinions of the authors. The authors are responsible for all content in their abstracts including accuracy of the facts, statements, citing resources, etc.

**Summary/Conclusion**

Our local cHL population demonstrates prognostic characteristics and outcomes consistent with global data. Elderly patients and Adv patients with iPET positivity continue to experience inferior outcomes, highlighting an unmet need for improved treatment strategies. As one of the largest real-world cHL analyses from our region, this study serves as an important benchmark for healthcare policy and future research in the field.

**Copyright Information:** (Online) ISSN: 2572-9241
©2026 The Author(s). HemaSphere is published by John Wiley & Sons Ltd on behalf of European Hematology Association. This is an open access Abstract Book distributed under the Attribution-NonCommercial-NoDerivs (CC BY-NC-ND), which allows third parties to download the articles and share them with others as long as they credit the author and the Abstract Book, but they cannot change the content in any way or use them commercially.
**Abstract Book Citations:** Authors, Title, HemaSphere, 2026;10:(S1):pages. The abstract book DOIs can be found on the "Author Information" page.
**Disclaimer:** Articles published in the journal HemaSphere exclusively reflect the opinions of the authors. The authors are responsible for all content in their abstracts including accuracy of the facts, statements, citing resources, etc.

### (PB3586) IMPACT OF INTERIM PET/CT AND DISEASE STAGING ON PROGRESSION AND TREATMENT TRAJECTORIES IN HODGKIN LYMPHOMA: A SINGLE CENTER COHORT STUDY.

**Topic:** 17. Hodgkin lymphoma - Clinical

Nikola Lemajic*[1], Vojin Vukovic[1, 2], Mihailo Smiljanic[1], Isidora Arsenovic[1], Andrijana Kojic[1], Andjela Nikolic[1], Tamara Bibic[1], Sofija Kozarac[1], Jelena Ivanović[1], Danijela Lekovic[1, 2], Darko Antic[1, 2]

*[1]Clinic for Hematology, University Clinical Center of Serbia, Belgrade, Serbia;[2]Faculty of Medicine, University of Belgrade, Belgrade, Serbia;*

### Background

Response-adapted therapy utilizing interim PET/CT (iPET) is the standard of care in classical Hodgkin Lymphoma (HL). While metabolic response is a powerful predictor, the interplay between initial disease stage and iPET Deauville scores (DS) remains complex, particularly regarding the stability of response. This study evaluates these dynamics in a single-center HL cohort.

### Aims

To evaluate the impact of iPET response in patients with all stages of HL treated with frontline ABVD.

### Methods

We retrospectively analysed 133 HL patients diagnosed and treated with ABVD in the frontline setting between January 2020 and January 2024. The follow-up cut off was January 2026. Patients were staged as limited stage, intermediate stage and advanced stage based on the initial Ann Arbor disease stage and accompanying risk factors. iPET and end-of-treatment (eotPET) metabolic responses were dichotomized into negative (DS 1–3) and positive (DS 4–5). The patients were further divided into 6 groups according to stage and iPET score. (limited stage DS 1–3, intermediate stage DS 1–3, advanced stage DS 1–3, limited stage DS 4–5 intermediate stage DS 4–5 and advanced stage DS 4–5). Statistical analysis was performed using Kaplan-Meier log-rank test, Mann-Whitney U test, Chi-squared test, Fisher's exact test and McNemar's test. Statistical analysis was performed using Easy R and Google Gemini.

### Results

In this cohort the median age was 32 years (range 18–67) with a slight male majority (54.1%). Disease stage at the start of treatment proved to be a reliable marker of iPET response. Patients with limited and intermediate stage disease had a higher probability of achieving a negative iPET when compared to patients with advanced stage disease (p=0.013). Among the 94 patients achieving a negative iPET, 13.8% experienced a "loss of response" by the end of treatment converting to DS 4–5 at eotPET. This was stage-dependent, occurring in 27.0% of advanced stage patients compared to 7.0% in intermediate and 0% in limited stage (p=0.009). In the group of patients with a positive iPET 45% successfully achieved a negative eotPET. The lowest probability of achieving a negative eotPET was in the iPET positive advanced stage group 36% (p=0.23). Escalation from ABVD to BEACOPPesc showed no statistical significance in regard to conversion from positive iPET to negative eotPET in our cohort. Median overall survival was not reached across any stage of HL but upon further subgroup analysis advanced stage HL patients with a positive iPET had a median progression free survival (PFS) of 19.9 months (p<0.001). Finally, an iPET of DS4-5 reliably predicted a significantly higher burden of subsequent salvage therapies compared to DS 1–3 with almost none of the DS 1–3 limited and intermediate stage patients requiring additional lines of treatment (p=0.001).

**Copyright Information:** (Online) ISSN: 2572-9241
©2026 The Author(s). HemaSphere is published by John Wiley & Sons Ltd on behalf of European Hematology Association. This is an open access Abstract Book distributed under the Attribution-NonCommercial-NoDerivs (CC BY-NC-ND), which allows third parties to download the articles and share them with others as long as they credit the author and the Abstract Book, but they cannot change the content in any way or use them commercially.
**Abstract Book Citations:** Authors, Title, HemaSphere, 2026;10:(S1):pages. The abstract book DOIs can be found on the "Author Information" page.
**Disclaimer:** Articles published in the journal HemaSphere exclusively reflect the opinions of the authors. The authors are responsible for all content in their abstracts including accuracy of the facts, statements, citing resources, etc.

**Summary/Conclusion**

While overall survival in HL remains robust across subgroups, an iPET DS 1–3 and advanced stage HL are powerful predictors of progression and increased salvage therapy requirement. In our patients a negative iPET did not guarantee sustained remission in advanced stage HL when treated with ABVD, with over a quarter of these patients losing their metabolic response by the end of treatment.

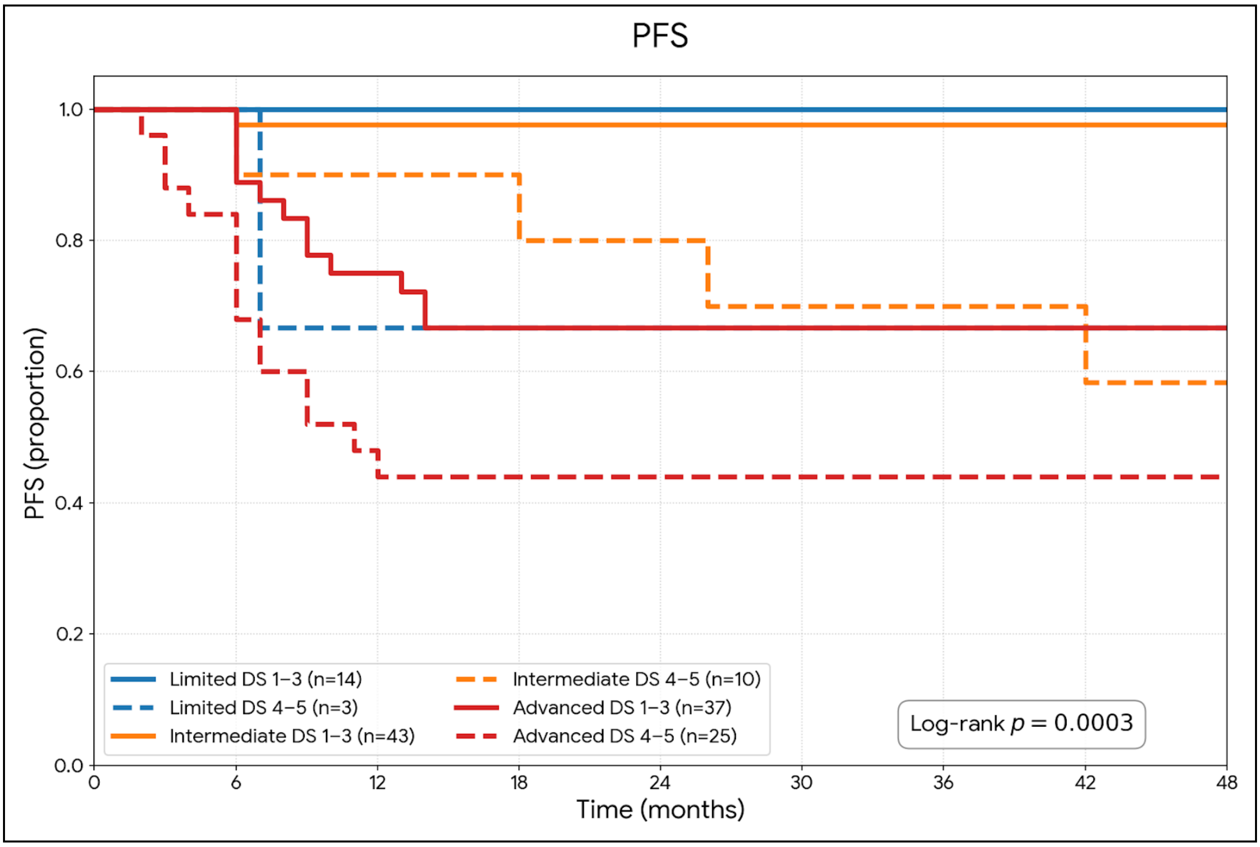

**Copyright Information:** (Online) ISSN: 2572-9241
©2026 The Author(s). HemaSphere is published by John Wiley & Sons Ltd on behalf of European Hematology Association. This is an open access Abstract Book distributed under the Attribution-NonCommercial-NoDerivs (CC BY-NC-ND), which allows third parties to download the articles and share them with others as long as they credit the author and the Abstract Book, but they cannot change the content in any way or use them commercially.
**Abstract Book Citations:** Authors, Title, HemaSphere, 2026;10:(S1):pages. The abstract book DOIs can be found on the "Author Information" page.
**Disclaimer:** Articles published in the journal HemaSphere exclusively reflect the opinions of the authors. The authors are responsible for all content in their abstracts including accuracy of the facts, statements, citing resources, etc.

### (PB3587) SEVERE AND LIFE-THREATENING POLYMORPHIC CUTANEOUS TOXICITY DURING BEGEV SALVAGE THERAPY FOR RELAPSED/REFRACTORY HODGKIN LYMPHOMA: A 9-CASE SERIES

**Topic:** 17. Hodgkin lymphoma - Clinical

Abdellah Seghiri[1], Ikram Sadki[1], Soumaya Barkhoukh[1], Zahida Aqodad[1], Samia Sabri[1], Houda Bachir[1], Siham Hamaz[1], Habiba Bennesser Alaoui*[1], Khalid Serraj Andaloussi[1]

*[1]Laboratory of Immuno-Hematology and Cell Therapy, Faculty of Medicine and Pharmacy of Oujda (FMPO), Mohammed First University, Oujda, Morocco., Department of Internal Medicine, Mohammed VI University Hospital of Oujda, Oujda, Morocco;*

### Background

The BeGEV regimen is widely used as salvage therapy in relapsed or refractory Hodgkin lymphoma prior to autologous stem cell transplantation. While bendamustine has been associated with cutaneous adverse events, the spectrum and severity of skin toxicity within BeGEV are not well characterized.

### Aims

To describe the clinical patterns, severity, management, and outcomes of cutaneous adverse events during BeGEV therapy.

### Methods

We retrospectively reviewed 25 patients receiving BeGEV at our institution between 2024–2026. Nine patients (9/25, 36%) developed cutaneous toxicity. Clinical presentation, timing, management, outcomes, and prior exposure to gemcitabine- and vinorelbine-based regimens (GDP or GVD) were analyzed. Three patients had previously received GVD or GDP without any cutaneous toxicity.

**Copyright Information:** (Online) ISSN: 2572-9241
©2026 The Author(s). HemaSphere is published by John Wiley & Sons Ltd on behalf of European Hematology Association. This is an open access Abstract Book distributed under the Attribution-NonCommercial-NoDerivs (CC BY-NC-ND), which allows third parties to download the articles and share them with others as long as they credit the author and the Abstract Book, but they cannot change the content in any way or use them commercially.
**Abstract Book Citations:** Authors, Title, HemaSphere, 2026;10:(S1):pages. The abstract book DOIs can be found on the "Author Information" page.
**Disclaimer:** Articles published in the journal HemaSphere exclusively reflect the opinions of the authors. The authors are responsible for all content in their abstracts including accuracy of the facts, statements, citing resources, etc.

### Results

Among the 25 patients receiving BeGEV, 9 (36%) developed cutaneous toxicity. The median age of affected patients was 32 years (range 23–56), with a female-to-male ratio of 5:4. Cutaneous manifestations were polymorphic: 1 case of DRESS, 3 cases of acute generalized exanthematous pustulosis (AGEP), 2 cases of maculopapular rash (RMP), 2 cases of Stevens-Johnson Syndrome (SJS), and 1 case of severe acneiform eruption. Most reactions (7/9, 78%) occurred after the second cycle of BeGEV, while 2 cases appeared earlier or later in treatment. Mild reactions were managed with topical therapy, whereas severe reactions required systemic corticosteroids **(CS)** (6/9) and hospitalization (6/9). BeGEV was discontinued in 7 patients. Two patients (22%) died due to severe cutaneous complications.

These findings highlight that cutaneous toxicity during BeGEV therapy can be frequent, polymorphic, and occasionally life-threatening, emphasizing the need for early recognition and prompt management.

**Table: 9 BeGEV Patients with Cutaneous Toxicity**

| Case | Age | Sex | Skin Lesion | Treatment | Outcome |
|---|---|---|---|---|---|
| 01 | 42 | F | DRESS | CS | Unfavorable |
| 02 | 31 | F | RMP | topical therapy | Favorable |
| 03 | 26 | M | Acneiform | topical therapy | Favorable |
| 04 | 23 | M | AGEP | CS | Favorable |
| 05 | 25 | F | RMP | topical therapy | Favorable |
| 06 | 52 | M | SJS | CS | Favorable |
| 07 | 39 | F | AGEP | CS | Favorable |
| 08 | 56 | F | SJS | CS | Unfavorable |
| 09 | 32 | F | AGEP | CS | Favorable |

### Summary/Conclusion

Cutaneous toxicity during BeGEV therapy can be polymorphic and occasionally life-threatening, including DRESS and epidermal necrolysis. Early recognition is crucial to guide management, prevent treatment interruption, and preserve transplant eligibility. Bendamustine appears to be the main trigger in these adverse events.

**Copyright Information:** (Online) ISSN: 2572-9241
©2026 The Author(s). HemaSphere is published by John Wiley & Sons Ltd on behalf of European Hematology Association. This is an open access Abstract Book distributed under the Attribution-NonCommercial-NoDerivs (CC BY-NC-ND), which allows third parties to download the articles and share them with others as long as they credit the author and the Abstract Book, but they cannot change the content in any way or use them commercially.
**Abstract Book Citations:** Authors, Title, HemaSphere, 2026;10:(S1):pages. The abstract book DOIs can be found on the "Author Information" page.
**Disclaimer:** Articles published in the journal HemaSphere exclusively reflect the opinions of the authors. The authors are responsible for all content in their abstracts including accuracy of the facts, statements, citing resources, etc.

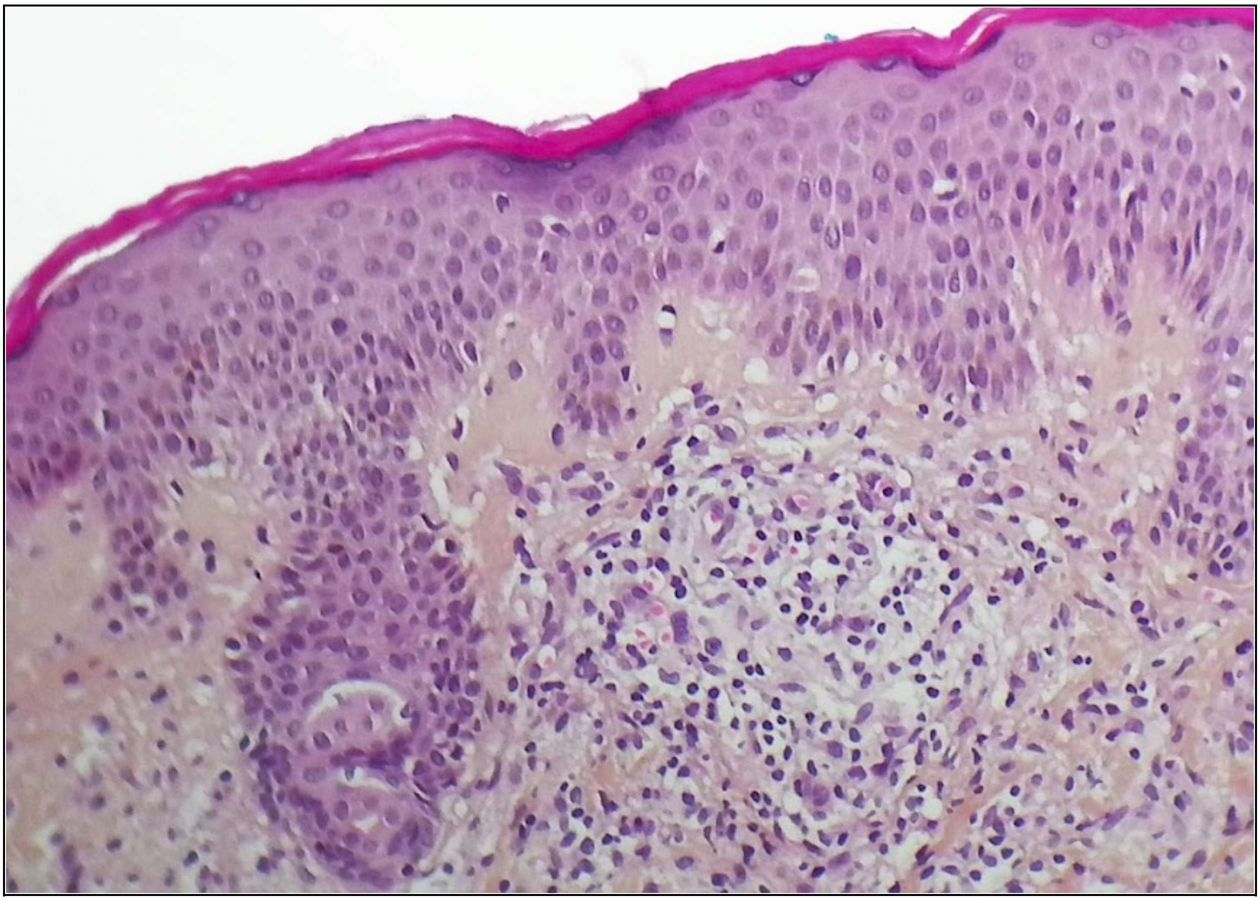

Figure 01 : Photomicrographs of the skin biopsy reveal a hyperkeratotic epidermis with spongiotic changes, neutrophilic exocytosis, and keratinocyte apoptosis (HES, ×200).

**Copyright Information:** (Online) ISSN: 2572-9241
©2026 The Author(s). HemaSphere is published by John Wiley & Sons Ltd on behalf of European Hematology Association. This is an open access Abstract Book distributed under the Attribution-NonCommercial-NoDerivs (CC BY-NC-ND), which allows third parties to download the articles and share them with others as long as they credit the author and the Abstract Book, but they cannot change the content in any way or use them commercially.
**Abstract Book Citations:** Authors, Title, HemaSphere, 2026;10:(S1):pages. The abstract book DOIs can be found on the "Author Information" page.
**Disclaimer:** Articles published in the journal HemaSphere exclusively reflect the opinions of the authors. The authors are responsible for all content in their abstracts including accuracy of the facts, statements, citing resources, etc.

### (PB3589) REAL-WORLD TOLERABILITY AND PRELIMINARY ANALISYS OF EFFICACY OF BRECADD IN ADVANCED-STAGE HODGKIN LYMPHOMA: A MULTICENTER BRAZILIAN EXPERIENCE

**Topic:** 17. Hodgkin lymphoma - Clinical

Talita Silveira*[1], Matheus Garcia[1], Giovanna Peruzini[1], Jayr Schimdt[1], Raquel Martins[2], Ruddy Dalfeor[3], Thiana Moura[4], Rafaela Samico[5], Nathalia Zing[6], Thais Fisher[1], Arthur Braga[1], Humberto Silva[1], Sérgio Fortier[7]

*[1]AC Camargo Cancer Center, São Paulo, Brazil; [2]Universidade Triangulo Mineiro, Uberaba, Brazil; [3]DASA oncologia, Rio de Janeiro, Brazil; [4]Hospital Cliom, Maceió, Brazil; [5]Hospital do Cancer de Muriaé, Muriaé, Brazil; [6]BP São Paulo, São Paulo, Brazil; [7]Santa Casa São Paulo, São Paulo, Brazil;*

### Background

Since the introduction of the ABVD and thereafter BEACOPP regimen, Hodgkin Lymphoma (HL) has achieved high cure rates. Since then, new therapeutic regimens have been investigated aiming to improve outcomes and reduce toxicity, particularly in advanced-stage HL. In this context, the HD21 trial comparing BrECADD regimen with BEACOPP, demonstrated lower toxicity, as well as improved 4-year progression-free survival (94.3% vs. 90.9%) and maintain overall survival benefit (98.6% vs. 98.2%). The main treatment-related adverse events (AEs) include anemia, thrombocytopenia, neutropenia, and neuropathy, with dose reductions required due to AEs in 34% of patients treated with BEACOPP and 21% of those receiving BrECADD. These results suggest that the BrECADD combination may preserve or even improve therapeutic efficacy, while reducing the incidence of adverse events and, consequently, the need for dose modifications[1].

### Aims

To describe the initial real-world data on the use of the BrECADD regimen for advanced-stage HL in Brazilian centers.

### Methods

This is an observational, retrospective study conducted across six private Brazilian referral centers in Onco-Hematology. A total of 22 patients diagnosed and treated at these institutions between September 2023 and January 2026 were included.

### Results

Among the 22 patients included, the first one was treated at september of 2023. Five had not yet completed the therapeutic protocol or realized final PET. The median age of the cohort was 36 years (range: 17–51 years), with 14 females and 8 males. Performance status was assessed using the Eastern Cooperative Oncology Group (ECOG) scale, with 100% of patients classified as ECOG 0 at treatment initiation. Regarding disease staging, most patients presented with advanced disease, with Ann Arbor stage IV observed in 68.2% (15 patients). The International Prognostic Score (IPS), 68.2% of patients had an IPS of 0–2, while 31.8% had an IPS ≥3. As preliminary results, 3/22 (13,63%) patients had iPET positive and realized 6 cycles of treatment. One of this patients had PET with Deauville 4 at the end of the treatment and was sent to radiotherapy ( now is waiting for final evaluation). No one patient had refractory disease or recidive (this is a very premature real life cohort to evaluate FUP). During treatment, febrile neutropenia was observed in 36,36% of patients (8 events in 5 patients). No clinically relevant peripheral neuropathy or systematic chemotherapy cycle delays were reported in the majority of patients. Hematologic toxicities, including anemia and/or thrombocytopenia, were observed in approximately half of the cohort, mostly of mild to moderate severity. No one required transfusion. Only two patients required dose adjustments of the therapeutic regimen due to hematologic toxicities.

**Copyright Information:** (Online) ISSN: 2572-9241
©2026 The Author(s). HemaSphere is published by John Wiley & Sons Ltd on behalf of European Hematology Association. This is an open access Abstract Book distributed under the Attribution-NonCommercial-NoDerivs (CC BY-NC-ND), which allows third parties to download the articles and share them with others as long as they credit the author and the Abstract Book, but they cannot change the content in any way or use them commercially.
**Abstract Book Citations:** Authors, Title, HemaSphere, 2026;10:(S1):pages. The abstract book DOIs can be found on the “Author Information” page.
**Disclaimer:** Articles published in the journal HemaSphere exclusively reflect the opinions of the authors. The authors are responsible for all content in their abstracts including accuracy of the facts, statements, citing resources, etc.

**Summary/Conclusion**

Preliminary data from this cohort suggest that the BrECADD regimen is well tolerated, with a low incidence of febrile neutropenia (36,36%) and a low requirement for dose adjustments (22.2%). Although 55.6% of patients experienced hematologic adverse events, such as anemia and thrombocytopenia, these toxicities did not lead to treatment interruption or systematic chemotherapy dose modifications. The favorable safety profile combined with high initial complete response rates reinforces the potential of BrECADD as a promising therapeutic option for patients with advanced-stage Hodgkin Lymphoma.

**Copyright Information:** (Online) ISSN: 2572-9241
©2026 The Author(s). HemaSphere is published by John Wiley & Sons Ltd on behalf of European Hematology Association. This is an open access Abstract Book distributed under the Attribution-NonCommercial-NoDerivs (CC BY-NC-ND), which allows third parties to download the articles and share them with others as long as they credit the author and the Abstract Book, but they cannot change the content in any way or use them commercially.
**Abstract Book Citations:** Authors, Title, HemaSphere, 2026;10:(S1):pages. The abstract book DOIs can be found on the "Author Information" page.
**Disclaimer:** Articles published in the journal HemaSphere exclusively reflect the opinions of the authors. The authors are responsible for all content in their abstracts including accuracy of the facts, statements, citing resources, etc.

### (PB3590) PREDICTIVE ROLE OF NEUTROPHIL TO LYMPHOCYTE (NLR) AND PLATELET TO LYMPHOCYTE RATIO (PLR) IN NEWLY DIAGNOSED HODGKIN LYMPHOMA

**Topic:** 17. Hodgkin lymphoma - Clinical

Andrijana Kojic*[1], Vojin Vukovic[1, 2], Isidora Arsenovic[1], Andjela Nikolic[1], Mihailo Smiljanic[1], Nikola Lemajić[1], Natasa Čolović[1, 2], Ivan Soldatovic[3], Tamara Bibic[1], Jelena Ivanović[1], Darko Aleksic[1], Danijela Lekovic[1, 2], Darko Antic[1, 2]

*[1]Clinic of Hematology, University Clinical Center of Serbia, Belgrade, Serbia;[2]Faculty of Medicine, University of Belgrade, Belgrade, Serbia; [3]Institute of Medical Statistics and Informatics, Belgrade, Serbia;*

### Background

Hodgkin lymphoma (HL) is a unique B-cell–derived lymphoid malignancy characterized by a disproportionately small population of malignant cells within a prominent inflammatory microenvironment. The neoplastic Reed–Sternberg cells in Hodgkin lymphoma typically constitute only 1–5% of the total tumor cellularity. These malignant cells coordinate a cytokine-rich microenvironment that promotes immune cell recruitment, immune evasion, and tumor survival. Neutrophil to lymphocyte (NLR) and platelet to lymphocyte ratio (PLR) demonstrated prognostic value in multiple studies on solid and less, hematological malignancies.

### Aims

To assess the role of NLR and PLR in PET/CT guided therapy of newly diagnosed HL.

### Methods

This is a retrospective study that included 101 patients newly diagnosed with Hodgkin lymphoma and treated at the Clinic of Hematology, University Clinical Center of Serbia in the period of January 2022 to October 2025. All records are collected from patients' history charts.
Response on interim PET/CT is defined as complete or partial metabolic and morphological remission assessed with Deauville score 1-3 after two cycles of chemotherapy.

### Results

A total of 101 patients were evaluated (53.8%, 56 male; 46.2%, 48 female), median age 31 years (18–66). The most common subtype was nodular sclerosis with 87% following mixed cellularity with 11%. Disease stage included 23.5% early favorable, 36.3% early unfavorable and 40.2% advanced disease. NLR and PLR were evaluated at baseline (NLR1; PLR1), after two cycles of chemotherapy at the interim PET/CT assessment (NLR2; PLR2), and at the end of treatment (NLR3; PLR3). Median values of NLR2 (1.65) and PLR2 (163.50) were statistically significantly lower than baseline values (NLR1 4.11, PLR1 243.12, $p < 0.001$). Median NLR3 (2.54) and PLR3 (209.68) remained lower than baseline, but slightly higher than NLR2 and PLR2, without statistical significance ($p > 0.5$). Partial or complete response on interim PET/CT was achieved in 89 patients (88.10%). Complete response at the end of treatment was achieved in 82 patients (79.6%). NLR and PLR values at interim and end-of-treatment were lower in patients achieving remission, however, did not differ significantly from those of the overall cohort regardless of therapy response ($p > 0.5$). Among patients who had early unfavorable or advanced disease (75%), 80% were treated with ABVD, 10.7% with escBEACOPP, and 9.3% with both regimens in escalated settings. There was no statistically significant difference in interim or end-of-treatment NLR and PLR values among patients with early unfavorable or advanced disease, regardless of the treatment regimen used.

**Copyright Information:** (Online) ISSN: 2572-9241
©2026 The Author(s). HemaSphere is published by John Wiley & Sons Ltd on behalf of European Hematology Association. This is an open access Abstract Book distributed under the Attribution-NonCommercial-NoDerivs (CC BY-NC-ND), which allows third parties to download the articles and share them with others as long as they credit the author and the Abstract Book, but they cannot change the content in any way or use them commercially.
**Abstract Book Citations:** Authors, Title, HemaSphere, 2026;10:(S1):pages. The abstract book DOIs can be found on the "Author Information" page.
**Disclaimer:** Articles published in the journal HemaSphere exclusively reflect the opinions of the authors. The authors are responsible for all content in their abstracts including accuracy of the facts, statements, citing resources, etc.

**Summary/Conclusion**

It is notable that only a limited number of studies have investigated these parameters in Hodgkin lymphoma, despite the pronounced inflammatory background of the disease. In our cohort, short follow-up precluded analysis of associations with PFS and OS. We observed a significant reduction in NLR and PLR during treatment and after its completion; however, this decrease was not associated with treatment response overall, or with the specific regimen used in patients with early unfavorable or advanced disease. This comparison was clinically justified, as guidelines endorse both regimens for these stages despite differences in toxicity. Given the limitations of this study, larger prospective studies with longer follow-up are warranted to better clarify their predictive and prognostic value in Hodgkin lymphoma.

**Copyright Information:** (Online) ISSN: 2572-9241
©2026 The Author(s). HemaSphere is published by John Wiley & Sons Ltd on behalf of European Hematology Association. This is an open access Abstract Book distributed under the Attribution-NonCommercial-NoDerivs (CC BY-NC-ND), which allows third parties to download the articles and share them with others as long as they credit the author and the Abstract Book, but they cannot change the content in any way or use them commercially.
**Abstract Book Citations:** Authors, Title, HemaSphere, 2026;10:(S1):pages. The abstract book DOIs can be found on the "Author Information" page.
**Disclaimer:** Articles published in the journal HemaSphere exclusively reflect the opinions of the authors. The authors are responsible for all content in their abstracts including accuracy of the facts, statements, citing resources, etc.

### (PB3592) THERAPEUTIC EFFICACY AND SAFETY OF THE TUNISIAN NATIONAL LH 2015 PROTOCOL FOR HODGKIN LYMPHOMA: A 9-YEAR RETROSPECTIVE MONOCENTRIC STUDY

**Topic:** 17. Hodgkin lymphoma - Clinical

Nesrine Ben Sayed*[1], Sabrine Hallabia[2], Amira Rahal[3], Abir Ezzine[4], Rihab Ben Sghaier[2], Wafa Chenbah[3], Ameni Ben Amor[3], Monia Guermazi[3], Haifa Regaieg[3], Kmira Zahra[3], Monia Zaier[5], Yosra Ben Youssef[6]

*[1]Farhat hached hospital, hematolgy department, Soussa, Tunisia; [2]Farhat hached hospital, hematolgy department, Sousse, Tunisia; [3]Farhat hached hospital, hematolgy department, SOUSSE, Tunisia; [4]saloul hospital, nuclear medecine, SOUSSE, Tunisia; [5]farhat hached hospital, hematology, SOUSSE, Tunisia; [6]farhat hached, hematology, SOUSSE, Tunisia;*

### Background

Hodgkin's lymphoma (HL) poses a significant challenge in hematology, particularly in regions with limited healthcare resources. In Tunisia, the LH 2015 protocol has been established to provide a standardised approach to managing HL, using risk stratification and treatment modalities such as ABVD and an escalated BEACOPP regimen.

### Aims

This retrospective study aims to evaluate the long-term efficacy and safety of the LH 2015 protocol in a cohort of 145 patients diagnosed with HL over nine years at Farhat Hached and Sahloul University Hospitals.

### Methods

The study focused on 145 patients diagnosed with HL from 2015 to 2024. Patients were stratified into three distinct risk groups based on clinical and radiological criteria. The treatment regimen involved induction chemotherapy, followed by consolidation radiotherapy (RT) for patients exhibiting bulky disease or PET-positive residual masses. The primary endpoints assessed included the complete remission (CR) rate, incidence of primary refractory disease, and relapse rates post-treatment.

### Results

The results indicated that 64.3% of the patients (n=92) achieved complete remission following first-line therapy, showing the protocol's effectiveness in a real-world setting. However, 34% (n=52) of patients demonstrated primary refractory disease, which is notably higher than rates reported in international clinical trials. Among those who achieved complete remission, 12.4% (n=18) experienced subsequent relapse, presenting an area for further investigation. In the high-risk subgroup (Group 3), the use of PET-CT was instrumental; 11 patients who displayed morphological remission on conventional CT were identified to have residual metabolic activity, leading to timely consolidation RT. Adverse effects were also recorded, with Grade 3/4 hematological toxicities observed in 28% of patients, predominantly in those receiving the BEACOPP-escalated regimen.

### Summary/Conclusion

The findings from this nine-year retrospective study highlight the LH 2015 protocol's ability to achieve substantial remission rates for Hodgkin Lymphoma in the Tunisian healthcare context. Nonetheless, the high primary refractory rate calls for a critical evaluation of treatment strategies and underscores the importance of integrating interim PET-CT (iPET) assessments to identify non-responders early. Furthermore, incorporating novel therapeutic agents, such as Brentuximab Vedotin or PD-1 inhibitors, may improve outcomes for high-risk patients. These insights underscore the need for continued research and adaptation of treatment protocols to improve patient care in Hodgkin Lymphoma management.

**Copyright Information:** (Online) ISSN: 2572-9241
©2026 The Author(s). HemaSphere is published by John Wiley & Sons Ltd on behalf of European Hematology Association. This is an open access Abstract Book distributed under the Attribution-NonCommercial-NoDerivs (CC BY-NC-ND), which allows third parties to download the articles and share them with others as long as they credit the author and the Abstract Book, but they cannot change the content in any way or use them commercially.
**Abstract Book Citations:** Authors, Title, HemaSphere, 2026;10:(S1):pages. The abstract book DOIs can be found on the "Author Information" page.
**Disclaimer:** Articles published in the journal HemaSphere exclusively reflect the opinions of the authors. The authors are responsible for all content in their abstracts including accuracy of the facts, statements, citing resources, etc.

### (PB3593) FRONTLINE ABVD IS STILL FEASIBLE IN ADVANCED STAGE HODGKIN LYMPHOMA AS EARLY STAGE: A RETROSPECTIVE REAL-WORLD COHORT STUDY FROM TURKEY

**Topic:** 17. Hodgkin lymphoma - Clinical

Derya Demirtas*[1], Elif Suyani[1], Alper Koc[2], Bilal Aygün[1], Zeynep Karabulut[1], Nurhilal Buyukkurt[1], Cagatay Unsal[1], Didar Yanardag Acik[1]

*[1]University Of Health Sciences, Adana City Training and Research Hospital, Department of Hematology, Adana, Türkiye;[2]Elazıg Fethi Sekin City Hospital, Hematology, Elazıg, Türkiye;*

### Background

Novel agents such as brentuximab vedotin and anti–PD-1 antibodies, together with PET-adapted intensified strategies, have improved outcomes in advanced-stage classical Hodgkin lymphoma (cHL). However, their applicability remains limited in many middle-income countries because of restricted drug availability, reimbursement barriers, and toxicity concerns. Therefore, real-world data on the effectiveness of ABVD (doxorubicin, bleomycin, vinblastine, and dacarbazine) remain essential.

### Aims

To evaluate the real-world effectiveness of first-line ABVD in advanced-stage cHL and compare outcomes with early-stage disease within the same cohort.

### Methods

We conducted a retrospective two-center study including 95 adult patients with newly diagnosed cHL treated with first-line ABVD between 2015 and 2025. Baseline characteristics, treatment response, progression-free survival (PFS), overall survival (OS), and mortality were compared between early-stage (I–II, n=33) and advanced-stage (III–IV, n=62) disease.

### Results

The median age was 38 years (range, 18–73), and 69.5% were male; 65% had advanced-stage. Complete response rates were high in early-stage (97%) and advanced-stage (95.2%). Median PFS (21 vs. 23 months; p=0.994) and OS (23 vs. 25 months; p=0.848) were similar. Five-year OS was 84.3% for early-stage and 98% for advanced-stage patients (p=0.292), while five-year PFS was 55.3% and 29.6% (p=0.526). Mortality rates were comparable.

### Summary/Conclusion

In this real-world cohort, first-line ABVD achieved high response rates and favorable long-term survival in advanced-stage cHL, supporting its continued use where access to novel or intensified regimens is limited.

**Copyright Information:** (Online) ISSN: 2572-9241
©2026 The Author(s). HemaSphere is published by John Wiley & Sons Ltd on behalf of European Hematology Association. This is an open access Abstract Book distributed under the Attribution-NonCommercial-NoDerivs (CC BY-NC-ND), which allows third parties to download the articles and share them with others as long as they credit the author and the Abstract Book, but they cannot change the content in any way or use them commercially.
**Abstract Book Citations:** Authors, Title, HemaSphere, 2026;10:(S1):pages. The abstract book DOIs can be found on the "Author Information" page.
**Disclaimer:** Articles published in the journal HemaSphere exclusively reflect the opinions of the authors. The authors are responsible for all content in their abstracts including accuracy of the facts, statements, citing resources, etc.

### (PB3594) THERAPEUTIC OUTCOMES OF ABVD VERSUS ESCALATED BEACOPP IN BULKY MEDIASTINAL HODGKIN LYMPHOMA: A SINGLE-CENTER TUNISIAN EXPERIENCE

**Topic:** 17. Hodgkin lymphoma - Clinical

Faten Kallel[1], Khouloud Mezghani*[1], Maha Charfi[1], Nour Siala[1], Mayeda Bourmech[2], Hanen Ben Salah[2], Rahma Mallek[1], Yousra Fakhfakh Derbel[1], Hanen Charfi[1], Imen Ben Amor[1], Aicha Ben Kahla[1], Ines Turki[1], Asma Koubaa[1], Imen Frikha[1], Slim Charfi[3], Moez Medhaffar[1], Moez Elloumi[1]

*[1]CHU Hedi Chaker, Hematology, Sfax, Tunisia;[2]CHU Habib Bourguiba, Radiotherapy, Sfax, Tunisia;[3]CHU Habib Bourguiba, Anatomopathology, Sfax, Tunisia;*

### Background

Bulky mediastinal disease (BMD) is one of the bad prognostic factors in Hodgkin lymphoma (HL).
In order to improve overall survival (OS) for patients with BMD, the Tunisian group changed the national protocol based on ABVD (MDH2002 in 2002) to BEACOPP escalated regimen (MDH2008 in 2008, then MDH2015 in 2015).

### Aims

Our study aims to compare therapeutic results in patients with BMD treated with ABVD versus BEACOPP regimen.

### Methods

This retrospective single-center study included adults aged 18–60 years diagnosed with HL and bulky mediastinum (mediastinal-thoracic ratio ≥0.35) treated between 2002 and 2024.
Patients treated under MDH2002 received 6–8 cycles of ABVD (P1).
MDH2008 and MDH2015 (P2) implemented BEACOPP-based intensification with interim radiologic evaluation after two cycles and de-escalation to ABVD in case of ≥75% response (partial response PR), and 4-6 cycles of BEACOPP if response <75%.
Complete response (CR), toxic deaths, OS and event-free survival (EFS) were analyzed using the Kaplan–Meier method and compared with the log-rank test.
A predefined subgroup analysis focused on localized bulky stages.

### Results

Eighty-three (83) patients were included. The following table resumes the therapeutic outcomes of different MDH versions.
The response difference between P1 versus P2 was statistically significant (p=0.02).
Toxic deaths were observed in both P1 (n=1) and P2 (n=1).

**Copyright Information:** (Online) ISSN: 2572-9241
©2026 The Author(s). HemaSphere is published by John Wiley & Sons Ltd on behalf of European Hematology Association. This is an open access Abstract Book distributed under the Attribution-NonCommercial-NoDerivs (CC BY-NC-ND), which allows third parties to download the articles and share them with others as long as they credit the author and the Abstract Book, but they cannot change the content in any way or use them commercially.
**Abstract Book Citations:** Authors, Title, HemaSphere, 2026;10:(S1):pages. The abstract book DOIs can be found on the "Author Information" page.
**Disclaimer:** Articles published in the journal HemaSphere exclusively reflect the opinions of the authors. The authors are responsible for all content in their abstracts including accuracy of the facts, statements, citing resources, etc.

**Summary/Conclusion**

Our study demonstrates that escalated BEACOPP-based regimens significantly improve response rates and survival outcomes compared to ABVD in patients with bulky mediastinal HL, with a 5-year OS at 85% versus 54–60% with ABVD.
These results are consistent with long-term follow-up data from the German Hodgkin Study Group (GHSG) HD9 and HD12 trials, which confirmed superior tumor control with escalated BEACOPP in advanced-stage HL, particularly in high-risk populations including those with bulky disease (approximately 80–85% vs 70–75% in high-risk populations) (1,2).
To further improve outcomes, integration of novel agents such as brentuximab vedotin or checkpoint inhibitors (nivolumab, pembrolizumab) into first-line therapy could enhance efficacy while reducing chemotherapy exposure, as suggested by phase II studies showing promising results in bulky disease (3,4).
Prospective studies incorporating these approaches are needed to personalize therapy and minimize long-term toxicity in bulky mediastinal HL.
**Références :**

1. Diehl V, Franklin J, Pfreundschuh M, et al. Standard and increased-dose BEACOPP chemotherapy compared with COPP-ABVD for advanced Hodgkin's disease. N Engl J Med. 2003;348(24):2386-2395.
2. Borchmann P, Haverkamp H, Diehl V, et al. Eight cycles of escalated-dose BEACOPP compared with four cycles of escalated-dose BEACOPP followed by four cycles of baseline-dose BEACOPP with or without radiotherapy in patients with advanced-stage Hodgkin's lymphoma: final analysis of the HD12 trial of the German Hodgkin Study Group. J Clin Oncol. 2011;29(32):4234-4242.
3. Allen PB, Savas H, Evens AM, et al. Pembrolizumab followed by AVD in untreated early unfavorable and advanced-stage classical Hodgkin lymphoma. Blood. 2021;138(16):1430-1440.
4. Ramchandren R, Domingo-Domènech E, Rueda A, et al. Nivolumab for newly diagnosed advanced-stage classic Hodgkin lymphoma: safety and efficacy in the phase II CheckMate 205 study. J Clin Oncol. 2019;37(23):1997-2007.

| | P1 (n=20) | | | P2 (n=63) | | |
|---|---|---|---|---|---|---|
| | Localized stage (n= 9) | Advanced Stage (n= 11) | **Total** | Localized stage (n= 21) | Advanced Stage (n= 42) | **Total** |
| **CR** | 6 | 5 | **11 (55%)** | 12 | 29 | **41 (65%)** |
| **PR** | 1 | 0 | **1 (5%)** | 7 | 5 | **12 (19%)** |
| **Primary failure** | 2 | 5 | **7 (35%)** | 1 | 8 | **9 (14%)** |
| **Toxic death** | 0 | 1 | **1 (5%)** | 1 | 0 | **1 (1.6%)** |
| **5-year OS** | 60% | 54.5% | **57%** | 85.6% | 85% | **85.3%** |
| **5-year EFS** | 40% | 36% | **38%** | 69.5% | 67.8% | **68.6%** |

Therapeutic results of different MDH versions.

Copyright Information: (Online) ISSN: 2572-9241
©2026 The Author(s). HemaSphere is published by John Wiley & Sons Ltd on behalf of European Hematology Association. This is an open access Abstract Book distributed under the Attribution-NonCommercial-NoDerivs (CC BY-NC-ND), which allows third parties to download the articles and share them with others as long as they credit the author and the Abstract Book, but they cannot change the content in any way or use them commercially.
Abstract Book Citations: Authors, Title, HemaSphere, 2026;10:(S1):pages. The abstract book DOIs can be found on the “Author Information” page.
Disclaimer: Articles published in the journal HemaSphere exclusively reflect the opinions of the authors. The authors are responsible for all content in their abstracts including accuracy of the facts, statements, citing resources, etc.

### (PB3595) HIGH DISCORDANCE BETWEEN CT AND PET ALTERS THERAPEUTIC STRATEGY IN HIGH-RISK HODGKIN LYMPHOMA: EVIDENCE FROM A PET-GUIDED REAL-WORLD COHORT

**Topic:** 17. Hodgkin lymphoma - Clinical

Nesrine Ben Sayed*[1], Sabrine Hallabia[1], Abir Ezzine[1], Amira Rahal[1], Roua Chokri[1], Rihab Ben Sghaier[1], Haifa Regaieg[1], Kmira Zahra[1], Wafa Chenbah[1], Nadia Sassi[1], Monia Zaier[1], Ben Youssef Yosra[1]

*[1]FARHAT HACHED HOSPITAL, Clinical Hematology, Sousse, Tunisia;*

### Background

In high-risk Hodgkin lymphoma (HL), accurate response assessment is essential to avoid undertreatment of refractory disease and unnecessary toxicity from overtreatment. While CT evaluates morphological changes, PET provides metabolic assessment and may significantly impact therapeutic decisions.

### Aims

In high-risk Hodgkin lymphoma (HL), accurate response assessment is essential to avoid undertreatment of refractory disease and unnecessary toxicity from overtreatment. While CT evaluates morphological changes, PET provides metabolic assessment and may significantly impact therapeutic decisions.

### Methods

Ninety-nine high-risk HL patients received BEACOPP-R–based therapy. An interim evaluation was performed after 2–3 cycles and repeated after 4–5 cycles in patients with suboptimal response. End-of-treatment assessment included CT and/or PET. Treatment de-escalation, and radiotherapy indication were based on imaging results.

### Results

At early interim assessment, CT (70%) showed CR/CRi in 28.8%, whereas PET (33.3%) demonstrated complete metabolic response (CMR) in 75.8%, revealing a substantial discrepancy in response evaluation. PET prompted therapeutic de-escalation in discordant cases. Among patients evaluated with both modalities at the end of chemotherapy (n=16), major discordance was observed in 15 cases (94%). PET detected occult progression in patients classified as stable disease, partial response, or equivocal CR on CT, and reclassified several CT-defined incomplete responses as metabolic CR. Overall end-of-chemotherapy response rate (≥75%) was 60%, with 38% refractory disease and 2% toxic mortality. Radiotherapy was indicated in 11% of patients based exclusively on PET-detected residual metabolic activity.

### Summary/Conclusion

CT substantially misclassified disease status compared with PET in high-risk HL. PET-guided assessment significantly altered therapeutic strategy, including de-escalation and radiotherapy indication. These findings strongly support systematic integration of PET into risk-adapted management algorithms for high-risk HL.

**Copyright Information:** (Online) ISSN: 2572-9241
©2026 The Author(s). HemaSphere is published by John Wiley & Sons Ltd on behalf of European Hematology Association. This is an open access Abstract Book distributed under the Attribution-NonCommercial-NoDerivs (CC BY-NC-ND), which allows third parties to download the articles and share them with others as long as they credit the author and the Abstract Book, but they cannot change the content in any way or use them commercially.
**Abstract Book Citations:** Authors, Title, HemaSphere, 2026;10:(S1):pages. The abstract book DOIs can be found on the “Author Information” page.
**Disclaimer:** Articles published in the journal HemaSphere exclusively reflect the opinions of the authors. The authors are responsible for all content in their abstracts including accuracy of the facts, statements, citing resources, etc.

### (PB3596) REFRACTORY/RELAPSED HODGKIN LYMPHOMA IN CHILDREN: EXPERIENCE OF THE PEDIATRIC CLINICAL HEMATOLOGY DEPARTMENT AT AZIZA OTHMANA HOSPITAL

**Topic:** 17. Hodgkin lymphoma - Clinical

Oumeyma Touati[1], Roua Hsasna[1], Emna Azza[1], Ben Othmen Aya[1], Yosr Ben Abdennabi[1], Marwa Bahri[1], Lamia Issaoui*[1]

[1]*Aziza Othmana Hospital, Tunis, Tunisia;*

### Background

Hodgkin lymphoma (HL) in children is highly curable in children with response rates between 80 and 90%. However, some patients have refractory disease or relapse (R/R) after first-line treatment, requiring more intensive follow-up treatment.

### Aims

The aim of our study was to describe the characteristics of children with R/R HL and to identify, through exploratory analysis, the factors associated with the R/R form.

### Methods

This is a retrospective descriptive study including patients aged 2 to 18 years old followed for R/R HL in the department from November 2021 to December 2025. The analysis of factors associated with the R/R form was performed using the chi-square test with a significance value of $p < 0.05$. Multivariate analysis was not performed due to the small sample size.

### Results

Among thirty-three patients monitored for HL during the study period, ten patients (30%) were refractory or relapsed: six patients had progressive disease at the end of treatment and four patients relapsed. Relapse was early in three patients, with a median relapse time of 4.5 months [3-16 months].
All patients received salvage chemotherapy. The IGEV protocol was used in 1 patient, but there was no response and the patient died. The ABVD-IEP protocol was used in 9 patients: 4 patients achieved complete response (CR) and received autologous hematopoietic stem cell transplantation (ASCT). Third-line treatment with brentuximab vedotin-bendamustine was administered in 5 patients, resulting in CR, which was consolidated by ASCT in 4 patients. One patient is awaiting fourth-line treatment with nivolumab. Overall, the CR rate after second-line treatment was 40% and the overall CR rate for R/R patients was 80%.
In a univariate analysis, the elements of the initial diagnosis that were significantly associated with the R/R form were: bulky disease ($p=0.049$), mediastinal enlargement ($p=0.035$), a CHIPS score ≥ 3 ($p=0.05$), and an inadequate response (partial or progression) after two courses of chemotherapy ($p=0.008$).

### Summary/Conclusion

In the era of immunotherapy, salvage treatment of R/R HL in children achieves satisfactory response rates, but at the cost of significant toxicity. Our population has a higher rate of R/R disease than reported in the literature, which requires further larger studies to accurately identify high-risk patients.

**Copyright Information:** (Online) ISSN: 2572-9241
©2026 The Author(s). HemaSphere is published by John Wiley & Sons Ltd on behalf of European Hematology Association. This is an open access Abstract Book distributed under the Attribution-NonCommercial-NoDerivs (CC BY-NC-ND), which allows third parties to download the articles and share them with others as long as they credit the author and the Abstract Book, but they cannot change the content in any way or use them commercially.
**Abstract Book Citations:** Authors, Title, HemaSphere, 2026;10:(S1):pages. The abstract book DOIs can be found on the “Author Information” page.
**Disclaimer:** Articles published in the journal HemaSphere exclusively reflect the opinions of the authors. The authors are responsible for all content in their abstracts including accuracy of the facts, statements, citing resources, etc.

### (PB3597) CLINICAL PROFILE, MANAGEMENT, AND EARLY OUTCOMES OF CLASSICAL HODGKIN LYMPHOMA: A REAL-WORLD DATA FROM A SINGLE-CENTER SERIES (2019–2024)

**Topic:** 17. Hodgkin lymphoma - Clinical

Amira Fahem[1], Amelia Ziane Khodja[1], Belkis Dekhili[1], Mounira Bitam[1], Nawal Ait Kaci[1], Naima Abdennebi[1], Fateh Boukhemia[1], Sabrina Akhrouf[1], Hanane Bouarab[1], Hanane Moussaoui[1], Rose Marie Hamladji[1], Redhouane Ahmed Nacer[1], Malek Benakli*[1]

[1]*Hematology and Bone Marrow Transplantation department, Pierre and Marie Curie Center, Algiers, Algeria;*

### Background

Classical Hodgkin lymphoma (cHL) is a highly curable malignancy. Survival depends on accurate staging and access to risk-adapted strategies, including PET-CT imaging, and immunotherapy. This study analyzes the demographic, clinical, and prognostic characteristics, along with first-line treatment patterns and early outcomes, of cHL patients (pts) managed at a single-center.

### Methods

This retrospective study included 315 pts diagnosed between January 2019 and December 2024. Data analyzed included age, sex, presenting symptoms, histopathology, Ann Arbor stage, GHSG classification, International Prognostic Score (IPS), imaging modalities, treatment protocols, and response rates.

### Results

The cohort showed a slight female predominance (n=171, 54.3%), with a peak incidence in young adults aged 25–34 years (n=103, 32.7%). A family history of malignancy was reported in 33% of pts (n=104), including hematologic malignancies in 5.1% (n=16). Superficial lymphadenopathy was the most common presenting feature (n=214, 67.9%). The nodular sclerosis subtype (subtype 2) was the most frequent histological finding (n=216, 68.5%). At presentation, 50.8% (n=160) had advanced-stage disease (III–IV). According to GHSG criteria, 58.7% (n=185) were classified as advanced stage, and 32.1% (n=101) were high-risk per IPS. Baseline PET-CT was performed in only 22.2% of pts (n=70), reflecting limited accessibility.
First-line therapy consisted of ABVD (n=176, 55.9%) or BEACOPP/escalated (n=121, 38.4%). Complete response (CR) was achieved in 32.1% (n=101) and partial response (PR) in 52.7% (n=166). Refractory disease was observed in 10.8% (n=34), and early mortality occurred in 2.5% (n=8).

### Summary/Conclusion

This study highlights a high prevalence of advanced-stage cHL at diagnosis alongside restricted access to PET-CT. Despite these constraints, early response rates remain encouraging. Improving early detection, expanding access to functional imaging, and ensuring the availability of innovative therapies (such as immunotherapy) are essential to optimizing patient outcomes in this setting.

**Copyright Information:** (Online) ISSN: 2572-9241
©2026 The Author(s). HemaSphere is published by John Wiley & Sons Ltd on behalf of European Hematology Association. This is an open access Abstract Book distributed under the Attribution-NonCommercial-NoDerivs (CC BY-NC-ND), which allows third parties to download the articles and share them with others as long as they credit the author and the Abstract Book, but they cannot change the content in any way or use them commercially.
**Abstract Book Citations:** Authors, Title, HemaSphere, 2026;10:(S1):pages. The abstract book DOIs can be found on the "Author Information" page.
**Disclaimer:** Articles published in the journal HemaSphere exclusively reflect the opinions of the authors. The authors are responsible for all content in their abstracts including accuracy of the facts, statements, citing resources, etc.

### (PB3598) THE USE OF PEMBROLIZUMAB AS MONOTHERAPY AND IN COMBINATION WITH CHEMOTHERAPY IN THE TREATMENT OF RELAPSED/REFRACTORY CLASSICAL HODGKIN LYMPHOMA

**Topic:** 17. Hodgkin lymphoma - Clinical

Bibigaisha Adusheva*[1], Vadim Kemaikin[1], Azat Karabekov[1], Aidana Shalkarbekova[1], Meiramgul' Yussupova[1], Akbota Tursyn[1], Olga Kolesnikova[1], Fariza Shokubayeva[1], Alexandr Kolesnev[1], Gulnur Mamyr[1], Rose Ibragimova[1]

*[1]NROC, Astana, Kazakhstan;*

### Background

According to the electronic oncology patients registry, 187 patients with classical Hodgkin lymphoma (cHL) were diagnosed in the Republic of Kazakhstan in 2024 and 180 patients in 12 months of 2025. Most patients with classical Hodgkin lymphoma (cHL) are treated with first-line combination chemotherapy. However, approximately 10–15% of patients with early-stage disease and 15–30% of those with advanced-stage disease will relapse or develop primary refractory disease [12,34],requiring the implementation of novel therapeutic approaches, including the integration of immune checkpoint inhibitors, such as Pembrolizumab, into the treatment of relapsed/refractory classical Hodgkin lymphoma (R/R cHL).

### Aims

To evaluate the efficacy of pembrolizumab as monotherapy in patients with relapsed/refractory classical Hodgkin lymphoma (R/R cHL), and to assess the rationale for combining immune checkpoint inhibitors with conventional chemotherapy to enhance antitumor activity in patients with lack of response to monotherapy.

### Methods

This retrospective single-center study included 24 patients with histologically confirmed classical Hodgkin lymphoma (cHL) who received pembrolizumab monotherapy at the LLP "National Scientific Oncology Center" (Astana, Kazakhstan).

The median follow-up was 23 months.

| | |
|---|---|
| **Number of patients, n** | **24** |
| **Median age, years (range)** | 31 (18 – 55) |
| **Sex** | Male n=9 (45%)<br>Female n=15 (55%) |
| **Ann-Arbor stage, n (%)** | II 6 (25%)<br>III 9 (37,5%)<br>IV 9 (37,5%) |
| **Bulky** | 6 (25%) |
| **Previous therapy** | 1-line: 1 (4%)<br>2 - lines: 5 (21%)<br>3 - lines: 14 (58%)<br>More than 4 lines : 4 (17%) |
| **Radiotherapy** | 7 (29%) |

**Copyright Information:** (Online) ISSN: 2572-9241
©2026 The Author(s). HemaSphere is published by John Wiley & Sons Ltd on behalf of European Hematology Association. This is an open access Abstract Book distributed under the Attribution-NonCommercial-NoDerivs (CC BY-NC-ND), which allows third parties to download the articles and share them with others as long as they credit the author and the Abstract Book, but they cannot change the content in any way or use them commercially.
**Abstract Book Citations:** Authors, Title, HemaSphere, 2026;10:(S1):pages. The abstract book DOIs can be found on the "Author Information" page.
**Disclaimer:** Articles published in the journal HemaSphere exclusively reflect the opinions of the authors. The authors are responsible for all content in their abstracts including accuracy of the facts, statements, citing resources, etc.

### Results

| | |
|---|---|
| Response evaluation after 2–4 cycles of pembrolizumab monotherapy<br>Pembrolizumab monotherapy,n(%) | 24 (100%) |
| Objective response – continued monotherapy (CR/PR)<br>Lack of response (SD/PD) | 12 (50%)<br>12 (50%) |
| Therapy escalation in case of lack of response, n(%)<br>Pembrolizumab<br>Pembrolizumab + Bendamustine<br>Pembrolizumab + ICE | 12 (100%)<br>3 (25%)<br>7 (58%)<br>2 (17%) |

Patients who achieved an objective response continued pembrolizumab monotherapy, while therapy escalation with the addition of chemotherapy was performed in 75% of patients who did not respond to pembrolizumab monotherapy. All of these patients subsequently achieved an objective response (Figure 1).

### Summary/Conclusion

Initially, the use of Pembrolizumab monotherapy may be accompanied by minimization of toxicity effects while achieving efficacy. However, in cases of a lack of response, an escalation strategy involving the addition the addition of chemotherapy may be justified, as confirmed by our observations. This phenomenon may be explained by the modulatory effect of pembrolizumab on the tumor microenvironment, which helps restore the sensitivity of tumor cells to cytostatic therapy.

**Copyright Information:** (Online) ISSN: 2572-9241
©2026 The Author(s). HemaSphere is published by John Wiley & Sons Ltd on behalf of European Hematology Association. This is an open access Abstract Book distributed under the Attribution-NonCommercial-NoDerivs (CC BY-NC-ND), which allows third parties to download the articles and share them with others as long as they credit the author and the Abstract Book, but they cannot change the content in any way or use them commercially.
**Abstract Book Citations:** Authors, Title, HemaSphere, 2026;10:(S1):pages. The abstract book DOIs can be found on the "Author Information" page.
**Disclaimer:** Articles published in the journal HemaSphere exclusively reflect the opinions of the authors. The authors are responsible for all content in their abstracts including accuracy of the facts, statements, citing resources, etc.

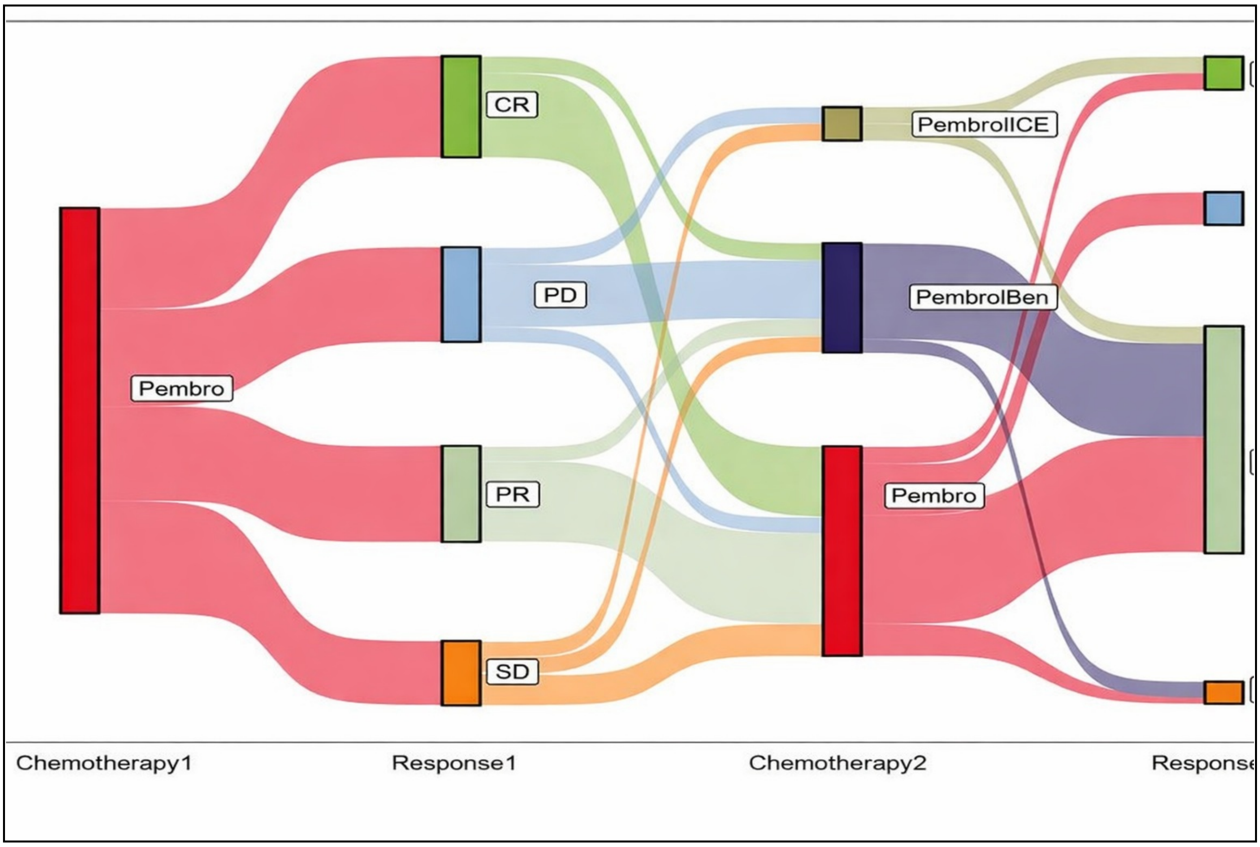

Copyright Information: (Online) ISSN: 2572-9241
©2026 The Author(s). HemaSphere is published by John Wiley & Sons Ltd on behalf of European Hematology Association. This is an open access Abstract Book distributed under the Attribution-NonCommercial-NoDerivs (CC BY-NC-ND), which allows third parties to download the articles and share them with others as long as they credit the author and the Abstract Book, but they cannot change the content in any way or use them commercially.
Abstract Book Citations: Authors, Title, HemaSphere, 2026;10:(S1):pages. The abstract book DOIs can be found on the "Author Information" page.
Disclaimer: Articles published in the journal HemaSphere exclusively reflect the opinions of the authors. The authors are responsible for all content in their abstracts including accuracy of the facts, statements, citing resources, etc.

### (PB3599) ANALYSIS OF THIRD-LINE TREATMENT OUTCOMES IN PATIENTS WITH RELAPSED/REFRACTORY MANTLE CELL LYMPHOMA FOLLOWING FRONTLINE BENDAMUSTINE/RITUXIMAB AND SECOND-LINE BTK INHIBITOR THERAPY

**Topic:** 18. Indolent and mantle-cell non-Hodgkin lymphoma - Clinical

Christine Ryan*[1], Melissa Larson[2], Yucai Wang[2], Stephen Hwang[2], Diego Villa[3], Laveniya Kugathasan[3], Anita Kumar[4], Ashlee Joseph[4], Imran Nizamuddin[5], Brad Kahl[5], Taylor Brooks[6], Brian Hill[6], Alexey Danilov[7], Georgios Pongas[8], Izidore Lossos[8], Patrick Reagan[9], Philip Young[10], Craig Portell[10], Jia Ruan[11], David Bond[12], Kami Maddocks[12], Firas Baidoun[13], Muhamad Alhaj Moustafa[13], Arash Velayati[14], Brian Greenwell[14], Evguenia Ouchveridze[15], Aung M. Tun[15], Rahul Bhansali[16], Stefan Barta[16], Manali Kamdar[17], Anna Jacob[17], Drew Gerber[18], Sabarish Ayyappan[19], Eric Mou[19], Zoey Harris[20], Javier Munoz[20], Anthony Stack[21], Marcus Messmer[21], Natalie Grover[22], Nazneen Khan[22], Lauren Banaszak[23], Priyanka Pophali[23], Mayur Narkhede[24], Amitkumar Mehta[24], Preetesh Jain[25], Michael L. Wang[25], Tamara K. Moyo[26], Nilanjan Ghosh[26], Alexander Stanisic[27], Reem Karmali[27], Matthew Maurer[2], James Cerhan[2], Jonathon Cohen[18], Peter Martin[11], Reid Merryman[1]

*[1]Dana-Farber Cancer Institute, Medical Oncology, Boston, MA, United States of America; [2]Mayo Clinic, Rochester, MN, United States of America; [3]BC Cancer Centre for Lymphoid Cancer and University of British Columbia, Vancouver, Canada; [4]Memorial Sloan-Kettering Cancer Center, New York, NY, United States of America; [5]Washington University in St. Louis, St. Louis, MO, United States of America; [6]Cleveland Clinic, Cleveland, OH, United States of America; [7]City of Hope, Duarte, CA, United States of America; [8]University of Miami, Miami, FL, United States of America; [9]University of Rochester, Rochester, NY, United States of America; [10]University of Virginia, Charlottesville, VA, United States of America; [11]Weill Cornell Medicine, New York, NY, United States of America; [12]Ohio State University, Columbus, OH, United States of America; [13]Mayo Clinic, Jacksonville, FL, United States of America; [14]Medical University of South Carolina, Charleston, SC, United States of America; [15]University of Kansas Medical Center, Kansas City, KS, United States of America; [16]University of Pennsylvania, Philadelphia, PA, United States of America; [17]University of Colorado, Aurora, CO, United States of America; [18]Emory University Winship Cancer Institute, Atlanta, GA, United States of America; [19]University of Iowa, Iowa City, IA, United States of America; [20]Mayo Clinic, Phoenix, AZ, United States of America; [21]Fox Chase Cancer Center, Philadelphia, PA, United States of America; [22]University of North Carolina, Chapel Hill, NC, United States of America; [23]University of Wisconsin, Madison, WI, United States of America; [24]University of Alabama, Birmingham, AL, United States of America; [25]MD Anderson Cancer Center, Houston, TX, United States of America; [26]Atrium Health Levine Cancer Institute, Wake Forest University School of Medicine, Charlotte, NC, United States of America; [27]Northwestern University, Chicago, IL, United States of America;*

### Background

MCL remains incurable and treatment options are limited for relapsed/refractory (R/R) disease. Sequential frontline (1L) bendamustine/rituximab (BR) and second-line (2L) BTK inhibitor (BTKi) remains a viable treatment approach given there is no significant overall survival (OS) benefit currently observed in the SHINE and ECHO studies. Real-world third-line (3L) outcomes following sequential BR and BTKi have not been robustly reported. The LEO consortium recently led a large multicenter retrospective study of patients (pts) with MCL treated with 1L BR (Wang et al., Blood Adv, 2026).

### Aims

We sought to describe outcomes of 2L BTKi and 3L treatments following 1L BR.

**Copyright Information:** (Online) ISSN: 2572-9241
©2026 The Author(s). HemaSphere is published by John Wiley & Sons Ltd on behalf of European Hematology Association. This is an open access Abstract Book distributed under the Attribution-NonCommercial-NoDerivs (CC BY-NC-ND), which allows third parties to download the articles and share them with others as long as they credit the author and the Abstract Book, but they cannot change the content in any way or use them commercially.
**Abstract Book Citations:** Authors, Title, HemaSphere, 2026;10:(S1):pages. The abstract book DOIs can be found on the "Author Information" page.
**Disclaimer:** Articles published in the journal HemaSphere exclusively reflect the opinions of the authors. The authors are responsible for all content in their abstracts including accuracy of the facts, statements, citing resources, etc.

### Methods

We leveraged the existing dataset from 8 LEO and 19 collaborating North American centers of pts with MCL who initiated 1L BR Jan 2014-Dec 2020. We analyzed pts who received 1L BR followed by 2L BTKi +/- rituximab or obinutuzumab. All 1L BR combinations, non-BTKi 2L treatments, and other 2L BTKi-based combinations were excluded. 3L treatments were categorized in a non-overlapping hierarchal approach. Event-free survival (EFS) was defined as time from index line treatment start to the first event (progression, relapse, re-treatment, or death from any cause).

### Results

249 pts were identified who received 1L BR followed by 2L BTKi. Patient characteristics at 2L were: 69% male, 95% white, median age 74 years (range 36-93), 82% age > 65 years. Disease characteristics were: 82% stage IV, 51% high-MIPI score, 31% Ki67 ≥ 50%, 55% TP53-aberrant (34/62 evaluable), 62% POD24. 2L BTKi was ibrutinib, acalabrutinib, or zanubrutinib in 59%, 29%, and 13% of pts, respectively.
The 2L BTKi overall response rate was 67%. With a median follow-up of 41 months (m) from 2L BTKi initiation, the median EFS was 11.5m (95% CI: 7.9-14.0) and the median OS was 26.4m (95% CI: 17.4-37.1).
At data cutoff 26% of pts were event-free following 2L BTKi treatment, 27% had died without receiving a 3L treatment (51/67 due to progressive disease [PD] and/or therapy-related complications), 42% (n=103) had proceeded to 3L treatment, and for 6%, no further data were available. 3L treatments were: chemotherapy (n=31), venetoclax-based (n=20), lenalidomide-based (n=15), bortezomib-based (n=11), covalent BTKi (n=8), anti-CD19 chimeric antigen receptor (CAR) T-cell therapy (n=7), and other (n=11, which included n=2 mosunetuzumab and n=1 pirtobrutinib).
With a median follow-up from 3L initiation of 33m, the median EFS was 3.0m (95% CI: 2.4-5.1). Median EFS by treatment (Figure 1) was longest with CAR T-cell therapy: 25.1m (95% CI: 10.4-NR; p=0.014) and shortest with chemotherapy: 1.6m (95% CI: 1.0-3.6). The median OS from 3L initiation was 12.3m (95% CI: 6.7-33.7). BTKi responders (complete or partial response) compared to nonresponders had significantly longer median EFS (5.2m vs 1.9m, p=0.016) and OS (33.7m vs 5.4m, p=0.032).

### Summary/Conclusion

In this large multicenter retrospective study, the median EFS of 2L BTKi following 1L BR was short, likely reflecting both an enrichment of pts with POD24 disease and poorer outcomes observed in the real-world setting. Notably ~1/4 of pts died from PD prior to receiving 3L treatment. Outcomes of 3L treatments were poor and non-responders to 2L BTKi are a particularly high-risk group. These results establish a benchmark for 3L treatment post 1L BR / 2L BTKi, demonstrate that more effective treatments for older pts with R/R MCL are needed post-BTKi, and suggest the importance of maximizing efficacy of early lines of therapy.

**Copyright Information:** (Online) ISSN: 2572-9241
©2026 The Author(s). HemaSphere is published by John Wiley & Sons Ltd on behalf of European Hematology Association. This is an open access Abstract Book distributed under the Attribution-NonCommercial-NoDerivs (CC BY-NC-ND), which allows third parties to download the articles and share them with others as long as they credit the author and the Abstract Book, but they cannot change the content in any way or use them commercially.
**Abstract Book Citations:** Authors, Title, HemaSphere, 2026;10:(S1):pages. The abstract book DOIs can be found on the “Author Information” page.
**Disclaimer:** Articles published in the journal HemaSphere exclusively reflect the opinions of the authors. The authors are responsible for all content in their abstracts including accuracy of the facts, statements, citing resources, etc.

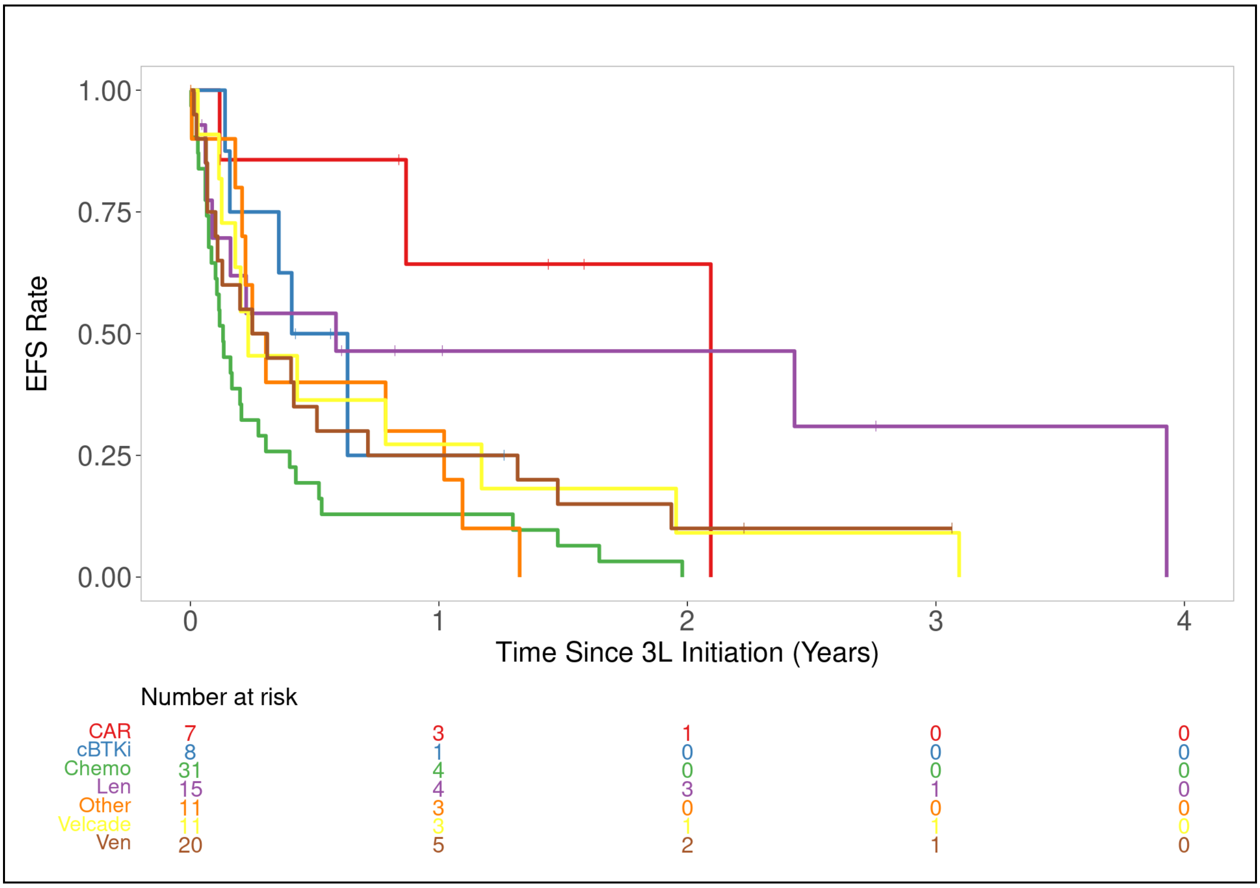

Figure 1

**Copyright Information:** (Online) ISSN: 2572-9241
©2026 The Author(s). HemaSphere is published by John Wiley & Sons Ltd on behalf of European Hematology Association. This is an open access Abstract Book distributed under the Attribution-NonCommercial-NoDerivs (CC BY-NC-ND), which allows third parties to download the articles and share them with others as long as they credit the author and the Abstract Book, but they cannot change the content in any way or use them commercially.
**Abstract Book Citations:** Authors, Title, HemaSphere, 2026;10:(S1):pages. The abstract book DOIs can be found on the "Author Information" page.
**Disclaimer:** Articles published in the journal HemaSphere exclusively reflect the opinions of the authors. The authors are responsible for all content in their abstracts including accuracy of the facts, statements, citing resources, etc.

### (PB3600) REAL-WORLD EFFECTIVENESS AND SAFETY OF ZANUBRUTINIB IN WALDENSTROM MACROGLOBULINEMIA: RESULTS FROM THE BELGIAN WIZARD STUDY

**Topic:** 18. Indolent and mantle-cell non-Hodgkin lymphoma - Clinical

Willem Daneels*[1, 2, 3], Jeroen Claes[4], Tom Feys[5], Caroline Pieters[6], Sandrine Dupont[6], Anke Verheyen[7]

*[1]Department of Hematology, Ghent University Hospital, Ghent, Belgium;[2]Department of Internal Medicine and Pediatrics, Ghent University, Ghent, Belgium;[3]Cancer Research Institute Ghent, Ghent, Belgium;[4]Cropland, Antwerp, Belgium;[5]Tom Feys Medical Writing, Izegem, Belgium;[6]BeOne Medicines, Ltd, Brussels, Belgium;[7]Novellas Healthcare, Brussels, Belgium;*

### Background

Zanubrutinib is a potent, highly selective, irreversible, second-generation Bruton tyrosine kinase (BTK) inhibitor. In the pivotal phase 3 ASPEN trial, zanubrutinib demonstrated high efficacy and favorable tolerability in patients with Waldenström macroglobulinemia (WM), establishing it as a standard BTK inhibitor in this setting. In November 2021, the European Medicines Agency approved zanubrutinib for adult patients with WM who had received at least one prior therapy or as first-line treatment in patients unsuitable for immunochemotherapy.

### Aims

The multicenter, observational WIZARD study collected Belgian real-world data on the efficacy and safety of zanubrutinib in adult WM patients.

### Methods

WIZARD was a retrospective and prospective, multicenter, observational study conducted in Belgium. Eligible participants were adult patients with symptomatic WM who had received zanubrutinib in accordance with Belgian reimbursement criteria, with treatment initiated between October 1, 2022 and September 1, 2024. The primary objective was to evaluate the real-world effectiveness of zanubrutinib, assessed by major response rate (MRR), overall response rate (ORR), best individual response, and progression-free survival (PFS). Secondary objectives included time to (patient best) response, (best) response duration, overall survival (OS), and time to next treatment. This abstract describes the efficacy and safety results in the overall study population and across selected subgroups.

### Results

Eighty-nine patients were included, with a median age of 76 years and a median of two prior treatment lines; 37 patients (41.6%) received zanubrutinib in first line, and 20 (22.5%) had previously received an ibrutinib-containing regimen. An *MYD88* mutation was confirmed in 56 patients (62.9%) (5 patients [5.6%] were *MYD88* wildtype; *MYD88* mutation status was not available/unknown in 28 patients [31.5%]).

The MRR and ORR for the overall study population were 64.0% (95% CI, 53.2%-73.9%) and 77.5% (95% CI, 67.4%-85.7%), respectively, with 23 patients (25.8%) achieving at least a very good partial response. In patients with prior ibrutinib treatment, the MRR was 45.0% and ORR was 60.0%. At 1 and 2 years, PFS rates were 83.1% and 67.1%, respectively, with corresponding OS rates of 89.8% and 81.4%. Efficacy outcomes were consistent across investigated subgroups (**Table**), with high response rates and comparable PFS and OS results in older patients (≥75 years) relative to their younger counterparts. Zanubrutinib also demonstrated similar effectiveness in treatment-naive and previously treated populations, with high response rates irrespective of *MYD88* mutation status. The treatment was well tolerated, with hematoma (n=5) and muscle cramps (n=4) as the most common adverse events (AEs). Grade ≥3 AEs were observed in 8.9% of patients, with 5.6% of patients discontinuing treatment due to AEs.

**Copyright Information:** (Online) ISSN: 2572-9241
©2026 The Author(s). HemaSphere is published by John Wiley & Sons Ltd on behalf of European Hematology Association. This is an open access Abstract Book distributed under the Attribution-NonCommercial-NoDerivs (CC BY-NC-ND), which allows third parties to download the articles and share them with others as long as they credit the author and the Abstract Book, but they cannot change the content in any way or use them commercially.
**Abstract Book Citations:** Authors, Title, HemaSphere, 2026;10:(S1):pages. The abstract book DOIs can be found on the "Author Information" page.
**Disclaimer:** Articles published in the journal HemaSphere exclusively reflect the opinions of the authors. The authors are responsible for all content in their abstracts including accuracy of the facts, statements, citing resources, etc.

**Summary/Conclusion**

WIZARD corroborates the efficacy and safety of zanubrutinib in patients with WM in a real-world Belgian setting, irrespective of age, treatment setting, and *MYD88* mutation status. As such, these findings are well in line with outcomes observed in the pivotal ASPEN trial.

**Table.** Efficacy Across Selected Subgroups in the WIZARD Study

| | n | MRR, % | ORR, % | 1-y PFS, % | 2-y PFS, % | 1-y OS, % | 2-y OS, % |
|---|---|---|---|---|---|---|---|
| Overall | 89 | 64.0 | 77.5 | 83.1 | 67.1 | 89.9 | 81.4 |
| Age | | | | | | | |
| ≤65 y | 13 | 61.5 | 76.9 | 100 | 74.1 | 100 | 100 |
| 66-75 y | 30 | 60.0 | 76.7 | 80 | 64.4 | 96.7 | 81.8 |
| ≥75 y | 46 | 67.4 | 78.3 | 80.3 | 66.7 | 82.5 | 77.9 |
| Tx status | | | | | | | |
| Tx naive | 37 | 67.6 | 81.1 | 78.4 | 53.7 | 86.5 | 75.2 |
| Previously treated | 52 | 61.5 | 75.0 | 86.4 | 74.5 | 92.2 | 85.9 |
| *MYD88* status | | | | | | | |
| *MYD88* mutation | 56 | 64.3 | 80.4 | 82.1 | 63.9 | 89.3 | 80.4 |
| *MYD88* wt/unknown | 33 | 63.6 | 72.7 | - | - | - | - |

Tx, treatment; wt, wild type; yr, year.

Copyright Information: (Online) ISSN: 2572-9241
©2026 The Author(s). HemaSphere is published by John Wiley & Sons Ltd on behalf of European Hematology Association. This is an open access Abstract Book distributed under the Attribution-NonCommercial-NoDerivs (CC BY-NC-ND), which allows third parties to download the articles and share them with others as long as they credit the author and the Abstract Book, but they cannot change the content in any way or use them commercially.
Abstract Book Citations: Authors, Title, HemaSphere, 2026;10:(S1):pages. The abstract book DOIs can be found on the "Author Information" page.
Disclaimer: Articles published in the journal HemaSphere exclusively reflect the opinions of the authors. The authors are responsible for all content in their abstracts including accuracy of the facts, statements, citing resources, etc.

### (PB3601) RETREATMENT IN PATIENTS WITH MANTLE CELL LYMPHOMA: A SWEDISH LYMPHOMA REGISTER STUDY

**Topic:** 18. Indolent and mantle-cell non-Hodgkin lymphoma - Clinical

Sandra Lockmer*[1], Oskar Gauffin[1], Karin Ekstrom Smedby[2], Mats Jerkeman[3], Ingrid Glimelius[1]

*[1]Uppsala University, Department of Immunology, Genetics and Pathology, Cancer Precision Medicine, Uppsala, Sweden; [2]Karolinska Institute, Department of Medicine Solna, Clinical Epidemiology Division, Stockholm, Sweden; [3]Lund University, Department of Clinical Sciences, Division of Oncology, Lund, Sweden;*

### Background

Mantle cell lymphoma (MCL) is a non-Hodgkin lymphoma usually with an aggressive clinical course characterized by repeated relapses. Therapies in later lines are often selected among drugs to which the patient is previously unexposed. Despite a rapidly evolving treatment landscape, the number of options in clinical practice are still limited and patients sometimes receive retreatment with the same or a similar substance. In this national population-based register study we investigate the use of retreatment among MCL patients and the following time to treatment failure.

### Aims

To explore retreatment as a management option in MCL.

### Methods

The study cohort included all MCL patients diagnosed 2000-2023 in the Swedish Lymphoma Register with a coverage above 95%. Some treatment information beyond first line is missing in this register but was completed within the MCL Complete project[1] by an extensive review of medical charts for those diagnosed 2006-2018.
All reported therapy lines and subsequent evaluations with their respective dates were inspected and considered for identification of retreated patients. Retreatment was defined as the initiation of an anti-lymphoma therapy already received in a previous treatment line. Combination regimens including one or more components to which the patient was naïve were not regarded as retreatments. Re-initiation of a given therapy after a pause shorter than three months was disregarded, as were surgery, radiotherapy and stem cell transplant.
Baseline clinical characteristics were extracted for comparison. The primary endpoint was time to failure after retreatment, defined as the start of retreatment to start of next line therapy or death, with censoring at February 2024. For patients with retreatment the median time to failure after retreatment and 95% confidence intervals (CI) were estimated both overall and stratified by therapy line.

### Results

Out of 2,619 MCL patients diagnosed 2000-2023, 651 (24.9%) had therapy information beyond primary therapy. Among these 651, 140 patients with retreatment were identified (25.1%). Baseline characteristics including age, sex, MIPI and Ann Arbor stage were comparable between retreated and not retreated patients. Rituximab-bendamustine was the most common retreatment regimen (n = 67, 47.9%) and most retreatments occurred in second line. Ibrutinib was used for retreatment in 7 patients. Median time from diagnosis to initiation of retreatment was 38.4 months. Median time to failure after retreatment was 8.1 months (95% CI, 6.3 – 10.0) overall and for retreatments in second line 9.4 months (95% CI, 6.6 - 13.1).

**Copyright Information:** (Online) ISSN: 2572-9241
©2026 The Author(s). HemaSphere is published by John Wiley & Sons Ltd on behalf of European Hematology Association. This is an open access Abstract Book distributed under the Attribution-NonCommercial-NoDerivs (CC BY-NC-ND), which allows third parties to download the articles and share them with others as long as they credit the author and the Abstract Book, but they cannot change the content in any way or use them commercially.
**Abstract Book Citations:** Authors, Title, HemaSphere, 2026;10:(S1):pages. The abstract book DOIs can be found on the "Author Information" page.
**Disclaimer:** Articles published in the journal HemaSphere exclusively reflect the opinions of the authors. The authors are responsible for all content in their abstracts including accuracy of the facts, statements, citing resources, etc.

### Summary/Conclusion

In our nationwide cohort, retreatment occurred in as many as 25% of MCL patients with available follow-up information. The time to failure after retreatment of 9.4 months for patients with retreatment in second line was comparable to the estimate of time to failure after all second line therapy of 8.9 months in the MCL Complete study[1]. In conclusion, retreated patients seem to have a prognosis in line with those who receive other treatments, as seen at least in the subgroup selected for retreatment in clinical practice.

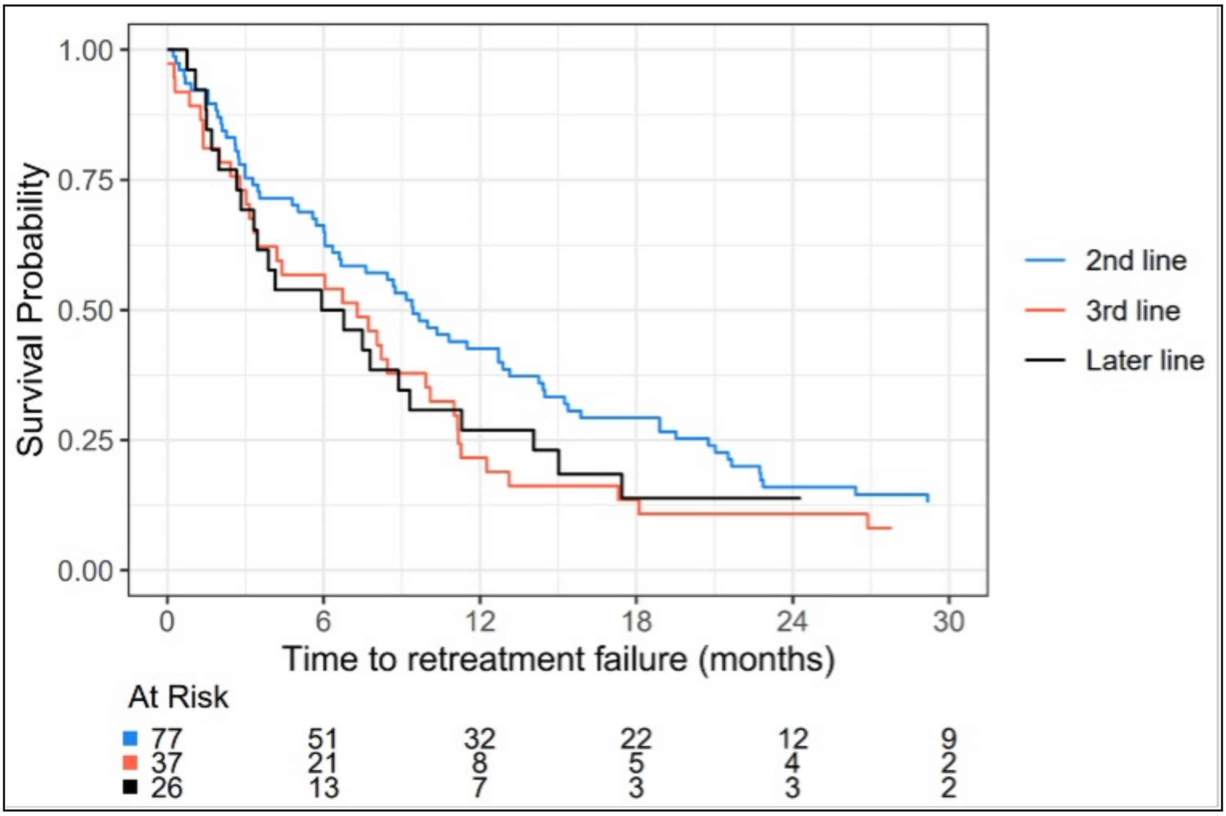

Figure 1. Time to failure after retreatment by therapy line.

**Copyright Information:** (Online) ISSN: 2572-9241
©2026 The Author(s). HemaSphere is published by John Wiley & Sons Ltd on behalf of European Hematology Association. This is an open access Abstract Book distributed under the Attribution-NonCommercial-NoDerivs (CC BY-NC-ND), which allows third parties to download the articles and share them with others as long as they credit the author and the Abstract Book, but they cannot change the content in any way or use them commercially.
**Abstract Book Citations:** Authors, Title, HemaSphere, 2026;10:(S1):pages. The abstract book DOIs can be found on the “Author Information” page.
**Disclaimer:** Articles published in the journal HemaSphere exclusively reflect the opinions of the authors. The authors are responsible for all content in their abstracts including accuracy of the facts, statements, citing resources, etc.

### (PB3602) TREATMENT OF HIGH-RISK WALDENSTROM MACROGLOBULINEMIA IN PATIENTS OVER 65 YEARS OF AGE

**Topic:** 18. Indolent and mantle-cell non-Hodgkin lymphoma - Clinical

Alexander Grachev*[1], Irina Galtseva[1], Anastasia Vinnikova[1], Elena Gribanova[1], Azaliya Valitova[1], Vyacheslav Ryzhko[1], Yulia Tsoi[1], Sofia Starchenko[1], Kulikov Sergey[1], Evgeniy Zvonkov[1]

[1]*National Medical Research Center of Hematology, Moscow, Russia;*

### Background

Waldenström's macroglobulinemia (WM) is currently an incurable disease. Despite the success of targeted therapies, the 5-year overall survival (OS) rates for patients in the very high-risk and high-risk groups are 36% and 47%, respectively. The low rate of complete responses (CR) to standard therapy and the relapsing course necessitate the search for new, effective treatment programs. Improving the effectiveness of therapy in patients over 65 years of age with unfavorable prognostic factors is a pressing issue.

### Aims

To study the efficacy of therapy using rituximab, bortezomib, bendamustine and dexamethasone (RBBd) in patients with WM.

### Methods

Thirty-four patients were included, 20 were men, with a median age of 68 years. According to R-IPSSWM, 19, 9, and 6 patients were in the intermediate, high, and very high risk groups, respectively. Hyperviscosity syndrome was present in 12 patients (35%), anemia in 31 (91%), Hb average – 83 g/L (60-120 g/L), platelets <100 thousand/µL in 11 (33%), hemolysis in 5 (15%), cryoglobulinemia in 8 (23.5%), PAP in 22 (65%), splenomegaly in 10 (29%), and B-symptoms in 24 (70%). LDH>250 U/L - in 11 (32%) patients, albumin <30 g/L - in 16 (49%), β-2-microglobulin >4 mg/L in 22 (64%) patients. Two patients had renal failure due to specific kidney damage. The average PIgM values were 37.5 g/L (3.6-77 g/L). MYD88 mutation - L265P was present in 29 (85%) and del-17p in 3 (8.8%) patients.

### Results

From December 2018 to August 2025, 34 patients received therapy according to the RBBd program. The response was assessed using modified IWWM-6 criteria. The overall response was 100%, partial response (PR) was 41%, very good partial response (VGPR) was 28.2%, and CR was 30.8% (Fig. 1). Of the two patients with renal failure at disease onset, renal function was restored in one. The 3- and 5-year OS was 90%. The 3- and 5-year progression-free survival rates were 85% and 75%, respectively.

### Summary/Conclusion

The RBBd therapy program is effective and safe for elderly patients with WM, as well as for low-risk patients under 65. This therapy achieves profound responses in more than half of patients and high rates of progression-free and overall survival. A study is ongoing to further evaluate the effectiveness of this treatment method.

**Copyright Information:** (Online) ISSN: 2572-9241
©2026 The Author(s). HemaSphere is published by John Wiley & Sons Ltd on behalf of European Hematology Association. This is an open access Abstract Book distributed under the Attribution-NonCommercial-NoDerivs (CC BY-NC-ND), which allows third parties to download the articles and share them with others as long as they credit the author and the Abstract Book, but they cannot change the content in any way or use them commercially.
**Abstract Book Citations:** Authors, Title, HemaSphere, 2026;10:(S1):pages. The abstract book DOIs can be found on the “Author Information” page.
**Disclaimer:** Articles published in the journal HemaSphere exclusively reflect the opinions of the authors. The authors are responsible for all content in their abstracts including accuracy of the facts, statements, citing resources, etc.

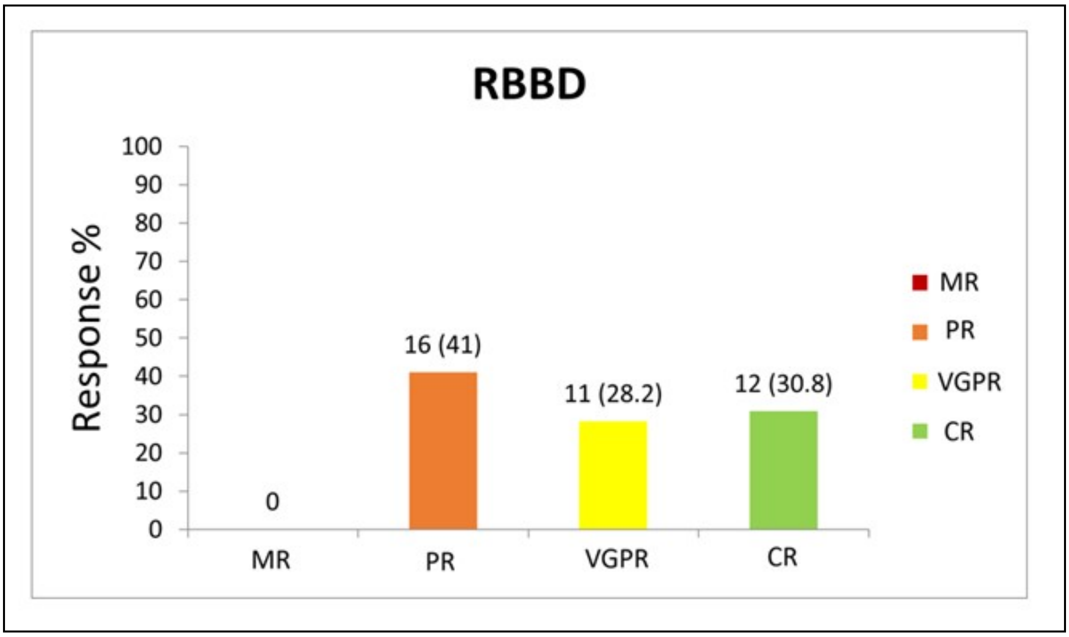

**Copyright Information:** (Online) ISSN: 2572-9241
©2026 The Author(s). HemaSphere is published by John Wiley & Sons Ltd on behalf of European Hematology Association. This is an open access Abstract Book distributed under the Attribution-NonCommercial-NoDerivs (CC BY-NC-ND), which allows third parties to download the articles and share them with others as long as they credit the author and the Abstract Book, but they cannot change the content in any way or use them commercially.
**Abstract Book Citations:** Authors, Title, HemaSphere, 2026;10:(S1):pages. The abstract book DOIs can be found on the "Author Information" page.
**Disclaimer:** Articles published in the journal HemaSphere exclusively reflect the opinions of the authors. The authors are responsible for all content in their abstracts including accuracy of the facts, statements, citing resources, etc.

### (PB3603) BREAST IMPLANT–ASSOCIATED LYMPHOMA: SURFACE TEXTURE AND SURVIVAL OUTCOMES IN A TEN-YEAR SYSTEMATIC REVIEW

**Topic:** 18. Indolent and mantle-cell non-Hodgkin lymphoma - Clinical

Kalysta Borges*[1], Cairo Borges Junior[1], Giulia Manuella Almeida[2], Sandrea Queiroz[1], Bianca Victória Almeida[1]

[1]*ONCOLÓGICA DO BRASIL, Santarém, Brazil;* [2]*SANTA CASA DE RIBEIRÃO PRETO, RIBEIRÃO PRETO, Brazil;*

### Background

Breast implant–associated lymphoma, particularly breast implant–associated anaplastic large cell lymphoma (BIA-ALCL), has been recognized as a distinct clinicopathological entity since 2016. Although the absolute incidence remains low, population-based evidence indicates increased risk related to implant surface texture. We performed a systematic review and meta-analysis (2016–2026) to quantify lymphoma risk and survival outcomes associated with breast implants.

### Aims

To systematically evaluate the incidence, implant surface–related risk, latency, and survival outcomes of breast implant–associated lymphomas, particularly BIA-ALCL, through a PRISMA-guided systematic review and meta-analysis of population-based and registry studies published between 2016 and 2026.

### Methods

A systematic search of PubMed/MEDLINE, Embase, Scopus, Web of Science, and the Cochrane Library was conducted. Eligible studies included cohort studies, registry analyses, and meta-analyses reporting standardized incidence ratios (SIRs), implant surface characteristics, or survival outcomes. A PRISMA 2020–compliant selection process was applied. Random-effects meta-analysis of SIRs was performed with subgroup analysis by implant surface (textured versus smooth). Pooled survival analysis with Kaplan–Meier reconstruction was conducted using published cohort and SEER data. Associations between implant texturization and lymphoma incidence were assessed using Pearson correlation, with 95% confidence intervals (CI).

### Results

The PRISMA flow diagram identified 2,100 unique records after duplicate removal, of which 38 studies met eligibility criteria and 24 were included in the meta-analysis, encompassing more than 17 million women with breast implants. A total of 1,170 cases of implant-associated lymphoma were reported. Overall, breast implants were associated with an increased relative risk of breast lymphoma, with a pooled standardized incidence ratio of 5.0 (95% CI 2.8–8.3), driven almost exclusively by BIA-ALCL. Subgroup analyses demonstrated that lymphoma occurrence was predominantly observed among recipients of textured breast implants, with pooled standardized incidence ratios ranging from 30 to 45 (95% CI 16–86), whereas exposure to smooth implants was not associated with a statistically significant excess risk. A positive correlation was observed between the degree of implant texturization and lymphoma incidence (Pearson $r = 0.72$; $p < 0.001$). Median latency from implantation to lymphoma diagnosis was seven years. Kaplan–Meier survival analysis demonstrated excellent prognosis for localized BIA-ALCL, with five-year disease-specific survival exceeding 95% (95% CI 92–98%) following implant removal and complete capsulectomy.

**Copyright Information:** (Online) ISSN: 2572-9241
©2026 The Author(s). HemaSphere is published by John Wiley & Sons Ltd on behalf of European Hematology Association. This is an open access Abstract Book distributed under the Attribution-NonCommercial-NoDerivs (CC BY-NC-ND), which allows third parties to download the articles and share them with others as long as they credit the author and the Abstract Book, but they cannot change the content in any way or use them commercially.
**Abstract Book Citations:** Authors, Title, HemaSphere, 2026;10:(S1):pages. The abstract book DOIs can be found on the "Author Information" page.
**Disclaimer:** Articles published in the journal HemaSphere exclusively reflect the opinions of the authors. The authors are responsible for all content in their abstracts including accuracy of the facts, statements, citing resources, etc.

**Summary/Conclusion**

This decade-long systematic review and meta-analysis confirm that breast implant–associated lymphoma remains exceedingly rare in absolute terms, despite a significantly increased relative risk driven by exposure to textured implants. PRISMA-guided synthesis, robust statistical associations, and Kaplan–Meier survival analysis demonstrate that BIA-ALCL is typically localized and associated with excellent prognosis when promptly treated surgically, supporting current recommendations against prophylactic implant removal in asymptomatic patients and regulatory restrictions on textured implants.

**Copyright Information:** (Online) ISSN: 2572-9241
©2026 The Author(s). HemaSphere is published by John Wiley & Sons Ltd on behalf of European Hematology Association. This is an open access Abstract Book distributed under the Attribution-NonCommercial-NoDerivs (CC BY-NC-ND), which allows third parties to download the articles and share them with others as long as they credit the author and the Abstract Book, but they cannot change the content in any way or use them commercially.
**Abstract Book Citations:** Authors, Title, HemaSphere, 2026;10:(S1):pages. The abstract book DOIs can be found on the "Author Information" page.
**Disclaimer:** Articles published in the journal HemaSphere exclusively reflect the opinions of the authors. The authors are responsible for all content in their abstracts including accuracy of the facts, statements, citing resources, etc.

### (PB3604) EFFICACY AND SAFETY OF NEMTABRUTINIB IN RELAPSED OR REFRACTORY FOLLICULAR LYMPHOMA: COHORT G OF THE PHASE 2 BELLWAVE-003 STUDY

**Topic:** 18. Indolent and mantle-cell non-Hodgkin lymphoma - Clinical

Wojciech Jurczak*[1], Ana Basquiera[2], Isabelle Fleury[3], Sujun Gao[4], Arpad Illes[5], Andras Masszi[6], Antonio Pinto[7], Sanne Lugthart[8], Omur Gokmen Sevindik[9], Iryna Kryachok[10], Elizabeth H. Phillips[11], Fei Li[12], Chan Y. Cheah[13], Yan Xu[14], Mohammed Farooqui[14], Jing Yang[14], Fangfang Lv[15]

*[1]Maria Sklodowska-Curie National Research Institute of Oncology, Kraków, Poland;[2]Hospital Privado Universitario de Córdoba, Córdoba, Argentina; [3]Hôpital Maisonneuve-Rosemont, Montréal, Canada;[4]First Hospital of Jilin University, Changchun, China; [5]Debreceni Egyetem Klinikai Központ, Debrecen, Hungary; [6]Országos Onkológiai Intézet, Budapest, Hungary; [7]Hematology-Oncology and Stem Cell Transplantation Unit, Istituto Nazionale Tumori, Fondazione G. Pascale, IRCCS, Naples, Italy; [8]University Hospital Bristol NHS Foundation Trust, Bristol, United Kingdom; [9]Istanbul Medipol University, International School of Medicine, Istanbul, Türkiye; [10]National Cancer Institute, Kyiv, Ukraine; [11]The Christie NHS Foundation Trust, Manchester, United Kingdom; [12]First Affiliated Hospital of Nanchang University, Nanchang, China;[13]Sir Charles Gairdner Hospital, Pathwest Laboratory Medicine WA, Linear Clinical Research and Hollywood Private Hospital, Nedlands, Australia; [14]Merck & Co., Inc., Rahway, United States of America; [15]Fudan University, Shanghai, China;*

### Background

Nemtabrutinib is a potent, noncovalent, reversible Bruton tyrosine kinase inhibitor (BTKi) administered orally (PO) once daily (QD) and has shown antitumor activity across B-cell malignancies, including in models harboring BTK mutations associated with resistance to covalent BTKis. BELLWAVE-003 (NCT04728893) is a phase 2 study evaluating nemtabrutinib in hematologic malignancies.

### Aims

To report efficacy and safety outcomes from cohort G of the BELLWAVE-003 study, comprising participants with relapsed or refractory (R/R) follicular lymphoma (FL) previously treated with chemoimmunotherapy and immunomodulatory agents, and who received nemtabrutinib at the recommended phase 2 dose (RP2D).

### Methods

BELLWAVE-003 is a multicenter, open-label, single-arm phase 2 study enrolling participants with R/R B-cell malignancies. Participants with R/R FL were required to have failed prior chemoimmunotherapy and immunomodulatory agents. Eligible participants received nemtabrutinib at the RP2D (65 mg PO QD) until unacceptable toxicity, disease progression, or withdrawal. The primary end point was objective response rate. Secondary end points included duration of response per Lugano 2014 criteria by blinded independent central review (BICR) and safety. Exploratory end points included progression-free survival per Lugano 2014 criteria by BICR and overall survival. The data cutoff date for this analysis was September 8, 2025.

**Copyright Information:** (Online) ISSN: 2572-9241
©2026 The Author(s). HemaSphere is published by John Wiley & Sons Ltd on behalf of European Hematology Association. This is an open access Abstract Book distributed under the Attribution-NonCommercial-NoDerivs (CC BY-NC-ND), which allows third parties to download the articles and share them with others as long as they credit the author and the Abstract Book, but they cannot change the content in any way or use them commercially.
**Abstract Book Citations:** Authors, Title, HemaSphere, 2026;10:(S1):pages. The abstract book DOIs can be found on the "Author Information" page.
**Disclaimer:** Articles published in the journal HemaSphere exclusively reflect the opinions of the authors. The authors are responsible for all content in their abstracts including accuracy of the facts, statements, citing resources, etc.

**Results**

A total of 58 participants with R/R FL were treated with nemtabrutinib at the RP2D. Median study follow-up was 17.0 months (range, 3.5-30.6). Median age was 59 years (range, 33-80); 27 participants (47%) were female, and 32 (55%) had Lugano stage IV disease. Participants had received a median of 4 (range, 1-11) prior lines of therapy. Median treatment duration was 3.8 months (range, 0.2-22.9), with 11 participants (19%) remaining on treatment at data cutoff. Objective response rate was 43% (95% CI, 30-57) by BICR and 35% (95% CI, 23-48) by investigator review. Median duration of response was 5.8 months (95% CI, 2.8-12.9). Median progression-free survival was 5.6 months by BICR and 5.3 months by investigator review. Median overall survival was not reached; the 12-month overall survival rate was 86.1% (95% CI, 71.5-93.5). All-cause adverse events (AEs) of any grade were reported in 56 participants (97%); the most common AEs (≥20%) were constipation (22%) and neutrophil count decreased (21%). Grade 3-5 all-cause AEs occurred in 25 participants (43%), most commonly anemia and neutropenia (each 7%) and decreased neutrophil count and decreased platelet count (each 5%). Dose reductions due to all-cause AEs were reported in 4 participants (7%), including asthenia, blood creatinine increased, platelet count decreased, and sepsis and septic shock (1 participant each). All-cause AEs led to discontinuation in 7 participants (12%) and 3 deaths (5%).

**Summary/Conclusion**

Nemtabrutinib showed clinically meaningful antitumor activity with a manageable safety profile in heavily pretreated participants with R/R FL, supporting ongoing clinical development of nemtabrutinib in this population.

**Copyright Information:** (Online) ISSN: 2572-9241
©2026 The Author(s). HemaSphere is published by John Wiley & Sons Ltd on behalf of European Hematology Association. This is an open access Abstract Book distributed under the Attribution-NonCommercial-NoDerivs (CC BY-NC-ND), which allows third parties to download the articles and share them with others as long as they credit the author and the Abstract Book, but they cannot change the content in any way or use them commercially.
**Abstract Book Citations:** Authors, Title, HemaSphere, 2026;10:(S1):pages. The abstract book DOIs can be found on the "Author Information" page.
**Disclaimer:** Articles published in the journal HemaSphere exclusively reflect the opinions of the authors. The authors are responsible for all content in their abstracts including accuracy of the facts, statements, citing resources, etc.

### (PB3605) INTENSIFICATION OF THERAPY FOR WALDENSTROM'S MACROGLOBULINEMIA IN PATIENTS UNDER 65 YEARS OF AGE WITH UNFAVORABLE PROGNOSTIC FACTORS

**Topic:** 18. Indolent and mantle-cell non-Hodgkin lymphoma - Clinical

Alexander Grachev*[1], Irina Galtseva[1], Anastasia Vinnikova[1], Elena Gribanova[1], Azaliya Valitova[1], Vyacheslav Ryzhko[1], Yulia Tsoi[1], Sofia Starchenko[1], Kulikov Sergey[1], Evgeniy Zvonkov[1]

[1]*National Medical Research Center of Hematology, Moscow, Russia;*

### Background

Waldenström's macroglobulinemia (WM) is heterogeneous in its treatment response and prognosis. The overall response rate with conventional treatment programs is 75-90%, with a complete response achieved in 3-7% of patients. Currently, an adapted treatment approach for young patients with unfavorable prognostic factors has not been developed.

### Aims

To evaluate the effectiveness of intensive induction immunochemotherapy in combination with targeted drugs and autologous hematopoietic stem cell transplantation (auto-HSCT).

### Methods

Forty-two patients were included. The average age was 51 years (25-65 years), 27 were men. Six patients belonged to the low-risk group according to R-IPSSWM, but they had extramedullary disease, which is an independent factor of unfavorable prognosis, 25 and 11 patients were in the intermediate and high-risk groups, respectively. Hyperviscosity syndrome was present in 19 patients (45%), anemia in 39 (92%), Hb - 81.5 g / L (44-132 g / L), platelets <100 thousand / µl in 8 (19%), hemolysis in 5 (12%), cryoglobulinemia in 5 (12%), PAP in 32 (76%), splenomegaly in 20 (48%), B-symptoms in 34 (81%). LDH>250 U/L - in 22 (52%) and albumin <30 g/L - in 18 (42%) patients, β-2-microglobulin >4 mg/L in 30 (71%) patients. Average PIgM values were 42.9 g/L (9.9-78 g/L). MYD88 mutation - L265P was present in 34 (82%) and del-17p in 4 (9.5%) patients.

### Results

From February 2017 to August 2025, 42 patients received the R-EPOCH\R-BAC regimen in combination with ibrutinib. auto-HSCT was performed in 26 patients. Five patients received the high-dose CEAM consolidation regimen, and 21 patients received a new regimen—a combination of the CEAM program with the anti-CD38 monoclonal antibody daratumumab (Dara-CEAM). The response was assessed using the modified IWWM-6 criteria. The overall response was 100%, with one patient having a minimal response, partial response in 31.7%, very good partial response in 34.2%, and complete response (CR) in 31.7% of patients, respectively. The 3- and 5-year overall survival were 97% and 93%, respectively. Three- and five-year progression-free survival rates were 89% and 80%, respectively. Patients who received immunochemotherapy with ibrutinib and subsequent high-dose consolidation under the Dara-CEAM program demonstrated superior progression-free survival compared to patients who did not receive auto-HSCT (Fig. 1).

### Summary/Conclusion

The R-EPOCH/R-BAC program with ibrutinib followed by high-dose consolidation with auto-HSCT is a highly effective and safe treatment option for high-risk WM. The use of high-dose consolidation in the Dara-CEAM program increases treatment efficacy. We are continuing the study to improve the reliability of the data.

**Copyright Information:** (Online) ISSN: 2572-9241
©2026 The Author(s). HemaSphere is published by John Wiley & Sons Ltd on behalf of European Hematology Association. This is an open access Abstract Book distributed under the Attribution-NonCommercial-NoDerivs (CC BY-NC-ND), which allows third parties to download the articles and share them with others as long as they credit the author and the Abstract Book, but they cannot change the content in any way or use them commercially.
**Abstract Book Citations:** Authors, Title, HemaSphere, 2026;10:(S1):pages. The abstract book DOIs can be found on the "Author Information" page.
**Disclaimer:** Articles published in the journal HemaSphere exclusively reflect the opinions of the authors. The authors are responsible for all content in their abstracts including accuracy of the facts, statements, citing resources, etc.

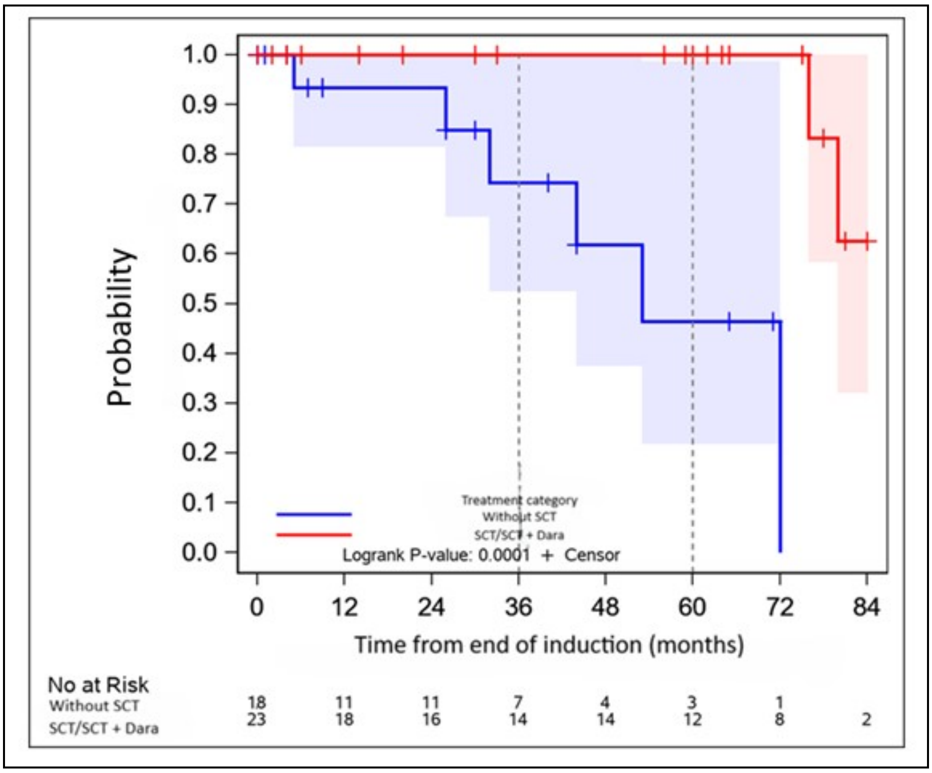

**Copyright Information:** (Online) ISSN: 2572-9241
©2026 The Author(s). HemaSphere is published by John Wiley & Sons Ltd on behalf of European Hematology Association. This is an open access Abstract Book distributed under the Attribution-NonCommercial-NoDerivs (CC BY-NC-ND), which allows third parties to download the articles and share them with others as long as they credit the author and the Abstract Book, but they cannot change the content in any way or use them commercially.
**Abstract Book Citations:** Authors, Title, HemaSphere, 2026;10:(S1):pages. The abstract book DOIs can be found on the "Author Information" page.
**Disclaimer:** Articles published in the journal HemaSphere exclusively reflect the opinions of the authors. The authors are responsible for all content in their abstracts including accuracy of the facts, statements, citing resources, etc.

### (PB3606) MRD-DRIVEN TRIPLET THERAPY WITH ORELABRUTINIB, POMALIDOMIDE, AND ZUBERITAMAB FOR NEWLY DIAGNOSED INTERMEDIATE/HIGH-RISK MANTLE CELL LYMPHOMA

**Topic:** 18. Indolent and mantle-cell non-Hodgkin lymphoma - Clinical

Ping Yang*[1], Chunyuan Li[1], Jiachen Liu[1], Ziyu Zhao[1], Shuozi Liu[1], Jing Wang[1], Yuan Yang[1], Hongmei Jing[1]

[1]*Peking University Third Hospital, Department of Hematology, Beijing, China;*

### Background

Bruton's tyrosine kinase inhibitor (BTKi)-based regimens have improved outcomes in mantle cell lymphoma (MCL), addressing the poor prognosis and toxicity associated with traditional immunochemotherapy. Current research focuses on chemo-free multi-agent combinations to deepen responses and enable fixed-duration strategies (fixed-cycle/MRD-guided). The OASIS2 trial demonstrated that adding venetoclax to ibrutinib and rituximab yields superior complete response (CR) and undetectable minimal residual disease (uMRD) rates, supporting the potential of BTKi + CD20 monoclonal antibody (mAb) + X regimens, particularly for high-risk MCL. Orelabrutinib exhibits potent efficacy with a favorable safety profile in relapsed/refractory MCL; pomalidomide provides immunomodulatory and pro-apoptotic effects; and zuberitamab, an innovative recombinant human-mouse chimeric anti-CD20 mAb, mediates tumor clearance via antibody-dependent cellular cytotoxicity (ADCC). We hypothesized that the triplet combination of orelabrutinib, pomalidomide, and zuberitamab (POZ) would synergistically inhibit tumor proliferation, achieve high uMRD rates, and prolong progression-free survival (PFS). Here, we present preliminary findings from the study (NCT07257510), evaluating this regimen in newly diagnosed intermediate/high-risk MCL with treatment de-escalation guided by post-induction MRD status.

### Methods

Eligible patients receive six cycles of induction therapy with the POZ regimen. Post-induction, patients achieving uMRD proceed to 18 cycles of maintenance therapy with orelabrutinib plus zuberitamab, while those with MRD positivity discontinue study treatment (Figure 1). The primary endpoint is the uMRD rate following induction therapy. Key secondary endpoints include CR rate, objective response rate (ORR), duration of response (DOR), PFS, overall survival (OS), and safety.

### Results

As of the data cutoff date (March 26, 2026), 10 patients were enrolled. The median age was 58.5 years (mean, 58.6 ± 6.8). According to MIPI scoring, 7 patients were low-risk and 3 were intermediate-risk. High proliferative activity (Ki-67 ≥30%) was observed in 6 patients. Among 6 patients evaluable for genetic profiling, TP53 mutations were detected in 3 (50%). With a median follow-up of 4.1 months (95% CI: 3.5-5.9), efficacy was assessed in 10 evaluable patients. The ORR was 100%, comprising 9 CRs (90%) and 1 partial response (PR). All patients remained on treatment with no reported disease progression or death. The safety profile was manageable. No treatment discontinuations occurred due to adverse events (AEs), and only one patient required a dose delay. The most common AEs were dermatologic: grade 1 rash (n=4) and grade 1 pruritus (n=1). Other reported AEs included grade 2 pulmonary infection (n=1), grade 3 leukopenia (n=2), and grade 4 neutropenia (n=1). No grade ≥5 AEs were observed.

### Summary/Conclusion

Although no patients have yet completed induction therapy and MRD data remain immature, this preliminary analysis demonstrates that the chemo-free POZ regimen induces deep and rapid responses (100% ORR, 90% CR) in newly diagnosed intermediate/high-risk MCL, including patients with high-risk features such as TP53 mutations. The safety profile is favorable and manageable. Continued follow-up is warranted to assess the primary endpoint of uMRD and long-term durability of response.

**Copyright Information:** (Online) ISSN: 2572-9241
©2026 The Author(s). HemaSphere is published by John Wiley & Sons Ltd on behalf of European Hematology Association. This is an open access Abstract Book distributed under the Attribution-NonCommercial-NoDerivs (CC BY-NC-ND), which allows third parties to download the articles and share them with others as long as they credit the author and the Abstract Book, but they cannot change the content in any way or use them commercially.
**Abstract Book Citations:** Authors, Title, HemaSphere, 2026;10:(S1):pages. The abstract book DOIs can be found on the “Author Information” page.
**Disclaimer:** Articles published in the journal HemaSphere exclusively reflect the opinions of the authors. The authors are responsible for all content in their abstracts including accuracy of the facts, statements, citing resources, etc.

**Figure 1. Study Design**

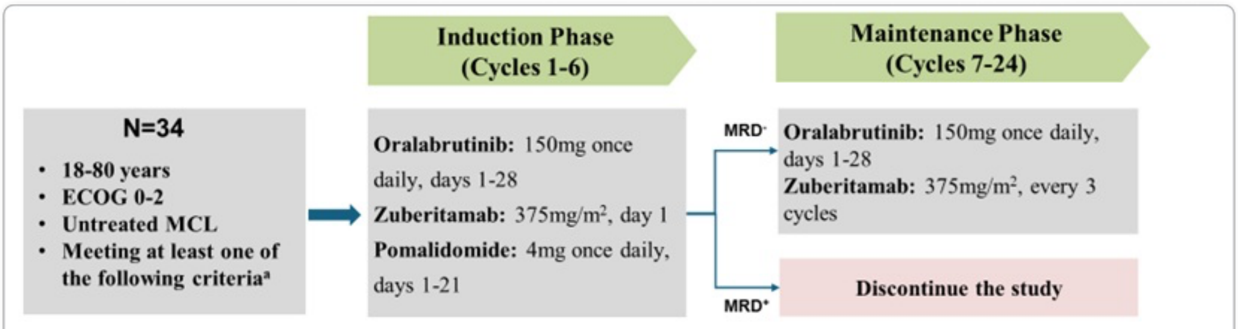

[a] MIPI intermediate-high risk, ki67≥30%, blastoid/pleomorphic, TP53 abnormality (mutation/deletion) or p53 >50%, large mass (maximum diameter ≥7.5cm), complex karyotype (≥3 chromosomal abnormalities (excluding t(11; 14))

**Copyright Information:** (Online) ISSN: 2572-9241
©2026 The Author(s). HemaSphere is published by John Wiley & Sons Ltd on behalf of European Hematology Association. This is an open access Abstract Book distributed under the Attribution-NonCommercial-NoDerivs (CC BY-NC-ND), which allows third parties to download the articles and share them with others as long as they credit the author and the Abstract Book, but they cannot change the content in any way or use them commercially.
**Abstract Book Citations:** Authors, Title, HemaSphere, 2026;10:(S1):pages. The abstract book DOIs can be found on the "Author Information" page.
**Disclaimer:** Articles published in the journal HemaSphere exclusively reflect the opinions of the authors. The authors are responsible for all content in their abstracts including accuracy of the facts, statements, citing resources, etc.

### (PB3607) REAL-WORLD OUTCOMES OF BISPECIFIC ANTIBODIES IN OLDER PATIENTS WITH RELAPSED/REFRACTORY (R/R) FOLLICULAR LYMPHOMA (FL)

**Topic:** 18. Indolent and mantle-cell non-Hodgkin lymphoma - Clinical

Jessica Allen*[1], Rahul Shah[2], Lei Feng[3], Lorenzo Falchi[4], Ayushi Chauhan[2], Sonia Godbole[5], Swetha Kambhampati[6], Ajay Major[7], Andrew Jallouk[8], Reid Merryman[9], Amy Ayers[2], Jennifer L. Crombie[9], Shakthi Bhaskar[8], Steven Bair[7], Alex F. Herrera[6], Yazeed Sawalha[5], Gilles Salles[4], Narendranath Epperla[1], Sairah Ahmed[2], Allison Bock[1]

*[1]University of Utah Huntsman Cancer Institute, Department of Hematology and Hematologic Malignancies, Salt Lake City, United States of America; [2]MD Anderson Cancer Center, Department of Lymphoma and Myeloma, Houston, United States of America; [3]MD Anderson Cancer Center, Department of Biostatistics, Houston, United States of America;[4]Memorial Sloan Kettering Cancer Center, Division of Hematologic Malignancies, New York, United States of America; [5]Ohio State University Cancer Center, Columbus, United States of America; [6]City of Hope, Department of Hematology & Hematopoietic Cell Transplantation, Duarte, United States of America; [7]University of Colorado, Aurora, United States of America; [8]Vanderbilt University Medical Center, Nashville, United States of America; [9]Dana Farber Cancer Institute, Boston, United States of America;*

### Background

Mosunetuzumab (mosun) and epcoritamab (epco) are bispecific antibodies (BsAbs) approved for R/R FL. BsAbs are appealing treatments for older patients (pts) due to low rates of cytokine release syndrome (CRS) and immune effector cell-associated neurotoxicity syndrome (ICANS), with favorable responses and survival outcomes in pivotal trials. However, real world outcomes remain limited, especially in older patients with higher-risk disease not well-represented in trials.

### Aims

To report the real-world efficacy and safety outcomes of BsAbs for older adult pts with R/R FL treated in the Collaborative United States Bispecifics Consortium (CUBIC).

### Methods

This is a multicenter retrospective study of adult pts with R/R FL treated with standard of caremosun or epco between 2/2023 and 2/2026 at nine centers. Outcomes included overall response rate (ORR), complete response rate (CRR), progression-free survival (PFS), and overall survival (OS). PFS and OS were estimated using the Kaplan-Meier method. Safety outcomes included incidence and grade (G) of CRS, ICANs, and infection. Analyses were stratified by age <65, ≥65, and ≥80 years (yrs).

**Copyright Information:** (Online) ISSN: 2572-9241
©2026 The Author(s). HemaSphere is published by John Wiley & Sons Ltd on behalf of European Hematology Association. This is an open access Abstract Book distributed under the Attribution-NonCommercial-NoDerivs (CC BY-NC-ND), which allows third parties to download the articles and share them with others as long as they credit the author and the Abstract Book, but they cannot change the content in any way or use them commercially.
**Abstract Book Citations:** Authors, Title, HemaSphere, 2026;10:(S1):pages. The abstract book DOIs can be found on the “Author Information” page.
**Disclaimer:** Articles published in the journal HemaSphere exclusively reflect the opinions of the authors. The authors are responsible for all content in their abstracts including accuracy of the facts, statements, citing resources, etc.

### Results

Of 99 pts with R/R FL treated with BsAbs, 60 (61%) pts were age ≥65 yrs and 12 (12%) ≥80 yrs. Of pts ≥65 yrs, median age was 71 (range 68–77). The majority of pts were treated with mosun (88%, n=53) and the remainder with epco (12%, n=7). The cohort was 53% male (n=32), 11% ECOG ≥2 (n=6), 66% stage IV disease (n=36), 72% FLIPI ≥3 (n=34), 25% bulky disease (n=13), median of 3 prior lines of treatment (range 1-14), 7% had prior CAR-T (n=4), and 40% (n=24) had progression of disease before 24 months (mo) following frontline treatment (POD24). There was a higher proportion of bulky disease in pts ≥80 (50% vs 17%, p=0.05).

Of 58 pts ≥65 yrs with available response data, CRR was 71% and ORR was 88%. Among the 11 pts ≥80 yrs with available response data, CRR was 73% and ORR was 82%.

At a median follow-up of 17 mo, the 1 and 2 yr OS rates in pts ≥65 were 98% and 87%, respectively. The 1 and 2 yr PFS rates were 77% and 54%, respectively. In pts ≥80, the 1 and 2 yr OS rates were 100%. The 1 yr PFS rate was 90% and not reached at 2yr. There was no difference in ORR, CRR, PFS, or OS among pts by age. There was no difference in PFS or OS by POD24 status, even when stratified by age (Figure 1).

Of 59 patients ≥65 yrs with complete safety data, CRS occurred in 32% of pts (n=19; G1 84%; G2 16%), with no ≥G3 events. Of these, 37% (n=7) required hospital admission. ICANS occurred in 5% of pts (n=3; G1 100%). Infections occurred in 31% (n=18) of pts. No pts required ICU admission. There was no difference in incidence or grade of CRS, ICANS, or infections between pts age <65 and ≥65 yrs. No deaths were due to CRS, ICANS, or infection. Two deaths were reported in pts ≥65 due to treatment-related cardiotoxicity.

### Summary/Conclusion

This real-world analysis of pts R/R FL treated with BsAbs included older pts with high-risk features, such as high FLIPI, POD24, and ECOG ≥2, representing a frailer population than typically included in trials. Despite this, the cohort had higher CRR and ORR than reported in trials, even in pts ≥80 yrs. The incidences of CRS and ICANS were comparable to pivotal trials, notably with no ≥G3 events. Infection rates were low, and none required ICU admission. This study supports the use of BsAbs for pts with R/R FL—including very elderly pts.

**Copyright Information:** (Online) ISSN: 2572-9241
©2026 The Author(s). HemaSphere is published by John Wiley & Sons Ltd on behalf of European Hematology Association. This is an open access Abstract Book distributed under the Attribution-NonCommercial-NoDerivs (CC BY-NC-ND), which allows third parties to download the articles and share them with others as long as they credit the author and the Abstract Book, but they cannot change the content in any way or use them commercially.
**Abstract Book Citations:** Authors, Title, HemaSphere, 2026;10:(S1):pages. The abstract book DOIs can be found on the “Author Information” page.
**Disclaimer:** Articles published in the journal HemaSphere exclusively reflect the opinions of the authors. The authors are responsible for all content in their abstracts including accuracy of the facts, statements, citing resources, etc.

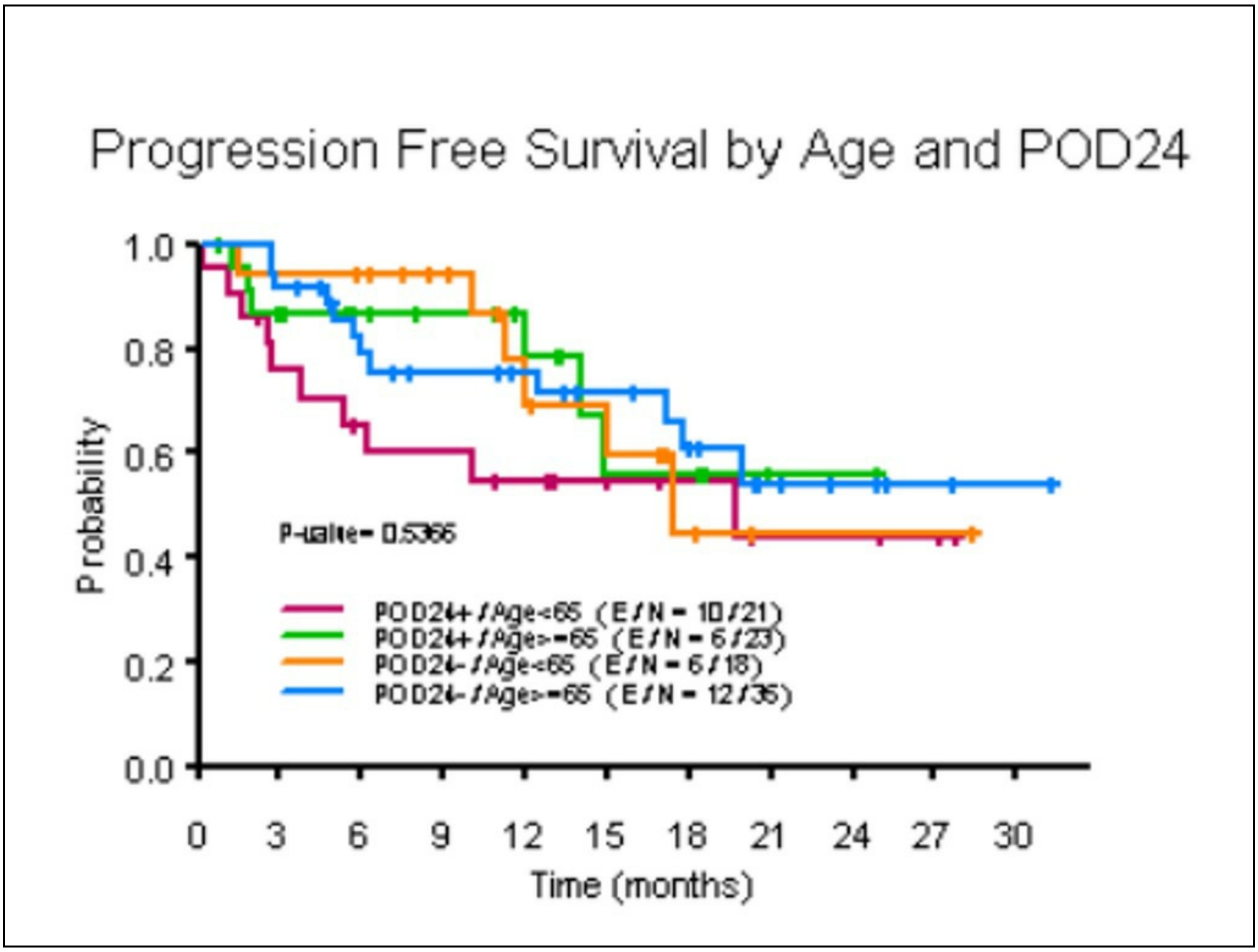

Figure 1: Progression-free survival by age and POD24 status

**Copyright Information:** (Online) ISSN: 2572-9241
©2026 The Author(s). HemaSphere is published by John Wiley & Sons Ltd on behalf of European Hematology Association. This is an open access Abstract Book distributed under the Attribution-NonCommercial-NoDerivs (CC BY-NC-ND), which allows third parties to download the articles and share them with others as long as they credit the author and the Abstract Book, but they cannot change the content in any way or use them commercially.
**Abstract Book Citations:** Authors, Title, HemaSphere, 2026;10:(S1):pages. The abstract book DOIs can be found on the “Author Information” page.
**Disclaimer:** Articles published in the journal HemaSphere exclusively reflect the opinions of the authors. The authors are responsible for all content in their abstracts including accuracy of the facts, statements, citing resources, etc.

### (PB3608) QUALITY OF LIFE OUTCOMES WITH TAFASITAMAB + LENALIDOMIDE AND RITUXIMAB FOR RELAPSED OR REFRACTORY FOLLICULAR LYMPHOMA (R/R FL): RESULTS FROM THE PHASE 3, DOUBLE-BLIND, PLACEBO-CONTROLLED INMIND STUDY

**Topic:** 18. Indolent and mantle-cell non-Hodgkin lymphoma - Clinical

Paolo Strati*[1], Christina Poh[2], Marek Trněný[3], Kai Hübel[4], Stefano Luminari[5, 6], Christian W. Scholz[7], Antonio Salar[8], Shankara Paneesha[9, 10], Bjorn Wahlin[11], Philomena Colucci[12], Michael Arbushites[12], Oscar Bortolami[13], Theresa Amoloja[12], Julie Wu[12], Laurie H. Sehn[14]

*[1]Division of Cancer Medicine, The University of Texas MD Anderson Cancer Center, Department of Lymphoma and Myeloma, Houston, TX, United States of America; [2]Fred Hutchinson Cancer Center/University of Washington, Seattle, WA, United States of America; [3]First Faculty of Medicine, Charles University, Prague, Czechia;[4]University of Cologne and Faculty of Medicine and University Hospital of Cologne, Cologne, Germany; [5]Azienda Unità Sanitaria Locale-Research Institute IRCCS di Reggio Emilia, Reggio Emilia, Italy; [6]Surgical, Medical and Dental Department of Morphological Sciences related to Transplant, Oncology and Regenerative Medicine, University of Modena and Reggio Emilia, Reggio Emilia, Italy; [7]Vivantes Klinikum Am Urban, Berlin, Germany; [8]Hospital del Mar-Medical Research Institute, Barcelona, Spain;[9]University Hospitals Birmingham NHS Foundation Trust, Birmingham, United Kingdom; [10]University of Birmingham, Birmingham, United Kingdom; [11]Karolinska University Hospital and Karolinska Institutet, Stockholm, Sweden; [12]Incyte Corporation, Wilmington, DE, United States of America; [13]Incyte Biosciences International Sàrl, Morges, Switzerland; [14]BC Cancer Centre for Lymphoid Cancer and The University of British Columbia, Vancouver, BC, Canada;*

### Background

Patients (pts) with FL experience remission and relapse, underscoring the need for effective therapies in the R/R setting. Understanding impact on quality of life (QoL) of novel agents that improve clinical outcomes in pts with R/R disease is important for evaluating their risk/benefit profile. Tafasitamab (tafa), an anti-CD19 mAb, is approved in combination with lenalidomide (len) + rituximab (R) for pts with R/R FL, based on the phase 3 inMIND study (NCT04680052). Results from inMIND showed that tafa+len+R prolonged progression-free survival versus placebo (pbo)+len+R (median 22 months vs 14 months, *P*<0.0001) in 548 pts with R/R FL, meeting its primary endpoint (Sehn L, *Lancet* 2026).

### Aims

We assessed QoL outcomes, a secondary endpoint of inMIND, to evaluate the risk/benefit profile of tafa+len+R in pts with R/R FL.

### Methods

Adults ≥18 years old with R/R FL and ≥1 prior systemic therapy, including an anti-CD20 mAb, were randomized to receive tafa+len+R (tafa arm) or pbo+len+R (pbo arm) for up to 12×28-day cycles. Pts completed the EORTC QLQ-C30 (including global health status/QoL scale, 5 functional scales [physical, role, emotional, cognitive, and social], 3 symptom scales [fatigue, presented; nausea/vomiting and pain, not presented], and 6 single items [not presented]), FACT-Lym (including well-being subscales physical, social/family, emotional, and functional [not presented], lymphoma subscale, trial outcome index, FACT-G total score, and total score [presented]), and EQ-5D-5L (including mobility, self-care, usual activities, pain/discomfort, and anxiety/depression dimensions [not presented], and a visual analog scale [VAS]) QoL questionnaires at baseline, on day 1 of each cycle, and at end of treatment (EOT; presented data includes pts with primary reason "completed" at EOT). Data were summarized with descriptive statistics, and mean change from baseline, over time, was plotted. Previously established minimally important difference (MID) thresholds were applied to mean change from baseline over time to determine whether observed changes in scores were clinically meaningful.

**Copyright Information:** (Online) ISSN: 2572-9241
©2026 The Author(s). HemaSphere is published by John Wiley & Sons Ltd on behalf of European Hematology Association. This is an open access Abstract Book distributed under the Attribution-NonCommercial-NoDerivs (CC BY-NC-ND), which allows third parties to download the articles and share them with others as long as they credit the author and the Abstract Book, but they cannot change the content in any way or use them commercially.
**Abstract Book Citations:** Authors, Title, HemaSphere, 2026;10:(S1):pages. The abstract book DOIs can be found on the "Author Information" page.
**Disclaimer:** Articles published in the journal HemaSphere exclusively reflect the opinions of the authors. The authors are responsible for all content in their abstracts including accuracy of the facts, statements, citing resources, etc.

### Results

Among 548 pts with R/R FL, baseline characteristics were similar between the tafa (n=273) and pbo (n=275) arms. Median number of treatment cycles received was 12 in the tafa arm and 11 in the pbo arm. Questionnaire completion rates were high for both arms and comparable from baseline through EOT (pts with ≥1 QoL assessment at each timepoint: tafa arm, >88%; pbo arm, >87%). Changes from baseline to EOT were comparable between treatment arms for EORTC QLQ-C30 global health status/QoL (mean [SD]: tafa arm, 2.1 [20.9]; pbo arm, 4.4 [21.6]), the FACT-Lym lymphoma subscale (mean [SD]: tafa arm, 2.0 [8.9]; pbo arm, 2.3 [7.4]), and the EQ-5D-5L VAS (mean [SD]: tafa arm, 2.8 [15.5]; pbo arm, 4.0 [17.8]) (**Figure**). Changes from baseline to EOT were also similar between treatment arms for the EORTC QLQ-C30 functional scales (physical, role, emotional, cognitive, and social) and the fatigue symptom scale, the FACT-Lym trial outcomes index, FACT-G total score, and FACT-Lym total score. There were no sustained changes exceeding MID thresholds from baseline to EOT for any QoL assessment in either arm.

### Summary/Conclusion

This study showed comparable QoL outcomes between the tafa and pbo arms over the course of treatment, with no sustained changes from baseline to EOT exceeding MID thresholds. This suggests that the addition of tafa to len+R resulted in clinical benefit without a negative impact on pt-reported QoL.

**Copyright Information:** (Online) ISSN: 2572-9241
©2026 The Author(s). HemaSphere is published by John Wiley & Sons Ltd on behalf of European Hematology Association. This is an open access Abstract Book distributed under the Attribution-NonCommercial-NoDerivs (CC BY-NC-ND), which allows third parties to download the articles and share them with others as long as they credit the author and the Abstract Book, but they cannot change the content in any way or use them commercially.
**Abstract Book Citations:** Authors, Title, HemaSphere, 2026;10:(S1):pages. The abstract book DOIs can be found on the "Author Information" page.
**Disclaimer:** Articles published in the journal HemaSphere exclusively reflect the opinions of the authors. The authors are responsible for all content in their abstracts including accuracy of the facts, statements, citing resources, etc.

**Figure. Mean change from baseline to EOT in EORTC QLQ-C30 global health status/QoL, FACT-Lym lymphoma subscale, and EQ-5D-5L VAS scores**

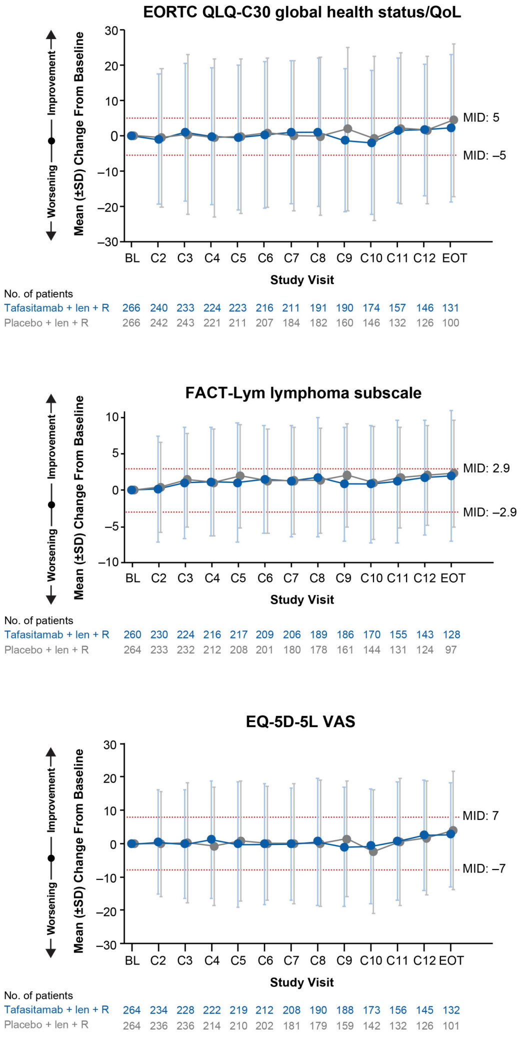

BL, baseline; C, cycle; EORTC QLQ-C30, European Organisation for Research and Treatment of Cancer Quality of Life Questionnaire-30 items; EOT, end of treatment; EQ-5D-5L VAS, EuroQoL 5-Dimension 5-Level visual analogue scale; FACT-Lym, Functional Assessment of Cancer Therapy-Lymphoma; len, lenalidomide; MID, minimally important difference; QoL, quality of life; R, rituximab; SD, standard deviation.

**Copyright Information:** (Online) ISSN: 2572-9241
©2026 The Author(s). HemaSphere is published by John Wiley & Sons Ltd on behalf of European Hematology Association. This is an open access Abstract Book distributed under the Attribution-NonCommercial-NoDerivs (CC BY-NC-ND), which allows third parties to download the articles and share them with others as long as they credit the author and the Abstract Book, but they cannot change the content in any way or use them commercially.
**Abstract Book Citations:** Authors, Title, HemaSphere, 2026;10:(S1):pages. The abstract book DOIs can be found on the "Author Information" page.
**Disclaimer:** Articles published in the journal HemaSphere exclusively reflect the opinions of the authors. The authors are responsible for all content in their abstracts including accuracy of the facts, statements, citing resources, etc.

### (PB3609) BRIDGING GAPS IN MANTLE CELL LYMPHOMA (MCL) MANAGEMENT: COMPARISON OFCOVALENT BTK INHIBITOR (CBTKI) USAGE AMONG EXPERTS AND HEALTHCARE PROFESSIONALS (HCPS)

**Topic:** 18. Indolent and mantle-cell non-Hodgkin lymphoma - Clinical

Kemi Obajimi*[1], Ana Marin Niebla[2], Georg Hess[3], Carlo Visco[4], Mats Jerkeman[5], Maria Ramos Lopes Gomes Da Silva[6], Ryan Topping[1], Timothy Quill[1]

*[1]Decera Clinical Education, Oncology, Reston, United States of America;[2]Hospital Universitario Vall d'Hebron, Barcolona, Spain; [3]University Medical School of the Johannes Gutenberg University, Mainz, Germany;[4]University of Verona, Verona, Italy; [5]Skane University Hospital, Lund, Sweden; [6]Instituto Portugues de Oncologia de Lisboa, Department of Hematology, Lisbon, Portugal;*

### Background

The use of cBTKis has transformed MCL treatment and these agents are recommended by clinical guidelines and experts for use in 1L and later settings. Approval of the noncovalent BTKi pirtobrutinib represents another significant advancement for patients who have previously received a cBTKi.

### Aims

In light of recent changes in the role of BTKis in MCL, this study compares patterns of BTKi use among HCPs with EU-based expert recommendations provided by an online decision support tool.

### Methods

In April 2025, an online decision support tool was developed with 5 EU-based experts in MCL. The tool provides treatment recommendations for various clinical scenarios, including newly diagnosed (ND) (n = 18) and relapsed/refractory (R/R) (n = 612) MCL, incorporating factors such as age, fitness, presence of high-risk (HR) features, history of cardiac disease, and prior therapies. HCPs entered specific patient characteristics and an intended treatment strategy. The tool then displayed expert recommendations for that scenario, and HCPs were asked whether the recommendations influenced their treatment choice. HCP-entered data vs expert recommendations were analyzed to identify common trends and divergence in current BTKi use in MCL.

**Copyright Information:** (Online) ISSN: 2572-9241
©2026 The Author(s). HemaSphere is published by John Wiley & Sons Ltd on behalf of European Hematology Association. This is an open access Abstract Book distributed under the Attribution-NonCommercial-NoDerivs (CC BY-NC-ND), which allows third parties to download the articles and share them with others as long as they credit the author and the Abstract Book, but they cannot change the content in any way or use them commercially.
**Abstract Book Citations:** Authors, Title, HemaSphere, 2026;10:(S1):pages. The abstract book DOIs can be found on the "Author Information" page.
**Disclaimer:** Articles published in the journal HemaSphere exclusively reflect the opinions of the authors. The authors are responsible for all content in their abstracts including accuracy of the facts, statements, citing resources, etc.

**Results**

HCPs across the globe entered 161 case scenarios into the online tool (119 ND and 42 R/R). In the ND MCL setting, for fit patients ± HR features, 3 of 5 experts recommended cBTKi-based therapy if the patient was ≤70 yr (TRIANGLE). For fit patients >70 yr with HR MCL, 4 experts recommended aggressive chemotherapy ± ASCT ± rituximab maintenance, and 1 chose a cBTKi-based regimen (ECHO). Among 13 HCPs, 31% selected a cBTKi-based regimen (TRIANGLE) for fit patients <65 yr without HR MCL. For fit patients 65-70 yr without HR MCL, ~40% of HCPs chose the ALTAMIRA regimen vs 45% who chose the ECHO regimen if HR features are present. Altogether, ~70% of HCPs selected a cBTKi-based therapy for fit patients with ND MCL without HR features.

In the 2L setting, if no prior exposure to a cBTKi (n = 18), HCPs selected cBTKi as monotherapy (21%) or with venetoclax ± anti-CD20 Ab (17%). In the same scenarios, experts selected ibrutinib monotherapy in 81% of cases and cBTKi + venetoclax in 18% of cases ± HR features. In the 3L+ setting, the experts recommended pirtobrutinib in 53% of cases or brexu-cel in 38% of cases if the patient had previously received a cBTKi. In these same scenarios, only 17% of HCPs selected pirtobrutinib and none selected brexu-cel. Zanubrutinib monotherapy, cBTKi + venetoclax ± anti-CD20 Ab, bendamustine/rituximab, R-BAC, and $R^2$ were selected by 8%-25% of HCPs.

In the ND MCL setting, 45% of HCPs indicated that the expert recommendations changed their choice, 23% indicated barriers to implementing the expert recommendations, 19% indicated that their intended choice matched expert recommendations, and 13% were still undecided vs 22%, 39%, 28% and 11%, respectively in the R/R MCL setting.

**Summary/Conclusion**

This analysis highlights differences between evolving expert recommendations and HCPs' use of BTKi-based therapy in MCL. Despite increasing expert support for regimens like ALTAMIRA, ECHO, ENRICH, BOVEN, and TRIANGLE for 1L cBTKi use and for pirtobrutinib in R/R MCL, integration of these regimens by HCPs remains low. Barriers to implementation of expert recommendations warrant further investigation. Detailed comparisons of expert and user responses from the online tool will be presented.

**Copyright Information:** (Online) ISSN: 2572-9241
©2026 The Author(s). HemaSphere is published by John Wiley & Sons Ltd on behalf of European Hematology Association. This is an open access Abstract Book distributed under the Attribution-NonCommercial-NoDerivs (CC BY-NC-ND), which allows third parties to download the articles and share them with others as long as they credit the author and the Abstract Book, but they cannot change the content in any way or use them commercially.
**Abstract Book Citations:** Authors, Title, HemaSphere, 2026;10:(S1):pages. The abstract book DOIs can be found on the "Author Information" page.
**Disclaimer:** Articles published in the journal HemaSphere exclusively reflect the opinions of the authors. The authors are responsible for all content in their abstracts including accuracy of the facts, statements, citing resources, etc.

### (PB3610) TREATMENT PATTERNS AND CLINICAL OUTCOMES IN CNS INVOLVEMENT OF MANTLE CELL LYMPHOMA (MCL) WITH PRIOR BTK INHIBITOR EXPOSURE; AN INTERNATIONAL RETROSPECTIVE STUDY.

**Topic:** 18. Indolent and mantle-cell non-Hodgkin lymphoma - Clinical

Katharine Lewis[1, 2], Elizabeth McKinnon[3], Marie-Kristin Tilch[4], Jonas Paludo[5], Georg Hess[6], Pavel Klener[7], Michael Williams[8], Mats Jerkeman[9], Diva Baggio[10], Satyen Gohil[10], Mark Bishton[11, 12], Ingrid Glimelius[13], Justin Desroches[5, 14], Musa Alzahrani[15], Martin Fehr[16, 17], Ana Marin Niebla[18], Toby A. Eyre[19], Diego Villa[20], Chan Y. Cheah*[1, 2]

*[1]Sir Charles Gairdner Hospital, Perth, Australia;[2]University of Western Australia, Perth, Australia;[3]The Kids Research Institute Australia, Perth, Australia; [4]University Medical Center Mainz, Department of Hematology and Clinical Oncology, Mainz, Germany; [5]Mayo Clinic, Division of Hematology, Rochester, United States of America;[6]University Medical School of the JOhannes Gutenberg-University, Mainz, Germany; [7]Všeobecná fakultní nemocnice, Prague, Czechia;[8]University of Virginia Comprehensive Cancer Center, Charlottesville, United States of America; [9]Skane University Hospital, Department of Oncology, Lund, Sweden; [10]University College London Hospitals, Department of Haematology, London, United Kingdom; [11]University of Nottingham, Nottingham, United Kingdom; [12]Nottingham University Hospitals NHS Trust, Nottingham, United Kingdom; [13]Cancer Precision Medicine, Uppsala University, Department of Immunology, Genetics and Pathology, Uppsala, Sweden; [14]BC Cancer Agency, Vancouver, Canada; [15]King Saud University, Department of Medicine, Riyadh, Saudi Arabia; [16]Canton Hospital St Gallen, St Gallen, Switzerland; [17]Swiss Cancer Institute, Switzerland, Switzerland; [18]Hospital Universitari Vall d'Hebron, Barcelona, Spain; [19]Oxford University Hospitals NHS Trust, Oxford, United Kingdom;[20]BC Cancer Centre for Lymphoid Cancer and Division of Medical Oncology, University of British Columbia, Vancouver, Canada;*

### Background

Development of central nervous system(CNS) involvement in mantle cell lymphoma (MCL) is rare, and results in poor clinical outcomes.  Data to guide management are scarce, and there is no standardised therapeutic approach.  Recent studies suggest that covalent Bruton's tyrosine kinase inhibitors (BTKi) may be more effective than chemotherapy, however contemporary systemic MCL regimens expose patients(pts) to BTKi earlier in therapy.  Pts with CNS MCL have therefore increasingly already been treated with a BTKi, warranting consideration of alternative therapies.

### Aims

To describe international treatment patterns and clinical outcomes for pts with CNS MCL following prior BTKi exposure.

### Methods

We performed an international, multicentre, retrospective study of outcomes and treatments for patients with CNS MCL.  Eligible pts were ≥18 years old with a diagnosis of CNS involvement by MCL, having previously received a BTKi as a component of systemic MCL therapy.

**Copyright Information:** (Online) ISSN: 2572-9241
©2026 The Author(s). HemaSphere is published by John Wiley & Sons Ltd on behalf of European Hematology Association. This is an open access Abstract Book distributed under the Attribution-NonCommercial-NoDerivs (CC BY-NC-ND), which allows third parties to download the articles and share them with others as long as they credit the author and the Abstract Book, but they cannot change the content in any way or use them commercially.
**Abstract Book Citations:** Authors, Title, HemaSphere, 2026;10:(S1):pages. The abstract book DOIs can be found on the "Author Information" page.
**Disclaimer:** Articles published in the journal HemaSphere exclusively reflect the opinions of the authors. The authors are responsible for all content in their abstracts including accuracy of the facts, statements, citing resources, etc.

**Results**

41 eligible pts were identified. Median age at diagnosis of CNS relapse was 64 years(range 45-79) and 32/41(78%) were male. At initial MCL diagnosis, 18/29(62%) had a Ki67 of ≥30%; 98% had advanced stage disease and 76% had marrow involvement. Median number of MCL therapies prior to CNS relapse was 3(range 1-8); 37% had received a prior autologous SCT. All pts had received a BTKi prior to CNS relapse;36/41(88%) ibrutinib, with 1 receiving subsequent pirtobrutinib, and 5/41(12%) acalabrutinib. 20/41(49%) received a BTKi as a component of 2$^{nd}$ line therapy. 29/41(71%) had received a BTKi as monotherapy.
23/41(56%) were still on MCL therapy at CNS progression, with 12 still taking a BTKi. Others had previously ceased their BTKi, mostly for progression(n=11) or toxicity(n=4). Median follow up from diagnosis of CNS relapse was 4.0 mths(range 0.5-55.6), with median time from BTKi initiation to CNS relapse of 11.4 mths(range 1.0-75.0) and median BTKi exposure prior to CNS relapse of 5.1 mths (range 0.2-75.0). 35/41 pts initiated therapy for CNS MCL; treatment was heterogeneous and included BTKi monotherapy(n=4), BTKi combination(n=7), chemotherapy(n=18), intrathecal therapy(n=4), bortezemib and rituximab(n=1), and radiotherapy(n=1). BTKi used post CNS relapse were ibrutinib(n=5), pirtobrutinib(n=4), zanubrutinib(n=1) and acalabrutinib(n=1).
11 pts(27%) received CAR-T as a component of CNS relapse therapy, with ORR 73%(n=7 CR; n=1 PR).
15/32 (47%) pts with available response assessment attained a CR to therapy, with remaining pts having PD (n=12,38%), PR (n=3,9%) or SD (n=2,6%). Median PFS and OS in the cohort from diagnosis of CNS MCL was 4.3 mths (95% CI 1.8-8.7) and 5.8 mths (95% CI 2.5-11.7) respectively. Median PFS and OS in pts receiving CAR-T was 23.7 mths (95% CI 7.4-NA) and 24.7 mths (95% CI 8.8-NA) respectively. 32 pts (78%) have died, 26 (81%) from lymphoma, 4 (13%) from treatment complications, (n=1 ICANS following CAR-T, n=2 infection [n=1 COVID-19 pneumonitis, n=1 pneumonia]

**Summary/Conclusion**

In this real-world dataset, treatment for CNS MCL was heterogeneous and clinical outcomes were generally poor. In line with other published CNS MCL cohorts, pts able to receive CAR-T cell therapy post CNS relapse appear to have more favourable outcomes, although this observation may be biased due to patient selection.
CAR-T cell therapy should be considered for suitable pts where available, but prospective studies are warranted to investigate the efficacy of this and other novel therapies in this rare entity.

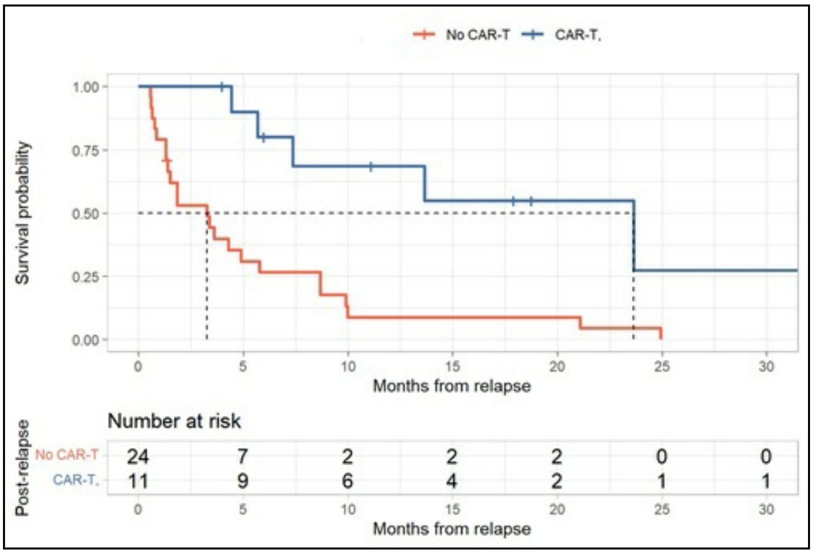

Kaplan-Meier survival plot of progression-free survival after CNS relapse, among non-palliative pts, stratified by CAR-T use subsequent to CNS relapse

**Copyright Information:** (Online) ISSN: 2572-9241
©2026 The Author(s). HemaSphere is published by John Wiley & Sons Ltd on behalf of European Hematology Association. This is an open access Abstract Book distributed under the Attribution-NonCommercial-NoDerivs (CC BY-NC-ND), which allows third parties to download the articles and share them with others as long as they credit the author and the Abstract Book, but they cannot change the content in any way or use them commercially.
**Abstract Book Citations:** Authors, Title, HemaSphere, 2026;10:(S1):pages. The abstract book DOIs can be found on the "Author Information" page.
**Disclaimer:** Articles published in the journal HemaSphere exclusively reflect the opinions of the authors. The authors are responsible for all content in their abstracts including accuracy of the facts, statements, citing resources, etc.

### (PB3611) UTILIZATION OF NOVEL AGENTS IN MANTLE CELL LYMPHOMA: RESULTS FROM A MULTI-SITE PROSPECTIVE COHORT STUDY (SUMMIT)

**Topic:** 18. Indolent and mantle-cell non-Hodgkin lymphoma - Clinical

Jakub Svoboda*[1], Jonathon Cohen[2], Chaitra Ujjani[3], David J. Andorsky[4], Andre Goy[5], Scott D. Ramsey[3], Victor Gonzalez[6], Francis Eusebio[7], Svea K. Wahlstrom[8], Samantha L. Thompson[9], Richard Hermann[10], Alan Yong[10], Dipen Patel[10], Anna Teschemaker[10], Essam Ibrahim[10], Michael L. Wang[11], Anita Kumar[12]

*[1]Department of Medicine, Hematology/Oncology Division, University of Pennsylvania, Philadelphia, Pennsylvania, United States of America; [2]Winship Cancer Institute, Emory University, Atlanta, Georgia, United States of America;[3]Fred Hutchinson Cancer Center, Seattle, Washington, United States of America; [4]Rocky Mountain Cancer Centers, US Oncology Research, Boulder, Colorado, United States of America; [5]John Theurer Cancer Center, Hackensack University Medical Center, Hackensack, New Jersey, United States of America; [6]Lymphoma Research Foundation, New York, New York, United States of America; [7]AstraZeneca, South San Francisco, California, United States of America;[8]AstraZeneca, Wilmington, Delaware, United States of America; [9]AstraZeneca, Cambridge, United Kingdom; [10]AstraZeneca, Gaithersburg, Maryland, United States of America; [11]The University of Texas MD Anderson Cancer Center, Houston, Texas, United States of America; [12]Department of Medicine, Lymphoma Service, Memorial Sloan Kettering Cancer Center, New York, New York, United States of America;*

### Background

Bruton tyrosine kinase inhibitors (BTKi), as monotherapy and in combination regimens, are approved for the treatment (tx) of adults with mantle cell lymphoma (MCL) in both front-line (1L) and relapse/refractory settings. As the MCL landscape evolves, evidence demonstrating patient (pt)-reported outcomes (PROs), quality of life (QoL), tx outcomes, and toxicity is needed to better inform pt-centered tx decisions.

### Aims

The aim of the SUMMIT study was to better understand the evolving real-world tx patterns and outcomes among pts with MCL who initiated a novel tx. Here, we report prospective cohort data from the MCL SUMMIT registry of pts with MCL who initiated 1L or second-line (2L) tx with BTKi monotherapy or combination therapy as part of the SUMMIT study (NCT03816683).

### Methods

The study enrolled adults with MCL across 34 US sites (Apr 2019-Mar 2022; data cutoff: Oct 24, 2024) who initiated a novel MCL tx including BTKi, B-cell lymphoma 2 inhibitors, immunomodulatory agents, or phosphoinositide 3-kinase inhibitors, as monotherapy or in combinations. The following endpoints were reported: overall survival (OS; defined as time from first index tx to death); time to next tx or death (TTNTD; defined as time from first index tx to start date of subsequent tx or death); and QoL (quarterly EQ5D VAS assessment). Outcomes were assessed for pts receiving the most common txs (n≥10): BTKi plus chemoimmunotherapy (BTKi+CIT) and BTKi monotherapy. All analyses were descriptive with no formal testing of hypotheses.

**Copyright Information:** (Online) ISSN: 2572-9241
©2026 The Author(s). HemaSphere is published by John Wiley & Sons Ltd on behalf of European Hematology Association. This is an open access Abstract Book distributed under the Attribution-NonCommercial-NoDerivs (CC BY-NC-ND), which allows third parties to download the articles and share them with others as long as they credit the author and the Abstract Book, but they cannot change the content in any way or use them commercially.
**Abstract Book Citations:** Authors, Title, HemaSphere, 2026;10:(S1):pages. The abstract book DOIs can be found on the “Author Information” page.
**Disclaimer:** Articles published in the journal HemaSphere exclusively reflect the opinions of the authors. The authors are responsible for all content in their abstracts including accuracy of the facts, statements, citing resources, etc.

**Results**

A total of 111/227 pts (1L, n=50; 2L, n=61) in the MCL SUMMIT registry receivedBTKi monotherapy (1L, n=17; 2L, n=48) or BTKi+CIT (1L, n=33; 2L, n=13). Median age was 72 years [yrs] for 1L BTKi (76% age ≥65 yrs), 68 yrs for 1L BTKi+CIT (82% age ≥65 yrs), 70 yrs for 2L BTKi (75% age ≥65 yrs), and 65 yrs for 2L BTKi+CIT (54% age ≥65 yrs). Race, ethnicity, and gender were similar for 1L BTKi±CIT and 2L BTKi±CIT. Most pts in the 1L (62%) and 2L (59%) cohorts were in the baseline (BL) MIPI medium- or low-risk groups. The proportions of pts who presented with bone marrow involvement, moderate to very bulky nodal mass (≥5 cm), and Ki-67 ≥30% at BL were 76%, 18%, and 22% for 1L BTKi+CIT pts, and 64%, 25%, and 44% for 2L BTKi+CIT pts, respectively.
Median OS was not reached (NR) for most cohorts, with follow-up ranging from 30.7–39.0 months (mo) across cohorts. Landmark 36-mo OS (95% CI) was 88.2% (60.6–96.9) for 1L BTKi, 87.0% (68.9–94.9) for 1L BTKi+CIT, 69.8% (53.7–81.3) for 2L BTKi, and 75.2% (40.7–91.4) for 2L BTKi+CIT (Fig 1A). Median (95% CI) TTNTD was NR for 1L BTKi, 36.9 (36.9–not calculable [NC]) mo for 1L BTKi+CIT, 33.6 (18.7–NC) mo for 2L BTKi, and 26.7 (5.8–NC) mo for 2L BTKi+CIT (Fig 1B). Mean (standard deviation) QoL numerically improved over time in the 1L BTKi (BL: 74.4 [18.23]; 36-mo: 76.8 [21.84]), 1L BTKi+CIT (BL: 81.7 [14.47]; 36-mo: 88.3 [2.89]), 2L BTKi (BL: 76.6 [17.69]; 36-mo: 79.3 [18.94]), and 2L BTKi+CIT (BL: 75.3 [23.36]; 36-mo: 80.0 [12.17]) cohorts.

**Summary/Conclusion**

In this prospective study of pts with MCL receiving BTKi±CIT, QoL numerically improved regardless of tx, providing some of the first longitudinal PRO/QoL data in this setting. Survival and treatment durability outcomes followed clinically expected, descriptive patterns by line of therapy. By integrating clinical effectiveness with PROs, these findings provide important context for evaluating survival outcomes alongside longitudinal QoL measures in the evolving MCL landscape.

**Copyright Information:** (Online) ISSN: 2572-9241
©2026 The Author(s). HemaSphere is published by John Wiley & Sons Ltd on behalf of European Hematology Association. This is an open access Abstract Book distributed under the Attribution-NonCommercial-NoDerivs (CC BY-NC-ND), which allows third parties to download the articles and share them with others as long as they credit the author and the Abstract Book, but they cannot change the content in any way or use them commercially.
**Abstract Book Citations:** Authors, Title, HemaSphere, 2026;10:(S1):pages. The abstract book DOIs can be found on the “Author Information” page.
**Disclaimer:** Articles published in the journal HemaSphere exclusively reflect the opinions of the authors. The authors are responsible for all content in their abstracts including accuracy of the facts, statements, citing resources, etc.

**Figure 1.** Kaplan-Meier estimates of (A) overall survival and (B) time to next treatment or death in patients receiving BTKi±CIT, stratified by line of therapy

**A**

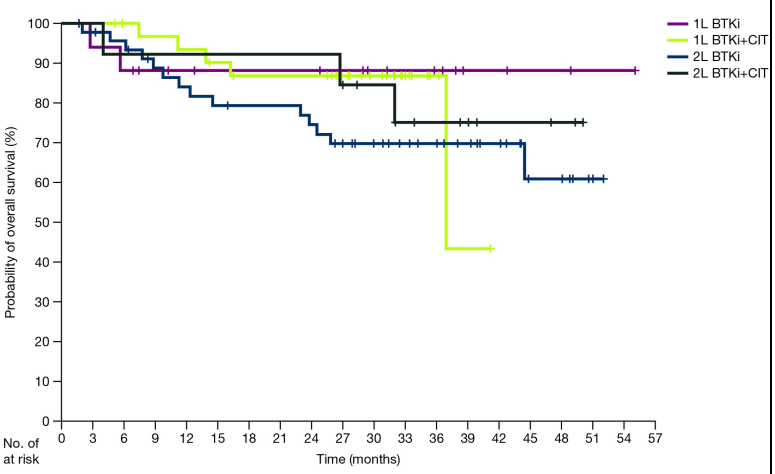

| No. of patients at risk | 0 | 6 | 12 | 18 | 24 | 30 | 36 | 42 | 48 | 54 |
|---|---|---|---|---|---|---|---|---|---|---|
| 1L BTKi | 17 | 15 | 12 | 11 | 11 | 8 | 6 | 3 | 2 | 0 |
| 1L BTKi+CIT | 33 | 31 | 29 | 25 | 25 | 15 | 3 | 0 | 0 | 0 |
| 2L BTKi | 48 | 43 | 36 | 33 | 31 | 24 | 19 | 12 | 5 | 0 |
| 2L BTKi+CIT | 13 | 12 | 12 | 12 | 12 | 9 | 6 | 3 | 2 | 0 |

**B**

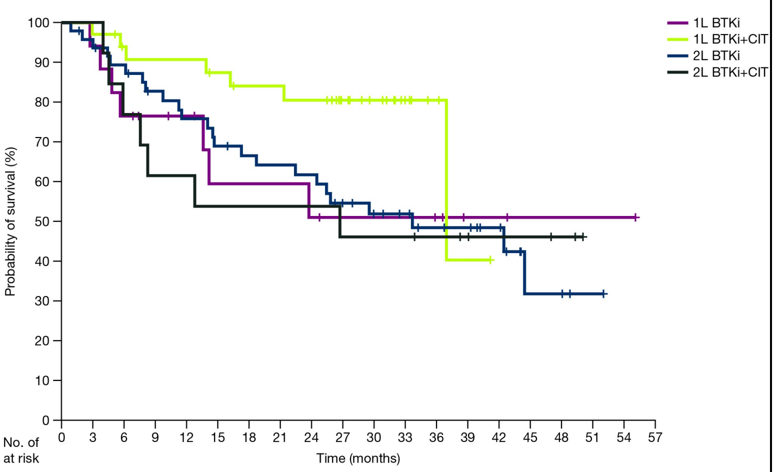

| No. of patients at risk | 0 | 6 | 12 | 18 | 24 | 30 | 36 | 42 | 48 | 54 |
|---|---|---|---|---|---|---|---|---|---|---|
| 1L BTKi | 17 | 13 | 10 | 7 | 6 | 5 | 4 | 2 | 1 | 0 |
| 1L BTKi+CIT | 33 | 29 | 28 | 24 | 23 | 13 | 3 | 0 | 0 | 0 |
| 2L BTKi | 48 | 41 | 33 | 28 | 26 | 18 | 13 | 9 | 2 | 0 |
| 2L BTKi+CIT | 13 | 10 | 8 | 7 | 7 | 6 | 5 | 3 | 2 | 0 |

1L, front-line; 2L, second-line; BTKi, Bruton tyrosine kinase inhibitor; BTKi+CIT, Bruton tyrosine kinase inhibitor plus chemoimmunotherapy.

**Copyright Information:** (Online) ISSN: 2572-9241
©2026 The Author(s). HemaSphere is published by John Wiley & Sons Ltd on behalf of European Hematology Association. This is an open access Abstract Book distributed under the Attribution-NonCommercial-NoDerivs (CC BY-NC-ND), which allows third parties to download the articles and share them with others as long as they credit the author and the Abstract Book, but they cannot change the content in any way or use them commercially.
**Abstract Book Citations:** Authors, Title, HemaSphere, 2026;10:(S1):pages. The abstract book DOIs can be found on the "Author Information" page.
**Disclaimer:** Articles published in the journal HemaSphere exclusively reflect the opinions of the authors. The authors are responsible for all content in their abstracts including accuracy of the facts, statements, citing resources, etc.

### (PB3612) ASSOCIATION OF MONOCLONAL PROTEIN WITH PLASMACYTIC DIFFERENTIATION AND POOR PROGNOSIS IN MARGINAL ZONE LYMPHOMA: A SINGLE-CENTER COHORT STUDY

**Topic:** 18. Indolent and mantle-cell non-Hodgkin lymphoma - Clinical

Wei Wang*[1], Yan Zhang[1], Jian Li[1]

[1]*Peking union medical college hospital, Beijing, China;*

### Background

Recent studies have demonstrated that detectable monoclonal protein (M-protein) at diagnosis is associated with inferior progression-free survival in patients with marginal zone lymphoma (MZL). However, these findings are predominantly based on Western cohorts, and the clinical characteristics of M-protein in Chinese MZL patients remain undefined. Moreover, the relationship between M-protein and a key pathological feature of MZL—plasmacytic differentiation—has not been systematically investigated.

### Aims

This study aims to characterize the clinical features of M-protein and explore its association with plasmacytic differentiation and prognosis in a Chinese MZL cohort.

### Methods

We retrospectively enrolled patients with MZL who received systemic therapy and underwent M-protein testing at baseline at Peking Union Medical College Hospital between January 2010 and October 2025. Patients were categorized into M-protein-positive and M-protein-negative groups. Clinicopathological characteristics, treatment modalities, and survival outcomes were compared between the two groups. The presence of M-protein was ascertained from serum protein electrophoresis(SPEP), immunofixation (SIFE), or an abnormal free kappa-tolambda (κ:λ) ratio on free light chain (FLC) assay. Plasmacytic differentiation was defined by morphological evidence and/or immunophenotyping demonstrating CD38- or CD138-positive cells with light chain restriction.

### Results

A total of 235 patients were enrolled, of whom 117 (49.8%) were M-protein positive. Baseline characteristics were generally balanced between the M-protein-positive and -negative groups, including age, sex, MZL subtype, Ann Arbor stage, elevated LDH, MZL-IPI risk distribution, histologic transformation, and treatment modalities. Patients with M-protein positivity had a numerically higher proportion of ECOG PS ≥2 (26.5% vs. 16.9%), although this difference did not reach statistical significance (p=0.076). The most striking difference between the two groups was the frequency of plasmacytic differentiation, which was significantly higher in the M-protein-positive group (38.1% vs. 16.2%, p<0.001)(Table 1). In univariable analysis for progression-free survival (PFS), M-protein positivity (p=0.046), ECOG PS ≥2 (p<0.001), histologic transformation (p=0.036), and receipt of anti-CD20 monotherapy (p=0.029) were identified as significant risk factors(Figure 1). However, in multivariable Cox regression analysis, only ECOG PS ≥2 (HR=1.773, 95%CI 1.04-3.024, p=0.035) and M-protein positivity (HR=1.60, 95%CI 1.021-2.505, p=0.040) remained independent predictors of inferior PFS. For overall survival (OS), ECOG PS ≥2 was the sole independent prognostic factor in both univariable and multivariable analyses (HR=6.181, 95%CI 2.493-15.325, p<0.001). Although M-protein-positive patients showed a trend toward worse OS, this did not reach statistical significance (p=0.071).

**Copyright Information:** (Online) ISSN: 2572-9241
©2026 The Author(s). HemaSphere is published by John Wiley & Sons Ltd on behalf of European Hematology Association. This is an open access Abstract Book distributed under the Attribution-NonCommercial-NoDerivs (CC BY-NC-ND), which allows third parties to download the articles and share them with others as long as they credit the author and the Abstract Book, but they cannot change the content in any way or use them commercially.
**Abstract Book Citations:** Authors, Title, HemaSphere, 2026;10:(S1):pages. The abstract book DOIs can be found on the “Author Information” page.
**Disclaimer:** Articles published in the journal HemaSphere exclusively reflect the opinions of the authors. The authors are responsible for all content in their abstracts including accuracy of the facts, statements, citing resources, etc.

### Summary/Conclusion

M-protein positivity at diagnosis was significantly associated with plasmacytic differentiation and was an independent predictor of inferior PFS in Chinese MZL patients receiving systemic therapy. These findings validate and extend previous observations from Western cohorts, highlighting the prognostic relevance of M-protein across diverse populations.

**Figure1. Baseline characteristics and survival by M protein and ECOG performance status**

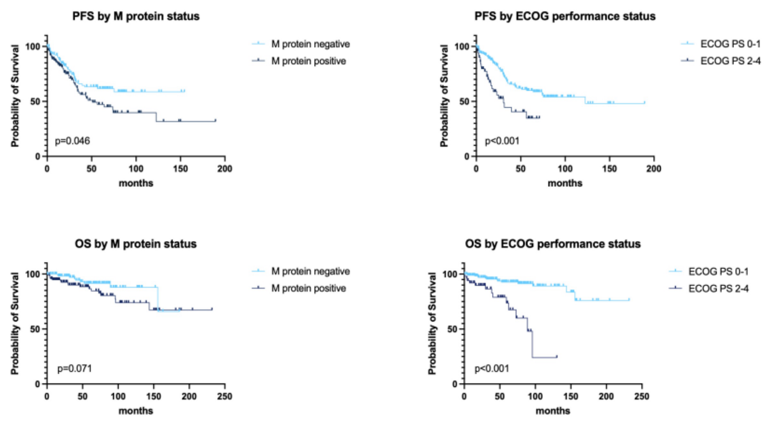

| Variable | M protein negative n=118 | M protein positive n=117 | P value |
|---|---|---|---|
| **Age mean ± SD** | 58.9 ± 13.6 | 60.4 ± 13.6 | 0.407 |
| **Sex, n(%)** | | | |
| male | 58(49.2%) | 53(45.3%) | 0.554 |
| female | 60(50.8%) | 64(54.7%) | |
| **Subtypes, n(%)** | | | |
| EMZL | 56(47.5%) | 47(40.2%) | 0.577 |
| NMZL | 7(5.9%) | 8(6.8%) | |
| SMZL | 46(39.0%) | 48(41.0%) | |
| dissMZL | 9(7.6%) | 14(12.0%) | |
| **ECOG PS, n(%)** | | | |
| 0-1 | 98(83.1%) | 86(73.5%) | 0.076 |
| 2-4 | 20(16.9%) | 31(26.5%) | |
| **Stage, n(%)** | | | |
| I-II | 13(11.0%) | 8(6.8%) | 0.261 |
| III-IV | 105(89.0%) | 109(93.2%) | |
| **LDH>ULN, n/total (%)** | 39/113(34.5%) | 44/117(37.6%) | 0.625 |
| **Plasmacytic differentiation, n/total (%)** | 17/105(16.2%) | 37/97(38.1%) | **<0.001** |
| **Large cell transformation, n/total(%)** | 14/117(12.0%) | 18/117(15.4%) | 0.447 |
| **MZL-IPI, n/total(%)** | | | |
| 0 | 17/113(15.0%) | 14/117(12.0%) | 0.731 |
| 1-2 | 75/113(66.4%) | 78/117(66.7%) | |
| 3-5 | 21/113(18.6%) | 25/117(21.3%) | |
| **First line treatment, n(%)** | | | |
| Anti-CD20+CHOP | 54(45.8%) | 46(39.2%) | 0.614 |
| BR | 6(5.1%) | 7(6.0%) | |
| Anti-CD20+COP | 19(16.1%) | 14(12.0%) | |
| Anti-CD20 monotherapy | 11(9.3%) | 14(12.0%) | |
| Anti-CD20+/-BTKi+/-Lenalidomied | 20(16.9%) | 22(18.8%) | |
| Others | 8(6.7%) | 14(12.0%) | |

**Copyright Information:** (Online) ISSN: 2572-9241
©2026 The Author(s). HemaSphere is published by John Wiley & Sons Ltd on behalf of European Hematology Association. This is an open access Abstract Book distributed under the Attribution-NonCommercial-NoDerivs (CC BY-NC-ND), which allows third parties to download the articles and share them with others as long as they credit the author and the Abstract Book, but they cannot change the content in any way or use them commercially.
**Abstract Book Citations:** Authors, Title, HemaSphere, 2026;10:(S1):pages. The abstract book DOIs can be found on the “Author Information” page.
**Disclaimer:** Articles published in the journal HemaSphere exclusively reflect the opinions of the authors. The authors are responsible for all content in their abstracts including accuracy of the facts, statements, citing resources, etc.

### (PB3613) REAL-WORLD EFFICACY OF ULTRA-LOW DOSE "BOOM BOOM" RADIOTHERAPY IN INDOLENT B-CELL LYMPHOMAS: PREDICTORS OF RESPONSE AND TIME TO NEXT TREATMENT

**Topic:** 18. Indolent and mantle-cell non-Hodgkin lymphoma - Clinical

Avishag Cohen*[1], Ron Yeffet[1], Erel Joffe[1, 2], Ori Grossberg[1, 2], Orit Gutfeld[1, 3], Chava Perry[1, 2], Irit Avivi Mazza[1, 2]

*[1]Grey Faculty of medicine, Tel Aviv University, Tel Aviv, Israel;[2]Hematology division, Tel Aviv Sourasky Medical center, Tel Aviv, Israel; [3]Radiotherapy department, Tel Aviv Sourasky Medical center, Tel Aviv, Israel;*

### Background

Ultra-low dose radiotherapy (LDR, 2 times 2 Gy), colloquially known as the "boom-boom" regimen, is an emerging, highly tolerable therapeutic modality for indolent lymphomas. While prospective trials have demonstrated its efficacy, real-world data characterizing predictive biomarkers for response and the impact of LDR on time to next treatment (TTNT) in both treatment-naïve and relapsed patients remain sparse.

### Aims

The current study evaluates the real-world outcomes and identifies prognostic markers for response to 4 Gy LDR in patients with Follicular Lymphoma (FL) and Marginal Zone Lymphoma (MZL).

### Methods

We conducted a real-world retrospective cohort analysis of patients with newly diagnosed or relapsed indolent lymphomas treated with 4.0 Gy LDR. We analyzed patient demographics, clinical characteristics, maximal complete metabolic response (CMR), and the need for subsequent systemic therapy. Univariate analysis was performed to identify baseline predictors for achieving CMR and maintaining freedom from subsequent therapy.

**Copyright Information:** (Online) ISSN: 2572-9241
©2026 The Author(s). HemaSphere is published by John Wiley & Sons Ltd on behalf of European Hematology Association. This is an open access Abstract Book distributed under the Attribution-NonCommercial-NoDerivs (CC BY-NC-ND), which allows third parties to download the articles and share them with others as long as they credit the author and the Abstract Book, but they cannot change the content in any way or use them commercially.
**Abstract Book Citations:** Authors, Title, HemaSphere, 2026;10:(S1):pages. The abstract book DOIs can be found on the "Author Information" page.
**Disclaimer:** Articles published in the journal HemaSphere exclusively reflect the opinions of the authors. The authors are responsible for all content in their abstracts including accuracy of the facts, statements, citing resources, etc.

**Results**

The cohort comprised of 65 patients (median age at LDR- 68.2 years). Histological subtypes included FL (43.1%), mucosa-associated lymphoid tissue (MALT) (33.8%), and nodal MZL (13.8%). The study consisted of 41 treatment-naïve patients (stage 1-4), and 24 previously treated patients who presented with non-localized relapse characterized by a predominant, symptomatic mass. Irradiated sites were primarily extranodal (57%) or superficial lymph nodes (40%), with a median lesion size of 2.8 cm (1.5-10) and a median pre-treatment SUVmax of 9.2 (3-26).
The overall response rate was 90.5%(n=59), with 69% (n=45) achieving CMR and 21.5% (n=14) achieving a partial response.
In univariate analysis, lymphoma subtype was the sole significant baseline predictor for achieving CMR (p=0.023). Specifically, patients with MALT histology achieved significantly higher CMR rates compared to those with FL (p=0.023). A higher burden of baseline comorbidities showed a borderline trend toward a lower probability of CMR (p=0.081).
With a median follow-up of 17 months, only 18.5% (n=12) of the total cohort required subsequent therapy; consolidative radiotherapy(n=6) or systemic therapy(n=6). Disease progression sites were evenly split between non-irradiated (n=6) and previously irradiated (n=6) sites. Achieving CMR emerged as the strongest predictor of freedom from subsequent therapy (p=0.014). While patients with progressive or stable disease consistently required further intervention (6 out of 6), only 4 of 45 patients with CMR (9%) needed additional treatment. Baseline demographics, tumor size, and SUVmax did not predict future need for subsequent therapy. In the relapsed sub-cohort, only 4 patients required subsequent therapy (median TTNT 16 months; 3 systemic, 1 radiation). Notably, Kaplan-Meier survival analysis revealed that the risk for further treatment decreased significantly after approximately 20 months.
A larger cohort will be presented at EHA.

**Summary/Conclusion**

Ultra-low dose "boom-boom" radiotherapy (4 Gy) is a highly effective modality, sparing a more intensified therapy, for both naïve and relapsed indolent B-cell lymphomas in real-world practice. MALT histology was associated with the highest rates of CMR. Furthermore, achieving CMR serves as a definitive prognostic marker for long-term disease control, translating into prolonged freedom from systemic interventions.

**Copyright Information:** (Online) ISSN: 2572-9241
©2026 The Author(s). HemaSphere is published by John Wiley & Sons Ltd on behalf of European Hematology Association. This is an open access Abstract Book distributed under the Attribution-NonCommercial-NoDerivs (CC BY-NC-ND), which allows third parties to download the articles and share them with others as long as they credit the author and the Abstract Book, but they cannot change the content in any way or use them commercially.
**Abstract Book Citations:** Authors, Title, HemaSphere, 2026;10:(S1):pages. The abstract book DOIs can be found on the "Author Information" page.
**Disclaimer:** Articles published in the journal HemaSphere exclusively reflect the opinions of the authors. The authors are responsible for all content in their abstracts including accuracy of the facts, statements, citing resources, etc.

### (PB3616) EPCORITAMAB WITH LENALIDOMIDE AND RITUXIMAB IN CHINESE PATIENTS WITH RELAPSED OR REFRACTORY FOLLICULAR LYMPHOMA: A SUBGROUP ANALYSIS FROM THE PHASE 3 EPCORE FL-1 TRIAL

**Topic:** 18. Indolent and mantle-cell non-Hodgkin lymphoma - Clinical

Rong Tao*[1], Jianzhen Shen[2], Xiaojing Yan[3], Jie Jin[4], Zhengzi Qian[5], Wenbin Qian[6], Hongmei Jing[7], Caixia Li[8], Wenyu Li[9], Huijing Wu[10], Xiaolei Wei[11], Xudong Wei[12], Gauri Sunkersett[13], Rastislav Moskal[13], Yukun Li[13], Xiaomeng Zhao[13], Brian Elliott[14], Huiqiang Huang[15]

*[1]Fudan University Cancer Hospital, Shanghai, China;[2]Fujian Medical University Union Hospital, Fuzhou, Fujian, China;[3]The First Affiliated Hospital of China Medical University, Shenyang, China; [4]The First Affiliated Hospital, Zhejiang University School of Medicine, Hangzhou, China; [5]Tianjin Medical University Cancer Institute and Hospital, Tianjin, China;[6]The Second Affiliated Hospital of Zhejiang University School of Medicine, Hangzhou, China; [7]Peking University Third Hospital, Beijing, China;[8]The First Affiliated Hospital of Soochow University, Jiangsu, China; [9]Guangdong Provincial People's Hospital of Southern Medical University, Guangzhou, China; [10]Hubei Cancer Hospital, Wuhan, China; [11]Nanfang Hospital, Southern Medical University, Guangzhou, China; [12]Henan Cancer Hospital, Zhengzhou, China; [13]AbbVie Inc., North Chicago, United States of America; [14]Genmab US Inc, Plainsboro, United States of America; [15]Sun Yat-sen University Cancer Center, Guangzhou, China;*

### Background

Second-line (2L) treatment options for relapsed/refractory follicular lymphoma (R/R FL) are often limited to chemoimmunotherapy or lenalidomide plus rituximab ($R^2$). Epcoritamab (E), a CD3×CD20 bispecific antibody, was approved as monotherapy for R/R FL after ≥2 lines of therapy (LOTs) and in combination with $R^2$ for R/R FL with fixed treatment duration. Chemotherapy-free E+$R^2$ was approved based on results from the pivotal Phase 3 EPCORE FL-1 trial (NCT05409066), which showed superiority of E+$R^2$ vs $R^2$ in patients (pts) with 2L+ FL (Falchi. *Lancet*. 2026;407:161-173).

### Aims

To report results of E+$R^2$ in a cohort of Chinese pts from EPCORE FL-1.

### Methods

Eligible adults with CD20+ R/R FL after ≥1 prior LOT who met GELF criteria/required treatment based on symptoms and/or disease burden were randomized to 12 cycles (C) of E+$R^2$ or $R^2$. Subcutaneous E was given as a 2- or 3-step-up dose (3SUD) regimen with full 48 mg doses thereafter (QW C1–3; Q4W C4–12). Oral lenalidomide was given once daily (Days 1–21) in C1–12. Intravenous rituximab was given QW in C1 and Q4W in C2–5. Mandatory cytokine release syndrome (CRS) prophylaxis was given during SUD. Dual primary endpoints were overall response rate (ORR) and progression-free survival (PFS) by independent review committee per Lugano criteria. Secondary endpoints included complete response rate (CRR), overall survival (OS), and safety.

**Copyright Information:** (Online) ISSN: 2572-9241
©2026 The Author(s). HemaSphere is published by John Wiley & Sons Ltd on behalf of European Hematology Association. This is an open access Abstract Book distributed under the Attribution-NonCommercial-NoDerivs (CC BY-NC-ND), which allows third parties to download the articles and share them with others as long as they credit the author and the Abstract Book, but they cannot change the content in any way or use them commercially.
**Abstract Book Citations:** Authors, Title, HemaSphere, 2026;10:(S1):pages. The abstract book DOIs can be found on the "Author Information" page.
**Disclaimer:** Articles published in the journal HemaSphere exclusively reflect the opinions of the authors. The authors are responsible for all content in their abstracts including accuracy of the facts, statements, citing resources, etc.

**Results**

At the 24May2025 cutoff (14.8-months median follow-up), in the intention-to-treat population (N=488; E+$R^2$ n=243, $R^2$ n=245) ORR was significantly improved with E+$R^2$ (95% [95% CI, 92–97]) vs $R^2$ (79% [95% CI, 74–84]; *P*<.0001). E+$R^2$ had superior PFS, with 79% reduction in the risk of disease progression or death (HR 0.21 [95% CI, 0.14–0.31]; *P*<.0001; **Figure A**).

In the Chinese cohort (n=64; 10.5-months median follow-up), 37 pts were randomized to E+$R^2$ and 27 to $R^2$. Pts in the E+$R^2$ vs $R^2$ arm had a median (range) age of 51 years (35–72) vs 52 years (30–86), Ann Arbor stage III–IV in 89% vs 85%, double refractory disease in 38% vs 44%, and POD24 in 46% vs 33%. Median (range) number of prior LOTs was 1 (1–5) in both arms. ORR was meaningfully improved with E+$R^2$ vs $R^2$ (95% [95% CI, 82–99] vs 78% [95% CI, 58–91]; *P*=.0203). Median PFS was longer with E+$R^2$ vs $R^2$ (HR 0.27 [95% CI, 0.08–0.94]; *P*=.0160; **Figure B**). In addition, CRR was higher with E+$R^2$ vs $R^2$ (81% [95% CI, 65–92] vs 63% [95% CI, 42–81]; *P*=.0156). Serious treatment-emergent adverse events (TEAEs) were reported in 51% of pts with E+$R^2$ and 19% with $R^2$. Among 18 pts receiving 3SUD E+$R^2$, 8 (44%) experienced CRS (all Grade 1). Seven of 10 CRS events occurred at time of first 48 mg E dose and all resolved. No immune effector cell-associated neurotoxicity syndrome events occurred with E+$R^2$. Grade 3/4 TEAEs occurred in 97% of pts treated with E+$R^2$ vs 73% with $R^2$. Rates of serious infection (35% vs 19%) and Grade 3/4 neutropenia (84% vs 62%) were higher with E+$R^2$ vs $R^2$. TEAEs led to one death with E+$R^2$ vs none with $R^2$. TEAEs led to any study drug discontinuation in 11% of pts treated with E+$R^2$ vs 4% treated with $R^2$.

**Summary/Conclusion**

In this subgroup analysis from EPCORE FL-1, E+$R^2$ led to meaningful improvements in ORR, CRR, and PFS vs $R^2$ in Chinese pts with 2L+ R/R FL. The safety profile was manageable, with no new safety signals. CRS events were all Grade 1 and the timing was predictable. These findings are consistent with those reported in the overall trial population and support E+$R^2$ as a new standard of care for Chinese pts with 2L+ FL.

**Copyright Information:** (Online) ISSN: 2572-9241
©2026 The Author(s). HemaSphere is published by John Wiley & Sons Ltd on behalf of European Hematology Association. This is an open access Abstract Book distributed under the Attribution-NonCommercial-NoDerivs (CC BY-NC-ND), which allows third parties to download the articles and share them with others as long as they credit the author and the Abstract Book, but they cannot change the content in any way or use them commercially.
**Abstract Book Citations:** Authors, Title, HemaSphere, 2026;10:(S1):pages. The abstract book DOIs can be found on the "Author Information" page.
**Disclaimer:** Articles published in the journal HemaSphere exclusively reflect the opinions of the authors. The authors are responsible for all content in their abstracts including accuracy of the facts, statements, citing resources, etc.

**Figure. PFS Per IRC Assessment in the ITT Population (A) and the Chinese Cohort (B)**

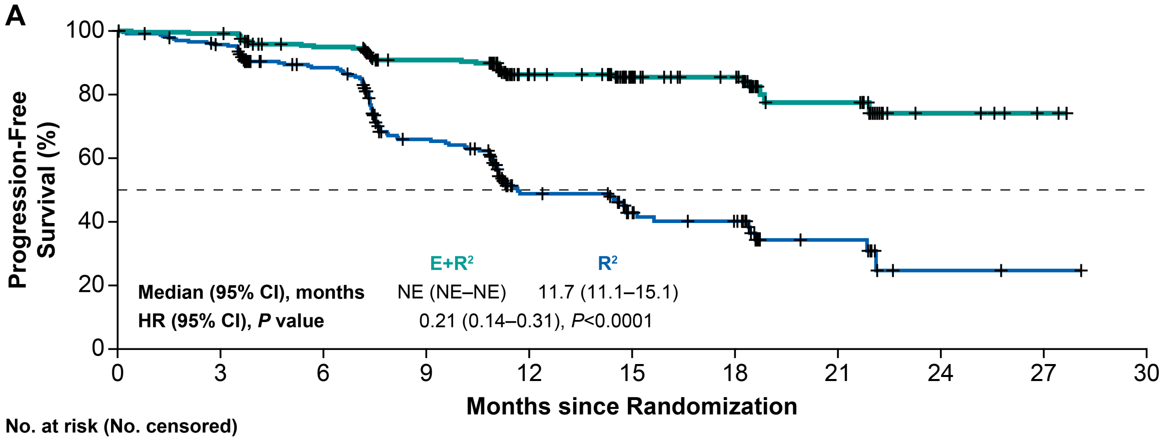

No. at risk (No. censored)

| | | | | | | | | | | | |
|---|---|---|---|---|---|---|---|---|---|---|---|
| E+$R^2$ | 243 (0) | 240 (1) | 218 (13) | 183 (39) | 112 (102) | 77 (136) | 65 (148) | 30 (179) | 6 (202) | 2 (206) | 0 (208) |
| $R^2$ | 245 (0) | 220 (15) | 180 (39) | 110 (67) | 58 (94) | 34 (112) | 28 (116) | 10 (131) | 2 (137) | 1 (138) | 0 (139) |

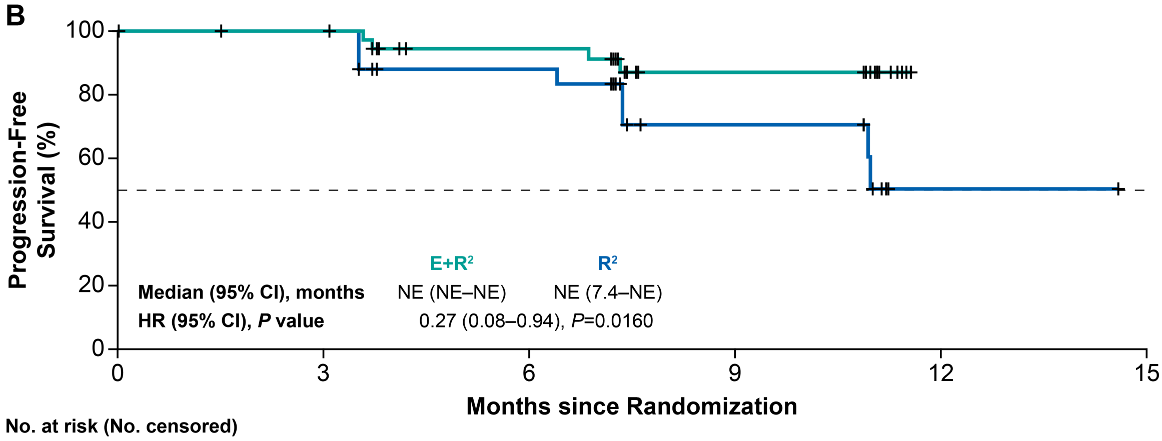

No. at risk (No. censored)

| | | | | | | |
|---|---|---|---|---|---|---|
| E+$R^2$ | 37 | 37 | 29 | 13 | 0 | 0 |
| $R^2$ | 27 | 25 | 19 | 9 | 1 | 0 |

Panel A was previously published and was reproduced with permission per CC BY 4.0 license (Falchi et al. *Lancet*. 2026;407:161-173).
E, epcoritamab; HR, hazard ratio; IRC, independent review committee; ITT, intention-to-treat; PFS, progression-free survival; $R^2$, epcoritamab plus lenalidomide and rituximab.

**Copyright Information:** (Online) ISSN: 2572-9241
©2026 The Author(s). HemaSphere is published by John Wiley & Sons Ltd on behalf of European Hematology Association. This is an open access Abstract Book distributed under the Attribution-NonCommercial-NoDerivs (CC BY-NC-ND), which allows third parties to download the articles and share them with others as long as they credit the author and the Abstract Book, but they cannot change the content in any way or use them commercially.
**Abstract Book Citations:** Authors, Title, HemaSphere, 2026;10:(S1):pages. The abstract book DOIs can be found on the "Author Information" page.
**Disclaimer:** Articles published in the journal HemaSphere exclusively reflect the opinions of the authors. The authors are responsible for all content in their abstracts including accuracy of the facts, statements, citing resources, etc.

### (PB3617) NAVIGATING THE POST–COVALENT BRUTON TYROSINE KINASE INHIBITOR LANDSCAPE IN MANTLE CELL LYMPHOMA: REAL-WORLD INSIGHTS ON TREATMENT PATTERNS, DISCONTINUATION, AND HEALTHCARE RESOURCE UTILIZATION

**Topic:** 18. Indolent and mantle-cell non-Hodgkin lymphoma - Clinical

Toby A. Eyre*[1], Swetha Challagulla[2], Dong Yuan[2], Catherine Fu[2], Keri Yang[2], Alvaro J. Alencar[3]

*[1]Oxford Cancer and Haematology Centre, Churchill Hospital, Headington, Oxford, United Kingdom;[2]BeOne Medicines, Ltd, San Carlos, CA, United States of America; [3]University of Miami Sylvester Comprehensive Cancer Center, Miami, FL, United States of America;*

### Background

Covalent Bruton tyrosine kinase inhibitors (cBTKis) have become standard therapy for relapsed/refractory mantle cell lymphoma (MCL) and are rapidly advancing to the first-line setting. However, real-world evidence on clinical and economic outcomes following cBTKi therapy is limited.

### Aims

To evaluate treatment patterns, time to treatment discontinuation (TTD), and healthcare resource utilization (HCRU) among patients with MCL previously treated with cBTKis

### Methods

This retrospective observational study used claims data from the Symphony Integrated Dataverse (Jan 2020-Aug 2025) to identify adults with MCL who received a cBTKi in any line of therapy (LOT) and, following discontinuation, initiated another treatment. Post-cBTKi regimens were categorized as B-cell lymphoma 2 inhibitor (BCL2i), cBTKi, noncovalent BTK inhibitor (ncBTKi), chimeric antigen receptor T-cell therapy (CAR-T), chemo±immunotherapy (C±IT), and other. TTD and HCRU (outpatient/inpatient/other medical, reported per-patient-per-month [PPPM]), were assessed for regimens received post cBTKi.

### Results

Of 10,519 total US patients with MCL, 571 were previously treated with cBTKis and received a subsequent therapy (median age, 72.0 years; ≥65 years, 77%; male, 74%; White non-Hispanic, 67%; Medicare, 62%). Regimens received in a subsequent LOT after cBTKi included cBTKis (38%), ncBTKis (32%), C±IT (11%), BCL2is (9%; monotherapy 7%), CAR-T (2%), and other (8%). Median TTD was longest with cBTKis (365 days), followed by ncBTKis (206 days), and shortest with BCL2is (140 days). Mean HCRU PPPM was lowest with cBTKis (outpatient, 1.21; inpatient, 0.20; other medical, 1.07) and highest with CAR-T (outpatient, 10.63; inpatient, 1.89; other medical, 1.55).

### Summary/Conclusion

In this real-world analysis, patients with MCL who previously received cBTKis were most frequently re-treated with cBTKis, highlighting the need for novel treatment options in the post-cBTKi setting. TTD was greatest in patients who received cBTKis or ncBTKis and shortest in those treated with BCL2is. HCRU burden was lowest for patients who received cBTKis and highest for those treated with CAR-T. Further research, including an understanding of the reasons for re-exposure to cBTKis, is warranted to confirm these findings.

**Copyright Information:** (Online) ISSN: 2572-9241
©2026 The Author(s). HemaSphere is published by John Wiley & Sons Ltd on behalf of European Hematology Association. This is an open access Abstract Book distributed under the Attribution-NonCommercial-NoDerivs (CC BY-NC-ND), which allows third parties to download the articles and share them with others as long as they credit the author and the Abstract Book, but they cannot change the content in any way or use them commercially.
**Abstract Book Citations:** Authors, Title, HemaSphere, 2026;10:(S1):pages. The abstract book DOIs can be found on the “Author Information” page.
**Disclaimer:** Articles published in the journal HemaSphere exclusively reflect the opinions of the authors. The authors are responsible for all content in their abstracts including accuracy of the facts, statements, citing resources, etc.

**Table. TTD and HCRU by MCL Treatment Regimens Received Post cBTKi**

| Outcomes | All N=571 | cBTKi n=215 | ncBTKi n=182 | C±IT n=62 | BCL2i n=53 | CAR-T n=12 |
|---|---|---|---|---|---|---|
| **Follow-up, median (IQR), days** | 294 (118-628) | 414 (206-762) | 189 (89-453) | 330 (116-727) | 256 (70-844) | 412 (216-912) |
| **TTD, median (95% CI), days** | 222 (181-281) | 365 (278-460) | 206 (173-304) | 169 (112-183) | 140 (100-315) | NA |
| **HCRU, mean (SD), PPPM** | | | | | | |
| Outpatient | 1.95 (2.82) | 1.21 (1.61) | 1.48 (2.19) | 2.19 (2.65) | 2.34 (2.70) | 10.63 (5.05) |
| Inpatient | 0.34 (0.91) | 0.20 (0.67) | 0.33 (0.75) | 0.29 (0.82) | 0.43 (1.22) | 1.89 (1.89) |
| Other medical services | 1.47 (2.62) | 1.07 (1.93) | 1.35 (2.54) | 2.54 (3.59) | 1.64 (2.82) | 1.55 (1.70) |

BCL2i, B-cell lymphoma 2 (BCL2) inhibitor; C±IT, chemo±immunotherapy; CAR-T, chimeric antigen receptor T-cell therapy; cBTKi, covalent Bruton tyrosine kinase inhibitor; HCRU, healthcare resource utilization; NA, not applicable; ncBTKi, noncovalent Bruton tyrosine kinase inhibitor; PPPM, per-patient-per-month; TTD, time to treatment discontinuation.

**Copyright Information:** (Online) ISSN: 2572-9241
©2026 The Author(s). HemaSphere is published by John Wiley & Sons Ltd on behalf of European Hematology Association. This is an open access Abstract Book distributed under the Attribution-NonCommercial-NoDerivs (CC BY-NC-ND), which allows third parties to download the articles and share them with others as long as they credit the author and the Abstract Book, but they cannot change the content in any way or use them commercially.
**Abstract Book Citations:** Authors, Title, HemaSphere, 2026;10:(S1):pages. The abstract book DOIs can be found on the "Author Information" page.
**Disclaimer:** Articles published in the journal HemaSphere exclusively reflect the opinions of the authors. The authors are responsible for all content in their abstracts including accuracy of the facts, statements, citing resources, etc.

### (PB3618) FIRST-LINE OBINUTUZUMAB PLUS CHEMOTHERAPY IN FOLLICULAR LYMPHOMA: A POLISH RESEARCH LYMPHOMA GROUP (PLRG) REAL-WORLD STUDY

**Topic:** 18. Indolent and mantle-cell non-Hodgkin lymphoma - Clinical

Ewa Paszkiewicz-Kozik*[1], Małgorzata Dębowska[2], Agnieszka Kołkowska-Leśniak[3], Agata Tyczyńska[4], Elżbieta Kalicińska[5], Magdalena Zapała[6], Michał Danecki[7], Ryszard Swoboda[8], Monika Joks[9], Marta Morawska[10], Łukasz Bołkun[11], Anna Kopinska[12], Ewa Wasik-Szczepanek[13], Dominika Maksylewicz[14], Anna Wiśniewska[15], Zuzanna Błach[16], Edyta Subocz[17], Joanna Drozd-Sokolowska[18], Łukasz Targoński[19], Marcin Szymański[19], Anna Dabrowska-Iwanicka[1], Beata Ostrowska[1], Katarzyna Domanska-Czyz[1], Monika Swierkowska[1], Ewa Mróz-Życińska[1], Agnieszka Druzd-Sitek[1], Agnieszka Giza[20], Magdalena Witkowska[21], Paweł Steckiewicz[22], Waldemar Sawicki[23], Zuzanna Dolata[24], Iuliia Vdovyn[25], Monika Długosz-Danecka[7], Małgorzata Raźny[14], Jaroslaw Dybko[16], Katarzyna Brzeźniakiewicz-Janus[25], Joanna Romejko-Jarosińska[1], Sebastian Giebel[8], Tomasz Wróbel[5], Jan Zaucha[4], Ewa Lech-Marańda[3], Jan Walewski[1]

*[1]Maria Skłodowska-Curie National Research Institute of Oncology, Department of Lymphoid Malignancies, Warszawa, Poland; [2]Nalecz Institute of Biocybernetics and Biomedical Engineering, Polish Academy of Sciences,, Warszawa, Poland;[3]Institute of Hematology and Transfusion Medicine, Department of Hematology, Warszawa, Poland; [4]Medical University of Gdańsk, Department of Hematology and Transplantology, Gdańsk, Poland; [5]Wrocław Medical University, Department and Clinic of Hematology, Cellular Therapies and Internal Medicine, Wrocław, Poland; [6]The University Hospital, Haematology and Internal Medicine Clinical Department, Krakow, Poland; [7]Maria Skłodowska-Curie National Research Institute of Oncology, Department of Clinical Oncology, Kraków, Poland; [8]Maria Sklodowska-Curie National Research Institute of Oncology, Department of Bone Marrow Transplantation and Oncohematology,, Gliwice, Poland; [9]Poznań University of Medical Sciences, Clinic of Hematology, Bone Marrow Transplantation and Cellular Therapy, Poznań, Poland; [10]Medical University of Lublin, Experimental Hematooncology Department, Lublin, Poland; [11]Medical University of Białystok, Department of Hematology, Białystok, Poland; [12]Medical University of Silesia, Department of Hematology and Bone Marrow Transplantation, Katowice, Poland;[13]Medical University in Lublin, Department of Hematooncology and Bone Marrow Transplantation, Lublin, Poland; [14]Ludwik Rydygier Hospital, Department of Hematology, Kraków, Poland; [15]Nicolas Copernicus State Hospital, Department of Oncology and Chemotherapy, Koszalin, Poland; [16]Lower Silesian Center of Oncology, Department of Hematology and Cellular Transplantation, Wrocław, Poland; [17]Warmian-Masurian Cancer Center of the Ministry of the Interior and Administration Hospital, Department of Hematology, Olsztyn, Poland; [18]Medical University of Warsaw, Department of Hematology, Transplantation and Internal Medicine, Warszawa, Poland; [19]Maria Skłodowska-Curie National Research Institute of Oncology, Department of Lymphoid Malignancies,, Warszawa, Poland; [20]Jagiellonian University Medical College, Faculty of Medicine, Department of Hematology, Kraków, Poland; [21]Medical University of Łódź, Department of Hematology, Łódź, Poland;[22]Holy Cross Cancer Center, Department of Hematology, Kielce, Poland; [23]Military Institute of Medicine-National Research Institute, Department of Internal Medicine and Hematology, Warszawa, Poland; [24]Medical University of Warsaw, Warszawa, Poland;[25]University of Zielona Góra, Multi-Specialist Hospital Gorzow Wielkopolski, Department of Hematology, Gorzów Wielkopolski, Poland;*

### Background

The final results of the GALLIUM study (Townsend et al.Hemasphere 2023) confirmed improved progression-free survival (PFS) and reduced progression rates within 24 months of initial treatment (POD24) with obinutuzumab-based chemotherapy (chth) followed with Obinutuzumab (O) maintenance compared with rituximab-based regimens. In Poland, O for induction therapy was reimbursed in Jan 2020 and since then has become widely used in first-line treatment for advanced follicular lymphoma.

### Aims

To assess the real-world effectiveness of different obinutuzumab-based induction treatment regimens.

**Copyright Information:** (Online) ISSN: 2572-9241
©2026 The Author(s). HemaSphere is published by John Wiley & Sons Ltd on behalf of European Hematology Association. This is an open access Abstract Book distributed under the Attribution-NonCommercial-NoDerivs (CC BY-NC-ND), which allows third parties to download the articles and share them with others as long as they credit the author and the Abstract Book, but they cannot change the content in any way or use them commercially.
**Abstract Book Citations:** Authors, Title, HemaSphere, 2026;10:(S1):pages. The abstract book DOIs can be found on the "Author Information" page.
**Disclaimer:** Articles published in the journal HemaSphere exclusively reflect the opinions of the authors. The authors are responsible for all content in their abstracts including accuracy of the facts, statements, citing resources, etc.

### Methods

We retrospectively collected data on consecutive patients (pts) who started front line chth with O within the first two years after reimbursement in PLRG oncohematology centers. A descriptive statistic was used to evaluate variables including demographic, pathologic, clinical, laboratory, type of treatment, response to the therapy, and causes of death of pts recorded in the analysis. Time-to-event variables were estimated using the Kaplan-Meier method.

### Results

Data of 261pts were analyzed. They started front line treatment in 21 PLRG Centers from Jan 2020 to Dec 2022. Five pts who received O under named patient access began the treatment in Aug 2018 at the earliest.
The median age at treatment initiation in the analyzed group was 54 years (range: 27-89), 41% were males. ECOG performance status was 0–1 in 85% of the cases. Clinical stage III/IV was observed in 91% of pts. The FLIPI score was low in 17%, intermediate in 33%, high in 50% of pts.
Thirty percent of pts had at least 3 months active surveillance before induction therapy. Median time to treatment start was 1.87 month (range:0-108).
Induction chemotherapy included: O-CVP (O, cyclophosphamide, vincristine, prednisolone); in 46.7%, O-CHOP (O-CVP plus doxorubicin) in 34.5%, O-B (O, bendamustine) in 18.8% of pts. Complete (CR) and partial response (PR) rates after O with chth were 74.3% and 20.9%, respectively. There was no difference in response rates between different regimens (p=0.268).Maintenance with O was started in 86.8% pts (n=224) with median number of administered cycles of 11(1-15). Thirty-five pts had shortened maintenance durationdue to SARS-CoV2 infection.
After a median 4.74 (95%CI:4.6-4.87) years of follow-up progression (PD) was reported in 17.6% (46 cases), including 9% of POD24 (23 pts). The rate of POD24 remained equal, regardless of the type of chth used(p=0.181). Five-year PFS was 62% (95% CI: 55–69%). There were no differences in PFS between frontline chth types(p=0.40). Five-year OS of the entire group was 77% (95%CI:71–82%). Five-year OS for pts with vs without POD24 were comparable, (68% vs 78%, p=0.098). During the observation 56 deaths were reported: 37.5% (21 cases) were related to SARS-CoV2 infection, 14% (8 cases) due to PD or tFL, 9% correlated with concomitant diseases (5 cases), one accident (1.8%), 7% secondary malignances (4 cases), 30% unknown (17 cases).

### Summary/Conclusion

Our results indicate that pts treated with O-based chemotherapy have low risk of POD24 and rate of PD overall, thus demonstrating consistent results with those of the GALLIUM Trial.

However, despite the low risk of progression-related deaths, the survival of our real-world series was apparently worse than in the original study inter alia due to high frequency of COVID-19. These results seem to support a recommendation of careful qualification for prolonged therapy with O, especially during periods of coexisting endemic viral infections.

**Copyright Information:** (Online) ISSN: 2572-9241
©2026 The Author(s). HemaSphere is published by John Wiley & Sons Ltd on behalf of European Hematology Association. This is an open access Abstract Book distributed under the Attribution-NonCommercial-NoDerivs (CC BY-NC-ND), which allows third parties to download the articles and share them with others as long as they credit the author and the Abstract Book, but they cannot change the content in any way or use them commercially.
**Abstract Book Citations:** Authors, Title, HemaSphere, 2026;10:(S1):pages. The abstract book DOIs can be found on the "Author Information" page.
**Disclaimer:** Articles published in the journal HemaSphere exclusively reflect the opinions of the authors. The authors are responsible for all content in their abstracts including accuracy of the facts, statements, citing resources, etc.

### (PB3620) THERAPY OF FOLLICULAR LYMPHOMA TAKING INTO ACCOUNT RISK FACTORS – A VECTOR FOR IMPROVING TREATMENT RESULTS

**Topic:** 18. Indolent and mantle-cell non-Hodgkin lymphoma - Clinical

Ekaterina Gitelzon*[1], Yana Mangasarova[1], Inzhela Israilova[1], Aminat Magomedova[1], Madina Bagova[1], Ekaterina Fastova[1], Oleg Margolin[1], Daria Belkina[1], Runisa Abdurashidova[1], Alexandra Khudovekova[1], Maria Gulieva[1], Alla Kovrigina[1], Tatiana Obukhova[1], Andrey B. Sudarikov[1], Svetlana Smirnova[1], Anna Garmash[1], Bidzhieva Malika[2], Valeriy Lapin[3], Varvara Bahtina[4], Sergey Kulikov[1], Evgeniy Zvonkov[1]

*[1] 1National Medical Research Center for Hematology, Moscow, Russian Federation, Moscow, Russia;[2]Clinical Scientific Center named after A. S. Loginov, Moscow, Russia; [3]Regional Clinical Hospital, Yaroslavl, Russia; [4]Regional Clinical Hospital, Krasnoyarsk, Russia;*

### Background

Follicular lymphoma (FL) is highly heterogeneous, encompassing a wide range of clinical, morphological, immunohistochemical, and molecular genetic presentations with distinct prognoses. Despite the rapid advancement of the pharmaceutical industry, the disease continues to recur, with varying relapse patterns (early/late, indolent/aggressive), and shorter remission durations with each line of therapy. Progression remains the primary cause of patient mortality. Predictors of decreased overall (OS), event-free survival (EFS), and early progression (progression of disease, POD24) in the nodal FL group with cytological type 1-3A include: intermediate/high risk according to the Personalized Predictive Index (PPI3, three risk groups, the factors: grade 3A, bulky, Ki-67>35%, short history of the disease <7 months) and absence of *BCL2* gene rearrangement. The integrated diagnostic approach to FL and the identified prognostic factors formed the basis for the development of a stratified differentiated therapy protocol "FL-2022" for patients with FL, aimed at improving treatment outcomes.

### Aims to determine the frequency of overall and complete antitumor response, to study the OS and EFS rates of patients with nodal FL who received therapy according to the "FL-2022" therapy.

### Methods

At the time of the first analysis of the data of the prospective multicenter study, 115 patients with nodal FL (grade 1-3A (n=90), transformed variant 3B (t3B) (n=11), 3A/3B with transformation to diffuse large B-cell lymphoma (DLBCL) (n=15) were included, who were treated from 2022 to 2025 at the National Medical Research Center of Hematology.

### Results

Fifty-seven patients with FL with the *BCL2* gene rearrangement (*BCL2-R+*) and 58 patients without it (*BCL2-R-*) received therapy according to the "FL-2022" protocol. All cases of т3B and with transformation to DLBCL were assigned to the *BCL2-R-* FL group. The median age of patients was 49.5 years (23-68). Therapy according to the R-B regimen was administered to 10 patients, R-CHOP – 25, R-CHOP-R-DHAP – 7, R-CHOP-R-DHAP-autoHSCT – 15, G-L-CHOP – 9, R-L- mNHL-BFM-90 – 35, G-L-CHOP + ibrutinib – 14. The initiation of the addition of ibrutinib to G-L-CHOP was associated with an attempt to improve the treatment outcomes of patients with *BCL2-R-* FL. The results of *BCL2-R+/-* FL therapy with a median follow-up of 28 months were as follows: 2-year OS in both groups was 100%, 2-year EFS was 100% and 79%, respectively. In the *BCL2-R-* FL group, on R-L-NHL-BFM-90 therapy, 68% (24/35) of patients achieved complete remission, 9% (3/35) - partial response, 23% (8/35) - progression; on G - L - CHOP +/- ibrutinib therapy, 91% (21/23) of patients achieved complete remission, 9% (2/23) - partial response.

**Copyright Information:** (Online) ISSN: 2572-9241
©2026 The Author(s). HemaSphere is published by John Wiley & Sons Ltd on behalf of European Hematology Association. This is an open access Abstract Book distributed under the Attribution-NonCommercial-NoDerivs (CC BY-NC-ND), which allows third parties to download the articles and share them with others as long as they credit the author and the Abstract Book, but they cannot change the content in any way or use them commercially.
**Abstract Book Citations:** Authors, Title, HemaSphere, 2026;10:(S1):pages. The abstract book DOIs can be found on the "Author Information" page.
**Disclaimer:** Articles published in the journal HemaSphere exclusively reflect the opinions of the authors. The authors are responsible for all content in their abstracts including accuracy of the facts, statements, citing resources, etc.

### Summary/Conclusion

A stratified differentiated therapy protocol “FL-2022” based on clinical, morphoimmunohistochemical and genetic (*BCL2-R+/-)* prognostic factors allows for an increase in 2-year OS and EFS, as well as a significant reduction in POD24 risks compared with the therapeutic approaches used until 2022. Anthracycline-containing regimens with the inclusion of modern targeted drugs in the therapy of *BCL2-R-* FL allow for complete remissions of the disease to be achieved in 91% of patients, including those with FL transformation to DLBCL, in contrast to high-dose pulse therapy (68% complete remissions), which allows us to recommend them as the most effective therapeutic option aimed at improving treatment outcomes.

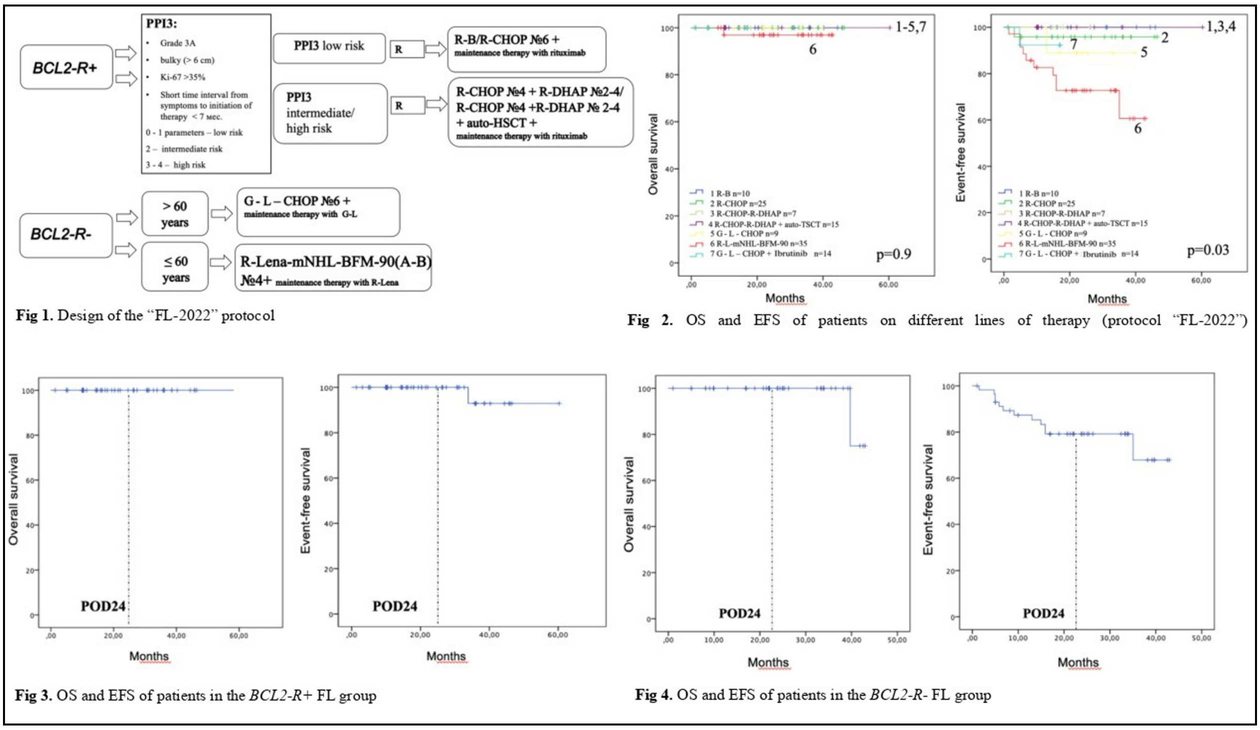

Figure 1. FL-2022 risk-adapted protocol and survival outcomes.

**Copyright Information:** (Online) ISSN: 2572-9241
©2026 The Author(s). HemaSphere is published by John Wiley & Sons Ltd on behalf of European Hematology Association. This is an open access Abstract Book distributed under the Attribution-NonCommercial-NoDerivs (CC BY-NC-ND), which allows third parties to download the articles and share them with others as long as they credit the author and the Abstract Book, but they cannot change the content in any way or use them commercially.
**Abstract Book Citations:** Authors, Title, HemaSphere, 2026;10:(S1):pages. The abstract book DOIs can be found on the “Author Information” page.
**Disclaimer:** Articles published in the journal HemaSphere exclusively reflect the opinions of the authors. The authors are responsible for all content in their abstracts including accuracy of the facts, statements, citing resources, etc.

### (PB3621) REAL-WORLD OUTCOMES OF LISOCABTAGENE MARALEUCEL IN PATIENTS WITH RELAPSED AND/OR REFRACTORY MANTLE CELL LYMPHOMA

**Topic:** 18. Indolent and mantle-cell non-Hodgkin lymphoma - Clinical

Sally Jones*[1], Aditi Saha[1], Ibrahim Muhsen[1], Caitlin Gribbin[2], Dorothy Rhee[3], Samuel Yamshon[4], Gaurav Varma[2], Lauren Shea[1], Firas Baidoun[1], Madiha Iqbal[5], Praveen Ramakrishnan Geethkumar[6], Vidiya Srikakulapu[6], Aishwarya Sannareddy[6], Nausheen Ahmed[7], Forat Lutfi[7], Javier Munoz[8], Kanishka Uttam Chandani[8], Yucai Wang[9], Jose Sandoval-Sus[10], Taha Al-Juhaishi[11], Nishant Tiwari[11], Silvina Odstrcil Bobillo[12], Narendranath Epperla[12], Amitkumar Mehta[1], Mohamed Kharfan-Dabaja[5], Julio Chavez[5]

*[1]University of Alabama at Birmingham, Birmingham, United States of America;[2]Weill Cornell Medicine, New York City, United States of America; [3]NewYork-Presbyterian Queens, Flushing, United States of America; [4]New York University Langone Health, New York City, United States of America; [5]Mayo Clinic, Jacksonville, United States of America; [6]Harold C. Simmons Comprehensive Cancer Center, The University of Texas Southwestern Medical Center, Dallas, United States of America; [7]The University of Kansas Medical Center, Kansas City, United States of America; [8]Mayo Clinic, Phoenix, United States of America; [9]Mayo Clinic, Rochester, United States of America; [10]Moffitt Cancer Center, Tampa, United States of America; [11]OU Health Stephenson Cancer Center, Oklahoma City, United States of America; [12]Huntsman Cancer Institute, University of Utah, Salt Lake City, United States of America;*

### Background

Lisocabtagene maraleucel (liso-cel) is a CD19-directed Chimeric Antigen Receptor (CAR) T-cell therapy, approved for treatment of relapsed and/or refractory (R/R) mantle cell lymphoma (MCL). Liso-cel demonstrated high efficacy in the TRANSCEND NHL 001 trial (n=88) with an overall response rate (ORR) of 83% and complete remission rate (CRR) of 72%. However, real-world evidence on the use of liso-cel in R/R MCL remains scarce.

### Aims

To assess the effectiveness and safety of liso-cel in adult patients (pts) with R/R MCL in real- world settings.

### Methods

This multicenter retrospective study included adult (≥18 years) MCL pts, from 9 USA institutions, who received liso-cel as standard-of-care therapy between 01/2023 to 12/2025. Outcomes of interest included progression free survival (PFS), overall survival (OS), ORR, CRR, and toxicities. Responses were assessed using Lugano criteria.

**Copyright Information:** (Online) ISSN: 2572-9241
©2026 The Author(s). HemaSphere is published by John Wiley & Sons Ltd on behalf of European Hematology Association. This is an open access Abstract Book distributed under the Attribution-NonCommercial-NoDerivs (CC BY-NC-ND), which allows third parties to download the articles and share them with others as long as they credit the author and the Abstract Book, but they cannot change the content in any way or use them commercially.
**Abstract Book Citations:** Authors, Title, HemaSphere, 2026;10:(S1):pages. The abstract book DOIs can be found on the "Author Information" page.
**Disclaimer:** Articles published in the journal HemaSphere exclusively reflect the opinions of the authors. The authors are responsible for all content in their abstracts including accuracy of the facts, statements, citing resources, etc.

### Results

Twenty-two pts were included, with a median age of 74 (range, 53-86) years at time of liso-cel infusion. Baseline characteristics are listed in Table 1. Median lines of therapy were 2 (range, 1-9). Bridging therapy was administered in 19 (86.3%) pts, including BTKi in 12 (54.5%) (noncovalent=10, covalent=2). Response was assessed after bridging therapy in 16 (72.7%) pts and 6 (37.5%) were in CR prior to liso-cel infusion. All but two pts received fludarabine and cyclophosphamide as lymphodepleting regimen. Median time from apheresis to infusion was 34 (range, 26-75) days.
All pts were evaluable for response. The ORR was 77.3% (17/22) with a CRR of 72.7% (16/22). In 5 (22.7%) pts there was evidence of disease progression post liso-cel. Median time to best response was 30.5 (range, 28-181) days. At a median follow-up of 10 months, 5 (22.7%) pts had died (progressive disease=4, sepsis=1). Thus far, there have been no relapses in pts who achieved CR. Six months Duration of Response, PFS, and OS rates are 70.1%, 71.3%, and 79.9% respectively (Figure 1).
Any grade Cytokine Release Syndrome (CRS) occurred in 15 (68.2%) pts (grade ≥3=1). Immune effector cell-associated neurotoxicity syndrome (ICANS) was reported in 10 pts (45.5%) with all being grade 1-2. Tocilizumab and corticosteroids were administered in 14 (63.6%) and 13 (59.1%) pts, respectively. Median duration of CRS and ICANS was 2 (range,1-5) and 5.5 (range,1-10) days, respectively. Cytopenia of any grade occurred in 20 (90.9%) pts (grade ≥3=8). Febrile neutropenia occurred in 8 (36.3%) pts. Infections occurred in 12 (54.5%) pts (bacterial=4, viral=10, and fungal=1) including 5 (22.7%) early infections (≤30 days) and 10 (45.5%) late infections (>30 days).

**Table 1. Baseline Patient, Disease and Treatment Characteristics**

| **Characteristics** | **N (%)** |
|---|---|
| Male gender | 13 (59.1) |
| Caucasian race | 21 (95.0) |
| ECOG PS 0-1 | 18 (81.8) |
| Stage IV | 21 (95.0) |
| CNS involvement at time of liso-cel infusion | 2 (9.1) |
| MIPI score | |
| Low | 5 (22.7) |
| Intermediate | 4 (18.2) |
| High | 13 (59.1) |
| Blastoid/Pleomorphic type | 6 (27.3) |
| del(17p)/*TP53* mutation | 5 (22.7) |
| Complex karyotype | 6 (27.3) |
| High Ki 67 (>30%) | 13 (59.1) |
| Prior BTKi | 22 (100) |
| Prior autologous stem cell transplant | 7 (31.8) |

### Summary/Conclusion

This study showed robust clinical activity with high ORR and CR rate of liso-cel in R/R heavily pretreated pts with R/R MCL, consistent with pivotal trial results. The safety profile was manageable, mostly with low-grade CRS and ICANS. Our results highlight a relatively high rate of late infections. These findings support the effectiveness and safety of liso-cel for R/R MCL in the real-world setting.

**Copyright Information:** (Online) ISSN: 2572-9241
©2026 The Author(s). HemaSphere is published by John Wiley & Sons Ltd on behalf of European Hematology Association. This is an open access Abstract Book distributed under the Attribution-NonCommercial-NoDerivs (CC BY-NC-ND), which allows third parties to download the articles and share them with others as long as they credit the author and the Abstract Book, but they cannot change the content in any way or use them commercially.
**Abstract Book Citations:** Authors, Title, HemaSphere, 2026;10:(S1):pages. The abstract book DOIs can be found on the “Author Information” page.
**Disclaimer:** Articles published in the journal HemaSphere exclusively reflect the opinions of the authors. The authors are responsible for all content in their abstracts including accuracy of the facts, statements, citing resources, etc.

### (PB3623) HEPATOBILIARY MALT LYMPHOMA: EXPANDING THE CLINICAL SPECTRUM OF A RARE EXTRANODAL MARGINAL ZONE LYMPHOMA

**Topic:** 18. Indolent and mantle-cell non-Hodgkin lymphoma - Clinical

Katarzyna Flegel[1], Dominika Witkowska[1], Benedikt Pelzer*[2]

*[1]Medical University of Warsaw, Transplantation and Nephrology Science Club, Warsaw, Poland;[2]Weill Cornell Medical College, Hematology, New York City, United States of America;*

### Background

Extranodal marginal zone lymphoma of mucosa-associated lymphoid tissue (MALT lymphoma) arising in the hepatobiliary system is exceedingly rare. Owing to the limited number of reported cases, knowledge regarding its clinical characteristics, associated conditions, and outcomes remains largely anecdotal.

### Aims

To comprehensively pool published cases of hepatic and biliary MALT lymphoma to characterize demographic features, anatomic distribution, infectious and autoimmune associations, and survival outcomes.

### Methods

A comprehensive non-systematic literature search was conducted to identify published cases of hepatic and biliary MALT lymphoma. Extracted variables included demographics, primary site and segmental involvement, autoimmune and infectious associations (HBV, HCV, *Helicobacter pylori*), and survival data. Descriptive analyses were performed, and comparisons between hepatic and biliary subgroups were conducted where data were available.

### Results

A total of 253 cases were identified with balanced sex distribution (Fig. A). Biliary MALT lymphoma patients were significantly older than hepatic MALT lymphoma patients (median 71 vs. 64 years; p=0.0001; Fig. B). While hepatic MALT lymphomas demonstrated a broader age range, including rare pediatric presentations, overall survival was favorable and did not differ significantly between subgroups (HR 1.86, 95% CI 0.38–9.03; p=0.34; Fig. C) although the number of events was small. Among hepatic cases, all liver segments were represented, with highest involvement of segments VI (n=29), VII (n=21), and VIII (n=19)(Fig. D); solitary lesions predominated (84%). In biliary cases, the gallbladder was most commonly affected (n=40), followed by the common bile duct (n=17) (Fig. E). Autoimmune comorbidities were frequent, particularly primary biliary cholangitis (PBC), primary Sjögren's syndrome (pSS), and primary sclerosing cholangitis (PSC), and were significantly more common in hepatic compared with biliary MALT lymphomas (p=0.013; Fig. F). Among patients with available data (n=137), viral hepatitis was documented in over 50% of cases (HBV 32.9%, HCV 19%) and was significantly enriched in hepatic MALT lymphomas (p<0.0001; Fig. G). Notably, although *H. pylori* infection is classically associated with gastric MALT lymphoma, 24.4% of patients with available data tested positive (Fig. H).

### Summary/Conclusion

To our knowledge, this represents the largest pooled analysis of hepatobiliary MALT lymphoma to date. Hepatic and biliary MALT lymphoma display distinct demographic and etiologic patterns, particularly regarding age at presentation and associations with autoimmune disease and viral hepatitis. Despite these differences, both subtypes demonstrate excellent prognosis. These findings refine the clinical characterization of this rare lymphoma entity and support the need for prospective multicenter registries.

**Copyright Information:** (Online) ISSN: 2572-9241
©2026 The Author(s). HemaSphere is published by John Wiley & Sons Ltd on behalf of European Hematology Association. This is an open access Abstract Book distributed under the Attribution-NonCommercial-NoDerivs (CC BY-NC-ND), which allows third parties to download the articles and share them with others as long as they credit the author and the Abstract Book, but they cannot change the content in any way or use them commercially.
**Abstract Book Citations:** Authors, Title, HemaSphere, 2026;10:(S1):pages. The abstract book DOIs can be found on the "Author Information" page.
**Disclaimer:** Articles published in the journal HemaSphere exclusively reflect the opinions of the authors. The authors are responsible for all content in their abstracts including accuracy of the facts, statements, citing resources, etc.

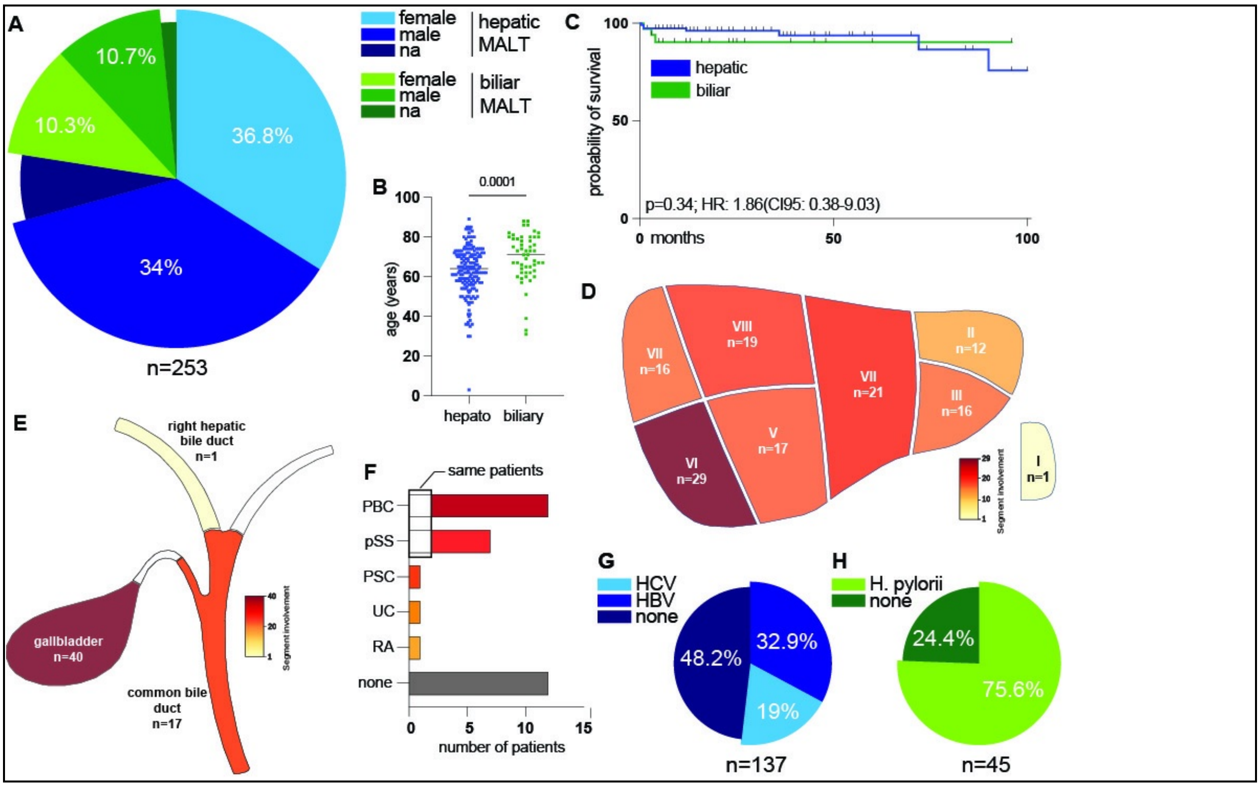

(A) Distribution of sex according to primary site (hepatic vs biliary MALT)(n = 253). (B) Age distribution at diagnosis by primary site. Scatter dot plot depicting individual patient ages (years) for hepatic and biliary MALT, with horizontal lines representing mean ± SD. Mann-Whitney test. (C) Kaplan–Meier overall survival analysis comparing hepatic and biliary MALT. Log-rank test. (D) Anatomic distribution of hepatic MALT lymphoma by liver segment (Couinaud classification). Color gradient reflects case density. (E) Distribution of biliary tract involvement. Color intensity reflects frequency of segment involvement. (F) Associated autoimmune or inflammatory conditions in the cohort. Bar graph showing the number of patients with primary biliary cholangitis (PBC), primary sclerosing cholangitis (PSC), primary Sjögren syndrome (pSS), ulcerative colitis (UC), rheumatoid arthritis (RA), or no associated condition. The shaded box highlights overlapping cases occurring in the same patients. (G) Hepatitis virus status in evaluated patients (n = 137). (H) Helicobacter pylori status in evaluated patients (n = 45).

**Copyright Information:** (Online) ISSN: 2572-9241
©2026 The Author(s). HemaSphere is published by John Wiley & Sons Ltd on behalf of European Hematology Association. This is an open access Abstract Book distributed under the Attribution-NonCommercial-NoDerivs (CC BY-NC-ND), which allows third parties to download the articles and share them with others as long as they credit the author and the Abstract Book, but they cannot change the content in any way or use them commercially.
**Abstract Book Citations:** Authors, Title, HemaSphere, 2026;10:(S1):pages. The abstract book DOIs can be found on the “Author Information” page.
**Disclaimer:** Articles published in the journal HemaSphere exclusively reflect the opinions of the authors. The authors are responsible for all content in their abstracts including accuracy of the facts, statements, citing resources, etc.

### (PB3624) BENEFITS OF RITUXIMAB MAINTENANCE AFTER FIRST-LINE BENDAMUSTINE-RITUXIMAB IN PATIENTS WITH MANTLE CELL LYMPHOMA: A META-ANALYSIS OF CLINICAL TRIALS AND OBSERVATIONAL STUDIES

**Topic:** 18. Indolent and mantle-cell non-Hodgkin lymphoma - Clinical

Julia Wang*[1], Dongni Yi[1], Yajuan Gao[1], Robin Klebig[1], Jonas Paludo[1], Javier Munoz[2], Muhamad Alhaj Moustafa[3], Madiha Iqbal[3], Julio Chavez[3], Yucai Wang[1], Jithma Abeykoon[1]

*[1]Mayo Clinic, Rochester, MN, United States of America;[2]Mayo Clinic, Phoenix, AZ, United States of America;[3]Mayo Clinic, Jacksonville, FL, United States of America;*

### Background

The role of rituximab maintenance therapy following first-line (1L) bendamustine-rituximab (BR) induction in mantle cell lymphoma (MCL) remains controversial. The randomized phase 2 MAINTAIN trial (a subgroup analysis within the StiL NHL7-2008 study) showed no significant benefit in progression-free survival (PFS) or overall survival (OS) with rituximab maintenance compared to observation after 1L BR. However, several retrospective observational studies and real-world analyses have suggested potential improvements in PFS and/or OS with maintenance rituximab in this setting. These conflicting findings highlight the need for a systematic synthesis of available evidence to clarify the clinical benefit of rituximab maintenance after 1L BR in MCL.

### Aims

We aimed to evaluate whether rituximab maintenance following 1L BR induction is associated with improved PFS and OS in patients with MCL through a systematic review and meta-analysis of randomized controlled trials (RCTs) and observational studies.

### Methods

We searched PubMed/MEDLINE, Embase, and abstracts from major hematology/oncology conferences (e.g., ASH, ASCO, EHA) from inception to February 2026 for studies evaluating rituximab maintenance versus no maintenance (observation) after 1L BR in MCL. Eligible studies included RCTs and observational cohort/registry studies reporting hazard ratios (HRs) with 95% confidence intervals (CIs) for PFS and/or OS, or data allowing HR estimation. Heterogeneity among studies was assessed with $I^2$ statistics. Pooled HRs with 95% CIs were calculated using the inverse-variance method. A random-effects model (DerSimonian-Laird) was employed to account for anticipated heterogeneity between studies. A sensitivity analyses with a common-effect (fixed-effect) model was also performed.

### Results

The search identified 238 relevant records. After screening, 6 studies met inclusion criteria (1 randomized trial [MAINTAIN] and 5 observational studies), encompassing 2,454 patients, of whom 1,201 (48.9%) received rituximab maintenance. All studies reported OS; PFS data were available from 4 studies (MAINTAIN plus 3 observational). Heterogeneity was low for PFS ($I^2$=0%, p=0.50) and moderate for OS ($I^2$=39%, p=0.15). Rituximab maintenance was associated with significantly improved PFS (pooled HR 0.55, 95% CI 0.48–0.64, p<0.001) as well as OS (pooled HR 0.63, 95% CI 0.54–0.74, p<0.001) (Figure). Sensitivity analysis using a common-effect model yielded nearly identical results.

**Copyright Information:** (Online) ISSN: 2572-9241
©2026 The Author(s). HemaSphere is published by John Wiley & Sons Ltd on behalf of European Hematology Association. This is an open access Abstract Book distributed under the Attribution-NonCommercial-NoDerivs (CC BY-NC-ND), which allows third parties to download the articles and share them with others as long as they credit the author and the Abstract Book, but they cannot change the content in any way or use them commercially.
**Abstract Book Citations:** Authors, Title, HemaSphere, 2026;10:(S1):pages. The abstract book DOIs can be found on the “Author Information” page.
**Disclaimer:** Articles published in the journal HemaSphere exclusively reflect the opinions of the authors. The authors are responsible for all content in their abstracts including accuracy of the facts, statements, citing resources, etc.

**Summary/Conclusion**

This meta-analysis provides evidence that rituximab maintenance after 1L BR induction in MCL is associated with statistically significant improvements in both PFS and OS, despite the negative findings of the MAINTAIN trial. These results, driven largely by observational data with low-to-moderate heterogeneity, support the incorporation of rituximab maintenance into routine clinical practice and future trial designs for patients with MCL receiving frontline BR.

**Figure. Forest plot of hazard ratios for PFS (A) and OS (B) with rituximab maintenance versus no maintenance after 1L BR in MCL**

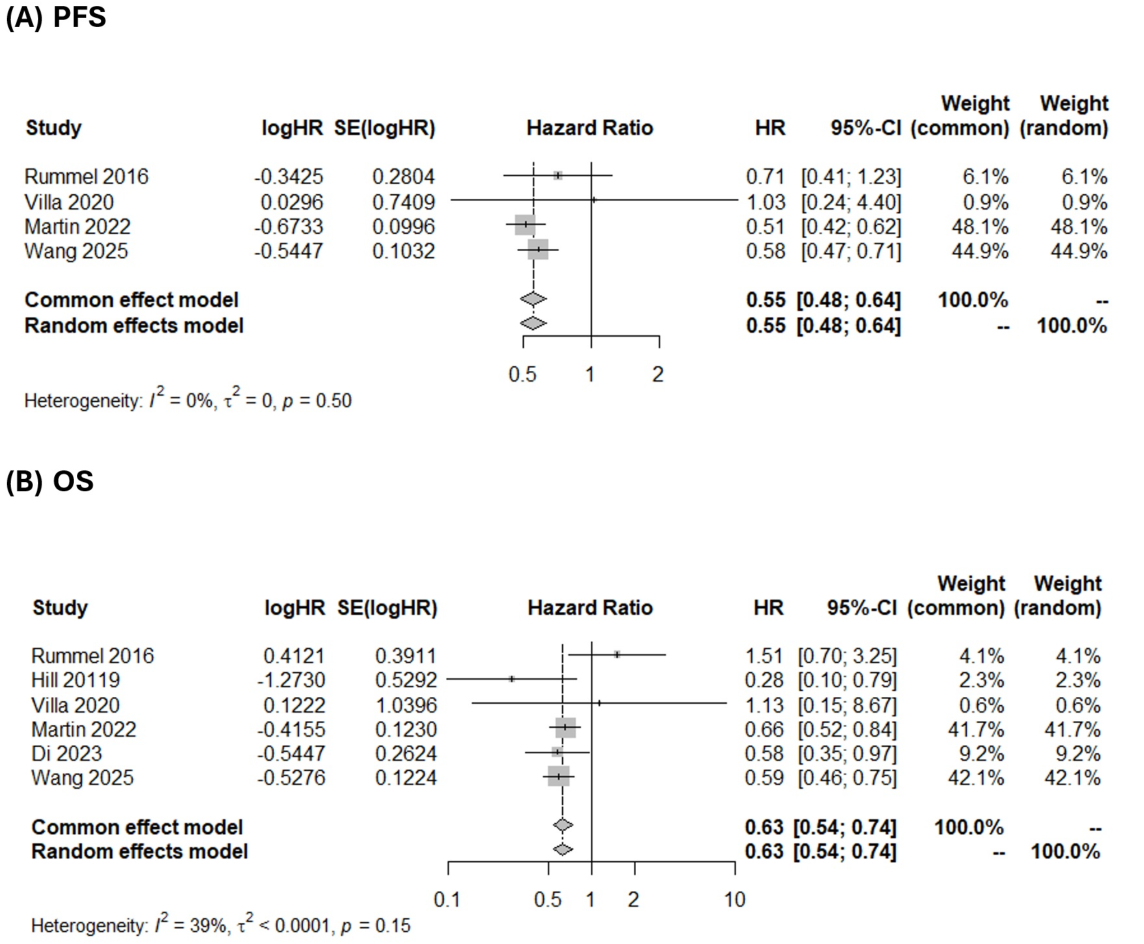

**Copyright Information:** (Online) ISSN: 2572-9241
©2026 The Author(s). HemaSphere is published by John Wiley & Sons Ltd on behalf of European Hematology Association. This is an open access Abstract Book distributed under the Attribution-NonCommercial-NoDerivs (CC BY-NC-ND), which allows third parties to download the articles and share them with others as long as they credit the author and the Abstract Book, but they cannot change the content in any way or use them commercially.
**Abstract Book Citations:** Authors, Title, HemaSphere, 2026;10:(S1):pages. The abstract book DOIs can be found on the "Author Information" page.
**Disclaimer:** Articles published in the journal HemaSphere exclusively reflect the opinions of the authors. The authors are responsible for all content in their abstracts including accuracy of the facts, statements, citing resources, etc.

### (PB3625) COMPARATIVE SAFETY AND EFFICACY OF CAR T-CELL THERAPY AND BISPECIFIC ANTIBODIES IN RELAPSED/REFRACTORY MANTLE CELL LYMPHOMA: A META-ANALYSIS OF CLINICAL TRIALS AND REAL-WORLD STUDIES

**Topic:** 18. Indolent and mantle-cell non-Hodgkin lymphoma - Clinical

Julia Wang*[1], Dongni Yi[1], Yajuan Gao[1], Robin Klebig[1], Jonas Paludo[1], Muhamad Alhaj Moustafa[2], Madiha Iqbal[2], Julio Chavez[2], Javier Munoz[3], Yucai Wang[1], Jithma Abeykoon[1]

*[1]Mayo Clinic, Rochester, MN, United States of America;[2]Mayo Clinic, Jacksonville, FL, United States of America; [3]Mayo Clinic, Phoenix, AZ, United States of America;*

### Background

Immunotherapies such as CD19-directed chimeric antigen receptor T-cell therapy (CAR-T) and CD3/CD20-directed bispecific antibodies (BsAbs) have shown high efficacy in relapsed/refractory (R/R) mantle cell lymphoma (MCL). Two CAR-T products, brexucabtagene autoleucel (brexu-cel) and lisocabtagene maraleucel (liso-cel), are FDA-approved, while two BsAbs, glofitamab and mosunetuzumab, are in clinical development. Comparative safety and efficacy data are limited, and direct head-to-head clinical trials are unlikely to be performed.

### Aims

We aimed to evaluate the comparative safety and efficacy of CAR-T versus BsAbs in R/R MCL through a systematic review and meta-analysis of clinical trials and real-world evidence (RWE) studies.

### Methods

We searched PubMed/MEDLINE, Embase, and abstracts from major hematology/oncology conferences (e.g., ASH, ASCO, EHA, ICML) from inception to February 2026 for studies of CD19 CAR-T (brexu-cel or liso-cel) or CD3/CD20 BsAbs in R/R MCL. Key outcomes included rates of cytokine release syndrome (CRS) and immune effector cell associated neurotoxicity syndrome (ICANS), overall response rate (ORR), complete response (CR) rate, and 12-month progression-free survival (PFS) and 12-month overall survival (OS). Pooled proportions with 95% confidence intervals (CIs) for adverse events and response rates, and pooled 12-month survival rates with 95% CIs for PFS and OS, were calculated using the inverse-variance method under a random-effects model (DerSimonian-Laird) to account for expected heterogeneity.

**Copyright Information:** (Online) ISSN: 2572-9241
©2026 The Author(s). HemaSphere is published by John Wiley & Sons Ltd on behalf of European Hematology Association. This is an open access Abstract Book distributed under the Attribution-NonCommercial-NoDerivs (CC BY-NC-ND), which allows third parties to download the articles and share them with others as long as they credit the author and the Abstract Book, but they cannot change the content in any way or use them commercially.
**Abstract Book Citations:** Authors, Title, HemaSphere, 2026;10:(S1):pages. The abstract book DOIs can be found on the "Author Information" page.
**Disclaimer:** Articles published in the journal HemaSphere exclusively reflect the opinions of the authors. The authors are responsible for all content in their abstracts including accuracy of the facts, statements, citing resources, etc.

### Results

The search yielded 368 records; after screening, 11 studies met eligibility and were included for meta-analysis: 3 CAR-T trials (ZUMA-2 cohorts 1 and 3; TRANSCEND NHL 001), 6 RWE studies on brexu-cel, and 2 BsAb trials (glofitamab; mosunetuzumab plus polatuzumab vedotin), totaling 1,369 patients.
Brexu-cel showed comparable CRS rates in trials versus RWE (94% vs 89%; grade ≥3 10% vs 11%), which were higher than for liso-cel (61%, grade ≥3 1%) and BsAbs (57%, grade ≥3 4%) in trials (P<0.01). ICANS rates with brexu-cel were also similar in trials versus RWE (71% vs 60%; grade≥3 29% vs 24%), higher than liso-cel (31%, grade ≥3 9%), while BsAbs had the lowest rates (7%, grade ≥3 0%) (P<0.01; Table).
No significant differences were observed in ORR (91% vs 89% vs 83% vs 86%) or CR rates (71% vs 77% vs 72% vs 78%) across brexu-cel trials, brexu-cel RWE, liso-cel trials, and BsAb trials, respectively. Corresponding 12-month PFS rates were 67.8% (95% CI 52.4–83.2), 57.3% (95% CI 50.3–64.4), 52.8% (95% CI 40.6–63.6), and 65.5% (95% CI 47.8–83.3), with no significant differences. The 12-month OS rates were 83.3% (95% CI 75.9–88.7), 75.4% (95% CI 71.9–78.5), 61.8% (95% CI 50.2–71.4), and 77.0% (95% CI 66.4–85.0), respectively, suggesting potentially slightly inferior OS with liso-cel relative to brexu-cel (P<0.05; Table).

### Summary/Conclusion

This meta-analysis showed comparable safety and efficacy for brexu-cel in RWE versus clinical trials. Relative to brexu-cel, liso-cel exhibited lower CRS and ICANS rates, similar ORR, CR rate, and 12-month PFS, but potentially slightly lower 12-month OS. Compared to CAR-T, BsAbs demonstrated lower CRS and ICANS rates, similar ORR and CR rate, and similar 12-month PFS and 12-month OS rates. These findings should be interpreted with caution, due to different patient populations and varied follow-up time across different studies.

**Table. Summary of CAR-T and BsAb safety and efficacy data in individual MCL studies and pooled results by meta-analyses**

| Group | Study | Publication | Infusion N | Safety N | Efficacy N [a] | Any Grade CRS | Grade ≥3 CRS | Any Grade ICANS | Grade ≥3 ICANS [b] | ORR | CR rate | Median follow-up (month) | 12m PFS | 12m PFS 95% CI | 12m OS | 12m OS 95% CI |
|---|---|---|---|---|---|---|---|---|---|---|---|---|---|---|---|---|
| Brexu-cel trial | Brexu-cel, ZUMA2 Cohort 1 | Wang 2022 [c] | 68 | 68 | 68 | 91% | 15% | 63% | 31% | 91% | 68% | 35.6 | 59.2% | 44.6–71.2% | 76.0% | 62.8–85.1% |
| Brexu-cel trial | Brexu-cel, ZUMA2 Cohort 3 | van Meerten 2025 | 86 | 86 | 86 | 95% | 6% | 78% | 27% | 91% | 73% | 15.5 | 75.0% | 64.5–83.4% | 90.0% | 80.7–94.4% |
| Brexu-cel RWE | Brexu-cel, US Lymphoma CART Consortium | Wang 2023 | 168 | 168 | 168 | 90% | 8% | 61% | 32% | 90% | 82% | 14.3 | 59.0% | 51.0–66.0% | 75.0% | 67.0–81.0% |
| Brexu-cel RWE | Brexu-cel, CIBMTR | Ahmed 2025 | 476 | 476 | 476 | 89% | 11% | 62% | 30% | 91% | 82% | 13.5 | 63.0% | 58.0–67.0% | 76.0% | 72.0–80.0% |
| Brexu-cel RWE | Brexu-cel, Europe EAP | Iacoboni 2022 | 33 | 33 | 33 | 91% | 3% | 64% | 36% | 91% | 79% | 10.1 | 51.0% | 31.0–83.0% | 61.0% | 41.0–92.0% |
| Brexu-cel RWE | Brexu-cel, DESCAR-T | Herbuax 2024 | 152 | 149 | 144 | 88% | 12% | 55% | 15% | 85% | 72% | 12.2 | 45.6% | 36.2–54.5% | NA | NA |
| Brexu-cel RWE | Brexu-cel, UK | O'Reilly 2024 | 83 | 83 | 83 | 93% | 12% | 55% | 23% | 87% | 81% | 13.3 | 62.0% | 49.0–73.0% | 74.0% | 62.0–83.0% |
| Brexu-cel RWE | Brexu-cel, Germany/Switzerland | Simon 2026 | 113 | 109 [d] | 85 | 85% | 13% | 54% | 15% | 88% | 61% | 17 | NA | NA | NA | NA |
| Liso-cel trial | Liso-cel, TRANSCEND NHL 001 | Wang 2024 | 88 | 88 | 83 | 61% | 1% | 31% | 9% | 83% | 72% | 16.1 | 52.8% | 40.6–63.6% | 61.8% | 50.2–71.4% |
| BsAb trial | Glofitamab | Phillips 2025 | 60 | 60 | 60 | 70% | 12% | 12% | 0% | 85% | 78% | 19.6 | 56.7% | 43.2–70.1% | 73.9% | 62.4–85.4% |
| BsAb trial | Mosunetuzumab + Polatuzumab vedotin | Budde 2025 | 42 | 42 | 42 | 43% | 0% | 2% | 0% | 88% | 79% | 15.9 | 74.8% | 60.2–89.4% | 83.1% | 71.7–94.5% |
| **Pooled results** | | | | | | | | | | | | | | | | |
| Brexu-cel trial | n=2 | | | | | 94% | 10% | 71% | 29% | 91% | 71% | | 67.8% | 52.4–83.2% | 83.3% | 75.9–88.7% |
| Brexu-cel RWE | n=5 | | | | | 89% | 11% | 60% | 24% | 89% | 77% | | 57.3% | 50.3–64.4% | 75.4% | 71.9–78.5% |
| Liso-cel trial | n=1 | | | | | 61% | 1% | 31% | 9% | 83% | 72% | | 52.8% | 40.6–63.6% | 61.8% | 50.2–71.4% |
| BsAb trial | n=4 | | | | | 57% | 4% | 7% | 0% | 86% | 78% | | 65.5% | 47.8–83.3% | 77.0% | 66.4–85.0% |

a. N used for ORR and CR rate calculations in individual studies.
b. ICANS or neurological events.
c. For PFS and OS in ZUMA-2 Cohort 1, due to lack of published 12-month data, 15-month estimates were used for this meta-analysis (Wang et al, ASH 2020).
d. 109 for CRS rate calculation and 107 for ICANS rate calculation.

**Copyright Information:** (Online) ISSN: 2572-9241
©2026 The Author(s). HemaSphere is published by John Wiley & Sons Ltd on behalf of European Hematology Association. This is an open access Abstract Book distributed under the Attribution-NonCommercial-NoDerivs (CC BY-NC-ND), which allows third parties to download the articles and share them with others as long as they credit the author and the Abstract Book, but they cannot change the content in any way or use them commercially.
**Abstract Book Citations:** Authors, Title, HemaSphere, 2026;10:(S1):pages. The abstract book DOIs can be found on the "Author Information" page.
**Disclaimer:** Articles published in the journal HemaSphere exclusively reflect the opinions of the authors. The authors are responsible for all content in their abstracts including accuracy of the facts, statements, citing resources, etc.

### (PB3626) EFFICACY OF SONROTOCLAX VERSUS PIRTOBRUTINIB IN POST–BRUTON TYROSINE KINASE INHIBITOR RELAPSED/REFRACTORY MANTLE CELL LYMPHOMA: AN INDIRECT COMPARISON

**Topic:** 18. Indolent and mantle-cell non-Hodgkin lymphoma - Clinical

Toby A. Eyre*[1], Swetha Challagulla[2], Kaijun Wang[2], Leyla Mohseninejad[3], Piotr Wojciechowski[4], Natalia Kut[4], Priscille Bourquelot[2], Keri Yang[2], Alvaro J. Alencar[5]

*[1]Oxford Cancer and Haematology Centre, Churchill Hospital, Headington, Oxford, United Kingdom;[2]BeOne Medicines, Ltd, San Carlos, CA, United States of America; [3]BeOne Medicines, Ltd, Schiphol, The Netherlands; [4]Clever-Access, Kraków, Poland; [5]University of Miami Sylvester Comprehensive Cancer Center, Miami, FL, United States of America;*

### Background

Sonrotoclax (BGB-11417-201) and pirtobrutinib (BRUIN MCL trial; NCT03740529) have demonstrated efficacy in separate single-arm post-Bruton tyrosine kinase inhibitor (BTKi) relapsed/refractory (R/R) mantle cell lymphoma (MCL) studies, but there are currently no head-to-head randomized trials that compare both therapies.

### Aims

To compare the efficacy of sonrotoclax versus pirtobrutinib in a post-BTKi R/R MCL setting in an unanchored matching-adjusted indirect comparison (MAIC).

### Methods

An unanchored MAIC compared individual patient-level data from the sonrotoclax trial (n=103 [efficacy set]; median overall survival (OS) follow-up, 15.2 months) with aggregate data from the pirtobrutinib trial (n=120 [US Food and Drug Administration efficacy cohort]; median OS follow-up, 9.3 months). Matching covariates were selected based on literature review and clinical expert input. The base-case model maximized covariate adjustment while maintaining an effective sample size (ESS) >50. Sensitivity analyses with adequate sample size (ESS >50) and alternative covariates were conducted to validate the base-case model results. Outcomes included overall response rate (ORR), duration of response (DOR), progression-free survival (PFS), and OS. Weighted Cox proportional hazards and logistic regression models were used for time-to-event and binary endpoints, respectively. Hazard ratios (HR) and odds ratios (OR) with 95% confidence intervals (95% CI) were reported.

### Results

In the base-case analysis, sonrotoclax showed a nonsignificant, numerically higher ORR (OR, 1.54; 95% CI, 0.80-2.97), as well as a numerically longer DOR (HR, 0.75; 95% CI, 0.37-1.54), versus pirtobrutinib. Consistent numerical improvements in the sonrotoclax cohort were observed for PFS (HR, 0.73; 95% CI, 0.48-1.11) and OS (HR, 0.66; 95% CI, 0.37-1.15). Sensitivity analyses using alternative covariate sets generated broadly consistent results (Table).

### Summary/Conclusion

Based on the data currently available, the MAIC findings suggest a nonsignificant trend of potential difference in efficacy outcomes between sonrotoclax and pirtobrutinib. Future studies with longer follow-up are warranted.

**Copyright Information:** (Online) ISSN: 2572-9241
©2026 The Author(s). HemaSphere is published by John Wiley & Sons Ltd on behalf of European Hematology Association. This is an open access Abstract Book distributed under the Attribution-NonCommercial-NoDerivs (CC BY-NC-ND), which allows third parties to download the articles and share them with others as long as they credit the author and the Abstract Book, but they cannot change the content in any way or use them commercially.
**Abstract Book Citations:** Authors, Title, HemaSphere, 2026;10:(S1):pages. The abstract book DOIs can be found on the “Author Information” page.
**Disclaimer:** Articles published in the journal HemaSphere exclusively reflect the opinions of the authors. The authors are responsible for all content in their abstracts including accuracy of the facts, statements, citing resources, etc.

**Table. MAIC Results Comparing Efficacy Outcomes With Sonrotoclax Versus Pirtobrutinib in Post-BTKi R/R MCL**

| **Model** | **ESS n (%)** | **ORR-IRC OR (95% CI) *P* value** | **DOR-IRC HR (95% CI) *P* value** | **PFS-IRC HR (95% CI) *P* value** | **OS HR (95% CI) *P* value** |
|---|---|---|---|---|---|
| **Base case[a-c]** | 55 (53) | 1.54 (0.80-2.97) .20 | 0.75 (0.37-1.54) .43 | 0.73 (0.48-1.11) .14 | 0.66 (0.37-1.15) .14 |
| **Sensitivity analysis 1[a,b]** | 97 (94) | 1.15 (0.67-1.97) .62 | 0.77 (0.44-1.36) .37 | 0.90 (0.64-1.25) .52 | 0.92 (0.59-1.44) .71 |
| **Sensitivity analysis 2[a]** | 102 (99) | 1.14 (0.67-1.94) .64 | 0.76 (0.43-1.32) .32 | 0.89 (0.64-1.23) .48 | 0.92 (0.59-1.43) .70 |

Covariates include: [a]sMIPI, no. of prior therapy lines; [b]PD to last BTKi; and [c]blastoid MCL.
BTKi, Bruton tyrosine kinase inhibitor; IRC, independent review committee; PD, progressive disease; sMIPI, simplified Mantle Cell Lymphoma International Prognostic Index.

**Copyright Information:** (Online) ISSN: 2572-9241
©2026 The Author(s). HemaSphere is published by John Wiley & Sons Ltd on behalf of European Hematology Association. This is an open access Abstract Book distributed under the Attribution-NonCommercial-NoDerivs (CC BY-NC-ND), which allows third parties to download the articles and share them with others as long as they credit the author and the Abstract Book, but they cannot change the content in any way or use them commercially.
**Abstract Book Citations:** Authors, Title, HemaSphere, 2026;10:(S1):pages. The abstract book DOIs can be found on the "Author Information" page.
**Disclaimer:** Articles published in the journal HemaSphere exclusively reflect the opinions of the authors. The authors are responsible for all content in their abstracts including accuracy of the facts, statements, citing resources, etc.

### (PB3627) GLOBAL TRENDS IN CLINICIAN PREPAREDNESS FOR FRONTLINE BTK INHIBITOR–BASED THERAPY IN MANTLE CELL LYMPHOMA: A LONGITUDINAL EDUCATIONAL OUTCOMES ANALYSIS

**Topic:** 18. Indolent and mantle-cell non-Hodgkin lymphoma - Clinical

Carmine Deluca*[1], Michael Beyer[1], Ben Johnson[1], Andrea Corti[1], Martin Dreyling[2], Toby A. Eyre[3], Christine Ryan[4], Kami Maddocks[5], Carlo Visco[6], Pier Luigi Zinzani[7]

*[1]PeerView Institute for Medical Education, New York, United States of America;[2]University Hospital, LMU, Munich, Germany; [3]Oxford University Hospitals Nhs Trust, Oxford, United Kingdom;[4]Dana-Farber Cancer Institute, Boston, United States of America; [5]The Ohio State University, Columbus, United States of America;[6]University of Verona Policlinico G.B. Rossi, Verona, Italy; [7]University of Bologna, Bologna, Italy, Bologna, Italy;*

### Background

The frontline management of mantle cell lymphoma (MCL) is evolving with increasing incorporation of covalent BTK inhibitors (BTKi) in combination regimens and a reconsideration of the role of autologous stem cell transplant (ASCT) in eligible patients. Recent clinical trial data have informed updates to treatment guidelines in Europe and the United States (Jerkeman M et al. *HemaSphere*. 2025;9:e70233; NCCN Guidelines B-Cell Lymphoma v1.2026; Dreyling M et al. *Lancet*. 2024;403:2293-2306).

### Aims

To evaluate global trends in clinician preparedness to apply frontline BTKi-based strategies through a longitudinal educational outcomes analysis.

### Methods

A total of 4,812 haematologists and haematologist-oncologists participated in live and enduring educational programmes between June 2024 and June 2025 across North America (n=2,340), Europe (n=1,179), and Rest of World (n=693). Identical pre- and post-activity assessment questions evaluated knowledge and application of evidence-aligned frontline treatment strategies. Paired analyses quantified changes in evidence-concordant responses. Logistic regression modelling evaluated the association between post-education status and selection of guideline-concordant strategies. A follow-up live poll (December 2025; n=318) further assessed preparedness for frontline BTKi-based regimens in specific clinical scenarios.

### Results

Selection of evidence-aligned frontline strategies increased by 36% relative to baseline (49% vs 85%). Post-education status was independently associated with a higher likelihood of selecting guideline-concordant strategies (odds ratio 5.0; p<0.001). The greatest relative improvement was observed among clinicians practising outside North America and Europe (33% vs 97%; 67% relative increase). Repeat participation was associated with greater gains in knowledge and skills (43% improvement with 1–2 engagements vs 48% with 3–4 engagements). Follow-up polling demonstrated increased readiness to incorporate BTKi-CIT combinations for both ASCT-ineligible (+24%, 46% vs 70%) and ASCT-eligible populations (+34%, 35% vs 69%), although baseline variability suggested persistent implementation gaps that may reflect regional system-level factors.

**Copyright Information:** (Online) ISSN: 2572-9241
©2026 The Author(s). HemaSphere is published by John Wiley & Sons Ltd on behalf of European Hematology Association. This is an open access Abstract Book distributed under the Attribution-NonCommercial-NoDerivs (CC BY-NC-ND), which allows third parties to download the articles and share them with others as long as they credit the author and the Abstract Book, but they cannot change the content in any way or use them commercially.
**Abstract Book Citations:** Authors, Title, HemaSphere, 2026;10:(S1):pages. The abstract book DOIs can be found on the “Author Information” page.
**Disclaimer:** Articles published in the journal HemaSphere exclusively reflect the opinions of the authors. The authors are responsible for all content in their abstracts including accuracy of the facts, statements, citing resources, etc.

**Summary/Conclusion**

In the context of rapid therapeutic evolution in frontline MCL management, longitudinal education was associated with substantial improvements in clinician readiness to implement BTKi-based strategies. Regional variability and residual gaps highlight the need for continued implementation-focused education to support harmonised adoption of contemporary treatment guidelines.

**Copyright Information:** (Online) ISSN: 2572-9241
©2026 The Author(s). HemaSphere is published by John Wiley & Sons Ltd on behalf of European Hematology Association. This is an open access Abstract Book distributed under the Attribution-NonCommercial-NoDerivs (CC BY-NC-ND), which allows third parties to download the articles and share them with others as long as they credit the author and the Abstract Book, but they cannot change the content in any way or use them commercially.
**Abstract Book Citations:** Authors, Title, HemaSphere, 2026;10:(S1):pages. The abstract book DOIs can be found on the "Author Information" page.
**Disclaimer:** Articles published in the journal HemaSphere exclusively reflect the opinions of the authors. The authors are responsible for all content in their abstracts including accuracy of the facts, statements, citing resources, etc.

### (PB3628) MUTATION STATUS OF THE CREBBP GENE IN AGGRESSIVE FOLLICULAR LYMPHOMA 3A-3B (14;18)-NEGATIVE.

**Topic:** 18. Indolent and mantle-cell non-Hodgkin lymphoma - Clinical

Anna Smolianinova*[1], Ivan Bolshakov[1], Yulia Sidorova[1], Bella Biderman[1], Nelli Gabeeva[1], Eduard Gemdzhian[1], Andrey B. Sudarikov[1], Evgeniy Zvonkov[1], Elena Parovichnikova[1]

*[1]National Medical Research Center for Hematology, 125167, Moscow, Russian Federation, Moscow, Russia;*

### Background

Follicular lymphoma (FL) is a heterogeneous disease. Currently, the diagnostic value of translocation (14;18)(q32;q21) as 100% FL marker is revised. Subgroups of (14;18)-negative FL have been identified, one of which is FL 3A-3B (14;18)-negative. Compared with the classical variant of the disease, FL 3A-3B (14;18)-negative is characterized by an aggressive clinical course, late germinal differentiation with a high frequency of rearrangements of the BCL6 gene [Magnoli F., et al. Front Med. 2019]. Mutations in the CREPB gene occur in many hematological tumors, however, it is the missense mutation in the CREBBP KAT domain (KATmiss) that is identified as a probable early driver mutation along with t(14;18) in classical FL. The effect of missense mutations and loss-of-function mutations in the KAT domain is fundamentally different, which theoretically explains the differences in the clinical course of diseases with different types of mutations. Mutations leading to knockout of the CREBBP gene (nonsense and frame shift mutations) cause a decrease of the protein level. In contrast, in case of missense mutation altered protein continues to be synthesized, which prevents compensatory hyperactivation of its EP300 parologue [Green M.R., et al. Blood. 2013, Dreval K., et al. Blood. 2023]. For FL 3A-3B (14;18)-negative, there are no data on the CREBBP mutation profile in the literature.

### Aims to determine the pattern of mutations in the CREBBP gene in patients with FL 3A-3B (14;18)-negative

### Methods

Targeted high-throughput sequencing of 22-30 exons of the CREBBP gene was performed in 25 tumor samples of FL 3A-3B (14;18)-negative, 69 samples of patients with FL 1-3B (14;18)-positive (Gr1-2 n=49, Gr3A n=17 Gr 3B n=3) and 10 samples of patients with diffuse large B-cell lymphoma de novo (DLBCL) were taken as a comparison group.

### Results mutations of the CREBBP gene were found in 3 (12%) patients with FL 3A-3B (14;18)-negative. In the group of FL 1-3B (14;18)-positive CREBBP mutations were detected in 38/70 (54%) patients and no mutations were found in the DLBCL group. In FL 1-3B (14;18)-positive, CREBBP missense mutations prevailed in the KAT domain, they were detected in 26/38 (68%) cases (Gr1-2 n=19 Gr3A n=7). No KATmiss mutation was found in any of the 25 FL 3A-3B (14;18)-negative. In 12/38 (32%) FL 1-3B (14;18)-negative and in 3/3 (100%) FL 3A-3B (14;18)-negative, CREBBP mutations were represented by nonsense mutations, frameshift mutations, or multiple mutations. Most of the CREBBP mutations occur in amino acid substitutions at positions D1435-S1436 (n=7), S1680 del (n=6), W1502-Y1503(n=5), R1446 (n=4) (**Figure 1**).

**Copyright Information:** (Online) ISSN: 2572-9241
©2026 The Author(s). HemaSphere is published by John Wiley & Sons Ltd on behalf of European Hematology Association. This is an open access Abstract Book distributed under the Attribution-NonCommercial-NoDerivs (CC BY-NC-ND), which allows third parties to download the articles and share them with others as long as they credit the author and the Abstract Book, but they cannot change the content in any way or use them commercially.
**Abstract Book Citations:** Authors, Title, HemaSphere, 2026;10:(S1):pages. The abstract book DOIs can be found on the "Author Information" page.
**Disclaimer:** Articles published in the journal HemaSphere exclusively reflect the opinions of the authors. The authors are responsible for all content in their abstracts including accuracy of the facts, statements, citing resources, etc.

### Summary/Conclusion

FL 3A-3B (14;18)-negative is characterized by a special pattern of CREBBP gene mutations, characterized by a low overall frequency of CREBBP mutations and the absence of KATmiss, that resembles DLBCL de novo. The data obtained may mean that FL 3A-3B (14;18)-negative has a different origin from classical FL.

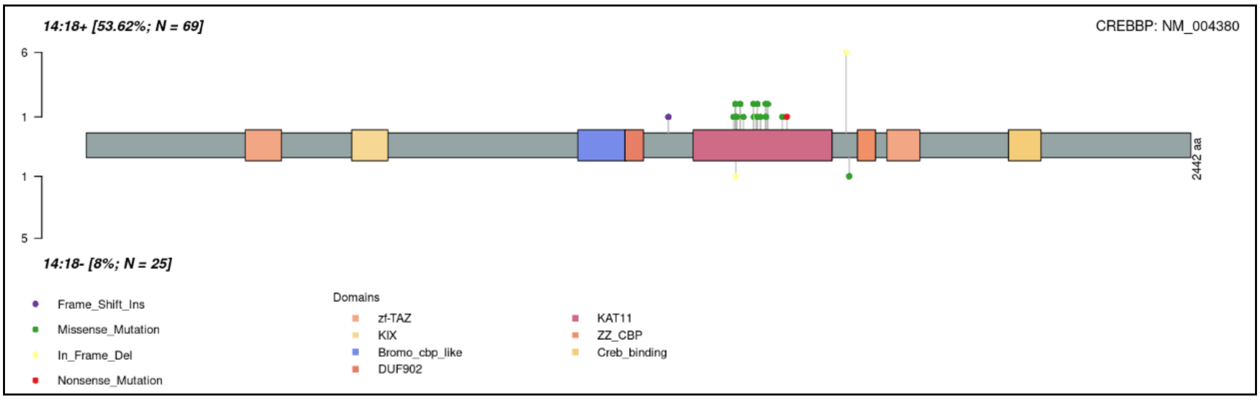

**Copyright Information:** (Online) ISSN: 2572-9241
©2026 The Author(s). HemaSphere is published by John Wiley & Sons Ltd on behalf of European Hematology Association. This is an open access Abstract Book distributed under the Attribution-NonCommercial-NoDerivs (CC BY-NC-ND), which allows third parties to download the articles and share them with others as long as they credit the author and the Abstract Book, but they cannot change the content in any way or use them commercially.
**Abstract Book Citations:** Authors, Title, HemaSphere, 2026;10:(S1):pages. The abstract book DOIs can be found on the "Author Information" page.
**Disclaimer:** Articles published in the journal HemaSphere exclusively reflect the opinions of the authors. The authors are responsible for all content in their abstracts including accuracy of the facts, statements, citing resources, etc.

### (PB3631) EXTRANODAL MARGINAL ZONE LYMPHOMA OF MUCOSA-ASSOCIATED LYMPHOID TISSUE OF THE LUNG: A SINGLE-CENTER RETROSPECTIVE ANALYSIS

**Topic:** 18. Indolent and mantle-cell non-Hodgkin lymphoma - Clinical

Yu Qiu*[1], Chunxia Zhang[1], Min Wang[1], Yayue Gao[1], Qianqian Ju[1], Chao Guo[1], Yuan Huang[1], Jingya Wang[1], Yang Li[1], Zi-Xian Liu[1], Ming Gong[1], Zhenling Li[1]

[1]*China-Japan Friendship Hospital, Beijing, China;*

### Background

Pulmonary mucosa-associated lymphoid tissue extranodal marginal zone lymphoma (MALT) is a rare indolent lymphoproliferative disease with atypical imaging features, and the optimal treatment pathway is not yet clear.

### Aims

This study aims to summarize the clinical characteristics and treatment outcomes of patients through retrospective cohort analysis to provide a theoretical basis for treatment selection.

### Methods

Retrospectively collect data on patients with a confirmed diagnosis of pulmonary MALT lymphoma at China-Japan Friendship Hospital since January 2010, including demographic characteristics, laboratory tests, imaging results, comorbidities, and treatment plans.

### Results

A total of 51 patients were included in this study, with females accounting for 68.6%, and the median age at diagnosis was 65 years (range 36-84). The main clinical manifestations included cough with sputum, dyspnea, weight loss, and fever, with a median time from symptom onset to diagnosis of 8.2 months. Patients who were asymptomatic and diagnosed through chest CT abnormalities during physical examination accounted for 33.3%. Regarding comorbidities, 14 patients had other pulmonary diseases (27.4%), 10 patients had autoimmune diseases (19.6%), and 7 patients had other tumors (13.7%). In terms of diagnostic methods, CT-guided lung puncture accounted for 49.0%, bronchoscopy for 25.5%, surgery for 17.6%, and others (bone marrow puncture, lymph node puncture) for 7.9%. The median LDH among patients was 172.5U/L (range 117-332), and 15 patients underwent M protein screening, with 10 showing positive immunofixation electrophoresis, predominantly IgM type. Among 29 cases, only 3 showed monoclonal B cells in bone marrow flow cytometry, and no lymphoma infiltration was observed in bone marrow smears and biopsies. 52.9% (27/51) of patients were in Ann Arbor stage IV, with high-risk MALT-IPI accounting for 13.7%. Pulmonary function tests were performed on 24 patients, with obstructive ventilatory dysfunction in 18 cases and diffuse ventilatory dysfunction in 13 cases. CT results were available for 32 patients, showing diverse imaging abnormalities, including consolidation (14/32), nodules (12/32), ground-glass opacities (11/32), masses (5/32), and stripes (4/32). PET-CT lesion SUVmax ranged from 3.9 to 24.2, with a median SUVmax of 5.9. Thirty patients received systemic treatment, with first-line treatments including R-CHOP-like regimens (46.7%), rituximab monotherapy, BR regimen, R2 regimen, R+BTKi, etc., with non-chemotherapy regimens accounting for 46.7%, and the ORR for R-CHOP-like regimens was 85.7%, while the ORR for non-chemotherapy regimens with evaluated efficacy was 50%. The median follow-up was 21 months, with 4 patients experiencing progression. Second-line treatments included R-CHOP and R+BTKi, with the ORR for the R+BTKi regimen being 66.7% (3/4). The median time to disease progression was 32 months (range 12-108). Among the patients with progression, only one patient died, and the cause of death was severe pneumonia (not related to disease progression).

**Copyright Information:** (Online) ISSN: 2572-9241
©2026 The Author(s). HemaSphere is published by John Wiley & Sons Ltd on behalf of European Hematology Association. This is an open access Abstract Book distributed under the Attribution-NonCommercial-NoDerivs (CC BY-NC-ND), which allows third parties to download the articles and share them with others as long as they credit the author and the Abstract Book, but they cannot change the content in any way or use them commercially.
**Abstract Book Citations:** Authors, Title, HemaSphere, 2026;10:(S1):pages. The abstract book DOIs can be found on the “Author Information” page.
**Disclaimer:** Articles published in the journal HemaSphere exclusively reflect the opinions of the authors. The authors are responsible for all content in their abstracts including accuracy of the facts, statements, citing resources, etc.

**Summary/Conclusion**

The clinical manifestations and imaging of patients with pulmonary MALT lymphoma lack characteristic features. Immunochemotherapy accounts for a certain proportion in the treatment of pulmonary MALT lymphoma, and there is still a need to explore more efficient and less toxic treatment regimens.

**Copyright Information:** (Online) ISSN: 2572-9241
©2026 The Author(s). HemaSphere is published by John Wiley & Sons Ltd on behalf of European Hematology Association. This is an open access Abstract Book distributed under the Attribution-NonCommercial-NoDerivs (CC BY-NC-ND), which allows third parties to download the articles and share them with others as long as they credit the author and the Abstract Book, but they cannot change the content in any way or use them commercially.
**Abstract Book Citations:** Authors, Title, HemaSphere, 2026;10:(S1):pages. The abstract book DOIs can be found on the “Author Information” page.
**Disclaimer:** Articles published in the journal HemaSphere exclusively reflect the opinions of the authors. The authors are responsible for all content in their abstracts including accuracy of the facts, statements, citing resources, etc.

### (PB3632) BRUMIZE: REAL-WORLD ZANUBRUTINIB USE AND PRELIMINARY SAFETY IN R/R MARGINAL ZONE LYMPHOMA IN EUROPE

**Topic:** 18. Indolent and mantle-cell non-Hodgkin lymphoma - Clinical

Côme Bommier*[1], Lucie Oberic[2], Serge Bologna[3], Kamal Bouabdallah[4], Alain Kentos[5], Griet Swennen[6], Laure Lebras[7], Omar Benbrahim[8], Camille Villesuzanne[9], Déborah Schwartz[10], Laura Dima[11], Sandrine Dupont[12], Aude Laroyenne[11], Sylvain Carras[13], Catherine Thieblemont[1]

*[1]APHP, Hôpital Saint Louis, Paris, France;[2]Oncopôle IUCT, Toulouse, France; [3]Hématologie Privée Nancéienne – HPN, Essey-lès-Nancy, France; [4]CHU Bordeaux, Bordeaux, France;[5]Pôle Hospitalier Jolimont, Haine-St Paul, Belgium;[6]ZOL Genk, Genk, Belgium; [7]Centre Léon Bérard, Lyon, France; [8]CHU Orléans, Orléans, France; [9]CH Brive, Brive, France; [10]Kappa Santé CRO, Paris, France; [11]BeOne Medicines France, Paris, France; [12]BeOne Medicines BELux, Brussels, Belgium; [13]CHU Grenoble, Grenoble, France;*

### Background

Zanubrutinib (zanu), a second-generation Bruton tyrosine kinase inhibitor (BTKi), has been available in Europe since 2022 as a treatment option for relapsed/refractory (R/R) marginal zone lymphoma (MZL), following demonstration of efficacy and a favourable safety profile in the phase 2, single arm MAGNOLIA trial (n=68). BRUMIZE was designed to complement MAGNOLIA and describe real-world (RW) use in Europe, capturing prevalent patients (pts) treated in a compassionate use setting and after reimbursement.

### Aims

To describe pt and disease characteristics and treatment patterns in pts initiating zanu for R/R MZL in France and Belgium.

### Methods

BRUMIZE is an ambispective, longitudinal, non-interventional, multicentre study conducted in 33 sites. Index date is zanu start (D0) regardless of retrospective (R) or prospective (P) enrolment. Pts are followed for 2 years (yrs) after the last prospective pt is enrolled, with visits at M3, M6, and every 6 months (mo) thereafter. This preliminary analysis reports baseline characteristics and early follow-up (FU) data after 9 mo of enrolment.

**Copyright Information:** (Online) ISSN: 2572-9241
©2026 The Author(s). HemaSphere is published by John Wiley & Sons Ltd on behalf of European Hematology Association. This is an open access Abstract Book distributed under the Attribution-NonCommercial-NoDerivs (CC BY-NC-ND), which allows third parties to download the articles and share them with others as long as they credit the author and the Abstract Book, but they cannot change the content in any way or use them commercially.
**Abstract Book Citations:** Authors, Title, HemaSphere, 2026;10:(S1):pages. The abstract book DOIs can be found on the “Author Information” page.
**Disclaimer:** Articles published in the journal HemaSphere exclusively reflect the opinions of the authors. The authors are responsible for all content in their abstracts including accuracy of the facts, statements, citing resources, etc.

### Results

Among the 90 enrolled pts (73R/17P), 19 (21.1%) had extranodal (E), 49 (54.4%) splenic (S), and 22 (24.4%) nodal (N) MZL. Median age was 74 yrs (IQR: 66.5–80.2; EMZL: 71.8; SMZL: 75.9; NMZL: 70.6), with 25.6% aged ≥80 yrs and 54.4% male. At D0, ECOG-PS was 0–1 for all EMZL/NMZL pts and 77.4% of SMZL pts. Cardiovascular (CV) comorbidities were present in 57.3% of pts (including 50.0% with hypertension).
Median time since diagnosis was 5.1 yrs (IQR: 2.1–9.3; EMZL: 5.3; SMZL: 3.8; NMZL: 6.8). Disease was bulky (>5 cm) in 20.3% (15/74) and bone marrow involvement occurred in 41.7% (25/60). For SMZL, HPLL score at diagnosis was A, B, C in 38.0%, 46.0%, 16.0%; for NMZL, FLIPI was 0–1, 2, 3–5 in 42.9%, 14.3%, 42.9%; for EMZL, MALT IPI score was low, intermediate, high in 36.8%, 31.6%, 31.6%. At D0, 83.0% of pts were stage III or IV (n=53 available) (EMZL: 69.2; SMZL: 83.3; NMZL: 93.8). LDH was elevated in 37.2% (n=51 tested), *TP53* mutated in 47.8% (n=23 tested), and Ki-67 >30% in 26.3% (n=19 tested).
Pts had received 1 (54.4%), 2 (30.0%), or ≥3 (15.6%) prior therapies. SMZL pts most often had 1 prior line (69.4%), while EMZL/NMZL had ≥2 (68.4%/59.1%). All pts were rituximab pretreated; 30.9% were refractory at D0. Latest therapy was rituximab monotherapy (45.6%) or combination (45.6%), lasting a median of 3.7 mo (IQR: 1.4–6.0), achieving CR/PR in 58.6% and progressive disease in 25.7% at 24 mo. Median time from end of latest therapy to zanu was 10.4 mo (IQR: 2.1–27.4; SMZL: 5.6; EMZL: 15.6; NMZL: 17.2).
Zanu was initiated at labelled dose in 92.0% (40.7% once daily, 59.3% twice daily) and reduced dose in 8.0%. At analysis (median FU: 6.1 mo, IQR: 2.2–16.6), 15.3% had dose reductions and 20.8% temporary interruptions.
Treatment-related adverse events (TRAEs) occurred in 38.9% (28/72 with FU), grade (G) ≥3 in 12.5%, and led to discontinuation in 8.3%. The most frequent were skin and subcutaneous disorders 11.1% (G≥3: 1.4%) and blood disorders 11.1% (G≥3: 5.5%). Three G1/2 vascular TRAEs were reported (2 haematomas, 1 hypertension).

### Summary/Conclusion

BRUMIZE represents a large European RW cohort enrolling pts with R/R MZL. Although pts were older and had a higher comorbidity burden than pts in MAGNOLIA, early safety profile is consistent with trial data. Ongoing analyses with extended FU will further evaluate RW effectiveness and long-term safety of zanu in this population.

**Copyright Information:** (Online) ISSN: 2572-9241
©2026 The Author(s). HemaSphere is published by John Wiley & Sons Ltd on behalf of European Hematology Association. This is an open access Abstract Book distributed under the Attribution-NonCommercial-NoDerivs (CC BY-NC-ND), which allows third parties to download the articles and share them with others as long as they credit the author and the Abstract Book, but they cannot change the content in any way or use them commercially.
**Abstract Book Citations:** Authors, Title, HemaSphere, 2026;10:(S1):pages. The abstract book DOIs can be found on the “Author Information” page.
**Disclaimer:** Articles published in the journal HemaSphere exclusively reflect the opinions of the authors. The authors are responsible for all content in their abstracts including accuracy of the facts, statements, citing resources, etc.

### (PB3633) EFFICACY, SAFETY, AND NGS ANALYSIS OF ORELABRUTINIB COMBINED WITH OBINUTUZUMAB IN UNTREATED MARGINAL ZONE LYMPHOMA

**Topic:** 18. Indolent and mantle-cell non-Hodgkin lymphoma - Clinical

Shanhu Qian[1], Honglan Qian*[1], Songfu Jiang[1], Yu Zhang[1], Shujuan Zhou[1], Yifen Shi[1], Yongyong MA[1]

[1] *The First Affiliated Hospital of Wenzhou Medical University, Department of Hematology, Wenzhou, China;*

### Background

Existing studies have demonstrated that combining BTK inhibitors with anti-CD20 monoclonal antibodies yields favorable outcomes in marginal zone lymphoma (MZL). As the first BTK inhibitor approved for MZL in China, orelabrutinib is expected to act synergistically with obinutuzumab.

### Aims

This study aims to evaluate the efficacy and safety of orelabrutinib in combination with obinutuzumab in patients with treatment-naïve MZL, and to explore the genetic mutation characteristics of MZL patients.

### Methods

This is a prospective, single-arm, phase II clinical trial. Untreated MZL at ECOG 0~2 criteria, in need of treatment, were eligible. All patients received 6-8 cycles of orelabrutinib (150 mg QD, days 1-21) and Obinutuzumab (1000 mg intravenously on days 1, 8, and 15 in cycle 1 and on day 1 in cycles 2-6), followed by 2 years of orelabrutinib monotherapy maintenance (150 mg once daily on days 1–28 of each cycle). The primary endpoint is the overall response rate (ORR). Secondary endpoints include complete response (CR) rate, partial response (PR) rate, and survival indicators such as duration of response (DOR), progression-free survival (PFS), and overall survival (OS). Safety is assessed by monitoring adverse events (AEs).

### Results

From January 2024 to December 2025, a total of 20 patients with MZL were enrolled. The median age at diagnosis was 64 years (range, 34-78), and 75% (15/20) were male. Among them, 75% (15/20) had mucosa-associated lymphoid tissue (MALT), 20% (4/20) had nodal marginal zone lymphoma (NMZL), and 5% (1/20) had splenic marginal zone lymphoma (SMZL). Most patients had an ECOG performance status of 0-1 (18/20) and Ann Arbor stage III-IV disease (12/20). Sixteen patients had an MZL-IPI score of 1-2. The sites of extranodal involvement in MALT included the stomach, parotid gland, ocular adnexa, and tonsil. Bone marrow involvement was present in five patients. Among fifteen efficacy-evaluable patients, the ORR was 93.3% (14/15), with a CR rate of 93.3%. Nine patients completed induction therapy and entered maintenance therapy. With a median follow-up of 9.8 months, the median DOR, PFS, and OS were not reached. The 1-year PFS rate was 93.8%, with an estimated 2-year PFS rate of 93.8%. The 1-year and 2-year OS rates were both 100%, with no deaths reported. Preliminary NGS analysis was performed on tumor tissue samples from six patients with MZL. A total of 47 mutated genes were identified, with missense mutations predominating (76%). Genetic alteration profiling showed that the frequently mutated genes were *IGLL5* (67%), *KMT2D* (50%), and *MYD88* (33%). *MYD88* mutations drive NF-κB signaling, providing a mechanistic rationale for orelabrutinib-based therapy. One patient harbored a TP53 mutation and achieved CR after treatment, indicating the applicability of this regimen in high-risk populations. Treatment was generally well tolerated, with only two treatment-related adverse events reported: thrombocytopenia and mild cutaneous bleeding. After one week of hematopoietic-stimulating therapy, the platelet count normalized, and the medication was resumed.

**Copyright Information:** (Online) ISSN: 2572-9241
©2026 The Author(s). HemaSphere is published by John Wiley & Sons Ltd on behalf of European Hematology Association. This is an open access Abstract Book distributed under the Attribution-NonCommercial-NoDerivs (CC BY-NC-ND), which allows third parties to download the articles and share them with others as long as they credit the author and the Abstract Book, but they cannot change the content in any way or use them commercially.
**Abstract Book Citations:** Authors, Title, HemaSphere, 2026;10:(S1):pages. The abstract book DOIs can be found on the "Author Information" page.
**Disclaimer:** Articles published in the journal HemaSphere exclusively reflect the opinions of the authors. The authors are responsible for all content in their abstracts including accuracy of the facts, statements, citing resources, etc.

**Summary/Conclusion**

The combination of Orelabrutinib and Obinutuzumab shows significant activity in MZL, with a manageable toxicity profile. NGS of tumor tissue is instrumental for elucidating the molecular pathogenesis of MZL and for guiding individualized therapeutic strategies. Future updates will incorporate additional follow-up data.

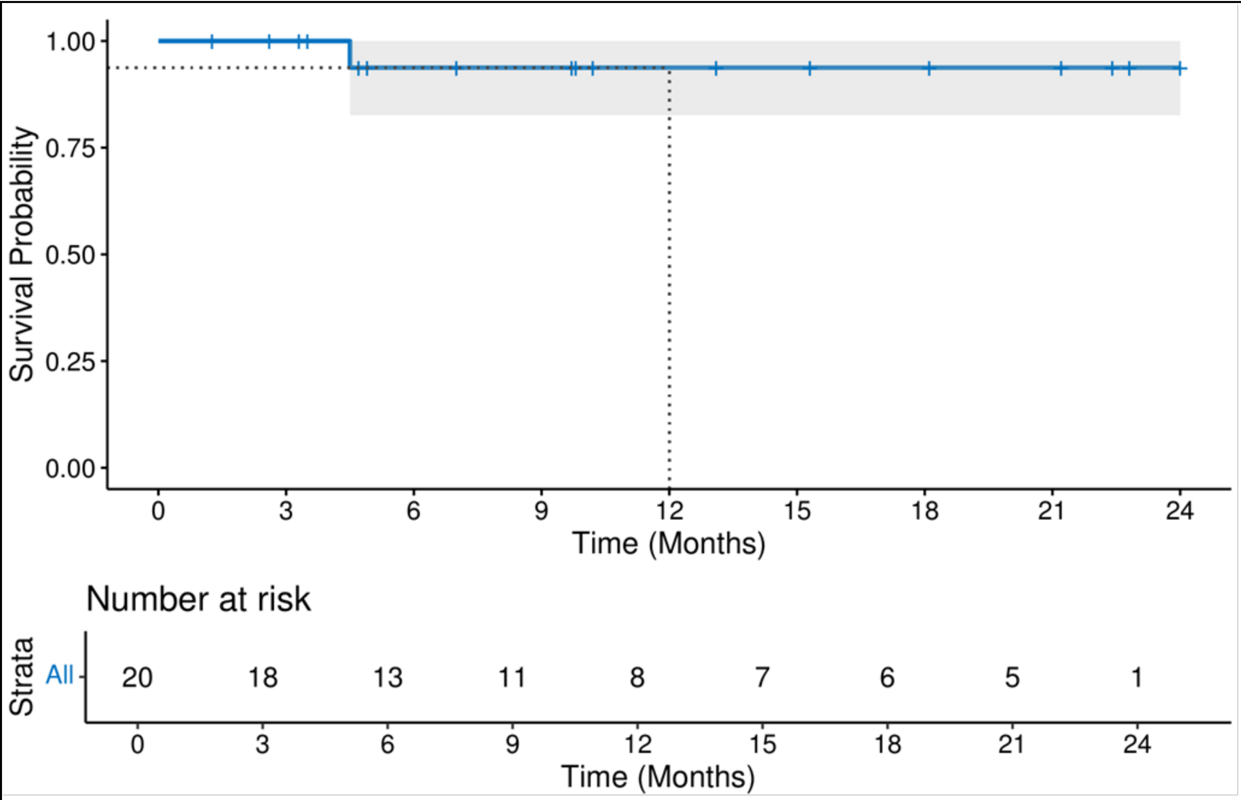

Fig. 1. Kaplan-Meier curve of PFS in patients receiving orelabrutinib combined with obinutuzumab.

**Copyright Information:** (Online) ISSN: 2572-9241
©2026 The Author(s). HemaSphere is published by John Wiley & Sons Ltd on behalf of European Hematology Association. This is an open access Abstract Book distributed under the Attribution-NonCommercial-NoDerivs (CC BY-NC-ND), which allows third parties to download the articles and share them with others as long as they credit the author and the Abstract Book, but they cannot change the content in any way or use them commercially.
**Abstract Book Citations:** Authors, Title, HemaSphere, 2026;10:(S1):pages. The abstract book DOIs can be found on the "Author Information" page.
**Disclaimer:** Articles published in the journal HemaSphere exclusively reflect the opinions of the authors. The authors are responsible for all content in their abstracts including accuracy of the facts, statements, citing resources, etc.

### (PB3634) OPTIMIZING DIAGNOSTIC STRATEGY IN ADULT LYMPHADENOPATHY: EXCISIONAL VERSUS CORE NEEDLE BIOPSY IN A DEDICATED LYMPHADENOPATHY CLINIC

**Topic:** 18. Indolent and mantle-cell non-Hodgkin lymphoma - Clinical

Marta Diaz-Lopez*[1], Alberto Martinez Llorens[1], Jose Gregorio Torres Quiroz[1], Sunil Lakhwani[1, 2], Patricia MacHado-MacHado[1]

*[1]Hospital Universitario de Canarias, Hematology and Hemotherapy, San Cristóbal de La Laguna, Spain;[2]University of La Laguna, Faculty of Medicine, San Cristobal de Laguna, Spain;*

### Background

Adult lymphadenopathy is a common clinical scenario with a broad differential diagnosis, including lymphoid malignancies, solid tumors, and benign conditions. Although several diagnostic techniques are available—fine-needle aspiration biopsy (FNAB), core needle biopsy (CNB), and excisional biopsy (EB)—the optimal initial strategy remains debated. Inadequate initial sampling may lead to sequential procedures and diagnostic delay, particularly in patients with suspected lymphoma. A structured hematology-led clinic may improve diagnostic efficiency and procedural selection.

### Aims

To compare the diagnostic yield of excisional biopsy versus core needle biopsy in a dedicated lymphadenopathy outpatient clinic.

### Methods

We conducted a retrospective observational study including consecutive adult patients evaluated at their first visit in a dedicated lymphadenopathy outpatient clinic within the Department of Hematology at our center between December 2023 and March 2025.

### Results

A total of 267 patients were included; 155 (58.1%) were female, and mean age was 50.4 years (range 15.9–89.3). All patients were referred for lymphadenopathy assessment, and four (1.5%) had concomitant splenomegaly. Superficial lymphadenopathy was detected on physical examination in 70.8%, and deep lymphadenopathy was identified by CT imaging in 39%.
Reactive lymphadenopathy was the final diagnosis in 60.7% (164) of patients. Nine patients (3,3%) were solid tumors. Overall, 96 patients (35.9%) were diagnosed with lymphoma, including follicular lymphoma (33.3%), diffuse large B-cell lymphoma (22.9%), Hodgkin lymphoma (19.8%), and other non-Hodgkin lymphomas (23.9%).
Forty patients (15%) had previously undergone FNAB; however, all required CNB or EB to establish a definitive diagnosis. CNB was performed in 32 patients (12%) and EB in 111 (41.6%). EB achieved a definitive diagnosis in 98.2% of cases (109/111), compared with 78.1% for CNB (25/32) being this difference statistically significant ($p<0.001$). Eight patients underwent both procedures. In five cases, CNB was non-diagnostic and subsequent EB established a definitive diagnosis. In two cases, EB was non-diagnostic (including one retrieving only adipose tissue), whereas CNB provided the diagnosis. In one patient, both procedures were diagnostic, establishing Hodgkin lymphoma. Overall, EB was associated with a higher diagnostic yield and reduced need for sequential procedures.

### Summary/Conclusion

In this real-world cohort, excisional biopsy showed superior diagnostic performance compared with core needle biopsy. Considering that more than one-third of patients were ultimately diagnosed with lymphoma, careful initial procedural selection within a specialized hematology clinic may be critical to ensure definitive diagnosis and avoid sequential non-diagnostic interventions.

**Copyright Information:** (Online) ISSN: 2572-9241
©2026 The Author(s). HemaSphere is published by John Wiley & Sons Ltd on behalf of European Hematology Association. This is an open access Abstract Book distributed under the Attribution-NonCommercial-NoDerivs (CC BY-NC-ND), which allows third parties to download the articles and share them with others as long as they credit the author and the Abstract Book, but they cannot change the content in any way or use them commercially.
**Abstract Book Citations:** Authors, Title, HemaSphere, 2026;10:(S1):pages. The abstract book DOIs can be found on the "Author Information" page.
**Disclaimer:** Articles published in the journal HemaSphere exclusively reflect the opinions of the authors. The authors are responsible for all content in their abstracts including accuracy of the facts, statements, citing resources, etc.

### (PB3635) ORELABRUTINIB IN CHRONIC LYMPHOCYTIC LEUKEMIA/SMALL LYMPHOCYTIC LYMPHOMA: A REAL-WORLD RETROSPECTIVE STUDY

**Topic:** 18. Indolent and mantle-cell non-Hodgkin lymphoma - Clinical

Xuewu Zhang[1], Haitao Meng[1], Gaixiang Xu[1], Min Yang[1], Wanzhuo Xie[1], Juying Wei[1], Liya MA[1], Xingnong Ye[1], Liping Mao[1], Wenyuan Mai[1], Chunmei Yang[1], Yanling Ren[1], Hongyan Tong[1], Yanan Zhu[1], Xiang Zhang[1], Jie Jin[1], Wenjuan Yu*[1]

[1]*The First Affiliated Hospital, Zhejiang University School of Medicine, Department of Hematology, Hangzhou, China;*

### Background

CLL/SLL are the most prevalent B-cell malignancy characterized by chronic B-cell receptor signaling. Bruton's tyrosine kinase inhibitors (BTKi) are pivotal in managing CLL/SLL. Previous real-world research has demonstrated that the rate at which patients discontinue ibrutinib stands at 29%. Orelabrutinib demonstrates superior kinase selectivity and enhanced safety profiles, with clinical trials indicating a reduced incidence of BTK inhibitor-related adverse events. Nevertheless, the availability of real-world data remains limited.

### Aims

This study aimed to evaluate the efficacy and safety of orelabrutinib for CLL/SLL patients in real-world.

### Methods

This study included patients with pathologically confirmed CLL/SLL who received orelabrutinib for ≥3 months. Clinical outcomes assessed included patient characteristics, hematological response rate, objective response rate (ORR), duration of treatment (DOT), progression-free survival (PFS), overall survival (OS), and safety.

### Results

From December 2021 to December 2024, a total of 75 patients (44 with CLL, 31 with SLL) were enrolled. The median age was 66 years (range, 44–90), and 73.3% were >60 years of age. Fifty-one patients (68.0%) were male. The majority had advanced-stage disease: 52.3% of CLL patients were at Binet stage C, and 61.3% of SLL patients were at Ann Arbor stage III/IV. Cytogenetic and molecular characteristics included TP53 mutation or del(17p) in 17.9% and unmutated IGHV status in 25.3%. Comorbidities were present in 54.7% of patients, including hypertension (39.0%) and anemia (22.0%).
The ORR was 86.6% (65/75). The median DOT was 30.8 months (range, 11.9–47.5). With a median follow-up of 30.8 months (range, 2.1–48.1), the median PFS was not reached; the 12- and 24-month PFS rates were 97.2% (95% CI, 90.1–99.0%) and 78.0% (95% CI, 69.0–88.2%), respectively. Death occurred in 5 patients (6.7%). The median OS was not reached, with 12- and 24-month OS rates of 93.3% (95% CI, 87.9–99.2%).
Treatment was generally well tolerated. Fifty-one patients (68.0%) experienced at least one AE of any grade, most of which were grade 1–2. The most common AEs were anemia (18/75, 24.0%), hypoalbuminemia (10/75, 13.3%), and neutropenia (5/75, 6.7%). Grade ≥3 AEs occurred in 6 patients, including lung infection (5/75, 6.7%) and neutropenia (1/75, 1.3%). No treatment-related deaths were reported.

### Summary/Conclusion

Orelabrutinib showed promising efficacy with durable responses and manageable safety in CLL/SLL patients, supporting its clinical benefit in real-world practice.

**Copyright Information:** (Online) ISSN: 2572-9241
©2026 The Author(s). HemaSphere is published by John Wiley & Sons Ltd on behalf of European Hematology Association. This is an open access Abstract Book distributed under the Attribution-NonCommercial-NoDerivs (CC BY-NC-ND), which allows third parties to download the articles and share them with others as long as they credit the author and the Abstract Book, but they cannot change the content in any way or use them commercially.
**Abstract Book Citations:** Authors, Title, HemaSphere, 2026;10:(S1):pages. The abstract book DOIs can be found on the “Author Information” page.
**Disclaimer:** Articles published in the journal HemaSphere exclusively reflect the opinions of the authors. The authors are responsible for all content in their abstracts including accuracy of the facts, statements, citing resources, etc.

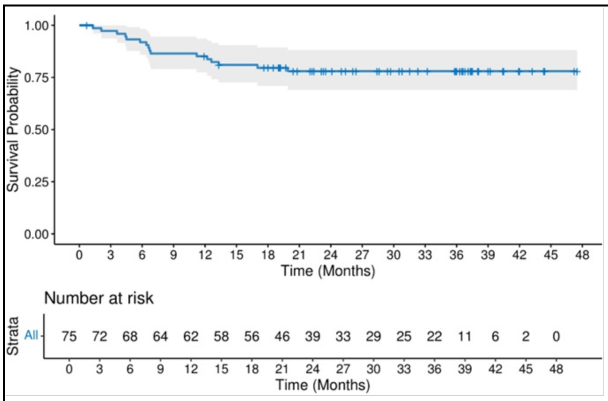

Fig. 1. PFS in patients treated with orelabrutinib

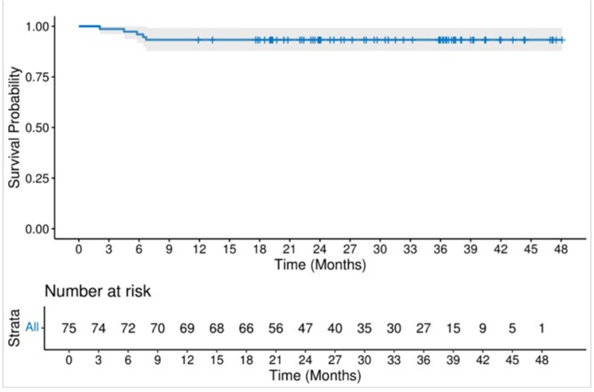

Fig. 2. OS in patients treated with orelabrutinib

**Copyright Information:** (Online) ISSN: 2572-9241
©2026 The Author(s). HemaSphere is published by John Wiley & Sons Ltd on behalf of European Hematology Association. This is an open access Abstract Book distributed under the Attribution-NonCommercial-NoDerivs (CC BY-NC-ND), which allows third parties to download the articles and share them with others as long as they credit the author and the Abstract Book, but they cannot change the content in any way or use them commercially.
**Abstract Book Citations:** Authors, Title, HemaSphere, 2026;10:(S1):pages. The abstract book DOIs can be found on the "Author Information" page.
**Disclaimer:** Articles published in the journal HemaSphere exclusively reflect the opinions of the authors. The authors are responsible for all content in their abstracts including accuracy of the facts, statements, citing resources, etc.

### (PB3636) CUTANEOUS T-CELL LYMPHOMA EPIDEMIOLOGY IN THE UNITED STATES: A REAL-WORLD DATA ANALYSIS OF ADMINISTRATIVE CLAIMS

**Topic:** 18. Indolent and mantle-cell non-Hodgkin lymphoma - Clinical

Pierluigi Porcu*[1], Chris Stuessy-Vidas[2], Mai Sato[2], Anthony Nguyen[2], Joe Stevens[2]

[1]*Lucille P. Markey Comprehensive Cancer Center, University of Kentucky College of Medicine, Department of Internal Medicine, Lexington, United States of America;* [2]*ZS Associates, New York, United States of America;*

### Background

Cutaneous T Cell lymphoma (CTCL) is a rare, heterogeneous group of non-Hodgkin lymphomas primarily affecting the skin, with limited real-world evidence describing epidemiology, patient demographics, and provider patterns across disease subtypes. Mycosis fungoides (MF), the most common subtype, and Sézary syndrome (SS), a leukemic and aggressive variant, are the most prevalent subtypes, together accounting for approximately 70–75% of all CTCL cases.

### Aims

To quantify real-world prevalence, incidence, demographic characteristics, and provider patterns of CTCL in the United States across mycosis fungoides (MF), Sézary syndrome (SS), and undefined CTCL.

### Methods

A retrospective observational study was conducted using the Komodo administrative claims database, representing approximately 95% of the U.S. population. Patients with CTCL were identified using ICD-10 diagnosis codes (MF: C84.0, SS: C84.1, undefined CTCL: C84.A) and required ≥2 CTCL-related claims. Annual prevalence and incidence were assessed from 2018–2023. Prevalence and incidence were quantified using patient counts, reflecting the rare nature of CTCL and the scale of the underlying database population. Patients were categorized into MF, SS, or undefined CTCL cohorts.

**Copyright Information:** (Online) ISSN: 2572-9241
©2026 The Author(s). HemaSphere is published by John Wiley & Sons Ltd on behalf of European Hematology Association. This is an open access Abstract Book distributed under the Attribution-NonCommercial-NoDerivs (CC BY-NC-ND), which allows third parties to download the articles and share them with others as long as they credit the author and the Abstract Book, but they cannot change the content in any way or use them commercially.
**Abstract Book Citations:** Authors, Title, HemaSphere, 2026;10:(S1):pages. The abstract book DOIs can be found on the “Author Information” page.
**Disclaimer:** Articles published in the journal HemaSphere exclusively reflect the opinions of the authors. The authors are responsible for all content in their abstracts including accuracy of the facts, statements, citing resources, etc.

**Results**

Annual CTCL prevalence increased from approximately 17,000 patients in 2018 to nearly 20,000 patients in 2023. Annual incidence remained stable at approximately 5,000 newly diagnosed patients per year. MF accounted for approximately 65% of patients with CTCL, SS for 5%, and undefined CTCL for 30%. CTCL predominantly affected older adults (57% between ages 65-88) and males comprised 55% of the CTCL population. Age and gender distributions were consistent across disease subtypes. Overall, CTCL care was highly concentrated in academic centers (~85%) and relied on a shared prescriber base across subtypes. MF and undefined CTCL patients were often managed by dermatologists (>70%) with 20% managed by hematologist/oncologists. In contrast, a higher proportion of patients with SS were managed by hematologist/oncologists (63%). These patterns are consistent with dermatologists typically managing earlier-stage disease.

| | MF | | SS | |
|---|---|---|---|---|
| **Year** | **Incident Patients (n)** | **Prevalent Patients (n)** | **Incident Patients (n)** | **Prevalent Patients (n)** |
| **2018** | 3,284 | 11,227 | 303 | 895 |
| **2019** | 3,141 | 11,790 | 319 | 918 |
| **2020** | 2,834 | 11,036 | 290 | 915 |
| **2021** | 2,988 | 12,086 | 325 | 1,008 |
| **2022** | 2,993 | 12,391 | 303 | 1,059 |
| **2023** | 2,900 | 12,426 | 316 | 1,100 |

**Summary/Conclusion**

This real-world analysis characterizes contemporary CTCL epidemiology and represents the largest U.S. CTCL cohort to date. The results demonstrated a sustained and increasing disease burden. CTCL care is centralized within academic centers and delivered through coordinated dermatology and hematology/oncology networks, with management patterns differing by subtype in alignment with established clinical practice.

This study was sponsored by Innate Pharma Inc.

**Copyright Information:** (Online) ISSN: 2572-9241
©2026 The Author(s). HemaSphere is published by John Wiley & Sons Ltd on behalf of European Hematology Association. This is an open access Abstract Book distributed under the Attribution-NonCommercial-NoDerivs (CC BY-NC-ND), which allows third parties to download the articles and share them with others as long as they credit the author and the Abstract Book, but they cannot change the content in any way or use them commercially.
**Abstract Book Citations:** Authors, Title, HemaSphere, 2026;10:(S1):pages. The abstract book DOIs can be found on the "Author Information" page.
**Disclaimer:** Articles published in the journal HemaSphere exclusively reflect the opinions of the authors. The authors are responsible for all content in their abstracts including accuracy of the facts, statements, citing resources, etc.

### (PB3637) IBRUTINIB MONOTHERAPY FOR RELAPSED AND REFRACTORY MANTLE CELL LYMPHOMA : A POLISH LYMPHOMA RESEARCH GROUP (PLRG) REAL-WORLD STUDY

**Topic:** 18. Indolent and mantle-cell non-Hodgkin lymphoma - Clinical

Joanna Romejko-Jarosińska*[1], Magdalena Witkowska[2], Krzysztof Baran[3], Jolanta Oleksiuk[4], Mateusz Wilk[5], Kamil Krzysztof Wdowiak[6], Bogusław MacHaliński[7], Anastazja Szlauer-Stefańska[8], Monika Joks[9], Joanna Drozd-Sokolowska[10], Waldemar Sawicki[11], Aneta Szudy-Szczyrek[12], Agnieszka Szymczyk[13], Karolina Kędziora[14], Barbara Kucharczyk[1], Jakub Gora[1], Michal Sekula[1], Beata Ostrowska[1], Katarzyna Domanska-Czyz[1], Łukasz Targoński[1], Agnieszka Giza[5], Lidia Gil[9], Jarosław Dybko[14], Iwona Hus[13], Jan MacIej Zaucha[3], Ewa Paszkiewicz-Kozik[1], Bartosz Puła[15, 16], Jan Walewski[1]

*[1]Maria sklodowska-Curie National Research Institute, Department of Lymphoid Malignacies, Warszawa, Poland;[2]Copernicus Memorial Hospital, Lodz, Poland, Department of Hematooncology, Lodz, Poland; [3]Medical University of Gdansk, University Clinical Center, Department of Hematology, Transplantology and Cellular Therapies, Gdansk, Poland; [4]Medical University of Bialystok, Department of Haematology, Bialystok, Poland; [5]Jagiellonian University Medical College, Faculty of Medicine, Department of Hematology, Krakow, Poland; [6]Medical University of Silesia, Faculty of Medical Sciences,, Department of Internal Diseases and Oncological Chemotherapy, Katowice, Poland; [7]Pomeranian Medical University, Department of Hematology and Bone Marrow Transplantation, Szczecin, Poland; [8]Maria Sklodowska-Curie National Research Institute of Oncology (branch in Gliwice), Bone Marrow Transplantation and Oncohematology Department, Gliwice, Poland; [9]Poznan University of Medical Science, Department of Hematology, Transplantation and Cellular Therapy, Poznan, Poland; [10]Medical University of Warsaw, Department of Hematology, Transplantation and Internal Medicine, Warszawa, Poland; [11]Military Institute of Medicine-National Research institute, Department of Internal Medicine and Hematology, Warszawa, Poland; [12]Medical University in Lublin, Department of Hematooncology and Bone Marrow Transplantation, Lublin, Poland; [13]National Institute of Medicine of the Ministry of the Interiorand Administration, Department of Hematology, Warszawa, Poland; [14]Lower Silesian Center of Oncology, Department of Hematology and Cellular Transplantation, Wroclaw, Poland; [15]Medical University of Lodz, Department of Hematology, Lodz, Poland; [16]Copernicus Memorial Hospital, Department of General Hematology and Internal Medicine, Lodz, Poland;*

### Background

Mantle cell lymphoma (MCL) is still incurable disease. Bruton kinase inhibitors (BTKi) are very active in naïve and relapse/refractory(R/R) MCL. Ibrutinib is only one of Bruton kinase inhibitors, which has been reimbursed for Polish patients (pts) with R/R MCL.

### Aims

Here, we evaluated clinical and pathological features, the efficacy and tolerability of ibrutinib in our patients with R/R MCL

### Methods

We retrospectively collected data from 172 R/R MCL patients treated with ibrutinib monotherapy out of clinical trials between 2015-2025 at fourteen hematooncology centers from the Polish Lymphoma Research Group.

**Copyright Information:** (Online) ISSN: 2572-9241
©2026 The Author(s). HemaSphere is published by John Wiley & Sons Ltd on behalf of European Hematology Association. This is an open access Abstract Book distributed under the Attribution-NonCommercial-NoDerivs (CC BY-NC-ND), which allows third parties to download the articles and share them with others as long as they credit the author and the Abstract Book, but they cannot change the content in any way or use them commercially.
**Abstract Book Citations:** Authors, Title, HemaSphere, 2026;10:(S1):pages. The abstract book DOIs can be found on the "Author Information" page.
**Disclaimer:** Articles published in the journal HemaSphere exclusively reflect the opinions of the authors. The authors are responsible for all content in their abstracts including accuracy of the facts, statements, citing resources, etc.

**Results**

Median age was 71 (range 37-93), with 58 pts more than 75. Male pts were 109 (63,3%). Blastoid and pleomorphic variants occurred in 13/125 (10,4%), and 17/125 (13,7%) pts, respectively. Four patients had cyclin D1 negative disease, 99/134 (74%) pts had Ki67>30%. High biologic MCL international prognostic index (MIPIb) was detected in 79 (45,9%) pts and TP53/ del17p in 16/60 (25%) pts, 95 patients (55%) belongs to high risk MCL at diagnosis. All patients received at least one chemotherapy with anti-CD20 monoclonal antibody. High dose chemotherapy with auto-HCT was performed in 33 pts (19,1%) and maintenance therapy in 57 pts (33%). Complete remission (CR) after first line treatment was achieved in 69 patients (40%). Median lines before ibrutinib were 1(range:1-8). Refractory status disease before ibrutinib treatmentwas diagnosed in 88 (51%) pts. Median 28 day-cycles of ibrutinib were 10 (range:1-142), 10 patients have received ibrutinib for more than 5 years. The best overall response rate was achieved in 100 pts (58,1%) with CR in 44 (25,6%) pts. With median follow up 12 months (range 1-135), probability of 2-year event free (EFS), 2- year progression free survival (PFS) and 2 year overall survival (OS) were 44% (95%C.I 36.2%,51,8), 58,8% (95%C.I.51%,66,6%) and 64% (95% C.I.:56,8%,71,8%), respectively. The number of lines, status disease has no impact on EFS, PFS and OS. High MIPIb (P=0.001 and p=0,004) and high risk MCL (p=0,001 and p=0,002), no CR after first line (p=0.02 and p=0.04) had an impact on EFS and PFS, and high risk MCL (p=0.0008) and high MIPIb (p=0.002) had an impact on OS. The reduction of doses was required in 27 pts (15,7%) due to hematologic (7pts) and non-hematologic toxicity (20pts). The most frequent adverse events were reported: atrial fibrillation 10 pts (5.8%), hypertension 17 pts (10%), thrombocytopenia in 6 patients (grade 3), infections in 43 pts (25%), diarrhea in 11 patients (6,4%). Fifty eight patients died. The was no death due to ibrutinib. The main cause death was progressive. Two patients were lost from follow up and 31 pts have received other therapies. Actually, 81 patients have been continuing ibrutinib monotherapy.

**Summary/Conclusion**

Our data confirmed that ibrutinib monotherapy is effective and safe for MCL patients. Patient with high risk MCL and MIPIb at diagnosis gain a little benefit ibrutinib monotherapy.

**Copyright Information:** (Online) ISSN: 2572-9241
©2026 The Author(s). HemaSphere is published by John Wiley & Sons Ltd on behalf of European Hematology Association. This is an open access Abstract Book distributed under the Attribution-NonCommercial-NoDerivs (CC BY-NC-ND), which allows third parties to download the articles and share them with others as long as they credit the author and the Abstract Book, but they cannot change the content in any way or use them commercially.
**Abstract Book Citations:** Authors, Title, HemaSphere, 2026;10:(S1):pages. The abstract book DOIs can be found on the "Author Information" page.
**Disclaimer:** Articles published in the journal HemaSphere exclusively reflect the opinions of the authors. The authors are responsible for all content in their abstracts including accuracy of the facts, statements, citing resources, etc.

### (PB3638) SCALABLE SEMI-SUPERVISED INTERVAL EXERCISE IMPROVES AEROBIC FITNESS IN INDOLENT NON-HODGKIN LYMPHOMA

**Topic:** 18. Indolent and mantle-cell non-Hodgkin lymphoma - Clinical

Courtney Wheatley-Guy*[1], Jordan Parks[1], Rebekah McCoy[1], Duke Butterfield[1], Matthew Buman[2], Allison Rosenthal[3], Lindsey Roeker*[4], Neil Kay[4], Bruce Johnson[5], Michael Gustafson[6]

*[1]Mayo Clinic Arizona, Cardiovascular Diseases, Scottsdale, United States of America;[2]Arizona State University, College of Health Solutions, Tempe, United States of America; [3]Mayo Clinic, Division of Hematology Medical Oncology, Phoenix, United States of America; [4]Mayo Clinic Rochester, Hematology, Rochester, United States of America;[5]Mayo Clinic Rochester, Cardiovascular Diseases, Rochester, United States of America; [6]Mayo Clinic Arizona, Laboratory Medicine and Pathology, Phoenix, United States of America;*

### Background

Exercise is an important adjunctive therapy in hematologic malignancies due to its potential to counter various causes of immunosuppression and support immune function; however, optimizing exercise dose and intensity are critical to achieve meaningful physiologic and immune benefits. Most exercise trials to date have required frequent supervision and fixed modalities, limiting scalability and accessibility, underscoring the need for pragmatic, remotely delivered programs capable of safely achieving vigorous exercise intensities, particularly for older patients with indolent disease.

### Aims

To evaluate the feasibility, tolerability, and functional efficacy of a semi-supervised, interval-based exercise training (EX) program compared with standard of care (SOC) in individuals with indolent non-Hodgkin lymphoma (iNHL) on or off treatment in a pilot trial.

### Methods

After providing informed written consent, participants with iNHL were randomized to EX or SOC (n: 14 vs. 13; age: 67±6 vs. 60±13 yrs; Male: 54% vs. 36%; BMI: 26.5 vs. 25.0 kg/m$^2$; Follicular lymphoma: 62% vs. 57%; On treatment 43% vs. 31%, EX vs. SOC). EX participants completed a 12-week, personalized, progressive vigorous interval-based aerobic (3 x 30-40 min sessions/week) program delivered via a mobile application with eight remote supervised sessions (22% of assigned workouts were supervised) whereas SOC received no exercise guidance. Aerobic fitness measured by peak oxygen consumption ($VO_{2PEAK}$) during graded maximal cardiopulmonary exercise testing, patient-reported physical activity and outcomes were assessed at baseline and 12±4-week post-intervention visits. Exercise dose was quantified using app-tracked session duration and heart rate–based intensity.

**Copyright Information:** (Online) ISSN: 2572-9241
©2026 The Author(s). HemaSphere is published by John Wiley & Sons Ltd on behalf of European Hematology Association. This is an open access Abstract Book distributed under the Attribution-NonCommercial-NoDerivs (CC BY-NC-ND), which allows third parties to download the articles and share them with others as long as they credit the author and the Abstract Book, but they cannot change the content in any way or use them commercially.
**Abstract Book Citations:** Authors, Title, HemaSphere, 2026;10:(S1):pages. The abstract book DOIs can be found on the "Author Information" page.
**Disclaimer:** Articles published in the journal HemaSphere exclusively reflect the opinions of the authors. The authors are responsible for all content in their abstracts including accuracy of the facts, statements, citing resources, etc.

**Results**

All EX participants completed the intervention with no intervention-related adverse events; three SOC participants withdrew. EX participants achieved a significantly higher aerobic exercise dose and reported an increase in vigorous physical activity (total aerobic load: 1658.7 ± 1124.4 vs. 553.6 ± 795.1 relative intensity minutes; Δvigorous activity: 56 [2, 110] vs. −61 [−130, 9] min/wk [95% CI], $p<0.05$ between group, EX vs. SOC). Despite relatively high baseline fitness, EX participants demonstrated improvements in aerobic fitness and exercise tolerance, including increases in $VO_{2PEAK}$, which were partially driven by weight loss, and oxygen uptake efficiency slope. Between-group differences favored EX for normative aerobic fitness ($\Delta VO_{2PEAK}$: 12.3 [6.0, 18]* vs. 0.4 [-6.4, 7.2] %pred†, *$p<0.05$ within-group; †$p<0.05$ between-group), while changes in relative (mL/min/kg) and absolute $VO_{2PEAK}$ (mL/min) were not significantly different between groups. Fatigue remained stable across groups, with transient increases during progression weeks in EX participants off treatment.

**Summary/Conclusion**

This randomized pilot trial demonstrates that a highly scalable, semi-supervised interval-based exercise program is safe, well tolerated, and capable of improving age-adjusted aerobic fitness and exercise tolerance in an older, relatively fit iNHL population. These findings support the feasibility of low-burden, remotely delivered exercise models and inform future trials targeting broader survivorship and subsequent evaluation of immune-related outcomes from biobanked pre and immediately post exercise samples at baseline and post-intervention.

**Copyright Information:** (Online) ISSN: 2572-9241
©2026 The Author(s). HemaSphere is published by John Wiley & Sons Ltd on behalf of European Hematology Association. This is an open access Abstract Book distributed under the Attribution-NonCommercial-NoDerivs (CC BY-NC-ND), which allows third parties to download the articles and share them with others as long as they credit the author and the Abstract Book, but they cannot change the content in any way or use them commercially.
**Abstract Book Citations:** Authors, Title, HemaSphere, 2026;10:(S1):pages. The abstract book DOIs can be found on the “Author Information” page.
**Disclaimer:** Articles published in the journal HemaSphere exclusively reflect the opinions of the authors. The authors are responsible for all content in their abstracts including accuracy of the facts, statements, citing resources, etc.

### (PB3639) A PROSPECTIVE, MULTICENTER, PHASE II STUDY OF ORELABRUTINIB COMBINED WITH RITUXIMAB IN PATIENTS WITH RELAPSED OR REFRACTORY MARGINAL ZONE LYMPHOMA

**Topic:** 18. Indolent and mantle-cell non-Hodgkin lymphoma - Clinical

Lixia Sheng*[1], Jiaping Wang[1], Hui Zeng[2], Shanhao Tang[1], Xibin Xiao[3], Yamin Tan[4], Qunyi Guo[5], Yanping Shao[5], Minchao Yan[3], Wenbin Qian[3], Yongcheng Sun[1], Ping Zhang[1], Guifang Ouyang[1]

*[1]The First Affiliated Hospital of Ningbo University, Department of Hematology, Ningbo, China;[2]The First Affiliated Hospital of Jiaxing University, Department of Hematology, Jiaxing, China; [3]The Second Affiliated Hospital, College of Medicine, Zhejiang University, Department of Hematology, Hangzhou, China; [4]Zhejiang Cancer Hospital, Department of Hematology, Hangzhou, China; [5]Taizhou Hospital Of Zhejiang Province, Department of Hematology, Taizhou, China;*

### Background

Relapsed or refractory Marginal Zone Lymphoma (R/R MZL) remains a clinical challenge. The Bruton tyrosine kinase (BTK) inhibitor orelabrutinib has demonstrated an overall response rate (ORR) of 58.9% as a monotherapy for R/R MZL, supporting further evaluation of combination strategies.

### Aims

This study aims to evaluate the efficacy and safety of orelabrutinib in combination with rituximab in patients with relapsed or refractory MZL.

### Methods

This is a prospective, multicenter, single-arm, phase II trial with a planned enrollment of 39 patients with MZL who relapsed after or were refractory to at least one prior line of systemic therapy. Patients received orelabrutinib 150 mg orally once daily plus rituximab 375 mg/m² intravenously on day 1 of each 21-day cycle for eight cycles, followed by maintenance therapy with either orelabrutinib or rituximab for up to 2 years at the investigator's discretion. The primary endpoint was ORR. Secondary endpoints included complete response (CR) rate, progression-free survival (PFS), overall survival (OS), and safety.

**Copyright Information:** (Online) ISSN: 2572-9241
©2026 The Author(s). HemaSphere is published by John Wiley & Sons Ltd on behalf of European Hematology Association. This is an open access Abstract Book distributed under the Attribution-NonCommercial-NoDerivs (CC BY-NC-ND), which allows third parties to download the articles and share them with others as long as they credit the author and the Abstract Book, but they cannot change the content in any way or use them commercially.
**Abstract Book Citations:** Authors, Title, HemaSphere, 2026;10:(S1):pages. The abstract book DOIs can be found on the "Author Information" page.
**Disclaimer:** Articles published in the journal HemaSphere exclusively reflect the opinions of the authors. The authors are responsible for all content in their abstracts including accuracy of the facts, statements, citing resources, etc.

### Results

Between December 2023 and January 2026, 21 patients were enrolled. The median age was 71 years (range, 51–82), and 57.1% were male. Histological subtypes included mucosa-associated lymphoid tissue lymphoma (MALT, 76.2%), nodal MZL (14.3%), and splenic MZL (9.5%). Most patients had ECOG performance status 0–1 (90.5%), Ann Arbor stage III–IV disease (57.1%), and an MZL-IPI score of 1–2 (57.1%).
Among 16 evaluable patients at interim assessment, the ORR was 100% (16/16), including a CR rate of 43.8% (7/16). At the end of induction, 14 patients were evaluable, with an ORR of 100% (14/14) and a CR rate of 71.4% (10/14), indicating deepening responses with continued treatment. Thirteen patients completed induction and entered maintenance therapy.
After a median follow-up of 18.3 months (range, 2.8–23.6), the median PFS and OS were not reached. The estimated 12-month PFS and OS rates were both 100%.
Among 16 patients with available sequencing data, 32 mutations were detected in 11 patients (68.8%), involving 18 distinct genes. The most frequently mutated genes were *TET2* (25%), *PPM1D* (19%), and *NOTCH2* (12%). No significant association was observed between baseline mutational status or variant allele frequency and treatment response.
Grade 1–2 adverse events occurred in 76.2% of patients, and grade 3–4 adverse events in 33.3%. The most common grade 1–2 events were infections and hematologic toxicities. Grade 3–4 events primarily included infections and neutropenia. No new safety signals were identified.

### Summary/Conclusion

In this interim analysis, orelabrutinib combined with rituximab demonstrated high response rates and manageable toxicity in patients with R/R MZL. Longer follow-up and completion of planned enrollment are warranted to confirm these findings.

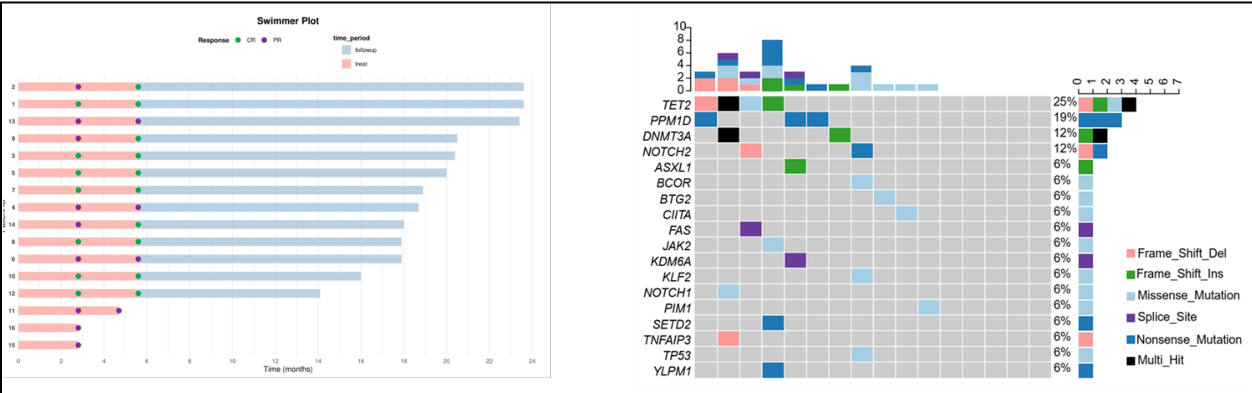

(A) Swimmer plot (B) Mutation plot

**Copyright Information:** (Online) ISSN: 2572-9241
©2026 The Author(s). HemaSphere is published by John Wiley & Sons Ltd on behalf of European Hematology Association. This is an open access Abstract Book distributed under the Attribution-NonCommercial-NoDerivs (CC BY-NC-ND), which allows third parties to download the articles and share them with others as long as they credit the author and the Abstract Book, but they cannot change the content in any way or use them commercially.
**Abstract Book Citations:** Authors, Title, HemaSphere, 2026;10:(S1):pages. The abstract book DOIs can be found on the "Author Information" page.
**Disclaimer:** Articles published in the journal HemaSphere exclusively reflect the opinions of the authors. The authors are responsible for all content in their abstracts including accuracy of the facts, statements, citing resources, etc.

### (PB3640) TNFRSF14 MUTATIONS ARE ASSOCIATED WITH INCREASED RISK OF HISTOLOGICAL TRANSFORMATION AND POOR PROGNOSIS IN FOLLICULAR LYMPHOMA

**Topic:** 18. Indolent and mantle-cell non-Hodgkin lymphoma - Clinical

Xia Zhao*[1], Zhihe Liu[1]

*[1]the affiliated hospital of Qingdao university, Qingdao, China;*

### Background

Histological transformation (HT) to an aggressive lymphoma represents a critical event in the clinical course of follicular lymphoma (FL), occurring in approximately 10-15% of patients and associated with markedly poor outcomes. Recent genomic studies have identified recurrent mutations in the TNFRSF14 gene in a substantial proportion of FL cases, with reported frequencies ranging from 18% to over 50%. The TNFRSF14 gene encodes the herpes virus entry mediator (HVEM), a key immune checkpoint regulator that modulates T-cell activation through interaction with B- and T-lymphocyte attenuator (BTLA). Loss-of-function mutations in TNFRSF14 are hypothesized to disrupt immune surveillance mechanisms and contribute to lymphomagenesis and disease progression, but their precise role in mediating histological transformation remains incompletely understood.

### Aims

This study aimed to investigate the association between TNFRSF14 mutations and the risk of histological transformation, as well as their impact on clinical outcomes in patients with FL.

### Methods

We analyzed a cohort of 32 patients with FL, including 15 patients harboring TNFRSF14 mutations (46.9%) and 17 patients with wild-type TNFRSF14 (53.1%). Baseline clinical characteristics, including age, sex, and Follicular Lymphoma International Prognostic Index (FLIPI) score, were recorded. The primary endpoint was the incidence of histological transformation to diffuse large B-cell lymphoma (DLBCL). Secondary endpoints included time to transformation (TTT), progression-free survival (PFS), and overall survival (OS). The association between TNFRSF14 mutation status and the rate of histological transformation was assessed using Fisher's exact test. Survival outcomes were compared between the two groups using the Mann-Whitney U test.

### Results

Baseline characteristics were generally comparable between the TNFRSF14-mutated and wild-type groups, with median ages of 57.5 and 54.2 years, respectively ($p = 0.277$). However, patients with TNFRSF14 mutations presented more frequently with high-risk FLIPI scores (40.0% vs. 11.8%). A significantly higher rate of histological transformation was observed in the TNFRSF14-mutated group compared to the wild-type group (46.7% [7/15] vs. 11.8% [2/17]; Odds Ratio = 6.56, $p = 0.049$). Among patients who experienced transformation, the mean time to transformation was 24.5 months in the mutated cohort versus 27.9 months in the wild-type cohort ($p = 0.795$). The presence of a TNFRSF14 mutation was associated with significantly worse survival outcomes. Median overall survival was substantially shorter in the mutated group compared to the wild-type group (54.3 vs. 104.6 months, $p = 0.0027$). A trend towards shorter median progression-free survival was also observed in the mutated cohort (35.0 vs. 65.9 months, $p = 0.070$). Mortality was higher in the TNFRSF14-mutated group (53.3% vs. 29.4%).

**Copyright Information:** (Online) ISSN: 2572-9241
©2026 The Author(s). HemaSphere is published by John Wiley & Sons Ltd on behalf of European Hematology Association. This is an open access Abstract Book distributed under the Attribution-NonCommercial-NoDerivs (CC BY-NC-ND), which allows third parties to download the articles and share them with others as long as they credit the author and the Abstract Book, but they cannot change the content in any way or use them commercially.
**Abstract Book Citations:** Authors, Title, HemaSphere, 2026;10:(S1):pages. The abstract book DOIs can be found on the "Author Information" page.
**Disclaimer:** Articles published in the journal HemaSphere exclusively reflect the opinions of the authors. The authors are responsible for all content in their abstracts including accuracy of the facts, statements, citing resources, etc.

**Summary/Conclusion**

The presence of TNFRSF14 mutations was strongly associated with an increased risk of histological transformation and significantly poorer overall survival in patients with FL.

**Copyright Information:** (Online) ISSN: 2572-9241
©2026 The Author(s). HemaSphere is published by John Wiley & Sons Ltd on behalf of European Hematology Association. This is an open access Abstract Book distributed under the Attribution-NonCommercial-NoDerivs (CC BY-NC-ND), which allows third parties to download the articles and share them with others as long as they credit the author and the Abstract Book, but they cannot change the content in any way or use them commercially.
**Abstract Book Citations:** Authors, Title, HemaSphere, 2026;10:(S1):pages. The abstract book DOIs can be found on the "Author Information" page.
**Disclaimer:** Articles published in the journal HemaSphere exclusively reflect the opinions of the authors. The authors are responsible for all content in their abstracts including accuracy of the facts, statements, citing resources, etc.

### (PB3641) CLINICAL CHARACTERISTICS AND OUTCOMES OF PATIENTS WITH LYMPHOPLASMACYTIC LYMPHOMA: A REAL-WORLD MULTI-CENTER STUDY FROM CHINA

**Topic:** 18. Indolent and mantle-cell non-Hodgkin lymphoma - Clinical

Xiao Gao*[1], Yuhong Ren[2], Peng Liu[2]

*[1]Department of Hematology, Zhongshan Hospital Minhang Campus, Fudan University, Shanghai, China;[2]Department of Hematology, Zhongshan Hospital, Fudan University, Shanghai, China;*

### Background

Lymphoplasmacytic lymphoma (LPL) is a rare B-cell malignancy, and real-world data on its clinical features and outcomes with novel agents remain limited, particularly in the Chinese population.

### Aims

This multicenter, real-world study aimed to characterize the clinical profile and evaluate the long-term outcomes of patients with LPL in China, with a focus on comparing the efficacy of different first-line treatment strategies.

### Methods

We retrospectively analyzed patients diagnosed with LPL across five Chinese centers between July 2009 and December 2024. Demographics, clinical characteristics, first-line treatment, response rate, and survival were collected.Survival outcomes were compared among different treatment groups using Kaplan-Meier methods.

### Results

A total of 149 patients were included. The median age was 67 years (range 35-91) with a male male-to-female ratio of 3.1:1. According to the IPSSWM, 21.6% were low-risk, 27.0% intermediate-risk, and 51.4% high-risk. The *MYD88 L265P* mutation was detected in 53.6% of tested patients. At data cutoff, 20 patients (13.4%) did not meet the criteria for treatment initiation and were under observation ("watch and wait"). Among the 129 patients who received first-line therapy, regimens included BTK inhibitors (BTKis)-based (n = 56, 43.4%), anti-CD20 monoclonal antibodies-based (n = 45, 34.9%), proteasome inhibitor (PI)-based (n = 17, 13.2%), and chemotherapy-based (n = 11, 8.5%). The best ORR was significantly higher in the BTKis group (69%) compared with the anti-CD20 (47%), PI (41%), and chemotherapy (36%) groups ($P = 0.05$). After a median follow-up of 47 months, the median PFS for the cohort was 24 months. The 5-year PFS rate was significantly improved in patients receiving BTKi-based therapy (73%) compared to those receiving anti-CD20-based (48%), PI-based (33%), and chemotherapy-based (27%) regimens ($P = 0.002$).

### Summary/Conclusion

This real-world study demonstrates that BTKi-based first-line therapy provides a significant PFS benefit and higher response rates compared with conventional therapy in patients with LPL.

**Table 1. Demographics and baseline characteristics.**

**Copyright Information:** (Online) ISSN: 2572-9241
©2026 The Author(s). HemaSphere is published by John Wiley & Sons Ltd on behalf of European Hematology Association. This is an open access Abstract Book distributed under the Attribution-NonCommercial-NoDerivs (CC BY-NC-ND), which allows third parties to download the articles and share them with others as long as they credit the author and the Abstract Book, but they cannot change the content in any way or use them commercially.
**Abstract Book Citations:** Authors, Title, HemaSphere, 2026;10:(S1):pages. The abstract book DOIs can be found on the "Author Information" page.
**Disclaimer:** Articles published in the journal HemaSphere exclusively reflect the opinions of the authors. The authors are responsible for all content in their abstracts including accuracy of the facts, statements, citing resources, etc.

| Characteristics | Patients |
|---|---|
| Median age, years (range) | 67 (35-91) |
| Sex | |
| Male | 113 (75.84%) |
| Female | 36 (24.16%) |
| ECOG | |
| 0-1 | 137 (94.48%) |
| ≥2 | 12 (8.28%) |
| IPSSWM score | |
| Low (0-1) | 32 (21.62%) |
| Intermediate (2) | 40 (27.03%) |
| High (>2) | 76 (51.35%) |
| IFE types | |
| IgM-κ | 104 (74.29%) |
| IgM-λ | 32 (22.86%) |
| IgM-κ, IgM-λ | 1 (0.71%) |
| IgG-κ | 1 (0.71%) |
| IgG-λ | 1 (0.71%) |
| IgA-κ | 1 (0.71%) |

**Copyright Information:** (Online) ISSN: 2572-9241
©2026 The Author(s). HemaSphere is published by John Wiley & Sons Ltd on behalf of European Hematology Association. This is an open access Abstract Book distributed under the Attribution-NonCommercial-NoDerivs (CC BY-NC-ND), which allows third parties to download the articles and share them with others as long as they credit the author and the Abstract Book, but they cannot change the content in any way or use them commercially.
**Abstract Book Citations:** Authors, Title, HemaSphere, 2026;10:(S1):pages. The abstract book DOIs can be found on the "Author Information" page.
**Disclaimer:** Articles published in the journal HemaSphere exclusively reflect the opinions of the authors. The authors are responsible for all content in their abstracts including accuracy of the facts, statements, citing resources, etc.

| | |
|---|---|
| BM involvement | 71 (47.97%) |
| *MYD88 L265P* mutation | 74 (53.62%) |
| *CXCR4* mutation | 3 (8.57%) |
| Laboratory values | |
| Median LDH (U/L) | 152.00 (26.00-987.00) |
| Median serum IgM (g/L) | 25.07 (0.16-126.00) |
| Serum β2-microglobulin (mg/L) | 4.56 (1.13-8308.00) |
| Median hemoglobin levels (g/L) | 93.00 (45.00-305.00) |
| Median platelet count ($10^9$/L) | 182.00 (14.00-578.00) |
| First-line therapy | |
| BTKis-based | 56 (43.41%) |
| Anti-CD20 monoclonal antibodies-based | 45 (34.88%) |
| Proteasome inhibitor-based | 17 (13.18%) |
| Chemotherapy-based | 11 (8.53%) |
| None (watch and wait) | 20 (13.42%) |

**Copyright Information:** (Online) ISSN: 2572-9241
©2026 The Author(s). HemaSphere is published by John Wiley & Sons Ltd on behalf of European Hematology Association. This is an open access Abstract Book distributed under the Attribution-NonCommercial-NoDerivs (CC BY-NC-ND), which allows third parties to download the articles and share them with others as long as they credit the author and the Abstract Book, but they cannot change the content in any way or use them commercially.
**Abstract Book Citations:** Authors, Title, HemaSphere, 2026;10:(S1):pages. The abstract book DOIs can be found on the "Author Information" page.
**Disclaimer:** Articles published in the journal HemaSphere exclusively reflect the opinions of the authors. The authors are responsible for all content in their abstracts including accuracy of the facts, statements, citing resources, etc.

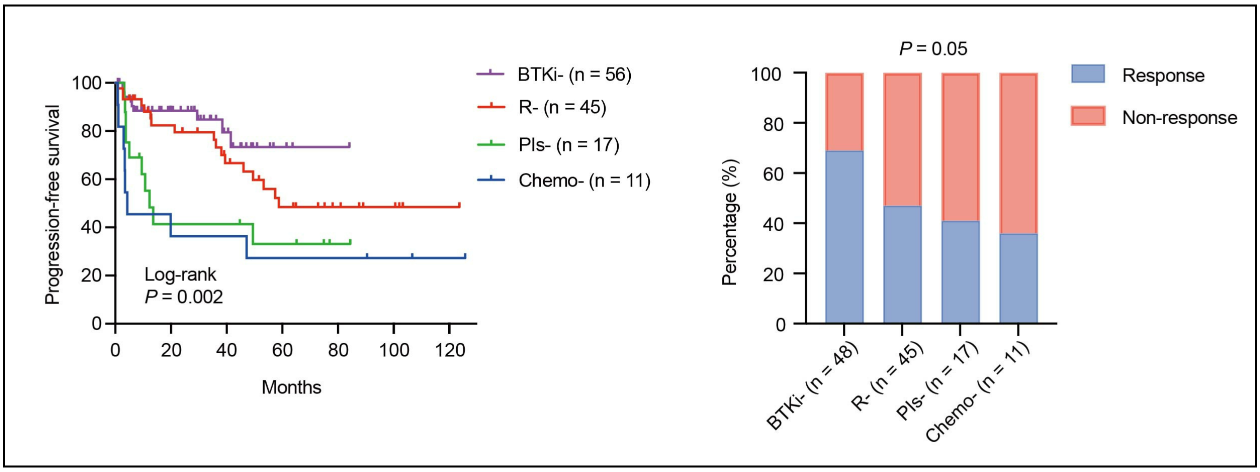

Progression-free survival (PFS) and overall response rate (ORR) in LPL patients according to first-line regimens.

**Copyright Information:** (Online) ISSN: 2572-9241
©2026 The Author(s). HemaSphere is published by John Wiley & Sons Ltd on behalf of European Hematology Association. This is an open access Abstract Book distributed under the Attribution-NonCommercial-NoDerivs (CC BY-NC-ND), which allows third parties to download the articles and share them with others as long as they credit the author and the Abstract Book, but they cannot change the content in any way or use them commercially.
**Abstract Book Citations:** Authors, Title, HemaSphere, 2026;10:(S1):pages. The abstract book DOIs can be found on the "Author Information" page.
**Disclaimer:** Articles published in the journal HemaSphere exclusively reflect the opinions of the authors. The authors are responsible for all content in their abstracts including accuracy of the facts, statements, citing resources, etc.

### (PB3642) SURVIVAL AND RISK STRATIFICATION IN LEUKEMIC-PHASE FOLLICULAR LYMPHOMA: OUTCOMES COMPARABLE TO CONVENTIONAL DISEASE

**Topic:** 18. Indolent and mantle-cell non-Hodgkin lymphoma - Clinical

Zheng Fan*[1], Da-Hui Li[2], Wen-Hao Zhang[2], Yu-Ting Xin[3], Jian-Bin Li[4], Dong Chen[1], Chuanxu Liu[2], Qian-Qian Long[5], Shuangyue Li[1], Yu Ruohan[1], Yu-Xiao Wang[1], Ying Lu[1], Rong Tao[2]

*[1]The Affiliated People's Hospital of Ningbo University, Department of Hematology, Ningbo, China;[2]Fudan University Shanghai Cancer centre, Department of Lymphoma and Medical Oncology, Shanghai, China; [3]Shenzhen Campus of Sun Yat-sen University, School of Public Health (Shenzhen), Shenzhen, China; [4]The Affiliated People's Hospital of Ningbo University, Medical Imaging and Nuclear Medicine Department, Ningbo, China; [5]Fudan University Shanghai Cancer centre, Department of Nuclear Medicine, Shanghai, China;*

### Background

Leukemic-phase follicular lymphoma (LP-FL), defined by circulating clonal lymphoma cells in peripheral blood, represents a rare presentation occurring in approximately 1–3% of follicular lymphoma cases. Although leukemic involvement has historically been considered an adverse feature, its prognostic impact in the contemporary immunochemotherapy era remains unclear. Available data are limited and largely derived from small series.

### Aims

To describe survival outcomes and evaluate clinical and laboratory prognostic factors in patients with LP-FL.

### Methods

We conducted a retrospective two-centre study including 63 patients diagnosed with LP-FL between 2017 and 2024, defined by more than 5×10^9/L circulating clonal lymphoma cells. Baseline clinical characteristics, FLIPI score, laboratory parameters including lactate dehydrogenase (LDH) and albumin, and PET metrics were collected. Progression-free survival (PFS) and overall survival (OS) were estimated using Kaplan–Meier analysis and Cox proportional hazards models. Early progression within 24 months (POD24) was evaluated using landmark analysis.

### Results

Median follow-up was 28.2 months. Median PFS was 49.2 months (95% CI, 34.5–not reached). Two-year PFS and OS were 68.6% and 98.1%, respectively; 5-year rates were 36.3% and 86.9%.High-risk FLIPI (3–5) was associated with shorter PFS (HR 2.44, 95% CI 1.06–5.62; p=0.0286). Elevated LDH predicted inferior PFS (HR 2.43; p=0.0383) and OS (HR 5.39; p=0.0305). POD24 occurred in 27% of patients and was associated with significantly worse subsequent OS (landmark p=0.038).Among exploratory composite indices, log1p(LDH/ALB) per interquartile-range increase was associated with shorter PFS (HR 2.03; p=0.046). Induction regimen (CHOP-based versus non-CHOP) did not significantly impact PFS or OS.

### Summary/Conclusion

LP-FL demonstrates heterogeneous outcomes in the modern treatment era. FLIPI and LDH remain clinically relevant prognostic factors, and early progression identifies patients at increased risk. These findings provide contemporary real-world data from one of the largest Asian series of LP-FL.

**Copyright Information:** (Online) ISSN: 2572-9241
©2026 The Author(s). HemaSphere is published by John Wiley & Sons Ltd on behalf of European Hematology Association. This is an open access Abstract Book distributed under the Attribution-NonCommercial-NoDerivs (CC BY-NC-ND), which allows third parties to download the articles and share them with others as long as they credit the author and the Abstract Book, but they cannot change the content in any way or use them commercially.
**Abstract Book Citations:** Authors, Title, HemaSphere, 2026;10:(S1):pages. The abstract book DOIs can be found on the "Author Information" page.
**Disclaimer:** Articles published in the journal HemaSphere exclusively reflect the opinions of the authors. The authors are responsible for all content in their abstracts including accuracy of the facts, statements, citing resources, etc.

### (PB3643) T-CELL CLONALITY OF UNCERTAIN SIGNIFICANCE: CLINICAL AND LABORATORY CORRELATION

**Topic:** 18. Indolent and mantle-cell non-Hodgkin lymphoma - Clinical

Phyo Thazin Myint*[1], Mohammed Saleh[1], Muhammad Hashim[2], Linnea Banker[2], Mridula Talari[1], Amanda Wheeler[3], Soumya Mikkilineni[2], Richard Hammer[2]

*[1]University of Missouri, Columbia, Columbia, United States of America;[2]University of Missouri, Columbia, Department of Pathology and Anatomical Sciences, Columbia, United States of America; [3]University of Missouri Healthcare, Columbia, United States of America;*

### Background

Oligoclonal T-cell expansions may occur as reactive responses to antigenic or inflammatory stimuli. T-cell clonality of uncertain significance (TCUS) is a clonal T-cell expansion, typically with a large granular lymphocyte count <0.5 × $10^9$/L, phenotypically similar to T- cell Large Granular Leukemia (T-LGL) with unclear clinical implications.

### Methods

Flow cytometry samples with TCUS clones from 2021-2024 were retrospectively reviewed at the University of Missouri with IRB approval. T-cell malignancies or T-LGL were excluded. For patients with multiple samples, the first was used as the index. Clinical, laboratory, and immunophenotypic data were obtained by chart review.

**Copyright Information:** (Online) ISSN: 2572-9241
©2026 The Author(s). HemaSphere is published by John Wiley & Sons Ltd on behalf of European Hematology Association. This is an open access Abstract Book distributed under the Attribution-NonCommercial-NoDerivs (CC BY-NC-ND), which allows third parties to download the articles and share them with others as long as they credit the author and the Abstract Book, but they cannot change the content in any way or use them commercially.
**Abstract Book Citations:** Authors, Title, HemaSphere, 2026;10:(S1):pages. The abstract book DOIs can be found on the "Author Information" page.
**Disclaimer:** Articles published in the journal HemaSphere exclusively reflect the opinions of the authors. The authors are responsible for all content in their abstracts including accuracy of the facts, statements, citing resources, etc.

### Results

Sixty-eight TCUS patients were identified (64.7% female). Primary reasons for flow cytometry were to exclude leukemia (23.5%) or lymphoma (17.6%). Mean values: hemoglobin 12.04 g/dL, WBC $9.65 \times 10^9/L$, lymphocytes $3.41 \times 10^9/L$, and platelets $212.7 \times 10^3/\mu L$. Concurrent malignancy was the most frequent association (52.9%; 30 hematologic, 6 solid). Recent infections occurred in 25 patients (36.8%), and autoimmune disease in 6 (8.9%). 5 patients had other conditions (3 ITP, 1 ulcerative colitis, 1 clonal CCUS), and 16 had no clear associated condition.

15 patients had repeat flow cytometry ≥3 months later; TCUS persisted in 10. 3 out of 5 non-persistent cases received hematologic malignancy treatment after TCUS identification, possibly contributing to clonal resolution. Notably, 5 patients had bacterial infection as the sole associated condition, representing the first reported link between bacterial infection and TCUS in humans.

From the immunophenotype perspective, among the 81 flow cytometry samples analyzed, the predominant phenotypes were CD8+ (71.6%), followed by double-positive CD4+/CD8+ (21%), CD4+ (3.7%), and CD4-/CD8- (2.5%), with associated CD56/57 expression in the majority.

**Table 1. Associated Conditions and TCUS clone %**

| Condition | N (% of cases) | Mean TCUS clone % of total lymphocytes (min-max) |
|---|---|---|
| Malignancy | 36 (52.9%) | 6.39 (0.5-32.3) |
| | | 5.2 (0.8-13.5) |
| Infection | 25 (36.8%) | |
| *Bacterial* | *14 (20.6%)* | |
| *Viral* | *10 (14.7%)* | |
| *Both* | *1 (1.5%)* | |
| Autoimmune | 6 (8.9%) | 5.67 (1-13.5) |
| Others | 5 (7.4%) | 3.6 (1-5.7) |
| No apparent association | 16 (23.5%) | 7.66 (0.1-24.4) |

### Summary/Conclusion

Unlike T-LGL, TCUS lacked consistent cytopenia or strong autoimmune associations, supporting a heterogeneous and context-dependent process rather than representing a uniform pre-T-LGL state. TCUS likely reflects oligoclonal or clonal immune-driven expansion from diverse triggers. This highlights the need for clinical correlation, including cytopenias and persistence over time. The novel link to bacterial infection also suggests a potential infectious trigger. Given the role of cytotoxic T cells in immune surveillance, future studies should evaluate whether CD8+T-cell clonality affects malignancy outcomes.

**Copyright Information:** (Online) ISSN: 2572-9241
©2026 The Author(s). HemaSphere is published by John Wiley & Sons Ltd on behalf of European Hematology Association. This is an open access Abstract Book distributed under the Attribution-NonCommercial-NoDerivs (CC BY-NC-ND), which allows third parties to download the articles and share them with others as long as they credit the author and the Abstract Book, but they cannot change the content in any way or use them commercially.
**Abstract Book Citations:** Authors, Title, HemaSphere, 2026;10:(S1):pages. The abstract book DOIs can be found on the "Author Information" page.
**Disclaimer:** Articles published in the journal HemaSphere exclusively reflect the opinions of the authors. The authors are responsible for all content in their abstracts including accuracy of the facts, statements, citing resources, etc.

### (PB3644) REAL-WORLD MULTIDIMENSIONAL COMPARISON OF FIRST-LINE IMMUNOCHEMOTHERAPY VERSUS ORELABRUTINIB-BASED THERAPY IN PULMONARY MALT LYMPHOMA: EFFICACY, SAFETY, AND PROGNOSTIC PERIPHERAL BIOMARKERS

**Topic:** 18. Indolent and mantle-cell non-Hodgkin lymphoma - Clinical

Zhihong Zou[1], Donghua Zhang[2], Yaya Wang[1], Jia Wei[2], Longlong Liu[1], Zhenqian Huang*[1]

*[1]The First Affiliated Hospital of Guangzhou Medical University, Hematology, Guangzhou, China;[2]Tongji Hospital, Tongji Medical College, Huazhong University of Science and Technology, Hematology, Wuhan, China;*

### Background

Pulmonary MALT lymphoma is clinically heterogeneous; conventional immunochemotherapy (IC) achieves high early response but incurs myelotoxicity and a marked risk of early progression (POD24). Next-generation, highly selective BTK inhibition offers a chemo-sparing alternative, yet frontline comparative effectiveness and practical biomarkers for regimen selection are lacking.

### Aims

To explore frontline comparative effectiveness and practical biomarkers for regimen selection.

### Methods

We performed a single-center retrospective review (2020–2025) of 53 consecutive pulmonary MALT patients treated front line with bendamustine-rituximab (BR, n=28), R-CHOP (n=7) or orelabrutinib-based therapy (n=17). Baseline clinical and laboratory features (including LDH, IFE, β2-microglobulin, D-dimer and infectious comorbidity) were recorded. Primary endpoints were overall response rate (ORR) and complete response (CR) at cycle-4. Secondary endpoints included on-therapy primary progression, grade ≥3 toxicities, infection incidence, and biomarker associations with systemic relapse.

### Results

Cohorts were well matched for age (median 59–63 years) and stage distribution (high prevalence of stage III–IV). At presentation, 11.3% had elevated LDH, 24.5% had abnormal IFE, 38% exhibited marked D-dimer elevation (>0.80 μg/mL FEU), and 28.3% had documented respiratory pathogen co-infections. At the cycle-4 assessment, ORR was 100% in the orelabrutinib group, 90.6% in BR, and 75.0% in R-CHOP (overall P=0.12). Cycle-4 CR rates were 41.2%, 43.8%, and 25.0%, respectively (P=0.58). Importantly, early primary progression occurred in 0% of patients treated with orelabrutinib compared with 6.2% in BR and 25.0% in R-CHOP (P=0.04), indicating superior early disease control with the targeted strategy. Safety profiles diverged: BR and R-CHOP cohorts experienced frequent grade ≥3 myelosuppression and opportunistic pulmonary infections, whereas the orelabrutinib cohort demonstrated markedly lower severe hematologic toxicity and no observed grade ≥3 cardiovascular or major hemorrhagic events in this series. Multivariate analysis revealed that baseline D-dimer elevation and β2-MG >3.0 mg/L were independent predictors of systemic relapse (adjusted hazard ratios significant), identifying a biomarker-defined subgroup with heightened relapse risk despite initial cytoreduction.

**Copyright Information:** (Online) ISSN: 2572-9241
©2026 The Author(s). HemaSphere is published by John Wiley & Sons Ltd on behalf of European Hematology Association. This is an open access Abstract Book distributed under the Attribution-NonCommercial-NoDerivs (CC BY-NC-ND), which allows third parties to download the articles and share them with others as long as they credit the author and the Abstract Book, but they cannot change the content in any way or use them commercially.
**Abstract Book Citations:** Authors, Title, HemaSphere, 2026;10:(S1):pages. The abstract book DOIs can be found on the “Author Information” page.
**Disclaimer:** Articles published in the journal HemaSphere exclusively reflect the opinions of the authors. The authors are responsible for all content in their abstracts including accuracy of the facts, statements, citing resources, etc.

**Summary/Conclusion**

In this real-world series, frontline orelabrutinib achieved rapid, robust responses comparable to IC while eliminating early progression and reducing severe toxicity. Elevated D-dimer and β2-MG delineate a biomarker-high subgroup at increased relapse risk despite cytoreduction, supporting biomarker-guided frontline selection. These findings warrant prospective validation of orelabrutinib-centered, risk-adaptive strategies and mechanistic studies to establish D-dimer and β2-MG as companion biomarkers.

**Copyright Information:** (Online) ISSN: 2572-9241
©2026 The Author(s). HemaSphere is published by John Wiley & Sons Ltd on behalf of European Hematology Association. This is an open access Abstract Book distributed under the Attribution-NonCommercial-NoDerivs (CC BY-NC-ND), which allows third parties to download the articles and share them with others as long as they credit the author and the Abstract Book, but they cannot change the content in any way or use them commercially.
**Abstract Book Citations:** Authors, Title, HemaSphere, 2026;10:(S1):pages. The abstract book DOIs can be found on the “Author Information” page.
**Disclaimer:** Articles published in the journal HemaSphere exclusively reflect the opinions of the authors. The authors are responsible for all content in their abstracts including accuracy of the facts, statements, citing resources, etc.

### (PB3645) FIRST-LINE KCD-R FOR WALDENSTROM MACROGLOBULINAEMIA IS ASSOCIATED WITH MORE SEVERE HYPOGAMMAGLOBULINAEMIA BUT NOT INFECTIONS REQUIRING IVIG OR HOSPITALISATION COMPARED TO BR OR ZANUBRUTINIB

**Topic:** 18. Indolent and mantle-cell non-Hodgkin lymphoma - Clinical

James Di Palma-Grisi*[1], Joseph Franz[2], Noa Biran[2], David H. Vesole[2], David Siegel[2], Pooja Phull[2], Harsh Parmar[2]

*[1]Hackensack University Medical Center, Hackensack, United States of America;[2]John Theurer Cancer Center, Hackensack, United States of America;*

### Background

Waldenström Macroglobulinaemia (WM) is a subtype of lymphoplasmacytic lymphoma characterized by the production of a monoclonal IgM protein. Standard first-line options include chemoimmunotherapy agents such as bendamustine-rituximab (BR), proteasome inhibitor–based therapies such as carfilzomib or bortezomib with cyclophosphamide, dexamethasone, and rituximab (KCD-R), and the BTK inhibitors. We report outcomes from a single referral center comparing these three first-line approaches.

### Aims

To determine whether there was any difference in treatment outcomes including survival, progression-free intervals, hypogammaglobulinaemia, and infectious adverse events.

### Methods

We performed retrospective chart review on an IRB-approved protocol of 82 patients with biopsy-confirmed WM diagnosed between April 2007 and August 2025. We identified the three most common first-line treatments in our centre - KCD-R, BR and Zanu (n = 58). We excluded 4 who went on to receive another of the three lines in later therapies. We included 3 in the KCD-R group who started KCD, KD-R, or R but proceeded to KCD-R within 30 days. We obtained baseline characteristics, molecular and pathology results, immunoglobulin testing at diagnosis and during treatment, and if they started IVIG for recurrent infection or required hospitalisation for infection. Outcomes were overall response rate (ORR) per the 6th International Workshop on WM, progression-free survival (PFS) and overall survival (OS) using the Kaplan-Meier method.

**Copyright Information:** (Online) ISSN: 2572-9241
©2026 The Author(s). HemaSphere is published by John Wiley & Sons Ltd on behalf of European Hematology Association. This is an open access Abstract Book distributed under the Attribution-NonCommercial-NoDerivs (CC BY-NC-ND), which allows third parties to download the articles and share them with others as long as they credit the author and the Abstract Book, but they cannot change the content in any way or use them commercially.
**Abstract Book Citations:** Authors, Title, HemaSphere, 2026;10:(S1):pages. The abstract book DOIs can be found on the "Author Information" page.
**Disclaimer:** Articles published in the journal HemaSphere exclusively reflect the opinions of the authors. The authors are responsible for all content in their abstracts including accuracy of the facts, statements, citing resources, etc.

### Results

Median age at treatment initiation was 68.8 years. Median follow-up was 58.5 months. Among 54 pts with available demographics, 51 were Caucasian, 2 were Asian, and 1 was African American. MYD88 testing was available for 48 pts, of whom 41 (85%) were positive. Baseline characteristics were largely balanced across groups (Table 1). Median number of cycles was 6 for KCD-R and 4 for BR. The ORR was 97% for KCD-R, 80% for BR, and 89% for zanubrutinib (p=0.20). Rates of very good partial response (VGPR) or better were 50%, 50%, and 67%, respectively (p=0.20). At 48 months, OS rate was 90% for KCD-R, 87% for BR, and 100% for zanubrutinib. Median PFS was 92.6 months for KCD-R and was not reached for zanubrutinib; in the BR group, median PFS was 86.9 months, with all PFS events attributable to death rather than disease progression (log-rank p=0.74) (Figure 1). Median IgG nadir was significantly lower in the KCD-R group than in the BR and zanubrutinib groups (206 mg/dL vs 473.5 mg/dL vs 334 mg/dL; p<0.01). Rates of IVIg initiation (24% vs 10% vs 30%; p=0.87) and hospitalisation for infection within 48 months of treatment (23.5% vs 20% vs 30%; p=0.46) were not significantly different across groups.

| | KCD-R (n=34) | BR (n=10) | Zanu (n=10) | p-value |
|---|---|---|---|---|
| Age (years) | 67.4 | 69.2 | 73.1 | 0.36 |
| Males | 70% | 59% | 50% | 0.65 |
| Bone Marrow Involvement | 28% | 60% | 35% | 0.3 |
| R-IPSSWM Stage 2 or greater | 8/23 = 35% | 3/9 = 33% | 4/9 = 44% | 0.9 |
| Median Haemoglobin (g/L) | 104 | 103 | 109 | 0.89 |
| Median Platelets (x10^3/uL) | 227 | 176 | 229 | 0.89 |
| Median IgM (mg/dL) | 2269 | 2239 | 4124 | 0.10 |

Table 1. Baseline characteristics, laboratory values and pathology at time of diagnosis.

### Summary/Conclusion

The lower bound of post-treatment IgG in the KCD-R group was significantly lower than the BR or Zanu groups, but this did not translate into higher rates infections requiring IVIg or hospitalisations. All three regimens demonstrated no statistically significant differences in survival, response, or infectious complications. Larger, prospective comparisons are needed to establish the optimal first-line approach for WM.

**Copyright Information:** (Online) ISSN: 2572-9241
©2026 The Author(s). HemaSphere is published by John Wiley & Sons Ltd on behalf of European Hematology Association. This is an open access Abstract Book distributed under the Attribution-NonCommercial-NoDerivs (CC BY-NC-ND), which allows third parties to download the articles and share them with others as long as they credit the author and the Abstract Book, but they cannot change the content in any way or use them commercially.
**Abstract Book Citations:** Authors, Title, HemaSphere, 2026;10:(S1):pages. The abstract book DOIs can be found on the "Author Information" page.
**Disclaimer:** Articles published in the journal HemaSphere exclusively reflect the opinions of the authors. The authors are responsible for all content in their abstracts including accuracy of the facts, statements, citing resources, etc.

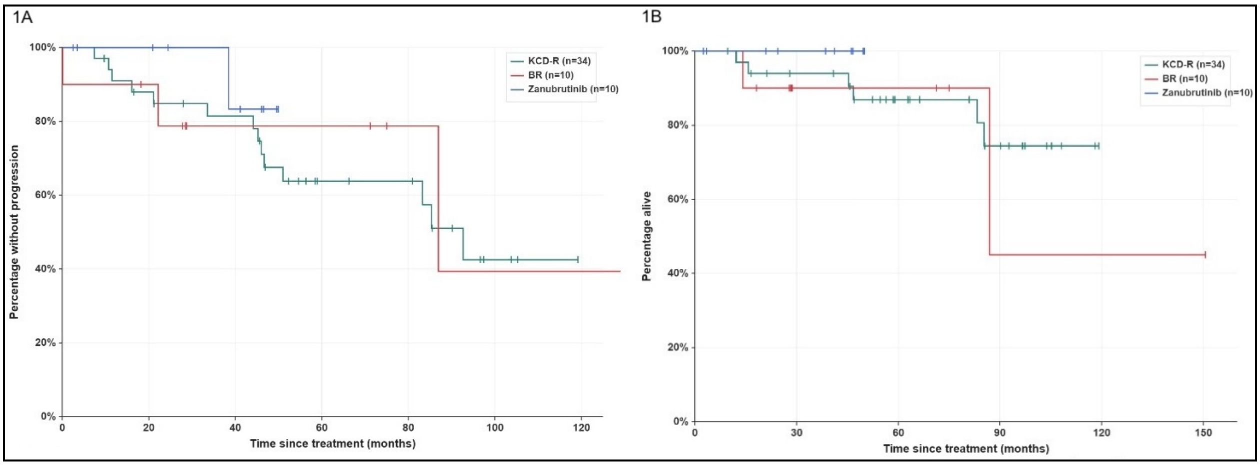

Figure 1. Outcomes of the three first-line treatment groups (KCD-R, BR and zanu). PFS per treatment group (1A). OS per treatment group (1B). 48 months was chosen as the most appropriate time period based on available data in the zanu group. Total follow-up data exceeded 120 months in the BR group.

**Copyright Information:** (Online) ISSN: 2572-9241
©2026 The Author(s). HemaSphere is published by John Wiley & Sons Ltd on behalf of European Hematology Association. This is an open access Abstract Book distributed under the Attribution-NonCommercial-NoDerivs (CC BY-NC-ND), which allows third parties to download the articles and share them with others as long as they credit the author and the Abstract Book, but they cannot change the content in any way or use them commercially.
**Abstract Book Citations:** Authors, Title, HemaSphere, 2026;10:(S1):pages. The abstract book DOIs can be found on the "Author Information" page.
**Disclaimer:** Articles published in the journal HemaSphere exclusively reflect the opinions of the authors. The authors are responsible for all content in their abstracts including accuracy of the facts, statements, citing resources, etc.

### (PB3646) LOW DOSE RADIOTHERAPY COMBINED WITH OBUTINIB IMPROVES THE COMPLETE RESPONSE RATE OF TREATMENT FOR LOCALIZED OCULAR ADNEXAL EXTRANODAL MARGINAL ZONE B-CELL LYMPHOMA

**Topic:** 18. Indolent and mantle-cell non-Hodgkin lymphoma - Clinical

Shaoxin Yang*[1], Chong Zhao[1], Jun Shi[1]

*[1]Shanghai Ninth People's Hospital, Shanghai JiaoTong University School of Medicine, Shanghai, China;*

### Background

Extranodal marginal zone B-cell lymphoma (EMZL) is the most common lymphoma of the ocular adnexal (OA). Although radiotherapy is still the first-line treatment for localized OAEMZL (stage I/II), the toxic effects of standard dose radiotherapy cannot be ignored. While reduced doses of radiotherapy can lessen these toxic effects, they also lower the complete response (CR) rate. We aim to use prospective trials to improve this CR rate while reducing the toxic effects of radiotherapy.

### Aims

To study the efficacy of OA-EMZL in early stage (stage I/II) treatment with  obutinib combined with low dose radiotherapy (4Gy).

### Methods

This is a single-arm, open clinical trial. Thirty-six patients were diagnosed with OA-EMZL through surgery. These patients had Ann Arbor stage I/II with residual lesions. They received low dose of radiation therapy, with each dose of 2 Gy administered twice, for a total of 4 Gy. Concurrently, the patients received oral obutinib, 150 mg daily, every 4 weeks as a course of treatment, and the efficacy and adverse reactions were evaluated. During the treatment process, the patients continued to take oral obutinib for four courses.

### Results

Four patients been involved with both eyes, while the remaining thirty-two patients had monocular involvement. Twenty-nine patients had lesions located in the orbit, one had lesions in the orbit and conjunctiva, and three had lesions in the orbit and lacrimal gland. Currently, twenty-seven patients have completed treatment, two patients withdrew from the study, while the remaining seven cases are still under treatment. Of all patients who completed treatment, twenty patients achieved complete response, and seven patients achieved a partial response. Additionally, the lesions in the patients still undergoing treatment have shrunk compared to before treatment. During treatment with obutinib, eight patients experienced leukopenia, eight patients experienced thrombocytopenia, seven patients experienced lymphocytopenia, and two patients experienced neutropenia. There was one case of pneumonia not requiring hospitalization, and two patients with ALT and AST levels more than twice the upper limit of normal. The above toxic effects are all of grade 1-2. There were no early toxic effects of radiotherapy, such as dermatitis, xerophthalmia, conjunctivitis, watering eyes in eight patients. Moreover, dry eye was not aggravated in one patient due to lacrimal gland resection.

### Summary/Conclusion

The combination of low dose radiotherapy and obutinib in the treatment of OA-EMZL not only improves the effectiveness of treatment but also significantly reduces the toxic effects of radiotherapy, providing a new approach for the treatment of localized OA-EMZL.

**Copyright Information:** (Online) ISSN: 2572-9241
©2026 The Author(s). HemaSphere is published by John Wiley & Sons Ltd on behalf of European Hematology Association. This is an open access Abstract Book distributed under the Attribution-NonCommercial-NoDerivs (CC BY-NC-ND), which allows third parties to download the articles and share them with others as long as they credit the author and the Abstract Book, but they cannot change the content in any way or use them commercially.
**Abstract Book Citations:** Authors, Title, HemaSphere, 2026;10:(S1):pages. The abstract book DOIs can be found on the “Author Information” page.
**Disclaimer:** Articles published in the journal HemaSphere exclusively reflect the opinions of the authors. The authors are responsible for all content in their abstracts including accuracy of the facts, statements, citing resources, etc.

### (PB3647) REAL-WORLD OUTCOMES OF ORELABRUTINIB-CONTAINING REGIMENS IN PREVIOUSLY UNTREATED PATIENTS WITH MARGINAL ZONE LYMPHOMA

**Topic:** 18. Indolent and mantle-cell non-Hodgkin lymphoma - Clinical

Xun Lai*[1], Zhenglei Shen[1], Jia Wei[1], Feng Li[1], Ming He[1], Yan Man[1], Kunmei Liu[1], Yunyan Sun[1], Jinyan Liu[1], Rui Zhang[1], Ci Duan[1]

*[1]Yunnan Provincial Cancer Hospital, Department of Dermatology, Kunming, China;*

### Background

Although immunochemotherapy has emerged as the preferred first-line therapy for marginal zone lymphoma (MZL), approximately 20% of patients (pts) develop disease progression within 24 months, which is associated with a median survival of only 3-5 years. Moreover, the considerable toxicity of conventional immunochemotherapy limits its clinical benefit. These unmet clinical needs underscore the necessity for more optimal first-line strategies for MZL. Orelabrutinib is a novel, potent, and irreversible Bruton tyrosine kinase inhibitor and has been approved for relapsed/refractory MZL in China.

### Aims

This real-world study aimed to evaluate the efficacy and safety of first-line orelabrutinib-containing regimens in pts with MZL.

### Methods

We retrospectively collected and analyzed clinical data from pts with MZL who received first-line orelabrutinib-containing regimens at the Yunnan Provincial Cancer Hospital between 2 June, 2023, and 24 December, 2025. Clinical outcomes were analyzed in the overall population, with subgroup analyses by treatment regimen, Ki-67 index (≥20 vs. <20), and primary site (extranodal vs. nodal MZL).

### Results

By data cut-off (February 6, 2026), 56 pts (26 males; median age 56 years [range 24-84]) were included, comprising 41 (73.2%) extranodal and 15 (26.8%) nodal MZL cases. The most common extranodal sites were the stomach (34.1%; 14/41), lung (17.1%; 7/41), and ocular adnexa (12.2%; 5/41). Of 56 pts, 16.1% presented with B symptoms, 25.0% had a Ki67 index of ≥20%, 12.5% had bone marrow involvement, and 57.1% had III-IV disease. Most pts (67.9%; 38/56) were classified as low risk based on MZL-IPI score. Pts most commonly received orelabrutinib plus anti-CD20 monoclonal antibody and chemotherapy (46.4%; 26/56), followed by orelabrutinib plus anti-CD20 monoclonal antibody (33.9%; 19/56), and orelabrutinib plus chemotherapy (19.6%; 11/56), with no significant differences in baseline characteristics across groups. In 50 efficacy-evaluable pts, the best complete response rate (CRR), overall response rate (ORR), and disease control rate were 74.0%, 86.0%, and 100.0%, respectively. Orelabrutinib plus an anti-CD20 monoclonal antibody and chemotherapy yielded a significantly higher CRR than orelabrutinib plus chemotherapy or orelabrutinib plus an anti-CD20 monoclonal antibody (87.5% vs. 80.0% vs. 50.0%; $P$=0.028). A similar trend was observed for ORR (95.8% vs. 80.0% vs. 75.0%) among the treatment groups, although the differences were not statistically significant. A numerically higher CRR (81.1% vs. 53.8%) and ORR (89.2% vs. 76.9%) were observed in the extranodal MZL subgroup compared with the nodal MZL subgroup. Pts with a Ki-67 index <20% achieved a CRR of 69.2% and an ORR of 80.8%, whereas those with a Ki-67 index ≥20% showed numerically higher response rates (CRR 75.0%; ORR 91.7%). With a median follow-up of 12.5 months (range 5.2-16.6), no disease progression or deaths occurred. Grade ≥3 adverse events were observed in 14.3% of patients (8/56), all of which were neutrophil count decreased.

**Copyright Information:** (Online) ISSN: 2572-9241
©2026 The Author(s). HemaSphere is published by John Wiley & Sons Ltd on behalf of European Hematology Association. This is an open access Abstract Book distributed under the Attribution-NonCommercial-NoDerivs (CC BY-NC-ND), which allows third parties to download the articles and share them with others as long as they credit the author and the Abstract Book, but they cannot change the content in any way or use them commercially.
**Abstract Book Citations:** Authors, Title, HemaSphere, 2026;10:(S1):pages. The abstract book DOIs can be found on the “Author Information” page.
**Disclaimer:** Articles published in the journal HemaSphere exclusively reflect the opinions of the authors. The authors are responsible for all content in their abstracts including accuracy of the facts, statements, citing resources, etc.

### Summary/Conclusion

First-line orelabrutinib-containing regimens demonstrated encouraging efficacy and acceptable safety for MZL and may represent a feasible therapeutic option that deserves further validation.

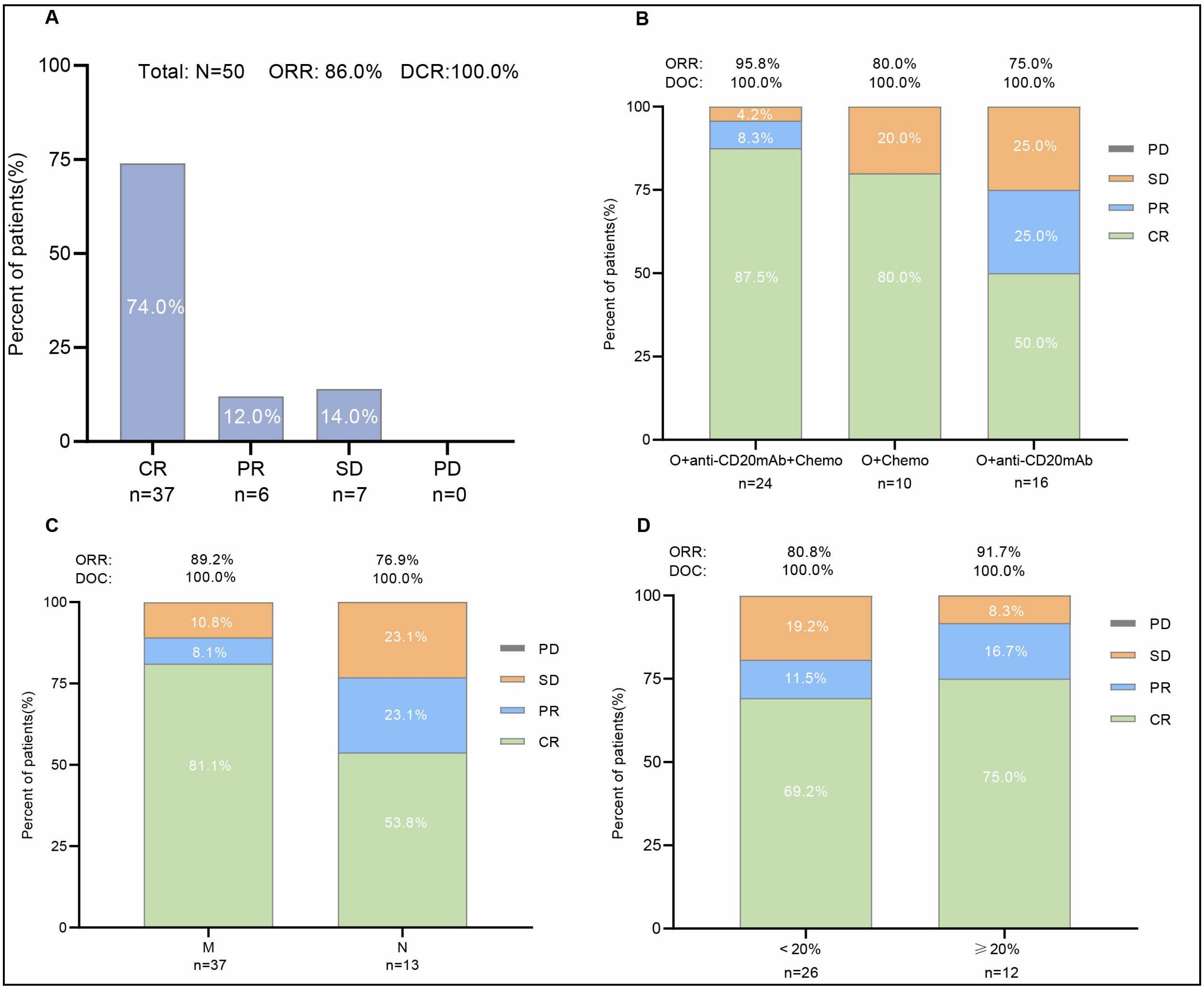

**Copyright Information:** (Online) ISSN: 2572-9241
©2026 The Author(s). HemaSphere is published by John Wiley & Sons Ltd on behalf of European Hematology Association. This is an open access Abstract Book distributed under the Attribution-NonCommercial-NoDerivs (CC BY-NC-ND), which allows third parties to download the articles and share them with others as long as they credit the author and the Abstract Book, but they cannot change the content in any way or use them commercially.
**Abstract Book Citations:** Authors, Title, HemaSphere, 2026;10:(S1):pages. The abstract book DOIs can be found on the "Author Information" page.
**Disclaimer:** Articles published in the journal HemaSphere exclusively reflect the opinions of the authors. The authors are responsible for all content in their abstracts including accuracy of the facts, statements, citing resources, etc.

### (PB3648) IDENTIFYING GAPS IN TREATMENT OF FOLLICULAR LYMPHOMA WITH BISPECIFICS THROUGH ONLINE CONTINUING MEDICAL EDUCATION

**Topic:** 18. Indolent and mantle-cell non-Hodgkin lymphoma - Clinical

Sarah Elizabeth Bomba*[1], Andrew Small[1], Jacob Cohen[1], Stephanie Baumhover[1], Tristin Abair[1]

[1]*Medscape Education, New York, United States of America;*

### Background

Follicular lymphoma (FL) is an indolent malignancy characterized by repeated cycles of remission and relapse, which over time can lead to treatment resistance and poses a significant challenge in patients who become refractory to standard-of-care therapies. The treatment landscape is currently undergoing a paradigm shift with the emergence of T-cell engaging bispecific antibodies, which offer an "off-the-shelf" immunotherapy by simultaneously targeting CD20 on malignant B-cells and CD3 on cytotoxic T-cells. As these therapies move from clinical trials into standard practice, hematologists/oncologists (hem/oncs) must navigate staying current on new data and treatment complexities, including the mitigation of unique toxicities. Given the rapid integration of these agents, hem/oncs may face knowledge and competence gaps around optimal patient selection, the nuances of step-up dosing, and the long-term management of patients receiving bispecific therapy.

### Aims

The objective of this study was to assess hem/oncs' knowledge and abilities related to the treatment of relapsed/refractory FL with bispecific antibodies, including pivotal trial data and clinical management, and to identify ongoing educational gaps to inform the development of future educational programs.

### Methods

An online, text-based CME survey instrument comprising 21 multiple-choice knowledge, practice-based, and confidence questions allowed participants to assess their clinical understanding and self-assessed learning needs related to bispecific antibodies and FL. The activity launched on March 13, 2025, and data were collected through January 2026. Hem/onc data was stratified by self-reported practice setting (community/non-academic vs. academic) and responses were analyzed by grouping questions into clinical themes and calculating the percentage of incorrect responses in each area.

### Results

2,644 total learners participated in this online CME activity within the data collection period, including 1472 Hem/Oncs. Specific ongoing educational gaps for community (n = 44) and academic (n=9) hem/oncs who answered all survey questions were identified through this initiative (see table 1). Regardless of practice setting, hem/oncs demonstrated similar gaps related to pivotal trial evidence (43%, 42%) and safety and toxicity management (38%, 38%); with marginally larger gaps identified among community clinicians related to clinical positioning and indications for bispecific antibodies impacting treatment selection (50%, 44%), as well as practical logistics (55%, 47%).

**Copyright Information:** (Online) ISSN: 2572-9241
©2026 The Author(s). HemaSphere is published by John Wiley & Sons Ltd on behalf of European Hematology Association. This is an open access Abstract Book distributed under the Attribution-NonCommercial-NoDerivs (CC BY-NC-ND), which allows third parties to download the articles and share them with others as long as they credit the author and the Abstract Book, but they cannot change the content in any way or use them commercially.
**Abstract Book Citations:** Authors, Title, HemaSphere, 2026;10:(S1):pages. The abstract book DOIs can be found on the "Author Information" page.
**Disclaimer:** Articles published in the journal HemaSphere exclusively reflect the opinions of the authors. The authors are responsible for all content in their abstracts including accuracy of the facts, statements, citing resources, etc.

**Summary/Conclusion**

This educational initiative yielded important insights into ongoing practice gaps related to bispecific antibodies for the treatment of FL and both the clinical data and practical considerations around their use with marginally great gaps seen among community hem/oncs likely attributed to less experience with these therapies in community practice. Further studies are planned to assess the effect of medical education initiatives in closing these clinical practice gaps.

Table 1: Questions Grouped by Educational Learning Theme

| Learning Themes | Descriptor | Incorrect Responses Community/Non-academic Hem/Oncs (%) | Incorrect Responses Academic Hem/Oncs (%) |
|---|---|---|---|
| Pivotal Evidence & Outcomes | Efficacy and safety benchmarks from pivotal trials | 43 | 42 |
| Clinical Positioning & Indications | Evidence- and guideline-based patient eligibility and treatment selection | 50 | 44 |
| Practical Logistics & Delivery | Operational requirements such as administration and step-up dosing | 55 | 47 |
| Safety & Toxicity Management | Identification, prevention, and management of adverse events | 38 | 38 |

**Copyright Information:** (Online) ISSN: 2572-9241
©2026 The Author(s). HemaSphere is published by John Wiley & Sons Ltd on behalf of European Hematology Association. This is an open access Abstract Book distributed under the Attribution-NonCommercial-NoDerivs (CC BY-NC-ND), which allows third parties to download the articles and share them with others as long as they credit the author and the Abstract Book, but they cannot change the content in any way or use them commercially.
**Abstract Book Citations:** Authors, Title, HemaSphere, 2026;10:(S1):pages. The abstract book DOIs can be found on the “Author Information” page.
**Disclaimer:** Articles published in the journal HemaSphere exclusively reflect the opinions of the authors. The authors are responsible for all content in their abstracts including accuracy of the facts, statements, citing resources, etc.

### (PB3649) DECIPHERING THE METABOLIC LINK TO MARGINAL ZONE LYMPHOMA THROUGH MENDELIAN RANDOMIZATION ANALYSIS

**Topic:** 18. Indolent and mantle-cell non-Hodgkin lymphoma - Clinical

Yupiao Feng*[1], Xinting Hu[2], Yutang Li[1], Hua Wang[2], Kang Lu[2], Xiangxiang Zhou[2]

*[1]Shandong Provincial Hospital, Shandong University, Jinan, China, Department of Hematology, Jinan, China;[2]Shandong Provincial Hospital Affiliated to Shandong First Medical University, Jinan, China, Department of Hematology, Jinan, China;*

### Background

Altered levels of plasma metabolites have been reported associated with the progression of marginal zone lymphoma (MZL). However, due to potential confounding factors, this relationship remains inconclusive.

### Aims

This study aims to clarify the causal connection between plasma metabolites and genes involved in MZL.

### Methods

A two-sample Mendelian Randomization (MR) approach was employed. Exposure data were derived from genome-wide association studies (GWAS) involving 1,400 plasma metabolites, and outcome data for MZL patients were sourced from the FinnGen database. The primary analytical method was inverse variance weighting (IVW), complemented by MR-Egger and weighted median approaches. Heterogeneity, pleiotropy, and sensitivity were systematically evaluated. Instrumental variables representing metabolites with causal links to MZL were subjected to SNP annotation to locate relevant genes. These genes were further analyzed using Kyoto Encyclopedia of Genes and Genomes (KEGG) and Gene Ontology (GO) enrichment analyses, integrating data from the Gene Expression Omnibus (GEO). Additionally, gene expression differences between MZL patients and healthy individuals were examined, and single-sample gene set enrichment analysis (ssGSEA) was performed.

### Results

The findings indicated that 5-methylthioadenosine (MTA) (OR = 0.31, 95%CI: 0.18-0.54, $p < 0.001$), succinylcarnitine (OR = 0.45, 95%CI: 0.29-0.70, $p < 0.001$), and the methionine to methionine sulfoxide ratio (OR = 0.33, 95%CI: 0.18-0.58, $p < 0.001$) may serve as protective plasma metabolites for MZL. Conversely, X-21471 (OR = 1.86, 95%CI: 1.33-2.59, $p < 0.001$) emerged as a potential risk metabolite. Sensitivity analyses confirmed the absence of significant heterogeneity or horizontal pleiotropy **(Figure 1A-B)**. SNP screening from significant metabolites led to the identification of 71 potential MZL-related genes. KEGG analysis highlighted pathways such as propanoate metabolism, bile secretion, and ABC transporters. GO analysis revealed associations with sulfur compound metabolic processes, basolateral plasma membrane components, and xenobiotic transmembrane transporter activity. Eight genes showed significant differential expression between MZL patients and controls. ssGSEA indicated enrichment in plasmacytoid dendritic cells and central memory $CD4^+$ T cells.

### Summary/Conclusion

This study identifies three plasma metabolites with protective effects against MZL and one associated with increased risk. The combination of genomic and metabolomic data offers novel insights into MZL pathogenesis. Further investigation is needed to elucidate the underlying biological mechanisms.

**Copyright Information:** (Online) ISSN: 2572-9241
©2026 The Author(s). HemaSphere is published by John Wiley & Sons Ltd on behalf of European Hematology Association. This is an open access Abstract Book distributed under the Attribution-NonCommercial-NoDerivs (CC BY-NC-ND), which allows third parties to download the articles and share them with others as long as they credit the author and the Abstract Book, but they cannot change the content in any way or use them commercially.
**Abstract Book Citations:** Authors, Title, HemaSphere, 2026;10:(S1):pages. The abstract book DOIs can be found on the "Author Information" page.
**Disclaimer:** Articles published in the journal HemaSphere exclusively reflect the opinions of the authors. The authors are responsible for all content in their abstracts including accuracy of the facts, statements, citing resources, etc.

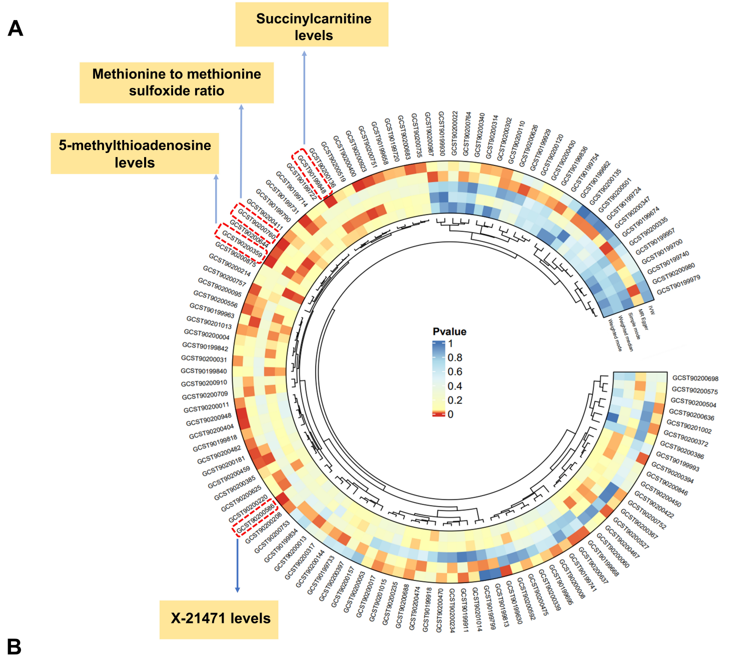

B

| exposure | nsnp | method | pval | OR(95% CI) |
|---|---|---|---|---|
| Succinylcarnitine levels | 28 | Weighted median | **0.008** | 0.441 (0.241 to 0.808) |
| Succinylcarnitine levels | 28 | Inverse variance weighted | **<0.001** | 0.453 (0.292 to 0.703) |
| Succinylcarnitine levels | 28 | Weighted mode | **0.019** | 0.363 (0.164 to 0.804) |
| 5−methylthioadenosine (mta) levels | 25 | Weighted median | **0.027** | 0.407 (0.183 to 0.903) |
| 5−methylthioadenosine (mta) levels | 25 | Inverse variance weighted | **<0.001** | 0.314 (0.182 to 0.542) |
| 5−methylthioadenosine (mta) levels | 25 | Weighted mode | 0.121 | 0.402 (0.132 to 1.222) |
| X−21471 levels | 26 | Weighted median | 0.257 | 1.340 (0.808 to 2.224) |
| X−21471 levels | 26 | Inverse variance weighted | **<0.001** | 1.859 (1.332 to 2.594) |
| X−21471 levels | 26 | Weighted mode | 0.297 | 1.305 (0.799 to 2.132) |
| Methionine to methionine sulfoxide ratio | 26 | Weighted median | **0.002** | 0.287 (0.131 to 0.632) |
| Methionine to methionine sulfoxide ratio | 26 | Inverse variance weighted | **<0.001** | 0.327 (0.185 to 0.577) |
| Methionine to methionine sulfoxide ratio | 26 | Weighted mode | 0.061 | 0.239 (0.057 to 1.001) |

**Copyright Information:** (Online) ISSN: 2572-9241
©2026 The Author(s). HemaSphere is published by John Wiley & Sons Ltd on behalf of European Hematology Association. This is an open access Abstract Book distributed under the Attribution-NonCommercial-NoDerivs (CC BY-NC-ND), which allows third parties to download the articles and share them with others as long as they credit the author and the Abstract Book, but they cannot change the content in any way or use them commercially.
**Abstract Book Citations:** Authors, Title, HemaSphere, 2026;10:(S1):pages. The abstract book DOIs can be found on the "Author Information" page.
**Disclaimer:** Articles published in the journal HemaSphere exclusively reflect the opinions of the authors. The authors are responsible for all content in their abstracts including accuracy of the facts, statements, citing resources, etc.

### (PB3650) RESPONSE-ADAPTED ORELABRUTINIB AND RITUXIMAB WITH OR WITHOUT LENALIDOMIDE FOR UNTREATED MARGINAL ZONE LYMPHOMA

**Topic:** 18. Indolent and mantle-cell non-Hodgkin lymphoma - Clinical

Haorui Shen[1], Chunling Wang[2], Mei Sun[3], Sanmei Wang[1], Lei Cao[1], Jujuan Wang[1], Yi Xia[1], Kaiyang Ding[4], Wei Wang[5], Lixia Sheng[6], Lei Fan*[1]

*[1]The First Affiliated Hospital of Nanjing Medical University, Jiangsu Province Hospital, Department of Hematology, Nanjing, China; [2]The Affiliated Huaian No.1 People's Hospital of Nanjing Medical University, Department of Hematology, Huai'an, China; [3]Northern Jiangsu People's Hospital, Department of Hematology, Yangzhou, China;[4]Anhui Province Cancer Hospital, Department of Hematology, Hefei, China; [5]The 2nd Affliated Hospital of Harbin Medical University, Department of Hematology, Harbin, China; [6]The First Affliated Hospital of Ningbo University, Department of Hematology, Ningbo, China;*

### Background

Marginal Zone Lymphoma (MZL) is a group of indolent lymphomas. Although rituximab monotherapy or rituximab-based immunochemotherapy is the standard frontline treatment for advanced MZL, its efficacy is often limited and safety concerns persist. Previous studies show that the BTK inhibitor (BTKi) ibrutinib combined with R and lenalidomide is effective for newly diagnosed MZL and follicular lymphoma, but it carries a high risk of severe (grade ≥3) rash. Orelabrutinib (O) is a next-generation BTKi that achieved an overall response rate (ORR) of 58.9% as monotherapy for relapsed/refractory MZL (R/R MZL) and is currently approved for R/R MZL in China. To improve response rates, reduce relapse, and minimize adverse events in newly diagnosed MZL, we conducted this study evaluating orelabrutinib combined with rituximab and lenalidomide.

### Aims

This open-label, multicenter, phase II study aims to evaluate the efficacy and safety of orelabrutinib and rituximab, with or without lenalidomide as response-adapted therapy for untreated marginal zone lymphoma. By tailoring treatment based on response, the approach seeks to minimize toxicity from lenalidomide exposure and improve overall clinical outcomes.

### Methods

The study plans to enroll 50 treatment-naïve MZL patients requiring therapy. Eligible patients will receive an induction therapy consisting of 6 cycles (21 days per cycle) of orelabrutinib (150 mg qd, days 1-21) and rituximab (375 mg/m² IV, day 1) (OR). After induction therapy, patients who achieve stable disease (SD) or better will proceed to maintenance treatment. Those with progressive disease (PD) will be withdrawn from the study. All patients entering the maintenance phase will receive treatment according to the response-adapted strategy: patients achieving complete response (CR) will continue to receive 6 cycles of OR, while those achieving partial response (PR) or SD will receive 6 cycles of lenalidomide (20 mg qd, days 1-14) in addition to OR ($OR^2$). The primary endpoint is the CR rate after 12 cycles. Secondary endpoints include ORR, best overall response (BOR), time to response (TTR), 2-year progression-free survival (PFS), 2-year overall survival (OS), and safety.

**Copyright Information:** (Online) ISSN: 2572-9241
©2026 The Author(s). HemaSphere is published by John Wiley & Sons Ltd on behalf of European Hematology Association. This is an open access Abstract Book distributed under the Attribution-NonCommercial-NoDerivs (CC BY-NC-ND), which allows third parties to download the articles and share them with others as long as they credit the author and the Abstract Book, but they cannot change the content in any way or use them commercially.
**Abstract Book Citations:** Authors, Title, HemaSphere, 2026;10:(S1):pages. The abstract book DOIs can be found on the "Author Information" page.
**Disclaimer:** Articles published in the journal HemaSphere exclusively reflect the opinions of the authors. The authors are responsible for all content in their abstracts including accuracy of the facts, statements, citing resources, etc.

### Results

As of January 29, 2026, 17 patients have been enrolled, with a median age of 67 years (range 44–76). 14 patients (82.4%) presented with Ann Arbor stage III–IV disease. Of evaluable patients, 46.2% (6/13) had a Ki67 index ≥20%. Of the 11 patients who underwent an interim assessment after 6 cycles, 10 (90.9%) achieved a CR and 1 (9.1%) had SD. The patient with SD subsequently received $OR^2$ during maintenance.

Regarding safety, all 17 patients (100%) experienced adverse events, including 2 (11.1%) with grade 3 anemia; all others were grade 1–2. The most common hematologic adverse events were anemia (6 patients, 35.3%), neutropenia (6 patients, 35.3%), and thrombocytopenia (5 patients, 29.4%). Only 1 patient developed grade 2 alanine aminotransferase elevation, and another patient developed a grade 1 rash. No atrial fibrillation (AF) or infection of any grade occurred.

### Summary/Conclusion

This phase II study demonstrates that induction therapy with orelabrutinib and rituximab may reduce lenalidomide-related toxicity. The adaptive response-guided treatment approach shows promising efficacy and a manageable safety profile in newly diagnosed MZL patients, with no grade 4–5 adverse events and no AF or infection reported.

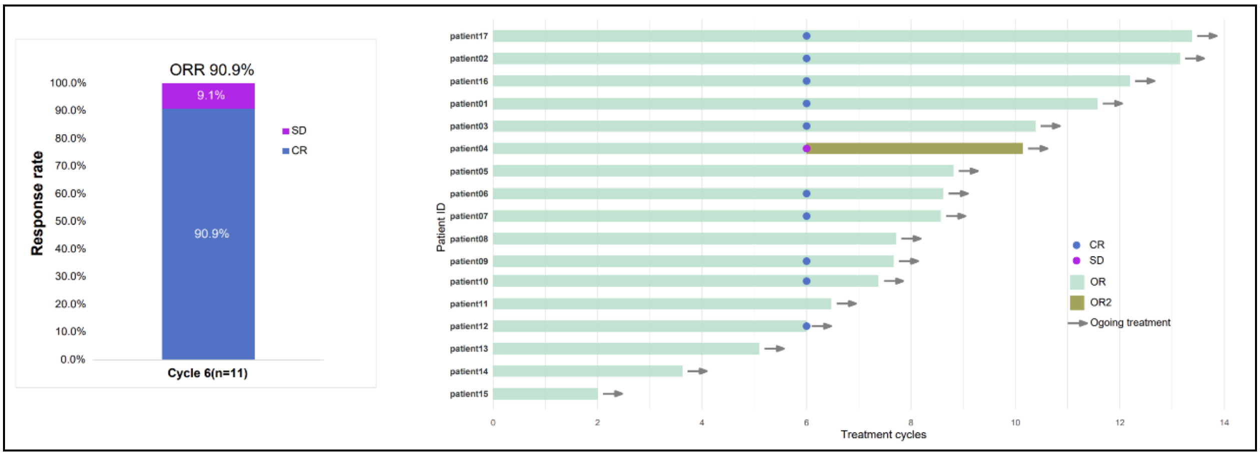

**Copyright Information:** (Online) ISSN: 2572-9241
©2026 The Author(s). HemaSphere is published by John Wiley & Sons Ltd on behalf of European Hematology Association. This is an open access Abstract Book distributed under the Attribution-NonCommercial-NoDerivs (CC BY-NC-ND), which allows third parties to download the articles and share them with others as long as they credit the author and the Abstract Book, but they cannot change the content in any way or use them commercially.
**Abstract Book Citations:** Authors, Title, HemaSphere, 2026;10:(S1):pages. The abstract book DOIs can be found on the “Author Information” page.
**Disclaimer:** Articles published in the journal HemaSphere exclusively reflect the opinions of the authors. The authors are responsible for all content in their abstracts including accuracy of the facts, statements, citing resources, etc.

### (PB3651) CLINICAL, PATHOLOGICAL, AND MOLECULAR LANDSCAPE AND PROGNOSTIC FACTORS OF DIFFUSE LARGE B-CELL LYMPHOMA TRANSFORMED FROM FOLLICULAR LYMPHOMA: A SINGLE-CENTER RETROSPECTIVE STUDY

**Topic:** 18. Indolent and mantle-cell non-Hodgkin lymphoma - Clinical

Rongjun MA[1], Fang Wang[2], Junhong Zhuo[1], Yuanbo Liu[1], Pan Zhou[1], Suqiong Zuo[1], Xiaohang Pei[1], Xiaoli Yuan*[1], Zunmin Zhu[1]

*[1]Henan Provincial People's Hospital, Zhengzhou City,Henan Province, China;[2]Zhengzhou Hospital of Traditional Chinese Medicine, Zhengzhou City,Henan Province, China;*

### Background

Histological transformation (HT) of follicular lymphoma (FL) into diffuse large B-cell lymphoma (t-DLBCL) represents a critical clinical event associated with rapid disease progression and poor outcomes. Despite the standard use of immunochemotherapy, the molecular drivers and clinical predictors for HT remain to be fully elucidated.

### Aims

To characterize the clinical, pathological, and genomic landscape of t-DLBCL and identify independent risk factors for transformation and survival.

### Methods

We retrospectively analyzed 34 t-DLBCL patients and 34 matched Grade 3A FL controls diagnosed between 2010 and 2024. The majority of t-DLBCL patients (82.4%) received G/R-CHOP-like regimens as first-line induction therapy. Clinical data, immunohistochemical (IHC) markers (CD21, CD30, Ki-67), and next-generation sequencing (NGS) using a 148-gene panel were evaluated. Survival outcomes were assessed using Kaplan-Meier and Cox proportional hazard models.

### Results

- **Clinical and Pathological Profiles (Table 1):** Among 209 FL patients, 34 experienced DLBCL transformation (16.3%), with 76.5% occurring at initial diagnosis. Compared to the control group, t-DLBCL patients had a significantly higher incidence of anemia (67.6% vs. 35.3%, P=0.015). IHC analysis revealed significantly higher CD30 positivity (68.4% vs. 28.6%, P=0.037) and a markedly elevated Ki-67 index (>50%: 70.6% vs. 48.4%, P=0.043) in the transformation group. Conversely, CD21 expression was significantly lower (73.1% vs. 96.8%, P=0.018), suggesting follicular dendritic cell network disruption.
- **Genomic Evolution (Figure 1):** NGS identified a dynamic mutational shift during HT. While KMT2D, FAS, and HIST1H1E were prevalent in both groups, HT was characterized by the de novo acquisition of high-frequency mutations in TP53 (67.7% vs. 0% pre-HT), BCL10, and BCL-6. Paired analysis confirmed clonal evolution with newly acquired hits in MYC, EZH2, and ARID1A post-transformation.
- **Survival and Prognostics:** The 5-year OS rate for the transformation group was 75.0%. Univariate analysis associated bone marrow (BM) involvement and elevated $\beta$2-microglobulin with inferior OS (P<0.05). Multivariate Cox regression identified BM involvement (HR 7.342, P=0.049) and FLIPI-2 score ≥3 (HR 15.631, P=0.033) as independent predictors of shortened PFS (Table 1).

**Copyright Information:** (Online) ISSN: 2572-9241
©2026 The Author(s). HemaSphere is published by John Wiley & Sons Ltd on behalf of European Hematology Association. This is an open access Abstract Book distributed under the Attribution-NonCommercial-NoDerivs (CC BY-NC-ND), which allows third parties to download the articles and share them with others as long as they credit the author and the Abstract Book, but they cannot change the content in any way or use them commercially.
**Abstract Book Citations:** Authors, Title, HemaSphere, 2026;10:(S1):pages. The abstract book DOIs can be found on the "Author Information" page.
**Disclaimer:** Articles published in the journal HemaSphere exclusively reflect the opinions of the authors. The authors are responsible for all content in their abstracts including accuracy of the facts, statements, citing resources, etc.

### Summary/Conclusion

HT in FL predominantly involves high-grade disease. Loss of CD21 and elevated Ki-67/CD30 expression are significant predictive markers for transformation. Genomic progression is driven by the acquisition of TP53, BCL-10, and BCL-6 mutations. BM involvement and FLIPI-2 ≥3 are critical independent prognostic factors for t-DLBCL.

Table 1: Key Differential and Prognostic Features in t-DLBCL

| **Feature** | **t-DLBCL (n=34)** | **Control (n=34)** | **P-value** |
|---|---|---|---|
| **Pathology** | | | |
| Ki-67 ≥50% | 70.6% | 48.4% | 0.043 |
| CD30 Positive | 68.4% | 28.6% | 0.037 |
| CD21 Positive | 73.1% | 96.8% | 0.018 |
| **Multivariate (PFS)** | **Hazard Ratio** | **95% CI** | **P-value** |
| FLIPI-2 ≥ 3 | 15.631 | 1.006–53.593 | 0.033 |
| BM Involvement | 7.342 | 1.066–10.326 | 0.049 |

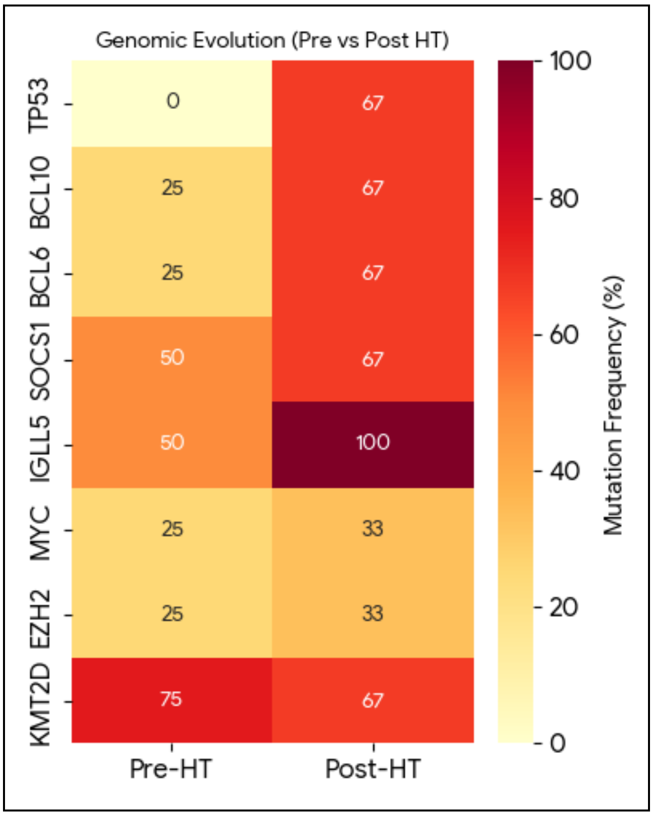

Figure 1: Genomic Evolution Heatmap

**Copyright Information:** (Online) ISSN: 2572-9241
©2026 The Author(s). HemaSphere is published by John Wiley & Sons Ltd on behalf of European Hematology Association. This is an open access Abstract Book distributed under the Attribution-NonCommercial-NoDerivs (CC BY-NC-ND), which allows third parties to download the articles and share them with others as long as they credit the author and the Abstract Book, but they cannot change the content in any way or use them commercially.
**Abstract Book Citations:** Authors, Title, HemaSphere, 2026;10:(S1):pages. The abstract book DOIs can be found on the "Author Information" page.
**Disclaimer:** Articles published in the journal HemaSphere exclusively reflect the opinions of the authors. The authors are responsible for all content in their abstracts including accuracy of the facts, statements, citing resources, etc.

### (PB3652) BASELINE CHARACTERISTICS AND CLINICAL FEATURES OF INDOLENT LYMPHOMA WITH CENTRAL NERVOUS SYSTEM INVOLVEMENT: A MULTICENTER RETROSPECTIVE STUDY OF 39 CASES

**Topic:** 18. Indolent and mantle-cell non-Hodgkin lymphoma - Clinical

Xixi Xiang*[1], Liang Wang[2], Li Gao[1], Wenzhe Yan[3], Wuping Li[4], Jun Fang[5], Guohui Cui[5], Yujia Huo[6], Xiaoxi Zhou[7], Wei Guo[8], Xutao Guo[9], Yafei Wang[10], Hua Wang[11], Ya Zhang[12], Fang Wei[13], Lijuan Cui[14], Hui Geng[15], Fangyi Fan[16], Qike Zhang[17], Wenqiang Xie[18], Hongling Peng[3], Xi Zhang[1]

*[1]Xinqiao Hospital of Army Medical University, Medical Center of Hematology, Chongqing, China;[2]Beijing Tongren Hospital, Capital Medical University, Department of Hematology, Beijing, China; [3]The Second Xiangya Hospital of Central South University, Department of Hematology, Changsha, China; [4]Jiangxi Cancer Hospital, Departments of Lymphatic and Hematological Oncology, Nanchang, China; [5]Affiliated Union Hospital�Huazhong University of Science and Technology, Department of Hematology, Wuhan, China; [6]Ruijin Hospital Affiliated to Shanghai Jiao Tong University School of Medicine, Department of Hematology, Shanghai, China; [7]Tongji Hospital, Tongji Medical College, Huazhong University of Science and Technology, Department of Hematology, Wuhan, China; [8]The First Hospital of Jilin University, Department of Hematology, Changchun, China; [9]Nanfang Hospital of Southern Medical University, Department of Hematology, Guangzhou, China;[10]Tianjin Medical University Cancer Institute and Hospital, Department of Hematology, Tianjin, China; [11]Sun Yat-sen University Cancer Center, Department of Hematological Oncology, Tianjin, China; [12]Shandong Provincial Hospital Affiliated to Shandong First Medical University, Department of Hematology, Jinan, China; [13]The First Hospital of Shanxi Medical University, Department of Hematology, Taiyuan, China; [14]General Hospital of Ningxia Medical University, Department of Hematology, Yinchuan, China;[15]Affiliated Hospital of Qinghai University, Department of Hematological Oncology, Xining, China; [16]The General Hospital of Western Theater Command, Department of Hematology, Chengdu, China; [17]Gansu Provincial Hospital, Department of Hematological Oncology, Lanzhou, China; [18]The First Affiliated Hospital of Guangxi Medical University, Department of Hematology, Nanning, China;*

### Background

Indolent lymphomas typically progress slowly, but CNS involvement presents complex clinical challenges. Limited multicenter data on baseline/clinical characteristics hinder standardized management strategies.

### Aims

To summarize baseline features, clinical involvement, and lab findings of indolent lymphoma with CNS involvement via multicenter retrospective analysis, supporting clinical decision-making and prognosis.

### Methods

We retrospectively collected clinical data from 39 indolent lymphoma patients with CNS involvement across 18 Chinese centers. We analyzed disease subtypes, Ki-67, primary sites, extranodal involvement, imaging/lab results, and prognostic scores.

**Copyright Information:** (Online) ISSN: 2572-9241
©2026 The Author(s). HemaSphere is published by John Wiley & Sons Ltd on behalf of European Hematology Association. This is an open access Abstract Book distributed under the Attribution-NonCommercial-NoDerivs (CC BY-NC-ND), which allows third parties to download the articles and share them with others as long as they credit the author and the Abstract Book, but they cannot change the content in any way or use them commercially.
**Abstract Book Citations:** Authors, Title, HemaSphere, 2026;10:(S1):pages. The abstract book DOIs can be found on the "Author Information" page.
**Disclaimer:** Articles published in the journal HemaSphere exclusively reflect the opinions of the authors. The authors are responsible for all content in their abstracts including accuracy of the facts, statements, citing resources, etc.

**Results**

Among 39 patients, MALT lymphoma was most common (43.6%, 17/39), followed by MCL and MZL (each 12.8%, 5/39). Ki-67 <30% in 73.3% (22/30) and ≥30% in 26.7% (8/30). Primary site most often CNS (35.9%, 14/39), then lymph node (28.2%, 11/39); 8 patients had ≥2 primary sites. Extranodal involvement: 44.1% (15/34) had one extranodal site, 55.9% (19/34) had ≥2. Lymph node involvement in 59.0% (23/39); hepatomegaly in 5.1% (2/39), splenomegaly in 23.1% (9/39), serous cavity effusion in 23.1% (9/39). Bone marrow (BM) involvement was documented in 43.6% (17/39), with abnormal BM smear in 65.4% (17/26), abnormal BM biopsy in 58.3% (14/24), and abnormal BM flow cytometry in 48.0% (12/25). Genetic abnormalities: chromosomal aberrations 11.1% (2/18), NGS abnormalities 88.9% (8/9), FISH abnormalities 50.0% (6/12). Ann Arbor stage (n=24): stage IV 79.2% (19/24), stage III/IV 87.5% (21/24). ECOG (n=34): 1-2 in 79.5% (27/34), 3-4 in 17.6% (6/34). MZL-IPI (n=18): moderate risk 66.7% (12/18), high 27.8% (5/18), low 5.6% (1/18). Among 38 patients with detailed CNS involvement sites : cerebral lobes (31.6%, 12/38), spinal cord/vertebral canal (21.1%, 8/38), visual pathway (15.8%, 6/38), ventricles/CSF (15.8%, 6/38); 18.4% (7/38) had ≥2 sites. CNS-related symptoms (n=32) were predominantly sensory disturbance (34.4%, 11/32) and visual disturbance (31.3%, 10/32); 13 patients (40.6%) presented with ≥2 symptoms. CSF flow cytometry abnormal in 50.0% (9/18); both tested for CSF cytokines abnormal. Lab findings: elevated LDH in 20.6% (7/34); abnormal neutrophil count in 10.5% (4/38), lymphocyte in 39.5% (15/38), platelet in 16.2% (6/37). B symptoms in 22.9% (8/35). Three patients HBV DNA positive.

**Summary/Conclusion**

Indolent lymphoma with CNS involvement is predominantly MALT subtype, often with low Ki-67 and CNS as primary site. Extranodal multifocal involvement, lymph node, and bone marrow infiltration are frequent; most have advanced stage, ECOG 1-2, and moderate-high MZL-IPI risk. CNS involvement most often affects spinal cord/vertebral canal and cerebral lobes, with visual/sensory disturbances. Common findings: abnormal CSF, lymphopenia/lymphocytosis, elevated LDH. Heterogeneous features and high risk necessitate individualized multidisciplinary management.

**Copyright Information:** (Online) ISSN: 2572-9241
©2026 The Author(s). HemaSphere is published by John Wiley & Sons Ltd on behalf of European Hematology Association. This is an open access Abstract Book distributed under the Attribution-NonCommercial-NoDerivs (CC BY-NC-ND), which allows third parties to download the articles and share them with others as long as they credit the author and the Abstract Book, but they cannot change the content in any way or use them commercially.
**Abstract Book Citations:** Authors, Title, HemaSphere, 2026;10:(S1):pages. The abstract book DOIs can be found on the "Author Information" page.
**Disclaimer:** Articles published in the journal HemaSphere exclusively reflect the opinions of the authors. The authors are responsible for all content in their abstracts including accuracy of the facts, statements, citing resources, etc.

### (PB3653) PROGNOSTIC DETERMINANTS IN EXTRANODAL MARGINAL ZONE LYMPHOMA

**Topic:** 18. Indolent and mantle-cell non-Hodgkin lymphoma - Clinical

Maha Charfi*[1], Malek Sayedi[2], Hela Ghedira[3], Slama Nader[4], Nesrine Ben Sayed[5], Wala Ben Kridis[6], Haifa Hafsa[7], Wafa Miled[2], Raoudha Mansouri[2], Rabeb Jaied[3], Wided Cherif[5], Zaineb Mlayah[4], Anna Chabaane[8], Moez Elloumi[1], Raihane Ben Lakhal[2], Fehmi Msadek[3], Mohamed Adnene Laatiri[4], Yosra Ben Youssef[5], Nour Ben Adejlil[8], Moez Mdhaffar[1]

*[1]Hedi Chaker hospital, hematology department, Sfax, Tunisia; [2]Aziza Othmana hospital, hematology department, tunis, Tunisia; [3]military hospital, hematology department, tunis, Tunisia; [4]Fattouma Bourguiba hospital, hematology department, Monastir, Tunisia; [5]Farhat Hached hospital, hematology department, sousse, Tunisia; [6]Hbib Bourguiba, oncology department, Sfax, Tunisia; [7]Mohamed Ben Sassi, hematology department, Gabes, Tunisia; [8]centre de greffe de moelle osseuse, hematology department, Tunis, Tunisia;*

### Background

Extranodal marginal zone lymphoma (EMZL) is an indolent lymphoma with heterogeneous clinical presentation and variable outcomes. While prognosis is generally favorable, a subset of patients experience early progression or treatment failure. Identifying prognostic factors in real-world cohorts remains crucial to refine risk stratification and guide therapeutic decisions.

### Aims

The aim of this study was to identify prognostic factors associated with treatment failure, progression-free survival, and overall survival in a cohort of patients with EMZL.

### Methods

We conducted a retrospective multicenter study including patients diagnosed with EMZL between January 2015 and December 2024 in six hematology centers and one oncology center in Tunisia. Clinical characteristics, disease distribution, treatment regimens, and outcomes were analyzed. Predictive factors of treatment failure, overall survival (OS), and progression-free survival (PFS) were assessed using univariate and multivariate analyses. The chi-square test is used to identify predictors of failure in univariate analysis and logistic regression in multivariate analysis.

### Results

Our study included 93 patients with ENMZL. Median age at diagnosis was 58 years, with a slight male predominance. Gastric involvement accounted for 52% of cases, while 48% were non-gastric EMZL. Localized disease (stage I–II) was observed in 72% of patients.

In univariate analysis, treatment failure was significantly associated with bulky disease (p=0.05), advanced stage (III–IV) (p=0.02), and treatment regimen (R-CLB vs R-CHOP, p=0.05). In multivariate analysis, treatment with R-CLB compared to R-CHOP remained the only independent predictor of treatment failure (p=0.05).

Regarding OS, univariate analysis identified age >70 years (p=0.01), bone marrow involvement (p=0.003), anemia (p=0.006), bulky disease (p=0.001), advanced stage (p=0.001), MALT-IPI (p<0.0001), MZL-IPI (p<0.0001), treatment failure (p=0.045), and POD24 (p=0.05) as adverse prognostic factors. In multivariate analysis, MZL-IPI (p<0.0001) remained independently associated with OS, while POD24 showed a strong trend (p=0.06).

For PFS, univariate analysis showed significant associations with bone marrow involvement, multiple nodal sites (>3), bulky disease, advanced stage, MALT-IPI, and MZL-IPI (all p≤0.05). In multivariate analysis, bone marrow involvement (p=0.003) and bulky disease (p=0.038) were independent predictors of inferior PFS.

**Copyright Information:** (Online) ISSN: 2572-9241
©2026 The Author(s). HemaSphere is published by John Wiley & Sons Ltd on behalf of European Hematology Association. This is an open access Abstract Book distributed under the Attribution-NonCommercial-NoDerivs (CC BY-NC-ND), which allows third parties to download the articles and share them with others as long as they credit the author and the Abstract Book, but they cannot change the content in any way or use them commercially.
**Abstract Book Citations:** Authors, Title, HemaSphere, 2026;10:(S1):pages. The abstract book DOIs can be found on the “Author Information” page.
**Disclaimer:** Articles published in the journal HemaSphere exclusively reflect the opinions of the authors. The authors are responsible for all content in their abstracts including accuracy of the facts, statements, citing resources, etc.

**Summary/Conclusion**

In this large EMZL cohort, bulky disease and bone marrow involvement emerged as the strongest predictors of disease progression, while MZL-IPI was the main determinant of overall survival. Early progression (POD24) identified a subgroup with particularly poor outcomes. These findings support the use of validated prognostic scores and early response assessment to guide risk-adapted therapeutic strategies in EMZL.

**Copyright Information:** (Online) ISSN: 2572-9241
©2026 The Author(s). HemaSphere is published by John Wiley & Sons Ltd on behalf of European Hematology Association. This is an open access Abstract Book distributed under the Attribution-NonCommercial-NoDerivs (CC BY-NC-ND), which allows third parties to download the articles and share them with others as long as they credit the author and the Abstract Book, but they cannot change the content in any way or use them commercially.
**Abstract Book Citations:** Authors, Title, HemaSphere, 2026;10:(S1):pages. The abstract book DOIs can be found on the "Author Information" page.
**Disclaimer:** Articles published in the journal HemaSphere exclusively reflect the opinions of the authors. The authors are responsible for all content in their abstracts including accuracy of the facts, statements, citing resources, etc.

### (PB3655) GOLSEEK-4: A PHASE 3, RANDOMIZED STUDY OF GOLCADOMIDE, A POTENTIAL, FIRST-IN-CLASS, ORAL CELMOD, + RITUXIMAB VS INVESTIGATOR'S CHOICE IN PATIENTS WITH R/R FL WHO RECEIVED ≥1 LINE OF SYSTEMIC THERAPY

**Topic:** 18. Indolent and mantle-cell non-Hodgkin lymphoma - Clinical

Muhit Ozcan*[1], Eliza Hawkes[2], Clémentine Sarkozy[3], Alex F. Herrera[4], Yuqin Song[5], Charalambos Andreadis[6], Alev Akyol[7], Parth Rao[7], Antonia Di Micco[8], Adrien Petel[8], Serena Perna[7], Franck Morschhauser[9]

*[1]Ankara University School of Medicine, Ankara, Türkiye;[2]Olivia Newton John Cancer Research Institute at Austin Health, Heidelberg, Australia; [3]Institut Curie, Hôpital Saint-Cloud, Université de Versailles, Saint-Quentin, France;[4]City of Hope Comprehensive Cancer Center, Duarte, United States of America; [5]Director of Department of Lymphoma, Peking University Cancer Hospital & Institute, Peking, China; [6]UCSF Health, San Francisco, United States of America;[7]Bristol Myers Squibb, Madison, United States of America; [8]Bristol Myers Squibb, Boudry, Switzerland;[9]University of Lille, Centre Hospitalier Universitaire de Lille, Lille, France;*

### Background

Follicular lymphoma (FL) is the most prevalent indolent non-Hodgkin lymphoma (NHL), typically diagnosed at advanced stage. While current standard of care (SOC; chemoimmunotherapy [CIT] and maintenance) can induce remissions in many patients (pts) with FL, the disease remains incurable, and subsequent relapses are associated with significantly shorter remissions and overall survival (OS) following relapses. Approved regimens for relapsed/refractory (R/R) FL, including non-cross-resistant CIT or rituximab (R)-lenalidomide (LEN) ($R^2$), provide suboptimal long-term benefit. T-cell-redirecting agents show improved efficacy in the third line (L) and later settings, however, they face tolerability and logistical issues. There remains an unmet need for effective, well-tolerated, and more convenient therapies for R/R FL after ≥1L of systemic therapy.
Golcadomide (GOLCA) is a potential, first-in-class, oral CELMoD designed for the treatment of lymphoma, with preferential distribution to lymphoid organs and enhanced activity in lymphoma cell lines. Golcadomide drives the closed, active conformation of cereblon to induce rapid and deep degradation of Ikaros and Aiolos, leading to direct cell killing and immunomodulatory activity.
In the Phase (Ph) 1/2 CC-99282-NHL-001 study, GOLCA (0.4 mg) + R resulted in promising objective response rate (ORR; 97%) and complete response rate (CRR; 78%), and tolerable safety in heavily pretreated R/R FL pts, including those receiving LEN or T-cell-redirecting therapies (Chavez et al., ASH 2025, #1006). These data informed the design of the GOLSEEK-4 Ph 3 trial.

### Aims

To evaluate the efficacy and safety of GOLCA + R vs investigator's choice (IC; $R^2$ or R-chemo) in pts with R/R FL after ≥1 prior line of systemic therapy.

**Copyright Information:** (Online) ISSN: 2572-9241
©2026 The Author(s). HemaSphere is published by John Wiley & Sons Ltd on behalf of European Hematology Association. This is an open access Abstract Book distributed under the Attribution-NonCommercial-NoDerivs (CC BY-NC-ND), which allows third parties to download the articles and share them with others as long as they credit the author and the Abstract Book, but they cannot change the content in any way or use them commercially.
**Abstract Book Citations:** Authors, Title, HemaSphere, 2026;10:(S1):pages. The abstract book DOIs can be found on the "Author Information" page.
**Disclaimer:** Articles published in the journal HemaSphere exclusively reflect the opinions of the authors. The authors are responsible for all content in their abstracts including accuracy of the facts, statements, citing resources, etc.

### Methods

GOLSEEK-4 (NCT06911502) is a global, randomized, Ph 3 study.
Eligible adults (≥18 years) had histologically-confirmed grade 1-3A R/R FL or classical FL, PET-positive measurable disease (Lugano criteria), ECOG PS 0–2 (3 if due to lymphoma), require treatment based on modified GELF, and ≥1 prior regimen, including anti-CD20 + alkylating agent. Prior T-cell-redirecting therapy is permitted. Excluded: composite DLBCL/FL, transformed NHL, CNS involvement, those who are R/R or intolerant to all options in IC arm.
Approximately 400 pts will be randomized 1:1 to GOLCA (0.4 mg QD, Days 1–14 per 28-day cycle) + R for 5 cycles, then GOLCA monotherapy for 7 cycles (12 cycles total) or IC ($R^2$ for 5 cycles then LEN monotherapy for 7 cycles, or R-CHOP or R-Bendamustine for 6 cycles). Randomization will be stratified by disease progression within 24 months (mo) vs >24 mo from initial CIT, prior lines (2L vs 3L+), and comparator IC regimen. Pts will be followed for at least 5 yrs. Recruitment began July 2025.
Primary endpoint: progression-free survival by independent review adjudication committee (IRAC). Key secondary endpoints: ORR by IRAC and overall survival. Additional secondary endpoints: investigator-assessed complete metabolic response, minimal residual disease via ctDNA at end of treatment, time to next treatment, and duration of response. Safety is an exploratory endpoint.

### Results

N/A.

### Summary/Conclusion

GOLSEEK-4 will evaluate the safety and efficacy of GOLCA + R as a fixed-duration, chemo-free, outpatient treatment for patients with R/R FL, who have received ≥1 line of systemic therapy as compared to IC of $R^2$ or R-chemo.
Acknowledgement: BMS ChatGPT4 was used to revise text with human input.

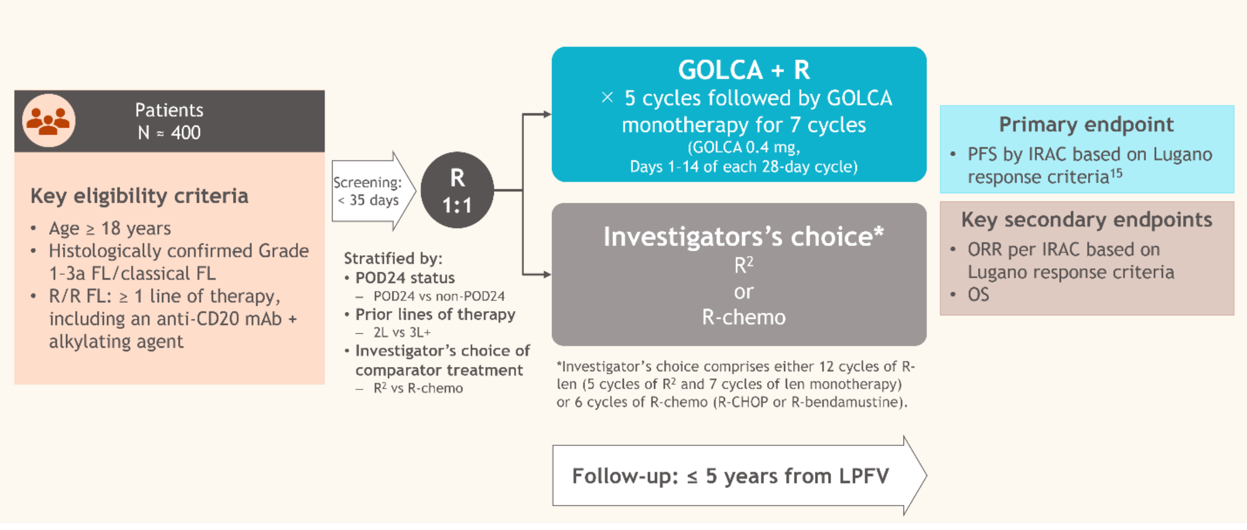

2L, second line; 3L+, third line and beyond; CD, cluster of differentiation; CMRR, complete metabolic response rate; DOR, duration of response; FL, follicular lymphoma; HRQoL, health-related quality of life; IRAC, Independent Radiology Adjudication Committee; len, lenalidomide; LPFV, last patient first visit; mAb, monoclonal antibody; ORR, overall response rate; OS, overall survival; PFS, progression-free survival; POD24, progression of disease within 24 months; R, rituximab; R-CHOP, rituximab, cyclophosphamide, doxorubicin, vincristine, and prednisone; R/R, relapsed/refractory.

Copyright Information: (Online) ISSN: 2572-9241
©2026 The Author(s). HemaSphere is published by John Wiley & Sons Ltd on behalf of European Hematology Association. This is an open access Abstract Book distributed under the Attribution-NonCommercial-NoDerivs (CC BY-NC-ND), which allows third parties to download the articles and share them with others as long as they credit the author and the Abstract Book, but they cannot change the content in any way or use them commercially.
Abstract Book Citations: Authors, Title, HemaSphere, 2026;10:(S1):pages. The abstract book DOIs can be found on the "Author Information" page.
Disclaimer: Articles published in the journal HemaSphere exclusively reflect the opinions of the authors. The authors are responsible for all content in their abstracts including accuracy of the facts, statements, citing resources, etc.

### (PB3656) ORELABRUTINIB PLUS ANTI-CD20 MONOCLONAL ANTIBODY WITH OR WITHOUT IMMUNOMODULATORY AGENT FOLLOWED BY ORELABRUTINIB MAINTENANCE THERAPY IN MARGINAL ZONE LYMPHOMA: A RETROSPECTIVE STUDY

**Topic:** 18. Indolent and mantle-cell non-Hodgkin lymphoma - Clinical

Ting Niu*[1, 2, 3], He Li[1], Jiazhuo Liu[1], Xiang Bing[1], Yongqian Jia[1], Yuhui Chen[1], Pazilaiti Tuohuti[1], Yutong Wang[1], Xinzhu Tang[1], Yu Wu[1], Jie Wang[1], Yuhuan Zheng[1], Jinrong Yang[1], Ailin Zhao[1], Yang Dai[1], Pu Kuang[1], Jie Huang[1], Yu Liu[2]

*[1]West China Hospital, Sichuan University, Department of Hematology, Chengdu, China;[2]West China Hospital, Sichuan University, State Key Laboratory of Biotherapy, Collaborative Innovation Center of Biotherapy, Chengdu, China; [3]West China Hospital, Sichuan University, National Facility for Translational Medicine (Sichuan), Chengdu, China;*

### Background

Marginal zone lymphoma (MZL) is the second most common subtype of indolent lymphoma. Anti-CD20 monoclonal antibody is the optimal strategy for patients with symptomatic, advanced MZL. Orelabrutinib, a novel, oral, and covalent next-generation Bruton tyrosine kinase (BTK) inhibitor with high target selectivity, has demonstrated a favorable efficacy and safety profile in MZL and has been approved in China for patients who have received at least one prior line of therapy. This study aimed to evaluate the efficacy and safety of orelabrutinib plus anti-CD20 monoclonal antibody with or without immunomodulatory agents in patients with MZL.

### Methods

This retrospective study included patients with MZL who received induction therapy consisting of orelabrutinib plus anti-CD20 monoclonal antibody with or without immunomodulatory agents (Lenalidomide or Pomalidomide) at West China Hospital of Sichuan University between September 2022 and October 2025. Patients could receive maintenance therapy with orelabrutinib. The primary endpoint was objective response rate (ORR). Secondary endpoints included complete response rate(CRR), progression-free survival (PFS), overall survival, and safety.

**Copyright Information:** (Online) ISSN: 2572-9241
©2026 The Author(s). HemaSphere is published by John Wiley & Sons Ltd on behalf of European Hematology Association. This is an open access Abstract Book distributed under the Attribution-NonCommercial-NoDerivs (CC BY-NC-ND), which allows third parties to download the articles and share them with others as long as they credit the author and the Abstract Book, but they cannot change the content in any way or use them commercially.
**Abstract Book Citations:** Authors, Title, HemaSphere, 2026;10:(S1):pages. The abstract book DOIs can be found on the "Author Information" page.
**Disclaimer:** Articles published in the journal HemaSphere exclusively reflect the opinions of the authors. The authors are responsible for all content in their abstracts including accuracy of the facts, statements, citing resources, etc.

**Results**

Of 44 enrolled patients, 36 (81.8%) were treatment-naïve and 8 (18.2%) had received≥1 prior therapy. The median age was 54 years (range, 46-62), and 70.5% (31/44) were female. MZL subtypes included extranodal (mucosa-associated lymphoid tissue, MALT) MZL in 84.1% (37/44), nodal MZL in 11.4% (5/44), and splenic MZL in 4.5% (2/44) of patients. Most patients had stage II-IV disease (65.9%, 29/44) and intermediate- or high-risk MZL-IPI scores (15.9%, 6/44). 13.6% of patients (6/44) had B symptoms, 15.9% (7/44) were positive for M protein, and 9% (4/44) had *helicobacter pylori* (*H. pylori*) infection. Bone marrow involvement was observed in 20.4% of patients (9/44), and Ki-67 index ≥ 20% was reported in 6.8% (3/44). 15 patients had received prior local therapy, including surgery (n=12), anti-*H. pylori* therapy (n=2), and both surgery and radiotherapy (n=1). 81.8% (36/44) received orelabrutinib combined with anti-CD20 monoclonal antibody, and 18.2% (8/44) received the combination with additional immunomodulatory agents.
At interim assessment (median 3 cycles,n=34), 19 (55.9%) patients achieved complete response (CR), 14 (41.2%) achieved partial response (PR), and 1 (2.9%) had stable disease (SD), and ORR of 96.9%. At the end of induction therapy (median 6 cycles,n=39), 27 (69.2%) patients achieved CR, 12 (30.8%) achieved PR, with an ORR of 100%. Among the 40 efficacy-evaluable patients, 67.5% (27/40) achieved CR, and the best ORR was 100% (40/40). In the first-line cohort (n=33), CRR was 66.7% (22/33) and ORR 100%; in the ≥2L cohort (n=7), CRR was 71.4% (5/7) and ORR 100%. 40.9% (18/44) undergoing maintenance treatment with orelabrutinib, and the median maintenance cycles was 3.5 (range, 1-22). By the data cut-off (Feb 10, 2026), and only one patient experienced disease progression. With a median follow-up of 7 months, the 12-month PFS rate was 100% and the 24-month PFS rate was 91.7%. 29(65.9%) experienced adverse events (AEs), and 14 (31.8%) had grade 3 or higher AEs. The most common ≥3 AE were neutropenia (5.8%) and leukopenia(5.8%). No deaths or BTKi-related AEs occurred.

**Summary/Conclusion**

Orelabrutinib plus rituximab preliminarily showed acceptable efficacy and safety in MZL, and the maintenance therapy was well tolerated. Additional follow-up data will be included in future updates.

**Copyright Information:** (Online) ISSN: 2572-9241
©2026 The Author(s). HemaSphere is published by John Wiley & Sons Ltd on behalf of European Hematology Association. This is an open access Abstract Book distributed under the Attribution-NonCommercial-NoDerivs (CC BY-NC-ND), which allows third parties to download the articles and share them with others as long as they credit the author and the Abstract Book, but they cannot change the content in any way or use them commercially.
**Abstract Book Citations:** Authors, Title, HemaSphere, 2026;10:(S1):pages. The abstract book DOIs can be found on the "Author Information" page.
**Disclaimer:** Articles published in the journal HemaSphere exclusively reflect the opinions of the authors. The authors are responsible for all content in their abstracts including accuracy of the facts, statements, citing resources, etc.

### (PB3657) EPIDEMIOLOGY AND OUTCOME OF MARGINAL ZONE LYMPHOMAS PATIENTS: A MULTICENTRIC STUDY FOR THE TUNISIAN INDOLENT LYMPHOMA STUDY GROUP

**Topic:** 18. Indolent and mantle-cell non-Hodgkin lymphoma - Clinical

Hela Ghedira*[1], Malek Sayadi[2], Maha Charfi[3], Slama Nader[4], Nesrine Ben Sayed[5], Wala Ben Kridis[6], Haifa Hafsa[7], Anna Chabaane[8], Rabeb Jaied[1], Wafa Miled[9], Raoudha Mansouri[9], Zaineb Mlayah[4], Wided Cherif[5], Fehmi Msadek[1], Raihane Ben Lakhal[2], Moez Elloumi[10], Mohamed Adnene Laatiri[4], Yosra Ben Youssef[5], Nour Ben Adejlil[8], Moez Medhaffar[10]

*[1]Military Hospital of Tunis, Hematology Department, Tunis, Tunisia;[2]Aziza Othmana Hospital, Hematology Department, Tunis, Tunisia; [3]Hedi Chaker Hospital, Sfax, Tunisia; [4]Fattouma Bourguiba Hospital, Hematology Department, Monastir, Tunisia; [5]Farhat Hached Hospital, Hematology Department, Sousse, Tunisia; [6]Habib Bourguiba Hospital, Sfax, Tunisia; [7]Mohamed Ben Sassi Hospital, Hematology Department, Gabes, Tunisia; [8]centre de greffe de moelle osseuse, Hematology and SCT Department, Tunis, Tunisia; [9]Aziza Othmana Hospital, Tunis, Tunisia; [10]Hedi Chaker Hospital, Hematology Department, Sfax, Tunisia;*

### Background

Marginal zone lymphomas (MZL) represents a heterogeneous and rare subgroup of non-Hodgkin lymphomas (NHL), accounting for approximately 5% to 10% of all cases. LZM comprises three main entities: splenic MZL (SMZL), nodal MZL (NMZL), and extranodal MZL of mucosa-associated lymphoid tissue, more commonly known as MALT lymphoma. Data are limited in developing countries regarding the clinicopathologic features and outcome of MZL.

### Aims

The objective of this study is to report on the clinicoepidemiologic features of MZL in Tunisia and their long-term outcome.

### Methods

We evaluated retrospectively all patients diagnosed with MZL from Jan 2015 to Dec 2024 across six hematology centers and one oncology center in Tunisia. Baseline characteristics, treatments and survival were collected.

**Copyright Information:** (Online) ISSN: 2572-9241
©2026 The Author(s). HemaSphere is published by John Wiley & Sons Ltd on behalf of European Hematology Association. This is an open access Abstract Book distributed under the Attribution-NonCommercial-NoDerivs (CC BY-NC-ND), which allows third parties to download the articles and share them with others as long as they credit the author and the Abstract Book, but they cannot change the content in any way or use them commercially.
**Abstract Book Citations:** Authors, Title, HemaSphere, 2026;10:(S1):pages. The abstract book DOIs can be found on the "Author Information" page.
**Disclaimer:** Articles published in the journal HemaSphere exclusively reflect the opinions of the authors. The authors are responsible for all content in their abstracts including accuracy of the facts, statements, citing resources, etc.

### Results

A total of 228 patients diagnosed with MZL were collected. The most frequent MZL was splenic, followed by MALT and nodal lymphoma with 43%, 41% and 16% of cases, respectively. The most frequent primary site locations for MALT lymphoma were gastric, pulmonary, ocular adnexal, parotid gland, with 48%, 17%, 14%, 7%, respectively.The median age for the entire cohort was 60 years (range 27-87). The median age for the MALT, splenic and nodal MZL cohort were 58 years (range 27-82), 62 years (range 28-87), 63 years (range 36-86), respectively (p=0.29). MZL-IPI were high in 7%, 47% and 11% of MALT, splenic and nodal lymphoma, respectively. Advanced-stage disease at diagnosis was found in 27%, 46%, 99%, and 78% of gastric MALT, non-gastric MALT, splenic, and nodal lymphomas, respectively.
Active surveillance was the initial management for 71 (31%) patients. The most prescribed therapies were rituximab-chlorambucil in the MALT (39%) and SMZL population (40%) and R-CHOP (70%) in the NMZL population. At the end of the first line, the overall response rate (ORR) was 79%, 71%, 64%, and 84% for gastric MALT, non-gastric MALT, splenic, and nodal lymphomas, respectively. At a median follow-up of 5 years, the overall survival (OS) was 79.4% for the entire cohort and 88.2%, 72.5% and 75% for the MALT, splenic MZL and nodal MZL, respectively (p=0.042). In the multivariate analysis, the independent predictors of OS were age >60 years (p=0.037), treatment failure (0.01), and bone marrow involvement (0.035). The 5-year progression-free survival (PFS) was 82.5% for the entire cohort and 87.1%, 78.8% and 80.6% for the MALT, and splenic MZL and nodal MZL, respectively (p=0.31). In the multivariate analysis, the independent predictors of PFS was bone marrow involvement (0.043). Transformed lymphoma occurred after a median delay of 23 months [13-54], cumulative incidence was 8.1% of the entire cohort (3.9%,11.6% and 8.6% in the MALT, splenic MZL and nodal MZL, respectively (p=0.35)).

### Summary/Conclusion

The clinical landscape was characterized by a predominance of SMZL and MALT subtypes. Gastric involvement was observed in 48% of cases. The patient cohort was relatively young, with a median age of 60 years. Active surveillance was the initial management in 31% of the cases. Rituximab-chlorambucil and R-CHOP were the most common treatment regimen for SMZL/MALT and NMZL, respectively. MZL represents a very heterogeneous category of NHL with excellent OS and PFS, especially in cases of MALT lymphoma.

**Copyright Information:** (Online) ISSN: 2572-9241
©2026 The Author(s). HemaSphere is published by John Wiley & Sons Ltd on behalf of European Hematology Association. This is an open access Abstract Book distributed under the Attribution-NonCommercial-NoDerivs (CC BY-NC-ND), which allows third parties to download the articles and share them with others as long as they credit the author and the Abstract Book, but they cannot change the content in any way or use them commercially.
**Abstract Book Citations:** Authors, Title, HemaSphere, 2026;10:(S1):pages. The abstract book DOIs can be found on the "Author Information" page.
**Disclaimer:** Articles published in the journal HemaSphere exclusively reflect the opinions of the authors. The authors are responsible for all content in their abstracts including accuracy of the facts, statements, citing resources, etc.

### (PB3658) ESTABLISHING REAL-WORLD SURVIVAL BENCHMARKS FOR MANTLE CELL LYMPHOMA PRIOR TO FRONTLINE BTK INHIBITORS: A MULTICENTRE STUDY

**Topic:** 18. Indolent and mantle-cell non-Hodgkin lymphoma - Clinical

Lawrence Cheng Kiat Ng*[1], Michelle Limei Poon[2], Joshua Loke[1], Xiu Hue Lee[1], Jing Yuan Tan[1], Shin Yeu Ong[1], Yunxin Chen[1], Chandramouli Nagarajan[1], Francesca Lorraine Wei Inng Lim[1], Aditi Manjeri Subramaniam[1], Yeow Tee Goh[1], Esther Hian Li Chan[2], Yogeshini Batumalai[2], Jayalakshmi .[2], Ya Hwee Tan[3], Esther Wei Yin Chang[3], Nagavalli Somasundaram[3], Jason Yongsheng Chan[3], Jianbang Chiang[3]

*[1]Singapore General Hospital, Haematology, Singapore, Singapore;[2]National Cancer Institute Singapore, Haematology, Singapore, Singapore; [3]National Cancer Centre Singapore, Medical Oncology, Singapore, Singapore;*

### Background

Mantle cell lymphoma (MCL) is a rare B-cell non-Hodgkin lymphoma with heterogeneous disease behaviour. Chemoimmunotherapy (CIT), autologous stem cell transplantation (ASCT) and rituximab maintenance (RM), remains a cornerstone of frontline treatment. Recent randomized controlled trials demonstrated the benefit of incorporating Bruton tyrosine kinase inhibitors (BTKi) in the frontline setting, representing a potentially practice-changing approach. Real-world outcome data from Southeast Asian populations remain limited.

### Aims

To describe the real-world characteristics and outcomes of MCL patients treated with CIT prior to the incorporation of BTKi in the frontline setting.

### Methods

This multicentre retrospective study included MCL patients diagnosed and treated at three cancer institutes in Singapore between January 2001 and June 2025. Clinical characteristics and treatment outcomes were extracted from medical records. Patients managed with observation alone, palliative-intent chemotherapy, non-chemotherapy or frontline BTKi were excluded.

**Copyright Information:** (Online) ISSN: 2572-9241
©2026 The Author(s). HemaSphere is published by John Wiley & Sons Ltd on behalf of European Hematology Association. This is an open access Abstract Book distributed under the Attribution-NonCommercial-NoDerivs (CC BY-NC-ND), which allows third parties to download the articles and share them with others as long as they credit the author and the Abstract Book, but they cannot change the content in any way or use them commercially.
**Abstract Book Citations:** Authors, Title, HemaSphere, 2026;10:(S1):pages. The abstract book DOIs can be found on the "Author Information" page.
**Disclaimer:** Articles published in the journal HemaSphere exclusively reflect the opinions of the authors. The authors are responsible for all content in their abstracts including accuracy of the facts, statements, citing resources, etc.

### Results

Eighty-four patients were included, with 42 in the high-intensity cohort (NORDIC, hyper-CVAD, or R-CHOP/R-DHAP) and 42 in the low-intensity cohort (BR/RBAC or R-CHOP). Baseline characteristics are summarized in Table 1.
In the high-intensity cohort, following induction therapy, complete response (CR), partial response (PR), and stable/progressive disease (SD/PD) occurred in 75%, 18%, and 8% of patients, respectively. After a median follow-up of 75 months, 55% progressed and 24% died, with similar rates of lymphoma- and non-lymphoma-related mortality, resulting in 5-year PFS and OS of 47% and 92%.
In the low-intensity cohort, following induction therapy, CR, PR, and SD/PD rates were 54%, 22%, and 24%. With a median follow-up of 65 months, 60% progressed and 40% died, most commonly from lymphoma-related causes, yielding 5-year PFS and OS of 40% and 70%.
On univariate analysis, elevated LDH was associated with inferior PFS (HR 2.05, p=0.02), and Ki-67 >30% with inferior OS (HR 2.8, p=0.03). RM was associated with improved PFS (HR 0.48, p=0.02) and OS (HR 0.32, p=0.02), while ASCT was associated with superior OS (HR 0.32, p=0.046). On multivariate analysis, RM remained independently associated with improved PFS (HR 0.41, p=0.02), and Ki-67 >30% remained an adverse prognostic factor for OS (HR 2.9, p=0.03).
Table 1.

| | | Intensive Therapies (n=42) | % | Non-intensive Therapies (n=42) | % |
|---|---|---|---|---|---|
| Median age | | 58 (26-74) | | 69 (41-89) | |
| Gender | Male | 22/42 | 52 | 21/42 | 50 |
| | Female | 20/42 | 48 | 21/42 | 50 |
| ECOG | 0-1 | 40/42 | 95 | 38/42 | 91 |
| | 2-3 | 2/42 | 5 | 4/42 | 9 |
| Stage | 1-2 | 4/42 | 10 | 3/42 | 7 |
| | 3-4 | 38/42 | 90 | 39/42 | 93 |
| Elevated LDH | | 29/42 | 69 | 24/41 | 59 |
| Elevated WBC | | 17/41 | 42 | 18/41 | 44 |
| Thrombocytopenia | | 10/41 | 24 | 11/41 | 27 |
| Ki67 | ≤30% | 17/34 | 50 | 23/38 | 61 |
| | >30% | 17/34 | 50 | 15/38 | 39 |
| Mipi-C | Low | 6/34 | 18 | 3/37 | 8 |
| | Low-int | 11/34 | 32 | 12/37 | 32 |
| | Int-High | 13/34 | 38 | 13/37 | 35 |
| | High | 4/34 | 12 | 9/37 | 24 |
| Treatment Regimens | NORDIC | 23/42 | 55 | | |
| | RCHOP/RDHAP | 7/42 | 17 | | |
| | HCVAD | 12/42 | 29 | | |
| | RCHOP-based | | | 13/42 | 31 |
| | BR/RBAC | | | 29/42 | 69 |
| ASCT consolidation | | 19/42 | 45 | 3/42 | 7 |
| Rituximab Maintenance | | 26/42 | 62 | 12/42 | 29 |

### Summary/Conclusion

This study establishes a Southeast Asian real-world survival benchmark for patients with MCL treated with CIT. Inferior survival outcomes were observed among patients receiving low-intensity therapy and those with elevated LDH or high Ki-67 indices, whereas rituximab maintenance conferred significant survival benefit. These findings provide an important reference for evaluating emerging frontline BTKi strategies and support prioritizing higher-risk patients for treatment intensification.

**Copyright Information:** (Online) ISSN: 2572-9241
©2026 The Author(s). HemaSphere is published by John Wiley & Sons Ltd on behalf of European Hematology Association. This is an open access Abstract Book distributed under the Attribution-NonCommercial-NoDerivs (CC BY-NC-ND), which allows third parties to download the articles and share them with others as long as they credit the author and the Abstract Book, but they cannot change the content in any way or use them commercially.
**Abstract Book Citations:** Authors, Title, HemaSphere, 2026;10:(S1):pages. The abstract book DOIs can be found on the “Author Information” page.
**Disclaimer:** Articles published in the journal HemaSphere exclusively reflect the opinions of the authors. The authors are responsible for all content in their abstracts including accuracy of the facts, statements, citing resources, etc.

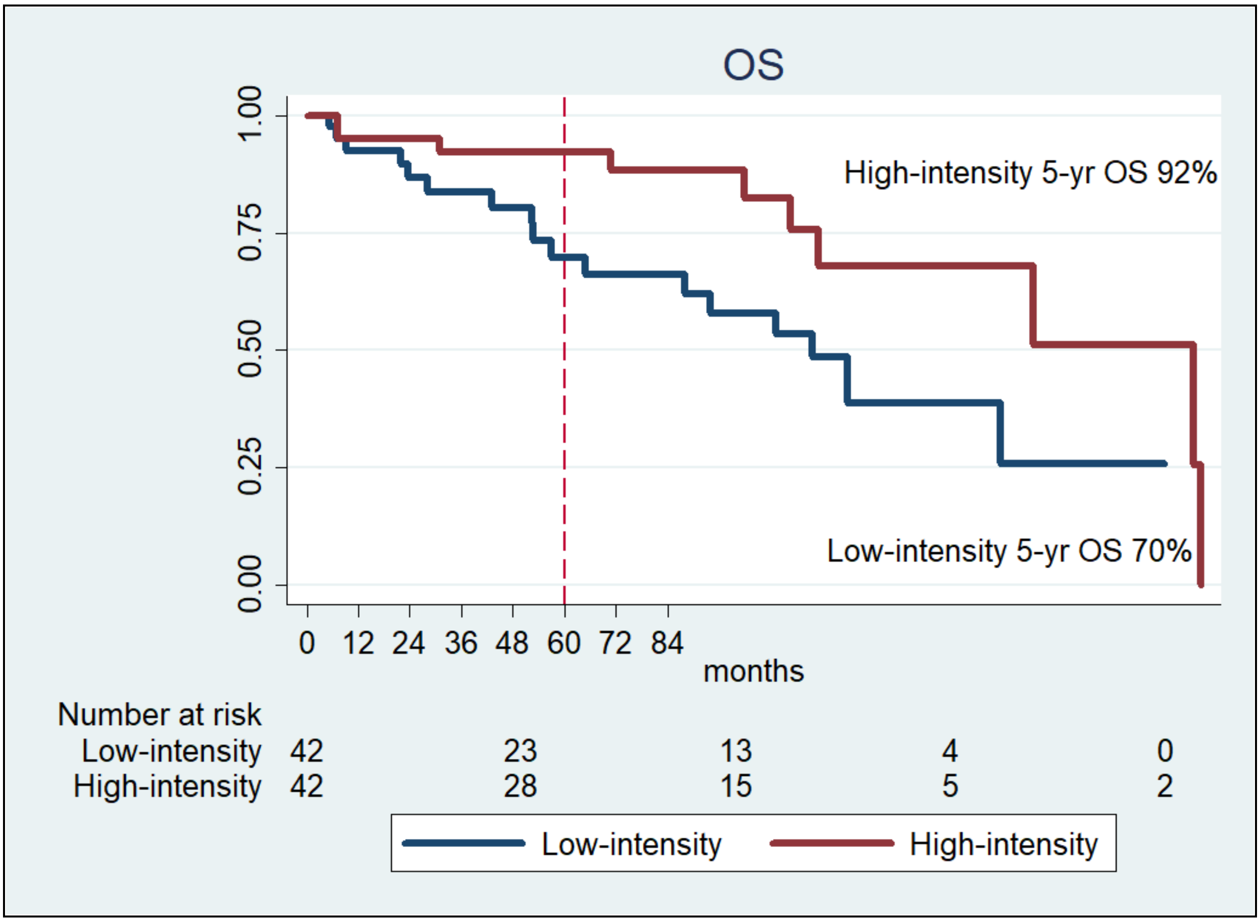

**Copyright Information:** (Online) ISSN: 2572-9241
©2026 The Author(s). HemaSphere is published by John Wiley & Sons Ltd on behalf of European Hematology Association. This is an open access Abstract Book distributed under the Attribution-NonCommercial-NoDerivs (CC BY-NC-ND), which allows third parties to download the articles and share them with others as long as they credit the author and the Abstract Book, but they cannot change the content in any way or use them commercially.
**Abstract Book Citations:** Authors, Title, HemaSphere, 2026;10:(S1):pages. The abstract book DOIs can be found on the "Author Information" page.
**Disclaimer:** Articles published in the journal HemaSphere exclusively reflect the opinions of the authors. The authors are responsible for all content in their abstracts including accuracy of the facts, statements, citing resources, etc.

### (PB3660) REAL-WORLD TREATMENT PATTERNS AND OUTCOMES IN ADVANCED-STAGE MYCOSIS FUNGOIDES: A 26-CASE RETROSPECTIVE COHORT STUDY FROM CHINA

**Topic:** 18. Indolent and mantle-cell non-Hodgkin lymphoma - Clinical

Shanshan He[1], Li Zeng[1], Xue Meng[1], Yanfeng MA[1], Yao Xie[2], Lin Wang[2], Liqun Zou[2], Ming Jiang*[1, 2]

[1]*Chengdu Shangjinnanfu Hospital, Chengdu, China;* [2]*West China Hospital of Sichuan University, Chengdu, China;*

### Background

Advanced-stage (IIB-IV) mycosis fungoides (MF) carries a poor prognosis, with no standardized treatment sequencing guidelines. Despite the emergence of novel agents, real-world data on their use across multiple lines of therapy remain limited.

### Aims

To characterize real-world features, treatment patterns, and outcomes in advanced-stage MF patients receiving ≥2 lines of therapy.

### Methods

We retrospectively analyzed 26 patients with stage IIB-IV MF who received ≥2 lines of systemic therapy (2003–2025). Baseline characteristics, treatment regimens (lines 2–7), response per mSWAT, PFS, and OS were collected. Descriptive statistics and Kaplan-Meier methods were applied.

### Results

Patient characteristics: Median age was 56 years (range 19-73); stage IIB in 13 (50%), III in 7 (27%), and IV in 6 (23%). Folliculotropic MF (FMF) accounted for 4 cases (15%), large cell transformation (LCT) in 14 (54%), and CD30 expression ≥10% in 11 (42%). Median lines of prior therapy: 3.5 (range 2-7).
Overall treatment patterns: 97 treatment lines were recorded. Most used agents: BV (81%, 21), HDAC inhibitors (58%, 15), golidocitinib (46%, 12), liposomal mitoxantrone (31%, 8),reflecting highly individualized sequencing in real-world practice.
BV treatment patterns and efficacy: 21 patients received BV (line 1: 2, line 2: 5, ≥line 3: 14); 17 as monotherapy. Median BV cycles: 7 (range 1-18). ORR was 80% (17/21), ORR4 (response lasting ≥4 months) was 71.4% (15/21), and median PFS was 12.0 months (95% CI 3.0-31.0). ORR4 was 100% (7/7) in CD30-high (≥10%) vs. 72.7% (8/11) in CD30-low patients; and 91.7% (11/12) in LCT-positive vs. 71.4% (5/7) in LCT-negative patients. Reasons for BV discontinuation: disease progression (9, 42.9%), transition to maintenance therapy after planned cycles (7, 33.3%). Among the 4 patients who received interferon maintenance post-BV, all remain progression-free with median PFS not reached (range 8-38 months).
Post-BV therapy: Among 11 patients progressing on BV, subsequent treatments included liposomal mitoxantrone (4), HDAC inhibitors (3), combination chemotherapy (2), and best supportive care (2). Median PFS on post-BV therapy was 5.0 months.
Other agents:HDAC inhibitors (n=15): ORR4 40% (6/15), median PFS 4.0 months (95% CI 1.0–8.0). Liposomal mitoxantrone (n=8; all ≥3rd line, median line 5): ORR 50% (4/8, all PR); survival data for liposomal mitoxantrone (median line 5) are immature due to limited follow-up in this late-line setting.
Golidocitinib in FMF: Of 4 FMF patients, 3 received second-line golidocitinib (all CD30<10%). All achieved sustained remission (duration: 43, 41, and 8 months; all progression-free at cutoff), with no grade ≥3 AEs leading to discontinuation. The one FMF patient not receiving golidocitinib died of progression after 4 lines within 2 years.
Survival outcomes: With a median follow-up of 63.4 months (range 9-228), median OS was not reached. The 3-year OS rate was 91% (95% CI 68.6%-97.7%) and the 5-year OS rate was 74.4% (95% CI 48.5%-88.6%). All 3 FMF patients receiving golidocitinib remain alive and progression-free.

**Copyright Information:** (Online) ISSN: 2572-9241
©2026 The Author(s). HemaSphere is published by John Wiley & Sons Ltd on behalf of European Hematology Association. This is an open access Abstract Book distributed under the Attribution-NonCommercial-NoDerivs (CC BY-NC-ND), which allows third parties to download the articles and share them with others as long as they credit the author and the Abstract Book, but they cannot change the content in any way or use them commercially.
**Abstract Book Citations:** Authors, Title, HemaSphere, 2026;10:(S1):pages. The abstract book DOIs can be found on the "Author Information" page.
**Disclaimer:** Articles published in the journal HemaSphere exclusively reflect the opinions of the authors. The authors are responsible for all content in their abstracts including accuracy of the facts, statements, citing resources, etc.

**Summary/Conclusion**

This real-world cohort with extended follow-up demonstrates: 1) BV is highly active in advanced MF, with post-BV interferon maintenance offering durable disease control; 2) Post-BV options remain limited with modest efficacy; 3) Golidocitinib produced durable responses in FMF, suggesting JAK inhibition warrants prospective study in this subtype.

**Copyright Information:** (Online) ISSN: 2572-9241
©2026 The Author(s). HemaSphere is published by John Wiley & Sons Ltd on behalf of European Hematology Association. This is an open access Abstract Book distributed under the Attribution-NonCommercial-NoDerivs (CC BY-NC-ND), which allows third parties to download the articles and share them with others as long as they credit the author and the Abstract Book, but they cannot change the content in any way or use them commercially.
**Abstract Book Citations:** Authors, Title, HemaSphere, 2026;10:(S1):pages. The abstract book DOIs can be found on the "Author Information" page.
**Disclaimer:** Articles published in the journal HemaSphere exclusively reflect the opinions of the authors. The authors are responsible for all content in their abstracts including accuracy of the facts, statements, citing resources, etc.

### (PB3661) ORELABRUTINIB WITH ANTI-CD20 MONOCLONAL ANTIBODIES IS HIGHLY EFFECTIVE AND WELL TOLERATED AS FIRST-LINE THERAPY FOR MARGINAL ZONE LYMPHOMA

**Topic:** 18. Indolent and mantle-cell non-Hodgkin lymphoma - Clinical

Na Yao*[1], Cheng-Ji Wang[1], Liang Wang[1]

[1]*Beijing Tongren Hospital, Capital Medical University, Beijing, China;*

### Background

Marginal zone lymphoma (MZL) is an indolent subtype of non-Hodgkin lymphoma with heterogeneous clinical features. First-line treatment typically involves anti-CD20 monoclonal antibodies with or without chemotherapy. Although Bruton's tyrosine kinase (BTK) inhibitors have shown efficacy in relapsed/refractory MZL, their role in frontline therapy remains underexplored.

### Aims

In this retrospective analysis, we report the outcomes of 25 treatment-naïve MZL patients treated with the CD20 monoclonal antibody combined with orelabrutinib, aiming to explore the potential role of this novel combination in frontline therapy of MZL.

### Methods

This single-center, retrospective study included 25 treatment-naïve MZL patients who received orelabrutinib (150 mg once daily) plus either rituximab or obinutuzumab for six cycles, followed by orelabrutinib maintenance for up to 6 months. The primary endpoint was overall response rate (ORR) per Lugano 2014 criteria. Secondary endpoints included progression-free survival (PFS), overall survival (OS), and safety.

### Results

The ORR was 96.0%, comprising 12 complete responses (CR) and 12 partial responses (PR), with one case of stable disease (SD). All 13 patients with ocular adnexal MZL (OAML) responded: 5 achieved CR and 8 PR. Regarding anti-CD20 agents, the rituximab subgroup (n=10) showed 5 CR and 5 PR, while the obinutuzumab subgroup (n=15) had 7 CR, 7 PR, and 1 SD, with no significant difference in CR rate (50.0% vs. 46.7%, p=1.00). Treatment-related adverse events occurred in 32.0% of patients; neutropenia (20.0%) and thrombocytopenia (12.0%) were most common. Grade 3–4 events occurred in 12.0% of patients, with no significant differences in safety profiles between the rituximab and obinutuzumab subgroups.

### Summary/Conclusion

CD20 antibodies combined with orelabrutinib may be a feasible low-toxicity first-line option for MZL, with favorable short-term efficacy and tolerability observed in our cohort. These preliminary findings warrant confirmation in larger prospective, randomized studies to establish long-term benefit and safety.

**Copyright Information:** (Online) ISSN: 2572-9241
©2026 The Author(s). HemaSphere is published by John Wiley & Sons Ltd on behalf of European Hematology Association. This is an open access Abstract Book distributed under the Attribution-NonCommercial-NoDerivs (CC BY-NC-ND), which allows third parties to download the articles and share them with others as long as they credit the author and the Abstract Book, but they cannot change the content in any way or use them commercially.
**Abstract Book Citations:** Authors, Title, HemaSphere, 2026;10:(S1):pages. The abstract book DOIs can be found on the "Author Information" page.
**Disclaimer:** Articles published in the journal HemaSphere exclusively reflect the opinions of the authors. The authors are responsible for all content in their abstracts including accuracy of the facts, statements, citing resources, etc.

### (PB3662) ACALABRUTINIB PLUS RITUXIMAB FOR THE TREATMENT OF UNTREATED, ELDERLY OR YOUNG WITH LOW- TO INTERMEDIATE- RISK MANTLE CELL LYMPHOMA: AN EXPLORATORY, MULTI-CENTER, SINGLE-ARM, PHASE II STUDY

**Topic:** 18. Indolent and mantle-cell non-Hodgkin lymphoma - Clinical

Qingqing Cai*[1], Yi Xia[1], Huiqiang Huang[1], Shuhua Yi[2], Ying Zhao[3], Yun Duan[4], Yirong Jiang[5], Runhui Zheng[6], Guowei Li[7], Bing Bai[1], Xiaoxiao Wang[1], Xiaojie Fang[1], Hang Yang[1], Man Nie[1], Yuchen Zhang[1], Yi Cao[1]

*[1]Sun Yat-sen University Cancer Center, Guangzhou, China; [2]Institute of Hematology and Blood Diseases Hospital, Chinese Academy of Medical Science and Peking Union Medical College, Tianjin, China; [3]The First People's Hospital of Foshan, Foshan, China; [4]Gansu Provincial Hospital, Department of Hematology, Lanzhou, China; [5]Dongguan People's Hospital, Dongguan, China; [6]The Fifth Affiliated Hospital of Guangzhou Medical University, Guangzhou, China; [7]Huizhou Central People's Hospital, Huizhou, China;*

### Background

Targeting B-cell receptor (BCR) signaling via Bruton's tyrosine kinase (BTK) inhibition benefits patients with mantle cell lymphoma (MCL). Acalabrutinib, a selective covalent BTK inhibitor, is effective in MCL and approved in many countries. Studies have suggested that acalabrutinib plus rituximab is promising in untreated elderly MCL. In the phase II CHESS study, we found that BTK inhibitor plus rituximab induction followed by shortened cytarabine-based chemotherapy offered a "less-chemo" frontline strategy. These results support exploring frontline "chemo-free" options in selected patients. We therefore initiated an exploratory phase II study of acalabrutinib plus rituximab as frontline therapy for elderly MCL patients and younger patients with low- to intermediate-risk disease.

### Aims

This multicenter, phase II study is planning to enroll 50 patients to assess the efficacy and safety of the combination of acalabrutinib plus rituximab.

### Methods

Key inclusion criteria are (1)Age ≥18 years; (2)Previously untreated histologically confirmed CD20+ mantle cell lymphoma; (3)Ann Arbor stage II-IV; (4)If young patients (<65 years) must meet all of criteria: a)Low to intermediate risk according to simplified MIPI (0-5); b)Ki67 < 50%; c)No TP53 mutation by NGS testing; d)Lesion diameter ≤5 cm; e)Non-blastoid, polymorphic disease. Treatment with acalabrutinib and rituximab will be continued until an unacceptable drug-related toxicity occurs or until treatment for 24 months or until disease progression, whichever occurs first. For young patients (<65 years) who couldn't get response after treatment of acalabrutinib plus rituximab for 12 months will be regarded as treatment failure and discontinue AR treatment. The primary endpoint is the complete response rate (CRR) at 12 months per the Lugano criteria. Secondary endpoints include objective response rate (ORR) at 12 months, duration of response (DOR), progression-free survival (PFS), overall survival (OS), and safety. Efficacy and safety will be summarized with descriptive statistics, while time-to-event endpoints will be analyzed with the Kaplan-Meier method. Enrollment is currently ongoing. Clinical trial identification: NCT06846489.

**Copyright Information:** (Online) ISSN: 2572-9241
©2026 The Author(s). HemaSphere is published by John Wiley & Sons Ltd on behalf of European Hematology Association. This is an open access Abstract Book distributed under the Attribution-NonCommercial-NoDerivs (CC BY-NC-ND), which allows third parties to download the articles and share them with others as long as they credit the author and the Abstract Book, but they cannot change the content in any way or use them commercially.
**Abstract Book Citations:** Authors, Title, HemaSphere, 2026;10:(S1):pages. The abstract book DOIs can be found on the "Author Information" page.
**Disclaimer:** Articles published in the journal HemaSphere exclusively reflect the opinions of the authors. The authors are responsible for all content in their abstracts including accuracy of the facts, statements, citing resources, etc.

### (PB3663) INCREASED EXPRESSION OF TIGIT(+)CD226(+)CD16(+)CD56(+) CELLS IN PATIENTS WITH AGVHD AFTER ALLOGENEIC HEMATOPOIETIC STEM CELL TRANSPLANTATION

**Topic:** 18. Indolent and mantle-cell non-Hodgkin lymphoma - Clinical

Yang Liu*[1], Tao Wu[1]

[1] *The 940th Hospital of Joint Logistics Support force of Chinese People's Liberation Army, hematology, Gansu, China;*

### Background

Allogeneic hematopoietic stem cell transplantation (allo-HSCT), as a mean to combat hematologic diseases, can effectively curb the rapid progression of the disease, improve the quality of patient survival, and prolong the survival of patients, and has become an indispensable part of the field of treatment of hematologic disorders after several decades of development. Despite the fact that allo-HSCT is currently used to combat a wide range of blood-related problems, severe post-transplantation complications remain the most important reason why some patients do not benefit from allo-HSCT, with graft-versus-host disease (GVHD) arguably being emblematic. The T cell immune receptor with Ig and ITIM domains (TIGIT), a "new rising emerging star" of the polio virus/nectin receptor family, was first published in 2009. Although studies related to TIGIT and hematologic disorders are constantly updated and some new drug trials targeting TIGIT are proceeding at a rapid pace, is TIGIT involved in the onset and progression of aGVHD? Can targeted therapies against TIGIT improve aGVHD and thus improve patient prognosis? These questions still need to be further explored.

### Aims

Acute graft-versus-host disease (aGVHD) is one of the most common complications after allogeneic hematopoietic stem cell transplantation (allo-HSCT).T cell immune receptor with Ig and ITIM domains (TIGIT), as an immune checkpoint-inhibitory receptor, can exert immunosuppressive effects by down-regulating the effector function of T cells after competing with CD226 for binding to the same ligand, CD155. And the binding of CD226 to CD155 exerts an immunoregulatory effect (in contrast to the function of TIGIT). Their role in aGVHD remains to be further investigated

### Methods

In this study, we divided the patients into non-aGVHD and aGVHD groups, and used multiparameter flow cytometry to detect the expression levels of TIGIT, CD226 and CD155 in peripheral blood and bone marrow lymphocytes (CD4+, CD8+, CD16+56+) at 30, 60 and 90 days post-transplantation. Then, the expression levels of TIGIT, CD226 and CD155 were compared within the aGVHD group at different times after transplantation

### Results

When comparing between non-aGVHD and aGVHD groups we found that at 30 days post-transplantation, peripheral blood TIGIT(+)CD226(+)CD16(+)CD56(+) cells expression levels were elevated in the aGVHD group compared to the non-aGVHD group. When comparing within the aGVHD group, there was no difference in the expression levels of TIGIT, CD226, and CD155 between the two groups, regardless of whether the primary disease, the time of aGVHD onset, and the type of aGVHD were the same or not.

**Copyright Information:** (Online) ISSN: 2572-9241
©2026 The Author(s). HemaSphere is published by John Wiley & Sons Ltd on behalf of European Hematology Association. This is an open access Abstract Book distributed under the Attribution-NonCommercial-NoDerivs (CC BY-NC-ND), which allows third parties to download the articles and share them with others as long as they credit the author and the Abstract Book, but they cannot change the content in any way or use them commercially.
**Abstract Book Citations:** Authors, Title, HemaSphere, 2026;10:(S1):pages. The abstract book DOIs can be found on the "Author Information" page.
**Disclaimer:** Articles published in the journal HemaSphere exclusively reflect the opinions of the authors. The authors are responsible for all content in their abstracts including accuracy of the facts, statements, citing resources, etc.

**Summary/Conclusion**

The peripheral blood TIGIT(+)CD226(+)CD16(+)CD56(+) cells expression level was elevated in the aGVHD group compared to the non-aGVHD group at 30 days after transplantation. It is suggested that TIGIT/CD226-CD155 may be involved in the development of aGVHD. In the next step, we will try to expand the case size and conduct a joint study with a multicenter, with the aim of more clearly elaborating the effect of TIGIT/CD226-CD155 in aGVHD.

**Copyright Information:** (Online) ISSN: 2572-9241
©2026 The Author(s). HemaSphere is published by John Wiley & Sons Ltd on behalf of European Hematology Association. This is an open access Abstract Book distributed under the Attribution-NonCommercial-NoDerivs (CC BY-NC-ND), which allows third parties to download the articles and share them with others as long as they credit the author and the Abstract Book, but they cannot change the content in any way or use them commercially.
**Abstract Book Citations:** Authors, Title, HemaSphere, 2026;10:(S1):pages. The abstract book DOIs can be found on the "Author Information" page.
**Disclaimer:** Articles published in the journal HemaSphere exclusively reflect the opinions of the authors. The authors are responsible for all content in their abstracts including accuracy of the facts, statements, citing resources, etc.

### (PB3664) RESULTS OF TREATMENT OF RELAPSE AND REFRACTORY (R/R) FOLLICULAR LYMPHOMA (FL) IN REAL CLINICAL PRACTICE IN THE RUSSIAN FEDERATION.

**Topic:** 18. Indolent and mantle-cell non-Hodgkin lymphoma - Clinical

Anna Smolianinova*[1], Lyubov Plastinina[1], Eduard Gemdzhian[1], Evgeniy Zvonkov[1], Vera Troitskaya[1], Elena Parovichnikova[1]

[1]*National Medical Research Center for Hematology, 125167, Moscow, Russian Federation, Moscow, Russia;*

### Background

FL is the most common indolent non-Hodgkin lymphoma in the world. The tumor is highly chemosensitive, but most patients do not achieve complete remission (CR). Data on the effectiveness of therapy and survival rates for patients with r/rFL in real clinical practice is limited. The main published real-world studies covered the period before the introduction of new drug classes such as bispecific antibodies (BsAbs) and CAR-T, and no such analysis has been conducted in Russia.

### Aims to characterize the real practice treatment patterns and outcomes in patients with r/rFL in Russia.

### Methods

This retrospective analysis used database of telemedicine consultations at National Medical Research Center for Hematology, Moscow conducted from 2019 to 2025. Medical cards of adult patients with FL who received 2+ line of therapy (LoT) were selected. Information about each included patient was obtained from local databases and cancer registries.

### Results

193 patients with r/rFL were selected from 71 cities in 27 regions of the Russia. The median age was 48 years (22-78 years). Stage III/IV was detected in 63% (118/187) of the patients, and PET-CT was performed in 40% (78/193) of the cases. Bone marrow biopsy was performed in 35% (67/193) of patients, and the bone marrow involvement detected in 79% (53/67). FL I/IIIA and 3B grades were observed in 92% (160/173) and 8% (13/173) of patients, respectively. The most frequent treatment options for lines 1-7 are presented in **Table. 1**. The overall response rate was 43% (78/181), 37% (36/97), 29% (14/48), 28% (4/18), and 9% (1/11) after LoT 2, 3, 4, 5, and 6, respectively. CR were achieved in 33% (59/181), 23% (22/97), 21% (9/48), 17% (3/18), and 9% (1/11) of patients after 2, 3, 4, 5, and 6 LoT, respectively. With each LoT, the median OS and PFS decreased (**Fig1**) and the proportion of patients with refractory disease increased from 39% in the 2L to 80% in the 6L. The OS of patients with failure within 12 months of starting 1LoT was significantly worse (median OS of 46 month) compared with relapses after 12-24 months and after 24 months (median OS not reached), p=0.01. Histological transformation (HT) was observed in 18% (35/193) of patients, with an average time to HT of 18 months (3-76 months). In addition, 57% (20/35) of HT cases occurred in the 12 months from the start of 1LoT. Patients with HT had a short OS (median 25 months), and HT within 12 month of starting 1LoT was associated with worse OS (median OS 14 months), p=0.01. mut/delTP53 was detected in biopsies with HT in 86% of patients (6/7). The most common cause of death was tumor (65% (64/82)).
Table 1

**Copyright Information:** (Online) ISSN: 2572-9241
©2026 The Author(s). HemaSphere is published by John Wiley & Sons Ltd on behalf of European Hematology Association. This is an open access Abstract Book distributed under the Attribution-NonCommercial-NoDerivs (CC BY-NC-ND), which allows third parties to download the articles and share them with others as long as they credit the author and the Abstract Book, but they cannot change the content in any way or use them commercially.
**Abstract Book Citations:** Authors, Title, HemaSphere, 2026;10:(S1):pages. The abstract book DOIs can be found on the “Author Information” page.
**Disclaimer:** Articles published in the journal HemaSphere exclusively reflect the opinions of the authors. The authors are responsible for all content in their abstracts including accuracy of the facts, statements, citing resources, etc.

| Therapy | 1LoT N=193 | 2LoT N=193 | 3LoT N=127 | 4LoT N=71 | 5LoT N=31 |
|---|---|---|---|---|---|
| CD20-CHOP | 76% | 16% | 6% | 2% | 0% |
| CD20-Benda | 10% | 30% | 17% | 21% | 16% |
| CD20-Platinum | 0% | 24% | 23% | 6% | 11% |
| CD20-Lena | 0% | 2% | 15% | 21% | 11% |
| Chemotherapy | 4% | 2% | 5% | 8% | 0% |
| Radiotherapy/surgery | 2% | 3% | 5% | 0% | 5% |
| R mono | 2% | 1% | 2% | 8% | 5% |
| R-NHL-BFM-90 | 3% | 1% | 2% | 8% | 5% |
| Autologous SCT | 0% | 12% | 6% | 0% | 0% |
| Experimental | 2% | 8% | 6% | 6% | 16% |
| BsAbs | 0% | 1% | 7% | 13% | 26% |
| PI3K | 0% | 0% | 4% | 4% | 5% |
| Palliative | 0% | 0% | 2% | 3% | 0% |

### Summary/Conclusion

Outcomes for r/r FL patients remain poor, the main cause of death is tumor progression. The median OS and PFS in our population of patients with r/rFL is shorter than in published studies. The most unfavorable outcomes were observed in patients with failures before 12 months and HT. With each LoT, the proportion of patients receiving BsAbs, CAR-T increased. However, in general, these drugs are currently used by a few patients with r/r FL, and it is necessary to introduce new options in real clinical practice more widely.

**Copyright Information:** (Online) ISSN: 2572-9241
©2026 The Author(s). HemaSphere is published by John Wiley & Sons Ltd on behalf of European Hematology Association. This is an open access Abstract Book distributed under the Attribution-NonCommercial-NoDerivs (CC BY-NC-ND), which allows third parties to download the articles and share them with others as long as they credit the author and the Abstract Book, but they cannot change the content in any way or use them commercially.
**Abstract Book Citations:** Authors, Title, HemaSphere, 2026;10:(S1):pages. The abstract book DOIs can be found on the "Author Information" page.
**Disclaimer:** Articles published in the journal HemaSphere exclusively reflect the opinions of the authors. The authors are responsible for all content in their abstracts including accuracy of the facts, statements, citing resources, etc.

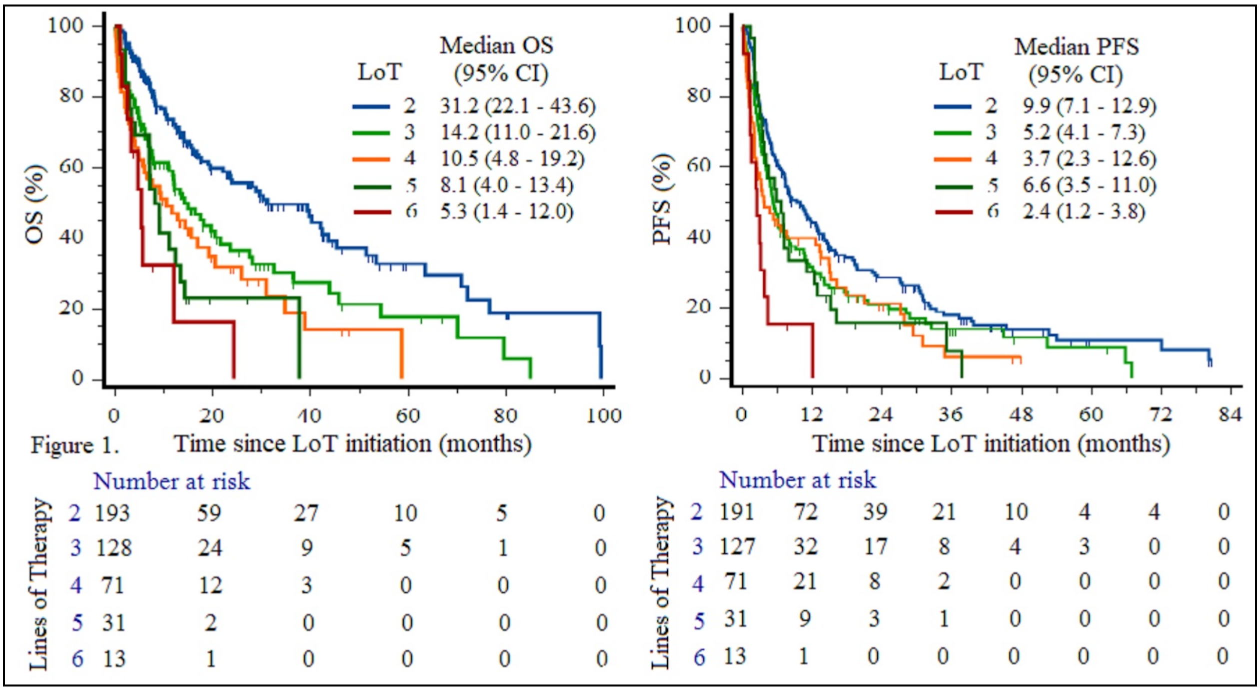

Figure 1.

**Copyright Information:** (Online) ISSN: 2572-9241
©2026 The Author(s). HemaSphere is published by John Wiley & Sons Ltd on behalf of European Hematology Association. This is an open access Abstract Book distributed under the Attribution-NonCommercial-NoDerivs (CC BY-NC-ND), which allows third parties to download the articles and share them with others as long as they credit the author and the Abstract Book, but they cannot change the content in any way or use them commercially.
**Abstract Book Citations:** Authors, Title, HemaSphere, 2026;10:(S1):pages. The abstract book DOIs can be found on the "Author Information" page.
**Disclaimer:** Articles published in the journal HemaSphere exclusively reflect the opinions of the authors. The authors are responsible for all content in their abstracts including accuracy of the facts, statements, citing resources, etc.

### (PB3665) PROGNOSTIC VALUE OF RED CELL DISTRIBUTION WIDTH (RDW) AT DIAGNOSIS IN FOLLICULAR LYMPHOMA.

**Topic:** 18. Indolent and mantle-cell non-Hodgkin lymphoma - Clinical

Marta Diaz-Lopez*[1], Chantal Le Chet-Luis[2], Dante Jorge Salamo[2], Belén Pérez-Pinilla[1], Patricia MacHado-MacHado[1], José María Raya Sánchez[1, 2], Sunil Lakhwani[1, 2]

*[1]Hospital Universitario de Canarias, Hematology and Hemotherapy, San Cristóbal de La Laguna, Spain;[2]University of La Laguna, Faculty of Medicine, San Cristobal de Laguna, Spain;*

### Background

Follicular lymphoma (FL) is the most common indolent non-Hodgkin lymphoma and is characterized by a heterogeneous clinical course. Although most patients achieve durable responses and prolonged survival, a subset experiences early progression and inferior outcomes. Prognostic scores such as FLIPI and FLIPI2 are widely used at diagnosis; however, a proportion of high-risk patients are not identified by these tools, highlighting the need for additional prognostic markers. Red cell distribution width (RDW), a routinely available parameter reflecting anisocytosis and systemic inflammation, has been associated with adverse outcomes in several malignancies, including lymphomas. However, its prognostic role in FL has not yet been well established.

### Aims

To assess whether RDW at diagnosis is associated with progression-free survival (PFS), overall survival (OS), response to first-line treatment, and POD24 (progression of disease within 24 months) in patients with FL.

### Methods

We conducted a retrospective single-center study including patients diagnosed and treated for FL between 2010 and 2017. PFS and OS were calculated from the start of first-line therapy. ROC curve analysis was used to determine the optimal RDW cutoff value for predicting PFS and OS.

**Copyright Information:** (Online) ISSN: 2572-9241
©2026 The Author(s). HemaSphere is published by John Wiley & Sons Ltd on behalf of European Hematology Association. This is an open access Abstract Book distributed under the Attribution-NonCommercial-NoDerivs (CC BY-NC-ND), which allows third parties to download the articles and share them with others as long as they credit the author and the Abstract Book, but they cannot change the content in any way or use them commercially.
**Abstract Book Citations:** Authors, Title, HemaSphere, 2026;10:(S1):pages. The abstract book DOIs can be found on the “Author Information” page.
**Disclaimer:** Articles published in the journal HemaSphere exclusively reflect the opinions of the authors. The authors are responsible for all content in their abstracts including accuracy of the facts, statements, citing resources, etc.

### Results

A total of 124 patients were included; 68 (54.8%) were male, and median age was 61 years (range 38–86). Stage IV disease was present in 66 patients (53.2%), mainly due to bone marrow involvement (60/124, 48.4%), and 51 (41.1%) had bulky disease. High-risk FLIPI and FLIPI2 scores were observed in 42/123 (34.1%) and 29/122 (23.8%) patients, respectively.
Complete response (CR) to first-line treatment was achieved in 95 patients (76.6%). Median PFS for the entire cohort was 121.1 months. After a median follow-up of 135.3 months, 50 patients (40.3%) had progressed after first-line therapy and 36 (29%) had died; median OS was not reached.
Median RDW value at diagnosis was 14.0% (range 11.6–28.4). The optimal cutoff value to discriminate both PFS and OS was 14.6%. Patients with RDW ≤14.6% had significantly longer PFS compared to those with RDW >14.6% (median PFS 172.2 vs 33.8 months; p=0.001; HR 0.45 [CI 95% 0.27-0.74]; **Figure 1A**). Similarly, OS was longer in patients with RDW ≤14.6% compared to patients with RDW >14.6% (median OS not reached vs 147.4 months; p=0.003; HR 0.39 [CI 95% 0.20-0.75]; **Figure 1B**).
We evaluated also POD24 and we found that 15 of 82 patients (18.3%) with RDW ≤ 14.6% were POD24-positive, compared to 19 of 42 patients (45.2%) with RDW > 14.6% (p =0.001). CR rate was 81.7% (67/82) for patients with RDW ≤ 14.6% versus 64.3% (27/42) for patients with RDW > 14.6% (p=0.032).
FLIPI and FLIPI2 demonstrated predictive value in the univariate analysis for PFS and for OS and we performed a multivariate model including RDW, FLIPI and FLIPI2. For PFS, RDW remains as an independent prognosis factor while FLIPI and FLIPI2 lost statistical significance. For OS, both RDW and FLIPI retained statistically significance in the multivariate model, while FLIPI2 did not.

### Summary/Conclusion

In our series, RDW at diagnosis is an independent prognosis factor for PFS an OS in FL and is also associated with CR rate and POD24. RDW may represent a simple, inexpensive, and widely available biomarker to improve risk stratification in FL. These findings warrant validation in larger multicenter studies

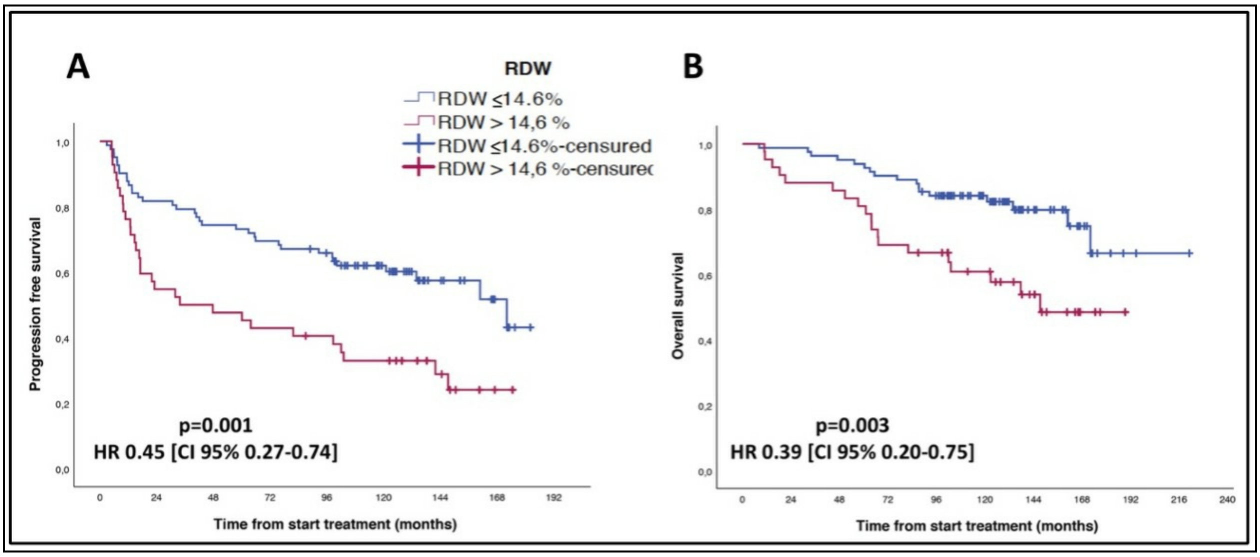

Figure 1A: PFS compared with RDW 14,6% cutoff. Figure 1B: OS compared with RDW 14,6% cutoff.

**Copyright Information:** (Online) ISSN: 2572-9241
©2026 The Author(s). HemaSphere is published by John Wiley & Sons Ltd on behalf of European Hematology Association. This is an open access Abstract Book distributed under the Attribution-NonCommercial-NoDerivs (CC BY-NC-ND), which allows third parties to download the articles and share them with others as long as they credit the author and the Abstract Book, but they cannot change the content in any way or use them commercially.
**Abstract Book Citations:** Authors, Title, HemaSphere, 2026;10:(S1):pages. The abstract book DOIs can be found on the "Author Information" page.
**Disclaimer:** Articles published in the journal HemaSphere exclusively reflect the opinions of the authors. The authors are responsible for all content in their abstracts including accuracy of the facts, statements, citing resources, etc.

### (PB3666) CLINICAL AND EVOLUTIONARY CHARACTERISTICS OF MARGINAL ZONE LYMPHOMA: A SINGLE-CENTER ALGERIAN EXPERIENCE

**Topic:** 18. Indolent and mantle-cell non-Hodgkin lymphoma - Clinical

Nassima Boulaziz*[1], Malika Allouda[1], Sabiha Gherras[1], Nadia Dali[1], Malek Benakli[2]

*[1]Hospital university Nedir Mohamed, Hematology department, Tizi-Ouzou, Algeria; [2]Pierre and Marie Curie Center, Hematology-Bone marrow transplantation, Algiers, Algeria;*

### Background

Marginal zone lymphoma (MZL) is a heterogeneous group of indolent B-cell lymphomas characterized by highly variable clinical presentations, progression, and outcomes.

### Aims

This study aimed to describe the clinical, prognostic, and evolutionary characteristics of MZL patients managed in our department.

### Methods

We conducted a retrospective descriptive study over a 15-year period (2010–2024). MZL diagnosis was confirmed via histopathology and immunohistochemistry. Out of 104 patients (pts), 74 received immunochemotherapy: R-Bendamustine (n=40), R-CHOP (n=26), R-COP (n=6), and R-Chlorambucil (n=2). Response assessment was completed by January 2026.

### Results

Among the 104 pts, 61 were men (58.7%) and 43 were women (41.3%), with a median age of 62 years (28–93). Histological distribution showed 48 pts (46%) with extranodal MZL (EMZL), 33 (31.7%) with splenic MZL (SMZL), and 23 (22.3%) with nodal MZL (NMZL). ECOG performance status < 2 in 94%. Lymphadenopathy and splenomegaly were observed in 34.6% of pts each; B symptoms were present in 47%, and bulky disease in 15%. At diagnosis, 47% of pts were stage IV and 12.5% were stage III. Bone marrow was the most frequent extranodal site (37.5%). According to the MALT-IPI, 39% were classified as intermediate-risk and 41% as high-risk. Histologic transformation to diffuse large B-cell lymphoma (DLBCL) occurred in 6 pts (5.8%). Among 72 evaluable pts: R-Bendamustine yielded a 65.8% complete response (CR) and 29% partial response (PR), with an overall response rate (ORR) of 94.8%. R-CHOP achieved an 80.7% CR and 15.4% PR (ORR 96.1%). Relapses were more frequent in the R-CHOP group (7 vs. 1), while the incidence of POD24 (progression of disease within 24 months) was similar. Grade 3–4 hematologic toxicities were common: neutropenia was higher with R-CHOP (65.4% vs. 34%), whereas thrombocytopenia was more frequent with R-Bendamustine (17.5% vs. 3.8%). After a median follow-up of 73 months (1–183), the 15-year OS was 82%, significantly higher in low/intermediate MALT-IPI groups (89% vs. 67% in high-risk; p=0.04). PFS was 68%, with a non-statistically significant trend favoring R-Bendamustine (85% vs. 68%). Multivariate analysis identified LDH and MALT-IPI as key factors influencing OS.

### Summary/Conclusion

MZL exhibits marked clinical and prognostic heterogeneity. Immunochemotherapy achieves excellent response rates; however, R-Bendamustine provides superior disease control and better hematologic tolerance compared to R-CHOP. MALT-IPI and LDH levels remain essential prognostic factors, highlighting the need for tailored risk stratification in MZL management.

**Copyright Information:** (Online) ISSN: 2572-9241
©2026 The Author(s). HemaSphere is published by John Wiley & Sons Ltd on behalf of European Hematology Association. This is an open access Abstract Book distributed under the Attribution-NonCommercial-NoDerivs (CC BY-NC-ND), which allows third parties to download the articles and share them with others as long as they credit the author and the Abstract Book, but they cannot change the content in any way or use them commercially.
**Abstract Book Citations:** Authors, Title, HemaSphere, 2026;10:(S1):pages. The abstract book DOIs can be found on the "Author Information" page.
**Disclaimer:** Articles published in the journal HemaSphere exclusively reflect the opinions of the authors. The authors are responsible for all content in their abstracts including accuracy of the facts, statements, citing resources, etc.

### (PB3667) HEPATITIS B CORE ANTIBODY POSITIVITY AS AN INDEPENDENT ADVERSE PROGNOSTIC FACTOR IN ELDERLY PATIENTS WITH FOLLICULAR LYMPHOMA

**Topic:** 18. Indolent and mantle-cell non-Hodgkin lymphoma - Clinical

Chunyuan Li*[1], Wei Wan[1], Ping Yang[1], Jiachen Liu[1], Ziyu Zhao[1], Shuozi Liu[1], Jing Wang[1], Yuan Yang[1], Hongmei Jing[1]

[1] *Peking University Third Hospital, Beijing, China;*

### Background

Follicular lymphoma (FL) is the most common indolent non-Hodgkin lymphoma, with significant heterogeneity in clinical presentation and prognosis.

### Aims

This study aimed to investigate the clinical characteristics of elderly FL patients and evaluate the prognostic impact of hepatitis B core antibody (anti-HBc) positivity.

### Methods

We retrospectively analyzed 493 FL patients treated at Peking University Third Hospital from January 2000 to July 2024. Among them, 193 patients were aged ≥60 years, and 141 elderly FL patients with complete baseline clinical data were included for analysis. Kaplan-Meier method, Cox regression model, and competing risk model were used for univariate and multivariate prognostic analyses.

### Results

The median age was 67 years (range: 60-88), with 11.6% of patients aged ≥80 years. According to simplified geriatric assessment (sCGA), 26.5% were classified as frail. FL grade 3A accounted for 31%, and 79.4% presented with Ann Arbor stage III-IV disease. With a median follow-up of 33 months (IQR: 18.5-55.6), the 2-year, 5-year, and 10-year progression-free survival (PFS) rates were 86.2%, 66.6%, and 21.4%, respectively; the corresponding overall survival (OS) rates were 89.8%, 76.1%, and 56.2%. Multivariate analysis identified FLIPI high-risk (HR=21.4, 95%CI 2.28-9.86, *P*=0.007), ECOG ≥2 (HR=19.0, 95%CI 2.90-33.57, *P*=0.002), Ki-67 high expression (HR=8.3, 95%CI 1.78-18.6, *P*=0.007), and **anti-HBc positivity (HR=7.1, 95%CI 1.74-28.68, *P*=0.006)** as independent risk factors for OS. Lymph node involvement ≥5 regions (HR=2.66, 95%CI 1.06-6.66, *P*=0.037) was an independent risk factor for PFS. Competing risk analysis further confirmed that anti-HBc positivity was an independent predictor of lymphoma-specific mortality (SHR=3.57, 95%CI 1.24-10.30, *P*=0.009).

### Summary/Conclusion

Anti-HBc positivity is an adverse prognostic factor for overall survival in elderly FL patients. Hepatitis B serological screening should be incorporated into the routine evaluation of elderly FL patients to optimize risk stratification and clinical management.

**Copyright Information:** (Online) ISSN: 2572-9241
©2026 The Author(s). HemaSphere is published by John Wiley & Sons Ltd on behalf of European Hematology Association. This is an open access Abstract Book distributed under the Attribution-NonCommercial-NoDerivs (CC BY-NC-ND), which allows third parties to download the articles and share them with others as long as they credit the author and the Abstract Book, but they cannot change the content in any way or use them commercially.
**Abstract Book Citations:** Authors, Title, HemaSphere, 2026;10:(S1):pages. The abstract book DOIs can be found on the "Author Information" page.
**Disclaimer:** Articles published in the journal HemaSphere exclusively reflect the opinions of the authors. The authors are responsible for all content in their abstracts including accuracy of the facts, statements, citing resources, etc.

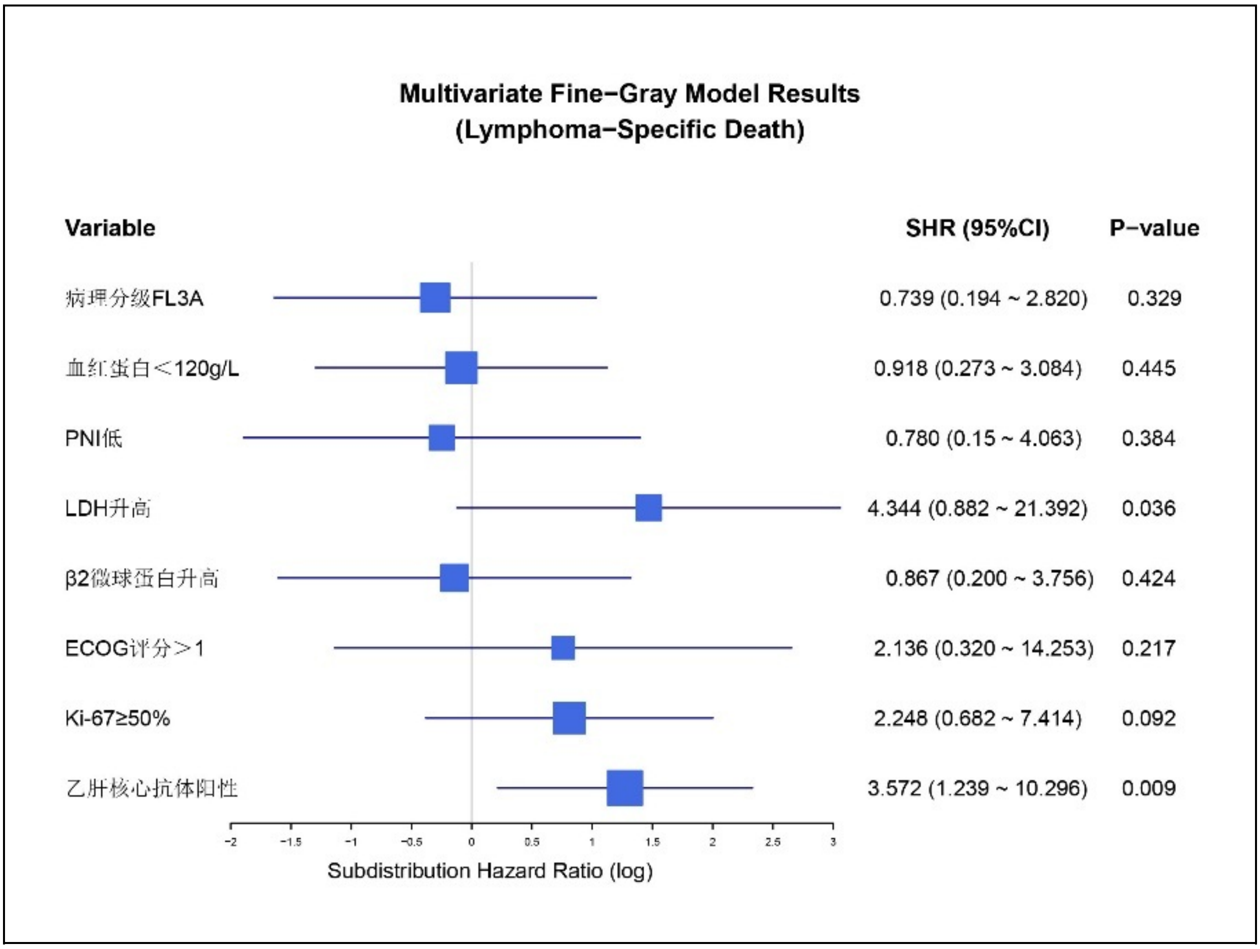

**Copyright Information:** (Online) ISSN: 2572-9241
©2026 The Author(s). HemaSphere is published by John Wiley & Sons Ltd on behalf of European Hematology Association. This is an open access Abstract Book distributed under the Attribution-NonCommercial-NoDerivs (CC BY-NC-ND), which allows third parties to download the articles and share them with others as long as they credit the author and the Abstract Book, but they cannot change the content in any way or use them commercially.
**Abstract Book Citations:** Authors, Title, HemaSphere, 2026;10:(S1):pages. The abstract book DOIs can be found on the “Author Information” page.
**Disclaimer:** Articles published in the journal HemaSphere exclusively reflect the opinions of the authors. The authors are responsible for all content in their abstracts including accuracy of the facts, statements, citing resources, etc.

### (PB3668) PRELIMINARY RESULTS OF A RETROSPECTIVE STUDY OF ORELABRUTINIB FOLLOWING WITH CHEMOTHERAPY FOR TREATMENT-NAIVE MARGINAL ZONE LYMPHOMA

**Topic:** 18. Indolent and mantle-cell non-Hodgkin lymphoma - Clinical

Kun Yang*[1], Yuping Gong[1], Ting Niu[1]

[1]*West China Hospital, Sichuan University, Department of Hematology, Chengdu, China;*

### Background

Marginal zone lymphoma (MZL) is the second most common subtype of indolent lymphoma. For MZL patients with treatment indications, anti-CD20 monoclonal antibody-based therapy is the currently accepted standard strategy. Orelabrutinib is a novel irreversible Bruton's tyrosine kinase (BTK) inhibitor that has demonstrated a high overall response rate (ORR) of 58.9% in patients with relapsed/refractory (r/r) MZL. When combined with anti-CD20 monoclonal antibodies, orelabrutinib preserves natural killer (NK) cell-mediated antibody-dependent cellular cytotoxicity (ADCC). In vitro studies have confirmed that the combination of the two agents significantly enhances tumor cell apoptosis. In a small-sample MZL study, the ORR of orelabrutinib plus rituximab reached 90%.
In addition, the presence of minimal residual disease (MRD) is closely associated with patient prognosis, and MRD negativity is significantly correlated with improved survival.

### Aims

This study aimed to systematically evaluate the efficacy and depth of response of orelabrutinib combined with anti-CD20 monoclonal antibody-based regimens in treatment-naive MZL(except gastric MALT).

### Methods

This was a single-arm, single-center retrospective study enrolling newly diagnosed MZL patients (except gastric MALT) at our hospital. All patients first received 4 courses of R-COP chemotherapy, then following orelabrutinib treatment for two yearsThe primary clinical endpoints included objective response rate, complete response rate at the end of combination therapy and three months, six months , one year, the end of two years after orelabrutinib treatment, as well as the incidence of adverse events, progression-free survival, and overall survival.

### Results

A total of 17 treatment-naive MZL patients (except gastric MALT) meeting the eligibility criteria were enrolled between February 2024 and January 2026. The median age was 54 years (range, 33–72 years); 9 patients (52.9%, 9/17) were male. Regarding pathological subtypes, mucosa-associated lymphoid tissue (MALT) lymphoma accounted for 76.5% (13/17), and splenic marginal zone lymphoma (SMZL) accounted for 23.5% (4/17). The main involved sites included the lung, parotid gland, gastrointestinal tract, spleen, and kidney. According to the Lugano staging system, 58.8% (10/17) of patients had stage III/IV disease; 35.2% (6/17) had an MZL-IPI score of 3–5. The median follow-up duration was 7.1 months (range, 1.2–12.8 months). During the induction therapy phase, efficacy was evaluated in 14 patients. The best complete response (CR) rate was 42.9% (6/14), and the best overall response rate (ORR) was 100% (14/14). During the orelabrutinib maintenance phase, efficacy was assessed in 9 patients, among whom 2 patients converted from PR to CR. No disease progression or death events have occurred to date. For safety, no treatment discontinuation or dose modification due to serious adverse events (AEs) was observed. The main adverse events included decreased neutrophil count, perioral herpes, hypogammaglobulinemia, *Candida albicans* pneumonia, and viral pneumonia, with an overall low incidence and favorable patient tolerability.

**Copyright Information:** (Online) ISSN: 2572-9241
©2026 The Author(s). HemaSphere is published by John Wiley & Sons Ltd on behalf of European Hematology Association. This is an open access Abstract Book distributed under the Attribution-NonCommercial-NoDerivs (CC BY-NC-ND), which allows third parties to download the articles and share them with others as long as they credit the author and the Abstract Book, but they cannot change the content in any way or use them commercially.
**Abstract Book Citations:** Authors, Title, HemaSphere, 2026;10:(S1):pages. The abstract book DOIs can be found on the "Author Information" page.
**Disclaimer:** Articles published in the journal HemaSphere exclusively reflect the opinions of the authors. The authors are responsible for all content in their abstracts including accuracy of the facts, statements, citing resources, etc.

### Summary/Conclusion

Chemotherapy following Orelabrutinib demonstrates favorable efficacy and safety in the treatment of treatment-naive marginal zone lymphoma. Given the small sample size and limited follow-up duration of this study, further expansion of the patient cohort and prolonged follow-up are warranted to validate the long-term efficacy and safety of this therapeutic regimen.

**Copyright Information:** (Online) ISSN: 2572-9241
©2026 The Author(s). HemaSphere is published by John Wiley & Sons Ltd on behalf of European Hematology Association. This is an open access Abstract Book distributed under the Attribution-NonCommercial-NoDerivs (CC BY-NC-ND), which allows third parties to download the articles and share them with others as long as they credit the author and the Abstract Book, but they cannot change the content in any way or use them commercially.
**Abstract Book Citations:** Authors, Title, HemaSphere, 2026;10:(S1):pages. The abstract book DOIs can be found on the "Author Information" page.
**Disclaimer:** Articles published in the journal HemaSphere exclusively reflect the opinions of the authors. The authors are responsible for all content in their abstracts including accuracy of the facts, statements, citing resources, etc.

### (PB3669) BEYOND STANDARD GELF: REDEFINING PROGNOSTIC RISK STRATIFICATION IN FOLLICULAR LYMPHOMA

**Topic:** 18. Indolent and mantle-cell non-Hodgkin lymphoma - Clinical

Efe Semercioğlu*[1], Taner Tan[2], Meltem Akay[2], Sinem Civriz Bozdağ[2]

*[1]Koç University, School of Medicine, Istanbul, Türkiye;[2]Koç University Hospital, Department of Hematology, Istanbul, Türkiye;*

### Background

The GELF (Groupe d'Etude des Lymphomes Folliculaires) criteria are widely used to determine treatment initiation in follicular lymphoma (FL), with current guidelines attributing equal prognostic weight to each component. However, emerging data question this uniformity. Grieve et al. proposed a distinction between "Hard GELF" (serous effusion, cytopenia, constitutional symptoms) associated with inferior overall survival (OS), and "Soft GELF."

### Aims

We aimed to evaluate the prognostic heterogeneity of individual GELF criteria and to further explore their relative impact on survival outcomes.

### Methods

We retrospectively analyzed patients aged ≥18 years diagnosed with FL (stage I–III A) between 2009 and 2025 at Koç University Hospital. Patients with stage III B, mixed histology, or transformation were excluded. Fifty-one eligible patients were included. GELF criteria were defined as ≥3 nodal areas ≥3 cm, any mass ≥7 cm, symptomatic splenomegaly, serous effusion (SE), constitutional symptoms (CSX), ≥1 cytopenia, or malignant lymphocytosis. Overall survival (OS) and progression-free survival (PFS) were estimated using the Kaplan–Meier method and compared using the log-rank test.

### Results

The cohort included 51 patients (median age 58 years [30–86]; 26 female). During a median follow-up of 54 months (0.5–172), 92.2% initiated treatment. In univariable analysis, serous effusion (p<0.001), bulky disease ≥7 cm (p=0.012), and ≥3 nodal areas (p=0.036) were significant predictors of inferior OS. In contrast, constitutional symptoms and cytopenia—classified as "Hard GELF" by Grieve et al.—were not associated with OS in our cohort (p=0.921 and p=0.273, respectively). Based on these findings, patients were stratified into three groups: Group A (high radiologic tumor burden: SE, ≥7 cm mass, or ≥3 nodal areas), Group B (clinical symptoms only), and Group C (no GELF criteria).
Kaplan–Meier analysis demonstrated significant separation in OS among groups (log-rank p=0.019) (Figure 1). Group A had significantly inferior OS compared with Group C (p=0.025) and showed a trend toward worse survival compared with Group B (p=0.082). Groups B and C had comparable OS (p=0.244). For progression-free survival (PFS), overall separation approached statistical significance (p=0.056). Group A had a median PFS of 34 months and demonstrated inferior outcomes compared with both Group C (p=0.061) and Group B (p=0.099), while no difference was observed between Groups B and C (p=0.656).

**Copyright Information:** (Online) ISSN: 2572-9241
©2026 The Author(s). HemaSphere is published by John Wiley & Sons Ltd on behalf of European Hematology Association. This is an open access Abstract Book distributed under the Attribution-NonCommercial-NoDerivs (CC BY-NC-ND), which allows third parties to download the articles and share them with others as long as they credit the author and the Abstract Book, but they cannot change the content in any way or use them commercially.
**Abstract Book Citations:** Authors, Title, HemaSphere, 2026;10:(S1):pages. The abstract book DOIs can be found on the "Author Information" page.
**Disclaimer:** Articles published in the journal HemaSphere exclusively reflect the opinions of the authors. The authors are responsible for all content in their abstracts including accuracy of the facts, statements, citing resources, etc.

**Summary/Conclusion**

Our findings suggest that GELF criteria may not carry equal prognostic weight. While prior studies have highlighted the potential impact of clinical symptoms and cytopenia on survival outcomes, our data indicate that radiologic tumor burden may play a more prominent role in determining adverse survival within our cohort. These observations underscore the heterogeneity among GELF components and suggest that a tumor burden–oriented risk stratification approach could be considered in the prognostic assessment of FL.

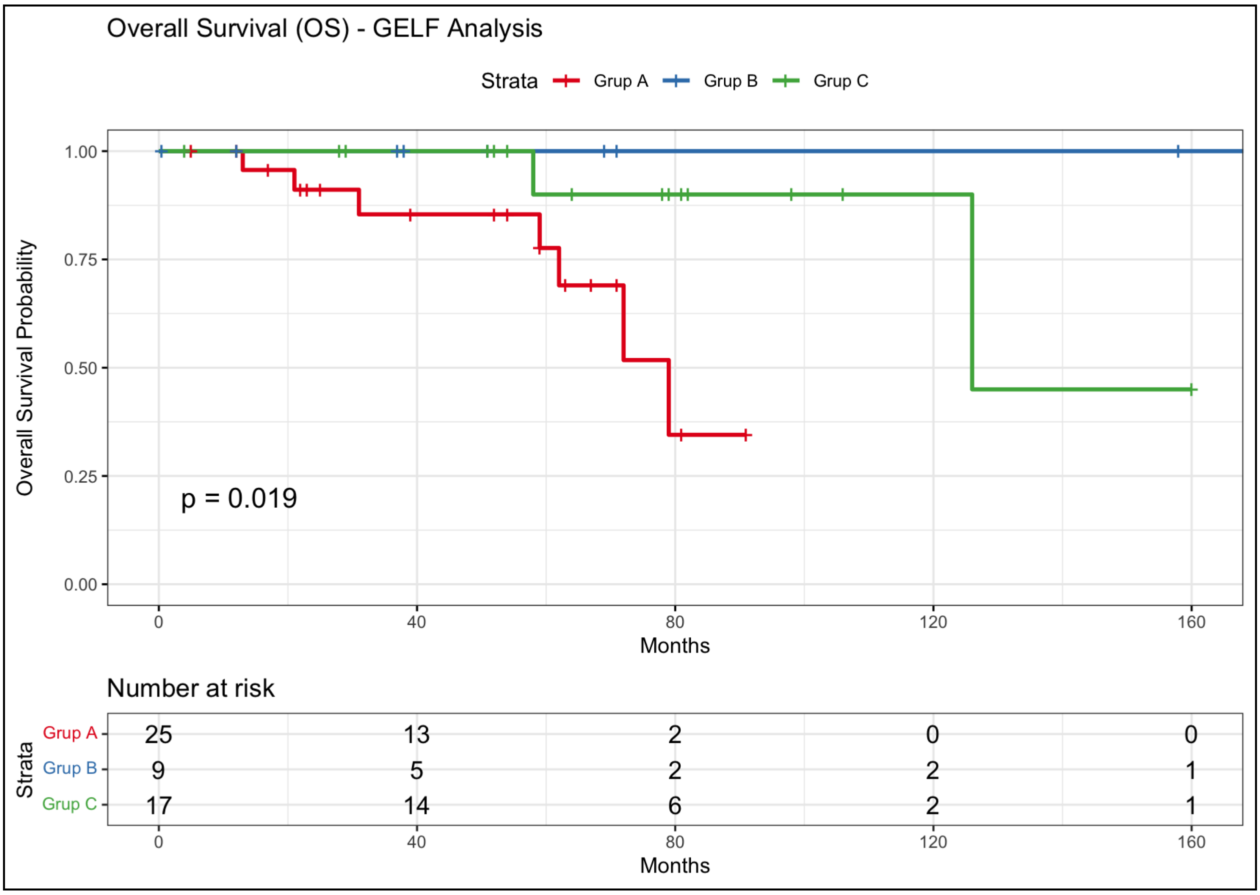

Figure 1. Kaplan–Meier overall survival (OS) curves according to GELF-based risk groups. Group A demonstrated significantly inferior OS compared with Groups B and C (log-rank p=0.019).

**Copyright Information:** (Online) ISSN: 2572-9241
©2026 The Author(s). HemaSphere is published by John Wiley & Sons Ltd on behalf of European Hematology Association. This is an open access Abstract Book distributed under the Attribution-NonCommercial-NoDerivs (CC BY-NC-ND), which allows third parties to download the articles and share them with others as long as they credit the author and the Abstract Book, but they cannot change the content in any way or use them commercially.
**Abstract Book Citations:** Authors, Title, HemaSphere, 2026;10:(S1):pages. The abstract book DOIs can be found on the “Author Information” page.
**Disclaimer:** Articles published in the journal HemaSphere exclusively reflect the opinions of the authors. The authors are responsible for all content in their abstracts including accuracy of the facts, statements, citing resources, etc.

### (PB3670) REAL-WORLD OUTCOMES OF R-CHOP PLUS RITUXIMAB MAINTENANCE AS FIRST-LINE THERAPY IN FOLLICULAR LYMPHOMA: A NATION-WIDE MULTICENTER 10-YEAR TUNISIAN STUDY

**Topic:** 18. Indolent and mantle-cell non-Hodgkin lymphoma - Clinical

Nesrine Ben Sayed*[1], Anna Chabaane[2], Wafa Miled[3], Jaied Rabeb[4], Maha Charfi[5], Zaineb Mlayah[6], Raoudha Mansouri[7], Wided Cherif[8], Malek Sayedi[7], Hela Ghedira[4], Slama Nader[6], Raihane Ben Lakhal[7], Yosra Ben Youssef[8], Med.Adnene Laatiri[6], Fehmi Msadek[4], Lamia Issaoui[7], Moez Elloumi[5], Moez Medhaffar[5]

*[1]Farhat hached hospital, hematolgy department, SOUSSE, Tunisia; [2]bone marrow transplantation center, hematolgy department, tunis, Tunisia; [3]Aziza Othmena hospital, tunis, Tunisia; [4]Military Hopital, hematolgy department, tunis, Tunisia; [5]Hedi chaker Hospital, hematolgy department, sfax, Tunisia; [6]monastir hsopital, hematolgy department, monastir, Tunisia; [7]Aziza Othmena hospital, hematolgy department, tunis, Tunisia; [8]Farhat hached hospital, hematolgy department, sousse, Tunisia;*

### Background

Follicular lymphoma (FL) is an indolent B-cell lymphoma with heterogeneous presentation and variable outcomes. Immunochemotherapy remains the backbone of frontline treatment in high tumour burden disease, with R-CHOP widely used despite the availability of alternative regimens.

### Aims

This study aims to evaluate the real-world efficacy and prognostic impact of R-CHOP as first-line therapy in a multicenter Tunisian cohort of patients with FL.

### Methods

This retrospective multicenter study included 157 patients diagnosed between January 2015 and December 2024 in six haematology and one oncology centre. Clinical characteristics, GELF classification, FLIPI/FLIPI2 scores, response rates, relapse patterns, and survival outcomes were analysed. Survival endpoints included overall survival (OS), relapse-free survival (RFS), and event-free survival (EFS).

### Results

Among the 157 patients, 134 (85%) received first-line immunochemotherapy, and 111 (83%) were treated with R-CHOP. Median age was 60 years, with 75% presenting with advanced-stage disease and 69% classified as having high tumor burden (GELF ≥1).In the R-CHOP group, the overall response rate (ORR) was 88%, including 61% complete responses. Two-thirds of responders achieved complete remission. Maintenance therapy was administered in the majority of responding patients.At 48 months, OS reached 95%, RFS 90%, and EFS 91%. Relapse occurred in 16% of responders, with a median time to relapse of 24.5 months. Early progression within 24 months (POD24) was observed in 11 patients and significantly impacted overall survival (p=0.005).Prognostic analysis within the R-CHOP-treated cohort demonstrated that FLIPI score significantly influenced treatment response (*p*=0.004) and OS (*p*=0.002), while GELF classification correlated with relapse risk (p=0.034). Advanced stage and type of chemotherapy were also associated with worse survival outcomes (p≤0.001).Histologic transformation occurred in 4 patients who received salvage therapy and underwent autologous stem cell transplantation.

**Copyright Information:** (Online) ISSN: 2572-9241
©2026 The Author(s). HemaSphere is published by John Wiley & Sons Ltd on behalf of European Hematology Association. This is an open access Abstract Book distributed under the Attribution-NonCommercial-NoDerivs (CC BY-NC-ND), which allows third parties to download the articles and share them with others as long as they credit the author and the Abstract Book, but they cannot change the content in any way or use them commercially.
**Abstract Book Citations:** Authors, Title, HemaSphere, 2026;10:(S1):pages. The abstract book DOIs can be found on the “Author Information” page.
**Disclaimer:** Articles published in the journal HemaSphere exclusively reflect the opinions of the authors. The authors are responsible for all content in their abstracts including accuracy of the facts, statements, citing resources, etc.

**Summary/Conclusion**

In this real-world multicenter cohort, the R-CHOP frontline regimen for FL showed high response rates and favorable 4-year survival despite a predominance of advanced-stage and high tumor burden disease. Prognostic scores, particularly FLIPI and POD24, have strong predictive value in patients treated with the R-CHOP regimen.

**Copyright Information:** (Online) ISSN: 2572-9241
©2026 The Author(s). HemaSphere is published by John Wiley & Sons Ltd on behalf of European Hematology Association. This is an open access Abstract Book distributed under the Attribution-NonCommercial-NoDerivs (CC BY-NC-ND), which allows third parties to download the articles and share them with others as long as they credit the author and the Abstract Book, but they cannot change the content in any way or use them commercially.
**Abstract Book Citations:** Authors, Title, HemaSphere, 2026;10:(S1):pages. The abstract book DOIs can be found on the "Author Information" page.
**Disclaimer:** Articles published in the journal HemaSphere exclusively reflect the opinions of the authors. The authors are responsible for all content in their abstracts including accuracy of the facts, statements, citing resources, etc.

### (PB3671) CHEMO-FREE FIRST-LINE THERAPY IN FRAIL PATIENTS WITH MARGINAL AND MANTLE CELL NON-HODGKIN LYMPHOMA: OUR EXPERIENCE

**Topic:** 18. Indolent and mantle-cell non-Hodgkin lymphoma - Clinical

Luca Pezzullo*[1], Antonella Malandrini[1, 2], Andrea Gigantiello[1, 2], Lorenzo Settembre[1, 2], Giovanni Signorile[1, 2], Valentina Giudice[2], Carmine Selleri[1, 2]

*[1]"San Giovanni diDio e Ruggi D'Aragona" Hospital, Hematology, Salerno, Italy;[2]University of Medicine, Hematology, Salerno, Italy;*

### Background

BKT inhibitors (iBKT) in the treatment of some non-Hodgkin's lymphomas are shifting the balance in the therapeutic approach to these diseases, increasingly moving towards chemotherapy-free treatment. The lymphomas that already have approval for the use of iBKT are mantle cell lymphoma (mNHL) and marginal lymphoma (MNHL) with good results but used only as monotherapy and from second line(1,2). In mNHL, studies in the first-line setting in combination with chemotherapy(3,4).  Have documented response rates and safety that will lead to changes in first-line treatments.
An unmet medical need is how to treat frail (often undertreated) elderly patients in the first line with iBTK and without (potentially less toxic) chemotherapy, to achieve maximum response with minimal toxicity and improve responses and quality of life in this patient cohort.

### Aims

The aim of our study is to treat frail patients diagnosed with mNHL or MNHL with a single dose of bendamustine 70 mg/m2 immediately discontinued, followed by one weekly cycle of rituximab 375 mg/m2, after which iBKT (zanubrutinib for MNHL and acalabrutinib for mNHL) was added concurrently with immunotherapy (Rituxmab 375 mg/m2, one dose every 28 days for 4 administrations). Subsequently, they were reevaluated and, if in response, the program includes iBTK and rituximab 375 mg/m2 every 2 months for 2 years or until unacceptable toxicity or disease recurrence.

### Methods

From September 2023 to February 2026, we treated 22 patients (7 mNHL and 15  MNHL) in our division. Nine females and 16 males had a median age of 78 years (range: 61-88 years) and a median performance status of 2 (range: 1-3). Sixteen patients (75%) were evaluable after 6 months of treatment.

**Copyright Information:** (Online) ISSN: 2572-9241
©2026 The Author(s). HemaSphere is published by John Wiley & Sons Ltd on behalf of European Hematology Association. This is an open access Abstract Book distributed under the Attribution-NonCommercial-NoDerivs (CC BY-NC-ND), which allows third parties to download the articles and share them with others as long as they credit the author and the Abstract Book, but they cannot change the content in any way or use them commercially.
**Abstract Book Citations:** Authors, Title, HemaSphere, 2026;10:(S1):pages. The abstract book DOIs can be found on the "Author Information" page.
**Disclaimer:** Articles published in the journal HemaSphere exclusively reflect the opinions of the authors. The authors are responsible for all content in their abstracts including accuracy of the facts, statements, citing resources, etc.

### Results

Two patients died: one due to mucor infection (after 1 month from the start of treatment) and one due to complications unrelated to treatment at month +3.
Of the 16 evaluable patients, the ORR at 6 months was 100%: 5 CR (30%); 5 VgPR (30%) and 6 PR (40%).
At 12 months, 15 patients were evaluable, with a confirmed ORR of 100%, with improved complete remissions: 11 CR (75%) and 4 RP (25%) (fig. 1).
No patients experienced grade >2 cardiac adverse events; 3/22 patients (14%) experienced grade >2 bleeding events that led to iBKT discontinuation with subsequent resumption at a 50% reduced dose; no patients experienced >2 hematological toxicity; no patients experienced CMV reactivation (from retrospective data from our center, approximately 25% of patients treated with R-Benda had developed CMV reactivation); 2 patients (9%) developed serious fungal infection (maxillary sinus mucormycosis with death and pulmonary aspergilloma treated with isavuconazole).
4/22 patients (18%) discontinued treatment: 2 due to death and 2 due to the development of a second neoplasm (1 breast carcinoma and 1 intestinal carcinoma).
No disease progression has been documented and the projected 30-months overall survival (OS) is 90% (whit a median follow-up of 13 monts). (figure 1)

### Summary/Conclusion

In conclusion, even if small cohort and with a short follow-up, the first-line chemotherapy-free treatment (combination of immunotherapy and iBKT) in frail patients with marginal and mantle cell lymphoma has proven to be very active with good compliance and an excellent quality of life.

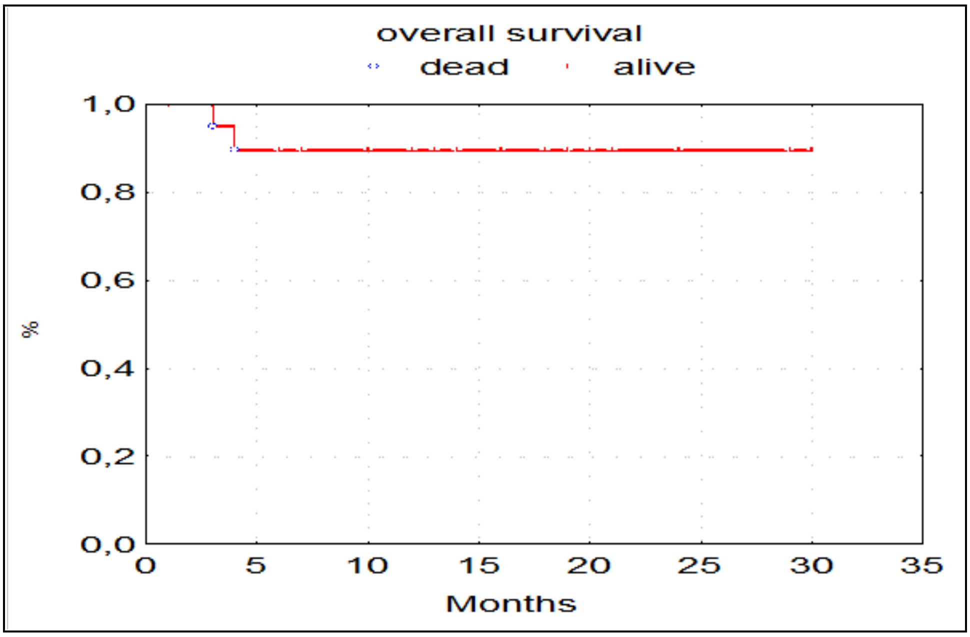

Figure1: Overall survival: Total Evaluable 22/22 patients: 2 patients died, one due to mucor mycosis infection and one due to causes not related to treatment No disease progression has been documented and the projected 30-month overall survival (OS) is 90% (with a median follow-up of 13 months).

**Copyright Information:** (Online) ISSN: 2572-9241
©2026 The Author(s). HemaSphere is published by John Wiley & Sons Ltd on behalf of European Hematology Association. This is an open access Abstract Book distributed under the Attribution-NonCommercial-NoDerivs (CC BY-NC-ND), which allows third parties to download the articles and share them with others as long as they credit the author and the Abstract Book, but they cannot change the content in any way or use them commercially.
**Abstract Book Citations:** Authors, Title, HemaSphere, 2026;10:(S1):pages. The abstract book DOIs can be found on the "Author Information" page.
**Disclaimer:** Articles published in the journal HemaSphere exclusively reflect the opinions of the authors. The authors are responsible for all content in their abstracts including accuracy of the facts, statements, citing resources, etc.

### (PB3672) EFFICACY AND SAFETY OF ORELABRUTINIB COMBINED WITH LOW-DOSE RADIOTHERAPY IN TREATMENT-NAIVE MUCOSA-ASSOCIATED LYMPHOID TISSUE LYMPHOMA

**Topic:** 18. Indolent and mantle-cell non-Hodgkin lymphoma - Clinical

Yamin Tan*[1], Mengyu Zhao[1], Xiaolin Yuan[1], Zhongzhu Tang[2], Peng Liu[3], Jianxin Xu[4], Chao Chen[5], Chun Han[6], Zhimin Ye[3]

*[1]Zhejiang Cancer Hospital, Department of Hematology, Hangzhou, China;[2]Tongde Hospital Zhejiang Provincial, Department of Radiotherapy, Hangzhou, China; [3]Zhejiang Cancer Hospital, Department of Radiation Oncology, Hangzhou, China;[4]Hospital of Chinese Medicine of Changxing County, Department of Oncology, Huzhou, China; [5]Zhejiang Cancer Hospital, Department of Head and Neck Surgery, Hangzhou, China; [6]Zhejiang Cancer Hospital, Department of Thyroid Surgery, Hangzhou, China;*

### Background

Mucosa-associated lymphoid tissue lymphoma (MALT) is an indolent B-cell malignancy frequently presenting with multi-site disease dissemination. Although radiotherapy remains the standard approach for localized (early-stage) disease, the substantial risks of radiation-induced tissue damage and procedure-related complications—especially when treating critical anatomical locations—limit its applicability. Orelabrutinib, a novel oral BTK inhibitor, offers improved target selectivity and fewer off-target effects, making it a promising alternative.

### Aims

To evaluate the efficacy and safety of orelabrutinib combined with low-dose radiotherapy in patients with treatment-naive MALT lymphoma.

### Methods

This is a prospective, single-arm, multicenter phase II clinical trial. Patients with untreated MALT who met ECOG criteria of 0–2 and in need of treatment were eligible. All participants will receive induction therapy: involved-site radiotherapy (ISRT) 4 Gy in 2 fractions (2 Gy daily), plus Orelabrutinib 150 mg orally once daily on days 1–28 of each 28-day cycle for 4–6 cycles. Patients achieving partial response (PR) or better entered orelabrutinib monotherapy maintenance (up to 24 cycles, 28 days per cycle). The primary endpoint is the complete response (CR) rate. Secondary endpoints include overall response rate (ORR), PR rate, progression-free survival (PFS), and overall survival (OS). Safety is assessed by monitoring adverse events (AEs).

### Results

From July 2025 to February 2026, a total of 7 patients with MALT lymphoma were enrolled. The median age at diagnosis was 52 years (range 37–76). All patients were female with an ECOG performance status of 0–1. Four patients had a MALT-International Prognostic Index score of ≥1. Extranodal involvement was predominantly observed in the parotid gland (42.9%) and thyroid gland (28.6%), with one patient each additionally involving the stomach and bladder. As of the data cutoff date, only one patient was evaluable and had achieved complete response. With a median follow-up of four months, the median PFS and OS were not reached; the estimated 6-months PFS and OS rates were both 100%. The treatment was well tolerated, with only three adverse events (AEs) observed: neutropenia, fatigue, and rash, all of which were grade 1–2. Notably, no radiotherapy-related toxicities or BTK inhibitor-associated cardiovascular adverse events were observed.

**Copyright Information:** (Online) ISSN: 2572-9241
©2026 The Author(s). HemaSphere is published by John Wiley & Sons Ltd on behalf of European Hematology Association. This is an open access Abstract Book distributed under the Attribution-NonCommercial-NoDerivs (CC BY-NC-ND), which allows third parties to download the articles and share them with others as long as they credit the author and the Abstract Book, but they cannot change the content in any way or use them commercially.
**Abstract Book Citations:** Authors, Title, HemaSphere, 2026;10:(S1):pages. The abstract book DOIs can be found on the “Author Information” page.
**Disclaimer:** Articles published in the journal HemaSphere exclusively reflect the opinions of the authors. The authors are responsible for all content in their abstracts including accuracy of the facts, statements, citing resources, etc.

**Summary/Conclusion**

Orelabrutinib combined with low-dose radiotherapy demonstrated promising efficacy and favorable safety in patients with treatment-naïve MALT lymphoma. Future studies will expand the cohort and investigate potential variations in efficacy according to distinct extranodal involvement sites.

**Copyright Information:** (Online) ISSN: 2572-9241
©2026 The Author(s). HemaSphere is published by John Wiley & Sons Ltd on behalf of European Hematology Association. This is an open access Abstract Book distributed under the Attribution-NonCommercial-NoDerivs (CC BY-NC-ND), which allows third parties to download the articles and share them with others as long as they credit the author and the Abstract Book, but they cannot change the content in any way or use them commercially.
**Abstract Book Citations:** Authors, Title, HemaSphere, 2026;10:(S1):pages. The abstract book DOIs can be found on the "Author Information" page.
**Disclaimer:** Articles published in the journal HemaSphere exclusively reflect the opinions of the authors. The authors are responsible for all content in their abstracts including accuracy of the facts, statements, citing resources, etc.

### (PB3673) THERAPEUTIC OUTCOMES OF YOUNG PATIENTS WITH MANTLE CELL LYMPHOMA: A TUNISIAN MULTICENTER REAL-WORLD STUDY

**Topic:** 18. Indolent and mantle-cell non-Hodgkin lymphoma - Clinical

Zaineb Mlayah*[1], Wided Cherif[2], Malek Sayedi[3], Jaied Rabeb[4], Maha Charfi[5], Miled Wafa[3], Anna Chabaane[6], Slama Nader[1], Nesrine Ben Sayed[2], Raoudha Mansouri[3], Hela Ghedira[4], Moez Medhaffar[5]

*[1]Fattouma Bourguiba Hospital, Hematology, Monastir, Tunisia; [2]Faraht Hached Hospital, Clinical Hematology, Sousse, Tunisia; [3]Aziza Othmana Hospital, Tunis, Tunisia; [4]Military Hospital, Clinical Hematology, Tunis, Tunisia; [5]hedi chaker Hospital, Clinical hematology, Sfax, Tunisia; [6]National Center of Bone Marrow transplantation, clinical hematology, Tunis, Tunisia;*

### Background

Intensive strategies with high-dose cytarabine (HD-AraC) followed by autologous stem cell transplantation (ASCT) and maintenance represent the standard of care for younger
patients with mantle cell lymphoma (MCL). However, their implementation may be limited in real-world settings.

### Aims

Analyze clinical characteristics, treatment strategies, and outcomes (PFS/OS) of patients ≤65 years in a multicenter Tunisian cohort.

### Methods

Between January 2015 and December 2025, 128 patients with MCL were included across five hematology centers. Sixty-nine patients (54%) were ≤65 years; 66 received treatment (3 early deaths). Descriptive analyses, Kaplan–Meier survival estimates, and uni-/multivariate Cox regression models were performed.

### Results

Median age was 56 years (30–65), with a male-to-female ratio of 2.23. B symptoms were present in 47%. Leukemic presentation was found in 23%. Ninety-sex percent had advanced-stage disease. According to simplified MIPI, 23% were low-risk, 25% intermediate-risk, and 21% high-risk. Elevated LDH was observed in 64% and PS ≥2 in 16%. Sixty-six received treatment (3 early deaths). Fifty patients received HD Cytarabine-based induction (19 alternating R-CHOP/R-DHAP; 31 R-DHAP ± C/Ox), while 16 received R CHOP alone. The overall response rate (ORR) was 84% (CR 51%, PR 33%). Only 29 (54%) underwent ASCT. Eighty-six percent achieved post-ASCT response (15 CR, 10 PR). High risk MIPI score and the absence of HD-AraC exposure were theunfavorouble predictors of treatment failure. Rituximab maintenance was administered to 36 patients. After a median follow-up of 63.5 months (12–128), median OS was not reached with 5-year OS at 60–65%. Sixteen relapse/progression occurred within 15 months. At relapse, salvage regimens mainly included R-bendamustine (4 responses/7 patients), RDHAP/RDHAC, HyperCVAD, and occasional ibrutinib, with heterogeneous responses. Median PFS was 78 months, with 5-year PFS of approximatively 45%. The exposure to cytarabine based regime, the low risk MIPI and the ASCT consolidation were significantly associated with improved OS (p=0,007 ; 0,007 and 0,021) respectively. Maintenance therapy provided a longer PFS, (p<0.001).

**Copyright Information:** (Online) ISSN: 2572-9241
©2026 The Author(s). HemaSphere is published by John Wiley & Sons Ltd on behalf of European Hematology Association. This is an open access Abstract Book distributed under the Attribution-NonCommercial-NoDerivs (CC BY-NC-ND), which allows third parties to download the articles and share them with others as long as they credit the author and the Abstract Book, but they cannot change the content in any way or use them commercially.
**Abstract Book Citations:** Authors, Title, HemaSphere, 2026;10:(S1):pages. The abstract book DOIs can be found on the "Author Information" page.
**Disclaimer:** Articles published in the journal HemaSphere exclusively reflect the opinions of the authors. The authors are responsible for all content in their abstracts including accuracy of the facts, statements, citing resources, etc.

**Summary/Conclusion**

In this real-world exposure to high-dose cytarabine (HD-AraC) and the MIPI score emerged as independent predictors of treatment failure. Consolidation with ASCT, as well as maintenance therapy, were associated with significantly improved survival outcomes. These findings underscore the pivotal role of intensive, risk-adapted strategies in optimizing disease control. However, ensuring equitable access to such approaches remains a major challenge in our resource-constrained setting.

**Copyright Information:** (Online) ISSN: 2572-9241
©2026 The Author(s). HemaSphere is published by John Wiley & Sons Ltd on behalf of European Hematology Association. This is an open access Abstract Book distributed under the Attribution-NonCommercial-NoDerivs (CC BY-NC-ND), which allows third parties to download the articles and share them with others as long as they credit the author and the Abstract Book, but they cannot change the content in any way or use them commercially.
**Abstract Book Citations:** Authors, Title, HemaSphere, 2026;10:(S1):pages. The abstract book DOIs can be found on the “Author Information” page.
**Disclaimer:** Articles published in the journal HemaSphere exclusively reflect the opinions of the authors. The authors are responsible for all content in their abstracts including accuracy of the facts, statements, citing resources, etc.

### (PB3674) PROSPECTIVE EXPLORATION OF ZUBERITAMAB, LENALIDOMIDE COMBINED WITH ORELABRUTINIB (HRO) IN THE TREATMENT OF NEWLY DIAGNOSED MARGINAL ZONE LYMPHOMA.

**Topic:** 18. Indolent and mantle-cell non-Hodgkin lymphoma - Clinical

Yu Qiu*[1], Chunxia Zhang[1], Min Wang[1], Yayue Gao[1], Qianqian Ju[1], Chao Guo[1], Yuan Huang[1], Jingya Wang[1], Yang Li[1], Zi-Xian Liu[1], Ming Gong[1], Zhenling Li[1]

[1]*China-Japan Friendship Hospital, Beijing, China;*

### Background

Marginal zone lymphoma (MZL) is a common B-cell non-Hodgkin lymphoma. Due to the high heterogeneity of MZL, there is no clear standard treatment regimen.

### Aims

This study aims to prospectively explore the efficacy and safety of a novel chemotherapy-free regimen combining the new CD20 monoclonal antibody zuberitamab, lenalidomide, and orelabrutinib (HRO) as a first-line treatment in newly diagnosed MZL patients with treatment indications at the China-Japan Friendship Hospital starting from Sep 2025.

### Methods

Eligible patients were adults aged ≥18 years with histologically confirmed newly diagnosed MZL. The treatment regimen includes 375mg/m² zuberitamab on days 1, 8, and 15 of the first cycle, followed by 375mg/m² zuberitamab on day 1 from the second cycle, lenalidomide 25mg on days 1-14, and orelabrutinib 150mg qd continuously orally, administered every 21 days for a total of 8 cycles. Subjects achieving PR or better after treatment will receive orelabrutinib monotherapy maintenance for a total of 2 years. The primary endpoint is the complete response rate (CRR), and secondary endpoints include overall response rate (ORR), progression-free survival (PFS), and overall survival (OS). Safety is assessed by monitoring adverse events (AE).

### Results

A total of 7 patients have been included in this study, with males accounting for 71.4% (5/7). The median age at diagnosis is 78 years (range 48-84). All 7 patients have mucosa-associated lymphoid tissue extranodal marginal zone lymphoma (MALT), with the main affected sites including the lung, intestine, and orbit. Among them, 1 patient has large cell transformation, 5 patients (71.4%) are at Ann Arbor stage IV, 42.9% of the patients have an MZL-IPI score of 1, and 42.9% have an MZL-IPI score of 2.
Median treatment of 5 cycles (2-7), 5 patients were evaluable for efficacy, ORR was 80%, with 20.0% (1/5) achieving CR, median OS and PFS were not reached. 57.1% (4/7) of patients experienced AE, all were graded 1-2, common AEs included rash, liver function impairment, lymphocyte reduction. No atrial fibrillation or bleeding related to BTK inhibitors occurred.

### Summary/Conclusion

The HRO regimen as first-line treatment for MZL, especially in elderly patients with high-risk features, is a safe and effective treatment strategy, and future research updates will include more follow-up data.

**Copyright Information:** (Online) ISSN: 2572-9241
©2026 The Author(s). HemaSphere is published by John Wiley & Sons Ltd on behalf of European Hematology Association. This is an open access Abstract Book distributed under the Attribution-NonCommercial-NoDerivs (CC BY-NC-ND), which allows third parties to download the articles and share them with others as long as they credit the author and the Abstract Book, but they cannot change the content in any way or use them commercially.
**Abstract Book Citations:** Authors, Title, HemaSphere, 2026;10:(S1):pages. The abstract book DOIs can be found on the "Author Information" page.
**Disclaimer:** Articles published in the journal HemaSphere exclusively reflect the opinions of the authors. The authors are responsible for all content in their abstracts including accuracy of the facts, statements, citing resources, etc.

### (PB3675) EXTRANODAL MARGINAL ZONE LYMPHOMA: CLINICAL FEATURES, PROGNOSTIC FACTORS AND TREATMENT OUTCOMES IN A TUNISIAN COHORT

**Topic:** 18. Indolent and mantle-cell non-Hodgkin lymphoma - Clinical

Maha Charfi*[1], Malek Sayadi[2], Hela Ghedira[3], Slama Nader[4], Nesrine Ben Sayed[5], Wala Ben Kridis[6], Haifa Hafsa[7], Wafa Miled[2], Raoudha Mansouri[2], Rabeb Jaied[3], Wided Cherif[5], Zaineb Mlayah[4], Anna Chabaane[8], Moez Elloumi[1], Raihane Ben Lakhal[2], Fehmi Msadek[3], Mohamed Adnene Laatiri[4], Yosra Ben Youssef[5], Nour Ben Adejlil[8], Moez Mdhaffar[1]

*[1]Hedi Chaker hospital, hematology department, Sfax, Tunisia; [2]Aziza Othmana hospital, hematology department, tunis, Tunisia; [3]military hospital, hematology department, tunis, Tunisia; [4]Fattouma Bourguiba hospital, hematology department, Monastir, Tunisia; [5]Farhat Hached hospital, hematology department, sousse, Tunisia; [6]Hbib Bourguiba, oncology department, Sfax, Tunisia; [7]Mohamed Ben Sassi, hematology department, Gabes, Tunisia; [8]centre de greffe de moelle osseuse, hematology department, Tunis, Tunisia;*

### Background

Extranodal marginal zone lymphoma (EMZL) is an indolent B-cell lymphoma that originates in mucosa-associated lymphoid tissue. The clinical presentation is heterogeneous depending on the primary site. While the overall prognosis is favorable, the disease's course and therapeutic strategies vary widely. Large, authentic case series are imperative to better define prognostic factors and optimize treatment strategies.

### Aims

This study aimed to describe the clinical characteristics, prognostic factors, and therapeutic outcomes of EMZL in a multicentric Tunisian cohort.

### Methods

We conducted a retrospective, descriptive, multicentric study of patients diagnosed with EMZL in six hematology centers and one oncology center in Tunisia between January 2015 and December 2024. We analyzed clinical presentation, primary site, stage, treatment modalities, response rates, and survival outcomes. We assessed prognostic factors for progression-free survival (PFS) and overall survival (OS) using univariate and multivariate analyses.

**Copyright Information:** (Online) ISSN: 2572-9241
©2026 The Author(s). HemaSphere is published by John Wiley & Sons Ltd on behalf of European Hematology Association. This is an open access Abstract Book distributed under the Attribution-NonCommercial-NoDerivs (CC BY-NC-ND), which allows third parties to download the articles and share them with others as long as they credit the author and the Abstract Book, but they cannot change the content in any way or use them commercially.
**Abstract Book Citations:** Authors, Title, HemaSphere, 2026;10:(S1):pages. The abstract book DOIs can be found on the “Author Information” page.
**Disclaimer:** Articles published in the journal HemaSphere exclusively reflect the opinions of the authors. The authors are responsible for all content in their abstracts including accuracy of the facts, statements, citing resources, etc.

### Results

A total of 93 patients with extranodal marginal zone lymphoma (EMZL) were included, representing 41% of all marginal zone lymphoma cases. The median age at diagnosis was 58 years (range: 27–82), with a slight male predominance (sex ratio = 1.21). The most frequent extranodal sites were the stomach (52%), lung (17%), ocular adnexa (14%), and parotid gland (7,5%), while less common localizations included the thyroid (3%), colon (1,5%), nasopharynx (1,5%), skin (1,5%), tonsil (1%), salivary glands (1%) and breast (1%). Localized disease (Ann Arbor stage I–II) was observed in 72% of patients. According to the MALT-IPI, 54% were classified as low risk.
All patients with gastric EMZL received Helicobacter pylori eradication therapy. First-line management consisted mainly of immunochemotherapy (67%), while a watchful waiting strategy was adopted in 15% of cases. The most frequently administered first-line regimens were CHOP/CHOP-like ± rituximab (26.8%), followed by R-CLB (25.8%); rituximab monotherapy and other regimens were used in 6.4% and 7.5% of cases, respectively.
The overall response rate was 81%, including 66% complete responses. After a median follow-up of 54 months, the 5-year progression-free survival (PFS) and overall survival (OS) rates were 87.1% and 88.2%, respectively. Second-line therapy was required in 22.5% of patients (n = 21), mainly consisting of rituximab–bendamustine (43%), followed by R-CHOP (28.5%), while other regimens accounted for 28.5% of cases.
In multivariate analysis, bulky disease (p = 0.038) and bone marrow involvement (p = 0.003) were independently associated with inferior PFS.

### Summary/Conclusion

Consistent with large international studies reporting 5-year OS rates of 85–95% and PFS rates of 70–90%, our multicenter cohort confirms the favorable long-term prognosis of EMZL. The predominance of localized disease (72%) and the high complete response rate (66%) align with published data. However, as previously reported, bulky disease and bone marrow involvement emerged as strong adverse prognostic factors. These results support risk-adapted therapeutic strategies that combine local and systemic approaches to maintain high disease control while minimizing treatment-related toxicity

**Copyright Information:** (Online) ISSN: 2572-9241
©2026 The Author(s). HemaSphere is published by John Wiley & Sons Ltd on behalf of European Hematology Association. This is an open access Abstract Book distributed under the Attribution-NonCommercial-NoDerivs (CC BY-NC-ND), which allows third parties to download the articles and share them with others as long as they credit the author and the Abstract Book, but they cannot change the content in any way or use them commercially.
**Abstract Book Citations:** Authors, Title, HemaSphere, 2026;10:(S1):pages. The abstract book DOIs can be found on the "Author Information" page.
**Disclaimer:** Articles published in the journal HemaSphere exclusively reflect the opinions of the authors. The authors are responsible for all content in their abstracts including accuracy of the facts, statements, citing resources, etc.

### (PB3676) REAL-WORLD RETROSPECTIVE STUDY OF ORELABRUTINIB-BASED REGIMENS IN CHINESE PATIENTS WITH MARGINAL ZONE LYMPHOMA

**Topic:** 18. Indolent and mantle-cell non-Hodgkin lymphoma - Clinical

Ziqian Wang*[1], Zi Wang[1], Yan Zhang[1], Yan Zhang[2], Mei Zhang[2], Dao-Bin Zhou[1], Wei Zhang[1]

*[1]Peking Union Medical College Hospital, Chinese Academy of Medical Science and Peking Union Medical College, Department of Hematology, Beijing, China; [2]Beijing Longfu Hospital, Department of Hematology, Beijing, China;*

### Background

Marginal zone lymphoma (MZL) is an indolent B-cell lymphoma subtype. Current standard treatment relies on rituximab-based immunochemotherapy, such as bendamustine or CHOP regimens. Although effective, chemotherapy causes notable toxicities including myelosuppression and increased infection risk. As most MZL patients are elderly with comorbidities and poor chemotherapy tolerance, chemo-free strategies represent a promising alternative. Orelabrutinib is a highly selective, irreversible second-generation Bruton tyrosine kinase inhibitor (BTKi) with sustained target coverage and reduced off-target effects.

### Aims

This retrospective real-world study evaluated the efficacy and safety of orelabrutinib-based regimens in Chinese patients with MZL.

### Methods

31 patients with histologically confirmed MZL treated at Peking Union Medical College Hospital from January 2022 to March 2025 were enrolled. All patients received orelabrutinib 150 mg once daily, as monotherapy or combined with CD20 monoclonal antibodies. Responses were evaluated by Lugano 2014 criteria and adverse events graded by CTCAE 5.0. Descriptive statistics, binomial confidence intervals and Kaplan–Meier analysis were applied. All patients gave written informed consent in accordance with the Declaration of Helsinki.

**Copyright Information:** (Online) ISSN: 2572-9241
©2026 The Author(s). HemaSphere is published by John Wiley & Sons Ltd on behalf of European Hematology Association. This is an open access Abstract Book distributed under the Attribution-NonCommercial-NoDerivs (CC BY-NC-ND), which allows third parties to download the articles and share them with others as long as they credit the author and the Abstract Book, but they cannot change the content in any way or use them commercially.
**Abstract Book Citations:** Authors, Title, HemaSphere, 2026;10:(S1):pages. The abstract book DOIs can be found on the “Author Information” page.
**Disclaimer:** Articles published in the journal HemaSphere exclusively reflect the opinions of the authors. The authors are responsible for all content in their abstracts including accuracy of the facts, statements, citing resources, etc.

### Results

Baseline clinical characteristics of the enrolled patients are summarized in Table 1. The median number of prior treatment lines was 6 (range: 1–14). The overall objective response rate (ORR) for the entire cohort was 96.8% (95% CI: 80.4–99.9%), with a complete response rate (CRR) of 87.1% (95% CI: 65.1–95.6%). First-line patients (n=19), mostly receiving orelabrutinib monotherapy, achieved a CRR of 94.7% (95% CI: 68.1–99.8%) and ORR of 100% (95% CI: 78.2–100%). Second-line patients (n=9) had both a CRR and ORR of 100% (95% CI: 63.1–100%), whereas third-line patients (n=3) achieved no CR with an ORR of 66.7% (95% CI: 9.4–99.2%).
Subgroup analysis revealed a CRR of 89.5% (95% CI: 61.7–98.4%) and ORR (95% CI: 69.8–99.8%) of 94.7% in MALT lymphoma, and 81.8% (95% CI: 44.4–97.5%) and 100% (95% CI: 69.2–100%) in splenic MZL, respectively. At a median follow-up of 25.4 months, all patients were alive and median overall survival was not reached (Figure 1).
Treatment-related adverse events occurred in 64.5% of patients, mostly grade 1-2, with grade 3-4 events in 9 patients. The most common TRAEs included thrombocytopenia (30.8%), anemia (26.9%), neutropenia (19.2%), decreased appetite (15.4%), and nausea (11.5%). No grade 5 events were reported. Importantly, no BTKi-related off-target toxicities such as atrial fibrillation or severe bleeding were observed.
Table 1. The Clinical Characteristics of Patients

| **Characteristics** | | **n (%)** |
|---|---|---|
| Age, years | Median (range) | 67 (36-79) |
| Gender | Male | 19 (61.3) |
| ECOG performance status | 0-1 | 24 (77.4) |
| | ≥2 | 7 (22.6) |
| Pathological subtype | MALT | 19 (61.3) |
| | SMZL | 11 (35.5) |
| | NMZL | 1 (3.2) |
| Ann Arbor stage | I-II | 6 (19.4) |
| | Ⅲ-IV | 25 (80.6) |
| MZL-IPI score | 0-2 | 28 (90.3) |
| | 3-5 | 3 (9.7) |
| Bone marrow involvement | Yes | 12 (38.7) |
| | No | 19 (61.3) |
| Hepatomegaly | Yes | 2 (6.5) |
| | No | 29 (93.5) |
| Splenomegaly | Yes | 13 (41.9) |
| | No | 18 (58.1) |
| Number of extranodal organs involved | ⊠2 | 17 (54.8) |
| | ≥2 | 14 (45.2) |
| B symptoms | Present | 8 (25.8) |
| | Absent | 23 (74.2) |

### Summary/Conclusion

Orelabrutinib-based regimens are effective and safe in Chinese MZL patients. Notably, first-line orelabrutinib monotherapy shows high efficacy, favorable safety and oral convenience, supporting its potential as a first-line chemo-free option. Further large-scale prospective trials are warranted.

**Copyright Information:** (Online) ISSN: 2572-9241
©2026 The Author(s). HemaSphere is published by John Wiley & Sons Ltd on behalf of European Hematology Association. This is an open access Abstract Book distributed under the Attribution-NonCommercial-NoDerivs (CC BY-NC-ND), which allows third parties to download the articles and share them with others as long as they credit the author and the Abstract Book, but they cannot change the content in any way or use them commercially.
**Abstract Book Citations:** Authors, Title, HemaSphere, 2026;10:(S1):pages. The abstract book DOIs can be found on the "Author Information" page.
**Disclaimer:** Articles published in the journal HemaSphere exclusively reflect the opinions of the authors. The authors are responsible for all content in their abstracts including accuracy of the facts, statements, citing resources, etc.

### (PB3677) EFFECTIVENESS AND SAFETY OF ORELABRUTINIB-BASED REGIMENS IN MARGINAL ZONE LYMPHOMA: A REAL-WORLD RETROSPECTIVE STUDY

**Topic:** 18. Indolent and mantle-cell non-Hodgkin lymphoma - Clinical

Tongjuan Li*[1], Zetong Hong[1], Yan Feng[1], Min Liu[1], Yaxian MA[1], Qing Yin[1], Zhaoyang Hong[2], Miao Zheng[1]

*[1]Department of Hematology, Tongji Hospital, Tongji Medical College, Huazhong University of Science and Technology, Wuhan, China; [2]Tongji Medical College, Huazhong University of Science and Technology, Wuhan, China;*

### Background

Marginal zone lymphoma (MZL) is an indolent non-Hodgkin lymphoma without a standardized first-line treatment. Orelabrutinib, a selective irreversible BTK inhibitor, has shown preliminary efficacy in MZL, but real-world data for first-line use remains limited. This study assessed real-world effectiveness and safety of orelabrutinib-containing regimens in treatment-naïve MZL patients.

### Methods

In this single-center retrospective study, 39 newly diagnosed MZL patients received orelabrutinib-based regimens at Tongji Hospital between January 2022 and September 2025. All patients completed ≥2 cycles of orelabrutinib 150 mg daily (28-day cycles) plus maintenance monotherapy. Patients with follow-up <3 months were excluded. Tumor response was evaluated by Lugano 2014 criteria and adverse events by NCI CTCAE v5.0. Primary endpoints were ORR and PFS; safety was the secondary endpoint.

### Results

The cohort included 23 females and 16 males with a median age of 58 years (range 33–77). MALT lymphoma was the predominant subtype (76.9%, 30/39), followed by nodal MZL (15.4%, 6/39). Advanced-stage disease (III–IV) accounted for 51.3%; 79.5% had normal LDH, 76.9% had comorbidities, and 6 patients had chronic HBV infection.

With a median follow-up of 16.5 months (range 3.7–31.0), 38 patients were evaluable for efficacy（Figure 1）. The ORR was 97.4% (95% CI 86.2–99.9), with CR rate 52.6% (20/38) and PR rate 44.7% (17/38); only 1 patient had SD. Among 34 patients receiving combination therapy, CR/PR/SD rates were 50.0%/47.1%/2.9%, while all 4 monotherapy patients achieved response (3 CR, 1 PR). The 24-month PFS rate was 95.7% (95% CI 87.7–100.0) and 24-month OS rate 100.0%. Subgroup analyses showed consistent CR rates across sex, age, stage, IPI, LDH, and histology.

For safety, 59.0% (23/39) had any-grade AEs and 17.9% (7/39) had grade ≥3 AEs. Common any-grade AEs were leukopenia (25.6%), thrombocytopenia (17.9%), and pneumonia (17.9%), with all pneumonia cases in combination therapy. Grade ≥3 AEs included pneumonia (17.9%), shingles (5.1%), and leukopenia (5.1%). Hemorrhagic events were mild, with 7.7% of patients experiencing grade 1-2 ecchymosis that resolved spontaneously without intervention. No grade ≥3 anemia or lymphopenia, and no atrial fibrillation, hypertension, hemorrhage, or other off-target AEs occurred. Two patients required dose adjustment due to rash and shingles with improvement. No HBV reactivation, serious AEs, or treatment-related deaths were reported.

### Summary/Conclusion

Orelabrutinib-based regimens showed high ORR, durable disease control, and favorable safety in first-line real-world MZL treatment. Satisfactory efficacy was maintained even in patients with advanced disease, elevated LDH, or comorbidities. Orelabrutinib monotherapy also demonstrated promising efficacy and tolerability, supporting its role as a valuable first-line option for MZL.

**Copyright Information:** (Online) ISSN: 2572-9241
©2026 The Author(s). HemaSphere is published by John Wiley & Sons Ltd on behalf of European Hematology Association. This is an open access Abstract Book distributed under the Attribution-NonCommercial-NoDerivs (CC BY-NC-ND), which allows third parties to download the articles and share them with others as long as they credit the author and the Abstract Book, but they cannot change the content in any way or use them commercially.
**Abstract Book Citations:** Authors, Title, HemaSphere, 2026;10:(S1):pages. The abstract book DOIs can be found on the “Author Information” page.
**Disclaimer:** Articles published in the journal HemaSphere exclusively reflect the opinions of the authors. The authors are responsible for all content in their abstracts including accuracy of the facts, statements, citing resources, etc.

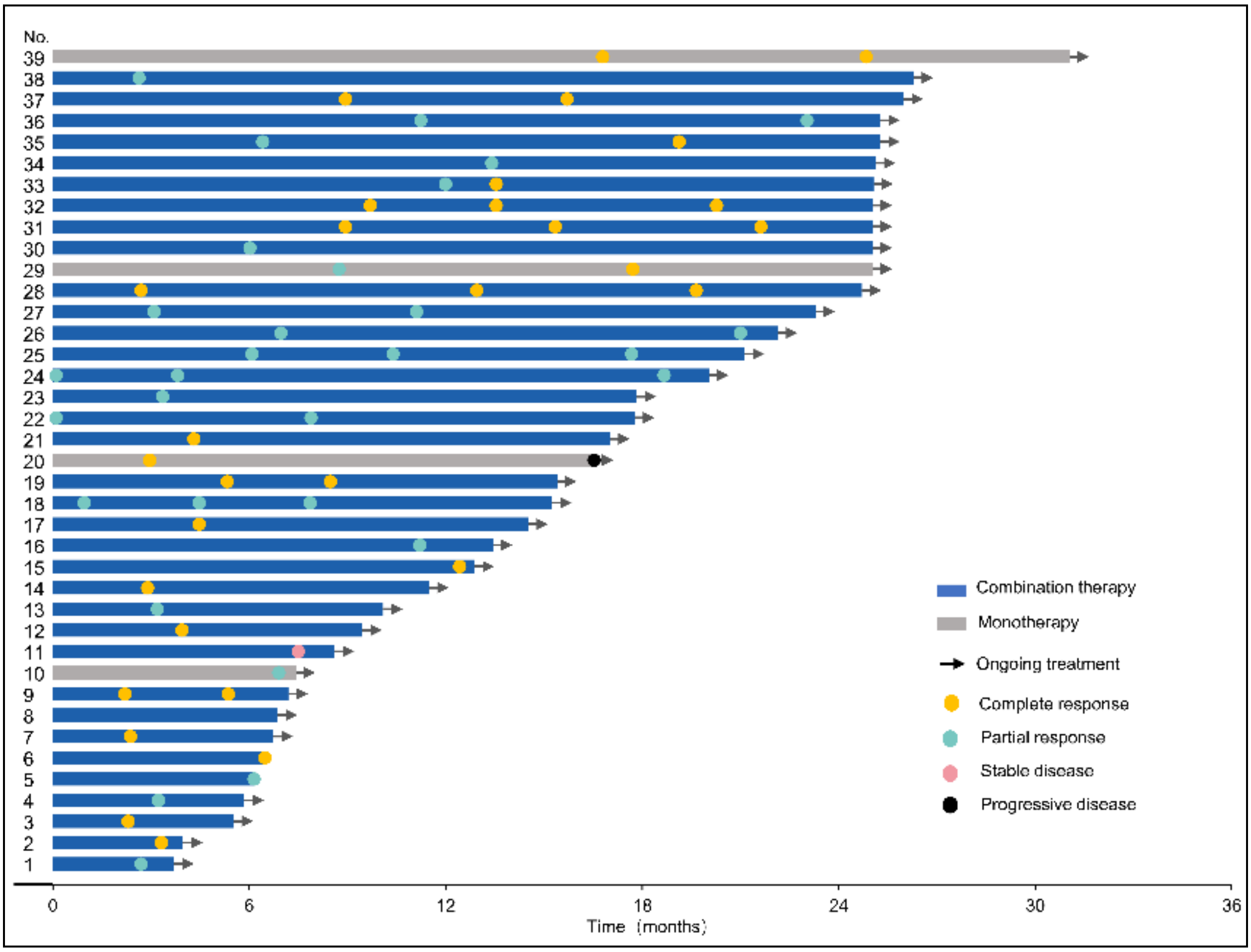

Figure 1. Swimmer plot of tumor response

**Copyright Information:** (Online) ISSN: 2572-9241
©2026 The Author(s). HemaSphere is published by John Wiley & Sons Ltd on behalf of European Hematology Association. This is an open access Abstract Book distributed under the Attribution-NonCommercial-NoDerivs (CC BY-NC-ND), which allows third parties to download the articles and share them with others as long as they credit the author and the Abstract Book, but they cannot change the content in any way or use them commercially.
**Abstract Book Citations:** Authors, Title, HemaSphere, 2026;10:(S1):pages. The abstract book DOIs can be found on the "Author Information" page.
**Disclaimer:** Articles published in the journal HemaSphere exclusively reflect the opinions of the authors. The authors are responsible for all content in their abstracts including accuracy of the facts, statements, citing resources, etc.

### (PB3678) A PROSPECTIVE STUDY ON THE EFFICACY AND SAFETY OF ORELABRUTINIB COMBINED WITH R-CHEMO REGIMEN FOLLOWED BY ORELABRUTINIB MAINTENANCE THERAPY IN MZL

**Topic:** 18. Indolent and mantle-cell non-Hodgkin lymphoma - Clinical

Zhirui Yang[1], Liren Qian[1], Wei Guan[1], Andi Zhang[1], Yong-Feng Su[1], Haitao Wang[2], Cuicui Feng[3], Hang Yu[1], Yu Liu[1], Haiyan Zhu*[1]

*[1]The Fifth Medical Center of Chinese PLA General Hospital, Beijing, China;[2]The Fourth Medical Center of Chinese PLA General Hospital, Beijing, China; [3]Beijing Anzhen Hospital,Capital Medical University, Beijing, China;*

### Background

Currently, there is no standardized treatment regimen with high-level evidence for newly diagnosed MZL. Even with immunochemotherapy, there are still some patients with poor prognosis. Therefore, whether the existing immunochemotherapy regimen can meet all the treatment needs of MZL remains to be explored, and further improvement in efficacy and benefit is still needed for newly diagnosed MZL patients.

### Aims

This study is a prospective, multi-center clinical study aimed at evaluating the efficacy and safety of orelabrutinib combined with immunochemotherapy regimens as a first-line treatment in newly diagnosed MZL patients with treatment indications. For MZL patients requiring immunochemotherapy, we adopt orelabrutinib combined with rituximab-based immunochemotherapy, followed by sequential orelabrutinib maintenance, with the goal of increasing the overall response rate of MZL, shortening the time to response, and achieving better PFS and OS.

### Methods

Patients received 1 cycle of rituximab-based immunochemotherapy (R-Chemo) regimen treatment. In the second course, orelabrutinib was added in combination with R-Chemo for treatment, and after the end of induction therapy, they entered the orelabrutinib monotherapy maintenance phase. The primary endpoint of the study is the complete response rate at the end of induction treatment (Eot CRR), and secondary endpoints include overall response rate (ORR), progression-free survival (PFS), and overall survival (OS). Safety is assessed by monitoring adverse events (AEs).

### Results

Currently, 7 patients have been enrolled in this study, with males accounting for 85.7% (6/7), median age at diagnosis is 71 years (58-86), among which 5 patients have nodal marginal zone lymphoma (NMZL). All patients are at Ann Arbor stage IV, 71.4% of patients have MZL-IPI score of 2. 3 patients received BR combined with orelabrutinib regimen, 2 patients received RCHOP combined with orelabrutinib regimen, one patient received RminiCHOP combined with orelabrutinib regimen, and another patient received RMTX combined with orelabrutinib regimen due to central nervous system involvement. All patients completed interim evaluations, ORR was 85.7%, CR rate was 42.8%. At the completion of 6 cycles of treatment, 6 patients were evaluable for efficacy, EOT ORR was 83.3%, EOT CR rate was 83.3%, median OS and PFS were not reached. 57.1% (4/7) of patients experienced AEs, among which 2 patients experienced grade 3 myelosuppression. No atrial fibrillation or bleeding related to BTK inhibitors occurred.

**Copyright Information:** (Online) ISSN: 2572-9241
©2026 The Author(s). HemaSphere is published by John Wiley & Sons Ltd on behalf of European Hematology Association. This is an open access Abstract Book distributed under the Attribution-NonCommercial-NoDerivs (CC BY-NC-ND), which allows third parties to download the articles and share them with others as long as they credit the author and the Abstract Book, but they cannot change the content in any way or use them commercially.
**Abstract Book Citations:** Authors, Title, HemaSphere, 2026;10:(S1):pages. The abstract book DOIs can be found on the "Author Information" page.
**Disclaimer:** Articles published in the journal HemaSphere exclusively reflect the opinions of the authors. The authors are responsible for all content in their abstracts including accuracy of the facts, statements, citing resources, etc.

**Summary/Conclusion**

Immunochemotherapy combined with Orelabrutinib regimen as first-line treatment for MZL is an effective treatment strategy, but attention should be paid to safety issues. Future research updates will include more follow-up data.

**Copyright Information:** (Online) ISSN: 2572-9241
©2026 The Author(s). HemaSphere is published by John Wiley & Sons Ltd on behalf of European Hematology Association. This is an open access Abstract Book distributed under the Attribution-NonCommercial-NoDerivs (CC BY-NC-ND), which allows third parties to download the articles and share them with others as long as they credit the author and the Abstract Book, but they cannot change the content in any way or use them commercially.
**Abstract Book Citations:** Authors, Title, HemaSphere, 2026;10:(S1):pages. The abstract book DOIs can be found on the "Author Information" page.
**Disclaimer:** Articles published in the journal HemaSphere exclusively reflect the opinions of the authors. The authors are responsible for all content in their abstracts including accuracy of the facts, statements, citing resources, etc.

### (PB3679) OUTCOMES OF FIRST-LINE BTK INHIBITOR- BASED THERAPY IN NEWLY DIAGNOSED MANTLE CELL LYMPHOMA

**Topic:** 18. Indolent and mantle-cell non-Hodgkin lymphoma - Clinical

Julia Zhuravleva*[1, 2], Elena Baryakh[1, 2], Liliia Shimanovskaya[1, 2], Tatiana Tolstykh[1, 2], Marat Mingalimov[1, 2], Dmitrii Lebedev[1, 2], Diana Ivanova[1, 2], Elena Misyurina[1, 2]

*[1]Moscow Clinical Science and Research Center 52, Moscow, Russia;[2]Sechenov First Moscow State Medical University, Moscow, Russia;*

### Background

Mantle cell lymphoma (MCL) is a unique subtype of non-Hodgkin lymphoma with a heterogeneous presentation and clinical course. Important steps in the development of treatment in MCL have been the use of BTK inhibitors that have been demonstrated to be safe and effective in patients with untreated MCL especially in the biologically high-risk subset. This has allowed targeted treatment modalities to become standard of care in first-line and maintenance therapy.

### Aims

To evaluate the efficacy and safety of first line BTK inhibitor-based therapy in newly diagnosed MCL.

### Methods

This retrospective study enrolled 30 patients with a histologically confirmed diagnosis of mantle cell lymphoma who were treated between 2018 and 2025. Descriptive statistics were used to report baseline disease and patient characteristics (Table 1). Treatment response was assessed by PET/CT according to the Lugano criteria. Overall survival and progression-free survival were analyzed using the Kaplan–Meier method.
**Table 1.**

**Copyright Information:** (Online) ISSN: 2572-9241
©2026 The Author(s). HemaSphere is published by John Wiley & Sons Ltd on behalf of European Hematology Association. This is an open access Abstract Book distributed under the Attribution-NonCommercial-NoDerivs (CC BY-NC-ND), which allows third parties to download the articles and share them with others as long as they credit the author and the Abstract Book, but they cannot change the content in any way or use them commercially.
**Abstract Book Citations:** Authors, Title, HemaSphere, 2026;10:(S1):pages. The abstract book DOIs can be found on the “Author Information” page.
**Disclaimer:** Articles published in the journal HemaSphere exclusively reflect the opinions of the authors. The authors are responsible for all content in their abstracts including accuracy of the facts, statements, citing resources, etc.

| Variable | N, % |
|---|---|
| **Gender**<br>Male<br>Female | 18 (60%)<br>12 (40%) |
| **Ann-Arbor disease staging (n=29)**<br>I<br>II<br>III<br>IV | 1 (3,3%)<br>5 (16,7%)<br>5 (16,7%)<br>19 (63,3%) |
| **Morhology (n=19)**<br>Blastoid<br>Others | 9 (47,4%)<br>10 (52,6%) |
| **Median age, years (range)** | 63 (44 – 85) |
| **Bone marrow involvement (n=28)**<br>Yes<br>No | 17 (60,7%)<br>11 (39,3%) |
| **Therapy regimen**<br>BTK+chemotherapy<br>BTK+chemo-free<br>BTK-mono | 24 (80%)<br>3 (10%)<br>3 (10%) |
| **Ki67 (n=20)**<br>>30%<br><30% | 12 (60%)<br>8 (40%) |
| **TP53 mut (n=16)**<br>Yes<br>No | 5 (31,2%)<br>11 (68,8%) |

### Results

The median follow-up was 26 months. The 2-year overall survival (OS) and progression-free survival (PFS) rates were 61.7% (95% CI:44.2-86.2) and 53.7% (95% CI: 37.2-77.5), respectively. The 2-year non-relapse mortality was 26.2% (95%: CI, 2.7-44.1). At the time of analysis, 17 of 30 patients were alive, including 11 in complete remission, 2 with stable disease, 2 with partial remission, and 1 with progressive disease. One patient has not yet undergone PET/CT evaluation.
Among 13 recorded deaths, disease progression was the leading cause (n=7, 53.8%), followed by infections (n=2, 15.4%) and comorbidities (n=2, 15.4%), while COVID-19 and secondary malignancy accounted for one case each (7.7%).

### Summary/Conclusion

Initial data demonstrate clinical activity of BTK inhibitors in the first-line treatment of MCL.However, outcomes were influenced by non-relapse mortality and real-world treatment heterogeneity. Longer follow-up is needed to optimize therapeutic strategies and refine patient selection

**Copyright Information:** (Online) ISSN: 2572-9241
©2026 The Author(s). HemaSphere is published by John Wiley & Sons Ltd on behalf of European Hematology Association. This is an open access Abstract Book distributed under the Attribution-NonCommercial-NoDerivs (CC BY-NC-ND), which allows third parties to download the articles and share them with others as long as they credit the author and the Abstract Book, but they cannot change the content in any way or use them commercially.
**Abstract Book Citations:** Authors, Title, HemaSphere, 2026;10:(S1):pages. The abstract book DOIs can be found on the "Author Information" page.
**Disclaimer:** Articles published in the journal HemaSphere exclusively reflect the opinions of the authors. The authors are responsible for all content in their abstracts including accuracy of the facts, statements, citing resources, etc.

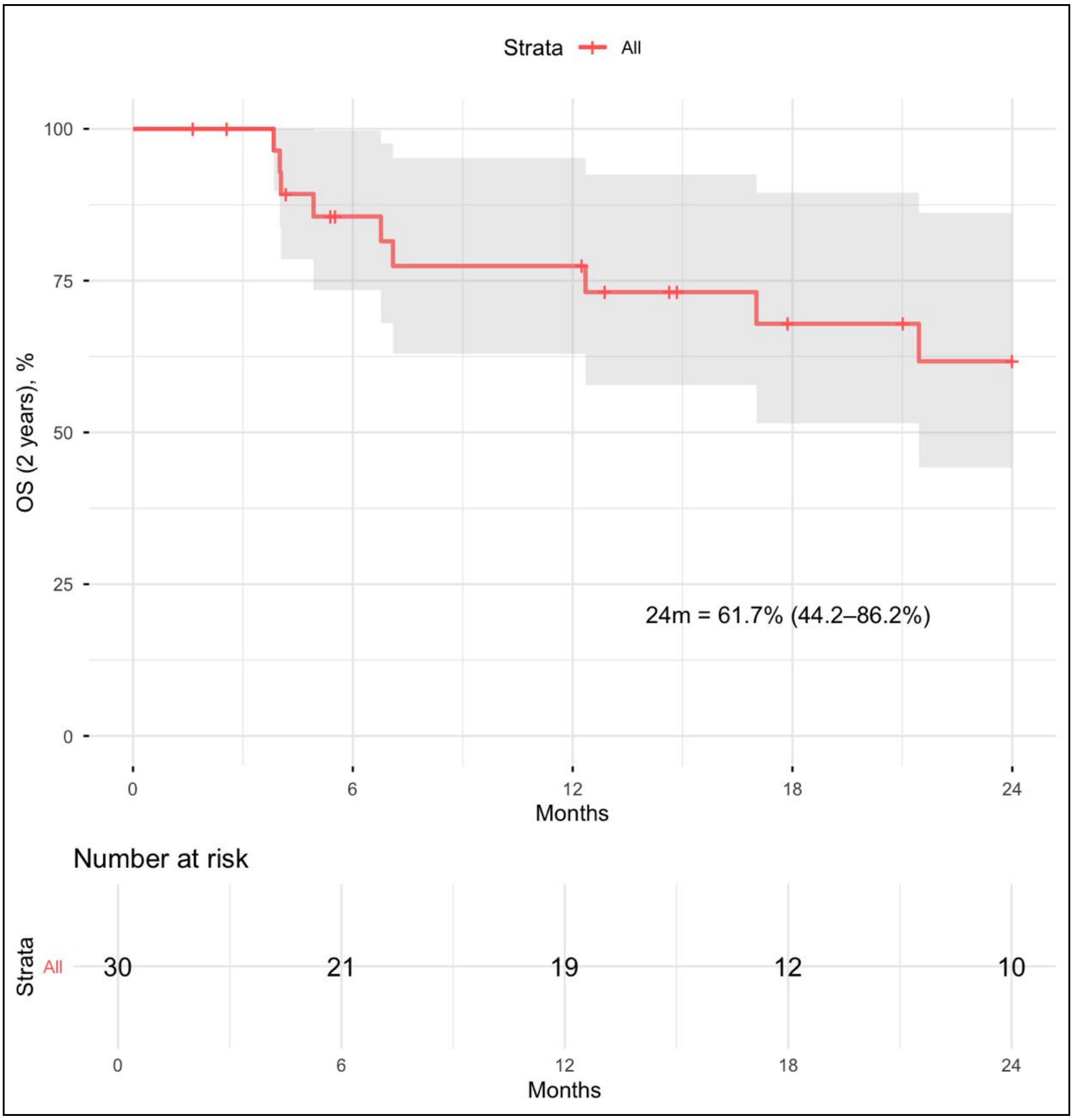

The 2-year overall survival

Copyright Information: (Online) ISSN: 2572-9241
©2026 The Author(s). HemaSphere is published by John Wiley & Sons Ltd on behalf of European Hematology Association. This is an open access Abstract Book distributed under the Attribution-NonCommercial-NoDerivs (CC BY-NC-ND), which allows third parties to download the articles and share them with others as long as they credit the author and the Abstract Book, but they cannot change the content in any way or use them commercially.
Abstract Book Citations: Authors, Title, HemaSphere, 2026;10:(S1):pages. The abstract book DOIs can be found on the "Author Information" page.
Disclaimer: Articles published in the journal HemaSphere exclusively reflect the opinions of the authors. The authors are responsible for all content in their abstracts including accuracy of the facts, statements, citing resources, etc.

### (PB3680) PRIMARY PULMONARY MARGINAL ZONE LYMPHOMA: A MULTICENTER EXPERIENCE WITH 14 PATIENTS TREATED WITH ORELABRUTINIB-BASED THERAPY

**Topic:** 18. Indolent and mantle-cell non-Hodgkin lymphoma - Clinical

Hongbing MA*[1], Ming Jiang[2], Ying Chen[3], Yun Zhan[3], Shisi Huang[3], Ping Liu[3], Rui Gao[3]

[1]*West China Hospital, Sichuan University, Hematology Department, Chengdu, China;*[2]*West China Hospital, Sichuan University, Department of Oncology, Chengdu, China;* [3]*The Affiliated Hospital of Guizhou Medical University, Guiyang, China;*

### Background

Pulmonary marginal zone lymphoma is a rare disease arising from bronchial-associated lymphoid tissue (BALT). Limited data exist regarding its clinical presentation, natural history, and optimal treatment. Current modalities include surgery, radiotherapy, and systemic therapy, but lung function preservation remains challenging with local approaches. Orelabrutinib is a novel oral, highly selective, irreversible Bruton's tyrosine kinase (BTK) inhibitor—the only BTK inhibitor approved for marginal zone lymphoma in China. This study evaluated the efficacy and safety of orelabrutinib-based therapy in patients with biopsy-proven primary pulmonary MALT lymphoma.

### Methods

We conducted a retrospective analysis of patients with biopsy-proven primary pulmonary MALT lymphoma treated with orelabrutinib-containing regimens (monotherapy or combination with rituximab or chemotherapy) at three centres in China. Primary endpoints included objective response rate (ORR), complete response (CR) rate, progression-free survival (PFS), overall survival (OS), and safety.

### Results

Between December 2018 and January 2026,14 patients with newly diagnosed primary pulmonary MALT lymphoma,including 4 (28.5%) males and 10 (71.4%) females with a median age of 57 years (range, 41-71). At the time of diagnosis, 3 (21.4%) were seen with B symptoms. According to the Ann Arbor system, 10 (71.4%) had stage I-II, and 4 (28.6%) stage IV disease; 8 (57.1%) had a bulky mass (≥7 cm) and 1 (7.1%) had bone marrow involvement. The main radiologic findings were pulmonary infiltrates (9 cases, 64.3%) and nodular lesions (5 cases, 35.7%). The lesions were localized and monolateral in 6 cases and multifocal and bilateral in 8 cases. Most lesions involved single lymph node regions (7 cases, 50.0%) and 1 extranodal organ (9 cases, 64.3%). Of 12 evaluable patients, all achieved objective response (ORR 100%), including 6 CRs and 6 PRs. CRs were observed in 4 of 5 patients (80%) receiving orelabrutinib plus rituximab, 1 of 1(100%) receiving monotherapy, and 0 of 5 (0%) receiving chemotherapy-based regimens; the single patient receiving orelabrutinib plus lenalidomide also achieved CR. With median follow-up of 15.6 months (range 6.8–17.9), no patient has progressed or died; all remain in remission. No grade ≥3 adverse events (AEs) were observed, or BTKi-related AEs occurred.

### Summary/Conclusion

Primary pulmonary marginal zone lymphoma is rarely reported due to scarcity of lymphoid tissue in normal adult lungs. Orelabrutinib-based therapy, particularly in combination with rituximab, achieved deep and durable remissions while preserving pulmonary function, offering an effective option for this rare lung-limited malignancy.

**Copyright Information:** (Online) ISSN: 2572-9241
©2026 The Author(s). HemaSphere is published by John Wiley & Sons Ltd on behalf of European Hematology Association. This is an open access Abstract Book distributed under the Attribution-NonCommercial-NoDerivs (CC BY-NC-ND), which allows third parties to download the articles and share them with others as long as they credit the author and the Abstract Book, but they cannot change the content in any way or use them commercially.
**Abstract Book Citations:** Authors, Title, HemaSphere, 2026;10:(S1):pages. The abstract book DOIs can be found on the "Author Information" page.
**Disclaimer:** Articles published in the journal HemaSphere exclusively reflect the opinions of the authors. The authors are responsible for all content in their abstracts including accuracy of the facts, statements, citing resources, etc.

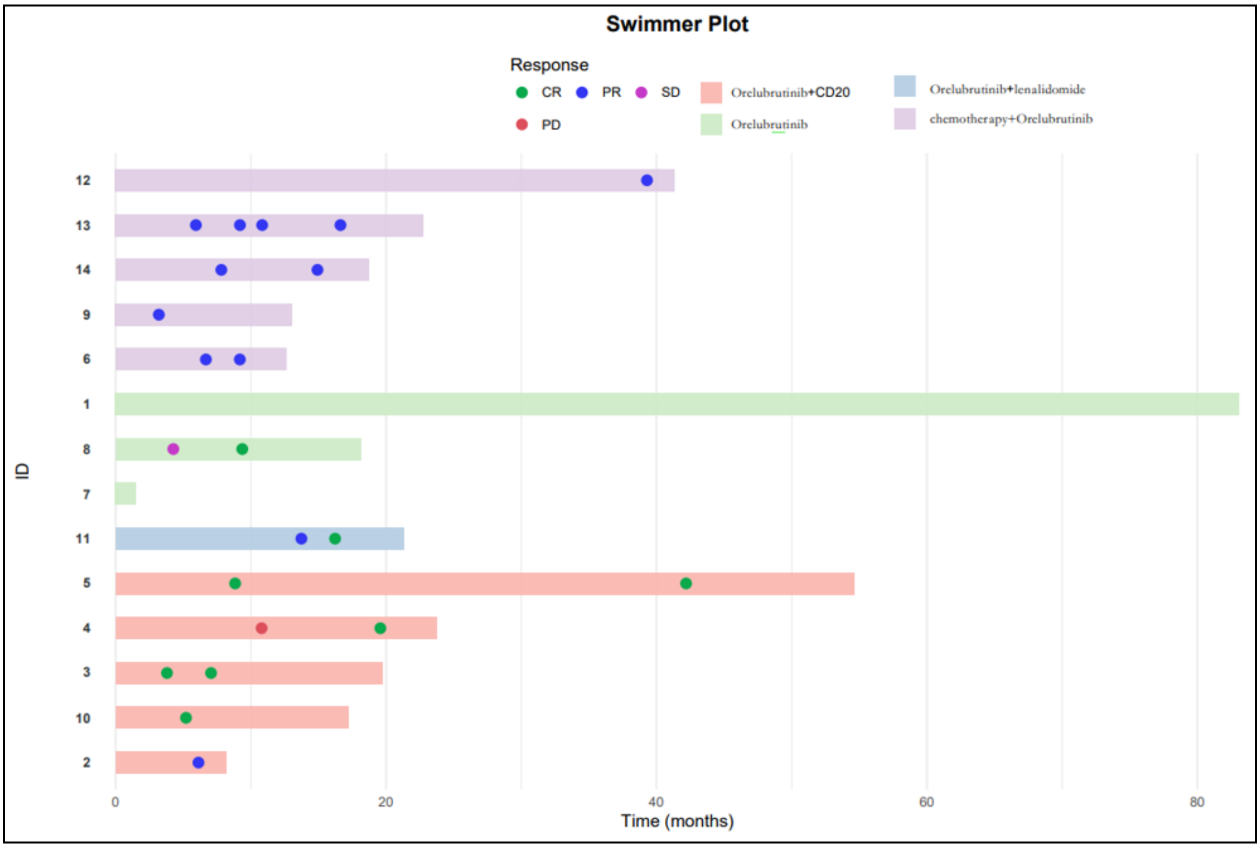

**Copyright Information:** (Online) ISSN: 2572-9241
©2026 The Author(s). HemaSphere is published by John Wiley & Sons Ltd on behalf of European Hematology Association. This is an open access Abstract Book distributed under the Attribution-NonCommercial-NoDerivs (CC BY-NC-ND), which allows third parties to download the articles and share them with others as long as they credit the author and the Abstract Book, but they cannot change the content in any way or use them commercially.
**Abstract Book Citations:** Authors, Title, HemaSphere, 2026;10:(S1):pages. The abstract book DOIs can be found on the "Author Information" page.
**Disclaimer:** Articles published in the journal HemaSphere exclusively reflect the opinions of the authors. The authors are responsible for all content in their abstracts including accuracy of the facts, statements, citing resources, etc.

### (PB3681) HIGH TREATMENT ATTRITION IN MANTLE CELL LYMPHOMA DESPITE PRESERVED RESPONSE: A REAL-WORLD SINGLE TERTIARY CANCER CENTER EXPERIENCE FROM INDONESIA

**Topic:** 18. Indolent and mantle-cell non-Hodgkin lymphoma - Clinical

Shafira Puspadina*[1], Noorwati Sutandyo[1], Hilman Tadjoedin[1], Dwi Hadisantoso[1], Nia Siregar[1]

[1]*Dharmais Cancer Hospital, Jakarta, Indonesia;*

### Background

Mantle cell lymphoma (MCL) is a rare and generally aggressive mature B-Cell lymphoma with heterogeneous clinical presentations and wide range of outcome. MCL in low-and middle-income countries presents unique challenges due to diagnostic delays, limited access to treatment, resource constraints, and high-cost treatment options. While newer clinical trials report favourable response rates with novel agents, real-world data from resource-limited settings are lacking due to diagnosis and treatment feasibility and continuity.

### Aims

To describe the clinical characteristics, treatment patterns, and attrition rate of patients with MCL at a national cancer center in Indonesia.

### Methods

We conducted a retrospective single-center study of 47 patients diagnosed with MCL between June 2020 and June 2025 from Dharmais Cancer Hospital in Indonesia. Baseline characteristics, first-line treatment patterns, treatment completion, response outcomes, and status at last follow-up were analysed. Treatment attrition was defined as failure to initiate or complete planned course of first-line systemic therapy.

### Results

The median age was 61 years (range 26-80), and 33 patients (70.2%) were male. Among subjects with complete pre-treatment staging, majority of the patients had advanced-stage disease (n=23, 57.5%). High risk MIPI was observed in 27 patients (57.4%), intermediate in 10 (21.2%), and was unknown in 10 patients (21.2%). After diagnosis fourteen patients (30%) patients did not initiate systemic therapy due to various reasons (patient's refusal, patient's death, and other reasons). Of the 47 patients, 32 patients (68%) were treated with systemic therapy, 11 (23%) were lost to follow-up, and 2 (4%) were managed with a watch-and-wait strategy. The most used first-line regimen was CHOP (n=14, 30%), followed by R-CHOP (n=9, 19%), and other regimens (n=7, 15%). Early discontinuation was common, with only 17 patients (36% of the total cohort, or 53% of those who started) completed their planned treatment. This resulted in an overall treatment attrition rate of 65%. Among the 21 patients with evaluable responses, the best response was partial response in 12 (57%) and complete response in 7 (33%). At the time of last follow-up, the number of patients lost to follow-up exceeded documented mortality.

**Copyright Information:** (Online) ISSN: 2572-9241
©2026 The Author(s). HemaSphere is published by John Wiley & Sons Ltd on behalf of European Hematology Association. This is an open access Abstract Book distributed under the Attribution-NonCommercial-NoDerivs (CC BY-NC-ND), which allows third parties to download the articles and share them with others as long as they credit the author and the Abstract Book, but they cannot change the content in any way or use them commercially.
**Abstract Book Citations:** Authors, Title, HemaSphere, 2026;10:(S1):pages. The abstract book DOIs can be found on the "Author Information" page.
**Disclaimer:** Articles published in the journal HemaSphere exclusively reflect the opinions of the authors. The authors are responsible for all content in their abstracts including accuracy of the facts, statements, citing resources, etc.

**Summary/Conclusion**

This study shows that at our hospital, a national cancer center in Indonesia, patients experience attrition to treatment at various points: at the time of diagnosis, at the start of treatment, or during the course of treatment. Most patients who received treatment were mostly treated with conventional regimens (CHOP) without biologic or novel agents, due to limited coverage under the national health insurance. While these patients still show some therapeutic response, the treatment provided is often inadequate. This shows structural barriers in our healthcare system as the primary obstacle, rather than true biological refractoriness. Without systemic support for access to diagnosis, comprehensive cancer treatment, continuity of care, and ensuring patient accompaniment through the treatment plan, the advances in MCL treatment with or without novel agents will remain out of reach for many in Indonesia.

**Copyright Information:** (Online) ISSN: 2572-9241
©2026 The Author(s). HemaSphere is published by John Wiley & Sons Ltd on behalf of European Hematology Association. This is an open access Abstract Book distributed under the Attribution-NonCommercial-NoDerivs (CC BY-NC-ND), which allows third parties to download the articles and share them with others as long as they credit the author and the Abstract Book, but they cannot change the content in any way or use them commercially.
**Abstract Book Citations:** Authors, Title, HemaSphere, 2026;10:(S1):pages. The abstract book DOIs can be found on the “Author Information” page.
**Disclaimer:** Articles published in the journal HemaSphere exclusively reflect the opinions of the authors. The authors are responsible for all content in their abstracts including accuracy of the facts, statements, citing resources, etc.

### (PB3682) BONE MARROW INVOLVEMENT PATTERNS IN MANTLE CELL LYMPHOMA: A MULTICENTER RETROSPECTIVE STUDY

**Topic:** 18. Indolent and mantle-cell non-Hodgkin lymphoma - Clinical

Yutong Wang*[1, 2], Li Wang[1], Hui Zhou[1], Xinzhu Tang[1], Ailin Zhao[1, 3, 4, 5], Ting Niu[1, 3, 4, 5]

*[1]West China Hospital, Sichuan University, Hematology Department, Chengdu, China;[2]West China School of Medicine, Sichuan University, Chengdu, China; [3]Institute of Hematology, West China Hospital, Sichuan University, Chengdu, China;[4]State Key Laboratory of Biotherapy, Collaborative Innovation Center of Biotherapy, West China Hospital, Sichuan University, Chengdu, China; [5]National Facility for Translational Medicine (Sichuan), West China Hospital, Sichuan University, Chengdu, China;*

### Background

Mantle Cell Lymphoma (MCL) is a heterogenous B-NHL with both indolent and aggressive characteristics. Bone marrow Involvement (BMI) is the most common extranodal infiltration but covert and easily ignored.

### Aims

To evaluate BMI patterns in MCL and their impact on prognosis and associated risk factors.

### Methods

This is a multicenter retrospective study continuously enrolled newly diagnosed classic MCL patients with BMI from January 2023 to December 2025. The primary endpoint is progression-free survival (PFS). Other endpoints include overall response rate (ORR) and complete response rate (CRR).

### Results

Overall, 2 centers enrolled 90 patients. 44 patients had extranodal involvement only in BM (BM-O), and 46 had extranodal involvement beyond BM (BM-B). No significant differences exist in baseline characteristics between two groups, except more splenomegaly (p=0.035), more monoclonal B lymphocytes detected by FC, and fewer areas of lymph node involvement (p=0.016) in BM-O (Table 1). Most Patients (98.8%) were confirmed with BMI by FC, among whom 38 patients were confirmed only by FC, and 14 BM-O patients were diagnosed exclusively by FC, despite their BM biopsy being negative. BM biopsy pathology is positive in 52.6% (47.5% in BM-B vs 57.9% in BM-O), and BMI founded by PET-CT was only in 24.7% patients (20.9% vs 29.4%). 23.3% patients had peripheral blood infiltration (PBI). The median follow-up time was 12 months. The 1-year PFS rate was 86.7%, with a CRR of 69.7% and ORR of 90.9%. Elevated LDH (HR 1.03, 95% CI 1.01-1.05, p = 0.017) and higher proportion of other cells in PB (1.51 1.04-2.20, p = 0.030) were related to shorter PFS, Ki-67≥30% (OR 5.02 1.00–25.32, p = 0.049), PBI (4.75 1.49-15.18, p = 0.009), high MIPI-c score (4.80 1.01-23.21, p = 0.048), and induction therapy without anti-CD20 (11.11 1.16-100.00, p=0.036) were related to lower CRR, and the number of involved lymph node regions (1.62 1.09-2.38, p = 0.016) and PBI (6.57 1.09-39.75, p = 0.040).

**Copyright Information:** (Online) ISSN: 2572-9241
©2026 The Author(s). HemaSphere is published by John Wiley & Sons Ltd on behalf of European Hematology Association. This is an open access Abstract Book distributed under the Attribution-NonCommercial-NoDerivs (CC BY-NC-ND), which allows third parties to download the articles and share them with others as long as they credit the author and the Abstract Book, but they cannot change the content in any way or use them commercially.
**Abstract Book Citations:** Authors, Title, HemaSphere, 2026;10:(S1):pages. The abstract book DOIs can be found on the "Author Information" page.
**Disclaimer:** Articles published in the journal HemaSphere exclusively reflect the opinions of the authors. The authors are responsible for all content in their abstracts including accuracy of the facts, statements, citing resources, etc.

| Characteristics | All Patients (n=90) | BM-B (n = 46) | BM-O (n = 44) | P |
|---|---|---|---|---|
| Age (years) | 64 (56, 69) | 65 (54, 70) | 64 (59, 69) | .734 |
| Male | 69/90 | 32/46 | 37/44 | .136 |
| ECOG[illegible]2 | 73/90 | 36/46 | 37/44 | .593 |
| B Symptoms | 20/90 | 11/46 | 9/44 | .802 |
| Splenomegaly | 46/88 | 19/46 | 27/42 | .035 |
| MIPI-c | | | | .802 |
| Low | 20/90 | 11/46 | 9/44 | |
| Low-Intermediate | 29/90 | 15/46 | 14/44 | |
| High-Intermediate | 20/90 | 11/46 | 9/44 | |
| High | 20/90 | 9/49 | 11/44 | |
| Blastoid/pleomorphic | 12/90 | 5/46 | 7/44 | .546 |
| Ki-67≥30% | 43/90 | 26/46 | 17/44 | .195 |
| Positive SOX-11 | 55/68 | 31/36 | 24/32 | .356 |
| Lymph Node Area Involvement | 13 (7, 15) | 14.0 (11.3, 15.8) | 11.5 (7.0, 14.0) | .016 |
| Diagnostic Evidence | | | | |
| FC | 84/85 | 41/41 | 43/44 | .999 |
| Histopathology | 41/78 | 19/40 | 22/38 | .375 |
| PET-CT | 19/77 | 9/43 | 10/34 | .434 |
| PB | 21/90 | 9/46 | 12/44 | .221 |
| BM Myelofibrosis | | | | |
| 0 | 45/78 | 26/40 | 19/38 | .661 |
| 1 | 30/78 | 13/40 | 17/38 | .353 |
| 2 | 3/78 | 1/40 | 2/38 | .610 |
| Proportion of Monoclonal B lymphocytes by FC | 7.5 (1.5, 22.8) | 3.4 (0.1, 10.8) | 11.7 (4.1, 39.0) | .007 |
| Restricted Light Chain Expression by FC | | | | .510 |
| κ | 32/85 | 17/41 | 15/44 | |
| λ | 42/85 | 20/41 | 22/44 | |
| HB (g/L) | 124 (111, 134) | 123 (114, 133) | 124 (93, 137) | .883 |
| WBC ($10^9$/L) | 6.8 (5.1, 10.2) | 6.4 (4.9, 8.1) | 7.1 (5.6, 13.07) | .189 |
| Lymphocyte ($10^9$/L) | 1.85 (1.24, 3.13) | 1.99 (1.21, 2.62) | 1.72 (1.34, 4.89) | .530 |
| PLT ($10^9$/L) | 153 (111, 202) | 159 (118, 200) | 149 (98, 201) | .405 |
| LDH (U/L) | 203 (166, 283) | 203 (178, 267) | 203 (163, 291) | .953 |
| Anti-CD20 Induction | 83/90 | 45/46 | 38/44 | .058 |
| BTKi Induction | 43/90 | 21/46 | 22/44 | .833 |
| Cytarabine Induction | 17/90 | 7/46 | 10/44 | .426 |
| Anti-CD20 Maintenance | 7/46 | 2/20 | 5/26 | .446 |
| BTKi Maintenance | 37/46 | 18/20 | 19/26 | .262 |

### Summary/Conclusion

FC is indispensable in MCL assessment; higher other cells in PB and LDH predict worse PFS, while PBI, no anti-CD20 induction, high Ki-67 and MIPI-c predict lower ORR and CRR.

**Copyright Information:** (Online) ISSN: 2572-9241
©2026 The Author(s). HemaSphere is published by John Wiley & Sons Ltd on behalf of European Hematology Association. This is an open access Abstract Book distributed under the Attribution-NonCommercial-NoDerivs (CC BY-NC-ND), which allows third parties to download the articles and share them with others as long as they credit the author and the Abstract Book, but they cannot change the content in any way or use them commercially.
**Abstract Book Citations:** Authors, Title, HemaSphere, 2026;10:(S1):pages. The abstract book DOIs can be found on the "Author Information" page.
**Disclaimer:** Articles published in the journal HemaSphere exclusively reflect the opinions of the authors. The authors are responsible for all content in their abstracts including accuracy of the facts, statements, citing resources, etc.

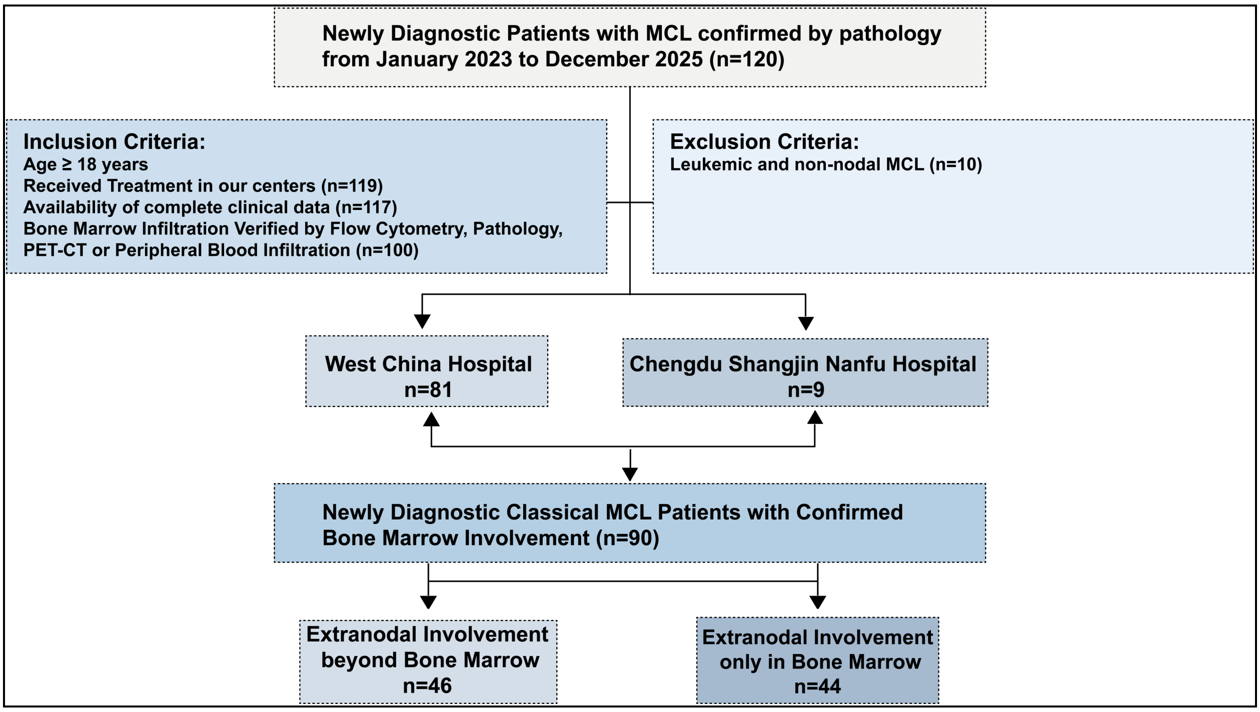

Figure 1 Flow Chart of the Study

**Copyright Information:** (Online) ISSN: 2572-9241
©2026 The Author(s). HemaSphere is published by John Wiley & Sons Ltd on behalf of European Hematology Association. This is an open access Abstract Book distributed under the Attribution-NonCommercial-NoDerivs (CC BY-NC-ND), which allows third parties to download the articles and share them with others as long as they credit the author and the Abstract Book, but they cannot change the content in any way or use them commercially.
**Abstract Book Citations:** Authors, Title, HemaSphere, 2026;10:(S1):pages. The abstract book DOIs can be found on the "Author Information" page.
**Disclaimer:** Articles published in the journal HemaSphere exclusively reflect the opinions of the authors. The authors are responsible for all content in their abstracts including accuracy of the facts, statements, citing resources, etc.

### (PB3683) PROGNOSTIC IMPACT OF FLIPI, FLIPI2, GELF SCORE, AND POD24 IN FOLLICULAR LYMPHOMA: A REAL-WORLD 10-YEAR MULTICENTER TUNISIAN STUDY

**Topic:** 18. Indolent and mantle-cell non-Hodgkin lymphoma - Clinical

Nesrine Ben Sayed*[1, 1], Wafa Miled[2], Jaied Rabeb[3], Anna Chabaane[4], Raoudha Mansouri[2], Maha Charfi[5], Slama Nader[6], Wided Cherif[7], Zaineb Mlayah[8], Hela Ghedira[3], Malek Sayedi[2], Med.Adnene Laatiri[8], Raihane Ben Lakhal[2], Nour Ben Adejlil[9], Yosra Ben Youssef[7], Fehmi Msadak[3], Moez Elloumi[10], Moez Medhaffar[11]

*[1]Farhat hached hospital, hematolgy department, SOUSSE, Tunisia; [2]Aziza Othmena hospital, hematolgy department, tunis, Tunisia; [3]Military Hopital, hematolgy department, tunis, Tunisia; [4]bone marrow transplantation center, tunis, Tunisia; [5]Hedi chaker Hospital, hematolgy department, sfax, Tunisia; [6]monastir hsopital, monastir, Tunisia; [7]Farhat hached hospital, hematolgy department, sousse, Tunisia; [8]monastir hsopital, hematolgy department, monastir, Tunisia; [9]bone marrow transplantation center, hematolgy department, tunis, Tunisia; [10]Hedi chaker Hospital, sfax, Tunisia; [11]Hedi chaker Hospital, hematolgy department, tunis, Tunisia;*

### Background

Follicular lymphoma (FL) is an indolent lymphoma characterized by heterogeneous clinical presentation and variable outcomes. Prognostic tools, including FLIPI, FLIPI2, GELF criteria, and early progression within 24 months (POD24), are essential for risk stratification.

### Aims

The aim was to analyse clinical presentation, treatment patterns, and prognostic impact of FLIPI, FLIPI2, GELF classification, and POD24.

### Methods

This Tunisian retrospective multicenter study included 157 patients diagnosed between January 2015 and December 2024 in six hematology and one oncology centre

### Results

Median age was 60 years (23–91), with a male predominance (59%). Diagnosis was mainly revealed by tumor syndrome (87.8%) and predominantly peripheral lymphadenopathy (87.7%). Advanced stage (III–IV) was observed in 75% of patients. High tumor burden (GELF ≥1) was present in 69%. Bone marrow infiltration was documented in 35% of evaluated cases, and LDH was elevated in 42%. First-line treatment consisted mainly of immunochemotherapy (85%), predominantly R-CHOP (83%). Overall response rate was 88%, including 61% complete responses. Rituximab maintenance therapy was administered in 60%. At 48 months, OS was 95%, RFS 90%, and EFS 91%. Relapse occurred in 16% of responders with a median time to relapse of 24.5 months. POD24 was observed in 11 patients. Prognostic analysis demonstrated that FLIPI was significantly associated with treatment response (p=0.004) and OS (p=0.002). FLIPI2 was significantly correlated with relapse risk (p=0.001). High tumor burden according to GELF criteria was associated with an increased relapse rate (p=0.034). Advanced stage negatively impacted response and survival (p=0.004 and p=0.05, respectively). POD24 emerged as the strongest adverse prognostic factor for OS (p=0.005). Histologic transformation occurred in 4 patients.

**Copyright Information:** (Online) ISSN: 2572-9241
©2026 The Author(s). HemaSphere is published by John Wiley & Sons Ltd on behalf of European Hematology Association. This is an open access Abstract Book distributed under the Attribution-NonCommercial-NoDerivs (CC BY-NC-ND), which allows third parties to download the articles and share them with others as long as they credit the author and the Abstract Book, but they cannot change the content in any way or use them commercially.
**Abstract Book Citations:** Authors, Title, HemaSphere, 2026;10:(S1):pages. The abstract book DOIs can be found on the “Author Information” page.
**Disclaimer:** Articles published in the journal HemaSphere exclusively reflect the opinions of the authors. The authors are responsible for all content in their abstracts including accuracy of the facts, statements, citing resources, etc.

**Summary/Conclusion**

In this Tunisian real-world cohort, FL was predominantly diagnosed at an advanced stage with high tumor burden. FLIPI, FLIPI2, GELF, and POD24 had significant prognostic value, with POD24 emerging as the most powerful predictor of inferior survival. Risk-adapted strategies remain essential to optimise outcomes.

**Copyright Information:** (Online) ISSN: 2572-9241
©2026 The Author(s). HemaSphere is published by John Wiley & Sons Ltd on behalf of European Hematology Association. This is an open access Abstract Book distributed under the Attribution-NonCommercial-NoDerivs (CC BY-NC-ND), which allows third parties to download the articles and share them with others as long as they credit the author and the Abstract Book, but they cannot change the content in any way or use them commercially.
**Abstract Book Citations:** Authors, Title, HemaSphere, 2026;10:(S1):pages. The abstract book DOIs can be found on the "Author Information" page.
**Disclaimer:** Articles published in the journal HemaSphere exclusively reflect the opinions of the authors. The authors are responsible for all content in their abstracts including accuracy of the facts, statements, citing resources, etc.

### (PB3684) CLINICAL PRESENTATION AND OUTCOMES OF SPLENIC MARGINAL ZONE LYMPHOMA: A MULTICENTER TUNISIAN STUDY

**Topic:** 18. Indolent and mantle-cell non-Hodgkin lymphoma - Clinical

Sayadi Malek*[1], Maha Charfi[2], Hela Ghedira[3], Slama Nader[4], Nesrine Ben Sayed[5], Miled Wafa[6], Haifa Hafsa[7], Raoudha Mansouri[6], Wala Ben Kridis[8], Jaied Rabeb[3], Wided Cherif[5], Zaineb Mlayah[4], Anna Chabaane[9], Moez Elloumi[8], Raihane Ben Lakhal[6], Fehmi Msadek[3], Med.Adnene Laatiri[4], Yosra Ben Youssef[5], Nour Ben Adejlil[9, 9], Moez Medhaffar[8]

*[1]Hôpital Aziza Othmana, CLINICAL HEMATOLOGY, Tunis, Tunisia;[2]Hôpital Universitaire Hédi Chaker, CLINICAL HEMATOLOGY, Sfax, Tunisia; [3]Hôpital Militaire, CLINICAL HEMATOLOGY, Tunis, Tunisia;[4]Hôpital Fattouma Bourguiba, Monastir, Tunisia; [5]Hôpital Farhat Hached, Sousse, Tunisia;[6]Hôpital Aziza Othmana, Tunis, Tunisia; [7]HOPITAL MOHAMED Sassi, CLINICAL HEMATOLOGY, gabes, Tunisia; [8]Hôpital Universitaire Hédi Chaker, Sfax, Tunisia;[9]CNGMO-Centre National de Greffe de la Moelle Osseuse, Tunis, Tunisia;*

### Background

Splenic marginal zone lymphoma (SMZL) is an indolent B-cell lymphoma characterized by splenomegaly, bone marrow infiltration, and frequent peripheral blood involvement. Although generally associated with a favorable prognosis, the clinical course is heterogeneous, and prognostic determinants remain insufficiently characterized, particularly in low- and middle-income countries.

### Aims the aim of our study was to analyze the epidemiological features, management strategies, and survival outcomes of patients diagnosed with SMZL in a multicenter Tunisian cohort.

### Methods

We performed a retrospective study including patients diagnosed with SMZL between January 2015 and december 2024 across 8 centers. Diagnosis was established based on histopathological, immunophenotypic, and clinical criteria. Baseline characteristics, laboratory findings, therapeutic approaches, and survival outcomes were collected. Overall survival (OS) and progression-free survival (PFS) were estimated using the Kaplan–Meier method. Prognostic factors were evaluated using Cox regression analysis.

**Copyright Information:** (Online) ISSN: 2572-9241
©2026 The Author(s). HemaSphere is published by John Wiley & Sons Ltd on behalf of European Hematology Association. This is an open access Abstract Book distributed under the Attribution-NonCommercial-NoDerivs (CC BY-NC-ND), which allows third parties to download the articles and share them with others as long as they credit the author and the Abstract Book, but they cannot change the content in any way or use them commercially.
**Abstract Book Citations:** Authors, Title, HemaSphere, 2026;10:(S1):pages. The abstract book DOIs can be found on the "Author Information" page.
**Disclaimer:** Articles published in the journal HemaSphere exclusively reflect the opinions of the authors. The authors are responsible for all content in their abstracts including accuracy of the facts, statements, citing resources, etc.

### Results

A total of 99 patients with SMZL were included (43% of zone marginal lymphoma). The median age at diagnosis was**62 years[28-87]**, with a sex-ratio of 1.6.
At presentation, B symptoms were present in 41% of patients, splenomegaly was observed in 85% of patients, anemia in 74% with 15 % of hemolytic anemia, thrombopenia in 36% and bone marrow involvement in 98%. Elevated LDH was reported in 37%. According to HPLL score, 19 % of patients were classified as high risk.
Initial management consisted of active surveillance in 32***% of cases, with a median observation period of 20 months [5-89]. Among the 89% of treated patients, the most frequently administered first-line treatment was Rituximab chlormbucil (40%), followed by RCHOP like regimen (32***%), ***followed by RCVP (18***%), Rituximab monotherapy (6%), Rituximab Bendamustine (1%); splenectomy was performed in 1%. Global response after first line therapy was 64%.
Second line tratment was heteregenous: RCHOP like (32%), RBendamustine (24%), Splenectomy (12%), Rituximab chlorombucil (8%), RCVP (4%) RGEMOX (4%), RDHAC (4%). Global response after second line therapy was 65%. Histologic transformation occurred in 3 patients. Three toxic death occured (after RCHOP, RBENDAMUSTINE and Splenectomy).
After a median follow-up of 54 months, the OS was 72.5 ***%, and the -year PFS was 78.8 %. In multivariate analysis, independent predictors of inferior PFSwas hight HPLL (p=0.009). Age>60 years and fail of treatment were significantly associated with shorter OS (p=0.037 and 0.01***).

### Summary/Conclusion

SMZL in our cohort demonstrated an overall indolent course with satisfactory long-term survival. However, a subset of patients exhibited adverse prognostic features associated with poorer outcomes. Identification of high-risk patients remains essential to optimize therapeutic decision-making in real-world practice.

**Copyright Information:** (Online) ISSN: 2572-9241
©2026 The Author(s). HemaSphere is published by John Wiley & Sons Ltd on behalf of European Hematology Association. This is an open access Abstract Book distributed under the Attribution-NonCommercial-NoDerivs (CC BY-NC-ND), which allows third parties to download the articles and share them with others as long as they credit the author and the Abstract Book, but they cannot change the content in any way or use them commercially.
**Abstract Book Citations:** Authors, Title, HemaSphere, 2026;10:(S1):pages. The abstract book DOIs can be found on the "Author Information" page.
**Disclaimer:** Articles published in the journal HemaSphere exclusively reflect the opinions of the authors. The authors are responsible for all content in their abstracts including accuracy of the facts, statements, citing resources, etc.

### (PB3685) ORELABRUTINIB PLUS VENETOCLAX IN PATIENTS WITH SPLENIC MARGINAL ZONE LYMPHOMA INTOLERANT TO RITUXIMAB

**Topic:** 18. Indolent and mantle-cell non-Hodgkin lymphoma - Clinical

Zhilin Gao*[1], Weiwei Tian[1], Yu Lian[1], Lifang Huang[2], Juanjuan Ti[1]

*[1]Shanxi Bethune Hospital, Shanxi Academy of Medical Sciences, Department of hematology, Taiyuan, China;[2]Tongji Hospital, Tongji Medical College, Huazhong University of Science and Technology, Wuhan, China;*

### Background

Rituximab-based regimen has ascertained to be effective in patients with marginal zone lymphoma (MZL). However, severe rituximab–related drug hypersensitivity reactions (e.g. laryngeal edema, anaphylactic shock, and urticaria) may still occur and can lead to discontinuation of rituximab-based first-line chemotherapy, which severely affect patient prognosis. In addition to premedication and desensitization, alternative treatment approaches should be explored in patients with rituximab intolerance. Orelabrutinib is a novel, oral, and covalent next-generation BTK inhibitor with high target selectivity and has been approved in China for the treatment of marginal zone lymphoma patients who have received at least one prior treatment. Besides, orelabrutinib plus venetoclax synergistically induce cell death in lymphoma by interfering with the crosstalk between the PI3K/AKT and p38/MAPK signaling.

### Aims

This study aimed to assess efficacy and safety of orelabrutinib plus venetoclax in patients with splenic marginal zone lymphoma (SMZL) who discontinued rituximab due to hypersensitivity reactions.

### Methods

This retrospective study enrolled patients with SMZL. Patients initially received rituximab-based chemoimmunotherapy as first-line treatment; however, severe hypersensitivity reactions were observed, including laryngeal edema with dyspnea (n=2), anaphylactic shock (n=2), and severe urticaria (n=1), rendering rituximab infusion intolerable. Conventional corticosteroid and anti-allergic management showed limited efficacy, and continued administration was considered potentially life-threatening. In addition, patients declined further rituximab-based regimen. Immunohistochemical analysis confirmed BCL2+ in all patients, and orelabrutinib (150 mg, once daily) plus venetoclax (100 mg, days 1-7) in a 28-day cycle was therefore initiated. The outcomes included objective response rate (ORR), progression-free survival (PFS), overall survival (OS), and safety.

### Results

As of February 9, 2026, 5 patients (male, 60%; median age, 64 years) were enrolled, most with Eastern Cooperative Oncology Group score of 2 (80%) and MZL-International Prognostic Index score of ≥3 (80%). B symptoms and *MYD88* mutation were both observed in 20% of patients, and splenomegaly occurred in all patients (100%). All patients completed 6 cycles of the orelabrutinib plus venetoclax regimen, with the ORR of 100% (CR: n=3; PR: n=2). No patient progression or died during a median follow-up of 8.6 (range 4.9-22.6) months with immature PFS and OS. Grade ≥3 adverse events occurred in 2/5 (40%) patients, including platelet count decreased (n=1) and lymphocyte count decreased (n=1).

**Copyright Information:** (Online) ISSN: 2572-9241
©2026 The Author(s). HemaSphere is published by John Wiley & Sons Ltd on behalf of European Hematology Association. This is an open access Abstract Book distributed under the Attribution-NonCommercial-NoDerivs (CC BY-NC-ND), which allows third parties to download the articles and share them with others as long as they credit the author and the Abstract Book, but they cannot change the content in any way or use them commercially.
**Abstract Book Citations:** Authors, Title, HemaSphere, 2026;10:(S1):pages. The abstract book DOIs can be found on the “Author Information” page.
**Disclaimer:** Articles published in the journal HemaSphere exclusively reflect the opinions of the authors. The authors are responsible for all content in their abstracts including accuracy of the facts, statements, citing resources, etc.

**Summary/Conclusion**

The combination of orelabrutinib and venetoclax resulted in promising efficacy and safety for SMZL intolerant to rituximab, despite being preliminary. More data will be updated from this ongoing study.

**Copyright Information:** (Online) ISSN: 2572-9241
©2026 The Author(s). HemaSphere is published by John Wiley & Sons Ltd on behalf of European Hematology Association. This is an open access Abstract Book distributed under the Attribution-NonCommercial-NoDerivs (CC BY-NC-ND), which allows third parties to download the articles and share them with others as long as they credit the author and the Abstract Book, but they cannot change the content in any way or use them commercially.
**Abstract Book Citations:** Authors, Title, HemaSphere, 2026;10:(S1):pages. The abstract book DOIs can be found on the "Author Information" page.
**Disclaimer:** Articles published in the journal HemaSphere exclusively reflect the opinions of the authors. The authors are responsible for all content in their abstracts including accuracy of the facts, statements, citing resources, etc.

### (PB3686) EFFICACY OF ULTRA-LOW-DOSE (ULD) RADIOTHERAPY IN MALT LYMPHOMA: REAL-WORLD DATA FROM AN UNIQUE BRAZILIAN CANCER CENTER

**Topic:** 18. Indolent and mantle-cell non-Hodgkin lymphoma - Clinical

Gabrielle Hyllen[1], Julia Balthazar[1], Matheus Garcia[1], Thais Fisher[1], Talita Maira Bueno Da Silveira*[1]

*[1]AC Camargo Cancer Center, São Paulo, Brazil;*

### Background

**BACKGROUND**

Extranodal marginal zone lymphomas of mucosa associated lymphoid tissue (MALT) is rare and account for 7-8% of all non-Hodgkin lymphomas. They are commonly associated with chronic antigenic stimulation due to infections or autoimmune conditions. The stomach is the most frequently affected organ because of its association with *H. pylori* infection. The initial treatment is usually the attempt to erradicate the infection, which can lead to remission in approximately 75% of cases. Moderate doses (24-30Gy) of radiotherapy (RT) are an option in cases of treatment failure, with high response rates. To reduce toxicity, ultra-low doses of radioteraphy have been tested, with good response rates. Na anterior trial showed a 83% complete response rate after 4Gy. Those who did not achieve a complete response recieved an additional dose of radiotherapy and thereafter achieved a complete response.

### Aims

This study aimed to describe the epidemiological characteristics and treatment outcomes of patients with newly diagnosed MALT lymphoma treated with ultra-low dose of RT in a Brazilian cancer center.

### Methods

We retrospectively collected data from patients diagnosed with MALT lymphoma between August 2016 and September 2025 who either did not present *H. pylori* infection or were refractory to its eradication and proceeded to ultra low dose radiotherapy.

### Results

A total of 16 patients were included. Most of the cases (93.75%) were gastric MALT lymphoma, while one case was a pulmonary BALT lymphoma. The median age at the diagnosis was 59.5 years (36-78) and 10 of 16 (62.5%) patients were male. Of the 16 patients, ten underwent PET imaging. Among these, the majority (9 patients) had localized disease and were classified as Ann Arbor stage I, whereas 1 patient presented with multifocal pulmonary BALT lymphoma. 11 (68.7%) patients had lactate dehydrogenase at the diagnosis, and the median level was 195 U/L (139-275). Of these 11 (90.9%) patients, 10 had low-risk MALT-IPI sore and 1 (9.1%) had an intermediate-risk. Of the 15 (93.7%) patients tested for *H. pylori*, 13 (86.7%) were negative. Only 1 patient had a positive t(11;18) status among the 5 (31.2%) patients who were tested. Most patients (75%) underwent empirical therapy to eradicate *H. pylori*. After 3 to 4 months, 13 patients (81.25%) were reassessed by endoscopy. 9 (69.23%) achieved complete response, 7.7% had partial endoscopic response, 15.38% had stable disease, and 7.7% experienced disease progression. An additional 20Gy of radiotherapy was administered to patients with stable disease, partial response or progressive disease. After a median follow-up of 16.5 months, 4 (25%) patients experienced disease progression, with a median time to progression of 10.5 months. No cases of death or histological transformation were observed in our cohort. One patient in partial response refused to add radiotherapy after 3 months of ULD and had CR after 6 months of the procedure.

**Copyright Information:** (Online) ISSN: 2572-9241
©2026 The Author(s). HemaSphere is published by John Wiley & Sons Ltd on behalf of European Hematology Association. This is an open access Abstract Book distributed under the Attribution-NonCommercial-NoDerivs (CC BY-NC-ND), which allows third parties to download the articles and share them with others as long as they credit the author and the Abstract Book, but they cannot change the content in any way or use them commercially.
**Abstract Book Citations:** Authors, Title, HemaSphere, 2026;10:(S1):pages. The abstract book DOIs can be found on the "Author Information" page.
**Disclaimer:** Articles published in the journal HemaSphere exclusively reflect the opinions of the authors. The authors are responsible for all content in their abstracts including accuracy of the facts, statements, citing resources, etc.

**Summary/Conclusion**

Ultra-low-dose RT showed excellent efficacy and disease control in newly diagnosed MALT lymphoma. Most patients achieved durable responses, and those with residual or progressive disease were successfully managed with additional RT. These real-world results support ultra-low-dose RT as a highly effective and well-tolerated treatment strategy, reinforcing its role as a frontline therapeutic option. And there is the suggestion of reevaluating this patients at least 6 months after de ULD.

**Copyright Information:** (Online) ISSN: 2572-9241
©2026 The Author(s). HemaSphere is published by John Wiley & Sons Ltd on behalf of European Hematology Association. This is an open access Abstract Book distributed under the Attribution-NonCommercial-NoDerivs (CC BY-NC-ND), which allows third parties to download the articles and share them with others as long as they credit the author and the Abstract Book, but they cannot change the content in any way or use them commercially.
**Abstract Book Citations:** Authors, Title, HemaSphere, 2026;10:(S1):pages. The abstract book DOIs can be found on the "Author Information" page.
**Disclaimer:** Articles published in the journal HemaSphere exclusively reflect the opinions of the authors. The authors are responsible for all content in their abstracts including accuracy of the facts, statements, citing resources, etc.

### (PB3687) ANALYSIS OF RISK FACTORS FOR INFUSION-RELATED REACTIONS IN PATIENTS WITH B-CELL LYMPHOMA DURING OBINUTUZUMAB TREATMENT

**Topic:** 18. Indolent and mantle-cell non-Hodgkin lymphoma - Clinical

Zi Wang*[1], Yan Zhang[1], Danqing Zhao[1], Chong Wei[1], Wei Wang[1], Dao-Bin Zhou[1], Wei Zhang[1]

*[1]Peking Union Medical College Hospital, Chinese Academy of Medical Science and Peking Union Medical College, Hematology,, Beijing, China;*

### Aims

This study aimed to investigate the risk factors associated with infusion-related reactions (IRRs) in patients with lymphoma receiving obinutuzumab treatment.

### Methods

A retrospective analysis of Peking Union Medical College Hospital patients (January 2021–December 2024) was conducted to examine various factors between patients experiencing IRRs (n = 44) and those without (n = 56). Parameters explored included gender, age, histological subtype, treatment status, Ann Arbor staging, extranodal/bone marrow involvement, bulky disease presence, splenomegaly status, serum lactate dehydrogenase (LDH), beta-2 microglobulin ($\beta_2$MG) concentrations and lymphocyte count. Univariate and multivariate analyses were performed.

### Results

A total of 100 patients with B-cell lymphoma (48 men, 52 women) with a mean age of 55.7 y (range: 44–67) received obinutuzumab for treatment at our hospital. Univariate analyses showed higher prevalence of treatment-naïve patients with bone marrow involvement and splenomegaly in the IRR group. Median LDH (235vs 195) and $\beta_2$MG (4 vs 3) levels pre-treatment were significantly elevated in the IRR group ($P < 0.05$). Multivariate analysis identified splenomegaly and pre-treatment LDH levels as potential risk factors for IRR occurrence.

### Summary/Conclusion

Potential risk factors for obinutuzumab-related IRRs include treatment-naïve status, bone marrow involvement, splenomegaly and elevated pre-treatment LDH and $\beta_2$MG levels. Special attention should be paid to splenomegaly and increased LDH levels, as well as to reducing or slowing the initial obinutuzumab dose to mitigate IRRs and enhance patient care. This research will assist in predicting and addressing IRRs to improve patient outcomes.

**Copyright Information:** (Online) ISSN: 2572-9241
©2026 The Author(s). HemaSphere is published by John Wiley & Sons Ltd on behalf of European Hematology Association. This is an open access Abstract Book distributed under the Attribution-NonCommercial-NoDerivs (CC BY-NC-ND), which allows third parties to download the articles and share them with others as long as they credit the author and the Abstract Book, but they cannot change the content in any way or use them commercially.
**Abstract Book Citations:** Authors, Title, HemaSphere, 2026;10:(S1):pages. The abstract book DOIs can be found on the "Author Information" page.
**Disclaimer:** Articles published in the journal HemaSphere exclusively reflect the opinions of the authors. The authors are responsible for all content in their abstracts including accuracy of the facts, statements, citing resources, etc.

### (PB3688) NODAL MARGINAL ZONE LYMPHOMA: RESULTS FROM A TUNISIAN MULTICENTER STUDY

**Topic:** 18. Indolent and mantle-cell non-Hodgkin lymphoma - Clinical

Slama Nader*[1], Malek Sayedi[2], Maha Charfi[3], Hela Ghedira[4], Nesrine Ben Sayed[5], Wala Ben Kridis[6], Haifa Hafsa[7], Wafa Miled[8], Raoudha Mansouri[9], Jaied Rabeb[10], Wided Cherif[11], Zaineb Mlayah[12], Anna Chabaane[13, 13], Mohamed Adnene Laatiri[12], Raihane Ben Lakhal[14], Moez Elloumi[15], Fahmi Msaddek[10], Yosra Ben Youssef[16], Moez Medhaffar[17]

*[1]Fattouma Bourguiba university hospital, hematology, Monastir, Tunisia; [2]Aziza Othmana hospital,, hematology, tunis, Tunisia; [3]Hedi Chaker hospital, hematology, Sfax, Tunisia; [4]military hospital, hematology, tunis, Tunisia; [5]Farhat Hached hospital, hematology, Sousse, Tunisia; [6]Hbib Bourguiba, oncology department, sfax, Tunisia; [7]Mohamed Ben Sassi, Gabes, Tunisia; [8]Aziza Othmana hospital, Hematology, tunis, Tunisia; [9]Aziza Othmana hospital, Tunis, Tunisia; [10]military hospital, hematology, Tunis, Tunisia; [11]Farhat Hached hospital, Hematology, sousse, Tunisia; [12]Fattouma Bourguiba hospital, hematology, Monastir, Tunisia; [13]National center of bone marrow transplantation, Hematology, Tunis, Tunisia; [14]Aziza Othmana hospital, hematology, Tunis, Tunisia; [15]Hedi Chaker hospital, Hematology, Sfax, Tunisia; [16]Farhat Hached hospital, Hematology, Sousse, Tunisia; [17]Hedi Chaker hospital, Hematology, sfax, Tunisia;*

### Background

Nodal marginal zone lymphoma (NMZL) is a rare indolent B-cell lymphoma with limited real-world data. We report epidemiological, clinical, radiological, and therapeutic outcomes from a Tunisian multicenter cohort.

### Methods

This retrospective study included patients diagnosed with marginal zone lymphoma between January 2015 and December 2024. Six university department of clinical hematology and one oncology department have participated in this study. NMZL was defined by exclusion of extranodal and splenic disease. Staging was based on Ann Arbor classification, and response assessment followed Lugano criteria.

### Results

Among 228 patients followed for MZL, NMZL represented a minority subgroup, comprising 36 patients (16% of the cohort). Median age was 63 years (36–86). Advanced-stage disease (III–IV) was noted in 50% of case, with bone marrow involvement in 89%. Clinical presentation was dominated by peripheral lymphadenopathy (75 % of cases). Peripheral bulky was noted in 5 cases (14% of the cohort).
Localized NMZL (n=8) was managed with watch-and-wait (n=2), radiotherapy (n=2), or immunochemotherapy (n=4) in bulky disease, achieving a 100% overall response rate (ORR).
For advanced NMZL (n=28), 15 asymptomatic patients were initially managed with observation, and treatment was subsequently initiated in 7 of them. In contrast, 13 symptomatic patients (meeting GELF criteria) were treated at diagnosis. For the 20 treated patients, first-line therapy mainly consisted of R-CHOP-like regimens utilized in 15 cases, yielding an ORR of 68%, with sustained responses in 42%. Relapses and progression were noted in 4 cases. Transformations were observed in one patient

Second-line treatments (R-bendamustine, R-GEMOX, R-miniCHOP) achieved an ORR of approximately 83%.

**Copyright Information:** (Online) ISSN: 2572-9241
©2026 The Author(s). HemaSphere is published by John Wiley & Sons Ltd on behalf of European Hematology Association. This is an open access Abstract Book distributed under the Attribution-NonCommercial-NoDerivs (CC BY-NC-ND), which allows third parties to download the articles and share them with others as long as they credit the author and the Abstract Book, but they cannot change the content in any way or use them commercially.
**Abstract Book Citations:** Authors, Title, HemaSphere, 2026;10:(S1):pages. The abstract book DOIs can be found on the "Author Information" page.
**Disclaimer:** Articles published in the journal HemaSphere exclusively reflect the opinions of the authors. The authors are responsible for all content in their abstracts including accuracy of the facts, statements, citing resources, etc.

**Summary/Conclusion**

NMZL in this cohort is characterized by advanced-stage presentation and frequent bone marrow involvement. Despite heterogeneous management, outcomes are favorable, particularly in localized disease. Standardization of treatment strategies may further improve outcomes.

**Copyright Information:** (Online) ISSN: 2572-9241
©2026 The Author(s). HemaSphere is published by John Wiley & Sons Ltd on behalf of European Hematology Association. This is an open access Abstract Book distributed under the Attribution-NonCommercial-NoDerivs (CC BY-NC-ND), which allows third parties to download the articles and share them with others as long as they credit the author and the Abstract Book, but they cannot change the content in any way or use them commercially.
**Abstract Book Citations:** Authors, Title, HemaSphere, 2026;10:(S1):pages. The abstract book DOIs can be found on the "Author Information" page.
**Disclaimer:** Articles published in the journal HemaSphere exclusively reflect the opinions of the authors. The authors are responsible for all content in their abstracts including accuracy of the facts, statements, citing resources, etc.

### (PB3689) REAL WORLD DATA ON TREATMENT OF RELAPSED/REFRACTORY INDOLENT LYMPHOMA WITH BISPECIFIC ANTIBODY MOSUNETUZUMAB – KROHEM EXPERIENCE

**Topic:** 18. Indolent and mantle-cell non-Hodgkin lymphoma - Clinical

Barbara Dreta*[1], Karla Misura Jakobac[2], Marija Petric[3], Marija Ivić[4], Neven Franjić[5], Goran Rincic[6], Ivan Krecak[7], Hrvoje Holik[8], Ivan Zekanovic[9], Dejan Strahija[10], Petra Bernes[11], Ana Kotris[12], Dino Dujmović[1], Sandra Bašić Kinda[1], Igor Aurer[1, 13]

*[1]UHC Zagreb, Department of Internal medicine, Division of hematology, Zagreb, Croatia;[2]UH Merkur, Zagreb, Croatia; [3]UHC Split, Split, Croatia; [4]UH Dubrava, Zagreb, Croatia; [5]UHC Rijeka, Rijeka, Croatia; [6]UHC "Sestre Milosrdnice", Zagreb, Croatia; [7]GH Šibenik, Šibenik, Croatia;[8]Dr Josip Bencevic General Hospital, Slavonski Brod, Croatia;[9]UH Zadar, Zadar, Croatia; [10]GH Varaždin, Varaždin, Croatia; [11]GH Pula, Pula, Croatia;[12]UHC Osijek, Osijek, Croatia; [13]University of Zagreb, School of medicine, Zagreb, Croatia;*

### Background

Mosunetuzumab, CD20xCD3 bispecific antibody showed high response rates and durable remissions in patients with follicular lymphoma in clinical trials. Until today there are limited real-world data reports on patients with indolent NHL treated with mosunetuzumab outside of clinical trials.

### Aims

Report effectives and safety of treatment with mosunetuzumab in R/R indolent NHL treated in routine clinical practice through national compassionate use program.

### Methods

We retrospectively collected data on patients with indolent NHL treated with at least one dose of mosunetuzumab until October 2025. Data was collected from 12 centers. Response was assessed with Lugano 2014. criteria. CRS and ICANS were graded according to ASCTC and other side effect with CTCAE 5.0 criteria. PFS and OS were estimated with Kaplan-Meier method.

### Results

A total of 15 patients were included in analysis. 14 pt with follicular lymphoma and 1 MZL. Median age was 71y (range 36-89), male 60%. FLIPI at diagnosis 2 -20%, 3 – 33%, 4 – 20%. Median number of previous therapies was 2, 40% had ASCT. POD24 were 33% of patients.
Response to treatment was evaluated with MSCT scan and ORR was 87%, CR 34%.
With median follow up of 10,4 mo, median OS was not reached, median PFS 16,2 mo, 12moOS 74% and 12moPFS 66%.
There was no significant difference in survival according to FLIPI and POD24 status.
CRS occurred in 47% pts. They were all gr 1-2 and occurred during first dose and first full dose of mosunetuzumab. There were no ICANS reported. Infusion related reactions had 3 pt, all grade 1. There were no TLS and no tumor flair reported.
Neutropenia gr 3-4 occurred in 6% and thrombocytopenia in 27%. There were infections in 47% of pts, gr 3-4 in 20%.
Hypogammaglobulinemia was reported in 67%.
Nine patients had treatment postponement, 6 due to infections, 1 neutropenia, 2 neurological complications and 1 trauma.
Progression of disease occurred in 3 patients and death occurred in 4 pts, one due to disease progression, one sepsis, one postoperative complication and one pulmonary embolism.

**Copyright Information:** (Online) ISSN: 2572-9241
©2026 The Author(s). HemaSphere is published by John Wiley & Sons Ltd on behalf of European Hematology Association. This is an open access Abstract Book distributed under the Attribution-NonCommercial-NoDerivs (CC BY-NC-ND), which allows third parties to download the articles and share them with others as long as they credit the author and the Abstract Book, but they cannot change the content in any way or use them commercially.
**Abstract Book Citations:** Authors, Title, HemaSphere, 2026;10:(S1):pages. The abstract book DOIs can be found on the “Author Information” page.
**Disclaimer:** Articles published in the journal HemaSphere exclusively reflect the opinions of the authors. The authors are responsible for all content in their abstracts including accuracy of the facts, statements, citing resources, etc.

### Summary/Conclusion

In this real-world data analysis of patients with R/R indolent NHL mosunetuzumab achieved durable responses comparable with clinical trials results. Toxicities were manageable. This results support use of mosunetuzumab as an effective outpatient immunotherapy option in routine clinical practice.

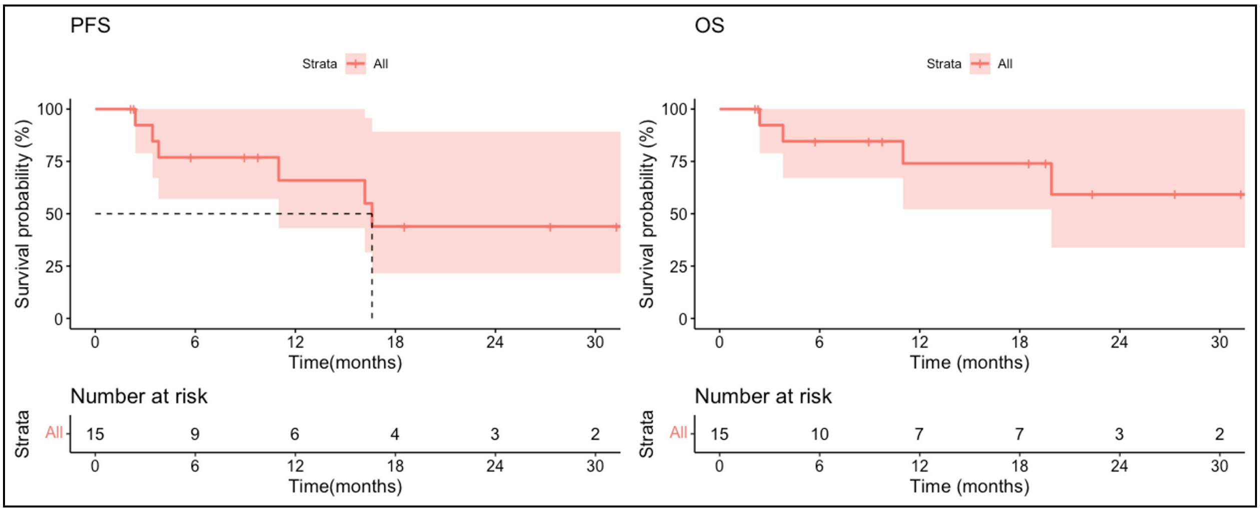

Overall survival curve

**Copyright Information:** (Online) ISSN: 2572-9241
©2026 The Author(s). HemaSphere is published by John Wiley & Sons Ltd on behalf of European Hematology Association. This is an open access Abstract Book distributed under the Attribution-NonCommercial-NoDerivs (CC BY-NC-ND), which allows third parties to download the articles and share them with others as long as they credit the author and the Abstract Book, but they cannot change the content in any way or use them commercially.
**Abstract Book Citations:** Authors, Title, HemaSphere, 2026;10:(S1):pages. The abstract book DOIs can be found on the "Author Information" page.
**Disclaimer:** Articles published in the journal HemaSphere exclusively reflect the opinions of the authors. The authors are responsible for all content in their abstracts including accuracy of the facts, statements, citing resources, etc.

### (PB3690) EARLY PROGRESSION WITHIN 24 MONTHS PREDICTS SURVIVAL ACROSS NODAL NON-HODGKIN LYMPHOMA INDEPENDENT OF HISTOLOGY AND PRIMARY LOCALIZATION

**Topic:** 18. Indolent and mantle-cell non-Hodgkin lymphoma - Clinical

Victor Tomacinschii*[1, 2], Maria Robu[1]

*[1]Nicolae Testemitanu State University of Medicine and Pharmacy, Department of Hematology, Chisinau, Moldova;[2]Institute of Oncology, Department of Hematology, Chisinau, Moldova;*

### Background

Non-Hodgkin lymphomas (NHL) represent a heterogeneous group of malignancies characterized by variable clinical behavior and survival outcomes. Early progression of disease within 24 months (POD24) has been consistently associated with inferior survival in several lymphoma subtypes; however, its prognostic impact across mixed nodal NHL populations treated in routine clinical practice remains less clearly characterized.

### Aims

To evaluate the impact of POD24 on overall survival (OS) and to explore its distribution across histologic subtypes and primary nodal disease localization in a real-world NHL cohort from Eastern Europe.

### Methods

This retrospective analysis included 118 patients diagnosed and treated at the Institute of Oncology, Republic of Moldova. Only patients diagnosed with primarily nodal NHL were included, while NHL with primary extranodal involvement were excluded. POD24 was defined as progression or relapse within 24 months from treatment initiation. OS was estimated using Kaplan-Meier survival curves and compared using log-rank test. Primary nodal localization was categorized as peripheral, mediastinal, or abdominal lymph node involvement.

### Results

The cohort included predominantly B-cell NHL (87.3%, n=103), while T-cell lymphomas accounted for 12.7% (n=15). The most frequent histologic subtypes were diffuse large B-cell lymphoma (28.8%, n=34), small B-cell lymphomas (20.3%, n=24), follicular lymphoma (9.3%, n=11), and mantle cell lymphoma (9.3%, n=11). POD24 status was evaluable in 115 patients, with early progression observed in 57 cases (49.6%). Median OS was 20 months in patients with POD24, whereas median OS was not reached in patients without POD24 ($p<0.001$). Death events occurred in 54/57 patients with POD24 compared with 6/58 patients without early progression. POD24 events were observed across all histologic entities and were comparable between peripheral lymph nodes (51.1%), mediastinal (42.1%), and abdominal lymph nodes (50.0%), without anatomical predominance.

### Summary/Conclusion

Early progression within 24 months occurred in 49.6% of nodal NHL patients and was associated with markedly inferior survival, with median OS of 20 months compared with patient without POD24 in whom the median OS was not reached. Similar POD24 rates were observed across peripheral, mediastinal and abdominal nodal localizations, as well as independently of cell origin (B- or T-cell NHL), indicating that POD24 serves as an adverse prognostic factor independent of histology or primary nodal site.

**Copyright Information:** (Online) ISSN: 2572-9241
©2026 The Author(s). HemaSphere is published by John Wiley & Sons Ltd on behalf of European Hematology Association. This is an open access Abstract Book distributed under the Attribution-NonCommercial-NoDerivs (CC BY-NC-ND), which allows third parties to download the articles and share them with others as long as they credit the author and the Abstract Book, but they cannot change the content in any way or use them commercially.
**Abstract Book Citations:** Authors, Title, HemaSphere, 2026;10:(S1):pages. The abstract book DOIs can be found on the "Author Information" page.
**Disclaimer:** Articles published in the journal HemaSphere exclusively reflect the opinions of the authors. The authors are responsible for all content in their abstracts including accuracy of the facts, statements, citing resources, etc.

### (PB3691) PROGNOSTIC DETERMINANTS IN SPLENIC MARGINAL ZONE LYMPHOMA

**Topic:** 18. Indolent and mantle-cell non-Hodgkin lymphoma - Clinical

Sayadi Malek*[1], Maha Charfi[2], Hela Ghedira[3], Slama Nader[4], Nesrine Bensayed[5], Wala Ben Kridis[6], Haifa Hafsa[7], Miled Wafa[8], Raoudha Mansouri[1], Jaied Rabeb[3], Wided Cherif[5], Zaineb Mlayah[4], Anna Chabaane[9], Moez Elloumi[2], Raihane Ben Lakhal[1], Fehmi Msadek[3], Med.Adnene Laatiri[4], Yosra Ben Youssef[5], Nour Ben Adejlil[9], Moez Medhaffar[2]

*[1]Hôpital Aziza Othmana, CLINICAL HEMATOLOGY, Tunis, Tunisia;[2]Hôpital Universitaire Hédi Chaker, CLINICAL HEMATOLOGY, Sfax, Tunisia; [3]Hôpital Militaire, CLINICAL HEMATOLOGY, Tunis, Tunisia;[4]Hôpital Fattouma Bourguiba, Monastir, Tunisia; [5]Hôpital Farhat Hached, Sousse, Tunisia;[6]Hôpital Régional Habib Bourguiba de Médenine, Medenine, Tunisia; [7]mohamed Sassi Hospital, CLINICAL HEMATOLOGY, gabes, Tunisia;[8]Hôpital Aziza Othmana, Tunis, Tunisia; [9]CNGMO-Centre National de Greffe de la Moelle Osseuse, Tunis, Tunisia;*

### Background

Splenic Marginal Zone Lymphoma (SMZL) represents a distinct subtype of indolent B-cell lymphoma accounting for a small proportion of non-Hodgkin lymphomas. Despite its generally favorable course, significant clinical heterogeneity exists, with a subset of patients experiencing early progression, transformation, or shortened survival. Although prognostic scores such as MZL-IPI have been proposed, their specific applicability in SMZL remains debated. Real-world data are needed to better define clinical and biological factors associated with adverse outcomes.

### Aims

The aim of this study was to identify prognostic factors associated with treatment failure, progression-free survival (PFS), and overall survival (OS) in patients with SMZL.

### Methods

We conducted a retrospective multicenter study including patients diagnosed with SMZL between January 2015 and December 2024 across 8 hematology centers. Clinical, laboratory, and molecular characteristics at diagnosis were collected, as well as treatment modalities and outcomes.
Predictors of treatment failure were analyzed using the chi-square test in univariate analysis and logistic regression in multivariate analysis. OS and PFS were estimated using the Kaplan–Meier method and compared using the log-rank test. Multivariate survival analyses were performed using Cox proportional hazards models.

**Copyright Information:** (Online) ISSN: 2572-9241
©2026 The Author(s). HemaSphere is published by John Wiley & Sons Ltd on behalf of European Hematology Association. This is an open access Abstract Book distributed under the Attribution-NonCommercial-NoDerivs (CC BY-NC-ND), which allows third parties to download the articles and share them with others as long as they credit the author and the Abstract Book, but they cannot change the content in any way or use them commercially.
**Abstract Book Citations:** Authors, Title, HemaSphere, 2026;10:(S1):pages. The abstract book DOIs can be found on the "Author Information" page.
**Disclaimer:** Articles published in the journal HemaSphere exclusively reflect the opinions of the authors. The authors are responsible for all content in their abstracts including accuracy of the facts, statements, citing resources, etc.

### Results

A total of 99 patients with SMZL were included (43% of zone marginal lymphoma). The median age at diagnosis was**62 years[28-87]**, with a sex-ratio of 1.6.
At presentation, B symptoms were present in 41% of patients, splenomegaly was observed in 85% of patients, anemia in 74% with 15 % of hemolytic anemia, thrombopenia in 36% and bone marrow involvement in 98%. Elevated LDH was reported in 37%. According to HPLL score, 19 % of patients were classified as high risk.
Initial management consisted of active surveillance in 32***% of cases, with a median observation period of 20 months [5-89]. the 89% of treated patients had*** systemic therapy (Rituximab chlorombucil, RCHOP like regimen, RBendamustine, RCVP, Splenectomy)
In univariate analysis, treatment failure was significantly associated with high score HPLL (p=0.004***), thrombocytopenia (p=0.07***). ***In multivariate analysis, age > 70 years and thrombocytopenia remained independent predictors of treatment failure (p=0.012, p=0.004***).
Regarding OS, univariate analysis identified a performans statu> 2 (p=0.052***), bulky (p=0.017), anemia*** (p=0.0048***), lymphopenia (p=0.052***), thrombopenia (p=0.02),clonal gammapathy (p=0.014***), high-risk HPLL (p=0.023***), and failure of treatment (p=0.037***) as adverse prognostic factors. In multivariate analysis, age>60 years(p=0.037***) and failure of treatment (p=0.01) remained independently associated with inferior OS.
For PFS, univariate analysis showed significant associations with performans status> 2 (p=0.032)***, elevated LDH (p=0.05) , and high score HPLL ( p=0.004). In multivariate analysis, high score HPLL (p=0.005***) emerged as an independent predictor of shorter PFS.

### Summary/Conclusion

In this SMZL cohort, high score HPLL emerged as the strongest predictors of adverse outcomes. These findings support the integration of clinical and biological parameters to refine risk-adapted therapeutic strategies in SMZL.

**Copyright Information:** (Online) ISSN: 2572-9241
©2026 The Author(s). HemaSphere is published by John Wiley & Sons Ltd on behalf of European Hematology Association. This is an open access Abstract Book distributed under the Attribution-NonCommercial-NoDerivs (CC BY-NC-ND), which allows third parties to download the articles and share them with others as long as they credit the author and the Abstract Book, but they cannot change the content in any way or use them commercially.
**Abstract Book Citations:** Authors, Title, HemaSphere, 2026;10:(S1):pages. The abstract book DOIs can be found on the "Author Information" page.
**Disclaimer:** Articles published in the journal HemaSphere exclusively reflect the opinions of the authors. The authors are responsible for all content in their abstracts including accuracy of the facts, statements, citing resources, etc.

### (PB3692) PROGNOSTIC DETERMINANTS IN NODAL MARGINAL ZONE LYMPHOMA

**Topic:** 18. Indolent and mantle-cell non-Hodgkin lymphoma - Clinical

Sayadi Malek*[1], Maha Charfi[2], Hela Ghedira[3], Slama Nader[4, 4], Nesrine Ben Sayed[5], Wala Ben Kridis[6], Haifa Hafsa[7], Miled Wafa[1], Raoudha Mansouri[1], Jaied Rabeb[3], Wided Cherif[5], Zaineb Mlayah[4], Anna Chabaane[8], Moez Elloumi[2], Raihane Ben Lakhal[1], Med.Adnene Laatiri[4], Nour Ben Adejlil[8], Yosra Ben Youssef[5], Fehmi Msadek[3], Moez Medhaffar[2]

*[1]Hôpital Aziza Othmana, CLINICAL HEMATOLOGY, Tunis, Tunisia;[2]Hôpital Universitaire Hédi Chaker, CLINICAL HEMATOLOGY, Sfax, Tunisia; [3]Hôpital Militaire, CLINICAL HEMATOLOGY, Tunis, Tunisia;[4]Hôpital Fattouma Bourguiba, Monastir, Tunisia; [5]Hôpital Farhat Hached, Sousse, Tunisia;[6]Hôpital Régional Habib Bourguiba de Médenine, Medenine, Tunisia; [7]HOPITAL MOHAMED Sassi, CLINICAL HEMATOLOGY, GABES, Tunisia;[8]CNGMO-Centre National de Greffe de la Moelle Osseuse, Tunis, Tunisia;*

### Background

Nodal Marginal Zone Lymphoma (NMZL) is a rare primary nodal indolent B-cell lymphoma characterized by clinical and biological heterogeneity. Unlike other marginal zone lymphoma subtypes, specific and validated prognostic models for NMZL remain limited, and therapeutic strategies are often extrapolated from broader indolent lymphoma data. Identification of robust prognostic determinants is therefore essential to improve risk stratification and guide personalized management.

### Aims

The aim of this study was to determine prognostic factors associated with treatment failure, progression-free survival (PFS), and overall survival (OS) in patients with NMZL.

### Methods

We retrospectively analyzed patients diagnosed with NMZL between January 2015 and December 2024 in 8 centers. Baseline clinical, laboratory, and molecular data were collected. Treatment modalities and outcomes were analyzed.
Univariate analysis of predictors of treatment failure was performed using the chi-square test, followed by multivariate logistic regression. OS and PFS were estimated using the Kaplan–Meier method and compared using the log-rank test. Cox regression models were used for multivariate survival analysis.

**Copyright Information:** (Online) ISSN: 2572-9241
©2026 The Author(s). HemaSphere is published by John Wiley & Sons Ltd on behalf of European Hematology Association. This is an open access Abstract Book distributed under the Attribution-NonCommercial-NoDerivs (CC BY-NC-ND), which allows third parties to download the articles and share them with others as long as they credit the author and the Abstract Book, but they cannot change the content in any way or use them commercially.
**Abstract Book Citations:** Authors, Title, HemaSphere, 2026;10:(S1):pages. The abstract book DOIs can be found on the “Author Information” page.
**Disclaimer:** Articles published in the journal HemaSphere exclusively reflect the opinions of the authors. The authors are responsible for all content in their abstracts including accuracy of the facts, statements, citing resources, etc.

### Results

**A total of 36 patients were included (16% of the overall cohort). The median age at diagnosis was 63 years (range, 36–86). Advanced-stage disease (stage III–IV) was observed in 50% of cases, with bone marrow involvement documented in 89%. The clinical presentation was predominantly characterized by peripheral lymphadenopathy (75%). Peripheral bulky disease was identified in 5 patients (14%).**
**Among patients with localized NMZL (n=8), management consisted of watch-and-wait (n=2), radiotherapy (n=2), or immunochemotherapy in cases with bulky disease (n=4), achieving a 100% overall response rate (ORR).**
**In advanced NMZL (n=28), 15 asymptomatic patients were initially managed with observation; treatment was subsequently initiated in 7 of them due to disease progression. Thirteen symptomatic patients meeting GELF criteria received upfront therapy at diagnosis. Among the 20 treated patients, first-line therapy predominantly consisted of R-CHOP–like regimens (n=15), resulting in an ORR of 68%, with durable responses observed in 42%.**
In univariate analysis, treatment failure was significantly associated with age>70 ans (p=0.019***) and bone marrow infiltration (p=0.034***). In multivariate analysis, no factors remained independently associated with treatment failure.
For OS, univariate analysis identified age >70 years (p=0.006***), advanced stage (p=0.08***), ***and bone marrow infiltration (p=0.024***) as adverse prognostic factors. In multivariate analysis, age>70 years (p=0.025***)*** was independent predictors of inferior OS.
Regarding PFS, univariate analysis demonstrated significant associations with B symptoms (p=0.044), Performans status>2 (p<0.0001), bone marrow infiltration(p=0.006)***,advanced stage (p=0.005) and high FLIPI score. Multivariate analysis identified high FLIPI score (p=0.05***) as an independent predictor of shorter PFS.

### Summary/Conclusion

In this NMZL cohort, advanced stage and high FLIPI score emerged as key determinants of high-risk subgroup outcome. These findings highlight the need for improved prognostic stratification to guide personalized therapeutic approaches in NMZL.

**Copyright Information:** (Online) ISSN: 2572-9241
©2026 The Author(s). HemaSphere is published by John Wiley & Sons Ltd on behalf of European Hematology Association. This is an open access Abstract Book distributed under the Attribution-NonCommercial-NoDerivs (CC BY-NC-ND), which allows third parties to download the articles and share them with others as long as they credit the author and the Abstract Book, but they cannot change the content in any way or use them commercially.
**Abstract Book Citations:** Authors, Title, HemaSphere, 2026;10:(S1):pages. The abstract book DOIs can be found on the "Author Information" page.
**Disclaimer:** Articles published in the journal HemaSphere exclusively reflect the opinions of the authors. The authors are responsible for all content in their abstracts including accuracy of the facts, statements, citing resources, etc.

### (PB3693) DEVELOPMENT OF SPLENIC B-CELL LYMPHOMA/LEUKEMIA WITH PROMINENT NUCLEOLI IN A PATIENT WITH PRIMARY MYELOFIBROSIS

**Topic:** 18. Indolent and mantle-cell non-Hodgkin lymphoma - Clinical

Abdulkadir Karismaz*[1], Rafet Eren[2], Sevil Sadri[1]

*[1]Istanbul Education And Research Hospital, istanbul, Türkiye;[2]biruni university hospital, istanbul, Türkiye;*

### Background

Primary myelofibrosis (PMF) is frequently associated with splenomegaly due to extramedullary hematopoiesis. However, the coexistence of a splenic B-cell lymphoid neoplasm may pose a significant diagnostic challenge, particularly in the absence of systemic lymphadenopathy.

### Aims

A 61-year-old woman had been followed since 2015 with a diagnosis of myeloproliferative neoplasm, most consistent with primary myelofibrosis/post–essential thrombocythemia myelofibrosis. She had been receiving ruxolitinib therapy for the preceding two years due to symptomatic splenomegaly and disease-related symptoms. Despite treatment, she developed progressive abdominal discomfort and early satiety. Physical examination revealed marked splenomegaly, while no peripheral lymphadenopathy was detected.
Laboratory findings were consistent with chronic MPN and showed no evidence of leukemic transformation or blast excess.
Baseline abdominal ultrasonography demonstrated massive splenomegaly, with the spleen measuring 295 mm in longitudinal diameter. The splenic parenchyma was homogeneous, and no focal lesions or abdominal lymphadenopathy were identified. Following four cycles of treatment, follow-up ultrasonography revealed a marked reduction in splenic size to 185 mm, consistent with a partial radiologic response.
Bone marrow biopsy revealed marked reticulin fibrosis compatible with the underlying myeloproliferative neoplasm, without increased blasts. In addition, a prominent lymphoid infiltration with an intrasinusoidal growth pattern was identified. In the absence of radiologic lymph node involvement, a primary splenic B-cell lymphoid neoplasm was favored.
Hairy cell leukemia was excluded. Although splenic marginal zone lymphoma was considered, the absence of typical lymphoid nodules and the presence of lymphoid cells with prominent nucleoli supported the diagnosis of splenic B-cell lymphoma/leukemia with prominent nucleoli, as defined in the WHO 2022 classification.

### Results

The patient received four cycles of rituximab plus bendamustine (R-B regimen). Following therapy, she demonstrated significant clinical improvement, accompanied by a substantial reduction in splenic size. No significant treatment-related adverse events were observed.

### Summary/Conclusion

This case underscores that disproportionate splenomegaly in patients with primary myelofibrosis, even under long-term ruxolitinib therapy, should prompt evaluation for a coexisting splenic B-cell lymphoid neoplasm. Careful integration of clinical, radiologic, and bone marrow findings is essential to ensure accurate diagnosis and appropriate management.

**Copyright Information:** (Online) ISSN: 2572-9241
©2026 The Author(s). HemaSphere is published by John Wiley & Sons Ltd on behalf of European Hematology Association. This is an open access Abstract Book distributed under the Attribution-NonCommercial-NoDerivs (CC BY-NC-ND), which allows third parties to download the articles and share them with others as long as they credit the author and the Abstract Book, but they cannot change the content in any way or use them commercially.
**Abstract Book Citations:** Authors, Title, HemaSphere, 2026;10:(S1):pages. The abstract book DOIs can be found on the “Author Information” page.
**Disclaimer:** Articles published in the journal HemaSphere exclusively reflect the opinions of the authors. The authors are responsible for all content in their abstracts including accuracy of the facts, statements, citing resources, etc.

### (PB3694) PROGNOSTIC FACTORS AND THERAPEUTIC OUTCOMES OF MANTLE CELL LYMPHOMA IN ELDERLY PATIENTS: A TUNISIAN MULTICENTER RETROSPECTIVE STUDY

**Topic:** 18. Indolent and mantle-cell non-Hodgkin lymphoma - Clinical

Wided Cherif*[1], Zaineb Mlayah[2], Jaied Rabeb[3], Maha Charfi[4], Malek Sayedi[5], Wafa Miled[5], Anna Chabaane[6], Slama Nader[2], Nesrine Ben Sayed[1], Raoudha Mansouri[5], Hela Ghedira[3], Moez Medhaffar[4]

*[1]Hôpital Farhat Hached, 1. Haematology department, Sousse, Tunisia;[2]5. Fattouma Bourguiba Hospital, 5. Haematology department, Monastir, Tunisia; [3]3. Military Hospital, 3. Haematology department, Tunis, Tunisia; [4]4. Hedi Chaker Hospital, 4. Haematology department, Sfax, Tunisia; [5]2. Aziza Othmana Hospital, 2. Haematology department, Tunis, Tunisia; [6]6. National Center of Bone Marrow transplantation, Haematology department,, Tunis, Tunisia;*

### Background

Mantle cell lymphoma (MCL) in elderly patients represents a therapeutic challenge because of the prognostic heterogeneity and the limit of therapeutic alternatives due to frequent comorbidities.

### Aims

We aimed to analyze prognostic factors and treatment outcomes in a Tunisian cohort of elderly patients with MCL.

### Methods

A retrospective multicenter descriptive study was conducted over a 10-year period (January 2015–December 2024). The frontline treatment was heterogenous depending on comorbidities status. The therapeutic reponse was based on Cheson criteria. Ethical concepts were respected.

### Results

Fifty-nine patients aged > 65 years with MCL were included. Five of whom died before treatment initiation. Median age was 72 years (66–97) with a male-to-female ratio of 2.33. Poor performance status (PS ≥2) was observed in 38% of patients. B symptoms were present in 48% of cases. Leukemic presentation was noted in 28%. According to the simplified MIPI score, 53% of patients were high risk. First-line therapy consisted of R-CHOP in 38 patients, cytarabine-based regimens in 8 patients, R-bendamustine (RB) in 3 patients, and R-mini-CHOP or R-CVP in 5 patients. The overall response rate (ORR) was 69% (34/54), including 20 complete responses (CR 41%) and 14 partial responses (PR 28%). In multivariate analysis, PS ≥2 and absence of cytarabine-based induction remained independent adverse prognostic factors, whereas high-risk MIPI showed only a non-significant trend (p = 0.08). Among the 15 patients with refractory or progressive disease, five received salvage therapy: four patients were treated with the RB regimen and one with HCVAD showing three additional responses. Overall, 37 patients achieved an ORR. Among them, 24 patients (65%) reached rituximab maintenance therapy. With a median follow-up of 56 months (9–126), the 5-year overall survival (OS) and the progression-free survival (PFS) were 50% and 59%, respectively. Median time to relapse was 17 months (6–72). The maintenance therapy was the independent factor of poorer PFS, p<0,0001.

### Summary/Conclusion

Therapeutic outcomes seems to be equal of international data. Improved prognostic stratification incorporating cytogenetic and molecular analyses, better adherence to therapeutic strategies (particularly wider use of maintenance therapy), and earlier introduction of BTK inhibitors at first relapse may improve outcomes in elderly patients with MCL.

**Copyright Information:** (Online) ISSN: 2572-9241
©2026 The Author(s). HemaSphere is published by John Wiley & Sons Ltd on behalf of European Hematology Association. This is an open access Abstract Book distributed under the Attribution-NonCommercial-NoDerivs (CC BY-NC-ND), which allows third parties to download the articles and share them with others as long as they credit the author and the Abstract Book, but they cannot change the content in any way or use them commercially.
**Abstract Book Citations:** Authors, Title, HemaSphere, 2026;10:(S1):pages. The abstract book DOIs can be found on the "Author Information" page.
**Disclaimer:** Articles published in the journal HemaSphere exclusively reflect the opinions of the authors. The authors are responsible for all content in their abstracts including accuracy of the facts, statements, citing resources, etc.

### (PB3695) FOLLICULAR LYMPHOMA IN TUNISIA: A DECADE-LONG MULTICENTER RETROSPECTIVE REAL-WORLD STUDY OF CLINICAL FEATURES AND OUTCOMES

**Topic:** 18. Indolent and mantle-cell non-Hodgkin lymphoma - Clinical

Nesrine Ben Sayed*[1], Jaied Rabeb[2], Wafa Miled[3], Anna Chabaane[2], Maha Charfi[4], Hela Ghedira[2], Zaineb Mlayah[5], Wided Cherif[1], Raoudha Mansouri[3], Slama Nader[5], Malek Sayedi[3], Fehmi Msadek[2], Med.Adnene Laatiri[5, 5], Nour Ben Adejlil[6], Yosra Ben Youssef[7], Lamia Issaoui[3], Moez Elloumi[4], Moez Medhaffar[4]

*[1]Farhat hached hospital, hematolgy department, SOUSSE, Tunisia; [2]Military Hopital, hematolgy department, tunis, Tunisia; [3]Aziza Othmena hospital, hematolgy department, tunis, Tunisia; [4]Hedi chaker Hospital, hematolgy department, sfax, Tunisia; [5]monastir hsopital, hematolgy department, monastir, Tunisia; [6]bone marrow transplantation center, hematolgy department, tunis, Tunisia; [7]Farhat hached hospital, Sousse, Tunisia;*

### Background

Follicular lymphoma (FL) is the second most common indolent non-Hodgkin lymphoma. Despite its frequency, real-world data from North African populations remains scarce.

### Aims

We conducted a retrospective study across six hematology departments and one oncology center (2015–2024), including 157 patients.

### Methods

We conducted a retrospective study across six hematology departments and one oncology center (2015–2024), including 157 patients.

### Results

Median age was 60 years (range: 23–91), with a 59% male predominance. Presentation was characterized by peripheral lymphadenopathy (87.7%), nodes >3 cm (56.8%), and bulky disease (22%). Most patients (75%) presented with advanced-stage disease (Ann Arbor III–IV). Bone marrow involvement and elevated LDH were observed in 35% and 42% of cases, respectively. High tumor burden (GELF criteria) was identified in 69%. High-risk FLIPI scores predominated (41%). First-line therapy consisted of immunochemotherapy in 85% of cases, R-CHOP (83%), followed by maintenance in 60%. A watch-and-wait strategy was applied in 15%. The overall response rate was 88% (61% CR). At a median follow-up of 48 months, 4-year OS, RFS, and EFS rates were 95%, 90%, and 91%, respectively. Relapse occurred in 16% of responders (median time: 24.5 months). Early progression within 24 months (POD24) was documented in 11 patients and was strongly associated with inferior survival ($p$=0.005). Multivariate analysis identified FLIPI score, clinical stage, chemotherapy type, and POD24 as independent predictors of survival.

### Summary/Conclusion

This Tunisian study confirms the efficacy of R-CHOP-based frontline therapy, despite a high prevalence of advanced-stage disease at diagnosis. POD24 and FLIPI score remain ~~a~~ critical prognostic, factors emphasizing the need for risk-stratified management.

**Copyright Information:** (Online) ISSN: 2572-9241
©2026 The Author(s). HemaSphere is published by John Wiley & Sons Ltd on behalf of European Hematology Association. This is an open access Abstract Book distributed under the Attribution-NonCommercial-NoDerivs (CC BY-NC-ND), which allows third parties to download the articles and share them with others as long as they credit the author and the Abstract Book, but they cannot change the content in any way or use them commercially.
**Abstract Book Citations:** Authors, Title, HemaSphere, 2026;10:(S1):pages. The abstract book DOIs can be found on the "Author Information" page.
**Disclaimer:** Articles published in the journal HemaSphere exclusively reflect the opinions of the authors. The authors are responsible for all content in their abstracts including accuracy of the facts, statements, citing resources, etc.

### (PB3696) CLINICOBIOLOGICAL FEATURES AND PROGNOSTIC IMPACT OF FIRST-LINE THERAPY IN MARGINAL ZONE LYMPHOMAS: A 44-PATIENT SINGLE-CENTER STUDY

**Topic:** 18. Indolent and mantle-cell non-Hodgkin lymphoma - Clinical

Souad Fennan*[1], Maha EL Amani[1], Zineb Afroukh[1], Imane Chajai[1], Otman Abdelmoula[1], Othman Doghmi[1], Amal Terfai[1], Elmaachi Nora[1], Naoufal Benlachgar[1], Soukaina Haidouri[1], Zoubida Tazi Mezalek[1]

*[1]ibn sina university hospital, clinical hematology department, rabat, Morocco;*

### Background

Marginal zone lymphoma (MZL) is an indolent B-cell lymphoma arising from post-germinal memory B cells. It includes three subtypes: extranodal (MALT), splenic, and nodal. Representing 5–10% of non-Hodgkin lymphomas, it shows marked clinical and biological heterogeneity. Its pathogenesis is often driven by chronic antigenic stimulation from infections or autoimmune disorders. Owing to its variable course and limited prospective data, management remains individualized and largely guided by retrospective evidence.

### Aims

The primary objective was to characterize the clinicobiological features of patients with MZL and evaluate the prognostic impact of first-line therapy on progression-free survival (PFS) and overall survival (OS). Secondary objectives were to describe subtype distribution and identify clinical and biological factors associated with disease progression.

### Methods

This retrospective single-center study was conducted at the Clinical Hematology Department of Ibn Sina University Hospital (January 2021–December 2025). Adults with histologically confirmed MZL were included; cases overlapping with other CD5-/CD10- small B-cell lymphomas were excluded. Response was assessed clinically, biologically (cytopenias, LDH), and radiologically by CT; gastric cases also underwent endoscopic and histologic evaluation. Analyses were performed using Jamovi 2.7.22. PFS and OS were estimated by Kaplan–Meier, with associations tested using chi-square ($p<0.05$). Adverse events were graded per CTCAE v5.0.

### Results

Forty-four patients were included (56.8% women), with a median age of 65 years (31–88). Digestive symptoms were the most frequent presentation (36.4%), followed by tumor syndrome (20.5%), constitutional symptoms (11.3%), ENT involvement (9.1%), and ocular manifestations (6.9%). Cytopenias occurred in 4.6%, and B symptoms were present in 36.4%.
According to Ann Arbor staging, 40.9% were stage I and 40.9% stage IV, while 9.1% were stage II and III each. MALT lymphoma predominated (70.7%), followed by NMZL (15.9%) and SMZL (13.6%), with diverse and sometimes multifocal extranodal sites.
First-line management included R-CHOP (31.8%), R-CVP (10%), R-chlorambucil (9.1%), R-bendamustine (4.5%), radiotherapy (6.8%), and watch-and-wait (20.5%); Helicobacter pylori eradication was performed when indicated. Among 35 treated patients, CR was achieved in 41.9%, PR in 18.6%, SD in 9.3%, and PD in 11.9%, yielding an ORR of 74.3%. Severe toxicities were rare (one case each of grade 4 neutropenia, neuropathy, and visual impairment).
After a median follow-up of 23.5 months, OS at 12 and 36 months was 91.9% and 86.5%, and PFS was 97.5% and 65.9%, respectively. Overall, 70.5% remained relapse-free, 18.2% relapsed, and 11.4% were resistant to Helicobacter pylori eradication. Relapse patterns included macrophage activation syndrome, splenic sequestration, hepatic infiltration, and transformation to diffuse large B-cell lymphoma. Age >65 years, PS 4, and malnutrition were significantly associated with relapse or progression

**Copyright Information:** (Online) ISSN: 2572-9241
©2026 The Author(s). HemaSphere is published by John Wiley & Sons Ltd on behalf of European Hematology Association. This is an open access Abstract Book distributed under the Attribution-NonCommercial-NoDerivs (CC BY-NC-ND), which allows third parties to download the articles and share them with others as long as they credit the author and the Abstract Book, but they cannot change the content in any way or use them commercially.
**Abstract Book Citations:** Authors, Title, HemaSphere, 2026;10:(S1):pages. The abstract book DOIs can be found on the “Author Information” page.
**Disclaimer:** Articles published in the journal HemaSphere exclusively reflect the opinions of the authors. The authors are responsible for all content in their abstracts including accuracy of the facts, statements, citing resources, etc.

**Summary/Conclusion**

Marginal zone lymphoma in our cohort was heterogeneous, mainly extranodal MALT with digestive involvement. Outcomes were favorable, but advanced age, poor performance status, and malnutrition increased relapse and progression risk. These findings emphasize geriatric and nutritional assessment, support individualized management, and call for larger prospective studies to refine risk stratification and therapy.

**Copyright Information:** (Online) ISSN: 2572-9241
©2026 The Author(s). HemaSphere is published by John Wiley & Sons Ltd on behalf of European Hematology Association. This is an open access Abstract Book distributed under the Attribution-NonCommercial-NoDerivs (CC BY-NC-ND), which allows third parties to download the articles and share them with others as long as they credit the author and the Abstract Book, but they cannot change the content in any way or use them commercially.
**Abstract Book Citations:** Authors, Title, HemaSphere, 2026;10:(S1):pages. The abstract book DOIs can be found on the "Author Information" page.
**Disclaimer:** Articles published in the journal HemaSphere exclusively reflect the opinions of the authors. The authors are responsible for all content in their abstracts including accuracy of the facts, statements, citing resources, etc.

### (PB3697) TYPE I CRYOGLOBULINEMIA ASSOCIATED WITH MARGINAL ZONE LYMPHOMA COMPLICATED BY THROMBOTIC MICROANGIOPATHY AND CRITICAL DISTAL ISCHEMIA AFTER RITUXIMAB

**Topic:** 18. Indolent and mantle-cell non-Hodgkin lymphoma - Clinical

Cristina Antón-Maldonado[1], Nataly Ibarra*[1], Amparo Santamaria[1], María Fernández López[1]

[1]*Hospital Universitario del Vinalopo, Hematologia y Hemoterapia, ELCHE, Spain;*

### Background

Type I cryoglobulinemia is a rare condition, usually associated with B-cell lymphoproliferative disorders producing monoclonal immunoglobulins, and is characterized by hyperviscosity phenomena and microvascular occlusion. It may present with severe distal ischemia and poses diagnostic and therapeutic challenges, particularly in the setting of anti-CD20 therapy.

### Aims

To describe a case of rapidly progressive distal ischemia secondary to type I cryoglobulinemia with thrombotic microangiopathy in a patient with marginal zone lymphoma receiving rituximab, highlighting the diagnostic approach and management.

### Methods

A 58-year-old man with a history of ischemic heart disease with coronary stents and low-grade B-cell non-Hodgkin lymphoma (splenic nodal marginal zone lymphoma with an IgM component) was treated with rituximab rituximab weekly ×4 doses.

### Results

After the last dose of rituximab, he developed severe pain and cyanosis of the right toes, with rapid progression to bilateral digital necrosis. Arterial Doppler ultrasound excluded thrombosis or macrovascular obstruction. Laboratory tests showed extreme thrombocytosis (up to 3,000,000/µL), progressive anemia, and elevated acute-phase reactants. PET-CT demonstrated absence of distal uptake in both feet, consistent with impaired microvascular flow. Skin biopsy revealed intravascular hyaline thrombi. Cryoglobulins were positive, establishing the diagnosis of type I cryoglobulinemia with thrombotic microangiopathy, likely paraneoplastic and precipitated by a rituximab flare phenomenon.
Given the progression of ischemia, intensive anticoagulation and high-dose corticosteroids were started, followed by plasmapheresis (5 sessions) and subsequent targeted therapy with zanubrutinib. Thrombocytosis progressively normalized and ischemic lesions stabilized. A right supracondylar lower-limb amputation was required, with good subsequent stump evolution.

### Summary/Conclusion

Type I cryoglobulinemia should be considered in patients with B-cell lymphomas presenting with rapidly progressive distal ischemia in the absence of macrovascular disease. Early multidisciplinary management and prompt therapy directed at the underlying clone are crucial to improve outcomes and limit irreversible tissue damage.

**Copyright Information:** (Online) ISSN: 2572-9241
©2026 The Author(s). HemaSphere is published by John Wiley & Sons Ltd on behalf of European Hematology Association. This is an open access Abstract Book distributed under the Attribution-NonCommercial-NoDerivs (CC BY-NC-ND), which allows third parties to download the articles and share them with others as long as they credit the author and the Abstract Book, but they cannot change the content in any way or use them commercially.
**Abstract Book Citations:** Authors, Title, HemaSphere, 2026;10:(S1):pages. The abstract book DOIs can be found on the "Author Information" page.
**Disclaimer:** Articles published in the journal HemaSphere exclusively reflect the opinions of the authors. The authors are responsible for all content in their abstracts including accuracy of the facts, statements, citing resources, etc.

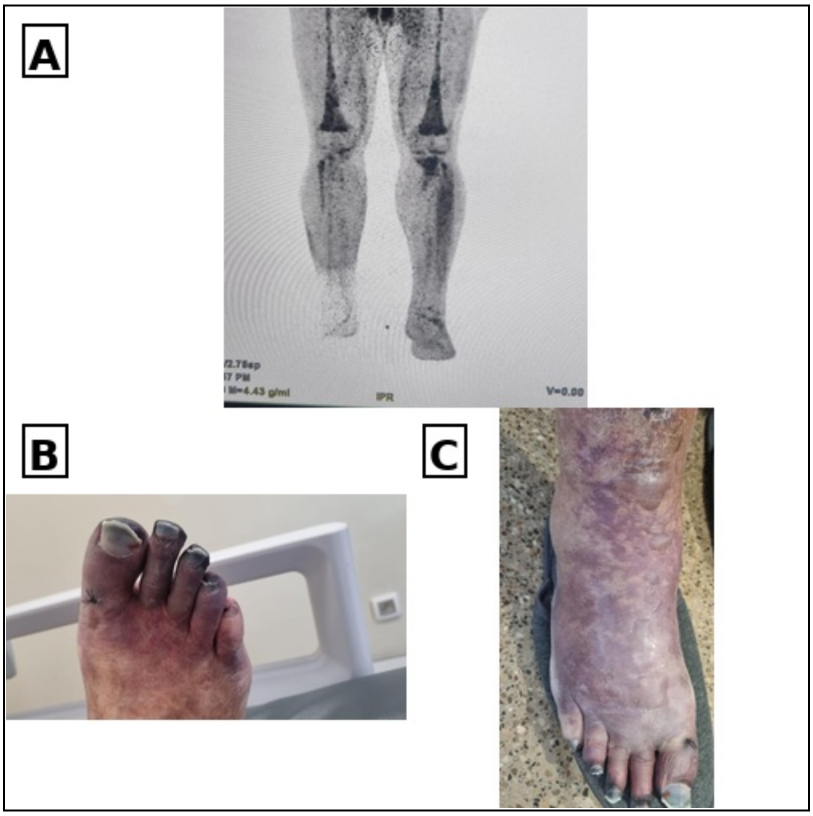

A) PET-CT scan, C) and C) Inferior limb distal ischemia

**Copyright Information:** (Online) ISSN: 2572-9241
©2026 The Author(s). HemaSphere is published by John Wiley & Sons Ltd on behalf of European Hematology Association. This is an open access Abstract Book distributed under the Attribution-NonCommercial-NoDerivs (CC BY-NC-ND), which allows third parties to download the articles and share them with others as long as they credit the author and the Abstract Book, but they cannot change the content in any way or use them commercially.
**Abstract Book Citations:** Authors, Title, HemaSphere, 2026;10:(S1):pages. The abstract book DOIs can be found on the "Author Information" page.
**Disclaimer:** Articles published in the journal HemaSphere exclusively reflect the opinions of the authors. The authors are responsible for all content in their abstracts including accuracy of the facts, statements, citing resources, etc.

### (PB3698) HISTOLOGICAL TRANSFORMATION OF MARGINAL ZONE LYMPHOMA INTO HIGH-GRADE LYMPHOMA

**Topic:** 18. Indolent and mantle-cell non-Hodgkin lymphoma - Clinical

Maha Charfi*[1], Imen Ben Amor[1], Faten Kallel[1], Yosra Fakhfakh[1], Asma Koubaa[1], Rahma Mallek[1], Imen Frikha[1], Moez Mdhaffar[1], Moez Elloumi[1]

[1]*Hedi Chaker hospital, hematology department, Sfax, Tunisia;*

### Background

This transformation can occur in all types of marginal zone lymphoma (MZL), including splenic MZL (S-MZL), lymph node MZL, and mucosa-associated lymphoid tissue (MALT) lymphoma. The most common form of transformation is diffuse large B-cell lymphoma (DLBCL). Transformation into Hodgkin lymphoma or Burkitt lymphoma is possible but rare.

### Aims

This study reports on the epidemiological, clinical, prognostic, and therapeutic characteristics of patients with MZL that has transformed into high-grade lymphoma.

### Methods

This is a descriptive, retrospective study conducted in the clinical hematology department of Hédi Chaker Hospital in Sfax between January 2015 and December 2024. Patients with MZL that had transformed into high-grade lymphoma were included. We reported the epidemiological, clinical, prognostic, and therapeutic characteristics of these patients.

### Results

During the study period, we collected eight cases of MZL that transformed into high-grade lymphoma, representing a prevalence of 17% among all MZL cases. The median time to transformation was 14 months after the initial MZL diagnosis.
The median patient age was 58 years, and the female-to-male ratio was 1.6.
Of the transformed MZL cases in our study, 50% were lymph node MZL, 25% were gastric MALT lymphoma, and 25% were S-MZL. Transformation was observed at the time of MZL diagnosis in three patients (37%), and the median time to transformation was 17 months (range, 13–62 months). The clinical and biological manifestations most frequently associated with the histological transformation of low-grade lymphomas were observed in our patients with the following frequencies: B symptoms (50%), performance status (PS) ≥ 2 (37%), disseminated disease (75%), and elevated LDH levels (12%). Treatment at the time of transformation included RCHOP in 75% of cases and RDHAC in 25%. The overall response rate was 75%. The median survival was 44 months. The five-year overall survival (OS) rate was 56%.

### Summary/Conclusion

The transformation rate of MZL in our study is similar to rates reported in the literature (11-14%), as is the median time to transformation (13 months). However, the distribution of cases according to the underlying type of MZL shows a predominance of transformation in nodal MZL. In contrast, the literature reports transformation in 61% of MALT lymphomas, 26% of nodal MZL, and 13% of S-MZL. The prognosis for MZL is poor, associated with shorter survival and a median survival of 36 months, similar to that observed in our series.

**Copyright Information:** (Online) ISSN: 2572-9241
©2026 The Author(s). HemaSphere is published by John Wiley & Sons Ltd on behalf of European Hematology Association. This is an open access Abstract Book distributed under the Attribution-NonCommercial-NoDerivs (CC BY-NC-ND), which allows third parties to download the articles and share them with others as long as they credit the author and the Abstract Book, but they cannot change the content in any way or use them commercially.
**Abstract Book Citations:** Authors, Title, HemaSphere, 2026;10:(S1):pages. The abstract book DOIs can be found on the "Author Information" page.
**Disclaimer:** Articles published in the journal HemaSphere exclusively reflect the opinions of the authors. The authors are responsible for all content in their abstracts including accuracy of the facts, statements, citing resources, etc.

### (PB3699) PROGNOSTIC VALUE OF RED CELL DISTRIBUTION WIDTH (RDW) AT DIAGNOSIS IN MANTLE CELL LYMPHOMA.

**Topic:** 18. Indolent and mantle-cell non-Hodgkin lymphoma - Clinical

Sunil Lakhwani*[1, 2], Roberto-Juan García-González[1], Marta Diaz-Lopez[2], Carolina De Bonis-Braun[2], María José Rodríguez Salazar[2], Taida Martín-Santos[2], José María Raya Sánchez[1, 2]

*[1]Universidad de La Laguna, Facultad de Medicina, La Laguna, Spain;[2]Hospital Universitario de Canarias, Hematology, La Laguna, Spain;*

### Background

Mantle cell lymphoma (MCL) is an uncommon and biologically heterogeneous B-cell non-Hodgkin lymphoma characterized by an aggressive clinical course and generally poor long-term outcomes despite therapeutic advances. Although prognostic tools such as the MIPI score and biological markers including Ki-67 are widely used, additional easily accessible biomarkers may help to refine risk stratification at diagnosis.
Red cell distribution width (RDW), a routinely available parameter reflecting anisocytosis and systemic inflammation, has emerged as a potential prognostic marker in several malignancies, including lymphomas. However, its prognostic role in MCL remains poorly defined.

### Aims

To assess whether RDW value at diagnosis is related to progression-free survival (PFS) to first-line treatment, as well as overall survival (OS) and response to first-line treatment in MCL patients.

### Methods

We performed a retrospective study of patients with MCL diagnosed and treated at our center between 2011 and 2020. Both PFS and OS were considered from the start of the treatment. We used a ROC curve to identify the RDW cutoff point with the highest sensitivity and specificity for PFS and OS.

### Results

We included 49 patients with MCL treated between 2011 and 2020. Of these, 37 were male (75,5%), mean age was 66 years (range 41-85), almost all had stage IV disease at diagnosis (48/49, 98%) and 8 patients (16.3%) were blastoid variant. Complete response (CR) to front-line treatment was achieved by 30/49 patients (61.2%) and median PFS of the whole group was 38.3 months. With a median follow-up of 124 months, 30/49 patients (61.2%) had died and median OS was 55.5 months.
Mean RDW value at diagnosis was 15.1% (range 12.6-26.8). Best cutoff value to discriminate PFS was 14.2%. However, we only found a trend towards statistical significance when comparing PFS of patients with RDW ≤14.2% versus patients with RDW >14.2% (median PFS 50.9 months VS 32.5 months, p=0.056; Figure 1A). The best cutoff value to discriminate OS was 16.3%, and patients with RDW ≤ 16.3% had a longer OS than patients with RDW > 16.3% (median OS 97.2 months VS 18.9 months, p=0.003; Figure 1B). Rate of CR was 76.9% (30/39) for patients with RDW ≤ 16.3% and 40% (4/10) for patients with RDW > 16.3% (p=0.033).
We performed a multivariate model for OS with those variables at diagnosis that were significant in the univariate analysis (LDH, Ki67>30% and RDW), but all three variables lost their statistical significance probably due to the small sample size.

**Copyright Information:** (Online) ISSN: 2572-9241
©2026 The Author(s). HemaSphere is published by John Wiley & Sons Ltd on behalf of European Hematology Association. This is an open access Abstract Book distributed under the Attribution-NonCommercial-NoDerivs (CC BY-NC-ND), which allows third parties to download the articles and share them with others as long as they credit the author and the Abstract Book, but they cannot change the content in any way or use them commercially.
**Abstract Book Citations:** Authors, Title, HemaSphere, 2026;10:(S1):pages. The abstract book DOIs can be found on the "Author Information" page.
**Disclaimer:** Articles published in the journal HemaSphere exclusively reflect the opinions of the authors. The authors are responsible for all content in their abstracts including accuracy of the facts, statements, citing resources, etc.

### Summary/Conclusion

Despite being a small and retrospective series, we found that the RDW at diagnosis is a prognostic factor, with better OS and CR rate in those patients with RDW ≤ 16.3%. These results need to be confirmed in multicentric studies with larger samples.

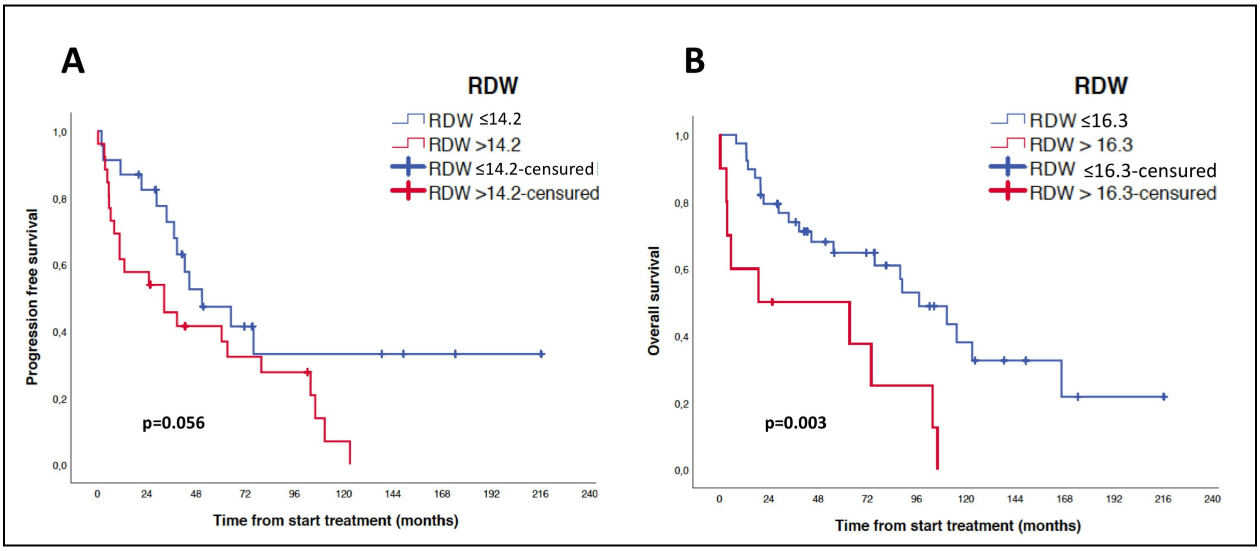

A. Progression free survival according to RDW value. B. Overall survival according to RDW value.

**Copyright Information:** (Online) ISSN: 2572-9241
©2026 The Author(s). HemaSphere is published by John Wiley & Sons Ltd on behalf of European Hematology Association. This is an open access Abstract Book distributed under the Attribution-NonCommercial-NoDerivs (CC BY-NC-ND), which allows third parties to download the articles and share them with others as long as they credit the author and the Abstract Book, but they cannot change the content in any way or use them commercially.
**Abstract Book Citations:** Authors, Title, HemaSphere, 2026;10:(S1):pages. The abstract book DOIs can be found on the "Author Information" page.
**Disclaimer:** Articles published in the journal HemaSphere exclusively reflect the opinions of the authors. The authors are responsible for all content in their abstracts including accuracy of the facts, statements, citing resources, etc.

### (PB3700) ASSOCIATION OF THE NUMBER OF P16INK4A-POSITIVE TUMOR CELLS WITH THE PRESENCE OF PATHOGENIC MISSENSE VARIANTS OF THE TP53 GENE IN DIFFUSE LARGE B-CELL LYMPHOMA

**Topic:** 19. Large B cell lymphomas - Clinical

Maria Berdnikova*[1], Dmitry Diakonov[1], Ekaterina Tregubova[1], Vitaliy Rosin[1], Elena Vaneeva[1], Anastasia Sokolova[1], Maria Loginova[1]

*[1]The Federal State-Financed Scientific Institution Kirov Research Institute of Hematology and Blood Transfusion under the Federal Medical Biological Agency, Kirov, Russia;*

### Background

Aberrations in the *TP53* tumor suppressor gene are among the most common genetic alterations in malignancies. The missense mutations of this gene leading to the accumulation of defective p53 protein inactivating the normal protein product in the nucleus, are often observed in diffuse large B-cell lymphoma (DLBCL). Decreased p53 functional activity disrupts critical cellular programs, including the induction of apoptosis, which contributes to tumor progression. An increased proportion of tumor cells stained in a reaction with p53 antibodies does not always correlate with p53 gene abnormalities. The search for additional immunohistochemical markers indicating the pathogenic status of *TP53* is relevant. Loss of p53 function leads to oncogenic stress, which may induce the expression of the p16INK4a protein, a product of the *CDKN2A* tumor suppressor gene. No data were found on the relationship between the number of p16INK4a-positive tumor cells and the presence of *TP53* gene mutations in DLBCL.

### Aims

To determine the association of the number of p16INK4a-positive tumor cells with the presence of pathogenic missense variants of the *TP53* gene in patients with DLBCL.

### Methods

The formalin-fixed paraffin embedded samples of tumor tissue of 85 patients with newly diagnosed DLBCL were included in the study. The mutation status of the *TP53* gene was determined by next-generation sequencing according to the standard protocols proposed by a group of researchers from the International Agency for Research on Cancer. The number of p16INK4a-positive tumor cells was studied using immunohistochemical and morphometric methods. The software SPSS Statistic 26 was used for statistical data processing (Mann-Whitney U test, ROC-analysis). Differences between indicators were considered statistically significant at $p<0.05$.

### Results

The pathogenic variants of the *TP53* gene were observed to be 17.6% (15/85) among the patient population, of which 86.7% (13/15) were missense variants (R282Q, Y220H, V157G, C176Y, K132N, M237I, I254S, R248W, V173G, A276G, E258K, R273S, H179Q). The relative number of p16INK4a-positive tumor cells ranged from 0.1% to 89.0% (median was 2.0% [0-6,5]). It was found that the content p16INK4a-positive karyocytes was significantly higher in the presence of *TP53* missense variants than in the absence of these disorders ($p = 0.012$): 9.2% [3.7-34.6] versus 1.0% [0-6.0]. The optimal cutoff point for p16INK4a-positive tumor cells was determined to be 3.6% (AUC = 0.719 ± 0.084; 95% CI = 0.554-0.885; sensitivity - 77%; specificity - 70%). The screening of the presence of pathogenic missense variants of the *TP53* gene is necessary when the content of p16INK4a-positive tumor cells is above the threshold (≥3.6%).

**Copyright Information:** (Online) ISSN: 2572-9241
©2026 The Author(s). HemaSphere is published by John Wiley & Sons Ltd on behalf of European Hematology Association. This is an open access Abstract Book distributed under the Attribution-NonCommercial-NoDerivs (CC BY-NC-ND), which allows third parties to download the articles and share them with others as long as they credit the author and the Abstract Book, but they cannot change the content in any way or use them commercially.
**Abstract Book Citations:** Authors, Title, HemaSphere, 2026;10:(S1):pages. The abstract book DOIs can be found on the “Author Information” page.
**Disclaimer:** Articles published in the journal HemaSphere exclusively reflect the opinions of the authors. The authors are responsible for all content in their abstracts including accuracy of the facts, statements, citing resources, etc.

**Summary/Conclusion**

A direct relationship was found between the above-threshold number of p16INK4a-positive cells and the presence of pathogenic missense variants of the *TP53* gene. A value of ≥3.6% can be used as a surrogate immunohistochemical marker to determine the pathogenic mutational status of this gene in patients with DLBCL.

**Copyright Information:** (Online) ISSN: 2572-9241
©2026 The Author(s). HemaSphere is published by John Wiley & Sons Ltd on behalf of European Hematology Association. This is an open access Abstract Book distributed under the Attribution-NonCommercial-NoDerivs (CC BY-NC-ND), which allows third parties to download the articles and share them with others as long as they credit the author and the Abstract Book, but they cannot change the content in any way or use them commercially.
**Abstract Book Citations:** Authors, Title, HemaSphere, 2026;10:(S1):pages. The abstract book DOIs can be found on the "Author Information" page.
**Disclaimer:** Articles published in the journal HemaSphere exclusively reflect the opinions of the authors. The authors are responsible for all content in their abstracts including accuracy of the facts, statements, citing resources, etc.

### (PB3701) THE USE OF RITUXIMAB IN THE TREATMENT OF NON-HODGKIN LYMPHOMAS IN PREGNANT WOMEN: MATERNAL AND INFANT OUTCOMES

**Topic:** 19. Large B cell lymphomas - Clinical

Inna Krasilnikova*[1], Yana Mangasarova[2, 3], Madina Bagova[2], Ekaterina Fastova[2], Ekaterina Gitelzon[2], Oleg Margolin[2], Runisa Abdurashidova[2], Aminat Magomedova[2], Anna Kravtsova[2], Darya Belkina[2], Sergey Kravchenko[2], Evgeniy Zvonkov[2, 3], Eugenia Polushkina[4], Roman Shmakov[5]

*[1]National Medical Research Center for Hematology, Moscow, Russia;[2] 1National Medical Research Center for Hematology, Moscow, Russian Federation, Moscow, Russia; [3]Federal State Budgetary Educational Institution of Higher Education "Russian University of Medicine" of the Ministry of Healthcare of the Russian Federation, Moscow, Russia; [4]National Medical Research Center for Obstetrics, Gynecology and Perinatology named after Academician V. I. Kulakov, Moscow, Russia; [5]Moscow Regional Research and Clinical Institute for Obstetrics and Gynecology named after V.I. Krasnopolsky, Moscow, Russia;*

### Background

Rituximab significantly improves the prognosis for Non-Hodgkin Lymphomas (NHL); however, its use during gestation is associated with risks due to its ability to cross the placental barrier and reduce B-lymphocyte levels in the fetus.

### Aims

To conduct a comparative analysis of the efficacy of NHL therapy in pregnant women (with and without rituximab) and to assess the health status of their infants in the neonatal period and in long-term follow-up.

### Methods

We analyzed the treatment outcomes of 23 pregnant women with newly diagnosed NHL and 24 of their children (including one set of twins). All mothers received chemotherapy (CT) during pregnancy. A comparison was made between two groups based on the treatment regimen:
• Group 1 - 11 children from mothers who received CT with rituximab;
• Group 2 - 13 children from mothers who received CT without rituximab.

**Copyright Information:** (Online) ISSN: 2572-9241
©2026 The Author(s). HemaSphere is published by John Wiley & Sons Ltd on behalf of European Hematology Association. This is an open access Abstract Book distributed under the Attribution-NonCommercial-NoDerivs (CC BY-NC-ND), which allows third parties to download the articles and share them with others as long as they credit the author and the Abstract Book, but they cannot change the content in any way or use them commercially.
**Abstract Book Citations:** Authors, Title, HemaSphere, 2026;10:(S1):pages. The abstract book DOIs can be found on the “Author Information” page.
**Disclaimer:** Articles published in the journal HemaSphere exclusively reflect the opinions of the authors. The authors are responsible for all content in their abstracts including accuracy of the facts, statements, citing resources, etc.

### Results

The study included 23 women with a median age of 33 years. At the start of CT, the median gestational age was 27 weeks. The histological diagnoses were: Primary Mediastinal B-cell Lymphoma–15 cases (65%), Diffuse Large B-cell Lymphoma–4 cases (17%), Follicular Lymphoma–2 cases (9%), and High-grade B-cell Lymphoma–2 cases (9%).
The median number of rituximab administrations before delivery was 3 (range 1–4). The patients received the following chemotherapy regimens: DA-EPOCH (n=14; 61%), CHOP (n=4; 17%), and VACOP-B (n=5; 22%).
First-line therapy efficacy was higher in the rituximab group: complete remission (CR) was achieved in 9 out of 11 women (82%). Among patients receiving CT without rituximab, CR after first-line therapy was documented in 5 out of 13 (38%).
A comparative characteristic of the 24 newborns, depending on whether the mother received rituximab therapy during pregnancy, is presented in Table 1.

**Table 1. Characteristics of Newborns from Women Who Received CT for Non-Hodgkin Lymphomas During Pregnancy.**

| Characteristic | Infants whose mothers received CT *with*rituximab during pregnancy (n=11) | Infants whose mothers received CT *without*rituximab during pregnancy (n=13) | p-value |
|---|---|---|---|
| Gestational age at delivery, Me(range), weeks | 36(30-38) | 33(31-39) | 0,037 |
| Birth weight, Me(range),g | 2600(1640-2865) | 2140(899-3665) | 0,374 |
| Birth length, Me(range),cm | 49(40-53) | 47(35-51) | 0,219 |
| Apgar score, 1 min, Me(range) | 7(3-8) | 7(1-8) | 0,966 |
| Apgar score, 5 min, Me(range) | 8(6-9) | 8(6-9) | 0,752 |
| NICU admission, n(%) | 4(36) | 8(61) | 0,414 |
| Perinatal infection, n(%) | 3(27) | 7(54) | 0,236 |
| **Condition at Birth** | | | |
| Satisfactory, n(%) | 6(55) | 4(31) | 0,178 |
| Moderate, n(%) | 1(9) | 3(23) | |
| Severe, n(%) | 4(36) | 6(46) | |

*Note: Me - Median; NICU - Neonatal Intensive Care Unit.*

Long-term health assessment of the children was conducted using a remote questionnaire specifically designed to evaluate physical development and the functional status of organs and systems. The results are shown in Figure 1.
In a single observation (an infant whose mother received a single dose of rituximab during pregnancy), a ventricular septal defect (VSD) was diagnosed at birth. Currently, the patient is under dynamic observation, with a trend towards spontaneous closure of the defect.

### Summary/Conclusion

Rituximab used during pregnancy significantly improves the efficacy of LPD therapy, while showing no significant adverse effects on infant health compared to chemotherapy regimens without rituximab.

**Copyright Information:** (Online) ISSN: 2572-9241
©2026 The Author(s). HemaSphere is published by John Wiley & Sons Ltd on behalf of European Hematology Association. This is an open access Abstract Book distributed under the Attribution-NonCommercial-NoDerivs (CC BY-NC-ND), which allows third parties to download the articles and share them with others as long as they credit the author and the Abstract Book, but they cannot change the content in any way or use them commercially.
**Abstract Book Citations:** Authors, Title, HemaSphere, 2026;10:(S1):pages. The abstract book DOIs can be found on the "Author Information" page.
**Disclaimer:** Articles published in the journal HemaSphere exclusively reflect the opinions of the authors. The authors are responsible for all content in their abstracts including accuracy of the facts, statements, citing resources, etc.

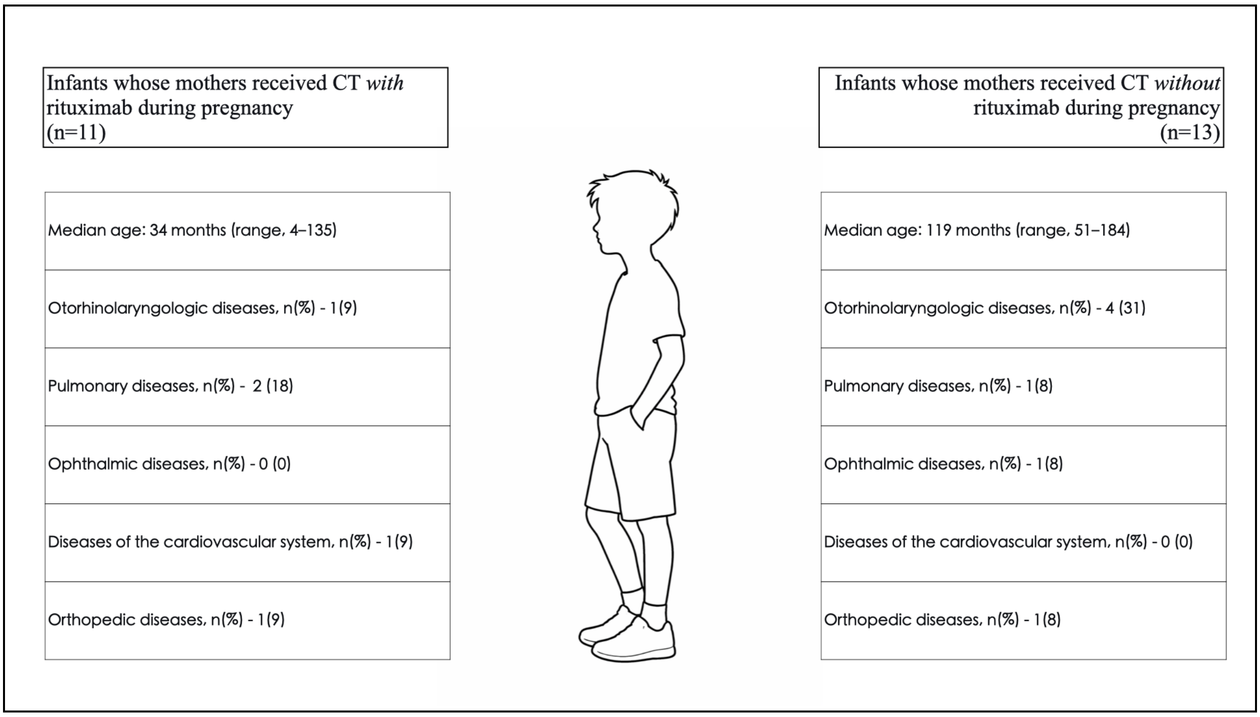

Figure 1. Structure of Acute and Chronic Diseases in Children Born to Women Who Received Chemotherapy for Non-Hodgkin Lymphomas During Pregnancy.

**Copyright Information:** (Online) ISSN: 2572-9241
©2026 The Author(s). HemaSphere is published by John Wiley & Sons Ltd on behalf of European Hematology Association. This is an open access Abstract Book distributed under the Attribution-NonCommercial-NoDerivs (CC BY-NC-ND), which allows third parties to download the articles and share them with others as long as they credit the author and the Abstract Book, but they cannot change the content in any way or use them commercially.
**Abstract Book Citations:** Authors, Title, HemaSphere, 2026;10:(S1):pages. The abstract book DOIs can be found on the "Author Information" page.
**Disclaimer:** Articles published in the journal HemaSphere exclusively reflect the opinions of the authors. The authors are responsible for all content in their abstracts including accuracy of the facts, statements, citing resources, etc.

### (PB3703) CHANGES IN THE UTILIZATION OF INNOVATIVE THERAPIES INCLUDING BISPECIFIC ANTIBODIES AND SURVIVAL OUTCOMES IN PATIENTS WITH RELAPSED/REFRACTORY DIFFUSE LARGE B-CELL LYMPHOMA AROUND 2024

**Topic:** 19. Large B cell lymphomas - Clinical

Haiwen Huang*[1], Shubang Xie[2], Longfei He[2], Kaiyu Sheng[2], Wanying Chen[2], Qian Cheng[2], Tao You[2], Zhou Jin[2], Depei Wu[2, 2]

*[1]The First Affiliated Hospital of Soochow University, Hematology, Suzhou, China;[2]The First Affiliated Hospital Of Soochow University, Hematology, Suzhou, China;*

### Background

Relapsed/refractory diffuse large B-cell lymphoma (r/r DLBCL) carries a poor prognosis, with limited efficacy from traditional salvage chemotherapy. The year 2024 marked a pivotal shift with the approval of bispecific antibodies (BsAbs). In the bispecific antibody era, it remains unclear whether treatment patterns impact the survival outcomes of patients with relapsed/refractory diffuse large B-cell lymphoma in the real-world setting.

### Aims to compare the adoption rate of innovative therapies (notably bispecific antibodies), therapeutic efficacy (including objective response rate and complete response rate), and survival outcomes (overall survival and event-free survival) between patients with relapsed/refractory diffuse large B-cell lymphoma (r/r DLBCL) who initiated third-line (L3) therapy before 2024 and those who initiated L3 therapy in 2024 or later, so as to clarify the impact of the post-2024 novel therapy-accessible era on the management and prognosis of advanced-line r/r DLBCL.

### Methods

This single-center prospective cohort study utilized data from a dedicated r/r DLBCL registry. Patients were divided into two cohorts: Group0 (initiating L3 therapy between 2020 and early 2024) and Group1 (initiating L3 therapy in 2024 or later). Key endpoints included rates of novel therapy use, objective response rate (ORR), complete response rate (CRR), overall survival (OS), and event-free survival (EFS).Subgroup analyses were pre-specified to evaluate survival outcomes stratified by bispecific antibody use.

### Results

A total of 84 patients were included. Baseline characteristics were balanced between groups. Group1 showed a significant increase in the utilization of novel therapies (93.2% vs. 77.8%, p=0.047), driven predominantly by a dramatic rise in BsAb use (54.5% vs. 7.7%, P<0.01). Consequently, the use of salvage chemotherapy alone declined. Efficacy was significantly improved in Group1, with higher CRR (40.0% vs. 13.8%, P=0.02) and ORR (50.0% vs. 25.0%, p=0.047). Survival outcomes were also superior, with a prolonged median EFS (12 vs. 5 months, p=0.006) and a trend toward improved OS(24 vs. 19 months, p=0.023).Stratified analysis by BsAb use further demonstrated the survival benefit of this novel therapy: among all patients receiving L3 treatment, those who received BsAbs achieved a markedly longer median EFS (12 vs. 7 months, p=0.027) and median OS (24 vs.16 months, p=0.018) compared with non-BsAb users. Even within the Post-2024 cohort, BsAb-treated patients had better survival outcomes than those who received other novel therapies without BsAbs (median EFS:19 vs. 13 months, p=0.028 ; median OS: 26 vs. 18 months, p=0.046 ).

**Copyright Information:** (Online) ISSN: 2572-9241
©2026 The Author(s). HemaSphere is published by John Wiley & Sons Ltd on behalf of European Hematology Association. This is an open access Abstract Book distributed under the Attribution-NonCommercial-NoDerivs (CC BY-NC-ND), which allows third parties to download the articles and share them with others as long as they credit the author and the Abstract Book, but they cannot change the content in any way or use them commercially.
**Abstract Book Citations:** Authors, Title, HemaSphere, 2026;10:(S1):pages. The abstract book DOIs can be found on the "Author Information" page.
**Disclaimer:** Articles published in the journal HemaSphere exclusively reflect the opinions of the authors. The authors are responsible for all content in their abstracts including accuracy of the facts, statements, citing resources, etc.

**Summary/Conclusion**

The initiation of L3 treatment in the post-2024 era, characterized by accessible BsAbs, is associated with a paradigm shift toward novel therapy adoption, leading to significantly improved response rates and survival. Stratified analysis confirmed that BsAb use was the key driver of these survival benefits, with patients receiving BsAbs achieving substantially longer EFS and OS compared to those without BsAb treatment. These real-world findings underscore the critical role of timely integration of innovative agents, particularly bispecific antibodies, in advanced-line r/r DLBCL management. Study limitations include its single-center design and sample size, warranting future multi-center validation and biomarker exploration.

**Copyright Information:** (Online) ISSN: 2572-9241
©2026 The Author(s). HemaSphere is published by John Wiley & Sons Ltd on behalf of European Hematology Association. This is an open access Abstract Book distributed under the Attribution-NonCommercial-NoDerivs (CC BY-NC-ND), which allows third parties to download the articles and share them with others as long as they credit the author and the Abstract Book, but they cannot change the content in any way or use them commercially.
**Abstract Book Citations:** Authors, Title, HemaSphere, 2026;10:(S1):pages. The abstract book DOIs can be found on the "Author Information" page.
**Disclaimer:** Articles published in the journal HemaSphere exclusively reflect the opinions of the authors. The authors are responsible for all content in their abstracts including accuracy of the facts, statements, citing resources, etc.

### (PB3704) LACK OF SURVIVAL BENEFIT WITH TEMOZOLOMIDE MAINTENANCE AFTER HIGH-DOSE METHOTREXATE TREATMENT IN PRIMARY CNS LYMPHOMA - A REAL-WORLD ANALYSIS FROM TWO SWEDISH CENTRES

**Topic:** 19. Large B cell lymphomas - Clinical

Jenni Niemi*[1], Nashif Chowdhury[1], Haris Babacic[2], Ingrid Glimelius[1], Mats Jerkeman[3], Mats Hellstrom[1]

*[1]Uppsala University, Department of Immunology, Genetics, and Pathology, Uppsala, Sweden; [2]Karolinska Institute, Department of Oncology and Pathology, Stockholm, Sweden; [3]Lund University, Lund, Sweden;*

### Background

Primary central nervous system lymphoma (PCNSL) is an aggressive subtype of diffuse large B-cell lymphoma. Due to poor clinical outcomes and high relapse rates, maintenance therapy with temozolomide is to be considered according to international clinical guidelines, although its efficacy is not proven. A recent randomized phase III trial conducted in Japan demonstrated that temozolomide maintenance failed to improve overall survival (OS) and progression-free survival (PFS) in patients newly diagnosed with PCNSL.

### Aims

To address the role of temozolomide maintenance in a real-world PCNSL cohort after induction therapy.

### Methods

We followed 110 consecutive PCNSL patients, diagnosed from 2009 to 2022 at two major hospitals in Sweden, Uppsala University Hospital (n=60) and Skåne University Hospital (n=50). Patients were followed from date of diagnosis to date of death or end of follow-up. Inclusion criteria consisted of histologically confirmed diagnoses of DLBCL localized exclusively to the CNS, obtained via biopsy or surgical resection.

### Results

The median follow-up time was 23.4 months (range, 0.33–182.2). Patients received a variety of treatments, most patients (n=84, 76.4%) were treated with high-dose methotrexate-containing (HD-MTX) induction chemotherapy; of these, 38 (35%) received temozolomide maintenance. As expected, median OS was markedly longer in HD-MTX-treated patients compared to patients who did not receive HD-MTX (38.2 vs. 2.2 months). Median OS did not differ between patients on temozolomide maintenance versus no maintenance (36.3 vs. 18.0 months, logrank test p=0.19). After adjustment for clinical confounders associated with OS in PCNSL (age >50 years, receipt of HD-MTX, completion of induction chemotherapy, first-line temozolomide induction, first-line autologous stem-cell transplantation, and radiotherapy), temozolomide maintenance was not associated with improved survival in the multivariable Cox model (HR 0.71, 95% CI 0.19–2.57).

### Summary/Conclusion

Our findings indicate that temozolomide maintenance does not improve post-induction OS in PCNSL. The lack of association with an OS benefit in this retrospective two-centre cohort study does not support routine use of temozolomide maintenance in PCNSL. Our results in combination with results from a Japanese phase III trial, prompt changing the current clinical guidelines.

**Copyright Information:** (Online) ISSN: 2572-9241
©2026 The Author(s). HemaSphere is published by John Wiley & Sons Ltd on behalf of European Hematology Association. This is an open access Abstract Book distributed under the Attribution-NonCommercial-NoDerivs (CC BY-NC-ND), which allows third parties to download the articles and share them with others as long as they credit the author and the Abstract Book, but they cannot change the content in any way or use them commercially.
**Abstract Book Citations:** Authors, Title, HemaSphere, 2026;10:(S1):pages. The abstract book DOIs can be found on the "Author Information" page.
**Disclaimer:** Articles published in the journal HemaSphere exclusively reflect the opinions of the authors. The authors are responsible for all content in their abstracts including accuracy of the facts, statements, citing resources, etc.

### (PB3705) AN ANATOMY-INFORMED CLASSIFICATION STRATIFIES PRIMARY TESTICULAR LYMPHOMA AND DISTINGUISHES IT FROM SYSTEMIC DLBCL WITH TESTICULAR INVOLVEMENT AT DIAGNOSIS: A RETROSPECTIVE COHORT STUDY

**Topic:** 19. Large B cell lymphomas - Clinical

Ying Shan*[1], Xiaoling Chen[2], Da-Hui Li[3], Wen-Hao Zhang[3], Dong Chen[1], Yuting Xin[4], Chuanxu Liu[3], Tiantian Wang[1], Xiaowei Shi[1], Fan Zhou[2], Ying Lu[1], Rong Tao[3]

*[1]The Affiliated People's Hospital of Ningbo University, haematology, Ningbo, China;[2]Shanghai Jing An District Zhabei Central Hospital, Hematologic & Oncology, Shanghai, China; [3]Fudan University Shanghai Cancer centre, Lymphoma and Medical Oncology, Shanghai, China; [4]Shenzhen Campus of Sun Yat-sen University, Shenzhen, China;*

### Background

Primary testicular lymphoma (PTL) is clinically heterogeneous and carries a meaningful risk of central nervous system (CNS) relapse. Conventional staging may not fully capture anatomy-driven spread along testicular lymphatic drainage or distinguish true PTL from systemic diffuse large B-cell lymphoma (DLBCL) that involves the testis at diagnosis.

### Aims

We evaluated an anatomy-informed PTL classification aligned with contiguous extension and lymphatic drainage, compared outcomes with systemic DLBCL with testicular involvement at diagnosis, and described CNS relapse.

### Methods

This retrospective cohort included patients with testicular lymphoma treated at participating centers between January 2018 and December 2025. Clinical, pathological, imaging, treatment, radiotherapy, CNS prophylaxis, and follow-up data were extracted from institutional electronic medical records. Patients were classified as PTL-OC (organ-confined PTL with allowed contiguous extension to epididymis/spermatic cord/tunica and no nodal or discontinuous extranodal disease), PTL-RN (PTL with regional nodal involvement limited to drainage-consistent retroperitoneal and/or iliac lymph node chains), PTL-DN (PTL with any discontinuous nodal involvement, excluding multi-organ extranodal dissemination involving ≥2 organ systems), or DLBCL-TI (treatment-naïve systemic DLBCL with testicular involvement at diagnosis). Outcomes included OS, PFS, and CNS relapse; survival was summarized using Kaplan–Meier estimates.

**Copyright Information:** (Online) ISSN: 2572-9241
©2026 The Author(s). HemaSphere is published by John Wiley & Sons Ltd on behalf of European Hematology Association. This is an open access Abstract Book distributed under the Attribution-NonCommercial-NoDerivs (CC BY-NC-ND), which allows third parties to download the articles and share them with others as long as they credit the author and the Abstract Book, but they cannot change the content in any way or use them commercially.
**Abstract Book Citations:** Authors, Title, HemaSphere, 2026;10:(S1):pages. The abstract book DOIs can be found on the "Author Information" page.
**Disclaimer:** Articles published in the journal HemaSphere exclusively reflect the opinions of the authors. The authors are responsible for all content in their abstracts including accuracy of the facts, statements, citing resources, etc.

**Results**

A total of 153 patients were analyzed, including PTL-OC (n=62), PTL-RN (n=24), PTL-DN (n=16), and DLBCL-TI (n=51). Baseline risk showed a clear gradient across strata: compared with PTL-OC/PTL-RN, PTL-DN and DLBCL-TI had substantially higher rates of LDH elevation (10.3% and 26.1% vs 56.3% and 64.7%) and a higher-risk IPI distribution (low IPI 0–2: 100.0% and 100.0% vs 68.8% and 15.7%), with B symptoms remaining uncommon in PTL-OC/PTL-RN (1.7%/0.0%) but more frequent in PTL-DN/DLBCL-TI (12.5%/17.6%). Treatment delivery was broadly similar across the three PTL subgroups, whereas radiotherapy and CNS prophylaxis were administered less frequently in DLBCL-TI than in PTL.

Long-term outcomes showed a consistent gradient across subgroups (Figure1). Five-year PFS was similar in PTL-OC and PTL-RN (74.0% [61.8–88.6] and 71.8% [54.6–94.4]), lower in PTL-DN (51.9% [27.7–97.2]), and lowest in DLBCL-TI (47.7% [32.3–70.3]); five-year OS followed the same pattern (83.7% [73.6–95.1], 86.6% [73.4–100.0], 72.1% [47.8–100.0], and 59.2% [41.0–85.5], respectively). In prespecified consolidated comparisons, Early PTL (PTL-OC+PTL-RN; n=86) achieved 5-year PFS/OS of 73.3% (63.1–85.3)/84.4% (76.1–93.6), compared with 51.9% (27.7–97.2)/72.1% (47.8–100.0) in PTL-DN. Overall PTL (PTL-OC+PTL-RN+PTL-DN; n=102) achieved 5-year PFS/OS of 70.6% (60.7–82.1)/82.6% (74.4–91.7), compared with 47.7% (32.3–70.3)/59.2% (41.0–85.5) in DLBCL-TI.

CNS relapse occurred in approximately 5% of PTL overall versus 10% in sDLBCL-TI, with low subgroup event rates within PTL (range 0% to 6.5%).

**Summary/Conclusion**

Drainage-consistent PTL forms a favorable phenotype in which organ-confined and regional-nodal disease show comparable long-term outcomes; non-drainage/discontinuous dissemination is the key inflection point for relapse risk and remains distinct from DLBCL-TI. CNS relapse occurred despite high prophylaxis uptake, indicating that CNS events remain clinically meaningful and should be prespecified in future validation efforts.

**Copyright Information:** (Online) ISSN: 2572-9241
©2026 The Author(s). HemaSphere is published by John Wiley & Sons Ltd on behalf of European Hematology Association. This is an open access Abstract Book distributed under the Attribution-NonCommercial-NoDerivs (CC BY-NC-ND), which allows third parties to download the articles and share them with others as long as they credit the author and the Abstract Book, but they cannot change the content in any way or use them commercially.
**Abstract Book Citations:** Authors, Title, HemaSphere, 2026;10:(S1):pages. The abstract book DOIs can be found on the “Author Information” page.
**Disclaimer:** Articles published in the journal HemaSphere exclusively reflect the opinions of the authors. The authors are responsible for all content in their abstracts including accuracy of the facts, statements, citing resources, etc.

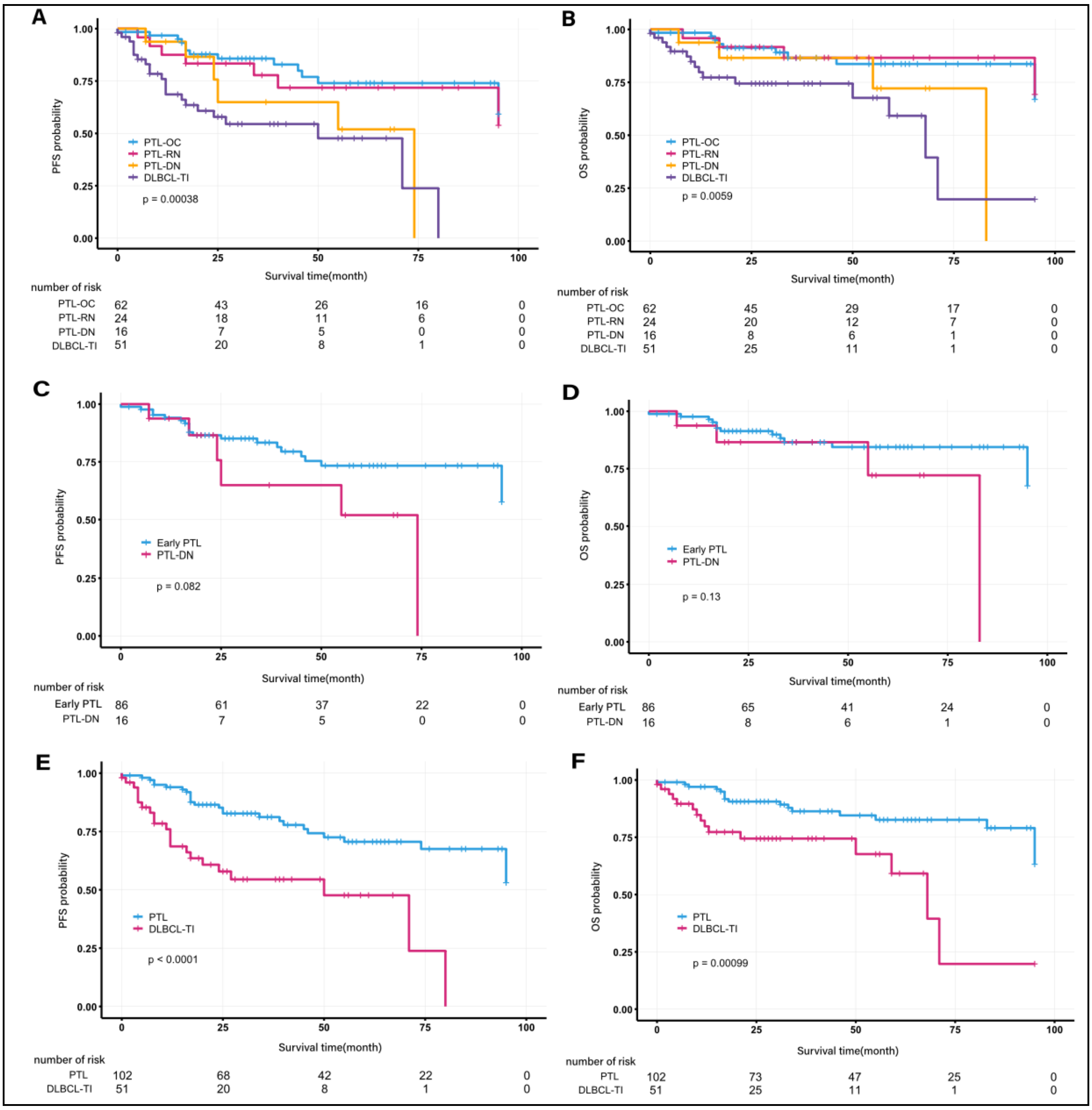

Figure1.Survival outcomes of patients: Kaplan-Meier curves comparing PFS and OS across disease groups. (A) PFS and (B) OS among the four groups: PTL-OC, PTL-RN, PTL-DN and DLBCL-TI.(C) PFS and (D) OS comparing Early PTL (PTL-OC + PTL-RN) versus PTL-DN. (E) PFS and (F) OS comparing PTL (PTL-OC + PTL-RN + PTL- DN) versus DLBCL-TI.Numbers at risk are shown below each plot. PFS, progression-free survival, OS, overall survival, PTL-OC, primary testicular lymphoma, organ- confined; PTL-RN, primary testicular lymphoma with regional nodal involvement; PTL-DN, primary testicular lymphoma with discontinuous nodal involvement; DLBCL-TI, diffuse large B-cell lymphoma with testicular involvement

**Copyright Information:** (Online) ISSN: 2572-9241
©2026 The Author(s). HemaSphere is published by John Wiley & Sons Ltd on behalf of European Hematology Association. This is an open access Abstract Book distributed under the Attribution-NonCommercial-NoDerivs (CC BY-NC-ND), which allows third parties to download the articles and share them with others as long as they credit the author and the Abstract Book, but they cannot change the content in any way or use them commercially.
**Abstract Book Citations:** Authors, Title, HemaSphere, 2026;10:(S1):pages. The abstract book DOIs can be found on the “Author Information” page.
**Disclaimer:** Articles published in the journal HemaSphere exclusively reflect the opinions of the authors. The authors are responsible for all content in their abstracts including accuracy of the facts, statements, citing resources, etc.

### (PB3706) DEVELOPMENT AND VALIDATION OF CTRS-AC FOR PREDICTING SEVERE CHEMOTHERAPY TOXICITY IN ELDERLY DIFFUSE LARGE B-CELL LYMPHOMA

**Topic:** 19. Large B cell lymphomas - Clinical

Mengyuan He[1], Cheng Zhang[2], Yutang Li[1], Tiange Lu[2], Mei Ding[2], Xiaoqian Liu[3], Ling Wang[4], Xiangxiang Zhou*[2]

*[1]Department of Hematology, Shandong Provincial Hospital, Shandong University, Jinan, China;[2]Department of Hematology, Shandong Provincial Hospital Affiliated to Shandong First Medical University, Jinan, China; [3]Department of Hematology, The Affiliated Yantai Yuhuangding Hospital of Qingdao University, Yantai, China; [4]Department of Hematology, Tai'an Central Hospital, Tai'an, China;*

### Background

Diffuse large B-cell lymphoma (DLBCL) is most commonly diagnosed in older adults, with a substantial proportion of patients aged 60 years or above at presentation. In this population, chemotherapy-related toxicity is common and frequently results in treatment modification. However, tools to accurately predict severe toxicity in routine clinical practice remain limited.

### Aims

The aim of this study was to investigate the risk factors for chemotherapy toxicity in elderly patients with DLBCL and to develop and validate a novel prognostic scoring system for the risk of chemotherapy toxicity.

### Methods

This multicenter retrospective study enrolled patients ≥60 years with DLBCL at Shandong Provincial Hospital (SPH) from January 1, 2014, to December 1, 2023. The study also collected data on 200 separate cases each from Tai'an Central Hospital (TCH) in Shandong and The Affiliated Yantai Yuhuangding Hospital (YYH) of Qingdao University as the external validation cohort. Baseline pre-chemotherapy data was collected, and followed up on chemotherapy sessions to document the occurrence of Grade 3 (severe), 4 (life-threatening or disabling), and 5 (death) adverse events, as defined by the National Cancer Institute Common Terminology Criteria for Adverse Events. A novel prognostic model for chemotherapy toxicity in elderly DLBCL patients was ultimately established via logistic regression.

**Copyright Information:** (Online) ISSN: 2572-9241
©2026 The Author(s). HemaSphere is published by John Wiley & Sons Ltd on behalf of European Hematology Association. This is an open access Abstract Book distributed under the Attribution-NonCommercial-NoDerivs (CC BY-NC-ND), which allows third parties to download the articles and share them with others as long as they credit the author and the Abstract Book, but they cannot change the content in any way or use them commercially.
**Abstract Book Citations:** Authors, Title, HemaSphere, 2026;10:(S1):pages. The abstract book DOIs can be found on the “Author Information” page.
**Disclaimer:** Articles published in the journal HemaSphere exclusively reflect the opinions of the authors. The authors are responsible for all content in their abstracts including accuracy of the facts, statements, citing resources, etc.

**Results**

Of the included 500 patients at SPH, using the published CTRS scoring system as a guide, 72.6% of patients were at low risk for chemotherapy toxicity scores, 23.6% were at intermediate risk, and 3.8% were at high risk. The investigators performed univariate and multivariate logistic regression and ultimately identified comorbidities ≤1 (OR 0.318, 95%CI 0.110–0.919, P=0.034), Ann Arbor stage Ⅰ-Ⅱ(OR 0.286, 95%CI 0.085–0.959, P=0.043), receipt of standard-dose chemotherapy or multiagent combination chemotherapy (OR 3.578, 95%CI 1.294–9.898, P=0.014), and a high CTRS score (low-risk, OR 0.074, 95%CI 0.011–0.515, P=0.009; intermediate-risk, OR 0.049, 95%CI 0.007–0.358, P=0.003) as independent predictive Factors. And therefore, a new prognostic stratification model, CTRS-AC, was developed for elderly DLBCL patients who developed grade 3-5 chemotherapy toxicity during chemotherapy. Risk score ranges from 0 to 27 points, categorizes elderly patients with DLBCL at risk for chemotherapy toxicity into three risk strata (low risk, 0 to 9 points; intermediate risk, 10 to 13 points; high risk, ≥14 points).
The area under the receiver operating characteristic curve indicated that the new score had higher discrimination (AUC values CTRS=0.85 and CTRS-AC=0.90 in the SPH training cohort, CTRS=0.72 and CTRS-AC=0.79 in the SPH validation cohort, CTRS=0.71 and CTRS-AC=0.77 in the TCH database, CTRS=0.68 and CTRS-AC=0.81 in the YYH database) (Figure 1). The Hosmer and Lemeshow test showed that the CTRS-AC model had high calibration and clinical benefit compared to the CTRS score, with a good fit and P > 0.05 for all models.

**Summary/Conclusion**

The results of the analysis demonstrated that pre-chemotherapy CTRS score, comorbidities, and Ann Arbor stage may be closely related to the prognosis of chemotherapy in elderly patients with DLBCL. We developed the CTRS-AC score, which demonstrated improved discrimination compared with the original CTRS and maintained consistent performance across independent validation cohorts. These findings support its potential utility for toxicity risk stratification prior to treatment initiation.

**Copyright Information:** (Online) ISSN: 2572-9241
©2026 The Author(s). HemaSphere is published by John Wiley & Sons Ltd on behalf of European Hematology Association. This is an open access Abstract Book distributed under the Attribution-NonCommercial-NoDerivs (CC BY-NC-ND), which allows third parties to download the articles and share them with others as long as they credit the author and the Abstract Book, but they cannot change the content in any way or use them commercially.
**Abstract Book Citations:** Authors, Title, HemaSphere, 2026;10:(S1):pages. The abstract book DOIs can be found on the “Author Information” page.
**Disclaimer:** Articles published in the journal HemaSphere exclusively reflect the opinions of the authors. The authors are responsible for all content in their abstracts including accuracy of the facts, statements, citing resources, etc.

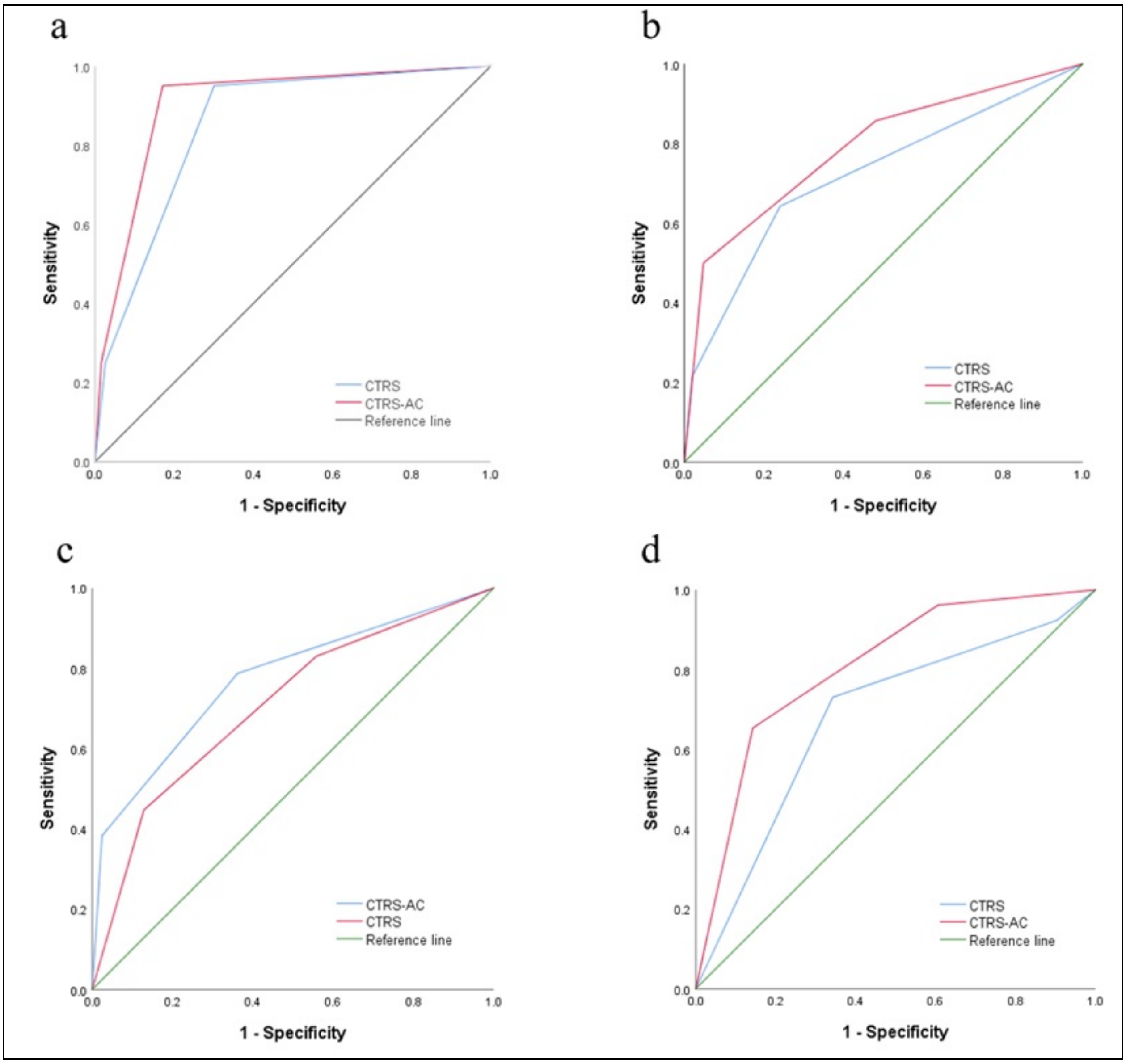

Receiver operating characteristic curves for CTRS versus CTRS-AC predicting chemotherapy toxicity in internal and external cohorts. a: ROC curve in the training cohort of SPH. b: ROC curve in the validation cohort of SPH. c: ROC curve in the TCH database. d: ROC curve in the YYH database.

**Copyright Information:** (Online) ISSN: 2572-9241
©2026 The Author(s). HemaSphere is published by John Wiley & Sons Ltd on behalf of European Hematology Association. This is an open access Abstract Book distributed under the Attribution-NonCommercial-NoDerivs (CC BY-NC-ND), which allows third parties to download the articles and share them with others as long as they credit the author and the Abstract Book, but they cannot change the content in any way or use them commercially.
**Abstract Book Citations:** Authors, Title, HemaSphere, 2026;10:(S1):pages. The abstract book DOIs can be found on the “Author Information” page.
**Disclaimer:** Articles published in the journal HemaSphere exclusively reflect the opinions of the authors. The authors are responsible for all content in their abstracts including accuracy of the facts, statements, citing resources, etc.

### (PB3707) THE IMPACT OF DIETETICS AND PHYSIOTHERAPY PREHABILITATION ON THE CAR-T PATIENT

**Topic:** 19. Large B cell lymphomas - Clinical

Joanna Sinnos*[1], Aidan Mooney[1], Bronagh McGoldrick[1], Juliet Morgan[1], Dhanasekara Koralage Damith Nirmal Dhanasekara[1], Helen Long[1], Wendy Ingram[1], Ceri Jones[1], Keith Wilson[1, 2]

[1]*University Hospital of Wales, Cardiff, Wales, Cardiff, United Kingdom;*[2]*Cardiff University School of Medicine, Cardiff, Wales, Cardiff, United Kingdom;*

### Background

The South Wales BMT Programme has a dedicated multidisciplinary prehabilitation service, including physiotherapy and dietetics, for patients embarking on cellular therapy – HSCT or CAR-T. Limited data exist on the utility of prehab in the haematology setting, particularly for CAR-T recipients who frequently need bridging therapy.

### Aims

To evaluate the impact of prehab in CAR-T recipients by assessing measures of muscle strength and mass between prehab and admission. A secondary aim was to compare physiotherapy and dietetic parameters to determine inter-measure consistency.

### Methods

47 patients who attended prehab prior to CAR-T between November 2021 and December 2024 were included. Differences in handgrip strength (HG), Bioelectrical Impedance Analysis (BIA) - phase angle (PhA) and fat free mass index (FFMI), 1 minute sit-to-stand (STS), 6-minute walk test (6MWT) and clinical frailty scale (CFS) were compared between prehab and admission.

### Results

The median (range) length of prehab was 3.8 (1-18.3) weeks, after which all parameters improved: STS-73% (16/22); 6MWT-55% (11/20); HG-48% (10/21); FFMI-44% (8/18); PhA-35% (6/17) and CFS-6% (2/34). At prehab, there was moderate to strong positive correlations between HG and 6MWT (p=0.0066), HG and STS (p=0.0025) and PhA and STS (p = 0.0186), with only PhA and STS maintaining a strong correlation on admission (p=0.0005). No statistically significant associations were noted between PhA and 6MWT, FFMI and 6MWT or FFMI and STS either at prehab or admission. A HG of <27 kg in males and <16 kg in females defines sarcopenia. BIA lacks strict cut-offs but PhA <4.5 is associated with malnutrition. Using these thresholds, PhA identified sarcopenia in 11 patients at prehab and 9 at admission, compared with 8 and 9 by HG, respectively, with overlap in 6 patients at prehab and 5 at admission.

**Copyright Information:** (Online) ISSN: 2572-9241
©2026 The Author(s). HemaSphere is published by John Wiley & Sons Ltd on behalf of European Hematology Association. This is an open access Abstract Book distributed under the Attribution-NonCommercial-NoDerivs (CC BY-NC-ND), which allows third parties to download the articles and share them with others as long as they credit the author and the Abstract Book, but they cannot change the content in any way or use them commercially.
**Abstract Book Citations:** Authors, Title, HemaSphere, 2026;10:(S1):pages. The abstract book DOIs can be found on the “Author Information” page.
**Disclaimer:** Articles published in the journal HemaSphere exclusively reflect the opinions of the authors. The authors are responsible for all content in their abstracts including accuracy of the facts, statements, citing resources, etc.

**Summary/Conclusion**

These data show that an MDT-led prehab programme is feasible for CAR-T recipients and proves valuable by improving measures of strength. Overall, 43.5% of patients improved, demonstrating the positive impact of prehab in this cohort. The strong correlation observed between PhA and STS was expected as PhA reflects cellular health and muscle quality, while STS assesses exercise capacity through lower limb strength. HG, a measure of muscle mass, was similarly positively correlated with STS and 6MWT which measures areobic capacity. PhA potentially identified more patients at risk of sarcopenia than HG, suggesting that BIA may be more sensitive than traditional measures. Caution is required, however, since HG can be affected by comorbidities (e.g. arthritis) which would not impact BIA. Although CFS demonstrated the smallest improvement (6%), the proportion classified as frail reduced from 20% to 14%. Potential limitations of this study include the short prehab-to-admission interval of 3.8 weeks, reducing the window for intervention. Additionally, the majority (87%) of CAR-T patients in our centre received bridging therapy which may have blunted improvement. It would therefore be interesting to compare our CAR-T and HSCT cohorts with the latter receiving a median of 11.5 weeks prehab and no bridging, being in remission. Future research should include larger datasets with measurements at diagnosis, evaluation of primary and bridging therapies and longitudinal follow up to confirm our findings and identify patients who may need targeted rehabilitation post CAR-T therapy.

**Copyright Information:** (Online) ISSN: 2572-9241
©2026 The Author(s). HemaSphere is published by John Wiley & Sons Ltd on behalf of European Hematology Association. This is an open access Abstract Book distributed under the Attribution-NonCommercial-NoDerivs (CC BY-NC-ND), which allows third parties to download the articles and share them with others as long as they credit the author and the Abstract Book, but they cannot change the content in any way or use them commercially.
**Abstract Book Citations:** Authors, Title, HemaSphere, 2026;10:(S1):pages. The abstract book DOIs can be found on the "Author Information" page.
**Disclaimer:** Articles published in the journal HemaSphere exclusively reflect the opinions of the authors. The authors are responsible for all content in their abstracts including accuracy of the facts, statements, citing resources, etc.

### (PB3708) EFFICACY AND TOXICITY OF CYTARABINE IN FIRST-LINE INDUCTION THERAPY FOR PRIMARY CENTRAL NERVOUS SYSTEM LYMPHOMA: A SYSTEMATIC REVIEW AND META-ANALYSIS

**Topic:** 19. Large B cell lymphomas - Clinical

Lu Cui*[1], Xi Quan[1], Zhiming Luo[1], Nan Zhang[1], Jian-Chuan Deng[1]

[1] *The Second Affiliated Hospital of Chongqing Medical University, Department of Hematology, Chongqing, China;*

### Background

Primary central nervous system lymphoma (PCNSL) accounts for 1% of all lymphomas and 3% of central nervous system neoplasms, 90% being diffuse large B-cell lymphoma. The blood-brain barrier impairs antilymphoma drug penetration, causing poor prognosis and no standardized first-line regimen. High-dose methotrexate (HD-MTX) is the mainstay of first-line induction therapy, but its monotherapy has suboptimal efficacy, with median overall survival (OS) of 16–38 months and progression-free survival (PFS) of 4–10 months. Cytarabine-containing regimens were explored to lift efficacy, yet the IELSG 20 trial reported 90% grade 3–4 hematological toxicity with limited CR and ORR rates. Other studies found no significant efficacy differences between cytarabine-containing and cytarabine-free regimens, creating a clinical dilemma in treatment selection.

### Aims

This systematic review and meta-analysis compared cytarabine-containing and cytarabine-free regimens in first-line induction therapy for newly diagnosed PCNSL, assessing CR rate, ORR, OS, PFS and grade 3–4 toxicity to clarify the efficacy and safety of cytarabine in this setting.

### Methods

PubMed, EMBASE and the Cochrane Library were searched up to September 30, 2024, for studies comparing the two regimens in newly diagnosed PCNSL. We included RCTs and high-quality retrospective studies with key outcomes, and excluded non-original research, small studies and those on relapsed disease. Two reviewers independently extracted data and assessed quality using the Cochrane tool and modified Newcastle-Ottawa Scale, with disagreements resolved by a third reviewer. Statistical analysis was performed in Review Manager 5.3 using OR and 95% CI. Heterogeneity and publication bias were evaluated by Chi-square, $I^2$ and funnel plots.

### Results

A total of 5390 records were identified, with 7 eligible studies (3 RCTs, 4 retrospective studies) enrolling 620 patients (300 cytarabine group, 320 control group) included after screening. All studies used HD-MTX-based regimens, with cytarabine dosed at 1–3 g/m² for 3–6 induction cycles. Quality assessment confirmed high study quality (NOS scores 7–8 for retrospective studies) with no significant publication bias for CR rate. After sensitivity analysis, the cytarabine group had a significantly higher CR rate (OR=1.74, 95%CI:1.09–2.75, P<0.01) and superior ORR (OR=2.80, 95%CI:1.66–4.70, P=0.0001). OS and PFS data are still under analysis; single-study data showed up to 90% grade 3–4 hematological toxicity in the cytarabine group. Subgroup analysis revealed varying relapse rates with different consolidation regimens: 35% for BEAM/BUCYE, 4% for TBC and 28% for other combinations.

**Copyright Information:** (Online) ISSN: 2572-9241
©2026 The Author(s). HemaSphere is published by John Wiley & Sons Ltd on behalf of European Hematology Association. This is an open access Abstract Book distributed under the Attribution-NonCommercial-NoDerivs (CC BY-NC-ND), which allows third parties to download the articles and share them with others as long as they credit the author and the Abstract Book, but they cannot change the content in any way or use them commercially.
**Abstract Book Citations:** Authors, Title, HemaSphere, 2026;10:(S1):pages. The abstract book DOIs can be found on the "Author Information" page.
**Disclaimer:** Articles published in the journal HemaSphere exclusively reflect the opinions of the authors. The authors are responsible for all content in their abstracts including accuracy of the facts, statements, citing resources, etc.

**Summary/Conclusion**

HD-MTX-based regimens with cytarabine significantly improve CR and ORR in newly diagnosed PCNSL, conferring clear short-term efficacy benefits and being well-suited for young, fit patients eligible for intensified chemotherapy to support subsequent consolidation therapy. However, these regimens carry high grade 3–4 hematological toxicity, necessitating close clinical toxicity monitoring. This study is limited by a small sample size; the impact of cytarabine on long-term PCNSL survival and optimal efficacy-toxicity balance strategies require validation in larger studies. Cytarabine dose reduction may optimize treatment tolerability, and first-line regimen selection for PCNSL should be individualized based on patient factors.

**Copyright Information:** (Online) ISSN: 2572-9241
©2026 The Author(s). HemaSphere is published by John Wiley & Sons Ltd on behalf of European Hematology Association. This is an open access Abstract Book distributed under the Attribution-NonCommercial-NoDerivs (CC BY-NC-ND), which allows third parties to download the articles and share them with others as long as they credit the author and the Abstract Book, but they cannot change the content in any way or use them commercially.
**Abstract Book Citations:** Authors, Title, HemaSphere, 2026;10:(S1):pages. The abstract book DOIs can be found on the "Author Information" page.
**Disclaimer:** Articles published in the journal HemaSphere exclusively reflect the opinions of the authors. The authors are responsible for all content in their abstracts including accuracy of the facts, statements, citing resources, etc.

### (PB3709) REAL WORLD DATA WITH POLATUZUMAB VEDOTIN TREATMENT IN THE FRONT LINE OF DLBCL – NATIONAL EXPERIENCE

**Topic:** 19. Large B cell lymphomas - Clinical

Lucka Boltezar*[1, 2], Andreja Eberl[3], Barbara Jezeršek Novaković[1, 2]

*[1]Institute of Oncology Ljubljana, Medical Oncology, Ljubljana, Slovenia;[2]Medical faculty University of Ljubljana, Ljubljana, Slovenia; [3]Institute of Oncology Ljubljana, Pharmacy, Ljubljana, Slovenia;*

### Background

Polatuzumab vedotin combined with cyclophosphamide, doxorubicin, and prednisone has demonstrated benefit in the POLARIX trial; however, it is not reimbursed for frontline treatment in all countries. Real-world data are emerging to evaluate its effectiveness outside clinical trials.

### Aims

The aim of this study was to describe real world data using polatuzumab vedotin frontline in DLBCL patients.

### Methods

We performed a retrospective study at the Institute of Oncology Ljubljana, the national referral center for high-grade lymphomas. In Slovenia, polatuzumab has been reimbursed since April 2023 for frontline treatment of patients with International Prognostic Index (IPI) scores 3–5. Patients treated since reimbursement were identified via pharmacy records. Demographic data, treatment modifications, time to treatment initiation, delays, outcomes, and survival were collected. Event-free survival (EFS) was defined as time from diagnosis to progression or death; overall survival (OS) as time from diagnosis to death.

**Copyright Information:** (Online) ISSN: 2572-9241
©2026 The Author(s). HemaSphere is published by John Wiley & Sons Ltd on behalf of European Hematology Association. This is an open access Abstract Book distributed under the Attribution-NonCommercial-NoDerivs (CC BY-NC-ND), which allows third parties to download the articles and share them with others as long as they credit the author and the Abstract Book, but they cannot change the content in any way or use them commercially.
**Abstract Book Citations:** Authors, Title, HemaSphere, 2026;10:(S1):pages. The abstract book DOIs can be found on the "Author Information" page.
**Disclaimer:** Articles published in the journal HemaSphere exclusively reflect the opinions of the authors. The authors are responsible for all content in their abstracts including accuracy of the facts, statements, citing resources, etc.

**Results**

Eighty patients with diffuse large B-cell lymphoma (DLBCL) were included. Median age was 67 years (range 29–86); 50% were female. Bulky disease was present in 43 patients and extranodal involvement in 69. IPI scores were 3 in 51.3%, 4 in 36.3%, and 5 in 12.5%. Median follow-up was 11 months (range 1–26).
Patients received a median of 8 cycles; polatuzumab was administered for 6 cycles followed by two rituximab monotherapies, as in POLARIX. Subtypes included ABC (32.5%), GCB (17.5%), unclassified (21.3%), and 28.7% other high-risk entities (including testicular and transformed lymphomas).
Median time from diagnosis to treatment was 20 days (range 0–77). Patients treated within 15 days had significantly higher IPI scores (p = 0.008); IPI 5 patients had the shortest time to treatment (median 9 days). Five patients received R-pola-CEP due to cardiac comorbidities and four R-mini-pola-CHP due to age. CNS prophylaxis was administered in 70% of patients, mainly intrathecal; 17 also received intravenous methotrexate. Two patients had CNS involvement at diagnosis.
Treatment delays occurred in 32 patients (40%), most commonly due to infections or neutropenia (75%), with a median delay of 7 days. Delays did not affect outcomes (p = 0.769). Ten patients discontinued treatment early, mainly due to progression or infectious complications.
At analysis, 81.3% achieved complete remission (65/80), 2.5% partial remission (2/80), 2.5% stable disease (2/80), and 5% progressive disease (4/80), 7 are still waiting for evaluation. Radiotherapy was applied in 20%. No CNS progression or relapse was observed. Three patients relapsed, all within 10 months after complete remission.
Twelve-month EFS was 90.0% (95% CI 85.9–94.1) and estimated 2-year EFS 85.5%. One-year and estimated 2-year OS were both 90.1%. In Cox regression model, IPI score and treatment delays had no significant impact on EFS or OS. Shorter time from diagnosis to treatment was associated with worse EFS (p = 0.026), reflecting higher-risk disease.

**Summary/Conclusion**

This real-world Slovenian cohort demonstrates favorable outcomes with frontline polatuzumab-containing therapy, also in high-risk subgroups, with no CNS relapse rates. Treatment delays did not adversely impact survival. Worse EFS in those with shorter time to treatment reflects underlying high-risk disease.

**Copyright Information:** (Online) ISSN: 2572-9241
©2026 The Author(s). HemaSphere is published by John Wiley & Sons Ltd on behalf of European Hematology Association. This is an open access Abstract Book distributed under the Attribution-NonCommercial-NoDerivs (CC BY-NC-ND), which allows third parties to download the articles and share them with others as long as they credit the author and the Abstract Book, but they cannot change the content in any way or use them commercially.
**Abstract Book Citations:** Authors, Title, HemaSphere, 2026;10:(S1):pages. The abstract book DOIs can be found on the “Author Information” page.
**Disclaimer:** Articles published in the journal HemaSphere exclusively reflect the opinions of the authors. The authors are responsible for all content in their abstracts including accuracy of the facts, statements, citing resources, etc.

### (PB3710) REAL-WORLD OUTCOMES OF POLATUZUMAB VEDOTIN, RITUXIMAB, CYCLOPHOSPHAMIDE, DOXORUBICIN AND PREDNISONE (POLA-R-CHP) IN HIV-ASSOCIATED LARGE B-CELL LYMPHOMA: A MULTICENTRE RETROSPECTIVE ANALYSIS

**Topic:** 19. Large B cell lymphomas - Clinical

James Paterson*[1], Bernard Maybury[2], Silvia Montoto[3], Marcel Teichert[4], Bastian von Tresckow[5], Elizabeth Nelmes[6], William Townsend[1], Aparna Chitharanjan[7], Vicky Calvert[8], Tatianna Fernandes[2], Aisling Barrett[9], Shireen Kassam[7], Wendy Osborne[8], Ramsha Nabeel[7], Alessia Dalla Pria[10], Pascal Migaud[11], Rebecca Auer[3], Andrea Kuhnl[7], Toby A. Eyre[9], Frank Post[7], Mark Bower[10], Kai Hübel[12], Kate Cwynarski[1]

*[1]University College Hospital London, Haematology, London, United Kingdom; [2]University Hospitals Birmingham NHS Foundation Trust, Haematology, Birmingham, United Kingdom; [3]Barts Health NHS Trust St Bartholomew's Hospital, London, United Kingdom; [4]Department of Hematology and Stem Cell Transplantation, West German Cancer Center and German Cancer consortium (DKTK partner site Essen), Essen, Germany; [5]West German Cancer Center and German Cancer Consortium (DKTK partner site Essen), University Hospital Essen, University of Duisburg-Essen, Essen, Germany; [6]BARKING, HAVERING AND REDBRIDGE UNIVERSITY HOSPITALS NHS TRUST, London, United Kingdom; [7]Kings College Hospital, London, United Kingdom; [8]The Newcastle upon Tyne Hospitals NHS Foundation Trust and Newcastle University, Newcastle, United Kingdom; [9]Oxford University Hospitals, Oxford, United Kingdom; [10]Chelsea and Westminster Hospital, National Centre for HIV Malignancy, London, United Kingdom; [11]St-Joseph Hospital, Berlin-Tempelhof., Department of Infectious Diseases, Berlin, Germany; [12]University of Cologne and Faculty of Medicine and University Hospital of Cologne, Cologne, Germany;*

**Background**

Historically, People Living with HIV (PLWH) have experienced lower response rates when treated with standard chemo-immunotherapy when compared to the general population [1]. Whilst contemporary data suggests that PLWH on effective antiretroviral therapy (ART) can achieve comparable outcomes [2,3], this cohort has often been excluded from pivotal clinical trials. Notably, the exclusion of PLWH from the landmark POLARIX study [4] in patients with diffuse large B cell lymphoma (DLBCL) has created a significant evidence gap, leaving clinicians and international guidelines hesitant to recommend frontline Pola-R-CHP in this population. This is especially important as non-germinal centre (non-GCB) B-cell DLBCL, where a significant benefit has been reported, is the most prevalent molecular subtype of DLBCL in PLWH. We present real-world data on the safety and efficacy of this regimen in a PLWH cohort.

**Aims**

To describe the clinical efficacy, safety and tolerability of Pola-R-CHP in PLWH with previously untreated DLBCL.

**Methods**

We conducted a retrospective multicentre analysis of 18 PLWH treated with Pola-R-CHP. Baseline characteristics included CD4+ counts, HIV viral load, ECOG, Charlson Comorbidity Index (CCI), stage, and International Prognostic Index (IPI). Safety was assessed by the incidence of febrile neutropenia (FN), Grade 3+ infections, peripheral neuropathy (PN), gastrointestinal (GI) toxicity and opportunistic infections. Clinical response was determined using the Lugano 2014 (Deauville) criteria.

**Copyright Information:** (Online) ISSN: 2572-9241
©2026 The Author(s). HemaSphere is published by John Wiley & Sons Ltd on behalf of European Hematology Association. This is an open access Abstract Book distributed under the Attribution-NonCommercial-NoDerivs (CC BY-NC-ND), which allows third parties to download the articles and share them with others as long as they credit the author and the Abstract Book, but they cannot change the content in any way or use them commercially.
**Abstract Book Citations:** Authors, Title, HemaSphere, 2026;10:(S1):pages. The abstract book DOIs can be found on the "Author Information" page.
**Disclaimer:** Articles published in the journal HemaSphere exclusively reflect the opinions of the authors. The authors are responsible for all content in their abstracts including accuracy of the facts, statements, citing resources, etc.

**Results**

The median age was 53 years (range, 33–64) with median CCI of 5. Baseline median CD4+ count of 155 x10^6/L (range, 19–323) and viral load 64 copies/ml (range, <20-1,288,250) were observed at diagnosis of DLBCL. The Overall Response Rate (ORR) was 83.3% (N=15/18), with 61.1% (N=11) achieving a Complete Response (CR) and 22.2% (n=4) achieving a Partial Response (PR). Three patients (17%) experienced progressive disease. At 12 months, the estimated progression free survival (PFS) was 85.7% (95% CI, 69.2–100%) and overall survival (OS) was 88.9% (95% CI, 75–100%). Regarding safety, febrile neutropenia occurred in 44.4% (N=8) of patients, and Grade 3+ infections were observed in 39% (N=7). Peripheral neuropathy was reported in 28% (N=5) of the cohort, the majority of which were Grade 1/2. No unexpected HIV-specific toxicities or loss of viral suppression were observed during the treatment period.

**Summary/Conclusion**

In this real-world multicentre cohort, Pola-R-CHP demonstrated high efficacy in PLWH, with response rates comparable to those observed in the POLARIX trial. While the incidence of febrile neutropenia and Grade 3+ infections were notable, this did not appear to significantly affect PFS and OS. These data suggest that Pola-R-CHP is a feasible and effective first-line option for PLWH, a population historically excluded from clinical trials.

**References:**

1. DeMario MD, et al. Semin Oncol. 1998;25(4):492-502.
2. Besson C, et al. Aids. 2017;31(18):2493-501.
3. Schommers P, et al. Haematologica. 2018;103(5):857-64.
4. Tilly H, et al. N Engl J Med. 2022;386(4):351-63

**Copyright Information:** (Online) ISSN: 2572-9241
©2026 The Author(s). HemaSphere is published by John Wiley & Sons Ltd on behalf of European Hematology Association. This is an open access Abstract Book distributed under the Attribution-NonCommercial-NoDerivs (CC BY-NC-ND), which allows third parties to download the articles and share them with others as long as they credit the author and the Abstract Book, but they cannot change the content in any way or use them commercially.
**Abstract Book Citations:** Authors, Title, HemaSphere, 2026;10:(S1):pages. The abstract book DOIs can be found on the "Author Information" page.
**Disclaimer:** Articles published in the journal HemaSphere exclusively reflect the opinions of the authors. The authors are responsible for all content in their abstracts including accuracy of the facts, statements, citing resources, etc.

### (PB3711) EFFICACY AND SAFETY OF ANTI-CD19 CHIMERIC ANTIGEN RECEPTOR T-CELL THERAPY IN SECONDARY CNS LYMPHOMAS REFRACTORY TO HIGH-DOSE METHOTREXATE (HD-MTX)-BASED CHEMOIMMUNOTHERAPY

**Topic:** 19. Large B cell lymphomas - Clinical

Piera Angelillo*[1], Edoardo Campodonico[2], Teresa Calimeri[1], Alice Di Rocco[3], Alessia Castellino[4], Andrea Assanelli[2], Lorenzo Lazzari[2], Maria Teresa Lupo Stanghellini[2], Maddalena Noviello[5], Livia Donzelli[3], Claudia Castellino[4], Andrea Ferrario[6], Melania Celli[7], Astrid Pavlovsky[8], Nicoletta Anzalone[9], Paolo Vezzulli[9], Federico Fallanca[10], Anna Chiara[11], Francesco Ancona[12], Paolo Fiore[1], Derna Busacchio[1], Vincenzo Cuzzupi[2], Federico Erbella[1], Elena Flospergher[1], Fabrizio Marino[1], Sarah Marktel[2], Sara Mastaglio[2], Alessia Orsini[13], Annalisa Ruggeri[2], Chiara Bonini[5, 14], Fabio Ciceri[2, 14], Andrés José María Ferreri[1, 14]

*[1]IRCCS San Raffaele Scientific Institute, Strategic Program on Lymphomas, Milan, Italy; [2]IRCCS San Raffaele Scientific Institute, Hematology and BMT Unit, Milan, Italy; [3]Sapienza Università di Roma, Hematology, Department of Translational and Precision Medicine, Roma, Italy; [4]AO Santa Croce e Carle di Cuneo, Hematology, Cuneo, Italy;[5]IRCCS San Raffaele Scientific Institute, Milan, Italy; [6]Azienda Socio Sanitaria Territoriale dei Sette Laghi, UOC Ematologia, Varese, Italy;[7]Ospedale Infermi, UO Ematologia, Rimini, Italy; [8]FUNDALEU and Centro de Hematologia Pavlovsky, Buenos Aires, Argentina;[9]IRCCS San Raffaele Scientific Institute, Neuroradiology Department, Milan, Italy; [10]IRCCS San Raffaele Scientific Institute, Nuclear Medicine Department, Milan, Italy; [11]IRCCS San Raffaele Scientific Institute, Radiotherapy Department, Milan, Italy; [12]IRCCS San Raffaele Scientific Institute, Cardiology Department, Milan, Italy; [13]IRCCS San Raffaele Scientific Institute, Immunohematology and Transfusion Medicine, Milan, Italy; [14]Vita-Salute San Raffaele University, Milano, Italy;*

### Background

Secondary CNS involvement in large B-cell lymphomas (SCNSL) is usually treated with HD-MTX-based therapy and thiotepa-conditioned autologous transplantation. However, most patients (pts) relapse or are refractory, and durable responses to salvage attempts are rare. Evidence supporting anti-CD19 CAR T-cell therapy in SCNSL pts remains limited, particularly regarding treatment eligibility and outcomes in HD-MTX–refractory pts.

### Aims

To evaluate CAR-T eligibility, treatment uptake, safety, and efficacy in HD-MTX-refractory SCNSL.

### Methods

Consecutive adults (18–75 years) with SCNSL referred to three Italian centers for salvage CAR-T between 2022 and 2025 were analyzed. Eligible pts had CNS and/or systemic disease refractory to HD-MTX. Pts received tailored holding therapy; those achieving CNS remission proceeded to CAR-T therapy.

**Copyright Information:** (Online) ISSN: 2572-9241
©2026 The Author(s). HemaSphere is published by John Wiley & Sons Ltd on behalf of European Hematology Association. This is an open access Abstract Book distributed under the Attribution-NonCommercial-NoDerivs (CC BY-NC-ND), which allows third parties to download the articles and share them with others as long as they credit the author and the Abstract Book, but they cannot change the content in any way or use them commercially.
**Abstract Book Citations:** Authors, Title, HemaSphere, 2026;10:(S1):pages. The abstract book DOIs can be found on the "Author Information" page.
**Disclaimer:** Articles published in the journal HemaSphere exclusively reflect the opinions of the authors. The authors are responsible for all content in their abstracts including accuracy of the facts, statements, citing resources, etc.

### Results

28 pts (median age 50 years, range 25–74) were evaluated. Histology included DLBCL (n=16), HGBCL (n=6), and PMBCL (n=6). CNS involvement occurred at diagnosis (n=6), first relapse (n=21), or second relapse (n=1). CNS sites were brain (n=15), meninges (n=5), brain plus meninges (n=2), cranial nerves (n=3), cranial nerves plus meninges (n=2), and eyes (n=1). Sixteen pts had concomitant systemic disease. Eight pts had received one line of therapy before CAR-T , 17 received two lines, and 3 more than two. Holding therapy was based on brain radiotherapy ± biologic agents (n=11), intrathecal chemotherapy for meningeal disease (n=7), or systemic chemotherapy for multifocal disease (n=10). Four pts progressed after holding therapy, were deemed ineligible for CAR-T, and died within 5–9 weeks. Twenty-four (86%) responsive pts proceeded to CAR-T, receiving steroid-free bridging therapy, Flu-Cy lymphodepletion, and CAR-T infusion (axi-cel n=18, liso-cel n=5, tisa-cel n=1).
CRS occurred in 21 pts (88%), was gr 1–2 in 19, gr 3 in one, and gr 4 in one (duration 0-18 days; median 4). Management included tocilizumab (n=19), dexamethasone (n=15), and anakinra (n=4). ICANS occurred in 14 (58%) pts: 12 had gr 1–2, one had gr 3 and one gr 5. All ICANS events were treated with dexamethasone; 2 also received anakinra, and 2 received anakinra and high-dose steroids. All pts but one recovered from ICANS, within 1-12 days (median 3); 2 required ICU admission. No tumor inflammation-associated neurotoxicity was seen. Late gr 3–4 cytopenias occurred in four pts; gr ≥3 ICAHT beyond 30 days occurred in 6, without major complications.
At one month post-CAR-T, 17 (71%) pts (95%CI=53-89) achieved CR and 3 PR. Two of the 3 PRs received radiotherapy on PET-positive residual lesions. All PRs converted to CR by 3 months, yielding a 3-month CR rate of 80% (95%CI=64-96). After a median follow-up of 14 (range 3-44) months, 17 (71%) pts remains alive and event-free, with a 2-year PFS of 54% (95%CI=46-62) for the whole series and 63% (95%CI=58-68) for CAR-T recipients (Figure) and a 2-year OS of 53% (95%CI=44-61) and 62% (95%CI=56-67), respectively.

### Summary/Conclusion

Tailored multimodal holding therapy enabled 86% (24/28) of HD-MTX–refractory SCNSL pts to safely receive CAR-T. After selection based on response to holding therapy, only 4% of pts experienced grade ≥4 ICANS and approximately two-thirds of pts were alive and event-free at two years. These outcomes compare favourably with CAR-T registry data and salvage strategies in prospective SCNSL trials, supporting the efficacy of CAR-T in fit, holding-responsive pts.

**Copyright Information:** (Online) ISSN: 2572-9241
©2026 The Author(s). HemaSphere is published by John Wiley & Sons Ltd on behalf of European Hematology Association. This is an open access Abstract Book distributed under the Attribution-NonCommercial-NoDerivs (CC BY-NC-ND), which allows third parties to download the articles and share them with others as long as they credit the author and the Abstract Book, but they cannot change the content in any way or use them commercially.
**Abstract Book Citations:** Authors, Title, HemaSphere, 2026;10:(S1):pages. The abstract book DOIs can be found on the “Author Information” page.
**Disclaimer:** Articles published in the journal HemaSphere exclusively reflect the opinions of the authors. The authors are responsible for all content in their abstracts including accuracy of the facts, statements, citing resources, etc.

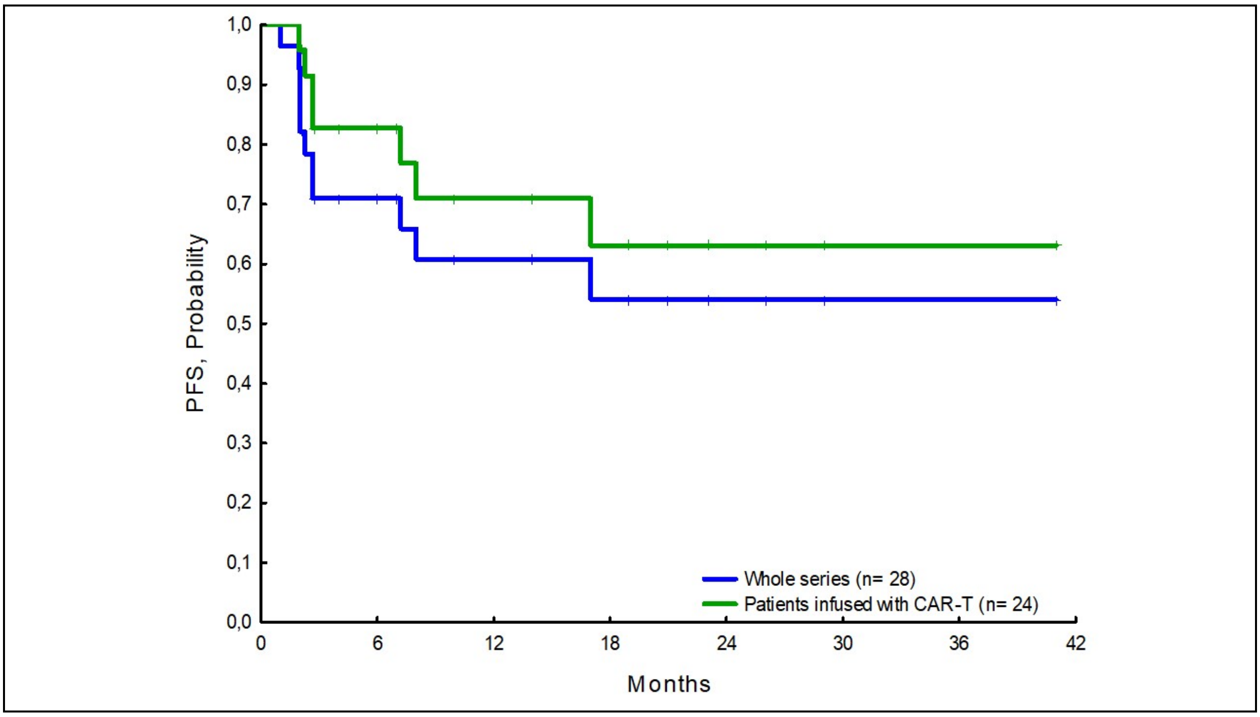

**Copyright Information:** (Online) ISSN: 2572-9241
©2026 The Author(s). HemaSphere is published by John Wiley & Sons Ltd on behalf of European Hematology Association. This is an open access Abstract Book distributed under the Attribution-NonCommercial-NoDerivs (CC BY-NC-ND), which allows third parties to download the articles and share them with others as long as they credit the author and the Abstract Book, but they cannot change the content in any way or use them commercially.
**Abstract Book Citations:** Authors, Title, HemaSphere, 2026;10:(S1):pages. The abstract book DOIs can be found on the "Author Information" page.
**Disclaimer:** Articles published in the journal HemaSphere exclusively reflect the opinions of the authors. The authors are responsible for all content in their abstracts including accuracy of the facts, statements, citing resources, etc.

### (PB3712) GLOBAL VARIATION IN CAR T CELL THERAPY PRACTICE PATTERNS FOR LBCL: QUANTITATIVE RESEARCH FINDINGS

**Topic:** 19. Large B cell lymphomas - Clinical

Yusuke Isshiki*[1], Gloria Iacoboni[2], David Qualls[3], Ajay Major[4], Lizamarie Bachier-Rodriguez[5], Anna Sureda[6]

*[1]Perlmutter Cancer Center, NYU Langone Health, New York City, United States of America;[2]Vall d'Hebron University Hospital, Barcelona, Spain; [3]Dana-Farber Cancer Institute, Boston, United States of America; [4]University of Colorado School of Medicine, Aurora, United States of America; [5]Blood and Marrow Transplant Group of Georgia, Atlanta, United States of America;[6]Institut Català d'Oncologia – L'Hospitalet, IDIBELL, Barcelona, Spain;*

### Background

Chimeric antigen receptor T cell (CAR T) therapy has transformed outcomes in hematologic malignancies. However, substantial variability exists in CAR T delivery and in prevention and management of associated toxicities, with potential implications for patient outcomes. We characterized physician practice patterns and quantified heterogeneity in adverse event (AE) prevention and management for CAR T therapy in large B cell lymphoma (LBCL).

### Methods

From September–December 2025, CAR T physicians were invited to complete an online questionnaire. Responses were obtained from 131 physicians across the United States (n=70), Europe (n=40), Canada (n=8), and Australia (n=3). Survey domains included physician and center characteristics and approaches to prophylaxis and management of cytokine release syndrome (CRS), immune effector cell–associated neurotoxicity syndrome (ICANS), cytopenia and infections.

### Results

The median annual LBCL CAR T volume was 30 patients (IQR 18–50). While physicians reported not using bridging therapy in a median of 10% of patients (IQR 5–20), 95% of physicians proceeded with CAR T infusion as soon as cells were available, rather than continuing bridging in partial responders. Prophylaxis practices were heterogeneous: 72%, 17%, and 71% reported use of dexamethasone, anakinra, and levetiracetam, respectively, in select patients.

Thresholds for tocilizumab initiation for CRS varied substantially: 14% administered tocilizumab at first fever, 55% after 24h of CRS, and 29% only after ≥72h of fever or progression to grade ≥2 CRS. Steroid use for CRS was also heterogeneous: 76% added steroids after an inadequate response to tocilizumab, 5% added steroids as early as first fever, and 27% added steroids in CRS occurring within 24 hours of infusion.

Approaches to ICANS management diverged markedly. Corticosteroids were initiated at grade 1 ICANS by 60% of respondents, whereas 38% waited until grade 2. For ICANS refractory to initial corticosteroids, 89% escalated steroid dose upon worsening grade, although 63% also considered escalation after 24h of persistent symptoms. Definitions of steroid-refractory ICANS varied, most commonly lack of improvement after 24h (36%), 48h (44%) or worsening grade despite steroids (66%). Anakinra was used as second-line therapy by 95% of respondents, while 19% each reported using intrathecal chemotherapy or siltuximab.
Nearly all clinicians used G-CSF for neutropenia (97%), with 32% using prophylactic G-CSF. IVIG use was heterogeneous: 56% administered IVIG in patients without recurrent infections, typically at IgG <4 g/L. In patients with recurrent infections, IVIG use was nearly universally (98%), though initiation thresholds varied.

**Copyright Information:** (Online) ISSN: 2572-9241
©2026 The Author(s). HemaSphere is published by John Wiley & Sons Ltd on behalf of European Hematology Association. This is an open access Abstract Book distributed under the Attribution-NonCommercial-NoDerivs (CC BY-NC-ND), which allows third parties to download the articles and share them with others as long as they credit the author and the Abstract Book, but they cannot change the content in any way or use them commercially.
**Abstract Book Citations:** Authors, Title, HemaSphere, 2026;10:(S1):pages. The abstract book DOIs can be found on the "Author Information" page.
**Disclaimer:** Articles published in the journal HemaSphere exclusively reflect the opinions of the authors. The authors are responsible for all content in their abstracts including accuracy of the facts, statements, citing resources, etc.

**Summary/Conclusion**

Global practice patterns for CAR T AE prevention and management are highly heterogeneous, with notable divergence from evidence supporting earlier, proactive intervention. These findings highlight the need for harmonized, evidence-aligned management algorithms and targeted educational initiatives to enable timely toxicity mitigation and optimize patient outcomes. Analyses stratified by geography and practice characteristics are ongoing and will be included in the final presentation.

**Copyright Information:** (Online) ISSN: 2572-9241
©2026 The Author(s). HemaSphere is published by John Wiley & Sons Ltd on behalf of European Hematology Association. This is an open access Abstract Book distributed under the Attribution-NonCommercial-NoDerivs (CC BY-NC-ND), which allows third parties to download the articles and share them with others as long as they credit the author and the Abstract Book, but they cannot change the content in any way or use them commercially.
**Abstract Book Citations:** Authors, Title, HemaSphere, 2026;10:(S1):pages. The abstract book DOIs can be found on the “Author Information” page.
**Disclaimer:** Articles published in the journal HemaSphere exclusively reflect the opinions of the authors. The authors are responsible for all content in their abstracts including accuracy of the facts, statements, citing resources, etc.

### (PB3713) BEYOND THE IPI: INFLAMMATION–NUTRITION-BASED INDICES IN THE RISK ASSESSMENT OF DIFFUSE LARGE B-CELL LYMPHOMA

**Topic:** 19. Large B cell lymphomas - Clinical

Zorica Cvetković*[1, 2], Snežana Pejić[3], Ilija Bukurecki[4], Miroslav Pavlovic[5], Anica Divac[4], Aleksandra Tomić[1], Mirjana Čolović Zvizdić[1], Marija Kaplarević[5], Nataša Stanisavljević[2, 4], Olivera Markovic[2, 5]

*[1]University Hospital Medical Center Zemun, Department of Hematology, Belgrade, Serbia;[2]Medical Faculty University of Belgrade, Belgrade, Serbia; [3]Department of Molecular Biology and Endocrinology, "Vinča" Institute of Nuclear Sciences, National Institute of the Republic of Serbia, University of Belgrade,, Belgrade, Serbia; [4]University Hospital Medical Center Bežanijska kosa, Department of Hematology, Belgrade, Serbia; [5]University Hospital Medical Center Zvezdara, Department of Hematology, Belgrade, Serbia;*

### Background

Diffuse large B-cell lymphoma (DLBCL), the most common aggressive subtype of non-Hodgkin lymphoma, remains refractory or relapsed (R/R) in 30–40% of patients despite advances in targeted therapy. Standard prognostic scores, including the International Prognostic Index (IPI), revised IPI (R-IPI), and NCCN-IP, fail to reliably identify patients at the highest risk. Increasing evidence indicates that inflammation and nutritional status are key determinants of tumor progression, immune response, and treatment outcomes, leading to the development of composite inflammation- and nutrition-based indices. These indices have demonstrated prognostic value for disease course and survival outcomes in DLBCL, reflecting tumor–host interactions more comprehensively than standard clinical models.

### Aims

To compare the discriminatory performance of inflammation- and nutrition-based indices versus IPI-derived models for R/R DLBCL and prediction of overall survival (OS) and event-free survival (EFS).

### Methods

We retrospectively analyzed 180 consecutive DLBCL, NOS patients treated with frontline R-CHOP or R-CHOP–like regimens at three University Clinical Centers (median age 67 years, IQR 59–73; F:M=1.29). Evaluated indices included: Hemoglobin–Albumin–Lymphocyte–Platelet (HALP) score; C-reactive protein–Albumin–Lymphocyte (CALLY) index; Prognostic Nutritional Index (PNI: 10×albumin + 0.005×lymphocyte); Platelet-to-Albumin Ratio (PAR); Platelet-to-Lymphocyte Ratio (PLR); Neutrophil-to-Lymphocyte Ratio (NLR); Monocyte-to-Lymphocyte Ratio (MLR); Systemic Immune-Inflammation Index (SII: platelets×neutrophils/lymphocytes); Systemic Inflammation Response Index (SIRI: neutrophils×monocytes/lymphocytes); and Pan-Immune-Inflammation Value (PIV: neutrophils×platelets×monocytes/lymphocytes). ROC analysis identified the optimal cutoffs with corresponding AUC, 95% CI, and p-values. Prognostic performance for OS, EFS, and R/R was assessed using Cox proportional hazards models; model fit was compared with Akaike Information Criterion (AIC).

**Copyright Information:** (Online) ISSN: 2572-9241
©2026 The Author(s). HemaSphere is published by John Wiley & Sons Ltd on behalf of European Hematology Association. This is an open access Abstract Book distributed under the Attribution-NonCommercial-NoDerivs (CC BY-NC-ND), which allows third parties to download the articles and share them with others as long as they credit the author and the Abstract Book, but they cannot change the content in any way or use them commercially.
**Abstract Book Citations:** Authors, Title, HemaSphere, 2026;10:(S1):pages. The abstract book DOIs can be found on the “Author Information” page.
**Disclaimer:** Articles published in the journal HemaSphere exclusively reflect the opinions of the authors. The authors are responsible for all content in their abstracts including accuracy of the facts, statements, citing resources, etc.

### Results

Primary nonresponse occurred in 53/180 patients (29.4%), with 17/127 (13.4%) relapsing over a median follow-up of 63 months. CALLY ≥6.5 (0.733, 0.659–0.808; p<0.001), PNI ≥43.4 (0.705, 0.628–0.782; p<0.001) and HALP ≥28.4 (0.663, 0.584–0.742; p<0.001) were stronger predictors than the other inflammation- and nutrition-based indices: PAR ≥9.2 (0.614, 0.532–0.696; p=0.011), SIRI ≥1.2 (0.613, 0.531–0.695; p=0.012), MLR ≥0.38 (0.612, 0.530–0.694; p=0.013), NLR ≥3.1 (0.604, 0.521–0.687; p=0.018), PLR ≥198 (0.601, 0.518–0.684; p=0.022), PIV ≥450 (0.601, 0.518–0.684; p=0.023), and SII ≥780 (0.596, 0.512–0.680; p=0.029). Among standard clinical prognostic scores, NCCN-IPI showed higher internal stability than IPI and R-IPI but exhibited lower discriminative power than the top-performing inflammation- and nutrition-based indices. Model fit rankings based on AIC were: OS— PNI (754.80) < CALLY (755.47) < NCCN-IPI (767.66) < HALP (768.69); EFS— CALLY (900.07) < PNI (905.87) < HALP (916.28) < NCCN-IPI (916.51); R/R— CALLY (682.15) < PNI (688.93) < NCCN-IPI (697.72) < HALP (698.40).

### Summary/Conclusion

Inflammation- and nutrition-based indices, particularly CALLY and PNI, reliably predict survival and R/R status in DLBCL and outperform standard clinical prognostic models, including NCCN-IPI. These non-invasive, inexpensive biomarkers may facilitate the timely identification of high-risk patients, guiding intensified and personalized monitoring and treatment.

**Copyright Information:** (Online) ISSN: 2572-9241
©2026 The Author(s). HemaSphere is published by John Wiley & Sons Ltd on behalf of European Hematology Association. This is an open access Abstract Book distributed under the Attribution-NonCommercial-NoDerivs (CC BY-NC-ND), which allows third parties to download the articles and share them with others as long as they credit the author and the Abstract Book, but they cannot change the content in any way or use them commercially.
**Abstract Book Citations:** Authors, Title, HemaSphere, 2026;10:(S1):pages. The abstract book DOIs can be found on the “Author Information” page.
**Disclaimer:** Articles published in the journal HemaSphere exclusively reflect the opinions of the authors. The authors are responsible for all content in their abstracts including accuracy of the facts, statements, citing resources, etc.

### (PB3714) REAL-WORLD OUTCOMES OF RELMACABTAGENE AUTOLEUCEL (RELMA-CEL) IN R/R LARGE B-CELL LYMPHOMA

**Topic:** 19. Large B cell lymphomas - Clinical

Zixun Yan*[1], Xiaoxi Zhou[2], Yan Gao[3], Wenbin Qian[4], Ping Li[5], Heng Mei[6], Depei Wu[7], Dehui Zou[8], Yao Liu[9], Hui-Lai Zhang[10], Ru Feng[11], Ying Lu[12], Zhifeng Li[13], Hongmei Jing[14], Yajun Li[15], Jianqiu Wu[16], Yongen Gu[17], Songyun Zhao[17], Huiqiang Huang[3], Weili Zhao[1]

*[1]Shanghai Institute of Hematology, State Key Laboratory of Medical Genomics, National Research Center for Translational Medicine at Shanghai, Ruijin Hospital Affiliated to Shanghai Jiao Tong University School of Medicine, Shanghai, China; [2]Department of Hematology, Tongji Hospital, Tongji Medical College, Huazhong University of Science and Technology, Wuhan, China; [3]Department of Medical Oncology, State Key Laboratory of Oncology in South China; Guangdong Provincial Clinical Research Center for Cancer, Sun Yat-sen University Cancer Center, Guangzhou, China; [4]Department of Hematology, the Second Affiliated Hospital, College of Medicine, Zhejiang University, Hangzhou, China; [5]Department of Hematology, Tongji Hospital of Tongji University, Shanghai, China; [6]Institute of Hematology, Union Hospital, Tongji Medical College, Huazhong University of Science and Technology, Wuhan, China; [7]Department of Hematology, The First Affiliated Hospital of Soochow University, National Clinical Research Center for Hematologic Diseases, Suzhou, China; [8]National Clinical Research Center for Blood Diseases, State Key Laboratory of Experimental Hematology, Blood Diseases Hospital & Institute of Hematology, Chinese Academy of Medical Sciences & Peking Union Medical College, Tianjin, China; [9]Department of Hematology Oncology, Chongqing Key Laboratory of Translational Research for Cancer Metastasis and Individualized Treatment, Chongqing University Cancer Hospital, Chongqing, China; [10]Department of Lymphoma, Tianjin Medical University Cancer Institute and Hospital, National Clinical Research Center for Cancer, Tianjin, China; [11]Department of Hematology, Nanfang Hospital of Southern Medical University, Guangzhou, China; [12]Department of Hematology, The Affiliated People's Hospital of Ningbo University, Ningbo, China; [13]Department of Hematology, The First Affiliated Hospital of Xiamen University and Institute of Hematology, School of Medicine, Xiamen University, Xiamen, China; [14]Department of Hematology, Peking University Third Hospital, Beijing, China; [15]Department of Lymphoma and Hematology, Hunan Cancer Hospital and The Affiliated Cancer Hospital of Xiangya School of Medicine, Central South University, Changsha, China; [16]Department of Medical Oncology, Jiangsu Cancer Hospital, Nanjing, China; [17]Department of Clinical Development, JW Therapeutics (Shanghai) Co. Ltd, Shanghai, China;*

### Background

Patients with relapsed or refractory (R/R) large B-cell lymphoma (LBCL) have poor prognoses and limited treatment options. Although the pivotal clinical trial demonstrated the efficacy and safety of Relmacabtagene Autoleucel (Relma-Cel) for patients with R/R LBCL,additional real-world evidence is needed to further characterize its clinical performance in routine practice.

### Aims

This real-world study(ClinicalTrials.gov:NCT06142175) aims to evaluate the efficacy and safety of relma-cel—an anti-CD19 chimeric antigen receptor (CAR) T-cell therapy approved by the China National Medical Products Administration (NMPA)—for the treatment of adult patients with R/R LBCL in the real-world setting.

**Copyright Information:** (Online) ISSN: 2572-9241
©2026 The Author(s). HemaSphere is published by John Wiley & Sons Ltd on behalf of European Hematology Association. This is an open access Abstract Book distributed under the Attribution-NonCommercial-NoDerivs (CC BY-NC-ND), which allows third parties to download the articles and share them with others as long as they credit the author and the Abstract Book, but they cannot change the content in any way or use them commercially.
**Abstract Book Citations:** Authors, Title, HemaSphere, 2026;10:(S1):pages. The abstract book DOIs can be found on the "Author Information" page.
**Disclaimer:** Articles published in the journal HemaSphere exclusively reflect the opinions of the authors. The authors are responsible for all content in their abstracts including accuracy of the facts, statements, citing resources, etc.

### Methods

Eligible patients were adults (≥18 years) with R/R LBCL who have been received commercially available relma-cel.Clinical data were collected from patients across the prescription stage of relma-cel, the pre-infusion period, the day of infusion, up to the last visit within 6 months post-infusion and/or the physician's final follow-up. Data acquisition was non-compulsory and passively obtained, based on information generated during patients' routine clinical visits.This study is an observational real-world study and does not involve any study-specific interventions in the diagnosis, treatment, or follow-up of patients' diseases due to the conduct of this research.

### Results

This study was conducted in 16 sites in China. As of September 15, 2025, 185 patients underwent leukapheresis and received relma-cel. The median age was 58 years (range,18–81 ), 31.4% of the patients were 65 years of age or older, and 54.6% were male. 162 (87.6%) of the patients had DLBCL-NOS, 41(22.2%) had stage III disease, and 105 (56.8%) had stage IV disease, 107 (57.8%) had relapsed disease, while 115 (62.2%) had refractory disease. 12 (6.5%) of the patients had an Eastern Cooperative Oncology Group performance status(ECOG PS) of 3 or more, 93(50.3%) had an IPI score of 3 or more, 94 (50.8%) had abnormal lactate dehydrogenase (LDH) levels, and 127 (68.6%) received bridging therapy.
In the Efficacy Analysis Set (EAS, primary analysis for efficacy) (n=179), the best objective response rate (ORR) was 75.4% (95% CI: 68.4%, 81.5%), the best complete response rate (CRR) was 54.7% (95% CI: 47.2%, 62.2%), and the best disease control rate (DCR) was 86.6% (95% CI: 80.7%, 91.2%). In the modified Intent-to-Treat analysis set (mITT, supplementary analysis for efficacy) (n=183), the best ORR was 73.8% (95% CI: 66.8%, 80.0%), the best CRR was 53.6% (95% CI: 46.0%, 60.9%), and the best DCR was 84.7% (95% CI: 78.7%, 89.6%) (Figure).
The median duration of response (DOR) was 13.4 months, the median progression-free survival (PFS) was 11.6 months, and the median overall survival (OS) was not reached.
Cytokine release syndrome (CRS) occurred in 130 (70.3%) patients, with 17 (9.2%) patients experienced grade 3 or worse. Neurotoxicity (NT) was observed in 33 (17.8%) patients, with 11 (5.9%) patients experienced grade3 or worse. No grade 5 CRS or NT events were reported.

### Summary/Conclusion

The study indicates that relma-cel is an effective treatment for adult patients with R/R LBCL with a favorable and manageable safety profile in the real-world setting.

**Copyright Information:** (Online) ISSN: 2572-9241
©2026 The Author(s). HemaSphere is published by John Wiley & Sons Ltd on behalf of European Hematology Association. This is an open access Abstract Book distributed under the Attribution-NonCommercial-NoDerivs (CC BY-NC-ND), which allows third parties to download the articles and share them with others as long as they credit the author and the Abstract Book, but they cannot change the content in any way or use them commercially.
**Abstract Book Citations:** Authors, Title, HemaSphere, 2026;10:(S1):pages. The abstract book DOIs can be found on the "Author Information" page.
**Disclaimer:** Articles published in the journal HemaSphere exclusively reflect the opinions of the authors. The authors are responsible for all content in their abstracts including accuracy of the facts, statements, citing resources, etc.

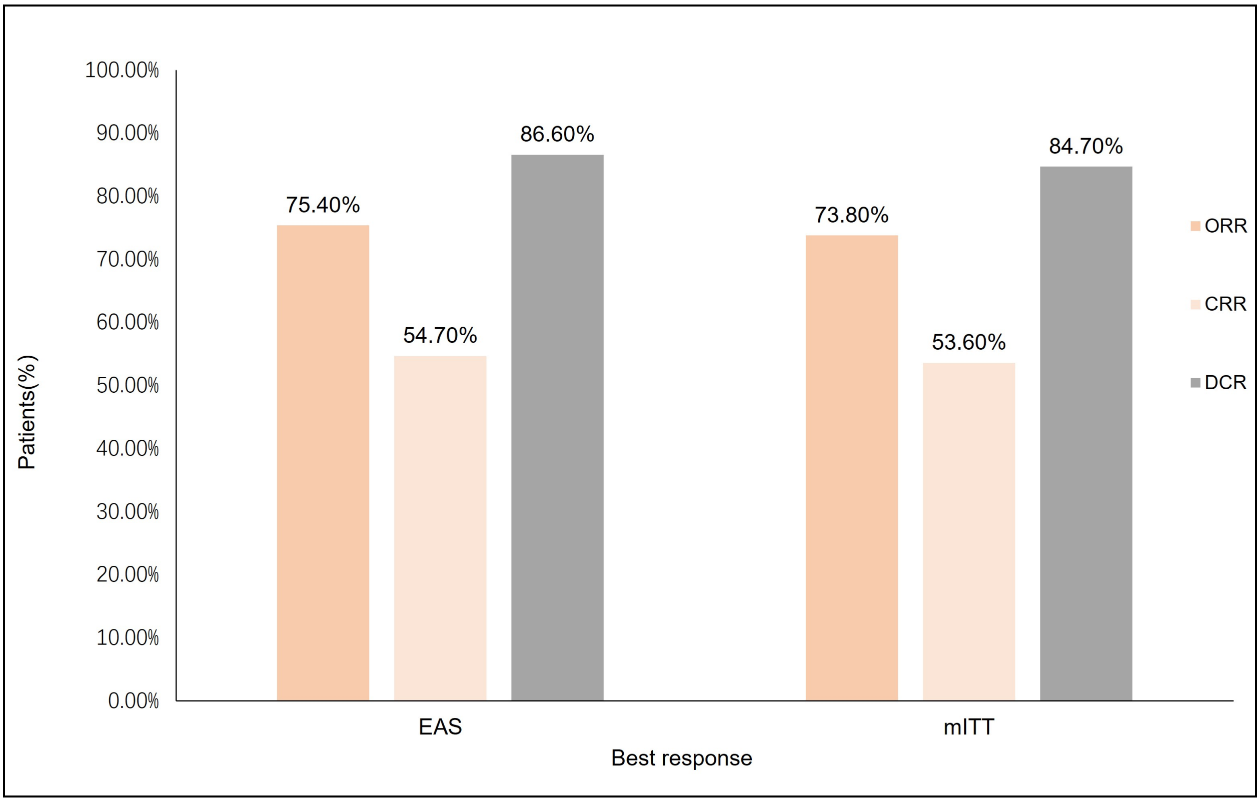

**Copyright Information:** (Online) ISSN: 2572-9241
©2026 The Author(s). HemaSphere is published by John Wiley & Sons Ltd on behalf of European Hematology Association. This is an open access Abstract Book distributed under the Attribution-NonCommercial-NoDerivs (CC BY-NC-ND), which allows third parties to download the articles and share them with others as long as they credit the author and the Abstract Book, but they cannot change the content in any way or use them commercially.
**Abstract Book Citations:** Authors, Title, HemaSphere, 2026;10:(S1):pages. The abstract book DOIs can be found on the "Author Information" page.
**Disclaimer:** Articles published in the journal HemaSphere exclusively reflect the opinions of the authors. The authors are responsible for all content in their abstracts including accuracy of the facts, statements, citing resources, etc.

### (PB3715) TREATMENT LANDSCAPE AND OUTCOME IN PATIENTS WITH STAGE I-II PRIMARY CUTANEOUS DIFFUSE LARGE B-CELL LYMPHOMA

**Topic:** 19. Large B cell lymphomas - Clinical

Diana Al-Sarayfi*[1], Sherida Woei-A-Jin[2], Marjolein Koldijk[3], Marcel Nijland[1], Mirian Brink[4]

*[1]University Medical Centre Groningen, University of Groningen, Department of Haematology, Groningen, The Netherlands; [2]University Hospitals Leuven, Department of General Medical Oncology, Leuven, Belgium;[3]University Medical Centre Groningen, University of Groningen, Department of Dermatology, Groningen, The Netherlands; [4]Netherlands Comprehensive Cancer Organisation (IKNL), Department of Research and Development, Utrecht, The Netherlands;*

### Background

Primary cutaneous diffuse large B-cell lymphoma, leg-type (PCDLBCL), is an aggressive B-cell lymphoma that predominantly arises in the lower legs of elderly patients. Due to its rarity, data are limited, and uniform treatment guidelines are lacking. First-line treatment generally involves radiotherapy or chemo-immunotherapy, but the optimal approach remains unclear.

### Aims

This population-based study aims to gain insight on outcomes of different treatment modalities among stage I-II PCDLBCL patients.

### Methods

All patients with stage I-II PCDLBCL ≥18 years diagnosed between 2014-2022 were identified from the Netherlands Cancer Registry, with survival follow-up until January 1st, 2025. Treatment strategies within all treatment lines were collected and categorized in radiotherapy (RT) or resection only, chemoimmunotherapy only (CHOP-like regimens), combined modality treatment (CMT) with RT and immunochemotherapy and other therapies. Primary endpoints were progression-free survival (PFS) and overall survival (OS). To estimate the effect of treatment modality on the risks of relapse and mortality, multivariable Cox proportional hazards regression was performed with adjustment for a priori selected clinically relevant confounders.

### Results

A total of 98 patients diagnosed with PCDLBCL were identified (median age 80 years; female 60%; 88% were stage I). First-line treatment was applied in 80% of patients. Most patients received RT or resection only (n=35, 36%), followed by CMT (n=22, 22%), chemoimmunotherapy only (n=21, 21%), and other therapies (n=20, 20%). For patients receiving treatment, overall response rate (ORR) was 68%, with 58% achieving complete remission. ORR was highest for chemoimmunotherapy only (86%), followed by 77% for CMT, and 51% for RT or resection only. A total of 22 patients experienced relapse or progression. Of those, only one patient relapsed in the central nervous system. Second-line treatment was given to 16 patients (16%). Among them radiotherapy monotherapy was most common (n=7, 44%), followed by anthracycline-based (n=3, 19%), autologous stem cell transplantation (n=2, 13%), and other therapies (n=4, 25%). With a median follow-up for the entire cohort of 43 months, the 3-year PFS and 3-year OS was 48% and 55%, respectively. The 3-year PFS estimate for stage I-II patients receiving RT or resection only, chemoimmunotherapy only, CMT or other therapies were similar (40%, 67%, 59% and 35%, respectively; p=0.78). The 3-year OS for patients receiving RT or resection only, chemoimmunotherapy only, CMT or other therapies were 51%, 71%, 63% and 35%, respectively (p=0.02). After adjusting for age (per year increment), sex, increased lactate dehydrogenase and performance score, treatment modality was not found to be associated with risk of relapse and mortality (Figure 1).

**Copyright Information:** (Online) ISSN: 2572-9241
©2026 The Author(s). HemaSphere is published by John Wiley & Sons Ltd on behalf of European Hematology Association. This is an open access Abstract Book distributed under the Attribution-NonCommercial-NoDerivs (CC BY-NC-ND), which allows third parties to download the articles and share them with others as long as they credit the author and the Abstract Book, but they cannot change the content in any way or use them commercially.
**Abstract Book Citations:** Authors, Title, HemaSphere, 2026;10:(S1):pages. The abstract book DOIs can be found on the "Author Information" page.
**Disclaimer:** Articles published in the journal HemaSphere exclusively reflect the opinions of the authors. The authors are responsible for all content in their abstracts including accuracy of the facts, statements, citing resources, etc.

### Summary/Conclusion

This population-based study among 98 patients with stage I-II PCDLBCL shows RT or resection only is the most applied regimen. No significant advantage of CMT over chemoimmunotherapy was observed. These real-world data provide valuable insights for clinical practice, showing RT or resection only as a tolerable treatment option in this elderly population.

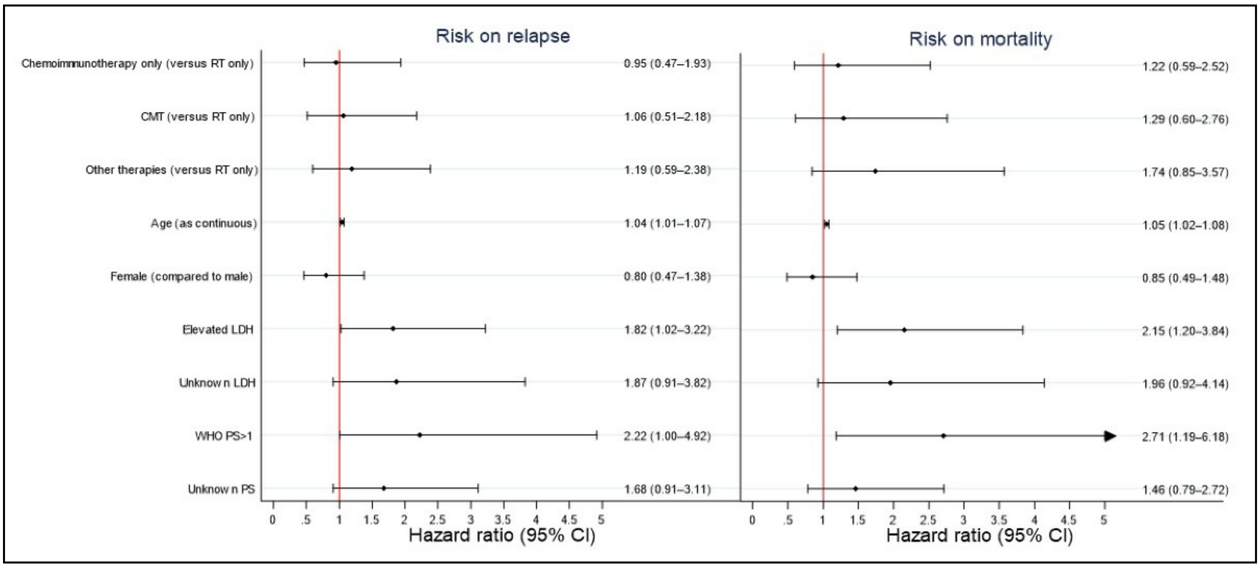

Figure 1

**Copyright Information:** (Online) ISSN: 2572-9241
©2026 The Author(s). HemaSphere is published by John Wiley & Sons Ltd on behalf of European Hematology Association. This is an open access Abstract Book distributed under the Attribution-NonCommercial-NoDerivs (CC BY-NC-ND), which allows third parties to download the articles and share them with others as long as they credit the author and the Abstract Book, but they cannot change the content in any way or use them commercially.
**Abstract Book Citations:** Authors, Title, HemaSphere, 2026;10:(S1):pages. The abstract book DOIs can be found on the "Author Information" page.
**Disclaimer:** Articles published in the journal HemaSphere exclusively reflect the opinions of the authors. The authors are responsible for all content in their abstracts including accuracy of the facts, statements, citing resources, etc.

### (PB3716) PET-INTEGRATED NOMOGRAMS FOR SURVIVAL PREDICTION IN PATIENTS WITH LARGE B-CELL LYMPHOMA TREATED WITH CAR T-CELL THERAPY

**Topic:** 19. Large B cell lymphomas - Clinical

Beatrice Casadei*[1], Marianna Gentilini[1, 2], Sara Proto[3], Claudio Malizia[4], Lisa Argnani[2], Cinzia Pellegrini[1], Giulia Gabrielli[1, 2], Riccardo Ussia[5], Andrea Paccagnella[6], Stella Blandino[1], Francesco De Felice[1], Margherita Ursi[1], Enrico Maffini[1], Cristina Nanni[7], Stefano Fanti[2, 7], Francesca Bonifazi[1], Andrea Farolfi[7], Pier Luigi Zinzani[1, 2]

*[1]IRCSS Azienda Ospedaliero-Universitaria di Bologna,, Bologna, Italy;[2]Dipartimento di Scienze Mediche e Chirurgiche, Università di Bologna,, Bologna, Italy; [3]Medina Nucleare, Alma Mater Studiorum Università di Bologna,, Bologna, Italy;[4]Medicina Nucleare, Azienda Ospedaliero-Universitaria di Modena,, Modena, Italy; [5]Medicina Nucleare, Ospedale S.S. Annunziata, ASL2 Abruzzo,, Chieti, Italy; [6]Medicina Nucleare, AUSL Romagna,, Cesena, Italy; [7]Nuclear Medicine, IRCCS Azienda Ospedaliero-Universitaria di Bologna, Bologna, Italy;*

### Background

CD19-directed chimeric antigen receptor T-cell (CART) therapy is effective in relapsed/refractory large B-cell lymphoma (R/R LBCL), however clinical outcomes remain heterogeneous. Reliable prognostic stratification prior to CART infusion is therefore crucial to optimise patient selection and management. ^18F-FDG PET/CT is the standard imaging modality for staging and response assessment in LBCL, providing quantitative metabolic parameters that reflect tumour burden and disease biology. When integrated with clinical and laboratory variables, these parameters may improve prognostic modelling.

### Aims

To develop PET-integrated nomograms for predicting progression-free survival (PFS) and overall survival (OS) in R/R LBCL patients (pts) treated with CART.

### Methods

This post-hoc analysis of a retro-prospective, single-center study included R/R LBCL pts treated with CART in routine clinical practice between August 2019 and November 2023. Baseline variables (n=35) included demographics, histology (DLBCL NOS vs high grade B cell lymphoma [HGBCL]), number and response to prior lines of treatment, bridging therapy (BT, yes or no), laboratory parameters (e.g. lactate dehydrogenase [LDH], C-reactive protein [CRP], hemoglobin [Hb], white blood cell [WBC] and lymphocyte [lym] count), and PET metrics (maximum and mean standardized uptake value, metabolic tumor volume [MTV], total lesion glycolysis [TLG], extranodal/bone involvement). Variable selection used adaptive LASSO with bootstrap stability selection (500 resamples). Multivariable Cox models were developed for PFS and OS. Model performance was assessed using Harrell's concordance index (C-index), time-dependent area under the curve (AUC), and calibration plots.

**Copyright Information:** (Online) ISSN: 2572-9241
©2026 The Author(s). HemaSphere is published by John Wiley & Sons Ltd on behalf of European Hematology Association. This is an open access Abstract Book distributed under the Attribution-NonCommercial-NoDerivs (CC BY-NC-ND), which allows third parties to download the articles and share them with others as long as they credit the author and the Abstract Book, but they cannot change the content in any way or use them commercially.
**Abstract Book Citations:** Authors, Title, HemaSphere, 2026;10:(S1):pages. The abstract book DOIs can be found on the "Author Information" page.
**Disclaimer:** Articles published in the journal HemaSphere exclusively reflect the opinions of the authors. The authors are responsible for all content in their abstracts including accuracy of the facts, statements, citing resources, etc.

**Results**

A total of 141 pts were analysed: 111 (78.7%) had DLBCL NOS and 30 HGBCL. Most pts had stage III/IV disease (73%), received a maximum of 2 previous line of treatment (73.7%), and were refractory to the last therapy (93%). BT was performed in 84% pts. CART products included axi-cel (n=109), tisa-cel (n=30), and liso-cel (n=2). With a median follow-up of 16.6 months, the median PFS and OS were 13.3 and 40.2 months, respectively. On univariate analysis, PET-derived parameters (MTV, TLG, bone involvement) and laboratory variables (LDH, CRP, ferritin, Hb, WBC and lym count) were associated with outcomes (p<0.05). In the multivariable OS model (C-index of 0.798, 95%CI 0.731–0.856), four independent predictors emerged: Hb (hazard ratio [HR] 0.72, 95%CI 061-084, p<0.001), WBC (HR 1.17, 95%CI 1.09-1.26, p<0.001) and lym_count (HR 0.41, 95%CI 0.22-0.75, p=0.004) as a protective factors, while bone involvement on PET (HR 2.36, 95%CI 1.28-4.36, p=0.006) as an adverse factor. In the PFS model (C-index 0.689, 95%CI 0.623–0.756), Hb remained protective (HR 0.78, 95%CI 0.68-0.89, p<0.001), while receiving BT (HR 2.53, 95%CI 1.14-5.63, p=0.02) was adverse. Time-dependent ROC showed AUCs at 12 months of 0.86 (OS) and 0.71 (PFS).

**Summary/Conclusion**

We developed PET-integrated nomograms that predict outcomes after CART in R/R LBCL. These models underscore the interplay between host marrow reserve (Hb and lym count) and metabolic tumour distribution, including bone involvement. The proposed tools may support trial design and individualized decision-making and warrant prospective multicentre validation.

**Copyright Information:** (Online) ISSN: 2572-9241
©2026 The Author(s). HemaSphere is published by John Wiley & Sons Ltd on behalf of European Hematology Association. This is an open access Abstract Book distributed under the Attribution-NonCommercial-NoDerivs (CC BY-NC-ND), which allows third parties to download the articles and share them with others as long as they credit the author and the Abstract Book, but they cannot change the content in any way or use them commercially.
**Abstract Book Citations:** Authors, Title, HemaSphere, 2026;10:(S1):pages. The abstract book DOIs can be found on the "Author Information" page.
**Disclaimer:** Articles published in the journal HemaSphere exclusively reflect the opinions of the authors. The authors are responsible for all content in their abstracts including accuracy of the facts, statements, citing resources, etc.

### (PB3717) SURVIVAL OF PATIENTS WITH DIFFUSE LARGE B-CELL LYMPHOMA DEPENDING ON THE NUMBER OF EZH2- AND PSTAT3-POSITIVE TUMOR CELLS

**Topic:** 19. Large B cell lymphomas - Clinical

Elena Vaneeva*[1], Vitaliy Rosin[1], Dmitry Diakonov[1], Natalia Loginova[1], Maria Berdnikova[1], Svetlana Samarina[1]

*[1]The Federal State-Financed Scientific Institution Kirov Research Institute of Hematology and Blood Transfusion under the Federal Medical Biological Agency, Kirov, Russia;*

### Background

As a nosological entity, diffuse large B-cell lymphoma (DLBCL) comprises over 18 tumor subtypes (WHO 2022 data) that share common pathogenetic mechanisms but differ in their clinical, phenotypic, molecular-genetic characteristics, therapeutic sensitivity, and prognosis. Approximately 30% of cases present with refractory-relapsed disease after first-line treatment. Consequently, the search for novel prognostic factors for DLBCL persists to more precisely identify patients at high risk of relapse. Methyltransferase EZH2 (Enhancer of zeste homolog 2) is the catalytic subunit of the repressive protein complex PRC2. It is an epigenetic regulator of the expression of many target genes. One of these is the transducer and activator of transcription pSTAT3. Its protein product controls the processes of cell proliferation, differentiation, and apoptosis. Excessive accumulation of EZH2 is detected in various solid and hematological tumors, including DLBCL. The indicated protein enhances the activation of pSTAT3 through methylation, thereby contributing to uncontrolled proliferation of tumor cells and inhibition of apoptosis. Research on the relationship between EZH2 and pSTAT3 markers with the prognosis of DLBCL has not been conducted.

### Aims

To evaluate the survival of DLBCL patients in relation to the level of EZH2- and pSTAT3-positive tumor cells.

### Methods

For the study, biopsies of tumor tissue and lymph nodes were used, obtained from 81 patients with a newly diagnosed DLBCL (median age - 61 years). All patients received standard first-line immunochemotherapy according to the R-CHOP regimen. The quantification of EZH2 and pSTAT3-positive tumor cells was performed using immunohistochemical and morphometric methods. Prognostically significant threshold values for these markers were calculated using ROC analysis: for EZH2, the level was 61%, and for pSTAT3, it was 68%. According to the established thresholds, all patients were divided into groups with above-threshold [≥61% (+) for EZH2; ≥68% (+) for pSTAT3] and below-threshold [<61% (-) for EZH2; <68% (-) for pSTAT3] content of positive tumor cells. Overall survival (OS) and progression-free survival (PFS) were calculated using the Kaplan-Meier method (log-rank test) and Cox regression analysis.

### Results

The highest OS (90%) was observed in the group of patients (n=20) with the simultaneous presence of tumor cells exhibiting subthreshold levels of EZH2 and pSTAT3. For the EZH2+/pSTAT3- and EZH2-/pSTAT3+ variants, it was 56% (n=16) and 76% (n=25), respectively. The lowest OS values (30%) were seen in patients (n=20) with the combination of EZH2+/pSTAT3+ (p=0.001; HR=2.1; 95% CI=1.45-3.04). A similar pattern was observed in the analysis of PFS. The proportion of patients without disease progression with the EZH2-/pSTAT3- variant was 75% (n=20), while it decreased to 56.3% (n=16) and 64% (n=25) in the presence of EZH2+/pSTAT3- and EZH2-/pSTAT3+ combinations, respectively. The lowest PFS (20%) was recorded in patients with simultaneous presence of above-threshold numbers of tumor cells expressing the studied proteins (n=20; p=0.001; HR=1.75; 95% CI=1.28-2.39).

**Copyright Information:** (Online) ISSN: 2572-9241
©2026 The Author(s). HemaSphere is published by John Wiley & Sons Ltd on behalf of European Hematology Association. This is an open access Abstract Book distributed under the Attribution-NonCommercial-NoDerivs (CC BY-NC-ND), which allows third parties to download the articles and share them with others as long as they credit the author and the Abstract Book, but they cannot change the content in any way or use them commercially.
**Abstract Book Citations:** Authors, Title, HemaSphere, 2026;10:(S1):pages. The abstract book DOIs can be found on the "Author Information" page.
**Disclaimer:** Articles published in the journal HemaSphere exclusively reflect the opinions of the authors. The authors are responsible for all content in their abstracts including accuracy of the facts, statements, citing resources, etc.

**Summary/Conclusion**

The combination of suprathreshold EZH2+/pSTAT3+ tumor cell levels is associated with poor OS and PFS in DLBCL patients treated with the R-CHOP regimen.

**Copyright Information:** (Online) ISSN: 2572-9241
©2026 The Author(s). HemaSphere is published by John Wiley & Sons Ltd on behalf of European Hematology Association. This is an open access Abstract Book distributed under the Attribution-NonCommercial-NoDerivs (CC BY-NC-ND), which allows third parties to download the articles and share them with others as long as they credit the author and the Abstract Book, but they cannot change the content in any way or use them commercially.
**Abstract Book Citations:** Authors, Title, HemaSphere, 2026;10:(S1):pages. The abstract book DOIs can be found on the "Author Information" page.
**Disclaimer:** Articles published in the journal HemaSphere exclusively reflect the opinions of the authors. The authors are responsible for all content in their abstracts including accuracy of the facts, statements, citing resources, etc.

### (PB3718) EFFICACY AND SAFETY OF AXICABTAGENE CILOLEUCEL (AXI-CEL) FOR THE TREATMENT OF RELAPSE/REFRACTORY NON-HODGKIN'S LYMPHOMA: REAL-WORLD DATA IN CHINESE POPULATION

**Topic:** 19. Large B cell lymphomas - Clinical

Li Wang*[1], Dehui Zou[2], Wenbin Qian[3], Ying Lu[4], Yuhua Li[5], Dongmei Ji[6], Xiaojian Zhu[7], Ping Li[8], Lei Zhang[9], Keshu Zhou[10], Hongmei Jing[11], Ting Niu[12], Chunyan Ji[13], Lanfang Li[14], Hua Yang[15], Fei Li[16], Wei Wang[17], Zhenyu Li[18], Zixun Yan[1], Jinghua Zhao[19], Depei Wu[20], Yu Hu[21], Weili Zhao[1]

*[1]Shanghai Institute of Hematology, State Key Laboratory of Medical Genomics, National Research Center for Translational Medicine at Shanghai, Ruijin Hospital affiliated to Shanghai Jiao Tong University School of Medicine, Shanghai, China; [2]State Key Laboratory of Experimental Hematology, Institute of Hematology & Blood Diseases Hospital, Chinese Academy of Medical Sciences and Peking Union Medical College, Tianjin, China; [3]Department of Hematology, The Second Affiliated Hospital, College of Medicine, Zhejiang University, Hangzhou, China; [4]Department of Hematology, The Affiliated People's Hospital of Ningbo University, Ningbo, China; [5]Department of Hematology, Zhujiang Hospital, Southern Medical University, Guangzhou, China; [6]Department of Medical Oncology, Fudan University Shanghai Cancer Center, Shanghai, China;[7]Department of Hematology, Tongji Hospital, Tongji Medical College, Huazhong University of Science and Technology, Wuhan, China; [8]Department of Hematology, Tongji Hospital, Tongji University School of Medicine, Shanghai, China; [9]Department of Oncology, The First Affiliated Hospital of Zhengzhou University, Zhengzhou, China; Lymphoma Diagnosis and Treatment Center of Henan Province, Zhengzhou, China; [10]Department of Hematology, Affiliated Cancer Hospital of Zhengzhou University, Henan Cancer Hospital, Zhengzhou, China; [11]Department of Hematology and Lymphoma Research Center, Peking University Third Hospital, Beijing, China; [12]Department of Hematology, West China Hospital, Sichuan University, Chengdu, China;[13]Department of Hematology, Qilu Hospital, Cheeloo College of Medicine, Shandong University, Jinan, China; [14]Department of Lymphoma, Tianjin Medical University Cancer Institute and Hospital, National Clinical Research Center of Cancer, Key Laboratory of Cancer Prevention and Therapy, Tianjin's Clinical Research Center for Cancer, the Sino-US Center for Lymphoma and Leukemia Research, Tianjin, China; [15]Department of Hematology, Hongkong University-Shenzhen Hospital, Shenzhen, China; [16]Department of Hematology, The First Affiliated Hospital of Nanchang University, Nanchang, China; [17]Department of Hematology, The Affiliated Hospital of Qingdao University, Qingdao, China; [18]Department of Hematology, The Affiliated Hospital of Xuzhou Medical University, Xuzhou, China; [19]Department of Clinical Development & Medical, Shanghai Fosun Kairos Biotechnology Co., Ltd., Shanghai, China,[20]The First Affiliated Hospital of Soochow University, National Clinical Research Center for Hematologic Diseases, Jiangsu Institute of Hematology, Suzhou, China; [21]Institute of Hematology, Union Hospital, Tongji Medical College, Huazhong University of Science and Technology, Wuhan, China;*

### Background

To better understand the efficacy and safety of commercial axi-cel in Chinese relapsed/refractory (R/R) non-Hodgkin's lymphoma (NHL) patients in a real-world setting, we conducted this multi-center, non-interventional study (ChiCTR2100047990). The accrual goal is 200 patients, and the primary endpoint is overall survival (OS).

### Aims

Here we report updated efficacy and safety outcomes of Chinese patients treated by commercial CAR-T products in real world after a median follow-up of 25.7 months.

**Copyright Information:** (Online) ISSN: 2572-9241
©2026 The Author(s). HemaSphere is published by John Wiley & Sons Ltd on behalf of European Hematology Association. This is an open access Abstract Book distributed under the Attribution-NonCommercial-NoDerivs (CC BY-NC-ND), which allows third parties to download the articles and share them with others as long as they credit the author and the Abstract Book, but they cannot change the content in any way or use them commercially.
**Abstract Book Citations:** Authors, Title, HemaSphere, 2026;10:(S1):pages. The abstract book DOIs can be found on the "Author Information" page.
**Disclaimer:** Articles published in the journal HemaSphere exclusively reflect the opinions of the authors. The authors are responsible for all content in their abstracts including accuracy of the facts, statements, citing resources, etc.

### Methods

We included R/R NHL patients treated with commercial axi-cel at 20 authorized treatment centers from Nov 2021 to Nov 2023. All the patients signed written informed consent. Responses were assessed by physicians using the 2014 Lugano Criteria. The primary outcome was overall survival (OS). Secondary outcomes included best objective response (ORR) and complete response (CR) rate, progression-free survival (PFS) and safety.

### Results

201 patients, including 171 with DLBCL and 11 with high-grade BCL, received axi-cel. The best CR rate and best ORR were 68.2% (95% CI, 61.2%-74.5%) and 83.1% (95% CI, 77.2%-88.0%). Response was ongoing in 70.8%, 64.2% and 52.5% of patients at 12, 24 and 36 months, respectively. At a median follow-up of 25.7 months, the median PFS was 27.8 months (95% CI, 15.1-not evaluable), with PFS rates of 61.9% at 12 months, 53.9% at 24 months, 42.7% at 36 months. The median OS was not reached, with OS rates of 79.0% at 12 months, 73.2% at 24 months, 70.8% at 36 months. Multivariate analysis showed that BTKi-containing combination therapy vs no combination therapy was associated with improved PFS (HR, 0.44; 95% CI, 0.26-0.75) and OS (HR, 0.34; 0.17-0.71). ECOG performance status 0-1 vs ≥ 2 was associated with improved PFS (HR, 0.39; 95% CI, 0.25-0.63) and OS (0.23; 0.13-0.41). Elevated lactate dehydrogenase (LDH) was negatively associated with OS (HR, 2.09; 95% CI, 1.19-3.68). No new safety signals were observed in the Chinese population. Cytokine release syndrome (CRS) of any grade occurred in 173 patients (86.1%), with 22 patients (10.9%) experiencing grade 3/4. Neurologic events (NE) of any grade occurred in 51 patients (25.4%), with 7 patients (3.5%) experiencing grade 3/4. No grade 5 CRS or NE appeared. We analyzed long-term AEs of interest in 156 patients completing > 12 months of follow-up. The most common axi-cel-related AEs included anemia (64 [41%]), leucocyte count decreased (54 [34.6%]), neutrophil count decreased (53 [34.0%]) and cytokine increased (48 [30.8%]). No secondary cancer was reported. The median cumulative cortisone-equivalent dose was 1333 mg (IQR 500-3692), which is similar to that in the ZUMA-1 cohort 6, but much lower than that in ZUMA-1 cohort 1+2. Corticosteroid use after 7 days of axi-cel treatment was associated with a trend for shorter PFS (HR, 1.67; 95% CI 0.97-2.88; P = .07), but no apparent trend for OS (HR, 1.61; 95% CI, 0.78-3.31).

### Summary/Conclusion

This analysis demonstrated axi-cel is effective and safe in Chinese relapsed/refractory B-NHL patients, with additional benefits from BTKi-containing combination therapy.

**Copyright Information:** (Online) ISSN: 2572-9241
©2026 The Author(s). HemaSphere is published by John Wiley & Sons Ltd on behalf of European Hematology Association. This is an open access Abstract Book distributed under the Attribution-NonCommercial-NoDerivs (CC BY-NC-ND), which allows third parties to download the articles and share them with others as long as they credit the author and the Abstract Book, but they cannot change the content in any way or use them commercially.
**Abstract Book Citations:** Authors, Title, HemaSphere, 2026;10:(S1):pages. The abstract book DOIs can be found on the "Author Information" page.
**Disclaimer:** Articles published in the journal HemaSphere exclusively reflect the opinions of the authors. The authors are responsible for all content in their abstracts including accuracy of the facts, statements, citing resources, etc.

Kaplan-Meier curves for progression-free survival and overall survival

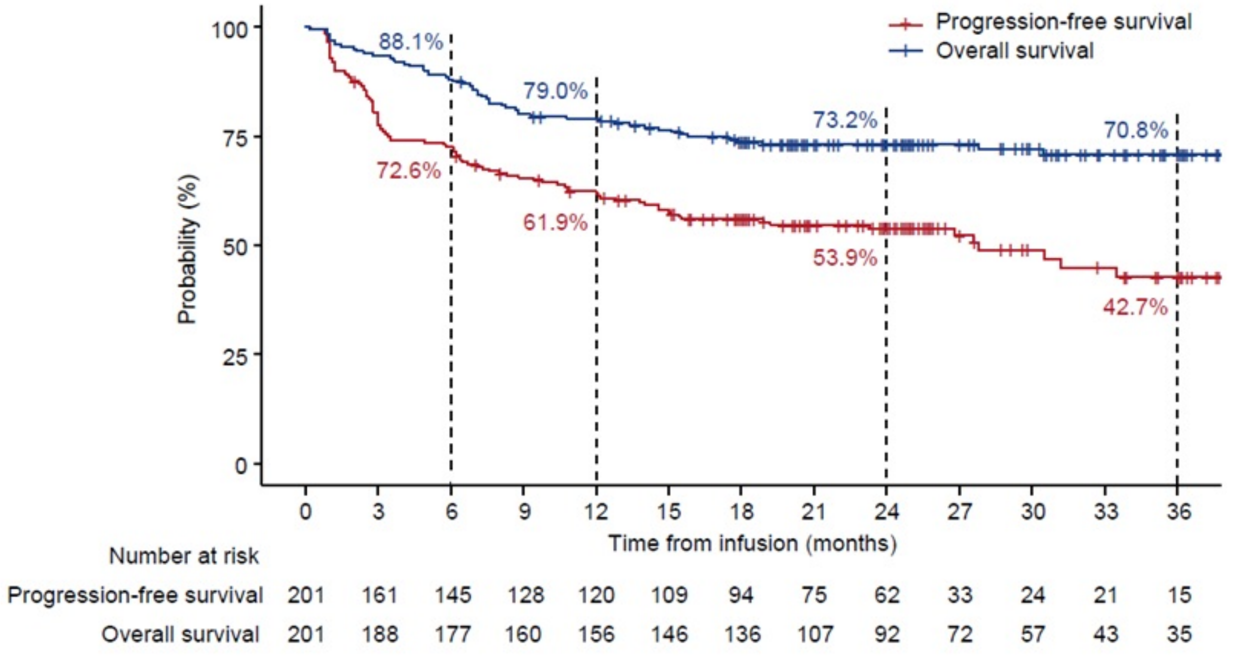

**Copyright Information:** (Online) ISSN: 2572-9241
©2026 The Author(s). HemaSphere is published by John Wiley & Sons Ltd on behalf of European Hematology Association. This is an open access Abstract Book distributed under the Attribution-NonCommercial-NoDerivs (CC BY-NC-ND), which allows third parties to download the articles and share them with others as long as they credit the author and the Abstract Book, but they cannot change the content in any way or use them commercially.
**Abstract Book Citations:** Authors, Title, HemaSphere, 2026;10:(S1):pages. The abstract book DOIs can be found on the "Author Information" page.
**Disclaimer:** Articles published in the journal HemaSphere exclusively reflect the opinions of the authors. The authors are responsible for all content in their abstracts including accuracy of the facts, statements, citing resources, etc.

### (PB3719) RIBOSOME BIOGENESIS–DRIVEN THERAPEUTIC RESISTANCE IN DLBCL: BIOLOGICAL MECHANISMS AND TARGETED INTERVENTION STRATEGIES

**Topic:** 19. Large B cell lymphomas - Clinical

Minxing Yue*[1], Ying Fang[1], Chaohui Bao[1], Jiaying Liu[1], Hai Fang[1], Weili Zhao[1], Li Wang[1]

[1]*Shanghai Institute of Hematology, State Key laboratory of Medical Genomics, National Research Center for Translational Medicine at Shanghai, Ruijin Hospital Affiliated to Shanghai Jiao Tong University School of Medicine, ShangHai, China;*

### Background

Ribosome biogenesis refers to the process of assembling new ribosomes, the intracellular organelles responsible for protein synthesis. Highly proliferative tumors often exhibit elevated rates of ribosome biogenesis. Whether increased ribosome biogenesis is associated with drug resistance, and how such resistance might be overcome, remain to be further elucidated.

### Aims

(1) To investigate the sensitivity to R-CHOP therapy and the mechanisms of resistance in ribosome biogenesis–high DLBCL; (2) To identify targeted therapeutic agents capable of overcoming ribosome biogenesis–high–associated resistance.

### Methods

Bulk RNA-seq data (n =400) and corresponding clinical information from patients with de novo DLBCL treated with R-CHOP were analyzed using a ribosome biogenesis signature (RiboSis). The optimal cutoff for the RiboSis score was determined by time-dependent ROC analysis, which stratified patients into RiboSis-high and RiboSis-low groups. Differential expression genes and pathway enrichment were analyzed according to the bulk RNA-seq. DB and U-2932 DLBCL cell lines were used to explore optimal therapeutic strategies for RiboSis-high patients.

### Results

Patients in the RiboSis-high group had significantly shorter progression-free survival and overall survival compared with those in the RiboSis-low group. RiboSis-high tumors showed enrichment of ribosome biogenesis and ribonucleoprotein complex biogenesis pathways, according to the bulk RNA-seq data.Polysome profiling demonstrated that selinexor, an XPO1 inhibitor, combined with cisplatin suppressed tumor growth by inhibiting ribosome biogenesis in tumor cells. Further mechanistic studies revealed that selinexor markedly reduced cytoplasmic ribosome production by blocking the nuclear export of ribosomal mRNAs as well as ribosomal large and small subunits. As a result, RiboSis-high tumor cells underwent cell cycle arrest and became more sensitive to chemotherapy.

### Summary/Conclusion

A previously defined RiboSis score identifies a DLBCL subset with hyperactive ribosome biogenesis, adverse genetics and poor survival. In DLBCL models, XPO1 inhibition with selinexor suppresses ribosome biogenesis programs and synergizes with DNA-damaging agents. These findings support RiboSis-high DLBCL as a rational therapeutic context for combining XPO1 inhibition with chemotherapy to improve clinical outcomes.

**Copyright Information:** (Online) ISSN: 2572-9241
©2026 The Author(s). HemaSphere is published by John Wiley & Sons Ltd on behalf of European Hematology Association. This is an open access Abstract Book distributed under the Attribution-NonCommercial-NoDerivs (CC BY-NC-ND), which allows third parties to download the articles and share them with others as long as they credit the author and the Abstract Book, but they cannot change the content in any way or use them commercially.
**Abstract Book Citations:** Authors, Title, HemaSphere, 2026;10:(S1):pages. The abstract book DOIs can be found on the "Author Information" page.
**Disclaimer:** Articles published in the journal HemaSphere exclusively reflect the opinions of the authors. The authors are responsible for all content in their abstracts including accuracy of the facts, statements, citing resources, etc.

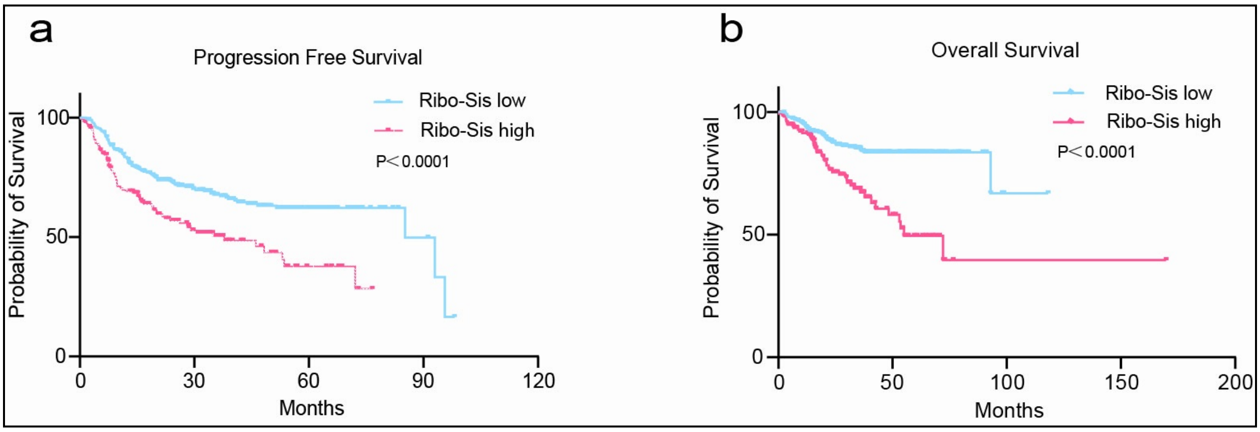

**Copyright Information:** (Online) ISSN: 2572-9241
©2026 The Author(s). HemaSphere is published by John Wiley & Sons Ltd on behalf of European Hematology Association. This is an open access Abstract Book distributed under the Attribution-NonCommercial-NoDerivs (CC BY-NC-ND), which allows third parties to download the articles and share them with others as long as they credit the author and the Abstract Book, but they cannot change the content in any way or use them commercially.
**Abstract Book Citations:** Authors, Title, HemaSphere, 2026;10:(S1):pages. The abstract book DOIs can be found on the "Author Information" page.
**Disclaimer:** Articles published in the journal HemaSphere exclusively reflect the opinions of the authors. The authors are responsible for all content in their abstracts including accuracy of the facts, statements, citing resources, etc.

### (PB3720) AXICABTAGENE CILOLEUCEL FOR THE TREATMENT OF RELAPSED/REFRACTORY DIFFUSE LARGE B-CELL LYMPHOMA IN BRAZIL: THE IMPACT OF CAR T-CELL THERAPY WAIT TIME AND TREATMENT SEQUENCING

**Topic:** 19. Large B cell lymphomas - Clinical

João De Holanda Farias*[1], Graeme Ball[2], Bradley Kievit[3], Blissett Rob[3], Jayr Schmidt[4]

*[1]Hospital Erasto Gaertner, Hematolgy, Curitiba, Brazil; [2]Kite, a Gilead Company, Santa Monica, United States of America;[3]Maple Health Group, New York, United States of America; [4]A.C. Camargo Cancer Center, Hematology, Sao Paulo, Brazil;*

### Background

Patients with relapsed/refractory (R/R) diffuse large B-cell lymphoma (DLBCL) who are medically eligible for chimeric antigen receptor T-cell (CAR T) treatment may experience reduced clinical benefit when treatment is delayed or if they receive non-CAR T treatments despite eligibility. In Brazil, prolonged real-world wait times may increase the risk of disease progression or death prior to infusion and misallocation to alternative therapies may further diminish health outcomes.

### Aims

This study assessed the impact of extended wait times and treatment sequencing on health outcomes in patients with R/R DLBCL in Brazil.

### Methods

A discrete event simulation was used to model patients with R/R DLBCL across treatment lines from progression after first-line (1L) through fourth-line (4L) therapy. A lifetime time horizon (50 years) and 5.0% annual discount rate was applied. Model inputs were sourced from clinical trials and published literature; efficacy was extrapolated using mixture cure models. The wait time analysis compared a 23-day wait time, consistent with trial conditions, against delays of up to 120 days, broadly informed by Brazilian real-world data in similar malignancies. Mortality during the waiting period was estimated from real-world data on death prior to second-line therapy. The treatment sequencing analysis explored scenarios in which 0–50% of CAR T–eligible patients were instead assigned to non–CAR T treatment pathways, contrary to international DLBCL treatment guideline recommendations. Key outcomes included life years [LYs], quality-adjusted life years [QALYs], and excess mortality at the individual and population level.

### Results

Assuming a 23-day wait time, the model estimated 6.24 LYs and 4.30 QALYs, with 6.40% mortality prior to infusion (Table 1). Extending the wait time to 120 days reduced projected LYs by 0.54 and QALYs by 0.37, while increasing pre-infusion mortality to 15.79%. An average 120-day wait time translated to 112 excess deaths annually, with eight additional deaths predicted for each week of delay. In the treatment sequencing analysis, life expectancy declined by 19.8 months at 50% misallocation to non-CAR T pathways compared with full CAR T access, and 10-year overall survival was reduced by 10% (Table 2). Nationally, an estimated 111 lives were projected to be lost within five years at a 50% misallocation rate.

**Copyright Information:** (Online) ISSN: 2572-9241
©2026 The Author(s). HemaSphere is published by John Wiley & Sons Ltd on behalf of European Hematology Association. This is an open access Abstract Book distributed under the Attribution-NonCommercial-NoDerivs (CC BY-NC-ND), which allows third parties to download the articles and share them with others as long as they credit the author and the Abstract Book, but they cannot change the content in any way or use them commercially.
**Abstract Book Citations:** Authors, Title, HemaSphere, 2026;10:(S1):pages. The abstract book DOIs can be found on the "Author Information" page.
**Disclaimer:** Articles published in the journal HemaSphere exclusively reflect the opinions of the authors. The authors are responsible for all content in their abstracts including accuracy of the facts, statements, citing resources, etc.

### Summary/Conclusion

This analysis demonstrates that both prolonged wait times and misallocation to non–CAR T treatment pathways significantly undermine the clinical value of CAR T therapy for patients with R/R DLBCL in Brazil. The findings underscore the urgent need for coordinated healthcare system strategies to optimize CAR T delivery and improve outcomes for patients with aggressive lymphoma.

**Table 1. Impact of wait times on health outcomes**

| Aspect | 23 days | 60 days | 120 days |
|---|---|---|---|
| LY | 6.24 | 5.88 | 5.70 |
| Incremental | - | -0.36 | -0.54 |
| QALYs | 4.30 | 4.06 | 3.93 |
| Incremental | - | -0.24 | -0.37 |
| % Deaths (wait time period) | 6.40% | 11.33% | 15.79% |
| % Statistically cured (1L progression to 2L progression) | 40.1% | 37.1% | 35.1% |

*1/2L, first/second line; LY, life year; QALY, quality-adjusted life year*

**Table 2. Survival rates, counts of patient outcomes based on suboptimal misallocation rates**

| Aspect | Misallocation rates | | | | | |
|---|---|---|---|---|---|---|
| | 0% | 10% | 20% | 30% | 40% | 50% |
| 1-year OS, % | 87.2% | 87.2% | 87.2% | 87.1% | 87.1% | 87.0% |
| 5-year OS, % | 53.8% | 52.0% | 50.1% | 48.2% | 46.4% | 44.5% |
| 10-year OS, % | 42.5% | 40.5% | 38.5% | 36.5% | 34.6% | 32.6% |
| Median OS, months | 76.7 | 72.8 | 68.8 | 64.9 | 60.9 | 56.9 |
| Reduction in life expectancy, months | - | 4.0 | 7.9 | 11.9 | 15.8 | 19.8 |

*CAR T, chimeric antigen receptor T-cell; OS, overall survival*

**Copyright Information:** (Online) ISSN: 2572-9241
©2026 The Author(s). HemaSphere is published by John Wiley & Sons Ltd on behalf of European Hematology Association. This is an open access Abstract Book distributed under the Attribution-NonCommercial-NoDerivs (CC BY-NC-ND), which allows third parties to download the articles and share them with others as long as they credit the author and the Abstract Book, but they cannot change the content in any way or use them commercially.
**Abstract Book Citations:** Authors, Title, HemaSphere, 2026;10:(S1):pages. The abstract book DOIs can be found on the "Author Information" page.
**Disclaimer:** Articles published in the journal HemaSphere exclusively reflect the opinions of the authors. The authors are responsible for all content in their abstracts including accuracy of the facts, statements, citing resources, etc.

### (PB3721) INDEPENDENT VALIDATION OF THE INTERNATIONAL METABOLIC PROGNOSTIC INDEX (IMPI) ON EFFICACY AND SAFETY OF CAR-T IN PATIENTS WITH AGGRESSIVE LARGE B-CELL LYMPHOMA

**Topic:** 19. Large B cell lymphomas - Clinical

Eugenio Galli*[1], Alessandro Corrente[2], Francesca Maria Portonera[3], Andrea Guarneri[4], Elena Romano Gargarella[3], Ilaria Pansini[1, 2], Marcello Viscovo[1, 2], Federica Sorà[1, 2], Maria Lucia Calcagni[3, 4], Patrizia Chiusolo[1, 2], Simona Sica[1, 2], Lucia Leccisotti[3, 4], Stefan Hohaus[1, 2]

*[1]Department of Laboratory and Hematological Sciences, Fondazione Policlinico Universitario A. Gemelli IRCCS, Rome, Italy, Rome, Italy; [2]Section of Hematology, Department of Radiological and Hematological Sciences, Università Cattolica del Sacro Cuore, Rome, Italy, Rome, Italy; [3]Section of Nuclear Medicine, Department of Radiological Sciences and Hematology, Università Cattolica del Sacro Cuore, Rome, Italy, Rome, Italy; [4]Nuclear Medicine Unit, Fondazione Policlinico Universitario A. Gemelli IRCCS, Rome, Italy;*

### Background

Chimeric antigen receptor T-cell (CAR-T) therapy is an established treatment for patients with relapsed/refractory large B-cell lymphoma (LBCL). The International Metabolic Prognostic Index (IMPI), integrating TMTV, age, and disease stage, was originally developed for newly diagnosed LBCL and has recently demonstrated prognostic value in the CAR-T setting.

### Aims

To investigate the prognostic significance of IMPI validating an externally validated TMTV cutoff in LBCL patients treated with CAR-T.

### Methods

We retrospectively evaluated 46 patients with LBCL treated with CAR-T. All patients underwent FDG-PET/CT within 1 month prior to infusion; stable disease was confirmed when bridging therapy was administered. TMTV was calculated using an AI-based automated segmentation algorithm (Lesion Scout with Auto ID, Siemens Healthineers; PERCIST-recommended liver-based threshold and SUVmax 4.0 for segmentation).
IMPI was applied using the CAR-T–specific TMTV cutoff ≥44.3 mL (CAR-IMPI). Responses were assessed clinically and radiologically every 3 months or at clinical progression. Progression-free survival (PFS), overall survival (OS), event-free survival (EFS), cytokine release syndrome (CRS), immune effector cell-associated neurotoxicity syndrome (ICANS), and ICU admission were analyzed.

**Copyright Information:** (Online) ISSN: 2572-9241
©2026 The Author(s). HemaSphere is published by John Wiley & Sons Ltd on behalf of European Hematology Association. This is an open access Abstract Book distributed under the Attribution-NonCommercial-NoDerivs (CC BY-NC-ND), which allows third parties to download the articles and share them with others as long as they credit the author and the Abstract Book, but they cannot change the content in any way or use them commercially.
**Abstract Book Citations:** Authors, Title, HemaSphere, 2026;10:(S1):pages. The abstract book DOIs can be found on the “Author Information” page.
**Disclaimer:** Articles published in the journal HemaSphere exclusively reflect the opinions of the authors. The authors are responsible for all content in their abstracts including accuracy of the facts, statements, citing resources, etc.

### Results

According to predefined CAR-IMPI risk categories (<1.07, 1.07–1.53, >1.53), 31 patients (67.4%) were classified as low-risk, 10 (21.7%) as intermediate-risk, and 5 (10.9%) as high-risk. Median PFS progressively decreased across risk groups, reaching 58.5 months in low-risk patients compared with 2.3 and 1.4 months in intermediate- and high-risk patients, respectively (p=0.074); corresponding 12-month PFS rates were 59%, 40%, and 0%.
CAR-IMPI was significantly associated with PFS (HR 1.69, 95% CI 1.18–2.42, p=0.004).
Consistently, intermediate- and high-risk patients showed significantly higher mortality compared with the low-risk group (HR 3.65, p=0.022 and HR 8.99, p=0.002; overall p=0.001), with 1-year OS rates of 78%, 56%, and 50%, respectively.
CAR-IMPI was associated with clinically relevant cytokine release syndrome (CRS grade ≥2), with lower incidence in low-risk patients (p=0.024). No statistically significant association was observed between CAR- IMPI and the occurrence of immune effector cell- associated neurotoxicity syndrome (ICANS) (p=0.27). ICU admission was <10% in low-risk patients and ~20–30% in intermediate/high-risk groups.

### Summary/Conclusion

CAR-IMPI effectively stratifies CAR-T–treated LBCL patients according to survival outcomes and risk of severe CRS, supporting its potential integration into pre-infusion risk assessment.

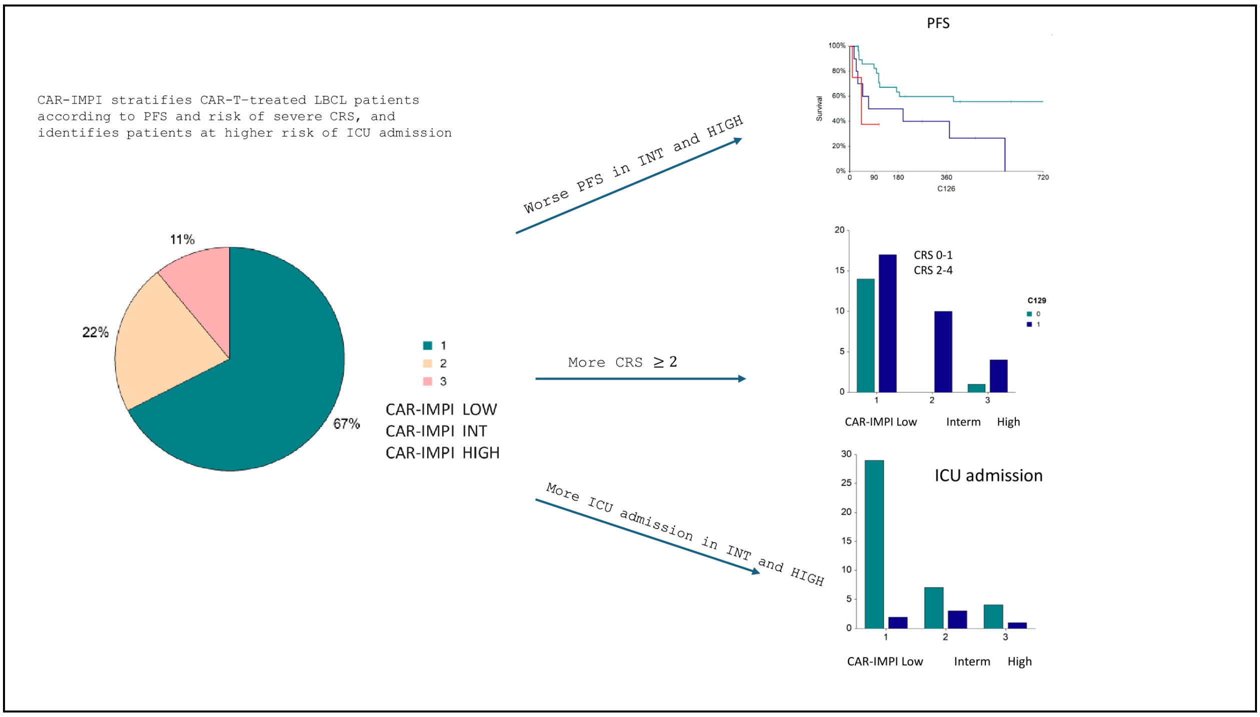

**Copyright Information:** (Online) ISSN: 2572-9241
©2026 The Author(s). HemaSphere is published by John Wiley & Sons Ltd on behalf of European Hematology Association. This is an open access Abstract Book distributed under the Attribution-NonCommercial-NoDerivs (CC BY-NC-ND), which allows third parties to download the articles and share them with others as long as they credit the author and the Abstract Book, but they cannot change the content in any way or use them commercially.
**Abstract Book Citations:** Authors, Title, HemaSphere, 2026;10:(S1):pages. The abstract book DOIs can be found on the "Author Information" page.
**Disclaimer:** Articles published in the journal HemaSphere exclusively reflect the opinions of the authors. The authors are responsible for all content in their abstracts including accuracy of the facts, statements, citing resources, etc.

### (PB3722) EARLY LONGITUDINAL HEALTH-RELATED QUALITY OF LIFE (HRQOL) CHANGES WITH TAFASITAMAB–LENALIDOMIDE IN TRANSPLANT-INELIGIBLE RELAPSED/REFRACTORY DIFFUSE LARGE B-CELL LYMPHOMA (R/R DLBCL): INTERIM PRO-MIND

**Topic:** 19. Large B cell lymphomas - Clinical

Alice Di Rocco*[1], Giovanni Caocci[2], Flaminia Bellisario[3], Maria Cristina Cox[4], Elsa Pennese[5], Francesco Piazza[6, 7, 8], Annalisa Chiarenza[9], Alberto Fabbri[10], Mario Lapecorella[11], Nicola Battaglia[11], Pier Luigi Zinzani[12], Antonio Pinto[13]

*[1]Institute of Hematology, Sapienza University of Roma, Department of Translational and Precision Medicine, Roma, Italy; [2]University of Cagliari, Department of Medical Sciences and Public Health, Cagliari, Italy; [3]UOSD Malattie Linfoproliferative Extramidollari, Fondazione Policlinico A. Gemelli IRCCS, Department of Laboratory and Hematological Sciences, Rome, Italy; [4]UOC Malattie Linfoproliferative, Fondazione Policlinico Tor Vergata, Rome, Italy; [5]Pescara Hospital, Pescara, Italy; [6]University of Padova, Department of Medicine, Padova, Italy; [7]Azienda Ospedale Università Padova, Hematology Unit, Padova, Italy; [8]Veneto Institute of Molecular Medicine, Padova, Italy; [9]University of Catania, Catania, Italy; [10]Senese University Hospital Company, UOC Ematologia Azienda Ospedaliero, Siena, Italy; [11]Incyte Biosciences Italy Srl, Milano, Italy; [12]Institute of Hematology, IRCCS Azienda Ospedaliero-Universitaria di Bologna, Department of Medical and Surgical Sciences, Bologna University School of Medicine, Bologna, Italy; [13]Istituto Nazionale Tumori IRCCS-Fondazione Pascale, Napoli, Italy;*

### Background

Real-world transplant-ineligible R/R DLBCL is frequently associated with functional impairment and symptom burden. Prospective longitudinal patient-reported outcomes (PROs) can clarify the timing and persistence of changes in HRQoL after treatment initiation.

### Aims

To describe baseline burden and early (month 3 [M3]) and later (month 6 [M6]) changes in selected HRQoL domains—Global Health Status (GHS), Physical Functioning (PF), Emotional Functioning (EF), and fatigue (EORTC QLQ-C30), plus neuropathy and worries about health/functioning (EORTC QLQ-NHL-HG29)—in a prospective Italian real-world cohort treated with tafasitamab–lenalidomide (Tafa–Len).

### Methods

PRO-MIND is a multicentre, prospective, observational Italian study (NCT06299553). HRQoL was assessed at baseline and scheduled follow-ups using QLQ-C30 and QLQ-NHL-HG29. For QLQ-C30, higher scores indicate better functioning (GHS, PF, EF) and worse symptoms (fatigue). For QLQ-NHL-HG29, higher scores indicate worse symptom/concern burden. Clinically meaningful improvements (CMI) thresholds were applied accordingly (C30: ≥10 points; HG29: ≥½ SD) (Osoba. *J Clin Oncol* 1998;16[1]:139-144; Norman. *Med Care* 2003;41[5]:582-592). Denominators reflect paired baseline and follow-up data. PROs stopped at progression per protocol; thus, change analyses describe the on-treatment evaluable set and are descriptive/exploratory.

Copyright Information: (Online) ISSN: 2572-9241
©2026 The Author(s). HemaSphere is published by John Wiley & Sons Ltd on behalf of European Hematology Association. This is an open access Abstract Book distributed under the Attribution-NonCommercial-NoDerivs (CC BY-NC-ND), which allows third parties to download the articles and share them with others as long as they credit the author and the Abstract Book, but they cannot change the content in any way or use them commercially.
Abstract Book Citations: Authors, Title, HemaSphere, 2026;10:(S1):pages. The abstract book DOIs can be found on the “Author Information” page.
Disclaimer: Articles published in the journal HemaSphere exclusively reflect the opinions of the authors. The authors are responsible for all content in their abstracts including accuracy of the facts, statements, citing resources, etc.

**Results**

At the interim data cut-off (July 25, 2025), 90 patients were enrolled. Patients were generally old and most had comorbidities (median age 77 years; stage IV 61.1%; primary refractory 57.8%; international prognostic index ≥3 64.8%; ≥3 comorbidities 66.2%). Efficacy at the same data cut-off showed an overall response rate of 53.0% in response-evaluable patients (n=66), and median progression-free survival was 10.4 months in the intention-to-treat population (n=90). Baseline questionnaires were available for 78 (QLQ-C30) and 76 (HG29) patients. PRO completion decreased over time (paired datasets: QLQ-C30 n=39 at M3 and n=19 at M6; HG29 n=35 at M3 and n=18 at M6). Baseline impairment was frequent; by M3, CMI were observed across functioning and symptom domains among evaluable patients. Mean (SD) baseline scores were GHS 61.9 (21.7), PF 71.4 (26.3), EF 75.6 (22.6), and fatigue 35.8 (27.1); neuropathy 14.7 (19.6) and worries 30.9 (21.9). Baseline clinically important problems were frequent for PF (57.7%), EF (37.2%), and fatigue (35.9%); clinically important neuropathy and worries were present in 26.3% and 25.0%, respectively. Among patients with paired baseline–M3 questionnaires, CMI were observed: GHS 30.8% (n=12/39) and PF 23.1% (n=9/39); and EF 28.2% (n=11/39) and fatigue 35.9% (n=14/39) **(Figure)**. HG29 domains also showed early CMI, notably worries 42.9% (n=15/35) and neuropathy 17.1% (n=6/35). At M6 (smaller denominators), CMI rates among patients with paired PRO data were: GHS 36.8% (n=7/19), PF 31.6% (n=6/19), EF 42.1% (n=8/19), and fatigue 31.6% (n=6/19); worries 33.3% (n=6/18) and neuropathy 22.2% (n=4/18).

**Summary/Conclusion**

In this older, comorbid, real-world R/R DLBCL cohort, baseline HRQoL burden was substantial, with frequent PF and fatigue problems and notable worries/neuropathy. CMI emerged by M3 and were generally consistent at M6 among evaluable patients treated with Tafa–Len.

**Copyright Information:** (Online) ISSN: 2572-9241
©2026 The Author(s). HemaSphere is published by John Wiley & Sons Ltd on behalf of European Hematology Association. This is an open access Abstract Book distributed under the Attribution-NonCommercial-NoDerivs (CC BY-NC-ND), which allows third parties to download the articles and share them with others as long as they credit the author and the Abstract Book, but they cannot change the content in any way or use them commercially.
**Abstract Book Citations:** Authors, Title, HemaSphere, 2026;10:(S1):pages. The abstract book DOIs can be found on the "Author Information" page.
**Disclaimer:** Articles published in the journal HemaSphere exclusively reflect the opinions of the authors. The authors are responsible for all content in their abstracts including accuracy of the facts, statements, citing resources, etc.

**Figure. Clinically Meaningful Improvement by Domain and Time Point**

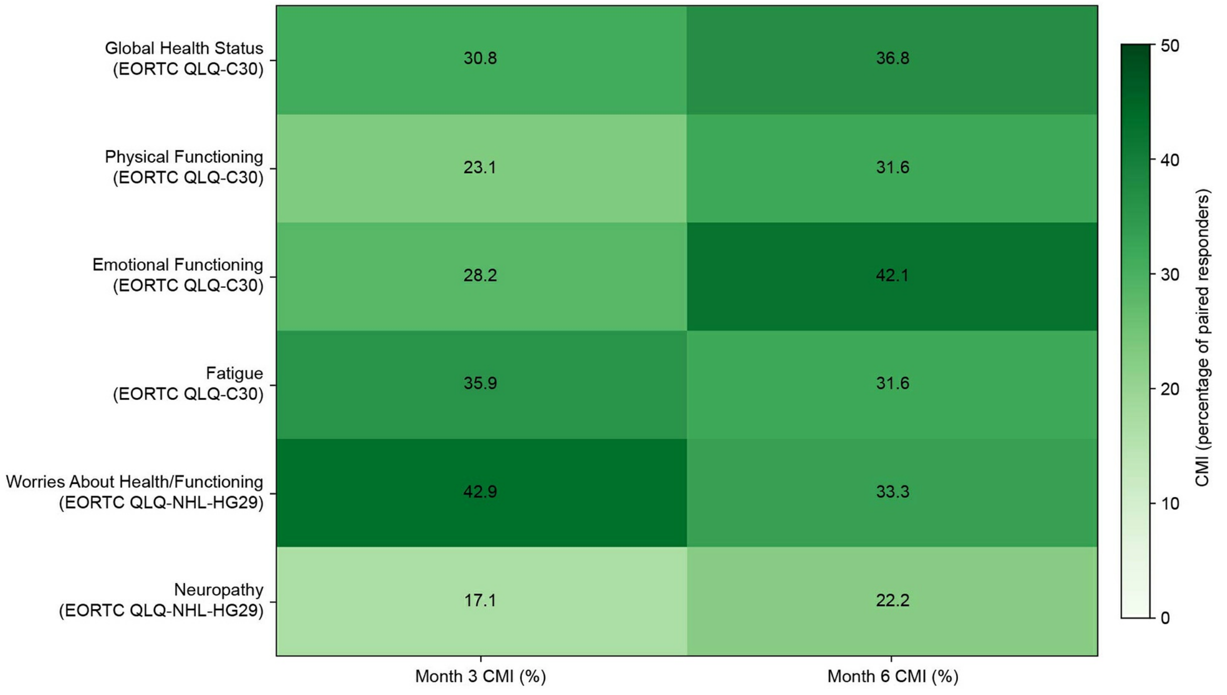

CMI, clinically meaningful improvement.

**Copyright Information:** (Online) ISSN: 2572-9241
©2026 The Author(s). HemaSphere is published by John Wiley & Sons Ltd on behalf of European Hematology Association. This is an open access Abstract Book distributed under the Attribution-NonCommercial-NoDerivs (CC BY-NC-ND), which allows third parties to download the articles and share them with others as long as they credit the author and the Abstract Book, but they cannot change the content in any way or use them commercially.
**Abstract Book Citations:** Authors, Title, HemaSphere, 2026;10:(S1):pages. The abstract book DOIs can be found on the "Author Information" page.
**Disclaimer:** Articles published in the journal HemaSphere exclusively reflect the opinions of the authors. The authors are responsible for all content in their abstracts including accuracy of the facts, statements, citing resources, etc.

### (PB3723) PRE-TREATMENT ANTIBIOTIC EXPOSURE AND OUTCOMES AFTER BISPECIFIC ANTIBODY THERAPY IN B-CELL NON-HODGKIN LYMPHOMA: A SINGLE-CENTRE RETROSPECTIVE STUDY

**Topic:** 19. Large B cell lymphomas - Clinical

Ana Cuervo Fonseca*[1], Lucía Medina de Alba[1], Jimena Martínez Fiel[1], Tycho Stephan Baumann[1], Antonia Rodriguez Izquierdo[1], Jose María Sánchez-Pina[1], Adolfo Jesus Saez Marin[1], Gonzalo Carreño-Tarragona[1], Almudena Zaratiegui[1], Cristian Gutierrez-Padilla[1], María Sánchez Tabernero[1], Miguel Navarro Sánchez[1], Antonio García Sánchez[1], Guillermo Ramos Moreno[1], Álvaro Riñón Basso[1], Natalia Vega-Larburu[1], Mariana González Fernández[1], Maria Calbacho[1], Ana Isabel Jimenez Ubieto[1], Joaquín Martinez-Lopez[1]

*[1]Hospital Universitario 12 de Octubre, Servicio de Hematología y Hemoterapia, Madrid, Spain;*

### Background

Bispecific antibodies (BsAb) targeting CD3 and CD20 have shown promising activity in relapsed or refractory B cell non-Hodgkin Lymphoma (B-NHL) but external immunomodulatory factors may influence treatment efficacy. In CAR-T therapy, prior antibiotic use has been associated with gut dysbiosis and impaired antitumor responses (García-Vicente et al., 2026), raising the question of whether asimilar effect may occur with BsAb.

### Aims

We aimed to evaluate the impact of antibiotic exposure within two months prior to BsAb therapy on clinical outcomes, including response rates, survival and treatment-related toxicities, in patients with B-cell NHL.

### Methods

Retrospective single-center study of B-cell NHL patients treated with BsAb at Hospital Universitario 12 de Octubre (Madrid, Spain) from Sep 2020 to Oct 2025. Clinical data were extracted from electronic records. Survival was estimated by Kaplan–Meier and compared with log-rank tests; multivariable Cox regression adjusted for relevant clinical variables was performed as well.

**Copyright Information:** (Online) ISSN: 2572-9241
©2026 The Author(s). HemaSphere is published by John Wiley & Sons Ltd on behalf of European Hematology Association. This is an open access Abstract Book distributed under the Attribution-NonCommercial-NoDerivs (CC BY-NC-ND), which allows third parties to download the articles and share them with others as long as they credit the author and the Abstract Book, but they cannot change the content in any way or use them commercially.
**Abstract Book Citations:** Authors, Title, HemaSphere, 2026;10:(S1):pages. The abstract book DOIs can be found on the “Author Information” page.
**Disclaimer:** Articles published in the journal HemaSphere exclusively reflect the opinions of the authors. The authors are responsible for all content in their abstracts including accuracy of the facts, statements, citing resources, etc.

### Results

Sixty-five patients were included, 16 (25%) received antibiotics within 60 days prior to BsAb. Demographic and baseline characteristics are shown in **Table 1**. Antibiotic exposure was associated with worse ECOG, higher HEMATOTOX and IPI/FLIPI scores

At a median follow-up of 10.6 months (IQR4.3–29.7) 15 deaths and 18 progression events were recorded. 12-month overral survival (OS) and progression-free survival (PFS) were 80% (IC 95%: 69-90) and 71% (IC95%: 59-83) respectively. Prior antibiotic exposure was associated with significantly poorer outcomes in OS (**Figure1**) and PFS (log-rank0.0002).

In multivariable Cox regression analysis adjusting for ECOG, antibiotic exposure, IPI and HEMATOTOX score, prior antibiotic exposure remained independently associated with worse OS (HR 4.23, 95% CI 1.04-17.16, p=0.04) and PFS (HR 6.84, 95% CI 2.2-21.25, p=0.001). ECOG was also independently associated with inferior survival outcomes. Regarding treatment toxicities, prior antibiotic exposure was associated with a higher incidence of ICANS (p=0.04) and ICU admission (p=0.029).

**Table 1.** Demographic and baseline characteristics.

| | **Total (n=65)** | **No prior antibiotic (n=49)** | **Prior antibiotic (n=16)** | **p-value** |
|---|---|---|---|---|
| **Demographic data** | | | | |
| •Age (years), median (IQR) | 62 (52-72.5) | 61 (52-68.5) | 66.5 (53-74.7) | 0.402 |
| •Sex female, n (%) | 34 (52.3 %) | 26 (53 %) | 8 (50 %) | 1 |
| **Lymphoma type, n (%)** | | | | 0.32 |
| •Diffuse large B-cell | 25 (38.5 %) | 17 (34.7%) | 8 (50%) | |
| •Follicular | 35 (53.9 %) | 28 (57.1%) | 7 (43.8 %) | |
| •Others | 5 (7.7 %) | 4 (8.2 %) | 1 (6.3 %) | |
| **Bispecific antibody, n (%)** | | | | 0.25 |
| •Epcoritamab | 16 (24.6 %) | 13 (26.5 %) | 3 (18.8 %) | |
| •Mosunetuzumab | 19 (29.2 %) | 16 (32.7 %) | 3 (18.8 %) | |
| •Glofitamab | 14 (21.5 %) | 11 (22.5 %) | 3 (18.8 %) | |
| •Odronextamab | 15 (23.1 %) | 8 (16.3 %) | 7 (43.8 %) | |
| **Other characteristics** | | | | |
| •Prior lines, median (IQR) | 2 (0-3) | 1 (0-2) | 2.5 (0.5-3) | 0.082 |
| •ECOG, median (IQR) | 0 (0-1) | 0 (0-0.50) | 1 (0-1) | 0.003 |
| •HEMATOTOX scale, median (IQR) | 1 (0-2) | 1 (0-2) | 1.5 (1-3.8) | 0.042 |
| •IPI, median (IQR) | 3 (2-3) | 3 (2-3) | 2 (1-2,8) | 0.05 |
| •Bulky disease, n (%) | 23 (35.4 %) | 19 (38.8 %) | 4 (25 %) | 0.38 |
| •Extranodal disease, n (%) | 26 (40 %) | 19 (38.8 %) | 7 (43.8 %) | 0.77 |

### Summary/Conclusion

Prior antibiotic exposure within 60 days before BsAb initiation was independently associated with worse OS and PFS in B-cell NHL, higher ICANS and ICU admission, suggesting microbiota disruption may affect immune-mediated toxicity. Although limited by sample size and baseline imbalances, these findings might support infection prevention and rational antibiotic use in this setting. Prospective validation in larger multicenter cohorts is warranted.

**Copyright Information:** (Online) ISSN: 2572-9241
©2026 The Author(s). HemaSphere is published by John Wiley & Sons Ltd on behalf of European Hematology Association. This is an open access Abstract Book distributed under the Attribution-NonCommercial-NoDerivs (CC BY-NC-ND), which allows third parties to download the articles and share them with others as long as they credit the author and the Abstract Book, but they cannot change the content in any way or use them commercially.
**Abstract Book Citations:** Authors, Title, HemaSphere, 2026;10:(S1):pages. The abstract book DOIs can be found on the "Author Information" page.
**Disclaimer:** Articles published in the journal HemaSphere exclusively reflect the opinions of the authors. The authors are responsible for all content in their abstracts including accuracy of the facts, statements, citing resources, etc.

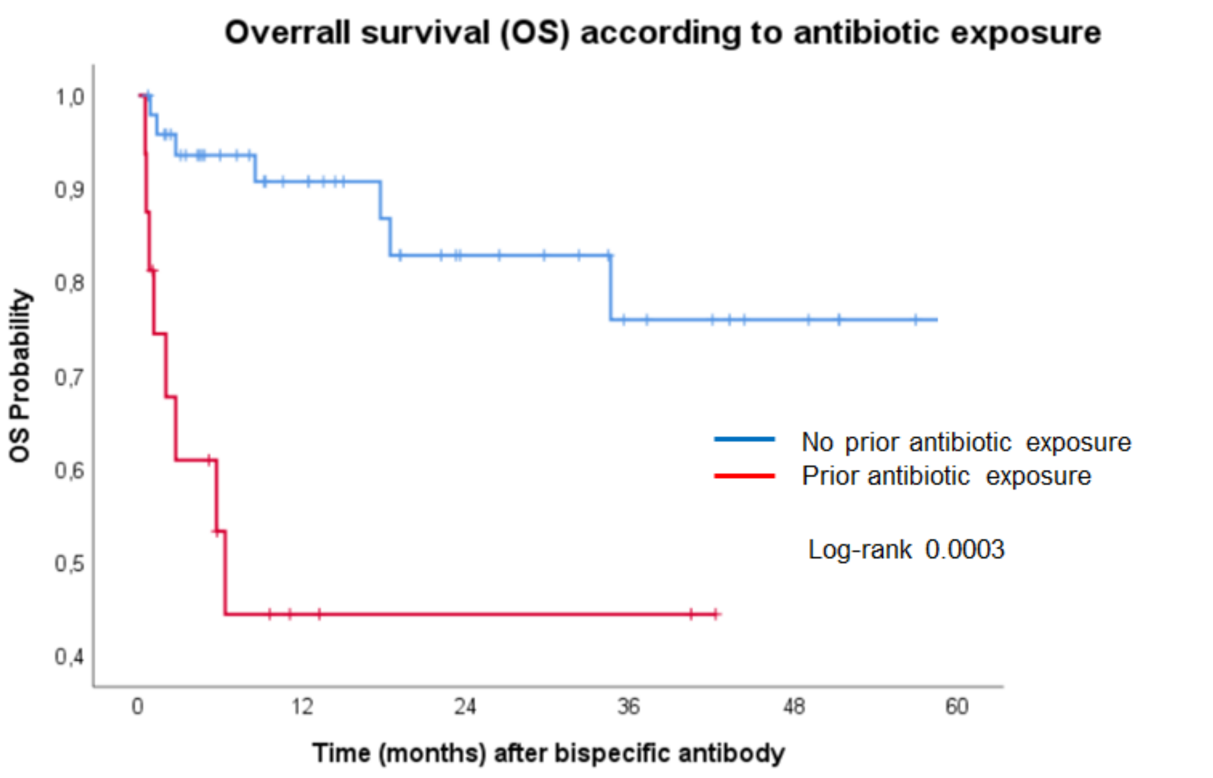

| | N | Median OS (months), CI 95% | 12 month OS (%), CI 95% | Median PFS (months), CI 95% | 12 month PFS (%), CI 95% |
|---|---|---|---|---|---|
| No prior antibiotic exposure | 49 | 73,74 (61.30 – 85.79) | 90.8 (82-99.6) | 64.76 (50.78 - 78.75) | 87.5 (77.5-97.5) |
| Prior antibiotic exposure | 16 | 20.28 (9.84 – 30.73) | 44.4 (17.7-71.1%) | 13.18 (4.38 – 21.98) | 23.7 (1-46.4) |

Kaplan Meier estimates of OS and PFS after BsAb therapy according to prior antibiotic exposure. Survival compared using log-rank test.

Copyright Information: (Online) ISSN: 2572-9241
©2026 The Author(s). HemaSphere is published by John Wiley & Sons Ltd on behalf of European Hematology Association. This is an open access Abstract Book distributed under the Attribution-NonCommercial-NoDerivs (CC BY-NC-ND), which allows third parties to download the articles and share them with others as long as they credit the author and the Abstract Book, but they cannot change the content in any way or use them commercially.
Abstract Book Citations: Authors, Title, HemaSphere, 2026;10:(S1):pages. The abstract book DOIs can be found on the “Author Information” page.
Disclaimer: Articles published in the journal HemaSphere exclusively reflect the opinions of the authors. The authors are responsible for all content in their abstracts including accuracy of the facts, statements, citing resources, etc.

### (PB3724) OBSERVATIONS OF CAR T AND CARNEG BYSTANDER T CELL DYNAMICS FOLLOWING EPCORITAMAB TREATMENT IN R/R DLBCL

**Topic:** 19. Large B cell lymphomas - Clinical

Kensuke Yanashima*[1], Yoshiki Furukawa[1], Shintaro Kinoshita[1], Midori Ishii[1], Kazuki Asazuma[1], Naoki Watanabe[1], Miyuki Tsutsui[1], Yutaka Tsukune[1], Yasuharu Hamano[1], Hajime Yasuda[1], Jun Ando[1, 2], Miki Ando[1]

*[1]Juntendo University School of Medicine, Department of Hematology, Tokyo, Japan;[2]Juntendo University School of Medicine, Division of Cell Therapy & Blood Transfusion Medicine, Tokyo, Japan;*

### Background

T cell engaging therapies including CD3 x CD20 bispecific antibodies (BsAb) have expanded therapeutic options even for patients relapsing after CD19 CAR T therapy. However, limited evidence exists concerning potential interactions between BsAb and CAR T cells. Furthermore, surrounding CAR-negative T ($CAR_{neg}$ T) cell status remains unclear. Our group previously reported that epcoritamab revived CAR T cells *in vivo* in 4 of 6 diffuse large B-cell lymphoma (DLBCL) patients who were administered epcoritamab following CD19 CAR T therapy (Yanashima et al. *EJHaem* 2025).

### Aims

In this study, we aimed to analyze the persistence of peripheral blood CAR T cells by qPCR in 15 DLBCL patients who received epcoritamab therapy following 3 commercial CD19 CAR T therapies, and to evaluate the association between CAR T persistence and clinical outcomes. In addition, we assessed T cell number, memory phenotype, and the expression levels of T cell exhaustion markers and cytolytic protein in both CAR-positive T ($CAR_{pos}$ T) cells and $CAR_{neg}$ T cells.

### Methods

Peripheral blood samples pre- and post-epcoritamab infusion were collected from 15 patients; 7 Tisa-cel, 3 Axi-cel, 5 Liso-cel treated patients. No patient received cytotoxic agents between CAR T and epcoritamab treatment. CAR copy numbers in peripheral blood were monitored by qPCR. Flow cytometric analyses were performed to compare memory phenotype, T cell exhaustion marker (PD-1, LAG-3, Tim-3), and cytolytic protein (perforin, granzyme B) expression between $CAR_{pos}$ T cells and $CAR_{neg}$ T cells.

### Results

CAR copy numbers increased from baseline in 6 of 15 patients. Of these all 6 achieved complete response or partial response. In a more detailed analysis, we assessed the association between transient peaks in CAR copy number and clinical outcomes. A transient peak at relapse was observed in 4 patients, and another peak following epcoritamab administration was observed in 9 patients. All patients who exhibited CAR copy number peaks both at lymphoma relapse and after epcoritamab administration achieved complete response (n = 3). In contrast, all patients without detectable CAR copy number peaks experienced progressive disease (n = 2). Flow cytometric analyses showed that exhaustion marker expression in both $CAR_{pos}$ T cells and $CAR_{neg}$ T cells was generally stable or decreased over time, irrespective of clinical outcome. By contrast, in patients achieving complete or partial response, perforin and granzyme B expression increased within the $CAR_{pos}$ T cell compartment. No clear association was observed between outcome and changes in $CAR_{neg}$ T cell frequency, exhaustion-marker expression, or cytolytic protein expression.

**Copyright Information:** (Online) ISSN: 2572-9241
©2026 The Author(s). HemaSphere is published by John Wiley & Sons Ltd on behalf of European Hematology Association. This is an open access Abstract Book distributed under the Attribution-NonCommercial-NoDerivs (CC BY-NC-ND), which allows third parties to download the articles and share them with others as long as they credit the author and the Abstract Book, but they cannot change the content in any way or use them commercially.
**Abstract Book Citations:** Authors, Title, HemaSphere, 2026;10:(S1):pages. The abstract book DOIs can be found on the "Author Information" page.
**Disclaimer:** Articles published in the journal HemaSphere exclusively reflect the opinions of the authors. The authors are responsible for all content in their abstracts including accuracy of the facts, statements, citing resources, etc.

**Summary/Conclusion**

These findings suggest that assessment of circulating CAR T cells may serve as a potential indicator of clinical outcome in patients treated with epcoritamab after CAR T therapy.

**Copyright Information:** (Online) ISSN: 2572-9241
©2026 The Author(s). HemaSphere is published by John Wiley & Sons Ltd on behalf of European Hematology Association. This is an open access Abstract Book distributed under the Attribution-NonCommercial-NoDerivs (CC BY-NC-ND), which allows third parties to download the articles and share them with others as long as they credit the author and the Abstract Book, but they cannot change the content in any way or use them commercially.
**Abstract Book Citations:** Authors, Title, HemaSphere, 2026;10:(S1):pages. The abstract book DOIs can be found on the "Author Information" page.
**Disclaimer:** Articles published in the journal HemaSphere exclusively reflect the opinions of the authors. The authors are responsible for all content in their abstracts including accuracy of the facts, statements, citing resources, etc.

### (PB3725) REAL-WORLD EXPERIENCE WITH POLATUZUMAB-R-BASED REGIMENS IN PREVIOUSLY UNTREATED LARGE B-CELL LYMPHOMA: A STUDY BY THE CROATIAN COOPERATIVE GROUP FOR HEMATOLOGIC DISEASES (KROHEM)

**Topic:** 19. Large B cell lymphomas - Clinical

Dino Dujmović*[1], Sandra Bašić Kinda[1], Barbara Dreta[1], Ida Hude[1], Zdravko Mitrović[2], Viktor Blaslov[3], Jasminka Sincic-Petricevic[4], Marin Kursar[5], Karla Misura Jakobac[5], Goran Rincic[6], Hrvoje Holik[7], Martina Morić Perić[8], Neven Franjić[9], Branimir Barišić[10], Petra Bernes[11], Ilenia Romić[12], Igor Aurer[1]

*[1]UHC Zagreb, Zagreb, Croatia;[2]University Hospital Dubrava, ZAgreb, Croatia; [3]UHC Split, Split, Croatia;[4]UHC Osijek, Osijek, Croatia; [5]University Hospital Merkur, Zagreb, Croatia;[6]UHC Sestre Milosrdnice, Zagreb, Croatia;[7]General Hospital Slavonski Brod, Slavonski Brod, Croatia; [8]General Hospital Zadar, Zadar, Croatia;[9]UHC Rijeka, Rijeka, Croatia; [10]University Hospital Sveti Duh, Zagreb, Croatia; [11]General Hospital Pula, Pula, Croatia;[12]General Hospital Dubrovnik, Dubrovnik, Croatia;*

### Background

For over two decades, R-CHOP has been the established standard of care for patients with diffuse large B-cell lymphoma (DLBCL).POLARIX trial recently demonstrated the superiority of polatuzumab vedotin in combination with R-CHP (Pola-R-CHP). However, experience with polatuzumab in daily clinical practice remains limited.

### Aims

To evaluate the real-world efficacy and safety of Polatuzumab Vedotin-based regimens in treatment-naive patients with diffuse large B-cell lymphoma (DLBCL).

### Methods

We conducted a retrospective, multicenter study across 16 Croatian centers, including patients treated between November 1, 2024, and November 1, 2025. To reflect routine clinical practice, inclusion was independent of age, International Prognostic Index (IPI) score, or ECOG performance status. Data were analyzed for overall response rate (ORR), complete remission (CR), progression-free survival (PFS), overall survival (OS), and toxicity profiles.

**Copyright Information:** (Online) ISSN: 2572-9241
©2026 The Author(s). HemaSphere is published by John Wiley & Sons Ltd on behalf of European Hematology Association. This is an open access Abstract Book distributed under the Attribution-NonCommercial-NoDerivs (CC BY-NC-ND), which allows third parties to download the articles and share them with others as long as they credit the author and the Abstract Book, but they cannot change the content in any way or use them commercially.
**Abstract Book Citations:** Authors, Title, HemaSphere, 2026;10:(S1):pages. The abstract book DOIs can be found on the "Author Information" page.
**Disclaimer:** Articles published in the journal HemaSphere exclusively reflect the opinions of the authors. The authors are responsible for all content in their abstracts including accuracy of the facts, statements, citing resources, etc.

**Results**

Total of 201 patients (106 males,95 females) were included, with a median age of 69 years (19-88). 68.1% (n=137) had Stage IV disease, 64.7% (n=130) had an IPI score of 3-5, and 38.3% (n=77) had a poor performance status (ECOG ≥ 2), including 29 patients with ECOG 3-4. Regarding histological subtypes, patients were classified as non-germinal center B-cell like (non-GCB, n=81), germinal center B-cell like (GCB, n=72), DLBCL NOS (n=27) high-grade B-cell lymphoma (n=12), and other subtypes (transformed FL n=3, PMBL n=6). 134 (66.7%) patients received Pola-R-CHP, while 67 (33.3%) received adapted regimens, including Pola-R-mini-CHP (n=24), Pola-R-CHEP (n=18), Pola-R-DA-EPOCH(n=12), and Pola-R-CEP (n=12). CNS prophylaxis was administered to 25 patients(23 high dose MTX).After a median follow-up of 7 months, the overall survival (OS) rate was 83.1% and the event-free survival (EFS) rate was 77.1%.163 patients were evaluable for response, while 38 patients are still in treatment. Among these 163 evaluable patients, the overall response rate (ORR) was 90.8% (148/163), with a complete remission (CR) rate of 65% (106/163).Prognostic analysis showed that IPI score provided strong stratification ($p < 0.001$); low-risk patients (IPI 0-1) reached 100% OS and EFS, the low-intermediate group (IPI 2) achieved 92.3% OS and 90.4% EFS, and the high-intermediate group (IPI 3) reached 94.1% OS and 84.3% EFS, while high-risk patients (IPI 4-5) had significantly lower outcomes (OS 66.3%; EFS 58.8%)( Image 1.) Age impacted OS (p=0.046) but not EFS, while ECOG status remained the strongest predictor ($p < 0.001$). Regarding safety, grade 3 or higher adverse events (AEs) occurred in 40.5% (n=81) of patients. The most frequent grade 3/4 hematological toxicities were neutropenia (33%), anemia (15.5%), and thrombocytopenia (12.5%). Febrile neutropenia was reported in 15.5% of cases, with a 1.5% fatal outcome rate. Non-hematological grade 3 or higher toxicities included infections (11%) and peripheral neuropathy (6%). Hospitalization due to AEs was required for 17.5% of patients, while 7% required polatuzumab dose reduction. One patient developed secondary AML.

**Summary/Conclusion**

We report one of the largest real-world experiences to date, confirming that Pola-R-based therapy is highly effective in a unselected patient population, including high-risk and "unfit" subgroups of patients.Our study supports the effectiveness and safety of these regimens in daily clinical practice, demonstrating their feasibility even in elderly patients requiring treatment adaptations.

**Copyright Information:** (Online) ISSN: 2572-9241
©2026 The Author(s). HemaSphere is published by John Wiley & Sons Ltd on behalf of European Hematology Association. This is an open access Abstract Book distributed under the Attribution-NonCommercial-NoDerivs (CC BY-NC-ND), which allows third parties to download the articles and share them with others as long as they credit the author and the Abstract Book, but they cannot change the content in any way or use them commercially.
**Abstract Book Citations:** Authors, Title, HemaSphere, 2026;10:(S1):pages. The abstract book DOIs can be found on the "Author Information" page.
**Disclaimer:** Articles published in the journal HemaSphere exclusively reflect the opinions of the authors. The authors are responsible for all content in their abstracts including accuracy of the facts, statements, citing resources, etc.

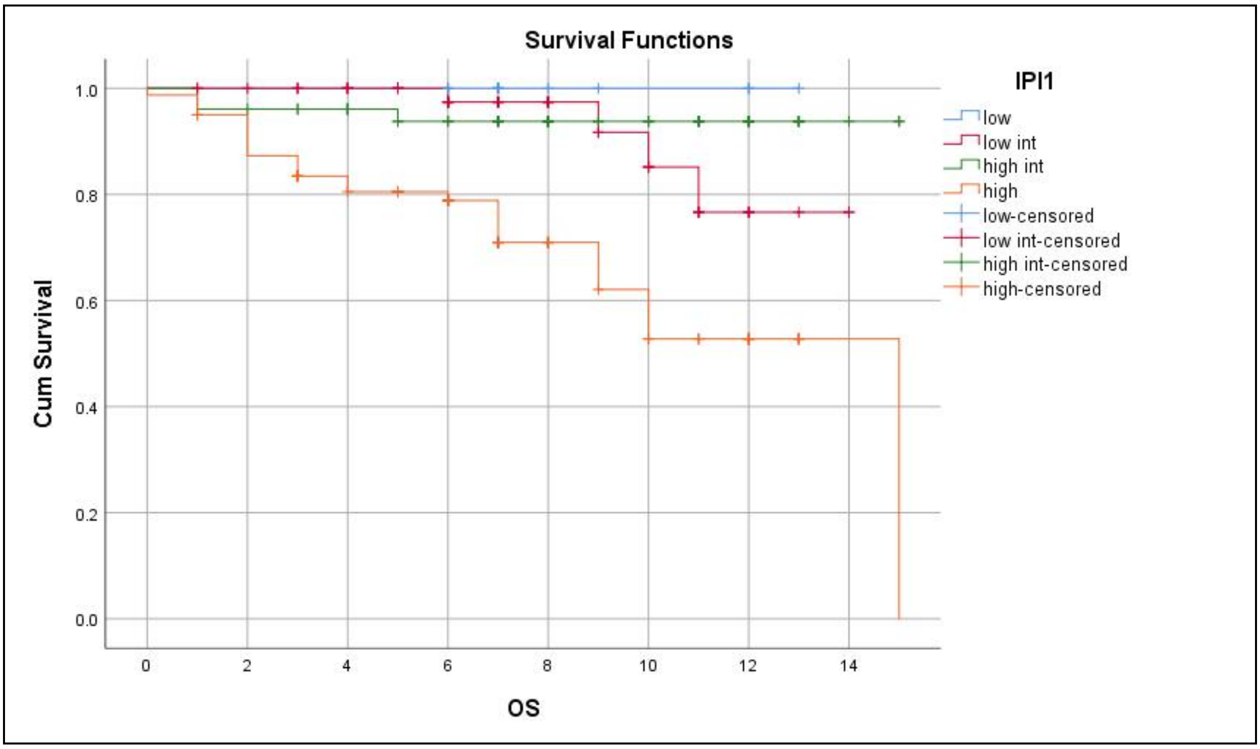

**Copyright Information:** (Online) ISSN: 2572-9241
©2026 The Author(s). HemaSphere is published by John Wiley & Sons Ltd on behalf of European Hematology Association. This is an open access Abstract Book distributed under the Attribution-NonCommercial-NoDerivs (CC BY-NC-ND), which allows third parties to download the articles and share them with others as long as they credit the author and the Abstract Book, but they cannot change the content in any way or use them commercially.
**Abstract Book Citations:** Authors, Title, HemaSphere, 2026;10:(S1):pages. The abstract book DOIs can be found on the "Author Information" page.
**Disclaimer:** Articles published in the journal HemaSphere exclusively reflect the opinions of the authors. The authors are responsible for all content in their abstracts including accuracy of the facts, statements, citing resources, etc.

### (PB3726) DIFFUSION MRI–DERIVED QUANTITATIVE METRICS IN DIFFUSE LARGE B-CELL LYMPHOMA

**Topic:** 19. Large B cell lymphomas - Clinical

Martine Nørskov*[1, 2], Elisabeth Cramer[3], Ulrich Lindberg[1], Derya Tireli[1, 2], Adam Hansen[2, 4], Jonathan Carlsen[2, 4], Peter Brown[3], Daniel Rinker[5], Atle Bjørnerud[6], Henrik Larsson[1, 2], Martin Hutchings[2, 3], Stig Cramer[1, 7]

*[1]Functional Imaging Unit, Department of Clinical Physiology and Nuclear Medicine, Copenhagen University Hospital - Rigshospitalet, Glostrup, Denmark; [2]University of Copenhagen, Department of Clinical Medicine, Copenhagen, Denmark; [3]Copenhagen University Hospital – Rigshospitalet, Department of Hematology, Copenhagen, Denmark; [4]Copenhagen University Hospital - Rigshospitalet, Department of Radiology, Copenhagen, Denmark; [5]Oslo University Hospital, Department of Neurology, Oslo, Norway; [6]Oslo University Hospital, Department of Physics and Computational Radiology, Oslo, Norway; [7]Amager and Hvidovre Hospital, Copenhagen University Hospital, Centre for Functional and Diagnostic Imaging and Research, Hvidovre, Denmark;*

### Background

Central nervous system (CNS) relapse in patients diagnosed with diffuse large B-cell lymphoma (DLBCL) poses a major clinical challenge. Current risk stratification relies on the CNS International Prognostic Index (CNS-IPI), which may result in both over- and undertreatment. This study investigated whether diffusion magnetic resonance imaging (MRI) metrics can detect early CNS microstructural changes associated with their CNS relapse risk.

### Aims

To characterize diffusion metric alterations in cerebral white matter and in cortical grey matter in patients diagnosed with DLBCL, and to assess their associations with CNS-IPI score. Secondary objectives were to explore relationships with individual CNS-IPI factors and key clinical risk factors and to compare diffusion measures between patients and healthy controls.

### Methods

In this single institution, cross-sectional study, we included sixty-three patients diagnosed with DLBCL (mean age = 64.94, SD = 13.91) and eleven healthy controls (mean age = 56.73, SD = 8.55), in a period of approximately two years. Both patients and healthy controls all underwent brain MRI including multi-shell diffusion imaging, utilizing restriction spectrum imaging (RSI) modelling. Cellularity index (CI), fractional anisotropy (FA), mean diffusivity (MD), kurtosis and neurite density (ND) in cerebral white matter and CI and MD in cortical gray matter, correlating these findings with CNS-IPI scores and clinical risk factors. Age was included as a covariant in many of the analyses given its significant association with diffusion parameters.

### Results

Patients with high CNS-IPI scores ≥4 demonstrated significantly elevated MD in both cortical grey matter and cerebral white matter and reduced CI and kurtosis in cerebral white matter compared to low/intermediate risk patients CNS-IPI <4. Relative to healthy controls, high CNS-IPI patients also exhibited reduced CI, FA, kurtosis and ND in cerebral white matter and significantly elevated MD in both cortical grey matter and cerebral white matter. The results regarding CNS-IPI and diffusion are presented uncorrected for age, as it is already part of the scoring tool, all subsequent analyses were performed with age as a covariant, due to a significant correlation between diffusion parameters and age. Kidney/adrenal involvement and Stage >2 exhibited significantly elevated cortical grey matter MD.

**Copyright Information:** (Online) ISSN: 2572-9241
©2026 The Author(s). HemaSphere is published by John Wiley & Sons Ltd on behalf of European Hematology Association. This is an open access Abstract Book distributed under the Attribution-NonCommercial-NoDerivs (CC BY-NC-ND), which allows third parties to download the articles and share them with others as long as they credit the author and the Abstract Book, but they cannot change the content in any way or use them commercially.
**Abstract Book Citations:** Authors, Title, HemaSphere, 2026;10:(S1):pages. The abstract book DOIs can be found on the “Author Information” page.
**Disclaimer:** Articles published in the journal HemaSphere exclusively reflect the opinions of the authors. The authors are responsible for all content in their abstracts including accuracy of the facts, statements, citing resources, etc.

### Summary/Conclusion

Diffusion MRI reveals subtle CNS microstructural alterations in DLBCL patients, most prominently in those at high risk for CNS relapse. These changes may reflect underlying brain vulnerability preceding clinical CNS involvement, warranting prospective investigation of diffusion metrics as predictive biomarkers.

**Copyright Information:** (Online) ISSN: 2572-9241
©2026 The Author(s). HemaSphere is published by John Wiley & Sons Ltd on behalf of European Hematology Association. This is an open access Abstract Book distributed under the Attribution-NonCommercial-NoDerivs (CC BY-NC-ND), which allows third parties to download the articles and share them with others as long as they credit the author and the Abstract Book, but they cannot change the content in any way or use them commercially.
**Abstract Book Citations:** Authors, Title, HemaSphere, 2026;10:(S1):pages. The abstract book DOIs can be found on the "Author Information" page.
**Disclaimer:** Articles published in the journal HemaSphere exclusively reflect the opinions of the authors. The authors are responsible for all content in their abstracts including accuracy of the facts, statements, citing resources, etc.

### (PB3727) INCIDENCE AND IMPACT OF MISDIAGNOSIS IN DIFFUSE LARGE B-CELL LYMPHOMA (DLBCL): AN ANALYSIS FROM LYMPHOMA COALITION'S 2024 GLOBAL PATIENT SURVEY ON LYMPHOMAS & CLL

**Topic:** 19. Large B cell lymphomas - Clinical

Lorna Warwick*[1], Shawn Sajkowski[1], Kathryn Perry[1], Mavis Lui[1], Natacha Bolaños[2]

*[1]Lymphoma Coalition, Etobicoke, Canada;[2]Lymphoma Coalition, Madrid, Spain;*

### Background

Diffuse large B-cell lymphoma (DLBCL) is the most prevalent B-cell lymphoma and due to its aggressive nature, a correct and timely diagnosis is critical to optimise prognosis. However, an accurate diagnosis remains challenging as symptoms can overlap with other unrelated illnesses.

### Aims

To examine the incidence of misdiagnosis DLBCL and its impact on diagnostic delays and healthcare utilisation across five European countries; Belgium, Bulgaria, France, Italy and the United Kingdom (UK).

### Methods

A 2024 cross-sectional, anonymous online global survey captured the lived experiences of people affected by lymphomas and CLL. People with lived experience of DLBCL (PWLE-DLBCL) reported whether they received an incorrect diagnosis prior to confirmation of DLBCL, the duration from symptom onset to diagnosis, and the number of medical consultations required. Data from five European countries, Belgium (n=69), Bulgaria (n=62), France (n=153), Italy (n=41) and the UK (n=177) were prioritised for further analysis.

**Copyright Information:** (Online) ISSN: 2572-9241
©2026 The Author(s). HemaSphere is published by John Wiley & Sons Ltd on behalf of European Hematology Association. This is an open access Abstract Book distributed under the Attribution-NonCommercial-NoDerivs (CC BY-NC-ND), which allows third parties to download the articles and share them with others as long as they credit the author and the Abstract Book, but they cannot change the content in any way or use them commercially.
**Abstract Book Citations:** Authors, Title, HemaSphere, 2026;10:(S1):pages. The abstract book DOIs can be found on the "Author Information" page.
**Disclaimer:** Articles published in the journal HemaSphere exclusively reflect the opinions of the authors. The authors are responsible for all content in their abstracts including accuracy of the facts, statements, citing resources, etc.

### Results

Globally, 1,354 PWLE-DLBCL responded to questions regarding prior misdiagnosis. **Of these, 45% were misdiagnosed at least once**. PWLE-DLBCL who were initially misdiagnosed experienced prolonged diagnostic timelines: 31% waited 3 months or more for a correct diagnosis compared to 14% who were diagnosed correctly. Furthermore, 69% of misdiagnosed PWLE-DLBCL had 3 or more medical consultations regarding symptoms versus only 35% who were initially diagnosed correctly.
Within countries examined in Europe, the UK reported the highest rate of DLBCL misdiagnosis (56%, n=99) followed by Bulgaria (48%, n=30), Italy (46%, n=19), Belgium (44%, n=30) and France (43%, n=65). Across all five countries, the misdiagnosed cohort had a higher percentage of patients who waited 3 months or more for a lymphoma diagnosis as well as a higher proportion who had 3 or more clinical visits before receiving their correct diagnosis (Table 1). Though highly varied, the most common misdiagnosis was another type of cancer (different hematological malignancy or solid tumour).

Table 1: Diagnostic delays and healthcare utilisation among misdiagnosed vs correctly diagnosed PWLE-DLBCL

| | Received Diagnosis 3 Months or Longer After Seeking Medical Attention | | Required 3 or More Doctor Visits Before Diagnosis | |
|---|---|---|---|---|
| | Initially Misdiagnosed (%) | Correct Initial Diagnosis (%) | Initially Misdiagnosed (%) | Correct Initial Diagnosis (%) |
| All DLBCL Respondents | 31 | 14 | 69 | 35 |
| Belgium | 17 | 0 | 53 | 24 |
| Bulgaria | 31 | 20 | 75 | 43 |
| France | 29 | 18 | 56 | 34 |
| Italy | 31 | 0 | 53 | 26 |
| United Kingdom | 39 | 22 | 63 | 24 |

### Summary/Conclusion

Our findings demonstrate that nearly half of PWLE-DLBCL experience an initial misdiagnosis, leading to significant diagnostic delays and increased healthcare utilisation for this population. Within Europe, DLBCL misdiagnosis appeared particularly pronounced in the UK. Misdiagnoses and delays impose significant clinical, psychological, and system-level burdens by postponing potentially life-saving treatment for patients, increasing the volume of avoidable clinical encounters on the healthcare system and placing amplified stress on patients and care partners. It is imperative that healthcare practitioners maintain a high index of clinical suspicion for lymphoma to expedite the appropriate diagnostic testing and treatment pathways as well as reduce excess pressure on healthcare resources and ultimately improve outcomes for patients with DLBCL.

**Copyright Information:** (Online) ISSN: 2572-9241
©2026 The Author(s). HemaSphere is published by John Wiley & Sons Ltd on behalf of European Hematology Association. This is an open access Abstract Book distributed under the Attribution-NonCommercial-NoDerivs (CC BY-NC-ND), which allows third parties to download the articles and share them with others as long as they credit the author and the Abstract Book, but they cannot change the content in any way or use them commercially.
**Abstract Book Citations:** Authors, Title, HemaSphere, 2026;10:(S1):pages. The abstract book DOIs can be found on the "Author Information" page.
**Disclaimer:** Articles published in the journal HemaSphere exclusively reflect the opinions of the authors. The authors are responsible for all content in their abstracts including accuracy of the facts, statements, citing resources, etc.

### (PB3728) A MACHINE LEARNING-ASSISTED PROGNOSTIC MODEL FOR PREDICTING OUTCOMES IN B-CELL LYMPHOMA PATIENTS WITH CONCOMITANT HEMOPHAGOCYTIC LYMPHOHISTIOCYTOSIS: CONSTRUCTION AND INTERNAL VALIDATION

**Topic:** 19. Large B cell lymphomas - Clinical

Luocheng Zhang*[1, 2], Yang Dai[1, 3, 4], Xushu Zhong[1], Yu Wu[1, 3, 4], Xiao'ou Huang[1], Jie Huang[1], Hongbing MA[1], Pu Kuang[1], Xu Sun[1, 2], Qiaolin Zhou[1, 2], Wenyi Liang[1, 2], Mengting Yang[1, 2], Zhuohang Zou[1, 2], Yushuang Xiao[1, 2], Yijun Wu[1, 3, 4], Ailin Zhao[1, 3, 4], Ting Niu[1, 3, 4]

*[1]West China Hospital, Sichuan University., Department of Hematology, Institute of Hematology, and Center for High Altitude Medicine, West China Hospital, Sichuan University., Chengdu, China; [2]West China Medical School, West China Hospital, Sichuan University, Chengdu, China, Chengdu, China; [3]State Key Laboratory of Biotherapy, Collaborative Innovation Center of Biotherapy, West China Hospital, Sichuan University., Chengdu, China; [4]National Facility for Translational Medicine (Sichuan), West China Hospital, Sichuan University., Chengdu, China;*

### Background

B-cell lymphoma with concomitant hemophagocytic lymphohistiocytosis (HLH) represents a complex condition in which hyperinflammatory manifestations often obscure the underlying malignancy, complicating both diagnosis and treatment. Current diagnostic criteria lack the specificity to effectively distinguish malignant from non-malignant HLH, and no prognostic model exists specifically for this patient population. This highlights the urgent need for a biomarker-based tool to differentiate malignancy-associated HLH from non-malignant forms, enable accurate risk stratification, and guide targeted therapeutic strategies.

### Aims

To differentiate non-malignant HLH from B-cell lymphoma-associated HLH using readily accessible biomarkers; to develop and internally validate a prognostic model for B-cell lymphoma with concomitant HLH; and to evaluate its utility in guiding treatment escalation and lymphoma-directed therapy.

### Methods

This retrospective cohort study included 102 patients with HLH-onset B-cell lymphoma (79 EBV-negative, 23 EBV-positive) and 80 patients with EBV-negative non-malignant HLH treated at West China Hospital between 2019 and 2025. HLH was diagnosed according to the HLH-2004 criteria, and B-cell lymphoma was confirmed histologically by biopsy or flow cytometry. Clinical and laboratory parameters were systematically collected, with missing values addressed using multiple imputation. The IPATS Score was developed through LASSO-penalized Cox regression for variable selection, supplemented by XGBoost analysis to capture nonlinear prognostic relationships. Model discrimination was evaluated using the C-index and time-dependent AUC, with performance compared against the OHI index and established clinical models.

**Copyright Information:** (Online) ISSN: 2572-9241
©2026 The Author(s). HemaSphere is published by John Wiley & Sons Ltd on behalf of European Hematology Association. This is an open access Abstract Book distributed under the Attribution-NonCommercial-NoDerivs (CC BY-NC-ND), which allows third parties to download the articles and share them with others as long as they credit the author and the Abstract Book, but they cannot change the content in any way or use them commercially.
**Abstract Book Citations:** Authors, Title, HemaSphere, 2026;10:(S1):pages. The abstract book DOIs can be found on the "Author Information" page.
**Disclaimer:** Articles published in the journal HemaSphere exclusively reflect the opinions of the authors. The authors are responsible for all content in their abstracts including accuracy of the facts, statements, citing resources, etc.

**Results**

Key biomarkers, including globulin, soluble CD25 (sCD25), ferritin, cytopenias, and HDL, effectively discriminated between B-cell lymphoma patients with concomitant HLH and non-malignant HLH. Building on these findings, the IPATS Score, which incorporates IL-6, PCT, TC, age, and sex, demonstrated strong predictive performance with a C-index of 0.70 for overall survival (OS). The model exhibited good calibration and consistent performance across different time points, with AUCs ranging from 0.71 at 30 days to 0.81 at 365 days. Moreover, further risk stratification using the IPATS Score revealed a significant association between higher predicted risk and the use of more intensive HLH-directed therapy, as well as improved survival outcomes following lymphoma-directed treatment.

**Summary/Conclusion**

By integrating readily accessible biomarkers, it facilitates the differentiation of malignant from non-malignant HLH. The IPATS Score provides a robust, practical tool for early risk stratification in B-cell lymphoma patients with concomitant HLH, which is reinforced by its strong predictive accuracy and the association with improved survival following lymphoma-targeted therapy.

**Copyright Information:** (Online) ISSN: 2572-9241
©2026 The Author(s). HemaSphere is published by John Wiley & Sons Ltd on behalf of European Hematology Association. This is an open access Abstract Book distributed under the Attribution-NonCommercial-NoDerivs (CC BY-NC-ND), which allows third parties to download the articles and share them with others as long as they credit the author and the Abstract Book, but they cannot change the content in any way or use them commercially.
**Abstract Book Citations:** Authors, Title, HemaSphere, 2026;10:(S1):pages. The abstract book DOIs can be found on the "Author Information" page.
**Disclaimer:** Articles published in the journal HemaSphere exclusively reflect the opinions of the authors. The authors are responsible for all content in their abstracts including accuracy of the facts, statements, citing resources, etc.

### (PB3729) EFFICACY AND SAFETY OF THE POLATUZUMAB, ACALABRUTINIB, RITUXIMAB, AND LENALIDOMIDE (POLA-AR2) REGIMEN AS FIRST-LINE THERAPY IN UNFIT OR FRAIL PATIENTS WITH DIFFUSE LARGE B-CELL LYMPHOMA

**Topic:** 19. Large B cell lymphomas - Clinical

Qian Wang[1, 2], Tao You[1, 2], Zhou Jin[1, 2], Depei Wu[1, 2], Haiwen Huang*[1, 2]

*[1]The First Affiliated Hospital Of Soochow University, Hematology, Suzhou, China;[2]National Clinical Research Center for Hematologic Diseases, The First Affiliated Hospital of Soochow University., SUZHOU, China;*

### Background

Elderly patients often present with multiple comorbidities due to age-related decline in physiological function, leading to significantly reduced tolerance to conventional cytotoxic chemotherapy. This frequently results in severe adverse events such as myelosuppression, necessitating treatment interruption or dose reduction, which compromises efficacy. Recent breakthroughs in molecularly targeted therapies, characterized by their tumor cell-specific cytotoxicity and reduced systemic toxicity, offer superior and better-tolerated treatment options for elderly and frail patients, substantially improving their quality of life and prognosis.

### Aims

To develop and evaluate superior, better-tolerated treatment options for elderly and frail patients, with the ultimate goal of substantially improving their quality of life and clinical prognosis.

### Methods

This study reports on 20 patients newly diagnosed with diffuse large B-cell lymphoma (DLBCL). All patients, comprehensively assessed as unfit or frail and deemed unsuitable for standard chemotherapy regimens, received first-line treatment with the Pola-AR2 regimen. Treatment was administered every 21 days for up to 6 cycles, followed by acalabrutinib maintenance therapy for one year. The dosing schedule was as follows: Polatuzumab vedotin 1.8 mg/kg intravenously on Day 1 of each cycle; Rituximab 375 mg/m² on Day 2 of each cycle; Acalabrutinib 100 mg orally twice daily from Days 1 to 21; Lenalidomide 25 mg orally once daily from Days 1 to 10. Treatment response was assessed by positron emission tomography/computed tomography (PET/CT) after four cycles. Patients achieving complete response (CR) or partial response (PR) after 4 cycles received two additional cycles followed by end-of-treatment PET/CT assessment. Patients not achieving response after 4 cycles discontinued the regimen and received second-line therapy.

### Results

Between July 2024 and February 2026, 19 patients were treated with the Pola-AR2 regimen and were evaluable for efficacy. The median age was 79 years (range: 67–88), with 9 (47.4%) males and 10 (52.6%) females. According to the Ann Arbor staging system, two patients (10.5%) had stage I–II disease, while 17 (89.5%) had stage III–IV disease. Based on the International Prognostic Index (IPI), four patients (21.1%) scored 1–2, and 15 (78.9%) scored 3–5. Histologically, 13 patients (68.4%) had the non-germinal center B-cell (non-GCB) subtype, and 6 (31.6%) had the GCB subtype. Ki-67 expression was <90% in 12 patients (63.2%) and ≥90% in 7 patients (36.8%). Extranodal involvement was observed in 17 patients (89.5%), and six patients (31.6%) exhibited double-expression lymphoma (Table 1).To date, all 19 patients have completed six cycles of treatment and undergone response evaluation. Among them, 12 patients (63.2%) achieved complete response (CR), five patients (26.3%) achieved partial response (PR), and two patients experienced disease progression during treatment and were switched to alternative regimens. The overall response rate (ORR) was 89.5%, and the complete response rate (CRR) was 63.2% (Fig. 1). No significant adverse events, such as febrile neutropenia, severe vomiting, prolonged neutropenia, or organ dysfunction, were observed in any patient.

**Copyright Information:** (Online) ISSN: 2572-9241
©2026 The Author(s). HemaSphere is published by John Wiley & Sons Ltd on behalf of European Hematology Association. This is an open access Abstract Book distributed under the Attribution-NonCommercial-NoDerivs (CC BY-NC-ND), which allows third parties to download the articles and share them with others as long as they credit the author and the Abstract Book, but they cannot change the content in any way or use them commercially.
**Abstract Book Citations:** Authors, Title, HemaSphere, 2026;10:(S1):pages. The abstract book DOIs can be found on the "Author Information" page.
**Disclaimer:** Articles published in the journal HemaSphere exclusively reflect the opinions of the authors. The authors are responsible for all content in their abstracts including accuracy of the facts, statements, citing resources, etc.

**Summary/Conclusion**

The Pola-AR2 regimen demonstrates promising efficacy and a favorable safety profile as first-line therapy for elderly and frail patients with newly diagnosed DLBCL. Larger prospective studies are warranted to validate these findings.

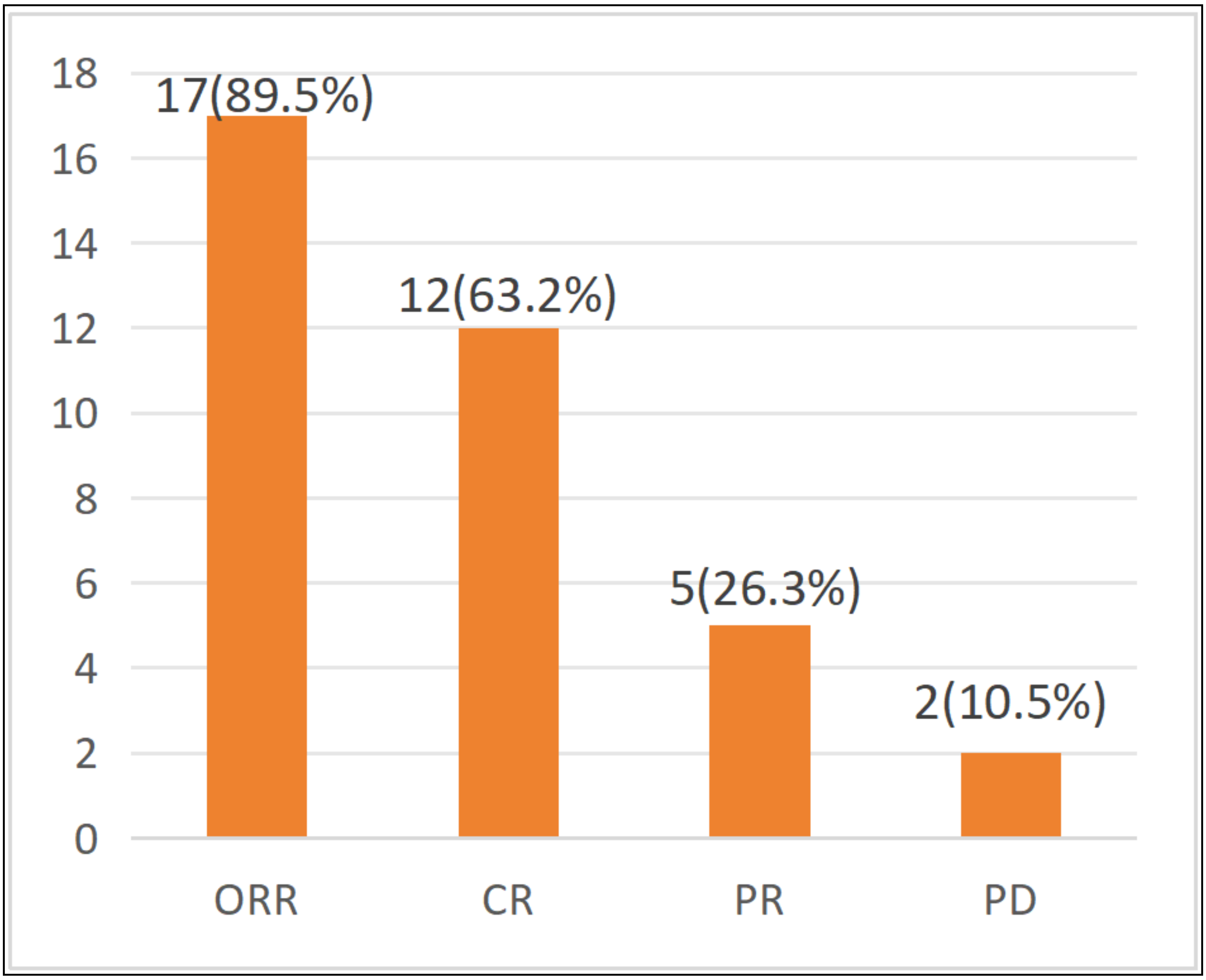

**Copyright Information:** (Online) ISSN: 2572-9241
©2026 The Author(s). HemaSphere is published by John Wiley & Sons Ltd on behalf of European Hematology Association. This is an open access Abstract Book distributed under the Attribution-NonCommercial-NoDerivs (CC BY-NC-ND), which allows third parties to download the articles and share them with others as long as they credit the author and the Abstract Book, but they cannot change the content in any way or use them commercially.
**Abstract Book Citations:** Authors, Title, HemaSphere, 2026;10:(S1):pages. The abstract book DOIs can be found on the "Author Information" page.
**Disclaimer:** Articles published in the journal HemaSphere exclusively reflect the opinions of the authors. The authors are responsible for all content in their abstracts including accuracy of the facts, statements, citing resources, etc.

### (PB3730) REAL-WORLD EFFICACY AND SAFETY OF POLATUZUMAB VEDOTIN-BASED REGIMENS IN TREATMENT-NAIVE DLBCL: A MULTICENTER STUDY FOCUSING ON BULKY DISEASE AND CD5 POSITIVITY

**Topic:** 19. Large B cell lymphomas - Clinical

Shu Zhao*[1], Wenhui Zhao[1], Lintao Bi[2], Libo Jiang[3], Xiaoling Li[4], Wei Wang[5], Xiaojing Xing[6], Hong Luo[7], Aijun Liao[8], Danhong Li[9], Ruiyang Sun[1], Qing-Yuan Zhang[1]

*[1]Harbin Medical University Cancer Hospital, Harbin, China;[2]China-Japan Union Hospital of Jilin University, Jilin, China;[3]The Second Affiliated Hospital of Qiqihar Medical University, Qiqihar, China; [4]Beidahuang Group General Hospital, Harbin, China; [5]The Second Affiliated Hospital of Harbin Medical University, Harbin, China;[6]Liaoning Cancer Hospital & Institute, Shenyang, China; [7]The First Hospital of Qiqihar, Qiqihar, China;[8]Shengjing Hospital of China Medical University, Shenyang, China;[9]Hongqi Hospital Affiliated to Mudanjiang Medical University, Mudanjiang, China;*

### Background

Diffuse large B-cell lymphoma (DLBCL) is a clinically and molecularly heterogeneous subtype of non-Hodgkin lymphoma. While Polatuzumab vedotin (Pola) plus R-CHP is a validated first-line therapy, real-world data regarding its efficacy in high-risk subgroups, particularly those with bulky disease or CD5 positivity, remains limited.

### Aims

To evaluate the clinical efficacy and tolerability of Pola-based regimens in patients with newly diagnosed DLBCL, with a focus on subgroups defined by bulky disease and CD5 positivity.

### Methods

A retrospective multicenter study enrolled newly diagnosed DLBCL patients from nine medical centers between April 1, 2023, and January 20, 2026. The majority (91.7%) received Pola-R-CHP, while the remaining 8.3% received Pola-R-CHP+Glofitamab. The primary endpoint was overall response rate (ORR) at the end of treatment. Secondary endpoints included complete response rate (CRR), progression-free survival (PFS), median overall survival (OS), and safety.

### Results

A total of 96 patients were included (median age 61 years, range 52–67), with a median of 6 treatment cycles (range 2–8). Baseline high-risk features included bulky disease (≥7.5cm) (17.7%, 17/96), CD5 positivity (32.1%, 26/81), Ann Arbor stage III/IV (70.8%), Ki-67 ≥80% (76.1%), and elevated lactate dehydrogenase (LDH) (53.1%). The ORR was 96.8%, with a CR rate of 63.4%. In subgroup analyses, ORR and CRR were 93.3% and 46.7% for patients with bulky disease, compared to 97.3% and 66.7% for those without. Among CD5-positive patients, ORR and CRR were 96.0% and 72.0%, versus 100% and 66.0% in CD5-negative patients. Among the 69 patients who completed six cycles of therapy, ORR was 98.6% and CR rate was 73.9%. The median follow-up was 11.98 months (range: 0.83–34.3 months). Median PFS was 31.3 months (95% CI: 21.6, not estimable [NE]), and median OS was 31.3 months (95% CI: 31.3, NE). In subgroup analyses, median PFS and OS were not reached in patients with bulky disease. Median PFS and OS in CD5-positive patients were 25.3 months and 31.1 months. The most common hematologic adverse events (AEs) were decreased lymphocyte count (75%), anemia (59.4%), neutropenia (58.3%), and thrombocytopenia (18.8%). Common non-hematologic AEs included elevated aminotransferases (43.8%), fatigue (37.5%), nausea (37.5%), vomiting (35.4%), and infection (32.3%). Most AEs were grade 1–2. Grade 3–4 AEs included decreased lymphocyte count (27.1%) and neutropenia (17.7%). No new safety signals were identified.

**Copyright Information:** (Online) ISSN: 2572-9241
©2026 The Author(s). HemaSphere is published by John Wiley & Sons Ltd on behalf of European Hematology Association. This is an open access Abstract Book distributed under the Attribution-NonCommercial-NoDerivs (CC BY-NC-ND), which allows third parties to download the articles and share them with others as long as they credit the author and the Abstract Book, but they cannot change the content in any way or use them commercially.
**Abstract Book Citations:** Authors, Title, HemaSphere, 2026;10:(S1):pages. The abstract book DOIs can be found on the “Author Information” page.
**Disclaimer:** Articles published in the journal HemaSphere exclusively reflect the opinions of the authors. The authors are responsible for all content in their abstracts including accuracy of the facts, statements, citing resources, etc.

**Summary/Conclusion**

Pola-based regimens demonstrate promising efficacy and acceptable safety in treatment-naive DLBCL patients. Subgroup analyses show comparable ORR, CRR, PFS and OS outcomes regardless of bulky disease status or CD5 expression, suggesting that Pola-based therapy may attenuate the adverse prognostic impact associated with these features. These findings support the use of Pola-based regimens as a valuable first-line treatment option. Larger, long-term studies are warranted for further validation.

**Copyright Information:** (Online) ISSN: 2572-9241
©2026 The Author(s). HemaSphere is published by John Wiley & Sons Ltd on behalf of European Hematology Association. This is an open access Abstract Book distributed under the Attribution-NonCommercial-NoDerivs (CC BY-NC-ND), which allows third parties to download the articles and share them with others as long as they credit the author and the Abstract Book, but they cannot change the content in any way or use them commercially.
**Abstract Book Citations:** Authors, Title, HemaSphere, 2026;10:(S1):pages. The abstract book DOIs can be found on the “Author Information” page.
**Disclaimer:** Articles published in the journal HemaSphere exclusively reflect the opinions of the authors. The authors are responsible for all content in their abstracts including accuracy of the facts, statements, citing resources, etc.

### (PB3731) REAL-WORLD OUTCOMES OF TAFA-LEN (TAFASITAMAB,LENALIDOMIDE) IN RELAPSED/REFRACTORY DIFFUSE LARGE B-CELL LYMPHOMA: INSIGHTS FROM THE POLISH LYMPHOMA RESEARCH GROUP (PLRG)

**Topic:** 19. Large B cell lymphomas - Clinical

Magdalena Olszewska-Szopa*[1], Magdalena Malicka[2], Anastazja Szlauer-Stefańska[3], Joanna Sciesinska[4], Joanna Drozd-Sokolowska[5], Anna Kopinska[6], Ewa Paszkiewicz-Kozik[7], Edyta Subocz[8], Mirosław Markiewicz[9], Ewa Wasik-Szczepanek[10], Agnieszka Giza[11], Agnieszka Kołkowska-Leśniak[12], Waldemar Sawicki[13], Artur Matyszewski[14], Monika Długosz-Danecka[15], Grzegorz Helbig[16], Grzegorz Basak[17], Tomasz Wróbel[1], Ewa Lech-Maranda[18], Anna Czyż[1]

*[1]Department and Clinic of Hematology, Cellular Therapies and Internal Medicine, Wroclaw Medical University, Wrocław, Poland; [2]Department of Hematology, Medical University of Lodz, Łódź, Poland;[3]Bone Marrow Transplantation and Oncohematology Department, Maria Sklodowska-Curie National Research Institute of Oncology (branch in Gliwice), Gliwice, Poland; [4]. Department of Hematology and Transplantology, Medical University of Gdansk, Gdańsk, Poland; [5]Department of Hematology, Oncology and Internal Medicine, Medical University of Warsaw, Warsaw, Warsaw, Poland; [6]Department of Hematology and Bone Marrow Transplantation, Faculty of Medicine in Katowice, Medical University of Silesia, Katowice, Katowice, Poland; [7]Maria Sklodowska-Curie National Research Institute of Oncology, Warsaw, Warsaw, Poland; [8]Department of Hematology, Warmian-Masurian Cancer Center of The Ministry Of The Interior And Administration's Hospital, Olsztyn, Poland; [9]Department of Hematology, Institute of Medical Sciences, College of Medical Sciences, University of Rzeszow, Rzeszów, Poland; [10]Department of Haematooncology and Bone Marrow Transplantation, Medical University in Lublin, Lublin, Poland; [11]Department of Hematology, Jagiellonian University Medical College, Kraków, Poland; [12]. Institute of Hematology and Transfusion Medicine, Warsaw, Warsaw, Poland; [13]Department of Internal Medicine and Hematology, Military Institute of Medicine-National Research Institute, Warsaw, Poland, Warsaw, Poland; [14]Department of Internal Medicine, Cardiology and Hypertensionr, L. Rydygier Province Hospital, Suwałki, Suwałki, Poland; [15]Maria Sklodowska-Curie National Research Institute of Oncology, Kraków, Poland;[16]Department of Hematology and Bone Marrow Transplantation, Faculty of Medicine in Katowice, Medical University of Silesia, Katowice, Poland; [17]Department of Hematology, Oncology and Internal Medicine, Medical University of Warsaw, Warsaw, Poland;[18]Institute of Hematology and Transfusion Medicine, Warsaw, Warsaw, Poland;*

### Background

This multicenter study conducted by the Polish Lymphoma Research Group (PLRG) evaluated real-world outcomes of tafasitamab plus lenalidomide (Tafa-Len) in patients with relapsed/refractory diffuse large B-cell lymphoma (R/R DLBCL). The regimen was given when T-cell–engaging therapies (TCE) were not available in second line for transplant-ineligible patients.

### Aims

To evaluate the efficacy of Tafa-Len in patients with R/R DLBCL in real-world clinical practice and to identify potential prognostic factors.

### Methods

In this retrospective analysis, 54 patients with R/R DLBCL treated with Tafa-Len in the second or subsequent lines were identified. Fourteen clinical and disease-related variables were analyzed as potential prognostic factors, including age, sex, ECOG performance status, DLBCL cell-of-origin subtype, clinical stage, IPI at diagnosis and at salvage, type of first-line failure (primary refractory disease vs relapse), number of prior treatment lines, refractoriness to the last therapy, disease status, bulky disease, and B symptoms at salvage.

**Copyright Information:** (Online) ISSN: 2572-9241
©2026 The Author(s). HemaSphere is published by John Wiley & Sons Ltd on behalf of European Hematology Association. This is an open access Abstract Book distributed under the Attribution-NonCommercial-NoDerivs (CC BY-NC-ND), which allows third parties to download the articles and share them with others as long as they credit the author and the Abstract Book, but they cannot change the content in any way or use them commercially.
**Abstract Book Citations:** Authors, Title, HemaSphere, 2026;10:(S1):pages. The abstract book DOIs can be found on the "Author Information" page.
**Disclaimer:** Articles published in the journal HemaSphere exclusively reflect the opinions of the authors. The authors are responsible for all content in their abstracts including accuracy of the facts, statements, citing resources, etc.

**Results**

The median age was 72 years (IQR 63–81). Primary refractory disease was present in 43 patients (81%). Patients had received a median of 2 prior lines of therapy (range 1–5). Tafa-Len was administered as second-line treatment in 22 patients with primary refractory disease (41%). Refractoriness to the last therapy was observed in 62% of patients. High-risk salvage IPI was present in 60% of patients, and bulky disease at salvage was reported in 18%.
The overall response rate (ORR) was 31%, including 15.5% complete responses (CR) and 15.5% partial responses (PR). ORR was significantly higher in patients with disease sensitive to the last therapy compared with those with refractory disease: CR, PR, and <PR were observed in 37%, 16%, and 47% of patients with sensitive disease versus 3%, 16%, and 81% in refractory cases (p=0.005). Moreover, in primary refractory disease the CR rate was lower than in relapsed cases (12% vs 30%), although the difference was not statistically significant (p=0.16).
After a median follow-up of 13 months (range 1–29), the median progression-free survival (PFS) was 3.7 months (IQR 1.7–10.9), and the median overall survival (OS) was 6.5 months (IQR 3.0–19.6). The only significant factor associated with shorter PFS was refractoriness to the last therapy line (HR 2.9, 95% CI 1.2–6.6). No significant prognostic factors for OS were identified.
Treatment-related toxicities, mainly neutropenia and infections led to at least 1 cycle delay/dose reduction in 19 patients.

**Summary/Conclusion**

In this PSM analysis of real-world data, Tafa-Len showed modest efficacy in relapsed DLBCL, with limited activity in patients refractory to the last line and a trend toward poorer outcomes in primary refractory disease. The regimen is intended for transplant-ineligible R/R DLBCL, and primary refractory cases were excluded from L-MIND. At that time, next-generation TCE therapies were not available in second line. As availability of CAR-T cell therapy and bispecific antibodies increases, TCE-based approaches should be prioritized early in primary refractory and early relapsing disease, consistent with outcomes from second-line CAR-T cell studies and the STARGLO trial. The precise role of Tafa-Len within current R/R DLBCL treatment pathways - which does not preclude CAR-T remains to be clearly defined, considering its meaningful efficacy and acceptable safety profile.

**Copyright Information:** (Online) ISSN: 2572-9241
©2026 The Author(s). HemaSphere is published by John Wiley & Sons Ltd on behalf of European Hematology Association. This is an open access Abstract Book distributed under the Attribution-NonCommercial-NoDerivs (CC BY-NC-ND), which allows third parties to download the articles and share them with others as long as they credit the author and the Abstract Book, but they cannot change the content in any way or use them commercially.
**Abstract Book Citations:** Authors, Title, HemaSphere, 2026;10:(S1):pages. The abstract book DOIs can be found on the “Author Information” page.
**Disclaimer:** Articles published in the journal HemaSphere exclusively reflect the opinions of the authors. The authors are responsible for all content in their abstracts including accuracy of the facts, statements, citing resources, etc.

### (PB3732) THE ROLE OF POLYMORPHISMS IN THE MYD88 AND CXCR4 GENES IN THE ETIOPATHOGENESIS OF THE DISEASE AND THEIR ASSOCIATIONS WITH PROGNOSIS IN PATIENTS WITH HODGKIN LYMPHOMA OR NON-HODGKIN LYMPHOMA

**Topic:** 19. Large B cell lymphomas - Clinical

Handan Haydaroglu Sahin*[1], Duriye PelIn Acel[2], Gulper Koyuncu[3]

*[1]Gaziantep University, Department of Hematology, Gaziantep, Türkiye;[2]Gaziantep University, Department of Internal Medicine, Gaziantep, Türkiye; [3]Gaziantep University, Department of Medical Biology, Gaziantep, Türkiye;*

### Background

Hodgkin (HL) and Non-Hodgkin lymphoma (NHL) are common hematopoietic malignancies worldwide. Many factors may underly HL and NHL, although these malignancies are known to be strongly associated with environmental and genetic. Over the past few years, mutations in the MYD88 and CXCR4 genes have been defined as drivers of many solid and hematological malignancies and therefore, identification of these mutations is of critical importance.

### Aims

This study aimed at determining the role of polymorphisms of the Myd88 and Cxcr4 genes in the etiopathogenesis of the disease, their potential associations with clinical parameters in patients with HL and NHL and treatment response in patients with these gene mutations.

### Methods

This study included 70 patients with HL and 70 patients with NHL who had been treated and were on follow up and 70 heathy controls. DNA was isolated from peripheral blood collected from each study participant. Genomic DNA isolated from blood underwent genotyping using real-time PCR and sequencing (Sanger sequencing) to identify specifically CXCR4 2228014-rs SNP and L265P SNP and MYD88 L265P SNP.

### Results

In patients with CXCR4 rs2228014 SNP, TT mutant genotype was identified in 13 patients HL (18.5%) and CC wild type genotype was identified in 57 (81.5%) patients with HL, whereas TT mutant genotype was identified in 11 (15.7%) patients with NHL and CC wild type genotype was identified in 59 (84.3%) patients with NHL. In patients with MYD88 L265P SNP mutations , CC mutant genotype was identified in 3 patients HL (4.2%) and TT wild type genotype was identified in 57 (95.7%) patients with HL whereas CC allele was identified in 5 (7.1%) patients with NHL and TT allele was identified in 65 (92.9%) patients with NHL. In conclusion, statistically significant associations were found between CC genotype of CXCR4 rs2228014 SNP and TT genotype of MYD88 L265P SNP and the occurrence of HL and NHL ($p<0.05$). Furthermore, significant differences were observed in disease progression between mutant CC genotype of MYD88 L265P ($\chi^2$=11.748, $p<0.001$) and mutant TT genotype of CXCR4 ($\chi^2$=28.475, $p<0.001$) in patients with NHL and mutant CC genotype of MYD88 L265P ($\chi^2$=9.055, $p$=0.015) and mutant TT genotype od CXCR4 ($\chi^2$=10.736, $p$=0.003) in patients with HL. The likelihood of disease progression was higher in the mutant MYDX88 and CXCR4 groups compared to the wild groups.

**Copyright Information:** (Online) ISSN: 2572-9241
©2026 The Author(s). HemaSphere is published by John Wiley & Sons Ltd on behalf of European Hematology Association. This is an open access Abstract Book distributed under the Attribution-NonCommercial-NoDerivs (CC BY-NC-ND), which allows third parties to download the articles and share them with others as long as they credit the author and the Abstract Book, but they cannot change the content in any way or use them commercially.
**Abstract Book Citations:** Authors, Title, HemaSphere, 2026;10:(S1):pages. The abstract book DOIs can be found on the "Author Information" page.
**Disclaimer:** Articles published in the journal HemaSphere exclusively reflect the opinions of the authors. The authors are responsible for all content in their abstracts including accuracy of the facts, statements, citing resources, etc.

**Summary/Conclusion**

Our study demonstrated that MYD88 L265P and CXCR4 rs2228014 polymorphisms might play a role in the pathogenesis of NHL and HL and these polymorphisms may be used as prognostic markers of clinical outcomes in patients with NHL and HL.

**Copyright Information:** (Online) ISSN: 2572-9241
©2026 The Author(s). HemaSphere is published by John Wiley & Sons Ltd on behalf of European Hematology Association. This is an open access Abstract Book distributed under the Attribution-NonCommercial-NoDerivs (CC BY-NC-ND), which allows third parties to download the articles and share them with others as long as they credit the author and the Abstract Book, but they cannot change the content in any way or use them commercially.
**Abstract Book Citations:** Authors, Title, HemaSphere, 2026;10:(S1):pages. The abstract book DOIs can be found on the "Author Information" page.
**Disclaimer:** Articles published in the journal HemaSphere exclusively reflect the opinions of the authors. The authors are responsible for all content in their abstracts including accuracy of the facts, statements, citing resources, etc.

### (PB3733) EFFICACY AND SAFETY OF EFBEMALENOGRASTIM ALFA IN THE TREATMENT OF NEUTROPENIA INDUCED BY R-CHOP-LIKE REGIMENS IN NEWLY DIAGNOSED NON-HODGKIN'S LYMPHOMA PATIENTS CLINICAL STUDY

**Topic:** 19. Large B cell lymphomas - Clinical

MA Jun[1], Donglu Zhao*[1], Hui-Lai Zhang[2], Qinhua Liu[3], Hong Cen[4], Lihong Liu[5], Liping Su[6], Hui Yu[7], Yao Liu[8], Haiyan Yang[9], Jin Zhang[10], Xiaotao Wang[11], Ou Bai[12], Heng Mei[13], Wuping Li[14], Yajun Li[15]

*[1]Harbin Institute of Hematology and Oncology, Harbin First Hospital, Harbin, China;[2]Tianjin Medical University Cancer Institute and Hospital, National Clinical Research Center for Cancer; Key Laboratory of Cancer Prevention and Therapy, Tianjin; Tianjin's Clinical Research Center for Cancer, Tianjin, China.Tianjin Medical University Cancer Institute and Hospital, Tianjin, China; [3]First Affiliated Hospital of Anhui Medical University, hefei, China;[4]Guangxi Medical University Affiliated Cancer Hospital, nanning, China; [5]The Fourth Hospital of Hebei Medical University, shijiazhuang, China;[6]Department of Hematology, Shanxi Tumor Hospital Affiliated to Shanxi Medical University, taiyuan, China; [7]The First Affiliated Hospital Of Zhengzhou University, Zhengzhou, China; [8]Chongqing University Cancer Hospital, Chongqing, China., chongqing, China; [9]Zhejiang Cancer Hospital, Hangzhou, China; [10]Run Run Shaw Hospital Affiliated to Medical College of Zhejiang University, Hangzhou, China;[11]The First Affiliated Hospital of Guilin Medical University,GuilinChina, Guilin, China; [12]Department of Haematology, The First Hospital of Jilin University, Changchun, China; [13]Union Hospital, Tongji Medical College, Huazhong University of Science and Technology, Wuhan, China; [14]Jiangxi Cancer Hospital, Nanchang, China;[15]Hunan Cancer Hospital and The Affiliated Cancer Hospital of Xiangya School of Medicine, Central South University, changsha, China;*

### Background

The R-CHOP-like regimen is currently the first-line treatment of choice for non-Hodgkin's lymphoma. However, myelosuppression is one of the main adverse reactions associated with this chemotherapy regimen, and severe cases may be accompanied by the occurrence of febrile neutropenia (FN). Efbemalenograstim a is anovelong-acting G-CSF approved by FDA in 2023, but there have been no reported data on its use in newly diagnosed non-Hodgkin's lymphoma patients to date.

### Aims

The study (ChiCTR2400080573) aims to evaluate the effectiveness and safety of Efbemalenograstim alfa in preventing neutropenia induced by R-CHOP-like treatment regimens in patients with non-Hodgkin's lymphoma.

### Methods

This was a multicenter, open-lable, randomized controlled, two-stage clinical study, in the first stage of the study enroll 20 patients who will undergo at least 6 cycles of chemotherapy, with efbemalenograstim alfa administered as primary prophylaxis. If ≤ 6 patients experience any events of special interest refer to neutrophil levels $\geq 20\times10^9$/L before the start of the second or third chemotherapy cycle (days -3 to 0) , the study will proceed to second stage. 108 patients will be randomized in 2:1. The experimental group will receive 20mg efbemalenograstim alfa within 24-48 hours after R-CHOP-like chemotherapy. In the control group, if the absolute neutrophil count (ANC) $\leq 1.0\times10^9$/L, patients may receive rhG-CSF(5μg/kg/day) until the ANC$\geq 5\times10^9$/L. Additionally, if FN or dose-limiting neutropenia occurs, the use of efbemalenograstim alfa for secondary prophylaxis is permitted based on institutional practice. The primary endpoint is the incidence of grade ≥3 neutropenia (ANC $<1.0\times10^9$/L) during the first cycle of chemotherapy. The safety profile was evaluated.

**Copyright Information:** (Online) ISSN: 2572-9241
©2026 The Author(s). HemaSphere is published by John Wiley & Sons Ltd on behalf of European Hematology Association. This is an open access Abstract Book distributed under the Attribution-NonCommercial-NoDerivs (CC BY-NC-ND), which allows third parties to download the articles and share them with others as long as they credit the author and the Abstract Book, but they cannot change the content in any way or use them commercially.
**Abstract Book Citations:** Authors, Title, HemaSphere, 2026;10:(S1):pages. The abstract book DOIs can be found on the "Author Information" page.
**Disclaimer:** Articles published in the journal HemaSphere exclusively reflect the opinions of the authors. The authors are responsible for all content in their abstracts including accuracy of the facts, statements, citing resources, etc.

**Results**

As of Nov 30, 2025, 92 newly diagnosed non-Hodgkin's lymphoma patients were enrolled. In stage 1, 22 patients were enrolled, with a median age of 62 years (range: 39-77). 3 patients were withdrawn from the study. The remaining 19 patients continued in the study, and no events of special interest were reported for any of these patients during the first phase.
In stage 2, 70 patients were randomized to the experimental group (prevention group n=47) and the control group (non-prevention group n= 23). The median age in the prevention group was 59 years (24-79), and 59.5 years (34-78) in controls. The primary endpoint was the incidence of grade ≥3 neutropenia (ANC <1.0 × $10^9$/L) in cycle 1, was 38.3% in the prevention group vs 73.91% in controls (P=0.0051) . Secondary endpoints the incidence of FN was 2.13% in the prevention group vs 17.39% in controls (P=0.0372); ANC nadir was 1.91×$10^9$/L (IQR 0.53-4.62) in the prevention group, 0.38×$10^9$/L (IQR 0.10-1.32) in controls; median recovery time to ANC ≥2.0×$10^9$/L was 2 (IQR 0-5) days in the prevention group vs 4(IQR 3-5) days ; In cycles 2-6, the incidence of ANC ≥ grade 3 was 42.22% (19/45) in the prevention group and 40% (8/20) in controls(P=0.8667), with no significant difference(non-prevention group patients were allowed to use Efbemalenograstim alfa for secondary prophylaxis according to institutional practice if who occurred FN or dose-limiting neutropenia).
Efbemalenograstim alfa was well-tolerated,with grade≥3 treatment-related adverse events (TRAE) in 8.51% of patients.

**Summary/Conclusion**

Using efbemalenograstim alfa for the primary prophylaxis of neutropenia induced by R-CHOP-like treatment regimens in patients with newly diagnosed non-Hodgkin's lymphoma is effective and safe.

**Copyright Information:** (Online) ISSN: 2572-9241
©2026 The Author(s). HemaSphere is published by John Wiley & Sons Ltd on behalf of European Hematology Association. This is an open access Abstract Book distributed under the Attribution-NonCommercial-NoDerivs (CC BY-NC-ND), which allows third parties to download the articles and share them with others as long as they credit the author and the Abstract Book, but they cannot change the content in any way or use them commercially.
**Abstract Book Citations:** Authors, Title, HemaSphere, 2026;10:(S1):pages. The abstract book DOIs can be found on the “Author Information” page.
**Disclaimer:** Articles published in the journal HemaSphere exclusively reflect the opinions of the authors. The authors are responsible for all content in their abstracts including accuracy of the facts, statements, citing resources, etc.

| **Efficacy** | **C1** | | |
|---|---|---|---|
| | **prevention group** | **non-prevention group** | P value |
| **N** | 47 | 23 | |
| **The incidence of grade ⩾3 neutropenia ,n(%)** | 18（38.3%） | 17（73.91%） | 0.0051 |
| **The incidence of FN** | 1 (2.13%) | 4 (17.39%) | 0.0372 |
| **The nadir of ANC, median (IQR)** | 1.91 (0.53-4.62) | 0.38 (0.10-1.32) | 0.0012 |
| **The time required to recover from nadir to 2.0×$10^9$/L , median (IQR), days** | 2 (0-5) | 4 (3-5) | 0.0225 |
| | **C2-C6** | | |
| | **prevention group** | **non-prevention group** | P value |
| **N** | 45 | 20 | |
| **The incidence of grade ⩾3 neutropenia ,n(%)** | 19 (42.22) | 8 (40.00) | 0.8667 |
| **Safety** | **prevention group** | **non-prevention group** | |
| **N** | 47 | 23 | |
| **Incidence of TEAE** | 44 (93.62) | 21 (91.30) | |
| **The incidence of grade ⩾3 TEAE** | 34 (72.34) | 20 (86.96) | |
| **Incidence of TRAE** | 28 (59.57) | 9 (39.13) | |
| **The incidence of grade ⩾3 TRAE** | 4 (8.51) | 0 (0.00) | |
| **The incidence of SAE** | 12 (25.53) | 5 (21.74) | |

The efficacy and safety of efbemalenograstim alfa in preventing neutropenia

**Copyright Information:** (Online) ISSN: 2572-9241
©2026 The Author(s). HemaSphere is published by John Wiley & Sons Ltd on behalf of European Hematology Association. This is an open access Abstract Book distributed under the Attribution-NonCommercial-NoDerivs (CC BY-NC-ND), which allows third parties to download the articles and share them with others as long as they credit the author and the Abstract Book, but they cannot change the content in any way or use them commercially.
**Abstract Book Citations:** Authors, Title, HemaSphere, 2026;10:(S1):pages. The abstract book DOIs can be found on the "Author Information" page.
**Disclaimer:** Articles published in the journal HemaSphere exclusively reflect the opinions of the authors. The authors are responsible for all content in their abstracts including accuracy of the facts, statements, citing resources, etc.

### (PB3734) OUTCOME OF RELAPSED OR REFRACTORY LARGE B-CELL LYMPHOMA ADMINISTERED WITH CAR T-CELL ALONE OR IN COMBINATION WITH ASCT: A PROPENSITY SCORE MATCHING COHORT STUDY

**Topic:** 19. Large B cell lymphomas - Clinical

Fan Yang*[1], Rui Liu[1], Zhonghua Fu[1], Danyang Li[1], Yuelu Guo[1], Lixia MA[1], Miaomiao Cao[1], Biping Deng[2], Shilin Gan[3], Xiaoyan Ke[1], Kai Hu[1]

*[1]Beijing Gobroad Hospital, Department of Lymphoma and Myeloma Research Center, Beijing, China; [2]Beijing Gobroad Boren Hospital, Cytology Laboratory, Beijing, China; [3]GoBroad Research Center, Beijing, China;*

### Background

Anti-CD19 chimeric antigen receptor T-cell therapy approved for relapsed or refractory large B-cell lymphoma (RR-LBCL) after at least two treatment lines.Autologous hematopoietic stem cell transplantation (ASCT) combined with CAR-T cell therapy is effective against multiple subtypes of RR-LBCL.

### Aims

This study conducted a retrospective analysis of the treatment efficacy and survival of the patients who underwent CD19 CAR-T therapy and ASCT combined with CAR-T cell therapy (ASCT+CAR-T) in RR-LBCL.

### Methods

This single-center, propensity score matching (PSM) cohort study analyzed 110 R/R LBCL patients. 27 patients received (ASCT) combined with CAR-T cell therapy, matched 1:3 with 83 patients receiving CART alone based on 9 baseline characteristics (Table 1). The two groups were well balanced across all baseline characteristics after PSM, and were appropriate for further comparative analysis of outcomes.Patients who had previously received CAR-T therapy or ASCT were excluded.
The conditioning regimen for the ASCT+CAR-T group was standard BEAM (bis-carmustine, etoposide, cytarabine, and melphalan) , while CAR-T cells were transfused within two days after transfusion of CD34+ HSCs in ASCT+CAR-T .

### Results

The dose of CD34+ hematopoietic stem cells (HSCs) ranged from 4.6 to 8.5×10^6/kg in ASCT+CAR-T group .The median numbers of CAR-T cell reinfusions were 2.2×10^6/kg (range: 0.3-4.5) and 2.8×10^6/kg (range: 0.4-3.5) in the CAR-T group and ASCT+CAR-T group, respectively.Stem cell engraftment in the ASCT+CAR-T group was unaffected.CAR T-cell expansion did not appear to differ in terms of therapeutic efficacy.In the ASCT+CART and the CART group, the incidences of grade ≥3 CRS was 12% vs. 6.8%, respectively, while the incidences of grade ≥3 ICANS was 0 vs. 1.4%, respectively.
A response at 3 month occurred in 66.7% of the patients in the ASCT+CART group and in 57.7% of those in the CART group (P=0.497) (with a complete response in 63% and 43.6%, respectively.(P=0.117).The median follow-up period was 31.9 months (95% CI: 0.23,71.62).The 3-year PFS and OS rates for the overall population were 41.46%(95% CI: 31.63,50.99) and 53.36%(95% CI: 44.66,64.80) respectively. Patients who received ASCT+CART had an improving trend in PFS [3-year PFS 57.72%(95% CI: 36.33,74.21)vs. 35.77%(95% CI: 24.87,46.77); P=0.067] ,and OS [3-year OS 74.07%(95% CI: 53.19,86.70) vs. 48.23%(95% CI: 35.60,59.76); P=0.058] compared to patients in the CAR-T group. (Fig.1A,B).

**Copyright Information:** (Online) ISSN: 2572-9241
©2026 The Author(s). HemaSphere is published by John Wiley & Sons Ltd on behalf of European Hematology Association. This is an open access Abstract Book distributed under the Attribution-NonCommercial-NoDerivs (CC BY-NC-ND), which allows third parties to download the articles and share them with others as long as they credit the author and the Abstract Book, but they cannot change the content in any way or use them commercially.
**Abstract Book Citations:** Authors, Title, HemaSphere, 2026;10:(S1):pages. The abstract book DOIs can be found on the "Author Information" page.
**Disclaimer:** Articles published in the journal HemaSphere exclusively reflect the opinions of the authors. The authors are responsible for all content in their abstracts including accuracy of the facts, statements, citing resources, etc.

### Summary/Conclusion

Compared to CAR-T therapy alone, the combination of ASCT and CAR-T therapy showed a trend of improved survival outcomes in patients with relapsed/refractory (R/R) large B-cell lymphoma. However, a larger study is required to confirm these findings.

Table 1 Baseline characteristics-PSM

| Variable | CAR-T (N=83) | ASCT+CAR-T (N=27) | Overall (N=110) | P value |
|---|---|---|---|---|
| Sex | | | | |
| Male | 50(60.2) | 15(55.6) | 65(59.1) | 0.822 |
| Female | 33(39.8) | 12(44.4) | 45(40.9) | |
| Age (years) | | | | |
| ≤60 | 71(85.5) | 23(85.2) | 94(85.5) | 1.000 |
| >60 | 12(14.5) | 4(14.8) | 16(14.5) | |
| Histologic subtype | | | | |
| DLBCL | 75(90.4) | 23(85.2) | 98(89.1) | 0.654 |
| GCB | 35(42.2) | 13(48.1) | 48(43.6) | 0.658 |
| Non-GCB | 48(57.8) | 14(51.9) | 62(56.4) | |
| HGBL | 3(3.6) | 2(7.4) | 5(4.5) | |
| PMBL | 5(6.0) | 2(7.4) | 7(6.4) | |
| IPI | | | | |
| <3 | 57(68.7) | 20(74.1) | 77(70.0) | 0.639 |
| ≥3 | 26(31.3) | 7(25.9) | 33(30.0) | |
| ECOG | | | | |
| <2 | 58(69.9) | 21(77.8) | 79(71.8) | 0.473 |
| ≥2 | 25(30.1) | 6(22.2) | 31(28.2) | |
| Disease stage | | | | |
| III or IV | 74(89.2) | 23(85.2) | 97(88.2) | |
| Cycles of therapy | | | | |
| ≤3 | 64(77.1) | 22(81.5) | 86(78.2) | 0.791 |
| >3 | 19(22.9) | 5(18.5) | 24(21.8) | |
| Previous local irradiation | | | | |
| Yes | 14(16.9) | 2(7.4) | 16(14.5) | 0.348 |
| No | 69(83.1) | 25(92.6) | 94(85.5) | |
| Disease status prior to CAR-T therapy | | | | |
| CR/PR | 46(55.4) | 18(66.7) | 64(58.2) | 0.372 |
| SD/PD | 37(44.6) | 9(33.3) | 46(41.8) | |

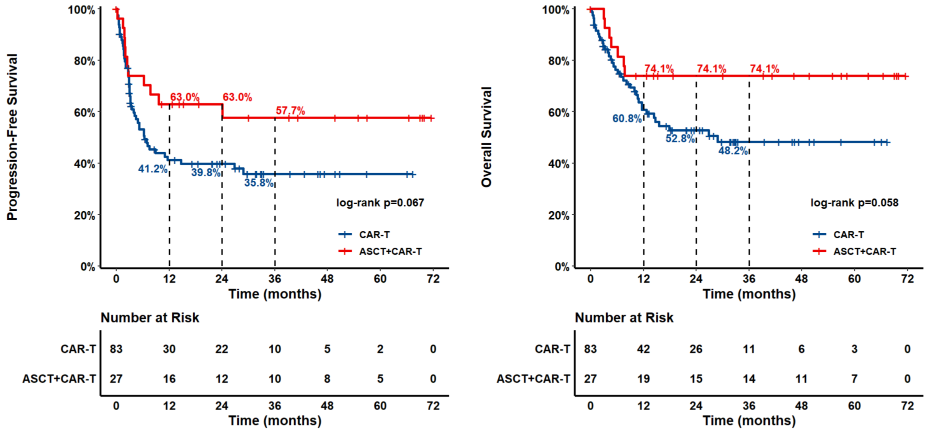

A B

Figure 1 Kaplan-Meier curves comparing progression-free survival (A), overall survival (B) between patients with relapsed/refractory large B-cell lymphoma (RR-LBCL) treated with CD19 CAR-T therapy versus ASCT combined with CAR-T (ASCT+CAR-T) by PSM.

**Copyright Information:** (Online) ISSN: 2572-9241
©2026 The Author(s). HemaSphere is published by John Wiley & Sons Ltd on behalf of European Hematology Association. This is an open access Abstract Book distributed under the Attribution-NonCommercial-NoDerivs (CC BY-NC-ND), which allows third parties to download the articles and share them with others as long as they credit the author and the Abstract Book, but they cannot change the content in any way or use them commercially.
**Abstract Book Citations:** Authors, Title, HemaSphere, 2026;10:(S1):pages. The abstract book DOIs can be found on the "Author Information" page.
**Disclaimer:** Articles published in the journal HemaSphere exclusively reflect the opinions of the authors. The authors are responsible for all content in their abstracts including accuracy of the facts, statements, citing resources, etc.

### (PB3735) A SYSTEMATIC ASSESSMENT OF DLBCL CELL-OF-ORIGIN CLASSIFICATION PERFORMANCE EVALUATING NON-INFERIORITY OF HANS IHC TO GEP

**Topic:** 19. Large B cell lymphomas - Clinical

Ash Alizadeh*[1], Anirban Basu[2], Shady Gendy[3], Brad Thomas[3], Manikanta Dasari[4], Girish Venkataraman[5]

*[1]Stanford University School of Medicine, Division of Oncology, Department of Medicine, Stanford Cancer Institute and Institute of Stem Cell Biology & Regenerative Medicine, Stanford, CA, United States of America; [2]University of Washington, The CHOICE Institute, Seattle, WA, United States of America; [3]AstraZeneca, Gaithersburg, MD, United States of America; [4]Indence Health, Kolkata, India; [5]University of Chicago Medicine, Department of Pathology, Chicago, IL, United States of America;*

### Background

Multiple international guidelines deem Cell-of-origin (COO) classification essential for diagnosis of diffuse large-B-cell lymphoma, not otherwise specified (DLBCL-NOS). Yet despite established prognostic value and potential predictive value for therapy selection, there is no consensus on the optimal method. Gene expression profiling (GEP) on fresh-frozen (FF) or formalin-fixed paraffin-embedded (FFPE) tissue is often viewed as the reference, but universal GEP is hampered by access and tissue constraints. In contrast, easy access to FFPE, a simpler platform, and a quick turnaround time have favored Immunohistochemistry (IHC) testing by Hans Algorithm (CD10/BCL6/MUM1), although its performance vs GEP remains debated, and prior comparisons are constrained by key methodological limitations. Reported accuracy varies widely across studies due to differences in study design and patient population, inclusion of heterogeneous pathologic subtypes (beyond pure DLBCL-NOS), frequent differential verification bias, particularly when GEP-unclassified cases are excluded, as well as reliance on a single GEP platform as the comparator despite documented platform and specimen variability.

### Aims

We established “ENHANSEMENT”, a novel meta-analytical framework for direct, case-level comparisons of COO classification performance in DLBCL-NOS by Hans IHC vs pooled GEP. The primary endpoint was to test non-inferiority (NI) of diagnostic accuracy for Hans vs GEP at a prespecified margin (10%). Secondary objectives evaluated non-Germinal Center B-Cell Like sensitivity, specificity, and positive/negative predictive value (PPV/NPV).

### Methods

We conducted a systematic literature review to identify studies reporting case-level COO by Hans and/or GEP in DLBCL-NOS, using PRISMA-DTA (Preferred Reporting Items for a Systematic Review and Meta-analysis of Diagnostic Test Accuracy Studies) guidance. Two complementary datasets were assembled: (i) studies comparing Hans IHC to GEP, and (ii) studies comparing GEP platforms; both datasets were reviewed for completeness by a consortium of domain experts. With no perfect gold standard, we used a Bayesian latent class model as the primary approach to jointly synthesize both evidence streams, treat true COO as latent, and estimate Hans and pooled GEP performance while accounting for study and platform-level heterogeneity. Hans was defined as non-inferior to GEP if the upper bound of the 95% credible interval (CrI) for the difference in performance metrics did not exceed the prespecified 10% NI margin, aligned with accepted FFPE/FF GEP variability, College of American Pathologists’ ≥90% concordance benchmark, diagnostic NI precedents and reviews reporting ~10% NI margins.

**Copyright Information:** (Online) ISSN: 2572-9241
©2026 The Author(s). HemaSphere is published by John Wiley & Sons Ltd on behalf of European Hematology Association. This is an open access Abstract Book distributed under the Attribution-NonCommercial-NoDerivs (CC BY-NC-ND), which allows third parties to download the articles and share them with others as long as they credit the author and the Abstract Book, but they cannot change the content in any way or use them commercially.
**Abstract Book Citations:** Authors, Title, HemaSphere, 2026;10:(S1):pages. The abstract book DOIs can be found on the “Author Information” page.
**Disclaimer:** Articles published in the journal HemaSphere exclusively reflect the opinions of the authors. The authors are responsible for all content in their abstracts including accuracy of the facts, statements, citing resources, etc.

### Results

19 studies (Fig.1) comprising 2,247 tumors were included: 16 IHC-GEP, 8 GEP-GEP, 4 both. Using latent truth as reference, accuracy was 90% (95%CrI:87-93) for Hans and 95% (93-96) for GEP. The difference of 4.5% (1-8) met the 10% NI margin. Secondary endpoints including specificity and PPV also met the NI margin, while sensitivity and NPV just exceeded the margin with difference of 6.5% (2-11) and 6.4% (2-11), respectively.

### Summary/Conclusion

In this largest meta-analysis of DLBCL-NOS classification methods, COO classification by Hans IHC is non-inferior to GEP at the prespecified margin for diagnostic accuracy, reinforcing the widely used and broadly accessible Hans IHC as a reliable method for guideline-recommended COO classification in DLBCL-NOS.

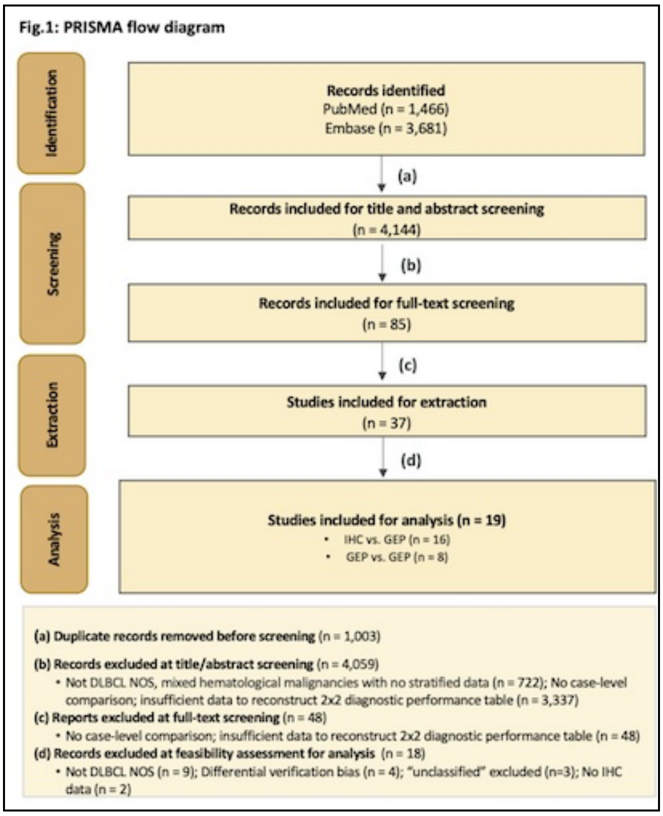

Fig.1: PRISMA flow diagram

**Copyright Information:** (Online) ISSN: 2572-9241
©2026 The Author(s). HemaSphere is published by John Wiley & Sons Ltd on behalf of European Hematology Association. This is an open access Abstract Book distributed under the Attribution-NonCommercial-NoDerivs (CC BY-NC-ND), which allows third parties to download the articles and share them with others as long as they credit the author and the Abstract Book, but they cannot change the content in any way or use them commercially.
**Abstract Book Citations:** Authors, Title, HemaSphere, 2026;10:(S1):pages. The abstract book DOIs can be found on the "Author Information" page.
**Disclaimer:** Articles published in the journal HemaSphere exclusively reflect the opinions of the authors. The authors are responsible for all content in their abstracts including accuracy of the facts, statements, citing resources, etc.

### (PB3736) PHASE 1 STUDY OF ARV-393, A PROTAC BCL6 DEGRADER, AS MONOTHERAPY IN PATIENTS WITH ADVANCED NON-HODGKIN LYMPHOMA (NHL) OR COMBINED WITH GLOFITAMAB IN PATIENTS WITH DIFFUSE LARGE B-CELL LYMPHOMA (DLBCL)

**Topic:** 19. Large B cell lymphomas - Clinical

Sarit Assouline*[1], Andrew Zelenetz[2], Jacob Haaber Christensen[3], Almudena Cascales Hernandez[4], Luis Malpica[5], Shalin Kothari[6], Dipenkumar Modi[7], Miguel Ángel Canales Albendea[8], Damian Cubillas[9], Alejandro Martín García-Sancho[10], Catherine Diefenbach[11], John Kuruvilla[12], Paolo F Caimi[13], Krish Patel[14], Sean Landrette[15], Eric Zhi[15], Yuanyuan Zhang[15], Roland Meier[15], Matthew Matasar[16], Martin Hutchings[17]

*[1]McGill University, Montreal, Canada;[2]Memorial Sloan Kettering Cancer Center, New York, United States of America;[3]Odense University Hospital, Odense, Denmark; [4]Hospital Clínico Universitario Virgen De La Arrixaca, Murcia, Spain;[5]The University of Texas MD Anderson Cancer Center, Houston, United States of America; [6]Yale School of Medicine and Yale Cancer Center, New Haven, United States of America; [7]Karmanos Cancer Institute, Wayne State University, Detroit, United States of America;[8]Clínica Universidad de Navarra, Pamplona, Spain; [9]START Center for Cancer Research, Madrid, Spain; [10]Hospital Universitario de Salamanca, Salamanca, Spain; [11]Laura and Isaac Perlmutter Cancer Center, New York University Grossman School of Medicine, New York, United States of America; [12]Princess Margaret Cancer Centre, Toronto, Canada; [13]Cleveland Clinic, Cleveland, United States of America; [14]Sarah Cannon Research Institute, Nashville, United States of America; [15]Arvinas Operations, Inc., New Haven, United States of America; [16]Rutgers Cancer Institute of New Jersey, New Brunswick, United States of America; [17]Rigshospitalet and University of Copenhagen, Copenhagen, Denmark;*

### Background

Despite recent advancements in NHL therapies, many patients experience disease progression or relapse. Agents with novel mechanisms of action and combination strategies are needed to improve clinical outcomes. B-cell lymphoma 6 (BCL6) is a master transcriptional regulator of immune cells, particularly of germinal center B cells, and an established oncogenic driver in NHL. ARV-393 is an oral PROteolysis TArgeting Chimera (PROTAC) BCL6 degrader that binds an E3 ubiquitin ligase and BCL6 to induce ubiquitination of BCL6 and its subsequent proteasomal degradation. ARV-393 monotherapy potently inhibited tumor growth and induced tumor regressions across NHL cell-derived xenograft (CDX) and patient-derived xenograft models, including models of DLBCL, transformed follicular lymphoma, and nodal T-follicular helper cell lymphoma (nTFHL). Furthermore, administration of ARV-393 with a CD20×CD3 bispecific antibody, glofitamab, demonstrated combinatorial antitumor activity in a humanized high-grade B-cell lymphoma CDX model, inducing deeper tumor growth inhibition and increased tumor regressions vs either monotherapy. These preclinical findings demonstrated single-agent ARV-393 antitumor activity across NHL subtypes and suggested mechanistic synergy with glofitamab.

### Aims

To clinically evaluate ARV-393 monotherapy in patients with NHL and ARV-393 in combination with glofitamab in patients with DLBCL, this global, multicenter, open-label, first-in-human, phase 1 dose escalation and optimization/expansion study (NCT06393738) is assessing the safety, tolerability, pharmacokinetics, pharmacodynamics, and preliminary antitumor activity of ARV-393 as monotherapy in relapsed or refractory (R/R) NHL or in combination with glofitamab in R/R DLBCL, with a primary objective of determining provisional doses for further exploration.

**Copyright Information:** (Online) ISSN: 2572-9241
©2026 The Author(s). HemaSphere is published by John Wiley & Sons Ltd on behalf of European Hematology Association. This is an open access Abstract Book distributed under the Attribution-NonCommercial-NoDerivs (CC BY-NC-ND), which allows third parties to download the articles and share them with others as long as they credit the author and the Abstract Book, but they cannot change the content in any way or use them commercially.
**Abstract Book Citations:** Authors, Title, HemaSphere, 2026;10:(S1):pages. The abstract book DOIs can be found on the “Author Information” page.
**Disclaimer:** Articles published in the journal HemaSphere exclusively reflect the opinions of the authors. The authors are responsible for all content in their abstracts including accuracy of the facts, statements, citing resources, etc.

**Methods**

Patients eligible for ARV-393 monotherapy are adults with confirmed R/R B-cell NHL who previously received ≥2 lines of systemic therapy (including rituximab), or nTFHL who previously received standard of care therapy. Patients eligible for ARV-393 in combination with glofitamab are adults with pathologically confirmed R/R DLBCL, DLBCL not otherwise specified, or large B-cell lymphoma arising from follicular lymphoma who were treated with ≥2 prior lines of systemic therapy. ARV-393 will be administered orally once daily in 28-day cycles alone or in combination with intravenous glofitamab during 21-day cycles. Approximately 255 patients will be enrolled across study cohorts.

**Results**

As of January 2026, enrollment is ongoing.

**Summary/Conclusion**

This study will provide data on the tolerability and preliminary antitumor activity of the PROTAC BCL6 degrader ARV-393 administered as monotherapy or in a chemotherapy-free combination with glofitamab.

**Copyright Information:** (Online) ISSN: 2572-9241
©2026 The Author(s). HemaSphere is published by John Wiley & Sons Ltd on behalf of European Hematology Association. This is an open access Abstract Book distributed under the Attribution-NonCommercial-NoDerivs (CC BY-NC-ND), which allows third parties to download the articles and share them with others as long as they credit the author and the Abstract Book, but they cannot change the content in any way or use them commercially.
**Abstract Book Citations:** Authors, Title, HemaSphere, 2026;10:(S1):pages. The abstract book DOIs can be found on the “Author Information” page.
**Disclaimer:** Articles published in the journal HemaSphere exclusively reflect the opinions of the authors. The authors are responsible for all content in their abstracts including accuracy of the facts, statements, citing resources, etc.

### (PB3737) CD5-POSITIVITY DOES NOT WORSEN OUTCOMES IN PATIENTS WITH DIFFUSE LARGE B CELL LYMPHOMAS TREATED WITH CAR-T CELLS: UPDATED RESULTS FROM THE MULTICENTER PROSPECTIVE CAR-T SIE STUDY

**Topic:** 19. Large B cell lymphomas - Clinical

Martina Pennisi*[1], Angelica Barone[1, 2], Silva Ljevar[1], Beatrice Casadei[3], Stefania Bramanti[4], Patrizia Chiusolo[5], Alice Di Rocco[6], Marcello Riva[7], Ilaria Cutini[8], Piera Angelillo[9], Massimo Martino[10], Maurizio Musso[11], Mirko Farina[12], Anna Maria Barbui[13], Barbara Botto[14], Francesca Patriarca[15], Giovanni Grillo[16], Mauro Krampera[17], Lucia Brunello[18], Alessia Castellino[19], Simone Ragaini[20], Manuel Gotti[21], Annalisa Chiappella[1], Anna Dodero[1], Anna Guidetti[1, 2], Federico Stella[1], Pier Luigi Zinzani[3, 22], Paolo Corradini[1, 2]

*[1]Istituto Nazionale dei Tumori-Fondazione IRCCS, Oncology and Hematology, Milano, Italy; [2]University of Milan, Milano, Italy; [3]IRCCS Azienda Ospedaliero-Universitaria di Bologna, Bologna, Italy; [4]Humanitas Research Hospital, Transplantation Unit Department of Oncology and Hematology, Rozzano, Italy; [5]Fondazione Policlinico Universitario Agostino Gemelli IRCCS, Ematologia e Trapianto, Dipartimento di Scienze Ematologiche e di laboratorio, Roma, Italy; [6]Università Sapienza, Hematology, Department of Translational and Precision Medicine, Roma, Italy; [7]San Bortolo Hospital, AULSS 8 Berica, Hematology Unit, Vicenza, Italy; [8]Ospedale Careggi, SOD Ematologia, AAD Trapianto di midollo osseo, Firenza, Italy; [9]IRCCS San Raffaele Scientific Institute, Milano, Italy; [10]Grande Ospedale Metropolitano "Bianchi-Melacrino-Morelli", Hematology and Stem Cell Transplantation and Cellular Therapies Unit (CTMO), Department of Hemato-Oncology and Radiotherapy, Reggio Calabria, Italy; [11]Dipartimento Oncologico "La Maddalena", UOC di Oncoematologia e TMO, Palermo, Italy; [12]University of Brescia, ASST Spedali Civili di Brescia, Unit of Blood Disease and Bone Marrow Transplantation, Brescia, Italy; [13]Azienda Socio-Sanitaria Territoriale Papa Giovanni XXIII, Bergamo, Italy; [14]AOU "Città della Salute e della Scienza di Torino", Division of Hematology, Torino, Italy; [15]Azienda Sanitaria Universitaria Integrata di Udine, Clinica Ematologica, Centro Trapianti e Terapie Cellulari "Carlo Melzi", Udine, Italy; [16]ASST Grande Ospedale Metropolitano Niguarda, Dipartimento di Ematologia e Trapianto di midollo, Milano, Italy; [17]University of Verona, Hematology and Bone Marrow Transplant Unit, Section of Biomedicine of Innovation, Department of Engineering for Innovative Medicine (DIMI), Verona, Italy; [18]AOU SS Antonio e Biagio e Cesare Arrigo, SCDU Ematologia, Alessandria, Italy; [19]AO S. Croce e Carle, Cuneo, Division of Hematology, Cuneo, Italy;[20]University Hospital A.O.U. "Città della Salute e della Scienza", Division of Hematology, Torino, Italy; [21]Fondazione IRCCS Policlinico San Matteo, Division of Hematology, Pavia, Italy; [22]Università di Bologna, Dipartimento di Scienze Mediche e Chirurgiche, Bologna, Italy;*

### Background

CD5-positivity occurs in 5-10% of patients (pts) with diffuse large B-cell lymphomas (DLBCL) and associates with worse outcomes after conventional immune-chemotherapy, likely due to distinct clinical and biological features, including common extranodal involvement. Data on the efficacy of anti-CD19 Chimeric Antigen Receptor (CAR) T-cell therapy in CD5+ DLBCL are lacking.

### Aims

Within the multicenter, prospective, observational CAR-T SIE Study, we aimed to compare outcomes between CD5+ and CD5- DLBCL pts treated with CAR-T cells.

**Copyright Information:** (Online) ISSN: 2572-9241
©2026 The Author(s). HemaSphere is published by John Wiley & Sons Ltd on behalf of European Hematology Association. This is an open access Abstract Book distributed under the Attribution-NonCommercial-NoDerivs (CC BY-NC-ND), which allows third parties to download the articles and share them with others as long as they credit the author and the Abstract Book, but they cannot change the content in any way or use them commercially.
**Abstract Book Citations:** Authors, Title, HemaSphere, 2026;10:(S1):pages. The abstract book DOIs can be found on the "Author Information" page.
**Disclaimer:** Articles published in the journal HemaSphere exclusively reflect the opinions of the authors. The authors are responsible for all content in their abstracts including accuracy of the facts, statements, citing resources, etc.

**Methods**

We included pts with de novo DLBCL, receiving either tisagenlecleucel (tisa-cel) or axicabtagene ciloleucel (axi-cel), for which CD5 status assessed on immunohistochemistry was available; we excluded high-grade B cell lymphomas, primary mediastinal B cell lymphoma, and DLBCL double expressor or transformed from indolent lymphomas. Few pts received lisocabtagene maraleucel (n=26) and were thereby excluded from the analysis. Primary endpoints included evaluation of overall (ORR) and complete response (CR), progression-free (PFS) and overall survival (OS), and toxicities, including cytokine release syndrome (CRS) and immune effector cell-associated neurotoxicity syndrome (ICANS).

**Results**

From 2019 to 2025, 282 pts were eligible for the analysis, stratified into CD5+ (n=43) and CD5- (n=239). CD5+ pts were slightly older (median age 63 [IQR 57-69] vs. 60 years [IQR 52-67], respectively; p=.051) and more frequently received tisa-cel (40% vs 21% in CD5+ vs CD5-, p=.01). Nonetheless, the two groups were balanced in terms of performance status, stage, bulky, extranodal and bone marrow disease, international prognostic index, prior lines of therapy, baseline LDH and C-reactive protein levels, relapsed vs. refractory status at leukapheresis, and response to bridging therapy.
CRS and ICANS rates were similar in CD5+ vs. CD5- pts (86% vs. 93%, p=.14 and 21% vs. 29%, p=.27, respectively). Any grade immune effector cells associated hematotoxicity (ICAHT) occurred more frequently in CD5+ pts (35% vs 18%, p=.023).
Median follow-up was 12.47 months (IQR 6.45-23.55). At 90 days after infusion, ORR and CR were similar between CD5+ and CD5- pts (ORR 61% vs 72%, p=.24; CR 61% vs 62%, p=.24). Survival outcomes were also comparable: 1- year PFS was 38.3% (95% CI 25.1-50.5%) vs. 46.8% (95% CI 40.4-54.3%) for CD5+ and CD5- patients, respectively (p=.25), while 1-year OS was 62.1% (95% CI 48-80.3%) and 67.3% (95% CI 60.8-74.6%) in CD5+ and CD5- patients, respectively (p=.46). Non-relapse mortality at 1 year was low (2.4% in CD5+ vs. 4.1% in CD5-, p=.98).
A multivariable analysis identified CAR-T product (tisa vs. axi, HR=2.10, 95%CI 1.39-3.15, p<.001) and bulky disease (HR=1.84, 95%CI 1.26-2.71, p=.003) as independent predictors of worse PFS, independent of CD5 status (p=0.795). Similarly, bulky disease (HR=2.67 (95%CI 1.67-4.26) p<.001 and CAR-T product [tisa vs. axi HR=1.80 (95% CI 1.11-2.92), p=.02] adversely impacted OS, while CD5 status was not prognostic (p=.93). A subgroup analysis conducted on 179 pts (148 CD5- and 31 CD5+), after excluding pts treated in 2nd line, showed comparable results.

**Summary/Conclusion**

Despite older age and more frequent tisa-cel use, which was consistently associated with worse outcomes in previous analyses, our real-world report shows comparable efficacy, safety and survival in CD5+ and CD5- patients receiving CAR-T cells, suggesting that CAR-T cell therapy might overcome the negative prognostic impact of CD5-positivity in patients with DLBCL.

**Copyright Information:** (Online) ISSN: 2572-9241
©2026 The Author(s). HemaSphere is published by John Wiley & Sons Ltd on behalf of European Hematology Association. This is an open access Abstract Book distributed under the Attribution-NonCommercial-NoDerivs (CC BY-NC-ND), which allows third parties to download the articles and share them with others as long as they credit the author and the Abstract Book, but they cannot change the content in any way or use them commercially.
**Abstract Book Citations:** Authors, Title, HemaSphere, 2026;10:(S1):pages. The abstract book DOIs can be found on the "Author Information" page.
**Disclaimer:** Articles published in the journal HemaSphere exclusively reflect the opinions of the authors. The authors are responsible for all content in their abstracts including accuracy of the facts, statements, citing resources, etc.

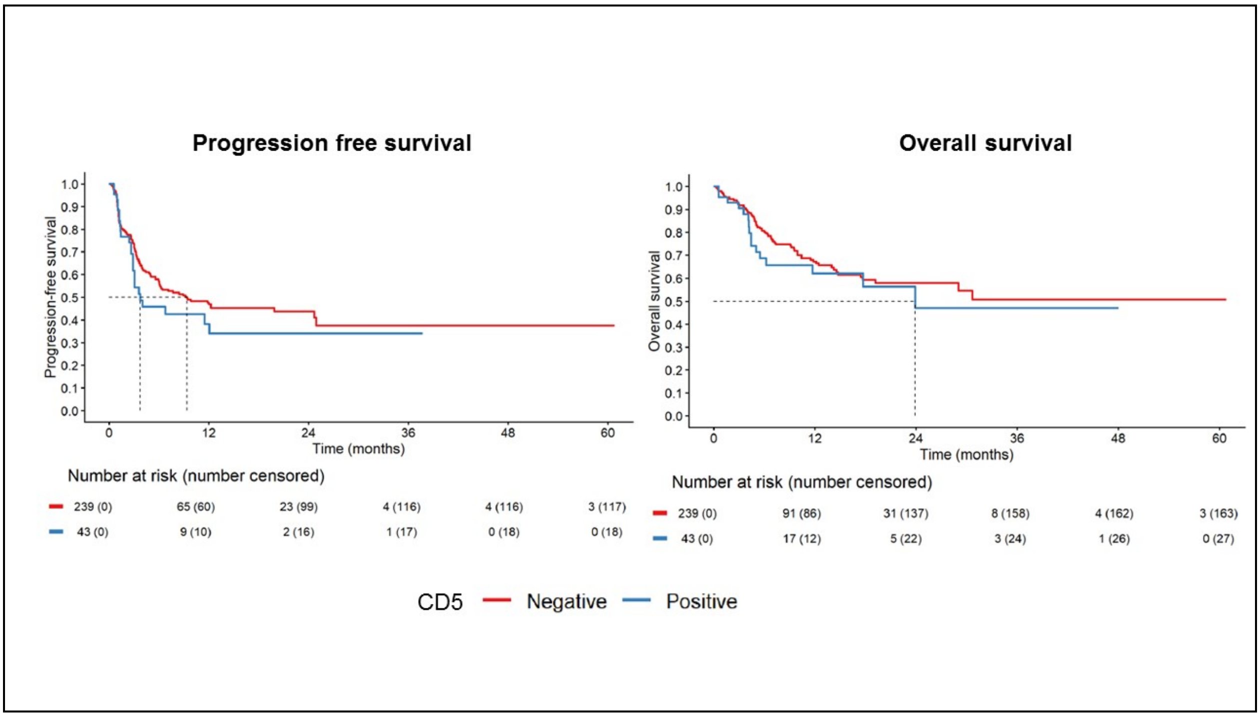

PFS and OS Kaplan Meier Curves for the overall CD5+ (blue line) vs. CD5- (red line) patients population

**Copyright Information:** (Online) ISSN: 2572-9241
©2026 The Author(s). HemaSphere is published by John Wiley & Sons Ltd on behalf of European Hematology Association. This is an open access Abstract Book distributed under the Attribution-NonCommercial-NoDerivs (CC BY-NC-ND), which allows third parties to download the articles and share them with others as long as they credit the author and the Abstract Book, but they cannot change the content in any way or use them commercially.
**Abstract Book Citations:** Authors, Title, HemaSphere, 2026;10:(S1):pages. The abstract book DOIs can be found on the "Author Information" page.
**Disclaimer:** Articles published in the journal HemaSphere exclusively reflect the opinions of the authors. The authors are responsible for all content in their abstracts including accuracy of the facts, statements, citing resources, etc.

### (PB3739) ZRTD, A CHEMO-LIGHT REGIMEN, CONFERS FAVORABLE EFFICACY AND SAFETY IN ELDERLY PATIENTS WITH NEWLY DIAGNOSED PRIMARY CENTRAL NERVOUS SYSTEM LYMPHOMA

**Topic:** 19. Large B cell lymphomas - Clinical

Xiaoping Zhang[1], Mingyu Zhao[1], Zheng Ge*[1]

*[1]Zhongda Hospital Southeast University, Institute of Hematology Southeast University, Department of Hematology, Nanjing, China;*

### Background

High-dose methotrexate (HD-MTX)-based regimens are first-line for newly diagnosed PCNSL but cause severe, intolerable toxicities in elderly/frail patients with comorbidities, creating an unmet need for safe alternatives. Zanubrutinib (a BTK inhibitor) was combined with rituximab, temozolomide, and dexamethasone to develop the ZRTD chemo-linght regimen for this cohort. This is the first single-center phase II study assessing ZRTD in HD-MTX-intolerant elderly PCNSL patients.

### Aims

To systematically evaluate the efficacy and safety of the ZRTD regimen in elderly patients with newly diagnosed PCNSL who are intolerant to HD-MTX-based therapy, and explore the benefit of maintenance therapy on clinical outcomes for this vulnerable population.

### Methods

Fourteen elderly patients with newly diagnosed PCNS DLBCL were enrolled (May 2022–October 2025) at Zhongda Hospital, with eligibility including histopathologic confirmation, radiologically assessable lesions, and HD-MTX intolerance; patients with uncontrolled bleeding, active infection, etc., were excluded. All provided informed consent, and the study was IRB-approved.
ZRTD regimen: zanubrutinib 160 mg PO BID, rituximab 375 mg/m² IV D1, temozolomide 150 mg/m² PO D1–5, dexamethasone 15 mg IV D1–5 (21-day cycle, 6–8 cycles); CR patients received 2-year maintenance with zanubrutinib or temozolomide.
Efficacy was assessed per RECIST v1.1/IPCG criteria (primary endpoint: ORR; secondary: PFS, OS, DOR, CR rate). Safety was evaluated via CTCAE v5.0. Statistics (GraphPad Prism 9.0/STATA 15) included Wilson/Brown method (95% CI for ORR) and Kaplan-Meier (survival distributions).

### Results

Median age was 74 years (67–83); 57.1% male. All had ECOG PS 3/4, median IELSG score 3 (2–5); 57.1% had deep brain tumors, 42.9% multifocal lesions, 21.4% CSF lymphoma cells, 79.4% normal baseline LDH.
Median follow-up: 16.5 months (6.9–44). ORR = 85.7% (12/14; CRR = 57.1% [8/14], PR = 28.6% [4/14]); 1 SD, 1 PD. 12-month PFS = 75.0% (95% CI:39.4%–91.5%), 12-month OS = 85.1% (95% CI:52.3%–96.1%). Seven patients had zanubrutinib maintenance with significantly prolonged DOR; 1 patient had durable CR for 44 months with zanubrutinib maintenance post-6 ZRTD cycles.
All patients completed 6–8 cycles; no treatment-related mortality or discontinuation due to TEAEs, no life-threatening bleeding/cardiac events. Most common all-grade TEAEs (>10%) were hematologic (neutropenia 50.0%, anemia 42.8%, thrombocytopenia 28.6%) and non-hematologic (hyperglycemia 42.8%, skin/nail infection 42.8%, pneumonitis 35.7%, insomnia 35.7%). All hematologic TEAEs were Grade 1/2 (no intervention needed). Grade 3/4 TEAEs were rare (pneumonitis 21.4%, hyperglycemia 14.3%, bleeding 7.14%) and managed with symptomatic treatment; 1 transient gross hematuria case resolved with temporary zanubrutinib hold (no recurrence).

**Copyright Information:** (Online) ISSN: 2572-9241
©2026 The Author(s). HemaSphere is published by John Wiley & Sons Ltd on behalf of European Hematology Association. This is an open access Abstract Book distributed under the Attribution-NonCommercial-NoDerivs (CC BY-NC-ND), which allows third parties to download the articles and share them with others as long as they credit the author and the Abstract Book, but they cannot change the content in any way or use them commercially.
**Abstract Book Citations:** Authors, Title, HemaSphere, 2026;10:(S1):pages. The abstract book DOIs can be found on the "Author Information" page.
**Disclaimer:** Articles published in the journal HemaSphere exclusively reflect the opinions of the authors. The authors are responsible for all content in their abstracts including accuracy of the facts, statements, citing resources, etc.

### Summary/Conclusion

The ZRTD regimen demonstrates high ORR/CRR, favorable survival, and a well-tolerated safety profile in HD-MTX-intolerant elderly patients with newly diagnosed PCNSL. Zanubrutinib maintenance further prolongs DOR, boosting clinical benefit for this population. As the first validation of a zanubrutinib-temozolomide-based chemo-lignht regimen in this specific cohort, ZRTD is a promising front-line option for elderly/frail PCNSL patients unable to tolerate HD-MTX chemotherapy. Larger multicenter trials and in-depth biomarker research (e.g., MGMT expression) are needed to validate these results and optimize patient selection for this chemo-linght approach.

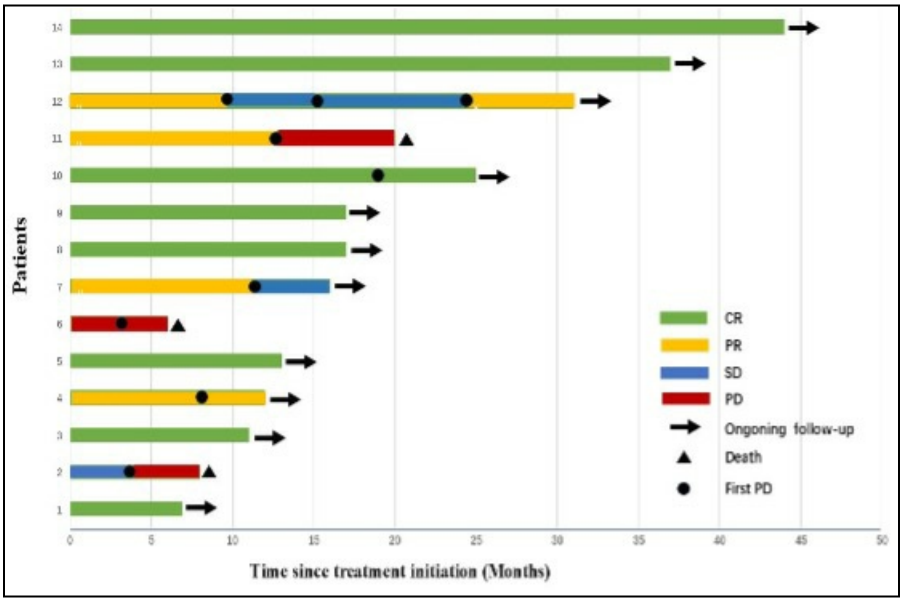

**Copyright Information:** (Online) ISSN: 2572-9241
©2026 The Author(s). HemaSphere is published by John Wiley & Sons Ltd on behalf of European Hematology Association. This is an open access Abstract Book distributed under the Attribution-NonCommercial-NoDerivs (CC BY-NC-ND), which allows third parties to download the articles and share them with others as long as they credit the author and the Abstract Book, but they cannot change the content in any way or use them commercially.
**Abstract Book Citations:** Authors, Title, HemaSphere, 2026;10:(S1):pages. The abstract book DOIs can be found on the "Author Information" page.
**Disclaimer:** Articles published in the journal HemaSphere exclusively reflect the opinions of the authors. The authors are responsible for all content in their abstracts including accuracy of the facts, statements, citing resources, etc.

### (PB3741) MOLECULAR FEATURES AND PROGNOSIS OF PRIMARY BONE DIFFUSE LARGE B-CELL LYMPHOMA VERSUS SECONDARY BONE DIFFUSE LARGE B-CELL LYMPHOMA

**Topic:** 19. Large B cell lymphomas - Clinical

Mengzhen Wang[1], Lei Shi[1], Bin Chu[1], Minqiu Lu[1], Shan Gao[1], Yuan Chen[1], Lijuan Fang[1], Qiuqing Xiang[1], Na Ji[1], Qi Yan[1], Li Bao*[1]

*[1]Beijing Jishuitan Hospital, Capital Medical University, Beijing, China;*

### Background

Diffuse large B-cell lymphoma (DLBCL) is the most prevalent aggressive non-Hodgkin lymphoma subtype with distinct clinical and molecular heterogeneity. Primary bone lymphoma (PBL) is rare, and PB-DLBCL patients usually have a favorable prognosis. Due to its rarity, PB-DLBCL has inconsistent inter-center findings and unclear molecular characteristics. We retrospectively analyzed clinical and molecular data of 177 newly diagnosed extranodal DLBCL (ENI-DLBCL) patients, including 43 PB-DLBCL cases.

### Aims

To identify unique clinical and molecular characteristics of newly diagnosed PB-DLBCL and secondary bone DLBCL versus ENI-DLBCL without bone involvement, and evaluate patients' survival status.

### Methods

This single-center retrospective study enrolled 43 newly diagnosed PB-DLBCL, 94 secondary bone DLBCL, and 40 ENI-DLBCL patients without bone involvement. Clinical data were recorded and analyzed. NGS sequencing and FISH testing were performed in 97 patients to identify LymphGen subtypes.

### Results

Baseline characteristics are shown in Table. PB-DLBCL patients were significantly younger. Prognostic indices varied significantly, with the secondary bone DLBCL group having the highest proportions of high-risk IPI, high-risk NCCN-IPI, and high-risk CNS-IPI. Treatment response was significantly associated with bone involvement status, with PB-DLBCL having the highest CRR (70.7%), followed by ENI-DLBCL without bone involvement (59.5%) and secondary bone DLBCL (43.5%) groups. PB-DLBCL had a higher prevalence of BN2 (35%) and MCD (26%) subtypes, while secondary bone DLBCL and ENI-DLBCL without bone involvement had higher MCD (25%, 32%) and A53 (17%, 18%) subtypes (Figure A). Median follow-up was 18.93 months. PB-DLBCL patients had significantly longer PFS (2-year PFS%: 87.4% *vs.* 48.0% and 58.2%; Figure B), but no significant difference in OS was observed (2-year OS%: 89.0% *vs.* 69.2% and 78.6%; Figure C).

Table. Patient demographics and baseline characteristics

| Characteristic | ENI-DLBCL | | | *p*-value |
|---|---|---|---|---|
| | PB-DLBCL N = 43 | Secondary bone DLBCL N = 94 | Without bone involvement N = 40 | |

**Copyright Information:** (Online) ISSN: 2572-9241
©2026 The Author(s). HemaSphere is published by John Wiley & Sons Ltd on behalf of European Hematology Association. This is an open access Abstract Book distributed under the Attribution-NonCommercial-NoDerivs (CC BY-NC-ND), which allows third parties to download the articles and share them with others as long as they credit the author and the Abstract Book, but they cannot change the content in any way or use them commercially.
**Abstract Book Citations:** Authors, Title, HemaSphere, 2026;10:(S1):pages. The abstract book DOIs can be found on the "Author Information" page.
**Disclaimer:** Articles published in the journal HemaSphere exclusively reflect the opinions of the authors. The authors are responsible for all content in their abstracts including accuracy of the facts, statements, citing resources, etc.

| | | | | |
|---|---|---|---|---|
| **Age** | | | | |
| Median (Min, Max) | 55 (20, 79) | 63 (15, 90) | 66 (16, 79) | 0.006 |
| **Gender, n (%)** | | | | 0.092 |
| Male | 30 (69.8%) | 50 (53.2%) | 19 (47.5%) | |
| **CNSL, n (%)** | 0 (0.0%) | 3 (3.2%) | 2 (5.0%) | 0.335 |
| **Lugano, n (%)** | | | | <0.001 |
| I-II | 8 (18.6%) | 0 (0.0%) | 5 (12.5%) | |
| III-IV | 35 (81.4%) | 94 (100.0%) | 35 (87.5%) | |
| **IPI, n (%)** | | | | <0.001 |
| Low | 5 (11.9%) | 3 (3.2%) | 4 (10.0%) | |
| Low-intermediate | 7 (16.7%) | 2 (2.2%) | 7 (17.5%) | |
| High-intermediate | 10 (23.8%) | 11 (11.8%) | 9 (22.5%) | |
| High | 20 (47.6%) | 77 (82.8%) | 20 (50.0%) | |
| **NCCN-IPI, n (%)** | | | | 0.007 |
| Low-intermediate | 5 (31.3%) | 4 (11.8%) | 5 (27.8%) | |
| High-intermediate | 11 (68.8%) | 16 (47.1%) | 6 (33.3%) | |
| High | 0 (0.0%) | 14 (41.2%) | 7 (38.9%) | |

**Copyright Information:** (Online) ISSN: 2572-9241
©2026 The Author(s). HemaSphere is published by John Wiley & Sons Ltd on behalf of European Hematology Association. This is an open access Abstract Book distributed under the Attribution-NonCommercial-NoDerivs (CC BY-NC-ND), which allows third parties to download the articles and share them with others as long as they credit the author and the Abstract Book, but they cannot change the content in any way or use them commercially.
**Abstract Book Citations:** Authors, Title, HemaSphere, 2026;10:(S1):pages. The abstract book DOIs can be found on the "Author Information" page.
**Disclaimer:** Articles published in the journal HemaSphere exclusively reflect the opinions of the authors. The authors are responsible for all content in their abstracts including accuracy of the facts, statements, citing resources, etc.

| | | | | |
|---|---|---|---|---|
| **CNS-IPI, n (%)** | | | | <0.001 |
| Low | 3 (8.6%) | 3 (4.4%) | 3 (9.1%) | |
| Intermediate | 15 (42.9%) | 6 (8.8%) | 10 (30.3%) | |
| High | 17 (48.6%) | 59 (86.8%) | 20 (60.6%) | |
| **COO, n (%)** | | | | 0.394 |
| GCB | 18 (43.9%) | 40 (44.0%) | 12 (31.6%) | |
| nonGCB | 23 (56.1%) | 51 (56.0%) | 26 (68.4%) | |
| **DEL/TEL, n (%)** | 12 (37.5%) | 18 (30.0%) | 7 (28.0%) | 0.692 |
| **DHL/THL, n (%)** | 0 (0.0%) | 9 (12.0%) | 1 (3.8%) | 0.078 |
| **Treatment, n (%)** | | | | |
| R-EPOCH | 20 (46.5%) | 34 (36.2%) | 5 (12.5%) | |
| R-CHOP | 14 (32.6%) | 36 (38.3%) | 25 (62.5%) | |
| EPOCH | 0 (0.0%) | 2 (2.1%) | 1 (2.5%) | |
| Pola-R-CHP | 8 (18.6%) | 13 (13.8%) | 7 (17.5%) | |
| CHOP | 1 (2.3%) | 4 (4.3%) | 1 (2.5%) | |
| BR | 0 (0.0%) | 4 (4.3%) | 0 (0.0%) | |
| Chemo-free | 0 (0.0%) | 1 (1.1%) | 1 (2.5%) | |

**Copyright Information:** (Online) ISSN: 2572-9241
©2026 The Author(s). HemaSphere is published by John Wiley & Sons Ltd on behalf of European Hematology Association. This is an open access Abstract Book distributed under the Attribution-NonCommercial-NoDerivs (CC BY-NC-ND), which allows third parties to download the articles and share them with others as long as they credit the author and the Abstract Book, but they cannot change the content in any way or use them commercially.
**Abstract Book Citations:** Authors, Title, HemaSphere, 2026;10:(S1):pages. The abstract book DOIs can be found on the "Author Information" page.
**Disclaimer:** Articles published in the journal HemaSphere exclusively reflect the opinions of the authors. The authors are responsible for all content in their abstracts including accuracy of the facts, statements, citing resources, etc.

| | | | | |
|---|---|---|---|---|
| **ASCT, n (%)** | 4 (9.5%) | 18 (19.1%) | 5 (12.5%) | 0.302 |
| **Efficacy, n (%)** | | | | 0.010 |
| CR | 29 (70.7%) | 37 (43.5%) | 22 (59.5%) | |
| PR | 7 (17.1%) | 29 (34.1%) | 13 (35.1%) | |
| SD | 4 (9.8%) | 7 (8.2%) | 2 (5.4%) | |
| PD | 1 (2.4%) | 12 (14.1%) | 0 (0.0%) | |

### Summary/Conclusion

PB-DLBCL has a higher prevalence of BN2 subtype and better prognosis.

**Copyright Information:** (Online) ISSN: 2572-9241
©2026 The Author(s). HemaSphere is published by John Wiley & Sons Ltd on behalf of European Hematology Association. This is an open access Abstract Book distributed under the Attribution-NonCommercial-NoDerivs (CC BY-NC-ND), which allows third parties to download the articles and share them with others as long as they credit the author and the Abstract Book, but they cannot change the content in any way or use them commercially.
**Abstract Book Citations:** Authors, Title, HemaSphere, 2026;10:(S1):pages. The abstract book DOIs can be found on the "Author Information" page.
**Disclaimer:** Articles published in the journal HemaSphere exclusively reflect the opinions of the authors. The authors are responsible for all content in their abstracts including accuracy of the facts, statements, citing resources, etc.

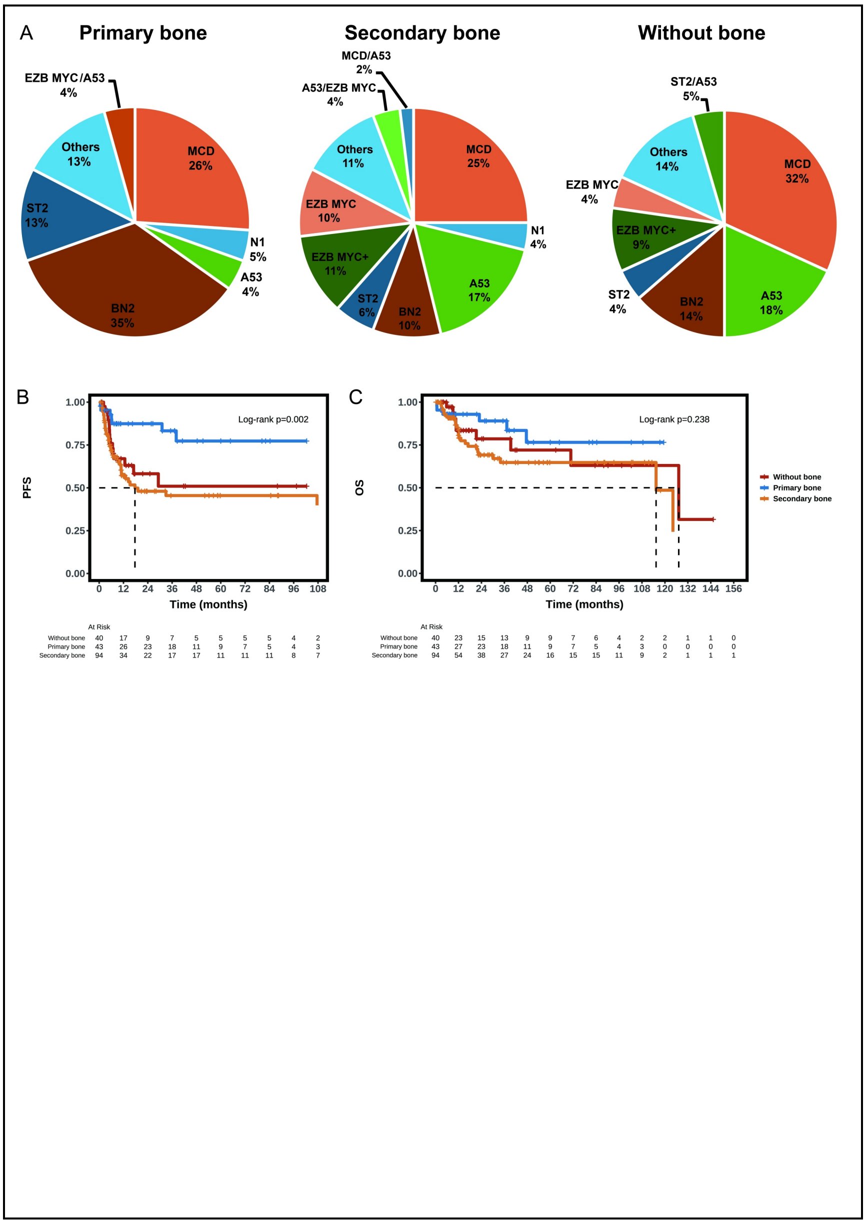

LymphGen subtypes(A) of patients with PB-DLBCL, secondary bone DLBCL, and ENI-DLBCL without bone involvement. PFS (B), and OS (C) of patients with PB-DLBCL, secondary bone DLBCL, and ENI-DLBCL without bone involvement.

**Copyright Information:** (Online) ISSN: 2572-9241
©2026 The Author(s). HemaSphere is published by John Wiley & Sons Ltd on behalf of European Hematology Association. This is an open access Abstract Book distributed under the Attribution-NonCommercial-NoDerivs (CC BY-NC-ND), which allows third parties to download the articles and share them with others as long as they credit the author and the Abstract Book, but they cannot change the content in any way or use them commercially.
**Abstract Book Citations:** Authors, Title, HemaSphere, 2026;10:(S1):pages. The abstract book DOIs can be found on the "Author Information" page.
**Disclaimer:** Articles published in the journal HemaSphere exclusively reflect the opinions of the authors. The authors are responsible for all content in their abstracts including accuracy of the facts, statements, citing resources, etc.

### (PB3742) ASSESSING PROGRESSION-FREE SURVIVAL (PFS) DURABILITY MILESTONES IN INTERIM PRO-MIND RELAPSED/REFRACTORY DIFFUSE LARGE B-CELL LYMPHOMA (R/R DLBCL) TREATED WITH TAFASITAMAB–LENALIDOMIDE

**Topic:** 19. Large B cell lymphomas - Clinical

Enrico Derenzini*[1], Carlo Visco[2], Guido Gini[3], Andrea Bernardelli[4], Eliana Valentina Liardo[5], Piero Maria Stefani[6], Marco Rossi[7], Caterina Patti[8], Mario Lapecorella[9], Nicola Battaglia[9], Antonio Pinto[10], Pier Luigi Zinzani[11]

*[1]IRCCS, Division of Oncohematology, European Institute of Oncology, Milan, Italy;[2]University of Verona, Hematology and Bone Marrow Transplant Unit, Section of Biomedicine of Innovation, Department of Engineering for Innovative Medicine, Verona, Italy; [3]Azienda Ospedaliero Universitaria delle Marche, Clinic of Hematology, Ancona, Italy;[4]Azienda Ospedaliera Universitaria Integrata di Verona, Hematology Unit, Department of Medicine, Verona, Italy; [5]IRCCS Istituto Romagnolo per lo studio dei Tumori (IRST) "Dino Amadori", Hematology Unit, Meldola, Italy; [6]Treviso Hospital, Structural Department of Internal Medicine, Treviso, Italy; [7]Azienda Ospedaliera Universitaria Renato Dulbecco, Hematology Unit, Onco-Hematology Department, Catanzaro, Italy; [8]Ospedali Riuniti Villa Sofia - Cervello (V. Cervello Hospital), Palermo, Italy;[9]Incyte Biosciences Italy Srl, Milano, Italy; [10]Istituto Nazionale Tumori IRCCS-Fondazione Pascale, Napoli, Italy; [11]Institute of Hematology, IRCCS Azienda Ospedaliero-Universitaria di Bologna, Department of Medical and Surgical Sciences, Bologna University School of Medicine, Bologna, Italy;*

### Background

Interim real-world analyses in R/R DLBCL are frequently characterized by short follow-up and heavy right-censoring, which can limit the interpretability of conventional Kaplan–Meier (KM) medians. Clinically, physicians often need simple and transparent estimates of durability at relevant time points early during evidence generation. A frequentist interim analysis of this PRO-MIND data cut was presented at SIE 2025. Here, we complement those results with Bayesian survival modeling to estimate clinically interpretable PFS durability probabilities at prespecified milestones, while quantifying uncertainty beyond KM summaries (Chen. *J Natl Cancer Inst.* 2015;107[9]:djv156; Chan. *Oncoimmunology.* 2018;7;8[10]:e1343774).

### Aims

To provide clinically interpretable interim estimates of PFS durability in PRO-MIND by reporting milestone probabilities of remaining progression free at 6, 9, and 12 months, with uncertainty quantification, and to assess robustness across modeling assumptions.

### Methods

PRO-MIND is a prospective, observational, real-world study of ASCT-ineligible adults with R/R DLBCL treated with tafasitamab-lenalidomide (NCT06299553). In this July 25, 2025, interim data cut-off (N=90), PFS was analyzed using a Bayesian Weibull model with a prespecified robust mixture meta-analytic predictive (MAP) prior (Bartoš. *BMC Med Res Methodol.* 2022;22[1]:238; Schmidli. *Biometrics.* 2014;70[4]:1023-1032), informed by historical IPD (Gutierrez. *Blood Adv.* 2025;9[19]:4924-4935), using a mixture weight w=0.5 to mitigate prior data conflict. Posterior milestone probabilities $P$ (PFS >6, 9, 12 months) and the posterior probability that the population median PFS exceeded 9 months were estimated. Sensitivity analyses included varying borrowing weights (w=0.2–0.8) and a piecewise exponential model (0–3, 3–6, 6–12, >12 months).

**Copyright Information:** (Online) ISSN: 2572-9241
©2026 The Author(s). HemaSphere is published by John Wiley & Sons Ltd on behalf of European Hematology Association. This is an open access Abstract Book distributed under the Attribution-NonCommercial-NoDerivs (CC BY-NC-ND), which allows third parties to download the articles and share them with others as long as they credit the author and the Abstract Book, but they cannot change the content in any way or use them commercially.
**Abstract Book Citations:** Authors, Title, HemaSphere, 2026;10:(S1):pages. The abstract book DOIs can be found on the "Author Information" page.
**Disclaimer:** Articles published in the journal HemaSphere exclusively reflect the opinions of the authors. The authors are responsible for all content in their abstracts including accuracy of the facts, statements, citing resources, etc.

**Results**

The interim cohort was high risk: median age 77 years; international prognostic index ≥3 64.8%; Ann Arbor stage III–IV 84.1%; primary refractory 57.8%. Median follow-up was 4.5 months with 34 PFS events; KM median PFS was 10.4 months. In the primary Bayesian model (Weibull + robust MAP, w=0.5), *P*=0.680 (95% CrI 0.610, 0.741) that PFS was >6 months, *P*=0.585 (95% CrI 0.507, 0.654) that PFS was >9 months, and *P*=0.507 (95% CrI 0.421, 0.584) that PFS was 12 months **(Figure)**. The posterior probability that the population median PFS exceeded 9 months was >0.975. Results were consistent in sensitivity analyses, including piecewise exponential modeling with *P* (PFS >12 months) 0.496 (95% CrI 0.394, 0.596) and across borrowing weights w=0.2–0.8.

**Summary/Conclusion**

In this early interim PRO-MIND real-world cohort of patients with high-risk R/R DLBCL, PFS durability with tafasitamab-lenalidomide can be communicated in practical clinical terms using milestone probabilities—68% at 6 months, 58.5% at 9 months, and 50.7% at 12 months—with explicit uncertainty and consistent findings across sensitivity models. These estimates provide pragmatic interim benchmarks when KM medians are limited by substantial censoring; ongoing follow-up will refine longer-term durability.

**Copyright Information:** (Online) ISSN: 2572-9241
©2026 The Author(s). HemaSphere is published by John Wiley & Sons Ltd on behalf of European Hematology Association. This is an open access Abstract Book distributed under the Attribution-NonCommercial-NoDerivs (CC BY-NC-ND), which allows third parties to download the articles and share them with others as long as they credit the author and the Abstract Book, but they cannot change the content in any way or use them commercially.
**Abstract Book Citations:** Authors, Title, HemaSphere, 2026;10:(S1):pages. The abstract book DOIs can be found on the "Author Information" page.
**Disclaimer:** Articles published in the journal HemaSphere exclusively reflect the opinions of the authors. The authors are responsible for all content in their abstracts including accuracy of the facts, statements, citing resources, etc.

**Figure. PFS Durability at 6, 9, and 12 Months**

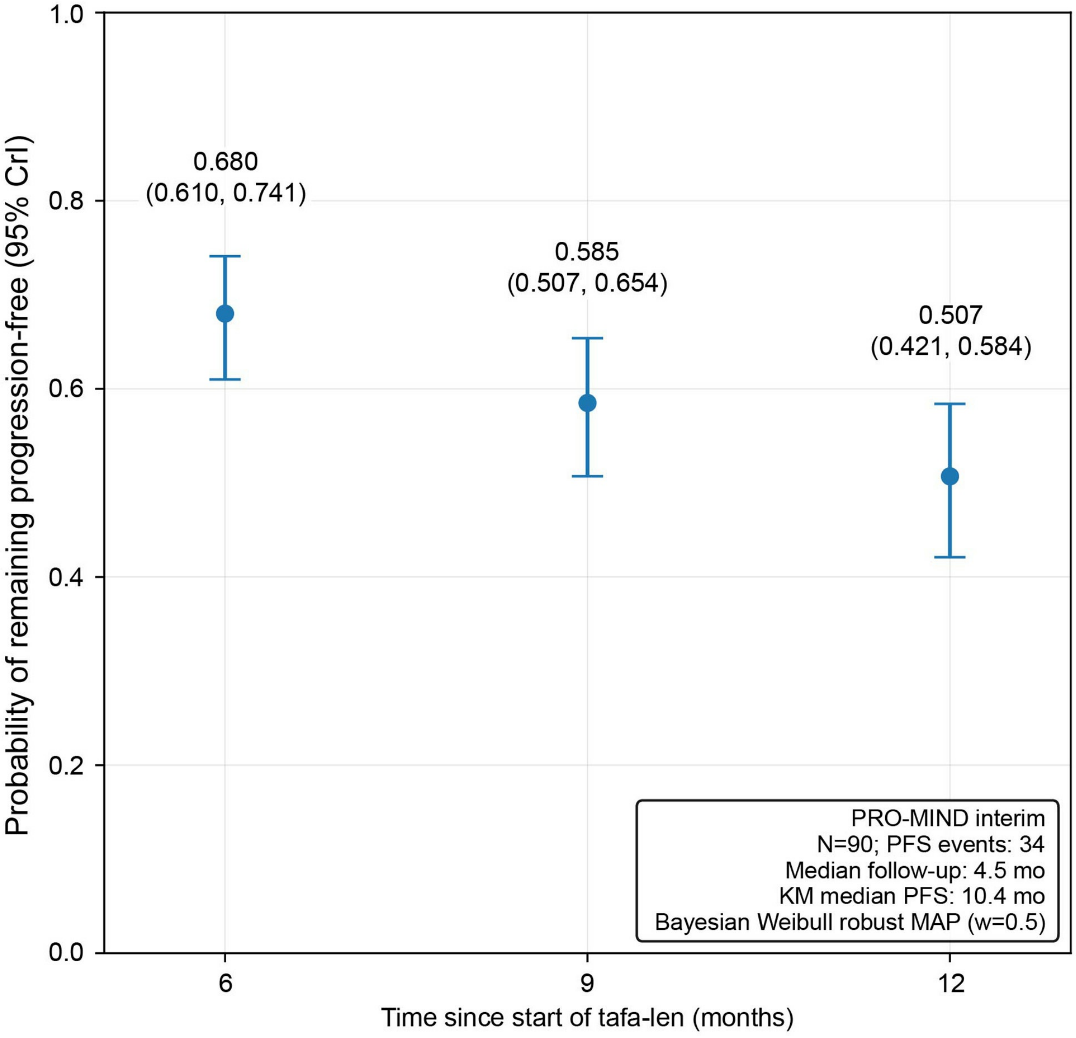

KM, Kaplan-Meier; MAP, meta-analytic predictive; mo, months, PFS, progression-free survival.

**Copyright Information:** (Online) ISSN: 2572-9241
©2026 The Author(s). HemaSphere is published by John Wiley & Sons Ltd on behalf of European Hematology Association. This is an open access Abstract Book distributed under the Attribution-NonCommercial-NoDerivs (CC BY-NC-ND), which allows third parties to download the articles and share them with others as long as they credit the author and the Abstract Book, but they cannot change the content in any way or use them commercially.
**Abstract Book Citations:** Authors, Title, HemaSphere, 2026;10:(S1):pages. The abstract book DOIs can be found on the "Author Information" page.
**Disclaimer:** Articles published in the journal HemaSphere exclusively reflect the opinions of the authors. The authors are responsible for all content in their abstracts including accuracy of the facts, statements, citing resources, etc.

### (PB3743) ORELABRUTINIB MAINTENANCE FOR DIFFUSE LARGE B-CELL LYMPHOMA PATIENTS RESPONDING TO R-CHOP

**Topic:** 19. Large B cell lymphomas - Clinical

Xing Guo[1], Fang Shu Guan[1], Jingwen Li[1], Jingsong He[1], Zhen Cai[1], Yi Zhao*[1]

[1] *The First Affiliated Hospital, School of Medicine, Zhejiang University, Bone Marrow Transplantation Center, Hangzhou, China;*

### Background

Diffuse large B-cell lymphoma (DLBCL) is the most common aggressive lymphoma, accounting for 25%-30% of all non-Hodgkin lymphomas (NHL), and is primarily treated with immuno-chemotherapy. Maintenance therapy has been proven to benefit patients with follicular lymphoma (FL) and mantle cell lymphoma (MCL), aiming to further eradicate residual disease after induction therapy and prolong remission duration. However, the benefit of maintenance therapy in DLBCL remains unclear.

### Aims

This study aims at evaluating the clinical effect of orelabrutinib maintenance therapy in responding DLBCL patients with R-CHOP treatment.

### Methods

This is a prospective, single-arm, multicenter clinical trial. Patients aged 18-75 years with an ECOG performance status of 0-2 who achieved complete response (CR) or partial response (PR) after 4-6 cycles of conventional R-CHOP-like therapy and were candidates for maintenance therapy were enrolled. The maintenance regimen consisted of orelabrutinib administered orally at 150 mg in 14-day cycles until disease progression or intolerable toxicity. The primary endpoint was progression-free survival (PFS). Secondary endpoints included the conversion rate from PR to CR, overall survival (OS), central nervous system (CNS) recurrence, and safety.

### Results

From October 2022 to March 2025, a total of 27 patients were enrolled. The median age at diagnosis was 64 years (range 40–75), with 63.0% of patients over 60 years old.  The majority of patients had non-GCB subtype (88.9%, 24/27) and double-expressor lymphoma (DEL, 63.0%, 17/27). High-risk features were common: 63.0% had intermediate-high risk CNS-IPI scores and 81.5% had Ki-67 ≥70%. Special site involvement included primary CNS (18.5%), breast (11.1%), and testicular (7.4%) DLBCL. At the end of induction therapy, the ORR was 100% (27/27), with a CRR of 81.5% (22/27). The median orelabrutinib maintenance duration was 20.1 months (range: 3.5-33.6), among the 5 PR patients, 3 PR patients got converted into a CR during orelabrutinib maintenance. Median follow-up was 29.6 months (range: 3.9-42.8), the median PFS and OS were not reached, and the 1-year and 2-year PFS rate were 96.3% (95% CI: 89.4-100%), 90.9% (95% CI: 79.5-100%), respectively. Notably, the PFS of patients who achieved CR at initial R-CHOP therapy was better than that of patients achieving PR (p=0.005). The 1-year and 2-year OS rate were both 100%. No CNS recurrence was observed during the study period. Treatment-emergent adverse events (TEAEs) occurred in 96.3% (26/27) of patients, with grade 1–2 TEAEs in 92.6% (26/27) and grade 3–4 TEAEs in 29.6% (8/27). The most common grade 1–2 events were hemoglobin decreased (63.0%), leukopenia (51.9%), and infection (37.0%). Grade 3–4 events were primarily hematologic: neutropenia (25.9%), leukopenia (18.5%), and thrombocytopenia (14.8%). Notably, no atrial fibrillation or clinically significant hemorrhage—both typical BTK inhibitor–associated toxicities—were observed."

**Copyright Information:** (Online) ISSN: 2572-9241
©2026 The Author(s). HemaSphere is published by John Wiley & Sons Ltd on behalf of European Hematology Association. This is an open access Abstract Book distributed under the Attribution-NonCommercial-NoDerivs (CC BY-NC-ND), which allows third parties to download the articles and share them with others as long as they credit the author and the Abstract Book, but they cannot change the content in any way or use them commercially.
**Abstract Book Citations:** Authors, Title, HemaSphere, 2026;10:(S1):pages. The abstract book DOIs can be found on the "Author Information" page.
**Disclaimer:** Articles published in the journal HemaSphere exclusively reflect the opinions of the authors. The authors are responsible for all content in their abstracts including accuracy of the facts, statements, citing resources, etc.

**Summary/Conclusion**

These findings suggest that orelabrutinib is an effective maintenance therapy that may deepen responses and prolong remission in aggressive DLBCL with controllable toxicity, offering a therapeutic strategy for high-risk patients vulnerable to relapse after standard immunochemotherapy.

**Copyright Information:** (Online) ISSN: 2572-9241
©2026 The Author(s). HemaSphere is published by John Wiley & Sons Ltd on behalf of European Hematology Association. This is an open access Abstract Book distributed under the Attribution-NonCommercial-NoDerivs (CC BY-NC-ND), which allows third parties to download the articles and share them with others as long as they credit the author and the Abstract Book, but they cannot change the content in any way or use them commercially.
**Abstract Book Citations:** Authors, Title, HemaSphere, 2026;10:(S1):pages. The abstract book DOIs can be found on the "Author Information" page.
**Disclaimer:** Articles published in the journal HemaSphere exclusively reflect the opinions of the authors. The authors are responsible for all content in their abstracts including accuracy of the facts, statements, citing resources, etc.

### (PB3744) TRANSFERRIN AS AN EARLY OUTCOME PREDICTOR IN PATIENTS WITH DIFFUSE LARGE B-CELL LYMPHOMA RECEIVING POLATUZUMAB VEDOTIN IN COMBINATION WITH RITUXIMAB, CYCLOPHOSPHAMIDE, DOXORUBICIN, AND PREDNISONE

**Topic:** 19. Large B cell lymphomas - Clinical

Ziyang Shi*[1, 2], Yuhong Ren[2], Jingli Zhuang[1, 2], Jing Li[2], Luya Cheng[2], Yang Ke[2], Ling Yuan[2], Zhixiang Cheng[2], Yian Zhang[2], Zhimei Wang[2], Xuejiao Zhang[1, 2], Weiguang Wang[2], Yue Wang[2], Lili Ji[2], Jiadai Xu[2], Peng Liu[1, 2]

*[1]Shanghai Geriatric Medical Center (Zhongshan Hospital Affiliated to Fudan University Minhang Campus), Shanghai, China;*
*[2]Zhongshan Hospital Fudan University, Department of Hematology, Shanghai, China;*

### Background

Polatuzumab vedotin in combination with rituximab, cyclophosphamide, doxorubicin, and prednisone (pola-R-CHP) regimen, as a new standard first-line treatment for diffuse large B-cell lymphoma (DLBCL), has a progression-free survival (PFS) benefit over conventional immunochemotherapy. However, the utility of established risk models like the International Prognostic Index (IPI) in patients receiving pola-R-CHP remains unvalidated. Emerging evidence suggests that tumor-associated iron dysregulation and systemic inflammation influence treatment outcomes in cancer. Transferrin, as an iron-transporting glycoprotein and negative acute-phase protein, reflects both processes, highlighting its potential as a prognostic biomarker.

### Aims

We aimed to determine whether transferrin levels can serve as an early outcome predictor in the era of pola-R-CHP.

### Methods

We retrospectively analyzed newly diagnosed DLBCL patients treated with pola-R-CHP at our center (April 2023-June 2025). Among routine laboratory indicators evaluated, transferrin was selected for further analysis based on its biological relevance and clinical availability. Baseline serum transferrin was measured via immunoturbidimetric assay (normal range: 2.0-3.6 g/L). For group comparisons, Mann-Whitney U test was used for continuous variables, and Chi-square test was used for categorical variables. PFS was measured from diagnosis to disease progression or death from any cause. The primary endpoint was PFS24, a binary measure of PFS at 24 months, defining early responders (alive and progression-free at 24 months) and non-responders. Survival analyses employed the Kaplan-Meier method with log-rank test, and the multivariate Cox proportional hazards model. The predictive value of transferrin and IPI was compared using receiver operating characteristic (ROC) curve with the DeLong test. Patients with missing data were omitted without imputation performed. All statistical analyses were conducted using R Studio (version 4.3.3). Written informed consent was obtained from all patients.

**Copyright Information:** (Online) ISSN: 2572-9241
©2026 The Author(s). HemaSphere is published by John Wiley & Sons Ltd on behalf of European Hematology Association. This is an open access Abstract Book distributed under the Attribution-NonCommercial-NoDerivs (CC BY-NC-ND), which allows third parties to download the articles and share them with others as long as they credit the author and the Abstract Book, but they cannot change the content in any way or use them commercially.
**Abstract Book Citations:** Authors, Title, HemaSphere, 2026;10:(S1):pages. The abstract book DOIs can be found on the "Author Information" page.
**Disclaimer:** Articles published in the journal HemaSphere exclusively reflect the opinions of the authors. The authors are responsible for all content in their abstracts including accuracy of the facts, statements, citing resources, etc.

**Results**

With a median follow-up of 11.1 months, 177 consecutive patients were included (median age 57, 57.6% male), among whom 149 (84.2%) were early responders and 28 (15.8%) were non-responders. Baseline transferrin was available for 169 (95.5%) patients. Non-responders had significantly higher rates of double-hit lymphoma (11.5% vs. 0.8%, P=0.015) and IPI score of 3-5 (82.1% vs. 51.4%, P=0.005), compared to early responders. Critically, median baseline transferrin was significantly higher in early responders (2.23 [IQR 1.94-2.46] vs. 1.85 [IQR 1.56-2.05], P<0.001). High transferrin (dichotomized at the median of 2.18 g/L) was associated with superior PFS in univariate analysis (Figure 1A-B; HR=0.080, 95% CI: 0.019-0.337, P<0.001), and remained an independent predictor in a multivariate model adjusting for five IPI components (Figure 1C; HR=0.100, 95% CI: 0.020-0.420, P=0.002). ROC analysis demonstrated that baseline transferrin had superior predictive accuracy for PFS24 compared to the IPI grouping (Figure 1D; AUC: 0.782 [95% CI: 0.702-0.861] versus 0.666 [95% CI: 0.583-0.748], P=0.018).

**Summary/Conclusion**

Serum transferrin is a promising, simplified biomarker for early risk stratification in DLBCL patients receiving pola-R-CHP. Higher transferrin levels at diagnosis identify patients with favorable prognosis. Further research is warranted to elucidate the biological mechanisms underlying this association.

**Copyright Information:** (Online) ISSN: 2572-9241
©2026 The Author(s). HemaSphere is published by John Wiley & Sons Ltd on behalf of European Hematology Association. This is an open access Abstract Book distributed under the Attribution-NonCommercial-NoDerivs (CC BY-NC-ND), which allows third parties to download the articles and share them with others as long as they credit the author and the Abstract Book, but they cannot change the content in any way or use them commercially.
**Abstract Book Citations:** Authors, Title, HemaSphere, 2026;10:(S1):pages. The abstract book DOIs can be found on the "Author Information" page.
**Disclaimer:** Articles published in the journal HemaSphere exclusively reflect the opinions of the authors. The authors are responsible for all content in their abstracts including accuracy of the facts, statements, citing resources, etc.

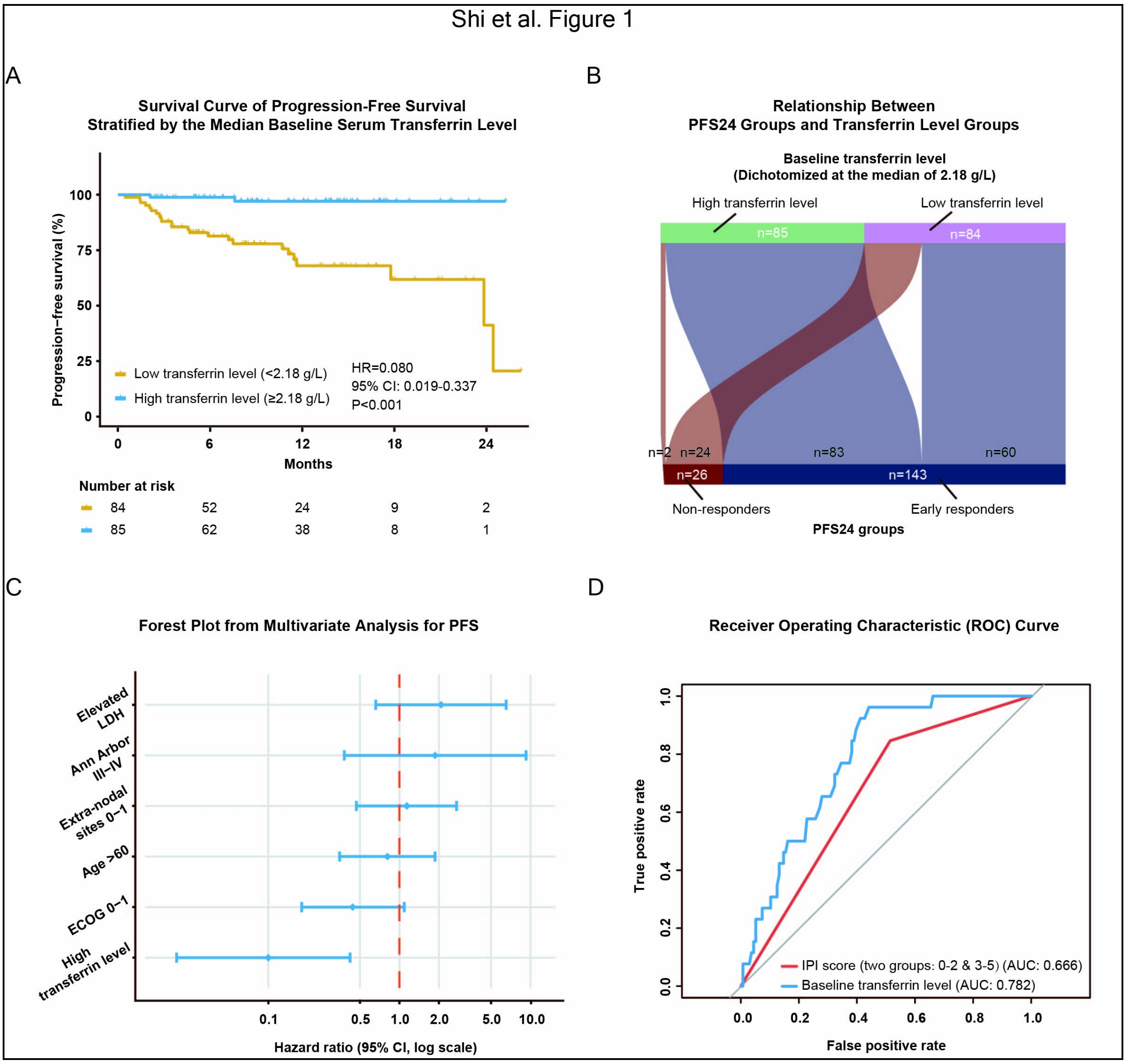

Figure 1 (A) Kaplan-Meier survival curve of PFS in patients treated with pola-R-CHP regimen, stratified by the median baseline serum transferrin level. (B) Relationship between PFS24 groups and transferrin level groups. The baseline transferrin level was dichotomized at the median of 2.18 g/L in this cohort. (C) Forest plot for PFS from multivariate Cox regression including baseline transferrin level and the five IPI components (age, serum LDH level, ECOG score, extra-nodal sites, and Ann Arbor stage). (D) ROC curves for PFS24 prediction using baseline transferrin levels (continuous) and IPI risk group (low [0-2] vs. high [3-5]). The grey line indicates the reference (AUC=0.5). PFS indicates progression-free survival; pola-R-CHP, polatuzumab vedotin in combination with rituximab, cyclophosphamide, doxorubicin, and prednisone; IPI, International Prognostic Index; LDH, lactate dehydrogenase; ECOG, Eastern Cooperative Oncology Group□ROC, receiver operating characteristic; and AUC, area under curve.

**Copyright Information:** (Online) ISSN: 2572-9241
©2026 The Author(s). HemaSphere is published by John Wiley & Sons Ltd on behalf of European Hematology Association. This is an open access Abstract Book distributed under the Attribution-NonCommercial-NoDerivs (CC BY-NC-ND), which allows third parties to download the articles and share them with others as long as they credit the author and the Abstract Book, but they cannot change the content in any way or use them commercially.
**Abstract Book Citations:** Authors, Title, HemaSphere, 2026;10:(S1):pages. The abstract book DOIs can be found on the "Author Information" page.
**Disclaimer:** Articles published in the journal HemaSphere exclusively reflect the opinions of the authors. The authors are responsible for all content in their abstracts including accuracy of the facts, statements, citing resources, etc.

### (PB3745) REAL-WORLD OUTCOMES OF PRIMARY CNS LYMPHOMA: A MULTICENTER ANALYSIS FROM EASTERN EUROPEAN AND CENTRAL ASIAN COHORTS

**Topic:** 19. Large B cell lymphomas - Clinical

Anastasiia Maliar*[1], Akmaral Jazyltayeva[2], Solomiia Hlukhovska[3], Nazarii Shokun[4], Ciprian Tomuleasa[5], Luana Conte[6], Ayazhan Umutbayeva[7], Anamaria Bancos[5], Raigul Ramazanova[7], Madina Valiyeva[7], Saule Gabbassova[2], Iryna Tytorenko[8], Alevtyna Burtna[8], Tetiana Kadnikova[8], Katya Moiseienko[8], Yana Stepanishyna[9], Tetiana Lymanets[10], Zoya Martina[11], Tymur Rudiuk[8], Zinaida Stupakova[12], Yan Pastushenko[8], Umut Yılmaz[13], Serhii Horbachov[14], Maxym Kushnir[15], Elif Birtaş Ateşoğlu[16], Iryna Kryachok[8], Massimo Federico[17]

*[1]National Cancer Institute, Department of Bone Marrow Transplantation, Kyiv, Ukraine;[2]Kazakh National Institute of Oncology and Radiology, Center for Hematology and Bone Marrow Transplantation, Almaty, Kazakhstan; [3]First Lviv Medical Union, Lviv, Ukraine; [4]University of Salento, Department of Experimental Medicine, Lecce, Italy;[5]Iuliu Hatieganu University of Medicine and Pharmacy, Department of Hematology, Cluj Napoca, Romania; [6]University of Palermo, Department of Physics and Chemistry, Palermo, Italy; [7]Kazakh National Medical University, Almaty, Kazakhstan;[8]National Cancer Institute, Kyiv, Ukraine; [9]Jules Bordet Institute, Brussels, Belgium; [10]PE "Poltava Regional Clinical Hospital n.a.M.V.Sklifosovsky PRC", Poltava, Ukraine; [11]Research Centre for Radiation Medicine, Academy of Medical Sciences of Ukraine, Kyiv, Ukraine; [12]National Research Center for Radiation Medicine, Hematology and Oncology of The National Academy of Medical Sciences of Ukraine, Kyiv, Ukraine; [13]Istanbul University-Cerrahpaşa, Istanbul, Türkiye; [14]Cherkasy Clinical Oncology Center, Cherkasy, Ukraine; [15]Non-profit municipal enterprise "City clinical hospital #4" Dnipro city council, Dnipro, Ukraine; [16]Yeditepe University Faculty of Medicine, Istanbul, Türkiye; [17]University of Modena and Reggio Emilia, Modena, Italy, Modena, Italy;*

### Background

Primary central nervous system lymphoma (PCNSL) represents an aggressive extranodal non-Hodgkin lymphoma variant. This subtype presents significant management challenges due to its inherent biological aggression. Existing data on real-world management and outcomes, particularly from Eastern European and Central Asian populations remains limited.

### Aims

To analyze the real-world management and outcomes of Primary CNS Lymphoma, one of the most aggressive and treatment-demanding lymphoma subtypes, through a multicenter collaborative study involving Ukraine, Romania, Kazakhstan, and Turkey.

### Methods

For the study, a total of 178 patients diagnosed with primary CNS lymphoma (PCNSL) were included between 2010 and 2026. Data collected included information on demographics, treatment, and response assessment; overall survival (OS) and progression-free survival (PFS) were estimated using Kaplan-Meier method.

**Copyright Information:** (Online) ISSN: 2572-9241
©2026 The Author(s). HemaSphere is published by John Wiley & Sons Ltd on behalf of European Hematology Association. This is an open access Abstract Book distributed under the Attribution-NonCommercial-NoDerivs (CC BY-NC-ND), which allows third parties to download the articles and share them with others as long as they credit the author and the Abstract Book, but they cannot change the content in any way or use them commercially.
**Abstract Book Citations:** Authors, Title, HemaSphere, 2026;10:(S1):pages. The abstract book DOIs can be found on the "Author Information" page.
**Disclaimer:** Articles published in the journal HemaSphere exclusively reflect the opinions of the authors. The authors are responsible for all content in their abstracts including accuracy of the facts, statements, citing resources, etc.

### Results

Of 178 patients, 112 (63%) were aged ≥60 years, with median age being 63 (28-80); 136 patients (76%) were from Ukraine, 31 (17%) from Kazakhstan, 6 (3.4%) from Romania and 5 (2.8%) from Turkey.
Involvement of deep brain structures was observed in 69 out of 125 cases (55%), whereas concurrent systemic involvement was present in 3 out of 122 cases (2.5%). Most of the study cohort had an ECOG performance status score of ≥2 (111/151, 74%), elevated LDH (54/96, 56%) and normal β2-microglobulin (31/41, 76%).
Information on induction therapy was available for 140 patients. R-MPV was the first-line regimen in most cases (91 patients, 65%). High-dose methotrexate (HD-Mtx) alone and HD-Mtx plus cytarabine (Ara-C) were the second most common regimens, each accounting for 6.4%, followed by radiotherapy (RT) in 8 patients (5.7%). Other regimens were administered in 18 cases (13%).104 patients were evaluable for response after induction therapy - CR was observed in most cases (44, 42%), PR in 26 (25%); PD was observed in 19 (18%) cases. Ara-C was the most common type of consolidation (34, 74%), while RT was administered in 3 cases (6.5%). Autologous stem cell transplantation (ASCT) was performed as consolidation in 18 patients.
78 patients (43.8%) experienced relapse. RT was the most commonly used salvage treatment (9, 26%), followed by temozolomide (7, 20%). R-MPV accounted for 11% of cases, followed by Ara-C plus HD-Mtx and MATRIX (each accounting for 8.6%). 24 patients were evaluable for response to salvage therapy. PD was the most common outcome (13, 54%); CR was observed in 5 patients (21%), PR in 3 (13%), and SD in 3 (13%).
After a median follow-up of 20 months(0.6-175 months), 3-year OS and PFS were 55% and 48%, respectively. Based on the response to induction, OS was 81% in the CR group, 70% in PR group, 50% in the SD group, and only 8% in the PD group (p<0.001). 3-year OS did not differ significantly between patients who received consolidation and those who did not (72% vs 71%, respectively; p=0.8). A similar trend was observed for PFS (68% vs 61%, p=0.4).

### Summary/Conclusion

This analysis provides valuable real-world data on PCNSL management and outcomes across Eastern European and Central Asian cohorts. The findings affirm the prognostic significance of induction therapy response for overall survival. While aggressive treatment approaches are essential for PCNSL, the role of consolidation needs further investigation.

**Copyright Information:** (Online) ISSN: 2572-9241
©2026 The Author(s). HemaSphere is published by John Wiley & Sons Ltd on behalf of European Hematology Association. This is an open access Abstract Book distributed under the Attribution-NonCommercial-NoDerivs (CC BY-NC-ND), which allows third parties to download the articles and share them with others as long as they credit the author and the Abstract Book, but they cannot change the content in any way or use them commercially.
**Abstract Book Citations:** Authors, Title, HemaSphere, 2026;10:(S1):pages. The abstract book DOIs can be found on the "Author Information" page.
**Disclaimer:** Articles published in the journal HemaSphere exclusively reflect the opinions of the authors. The authors are responsible for all content in their abstracts including accuracy of the facts, statements, citing resources, etc.

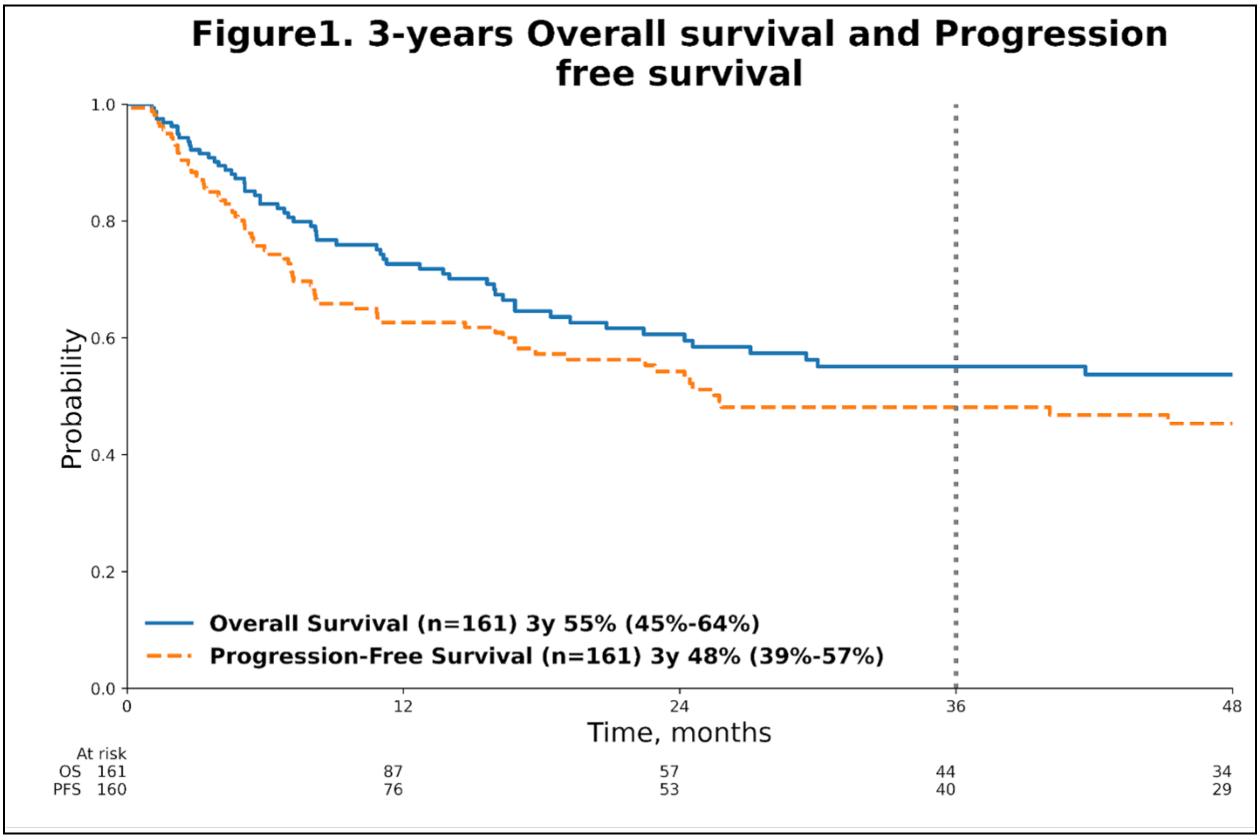

**Copyright Information:** (Online) ISSN: 2572-9241
©2026 The Author(s). HemaSphere is published by John Wiley & Sons Ltd on behalf of European Hematology Association. This is an open access Abstract Book distributed under the Attribution-NonCommercial-NoDerivs (CC BY-NC-ND), which allows third parties to download the articles and share them with others as long as they credit the author and the Abstract Book, but they cannot change the content in any way or use them commercially.
**Abstract Book Citations:** Authors, Title, HemaSphere, 2026;10:(S1):pages. The abstract book DOIs can be found on the "Author Information" page.
**Disclaimer:** Articles published in the journal HemaSphere exclusively reflect the opinions of the authors. The authors are responsible for all content in their abstracts including accuracy of the facts, statements, citing resources, etc.

### (PB3746) CLINICAL FEATURES AND OUTCOMES OF METHOTREXATE ASSOCIATED DLBCL- AUSTRALIAN STUDY

**Topic:** 19. Large B cell lymphomas - Clinical

Vibooshini Ganeshalingam*[1, 2, 3], Jacques Malherbe[4], Harry Hambleton[5], Fiona Swain[3, 6], Chan Y. Cheah[5, 7, 8], Allison Barraclough[4], Pietro Di Ciaccio[9, 10, 11, 12], Maya Latimer[9], Costas K. Yannakou[13, 14], Charmaine Tan[13], Joel Wight[2, 15]

*[1]Pathology Queensland, Haematology, Woolloongabba, Australia; [2]Townsville University Hospital, Douglas, Australia; [3]Princess Alexandra Hospital, Haematology, Woolloongabba, Australia; [4]Fiona Stanley Hospital, Perth, Australia; [5]Sir Charles Gairdner Hospital, Perth, Australia; [6]Frazer Institute, University of Queensland, Woolloongabba, Brisbane, Australia; [7]Linear Clinical Research, Nedlands, Australia; [8]University of Western Australia, Perth, Australia; [9]The Canberra Hospital, Canberra, Australia; [10]Australasian lymphoma Alliance, Melbourne, Australia; [11]School of medicine, Australian National University, Canberra, Australia; [12]School of Medicine, Macquarie University, Macquarie Park, Australia; [13]Epworth Health Care, Richmond, Victoria, Australia; [14]Department of clinical pathology, Faculty of Medicine, Dentistry and Health Science, University of Melbourne, Melbourne, Australia; [15]James Cook University, Douglas, Australia;*

### Background

Methotrexate-associated diffuse large B-cell lymphoma (MTX-DLBCL) arises in the setting of chronic immunosuppression for autoimmune disease. Evidence guiding prognosis and treatment in MTX associated DLBCL (MTX-DLBCL), particularly in Western populations, remains limited.

### Aims

We aimed to evaluate clinical characteristics, treatment patterns, and survival predictors in a multicentre cohort of patients with MTX-associated DLBCL.

### Methods

We performed a retrospective analysis of patients diagnosed with MTX-associated DLBCL across six tertiary centres in Australia. Baseline clinicopathologic features were collected. Overall survival (OS) and progression-free survival (PFS) were estimated using Kaplan–Meier methods. Univariable Cox proportional hazards models were used to evaluate predictors of outcome.

**Copyright Information:** (Online) ISSN: 2572-9241
©2026 The Author(s). HemaSphere is published by John Wiley & Sons Ltd on behalf of European Hematology Association. This is an open access Abstract Book distributed under the Attribution-NonCommercial-NoDerivs (CC BY-NC-ND), which allows third parties to download the articles and share them with others as long as they credit the author and the Abstract Book, but they cannot change the content in any way or use them commercially.
**Abstract Book Citations:** Authors, Title, HemaSphere, 2026;10:(S1):pages. The abstract book DOIs can be found on the “Author Information” page.
**Disclaimer:** Articles published in the journal HemaSphere exclusively reflect the opinions of the authors. The authors are responsible for all content in their abstracts including accuracy of the facts, statements, citing resources, etc.

### Results

Forty-one patients were included. Median age at diagnosis was 68 years (range 29–87), and 73% presented with advanced-stage disease. Extranodal involvement at more than one site was observed in 49%, and CNS involvement in 10%. Distribution of NCCN-IPI between low/low-intermediate and high-intermediate/high were 36% and 64%. Higher ECOG (2-3) were seen in 17% of the cohort. EBER-ISH positivity was detected in 21% of evaluable cases.
Most patients (83%) received RCHOP or RCHOP–like chemotherapy with MTX withdrawal; four patients were treated with MTX withdrawal alone, three of whom achieved complete response. Cohort staging was distributed between limited-stage (n=2) and Stage III (n=1), with one instance of progressive Stage IV disease. Extranodal involvement at >1site was observed in two patients; no subjects presented with CNS involvement. After a median follow-up of 38.4 months, estimated PFS and OS were 80% (95%Cl 62-90%) and 92% (95% Cl 77 -97%). 3-year OS was 92% (95% CI 77 –97%), 3- year PFS was 80% (95% Cl 62–90%) and median PFS was 82.4 months.
NCCN-IPI risk category was associated with OS (p = 0.025), with no deaths observed among patients with NCCN-IPI <4. Extranodal involvement at more than one site was associated with inferior PFS (HR 5.56; 95% CI 1.2–25.5; p = 0.027). Higher NCCN-IPI demonstrated a strong trend toward inferior PFS (HR 3.61; p = 0.061). ECOG performance status and CNS involvement showed a non-significant trend toward inferior OS (HR 7.6; 95% CI 0.7–84.5; p = 0.09) (HR 3.7; 95% Cl 0.87–15.72; p=0.07). EBER status was associated with neither OS nor PFS.

### Summary/Conclusion

MTX-associated DLBCL in this multicentre cohort demonstrated favourable overall outcomes despite presenting with high-risk features such as frequent extranodal disease. Conventional risk stratification using NCCN-IPI retained prognostic relevance. Conventional treatment appears effective though withdrawal of MTX alone as a definitive therapy may be an under-utilised strategy. Prospective studies are required.

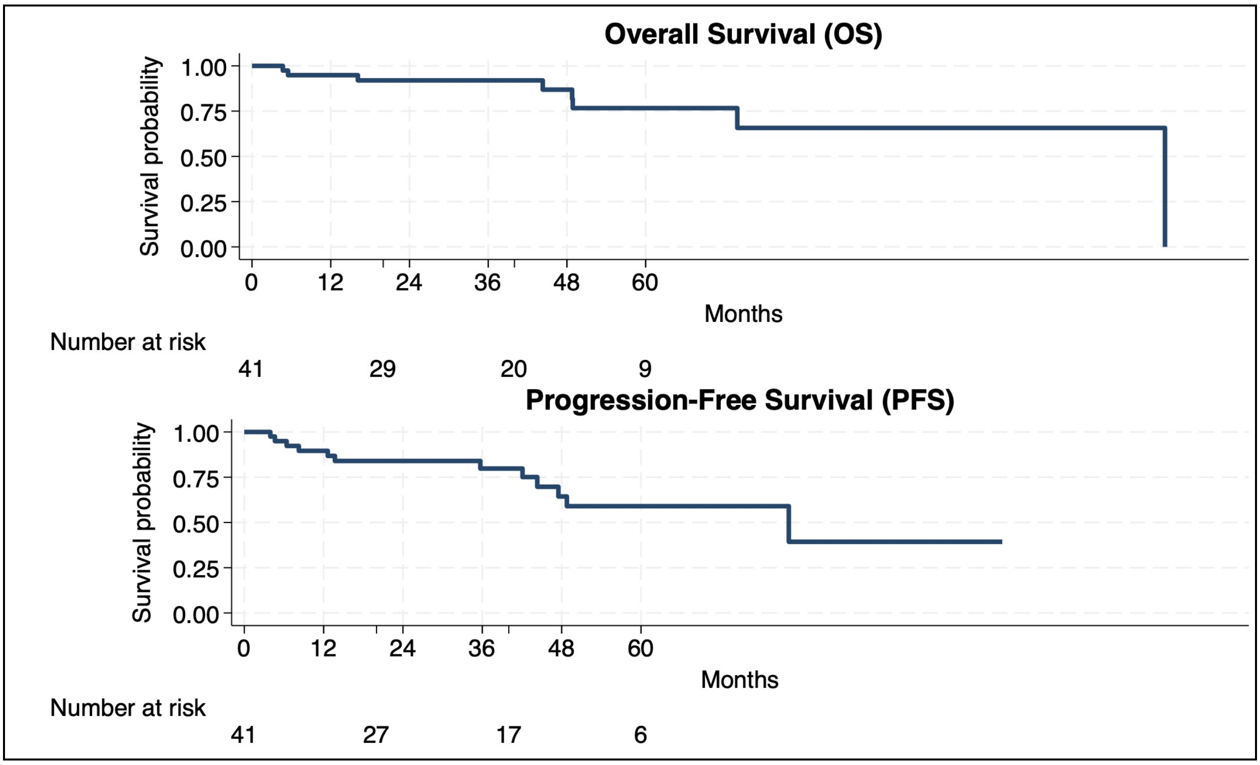

**Copyright Information:** (Online) ISSN: 2572-9241
©2026 The Author(s). HemaSphere is published by John Wiley & Sons Ltd on behalf of European Hematology Association. This is an open access Abstract Book distributed under the Attribution-NonCommercial-NoDerivs (CC BY-NC-ND), which allows third parties to download the articles and share them with others as long as they credit the author and the Abstract Book, but they cannot change the content in any way or use them commercially.
**Abstract Book Citations:** Authors, Title, HemaSphere, 2026;10:(S1):pages. The abstract book DOIs can be found on the "Author Information" page.
**Disclaimer:** Articles published in the journal HemaSphere exclusively reflect the opinions of the authors. The authors are responsible for all content in their abstracts including accuracy of the facts, statements, citing resources, etc.

### (PB3747) COMPARISON OF EFFICACY BETWEEN POLA-R-CHP AND TUCIDINOSTAT-R-CHOP IN PREVIOUSLY UNTREATED PATIENTS WITH DOUBLE-EXPRESSOR DIFFUSE LARGE B-CELL LYMPHOMA: A MATCHING-ADJUSTED INDIRECT COMPARISON (MAIC)

**Topic:** 19. Large B cell lymphomas - Clinical

Yuqin Song*[1], Yukun Lan[2], Qian Zhang[2], Lukas Enderlein[3], Yanwen Jiang[4], Mark Yan[5], Lisa Musick[4], Connie Lee Batlevi[4], Adriano Sa[3], Yunyue Ju[2]

*[1]Key Laboratory of Carcinogenesis and Translational Research (Ministry of Education), Peking University Cancer Hospital and Institute, Beijing, China; [2]Shanghai Roche Pharmaceuticals Ltd., Shanghai, China; [3]F. Hoffmann-La Roche Ltd., Basel, Swaziland; [4]Genentech, Inc, South San Francisco, United States of America; [5]F. Hoffmann-La Roche Ltd., Mississauga, Canada;*

### Background

Double-Expressor Diffuse Large B-Cell Lymphoma (DE-DLBCL) is an intermediate-risk molecular subtype associated with poor prognosis. In the phase III POLARIX trial (NCT03274492), polatuzumab vedotin plus R-CHP (Pola-R-CHP) demonstrated superior progression-free survival (PFS) versus R-CHOP in previously untreated DLBCL, including DE subgroup. Similarly, tucidinostat (an HDAC inhibitor) combined with R-CHOP (Tuci-R-CHOP) showed promising efficacy in phase III DEB trial (NCT04231448) for untreated DE patients.

### Aims

Given the absence of head-to-head trials, we conducted a MAIC to evaluate the comparative efficacy between Pola-R-CHP and Tuci-R-CHOP in previously untreated DE-DLBCL.

### Methods

To adjust for cross-trial difference in the DE-DLBCL subgroup, an unanchored MAIC was performed. Individual patient data (IPD; N=115 global pts, N=35 Asian pts, N=15 Chinese pts) from the Pola-R-CHP arm of POLARIX were reweighted to match the aggregate data (AgD; N=211) of the Tuci-R-CHOP arm of DEB trial. Matching variables included gender, International Prognostic Index, and bulky disease. IPD for Tuci-R-CHOP were reconstructed from published Kaplan–Meier curves (Zhao et al. ASH 2025) using the algorithm by Liu et al. (2021), achieving high accuracy as confirmed by root mean square error and mean/max absolute error. The primary endpoint was PFS, secondary endpoint included CRR, OS, and EFS. Reweighted treatment effects are reported as hazard ratios (HR) for time-to-event outcomes and odds ratios (OR) for CRR, with 95% confidence intervals (CIs) and p-values.

**Copyright Information:** (Online) ISSN: 2572-9241
©2026 The Author(s). HemaSphere is published by John Wiley & Sons Ltd on behalf of European Hematology Association. This is an open access Abstract Book distributed under the Attribution-NonCommercial-NoDerivs (CC BY-NC-ND), which allows third parties to download the articles and share them with others as long as they credit the author and the Abstract Book, but they cannot change the content in any way or use them commercially.
**Abstract Book Citations:** Authors, Title, HemaSphere, 2026;10:(S1):pages. The abstract book DOIs can be found on the “Author Information” page.
**Disclaimer:** Articles published in the journal HemaSphere exclusively reflect the opinions of the authors. The authors are responsible for all content in their abstracts including accuracy of the facts, statements, citing resources, etc.

### Results

In the global DE population, after weighting, the effective sample size (ESS) was 114.2. Pola-R-CHP significantly improved CRR compared to Tuci-R-CHOP (84.0% vs 73.0%; p=0.019) and significantly improved overall EFS (HR=0.53; 95% CI: 0.36–0.77; p<0.001), with 24-month landmark rates of 77.6% vs 59.6%. The treatment also demonstrated favourable numerical trends in overall PFS (HR=0.67; p=0.054) and overall OS (HR=0.67; p=0.114), with corresponding 24-month snapshot estimates of 78.1% vs 69.5% and 88.9% vs 81.3%, respectively.
Asian and Chinese subgroups analyses demonstrated efficacy signals consistent with the global results. In the Asian subgroup (ESS=32.3), Pola-R-CHP showed favourable trends across all endpoints, including CRR (OR=1.27) and overall EFS (HR=0.69). In the Chinese subgroup, despite a limited sample size (N=15), the MAIC weighting achieved a high ESS retention rate of 97.3% (ESS=14.6), indicating strong baseline comparability. Pola-R-CHP achieved a numerically higher CRR (92.1% vs 73.0%). Notably, no deaths occurred in the Pola-R-CHP Chinese cohort (24-month OS: 100%), contrasting with the 81.3% rate in the comparator arm. Kaplan–Meier inspection indicated that survival in the Chinese and Asian subgroups was consistent with the results observed in the global analysis.

### Summary/Conclusion

This analysis provides the first comparative evidence between Pola-R-CHP and Tuci-R-CHOP in untreated DE-DLBCL. The global analysis demonstrates that Pola-R-CHP offers statistically significant improvements in CRR and EFS, with consistent favourable trends in PFS and OS. Limitations include cross-trial heterogeneity, ethnic differences, and small Asian/Chinese sample sizes. Nevertheless, efficacy signals were consistent across global, Asian, and Chinese subgroups. Collectively, these data support Pola-R-CHP as a highly effective first-line treatment option for this molecular subtype.

| Efficacy | Category | Tuci-R-CHOP at 2025 ASH | A<br>Tuci-R-CHOP reconstruction data* | B<br>Weighted Pola-R-CHP Global subgroup | Weighted OR/HR<br>A vs. B | C<br>Weighted Pola-R-CHP Asian subgroup | Weighted OR/HR<br>A vs. C | D<br>Weighted Pola-R-CHP Chinese subgroup# |
|---|---|---|---|---|---|---|---|---|
| Effective Sample Size (ESS) | | 211 | 211 | 114.2 | | 32.3 | | 14.6 |
| Median follow up time (month) | | 44.9 | 44.9 | 72.9 | | | | 48.2 |
| CRR | | 73.0%<br>(66.6%, 78.5%) | 73.0%<br>(66.6%, 78.5%) | 84.0%<br>(76.7%, 89.4%) | OR=1.949<br>(1.116, 3.402)<br>P=0.019 | 77.4%<br>(60.9%, 88.3%) | OR=1.268<br>(0.513, 3.132)<br>P=0.607 | 92.1%<br>(68.4%, 98.4%) |
| PFS | Median/M | NR<br>(NE, NE) | NR<br>(NE, NE) | 78.1<br>(NE, NE) | HR=0.669<br>(0.444, 1.006) | 78.1<br>(33.3, NE) | HR=0.868<br>(0.465, 1.619) | NR<br>(20.2, NE) |
| | 24M Rate | 69.3%<br>(61.7%, 75.6%) | 69.5%<br>(61.9%, 75.9%) | 78.1%<br>(69.4%, 84.6%) | P=0.054 | 69.9%<br>(50.7%, 82.8%) | P=0.657 | 71.6%<br>(44.1%, 88.2%) |
| OS | Median/M | NR<br>(NE, NE) | NR<br>(NE, NE) | NR<br>(NE, NE) | HR=0.670<br>(0.407, 1.101) | NR<br>(78.1, NE) | HR=0.686<br>(0.300,1.569) | NR<br>(NE, NE) |
| | 24M Rate | 81.4%<br>(75.3%, 86.1%) | 81.3%<br>(75.3%, 86.1%) | 88.9%<br>(82.0%, 93.3%) | P=0.114 | 86.2%<br>(69.0%, 94.2%) | P=0.372 | 100.0%<br>(100.0%, 100.0%) |
| $EFS_{eff}$ | Median/M | NR<br>(35.3, NE) | NR<br>(27.4, NE) | 78.1<br>(NE, NE) | HR=0.526<br>(0.359, 0.770) | 78.1<br>(33.3, NE) | HR=0.688<br>(0.366, 1.219) | NR<br>(20.2, NE) |
| | 24M Rate | 60.3%<br>(53.0%, 66.9%) | 59.6%<br>(52.2%, 66.2%) | 77.6%<br>(68.9%, 84.1%) | P=0.001 | 69.9%<br>(50.7%, 82.8%) | P=0.188 | 71.6%<br>(44.1%, 88.2%) |

* Individual patient data for the Tuci-R-CHOP arm were reconstructed from published Kaplan–Meier curves presented at ASH 2025, and minor discrepancies were assessed and deemed acceptable.
### The Chinese subgroup results are presented descriptively due to the limited sample size, but the landmark analysis findings are consistent with the Asian and global populations

Image. The weighted efficacy comparative analysis between Pola-R-CHP vs. Tuci-R-CHOP

Copyright Information: (Online) ISSN: 2572-9241
©2026 The Author(s). HemaSphere is published by John Wiley & Sons Ltd on behalf of European Hematology Association. This is an open access Abstract Book distributed under the Attribution-NonCommercial-NoDerivs (CC BY-NC-ND), which allows third parties to download the articles and share them with others as long as they credit the author and the Abstract Book, but they cannot change the content in any way or use them commercially.
**Abstract Book Citations:** Authors, Title, HemaSphere, 2026;10:(S1):pages. The abstract book DOIs can be found on the "Author Information" page.
**Disclaimer:** Articles published in the journal HemaSphere exclusively reflect the opinions of the authors. The authors are responsible for all content in their abstracts including accuracy of the facts, statements, citing resources, etc.

### (PB3748) IMPACT OF PRIOR COVALENT BTK INHIBITOR EXPOSURE ON SURVIVAL AFTER RICHTER TRANSFORMATION IN PATIENTS WITH CLL

**Topic:** 19. Large B cell lymphomas - Clinical

Truman Koh*[1], Farrukh Awan[1]

[1]*University of Texas Southwestern Medical Center at Dallas, Department of Internal Medicine, Division of Hematology and Oncology, Dallas, United States of America;*

### Background

Covalent Bruton tyrosine kinase inhibitors (BTKi) are widely used in chronic lymphocytic leukemia (CLL), yet their impact on outcomes following transformation to diffuse large B-cell lymphoma (DLBCL)-type Richter transformation (RT) remains unclear.

### Aims

To evaluate the association between prior covalent BTKi exposure and survival outcomes following DLBCL-type RT.

### Methods

We conducted a single-center retrospective analysis, with appropriate IRB approval, of patients with documented CLL/SLL preceding biopsy-proven DLBCL-type RT. Hodgkin-variant RT was excluded. The primary endpoint was overall survival (OS) from RT diagnosis. Secondary endpoints included progression-free survival (PFS) and overall response rate (ORR). Progression required confirmation ≥14 days from RT diagnosis. Survival was estimated using Kaplan–Meier methodology and compared using log-rank testing. Hazard ratios (HR) were calculated using the Mantel–Haenszel methods.

### Results

Fifty-three patients met the inclusion criteria, including 22 with prior covalent BTKi exposure and 31 BTKi-naïve patients. Median follow-up for the overall cohort was 14.4 months.
Median OS from RT diagnosis was significantly shorter in patients with prior BTKi exposure compared to BTKi-naïve patients (14.4 vs 27.0 months; HR 2.46, 95% CI 1.06–5.71; log-rank p=0.036). Median PFS was 5.9 months in the prior BTKi group and 9.9 months in BTKi-naïve patients (HR 2.03, 95% CI 0.997–4.13; log-rank p=0.051). Best ORR to RT-directed therapy was 41% (9/22) in the prior BTKi cohort and 45% (14/31) in BTKi-naïve patients. Complete response rates were 32% and 42%, respectively. Additional exploratory analyses evaluating clinical and cytogenetic predictors of survival are ongoing.

### Summary/Conclusion

In this real-world cohort of DLBCL-type Richter transformation, prior covalent BTKi exposure was associated with significantly inferior overall survival and a trend toward shorter progression-free survival following RT diagnosis. These findings suggest that antecedent BTKi therapy may adversely influence post-transformation outcomes and underscore the need for improved therapeutic strategies in this high-risk population.

**Copyright Information:** (Online) ISSN: 2572-9241
©2026 The Author(s). HemaSphere is published by John Wiley & Sons Ltd on behalf of European Hematology Association. This is an open access Abstract Book distributed under the Attribution-NonCommercial-NoDerivs (CC BY-NC-ND), which allows third parties to download the articles and share them with others as long as they credit the author and the Abstract Book, but they cannot change the content in any way or use them commercially.
**Abstract Book Citations:** Authors, Title, HemaSphere, 2026;10:(S1):pages. The abstract book DOIs can be found on the "Author Information" page.
**Disclaimer:** Articles published in the journal HemaSphere exclusively reflect the opinions of the authors. The authors are responsible for all content in their abstracts including accuracy of the facts, statements, citing resources, etc.

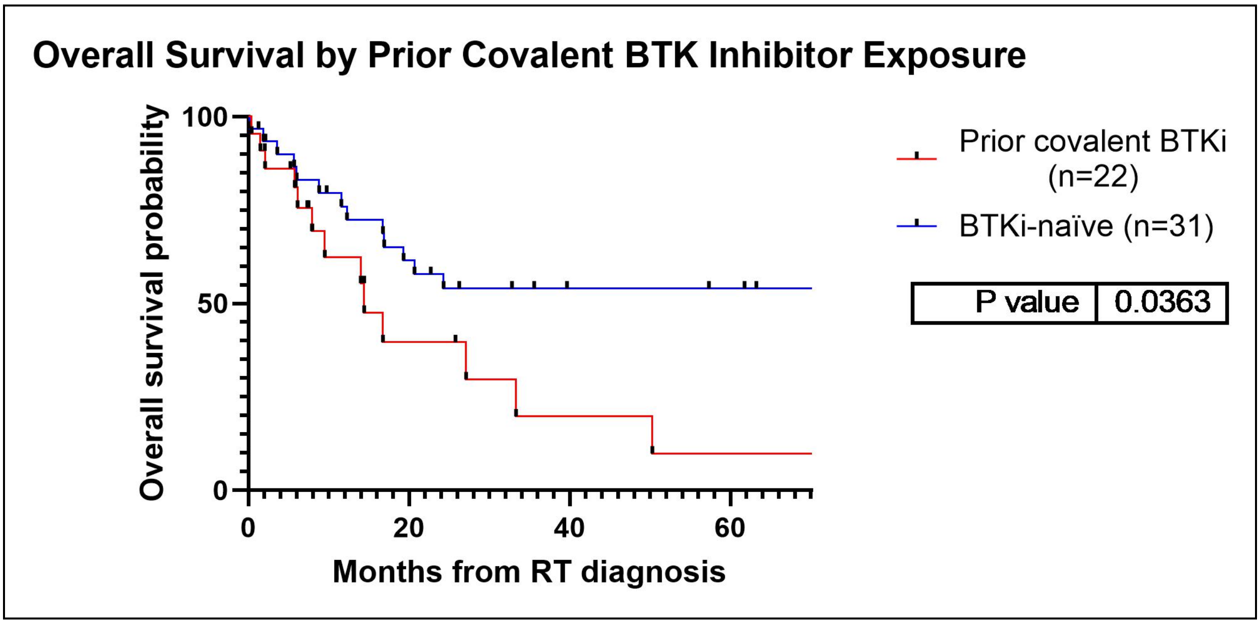

Kaplan–Meier overall survival from time of Richter transformation (RT) diagnosis stratified by prior covalent BTK inhibitor exposure. Tick marks indicate censored observations. Survival distributions were compared using the log-rank (Mantel–Cox) test (p=0.0363). Prior covalent BTKi (n=22); BTKi-naïve (n=31).

**Copyright Information:** (Online) ISSN: 2572-9241
©2026 The Author(s). HemaSphere is published by John Wiley & Sons Ltd on behalf of European Hematology Association. This is an open access Abstract Book distributed under the Attribution-NonCommercial-NoDerivs (CC BY-NC-ND), which allows third parties to download the articles and share them with others as long as they credit the author and the Abstract Book, but they cannot change the content in any way or use them commercially.
**Abstract Book Citations:** Authors, Title, HemaSphere, 2026;10:(S1):pages. The abstract book DOIs can be found on the "Author Information" page.
**Disclaimer:** Articles published in the journal HemaSphere exclusively reflect the opinions of the authors. The authors are responsible for all content in their abstracts including accuracy of the facts, statements, citing resources, etc.

### (PB3749) BASELINE SPLEEN VOLUME IS INDEPENDENTLY ASSOCIATED WITH SURVIVAL AFTER CD19-DIRECTED CAR T-CELL THERAPY IN R/R DLBCL

**Topic:** 19. Large B cell lymphomas - Clinical

Alexander Pohlmann[1], Philipp Schindler[2], Christian Römer[2], Akhil Behringer[1], Valeria Carubin[1], Georg Evers[1], Cyrus Khandanpour[3], Julia Marx[1], Christian Reicherts[1], Matthias Stelljes[1], Evgenii Shumilov[1], Georg Lenz[1], Lucas Brehm*[1]

*[1]University Hospital Münster, Department of Medicine A, Münster, Germany;[2]University Hospital Münster, Department of Radiology, Münster, Germany; [3]University Hospital Oldenburg, Department of Oncology and Hematology, Oldenburg, Germany;*

### Background

The introduction of CD19-directed chimeric antigen receptor (CAR) T-cell therapy has transformed the treatment landscape of relapsed or refractory (r/r) diffuse large B-cell lymphoma (DLBCL). Nevertheless, long-term disease control remains limited for the majority of patients treated with CAR T-cell therapy, underscoring the need for pretreatment risk stratification.
Given that CAR T-cell efficacy is determined not only by tumor-intrinsic characteristics, host-related factors may also contribute to the observed heterogeneity in outcomes. The spleen, as the largest secondary lymphoid organ and a key regulator of immune cell trafficking, represents a biologically plausible but underexplored imaging marker in this setting.

### Aims

This study aimed to determine whether baseline spleen volume (SV) is independently associated with survival outcomes in patients with r/r DLBCL treated with CD19-directed CAR T-cell therapy.

### Methods

We retrospectively analyzed 88 r/r DLBCL patients between September 2019 and July 2025 with baseline SV available. Patients received one of the three commercial CAR T-cell products axicabtagene ciloleucel (axi-cel), lisocabtagene maraleucel (liso-cel), or tisagenlecleucel (tisa-cel). SV was quantified on the most recent cross-sectional imaging (CT, PET-CT, or MRI) prior to CAR T-cell therapy. SV was analyzed as a continuous variable (per 100 mL), without dichotomization or prespecified cutoffs. Cox and Fine-Gray analyses were performed for the assessment of associations between SV and clinical outcomes. Non-linearity was evaluated using restricted cubic splines.

### Results

With a median follow-up of 24.9 months, higher SV was associated with inferior OS (HR 1.16 per 100 mL; 95% CI 1.03–1.31; p=0.016) and remained independently associated with OS after multivariable adjustment for age, ECOG, remission status prior to CAR T-cell therapy, bulky disease, and LDH (aHR 1.14; 95% CI 1.01–1.28; p=0.030). These findings remained robust in two sensitivity analyses with additional adjustment for BMI and, separately, splenic lymphoma involvement.
Higher SV was associated with a higher cumulative incidence of relapse/progression (sHR 1.10 per 100 mL; 95% CI 1.00–1.21; p=0.041). Among patients with relapse/progression (n=40), higher baseline SV was associated with inferior post-progression survival (PPS; HR 1.28; 95% CI 1.09–1.51; p=0.003). Spline modeling showed that the mortality risk rises steadily as SV increases, without evidence of a specific volume cutoff (p for non-linearity=0.609).

**Copyright Information:** (Online) ISSN: 2572-9241
©2026 The Author(s). HemaSphere is published by John Wiley & Sons Ltd on behalf of European Hematology Association. This is an open access Abstract Book distributed under the Attribution-NonCommercial-NoDerivs (CC BY-NC-ND), which allows third parties to download the articles and share them with others as long as they credit the author and the Abstract Book, but they cannot change the content in any way or use them commercially.
**Abstract Book Citations:** Authors, Title, HemaSphere, 2026;10:(S1):pages. The abstract book DOIs can be found on the "Author Information" page.
**Disclaimer:** Articles published in the journal HemaSphere exclusively reflect the opinions of the authors. The authors are responsible for all content in their abstracts including accuracy of the facts, statements, citing resources, etc.

**Summary/Conclusion**

Baseline spleen volume is a readily available imaging-derived marker for patients with r/r DLBCL undergoing CAR T-cell therapy. Higher SV was independently associated with inferior OS, increased risk of relapse/progression, and inferior post-progression survival. Spleen volume may complement established pretreatment risk assessment and support refined risk stratification before CAR T-cell therapy.

**Copyright Information:** (Online) ISSN: 2572-9241
©2026 The Author(s). HemaSphere is published by John Wiley & Sons Ltd on behalf of European Hematology Association. This is an open access Abstract Book distributed under the Attribution-NonCommercial-NoDerivs (CC BY-NC-ND), which allows third parties to download the articles and share them with others as long as they credit the author and the Abstract Book, but they cannot change the content in any way or use them commercially.
**Abstract Book Citations:** Authors, Title, HemaSphere, 2026;10:(S1):pages. The abstract book DOIs can be found on the "Author Information" page.
**Disclaimer:** Articles published in the journal HemaSphere exclusively reflect the opinions of the authors. The authors are responsible for all content in their abstracts including accuracy of the facts, statements, citing resources, etc.

### (PB3750) EFFICACY AND SAFETY OF BISPECIFIC ANTIBODIES IN RELAPSED OR REFRACTORY POST TRANSPLANTATION LYMPHOPROLIFERATIVE DISORDERS : A RETROSPECTIVE COHORT STUDY FROM THE FRENCH REGISTRY KVIROGREF.

**Topic:** 19. Large B cell lymphomas - Clinical

Elise Bandet[1], Antoine Tichadou[2], Elise Toussaint[3], Emilie Corvilain[4], Pierre Sesques[5], Justine De Croocq[6], Morgane Cheminant[7], Laure Ricard[8], Veronique Morel Malek[1], Elise Corre[8], Damien Roos-Weil[1], Sylvain Choquet[1], Marine Baron*[1]

*[1]Hôpital Pitié-Salpétrière, Paris, France;[2]Assistance Publique-Hôpitaux de Marseille, Marseille, France;[3]Hôpitaux Universitaires de Strasbourg, Strasbourg, France; [4]Hopital Saint Louis, Paris, France;[5]CHU Lyon-Sud, Lyon, France; [6]Cochin hospital, Paris, France; [7]APHP, Necker, paris, France;[8]Hôpital Saint Antoine, paris, France;*

### Background

Post transplant lymphoproliferative disorder (PTLD) is an uncommon but life-threatening complication of organ transplant. First line treatment (tt) usually combines reduction of immunosuppression, anti-CD20 monoclonal antibodies and anthracycline containing chemotherapy, leading to durable remissions in upwards 60% of patients (pts). However, relapsed or refractory (RR) PTLD is an unmet medical need in frail and comorbid pts, and novel innovative therapies with good safety profile are necessary. EBV-specific cytotoxic T lymphocytes are restricted to EBV+ disease while nearly half of PTLD cases are EBV-negative. CD20xCD3 bispecific antibodies (BsAb) are increasingly used in RR B-cell malignancies in immunocompetent pts because of their efficacy, manageable toxicity, and off-the-self availability. Data in PTLD are scarce, with concerns regarding impaired T-cell function and lack of response, graft rejection and infections.

### Aims

To assess the efficacy and safety of BsAb in PTLD, we conducted a retrospectively multicentric review of RR pts treated with at least one full dose of BsAb among the French Kvirogref registry.

### Methods

Primary end-points included response rates, overall survival (OS) and progression-free survival (PFS). Secondary end-points included toxicity and graft function.

**Copyright Information:** (Online) ISSN: 2572-9241
©2026 The Author(s). HemaSphere is published by John Wiley & Sons Ltd on behalf of European Hematology Association. This is an open access Abstract Book distributed under the Attribution-NonCommercial-NoDerivs (CC BY-NC-ND), which allows third parties to download the articles and share them with others as long as they credit the author and the Abstract Book, but they cannot change the content in any way or use them commercially.
**Abstract Book Citations:** Authors, Title, HemaSphere, 2026;10:(S1):pages. The abstract book DOIs can be found on the “Author Information” page.
**Disclaimer:** Articles published in the journal HemaSphere exclusively reflect the opinions of the authors. The authors are responsible for all content in their abstracts including accuracy of the facts, statements, citing resources, etc.

**Results**

Ten pts were identified : 5 were males, median age was 52 years (range 18-71). Transplanted organs were kidney (8), lung (1) and liver (1). All pts had late-onset PTLD diagnosis, with a median of 9 years from transplantation, and EBV-negative monomorphic CD20+ diseases. Median previous lines of tt were 3 (range 1-9), and 7 pts were refractory to their last tt. Frontline, all pts received rituximab and anthracycline-based tt (CHOP for 9 pts and ACVBP for 1). Five pts had prior CAR T-cell therapy. Median delay from PTLD diagnosis to BsAb initiation was 11 months (range 4-200). At tt start, all pts had stage IV disease without CNS involvement; 9 had elevated LDH. For rejection prophylaxis, 4 pts were receiving calcineurin inhibitors and 8 corticosteroids; none were on anti-metabolites. Median lymphocyte count was 500 cells /µL (range 210-1,610).
With a median follow-up of 21 months, 6 pts received glofitamab and 4 epcoritamab. Lenalidomide was given upfront in 2 pts, and added secondarily in 2; one of them also received ibrutinib. Median number of cycles was 3 (range 1-12). At last follow-up, all pts discontinued tt, mostly for progression. Overall response rate was 30% (2 CR, 1 PR, 1 SD, 6 PD). The 2 CR pts remain alive and in remission after 17 and 21 months (after 11 full-dose cycles of epcoritamab and 10 of glofitamab respectively); the first one received lenalidomide at the same time during 4 months and the other one no other anti-lymphoma tt. Their baseline lymphocyte count was 1.6 and 0.7 cells/µL, and neither was receiving calcineurin inhibitors. The PR pt progressed after 3 months and the SD pt died from pneumonia 1 month later. Median PFS and OS were 2 and 6 months.
CRS occurred in 6 pts during the cycle 1; 3 received tocilizumab. No grade ≥3 CRS or neurotoxicity was observed, and 7 pts developed infections, mainly bacterial (1 patient died from pneumonia, while others were successfully managed). No graft rejection occurred.

**Summary/Conclusion**

Despite underlying immunosuppression, BsAb induced durable response > 12 months in 20% cases of 10 heavily pretreated RR PTLD pts. Although numerically lower than in immunocompetent pts, BsAb may be a therapeutic option without new safety signals.

**Copyright Information:** (Online) ISSN: 2572-9241
©2026 The Author(s). HemaSphere is published by John Wiley & Sons Ltd on behalf of European Hematology Association. This is an open access Abstract Book distributed under the Attribution-NonCommercial-NoDerivs (CC BY-NC-ND), which allows third parties to download the articles and share them with others as long as they credit the author and the Abstract Book, but they cannot change the content in any way or use them commercially.
**Abstract Book Citations:** Authors, Title, HemaSphere, 2026;10:(S1):pages. The abstract book DOIs can be found on the “Author Information” page.
**Disclaimer:** Articles published in the journal HemaSphere exclusively reflect the opinions of the authors. The authors are responsible for all content in their abstracts including accuracy of the facts, statements, citing resources, etc.

### (PB3751) FRONTLINE POLATUZUMAB-BASED THERAPY IN HIGH-RISK DLBCL: REAL-WORLD OUTCOMES AND EXPLORATORY ANALYSIS OF CUMULATIVE RISK BURDEN

**Topic:** 19. Large B cell lymphomas - Clinical

Qing Zhang*[1], Xingchen Liu[1], Xueli Jiao[1], Qianying Pan[1], Fenghua Gao[1], Hao Guo[1], Jingyi Yang[1], Quande Lin[1], Lihua Dong[1], Jianwei Du[1], Zhihua Yao[1], Lina Zhang[1], Wenli Zuo[1], Xue Gao[1], Bingjie Ding[1], Hongqiang Guo[1]

[1]*the Affiliated Cancer Hospital of Zhengzhou University & Henan Cancer Hospital, Zhengzhou, China;*

### Background

Patients with diffuse large B-cell lymphoma (DLBCL) who harbor adverse prognostic features, including TP53 abnormalities, double-expressor lymphoma (DEL), and high tumor burden, such as bulky disease, represent a clinically challenging population and are associated with inferior outcomes. Although the POLARIX trial demonstrated significantly improved progression-free survival with Pola-R-CHP in an unselected frontline DLBCL population, real-world data specifically addressing biologically high-risk subgroups remain limited. Therefore, we evaluated the efficacy of frontline polatuzumab-based regimens in high-risk DLBCL and further assessed the impact of cumulative adverse risk features on clinical outcomes.

### Aims

To assess the efficacy and safety of frontline polatuzumab-based therapy in high-risk DLBCL and to examine whether cumulative risk burden influences disease control and early survival outcomes.

### Methods

This single-center, real-world cohort study enrolled 103 newly diagnosed DLBCL patients treated with frontline polatuzumab-based regimens. Adverse risk features were prespecified and included TP53 aberrations, double-expressor lymphoma (DEL), and bulky disease (≥7.5 cm).
The primary endpoint was overall response rate (ORR). Secondary endpoints included complete response (CR), progression-free survival (PFS), overall survival (OS), and safety. Survival outcomes were estimated using the Kaplan–Meier method. An exploratory cumulative risk burden analysis was conducted comparing patients with ≥2 versus <2 high-risk features, defined by the three prespecified risk factors (TP53 abnormalities, DEL, and bulky disease).

**Copyright Information:** (Online) ISSN: 2572-9241
©2026 The Author(s). HemaSphere is published by John Wiley & Sons Ltd on behalf of European Hematology Association. This is an open access Abstract Book distributed under the Attribution-NonCommercial-NoDerivs (CC BY-NC-ND), which allows third parties to download the articles and share them with others as long as they credit the author and the Abstract Book, but they cannot change the content in any way or use them commercially.
**Abstract Book Citations:** Authors, Title, HemaSphere, 2026;10:(S1):pages. The abstract book DOIs can be found on the "Author Information" page.
**Disclaimer:** Articles published in the journal HemaSphere exclusively reflect the opinions of the authors. The authors are responsible for all content in their abstracts including accuracy of the facts, statements, citing resources, etc.

### Results

**Baseline characteristics**

The median age was 63 years (range, 11–97); 72.8% had stage III–IV disease, and 65.0% had elevated LDH. High-risk features included TP53 abnormalities (52.4%), DEL (52.4%), double-hit lymphoma (4.9%), and bulky disease (33.0%).

**Overall efficacy**

Among 88 response-evaluable patients, ORR was 88.6% (78/88), with a CR rate of 65.9% (58/88) and a disease control rate of 96.6% (85/88). With a median follow-up of 10.1 months, median PFS and OS were not reached, the estimated 12-month PFS and OS rates were 87.5% and 98.5%, respectively.

**Subgroup analyses**

Clinical activity was consistent across high-risk subgroups. The ORR was 88.9% (40/45) in TP53-abnormal patients, 91.1% (41/45) in DEL patients, and 75.0% (21/28) in patients with bulky disease. The estimated 12-month PFS rates were 85.6%, 81.6%, and 81.1%, respectively.

**Cumulative risk burden analysis**

Patients with ≥2 high-risk features (n=24) had an ORR of 87.5%, compared with 89.1% in those with <2 factors (P=1.000). The estimated 12-month PFS rate was lower in the ≥2 group (76.1% vs 92.1%, P=0.825).

**Safety**

The treatment was well tolerated, with grade ≥3 neutropenia in 12.0%, anemia in 10.0%, and infections in 22.0%. Peripheral neuropathy occurred in 4%, all grade 1–2.

### Summary/Conclusion

In this real-world cohort enriched with high-risk features, frontline polatuzumab-based therapy achieved high response rates with manageable toxicity. Clinical activity was sustained in TP53-abnormal and DEL subgroups. Although ORR was similar across cumulative risk groups, PFS was numerically lower in patients with ≥2 high-risk features. While this difference did not reach statistical significance, it suggests that cumulative risk burden may still influence sustained disease control.

**Copyright Information:** (Online) ISSN: 2572-9241
©2026 The Author(s). HemaSphere is published by John Wiley & Sons Ltd on behalf of European Hematology Association. This is an open access Abstract Book distributed under the Attribution-NonCommercial-NoDerivs (CC BY-NC-ND), which allows third parties to download the articles and share them with others as long as they credit the author and the Abstract Book, but they cannot change the content in any way or use them commercially.
**Abstract Book Citations:** Authors, Title, HemaSphere, 2026;10:(S1):pages. The abstract book DOIs can be found on the “Author Information” page.
**Disclaimer:** Articles published in the journal HemaSphere exclusively reflect the opinions of the authors. The authors are responsible for all content in their abstracts including accuracy of the facts, statements, citing resources, etc.

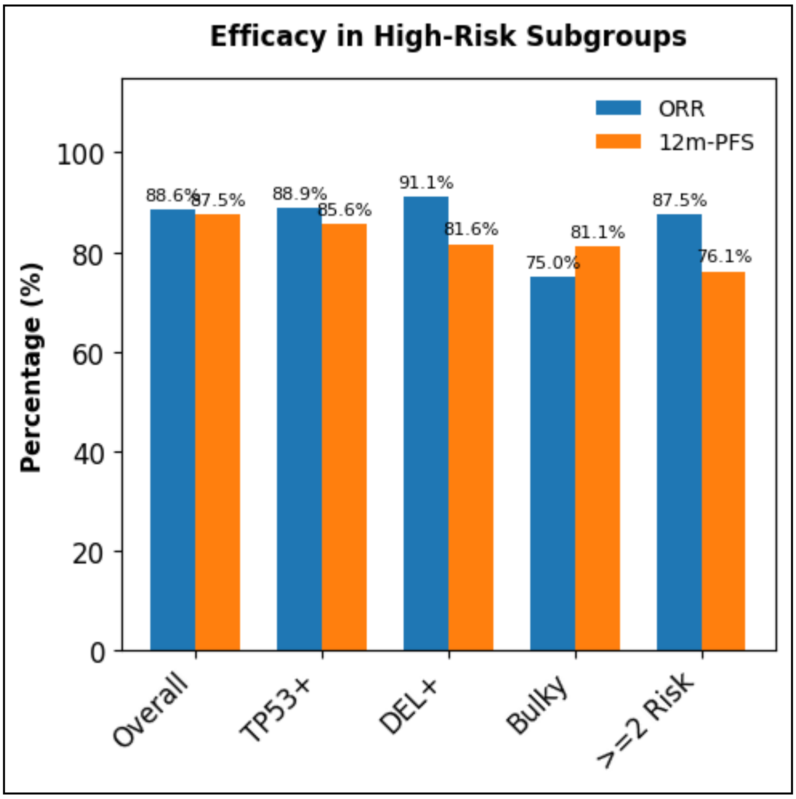

Figure 1 . Efficacy metrics across high-risk DLBCL subgroups. Bars represent the overall response rate (ORR, left axis) and the estimated 12-month progression-free survival (PFS, right axis) for each cohort. ≥2 Risks denotes patients harboring at least two of the following: TP53 abnormality, DEL, or bulky disease.

**Copyright Information:** (Online) ISSN: 2572-9241
©2026 The Author(s). HemaSphere is published by John Wiley & Sons Ltd on behalf of European Hematology Association. This is an open access Abstract Book distributed under the Attribution-NonCommercial-NoDerivs (CC BY-NC-ND), which allows third parties to download the articles and share them with others as long as they credit the author and the Abstract Book, but they cannot change the content in any way or use them commercially.
**Abstract Book Citations:** Authors, Title, HemaSphere, 2026;10:(S1):pages. The abstract book DOIs can be found on the "Author Information" page.
**Disclaimer:** Articles published in the journal HemaSphere exclusively reflect the opinions of the authors. The authors are responsible for all content in their abstracts including accuracy of the facts, statements, citing resources, etc.

### (PB3752) GLOFITAMAB IN RELAPSED/REFRACTORY PRIMARY CNS LYMPHOMA: A COMPARATIVE ANALYSIS WITH HISTORICAL CONTROLS

**Topic:** 19. Large B cell lymphomas - Clinical

Jingrui Sui*[1], Junqing Xu[1]

*[1]Qingdao University affiliated Yantai Yuhuangding Hospital, Hematology, Yantai, China;*

### Background

Primary Central Nervous System Lymphoma (PCNSL) is a rare and aggressive form of extranodal non-Hodgkin lymphoma that primarily affects the brain, eyes, and leptomeninges. Patients with relapsed or refractory (R/R) disease typically face a poor prognosis, and their treatment options are often constrained by the blood-brain barrier. Glofitamab, a bispecific antibody that targets CD20 and CD3 T-cells, has shown significant efficacy in systemic B-cell lymphomas.

### Aims

This study aims to assess the real-world safety and efficacy of Glofitamab in patients with R/R PCNSL and to compare these outcomes with historical controls treated with conventional therapies.

### Methods

We conducted a retrospective analysis of patients with relapsed/refractory primary central nervous system lymphoma (R/R PCNSL) who received Glofitamab from February 2025 to October 2025. Patients were administered Glofitamab according to standard dosing schedules. A comparative cohort of patients treated with conventional salvage regimens (such as high-dose methotrexate re-challenge, BTK inhibitors, immunomodulatory drugs, or whole-brain radiotherapy) was identified for outcome comparison during the same time period. The data collected encompassed patient demographics, prior lines of therapy, baseline central nervous system involvement, toxicity profiles (specifically Cytokine Release Syndrome [CRS] and Immune Effector Cell-Associated Neurotoxicity Syndrome [ICANS]), response rates (assessed using CNS-specific response criteria, including MRI and cerebrospinal fluid analysis), and survival outcomes.

### Results

A total of 8 patients were identified in the Glofitamab cohort, while 11 patients were included in the historical control cohort. The median age of participants was 63.7 years, and they had received a median of 3.2 prior lines of therapy (range 2.0-4.0), with 63.2% having prior exposure to BTK inhibitors. At baseline, 89.5% of patients exhibited multifocal parenchymal disease.
In the Glofitamab cohort, CRS occurred in 25% of patients, all of whom were classified as Grade 1-2; no cases of Grade ≥3 CRS were reported. No cases of ICANS were reported. All CRS were reversible with supportive care and corticosteroids. The overall response rate (ORR) in the Glofitamab cohort was 87.5%, accompanied by a complete response (CR) rate of 62.5%. Notably, responses were observed in patients with bulky disease and those who had previously failed BTK inhibitors. In contrast, the historical control cohort exhibited an ORR of 45.5% and a CR rate of 9.1%. With a median follow-up of 9.3 months, the median progression-free survival (PFS) for patients treated with Glofitamab was not reached, whereas the control group had a median PFS of 3.7 months. Additionally, the median overall survival (OS) for the Glofitamab cohort was not reached, in comparison to 6.3 months for the controls.

**Copyright Information:** (Online) ISSN: 2572-9241
©2026 The Author(s). HemaSphere is published by John Wiley & Sons Ltd on behalf of European Hematology Association. This is an open access Abstract Book distributed under the Attribution-NonCommercial-NoDerivs (CC BY-NC-ND), which allows third parties to download the articles and share them with others as long as they credit the author and the Abstract Book, but they cannot change the content in any way or use them commercially.
**Abstract Book Citations:** Authors, Title, HemaSphere, 2026;10:(S1):pages. The abstract book DOIs can be found on the "Author Information" page.
**Disclaimer:** Articles published in the journal HemaSphere exclusively reflect the opinions of the authors. The authors are responsible for all content in their abstracts including accuracy of the facts, statements, citing resources, etc.

**Summary/Conclusion**

This retrospective series provides preliminary evidence that Glofitamab demonstrates clinical activity in R/R PCNSL and maintains a manageable safety profile. In comparison to historical controls treated with conventional salvage regimens, Glofitamab was associated with significantly higher response rates and improved survival outcomes. These findings indicate that Glofitamab may represent a promising therapeutic option for this challenging patient population. Prospective randomized trials are necessary to validate these findings.

**Copyright Information:** (Online) ISSN: 2572-9241
©2026 The Author(s). HemaSphere is published by John Wiley & Sons Ltd on behalf of European Hematology Association. This is an open access Abstract Book distributed under the Attribution-NonCommercial-NoDerivs (CC BY-NC-ND), which allows third parties to download the articles and share them with others as long as they credit the author and the Abstract Book, but they cannot change the content in any way or use them commercially.
**Abstract Book Citations:** Authors, Title, HemaSphere, 2026;10:(S1):pages. The abstract book DOIs can be found on the "Author Information" page.
**Disclaimer:** Articles published in the journal HemaSphere exclusively reflect the opinions of the authors. The authors are responsible for all content in their abstracts including accuracy of the facts, statements, citing resources, etc.

### (PB3753) ODRONEXTAMAB FOR RELAPSED/REFRACTORY LARGE B-CELL LYMPHOMA BEFORE AUTOLOGOUS CHIMERIC ANTIGEN RECEPTOR T CELL (CART) THERAPIES

**Topic:** 19. Large B cell lymphomas - Clinical

Mengyang Di*[1, 2], Mazyar Shadman[1], Mary W Redman[1], Stephen Smith[1, 2], Chaitra Ujjani[1, 2], Ryan Lynch[1, 2], Paul Martin[1, 2], Jennifer Huang[1], Jordan Gauthier[1], Alexandre Hirayama[1], Houston E Warren[1], Emily Liang[1], Neena Kennedy[1, 2], Brian Till[1, 2], Ajay Gopal[1, 2]

*[1]Fred Hutchinson Cancer Center, Seattle, United States of America;[2]University of Washington, Seattle, United States of America;*

### Background

Autologous CART has been approved for relapsed/refractory (r/r) large B cell lymphoma (LBCL). However, the logistical challenges of CART manufacturing, need for caregiver support, and insurance approval often lead to a substantial delay in therapy administration. As a result, ~50% CART candidates with LBCL are unable to receive this potentially lifesaving therapy, primarily due to uncontrolled disease progression (Marie Hu et al, ASTCT abstract 2023; Minoo et al, ASTCT abstract 2023). Survival outcomes in these patients are often dismal. Although bridging therapy (BT) has been used, the efficacy of most bridging regimens is disappointing. The overall response rate (ORR) was only 20-30% even with the use of some novel agents, e.g., polatuzumab vedotin (Berthge et al, Blood 2022).

Bispecific T cell engagers (TCE) targeting CD20 x CD3 have emerged as highly effective therapies in heavily treated r/r LBCL. For instance, odronextamab (odron) provided an ORR of 52% with 32% complete response (CR) rate based on the ELM2 trial. These data suggest a great potential of using odron as BT to increase the chance of successful CART administration. It may also help improve efficacy of CART through reducing the tumor burden. On the other hand, despite emerging real-world data suggest similar outcomes following CART regardless of TCE before CART (Crochet et al, Blood 2024), there remains the hypothetical concern about development of T-cell exhaustion during TCE. The latter could theoretically increase the chance of CART manufacturing failure and decrease CART efficacy.

### Aims

In this trial, we aim to examine the safety and efficacy of odron as BT in LBCL.

**Copyright Information:** (Online) ISSN: 2572-9241
©2026 The Author(s). HemaSphere is published by John Wiley & Sons Ltd on behalf of European Hematology Association. This is an open access Abstract Book distributed under the Attribution-NonCommercial-NoDerivs (CC BY-NC-ND), which allows third parties to download the articles and share them with others as long as they credit the author and the Abstract Book, but they cannot change the content in any way or use them commercially.
**Abstract Book Citations:** Authors, Title, HemaSphere, 2026;10:(S1):pages. The abstract book DOIs can be found on the “Author Information” page.
**Disclaimer:** Articles published in the journal HemaSphere exclusively reflect the opinions of the authors. The authors are responsible for all content in their abstracts including accuracy of the facts, statements, citing resources, etc.

**Methods**

This phase II trial (NCT06784726) has been open since June 2025. It includes adult patients with LBCL who are candidates for commercial anti-CD19 CART. Disease involving the central nervous system, or prior receipt of CART or allogeneic stem cell transplant are exclusionary criteria.

Eligible participants receive at least two cycles of odron before leukapheresis based on the standard dosing schedule (Figure 1). Participants who experience CR after C2 of odron may opt out of CART and continue odron for up to a total of 12 months. Decision of opting out of CART should be made before leukapheresis based on participant preference and clinical discretion. For participants moving forward with CART, additional cycles of odron may be given.

The primary endpoint is failure to receive leukapheresis due to progression or adverse events secondary to odron, or requiring other BT before leukapheresis due to lack of response. We will examine successful receipt of CART, other efficacy endpoints, and safety. We will also explore the impact of odron as BT on T-cell function.

We plan to enroll 27 participants and the study is designed to rule out a >50% rate of failure to receive leukapheresis (primary endpoint). The analyses of the trial results will be primarily descriptive.

**Results**

NA for TIP

**Summary/Conclusion**

To our knowledge, this is the first clinical trial that examines the use of TCE as BT to CART in LBCL. It will provide important data on safety and efficacy of this bridging strategy. Furthermore, it helps answer the critical question about the potential impact of TCE before leukapheresis on T-cell fitness and CART efficacy. The results of this trial have the potential of transforming the current bridging practice and of improving outcomes in this challenging to treat population.

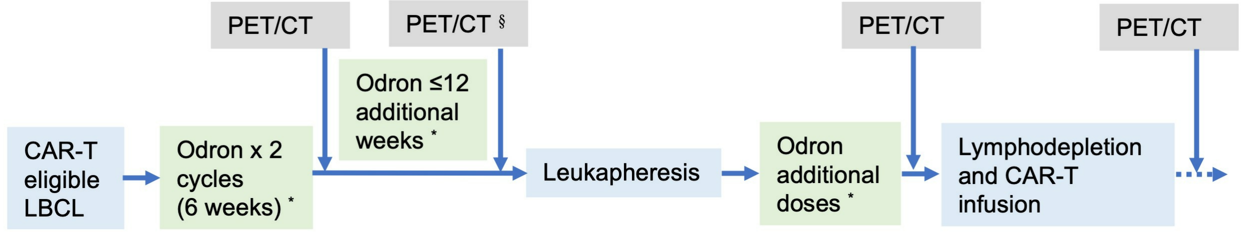

**Figure 1. Study schema of the odron trial.**
* Based on the standard dosing schedule of odron: 0.2 mg C1D1, 0.5 mg C1D2, 2 mg on C1D8 and C1D9, 10 mg on C1D15 and C1D16, 160 mg weekly C2-C4, then 320 mg biweekly (please see ELM2 trial).
§ PET/CT will be obtained if patients receive additional doses before leukapheresis.

**Copyright Information:** (Online) ISSN: 2572-9241
©2026 The Author(s). HemaSphere is published by John Wiley & Sons Ltd on behalf of European Hematology Association. This is an open access Abstract Book distributed under the Attribution-NonCommercial-NoDerivs (CC BY-NC-ND), which allows third parties to download the articles and share them with others as long as they credit the author and the Abstract Book, but they cannot change the content in any way or use them commercially.
**Abstract Book Citations:** Authors, Title, HemaSphere, 2026;10:(S1):pages. The abstract book DOIs can be found on the “Author Information” page.
**Disclaimer:** Articles published in the journal HemaSphere exclusively reflect the opinions of the authors. The authors are responsible for all content in their abstracts including accuracy of the facts, statements, citing resources, etc.

### (PB3754) POLATUZUMAB VEDOTIN BASED REGIMENS AS FIRST-LINE THERAPY FOR LARGE B-CELL LYMPHOMA: REAL-WORLD DATA FROM CLINICAL PRACTICE IN THE RUSSIAN FEDERATION

**Topic:** 19. Large B cell lymphomas - Clinical

Gayane Tumyan*[1], Anastasia Semenova[1], Yulia Ermakova[2], Alina Chuprakova[3], Alexandra Chernova[4], Maria Frolova[5], Natalya Mikhaylova[6], Liudmila Fedorova[6], Olga Serdyuk[7], Svetlana Samarina[8], Nadezhda Nikolaeva[9], Irina Lysenko[9], Leisan Mukharliamova[10], Gulnara Khusainova[10], Nadezhda Medvedeva[11], Yana Mangasarova[12], Alexey Kuvshinov[13], Daniil Zaytcev[13], Andrey Novitsky[14], Elena Karyagina[14], Vera Denisova[15], Tatiana Pospelova[16], Irina Nechunaeva[16], Ksenia Deduhina[17], Nelli Gabeeva[12], Evgeniy Zvonkov[12], Andrey Vorobyev[18], Irina Poddubnaya[19], Olga Samoilova[18], Yulia Batukhtina[20], Ruslan Zukov[20], Elena Babich[21], Tuiara Aleksandrova[22], Irina Verhoglyad[23], Natalya Glonina[23], Irina Kudinova[24], Ekaterina Ukrainchenko[25], Tatyana Sycheva[26], Natalia Ipatova[27], Andrey Proydakov[28]

*[1]The Blokhin Russian Cancer Research Center, Moscow, Russia;[2]Samara Regional Clinical Oncological Dispensary, Samara, Russia; [3]Kaliningrad Central City Clinical Hospital, Kaliningrad, Russia; [4]Salekhard District Clinical Hospital, Salekhard, Russia; [5]Vologda Regional Clinical Hospital, Vologda, Russia; [6]RM Gorbacheva Research Institute, Saint Petersburg, Russia;[7]Krasnodar Clinical Oncology Dispensary No. 1, Krasnodar, Russia; [8]Kirov Research Institute of Hematology and Blood Transfusion of the Federal Medical and Biological Agency, Kirov, Russia; [9]Rostov-on-Don National Medical Research Center of Oncology, Rostov-on-Don, Russia; [10]Kazan Republican Clinical Oncology Dispensary, Kazan, Russia; [11]Saint Petersburg City Clinical Hospital No. 31, Saint Petersburg, Russia; [12]Federal Hematology Research Center, Moscow, Russia; [13]Russian Research Institute of Hematology and Transfusion Medicine, Saint Petersburg, Russia; [14]Saint Petersburg City Hospital No. 15, Saint Petersburg, Russia; [15]Research Institute of Fundamental and Clinical Immunology, Novosibirsk, Russia;[16]Novosibirsk Regional Clinical Hospital, Novosibirsk, Russia; [17]Chita Zabaykalsky Regional Oncology Dispensary, Chita, Russia; [18]Nizhny Novgorod Regional Clinical Hospital, Nizhny Novgorod, Russia; [19]Russian Medical Academy of Continuous Professional Education, Moscow, Russia; [20]Krasnoyarsk Regional Oncology Dispensary, Krasnoyarsk, Russia;[21]KhantyMansiysk Regional Clinical Hospital, KhantyMansiysk, Russia; [22]Yakutsk Regional Clinical Hospital No. 1, Yakutsk, Russia;[23]Khabarovsk Regional Clinical Hospital, Khabarovsk, Russia; [24]Barnaul Regional Clinical Hospital, Barnaul, Russia; [25]Saint Petersburg City Aleksandrovskaya Hospital, Saint Petersburg, Russia; [26]Astrakhan City Aleksandro-Mariinskaya Hospital, Astrakhan City, Russia;[27]Republican Clinical Hospital, Izhevsk, Russia; [28]Komi Republican Clinical Oncology Dispensary, Syktyvkar, Russia;*

### Background

Based on the results of the POLARIX phase III clinical trial, polatuzumab vedotin in combination with rituximab, cyclophosphamide, doxorubicin, and prednisone was approved for frontline diffuse large B-cell lymphoma (DLBCL) treatment in Russian Federation in 2022. With the accumulating real-world experience of using polatuzumab vedotin based regimens in the first-line setting, further investigation of practical application is warranted. Thus the aim of our study was to assess the efficacy and safety of Pola-based treatment in first line LBCL setting in routine clinical practice.

### Aims

The primary endpoints were objective response rate (ORR), complete response rate (CR), and progression-free survival (PFS). Secondary endpoints were: treatment completion rate, overall survival (OS).

**Copyright Information:** (Online) ISSN: 2572-9241
©2026 The Author(s). HemaSphere is published by John Wiley & Sons Ltd on behalf of European Hematology Association. This is an open access Abstract Book distributed under the Attribution-NonCommercial-NoDerivs (CC BY-NC-ND), which allows third parties to download the articles and share them with others as long as they credit the author and the Abstract Book, but they cannot change the content in any way or use them commercially.
**Abstract Book Citations:** Authors, Title, HemaSphere, 2026;10:(S1):pages. The abstract book DOIs can be found on the "Author Information" page.
**Disclaimer:** Articles published in the journal HemaSphere exclusively reflect the opinions of the authors. The authors are responsible for all content in their abstracts including accuracy of the facts, statements, citing resources, etc.

### Methods

This retrospective, observational multicenter study included 108 adult patients (pts) with LBCL who received first-line polatuzumab vedotin–based therapy between 2022 and 2026 in 27 centers in Russia. The median age was 65 years (range: 22–87), and 61 pts (56.4%) were male. Histological subtypes included 83 pts (76,8%) DLBCL NOS, with non-GCB subtype in 67% and GCB subtype in 33%; newly diagnosed transformations of indolent B-cell lymphomas - 12 (11%); high-grade B-cell lymphoma - 7 (6.4%); other rare variants - 6 (5,8%). At diagnosis, 93 pts (86.1%) had stage III–IV disease, 56 (51.8%) presented with B-symptoms, 68 (62.9%) - ECOG >= 2, 55 (50,9%) – bulky disease (≥7.5 cm). Based on IPI, 85 pts (78.7%) were classified as intermediate-high or high risk (IPI 3–5). Treatment regimens included: Pola-R-CHP - 93 pts (86.1%), Pola-R-miniCHP - 5 (4.7%), Pola-R-EPOCH - 10 (9.2%). Multivariate analysis for OS and PFS was performed using Cox proportional hazards regression.

### Results

As of the data cut-off (February 2026), the median number of treatment cycles was 6 (range: 1–6). The full course of treatment was completed in 94 pts (87%). Among pts who completed treatment, 80% (76/94) underwent end-of-treatment assessment via PET/CT. A complete metabolic response (CMR; DC1–3) was achieved in 84.2% (64/76). Interim assessment after 3 cycles was conducted in 85 pts (78.7%), with 64 evaluated by PET/CT: complete responses (CR) were 61,1% (52/85), including 49 CMR. Among 30 pts with partial response at interim, 63% (19/30) converted to CR before therapy completion. The hematological toxicity rates were: neutropenia 62.9%, leukopenia 43.5%, anemia 39.8%, thrombocytopenia 2.7%. Non-hematologic adverse events: nausea 27.7%, diarrhea 8.3%, infections 9.2%, peripheral neuropathy 3.7%. Grade ≥3 adverse events occurred in 3.3% (36/108), mainly due to neutropenia and febrile neutropenia (19.4%). Dose reductions due to hematologic toxicity were required in 13 patients (12%). At the time of analysis 97 pts (89.8%) were alive, 11 (10.1%) had died: 4 from disease progression and 7 from disease unrelated causes. Disease progression occurred in 14 pts (12.9%). At median follow-up 17 months, median PFS and OS were not reached, 1-year PFS – 81,8%, 1-year OS – 89.5%.

### Summary/Conclusion

This multicenter real-world study of polatuzumab vedotin–based treatment in Russia demonstrate high efficacy and an acceptable safety profile in a predominantly high-risk previously untreated LBCL population. The clinical outcomes are align with global trial data, supporting the regimen's consistency and effectiveness in routine practice.

**Copyright Information:** (Online) ISSN: 2572-9241
©2026 The Author(s). HemaSphere is published by John Wiley & Sons Ltd on behalf of European Hematology Association. This is an open access Abstract Book distributed under the Attribution-NonCommercial-NoDerivs (CC BY-NC-ND), which allows third parties to download the articles and share them with others as long as they credit the author and the Abstract Book, but they cannot change the content in any way or use them commercially.
**Abstract Book Citations:** Authors, Title, HemaSphere, 2026;10:(S1):pages. The abstract book DOIs can be found on the "Author Information" page.
**Disclaimer:** Articles published in the journal HemaSphere exclusively reflect the opinions of the authors. The authors are responsible for all content in their abstracts including accuracy of the facts, statements, citing resources, etc.

### (PB3755) REAL-WORLD OUTCOMES OF CAR-T THERAPY IN PATIENTS WITH RELAPSED/REFRACTORY DLBCL AFTER ≥3 PRIOR LINES OF TREATMENT

**Topic:** 19. Large B cell lymphomas - Clinical

Rui Liu*[1], Zhonghua Fu[1], Yuelu Guo[1], Miaomiao Cao[1], Lixia MA[1], Fan Yang[1], Biping Deng[2], Alex Chang[3], Xiaoyan Ke[1], Kai Hu[1]

*[1]Beijing GoBroad Hospital, Department of Lymphoma and Myeloma Research Center, Beijing, China; [2]Beijing GoBroad Boren Hospital, Cytology Laboratory, Beijing, China; [3]Shanghai YaKe Biotechnology Ltd., Engineering Research Center of Gene Technology, Ministry of Education, Institute of Genetics, School of Life Sciences, Shanghai, China;*

### Background

Patients with relapsed/refractory diffuse large B-cell lymphoma (r/r DLBCL) who progress after ≥3 prior lines of therapy have extremely poor outcomes, and therapeutic options remain limited. We evaluated the real-world efficacy and safety of CD19-directed CAR-T therapy in patients without previous exposure to CAR-T.

### Aims

To evaluate the real-world efficacy and safety of CD19-directed CAR-T therapy in patients with relapsed or refractory diffuse large B-cell lymphoma (r/r DLBCL) who had received ≥3 prior lines of therapy and had no previous exposure to CAR-T, and to characterize clinical outcomes in heavily pretreated and high-risk subgroups.

### Methods

We retrospectively analyzed consecutive r/r DLBCL patients who received CAR-T therapy at our center between March 2019 and July 2025 after ≥3 prior treatment lines. A total of 178 patients were included. The median age was 56 years (range, 13–86), and 86/178 (48.3%) were male. High-risk features were common: 86/178 (48.3%) had an IPI ≥3, 18/178 (10.1%) had double-hit or triple-hit genetics, 19/178 (10.7%) had relapsed after autologous transplantation, and 163/178 (91.6%) had stage III–IV disease. Prior therapy consisted of 3 lines in 64/178 (36.0%) and >3 lines in 114/178 (64.0%). Bridging therapy was administered in 116/178 (65.2%) patients, with a median of 2 cycles (range, 1–9). Among these, 4/178 (2.2%) received CD20×CD3 bispecific antibodies and 37/178 (20.8%) received polatuzumab-based regimens.

### Results

*Efficacy:*
The 3-month overall response rate (ORR) was 45% (80/178), including a complete response (CR) rate of 34% (61/178). Best ORR was 57% (102/178) and best CR rate was 43% (76/178). With a median follow-up of 29.42 months, median progression-free survival (PFS) was 4.41 months and median overall survival (OS) was 18.77 months. The 1- and 2-year PFS rates were 38% and 35%, respectively; corresponding OS rates were 58% and 48%.
*Safety:*
Cytokine release syndrome (CRS) occurred in 126/178 patients (71%), with grade ≥3 CRS in 12/178 (7%). Immune effector cell–associated neurotoxicity syndrome (ICANS) occurred in 8/178 (4%), including 4/178 (2%) with grade ≥3 events.

**Copyright Information:** (Online) ISSN: 2572-9241
©2026 The Author(s). HemaSphere is published by John Wiley & Sons Ltd on behalf of European Hematology Association. This is an open access Abstract Book distributed under the Attribution-NonCommercial-NoDerivs (CC BY-NC-ND), which allows third parties to download the articles and share them with others as long as they credit the author and the Abstract Book, but they cannot change the content in any way or use them commercially.
**Abstract Book Citations:** Authors, Title, HemaSphere, 2026;10:(S1):pages. The abstract book DOIs can be found on the “Author Information” page.
**Disclaimer:** Articles published in the journal HemaSphere exclusively reflect the opinions of the authors. The authors are responsible for all content in their abstracts including accuracy of the facts, statements, citing resources, etc.

**Summary/Conclusion**

In heavily pretreated, CAR-T–naïve r/r DLBCL patients, CAR-T therapy produced meaningful and durable clinical responses with manageable toxicity. These real-world data support the effectiveness of CAR-T therapy even in patients with extensive prior treatment exposure and high-risk disease features.

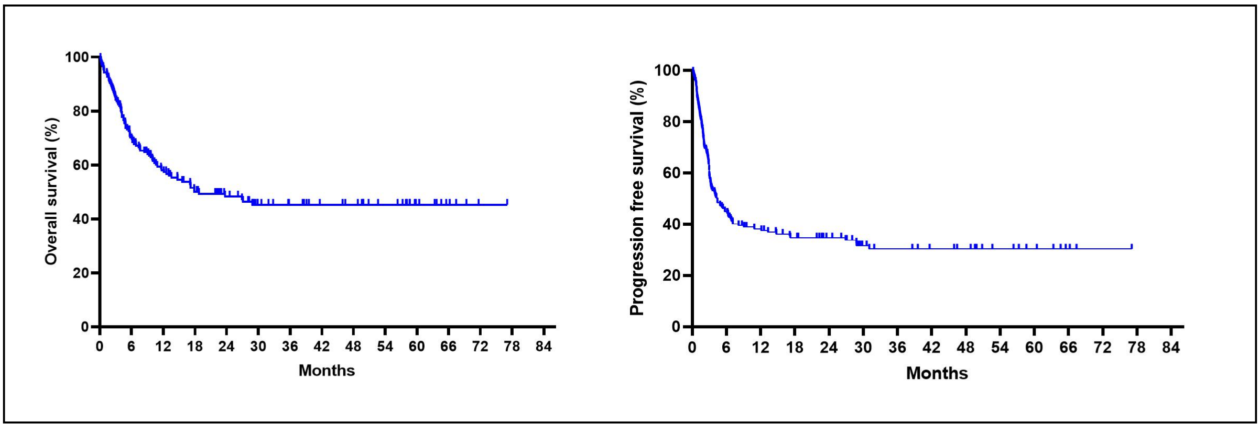

**Copyright Information:** (Online) ISSN: 2572-9241
©2026 The Author(s). HemaSphere is published by John Wiley & Sons Ltd on behalf of European Hematology Association. This is an open access Abstract Book distributed under the Attribution-NonCommercial-NoDerivs (CC BY-NC-ND), which allows third parties to download the articles and share them with others as long as they credit the author and the Abstract Book, but they cannot change the content in any way or use them commercially.
**Abstract Book Citations:** Authors, Title, HemaSphere, 2026;10:(S1):pages. The abstract book DOIs can be found on the "Author Information" page.
**Disclaimer:** Articles published in the journal HemaSphere exclusively reflect the opinions of the authors. The authors are responsible for all content in their abstracts including accuracy of the facts, statements, citing resources, etc.

### (PB3756) COMPARATIVE EFFICACY AND SAFETY OF CD19-DIRECTED THERAPIES IN RELAPSED/REFRACTORY LARGE B-CELL LYMPHOMA

**Topic:** 19. Large B cell lymphomas - Clinical

Mengjiao Li[1, 2], Ling Qiu[1], Yanling Li[1, 2], Caiyang Li[1, 3], Jiajia Li[1, 2], Ying He[1], Dan Chen[1], Shihui Ren[1], Hao Yao*[1, 3, 4, 5], Fangyi Fan[1, 2]

*[1]Department of Hematology, The General Hospital of Western Theater Command, Chengdu, China;[2]Department of Clinical Medicine, North Sichuan Medical College, Nanchong, China; [3]Institute of Basic Medicine, North Sichuan Medical College, Nanchong, China; [4]College of Medicine, Southwest Jiaotong University, Chengdu, China; [5]Tissue Stress Injury and Functional Repair Key Laboratory of Sichuan Province, Chengdu, China;*

### Background

CD19-directed therapies—including chimeric antigen receptor (CAR) T-cell therapy, antibody–drug conjugates (ADCs), and monoclonal antibodies (mAbs)—have substantially expanded treatment options for relapsed/refractory large B-cell lymphoma (R/R LBCL). However, their relative efficacy and safety profiles remain unclear due to the absence of direct comparative trials.

### Aims

This meta-analysis aimed to compare the efficacy and safety of CAR T-cell therapy, ADCs, and mAbs in patients with R/R LBCL and to explore clinical factors associated with treatment response.

### Methods

A systematic search of PubMed, Embase, and the Cochrane Library was conducted to identify studies reporting outcomes of CD19-directed therapies in R/R LBCL. Twenty-one studies met inclusion criteria (13 CAR T-cell, 4 ADC, 4 mAb). Random-effects models were applied to estimate pooled complete response (CR), overall response rate (ORR), and grade ≥3 toxicity rates. Subgroup analyses and meta-regression were performed to evaluate baseline modifiers, and sensitivity analyses assessed the robustness of pooled estimates.

### Results

Among 21 studies, CAR T-cell therapy demonstrated the highest pooled CR rate (0.43; 95% CI, 0.35–0.51), significantly exceeding ADCs (0.22) and mAbs (0.23) ($P<0.0001$ for subgroup difference). ORR was numerically higher with CAR T-cell therapy but did not significantly differ across modalities ($P=0.3866$).
Safety profiles differed between treatment strategies. ADCs were associated with the highest incidence of grade ≥3 hematologic toxicity (0.50), whereas CAR T-cell therapy showed the greatest infection rate (0.35). CAR T-specific toxicities included cytokine release syndrome (CRS, 0.12) and immune effector cell-associated neurotoxicity syndrome (ICANS, 0.16).
Meta-regression analysis identified prior autologous stem-cell transplantation (ASCT) exposure as the only factor significantly associated with CR rate, with lower prior ASCT exposure correlating with higher CR rates. Age, disease stage, double-/triple-hit status, and refractory status were not significantly associated with response. Sensitivity analyses confirmed the stability of pooled CR estimates.

**Copyright Information:** (Online) ISSN: 2572-9241
©2026 The Author(s). HemaSphere is published by John Wiley & Sons Ltd on behalf of European Hematology Association. This is an open access Abstract Book distributed under the Attribution-NonCommercial-NoDerivs (CC BY-NC-ND), which allows third parties to download the articles and share them with others as long as they credit the author and the Abstract Book, but they cannot change the content in any way or use them commercially.
**Abstract Book Citations:** Authors, Title, HemaSphere, 2026;10:(S1):pages. The abstract book DOIs can be found on the “Author Information” page.
**Disclaimer:** Articles published in the journal HemaSphere exclusively reflect the opinions of the authors. The authors are responsible for all content in their abstracts including accuracy of the facts, statements, citing resources, etc.

**Summary/Conclusion**

CAR T-cell therapy achieves the deepest responses among CD19-directed treatments in R/R LBCL but is associated with distinct toxicity risks. Prior ASCT exposure appears to be a key modifier of treatment response and should be considered when selecting CD19-directed strategies. Given the indirect nature of comparisons and heterogeneity across studies, prospective head-to-head trials are warranted to define the optimal therapeutic sequencing in R/R LBCL.

**Copyright Information:** (Online) ISSN: 2572-9241
©2026 The Author(s). HemaSphere is published by John Wiley & Sons Ltd on behalf of European Hematology Association. This is an open access Abstract Book distributed under the Attribution-NonCommercial-NoDerivs (CC BY-NC-ND), which allows third parties to download the articles and share them with others as long as they credit the author and the Abstract Book, but they cannot change the content in any way or use them commercially.
**Abstract Book Citations:** Authors, Title, HemaSphere, 2026;10:(S1):pages. The abstract book DOIs can be found on the "Author Information" page.
**Disclaimer:** Articles published in the journal HemaSphere exclusively reflect the opinions of the authors. The authors are responsible for all content in their abstracts including accuracy of the facts, statements, citing resources, etc.

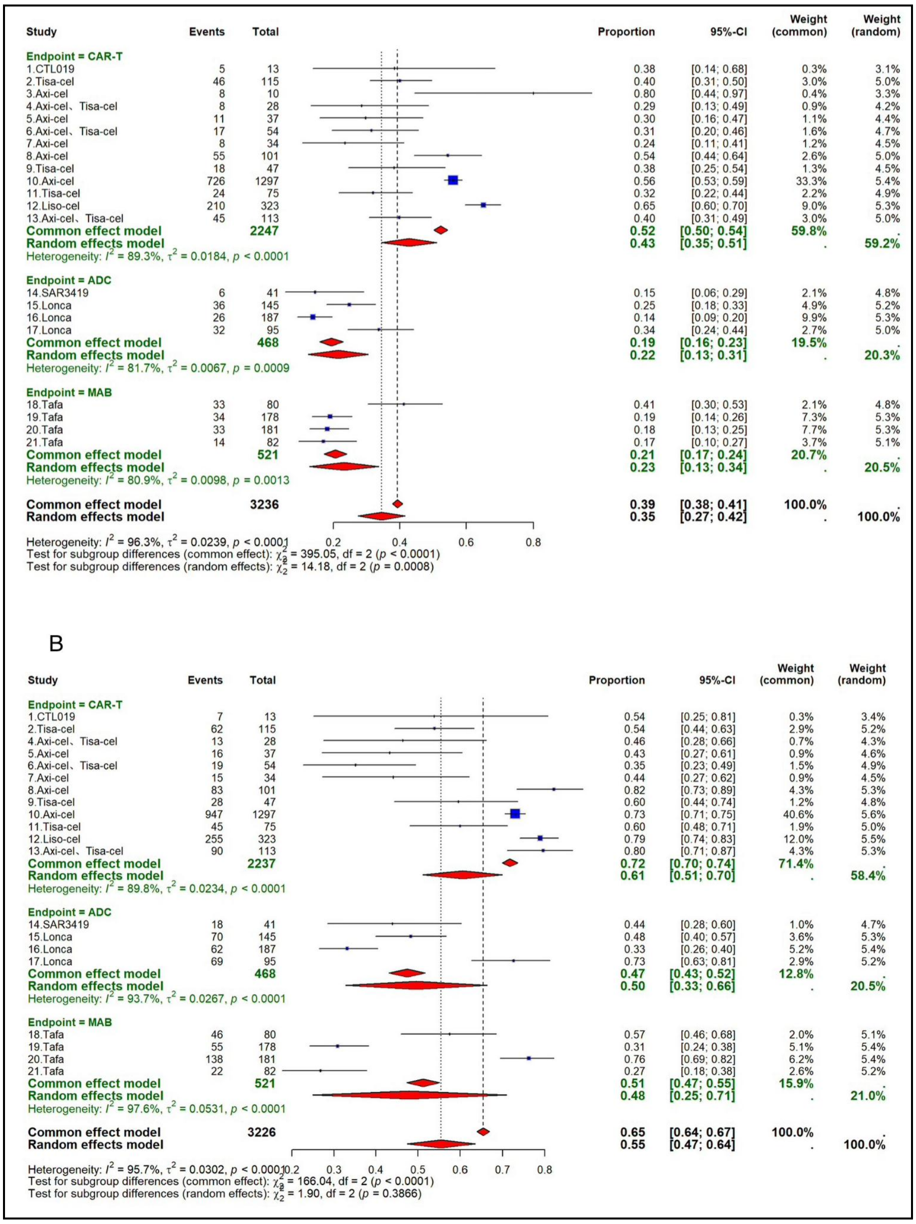

Figure . Comparative pooled response outcomes of CD19-directed therapies in relapsed/refractory large B-cell lymphoma. (A) Forest plot of complete response (CR) rates across CAR T-cell therapy, antibody–drug conjugates (ADCs), and monoclonal antibodies (mAbs). CAR T-cell therapy achieved the highest pooled CR rate and demonstrated a statistically significant advantage over ADCs and mAbs (P<0.0001 for subgroup comparison), highlighting its superior depth of response among CD19-directed strategies. (B) Forest plot of overall response rates (ORR). Although ORR differences were less pronounced and did not reach statistical significance (P=0.3866), CAR T-cell therapy consistently showed numerically higher response proportions across studies. Squares represent study-specific estimates weighted by inverse variance; horizontal lines denote 95% confidence intervals; diamonds indicate pooled effects derived from random-effects models. Substantial between-study heterogeneity was observed.

**Copyright Information:** (Online) ISSN: 2572-9241
©2026 The Author(s). HemaSphere is published by John Wiley & Sons Ltd on behalf of European Hematology Association. This is an open access Abstract Book distributed under the Attribution-NonCommercial-NoDerivs (CC BY-NC-ND), which allows third parties to download the articles and share them with others as long as they credit the author and the Abstract Book, but they cannot change the content in any way or use them commercially.
**Abstract Book Citations:** Authors, Title, HemaSphere, 2026;10:(S1):pages. The abstract book DOIs can be found on the “Author Information” page.
**Disclaimer:** Articles published in the journal HemaSphere exclusively reflect the opinions of the authors. The authors are responsible for all content in their abstracts including accuracy of the facts, statements, citing resources, etc.

### (PB3757) EDUCATIONAL OUTCOMES OF LIVE AND ENDURING CME/CE FOR HEMATOLOGY/ONCOLOGY CARE TEAMS MANAGING LYMPHOMAS WITH BISPECIFIC ANTIBODIES VIA PRE-/POST- ASSESSMENT

**Topic:** 19. Large B cell lymphomas - Clinical

Sarah Elizabeth Bomba*[1], Margie Stefan[1], Stephanie Baumhover[1], Sanneke Koekkoek[1], Tristin Abair[1], Chan Cheah[2]

*[1]Medscape Education, New York, United States of America; [2]Sir Charles Gairdner Hospital, Perth, Australia;*

### Background

As translational and clinical research related to bispecific antibodies (BsAbs) for the treatment of lymphomas continues to accelerate at an unprecedented pace, the hematology/oncology (hem/onc) care team may find it challenging to stay up to date on new discoveries and evolving standards of care. Major hematology/oncology annual meetings offer an opportunity to present live continuing medical education (CME/CE) activities to a broad cross-section of care team learners. The reach of these in-person programs is multiplied by converting recordings into accredited, online CME/CE activities available to interprofessional clinician audiences for on-demand learning.

### Aims

The objective of this study was to assess the educational impact of 6 live educational offerings throughout 2025 focused on bispecific antibodies in follicular lymphoma (FL) and diffuse large B-cell lymphoma (DLBCL) plus their enduring content on the clinical knowledge, competencies, and confidence of hem/onc care team members.

### Methods

Medscape Oncology's 2025 lymphoma bispecific antibody portfolio included 6 live and enduring CME/CE programs at or directly after major congresses. These included 5, 60-90 minute Satellite Symposia at Oncology Nursing Society (ONS), American Society of Clinical Oncology (ASCO), Journal of the Advanced Practitioner in Oncology (JADPRO), and American Society of Hematology (ASH) annual meetings, with endurings posted online within three weeks and one Live Webinar streamed after the European Hematology Association (EHA) annual meeting. The curriculum provided audience-tailored, disease-specific, and integrated programming for bispecific antibodies in FL and DLBCL.
Participation captured live (in-person, virtual, social media) and enduring material views. Pre- and post-education assessments measured the impact based on Moore's Level of knowledge, competency, and confidence. Data were aggregated across activities and stratified by learning objective theme. Relative changes in correct responses and confidence levels (4 or 5 on a 5-point Likert scale) were analyzed. A McNemar test assessed significant changes, with $P$ values < .05 considered statistically significant. Data collection concluded in February 2026.

**Copyright Information:** (Online) ISSN: 2572-9241
©2026 The Author(s). HemaSphere is published by John Wiley & Sons Ltd on behalf of European Hematology Association. This is an open access Abstract Book distributed under the Attribution-NonCommercial-NoDerivs (CC BY-NC-ND), which allows third parties to download the articles and share them with others as long as they credit the author and the Abstract Book, but they cannot change the content in any way or use them commercially.
**Abstract Book Citations:** Authors, Title, HemaSphere, 2026;10:(S1):pages. The abstract book DOIs can be found on the "Author Information" page.
**Disclaimer:** Articles published in the journal HemaSphere exclusively reflect the opinions of the authors. The authors are responsible for all content in their abstracts including accuracy of the facts, statements, citing resources, etc.

### Results

The educational portfolio showed a profound reach, with a total of 46,372 HCP learners (6,402 live; 39,970 enduring) at the close of data collection. Notably, 1,747 were hem/oncs learners. Multiple questions were used to aggregate data thematically by learning objective across the activities and by target audience (n=71 to 10,712). Statistically significant relative percentage increases were observed across all target audiences (Table 1). Statistically significant improvements were noted in areas related to:

- Knowledge: Improved understanding of key clinical data, including safety, efficacy, and guideline-recommended use for BsAbs
- Competence: Enhanced ability to apply evidence-based strategies for treatment selection, sequencing, and adverse event management for BsAbs
- Confidence: Increased confidence in assessing clinical evidence and tailoring individual BsAb therapy

### Summary/Conclusion

Participation in live or recorded CME-/CE-accredited activities on bispecific antibodies in FL and DLBCL presented throughout 2025 resulted in statistically significant improvements in knowledge, competency, and confidence across care team members. These activities have been observed to help address educational gaps, but the effect of these activities on clinical practice requires further study.

**Table 1: Assessment of pre- and post- responses by target audiences by learning theme**

| | Hem/Oncs (n=71-177) | | | | Pharmacists (n=415-750) | | | | Nurses (n=6,355-10,712) | | | | NP (n=483-1,313) | | | |
|---|---|---|---|---|---|---|---|---|---|---|---|---|---|---|---|---|
| Learning Objective Theme | Pre (%) | Post (%) | Relative change (%) | P-value | Pre (%) | Post (%) | Relative change (%) | P-value | Pre (%) | Post (%) | Relative change (%) | P-value | Pre (%) | Post (%) | Relative change (%) | P-value |
| Knowledge | 57 | 75 | 32 | <.001 | 37 | 57 | 54 | <.001 | 37 | 55 | 49 | <.001 | 42 | 61 | 45 | <.001 |
| Competence | 55 | 75 | 36 | <.01 | 33 | 53 | 61 | <.001 | 38 | 58 | 53 | <.001 | 41 | 67 | 63 | <.001 |
| Confidence | 25 | 31 | 24 | <.05 | 10 | 13 | 30 | <.001 | 14 | 18 | 29 | <.001 | 18 | 24 | 33 | <.001 |

**Copyright Information:** (Online) ISSN: 2572-9241
©2026 The Author(s). HemaSphere is published by John Wiley & Sons Ltd on behalf of European Hematology Association. This is an open access Abstract Book distributed under the Attribution-NonCommercial-NoDerivs (CC BY-NC-ND), which allows third parties to download the articles and share them with others as long as they credit the author and the Abstract Book, but they cannot change the content in any way or use them commercially.
**Abstract Book Citations:** Authors, Title, HemaSphere, 2026;10:(S1):pages. The abstract book DOIs can be found on the "Author Information" page.
**Disclaimer:** Articles published in the journal HemaSphere exclusively reflect the opinions of the authors. The authors are responsible for all content in their abstracts including accuracy of the facts, statements, citing resources, etc.

### (PB3758) A NOVEL PROGNOSTIC INDEX MODEL INCLUDE IL-6 PREDICTS THE CLINICAL OUTCOMES FOR NEWLY DIAGNOSED DIFFUSE LARGE B-CELL LYMPHOMA

**Topic:** 19. Large B cell lymphomas - Clinical

Shuhan Tang[1], Jing Xia[1], Jun Tao*[1], Li Gao[1]

[1]*hospital, chongqing, China;*

**Background**

Diffuse large B-cell lymphoma (DLBCL) is the most common aggressive non-Hodgkin lymphoma with marked heterogeneity and suboptimal prognostic stratification using conventional models such as the International Prognostic Index (IPI). Lymphocyte cytokines are closely associated with DLBCL pathogenesis and progression, yet their integration into clinical prognostic models remains underexplored.

**Aims**

This study aimed to identify cytokine-related independent prognostic factors for newly diagnosed DLBCL, develop a novel nomogram prognostic model integrating cytokines and clinical variables, and validate its predictive performance against the traditional IPI.

**Methods**

A total of 148 newly diagnosed DLBCL patients treated at a single center between January 2021 and September 2023 were enrolled. Baseline serum levels of seven lymphocyte cytokines (IL-2, IL-4, IL-6, IL-10, TNF-α, TNF-β, IL-17A) were measured, and their optimal cutoff values were determined by receiver operating characteristic (ROC) analysis. Kaplan-Meier survival curves and log-rank tests were used for survival analysis, while Cox proportional hazards models were applied to identify univariate and multivariate prognostic factors. A nomogram was constructed using the R rms package with independent prognostic factors, and its predictive efficacy was evaluated by Harrell's concordance index (C-index), area under the ROC curve (AUC), calibration plots, and time-dependent ROC analysis, with internal validation via 1000 bootstrap samples.

**Results**

The median follow-up was 16.4 months, with a 3-year overall survival (OS) rate of 51.3% and progression-free survival (PFS) rate of 63.7% for the cohort. ROC analysis established the optimal cutoff for IL-6 at 22.550 pg/ml, and multivariate Cox analysis identified ECOG performance status, extranodal involvement sites, and IL-6 level as key factors for DLBCL prognosis (all $P<0.05$). The constructed nomogram incorporating these three factors exhibited superior predictive power, with C-indices of 0.774 (95% CI: 0.695-0.848) for OS and 0.760 (95% CI: 0.677-0.844) for PFS—both higher than those of the IPI. Calibration plots confirmed good consistency between predicted and actual 1- and 3-year survival probabilities. Time-dependent ROC analysis showed that the AUCs of the nomogram for OS and PFS were consistently higher and more stable than the IPI across follow-up time points. Risk stratification by the nomogram divided patients into low-, intermediate-, and high-risk groups, with significant differences in 3-year PFS (41.2%, 35.1%, 26.7%, $P<0.05$) and 2-year OS (90.1%, 80.9%, 18.8%, $P<0.001$).

**Copyright Information:** (Online) ISSN: 2572-9241
©2026 The Author(s). HemaSphere is published by John Wiley & Sons Ltd on behalf of European Hematology Association. This is an open access Abstract Book distributed under the Attribution-NonCommercial-NoDerivs (CC BY-NC-ND), which allows third parties to download the articles and share them with others as long as they credit the author and the Abstract Book, but they cannot change the content in any way or use them commercially.
**Abstract Book Citations:** Authors, Title, HemaSphere, 2026;10:(S1):pages. The abstract book DOIs can be found on the "Author Information" page.
**Disclaimer:** Articles published in the journal HemaSphere exclusively reflect the opinions of the authors. The authors are responsible for all content in their abstracts including accuracy of the facts, statements, citing resources, etc.

**Summary/Conclusion**

We developed a novel DLBCL prognostic nomogram integrating IL-6, ECOG performance status, and extranodal involvement, which demonstrates better prognostic stratification and predictive accuracy than the traditional IPI. This model can effectively identify high-risk DLBCL patients, providing a reliable tool for clinical risk assessment and personalized therapeutic decision-making. IL-6 may serve as a potential biomarker and therapeutic target for DLBCL.

**Copyright Information:** (Online) ISSN: 2572-9241
©2026 The Author(s). HemaSphere is published by John Wiley & Sons Ltd on behalf of European Hematology Association. This is an open access Abstract Book distributed under the Attribution-NonCommercial-NoDerivs (CC BY-NC-ND), which allows third parties to download the articles and share them with others as long as they credit the author and the Abstract Book, but they cannot change the content in any way or use them commercially.
**Abstract Book Citations:** Authors, Title, HemaSphere, 2026;10:(S1):pages. The abstract book DOIs can be found on the "Author Information" page.
**Disclaimer:** Articles published in the journal HemaSphere exclusively reflect the opinions of the authors. The authors are responsible for all content in their abstracts including accuracy of the facts, statements, citing resources, etc.

### (PB3759) RED CELL DISTRIBUTION WIDTH, SERUM ALBUMIN, AND LACTATE DEHYDROGENASE AS A COMPOSITE PROGNOSTIC SCORE (RAL) IN DIFFUSE LARGE B-CELL LYMPHOMA: A MULTICENTER RETROSPECTIVE COHORT STUDY

**Topic:** 19. Large B cell lymphomas - Clinical

Dragana Grohovac*[1], Vlatka Periša[2, 3], Nika Pušeljić[2], Marta Poje[2], Lucija Todić[2], Aristea Pavešić Radonja[4, 5], Marta Žuvić[6]

*[1]Clinical hospital center Rijeka, Hematology, Rijeka, Croatia;[2]Clinical hospital center Osijek, Hematology, Osijek, Croatia; [3]Faculty of medicine, University Josip Juraj Strossmayer Osijek, Internal medicine, Osijek, Croatia;[4]Clinical hospital center Rijeka, Psychiatry, Rijeka, Croatia; [5]Faculty of medicine Rijeka, University of Rijeka, Psychiatry, Rijeka, Croatia;[6]Faculty of Biotechnology and Drug Development, University of Rijeka, Rijeka, Croatia;*

### Background

Diffuse large B-cell lymphoma (DLBCL) is the most common aggressive lymphoma, yet outcomes remain highly heterogeneous despite modern immunochemotherapy. Established prognostic models (IPI, R-IPI, NCCN-IPI) rely mainly on clinical disease-burden parameters; however, substantial variability remains within prognostic strata, suggesting that additional objective biomarkers may improve risk stratification.

Red cell distribution width (RDW), serum albumin, and lactate dehydrogenase (LDH) are routinely available laboratory parameters reflecting systemic inflammation, nutritional and metabolic status, and tumor burden. Although each has been individually associated with survival in DLBCL, their combined prognostic significance has not been systematically evaluated. The proposed RAL score integrates RDW, albumin, and LDH as a composite marker of tumor burden and host inflammatory–nutritional status.

### Aims

The aim of this study was to evaluate the prognostic value of the pretreatment RAL score in patients with DLBCL treated with curative intent, across multiple clinical endpoints, including overall survival (OS), progression-free survival (PFS) and time to next systemic anti-lymphoma therapy or death (TTNTD).

### Methods

We conducted a retrospective cohort study of 271 consecutive patients with newly diagnosed DLBCL treated with curative intent between 2016 and 2025 at two tertiary centers ( Clinical Hospital Centers Rijeka and Osijek ). Baseline RDW, serum albumin, and LDH values obtained at diagnosis were used to calculate the RAL score. RDW ≥15%, albumin <35 g/L, and LDH/ULN >1.0 were defined as abnormal; one point was assigned for each parameter (range 0–3). Clinical and survival data were extracted from institutional databases. Endpoints included OS, PFS and TTNTD. Survival was estimated using the Kaplan–Meier method and compared with the log-rank test; Cox proportional hazards models were applied for multivariable analyses.

**Copyright Information:** (Online) ISSN: 2572-9241
©2026 The Author(s). HemaSphere is published by John Wiley & Sons Ltd on behalf of European Hematology Association. This is an open access Abstract Book distributed under the Attribution-NonCommercial-NoDerivs (CC BY-NC-ND), which allows third parties to download the articles and share them with others as long as they credit the author and the Abstract Book, but they cannot change the content in any way or use them commercially.
**Abstract Book Citations:** Authors, Title, HemaSphere, 2026;10:(S1):pages. The abstract book DOIs can be found on the “Author Information” page.
**Disclaimer:** Articles published in the journal HemaSphere exclusively reflect the opinions of the authors. The authors are responsible for all content in their abstracts including accuracy of the facts, statements, citing resources, etc.

### Results

In this real-world cohort a total of 271 patients were included. The median age was 68 years (IQR 61–76).  Higher RAL scores were significantly associated with adverse baseline characteristics, including advanced stage, higher ECOG, B symptoms, extranodal involvement, bone marrow infiltration, elevated C-reactive protein, lower hemoglobin, and lower complete remission rates (all $p \leq 0.006$).
RAL significantly predicted overall survival (OS) (log-rank $p<0.001$); each 1-point increase was associated with increased mortality risk (HR 1.57, 95% CI 1.25–1.97; $p<0.001$). RAL also predicted PFS ($p=0.003$) and TTNTD ($p<0.00001$), with a a pronounced stepwise increase in risk, particularly in the highest RAL category.
In multivariable stepwise Cox analysis including NCCN-IPI, RAL remained independently associated with OS (HR 1.29, 95% CI 1.02–1.64; $p=0.032$).

### Summary/Conclusion

The RAL score in patients with newly diagnosed DLBCL treated with curative intent is strongly associated with markers of aggressive disease biology and predicts inferior clinical outcomes, including overall survival, progression-free survival, and time to next treatment or death. In multivariable analysis incorporating established prognostic indices, RAL retained independent association with overall survival alongside NCCN-IPI, suggesting complementary prognostic value. These findings support RAL as a readily available, inflammation-based biomarker that may refine risk stratification in routine clinical practice; however, prospective validation is warranted.

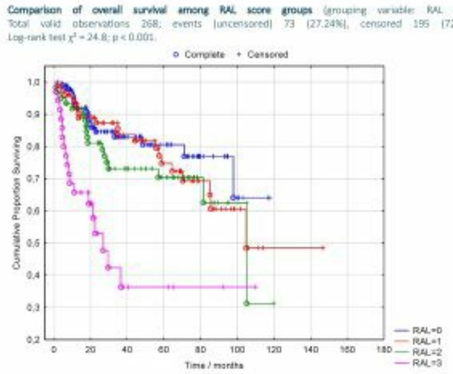

Comparison of overall survival among RAL score groups (grouping variable: RAL score) Total valid observations 268; events (uncensored) 73 (27.24%), censored 195 (72.76%) Log-rank test $\chi^2 = 24.8$; $p < 0.001$.

**Copyright Information:** (Online) ISSN: 2572-9241
©2026 The Author(s). HemaSphere is published by John Wiley & Sons Ltd on behalf of European Hematology Association. This is an open access Abstract Book distributed under the Attribution-NonCommercial-NoDerivs (CC BY-NC-ND), which allows third parties to download the articles and share them with others as long as they credit the author and the Abstract Book, but they cannot change the content in any way or use them commercially.
**Abstract Book Citations:** Authors, Title, HemaSphere, 2026;10:(S1):pages. The abstract book DOIs can be found on the “Author Information” page.
**Disclaimer:** Articles published in the journal HemaSphere exclusively reflect the opinions of the authors. The authors are responsible for all content in their abstracts including accuracy of the facts, statements, citing resources, etc.

### (PB3760) NOVEL CLGAB VS BEAM-L CONDITIONING IN HIGH-RISK LYMPHOMA ASCT: A PROSPECTIVE STUDY ON LONG-TERM OUTCOMES

**Topic:** 19. Large B cell lymphomas - Clinical

Xiangli Chen*[1], Yuzu Zang[1], Wenyi Lu[1], Pingfan Lu[1], Fei Ge[1], Xiaona Niu[1]

[1]*Henan Provincial People's Hospital, Department of Hematology, Zhengzhou City, China;*

**Background**

Despite autologous stem cell transplantation (ASCT), relapse remains a major hurdle for patients with high-risk, relapsed/refractory (R/R) lymphoma, underscoring the need for more effective conditioning. We designed the novel CLGAB regimen to enhance tumor cytoreduction. This study prospectively compared the long-term efficacy and safety of CLGAB versus the BEAM-L regimen.

**Methods**

In this prospective, controlled study, 48 consecutive patients scheduled for ASCT between Jan-2020 and Aug-2023 were assigned to either the CLGAB (n=18) or BEAM-L (n=30) group. Conditioning comprised: CLGAB: cladribine 5mg/m²/d (days -7 to -3), gemcitabine 1250mg/m² (day -7), cytarabine 1500mg/m² (days -6 to -5), busulfan 0.8mg/kg q6h (days -4 to -2). BEAM-L: bendamustine 200mg/m²/d (days -7 to -6) or semustine 300mg/m² (day -7), plus etoposide 100mg/m² q12h, cytarabine 200mg/m² q12h (both days -6 to -3), and melphalan 140mg/m² (day -2). Primary endpoints were disease-free survival (DFS) and overall survival (OS).

**Results**

At a median follow-up of ~3 years, the 4-year DFS was significantly higher with CLGAB (88.9% vs 43.0%; HR 0.25, 95% CI 0.07-0.86, P<0.05). A favorable trend was observed for 4-year OS (88.5% vs 69.7%; HR 0.42, 95% CI 0.10-1.74, P=0.220). In the DE-DLBCL subgroup, relapse rate was markedly lower with CLGAB (11.1% vs 50.0%, P<0.05). Regarding safety, CLGAB facilitated faster hematopoietic recovery: shorter duration of grade IV myelosuppression (median 0.5 vs 2.5 days, P=0.028) and accelerated platelet engraftment (10.1±2.9 vs 11.7±2.6 days, P=0.035). CLGAB was associated with significantly lower incidences of nausea/vomiting (61.1% vs 83.3%, P=0.009) and fever (55.6% vs 83.3%, P=0.049). The incidence of grade ≥3 non-hematologic adverse events was comparable between groups, with no treatment-related mortality.

**Summary/Conclusion**

The novel CLGAB conditioning regimen significantly improves long-term DFS and accelerates engraftment compared to BEAM-L, while demonstrating a favorable safety profile in ASCT for high-risk/R/R lymphoma. Its superior efficacy in DE-DLBCL highlights CLGAB as a promising optimized conditioning strategy worthy of further validation.

**Copyright Information:** (Online) ISSN: 2572-9241
©2026 The Author(s). HemaSphere is published by John Wiley & Sons Ltd on behalf of European Hematology Association. This is an open access Abstract Book distributed under the Attribution-NonCommercial-NoDerivs (CC BY-NC-ND), which allows third parties to download the articles and share them with others as long as they credit the author and the Abstract Book, but they cannot change the content in any way or use them commercially.
**Abstract Book Citations:** Authors, Title, HemaSphere, 2026;10:(S1):pages. The abstract book DOIs can be found on the "Author Information" page.
**Disclaimer:** Articles published in the journal HemaSphere exclusively reflect the opinions of the authors. The authors are responsible for all content in their abstracts including accuracy of the facts, statements, citing resources, etc.

### (PB3761) FRONTLINE POLA-BASED THERAPY IN NEWLY DIAGNOSED DLBCL WITH HIGH-RISK EXTRANODAL INVOLVEMENT FOR CNS RELAPSE: A REAL-WORLD STUDY

**Topic:** 19. Large B cell lymphomas - Clinical

Fenghua Gao*[1], Hao Guo[1], Xingcheng Liu[1], Qing Zhang[1], Qianying Pan[1], Xueli Jiao[1], Jingyi Yang[2], Quande Lin[1], Jianwei Du[1], Lihua Dong[1], Keshu Zhou[1]

*[1]the Affiliated Cancer Hospital of Zhengzhou University & Henan Cancer Hospital, Department of Hematology, Zhengzhou, China; [2]the Affiliated Cancer Hospital of Zhengzhou University & Henan Cancer Hospital, Zhengzhou, China;*

### Background

Central nervous system (CNS) relapse remains a devastating complication of DLBCL with extremely poor prognosis. Certain extranodal sites—including immune-privileged sites (CNS, testis) and kidney/adrenal, breast, and uterus—are associated with higher risk of CNS relapse and inferior outcomes, and are considered high-risk locations for CNS dissemination. The POLARIX trial established pola-R-CHP as frontline standard for DLBCL. However, patients with high-risk extranodal involvement were underrepresented in prospective trials, and efficacy of pola-based regimens in this population remains largely unknown.

### Aims

To evaluate efficacy and safety of frontline pola-based regimens in newly diagnosed DLBCL with extranodal involvement at high risk for CNS relapse in real-world practice.

### Methods

This retrospective study included consecutive newly diagnosed DLBCL patients with involvement of high-risk extranodal sites for CNS relapse (CNS/testis, kidney/adrenal, breast, uterus) who received ≥1 cycle of frontline pola-based therapy (Aug 2023–Dec 2025). Responses were assessed per Lugano criteria. PFS and OS were estimated by Kaplan-Meier method. AEs were graded per CTCAE v5.0.

**Copyright Information:** (Online) ISSN: 2572-9241
©2026 The Author(s). HemaSphere is published by John Wiley & Sons Ltd on behalf of European Hematology Association. This is an open access Abstract Book distributed under the Attribution-NonCommercial-NoDerivs (CC BY-NC-ND), which allows third parties to download the articles and share them with others as long as they credit the author and the Abstract Book, but they cannot change the content in any way or use them commercially.
**Abstract Book Citations:** Authors, Title, HemaSphere, 2026;10:(S1):pages. The abstract book DOIs can be found on the "Author Information" page.
**Disclaimer:** Articles published in the journal HemaSphere exclusively reflect the opinions of the authors. The authors are responsible for all content in their abstracts including accuracy of the facts, statements, citing resources, etc.

**Results**

Among 218 newly diagnosed DLBCL patients treated with frontline pola-containing regimens, 40 met eligibility criteria:8 with immune-privileged sites (1 CNS, 7 testis) and 32 with other high-risk sites (16 kidney/adrenal, 9 breast, 7 uterus); 80% had ≥2 extranodal sites. Median age was 63.5 years (range 34–87); 47.5% ≥65 years; 42.5% male. Most had advanced stage (85%), elevated LDH (70%), ECOG 1–2 (85%), and intermediate/high-risk IPI (87.5%). Non-GCB subtype (62.5%) and double-expressor (30%) were common.
Frontline regimens included pola-R-CHP in 22 patients and other pola-based combinations in 18 patients (pola-R plus BTK inhibitor, n=8; pola-based regimens combined with methotrexate, n=5; pola-R plus immunomodulatory drugs, n=5). At data cut-off (9 February 2026), the median number of administered cycles was 5 (range 1–7).
Among 30 response-evaluable patients at interim assessment, the complete response (CR) rate was 60.0% (18/30) and the overall response rate (ORR) was 96.7% (29/30). At end-of-combination therapy assessment (n=23), the CR rate was 73.9% (17/23) and ORR was 87.0% (20/23). Notably, all three patients bridged to first-line CAR T-cell therapy achieved CR.Subgroup analyses showed no significant differences in response rates according to cell-of-origin subtype, extranodal site category (immune-privileged vs other high-risk extranodal sites), or number of extranodal sites involved (<2 vs ≥2).With a median follow-up of 9 months, median PFS and OS were not reached. The estimated 12-month PFS and OS rates were 78.8% (±8.6%) and 96.4% (±3.5%), respectively.
All patients received intrathecal methotrexate prophylaxis during treatment, and no CNS relapse was observed at last follow-up.
Pola-based regimens were generally well tolerated. The most common grade 3-4 AEs were pulmonary infections (37.5%), anemia (15.0%), and neutropenia (12.5%).

**Summary/Conclusion**

In this real-world cohort of newly diagnosed DLBCL involving high-risk extranodal sites for CNS relapse, frontline pola-based regimens achieved high response rates and encouraging early survival with manageable toxicity. The absence of CNS relapse during follow-up suggests that pola-based therapy combined with intrathecal prophylaxis may represent an effective frontline strategy for this high-risk population and warrants further prospective evaluation.

**Copyright Information:** (Online) ISSN: 2572-9241
©2026 The Author(s). HemaSphere is published by John Wiley & Sons Ltd on behalf of European Hematology Association. This is an open access Abstract Book distributed under the Attribution-NonCommercial-NoDerivs (CC BY-NC-ND), which allows third parties to download the articles and share them with others as long as they credit the author and the Abstract Book, but they cannot change the content in any way or use them commercially.
**Abstract Book Citations:** Authors, Title, HemaSphere, 2026;10:(S1):pages. The abstract book DOIs can be found on the “Author Information” page.
**Disclaimer:** Articles published in the journal HemaSphere exclusively reflect the opinions of the authors. The authors are responsible for all content in their abstracts including accuracy of the facts, statements, citing resources, etc.

### (PB3763) COMPARISON OF ORELABRUTINIB, RITUXIMAB, AND METHOTREXATE (OR-MTX) VERSUS RITUXIMAB AND METHOTREXATE (R-MTX) IN FIRST-LINE TREATMENT OF PRIMARY CNS LYMPHOMA: A RETROSPECTIVE ANALYSIS

**Topic:** 19. Large B cell lymphomas - Clinical

Fang Shu Guan*[1], Xing Guo[1], He Jingsong[1], Zhen Cai[1], Yi Zhao[1]

[1] *The First Affiliated Hospital, School of Medicine, Zhejiang University, Bone Marrow Transplantation Center, Hangzhou, China;*

### Background

Primary central nervous system lymphoma (PCNSL) is an aggressive and challenging extranodal lymphoma. While high-dose methotrexate (HD-MTX)-based regimens remain the backbone of first-line treatment, outcomes vary considerably, necessitating the exploration of novel combination strategies. Bruton's tyrosine kinase (BTK) inhibitors, such as orelabrutinib, are active in PCNSL.

### Aims

This study aimed to compare the efficacy of the obutinib-containing OR-MTX regimen (Orelabrutinib, Rituximab, and MTX) versus the standard R-MTX (Rituximab and MTX) in newly diagnosed PCNSL.

### Methods

In this retrospective cohort study, we analyzed data from consecutive patients newly diagnosed with PCNSL between January 1, 2013, and August 31, 2025. Patients receiving first-line OR-MTX (n=53) or R-MTX (n=64) were included. Survival differences were assessed using Kaplan-Meier analysis and the log-rank test.

### Results

Baseline characteristics, including age, sex, and IELSG risk group distribution, were comparable between the two groups.The median age was 64 years in the OR-MTX group and 60.5 years in the R-MTX group. Thirty (56.6%) patients in the OR-MTX group and 44 (68.8%) patients in the R-MTX group had pathologic subtype of non-germinal center B-cell-like (non-GCB) DLBCL, respectively. High-risk disease features were prevalent: the majority of patients in both groups had intermediate-high risk IELSG scores (84.9% vs. 71.8%). However, a significantly higher proportion of patients in the OR-MTX group subsequently underwent autologous stem cell transplantation (ASCT) (30.2% vs. 10.9%, P=0.009). At the end of induction therapy, the OR-MTX group demonstrated a significantly higher complete response (CR) rate (67.9% vs. 35.9%, OR 4.89, 95% CI 2.30-10.36, P=0.001) and objective response rate (ORR) (88.7% vs. 67.2%, OR 3.83, 95% CI 1.53-9.57, P=0.006) compared to the R-MTX group . After a median follow-up of 41.5 months (95% CI 32.1-50.8; 31.6 months in the OR-MTX group vs. 74.2 months in the R-MTX group), patients treated with OR-MTX achieved a significantly superior PFS (median PFS: not reached) compared to those receiving R-MTX [median PFS: 10.6 (95% CI 5.3-15.9) months, P<0.001]. Although a trend favoring OR-MTX was observed, no statistically significant difference in OS was detected between the two groups at the time of analysis [median OS: not reached vs 36.2 (95% CI 11.8-60.7) months, P=0.126].

**Copyright Information:** (Online) ISSN: 2572-9241
©2026 The Author(s). HemaSphere is published by John Wiley & Sons Ltd on behalf of European Hematology Association. This is an open access Abstract Book distributed under the Attribution-NonCommercial-NoDerivs (CC BY-NC-ND), which allows third parties to download the articles and share them with others as long as they credit the author and the Abstract Book, but they cannot change the content in any way or use them commercially.
**Abstract Book Citations:** Authors, Title, HemaSphere, 2026;10:(S1):pages. The abstract book DOIs can be found on the "Author Information" page.
**Disclaimer:** Articles published in the journal HemaSphere exclusively reflect the opinions of the authors. The authors are responsible for all content in their abstracts including accuracy of the facts, statements, citing resources, etc.

**Summary/Conclusion**

In summary, this retrospective analysis demonstrates that incorporating the BTK inhibitor orelabrutinib into the R-MTX backbone (OR-MTX regimen) significantly enhances therapeutic outcomes for patients with newly diagnosed PCNSL. The regimen yielded superior early response rates, with significantly higher CR rates and ORR at the end of induction therapy, and conferred a marked PFS advantage over standard R-MTX therapy. While the observed benefit in OS did not reach statistical significance at this analysis, the strong trend is encouraging.These findings provide compelling real-world evidence supporting the integration of orelabrutinib into the first-line treatment paradigm for PCNSL. This strategy represents a promising advancement for managing this aggressive disease. Further validation through prospective, randomized controlled trials with longer follow-up is warranted to definitively establish the long-term survival benefit and optimize the treatment algorithm for patients with PCNSL.

**Copyright Information:** (Online) ISSN: 2572-9241
©2026 The Author(s). HemaSphere is published by John Wiley & Sons Ltd on behalf of European Hematology Association. This is an open access Abstract Book distributed under the Attribution-NonCommercial-NoDerivs (CC BY-NC-ND), which allows third parties to download the articles and share them with others as long as they credit the author and the Abstract Book, but they cannot change the content in any way or use them commercially.
**Abstract Book Citations:** Authors, Title, HemaSphere, 2026;10:(S1):pages. The abstract book DOIs can be found on the "Author Information" page.
**Disclaimer:** Articles published in the journal HemaSphere exclusively reflect the opinions of the authors. The authors are responsible for all content in their abstracts including accuracy of the facts, statements, citing resources, etc.

### (PB3764) GLOFITAMAB DEMONSTRATES SUPERIOR EFFICACY COMPARED WITH SECOND-DIFFERENT-TARGET CAR-T AS SALVAGE THERAPY AFTER CD19 CAR-T FAILURE IN R/R B-CELL NON-HODGKIN LYMPHOMA

**Topic:** 19. Large B cell lymphomas - Clinical

Lixia MA*[1], Rui Liu[1], Zhonghua Fu[1], Fan Yang[1], Yuelu Guo[1], Miaomiao Cao[1], Yang Lei[1], Yimeng Dou[1], Biping Deng[2], Alex Chang[3], Xiaoyan Ke[1], Kai Hu[1]

*[1]Beijing GoBroad Hospital, Department of Lymphoma and Myeloma Research Center, Beijing, China; [2]Beijing GoBroad Boren Hospital, Cytology Laboratory, Beijing, China; [3]Shanghai YaKe Biotechnology Ltd., Engineering Research Center of Gene Technology, Ministry of Education, Institute of Genetics, School of Life Sciences, Shanghai, China;*

**Background**

Although CD19-directed CAR-T therapy has significantly improved outcomes in relapsed/refractory B-cell non-Hodgkin lymphoma (R/R B-NHL), a substantial proportion of patients relapse after treatment and have poor prognosis, with no established standard salvage strategy. CD3×CD20 bispecific antibodies and Second-different-target CAR-T therapies have both shown activity in this setting. We conducted a retrospective study to compare the efficacy and survival outcomes of glofitamab versus Second-different-target CAR-T therapy in patients progressing after CD19 CAR-T.

**Aims**

To compare the efficacy, survival outcomes, and safety of glofitamab versus Second-different-target CAR-T therapy as salvage treatment in patients with R/R B-NHL who progressed after CD19 CAR-T therapy.

**Methods**

We retrospectively analyzed R/R B-NHL patients treated at Beijing GoBroad Hospital between January 2019 and October 2025 who experienced disease progression after CD19 CAR-T therapy. Among them, 77 patients received second-different-target CAR-T therapy (Group A), and 19 received glofitamab (Group B). Clinical responses, survival outcomes, and safety were evaluated.

**Results**

Baseline characteristics were generally comparable, although early failure (<6 months after CD19 CAR-T) was more frequent in Group A.In Group A, the objective response rate (ORR) was 37.7% (29/77), including complete response (CR) in 23.4% (18/77). In Group B, ORR was 73.7% (14/19), including CR in 42.1% (8/19).Any-grade CRS occurred in 68.9% vs 36.8%; grade ≥3 CRS and ICANS only in Group A.Median progression-free survival (PFS) was 6.28 months in Group A and 5.98 months in Group B, with 1-year PFS rates of 32.5% and 46.8%, respectively. Median overall survival (OS) was 7.13 versus 12.26 months, with 1-year OS rates of 39.0% and 56.0%.
Among patients receiving non-CD19 CAR-T, early versus late progression had median PFS of 5.26 versus 9.76 months (p=0.213) and median OS of 5.56 months versus not reached (p=0.008); among glofitamab recipients, early versus late progression had median PFS of 5.52 versus 5.98 months (p=0.914) and median OS of 7.23 versus not reached (p=0.528).

**Copyright Information:** (Online) ISSN: 2572-9241
©2026 The Author(s). HemaSphere is published by John Wiley & Sons Ltd on behalf of European Hematology Association. This is an open access Abstract Book distributed under the Attribution-NonCommercial-NoDerivs (CC BY-NC-ND), which allows third parties to download the articles and share them with others as long as they credit the author and the Abstract Book, but they cannot change the content in any way or use them commercially.
**Abstract Book Citations:** Authors, Title, HemaSphere, 2026;10:(S1):pages. The abstract book DOIs can be found on the “Author Information” page.
**Disclaimer:** Articles published in the journal HemaSphere exclusively reflect the opinions of the authors. The authors are responsible for all content in their abstracts including accuracy of the facts, statements, citing resources, etc.

| Characteristic | Second-different-target CAR-T Salvage therapy（n=77）(A group） | BsAb (CD3-CD20) Salvage therapy（n=19) (B group） |
|---|---|---|
| Age | | |
| Median(min:mas) | 51(20-82) | 55(26-68) |
| Age>60y | 25(32.5%) | 4(21.1%) |
| Sex,male | 39(50.6%) | 15(78.9%) |
| ECOG | | |
| 0-1 | 51(66.2%) | 16(84.2%) |
| ≥2 | 26(33.8%) | 3(15.8%) |
| Ann Arbor stage | | |
| Ⅰ-Ⅱ | 5(6.5%) | 2(10.5%) |
| Ⅲ-Ⅳ | 72(93.5%) | 17(89.5%) |
| IPI | | |
| 0-1 | 16(20.8%) | 5(26.3%) |
| 2 | 14(18.2%) | 2(10.5%) |
| 3 | 28(36.4%) | 8(42.1%) |
| 4-5 | 19(24.7%) | 4(21.1%) |
| Histology | | |
| DLBCL | 57(74.0%) | 13(68.4%) |
| FL/tFL | 4(5.2%) | 3(15.8%) |
| BL | 5(6.5%) | 1(5.3%) |
| SCNSL | 5(6.5%) | 2(10.5%) |
| MCL | 3(3.9%) | |
| PMBL | 2(2.6%) | |
| Richter | 1(1.3%) | |
| Best response after CD19 CART | | |
| CR | 16(20.8%) | 6(31.6%) |
| PR | 22(28.6%) | 2(10.5%) |
| SD | 11(14.3%) | |
| PD | 27(35.1%) | 11(57.9%) |
| Not evaluated | 1(1.3%) | |
| Failure post-CD19 CART | | |
| Early failure(<6 months) | 61(79.2%) | 9(47.4%) |
| Late failure(≥6 months) | 16(20.8%) | 10(52.6%) |
| The median time from CD19 CART treatment to PD | 2.8m（range:0.46~21.27m） | 4.9m（range：0.5~38.2m） |
| Other Targets CART | | |
| CD20 CART | 58(75.3%) | |
| CD22 CART | 15(19.5%) | |
| CD79b CART | 3(3.9%) | |
| CD70 CART | 1(1.3%) | |

**Copyright Information:** (Online) ISSN: 2572-9241
©2026 The Author(s). HemaSphere is published by John Wiley & Sons Ltd on behalf of European Hematology Association. This is an open access Abstract Book distributed under the Attribution-NonCommercial-NoDerivs (CC BY-NC-ND), which allows third parties to download the articles and share them with others as long as they credit the author and the Abstract Book, but they cannot change the content in any way or use them commercially.
**Abstract Book Citations:** Authors, Title, HemaSphere, 2026;10:(S1):pages. The abstract book DOIs can be found on the "Author Information" page.
**Disclaimer:** Articles published in the journal HemaSphere exclusively reflect the opinions of the authors. The authors are responsible for all content in their abstracts including accuracy of the facts, statements, citing resources, etc.

### Summary/Conclusion

In patients with R/R B-NHL progressing after CD19 CAR-T, glofitamab demonstrated higher response rates, favorable survival trends, and improved safety compared with Second-different-target CAR-T therapy. Second-different-target CAR-T remains a potential option, particularly in patients with late relapse or CD20-negative disease. Prospective studies are warranted to define optimal sequencing strategies after CD19 CAR-T failure.

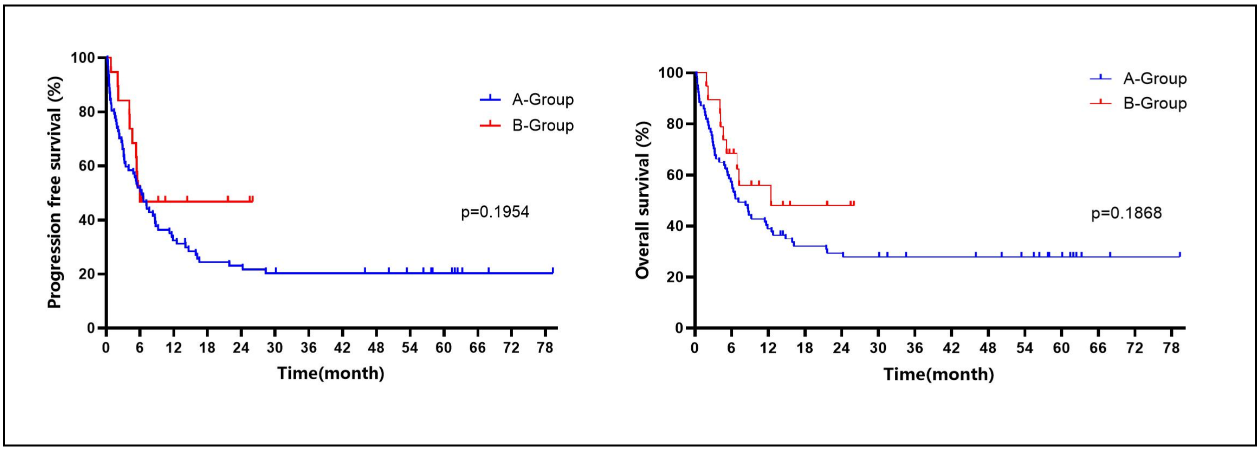

**Copyright Information:** (Online) ISSN: 2572-9241
©2026 The Author(s). HemaSphere is published by John Wiley & Sons Ltd on behalf of European Hematology Association. This is an open access Abstract Book distributed under the Attribution-NonCommercial-NoDerivs (CC BY-NC-ND), which allows third parties to download the articles and share them with others as long as they credit the author and the Abstract Book, but they cannot change the content in any way or use them commercially.
**Abstract Book Citations:** Authors, Title, HemaSphere, 2026;10:(S1):pages. The abstract book DOIs can be found on the "Author Information" page.
**Disclaimer:** Articles published in the journal HemaSphere exclusively reflect the opinions of the authors. The authors are responsible for all content in their abstracts including accuracy of the facts, statements, citing resources, etc.

### (PB3766) EPCOREAL: A PROSPECTIVE OBSERVATIONAL TRIAL-IN-PROGRESS OF EPCORITAMAB IN PATIENTS WITH RELAPSED/REFRACTORY DIFFUSE LARGE B-CELL LYMPHOMA AND FOLLICULAR LYMPHOMA

**Topic:** 19. Large B cell lymphomas - Clinical

Christian Buske*[1], Daniel Maria Mayer[2], Thamer Sliwa[3], Anthea Peters[4], Ronit Gurion[5], Shlomzion Aumann[6], Christopher Brown[7], Anthony Wang[7], Pearly Lee[7], Jieying Jiang[7], Theodoros Vassilakopoulos[8]

*[1]University Hospital of Ulm, Institute of Experimental Cancer Research, Ulm, Germany;[2]Hospitaller Order of Saint John of God Hospital Graz, Department of Internal Medicine, Graz, Austria; [3]Hanusch Hospital, 3rd Medical Department, Hematology and Oncology, Vienna, Austria; [4]University of Alberta, Cross Cancer Institute, Edmonton, Canada;[5]Rabin Medical Center, Institute of Hematology, Davidoff Cancer Center, Petah-Tikva, Israel; [6]Hebrew University of Jerusalem, Department of Hematology, Hadassah Medical Center and Faculty of Medicine, Jerusalem, Israel; [7]AbbVie Inc., North Chicago, United States of America; [8]National and Kapodistrian University of Athens, Department of Haematology and Bone Marrow Transplantation, Laikon General Hospital, Athens, Greece;*

### Background

Epcoritamab is a subcutaneously administered T-cell engaging CD3xCD20 bispecific antibody approved as monotherapy for the treatment of relapsed/refractory (R/R) diffuse large B-cell lymphoma (DLBCL) and follicular lymphoma (FL) after ≥ 2 lines of therapy. In the phase 1/2 EPCORE NHL-1 trial (NCT03625037), epcoritamab monotherapy showed deep and durable responses and a manageable safety profile in patients with R/R B-cell non-Hodgkin lymphoma. There remains, however, a need for structured real-world data on epcoritamab treatment and its impact on disease outcomes, quality of life, and healthcare utilization for patients with R/R DLBCL and R/R FL. Here, we describe the study design for EpcoReal (NCT06830759), a prospective observational multicenter trial evaluating outcomes of epcoritamab in patients with third-line or later (3L+) (D)LBCL and 3L+ FL in the real-world setting.

### Aims

The aim of the EpcoReal trial is to evaluate real-world outcomes of epcoritamab in patients with R/R (D)LBCL and R/R FL, allowing us to gain a better understanding of its value and impact on unmet needs.

### Methods

This global study evaluates structured real-world data associated with epcoritamab treatment, including efficacy, treatment patterns, healthcare resource utilization data, and quality of life. Eligible patients are adults (≥ 18 years of age) with either 3L+ (D)LBCL or 3L+ FL whose clinician has decided that treatment with epcoritamab is warranted prior to and independently of patient enrollment in the study. Approximately 700 patients will be enrolled (400 patients with 3L+ [D]LBCL and 300 patients with 3L+ FL) from sites around the world. The primary endpoint of the study is overall response determined by Lugano criteria as assessed by the investigator. Secondary endpoints include complete response (CR), duration of response/complete response (DoR/DoCR), progression-free survival (PFS), overall survival (OS), time to progression (TTP), and time to next treatment (TTNT). Exploratory endpoints include changes in quality of life (measured by the Functional Assessment of Cancer Therapy-Lymphoma [FACT-Lym] and European Organization for Research and Treatment of Cancer Quality of Life Questionnaire-Core [EORTC-QLQ-C30]), real-world healthcare resource utilization, and treatment patterns in clinical care.

### Results

The study is open for enrollment, with Austria enrolling the first patient in March 2025. As of February 2026, 69 patients have been enrolled across 10 countries. Updated information will be presented.

**Copyright Information:** (Online) ISSN: 2572-9241
©2026 The Author(s). HemaSphere is published by John Wiley & Sons Ltd on behalf of European Hematology Association. This is an open access Abstract Book distributed under the Attribution-NonCommercial-NoDerivs (CC BY-NC-ND), which allows third parties to download the articles and share them with others as long as they credit the author and the Abstract Book, but they cannot change the content in any way or use them commercially.
**Abstract Book Citations:** Authors, Title, HemaSphere, 2026;10:(S1):pages. The abstract book DOIs can be found on the "Author Information" page.
**Disclaimer:** Articles published in the journal HemaSphere exclusively reflect the opinions of the authors. The authors are responsible for all content in their abstracts including accuracy of the facts, statements, citing resources, etc.

### Summary/Conclusion

This study will start to address the knowledge gap in real-world data on the impact of epcoritamab treatment on disease outcomes, quality of life, and healthcare resource utilization in patients with R/R DLBCL and R/R FL.

Figure. Countries With Planned or Active EpcoReal Enrollment Sites

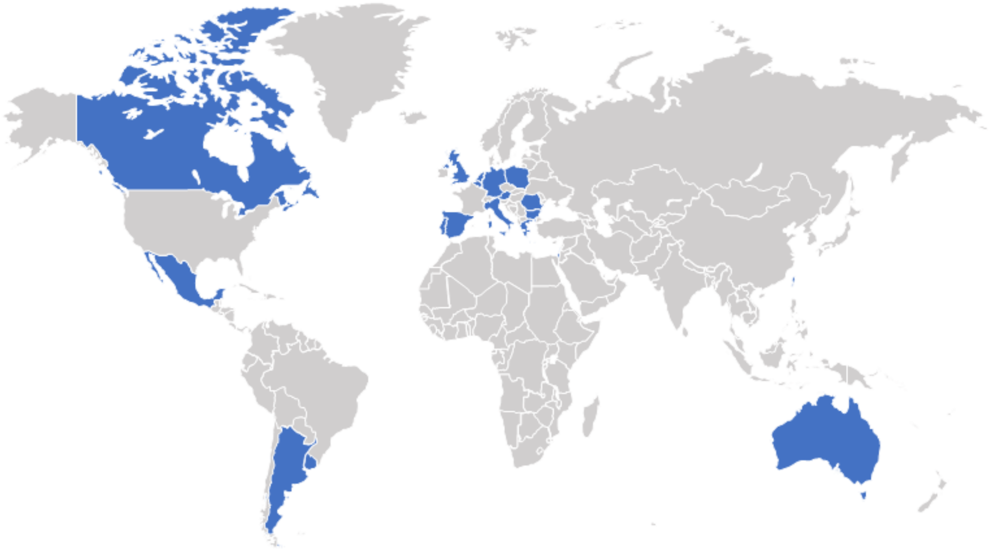

Copyright Information: (Online) ISSN: 2572-9241
©2026 The Author(s). HemaSphere is published by John Wiley & Sons Ltd on behalf of European Hematology Association. This is an open access Abstract Book distributed under the Attribution-NonCommercial-NoDerivs (CC BY-NC-ND), which allows third parties to download the articles and share them with others as long as they credit the author and the Abstract Book, but they cannot change the content in any way or use them commercially.
Abstract Book Citations: Authors, Title, HemaSphere, 2026;10:(S1):pages. The abstract book DOIs can be found on the "Author Information" page.
Disclaimer: Articles published in the journal HemaSphere exclusively reflect the opinions of the authors. The authors are responsible for all content in their abstracts including accuracy of the facts, statements, citing resources, etc.

### (PB3767) GLOFITAMAB IN CNS LYMPHOMA: VERSATILITY FOR RECURRENT AND TREATMENT-NAIVE DISEASE.

**Topic:** 19. Large B cell lymphomas - Clinical

Daria Koroleva*[1], Evgeniy Zvonkov[1], Olga Shchetsova[1], Tatiana Obukhova[2], Galina Yatsyk[3], Elena Nikulina[4], Vera Troitskaya[5], Andrey B. Sudarikov[4], Elena Parovichnikova[5]

*[1]National Medical Research Center of Hematology, Department of Hematology and Chemotherapy of Lymphomas with a Bone Marrow and Hematopoietic Stem Cell Transplant Unit, Moscow, Russia; [2]National Medical Research Center of Hematology, Karyology laboratory, Moscow, Russia; [3]National Medical Research Center of Hematology, Magnetic resonance imaging room, Moscow, Russia; [4]National Medical Research Center of Hematology, Department of Molecular Hematology, Moscow, Russia; [5]National Medical Research Center of Hematology, Moscow, Russia;*

### Background

The results of treatment of patients with primary central nervous system lymphoma (PCNSL), especially in relapse, unmet when using standard chemotherapy (CT) programs. Data on the use of glofitamab in relapsed PCNSL are limited and generally absent in first-line settings. The high cost of glofitamab is one of the factors that also limits its use. It has been reported, that nivolumab dose reduction from 240 mg to 40 mg did not hinder the efficacy of treatment in Hodgkin lymphoma. Despite the assumption that reducing the dose of glofitamab to the minimum effective (2.5 mg) will also not affect the results of lymphoma treatment, this approach have not been studied yet. Our own successful experience with the use of glofitamab in relapses of PCNSL created all the grounds for its integration into the first line.

### Aims

To share the experience of treating patients with PCNSL in relapse and first line by adding glofitamab at a dose of 2.5 mg to various treatment regimens.

### Methods

Between 2025 and 2026, 8 patients with PCNSL (5 – relapsed and 3 treatment-naïve (TN)) were enrolled in the study. Detailed patient characteristics are presented in Table 1. For relapsed patients, glofitamab 2.5 mg was used in combination with various second-line CT regimens (Table 1). The median number of administrations was 4 (range 3–5).
For TN pts, glofitamab 2.5 mg was integrated into the induction therapy regimen of R-MPV with lenalidomide and ibrutinib (a total of 4 administrations, on day 7 of each course).

### Results

All 5 patients with relapsed PCNSL achieved a complete response after receiving glofitamab in combination with various chemotherapy regimens. The maximum follow-up periods were 12 months, 9 months, and 1 month in 3 patients. Remission consolidation was achieved in 4 cases: 3 patients underwent repeated autologous HCT, and 1 patient received penta-CAR-T cell therapy. In one case, due to comorbidities, the patient received only one course of platinum-based CT and three glofitamab infusions; however, the remission still maintained for 12 months.
In all patients with TN PCNSL, the addition of glofitamab to the RIL-MPV program demonstrated rapid and deep antitumor responses after just one course of chemotherapy (Fig. 1). They are currently continuing treatment and are scheduled for auto-HCT. None of the 8 patients developed CRS or ICANS .

**Copyright Information:** (Online) ISSN: 2572-9241
©2026 The Author(s). HemaSphere is published by John Wiley & Sons Ltd on behalf of European Hematology Association. This is an open access Abstract Book distributed under the Attribution-NonCommercial-NoDerivs (CC BY-NC-ND), which allows third parties to download the articles and share them with others as long as they credit the author and the Abstract Book, but they cannot change the content in any way or use them commercially.
**Abstract Book Citations:** Authors, Title, HemaSphere, 2026;10:(S1):pages. The abstract book DOIs can be found on the “Author Information” page.
**Disclaimer:** Articles published in the journal HemaSphere exclusively reflect the opinions of the authors. The authors are responsible for all content in their abstracts including accuracy of the facts, statements, citing resources, etc.

| № | Age | Diagnosis | IELSG risk group | FISH | mut *MYD88* | mut *CD79b* | Time until relapse | Second line therapy | Consolidation |
|---|---|---|---|---|---|---|---|---|---|
| Primary | | | | | | | | | |
| №1 | 39 | DHL | I | *c-Myc/BCL6* | not found | not found | - | - | |
| №2 | 47 | PCNCL | H | t *BCL6* | + | + | - | - | |
| №3 | 61 | PCNSL | I | not found | + | + | - | - | |
| Relapse | | | | | | | | | |
| №1 | 44 | PCNSL | I | no data | no data | no data | refractory | Ibru/Len/Veneto/ Procarbazine/Nivo-Pola-DHAP-E + Glofi №3 | Penta CAR-T (CD19/CD20 CD79b/CD70/ PSMA |
| №2 | 67 | PCNSL | I | t *BCL6* | no data | no data | + 3 mo | Nivo-DHAP-E + Glofi№3 | - |
| №3 | 49 | PCNSL | I | t *BCL6, trisomy 18* | not found | | +26 mo | G-DHAP+Poma+Veneto +Glofi№5 | Auto-HCT/TV |
| №4 | 38 | PCNSL | L | t *BCL6* | not found | | +38 mo | G-DHAP+Poma+Veneto +Glofi№5 | Auto-HCT/TV |
| №5 | 49 | PCNSL | L | *trisomy 18* | + | | +13 mo | G-DHAP+Poma+Veneto +Glofi№5 | Auto-HCT/TV |

### Summary/Conclusion

Integrating glofitamab into standard treatment regimens for both relapsed and TN PCNSL patients has yielded remarkable results. Based on one of the most prognostically unfavorable cohorts of PCNSL patients, it can be concluded that glofitamab maintains its high efficacy with a minimum dose of 2.5 mg. These findings can be safely extrapolated to the treatment of patients with other with ultra-high-risk B-cell lymphomas. However, to draw final conclusions, it is necessary to increase the number of patients and the duration of observation periods.

**Copyright Information:** (Online) ISSN: 2572-9241
©2026 The Author(s). HemaSphere is published by John Wiley & Sons Ltd on behalf of European Hematology Association. This is an open access Abstract Book distributed under the Attribution-NonCommercial-NoDerivs (CC BY-NC-ND), which allows third parties to download the articles and share them with others as long as they credit the author and the Abstract Book, but they cannot change the content in any way or use them commercially.
**Abstract Book Citations:** Authors, Title, HemaSphere, 2026;10:(S1):pages. The abstract book DOIs can be found on the "Author Information" page.
**Disclaimer:** Articles published in the journal HemaSphere exclusively reflect the opinions of the authors. The authors are responsible for all content in their abstracts including accuracy of the facts, statements, citing resources, etc.

Fig. 1 MRI data in patients with TN PCNSL at initially and during therapy.

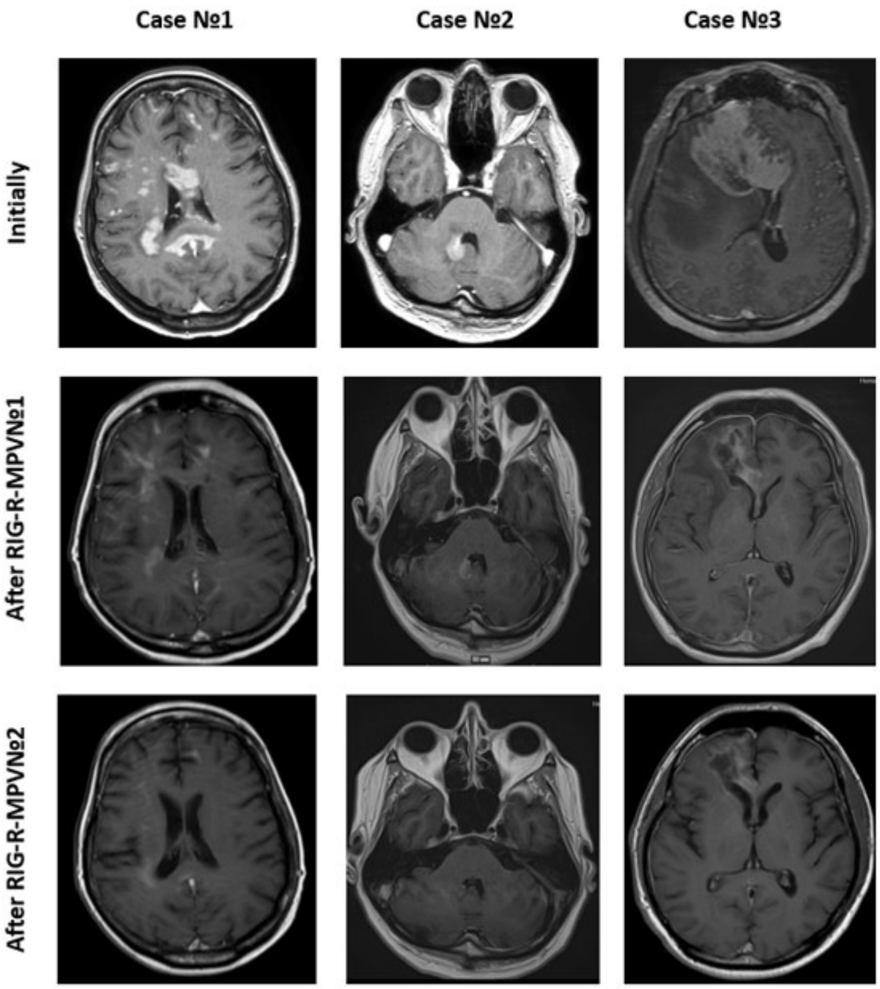

Copyright Information: (Online) ISSN: 2572-9241
©2026 The Author(s). HemaSphere is published by John Wiley & Sons Ltd on behalf of European Hematology Association. This is an open access Abstract Book distributed under the Attribution-NonCommercial-NoDerivs (CC BY-NC-ND), which allows third parties to download the articles and share them with others as long as they credit the author and the Abstract Book, but they cannot change the content in any way or use them commercially.
Abstract Book Citations: Authors, Title, HemaSphere, 2026;10:(S1):pages. The abstract book DOIs can be found on the "Author Information" page.
Disclaimer: Articles published in the journal HemaSphere exclusively reflect the opinions of the authors. The authors are responsible for all content in their abstracts including accuracy of the facts, statements, citing resources, etc.

### (PB3768) DELAYED T-CELL ACTIVATION AND STAGE-SPECIFIC IMMUNE REPROGRAMMING AFTER CRYOABLATION IN HEMATOLOGIC MALIGNANCIES: INSIGHTS FROM SINGLE-CELL SEQUENCING AND ANIMAL MODELS

**Topic:** 19. Large B cell lymphomas - Clinical

Muyun Wu[1], Xing Cui*[2]

*[1]Shandong University of Traditional Chinese Medicine, Jinan, China;[2]The Second Affiliated Hospital of Shandong University of Traditional Chinese Medicine, Jinan, China;*

### Background

Cryoablation induces tumor necrosis and antigen release and may reshape systemic immunity in hematologic malignancies. However, the temporal pattern of T-cell responses after cryoablation remains unclear.

### Aims

To characterize the temporal dynamics of T-cell responses after cryoablation in hematologic malignancies and to determine whether a transient immune remodeling or delayed activation phase occurs.

### Methods

Peripheral blood mononuclear cells (PBMCs) were collected from two lymphoma patients at three time points: 1 day before cryoablation (-1d), 2 days after (d2), and 60 days after (d60). Single-cell RNA sequencing (10x Genomics) was performed to analyze immune cell composition and T-cell subsets. Flow cytometry was used to assess $CD3^+$, $CD8^+$ T cells and activation markers, as well as cytokine expression.

A 5TMM multiple myeloma tumor-bearing BALB/c mouse model was established. Cryoablation was performed on day 10 post-inoculation. Spleen and lymph nodes were harvested on days 2 and 5 for flow cytometric evaluation of T-cell activation phenotypes.

**Copyright Information:** (Online) ISSN: 2572-9241
©2026 The Author(s). HemaSphere is published by John Wiley & Sons Ltd on behalf of European Hematology Association. This is an open access Abstract Book distributed under the Attribution-NonCommercial-NoDerivs (CC BY-NC-ND), which allows third parties to download the articles and share them with others as long as they credit the author and the Abstract Book, but they cannot change the content in any way or use them commercially.
**Abstract Book Citations:** Authors, Title, HemaSphere, 2026;10:(S1):pages. The abstract book DOIs can be found on the "Author Information" page.
**Disclaimer:** Articles published in the journal HemaSphere exclusively reflect the opinions of the authors. The authors are responsible for all content in their abstracts including accuracy of the facts, statements, citing resources, etc.

### Results

**1. Early T-cell subset changes after cryoablation in lymphoma indicate transient immune suppression**
Single-cell RNA-seq UMAP analysis (Figs. A–C) demonstrated a clear time-dependent remodeling of peripheral T cells after cryoablation. At −1d, T cells exhibited high heterogeneity with dispersed clusters and well-defined subsets. At d2, clusters became highly concentrated and heterogeneity markedly decreased, with no evident activation signatures. By d60, clustering returned to a dispersed pattern, heterogeneity was restored, indicating full recovery from suppression and transition to enhanced immune activation.
Integrated single-cell and flow cytometry analyses (Figs. D–F) revealed characteristic early restructuring at d2, with increased proportions of $CD4^+$ and $CD8^+$ naïve T cells and limited effector expansion, suggesting entry into a “reloading” and antigen-priming stage. At d60, pronounced immune conversion occurred, characterized by significant $CD8^+$ effector T-cell expansion, enhanced cytotoxicity, and a marked reduction in Treg cells, forming an “effector enhancement–suppression release” pattern. Overall, the immune response followed a dynamic trajectory of early remodeling, delayed activation, and late reinforcement.
Cytokine profiling (Fig. F) showed distinct temporal dynamics. No cytokine surge was observed at d2 compared with −1d; IFN-γ transiently declined, indicating absence of acute cytokine release. Subsequently, inflammatory and effector cytokines increased gradually. IFN-γ and TNF-α rose most prominently and peaked at the middle-to-late stage, consistent with progressive Th1-type antitumor immunity.
**2. Asynchronous T-cell activation in myeloma-bearing mice supports transient immune suppression**
Flow cytometry of lymph nodes and spleens on days 2 and 5 post-ablation (Figs. G–H) revealed progressive increases in $CD8^+$ T cells in the ablation group, with significantly higher proportions on day 5 than day 2. In contrast, sham-operated mice showed only slight time-dependent increases, markedly lower than the ablation group. This intergroup difference confirms delayed immune activation following a transient immunosuppressive phase after cryoablation.

### Summary/Conclusion

Cryoablation induces a transient early immunosuppressive remodeling of T cells, followed by delayed but enhanced antitumor immune activation characterized by $CD8^+$ effector expansion and Th1-type cytokine upregulation.

**Copyright Information:** (Online) ISSN: 2572-9241
©2026 The Author(s). HemaSphere is published by John Wiley & Sons Ltd on behalf of European Hematology Association. This is an open access Abstract Book distributed under the Attribution-NonCommercial-NoDerivs (CC BY-NC-ND), which allows third parties to download the articles and share them with others as long as they credit the author and the Abstract Book, but they cannot change the content in any way or use them commercially.
**Abstract Book Citations:** Authors, Title, HemaSphere, 2026;10:(S1):pages. The abstract book DOIs can be found on the “Author Information” page.
**Disclaimer:** Articles published in the journal HemaSphere exclusively reflect the opinions of the authors. The authors are responsible for all content in their abstracts including accuracy of the facts, statements, citing resources, etc.

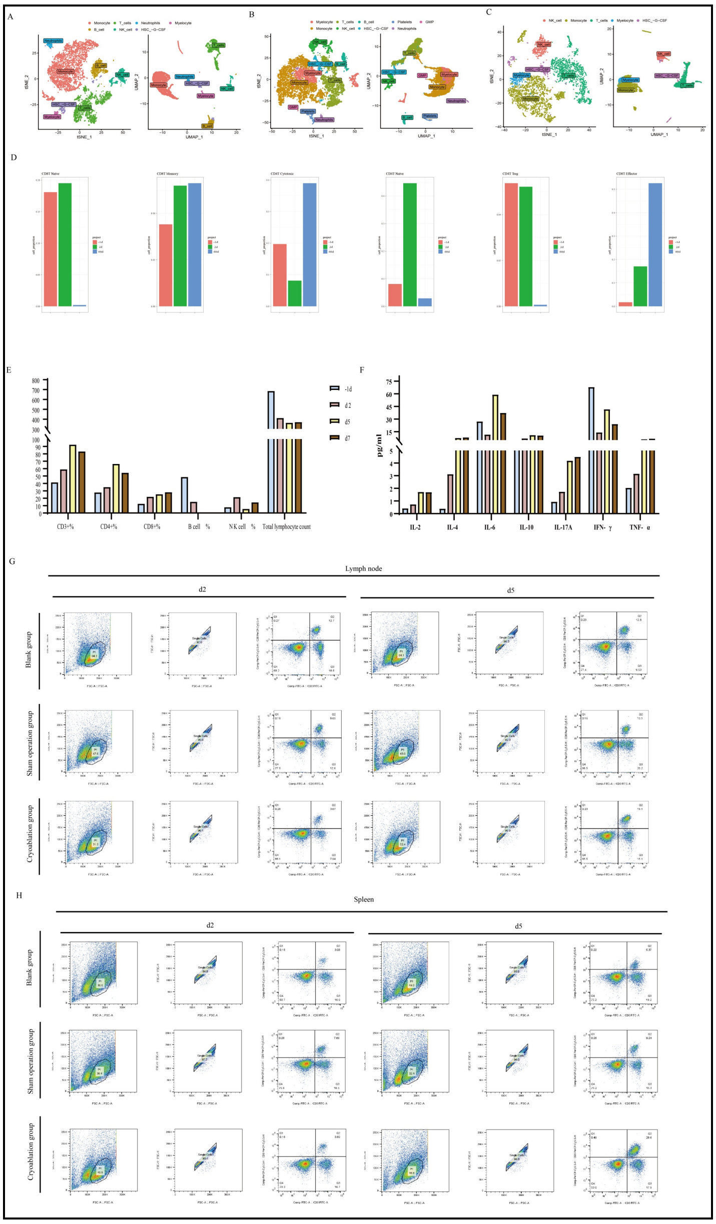

**Copyright Information:** (Online) ISSN: 2572-9241
©2026 The Author(s). HemaSphere is published by John Wiley & Sons Ltd on behalf of European Hematology Association. This is an open access Abstract Book distributed under the Attribution-NonCommercial-NoDerivs (CC BY-NC-ND), which allows third parties to download the articles and share them with others as long as they credit the author and the Abstract Book, but they cannot change the content in any way or use them commercially.
**Abstract Book Citations:** Authors, Title, HemaSphere, 2026;10:(S1):pages. The abstract book DOIs can be found on the "Author Information" page.
**Disclaimer:** Articles published in the journal HemaSphere exclusively reflect the opinions of the authors. The authors are responsible for all content in their abstracts including accuracy of the facts, statements, citing resources, etc.

### (PB3769) TNFRSF14 MUTATIONS ARE ASSOCIATED WITH INCREASED RELAPSE RISK AND POOR SURVIVAL IN DIFFUSE LARGE B-CELL LYMPHOMA

**Topic:** 19. Large B cell lymphomas - Clinical

Xia Zhao*[1], Zhihe Liu[1]

[1]*the affiliated hospital of Qingdao university, Qingdao, China;*

### Background

Diffuse large B-cell lymphoma (DLBCL) is an aggressive malignancy, with a substantial number of patients experiencing relapse or refractory disease. The tumor microenvironment and immune escape mechanisms are critical to its pathogenesis. TNFRSF14 (HVEM), a member of the tumor necrosis factor receptor superfamily, plays a dual role in immune regulation. While inactivating mutations in TNFRSF14 have been reported in 20-30% of germinal center B-cell-like (GCB) DLBCL and are linked to immune evasion, their direct association with clinical outcomes like relapse remains to be fully elucidated.

### Aims

This study aimed to investigate the association between TNFRSF14 mutations and the risk of relapse and progression-free survival (PFS) in patients with DLBCL.

### Methods

We analyzed a simulated cohort of 35 DLBCL patients, comprising 13 with TNFRSF14 mutations and 22 with wild-type TNFRSF14. The two groups were compared for relapse rates, PFS. Statistical analyses included the chi-square test for relapse rates, and the Kaplan-Meier method with log-rank test for survival analysis. A Cox proportional hazards model was used to calculate hazard ratios (HR).

### Results

Patients with TNFRSF14 mutations demonstrated a higher relapse rate compared to those with the wild-type, although this difference was not statistically significant (61.5% vs. 27.3%; p = 0.1005; Odds Ratio: 4.27). The presence of a TNFRSF14 mutation was significantly associated with poorer survival outcomes. The median progression-free survival (PFS) for the mutant group was 23.0 months, while it was not reached in the wild-type group (log-rank p = 0.0496; HR: 2.78, 95% CI: 0.96-8.05). Furthermore, all patients with TNFRSF14 mutations were classified as the GCB subtype, compared to 54.5% of the wild-type group.

### Summary/Conclusion

Our results indicate that TNFRSF14 mutations are associated with a significantly higher risk of disease progression and mortality in DLBCL patients.

**Copyright Information:** (Online) ISSN: 2572-9241
©2026 The Author(s). HemaSphere is published by John Wiley & Sons Ltd on behalf of European Hematology Association. This is an open access Abstract Book distributed under the Attribution-NonCommercial-NoDerivs (CC BY-NC-ND), which allows third parties to download the articles and share them with others as long as they credit the author and the Abstract Book, but they cannot change the content in any way or use them commercially.
**Abstract Book Citations:** Authors, Title, HemaSphere, 2026;10:(S1):pages. The abstract book DOIs can be found on the "Author Information" page.
**Disclaimer:** Articles published in the journal HemaSphere exclusively reflect the opinions of the authors. The authors are responsible for all content in their abstracts including accuracy of the facts, statements, citing resources, etc.

### (PB3770) A PROSPECTIVE, SINGLE-ARM, PHASE II TRIAL OF THIOTEPA, PIRTOBRUTINIB AND SINTILIMAB (TPP) IN PATIENTS WITH UNFIT OR RELAPSED/REFRACTORY PRIMARY OR SECONDARY CENTRAL NERVOUS SYSTEM LYMPHOMA

**Topic:** 19. Large B cell lymphomas - Clinical

Li Cong*[1], Xiyue Xu[1], Tao Lei[1], Haifeng Yu[1], Xi Chen[1], Shuailing Peng[1], Shuiyun Han[1], Jieyu Xu[1], Haiyan Yang[1]

[1]*Zhejiang Cancer Hospital, Hangzhou Institute of Medicine (HIM), Chinese Academy of Sciences, Hangzhou, China;*

### Background

Primary or Secondary central nervous system lymphoma (PCNSL/SCNSL) and those with relapsed or refractory (r/r) disease, have limited treatment choices and extremely poor outcomes, with a median OS of less than six months. Palliative whole brain radiotherapy (WBRT), enrollment in clinical trials, or chemo-free therapies are recommended. There remains an unmet need for effective and tolerable therapeutic strategies for this vulnerable patient population.

### Aims

This clinical trial (NCT07416890) aims to evaluate the efficacy and safety of a novel triplet regimen combining Thiotepa, Pirtobrutinib and Sintilimab in patients with unfit or r/r PCNSL/SCNSL. The objective is to assess the BBB penetration of pirtobrutinib by measuring its concentration in paired CSF and plasma samples.

### Methods

Eligible patients were adults with histologically confirmed PCNSL or SCNSL without systemic involvement and KPS ≥40. Patients were deemed unfit/frail for intensive chemotherapy per sGA, including those with MTX contraindications or refusing high-dose MTX chemotherapy.

Patients received up to six 21-day cycles of TPP induction: IV Thiotepa 40 mg/m² day 1, IV Sintilimab 200 mg day 1, oral Pirtobrutinib 200 mg once daily days 1–21. Responders (PR/CR) received consolidation (ASCT or WBRT) or maintenance with Sintilimab + Pirtobrutinib. Primary endpoint: ORR. Secondary endpoints: CR, PFS, OS, safety. For PK analysis, paired CSF/plasma samples were collected 2–6 h after Pirtobrutinib dosing. Concentrations were quantified by validated LC-MS/MS and corrected CSF/plasma ratios were calculated.

**Copyright Information:** (Online) ISSN: 2572-9241
©2026 The Author(s). HemaSphere is published by John Wiley & Sons Ltd on behalf of European Hematology Association. This is an open access Abstract Book distributed under the Attribution-NonCommercial-NoDerivs (CC BY-NC-ND), which allows third parties to download the articles and share them with others as long as they credit the author and the Abstract Book, but they cannot change the content in any way or use them commercially.
**Abstract Book Citations:** Authors, Title, HemaSphere, 2026;10:(S1):pages. The abstract book DOIs can be found on the "Author Information" page.
**Disclaimer:** Articles published in the journal HemaSphere exclusively reflect the opinions of the authors. The authors are responsible for all content in their abstracts including accuracy of the facts, statements, citing resources, etc.

### Results

Between Aug 2025 and Jan 2026, 7 patients were enrolled. Median age was 71 (range, 32–80); 4 were male. Histologies: PCNSL (n=6), SCNSL (n=1). Median baseline KPS was 50 (40–70). Treatment was generally tolerable. The most common AEs were cytopenias (thrombocytopenia, n=2; neutropenia, n=2), manageable with supportive care. No unexpected safety signals were identified. Baseline characteristics and treatment details are shown in Table1.
Pharmacokinetic data were available from 6 paired plasma/CSF samples. The mean plasma concentration of pirtobrutinib was 6380 ng/mL (range, 2060–13600). The mean CSF concentration was 164.5 ng/mL (range, 33.6–413) ,The mean corrected CSF/plasma ratio was 0.645(range, 0.351–1.094) (Figure 1), confirming measurable BBB penetration.
Table1.Patients baseline characteristics and details of TPP

| ID | Age | Diagnosis | KPS score | prior chemotherapy regimens | Efficiency | cycles of TPP | survival outcome |
|---|---|---|---|---|---|---|---|
| 1 | 32 | SCNSL | 40 | RCHOP□RICEO□GGEMOX□Pola+ bendamustine | Not evaluated□symptoms significantly improved | 2 | alive |
| 2 | 80 | PCNSL | 70 | no | Not evaluated (symptoms significantly improved) | 2 | alive |
| 3 | 71 | PCNSL | 70 | RMTT | CR | 5 | alive |
| 4 | 66 | PCNSL | 40 | no | CR | 5+ | death(2026.1.17) |
| 5 | 81 | PCNSL | 70 | no | PR | 3+ | alive |
| 6 | 68 | PCNSL | 50 | RMTT□RMB□PD-1 inhibitor +Pomalidomide+thieope+Rituximab□ Tinapridecin+Pemetrexed | PD | 2 | alive |
| 7 | 72 | PCNSL | 40 | no | Not evaluated□symptoms significantly improved | 2 | death(2025.11.15) |

### Summary/Conclusion

Preliminary results from this ongoing study indicate that the TPP regimen is feasible and exhibits a manageable safety profile in unfit/frail or r/r CNS lymphoma patients. Pharmacokinetic analysis confirms that pirtobrutinib achieves measurable concentrations in the CSF compartment. These early findings support the potential of this chemo-immunotherapy combination as a promising therapeutic option for this high-risk population. Updated efficacy and survival data will be presented.

**Copyright Information:** (Online) ISSN: 2572-9241
©2026 The Author(s). HemaSphere is published by John Wiley & Sons Ltd on behalf of European Hematology Association. This is an open access Abstract Book distributed under the Attribution-NonCommercial-NoDerivs (CC BY-NC-ND), which allows third parties to download the articles and share them with others as long as they credit the author and the Abstract Book, but they cannot change the content in any way or use them commercially.
**Abstract Book Citations:** Authors, Title, HemaSphere, 2026;10:(S1):pages. The abstract book DOIs can be found on the "Author Information" page.
**Disclaimer:** Articles published in the journal HemaSphere exclusively reflect the opinions of the authors. The authors are responsible for all content in their abstracts including accuracy of the facts, statements, citing resources, etc.

### (PB3771) THE SOMATIC MUTATION PROFILE OF RUSSIAN PATIENTS WITH DLBCL: PRELIMINARY NGS FINDINGS FROM A SINGLE-CENTER STUDY

**Topic:** 19. Large B cell lymphomas - Clinical

Daria Belkina[1], Bella Biderman[1], Aminat Magomedova[1], Yana Mangasarova[1], Andrey B. Sudarikov[1], Nelli Gabeeva[1], Evgeniy Zvonkov*[1]

*[1]NATIONAL MEDICAL RESEARCH CENTER FOR HEMATOLOGY, Moscow, Russia;*

### Background

Diffuse large B-cell lymphoma (DLBCL) is a heterogeneous group of lymphoid tumors with different clinical, molecular-genetic, morphological and cytogenetic manifestations and varying response to therapy. The role of somatic mutations (SM) of various genes in patients with DLBCL is being actively studied. Data on the association of SM with response to therapy are limited, which makes studies of SM important for understanding the causes of resistance.

### Aims

To evaluate SM in patients with DLBCL by high-throughput sequencing (NGS) to assess the prognostic and clinical significance of the detected mutations.

### Methods

The study included 70 patients with DLBCL, aged 19–90 years (Me = 58), 38 f/32 m, attended to the National Medical Research Center for Hematology (2014-2025). NGS panel targeting(*STAT6, DTX1, BTG1, DDX3X, CD70, GNA13, TNFRSF14, NOTCH2, NOTCH1, SGK1, CD79B, MYD88, TET2, TP53, PIM1, CCND3, EZH2, CREBBP, TBL1XR1, MPEG1,* and *ID3* genes was obtained from “Parseq” (Russia). Only SM were included in the analysis (assessed by VAF level). The pathogenicity of the identified variants was evaluated using open-access databases.

### Results

Pathogenic or likely pathogenic SM were identified in 37 (53%) patients. Most frequen mutations were found in the genes: *TP53* — 17 (27%), *TET2* — 9 (14%), *CD79B* — 9 (14%), *MYD88* — 6 (9%), *NOTCH2* — 6 (9%), *CREBBP* — 3 (4%), *NOTCH1* — 3 (4%), *EZH2* — 2 (3%), *DDX3X* — 2 (3%), *PIM1* — 2 (3%), *TBL1XR1* — 2 (3%), *CCND3* — 1 (1%), *CD70* — 1 (1%). Mutations in one gene were detected a 24 (71%) cases, two genes — 9 (28%), three genes — 4 (1%).In eight genes (*STAT6, DTX1, BTG1, GNA13, TNFRSF14, SGK1, MPEG1, ID3*) only variants of uncertain significance were observed; in the *PIM1* gene their number varied from 1 to 26.
Based on the obtained data, we divided patients into clusters similar to the LymphGen system. 15 patients (40%) were assigned to cluster *A53* (main gene *TP53*), 8 (24%) to *MCD (MYD88/CD79B*), 4 (10%) to *BN2 (NOTCH2)*, 3 (8%) to *ST2 (TET2)*, 2 (5%) to *EZB (EZH2)*, 2 (5%) to *N1 (NOTCH1).* In 3 (5%) patients, only minor mutations were identified, which did not allow unambiguous classification of these patients.Young patients were prescribed high-intensity therapy, whereas elderly patients and individuals with severe somatic disorders were treated according to the CHOP+X protocol, in accordance with clinical trial data.
The majority of patients with pathogenic mutations belonging to molecular clusters with an extremely unfavorable prognosis (*A53, MCD, N1*) received R-m-NHL-BFM-90 therapy — 30 of 37. Relapse was registered in 2 patients of the A53 cluster: 1 case — with involvement of an immune-privileged organ (testis), the 2nd — after a forced switch to R-CHOP due to severe sepsis. The remaining patients of the *A53* cluster, as well as *MCD* and *N1*, achieved complete remission. Also 3 patients from the *A53* cluster are undergoing treatment and have not yet completed interim restaging.

**Copyright Information:** (Online) ISSN: 2572-9241
©2026 The Author(s). HemaSphere is published by John Wiley & Sons Ltd on behalf of European Hematology Association. This is an open access Abstract Book distributed under the Attribution-NonCommercial-NoDerivs (CC BY-NC-ND), which allows third parties to download the articles and share them with others as long as they credit the author and the Abstract Book, but they cannot change the content in any way or use them commercially.
**Abstract Book Citations:** Authors, Title, HemaSphere, 2026;10:(S1):pages. The abstract book DOIs can be found on the “Author Information” page.
**Disclaimer:** Articles published in the journal HemaSphere exclusively reflect the opinions of the authors. The authors are responsible for all content in their abstracts including accuracy of the facts, statements, citing resources, etc.

**Summary/Conclusion**

We discovered a wide spectrum of pathogenic SM in patients with DLBCL and stratified them according to the LymphGene molecular classification. High-intensity therapy with the R-m-NHL-BFM-90 regimen demonstrated high efficacy even in patients with the most unfavorable prognostic clusters (A53, MCD, N1).Further studies of the molecular genetic profile are planned to clarify the prognostic significance of the integrated molecular-clinical approach in DLBCL.

**Copyright Information:** (Online) ISSN: 2572-9241
©2026 The Author(s). HemaSphere is published by John Wiley & Sons Ltd on behalf of European Hematology Association. This is an open access Abstract Book distributed under the Attribution-NonCommercial-NoDerivs (CC BY-NC-ND), which allows third parties to download the articles and share them with others as long as they credit the author and the Abstract Book, but they cannot change the content in any way or use them commercially.
**Abstract Book Citations:** Authors, Title, HemaSphere, 2026;10:(S1):pages. The abstract book DOIs can be found on the "Author Information" page.
**Disclaimer:** Articles published in the journal HemaSphere exclusively reflect the opinions of the authors. The authors are responsible for all content in their abstracts including accuracy of the facts, statements, citing resources, etc.

### (PB3773) PROGNOSTIC IMPACT OF PRE-TRANSPLANT DISEASE STATUS AND DEAUVILLE SCORE ON OUTCOME AFTER AUTOLOGOUS STEM CELL TRANSPLANT IN RELAPSED/REFRACTORY DIFFUSE LARGE B CELL LYMPHOMA

**Topic:** 19. Large B cell lymphomas - Clinical

Pei Shin Teo*[1], Evelyn Aun[1], Xinxin Huang[2], Esther Hian Li Chan[1], Liang Piu Koh[1], Lip Kun Tan[1], Edwin Thong[1], Jing Yuan Tan[2], Francesca Lorraine Wei Inng Lim[2], Yunxin Chen[2], Shin Yeu Ong[2], Chandramouli Nagarajan[2], Cheng Kiat Lawrence Ng[2], Michelle Limei Poon[1], Victor Ling[1]

*[1]National University Hospital, Haematology, Singapore, Singapore;[2]Singapore General Hospital, Haematology, Singapore, Singapore;*

### Background

Autologous stem cell transplant (ASCT) remains an established consolidative strategy for transplant-eligible patients with relapsed/refractory diffuse large B cell lymphoma (DLBCL). While CAR-T cell therapy has emerged as a preferred option for early relapse, ASCT remains crucial in late relapse and in settings with limited access to cellular therapies.

### Aims

This study evaluated the prognostic impact of pre-transplant disease status, Deauville score (DS), and relapse timing on post-ASCT outcomes in a real-world two-centre cohort.

### Methods

We conducted a retrospective study of 149 patients with relapsed/refractory DLBCL who underwent ASCT after salvage therapy at Haematology Department, National University Hospital and Singapore General Hospital, Singapore, between 2010 and 2024. Clinical outcomes were assessed using Kaplan-Meier survival analysis and Cox regression models. Subgroup analyses were performed according to pre-transplant DS, disease status and relapse timing.

**Copyright Information:** (Online) ISSN: 2572-9241
©2026 The Author(s). HemaSphere is published by John Wiley & Sons Ltd on behalf of European Hematology Association. This is an open access Abstract Book distributed under the Attribution-NonCommercial-NoDerivs (CC BY-NC-ND), which allows third parties to download the articles and share them with others as long as they credit the author and the Abstract Book, but they cannot change the content in any way or use them commercially.
**Abstract Book Citations:** Authors, Title, HemaSphere, 2026;10:(S1):pages. The abstract book DOIs can be found on the "Author Information" page.
**Disclaimer:** Articles published in the journal HemaSphere exclusively reflect the opinions of the authors. The authors are responsible for all content in their abstracts including accuracy of the facts, statements, citing resources, etc.

**Results**

Among 149 patients, 58.4% were male; with median age at transplant of 57 years (range: 17–82). Median overall survival (OS) was not reached at a median follow-up of 5.7 years, while median progression-free survival (PFS) was 7.9 years. 5-year OS was 67.3% (95% CI 58.8–74.4) and 5-year PFS was 53.2%(95% CI 44.6–61.1). Non-relapse mortality was 5.5% at 5 years (95% CI 2.6–10.1). Prior to ASCT, 70.5% achieved complete remission (DS 1–3), 24.2% were in partial remission, 1.3% had refractory or progressive disease (DS 4–5). 5-year OS was numerically better in patients transplanted in complete remission than partial remission (69.6% [95% CI 59.1–77.9] vs 57.6%[95% CI 39.7–71.9], p=0.100), with a similar trend for PFS (58.0%[95% CI 47.6–67.1] vs 41.3% [95% CI 25.2–56.7], p=0.110).
Among patients with DS ≤3, no significant difference in outcome was observed between DS 1–2 and DS 3. 5-year OS was 68.2% (95% CI 53.6–79.0) for DS 1–2 vs 78.4% (95% CI 51.6–91.4) for DS 3 (p=0.934). Corresponding 5-year PFS were 57.6% (95% CI 43.6–69.2) for DS 1–2 vs 67.5% (95% CI 40.5–84.3) for DS 3 (p=0.694).
Among patients with early relapse (<12 months, n=66), 5-year OS was 61.5% (95% CI 48.1–72.4), compared with 72.0% (95%CI 60.6–80.6) in those with late relapse (>12 months; p=0.492). Corresponding five-year PFS were 51.8% (95% CI 38.9–63.2) and 54.3% (95% CI 42.5–64.7) respectively (p=0.905), though these comparisons were likely underpowered.
On multivariable analysis, CNS involvement (n=22, HR 2.39, 95% CI 1.26–4.55, p=0.008), increasing age (HR 1.03 per year increment, 95% CI 1.01–1.06, p=0.01), and partial remission compared to complete remission (HR 1.80, 95% CI 1.00–3.24, p=0.05) were independently associated with inferior OS. CNS involvement (HR 1.86, 95% CI 1.02–3.38, p=0.042) and increasing age (HR 1.03 per year increment, 95% CI 1.01–1.05, p=0.009) were independently associated with inferior PFS.

**Summary/Conclusion**

This real-world two-centre cohort confirms the continued role of ASCT in relapsed/refractory DLBCL, with a trend towards better survival in late relapse. Pre-transplant remission depth was a key determinant of outcome, with partial remission and CNS involvement linked to poorer outcomes. Among patients with metabolic response (DS ≤3), outcomes were similar between DS 1-2 and DS 3, supporting DS 3 as an adequate threshold for ASCT. These findings emphasise achieving complete remission before ASCT and support risk-adapted approaches, including further salvage or alternative cellular therapies for suboptimal responders.

**Copyright Information:** (Online) ISSN: 2572-9241
©2026 The Author(s). HemaSphere is published by John Wiley & Sons Ltd on behalf of European Hematology Association. This is an open access Abstract Book distributed under the Attribution-NonCommercial-NoDerivs (CC BY-NC-ND), which allows third parties to download the articles and share them with others as long as they credit the author and the Abstract Book, but they cannot change the content in any way or use them commercially.
**Abstract Book Citations:** Authors, Title, HemaSphere, 2026;10:(S1):pages. The abstract book DOIs can be found on the “Author Information” page.
**Disclaimer:** Articles published in the journal HemaSphere exclusively reflect the opinions of the authors. The authors are responsible for all content in their abstracts including accuracy of the facts, statements, citing resources, etc.

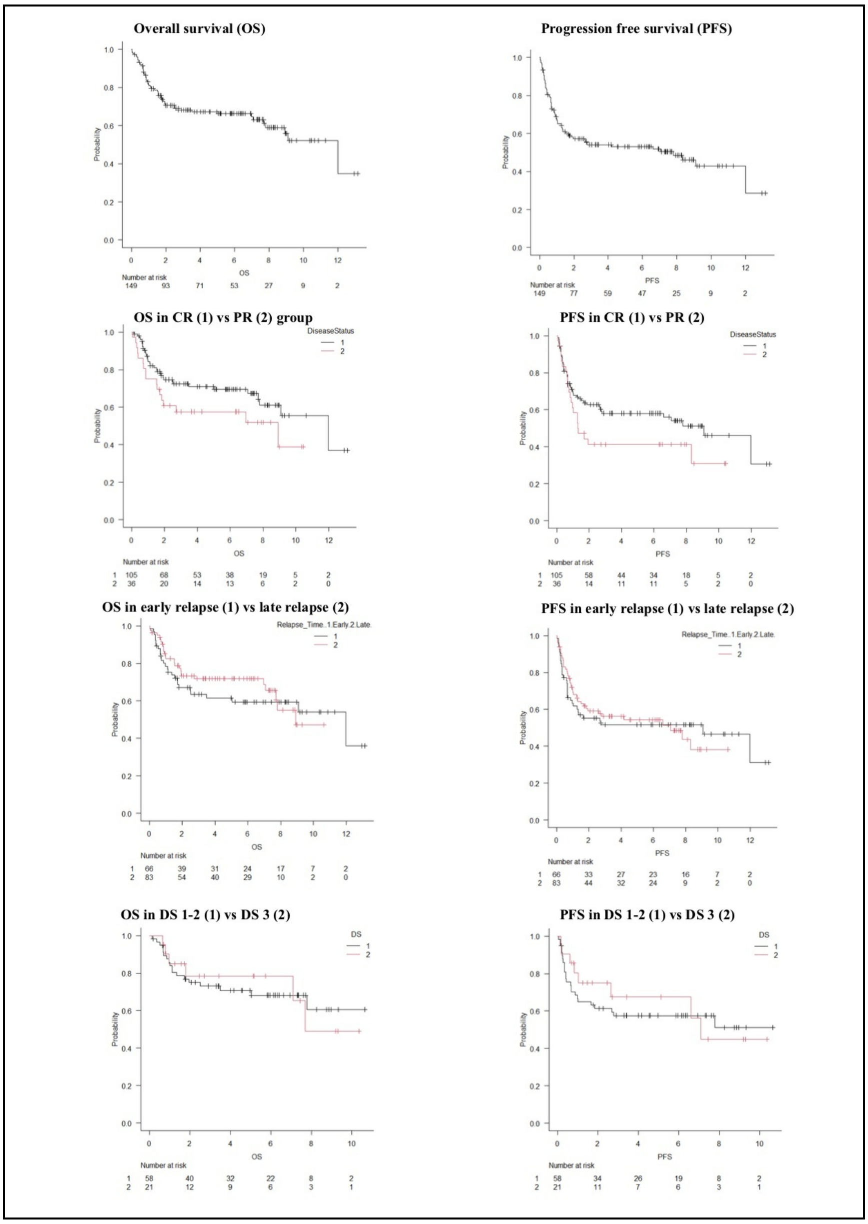

OS = overall survival; PFS = progression-free survival; CR = complete remission (DS 1–3); PR = partial remission (DS 4-5); DS = Deauville score. Survival estimates were calculated using the Kaplan–Meier method and compared using the log-rank test. 5-year survival rates (with 95% confidence intervals) are shown for each group.

**Copyright Information:** (Online) ISSN: 2572-9241
©2026 The Author(s). HemaSphere is published by John Wiley & Sons Ltd on behalf of European Hematology Association. This is an open access Abstract Book distributed under the Attribution-NonCommercial-NoDerivs (CC BY-NC-ND), which allows third parties to download the articles and share them with others as long as they credit the author and the Abstract Book, but they cannot change the content in any way or use them commercially.
**Abstract Book Citations:** Authors, Title, HemaSphere, 2026;10:(S1):pages. The abstract book DOIs can be found on the "Author Information" page.
**Disclaimer:** Articles published in the journal HemaSphere exclusively reflect the opinions of the authors. The authors are responsible for all content in their abstracts including accuracy of the facts, statements, citing resources, etc.

### (PB3774) REAL-WORLD OUTCOMES OF PRIMARY MEDIASTINAL B-CELL LYMPHOMA IN THE ERA OF NOVEL AGENTS: A SINGLE-CENTER RETROSPECTIVE ANALYSIS

**Topic:** 19. Large B cell lymphomas - Clinical

Xue Yang*[1], Luya Cheng[1], Lili Ji[1], Zheng Wei[1], Weiguang Wang[1], Peng Liu[1]

[1]*Zhongshan Hospital, Fudan University, Shanghai, China;*

### Background

Primary mediastinal B-cell lymphoma (PMBCL) is a rare and distinct subtype of diffuse large B-cell lymphoma. While standard immunochemotherapies demonstrate high cure rates, optimal frontline therapy remains debated, particularly regarding the integration of novel agents and the management of primary refractory disease.

### Aims

To evaluate the clinical characteristics, treatment patterns, and outcomes of PMBCL patients, including the efficacy of emerging frontline and salvage regimens.

### Methods

We retrospectively analyzed 62 PMBCL patients treated at a single institution between December 2015 and October 2025. Clinicopathological features, treatment modalities, and Lugano 2014 PET/CT responses were evaluated. Overall survival (OS) and progression-free survival (PFS) were estimated using the Kaplan-Meier method.

### Results

The median age was 30 years (range, 15–63), with a female predominance (54.5%). At diagnosis, 61.3% had bulky disease (≥10 cm), 87.1% presented with Ann Arbor stage I-II, and 83.9% had an aaIPI score of 0-1. Elevated LDH was observed in 77.4%. Pleural and pericardial effusions were present in 48.4% and 53.2% of patients, respectively. Frontline regimens included R-CHOP (n=15), R2-CHOP (n=5), DA-EPOCH-R (n=25), and Pola-R-CHP (n=17). Consolidative radiotherapy was administered to 16.1% and autologous stem cell transplantation (ASCT) to 27.4%. Among 58 patients evaluable by interim PET-CT, 31% achieved a complete metabolic response (CMR) and 69% a partial metabolic response (PMR). At the end of treatment (EoT), 60 patients were evaluable. The overall response rate (ORR) was 93.3%, including 44 CMR (73.3%), 12 PMR (20.0%), and 4 progressive diseases (PD; 6.7%). By regimen, ORR was 86.7% for R-CHOP (8 CMR, 5 PMR, 2 PD), 91.3% for DA-EPOCH-R (16 CMR, 5 PMR, 2 PD among 23 evaluable), 100% for R2-CHOP (4 CMR, 1 PMR), and 100% for Pola-R-CHP (16 CMR, 1 PMR). With a median follow-up of 33.1 months (range 3.6–122.4), the 3-year OS and PFS rates were 97.1% and 93.1%, respectively. Among the four primary refractory patients, salvage therapies showed notable efficacy: one achieved CMR with Brentuximab vedotin (BV) plus bendamustine followed by DA-EPOCH-R and ASCT; another reached CMR after only three cycles of a Glofitamab-GemOx regimen. A third patient (Ann Arbor stage IV, treated with DA-EPOCH-R) achieved CMR via CAR-T therapy (bridged with BV plus PD-1 inhibitor, and BR) but ultimately succumbed to therapy-related acute myeloid leukemia (AML M5b) and COVID-19 infection 17 months post-CAR-T. The remaining patient (Ann Arbor stage IV, treated with R-CHOP followed by consolidative ASCT and achieving CMR) was subsequently diagnosed with acute leukemia and lost to follow-up.

**Copyright Information:** (Online) ISSN: 2572-9241
©2026 The Author(s). HemaSphere is published by John Wiley & Sons Ltd on behalf of European Hematology Association. This is an open access Abstract Book distributed under the Attribution-NonCommercial-NoDerivs (CC BY-NC-ND), which allows third parties to download the articles and share them with others as long as they credit the author and the Abstract Book, but they cannot change the content in any way or use them commercially.
**Abstract Book Citations:** Authors, Title, HemaSphere, 2026;10:(S1):pages. The abstract book DOIs can be found on the “Author Information” page.
**Disclaimer:** Articles published in the journal HemaSphere exclusively reflect the opinions of the authors. The authors are responsible for all content in their abstracts including accuracy of the facts, statements, citing resources, etc.

**Summary/Conclusion**

Rituximab-based frontline immunochemotherapies yield excellent survival in PMBCL, with early treatment failures accounting for observed relapses. First-line Pola-R-CHP regimen demonstrated a highly promising 100% ORR, warranting longer follow-up for durability. Notably, the rapid and deep response to Glofitamab-GemOx in a primary refractory PMBCL patient highlights the potential of bispecific antibodies as highly effective salvage agents in this challenging setting. These findings support the continued evaluation of novel frontline and salvage strategies in PMBCL.

**Copyright Information:** (Online) ISSN: 2572-9241
©2026 The Author(s). HemaSphere is published by John Wiley & Sons Ltd on behalf of European Hematology Association. This is an open access Abstract Book distributed under the Attribution-NonCommercial-NoDerivs (CC BY-NC-ND), which allows third parties to download the articles and share them with others as long as they credit the author and the Abstract Book, but they cannot change the content in any way or use them commercially.
**Abstract Book Citations:** Authors, Title, HemaSphere, 2026;10:(S1):pages. The abstract book DOIs can be found on the "Author Information" page.
**Disclaimer:** Articles published in the journal HemaSphere exclusively reflect the opinions of the authors. The authors are responsible for all content in their abstracts including accuracy of the facts, statements, citing resources, etc.

### (PB3775) CLINICAL AND ECONOMIC OUTCOMES BY RISK GROUP AMONG FIRST-LINE PATIENTS WITH LARGE B-CELL LYMPHOMA IN THE UNITED STATES-SEER-MEDICARE DATA ANALYSIS

**Topic:** 19. Large B cell lymphomas - Clinical

Yi Lin*[1], Liana Nikolaenko[2], Elyse Swallow[3], Madhu Palivela[2], Jinlin Song[3], Markqayne Ray[2], Sakshi Sethi[3], David Cobley[2], Matthew Mattera[3], Hil Hsu[2], Shravanthi Seshasayee[3], Jean A. Yared[4]

*[1]Mayo Clinic, Rochester, United States of America; [2]Kite, a Gilead Company, Santa Monica, United States of America; [3]Analysis Group, Inc., Boston, United States of America; [4]University of Maryland, College Park, United States of America;*

### Background

Large B-cell Lymphoma (LBCL) is curable in over 60% of patients treated with first-line (1L) chemoimmunotherapy, most commonly R-CHOP (cyclophosphamide, doxorubicin, vincristine, prednisone, and rituximab). However, outcomes remain poor for patients with high-risk disease, typically defined by an International Prognostic Index (IPI) score of 4–5, underscoring a persistent unmet need in this subgroup. Contemporary real-world evidence describing the incremental clinical and economic burden associated with high-risk LBCL is limited, highlighting the need for updated evaluation.

### Aims

To assess the incremental clinical and economic burden of real-world 1L patients with high-risk LBCL compared with those with low- or intermediate-risk LBCL among Medicare beneficiaries in the United States (US).

### Methods

Data from the retrospective US Surveillance, Epidemiology, and End Results (SEER)-Medicare linked database (2009-2022) were used to identify patients with high-risk (IPI 4–5) and low- or intermediate-risk (IPI ≤ 3) LBCL. All patients received treatment for LBCL following diagnosis. The index date was defined as initiation of 1L treatment. Baseline was the 12 months prior to index, and follow-up extended from index until Medicare disenrollment, end of data availability, or death, whichever occurred first. Outcomes included: Overall survival (OS) evaluated using Kaplan-Meier analysis, all-cause healthcare resource utilization (HCRU), and costs (2025 US dollars). OS was compared between cohorts using an unadjusted Cox proportional hazards model and HCRU and costs using generalized linear models. Results are reported as hazard ratio (HR), incidence rate ratios (IRRs), or cost differences; adjusted estimates from multivariable models will be presented in the poster.

### Results

A total of 103 patients were included in the high-risk cohort and 235 in the low- or intermediate-risk cohort. Mean (standard deviation [SD]) age at index was 75.9 (6.4) years among high-risk patients and 75.7 (6.5) years among low- or intermediate-risk patients; 52.4% and 47.2% were male, respectively. Mean National Cancer Institute Comorbidity Index score was higher in the high-risk cohort (0.9 [SD=0.7] vs. 0.7 [0.6]; p=0.02). Mean (SD) follow-up duration was 3.5 (3.3) years and 4.9 (3.2) years, respectively. During follow-up, median (95% confidence interval) OS was 2.8 (1.6–6.5) years in the high-risk cohort compared with 8.7 (7.1–10.2) years in the low- or intermediate-risk cohort (HR=2.1; p<0.001; **Fig 1**). High-risk patients had 2.7-fold higher inpatient admissions (p<0.001) with mean admissions per person per year (PPPY) of 3.8 (SD=5.1) vs 1.8 (3.9). Mean emergency room visits PPPY were 1.5 (2.4) and 1.2 (2.9), respectively (IRR=1.4; p=0.069), while mean outpatient visits PPPY were 37.0 (20.9) and 32.2 (18.4) (IRR=1.2; p=0.024), respectively. Total healthcare costs PPPY were significantly higher among high-risk patients with an unadjusted cost difference of $45,619 (p<0.001), driven primarily by higher medical costs ($43,272; p<0.001).

**Copyright Information:** (Online) ISSN: 2572-9241
©2026 The Author(s). HemaSphere is published by John Wiley & Sons Ltd on behalf of European Hematology Association. This is an open access Abstract Book distributed under the Attribution-NonCommercial-NoDerivs (CC BY-NC-ND), which allows third parties to download the articles and share them with others as long as they credit the author and the Abstract Book, but they cannot change the content in any way or use them commercially.
**Abstract Book Citations:** Authors, Title, HemaSphere, 2026;10:(S1):pages. The abstract book DOIs can be found on the "Author Information" page.
**Disclaimer:** Articles published in the journal HemaSphere exclusively reflect the opinions of the authors. The authors are responsible for all content in their abstracts including accuracy of the facts, statements, citing resources, etc.

### Summary/Conclusion

In this real-world study, patients with high-risk LBCL receiving 1L therapy experienced markedly poorer OS, as well as substantially higher HCRU and costs, compared with patients with low- or intermediate-risk LBCL. These findings highlight the critical unmet need and reinforce the need for more effective 1L treatment options for patients with high-risk LBCL.

**Figure 1. Overall survival among the 1L high–risk and low– or intermediate–risk patients with LBCL**

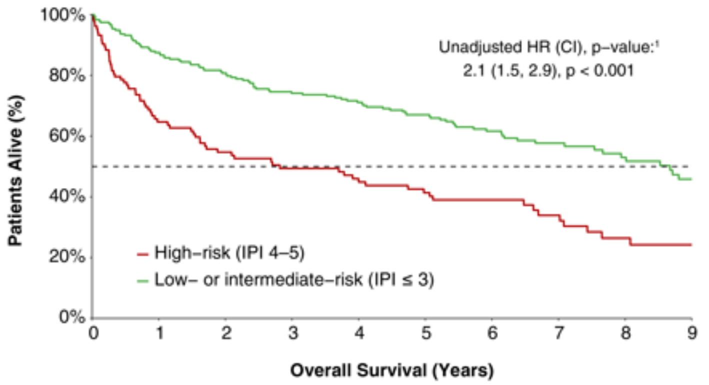

| | | | | | | | | | | |
|---|---|---|---|---|---|---|---|---|---|---|
| High–risk (IPI 4–5) | 103 | 65 | 53 | 45 | 41 | 35 | 24 | 19 | 12 | <11 |
| Low– or intermediate–risk (IPI ≤ 3) | 235 | 200 | 172 | 153 | 140 | 127 | 85 | 58 | 42 | 29 |

Patients at risk

Abbreviations: 1L: first line; CI: confidence interval; HR: hazard ratio; IPI: international prognostic index; LBCL: large B-cell lymphoma. Notes: [1] The proportional hazards assumption was violated for the risk coefficient in the unadjusted Cox proportional hazards model (Schoenfeld Residuals Test: p-value = 0.023). Consequently, the hazard ratio should be interpreted as the average risk effect over the entire time period, rather than a constant effect.

**Copyright Information:** (Online) ISSN: 2572-9241
©2026 The Author(s). HemaSphere is published by John Wiley & Sons Ltd on behalf of European Hematology Association. This is an open access Abstract Book distributed under the Attribution-NonCommercial-NoDerivs (CC BY-NC-ND), which allows third parties to download the articles and share them with others as long as they credit the author and the Abstract Book, but they cannot change the content in any way or use them commercially.
**Abstract Book Citations:** Authors, Title, HemaSphere, 2026;10:(S1):pages. The abstract book DOIs can be found on the "Author Information" page.
**Disclaimer:** Articles published in the journal HemaSphere exclusively reflect the opinions of the authors. The authors are responsible for all content in their abstracts including accuracy of the facts, statements, citing resources, etc.

### (PB3776) THE OUTCOME OF CAR-T THERAPY FOR CENTRAL NERVOUS SYSTEM LYMPHOMA FOLLOWING FAILED AUTOLOGOUS HEMATOPOIETIC STEM CELL TRANSPLANTATION

**Topic:** 19. Large B cell lymphomas - Clinical

Rui Liu*[1], Zhonghua Fu[1], Fan Yang[1], Miaomiao Cao[1], Lixia MA[1], Yuelu Guo[1], Biping Deng[2], Alex Chang[3], Xiaoyan Ke[1], Kai Hu[1]

*[1]Beijing GoBroad Hospital, Department of Lymphoma and Myeloma Research Center, Beijing, China; [2]Beijing GoBroad Boren Hospital, Cytology Laboratory, Beijing, China; [3]Shanghai YaKe Biotechnology Ltd., Engineering Research Center of Gene Technology, Ministry of Education, Institute of Genetics, School of Life Sciences, Shanghai, China;*

### Background

Relapsed or refractory central nervous system lymphoma (R/R CNSL) remains a major clinical challenge. High-dose chemotherapy followed by autologous stem cell transplantation (ASCT) achieves a median progression-free survival (PFS) of 41.4 months and overall survival (OS) of 58.6 months in primary CNS lymphoma (PCNSL). However, outcomes after ASCT failure are rarely reported. Chimeric antigen receptor T-cell (CAR-T) therapy has shown substantial efficacy in relapsed or refractory large B-cell lymphoma and has demonstrated promising activity in R/R CNSL. This study aimed to evaluate the efficacy of CAR-T therapy and identify factors associated with long-term survival in patients with ASCT-refractory R/R CNSL.

### Aims

To evaluate the efficacy and safety of CAR-T therapy and to identify factors associated with long-term survival in patients with ASCT-refractory R/R CNSL.

### Methods

We conducted a single-center, retrospective analysis of patients with R/R CNSL treated with CAR-T therapy at Beijing GoBroad Hospital between October 2021 and September 2025. A total of 27 patients were included: 52% were female, the median age was 52 years (range 29–68), 56% (15/27) had PCNSL, and 44% (12/27) had secondary CNS lymphoma (SCNSL). Histologies among SCNSL were DLBCL (n=11) and HGBL (n=1).
Overall, 56% (15/27) had received ≥3 prior regimens. Additionally, 44% (12/27) had an International Prognostic Index (IPI) ≥3, and 93% (25/27) were refractory to high-dose methotrexate (HD-MTX), with 13 patients progressing within 6 months of MTX therapy. BTK inhibitor resistance was observed in 70% (19/27).
Patterns of CNS involvement were as follows: 19 with parenchymal disease, 7 with both parenchymal and cerebrospinal fluid (CSF) involvement, and 1 with isolated CSF involvement.
Patients received a median of 2 cycles (range 0–4) of bridging therapy. All CAR-T products targeted CD19, including 9 murine and 16 humanized constructs. The median infusion dose was $1.4 \times 10^6$ CAR-T cells/kg (range 0.05–10.01). Prior to CAR-T infusion, disease status was CR in 19 patients, PR in 3, SD in 1, and PD in 3.

**Copyright Information:** (Online) ISSN: 2572-9241
©2026 The Author(s). HemaSphere is published by John Wiley & Sons Ltd on behalf of European Hematology Association. This is an open access Abstract Book distributed under the Attribution-NonCommercial-NoDerivs (CC BY-NC-ND), which allows third parties to download the articles and share them with others as long as they credit the author and the Abstract Book, but they cannot change the content in any way or use them commercially.
**Abstract Book Citations:** Authors, Title, HemaSphere, 2026;10:(S1):pages. The abstract book DOIs can be found on the "Author Information" page.
**Disclaimer:** Articles published in the journal HemaSphere exclusively reflect the opinions of the authors. The authors are responsible for all content in their abstracts including accuracy of the facts, statements, citing resources, etc.

**Results**

With a median follow-up of 23.8 months, the 3-month overall response rate (ORR) was 85% (23/27), and the complete response rate (CRR) was 81% (22/27). Median PFS and OS were not reached.
The 1-year PFS rate was 62%, which remained unchanged at 2 years. The 1-year OS rate was 83%, decreasing slightly to 77% at 2 years.
Any-grade cytokine release syndrome (CRS) occurred in 67% (18/27), with one patient experiencing grade ≥3 CRS. Immune effector cell–associated neurotoxicity syndrome (ICANS) occurred in 11% (3/27), with no grade ≥3 events reported.
The median interval from ASCT failure to CAR-T infusion was 9.73 months (range 1.87–97.08).

**Summary/Conclusion**

These findings support CAR-T therapy as an effective salvage option for ASCT-refractory R/R CNSL, providing a potential new standard of care in this high-risk population.

**Copyright Information:** (Online) ISSN: 2572-9241
©2026 The Author(s). HemaSphere is published by John Wiley & Sons Ltd on behalf of European Hematology Association. This is an open access Abstract Book distributed under the Attribution-NonCommercial-NoDerivs (CC BY-NC-ND), which allows third parties to download the articles and share them with others as long as they credit the author and the Abstract Book, but they cannot change the content in any way or use them commercially.
**Abstract Book Citations:** Authors, Title, HemaSphere, 2026;10:(S1):pages. The abstract book DOIs can be found on the "Author Information" page.
**Disclaimer:** Articles published in the journal HemaSphere exclusively reflect the opinions of the authors. The authors are responsible for all content in their abstracts including accuracy of the facts, statements, citing resources, etc.

### (PB3777) A PHASE 1/2, OPEN-LABEL, MULTICENTER STUDY OF UB-VV400 IN COMBINATION WITH RAPAMYCIN IN RELAPSED OR REFRACTORY B-CELL MALIGNANCIES

**Topic:** 19. Large B cell lymphomas - Clinical

Jacob Garcia*[1], Christine Dehner[1], Jeff Teoh[1]

[1]*Umoja Biopharma, Seattle, WA, United States of America;*

### Background

Autologous, ex vivo-manufactured chimeric antigen receptor (CAR) T cells have demonstrated clinical activity in hematologic malignancies.  These products have gained approvals in the relapsed/refractory (R/R) setting in multiple B-cell malignancies (BCMs), including large B-cell lymphoma (LBCL) and mantle cell lymphoma (MCL).  However, challenges in product availability due to limited manufacturing capacity, the need for apheresis and lymphodepletion, failure to prior ex vivo CAR T therapy, and the level of patient fitness needed to wait for and receive ex vivo autologous CAR T therapy all pose significant challenges to the field, presenting significant unmet clinical need.
UB-VV400 is a third-generation, self-inactivating, replication-incompetent lentiviral vector (LVV) investigational drug product comprising an envelope with cocal virus fusion glycoprotein (cocal) and surface engineered with a membrane-bound multidomain fusion (MDF) protein.  The MDF protein contains T-cell adhesion, activation, and costimulation components that promote both T-cell tropism and activation signals thought to be critical for effective CAR T-cell generation.  UB-VV400 is designed to generate CD22-directed CAR T cells in a patient, thus addressing the delivery limitations that exist for currently available autologous ex vivo CD19-directed CAR T therapies.  CD22 is an attractive therapeutic target because, like CD19, its expression is restricted to B cells, including both normal and malignant B cells. Moreover, CD22 offers an alternative target for patients who progress or relapse after exposure to CD19-directed therapies. UB-VV400 is designed to transduce T cells in vivo to generate CAR T cells that bind to CD22 antigen to mediate cell killing and express the rapamycin-activated cytokine receptor (RACR) system which, in the presence of rapamycin, provides specific pro-survival and expansion signals to transduced cells.

### Methods

Study UB-VV400-03 is a Phase 1/2, open-label, multicenter study of UB-VV400 administered intravenously (IV) in combination with rapamycin in adult participants with R/R CD22-positive BCMs, including LBCL and MCL. In Phase 1, dose escalation will proceed independently for each disease cohort using a Bayesian optimal interval (BOIN) design.  Confirmation of CD22 expression will be required for all participants.  Major eligibility criteria include adults with R/R LBCL or MCL who provide informed consent and who have standard organ function, measurable disease according to Lugano 2014, ECOG 0 or 1, and no prior allogeneic transplant.  Primary objectives for Phase 1 include determining the safety profile, maximum tolerated/administered dose, and recommended Phase 2 dose of UB-VV400 + rapamycin.  The primary objective in Phase 2 is to assess the antitumor activity (overall response rate); durability of response and pharmacokinetics (PK) will be assessed as a secondary objective.  In both parts of the study, pharmacodynamics will be evaluated as exploratory objectives.

**Copyright Information:** (Online) ISSN: 2572-9241
©2026 The Author(s). HemaSphere is published by John Wiley & Sons Ltd on behalf of European Hematology Association. This is an open access Abstract Book distributed under the Attribution-NonCommercial-NoDerivs (CC BY-NC-ND), which allows third parties to download the articles and share them with others as long as they credit the author and the Abstract Book, but they cannot change the content in any way or use them commercially.
**Abstract Book Citations:** Authors, Title, HemaSphere, 2026;10:(S1):pages. The abstract book DOIs can be found on the “Author Information” page.
**Disclaimer:** Articles published in the journal HemaSphere exclusively reflect the opinions of the authors. The authors are responsible for all content in their abstracts including accuracy of the facts, statements, citing resources, etc.

### (PB3778) EFFICACY ANALYSIS OF A FOUR-DRUG REGIMEN IN NEWLY DIAGNOSED PRIMARY CENTRAL NERVOUS SYSTEM LYMPHOMA

**Topic:** 19. Large B cell lymphomas - Clinical

Shaojie Wu*[1], Yanni Deng[1], Sanfang Tu[1], Rui Huang[1], Chaoyang Song[1], Zhiming Li[2], Yuhua Li[1]

*[1]Department of Hematology, ZhuJiang Hospital of Southern Medical University, Guangzhou, China;[2]Sun Yat-sen University Cancer Center, Department of Medical Oncology, Guangzhou, China;*

### Background

Primary central nervous system systemlymphoma (PCNSL) is a rare type of extranodal non-Hodgkin lymphoma with poor prognosis and high recurrence rate. Traditional treatment regimens are mainly high-dose methotrexate-based combination chemotherapy, but these regimens are often accompanied by high toxicity and side effects and limited efficacy, with a median overall survival (OS) of only about 1.3 years and a 5-year OS rate of about 30%, and there is a huge unmet clinical need. In recent years, with the advancement of molecular targeted therapy, the therapeutic potential of Bruton's tyrosinekinase (BTK) inhibitors in relapsed and refractory PCNSL has gradually emerged, and its efficacy and safety have been preliminarily verified. However, there are few reports on relevant clinical studies in treatment-naïve PCNSL. Based on this, this study evaluated the clinical efficacy and safety of orelabrutinib-containing regimen in the first-line treatment of treatment-naïve PCNSL patients through retrospective analysis.

### Aims

To evaluate the clinical efficacy and safety of the four-drug regimen (RMTO) in the first-line treatment of newly diagnosed primary central nervous system lymphoma (PCNSL).

### Methods

A retrospective analysis was conducted on the clinical data of 22 patients with PCNSL admitted to Zhujiang Hospital, Southern Medical University, from September 2021 to September 2025. All patients received induction therapy with the four-drug RMTO regimen, consisting of rituximab (375 mg/m²), high-dose methotrexate (HD-MTX, 3–3.5 g/m²), temozolomide (150 mg/m²), and orelabrutinib (150 mg qd), followed by autologous hematopoietic stem cell transplantation (AHSCT) as consolidation therapy. First-line treatment response, overall survival (OS), and progression-free survival (PFS) were analyzed. Survival curves were plotted using the Kaplan-Meier method.

### Results

The median follow-up time for the 22 PCNSL patients was 20 months. As of the analysis cutoff, the median OS and median PFS were not reached. The 12-month OS and PFS rates were 100% and 84.4%, respectively（Figure A and B). Among the 21 patients evaluated after 4 cycles of induction therapy, the objective response rate (ORR) was 100%, with a complete response (CR) rate of 80.95%. Among the 14 patients evaluated after 6 cycles, the ORR was 100%, with a CR rate of 92.86%(Figure D). The safety profile was favorable: 36.4% (8/22) of patients experienced CTCAE grade 1–2 hematological toxicity, and non-hematological toxicities were mainly mild nausea, vomiting, and elevated transaminases. No treatment-related deaths occurred(Figure C). The median OS and PFS for patients who received AHSCT consolidation were not reached.

**Copyright Information:** (Online) ISSN: 2572-9241
©2026 The Author(s). HemaSphere is published by John Wiley & Sons Ltd on behalf of European Hematology Association. This is an open access Abstract Book distributed under the Attribution-NonCommercial-NoDerivs (CC BY-NC-ND), which allows third parties to download the articles and share them with others as long as they credit the author and the Abstract Book, but they cannot change the content in any way or use them commercially.
**Abstract Book Citations:** Authors, Title, HemaSphere, 2026;10:(S1):pages. The abstract book DOIs can be found on the "Author Information" page.
**Disclaimer:** Articles published in the journal HemaSphere exclusively reflect the opinions of the authors. The authors are responsible for all content in their abstracts including accuracy of the facts, statements, citing resources, etc.

### Summary/Conclusion

The RMTO four-drug regimen demonstrates a high response rate and favorable safety profile in patients with PCNSL. Consolidation with AHSCT following induction therapy may further improve treatment outcomes.

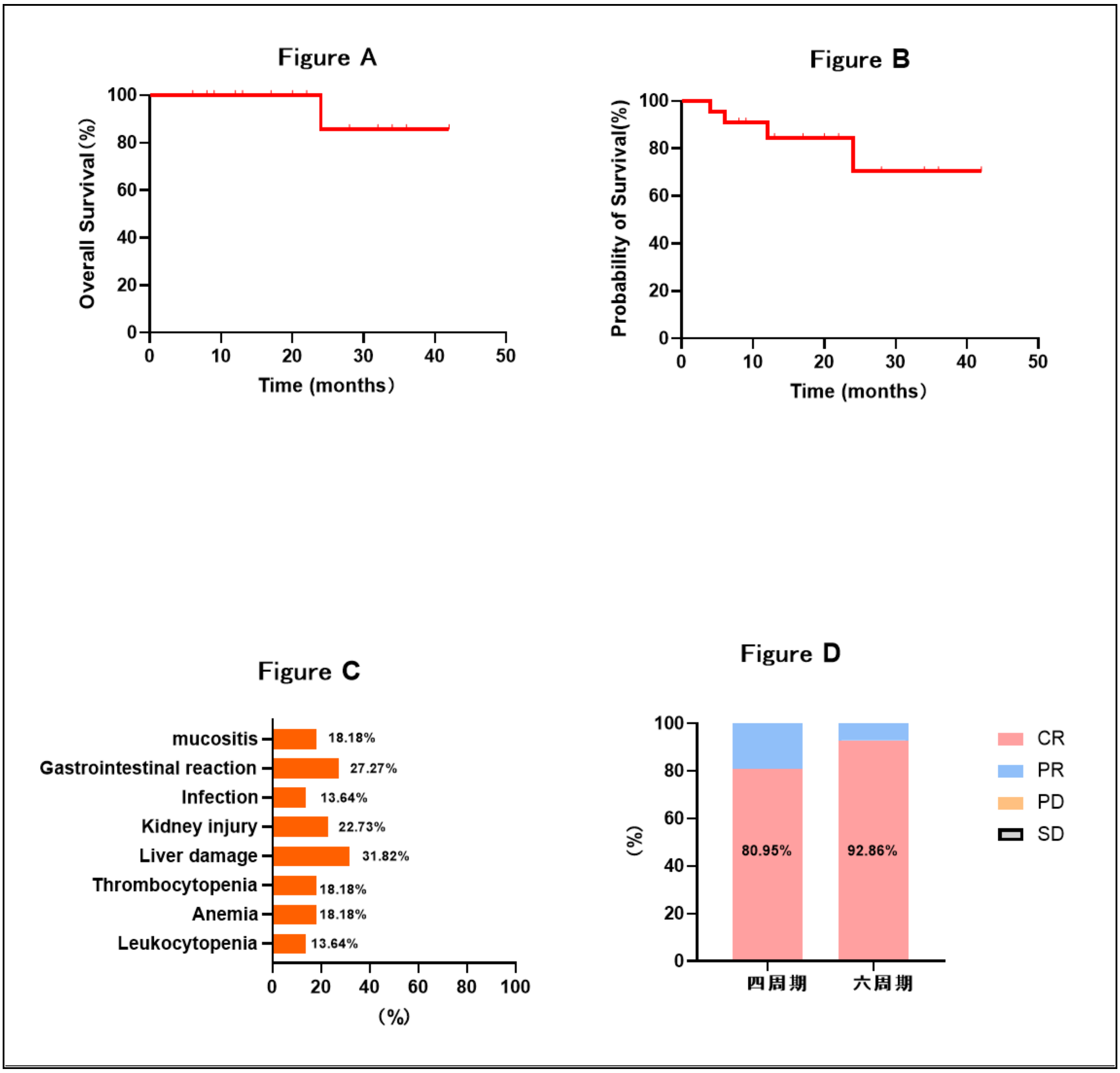

**Copyright Information:** (Online) ISSN: 2572-9241
©2026 The Author(s). HemaSphere is published by John Wiley & Sons Ltd on behalf of European Hematology Association. This is an open access Abstract Book distributed under the Attribution-NonCommercial-NoDerivs (CC BY-NC-ND), which allows third parties to download the articles and share them with others as long as they credit the author and the Abstract Book, but they cannot change the content in any way or use them commercially.
**Abstract Book Citations:** Authors, Title, HemaSphere, 2026;10:(S1):pages. The abstract book DOIs can be found on the "Author Information" page.
**Disclaimer:** Articles published in the journal HemaSphere exclusively reflect the opinions of the authors. The authors are responsible for all content in their abstracts including accuracy of the facts, statements, citing resources, etc.

### (PB3779) PERIPHERAL T-CELL IMMUNOPHENOTYPING UNDER EPCORITAMAB EXPOSURE IN AGGRESSIVE B-CELL LYMPHOMAS: A MULTICENTER REAL-WORLD STUDY

**Topic:** 19. Large B cell lymphomas - Clinical

Francesco Gaudio*[1, 2], Clorinda Derosa[2], Giuseppina Greco[3], Pierluigi Masciopinto[4], Pamela Pinto[2], Bruna Daraia[2], Angela Moramarco[2], Flavia Carbone[2], Angelica Clemente[2], Anna Mele[3], Vincenzo Pavone[3], Stefano Martinotti[1, 2]

*[1]Department of Medicine and Surgery, LUM University "Giuseppe Degennaro", casamassima, Italy; [2]Ecclesiastical Entity Regional Generale Hospital "F. Miulli", Acquaviva delle Fonti (BA), Italy; [3]Hematology and Transplant Unit, "Cardinal Panico" Hospital, Tricase (LE), Italy; [4]Hematology and Cell Therapy Unit, IRCCS Istituto Tumori "Giovanni Paolo II", Bari, Italy;*

### Background

Epcoritamab, a CD3×CD20 bispecific antibody, relies on T-cell engagement to mediate cytotoxic activity against malignant B cells. Because of this mechanism, examining the peripheral composition of T-cell subsets, specifically CD3, CD4, and CD8, offers a valuable window into the early immunologic remodeling that occurs during therapy and can provide biologically meaningful insights applicable to real-world practice.

### Aims

To characterize peripheral T-cell immunophenotyping at baseline and during the early phases of treatment in a multicenter, real-world setting, and to contextualize these findings within the clinical profile of the treated cohort.

### Methods

This multicenter retrospective study included consecutive patients with aggressive B-cell lymphomas treated with standard step-up dosing of epcoritamab. Clinical characteristics (N=19): The cohort comprised 15 males and 4 females, with a median age at diagnosis of 58 years (IQR 45–67.5). Advanced disease was common, with 84% (16/19) presenting with Ann Arbor stage IV, 21% (4/19) with bulky disease, and 95% (18/19) with extranodal involvement. Patients had received a median of 3 prior treatment lines (IQR 2–4); 37% (7/19) had undergone CAR-T therapy, 16% (3/19) autologous HSCT, and 5% (1/19) allogeneic HSCT. Peripheral immunophenotyping: Blood samples were collected at baseline (pre-epcoritamab) and during early on-treatment. Flow cytometry quantified CD3+ T cells (%, cells/µL), CD4+, CD8+, and the CD4/CD8 ratio. When available, additional populations—including CD19+, CD3−CD56+ NK cells, and CD3+CD56+ activated T cells were also described. Clinical outcome reported: The final metabolic response was assessed by PET/CT according to routine clinical practice.

### Results

At baseline, patients exhibited high peripheral CD3+ T-cell availability, indicating a substantial effector reservoir before treatment initiation. Early on-treatment samples revealed a clear cytotoxic shift, characterized by an increase in CD8+ T-cell representation and a corresponding reduction in the CD4/CD8 ratio in a subset of patients an immunophenotypic pattern compatible with early effector/memory-like remodeling. Where additional immunophenotypic data were available, patients showed marked B-cell depletion (CD19+), consistent with prior anti-CD20 exposure, along with a frequent reduction in NK cells (CD3−CD56+). The presence of CD3+CD56+ activated T cells in some samples further supported the emergence of an activated T-cell milieu during therapy. Treatment response: Final PET-based evaluation demonstrated an ORR of 42% (8/19), including CR 26% (5/19), PR 16% (3/19), SD 11% (2/19), and PD 47% (9/19).

**Copyright Information:** (Online) ISSN: 2572-9241
©2026 The Author(s). HemaSphere is published by John Wiley & Sons Ltd on behalf of European Hematology Association. This is an open access Abstract Book distributed under the Attribution-NonCommercial-NoDerivs (CC BY-NC-ND), which allows third parties to download the articles and share them with others as long as they credit the author and the Abstract Book, but they cannot change the content in any way or use them commercially.
**Abstract Book Citations:** Authors, Title, HemaSphere, 2026;10:(S1):pages. The abstract book DOIs can be found on the "Author Information" page.
**Disclaimer:** Articles published in the journal HemaSphere exclusively reflect the opinions of the authors. The authors are responsible for all content in their abstracts including accuracy of the facts, statements, citing resources, etc.

**Summary/Conclusion**

In this multicenter real-world cohort, treatment with epcoritamab was accompanied by distinct peripheral T-cell remodeling, marked by robust CD3+ availability and a prominent, early rise in CD8+ T-cell predominance. These changes support the biologic premise of CD3×CD20 bispecific engagement and reflect an activated T-cell environment emerging soon after treatment initiation. Standardized CD3/CD4/CD8 profiling thus represents a simple, reproducible, and informative approach for capturing immune activation dynamics in patients receiving T-cell–engaging bispecific antibodies.

**Copyright Information:** (Online) ISSN: 2572-9241
©2026 The Author(s). HemaSphere is published by John Wiley & Sons Ltd on behalf of European Hematology Association. This is an open access Abstract Book distributed under the Attribution-NonCommercial-NoDerivs (CC BY-NC-ND), which allows third parties to download the articles and share them with others as long as they credit the author and the Abstract Book, but they cannot change the content in any way or use them commercially.
**Abstract Book Citations:** Authors, Title, HemaSphere, 2026;10:(S1):pages. The abstract book DOIs can be found on the “Author Information” page.
**Disclaimer:** Articles published in the journal HemaSphere exclusively reflect the opinions of the authors. The authors are responsible for all content in their abstracts including accuracy of the facts, statements, citing resources, etc.

### (PB3780) WHAT DRIVES EARLY MORTALITY IN PATIENTS OVER 75 WITH DIFFUSE LARGE B-CELL LYMPHOMA?

**Topic:** 19. Large B cell lymphomas - Clinical

Mónica Fernández Pérez*[1], Ana Vega Gonzalez De Viñaspre[1], Ariane Unamunzaga Cilaurren[1]

[1]*Hospital Universitario Araba, Vitoria, Spain;*

### Background

Diffuse large B-cell lymphoma (DLBCL) is the most common form of adult non-Hodgkin lymphoma (NHL). Numerous factors have been related to the prognosis of DLBCL, but none of them have sufficient individual prognostic effect to condition clinical decisions. The patients with DLBCL suppose a therapeutic challenge due to comorbidities, presenting an increased risk of early death.

### Aims

The main objective is to identify the variables that predict the risk of early death or complications in patients with DLBCL.

### Methods

This is a retrospective, unicentric study of the University Hospital of Araba (HUA). Patients diagnosed with DLBCL between 1/01/2015-31/12/2025 and who died at the time of the study were selected. The variables that were collected, through the retrospective review of the clinical history, in a data collection notebook (CRD) were: age, gender, histological type and comorbidities. As well as the scales of fragility and comorbidity: International Prognostic Index (IPI), Charlson, Cumulative Illness Rating Scale (CIRS), Barthel and body mass index (BMI), analytical parameters such as fibrinogen, albumin and lymphocytes and the treatment received.

### Results

We included 156 patients who met the selection criteria. The median age of the included patients was 82 years. Of these, 43.58% died in the first year after diagnosis. The histological type of deceased patients was 53% for non-classificable patients, 20.6% germinal center B-cell-like and 26.4% for non-germinal center B-cell-like. Assessing the multipathology of the patients who died one year after diagnosis, it was observed that they presented in a higher percentage vascular disease ($p<0.001$), diabetes mellitus (p 0.631), chronic obstructive pulmonary disease (COPD) ($p<0.024$), heart failure (p 0.000), dementia (p 0.000), arterial disease (p 0.083), chronic kidney disease (p 0.036) and high BMI (25-35 vs 16-24) (p 0.040). Taking into account fragility parameters, an increased risk of death was observed in patients with elevated IPI (IPI: 4-5 vs 1-3) (p 0.003) and with elevated Charlson (5-8 vs 1-4) (p 0.000). As well as in patients with high CIRS (15-30 vs 0-15) ($p = 0.000$) and with a lower Barthel (<60 vs 60-100) ($p = 0.000$). Reviewing the analytical values of the patients, it was observed how patients with hyperfibrinogenemia (>500) had a worse prognosis (p 0.000), as well as those with hypoalbuminemia (2-3.5 vs 3.6-4.5) ($p <0.001$) and lymphopenia (0-1000 vs 1100-5000) (p 0.253). In 1st line, the treatment with the best response in less fragile patients was R-COP, followed by R-mini-CHOP.

### Summary/Conclusion

The population over 75 years of age with DLBCL is very heterogeneous. In our study we found that the fragility of patients has an impact on the therapeutic decision and on the risk of death one year after diagnosis, so it is necessary to carry out an adequate geriatric assessment to adapt the treatment to the clinical situation of the patients.

**Copyright Information:** (Online) ISSN: 2572-9241
©2026 The Author(s). HemaSphere is published by John Wiley & Sons Ltd on behalf of European Hematology Association. This is an open access Abstract Book distributed under the Attribution-NonCommercial-NoDerivs (CC BY-NC-ND), which allows third parties to download the articles and share them with others as long as they credit the author and the Abstract Book, but they cannot change the content in any way or use them commercially.
**Abstract Book Citations:** Authors, Title, HemaSphere, 2026;10:(S1):pages. The abstract book DOIs can be found on the "Author Information" page.
**Disclaimer:** Articles published in the journal HemaSphere exclusively reflect the opinions of the authors. The authors are responsible for all content in their abstracts including accuracy of the facts, statements, citing resources, etc.

| | | | Deceased | NO Deceased | Statistical significance |
|---|---|---|---|---|---|
| HISTOLOGICAL TYPE | Non-classificable | | 54% | 46% | p>0,05 |
| | Germinal center | | 43,75% | 56,25% | |
| | Non-Germinal center | | 39,13% | 65,21% | |
| Comorbidities | Vascular disease | | 70,37% | 29,63% | P<0,001 |
| | Diabetes mellitus | | 47,82% | 52,17% | P<0,631 |
| | Chronic obstructive pulmonary disease (COPD) | | 66,66% | 33,34% | P 0,024 |
| | Heart failure | | 72,72% | 27,27% | P 0,000 |
| | Dementia | | 92% | 8% | P 0,000 |
| | Arterial disease | | 49,15% | 50,85% | P 0,083 |
| | Chronic kidney disease | | 72,72% | 27,27% | P 0,036 |
| | Body mass index (BMI) | 16-24 | 31,25% | 68,75% | P 0,040 |
| | | 25-35 | 52,17% | 47,83% | |
| Fragility parameters | IPI | 1-3 | 23,68% | 76,31% | P 0,003 |
| | | 4-5 | 65% | 35% | |
| | Charlson | 1-4 | 30,50% | 69,50% | P 0,000 |
| | | 5-8 | 84,22% | 15,78% | |
| | CIRS | 0-15 | 6,82% | 93,18% | P 0,000 |
| | | 16-30 | 9,18% | 8,82% | |
| | Barthel | <60 | 89,48% | 10,52% | P 0,000 |
| | | 60-100 | 28,81% | 71,19% | |
| Analytical values | Fibrinogen | 0-500 | 20,93% | 79,07% | P 0,000 |
| | | 500-700 | 74,28% | 25,72% | |
| | Albumin | 2-3,5 | 69,23% | 30,76% | P <0,001 |
| | | 3,6-4,5 | 30,76% | 69,23% | |
| | Lymphocytes | 0-1000 | 70% | 30% | P 0,253 |
| | | 1100-5000 | 34,48% | 65,52% | |

**Copyright Information:** (Online) ISSN: 2572-9241
©2026 The Author(s). HemaSphere is published by John Wiley & Sons Ltd on behalf of European Hematology Association. This is an open access Abstract Book distributed under the Attribution-NonCommercial-NoDerivs (CC BY-NC-ND), which allows third parties to download the articles and share them with others as long as they credit the author and the Abstract Book, but they cannot change the content in any way or use them commercially.
**Abstract Book Citations:** Authors, Title, HemaSphere, 2026;10:(S1):pages. The abstract book DOIs can be found on the "Author Information" page.
**Disclaimer:** Articles published in the journal HemaSphere exclusively reflect the opinions of the authors. The authors are responsible for all content in their abstracts including accuracy of the facts, statements, citing resources, etc.

### (PB3782) LABORATORY-IPI (LAB-IPI) IN PREVIOUSLY UNTREATED DIFFUSE LARGE B-CELL LYMPHOMA (DLBCL): A HELLENIC DATABASE VALIDATION

**Topic:** 19. Large B cell lymphomas - Clinical

Theodoros Vassilakopoulos*[1], Alexia Piperidou[1], Athanasios Liaskas[1], Panagiota Maria Arapaki[1], Christina Apostolopoulou[2], Theodora Triantafyllou[2], Christina Kalpadaki[3], Sotirios Sachanas[4], Sotirios Tsakatikas[2], Aikaterini Bitsani[1], Maria Dimou[1], Maria Anastasia Ioakeim[1], Marina Siakantaris[1], Gerassimos Pangalis[4], Eirini Katodritou[5], Maria Angelopoulou[1], Vasiliki Pappa[2], Panagiotis Panagiotidis[1], Papageorgiou Sotirios[2]

*[1]National and Kapodistrian University of Athens, Department of Hematology and Bone Marrow Transplantation, Athens, Greece; [2]Second Department of Internal Medicine, Propaedeutic, Hematology Unit, Attikon University Hospital, Athens, Greece; [3]University of Crete School of Medicine & University Hospital of Heraklion, heraklion, Greece;[4]Department of Haematology, Athens Medical Center, Psychikon Branch, Athens, Greece, athens, Greece; [5]Theagenio Cancer Hospital, Hematology-Oncology, thessaloniki, Greece;*

### Background

The international prognostic index (IPI) is the standard tool for prognostication in patients with DLBCL. Recently Martin-Moro et al published a simplified, more convenient and more objective IPI version, the LAB-IPI, including elevated serum LDH (1 point), anemia (1 point) and elevated serum $\beta_2$-microglobulin (1 point if up to 2x; 2 points if >2x). This important finding warrants further validation.

### Aims

We sought to validate the LAB-IPI by analyzing data from the developing Hellenic database.

### Methods

Among 1094 DLBCL pts treated with immunochemotherapy (ICT) and registered in our database with available serum $\beta_2$-microglobulin data, 1080 had full LAB-IPI data and 1047 had available data on outcome. Freedom From Progression (FFP) was defined as the time between treatment initiation and death during treatment, primary refractoriness or relapse. Progression Free Survival (PFS) was defined as FFP plus death of any cause in first complete remission.

### Results

The LAB-IPI was highly effective in predicting the 5-year FFP with rates 88.0%, 76.6%, 60.4% and 32.3% for patients with low (L – 0 points), low-intermediate (LI – 1-2 points), high-intermediate (HI – 3 points) and high-risk LAB-IPI (H- 4 points) (p<0.001). The corresponding rates in the original LAB-IPI publication were 87%, 69%, 55% and 37% for event-free survival. The frequency of L, LI, HI and H groups was 15%, 48%, 21% and 15% respectively. The 5-year PFS rates were 3-8% lower than FFP, while the 5-year overall survival was 87.8%, 59.0%, 40.7% and 23.1% (p<0.001). LAB-IPI was highly effective in predicting FFP both in GCB and non-GCB subgroups, patients younger or older than 70 years old and those with normal or impaired renal function.

### Summary/Conclusion

The analysis of the Hellenic dataset confirmed the value of LAB-IPI in previously untreated DLBCL. Furthermore LAB-IPI was highly applicable in clinically relevant subgroups providing an objective and easily available prognostic tool.

**Copyright Information:** (Online) ISSN: 2572-9241
©2026 The Author(s). HemaSphere is published by John Wiley & Sons Ltd on behalf of European Hematology Association. This is an open access Abstract Book distributed under the Attribution-NonCommercial-NoDerivs (CC BY-NC-ND), which allows third parties to download the articles and share them with others as long as they credit the author and the Abstract Book, but they cannot change the content in any way or use them commercially.
**Abstract Book Citations:** Authors, Title, HemaSphere, 2026;10:(S1):pages. The abstract book DOIs can be found on the "Author Information" page.
**Disclaimer:** Articles published in the journal HemaSphere exclusively reflect the opinions of the authors. The authors are responsible for all content in their abstracts including accuracy of the facts, statements, citing resources, etc.

### (PB3783) FIRST-LINE PRIMZ-D CHEMOIMMUNOTHERAPY FOR PRIMARY CNS LYMPHOMA: REAL-WORLD EFFICACY IN A SINGLE-CENTER COHORT

**Topic:** 19. Large B cell lymphomas - Clinical

Fei Xiao[1], Yi Fang[1], Minyue Zhang[1], Yanni Hua[1], Xin Li[1], Shasha Lu[1], Lijing Shen[1], Honghui Huang*[2]

*[1]Renji Hospital, Shanghai Jiaotong University School of Medicine, Department of Hematology, Shanghai, China;[2]Renji Hospital, Shanghai Jiao Tong University School of Medicine, Department of hematology, Shanghai, China;*

### Background

Primary central nervous system lymphoma (PCNSL) is a rare and aggressive extranodal non-Hodgkin lymphoma. High-dose methotrexate (HD-MTX)–based chemoimmunotherapy remains the standard first-line induction backbone; however, long-term disease control remains suboptimal. Novel agents, including immunomodulatory drugs (IMiDs) such as pomalidomide and Bruton tyrosine kinase inhibitors (BTKis) such as zanubrutinib, have demonstrated meaningful central nervous system (CNS) penetration and single-agent activity in relapsed/refractory disease. The clinical value of integrating these agents with HD-MTX–based induction in the first-line setting warrants evaluation in routine practice.

### Aims

To report the real-world efficacy, safety, and survival outcomes of a five-drug combination regimen, PRiMZ-d (pomalidomide, rituximab, HD-MTX, zanubrutinib, and dexamethasone), as first-line induction therapy followed by consolidative autologous stem cell transplantation (ASCT) in patients with newly diagnosed PCNSL.

### Methods

We conducted a retrospective analysis of consecutive patients with newly diagnosed PCNSL treated at our center between March 2024 and October 2025. PRiMZ-d was administered every 21 days for up to six cycles in routine clinical practice. Consolidative ASCT was considered for eligible responders based on physician assessment and patient preference; maintenance therapy was optional. The primary endpoint was overall response rate (ORR). Secondary endpoints included complete response (CR) rate, progression-free survival (PFS), and overall survival (OS). Responses were assessed according to IPCG criteria. Survival outcomes were estimated using the Kaplan–Meier method. Where tissue was available, next-generation sequencing (NGS) was performed to characterize molecular subtypes (MCD and non-MCD); subtype-specific outcomes were summarized descriptively.

### Results

Twelve patients were included (median age 49 years [range, 34–74]; 55% male). All patients had histologically confirmed diffuse large B-cell lymphoma (DLBCL). NGS was performed in 10 patients; 6 were classified as MCD and 4 as non-MCD. One patient was excluded from the efficacy-evaluable population due to a significant treatment delay unrelated to toxicity, leaving 11 efficacy-evaluable patients.

In the efficacy-evaluable cohort (n=11), ORR was 91% (10/11) and the CR rate was 82% (9/11). Nine patients (82%) successfully proceeded to consolidative ASCT. At a median follow-up of 13.5 months (range, 3.9–23.2), the median PFS was 18.7 months and the 12-month PFS rate was 79.5%. Median OS was not reached, and no deaths were reported during the observation period. A sensitivity analysis including all 12 treated patients (intention-to-treat) showed consistent findings, with a median PFS of 16.4 months.

Among NGS-evaluated patients (n=10), subtype-specific PFS outcomes were summarized descriptively given the limited sample size (median PFS 12.5 months for MCD; median not reached for non-MCD). The regimen was generally well tolerated, and no treatment-related mortality was observed.

**Copyright Information:** (Online) ISSN: 2572-9241
©2026 The Author(s). HemaSphere is published by John Wiley & Sons Ltd on behalf of European Hematology Association. This is an open access Abstract Book distributed under the Attribution-NonCommercial-NoDerivs (CC BY-NC-ND), which allows third parties to download the articles and share them with others as long as they credit the author and the Abstract Book, but they cannot change the content in any way or use them commercially.
**Abstract Book Citations:** Authors, Title, HemaSphere, 2026;10:(S1):pages. The abstract book DOIs can be found on the "Author Information" page.
**Disclaimer:** Articles published in the journal HemaSphere exclusively reflect the opinions of the authors. The authors are responsible for all content in their abstracts including accuracy of the facts, statements, citing resources, etc.

### Summary/Conclusion

This real-world case series demonstrates that PRiMZ-d is a highly active first-line induction regimen for PCNSL, yielding deep responses and enabling successful bridging to ASCT. These findings support the rationale for future prospective, controlled clinical trials to validate this approach.

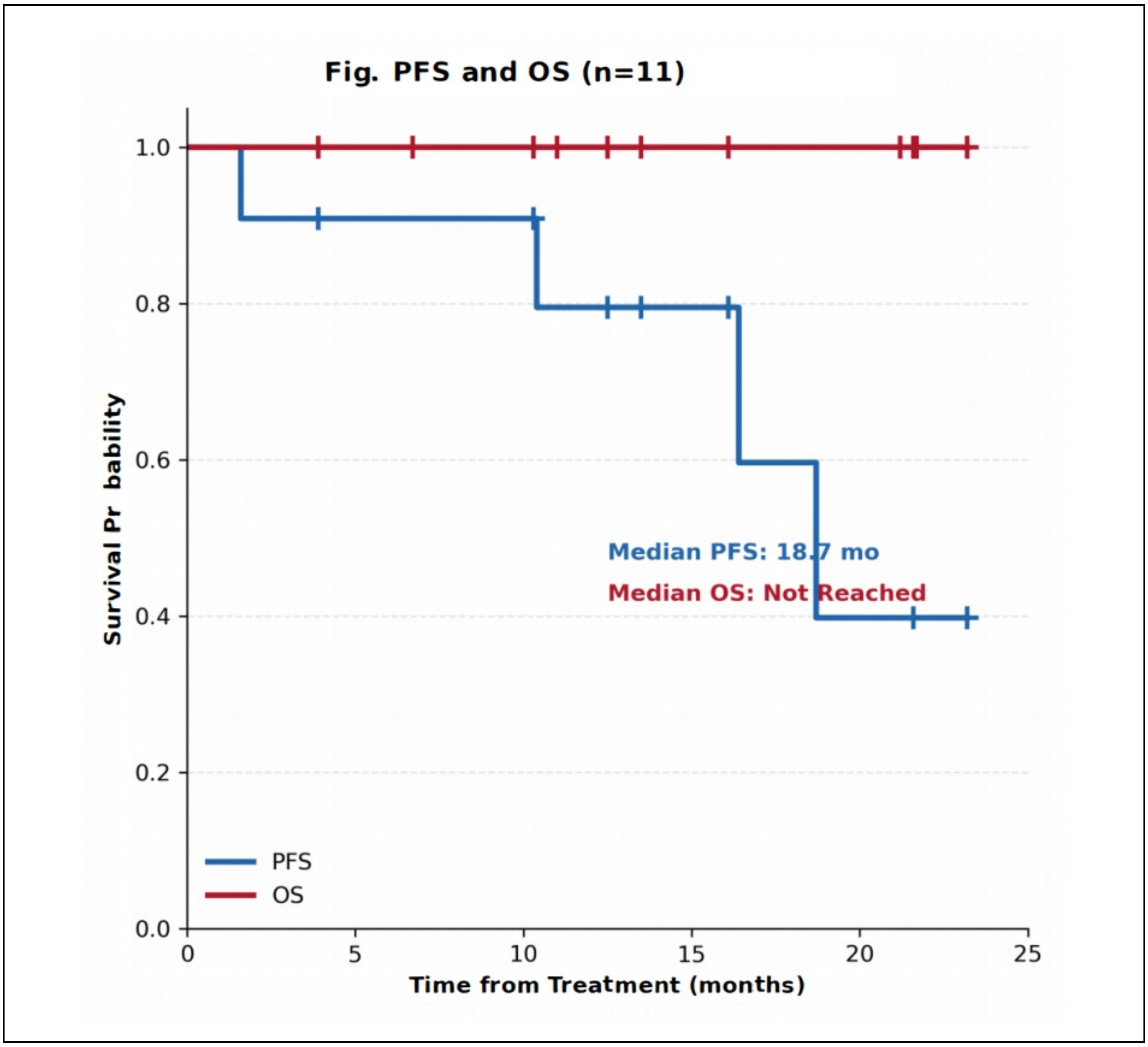

Figure. Kaplan-Meier estimates of progression-free survival (PFS) and overall survival (OS) in the efficacy-evaluable population (n=11) treated with first-line PRiMZ-d. PFS (blue) and OS (red) for the main cohort.

**Copyright Information:** (Online) ISSN: 2572-9241
©2026 The Author(s). HemaSphere is published by John Wiley & Sons Ltd on behalf of European Hematology Association. This is an open access Abstract Book distributed under the Attribution-NonCommercial-NoDerivs (CC BY-NC-ND), which allows third parties to download the articles and share them with others as long as they credit the author and the Abstract Book, but they cannot change the content in any way or use them commercially.
**Abstract Book Citations:** Authors, Title, HemaSphere, 2026;10:(S1):pages. The abstract book DOIs can be found on the "Author Information" page.
**Disclaimer:** Articles published in the journal HemaSphere exclusively reflect the opinions of the authors. The authors are responsible for all content in their abstracts including accuracy of the facts, statements, citing resources, etc.

### (PB3784) REAL-WORLD OUTCOMES AND HEALTHCARE DISPARITIES IN DIFFUSE LARGE B-CELL LYMPHOMA: A 7-YEAR RETROSPECTIVE COHORT STUDY IN A BRAZILIAN PUBLIC UNIVERSITY HOSPITAL

**Topic:** 19. Large B cell lymphomas - Clinical

Carolina Ribeiro de Castro Pires*[1], Sergio Shusterschitz[1], Thamiris Silva Soares[1], Pedro Henrique Fernandes Do Carmo Las Casas[1], Frederico Lisboa Nogueira[1]

[1]*Universidade Federal de Minas Gerais, Belo Horizonte, Brazil;*

### Background

Diffuse large B-cell lymphoma (DLBCL) is a heterogeneous disease where outcomes are heavily influenced by biological factors and healthcare access. In low- and middle-income countries, structural barriers often lead to diagnostic delays and advanced disease at presentation, potentially widening global survival disparities. Furthermore, the limited access to novel therapies, remains a critical challenge in achieving optimal outcomes in these regions.

### Aims

To describe the epidemiological profile, treatment response, and survival outcomes — progression-free survival (PFS) and overall survival (OS) — of DLBCL patients at a Brazilian public university hospital, while identifying prognostic factors for death. For the relapsed/refractory (R/R) population, the study aims to describe the access to therapies available at our institution and their outcomes.

### Methods

This retrospective cohort study included adult patients with a primary diagnosis of DLBCL who initiated first-line treatment at Hospital das Clínicas – Universidade Federal de Minas Gerais between 2018 and 2024. Survival was analyzed using Kaplan-Meier curves and prognostic factors with Cox regression models (variables with p < 0,05 were included in the multivariate analysis).

### Results

Sixty-nine patients were included (median age 57 years, IQ 41–71). Most presented with advanced Lugano stage III-IV (79.7%), R-IPI ≥ 3 (60.9%), and 34.7% had an ECOG performance status ≥ 3, with a median Charlson comorbidity index of 3 (2–6). Median time for immunohistochemistry (IHC) results was 22 days (range 2–157). Non-germinal center (NGCB) origin was found in 49.3%. Notably, 24.6% of cases were non-classifiable by the Hans algorithm due to incomplete panels, and FISH for MYC rearrangement was not performed in any patient. Primary treatment was R-CHOP (59.4%) or R-mini-CHOP (21.7%). In the evaluable population (n = 63), the overall response rate was 74.6%. With a median follow-up of 43.3 months, median PFS and OS were not reached. The 36-month PFS and OS were 55.0% (95% CI 41,8 – 66,5) and 59.3% (95% CI 46,0 – 70,4), respectively. Total mortality was 37.7%, primarily driven by disease progression (53.8%) and infection (34.6%). Multivariate analysis identified ≥ 2 extra nodal sites (HR 6.23; 95% CI 2,14 – 18,08, p < 0.001) and R-mini-CHOP (HR 4.39; 95% CI 1,42 – 13,55, p 0.010) as independent predictors of worse OS. In the R/R setting (30.4%), 80.9% occurred early (refractory or < 12 months). Among treatment-eligible R/R patients, 70.6% received platinum-based salvage chemotherapy (GDP, ICE or DHAP), 17.6% received lower-intensity chemotherapy (mostly Gemox) and 11,8% received radiotherapy. The median OS after second-line initiation was only 4.2 months (95% CI 1,73 – 5,7). In early R/R cases, the response rate was 0%; none underwent autologous stem cell transplantation (ASCT) and the mortality rate was 100%.

**Copyright Information:** (Online) ISSN: 2572-9241
©2026 The Author(s). HemaSphere is published by John Wiley & Sons Ltd on behalf of European Hematology Association. This is an open access Abstract Book distributed under the Attribution-NonCommercial-NoDerivs (CC BY-NC-ND), which allows third parties to download the articles and share them with others as long as they credit the author and the Abstract Book, but they cannot change the content in any way or use them commercially.
**Abstract Book Citations:** Authors, Title, HemaSphere, 2026;10:(S1):pages. The abstract book DOIs can be found on the “Author Information” page.
**Disclaimer:** Articles published in the journal HemaSphere exclusively reflect the opinions of the authors. The authors are responsible for all content in their abstracts including accuracy of the facts, statements, citing resources, etc.

**Summary/Conclusion**

Our cohort highlights a high prevalence of high-risk DLBCL and poor performance status at diagnosis, likely reflecting systemic barriers to early care. While frontline outcomes were comparable to literature, the dismal prognosis in early R/R cases reveals a gap in the Brazilian public health system: the lack of access to novel therapies, such as CAR-T cell and bispecific antibodies. These results emphasize the urgent need for improved diagnostic resources, including timely and standardized IHC panels, and the incorporation of advanced second-line therapies to mitigate survival disparities in developing regions.

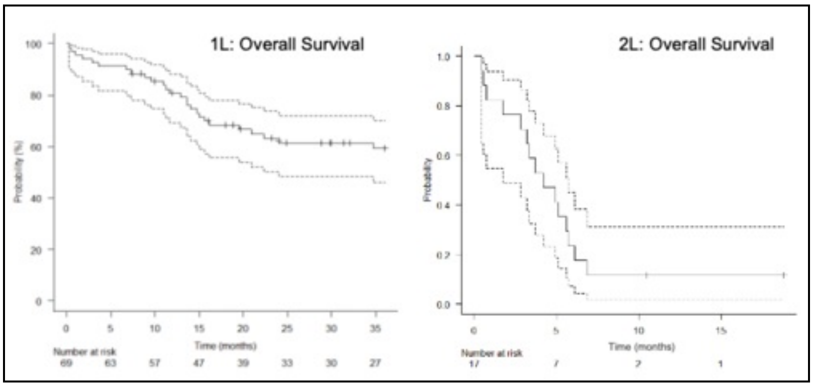

Kaplan-Meier estimates for OS according to treatment phase: (A) Frontline setting; (B) R/R setting.

**Copyright Information:** (Online) ISSN: 2572-9241
©2026 The Author(s). HemaSphere is published by John Wiley & Sons Ltd on behalf of European Hematology Association. This is an open access Abstract Book distributed under the Attribution-NonCommercial-NoDerivs (CC BY-NC-ND), which allows third parties to download the articles and share them with others as long as they credit the author and the Abstract Book, but they cannot change the content in any way or use them commercially.
**Abstract Book Citations:** Authors, Title, HemaSphere, 2026;10:(S1):pages. The abstract book DOIs can be found on the "Author Information" page.
**Disclaimer:** Articles published in the journal HemaSphere exclusively reflect the opinions of the authors. The authors are responsible for all content in their abstracts including accuracy of the facts, statements, citing resources, etc.

### (PB3785) VALUE OF THE IL-1B/IL-10 RATIO IN PREDICTING RESPONSE TO R-CHOP THERAPY IN PATIENTS WITH DLBCL

**Topic:** 19. Large B cell lymphomas - Clinical

Lijuan Li*[1], Liansheng Zhang[1], Jianing Yu[1]

*[1]Lanzhou University Second Hospital, Department of Hematology, lanzhou, China;*

### Background

Diffuse large B-cell lymphoma (DLBCL) comprises a heterogeneous group of diseases. Although the R-CHOP regimen is curative for the majority of patients, a subset exhibits primary resistance. The immune microenvironment plays a crucial role in treatment response, with IL-1β and IL-10 representing pro-inflammatory and anti-inflammatory signals, respectively. Their balance may influence anti-tumor immunity.

### Aims

This study aims to investigate the predictive value of the pretreatment peripheral blood IL-1β/IL-10 ratio for response to R-CHOP therapy in patients with DLBCL.

### Methods

Thirty-two patients with newly diagnosed DLBCL were retrospectively enrolled. Clinical data and pretreatment peripheral blood samples were collected. Serum levels of IL-1β and IL-10 were measured using flow cytometry and Cytometric Bead Array (CBA), and the IL-1β/IL-10 ratio was calculated. The primary outcome was the achievement of complete remission (CR) after 4-6 cycles of R-CHOP therapy. The optimal cut-off value was determined using receiver operating characteristic (ROC) curve analysis. The association between the ratio and response was analyzed using Fisher's exact test, logistic regression, and ROC curve analysis.

### Results

The IL-1β/IL-10 ratio ranged from 0.00 to 1.19 (median 0.10). The optimal cut-off value was 0.3, dividing patients into a high-ratio group (≥0.3, n=8) and a low-ratio group (<0.3, n=24). The CR rate in the high-ratio group was 87.5% (7/8), which was significantly higher than the 33.3% (8/24) observed in the low-ratio group (Fisher's exact test, P=0.013; Odds Ratio [OR] = 12.85, 95% CI 1.30–666.42). The Risk Ratio (RR) was 2.62 (95% CI 1.41–4.90), with an absolute risk difference of 0.54. The area under the ROC curve (AUC) was 0.68 (95% CI 0.48–0.88), suggesting moderate predictive performance.

### Summary/Conclusion

The pretreatment peripheral blood IL-1β/IL-10 ratio is significantly associated with response to R-CHOP therapy in patients with DLBCL. Patients with a high ratio have a higher probability of achieving CR. This ratio may serve as a simple, immune-related biomarker to identify patients more sensitive to R-CHOP, although its predictive value requires further validation in larger prospective cohorts.

**Copyright Information:** (Online) ISSN: 2572-9241
©2026 The Author(s). HemaSphere is published by John Wiley & Sons Ltd on behalf of European Hematology Association. This is an open access Abstract Book distributed under the Attribution-NonCommercial-NoDerivs (CC BY-NC-ND), which allows third parties to download the articles and share them with others as long as they credit the author and the Abstract Book, but they cannot change the content in any way or use them commercially.
**Abstract Book Citations:** Authors, Title, HemaSphere, 2026;10:(S1):pages. The abstract book DOIs can be found on the "Author Information" page.
**Disclaimer:** Articles published in the journal HemaSphere exclusively reflect the opinions of the authors. The authors are responsible for all content in their abstracts including accuracy of the facts, statements, citing resources, etc.

### (PB3786) LATE-ONSET NEUTROPENIA AND HYPOGAMMAGLOBULINEMIA IN DLBCL PATIENTS TREATED WITH THE R-DA-EPOCH REGIMEN

**Topic:** 19. Large B cell lymphomas - Clinical

Marko Lucijanic*[1], Rafaela Filipan[2], Martina Sedinić Lacko[3], Marija Ivić[3], Zdravko Mitrović[3], Ozren Jakšić[3]

*[1]Klinička Bolnica Dubrava, Hematology department, Zagreb, Croatia;[2]Medicinski Fakultet, Zagreb, Croatia;[3]University Hospital Dubrava, Hematology department, Zagreb, Croatia;*

### Background

Although late-onset neutropenia (LON) and hypogammaglobulinemia are recognized long-term complications of rituximab therapy, their patterns and clinical impact within the context of intensive, dose-adjusted regimens such as R-DA-EPOCH for aggressive B-cell lymphomas have not been fully elucidated.

### Aims

To investigate the occurrence and predictors of LON and hypogammaglobulinemia, as well as their association with infectious complications, in DLBCL patients achieving remission after first-line R-DA-EPOCH therapy.

### Methods

We retrospectively analyzed a cohort of newly diagnosed DLBCL patients with unfavorable clinical features treated with the R-DA-EPOCH regimen at our institution between 2005 and 2019 who achieved remission upon treatment completion. Selected subsets of patients underwent auto-SCT or radiotherapy as part of their first-line therapy. The protocol was administered according to the original recommendations, with mandatory G-CSF prophylaxis following each cycle. ANC and IgG levels were recorded at baseline, at the end of treatment (EOT), and at 6 and 12 months post-treatment. Neutropenia was defined as an ANC<1.5x$10^9$/L, and hypogammaglobulinemia as an IgG<5g/L.

**Copyright Information:** (Online) ISSN: 2572-9241
©2026 The Author(s). HemaSphere is published by John Wiley & Sons Ltd on behalf of European Hematology Association. This is an open access Abstract Book distributed under the Attribution-NonCommercial-NoDerivs (CC BY-NC-ND), which allows third parties to download the articles and share them with others as long as they credit the author and the Abstract Book, but they cannot change the content in any way or use them commercially.
**Abstract Book Citations:** Authors, Title, HemaSphere, 2026;10:(S1):pages. The abstract book DOIs can be found on the "Author Information" page.
**Disclaimer:** Articles published in the journal HemaSphere exclusively reflect the opinions of the authors. The authors are responsible for all content in their abstracts including accuracy of the facts, statements, citing resources, etc.

### Results

Among the 53 analyzed patients, 25 (47.2%) were female. The median age was 64 years. The median R-IPI score was 3, with 3 (5.7%), 21 (39.6%), and 29 (54.7%) patients categorized into very good, good, and poor risk groups, respectively. Overall, 44 (83%) patients achieved complete remission, while 9 (17%) achieved partial remission.
The severity of ANC and IgG derangements at baseline, EOT, and at 6 and 12 months post-treatment are presented in Figure 1. The lowest ANC was observed 6 months after treatment completion, with 11 (22.4%) patients exhibiting neutropenia (mild, moderate, and severe in 7, 2, and 2 patients, respectively). There was no statistically significant clustering of patients developing neutropenia at different time points. In multivariate regression analysis, independent predictors of LON at 6 months post-treatment were lower leukocyte count at diagnosis, absence of infective complications during the initial treatment period, and a higher number of dose-escalation cycles.
The lowest IgG concentrations were observed at EOT, with gradual recovery over the following 12 months. Hypogammaglobulinemia at EOT developed in 8 patients (33% of those with available data). In contrast to neutropenia patterns, patients with hypogammaglobulinemia at EOT were more likely to remain hypogammaglobulinemic at later time points. No independent multivariate predictors of hypogammaglobulinemia at treatment completion were identified.
A total of 39/53 (73.6%) patients experienced post-treatment infectious complications, primarily involving the respiratory tract (56.6%), urinary tract (34%), and other sites (28.3%); most events were mild to moderate. Neutropenia at any time point was not associated with a higher frequency of infections, whereas hypogammaglobulinemia at EOT was significantly associated with an increased incidence of respiratory infections (P=0.040).

### Summary/Conclusion

In first-line DLBCL patients who achieved remission post R-DA-EPOCH, neutropenia and hypogammaglobulinemia exhibit distinct peak timings and occur in different patient subsets. Although both are prevalent, they are primarily mild to moderate. Only post-treatment hypogammaglobulinemia correlates significantly with an increased frequency of respiratory infections

**Copyright Information:** (Online) ISSN: 2572-9241
©2026 The Author(s). HemaSphere is published by John Wiley & Sons Ltd on behalf of European Hematology Association. This is an open access Abstract Book distributed under the Attribution-NonCommercial-NoDerivs (CC BY-NC-ND), which allows third parties to download the articles and share them with others as long as they credit the author and the Abstract Book, but they cannot change the content in any way or use them commercially.
**Abstract Book Citations:** Authors, Title, HemaSphere, 2026;10:(S1):pages. The abstract book DOIs can be found on the "Author Information" page.
**Disclaimer:** Articles published in the journal HemaSphere exclusively reflect the opinions of the authors. The authors are responsible for all content in their abstracts including accuracy of the facts, statements, citing resources, etc.

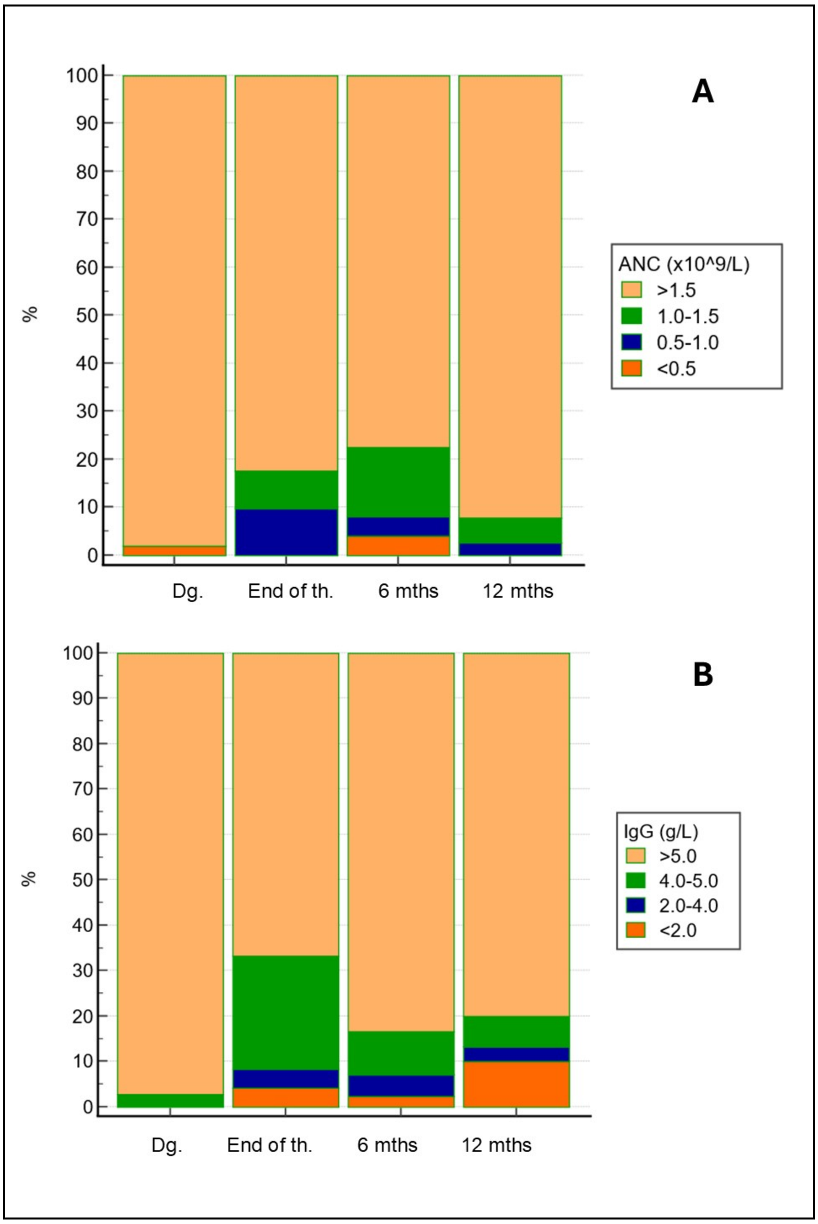

**Copyright Information:** (Online) ISSN: 2572-9241
©2026 The Author(s). HemaSphere is published by John Wiley & Sons Ltd on behalf of European Hematology Association. This is an open access Abstract Book distributed under the Attribution-NonCommercial-NoDerivs (CC BY-NC-ND), which allows third parties to download the articles and share them with others as long as they credit the author and the Abstract Book, but they cannot change the content in any way or use them commercially.
**Abstract Book Citations:** Authors, Title, HemaSphere, 2026;10:(S1):pages. The abstract book DOIs can be found on the "Author Information" page.
**Disclaimer:** Articles published in the journal HemaSphere exclusively reflect the opinions of the authors. The authors are responsible for all content in their abstracts including accuracy of the facts, statements, citing resources, etc.

### (PB3787) REAL WORLD DATA ON CD20CD3 BISPECIFIC ANTIBODIES IN RELAPSED/REFRACTORY LARGE B CELL LYMPHOMA –KROHEM

**Topic:** 19. Large B cell lymphomas - Clinical

Barbara Dreta*[1], Karla Misura Jakobac[2], Antonija Miljak[3], Marija Ivić[4], Neven Franjić[5], Goran Rincic[6], Ivan Krecak[7], Hrvoje Holik[8], Dejan Strahija[9], Petra Bernes[10], Ivan Zekanovic[11], Ana Kotris[12], Sandra Bašić Kinda[13], Dino Dujmović[13], Igor Aurer[13, 14]

*[1]UHC Zagreb, Department of Internal medicine, Division of hematology, Zagreb, Croatia;[2]UH Merkur, Zagreb, Croatia; [3]UHC Split, Split, Croatia; [4]UH Dubrava, Zagreb, Croatia; [5]UHC Rijeka, Rijeka, Croatia; [6]UHC "Sestre Milosrdnice", Zagreb, Croatia; [7]GH Šibenik, Šibenik, Croatia; [8]Dr Josip Bencevic General Hospital, Slavonski Brod, Croatia; [9]GH Varaždin, Varaždin, Croatia; [10]GH Pula, Pula, Croatia; [11]UH Zadar, Zadar, Croatia; [12]UHC Osijek, Osijek, Croatia; [13]UHC Zagreb, Zagreb, Croatia; [14]University of Zagreb, School of medicine, Zagreb, Croatia;*

### Background

Treatment of relapse/refractory large B cell lymphoma (LBCL) in recent years showed great improvement with introduction of T cell engaging therapies. Bispecific antibodies have shown benefit in patient survival in clinical trials. There are limited data for patients treated with glofitamab and epcoritamab outside of clinical trials.

### Aims

Report effectives and safety of treatment with bispecific antibodies in R/R LBCL treated in routine clinical practice through national compassionate use program.

### Methods

We retrospectively collected data on patients with LBCL treated with at least one dose of glofitamab or epcoritamab until October 2025. Response was assessed with Lugano 2014. criteria. CRS and ICANS were graded according to ASCTC and other side effect with CTCAE 5.0 criteria. PFS and OS were estimated with Kaplan-Meier method.

### Results

We included 40 consecutive patients in analysis. Median age was 66y (range 37-80), male 60%. Median number of previous therapies was 3, 27.5% previously had tisagenlecleucel and 20% had ASCT. 82% were primary refractory or relapsed in 12 months after first line. At time of relapse IPI was 1- 2,5%, 2-22,5%, 3-20%, 4-35%, 5-7,5%, and 75% of patients had extra nodal disease.
Epcoritamab treatment had 9 pt and glofitamab 31 pt. There was no significant difference regarding patient characteristics although all patient treated after tisagenlecleucel had glofitamab. (Table 1)
For all patients ORR was 45%, CR 27%, glofitamab ORR 45%, CR 29%, and epcoritamab ORR 44%, CR 22%.With median follow up of 4,9 months, median OS was 4,6 mo, 6moOS 45%, median PFS 3,2mo, 6moPFS 42%.Patients who achieved CR had durable response with median OS 22,9mo.Patients with extra nodal disease at time of relapse had significantly worse OS (p 0.02) as patients with CD20 negative tumor biopsy at time of relapse (p 0.02).
There was no difference in survival according to bispecific antibody used, line of treatment, value of IPI at relapse and previous treatment with CAR-T cells.
CRS occurred in 45% pts treated with glofitamab and 55% with epcoritamab. They were all gr 1-2 except 1 pt with gr 4 on glofitamab. There was 1 pt with ICANS gr 1 on epcoritamab.
Tumor flair had 3 pt on glofitamab and 1 pt on epcoritamab. Neutropenia gr 3-4 occurred in 35% of patients and thrombocytopenia in 25%. There were infections in 45% pts, gr 3-4 in 32%.

**Copyright Information:** (Online) ISSN: 2572-9241
©2026 The Author(s). HemaSphere is published by John Wiley & Sons Ltd on behalf of European Hematology Association. This is an open access Abstract Book distributed under the Attribution-NonCommercial-NoDerivs (CC BY-NC-ND), which allows third parties to download the articles and share them with others as long as they credit the author and the Abstract Book, but they cannot change the content in any way or use them commercially.
**Abstract Book Citations:** Authors, Title, HemaSphere, 2026;10:(S1):pages. The abstract book DOIs can be found on the "Author Information" page.
**Disclaimer:** Articles published in the journal HemaSphere exclusively reflect the opinions of the authors. The authors are responsible for all content in their abstracts including accuracy of the facts, statements, citing resources, etc.

Discontinuation of treatment had 23 pts on glofitamab due to disease progression 19, toxicities 3 and 1 SPM. With epcoritamab 7 pts discontinued treatment, 4 toxicities and 3 disease progressions.
Deaths occurred in 28 pts, 24 disease progression, 3 sepsis and 1 neurological disease.

Table 1.

| | **Epcoritamab** | | **Glofitamab** | |
|---|---|---|---|---|
| N pt | 9 | | 31 | |
| Age (range) | 69 (61-80) | | 66 (37-80) | |
| | N | % | N | % |
| Male | 4 | 44 | 20 | 65 |
| Primary ref | 7 | 78 | 26 | 84 |
| Ekstranodal | 7 | 78 | 23 | 74 |
| CAR-T | 1 | 11 | 10 | 32 |
| Line of treatment | | | | |
| 2 | 0 | 0 | 0 | 0 |
| 3 | 4 | 44 | 18 | 58 |
| 4 | 2 | 22 | 9 | 29 |
| 5 | 3 | 33 | 4 | 13 |
| IPI 0-2 | 1 | 11 | 9 | 29 |
| IPI>2 | 7 | 78 | 18 | 58 |

**Copyright Information:** (Online) ISSN: 2572-9241
©2026 The Author(s). HemaSphere is published by John Wiley & Sons Ltd on behalf of European Hematology Association. This is an open access Abstract Book distributed under the Attribution-NonCommercial-NoDerivs (CC BY-NC-ND), which allows third parties to download the articles and share them with others as long as they credit the author and the Abstract Book, but they cannot change the content in any way or use them commercially.
**Abstract Book Citations:** Authors, Title, HemaSphere, 2026;10:(S1):pages. The abstract book DOIs can be found on the "Author Information" page.
**Disclaimer:** Articles published in the journal HemaSphere exclusively reflect the opinions of the authors. The authors are responsible for all content in their abstracts including accuracy of the facts, statements, citing resources, etc.

| | | | | |
|---|---|---|---|---|
| IPI UNK | 1 | 11 | 4 | 13 |
| CD20 pos | 8 | 89 | 18 | 58 |
| CD20 neg | 1 | 11 | 2 | 6 |
| **COO** | | | | |
| GCB | 3 | 33 | 9 | 29 |
| nonGCB | 2 | 22 | 14 | 45 |
| UNK | 4 | 44 | 8 | 26 |
| **IHC myc/bcl2 expression** | | | | |
| myc/bcl2 ex | 2 | 22 | 13 | 42 |
| myc/bcl2 neg | 4 | 44 | 17 | 55 |
| UNK | 4 | 44 | 1 | 3 |

### Summary/Conclusion

In this real-world data analysis of patients with R/R LBCL bispecific antibodies showed clinical activity with manageable toxicity. These findings support feasibility of bispecific antibody treatment in routine clinical practice although showing need for further treatment improvement for this high-risk group of LBCL patients.

**Copyright Information:** (Online) ISSN: 2572-9241
©2026 The Author(s). HemaSphere is published by John Wiley & Sons Ltd on behalf of European Hematology Association. This is an open access Abstract Book distributed under the Attribution-NonCommercial-NoDerivs (CC BY-NC-ND), which allows third parties to download the articles and share them with others as long as they credit the author and the Abstract Book, but they cannot change the content in any way or use them commercially.
**Abstract Book Citations:** Authors, Title, HemaSphere, 2026;10:(S1):pages. The abstract book DOIs can be found on the "Author Information" page.
**Disclaimer:** Articles published in the journal HemaSphere exclusively reflect the opinions of the authors. The authors are responsible for all content in their abstracts including accuracy of the facts, statements, citing resources, etc.

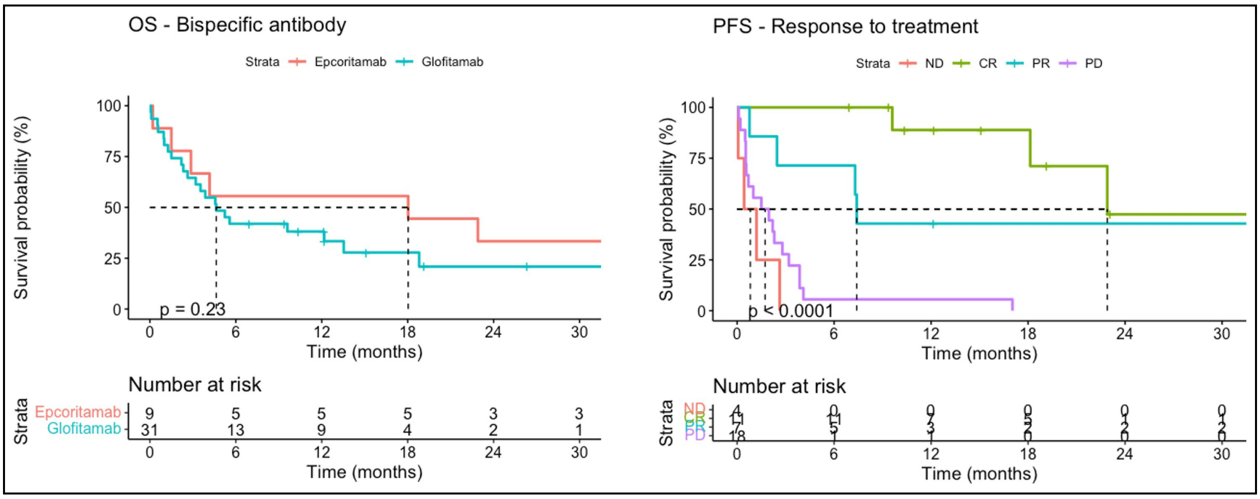

Survival curves - according to Bispecifics antibody and response to treatment

**Copyright Information:** (Online) ISSN: 2572-9241
©2026 The Author(s). HemaSphere is published by John Wiley & Sons Ltd on behalf of European Hematology Association. This is an open access Abstract Book distributed under the Attribution-NonCommercial-NoDerivs (CC BY-NC-ND), which allows third parties to download the articles and share them with others as long as they credit the author and the Abstract Book, but they cannot change the content in any way or use them commercially.
**Abstract Book Citations:** Authors, Title, HemaSphere, 2026;10:(S1):pages. The abstract book DOIs can be found on the "Author Information" page.
**Disclaimer:** Articles published in the journal HemaSphere exclusively reflect the opinions of the authors. The authors are responsible for all content in their abstracts including accuracy of the facts, statements, citing resources, etc.

### (PB3788) EACH DAY OF TREATMENT DELAY AFTER DIAGNOSIS OF DIFFUSE LARGE B-CELL LYMPHOMA INCREASES THE HAZARD OF DEATH BY 1.5%: A SINGLE-CENTER RETROSPECTIVE STUDY

**Topic:** 19. Large B cell lymphomas - Clinical

Vojin Vukovic*[1, 2], Lena Arandjelovic[1], Zoran Bukumiric[3], Sofija Pendic[3], Tamara Bibic[1], Sofija Kozarac[1], Jelena Ivanović[1], Darko Aleksic[1], Darko Antic[1, 2]

*[1]Clinic of Hematology, University Clinical Center of Serbia, Belgrade, Serbia;[2]Medical Faculty, University of Belgrade, Belgrade, Serbia; [3]Medical Faculty, University of Belgrade, Institut for Medical Statistics and Informatics, Belgrade, Serbia;*

### Background

Different pretreatment intervals of patients (pts) with diffuse large B-cell lymphoma (DLBCL), particularly diagnosis-to-treatment interval (DTI) may influence treatment outcome, although published data are conflicting.

### Aims

To investigate association of time from the first contact with health system to biopsy (contact-to-biopsy interval [CBI]) and DTI with survival of DLBCL pts treated with anthracycline-based therapy.

### Methods

We retrospectively collected dates of first contact with health system, biopsy, histopathologic diagnosis (WHO 2016), and treatment initiation, along with clinical and laboratory data from medical records of patients treated from 2020-2023. Analyses were performed using SPSS 21.0.

**Copyright Information:** (Online) ISSN: 2572-9241
©2026 The Author(s). HemaSphere is published by John Wiley & Sons Ltd on behalf of European Hematology Association. This is an open access Abstract Book distributed under the Attribution-NonCommercial-NoDerivs (CC BY-NC-ND), which allows third parties to download the articles and share them with others as long as they credit the author and the Abstract Book, but they cannot change the content in any way or use them commercially.
**Abstract Book Citations:** Authors, Title, HemaSphere, 2026;10:(S1):pages. The abstract book DOIs can be found on the “Author Information” page.
**Disclaimer:** Articles published in the journal HemaSphere exclusively reflect the opinions of the authors. The authors are responsible for all content in their abstracts including accuracy of the facts, statements, citing resources, etc.

**Results**

The cohort included 158 newly diagnosed DLBCL pts (M/F 1.14). Most had DLBCL NOS (87.3%), while remaining pts were diagnosed with PMBCL (10), TCHRBCL (7), and gray zone lymphoma (3). Median age was 59 years. Advanced Ann Arbor stage was present in 47.1%, B symptoms in 56.7%, extranodal involvement in 65%, bulky disease (≥7 cm) in 45.9%, and ECOG >1 in 23.6%. According to IPI, 45.8% were low-, 38% intermediate-, and 15.4% high-risk. NonGCB subtype was present in 58.8% of DLBCL NOS cases. Laboratory characteristics are summarized in Table 1.
Median CBI was 23 days (range, 1-934) and median DTI 22 days (range, 1-130). All pts received anthracycline-based first-line therapy. Overall response rate was 87.8% (CR 75.6%, PR 12.2%). Median follow-up was 38.5 months; 38 pts died. Median PFS and OS were not reached; 3-year PFS and OS were 73% and 76%, respectively.
Univariable robust regression showed no association between clinical, laboratory and biological characteristics and CBI, while there was significant association between DTI and age (p=0.028), Ann Arbor (p<0.001), bulky (p<0.001), and IPI (p=0.007). In multivariable robust regression model, only the presence of bulky disease remained statistically significant predictor of a shorter DTI (B=-8,840; p=0,002).
In univariable Cox regression, shorter PFS was associated with B symptoms, poor ECOG, higher IPI, low absolute lymphocyte count (ALC), elevated β2-microglobulin, and CRP, while CBI and DTI were not significant. Multivariable models including clinical variables (B symptoms, bulky, IPI, and ALC) with either CBI or DTI identified ALC as the only independent predictor of PFS (p=0.007, and p=0.03, respectively in both multivariable models with either CBI, or DTI).
Predictors of shorter OS in univariable Cox regression analysis were age (p=0.006), presence of B symptoms (p=0.029), bulky disease (p=0.005), poor ECOG (p=0.001), while other parameters including CBI, and DTI were not significant.
Multivariable Cox regression model consisting of IPI, B symptoms, bulky disease, and CBI was performed, resulting with no significant independent predictors of OS. However, when DTI was incorporated into multivariable Cox regression analysis with same clinical and laboratory parameters (B symptoms, bulky, and IPI), DTI emerged as the only independent predictor of OS (HR 1,015, 95%CI 1,0001-1,028; p=0.032). In other words, with each day of treatment delay hazard of death increased by 1.5%.

**Summary/Conclusion**

Treatment delay can significantly impact treatment outcomes in DLBCL pts, but larger, well-designed studies are warranted to confirm the place of DTI and other intervals within pretreatment period in prognosis of DLBCL pts. Patients with bulky disease are more likely to start treatment earlier.

**Table 1.** Laboratory characteristics of patients in cohort

| Parameter | median | range |
|---|---|---|
| Hemoglobin (g/L) | 130.5 | 75-177 |
| White blood cells (x10$^9$/L) | 6.7 | 1.1-19.6 |
| Neutrophils (x10$^9$/L) | 4.3 | 0.8-17.0 |
| Lymphocytes (x10$^9$/L) | 1.3 | 0.0-4.6 |
| Platelets (x10$^9$/L) | 260 | 54-632 |
| LDH (xULN) | 0.9 | 0.3-24.1 |
| β2 microglobulin (mg/L) | 2.53 | 0.8-21.42 |
| ESR (mm/h) | 22 | 2-150 |
| C-reactive protein (mg/L) | 8.0 | 0.1-255.5 |

LDH – lactate dehydrogenase, ULN – upper limit of normal range, ESR – erythrocyte sedimentation rate

**Copyright Information:** (Online) ISSN: 2572-9241
©2026 The Author(s). HemaSphere is published by John Wiley & Sons Ltd on behalf of European Hematology Association. This is an open access Abstract Book distributed under the Attribution-NonCommercial-NoDerivs (CC BY-NC-ND), which allows third parties to download the articles and share them with others as long as they credit the author and the Abstract Book, but they cannot change the content in any way or use them commercially.
**Abstract Book Citations:** Authors, Title, HemaSphere, 2026;10:(S1):pages. The abstract book DOIs can be found on the "Author Information" page.
**Disclaimer:** Articles published in the journal HemaSphere exclusively reflect the opinions of the authors. The authors are responsible for all content in their abstracts including accuracy of the facts, statements, citing resources, etc.

### (PB3789) OUTCOMES AND OPTIMAL SEQUENCING OF SALVAGE THERAPIES FOLLOWING CD19-DIRECTED CAR-T FAILURE IN RELAPSED/REFRACTORY LARGE B-CELL LYMPHOMA: A SINGLE-CENTER REAL-WORLD ANALYSIS

**Topic:** 19. Large B cell lymphomas - Clinical

Sheng-Yu Tian[1], Xindi Liu[1], He-Nan Wang[1], Jia Cong[1], Liang Wang*[1]

[1]*Beijing Tongren Hospital, Capital Medical University, Beijing, China;*

### Background

While chimeric antigen receptor T-cell (CAR-T) therapy has transformed the treatment landscape for relapsed/refractory large B-cell lymphoma (R/R LBCL), 40-60% of patients experience progression within 6 months. Current guidelines offer limited recommendations for post-CAR-T salvage strategies, and real-world evidence regarding optimal treatment sequencing remains scarce.

### Aims

In this retrospective study, we aimed to demonstrate the outcomes of patients who failed prior CAR-T cell therapy, and explore the optimal salvage therapies in the real-world setting.

### Methods

We conducted a retrospective analysis of 71 R/R LBCL patients who received investigational CD19 or CD19/CD22 CAR-T products at Beijing Tongren Hospital between April 2022 and December 2024. Among 36 patients with post-CAR-T progression/relapse, 29 received salvage therapy and were included in this analysis. Treatment trajectories, overall survival (OS), duration of response (DOR), objective response rate (ORR), and complete response (CR) rate were evaluated. Prognostic factors were identified using Cox regression analysis.

### Results

The cohort demonstrated an ORR of 80.3% and CR rate of 46.5% to initial CAR-T therapy. Following failure, 58 salvage treatment episodes were administered across 29 patients, utilizing bispecific antibodies (BsAb, n=9), second CAR-T infusion (n=10), radiotherapy (n=9), polatuzumab vedotin (Pola, n=9), immune checkpoint inhibitors (ICI, n=8), and other agents (n=13). Median OS from progression was 7.0 months, with overall salvage ORR of 41.4% and CR rate of 12.1%. Notably, Pola-based regimens achieved the highest ORR (66.7%), while second CAR-T infusion—particularly with CD19/CD22 dual-targeting products—demonstrated superior CR rates (60.0% vs. 7.5% for other therapies; p=0.010). Among 5 patients switching from CD19 to CD19/CD22 CAR-T, 4 achieved response (3 CR, 1 PR), whereas 5 patients re-treated with identical products showed no response. Univariate analysis identified early relapse (<60 days post-infusion) as the sole independent predictor of inferior OS (HR 0.44; 95% CI 0.21-0.95; p=0.038).

### Summary/Conclusion

This study highlights the therapeutic potential of antigen-switching second CAR-T and Pola-based salvage strategies in the post-CAR-T setting. The marked efficacy of CD19/CD22 dual-target CAR-T as salvage therapy suggests that antigen heterogeneity or loss may represent a key resistance mechanism amenable to target modification. Conversely, early relapse portends dismal outcomes regardless of salvage approach, underscoring the unmet need for novel interventions in this high-risk population. These findings support the integration of biomarker-driven salvage strategies and prospective validation of sequential immunotherapy approaches in R/R LBCL.

**Copyright Information:** (Online) ISSN: 2572-9241
©2026 The Author(s). HemaSphere is published by John Wiley & Sons Ltd on behalf of European Hematology Association. This is an open access Abstract Book distributed under the Attribution-NonCommercial-NoDerivs (CC BY-NC-ND), which allows third parties to download the articles and share them with others as long as they credit the author and the Abstract Book, but they cannot change the content in any way or use them commercially.
**Abstract Book Citations:** Authors, Title, HemaSphere, 2026;10:(S1):pages. The abstract book DOIs can be found on the “Author Information” page.
**Disclaimer:** Articles published in the journal HemaSphere exclusively reflect the opinions of the authors. The authors are responsible for all content in their abstracts including accuracy of the facts, statements, citing resources, etc.

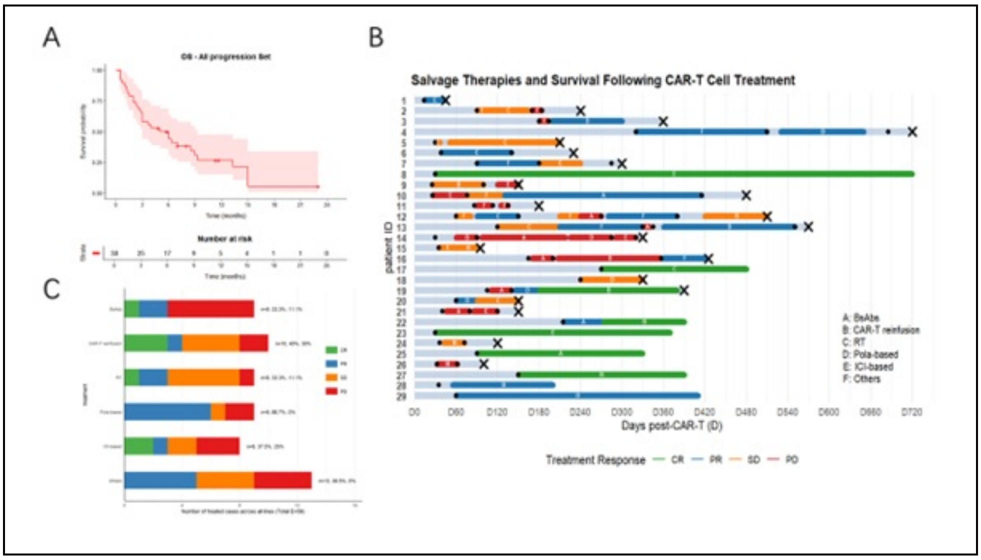

**Copyright Information:** (Online) ISSN: 2572-9241
©2026 The Author(s). HemaSphere is published by John Wiley & Sons Ltd on behalf of European Hematology Association. This is an open access Abstract Book distributed under the Attribution-NonCommercial-NoDerivs (CC BY-NC-ND), which allows third parties to download the articles and share them with others as long as they credit the author and the Abstract Book, but they cannot change the content in any way or use them commercially.
**Abstract Book Citations:** Authors, Title, HemaSphere, 2026;10:(S1):pages. The abstract book DOIs can be found on the "Author Information" page.
**Disclaimer:** Articles published in the journal HemaSphere exclusively reflect the opinions of the authors. The authors are responsible for all content in their abstracts including accuracy of the facts, statements, citing resources, etc.

### (PB3790) COMPARISON OF THE EFFICACY OF ZANUBRUTINIB PLUS RITUXIMAB-BASED REGIMEN AS FIRST-LINE TREATMENT IN PATIENTS WITH PREVIOUSLY UNTREATED DIFFUSE LARGE B-CELL LYMPHOMA

**Topic:** 19. Large B cell lymphomas - Clinical

Mingyu Zhao[1], Ziying Jian[1], Xiaoping Zhang[1], Zheng Ge*[1]

[1]*Zhongda Hospital Southeast University, Institute of Hematology Southeast University, Department of Hematology, Nanjing, China;*

### Background

Diffuse large B-cell lymphoma (DLBCL), the most common aggressive non-Hodgkin lymphoma, is clinically and molecularly heterogeneous. While standard R-CHOP regimen achieves durable remissions in a substantial proportion of patients, up to 40%patients experience primary refractoriness or relapse. Recent insights into DLBCL pathogenesis highlight B-cell receptor (BCR) signaling, particularly Bruton's tyrosine kinase (BTK), drives tumor survival in DLBCL subsets. Zanubrutinib, a selective irreversible BTK inhibitor, shows promise in relapsed/refractory settings. However, data on frontline Zanubrutinib plus Rituximab-based chemotherapy (ZR+X regimen) as treatment for previously untreated DLBCL remain limited.

### Aims

To evaluate the efficacy of frontline ZR+X regimen in previously untreated DLBCL patients, and to identify predictive biomarkers.

### Methods

The retrospective study included 262 patients with newly diagnosed DLBCL treated in our center between January 1, 2020 and December 31, 2025, stratified by frontline regimen: ZR+X (n=37) vs. non-ZR+X (n=225). Among ZR+X group, 7 patients received ZR+ Lenalidomide regimen, 7 patinets exposed to ZR-CDOP and 4 for ZR-miniCDOP, 5 for ZR+ CHOP, 3 for ZR+ MA etc. Clinical and laboratory data, including age, sex, Ann Arbor stage, International Prognostic Index (IPI), lactate dehydrogenase (LDH), neutrophil-to-lymphocyte ratio (NLR), and $\beta_2$-microglobulin ($\beta_2$-MG) levels, were collected. The primary endpoints were progression-free survival (PFS) and overall survival (OS). The optimal cutoff value for $\beta_2$-MG was determined using maximally selected rank statistics. Data processing and analysis were performed using R version 4.3.3, R stuido, along with Zstats 1.0.

### Results

Baseline analysis revealed that patients in the ZR+X group were significantly older (median age: 72 vs. 66 years, P=0.002), had higher IPI scores (median: 4 vs. 3, P<0.001), and higher $\beta_2$-MG levels (median: 5 vs. 3, P=0.026) compared to non-ZR+X group. The ZR+X group also had a higher proportion of patients with advanced-stage (stage IV) disease (86.36% vs. 60.61%, P=0.076). The ZR+X group had significantly shorter OS (median: 13.57 vs. 16.37 months, log-rank P=0.013) and PFS (median: 11.20 vs. 13.83 months, log-rank P=0.013) compared to the non-ZR+X group. Univariate Cox regression confirmed that high $\beta_2$-MG expression (HR=2.88, 95% CI: 1.59-5.23, P<0.001), and elevated LDH were independent poor prognostic factors. Conversely, low-risk and intermediate-risk IPI were independent protective factors for survival. For the entire elderly DLBCL population, Zanubrutinib does not improve prognosis.

**Copyright Information:** (Online) ISSN: 2572-9241
©2026 The Author(s). HemaSphere is published by John Wiley & Sons Ltd on behalf of European Hematology Association. This is an open access Abstract Book distributed under the Attribution-NonCommercial-NoDerivs (CC BY-NC-ND), which allows third parties to download the articles and share them with others as long as they credit the author and the Abstract Book, but they cannot change the content in any way or use them commercially.
**Abstract Book Citations:** Authors, Title, HemaSphere, 2026;10:(S1):pages. The abstract book DOIs can be found on the "Author Information" page.
**Disclaimer:** Articles published in the journal HemaSphere exclusively reflect the opinions of the authors. The authors are responsible for all content in their abstracts including accuracy of the facts, statements, citing resources, etc.

### Summary/Conclusion

Our finding shows that the ZR+X regimen was administered to a cohort of patients with more adverse baseline prognostic features clinically, and observed inferior survival outcomes may reflect the selection bias inherent to real-world study. The strong prognostic impact of β2-MG highlights potential utility for risk stratification. In the new era of targeted therapy for BTK, ZR+X regimen has emerged as a hot research treatment strategy. However, our study suggests another possibility: for patients with high baseline β2-MG levels, advanced age, and high IPI scores, even treated with the ZR+X regimen may fail to improve treatment outcomes in this patient population. Multicenter, large-sample studies are needed to validate.

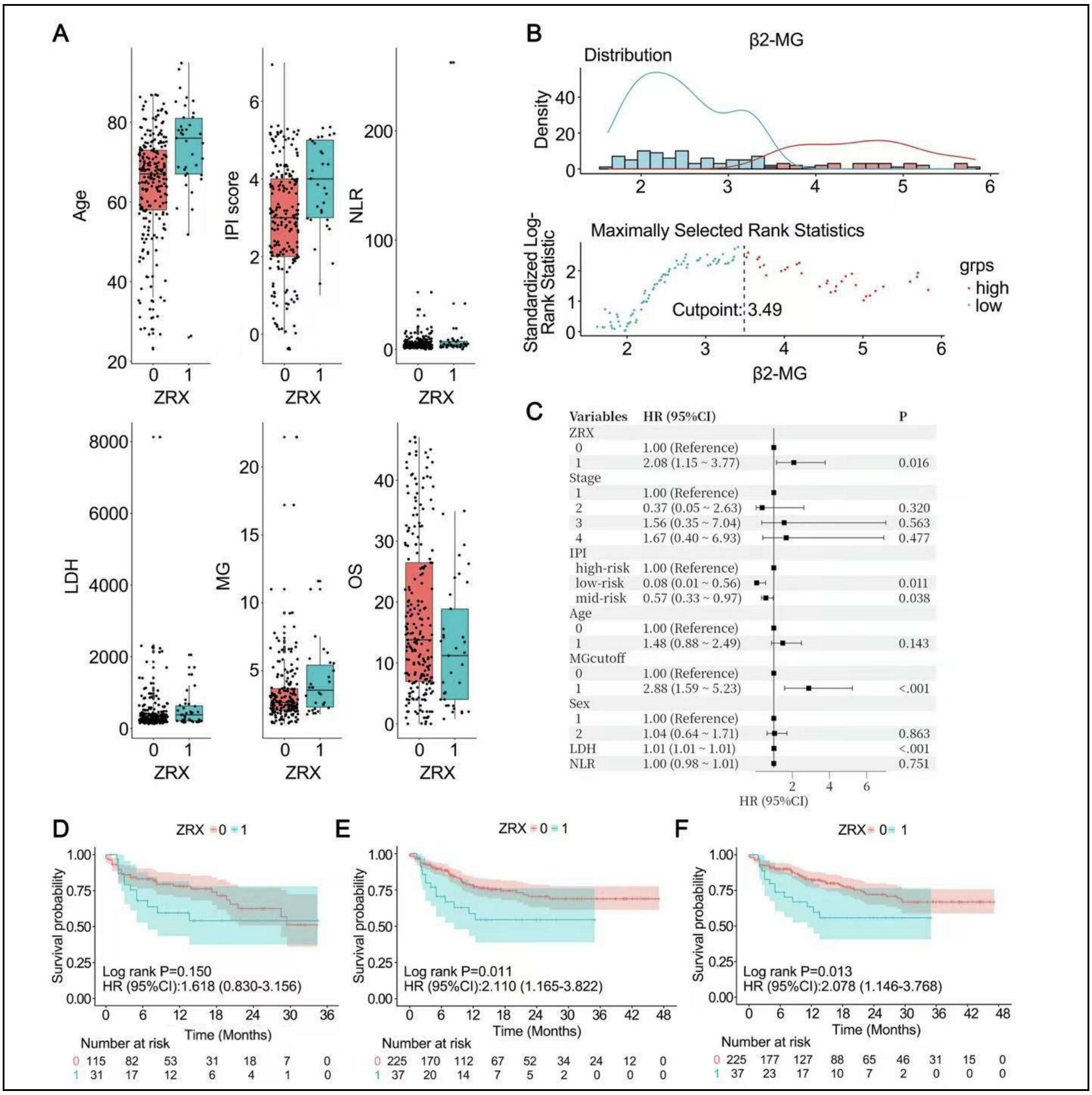

**Copyright Information:** (Online) ISSN: 2572-9241
©2026 The Author(s). HemaSphere is published by John Wiley & Sons Ltd on behalf of European Hematology Association. This is an open access Abstract Book distributed under the Attribution-NonCommercial-NoDerivs (CC BY-NC-ND), which allows third parties to download the articles and share them with others as long as they credit the author and the Abstract Book, but they cannot change the content in any way or use them commercially.
**Abstract Book Citations:** Authors, Title, HemaSphere, 2026;10:(S1):pages. The abstract book DOIs can be found on the "Author Information" page.
**Disclaimer:** Articles published in the journal HemaSphere exclusively reflect the opinions of the authors. The authors are responsible for all content in their abstracts including accuracy of the facts, statements, citing resources, etc.

### (PB3791) TREATMENT OUTCOMES FOR PATIENTS WITH PRIMARY CNS LYMPHOMA. DATA FROM A SINGLE-CENTER STUDY

**Topic:** 19. Large B cell lymphomas - Clinical

Daria Koroleva*[1, 1], Evgeniy Zvonkov[1], Olga Shchetsova[1], Andrey Gubkin[2], Alla Kovrigina[1], Galina Yatsyk[1], Svetlana Fedorova[1], Galstyan Gennady[1], Elena Nikulina[1], Andrey B. Sudarikov[1], Elena Parovichnikova[1]

*[1]National Medical Research Center of Hematology, Moscow, Russia;[2]Moscow Clinical Research Center named after A.S. Loginova, Moscow, Russia;*

### Background

Survival of pts with PCNSL on standard CT regimens does not exceed 10%. The use of HD consolidation has significantly increased the OS and EFS of pts with PCNSL to 70-80%, but the search for induction therapy (with or without targeted drug integration) and consolidation approaches (with or without thiotepa-containing regimens) continues.

### Aims

To present long-term treatment outcomes for pts with PCNSL for the period from 2015 to 2025.

### Methods

In the period from 2015 to 2025, 90 patients with PCNSL were treated at the NMRC of Hematology («CNS-2015» n = 29, «CNS-2021» n = 39, and «CNS-2025» n = 22) (Table 1). The mean age was 51 (18-75) years. The m:f ratio was 50 (56%):40 (44%). ECOG scale was ≥1 point in 43 (48%). Solitary and multifocal lesions were detected in 57 (63%) and 33 (37%), respectively. Intraocular involvement and neuroleukemia were diagnosed in 11 (12%) and 27/69 (39%) cases, respectively. Distribution by risk group according to the IELSG scale: low-risk group – 21 (23%), intermediate-risk group – 52 (58%), high-risk group – 17 (19%). Mutations in the *MYD88* gene were detected in 37 (63%) of 59 examined patients. In 7 cases (8%) , the diagnosis was established by biopsy-free diagnostics based on the detection of *MYD88* mutations in the cerebrospinal fluid.

**Table 1. Treatment regimens according to the CNS-2015, CNS-2021, CNS-2025 protocols.**

| Schemes | CNS-2015 | CNS-2021 | CNS-2025 |
|---|---|---|---|
| Induction | R-MPV (rituximab, methotrexate, vincristine, procarbazine) | R-MPV + lenalidomide | R-MPV + lenalidomide + ibrutinib |
| Consolidation | TBC (thiotepa, busulfan, cyclophosphamide) | **B**BC (carmustine, busulfan, cyclophosphamide) | TBC/**BBC** |
| Maintenance | Temozolomide | Nivolumab | - |

**Copyright Information:** (Online) ISSN: 2572-9241
©2026 The Author(s). HemaSphere is published by John Wiley & Sons Ltd on behalf of European Hematology Association. This is an open access Abstract Book distributed under the Attribution-NonCommercial-NoDerivs (CC BY-NC-ND), which allows third parties to download the articles and share them with others as long as they credit the author and the Abstract Book, but they cannot change the content in any way or use them commercially.
**Abstract Book Citations:** Authors, Title, HemaSphere, 2026;10:(S1):pages. The abstract book DOIs can be found on the “Author Information” page.
**Disclaimer:** Articles published in the journal HemaSphere exclusively reflect the opinions of the authors. The authors are responsible for all content in their abstracts including accuracy of the facts, statements, citing resources, etc.

### Results

In the total group of 90 pts, 5-year EFS and OS were 67% and 78% (Fig. 1). After the end of induction, CR of the disease was achieved in 89 (99%) of 90 pts, and early mortality from septic shock occurred in 1 (1%) case. Auto-HCT was performed in 83 cases (92%), no early mortality associated with the transplantation was observed. Relapses occurred in 20 (24%) pts (5 (22%) in the TBC group, 15 (68%) in the BBC group). With a median follow-up of 23.8 months, 5-y EFS was 69% (55% BBC, 81% TBC) and OS was 82% (88% BBC, 80% TBC). In 40 pts from the high-risk group according to the IELSG who received the TBC regimen, the 5-y EFS and OS were 63% and 53%, respectively.
Auto-HCT failed in 7 (8%) pts: "CNS-2015" - 1 (patient refusal), "CNS-2021" - 5 (2 - autologous stem cell collection failure, 1 - early mortality, 2 - comorbidity), and "CNS-2025" - 1 (comorbidity). Of the 7 pts, 3 are alive in CR (1 - after the induction phase of the "CNS-2015" protocol, 2 - after the induction phase of the "CNS-2021" protocol on maintenance therapy with lenalidomide) and 4 died (1 - early mortality, 2 - progression, 1 - other causes).

### Summary/Conclusion

The analysis revealed an eightfold increase in 5-y EFS rates in pts with PCNSL compared to retrospective data. Thiotepa-based conditioning regimens increased EFS rates by 26% compared to carmustine-containing regimens. Minimal results, even with thiotepa-based HDT, were achieved in the high-risk group according to IELSG criteria. For this group of pts, the development of a new treatment strategy integrating bispecific monoclonal antibodies into first-line therapy is advisable.

**Fig. 1. 5-year EFS and OS in the general group of 90 patients with PCNSL.**

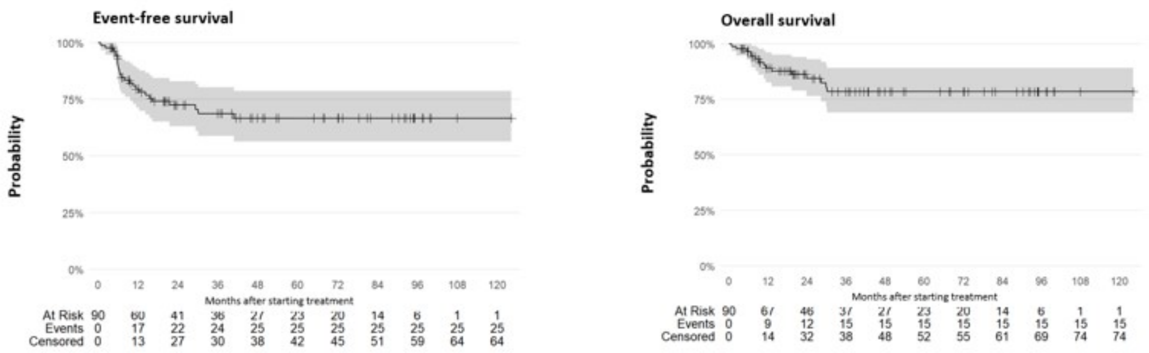

**Copyright Information:** (Online) ISSN: 2572-9241
©2026 The Author(s). HemaSphere is published by John Wiley & Sons Ltd on behalf of European Hematology Association. This is an open access Abstract Book distributed under the Attribution-NonCommercial-NoDerivs (CC BY-NC-ND), which allows third parties to download the articles and share them with others as long as they credit the author and the Abstract Book, but they cannot change the content in any way or use them commercially.
**Abstract Book Citations:** Authors, Title, HemaSphere, 2026;10:(S1):pages. The abstract book DOIs can be found on the "Author Information" page.
**Disclaimer:** Articles published in the journal HemaSphere exclusively reflect the opinions of the authors. The authors are responsible for all content in their abstracts including accuracy of the facts, statements, citing resources, etc.

### (PB3792) UPFRONT POLA-R-CHP IN DLBCL: A META-ANALYSIS INCLUDING RCT AND RWS

**Topic:** 19. Large B cell lymphomas - Clinical

Salvatore Perrone[1], Giada Pacitto*[2], Natalia Cenfra[2, 2], Sebastian Monachetti[1], Matteo Molica[3], Diana Giannarelli[4], Simone Ferrero[5]

*[1]Sapienza university of Rome, Department of Hemtology, Rome, Italy;[2]Santa Maria Goretti Hospital, Department of Hematology, Latina, Italy; [3]Renato Rubecco University Hospital, Department of Hematology-Oncology, Catanzaro, Italy;[4]Policlinico universitario Agostino Gemelli, Biostatistical unit, comprehensive cancer center, Rome, Italy; [5]University of Torno, Hematology Division, Torino, Italy;*

### Background

**Background:** The Pola-R-CHP regimen is the new standard of care for untreated diffuse large B-cell lymphoma (DLBCL) following the POLARIX trial.

### Aims

This study evaluates whether clinical trial (RCT) efficacy and safety are reproducible in real-world evidence (RWE) settings

### Methods

**Methods:** A systematic review and meta-analysis compared outcomes between RCTs and RWE studies. Primary endpoints were complete response (CR) rate, progression-free survival (PFS), and overall survival (OS). Meta-regression explored heterogeneity using age, International Prognostic Index (IPI), and activated B-cell-like (ABC) subtype as moderators

### Results

**Results:** Data from 2 RCTs and 10 RWE studies (3,472 patients) were integrated. The pooled CR rate was 78% (95% CI: 74%–82%), with RWE studies showing a significantly higher CR (80%) than RCTs (69%, P<0.001). The pooled 1-year PFS was 85%, significantly higher in RWE (86%) than RCTs (81%, P=0.02). Pooled 1-year OS was 91%, with no significant difference between RWE (90%) and RCTs (94%, P=0.35). Despite high heterogeneity (I 2 74%–91%), meta-regression confirmed that age, IPI score, and ABC subtype did not significantly influence treatment effects, though trends for improved CR and PFS were noted.

### Summary/Conclusion

**Conclusions:** This meta-analysis confirms that Pola-R-CHP outcomes in real-world practice are consistent with, and occasionally exceed, those observed in clinical trials. Despite inherent heterogeneity in RWE, the findings support Pola-R-CHP as a robust frontline therapy for a broad DLBCL population, regardless of baseline clinical characteristics.

**Copyright Information:** (Online) ISSN: 2572-9241
©2026 The Author(s). HemaSphere is published by John Wiley & Sons Ltd on behalf of European Hematology Association. This is an open access Abstract Book distributed under the Attribution-NonCommercial-NoDerivs (CC BY-NC-ND), which allows third parties to download the articles and share them with others as long as they credit the author and the Abstract Book, but they cannot change the content in any way or use them commercially.
**Abstract Book Citations:** Authors, Title, HemaSphere, 2026;10:(S1):pages. The abstract book DOIs can be found on the “Author Information” page.
**Disclaimer:** Articles published in the journal HemaSphere exclusively reflect the opinions of the authors. The authors are responsible for all content in their abstracts including accuracy of the facts, statements, citing resources, etc.

### (PB3793) REAL-WORLD OUTCOMES OF GLOFITAMAB-BASED REGIMENS IN DLBCL PATIENTS WITH EXTRANODAL INVOLVEMENT

**Topic:** 19. Large B cell lymphomas - Clinical

Yuhong Ren*[1], Yang Ke[1], Zhixiang Cheng[1], Ling Yuan[1], Weiguang Wang[1], Jingli Zhuang[1], Luya Cheng[1], Yue Wang[1], Jiadai Xu[1], Xuejiao Zhang[1], Jing Li[1], Peng Liu[1]

*[1]Zhongshan Hospital Affiliated to Fudan University, Department of Hematology, Shanghai, China;*

### Background

Diffuse large B-cell lymphoma (DLBCL) with extranodal involvement occurs in approximately 40% of patients and exhibits clinical and prognostic heterogeneity. Glofitamab, a bispecific antibody targeting CD20 and CD3, showed efficacy in refractory or relapsed DLBCL either as monotherapy or in combination with chemotherapies. However, little evidence was provided concerning the extranodal subgroups. Here, we analyzed the efficacy and safety of glofitamab-based regimens in DLBCL patients with different extranodal involvement in a real-world perspective.

### Aims

In this real-world study, we aim to investigate safety and efficacy of glofitamab-based regimens in DLBCL patients with extranodal involvement.

### Methods

DLCBL patients aged over 18 years old with at least extranodal site involvement were enrolled. All patients received glofitamab-based regimens as either induction or salvage treatment. Glofitamab was administered intravenously as step-up doses from 2.5 mg on day 8, 10 mg on day 15 in cycle 1, followed by a dose of 30 mg on day 1 in cycle 2 and afterwards. Twenty-one days was a cycle. Whole body 18F-FDG PET/CT scan or contrast-enhanced CT scan was scheduled every 2 to 4 cycles per physician's choice. The primary end point was overall response rate (ORR). The secondary end points were safety, complete response (CR) rate and progression-free survival (PFS).

### Results

From March 1, 2024, to January 31, 2026, altogether 39 consecutive DLBCL patients were enrolled in this study. The median age was 67 (range: 32-94), with 25 (64.1%) patients aged ≥ 65. Sixteen (41.0%) patients were female. Twenty (51.3%) patients had ≥ 3 extranodal sites, among which mediastinum was the most common extranodal site followed by gastrointestinal tract, bone and lungs at presentation. Two patients received glofitamab-based regimens in the first line, and 37 patients were treated as salvage. Regimens were composed of glofitamab monotherapy (38.5%), glofitamab-chemotherapy (53.8%) and glofitamab-bruton tyrosine kinase inhibitor (7.7%). Four (10.3%) patients received prior CD19 CAR-T, and no patients received prior stem cell transplantation. At the cut-off date February 26, 2026, the median treatment cycle was 4 (range: 1-12). Altogether 30 patients had tumor assessment results that the ORR was 70.0% and CR rate was 43.4%. After a median follow-up of 4.6 (95% CI: 2.825-6.965) months of PFS, the PFS rate of 6 months was 48.5% (95% CI:31.7-74.2) and the median PFS was 4.8 (95% CI: 3.7-NA) months. PFS of the four most common sites was shown in **Figure 1**. Eighteen (46.2%) patients had cytokine release syndrome, among which 17 were grade 1 to 2, and 1 was grade 3. One (2.6%) patient had grade 1 immune effector cell-associated neurotoxicity syndrome after cycle 1 day 15 (glofitamab 10 mg).

**Copyright Information:** (Online) ISSN: 2572-9241
©2026 The Author(s). HemaSphere is published by John Wiley & Sons Ltd on behalf of European Hematology Association. This is an open access Abstract Book distributed under the Attribution-NonCommercial-NoDerivs (CC BY-NC-ND), which allows third parties to download the articles and share them with others as long as they credit the author and the Abstract Book, but they cannot change the content in any way or use them commercially.
**Abstract Book Citations:** Authors, Title, HemaSphere, 2026;10:(S1):pages. The abstract book DOIs can be found on the "Author Information" page.
**Disclaimer:** Articles published in the journal HemaSphere exclusively reflect the opinions of the authors. The authors are responsible for all content in their abstracts including accuracy of the facts, statements, citing resources, etc.

### Summary/Conclusion

Glofitamab-based regimens showed efficacy in DLBCL patients with extranodal involvement and exhibited manageable toxicities. Ongoing treatment and follow-up will be further updated.

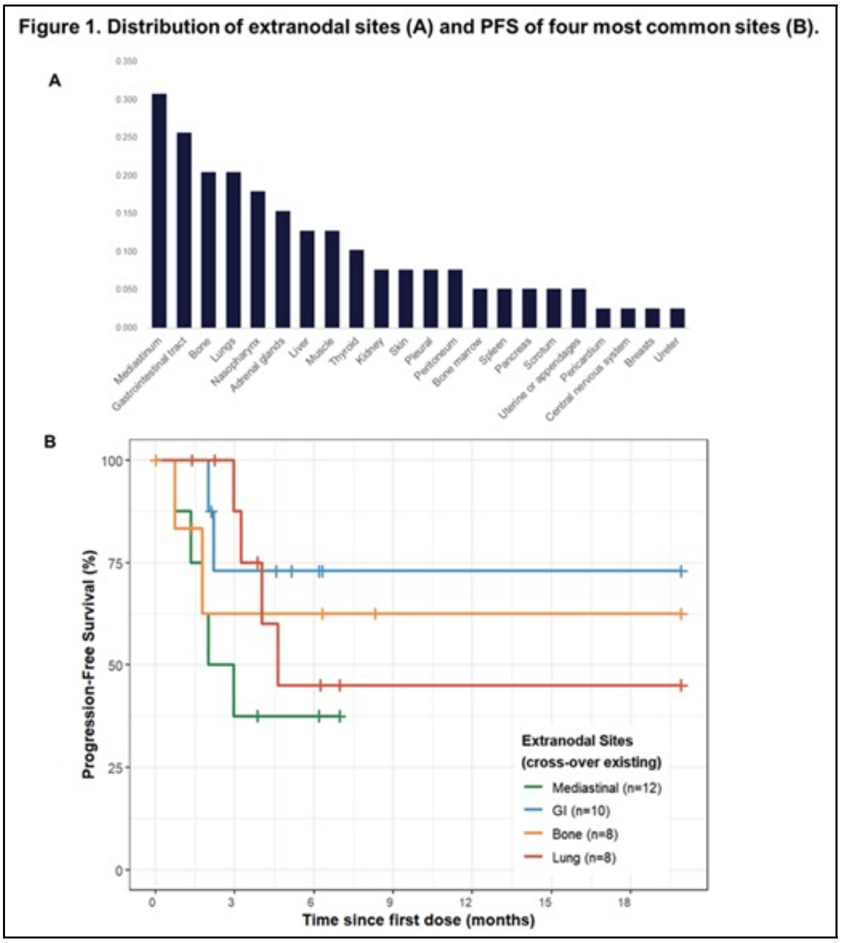

Figure 1. Distribution of extranodal sites (A) and PFS of four most common sites (B).

**Copyright Information:** (Online) ISSN: 2572-9241
©2026 The Author(s). HemaSphere is published by John Wiley & Sons Ltd on behalf of European Hematology Association. This is an open access Abstract Book distributed under the Attribution-NonCommercial-NoDerivs (CC BY-NC-ND), which allows third parties to download the articles and share them with others as long as they credit the author and the Abstract Book, but they cannot change the content in any way or use them commercially.
**Abstract Book Citations:** Authors, Title, HemaSphere, 2026;10:(S1):pages. The abstract book DOIs can be found on the "Author Information" page.
**Disclaimer:** Articles published in the journal HemaSphere exclusively reflect the opinions of the authors. The authors are responsible for all content in their abstracts including accuracy of the facts, statements, citing resources, etc.

### (PB3794) EARLY SEVERE INFECTIONS AND EARLY MORTALITY IN NEWLY DIAGNOSED DIFFUSE LARGE B-CELL LYMPHOMA: A SINGLE-CENTER COHORT FROM ROMANIA

**Topic:** 19. Large B cell lymphomas - Clinical

Cosmin Minciuna*[1, 2]

*[1]Regional Institute of Oncology Iasi, Hematology, Iasi, Romania;[2]"Grigore T. Popa" University of Medicine and Pharmacy, Iasi, Romania;*

### Background

Delayed access to effective therapy and infection-related complications can pose a significant threat when aiming to cure Diffuse large B-cell lymphoma (DLBCL).

### Aims

Our goal is to identify the cases where ICU admission was nedeed within 60 days from the initial diagnosis, the early mortality rate (≤90 days from the first admission in our hospital) and to clarify the leading cause which determined early death of the patients in a real-world DLBCL cohort.

### Methods

We performed a retrospective single-center study using electronic records of patients diagnosed with DLBCL. We have identified 418 patients that have been diagnosed in our center between October 2019 and October 2025. Baseline variables were extracted with rule-based parsing (including Charlson Comorbidity Index, stage of the disease at diagnosis, age, sex, IPI). Time-zero was defined as the date of first hospital admission recorded. Data on chemotherapy, treatment complications, ICU admission and survival status were then used to complete the analysis of our cohort and identify the patients who met our endpoints (ICU admission within 60 days / 90-day mortality). First-line treatment was classified as standard immunochemotherapy (R-CHOP) and frail-adapted regimens (miniR-CHOP, miniR-CEOP or R-CVP).

### Results

Mean age was 62.6 years (range 17–91); 44.7% of the patients were male. CCI (age-adjusted, lymphoma excluded) mean was 3.16 (range 0–12). Any chemotherapy was documented in 95.0% of the cases with 5.0% of the patients unable to administer treatment due to poor condition at the diagnosis. Frailty status, using CCI, was identified in 22.2% of the patients. 15.3% of the patients managed to receive frail-adapted regimens. **ICU admission within 60 days occurred in 6.0% of the cohort. Early death within 90 days occurred in 61/418 (14.6%).** Among standard-therapy early deaths (n=25), 15 (60.0%) were identified as infection-related.

### Summary/Conclusion

Early mortality within 90 days after first admission identifies a clinically distinct, high-risk window in real-world setting for DLBCL patients. A substantial proportion of early deaths occurred in the context of documented infection and/or ICU-level care, including among patients receiving standard immunochemotherapy. These cases were associated with higher comorbidity burden. The time elapsed from the first symptom to the initial diagnosis and further to the first course of the chemotherapy plays an essential role in preserving the curative status of DLBCL. Targeted early supportive-care bundles (infection screening, frail adapted treatment, and proactive antimicrobial/hematologic support in selected patients) are key factors to reduce preventable early deaths and improve real-world outcomes.

**Copyright Information:** (Online) ISSN: 2572-9241
©2026 The Author(s). HemaSphere is published by John Wiley & Sons Ltd on behalf of European Hematology Association. This is an open access Abstract Book distributed under the Attribution-NonCommercial-NoDerivs (CC BY-NC-ND), which allows third parties to download the articles and share them with others as long as they credit the author and the Abstract Book, but they cannot change the content in any way or use them commercially.
**Abstract Book Citations:** Authors, Title, HemaSphere, 2026;10:(S1):pages. The abstract book DOIs can be found on the "Author Information" page.
**Disclaimer:** Articles published in the journal HemaSphere exclusively reflect the opinions of the authors. The authors are responsible for all content in their abstracts including accuracy of the facts, statements, citing resources, etc.

### (PB3795) GLOFITAMAB-BASED THERAPY IN PRIMARY REFRACTORY OR EARLY RELAPSED DIFFUSE LARGE B-CELL LYMPHOMA: A MULTICENTER REAL-WORLD STUDY IN CHINA

**Topic:** 19. Large B cell lymphomas - Clinical

Xia Zhao*[1], Zhihe Liu[1], Junqing Xu[2], Minjie Li[3], Zhao Li[4]

*[1]the affiliated hospital of Qingdao university, Qingdao, China;[2]The Affiliated Yantai Yuhuangding Hospital of Qingdao University, Yantai, China; [3]Linyi Cancer Hospital, Linyi, China; [4]Qilu Hospital of Shandong University (Qingdao), Qingdao, China;*

### Background

Patients with primary refractory (pRef) or early relapsed (eRel) diffuse large B-cell lymphoma (DLBCL) face a poor prognosis, representing a critical unmet medical need. Glofitamab, a CD20×CD3 T-cell engaging bispecific antibody, has shown promising efficacy in relapsed/refractory DLBCL. However, real-world data specifically from high-risk Chinese patients remain scarce.

### Aims

This multicenter, retrospective study sought to assess the efficacy and safety of glofitamab-based therapies in Chinese patients with pRef or eRel DLBCL within a real-world clinical setting.

### Methods

We retrospectively reviewed data from 39 patients with pRef or eRel DLBCL treated with glofitamab-based regimens at four medical centers across China, between March 2024 and December 2025. pRef was defined as failure to achieve a complete response (CR) or disease progression within 3 months after first-line therapy. eRel was defined as relapse occurring within 12 months after initial therapy. The primary endpoint was the overall response rate (ORR), and secondary endpoints included the CR rate and the safety profile.

### Results

A total of 39 patients were included, with a median age of 65 years (range: 29–82), and 64.1% were male. Among these, 23 patients (59.0%) had pRef disease, and 16 (41.0%) had eRel disease. The majority presented with advanced-stage disease (stage III/IV) (74.4%), high-risk International Prognostic Index (IPI) scores (≥3) (59.0%), and a non-GCB subtype (64.1%). Additionally, 43.6% of patients were diagnosed with double-expression lymphoma. The median number of prior therapy lines was 3 (range: 2–8). Nine patients (23.1%) received glofitamab monotherapy, while 30 patients (76.9%) were treated with combination therapies, most commonly including GemOx (n=15). After a median of 6 cycles, the overall best ORR was 61.5% (24/39), with a CR rate of 43.6% (17/39). In the pRef subgroup, the best ORR and CR rates were 60.9% and 47.8%, respectively, while in the eRel subgroup, these rates were 62.5% and 37.5%, respectively. Patients with DEL-negative status exhibited a higher best ORR (68.2% vs 52.9%) and best CR rate (50.0% vs 35.3%) compared to DEL-positive patients. The most common adverse events were fever (41.0%) and tumor flare (20.5%), with two treatment-related deaths (5.1%).

### Summary/Conclusion

This real-world study of high-risk Chinese patients with pRef or eRel DLBCL demonstrated that glofitamab-based therapy provides substantial efficacy and a manageable safety profile. These findings support the potential of glofitamab as a valuable treatment option for this challenging patient population, with DEL status possibly serving as a factor influencing therapeutic response.

**Copyright Information:** (Online) ISSN: 2572-9241
©2026 The Author(s). HemaSphere is published by John Wiley & Sons Ltd on behalf of European Hematology Association. This is an open access Abstract Book distributed under the Attribution-NonCommercial-NoDerivs (CC BY-NC-ND), which allows third parties to download the articles and share them with others as long as they credit the author and the Abstract Book, but they cannot change the content in any way or use them commercially.
**Abstract Book Citations:** Authors, Title, HemaSphere, 2026;10:(S1):pages. The abstract book DOIs can be found on the "Author Information" page.
**Disclaimer:** Articles published in the journal HemaSphere exclusively reflect the opinions of the authors. The authors are responsible for all content in their abstracts including accuracy of the facts, statements, citing resources, etc.

### (PB3796) PROGNOSTIC VALUE OF SERIAL CBC-DERIVED INDICES FROM LEUKAPHERESIS TO DAY 30 IN THIRD-LINE AXICABTAGENE CILOLEUCEL FOR DLBCL

**Topic:** 19. Large B cell lymphomas - Clinical

Chokri Ben Lamine*[1, 2], Ibrahim Alredaini[1, 3], Salwa Alsuhaibani[4], Almaha Alhnyshil[3], Luluah Altukhaifi[1], Abdullah Al-Amer[1], Ali Alahmari[1], Ahmed Alnughmush[1], Alfadel Alshaibani[1], Saud Haily[1], Ayman Saad[1], Walid Rasheed[1], Syed Osman Ahmed[1], Abdulwahab Albabtain[1], Hazzaa Alzahrani[1], Aljurf Mahmoud[1], Riad El Fakih[1, 2]

*[1]King Faisal Specialist Hospital & Research Centre, Adult Hematology, Stem cell transplnatation and Cellular Therapy, Riyadh, Saudi Arabia; [2]Al Faisal University, Riyadh, Saudi Arabia;[3]King Fahd Specialist Hospital, Buraydah, Qassim, Saudi Arabia; [4]Prince Sultan Cardiac Center, Clinical Pharmacy Department, Qassim, Saudi Arabia;*

### Background

CD19-directed chimeric antigen receptor T-cell therapy (CAR-T) improves outcomes in relapsed/refractory diffuse large B-cell lymphoma (DLBCL), but durable disease control remains variable. Complete blood count (CBC)–derived indices are inexpensive and universally available and could enable pragmatic risk stratification across the CAR-T pathway.

### Aims

To describe real-world outcomes of axicabtagene ciloleucel (axi-cel) in DLBCL and test whether pre-lymphodepletion CBC-derived indices—neutrophil-to-lymphocyte ratio (NLR), platelet-to-lymphocyte ratio (PLR), and systemic immune-inflammation index (SII)—are associated with day-30 response and survival, while characterizing serial marker behavior from leukapheresis to day 30.

### Methods

Single-center retrospective cohort of adults treated with axi-cel (N=55). Absolute lymphocyte count (ALC), absolute neutrophil count (ANC), and platelets were captured at pre-apheresis, pre-lymphodepletion, and day 30 (±7 days) when available; NLR, PLR, and SII were derived at pre-lymphodepletion (available in 54 patients). Day-30 positron emission tomography (PET) response was categorized as complete response (CR)/partial response (PR) versus stable disease (SD)/progressive disease (PD). Progression-free survival (PFS) and overall survival (OS) were estimated by Kaplan–Meier. Associations were explored using univariable Cox regression (PFS/OS) and logistic regression (response).

**Copyright Information:** (Online) ISSN: 2572-9241
©2026 The Author(s). HemaSphere is published by John Wiley & Sons Ltd on behalf of European Hematology Association. This is an open access Abstract Book distributed under the Attribution-NonCommercial-NoDerivs (CC BY-NC-ND), which allows third parties to download the articles and share them with others as long as they credit the author and the Abstract Book, but they cannot change the content in any way or use them commercially.
**Abstract Book Citations:** Authors, Title, HemaSphere, 2026;10:(S1):pages. The abstract book DOIs can be found on the “Author Information” page.
**Disclaimer:** Articles published in the journal HemaSphere exclusively reflect the opinions of the authors. The authors are responsible for all content in their abstracts including accuracy of the facts, statements, citing resources, etc.

### Results

Disease activity prior to lymphodepletion was high, with PD on pre-lymphodepletion PET in 43/55 (78.2%). Day-30 PET was available in 53/55: CR 23, PR 20, SD 1, PD 9, yielding an objective response rate (ORR) of 43/53 (81.1%) and CR rate of 23/53 (43.4%). Cytokine release syndrome (CRS) grades (N=53) were mainly grade 1–2 (67.9% and 26.4%, respectively), with grade 3–4 in 5.7%. Immune effector cell–associated neurotoxicity syndrome (ICANS) occurred in 20/55 (36.3%); ICU admission occurred in 10/55 (18.1%), and mechanical ventilation (MV) in 4/55 (7.3%). PFS probabilities were 48% at 6 months and 40% at 12 months; OS probabilities were 81% at 6 months and 71% at 12 months. Pre-lymphodepletion indices were: median NLR 2.63 (IQR 1.62–6.23), PLR 245 (156–350), and SII 560 (267–1274). In univariable analyses, NLR/PLR/SII were not significantly associated with PFS (p=0.300/0.582/0.435) or OS (NLR hazard ratio [HR] 1.011, 95% CI 0.978–1.046; p=0.513; PLR p=0.240; SII p=0.143) and were not associated with ORR (NLR odds ratio [OR] 0.95, 95% CI 0.81–1.13; p=0.569; SII p=0.724). Serial profiling showed a marked post-infusion cytopenic shift by day 30, emphasizing timepoint-specific interpretation of CBC-derived indices.

### Summary/Conclusion

In this real-world third-line axi-cel cohort with high pre-infusion disease activity, early metabolic response at day 30 was frequent and CRS was largely low grade, while ICU-level care remained clinically relevant. CBC-derived indices are feasible to capture longitudinally, but single pre-lymphodepletion composites (NLR/PLR/SII) were not prognostic for response or survival on univariable testing. These findings discourage isolated CBC-index–based decision-making and support trajectory-based models (pre-apheresis → pre-lymphodepletion → day 30) integrated with disease burden markers to develop workflow-ready risk stratification in CAR-T programs.

**Figure1. Kaplan–Meier Curve for Progression-Free Survival (PFS) from CAR-T Infusion**

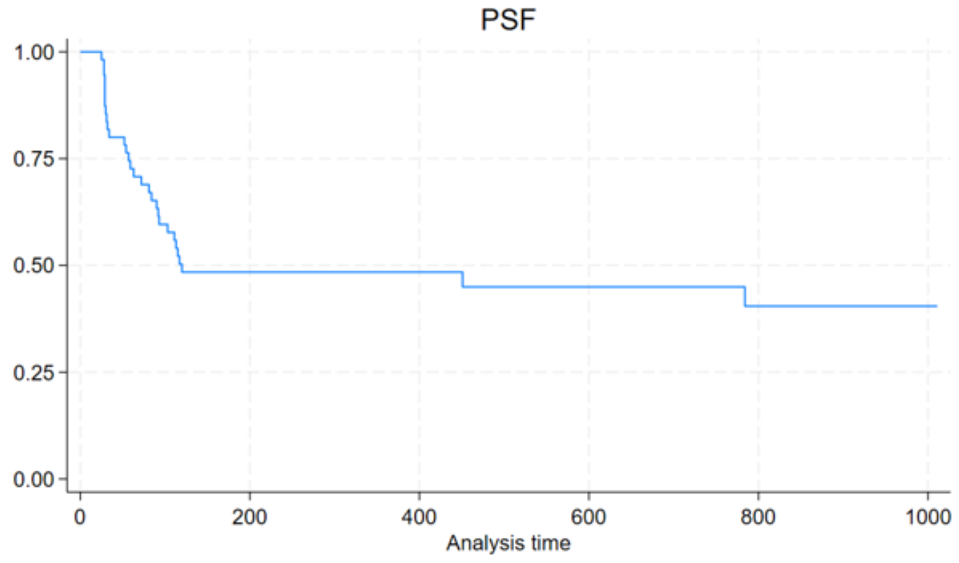

Figure1. Kaplan–Meier Curve for Progression-Free Survival (PFS) from CAR-T Infusion

**Copyright Information:** (Online) ISSN: 2572-9241
©2026 The Author(s). HemaSphere is published by John Wiley & Sons Ltd on behalf of European Hematology Association. This is an open access Abstract Book distributed under the Attribution-NonCommercial-NoDerivs (CC BY-NC-ND), which allows third parties to download the articles and share them with others as long as they credit the author and the Abstract Book, but they cannot change the content in any way or use them commercially.
**Abstract Book Citations:** Authors, Title, HemaSphere, 2026;10:(S1):pages. The abstract book DOIs can be found on the "Author Information" page.
**Disclaimer:** Articles published in the journal HemaSphere exclusively reflect the opinions of the authors. The authors are responsible for all content in their abstracts including accuracy of the facts, statements, citing resources, etc.

### (PB3797) IMMUNOHISTOCHEMICAL PRACTICE IN THE DIAGNOSIS OF DIFFUSE LARGE B-CELL LYMPHOMA IN AUSTRALIA

**Topic:** 19. Large B cell lymphomas - Clinical

Vibooshini Ganeshalingam*[1, 2], Cameron Wellard[3], Eliza Chung[3], Alex Bew[4], Annmarie Bosco[5], Chan Y. Cheah[6], Tania Cushion[7], Elliot Fox[4, 8], Eliza Hawkes[3, 7], Isaac Hui[9], Arina Martynchyk[7], Jack McHenry[8], Stephen Opat[9, 10], Ross Salvaris[9], Niamh Waters[7], Joel Wight[4, 8]

*[1]Princess Alexandra Hospital, Woolloongabba, Brisbane, Australia;[2]Townsville University Hospital, Douglas, Townsville, Australia; [3]School of Public Health and Preventive Medicine, Monash University, Melbourne, Australia;[4]James Cook University, Douglas, Australia; [5]Prince of Wales Hospital, Randwick, Australia;[6]Sir Charles Gairdner Hospital, Perth, Australia;[7]Olivia Newton John Cancer Research Institute at Austin Health, Heidelberg, Australia; [8]Townsville University Hospital, Douglas, Australia; [9]Monash Health, Melbourne, Australia; [10]Department of Medicine, School of Clinical Sciences at Monash Health, Monash University, Clayton, Australia;*

### Background

Diffuse large B-cell lymphoma (DLBCL) is a heterogeneous group of aggressive lymphomas with an array of biological subtypes classified by immunohistochemistry (IHC). Despite published guidelines, approaches to IHC vary by jurisdiction.

### Aims

This study aimed to evaluate IHC testing practices in Australian DLBCL patients using national registry data and benchmark against 5$^{th}$ edition WHO-Haem 2022, National comprehensive care management (NCCN), European haematology association (EHA), Spanish society of medical oncology and Oncology group for treatment and study of lymphomas (SEOM-GOTEL) and Australasian lymphoma alliance (ALA) criteria.

### Methods

This national Lymphoma and Related Diseases (LaRDR) study analysed prospectively recruited adult DLBCL cases between 2016-2025. Source histopathology reports and clinical data entered into the registry were reviewed for IHC results. These were compared against a reference panel developed from above listed guidelines and expert consensus.

### Results

647 eligible patients were included. 15% met NCCN recommended panel, 46% met EHA panel, 39% SEOM-GOTEL, and 2% ALA criteria.
CD20 expression was reported in 96%, and cell-of-origin (COO) markers in 86-92% with COO assignment reported in 88%. Markers required for excluding differential diagnoses were frequently missing; 52% reported the follicular markers CD21/CD23 and markers for Mantle Cell Lymphoma (MCL) were unreported in 20%. Prognostic markers e.g., EBER-ISH were reported in 55% of cases. There were no significant differences in percentage of complete IHC panel in cases with surgical/excisional biopsies and those with core biopsies, suggesting lack of tissue was not a factor.

Table 1: Rates of reporting and positivity in registry patients

**Copyright Information:** (Online) ISSN: 2572-9241
©2026 The Author(s). HemaSphere is published by John Wiley & Sons Ltd on behalf of European Hematology Association. This is an open access Abstract Book distributed under the Attribution-NonCommercial-NoDerivs (CC BY-NC-ND), which allows third parties to download the articles and share them with others as long as they credit the author and the Abstract Book, but they cannot change the content in any way or use them commercially.
**Abstract Book Citations:** Authors, Title, HemaSphere, 2026;10:(S1):pages. The abstract book DOIs can be found on the "Author Information" page.
**Disclaimer:** Articles published in the journal HemaSphere exclusively reflect the opinions of the authors. The authors are responsible for all content in their abstracts including accuracy of the facts, statements, citing resources, etc.

| | Total evaluable (n=647) | | | |
|---|---|---|---|---|
| | Not reported (%) | Reported (%) | | |
| | | Total | Positive | Negative |
| **Standard IHC panel** | | | | |
| CD20 | 26 (4) | 621 (96) | 615 | 6 |
| CD3 | 172 (26) | 475 (74) | 5 | 470 |
| CD5 | 169 (26) | 478 (74) | 42 | 436 |
| CD10 | 50 (8) | 597 (92) | 289 | 308 |
| CD23 | 395 (61) | 252 (39) | 54 | 198 |
| CD21 | 478 (74) | 169 (26) | 41 | 128 |
| MUM1 | 92 (14) | 555 (86) | 364 | 191 |
| BCL2 | 73 (11) | 574 (89) | 477 | 97 |
| BCL6 | 68 (12) | 579 (89) | 515 | 64 |
| MYC | 117 (18) | 530 (82) | 350 | 179 |
| EBER-ISH | 289 (45) | 358 (55) | 26 | 332 |
| **Markers for specific conditions** | | | | |
| CD30 | 373 (58) | 274 (42) | 69 | 205 |
| PAX5 | 482 (74) | 165 (26) | 162 | 3 |
| CD79a | 611 (94) | 36 (6) | 33 | 3 |
| CD22 | 600 (93) | 47 (7) | 44 | 3 |
| ALK | 575 (89) | 72 (11) | 2 | 70 |
| Cyclin D1 | 364 (56) | 283 (44) | 8 | 275 |
| SOX11 | 614 (95) | 33 (5) | 1 | 32 |
| Oct-02 | 631(98) | 16 (2) | 15 | 1 |
| BOB1 | 635 (98) | 12 (2) | 11 | 1 |
| FOXp1 | 645 (>99) | 2 (<1) | 1 | 1 |
| GCET1 | 645 (>99) | 2 (<1) | 1 | 1 |
| LMO2 | 643 (>99) | 3 (<1) | 3 | 0 |
| CD38 | 601 (93) | 46 (7) | 31 | 15 |
| CD138 | 605 (94) | 42 (6) | 12 | 30 |
| Cyclin D2 | 647 (100) | 0 (0) | 0 | 0 |

### Summary/Conclusion

Suboptimal uptake of comprehensive IHC panels for DLBCL diagnosis exists in Australia, impacting diagnostic accuracy and treatment selection. Standardized reporting and expert review in the context of multidisciplinary team meetings are needed to improve diagnostic fidelity.

**Copyright Information:** (Online) ISSN: 2572-9241
©2026 The Author(s). HemaSphere is published by John Wiley & Sons Ltd on behalf of European Hematology Association. This is an open access Abstract Book distributed under the Attribution-NonCommercial-NoDerivs (CC BY-NC-ND), which allows third parties to download the articles and share them with others as long as they credit the author and the Abstract Book, but they cannot change the content in any way or use them commercially.
**Abstract Book Citations:** Authors, Title, HemaSphere, 2026;10:(S1):pages. The abstract book DOIs can be found on the "Author Information" page.
**Disclaimer:** Articles published in the journal HemaSphere exclusively reflect the opinions of the authors. The authors are responsible for all content in their abstracts including accuracy of the facts, statements, citing resources, etc.

### (PB3798) CLINICAL FEATURES AND OUTCOMES OF PEDIATRIC DLBCL: A SINGLE-CENTER EXPERIENCE

**Topic:** 19. Large B cell lymphomas - Clinical

Maha Charfi*[1], Rahma Mallek[1, 1, 1], Fakhfekh Yousra[1], Faten Kallel[1], Imen Ben Amor[1], Asma Koubaa[1], Imen Frikha[1], Moez Medhaffar[1], Moez Elloumi[1]

[1]*Hedi Chaker Hospital, Hematology departement, Sfax, Tunisia;*

### Background

Non-Hodgkin lymphomas (NHL) in children differ markedly from adult NHL in terms of clinical presentation, biological features, and disease behavior. One of the main challenges in pediatric NHL is to optimize cure rates while minimizing long-term treatment-related toxicity. Diffuse large B-cell lymphoma (DLBCL) accounts for approximately 10–20% of pediatric NHL and is associated with a significantly better prognosis than in adults.

### Aims

This study aimed to describe the epidemiological, clinical, and therapeutic characteristics of pediatric and adolescent patients with DLBCL treated at our center.

### Methods

We conducted a retrospective study including patients under 18 years of age diagnosed with DLBCL and treated at the Department of Clinical Hematology, Hédi Chaker Hospital, between January 2001 and December 2024. Epidemiological, clinical, and therapeutic data were collected using a standardized form. Patients under 16 years were treated according to the LMB 96 or LMB 02 protocols, while those aged 16 years or older received anthracycline-based regimens (CHOP or ACVBP), with or without rituximab.

### Results

Twenty one patients were included. The median age at diagnosis was 16 years (range: 5–18), with a sex ratio of 1.33. Peripheral tumor syndrome was present in 44% of patients, and B symptoms were reported in 44%. Elevated LDH levels were observed in 83% of cases. Advanced-stage disease was found in 56% of patients, including stage IV disease in 50%. Extranodal involvement was frequent, with ear, nose, and throat  and pulmonary localizations observed in 29% and 17% of patients, respectively. Primary mediastinal DLBCL was identified in five patients.
Treatment consisted mainly of LMB-based protocols (68%), while 21% received R-CHOP and 11% ACVBP. Complete remission at the end of treatment was achieved in 71% of patients. Treatment failure occurred in 6%, and one patient died during therapy. Relapse was observed in 11% of cases. The 5-year overall survival rate was 78%.

### Summary/Conclusion

In contrast to published pediatric series reporting advanced-stage disease in approximately 40–50% of cases and 5-year overall survival rates of 85–90%, our cohort was characterized by a higher proportion of advanced-stage and extranodal disease (56% and 50%, respectively) and a lower 5-year overall survival of 78%. These differences may reflect delayed diagnosis, disease burden at presentation, and treatment heterogeneity. Improved protocol standardization, earlier diagnosis, and prolonged follow-up are essential to optimize outcomes and reduce long-term toxicity in pediatric DLBCL.

**Copyright Information:** (Online) ISSN: 2572-9241
©2026 The Author(s). HemaSphere is published by John Wiley & Sons Ltd on behalf of European Hematology Association. This is an open access Abstract Book distributed under the Attribution-NonCommercial-NoDerivs (CC BY-NC-ND), which allows third parties to download the articles and share them with others as long as they credit the author and the Abstract Book, but they cannot change the content in any way or use them commercially.
**Abstract Book Citations:** Authors, Title, HemaSphere, 2026;10:(S1):pages. The abstract book DOIs can be found on the "Author Information" page.
**Disclaimer:** Articles published in the journal HemaSphere exclusively reflect the opinions of the authors. The authors are responsible for all content in their abstracts including accuracy of the facts, statements, citing resources, etc.

### (PB3799) DIAGNOSTIC YIELD OF ENDOSCOPIC BIOPSIES AND CORRELATION BETWEEN ENDOSCOPIC FEATURES AND HISTOLOGICAL SUBTYPES IN PRIMARY GASTRIC LYMPHOMAS

**Topic:** 19. Large B cell lymphomas - Clinical

Ameni Yahia*[1], Sarra Boukhriss[1], Imen Jemni[2], Ahlem Slama[1], Bizid Inaam[1], Wiem Boufrikha[1], Zaineb Mlayah[1], Slama Nader[1], Mohamed Adnene Laatiri[1]

*[1]Fattouma Bourguiba University Hospital, Hematology Department, Monastir, Tunisia; [2]Fattouma Bourguiba University Hospital, Gastroenterology, Monastir, Tunisia;*

### Background

The diagnosis of primary gastric lymphomas primarily relies on upper gastrointestinal endoscopy with multiple biopsies. However, endoscopic presentations are highly variable and often non-specific, potentially delaying diagnosis, particularly depending on the histological subtype.

### Aims

To evaluate the diagnostic yield of endoscopic biopsies and to analyze the correlation between endoscopic features and histological subtypes of primary gastric lymphomas.

### Methods

We conducted a retrospective study including patients with histologically confirmed primary gastric lymphoma over an 11-year period. Collected data included endoscopic characteristics, number and diagnostic yield of biopsies, as well as histological and immunohistochemical findings. Endoscopic features were compared according to histological subtype: diffuse large B-cell lymphoma (DLBCL) and MALT lymphoma. Statistical analysis was performed to assess associations between endoscopic and histological variables.

### Results

A total of 46 patients were included: 35 with DLBCL and 11 with MALT lymphoma.
At the first endoscopy, gastric biopsies established the diagnosis in 78.7% of cases. The diagnostic yield increased to 94% after a second endoscopic examination. The median number of biopsy fragments required for diagnosis was 9 (range: 5–19).
Endoscopic features differed significantly according to histological subtype. DLBCL was mainly characterized by an ulcerative-proliferative (ulcero-budding) pattern, observed in 68.6% of cases and absent in MALT lymphoma ($p < 10^{-3}$). Conversely, MALT lymphoma more frequently presented with ulcerative lesions (72.7% vs 28.6%; $p = 0.014$), particularly multiple ulcers (54.5% vs 17.1%; $p = 0.022$). Thickened gastric folds and parietal infiltration were more frequent in MALT lymphoma, although without statistical significance. Lesion location did not significantly differ between the two groups.

### Summary/Conclusion

Endoscopic biopsies are a highly effective diagnostic tool for primary gastric lymphomas, with improved yield after repeat procedures. Endoscopic features significantly differ according to histological subtype: DLBCL typically presents with more aggressive, proliferative lesions, whereas MALT lymphoma more often manifests as superficial and ulcerative lesions. Recognizing these correlations may help optimize diagnostic strategies and reduce delays in management.

**Copyright Information:** (Online) ISSN: 2572-9241
©2026 The Author(s). HemaSphere is published by John Wiley & Sons Ltd on behalf of European Hematology Association. This is an open access Abstract Book distributed under the Attribution-NonCommercial-NoDerivs (CC BY-NC-ND), which allows third parties to download the articles and share them with others as long as they credit the author and the Abstract Book, but they cannot change the content in any way or use them commercially.
**Abstract Book Citations:** Authors, Title, HemaSphere, 2026;10:(S1):pages. The abstract book DOIs can be found on the "Author Information" page.
**Disclaimer:** Articles published in the journal HemaSphere exclusively reflect the opinions of the authors. The authors are responsible for all content in their abstracts including accuracy of the facts, statements, citing resources, etc.

### (PB3800) HIGH STEM CELL MOBILIZATION FAILURE RATE WITH MITOXANTRONE LIPOSOME-CONTAINING SALVAGE CHEMOTHERAPY IN RELAPSED LYMPHOMA

**Topic:** 19. Large B cell lymphomas - Clinical

Xia Zhao*[1], Zhihe Liu[1]

[1]*the affiliated hospital of Qingdao university, Qingdao, China;*

### Background

Autologous stem cell transplantation (ASCT) is a crucial consolidation therapy for patients with relapsed/refractory (R/R) lymphoma. The efficacy of salvage chemotherapy is critical for bridging to ASCT, but its impact on subsequent peripheral blood stem cell (PBSC) mobilization is a significant concern. Liposomal mitoxantrone (L-MIT) has shown promise in R/R lymphoma, but its effect on hematopoietic stem cell reserves is not well-defined. Published data suggests that certain agents, including conventional mitoxantrone, can impair PBSC collection, with failure rates varying widely depending on prior treatments and mobilization regimens.

### Aims

To evaluate the impact of L-MIT (20mg/m²) containing second-line chemotherapy on the efficiency of PBSC mobilization in patients with R/R lymphoma.

### Methods

We retrospectively analyzed 29 patients with first R/R lymphoma in our hospital. Patients received L-MIT (20mg/m²) as part of their salvage regimen (CMOP±R, n=6; GVM±R, n=8; MINE±R, n=15). PBSC mobilization with G-CSF was attempted after 2 cycles (n=15) or 4 cycles (n=14) of chemotherapy. Mobilization failure was defined as the inability to collect a minimum of $2.0 \times 10^6$ CD34+ cells/kg. Patient characteristics, treatment details, and mobilization outcomes were collected and analyzed.

### Results

The overall response rate (ORR) to the L-MIT containing regimens was 72.4% (CR 41.4%). However, a significant disparity in mobilization success was observed based on the timing of collection. Among patients who attempted mobilization after 2 cycles, the failure rate was 20% (3/15), with a median CD34+ yield of $3.8 \times 10^6$ cells/kg. In contrast, patients attempting mobilization after 4 cycles experienced a failure rate of 85.7% (12/14), with a median CD34+ yield of only $1.2 \times 10^6$ cells/kg ($p<0.001$). The number of chemotherapy cycles was the strongest predictor of mobilization failure (OR 28.5, 95% CI 5.2-156.3). The most common grade 3-4 toxicities were neutropenia (89.7%) and thrombocytopenia (48.3%).

### Summary/Conclusion

Although salvage chemotherapy with liposomal mitoxantrone at 20mg/m² is highly effective for R/R lymphoma, it significantly depletes the hematopoietic stem cell reserve in a cycle-dependent manner. This leads to a very high failure rate in PBSC mobilization after 4 cycles. For patients eligible for transplantation, we recommend limiting induction to 2 cycles before stem cell collection to increase the likelihood of a successful harvest for ASCT.

**Copyright Information:** (Online) ISSN: 2572-9241
©2026 The Author(s). HemaSphere is published by John Wiley & Sons Ltd on behalf of European Hematology Association. This is an open access Abstract Book distributed under the Attribution-NonCommercial-NoDerivs (CC BY-NC-ND), which allows third parties to download the articles and share them with others as long as they credit the author and the Abstract Book, but they cannot change the content in any way or use them commercially.
**Abstract Book Citations:** Authors, Title, HemaSphere, 2026;10:(S1):pages. The abstract book DOIs can be found on the "Author Information" page.
**Disclaimer:** Articles published in the journal HemaSphere exclusively reflect the opinions of the authors. The authors are responsible for all content in their abstracts including accuracy of the facts, statements, citing resources, etc.

### (PB3801) GLOFITAMAB IN HEAVILY PRETREATED RELAPSED/REFRACTORY B-CELL LYMPHOMA: A REAL-WORLD SINGLE-CENTER ANALYSIS OF EFFICACY, PROGNOSTIC FACTORS, AND COMBINATION STRATEGIES

**Topic:** 19. Large B cell lymphomas - Clinical

Jia Cong[1], Cheng-Ji Wang[1], Liang Wang*[1]

[1]*Beijing Tongren Hospital, Capital Medical University, Beijing, China;*

### Background

Glofitamab, a novel CD20×CD3 bispecific antibody with a unique 2:1 configuration, has demonstrated promising efficacy in relapsed/refractory (R/R) B-cell lymphoma. However, real-world evidence from Chinese patients with high-burden disease remains limited.

### Aims

This study evaluated the effectiveness, safety, and prognostic factors of glofitamab-based regimens in a consecutive single-center cohort.

### Methods

We retrospectively analyzed 32 R/R B-cell lymphoma patients who received glofitamab-containing therapy at Beijing Tongren Hospital between February 2024 and November 2025. Patients received glofitamab as salvage therapy (n=25), bridging therapy for CAR-T (n=6), or consolidation (n=1). Treatment comprised glofitamab monotherapy (37.5%) or combination regimens including GemOx, polatuzumab vedotin, selinexor, and others. Endpoints included progression-free survival (PFS), overall survival (OS), objective response rate (ORR), and safety assessed per CTCAE v5.0. Prognostic factors were identified using univariate and multivariate Cox regression with Firth correction.

### Results

Median age was 60.5 years; 87.5% had advanced-stage disease, 59.4% had ECOG >1, and 46.9% had prior CAR-T therapy. With median follow-up of 414 days, median PFS was 158 days and median OS was 319 days; 180-day PFS and OS rates were 39.1% and 72.2%, respectively. In evaluable salvage therapy patients (n=21), ORR was 47.6% with CRR of 14.3%; median duration of response was 204 days. All 6 bridging therapy patients achieved partial response. Multivariate analysis identified monotherapy as an independent adverse prognostic factor for PFS (HR=4.09, p=0.010). Prior CAR-T exposure, bendamustine treatment, and increased treatment lines were associated with inferior outcomes. Grade 1-2 cytokine release syndrome occurred in 28.1% with no grade ≥3 CRS or ICANS. Hematologic toxicity (78.1%) and infections were predominant; 2 patients died from severe infections.

### Summary/Conclusion

In this high-risk, heavily pretreated real-world cohort, glofitamab demonstrated meaningful antitumor activity but single-agent efficacy was limited. Combination regimens showed superior disease control and manageable safety profiles. These findings support integrating glofitamab earlier in treatment sequences and highlight the importance of combination strategies and vigilant infection monitoring in real-world practice.

**Copyright Information:** (Online) ISSN: 2572-9241
©2026 The Author(s). HemaSphere is published by John Wiley & Sons Ltd on behalf of European Hematology Association. This is an open access Abstract Book distributed under the Attribution-NonCommercial-NoDerivs (CC BY-NC-ND), which allows third parties to download the articles and share them with others as long as they credit the author and the Abstract Book, but they cannot change the content in any way or use them commercially.
**Abstract Book Citations:** Authors, Title, HemaSphere, 2026;10:(S1):pages. The abstract book DOIs can be found on the "Author Information" page.
**Disclaimer:** Articles published in the journal HemaSphere exclusively reflect the opinions of the authors. The authors are responsible for all content in their abstracts including accuracy of the facts, statements, citing resources, etc.

### (PB3802) OUTPATIENT ADMINISTRATION OF R-ICE IN RELAPSED/REFRACTORY B-CELL LYMPHOMA: A REAL-WORLD SINGLE-CENTER STUDY

**Topic:** 19. Large B cell lymphomas - Clinical

Francisca Ribeiro Soares*[1], Ana Silva Martins[1], Rita Gerivaz[1], Arianda Mateus[1], Ana Raquel Vicente Dias[1], Ana Rebelo[1], Ana Tomé[1], Joana Caldas[1], Patricia Ribeiro[1]

[1]*ULS São José – Hospital S.A. Capuchos, Department of Hematology and Bone Marrow Transplant, Lisbon, Portugal;*

### Background

R-ICE is a standard salvage regimen for transplant-eligible patients with relapsed or refractory lymphomas. However, its administration has traditionally been restricted to inpatient setting, which has the drawback of increasing treatment burden and augmenting healthcare resources utilization. Despite R-ICE widespread use, contemporary data supporting outpatient delivery remains limited.

### Aims

To evaluate the efficacy and safety of outpatient R-ICE in transplant-eligible patients.

### Methods

We conducted a single-center retrospective analysis of transplant-eligible patients treated with outpatient fractionated ICE with or without rituximab over a 10-year period, between January 2015 and December 2025. Patients without documented cause of death or who were lost to follow-up without response assessment were excluded from the analysis. The R-ICE outpatient protocol consisted of ifosfamide 2000 mg/m² infused over 2 h daily on days 1–3; mesna 400 mg i.v. immediately after, and at 4 and 8 hours after ifosfamide; carboplatin 300 mg/m² i.v. on day 1; and etoposide 80 mg/m² i.v. daily on days 1–3, with or without rituximab 375 mg/m² i.v. on day 1. Filgrastim from days 6 to 10 was included. Outpatient ICE cycles were administered every 21 days. Primary outcome was overall response rate (ORR). Secondary outcomes were toxicity grade 3-4 according to CTCAE (v5.0), Treatment-related mortality, Overall Survival (OS) and Progression-Free Survival (PFS). Data was collected from clinical records and analysed with IBM® SPSS *v.23*.

### Results

88 patients (39.8% female and 60,2% male) with a median age of 59 years old (range 20-74) at ICE treatment were included. Diagnosis were diffuse large B-cell lymphoma (DLBCL) in 61.4%; follicular lymphoma in 23.9%, of which 52.4% were POD24; transformed follicular lymphoma in 9.1% of cases. R-ICE was administered as 2nd-line therapy in 99.6% of patients, with prior R-CHOP exposure in 93.1%. The median number of R-ICE cycles was 4 (range 1-8). The overall response rate (ORR) was 62.9%, with complete response in 50.6% of patients. Seven patients discontinued treatment due to toxicity, thus response was not formally evaluated. 31.8% proceeded to Autologous Stem Cell Transplantation (ASCT). Reasons for not proceding to transplant were progressive disease in 66.7%, inadequate peripheral blood stem cell mobilization in 15% and loss eligibility in 8.3%. With a median follow-up time of 63 months (range 4-223), the estimated median PFS was 8 months [95%CI 1.301-14.699] and estimated median OS was 30 months [95%CI 5.511;54.489]. 2 patients died during treatment due to infection and 1 died due to toxicity. Grade 4 toxicity was documented in 44.3% of patients as hematologic toxicity, in 4.5% as febrile neutropenia and in 2.3% as non-neutropenic infection. For 41.1% of patients, medical appointments were only 1 per protocol, without any need of emergency department visits.

**Copyright Information:** (Online) ISSN: 2572-9241
©2026 The Author(s). HemaSphere is published by John Wiley & Sons Ltd on behalf of European Hematology Association. This is an open access Abstract Book distributed under the Attribution-NonCommercial-NoDerivs (CC BY-NC-ND), which allows third parties to download the articles and share them with others as long as they credit the author and the Abstract Book, but they cannot change the content in any way or use them commercially.
**Abstract Book Citations:** Authors, Title, HemaSphere, 2026;10:(S1):pages. The abstract book DOIs can be found on the "Author Information" page.
**Disclaimer:** Articles published in the journal HemaSphere exclusively reflect the opinions of the authors. The authors are responsible for all content in their abstracts including accuracy of the facts, statements, citing resources, etc.

**Summary/Conclusion**

The efficacy outcomes observed in our outpatient cohort were comparable to those reported with inpatient protocols in previous studies, supporting that ambulatory administration does not compromise treatment effectiveness. The retrospective design of the study, including a time when there were no alternative curative options other than ASCT in second line, may have contributed to higher toxicity outcomes due to patient selection bias. Importantly, our study expands previous outpatient experiences by providing contemporary real-world safety data.

**Copyright Information:** (Online) ISSN: 2572-9241
©2026 The Author(s). HemaSphere is published by John Wiley & Sons Ltd on behalf of European Hematology Association. This is an open access Abstract Book distributed under the Attribution-NonCommercial-NoDerivs (CC BY-NC-ND), which allows third parties to download the articles and share them with others as long as they credit the author and the Abstract Book, but they cannot change the content in any way or use them commercially.
**Abstract Book Citations:** Authors, Title, HemaSphere, 2026;10:(S1):pages. The abstract book DOIs can be found on the "Author Information" page.
**Disclaimer:** Articles published in the journal HemaSphere exclusively reflect the opinions of the authors. The authors are responsible for all content in their abstracts including accuracy of the facts, statements, citing resources, etc.

### (PB3803) TREATMENT OUTCOMES FOR PATIENTS WITH PRIMARY TESTICULAR LYMPHOMA FOLLOWING THE PTL-2022 PROTOCOL

**Topic:** 19. Large B cell lymphomas - Clinical

Daria Koroleva*[1], Olga Shchetsova[1], Nelli Gabeeva[1], Darima Badmazhapova[1], Alla Kovrigina[2], Bella Biderman[3], Elena Nikulina[3], Andrey B. Sudarikov[3], Galstyan Gennady[4], Tatiana Obukhova[5], Evgeniy Zvonkov[1]

*[1]National Medical Research Center of Hematology, Department of Hematology and Chemotherapy of Lymphomas with a Bone Marrow and Hematopoietic Stem Cell Transplant Unit, Moscow, Russia; [2]National Medical Research Center of Hematology, Pathology Department, Moscow, Russia; [3]National Medical Research Center of Hematology, Department of Molecular Hematology, Moscow, Russia; [4]National Medical Research Center of Hematology, Department of Resuscitation and Intensive Care, Moscow, Russia; [5]National Medical Research Center of Hematology, Karyology laboratory, Moscow, Russia;*

### Background

Primary testicular lymphoma (PTL) belongs to the prognostically unfavorable group of extranodal DLBCL of immune-privileged organs and the MCD/C5/MYD88 genetic subtype. The effectiveness of standard treatment protocols is extremely low, especially in advanced stages of the disease with a high risk of CNS relapse. The PTL-2022 protocol, developed in 2022 at the National Medical Research Center for Hematology, involved categorizing patients according to their age and the presence of CNS lesions. Additionally, it incorporated intensified induction therapy using targeted agents and high-dose consolidation treatment for patients younger than 65 years (see Fig. 1).

### Aims

To present the interim results of therapy for patients with PTL according to the "PTL-2022" protocol.

### Methods

In the period from 2021 to 2026, 12 patients with PTL were included in the protocol "PTL-2022". The average age of patients was 57 (43-75) years, 5 (42%) were over 60 years old. Diagnostic method: orchiectomy - 10 cases (83%), biopsy - 2 cases (17%). Ann Arbor stages: IE - 2 (17%), IIE - 1 (8%), IV - 9 (75%) patients. Mutations in the *MYD88* and *TP53* genes were detected in 8/12 (67%) and 4/12 (33%) patients, respectively. CNS involvement was detected in 6 (50%) patients: 1 - parenchymal lesion, 5 - neuroleukemia (1 - flow cytometry, 4 - mut*MYD88*).

### Results

Of the 12 patients, 11 completed treatment, and 1 is continuing therapy. Of these 11 patients, 5 received the R-mNHL-BFM-90 regimen with ibrutinib and lenalidomide followed by autologous HSCT, 4 received the R-mNHL-BFM-90/R-CHOP regimen with ibrutinib and lenalidomide followed by autologous HSCT, and 2 received the R-CHOP regimen with ibrutinib and lenalidomide. All 11 patients achieved CR, including a patient with combined brain and testicular involvement. Of the 11 patients, 2 (18%) with mutations in the *TP53* gene developed early relapses of the disease (+4, +5 months): 1 with a relapse in the pelvis subsequently died from progression, 1 with a relapse in the central nervous system achieved a second CR against the background of therapy with a glofitamab, nivolumab with venetoclax and pomalidomide

**Copyright Information:** (Online) ISSN: 2572-9241
©2026 The Author(s). HemaSphere is published by John Wiley & Sons Ltd on behalf of European Hematology Association. This is an open access Abstract Book distributed under the Attribution-NonCommercial-NoDerivs (CC BY-NC-ND), which allows third parties to download the articles and share them with others as long as they credit the author and the Abstract Book, but they cannot change the content in any way or use them commercially.
**Abstract Book Citations:** Authors, Title, HemaSphere, 2026;10:(S1):pages. The abstract book DOIs can be found on the "Author Information" page.
**Disclaimer:** Articles published in the journal HemaSphere exclusively reflect the opinions of the authors. The authors are responsible for all content in their abstracts including accuracy of the facts, statements, citing resources, etc.

**Summary/Conclusion**

The initial results of the «PTL-2022» protocol demonstrated high efficacy and safety. A high rate of CNS involvement (50%) was observed at disease onset. Early relapses occurred in 50% of patients (2/4) with *TP53* gene mutations, necessitating improved therapeutic approaches for this subgroup (CAR-T cell therapy consolidation or early integration of bispecific antibodies).

**Fig 1. Scheme of the protocol "PTL-2022".**

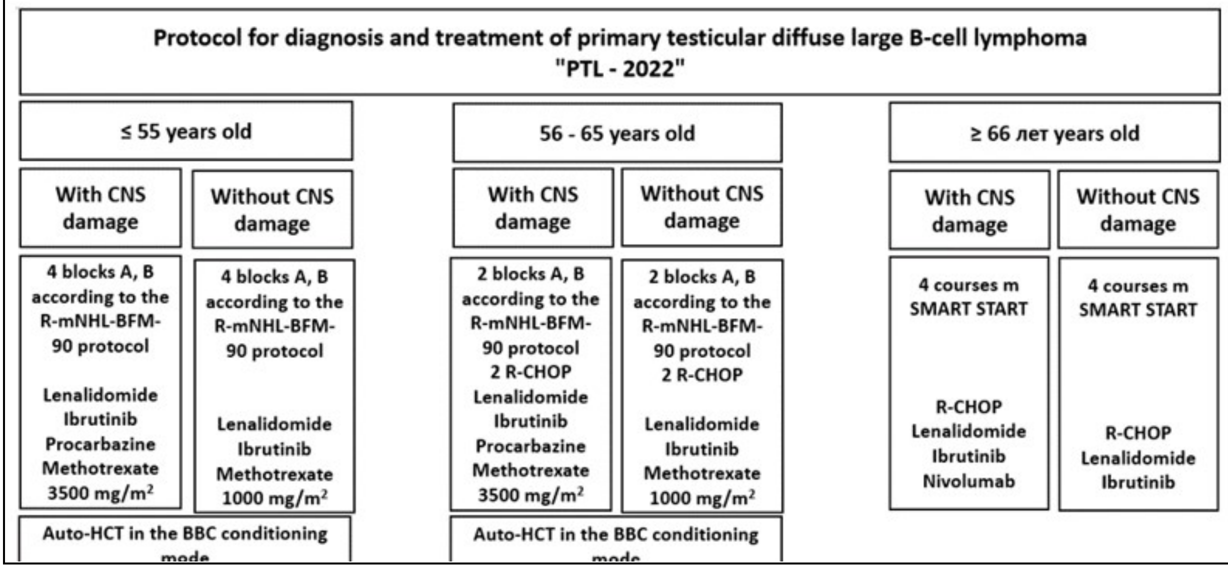

**Copyright Information:** (Online) ISSN: 2572-9241
©2026 The Author(s). HemaSphere is published by John Wiley & Sons Ltd on behalf of European Hematology Association. This is an open access Abstract Book distributed under the Attribution-NonCommercial-NoDerivs (CC BY-NC-ND), which allows third parties to download the articles and share them with others as long as they credit the author and the Abstract Book, but they cannot change the content in any way or use them commercially.
**Abstract Book Citations:** Authors, Title, HemaSphere, 2026;10:(S1):pages. The abstract book DOIs can be found on the "Author Information" page.
**Disclaimer:** Articles published in the journal HemaSphere exclusively reflect the opinions of the authors. The authors are responsible for all content in their abstracts including accuracy of the facts, statements, citing resources, etc.

### (PB3804) REAL-WORLD EFFECTIVENESS AND SAFETY OF EPCORITAMAB IN RELAPSED/REFRACTORY AGGRESSIVE B-CELL LYMPHOMAS: A MULTICENTER ITALIAN STUDY

**Topic:** 19. Large B cell lymphomas - Clinical

Francesco Gaudio*[1, 2], Pierluigi Masciopinto[3], Giuseppina Greco[4], Pamela Pinto[2], Bruna Daraia[2], Francesca Ciardo de Marti[4], Giacomo Loseto[3], Attilio Guarini[3], Anna Mele[4], Vincenzo Pavone[4]

*[1]Department of Medicine and Surgery, LUM University "Giuseppe Degennaro", casamassima, Italy; [2]Ecclesiastical Entity Regional Generale Hospital "F. Miulli", Acquaviva delle Fonti (BA), Italy; [3]Hematology and Cell Therapy Unit, IRCCS Istituto Tumori "Giovanni Paolo II", Bari, Italy; [4]Hematology and Transplant Unit, "Cardinal Panico" Hospital, Tricase (LE), Italy;*

### Background

Epcoritamab, a subcutaneous CD3×CD20 bispecific antibody, has shown activity in relapsed/refractory (R/R) diffuse large B-cell lymphoma (DLBCL). Real-world evidence in heterogeneous, heavily pretreated populations remains limited.

### Aims

To evaluate real-world effectiveness and safety of epcoritamab in patients with aggressive B-cell lymphomas treated in a multicenter Italian setting.

### Methods

This retrospective multicenter study included adults with DLBCL/HGBL who initiated epcoritamab between July 2023 and July 2025. Clinical characteristics, final metabolic response (PET-CT), adverse events, and outcomes were extracted from electronic records. Progression-free survival (PFS) was defined from treatment start to progression/relapse or death; overall survival (OS) to death. Kaplan–Meier analysis was applied.

### Results

Nineteen patients were included (79% male; median age 58 years). Stage IV disease was present in 84%, bulky disease in 21%, and extranodal involvement in 95%. Median prior lines were 3 (IQR 2–4); 37% had received CAR-T therapy and 16% autologous HSCT.
Final PET-CT response showed an overall response rate (ORR) of 42%, with 26% CR and 16% PR; 11% SD and 47% PD. Median follow-up was 9.2 months. Median PFS was 10.0 months, with 6-month PFS 62.5% (10 events). Median OS was 20.4 months, with 6-month OS 71.1% (8 deaths). Safety was manageable: cytokine-release syndrome occurred in 47% (all grade 1–2), ICANS in 26%, and infections in 26%; persistent cytopenias were uncommon.

### Summary/Conclusion

In this multicenter Italian real-world cohort, epcoritamab demonstrated meaningful activity in heavily pretreated aggressive B-cell lymphomas, with ORR 42%, durable responses, and median PFS and OS of 10.0 and 20.4 months, respectively. Toxicities were consistent with the known profile, supporting epcoritamab as an effective real-world option beyond clinical trials.

**Copyright Information:** (Online) ISSN: 2572-9241
©2026 The Author(s). HemaSphere is published by John Wiley & Sons Ltd on behalf of European Hematology Association. This is an open access Abstract Book distributed under the Attribution-NonCommercial-NoDerivs (CC BY-NC-ND), which allows third parties to download the articles and share them with others as long as they credit the author and the Abstract Book, but they cannot change the content in any way or use them commercially.
**Abstract Book Citations:** Authors, Title, HemaSphere, 2026;10:(S1):pages. The abstract book DOIs can be found on the “Author Information” page.
**Disclaimer:** Articles published in the journal HemaSphere exclusively reflect the opinions of the authors. The authors are responsible for all content in their abstracts including accuracy of the facts, statements, citing resources, etc.

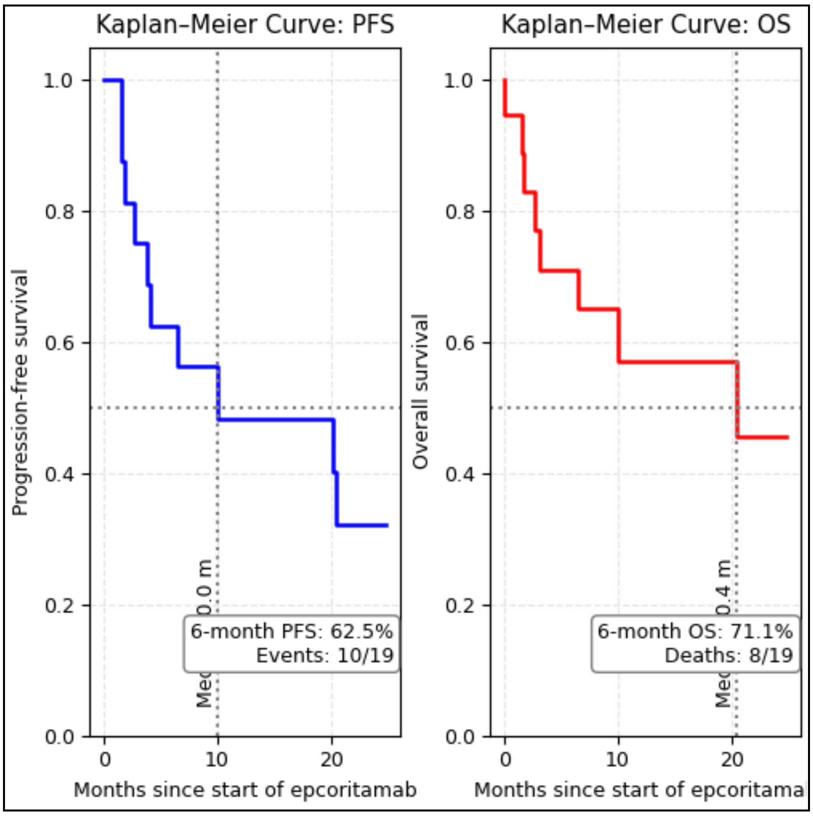

Figure 1 — Kaplan–Meier Curves (PFS & OS)

**Copyright Information:** (Online) ISSN: 2572-9241
©2026 The Author(s). HemaSphere is published by John Wiley & Sons Ltd on behalf of European Hematology Association. This is an open access Abstract Book distributed under the Attribution-NonCommercial-NoDerivs (CC BY-NC-ND), which allows third parties to download the articles and share them with others as long as they credit the author and the Abstract Book, but they cannot change the content in any way or use them commercially.
**Abstract Book Citations:** Authors, Title, HemaSphere, 2026;10:(S1):pages. The abstract book DOIs can be found on the "Author Information" page.
**Disclaimer:** Articles published in the journal HemaSphere exclusively reflect the opinions of the authors. The authors are responsible for all content in their abstracts including accuracy of the facts, statements, citing resources, etc.

### (PB3805) CLINICOPATHOLOGIC SPECTRUM AND OUTCOMES OF PRIMARY OVARIAN LYMPHOMA: A RETROSPECTIVE CASE SERIES

**Topic:** 19. Large B cell lymphomas - Clinical

Ying Shan*[1], Xiaoling Chen[2], Da-Hui Li[3], Wen-Hao Zhang[3], Dong Chen[3], Yuting Xin[4], Chuanxu Liu[3], Shuangyue Li[1], Tiantian Wang[1], Xiaowei Shi[1], Peipei Ye[1], Fan Zhou[2], Ying Lu[1], Rong Tao[3]

*[1]The Affiliated People's Hospital of Ningbo University, haematology, Ningbo, China;[2]Shanghai Jing An District Zhabei Central Hospital, Hematologic & Oncology, Shanghai, China; [3]Fudan University Shanghai Cancer centre, Lymphoma and Medical Oncology, Shanghai, China; [4]Shenzhen Campus of Sun Yat-sen University, Shenzhen, China;*

### Background

Primary ovarian lymphoma (POL) is rare and often presents as an adnexal mass, creating diagnostic and management challenges.

### Aims

To describe the clinicopathologic spectrum, treatment patterns and outcomes in contemporary practice, including the use of modern systemic and salvage approaches where applied.

### Methods

We performed a retrospective cohort study of women diagnosed with ovarian-dominant lymphoma presentation between January 2018 and December 2023. Cases were identified if clinical records and survival follow-up were available.

### Results

13 women with POL were identified. Median age was 44 years (range 13–69) and 69.2% were premenopausal. LDH was elevated in 9/13 (69.2%) and CA-125 in 8/10 (80.0%); CEA and CA19-9 were negative in all evaluable cases. Histology was heterogeneous: DLBCL 46.2%, extranodal NK/T-cell lymphoma 23.1%, Burkitt lymphoma 15.4%, marginal zone lymphoma 1/13, and follicular lymphoma 1/13. Median follow-up was 28 months (range 0.5–84). Three deaths occurred, all in patients without timely subtype-appropriate systemic therapy. Estimated 5-year OS was approximately 76.92% (95% CI, 57.11%-100%).

### Summary/Conclusion

In this contemporary POL series, clinicopathologic heterogeneity was prominent, and laboratory profiles frequently overlapped with ovarian malignancy work-up. Outcomes were favourable among patients receiving timely, subtype-appropriate systemic treatment after a surgical-first diagnostic pathway.

**Copyright Information:** (Online) ISSN: 2572-9241
©2026 The Author(s). HemaSphere is published by John Wiley & Sons Ltd on behalf of European Hematology Association. This is an open access Abstract Book distributed under the Attribution-NonCommercial-NoDerivs (CC BY-NC-ND), which allows third parties to download the articles and share them with others as long as they credit the author and the Abstract Book, but they cannot change the content in any way or use them commercially.
**Abstract Book Citations:** Authors, Title, HemaSphere, 2026;10:(S1):pages. The abstract book DOIs can be found on the “Author Information” page.
**Disclaimer:** Articles published in the journal HemaSphere exclusively reflect the opinions of the authors. The authors are responsible for all content in their abstracts including accuracy of the facts, statements, citing resources, etc.

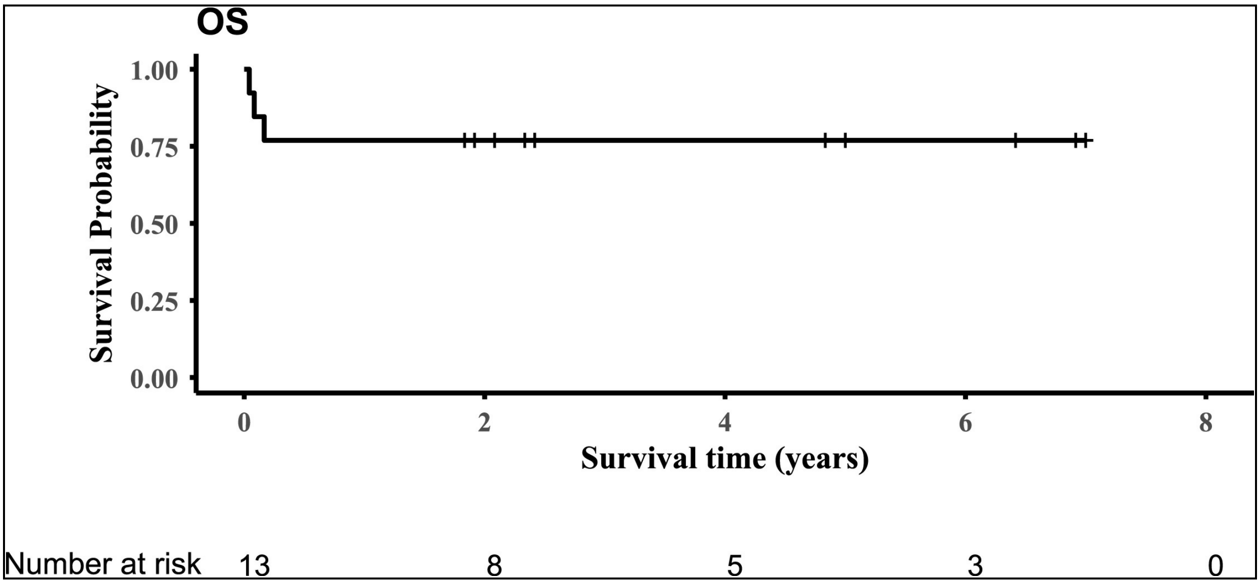

Figure 2. Overall Survival of POL Patients

**Copyright Information:** (Online) ISSN: 2572-9241
©2026 The Author(s). HemaSphere is published by John Wiley & Sons Ltd on behalf of European Hematology Association. This is an open access Abstract Book distributed under the Attribution-NonCommercial-NoDerivs (CC BY-NC-ND), which allows third parties to download the articles and share them with others as long as they credit the author and the Abstract Book, but they cannot change the content in any way or use them commercially.
**Abstract Book Citations:** Authors, Title, HemaSphere, 2026;10:(S1):pages. The abstract book DOIs can be found on the "Author Information" page.
**Disclaimer:** Articles published in the journal HemaSphere exclusively reflect the opinions of the authors. The authors are responsible for all content in their abstracts including accuracy of the facts, statements, citing resources, etc.

### (PB3806) ATYPICAL EXTRANODAL DIFFUSE LARGE B-CELL LYMPHOMA: MAPPING RARE SITES AND QUANTIFYING DIAGNOSTIC COMPLEXITY IN A REAL-WORLD COHORT

**Topic:** 19. Large B cell lymphomas - Clinical

Mihaela-Sabrina Herdea*[1], Adela-Sara Ionescu[1], Ana-Maria Petraru[1], Gratiela-Elena Dobre[1], Oana Dragoi Cozmaciuc[1], Florina Oana Gianina Pătrînoiu[1], Viola Maria Popov[1], Mihaela Andreescu[1, 2]

*[1]Colentina Clinical Hospital, Hematology, Bucharest, Romania;[2]Titu Maiorescu University, Hematology, Bucharest, Romania;*

### Background

Diffuse large B-cell lymphoma (DLBCL) may present in extranodal sites, but involvement of rare anatomical locations remains uncommon and may delay diagnosis, mimicking inflammatory/infectious diseases or solid tumor conditions and leading to complex diagnostic pathways.

### Aims

To study diagnostic delays, clinical heterogeneity and the utility of a Diagnostic Complexity Score (DCS) for identifying patients at risk of delayed diagnosis in atypical extranodal DLBCL.

### Methods

We retrospectively analyzed 35 patients with DLBCL arising in rare extranodal sites. Demographic, clinical, laboratory, pathological and imaging data were collected, including stage, LDH, IPI, ECOG score, cell-of-origin subtype, Ki67 and therapy. Diagnostic timelines (symptom onset, biopsy, first hematology visit and treatment start) were analyzed in a subset with complete data.
To quantify real-world diagnostic burden, we developed DCS integrating: multi-modality imaging (CT/RMN/PET-CT), involvement of ≥2 specialties prior to hematology referral, repeat biopsy, symptom-to-biopsy interval >60 days, and anatomically deceptive presentations. One point was assigned for each imaging modality performed and one point for each additional parameter as previously mentioned, resulting in a maximum DCS of 7 if all criteria were present for a given patient. Trends between DCS and Ki67/IPI/LDH were explored descriptively.

### Results

The most frequent sites were located in the oral cavity and adjacent structures (tongue, gingiva, cavum and buccal mucosa) 26% and the bone (long bones, cranial bones, facial bones and bone marrow) 26%, while others had anorectal involvement 14%, primary cerebral involvement 11%, orbit/ocular-adnexal 6%, thyroid 3%, retroperitoneal/mesenteric fat/adrenal 9%, breast 6%, subcutaneous/soft tissue/scalp 6%, intravascular 3%.
Median age at diagnosis was 65 years (95% CI: 57–67). LDH was elevated above the sex-specific institutional upper limit of normal (214 U/L for females and 225 U/L for males) in 51.4% of patients, median Ki67 was 80% (95% CI: 75–90%) and 37.14% of patients had non-germinal center B-cell cell-of-origin. First-line therapy included R-CHOP for 48.57%, Pola-R-CHP for 20% and only 31.43%received other therapies.
Among patients with complete timelines(27), median symptom-to-biopsy interval was 90 days and biopsy-to-treatment interval was 42 days. DCS ranged 2–6(median4,4). Higher DCS was observed in craniofacial and ocular disease and in physically active patients with bone involvement at the joint level, where symptoms were frequently attributed to post-procedural, inflammatory, mechanical stress or site-specific conditions. Higher DCS scores were observed in several cases with high Ki67 (≥80%) and higher IPI (≥3).
Interim PET-CT was available for 19 of 35 patients after 2–4 cycles of therapy, with an overall response rate (ORR) 78.9% (CR 42.1%, PR 36.8%), with stable disease in 5.3% and progression in 15.8%.

**Copyright Information:** (Online) ISSN: 2572-9241
©2026 The Author(s). HemaSphere is published by John Wiley & Sons Ltd on behalf of European Hematology Association. This is an open access Abstract Book distributed under the Attribution-NonCommercial-NoDerivs (CC BY-NC-ND), which allows third parties to download the articles and share them with others as long as they credit the author and the Abstract Book, but they cannot change the content in any way or use them commercially.
**Abstract Book Citations:** Authors, Title, HemaSphere, 2026;10:(S1):pages. The abstract book DOIs can be found on the "Author Information" page.
**Disclaimer:** Articles published in the journal HemaSphere exclusively reflect the opinions of the authors. The authors are responsible for all content in their abstracts including accuracy of the facts, statements, citing resources, etc.

**Summary/Conclusion**

Atypical extranodal DLBCL shows marked clinical heterogeneity and frequent diagnostic delays, particularly in hard-to-biopsy sites. The proposed Diagnostic Complexity Score (DCS) captures real-world diagnostic pathways, identifies patients at risk of delayed biopsy, and may facilitate earlier referral and optimized management.
Ongoing follow-up will expand the cohort and implement the DCS prospectively, supporting early recognition, streamlined management of complex extranodal presentations, and evaluation of outcomes, including overall survival.

**Copyright Information:** (Online) ISSN: 2572-9241
©2026 The Author(s). HemaSphere is published by John Wiley & Sons Ltd on behalf of European Hematology Association. This is an open access Abstract Book distributed under the Attribution-NonCommercial-NoDerivs (CC BY-NC-ND), which allows third parties to download the articles and share them with others as long as they credit the author and the Abstract Book, but they cannot change the content in any way or use them commercially.
**Abstract Book Citations:** Authors, Title, HemaSphere, 2026;10:(S1):pages. The abstract book DOIs can be found on the “Author Information” page.
**Disclaimer:** Articles published in the journal HemaSphere exclusively reflect the opinions of the authors. The authors are responsible for all content in their abstracts including accuracy of the facts, statements, citing resources, etc.

### (PB3807) A ANALYSIS OF FIRST-LINE TREATMENT WITH POLATUZUMAB VEDOTIN-RITUXIMAB-CYCLOPHOSPHAMIDE, DOXORUBICIN AND PREDNISONE (POLA-R-CHP) FOR PREVIOUSLY UNTREATED DIFFUSE LARGE B-CELL LYMPHOMA IN CHINA.

**Topic:** 19. Large B cell lymphomas - Clinical

### Background

Polatuzumab vedotin plus rituximab, cyclophosphamide, doxorubicin, and prednisone (Pola-R-CHP) has showed a significant progression-free survival (PFS) benefit versus rituximab, cyclophosphamide, doxorubicin, vincristine, and prednisone (R-CHOP) in patients with previously untreated intermediate- or high-risk diffuse large B-cell lymphoma (DLBCL) in POLARIX study.

### Methods

In this study, we retrospectively evaluated 82 previously untreated diffuse large B-cell lymphoma patients who received Pola-R-CHP as first-line treatment from January 2023 to December 2025 in our medical center.

### Results

Until February 1, 2026, the median follow-up time was 13 months (range:2-34 months). Data from four patients were missing. 71(90.9%) patients achieved overall response (ORR) by using pola-R-CHP, with 63 complete response (CR) patients (80.7%) and 8 partial response (PR) patients (10.2%). The 2-year overall survival (OS) rate was 93.7%. The 2-year progression free survival (PFS) rate was 76.7%. Multivariate regression analysis showed that IPI score ≥3, stage, B symptoms, age ≥60 years, LDH level more than twice the upper limit of normal, and immunophenotype as double-expressor were not associated with survival outcomes. For patients with diffuse large B-cell lymphoma receiving POLA-R-CHP as first-line treatment, there was no statistically significant difference in overall survival or PFS between the immunophenotype double-expressor and non-double-expressor groups. For the 28 patients with non-double-expressor status, the 2-year OS rate was 94.4% and the 2-year PFS rate was 86%; for the 44 patients with double-expressor status, the 2-year OS rate was 92.3% and the 2-year PFS rate was 74.1%. Among the 78 patients, 2 developed *Pneumocystis jirovecii* pneumonia. One patient recovered after treatment, while the other died from the pneumonia. No other severe adverse events were observed.

### Summary/Conclusion

Our study demonstrates that POLA-R-CHP is a safe and effective first-line treatment for patients with newly diagnosed DLBCL. The regimen was well-tolerated in frail patients, and that those with immunophenotype double-expressor could derive benefit from it.

**Copyright Information:** (Online) ISSN: 2572-9241
©2026 The Author(s). HemaSphere is published by John Wiley & Sons Ltd on behalf of European Hematology Association. This is an open access Abstract Book distributed under the Attribution-NonCommercial-NoDerivs (CC BY-NC-ND), which allows third parties to download the articles and share them with others as long as they credit the author and the Abstract Book, but they cannot change the content in any way or use them commercially.
**Abstract Book Citations:** Authors, Title, HemaSphere, 2026;10:(S1):pages. The abstract book DOIs can be found on the "Author Information" page.
**Disclaimer:** Articles published in the journal HemaSphere exclusively reflect the opinions of the authors. The authors are responsible for all content in their abstracts including accuracy of the facts, statements, citing resources, etc.

### (PB3808) ASSOCIATION OF PRETREATMENT NEUTROPHIL-TO-LYMPHOCYTE RATIO WITH CLINICAL OUTCOMES IN DIFFUSE LARGE B CELL LYMPHOMA: A RETROSPECTIVE ANALYSIS OF R-CHOP TREATED PATIENTS

**Topic:** 19. Large B cell lymphomas - Clinical

Bonnie R K Singh*[1], Raghuveer Prabhu[1], Narayanankutty Warrier[1], Prasanth Parameswaran[1], Sreedharan P S[1], Sajeevan K V[1], Arunmohan P V[1], Akhil P Santhosh[1], Uma Maheshwari P[1]

[1]*MVR Cancer Centre and Research Institute, Medical Oncology, Calicut, India;*

### Background

The pretreatment neutrophil-to-lymphocyte ratio (NLR) has been proposed as a potential inflammatory biomarker with prognostic relevance in several haematological malignancies, including diffuse large B-cell lymphoma (DLBCL). However, its independent prognostic value beyond established clinical risk models such as the International Prognostic Index (IPI) remains uncertain.

### Aims

To evaluate the association between baseline NLR and progression-free survival (PFS) in patients with de novo DLBCL, identify an NLR cutoff predictive of PFS, and determine whether NLR retains prognostic significance independent of IPI risk category.

### Methods

We did a retrospective review of data of de-novo DLBCL patients aged 18-70 years, treated with 6 cycles of rituximab, cyclophosphamide, doxorubicin, vincristine and prednisolone (R-CHOP combination regimen), from 2018 to 2021. Baseline NLR was analysed as both a continuous and categorical variable. The cutoff value of NLR (2.73) was derived from the cohort median due to the limited number of disease relapse / progression events precluding reliable ROC-based optimization. PFS was assessed using Kaplan–Meier methodology and compared by log-rank testing. Cox proportional hazards regression was performed for univariate and multivariate analyses adjusting for IPI risk category. All statistical analysis was performed using Software R studio (V. 4.4.3).

### Results

A total of 122 patients with DLBCL were finally analysed for our preferred objectives, with 85 of them aged <=60yrs. Among them, 27 patients were found to have a disease relapse / progression following treatment with R-CHOP regimen. Correlation analysis using Pearson's test (r= -0.094, 95% CI: -0.266 to 0.084; p= 0.30) and Spearman's test ($\rho$ = -0.016, p = 0.86), did not demonstrate a meaningful linear or monotonic association between baseline NLR and raw PFS duration. In univariate Cox regression analysis, baseline NLR as a continuous variable was significantly associated with inferior PFS (HR 1.105; 95% CI 1.015–1.204; p = 0.021), corresponding to a 10.5% increase in progression risk per unit increase in NLR. Kaplan–Meier analysis using the cutoff of 2.73 demonstrated significantly inferior PFS in the high NLR group compared with the low NLR group (20 vs. 7 progression events; log-rank $\chi^2$ = 7.1, p = 0.0077). In multivariate analysis adjusting for IPI risk category, baseline NLR was not independently associated with PFS (HR 1.061; 95% CI 0.965–1.168; p = 0.22). In contrast, intermediate-risk (61 patients; HR 4.73; 95% CI 1.58–14.13; p = 0.005) and high-risk IPI categories (6 patients; HR 14.73; 95% CI 3.63–59.74; p < 0.001) remained strong independent predictors of inferior PFS.

**Copyright Information:** (Online) ISSN: 2572-9241
©2026 The Author(s). HemaSphere is published by John Wiley & Sons Ltd on behalf of European Hematology Association. This is an open access Abstract Book distributed under the Attribution-NonCommercial-NoDerivs (CC BY-NC-ND), which allows third parties to download the articles and share them with others as long as they credit the author and the Abstract Book, but they cannot change the content in any way or use them commercially.
**Abstract Book Citations:** Authors, Title, HemaSphere, 2026;10:(S1):pages. The abstract book DOIs can be found on the "Author Information" page.
**Disclaimer:** Articles published in the journal HemaSphere exclusively reflect the opinions of the authors. The authors are responsible for all content in their abstracts including accuracy of the facts, statements, citing resources, etc.

**Summary/Conclusion**

Baseline NLR is significantly associated with inferior progression-free survival in patients with de novo DLBCL treated with R-CHOP when analysed independently. However, its prognostic value is not independent of IPI risk category. These findings suggest that NLR may reflect disease-related inflammatory burden captured within established prognostic indices rather than serving as an independent risk marker. Further prospective validation is warranted to clarify the incremental utility of NLR in contemporary risk stratification models.

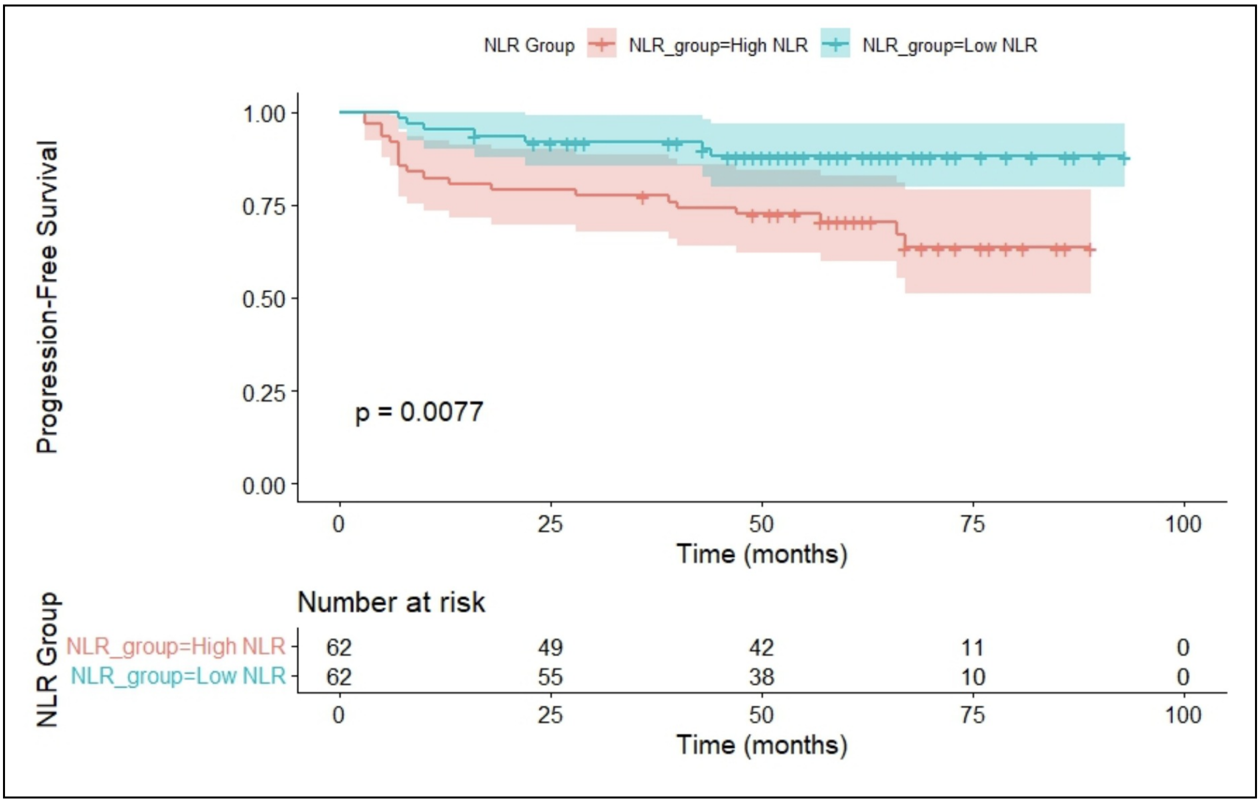

Kaplan-Meier curve showing significantly poorer PFS in patients with high baseline NLR compared with low baseline NLR

**Copyright Information:** (Online) ISSN: 2572-9241
©2026 The Author(s). HemaSphere is published by John Wiley & Sons Ltd on behalf of European Hematology Association. This is an open access Abstract Book distributed under the Attribution-NonCommercial-NoDerivs (CC BY-NC-ND), which allows third parties to download the articles and share them with others as long as they credit the author and the Abstract Book, but they cannot change the content in any way or use them commercially.
**Abstract Book Citations:** Authors, Title, HemaSphere, 2026;10:(S1):pages. The abstract book DOIs can be found on the "Author Information" page.
**Disclaimer:** Articles published in the journal HemaSphere exclusively reflect the opinions of the authors. The authors are responsible for all content in their abstracts including accuracy of the facts, statements, citing resources, etc.

### (PB3809) OPTIMIZING MATRIX-BASED THERAPY THROUGH CHEMO-FREE PROD INDUCTION IN UNFIT PATIENTS WITH CENTRAL NERVOUS SYSTEM LYMPHOMA (CNSL): A REAL-WORLD RETROSPECTIVE STUDY

**Topic:** 19. Large B cell lymphomas - Clinical

Fang Bao*[1], Sen Li[1], Lingli Wang[1], Wei Wan[1], Qihui Li[1], Hongmei Jing[1]

[1]*Peking University Third Hospital, Beijing, China;*

### Background

CNSL is an aggressive subtype of non-Hodgkin lymphoma, with no established standard-of-care therapy. For patients (pts) with primary CNSL (PCNSL), the full-dose MATRix regimen (high-dose methotrexate [HD-MTX], HD-cytarabine, thiotepa, and rituximab) has shown promising long-term outcomes, with a 7-year overall survival (OS) of 70% when followed by consolidation therapy. However, this treatment approach carries substantial hematologic and infectious risks, restricting its applicability in unfit pts. In recent years, second-generation Bruton's tyrosine kinase inhibitors (BTKis), such as orelabrutinib, have emerged as an attractive therapeutic approach in CNSL due to their superior blood-brain barrier penetration and reduced off-target effects. Incorporating BTKis into established therapies may enhance therapeutic efficacy while mitigating toxicity, particularly in unfit pts.

### Aims

We aimed to explore a sequential strategy in which pomalidomide, rituximab, orelabrutinib, and dexamethasone (PROD) induction was used to modulate disease burden and optimize patient condition prior to MATRix, thereby improving the feasibility and tolerability while maintaining disease control in unfit CNSL patients.

### Methods

Between January 21, 2023, and January 30, 2026, 13 pts with CNSL who received PROD regimen on a 21-day cycle were included in this retrospective study. Pts who tolerated the PROD subsequently received MATRix regimen, followed by consolidation with autologous stem cell transplantation (ASCT) or maintenance therapy with orelabrutinib. Baseline characteristics, subsequent treatment regimens, and response assessment outcomes of pts were collected retrospectively.

### Results

Of the 13 pts (11 males; median age 62 years), 11 were diagnosed with PCNSL and 2 with secondary CNSL. Nine pts were treatment-naïve, while 4 had received 1–2 lines of prior chemotherapy-based therapy. Most pts had non-GCB subtype (53.8%), multifocal disease (76.9%), and deep-seated intracranial lesions (61.5%). Per MSKCC scoring system, 61.5% of pts were classified as intermediate risk, 23.1% as high risk, and 15.4% as low risk. All pts initially presented with poor physical conditions or a substantial burden of comorbidities, including gynecologic malignancies, severe infections, and pulmonary or renal impairment. At data cutoff, all pts had completed ≥2 PROD cycles, and 8 pts received 1–3 cycles of MATRix during induction. Seven pts (53.8%) achieved complete response (CR) and 6 (46.2%) had partial response. Following induction therapy, 7 pts (53.8%) underwent consolidation with ASCT and achieved CR, of whom 6 subsequently received orelabrutinib as maintenance therapy and have maintained CR to date. Overall, 8 (61.5%) pts had CR as their best response, yielding an objective response rate of 100.0%. After a median follow-up of 11.8 months (range, 0.9–34.4), the 18-months progress-free survival and 24-months OS were 88.9% (95% CI, 70.6–100.0) and 100.0%, respectively. Four pts (30.8%) experienced adverse events associated with PROD, with grade 3 events occurred in 2 pts (15.4%).

**Copyright Information:** (Online) ISSN: 2572-9241
©2026 The Author(s). HemaSphere is published by John Wiley & Sons Ltd on behalf of European Hematology Association. This is an open access Abstract Book distributed under the Attribution-NonCommercial-NoDerivs (CC BY-NC-ND), which allows third parties to download the articles and share them with others as long as they credit the author and the Abstract Book, but they cannot change the content in any way or use them commercially.
**Abstract Book Citations:** Authors, Title, HemaSphere, 2026;10:(S1):pages. The abstract book DOIs can be found on the "Author Information" page.
**Disclaimer:** Articles published in the journal HemaSphere exclusively reflect the opinions of the authors. The authors are responsible for all content in their abstracts including accuracy of the facts, statements, citing resources, etc.

**Summary/Conclusion**

The novel four-drug PROD regimen showed promising effectiveness in unfit pts with CNSL, allowing those with severe comorbidities or complications to receive subsequent HD-MTX-based treatment. This strategy may shorten the duration of HD-MTX treatment, reduce toxicity, and improve tolerability. Subsequent ASCT consolidation and BTKi maintenance may further contribute to durable disease control

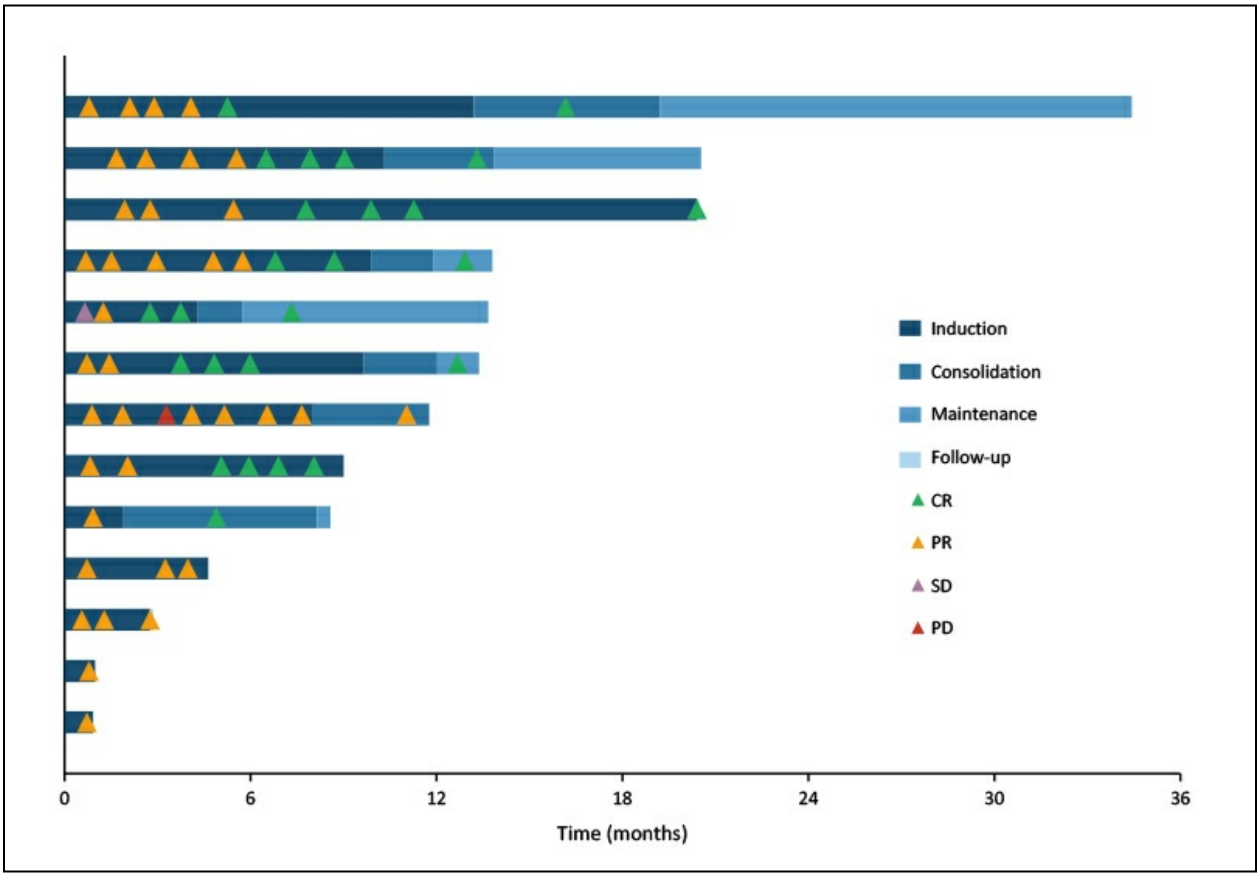

**Copyright Information:** (Online) ISSN: 2572-9241
©2026 The Author(s). HemaSphere is published by John Wiley & Sons Ltd on behalf of European Hematology Association. This is an open access Abstract Book distributed under the Attribution-NonCommercial-NoDerivs (CC BY-NC-ND), which allows third parties to download the articles and share them with others as long as they credit the author and the Abstract Book, but they cannot change the content in any way or use them commercially.
**Abstract Book Citations:** Authors, Title, HemaSphere, 2026;10:(S1):pages. The abstract book DOIs can be found on the "Author Information" page.
**Disclaimer:** Articles published in the journal HemaSphere exclusively reflect the opinions of the authors. The authors are responsible for all content in their abstracts including accuracy of the facts, statements, citing resources, etc.

### (PB3810) CLINICAL AND TREATMENT-RELATED PROGNOSTIC FACTORS IN DIFFUSE LARGE B-CELL LYMPHOMA: A SINGLE-CENTER EXPERIENCE

**Topic:** 19. Large B cell lymphomas - Clinical

Maha Charfi*[1], Zeineb Amouri[1], Rahma Mallek[1], Faten Kallel[1], Imen Ben Amor[1], Yosra Fakhfakh[1], Asma Koubaa[1], Imen Frikha[1], Moez Mdhaffar[1], Moez Elloumi[1]

[1]*Hedi Chaker hospital, hematology department, Sfax, Tunisia;*

### Background

Diffuse large B-cell lymphoma (DLBCL) is a heterogeneous disease with variable clinical and biological characteristics and outcomes. While the International Prognostic Index (IPI) is the most widely used prognostic tool, other clinical, biological, and treatment-related factors may also affect survival. Identifying these prognostic determinants is essential to optimize risk stratification and therapeutic strategies.

### Aims

This study aims to analyze the prognostic factors associated with outcomes in DLBCL patients treated at our center.

### Methods

This retrospective study included patients aged 16 years and older who were diagnosed with DLBCL and treated according to the LNH2013 (R-CHOP-based) protocol at the Department of Hematology, Hedi Chaker University Hospital, between January 2013 and December 2023. Clinical, biological, therapeutic, and outcome data were collected. Prognostic factors, including age, sex, B symptoms, performance status (PS), lactate dehydrogenase (LDH) level, disease stage, international prognostic index (IPI) and advanced international prognostic index (aaIPI) scores, interim response, and end-of-treatment response, were evaluated using univariate and multivariate analyses.

### Results

A total of 130 patients were included. The median age was 50 years (range: 17–85), with a male predominance (58.5%). B symptoms and PS ≥2  were observed in 62.5% and 29% of cases, respectively. Advanced-stage disease and elevated LDH level were present in 58% and 50% of patients. High IPI and aaIPI ≥2 were observed in 29% and 42.3% of patients, respectively. The overall response rate after first-line treatment was 75%, including 62.5% complete responses. With a median follow-up of 8 years, 5-year OS and EFS were 82.4% and 57.5% respectively.
Multivariate analysis revealed that sex ($p=0.03$), advanced stage ($p<0.01$), aaIPI ≥2 ($p=0.008$), interim response ($p=0.025$), and end-of-treatment response ($p<0.01$) were independently associated with EFS. For OS, only IPI score ($p=0.03$) and response at the end of treatment ($p<0.001$) remained significant.

### Summary/Conclusion

In line with findings from large international cohorts, advanced-stage disease, elevated prognostic scores (IPI/aaIPI), and poor treatment response were associated with poorer survival outcomes in DLBCL patients. Our findings confirm the continued prognostic value of simple clinical scores and early response assessment in the rituximab era, supporting their use to guide risk-adapted therapeutic strategies in real-world practice.

**Copyright Information:** (Online) ISSN: 2572-9241
©2026 The Author(s). HemaSphere is published by John Wiley & Sons Ltd on behalf of European Hematology Association. This is an open access Abstract Book distributed under the Attribution-NonCommercial-NoDerivs (CC BY-NC-ND), which allows third parties to download the articles and share them with others as long as they credit the author and the Abstract Book, but they cannot change the content in any way or use them commercially.
**Abstract Book Citations:** Authors, Title, HemaSphere, 2026;10:(S1):pages. The abstract book DOIs can be found on the “Author Information” page.
**Disclaimer:** Articles published in the journal HemaSphere exclusively reflect the opinions of the authors. The authors are responsible for all content in their abstracts including accuracy of the facts, statements, citing resources, etc.

### (PB3811) POOR PERFORMANCE STATUS MAY PRECLUDE THE USE OF RITUXIMAB, METHOTREXATE, PROCARBAZINE, AND VINCRISTINE IN INDUCTION THERAPY OF PRIMARY CNS DLBCL: A SINGLE-CENTER EXPERIENCE

**Topic:** 19. Large B cell lymphomas - Clinical

Vojin Vukovic*[1, 2], Teodora Karan-Đurašević[3], Tamara Bibic[1], Sofija Kozarac[1], Jelena Ivanović[1], Darko Aleksic[1], Snjezana Bogicevic[4], Milena Todorovic Balint[1, 2], Darko Antic[1, 2]

*[1]Clinic of Hematology, University Clinical Center of Serbia, Belgrade, Serbia;[2]Medical Faculty, University of Belgrade, Belgrade, Serbia; [3]Institute of Molecular Genetics and Genetic Engineering, University of Belgrade, Belgrade, Serbia;[4]Department of Radiation Oncology, Institute for Oncology and Radiology of Serbia, Belgrade, Belgrade, Serbia;*

### Background

Mainstay of induction treatment of patients with primary diffuse large B-cell lymphoma of central nervous system (PCNSL) for last few decades have been high-dose methotrexate (HD-MTX) – based regimens followed by consolidation with either whole brain radiotherapy (WBRT) or autologous hematopoietic stem cell transplantation (ASCT). Combination of rituximab, HD-MTX, procarbazine, and vincristine (RMPV), developed by group around Memorial Sloan Kettering Cancer Center (MSKCC), represents one of the most used protocols for induction nowadays. Published real-world experience with RMPV induction is limited, and patients with very poor performance status (PS) are markedly underrepresented, particularly in clinical trials.

### Aims

To evaluate outcomes of unselected immunocompetent PCNSL patients treated with RMPV induction, including a substantial proportion with poor PS.

### Methods

We retrospectively analysed data of 21 consecutive PCNSL patients treated with RMPV in induction phase at Clinic of Hematology, University Clinical Center of Serbia in 2017-2024 period. Data were collected from patients' medical records. Analyses were performed using SPSS 21.0.

**Copyright Information:** (Online) ISSN: 2572-9241
©2026 The Author(s). HemaSphere is published by John Wiley & Sons Ltd on behalf of European Hematology Association. This is an open access Abstract Book distributed under the Attribution-NonCommercial-NoDerivs (CC BY-NC-ND), which allows third parties to download the articles and share them with others as long as they credit the author and the Abstract Book, but they cannot change the content in any way or use them commercially.
**Abstract Book Citations:** Authors, Title, HemaSphere, 2026;10:(S1):pages. The abstract book DOIs can be found on the "Author Information" page.
**Disclaimer:** Articles published in the journal HemaSphere exclusively reflect the opinions of the authors. The authors are responsible for all content in their abstracts including accuracy of the facts, statements, citing resources, etc.

**Results**

Median age was 61 years; 71.4% were male. Poor ECOG PS (3–4) was present in 52.4% (23.8% ECOG 4; median ECOG 3; median KPS 40%). Most patients had non-GCB subtype (80%) with high Ki-67 (median 80%). Deep brain involvement was observed in 71.4%, and multiple lesions in 23.8%. According to MSKCC and ABLE scores, 47.6% and 81% were high risk, respectively. Clinical and laboratory characteristics are summarized in table 1.
Diagnosis was established after stereotactic biopsy, surgical biopsy/partial tumor resection, maximal tumor resection, and total tumor extirpation in 28.6%, 19%, 14.2%, and 38% of cases, respectively.
Median number of RMPV cycles was 5 (range: 1-6). Consolidation was administered in 57.1% (WBRT in 11 patients, including 5 with reduced dose 23.4 Gy; ASCT in 1). Overall response rate was 70% (CR 40%, PR 30%). Completion of induction and delivery of consolidation were the only predictors of response (p=0.007 and p<0.001).
After median follow-up of 61 months, 52.4% of patients died. Median progression-free survival (PFS) was 58 months and overall survival (OS) 67 months. Five of 14 responders relapsed after median PFS of 37 months.
Kaplan-Meier analysis revealed that patients with incompleted RMPV induction, without administered consolidation, those who did not achieved response, as well as patients presenting with ECOG PS 4 exhibited strikingly shorter PFS (p<0.001, p<0.001, p<0.001, and p=0.027, respectively, Log-Rank test) and OS (p<0.001, p<0.001, p<0.001, and p=0.009). Of note, patients with ECOG 4 had median PFS and OS 2 and 3 months, respectively, compared to ECOG PS 0-3 with median PFS and OS of 60 months and not reached, respectively. Furthermore, patients who experienced total or maximal tumor reduction at diagnosis were more likely to survive longer (p=0.008, Log-Rank test), and without disease progression (borderline significance, p=0.088, Log-Rank test).

**Summary/Conclusion**

Patients who present with poor performance status may not be suitable candidates for standard induction regimens such as RMVP, but rather for less intensive or novel therapeutic approaches. Total or maximal tumor resection may be beneficial for prolonged survival.

**Copyright Information:** (Online) ISSN: 2572-9241
©2026 The Author(s). HemaSphere is published by John Wiley & Sons Ltd on behalf of European Hematology Association. This is an open access Abstract Book distributed under the Attribution-NonCommercial-NoDerivs (CC BY-NC-ND), which allows third parties to download the articles and share them with others as long as they credit the author and the Abstract Book, but they cannot change the content in any way or use them commercially.
**Abstract Book Citations:** Authors, Title, HemaSphere, 2026;10:(S1):pages. The abstract book DOIs can be found on the "Author Information" page.
**Disclaimer:** Articles published in the journal HemaSphere exclusively reflect the opinions of the authors. The authors are responsible for all content in their abstracts including accuracy of the facts, statements, citing resources, etc.

**Table 1.** Clinical, laboratory and histology characteristics of the cohort

| Characteristic | N (%) | Median, range |
|---|---|---|
| Gender | | |
| male | 15 (71.4) | |
| female | 6 (28.6) | |
| Age (years) | | 61 (44-73) |
| Symptoms | | |
| focal neurological deficit | 18 (85.7) | |
| signs of increased intracranial pressure | 9 (42.9) | |
| cognitive and behavioral changes | 5 (23.8) | |
| ocular symptoms | 6 (28.6) | |
| seizures | 4 (19) | |
| B symptoms | 2 (9.5) | |
| ECOG PS | | 3 (0-4) |
| 0-2 | 10 (47.6) | |
| 3-4 | 11 (52.4) | |
| Karnofsky PS (%) | | 40 (20-100) |
| COO subtype | 20 (95.2) | |
| nonGCB | 16 (80) | |
| GCB | 4 (20) | |
| Ki-67 (%) | | 80 (IQR 60-90) |
| Distibution of lesions | | |
| Unifocal disease | 16 (76.2) | |
| Multifocal disease | 5 (23.8) | |
| Deep brain structures involvement | | |
| yes | 15 (71.4) | |
| no | 6 (28.6) | |
| LDH | | |
| ≤ULN | 15 (71.4) | |
| >ULN | 6 (28.6) | |
| β2 microglobulin (mg/L) | 13 (61.9) | 2.39 (IQR 1.81-2.99) |
| Prognostic models | | |
| MSKCC | 21 (100) | |
| 0 | 3 (14.3) | |
| 1 | 8 (38.1) | |
| 2 | 10 (47.6) | |
| ABLE | 16 (76.2) | |
| 0 | 3 (18.7) | |
| 1 | 0 (0) | |
| ≥2 | 13 (81.3) | |

ECOG PS – Eastern Cooperative Oncology Group performans status; COO – cell of ori[illegible]; LDH – lactate dehydrogenase; MSKCC – Memorial Sloan Kettering Cancer Center skor; ABLE – ABLE score, IQR – interquartile range

**Copyright Information:** (Online) ISSN: 2572-9241
©2026 The Author(s). HemaSphere is published by John Wiley & Sons Ltd on behalf of European Hematology Association. This is an open access Abstract Book distributed under the Attribution-NonCommercial-NoDerivs (CC BY-NC-ND), which allows third parties to download the articles and share them with others as long as they credit the author and the Abstract Book, but they cannot change the content in any way or use them commercially.
**Abstract Book Citations:** Authors, Title, HemaSphere, 2026;10:(S1):pages. The abstract book DOIs can be found on the "Author Information" page.
**Disclaimer:** Articles published in the journal HemaSphere exclusively reflect the opinions of the authors. The authors are responsible for all content in their abstracts including accuracy of the facts, statements, citing resources, etc.

### (PB3812) REAL-WORLD EXPERIENCE WITH R-IDA-RAM IN PREDOMINANTLY SECONDARY CNS LYMPHOMA: AN IRISH SINGLE-CENTRE STUDY

**Topic:** 19. Large B cell lymphomas - Clinical

Jimmy William[1], Sarah Connor*[1], Roseann O' Doherty[1], Michael Fay[1], Su Maung[1], Peter O'gorman[1], Fionnuala Ní Aínle[1], Barry Kevane[1], Maria Eduarda Dos Santos Couto[1], Anne Fortune[1]

[1]*Mater Misericordiae University Hospital, Haematology, Dublin, Ireland;*

**Background**

Secondary central nervous system lymphoma (SCNSL) is a devastating complication of diffuse large B-cell lymphoma (DLBCL), with a median overall survival (OS) historically only 2–6 months following CNS relapse.[1] Intensive CNS-penetrating regimens like MATRix improve outcomes but are toxic and less feasible in practice. R-IDARAM, combining rituximab, idarubicin, cytarabine, high-dose methotrexate, dexamethasone, and intrathecal therapy, offers effective CNS penetration with encouraging activity in small series.[2-4]
In prospective data, intensive approaches such as MARIETTA demonstrated 2-year PFS up to 70% in treatment-naïve SCNSL, but outcomes are markedly inferior in relapsed or concomitant disease, with 2-year PFS closer to 14–40%.[5] Long-term survival beyond 5 years remains rare outside selected transplant-eligible patients.

**Aims**

To report real-world outcomes with R-IDA-RAM in predominantly secondary CNS lymphoma and compare with published MARIETTA trial data.

**Methods**

We retrospectively analysed 10 patients with CNS lymphoma (9 secondary, 1 primary) treated with R-IDARAM [Fig. 1] between 2015 and 2025. Data collected included demographics, prior therapies, treatment cycles, and toxicities. Progression-free survival (PFS) and overall survival (OS) were estimated by the Kaplan–Meier method with 95% confidence intervals from bootstrap resampling, as of August 1, 2025.

**Results**

The median age at CNS lymphoma diagnosis was 69 years (range, 29–78), and 7 of 10 patients were male. Nine patients (90%) had SCNSL, and one had primary CNS lymphoma (PCNSL).
Patients received a median of three R-IDARAM cycles (range, 2–6). Among SCNSL patients, 78% (n=8) had received one prior regimen (R-CHOP), while 22% (n=2) had ≥3 lines (including R-ICE and R-DHAP). The PCNSL patient received frontline R-IDA-RAM followed by consolidative WBRT. One SCNSL patient also received consolidative WBRT.

Median PFS was 8 months (95% CI: 5–31) and median OS was 19 months (95% CI: 5–47). At 12 months, PFS and OS were 50% (95% CI: 20–80%); at 24 months, 30% (95% CI: 0–60%); and at 60 months, PFS was 10% (95% CI: 0–30%) and OS 15% (95% CI: 0–40%).
Two patients (20%) remain alive and progression-free at ~2.6 and ~5.7 years, paralleling prior R-IDARAM data. Toxicity was primarily hematologic and manageable; no significant long-term neurotoxicity was observed.

**Copyright Information:** (Online) ISSN: 2572-9241
©2026 The Author(s). HemaSphere is published by John Wiley & Sons Ltd on behalf of European Hematology Association. This is an open access Abstract Book distributed under the Attribution-NonCommercial-NoDerivs (CC BY-NC-ND), which allows third parties to download the articles and share them with others as long as they credit the author and the Abstract Book, but they cannot change the content in any way or use them commercially.
**Abstract Book Citations:** Authors, Title, HemaSphere, 2026;10:(S1):pages. The abstract book DOIs can be found on the "Author Information" page.
**Disclaimer:** Articles published in the journal HemaSphere exclusively reflect the opinions of the authors. The authors are responsible for all content in their abstracts including accuracy of the facts, statements, citing resources, etc.

**Summary/Conclusion**

In this real-world cohort of relapsed SCNSL, R-IDARAM achieved a median PFS of 8 months and median OS of 19 months, with a 2-year OS of 30% despite patients being older, heavily pretreated, and not undergoing transplantation.  While outcomes were somewhat lower than those reported in the MARIETTA trial (median age 58, intensive MATRix/R-ICE induction, and ASCT consolidation), a 20% long-term PFS plateau was observed, consistent with historical SCNSL patterns.
These findings support R-IDARAM as a feasible option in less fit or transplant-ineligible patients, though prospective studies integrating modern strategies (e.g., CAR-T, ASCT) are needed to optimize outcomes.

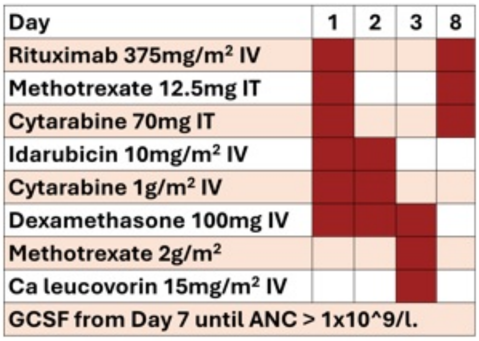

**Figure 1: R-IDA-RAM chemotherapy schedule*.**

** Treatment cycles are repeated every 21 days with interim reassessment after cycle two.*
** Up to four cycles may be administered depending on response and tolerability.*
** Cranial radiotherapy (40 Gy in 20 fractions) may also be delivered when feasible.*

**Copyright Information:** (Online) ISSN: 2572-9241
©2026 The Author(s). HemaSphere is published by John Wiley & Sons Ltd on behalf of European Hematology Association. This is an open access Abstract Book distributed under the Attribution-NonCommercial-NoDerivs (CC BY-NC-ND), which allows third parties to download the articles and share them with others as long as they credit the author and the Abstract Book, but they cannot change the content in any way or use them commercially.
**Abstract Book Citations:** Authors, Title, HemaSphere, 2026;10:(S1):pages. The abstract book DOIs can be found on the "Author Information" page.
**Disclaimer:** Articles published in the journal HemaSphere exclusively reflect the opinions of the authors. The authors are responsible for all content in their abstracts including accuracy of the facts, statements, citing resources, etc.

### (PB3813) PRIMARY CENTRAL NERVOUS SYSTEM LYMPHOMA IN LATIN AMERICA: REAL-WORLD CLINICOPATHOLOGICAL CHARACTERISTICS AND SURVIVAL OUTCOMES FROM A NATIONAL REFERENCE CENTER

**Topic:** 19. Large B cell lymphomas - Clinical

Luis Felipe Rubalcava Lara*[1], Amairani Carpintero-Díaz[1], Andy Mejía-Castro[1], Juan Reyes-Islas[1], Maria Belem Andrino-Ruiz[1], Abi Saucedo-Jardon[1], Myrna Candelaria Hernandez[1], Omar Eduardo Fernandez Vargas[1], Amaya Llorente-Chávez[1], Karla Sánchez Hernández[1], Irma Karen Pellon Tellez[1]

[1]*Instituto Nacional de Cancerología - México, Mexico City, Mexico;*

### Background

Primary central nervous system lymphoma (PCNSL) is a rare and aggressive extranodal non-Hodgkin lymphoma. Data from Latin American populations are scarce, particularly regarding real-world clinical presentation, treatment strategies, and survival outcomes.

### Aims

To describe the clinicopathological characteristics, treatment patterns, and survival outcomes of patients with PCNSL treated at a national cancer referral center in Mexico.

### Methods

We conducted a retrospective cohort study including patients diagnosed with PCNSL; **between 2010 to 2025.** Twenty-five patients were identified. Clinical, laboratory, pathological, treatment, and outcome data were collected. Overall survival (OS) was estimated using the Kaplan–Meier method, with median follow-up calculated using the inverse Kaplan–Meier approach.

**Copyright Information:** (Online) ISSN: 2572-9241
©2026 The Author(s). HemaSphere is published by John Wiley & Sons Ltd on behalf of European Hematology Association. This is an open access Abstract Book distributed under the Attribution-NonCommercial-NoDerivs (CC BY-NC-ND), which allows third parties to download the articles and share them with others as long as they credit the author and the Abstract Book, but they cannot change the content in any way or use them commercially.
**Abstract Book Citations:** Authors, Title, HemaSphere, 2026;10:(S1):pages. The abstract book DOIs can be found on the “Author Information” page.
**Disclaimer:** Articles published in the journal HemaSphere exclusively reflect the opinions of the authors. The authors are responsible for all content in their abstracts including accuracy of the facts, statements, citing resources, etc.

### Results

The mean age at diagnosis was 43.6 years (± SD 13.47 years); most of our patients were male (56%), and 56% had an ECOG performance status ≥2 at diagnosis. Seven patients (28%) were HIV-positive. Mean baseline laboratory values included hemoglobin 13.9 ± 2.47 g/dL, white blood cell counts 7.7 ± 2.91 $\times 10^9$/L, absolute neutrophil count 5.57 ± 2.86 $\times 10^9$/L, and platelet count 268.0 ± 75.89 $\times 10^9$/L.
Clinically, neurological symptoms were present at diagnosis in 96% of patients. Parenchymal cerebral involvement was the predominant site of disease in all but one patient, who presented with isolated spinal cord disease. Fifteen patients (60%) had isolated parenchymal disease, while 10 (40%) presented with combined parenchymal and leptomeningeal involvement; no cases of isolated leptomeningeal disease were identified.
Diagnostically, cerebrospinal fluid (CSF) analysis with flow cytometry, using CD20, CD10, CD5, BCL2, BCL6 and MYC was performed in 21 patients (84%), with 10 (47.6%) patients testing positive for infiltration. Histopathological confirmation was obtained in 24 patients, all showing diffuse large B-cell lymphoma histology; four cases (16.7%) were classified as germinal center subtype by the Hans algorithm. Eighty percent of cases exhibited a non–germinal center phenotype; with only 16.7% classified as germinal center subtype by the Hans algorithm.
One patient without an accessible lesion was diagnosed based on CSF flow cytometry.
All patients received treatment based on high-dose methotrexate, with dosing between 1.5 g/ and 3.5 g/. Fourteen patients (56%) received consolidation radiotherapy, with a median dose of 38 Gy (range, 20–50 Gy). Only one patient underwent autologous hematopoietic cell transplantation and remained in complete response at the last follow-up.
With a median follow-up of 30.1 months (95% CI, 13.9–66.5), median OS was not reached. Six- and twelve-month OS rates were 90.9% and 81.0%, respectively. Six deaths occurred: two due to infection, two due to disease progression, one due to hepatic failure, and one due to unrelated causes.

### Summary/Conclusion

In this real-world cohort, PCNSL predominantly affected younger patients and frequently presented with poor performance status and parenchymal brain involvement. Survival outcomes were comparable to international reports despite limited use of autologous transplantation, highlighting the need to optimize access to intensive consolidation strategies in resource-limited settings.

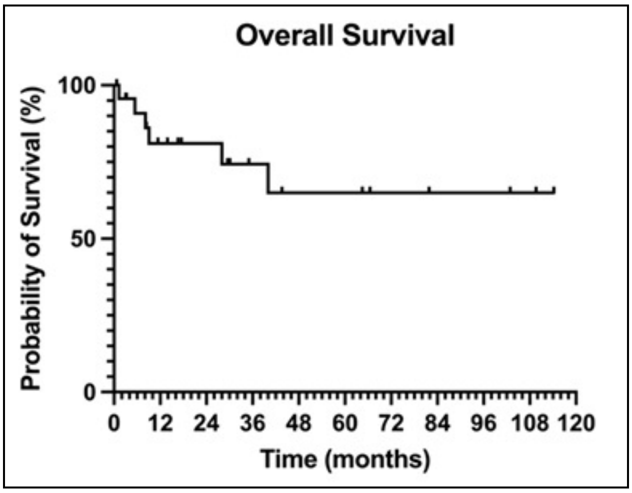

Overall survival of PCNSL

Copyright Information: (Online) ISSN: 2572-9241
©2026 The Author(s). HemaSphere is published by John Wiley & Sons Ltd on behalf of European Hematology Association. This is an open access Abstract Book distributed under the Attribution-NonCommercial-NoDerivs (CC BY-NC-ND), which allows third parties to download the articles and share them with others as long as they credit the author and the Abstract Book, but they cannot change the content in any way or use them commercially.
Abstract Book Citations: Authors, Title, HemaSphere, 2026;10:(S1):pages. The abstract book DOIs can be found on the "Author Information" page.
Disclaimer: Articles published in the journal HemaSphere exclusively reflect the opinions of the authors. The authors are responsible for all content in their abstracts including accuracy of the facts, statements, citing resources, etc.

### (PB3814) EFFICACY OF BTKI PLUS IMMUNOCHEMOTHERAPY IN HIGH-RISK DLBCL: A RETROSPECTIVE ANALYSIS

**Topic:** 19. Large B cell lymphomas - Clinical

Wenjin Li*[1], Lichan Xiao[1], Qiurong Liu[1]

[1]*Pingxiang People`s Hospital, Hematology, Pingxiang, China;*

### Background

Diffuse large B-cell lymphoma (DLBCL) is an aggressive lymphoma with high heterogeneity. High-risk DLBCL patients (e.g., those with advanced stage, high International Prognostic Index [IPI] scores, or elevated central nervous system [CNS] relapse risk) have poor outcomes with conventional immunochemotherapy. The combination of BTK inhibitors (BTKi) with immunochemotherapy represents a novel approach for high-risk DLBCL, but clinical data in this population are limited. This retrospective study by Professor Li Wenjin's team at Pingxiang People's Hospital evaluated the efficacy and survival of BTKi-based regimens in high-risk DLBCL patients.

### Aims

To retrospectively analyze the efficacy, survival, and clinical characteristics of high-risk DLBCL patients treated with BTKi combined with immunochemotherapy, and to assess the clinical value of this regimen in this setting.

### Methods

This retrospective study enrolled 23 high-risk DLBCL patients who received BTKi plus immunochemotherapy in the Hematology Department of Pingxiang People's Hospital from September 2020 to December 2025. High-risk was defined based on IPI, NCCN-IPI, or CNS-IPI scores. Baseline data, treatment regimens, efficacy, and survival outcomes were collected. Eighteen patients received orelabrutinib-based regimens and five received zanubrutinib-based regimens. Efficacy was assessed using the 2014 Lugano criteria (ORR, CR, DCR). Survival endpoints were PFS and OS.

**Copyright Information:** (Online) ISSN: 2572-9241
©2026 The Author(s). HemaSphere is published by John Wiley & Sons Ltd on behalf of European Hematology Association. This is an open access Abstract Book distributed under the Attribution-NonCommercial-NoDerivs (CC BY-NC-ND), which allows third parties to download the articles and share them with others as long as they credit the author and the Abstract Book, but they cannot change the content in any way or use them commercially.
**Abstract Book Citations:** Authors, Title, HemaSphere, 2026;10:(S1):pages. The abstract book DOIs can be found on the "Author Information" page.
**Disclaimer:** Articles published in the journal HemaSphere exclusively reflect the opinions of the authors. The authors are responsible for all content in their abstracts including accuracy of the facts, statements, citing resources, etc.

### Results

The cohort (median age 73 years, range 33–96; 56.5% male) exhibited prominent high-risk features: 82.6% had Ann Arbor stage III/IV, 43.5% had ECOG PS ≥2, 34.8% had B symptoms, 73.9% had extranodal involvement, 34.8% had bulky disease, and 21.7% had bone marrow involvement. Risk stratification: 73.9% were IPI high-intermediate/high, 86.4% (22 evaluable) were NCCN-IPI high-intermediate/high, and 52.2% were CNS-IPI high-intermediate/high. Molecularly, TP53 mutations were detected in 75.0% (12/16), and MYC/BCL-2 double expression in 59.1% (13/22).
The overall ORR was 78.3% (18/23, 95%CI: 56.3–92.5), with a CR rate of 60.9% (14/23, 95%CI: 38.5–80.3) and DCR of 87.0% (20/23, 95%CI: 66.4–97.2). Subgroup analysis: orelabrutinib group (n=18) showed ORR 77.8%, CR 61.1%, DCR 88.9%; zanubrutinib group (n=5) showed ORR 80.0%, CR 60.0%, DCR 80.0%. After a median follow-up of 36 months (range 7–58), median PFS was 43.0 months (95%CI: 8.0–NR), with 12- and 24-month PFS rates of 62.5% (95%CI: 43.8–89.1) and 56.2% (95%CI: 37.3–84.8). Median OS was 43.0 months (95%CI: 24.0–NR), with 12- and 24-month OS rates of 68.6% (95%CI: 50.6–93.0) and 62.4% (95%CI: 43.7–89.1, Table 1).

**Table 1. Efficacy Outcomes of BTKi Combined with Immunochemotherapy in High-Risk DLBCL Patients**

| Efficacy endpoints (n, %) | Overall (n=23) | Group A (n=18) | Group B (n=5) |
|---|---|---|---|
| ORR | 18 (78.3) | 14 (77.8) | 4 (80.0) |
| CR | 14 (60.9) | 11 (61.1) | 3 (60.0) |
| PR | 4 (17.4) | 3 (16.7) | 1 (20.0) |
| SD | 2 (8.7) | 2 (11.1) | 0 (0.0) |
| DCR | 20 (87.0) | 16 (88.9) | 4 (80.0) |
| PD | 1 (4.3) | 0 (0.0) | 1 (20.0) |
| NE | 2 (8.7) | 2 (11.1) | 0 (0.0) |
| ORR, 95% CI | 56.3–92.5 | 52.4–93.6 | 28.4–99.5 |
| CR, 95% CI | 38.5–80.3 | 35.7–82.7 | 14.7–94.7 |
| DCR, 95% CI | 66.4–97.2 | 65.3–98.6 | 28.4–99.5 |

Group A: Orelabrutinib-based regimen.
Group B: Zanubrutinib-based regimen.

### Summary/Conclusion

BTKi combined with immunochemotherapy demonstrates promising efficacy and favorable survival outcomes in high-risk DLBCL patients, with acceptable tolerability. This regimen may represent a valuable therapeutic option for this difficult-to-treat population. Prospective studies with larger cohorts are warranted to confirm these findings.

**Copyright Information:** (Online) ISSN: 2572-9241
©2026 The Author(s). HemaSphere is published by John Wiley & Sons Ltd on behalf of European Hematology Association. This is an open access Abstract Book distributed under the Attribution-NonCommercial-NoDerivs (CC BY-NC-ND), which allows third parties to download the articles and share them with others as long as they credit the author and the Abstract Book, but they cannot change the content in any way or use them commercially.
**Abstract Book Citations:** Authors, Title, HemaSphere, 2026;10:(S1):pages. The abstract book DOIs can be found on the "Author Information" page.
**Disclaimer:** Articles published in the journal HemaSphere exclusively reflect the opinions of the authors. The authors are responsible for all content in their abstracts including accuracy of the facts, statements, citing resources, etc.

### (PB3815) INITIAL OUTCOMES OF GLOFITAMAB-BASED REGIMENS IN RELAPSED OR REFRACTORY B-CELL LYMPHOMA: A REAL-WORLD ANALYSIS

**Topic:** 19. Large B cell lymphomas - Clinical

Lingke Zhang*[1], Wei Wan[1], Ping Yang[1], Hongmei Jing[1]

[1]*Peking University Third Hospital, Hematology, Beijing, China;*

### Background

Relapsed or refractory (R/R) diffuse large B-cell lymphoma (DLBCL) and high-grade B-cell lymphoma (HGBL) pose significant clinical challenges, with limited therapeutic options available. Bispecific antibodies, such as Glofitamab, have emerged as promising immunotherapeutic agents; however, real-world data on their efficacy across diverse patient populations remain limited.

### Aims

To evaluate the baseline clinical characteristics and treatment responses of patients with R/R B-cell lymphoma treated with Glofitamab-based regimens in a real-world setting.

### Methods

A retrospective analysis was performed on 26 patients with B-cell lymphoma (84.6% DLBCL, 11.5% HGBL, and 3.8% tFL-DLBCL). Baseline demographics, disease burden (Ann Arbor stage, LDH levels, bulky disease), and prior treatment histories were collected. Efficacy was assessed according to the best overall response.

### Results

The median age was 62 years (range: 22–79). Most patients presented with advanced-stage disease (Stage III–IV: 84.6%) and high risk (median IPI score: 3). Extranodal involvement was noted in 65.4% of cases, and 42.3% had bulky disease (≥7 cm). The cohort was heavily pre-treated, with a median of two prior lines of therapy (range: 1–4). Treatment regimens primarily comprised Glofitamab monotherapy or Glofitamab in combination with chemotherapy (e.g., Gemox, Pola-RCHP). Comorbidities were present in 96.2% of patients.
At analysis, the overall response rate (ORR) was 76.9% (20/26), with a high complete response rate (CRR/CMR) of 73.1% (19/26). Six patients (23.1%) experienced disease progression (PD) during follow-up. After a median follow-up of 10.2 months, neither median progression-free survival (PFS) nor median overall survival (OS) was reached (NR). The 12-month PFS rate was 81.7%, and the 12-month OS rate was 95.0%.
**Table: Baseline Characteristics of Patients (N=26)**

**Copyright Information:** (Online) ISSN: 2572-9241
©2026 The Author(s). HemaSphere is published by John Wiley & Sons Ltd on behalf of European Hematology Association. This is an open access Abstract Book distributed under the Attribution-NonCommercial-NoDerivs (CC BY-NC-ND), which allows third parties to download the articles and share them with others as long as they credit the author and the Abstract Book, but they cannot change the content in any way or use them commercially.
**Abstract Book Citations:** Authors, Title, HemaSphere, 2026;10:(S1):pages. The abstract book DOIs can be found on the “Author Information” page.
**Disclaimer:** Articles published in the journal HemaSphere exclusively reflect the opinions of the authors. The authors are responsible for all content in their abstracts including accuracy of the facts, statements, citing resources, etc.

| Characteristics | | Median (Range) / N | Percentage (%) |
|---|---|---|---|
| Age (years) | | 62.0 (22 - 79) | |
| Gender (Male/Female) | Male | 10 | 38.5% |
| | Female | 16 | 61.5% |
| Diagnosis | DLBCL | 22 | 84.6% |
| | HGBL (High-grade B-cell lymphoma) | 3 | 11.5% |
| | tFL-DLBCL | 1 | 3.8% |
| ECOG performance status | 0-1 | 23 | 88.5% |
| | 2-4 | 3 | 11.5% |
| Ann Arbor Stage | I-II | 3 | 11.5% |
| | III-IV | 22 | 84.6% |
| | Unknown | 1 | 3.8% |
| Prior lines of therapy | | 2 (1 - 4) | |
| Bulky disease (≥7cm) | Yes | 11 | 42.3% |
| | No | 10 | 38.5% |
| | Unknown | 5 | 19.2% |
| Extranodal involvement | Yes | 17 | 65.4% |
| | No | 2 | 7.7% |
| | Unknown | 7 | 26.9% |
| IPI Score | 1 | 3 | 11.5% |
| | 2 | 5 | 19.2% |
| | 3 | 8 | 30.8% |
| | 4-5 | 10 | 38.5% |
| LDH (U/L) | Normal | 12 | 46.2% |
| | Elevated | 14 | 53.8% |
| Comorbidities | Yes | 25 | 96.2% |
| | No | 1 | 3.8% |

### Summary/Conclusion

Glofitamab-based regimens demonstrate potent clinical activity in patients with high-risk, advanced-stage R/R B-cell lymphoma The high complete response rate (73.1%) in this heavily pre-treated real-world cohort indicates that Glofitamab is a highly effective salvage option, even for patients who have failed multiple prior lines of chemoimmunotherapy and those with comorbidities. Overall security is manageable, the most frequently observed adverse event was CRS, while infections necessitate careful monitoring.

**Copyright Information:** (Online) ISSN: 2572-9241
©2026 The Author(s). HemaSphere is published by John Wiley & Sons Ltd on behalf of European Hematology Association. This is an open access Abstract Book distributed under the Attribution-NonCommercial-NoDerivs (CC BY-NC-ND), which allows third parties to download the articles and share them with others as long as they credit the author and the Abstract Book, but they cannot change the content in any way or use them commercially.
**Abstract Book Citations:** Authors, Title, HemaSphere, 2026;10:(S1):pages. The abstract book DOIs can be found on the “Author Information” page.
**Disclaimer:** Articles published in the journal HemaSphere exclusively reflect the opinions of the authors. The authors are responsible for all content in their abstracts including accuracy of the facts, statements, citing resources, etc.

### (PB3816) CLINICAL CHARACTERISTICS AND TREATMENT ANALYSIS OF 7 CASES OF INTRAVASCULAR LARGE B-CELL LYMPHOMA

**Topic:** 19. Large B cell lymphomas - Clinical

Yuanbo Liu[1], Xiaoli Yuan*[2, 2]

*[1]Henan Provincial People's Hospital，People's Hospital of Zhengzhou University, Zhengzhou City,Henan Province, China;[2]Henan Provincial People's Hospital, Zhengzhou City,Henan Province，People's Hospital of Zhengzhou University, China;*

### Background

Intravascular large B-cell lymphoma is a rare type of lymphoma. It progresses rapidly and has a poor prognosis for patients. Currently, its treatment still follows the non-specific type of Diffuse Large B-cell Lymphoma, but the outcome is poor.

### Aims

To investigate the clinical characteristics of patients with intravascular large B-cell lymphoma

### Methods

A retrospective analysis was conducted on 7 patients with intravascular large-B-cell lymphoma admitted to the People's Hospital of Henan Province (People's Hospital of Zhengzhou University) from January 2017 to January 2026. Clinical data, treatment outcomes, and clinical outcomes were collected, and the clinical characteristics of the patients were analyzed.

**Copyright Information:** (Online) ISSN: 2572-9241
©2026 The Author(s). HemaSphere is published by John Wiley & Sons Ltd on behalf of European Hematology Association. This is an open access Abstract Book distributed under the Attribution-NonCommercial-NoDerivs (CC BY-NC-ND), which allows third parties to download the articles and share them with others as long as they credit the author and the Abstract Book, but they cannot change the content in any way or use them commercially.
**Abstract Book Citations:** Authors, Title, HemaSphere, 2026;10:(S1):pages. The abstract book DOIs can be found on the "Author Information" page.
**Disclaimer:** Articles published in the journal HemaSphere exclusively reflect the opinions of the authors. The authors are responsible for all content in their abstracts including accuracy of the facts, statements, citing resources, etc.

### Results

Of the 7 patients, 3 were male and 4 were female, with a median age of 72 years (43-76 years) at the time of diagnosis. All patients had Ann Arbor stage IV disease, with 4 patients exhibiting B symptoms. Lactate dehydrogenase levels were elevated in 7 patients, and the IPI scores were all ≥3. All patients primarily exhibited extranodal involvement. FISH testing was performed in 4 cases, and 1 patient showed a positive C-MYC break. Six patients received immunochemotherapy based on CD20 monoclonal antibodies for the first 4 cycles. As of February 2026, the median follow-up time was 10 months (1-45 months), with 2 patients (2/6) achieving complete remission (CR), 1 patient (1/6) achieving stable disease (SD), and 3 patients (3/6) experiencing progressive disease (PD). CR and SD patients received Pola-R-CHP-like regimens. All 3 PD patients discontinued treatment.

| Case No. | Age (y⊠ | Gender | Diagnosis Date | Type | FISH | Extranodal Involvement | Treatment Regimen | Assessment after 4 cycles |
|---|---|---|---|---|---|---|---|---|
| Case 1 | 72 | Female | February 2017 | GCB | / | Skin | Untreated | / |
| Case 2 | 76 | Male | May 2022 | GCB | / | Stomach, Muscle, Bone, CNS | R-CHOP × 4 | PD |
| Case 3 | 75 | Female | June 2022 | GCB | / | Liver, skin | R-CHOP × 4 | PD |
| Case 4 | 76 | Male | April 2025 | nonGCB | CMYC positive | Prostate | PolaRCHP + Z×4;<br>PolaRCHP + Z× 2,<br>ZR + MTX + Z× 2 | CR |
| Case 5 | 74 | Female | July 2025 | nonGCB | Negative | Bone marrow | PolaRCHP × 4<br>PolaRCHP × 2<br>R + MTX × 2 | CR |
| Case 6 | 66 | Female | December 2025 | nonGCB | Negative | Adrenal gland, Bone | R-CHOP × 4 | PD |
| Case 7 | 43 | Male | January 2026 | nonGCB | Negative | CNS | PolaRCHP × 1 | SD |

**Note: R-CHOP Rituximab + Cyclophosphamide + Doxorubicin + Vincristine + Prednisone; Pola+R-CHP = Polatuzumab + Rituximab + Cyclophoshamide + Doxorubicin + Prednisone; Z = Zanubrutinib; MTX = Methotrexate; FISH = Fluorescence in Situ Hybridization; CR = Complete Desponse; SD = Stable Disease; PD = Progressive Disease.**

### Summary/Conclusion

Intravascular large B-cell lymphoma mainly present withextranodal organ involvement as the primary manifestation. The disease progresses rapidly, and the prognosis is poor. Patients do not respond well to R-CHOP regimens. Novel targeted immunotherapy agents, such as vibostolimab and zebinutuzumab, may potentially improve patient outcomes.

**Copyright Information:** (Online) ISSN: 2572-9241
©2026 The Author(s). HemaSphere is published by John Wiley & Sons Ltd on behalf of European Hematology Association. This is an open access Abstract Book distributed under the Attribution-NonCommercial-NoDerivs (CC BY-NC-ND), which allows third parties to download the articles and share them with others as long as they credit the author and the Abstract Book, but they cannot change the content in any way or use them commercially.
**Abstract Book Citations:** Authors, Title, HemaSphere, 2026;10:(S1):pages. The abstract book DOIs can be found on the "Author Information" page.
**Disclaimer:** Articles published in the journal HemaSphere exclusively reflect the opinions of the authors. The authors are responsible for all content in their abstracts including accuracy of the facts, statements, citing resources, etc.

### (PB3817) REAL-WORLD ANALYSIS OF AXICABTAGENE CILOLEUCEL (AXI-CEL) AS FIRST-LINE CONSOLIDATION THERAPY FOR B-CELL LYMPHOMAS WITH HIGH-RISK FACTORS A SINGLE-CENTER EXPERIENCE

**Topic:** 19. Large B cell lymphomas - Clinical

Jieming Zhang*[1], Mengjie Ding[1], Feifei Nan[1], Yu Chang[1], Lei Zhang[1], Mingzhi Zhang[1], Xudong Zhang[1]

[1]*First Affiliated Hospital of Zhengzhou University, Zhengzhou, China;*

### Background

Approximately 60% of patients with large B-cell lymphoma (LBCL) achieve cure with standard first-line (1L) therapies. However, 10% of patients remain refractory to 1L treatment, and 30% of responders experienced relapse within two years. Axicabtagene ciloleucel (axi-cel), an autologous anti-CD19 chimeric antigen receptor (CAR) T cell therapy, is approved for the treatment of patients with relapsed/refractory LBCL.

### Aims

This study aims to explore the efficacy and safety of axi-cel as consolidation in 1L treatment responders with high risk of relapse.

### Methods

This real-world case series study enrolled patients with B-cell lymphoma, who received axi-cel as consolidation therapy after achieving CR or partial response (PR) to 1L treatment. Efficacy endpoints included complete metabolic response (CMR), 18-month overall survival (OS) rate, and progression-free survival (PFS) rate. Safety endpoints comprised the incidence of adverse events (AEs) including cytokine release syndrome (CRS), leukopenia, thrombocytopenia, and abnormal liver function. Additionally, this study explored the impact of post-infusion maintenance therapy on CAR-T cell expansion.

### Results

A total of 5 patients were enrolled, with a median age of 66 years and a male-to-female ratio of 3:2. The lymphoma subtypes included 2 cases of non germinal center B-cell-like DLBCL (DLBCL-non-GCB), 2 cases of germinal center B-cell-like DLBCL (DLBCL-GCB), and 1 case of mantle cell lymphoma (MCL). DLBCL patients had at least one of the following high-risk factors: International Prognostic Index (IPI) score≥4 /age-adjusted IPI (aaIPI) ≥2, double expression, or Ki-67 >90%. High-risk features in the MCL patient included advanced age and Ki-67 >30%. All 4 DLBCL patients received R-CHOP or R-CHOP-like regimens as 1L therapy, while the MCL patient received bendamustine and rituximab (BR) . Before lymphodepletion, 2 patients had achieved CR and 3 achieved PR. With a median follow-up of 21 months since infusion, the best CMR rate was 100 %, and all patients achieved CMR within one month after infusion. The 18-month OS and PFS rates were both 100%. The median peak CAR-T cell counts were 74.65 cells/μL, and the median CAR-T cell counts at 30 days post-infusion were 7.26 cells/μL. Following infusion, 3 patients received IL-2 monotherapy (n=1) or PD-1 inhibitor combined with IL-2 (n=2). At 6 months post-infusion, CAR-T cells were detectable in the peripheral blood of all 3 treated patients, whereas only one of the 2 untreated patients had detectable CAR-T cells. CRS occurred in 100% of patients (including 1 case of capillary leak syndrome), with no grade ≥3 CRS and no incidence of immune effector cell-associated neurotoxicity syndrome (ICANS). The median time from CAR-T cell infusion to the onset of CRS was 4 days, and the median time from CRS onset to resolution was 3 days. After infusion, the incidence rates of grade ≥3 leukopenia, grade ≥3 thrombocytopenia, and liver dysfunction were 60%, 20%, and 60%, respectively.

**Copyright Information:** (Online) ISSN: 2572-9241
©2026 The Author(s). HemaSphere is published by John Wiley & Sons Ltd on behalf of European Hematology Association. This is an open access Abstract Book distributed under the Attribution-NonCommercial-NoDerivs (CC BY-NC-ND), which allows third parties to download the articles and share them with others as long as they credit the author and the Abstract Book, but they cannot change the content in any way or use them commercially.
**Abstract Book Citations:** Authors, Title, HemaSphere, 2026;10:(S1):pages. The abstract book DOIs can be found on the "Author Information" page.
**Disclaimer:** Articles published in the journal HemaSphere exclusively reflect the opinions of the authors. The authors are responsible for all content in their abstracts including accuracy of the facts, statements, citing resources, etc.

**Summary/Conclusion**

Consolidation therapy with CAR-T following 1L treatment demonstrated encouraging rates of CMR, PFS, and OS in B-cell lymphoma patients with high risk of relapse. The safety profile was manageable. Administration of PD-1 inhibitors and/or IL-2 post-infusion may support sustained expansion and persistence of CAR-T cells.

**Copyright Information:** (Online) ISSN: 2572-9241
©2026 The Author(s). HemaSphere is published by John Wiley & Sons Ltd on behalf of European Hematology Association. This is an open access Abstract Book distributed under the Attribution-NonCommercial-NoDerivs (CC BY-NC-ND), which allows third parties to download the articles and share them with others as long as they credit the author and the Abstract Book, but they cannot change the content in any way or use them commercially.
**Abstract Book Citations:** Authors, Title, HemaSphere, 2026;10:(S1):pages. The abstract book DOIs can be found on the "Author Information" page.
**Disclaimer:** Articles published in the journal HemaSphere exclusively reflect the opinions of the authors. The authors are responsible for all content in their abstracts including accuracy of the facts, statements, citing resources, etc.

### (PB3818) REAL-WORLD OUTCOMES OF SECOND-LINE AXICABTAGENE CILOLEUCEL (AXI-CEL) THERAPY IN PATIENTS WITH RELAPSED OR REFRACTORY LARGE B-CELL LYMPHOMA IN A CANADIAN CENTRE

**Topic:** 19. Large B cell lymphomas - Clinical

Catherine Sohier-Poirier*[1], Christopher Lemieux[1]

[1]*CHU de Québec-Université Laval, Québec, Canada;*

### Background

CD19-targeted chimeric antigen receptor (CAR) T-cell therapy has become standard of care second-line treatment for relapsed or refractory large B-cell lymphoma (R/R LBCL) in eligible patients. However, there are limited studies reporting real-world outcomes within a universal health care system in the second-line setting.

### Aims

The objective of this study was to assess real-world efficacy and safety of Axi-cel as second-line therapy for R/R LBCL.

### Methods

We retrospectively analyzed data for all adult patients with R/R LBCL who received Axi-cel infusion as second-line therapy at our centre between July 2023 and November 2025. Response was assessed by positron emission tomography-computed tomography (PET-CT) according to Lugano criteria at one month and three months post-infusion. Cytokine release syndrome (CRS) and immune effector cell-associated neurotoxicity syndrome (ICANS) were evaluated according to American Society for Transplantation and Cellular Therapy (ASTCT) guidelines.

### Results

Forty patients who received Axi-cel as second-line therapy were included in this analysis. Most patients had diffuse large B-cell lymphoma (DLBCL) (73%), followed by high-grade B-cell lymphoma with MYC and BCL2 rearrangements (10%). CAR-T was administered for primary refractory disease in 73% of the cohort. Median age was 63 years old at time of CAR-T infusion. Twenty-nine (73%) patients received bridging therapy prior to Axi-cel. Bridging therapy consisted of chemoimmunotherapy (n = 15, 52%), radiation (n = 10, 34%) or steroids (n = 4, 14%). Four patients did not have repeat PET-CT prior to CAR-T infusion to assess response to bridging therapy. Twelve (48%) patients responded to bridging therapy, consisting mainly of partial metabolic responses (PMR) (n = 11, 44%).
Median follow-up was 12 months. Following CAR-T infusion, 80% of patients obtained a response, with 70% of patients achieving a complete metabolic response (CMR). Most patients who had a PMR at initial assessment one month after Axi-cel (n = 9) eventually obtained a CMR (n = 5, 56%). Of the four patients whose best response was a PMR, three relapsed in the following months and one has not relapsed with two years of follow-up. Median progression-free survival (PFS) was 17 months. Median overall survival (OS) was not reached. At the time of this analysis, most patients in the cohort were still alive (n = 28, 70%) and had not relapsed (n = 23, 58%). The most common cause of death was progressive disease or relapse (n = 7, 58%). Only one patient died in remission.
All patients developed CRS with three (7.5%) patients experiencing grade ≥ 3 CRS. Twenty-six (65%) patients developed ICANS with eight (20%) patients experiencing grade ≥ 3 ICANS. Age, bridging therapy, bulky disease or refractory status did not impact incidence of CRS or ICANS. Eighteen (45%) patients had prolonged grade ≥ 3 cytopenias between 30 and 90 days after infusion. Eight (23%) patients had late grade ≥ 3 cytopenias persisting more than 90 days after CAR-T infusion.

**Copyright Information:** (Online) ISSN: 2572-9241
©2026 The Author(s). HemaSphere is published by John Wiley & Sons Ltd on behalf of European Hematology Association. This is an open access Abstract Book distributed under the Attribution-NonCommercial-NoDerivs (CC BY-NC-ND), which allows third parties to download the articles and share them with others as long as they credit the author and the Abstract Book, but they cannot change the content in any way or use them commercially.
**Abstract Book Citations:** Authors, Title, HemaSphere, 2026;10:(S1):pages. The abstract book DOIs can be found on the "Author Information" page.
**Disclaimer:** Articles published in the journal HemaSphere exclusively reflect the opinions of the authors. The authors are responsible for all content in their abstracts including accuracy of the facts, statements, citing resources, etc.

**Summary/Conclusion**

In this single-centre retrospective study, response rates, PFS and toxicities were consistent with results obtained in the pivotal ZUMA-7 trial. These findings add to the growing evidence confirming the efficacy and safety of Axi-cel as second-line therapy for R/R LBCL in the real-world setting.

**Copyright Information:** (Online) ISSN: 2572-9241
©2026 The Author(s). HemaSphere is published by John Wiley & Sons Ltd on behalf of European Hematology Association. This is an open access Abstract Book distributed under the Attribution-NonCommercial-NoDerivs (CC BY-NC-ND), which allows third parties to download the articles and share them with others as long as they credit the author and the Abstract Book, but they cannot change the content in any way or use them commercially.
**Abstract Book Citations:** Authors, Title, HemaSphere, 2026;10:(S1):pages. The abstract book DOIs can be found on the "Author Information" page.
**Disclaimer:** Articles published in the journal HemaSphere exclusively reflect the opinions of the authors. The authors are responsible for all content in their abstracts including accuracy of the facts, statements, citing resources, etc.

### (PB3819) COMPARATIVE PATIENT-REPORTED OUTCOMES IN RELAPSED/REFRACTORY DIFFUSE LARGE B-CELL LYMPHOMA TREATED WITH BISPECIFIC ANTIBODIES VERSUS CHEMOTHERAPY: A STRUCTURED REVIEW OF PUBLISHED EVIDENCE

**Topic:** 19. Large B cell lymphomas - Clinical

Lin Alokeh*[1]

[1]*Lotus Med, Hematology, București, Romania;*

### Background

Patients with relapsed/refractory diffuse large B-cell lymphoma (R/R DLBCL) have limited therapeutic options, high symptom burden, and impaired health-related quality of life (HRQoL). Recently, CD20×CD3 bispecific antibodies, including epcoritamab and glofitamab, have demonstrated promising efficacy in heavily pretreated populations and transplant-ineligible patients, with emerging data on patient-reported outcomes and quality of life compared with conventional chemotherapy regimens.

### Aims

To compare patient-reported outcomes, HRQoL, and clinical outcomes in R/R DLBCL patients treated with bispecific antibodies versus chemotherapy based on available clinical trials and real-world evidence.

### Methods

We conducted a literature-based qualitative synthesis of published clinical trials and real-world studies reporting patient-reported outcomes in adult patients with R/R DLBCL. A total of 8 articles were included, of which 4 were clinical studies evaluating bispecific antibodies, and the remaining 4 reported outcomes from chemotherapy-based regimens.  Data extraction focused on overall response rates (ORR), survival outcomes, symptom burden, and PRO measures. Reported PROs were assessed using validated instruments, primarily EORTC QLQ-C30 and EQ-5D-5L, as described in the included studies, without primary patient-level data collection

### Results

Bispecific antibodies demonstrated favorable overall and complete response rates with durable clinical responses in heavily pretreated R/R DLBCL populations. Evidence from clinical trials showed improved overall survival with glofitamab plus GemOx compared with chemotherapy. Reported patient-reported outcomes indicated maintenance or improvement in HRQoL, functional status, and symptom burden with bispecific antibodies. In contrast, chemotherapy-based regimens were associated with transient deterioration in quality of life and higher treatment-related burden.

### Summary/Conclusion

Available evidence suggests that bispecific antibodies provide meaningful clinical benefit with favorable patient-reported outcomes compared with chemotherapy in R/R DLBCL. Literature synthesis indicates consistent HRQoL preservation and symptom improvement with bispecific therapies in heavily pretreated and transplant-ineligible patients. Further prospective head-to-head studies using standardized patient-reported outcome instruments are warranted to strengthen comparative evidence.

**Copyright Information:** (Online) ISSN: 2572-9241
©2026 The Author(s). HemaSphere is published by John Wiley & Sons Ltd on behalf of European Hematology Association. This is an open access Abstract Book distributed under the Attribution-NonCommercial-NoDerivs (CC BY-NC-ND), which allows third parties to download the articles and share them with others as long as they credit the author and the Abstract Book, but they cannot change the content in any way or use them commercially.
**Abstract Book Citations:** Authors, Title, HemaSphere, 2026;10:(S1):pages. The abstract book DOIs can be found on the "Author Information" page.
**Disclaimer:** Articles published in the journal HemaSphere exclusively reflect the opinions of the authors. The authors are responsible for all content in their abstracts including accuracy of the facts, statements, citing resources, etc.

### (PB3820) A PROSPECTIVE, CONTROLLED STUDY OF ORELABRUTINIB COMBINED WITH POLA-R VERSUS POLA-R-MINICHP IN THE FIRST-LINE TREATMENT OF ELDERLY PATIENTS WITH DIFFUSE LARGE B-CELL LYMPHOMA

**Topic:** 19. Large B cell lymphomas - Clinical

Xing Zhong*[1], Wuping Li[1], Zhiqiang Peng[1], Nasha Yu[1], Weiming Zhang[1], Xiangxiang Song[1], Qiang Ding[1], Yan Huang[1]

*[1]Jiangxi Cancer Hospital, The Second Affiliated Hospital of Nanchang Medical College, Departments of Lymphatic and Hematological Oncology, Nanchang, China;*

### Background

Diffuse large B-cell lymphoma (DLBCL) is a highly aggressive subtype of non-Hodgkin lymphoma in adults, with significant prognostic variability among patients. For elderly patients, those with advanced-stage disease, high-risk molecular features, or extranodal involvement, conventional chemotherapy regimens demonstrate limited efficacy and are often poorly tolerated. While the Pola-R-CHP regimen has shifted the treatment paradigm for first-line DLBCL, chemotherapy tolerability remains a challenge, particularly in the elderly population. As key targeted therapies, Bruton's tyrosine kinase inhibitors (BTKi) have led to combination treatment models that overcome some limitations of traditional therapy, offering new avenues for treatment, especially for elderly DLBCL patients. Objective: This study aims to investigate the clinical value of the novel BTKi orelabrutinib in combination with Pola-R for the treatment of elderly patients with DLBCL, thereby providing additional practical insights for individualized clinical management of DLBCL.

### Methods

This is a prospective, multicenter, single-arm study utilizing a historical control design. The study enrolls elderly patients with previously untreated DLBCL. Patients in the experimental group receive 6 cycles of orelabrutinib in combination with Pola-R, followed by 2 cycles of rituximab monotherapy. Patients achieving at least a partial response (PR) subsequently receive 6 months of orelabrutinib maintenance monotherapy. Efficacy is assessed using the 2014 Lugano criteria. The primary endpoint is the complete remission rate (CRR) at the end of induction therapy. Secondary endpoints include objective response rate (ORR), disease control rate (DCR), progression-free survival (PFS), overall survival (OS), and safety profile.

**Copyright Information:** (Online) ISSN: 2572-9241
©2026 The Author(s). HemaSphere is published by John Wiley & Sons Ltd on behalf of European Hematology Association. This is an open access Abstract Book distributed under the Attribution-NonCommercial-NoDerivs (CC BY-NC-ND), which allows third parties to download the articles and share them with others as long as they credit the author and the Abstract Book, but they cannot change the content in any way or use them commercially.
**Abstract Book Citations:** Authors, Title, HemaSphere, 2026;10:(S1):pages. The abstract book DOIs can be found on the "Author Information" page.
**Disclaimer:** Articles published in the journal HemaSphere exclusively reflect the opinions of the authors. The authors are responsible for all content in their abstracts including accuracy of the facts, statements, citing resources, etc.

### Results

As of February 2026, a total of 7 patients with DLBCL have been enrolled in this study. The age range is 79 to 87 years, with both mean and median age of 83 years. The cohort includes 3 males (42.9%) and 4 females (57.1%), with a male-to-female ratio of 0.75:1. Regarding disease stage, 5 patients (71.4%) had Ann Arbor Stage III/IV disease; none presented with B symptoms. Extranodal involvement was observed in 3 patients (42.9%), primarily affecting the gastrointestinal tract, pancreas, and kidney. Three patients presented with bulky disease, and one patient had bone marrow (BM) involvement. Efficacy analysis revealed an overall response rate (ORR) of 71.4% (5/7, 95% CI: 29.0–96.3). The complete remission (CR) rate was 42.9% (3/7, 95% CI: 9.9–81.6), and the partial remission (PR) rate was 28.6% (2/7). One patient (14.3%) achieved stable disease (SD) as the best response. The disease control rate (DCR) was 71.4% (5/7, 95% CI: 29.0–96.3). Progressive disease (PD) was observed in one patient (14.3%), and response was not evaluable (NE) in one patient (14.3%). Median PFS and median OS were not reached. Regarding safety, the incidence of adverse events was low, and all were Grade 1 in severity; these included pneumonia in 3 patients and bleeding in 1 patient.

| | **Overall(n=7)** |
|---|---|
| ORR | 5 (71.4, 29.0-96.3) |
| CR | 3 (42.9, 9.9-81.6) |
| PR | 2 (28.6) |
| SD | 0 (0.0) |
| DCR | 5 (71.4, 29.0-96.3) |
| PD | 1 (14.3) |
| NE | 1 (14.3) |

### Summary/Conclusion

The combination of orelabrutinib and Pola-R demonstrates promising efficacy and a favorable safety profile in the first-line treatment of elderly patients with DLBCL. This regimen could be considered a valuable clinical treatment option for those who are unfit for or unwilling to receive conventional chemotherapy.

**Copyright Information:** (Online) ISSN: 2572-9241
©2026 The Author(s). HemaSphere is published by John Wiley & Sons Ltd on behalf of European Hematology Association. This is an open access Abstract Book distributed under the Attribution-NonCommercial-NoDerivs (CC BY-NC-ND), which allows third parties to download the articles and share them with others as long as they credit the author and the Abstract Book, but they cannot change the content in any way or use them commercially.
**Abstract Book Citations:** Authors, Title, HemaSphere, 2026;10:(S1):pages. The abstract book DOIs can be found on the "Author Information" page.
**Disclaimer:** Articles published in the journal HemaSphere exclusively reflect the opinions of the authors. The authors are responsible for all content in their abstracts including accuracy of the facts, statements, citing resources, etc.

### (PB3821) CLINICAL PRACTICE PATTERNS AND UNMET NEEDS IN DLBCL MANAGEMENT IN EGYPT: RESULTS OF A NATIONWIDE CROSS-SECTIONAL SURVEY

**Topic:** 19. Large B cell lymphomas - Clinical

Mervat Mattar[1], Ashraf Elghandour[2], Gamal Eldin Fathy[3], Manal Elsorady[2], Mohamed Abdelkader Morad[4], Mohamed Kaooh[5], Mohamed Khattab[6], Mohamed Moussa[7], Nourhan Badr[6], Rafaat Abdelfattah[8], Rania Afifi[9], Mohamed Samra*[10]

*[1]Professor of Hematology, Cairo University, Cairo, Egypt; [2]Professor of Hematology and Bone Marrow Transplant, Alexandria University, Alexandria, Egypt; [3]Consultant of hematology and Bone marrow transplantation, Nasser Institute, Cairo, Egypt; [4]Clinical Hematology Unit, Internal Medicine Department, Kasr Alainy, Faculty of Medicine, Cairo University, Cairo, Egypt; [5]Medical Lead, AstraZeneca Company, Cairo, Egypt; [6]Medical Advisor, AstraZeneca Company, Cairo, Egypt; [7]Professor of Hematology and Bone marrow transplantation, Ain Shams University, Cairo, Egypt; [8]Consultant of hematology and Bone marrow transplantation, Air force hospital, Cairo, Egypt; [9]Professor of Hematology, Helwan University, Cairo, Egypt; [10]Professor of Medical Oncology and Hematology, National Cancer Institute, Cairo, Egypt;*

### Background

Diffuse large B-cell lymphoma (DLBCL) is the most common subtype of non-Hodgkin lymphoma (NHL), representing 25-30% of all NHL cases worldwide. Despite the heterogeneity of DLBCL and the availability of targeted therapies and CAR T-cell therapy, data on treatment practices, outcomes, and obstacles in middle-income countries remain limited.

### Aims

This is the first nationwide, cross-sectional survey-based study aiming to reflect real-world practices, treatment patterns, diagnostic utilization, and barriers in the management of DLBCL across all healthcare sectors in Egypt.

### Methods

A nationwide survey was conducted between December 2025 and February 2026, targeting hematologists and medical oncologists across university hospitals, insurance facilities, and private practice settings in Egypt. The 22-item questionnaire addressed the number of DLBCL cases per month, referral pathways, subtype testing and prevalence, risk stratification, treatment modalities, and barriers to optimal management. The survey was anonymous, and no identifiable data was collected.

**Copyright Information:** (Online) ISSN: 2572-9241
©2026 The Author(s). HemaSphere is published by John Wiley & Sons Ltd on behalf of European Hematology Association. This is an open access Abstract Book distributed under the Attribution-NonCommercial-NoDerivs (CC BY-NC-ND), which allows third parties to download the articles and share them with others as long as they credit the author and the Abstract Book, but they cannot change the content in any way or use them commercially.
**Abstract Book Citations:** Authors, Title, HemaSphere, 2026;10:(S1):pages. The abstract book DOIs can be found on the “Author Information” page.
**Disclaimer:** Articles published in the journal HemaSphere exclusively reflect the opinions of the authors. The authors are responsible for all content in their abstracts including accuracy of the facts, statements, citing resources, etc.

**Results**

A total of 82 physicians with extensive experience in DLBCL management completed the survey, with 68% reported managing 1–7 new DLBCL cases per month. Internal Medicine and General Practice were the most common referral sources. Around 45% of physicians indicated that 25-50% of DLBCL cases involve young, fit patients, while most of them (79%) reported that >25% of the cases are old, frail patients. Cell-of-origin (COO) testing was never or rarely performed by 71%; the leading reason for non-use was that the first-line therapy is standardized across all centers, regardless of disease subtype. Among physicians conducting COO testing, two-thirds estimated that non-GCB DLBCL accounts for about 40% of the cases, and GCB DLBCL cases are approximately 60%. Additionally, half of them reported that ABC DLBCL accounts for ≥70% of cases. For patients with non-GCB DLBCL, R-CHOP was the preferred first-line (1L) therapy because most physicians consider all patients eligible for it. Around 18% reported using Pola-R-CHP for up to 10% of their patients. R-DHAP was the primary second-line option for 67% of participants, followed by R-ICE (57%). About 68% mentioned salvage chemotherapy as the most used third-line therapy or beyond and some of them indicated that bispecifics may be a good third-line option. Half of the participants mentioned that more than 50% of the patients experience relapse after the 1L therapy, and a majority reported that relapse takes >12 months. Around a third of the physicians believed that management would not differ by disease subtype due to limited treatment options. The most common obstacles to achieving optimal management were access to novel therapies (73%), financial barriers (39%), and diagnostic limitations (37%).

**Summary/Conclusion**

This is the first study to provide insights into DLBCL management in real-world clinical settings across Egypt, as well as diagnostic and therapeutic gaps. Despite the high prevalence of non-GCB DLBCL, which is more aggressive with a high relapse rate, as well as the existence of international guideline recommendations for risk-adapted treatment, the majority of Egyptian DLBCL patients are managed with the same approach. This is driven by limited integration of COO testing and access to novel therapies. These findings call for expanded access to advanced diagnostics and innovative treatments to bridge the gap between guideline recommendations and clinical practice.

**Copyright Information:** (Online) ISSN: 2572-9241
©2026 The Author(s). HemaSphere is published by John Wiley & Sons Ltd on behalf of European Hematology Association. This is an open access Abstract Book distributed under the Attribution-NonCommercial-NoDerivs (CC BY-NC-ND), which allows third parties to download the articles and share them with others as long as they credit the author and the Abstract Book, but they cannot change the content in any way or use them commercially.
**Abstract Book Citations:** Authors, Title, HemaSphere, 2026;10:(S1):pages. The abstract book DOIs can be found on the "Author Information" page.
**Disclaimer:** Articles published in the journal HemaSphere exclusively reflect the opinions of the authors. The authors are responsible for all content in their abstracts including accuracy of the facts, statements, citing resources, etc.

### (PB3822) REAL-LIFE EXPERIENCE WITH THE POLA-R-CHP REGIMEN IN DIFFUSE LARGE B-CELL LYMPHOMA (DLBCL): A RETROSPECTIVE ANALYSIS

**Topic:** 19. Large B cell lymphomas - Clinical

Luca Scalise*[1], Marco Rossi[1], Luca Pezzullo[2], Carmine Selleri[2], Domenico Pastore[3], Giuseppe Pietrantuono[4], Giovanna Mansueto[4], Massimo Gentile[5], Enrica Antonia Martino[5]

*[1]Azienda ospedaliera Renato Dulbecco, Catanzaro, Italy;[2]San giovanni di Dio e Ruggi s Aragona Hospital, salerno, Italy;[3]ASL Brindisi Ospedale Perrino, BRINDISI, Italy; [4]Ematologia e Trapianto di cellule staminali IRCCS CROB, Rionero In Vulture, Italy; [5]Dipartimento Oncoematologico , A.O COSENZA, COSENZA, Italy;*

### Background

Historically, R-CHOP regimen has been the standard first line treatment for diffuse large B-cell lymphoma (DLBCL). The introduction of polatuzumab vedotin in combination with rituximab, cyclophosphamide, doxorubicin, and prednisone (POLA-R-CHP) has shown a progression-free survival benefit in the POLARIX trial, particularly among patients with high IPI score. However, real-world data on efficacy and toxicity profile of this combination regimen remain limited.

### Methods

A retrospective analysis was conducted on 40 patients diagnosed with DLBCL and treated with the POLA-R-CHP regimen at five Hematology Centers between January 2024 and Dicember 2025. For each patient, demographic, clinical-biological, and therapeutic data were collected, including age, sex, ECOG performance status, stage, IPI, histologic subtype, metabolic response (interim and end of treatment PET CT scan), toxicity (≥ grade III hematologic and non-hematologic), relapse events, and vital status at last follow-up.

### Results

The median age at diagnosis was 69 years (range 26–84); 31 patients (77%) were male. Advanced stages (III–IV) were predominant (78%), with a mean IPI of 3. The most frequent subtype was ABC (activated B-cell-like). At the end of treatment, 29 patients (72%) achieved complete response (CR), 6 partial response (PR), and 5 (8%) had progressive disease. 5 relapses occurred during the observation period; 4 of these patients received CAR-T therapy as second-line treatment, and 1 developed CNS relapse. There were 5 deaths (8%): one due to thromboembolic toxicity, one to sepsis, and three related to disease progression. The estimated 1-year PFS (29/40) was 73,1% (95% CI 57.5–90.6). The most common ≥ grade III toxicity was neutropenia. No grade III/IV neuropathy or other polatuzumab-related severe toxicity was observed.

### Summary/Conclusion

POLA-R-CHP regimen proved to be effective and well tolerated, even in elderly and comorbid patients. The high complete response rate and 1-year PFS support the robustness of the POLARIX trial results in a real-world setting. The favorable tolerability profile of polatuzumab was confirmed. Longer follow-up and larger cohorts are warranted to assess response durability and to better characterize late-onset toxicities.

**Copyright Information:** (Online) ISSN: 2572-9241
©2026 The Author(s). HemaSphere is published by John Wiley & Sons Ltd on behalf of European Hematology Association. This is an open access Abstract Book distributed under the Attribution-NonCommercial-NoDerivs (CC BY-NC-ND), which allows third parties to download the articles and share them with others as long as they credit the author and the Abstract Book, but they cannot change the content in any way or use them commercially.
**Abstract Book Citations:** Authors, Title, HemaSphere, 2026;10:(S1):pages. The abstract book DOIs can be found on the “Author Information” page.
**Disclaimer:** Articles published in the journal HemaSphere exclusively reflect the opinions of the authors. The authors are responsible for all content in their abstracts including accuracy of the facts, statements, citing resources, etc.

### (PB3823) PD-1/CD20 DUAL-TARGET THERAPY IN UNFIT DIFFUSE LARGE B-CELL LYMPHOMA: A REAL-WORLD PILOT STUDY

**Topic:** 19. Large B cell lymphomas - Clinical

Shaozhen Chen*[1], Chang Wang[1], Jingjing Wen[1], Jianyang Lin[1], Ying Chen[1], Daoguang Chen[1], Hongming He[1], Jiesong Wang[1]

[1]*Fujian Medical University Cancer Hospital& Fujian Cancer Hospital, Department of Lymphoma & head and neck oncology, Fuzhou, China;*

### Background

Patients with diffuse large B-cell lymphoma (DLBCL) who are considered unfit for intensive chemotherapy have poor tolerance to standard regimens, underscoring the need for effective, less toxic alternatives.

### Aims

This real-world study aimed to evaluate the efficacy and safety of combining a PD-1 inhibitor with a CD20 monoclonal antibody as a chemotherapy-sparing treatment for unfit patients with DLBCL.

### Methods

This retrospective cohort study analyzed 7 consecutive adult patients with histologically confirmed diffuse large B-cell lymphoma (DLBCL) who were deemed unfit for standard intensive chemotherapy (Colorectal Cancer Intrinsic Subtypes, CRIS Scoring ≥6, ECOG performance status ≥2, or major organ dysfunction). All patients received PD-1 inhibitor (pembrolizumab 200 mg intravenously every 3 weeks) combined with CD20 monoclonal antibody (rituximab 375 mg/m² intravenously every 3 weeks) at our institution between January 1, 2023, and December 30, 2024. Treatment response was evaluated using Lugano criteria, with progression-free survival (PFS) and overall survival (OS) calculated via Kaplan-Meier analysis. Safety profiles were graded by CTCAE v5.0.

### Results

This real-world pilot study evaluated PD-1/CD20 dual-target therapy in 7 unfit DLBCL patients (median age: 70 years; 85.7% female). Baseline biomarker analysis showed a median CRIS score of 7.0 (96% CI: 6.0–9.0), with notably elevated PD-L1 expression (median CPS: 80; range: 0–90; 71.4% with CPS ≥80). Patients received a median cumulative PD-1 inhibitor dose of 2200 mg (95% CI: 1200–2800 mg) over 251 days (95% CI: 121–576 days), achieving an 85.7% objective response rate (5 complete responses, 1 partial response). A single progression event occurred in a patient with PD-L1 CPS of 0, preventing median PFS estimation. All patients remained alive at the February 28, 2025, data cutoff, with median OS undefined. Safety analysis revealed hematologic toxicities (28.6% grade 2, 28.6% grade 3, 14.3% grade 4) and grade 1 hepatic abnormalities (14.3%), all managed supportively without treatment-related mortality.

### Summary/Conclusion

PD-1/CD20 dual-target therapy demonstrated promising efficacy (85.7% response rate) and manageable safety in unfit DLBCL, patients, highlighting its potential as a chemotherapy-sparing regimen. Larger prospective studies are warranted to validate these findings.

**Copyright Information:** (Online) ISSN: 2572-9241
©2026 The Author(s). HemaSphere is published by John Wiley & Sons Ltd on behalf of European Hematology Association. This is an open access Abstract Book distributed under the Attribution-NonCommercial-NoDerivs (CC BY-NC-ND), which allows third parties to download the articles and share them with others as long as they credit the author and the Abstract Book, but they cannot change the content in any way or use them commercially.
**Abstract Book Citations:** Authors, Title, HemaSphere, 2026;10:(S1):pages. The abstract book DOIs can be found on the "Author Information" page.
**Disclaimer:** Articles published in the journal HemaSphere exclusively reflect the opinions of the authors. The authors are responsible for all content in their abstracts including accuracy of the facts, statements, citing resources, etc.

Table1: Clinical characteristics of the study cohort.

| Characteristics | n=7 |
|---|---|
| Age,years(medine, 95%CI) | 70.0 (46.0-80.0) |
| Sex | |
| Male | 1(14.3%) |
| Female | 6(85.7%) |
| Stage | |
| II | 2(25%) |
| III | 3(37.5%) |
| IV | 3(37.5%) |
| CRIS Scoring(medine, 95%CI) | 7.0(5.0-9.0) |
| PD-L1 CPS Evaluation(medine, 95%CI) | 80.0(1.0-90.0) |
| Ki67% (medine, 95%CI) | 85.0(80.0-90.0) |
| WBC, 10^9/L (medine, 95%CI) | 3.10(2.98-5.33) |
| ANE, 10^9/L (medine, 95%CI) | 1.97(1.25-4.28) |
| HGB, g/L (medine, 95%CI) | 114.0(107.0-124.0) |
| PLT, 10^9/L (medine, 95%CI) | 216.0(120.0-349.0) |
| PD-1 Exposure,mg（medine,95%CI） | 2200.0(1200.0-2800.0) |
| The duration of PD-1 administration, days(medine,95%CI) | 251.0(121.0-576.0) |
| Response | |
| CR | 5(71.4%) |
| PR | 1(14.3%) |
| PD | 1(14.3%) |
| Follow up time,days(medine, 95%CI) | 308.0(162.0-676.00) |

Abbreviation: CI, Confidence interval; CRIS, Colorectal cancer intrinsic subtypes; CPS, Combined positive score; WBC, White blood cell; ANE, Absolute neutrophil count; HGB, Hemoglobin; PLT, Platelet; CR, complete response; PR, partial response; PD, progressive disease.

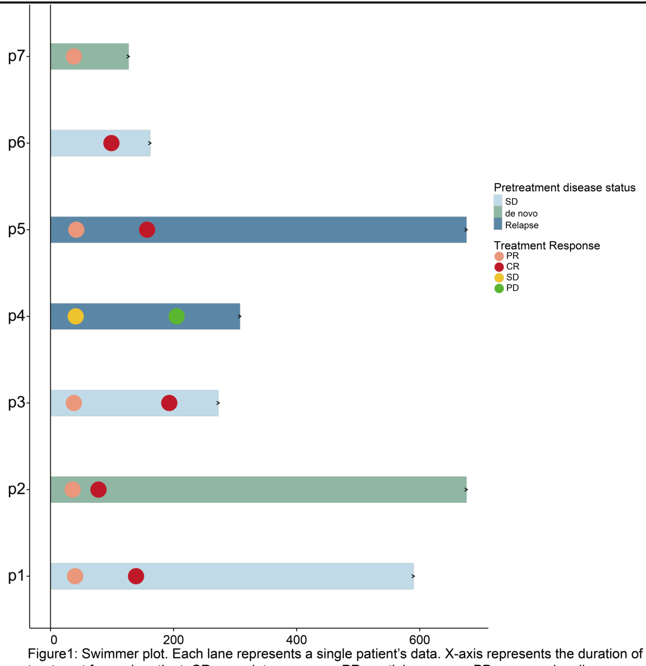

Figure1: Swimmer plot. Each lane represents a single patient's data. X-axis represents the duration of treatment for each patient. CR, complete response; PR, partial response; PD, progressive disease; SD, stable disease.

**Copyright Information:** (Online) ISSN: 2572-9241
©2026 The Author(s). HemaSphere is published by John Wiley & Sons Ltd on behalf of European Hematology Association. This is an open access Abstract Book distributed under the Attribution-NonCommercial-NoDerivs (CC BY-NC-ND), which allows third parties to download the articles and share them with others as long as they credit the author and the Abstract Book, but they cannot change the content in any way or use them commercially.
**Abstract Book Citations:** Authors, Title, HemaSphere, 2026;10:(S1):pages. The abstract book DOIs can be found on the "Author Information" page.
**Disclaimer:** Articles published in the journal HemaSphere exclusively reflect the opinions of the authors. The authors are responsible for all content in their abstracts including accuracy of the facts, statements, citing resources, etc.

### (PB3824) CLINICAL OUTCOMES AND SURVIVAL PREDICTORS IN PRIMARY EXTRANODAL NON-HODGKIN LYMPHOMA: A SINGLE-CENTER EXPERIENCE

**Topic:** 19. Large B cell lymphomas - Clinical

Mihailo Smiljanic*[1], Nikola Lemajić[1], Andjela Nikolic[1], Andrijana Kojic[1], Isidora Arsenovic[1], Danijela Lekovic[1, 2], Darko Antic[1, 2]

*[1]Clinic of Hematology, University Clinical Centre of Serbia, Belgrade, Serbia;[2]Faculty of Medicine, University of Belgrade, Belgrade, Serbia;*

### Background

Primary extranodal lymphomas (PENLs) are heterogeneous group of malignancies with diverse clinical presentations and geographical distribution. PENLs constitutes up to 24% of all lymphomas and 24-48% of all Non Hodgkin Lymphomas (NHL) in western studies, compared to approximately18% in recent Asian studies. Contemporary definition categorize PENL as lymphoma with dominant extranodal involvement without significant lymphadenopathy. The correlation between PENL anatomical site of involvement, histological subtypes and various treatment options is a subject of ongoing research.

### Aims

In this study we analyzed epidemiological data, clinicopathological features, treatment efficacy and survival outcomes in order to identify relevant prognostic factors.

### Methods

We performed a retrospective analysis of 116 PENL patients (pts) diagnosed and treated at our institution, between January 2022 and January 2026. Analyzed data included epidemiological data, histology, specific anatomical localizations, treatment modalities and clinical risk factors. We used Kaplan-Meier estimation for Overall Survival (OS) and Progression-Free Survival (PFS) analysis, as well as a multivariate Cox proportional hazards model to determine independent prognostic factor significance. AI (Gemini) was used for data analysis only.

**Copyright Information:** (Online) ISSN: 2572-9241
©2026 The Author(s). HemaSphere is published by John Wiley & Sons Ltd on behalf of European Hematology Association. This is an open access Abstract Book distributed under the Attribution-NonCommercial-NoDerivs (CC BY-NC-ND), which allows third parties to download the articles and share them with others as long as they credit the author and the Abstract Book, but they cannot change the content in any way or use them commercially.
**Abstract Book Citations:** Authors, Title, HemaSphere, 2026;10:(S1):pages. The abstract book DOIs can be found on the “Author Information” page.
**Disclaimer:** Articles published in the journal HemaSphere exclusively reflect the opinions of the authors. The authors are responsible for all content in their abstracts including accuracy of the facts, statements, citing resources, etc.

### Results

Mean age was 62.9 years, M:F ratio 0.92:1. The most frequent was Diffuse Large B-cell Lymphoma (DLBCL, n=70, 60%), followed by Marginal Zone Lymphoma (MZL, n=27, 23%) and Follicular Lymphoma (FL, n=11, 9%). The most frequent extranodal sites were the gastrointestinal tract (n=30, 26%) with predominant gastric involvement (n=20), followed by lung/pleura (n=12), spleen/liver (n=11), and orbit (n=10).
Overall Mean OS and PFS was 27.5 and 24.6 months respectively.
We confirmed histology as the most powerful outcome predictor (PFS, p= .0090). Indolent MZL demonstrated a superior mean PFS of 31.4 months over aggressive subtypes such as Mantle Cell Lymphoma and T-NHL (mean PFS 15.0 and 5.5 months respectively). Multivariate Cox model confirmed histological subtype as sole independent prognostic factor (PFS, HR 2.84;p= .0003). Anatomical localization did not independently correlate with OS (p= .1817) or PFS (p= .1115), nor did age (>65 vs ≤65), gender, use of anthracycline or risk factor presence (prior malignancy, autoimmune history).
Paradoxically, pts with lung/pleura and salivary gland involvement had the best outcomes (mean OS 31.8 and 31.2 months respectively), while pts with skin and muscle involvement had the worst outcome (mean OS 11.5 and 16.7 months respectively). This should be observed in terms of low grade NHL involvement.
Therapy selection significantly impacted OS in DLBCL subgroup (p= .0338), survival improvement was associated with integration of anthracycline-based chemotherapy and consolidative radiotherapy. Combined modality (chemo+ radiotherapy) treatment achieved 100% survival in the MZL subgroup. Risk factors, such as prior malignancies, were associated with higher mortality (33.3%), but no statistical significance (p= .1590) was reached.

### Summary/Conclusion

Our data confirmed PENL histological subtype, as the primary determinant of survival and disease progression. Although PENL can arise in nearly any organ system, the site of involvement is not an independent prognostic factor. Clinical management should prioritize aggressive, subtype-specific therapy based on histopathological grading rather than anatomical staging.

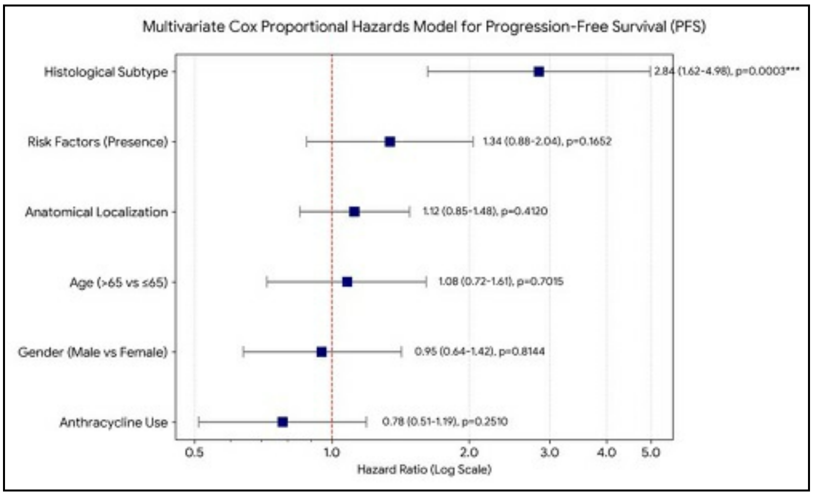

Forest Plot for the multivariate Cox analysis (n=116). The Histological subtype (HR = 2.84, p = .0003), it is the statistically significant predictor of disease progression.

**Copyright Information:** (Online) ISSN: 2572-9241
©2026 The Author(s). HemaSphere is published by John Wiley & Sons Ltd on behalf of European Hematology Association. This is an open access Abstract Book distributed under the Attribution-NonCommercial-NoDerivs (CC BY-NC-ND), which allows third parties to download the articles and share them with others as long as they credit the author and the Abstract Book, but they cannot change the content in any way or use them commercially.
**Abstract Book Citations:** Authors, Title, HemaSphere, 2026;10:(S1):pages. The abstract book DOIs can be found on the "Author Information" page.
**Disclaimer:** Articles published in the journal HemaSphere exclusively reflect the opinions of the authors. The authors are responsible for all content in their abstracts including accuracy of the facts, statements, citing resources, etc.

### (PB3825) ZANUBRUTINIB MAINTENANCE AFTER AUTOLOGOUS STEM CELL TRANSPLANTATION IN HIGH-RISK DIFFUSE LARGE B-CELL LYMPHOMA: A PROSPECTIVE SINGLE-ARM STUDY

**Topic:** 19. Large B cell lymphomas - Clinical

Yanli Lai*[1]

*[1]The First Affiliated Hospital of Ningbo University, hematology, ningbo, China;*

### Background

High-risk diffuse large B-cell lymphoma (DLBCL), characterized by adverse clinical and/or biologic features, remains associated with a substantial risk of relapse despite achieving remission with frontline immunochemotherapy and consolidation with autologous stem cell transplantation (ASCT). Relapse after ASCT remains a clinically relevant challenge, and no standard post-ASCT maintenance approach has been established in DLBCL. Zanubrutinib, a next-generation Bruton's tyrosine kinase (BTK) inhibitor, has demonstrated clinical activity across B-cell malignancies with a generally favorable tolerability profile, providing a rationale for evaluation as post-ASCT maintenance in high-risk DLBCL.

### Aims

To evaluate the feasibility and preliminary efficacy of Zanubrutinib maintenance following ASCT in adults with newly diagnosed high-risk DLBCL.

### Methods

This prospective, single-arm study enrolled adults with newly diagnosed DLBCL considered high-risk if they met ≥1 of the following criteria: (1) immunohistochemistry consistent with double expression (BCL2 ≥50% and MYC ≥40%) or p53 overexpression; (2) fluorescence in situ hybridization demonstrating double-hit/triple-hit lymphoma (MYC rearrangement with BCL2 and/or BCL6 rearrangement) and/or TP53 mutation identified by molecular testing; and/or (3) International Prognostic Index (IPI) ≥3. Patients who achieved ≥partial response (PR) after R-CHOP–like therapy proceeded to ASCT, followed by Zanubrutinib maintenance at 160 mg orally twice daily for 12 months. The primary endpoint was progression-free survival (PFS), defined as time from ASCT to disease progression or death.

**Copyright Information:** (Online) ISSN: 2572-9241
©2026 The Author(s). HemaSphere is published by John Wiley & Sons Ltd on behalf of European Hematology Association. This is an open access Abstract Book distributed under the Attribution-NonCommercial-NoDerivs (CC BY-NC-ND), which allows third parties to download the articles and share them with others as long as they credit the author and the Abstract Book, but they cannot change the content in any way or use them commercially.
**Abstract Book Citations:** Authors, Title, HemaSphere, 2026;10:(S1):pages. The abstract book DOIs can be found on the "Author Information" page.
**Disclaimer:** Articles published in the journal HemaSphere exclusively reflect the opinions of the authors. The authors are responsible for all content in their abstracts including accuracy of the facts, statements, citing resources, etc.

**Results**

A total of 15 patients were enrolled, including 8 males (53.3%)  7 females (46.7%), with a median age of 59 years (range, 25–75). ECOG performance status was 0–1 in 8 patients (53.3%) and ≥2 in 7 (46.7%). Most patients had high-risk disease by IPI, with scores of 3–5 in 10 patients (66.7%), score 2 in 3 (20.0%), and scores 0–1 in 2 (13.3%). The majority presented with advanced-stage disease (Ann Arbor stage III–IV: 13/15, 91.7%), and extranodal involvement was common (>1 extranodal site: 8/15, 53.3%). cell-of-origin was predominantly non-GCB (11/15, 73.3%), with GCB in 4/15 (26.7%). Overall, high-risk designation was primarily driven by adverse clinical factors (e.g., IPI ≥3 and advanced-stage disease), with additional biologic high-risk features observed in a subset, including double-expressor status in 4/15 (26.7%), double-hit lymphoma in 1/15 (6.7%), and TP53 mutation in 1/15 (6.7%) (features not mutually exclusive).
At the end of induction therapy, 12 patients (80.0%) achieved complete remission (CR) and 3 (20.0%) achieved PR before proceeding to ASCT. Of the 15 patients who received Zanubrutinib maintenance after ASCT, 14 (93.3%) completed the planned 12-month maintenance, and 1 patient remained on treatment at the data cutoff. With a median follow-up of 18.3 months from ASCT, one patient experienced relapse at 6 months from ASCT and subsequently died. The Kaplan–Meier–estimated 2-year PFS rate was 92.86% (95% CI, 59.08–98.96). At the data cutoff, 14/15 patients were alive, with one death following relapse. Zanubrutinib maintenance was generally well tolerated, and no unexpected safety signals were observed.

**Summary/Conclusion**

Zanubrutinib maintenance following ASCT was feasible and showed an encouraging signal of prolonged PFS in high-risk DLBCL. Further studies are needed to validate benefit and define optimal patient selection and safety.

**Copyright Information:** (Online) ISSN: 2572-9241
©2026 The Author(s). HemaSphere is published by John Wiley & Sons Ltd on behalf of European Hematology Association. This is an open access Abstract Book distributed under the Attribution-NonCommercial-NoDerivs (CC BY-NC-ND), which allows third parties to download the articles and share them with others as long as they credit the author and the Abstract Book, but they cannot change the content in any way or use them commercially.
**Abstract Book Citations:** Authors, Title, HemaSphere, 2026;10:(S1):pages. The abstract book DOIs can be found on the “Author Information” page.
**Disclaimer:** Articles published in the journal HemaSphere exclusively reflect the opinions of the authors. The authors are responsible for all content in their abstracts including accuracy of the facts, statements, citing resources, etc.

### (PB3826) REAL-WORLD OUTCOMES AND TOXICITY MANAGEMENT OF SECOND-LINE AXICABTAGENE CILOLEUCEL IN LARGE B-CELL LYMPHOMA: A SINGLE-CENTER EXPERIENCE

**Topic:** 19. Large B cell lymphomas - Clinical

Carola Diaz Aizpun*[1], Francisco Cabrera Ruiz[1], Marta Luque Garrido[1], Alberto Doblas Marquez[1], Manuel Espeso de Haro[1]

[1]*Hospital Regional Universitario de Malaga, Malaga, Spain;*

### Background

Axicabtagene ciloleucel (axi-cel), an anti-CD19 chimeric antigen receptor T-cell (CAR-T) therapy, has become a standard second-line option for patients with relapsed or refractory diffuse large B-cell lymphoma (DLBCL) with early relapse <12 months or refractory to first-line chemoimmunotherapy. Although pivotal trials demonstrated superior efficacy compared with standard of care, real-world evidence is essential to better characterize outcomes and optimize toxicity management.

### Aims

To assess clinical efficacy and treatment-related toxicities of second-line axi-cel in patients with DLBCL treated at our center.

### Methods

We performed a retrospective analysis of consecutive adult patients with early relapse (<12 months) or refractory DLBCL treated with axi-cel as second-line therapy between 2024 and 2025. Baseline characteristics, response rates and CAR-T–related toxicities were evaluated. Cytokine release syndrome (CRS) and immune effector cell–associated neurotoxicity syndrome (ICANS) were graded per ASTCT criteria.

### Results

A total of 22 patients were included, with a median age at diagnosis of 54 years (range 24 - 79). The general and baseline characteristics prior to CART treatment are described in Table 1.

The overall response rate at 3 months was 68% (n = 13) of the 19 patients evaluated, of whom 84% (n = 11) had a complete metabolic response (CMR). At 6 months, among the 15 evaluable patients, the complete response rate was 60% (n = 9).

Regarding early toxicity, 100% of patients experienced some degree of CRS. Most patients experienced grade 1 CRS, only 19% experienced grade 2, and there were no grade ≥3 events. In our early intervention protocol, all patients with persistent grade 1 CRS for 24-48 hours (adapted to the risk profile) or grade 2 CRS receive at least one dose of tocilizumab associated with 10 mg dexamethasone, up to a maximum of 2 doses.

Thirty-eight per cent of patients (n=8) developed ICANS. 5 patients (63%) were grade 1 and 1 patient (12%) was grade 2. The 2 patients who developed grade 3 ICANS were patients diagnosed with PCNSL. There were no grade 4-5 events. Upon onset of neurotoxicity, dexamethasone 10 mg every 24 hours was administered until resolution. Treatment with dexamethasone 10 mg every 6 hours was administered in patients with grade 2, and Anakinra was added in patients with grade 3. All events were resolved without consequences. No treatment-related mortality was observed, with a 30-day mortality rate of 0%.

**Copyright Information:** (Online) ISSN: 2572-9241
©2026 The Author(s). HemaSphere is published by John Wiley & Sons Ltd on behalf of European Hematology Association. This is an open access Abstract Book distributed under the Attribution-NonCommercial-NoDerivs (CC BY-NC-ND), which allows third parties to download the articles and share them with others as long as they credit the author and the Abstract Book, but they cannot change the content in any way or use them commercially.
**Abstract Book Citations:** Authors, Title, HemaSphere, 2026;10:(S1):pages. The abstract book DOIs can be found on the “Author Information” page.
**Disclaimer:** Articles published in the journal HemaSphere exclusively reflect the opinions of the authors. The authors are responsible for all content in their abstracts including accuracy of the facts, statements, citing resources, etc.

| BASELINE CHARACTHERISTICS | N (%) |
|---|---|
| SEX | |
| - MALE | 16 (73) |
| - FEMALE | 6 (27) |
| LYMPHOMA SUBTIPE | |
| - HGBCL | 6 (27) |
| - DLBCL | 8 (36) |
| - PML | 3 (14) |
| - PCNSL | 3(14) |
| - tFL | 2 (9) |
| STAGE | |
| - I | 0 |
| - II | 2 (10) |
| - III | 2 (10) |
| - IV | 17 (80) |
| DISEASE STATUS | |
| - REFRACTORY | 16 (73) |
| - EARLY RELAPSE <12 MONTHS | 6 (27) |
| HOLDING | 12 (54) |
| BRIDGING | 21 (95) |
| STATUS PRE-CART | |
| - Progression | 9 (41) |
| - Stable Disease | 3 (14) |
| - Partial Metabolic Response | 9 (41) |
| - Complete Metabolic Response | 1(4) |

### Summary/Conclusion

In this real-world single-center experience, second-line axi-cel demonstrated high response rates, consistent with pivotal clinical trials. Although CRS was frequent, no severe toxicities were reported. With early recognition and intervention, we report a lower rate of overall and severe ICANS. Early intervention protocols and close monitoring allowed for effective control of toxicity. Limitations include a small sample size and the need for further follow-up.

**Copyright Information:** (Online) ISSN: 2572-9241
©2026 The Author(s). HemaSphere is published by John Wiley & Sons Ltd on behalf of European Hematology Association. This is an open access Abstract Book distributed under the Attribution-NonCommercial-NoDerivs (CC BY-NC-ND), which allows third parties to download the articles and share them with others as long as they credit the author and the Abstract Book, but they cannot change the content in any way or use them commercially.
**Abstract Book Citations:** Authors, Title, HemaSphere, 2026;10:(S1):pages. The abstract book DOIs can be found on the “Author Information” page.
**Disclaimer:** Articles published in the journal HemaSphere exclusively reflect the opinions of the authors. The authors are responsible for all content in their abstracts including accuracy of the facts, statements, citing resources, etc.

### (PB3827) PATIENT CHARACTERISTICS AND OUTCOMES OF POLA-R-CHP AND R-CHOP/R-MINICHOP IN PATIENTS WITH NEWLY DIAGNOSED DLBCL: A SINGLE-CENTER EXPERIENCE IN TAIWAN

**Topic:** 19. Large B cell lymphomas - Clinical

Ling-Jung Chiu[1], Ming-Chung Wang*[1], Hung-Lin Liu[1], Ming-Chun MA[1]

[1]*Kaohsiung Chang Gung Memorial Hospital, Division of Hematology and Oncology, Niaosong District, Taiwan;*

### Background

In Taiwan, real-world evidence comparing Pola-R-CHP with R-CHOP and the dose-attenuated R-miniCHOP regimen in the post-POLARIX era remains limited.

### Aims

To describe the patient characteristics and outcomes of Pola-R-CHP and R-CHOP/R-miniCHOP in patients with newly diagnosed diffuse large B-cell lymphoma (DLBCL) in Taiwan.

### Methods

We conducted a retrospective cohort study of adult patients with DLBCL who received first-line therapy at the largest medical center in Southern Taiwan. Patients were included if they initiated either (1) Pola-R-CHP between July 2023 (when polatuzumab vedotin was introduced in Taiwan) and December 31, 2025, or (2) R-CHOP or R-miniCHOP between December 2021 and December 2022. Follow-up began at treatment initiation and continued until disease progression, the last clinical visit, or the end of the study period (December 31, 2025). The primary objectives were to describe patient characteristics, complete response, overall response, and progression-free survival (PFS) rates at 12 and 18 months between the Pola-R-CHP and R-CHOP/R-miniCHOP groups.

### Results

We included 55 patients in this study (Pola-R-CHP: n = 15; R-CHOP/R-miniCHOP: n = 40). With the exception of double-expressor status, advanced Ann Arbor stage (III–IV), and elevated baseline LDH, which were comparable between groups, patients in the Pola-R-CHP cohort were more likely to be male (73.3% vs. 42.5%; p = 0.04), have a non-germinal center B-cell subtype (86.7% vs. 45.0%; p = 0.015), and present with an Eastern Cooperative Oncology Group performance status ≥2 (86.6% vs. 57.5%; p = 0.05). Nevertheless, Pola-R-CHP demonstrated a trend toward improved outcomes, including higher complete response rates (86.6% vs. 77.5%; p = 0.452) and superior 12-month (88.9% vs. 75%; p = 0.159) and 18-month PFS rates (88.9% vs. 69.9%). The PFS rates in the high-risk subgroup International Prognostic Index score of 3–5 and Ann Arbor stage III–IV) were consistent with the overall findings.

### Summary/Conclusion

In Taiwan, Pola-R-CHP was more often used as first-line therapy in DLBCL patients with poorer prognostic features than those receiving R-CHOP or R-miniCHOP, yet it was still associated with better outcomes, most notably in high-risk patients. Our findings supported Pola-R-CHP as a preferred frontline option for DLBCL patients in routine practice.

**Copyright Information:** (Online) ISSN: 2572-9241
©2026 The Author(s). HemaSphere is published by John Wiley & Sons Ltd on behalf of European Hematology Association. This is an open access Abstract Book distributed under the Attribution-NonCommercial-NoDerivs (CC BY-NC-ND), which allows third parties to download the articles and share them with others as long as they credit the author and the Abstract Book, but they cannot change the content in any way or use them commercially.
**Abstract Book Citations:** Authors, Title, HemaSphere, 2026;10:(S1):pages. The abstract book DOIs can be found on the "Author Information" page.
**Disclaimer:** Articles published in the journal HemaSphere exclusively reflect the opinions of the authors. The authors are responsible for all content in their abstracts including accuracy of the facts, statements, citing resources, etc.

Table 1. Baseline Characteristics and Treatment Outcomes of Pola-R-CHP and R-CHOP or R-miniCHOP

| Variable | Pola-R-CHP (N=15) | R-CHOP or R-miniCHOP (N=40) | P-value |
|---|---|---|---|
| **Demographics** | | | |
| Age, mean ± SD (years) | 63.1±10 | 60.5±12 | 0.457 |
| Male, n (%) | 11 (73.3%) | 17 (42.5%) | 0.040 |
| **Disease Characteristics, n (%)** | | | |
| Non-GCB subtype | 13 (86.7%) | 18 (45.0%) | 0.015 |
| Double expressor | 11 (73.3%) | 16 (40.0%) | 0.024 |
| IPI 3–5 | 11(73.3%) | 22(55%) | 0.216 |
| Ann Arbor Stage III–IV | 13 (86.7%) | 26 (65.0%) | 0.117 |
| ECOG performance status ≥ 2 | 13 (86.6%) | 23 (57.5%) | 0.050 |
| Elevated LDH | 12 (80.0%) | 26 (65.8%) | 0.354 |
| Number of extranodal sites | 9 (60%) | 14(35%) | 0.086 |
| **Treatment Response, n (%)** | | | |
| Complete Response | 13 (86.6%) | 31 (77.5%) | 0.452 |
| Overall Response | 14 (93.3%) | 36 (90.0%) | 0.730 |
| **Survival Estimates, %** | | | |
| 12-month PFS (All patients) | 88.9% | 75% | 0.159 |
| 18-month PFS (All patients) | 88.9% | 69.9% | |
| 12-month PFS ( IPI 3–5) | 87.5% | 59.1% | 0.058 |
| 18-months PFS ( IPI 3–5) | 87.5% | 50% | |
| 12-month PFS (Ann Arbor Stage III-IV) | 87.5% | 65.4% | 0.07 |
| 18-month PFS (Ann Arbor Stage III-IV) | 87.5% | 57.7% | |

Abbreviations: LDH = Lactate Dehydrogenase; ECOG PS = Eastern Cooperative Oncology Group; PFS = Progression-free survival; GCB = Germinal center B-cell; IPI = International Prognostic Index.

**Copyright Information:** (Online) ISSN: 2572-9241
©2026 The Author(s). HemaSphere is published by John Wiley & Sons Ltd on behalf of European Hematology Association. This is an open access Abstract Book distributed under the Attribution-NonCommercial-NoDerivs (CC BY-NC-ND), which allows third parties to download the articles and share them with others as long as they credit the author and the Abstract Book, but they cannot change the content in any way or use them commercially.
**Abstract Book Citations:** Authors, Title, HemaSphere, 2026;10:(S1):pages. The abstract book DOIs can be found on the "Author Information" page.
**Disclaimer:** Articles published in the journal HemaSphere exclusively reflect the opinions of the authors. The authors are responsible for all content in their abstracts including accuracy of the facts, statements, citing resources, etc.

### (PB3828) SAFETY AND EFFICACY OF POLATUZUMAB VEDOTIN-BASED CHEMOIMMUNOTHERAPY IN ELDERLY LIVER-TRANSPLANT RECIPIENTS WITH MONOMORPHIC DLBCL-TYPE PTLD

**Topic:** 19. Large B cell lymphomas - Clinical

Fei Xiao[1], Lan Xu[1], Yanni Hua[1], Xin Li[1], Hua Zhong[1], Honghui Huang*[1]

*[1]Renji Hospital, Shanghai Jiao Tong University School of Medicine, Department of hematology, Shanghai, China;*

### Background

Post-transplant lymphoproliferative disorder (PTLD) following liver transplantation (LT) is a life-threatening complication. Managing elderly recipients represents a significant clinical challenge due to comorbidities, frailty, and the need to balance lymphoma control with graft preservation. While rituximab monotherapy is a common first-line approach, responses are often inadequate in aggressive subtypes. Polatuzumab vedotin (Pola)-based regimens have demonstrated efficacy in diffuse large B-cell lymphoma (DLBCL), yet real-world data regarding their safety—specifically graft toxicity and infection risk—and efficacy in elderly LT recipients remain scarce.

### Aims

To evaluate the safety, tolerability, and systemic efficacy of Pola-based chemoimmunotherapy in elderly liver-transplant recipients with monomorphic DLBCL-type PTLD, and to assess graft outcomes during antibody-drug conjugate treatment.

### Methods

We retrospectively analyzed three consecutive elderly/older LT recipients (age ≥65 years) with histologically confirmed monomorphic DLBCL-type PTLD treated at a single center. All patients had received prior rituximab-based induction with inadequate response or early progression before switching to Pola-based regimens (Pola combined with dose-adjusted chemotherapy). Responses were assessed by PET-CT according to Lugano criteria. Safety was evaluated per CTCAE v5.0, with a specific focus on adverse events of special interest (AESI) including peripheral neuropathy, cytopenias, severe infections, and hepatic graft dysfunction.

### Results

Three male liver-transplant recipients (median age 75 years; range 65–82) with EBER-negative monomorphic DLBCL-type PTLD were included. All patients presented with advanced stage disease and/or high-risk molecular features, such as *MYD88* mutation, or *TP53* mutation. Following sub-optimal responses to prior rituximab-containing lines, all patients received Pola-based chemoimmunotherapy (Pola-R-miniCHP or Pola-R-CDP).
Efficacy: The overall response rate (ORR) and complete metabolic response (CMR) rate were both 100% (3/3). Patient 1 (82 years, non-GCB) and Patient 2 (75 years, GCB) achieved durable CMR with a progression-free survival (PFS) of 10.4+ months and 19.5+ months, respectively, at the data cutoff. Patient 3 (65 years, non-GCB with TP53 mutation) achieved systemic CMR; however, disease progression occurred at 9.2 months post-treatment, highlighting a divergent response in high-risk molecular subtypes.
Safety: The regimen was well-tolerated in this elderly cohort. Grade 3-4 neutropenia occurred in 3/3 patients but was manageable with growth factor support. No grade ≥3 peripheral neuropathy was observed. Importantly, no events of acute graft rejection, treatment-related graft failure, or irreversible hepatotoxicity were recorded. Infections were managed with standard antimicrobials, and no treatment-related mortality occurred.

**Copyright Information:** (Online) ISSN: 2572-9241
©2026 The Author(s). HemaSphere is published by John Wiley & Sons Ltd on behalf of European Hematology Association. This is an open access Abstract Book distributed under the Attribution-NonCommercial-NoDerivs (CC BY-NC-ND), which allows third parties to download the articles and share them with others as long as they credit the author and the Abstract Book, but they cannot change the content in any way or use them commercially.
**Abstract Book Citations:** Authors, Title, HemaSphere, 2026;10:(S1):pages. The abstract book DOIs can be found on the “Author Information” page.
**Disclaimer:** Articles published in the journal HemaSphere exclusively reflect the opinions of the authors. The authors are responsible for all content in their abstracts including accuracy of the facts, statements, citing resources, etc.

### Summary/Conclusion

Pola-based chemoimmunotherapy achieved a 100% CMR rate with manageable toxicity and preserved liver graft function in elderly LT recipients with PTLD, supporting its feasibility in frail transplant recipients who are not candidates for intensive chemotherapy and showing no apparent signals of graft rejection. This analysis is limited by the small sample size, and larger prospective studies are needed to confirm these findings. Separately, biologically high-risk disease (e.g., TP53-altered PTLD) remains an unmet need and warrants further therapeutic optimization and exploration of novel strategies.

**Table . Patient Characteristics, Treatment History, and Clinical Outcomes**

| Pt | Age (y) | Pathology / Key Biology | Prior Therapy (Pre-Pola) | Pola-based Regimen | Best Response | PFS (months) | Graft Outcome |
|---|---|---|---|---|---|---|---|
| **1** | 82 | non-GCB; *MYD88* mut | Rituximab monotherapy | Pola-R-miniCHP ×5 | CMR | 10.4+ (Ongoing) | Stable |
| **2** | 75 | GCB; BCL6 rearr | Rituximab monotherapy | Pola-R-miniCHP ×3 | CMR | 19.5+ (Ongoing) | Stable |
| **3** | 65 | non-GCB; *TP53*/ *PIM1* mut | R-CDOP | Pola-R-CDP ×5 | CMR | 9.2 (CNS Relapse) | Stable |

*Abbreviations: PFS is calculated from the initiation of Pola-based therapy. R, rituximab; miniCHP, dose-adjusted cyclophosphamide, doxorubicin, prednisone; CDP, cyclophosphamide, liposomal doxorubicin, prednisone.*

**Copyright Information:** (Online) ISSN: 2572-9241
©2026 The Author(s). HemaSphere is published by John Wiley & Sons Ltd on behalf of European Hematology Association. This is an open access Abstract Book distributed under the Attribution-NonCommercial-NoDerivs (CC BY-NC-ND), which allows third parties to download the articles and share them with others as long as they credit the author and the Abstract Book, but they cannot change the content in any way or use them commercially.
**Abstract Book Citations:** Authors, Title, HemaSphere, 2026;10:(S1):pages. The abstract book DOIs can be found on the “Author Information” page.
**Disclaimer:** Articles published in the journal HemaSphere exclusively reflect the opinions of the authors. The authors are responsible for all content in their abstracts including accuracy of the facts, statements, citing resources, etc.

### (PB3829) PRIMARY CENTRAL NERVOUS SYSTEM LYMPHOMA: A SINGLE-CENTER EXPERIENCE

**Topic:** 19. Large B cell lymphomas - Clinical

Maha Charfi*[1], Khedija Kharrat[1], Rahma Mallek[1], Faten Kallel[1], Mayeda Bourmech[2], Asma Koubaa[1], Imen Ben Amor[1], Yosra Fakhfakh[1], Fatma Dhouib[2], Imen Frikha[1], Moez Mdhaffar[1], Hanen Ben Salah[2], Moez Elloumi[1]

*[1]Hedi Chaker hospital, hematology department, Sfax, Tunisia; [2]Hbib Bourguiba, radiotherapy department, Sfax, Tunisia;*

### Background

Primary central nervous system lymphoma (PCNSL) is a rare and aggressive malignancy, accounting for approximately 1–2% of non-Hodgkin lymphomas and 2–6% of primary intracranial tumors. It most commonly corresponds to diffuse large B-cell lymphoma confined to the central nervous system, including the brain parenchyma, meninges, cerebrospinal fluid, or eyes, without initial systemic involvement. Its incidence has increased in recent decades, particularly among immunocompromised patients and immunocompetent individuals over 60 years of age.

### Aims

The aim of this study is to describe clinical presentation, treatment modalities and outcomes in patients with PCNSL in our center.

### Methods

We conducted a retrospective descriptive study in the Department of Clinical Hematology at Hedi Chaker University Hospital between June 2010 and December 2023. All patients diagnosed with PCNSL were included. Clinical presentation, imaging findings, treatment modalities, and outcomes were analyzed.

### Results

Fifteen patients were included, with a median age of 55 years (range: 31–75) and a slight female predominance (53%). B symptoms were uncommon, reported in 3 patients (20%), and the main presenting feature was hemiparesis (53%). Brain MRI revealed lesions predominantly involving the frontoparietal and/or temporal regions. Histological diagnosis was obtained in all cases via stereotactic biopsy, which confirmed the LBGC subtype in every patient. Cerebrospinal fluid examination, available for 8 patients, showed no evidence of lymphomatous involvement. Fourteen patients received high-dose methotrexate–based induction chemotherapy and the most commonly used regimen was RMPV (64%). while one patient died before treatment initiation. The overall response rate after induction was 78.5%, including complete remission in five patients (45%). Treatment-related mortality during induction occurred in 3 patients (21%). Consolidation consisted of chemotherapy alone in 6 patients and combined chemotherapy and radiotherapy in 5 patients. For patients who received whole brain radiotherapy with doses ranged from 30 to 50 Gy. Disease progression during treatment was observed in 6 patients. Relapse occurred in four patients, with a median time to relapse of 2 years (range: 8 months–6 years). Only one patient received salvage chemotherapy, achieving a favorable response. Median overall survival and progression-free survival were 13 and 12 months, respectively.

**Copyright Information:** (Online) ISSN: 2572-9241
©2026 The Author(s). HemaSphere is published by John Wiley & Sons Ltd on behalf of European Hematology Association. This is an open access Abstract Book distributed under the Attribution-NonCommercial-NoDerivs (CC BY-NC-ND), which allows third parties to download the articles and share them with others as long as they credit the author and the Abstract Book, but they cannot change the content in any way or use them commercially.
**Abstract Book Citations:** Authors, Title, HemaSphere, 2026;10:(S1):pages. The abstract book DOIs can be found on the "Author Information" page.
**Disclaimer:** Articles published in the journal HemaSphere exclusively reflect the opinions of the authors. The authors are responsible for all content in their abstracts including accuracy of the facts, statements, citing resources, etc.

**Summary/Conclusion**

Despite an acceptable global therapeutic response (78.5%), outcomes in our cohort remained inferior to those reported in the literature. Complete remission was achieved in 45% of patients, also slightly below reported rates. Disease progression occurred in 6 patients and relapse in 4, contributing to a median overall survival of 13 months, markedly lower than the 25–60 months described in published series. According to current evidence, improving results will require better optimization of high-dose methotrexate–based induction regimens, enhanced supportive care to reduce treatment-related mortality which reached 21% in our cohort compared with the usual 5–10% and broader use of intensive consolidation strategies such as autologous stem cell transplantation to improve survival rates.

**Copyright Information:** (Online) ISSN: 2572-9241
©2026 The Author(s). HemaSphere is published by John Wiley & Sons Ltd on behalf of European Hematology Association. This is an open access Abstract Book distributed under the Attribution-NonCommercial-NoDerivs (CC BY-NC-ND), which allows third parties to download the articles and share them with others as long as they credit the author and the Abstract Book, but they cannot change the content in any way or use them commercially.
**Abstract Book Citations:** Authors, Title, HemaSphere, 2026;10:(S1):pages. The abstract book DOIs can be found on the "Author Information" page.
**Disclaimer:** Articles published in the journal HemaSphere exclusively reflect the opinions of the authors. The authors are responsible for all content in their abstracts including accuracy of the facts, statements, citing resources, etc.

### (PB3830) IMPACT OF 18F-FDG PET/CT ON STAGING AND PROGNOSIS IN DIFFUSE LARGE B-CELL LYMPHOMA: A TUNISIAN REAL-WORLD EXPERIENCE

**Topic:** 19. Large B cell lymphomas - Clinical

Aya Zaouali Dridi*[1], Zaineb Mlayah[1], Moetez Ammar[1], Jamila Mizouni[1], Bizid Inaam[1], Wiem Boufrikha[1], Nader Slama[1], Mohamed Adnene Laatiri[1], Sarra Boukhriss[1]

[1]*Fattouma Bourguiba University Hospital, Monastir, Tunisia;*

### Background

Diffuse large B-cell lymphoma (DLBCL) is the most common aggressive non-Hodgkin lymphoma (NHL) and remains potentially curable. 18F-FDG PET/CT plays a pivotal role in staging and response assessment, refining prognostic stratification and therapeutic decision-making.

### Aims

To assess the impact of 18F-FDG PET/CT on initial staging, interim evaluation, and end-of-treatment response in DLBCL patients, and to explore its implications for potential revision of the Tunisian national protocol.

### Methods

This retrospective study included adult DLBCL patients treated at the Clinical Hematology Department, Fattouma Bourguiba University Hospital, Monastir, between January 2018 and December 2024. All patients received treatment according to the Tunisian NHL-2013 protocol and underwent at least one PET/CT evaluation.
Survival analyses were performed using the Kaplan–Meier method and Cox proportional hazards regression.

### Results

Fifty-seven patients were included (mean age 49 years; M/F ratio 1.2). Peripheral lymphadenopathy was observed in 37% (n=21), and the non–germinal center (non-GCB) phenotype predominated (51%, n=29).
Baseline PET/CT detected additional nodal and extranodal sites compared with CT, leading to disease upstaging in nine patients (16%) and therapeutic group modification in two (4%). Stage IV disease was present in 47% (n=27), and low–intermediate aaIPI risk predominated (53%, n=30).
Interim PET/CT (n=21) was discordant with CT in three cases (14%). End-of-treatment PET/CT reclassified response in nine patients (27%), including detection of progression not identified on CT.
After first-line therapy, 40 patients (70%) achieved remission, while 15 (26%) required salvage therapy. Five-year overall survival (OS) and progression-free survival (PFS) were 79% and 67%, respectively.
Factors significantly associated with inferior OS were age >60 years (48% vs 97%, p=0.007), smoking (58% vs 92%, p=0.042), advanced stage (62% vs 95%, p=0.04), elevated LDH (61% vs 90%, p=0.009), and non-GCB phenotype (64% vs 100%, p=0.027).
On multivariate analysis, end-of-treatment complete metabolic response (CMR) remained independently associated with improved survival and a markedly reduced risk of progression or relapse (HR 0.009; 95% CI 0.001–0.107; p<0.001), predicting both OS and PFS.

**Copyright Information:** (Online) ISSN: 2572-9241
©2026 The Author(s). HemaSphere is published by John Wiley & Sons Ltd on behalf of European Hematology Association. This is an open access Abstract Book distributed under the Attribution-NonCommercial-NoDerivs (CC BY-NC-ND), which allows third parties to download the articles and share them with others as long as they credit the author and the Abstract Book, but they cannot change the content in any way or use them commercially.
**Abstract Book Citations:** Authors, Title, HemaSphere, 2026;10:(S1):pages. The abstract book DOIs can be found on the “Author Information” page.
**Disclaimer:** Articles published in the journal HemaSphere exclusively reflect the opinions of the authors. The authors are responsible for all content in their abstracts including accuracy of the facts, statements, citing resources, etc.

**Summary/Conclusion**

In this real-world Tunisian cohort, 18F-FDG PET/CT significantly improved staging accuracy, response assessment, and prognostic stratification in DLBCL. End-of-treatment CMR emerged as a strong independent prognostic marker, supporting the integration of PET/CT into routine assessment and informing future optimization of national treatment strategies.

**Copyright Information:** (Online) ISSN: 2572-9241
©2026 The Author(s). HemaSphere is published by John Wiley & Sons Ltd on behalf of European Hematology Association. This is an open access Abstract Book distributed under the Attribution-NonCommercial-NoDerivs (CC BY-NC-ND), which allows third parties to download the articles and share them with others as long as they credit the author and the Abstract Book, but they cannot change the content in any way or use them commercially.
**Abstract Book Citations:** Authors, Title, HemaSphere, 2026;10:(S1):pages. The abstract book DOIs can be found on the "Author Information" page.
**Disclaimer:** Articles published in the journal HemaSphere exclusively reflect the opinions of the authors. The authors are responsible for all content in their abstracts including accuracy of the facts, statements, citing resources, etc.

### (PB3831) THERAPY OF PARVOVIRUS B19 INFECTION IN PATIENTS WITH B-CELL LYMPHOMAS

**Topic:** 19. Large B cell lymphomas - Clinical

Aleksey Zubarev[1], Anton Luchkin[1], Anna Smolianinova[1], Anastasia Abramova[1], Svetlana Tatarnicova[1], Irina Fevraleva[1], Anna Garmash[1], Alina Kohno[1], Evgeniy Zvonkov[1], Elena Parovichnikova[1]

[1]*National Medical Research Center for Hematology, Ministry of Health of Russia, Moscow, Russia;*

### Background

The etiological role of parvovirus B19 (B19V) infection in the development of pure red cell aplasia (PRCA) in immunocompromised patients, including patients with B-cell lymphomas, is well established [J. Escobar-Sevilla, 2020]. The use of anti-CD20 antibodies, corticosteroids, and chemotherapy (bendamustine, etc.) are considered factors predisposing to B19V reactivation. In addition to erythroid lineage suppression, there is evidence that other hematopoietic lines may be suppressed in B19V infection [F. Simões, 2024].

### Aims

To present a case series of B19V infection in patients with B-cell lymphomas.

### Methods

We diagnosed 4 cases of B19V infection in patients undergoing therapy of B-cell lymphomas: follicular lymphoma, marginal zone lymphoma, Hodgkin lymphoma, and DLBCL (Table 1). B19V DNA was detected by PCR in blood and bone marrow (BM) cells. In one case, B19V DNA was detected in the BM but not in the blood. Other obvious causes of cytopenia were excluded, and all patients were in remission of lymphoma. Therapy with intravenous immunoglobulin (IVIG) was administered at a dose of 0.4 g/kg, and the effect was assessed after one month. If viral replication persisted, treatment courses were repeated.

### Results

Among the 4 patients with B19V infection, 3 presented with PRCA, and Patient №2 presented with pancytopenia and a normocellular bone marrow. One patient in the chemotherapy course used rituximab, and 2 patients used rituximab with bendamustine. IVIG therapy was initiated in all patients. After the first course, Patient №2 achieved complete normalization of blood counts. Patients №1 and №3 showed an increase in hemoglobin above 100 g/L, and Patient №4 showed an increase above 80 g/L without transfusion dependence. After discontinuation of IVIG therapy, Patient №1 experienced a relapse of PRCA, while blood counts remained stable in the other patients. All patients had persistent B19V replication in the BM, necessitating repeated IVIG treatment (median follow-up time: 7 months).

### Summary/Conclusion

B19V infection is one of the causes of cytopenia in patients with B-cell lymphomas. Primarily, B19V leads to PRCA, but it can also cause trilineage cytopenia. The B19V test must also be performed in BM, as a negative result in blood may be a false negative. It can be assumed that agents causing profound B-cell and T-cell depletion (rituximab, bendamustine, etc.) may increase the risk of B19V reactivation, but this hypothesis requires confirmation in larger patient cohorts. Timely IVIG therapy can correct cytopenic syndrome, but viral reactivation monitoring is necessary.

**Copyright Information:** (Online) ISSN: 2572-9241
©2026 The Author(s). HemaSphere is published by John Wiley & Sons Ltd on behalf of European Hematology Association. This is an open access Abstract Book distributed under the Attribution-NonCommercial-NoDerivs (CC BY-NC-ND), which allows third parties to download the articles and share them with others as long as they credit the author and the Abstract Book, but they cannot change the content in any way or use them commercially.
**Abstract Book Citations:** Authors, Title, HemaSphere, 2026;10:(S1):pages. The abstract book DOIs can be found on the "Author Information" page.
**Disclaimer:** Articles published in the journal HemaSphere exclusively reflect the opinions of the authors. The authors are responsible for all content in their abstracts including accuracy of the facts, statements, citing resources, etc.

| Parvovirus B19 infection in patients with B-cell lymphomas (n=4) | | | | |
|---|---|---|---|---|
| **Patients** | №1, ♀, 50 y/o | №2, ♀, 50 y/o | №3, ♂, 25 y/o | №4, ♀, 35 y/o |
| **Diagnosis** | Follicular lymphoma | Marginal zone lymphoma | Hodgkin lymphoma | DLBCL |
| **Prior therapy** | 6 cycles of "RB" | 1 cycle of "R-EPOCH", 1 cycle of "R-BAC" | 7 cycles of "BEACOPP-14" | 4 cycles of "R-EPOCH", 3 cycles of "R-DHAP", auto-HSCT (+11 months), EBRT |
| **Status at examination** | Complete remission | Complete remission | Complete remission | Complete remission |
| **Hb (g/L)** | 84 | 91 | 37 | 68 |
| **RET (x10⁹/L)** | 11,7 | 58,2 | 1,3 | 7,6 |
| **PLT (x10⁹/L)** | 479 | 57 | 489 | 200 |
| **NEUT (x10⁹/L)** | 2,13 | 1,12 | 6,95 | 2,52 |
| **Transfusion dependence** | YES | NO | YES | YES |
| **Bone marrow histology** | PRCA | Normocellular | PRCA | PRCA |
| **Immunoglobulin levels** | NORMAL | ↓IgM (0,24 g/L) | NORMAL | ↓IgA (0,55 g/L) |
| **B19V replication: peripheral blood / bone marrow** | YES/YES | NO/YES | YES/YES | YES/YES |
| **Response after 1st course of IVIG (0.4 g/kg)** | Partial remission *(Hb>100 g/L)* | Complete remission *(Hb>120 g/L, PLT>150x10⁹/L, NEUT>1.5x10⁹/L)* | Partial remission *(Hb>100 g/L)* | Hematologic improvement *(Hb>80 g/L), no transfusion dependence* |
| **Total dose of IVIG (g)** | 120 (6 courses) | 100 (5 courses) | 40 (2 courses) | 60 (3 courses) |
| **B19V elimination from bone marrow** | NO | YES (after 1st course) | NO | NO |
| **B19V replication relapse** | - | YES (after 4st course) | - | - |
| **Relapse of cytopenia, transfusion dependence** | YES | NO | NO | NO |

Table 1. Characteristics of the patient group with B-cell lymphomas and diagnosed B19V infection Hb — hemoglobin, PLT — platelets, NEUT — neutrophils, RET — reticulocytes, PRCA — pure red cell aplasia, EBRT — external beam radiation therapy, "RB" — rituximab + bendamustine, "R-EPOCH" — rituximab + etoposide + prednisone + vincristine + cyclophosphamide + doxorubicin, "R-BAC" — rituximab + bendamustine + cytarabine, "R-DHAP" — rituximab + dexamethasone + cytarabine + cisplatin, "BEACOPP-14" — cyclophosphamide + doxorubicin + etoposide + procarbazine + prednisone + vincristine + bleomycin.

**Copyright Information:** (Online) ISSN: 2572-9241
©2026 The Author(s). HemaSphere is published by John Wiley & Sons Ltd on behalf of European Hematology Association. This is an open access Abstract Book distributed under the Attribution-NonCommercial-NoDerivs (CC BY-NC-ND), which allows third parties to download the articles and share them with others as long as they credit the author and the Abstract Book, but they cannot change the content in any way or use them commercially.
**Abstract Book Citations:** Authors, Title, HemaSphere, 2026;10:(S1):pages. The abstract book DOIs can be found on the "Author Information" page.
**Disclaimer:** Articles published in the journal HemaSphere exclusively reflect the opinions of the authors. The authors are responsible for all content in their abstracts including accuracy of the facts, statements, citing resources, etc.

### (PB3832) CLINICAL CHARACTERISTICS AND THERAPEUTIC OUTCOMES OF REFRACTORY/RELAPSED DIFFUSE LARGE B-CELL LYMPHOMA

**Topic:** 19. Large B cell lymphomas - Clinical

Maha Charfi*[1], Aicha Ben Kahla[1], Zeineb Amouri[1], Faten Kallel[1], Imen Ben Amor[1], Yousra Fakhfakh Derbel[1], Rahma Mallek[1], Turki Ines[1], Asma Koubaa[1], Nour Siala[1], Hanen Charfi[1], Moez Medhaffar[1, 1], Moez Elloumi[1]

[1]*hedi chaker hospital, hematology, sfax, Tunisia;*

### Background

Diffuse large B-cell lymphoma (DLBCL) is an aggressive non-Hodgkin lymphoma. Despite therapeutic advances, 10–15% of patients present with refractory/relapsed (R/R) disease, which is associated with a poor prognosis.

### Aims

Analyze the epidemiological and clinical characteristics and to evaluate the therapeutic outcomes of patients with R/R DLBCL.

### Methods

We conducted a retrospective study including R/R cases of DLBCL diagnosed and followed up at the Clinical Hematology Department of Hedi Chaker University Hospital in Sfax between January 2013 and December 2023. All patients were treated in first line according to the LNH 2013 protocol (R-CHOP ± autologous stem cell transplantation). Salvage therapy was based on rituximab combined with chemotherapy (DHAC or GEMOX) followed by autologous hematopoietic stem cell transplantation for patients who responded and were eligible for transplantation. We analyzed epidemiological and clinical characteristics, therapeutic response, and overall survival (OS) of R/R DLBCL cases.

### Results

We identified 39 cases of relapsed/refractory DLBCL among 130 DLBCL cases, representing 30 % during the same study period. Fourteen % of cases were refractory and 16% of cases have relapsed with median delay to relapse of 20 months. The median age was 62 years and the sex-ratio was 2,1.

Table : Clinical and Prognostic Characteristics at Initial Diagnosis

| Characteristics | Number of relapsed/refractory cases (%) | Number of cases in persistant complete remission (%) n= 91 | P |
|---|---|---|---|
| **Symptoms B** | 21/34 (62) | 60 (66) | 0,720 |
| **PS ≥ 2** | 9/38 (23) | 29 (32) | 0,078 |
| **Stage III–IV** | 27/38 (71) | 48 (53) | **0,047** |
| **Elevated LDH** | 15/38 (39) | 50 (55) | 0,04 |
| **IPIaa high risk ≥ 2** | 15/38 (39) | 39 (46) | 0,010 |

All patients received R-CHOP as first-line therapy. Salvage treatment was administered to 32 patients (82%), consisting of RDHAC in 30 patients and RGEMOX in 2 patients. Supportive care was indicated for 7 patients. The overall response rate to second-line treatment was 28%. Eleven patients underwent autologous stem cell transplantation. The 5-year overall survival (OS) rate was 13%

**Copyright Information:** (Online) ISSN: 2572-9241
©2026 The Author(s). HemaSphere is published by John Wiley & Sons Ltd on behalf of European Hematology Association. This is an open access Abstract Book distributed under the Attribution-NonCommercial-NoDerivs (CC BY-NC-ND), which allows third parties to download the articles and share them with others as long as they credit the author and the Abstract Book, but they cannot change the content in any way or use them commercially.
**Abstract Book Citations:** Authors, Title, HemaSphere, 2026;10:(S1):pages. The abstract book DOIs can be found on the “Author Information” page.
**Disclaimer:** Articles published in the journal HemaSphere exclusively reflect the opinions of the authors. The authors are responsible for all content in their abstracts including accuracy of the facts, statements, citing resources, etc.

**Summary/Conclusion**

The frequency of relapsed/refractory DLBCL in our series (30%) is higher than the data reported in the literature (10–15%). Analysis of relapsed/refractory cases identified the advanced stage (p=0,047) as predictive factor of R/R disease.
The 5-year OS rate in our study (13%) remains inferior to other series (39–51%). These results highlight the need for more intensive therapeutic approaches and novel strategies including CAR-T cell therapy and bispecific antibodies, which have significantly improved the prognosis of this disease.

**Copyright Information:** (Online) ISSN: 2572-9241
©2026 The Author(s). HemaSphere is published by John Wiley & Sons Ltd on behalf of European Hematology Association. This is an open access Abstract Book distributed under the Attribution-NonCommercial-NoDerivs (CC BY-NC-ND), which allows third parties to download the articles and share them with others as long as they credit the author and the Abstract Book, but they cannot change the content in any way or use them commercially.
**Abstract Book Citations:** Authors, Title, HemaSphere, 2026;10:(S1):pages. The abstract book DOIs can be found on the "Author Information" page.
**Disclaimer:** Articles published in the journal HemaSphere exclusively reflect the opinions of the authors. The authors are responsible for all content in their abstracts including accuracy of the facts, statements, citing resources, etc.

### (PB3833) PRIMARY GASTRIC LYMPHOMAS: CLINICAL AND DIAGNOSTIC INSIGHTS FROM A TUNISIAN COHORT

**Topic:** 19. Large B cell lymphomas - Clinical

Ameni Yahia*[1], Sarra Boukhriss[1], Imen Jemni[2], Kmar Mrad[1], Bizid Inaam[1], Wiem Boufrikha[1], Zaineb Mlayah[1], Slama Nader[1], Mohamed Adnene Laatiri[1]

*[1]Fattouma Bourguiba University Hospital, Hematology Department, Monastir, Tunisia; [2]Fattouma Bourguiba University Hospital, Gastroenterology, Monastir, Tunisia;*

### Background

Although the stomach is the most commonly involved extranodal site in non-Hodgkin lymphomas, primary gastric lymphomas remain a rare entity. Their clinical and histological heterogeneity makes them a complex disease. Data from North Africa remain limited.

### Aims

The aim of our study was to describe the epidemiological, clinical, biological, and diagnostic characteristics of patients with gastric lymphoma in a Tunisian cohort.

### Methods

This is a retrospective study including patients diagnosed with gastric lymphoma and followed at the Fattouma Bourguiba University Hospital in Monastir over an 11-year period, from January 2013 to December 2023.

### Results

Forty-six cases of gastric lymphoma were identified among 191 malignant gastric tumors (24.1%). The mean age was 55 ± 14 years, with a slight female predominance (sex ratio = 0.91). A history of gastroduodenal ulcer and dysimmune diseases was found in 10.8% of patients each. A delay in consultation of more than three months was observed in 60.8% of patients.
Epigastric pain was the predominant symptom (84.4%). Other gastrointestinal manifestations included vomiting (21.7%), dyspepsia (13%), dysphagia (6.5%), and abdominal distension (6.5%). Systemic symptoms were mainly represented by weight loss (41.3%). An inaugural complication revealed the disease in 10.9% of cases, predominantly gastrointestinal bleeding (8.7%).
At diagnosis, 71.7% of patients had a preserved performance status (PS ≤ 1). Clinical examination was normal in 56.5% of cases. Anemia was present in 50% of patients, and elevated LDH levels were observed in 23.9% of cases. The most frequent endoscopic pattern was ulcerative-proliferative (52.2%), exclusively observed in patients with diffuse large B-cell lymphoma (DLBCL), followed by ulcerative lesions (39%).
Histologically, DLBCL was the predominant subtype (76.1%), followed by MALT lymphoma (23.9%). *Helicobacter pylori* infection was detected in 79% of patients. After staging workup, 65.2% of patients were diagnosed at a localized stage, whereas 34.8% had advanced disease, including 11 cases classified as stage IV.

### Summary/Conclusion

Primary gastric lymphomas represent a rare and heterogeneous entity. Diagnosis is often delayed due to nonspecific symptoms, although the majority of patients are identified at a localized stage. Early and multidisciplinary diagnostic management remains essential.

**Copyright Information:** (Online) ISSN: 2572-9241
©2026 The Author(s). HemaSphere is published by John Wiley & Sons Ltd on behalf of European Hematology Association. This is an open access Abstract Book distributed under the Attribution-NonCommercial-NoDerivs (CC BY-NC-ND), which allows third parties to download the articles and share them with others as long as they credit the author and the Abstract Book, but they cannot change the content in any way or use them commercially.
**Abstract Book Citations:** Authors, Title, HemaSphere, 2026;10:(S1):pages. The abstract book DOIs can be found on the "Author Information" page.
**Disclaimer:** Articles published in the journal HemaSphere exclusively reflect the opinions of the authors. The authors are responsible for all content in their abstracts including accuracy of the facts, statements, citing resources, etc.

### (PB3834) R-MIADD FOR SECONDARY CNS INVOLVEMENT IN DIFFUSE LARGE B-CELL LYMPHOMA: A SINGLE-CENTER EXPERIENCE

**Topic:** 19. Large B cell lymphomas - Clinical

Deonne Thaddeus Gauiran[1], Rene Amadore*[1], Jerome Anopol[1], Edessa Casalla[1], Victor Alfred Catambing[1]

[1]*University of the Philippines - Philippine General Hospital, Division of Hematology, Department of Medicine, Manila, Philippines;*

**Background**

Secondary central nervous system lymphoma (SCNSL) is a rare yet devastating complication of diffuse large B-cell lymphoma (DLBCL), associated with dismal outcomes despite modern immunochemotherapy. No standard induction regimen has been established for patients with overt CNS involvement, particularly in resource-constrained settings where access to intensive consolidation strategies may be limited.

**Aims**

To describe the feasibility, safety, and clinical outcomes of the R-MIADD regimen in adult patients with DLBCL and secondary CNS involvement.

**Methods**

We conducted a retrospective descriptive case series of four adult patients with histologically confirmed DLBCL and CNS involvement treated with R-MIADD (rituximab, high-dose methotrexate, ifosfamide, cytarabine, liposomal doxorubicin, and dexamethasone). Clinical characteristics, treatment delivery, toxicities, and outcomes were reviewed. Systemic responses were assessed using Lugano criteria where applicable, while CNS responses were evaluated by serial MRI with clinical correlation. Toxicities were graded according to CTCAE v5.0.

**Copyright Information:** (Online) ISSN: 2572-9241
©2026 The Author(s). HemaSphere is published by John Wiley & Sons Ltd on behalf of European Hematology Association. This is an open access Abstract Book distributed under the Attribution-NonCommercial-NoDerivs (CC BY-NC-ND), which allows third parties to download the articles and share them with others as long as they credit the author and the Abstract Book, but they cannot change the content in any way or use them commercially.
**Abstract Book Citations:** Authors, Title, HemaSphere, 2026;10:(S1):pages. The abstract book DOIs can be found on the "Author Information" page.
**Disclaimer:** Articles published in the journal HemaSphere exclusively reflect the opinions of the authors. The authors are responsible for all content in their abstracts including accuracy of the facts, statements, citing resources, etc.

### Results

Three patients had stage IV DLBCL at diagnosis, while one had stage II disease. CNS involvement occurred at relapse following R-CHOP–based therapy in three patients (including the stage II patient who relapsed with brain involvement) and was present at diagnosis in one patient. CNS disease involved the brain parenchyma and/or spinal cord. R-MIADD was successfully delivered in all patients. Three patients achieved complete CNS response, while one achieved partial CNS response with marked neurologic improvement, progressing from non-ambulatory to ambulatory status after six cycles, but was subsequently lost to follow-up. Systemic disease control was achieved in all evaluable patients. Hematologic toxicities were frequent, including grade 3–4 neutropenia in all cases. Non-hematologic toxicities were limited and reversible. One patient discontinued therapy after three cycles due to reversible ifosfamide-related neurotoxicity and proceeded to autologous stem cell transplantation. No treatment-related mortality occurred. Clinical characteristics and outcomes are summarized in Table 1.

Table 1. Clinical Characteristics and Outcomes

| **Variable** | **Case 1** | **Case 2** | **Case 3** | **Case 4** |
|---|---|---|---|---|
| Stage at initial diagnosis | IV | II | IV | IV |
| Main CNS site | Brainstem/ cerebellum | Spinal cord | Cerebellum/ midbrain | Pons |
| Best CNS response | CR | PR | CR | CR |
| Best systemic response | CR | CR | CR | CR |
| R-MIADD cycles received | 3 | 6 | 6 | 6 |
| Key clinical outcome | Switched to auto-HSCT | Functional recovery; lost to follow-up | Isolated CNS relapse → WBRT + auto-HSCT | Durable remission |
| Treatment-related mortality | No | No | No | No |

### Summary/Conclusion

R-MIADD was feasible and demonstrated meaningful CNS and systemic activity with manageable toxicity in patients with SCNSL. Clinically relevant responses were observed across heterogeneous CNS presentations and were accompanied by functional neurologic improvement. The regimen was deliverable in a real-world setting. These findings suggest that CNS-directed combination therapy may offer a potential therapeutic option for selected patients, particularly in settings where immediate transplant consolidation is not feasible, and support further prospective evaluation of CNS-penetrant strategies in this high-risk population.

**Copyright Information:** (Online) ISSN: 2572-9241
©2026 The Author(s). HemaSphere is published by John Wiley & Sons Ltd on behalf of European Hematology Association. This is an open access Abstract Book distributed under the Attribution-NonCommercial-NoDerivs (CC BY-NC-ND), which allows third parties to download the articles and share them with others as long as they credit the author and the Abstract Book, but they cannot change the content in any way or use them commercially.
**Abstract Book Citations:** Authors, Title, HemaSphere, 2026;10:(S1):pages. The abstract book DOIs can be found on the "Author Information" page.
**Disclaimer:** Articles published in the journal HemaSphere exclusively reflect the opinions of the authors. The authors are responsible for all content in their abstracts including accuracy of the facts, statements, citing resources, etc.

### (PB3835) PRIMARY EXTRANODAL DIFFUSE LARGE B-CELL LYMPHOMA: 76 CASES FROM NORTHERN MOROCCO IN THE RITUXIMAB ERA

**Topic:** 19. Large B cell lymphomas - Clinical

Siham Belkadi*[1], Imane El Boutahiri[1], Oumayma Hari[1], Safae Regragui[1]

[1]*University Hospital Center Mohammed VI, Clinical Hematology Service, Tangier, Morocco;*

### Background

Primary extranodal diffuse large B-cell lymphomas (PE-DLBCL) represent a subgroup of DLBCL whose definition remains controversial. Two definitions coexist: the first describes clinically dominant extranodal masses without nodal involvement, while the second allows minor nodal involvement at diagnosis.
Published series estimate that approximately 30–40% of DLBCL are initially extranodal, with survival varying according to the organ of origin independent of the IPI score, which considers ≥2 extranodal sites as poor prognosis, a factor that does not apply to DLBCL-PE with a single initial site.

### Aims

This study aims to evaluate clinico-pathological features and outcomes (PFS/OS) of 76 PE-DLBCL cases from Northern Morocco in the rituximab era.

### Methods

We conducted a retrospective, single-center study at Tangier University Hospital Center (CHU) from 2018 to 2022. Seventy-six patients diagnosed with histopathologically and radiologically confirmed PE-DLBCL were included.
Data extracted from medical records included primary tumor location, histological subtype, treatments administered (chemotherapy, radiotherapy, others), as well as short- and long-term outcomes, particularly overall survival (OS) and progression-free survival (PFS).

### Results

Of the 76 patients, 52 (68%) were men and 24 (32%) women, with a median age of 54 years. Primary sites were mostly gastrointestinal (31%), followed by ENT (24%) and bone (12%).
Histopathologically, 37% were non-germinal center type, 14.7% germinal center type, and Ki67 expression exceeded 70% in 25% of cases.
The treatments administered were mainly R-CHOP, with 4 patients receiving radiotherapy. The initial treatment response rate was 77.77%, and 13.88% of patients experienced relapse or progression after treatment. The median OS was 43.27 months, and the median PFS was 47.22 months.

### Summary/Conclusion

This is the first large North African PE-DLBCL series, highlighting high frequency of gastrointestinal sites consistent with published series. Standard R-CHOP yields favorable responses in most patients, but median PFS (47 months) and OS (43 months) indicate reserved prognosis.

**Copyright Information:** (Online) ISSN: 2572-9241
©2026 The Author(s). HemaSphere is published by John Wiley & Sons Ltd on behalf of European Hematology Association. This is an open access Abstract Book distributed under the Attribution-NonCommercial-NoDerivs (CC BY-NC-ND), which allows third parties to download the articles and share them with others as long as they credit the author and the Abstract Book, but they cannot change the content in any way or use them commercially.
**Abstract Book Citations:** Authors, Title, HemaSphere, 2026;10:(S1):pages. The abstract book DOIs can be found on the "Author Information" page.
**Disclaimer:** Articles published in the journal HemaSphere exclusively reflect the opinions of the authors. The authors are responsible for all content in their abstracts including accuracy of the facts, statements, citing resources, etc.

### (PB3836) COMPLETE METABOLIC RESPONSE TO POLATUZUMAB VEDOTIN AND GLOFITAMAB IN PRIMARY REFRACTORY DOUBLE-EXPRESSOR DIFFUSE LARGE B-CELL LYMPHOMA WITH EXTENSIVE EXTRANODAL DISEASE AND ECOG 4

**Topic:** 19. Large B cell lymphomas - Clinical

Ayesha Alaleeli*[1], Reem Alsaadi[1], Sara Saad[1], Anas Hanini[1], Carlos Barrera[1], Yaser Alkhatib[1], Fatin Sammour[1], Ahmad Raza[1], Stephen Grobmyer[1], Ajlan Al Zaki[1, 2]

*[1]Cleveland Clinic Abu Dhabi, Abu Dhabi, United Arab Emirates;[2]Khalifa University, Abu Dhabi, United Arab Emirates;*

### Background

Diffuse large B-cell lymphoma is one of the most common types of non-Hodgkin lymphoma, accounting for approximately 30-40% of cases. Although MYC, BCL2, and BCL6 rearrangements are known to be associated with a poor prognosis, co-overexpression of MYC, BCL2 and/or BCL6 proteins, also known as double-expressors (DE-DLBCL) or triple-expressors (TE-DLBCL), is much more common, occurring in 20–30% of cases, and still carries a poor prognosis compared to non-double-expressor DLBCL, frequently displaying resistance to standard R-CHOP chemotherapy.

### Aims

To describe the clinical course, progression and treatment response in a primary resistant DE-DLBCL patient receiving Polatuzumab vedotin and Glofitamab, with severe extranodal involvement and poor baseline functional status.

### Methods

We present the case of a 70-year-old male patient with CD20-positive DLBCL who had a high proliferative index (Ki-67 ~90%), overexpression of MYC (90%) and BCL2 (80–90%). Ascites, peritoneal deposits, pleural effusions, widespread nodal disease, and significant stomach wall involvement were all seen on the initial staging PET/CT scan. A bone marrow biopsy revealed only 0.3% involvement of clonal B cells.
At treatment initiation, the patient was bed-bound (ECOG performance status 4) with severe cancer-related cachexia that required nasogastric tube feeding.
Due to frailty and risk of adverse effects, the patient underwent one cycle of DA-R-EPOCH and two cycles of R-mini-CHOP. Mid-treatment PET/CT demonstrated a mixed metabolic response with persistent peritoneal disease and slight progression in gastric involvement. Refractory lymphoma was confirmed by a second biopsy (Fig. 1A).
CAR T-cell and other conventional chemotherapy were considered, but the patient was deemed unsuitable for these approaches due to severe cachexia and poor performance status.
After receiving obinutuzumab pretreatment, Polatuzumab vedotin was initiated, followed by step-up dosing of Glofitamab. PET/CT was subsequently performed to evaluate response using Lugano criteria.

**Copyright Information:** (Online) ISSN: 2572-9241
©2026 The Author(s). HemaSphere is published by John Wiley & Sons Ltd on behalf of European Hematology Association. This is an open access Abstract Book distributed under the Attribution-NonCommercial-NoDerivs (CC BY-NC-ND), which allows third parties to download the articles and share them with others as long as they credit the author and the Abstract Book, but they cannot change the content in any way or use them commercially.
**Abstract Book Citations:** Authors, Title, HemaSphere, 2026;10:(S1):pages. The abstract book DOIs can be found on the "Author Information" page.
**Disclaimer:** Articles published in the journal HemaSphere exclusively reflect the opinions of the authors. The authors are responsible for all content in their abstracts including accuracy of the facts, statements, citing resources, etc.

**Results**

Despite aggressive, chemo-refractory disease with extensive extranodal involvement and profound baseline functional status impairment, the patient demonstrated rapid clinical improvement following initiation of Polatuzumab and Glofitamab.
Treatment was well tolerated, with no cytokine release syndrome or ICANS. A delayed cutaneous hypersensitivity reaction occurred after cycle 3 necessitating temporary treatment interruption, which was successfully managed with corticosteroids, allowing re-initiation of step-up dosing of Glofitamab. Notably, marked functional recovery was observed, including successful removal of the nasogastric tube.
PET/CT performed after the second cycle of Glofitamab and the third cycle of Polatuzumab vedotin showed complete metabolic response, with interval resolution of hypermetabolic disease in the chest, abdomen, and pelvis (DS 1) (Fig. 1B). The patient achieved complete symptom resolution and remains in ongoing remission at the latest follow-up.

**Summary/Conclusion**

This case demonstrates the potential efficacy and tolerability of Polatuzumab vedotin combined with Glofitamab in aggressive refractory DLBCL with extensive extranodal involvement. The rapid achievement of a complete metabolic response using antibody-based immunotherapy, even in the presence of high-risk tumor biology and severe baseline frailty, supports their expanding role in treating patients who are unfit for autologous transplant or intensive salvage strategies.

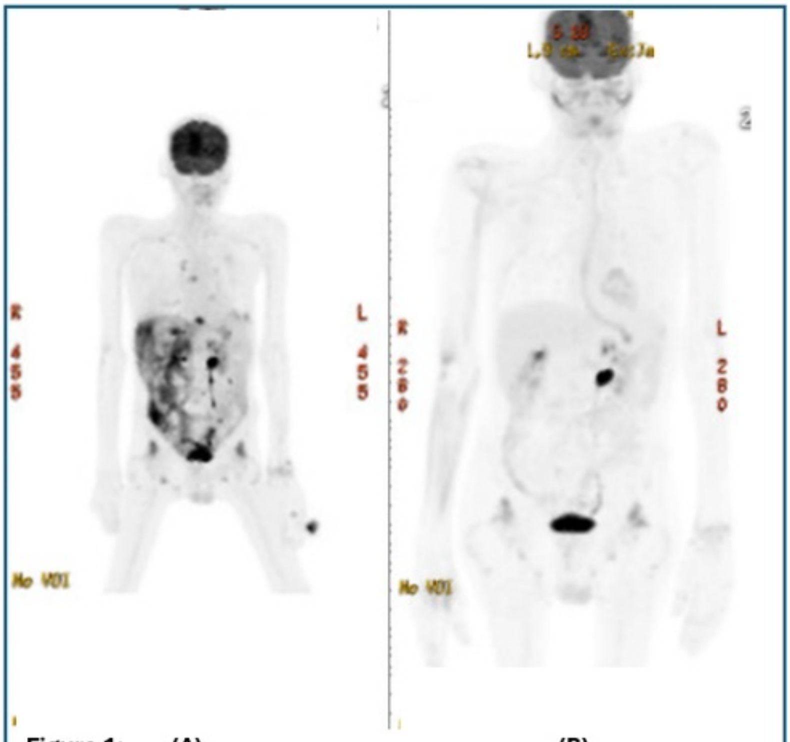

Figure 1: (A) (B)

**Copyright Information:** (Online) ISSN: 2572-9241
©2026 The Author(s). HemaSphere is published by John Wiley & Sons Ltd on behalf of European Hematology Association. This is an open access Abstract Book distributed under the Attribution-NonCommercial-NoDerivs (CC BY-NC-ND), which allows third parties to download the articles and share them with others as long as they credit the author and the Abstract Book, but they cannot change the content in any way or use them commercially.
**Abstract Book Citations:** Authors, Title, HemaSphere, 2026;10:(S1):pages. The abstract book DOIs can be found on the "Author Information" page.
**Disclaimer:** Articles published in the journal HemaSphere exclusively reflect the opinions of the authors. The authors are responsible for all content in their abstracts including accuracy of the facts, statements, citing resources, etc.

### (PB3837) PROGNOSTIC IMPACT OF BASELINE WEIGHT LOSS ON SURVIVAL OUTCOMES IN DIFFUSE LARGE B-CELL LYMPHOMA:A SINGLE-CENTER STUDY IN TUNISIA

**Topic:** 19. Large B cell lymphomas - Clinical

Daami Mayssa*[1], Zaineb Mlayah[1], Wiem Boufrikha[1], Bizid Inaam[1], Slama Nader[1], Sarra Boukhriss[1], Mohamed Adnene Laatiri[1]

[1]*Fattouma Bourguiba University Hospital, clinical hematology, MONASTIR, Tunisia;*

### Background

Unintentional weight loss is a major diagnostic criterion of malnutrition according to French National Authority for Health (HAS) guidelines and reflects impaired nutritional status in patients with hematologic malignancies. In diffuse large B-cell lymphoma (DLBCL), its independent prognostic significance remains insufficiently defined.

### Aims

Evaluate the prevalence of weight loss at diagnosis and its impact on overall survival (OS), relapse-free survival (RFS), and progression-free survival (PFS).

### Methods

This retrospective single-center study included adult patients diagnosed with DLBCL between 2020 and 2024 at the Department of Clinical Hematology, Fattouma Bourguiba University Hospital, Monastir. Weight loss was defined as ≥5% body weight reduction within 1 month or ≥10% within 6 months prior to diagnosis. Survival outcomes were estimated using the Kaplan–Meier method and compared using the log-rank test.

### Results this study included 55 patients. At diagnosis, 34 patients (61.8%) met criteria for weight loss. Three-year OS was significantly lower in patients with weight loss compared to those without (48.4% vs 100%; p=0.006). Similarly, three-year PFS was significantly reduced (59.7% vs 95.2%; p=0.020). Although patients with weight loss had inferior three-year RFS (41.2% vs 75.0%), this difference did not reach statistical significance (p=0.120).

### Summary/Conclusion

Consistent with the literature, baseline weight loss was significantly associated with inferior OS and PFS in DLBCL. These findings support the integration of early nutritional assessment into initial risk stratification.

**Copyright Information:** (Online) ISSN: 2572-9241
©2026 The Author(s). HemaSphere is published by John Wiley & Sons Ltd on behalf of European Hematology Association. This is an open access Abstract Book distributed under the Attribution-NonCommercial-NoDerivs (CC BY-NC-ND), which allows third parties to download the articles and share them with others as long as they credit the author and the Abstract Book, but they cannot change the content in any way or use them commercially.
**Abstract Book Citations:** Authors, Title, HemaSphere, 2026;10:(S1):pages. The abstract book DOIs can be found on the "Author Information" page.
**Disclaimer:** Articles published in the journal HemaSphere exclusively reflect the opinions of the authors. The authors are responsible for all content in their abstracts including accuracy of the facts, statements, citing resources, etc.

### (PB3838) SUCCESSFUL USE OF CAR-T WITH BRIDGE RADIOTHERAPY IN A PATIENT WITH EARLY RELAPSE OF REFRACTORY DLBCL AFTER ALLO-HSCT. CLINICAL CASE.

**Topic:** 19. Large B cell lymphomas - Clinical

Katsiaryna Zharkova*[1], Natalya Konoplya[1], Tatyana Savich[1], Alena Stsepanovich[1], Tatyana Doroshenko[1], Sergey Poliakov[1], Anna Smolianinova[2], Evgeniy Zvonkov[2], Ihar Severin[1], Yuliya Mareiko[3], Elena Parovichnikova[2], Svetlana Tatarnicova[2], Eldar Saridze[1]

*[1]N.N. Alexandrov National Cancer Centre of Belarus, Minsk, Belarus;[2]National medical research center for hematology, Moscow, Russian Federation, Moscow, Russia; [3]Centre for pediatric oncology, hematology and immunology, Minsk, Belarus;*

### Background

To the best of our knowledge there are few reports describing the clinical efficacy of anti-CD19 CAR-T therapy in patients with refractory DLBCL with early relapse following allo-HSCT.

**Copyright Information:** (Online) ISSN: 2572-9241
©2026 The Author(s). HemaSphere is published by John Wiley & Sons Ltd on behalf of European Hematology Association. This is an open access Abstract Book distributed under the Attribution-NonCommercial-NoDerivs (CC BY-NC-ND), which allows third parties to download the articles and share them with others as long as they credit the author and the Abstract Book, but they cannot change the content in any way or use them commercially.
**Abstract Book Citations:** Authors, Title, HemaSphere, 2026;10:(S1):pages. The abstract book DOIs can be found on the "Author Information" page.
**Disclaimer:** Articles published in the journal HemaSphere exclusively reflect the opinions of the authors. The authors are responsible for all content in their abstracts including accuracy of the facts, statements, citing resources, etc.

### Results

Patient A., 35 years old, was diagnosed with diffuse large B-cell lymphoma/ high grade B-cell lymphoma with MYC and BCL2 rearrangements, skeletal bone involvement (Th12 vertebra with involvement of massive paravertebral conglomerate at the level of Th10 - L2, prolapsing into the spinal canal, involvement of the diaphysis of the right humerus), involvement of lymph nodes on both sides of the diaphragm, bone marrow, neuroleukemia, IVB stage Ann Arbor, hight-risk IPI in January 2025. The patient had severe neurological deficit.
After three lines of chemotherapy, including auto-HSCT, remission was not achieved. On September 9, 2025, a haplo-HSCT with post-transplantcyclophosphamide was performed from a related donor (sister), with $6.0x10^6$/kg CD34+ cells. Conditioning was performed according to the fludarabine + busulfan protocol. MRI on day +16 after allo-HSCT revealed progression. Complete donor chimerism (blood and bone marrow) was detected on day +30. The only treatment currently available was CAR-T immune cell therapy with sister as a donor of lymphocytes.
In the early post-transplant period, the patient received immunosuppressive therapy with 400 mg of cyclosporine and 3000 mg of mycophenolate mofetil daily. Further GVHD prophylaxis at this dose would render the planned CAR-T therapy ineffective. An attempt was made to gradually discontinue immunosuppressive therapy based on monitoring for acute GVHD. When cyclosporine and MMF were reduced to 200 mg and 1000 mg respectively, symptoms of grade 3 cutaneous GVHD and grade 1 intestinal GVHD developed. Short course methylprednisolone 500 mg was additionally administered. Subsequently, the patient was receiving the above-mentioned immunosuppressive therapy doses without signs of active GVHD. Cyclosporine levels ranged from 95 to 319 ng/ml, with an average level of 144.
For the purpose of cytoreduction, weakening of clinical symptoms and prevention of further development of neurological deficit, bridge therapy was performed in the form of radiation therapy applied to the fastest growing and symptomatic recurrent lesion with a total dose of 30 Gy in 10 fractions. In a week patient received lymphodepleting chemotherapy with cyclophophamide (500mg/m²) and fludarabine (30mg/m²) on days -5 to -3. Anti-CD19 CAR-T cells were infused at the dose $1,15X10^6$/kg on day 0 (+ 55 day after allo-HSCT).
The patient developed cytokine release syndrome (CRS) Grade II, from day +7 to day +9 and required oxygen support. CT scans revealed left-sided hydrothorax, which was successfully resolved by puncture. Immune effector cell-associated neurotoxicity syndrome (ICANS) Grade II (ICE III) was observed from day +10 to day + 14, and was relieved by administering dexamethasone and anakinra. Peak CAR-T cell concentration in peripheral blood was measured on day + 12 (661 cells/µl) with further persistence up to the last measurement in 4 months.
FDG-PET scan on day 30 revealed complete metabolic response (Deauville Score 2).

### Summary/Conclusion

This clinical case demonstrated the efficacy of CAR-T cell therapy in a patient under the immunosuppressive drugs regime without the development of pronounced manifestations of GVHD in the early post-transplant period. Bridging radiotherapy proved to be an efficient tool to control tumor progression.

**Copyright Information:** (Online) ISSN: 2572-9241
©2026 The Author(s). HemaSphere is published by John Wiley & Sons Ltd on behalf of European Hematology Association. This is an open access Abstract Book distributed under the Attribution-NonCommercial-NoDerivs (CC BY-NC-ND), which allows third parties to download the articles and share them with others as long as they credit the author and the Abstract Book, but they cannot change the content in any way or use them commercially.
**Abstract Book Citations:** Authors, Title, HemaSphere, 2026;10:(S1):pages. The abstract book DOIs can be found on the "Author Information" page.
**Disclaimer:** Articles published in the journal HemaSphere exclusively reflect the opinions of the authors. The authors are responsible for all content in their abstracts including accuracy of the facts, statements, citing resources, etc.

### (PB3839) PREVALENCE OF SARCOPENIA AND ITS PROGNOSTIC IMPACT ON SURVIVAL IN DIFFUSE LARGE B-CELL LYMPHOMA:A SINGLE-CENTER STUDY FROMTUNISIA

**Topic:** 19. Large B cell lymphomas - Clinical

Daami Mayssa*[1], Zaineb Mlayah[1], Wiem Boufrikha[1], Bizid Inaam[1], Slama Nader[1], Sarra Boukhriss[1], Mohamed Adnene Laatiri[1]

*[1]Fattouma Bourguiba University Hospital, clinical hematology, MONASTIR, Tunisia;*

### Background

Body composition has emerged as a relevant host-related determinant of outcomes in oncology. Sarcopenia, defined as reduced skeletal muscle mass assessed by computed tomography, has been associated with inferior survival in several solid and hematologic malignancies. However, its prevalence and prognostic significance in diffuse large B-cell lymphoma (DLBCL) remain inconsistently reported.

### Aims

To determine the prevalence of CT-defined sarcopenia at diagnosis and to evaluate its impact on overall survival (OS), progression-free survival (PFS), and relapse-free survival (RFS) in patients with DLBCL.

### Methods

This retrospective single-center study included 55 adult patients diagnosed with DLBCL between 2020 and 2024 at the Department of Clinical Hematology, Fattouma Bourguiba University Hospital, Monastir. Sarcopenia was assessed on baseline computed tomography at the L3 level using skeletal muscle index (SMI). Sarcopenia was defined as SMI $<52.4$ cm²/m² in men and $<38.5$ cm²/m² in women. Overall survival (OS), progression-free survival (PFS), and relapse-free survival (RFS) were estimated using the Kaplan–Meier method and compared by log-rank test.

### Results

Fifty-five patients were included in the analysis. Radiological sarcopenia at diagnosis was identified in 12 patients (21.8%), including 9 men and 3 women, according to predefined sex-specific SMI thresholds.
Three-year overall survival (OS) was 71.7% in sarcopenic patients compared with 78.6% in non-sarcopenic patients ($p=0.931$).
Three-year relapse-free survival (RFS) was 49.4% in sarcopenic patients versus 64.9% in non-sarcopenic patients ($p=0.756$).
Three-year progression-free survival (PFS) was 73.6% in sarcopenic patients compared with 90.9% in non-sarcopenic patients ($p=0.427$).

### Summary/Conclusion

Although prior studies have reported sarcopenia as an adverse prognostic factor in DLBCL, our findings did not demonstrate a statistically significant association with survival outcomes. The relatively low prevalence observed and limited sample size may partly explain this discrepancy. These results underscore the need for larger prospective studies to clarify the independent prognostic role of body composition in DLBCL.

**Copyright Information:** (Online) ISSN: 2572-9241
©2026 The Author(s). HemaSphere is published by John Wiley & Sons Ltd on behalf of European Hematology Association. This is an open access Abstract Book distributed under the Attribution-NonCommercial-NoDerivs (CC BY-NC-ND), which allows third parties to download the articles and share them with others as long as they credit the author and the Abstract Book, but they cannot change the content in any way or use them commercially.
**Abstract Book Citations:** Authors, Title, HemaSphere, 2026;10:(S1):pages. The abstract book DOIs can be found on the "Author Information" page.
**Disclaimer:** Articles published in the journal HemaSphere exclusively reflect the opinions of the authors. The authors are responsible for all content in their abstracts including accuracy of the facts, statements, citing resources, etc.

### (PB3840) INTENSIVE THERAPY FOR HIGH-RISK B-CELL LYMPHOMAS. A 10-YEAR SINGLE-CENTER EXPERIENCE

**Topic:** 20. Other aggressive lymphomas

Nelli Gabeeva*[1], Daria Koroleva[2], Anna Smolianinova[2], Chabaeva Julia[2], Tatiana Obukhova[2], Alla Kovrigina[2], Evgeniy Zvonkov[2]

*[1]NATIONAL MEDICAL RESEARCH CENTER FOR HEMATOLOGY, Moscow, Russia;[2]NATIONAL MEDICAL RESEARCH CENTER FOR HEMATOLOGY Moscow, Russia, Moscow, Russia;*

### Background

Despite the superior efficacy of intensive chemotherapy regimens (ICT) compared with the standard R-CHOP regimen in the first-line treatment of aggressive B-cell lymphomas (ABL), their broader application is limited by high toxicity. The identification of optimal chemotherapy (CT) that combine high efficacy with acceptable tolerability remains an unmet clinical need. In current treatment of ABL, targeted agents are being increasingly used, raising the question of their integration into ICT.

### Aims

To report 10-year long-term outcomes of ICT based on modified pediatric NHL-BFM-90 protocol (blocks A and B) with rituximab (R-mNHL-BFM-90) adapted for adult patients with newly diagnosed ABL.

### Methods

Between 2015 and 2025, 435 pts with ABL received treatment in our department. The analysis included 312 pts with newly diagnosed ABL: diffuse large B-cell lymphoma (DLBCL) – 171 (54.8%), primary mediastinal lymphoma (PMBCL) – 41 (13%), follicular lymphoma grade 3A+B (FL3A+B)–35 (11.2%), Burkitt lymphoma (BL) – 28 (9%), high-grade B-cell lymphoma with MYC/BCL2/BCL6 rearrangement (HGBL MYC/BCL2/BCL6)–20 (6.5%), HGBL NOS–7 (2.3%), gray-zone lymphoma (GZL)–7 (2.3%), plasmablastic lymphoma (PBL) – 3 (0.9%). Among DLBCL the non-GCB subtype accounted for 102/171 (59.6%), GCB-56/171 (32.7%), unclassified-13/171 (7.6%). Double-expressor lymphoma - 21 pts (6.7%). TP53 gene mutations were detected in 24.7% pts. According to the protocols, 243/312 ps (78%) received ICT. The median age 48 y. (range, 18–70). Among them, 181 pts (74.5%) received 4 cycles of R-mNHL-BFM-90, 4 pts (1.6%)-2 cycles, and 3 pts (1.2%)-6 cycles. Hybrid treatment regimens consists of 2 cycles of R-mNHL-BFM-90 combined with R-CHOP or EPOCH or BEACOPP received 55 pts (22.6%). ICT combined with mono- or multi-targeted agents (lenalidomide/ibrutinib/ /venetoclax) and/or polatuzumab vedotin or bispecific monoclonal antibody (glofitamab) was administered to 114/243 pts (47%). Non-intensive therapy (non-ICT) protocols, including R-CHOP, R-EPOCH/HMA with/without targeted agents, were given to 69/312 patients (22%).

### Results

Following ICT, complete remission (CR) was achieved in 211/243 patients (87%). Relapse/refractoriness (R/R) occurred in 30/243 patients (12.3%), death in remission from AML three years after treatment completion in 1/243 patient (0.4%), and early death during therapy in 1/243 patient (0.4%) due to tumor lysis syndrome. Among the 30 R/R patients, 14 (46.6%) remain in remission (7 after chemotherapy ± targeted therapy, 6 after CAR-T, and 1 after allo-HSCT). CAR-T therapy was administered as second or third-line treatment in 3 pts, after multiple lines and allo-HSCT in 1 pt, and after 2 cycles of first-line therapy in 2 pts. At a median follow-up of 48 months, the 5-year event-free survival (EFS) and overall survival (OS) rates were 85% and 92%, respectively (Fig. 1). In the non-ICT group, CR was achieved in 39/69 (56.5%). R/R occurred in 26/69 (37.6%), early death on therapy 2/69 (3%), death from AML or MDS 2 (3%). At a median follow-up of 70 months, the 5-year EFS and OS rates were 58% and 68%.

**Copyright Information:** (Online) ISSN: 2572-9241
©2026 The Author(s). HemaSphere is published by John Wiley & Sons Ltd on behalf of European Hematology Association. This is an open access Abstract Book distributed under the Attribution-NonCommercial-NoDerivs (CC BY-NC-ND), which allows third parties to download the articles and share them with others as long as they credit the author and the Abstract Book, but they cannot change the content in any way or use them commercially.
**Abstract Book Citations:** Authors, Title, HemaSphere, 2026;10:(S1):pages. The abstract book DOIs can be found on the "Author Information" page.
**Disclaimer:** Articles published in the journal HemaSphere exclusively reflect the opinions of the authors. The authors are responsible for all content in their abstracts including accuracy of the facts, statements, citing resources, etc.

### Summary/Conclusion

Analysis of a 10-year experience treating ABL using R-mNHL-BFM-90 demonstrated the feasibility of ICT de-escalation without loss of efficacy, achieved through a reduction in the number of cycles and the use of hybrid treatment regimens. The integration of mono- and multi-targeted therapies into a methotrexate-containing ICT was shown to be possible.

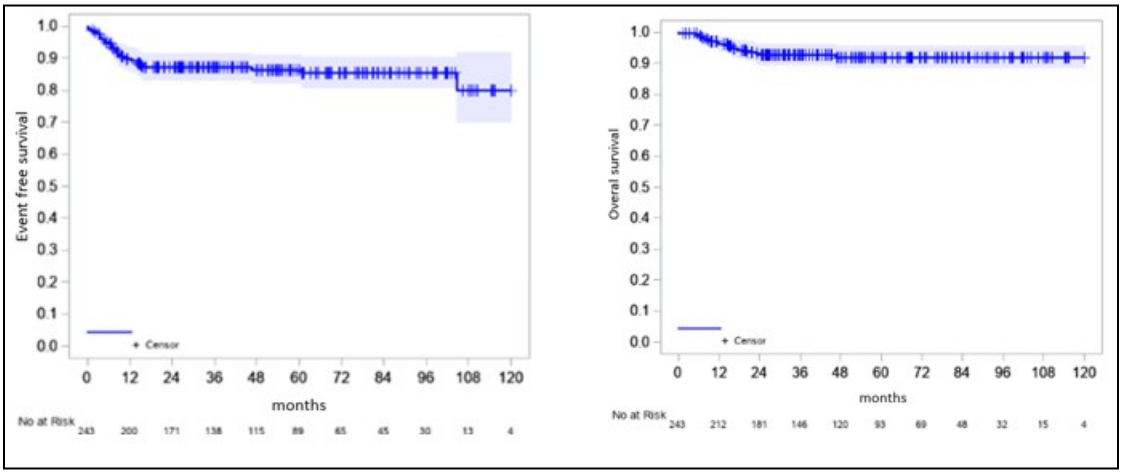

Five-year EFS and OS in patients with ABL treated with ICT.

**Copyright Information:** (Online) ISSN: 2572-9241
©2026 The Author(s). HemaSphere is published by John Wiley & Sons Ltd on behalf of European Hematology Association. This is an open access Abstract Book distributed under the Attribution-NonCommercial-NoDerivs (CC BY-NC-ND), which allows third parties to download the articles and share them with others as long as they credit the author and the Abstract Book, but they cannot change the content in any way or use them commercially.
**Abstract Book Citations:** Authors, Title, HemaSphere, 2026;10:(S1):pages. The abstract book DOIs can be found on the "Author Information" page.
**Disclaimer:** Articles published in the journal HemaSphere exclusively reflect the opinions of the authors. The authors are responsible for all content in their abstracts including accuracy of the facts, statements, citing resources, etc.

### (PB3841) EFFICACY AND PROGNOSTIC ANALYSIS OF FIRST - LINE BTK INHIBITOR THERAPY IN MANTLE CELL LYMPHOMA: A REAL - WORLD MULTICENTER STUDY

**Topic:** 20. Other aggressive lymphomas

Haiwen Huang*[1], Longfei He[1], Tao You[1], Zhou Jin[1], Wanying Chen[1], Qian Cheng[1], Kaiyu Sheng[1], Shubang Xie[1]

[1] *The First Affiliated Hospital Of Soochow University, Hematology, Suzhou, China;*

### Background

Mantle cell lymphoma (MCL) is an incurable, aggressive B-cell malignancy. While intensive chemoimmunotherapy followed by autologous stem cell transplantation (ASCT) is standard for young, fit Western patients, optimal first-line strategies in Asian populations remain unclear. Real-world data are limited. Bruton's tyrosine kinase inhibitors (BTKi) have reshaped front-line therapy, yet their real-world effectiveness and impact on outcomes need further study.

### Aims

To describe initial treatment patterns and survival outcomes in real-world MCL patients, focusing on overall survival (OS), progression-free survival (PFS), and multivariate prognostic analysis.

### Methods

This retrospective study included 235 newly diagnosed MCL patients from four Chinese hematology centers (2017–2025). Statistical analyses included: Chi-square/Fisher's exact tests for categorical variables; Kaplan-Meier and log-rank tests for OS/PFS; Cox regression for independent prognostic factors.

### Results

Median age was 61 years; 9.4% had ECOG PS ≥2; 73.2% had stage III–IV disease; 18.7% were MIPI high-risk; 30.6% had elevated LDH; 52.3% had bone marrow involvement; 59.6% had Ki-67 ≥30%. Treatment: 43.8% received BTKi; 92.8% received rituximab; 26.0% received intermediate-dose cytarabine (most common chemotherapy), 25.5% CHOP-like, 7.7% bendamustine-based; 26.0% underwent ASCT.

After median follow-up of 29.83 months, median PFS was 58.23 months; median OS was not reached (over 50% alive as of September 30, 2025). ORR was 75.3%; CR 50.6%; 2-/3-year OS: 82.9%/78.6%; 2-/3-year PFS: 73.8%/67.5%. BTKi and non-BTKi groups were balanced in age, MIPI, stage, LDH, Ki-67 (all P>0.05). BTKi group had lower cytarabine use (10.7% vs. 37.9%, P<0.001) but higher 3-year OS (86.5% vs. 73.3%, P=0.004) and numerically higher 3-year PFS (79.0% vs. 61.1%, P=0.088). Univariate Cox analysis: rituximab, ECOG PS, Ki-67, MIPI affected PFS/OS. Multivariate Cox: BTKi was independent protective for OS (HR=0.238, 95% CI 0.063–0.902, P=0.035); Ki-67≥30% was adverse for OS (HR=3.261, 95% CI 1.017–10.452, P=0.047). No survival benefit was observed for cytarabine regimens (3-year OS: 80.2% vs. 78.0%, P=0.318; PFS: 67.1% vs. 67.8%, P=0.878) or ASCT (3-year OS: 76.1% vs. 79.6%, P=0.318; PFS: 67.2% vs. 68.0%, P=0.991). Patients receiving intensive therapies had higher baseline risk (age, high-risk MIPI, Ki-67≥30%), indicating selection bias.In subgroup analysis by MIPI risk, the 3-year OS (81.9% vs. 80.1%, P=0.723) and 3-year PFS (69.7% vs. 71.8%, P=0.733) were comparable between low- and intermediate-risk groups, suggesting a potential attenuation of the traditional MIPI scoring system's prognostic discriminatory power in the era of BTKi therapy.

**Copyright Information:** (Online) ISSN: 2572-9241
©2026 The Author(s). HemaSphere is published by John Wiley & Sons Ltd on behalf of European Hematology Association. This is an open access Abstract Book distributed under the Attribution-NonCommercial-NoDerivs (CC BY-NC-ND), which allows third parties to download the articles and share them with others as long as they credit the author and the Abstract Book, but they cannot change the content in any way or use them commercially.
**Abstract Book Citations:** Authors, Title, HemaSphere, 2026;10:(S1):pages. The abstract book DOIs can be found on the "Author Information" page.
**Disclaimer:** Articles published in the journal HemaSphere exclusively reflect the opinions of the authors. The authors are responsible for all content in their abstracts including accuracy of the facts, statements, citing resources, etc.

**Summary/Conclusion**

Adding BTKi to first-line therapy is associated with improved OS in newly diagnosed MCL and is an independent protective factor, though its effect on PFS needs validation. High Ki-67 remains a poor prognostic factor.  No additional benefit was seen for cytarabine regimens or ASCT, but higher risk in these groups suggests selection bias; prospective studies are needed. Findings support prioritizing BTKi in initial MCL therapy.The study also observed comparable long-term outcomes between low- and intermediate-risk MIPI groups in the context of BTKi treatment, implying that the predictive utility of the conventional MIPI risk stratification may be diminished, highlighting the need for novel prognostic models incorporating contemporary therapeutic factors.

**Copyright Information:** (Online) ISSN: 2572-9241
©2026 The Author(s). HemaSphere is published by John Wiley & Sons Ltd on behalf of European Hematology Association. This is an open access Abstract Book distributed under the Attribution-NonCommercial-NoDerivs (CC BY-NC-ND), which allows third parties to download the articles and share them with others as long as they credit the author and the Abstract Book, but they cannot change the content in any way or use them commercially.
**Abstract Book Citations:** Authors, Title, HemaSphere, 2026;10:(S1):pages. The abstract book DOIs can be found on the "Author Information" page.
**Disclaimer:** Articles published in the journal HemaSphere exclusively reflect the opinions of the authors. The authors are responsible for all content in their abstracts including accuracy of the facts, statements, citing resources, etc.

### (PB3842) AZACITIDINE-CHEMOTHERAPY COMBINATIONS FOR T-CELL LYMPHOMAS

**Topic:** 20. Other aggressive lymphomas

Arvid Cederlund*[1], Torsten Dahlén[1], Maria Ljungqvist[1], Christopher Melén[1], Erik Boberg[1], Kristina Sonnevi[1], Bjorn Wahlin[1]

[1]*Karolinska Institutet, Stockholm, Sweden;*

### Background

Peripheral T-cell lymphoma (PTCL) has poor prognosis, especially when relapsed. Epigenetic modulation using azacitidine, particularly in patients with the PTCL subtypes T follicular helper or angioimmunoblastic T cell lymphoma (TFHL; AITL), may induce clinical response. Emerging evidence from a small number of studies suggests that combining azacitidine and chemotherapy further improves outcome.

### Aims

This study reports the Karolinska single-centre experience using azacitidine in PTCLs.

### Methods

All PTCL patients treated with azacitidine at Karolinska were included in this work. Responses to therapy were recorded and progression-free survival (PFS) calculated from the day of the first azacitidine dose to the day of progression or death; patients were censored at last follow-up (2 February 2026). Azacitidine was administered subcutaneously at 100 mg/m$^2$ (maximum 200 mg) on days 1–5 of each course. When chemotherapy was combined with azacitidine, it was started 3-5 days after the initiation of azacitidine. Azacitidine was delivered every 4$^{th}$ week as monotherapy, otherwise according to the schedule of the accompanying chemotherapy regimen.

**Copyright Information:** (Online) ISSN: 2572-9241
©2026 The Author(s). HemaSphere is published by John Wiley & Sons Ltd on behalf of European Hematology Association. This is an open access Abstract Book distributed under the Attribution-NonCommercial-NoDerivs (CC BY-NC-ND), which allows third parties to download the articles and share them with others as long as they credit the author and the Abstract Book, but they cannot change the content in any way or use them commercially.
**Abstract Book Citations:** Authors, Title, HemaSphere, 2026;10:(S1):pages. The abstract book DOIs can be found on the "Author Information" page.
**Disclaimer:** Articles published in the journal HemaSphere exclusively reflect the opinions of the authors. The authors are responsible for all content in their abstracts including accuracy of the facts, statements, citing resources, etc.

### Results

Since 2017, 30 PTCL patients had been treated with azacitidine (24 with TFHL/AITL subtypes and 6 with other PTCLs). Ten patients were women. Age ranged from 35 to 86 years (median 68). Most patients had widespread symptomatic disease at the initiation of azacitidine (22 had stage IV, 6 stage III, and B symptoms were present in 19 patients).

Azacitidine-chemotherapy in first line

Seven patients received azacitidine in first line, all had TFHL/AITL, and azacitidine was combined with CHOP-like or Hyper-CVAD/MTX-HIDAC regimens; two patients underwent consolidation with BEAM and autologous stem-cell transplantation. These patients had a median age of 71 years (range 56-82) and presented with clinically severe disease, including hyperinflammatory features, CNS involvement, or extensive extranodal lymphoma. No additional toxicity was attributable to the combination of azacitidine and chemotherapy. The response rate was 100% and the 2-year PFS was 83% (Figure). No patient experienced lymphoma progression; one patient died from a pre-existing heart failure unrelated to lymphoma or to therapy.

Azacitidine-chemotherapy in second or third line

Among the 23 patients treated in later lines, 17 had refractory disease or relapse within 120 days and eight had concurrent malignancies (myelodysplastic syndrome [n=2], B-cell derived lymphoid malignancies [n=4], metastasized solid cancers [n=2]). Six elderly patients received azacitidine monotherapy until progression: five attained a response, but these were of short duration and all patients died from lymphoma within a year. The remaining 17 patients received in 2$^{nd}$ (n=12) or 3$^{rd}$ (n=5) line azacitidine combined with chemotherapy such as GemOx, ICE, MTX-HIDAC, brentuximab-vedotin and bendamustine. No excess toxicity from combination therapy was observed. Responses were achieved in 94%, and five patients were consolidated with allogeneic stem cell transplantation. The 2-year PFS rate was 26%, improving to 47% when patients with concurrent malignancies were excluded (Figure).

### Summary/Conclusion

We conclude that the addition of azacitidine to chemotherapy was well tolerated and appeared to improve outcome in AITL/TFHL. We will continue to explore azacitidine-chemotherapy in T-cell lymphoma.

**Copyright Information:** (Online) ISSN: 2572-9241
©2026 The Author(s). HemaSphere is published by John Wiley & Sons Ltd on behalf of European Hematology Association. This is an open access Abstract Book distributed under the Attribution-NonCommercial-NoDerivs (CC BY-NC-ND), which allows third parties to download the articles and share them with others as long as they credit the author and the Abstract Book, but they cannot change the content in any way or use them commercially.
**Abstract Book Citations:** Authors, Title, HemaSphere, 2026;10:(S1):pages. The abstract book DOIs can be found on the “Author Information” page.
**Disclaimer:** Articles published in the journal HemaSphere exclusively reflect the opinions of the authors. The authors are responsible for all content in their abstracts including accuracy of the facts, statements, citing resources, etc.

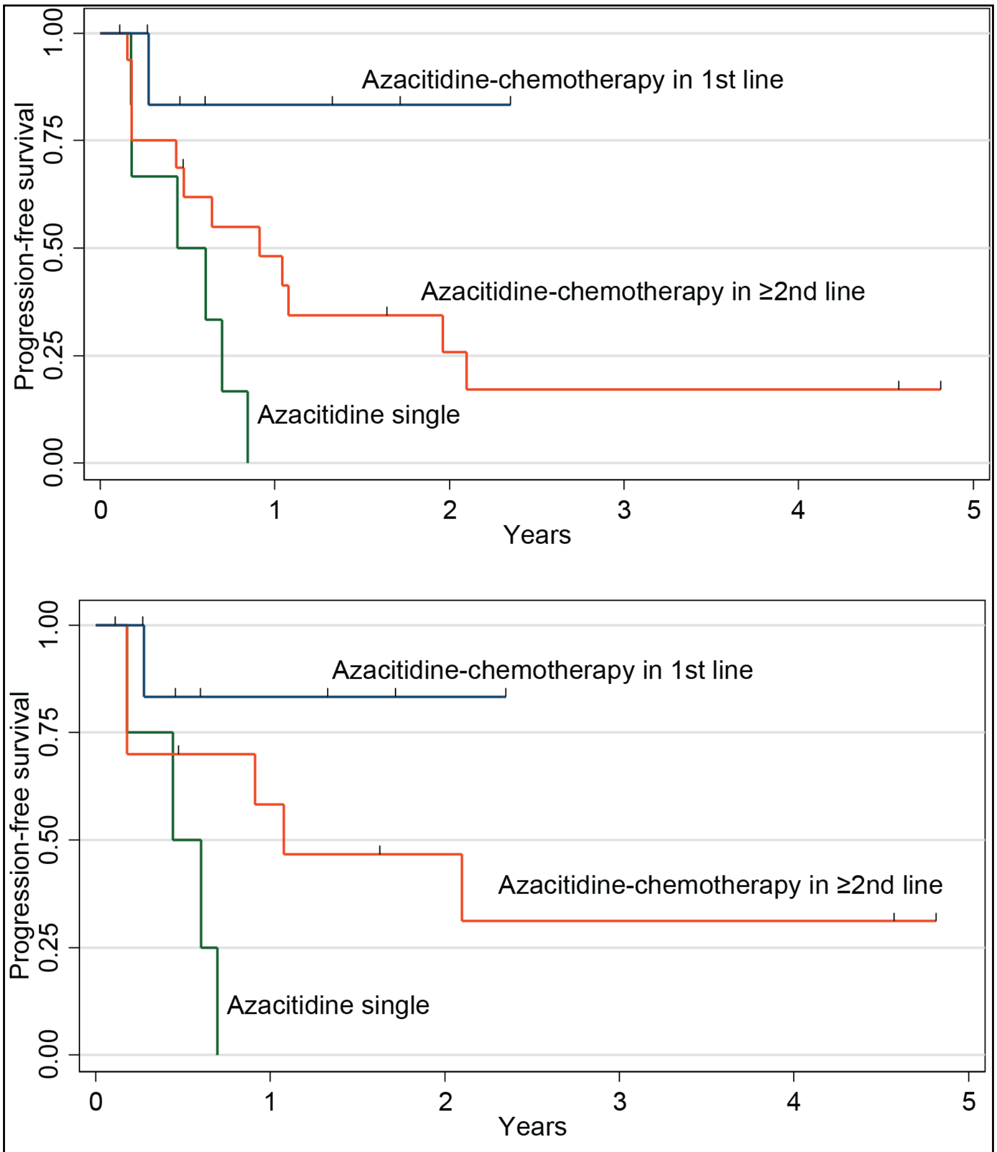

Progression-free survival after therapy with azacitidine in (above) all 30 patients and (below) in 22 patients without other concurrent malignancy.

**Copyright Information:** (Online) ISSN: 2572-9241
©2026 The Author(s). HemaSphere is published by John Wiley & Sons Ltd on behalf of European Hematology Association. This is an open access Abstract Book distributed under the Attribution-NonCommercial-NoDerivs (CC BY-NC-ND), which allows third parties to download the articles and share them with others as long as they credit the author and the Abstract Book, but they cannot change the content in any way or use them commercially.
**Abstract Book Citations:** Authors, Title, HemaSphere, 2026;10:(S1):pages. The abstract book DOIs can be found on the "Author Information" page.
**Disclaimer:** Articles published in the journal HemaSphere exclusively reflect the opinions of the authors. The authors are responsible for all content in their abstracts including accuracy of the facts, statements, citing resources, etc.

### (PB3843) INTEGRATING CLINICAL, GENETIC, AND IMAGING PARAMETERS FOR RISK STRATIFICATION IN ADULT BURKITT'S LYMPHOMA: A SINGLE-CENTER REAL-WORLD COHORT STUDY

**Topic:** 20. Other aggressive lymphomas

Ji-Nuo Wang*[1], Xiaoyu Cao[1], Fei Cheng[1], Ruyi Xu[1], Xi Huang[1], Xiaoyan Han[1], Yi Zhao[1], Zhen Cai[1], Jian Yu[1], Wenjun Wu[1]

[1] *The First Affiliated Hospital. Zhejiang University School of Medicine, Hangzhou, China;*

### Background

Adult Burkitt's lymphoma (aBL) is a highly aggressive B-cell neoplasm. While clinical models like the BL-IPI effectively risk-stratify patients, the prognostic equivalence of various extranodal sites remains contentious. Furthermore, the independent value of tumor bulk versus metabolic burden (LDH), the impact of recurrent genetic alterations, and the role of interim PET-CT (iPET-CT) in real-world settings require further validation to refine treatment adaptation strategies.

### Aims

This study aimed to systematically evaluate the clinical characteristics, treatment patterns, and survival outcomes of aBL in a real-world cohort. We sought to identify independent prognostic factors by analyzing site-specific organ involvement, LDH-defined tumor burden, and mutational profiles, while assessing the utility of iPET-CT response to inform refined risk-stratification and individualized therapy.

### Methods

This retrospective study included 100 adult BL patients treated at the First Affiliated Hospital, Zhejiang University School of Medicine since January 2019 to July 2025. Clinical, laboratory, and pathological data were analyzed. Targeted next-generation sequencing (255-gene panel) was performed on 38 patients. Survival outcomes were estimated via the Kaplan-Meier method, and independent prognostic factors were identified using multivariate Cox proportional hazards regression.

### Results

The cohort (median age 58; 74% male) showed that both IPI and BL-IPI effectively discriminated outcomes. High tumor burden, defined as LDH >2×ULN (>500 U/L), was significantly associated with inferior survival (P<0.001), whereas bulky disease (>7 cm) was not (P=0.654). Univariate analysis linked central nervous system and bone marrow involvement to poor prognosis (P<0.001). Crucially, multivariate analysis identified involvement of the gastrointestinal tract (HR 2.48, P=0.011), liver/spleen (HR 2.11, P=0.025), and lung (HR 2.48, P=0.042) as independent adverse prognostic factors; pancreatic or adrenal involvement showed no significance.

Genetic profiling revealed TP53 mutations (39.5%) as a potent independent risk factor (HR 18.87, P=0.007). Other recurrent mutations included IGLL5 (34.2%), DDX3X (31.6%), CCND3 (26.3%), and ID3 (26.3%). While CCND3 correlated with worse survival in univariate analysis (P=0.009), it was not independent in multivariate models. HIV (23%) and EBV (42.7%) status did not significantly influence survival. Regarding therapy, no significant OS difference was observed between R-DA-EPOCH, R-Hyper-CVAD, and R-CHOP-based regimens (P>0.05), though R-CHOP showed a trend toward inferior median OS (26.17 months vs. NR). In 71 evaluable patients, iPET-CT strongly predicted long-term survival (P<0.001): median OS was NR for CR (n=35), 44.97 months for PR (n=27), and 7.03 months for SD/PD (n=9).

**Copyright Information:** (Online) ISSN: 2572-9241
©2026 The Author(s). HemaSphere is published by John Wiley & Sons Ltd on behalf of European Hematology Association. This is an open access Abstract Book distributed under the Attribution-NonCommercial-NoDerivs (CC BY-NC-ND), which allows third parties to download the articles and share them with others as long as they credit the author and the Abstract Book, but they cannot change the content in any way or use them commercially.
**Abstract Book Citations:** Authors, Title, HemaSphere, 2026;10:(S1):pages. The abstract book DOIs can be found on the "Author Information" page.
**Disclaimer:** Articles published in the journal HemaSphere exclusively reflect the opinions of the authors. The authors are responsible for all content in their abstracts including accuracy of the facts, statements, citing resources, etc.

**Summary/Conclusion**

Our data suggest that specific extranodal sites (GI, liver/spleen, lung), rather than tumor bulk, are independent prognosticators in aBL. TP53 mutations identify an ultra-high-risk subset, and iPET-CT response serves as a critical marker for survival. These findings advocate for a multidimensional risk-stratification model integrating site-specific involvement, molecular signatures, and metabolic response to guide individualized therapy.

**Copyright Information:** (Online) ISSN: 2572-9241
©2026 The Author(s). HemaSphere is published by John Wiley & Sons Ltd on behalf of European Hematology Association. This is an open access Abstract Book distributed under the Attribution-NonCommercial-NoDerivs (CC BY-NC-ND), which allows third parties to download the articles and share them with others as long as they credit the author and the Abstract Book, but they cannot change the content in any way or use them commercially.
**Abstract Book Citations:** Authors, Title, HemaSphere, 2026;10:(S1):pages. The abstract book DOIs can be found on the "Author Information" page.
**Disclaimer:** Articles published in the journal HemaSphere exclusively reflect the opinions of the authors. The authors are responsible for all content in their abstracts including accuracy of the facts, statements, citing resources, etc.

### (PB3844) REAL-WORLD OUTCOMES OF BELINOSTAT IN RELAPSED REFRACTORY T-CELL LYMPHOMAS: A REPORT FROM THE LYMPHOMA STUDY GROUP OF TURKISH SOCIETY OF HEMATOLOGY

**Topic:** 20. Other aggressive lymphomas

Elif Birtaş Ateşoğlu*[1], Meral Ulukoylu Menguc[1], Fatma Arikan[2], Umut Yılmaz[3], Pelin Aytan[4], Huseyin Saffet Bekoz[5], Fazıl Cagrı Hunutlu[6], Fatma Keklik Karadag[7], Gulcin Miyase Sonmez[8], Burak Deveci[9], Fatma Aykas[10], Figen Atalay[1], Ayşe Uysal[11], Bugse Gulec Zengin[3], Turhan Koksal[12], Hulya Odluyurt[13], Deniz Goren[14], Turgay Ulas[15], Emel Isleyen[16], Esra Terzi Demirsoy[17], Metban Mastanzade[18], Esin Oguz Kozan[4], Selin Küçükyurt[19], Süreyya Yiğit Kaya[5], Vildan Özkocaman[6], Zehra Akşİt[7], Selami Toprak[8], Mustafa Cabuk[20], Volkan Karakus[10], Ahmet Emre Eşkazan[3], Mehmet Sonmez[12], Melda Comert[21], Gülsüm Özet[16], Özgür Mehtap[17], Mustafa Nuri Yenerel[18], Ahmet Kursad Gunes[19], Ayse Tulin Tuglular[22], Güray Saydam[7], Fahir Özkalemkaş[6], Isik Atagunduz[2], Tayfur Toptas[2]

*[1]Yeditepe University School of Medicine, Hematology, Istanbul, Türkiye;[2]Marmara University School of Medicine, Hematology, Istanbul, Türkiye; [3]Istanbul University Cerrahpaşa School of Medicine, Hematology, Istanbul, Türkiye;[4]Mersin University School of Medicine, Hematology, Mersin, Türkiye; [5]Medipol University School of Medicine, Hematology, Istanbul, Türkiye;[6]Uludag University Faculty of Medicine, Hematology, Bursa, Türkiye; [7]Ege University School of Medicine, Hematology, Izmir, Türkiye; [8]Ankara University School of Medicine, Hematology, Ankara, Türkiye;[9]Medstar Antalya Hospital, Hematology, Antalya, Türkiye; [10]Antalya Research and Training Hospital, Hematology, Antalya, Türkiye; [11]Fırat University School of Medicine, Hematology, Elazıg, Türkiye; [12]Karadeniz Teknik University School of Medicine, Hematology, Trabzon, Türkiye;[13]Gulhane Training and Research Hospital, Hematology, Ankara, Türkiye; [14]Halic University School of Medicine, Hematology, Istanbul, Türkiye;[15]Ankara Oncology Hospital, Hematology, Ankara, Türkiye; [16]Ankara Bilkent City Hospital, Ankara, Türkiye; [17]Kocaeli University School of Medicine, Hematology, Kocaeli, Türkiye; [18]Istanbul University Capa School of Medicine, Hematology, Istanbul, Türkiye;[19]Ankara Etlik City Hospital, Hematology, Ankara, Türkiye; [20]Antalya University of Health Sciences, Antalya, Türkiye;[21]University of Health Sciences Gülhane Training and Research Hospital, Hematology, Ankara, Türkiye; [22]Acıbadem Fulya Hospital, Hematology, Istanbul, Türkiye;*

### Background

T-cell lymphomas (TCL) comprise a rare group of non-Hodgkin lymphomas, and the outcomes are usually dismal. First-line therapy typically includes an anthracycline-based regimen. Unfortunately, this approach yields a low rate of complete responses, with a five-year survival rate of approximately 35% . Although treatment options are evolving, current approaches include salvage chemotherapy followed by autologous or allogeneic stem cell transplantation (auto-SCT or allo-SCT), as well as single-agent novel therapies, followed by allo-SCT. Belinostat is a pan-HDAC inhibitor approved for relapsed refractory peripheral T-cell lymphomas (PTCLs).

### Aims

In this retrospective study, we analyzed the real-world outcomes of single-agent Belinostat in Turkish patients with relapsed refractory (RR) TCLs.

### Methods

Fifty-nine patients with RR TCLs from 19 centers across Turkey were analyzed retrospectively . Clinical and follow-up data were obtained from medical records. Patients with RR TCLs who received at least one course of belinostat were included in the study. Patients who received belinostat for less than one course or those who received belinostat in combination with chemotherapy were excluded. Response to belinostat was assessed according to the 2014 Lugano Response Criteria for NHLs .

**Copyright Information:** (Online) ISSN: 2572-9241
©2026 The Author(s). HemaSphere is published by John Wiley & Sons Ltd on behalf of European Hematology Association. This is an open access Abstract Book distributed under the Attribution-NonCommercial-NoDerivs (CC BY-NC-ND), which allows third parties to download the articles and share them with others as long as they credit the author and the Abstract Book, but they cannot change the content in any way or use them commercially.
**Abstract Book Citations:** Authors, Title, HemaSphere, 2026;10:(S1):pages. The abstract book DOIs can be found on the "Author Information" page.
**Disclaimer:** Articles published in the journal HemaSphere exclusively reflect the opinions of the authors. The authors are responsible for all content in their abstracts including accuracy of the facts, statements, citing resources, etc.

**Results**

A total of 36 male and 23 female relapsed refractory patients with TCL were enrolled in the study, with a median age of 62 years . Most patients were diagnosed with TCL after 2019. The most frequent types of TCLs included peripheral T-cell lymphoma, not otherwise specified (PTCL, NOS), and angioimmunoblastic T-cell lymphoma (AITL). The majority of patients had advanced-stage disease. The International Prognostic Index (IPI) score was 1 for 24% of patients, 2 for 17%, 3 for 51%, and 4 for 8.5%. Anthracycline-containing chemotherapy, with or without etoposide, was the most preferred first-line therapy. The median number of previous lines of therapy before Belinostat was 2.5 , and the median number of Belinostat cycles was 2 . Ten patients (17%) proceeded to allo-SCT after Belinostat.
The overall response rate for Belinostat was 32% in evaluable patients. At the time of data analysis, 46% of patients were alive. The cause of death was disease progression in 72% of cases . The median follow-up period was 9 months . The median PFS was 3 months . The median OS was 12 months . Belinostat was well tolerated, with TEAEs primarily manifesting as mild (grade 1 or 2). The main adverse events included cytopenias, particularly neutropenia, and transaminitis .

**Summary/Conclusion**

The prevalence of specific TCL subtypes, particularly PTCL, NOS and AITL highlights the heterogeneity within TCLs. Our assessment of Belinostat revealed an overall response rate of 32%, indicating its potential efficacy in managing relapsed refractory T-cell lymphoma, although the response rate can be considered modest when contrasted with therapies used in other lymphomas . The median PFS of 3 months and OS of 12 months illustrates the aggressive nature of this malignancy .Adverse events associated with Belinostat were primarily mild, with notable instances of cytopenias and transaminitis, aligning with the established safety profile of histone deacetylase inhibitors.
Finally , while Belinostat exhibits certain therapeutic potential, further exploration through larger, randomized controlled trials is essential to delineate its role within the treatment spectrum of TCLs.

**Copyright Information:** (Online) ISSN: 2572-9241
©2026 The Author(s). HemaSphere is published by John Wiley & Sons Ltd on behalf of European Hematology Association. This is an open access Abstract Book distributed under the Attribution-NonCommercial-NoDerivs (CC BY-NC-ND), which allows third parties to download the articles and share them with others as long as they credit the author and the Abstract Book, but they cannot change the content in any way or use them commercially.
**Abstract Book Citations:** Authors, Title, HemaSphere, 2026;10:(S1):pages. The abstract book DOIs can be found on the “Author Information” page.
**Disclaimer:** Articles published in the journal HemaSphere exclusively reflect the opinions of the authors. The authors are responsible for all content in their abstracts including accuracy of the facts, statements, citing resources, etc.

### (PB3845) REAL-WORLD EFFECTIVENESS AND SAFETY OF TOTAL SKIN ELECTRON BEAM THERAPY IN ADVANCED CUTANEOUS T-CELL LYMPHOMAS: A REAL-WORLD SINGLE-CENTER EXPERIENCE.

**Topic:** 20. Other aggressive lymphomas

Carmen Amorós Pérez*[1], Paola Antonini[2], Laura Giménez[3], Mariam Ibáñez Company[1], Amparo González[2], Jorge Magdaleno[3], Maria Jose Lis[1], Margarita Monzón Torres[1], Paula Sánchez[1], Rosa Collado Nieto[1], Karla Javier[1], José Ángel García-García[4], Esther Roselló[4], Esther Diez-Recio[3], Carmen García-Mora[2], Josefa Marco Buades[1]

*[1]Hematology and Hemotherapy Department. Consorcio Hospital General Universitario de Valencia.Hematology and Hemotherapy Research Group. Fundación Investigación Hospital General de Valencia., Hematology, Lymphoma unit, Valencia, Spain; [2]Consorcio Hospital General Universitario de Valencia, Radiotherapy Oncology, Valencia, Spain;[3]Consorcio Hospital General Universitario de Valencia, Dermatology, Valencia, Spain; [4]Consorcio Hospital General Universitario de Valencia, Pathology, Valencia, Spain;*

### Background

Primary cutaneous T-cell lymphomas (CTCL), specifically mycosis fungoides (MF) and Sézary syndrome (SS), are highly radiosensitive malignancies, accounting for 70–80% of cutaneous lymphomas. Relapses are frequent despite standard therapies, highlighting the need for durable strategies. Total skin electron beam therapy (TSEBT) delivers uniform skin irradiation with minimal deep tissue exposure and shows high efficacy even at low or condensed doses.

### Aims

We evaluated efficacy, safety, and durability of TSEBT (Stanford technique) in MF/SS, stratified by stage, emphasizing stage-dependent responses and multimodal strategies.

### Methods

Single-center retrospective study of 87 consecutive CTCL patients (67% male, mean age 60 years) treated with TSEBT at Hospital General de Valencia (2010–2023), minimum follow-up 2 years. Most received standard doses (30–36 Gy) via the Stanford technique using a linear accelerator (6 MeV electrons, 1–3 Gy/session). Results were correlated with prospective low-dose (12 Gy) MF/SS cohorts. We assessed response (complete, partial, palliative, progression), disease-free survival (DFS), progression-free survival (PFS), overall response rate (ORR) per EORTC criteria, and adverse events graded by radiation dermatitis severity and management. Survival outcomes were compared by stage (IA–IIA vs IIB–IV) and bexaroten maintenance. Analyses used SPSS Statistics (IBM Corp., Armonk, NY) and Python (Lifelines, SciPy, Matplotlib). DFS, PFS, and OS were estimated by Kaplan–Meier, compared with log-rank test; hazard ratios (HR) with 95% CI were calculated using Cox models.

**Copyright Information:** (Online) ISSN: 2572-9241
©2026 The Author(s). HemaSphere is published by John Wiley & Sons Ltd on behalf of European Hematology Association. This is an open access Abstract Book distributed under the Attribution-NonCommercial-NoDerivs (CC BY-NC-ND), which allows third parties to download the articles and share them with others as long as they credit the author and the Abstract Book, but they cannot change the content in any way or use them commercially.
**Abstract Book Citations:** Authors, Title, HemaSphere, 2026;10:(S1):pages. The abstract book DOIs can be found on the "Author Information" page.
**Disclaimer:** Articles published in the journal HemaSphere exclusively reflect the opinions of the authors. The authors are responsible for all content in their abstracts including accuracy of the facts, statements, citing resources, etc.

**Results**

The global ORR was 97%, CR 74%. Response was significantly stage-dependent: IA-IIA, (n=35) achieved a 100%CR rate, while IIB-IV (n=52) achieved a 58% CR rate. Patients with stage I–II disease (n = 67) did not reach median DFS during follow-up, indicating that >50% remained disease-free at study end. For stage III–IV patients (n = 20), median DFS was 365.2 days (12.0 months) [95% CI: 182.6–1095.8], with statistically significant differences between groups (log-rank p = 0.0017). Median PFS for advanced-stage was significantly shorter than for early-stage disease (95% CI: 6–36 months; p=0.004). In erythrodermic cases, the integration of subsequent systemic therapy (e.g., mogalizumab, methotrexate, or low-dose bexarotene) significantly extended the time to next treatment (TNT) to 30 moths compared to 6 months for those without (p=0.001). Maintenace with low-dose bexarotene showed a trend toward improved disease control (median DFS 27 months). Toxcicity was manageable: 96% of adverse events were mild (grade 1), primarily alopecia, nail distrophy, and fatigue (Figure 1).

**Summary/Conclusion**

TSEBT is a highly effective modality for CTCL with durable responses, particularly in early-stage MF/SS, and Standard doses (30–36 Gy) were administered in most cases, using the Standford technique with a linear accelerator delivering 6 MeV electrons, at dose rates of 1–3 Gy per session  shows prolonged long-term safety. The transition toward low-dose regimens (12Gy) is supported, to minimize cumulative toxicity and facilitate repeatable interventions in this malignancies. Despite requiring specialized equipment available in only a few centers, TSEBT serves as an essential debulking tool that must be integrated into multimodal hematological strategies.

**Copyright Information:** (Online) ISSN: 2572-9241
©2026 The Author(s). HemaSphere is published by John Wiley & Sons Ltd on behalf of European Hematology Association. This is an open access Abstract Book distributed under the Attribution-NonCommercial-NoDerivs (CC BY-NC-ND), which allows third parties to download the articles and share them with others as long as they credit the author and the Abstract Book, but they cannot change the content in any way or use them commercially.
**Abstract Book Citations:** Authors, Title, HemaSphere, 2026;10:(S1):pages. The abstract book DOIs can be found on the "Author Information" page.
**Disclaimer:** Articles published in the journal HemaSphere exclusively reflect the opinions of the authors. The authors are responsible for all content in their abstracts including accuracy of the facts, statements, citing resources, etc.

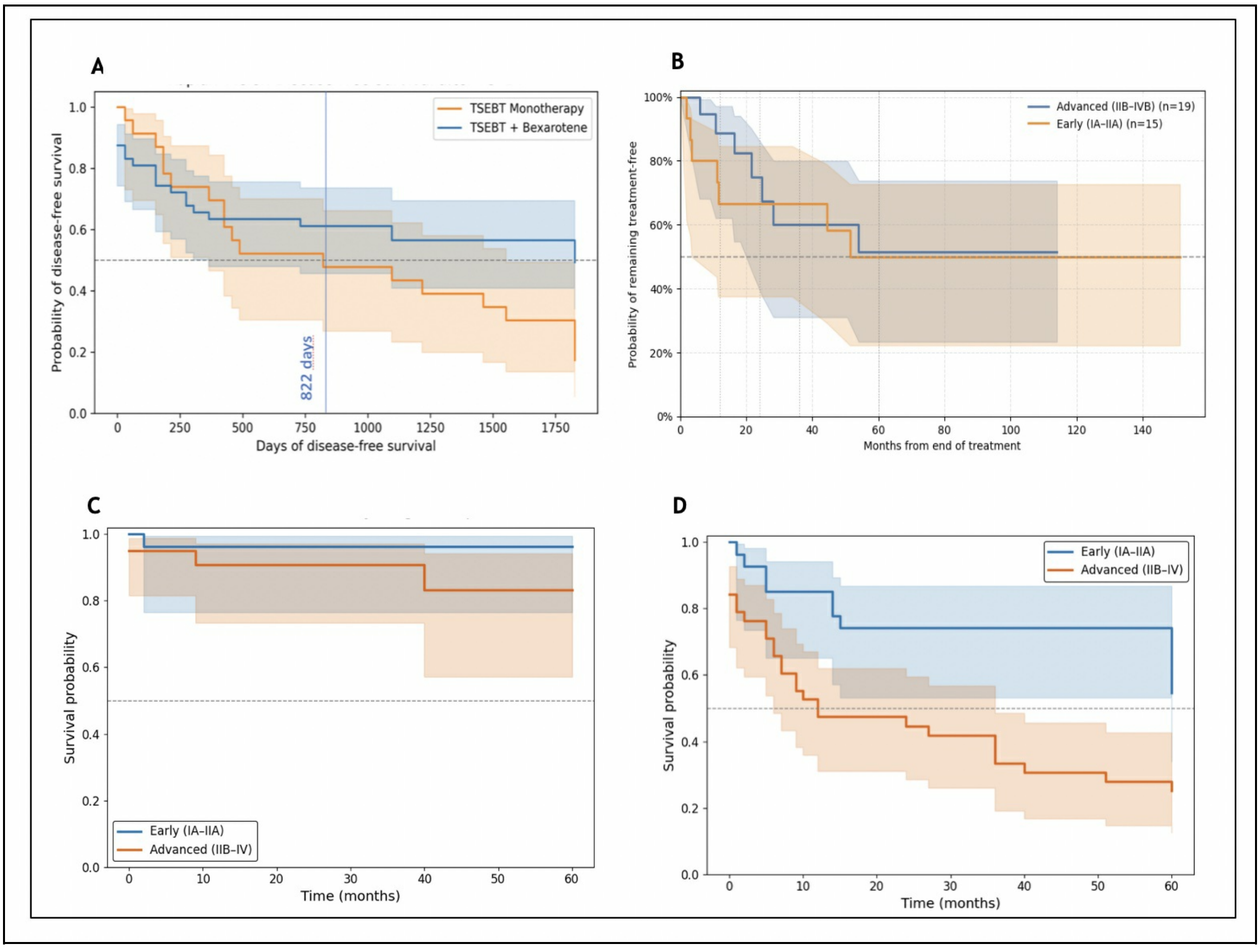

Figure 1. Impact of maintenance therapy and disease stage on survival outcomes following TSEBT. (A) Disease-free survival (DFS) according to maintenance bexarotene. The blue curve represents TSEBT + bexarotene and the orange curve TSBET alone. (B) Time to next treatment (TTNT) stratified by stage at TSBET initiation. (C) Overall survival (OS) for early-stage mycosis fungoides (IA-IIA) versus advanced-stage disease (IIB-IV). (D) Progression-free survival (PFS) by stage; early-stage patients had significantly longer PFS.

**Copyright Information:** (Online) ISSN: 2572-9241
©2026 The Author(s). HemaSphere is published by John Wiley & Sons Ltd on behalf of European Hematology Association. This is an open access Abstract Book distributed under the Attribution-NonCommercial-NoDerivs (CC BY-NC-ND), which allows third parties to download the articles and share them with others as long as they credit the author and the Abstract Book, but they cannot change the content in any way or use them commercially.
**Abstract Book Citations:** Authors, Title, HemaSphere, 2026;10:(S1):pages. The abstract book DOIs can be found on the "Author Information" page.
**Disclaimer:** Articles published in the journal HemaSphere exclusively reflect the opinions of the authors. The authors are responsible for all content in their abstracts including accuracy of the facts, statements, citing resources, etc.

### (PB3847) EXTRANODAL SITES AND NUMBER AS PROGNOSTIC INDICATORS IN ANGIOIMMUNOBLASTIC T-CELL LYMPHOMA WITH FOCUS ON LIVER, SPLEEN, AND BONE MARROW

**Topic:** 20. Other aggressive lymphomas

Mengmeng Dong*[1], Yi Zhong[2], Fang Yu[2], Yi Zhao[2], Lixia Zhu[2], Fengping Zhou[3], Linlin Huang[2], Xiujin Ye[2], Zhen Cai[2], Jin Zhang[3], Ye Baodong[4], Qingming Wang[5], He Jingsong[1]

*[1]The First Affiliated Hospital, Zhejiang University, School of Medicine, Bone Marrow Transplantation Center, Hangzhou, China; [2]The First Affiliated Hospital, Zhejiang University, School of Medicine, Hangzhou, China;[3]Sir Run Run Shaw Hospital, affiliated with the Zhejiang University School of Medicine, Hangzhou, China; [4]The First Affiliated Hospital of Zhejiang Chinese Medical University, Hangzhou, China; [5]The Second Affiliated Hospital of Nanchang University, Hangzhou, China;*

### Background

Angioimmunoblastic T-cell lymphoma (AITL), a distinct subtype of peripheral T-cell lymphoma (PTCL), predominantly affects the elderly and is characterized by aggressive yet heterogeneous clinical course. Patients often present with progressive lymphadenopathy, hepatosplenomegaly, B symptoms, skin lesions, anemia, and hypergammaglobulinemia. Approximately one-quarter of patients exhibit extranodal involvement, with spleen, liver, lung, and skin being the most common sites. Notably, rash (13.1%) and effusions/ascites (14.1%) represent unique autoimmune manifestations in AITL. However, the prognostic significance of specific extranodal involvement sites and their cumulative number remains poorly defined.

### Aims

This study aims to comprehensively investigate the association between extranodal involvement patterns—both anatomical sites and quantity—with progression-free survival (PFS) and overall survival (OS) in AITL, to enhance clinical risk stratification and guide therapeutic decisions.

### Methods

We retrospectively analyzed 350 patients with AITL from four Chinese medical centers between August 2015 and November 2023. Univariate analysis, LASSO regression, multivariate Cox regression, and Kaplan-Meier (K-M) curve analyses were performed to evaluate the impact of extranodal involvement sites and number on PFS and OS.

### Results

In this large multicenter cohort, univariate analysis revealed that liver, spleen, and bone marrow involvement were significantly associated with inferior PFS (HR [95% CI]: 1.72 [1.06-2.79], P=0.0278; 1.49 [1.11-2.00], P=0.0075; and 1.83 [1.41-2.38], P<0.0001, respectively) and OS (1.59 [1.00-2.52], P=0.0488; 2.07 [1.47-2.92], P<0.0001; and 2.25 [1.69-2.99], P<0.0001, respectively). Importantly, the total number of extranodal sites also correlated with survival outcomes, with ≥2 extranodal sites demonstrating strong prognostic significance for both PFS and OS (OS: HR 2.22 [1.59-3.11], P<0.0001). LASSO regression incorporating all involved sites identified liver, spleen, and bone marrow as key risk factors retained in models for both PFS and OS. After adjusting for gender, age, ECOG performance status, β2-microglobulin, and lactate dehydrogenase in multivariate Cox analysis, liver involvement and ≥2 extranodal sites remained independent predictors for PFS, while spleen involvement, bone marrow involvement, and ≥2 extranodal sites were independently associated with OS. K-M curves further confirmed significantly worse PFS and OS in patients with spleen or bone marrow involvement, as well as those with ≥2 extranodal sites (all P<0.05).

**Copyright Information:** (Online) ISSN: 2572-9241
©2026 The Author(s). HemaSphere is published by John Wiley & Sons Ltd on behalf of European Hematology Association. This is an open access Abstract Book distributed under the Attribution-NonCommercial-NoDerivs (CC BY-NC-ND), which allows third parties to download the articles and share them with others as long as they credit the author and the Abstract Book, but they cannot change the content in any way or use them commercially.
**Abstract Book Citations:** Authors, Title, HemaSphere, 2026;10:(S1):pages. The abstract book DOIs can be found on the "Author Information" page.
**Disclaimer:** Articles published in the journal HemaSphere exclusively reflect the opinions of the authors. The authors are responsible for all content in their abstracts including accuracy of the facts, statements, citing resources, etc.

### Summary/Conclusion

Liver, spleen, and bone marrow involvement are adverse prognostic factors for both PFS and OS in AITL. An increasing number of extranodal sites correlates with poorer outcomes, with ≥2 extranodal sites serving as an independent predictor of inferior survival. These findings underscore the critical importance of comprehensive extranodal assessment in refining prognostic stratification and guiding clinical management for AITL patients.

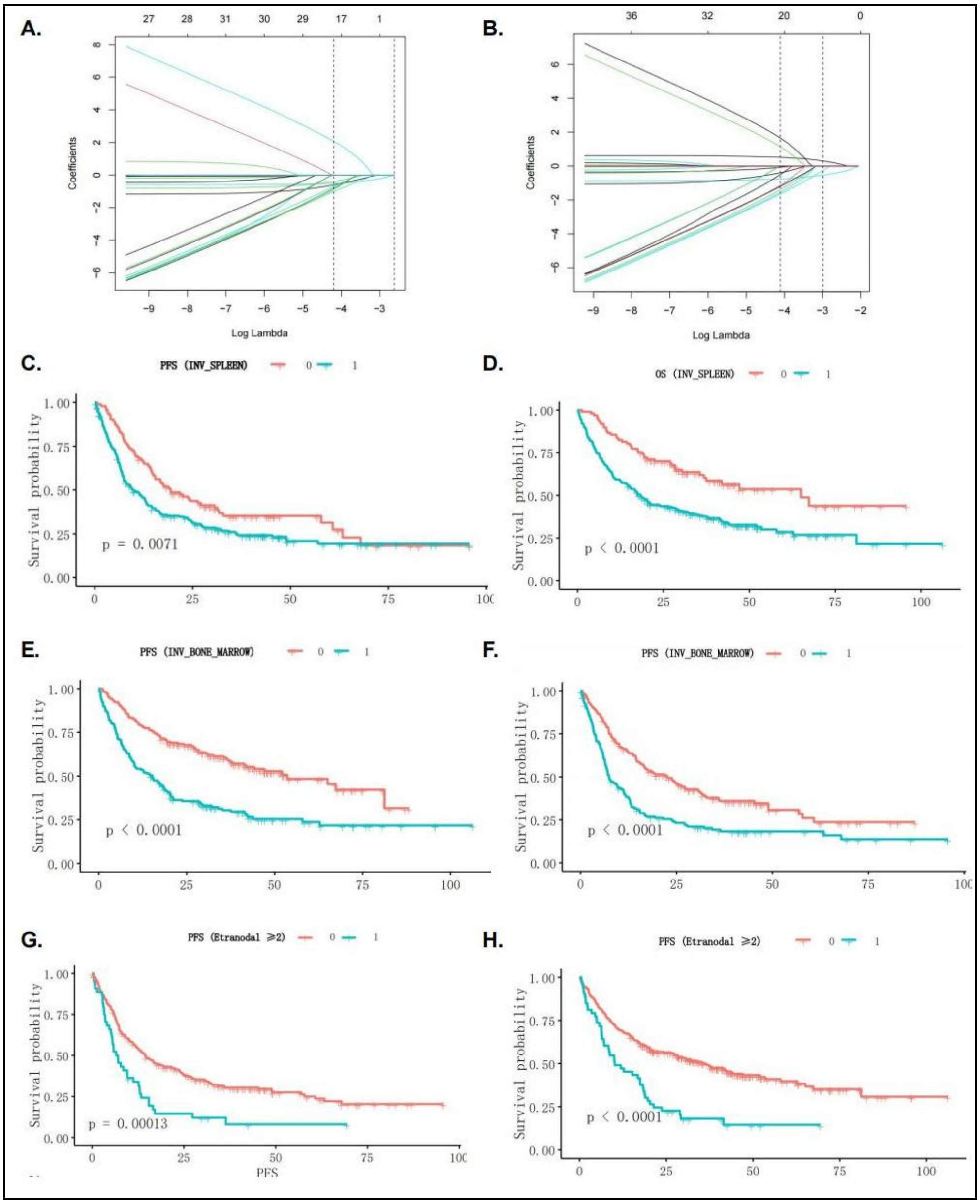

Figure. Regression analysis and Kaplan-Meier curves for extranodal involvement sites associated with PFS and OS in patients. LASSO regression analysis was performed using involved sites as risk factors for PFS (A) and OS (B). Kaplan-Meier curves indicated that patients with spleen involvement (PFS in C, OS in D), bone marrow involvement (PFS in E, OS in F), and extranodal sites ≥2 (PFS in G, OS in H) had worse PFS and OS (C-H).

**Copyright Information:** (Online) ISSN: 2572-9241
©2026 The Author(s). HemaSphere is published by John Wiley & Sons Ltd on behalf of European Hematology Association. This is an open access Abstract Book distributed under the Attribution-NonCommercial-NoDerivs (CC BY-NC-ND), which allows third parties to download the articles and share them with others as long as they credit the author and the Abstract Book, but they cannot change the content in any way or use them commercially.
**Abstract Book Citations:** Authors, Title, HemaSphere, 2026;10:(S1):pages. The abstract book DOIs can be found on the "Author Information" page.
**Disclaimer:** Articles published in the journal HemaSphere exclusively reflect the opinions of the authors. The authors are responsible for all content in their abstracts including accuracy of the facts, statements, citing resources, etc.

### (PB3848) IMPACT OF FACILITY TYPE ON OUTCOMES FOR ELDERLY PATIENTS (≥75) WITH BURKITT LYMPHOMA: REAL-WORLD DATA OF ACADEMIC VS. COMMUNITY CANCER PROGRAMS

**Topic:** 20. Other aggressive lymphomas

Luis Felipe Rubalcava Lara*[1], Alfredo Reinaldo Quiroz Astigarraga[2], Carolina Velez-Mejia[3], Joel Michalek[3], Qianqian Liu[3], Adolfo Diaz Duque[3]

*[1]Instituto Nacional de Cancerología - México, Mexico City, Mexico;[2]Hospital Central del Instituto de Previsión Social, Asuncion, Paraguay; [3]Mays Cancer Center, San Antonio, United States of America;*

### Background

Burkitt lymphoma (BL) is an aggressive B-cell malignancy characterized by rapid proliferation. While highly curable in children and young adults, prognosis remains poor in the elderly due to poor tolerance for intensive chemotherapy and higher baseline comorbidities which presents as a significant treatment challenging. This is the first real-world study to compare demographic characteristics, treatment patterns, and survival outcomes for patients aged 75 and older treated at Academic Cancer Programs (ACP) versus Community Cancer Programs (CCP).

### Methods

A retrospective analysis using the National Cancer Database was conducted on 1,618 patients (ACP n=837; CCP n=781) diagnosed with BL between 2004 and 2022 was carried out. ACP included academic and National Cancer Institute-designated research centers; CCP included community, comprehensive community, and integrated network cancer programs. Kaplan-Meier and Cox regression models were used to compare overall survival, adjusting for: age, race/ethnicity, insurance status, Charlson-Deyo comorbidity score, and distance from treating facility.

### Results

ACP demonstrated significantly superior overall survival (OS) outcomes compared to CCP [p=0.004].
Patients at ACP were slightly younger with a median age of 80 vs 81 years [p=0.001]. No difference in sex, race or ethnicity was noted in both groups. Although most patients had Medicare, almost 10% for both groups had private insurance. Patients treated at ACP were more likely from the metropolitan area, 83% vs 77% in CCP [p=0.016], with the median distance in miles was 9.2 vs 8.1, respectively.
ACP had a higher proportion of Stage IV disease, 50% vs 43%, [p=0.029]. Treatment was more frequently administered at ACP with 58% vs 52% [p=0.001], and when administered it was initiated faster than CCP with median of 12 vs 14 days [p=0.018]. Median follow-up time, measured as the interval from diagnosis to last contact, was longer in the ACP cohort with 5.4 months vs 3.1 months, [P<0.001].
Two, five, and ten-year survival probabilities were higher at ACP than CCP: 36% vs 28%, 26% vs 21%, and 14% vs 11%, respectively. The adjusted median OS was 0.48 years for ACP and 0.27 years for CCP.

**Copyright Information:** (Online) ISSN: 2572-9241
©2026 The Author(s). HemaSphere is published by John Wiley & Sons Ltd on behalf of European Hematology Association. This is an open access Abstract Book distributed under the Attribution-NonCommercial-NoDerivs (CC BY-NC-ND), which allows third parties to download the articles and share them with others as long as they credit the author and the Abstract Book, but they cannot change the content in any way or use them commercially.
**Abstract Book Citations:** Authors, Title, HemaSphere, 2026;10:(S1):pages. The abstract book DOIs can be found on the "Author Information" page.
**Disclaimer:** Articles published in the journal HemaSphere exclusively reflect the opinions of the authors. The authors are responsible for all content in their abstracts including accuracy of the facts, statements, citing resources, etc.

### Summary/Conclusion

Among patients aged ≥75 years with BL, treatment at ACP was associated with a statistically significant and clinically meaningful improvement in OS compared with CCP (log-rank p=0.004), despite a higher proportion of advanced-stage disease at ACP. Patients treated at ACP were more likely to receive therapy and experienced shorter time to initiation of systemic treatment. These findings highlight the persistent vulnerability of elderly patients with BL and underscore the need for system-level strategies, including early referral and coordinated academic–community care pathways, to optimize timely treatment delivery and supportive care for this high-risk population.

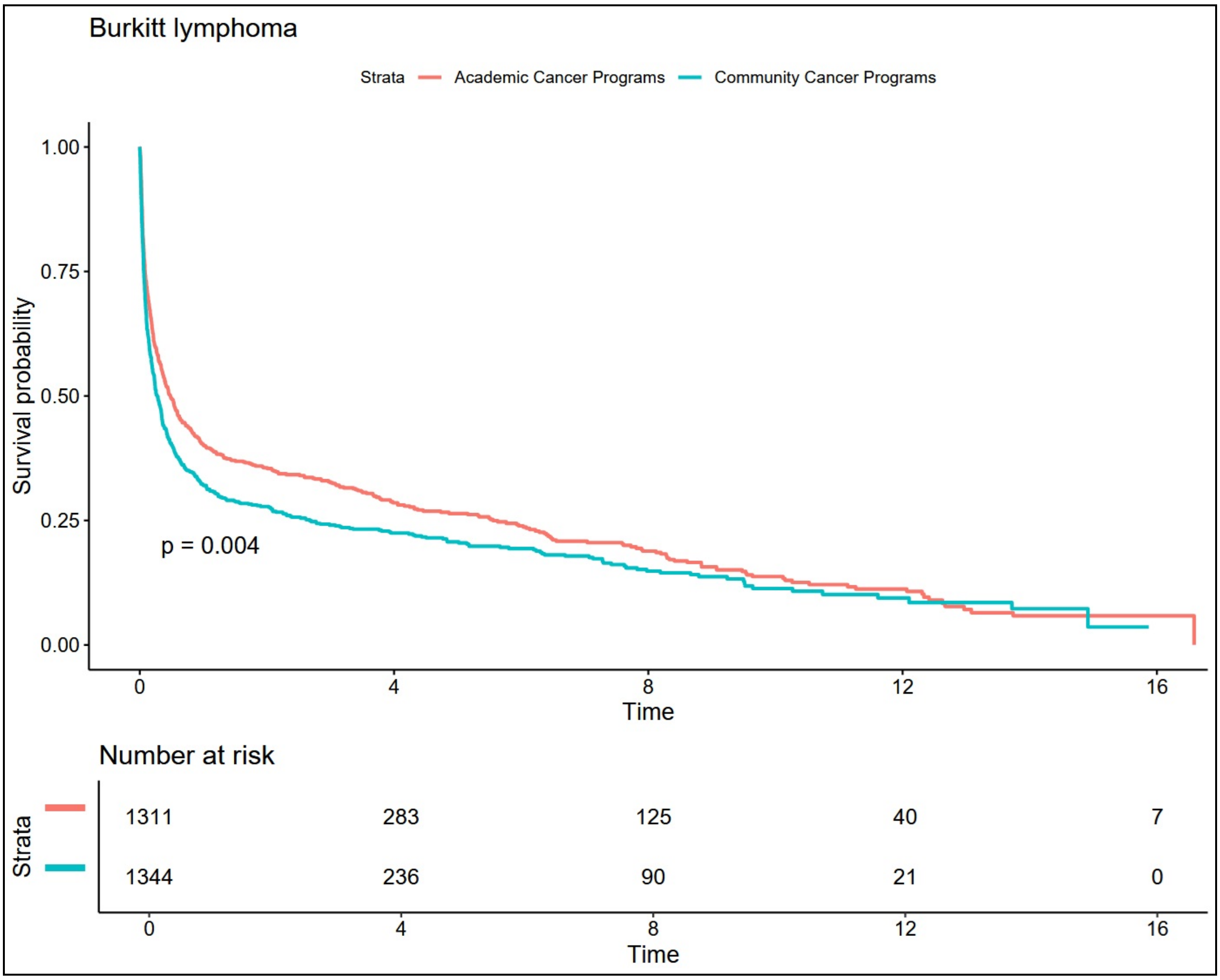

Survival probability of patients with Burkitt lymphoma >75 years treated at ACP show an improvement over CCP

Copyright Information: (Online) ISSN: 2572-9241
©2026 The Author(s). HemaSphere is published by John Wiley & Sons Ltd on behalf of European Hematology Association. This is an open access Abstract Book distributed under the Attribution-NonCommercial-NoDerivs (CC BY-NC-ND), which allows third parties to download the articles and share them with others as long as they credit the author and the Abstract Book, but they cannot change the content in any way or use them commercially.
Abstract Book Citations: Authors, Title, HemaSphere, 2026;10:(S1):pages. The abstract book DOIs can be found on the “Author Information” page.
Disclaimer: Articles published in the journal HemaSphere exclusively reflect the opinions of the authors. The authors are responsible for all content in their abstracts including accuracy of the facts, statements, citing resources, etc.

### (PB3849) DISTINCT CLINICAL FEATURES BUT COMPARABLE SURVIVAL BETWEEN PEDIATRIC AND ADULT PATIENTS WITH SYSTEMIC ANAPLASTIC LARGE CELL LYMPHOMA

**Topic:** 20. Other aggressive lymphomas

Sun-Hye Ko*[1], Tameem Almahmoud[2], Wafaa Aljizani[2], Chathuri Abeyakoon[1], Sita Bhella[1], Robert Kridel[1], Vishal Kukreti[1], Anca Prica[1], Abi Vijenthira[1], Chloe Yang[1], Michael Crump[1], John Kuruvilla[1], Ali Sakhdari[3], Angela Punnett[2], Tomohiro Aoki[1, 4]

*[1]Princess Margaret Cancer Centre, University Health Network, Medical Oncology and Hematology, Toronto, Canada;[2]The Hospital for Sick Children, Division of Haematology/Oncology, Department of Paediatrics, Toronto, Canada; [3]University Health Network, Laboratory Medicine and Pathobiology department, Toronto, Canada; [4]University of Toronto, Department of Immunology, Toronto, Canada;*

### Background

Anaplastic large cell lymphoma (ALCL) is a subtype of peripheral T-cell lymphoma characterized by age-related differences in clinical presentation and outcomes. While pediatric ALCL is often perceived to have more favorable survival than adult disease, the factors underlying these differences remain incompletely defined.

### Aims

This study compared clinical characteristics and survival outcomes between pediatric and adult patients with ALCL and identified prognostic factors associated with progression and survival within each age group.

### Methods

We conducted a retrospective cohort study of patients with CD30-positive systemic ALCL diagnosed between January 1, 2010 and December 31, 2024 at Princess Margaret Cancer Centre and The Hospital for Sick Children. Patients were stratified by age at diagnosis into pediatric (<18 years) and adult (≥18 years) groups. Lansky Performance Scale scores were converted to Eastern Cooperative Oncology Group performance status (ECOG PS). The age-adjusted International Prognostic Index (aaIPI) was used to standardize risk assessment. Treatment patterns, response, progression-free survival (PFS), and overall survival (OS) were analyzed using Kaplan–Meier methods and Cox proportional hazards models.

**Copyright Information:** (Online) ISSN: 2572-9241
©2026 The Author(s). HemaSphere is published by John Wiley & Sons Ltd on behalf of European Hematology Association. This is an open access Abstract Book distributed under the Attribution-NonCommercial-NoDerivs (CC BY-NC-ND), which allows third parties to download the articles and share them with others as long as they credit the author and the Abstract Book, but they cannot change the content in any way or use them commercially.
**Abstract Book Citations:** Authors, Title, HemaSphere, 2026;10:(S1):pages. The abstract book DOIs can be found on the "Author Information" page.
**Disclaimer:** Articles published in the journal HemaSphere exclusively reflect the opinions of the authors. The authors are responsible for all content in their abstracts including accuracy of the facts, statements, citing resources, etc.

### Results

Eighty-seven patients were included (31 pediatric, 56 adult). Pediatric patients more frequently had ALK-positive disease (90% vs 46%, p<0.001), were male (74% vs 46%, p=0.014), and had B symptoms (74% vs 32%, p<0.001). Adults more often presented with high aaIPI (32% vs 9.7%, p=0.020), elevated ECOG PS (23% vs 0%, p=0.024), and less frequent skin involvement (11% vs 29%, p=0.042). These differences persisted within the ALK-positive subgroup (n=54). First-line therapy differed by age group: pediatric patients mainly received non-anthracycline–based protocols (ALCL-99, 74%; ANHL0131, 9.7%), whereas adults predominantly received anthracycline-based CHOP/CHOEP (71%), with 13% receiving brentuximab vedotin–containing regimens.
With a median follow-up of 63.0 months (range, 23.0–98.0), 28 patients experienced disease progression (32% in both groups), and 18 patients died (4 pediatric and 14 adult), most due to disease progression (75.0% in pediatric and 64.3% in adults).
In the overall cohort, 7-year PFS was 65.7% in pediatric patients and 64.0% in adults (p=0.965), and 7-year OS was 85.8% vs 73.8% (p=0.242). No significant differences were observed in PFS (HR 0.99; p=0.97) or OS (HR 1.92; p=0.25). Similarly, within the ALK-positive subgroup, 7-year PFS (65.6% vs 74.8%; p=0.467) and OS (88.6% vs 79.0%; p=0.404) did not differ significantly.
Among adults, high aaIPI (HR 2.94; p=0.001) and visceral involvement (HR 3.94; p=0.012) were associated with inferior OS, and radiation as initial therapy with worse PFS (HR 28.8; p<0.001), whereas ALK status was not prognostic. In pediatric patients, high aaIPI (HR 11.1; p=0.017) and bone involvement (HR 10.5; p=0.042) were associated with inferior OS, and high aaIPI was also associated with worse PFS (HR 6.90; p=0.009); ALK status was not associated with outcomes.

### Summary/Conclusion

Despite distinct age-related differences in clinical presentation, survival outcomes were comparable between pediatric and adult patients with systemic ALCL, including within the ALK-positive subgroup. Prognostic factors differed by age group, suggesting potential biological and clinical heterogeneity that warrants further investigation in larger cohorts.

**Copyright Information:** (Online) ISSN: 2572-9241
©2026 The Author(s). HemaSphere is published by John Wiley & Sons Ltd on behalf of European Hematology Association. This is an open access Abstract Book distributed under the Attribution-NonCommercial-NoDerivs (CC BY-NC-ND), which allows third parties to download the articles and share them with others as long as they credit the author and the Abstract Book, but they cannot change the content in any way or use them commercially.
**Abstract Book Citations:** Authors, Title, HemaSphere, 2026;10:(S1):pages. The abstract book DOIs can be found on the "Author Information" page.
**Disclaimer:** Articles published in the journal HemaSphere exclusively reflect the opinions of the authors. The authors are responsible for all content in their abstracts including accuracy of the facts, statements, citing resources, etc.

| Characteristic | Overall N = 87 | Pediatric N = 31 | Adult N = 56 | *P*-value |
|---|---|---|---|---|
| Sex, male | 49 (56%) | 23 (74%) | 26 (46%) | 0.014 |
| Age | 30 (12-56) | 10 (5, 13) | 51 (31, 64) | <0.001 |
| ALK, positive | 54 (62%) | 28 (90%) | 26 (46%) | <0.001 |
| Stage at diagnosis | | | | <0.001 |
| stage1 | 18 (21%) | 3 (9.7%) | 15 (27%) | |
| stage2 | 15 (17%) | 6 (19%) | 9 (16%) | |
| stage3 | 23 (26%) | 17 (55%) | 6 (11%) | |
| stage4 | 31 (36%) | 5 (16%) | 26 (46%) | |
| B symptoms, yes | 41 (47%) | 23 (74%) | 18 (32%) | <0.001 |
| ECOG PS | 9 (11%) | 0 (0%) | 9 (17%) | 0.024 |
| aaIPI≥3 | 21 (24%) | 3 (9.7%) | 18 (32%) | 0.020 |
| Extranodal involvement, yes | 55 (63%) | 18 (58%) | 37 (66%) | 0.493 |
| Bone marrow involvement, yes | 4 (4.8%) | 1 (3.6%) | 3 (5.5%) | >0.999 |
| CNS involvement, yes | 1 (1.2%) | 0 (0%) | 1 (1.8%) | >0.999 |
| Skin involvement, yes | 15 (17%) | 9 (29%) | 6 (11%) | 0.042 |
| Bone involvement, yes | 19 (22%) | 7 (23%) | 12 (21%) | >0.999 |
| Visceral involvement, yes | 25 (29%) | 7 (23%) | 18 (32%) | 0.460 |
| Chemotherapy[1] | | | | <0.001 |
| ALCL-99 | 23 (26%) | 23 (74%) | 0 (0%) | |
| ANHL0131 | 3 (3.4%) | 3 (9.7%) | 0 (0%) | |
| CHOP/CHOEP | 40 (46%) | 3 (9.7%) | 40 (71%) | |
| Brentuximab±CHP | 7 (8.0%) | 0 (0%) | 7 (13%) | |
| GDP | 1 (1.1%) | 0 (0%) | 1 (1.8%) | |

[1] Chemotherapy as first line treatment aaIPI, age-adjusted International Prognostic Index; ECOG PS, Eastern Cooperative Oncology Group performance status.

Copyright Information: (Online) ISSN: 2572-9241
©2026 The Author(s). HemaSphere is published by John Wiley & Sons Ltd on behalf of European Hematology Association. This is an open access Abstract Book distributed under the Attribution-NonCommercial-NoDerivs (CC BY-NC-ND), which allows third parties to download the articles and share them with others as long as they credit the author and the Abstract Book, but they cannot change the content in any way or use them commercially.
Abstract Book Citations: Authors, Title, HemaSphere, 2026;10:(S1):pages. The abstract book DOIs can be found on the "Author Information" page.
Disclaimer: Articles published in the journal HemaSphere exclusively reflect the opinions of the authors. The authors are responsible for all content in their abstracts including accuracy of the facts, statements, citing resources, etc.

### (PB3851) GOLIDOCITINIB AND LIPOSOMAL MITOXANTRONE FOR RELAPSED/REFRACTORY PERIPHERAL T-CELL LYMPHOMA: PRELIMINARY SAFETY AND EFFICACY FROM A MULTI-CENTER RETROSPECTIVE STUDY

**Topic:** 20. Other aggressive lymphomas

Qingqing Cai*[1], Jianbo Liu[2], Zhijun Wuxiao[3], Runhui Zheng[2], Yi Xia[1], Shenrui Bai[1], Xueyi Pan[4], Min Zhong[5], Boyun Shi[2], Danyun Yuan[2], Man Nie[1], Yuchen Zhang[1]

*[1]Department of Medical Oncology, State Key Laboratory of Oncology in South China, Collaborative Innovation Center for Cancer Medicine, Sun Yat-sen University Cancer Center, Guangzhou, People's Republic of China, Guangzhou, China; [2]The Fifth Affiliated Hospital of Guangzhou Medical University, Guangzhou, China; [3]The First Affiliated Hospital of Hainan Medical University, Guangzhou, China; [4]The First Affiliated Hospital of Guangdong Pharmaceutical University,, Guangzhou, China;[5]The Second Affiliated Hospital of Hainan Medical University, haikou, China;*

### Background

Relapsed or refractory (R/R) peripheral T-cell lymphoma (PTCL) remains a clinically challenging entity with a poor prognosis and no well-established standard of care, representing a significant unmet medical need. Golidocitinib is an oral, highly selective Janus kinase 1 (JAK1) inhibitor. Preclinical evidence has demonstrated that chemical inhibition of the signal transducer and activator of transcription 3 (STAT3) pathway can potentiate the cytotoxic effects of anthracycline-based chemotherapy in non-Hodgkin lymphoma (NHL). Against this background, the present study aimed to evaluate the synergistic antitumor potential of combining golidocitinib (JAK1 inhibitor) with liposomal mitoxantrone in patients with R/R PTCL.

### Aims

To evaluate the safety and efficacy of golidocitinib combined with liposomal mitoxantrone in patients with relapsed/refractory peripheral T-cell lymphoma.

### Methods

This is a single-center retrospective study enrolling patients with R/R PTCL from July 2024 to October 2025. Patients received golidocitinib (150mg once daily) in combination with liposomal mitoxantrone. Safety was assessed per CTCAE v5.0. Tumor response was evaluated by PET-CT according to the Lugano 2014 criteria.

### Results

As of the data cut-off date, a total of 23 patients were enrolled. Baseline characteristics indicated a high-risk and heavily pretreated population. The median age was 57 years (range: 34-76), 82.6% (19/23) had stage III/IV disease, and 43.4% (10/23) had an IPI score ≥3. Patients had received a median of 1 prior line of therapy (range: 1–6). Histological subtypes included AITL (n=8), NKTCL (n=9), PTCL-NOS (n=3), MEITL (n=2), and ALK+ ALCL (n=1). All 23 patients were evaluable for efficacy. The combination demonstrated robust antitumor activity with an ORR of 91.3% (21/23) and a CR rate of 30.4% (7/23). Notably, in the NKTCL subgroup, the response rate was 88.9% (8/9) with a CR rate of 33.3% (3/9). Responses were rapid and durable, with a median PFS of 8 months. The regimen was generally well-tolerated. The main adverse events were hematological toxicities and infections. Grade ≥3 neutropenia occurred in 56.5% of patients, and Grade ≥3 thrombocytopenia in 47.8%. Pulmonary infection was reported in 56.5% of patients (including 3 patients with pre-existing infection prior to treatment), and CMV reactivation occurred in 30.4% of patients, all of which resolved with supportive care.

**Copyright Information:** (Online) ISSN: 2572-9241
©2026 The Author(s). HemaSphere is published by John Wiley & Sons Ltd on behalf of European Hematology Association. This is an open access Abstract Book distributed under the Attribution-NonCommercial-NoDerivs (CC BY-NC-ND), which allows third parties to download the articles and share them with others as long as they credit the author and the Abstract Book, but they cannot change the content in any way or use them commercially.
**Abstract Book Citations:** Authors, Title, HemaSphere, 2026;10:(S1):pages. The abstract book DOIs can be found on the “Author Information” page.
**Disclaimer:** Articles published in the journal HemaSphere exclusively reflect the opinions of the authors. The authors are responsible for all content in their abstracts including accuracy of the facts, statements, citing resources, etc.

**Summary/Conclusion**

The combination of golidocitinib and liposomal mitoxantrone demonstrates a manageable safety profile and promising preliminary efficacy in patients with R/R PTCL. A prospective Phase II study is ongoing to further validate these encouraging findings.

**Copyright Information:** (Online) ISSN: 2572-9241
©2026 The Author(s). HemaSphere is published by John Wiley & Sons Ltd on behalf of European Hematology Association. This is an open access Abstract Book distributed under the Attribution-NonCommercial-NoDerivs (CC BY-NC-ND), which allows third parties to download the articles and share them with others as long as they credit the author and the Abstract Book, but they cannot change the content in any way or use them commercially.
**Abstract Book Citations:** Authors, Title, HemaSphere, 2026;10:(S1):pages. The abstract book DOIs can be found on the "Author Information" page.
**Disclaimer:** Articles published in the journal HemaSphere exclusively reflect the opinions of the authors. The authors are responsible for all content in their abstracts including accuracy of the facts, statements, citing resources, etc.

### (PB3852) PROGNOSTIC FACTORS AND REAL-WORLD OUTCOMES OF ADULT T-CELL LEUKEMIA/LYMPHOMA IN AN HTLV-1 ENDEMIC REGION: A 10-YEAR SINGLE-CENTER STUDY

**Topic:** 20. Other aggressive lymphomas

Leila Cysique-Foinlan*[1], Charlotte Holuigue[1], Louis Guezet[1], Karel Edom[1], Olivia Hippomene-Jerier[1], Livia Claude[1], Veronique Baccini[1], Philippe Helias[1]

[1]*University Hospital of Guadeloupe, Les abymes, France;*

### Background

Adult T-cell leukemia/lymphoma (ATLL) remains one of the most aggressive peripheral T-cell neoplasms worldwide. In endemic areas, early mortality appears particularly high, yet determinants of ultra-early death are insufficiently characterized. Identifying readily available prognostic markers at diagnosis may enable rapid risk-adapted strategies.

### Methods

We conducted a retrospective single-center study including all consecutive ATLL diagnoses between January 2015 and December 2024 at the University Hospital of Guadeloupe, an HTLV-1 endemic area. Clinical and biological characteristics at diagnosis were collected to (1) describe real-world management and (2) identify prognostic factors for overall survival (OS). Survival was estimated using the Kaplan–Meier method. Prognostic variables were evaluated using univariate and multivariate Cox proportional hazards models.

### Results

Fifty-seven patients were included. Acute (60%) and lymphoma (29%) subtypes predominated, while no smoldering forms were observed. Median age at diagnosis was 61 years (range, 27–93). Among evaluable cases, 14 (28%) expressed CD30.
Median OS was 2.7 months (range, 0.03–132.4) underscoring the extreme aggressiveness of ATLL in this setting. Deaths were mainly related to disease progression and infectious complications. Forty-five patients (79%) received first-line therapy: antiviral therapy alone (57%), combination chemotherapy (27%), or combined antiviral-chemotherapy (16%). Only one patient underwent upfront allogeneic stem cell transplantation. Notably, twelve patients (21%) died before treatment initiation.
First-line response emerged as the major determinant of outcome (median OS 1.3 vs 12.5 months in non-responders versus responders; $p<0.001$). Among 24 relapsing patients, 16 received salvage therapy.
At diagnosis, several biological parameters were significantly associated with inferior OS in univariate analysis, including >10% circulating ATL cells, hypercalcemia, elevated transaminases, and LDH >3×ULN. In multivariate analysis, hypercalcemia (HR X.X), ALT >25 IU/L (HR X.X), and LDH >3×ULN (HR X.X) remained independent predictors of mortality. Importantly, these markers are universally available and allow immediate bedside risk stratification.
Fifteen patients survived more than 12 months. Five patients (7%) were alive at last follow-up (October 2025), including one transplanted patient and two HIV-coinfected patients. Notably, none of the long-term survivors had acute subtype disease.

**Copyright Information:** (Online) ISSN: 2572-9241
©2026 The Author(s). HemaSphere is published by John Wiley & Sons Ltd on behalf of European Hematology Association. This is an open access Abstract Book distributed under the Attribution-NonCommercial-NoDerivs (CC BY-NC-ND), which allows third parties to download the articles and share them with others as long as they credit the author and the Abstract Book, but they cannot change the content in any way or use them commercially.
**Abstract Book Citations:** Authors, Title, HemaSphere, 2026;10:(S1):pages. The abstract book DOIs can be found on the "Author Information" page.
**Disclaimer:** Articles published in the journal HemaSphere exclusively reflect the opinions of the authors. The authors are responsible for all content in their abstracts including accuracy of the facts, statements, citing resources, etc.

**Summary/Conclusion**

In this contemporary endemic cohort, aggressive ATLL subtypes predominated and early mortality was strikingly high despite widespread use of antiviral therapy. One in five patients died before treatment initiation and response to first-line treatment emerged as the major determinant of survival. Simple baseline biological parameters—particularly liver dysfunction and elevated LDH—may enable early risk stratification in routine practice. The presence of long-term survivors among HIV-coinfected patients, although hypothesis-generating, raises questions regarding a potential impact of modern antiretroviral therapy on disease control and warrants further investigation.

**Copyright Information:** (Online) ISSN: 2572-9241
©2026 The Author(s). HemaSphere is published by John Wiley & Sons Ltd on behalf of European Hematology Association. This is an open access Abstract Book distributed under the Attribution-NonCommercial-NoDerivs (CC BY-NC-ND), which allows third parties to download the articles and share them with others as long as they credit the author and the Abstract Book, but they cannot change the content in any way or use them commercially.
**Abstract Book Citations:** Authors, Title, HemaSphere, 2026;10:(S1):pages. The abstract book DOIs can be found on the "Author Information" page.
**Disclaimer:** Articles published in the journal HemaSphere exclusively reflect the opinions of the authors. The authors are responsible for all content in their abstracts including accuracy of the facts, statements, citing resources, etc.

### (PB3853) EFFICACY AND SAFETY OF LIPOSOMAL MITOXANTRONE–CONTAINING REGIMENS IN ELDERLY PATIENTS WITH NEWLY DIAGNOSED ADVANCED ANGIOIMMUNOBLASTIC T-CELL LYMPHOMA

**Topic:** 20. Other aggressive lymphomas

Lin Shen[1], Jifeng Jiang*[1], Xiaoqiang Fan[2], Zhongyuan Feng[2], Min Wu[1], Mingyue Chen[1], Wensi Qian[1], Pingping Chen[1]

*[1]Huadong Hospital Affiliated with Fudan University, Shanghai, China;[2]Shanghai Changzheng Hospital, Shanghai, China;*

### Background

Angioimmunoblastic T-cell lymphoma (AITL) mainly affects older patients and is often diagnosed at an advanced stage with high-risk clinical features. Outcomes with conventional chemotherapy regimens such as CHOP or CHOPE remain generally unsatisfactory, highlighting the unmet need for more effective and safer therapeutic strategies.

### Aims

To evaluate the efficacy and safety of regimens containing liposomal mitoxantrone in elderly patients with newly diagnosed advanced angioimmunoblastic T-cell lymphoma.

### Methods

We conducted a retrospective analysis of 24 elderly patients aged 60 years or older who were newly diagnosed with AITL and received treatment between November 2017 and December 2022 at two centers in Shanghai, China: Huadong Hospital affiliated with Fudan University and Shanghai Changzheng Hospital. Treatment responses, progression-free survival (PFS), overall survival (OS), and treatment-related toxicities were evaluated.
Patients received combination chemotherapy every 21 days for six planned cycles. The CMOP regimen consisted of cyclophosphamide 500–750 mg/m² intravenously on day 1, liposomal mitoxantrone 12–18 mg/m² intravenously on day 1, vindesine 3 mg intravenously on day 1, and methylprednisolone 80 mg intravenously on days 1–5. In anthracycline-based regimens (CHOP or CHOP-like), doxorubicin 35–50 mg/m² or epirubicin 45–70 mg/m² was administered intravenously on day 1 instead of liposomal mitoxantrone, while the doses of cyclophosphamide, vindesine, and methylprednisolone were consistent with those used in the CMOP regimen. Dose modifications were permitted according to patient tolerance and clinical condition. Survival outcomes were estimated using the Kaplan–Meier method and compared using the log-rank test.

### Results

The median age was 64.5 years. Twenty-three patients (96.0%) had Ann Arbor stage III–IV disease and 22 patients (92.0%) had IPI ≥3. The complete response rate was significantly higher in the liposomal mitoxantrone group compared with the non–liposomal mitoxantrone group (76.9% vs 9.1%, P=0.001). At a median follow-up of 23.6 months, the median progression-free survival (PFS) for the entire cohort was 12 months, while median overall survival (OS) was not reached. The 2-year PFS and OS rates were 26.1% and 81.0%, respectively. Patients receiving liposomal mitoxantrone achieved significantly longer PFS compared with those not receiving liposomal mitoxantrone (20 vs 6 months, P=0.0135). Overall survival was also significantly improved in the liposomal mitoxantrone group (median not reached vs approximately 15 months, P=0.0123). Grade 3–4 hematologic toxicities occurred in 46.2% versus 72.7% of patients, and infection rates were 30.8% versus 72.7%, with no statistically significant differences between groups.

**Copyright Information:** (Online) ISSN: 2572-9241
©2026 The Author(s). HemaSphere is published by John Wiley & Sons Ltd on behalf of European Hematology Association. This is an open access Abstract Book distributed under the Attribution-NonCommercial-NoDerivs (CC BY-NC-ND), which allows third parties to download the articles and share them with others as long as they credit the author and the Abstract Book, but they cannot change the content in any way or use them commercially.
**Abstract Book Citations:** Authors, Title, HemaSphere, 2026;10:(S1):pages. The abstract book DOIs can be found on the “Author Information” page.
**Disclaimer:** Articles published in the journal HemaSphere exclusively reflect the opinions of the authors. The authors are responsible for all content in their abstracts including accuracy of the facts, statements, citing resources, etc.

### Summary/Conclusion

In elderly patients with advanced AITL, CMOP regimens containing liposomal mitoxantrone were associated with higher response rates and significantly prolonged survival without an apparent increase in major toxicities. These findings suggest that liposomal mitoxantrone–containing regimens may represent an effective and tolerable therapeutic option for elderly patients with advanced disease.

Table 1. Baseline clinical characteristics of Patients (n = 24)

| Characteristic | No. | % |
|---|---|---|
| Age, years | | |
| Median (Range) | 64.5(60-72) | |
| >65 | 10 | 47.6 |
| Male sex | 16 | 66.7 |
| ECOG PS ≥ 2 | 23 | 96.0 |
| Stages III to IV | 23 | 96.0 |
| CNS involvement | 4 | 16.7 |
| BM involvement | 12 | 50.0 |
| B symptoms | 19 | 79.2 |
| Hemolytic anemia | 13 | 54.2 |
| EBER Positive | 17 | 70.8 |
| With HLH | 6 | 25.0 |
| Elevated LDH | 20 | 83.3 |
| With ASCT | 9 | 37.5 |
| IDH2 mutation | 14 | 58.3 |
| TET2 mutation | 19 | 79.2 |
| DNMT3A mutation | 20 | 83.3 |
| RHOA mutation | 15 | 62.5 |
| KMT2C mutation | 17 | 78.8 |
| IPI ≥ 3 | 22 | 92.0 |
| PIT | | |
| 1 | 0 | 0 |
| 2 | 5 | 20.8 |
| 3 | 9 | 37.5 |
| 4 | 10 | 41.7 |

Abbreviations: BM, bone marrow; HLH, hemophagocytic lymphohistiocytosis; IPI, International Prognostic Index.

Table 2. Baseline differences between Lipo-MIT and non–Lipo-MIT groups

| Characteristic | Regimens with Lipo-MIT | | Regimens without Lipo-MIT | | |
|---|---|---|---|---|---|
| | No. | % | No. | % | p |
| Age, years | | | | | |
| Median(Range) | 65(60-72) | | 64(61-70) | | 0.187 |
| > 65 | 6 | 64.2 | 5 | 45.5 | |
| Male sex | 10 | 76.9 | 6 | 54.5 | 0.390 |
| ECOG PS ≥ 2 | 13 | 100 | 10 | 91.1 | 0.460 |
| Stages III to IV | 12 | 93.2 | 11 | 100 | 1.000 |
| CNS involvement | 2 | 15.4 | 2 | 18.2 | 1.000 |
| BM involvement | 8 | 61.5 | 4 | 36.4 | 0.414 |
| B symptoms | 10 | 76.9 | 9 | 81.8 | 1.000 |
| Hemolytic anemia | 6 | 46.2 | 7 | 63.6 | 0.444 |
| EBER Positive | 9 | 69.2 | 8 | 72.7 | 1.000 |
| With HLH | 5 | 38.5 | 4 | 36.4 | 1.000 |
| Elevated LDH | 9 | 69.2 | 11 | 100 | 0.098 |
| With ASCT | 5 | 38.5 | 4 | 36.4 | 1.000 |
| IDH2 mutation | 7 | 53.8 | 7 | 63.6 | 0.697 |
| TET2 mutation | 12 | 92.3 | 7 | 63.6 | 0.142 |
| DNMT3A mutation | 12 | 92.3 | 7 | 63.6 | 0.300 |
| RHOA mutation | 9 | 69.2 | 6 | 54.5 | 0.675 |
| KMT2C mutation | 12 | 92.3 | 9 | 69.2 | 1.000 |
| IPI ≥ 3 | 11 | 84.6 | 11 | 100 | 0.482 |
| PIT | | | | | 0.288 |
| 1 | 0 | 0 | 0 | 0 | |
| 2 | 4 | 30.8 | 1 | 9.1 | |
| 3 | 3 | 23.1 | 6 | 54.5 | |
| 4 | 6 | 46.2 | 4 | 36.4 | |

Abbreviations:
BM, bone marrow; HLH, hemophagocytic lymphohistiocytosis; IPI, International Prognostic Index; ASCT, autologous hematopoietic cell transplantation; Lipo-MIT: liposomal mitoxantrone.

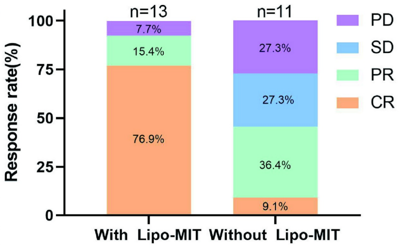

**Figure 1. Best overall response with and without liposomal mitoxantrone. Response distribution in patients receiving liposomal mitoxantrone (n = 13) or not (n = 11).CR, complete response; PR, partial response; SD, stable disease; PD, progressive disease.**

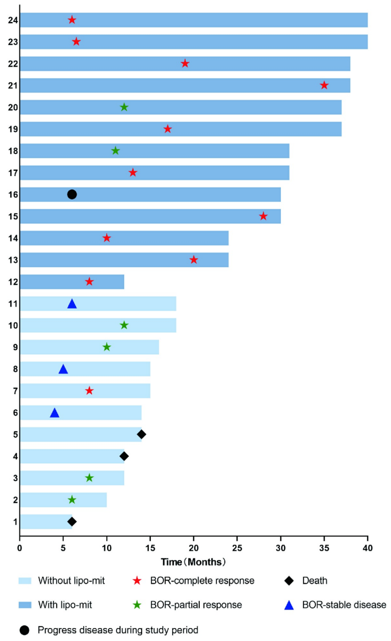

**Figure 2. Swimmer plot**

Table 3. Treatment-related toxicities in patients receiving regimens with versus without liposomal mitoxantrone (Lipo-MIT).

| | Regimens with Lipo-MIT | | Regimens without Lipo-MIT | | |
|---|---|---|---|---|---|
| Toxicity | No. | % | No. | % | p |
| Hematologic Toxicity (Grade 3-4) | 6 | 46.2 | 8 | 72.7 | 0.24 |
| Infections | 4 | 30.8 | 8 | 72.7 | 1 |
| Cardiovascular Events | 0 | 0 | 2 | 20.0 | 0.178 |

Toxicities are reported as number (percentage). Grade 3–4 hematologic toxicity was defined according to the Common Terminology Criteria for Adverse Events (CTCAE 5.0) .

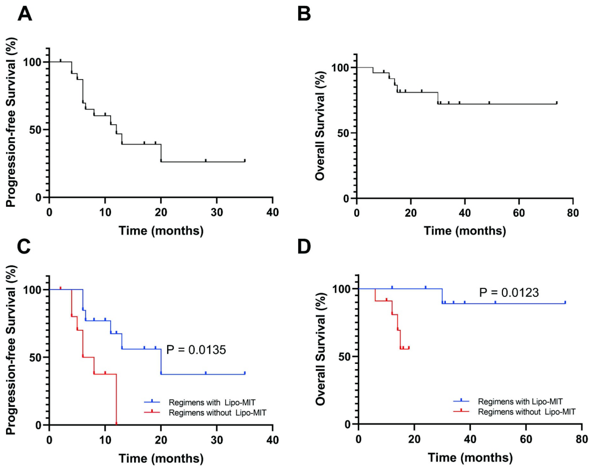

**Figure 3. Kaplan–Meier curves for progression-free survival (PFS) and overall survival (OS).**
**(A) PFS in the overall cohort. (B) OS in the overall cohort.**
**(C) PFS in treatment regimens group with Lipo-MIT versus without Lipo-MIT.**
**(D) OS in treatment regimens group with Lipo-MIT versus without Lipo-MIT.**

**Copyright Information:** (Online) ISSN: 2572-9241
©2026 The Author(s). HemaSphere is published by John Wiley & Sons Ltd on behalf of European Hematology Association. This is an open access Abstract Book distributed under the Attribution-NonCommercial-NoDerivs (CC BY-NC-ND), which allows third parties to download the articles and share them with others as long as they credit the author and the Abstract Book, but they cannot change the content in any way or use them commercially.
**Abstract Book Citations:** Authors, Title, HemaSphere, 2026;10:(S1):pages. The abstract book DOIs can be found on the "Author Information" page.
**Disclaimer:** Articles published in the journal HemaSphere exclusively reflect the opinions of the authors. The authors are responsible for all content in their abstracts including accuracy of the facts, statements, citing resources, etc.

### (PB3854) SERUM INTERLEUKIN-6 (IL-6) MONITORING IN IDIOPATHIC MULTICENTRIC CASTLEMAN DISEASE: IMPORTANT VALUE IN PATIENTS RECEIVING NON-ANTI-IL-6 THERAPY

**Topic:** 20. Other aggressive lymphomas

Zi-Han Yang*[1], Yu-Han Gao[1], Si-Yuan Li[1], Miao-Yan Zhang[1], An-an Li[1], Hao-Yi Xu[1], Lu Zhang[1], Jian Li[1]

*[1]Peking Union Medical College Hospital, Department of Hematology, Beijing, China;*

### Background

Idiopathic multicentric Castleman disease (iMCD) is a rare hematologic disorder characterized by a systemic cytokine storm. Interleukin-6 (IL-6) is central to iMCD pathogenesis and IL-6-targeted therapy is the preferred first-line treatment; however, serum IL-6 is not recommended for assessing treatment response due to the influence of IL-6 inhibitors on serum IL-6 examination. In fact, the accessibility of IL-6 inhibitors in real-world practice remains limited, and there is no recommendation for monitoring serum IL-6 in patients receiving non-IL-6 inhibition.

### Aims

We aimed to evaluate the clinical utility of serum IL-6 monitoring at baseline and longitudinally in iMCD, with emphasis on patients receiving non-IL-6-targeted therapy. We also aimed to determine whether early IL-6 changes predict subsequent biochemical response and imminent exacerbation.

### Methods

We conducted a single-center retrospective cohort study at Peking Union Medical College Hospital (PUMCH) including consecutively treated iMCD patients diagnosed according to the Castleman Disease Collaborative Network (CDCN) criteria with available serum IL-6 monitoring (July 2016–June 2025). Patients with missing baseline IL-6 or key laboratories (hemoglobin, platelet count, albumin, serum creatinine, C-reactive protein (CRP), immunoglobulin G (IgG)) or missing follow-up IL-6 and key labs were excluded. Patients treated with regimens containing siltuximab or tocilizumab were classified into the 'anti-IL-6 therapies' group, whereas patients who did not receive any IL-6-targeted drug were assigned to the 'non-anti-IL-6 therapies' group. Serum IL-6 was measured by chemiluminescent immunoassay (LK6P1; Siemens) in the central laboratory of PUMCH. Biochemical response was assessed according to CDCN criteria.

### Results

Among 149 patients (685 IL-6 measurements; 76 male/73 female; median age 45 years), 34 received anti-IL-6 therapy and 115 received non-anti-IL-6 therapy which further demonstrated that IL-6 targeted therapy was not the mainstream treatment in the real world. Baseline IL-6 differed across clinical and pathologic subtypes and correlated negatively with hemoglobin and albumin, and positively with platelet count, C-reactive protein (CRP), and immunoglobulin G (IgG). Baseline IL-6 did not predict biochemical response. IL-6 dynamics were uninformative in the anti-IL-6 group, with IL-6 increasing above baseline at follow-up. In contrast, in the non-anti-IL-6 group, IL-6 declined significantly after treatment, with greater decreases in responders than in non-responders. These longitudinal response-associated patterns remained qualitatively unchanged after excluding iMCD-TAFRO. Among initial responders, rising IL-6 preceded subsequent disease progression, whereas persistently suppressed IL-6 was observed in sustained remission. Early IL-6 change predicted subsequent biochemical response; at 1 month, a $\Delta$logIL-6 threshold of –0.16 achieved 91.7% sensitivity and 92.3% specificity.

**Copyright Information:** (Online) ISSN: 2572-9241
©2026 The Author(s). HemaSphere is published by John Wiley & Sons Ltd on behalf of European Hematology Association. This is an open access Abstract Book distributed under the Attribution-NonCommercial-NoDerivs (CC BY-NC-ND), which allows third parties to download the articles and share them with others as long as they credit the author and the Abstract Book, but they cannot change the content in any way or use them commercially.
**Abstract Book Citations:** Authors, Title, HemaSphere, 2026;10:(S1):pages. The abstract book DOIs can be found on the "Author Information" page.
**Disclaimer:** Articles published in the journal HemaSphere exclusively reflect the opinions of the authors. The authors are responsible for all content in their abstracts including accuracy of the facts, statements, citing resources, etc.

**Summary/Conclusion**

Baseline serum IL-6 level does not predict treatment response, but is useful for assessing baseline disease characteristics. Monitoring IL-6 trajectories is of limited value during anti-IL-6 therapy (the guideline recommended first choice), but is informative in patients receiving non-anti-IL-6 regimens, which are commonly used in the real-world. This study support incorporating IL-6 monitoring into baseline disease characterization of iMCD, response assessment and early prediction for non-anti-IL-6 inhibition.

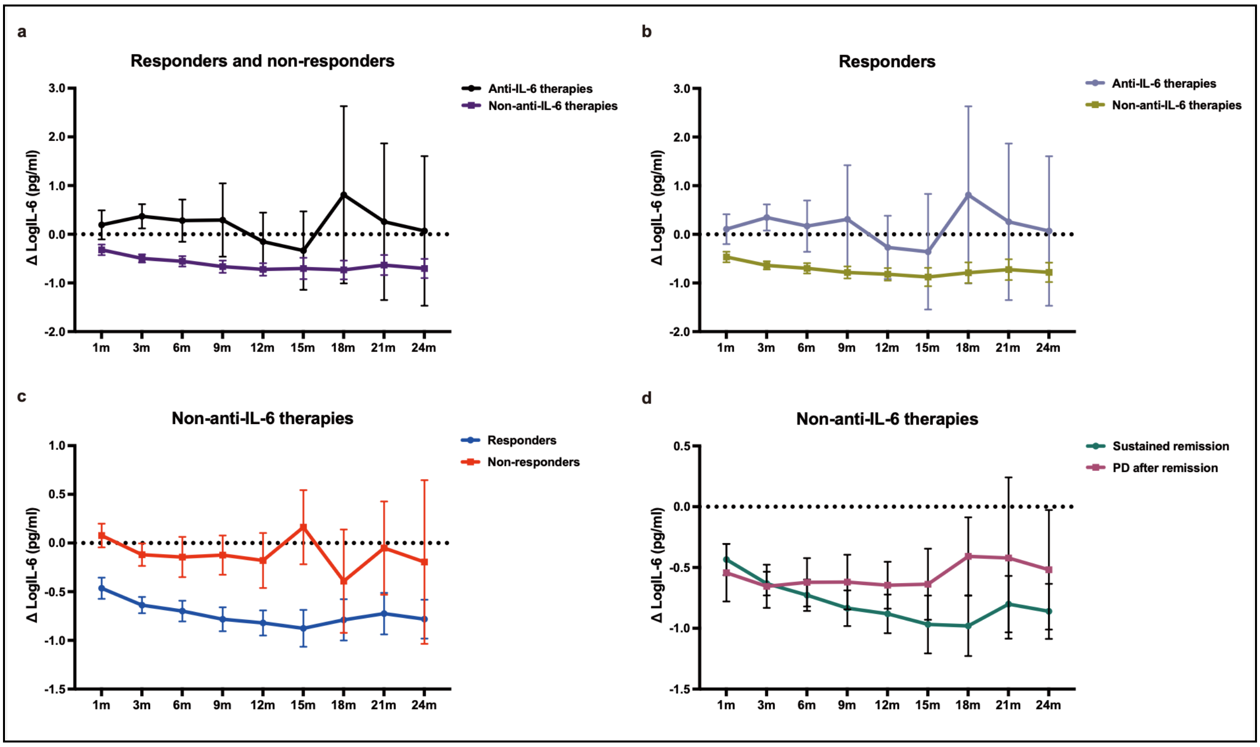

Trajectories of serum IL-6 levels under anti-IL-6 and non-anti-IL-6 therapies. (a) Longitudinal change in IL-6 (ΔlogIL-6) for all treated iMCD patients. Anti-IL-6 therapy resulted in a modest increase in IL-6 levels above baseline (positive ΔlogIL-6) at most follow-up points. In contrast, non-anti-IL-6 therapy led to a significant decrease from baseline (negative ΔlogIL-6) across the follow-up points. (b) Among responders only, anti-IL-6 therapies again caused an overall increase in IL-6 relative to baseline, whereas non-anti-IL-6 therapies exhibited a significant and sustained decrease. (c) In patients receiving non-anti-IL-6 therapies, IL-6 levels decreased more substantially in responders than in non-responders, with statistically significant differences emerging as early as 1 month and persisting through 15 months. (d) Among patients who initially responded to non-anti-IL-6 therapies, those who maintained sustained remission continued to show suppressed IL-6 levels, whereas those who later experienced PD exhibited an early post-treatment IL-6 decline followed by a rebound. PD, progressive disease; IL-6, interleukin-6; ΔlogIL-6, the change in log10-transformed IL-6 relative to baseline.

**Copyright Information:** (Online) ISSN: 2572-9241
©2026 The Author(s). HemaSphere is published by John Wiley & Sons Ltd on behalf of European Hematology Association. This is an open access Abstract Book distributed under the Attribution-NonCommercial-NoDerivs (CC BY-NC-ND), which allows third parties to download the articles and share them with others as long as they credit the author and the Abstract Book, but they cannot change the content in any way or use them commercially.
**Abstract Book Citations:** Authors, Title, HemaSphere, 2026;10:(S1):pages. The abstract book DOIs can be found on the “Author Information” page.
**Disclaimer:** Articles published in the journal HemaSphere exclusively reflect the opinions of the authors. The authors are responsible for all content in their abstracts including accuracy of the facts, statements, citing resources, etc.

### (PB3855) FRONTLINE MGZL: HIGH EVENT RATES WITH HL-LIKE AND AGGRESSIVE B-CELL REGIMENS; EARLY FEASIBILITY OF NIVOLUMAB-BASED, TRANSPLANT-SPARING R-DA-EPOCH + DACARBAZINE

**Topic:** 20. Other aggressive lymphomas

Runisa Abdurashidova*[1], Yana Mangasarova[1, 2], Tanzila Ushaeva[1], Aminat Magomedova[1], Anna Kravtsova[1], Raisa Dorzhieva[1], Daria Belkina[1], Ekaterina Fastova[1], Ekaterina Gitelzon[1], Oleg Margolin[1], Madina Bagova[1], Inna Krasilnikova[1], Irina Shupletsova[1], Alla Kovrigina[1, 3], Evgeniy Zvonkov[1, 2]

*[1] 1National Medical Research Center for Hematology, Moscow, Russian Federation, Moscow, Russia;[2]Russian University of Medicine, Moscow, Russia; [3]2I.M. Sechenov First Moscow State Medical University (Sechenov University), Moscow, Russia;*

### Background

Mediastinal gray zone lymphoma (MGZL) is a rare lymphoma in the mediastinum with features of both classical Hodgkin lymphoma (HL) and primary mediastinal large B-cell lymphoma (PMBCL). Because it is rare, we do not have many clinical trials, and there is no standard first-line treatment. In real life, doctors often use regimens developed for HL or for aggressive B-cell lymphomas, but results are often not good enough. R-DA-EPOCH plus dacarbazine with autologous stem cell transplantation (auto-HSCT) may improve disease control, but auto-HSCT can cause long-term side effects, which matters because many MGZL patients are young. Adding PD-1 blockade (nivolumab) may allow us to avoid transplantation while keeping good disease control. This is supported by frequent immune-escape features in the mediastinal HL/PMBCL spectrum.

### Aims

To compare event-free survival (EFS) between first-line treatment strategies for MGZL in a single-center real-world cohort and to report early outcomes of a nivolumab-based, transplant-sparing approach.

### Methods

We retrospectively analyzed consecutive adult patients with MGZL treated at the National Medical Research Center for Hematology (Moscow) between 2012 and 2025. Diagnosis was confirmed by institutional pathology review according to the WHO classification valid at the time of diagnosis. EFS events were progression, relapse, or death. Patients were grouped by first-line strategy: HL-like regimens (n=9; ABVD, BV-AVD, R-BEACOPP-14 ± radiotherapy), aggressive B-cell-type regimens (n=15; EPOCH-based regimens without dacarbazine and/or intensive regimens including R-mNHL-BFM-90), R-DA-EPOCH + dacarbazine with consolidative auto-HSCT (n=14), and R-DA-EPOCH + dacarbazine + nivolumab (fixed 40 mg on day 1 of each cycle) without auto-HSCT (n=6). Response was assessed by PET-CT using Lugano 2014. EFS was estimated with Kaplan–Meier and compared by the log-rank test. Median follow-up was calculated with reverse Kaplan–Meier. Groups were broadly comparable in key baseline clinical characteristics.

### Results

We included 44 patients (median age 35 years; IQR 28–41; range 18–63). EFS differed between the four strategies (log-rank p=0.006) (Figure 1). At 5 years, EFS was 55.6% in the HL-like group (n=9) and 46.7% in the aggressive B-cell-type group (n=15). No EFS events were seen after R-DA-EPOCH + dacarbazine with auto-HSCT (0/14) or after R-DA-EPOCH + dacarbazine + nivolumab without auto-HSCT (0/6). Median follow-up (reverse Kaplan–Meier) was 61 months (95% CI 32–NR), 79 months (63–NR), 33 months (21–63), and 15 months (13–NR), respectively. Follow-up was shorter in the transplant-sparing nivolumab group. Nivolumab was well tolerated; no grade ≥3 immune-related adverse events were observed.

**Copyright Information:** (Online) ISSN: 2572-9241
©2026 The Author(s). HemaSphere is published by John Wiley & Sons Ltd on behalf of European Hematology Association. This is an open access Abstract Book distributed under the Attribution-NonCommercial-NoDerivs (CC BY-NC-ND), which allows third parties to download the articles and share them with others as long as they credit the author and the Abstract Book, but they cannot change the content in any way or use them commercially.
**Abstract Book Citations:** Authors, Title, HemaSphere, 2026;10:(S1):pages. The abstract book DOIs can be found on the “Author Information” page.
**Disclaimer:** Articles published in the journal HemaSphere exclusively reflect the opinions of the authors. The authors are responsible for all content in their abstracts including accuracy of the facts, statements, citing resources, etc.

**Summary/Conclusion**

In this real-world MGZL cohort, HL-like and aggressive B-cell-type regimens had high event rates. An EPOCH-based strategy with dacarbazine showed excellent short- to mid-term disease control. Early results suggest that adding nivolumab may allow a transplant-sparing approach, but longer follow-up is needed to confirm durability and safety.

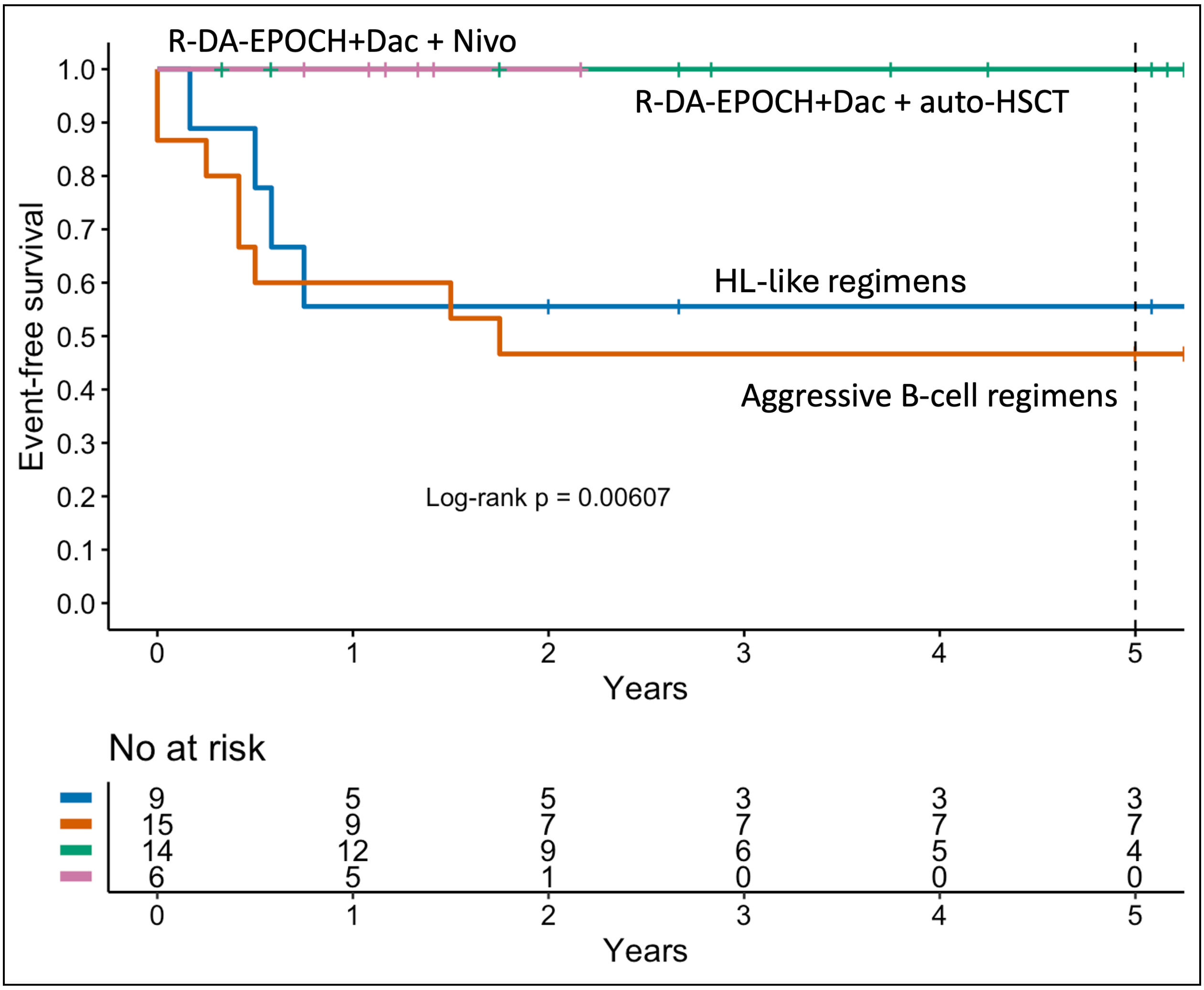

Figure 1. EFS in mediastinal gray zone lymphoma by frontline strategy.

**Copyright Information:** (Online) ISSN: 2572-9241
©2026 The Author(s). HemaSphere is published by John Wiley & Sons Ltd on behalf of European Hematology Association. This is an open access Abstract Book distributed under the Attribution-NonCommercial-NoDerivs (CC BY-NC-ND), which allows third parties to download the articles and share them with others as long as they credit the author and the Abstract Book, but they cannot change the content in any way or use them commercially.
**Abstract Book Citations:** Authors, Title, HemaSphere, 2026;10:(S1):pages. The abstract book DOIs can be found on the "Author Information" page.
**Disclaimer:** Articles published in the journal HemaSphere exclusively reflect the opinions of the authors. The authors are responsible for all content in their abstracts including accuracy of the facts, statements, citing resources, etc.

### (PB3856) PRELIMINARY EFFICACY AND SAFETY OF THE PMOED REGIMEN AS INDUCTION THERAPY FOR NEWLY DIAGNOSED NATURAL KILLER/T-CELL LYMPHOMA

**Topic:** 20. Other aggressive lymphomas

Hanshan Yang[1], Xixi Xiang[1], Jiali Li[1], Li Gao*[1]

[1] *The Second Affiliated Hospital of Army Medical University, Chongqing, China;*

### Background

Natural killer/T-cell lymphoma (NKTCL) is an aggressive malignancy with a strong association with Epstein-Barr virus, prevalent in Asia. Current standard induction regimens, primarily asparaginase-based, achieve promising outcomes, yet a subset of patients exhibit refractory disease or relapse, highlighting an unmet need for more effective therapies. Mitoxantrone liposome, a novel liposomal formulation, offers a potentially improved therapeutic index with reduced cardiac toxicity compared to conventional anthracyclines.

### Aims

This study was designed to evaluate the preliminary efficacy and safety of a novel induction regimen combining mitoxantrone liposome, pegaspargase, vincristine, etoposide, and dexamethasone (PMOED) in patients with newly diagnosed NKTCL.

### Methods

Newly diagnosed NKTCL patients over the age of 18 years received PMOED regimen. The treatment consisted of pegaspargase (2000 IU/m², day 1), mitoxantrone liposome (20 mg/m², days 1-3), vincristine (1.4 mg/m², days 1-2), etoposide (60 mg/m², days 2-4), and dexamethasone (10 mg, days 1-5).The primary endpoint was the complete response rate(CRR), and the secondary endpoint was objective response rate(ORR) and safety.

### Results

From June 2022 to February 2026, a total of 22 newly diagnosed NKTCL patients received induction chemotherapy with PMOED regimen. The median age was 41 years (range, 21–75), including 15 males and 7 females. 14 patients had early-stage disease and 8 had advanced-stage disease. 10 patients were low-intermediate risk and 12 were high-risk. Four patients had bulky disease, and three presented with hemophagocytic syndrome. After three cycles of induction therapy, eighteen patients were evaluable for response. Among them, fourteen achieved a CR, two a partial response (PR), and two progressive disease (PD), resulting in an ORR of 88.9% (16/18) and a CRR of 77.8% (14/18).With a median follow-up time of 15.8 months (range 1.2-32.6), the median PFS and OS were not reached. Of the 16 patients who responded to treatment (achieving PR or better), one underwent autologous hematopoietic stem cell transplantation (auto-HSCT) and three received allogeneic transplantation (allo-HSCT). Additionally, eight patients (40%) received radiotherapy, comprising six with early-stage and two with advanced-stage disease. No grade III-IV hematologic or non-hematologic adverse events were observed in any of the patients.

### Summary/Conclusion

The combination of mitoxantrone liposome with the POED regimen demonstrates efficacy and a manageable safety profile as induction chemotherapy for NKTCL.

**Copyright Information:** (Online) ISSN: 2572-9241
©2026 The Author(s). HemaSphere is published by John Wiley & Sons Ltd on behalf of European Hematology Association. This is an open access Abstract Book distributed under the Attribution-NonCommercial-NoDerivs (CC BY-NC-ND), which allows third parties to download the articles and share them with others as long as they credit the author and the Abstract Book, but they cannot change the content in any way or use them commercially.
**Abstract Book Citations:** Authors, Title, HemaSphere, 2026;10:(S1):pages. The abstract book DOIs can be found on the "Author Information" page.
**Disclaimer:** Articles published in the journal HemaSphere exclusively reflect the opinions of the authors. The authors are responsible for all content in their abstracts including accuracy of the facts, statements, citing resources, etc.

### (PB3857) SAFETY AND EFFECTIVENESS OF BRENTUXIMAB VEDOTIN IN CHINESE PATIENTS WITH CD30-POSITIVE NON-NODAL PERIPHERAL T-CELL LYMPHOMAS: A SUBGROUP ANALYSIS OF BRAVE STUDY

**Topic:** 20. Other aggressive lymphomas

Shu Cheng*[1], Ou Bai[2], Luqun Wang[3], Lihong Liu[4], Chuanxu Liu[5], Liping Su[6], Zengjun Li[7], Wei Xu[8], Bing Xu[9], Ying Lu[10], Jianli Wang[11], Ziheng Guo[11], Weili Zhao[1]

*[1]Shanghai Institute of Hematology, State Key Laboratory of Medical Genomics, National Research Center for Translational Medicine at Shanghai, Ruijin Hospital affiliated to Shanghai Jiao Tong University School of Medicine, Shanghai, China; [2]Department of Lymphoma, the First Bethune Hospital of Jilin University, Changchun, China;[3]Department of Hematology, Qilu Hospital of Shandong University, Jinan, China; [4]Department of Hematology, The Fourth Hospital of Hebei Medical University, Hebei Tumor Hospital, Shijiazhuang, China; [5]Department of Lymphoma, Fudan University Shanghai Cancer Center, Shanghai, China; [6]Department of Hematology, Shanxi Cancer Hospital, Taiyuan, China;[7]Department of Hematology, Shandong Cancer Hospital & Institute, Jinan, China; [8]Department of Hematology, Jiangsu Province Hospital, The First Affiliated Hospital of Nanjing Medical University, Nanjing, China; [9]Department of Hematology, The first affiliated hospital of Xiamen university, Xiamen, China; [10]Department of Hematology, The Affiliated People's Hospital of Ningbo University, Ningbo, China;[11]Takeda (China) International Trading Co., Ltd., Shanghai, China;*

### Background

Brentuximab vedotin (BV) plus CHP has been recommended as a first-line treatment for CD30-positive peripheral T-cell lymphomas (CD30+ PTCL) based on the ECHELON-2 study. However, evidence remains limited for rare subtypes of CD30-positive non-nodal PTCL, including NK/T-cell lymphoma (NKTCL), cutaneous T-cell lymphoma (CTCL), and adult T-cell leukemia/lymphoma (ATLL).

### Aims

To evaluate the real-world safety and effectiveness of BV in newly diagnosed (ND) Chinese patients (pts) with these rare CD30-positive non-nodal PTCL subtypes.

### Methods

BRAVE (NCT04837222) is a multicenter, prospective, observational study, which enrolled adult pts with CD30+ PTCL treated with BV-based regimens across 30 centers in China. The primary endpoint was serious adverse events (SAEs), and secondary endpoints included adverse events (AEs), adverse drug reactions (ADRs), BV exposure, objective response rate (ORR), complete response (CR), time to response (TTR), time to CR (TTCR), and progress-free survival (PFS). Safety was assessed in all treated ND pts with efficacy analysis in response-evaluable pts. This is an interim analysis.

**Copyright Information:** (Online) ISSN: 2572-9241
©2026 The Author(s). HemaSphere is published by John Wiley & Sons Ltd on behalf of European Hematology Association. This is an open access Abstract Book distributed under the Attribution-NonCommercial-NoDerivs (CC BY-NC-ND), which allows third parties to download the articles and share them with others as long as they credit the author and the Abstract Book, but they cannot change the content in any way or use them commercially.
**Abstract Book Citations:** Authors, Title, HemaSphere, 2026;10:(S1):pages. The abstract book DOIs can be found on the "Author Information" page.
**Disclaimer:** Articles published in the journal HemaSphere exclusively reflect the opinions of the authors. The authors are responsible for all content in their abstracts including accuracy of the facts, statements, citing resources, etc.

**Results**

A total of 26 ND pts with CD30-positive non-nodal PTCL were enrolled, including 11 NKTCL, 9 CTCL (6 mycosis fungoides, 1 Sézary syndrome and 2 primary cutaneous ALCL), and 6 ATLL. Median age was 44.0, 59.0 and 57.0 years in NKTCL, CTCL and ATLL, respectively. Most pts had an Eastern Cooperative Oncology Group performance status (ECOG PS) ≤1 (72.8%, 87.5% and 66.7% in NKTCL, CTCL, and ATLL subgroups, respectively). Advanced-stage (Ann Arbor III–IV) disease was present in 18.2%, 66.7%, and 83.4% patients, respectively. The proportion of patients with a low-to-intermediate IPI score (0–3) was 81.9%, 57.2%, and 60.0%, respectively. Median BV cycles were 4.0 in NKTCL, 3.0 in CTCL, and 3.5 in ATLL. Median dosage intensity was 1.48, 1.63, and 1.65 mg/kg in each cycle, respectively.

Totally, any-grade AEs were reported in 76.9% of patients, with SAEs observed in 19.2% of pts. The SAE included one case of pneumonia, three events of lymphoma progression, and one death of undetermined cause in a patient with ATLL. No BV dose reductions were reported and no new safety signals were identified.

Efficacy-evaluable pts consisted of 9 NKTCL, 7 CTCL, and 4 ATLL. ORR/CR rate were 55.6%/33.3% in NKTCL, 42.9%/28.6% in CTCL, and 75.0%/75.0% in ATLL according to 2014 Lugano criteria. Median TTR was 2.10, 2.63 and 2.40 months, and median TTCR was 2.89, 2.87, and 2.69 months, respectively. At a median follow-up of 8.84 months, median PFS was 4.4 months in NKTCL, 5.8 months in CTCL, and was not reached in ATLL.

**Summary/Conclusion**

This interim analysis of the BRAVE study provides the first prospective real-world evidence on frontline BV in Chinese patients with rare non-nodal CD30-positive PTCL. BV showed manageable safety profile and encouraging effectiveness, with rapid responses observed across subtypes. These findings add valuable real-world information in a rare and under-studied disease setting, although interpretation is limited due to small subgroup samples and short follow-up time.

**Copyright Information:** (Online) ISSN: 2572-9241
©2026 The Author(s). HemaSphere is published by John Wiley & Sons Ltd on behalf of European Hematology Association. This is an open access Abstract Book distributed under the Attribution-NonCommercial-NoDerivs (CC BY-NC-ND), which allows third parties to download the articles and share them with others as long as they credit the author and the Abstract Book, but they cannot change the content in any way or use them commercially.
**Abstract Book Citations:** Authors, Title, HemaSphere, 2026;10:(S1):pages. The abstract book DOIs can be found on the “Author Information” page.
**Disclaimer:** Articles published in the journal HemaSphere exclusively reflect the opinions of the authors. The authors are responsible for all content in their abstracts including accuracy of the facts, statements, citing resources, etc.

**Table 1: Baseline character, safety and effectiveness results**

| | | ND | |
|---|---|---|---|
| **Safety set** | **NKTCL (N=11)** | **CTCL (N=9)** | **ATLL (N=6)** |
| **Demographics** | | | |
| Median age (Q1, Q3), years | 44.0 (30.0 58.0) | 59.0 (39.0 66.0) | 57.0 (49.0 75.0) |
| Gender, male, n (%) | 7 (63.6) | 6 (66.7) | 4 (66.7) |
| **Disease characterization** | | | |
| ECOG 0~1, n (%) | 8 (72.8) | 7 (87.5) | 4 (66.7) |
| IPI score 0~3, n (%) | 9 (81.9) | 4 (57.2) | 3 (60.0) |
| Ann Arbor staging III~IV, n (%) | 2 (18.2) | 6 (66.7) | 5 (83.4) |
| **Safety, n (%)** | **NKTCL (N=11)** | **CTCL (N=9)** | **ATLL (N=6)** |
| Any AE | 10 (90.9) | 4 (44.4) | 6 (100) |
| SAEs | 2 (18.2) | 2 (22.2) | 1 (16.7) |
| grade ≥3 AEs | 3 (27.3) | 2 (22.2) | 1 (16.7) |
| AEs leading to dose reduction of BV | 0 | 0 | 0 |
| AEs resulting in discontinuation of BV | 0 | 1 (11.1) | 0 |
| AEs leading to withdrawal from the study | 1 (9.1) | 2 (22.2) | 1 (16.7) |
| AE leading to death | 2 (18.2) | 2 (22.2) | 1 (16.7) |
| Any ADR | 7 (63.6) | 2 (22.2) | 4 (66.7) |
| Serious ADRs | 1 (9.1) | 0 | 0 |
| grade ≥3 ADRs | 2 (18.2) | 0 | 0 |
| ADRs leading to dose reduction of BV | 0 | 0 | 0 |
| ADRs resulting in discontinuation of BV | 0 | 0 | 0 |
| ADRs leading to withdrawal from the study | 0 | 0 | 0 |
| ADRs leading to death | 1 (9.1) | 0 | 0 |
| **BV exposure** | | | |
| Median cycles of BV (IQR) | 4.0 (2.0, 4.0) | 3.0 (2.0, 6.0) | 3.5 (1.0, 4.0) |
| Dosing intensity, mg/kg (IQR) | 1.48 (1.38, 1.82) | 1.63 (1.52, 1.81) | 1.65 (1.58, 1.72) |
| **Effectiveness** | NKTCL (N=9) | CTCL (N=7) | ATLL (N=4) |
| ORR, n (%) | 5 (55.6) | 3 (42.9) | 3 (75) |
| CR, n (%) | 3 (33.3) | 2 (28.6) | 3 (75) |
| TTR, median (IQR), months | 2.10 (1.45, 2.89) | 2.63 (1.87, 3.88) | 2.40 (1.15, 2.69) |
| TTCR, median (IQR), months | 2.89 (2.10, 6.93) | 2.87 (1.87, 3.88) | 2.69 (2.40, 2.73) |
| DOCR, median (95% CI), months | NA (NA, NA) | NA (NA, NA) | NA (NA, NA) |
| TTNT, median (95% CI), months | NA (1.3, NA) | NA (1.8, NA) | 2.8 (0.4, NA) |
| PFS | | | |
| Median (95% CI), months | 4.4 (1.0, NA) | 5.8 (1.7, NA) | NA (0.4, NA) |
| OS | | | |
| Median (95% CI), months | NA (1.3, NA) | 10.0 (3.1, NA) | NA (0.4, NA) |
| Median follow-up (95% CI), months | 9.1 (2.7, 12.2) | 7.9 (3.5, NA) | 11.9 (6.1, NA) |
| 1-year OS rate (95% CI) | 77.8 (36.5, 93.9) | 42.9 (1.1, 85.3) | 75.0 (12.8, 96.1) |

ND, newly diagnosed; NKTCL, natural killer/T-cell lymphoma; CTCL, cutaneous T-cell lymphoma; ATLL, adult T-cell leukemia/lymphoma; ECOG PS, Eastern Cooperative Oncology Group performance status; IPI, International Prognostic Index; AEs, adverse events; SAEs, serious adverse events; BV, brentuximab vedotin; ADR, adverse drug reactions; IQR, interquartile range; ORR, objective response rate; CR, complete response; TTR, time to response; TTCR, time to complete response; DOCR, duration of complete response; CI, confidence interval; NA, not applicable; TTNT, time to next treatment; PFS, progression-free survival; OS, overall survival.

**Copyright Information:** (Online) ISSN: 2572-9241
©2026 The Author(s). HemaSphere is published by John Wiley & Sons Ltd on behalf of European Hematology Association. This is an open access Abstract Book distributed under the Attribution-NonCommercial-NoDerivs (CC BY-NC-ND), which allows third parties to download the articles and share them with others as long as they credit the author and the Abstract Book, but they cannot change the content in any way or use them commercially.
**Abstract Book Citations:** Authors, Title, HemaSphere, 2026;10:(S1):pages. The abstract book DOIs can be found on the "Author Information" page.
**Disclaimer:** Articles published in the journal HemaSphere exclusively reflect the opinions of the authors. The authors are responsible for all content in their abstracts including accuracy of the facts, statements, citing resources, etc.

### (PB3858) ASSOCIATION BETWEEN SHORT DIAGNOSIS-TO-TREATMENT INTERVAL AND POOR PROGNOSIS IN NEWLY DIAGNOSED AGGRESSIVE ADULT T-CELL LEUKEMIA/LYMPHOMA (ATL)

**Topic:** 20. Other aggressive lymphomas

Kikuaki Yoshida*[1], Yuto Amano[2], Shotaro Tabata[1], Hideki Kosako[1], Ken Tanaka[1, 2], Hiroki Hosoi[1], Shogo Murata[1], Takashi Sonoki[1]

*[1]Wakayama Medical University, Department of Hematology/Oncology, Wakayama, Japan;[2]Kinan Hospital, Department of Hematology, Wakayama, Japan;*

### Background

Adult T-cell Leukemia/lymphoma (ATL) is a peripheral T-cell lymphoma caused by human T-cell leukemia virus type 1 (HTLV-1) and is endemic in several regions, including southwestern Japan. While the association between diagnosis-to-treatment interval (DTI) and prognosis has been reported in other lymphoma subtypes, this relationship has not been evaluated in ATL.

### Aims

To assess the duration of DTI and its association with overall survival (OS) in newly diagnosed ATL.

### Methods

We retrospectively analyzed consecutive patients with aggressive ATL (acute, lymphoma, or unfavorable chronic types per the Shimoyama classification) treated at Wakayama Medical University Hospital and Kinan Hospital from January 2003 to March 2025. DTI was defined as the number of days between the diagnostic biopsy and the start of chemotherapy.

### Results

A total of 80 patients were included in the analysis. The median age was 68 years (range, 31–89). There were 40 male and 40 female patients. Elevated LDH levels were observed in 69 patients (86%), and 51 patients (73%) had an ECOG-PS of 0–1. The most common clinical subtype was acute (51 patients), followed by lymphoma (23 patients) and unfavorable chronic (6 patients). Based on the simplified prognostic index for acute- and lymphoma-type ATL (sATL-PI), 15 patients (19%) were categorized as having high risk. The most common first-line regimen was CHOP (52%), followed by mLSG15 (24%). Allogeneic hematopoietic stem cell transplantation was performed in 27 patients (34%) during the clinical course. With a median follow-up duration of 55 months, the median progression-free survival and OS for the entire cohort were 7.8 months and 12.3 months, respectively. During the study period, 68 patients died; the most common cause of death was ATL (35 patients), followed by infections (10 patients). The median DTI was 12 days. Using 7 days as the cutoff value, patients were classified into the short DTI group (35 patients) and the long DTI group (45 patients). Regarding baseline characteristics, the short DTI group included a higher proportion of patients with the acute subtype. In the OS analysis, patients in the short DTI group had significantly worse OS (HR, 1.68; 95% CI, 1.02–2.76). When stratified by clinical subtype, the short DTI group was significantly associated with inferior OS in patients with the acute (HR, 2.31; 95% CI, 1.18–4.54) and lymphoma subtypes (HR,3.25; 95% CI, 1.01–10.5). In a multivariate analysis that included high-risk sATL-PI status and allogeneic hematopoietic stem cell transplantation as covariates, a short DTI remained independently associated with inferior OS.

**Copyright Information:** (Online) ISSN: 2572-9241
©2026 The Author(s). HemaSphere is published by John Wiley & Sons Ltd on behalf of European Hematology Association. This is an open access Abstract Book distributed under the Attribution-NonCommercial-NoDerivs (CC BY-NC-ND), which allows third parties to download the articles and share them with others as long as they credit the author and the Abstract Book, but they cannot change the content in any way or use them commercially.
**Abstract Book Citations:** Authors, Title, HemaSphere, 2026;10:(S1):pages. The abstract book DOIs can be found on the “Author Information” page.
**Disclaimer:** Articles published in the journal HemaSphere exclusively reflect the opinions of the authors. The authors are responsible for all content in their abstracts including accuracy of the facts, statements, citing resources, etc.

**Summary/Conclusion**

Our study demonstrated that short DTI may be associated with unfavorable survival outcomes in patients with aggressive ATL. These results indicate that DTI could be a useful marker of disease aggressiveness and support the consideration of DTI in the interpretation of clinical trial results and in the planning of future studies in aggressive ATL.

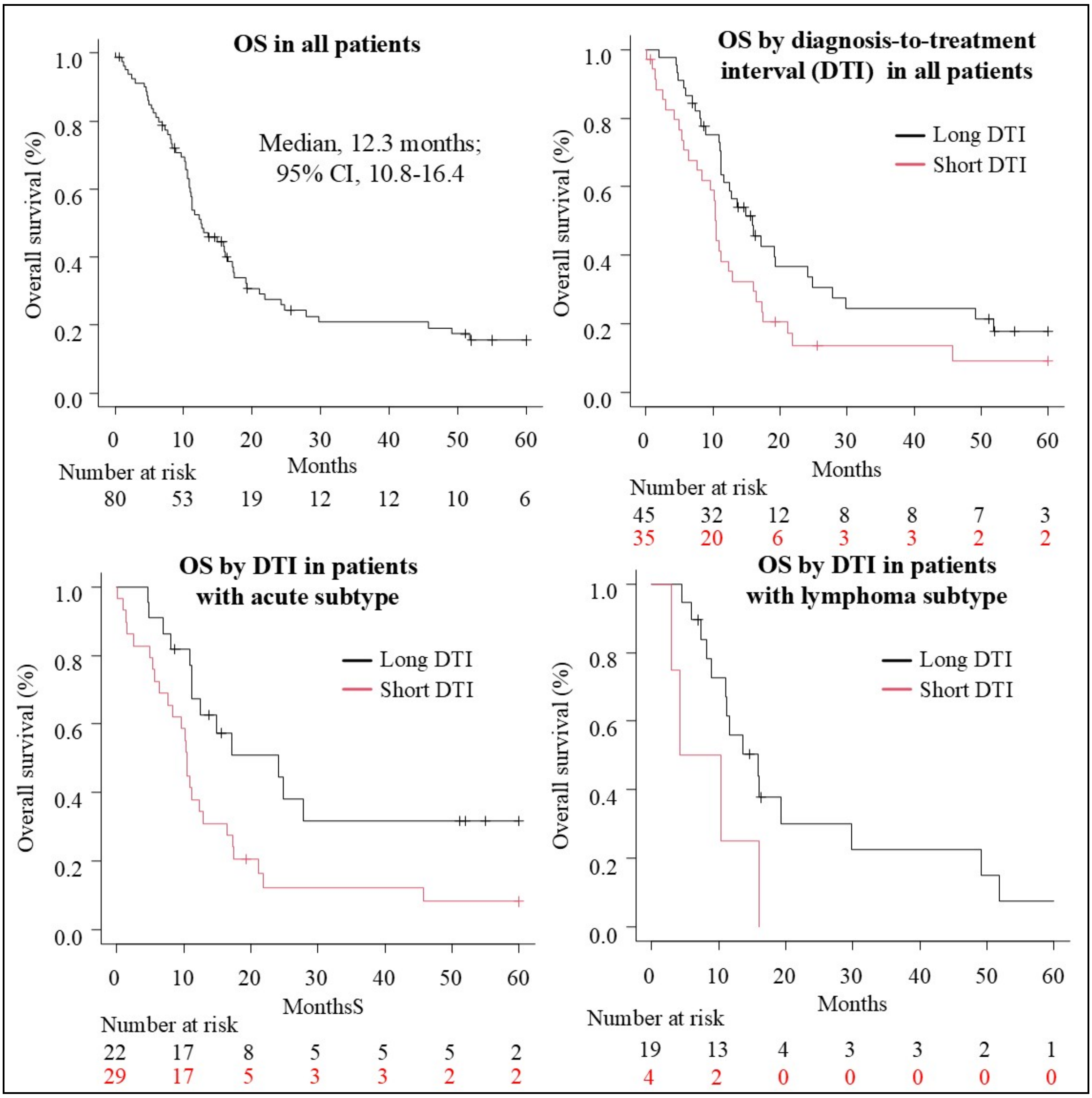

**Copyright Information:** (Online) ISSN: 2572-9241
©2026 The Author(s). HemaSphere is published by John Wiley & Sons Ltd on behalf of European Hematology Association. This is an open access Abstract Book distributed under the Attribution-NonCommercial-NoDerivs (CC BY-NC-ND), which allows third parties to download the articles and share them with others as long as they credit the author and the Abstract Book, but they cannot change the content in any way or use them commercially.
**Abstract Book Citations:** Authors, Title, HemaSphere, 2026;10:(S1):pages. The abstract book DOIs can be found on the "Author Information" page.
**Disclaimer:** Articles published in the journal HemaSphere exclusively reflect the opinions of the authors. The authors are responsible for all content in their abstracts including accuracy of the facts, statements, citing resources, etc.

### (PB3859) CLINICAL AND PATHOLOGICAL HETEROGENEITY WITH DISTINCT GENOMIC FEATURES IMPACTS PROGNOSIS IN MONOMORPHIC EPITHELIOTROPIC INTESTINAL T-CELL LYMPHOMA: A MULTICENTER RETROSPECTIVE COHORT STUDY FROM CHINA

**Topic:** 20. Other aggressive lymphomas

Ji-Nuo Wang*[1], Fei Cheng[1], Teng Yu[2], Feiteng Huang[3], Junyu Zhang[4], Yi Zhao[1], Qianqian Cai[5], Zhaoming Wang[1], Wenbin Qian[2], Jin Zhang[3], Wenjun Wu[1], Zhen Cai[1]

*[1]The First Affiliated Hospital. Zhejiang University School of Medicine, Hangzhou, China;[2]The Second Affiliated Hospital, Zhejiang University School of Medicine, Hangzhou, China; [3]Sir Run Run Shaw Hospital, Zhejiang University School of Medicine, Hangzhou, China; [4]Lishui Central Hospital, Lishui, China;[5]Ningbo Medical Center Lihuili Hospital, Ningbo, China;*

### Background

Monomorphic epitheliotropic intestinal T-cell lymphoma (MEITL) is a rare and highly aggressive intestinal T-cell lymphoma with extremely poor prognosis. Most molecular data are derived from Western populations, and the clinicopathological and genomic heterogeneity of MEITL in Asian patients remains incompletely characterized.

### Aims

To comprehensively characterize the clinical, pathological, and genomic features of MEITL in a multicenter Chinese cohort and to identify prognostic determinants with potential therapeutic implications.

### Methods

We retrospectively analyzed 44 patients with pathologically confirmed MEITL diagnosed between 2013 and 2024 across five tertiary centers in China. Centralized pathology review classified cases as typical or atypical based on morphology (e.g., monomorphism, epitheliotropism, necrosis). Targeted next-generation sequencing (255-gene panel) was performed in 28 patients. Overall survival (OS) was estimated using Kaplan–Meier analysis and compared by log-rank test.

### Results

The median age was 61 years, with male predominance (68.2%). Small intestinal involvement was present in 97.7%, and intestinal perforation or obstruction occurred in 54.6% at diagnosis. Median OS for the entire cohort was 3.0 months, with a 1-year OS rate of approximately 10%.
Atypical morphology was identified in 29.5% of cases and was significantly associated with non-monomorphic cytology, necrosis, starry-sky pattern, and angiotropism (all $P < 0.001$). Hypoalbuminemia was more frequent in atypical MEITL (76.9% vs 38.7%, $P = 0.045$). Atypical cases showed a trend toward inferior OS (median 2.0 vs 3.5 months, $P = 0.24$) and were less likely to receive salvage therapy (P = 0.049).
NGS revealed frequent alterations in SETD2 (71.4%) and recurrent activation of the JAK/STAT pathway, including JAK3 (46.4%) and STAT5B (25%), the latter occurring at a substantially lower frequency than reported in Western cohorts. STAT5B and TP53 mutations were not associated with adverse survival ($P = 0.32$ and $P = 0.22$, respectively). Unexpectedly, SETD2 alterations were associated with significantly prolonged OS (median 8.5 vs 2.5 months, $P = 0.0031$), whereas NRAS/KRAS mutations showed a trend toward inferior survival (median 2.0 vs 6.0 months, $P = 0.07$).
Among 36 treated patients, regimens incorporating histone deacetylase inhibitors and/or hypomethylating agents showed a trend toward improved OS compared with conventional chemotherapy alone (median OS 10.0 vs 4.0 months, P = 0.12).

**Copyright Information:** (Online) ISSN: 2572-9241
©2026 The Author(s). HemaSphere is published by John Wiley & Sons Ltd on behalf of European Hematology Association. This is an open access Abstract Book distributed under the Attribution-NonCommercial-NoDerivs (CC BY-NC-ND), which allows third parties to download the articles and share them with others as long as they credit the author and the Abstract Book, but they cannot change the content in any way or use them commercially.
**Abstract Book Citations:** Authors, Title, HemaSphere, 2026;10:(S1):pages. The abstract book DOIs can be found on the "Author Information" page.
**Disclaimer:** Articles published in the journal HemaSphere exclusively reflect the opinions of the authors. The authors are responsible for all content in their abstracts including accuracy of the facts, statements, citing resources, etc.

**Summary/Conclusion**

MEITL in Chinese patients exhibits distinct clinicopathological and molecular features compared with Western populations. Morphological atypia and RAS pathway activation may define a high-risk subgroup, while SETD2 alterations identify a biologically distinct subset with relatively favorable outcomes. These findings highlight marked population-specific heterogeneity and support integrated morphological–genomic stratification to guide precision therapeutic strategies in MEITL.

**Copyright Information:** (Online) ISSN: 2572-9241
©2026 The Author(s). HemaSphere is published by John Wiley & Sons Ltd on behalf of European Hematology Association. This is an open access Abstract Book distributed under the Attribution-NonCommercial-NoDerivs (CC BY-NC-ND), which allows third parties to download the articles and share them with others as long as they credit the author and the Abstract Book, but they cannot change the content in any way or use them commercially.
**Abstract Book Citations:** Authors, Title, HemaSphere, 2026;10:(S1):pages. The abstract book DOIs can be found on the "Author Information" page.
**Disclaimer:** Articles published in the journal HemaSphere exclusively reflect the opinions of the authors. The authors are responsible for all content in their abstracts including accuracy of the facts, statements, citing resources, etc.

### (PB3860) LATE HIV PRESENTATION IN PATIENTS WITH HIV-ASSOCIATED LYMPHOMA: DIAGNOSTIC AND MANAGEMENT CHALLENGES – A MULTICENTER RETROSPECTIVE STUDY BY THE POLISH LYMPHOMA RESEARCH GROUP

**Topic:** 20. Other aggressive lymphomas

Agnieszka Giza*[1], Magdalena Zapała[2], Joanna Drozd-Sokolowska[3], Magdalena Witkowska[4], Jan Zaucha[5], Małgorzata Sobczyk-Kruczelnicka[6], Anastazja Szlauer-Stefańska[7], Anna Kopinska[8], Magdalena Zawartko[9], Magdalena Witak- Jędra[10], Kamil Wiśniewski[11], Kamil Krzysztof Wdowiak[12], Jarosław Grzyb[13], Anna Parfieniuk-Kowerda[14], Natalia Leończuk[15], Alicja Wandałowicz[16], Monika Joks[17], Justyna Rybka[18], Judyta Strzala[19], Agnieszka Szeremet[20], Aleksandra Szymczak[21], Marcin Czarnecki[21], Marta Głuchowska[2], Jaroslaw Dybko[20], Dagmara Zimowska-Curyło[1], Ewa Lech-Marańda[11], Tomasz Sacha[1], Jan MacIej Zaucha[5], Miłosz Parczewski[22], Monika Bociąga-Jasik[23]

*[1]Department of Hematology, Jagiellonian University Medical College, Faculty of Medicine, Krakow, Poland;[2]Haematology and Internal Medicine Clinical Department, The University Hospital in Krakow, Haematology and Internal Medicine Clinical Department, The University Hospital, Krakow, Poland; [3]Department of Hematology, Transplantation and Internal Medicine, University Clinical Centre, Medical University of Warsaw, Warsaw, Poland; [4]Department of Hematooncology, Copernicus Memorial Hospital, Department of Hematology, Medical University of Lodz, Lodz, Poland; [5]Department of Haematology and Transplantology, Medical University of Gdansk, Department of Hematology, Transplantology and Cellular Therapies, University Clinical Center, Gdansk, Poland; [6]Maria Sklodowska-Curie National Research Institute of Oncology, Gliwice Branch, Gliwice, Poland; [7]Maria Sklodowska-Curie National Research Institute of Oncology, Gliwice Branch., Gliwice, Poland;[8]Department of Hematology and Bone Marrow Transplantation, Medical University of Silesia Katowice, Katowice, Poland; [9]Department of Hematology, Provincial Hospital, Szczecin, Poland; [10]Department of Infectious, Tropical Diseases and Acquired Immune Deficiency, Provincial Hospital, Szczecin, Poland; [11]Department of Hematology, Institute of Hematology and Transfusion Medicine, Warsaw, Poland; [12]Internal Medicine and Oncology Department, Medical University of Silesia, Katowice, Poland; [13]Hematology Clinic; Fryderyk Chopin University Clinical Hospital in Rzeszow, Rzeszow, Poland; [14]Department of Infectious Diseases and Hepatology, Medical University of Bialystok, Bialystok, Poland; [15]Department of Haematology, Internal Medicine and Angiology with Haematopoietic Cell Transplantation Unit, Medical University of Bialystok, Bialystok, Poland; [16]. Department of Infectious Diseases and Hepatology, Medical University of Bialystok, Bialystok, Poland; [17]Department of Hematology and Bone Marrow Transplantation, Poznan University of Medical Sciences, Poznan, Poland; [18]Department of Hematology, Cellular Therapies, and Internal Medicine, Wroclaw Medical University, Wroclaw, Poland; [19]Department of Hematology and Bone Marrow Transplantations, Polish Red Cross Maritime Hospital Gdynia, Gdynia, Poland; [20]Lower Silesia Centre for Oncology, Pulmonology and Hematology in Wroclaw, Wroclaw, Poland; [21]Department of Infectious Diseases, Liver Diseases and Acquired Immune Deficiencies, Wroclaw Medical University, Wroclaw, Poland; [22]Department of Infectious, Tropical Diseases and Immune Deficiency, Pomeranian Medical University, Szczecin, Poland; [23]Department of Infectious Diseases, Jagiellonian University Medical College, Faculty of Medicine and Clinic of Infectious Diseases Clinical Department, The University Hospital in Krakow, Krakow, Poland;*

### Background

Despite widespread access to combination antiretroviral therapy (ART), HIV-related lymphoma (HRL) remains the most common malignancy and a leading cause of mortality in HIV patients. Key risk factors for aggressive lymphomas include low CD4 T-cell count and uncontrolled HIV-1 viral load (VL).

### Aims

To evaluate clinical and immunovirological features and their impact on treatment response and survival in HIV-related lymphoma.

**Copyright Information:** (Online) ISSN: 2572-9241
©2026 The Author(s). HemaSphere is published by John Wiley & Sons Ltd on behalf of European Hematology Association. This is an open access Abstract Book distributed under the Attribution-NonCommercial-NoDerivs (CC BY-NC-ND), which allows third parties to download the articles and share them with others as long as they credit the author and the Abstract Book, but they cannot change the content in any way or use them commercially.
**Abstract Book Citations:** Authors, Title, HemaSphere, 2026;10:(S1):pages. The abstract book DOIs can be found on the "Author Information" page.
**Disclaimer:** Articles published in the journal HemaSphere exclusively reflect the opinions of the authors. The authors are responsible for all content in their abstracts including accuracy of the facts, statements, citing resources, etc.

### Methods

HRL adult patients treated across 10 Polish hematological centers between 2007 and 2025 were included in this retrospective analysis. Clinical, treatment, and virological data were collected in collaboration with infectious disease departments. Survival outcomes were analyzed using Kaplan–Meier methods.

### Results

Ninety-six patients were included, 80.2% males, median age at HRL diagnosis 41 years (range: 23–65). Most patients had high-risk features at diagnosis: Lugano stage IV (69%), CNS involvement (13%), and bone marrow involvement (21.3%). At the time of HRL diagnosis median CD4 count was 122 cells/µL (range: 4,6-1612,00); 60.8% had CD4 <350 cells/µL. Median CD4 nadir was 111 cells/µL (IQR 28.5–239.6); 68.6% had CD4 nadir <200 cells/µL, including 42.9% <100 and 31.4% <50 cells/µL. Among patients with available CD4 data, 84.8% had CD4 <350 cells/µL at HRL diagnosis - late presenters, including 30.4% with very advanced HIV disease (CD4 <50 cells/µL). Median HIV VL was 26 050 copies/mL, with detectable HIV viremia in 77% cases. HIV and lymphoma diagnoses were concurrent in 53% of patients; thus, the majority initiated antiretroviral therapy (ART) concurrently with lymphoma treatment. The longest HIV-to-lymphoma interval was 312 months.
Non-Hodgkin lymphoma (NHL) was diagnosed in 88.5% (85/96) and Hodgkin lymphoma (HL) in 11.5% (11/96) of patients. Kaposi's sarcoma was diagnosed during lymphoma treatment in 6/86 (5.3%) patients. After the first-line treatment, complete response (CR) rate was 49.9% and varied by subtype: DLBCL 38.3%, BL 68.8%, PBL 18.2%, HL 72.8% (Table 1). Thirty-nine patients (40.6%) died, mainly from lymphoma progression (71.8%, including 4 with hemophagocytic lymphohistiocytosis) or infections (20.5%). After a median follow-up of 38.9 months, median OS was 55.3 months (95% CI 21.7–NR) and median PFS 28.8 months (95% CI 8.8–NR). The 12-, 24-, and 36-month OS rates were 65.7% (95% CI 53.8–75.2), 60.6% (95% CI 48.2–70.9), and 58.5% (95% CI 45.9–69.1), respectively. Stratification into four CD4 categories (<50, 50–200, 200–350, >350 cells/µL) showed a trend toward differences in PFS (log-rank $\chi^2$=6.52, df=3, P=0.089), while OS differences were not statistically significant (P=0.17).

**Table 1. Histopathological diagnosis with remission rates to 1st line therapy.**

| | DLBCL N=48 (50,0%) | | BL N=16 (16,7%) | | PBL N=12 (12,5%) | | ALCL N=2 (2,1%), PTCL N=1 (1,1%), AITL N=1 (1,1%) | | HODGKIN N=11 (11,5%) | | TOTAL N=96 | |
|---|---|---|---|---|---|---|---|---|---|---|---|---|
| | N | % | N | % | N | % | N | % | N | % | N | % |
| CR | 18 | 38.3 | 11 | 68.8 | 2 | 16.7 | 3 | 75.0 | 8 | 72.8 | 45 | 49.9 |
| PR | 5 | 10.6 | - | - | 2 | 16.7 | - | - | - | - | 7 | 7.3 |
| SD | 1 | 2.1 | - | - | 1 | 8.3 | 1 | 25.0 | - | - | 3 | 3.1 |
| NR | 10 | 21,3 | 1 | 6,2 | 3 | 25,0 | - | - | 2 | 18,2 | 17 | 17,7 |
| NO DATA | 14 | 29,2 | 4 | 25,0 | 4 | 33,3 | - | - | 1 | 9,1 | 24 | 25,0 |
| DEATH | 22 | 46,8 | 2 | 13,3 | 6 | 50,0 | 1 | 25,0 | 3 | 27,3 | 39 | 40,6 |

### Summary/Conclusion

This multicenter cohort was characterized by a high burden of advanced HIV disease at HRL diagnosis, with the majority of patients being late presenters, and a substantial proportion of patients was diagnosed with HIV concurrently with lymphoma. Late diagnosis of HIV infection in patients with HRL is associated with poor prognosis. The impact of CD4 T cell count at HRL diagnosis on patient outcomes warrants further research.

**Copyright Information:** (Online) ISSN: 2572-9241
©2026 The Author(s). HemaSphere is published by John Wiley & Sons Ltd on behalf of European Hematology Association. This is an open access Abstract Book distributed under the Attribution-NonCommercial-NoDerivs (CC BY-NC-ND), which allows third parties to download the articles and share them with others as long as they credit the author and the Abstract Book, but they cannot change the content in any way or use them commercially.
**Abstract Book Citations:** Authors, Title, HemaSphere, 2026;10:(S1):pages. The abstract book DOIs can be found on the "Author Information" page.
**Disclaimer:** Articles published in the journal HemaSphere exclusively reflect the opinions of the authors. The authors are responsible for all content in their abstracts including accuracy of the facts, statements, citing resources, etc.

### (PB3861) FRONTLINE BV-CHP VERSUS CHOP/CHOEP IN PERIPHERAL T-CELL LYMPHOMA: A MULTICENTER REAL-WORLD COHORT STUDY

**Topic:** 20. Other aggressive lymphomas

Shlomzion Aumann*[1], Noy Havusha[1], Dahlia Greidinger[2], Nurit Horesh[3], Uri Abadi[4], Nir Weigert[5], Reut Harel[6], Tamar Tadmor[7], Tzvika Porges[8], Ronit Gurion[9], Boaz Nachmias[1]

*[1]Hadassah Medical Center and Faculty of Medicine, Hebrew University of Jerusalem, Department of Hematology, Jerusalem, Israel; [2]Tel-Aviv Sourasky Medical Center Affiliated to Sackler Faculty of Medicine, Tel Aviv University, Hematology, Tel Aviv, Israel; [3]Division of Hematology, Rambam Health Care Campus and the Ruth and Bruce Rappaport Faculty of Medicine, Technion-Israel Institute of Technology, Hematology, Haifa, Israel; [4]Hematology Unit, Meir Medical Center, Kfar Saba, and Sackler Faculty of Medicine, Tel Aviv University, Tel Aviv, Israel; [5]Shaare Zedek Medical Center, Hematology, Jerusalem, Israel; [6]Emek Medical Center, Hematology, Afula, Israel; [7]Hematology Division, Bnai Zion Medical Center and the Ruth and Bruce Rappaport Faculty of Medicine, Technion-Israel Institute of Technology, Haifa, Israel; [8]Soroka University Medical Center and Faculty of Health Sciences, Ben-Gurion University of the Negev, Hematology, Beer Sheva, Israel; [9]Institute of Hematology, Davidoff Cancer Center, Rabin Medical Center (Beilinson), Petah Tikva, and Sackler Faculty of Medicine, Tel Aviv University, Tel Aviv, Israel;*

### Background

The phase III ECHELON-2 trial established brentuximab vedotin plus CHP (BV-CHP) as a frontline standard for CD30-positive peripheral T-cell lymphoma (PTCL), although benefit was largely driven by ALCL and the comparator was CHOP rather than CHOEP. Real-world data comparing BV-CHP with contemporary chemotherapy practice are limited.

### Aims

To evaluate the efficacy and safety of frontline BV-CHP versus CHOP/CHOEP in a multicenter real-world PTCL cohort.

### Methods

We retrospectively analyzed 100 consecutive patients with PTCL treated between 2012 and 2025 across nine Israeli centers. The BV-CHP cohort (n=67) consisted of patients with documented CD30 expression ≥1% and was treated between 2019 and 2025. The control cohort (n=33) received CHOP (n=19) or CHOEP (n=14), primarily reflecting pre-BV-CHP practice; CD30 status was systematically documented in the BV-CHP era but was available for 15 (45%) controls treated earlier. Early Event Rate (EER) was defined as primary refractory disease or relapse within 12 months of diagnosis. Survival was estimated using Kaplan–Meier methodology. Multivariable Cox proportional hazards and logistic regression models were adjusted for age and IPI.

**Copyright Information:** (Online) ISSN: 2572-9241
©2026 The Author(s). HemaSphere is published by John Wiley & Sons Ltd on behalf of European Hematology Association. This is an open access Abstract Book distributed under the Attribution-NonCommercial-NoDerivs (CC BY-NC-ND), which allows third parties to download the articles and share them with others as long as they credit the author and the Abstract Book, but they cannot change the content in any way or use them commercially.
**Abstract Book Citations:** Authors, Title, HemaSphere, 2026;10:(S1):pages. The abstract book DOIs can be found on the "Author Information" page.
**Disclaimer:** Articles published in the journal HemaSphere exclusively reflect the opinions of the authors. The authors are responsible for all content in their abstracts including accuracy of the facts, statements, citing resources, etc.

**Results**

At a median follow-up of 21.5 months (18.1 BV-CHP, 22.2 control), the control cohort was younger (median 46 vs 63 years, p<0.01). Median IPI was 2.0 in both groups (p=0.41), although high-risk IPI (3–5) was numerically more frequent in the BV-CHP cohort (47% vs 34%, p=0.23).
In the overall cohort, BV-CHP was associated with improved progression-free survival (PFS HR 0.44, 95% CI 0.28–0.69, p<0.001; Figure 1) and overall survival (OS HR 0.52, 95% CI 0.31–0.88, p=0.01). EER was lower with BV-CHP compared with the overall control cohort (31.3% vs 63.6%, OR 0.26, p=0.003). Exploratory analyses suggested lower early failure rates with BV-CHP across major histologies, including PTCL-NOS and AITL, although subgroup sizes were limited.
Outcomes differed according to CD30 expression. CD30-negative control patients experienced particularly poor early outcomes (EER 83.3%, 15/18), whereas CD30-positive control patients had an EER of 40.0% (6/15). In a CD30-positive-restricted comparison (BV-CHP n=67; CD30-positive controls n=15), BV-CHP demonstrated a numerically lower EER than CD30-positive controls (31.3% vs 40.0%, OR 0.68, p=0.55). Correspondingly, treatment effects were attenuated in CD30-restricted survival analyses (PFS HR 0.71, 95% CI 0.38–1.34; OS HR 0.84, 95% CI 0.36–1.95).
Grade 3–4 hematologic toxicity was comparable between groups (53.7% vs 51.5%, p=0.83). Grade 3–4 infections occurred in 26.9% vs 18.2% (p=0.33). Grade 3–4 peripheral neuropathy was infrequent (6.0% vs 3.0%, p=0.51).

**Summary/Conclusion**

In this multicenter real-world cohort, BV-CHP was associated with favorable survival outcomes in the overall analysis. However, treatment effects were attenuated when restricted to CD30-positive patients, suggesting a more modest relative difference versus CHOP/CHOEP within this subgroup. CD30-negative PTCL treated with standard chemotherapy demonstrated particularly poor early outcomes. These findings underscore the biological relevance of CD30 expression and highlight the need for continued evaluation of optimal frontline strategies in PTCL.

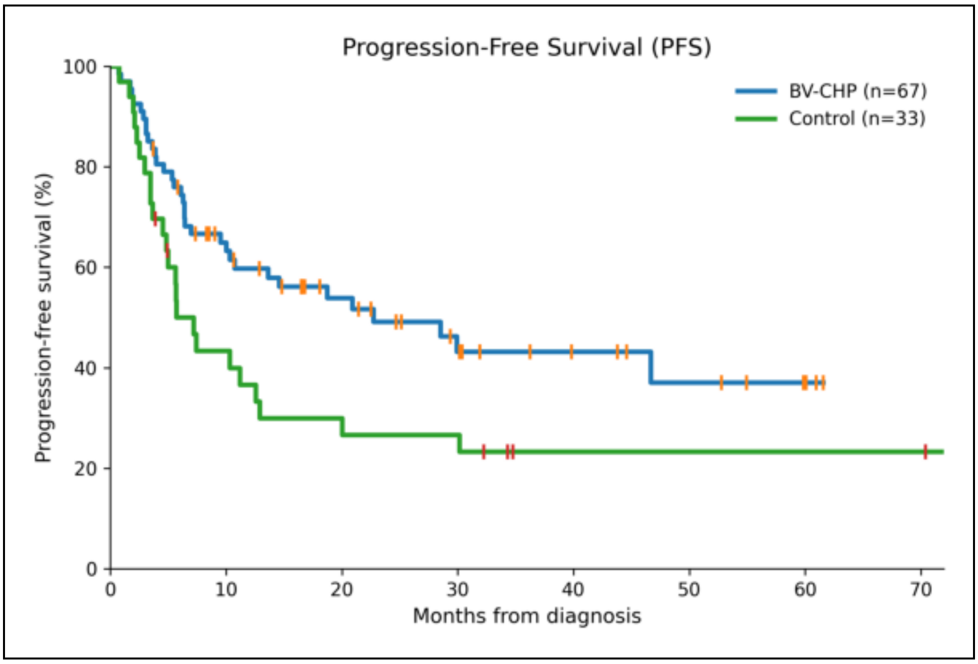

Figure 1. Kaplan–Meier progression-free survival by frontline treatment. Kaplan–Meier estimates of PFS for BV-CHP versus CHOP/CHOEP historical controls. Tick marks indicate censored observations.

**Copyright Information:** (Online) ISSN: 2572-9241
©2026 The Author(s). HemaSphere is published by John Wiley & Sons Ltd on behalf of European Hematology Association. This is an open access Abstract Book distributed under the Attribution-NonCommercial-NoDerivs (CC BY-NC-ND), which allows third parties to download the articles and share them with others as long as they credit the author and the Abstract Book, but they cannot change the content in any way or use them commercially.
**Abstract Book Citations:** Authors, Title, HemaSphere, 2026;10:(S1):pages. The abstract book DOIs can be found on the "Author Information" page.
**Disclaimer:** Articles published in the journal HemaSphere exclusively reflect the opinions of the authors. The authors are responsible for all content in their abstracts including accuracy of the facts, statements, citing resources, etc.

### (PB3862) SAFETY AND EFFICACY OF GOLIDOCITINIB IN COMBINATION WITH AZACITIDINE AND CHIDAMIDE FOR THE TREATMENT OF PERIPHERAL T-CELL LYMPHOMA: AN OPEN-LABEL, SINGLE-ARM STUDY

**Topic:** 20. Other aggressive lymphomas

Yaohui Huang[1], Mingci Cai[1], Li Wang[1], Pengpeng Xu[1], Weili Zhao[1], Shu Cheng*[1]

*[1]Shanghai Institute of Hematology, State Key Laboratory of Medical Genomics, National Research Center for Translational Medicine at Shanghai, Ruijin Hospital, Shanghai Jiao Tong University School of Medicine, shanghai, China;*

### Background

Peripheral T-cell lymphomas (PTCL) are aggressive non-Hodgkin lymphomas with poor outcomes. Traditional frontline therapy, such as anthracycline-based chemotherapy, offers limited clinical benefit, particularly in elderly patients or those with severe comorbidities. Golidocitinib, a selective oral JAK1 inhibitor, has shown reasonable antitumor activity with manageable safety in relapsed/refractory PTCL. Hypomethylating agents (azacitidine) and HDAC inhibitors (chidamide) also demonstrate clinical activity in practice. Preclinical studies indicated synergistic effects of the triplet combination, providing a rationale for a chemo-free approach in these frail patients.

### Aims

To evaluate the safety and efficacy of golidocitinib in combination with azacytidine and chidamide in patients with PTCL who are ineligible for standard chemotherapy or transplantation.

### Methods

Phase 1 employed a 3+3 dose-escalation design, exploring golidocitinib at two dose levels of 150 mg QOD or 150 mg QD to determine the RP2D. Phase 2 explored the safety and efficacy of golidocitinib RP2D in combination with chidamide 20mg twice a week and azacytidine 100mg D1-7 in PTCL. The primary endpoint was overall response rate (ORR) after at least 6 cycles according to 2024 Lugano criteria (NCT07081607).

### Results

Between Aug 1, 2025, and Feb 28, 2026, 11 patients, including 10 nodal T follicular helper cell lymphoma, angioimmunoblastic type (nTFHL-AI) and 1 PTCL-NOS, were enrolled in the study. Of the 11 patients, 9 patients were newly diagnosed and 2 in relapsed disease. The median age was 74 years (range, 68-84); 8 patients (72.7%) were male. 8 patients (72.7%) were in Lugano stage IV disease, 6 (54.5%) had ECOG performance status ≥2, and 9 (81.8%) with an international prognostic index (IPI) score ≥3. Among the 11 patients, 10 had the following comorbidities at baseline, considered ineligible for intensive treatment, including 7 with cardiovascular comorbidities (2 with arrhythmia and 5 with coronary heart disease), 2 with renal insufficiency, and 1 with cachexia. At data cutoff (Feb 28, 2026), with a median follow-up of 7.1 months, eight patients were evaluable for response assessment. Four patients achieved complete response (CR), with three partial response (PR) and one stable disease (SD), yielding an ORR of 87.5% (7/8) and a CR rate of 50% (4/8) after 3 cycles. Among the four patients who completed end-of-treatment assessment (6 cycles), two achieved CR (50%), and two had progressive disease (PD).
The most common grade ≥3 treatment-related adverse events were hematologic toxicities, consisting of 4 (36.4%) grade 3 leukopenia, 4 (36.4%) grade 3-4 thrombocytopenia, and 2 (18.2%) anemia. Grade 3 pulmonary infection and grade 3 gastrointestinal bleeding were separately observed in one patient (9.1%). Grade 1-2 cytomegalovirus infection occurred in six patients (54.5%) and resolved with antiviral therapy.

**Copyright Information:** (Online) ISSN: 2572-9241
©2026 The Author(s). HemaSphere is published by John Wiley & Sons Ltd on behalf of European Hematology Association. This is an open access Abstract Book distributed under the Attribution-NonCommercial-NoDerivs (CC BY-NC-ND), which allows third parties to download the articles and share them with others as long as they credit the author and the Abstract Book, but they cannot change the content in any way or use them commercially.
**Abstract Book Citations:** Authors, Title, HemaSphere, 2026;10:(S1):pages. The abstract book DOIs can be found on the "Author Information" page.
**Disclaimer:** Articles published in the journal HemaSphere exclusively reflect the opinions of the authors. The authors are responsible for all content in their abstracts including accuracy of the facts, statements, citing resources, etc.

**Summary/Conclusion**

Golidocitinib combined with azacitidine and chidamide showed encouraging efficacy with manageable toxicity in frail PTCL patients. These preliminary results support further evaluation of this chemo-free regimen in this population.

**Copyright Information:** (Online) ISSN: 2572-9241
©2026 The Author(s). HemaSphere is published by John Wiley & Sons Ltd on behalf of European Hematology Association. This is an open access Abstract Book distributed under the Attribution-NonCommercial-NoDerivs (CC BY-NC-ND), which allows third parties to download the articles and share them with others as long as they credit the author and the Abstract Book, but they cannot change the content in any way or use them commercially.
**Abstract Book Citations:** Authors, Title, HemaSphere, 2026;10:(S1):pages. The abstract book DOIs can be found on the "Author Information" page.
**Disclaimer:** Articles published in the journal HemaSphere exclusively reflect the opinions of the authors. The authors are responsible for all content in their abstracts including accuracy of the facts, statements, citing resources, etc.

### (PB3863) OVERALL SURVIVAL DIFFERENCES IN ELDERLY PATIENTS WITH ANGIOIMMUNOBLASTIC T CELL LYMPHOMA TREATED AT ACADEMIC VS COMMUNITY CANCER PROGRAMS

**Topic:** 20. Other aggressive lymphomas

Henry Idrobo*[1], Laura Reyes Uribe[2], Joel Michalek[3], Qianqian Liu[3], Adolfo Diaz Duque[3]

*[1]Universidad Tecnológica de Pereira, Clínica Central del Eje, Pereira, Colombia;[2]DHR Health, Edinburg, United States of America;[3]UT Health San Antonio / Mays Cancer Center, San Antonio, United States of America;*

### Background

Angioimmunoblastic T cell lymphoma (AITL) is a distinct and aggressive subtype of peripheral T cell lymphoma characterized by immune dysregulation and frequent presentation with advanced stage disease. It predominantly affects older adults and is associated with inferior survival outcomes compared with many B cell lymphomas, despite advances in clinical practice. Although therapeutic advances and supportive care strategies have improved outcomes across lymphoma subtypes, patients aged 75 years and older remain underrepresented in clinical trials, resulting in limited real-world data to guide management in this population. Advanced age is often accompanied by higher comorbidity burden and barriers to specialized care that may influence outcomes independently of disease biology. The impact of treatment facility type on outcomes in elderly patients with AITL is not well defined.

### Aims

### Primary Aim

To determine whether treatment facility type (Academic Cancer Programs vs Community Cancer Programs) is associated with differences in overall survival (OS) among patients aged ≥75 years diagnosed with angioimmunoblastic T-cell lymphoma (AITL) in a national cohort.

### Methods

We conducted a retrospective analysis of patients aged 75 years and older diagnosed with AITL in the US between 2004 and 2022 using the National Cancer Database. Demographic, socioeconomic, clinical, treatment, and survival characteristics were compared between patients treated at Academic Cancer Programs (ACP) and Community Cancer Programs (CCP). Kaplan-Meier and Cox regression analyses were used to compare overall survival (OS) between the two cohorts. Variables used for adjustment included age, ethnicity, insurance status, distance from hospital, and Charlson-Deyo comorbidity score.

### Results

A total of 2,117 patients aged 75 and older with AITL were identified including 1,112 treated at ACP and 1,005 treated at CCP. Median age was 80 years in both cohorts, and most patients presented with advanced stage. The majority were insured through Medicare, and baseline comorbidity burden was substantial and similar across treatment settings. Treatment patterns differed by facility type. Patients treated at ACP were more likely to receive treatment, while a high proportion of patients treated at CCP (45%) had no treatment given or had missing or unknown treatment status. Time from diagnosis to initiation of chemotherapy was significantly shorter at ACP compared with CCP.
Overall survival (OS) favored patients treated at ACP, with higher two-year (37% vs 30%), five-year (24% vs 17%), and ten-year (11% vs 5%) survival compared with CCP.

**Copyright Information:** (Online) ISSN: 2572-9241
©2026 The Author(s). HemaSphere is published by John Wiley & Sons Ltd on behalf of European Hematology Association. This is an open access Abstract Book distributed under the Attribution-NonCommercial-NoDerivs (CC BY-NC-ND), which allows third parties to download the articles and share them with others as long as they credit the author and the Abstract Book, but they cannot change the content in any way or use them commercially.
**Abstract Book Citations:** Authors, Title, HemaSphere, 2026;10:(S1):pages. The abstract book DOIs can be found on the "Author Information" page.
**Disclaimer:** Articles published in the journal HemaSphere exclusively reflect the opinions of the authors. The authors are responsible for all content in their abstracts including accuracy of the facts, statements, citing resources, etc.

**Summary/Conclusion**

In this national cohort of patients aged 75 years and older with AITL, OS differed significantly by treatment facility type, with a statistically significant and clinically meaningful survival advantage observed among patients treated at ACP. These findings underscore persistent disparities in treatment delivery and early mortality in this vulnerable population and support efforts to strengthen referral pathways to ACP or develop structured partnerships between CCP and ACP. Such strategies may facilitate timely access to specialized care, multidisciplinary expertise, and clinical trials for older adults with aggressive T-cell lymphomas.

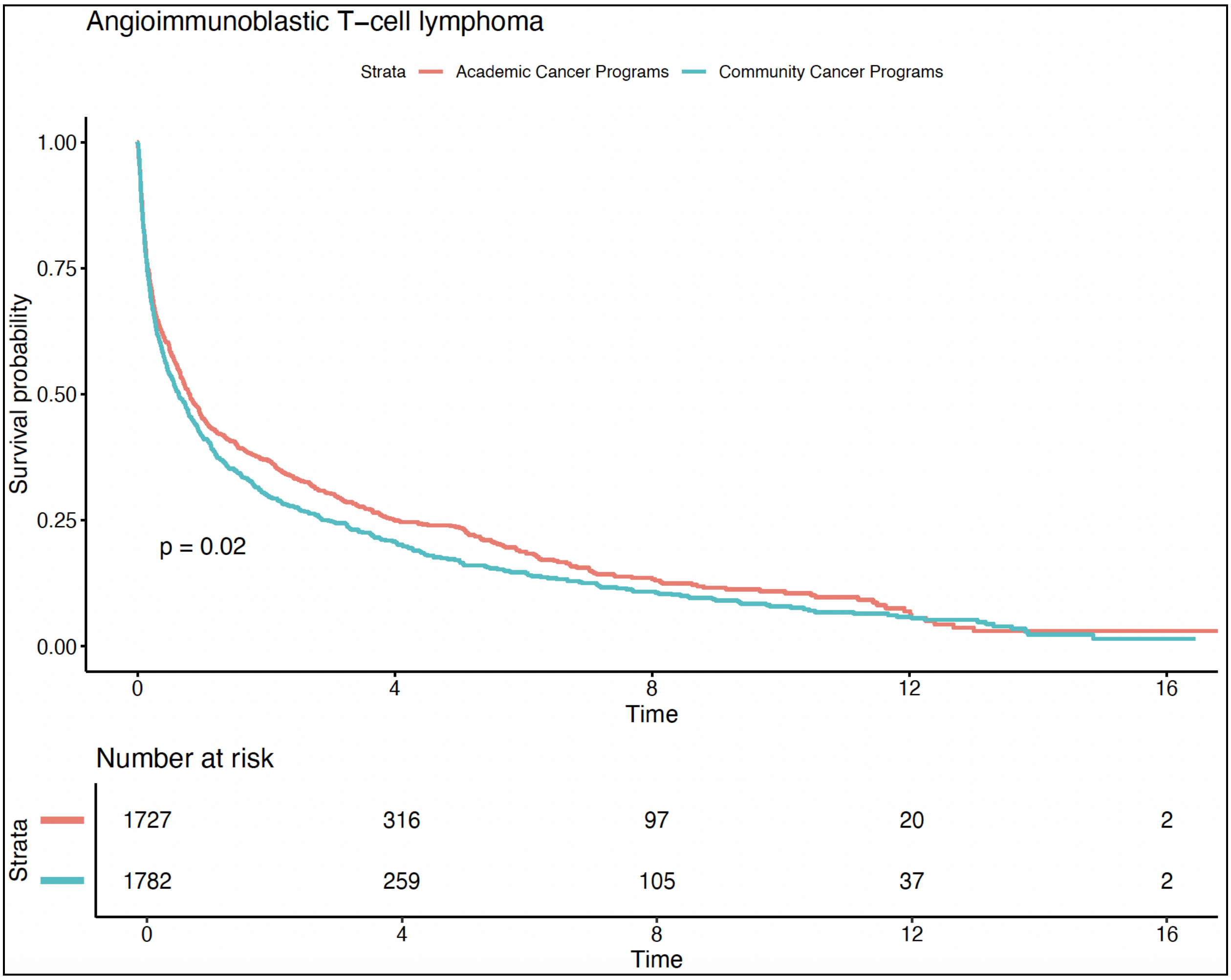

**Copyright Information:** (Online) ISSN: 2572-9241
©2026 The Author(s). HemaSphere is published by John Wiley & Sons Ltd on behalf of European Hematology Association. This is an open access Abstract Book distributed under the Attribution-NonCommercial-NoDerivs (CC BY-NC-ND), which allows third parties to download the articles and share them with others as long as they credit the author and the Abstract Book, but they cannot change the content in any way or use them commercially.
**Abstract Book Citations:** Authors, Title, HemaSphere, 2026;10:(S1):pages. The abstract book DOIs can be found on the "Author Information" page.
**Disclaimer:** Articles published in the journal HemaSphere exclusively reflect the opinions of the authors. The authors are responsible for all content in their abstracts including accuracy of the facts, statements, citing resources, etc.

### (PB3864) A MULTICENTER, OPEN-LABEL, PHASE 3, RANDOMIZED TRIAL OF DUVELISIB VS INVESTIGATOR'S CHOICE (GEMCITABINE OR BENDAMUSTINE) IN RELAPSED/REFRACTORY NODAL T-CELL LYMPHOMA WITH T-FOLLICULAR HELPER PHENOTYPE

**Topic:** 20. Other aggressive lymphomas

Kate Cwynarski*[1], Eva Domingo[2], David Sidransky[3], Ohad Bentur[4], Pier Luigi Zinzani[5]

*[1]University College London Hospital (UCLH), Department of Hematology, London, United Kingdom;[2]Catalan Institute of Oncology, Hospital Duran i Reynals, IDIBELL, Barcelona, Spain; [3]Johns Hopkins University, Baltimore, United States of America; [4]Secura Bio, Inc., Berkeley Heights, United States of America;[5]Institute of Hematology "Seràgnoli", University of Bologna, Bologna, Italy;*

### Background

Peripheral T cell lymphomas (PTCL) are a rare, heterogeneous group of aggressive lymphomas. The current World Health Organization classification of lymphoid neoplasms groups PTCL subtypes angioimmunoblastic T-cell lymphoma (AITL), PTCL-NOS with T-follicular helper (TFH) phenotype, and follicular T-cell lymphoma under the category of nodal TFH lymphoma (nTFHL). nTFHL entities have similarities in clinical, immunophenotypic, and genetic characteristics.
Most patients diagnosed with PTCL will develop relapsed/refractory (R/R) disease. Currently, the only agent approved in the EU/UK for treatment of R/R PTCL is brentuximab vedotin for anaplastic large-cell lymphoma. Novel agents such as romidepsin, belinostat, and pralatrexate have received accelerated approvals outside the EU/UK, but to date, none have received full approval. As such, there are no novel agents approved and available for these patients in EU/UK.
Duvelisib (DUV) is an oral inhibitor of phosphatidylinositol 3-kinase-δ/γ. US indications: adults with R/R chronic lymphocytic leukemia (CLL) or small lymphocytic lymphoma (SLL) after ≥2 prior lines of systemic therapy. EU/UK indications: adults with R/R CLL after ≥2 prior therapies and for follicular lymphoma refractory to ≥2 prior systemic therapies. The use of DUV in PTCL is investigational.
The phase 2 PRIMO study evaluated DUV in R/R PTCL (NCT03372057). Outcomes included objective response rate (ORR) 48.0%, median progression-free survival (mPFS) 3.4 mo, and median overall survival (mOS) 12.4 mo. In patients with AITL (a subtype of nTFHL), outcomes were ORR 62.2%, mPFS 8.3 mo, and mOS 18.1 mo.
Outcomes in the AITL subgroup of the PRIMO study provided rationale to initiate the TERZO™ study (NCT06522737; EU CT: 2024-516605-23-00), which is evaluating DUV versus investigator's choice of gemcitabine (GEM) or bendamustine (BEN), two standard of care regimens commonly used in patients with R/R nTFHL.

### Methods

TERZO is a multicenter, open-label, phase 3, randomized study expected to enroll 124 patients with R/R nTFHL who have progressed on ≥1 line of systemic anticancer therapy. Study duration will be 48 months; patients are randomized 1:1 and stratified by number of prior therapies and AITL score.
Arm 1: DUV 75 mg orally twice daily (BID) for cycles 1 and 2, followed by DUV 25 mg BID for cycles ≥3, in 28-day cycles. Arm 2: either GEM 1200 mg/m$^2$ IV on Day 1, 8, and 15 of each 28-day cycle (≤6 cycles) or BEN 90-120 mg/m$^2$ IV on Day 1 and 2 of each 21-day cycle (≤6 cycles).
Primary endpoint is Independent Review Committee-assessed PFS, a key secondary endpoint is OS, and additional secondary endpoints include investigator (INV)-assessed PFS, ORR, complete response rate, duration of response, safety, quality-of-life, percent of patients proceeding to stem cell transplant (SCT), INV-assessed PFS in patients proceeding to SCT, and PK parameters derived from blood concentrations of DUV and its metabolites. Eligibility criteria include adults with R/R nTFHL, ≥1 prior line of systemic therapy, measurable disease, ECOG performance status ≤2, and no prior history of allogeneic SCT or PI3K inhibitor use.

**Copyright Information:** (Online) ISSN: 2572-9241
©2026 The Author(s). HemaSphere is published by John Wiley & Sons Ltd on behalf of European Hematology Association. This is an open access Abstract Book distributed under the Attribution-NonCommercial-NoDerivs (CC BY-NC-ND), which allows third parties to download the articles and share them with others as long as they credit the author and the Abstract Book, but they cannot change the content in any way or use them commercially.
**Abstract Book Citations:** Authors, Title, HemaSphere, 2026;10:(S1):pages. The abstract book DOIs can be found on the "Author Information" page.
**Disclaimer:** Articles published in the journal HemaSphere exclusively reflect the opinions of the authors. The authors are responsible for all content in their abstracts including accuracy of the facts, statements, citing resources, etc.

### Summary/Conclusion

DUV monotherapy has demonstrated activity in R/R PTCL, with more pronounced effects in AITL. TERZO will test the hypothesis that DUV monotherapy is associated with improved outcomes versus GEM or BEN in patients with R/R nTFHL. TERZO is the only phase 3 study dedicated to nTFHL in the EU/UK, designed to address this serious unmet need.

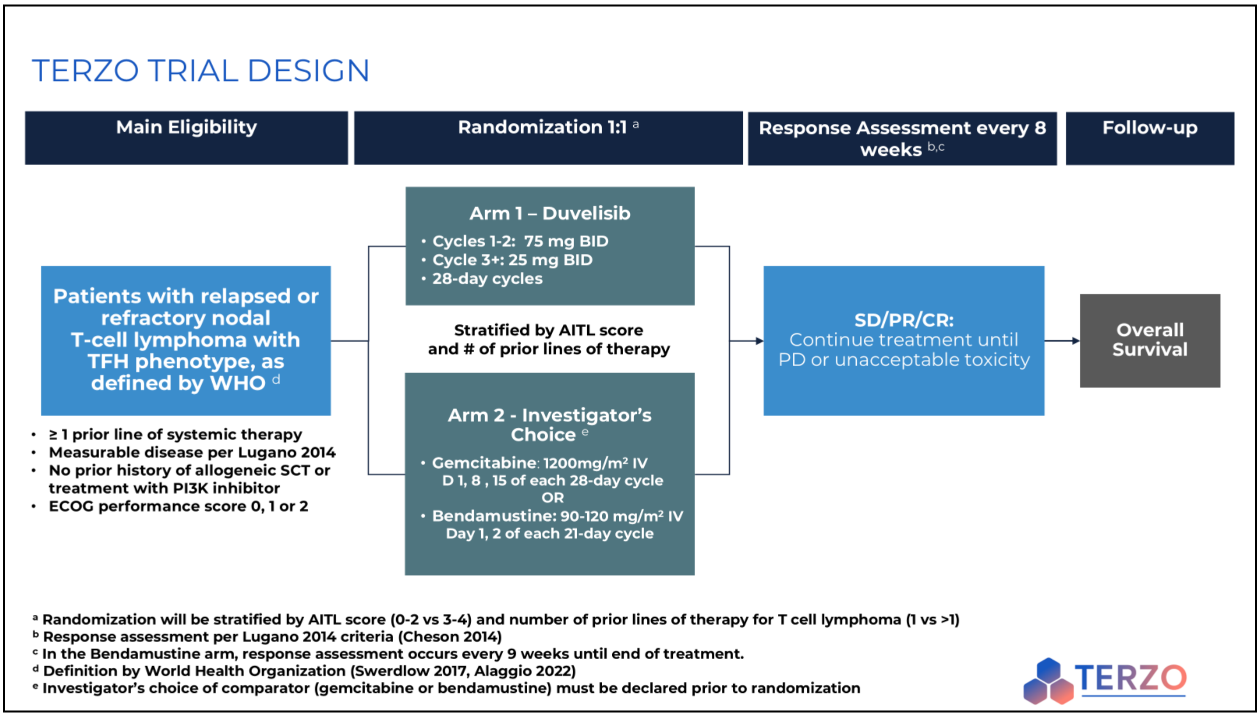

**Copyright Information:** (Online) ISSN: 2572-9241
©2026 The Author(s). HemaSphere is published by John Wiley & Sons Ltd on behalf of European Hematology Association. This is an open access Abstract Book distributed under the Attribution-NonCommercial-NoDerivs (CC BY-NC-ND), which allows third parties to download the articles and share them with others as long as they credit the author and the Abstract Book, but they cannot change the content in any way or use them commercially.
**Abstract Book Citations:** Authors, Title, HemaSphere, 2026;10:(S1):pages. The abstract book DOIs can be found on the "Author Information" page.
**Disclaimer:** Articles published in the journal HemaSphere exclusively reflect the opinions of the authors. The authors are responsible for all content in their abstracts including accuracy of the facts, statements, citing resources, etc.

### (PB3867) REAL-WORLD EFFICACY AND PROGNOSTIC LANDSCAPE OF BRENTUXIMAB VEDOTIN (BV) IN DIVERSE LYMPHOMA SUBTYPES: A MULTICENTER STUDY

**Topic:** 20. Other aggressive lymphomas

Yun Liang[1], Xian Li*[1], Wenbin Qian[1], Zifeng Wang[2], Weiying Feng[3]

*[1]The Second Affiliated Hospital, College of Medicine, Zhejiang University, Department of Hematology, Hangzhou, China; [2]Shangrao People's Hospital, Department of Hematology, Shangrao, China;[3]Shaoxing people's Hospital, Department of Hematology, Shaoxing, China;*

### Background

Brentuximab Vedotin (BV) has transformed the therapeutic landscape for CD30-positive lymphomas. However, real-world data describing its performance across diverse pathological subtypes and disease states remain essential to optimize clinical practice outside of controlled trials.

### Aims

To characterize the real-world clinical status, efficacy, safety, and survival outcomes of patients with multiple lymphoma subtypes treated with BV and to identify independent prognostic factors.

### Methods

This retrospective multicenter study evaluated patients with various lymphoma subtypes who received BV between June 1, 2020, and June 1, 2025. The study included both newly diagnosed (ND) and relapsed/refractory (R/R) patients. Endpoints included overall response rate (ORR), complete response (CR), overall survival (OS), event-free survival (EFS), and safety. Survival was estimated via Kaplan-Meier method; prognostic factors were identified using Cox proportional hazards models. Median follow-up was 13.3 months (range: 0.2–54.0 months).

**Copyright Information:** (Online) ISSN: 2572-9241
©2026 The Author(s). HemaSphere is published by John Wiley & Sons Ltd on behalf of European Hematology Association. This is an open access Abstract Book distributed under the Attribution-NonCommercial-NoDerivs (CC BY-NC-ND), which allows third parties to download the articles and share them with others as long as they credit the author and the Abstract Book, but they cannot change the content in any way or use them commercially.
**Abstract Book Citations:** Authors, Title, HemaSphere, 2026;10:(S1):pages. The abstract book DOIs can be found on the "Author Information" page.
**Disclaimer:** Articles published in the journal HemaSphere exclusively reflect the opinions of the authors. The authors are responsible for all content in their abstracts including accuracy of the facts, statements, citing resources, etc.

### Results

The study enrolled 141 patients: 64 (45.4%) ND and 77 (54.6%) R/R cases. Pathological distribution included peripheral T-cell lymphoma (PTCL, n=58), classical Hodgkin lymphoma (cHL, n=57), other B-cell lymphomas (BCL, n=14), cutaneous T-cell lymphoma (CTCL, n=7), and extranodal NK/T-cell lymphoma (ENKTL, n=5). Treatment intensity: 51.1% received 1–4 cycles and 39.7% received 5–6 cycles.
For the total population (N=141), BV demonstrated robust activity with an ORR of 81.6% and a CR rate of 48.2%. Survival outcomes were highly favorable, with 2-year OS and EFS rates of 82.5% and 83.5%, respectively. Median OS and EFS for the entire cohort were not reached (NR).
Subgroup analysis revealed that cHL and CTCL achieved the most outstanding clinical outcomes. ND-cHL patients (n=21) achieved 100% OS and 100% EFS at 2 years, while R/R-cHL (n=36) maintained a 2-year OS of 85.2% and a 2-year EFS of 85.4%. CTCL patients (n=7) achieved a 100% ORR and 100% OS/EFS across both settings. As the largest cohort (n=58), PTCL derived significant benefit: ND-PTCL (n=34) reached an 85.3% ORR and 85.8% 2-year OS; R/R-PTCL (n=24) achieved a 79.2% ORR and 76.7% 2-year OS. Conversely, survival was poorest in the ENKTL group, though results were limited by the small sample size (ND n=1, median OS 0.6 months; R/R n=4, median OS 3.3 months).
Multivariate analysis identified age at diagnosis as an independent risk factor for OS (HR=1.04,95%CI:1.00–1.08,p=0.03). Furthermore, a longer EFS during BV therapy significantly correlated with superior OS (HR=0.66,95%CI:0.52–0.85,p<0.005). Baseline ECOG ≥ 1 was also a significant predictor of increased mortality risk (HR=6.29,p=0.03).
Safety analysis demonstrated a manageable profile. Common all-grade adverse events included anemia (58.9%), neutropenia (51.1%), and febrile neutropenia (29.1%). Grade ≥ 3 events were relatively infrequent, primarily involving infections (6.4%) and anemia (5.0%).

### Summary/Conclusion

BV demonstrates robust real-world efficacy and a manageable safety profile across the total population. Optimal clinical benefits are achieved in cHL and CTCL, particularly in the ND setting where 100% survival was observed. PTCL patients also derive substantial benefit. Achieving early disease control and addressing baseline factors like age and performance status remain critical for maximizing long-term survival in this real-world setting.

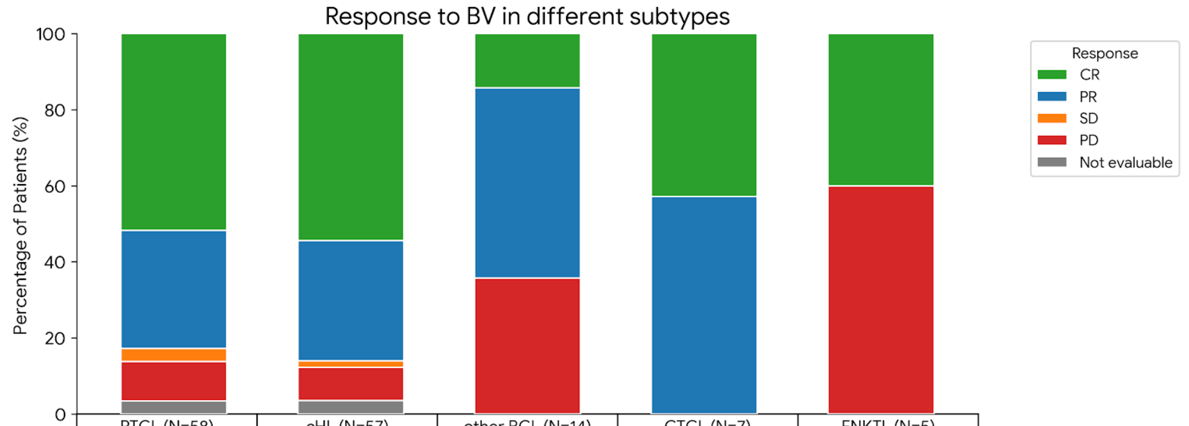

| | PTCL (N=58) | cHL (N=57) | other BCL (N=14) | CTCL (N=7) | ENKTL (N=5) |
|---|---|---|---|---|---|
| CR | 51.7% | 54.4% | 14.3% | 42.9% | 40.0% |
| PR | 31.0% | 31.6% | 50.0% | 57.1% | 0.0% |
| SD | 3.4% | 1.8% | 0.0% | 0.0% | 0.0% |
| PD | 10.3% | 8.8% | 35.7% | 0.0% | 60.0% |
| Not evaluable | 3.4% | 3.5% | 0.0% | 0.0% | 0.0% |

**Copyright Information:** (Online) ISSN: 2572-9241
©2026 The Author(s). HemaSphere is published by John Wiley & Sons Ltd on behalf of European Hematology Association. This is an open access Abstract Book distributed under the Attribution-NonCommercial-NoDerivs (CC BY-NC-ND), which allows third parties to download the articles and share them with others as long as they credit the author and the Abstract Book, but they cannot change the content in any way or use them commercially.
**Abstract Book Citations:** Authors, Title, HemaSphere, 2026;10:(S1):pages. The abstract book DOIs can be found on the "Author Information" page.
**Disclaimer:** Articles published in the journal HemaSphere exclusively reflect the opinions of the authors. The authors are responsible for all content in their abstracts including accuracy of the facts, statements, citing resources, etc.

### (PB3868) TREATMENT OF MEDIASTINAL GRAY ZONE LYMPHOMA WITH HIGH-DOSE THERAPY AND IMMUNE CHECK-POINT INHIBITORS AFTER RELAPSE ON FIRST LINE CHEMO-IMMUNOTHERAPY: SURVIVAL UPDATE

**Topic:** 20. Other aggressive lymphomas

Maher Salamoon*[1]

[1]*al bairouni university cancer center, hematology/oncology, Damascus, Syrian Arab Republic;*

### Background

Mediastinal gray zone lymphoma (MGZL) is a rare non-Hodgkin lymphoma occurring predominantly in young men. This disease is situated at the border between mediastinal classical nodular sclerosis Hodgkin disease and primary mediastinal diffuse large B-cell lymphoma, with different responses to the first-line treatment schedules. Gray zone lymphoma also expresses programmed cell death ligand-1 (PD-L1), making it a probable target for immune checkpoint inhibitors.

### Aims

To evaluate the role of high-dose chemotherapy combined with both nivolumab and ipilimumab in progressed gray zone lymphoma and the impact on both response rate and progression-free survival.

### Methods

We included 12 patients known to have mediastinal gray zone lymphoma confirmed by a reference lab. All patients progressed during and/or after the completion of first-line chemotherapy including anti-CD20 monoclonal antibodies. Patients were aged 18–38 (median, 27 years); 3 were female and 9 were male. PET-CT demonstrated high SUV after the first-line therapy, ranging between 7.8 and 18 (median, 11.3). All patients were treated with 6 cycles of the new chemotherapy regimen ACEP (day 1: doxorubicin 75 mg/m2 + cyclophosphamide 1,200 mg/m² + etoposide 300 mg/m2; days 1–5: prednisolone 60 mg/m2) followed by 4 cycles of ifosfamide (day 1: ifosfamide 4 g/m2), given after the completion of the first 6 cycles of ACEP. Nivolumab 240 mg and ipilimumab 10 mg/kg were given every 3 weeks for a total of 10 cycles

### Results

We documented partial responses in nine patients (75%), and complete responses were documented in three patients (25%) at the end of the third cycle. After the end of therapy, complete response wase documented in six patients (50%). Progression was documented in 2 patients after the end of study; 1 patient only progressed at the end of the 6 cycles. The progression-free survival at 36 months was 86% (95% CI: 75–90). The main toxicities included profound neutropenia and renal toxicity. the overall survival after the same period is 86% as well.

### Summary/Conclusion

Chemotherapy with a dose-dense schedule and immune checkpoint inhibitors seems to offer an effective management in patients with gray zone lymphoma demonstrating early progression on first-line therapies.

**Copyright Information:** (Online) ISSN: 2572-9241
©2026 The Author(s). HemaSphere is published by John Wiley & Sons Ltd on behalf of European Hematology Association. This is an open access Abstract Book distributed under the Attribution-NonCommercial-NoDerivs (CC BY-NC-ND), which allows third parties to download the articles and share them with others as long as they credit the author and the Abstract Book, but they cannot change the content in any way or use them commercially.
**Abstract Book Citations:** Authors, Title, HemaSphere, 2026;10:(S1):pages. The abstract book DOIs can be found on the "Author Information" page.
**Disclaimer:** Articles published in the journal HemaSphere exclusively reflect the opinions of the authors. The authors are responsible for all content in their abstracts including accuracy of the facts, statements, citing resources, etc.

### (PB3869) THE EFFICACY AND SAFETY OF THE IVD/EM REGIMEN FOR PATIENTS WITH T-CELL LYMPHOBLASTIC LYMPHOMA/LEUKEMIA

**Topic:** 20. Other aggressive lymphomas

Ziteng Xie[1], Yue Song*[1], Yu Chang[1], Lei Zhang[1]

[1] *The First Affiliated Hospital of Zhengzhou University, Zhengzhou, Henan, China;*

### Background

T-cell lymphoblastic lymphoma/leukemia (T-LBL/ALL) is a relatively rare hematological malignancy. Its high recurrence rate is an important factor limiting the long-term survival of patients. Currently, hematopoietic stem cell transplantation (HSCT) is the primary treatment option following remission, but it is limited by the patient's physical condition and financial situation. So there remains a substantial unmet clinical need for effective consolidation and maintenance therapy in patients ineligible for transplantation.

### Aims

To evaluate the efficacy and safety of the IVD/EM (ifosfamide, vincristine, and dexamethasone/etoposide and mitoxantrone) regimen as post-remission treatment for patients with T-LBL/ALL who cannot receive HSCT.

### Methods

1. This retrospective analysis included 68 patients who achieved remission after induction therapy, among whom 24 received IVD/EM maintenance treatment and 44 received HSCT.
2. The primary endpoints of the study were PFS and OS, while secondary endpoints included CIR and NRM.

Table . Alternating IVD/EM Treatment Regimen

| Regimen | Drug | Dosage | Administration | Schedule | Remarks |
|---|---|---|---|---|---|
| IVD | Ifosfamide | 2.0g | ivgtt | d1-3 | |
| | Vincristine | 2mg | ivgtt | d1 | |
| | Dexamethasone | 6mg/m$^2$ | po | d1-5 | In conjunction with the intrathecal chemotherapy. Regimens are alternated every 3 months for up to 2 years or until disease progression. |
| EM | Etoposide | 100mg | ivgtt | d1-3 | |
| | Mitoxantrone | 10mg | ivgtt | d1 | |

Concurrent oral administration of 6-MP at a dosage of 30 mg/m² once daily and MTX at a dosage of 20 mg/m² once weekly is indicated. Both agents are withheld during periods of concurrent systemic chemotherapy.

**Copyright Information:** (Online) ISSN: 2572-9241
©2026 The Author(s). HemaSphere is published by John Wiley & Sons Ltd on behalf of European Hematology Association. This is an open access Abstract Book distributed under the Attribution-NonCommercial-NoDerivs (CC BY-NC-ND), which allows third parties to download the articles and share them with others as long as they credit the author and the Abstract Book, but they cannot change the content in any way or use them commercially.
**Abstract Book Citations:** Authors, Title, HemaSphere, 2026;10:(S1):pages. The abstract book DOIs can be found on the "Author Information" page.
**Disclaimer:** Articles published in the journal HemaSphere exclusively reflect the opinions of the authors. The authors are responsible for all content in their abstracts including accuracy of the facts, statements, citing resources, etc.

### Results

**Baseline and treatment**

The median age was 23 (range, 8-73) years, and the male-to-female ratio was 2.4:1. There were no statistically significant differences ($P > 0.05$) between the two groups.

**Efficacy**

1. Compared with the transplantation group, the 1-year, 3-year, and 5-year progression-free survival (PFS) rates of the IVD/EM group were 70.8% *vs* 75.0%, 54.2% *vs* 67.5%, and 47.4% *vs* 67.3%, respectively. The 1-year, 3-year, and 5-year overall survival (OS) rates were 87.5% *vs* 77.3%, 57.8% *vs* 72.7%, and 57.8% *vs* 68.7%, respectively. There was no statistically significant difference in the PFS and OS rates between the two groups ($P$=0.22; $P$=0.49).
2. Although the recurrence rate of the IVD/EM group was higher than that of the transplantation group ($P$=0.01), it significantly reduced the non-recurrence mortality rate ($P$=0.03).

**Safety**

1. The adverse reactions in the IVD/EM group were mainly grade 1-2, and no treatment-related deaths were found.
2. The most common hematology events include anemia (85.19%) and leukopenia (85.19%). Nonhematologic adverse reactions included pulmonary infection (70.37%), liver and kidney function damage (44.44%), and gastrointestinal reactions (22.22%).

### Summary/Conclusion

1. The IVD/EM regimen may serve as an effective treatment option for patients with T-LBL/ALL who are ineligible for transplantation and also reduced the non-recurrence mortality rate compared with HSCT.
2. The IVD/EM regimen has shorter hospitalization times and less financial burden, making it more accessible.
3. Blood tests should be conducted during the treatment process, and symptomatic treatment should be given in a timely manner.

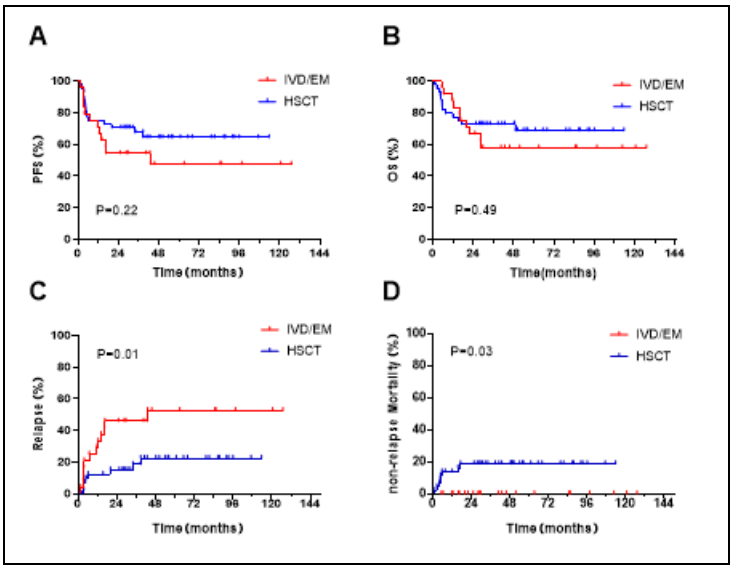

**Copyright Information:** (Online) ISSN: 2572-9241
©2026 The Author(s). HemaSphere is published by John Wiley & Sons Ltd on behalf of European Hematology Association. This is an open access Abstract Book distributed under the Attribution-NonCommercial-NoDerivs (CC BY-NC-ND), which allows third parties to download the articles and share them with others as long as they credit the author and the Abstract Book, but they cannot change the content in any way or use them commercially.
**Abstract Book Citations:** Authors, Title, HemaSphere, 2026;10:(S1):pages. The abstract book DOIs can be found on the "Author Information" page.
**Disclaimer:** Articles published in the journal HemaSphere exclusively reflect the opinions of the authors. The authors are responsible for all content in their abstracts including accuracy of the facts, statements, citing resources, etc.

### (PB3870) TRIAL IN PROGRESS: A PHASE 3, MULTIREGIONAL, OPEN-LABEL, RANDOMIZED STUDY OF TIRABRUTINIB VS RITUXIMAB AND TEMOZOLOMIDE IN RELAPSED/REFRACTORY PRIMARY CENTRAL NERVOUS SYSTEM LYMPHOMA (IGNITE)

**Topic:** 20. Other aggressive lymphomas

Lakshmi Nayak*[1], Christian Grommes[2], Charles Psoinos[3], Akira Takazawa[4], Tracy Batchelor[5]

*[1]Dana-Farber Cancer Institute, Boston, United States of America; [2]Memorial Sloan Kettering Cancer Center, New York, United States of America; [3]Deciphera Pharmaceuticals, LLC, Waltham, United States of America; [4]ONO PHARMA USA, INC., Cambridge, United States of America; [5]Brigham and Women's Hospital, Boston, United States of America;*

### Background

Primary central nervous system lymphoma (PCNSL) is a rare, highly aggressive non-Hodgkin lymphoma localized to the brain, spinal cord, cerebrospinal fluid, and/or eyes. Although patients (pts) generally respond to standard high-dose methotrexate (HD-MTX) induction, 10%–30% of pts have refractory disease. In a 1002-pt database, median progression-free survival (PFS) was <1 yr, and 2-yr PFS was 36%. Treatment options are limited in the US and there are no approved therapies in the EU for relapsed/refractory (R/R) PCNSL. Recommended treatment includes high-dose chemotherapy (HDC) ± autologous stem cell transplantation (ASCT), whole brain radiation therapy (WBRT), or better-tolerated regimens (temozolomide [TMZ] or lenalidomide ± rituximab [RTX], RTX, pomalidomide, ibrutinib, or pemetrexed). Few pts with R/R PCNSL get HDC-ASCT or WBRT, and TMZ is recommended per NCCN (± RTX) and EHA-ESMO guidelines. Current treatments are associated with high toxicity, limited efficacy, and short duration of response (DOR). Tirabrutinib is a highly potent, selective second-generation Bruton tyrosine kinase inhibitor (BTKi) approved in Japan, Taiwan, and South Korea for R/R PCNSL. Primary results from the PROSPECT phase 2 US study (NCT04947319) showed a 67% overall response rate (ORR) with tirabrutinib monotherapy in pts with R/R PCNSL, and tirabrutinib was generally well tolerated without major cardiac toxicity. Most common any-grade treatment-related adverse events were anemia (19%), maculopapular rash (17%), fatigue, lymphocyte count and neutrophil count decreased, pruritus, and rash (all 15%).

### Aims

To confirm the clinical benefit of tirabrutinib vs standard RTX-TMZ combination therapy in R/R PCNSL.

**Copyright Information:** (Online) ISSN: 2572-9241
©2026 The Author(s). HemaSphere is published by John Wiley & Sons Ltd on behalf of European Hematology Association. This is an open access Abstract Book distributed under the Attribution-NonCommercial-NoDerivs (CC BY-NC-ND), which allows third parties to download the articles and share them with others as long as they credit the author and the Abstract Book, but they cannot change the content in any way or use them commercially.
**Abstract Book Citations:** Authors, Title, HemaSphere, 2026;10:(S1):pages. The abstract book DOIs can be found on the "Author Information" page.
**Disclaimer:** Articles published in the journal HemaSphere exclusively reflect the opinions of the authors. The authors are responsible for all content in their abstracts including accuracy of the facts, statements, citing resources, etc.

### Methods

This phase 3, multiregional, open-label, randomized study (NCT07104032; IGNITE) will enroll ~132 adults with R/R PCNSL. Pts must have confirmed R/R B-cell PCNSL with ≥1 prior HD-MTX–based therapy for PCNSL, ≥1 bi-dimensionally measurable brain lesion with a diameter ≥1 cm on MRI without systemic lymphoma, and an Eastern Cooperative Oncology Group performance status ≤2. Pts with intraocular PCNSL and no brain lesions, non–B-cell PCNSL, systemic lymphoma presence, prior BTKi treatment, and those refractory to TMZ ± RTX–containing regimens in their last PCNSL treatment are ineligible. Pts will be randomized 2:1 to tirabrutinib 480 mg once daily or RTX-TMZ combination (intravenous RTX 375 mg/m$^2$ on day [D]1 and oral TMZ 150 mg/m$^2$/day on D1–5 from cycle [C]1 through 6). After C1, TMZ can be modified to 200 mg/m$^2$ in absence of grade ≥2 toxicity or 100 mg/m$^2$ if there is toxicity, at the investigator's discretion. Randomization will be stratified by age (<70 vs ≥70 yrs) and R/R status from last therapy (relapsed vs refractory). Study drug will be given in 28-day cycles until treatment end (6 cycles in the RTX-TMZ arm), disease progression assessed by blinded independent review committee (BIRC) per International PCNSL Collaborative Group (IPCG) criteria, unacceptable toxicity, or consent withdrawal. The primary endpoint is PFS by BIRC per IPCG criteria. Key secondary endpoints are ORR by BIRC and overall survival. Other secondary endpoints include complete response rate, best overall response, time to response, time to complete response, DOR, and disease-free survival, all by BIRC. Tumor response will be assessed at screening, C2D1, C3D1, then every 2 cycles through C25D1, and every 4 cycles thereafter per IPCG criteria. Previously presented at ASH 2025, Orlando, FL, USA.

**Copyright Information:** (Online) ISSN: 2572-9241
©2026 The Author(s). HemaSphere is published by John Wiley & Sons Ltd on behalf of European Hematology Association. This is an open access Abstract Book distributed under the Attribution-NonCommercial-NoDerivs (CC BY-NC-ND), which allows third parties to download the articles and share them with others as long as they credit the author and the Abstract Book, but they cannot change the content in any way or use them commercially.
**Abstract Book Citations:** Authors, Title, HemaSphere, 2026;10:(S1):pages. The abstract book DOIs can be found on the "Author Information" page.
**Disclaimer:** Articles published in the journal HemaSphere exclusively reflect the opinions of the authors. The authors are responsible for all content in their abstracts including accuracy of the facts, statements, citing resources, etc.

### (PB3871) DESIGN, SYNTHESIS AND PHARMACOLOGICAL EVALUATION OF 3H-SPIRO[BENZOFURAN-2,4'-PIPERIDINE] IRAK4 INHIBITORS FOR THE TREATMENT OF DIFFUSE LARGE B-CELL LYMPHOMA

**Topic:** 20. Other aggressive lymphomas

Hua Xie*[1, 2], Zhiwei Chen[1], Qi Zhang[1], Q-I Zhang[2], Jian Ding[1], Wenhu Duan[1]

*[1]Shanghai Institute of Materia Medica, Chinese Academy of Sciences, shanghai, China;[2]Zhongshan Institute for Drug Discovery, Shanghai Institute of Materia Medica, Chinese Academy of Sciences, zhongshan, China;*

### Background

Diffuse large B-cell lymphoma (DLBCL) is the most common subtype of non-Hodgkin lymphoma, which can be classified into two main subtypes: germinal center B-cell-like type (GCB) DLBCL and activated B-cell-like type (ABC) DLBCL. Approximately 29% of ABC DLBCL patients harbor myeloid differentiation factor 88 (MYD88) L265P mutation, which causes constitutive activation of the NF-κB signaling pathway and is associated with poorer survival in ABC DLBCL patients.
Interleukin-1 receptor-associated kinase 4 (IRAK4) is a serine/threonine protein kinase that plays a critical role in MYD88-mediated NF-κB activation. Mutated MyD88 spontaneously recruits IRAK4, IRAK1, and TRAF6 to form the myddosome complex, triggering NF-κB activation and promoting the survival of DLBCL cells. Given that both IRAK4 shRNA and IRAK4 inhibitors could selectively decrease the survival of MYD88 L265P DLBCL cell lines, inhibition of IRAK4 is an attractive approach for the treatment of DLBCL.

### Aims

In this study, we design and synthesize a series of 3*H*-spiro[benzofuran-2,4'-piperidine] IRAK4 inhibitors and evaluate their inhibitory potency against IRAK4 and their antiproliferative effects against DLBCL. We aim to identify promising lead compounds for therapeutic intervention in DLBCL.

### Methods

IRAK4 inhibitory activity was evaluated using the Z'-LYTE kinase assay, and kinase selectivity profiling was performed by enzyme-linked-immunosorbent assay (ELISA). Antiproliferative effects were assessed using cell viability and apoptosis assays. NF-κB pathway inhibition was analyzed by Western blot analysis. Pharmacokinetic properties were evaluated in rat models.

### Results

We designed and synthesized a series of 3*H*-spiro[benzofuran-2,4'-piperidine] derivatives and identified compound **13** as a candidate with a novel structural scaffold. Compound **13** exhibited potent inhibitory activity against IRAK4, with an $IC_{50}$ value of 7.5 nM. At the cellular level, compound **13** displayed significant antiproliferative activity in MYD88 L265P-mutant DLBCL OCI-LY 10 cells, with an $IC_{50}$ of 0.86 μM, while its inhibitory effect on normal GM00637 cells was markedly weaker ($IC_{50}$ > 50 μM). Mechanistic investigations further revealed that compound **13** markedly suppressed activation of the downstream NF-κB signaling pathway in both a time- and dose-dependent manner. Apoptosis assays demonstrated that compound **13** induced time- and dose-dependent apoptosis in OCI-LY10 cells, accompanied by cleavage of PARP and caspase-3. Moreover, pharmacokinetic studies showed that compound **13** possessed favorable pharmacokinetic properties and demonstrated oral bioavailability. Collectively, these findings indicate that compound **13** represents a promising novel IRAK4 inhibitor for further preclinical development for the treatment of DLBCL.

**Copyright Information:** (Online) ISSN: 2572-9241
©2026 The Author(s). HemaSphere is published by John Wiley & Sons Ltd on behalf of European Hematology Association. This is an open access Abstract Book distributed under the Attribution-NonCommercial-NoDerivs (CC BY-NC-ND), which allows third parties to download the articles and share them with others as long as they credit the author and the Abstract Book, but they cannot change the content in any way or use them commercially.
**Abstract Book Citations:** Authors, Title, HemaSphere, 2026;10:(S1):pages. The abstract book DOIs can be found on the “Author Information” page.
**Disclaimer:** Articles published in the journal HemaSphere exclusively reflect the opinions of the authors. The authors are responsible for all content in their abstracts including accuracy of the facts, statements, citing resources, etc.

### Summary/Conclusion

Our study identified compound **13** as a potent, selective, and orally bioavailable IRAK4 inhibitor. Compound **13** exhibited enhanced IRAK4 inhibitory potency, increased antiproliferative activity against MYD88 L265P DLBCL cells, and more favorable pharmacokinetic properties. These findings indicate that compound **13** is a potential therapeutic agent for the treatment of DLBCL and deserves further preclinical development.

**Copyright Information:** (Online) ISSN: 2572-9241
©2026 The Author(s). HemaSphere is published by John Wiley & Sons Ltd on behalf of European Hematology Association. This is an open access Abstract Book distributed under the Attribution-NonCommercial-NoDerivs (CC BY-NC-ND), which allows third parties to download the articles and share them with others as long as they credit the author and the Abstract Book, but they cannot change the content in any way or use them commercially.
**Abstract Book Citations:** Authors, Title, HemaSphere, 2026;10:(S1):pages. The abstract book DOIs can be found on the "Author Information" page.
**Disclaimer:** Articles published in the journal HemaSphere exclusively reflect the opinions of the authors. The authors are responsible for all content in their abstracts including accuracy of the facts, statements, citing resources, etc.

### (PB3872) REAL-WORLD EFFICACY AND SAFETY ANALYSIS OF MITOXANTRONE HYDROCHLORIDE LIPOSOME IN THE TREATMENT OF PERIPHERAL T-CELL LYMPHOMA

**Topic:** 20. Other aggressive lymphomas

Xiaoqing Li*[1], Qingli Ji[1], Xinwen Jiang[1], Xinrui Zhang[1], Chen Cao[1], Ran Wang[1], Sai MA[1], Minran Zhou[1], Chunyan Chen[1]

*[1]Qilu Hospital, Shandong University, Jinan, China;*

### Background

Peripheral T-cell lymphoma (PTCL) constitutes a highly heterogeneous group of aggressive non-Hodgkin lymphomas. Mitoxantrone hydrochloride liposome, a modified formulation of conventional mitoxantrone, enables targeted enrichment in tumor tissues, enhancing anti-tumor activity while reducing systemic toxicity. Clinical data on mitoxantrone liposome-containing regimens for treating PTCL remain limited.

### Aims

To evaluate the efficacy and safety of mitoxantrone hydrochloride liposome in patients with PTCL in a real-world setting.

### Methods

Data from 42 PTCL patients treated with mitoxantrone liposome (either as monotherapy or in combination) at Qilu Hospital of Shandong University between January 2022 and February 2026 were retrospectively collected. The primary efficacy endpoint was the objective response rate (ORR), comprising complete response (CR) and partial response (PR). The secondary endpoint was progression-free survival (PFS). Safety was assessed according to the Common Terminology Criteria for Adverse Events (CTCAE) version 5.0.

### Results

The median age of the 42 patients was 59 years (range 22-79), with 29 males (69.0%). Pathological subtypes included PTCL-not otherwise specified (14 cases, 33.3%), angioimmunoblastic T-cell lymphoma (14 cases, 33.3%), NK/T-cell lymphoma (11 cases, 26.2%), and anaplastic large cell lymphoma (3 cases, 7.1%). Stage III-IV disease was present in 33 patients (78.6%), and 19 patients (45.2%) had an International Prognostic Index (IPI) score ≥3. Twenty-five patients (59.5%) had received ≥4 prior lines of therapy. The median number of mitoxantrone liposome treatment cycles was 3 (range 1-7), with 37 patients (88.1%) receiving combination therapy.

In the overall population, the ORR was 38.1% (16/42). Among the 11 treatment-naïve patients and the 31 relapsed/refractory (R/R) patients, the ORRs were 72.7% (8/11) and 25.8% (8/31), respectively. Within the R/R group, 5 patients received monotherapy (3 evaluable for efficacy; ORR 33.3% [1/3]), and 26 received combination therapy (23 evaluable; ORR 30.4% [7/23]). The median PFS was 4.0 months (range 1-12) in the treatment-naïve group and 2.5 months (range 1-7) in the R/R group. Regarding safety, treatment-related adverse events occurred in 83.3% (35/42) of patients, with grade ≥3 events observed in 57.1% (24/42). The most common grade ≥3 events were neutropenia and leukopenia. No unexpected serious adverse events were observed.

**Copyright Information:** (Online) ISSN: 2572-9241
©2026 The Author(s). HemaSphere is published by John Wiley & Sons Ltd on behalf of European Hematology Association. This is an open access Abstract Book distributed under the Attribution-NonCommercial-NoDerivs (CC BY-NC-ND), which allows third parties to download the articles and share them with others as long as they credit the author and the Abstract Book, but they cannot change the content in any way or use them commercially.
**Abstract Book Citations:** Authors, Title, HemaSphere, 2026;10:(S1):pages. The abstract book DOIs can be found on the “Author Information” page.
**Disclaimer:** Articles published in the journal HemaSphere exclusively reflect the opinions of the authors. The authors are responsible for all content in their abstracts including accuracy of the facts, statements, citing resources, etc.

**Summary/Conclusion**

This real-world study demonstrates that treatment regimens containing mitoxantrone liposome exhibit promising anti-tumor activity in PTCL patients, with an ORR of 72.7% in treatment-naïve patients and a generally manageable safety profile. For R/R patients, combination therapy may offer greater benefit. These findings warrant further validation through extended follow-up and larger sample sizes.

**Copyright Information:** (Online) ISSN: 2572-9241
©2026 The Author(s). HemaSphere is published by John Wiley & Sons Ltd on behalf of European Hematology Association. This is an open access Abstract Book distributed under the Attribution-NonCommercial-NoDerivs (CC BY-NC-ND), which allows third parties to download the articles and share them with others as long as they credit the author and the Abstract Book, but they cannot change the content in any way or use them commercially.
**Abstract Book Citations:** Authors, Title, HemaSphere, 2026;10:(S1):pages. The abstract book DOIs can be found on the "Author Information" page.
**Disclaimer:** Articles published in the journal HemaSphere exclusively reflect the opinions of the authors. The authors are responsible for all content in their abstracts including accuracy of the facts, statements, citing resources, etc.

### (PB3874) MINE REGIMEN COMBINED WITH TARGETED AGENTS IN RELAPSED/REFRACTORY ANGIOIMMUNOBLASTIC T-CELL LYMPHOMA: A PRELIMINARY EXPLORATORY RETROSPECTIVE STUDY INTEGRATED WITH NEXT-GENERATION SEQUENCING

**Topic:** 20. Other aggressive lymphomas

Yuelu Guo*[1, 1], Lixia MA[1], Miaomiao Cao[1], Jinping Deng[1], Bian Wei[1], Fan Yang[1], Rui Liu[1], Zhonghua Fu[1], Kai Hu[1]

[1]*Beijing Gobroad Hospital, Department of Lymphoma and Myeloma Research Center, Beijing, China;*

### Background

Angioimmunoblastic T-cell lymphoma (AITL) is an aggressive peripheral T-cell lymphoma subtype with extremely poor prognosis in relapsed/refractory (rr) setting, requiring novel therapeutic strategies. The MINE regimen (ifosfamide, mitoxantrone, etoposide) is a salvage option, but data on its combination with targeted agents (azacitidine, chidamide, golidocitinib, tazemetostat, enasidenib, ruxolitinib) in rrAITL remain limited.

### Aims

This study aimed to explore the clinical value of MINE plus targeted therapy in rrAITL and identify predictive biomarkers via next-generation sequencing (NGS).

### Methods

This single-center retrospective study enrolled 9 rrAITL patients treated with MINE plus targeted agents between February 2024 and January 2026. Baseline NGS (119 genes: RHOA, CD28, TET2, DNMT3A, IDH2, TET1/TET3, STAT3, KMT2D, TP53, etc.) was performed on tumor or peripheral blood samples. Targeted agents were selected per molecular profiles: epigenetic drugs (azacitidine, chidamide) for TET2/DNMT3A mutations; JAK/STAT inhibitors (golidocitinib, ruxolitinib) for RHOA-driven JAK-STAT activation; enasidenib for IDH2 mutations. Efficacy was assessed by Lugano 2014 criteria (primary endpoints: ORR, CRR; secondary endpoints: PFS, DOR, OS). Safety was evaluated for ≥ grade 3 treatment-related adverse events (TRAEs).

**Copyright Information:** (Online) ISSN: 2572-9241
©2026 The Author(s). HemaSphere is published by John Wiley & Sons Ltd on behalf of European Hematology Association. This is an open access Abstract Book distributed under the Attribution-NonCommercial-NoDerivs (CC BY-NC-ND), which allows third parties to download the articles and share them with others as long as they credit the author and the Abstract Book, but they cannot change the content in any way or use them commercially.
**Abstract Book Citations:** Authors, Title, HemaSphere, 2026;10:(S1):pages. The abstract book DOIs can be found on the "Author Information" page.
**Disclaimer:** Articles published in the journal HemaSphere exclusively reflect the opinions of the authors. The authors are responsible for all content in their abstracts including accuracy of the facts, statements, citing resources, etc.

### Results

Median age was 56 (32–71) years; 7 (77.8%) had stage IV, 2 (22.2%) stage III disease. 77.8% (7/9) were male.High/intermediate-high IPI was noted in 8 (88.9%) patients, high PIT in 6 (66.7%). Coinfection rates: CMV 55.6% (5/9), EBV 77.8% (7/9), HHV6 22.2% (2/9), HHV8 11.1% (1/9). Median prior therapy lines: 3 (2–6). With median follow-up of 134 (23–292) days, ORR was 77.8% (7/9), CRR 55.6% (5/9), median DOR 86 days. Median PFS and OS were both 134 days (data immature due to short follow-up and early events). NGS showed frequent mutations: TET2 77.8% (7/9), RHOA 66.7% (6/9), DNMT3A 55.6% (5/9), IDH2 33.3% (3/9), STAT3/PLCG1/TP53 11.1% (1/9 each). Two non-responders (disease progression/death) had 5 and 7 mutations, respectively; 7 responders (≥PR) had median 2 mutations (0–3). Mean mutations: 6 in non-responders vs 1.2 in responders. Grade 3–4 TRAEs: neutropenia 55.6% (5/9), thrombocytopenia 33.3% (3/9), anemia 44.4% (4/9). Death causes: infection 44.4% (4/9), gastrointestinal bleeding 11.1% (1/9).

| | Age | Gender | Ann-anbor | Group | bone marrow infiltration | IPI score | PIT score | gene mutation(NGS) | viral infection |
|---|---|---|---|---|---|---|---|---|---|
| Pt.01 | 71 | male | III | A | No | 2 | 3 | RHOA,TET2,DNMT3A,IDH2 | |
| Pt.02 | 65 | female | IV | B | No | 3 | 3 | RHOA,TET2,DNMT3A | EBV |
| Pt.03 | 58 | male | IV | A | No | 3 | 2 | TET2,DNMT3A,IDH2,TP53, | CMV,EBV |
| Pt.04 | 57 | male | IV | B | Yes | 3 | 3 | RHOA,TET2 | CMV,HHV6,HHV8,EBV |
| Pt.05 | 56 | male | IV | A | No | 3 | 3 | RHOA,TET2 | CMV,EBV |
| Pt.06 | 52 | female | IV | B | No | 3 | 2 | | EBV |
| Pt.07 | 47 | male | IV | B | Yes | 3 | 3 | RHOA,TET2,DNMT3A,IDH2 | CMV,HHV6,EBV |
| Pt.08 | 43 | male | IV | B | Yes | 3 | 3 | TET2,DNMT3A | CMV,EBV |
| Pt.09 | 32 | male | III | B | No | 3 | 2 | | |

### Summary/Conclusion

To our knowledge, this is the first study showing MINE plus personalized targeted therapy yields promising efficacy (ORR 77.8%) and manageable toxicity in rrAITL. High tumor mutational burden was associated with early progression and poor outcome, suggesting a potential predictive biomarker for treatment selection. These preliminary findings warrant validation in larger prospective studies.

**Copyright Information:** (Online) ISSN: 2572-9241
©2026 The Author(s). HemaSphere is published by John Wiley & Sons Ltd on behalf of European Hematology Association. This is an open access Abstract Book distributed under the Attribution-NonCommercial-NoDerivs (CC BY-NC-ND), which allows third parties to download the articles and share them with others as long as they credit the author and the Abstract Book, but they cannot change the content in any way or use them commercially.
**Abstract Book Citations:** Authors, Title, HemaSphere, 2026;10:(S1):pages. The abstract book DOIs can be found on the "Author Information" page.
**Disclaimer:** Articles published in the journal HemaSphere exclusively reflect the opinions of the authors. The authors are responsible for all content in their abstracts including accuracy of the facts, statements, citing resources, etc.

### (PB3875) CELL CYCLE–FOCUSED CHEMICAL SCREEN REVEALS MITOTIC KINASE INHIBITORS THAT ENHANCE THE EFFICACY OF A CD30-TARGETED ANTIBODY–DRUG CONJUGATE

**Topic:** 20. Other aggressive lymphomas

Yuto Mori*[1], Keito Suto[1], Norio Takei[2], Keito Yokoyama[1], Masahiro Chiba[1], Takashi Ishio[1], Hideki Goto[1], Tomoyuki Endo[1], Satoko Otsuguro[3], Katsumi Maenaka[3, 4], Takanori Teshima[1], Masao Nakagawa[1]

*[1]Hokkaido University Faculty of Medicine, Department of Hematology, Sapporo, Japan;[2]Hokkaido University Faculty of Medicine, Institute for Animal Experimentation, Sapporo, Japan; [3]Hokkaido University Faculty of Pharmaceutical Science, Center for Research and Education on Drug Discovery, Sapporo, Japan; [4]Hokkaido University, Global Station for Biosurfaces and Drug Discovery, Sapporo, Japan;*

### Background

Brentuximab vedotin (BV), an antibody–drug conjugate (ADC) composed of an anti-CD30 monoclonal antibody conjugated to the microtubule-disrupting drug monomethyl auristatin E (MMAE), is widely used as a key therapeutic agent for the treatment of peripheral T-cell lymphoma (PTCL). Nevertheless, most patients fail to achieve durable remission, highlighting the need for improved BV-based strategies. We previously performed a genome-wide loss-of-function CRISPR screen and found that knockout of *MAD2L1BP* and *ANAPC15* genes, which promote mitotic progression, increased BV sensitivity in CD30-positive PTCL cells (Suto, et al. Leukemia. 2025), though inhibitors targeting MAD2L1BP or ANAPC15 have not yet been developed. Given the link between mitotic regulation and BV efficacy, we hypothesized that pharmacological targeting of cell cycle regulators could provide a therapeutically actionable approach to enhance BV activity.

### Aims

To identify therapeutically actionable cell cycle regulators that enhance BV efficacy in CD30-positive PTCL.

### Methods

We established a focused compound library of 118 cell cycle-related agents. Two CD30-positive PTCL cell lines—the ALK-positive anaplastic large cell lymphoma (ALK+ ALCL) line DEL and the adult T-cell leukemia/lymphoma (ATLL) line ED40515(–)—were treated with BV and the drug panel for 72 hours. Cell viability was assessed, and combinatorial effects were analyzed using the Bliss independence model.

**Copyright Information:** (Online) ISSN: 2572-9241
©2026 The Author(s). HemaSphere is published by John Wiley & Sons Ltd on behalf of European Hematology Association. This is an open access Abstract Book distributed under the Attribution-NonCommercial-NoDerivs (CC BY-NC-ND), which allows third parties to download the articles and share them with others as long as they credit the author and the Abstract Book, but they cannot change the content in any way or use them commercially.
**Abstract Book Citations:** Authors, Title, HemaSphere, 2026;10:(S1):pages. The abstract book DOIs can be found on the "Author Information" page.
**Disclaimer:** Articles published in the journal HemaSphere exclusively reflect the opinions of the authors. The authors are responsible for all content in their abstracts including accuracy of the facts, statements, citing resources, etc.

### Results

The screen identified three compounds—volasertib, GSK461364, and onvansertib—that exhibited strong synergy with BV in both cell lines. Notably, all three agents were polo-like kinase 1 (PLK1) inhibitors, supporting biological coherence. To validate these findings, we performed cell proliferation assays in four CD30-positive PTCL cell lines. BV combined with each PLK1 inhibitor demonstrated synergistic effects across all four cell lines. Among the three PLK1 inhibitors, volasertib was selected for further experiments given its advanced clinical development and established safety profile.
To elucidate the mechanism underlying the combinatorial effects, we performed cell cycle analyses and found that the proportion of cells in the G2/M phase significantly increased upon treatment with BV and volasertib. Western-blot analysis also revealed elevated expression of M phase markers, cyclinB1 and phospho-Histone H3 (Ser10), in cells treated with the combination, indicating enhanced mitotic arrest. In addition, the DNA damage marker phospho-histone H2A.X (Ser139) and the apoptosis marker cleaved caspase-3 were increased, suggesting that mitotic arrest led to DNA damage and apoptotic cell death. Inhibition of Mps1 abolished the combinatorial induction of mitotic arrest markers, establishing that SAC activation is essential for the synergistic cytotoxicity of BV and volasertib.
In vivo, the combination of BV and volasertib suppressed tumor growth more than either monotherapy in the DEL xenograft model. Similarly, in the ALK-negative ALCL PDX model, the combination resulted in significantly greater tumor reduction compared with individual treatments.

### Summary/Conclusion

Our findings highlight the therapeutic potential of combining BV with PLK1 inhibitors—particularly volasertib—as a rational strategy to enhance cytotoxicity in CD30-positive PTCL. This study provides a mechanistic and preclinical foundation for future clinical evaluation of this combination therapy.

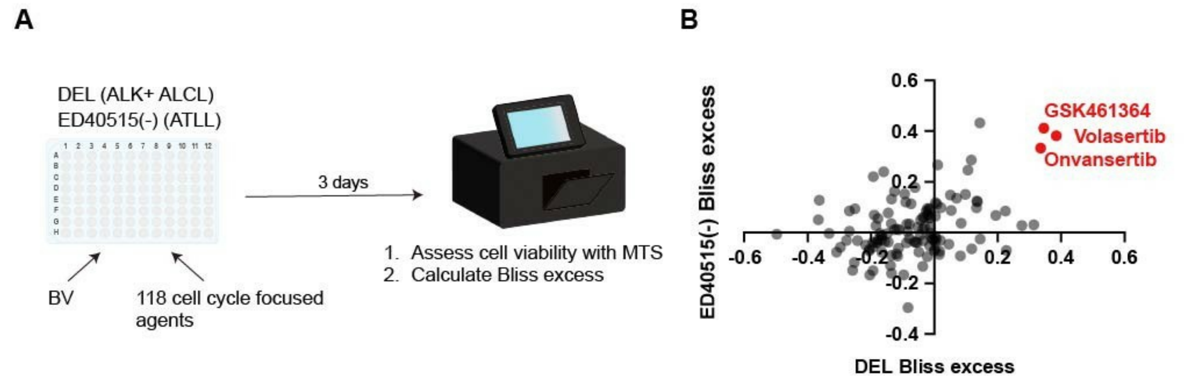

(A) Schematic diagram of the drug screen. Two PTCL cell lines, DEL (ALK+ ALCL) and ED40515(−) (ATLL), were treated with BV (0.03 nM) and 118 cell cycle associated agents (10 nM) for 72 h. Then we evaluated cell viability using MTS and calculated Bliss excess score.

(B) Plot showed Bliss excess scores of each agents in the two cell lines.

**Copyright Information:** (Online) ISSN: 2572-9241
©2026 The Author(s). HemaSphere is published by John Wiley & Sons Ltd on behalf of European Hematology Association. This is an open access Abstract Book distributed under the Attribution-NonCommercial-NoDerivs (CC BY-NC-ND), which allows third parties to download the articles and share them with others as long as they credit the author and the Abstract Book, but they cannot change the content in any way or use them commercially.
**Abstract Book Citations:** Authors, Title, HemaSphere, 2026;10:(S1):pages. The abstract book DOIs can be found on the “Author Information” page.
**Disclaimer:** Articles published in the journal HemaSphere exclusively reflect the opinions of the authors. The authors are responsible for all content in their abstracts including accuracy of the facts, statements, citing resources, etc.

### (PB3876) HIGH-DOSE VITAMIN C SUPPRESSES JAK-STAT/IFN-Γ SIGNALING AND IMPROVES SURVIVAL IN HEMOPHAGOCYTIC LYMPHOHISTIOCYTOSIS

**Topic:** 20. Other aggressive lymphomas

Kai Qing*[1], Shishuang Wu[1], Jiayi Ren[1, 2], Xinyuan Zhao[3], Yuting Tao[2], Kai Xue[1]

*[1]Ruijin Hospital School of Medicine, Shanghai Jiao Tong University, Shanghai Institute of Hematology, State Key Laboratory of Medical Genomics, National Research Center for Translational Medicine at Shanghai, Shanghai, China; [2]Ruijin Hospital School of Medicine, Shanghai Jiao Tong University, Shanghai, China; [3]Fudan University, Institute of Brain Science, Shanghai, China;*

### Background

Hemophagocytic lymphohistiocytosis (HLH) is a life-threatening hyperinflammatory syndrome driven by excessive interferon-gamma (IFN-γ) production, which activates JAK-STAT signaling and triggers macrophage activation with subsequent cytokine storm (IL-6, TNF-α, IFN-γ). Current therapies have limited efficacy and significant toxicity. High-dose Vitamin C possesses immunomodulatory and anti-inflammatory properties and can suppress pro-inflammatory signaling. We hypothesized that high-dose vitamin C could attenuate HLH pathogenesis through JAK-STAT pathway inhibition and macrophage modulation.

### Aims

To evaluate the therapeutic efficacy and mechanisms of high-dose vitamin C in HLH, focusing on survival improvement, inflammation attenuation, and signaling pathway suppression.

### Methods

HLH was induced in mice using sequential polyinosinic-polycytidylic acid (Poly I:C) and lipopolysaccharide (LPS) administration. Mice received high-dose vitamin C (4 g/kg every 8 hours, initiated 3 hours post-LPS) or phosphate-buffered saline as control. Survival was monitored. Splenic expression of pro-inflammatory cytokines (IL-6, IL-1β, TNF-α, and IFN-γ) was quantified by quantitative PCR. Western blot analysis assessed phosphorylation of JAK2, STAT1, and IκB-α. Flow cytometry characterized Ly6C-high and Ly6C-low macrophage subsets. In vitro, bone marrow-derived macrophages stimulated with IFN-γ and LPS were used to evaluate vitamin C-mediated cytoprotection under inflammatory stress.

### Results

Administration of high-dose vitamin C significantly improved survival in HLH model mice compared to controls (Figure 1E), concomitant with attenuated hepatic and splenic injury and reduced splenomegaly evidenced by decreased spleen-to-body weight ratios (Figure 1A, C). Mechanistically, vitamin C downregulated splenic mRNA expression of pro-inflammatory cytokines IL-6, IL-1β, TNF-α, and IFN-γ (Figure 1F), and inhibited phosphorylation of JAK2, STAT1, and IκB-α (Figure 6I), demonstrating suppression of JAK-STAT and NF-κB signaling. Immunofluorescence revealed increased splenic macrophage abundance (Figure 1D, G), whereas flow cytometric analysis demonstrated reduced proportions of both Ly6C-high and Ly6C-low macrophage subsets (Figure 1B, H), suggesting modulation of inflammatory monocyte dynamics. In vitro, vitamin C protected bone marrow-derived macrophages from cell death induced by IFN-γ and LPS stimulation, enhancing cellular resilience without depleting effector cells.

Copyright Information: (Online) ISSN: 2572-9241
©2026 The Author(s). HemaSphere is published by John Wiley & Sons Ltd on behalf of European Hematology Association. This is an open access Abstract Book distributed under the Attribution-NonCommercial-NoDerivs (CC BY-NC-ND), which allows third parties to download the articles and share them with others as long as they credit the author and the Abstract Book, but they cannot change the content in any way or use them commercially.
**Abstract Book Citations:** Authors, Title, HemaSphere, 2026;10:(S1):pages. The abstract book DOIs can be found on the “Author Information” page.
**Disclaimer:** Articles published in the journal HemaSphere exclusively reflect the opinions of the authors. The authors are responsible for all content in their abstracts including accuracy of the facts, statements, citing resources, etc.

### Summary/Conclusion

High-dose vitamin C improves survival in HLH by attenuating systemic hyperinflammation and organ damage through suppression of JAK-STAT/NF-κB signaling, downregulation of IFN-γ-driven cytokines, and modulation of macrophage resilience. These mechanistic findings establish a foundation for prospective clinical trials, supporting vitamin C as an ultra-early, chemotherapy-free bridging strategy to definitive therapy in HLH.

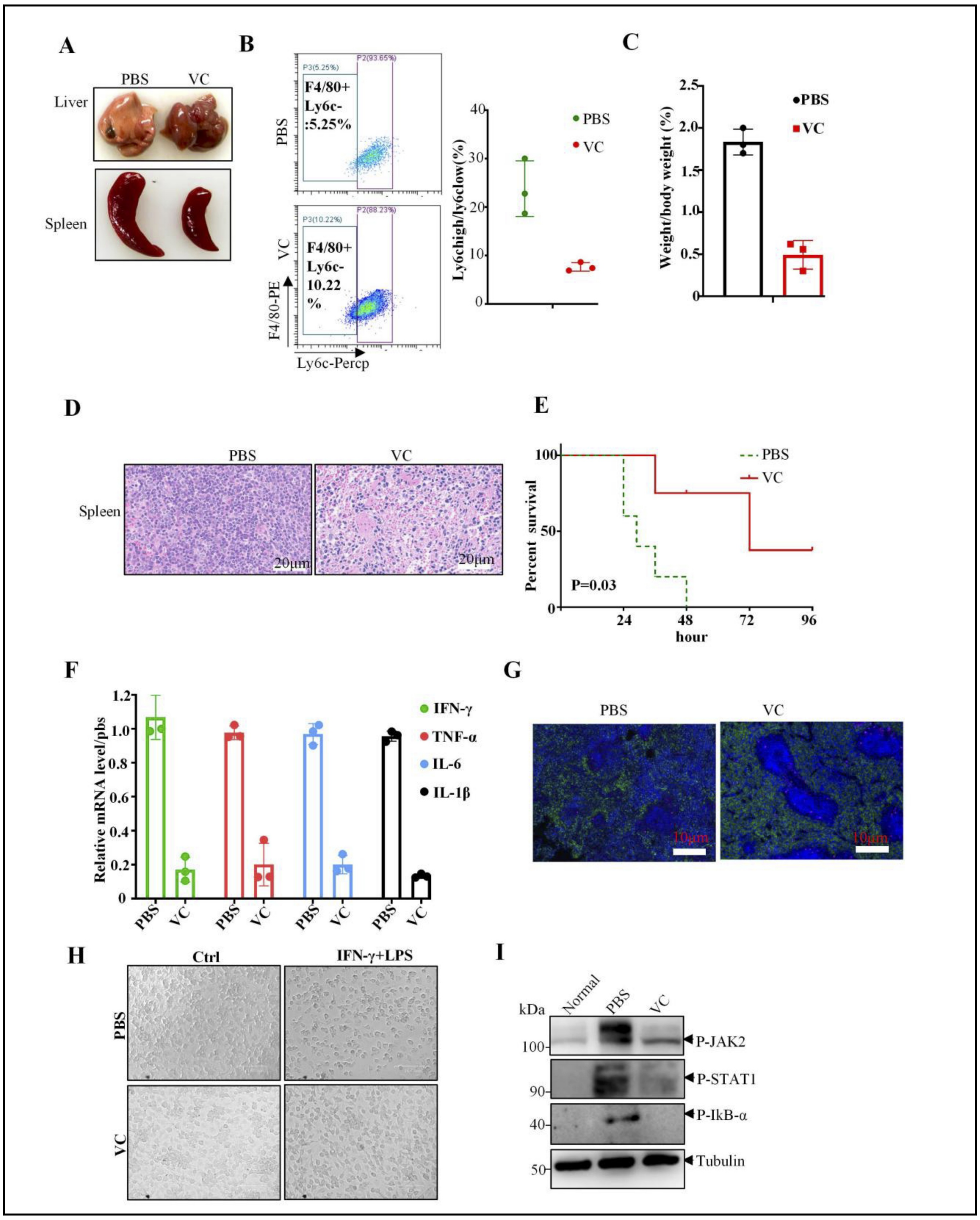

Figure1. Therapeutic effects of VC on HLH model mice. A. Illustrative diagrams of liver and kidney. B. Proportions of Ly6Chi and Ly6Clow macrophage subsets in the spleen. C. Ratio of spleen weight to body weight. D. Distribution of macrophages within the spleen. E. Survival rate of mice. F. mRNA expression levels of pro-inflammatory cytokines in the spleen. G. Representative immuno-fluorescence images of macrophages. H. Macrophage distribution profile. I. Western blot analysis of key molecules in the JAK signaling pathway.

**Copyright Information:** (Online) ISSN: 2572-9241
©2026 The Author(s). HemaSphere is published by John Wiley & Sons Ltd on behalf of European Hematology Association. This is an open access Abstract Book distributed under the Attribution-NonCommercial-NoDerivs (CC BY-NC-ND), which allows third parties to download the articles and share them with others as long as they credit the author and the Abstract Book, but they cannot change the content in any way or use them commercially.
**Abstract Book Citations:** Authors, Title, HemaSphere, 2026;10:(S1):pages. The abstract book DOIs can be found on the "Author Information" page.
**Disclaimer:** Articles published in the journal HemaSphere exclusively reflect the opinions of the authors. The authors are responsible for all content in their abstracts including accuracy of the facts, statements, citing resources, etc.

### (PB3877) DO BORTEZOMIB-CONTAINING THERAPIES IMPROVE THE OUTCOMES IN PLASMABLASTIC LYMPHOMA?

**Topic:** 20. Other aggressive lymphomas

Denis Cetin[1], Fatma Keklik Karadag[1], Derya Demir[2], Ajda Gunes[1], Nazan Ozsan[1], Nur Soyer[1], Fahri Sahin[1], Mahmut Töbü[1], Filiz Vural[1], Mine Hekimgil[1], Guray Saydam*[1]

*[1]Ege University, Hematology, Izmir, Türkiye; [2]Ege University, Pathology, Izmir, Türkiye;*

### Background

Plasmablastic lymphoma (PBL), which is known as CD20-negative aggressive B-cell lymphoma, is rare and characterized by poor survival outcomes. Although the association between PBL and immunodeficiency conditions such as HIV and solid organ transplantation is well known, it would be seen in patients with immunocompetent. However, unusual immunophenotypic profile has been reported; PBL is typically positive for the plasma cell markers CD38, CD138, and MUM1, and upregulation of Ki67. Patients are generally treated with combination chemotherapy; however, there is no established standard of care.

### Aims

Given the rarity of PBL and the diagnostic challenges it presents, data regarding its clinical characteristics and optimal treatment strategies remain limited. We aimed to evaluate the clinical and pathological features, treatment approaches, and prognostic outcomes of patients diagnosed with PBL at our institution, with a particular focus on the impact of bortezomib-containing regimens.

### Methods

We conducted a retrospective analysis of patients diagnosed with PBL between 2010 and 2025 at Ege University. We evaluated demographic data, clinical features, management aspects, and outcomes of patients. All cases were examined based on morphological and immunohistochemical features according to the World Health Organization classification. We compare the patients who were treated with bortezomib-containing regimens and those who were not.

### Results

Overall, 37 patients were identified, with an average age of 63 (range, 30–83) years. The male-to-female ratio was 27/10, and 5 patients were HIV positive, while one had a history of kidney transplantation. Extranodal sites included the gastrointestinal tract ($n$ = 10), bone marrow ($n$ = 4), skeletal bone ($n$ = 4), head/neck ($n$ = 3), soft tissue ($n$ = 3), kidney/adrenals ($n$ = 2), and testicles ($n$ = 1). Seventeen patients received bortezomib-containing regimens [bortezomib plus CHOP (cyclophosphamide, doxorubicin, vincristine, and prednisone) or CHOP-like regimens, hyper-CVAD, and EPOCH (etoposide, prednisone, vincristine, cyclophosphamide, and doxorubicin)], and 20 were treated without bortezomib. The overall response rate (ORR) was 52.9% (9/17) in patients treated with bortezomib-containing regimens, while it was 50% (10/10) in patients treated without bortezomib. Median overall survival (OS) was 20 months (range, 1-167). There was no association between OS rates and HIV status, bortezomib-containing regimen, bulky disease, and EBER status.

**Copyright Information:** (Online) ISSN: 2572-9241
©2026 The Author(s). HemaSphere is published by John Wiley & Sons Ltd on behalf of European Hematology Association. This is an open access Abstract Book distributed under the Attribution-NonCommercial-NoDerivs (CC BY-NC-ND), which allows third parties to download the articles and share them with others as long as they credit the author and the Abstract Book, but they cannot change the content in any way or use them commercially.
**Abstract Book Citations:** Authors, Title, HemaSphere, 2026;10:(S1):pages. The abstract book DOIs can be found on the “Author Information” page.
**Disclaimer:** Articles published in the journal HemaSphere exclusively reflect the opinions of the authors. The authors are responsible for all content in their abstracts including accuracy of the facts, statements, citing resources, etc.

**Summary/Conclusion**

PBL originates from activated B cells with plasmacytic differentiation, and bortezomib has been considered a promising therapeutic agent due to its activity against plasma cells. Although recent studies have reported favorable outcomes with the bortezomib-EPOCH regimen, including a 5-year OS rate of up to 65%, this benefit was not confirmed in our real-world cohort. However, the small sample size may have limited the statistical power of our analysis. Larger, multicenter studies are warranted to clarify the role of bortezomib in the treatment of PBL.

**Copyright Information:** (Online) ISSN: 2572-9241
©2026 The Author(s). HemaSphere is published by John Wiley & Sons Ltd on behalf of European Hematology Association. This is an open access Abstract Book distributed under the Attribution-NonCommercial-NoDerivs (CC BY-NC-ND), which allows third parties to download the articles and share them with others as long as they credit the author and the Abstract Book, but they cannot change the content in any way or use them commercially.
**Abstract Book Citations:** Authors, Title, HemaSphere, 2026;10:(S1):pages. The abstract book DOIs can be found on the "Author Information" page.
**Disclaimer:** Articles published in the journal HemaSphere exclusively reflect the opinions of the authors. The authors are responsible for all content in their abstracts including accuracy of the facts, statements, citing resources, etc.

### (PB3878) GOLIDOCITINIB-BASED COMBINATION THERAPY AS FIRST-LINE TREATMENT FOR PTCL: A SINGLE-CENTER RETROSPECTIVE ANALYSIS (JACKPOT55)

**Topic:** 20. Other aggressive lymphomas

Wenyu Li*[1], Ling Huang[1]

*[1]GuangDong Provincial People's Hospital, GuangDong, China;*

### Background

Peripheral T-cell lymphoma (PTCL), except for the anaplastic large cell lymphoma (ALCL) subtype, is characterized by a poor prognosis and low overall survival rate. Golidocitinib, a selective JAK1 tyrosine-kinase inhibitor, was granted conditional approval in China for the treatment of patients with relapsed or refractory PTCL (r/r PTCL), based on its promising anti-tumor activity in a phase 2 pivotal study (JACKPOT8 Part B). Here we explored anti-tumor efficacy and safety of golidocitinib in combination with other agents as first-line therapy in patients with PTCL.

### Aims

To evaluate the efficacy and safety of golidocitinib combination with CHOP-like chemotherapy (Go-CHOP) as first-line treatment for patients with PTCL. After completion of Go-CHOP induction phase, patients will continue with golidocitinib monotherapy as maintenance treatment until discontinuation criteria were met.

### Methods

This is a real-world study. Between July 2024 and January 2026, 21 adult patients (>18 years old) with PTCL who received first-line golidocitinib plus CHOP-like chemotherapy were identified at Guangdong Provincial People's Hospital. Patient demographics, clinical characteristics and treatment modalities were summarized.

### Results

Among 21 patients enrolled into the study, the median age was 63 years (range, 40-74), 9 patients (42.9%) were male, 7 (33.3%) had ECOG PS of 0-1, 18 (85.7%) had stage III-IV disease, and 18 (85.7%) had an IPI score of 3-5. Seven patients (33.3%) had bone marrow involvement before treatment, and 18 (85.7%) had extranodal involvement (with 17 patients having ≥2 extranodal involvement). One patient (4.8%) was diagnosed with EATL, 13 patients (61.9%) were diagnosed with AITL, 4 patients (19%) were diagnosed with PTCL-NOS. ALL patients received golidocitinib plus CHOP-like chemotherapy as first-line treatment. One patient (5.0%) received golidocitinib 150 mg every day (QD), 5 patients (23.8%) received golidocitinib 150 mg QD 2 week-on and 1 week-off and 15 patients (71.4%) received golidocitinib 150 mg every other day (QOD) 2 week-on and 1 week-off.
Among 19 efficacy-evaluable patients, 13 achieved complete response (CR) and 4 achieved partial response (PR) at mid-term evaluation, yielding an overall response rate (ORR) of 89.5% and a CR rate of 68.4%. 10 patients (47.6%) were still ongoing with the treatment, and the longest duration of treatment was longer than 18 months. Treatment-related adverse events (TRAEs) were observed in 16 patients (76.2%), with the most common adverse events (7, 33.3%) of cytomegalovirus reactivation, followed by leukopenia, febrile neutropenia, and thrombocytopenia (4 each, 19%), infectious pneumonia (3,14.3%), and rash, abnormal liver function and herpes simplex (1 each, 4.8%). One patient permanently withdrew from treatment to receive stem cell transplant.

**Copyright Information:** (Online) ISSN: 2572-9241
©2026 The Author(s). HemaSphere is published by John Wiley & Sons Ltd on behalf of European Hematology Association. This is an open access Abstract Book distributed under the Attribution-NonCommercial-NoDerivs (CC BY-NC-ND), which allows third parties to download the articles and share them with others as long as they credit the author and the Abstract Book, but they cannot change the content in any way or use them commercially.
**Abstract Book Citations:** Authors, Title, HemaSphere, 2026;10:(S1):pages. The abstract book DOIs can be found on the "Author Information" page.
**Disclaimer:** Articles published in the journal HemaSphere exclusively reflect the opinions of the authors. The authors are responsible for all content in their abstracts including accuracy of the facts, statements, citing resources, etc.

**Summary/Conclusion**

Golidocitinib combined with CHOP-like chemotherapy regimens demonstrated encouraging anti-tumor efficacy and clinically manageable safety profile in patients with newly diagnosed PTCL. Prospective studies to further validate these findings are warranted.

**Copyright Information:** (Online) ISSN: 2572-9241
©2026 The Author(s). HemaSphere is published by John Wiley & Sons Ltd on behalf of European Hematology Association. This is an open access Abstract Book distributed under the Attribution-NonCommercial-NoDerivs (CC BY-NC-ND), which allows third parties to download the articles and share them with others as long as they credit the author and the Abstract Book, but they cannot change the content in any way or use them commercially.
**Abstract Book Citations:** Authors, Title, HemaSphere, 2026;10:(S1):pages. The abstract book DOIs can be found on the "Author Information" page.
**Disclaimer:** Articles published in the journal HemaSphere exclusively reflect the opinions of the authors. The authors are responsible for all content in their abstracts including accuracy of the facts, statements, citing resources, etc.

### (PB3879) TREATMENT OUTCOMES AND RELAPSE DYNAMICS IN BURKITT LYMPHOMA: REAL-WORLD DATA FROM A NATIONAL LATIN AMERICAN REFERENCE CENTER

**Topic:** 20. Other aggressive lymphomas

Amaya Llorente-Chávez*[1], Andy Mejía-Castro[1], Amairani Carpintero-Díaz[1], Rodrigo Figueroa-Méndez[2], Diana Laura de la Torre-Torres[1], Karla López-Cisneros[1], Cindy Bracamontes-Nolasco[1], Karla Sánchez Hernández[1], Luis Felipe Rubalcava Lara[1], Irma Karen Pellon Tellez[1]

*[1]Instituto Nacional de Cancerología, Hematology, Mexico City, Mexico;[2]Instituto Nacional de Ciencias Médicas y Nutrición Salvador Zubirán, Mexico City, Mexico;*

### Background

Burkitt lymphoma (BL) is a highly aggressive mature B-cell neoplasm characterized by rapid proliferation, extranodal involvement, and  treatment-related morbidity.

### Aims

Describe clinical characteristics and outcomes of patients with BL in a national Latin American reference center.

### Methods

We conducted a retrospective cohort study including patients (≥18 years) diagnosed with BL between 2011 to 2025. Overall survival (OS), and relapse-free survival (RFS) were estimated using Kaplan-Meier method.

**Copyright Information:** (Online) ISSN: 2572-9241
©2026 The Author(s). HemaSphere is published by John Wiley & Sons Ltd on behalf of European Hematology Association. This is an open access Abstract Book distributed under the Attribution-NonCommercial-NoDerivs (CC BY-NC-ND), which allows third parties to download the articles and share them with others as long as they credit the author and the Abstract Book, but they cannot change the content in any way or use them commercially.
**Abstract Book Citations:** Authors, Title, HemaSphere, 2026;10:(S1):pages. The abstract book DOIs can be found on the “Author Information” page.
**Disclaimer:** Articles published in the journal HemaSphere exclusively reflect the opinions of the authors. The authors are responsible for all content in their abstracts including accuracy of the facts, statements, citing resources, etc.

**Results**

A total of 67 patients were included, of whom 65.7% were male. Regarding functional status, 59.7% had an ECOG of 0–1, while 40% had ≥2.
At diagnosis, 53.7% presented with B symptoms, and tumor lysis syndrome (TLS) in 13.4%. The median lactate dehydrogenase level was 450 IU/L (IQR: 251–952), median β2-microglobulin was 3.5 mg/L (IQR: 2.4–4.9), and lymphopenia was present in 22.4%.
Stage IV disease predominated in 83.6%, whereas only 12% had early stage disease (I-II). The median number of extranodal sites was 3 (IQR: 1–4), and bulky disease was documented in 29.9%. Extranodal involvement included bone marrow in 34%, gastrointestinal tract in 58.2%, and central nervous system (CNS) in 31%. CNS disease was classified as leptomeningeal in 16.4%, parenchymal in 3%, and combined involvement in 10%. The median BLIPI score was 1 (IQR, 1–2), with 20.3% classified as low risk, 35.8% as intermediate risk, and 43.3% as high risk. The median IPI score was 3 (IQR, 2–4).
All cases exhibited a "starry-sky" pattern on morphology, with a median Ki-67 index of 95% (IQR: 90-99). Immunophenotypically, lymph nodes expressed CD10 (92.5%), CD20 (94%), BCL6 (84%), and MYC (58%). FISH analysis was scarce at our center, with MYC rearrangements confirmed in only 23.9% of cases, predominantly t(8;14). EBER positivity was observed in 22.4%.
Concomitant HIV infection was identified in 43.3%. The median viral load at BL diagnosis was 118,048 copies/mL (IQR: 22,533-401,258), with only 7.5% achieving undetectable levels. The median CD4 count was 177 cells/μL (IQR: 100–350), and only 17.9% were receiving antiretroviral therapy (ART) prior to BL.
First-line treatment included R-DA-EPOCH in 43.3%, R-CODOX/M-IVAC in 26.9%, HCVAD in 11.9%, and alternative regimens in 17.9%. The median number of chemotherapy cycles was 4 (IQR: 2–6). Treatment interruptions ocurred in approximately one-third of cases, primarily due to inaceptable toxicity or death. Hematologic toxicity occurred in 70.1%, non-hematologic toxicity in 26.9%, and infections in 59.7%.
Final PET-CT demonstrated complete response in 44.8%, partial response (PR) in 9%, and progressive disease in 9%. Relapse occurred in 17.9%, predominantly at extranodal sites, particularly the CNS.
Median OS was 17 months (IQR: 5–68), and overall mortality was 20.9%; mainly attributable to infections and disease progression. Non-hematologic toxicity (p=0.042) and interim PET-CT PR (p=0.001) were associated with mortality.
Median RFS was 12 months (IQR: 5–67), and relapsed patients had inferior OS (median 9.5 months). Variables associated with relapse included TLS (p=0.047), ART other than NNRTI-based therapy (p = 0.046), CNS infiltration (p = 0.048), HIV (p = 0.05), and PR on interim PET-CT (p = 0.013).

**Summary/Conclusion**

BL presented predominantly as advanced-stage disease with high extranodal burden, substantial treatment-related toxicity, and modest survival outcomes. CNS involvement, HIV, TLS, and suboptimal treatment responses were key determinants of prognosis.

**Copyright Information:** (Online) ISSN: 2572-9241
©2026 The Author(s). HemaSphere is published by John Wiley & Sons Ltd on behalf of European Hematology Association. This is an open access Abstract Book distributed under the Attribution-NonCommercial-NoDerivs (CC BY-NC-ND), which allows third parties to download the articles and share them with others as long as they credit the author and the Abstract Book, but they cannot change the content in any way or use them commercially.
**Abstract Book Citations:** Authors, Title, HemaSphere, 2026;10:(S1):pages. The abstract book DOIs can be found on the "Author Information" page.
**Disclaimer:** Articles published in the journal HemaSphere exclusively reflect the opinions of the authors. The authors are responsible for all content in their abstracts including accuracy of the facts, statements, citing resources, etc.

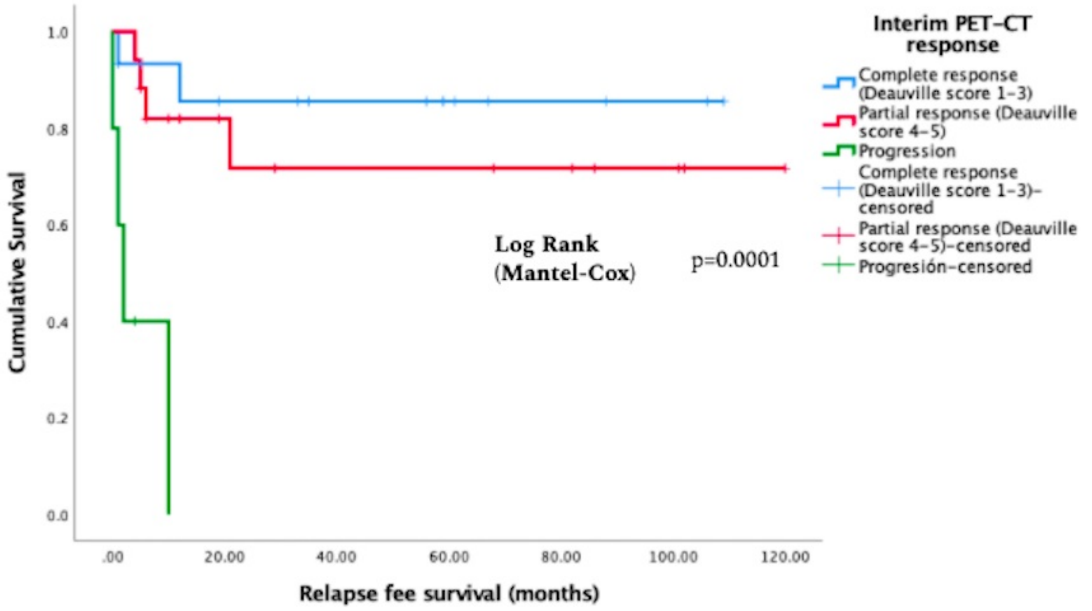

**Number at risk**

| | | | | | | | |
|---|---|---|---|---|---|---|---|
| Complete response | 15 | 10 | 8 | 6 | 3 | 2 | 1 |
| Partial response | 17 | 8 | 6 | 5 | 4 | 2 | 1 |
| Progression | 5 | 0 | 0 | 0 | 0 | 0 | 0 |

**Copyright Information:** (Online) ISSN: 2572-9241
©2026 The Author(s). HemaSphere is published by John Wiley & Sons Ltd on behalf of European Hematology Association. This is an open access Abstract Book distributed under the Attribution-NonCommercial-NoDerivs (CC BY-NC-ND), which allows third parties to download the articles and share them with others as long as they credit the author and the Abstract Book, but they cannot change the content in any way or use them commercially.
**Abstract Book Citations:** Authors, Title, HemaSphere, 2026;10:(S1):pages. The abstract book DOIs can be found on the "Author Information" page.
**Disclaimer:** Articles published in the journal HemaSphere exclusively reflect the opinions of the authors. The authors are responsible for all content in their abstracts including accuracy of the facts, statements, citing resources, etc.

### (PB3880) BRENTUXIMAB VEDOTIN IN COMBINATION WITH CHEMOTHERAPY FOR PEDIATRIC RELAPSED/REFRACTORY ALK+ALCL PATIENTS IN CHINA

**Topic:** 20. Other aggressive lymphomas

Qing Zhu[1], Ying Liu[1], Yang Liu[1], Pei Zhang[1], Yali Peng[1], Guomin Wang[1, 1], Qinlong Zheng[1], Chunju Zhou[1], Yonghong Zhang*[1]

[1]*Beijing Gobroad Boren Hospital, Beijing, China;*

### Background

ALCL accounts for 10%–15% of pediatric NHL, with 90% being ALK+ALCL. Over 30% of pediatric ALK+ALCL patients experience relapse following first-line chemotherapy. ALK+ALCL lacks specific immune markers and is typically characterized by CD30 positivity and aberrant ALK gene activation. Brentuximab vedotin (BV), an anti-CD30 targeted antibody-drug conjugate, has demonstrated favorable efficacy and safety in R/R CD30-positive lymphomas; however, data on its use in pediatric R/R-ALK+ALCL remain scarce.

### Aims

To investigate the clinical efficacy and safety of BV combined with chemotherapy in pediatric patients with R/R-ALK+ALCL in a real-world setting.

### Methods

We retrospectively analyzed clinical data of 17 pediatric patients with R/R-ALK+ALCL treated at our institution from January 2020 to December 2025. Pathological diagnoses were confirmed by multidisciplinary expert consultation. Staging was performed using IPNHLSS. Assessment of bone marrow and central nervous system involvement integrated cytomorphology, flow cytometry immunophenotyping,and quantitative ALK gene detection. Risk stratification was based on pathological subtype, gene mutation profile, treatment reaction and time from diagnosis to progression/relapse. Primary treatment regimens consisted of BV+CHP or BV + ALCL99(BV dose: 1.8 mg/kg per administration). Patients achieving mCR or iCR received maintenance therapy with VBL+ALK inhibitor or allo-HSCT. The follow-up cutoff date was December 31, 2025.

**Copyright Information:** (Online) ISSN: 2572-9241
©2026 The Author(s). HemaSphere is published by John Wiley & Sons Ltd on behalf of European Hematology Association. This is an open access Abstract Book distributed under the Attribution-NonCommercial-NoDerivs (CC BY-NC-ND), which allows third parties to download the articles and share them with others as long as they credit the author and the Abstract Book, but they cannot change the content in any way or use them commercially.
**Abstract Book Citations:** Authors, Title, HemaSphere, 2026;10:(S1):pages. The abstract book DOIs can be found on the "Author Information" page.
**Disclaimer:** Articles published in the journal HemaSphere exclusively reflect the opinions of the authors. The authors are responsible for all content in their abstracts including accuracy of the facts, statements, citing resources, etc.

**Results**

Among the 17 patients (11 males, 6 females), the median age at onset was 9.93 years.The median time from initial diagnosis to disease progression/relapse was 7.47 months. All patients had undergone multiple lines of therapy after relapse, including VBL monotherapy, ALK inhibitor monotherapy, VBL+ALK inhibitor,second-line chemotherapy combined with targeted therapy,allo-HSCT, and radiotherapy, but failed to achieve sustained CR or relapsed after temporary remission.
Re-staging before BV treatment: 1 case of stage I, 4 cases of stage III, and 12 cases of stage IV(10 with isolated BM, 2 with BM + CNS involvement). All 17 patients were classified as high-risk. Treatment distribution: 13 patients received BV+CHP, and 4 received BV+modified ALCL99. The median number of BV cycles was 4. With a median follow-up of 21.9 months, the ORR was 100%, including 47.0% mCR (8/17)and 47.0% iCR(8/17)and 5.8% PR(1/17).all patients continued maintenance therapy with VBL+ALK inhibitor, and 3 underwent allo-HSCT. Follow-up outcomes: Two patients experienced early relapse after BV treatment. One patient achieved CR following allo-HSCT,but ultimately died of infection. Another patient achieved a second CR with VBL combined with a novel ALK inhibitor.At the last follow-up, 4 patients were off therapy for observation, and 14 continued maintenance therapy with VBL +ALK inhibitor (92.8% mCR, 7.1% iCR). The 2-year PFS after BV treatment was 0.88 (95%CI: 0.60–0.97), and the 2-year OS was 0.93 (95%CI: 0.62–0.99).
Regarding safety:adverse included fever, rash, nausea, neutropenia, and peripheral neuritis. All adverse events were graded according to NCI CTCAE version 5.0, with no grade ≥3 adverse events reported.

**Summary/Conclusion**

Brentuximab vedotin combined with chemotherapy achieves a 100% ORR in pediatric patients with R/R-ALK+ALCL, with significant short-term efficacy and long-term survival benefits, as well as a favorable safety profile. This regimen provides a novel therapeutic option for heavily pretreated pediatric patients with R/R-ALK+ALCL.

**Copyright Information:** (Online) ISSN: 2572-9241
©2026 The Author(s). HemaSphere is published by John Wiley & Sons Ltd on behalf of European Hematology Association. This is an open access Abstract Book distributed under the Attribution-NonCommercial-NoDerivs (CC BY-NC-ND), which allows third parties to download the articles and share them with others as long as they credit the author and the Abstract Book, but they cannot change the content in any way or use them commercially.
**Abstract Book Citations:** Authors, Title, HemaSphere, 2026;10:(S1):pages. The abstract book DOIs can be found on the “Author Information” page.
**Disclaimer:** Articles published in the journal HemaSphere exclusively reflect the opinions of the authors. The authors are responsible for all content in their abstracts including accuracy of the facts, statements, citing resources, etc.

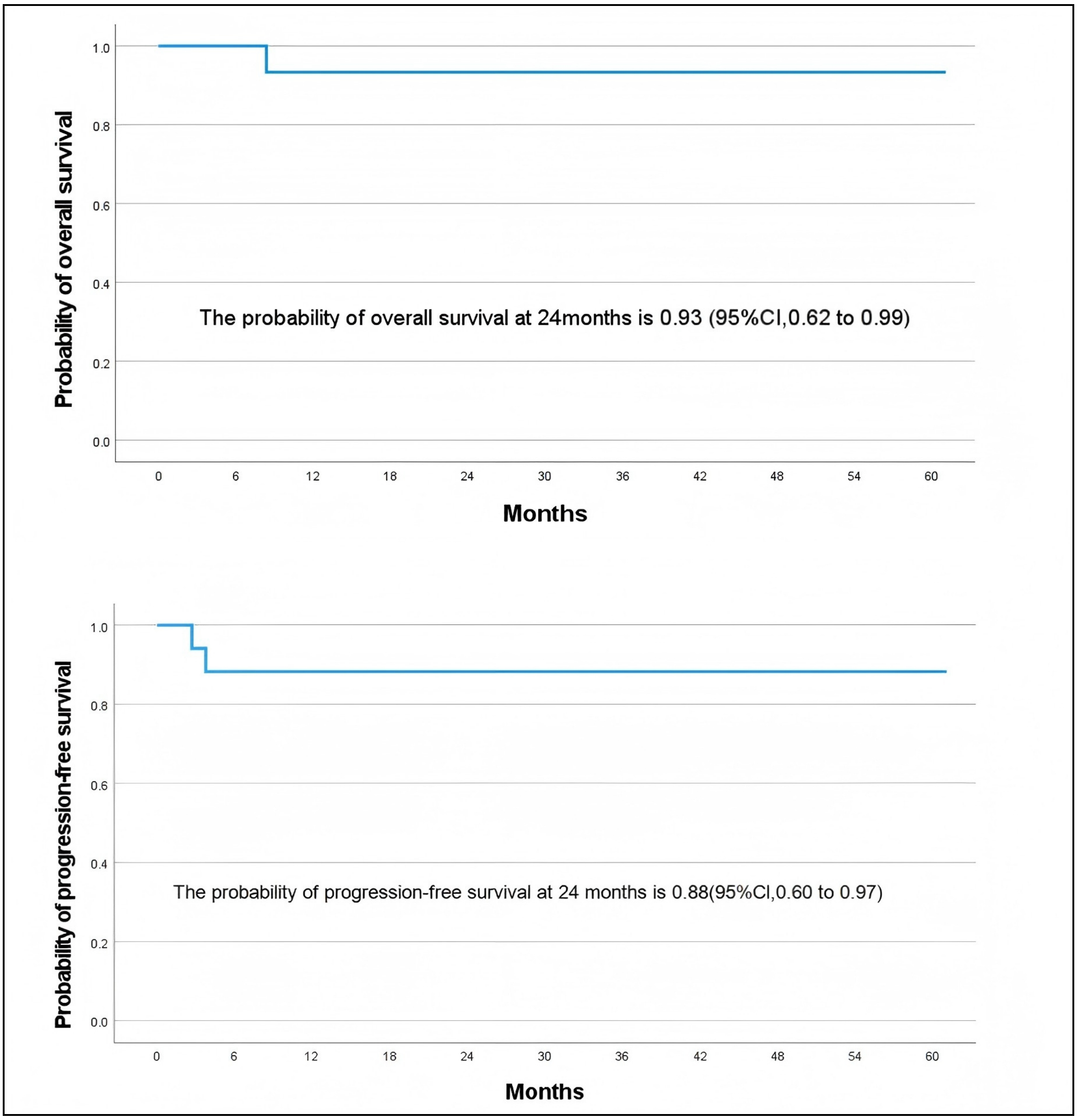

Kaplan-Meier curves of Progression-Free Survival(PFS) and overall survival (OS) in pediatric patients with ALK+ anaplastic large cell lymphoma (ALK+ALCL). The 24-month EFS probability is 0.88 (95%CI: 0.60 to 0.97), and the 24-month OS probability is 0.93 (95%CI: 0.62 to 0.99).

**Copyright Information:** (Online) ISSN: 2572-9241
©2026 The Author(s). HemaSphere is published by John Wiley & Sons Ltd on behalf of European Hematology Association. This is an open access Abstract Book distributed under the Attribution-NonCommercial-NoDerivs (CC BY-NC-ND), which allows third parties to download the articles and share them with others as long as they credit the author and the Abstract Book, but they cannot change the content in any way or use them commercially.
**Abstract Book Citations:** Authors, Title, HemaSphere, 2026;10:(S1):pages. The abstract book DOIs can be found on the "Author Information" page.
**Disclaimer:** Articles published in the journal HemaSphere exclusively reflect the opinions of the authors. The authors are responsible for all content in their abstracts including accuracy of the facts, statements, citing resources, etc.

### (PB3881) RUXOLITINIB PENETRATES BLOOD BRAIN BARRIER AND REDUCES THE CYTOKINE STORM IN PATIENTS WITH HEMOPHAGOCYTIC LYMPHOHISTIOCYTOSIS

**Topic:** 20. Other aggressive lymphomas

Kai Qing*[1], Shishuang Wu[1], Fang Jie[2], Wenjun Ji[3], Jiayi Ren[1], Yuting Tao[4], Kai Xue[1]

*[1]Ruijin Hospital School of Medicine, Shanghai Jiao Tong University, Shanghai Institute of Hematology, State Key Laboratory of Medical Genomics, National Research Center for Translational Medicine at Shanghai, Shanghai, China; [2]Ruijin Hospital School of Medicine, Shanghai Jiao Tong University, Department of Pharmacy, Shanghai, China; [3]the Affiliated Taizhou People's Hospital of Nanjing Medical University, Department of Pharmacy, Taizhou, China; [4]Liquan Hospital, Department of Pharmacy, Shanghai, China;*

### Background

Hemophagocytic lymphohistiocytosis (HLH) with CNS involvement causes high mortality. However, owing to its severity and complexity, HLH is frequently subject to clinical misdiagnosis and missed diagnosis. The median survival for untreated HLH patients is ≤ 2 months. CNS involvement occurs in 30-73% patients and represents an independent risk factor for death. Cerebrospinal fluid cytokines including sCD25, IL-10, IL-18, IFN-γ and CXCL9 are relevant to CNS-HLH. JAK/STAT pathway modulates these inflammatory factors. Ruxolitinib, a JAK1/2 inhibitor, has been recommended for HLH, but conventional studies reported limited BBB penetration.

### Aims

To evaluate BBB permeability of ruxolitinib and its CSF concentrations in HLH patients with and without CNS involvement.

### Methods

We analyzed 17 CSF samples from 10 secondary HLH patients (2 CNS+, 8 CNS-) receiving oral ruxolitinib (20 mg/day) at Ruijin Hospital (Feb-Aug 2025). Diagnosis required neurological symptoms, CSF abnormalities, or radiographic lesions. Ruxolitinib concentrations were measured by LC-MS/MS. BBB penetration was calculated as CSF/plasma ratio. Pearson correlation analyzed CSF-plasma associations ($P<0.05$ significant).

**Copyright Information:** (Online) ISSN: 2572-9241
©2026 The Author(s). HemaSphere is published by John Wiley & Sons Ltd on behalf of European Hematology Association. This is an open access Abstract Book distributed under the Attribution-NonCommercial-NoDerivs (CC BY-NC-ND), which allows third parties to download the articles and share them with others as long as they credit the author and the Abstract Book, but they cannot change the content in any way or use them commercially.
**Abstract Book Citations:** Authors, Title, HemaSphere, 2026;10:(S1):pages. The abstract book DOIs can be found on the "Author Information" page.
**Disclaimer:** Articles published in the journal HemaSphere exclusively reflect the opinions of the authors. The authors are responsible for all content in their abstracts including accuracy of the facts, statements, citing resources, etc.

### Results

We included 10 secondary HLH patients (2 with CNS involvement, 8 without). Two CNS-HLH patients presented with active neuroinflammation (median ferritin: 5120.5 ng/ml; sCD25: 4772.5 pg/mL). CSF showed elevated protein (1915.12 mg/L) and sCD25 (124 pg/mL and 70 pg/mL respectively). During active disease, BBB permeability reached 15.98% (CSF: 5.05 ng/ml) and 14.04% (CSF: 4.01 ng/ml); post-treatment values decreased to 9.15% and 7.72%, correlating with clinical stabilization (Figure 1).
Comparative analysis (CNS-involved n=2 vs non-CNS n=8) showed median ages of 41.5 vs 54.5 years. Both cohorts exhibited persistent high fever (>38.5°C). The CNS group maintained higher inflammatory loads (ferritin: 5120.5 vs 4074.34 ng/ml; sCD25: 1971.5 vs 1734.36 pg/ml).
The LC-MS/MS analysis of ruxolitinib facilitated the assessment of its BBB permeability. The value of ruxolitinib $C_{min}$ was 3.86 ± 0.47 ng/ml in CSF and 34.97 ± 3.60 ng/ml in the plasma. The BBB permeability of ruxolitinib was 11.33 ± 0.89% (between 5.31% and 18.08%) (Table 1).

| Sample | BBB penetration of ruxolitinib (%) |
|---|---|
| 1 | 9.76 |
| 2 | 7.72 |
| 3 | 14.04 |
| 4 | 18.08 |
| 5 | 15.84 |
| 6 | 11.59 |
| 7 | 15.98 |
| 8 | 7.22 |
| 9 | 12.30 |
| 10 | 9.15 |
| 11 | 8.20 |
| 12 | 7.12 |
| 13 | 11.25 |
| 14 | 5.31 |
| 15 | 15.80 |
| 16 | 11.56 |
| Mean±SD | 11.31±0.95 |

BBB permeability analysis (Figure 2) revealed mean CSF ruxolitinib of 3.86 ± 0.47 ng/ml vs plasma 34.97 ± 3.60 ng/ml. Overall BBB penetration was 11.33 ± 0.89% (range 5.31%-18.08%) .Significant CSF-plasma correlation was observed (r = 0.637, P = 0.008) (Figure 3). Median penetration was higher in CNS-involved patients (15.01% vs 12.82%), with CSF concentrations reaching therapeutic thresholds (4.53 ng/ml) for JAK/STAT inhibition.

### Summary/Conclusion

Ruxolitinib demonstrates meaningful BBB penetration (5.31%-18.08%) with higher permeability during active CNS inflammation. CSF concentrations reach therapeutic thresholds, supporting high-dose oral ruxolitinib as an alternative when intrathecal therapy is contraindicated by thrombocytopenia. Prospective studies are needed to validate optimal dosing.

**Copyright Information:** (Online) ISSN: 2572-9241
©2026 The Author(s). HemaSphere is published by John Wiley & Sons Ltd on behalf of European Hematology Association. This is an open access Abstract Book distributed under the Attribution-NonCommercial-NoDerivs (CC BY-NC-ND), which allows third parties to download the articles and share them with others as long as they credit the author and the Abstract Book, but they cannot change the content in any way or use them commercially.
**Abstract Book Citations:** Authors, Title, HemaSphere, 2026;10:(S1):pages. The abstract book DOIs can be found on the "Author Information" page.
**Disclaimer:** Articles published in the journal HemaSphere exclusively reflect the opinions of the authors. The authors are responsible for all content in their abstracts including accuracy of the facts, statements, citing resources, etc.

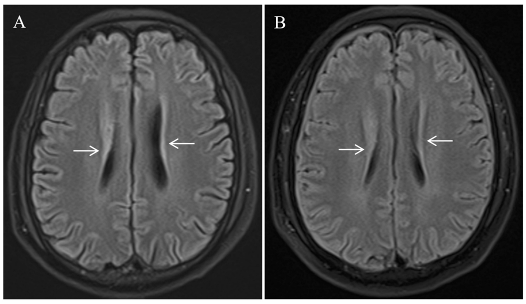

Figure 1

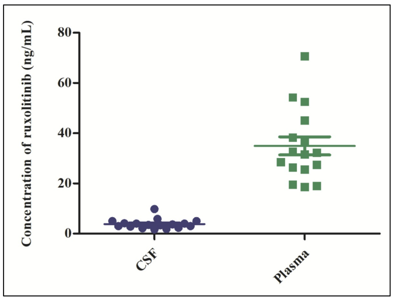

Figure 2

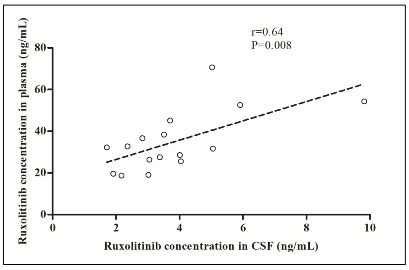

Figure 3

Figure 1. Brain MRI scans at symptom onset (A) and after symptom improvement (B) of case 1. white arrows point to the patchy hyperintensity.Figure 2. Ruxolitinib level in CSF and plasma of patients with HLH (samples=17, n=10).FIGURE 3|Correlation between ruxolitinib concentration in CSF and plasma in patients with secondary HLH (samples=16, n=10); Person correlation coefficients were employed to analyze the correlation of ruxolitinib concentration in CSF and plasma (r=0.637, P=0.008).

**Copyright Information:** (Online) ISSN: 2572-9241
©2026 The Author(s). HemaSphere is published by John Wiley & Sons Ltd on behalf of European Hematology Association. This is an open access Abstract Book distributed under the Attribution-NonCommercial-NoDerivs (CC BY-NC-ND), which allows third parties to download the articles and share them with others as long as they credit the author and the Abstract Book, but they cannot change the content in any way or use them commercially.
**Abstract Book Citations:** Authors, Title, HemaSphere, 2026;10:(S1):pages. The abstract book DOIs can be found on the "Author Information" page.
**Disclaimer:** Articles published in the journal HemaSphere exclusively reflect the opinions of the authors. The authors are responsible for all content in their abstracts including accuracy of the facts, statements, citing resources, etc.

### (PB3882) PRELIMINARY RESULTS OF INDUCTION THERAPY USING MITOXANTRONE LIPOSOMES,ETOPOSIDE,AND METHYLPREDNISOLONE FOR LYMPHOMA-ASSOCIATED HEMOPHAGOCYTIC LYMPHOHISTIOCYTOSIS

**Topic:** 20. Other aggressive lymphomas

Lu Zhang*[1], Bo Zhang[1], Liang Tang[1], Yanxue Yin[1], Tao Guo[1]

[1] *Union Hospital,Tongji Medical College, Huazhong University of Science and Technology, Wuhan, China;*

### Background

Hemophagocytic lymphohistiocytosis (HLH) is a group of hyperinflammatory syndromes caused by primary or secondary immune dysfunction. HLH occurring in the context of lymphoma diagnosis and treatment is collectively referred to as lymphoma-associated HLH (LA-HLH), the incidence of which increases with age.The prognosis of LA-HLH is extremely poor, with a 5-year overall survival rate of only about 15% and a median survival time of less than 2 months. Mitoxantrone hydrochloride liposome (Lipo-MIT) is a nano-drug that has been approved as the first treatment option for relapsed or refractory peripheral T-cell lymphoma (R/R PTCL).

### Aims

This study was designed to evaluate the effectiveness and safety of combining Lipo-MIT with etoposide, methylprednisolone (MEP) in newly diagnosed adult LA-HLH.

### Methods

This was a prospective, multi-center, single-arm, exploratory study (ChiCTR2300078813). LA-HLH patients aged 18-70 years were eligible for enrollment. All patients received induction therapy with Lipo-MIT 20 mg/m$^2$/d d1;etoposide 100 mg/m$^2$/d d1; methylprednisolone 2mg/kg/d d1–3, 0.75mg/kg/d d4–7, 0.25mg/kg/d d8–10, and 0.1mg/kg/d until the next cycle.This MEP regimen will be administered for a total of 1- 2 cycles. The primary endpoint of the study was overall response rate (ORR), with secondary endpoints including complete response (CR), partial response (PR), overall survival (OS), and safety.

### Results

As of December 3,2025, 16 patients eligible for enrollment were treated with the MEP regimen. Among enrolled patients, the median age was 56 years (range 19-65), 56.3% were male.The underlying lymphoma diagnoses included six cases of diffuse large B-cell lymphoma, ten cases of NK/T and T-cell lymphoma. Among the patients with lymphoma,68.8% were in Ann Arbor stage III–IV. All patients newly diagnosed with LA-HLH received one cycle of the MEP regimen, with a median dose of 19.5 mg/m² (range,10.7-20.8) of Lipo-MIT. A total of 14 patients were evaluable for response, The CR rate and ORR were 28.6% (4/14) and 92.9% (13/14), respectively.With a median follow-up of 2.8 months(range: 0.7-35.5 months), the median PFS and OS were not reached.The estimated 1-year PFS and OS rates were 66.7% and 58.5%, respectively. The most common grade 3/4 treatment-related adverse events (TRAEs) with an incidence ≥10% were leucopenia (50%), neutropenia (62.5%), thrombocytopenia(50%), anemia (62.5%), pneumonia(12.5%).Given that persistent cytopenia is a cardinal manifestation of active HLH, it was challenging to accurately attribute hematologic toxicity specifically to the MEP regimen.

**Copyright Information:** (Online) ISSN: 2572-9241
©2026 The Author(s). HemaSphere is published by John Wiley & Sons Ltd on behalf of European Hematology Association. This is an open access Abstract Book distributed under the Attribution-NonCommercial-NoDerivs (CC BY-NC-ND), which allows third parties to download the articles and share them with others as long as they credit the author and the Abstract Book, but they cannot change the content in any way or use them commercially.
**Abstract Book Citations:** Authors, Title, HemaSphere, 2026;10:(S1):pages. The abstract book DOIs can be found on the "Author Information" page.
**Disclaimer:** Articles published in the journal HemaSphere exclusively reflect the opinions of the authors. The authors are responsible for all content in their abstracts including accuracy of the facts, statements, citing resources, etc.

### Summary/Conclusion

Collectively, these preliminary findings suggest that the MEP regimen represents a safe and effective therapeutic strategy for patients with LA-HLH. This study is ongoing and updated results will be presented in the future.

**Table 1. Patients clinical characteristies**

| characteristics, n(%) | N=16 |
|---|---|
| Median age (range), years, | 56 (19-65) |
| Gender (Male), n (%) | 9(56.3) |
| Disease Stage III-IV, n (%) | 11 (68.8) |
| Fever, n(%) | 8 (50) |
| Splenomegaly, n(%) | 3 (18.8) |
| Neutropenia, n(%) | 7 (43.8) |
| Anemia | 15 (93.8) |
| Thrombocytopenia | 15 (93.8) |
| Elevated bilirubin | 11 (68.8) |
| Elevated alanine aminotransferase | 10 (62.5) |
| Hypertriglyceridemia | 10 (62.5) |
| Elevated sCD25 | 14 (87.5) |
| Elevated ferritin | 15 (93.8) |
| Lymphoma Subtype, n (%) | |
| DLBDL | 6 (50) |
| NKTCL | 7 (37.5) |
| PTCL | 3 (12.5) |

**Table 1. The overall efficacy results**

| Response, n (%) | N=14 |
|---|---|
| CR | 4(28.6) |
| PR | 9(64.3) |
| NR | 1(7.1) |
| ORR | 1(92.9) |

**Table 3. Incidence of Treatment-Related Adverse Events**

| Adverse Event, n(%) | Grade 1-2 | Grade 3-4 | All Grades |
|---|---|---|---|
| Hematologic Toxicity | | | |
| Leukopenia | 3(18.8) | 8(50) | 11(68.8) |
| Thrombocytopenia | 3(18.8) | 8(50) | 10(68.8) |
| Neutropenia | 0(0.0) | 10(62.5) | 10(62.5) |
| Anemia | 2(12.5) | 10(62.5) | 14(75) |
| Non-hematologic Toxicity | | | |
| Increased ALT | 4( 25) | 0(0.0) | 4( 25) |
| Hyperbilirubinemia | 3(18.8 ) | 0(0) | 3(18.8 ) |
| Pulmonary Infection | 1(6.3) | 2(12.5) | 3(18.8) |
| Fever | 1(6.3) | 1(6.3) | 2(12.5) |

**Copyright Information:** (Online) ISSN: 2572-9241
©2026 The Author(s). HemaSphere is published by John Wiley & Sons Ltd on behalf of European Hematology Association. This is an open access Abstract Book distributed under the Attribution-NonCommercial-NoDerivs (CC BY-NC-ND), which allows third parties to download the articles and share them with others as long as they credit the author and the Abstract Book, but they cannot change the content in any way or use them commercially.
**Abstract Book Citations:** Authors, Title, HemaSphere, 2026;10:(S1):pages. The abstract book DOIs can be found on the "Author Information" page.
**Disclaimer:** Articles published in the journal HemaSphere exclusively reflect the opinions of the authors. The authors are responsible for all content in their abstracts including accuracy of the facts, statements, citing resources, etc.

### (PB3883) REAL-WORLD OUTCOMES OF MITOXANTRONE HYDROCHLORIDE LIPOSOME COMBINED WITH ETOPOSIDE, METHYLPREDNISOLONE IN THE TREATMENT OF LYMPHOMA-ASSOCIATED HEMOPHAGOCYTIC LYMPHOHISTIOCYTOSIS

**Topic:** 20. Other aggressive lymphomas

Jianbo Liu[1], Boyun Shi[1], Xiaodan Luo[1], Danyun Yuan[1], Sihui Chen[1], Ying Luo[1], Runhui Zheng[1], Huo Tan*[1]

[1] *The Fifth Affiliated Hospital of Guangzhou Medical University, Guangzhou, China;*

### Background

Hemophagocytic lymphohistiocytosis (HLH) is a type of hyperinflammatory syndrome (uncontrolled inflammatory response) caused by primary or secondary immune abnormalities. HLH is a rapidly progressive and highly fatal disease, and the median survival time of untreated patients with HLH is no more than 2 months. The prognosis for lymphoma-associated HLH (LA-HLH) is very poor, so more effective treatment options need to be sought. Although DEP regimen (liposomal doxorubicin, etoposide and methylprednisolone) can be used as the initial induction therapy for LA-HLH, and can also be used as the salvage therapy for refractory patients who fail to respond to HLH-94 regimen, however, the therapeutic efficacy still requires further improvement. Mitoxantrone hydrochloride liposome (Lipo-MIT) is a novel nano-drug with lower cardiotoxicity than traditional anthracyclines, and has shown certain efficacy and safety in lymphoma.

### Aims

To evaluate the efficacy and safety of mitoxantrone hydrochloride liposome in the treatment of LA-HLH.

### Methods

In this real-world study, we enrolled HLH patients aged 18-80 years who met both the HLH-2004 diagnostic criteria and lymphoma criteria. Patients received MEP regimen (mitoxantrone liposome 10-20 mg/m$^2$ d1; Etoposide 50-100 mg/m$^2$ d1; Methylprednisolone 2 mg/kg/day d1-3, 0.75 mg/kg/day d4-8 ,0.25 mg/kg/day d9-10, 0.15 mg/kg/day d11-15 ) 3 to 4 weeks per cycle for 1-2 cycles. The primary endpoint was objective response rate (ORR). Secondary endpoints included complete remission rate (CR), partial response rate (PR), progression-free survival (PFS), overall survival (OS), and safety.

### Results

As of December, 2025, a total of 18 patients with LA-HLH were enrolled, including 12 males and 6 females, with a median age of 48.5 years (range, 24-72 years). Among them, the lymphoma subtype included DLBCL (n=2), AITL (n=5), NKTCL/PTCL-NOS (n=11). 7 cases were stage III-IV. 11 cases had IPI score≥3 and relapsed/refractory LA-HLH (n=13). The median dose of Lipo-MIT was 15.67mg/m$^2$ (range: 10.3-19.6mg/m$^2$). Of these, 17 patients were evaluable efficacy. The ORR of HLH was 82.3% (14/17), of which 13.6% (3/17) achieved CR and 64.7% (11/17) achieved PR. The ORR of the newly diagnosed HLH (n=5) was 100%, 1 case achieved CR and 4 achieved PR. The ORR of the refractory HLH group (n=12) was 66.7%, with 2 and 6 patients achieving CR and PR, respectively. In the 11 NKTCL/ PTCL-NOS subgroup, 2 cases achieved CR, 8 cases PR and 1 case PD. In the 2 DLBCL subgroups, 1 patient achieved CR and 1 patient achieved PR. Two patients in the 4 AITL subgroup achieved PR. Other efficacy indicators such as PFS and OS will be reported after a long-term follow-up. The hematological grade ≥3 TRAEs including neutropenia (44.4%), leukopenia (50.0%), thrombocytopenia (66.7%), and anemia (66.7%). The common non-hematological grade ≥3 TRAEs were fever and infection. It should be noted that cytopenia is a hallmark of active HLH, making precise attribution to therapy challenging.

**Copyright Information:** (Online) ISSN: 2572-9241
©2026 The Author(s). HemaSphere is published by John Wiley & Sons Ltd on behalf of European Hematology Association. This is an open access Abstract Book distributed under the Attribution-NonCommercial-NoDerivs (CC BY-NC-ND), which allows third parties to download the articles and share them with others as long as they credit the author and the Abstract Book, but they cannot change the content in any way or use them commercially.
**Abstract Book Citations:** Authors, Title, HemaSphere, 2026;10:(S1):pages. The abstract book DOIs can be found on the “Author Information” page.
**Disclaimer:** Articles published in the journal HemaSphere exclusively reflect the opinions of the authors. The authors are responsible for all content in their abstracts including accuracy of the facts, statements, citing resources, etc.

### Summary/Conclusion

The MEP regimen showed good tolerance and clinically meaningful efficacy in the treatment of LA-HLH.

**Table 1. Patients clinical characteristics**

| characteristics | ALL(N=18) |
|---|---|
| General | |
| Median age (range), years | 48.5(24-72) |
| Gender (male), n (%) | 12(66.7) |
| Ann Arbor Stage III–IV, n (%) | 7(38.9) |
| IPI score≥3, n (%) | 11(61.1) |
| ECOG-PS≥2, n (%) | 6(33.3) |
| Clinical features | |
| Fever, n (%) | 13(72.2) |
| Splenomegaly, n (%) | 7(38.9) |
| Neutropenia, n (%) | 8(44.4) |
| Anemia, n (%) | 17(94.4) |
| Thrombocytopenia, n (%) | 15(83.3) |
| Ferritin↑, n (%) | 16(88.9) |
| Hypofibrinogen, n (%) | 10(55.5) |
| EBV+, n (%) | 13(72.2) |
| HLH onset | |
| At lymphoma diagnosis | 5(27.8) |
| At lymphoma relapse | 13(72.2) |

**Table2 The overall optimun efficacy results**

| Efficacy, n (%) | ALL N=17 | NKTCL N=7 | PTCL-NOS N=4 | AITL N=4 | DLBDL N=2 |
|---|---|---|---|---|---|
| CR | 3(17.6) | 1(14.3) | 1(25.0) | 0 | 1(50.0) |
| PR | 11(64.7) | 5(71.4) | 3(75.0) | 2(50.0) | 1(50.0) |
| SD | 1(5.9) | 0 | 0 | 1(25.0) | 0 |
| ORR | 14(82.3) | 6(81.8) | 4(100.0) | 2(50.0) | 2(100.0) |

**Table3 Treatment-related adverse events**

| TRAEs，n(%) | Grades 1-2 | Grades 3-4 | Any Grades |
|---|---|---|---|
| Hematological toxicities | | | |
| Neutropenia | 5(27.8) | 8(44.4) | 13(72.2) |
| Leukopenia | 6(33.3) | 9(50.0) | 15(83.3) |
| Thrombocytopenia | 4(22.2) | 12(66.7) | 16(88.9) |
| Anemia | 5(27.8) | 12(66.7) | 17(94.4) |
| Nonhematological toxicities | | | |
| Infection | 4(22.2) | 2(11.1) | 6(33.3) |
| fever | 4(22.2) | 2(11.1) | 6(33.3) |
| fatigue | 2(11.1) | 0 | 2(11.1) |
| Nosebleed | 0 | 1(5.5) | 1(5.5) |

**Copyright Information:** (Online) ISSN: 2572-9241
©2026 The Author(s). HemaSphere is published by John Wiley & Sons Ltd on behalf of European Hematology Association. This is an open access Abstract Book distributed under the Attribution-NonCommercial-NoDerivs (CC BY-NC-ND), which allows third parties to download the articles and share them with others as long as they credit the author and the Abstract Book, but they cannot change the content in any way or use them commercially.
**Abstract Book Citations:** Authors, Title, HemaSphere, 2026;10:(S1):pages. The abstract book DOIs can be found on the "Author Information" page.
**Disclaimer:** Articles published in the journal HemaSphere exclusively reflect the opinions of the authors. The authors are responsible for all content in their abstracts including accuracy of the facts, statements, citing resources, etc.

### (PB3884) ANALYSIS OF TREATMENT OUTCOMES AND PROGNOSIS IN 52 CASES OF ANAPLASTIC LARGE CELL LYMPHOMA WITH LYMPHOMA KINASE MUTATION

**Topic:** 20. Other aggressive lymphomas

Wen Zhang*[1], Mengyu Ruan[1], Zhuoxin Zhang[1], Wei Liu[1], Tingyu Wang[1], Weiwei Sui[1], Wenjie Xiong[1], Yi Wang[1], Qi Wang[1], Wenyang Huang[1], Lugui Qiu[1], Dehui Zou[1]

*[1]State Key Laboratory of Experimental Hematology, National Clinical Research Center for Blood Diseases, Haihe Laboratory of Cell Ecosystem, Institute of Hematology& Blood Diseases Hospital, Chinese Academy of Medical Sciences & Peking Union Medical College, tianjin, China;*

### Background

Anaplastic large cell lymphoma (ALCL) is a rare subtype of peripheral T-cell lymphoma that originates in the lymph nodes. Compared to ALK-ALCL, ALK+ALCL is more commonly observed in children and young men, responds better to chemotherapy and generally has favourable outcomes. However, the factors that influence treatment efficacy, and whether patients with this subtype may benefit from autologous stem cell transplantation, remain controversial.

### Aims

To analyze the clinical characteristics of anaplastic large cell lymphoma (ALCL) with anaplastic lymphoma kinase (ALK) positivity, and to explore the treatment strategies, treatment outcomes, and prognostic factors.

### Methods

The clinical data and survival information of 52 patients with ALK+ ALCL who were treated to the Institute of Hematology, Chinese Academy of Medical Sciences between May 2011 and November 2024 were retrospectively analyzed.

### Results

The median age at onset for the 52 patients was 25.5 years (range: 11–64 years), with a male-to-female ratio of 2.3:1. At initial diagnosis, 42 patients (81.1%) presented with Ann Arbor stage III–IV disease, 31 (59.6%) had extranodal involvement, and 11 (21.2%) had an IPI score in the intermediate-high/high-risk group (3–5 points). The first-line regimen was EDOCH/EPOCH/CHOPE in 36 patients (69.2%), BV+CHP in 11 patients (21.2%), and CHOP in 5 patients (9.6%). The overall complete response (CR) rate was 75%, and the overall response rate (ORR) was 88.5%. The median follow-up duration was 45.7 months. The 5-year progression-free survival (PFS) and overall survival (OS) rates were 67.6% and 91.1%, respectively. Induction therapy failure occurred in 15 patients and was an adverse factor affecting OS (P=0.017), with 5-year and 10-year OS rates of 85.6% and 57.0%, respectively. Subgroup analysis revealed that among IPI 0-2 patients, the 5-year PFS rate for the EDOCH/EPOCH/CHOPE regimen significantly exceeded that of the CHOP regimen (73.1% vs 30%, P=0.028). Among IPI ≥3 patients, the 5-year OS rates for those receiving first-line ASCT versus those not receiving first-line ASCT were 100% and 80%, respectively, and 5-year PFS rates of 80% and 50%, respectively (P=0.36; P=0.25). Among patients with intermediate PR, the 5-year OS rates for those receiving first-line ASCT versus those not receiving first-line ASCT were 100% and 60%, respectively, and the 5-year PFS rates were 100% and 48% (P=0.24; P=0.088). Among 15 relapsed/refractory patients, 12 who achieved CR/PR after second- or third-line therapy followed by ASCT had a 2-year OS rate of 100%. The 2-year OS rate for the remaining 3 patients who did not receive ASCT was 66.7% (P=0.00041).

**Copyright Information:** (Online) ISSN: 2572-9241
©2026 The Author(s). HemaSphere is published by John Wiley & Sons Ltd on behalf of European Hematology Association. This is an open access Abstract Book distributed under the Attribution-NonCommercial-NoDerivs (CC BY-NC-ND), which allows third parties to download the articles and share them with others as long as they credit the author and the Abstract Book, but they cannot change the content in any way or use them commercially.
**Abstract Book Citations:** Authors, Title, HemaSphere, 2026;10:(S1):pages. The abstract book DOIs can be found on the “Author Information” page.
**Disclaimer:** Articles published in the journal HemaSphere exclusively reflect the opinions of the authors. The authors are responsible for all content in their abstracts including accuracy of the facts, statements, citing resources, etc.

### Summary/Conclusion

ALK+ALCL predominantly affects males, with most cases occurring before age 30. At initial diagnosis, the majority are advanced-stage (stage III/IV). Pathologically, it is characterized by CD30 and EMA positivity. Induction chemotherapy yields high response rates. First-line treatment regimens should be individualized based on risk factors. EDOCH/EPOCH/CHOPE regimens demonstrate superiority over CHOP in maintaining remission. Overall survival is generally favorable. First-line autologous stem cell transplantation (ASCT) improves survival in IPI intermediate-high/high-risk patients and those with poor response to intermediate-stage therapy. Salvage ASCT significantly enhances long-term survival in relapsed/refractory patients.

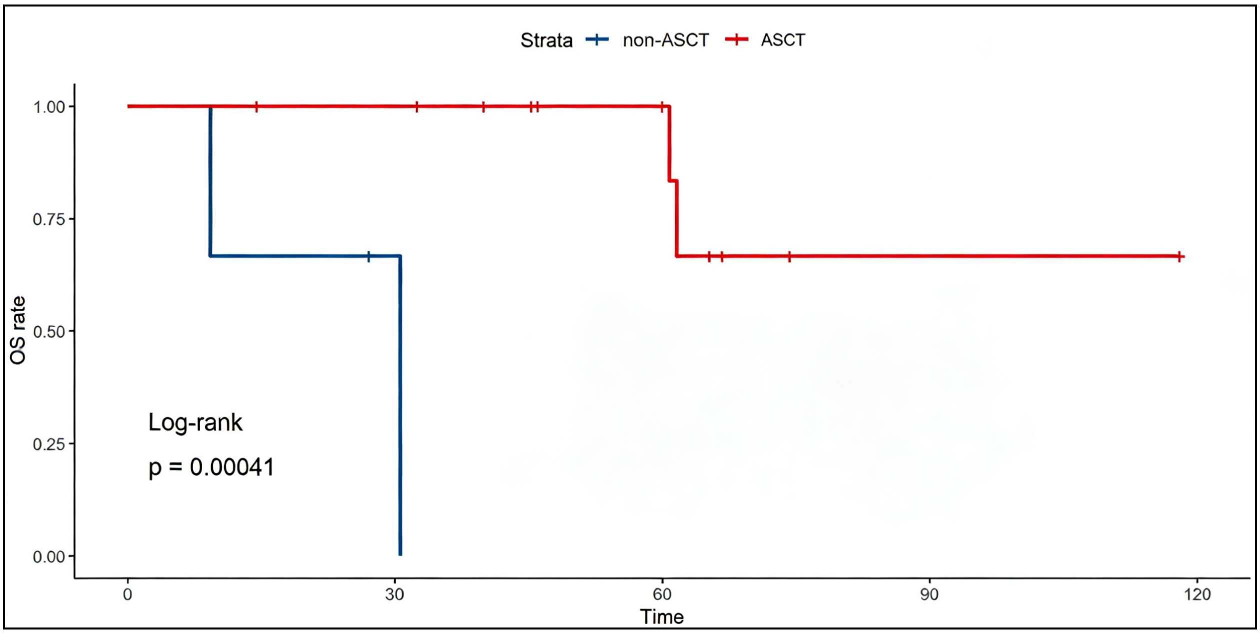

ASCT improves survival in patients with induction therapy failure.

**Copyright Information:** (Online) ISSN: 2572-9241
©2026 The Author(s). HemaSphere is published by John Wiley & Sons Ltd on behalf of European Hematology Association. This is an open access Abstract Book distributed under the Attribution-NonCommercial-NoDerivs (CC BY-NC-ND), which allows third parties to download the articles and share them with others as long as they credit the author and the Abstract Book, but they cannot change the content in any way or use them commercially.
**Abstract Book Citations:** Authors, Title, HemaSphere, 2026;10:(S1):pages. The abstract book DOIs can be found on the "Author Information" page.
**Disclaimer:** Articles published in the journal HemaSphere exclusively reflect the opinions of the authors. The authors are responsible for all content in their abstracts including accuracy of the facts, statements, citing resources, etc.

### (PB3885) IMPACT OF MODERN TREATMENT OPTIONS IN PATIENTS WITH HIV-ASSOCIATED LYMPHOMAS

**Topic:** 20. Other aggressive lymphomas

Nathalie Lehnen*[1], Leonie Neumann[1], Marie José Kersten[2], Igor Aurer[3], Marcus Hentrich[4], Jose Tomás Navarro Ferrando[5], Andrés José María Ferreri[6], Kai Hübel[1]

*[1]University of Cologne and Faculty of Medicine and University Hospital of Cologne, Cologne, Germany;[2]Department of Hematology, Amsterdam University Medical Centers, Cancer Center Amsterdam, Amsterdam, The Netherlands; [3]Department of Internal Medicine, University Hospital Centre, Zagreb and Medical School, University of Zagreb, Zagreb, Croatia; [4]Department of Hematology and Oncology, Red Cross Hospital Munich, Ludwig Maximilian University Munich, Munich, Germany; [5]Department of Hematology, Institut Català d'Oncologia, Josep Carreras Leukaemia Research Institute, Universitat Autònoma de Barcelona, Barcelona, Spain; [6]University Vita-Salute San Raffaele, Strategic Program on Lymphomas, IRCCS San Raffaele Scientific Institute, Milano, Italy;*

### Background

People living with human immunodeficiency virus (PLWH) have an increased risk to develop Hodgkin and Non-Hodgkin lymphoma. However, the vast majority of clinical trials exclude HIV-positive patients; therefore only limited data on efficacy and tolerability of modern treatment options such as T-cell-redirecting therapies or checkpoint-inhibitors (CPI) in HIV-associated lymphomas are available. It is still unclear if these treatments can be used as standard of care in patients with sustained immunological impairment like PLWH.

### Aims

We assessed the outcome of new treatment approaches in HIV-associated lymphomas.

### Methods

In this retrospective analysis we screened major data bases (Embase, Medline) between 2015 and 2025. In total, we identified 1442 clinical trials (including prospective and retrospective studies and case series) for "HIV lymphomas"; however, finally a total of 41 trials qualified for evaluation. HIV lymphomas were grouped by histology (diffuse large B cell lymphoma, DLBCL; Burkitt lymphoma, BL; primary effusion lymphoma, PEL; plasmablastic lymphoma, PBL; classical Hodgkin lymphoma, cHL) and by treatment regimen (CAR-T-cells; polatuzumab vedotin/bendamustine/rituximab, PBR; bispecific antibodies, glofitamab; pembrolizumab or nivolumab, CPI; brentuximab vedotin, BV).

**Copyright Information:** (Online) ISSN: 2572-9241
©2026 The Author(s). HemaSphere is published by John Wiley & Sons Ltd on behalf of European Hematology Association. This is an open access Abstract Book distributed under the Attribution-NonCommercial-NoDerivs (CC BY-NC-ND), which allows third parties to download the articles and share them with others as long as they credit the author and the Abstract Book, but they cannot change the content in any way or use them commercially.
**Abstract Book Citations:** Authors, Title, HemaSphere, 2026;10:(S1):pages. The abstract book DOIs can be found on the "Author Information" page.
**Disclaimer:** Articles published in the journal HemaSphere exclusively reflect the opinions of the authors. The authors are responsible for all content in their abstracts including accuracy of the facts, statements, citing resources, etc.

### Results

We identified a total of 38 patients (mostly DLBCL) receiving CAR-T-cells. In these patients, 41% achieved a complete remission (CR) and 18% a partial remission (PR). Overall survival at 6 months was 70% (12 months: 25%; 24 months: 10%). Cytokine release syndrome was documented in 17 patients (only grade 1-2), 8 patients developed immune effector cell associated neurotoxicity syndrome (grade 2: 3; grade 3-4: 5). Only 2 patients with DLBCL received PBR, 1 patient responded with CR, both are alive at 6 months. Glofitamab was used in 4 patients with DLBCL, 2 patients achieved a CR, and all were alive at 6 months. In total 51 patients with cHL were treated with CPI, 66% achieved a CR (PR: 24%), and the 6-months OS was 91% (12 months: 91%; 24 months: 67%). A total of 12 patients with DLBCL received CPI; however, only 2 patients achieved a CR (PR: 2). In PEL, CPI achieved a CR in 1/7 patients (PR: 3/7), all patients were alive at 6 months. In PBL, 1/2 patients responded (PR) to CPI. BV was used in 63 patients with cHL; 71% achieved a CR (PR: 10%), 92% of patients were alive after 6 months (12 months: 88%; 24 months: 87%). In PEL, the use of BV was reported in 4 patients (CR: 50%), all 4 patients are alive at 12 months. More detailed analysis and a comparison to HIV negative lymphoma patients will be presented at the congress.

### Summary/Conclusion

These results indicate that modern treatment options also improve the outcome of patients with HIV-associated lymphomas. However, more data are needed. The inclusion of PLWH who have a lymphoma in clinical trials should be mandatory.

**Copyright Information:** (Online) ISSN: 2572-9241
©2026 The Author(s). HemaSphere is published by John Wiley & Sons Ltd on behalf of European Hematology Association. This is an open access Abstract Book distributed under the Attribution-NonCommercial-NoDerivs (CC BY-NC-ND), which allows third parties to download the articles and share them with others as long as they credit the author and the Abstract Book, but they cannot change the content in any way or use them commercially.
**Abstract Book Citations:** Authors, Title, HemaSphere, 2026;10:(S1):pages. The abstract book DOIs can be found on the "Author Information" page.
**Disclaimer:** Articles published in the journal HemaSphere exclusively reflect the opinions of the authors. The authors are responsible for all content in their abstracts including accuracy of the facts, statements, citing resources, etc.

### (PB3886) AGGRESSIVE PRIMARY GASTROINTESTINAL LYMPHOMAS: ARE WE MANAGING THEM OPTIMALLY?

**Topic:** 20. Other aggressive lymphomas

Anaghim Aloui*[1], Wafa Sakli[1], Amine Ben Moussa[1], Jaied Rabeb[1], Hela Ghedira[1], Sami Zriba[1], Fehmi Msadek[1]

[1]*The Principal Military Hospital of Instruction of Tunis (HMPIT)- Tunisia, Hematology, Tunis, Tunisia;*

### Background

Primary gastrointestinal lymphomas (PGIL) refers to a group of malignancies that develops in the gastrointestinal tract with a steadily increasing incidence. Diverse clinical presentations and histological types are possible. While MALT lymphomas are well-characterized and often indolent, non-MALT subtypes, including diffuse large B-cell lymphoma (DLBCL), mantle cell lymphoma (MCL), Burkitt lymphoma (BL), follicular lymphoma (FL) and T-cell lymphomas (TCL), are less common, more aggressive, and therapeutically challenging. The management remains controversial, requires a multidisciplinary team and is subtype-dependent.

### Aims

The aim of this study is to analyze the clinical characteristics of non-MALT PGIL and to evaluate the outcomes of the different treatment approaches.

### Methods

We have conducted a 15-year retrospective study from January 2008 to December 2023 in the clinical hematology department of the Military Teaching hospital in Tunis, Tunisia. Data related to the patients were assembled. Patients with MALT lymphomas were excluded. The PGIL diagnosis was based on the 2008 WHO classification. We analyzed clinicopathological characteristics, therapeutic modalities and final outcomes in patients diagnosed with primary gastrointestinal lymphomas. The staging was defined according to the Lugano International Conference classification, which was proposed as a modified version of the Ann Arbor criteria for gastrointestinal lymphoma.

### Results

Our study included 45 patients. The mean age was 62.7 with a male predominance (gender ratio= 1.64). Histologically, DLBCL was the predominant subtype, accounting for 77.8% of the cases. According to the Ann Arbor modified staging system, 48.9% of the patients were diagnosed at a localized stage while 51.2% of them presented with an advanced disease. All patients received systemic chemotherapy (CT) as first-line treatment, and the most administered regimen was R-CHOP given in 59.9% of cases. The other patients were given alternative protocols mainly based on age, performance status and the histological subtype, with an adherence to the planned CT schedule in 56.5% of cases. Treatment related toxicity occurred in 78.3% of patients, predominantly infectious. At the end of treatment evaluation, complete remission was achieved in 85% of cases, while partial remission was observed in 4.7% of them. We noted that 10.3% of patients had a primary refractory disease and only 8.9% of the patients underwent autologous stem cell transplantation after the first line treatment. Relapse exclusively occurred in the DLBCL subgroup affecting 18.8% of these patients. At a median follow-up of 48 months the overall survival rate was 79.5% and the relapse-free survival rate was 73.9%.

**Copyright Information:** (Online) ISSN: 2572-9241
©2026 The Author(s). HemaSphere is published by John Wiley & Sons Ltd on behalf of European Hematology Association. This is an open access Abstract Book distributed under the Attribution-NonCommercial-NoDerivs (CC BY-NC-ND), which allows third parties to download the articles and share them with others as long as they credit the author and the Abstract Book, but they cannot change the content in any way or use them commercially.
**Abstract Book Citations:** Authors, Title, HemaSphere, 2026;10:(S1):pages. The abstract book DOIs can be found on the "Author Information" page.
**Disclaimer:** Articles published in the journal HemaSphere exclusively reflect the opinions of the authors. The authors are responsible for all content in their abstracts including accuracy of the facts, statements, citing resources, etc.

**Summary/Conclusion**

Aggressive PGIL subtypes present distinct characteristics, showing different clinical features, response to treatment and survival. DLBCL is the most common type. Standard chemotherapy is highly effective in PGIL achieving high remission rates, but infectious toxicity was common. Relapses were mainly observed in DLBCL, highlighting the importance of a long and well-tailored follow-up especially for high-risk patients.

**Copyright Information:** (Online) ISSN: 2572-9241
©2026 The Author(s). HemaSphere is published by John Wiley & Sons Ltd on behalf of European Hematology Association. This is an open access Abstract Book distributed under the Attribution-NonCommercial-NoDerivs (CC BY-NC-ND), which allows third parties to download the articles and share them with others as long as they credit the author and the Abstract Book, but they cannot change the content in any way or use them commercially.
**Abstract Book Citations:** Authors, Title, HemaSphere, 2026;10:(S1):pages. The abstract book DOIs can be found on the "Author Information" page.
**Disclaimer:** Articles published in the journal HemaSphere exclusively reflect the opinions of the authors. The authors are responsible for all content in their abstracts including accuracy of the facts, statements, citing resources, etc.

### (PB3887) IDENTIFYING KNOWLEDGE GAPS IN PRIMARY CENTRAL NERVOUS SYSTEM LYMPHOMA THROUGH ONLINE CONTINUING MEDICAL EDUCATION

**Topic:** 20. Other aggressive lymphomas

Sarah Elizabeth Bomba*[1], Andrew Small[1], Stephanie Baumhover[1], Tristin Abair[1]

[1]*Medscape Education, New York, United States of America;*

### Background

Primary central nervous system lymphoma (PCNSL) is a distinct, aggressive extranodal non-Hodgkin lymphoma characterized by unique therapeutic challenges due to its localization within the CNS and the restrictive nature of the blood-brain barrier. Recent advancements in the characterization of molecular drivers, alongside the refinement of prognostic models, have transitioned the field toward more personalized management strategies. While high-dose methotrexate-based induction remains the standard of care, the evolving treatment landscape now incorporates autologous stem cell rescue for consolidation and targeted strategies for relapsed/refractory (R/R) disease, including BTK inhibitors and CD19-targeted CAR-T cell therapies. Hematologist/oncologists, neuro-oncologists, and neurologists are central to navigating the complex diagnostic workup and management of these patients. Given the rarity of PCNSL, specialists may have unidentified gaps in general PCNSL disease knowledge, as well as current and emerging approaches.

### Aims

The objective of this study was to assess the knowledge and abilities of Hem/Oncs and Neurologists related to PCNSL disease understanding, current management, and emerging approaches, as well as to highlight ongoing educational gaps to guide the development of future education.

### Methods

An online, text-based CME survey instrument comprising 21 multiple-choice knowledge, practice-based, and confidence questions allowed participants to assess their clinical understanding and self-assessed learning needs related to PCNSL. The activity launched on September 18, 2024, and data were collected through January 2026. Hem/Oncs and Neurologists responses were analyzed by grouping questions into clinical themes and calculating the percentage of incorrect responses in each area.

### Results

4,899 total learners participated in this online CME activity within the data collection period, including 947 Hem/Oncs and 875 Neurologists. Specific ongoing educational gaps among Hem/Oncs (n = 74) and neurologists (n=148) who answered all questions in the survey were identified through this initiative (see table 1). Both Hem/Oncs and Neurologists demonstrated gaps in general PCNSL disease awareness (38%, 54%); knowledge of diagnostic guidelines and clinical application (45%, 61%); knowledge of induction and consolidation strategies (31%, 34%), and the data for novel therapies and their associated management (48, 62%).

### Summary/Conclusion

This educational initiative yielded important insights into ongoing practice gaps related to PCNSL disease education, guideline-directed diagnostic and treatment strategies, and novel PCNSL therapies and their management. Further studies are planned to assess the effect of medical education initiatives in closing these clinical practice gaps.

**Copyright Information:** (Online) ISSN: 2572-9241
©2026 The Author(s). HemaSphere is published by John Wiley & Sons Ltd on behalf of European Hematology Association. This is an open access Abstract Book distributed under the Attribution-NonCommercial-NoDerivs (CC BY-NC-ND), which allows third parties to download the articles and share them with others as long as they credit the author and the Abstract Book, but they cannot change the content in any way or use them commercially.
**Abstract Book Citations:** Authors, Title, HemaSphere, 2026;10:(S1):pages. The abstract book DOIs can be found on the "Author Information" page.
**Disclaimer:** Articles published in the journal HemaSphere exclusively reflect the opinions of the authors. The authors are responsible for all content in their abstracts including accuracy of the facts, statements, citing resources, etc.

Table 1: Questions Grouped by Educational Learning Theme

| Learning Themes | Incorrect Responses (%) Hem/Oncs | Incorrect Responses (%) Neurologists |
|---|---|---|
| PCNSL Disease Awareness | 38 | 54 |
| Diagnostic Guidelines and Clinical Application | 45 | 61 |
| Induction and Consolidation Strategies | 31 | 34 |
| Novel Therapies and R/R PCNSL Management | 48 | 62 |

**Copyright Information:** (Online) ISSN: 2572-9241
©2026 The Author(s). HemaSphere is published by John Wiley & Sons Ltd on behalf of European Hematology Association. This is an open access Abstract Book distributed under the Attribution-NonCommercial-NoDerivs (CC BY-NC-ND), which allows third parties to download the articles and share them with others as long as they credit the author and the Abstract Book, but they cannot change the content in any way or use them commercially.
**Abstract Book Citations:** Authors, Title, HemaSphere, 2026;10:(S1):pages. The abstract book DOIs can be found on the "Author Information" page.
**Disclaimer:** Articles published in the journal HemaSphere exclusively reflect the opinions of the authors. The authors are responsible for all content in their abstracts including accuracy of the facts, statements, citing resources, etc.

### (PB3888) GENOMIC SIGNATURES GUIDED COMBINATION THERAPY OF TAZEMETOSTAT PLUS LINPERLISIB OR GOLIDOCITINIB IN RELAPSED AND REFRACTORY PERIPHERAL T-CELL LYMPHOMA PATIENTS

**Topic:** 20. Other aggressive lymphomas

Shu Cheng*[1], Mingci Cai[1], Yaohui Huang[1], Li Wang[1], Pengpeng Xu[1], Weili Zhao[1]

*[1]Ruijin Hospital, Shanghai Jiaotong University School Of Medicine, Shanghai, China;*

### Background

Peripheral T-cell lymphomas (PTCL) are aggressive, heterogeneous T cell neoplasms lacking effective treatments[1]. Patients (pts) with distinct gene signatures respond differently to therapies[1]. Enhancer of zeste homolog 2 (EZH2) is frequently upregulated in patients with PTCL, and the patients with a higher EZH2 expression usually exhibited a poorer survival rate[2]. Therefore, EZH2 may be a prognostic marker in patients with PTCL, particularly in those with PTCL-NOS[2]. A phase 2 study showed Tazemetostat (EZH2 inhibitor) plus Amdizalisib (PI3K inhibitors) achieved objective response rate (ORR) 60.7% in R/R PTCL, remarkably with 100% complete response rate(CRR) in 5 patients with TET2/RHOA co-mutations[1]. Primary data has shown JAK inhibitors can block the oncogenic effects of EZH2 by inhibiting its phosphorylation[3]. Golidocitinib is a selective JAK-1 tyrosine-kinase inhibitor with encouraging anti-tumour activity in heavily pre-treated patients with R/R PTCL[4]. The potential for clinical benefit is expected from combining Tazemetostat with PI3K inhibitors or JAK-1 inhibitors in different R/R PTCL molecular typing.

### Aims

In this study we will explore the efficacy and safety of Tazemetostat plus Linperlisib (PI3K inhibitors) or Golidocitinib (JAK-1 inhibitors) in pts with distinct gene signatures of R/R PTCL.

### Methods

According to genomic classification, the study has 2 arms. Arm1: patients with co-mutation of TET2 and RHOA will receive Tazemetostat plus linperlisib. Arm2: patients with non-co-mutation of TET2 and RHOA will receive Tazemetostat plus Golidocitinib.Patients eligible for transplantation who achieved CR/PR after 3 cycles of treatment will received transplantion subsequently. Otherwise, patients will receive maintenance with the previously regimen until disease progression or intolerable toxicity.

This study includes 2 phases. Bayesian optimal interval (BOIN) design was used to determine recommended Phase 2 doses (RP2D). Tazemetostat doses were preset at 800mg BID with linperlisib 60mg QD or 80mg QD in Arm 1, and Golidocitinib 150 mg QOD or 150 mg QD in Arm 2. In phase 2, Bayesian optimal Phase 2 (BOP2) design was used to determine ORR as the primary endpoint. If ≥ 14 objective responses were observed (24 patients were included), the null hypothesis was rejected, and the treatment (Tazemetostat plus linperlisib/ Golidocitinib) was considered acceptable; otherwise, the treatment was considered unacceptable. If ≤ 13 objective responses were observed, the null hypothesis could not be rejected, and it was concluded that there was insufficient evidence of efficacy for this treatment strategy. Based on preliminary efficacy results, this study may increase the sample size for further exploration later.

### Summary/Conclusion

**Copyright Information:** (Online) ISSN: 2572-9241
©2026 The Author(s). HemaSphere is published by John Wiley & Sons Ltd on behalf of European Hematology Association. This is an open access Abstract Book distributed under the Attribution-NonCommercial-NoDerivs (CC BY-NC-ND), which allows third parties to download the articles and share them with others as long as they credit the author and the Abstract Book, but they cannot change the content in any way or use them commercially.
**Abstract Book Citations:** Authors, Title, HemaSphere, 2026;10:(S1):pages. The abstract book DOIs can be found on the "Author Information" page.
**Disclaimer:** Articles published in the journal HemaSphere exclusively reflect the opinions of the authors. The authors are responsible for all content in their abstracts including accuracy of the facts, statements, citing resources, etc.

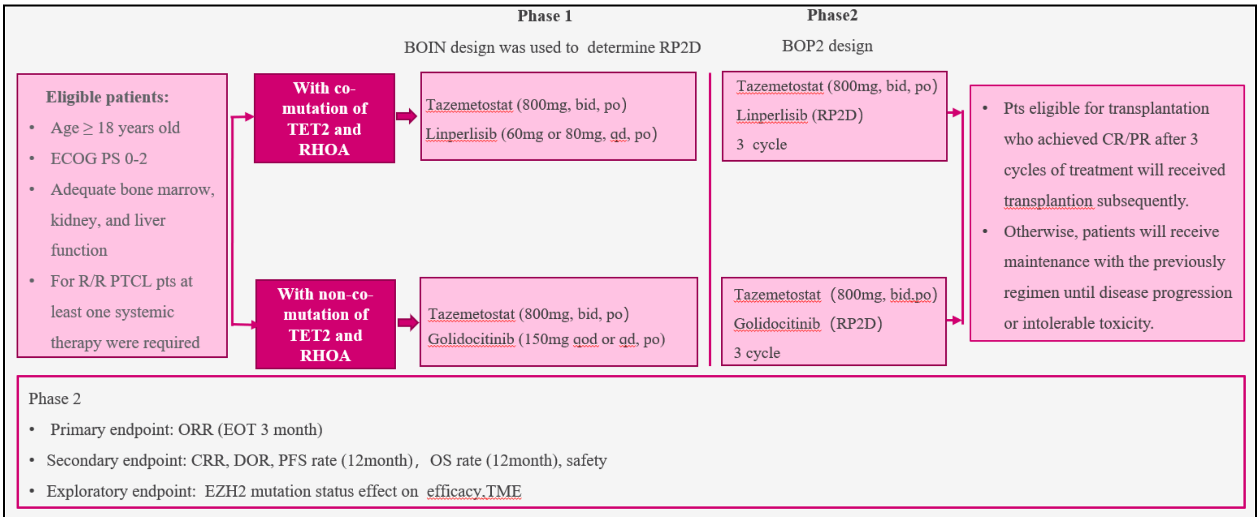

RP2D, recommended Phase 2 doses; ORR, objective response rate; CRR, complete response rate; PFS, progression free survival ; OS, overall survival; TME, Tumor Microenvironment.

**Copyright Information:** (Online) ISSN: 2572-9241
©2026 The Author(s). HemaSphere is published by John Wiley & Sons Ltd on behalf of European Hematology Association. This is an open access Abstract Book distributed under the Attribution-NonCommercial-NoDerivs (CC BY-NC-ND), which allows third parties to download the articles and share them with others as long as they credit the author and the Abstract Book, but they cannot change the content in any way or use them commercially.
**Abstract Book Citations:** Authors, Title, HemaSphere, 2026;10:(S1):pages. The abstract book DOIs can be found on the “Author Information” page.
**Disclaimer:** Articles published in the journal HemaSphere exclusively reflect the opinions of the authors. The authors are responsible for all content in their abstracts including accuracy of the facts, statements, citing resources, etc.

### (PB3889) FOLLICULAR MYCOSIS FUNGOIDES: CLINICAL CHARACTERISTICS, TREATMENT PATTERNS AND SURVIVAL OUTCOMES IN A CHINESE SINGLE-CENTRE COHORT OF 19 PATIENTS

**Topic:** 20. Other aggressive lymphomas

Jiawen Luo*[1], Liqun Zou[1], Kexing Ren[1], Lin Wang[1], Mo Cheng[1], Ming Jiang[1], Yao Xie[1], Xingwei Zhang[1], Li Xie[1], Na Li[1], Chunli Yang[1], Xi Chen[1], You Feng[1], Tao Hai[1]

[1]*West China Hospital of Sichuan University, Chengdu, China;*

### Background

Follicular mycosis fungoides (FMF) represents a distinct clinicopathological variant of mycosis fungoides (MF), characterised by marked phenotypic heterogeneity and considerable variability in prognosis. Significant differences have been reported in clinicopathological presentation, treatment response and prognostic determinants among different ethnic populations. Currently, clinical studies focusing on Chinese patients with FMF remain scarce. There is therefore an urgent need for relevant clinical data to inform diagnostic, therapeutic and prognostic stratification strategies in FMF.

### Aims

To retrospectively analyse baseline clinical characteristics, treatment selection and long-term clinical outcomes in a cohort of Chinese patients with FMF, and to identify key prognostic factors.

### Methods

A single-centre retrospective review was conducted of 19 patients diagnosed with FMF between 2009 and 2025 in West China Hospital of Sichuan University. Clinical features, therapeutic approaches and long-term outcomes were evaluated. Cox proportional hazards models were used to identify independent prognostic factors for overall survival (OS; defined from initial MF diagnosis to all-cause mortality) and progression-free survival (PFS; defined from diagnosis to first documented disease progression or death from any cause).

### Results

The median follow-up time was 38 months (range 7-121 months). The median age at diagnosis was 32 years (range 12-70 years), with 84.2% of patients aged <60 years. Clinically, 63.2% of patients presented with early-stage disease and 36.8% with advanced-stage disease. The head and neck region was the most frequently involved anatomical site (89.5%), followed by the trunk (63.2%) and extremities (47.4%). The predominant morphological features was papules (52.6%). In terms of treatment, IFN-α was the most commonly used systemic agent (94.7%), while NB-UVB phototherapy was the primary skin-directed treatment (42.1%). The median duration of all treatments was 5 months (range 1-50 months), with a median duration of IFN-α therapy of 9 months (range 1-50 months) and of chemotherapy 3 months (range 1-42 months). At the last follow-up, the overall clinical response rate was 73.7% (10.5% CR, 63.2% PR). Progressive disease was documented in 5.3% of patients; 15.8% died from FMF and 5.3% from other causes. Prognostic analysis revealed that early phototherapy was a protective factor for PFS (HR 0.2202, P=0.0458), significantly improving PFS but without a statistically significant effect on OS (P >0.05). Advanced disease stage showed a trend towards reduced PFS (HR 5.708, P=0.0970), though it did not significantly affect OS (P >0.05). Other factors did not significantly influence OS or PFS (all P >0.05).

**Copyright Information:** (Online) ISSN: 2572-9241
©2026 The Author(s). HemaSphere is published by John Wiley & Sons Ltd on behalf of European Hematology Association. This is an open access Abstract Book distributed under the Attribution-NonCommercial-NoDerivs (CC BY-NC-ND), which allows third parties to download the articles and share them with others as long as they credit the author and the Abstract Book, but they cannot change the content in any way or use them commercially.
**Abstract Book Citations:** Authors, Title, HemaSphere, 2026;10:(S1):pages. The abstract book DOIs can be found on the "Author Information" page.
**Disclaimer:** Articles published in the journal HemaSphere exclusively reflect the opinions of the authors. The authors are responsible for all content in their abstracts including accuracy of the facts, statements, citing resources, etc.

### Summary/Conclusion

This Chinese cohort of follicular mycosis fungoides presented with a notably younger age at onset, a feature that may be influenced by ethnic factors or the relatively limited sample size. Early initiation of phototherapy was identified as a significant protective factor for PFS, underscoring its value as a strategic intervention in early-stage disease to improve clinical outcomes. Advanced disease stage at presentation exhibited a trend toward reduced PFS, indicating its potential role as a prognostic marker in future larger-scale studies. In this analysis, other factors did not demonstrate statistically significant associations with survival outcomes, which may be attributable to the constraints inherent in a single-centre study with a modest cohort size.

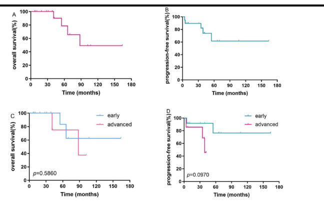

Figure 1. Kaplan-Meier survival curves for patients with FMF. (A) OS for the overall study population. (B) PFS for the overall study population (C) OS for patients with FMF stratified by stage. (D) PFS for patients with FMF stratified by stage. FMF: follicular mycosis fungoides; OS, overall survival; PFS, progression-free survival.

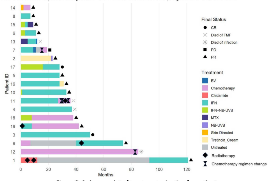

Figure 2. Swimmer plot of treatment selection for patients
Skin-directed: Acitretin+IFN+NB-UVB

**Table 1. Baseline characteristics**

| | Patients (N=19) |
|---|---|
| **Median age at diagnosis** | 32(12-70) |
| | NO. (%) |
| **Age group** | |
| ≥60 | 3(15.8) |
| <60 | 16(84.2) |
| **Sex** | |
| Male | 11(57.9) |
| Female | 8(42.1) |
| **Stage** | |
| Early | 12(63.2) |
| Advanced | 7(36.8) |
| **Anatomical Area** | |
| Head and neck | 17(89.5) |
| Trunk | 12(63.2) |
| Extremities | 9(47.4) |
| **Morphologic Features of Lesions** | |
| Patch | 8(42.1) |
| Papule | 10(52.6) |
| Plaque | 7(36.8) |
| Phyma | 2(10.5) |
| Nodule | 3(15.8) |
| Folliculocentric lesions | 15(78.9) |
| Alopecia | 7(36.8) |
| **LDH** | |
| Elevated | 7(36.8) |
| Normal | 6(31.6) |
| Untested | 6(31.6) |
| **Large cell transformation** | |
| Yes | 2(11.8) |
| No | 15(88.2) |

Folliculocentric lesions: including follicular papule and acneiform lesion, etc.

**Table 2. Treatments**

| **Skin directed therapies** | NO. (%) |
|---|---|
| NB-UVB | 8(42.1) |
| TSEBI or other radiation therapy | 4(21.1) |
| Topical corticosteroids | 5(26.3) |
| Tretinoin Cream | 3(15.8) |
| **Systemic treatments** | |
| IFN-α | 18(94.7) |
| MTX | 2(10.5) |
| Acitretin | 1(5.3) |
| Chidamide | 4(21.1) |
| BV | 2(10.5) |
| Golidocitinib | 3(15.8) |
| GemOx | 1(5.3) |
| CHOP | 1(5.3) |
| CHP | 1(5.3) |
| GDP | 1(5.3) |
| COMP | 1(5.3) |

NB-UVB: narrow band ultraviolet B radiation, INF-α: interferon α-1b, BV: Brentuximab Vedotin, COMP: CTX+VCR+MiL+P, CHOP: CTX+DOX+VCR+P, GDP: GEM+DDP+DXM, GemOx: Gem+L-OHP, CHP: CTX+DOX+P

**Table 3, Long-term outcome of the patients**

| **Median follow-up time (months)** | 38(7-121) |
|---|---|
| **Duration of treatments(months)** | **median(range)** |
| All treatments | 5(1-50) |
| IFN | 9(1-50) |
| Chemotherapy | 3(1-42) |
| **Clinical Status at Last Follow-up, No. (%)** | |
| CR | 2(10.5) |
| PR | 12(63.2) |
| PD | 1(5.3) |
| Died of FMF | 3(15.8) |
| Died of other causes | 1(5.3) |

CR: complete response, clearance of almost all skin symptoms >90%, PR: partial response, 50% clearance of skin symptoms, PD: progressive disease

**Table 4. Prognosis Factors of FMF Associated With OS, and PFS**

| | | OS | | PFS | |
|---|---|---|---|---|---|
| Characteristics | No. (%) | HR | *P* value | HR | *P* value |
| **Sex** | | | | | |
| Male sex | 11(57.9) | 1(reference) | / | 1(reference) | / |
| Female sex | 8(42.1) | 1.187(0.1626-8.664) | 0.8658 | 0.4739(0.1143-1.964) | 0.3032 |
| **Age group** | | | | | |
| Age < 60 | 16(84.2) | 1(reference) | / | 1(reference) | / |
| Age≥60 | 3(15.8) | 6.040(0.2077-175.6) | 0.2956 | 1.657(0.2661-10.32) | 0.5883 |
| **Stage** | | | | | |
| Early | 12(63.2) | 1(reference) | / | 1(reference) | / |
| Advanced | 7(36.8) | 1.776(0.2249-14.02) | 0.5860 | 5.708(0.7298-44.65) | 0.0970 |
| **Alopecia** | | | | | |
| No | 12(63.2) | 1(reference) | / | 1(reference) | / |
| Yes | 7(36.8) | 4.364(0.5233-36.38) | 0.1733 | 1.243(0.2816-5.485) | 0.7743 |
| **LDH** | | | | | |
| Normal | 6(31.6) | 1(reference) | / | 1(reference) | / |
| Elevated | 7(36.8) | 3.796(0.2711-53.14) | 0.3219 | 2.106(0.3290-13.48) | 0.4317 |
| **LCT** | | | | | |
| N-LCT | 17(89.5) | 1(reference) | / | 1(reference) | / |
| LCT | 2(10.5) | 6.040(0.2077-175.6) | 0.2956 | 0.9365(0.1185-7.401) | 0.9504 |
| **Phototherapy in early stage** | | | | | |
| No | 12(63.2) | 1(reference) | / | 1(reference) | / |
| Yes | 7(36.8) | 0.2764(0.02538-3.009) | 0.2911 | 0.2202(0.04988-0.9724) | 0.0458 |
| **MTX in early stage** | | | | | |
| No | 17(89.5) | 1(reference) | / | 1(reference) | / |
| Yes | 2(10.5) | 0.2951(0.01977-4.405) | 0.3762 | 0.3079(0.03895-2.433) | 0.2640 |

**Copyright Information:** (Online) ISSN: 2572-9241
©2026 The Author(s). HemaSphere is published by John Wiley & Sons Ltd on behalf of European Hematology Association. This is an open access Abstract Book distributed under the Attribution-NonCommercial-NoDerivs (CC BY-NC-ND), which allows third parties to download the articles and share them with others as long as they credit the author and the Abstract Book, but they cannot change the content in any way or use them commercially.
**Abstract Book Citations:** Authors, Title, HemaSphere, 2026;10:(S1):pages. The abstract book DOIs can be found on the "Author Information" page.
**Disclaimer:** Articles published in the journal HemaSphere exclusively reflect the opinions of the authors. The authors are responsible for all content in their abstracts including accuracy of the facts, statements, citing resources, etc.

### (PB3891) CANADIAN PROVINCE PRESENTATION AND TREATMENT RELATED OUTCOMES OF ANGIOIMMUNOBLASTIC T-CELL LYMPHOMA: A RETROSPECTIVE CHART REVIEW

**Topic:** 20. Other aggressive lymphomas

Montana Mellor*[1], Brooke Roeges[1], Shirmin Kader[2], Waleed Sabry[3, 4], Mohamed Elemary[3, 4], Prosanta Mondal[5], Ibraheem Othman[1, 6]

*[1]University of Saskatchewan, College of Medicine, Regina, Canada;[2]Saskatchewan Health Agency, Regina, Canada; [3]Saskatoon Cancer Centre, Medical Oncology and Hematology, Saskatoon, Canada;[4]University of Saskatchewan, College of Medicine, Saskatoon, Canada; [5]University of Saskatchewan, Department of Epidemiology and Community Health, Saskatoon, Canada; [6]Allan Blair Cancer Centre, Medical Oncology and Hematology, Regina, Canada;*

### Background

We conducted a retrospective population-based analysis of 30 patients diagnosed with AITL in Saskatchewan between 2010 and 2024. Patient demographics, clinical characteristics, laboratory findings, pathological features, treatment regimens, and survival outcomes were analyzed. Immunohistochemical markers were evaluated according to established diagnostic criteria for T follicular helper cell (TFH) markers. Epstein-Barr virus (EBV) status was assessed by in situ hybridization. Treatment responses and survival outcomes were documented with follow-up data.

### Aims

This study aimed to characterize the clinical features, treatment patterns, and outcomes of AITL patients in Saskatchewan, Canada.

### Methods

We conducted a retrospective population-based analysis of 30 patients diagnosed with AITL in Saskatchewan between 2010 and 2024. Patient demographics, clinical characteristics, laboratory findings, pathological features, treatment regimens, and survival outcomes were analyzed. Immunohistochemical markers were evaluated according to established diagnostic criteria for T follicular helper cell (TFH) markers. Epstein-Barr virus (EBV) status was assessed by in situ hybridization. Treatment responses and survival outcomes were documented with follow-up data.

### Results

The study population had a mean age of 66, with a slight female predominance, differing from international studies showing male predominance. Most patients presented with lymph node involvement as the initial biopsy site. Immunophenotyping revealed CD3 positivity in 84 % , CD4 positivity in 63%, and CD10 positivity in 42% of tested samples. Expression of specific TFH markers was lower than expected with PD-1 observed in 32% of cases and CXCL13 in 16 % of tested cases. EBV positivity was 35% aligning with global reports. Mean hemoglobin was 106  and mean platelet count of 243 × $10^9$/L. Front-line regimens predominantly consisted of CHOP (40%) and CHOEP (35%). Novel targeted agents, including histone deacetylase (HDAC) inhibitors and monoclonal antibodies such as brentuximab, were utilized in 15% of cases. Consolidative bone marrow transplantation was pursued in 30% of patients (75% autologous, 25% allogeneic). The mean International Prognostic Index (IPI) was 2.7. The overall cohort mortality rate was 77%. Notably, the 5-year OS was markedly superior in transplanted patients (84.2%) compared to non-transplanted patients (37.1%).

**Copyright Information:** (Online) ISSN: 2572-9241
©2026 The Author(s). HemaSphere is published by John Wiley & Sons Ltd on behalf of European Hematology Association. This is an open access Abstract Book distributed under the Attribution-NonCommercial-NoDerivs (CC BY-NC-ND), which allows third parties to download the articles and share them with others as long as they credit the author and the Abstract Book, but they cannot change the content in any way or use them commercially.
**Abstract Book Citations:** Authors, Title, HemaSphere, 2026;10:(S1):pages. The abstract book DOIs can be found on the "Author Information" page.
**Disclaimer:** Articles published in the journal HemaSphere exclusively reflect the opinions of the authors. The authors are responsible for all content in their abstracts including accuracy of the facts, statements, citing resources, etc.

**Summary/Conclusion**

This provincial analysis confirms the aggressive clinical course and high baseline mortality of AITL in the real-world setting. While presentation age, frontline treatment patterns, and EBV association aligned with established literature, our cohort revealed a distinct slight female predominance and lower expression of defining TFH markers. The high utilization of cellular therapy reflects modern treatment paradigms, and the striking survival advantage observed in the transplanted cohort underscores the critical role of consolidation in eligible patients. These findings highlight the ongoing need for improved frontline integration of novel targeted and immunomodulatory agents to overcome the poor outcomes of this challenging malignancy.

**Copyright Information:** (Online) ISSN: 2572-9241
©2026 The Author(s). HemaSphere is published by John Wiley & Sons Ltd on behalf of European Hematology Association. This is an open access Abstract Book distributed under the Attribution-NonCommercial-NoDerivs (CC BY-NC-ND), which allows third parties to download the articles and share them with others as long as they credit the author and the Abstract Book, but they cannot change the content in any way or use them commercially.
**Abstract Book Citations:** Authors, Title, HemaSphere, 2026;10:(S1):pages. The abstract book DOIs can be found on the "Author Information" page.
**Disclaimer:** Articles published in the journal HemaSphere exclusively reflect the opinions of the authors. The authors are responsible for all content in their abstracts including accuracy of the facts, statements, citing resources, etc.

### (PB3892) CORRELATION BETWEEN THE CD4+/CD8+ RATIO AND IMMUNOPHENOTYPIC PARAMETERS IN MYCOSIS FUNGOIDES AND SÉZARY SYNDROME: A MULTICENTER RETROSPECTIVE ANALYSIS

**Topic:** 20. Other aggressive lymphomas

Hadhami Touaiti*[1], Manel Kasdallah[1], Mbarka Barmat[1], Hedia Cherni[1], Ines Safra[1]

[1]*Institut Pasteur de Tunis, Hematology, Tunis, Tunisia;*

### Background

Primary cutaneous T/NK-cell lymphomas account for approximately 70–82% of all cutaneous lymphomas. Mycosis fungoides (MF), the predominant clinicopathological entity, is characterized by a monoclonal proliferation of CD4+ T lymphocytes with cutaneous tropism. Sézary syndrome (SS) represents a rare but particularly aggressive leukemic variant of this disease. Despite the major diagnostic contribution of immunophenotyping by flow cytometry, correlations between the various immunophenotypic parameters remain insufficiently documented in the scientific literature.

### Aims

The present study aimed to analyze these correlations within a cohort of patients with MF and SS.

### Methods

This multicenter retrospective study was conducted at the Institut Pasteur of Tunis between 2023 and 2025. The CD4+/CD8+ ratio, the percentage of CD4+CD7− cells, and the mean fluorescence intensities (MFI) of SSC, CD45, sCD3, CD4, CD5, and CD2 were analyzed by flow cytometry.

### Results

The study included 18 patients (16 MF, 2 SS) with a mean age of 56 ± 18 years and a male-to-female ratio of 1.8. The median CD4+/CD8+ ratio was 3.4 [0.9–7.6], and the median CD4+CD7− phenotypic gap was 17% [11–27.75%]. MFI analysis showed the following values: SSC (26.76 ± 4.47), CD45 (7.94 ± 9.2), CD4+ (median 1.66 [1.32–2.83]), and CD7+ (5 ± 2). Correlation analyses revealed statistically significant associations between the CD4+/CD8+ ratio and CD4+ MFI ($p < 0.01$, Pearson correlation coefficient $r = 0.9$), as well as between the CD4+/CD8+ ratio and the CD4+CD7− phenotypic gap ($p < 0.01$, $r = 0.8$). A significant correlation was also identified between the CD4+CD7− phenotypic gap and CD4 MFI ($p < 0.01$, $r = 0.8$), and between the CD4+CD7− phenotypic gap and CD7+ MFI ($p = 0.011$, $r = -0.6$).

### Summary/Conclusion

This study highlights significant correlations between the CD4+/CD8+ ratio, the CD4+CD7− phenotypic gap, and CD4 expression. These associations may improve disease monitoring and therapeutic assessment in cutaneous T-cell lymphomas.

**Copyright Information:** (Online) ISSN: 2572-9241
©2026 The Author(s). HemaSphere is published by John Wiley & Sons Ltd on behalf of European Hematology Association. This is an open access Abstract Book distributed under the Attribution-NonCommercial-NoDerivs (CC BY-NC-ND), which allows third parties to download the articles and share them with others as long as they credit the author and the Abstract Book, but they cannot change the content in any way or use them commercially.
**Abstract Book Citations:** Authors, Title, HemaSphere, 2026;10:(S1):pages. The abstract book DOIs can be found on the “Author Information” page.
**Disclaimer:** Articles published in the journal HemaSphere exclusively reflect the opinions of the authors. The authors are responsible for all content in their abstracts including accuracy of the facts, statements, citing resources, etc.

### (PB3893) EFFICACY AND SAFETY OF BRENTUXIMAB VEDOTIN WITH CHIDAMIDE IN CD30-POSITIVE CUTANEOUS T-CELL LYMPHOMA: A MULTICENTER, OPEN-LABEL, SINGLE-COHORT STUDY PROTOCOL IN PROGRESS

**Topic:** 20. Other aggressive lymphomas

Qian Wang*[1], Qinyuan Zhu[2], Ziqi Liu[2], Shanglin Jin[2], Hui Yang[1], Yimin Ren[1], Qiong Huang[2], Tianling Ding[1], Wenyu Wu[2], Tong Chen[1]

*[1]Department of Hematology, Huashan Hospital Affiliated to Fudan University, No. 12, Urumqi, China, Shanghai, China;*
*[2]Department of Dermatology, Huashan Hospital Affiliated to Fudan University, No. 12, Urumqi, China, Shanghai, China;*

### Background

Cutaneous T-cell lymphoma (CTCL) is an incurable malignancy of skin-tropic malignant CD4+ T cells, with mycosis fungoides (MF; indolent cutaneous plaques/tumors) and Sézary syndrome (SS) as predominant subtypes. Phototherapy and topical drugs are recommended for early-stage diseases. However, patients with advanced-stage diseases experienced rapid progression, with poor responses to current systemic treatments. Brentuximab vedotin (BV), an antibody-drug conjugate targeting CD30, approved by China Food and Drug Administration (CFDA) for the treatment of CD30-positive CTCL in 2021. BV paved a way for the treatment of patients with CTCL with favourable clinical outcomes in advanced MF. Moreover, chidamide, a histone deacetylase inhibitor, has reported efficacy in T-cell lymphomas. However, prospective data on BV+chidamide combination therapy in patients with advanced or relapsed/refractory CD30-positive CTCL are limited.

### Aims

This study aims to evaluate the efficacy and safety of BV+Chidamide combination therapy in patients with CD30-positive MF and SS.

### Methods

This is a prospective, single-arm, open-label, multicenter clinical trial. Between May 2026 to November 2028, adult patients (aged:18–69 years) with CD30-positive CTCL (MF:stage IIA–IVB) or SS, received at least one prior systemic therapy, ECOG performance status ≤2, radiographically detectable lesions, men and women using highly effective contraception or women with negative pregnancy test will be enrolled. Patients previously treated with BV or chidamide will be excluded. Eligible patients will receive BV (1.8 mg/kg intravenously every 3 weeks) in combination with chidamide (20 mg orally twice-weekly on days 1, 4, 8, and 11) in 21-day cycles. Initiation of treatment will be done if the hematologic parameters are within range (absolute neutrophil:≥$1.0\times10^9$/ L, platelet:≥$50\times10^9$/ L, and hemoglobin:≥75 g/L). Initiation of treatment will be delayed if grade ≥3 non-hematologic toxicities occur. All patients will receive an initial 3 cycles of BV+Chidamide combination therapy and continue treatment for up to 8 cycles on achieving partial response (PR) or higher efficacy. Discontinuation of the treatment will be done in case of stable disease (SD) or lower efficacy after 3 cycles of treatment. Follow-up will be performed for adverse events (AEs). Survival follow-up will be conducted every three months. Primary endpoints (EPs) will include objective response rate lasting at least four months (ORR4). Secondary EPs include disease control rate (DCR), objective response rate (ORR), time to response (TTR), time to progression (TTP), duration of response (DoR), survival, quality of life and safety. Moreover, exploratory EPs will include relationship between CD30 expression and therapeutic efficacy. All primary and secondary efficacy EPs analyses will be based on the full analysis set (FAS) and 95% confidence-intervals will be calculated.

**Copyright Information:** (Online) ISSN: 2572-9241
©2026 The Author(s). HemaSphere is published by John Wiley & Sons Ltd on behalf of European Hematology Association. This is an open access Abstract Book distributed under the Attribution-NonCommercial-NoDerivs (CC BY-NC-ND), which allows third parties to download the articles and share them with others as long as they credit the author and the Abstract Book, but they cannot change the content in any way or use them commercially.
**Abstract Book Citations:** Authors, Title, HemaSphere, 2026;10:(S1):pages. The abstract book DOIs can be found on the “Author Information” page.
**Disclaimer:** Articles published in the journal HemaSphere exclusively reflect the opinions of the authors. The authors are responsible for all content in their abstracts including accuracy of the facts, statements, citing resources, etc.

**Summary/Conclusion**

This study will address a critical need for real-world evidence to understand the efficacy and safety ofBV+Chidamide combination therapy in patients with CD30-positive MF and SS in China. By elucidating these factors, we aim to contribute substantial evidence to the field in ultimately guiding clinicians in making informed decisions tailored to the needs of Chinese patients with CD30-positive MF or SS.

**Copyright Information:** (Online) ISSN: 2572-9241
©2026 The Author(s). HemaSphere is published by John Wiley & Sons Ltd on behalf of European Hematology Association. This is an open access Abstract Book distributed under the Attribution-NonCommercial-NoDerivs (CC BY-NC-ND), which allows third parties to download the articles and share them with others as long as they credit the author and the Abstract Book, but they cannot change the content in any way or use them commercially.
**Abstract Book Citations:** Authors, Title, HemaSphere, 2026;10:(S1):pages. The abstract book DOIs can be found on the "Author Information" page.
**Disclaimer:** Articles published in the journal HemaSphere exclusively reflect the opinions of the authors. The authors are responsible for all content in their abstracts including accuracy of the facts, statements, citing resources, etc.

### (PB3894) SPATIAL AND TEMPORAL HETEROGENEITY OF CD30 EXPRESSION IN T-CELL LYMPHOMAS

**Topic:** 20. Other aggressive lymphomas

Leonie Oevel*[1, 2], Clara Neuwirth[3], Sarah Bernhardt[3], Andreas Wilfer[1], Franziska Schümann[3], Elisabeth Groß[3], Marcus Bauer[1, 2], Michael Heuser[3], Claudia Wickenhauser[1, 2], Thomas Weber[3]

*[1]University Hospital Halle (Saale), University Medicine Halle, Institute of Pathology, Halle (Saale), Germany; [2]Martin Luther University Halle-Wittenberg, Institute of Pathology, Section Immunopathology, Halle (Saale), Germany; [3]University Hospital Halle (Saale), Martin-Luther-University Halle-Wittenberg, Department of Internal Medicine IV, Oncology and Hematology, Halle (Saale), Germany;*

### Background

T-cell lymphomas are heterogeneous, predominantly aggressive malignancies with poor prognosis. The CD30-targeting antibody–drug conjugate brentuximab vedotin (BV) improved progression-free and overall survival in the phase III ECHELON-2 trial in CD30-expressing (≥10%) peripheral T-cell lymphomas, with benefit largely driven by anaplastic large cell lymphoma (ALCL), while no clear benefit was observed in non-ALCL subgroups. BV has shown benefit in CD30-positive cutaneous T-cell lymphomas, whereas prospective randomized data are lacking for PTCL with low-level CD30 expression (≥1%). Accordingly, CD30 thresholds for BV therapy remain undefined, and reliable CD30 assessment is critical.

### Aims

To analyze spatial and temporal variability of immunohistochemically assessed CD30 expression in T-cell lymphomas.

### Methods

We analyzed 162 formalin-fixed, paraffin-embedded samples from 126 patients with T-cell lymphomas. CD30 expression was assessed by immunohistochemistry (clone BerH2) using semi-quantitative H-scores and digital image analysis. Multispectral immunofluorescence was performed in selected cases. Longitudinal, tissue-based, and survival analyses were conducted.

### Results

CD30 expression varied substantially across lymphoma entities. ALCL showed uniformly high expression, whereas non-ALCL PTCL displayed low and heterogeneous CD30 levels. At the time of primary diagnosis CD30 expression was significantly lower in bone marrow manifestations (n = 27) compared with lymph node manifestations (n = 39) and manifestations in other tissues (n = 17). Multispectral immunofluorescence confirmed predominant CD30 expression in $CD3^+$ tumor T cells, with minimal contribution from non-neoplastic cells. Longitudinal analyses showed mainly stable or decreasing CD30 expression, with increases observed in two PTCL-NOS cases and one transformed mycosis fungoides. CD30 expression was not consistently associated with clinicopathological features. Using a ≥1% cutoff, CD30 positivity correlated with inferior overall survival, while no prognostic impact was observed using a ≥10% cutoff.

### Summary/Conclusion

CD30 expression in T-cell lymphomas shows pronounced spatial and temporal heterogeneity, particularly in non-ALCL entities. Bone marrow specimens may be suboptimal for therapeutic decision-making. Re-biopsy may be informative in selected subtypes and supports harmonized CD30 assessment strategies for CD30-targeted therapies.

**Copyright Information:** (Online) ISSN: 2572-9241
©2026 The Author(s). HemaSphere is published by John Wiley & Sons Ltd on behalf of European Hematology Association. This is an open access Abstract Book distributed under the Attribution-NonCommercial-NoDerivs (CC BY-NC-ND), which allows third parties to download the articles and share them with others as long as they credit the author and the Abstract Book, but they cannot change the content in any way or use them commercially.
**Abstract Book Citations:** Authors, Title, HemaSphere, 2026;10:(S1):pages. The abstract book DOIs can be found on the "Author Information" page.
**Disclaimer:** Articles published in the journal HemaSphere exclusively reflect the opinions of the authors. The authors are responsible for all content in their abstracts including accuracy of the facts, statements, citing resources, etc.

### (PB3895) CD30-INDEPENDENT EFFICACY OF BRENTUXIMAB VEDOTIN IN ADVANCED CUTANEOUS T-CELL LYMPHOMA: INSIGHTS FROM A REAL-WORLD COHORT

**Topic:** 20. Other aggressive lymphomas

Mo Cheng*[1], Liqun Zou[1], Kexing Ren[1], Lin Wang[2], Jiawen Luo[2], Ming Jiang[1], Li Xie[1], Na Li[3], Chunli Yang[1], Xi Chen[1], You Feng[3], Tao Hai[1], Yao Xie[2], Xingwei Zhang[2]

*[1]Department of Head and Neck Oncology, Cancer Center, West China Hospital, Sichuan University, Chengdu, China., Department of Head and Neck Oncology, Cancer Center, West China Hospital, Sichuan University, Chengdu, China., Chengdu, China; [2]West China Hospital of Sichuan University, chengdu, China;[3]West China Hospital of Sichuan Uniersity, chengdu, China;*

### Background

Cutaneous T-cell lymphomas (CTCL) are a heterogeneous group of non-Hodgkin lymphomas that typically pursue an aggressive disease course, have limited therapeutic options, and respond poorly to existing treatments. Brentuximab vedotin (BV), an anti-CD30 antibody-drug conjugate, has been shown to be efficacious in CD30-positive lymphomas. Real-world data regarding its application in CTCL are limited.

### Aims

To assess the real-world treatment patterns, efficacy, and safety of BV in patients with CTCL.

### Methods

In this retrospective analysis, we enrolled patients with CTCL who received BV at West China Hospital, Sichuan University (n=20). Baseline demographics and clinical characteristics were collected. Response rates were evaluated based on best overall response, and survival outcomes, including PFS and OS, were estimated using the K-M method.

### Results

The median age was 58 years (IQR: 42–68), with 55% female and 45% male. The most common subtype was MF (80%), followed by pc-ALCL (15%) and LyP (5%). Disease stages ranged from IB to IVB, with 30% of patients presenting with stage IVB. CD30 expression was ≥10% in 70% of patients. BV was administered as monotherapy in 75% of patients, while 5 patients (25%) received combination therapy, including BV-CHP, BV-EPOCH, and BV plus chidamide. The median number of BV cycles was 9 (range: 2–16). The overall response rate was 85%, including 3 patients (15%) achieving CR and 14 patients (70%) achieving PR. After a median follow-up of 24.0 months (IQR: 15.3–34.1), mPFS was 9.7 months (95% CI: 4.7–18.5), with a 12-month PFS rate of 44.4%. mOS was not reached. No significant difference in PFS was observed between patients stratified by CD30 expression level. mPFS was 7.1 months (95% CI: 3.0–NE) in patients with CD30 expression ≥10% and 11.9 months (95% CI: 4.7–NE) in those with CD30 expression <10%. TEAEs occurred in 15 patients (75%), with grade ≥3 AEs observed in 5 patients (25%). The most common involvement was hematologic (55%).
Table1 Baseline characteristics

**Copyright Information:** (Online) ISSN: 2572-9241
©2026 The Author(s). HemaSphere is published by John Wiley & Sons Ltd on behalf of European Hematology Association. This is an open access Abstract Book distributed under the Attribution-NonCommercial-NoDerivs (CC BY-NC-ND), which allows third parties to download the articles and share them with others as long as they credit the author and the Abstract Book, but they cannot change the content in any way or use them commercially.
**Abstract Book Citations:** Authors, Title, HemaSphere, 2026;10:(S1):pages. The abstract book DOIs can be found on the "Author Information" page.
**Disclaimer:** Articles published in the journal HemaSphere exclusively reflect the opinions of the authors. The authors are responsible for all content in their abstracts including accuracy of the facts, statements, citing resources, etc.

| Patients (N = 20) | |
|---|---|
| **Median age, years (IQR)** | 58 (42–68) |
| **Age group** | |
| <60 | 10 (50%) |
| ≥60 | 10 (50%) |
| **Sex** | |
| Male | 9 (45%) |
| Female | 11 (55%) |
| **Subtype of CTCL** | |
| MF | 16 (80%) |
| pc-ALCL | 3 (15%) |
| LyP | 1 (5%) |
| **Stage (EORTC/ISCL 2007)** | |
| IB | 2 (10%) |
| IIB | 3 (15%) |
| III | 4 (20%) |
| IVA1 | 3 (15%) |
| IVA2 | 2 (10%) |
| IVB | 6 (30%) |
| **CD30 expression** | |
| <10% | 6 (30%) |
| ≥10% | 14 (70%) |
| **Systemic therapy prior to BV** | |
| previously untreated | 3 (15%) |
| 1 previous systemic | 12 (60%) |
| ≥ 2 previous systemic | 5 (25%) |
| **Therapeutic agents** | |
| Methotrexate | 8 (40%) |
| Interferon | 14 (70%) |
| Phototherapy | 7 (35%) |
| Radiotherapy | 4 (20%) |
| Chidamide | 6 (30%) |
| Intravenous chemotherapy | 3 (15%) |
| **BV therapy** | |
| BV monotherapy | 15 (75%) |
| Combination therapy | 5 (25%) |
| BV-CHP | 3 (15%) |
| BV-EPOCH | 1 (5%) |
| BV + Chidamide | 1 (5%) |

Table2 Therapeutic outcome

**Copyright Information:** (Online) ISSN: 2572-9241
©2026 The Author(s). HemaSphere is published by John Wiley & Sons Ltd on behalf of European Hematology Association. This is an open access Abstract Book distributed under the Attribution-NonCommercial-NoDerivs (CC BY-NC-ND), which allows third parties to download the articles and share them with others as long as they credit the author and the Abstract Book, but they cannot change the content in any way or use them commercially.
**Abstract Book Citations:** Authors, Title, HemaSphere, 2026;10:(S1):pages. The abstract book DOIs can be found on the "Author Information" page.
**Disclaimer:** Articles published in the journal HemaSphere exclusively reflect the opinions of the authors. The authors are responsible for all content in their abstracts including accuracy of the facts, statements, citing resources, etc.

| | |
|---|---|
| **Patients (N = 20)** | |
| **Median number of BV cycles (range)** | 9 (2–16) |
| **BV dosing** | |
| 1.8 mg/kg Q3W | 18 (90%) |
| Dosing modification during BV | 2 (10%) |
| **Best overall response** | |
| CR | 3 (15%) |
| PR | 14 (70%) |
| SD | 2 (10%) |
| PD | 1 (5%) |
| **Subsequent therapy** | 16 (80%) |
| **Median Follow-up (months) (IQR)** | 24.0 (15.3-34.1) |
| **Progression-free survival** | |
| Median progression-free survival (months) | 9.7 (95%CI: 4.7-18.5) |
| 12-months rate (%) | 44.4% (95%CI: 27.1-72.9) |
| **Overall survival** | |
| Median overall survival | Not reached |

Table3 Adverse events

| | |
|---|---|
| **Adverse events (N = 20)** | |
| **Any grade** | 15 (75%) |
| **≥ Grade 3** | 5 (25%) |
| **Involvement of organ systems** | |
| Hematologic | 11 (55%) |
| Skin | 2 (10%) |
| Liver/Pancreatic | 5 (25%) |
| Neurologic | 5 (25%) |
| Other | 2 (10%) |

### Summary/Conclusion

BV demonstrated high overall response rates and acceptable durability in a real-world CTCL patients with diverse prior treatments and disease stages, with efficacy observed regardless of CD30 expression level.

**Copyright Information:** (Online) ISSN: 2572-9241
©2026 The Author(s). HemaSphere is published by John Wiley & Sons Ltd on behalf of European Hematology Association. This is an open access Abstract Book distributed under the Attribution-NonCommercial-NoDerivs (CC BY-NC-ND), which allows third parties to download the articles and share them with others as long as they credit the author and the Abstract Book, but they cannot change the content in any way or use them commercially.
**Abstract Book Citations:** Authors, Title, HemaSphere, 2026;10:(S1):pages. The abstract book DOIs can be found on the “Author Information” page.
**Disclaimer:** Articles published in the journal HemaSphere exclusively reflect the opinions of the authors. The authors are responsible for all content in their abstracts including accuracy of the facts, statements, citing resources, etc.

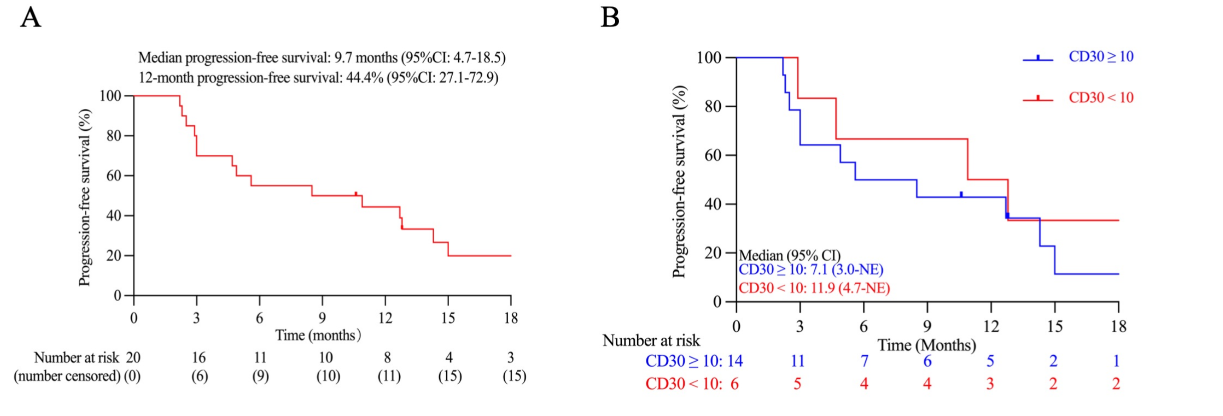

**Figure 1: Progression-free survival in patients with CTCL treated with brentuximab vedotin.**
(A) PFS in the total cohort (N=20). (B) PFS stratified by CD30 expression level.

Figure 1: Progression-free survival in patients with CTCL treated with brentuximab vedotin. (A) PFS in the total cohort (N=20). (B) PFS stratified by CD30 expression level.

**Copyright Information:** (Online) ISSN: 2572-9241
©2026 The Author(s). HemaSphere is published by John Wiley & Sons Ltd on behalf of European Hematology Association. This is an open access Abstract Book distributed under the Attribution-NonCommercial-NoDerivs (CC BY-NC-ND), which allows third parties to download the articles and share them with others as long as they credit the author and the Abstract Book, but they cannot change the content in any way or use them commercially.
**Abstract Book Citations:** Authors, Title, HemaSphere, 2026;10:(S1):pages. The abstract book DOIs can be found on the "Author Information" page.
**Disclaimer:** Articles published in the journal HemaSphere exclusively reflect the opinions of the authors. The authors are responsible for all content in their abstracts including accuracy of the facts, statements, citing resources, etc.

### (PB3896) SQLE UPREGULATION AND ALTERED CHOLESTEROL HOMEOSTASIS SIGNATURE IN CRIZOTINIB-RESISTANT ALK-POSITIVE ANAPLASTIC LARGE CELL LYMPHOMA

**Topic:** 20. Other aggressive lymphomas

Wenyu Shi*[1], Beiqi Gong[1], Yaping Zhang[1]

[1]*Affiliated Hospital of Nantong University, Nantong, China;*

### Background

Anaplastic large cell lymphoma (ALCL) is a peripheral T-cell neoplasm, and 50–60% of cases harbor ALK rearrangements (most commonly NPM-ALK). Although crizotinib improves outcomes in ALK+ ALCL, acquired resistance remains frequent and may occur without secondary ALK mutations, suggesting non-kinase bypass mechanisms. Metabolic rewiring has been implicated in targeted-therapy resistance; however, the contribution of cholesterol homeostasis and ferroptosis-related programs in ALK inhibitor-resistant ALCL is unclear.

### Aims

This study aimed to characterize the transcriptional and metabolic adaptations driving crizotinib resistance in ALK+ ALCL. Specifically, the objectives were to establish stable crizotinib-resistant (CrizR) models, to delineate the resistance-associated transcriptional landscape via high-throughput RNA sequencing, and to interrogate alterations in cholesterol homeostasis and ferroptosis pathways. Ultimately, the study sought to nominate candidate metabolic vulnerabilities, such as sterol biosynthesis regulators, for potential therapeutic targeting.

### Methods

Crizotinib-resistant (CrizR) sublines were generated from SR786 and Karpas299 cells through long-term exposure to escalating drug concentrations. Resistance was quantified via CCK-8 dose-response assays and IC50 determination. Bulk RNA-seq was performed on parental and matched CrizR cells (n=3 biological replicates per condition). Differential expression analysis was conducted using DESeq2 (adjusted $P<0.05$, $|\log2FC|\geq1$), and pathway enrichment was analyzed using GSEA with MSigDB Hallmark gene sets to identify dysregulated biological processes.

### Results

CrizR populations exhibited significant resistance, with IC50 values increasing from 23.02 nM to 521.3 nM in SR786 (22.7-fold) and from 51.19 nM to 1332 nM in Karpas299 (16.9-fold). Transcriptomic profiling revealed broad reprogramming, and GSEA showed a significant alteration of the HALLMARK_CHOLESTEROL_HOMEOSTASIS signature between CrizR and parental cells (NES = -2.053; P = 6.20e-03; FDR = 2.81e-03). Differential expression analysis highlighted SQLE (Squalene Epoxidase), a rate-limiting enzyme in sterol biosynthesis, as a top-upregulated gene (log2FC = 4.889; P = 1.16e-04), alongside increased expression of the cholesterol exporter ABCA1 (log2FC = 1.650; P = 1.18e-04). Conversely, the cystine transporter SLC7A11, a critical suppressor of ferroptosis, was significantly downregulated (log2FC = -2.427; P = 1.14e-03), suggesting a distinct metabolic stress state accompanying resistance.

**Copyright Information:** (Online) ISSN: 2572-9241
©2026 The Author(s). HemaSphere is published by John Wiley & Sons Ltd on behalf of European Hematology Association. This is an open access Abstract Book distributed under the Attribution-NonCommercial-NoDerivs (CC BY-NC-ND), which allows third parties to download the articles and share them with others as long as they credit the author and the Abstract Book, but they cannot change the content in any way or use them commercially.
**Abstract Book Citations:** Authors, Title, HemaSphere, 2026;10:(S1):pages. The abstract book DOIs can be found on the "Author Information" page.
**Disclaimer:** Articles published in the journal HemaSphere exclusively reflect the opinions of the authors. The authors are responsible for all content in their abstracts including accuracy of the facts, statements, citing resources, etc.

**Summary/Conclusion**

Crizotinib resistance in ALK+ ALCL is characterized by reproducible transcriptional reprogramming of cholesterol homeostasis, marked by significant SQLE upregulation. The concurrent downregulation of SLC7A11 suggests that resistant cells may harbor specific vulnerabilities related to lipid peroxidation handling. These findings nominate SQLE-mediated sterol metabolism as a promising candidate for functional validation and highlight the potential of targeting metabolic dependencies to overcome crizotinib resistance in ALCL.

**Copyright Information:** (Online) ISSN: 2572-9241
©2026 The Author(s). HemaSphere is published by John Wiley & Sons Ltd on behalf of European Hematology Association. This is an open access Abstract Book distributed under the Attribution-NonCommercial-NoDerivs (CC BY-NC-ND), which allows third parties to download the articles and share them with others as long as they credit the author and the Abstract Book, but they cannot change the content in any way or use them commercially.
**Abstract Book Citations:** Authors, Title, HemaSphere, 2026;10:(S1):pages. The abstract book DOIs can be found on the “Author Information” page.
**Disclaimer:** Articles published in the journal HemaSphere exclusively reflect the opinions of the authors. The authors are responsible for all content in their abstracts including accuracy of the facts, statements, citing resources, etc.

### (PB3897) ALK-POSITIVE VERSUS ALK-NEGATIVE ANAPLASTIC LARGE CELL LYMPHOMA: CLINICAL FEATURES, PROGNOSTIC FACTORS, AND OUTCOMES IN MEXICAN POPULATION

**Topic:** 20. Other aggressive lymphomas

Amaya Llorente-Chávez*[1], Amairani Carpintero-Díaz[1], Mistral Castellanos-Mares[1], Rodrigo Figueroa-Méndez[2], Alejandra Hernández-Galicia[1], Luis Felipe Rubalcava Lara[1], Maria Silvia Rivas[1], Irma Karen Pellon Tellez[1]

*[1]Instituto Nacional de Cancerología, Hematology, Mexico City, Mexico;[2]Instituto Nacional de Ciencias Médicas y Nutrición Salvador Zubirán, Mexico City, Mexico;*

### Background

Anaplastic large cell lymphoma (ALCL) is a rare subtype of peripheral T-cell lymphoma classified into ALK-positive and ALK-negative. Data regarding incidence and outcomes in Latin American populations remain limited.

### Aims

To compare clinical characteristics and outcomes such as overall survival (OS) and progression-free survival (PFS) between ALK-positive and ALK-negative ALCL patients treated at a national reference center.

### Methods

We conducted a retrospective cohort study including adults (≥18 years) diagnosed with ALCL between 2011 and 2025. OS, and PFS were estimated using Kaplan–Meier methods.

**Copyright Information:** (Online) ISSN: 2572-9241
©2026 The Author(s). HemaSphere is published by John Wiley & Sons Ltd on behalf of European Hematology Association. This is an open access Abstract Book distributed under the Attribution-NonCommercial-NoDerivs (CC BY-NC-ND), which allows third parties to download the articles and share them with others as long as they credit the author and the Abstract Book, but they cannot change the content in any way or use them commercially.
**Abstract Book Citations:** Authors, Title, HemaSphere, 2026;10:(S1):pages. The abstract book DOIs can be found on the "Author Information" page.
**Disclaimer:** Articles published in the journal HemaSphere exclusively reflect the opinions of the authors. The authors are responsible for all content in their abstracts including accuracy of the facts, statements, citing resources, etc.

### Results

Seventy-five patients with ALCL were included; 62.7% (n=47) were ALK-negative and 37.3% (n=28) ALK-positive. ALK-positive patients were younger (35 ± 14 years) than ALK-negative (51 ± 17 years), with a female predominance. The majority of patients had ECOG 0–1, and the median IPI score was 2 (1-3).
Advanced-stage disease (stage III–IV) was observed in 68%, bulky disease in 23% (10/28 ALK-positive, 9/47 ALK-negative), and B symptoms in 57.3%. Regarding extranodal involvement, the skin was the most affected site (37.3%; 9/28 ALK-positive, 19/47 ALK-negative), followed by bone marrow (17.3%; 3/28 ALK-positive, 10/47 ALK-negative) and central nervous system (2.7%).
ALK-positive cases commonly expressed CD5, granzyme B, perforin, and CD45; CD3 was present in one-third, and CD30 in 86%. ALK-negative mostly expressed CD3, CD4, CD5, CD45, and granzyme B, with CD30 positivity in 97%.
Anthracycline-based chemotherapy was administered as first-line treatment in 75%. Among those with ALK-positive, etoposide was incorporated into the regimen in 85.7%. Brentuximab vedotin was administered in 25%, and radiotherapy was employed in 45.3%. The overall response rate was 57%. Complete response after first-line therapy (CR1) was achieved in 40%; including 50% (14/28) of those with ALK-positive and 34% (16/47) with ALK-negative.
Relapse or progression (R/R) occurred in 36%; including 28.6% (8/28) in ALK-positive and 40.4% (19/47) in ALK-negative. Primary refractory disease ocurred in 10.7% (3/28) of ALK-positive and 14.9% (7/47) of ALK-negative.
Only 12% (n = 9) underwent hematopoietic stem cell transplantation (HSCT), predominantly autologous; 6/9 ALK-negative, and 3/9 ALK-positive. Among transplanted patients, 33% had HSCT in CR1, while 66% because of R/R disease. During follow-up, progression was observed in 44.4%.
Overall mortality was 28%. Median OS was 2.5 years (IQR: 0-6) for ALK-positive and 1 year (IQR: 0-5) for ALK-negative, p=0.94. Respectively, median PFS was 14 months (IQR: 2-65), and 8 months (IQR: 4-60), p=0.85.
Favorable OS was associated with ECOG 0–1 (p=0.002), absence of adenomegaly (p=0.001), albumin >3.5 g/dL (p=0.003), low/low-intermediate IPI (p=0.008), and CR1 (p=0.0001). Improved PFS was associated with brentuximab vedotin exposure (p=0.025) and CR1 (p=0.001).
ALK-negative patients were older (p=0.0001) and had ECOG$\geq$ 2 (p=0.038), whereas ALK-positive more commonly presented with B symptoms (p=0.001) and higher median leukocyte counts (p=0.038).

### Summary/Conclusion

Our findings confirm the aggressive behavior of systemic ALCL. The disease predominantly presented at an advanced stage and was associated with modest survival outcomes, particularly among ALK-negative cases. These results reflect delayed presentation, underlying biological variability, and disparities in treatment access. Performance status, disease burden, and response emerged as the principal prognostic factors.

**Copyright Information:** (Online) ISSN: 2572-9241
©2026 The Author(s). HemaSphere is published by John Wiley & Sons Ltd on behalf of European Hematology Association. This is an open access Abstract Book distributed under the Attribution-NonCommercial-NoDerivs (CC BY-NC-ND), which allows third parties to download the articles and share them with others as long as they credit the author and the Abstract Book, but they cannot change the content in any way or use them commercially.
**Abstract Book Citations:** Authors, Title, HemaSphere, 2026;10:(S1):pages. The abstract book DOIs can be found on the "Author Information" page.
**Disclaimer:** Articles published in the journal HemaSphere exclusively reflect the opinions of the authors. The authors are responsible for all content in their abstracts including accuracy of the facts, statements, citing resources, etc.

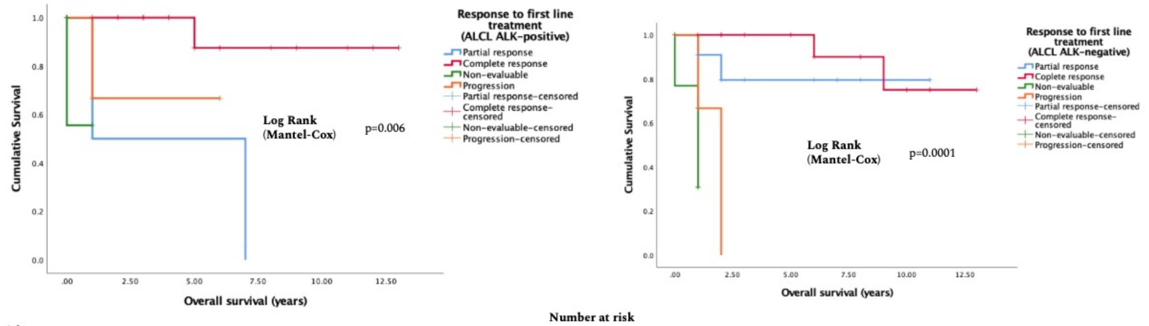

Number at risk

| | | | | | | | |
|---|---|---|---|---|---|---|---|
| Partial Response | 2 | 1 | 1 | 1 | 0 | 0 | 0 |
| Complete response | 13 | 12 | 9 | 5 | 4 | 2 | 1 |
| Non-evaluable | 9 | 1 | 0 | 0 | 0 | 0 | 0 |
| Progression | 3 | 1 | 1 | 1 | 0 | 0 | 0 |

Number at risk

| | | | | | | | |
|---|---|---|---|---|---|---|---|
| Partial Response | 11 | 8 | 5 | 4 | 2 | 1 | 0 |
| Complete response | 15 | 13 | 10 | 8 | 6 | 3 | 1 |
| Non-evaluable | 1 | 0 | 0 | 0 | 0 | 0 | 0 |
| Progression | 6 | 1 | 0 | 0 | 0 | 0 | 0 |

**Copyright Information:** (Online) ISSN: 2572-9241
©2026 The Author(s). HemaSphere is published by John Wiley & Sons Ltd on behalf of European Hematology Association. This is an open access Abstract Book distributed under the Attribution-NonCommercial-NoDerivs (CC BY-NC-ND), which allows third parties to download the articles and share them with others as long as they credit the author and the Abstract Book, but they cannot change the content in any way or use them commercially.
**Abstract Book Citations:** Authors, Title, HemaSphere, 2026;10:(S1):pages. The abstract book DOIs can be found on the "Author Information" page.
**Disclaimer:** Articles published in the journal HemaSphere exclusively reflect the opinions of the authors. The authors are responsible for all content in their abstracts including accuracy of the facts, statements, citing resources, etc.

### (PB3898) SEQUENTIAL POST-TRANSPLANT LYMPHOPROLIFERATIVE DISORDERS (PTLDS) WITH DISTINCT HISTOPATHOLOGICAL FEATURES: COMPREHENSIVE CLINICAL AND MOLECULAR ANALYSIS

**Topic:** 20. Other aggressive lymphomas

Marketa Rackova*[1], Andrea Rennerova[1], Markéta Kalinová[2], Petr Hubacek[3], Vit Campr[4], Janez Jazbec[5], Anna Fuereder[6], Simon Bomken[7], Dawid Przystupski[8], Francesco Ceppi[9], Lucie Sramkova[1], Leona Rezkova Reznickova[1], Eva Fronkova[1], Andishe Attarbaschi[6]

*[1]Department of Paediatric Haematology and Oncology, Second Faculty of Medicine, Charles University, and University Hospital Motol, Prague, Czechia; [2]Central Laboratories, University Hospital Kralovske Vinohrady, Prague, Czechia; [3]Department of Medical Microbiology, Second Faculty of Medicine, Charles University, and University Hospital Motol, Prague, Czechia; [4]Department of Pathology and Molecular Medicine, Second Faculty of Medicine, Charles University, and University Hospital Motol, Prague, Czechia; [5]Division of Pediatric Hematology and Oncology, University Medical Center Ljubljana, Ljubljana, Slovenia; [6]Department of Pediatric Hematology and Oncology, St. Anna Children's Hospital, Medical University of Vienna, Vienna, Austria; [7]Department of Paediatric Haematology and Oncology, Newcastle Upon Tyne Hospitals NHS Foundation Trust, Newcastle Upon Tyne, United Kingdom; [8]Department of Paediatric Bone Marrow Transplantation, Haematology and Oncology, Wroclaw Medical University, Wroclaw, Poland; [9]Division of Pediatrics, Department of Woman, Mother-Child, Pediatric Hematology-Oncology Unit, University Hospital Lausanne, Lausanne, Switzerland;*

### Background

Post-transplant lymphoproliferative disorders (PTLDs) comprise a heterogeneous group of lymphoid proliferations that share some pathogenetic features with sporadic non-Hodgkin lymphomas (NHLs). Cases of patients developing multiple PTLDs with distinct morphologies offer a unique real-life model of lymphomagenesis. Due to their rarity, only isolated cases have been reported to date, without any systematic analysis of larger patient cohorts.

### Aims

To characterize cases of pediatric transplant recipients who developed multiple PTLDs with distinct histopathology aiming to: (i) identify potential risk factors for developing multiple PTLDs, (ii) assess clonal relatedness between sequential PTLDs in the same patient, and (iii) explore molecular mechanisms behind their different morphology.

### Methods

Clinical data were retrospectively collected from pediatric patients treated across iBFM centers who developed ≥2 distinct PTLDs. Available paired biopsy samples were analyzed for IG/TR rearrangements and subjected to whole-exome sequencing (WES) to determine clonal relationships and mutational landscapes.

**Copyright Information:** (Online) ISSN: 2572-9241
©2026 The Author(s). HemaSphere is published by John Wiley & Sons Ltd on behalf of European Hematology Association. This is an open access Abstract Book distributed under the Attribution-NonCommercial-NoDerivs (CC BY-NC-ND), which allows third parties to download the articles and share them with others as long as they credit the author and the Abstract Book, but they cannot change the content in any way or use them commercially.
**Abstract Book Citations:** Authors, Title, HemaSphere, 2026;10:(S1):pages. The abstract book DOIs can be found on the "Author Information" page.
**Disclaimer:** Articles published in the journal HemaSphere exclusively reflect the opinions of the authors. The authors are responsible for all content in their abstracts including accuracy of the facts, statements, citing resources, etc.

### Results

Data from 9 patients who fulfilled the inclusion criteria were analyzed. All 9 patients were solid organ transplant recipients, heart transplant recipients were the most prevalent (n=4). Median time from transplantation to the first PTLD development was 1.87 years, the median interval between the first and subsequent PTLD was 1.45 years. Histological subtypes differed, however the first PTLD was polymorphic or early-lesion PTLD in 7/9 patients, while the subsequent PTLD was monomorphic in 8/9 patients. Eight of the nine PTLDs were EBV positive and of B-cell origin, typically arising at a different anatomical location than the prior lesion. Despite recurrence, the subsequent PTLDs generally responded well to standard treatment. The only exception was a patient who presented with ALK-negative anaplastic large cell lymphoma (ALK-ALCL) after two episodes of polymorphic PTLD and who died of progressive disease.
A detailed molecular analysis was performed in two patients. In the first patient, who initially developed polymorphic PTLD, followed by diffuse large B-cell lymphoma (DLBCL), identical IGH gene rearrangement was detected in both biopsy samples indicating clonal origin of these diseases. The DLBCL sample had higher mutational burden and was notably enriched for pathogenic variants in *KMT2C*, *FAS* and *LRP1B* - genes previously associated with DLBCL. In the second patient, we analyzed a biopsy sample of one of his polymorphic PTLD and a subsequently developed ALK-ALCL. No shared TR rearrangement was identified between these samples, the WES analysis is currently ongoing. No germline variants predisposing to lymphoid malignancy were identified in either of these patients.

### Summary/Conclusion

Though multiple mechanisms probably play a role in the development of sequential PTLDs, our findings demonstrate that both lesions can originate from the same B-cell clone, as shown in one of our patients. Morphological progression may be driven by the acquisition of additional pathogenic variants. Analysis of further cases could uncover novel mechanisms that might also be relevant to the pathogenesis of sporadic non-Hodgkin lymphomas.

*Supported by the Ministry of Health of the Czech Republic in cooperation with the Czech Health Research Council under project No. NW25J-03-00076.*

**Copyright Information:** (Online) ISSN: 2572-9241
©2026 The Author(s). HemaSphere is published by John Wiley & Sons Ltd on behalf of European Hematology Association. This is an open access Abstract Book distributed under the Attribution-NonCommercial-NoDerivs (CC BY-NC-ND), which allows third parties to download the articles and share them with others as long as they credit the author and the Abstract Book, but they cannot change the content in any way or use them commercially.
**Abstract Book Citations:** Authors, Title, HemaSphere, 2026;10:(S1):pages. The abstract book DOIs can be found on the “Author Information” page.
**Disclaimer:** Articles published in the journal HemaSphere exclusively reflect the opinions of the authors. The authors are responsible for all content in their abstracts including accuracy of the facts, statements, citing resources, etc.

### (PB3899) THE EPIDEMIOLOGICAL BURDEN OF HEMATOLOGICAL NEOPLASMS AMONG PEDIATRIC POPULATION IN SPAIN, 2009-2018

**Topic:** 20. Other aggressive lymphomas

Aina Romaguera*[1, 2, 3], Jan Trallero[2, 3, 4], Rafael Marcos-Gragera[2, 3, 4, 5], María Isabel Palacios[6], Marta Muncunill[7], Antonia Sánchez-Gil[8], Alba Gasque[9], Clàudia Pla[3, 10], Noura Jeghalef el Karoui[11], Adela Cañete[12], Patricia Camporro Burguillo[13], Xitama Álvarez[14], Montserrat Garrido[14], Fernando Almela[15], Sonia Gil[16], Arantza Lopez-de-Munain[17], Marta De la Cruz[17], Silvia Sanclemente[17], Óscar Mendoza-García[18], Marta Solans[4, 5], Arantza Sanvisens[2, 3]

*[1]University of Girona, Department of Computer Science, Applied Mathematics and Statistics, University of Girona, Girona, Spain., Girona, Spain; [2]Catalan Institute of Oncology, Girona Biomedical Research Institute Dr. Josep Trueta (IDIBGI-CERCA), Spain., Epidemiology Unit and Girona Cancer Registry, Girona, Spain; [3]Josep Carreras Leukaemia Research Institute, Girona, Spain; [4]University of Girona, Research Group on Statistics, Econometrics and Health (GRECS), University of Girona, Spain., Girona, Spain; [5]CIBER of Epidemiology and Public Health (CIBERESP), Madrid, Spain; [6]Directorate General of Public Health, Consumer Affairs and Care, La Rioja, Epidemiology and Health Prevention Service, Logroño, Spain; [7]Mallorca Cancer Registry, Palma de Mallorca, Spain; [8]Murcia Cancer Registry, Murcia, Spain; [9]Navarre Institute of Public and Occupational Health, Navarre, Spain; [10]Hospital Universitari Sant Joan de Reus, Institut de Recerca Biomèdica Catalunya Sud, Cancer Epidemiology and Prevention Service, Reus, Spain; [11]Registry of childhood tumors of the Valencian Community, València, Spain; [12]Spanish Registry of Childhood Tumors (RETI-SEHOP), València, Spain; [13]Public Health Directorate, Cancer Registry of Asturias., Oviedo, Spain; [14]Canarias Cancer Registry, Las Palmas de Gran Canaria, Spain; [15]Castellón Tumor Registry, Castellón, Spain; [16]Department of Health, Castilla y León Cancer Registry, Valladolid, Spain; [17]Basque Government, Basque Country Cancer Registry, Vitoria-Gasteiz, Spain; [18]Escuela Andaluza de Salud Pública (EASP), Granada, Spain;*

### Background

Hematological malignancies (HM) are the most common cancers in children worldwide and are classically divided into leukemias and lymphomas.

### Aims

To analyze the incidence rates, trends, projections for 2026, and survival estimates of the HM in the Spanish pediatric population.

### Methods

All primary HM (<15 years of age) were collected from the population-based Spanish Network of Cancer Registries (REDECAN) and the Spanish Pediatric Tumor Registry (RETI) databases, which cover 64.5% of the Spanish pediatric population, from 2009 to 2018. Tumor cases were coded according to the International Classification of Diseases for Oncology, 3rd edition (ICD-O-3), and grouped based on the International Classification of Childhood Cancer 3rd edition (ICCC-3): 1) Leukemias (lymphoid leukemias, myeloid leukemias, and others), 2) Lymphomas (Hodgkin lymphomas, non-Hodgkin lymphomas, and others).
Crude rates (CR) and age-standardized incidence rates (ASRe) (using the European 2013 standard population) were calculated and expressed as cases per 1,000,000 children-years. Incidence trends were estimated using *Poisson* generalized linear models and expressed as the annual percentage change (APC). Incidence projections for 2026 were expressed as number of cases, CR, and ASRe for the entire Spanish pediatric population. Observed survival (OS) at 5-year was calculated using the *Kaplan-Meier* estimator.

**Copyright Information:** (Online) ISSN: 2572-9241
©2026 The Author(s). HemaSphere is published by John Wiley & Sons Ltd on behalf of European Hematology Association. This is an open access Abstract Book distributed under the Attribution-NonCommercial-NoDerivs (CC BY-NC-ND), which allows third parties to download the articles and share them with others as long as they credit the author and the Abstract Book, but they cannot change the content in any way or use them commercially.
**Abstract Book Citations:** Authors, Title, HemaSphere, 2026;10:(S1):pages. The abstract book DOIs can be found on the "Author Information" page.
**Disclaimer:** Articles published in the journal HemaSphere exclusively reflect the opinions of the authors. The authors are responsible for all content in their abstracts including accuracy of the facts, statements, citing resources, etc.

**Results**

A total of 3,078 cases were retrieved, with a mean age at diagnosis of 6.4 (standard deviation ± 4.3) years. More than half of the diagnoses were in boys (60%). Leukemias (68.8%) were more common than lymphomas (31.2%). Among leukemias, the most common histotype was lymphoid leukemias (77.2%), while among lymphomas it was Hodgkin lymphomas (HL) (32.6%). The overall ASRe was 69.2 cases per 1,000,000 children-years [95%CI: 66.8;72.3], with a boys:girls ratio of 1.4. Incidence trends were stable, with 447 new HM cases projected for 2026. The OS for the entire pediatric population was 87.5%, which was similar for both sexes but differed notably according to histotype. Lymphomas had a better prognosis than leukemias (94.8% and 84.2%, respectively). Myeloid leukemia had the worst prognosis (70.0%), while HL had the best (97.2%).

**Summary/Conclusion**

This study provides an overview of the incidence and survival of HM in Spain in children, which is crucial for public health authorities and clinicians, as well as for improving understanding of these diseases in such vulnerable demographicgroup.

**Copyright Information:** (Online) ISSN: 2572-9241
©2026 The Author(s). HemaSphere is published by John Wiley & Sons Ltd on behalf of European Hematology Association. This is an open access Abstract Book distributed under the Attribution-NonCommercial-NoDerivs (CC BY-NC-ND), which allows third parties to download the articles and share them with others as long as they credit the author and the Abstract Book, but they cannot change the content in any way or use them commercially.
**Abstract Book Citations:** Authors, Title, HemaSphere, 2026;10:(S1):pages. The abstract book DOIs can be found on the “Author Information” page.
**Disclaimer:** Articles published in the journal HemaSphere exclusively reflect the opinions of the authors. The authors are responsible for all content in their abstracts including accuracy of the facts, statements, citing resources, etc.

### (PB3900) REAL-WORLD TOXICITY AND DOSE-LEVEL DYNAMICS OF R-DA-EPOCH IN AGGRESSIVE B-CELL LYMPHOMAS

**Topic:** 20. Other aggressive lymphomas

Marta Rodríguez-Quecedo*[1], Gabriela Abreu-Nazco[1], Irati Uriarte-Gómez[1], Eukene Gainza-González[1], Garazi Letamendi-Madariaga[1], Francisco Javier Goikolea-Ugarte[1], Mercedes Cárdenas-Sierra[1], Jose Luis Suárez-Laguna[1]

[1]*Hospital Universitario Galdakao-Usansolo, Usansolo, Spain;*

### Background

R-DA-EPOCH (rituximab plus dose-adjusted EPOCH: etoposide, prednisone, vincristine, cyclophosphamide, doxorubicin) is widely used for aggressive B-cell lymphomas such as high-grade B-cell lymphoma (HGBL) and primary mediastinal B-cell lymphoma (PMBL). In routine practice, escalation is constrained by infectious, hematologic and neurotoxic adverse events; dose-level real-world data are limited.

### Aims

To describe dose-level distribution, escalation/de-escalation dynamics and toxicity patterns of R-DA-EPOCH at a tertiary-care hospital.

### Methods

Single-centre retrospective cohort of patients starting R-DA-EPOCH (Jan 2019-Feb 2026). One lead-in R-CHOP (rituximab, cyclophosphamide, doxorubicin, vincristine, prednisone) cycle was allowed when diagnosis was pending and was not assigned to a dose level. Dose levels were adjusted per cycle based on complete blood counts twice weekly in weeks 2-3: escalated if absolute neutrophil count (ANC) nadir >0.5×10^9/L; maintained if ANC nadir <0.5×10^9/L in 1-2 measurements; de-escalated if ANC nadir <0.5×10^9/L in ≥3 measurements or platelet nadir <25×10^9/L. Toxicities (infectious complications, febrile neutropenia (FN), peripheral neuropathy (PNP), gastrointestinal, non-febrile hematologic, cardiovascular) were collected from clinical records. Reported events correspond to episodes requiring medical intervention. FN was defined as temperature ≥38.3°C with ANC <500/µL (or expected to fall below). Infectious complications were recorded separately and could co-occur with FN. Events were counted as discrete episodes; multiple events could occur in one cycle. Analyses were performed per dose level; patients could contribute to multiple levels.

### Results

Twenty-two patients were included (54.5% male), median age 47 (range 20-72); three (13.6%) were ≥60 years. Diagnoses: HGBL n=11, PMBL n=10, mediastinal gray-zone lymphoma n=1; 5/22 (22.7%) received lead-in R-CHOP. Advanced stage (III-IV) was present in 14/22 (63.6%). International Prognostic Index (IPI) was available for 21 patients: low (0-1) 8/21 (38.1%), intermediate (2-3) 7/21 (33.3%), high (4-5) 6/21 (28.6%). A total of 118 R-DA-EPOCH cycles were delivered; dose-level exposure and overall adverse-event density per cycle are shown in Figure 1. Maximum dose level achieved was: 1 (n=2), 2 (n=5), 3 (n=5), 4 (n=8), 5 (n=1), 6 (n=1). Across all cycles, the most frequent toxicities were infectious complications (24 episodes; 0.21/cycle) and FN (20; 0.17/cycle). Gastrointestinal toxicity occurred in 16 episodes (0.14/cycle), mainly mucositis and nausea. Vincristine-associated PNP occurred in 17 episodes (0.15/cycle) and tended to cluster at higher dose levels. Non-febrile hematologic events were uncommon (4; 0.03/cycle) and cardiovascular toxicity was rare (2; 0.02/cycle) and generally not dose-limiting. In patients ≥60 years (n=3), no escalation beyond level 3 was achieved; all experienced significant infections requiring hospitalization, including one septic shock episode.

**Copyright Information:** (Online) ISSN: 2572-9241
©2026 The Author(s). HemaSphere is published by John Wiley & Sons Ltd on behalf of European Hematology Association. This is an open access Abstract Book distributed under the Attribution-NonCommercial-NoDerivs (CC BY-NC-ND), which allows third parties to download the articles and share them with others as long as they credit the author and the Abstract Book, but they cannot change the content in any way or use them commercially.
**Abstract Book Citations:** Authors, Title, HemaSphere, 2026;10:(S1):pages. The abstract book DOIs can be found on the "Author Information" page.
**Disclaimer:** Articles published in the journal HemaSphere exclusively reflect the opinions of the authors. The authors are responsible for all content in their abstracts including accuracy of the facts, statements, citing resources, etc.

**Summary/Conclusion**

Adverse-event density increased at higher dose levels, with a marked burden at level 4 despite fewer administered cycles, driven mainly by infectious/FN events and neuropathy. Escalation beyond level 3 was not achieved in the small ≥60-year subgroup. Findings support infection monitoring and early neuropathy surveillance to guide individualized escalation.

| Dose level | Cycles (n) | Events (n) | Events/cycle |
|---|---|---|---|
| 1 | 33 | 17 | 0.52 |
| 2 | 35 | 18 | 0.51 |
| 3 | 34 | 28 | 0.82 |
| 4 | 13 | 15 | 1.15 |
| 5 | 2 | NR | NR |
| 6 | 1 | NR | NR |

NR: Non reported

Figure 1. Dose-level exposure and overall adverse-event density per cycle

**Copyright Information:** (Online) ISSN: 2572-9241
©2026 The Author(s). HemaSphere is published by John Wiley & Sons Ltd on behalf of European Hematology Association. This is an open access Abstract Book distributed under the Attribution-NonCommercial-NoDerivs (CC BY-NC-ND), which allows third parties to download the articles and share them with others as long as they credit the author and the Abstract Book, but they cannot change the content in any way or use them commercially.
**Abstract Book Citations:** Authors, Title, HemaSphere, 2026;10:(S1):pages. The abstract book DOIs can be found on the "Author Information" page.
**Disclaimer:** Articles published in the journal HemaSphere exclusively reflect the opinions of the authors. The authors are responsible for all content in their abstracts including accuracy of the facts, statements, citing resources, etc.

### (PB3901) PRESENTATION AND CLINICAL OUTCOMES IN ANAPLASTIC LARGE CELL LYMPHOMA PATIENTS DIAGNOSED IN SASKATCHEWAN, CANADA: A POPULATION-BASED COHORT STUDY

**Topic:** 20. Other aggressive lymphomas

Brooke Roeges*[1, 2], Montana Mellor[1, 2], Shirmin Kader[2], Mohamed Elemary[3], Waleed Sabry[3], Prosanta Mondal[4], Ibraheem Othman[5]

*[1]University of Saskatchewan, College of Medicine, Regina, Canada; [2]Saskatchewan Health Authority, Research Department, Regina, Canada; [3]Saskatoon Cancer Center, Medical Oncology & Hematology, Saskatoon, Canada; [4]University of Saskatchewan, Community Health & Epidemiology, Saskatoon, Canada; [5]Allan Blair Cancer Center, Medical Oncology & Hematology, Regina, Canada;*

### Background

Anaplastic large cell lymphoma (ALCL) is a rare subtype of peripheral T-cell lymphoma accounting for 3-5% of all non-Hodgkin's lymphomas, with distinct clinical and prognostic differences between ALK-positive and ALK-negative subtypes. The incidence of ALCL is approximately 0.25 cases per 100,000 people annually, with ALK-positive cases typically demonstrating superior outcomes compared to ALK-negative.

### Aims

Primary:
1) To describe the presentation, clinical characteristics, treatment, and survival outcomes of patients diagnosed with ALCL in Saskatchewan.
Secondary:
1) To determine the proportion of ALK-positive versus ALK-negative ALCL in Saskatchewan
2) To explore potential geographic or demographic patterns, including the observed rural predominance

### Methods

We conducted a retrospective population-based analysis of all ALCL cases diagnosed in Saskatchewan between 2010 and 2024. Patient demographics, clinical characteristics, pathological features, treatment regimens, and survival outcomes were analyzed using data from the Saskatchewan Cancer Registry, electronic medical records, and pathology reports. Statistical analysis included descriptive statistics for demographic and clinical variables, with survival analysis performed using appropriate methods.

### Results

We identified 31 ALCL patients with a mean age of 47.3 ± 26.5 years. Notably, 64.5% of systemic cases were ALK-positive, representing a substantial epidemiological anomaly compared to standard unselected adult cohorts where ALK-negative disease typically predominates. The majority of patients (71.0%) presented with primary nodal involvement, while 29.0% exhibited extranodal manifestations. Strikingly, 83.9% of the cohort resided in rural agricultural zones. First-line treatment reflected standard-of-care: 71.0% received intensive anthracycline-based chemotherapy (CHOEP: 27.3%; CHOP: 18.2%). Consolidative stem cell transplantation was realized in 16.1% of patients (80% autologous, 20% allogeneic). The overall mortality rate for systemic disease was 41.9%, aligning with expected survival curves for high-risk, pre-brentuximab PTCL cohorts.

**Copyright Information:** (Online) ISSN: 2572-9241
©2026 The Author(s). HemaSphere is published by John Wiley & Sons Ltd on behalf of European Hematology Association. This is an open access Abstract Book distributed under the Attribution-NonCommercial-NoDerivs (CC BY-NC-ND), which allows third parties to download the articles and share them with others as long as they credit the author and the Abstract Book, but they cannot change the content in any way or use them commercially.
**Abstract Book Citations:** Authors, Title, HemaSphere, 2026;10:(S1):pages. The abstract book DOIs can be found on the “Author Information” page.
**Disclaimer:** Articles published in the journal HemaSphere exclusively reflect the opinions of the authors. The authors are responsible for all content in their abstracts including accuracy of the facts, statements, citing resources, etc.

**Summary/Conclusion**

This Saskatchewan population-based study reveals several notable findings that contribute to our understanding of ALCL epidemiology and outcomes. The unusually high proportion of ALK-positive cases (64.52%) compared to typical adult cohorts suggests potential regional variations in ALCL biology or referral patterns. The striking rural predominance (83.87%) represents a unique geographic finding that warrants further investigation into potential environmental, genetic, or healthcare access factors.
The mortality rate of 41.94% falls between expected outcomes for ALK-positive (typically 10-30% mortality) and ALK-negative (50-65% mortality) ALCL, consistent with the mixed population studied. Treatment patterns reflected standard-of-care approaches with CHOP-based regimens, though the study period likely preceded widespread adoption of brentuximab vedotin.
These findings highlight the importance of population-based studies in understanding real-world ALCL outcomes and the rural predominance observed in Saskatchewan suggest that geographic and demographic factors may influence disease presentation and outcomes. Future research should focus on investigating the unique epidemiological patterns observed in this Canadian prairie population.

**Copyright Information:** (Online) ISSN: 2572-9241
©2026 The Author(s). HemaSphere is published by John Wiley & Sons Ltd on behalf of European Hematology Association. This is an open access Abstract Book distributed under the Attribution-NonCommercial-NoDerivs (CC BY-NC-ND), which allows third parties to download the articles and share them with others as long as they credit the author and the Abstract Book, but they cannot change the content in any way or use them commercially.
**Abstract Book Citations:** Authors, Title, HemaSphere, 2026;10:(S1):pages. The abstract book DOIs can be found on the “Author Information” page.
**Disclaimer:** Articles published in the journal HemaSphere exclusively reflect the opinions of the authors. The authors are responsible for all content in their abstracts including accuracy of the facts, statements, citing resources, etc.

### (PB3902) GOLIDOCITINIB MITOXANTRONE HYDROCHLORIDE LIPOSOME CHIDAMIDE AND PREDNISONE (GO-MCP) IN RELAPSED/REFRACTORY PERIPHERAL T-CELL LYMPHOMA:A REAL-WORLD RETROSPECTIVE STUDY

**Topic:** 20. Other aggressive lymphomas

Luxuan Xu[1], Ziying Jian[1], Zheng Ge*[1]

[1]*Zhongda Hospital Southeast University, Institute of Hematology Southeast University, Nanjing, China;*

### Background

Peripheral T-cell lymphomas (PTCLs) are heterogeneous aggressive malignancies with poor outcomes.Patients are easyly progressed to relapsed/refractory(R/R) diseases. The 3-year survival rate of R/R PTCLs was very low. Golcitinib, an oral selective JAK1 inhibitor, has demonstrated promising therapeutic efficacy in R/R PTCLs in clinical trials. Accordingly, we designed the Go-MCP regimen, which combines golidocitinib with mitoxantrone hydrochloride liposome, chidamide, and prednisone, and conducted a observational study to evaluate its efficacy and safety .

### Aims

To evaluate the efficacy, safety of Go-MCP regiment in patients with peripheral T-cell lymphoma, and provide a new treatment option.

### Methods

Enrolled patients received the Go-MCP regimen: golidocitinib 150 mg once daily, mitoxantrone hydrochloride liposome 12mg/m² on day 1, chidamide 15 mg twice weekly, prednisone 100 mg on days 1–5, in 21-day cycles. All recruited patients received ≥1 cycle of Go-MCP therapy. Response was assessed every 3–4 cycles via PET-CT or other appropriate imaging based on disease sites. Statistical analyses were conducted using R studio.

### Results

Seven patients (2 male, 5 female) were enrolled,one discontinued due to herpes zoster. Histologic subtypes included PTCL-NOS (n=3), angioimmunoblastic T-cell lymphoma (n=2) and unclassifiable peripheral T-cell lymphoma (n=2).
At data cutoff, mean OS was 107 days (SD 53.5, range 39–190), and mean PFS was 105 days (SD 55.1, range 0–190). Patients received a median of 3.57 cycles (range 1–7) of standard-intensity Go-MCP.
Of note, one PTCL patient with CNS relapse after five cycles of CHOP (cyclophosphamide, doxorubicin, vincristine, and prednisone) and subsequent progression on second-line methotrexate/temozolomide with intrathecal therapy achieved CR on PET-CT after 7 cycles of Go-MCP.This result indicated promising efficency in CNS relapse patients and in mitoxantrone exposure patients.Two enrolled patients have proceeded to autologous stem cell transplantation following Go-MCP treatment, with the longest progression-free survival (PFS) reaching 190 days to date, demonstrating deep remission of Go-MCP regimen.
The dominant treatment-related adverse events were hematologic toxicity and infections, but all adverse events were controllable and there were no fatal adverse events. Anemia and myelosuppression occurred in all patients. Grade 3 infections occurred in five patients (71.4%, including three of pulmonary infection, two of skin infection, and one of abdominal infection); No hemolysis or other hematolymphatic disorders were observed.

**Copyright Information:** (Online) ISSN: 2572-9241
©2026 The Author(s). HemaSphere is published by John Wiley & Sons Ltd on behalf of European Hematology Association. This is an open access Abstract Book distributed under the Attribution-NonCommercial-NoDerivs (CC BY-NC-ND), which allows third parties to download the articles and share them with others as long as they credit the author and the Abstract Book, but they cannot change the content in any way or use them commercially.
**Abstract Book Citations:** Authors, Title, HemaSphere, 2026;10:(S1):pages. The abstract book DOIs can be found on the “Author Information” page.
**Disclaimer:** Articles published in the journal HemaSphere exclusively reflect the opinions of the authors. The authors are responsible for all content in their abstracts including accuracy of the facts, statements, citing resources, etc.

**Summary/Conclusion**

In conclusion, the Go-MCP regimen demonstrates preliminary efficacy and a generally manageable safety profile in patients with R/R PTCL, but larger prospective studies with extended follow-up are needed to confirm its long-term therapeutic value.

**Copyright Information:** (Online) ISSN: 2572-9241
©2026 The Author(s). HemaSphere is published by John Wiley & Sons Ltd on behalf of European Hematology Association. This is an open access Abstract Book distributed under the Attribution-NonCommercial-NoDerivs (CC BY-NC-ND), which allows third parties to download the articles and share them with others as long as they credit the author and the Abstract Book, but they cannot change the content in any way or use them commercially.
**Abstract Book Citations:** Authors, Title, HemaSphere, 2026;10:(S1):pages. The abstract book DOIs can be found on the "Author Information" page.
**Disclaimer:** Articles published in the journal HemaSphere exclusively reflect the opinions of the authors. The authors are responsible for all content in their abstracts including accuracy of the facts, statements, citing resources, etc.

### (PB3903) TUMOR MICROENVIRONMENT–ASSOCIATED GENE EXPRESSION AND IMMUNE INFILTRATION PATTERNS IN DIFFUSE LARGE B-CELL AND HODGKIN LYMPHOMAS PATIENTS

**Topic:** 20. Other aggressive lymphomas

Magdalena Witkowska*[1], Dariusz Jarych[2], Sława Glińska[3], Edyta Paradowska[2], Agata Majchrzak[4], Michał Juraniec[2], Tadeusz Robak[5], Paweł Robak[5], Agnieszka Wierzbowska[5]

*[1]dical university in lodz, department of hematology, łódź, Poland;[2]Polish Academy of Sciences, Łódź, Laboratory of Virology, Institute of Medical Biology, łódź, Poland; [3]university of lodz, Laboratory of Microscopic Imaging and Specialized Biological Techniques, łódź, Poland; [4]M. Copernicus Regional Multidisciplinary Center of Oncology and Traumatology in Łódź, Department of General Hematology and Internal Medicine, łódź, Poland; [5]Medical University of Lodz, Department of Haematology, łódź, Poland;*

### Background

Lymphoma represent complex ecosystems comprising cancer cells and a multitude of non-cancerous cells, embedded in an altered extracellular matrix. Researches showed that the role of the tumor microenvironment (TME) in the progression and immunosuppression of lymphomas, have increased considerably in recent years, allowing for a better understanding of the disease. TME critically influences lymphoma biology through interactions between malignant cells, immune infiltrates, and vascular–stromal signaling pathways, including ANGPT/TIE, CXCL12–CXCR4, NOTCH, SLIT/ROBO, and SPP1. Data integrating gene expression in FFPE lymphoma tissue with immune cell profiles remain limited.

### Aims

The main aim of this study was to assess the expression of genes related to angiogenesis, migration, adhesion, and immune regulation (*ANGPT1, CD44, CXCL12, CXCR4, JAG1, NOTCH1, ROBO4, SLIT2, SPP1, TEK*) in FFPE lymphoma tissue and their associations with peripheral blood immune cell subsets in diffuse large B-cell lymphoma (DLBCL), Hodgkin lymphoma (HL), and other lymphoma subtypes.

### Methods

FFPE samples from 27 patients (14 DLBCL, 9 HL, and other lymphomas including MCL, MZL, and THRLBCL) were analyzed. RNA was isolated from microtome-cut FFPE sections using the AllPrep DNA/RNA FFPE Kit (Qiagen) and reverse-transcribed with the High Capacity cDNA Reverse Transcription Kit (Thermo Fisher Scientific). Gene expression was quantified using TaqMan Gene Expression Assays on a QuantStudio™ 5 PCR System. Raw Ct values were analyzed with QuantStudio™ Design and Analysis Software v1.6.1. Statistical analyses were performed in Statistica v13.3.721.0 (TIBCO). Group comparisons were conducted using the Mann–Whitney U test, and correlations were assessed with Spearman's rank correlation.

**Copyright Information:** (Online) ISSN: 2572-9241
©2026 The Author(s). HemaSphere is published by John Wiley & Sons Ltd on behalf of European Hematology Association. This is an open access Abstract Book distributed under the Attribution-NonCommercial-NoDerivs (CC BY-NC-ND), which allows third parties to download the articles and share them with others as long as they credit the author and the Abstract Book, but they cannot change the content in any way or use them commercially.
**Abstract Book Citations:** Authors, Title, HemaSphere, 2026;10:(S1):pages. The abstract book DOIs can be found on the "Author Information" page.
**Disclaimer:** Articles published in the journal HemaSphere exclusively reflect the opinions of the authors. The authors are responsible for all content in their abstracts including accuracy of the facts, statements, citing resources, etc.

### Results

HL patients exhibited higher CD3+ T-cell proportions than DLBCL patients (median 78.2% vs. 73.5%, p=0.044). DLBCL showed a trend toward higher CD19+ levels compared with HL (p=0.066) and significantly higher CD19+ levels than other lymphoma subtypes (p=0.016).

DLBCL demonstrated higher *ANGPT1* (p=0.011) and *ROBO4* (p=0.038) expression compared with other lymphomas, with a trend toward increased *JAG1* expression (p=0.074). In HL, *ANGPT1* expression was higher than in other lymphomas (p=0.021), accompanied by higher TEK expression (p=0.037) and a trend toward increased *CXCL12* expression (p=0.053).

In DLBCL, CD8+ T-cell levels negatively correlated with *TEK* expression (R = −0.62, p=0.043). In HL, negative correlations were observed between CD3+ cells and *CXCR4* (R = −0.69, p=0.038), CD3+ cells and *ROBO4* (R = −0.74, p=0.035), and between CD8+ cells and *SPP1* expression (R = −0.89, p=0.0068).

### Summary/Conclusion

Distinct immune–molecular profiles characterize lymphoma subtypes. Elevated *ANGPT1* and *ROBO4* expression in DLBCL suggests enhanced angiogenic and vascular signaling potentially linked to reduced cytotoxic T-cell presence. HL displays pronounced T-cell enrichment with increased *ANGPT1, TEK*, and *CXCL12* expression, indicating active vascular–stromal signaling within an immune-rich TME. These findings highlight subtype-specific regulation of angiogenic and migratory pathways as key modulators of immune architecture and potential therapeutic targets.

**Copyright Information:** (Online) ISSN: 2572-9241
©2026 The Author(s). HemaSphere is published by John Wiley & Sons Ltd on behalf of European Hematology Association. This is an open access Abstract Book distributed under the Attribution-NonCommercial-NoDerivs (CC BY-NC-ND), which allows third parties to download the articles and share them with others as long as they credit the author and the Abstract Book, but they cannot change the content in any way or use them commercially.
**Abstract Book Citations:** Authors, Title, HemaSphere, 2026;10:(S1):pages. The abstract book DOIs can be found on the “Author Information” page.
**Disclaimer:** Articles published in the journal HemaSphere exclusively reflect the opinions of the authors. The authors are responsible for all content in their abstracts including accuracy of the facts, statements, citing resources, etc.

### (PB3904) EPIDEMIOLOGICAL, CLINICAL, BIOLOGICAL CHARACTERISTICS AND THERAPEUTIC OUTCOMES OF BURKITT LYMPHOMA: A SINGLE-CENTER STUDY IN TUNISIA

**Topic:** 20. Other aggressive lymphomas

Daami Mayssa*[1], Bizid Inaam[1], Wiem Boufrikha[1], Ameni Yahia[1], Zaineb Mlayah[1], Slama Nader[1], Mohamed Adnene Laatiri[1], Sarra Boukhriss[1]

[1]*Fattouma Bourguiba University Hospital, clinical hematology, MONASTIR, Tunisia;*

### Background

Burkitt lymphoma (BL) is a highly aggressive mature B-cell lymphoma characterized by rapid tumor proliferation driven by MYC dysregulation. Despite significant improvements in prognosis with intensive polychemotherapy, the disease frequently presents at an advanced stage and carries a high risk of early complications, which may adversely affect overall outcomes and survival.

### Aims

To describe the epidemiological, clinical, biological characteristics, tumor burden parameters, and therapeutic outcomes of patients with BL treated at our center.

### Methods

We conducted a retrospective, descriptive, single-center study including patients diagnosed with BL at the Department of Clinical Hematology in Monastir, Tunisia, between January 2015 and December 2025.
Statistical analysis was performed using SPSS version 25. Survival rates were estimated using the Kaplan–Meier method.

**Copyright Information:** (Online) ISSN: 2572-9241
©2026 The Author(s). HemaSphere is published by John Wiley & Sons Ltd on behalf of European Hematology Association. This is an open access Abstract Book distributed under the Attribution-NonCommercial-NoDerivs (CC BY-NC-ND), which allows third parties to download the articles and share them with others as long as they credit the author and the Abstract Book, but they cannot change the content in any way or use them commercially.
**Abstract Book Citations:** Authors, Title, HemaSphere, 2026;10:(S1):pages. The abstract book DOIs can be found on the "Author Information" page.
**Disclaimer:** Articles published in the journal HemaSphere exclusively reflect the opinions of the authors. The authors are responsible for all content in their abstracts including accuracy of the facts, statements, citing resources, etc.

**Results**

Twenty-five patients were included. The median age at diagnosis was 23 years (range, 3–72), with 10 patients (40%) aged over 18 years. The male-to-female ratio was 2.12. The most common presenting symptom was abdominal pain (40%). B symptoms were observed in five patients (20%).
Initial CT imaging was performed in all patients. Gastrointestinal involvement was the most frequent site (n=10), including five cases of cecal localization, followed by nodal (n=7), hepatic (n=3), renal (n=3), and central nervous system involvement (n=2). Bulky disease was present in eight patients (32%), with tumor size ranging from 10 to 20 cm. Most patients had advanced disease according to the Murphy classification (stage III–IV: 76%). Bone marrow involvement with leukemic presentation was observed in 10 patients (40%). Elevated LDH levels were observed in 18 patients (72%). Six patients presented with biological tumor lysis syndrome (TLS), including four cases of clinical TLS.
Cytogenetic analysis was performed in all patients. Five patients had abnormal karyotypes. The t(8;14) translocation was identified in two patients. One patient presented multiple chromosomal abnormalities, including inv(2;8), inv(12;8), and del(6), with structural abnormalities involving chromosomes 2, 6, 8, and 12. Another patient had abnormalities involving chromosomes 1 and 7. One patient exhibited hyperdiploidy (51 chromosomes) associated with abnormalities of chromosomes 9 and 18. Molecular testing was not performed due to limited resources.
Risk group distribution was as follows: one patient was classified as group A, eleven as group B, and thirteen as group C. All patients received intensive polychemotherapy. Patients younger than 18 years were treated according to the LMB01 protocol, while those older than 18 years received the LMB02 protocol adapted to risk stratification. At day 8 evaluation after the first COP course (cyclophosphamide, vincristine, prednisone), a good therapeutic response (defined as a reduction in tumor mass >20%) was observed in 19 patients (76%). At the end-of-treatment evaluation, complete remission was achieved in 86% of patients. Relapse occurred in seven patients.
The estimated 5-year overall survival (OS) was 72,2 %, and the 3-year relapse-free survival (RFS) was 67.5%.

**Summary/Conclusion**

Our series confirms the predominance of advanced-stage BL at diagnosis. The 5-year OS of 72,2 % is consistent with outcomes reported in adult LMB-based series. Initial tumor burden and early metabolic complications remain major determinants of prognosis.

**Copyright Information:** (Online) ISSN: 2572-9241
©2026 The Author(s). HemaSphere is published by John Wiley & Sons Ltd on behalf of European Hematology Association. This is an open access Abstract Book distributed under the Attribution-NonCommercial-NoDerivs (CC BY-NC-ND), which allows third parties to download the articles and share them with others as long as they credit the author and the Abstract Book, but they cannot change the content in any way or use them commercially.
**Abstract Book Citations:** Authors, Title, HemaSphere, 2026;10:(S1):pages. The abstract book DOIs can be found on the “Author Information” page.
**Disclaimer:** Articles published in the journal HemaSphere exclusively reflect the opinions of the authors. The authors are responsible for all content in their abstracts including accuracy of the facts, statements, citing resources, etc.

### (PB3905) PRIMARY GASTROINTESTINAL LYMPHOMAS: A CLINICOPATHOLOGIC ANALYSIS OF A SINGLE-CENTER COHORT

**Topic:** 20. Other aggressive lymphomas

Wafa Sakli*[1], Anaghim Aloui[1], Jaied Rabeb[1], Amine Ben Moussa[1], Sami Zriba[1], Hela Ghedira[1], Fehmi Msadek[1]

[1]*The Principal Military Hospital of Instruction of Tunis (HMPIT), Hematology, Tunis, Tunisia;*

### Background

Primary gastrointestinal lymphomas (PGIL) represent the most common type of extranodal non-Hodgkin lymphoma (NHL). Their clinical presentation and outcomes are heterogeneous, largely determined by histological subtype and disease stage.

### Aims

To analyze the epidemiological, clinical, and histopathological features, as well as the treatment outcomes, of patients diagnosed with PGIL at our center.

### Methods

We conducted a retrospective single-center study at the Military Teaching Hospital of Tunis including patients diagnosed with PGIL between January 2008 and December 2023. Collected data included patient demographics, tumor location, histological subtype, stage (according to the Lugano International Conference classification), laboratory data, treatment modalities, and clinical outcomes.

### Results

A total of 49 patients with PGIL were identified among 387 cases of NHL during the study period (12.6%). The median age at diagnosis was 63 years (range, 35–88), with a male predominance (sex ratio 1.45). The median time from symptom onset to diagnosis was 4 months. Performance status ≥2 was observed in 31.7% of patients. Peripheral lymphadenopathy was present in 17% of cases, splenomegaly in 22.6%, and hepatomegaly in 16%. The stomach was the most frequently involved site (53.6%), followed by ileal and small intestinal localizations. Concomitant extranodal involvement was observed in 32% of patients. Anemia was present in 40% of patients (median hemoglobin level 11.1 g/dL). Elevated LDH levels and hypoalbuminemia were observed in 20% and 8% of cases, respectively. Diffuse large B-cell lymphoma (DLBCL) was the predominant histological subtype (71.4%), followed by marginal zone lymphoma (n=4), mantle cell lymphoma (n=4), T-cell lymphoma (n=3), follicular lymphoma (n=1), and Burkitt lymphoma (n=2). Advanced stage disease was observed in 53.2% of patients. Regarding treatment, R-CHOP was the most frequently administered regimen (71.7%). Complete remission was achieved in 75.4% of patients, while primary treatment failure occurred in 12.1%. At last follow-up, 81.4% of patients were alive, of whom 77.8% were in complete remission.

### Summary/Conclusion

This real-world study highlights the predominance of aggressive histological subtypes and the high proportion of advanced-stage diagnoses in our setting. These findings emphasize the need for earlier diagnosis and optimization of treatment strategies adapted to the regional epidemiological context. Further prospective multicenter studies are warranted to better define prognostic factors and improve outcomes in North African patients with PGIL.

**Copyright Information:** (Online) ISSN: 2572-9241
©2026 The Author(s). HemaSphere is published by John Wiley & Sons Ltd on behalf of European Hematology Association. This is an open access Abstract Book distributed under the Attribution-NonCommercial-NoDerivs (CC BY-NC-ND), which allows third parties to download the articles and share them with others as long as they credit the author and the Abstract Book, but they cannot change the content in any way or use them commercially.
**Abstract Book Citations:** Authors, Title, HemaSphere, 2026;10:(S1):pages. The abstract book DOIs can be found on the "Author Information" page.
**Disclaimer:** Articles published in the journal HemaSphere exclusively reflect the opinions of the authors. The authors are responsible for all content in their abstracts including accuracy of the facts, statements, citing resources, etc.

### (PB3906) BRENTUXIMAB VEDOTIN COMBINED WITH CHIDAMIDEIN AND MITOXANTRONE HYDROCHLORIDE LIPOSOME OR GOLIDOCITINIB IN RELAPSED/REFRACTORY CD30-POSITIVE CUTANEOUS T-CELL LYMPHOMA: A REAL-WORLD STUDY DESIGN

**Topic:** 20. Other aggressive lymphomas

Qian Wang*[1], Qinyuan Zhu[2], Ziqi Liu[2], Shanglin Jin[2], Hui Yang[1], Yimin Ren[1], Qiong Huang[2], Tianling Ding[1], Wenyu Wu[2], Tong Chen[1]

[1]*Department of Hematology, Huashan Hospital Affiliated to Fudan University, No. 12, Urumqi, China, Shanghai, China;*
[2]*Department of Dermatology, Huashan Hospital Affiliated to Fudan University, No. 12, Urumqi, China, Shanghai, China;*

### Background

Cutaneous T-cell lymphomas (CTCLs), characterized by recurrent and progressive course, are a group of rare and heterogenous diseases and associated with high symptoms burden. Mycosis fungoides and Sézary syndrome (MF/SS) are the most common subtypes, accounting for 70%. For advanced MF/SS patients, systemic therapies are often used including brentuximab vedotin (BV), liposomal doxorubicin, and HDAC inhibitors. Among these, BV, a CD30-directed ADC, combined with chidamide, an HDAC inhibitor, has been proven to have a synergistic effect and recommended in relapsed/refractory (R/R) settings. Additionally, BV in combination with mitoxantrone liposomal (MIT-LIP) or golidocitinib, a selective-JAK1 inhibitor, demonstrates favorable overall response rates (ORR). These findings indicate promising efficacy for the combination therapy containing BV and chidamidein with MIT-LIP or golidocitinib in R/R MF/SS patients.

### Aims

This study aims to describe an ongoing real-world (RW) study evaluating the effectiveness and safety of combination therapy with BV and chidamidein, in combination with either MIT-LIP or golidocitinib for R/R CD30+ Chinese lymphoma patients.

### Methods

This is a prospective, observational, multicenter, RW study conducted in China between May 2026 to November 2028. Patients aged 18-69 years, with a confirmed diagnosis of R/R CD30+ MF/SS at stages IIB-IVB and an Eastern Cooperative Oncology Group performance status of ≤2, were eligible. R/R patients are defined as those who received ≥3 prior courses of systemic anti-tumor therapy without response, or with progression/relapses. Those with stage IIB disease are required to have extensive/multicentric tumoral involvement and either failed prior local radiotherapy, relapsed after it, or tumors in locations unsuitable for radiotherapy. Patients have already started combination therapy for BV plus chidamidein, in combination with either MIT-LIP or golidocitinib before the enrollment and the treatment has not exceeded 28 days. The primary endpoint is ORR lasting ≥4 months (ORR4). The secondary endpoints are the ORR (including complete response [CR]), response rate for each component (skin, blood, lymph nodes, and viscera), disease control rate (DCR), time to response, time to progression, duration of response (DOR), time to next treatment, progression-free survival (PFS), overall survival (OS), disease-free survival (DFS), quality of life, and safety. The relationship between CD30 expression and efficacy is explored as well. All the efficacy analyses will be based on the full analysis set (FAS), defined as all enrolled patients with confirmed MF/SS who received at least one dose of the study drug. The safety assessments will utilize the safety analysis set including same population in the FAS. The ORR4, ORR, CR, and DCR are summarized as proportions with two-sided 95% confidence intervals (CIs), with no data imputation. Time-to-event endpoints, including DOR, PFS, and OS, are estimated using the Kaplan-Meier method, with the median and two-sided 95% CIs reported. Safety is assessed using descriptive statistics.

**Copyright Information:** (Online) ISSN: 2572-9241
©2026 The Author(s). HemaSphere is published by John Wiley & Sons Ltd on behalf of European Hematology Association. This is an open access Abstract Book distributed under the Attribution-NonCommercial-NoDerivs (CC BY-NC-ND), which allows third parties to download the articles and share them with others as long as they credit the author and the Abstract Book, but they cannot change the content in any way or use them commercially.
**Abstract Book Citations:** Authors, Title, HemaSphere, 2026;10:(S1):pages. The abstract book DOIs can be found on the "Author Information" page.
**Disclaimer:** Articles published in the journal HemaSphere exclusively reflect the opinions of the authors. The authors are responsible for all content in their abstracts including accuracy of the facts, statements, citing resources, etc.

**Summary/Conclusion**

This study is designed to provide robust evidence for the combination therapy (BV plus chidamidein and MIT-LIP or golidocitinib) in RW settings, potentially offering a valuable therapeutic strategy in patients with R/R CD30+ CTCL. The study is recruiting, with preliminary results available in subsequent reports.

**Copyright Information:** (Online) ISSN: 2572-9241
©2026 The Author(s). HemaSphere is published by John Wiley & Sons Ltd on behalf of European Hematology Association. This is an open access Abstract Book distributed under the Attribution-NonCommercial-NoDerivs (CC BY-NC-ND), which allows third parties to download the articles and share them with others as long as they credit the author and the Abstract Book, but they cannot change the content in any way or use them commercially.
**Abstract Book Citations:** Authors, Title, HemaSphere, 2026;10:(S1):pages. The abstract book DOIs can be found on the "Author Information" page.
**Disclaimer:** Articles published in the journal HemaSphere exclusively reflect the opinions of the authors. The authors are responsible for all content in their abstracts including accuracy of the facts, statements, citing resources, etc.

### (PB3907) BURKITT'S LYMPHOMA IN CHILDREN: A REPORT OF 35 CASES - EXPERIENCE OF THE PEDIATRIC CLINICAL HEMATOLOGY DEPARTMENT OF AZIZA OTHMANA HOSPITAL.

**Topic:** 20. Other aggressive lymphomas

Ons Dagdoug*[1], Marwa Bahri[1], Roua Hsasna[1], Emna Azza[1], Ben Othmen Aya[1], Yosr Ben Abdennabi[1], Lamia Issaoui[1]

*[1]Aziza Othmena Hospital, Hematology clinic pediatric departement, Tunis, Tunisia;*

**Background**

The occurrence of Burkitt's lymphoma (BL) is frequent in the paediatric population (50%). Intensive chemotherapy remains the primary treatment, exposing patients to significant toxicity.

**Aims**

To describe the epidemiological characteristics, therapeutic outcomes and chemotoxicity of BL treatment.

**Methods**

All children diagnosed with Burkitt's lymphoma between January 2005 and May 2025 at the Pediatric Clinical Hematology Department of Aziza Othmana Hospital in Tunis were included.

**Copyright Information:** (Online) ISSN: 2572-9241
©2026 The Author(s). HemaSphere is published by John Wiley & Sons Ltd on behalf of European Hematology Association. This is an open access Abstract Book distributed under the Attribution-NonCommercial-NoDerivs (CC BY-NC-ND), which allows third parties to download the articles and share them with others as long as they credit the author and the Abstract Book, but they cannot change the content in any way or use them commercially.
**Abstract Book Citations:** Authors, Title, HemaSphere, 2026;10:(S1):pages. The abstract book DOIs can be found on the "Author Information" page.
**Disclaimer:** Articles published in the journal HemaSphere exclusively reflect the opinions of the authors. The authors are responsible for all content in their abstracts including accuracy of the facts, statements, citing resources, etc.

### Results

Thirty-five patients were included with a median age at diagnosis of 9 years [2-17] and a male-to-female sex ratio of 3.37.
The most common presenting symptom was lymph node involvement in 14 cases (40%), and multifocal involvement was observed in 21 patients (60%).
The main sites of involvement were lymph nodes (51%), bone marrow (34%), abdomen (31%), and neuromeningeal (28%).
According to the Murphy classification, patients were distributed as follows: stage I (6%), stage II (20%), stage III (14%), and stage IV (60%).
A pathological karyotype was identified in 6 patients (17%).
The median LDH level was 454 [167–6556], and tumour lysis syndrome occurred in four patients (two cases were spontaneous).

Treatment was based on the LMB 03 protocol (without immunotherapy) stratified by risk group: 15 patients (43%) in group B and 20 (57%) in group C.
Outcomes and toxicity are summarized in the table below:

| | Group B (n=15) | Group C (n=20) |
|---|---|---|
| Toxicity | | |
| Grade III to IV mucositis | 3 (20%) | 19 (95%) |
| Anal ulceration | | 3 (15%) |
| Diarrhea | | 6 (30%) |
| Renal toxicity | | 1 (5%) |
| Cytolysis (5N) | 4 (26%) | 4 20%) |
| Press syndrome | 1 (6%) | |
| Peripheral neuropathy | | 2 (10%) |
| Toxic death | | 1 (5%) |
| Outcomes | | |
| Day 7 Evaluation | CR 11 (73%) | CR 10 (50%) |
| Progression | | 5 (25%) |
| Death | | 6 (30%) |
| Complete remission (CR) | 100% | 15 (75%) |
| Progression-free survival | 100% | 15 (75%) |
| Relapse-free survival | 100% | 20 (100%) |
| Event-free survival | 100% | 16 (65%) |
| Overall survival | 100% | 14 (70%) |

Among the 35 patients, 28 (80%) had a favorable outcome, 6 (17%) died (1 from toxicity, 5 from disease progression), and 1 (3%) developed myelodysplastic syndrome.

### Summary/Conclusion

Burkitt lymphoma in children has a good prognosis when treated early with intensive chemotherapy, offering satisfactory results with an acceptable benefit-risk ratio.
Currently, rituximab is being added to pediatric protocols, and favorable therapeutic results are expected in the future.

**Copyright Information:** (Online) ISSN: 2572-9241
©2026 The Author(s). HemaSphere is published by John Wiley & Sons Ltd on behalf of European Hematology Association. This is an open access Abstract Book distributed under the Attribution-NonCommercial-NoDerivs (CC BY-NC-ND), which allows third parties to download the articles and share them with others as long as they credit the author and the Abstract Book, but they cannot change the content in any way or use them commercially.
**Abstract Book Citations:** Authors, Title, HemaSphere, 2026;10:(S1):pages. The abstract book DOIs can be found on the "Author Information" page.
**Disclaimer:** Articles published in the journal HemaSphere exclusively reflect the opinions of the authors. The authors are responsible for all content in their abstracts including accuracy of the facts, statements, citing resources, etc.

### (PB3908) BURKITT LYMPHOMA: A RETROSPECTIVE STUDY OF 51 CASES

**Topic:** 20. Other aggressive lymphomas

Nizar Dahmaoui*[1], Sophia Berrada[1], Mariem Cheikhna[1], Sara Naim[1], Meryem Quachouh[1], Benmoussa Amine[1], Mouna Lamchahab[1], Siham Cherkaoui[1], Mohamed Rachid[1], Abdellah Madani[1]

*[1]hopital 20 aout 1953 CHU Ibn rochd, Service d'hématologie clinique et d'oncologie pédiatrique, Casablanca, Morocco;*

### Background

Burkitt lymphoma is a rare, high-grade B-cell lymphoma in adults, characterized by rapid tumor proliferation and often an advanced presentation at diagnosis. Despite the effectiveness of intensive treatment protocols, this hematological malignancy remains associated with significant morbidity and mortality, related to the severity of the disease and treatment complications. Data from adult series in real-world practice remain limited.

### Aims

To determine the epidemiological profile of patients with Burkitt lymphoma followed at the clinical hematology and pediatric oncology department of the Ibn Rochd University Hospital in Casablanca

**Copyright Information:** (Online) ISSN: 2572-9241
©2026 The Author(s). HemaSphere is published by John Wiley & Sons Ltd on behalf of European Hematology Association. This is an open access Abstract Book distributed under the Attribution-NonCommercial-NoDerivs (CC BY-NC-ND), which allows third parties to download the articles and share them with others as long as they credit the author and the Abstract Book, but they cannot change the content in any way or use them commercially.
**Abstract Book Citations:** Authors, Title, HemaSphere, 2026;10:(S1):pages. The abstract book DOIs can be found on the "Author Information" page.
**Disclaimer:** Articles published in the journal HemaSphere exclusively reflect the opinions of the authors. The authors are responsible for all content in their abstracts including accuracy of the facts, statements, citing resources, etc.

### Methods

This was a single-center retrospective study conducted at the Ibn Rochd University Hospital in Casablanca between January 2014 and December 2024, including adult patients (≥18 years) treated for Burkitt lymphoma. Staging was performed according to the Murphy classification, and prognostic stratification according to the LMBA02 protocol (groups B and C). All patients were treated according to the LMBA02 protocol. Therapeutic responses, early mortality, toxic complications, and overall survival were analyzed. Overall survival was estimated using the Kaplan-Meier method.

### Results

Fifty-one patients were included, with a median age of 35.5 years [18–71] and a male-to-female ratio of 3.25. HIV infection was present in 9 patients (17%). The predominant tumor sites were gastrointestinal and peritoneal, and 65% of patients were diagnosed at an advanced stage (Murphy's stage III–IV). Thirty-four patients (66.7%) were classified as group B and 17 (33.3%) as group C. Early mortality was observed in 5 patients (10%), mainly related to tumor lysis syndrome and infectious complications. At the end of treatment, 26 patients (51%) were in complete remission. Relapses and treatment failures were observed in 11 patients (22%). After a median follow-up of 19 months, the 10-year overall survival rate was 46%. Toxic deaths accounted for 12% of cases, predominantly due to infectious complications in 4 patients and hemorrhagic complications in 2 patients.

**Copyright Information:** (Online) ISSN: 2572-9241
©2026 The Author(s). HemaSphere is published by John Wiley & Sons Ltd on behalf of European Hematology Association. This is an open access Abstract Book distributed under the Attribution-NonCommercial-NoDerivs (CC BY-NC-ND), which allows third parties to download the articles and share them with others as long as they credit the author and the Abstract Book, but they cannot change the content in any way or use them commercially.
**Abstract Book Citations:** Authors, Title, HemaSphere, 2026;10:(S1):pages. The abstract book DOIs can be found on the "Author Information" page.
**Disclaimer:** Articles published in the journal HemaSphere exclusively reflect the opinions of the authors. The authors are responsible for all content in their abstracts including accuracy of the facts, statements, citing resources, etc.

**Summary/Conclusion**

Despite the effectiveness of the LMBA02 protocol, adult Burkitt lymphoma remains associated with early mortality and significant toxicity, highlighting the need to optimize initial management and supportive care.

**Copyright Information:** (Online) ISSN: 2572-9241
©2026 The Author(s). HemaSphere is published by John Wiley & Sons Ltd on behalf of European Hematology Association. This is an open access Abstract Book distributed under the Attribution-NonCommercial-NoDerivs (CC BY-NC-ND), which allows third parties to download the articles and share them with others as long as they credit the author and the Abstract Book, but they cannot change the content in any way or use them commercially.
**Abstract Book Citations:** Authors, Title, HemaSphere, 2026;10:(S1):pages. The abstract book DOIs can be found on the "Author Information" page.
**Disclaimer:** Articles published in the journal HemaSphere exclusively reflect the opinions of the authors. The authors are responsible for all content in their abstracts including accuracy of the facts, statements, citing resources, etc.

### (PB3909) FOLLICULAR LYMPHOMA: REAL-WORLD DATA ON PRESENTATION, MANAGEMENT, AND SURVIVAL FROM A MOROCCAN TERTIARY CARE CENTER

**Topic:** 20. Other aggressive lymphomas

Zineb Afroukh*[1], Amal Terfai[1], Imane Chajai[1], Souad Fennan[1], Otman Abdelmoula[1], Maha EL Amani[1], Othman Doghmi[1], Elmaachi Nora[1], Naoufal Benlachgar[1], Soukaina Haidouri[1], Zoubida Tazi Mezalek[1]

[1]*Ibn Sina University Hospital, rabat, Morocco;*

### Background

Follicular lymphoma is the second most common non-Hodgkin lymphoma in adults. It is an indolent B-cell lymphoma that progresses slowly and recurs, with a risk of transformation of 2 to 3% per year. Despite improved outcomes with immunotherapy using anti-CD20 antibodies, prognostic heterogeneity persists. POD24 is a major prognostic marker for identifying a high-risk subgroup.

### Aims

To describe the initial characteristics of patients with follicular lymphoma, the first-line and subsequent treatments received, and their efficacy by evaluating responses and POD24, as well as progression-free survival and overall survival.

### Methods

This is a retrospective descriptive study from the Clinical Hematology Department of Ibn Sina University Hospital, including patients with follicular lymphoma diagnosed between January 2016 and July 2025. Patients who were lost to follow-up or had missing data were excluded.
Clinical, biological, radiological, and therapeutic data were collected.

### Results

Thirty-six patients were included. The median age was 55 years, with a sex ratio (M/F) of 0.9. At least one GELF criterion (clinical and/or biological) was found in 61% of patients. Staging according to the Ann Arbor classification showed 72.2% of patients to be in advanced stages.
The FLIPI, FLIPI-2, and PRIMA-PI prognostic scores were used to stratify patients into three risk groups : low risk (FLIPI: 52.7%, FLIPI2: 50%, PRIMA-PI: 89%), intermediate risk (FLIPI: 27.9%, FLIPI2: 13.8%, PRIMA-PI: 8.3%) and high risk (FLIPI: 19.4%, FLIPI2: 36.2%, PRIMA-PI: 2.7%). The first-line therapeutic approach consisted of surveillance in 6.2% of cases, radiotherapy in 2.1% of cases, or systemic treatment (immunochemotherapy) in 91.7% of cases. The first-line treatment protocols were as follows: RCHOP (94.6% of cases), R-MTX-CHOP (2.7% of cases) and Rituximab (2.7% of cases). Regarding the efficacy of first-line treatment, 58.3% of patients had a complete response, 16.7% of patients had a partial response, 11.2% of patients had stable disease, and 13.8% of patients progressed. Rituximab maintenance therapy was administered in 75% of cases.
Over a median follow-up period of 24.5 months, 31.3% of patients relapsed or were refractory, with a median relapse time of 13 months. POD24 was observed in 22.9% of patients. Median progression-free survival was estimated at 29 months, while overall survival was not reached

### Summary/Conclusion

This study reports a high proportion of advanced stages and a significant proportion of early progression (POD24), hence the need to optimize the management of high-risk patients.

**Copyright Information:** (Online) ISSN: 2572-9241
©2026 The Author(s). HemaSphere is published by John Wiley & Sons Ltd on behalf of European Hematology Association. This is an open access Abstract Book distributed under the Attribution-NonCommercial-NoDerivs (CC BY-NC-ND), which allows third parties to download the articles and share them with others as long as they credit the author and the Abstract Book, but they cannot change the content in any way or use them commercially.
**Abstract Book Citations:** Authors, Title, HemaSphere, 2026;10:(S1):pages. The abstract book DOIs can be found on the "Author Information" page.
**Disclaimer:** Articles published in the journal HemaSphere exclusively reflect the opinions of the authors. The authors are responsible for all content in their abstracts including accuracy of the facts, statements, citing resources, etc.

### (PB3910) EPIDEMIOLOGICAL AND CLINICAL CHARACTERISTICS OF BURKITT LYMPHOMA: A SINGLE-CENTERRETROSPECTIVE STUDY

**Topic:** 20. Other aggressive lymphomas

Cyrine Michani[1], Rym Cherni*[1], Ghassen Saidi[1], Selmi Amany[1], Meriem Achour[1], Hend Ben Neji[1], Raihane Ben Lakhal[1]

*[1]Aziza Othmana Hospital, department of hematology for adults, tunis, Tunisia;*

### Background

Burkitt lymphoma (BL) remains one of the most aggressive B-cell malignancies, defined by its extreme proliferation and hallmark MYC gene rearrangements. In adults, the rarity of the disease and its fulminant presentation make it a true hematologic emergency that requires immediate diagnostic workup..

### Aims

Analyse the epidemiological, clinical, and biological features of adult LB patients managed in our center.

### Methods

We conducted a retrospective descriptive study in the Adult Clinical Hematology Department of Aziza Othmana Hospital in Tunis. The study included all adult patients diagnosed with BL covering the period between 2012 and 2024. Clinical data, histopathological findings, and cytogenetic profiles were reviewed and analyzed using Jamovi software.

### Results

The cohort included 27 patients. The median age at diagnosis was 36 years (range 18–75), with a male predominance (M/F ratio1.7). Human immunodeficiency virus infection was present in 2 patients (7.4%).
Clinically, B-symptoms were present in one-third of the patients. The most frequent presentations were peripheral lymphadenopathy and abdominal pain, both occurring in 44.4% of cases, while a bulky tumor syndrome was noted in 40.7%. Diagnosis relied mainly on lymph node (37%) and digestive (25.9%) biopsies.
Biologically, the median Ki-67 index reached 99%, confirming the high proliferative nature of these tumors. Immunohistochemistry showed c-MYC positivity in only 57.7% of cases, and the t(8;14) translocation was present in 36.8% of the patients tested.77.8% of our patients presented with advanced stage IV disease. Bone marrow involvement was particularly high at 60%, and 18.5% of the cohort already had neuromeningeal involvement at the time of diagnosis.

**Copyright Information:** (Online) ISSN: 2572-9241
©2026 The Author(s). HemaSphere is published by John Wiley & Sons Ltd on behalf of European Hematology Association. This is an open access Abstract Book distributed under the Attribution-NonCommercial-NoDerivs (CC BY-NC-ND), which allows third parties to download the articles and share them with others as long as they credit the author and the Abstract Book, but they cannot change the content in any way or use them commercially.
**Abstract Book Citations:** Authors, Title, HemaSphere, 2026;10:(S1):pages. The abstract book DOIs can be found on the “Author Information” page.
**Disclaimer:** Articles published in the journal HemaSphere exclusively reflect the opinions of the authors. The authors are responsible for all content in their abstracts including accuracy of the facts, statements, citing resources, etc.

**Summary/Conclusion**

Our findings confirm that BL in our center typically affects young adults who present with a high tumor burden and advanced-stage disease. The high frequency of bone marrow and CNS involvement at diagnosis highlights the aggressive clinical course in our setting..t hese results underscore the vital importance of rapid diagnostic pathways and comprehensive staging to optimize the management of this high-risk population.

**Copyright Information:** (Online) ISSN: 2572-9241
©2026 The Author(s). HemaSphere is published by John Wiley & Sons Ltd on behalf of European Hematology Association. This is an open access Abstract Book distributed under the Attribution-NonCommercial-NoDerivs (CC BY-NC-ND), which allows third parties to download the articles and share them with others as long as they credit the author and the Abstract Book, but they cannot change the content in any way or use them commercially.
**Abstract Book Citations:** Authors, Title, HemaSphere, 2026;10:(S1):pages. The abstract book DOIs can be found on the "Author Information" page.
**Disclaimer:** Articles published in the journal HemaSphere exclusively reflect the opinions of the authors. The authors are responsible for all content in their abstracts including accuracy of the facts, statements, citing resources, etc.

### (PB3911) THERAPEUTIC OUTCOMES IN ADULTS WITH BURKITT LYMPHOMA TREATED WITH THE LMB-02PROTOCOL: A SINGLE-CENTER EXPERIENCE

**Topic:** 20. Other aggressive lymphomas

Ghassen Saidi[1], Rym Cherni*[1], Cyrine Michani[1], Selmi Amany[1], Meriem Achour[1], Hend Ben Neji[1], Raihane Ben Lakhal[1]

[1]*Aziza Othmana Hospital, department of hematology for adults, tunis, Tunisia;*

### Background

Burkitt lymphoma (BL) is a highly aggressive B-cell malignancy requiring intensive chemotherapy. However, data regarding treatment outcomes in adult patients remain limited.

### Aims

To evaluate treatment response, survival outcomes, and causes of mortality in adult patients with BL treated with the LMB-02 protocol.

### Methods

We conducted a retrospective study including adult patients diagnosed with BL and treated with the LMB-02 protocol at Aziza Othmana Hospital in Tunis between 2012 and 2024.
Response was assessed after the initial COP course (cyclophosphamide, vincristine, and prednisolone) then after the first consolidation course and at the end of treatment.
Overall survival (OS) was estimated using the Kaplan–Meier method.

### Results

Twenty-seven patients were included. The median age was 36 years (range 18–75). The median time from diagnosis to initiation of the first COP course was 2 days (range 0–35).
According to risk stratification, 11 (40.7%) patients were classified in group B and 16 (59.3%) in group C.
Tumor lysis syndrome occurred in 12 patients (44.4%) following the COP course, and 14 patients (51.9%) received rasburicase.
After the initial COP course, an adequate response was achieved in 18 patients (66.7%).
Three patients initially classified in group B were upgraded to group C due to insufficient response. Seven patients (25.9%) received Rituximab during the induction courses.
At final evaluation, complete remission was achieved in 13 patients (48.1%), while 4 patients (14.8%) achieved partial remission or were lost to follow-up. Overall mortality was 37% (10/27), and all deceased patients belonged to group C. Causes of death were equally attributed to treatment-related toxicity (50%, n=5) and disease progression (50%, n=5).
Treatment-related deaths occurred during induction (n=3) and consolidation courses (n=2).
The estimated overall survival was 78% [95% CI: 64–95%] at 3 months and 67% [95% CI: 51–87%] at 1 year.

**Copyright Information:** (Online) ISSN: 2572-9241
©2026 The Author(s). HemaSphere is published by John Wiley & Sons Ltd on behalf of European Hematology Association. This is an open access Abstract Book distributed under the Attribution-NonCommercial-NoDerivs (CC BY-NC-ND), which allows third parties to download the articles and share them with others as long as they credit the author and the Abstract Book, but they cannot change the content in any way or use them commercially.
**Abstract Book Citations:** Authors, Title, HemaSphere, 2026;10:(S1):pages. The abstract book DOIs can be found on the "Author Information" page.
**Disclaimer:** Articles published in the journal HemaSphere exclusively reflect the opinions of the authors. The authors are responsible for all content in their abstracts including accuracy of the facts, statements, citing resources, etc.

**Summary/Conclusion**

Intensive chemotherapy with the LMB-02 protocol achieves favorable response rates in adult BL. Early response assessment after the COP prephase appears useful for risk stratification. However, treatment-related mortality remains significant, particularly during induction and consolidation, and was observed exclusively in high-risk (group C) patients, highlighting the importance of optimized supportive care in this population.

**Copyright Information:** (Online) ISSN: 2572-9241
©2026 The Author(s). HemaSphere is published by John Wiley & Sons Ltd on behalf of European Hematology Association. This is an open access Abstract Book distributed under the Attribution-NonCommercial-NoDerivs (CC BY-NC-ND), which allows third parties to download the articles and share them with others as long as they credit the author and the Abstract Book, but they cannot change the content in any way or use them commercially.
**Abstract Book Citations:** Authors, Title, HemaSphere, 2026;10:(S1):pages. The abstract book DOIs can be found on the "Author Information" page.
**Disclaimer:** Articles published in the journal HemaSphere exclusively reflect the opinions of the authors. The authors are responsible for all content in their abstracts including accuracy of the facts, statements, citing resources, etc.

### (PB3912) SINGLE-CELL TRANSCRIPTOMIC PROFILING OF PEDIATRIC T-LYMPHOBLASTIC LYMPHOMA REVEALS RELAPSE-SPECIFIC PROGRAMS AND CANDIDATE THERAPEUTIC TARGETS

**Topic:** 21. Lymphoma biology & translational research

Vlada Tuliavko*[1], Egor Volchkov[1, 2, 3], Alexandr Ikonnikov[1], Larisa Karpova[4], Roman Glushak[5], Matthew Beliakov[6], Veronica Samusik[7], Valeria Zhmurina[8], Dmitry Abramov[2], Natalia Miakova[2]

*[1]Research Institute of Molecular and Cellular Medicine, Peoples' Friendship University of Russia named after Patrice Lumumba (RUDN University), Moscow, Russia; [2]National Medical Research Center for Pediatric Hematology, Oncology and Immunology, named by Dmitry Rogachev, Moscow, Russia; [3]Hospital named by Sergey Botkin, Moscow, Russia; [4]European University at Saint Petersburg, Saint Petersburg, Russia; [5]Moscow Institute of Physics and Technology, Moscow, Russia;[6]Saint Petersburg State University, Saint Petersburg, Russia; [7]Saint Petersburg State Chemical and Pharmaceutical University, Saint Petersburg, Russia; [8]Lopukhin Federal Research and Clinical Center of Physical-Chemical Medicine of Federal Medical Biological Agenc, Moscow, Russia;*

### Background

Pediatric T-lymphoblastic lymphoma (T-LBL) is an aggressive malignancy arised from immature T-cell precursors. While frontline therapy is effective for many patients, relapsed disease is associated with an extremely poor prognosis and high mortality. Mechanisms underlying relapse and therapy resistance remain insufficiently understood.

### Aims

To characterize interpatient and intratumoral heterogeneity in pediatric T-LBL using single-cell RNA-sequencing and to identify relapse-specific transcriptional programs, biomarkers, and candidate therapeutic targets.

### Methods

Five pediatric T-LBL samples representing remission (#2) and progression/relapse (#3) were analyzed using the Chromium Single Cell 3' Gene Expression platform (10x Genomics). After quality control and integration, unsupervised clustering and differential expression analyses were performed. Gene co-expression modules were identified to define relapse-associated programs. Functional enrichment analysis assessed pathway activation. Drug response prediction was performed using ScTherapy based on tumor transcriptomic profiles.

### Results

Single-cell analysis revealed pronounced interpatient transcriptional heterogeneity, whereas individual tumors demonstrated relative intratumoral homogeneity, suggesting dominance of major malignant clones within each patient. Two biologically distinct relapse-associated molecular programs were identified. The first subtype was characterized by a phenotypically plastic immature T-cell population with activation of NOTCH-signaling and persistent *DNTT* expression, indicating developmental plasticity and a potentially therapy-resistant cellular state. The second subtype consisted of highly proliferative malignant cells exhibiting transcriptional features of activated mature effector T cells, consistent with an aggressive disease phenotype. A relapse-associated gene module including *CDKN2A*, *IKZF2*, *PPM1H*, and *TRAC* was identified. Within this network, *CDKN2A* demonstrated strong dysregulation and central network connectivity, supporting its role as a potential biomarker of relapse biology. Transcriptome-guided drug prediction identified such possible effective candidates as proteasome inhibitors and histone deacetylase inhibitors (HDAC). Additional possible targets associated with relapse included CDH2, MYC, and CDKN2A, as well as combinatorial MYC-CDKN2A transcription programs.

**Copyright Information:** (Online) ISSN: 2572-9241
©2026 The Author(s). HemaSphere is published by John Wiley & Sons Ltd on behalf of European Hematology Association. This is an open access Abstract Book distributed under the Attribution-NonCommercial-NoDerivs (CC BY-NC-ND), which allows third parties to download the articles and share them with others as long as they credit the author and the Abstract Book, but they cannot change the content in any way or use them commercially.
**Abstract Book Citations:** Authors, Title, HemaSphere, 2026;10:(S1):pages. The abstract book DOIs can be found on the "Author Information" page.
**Disclaimer:** Articles published in the journal HemaSphere exclusively reflect the opinions of the authors. The authors are responsible for all content in their abstracts including accuracy of the facts, statements, citing resources, etc.

### Summary/Conclusion

Single-cell transcriptomic profiling of pediatric T-LBL revealed pronounced interpatient heterogeneity but relative intratumoral homogeneity. Two biologically distinct relapse programs were identified: a plastic NOTCH-associated immature subtype and an aggressive proliferative mature-like subtype. CDKN2A emerged as a candidate biomarker. Transcriptome-based drug prediction identified both shared and individualized therapeutic candidates. These findings provide a molecular framework for stratifying and precision-treating relapsed pediatric T-LBL.

**Copyright Information:** (Online) ISSN: 2572-9241
©2026 The Author(s). HemaSphere is published by John Wiley & Sons Ltd on behalf of European Hematology Association. This is an open access Abstract Book distributed under the Attribution-NonCommercial-NoDerivs (CC BY-NC-ND), which allows third parties to download the articles and share them with others as long as they credit the author and the Abstract Book, but they cannot change the content in any way or use them commercially.
**Abstract Book Citations:** Authors, Title, HemaSphere, 2026;10:(S1):pages. The abstract book DOIs can be found on the “Author Information” page.
**Disclaimer:** Articles published in the journal HemaSphere exclusively reflect the opinions of the authors. The authors are responsible for all content in their abstracts including accuracy of the facts, statements, citing resources, etc.

### (PB3913) T-CELL ACTIVATION AND ANTITUMOR ACTIVITY OF MOSUNETUZUMAB PLUS LENALIDOMIDE IN HUMANIZED MICE AND FOLLICULAR LYMPHOMA PATIENT-DERIVED T-CELLS

**Topic:** 21. Lymphoma biology & translational research

Hayato Mizuta*[1], Koh Furugaki[1], Sei Shu[1], Satoko Takagi[1], Yoriko Yamashita-Kashima[1]

[1]*Chugai Pharmaceutical Co., Ltd., Yokohama, Japan;*

### Background

Mosunetuzumab (Mosun) is a CD20×CD3 T-cell engaging bispecific antibody approved in multiple regions including the EU, US, and Japan for relapsed/refractory follicular lymphoma (R/R FL) after ≥2 prior lines of therapy[1,2]. Lenalidomide (Len) is an immunomodulatory drug with broad regulatory effects on immune cell functions, including T-cell activity[3]. Several phase II and III clinical trials investigating Mosun+Len in untreated and R/R FL are currently ongoing, with preliminary results being highly encouraging[4-7]. We have shown Mosun+Len activity in vitro in healthy donor-derived CD8+ T-cells with human FL cell lines[8], however, its effect on CD4+ T-cell activation remains unclear. Furthermore, the effect of the combination and its underlying mechanisms in mouse models and FL patient-derived models is not fully understood.

### Aims

To investigate whether Len enhances Mosun induced T-cell activation and antitumor efficacy in a mouse model and in vitro using T-cells derived from patients with FL and healthy donors.

### Methods

In vivo antitumor activity of Mosun+Len was assessed in a humanized NOD/Shi-Scid, IL-2Rγ null (huNOG) mouse model established by irradiation followed by transplantation of human CD34+ cells. After confirming chimerism, Minami-1 cells, a human FL cell line, were implanted subcutaneously, and mice were randomized to Vehicle, Len alone, Mosun alone, or Mosun+Len groups. For patient-derived cell-based assays, T-cells purified from the peripheral blood mononuclear cells (PBMCs) of a patient with FL were cocultured with Minami-1 cells at an effector to target (E:T) ratio of 5:1 and treated with Mosun alone or Mosun+Len. After 7 days, T cell–dependent cellular cytotoxicity (TDCC), cytotoxic granule expression, CD8+ T-cell counts, and cytokine levels in culture supernatants were measured by flow cytometry and multiplex immunoassay. For CD4+T-cell activation assay, T-cells purified from healthy donor-derived PBMCs were cocultured with Minami-1 cells at an E:T ratio of 10:1 and treated with vehicle, Len alone, Mosun alone, or Mosun+Len. After 2 days, the proportions of CD69+ and CD25+ cells were assessed in CD8+ and CD4+ T-cells by flow cytometry.

### Results

In huNOG mice, Mosun alone induced significant antitumor activity compared with vehicle control, and the Mosun+Len combination achieved an even further increase in antitumor efficacy compared with both Len alone and Mosun alone. In vitro, Mosun+Len significantly enhanced TDCC activity compared to Mosun alone in T-cells derived from a patient with FL. Consistently, CD8+ T-cell counts and the proportion of granzyme B expressing CD8+ T-cells were significantly higher with Mosun+Len than with Mosun alone. Mosun+Len, also increased IFN-γ production compared with Mosun alone. In addition, cotreatment with Mosun+Len significantly enhanced expression of CD69 and CD25 in both CD4+ and CD8+ T-cells compared to other groups.

**Copyright Information:** (Online) ISSN: 2572-9241
©2026 The Author(s). HemaSphere is published by John Wiley & Sons Ltd on behalf of European Hematology Association. This is an open access Abstract Book distributed under the Attribution-NonCommercial-NoDerivs (CC BY-NC-ND), which allows third parties to download the articles and share them with others as long as they credit the author and the Abstract Book, but they cannot change the content in any way or use them commercially.
**Abstract Book Citations:** Authors, Title, HemaSphere, 2026;10:(S1):pages. The abstract book DOIs can be found on the "Author Information" page.
**Disclaimer:** Articles published in the journal HemaSphere exclusively reflect the opinions of the authors. The authors are responsible for all content in their abstracts including accuracy of the facts, statements, citing resources, etc.

### Summary/Conclusion

Mosun+Len induced significantly higher antitumor activity compared to Mosun alone. In FL patient–derived T-cells, Mosun+Len combination increased the number of CD8+ T-cells and expression of granzyme B. These findings support the rationale for combining Mosun with Len in treating FL.

**Copyright Information:** (Online) ISSN: 2572-9241
©2026 The Author(s). HemaSphere is published by John Wiley & Sons Ltd on behalf of European Hematology Association. This is an open access Abstract Book distributed under the Attribution-NonCommercial-NoDerivs (CC BY-NC-ND), which allows third parties to download the articles and share them with others as long as they credit the author and the Abstract Book, but they cannot change the content in any way or use them commercially.
**Abstract Book Citations:** Authors, Title, HemaSphere, 2026;10:(S1):pages. The abstract book DOIs can be found on the "Author Information" page.
**Disclaimer:** Articles published in the journal HemaSphere exclusively reflect the opinions of the authors. The authors are responsible for all content in their abstracts including accuracy of the facts, statements, citing resources, etc.

### (PB3914) EXPRESSION OF THE HLA CLASS I ANTIGEN PRESENTATION PATHWAY ASSOCIATES WITH SENSITIVITY TO PD-L1 BLOCKADE IN TREATMENT-NAÏVE DIFFUSE LARGE B-CELL LYMPHOMA (DLBCL)

**Topic:** 21. Lymphoma biology & translational research

Kate Manos*[1, 2], Melinda Burgess[3], Sarah Kempe[4], Valentine Murigneux[3], Rakin Chowdhury[5], Geoff Chong[2, 6], Sze Ting Lee[6], Charmaine Smith[6], William Renwick[7], Piers Blombery[8], Niles Nelson[9], Allison Barraclough[10], Joel Wight[11, 12], Colm Keane[3, 5], Eliza Hawkes[6, 13]

*[1]Flinders Medical Centre, Adelaide, Australia;[2]University of Melbourne, Melbourne, Australia;[3]University of Queensland, Brisbane, Australia;[4]QIMR Berghofer, Brisbane, Australia;[5]Princess Alexandra Hospital, Brisbane, Australia;[6]Olivia Newton John Cancer Research Institute at Austin Health, Melbourne, Australia;[7]Western Health, Melbourne, Australia;[8]Peter MacCallum Cancer Centre, Melbourne, Australia;[9]Royal Hobert Hospital, Hobert, Australia;[10]Fiona Stanley Hospital, Perth, Australia;[11]Townsville University Hospital, Townsville, Australia;[12]James Cook University, Townsville, Australia;[13]Monash University, Melbourne, Australia;*

### Background

Effective PD-1/PD-L1 blockade requires intact tumour antigen presentation via HLA class I (HLA-I). Although checkpoint inhibitors have shown limited activity in relapsed/refractory DLBCL, our phase II trial of anti–PD-L1, avelumab, with rituximab priming (AvR) in untreated DLBCL demonstrated metabolic responses across histological subgroups, independent of PD-L1 status (Manos 2023). Here, we evaluated the HLA-I antigen presentation pathway as a potential determinant of PD-L1 sensitivity.

### Aims

To evaluate HLA-I pathway expression and integrity as a predictor of response to PD-L1 blockade in untreated DLBCL.

### Methods

Patients received 2 cycles of AvR followed by R-CHOP. In this analysis, we used a more sensitive quantitative reduction of ≥75% in PET total lesion glycolysis (TLG75) to AvR to define response. Gene expression profiling (n=21) was performed using the NanoString PanCancer Immune Panel. Whole-exome sequencing (n=16) assessed structural variants and HLA genotype. Molecular subtypes were assigned using LymphGen. Comparisons used non-parametric tests with Spearman correlation and Cox regression.

**Copyright Information:** (Online) ISSN: 2572-9241
©2026 The Author(s). HemaSphere is published by John Wiley & Sons Ltd on behalf of European Hematology Association. This is an open access Abstract Book distributed under the Attribution-NonCommercial-NoDerivs (CC BY-NC-ND), which allows third parties to download the articles and share them with others as long as they credit the author and the Abstract Book, but they cannot change the content in any way or use them commercially.
**Abstract Book Citations:** Authors, Title, HemaSphere, 2026;10:(S1):pages. The abstract book DOIs can be found on the “Author Information” page.
**Disclaimer:** Articles published in the journal HemaSphere exclusively reflect the opinions of the authors. The authors are responsible for all content in their abstracts including accuracy of the facts, statements, citing resources, etc.

**Results**

Metabolic response (TLG75) was observed in 48% (10/21; 3 PMBCL, 5 non-GCB, 2 GCB).
A composite antigen processing machinery (APM) score, derived from HLA-I pathway gene expression (HLA-A/B/C, TAP1/2, TAPBP, PSMB8/9/10, NLRC5), was strongly associated with TLG75 ($p=0.010$) and depth of metabolic response (Spearman $\rho=0.62$, $p=0.003$), with a trend toward improved progression-free survival (HR 0.47, $p=0.08$). In contrast, HLA-I expression alone demonstrated only modest correlation with response ($\rho=0.46$, $p=0.04$) and did not retain independent predictive value in multivariable analysis.
Given the role of interferon-γ (IFN-γ) signalling in regulating HLA-I genes, we assessed an IFN-γ transcriptional score. IFN-γ score correlated with APM score ($\rho=0.60$, $p=0.004$) but showed only borderline association with metabolic response ($\rho=0.42$, $p=0.057$). While IFN-γ activity and APM were biologically linked, the APM score demonstrated a stronger association with response, indicating that coordinated antigen processing, rather than IFN-γ-driven inflammation alone, underlies response.
APM score did not associate with cell of origin, histology or EBV status. The ST2 subgroup was enriched for responders (5/6) and trended toward a higher APM score ($p=0.09$). HLA-I pathway mutations (including B2M, HLA-I, APM and IFN pathway regulatory genes) were identified in 56% of cases but did not associate with APM score or response. Host HLA genotype, evolutionary divergence and homozygosity were likewise non-predictive.
Our prior work identified the cancer testis antigen *PRAME* as a marker of a $CD8^{+}$ T-cell–inflamed, checkpoint-regulated microenvironment associated with PD-L1 sensitivity (Manos ASH 2025). The APM score predicted metabolic response independent of PRAME expression, including within PRAME-high tumours ($p=0.020$), suggesting complementary roles for an inflamed microenvironment and preserved tumour-intrinsic antigen processing in PD-L1 sensitivity.

**Summary/Conclusion**

APM pathway expression emerged as a tumour-intrinsic correlate of PD-L1 efficacy, independent of HLA-I expression and PRAME-defined immune contexture. Structural HLA pathway alterations were not predictive. These data suggest that coordinated transcriptional competence of the antigen presentation pathway, rather than HLA genotype or isolated mutations, underpins checkpoint sensitivity in untreated DLBCL.

**Copyright Information:** (Online) ISSN: 2572-9241
©2026 The Author(s). HemaSphere is published by John Wiley & Sons Ltd on behalf of European Hematology Association. This is an open access Abstract Book distributed under the Attribution-NonCommercial-NoDerivs (CC BY-NC-ND), which allows third parties to download the articles and share them with others as long as they credit the author and the Abstract Book, but they cannot change the content in any way or use them commercially.
**Abstract Book Citations:** Authors, Title, HemaSphere, 2026;10:(S1):pages. The abstract book DOIs can be found on the "Author Information" page.
**Disclaimer:** Articles published in the journal HemaSphere exclusively reflect the opinions of the authors. The authors are responsible for all content in their abstracts including accuracy of the facts, statements, citing resources, etc.

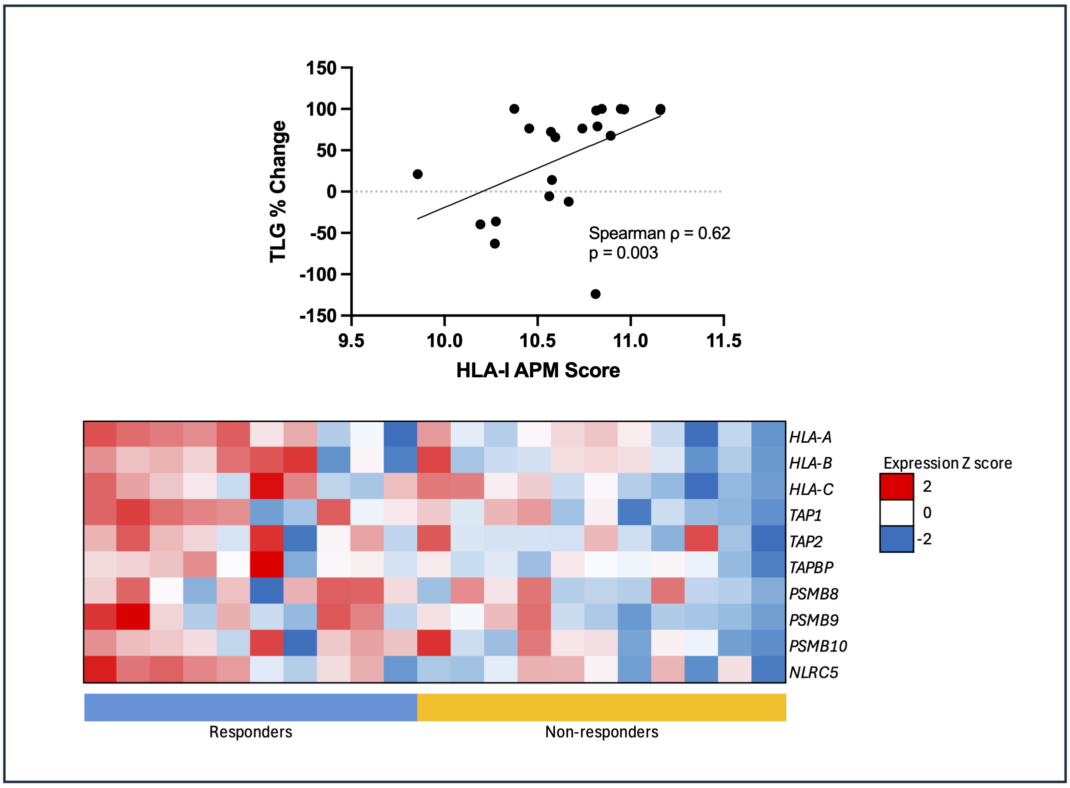

**Copyright Information:** (Online) ISSN: 2572-9241
©2026 The Author(s). HemaSphere is published by John Wiley & Sons Ltd on behalf of European Hematology Association. This is an open access Abstract Book distributed under the Attribution-NonCommercial-NoDerivs (CC BY-NC-ND), which allows third parties to download the articles and share them with others as long as they credit the author and the Abstract Book, but they cannot change the content in any way or use them commercially.
**Abstract Book Citations:** Authors, Title, HemaSphere, 2026;10:(S1):pages. The abstract book DOIs can be found on the "Author Information" page.
**Disclaimer:** Articles published in the journal HemaSphere exclusively reflect the opinions of the authors. The authors are responsible for all content in their abstracts including accuracy of the facts, statements, citing resources, etc.

### (PB3915) NEUTROPHIL–DLBCL CROSSTALK REVEALS AN ANTI-TUMOR ROLE FOR CATHELICIDIN

**Topic:** 21. Lymphoma biology & translational research

Nataliia Pavliuchenko*[1], Gema Gorjón-de-Pablo[1], Nuria Profitos-Peleja[1], Gaël Roué[1]

[1]*Josep Carreras Leukaemia Research Institute, Lymphoma Translational Group, Barcelona, Spain;*

### Background

Diffuse large B-cell lymphoma (DLBCL) is the most common subtype of non-Hodgkin lymphoma in western countries, accounting for approximately 30–40% of cases worldwide. Clinically, DLBCL is classified into germinal center B-cell–like (GCB) and activated B-cell–like (ABC) subtypes based on gene expression profiling, additionally, they differ in pathogenesis and clinical outcome. DLBCL most commonly develops in lymph nodes, but up to 30–40% of cases show extranodal involvement, reflecting biological heterogeneity. Despite the well-established prognostic relevance of the tumor microenvironment (TME) in DLBCL, the cellular mechanisms through which malignant B cells derive a proliferative advantage from immune interactions remain poorly understood. Consistently, clinical immune parameters such as the neutrophil-to-lymphocyte ratio (NLR) correlate with patient outcomes, yet the biological basis of this association remains largely unknown. This represents a critical knowledge gap that limits the development of rational TME-targeting therapies.

### Aims

To identify and evaluate the impact of neutrophil-secreted factors on the proliferation and survival of malignant DLBCL tumors.

### Methods

Primary human neutrophils from healthy donors were co-cultured with DLBCL cell lines (SU-DHL-5, Karpas 422), and conditioned media were analyzed by LC–MS/MS. DLBCL proliferation was assessed by histone H3-pS10 expression using flow cytometry. For in ovo studies, DLBCL cells with or without prior neutrophil co-culture were inoculated into the chick chorioallantoic membrane (CAM) model. 3D multicellular spheroids incorporating DLBCL cells, StromalNKTert stromal cells, and monocyte-derived M2-like macrophages were treated with LL-37 and analyzed using CellTiter-Glo. Cell proliferation and mitochondrial membrane potential following LL-37 exposure were evaluated by MTT assay and DiOC staining with flow cytometry.

### Results

Exposure of DLBCL cells to primary human neutrophils isolated from healthy donors resulted a significant reduction in tumor mitotic index in vitro, as evidenced by downregulation of H3-pS10, suggesting the release of anti-proliferative factors by neutrophils. Using an immunocompetent CAM model of DLBCL xenograft, we further demonstrate that pre-conditioning the DLBCL graft with neutrophils triggers a remarkable (~80%) tumor engraftment, independently of reactive oxygen species production and NETosis induction. To uncover potential antitumoral factors secreted by neutrophils, we employed LC–MS/MS to carry out a proteomic profiling of conditioned media from neutrophil–DLBCL co-cultures, leading to the identification of the antimicrobial peptide LL-37 (cathelicidin) among the most abundant secreted proteins. In vitro, exogenous LL-37 exerted a dose-dependent antitumor activity in both LN- or pleural effusion-derived GCB-DLBCL cell lines, without affecting healthy peripheral blood mononuclear cells. Consistently, LL-37 displayed selective, dose-dependent anti-lymphoma activity in DLBCL 3D multicellular spheroids. Mechanistically, the peptide was shown to induce mitochondrial dysfunction leading to apoptosis of DLBCL cells.

**Copyright Information:** (Online) ISSN: 2572-9241
©2026 The Author(s). HemaSphere is published by John Wiley & Sons Ltd on behalf of European Hematology Association. This is an open access Abstract Book distributed under the Attribution-NonCommercial-NoDerivs (CC BY-NC-ND), which allows third parties to download the articles and share them with others as long as they credit the author and the Abstract Book, but they cannot change the content in any way or use them commercially.
**Abstract Book Citations:** Authors, Title, HemaSphere, 2026;10:(S1):pages. The abstract book DOIs can be found on the “Author Information” page.
**Disclaimer:** Articles published in the journal HemaSphere exclusively reflect the opinions of the authors. The authors are responsible for all content in their abstracts including accuracy of the facts, statements, citing resources, etc.

**Summary/Conclusion**

Collectively, these findings highlight a previously unrecognized anti-tumor role of neutrophils in DLBCL and suggest that neutrophil-derived factors such as cathelicidin may represent promising therapeutic tools to counteract stroma-mediated lymphoma growth.

**Copyright Information:** (Online) ISSN: 2572-9241
©2026 The Author(s). HemaSphere is published by John Wiley & Sons Ltd on behalf of European Hematology Association. This is an open access Abstract Book distributed under the Attribution-NonCommercial-NoDerivs (CC BY-NC-ND), which allows third parties to download the articles and share them with others as long as they credit the author and the Abstract Book, but they cannot change the content in any way or use them commercially.
**Abstract Book Citations:** Authors, Title, HemaSphere, 2026;10:(S1):pages. The abstract book DOIs can be found on the "Author Information" page.
**Disclaimer:** Articles published in the journal HemaSphere exclusively reflect the opinions of the authors. The authors are responsible for all content in their abstracts including accuracy of the facts, statements, citing resources, etc.

### (PB3916) POLATUZUMAB VEDOTIN ENHANCES T-CELL INFILTRATION INTO TUMORS AND DEMONSTRATES COMBINATORIAL EFFICACY WITH CD20-CD3 BISPECIFIC ANTIBODY IN A SYNGENEIC B-CELL MALIGNANCY MOUSE MODEL

**Topic:** 21. Lymphoma biology & translational research

Mayu Tomita*[1], Sei Shu[1], Natsumi Kawasaki[1], Satoko Takagi[1], Shigeki Yoshiura[1], Yoriko Yamashita-Kashima[1]

[1]*Chugai Pharmaceutical Co., Ltd., Product Research Department, Yokohama, Japan;*

### Background

Polatuzumab vedotin (Pola) is an antibody-drug conjugate that combines an anti-CD79b antibody with the cytotoxic agent, monomethyl auristatin E (MMAE), and has received regulatory approval in several countries for diffuse large B-cell lymphoma (DLBCL) treatment. Clinical data supporting the safety and efficacy of mosunetuzumab plus Pola in relapsed or refractory DLBCL have been reported (Lihua E Budde et al., J Clin Oncol. 2025;43:3799-3811). Our previous investigations demonstrated that Pola facilitates infiltration of innate immune cells, specifically macrophages and NK cells, into the tumor microenvironment, contributing to its anti-tumor activity (Tomita M et al., eJHaem, 2026; 7:e70219). However, comprehensive research on Pola's effects on the adaptive immune system remains limited, and the potential synergistic mechanisms with T cell-activating therapies warrant further investigation.

### Aims

The study aims to investigate Pola's influence on the tumor microenvironment using a syngeneic mouse model, and assess the potential synergistic effect when combined with CD20-CD3 bispecific antibodies.

### Methods

We utilized DBA/2 mice implanted with L1210-hCD79b-mCD20 cells, a murine B-lymphocytic leukemia cell line genetically engineered to express human-mouse chimeric CD79b containing the Pola-binding domain and murine CD20. Following tumor establishment, mice received intravenous control IgG (2 mg/kg) or Pola (2 mg/kg) on Day 1. Tumor-infiltrating immune cell populations were quantitatively analyzed via flow cytometry. A multiplex immunoassay was utilized to quantify humoral factors in tumors following Pola administration. Additionally, we evaluated the combinatorial efficacy of Pola (2 mg/kg) with a murine CD20-murine CD3 bispecific antibody (mCD20-mCD3 mAb, 0.3 mg/kg) treated on Day 1 in a mouse model implanted with L1210-hCD79b-mCD20 cells.

### Results

In the L1210-hCD79b-mCD20 syngeneic mouse model, Pola treatment significantly enhanced tumor infiltration by multiple immune cell populations compared with control IgG on Day 4. This included not only innate immune cells such as macrophages, NK cells, and dendritic cells but also CD8+ cytotoxic T cells, CD4+Foxp3- helper T cells, and CD4+Foxp3+ regulatory T cells. Concurrently, Pola treatment increased intratumoral levels of CXCL10, IFN-γ, CCL5, and CCL2, which could convert the immunosuppressive tumor microenvironment. Based on the hypothesis that Pola-mediated enhancement of immune infiltration would improve therapeutic efficacy, we evaluated Pola + mCD20-mCD3 mAb combination therapy in mice. In the L1210-hCD79b-mCD20 syngeneic mouse model, combining Pola with the mCD20-mCD3 mAb resulted in significantly smaller tumor volumes on Day 8 compared to each monotherapy (mean tumor volume: control group 776 $mm^3$, mCD20-mCD3 group 185 $mm^3$, Pola group 224 $mm^3$, combination group 64 $mm^3$).

**Copyright Information:** (Online) ISSN: 2572-9241
©2026 The Author(s). HemaSphere is published by John Wiley & Sons Ltd on behalf of European Hematology Association. This is an open access Abstract Book distributed under the Attribution-NonCommercial-NoDerivs (CC BY-NC-ND), which allows third parties to download the articles and share them with others as long as they credit the author and the Abstract Book, but they cannot change the content in any way or use them commercially.
**Abstract Book Citations:** Authors, Title, HemaSphere, 2026;10:(S1):pages. The abstract book DOIs can be found on the "Author Information" page.
**Disclaimer:** Articles published in the journal HemaSphere exclusively reflect the opinions of the authors. The authors are responsible for all content in their abstracts including accuracy of the facts, statements, citing resources, etc.

**Summary/Conclusion**

In our mouse model, Pola treatment enhanced tumor infiltration by various immune cell populations, including CD8+ T cells that are critical for anti-tumor immunity. When combined with an mCD20-mCD3 mAb, we observed superior anti-tumor effects compared with either monotherapy. These findings suggest that Pola not only exerts direct cytotoxic effects on tumor cells but also modulates the tumor immune microenvironment, which may potentially enhance the efficacy of T cell-engaging therapies.

**Copyright Information:** (Online) ISSN: 2572-9241
©2026 The Author(s). HemaSphere is published by John Wiley & Sons Ltd on behalf of European Hematology Association. This is an open access Abstract Book distributed under the Attribution-NonCommercial-NoDerivs (CC BY-NC-ND), which allows third parties to download the articles and share them with others as long as they credit the author and the Abstract Book, but they cannot change the content in any way or use them commercially.
**Abstract Book Citations:** Authors, Title, HemaSphere, 2026;10:(S1):pages. The abstract book DOIs can be found on the "Author Information" page.
**Disclaimer:** Articles published in the journal HemaSphere exclusively reflect the opinions of the authors. The authors are responsible for all content in their abstracts including accuracy of the facts, statements, citing resources, etc.

### (PB3917) MULTIMODAL SINGLE CELL SPATIAL TRANSCRIPTOMIC AND PROTEOMIC ANALYSIS DETECTS CAR-T CELLS WITHIN THE LBCL TUMOUR MICROENVIRONMENT FOLLOWING CD19 CAR-T IMMUNOTHERAPY

**Topic:** 21. Lymphoma biology & translational research

Helen Cashman*[1], Alexandre Hirayama[2], Jocelyn Wright[3], Lijia Yu[4], Jean Yang[4], Chanukya Colonne[1, 5], Akira Nguyen-Shaw[1], Salvatore Fiorenza[1], Eva Shteinman[6], Samson Dowland[7], Jordan Gauthier[2], Kirsten McIlroy[5], Anthony Gill[1, 5], Cameron Turtle[1, 3, 5]

*[1]University of Sydney, Sydney Medical School, Faculty of Medicine and Health, Camperdown, Australia;[2]Fred Hutchinson Cancer Center, Clinical Research Division, Seattle, United States of America; [3]Fred Hutchinson Cancer Center, Translational Science and Therapeutics Division, Seattle, United States of America; [4]University of Sydney, Sydney Precision Data Science Centre, Faculty of Science, Sydney, Australia; [5]Royal North Shore Hospital, St. Leonards, Australia;[6]University of Sydney, Melanoma Institute Australia, Sydney, Australia; [7]University of Sydney, Histology Facility, Charles Perkins Centre, Sydney, Australia;*

### Background

Large B-cell lymphoma (LBCL) encompasses numerous B-cell lymphoma histologies with an aggressive clinical phenotype and poor outcomes in the refractory and multiply relapsed setting. Over half of patients do not demonstrate durable remissions with CD19 CAR-T immunotherapy. Understanding the immune milieu of the LBCL tumour microenvironment (TME), including the spatial context of infused CAR-T cells, is essential to informing the development of novel immunotherapies. CAR-T cells *in situ* have remained relatively uncharacterised due to the absence of comprehensive spatially resolved detection methods. Our study addresses this gap by applying two complementary spatial platforms, spatial transcriptomics (ST) and high-plex immunofluorescence, to provide a novel means of investigating mechanisms of CAR-T resistance.

### Aims

We aimed to characterise the LBCL TME in patients treated with CD19 CAR-T immunotherapy to resolve lymphoma tissue architecture and detect CAR-T cells in situ.

### Methods

We applied spatial multiomic techniques across complementary platforms to profile the immune composition of the LBCL TME at subcellular resolution. Formalin-fixed paraffin-embedded (FFPE) excision or core lymphoid tissue biopsy samples were analysed using the Phenocycler-Fusion (PCF) instrument (Akoya, now Quanterix) using a 52-marker panel including CAR-T specific antibodies. FFPE tissue sections were also analysed using the Xenium Prime 5000-gene ST panel plus 100 custom probes including unique CAR-T transcript detection. Regions of interest for focussed analysis were selected.

**Copyright Information:** (Online) ISSN: 2572-9241
©2026 The Author(s). HemaSphere is published by John Wiley & Sons Ltd on behalf of European Hematology Association. This is an open access Abstract Book distributed under the Attribution-NonCommercial-NoDerivs (CC BY-NC-ND), which allows third parties to download the articles and share them with others as long as they credit the author and the Abstract Book, but they cannot change the content in any way or use them commercially.
**Abstract Book Citations:** Authors, Title, HemaSphere, 2026;10:(S1):pages. The abstract book DOIs can be found on the “Author Information” page.
**Disclaimer:** Articles published in the journal HemaSphere exclusively reflect the opinions of the authors. The authors are responsible for all content in their abstracts including accuracy of the facts, statements, citing resources, etc.

**Results**

26 biopsies from 17 patients and 7 control tonsil sections were analysed by PCF. For the PCF analysis, 15 pt biopsies alongside tonsil controls were included in downstream analysis after histological quality control review. Biopsies were taken pre-CAR-T infusion (n=7) or post-infusion (n=8), with 4 paired samples. Best response was CR in 6 pts, and non-CR in 9 pts (PR=2, PD=5, SD=2). Post-infusion samples were taken between D+2 to D+69. Preliminary qualitative assessment revealed *in situ* detection of CAR-T cells in 4/7 post-infusion patients using the custom CAR-T antibody (Figure 1A; CAR-T cells shown in yellow).
ST analysis was also performed on 8 pts from this sample group. 2 pt biopsies were pre-infusion and 6 were post-infusion. 4 pts had CR as best response and 4 pts had non-CR as best response (PR=1, PD=1, SD=2). CAR-T cells were identified *in situ* using eight custom probes targeting regions of the CAR-T construct (Figure 1B; distinct CAR-T transcripts are visualised as differently coloured dots). Regions enriched with CAR-T cells as seen by PCF also demonstrated cells with CAR- T transcripts by ST analysis. No clear relationship was observed between the total number of CAR-T cells detected and the sampling timepoint (early vs late).
Quantitative single-cell analyses across both spatial platforms are ongoing to define clinically relevant structural and functional features of the TME. Further spatial and transcriptomic characterisation is underway, with full results to be presented.

**Summary/Conclusion**

Using integrated spatial proteomics and transcriptomics, we demonstrate a new capability to visualise and characterise CAR-T cells within the LBCL TME. This multiomic approach will provide crucial insights into LBCL TME architecture in patients receiving CD19 CAR-T therapy and will guide the development of future targeted interventions.

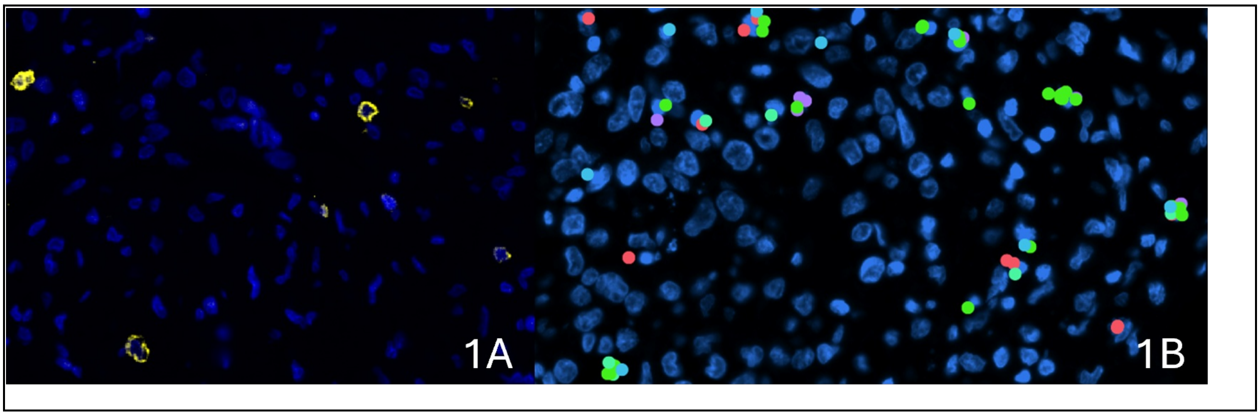

Figure 1. Detection of CAR-T cells in LBCL tissue using Phenocycler-Fusion (A; CAR-T cells shown in yellow) and Xenium (B; distinct CAR-T transcripts visualised as differently coloured dots).

**Copyright Information:** (Online) ISSN: 2572-9241
©2026 The Author(s). HemaSphere is published by John Wiley & Sons Ltd on behalf of European Hematology Association. This is an open access Abstract Book distributed under the Attribution-NonCommercial-NoDerivs (CC BY-NC-ND), which allows third parties to download the articles and share them with others as long as they credit the author and the Abstract Book, but they cannot change the content in any way or use them commercially.
**Abstract Book Citations:** Authors, Title, HemaSphere, 2026;10:(S1):pages. The abstract book DOIs can be found on the “Author Information” page.
**Disclaimer:** Articles published in the journal HemaSphere exclusively reflect the opinions of the authors. The authors are responsible for all content in their abstracts including accuracy of the facts, statements, citing resources, etc.

### (PB3918) TRANSFERRIN RECEPTOR 1-TARGETED NANOCARRIER FOR METHOTREXATE DELIVERY ENHANCES BLOOD-BRAIN BARRIER PENETRATION AND THERAPEUTIC EFFICACY IN PRIMARY CENTRAL NERVOUS SYSTEM LYMPHOMA

**Topic:** 21. Lymphoma biology & translational research

Qing Yang*[1], Qian Liang[2], Zhenzhen Yang[1], Yue Zhang[1], Siyu Qian[1], Zeyuan Wang[1], Wanyue Zhao[1], Mingzhi Zhang[1], Xudong Zhang[1], Chen Qingjiang[1, 2]

*[1]The First Affiliated Hospital of Zhengzhou University, Zhengzhou, China;[2]Henan Academy of Innovations in Medical Science, Zhengzhou, China;*

### Background

Primary central nervous system lymphoma (PCNSL) is a highly aggressive extranodal non-Hodgkin lymphoma confined to immune-privileged sites. Although high-dose methotrexate (HD-MTX)-based chemotherapy remains the standard first-line treatment, clinical outcomes remain unsatisfactory owing to dose-limiting toxicities and the blood-brain barrier (BBB), which severely compromises effective drug delivery to lesions. Thus, there is an urgent need for a targeted delivery system capable of efficient BBB transcytosis and selective tumor accumulation. Transferrin receptor 1 (TfR1) is highly expressed on the BBB and in numerous malignant tumors, representing a promising target for mediating BBB penetration. Heavy chain ferritin (HFn) can specifically bind to TfR1 without perturbing its function. HFn self-assembles into a spherical nanocage that that enables stable encapsulation of chemotherapeutic agents. TfR1-mediated transcytosis further enhances tumor penetration. Based on these features, we developed an HFn-based nanocarrier for targeted methotrexate delivery (MTX@HFn).

### Aims

This study aimed to evaluate the anti-tumor activity of MTX@HFn against PCNSL cells and its therapeutic efficacy in vivo, with the goal of establishing a novel treatment paradigm with improved efficacy and reduced toxicity for PCNSL.

### Methods

TfR1 expression in PCNSL cell lines was quantified by western blot and flow cytometry, using healthy donor PBMCs as controls. MTX@HFn and Cy5.5-MTX@HFn were encapsulated for therapeutic studies and drug tracking, respectively. The in vitro BBB penetration of HFn-based nanocarrier was evaluated using a transwell BBB model. PCNSL cells were treated with free MTX or MTX@HFn for 24, 48, and 72 hours. Cell viability was assessed by CCK-8 assay, while cell cycle and apoptosis were analyzed by flow cytometry. Specialized pre-coated round-bottom plates were employed to generate multicellular spheroids. Drug penetration capacity was evaluated by visualizing cytotoxicity using the Calcein/PI assay. Therapeutic efficacy was further examined in an orthotopic xenograft mouse model of PCNSL. IVIS Lumina II system was used to evaluate tumor progression and drug distribution. Outcomes were further quantified by survival analysis.

**Copyright Information:** (Online) ISSN: 2572-9241
©2026 The Author(s). HemaSphere is published by John Wiley & Sons Ltd on behalf of European Hematology Association. This is an open access Abstract Book distributed under the Attribution-NonCommercial-NoDerivs (CC BY-NC-ND), which allows third parties to download the articles and share them with others as long as they credit the author and the Abstract Book, but they cannot change the content in any way or use them commercially.
**Abstract Book Citations:** Authors, Title, HemaSphere, 2026;10:(S1):pages. The abstract book DOIs can be found on the "Author Information" page.
**Disclaimer:** Articles published in the journal HemaSphere exclusively reflect the opinions of the authors. The authors are responsible for all content in their abstracts including accuracy of the facts, statements, citing resources, etc.

### Results

Both western blot and flow cytometry results indicated PCNSL cell lines exhibited significantly higher TfR1 expression relative to healthy PBMCs. HFn-based nanocarrier effectively traversed both the mouse and human BBB while maintaining its intact nanocarrier structure. Consistent with these findings, in vitro, MTX@HFn treatment for 48 and 72 hours elicited enhanced cytotoxicity compared to free MTX, accompanied by marked S-phase arrest and increased apoptosis. Notably, Calcein/PI staining of multicellular spheroids revealed that MTX@HFn induced apoptosis specifically within the avascular core, demonstrating superior tumor penetration. In vivo, the bioluminescence (BLI) signal confirmed that MTX@HFn significantly reduced intracranial tumor burden relative to both PBS group and free MTX group. Near infrared fluorescence (NIRF) imaging showed Cy5.5-MTX@HFn accumulation in the tumor-bearing brain. MTX@HFn treatment significantly prolonged overall survival in PCNSL-bearing mice.

### Summary/Conclusion

MTX@HFn represents a promising nanotherapy that combines efficient BBB traversal with targeted drug delivery. This strategy offers a novel treatment paradigm with enhanced efficacy and reduced toxicity for the management of PCNSL.

**Copyright Information:** (Online) ISSN: 2572-9241
©2026 The Author(s). HemaSphere is published by John Wiley & Sons Ltd on behalf of European Hematology Association. This is an open access Abstract Book distributed under the Attribution-NonCommercial-NoDerivs (CC BY-NC-ND), which allows third parties to download the articles and share them with others as long as they credit the author and the Abstract Book, but they cannot change the content in any way or use them commercially.
**Abstract Book Citations:** Authors, Title, HemaSphere, 2026;10:(S1):pages. The abstract book DOIs can be found on the "Author Information" page.
**Disclaimer:** Articles published in the journal HemaSphere exclusively reflect the opinions of the authors. The authors are responsible for all content in their abstracts including accuracy of the facts, statements, citing resources, etc.

### (PB3919) HIGH-DEFINITION SPATIAL TRANSCRIPTOMICS AND SINGLE-CELL ATLAS OF PRIMARY BREAST DLBCL REVEAL FUNCTIONAL COMPARTMENTALIZATION AND SPECIALIZED IMMUNE

**Topic:** 21. Lymphoma biology & translational research

Chaowei Zhang*[1, 2], Yuxuan Han[1, 2], Weiyi Yao[1, 2], Qing Hong[1, 2], Xinyu Tang[1, 2], Yanan Zhu[1, 2], Na Chen[1]

*[1]Beijing Luhe Hospital, Capital Medical University, Beijing, China;[2]Shandong First Medical University, Jinan, China;*

### Background

Primary Breast Diffuse Large B-cell Lymphoma (PB-DLBCL) is a rare and aggressive extranodal malignancy. Although single-cell RNA sequencing (scRNA-seq) has begun to unravel its cellular heterogeneity, the spatial organization and functional interactions within the tumor microenvironment (TME) remain poorly understood. Traditional genomic profiling lacks the spatial context necessary to understand how malignant B cells exploit specific anatomical niches for survival and immune evasion.

### Aims

This study aims to construct a high-resolution multidimensional spatial atlas of PB-DLBCL to decode the "spatial compartmentalization" of malignant B-cell subpopulations and identify the functional micro-niches that drive tumor progression and treatment resistance.

### Methods

We integrated high-definition spatial transcriptomics (Visium HD) from three treatment-naive PB-DLBCL patient tissues with scRNA-seq data comprising 23,536 cells. The computational workflow included Harmony for batch correction, Leiden clustering for subpopulation identification, and Robust Cell Type Decomposition (RCTD) to map single-cell resolution transcriptomic signatures onto the spatial tissue architecture. Functional enrichment (GO/KEGG) and protein-protein interaction (PPI) network analyses were utilized to define spatial functional modules.

### Results

We identified 23 distinct B-cell subpopulations organized into six unique Spatial Functional Modules, unveiling a highly ordered "Spatial Compartmentalization" rather than chaotic cell accumulation:

1. The Tumor Core (Module I): Acts as a "survival niche." Cluster 5 (BCL2+/ETV6+) maintains stemness and anti-apoptotic capacity, while Cluster 3 (IL10+) facilitates immune evasion by constructing an immunosuppressive milieu within the core.
2. The High-Proliferation Zone (Module II): Driven by an "S-phase engine" (Clusters 7/4) and metabolic reprogramming. Notably, we identified an atypical "Killer B cell" subpopulation (Cluster 11) expressing cytotoxic molecules (GZMB/NKG7), reflecting intense immune editing.
3. The Immune Synapse Zone (Module III): Features a dynamic "Recruitment-Expansion-Differentiation" axis. Cluster 9, located near dendritic cells (DCs), overexpresses CCL17 (TARC) to actively recruit T cells, promoting local B-DC-T tripartite interactions and clonal expansion.
4. The Peripheral Adaptation Zones (Modules IV-VI): B cells in myeloid-rich regions (Module IV) activate the Unfolded Protein Response (UPR) via HSP genes (HSPA6/DNAJB1) to combat proteotoxic stress. At the lymphocyte infiltration front (Module VI), B cells utilize RAGE-mediated inflammatory signaling and form stable immunological synapses with CD4+ T cells.

**Copyright Information:** (Online) ISSN: 2572-9241
©2026 The Author(s). HemaSphere is published by John Wiley & Sons Ltd on behalf of European Hematology Association. This is an open access Abstract Book distributed under the Attribution-NonCommercial-NoDerivs (CC BY-NC-ND), which allows third parties to download the articles and share them with others as long as they credit the author and the Abstract Book, but they cannot change the content in any way or use them commercially.
**Abstract Book Citations:** Authors, Title, HemaSphere, 2026;10:(S1):pages. The abstract book DOIs can be found on the "Author Information" page.
**Disclaimer:** Articles published in the journal HemaSphere exclusively reflect the opinions of the authors. The authors are responsible for all content in their abstracts including accuracy of the facts, statements, citing resources, etc.

### Summary/Conclusion

This study provides the first high-resolution spatial landscape of PB-DLBCL, demonstrating that the TME is a highly structured ecosystem. Malignant B cells exhibit profound functional plasticity, performing distinct roles—from survival and suppression in the core to recruitment and stress adaptation in the periphery—based on their spatial micro-niches. These findings identify novel spatially-defined therapeutic targets, such as the CCL17/CCR4 recruitment axis and BCL2-mediated survival niches, providing a blueprint for precision medicine in extranodal lymphomas.

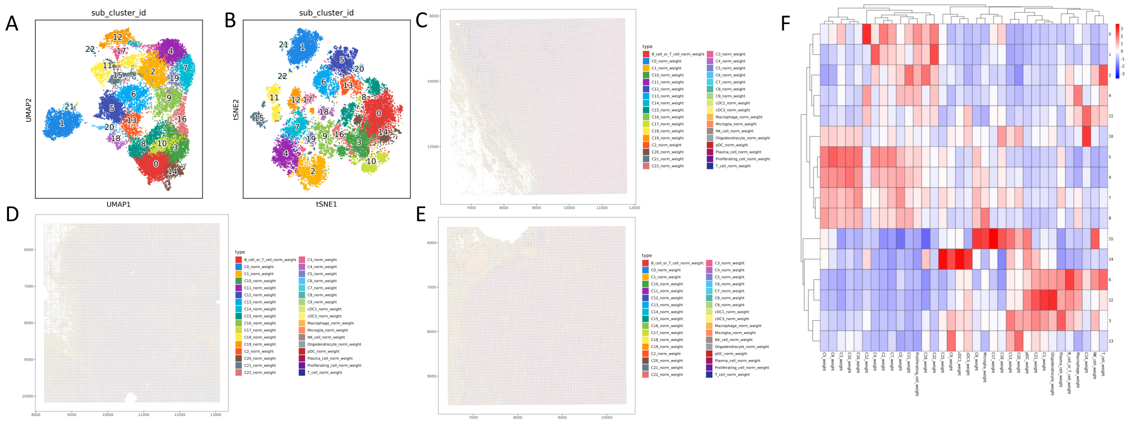

Figure Legend
(A, B) UMAP and t-SNE projections colored by unsupervised clustering results (sub_cluster_id). All cells are categorized into 23 distinct subpopulations (Cluster 0–22), revealing profound cellular heterogeneity within the tumor microenvironment. (C–E) Spatial scatter pie charts visualizing the cell-type composition across three independent patient samples—(C) 52301056_13, (D) 202202361_A4, and (E) S2302081—as resolved by the RCTD algorithm. Each capture spot (square) is represented by a pie chart, where distinct colors indicate the relative proportional abundance of various cell types within that specific spatial location. (F) Heatmap illustrating the spatial abundance of cell types derived from the integrated analysis of all three samples. Rows represent the cluster IDs defined by spatial transcriptomic clustering, and columns correspond to the cell types identified via single-cell RNA-seq. The color intensity reflects the proportion of each cell type within the respective spatial clusters.

**Copyright Information:** (Online) ISSN: 2572-9241
©2026 The Author(s). HemaSphere is published by John Wiley & Sons Ltd on behalf of European Hematology Association. This is an open access Abstract Book distributed under the Attribution-NonCommercial-NoDerivs (CC BY-NC-ND), which allows third parties to download the articles and share them with others as long as they credit the author and the Abstract Book, but they cannot change the content in any way or use them commercially.
**Abstract Book Citations:** Authors, Title, HemaSphere, 2026;10:(S1):pages. The abstract book DOIs can be found on the "Author Information" page.
**Disclaimer:** Articles published in the journal HemaSphere exclusively reflect the opinions of the authors. The authors are responsible for all content in their abstracts including accuracy of the facts, statements, citing resources, etc.

### (PB3920) GENE EXPRESSION PROFILING OF EBV-POSITIVE VERSUS EBV-NEGATIVE HIV-ASSOCIATED DIFFUSE LARGE B-CELL LYMPHOMA

**Topic:** 21. Lymphoma biology & translational research

Aleix Mendez-Lopez*[1], Gianfranco Lapietra[2], Miriam Verdu-Bou[3], Gustavo Tapia Melendo[4], Silvia Montoto[5], Marie Calaminici[5], Armando Lopez Guillermo[6], Antonio Martinez[7], Miguel Alcoceba Sánchez[8], Joaquin Gomez Espuch[9], María José Terol[10], Carolina Martinez[11], Mariana Bastos-Oreiro[12], Francisco Díaz Crespo[13], Eva Gonzalez Barca[14], Ana Muntañola Prat[15], Ferran Vall-Llovera Calmet[16], Pau Abrisqueta[17], Jose Maria Ribera[18], Juan-Manuel Sancho Cia[19], Jose Tomás Navarro Ferrando[20]

*[1]Institut Català d'Oncologia, Hematology, Badalona, Spain;[2]Josep Carreras Leukaemia Research Institute, Lymphoid Neoplasms Group, Badalona, Spain; [3]Institut d'Investigacions Biomèdiques August Pi i Sunyer (IDIBAPS), Barcelona, Spain; [4]Hospital Universitari Germans Trias i Pujol, Department of Pathology, Badalona, Spain;[5]St Bartholomew's Hospital, London, United Kingdom; [6]Fcrb-idibpas/hospital Clinic Bcn, Barcelona, Spain; [7]Hospital Clinic, IDIBAPS, Barcelona, Spain; [8]University Hospital of Salamanca (HUS/IBSAL), CIBERONC and Cancer Research Institute of Salamanca-IBMCC (USAL-CSIC), Salamanca(SP), Department of Hematology,, Salamanca, Spain; [9]Hospital U. Virgen de la Arrixaca, Murcia, Spain; [10]Hosp. Clinico De Valencia, Department of Hematology, Valencia, Spain; [11]INCLIVA Instituto de Investigación Sanitaria, Departamento de Patologia, Valencia, Spain; [12]Gregorio Marañon, Madrid, Spain; [13]10Hospital Universitario Gregorio Marañón, Madrid, Spain; [14]Ico Hospitalet-Duran I Reynals, Serv Hematologia, Barcelona, Spain;[15]Hospital de la Santa Creu i Sant Pau, Hematología Clínica, Barcelona, Spain; [16]Hospital Universitari Mútua de Terrassa, Terrassa, Spain; [17]Hospital Universitario Vall d'Hebron, Hematology, Barcelona, Spain; [18]ICO-Hospital Germans Trias i Pujol, Badalona, Spain; [19]ICO-Hospital Germans Trias i Pujol, Hematologia, Badalona, Spain; [20]Institut Català d'Oncologia. Josep Carreras Leukaemia Research Institute, Badalona, Spain;*

### Background

People living with HIV (PWH) have an increased risk of developing aggressive non-Hodgkin lymphomas, with diffuse large B-cell lymphoma (DLBCL) being the most common subtype. Epstein–Barr virus (EBV) is detected in 30–40% of HIV-associated DLBCL cases and it seems to be associated with poorer outcomes, similarly to what has been reported in the general population. However, the biological landscape of EBV+ HIV-associated DLBCL remains largely unexplored.

### Aims

We compared tumor gene expression profiles between EBV+ and EBV- HIV-associated DLBCL with the aim of elucidating biological differences and identifying distinct immunological signatures.

### Methods

Total mRNA was extracted from formalin-fixed paraffin-embedded (FFPE) of 47 HIV-associated DLBCL cases selected retrospectively based on tissue availability and RNA quality. EBV detection was performed at diagnosis by in situ hybridization using EBER-targeting probes. Gene expression profiling was performed using the NanoString nCounter Human PanCancer IO 360 panel supplemented with an additional 55 gene. All bioinformatics analyses were performed in R (v4.3.3) using NanoStringNCTools for data import, quality control, and normalization; limma for differential expression; and fgsea for gene set enrichment analysis with MSigDB Hallmark gene sets retrieved via msigdbr. Differential expression and gene set enrichment analysis were considered significant at Benjamini–Hochberg–adjusted P value (adj.P) < 0.05; effect sizes were summarized as log Fold Change (logFC) and normalized enrichment scores (NES).

**Copyright Information:** (Online) ISSN: 2572-9241
©2026 The Author(s). HemaSphere is published by John Wiley & Sons Ltd on behalf of European Hematology Association. This is an open access Abstract Book distributed under the Attribution-NonCommercial-NoDerivs (CC BY-NC-ND), which allows third parties to download the articles and share them with others as long as they credit the author and the Abstract Book, but they cannot change the content in any way or use them commercially.
**Abstract Book Citations:** Authors, Title, HemaSphere, 2026;10:(S1):pages. The abstract book DOIs can be found on the "Author Information" page.
**Disclaimer:** Articles published in the journal HemaSphere exclusively reflect the opinions of the authors. The authors are responsible for all content in their abstracts including accuracy of the facts, statements, citing resources, etc.

### Results

Overall, 13 cases (27.6%) were classified as EBV+ DLBCL. Comparative gene expression analysis between EBV + and EBV- cases revealed 17 differentially expressed genes (Fig. 1). EBV+ tumors showed higher expression of chemokines linked to immune recruitment/activation, including *CCL22* (logFC=1.98; adj.P=0.037), *CXCL13* (logFC=1.86; adj.P=0.036) and *XCL1/2* (logFC=1.49; adj.P=0.020), together with interferon/antiviral response genes such as *IRF1* (logFC=1.14; adj.P=0.020), *GBP1* (logFC=1.34; adj.P=0.036), *GBP4* (logFC=1.32; adj.P=0.036) and *APOL6* (logFC=1.05; adj.P=0.036). These cases also showed a higher expression of immune regulatory/checkpoint-associated markers, including *CTLA4* (logFC=1.12; adj.P=0.049) and *CD244* (logFC=1.09; adj.P=0.049), with concordant upregulation of *CD274/PD-L1* (logFC=0.92; adj.P=0.036). Moreover, EBER+ tumors displayed reduced expression of cellular maturation antigens *PAX5* (logFC=–1.14; adj.P=0.036), *LMO2* (logFC=–1.54; adj.P=0.036) and *DTX1* (logFC=–1.68; adj.P=0.036).

Consistently, gene set enrichment analysis demonstrated significant enrichment of interferon-mediated inflammatory programs in EBER+ tumors, including interferon-α (NES=2.15; adj.P=$5.8\times10^{-4}$) and interferon-γ responses (NES=2.30; adj.P=$5.8\times10^{-4}$), as well as allograft rejection (NES=1.79; adj.P=$7.0\times10^{-4}$) and complement system-related genes (NES=1.95; adj.P=0.0027). Leading-edge genes included *IRF1*, *GBP* family members, *CXCL13/CCL22*, and *CD274* (*PD-L1*), supporting an interferon-driven, immune-inflamed phenotype in EBV+ HIV-associated DLBCL.

### Summary/Conclusion

Compared to EBV- cases, EBV+ HIV-associated DLBCL were characterized by an enhanced immature phenotype and an increased immunologically active gene expression profile, suggesting more aggressive microenvironment biology.

**Copyright Information:** (Online) ISSN: 2572-9241
©2026 The Author(s). HemaSphere is published by John Wiley & Sons Ltd on behalf of European Hematology Association. This is an open access Abstract Book distributed under the Attribution-NonCommercial-NoDerivs (CC BY-NC-ND), which allows third parties to download the articles and share them with others as long as they credit the author and the Abstract Book, but they cannot change the content in any way or use them commercially.
**Abstract Book Citations:** Authors, Title, HemaSphere, 2026;10:(S1):pages. The abstract book DOIs can be found on the “Author Information” page.
**Disclaimer:** Articles published in the journal HemaSphere exclusively reflect the opinions of the authors. The authors are responsible for all content in their abstracts including accuracy of the facts, statements, citing resources, etc.

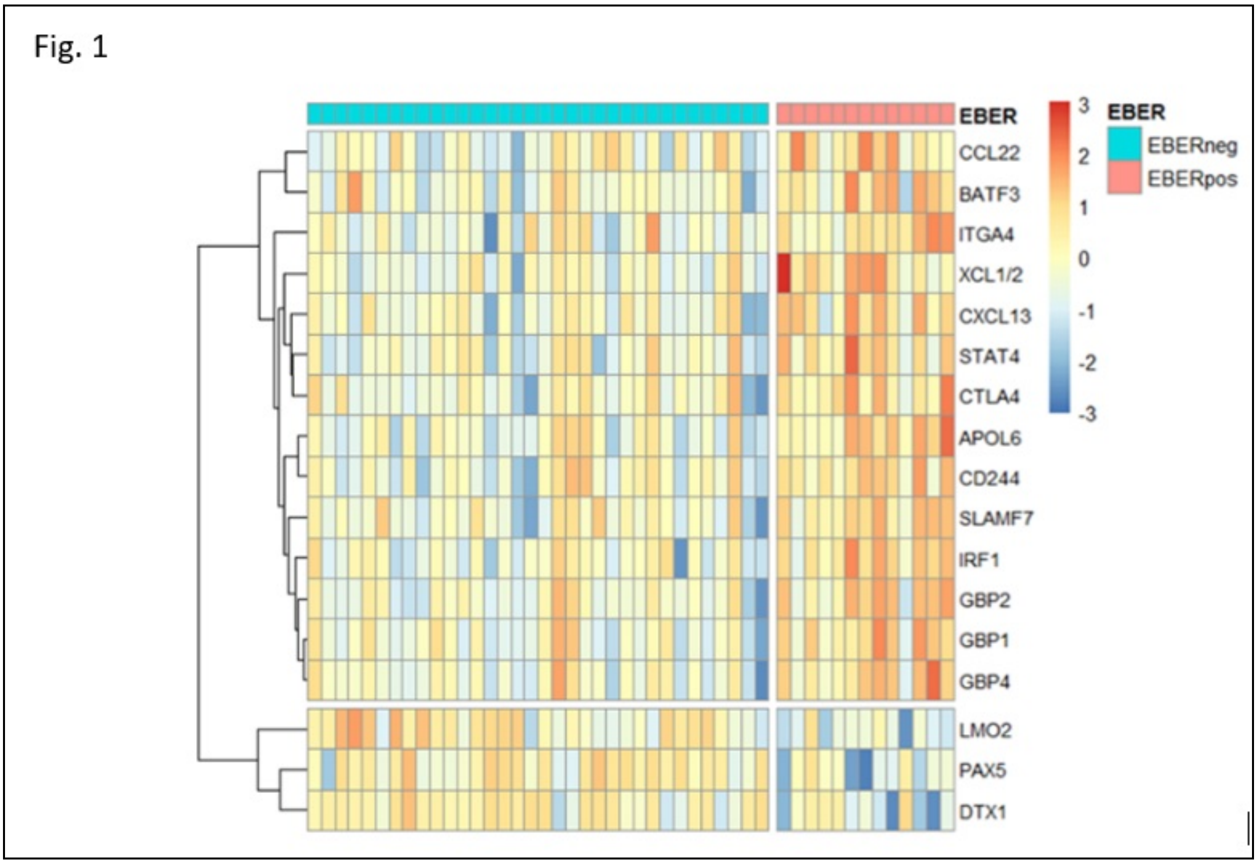

Heatmap showing gene expression profiles stratified by EBV status.

**Copyright Information:** (Online) ISSN: 2572-9241
©2026 The Author(s). HemaSphere is published by John Wiley & Sons Ltd on behalf of European Hematology Association. This is an open access Abstract Book distributed under the Attribution-NonCommercial-NoDerivs (CC BY-NC-ND), which allows third parties to download the articles and share them with others as long as they credit the author and the Abstract Book, but they cannot change the content in any way or use them commercially.
**Abstract Book Citations:** Authors, Title, HemaSphere, 2026;10:(S1):pages. The abstract book DOIs can be found on the "Author Information" page.
**Disclaimer:** Articles published in the journal HemaSphere exclusively reflect the opinions of the authors. The authors are responsible for all content in their abstracts including accuracy of the facts, statements, citing resources, etc.

### (PB3921) UNLEASHING BCL2 INHIBITION IN FOLLICULAR LYMPHOMA WITH ZANUBRUTINIB: A TRANSLATIONAL TRIAL WITH MECHANISTIC INSIGHTS FROM SINGLE CELL ANALYSIS

**Topic:** 21. Lymphoma biology & translational research

Pin Lu[1, 2], Tingwei Guo[2], Yunyun Zhou[3], Miraan Jhaveri[1], Bryant Schultz[4], Johanna James[4], Yinfei Tan[5], Juehua Gao[6], Denise Connolly[1, 4], Rashmi Khanal[7], Shazia Nakhoda[7], Marcus Messmer[7], Y. Lynn Wang*[5]

*[1]Fox Chase Cancer Center, Cancer Signaling and Microenvironment Program, Philadelphia, United States of America;[2]Fox Chase Cancer Center, Department of Pathology, Philadelphia, United States of America; [3]Fox Chase Cancer Center, Biostatistics and Bioinformatics, Philadelphia, United States of America; [4]Fox Chase Cancer Center, Biosample Repository Facility, Philadelphia, United States of America; [5]Fox Chase Cancer Center, Pathology, Philadelphia, United States of America; [6]Northwestern University Feinberg School of Medicine, Chicago, United States of America;[7]Fox Chase Cancer Center, Department of Hematology/Oncology, Philadelphia, United States of America;*

### Background

Follicular lymphoma (FL), the most common indolent non-Hodgkin lymphoma, remains incurable. Despite initial treatment responses, many patients develop relapsed/refractory disease. While FL carries BCL2 translocations and displays elevated B-cell receptor (BCR) signaling, single-agent targeted therapies such as BTK inhibitors (BTKi) or BCL2 inhibitors (BCL2i), have shown limited efficacy.

### Aims

We hypothesized that the tumor microenvironment (TME) contributes to the activation of alternative survival mechanisms, and dual inhibition of BTK and BCL2 with zanubrutinib (Zanu) and sonrotoclax (Sonro) may overcome TME-mediated resistance and improve therapeutic response. In this study, we aim to investigate the combination of BCL2i and BTKi in a "translational trial" using our personalized iNHL-TME models built with tumor biopsies. We also aim to employ a single-cell sequencing strategy to explore the potential transcriptomic mechanisms of drug resistance in the tumor B-cells.

### Methods

We leveraged a novel ex vivo co-culture model (iNHL-TME) that mimics the lymph node microenvironment to test this combination in primary FL tumors, a disease that has historically been difficult to model.

### Results

Among nine biopsy samples, responses to single drug varied. Four responded to zanu and a different four responded to sonro, and one showed responsiveness to both single agents. With the drug combination, seven of the nine samples demonstrated enhanced cell killing while the remaining two already had high sensitivity to single agent sonro. Overall, eight of nine samples showed sensitivity or improved sensitivity to the dual inhibition. To understand potential resistance mechanisms, we performed single-cell RNA sequencing on the FL biopsy, which showed suboptimal responses to single agent or the combination. The study revealed four distinct B-cell states characterized by BCR signaling, ER stress, NF-κB activation, or heat-shock response. As expected, zanu suppressed BCR signaling, and sonro induced apoptosis and the activation of TP53 pathways. Interestingly, in this FL with sub-optimal responses, sonro and the combination upregulated cytoprotective NF-κB signaling, suggesting that a subset of tumor B cells may survive the treatment by activating alternative survival pathways (See Figure).

**Copyright Information:** (Online) ISSN: 2572-9241
©2026 The Author(s). HemaSphere is published by John Wiley & Sons Ltd on behalf of European Hematology Association. This is an open access Abstract Book distributed under the Attribution-NonCommercial-NoDerivs (CC BY-NC-ND), which allows third parties to download the articles and share them with others as long as they credit the author and the Abstract Book, but they cannot change the content in any way or use them commercially.
**Abstract Book Citations:** Authors, Title, HemaSphere, 2026;10:(S1):pages. The abstract book DOIs can be found on the "Author Information" page.
**Disclaimer:** Articles published in the journal HemaSphere exclusively reflect the opinions of the authors. The authors are responsible for all content in their abstracts including accuracy of the facts, statements, citing resources, etc.

**Summary/Conclusion**

This study integrates functional drug testing with high-resolution molecular profiling.  Functional phenotypic studies suggest that the combination of BTK and BCL2 inhibitors deliver effective cell killing. The mechanistic study also suggests that most tumor cells are under drug-induced stress and undergo apoptosis. However, a subpopulation of tumor cells may survive by upregulating cytoprotective pathways. Further studies are needed to verify these results and to determine if this upregulation would lead to tumor persistence and resistance.

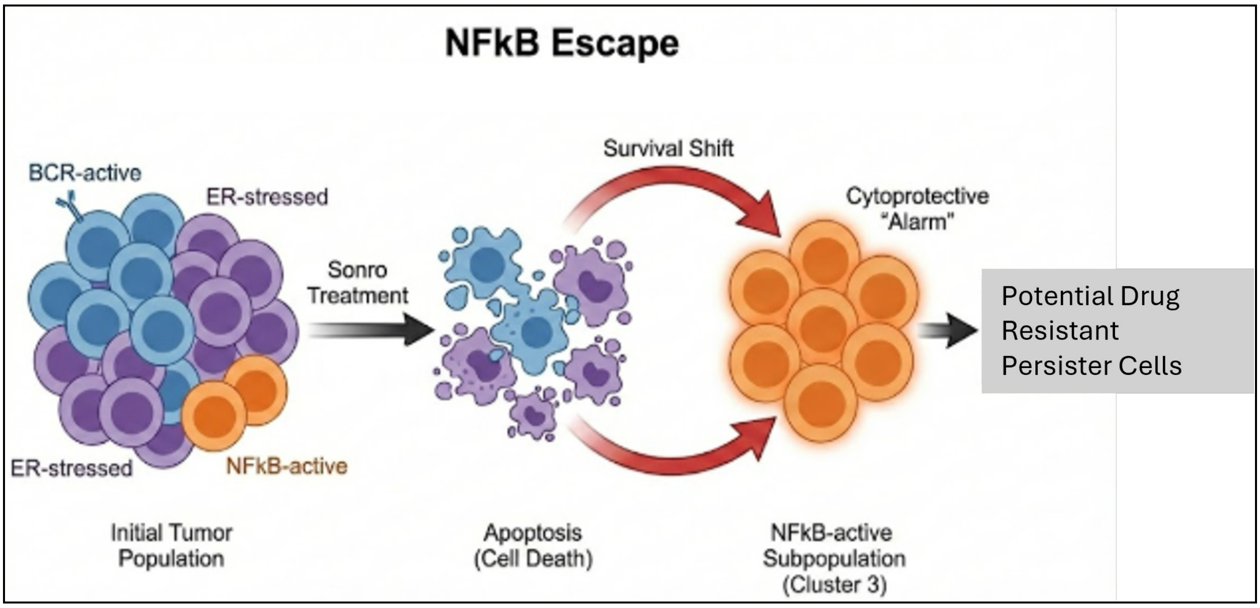

NFkB Escape Mechanism

**Copyright Information:** (Online) ISSN: 2572-9241
©2026 The Author(s). HemaSphere is published by John Wiley & Sons Ltd on behalf of European Hematology Association. This is an open access Abstract Book distributed under the Attribution-NonCommercial-NoDerivs (CC BY-NC-ND), which allows third parties to download the articles and share them with others as long as they credit the author and the Abstract Book, but they cannot change the content in any way or use them commercially.
**Abstract Book Citations:** Authors, Title, HemaSphere, 2026;10:(S1):pages. The abstract book DOIs can be found on the "Author Information" page.
**Disclaimer:** Articles published in the journal HemaSphere exclusively reflect the opinions of the authors. The authors are responsible for all content in their abstracts including accuracy of the facts, statements, citing resources, etc.

### (PB3922) THE EPSTEIN-BARR VIRUS LMP1 PROMOTES B CELL LYMPHOMA BY ENHANCING AICDA VIA AP-1 SIGNALLING

**Topic:** 21. Lymphoma biology & translational research

Lungile Mapekula*[1], Shaheen Mowla[1]

[1]*University of Cape Town, Pathology, Cape Town, South Africa;*

### Background

Infection with the Epstein-Barr virus (EBV) is a ubiquitous feature in human populations and a well-recognized co-factor in the development of several aggressive neoplasms. The virus elicits its oncogenic potential through the action of several viral proteins including the EBV encoded latent membrane protein 1 (LMP1), the latter being a well described oncoprotein in the context of B cell lymphoma development.

### Aims

The current study aimed to elucidate the role of LMP1 in the dysregulation of*Activation-induced cytidine deaminase* (*AICDA*), a major driver of B cell lymphoma.

### Methods

Dual luciferase reporter assays were employed to evaluate the effect of LMP1 and activator protein 1 (AP-1) transcription factor family members, c-JUN and JunB, on two *AICDA* enhancer regions: R2 (located within intron 1; +121 to +2221 bp downstream of the transcription start site) and R4 (~ 8 kB upstream of transcription start site). LMP1 was ectopically in the EBV-negative Diffuse Large B-cell Lymphoma cell line (U2932) using lipid-based transfection methods. Western blot analyses were used to verify changes in AICDA, c-MYC and phosphorylated gamma H2AX (gH2AX), a marker of DNA double strand breaks. The PROMO software was used to predict transcription factor (TF) binding sites within the *AICDApR4* DNA sequence. Sequential deletion constructs of the *AICDA* promoter R4 region were generated by PCR and cloning. Putative AP-1 binding sites were mutated using site-directed mutagenesis followed by Sanger sequencing to confirm the mutations. Chromatin immunoprecipitation (ChIP) assays were used to evaluate the recruitment of phosphorylated c-Jun to relevant regions within R4 in response to LMP1 signalling.

### Results

The transcriptional activity of both *AICDA* enhancer elements R2 and R4 are significantly increased in lymphoma cells expressing LMP1 relative to parental control cells. Ectopic expression of LMP1 in the EBV-negative U2932 DLBCL cell line led to enhanced AICDA protein expression, as well as gH2AX, a known mediator of AICDA-induced genomic instability. c-MYC, which is strongly correlated with aggressive lymphomas, was also enhanced. By generating sequential deletions of the *AICDApR4* region, the minimal promoter region responsible for mediating the LMP1-induced expression was mapped, with the latter containing binding sites for STAT4, FOXP3 and AP-1. The role of four AP-1 sites was interrogated due to the established relationship between LMP1 and AP-1, and were found to be important in mediating the increase in activity of *AICDApR4.* Finally, ChIP assays demonstrated significant enrichment of phosphorylated-c-JUN proteins at these regions of the *AICDA*pR4 region in the presence of LMP1, confirming LMP-1 mediated recruitment of AP-1 to the *AICDA* promoter.

**Copyright Information:** (Online) ISSN: 2572-9241
©2026 The Author(s). HemaSphere is published by John Wiley & Sons Ltd on behalf of European Hematology Association. This is an open access Abstract Book distributed under the Attribution-NonCommercial-NoDerivs (CC BY-NC-ND), which allows third parties to download the articles and share them with others as long as they credit the author and the Abstract Book, but they cannot change the content in any way or use them commercially.
**Abstract Book Citations:** Authors, Title, HemaSphere, 2026;10:(S1):pages. The abstract book DOIs can be found on the "Author Information" page.
**Disclaimer:** Articles published in the journal HemaSphere exclusively reflect the opinions of the authors. The authors are responsible for all content in their abstracts including accuracy of the facts, statements, citing resources, etc.

**Summary/Conclusion**

This study demonstrated that the EBV-encoded LMP1 oncogenic protein enhances the expression of the lymphoma driving factor *AICDA* through AP-1-dependent transcriptional regulation, likely via the kinase (ERK)-MAPK pathway. The use of approved AP-1 inhibitors, such as T-5224, which blocks AP-1 binding to DNA, may be a useful addition in B-cell lymphoma therapy, and other EBV-driven malignancies. Future work could use pre-clinical laboratory investigations to interrogate this further.

**Copyright Information:** (Online) ISSN: 2572-9241
©2026 The Author(s). HemaSphere is published by John Wiley & Sons Ltd on behalf of European Hematology Association. This is an open access Abstract Book distributed under the Attribution-NonCommercial-NoDerivs (CC BY-NC-ND), which allows third parties to download the articles and share them with others as long as they credit the author and the Abstract Book, but they cannot change the content in any way or use them commercially.
**Abstract Book Citations:** Authors, Title, HemaSphere, 2026;10:(S1):pages. The abstract book DOIs can be found on the "Author Information" page.
**Disclaimer:** Articles published in the journal HemaSphere exclusively reflect the opinions of the authors. The authors are responsible for all content in their abstracts including accuracy of the facts, statements, citing resources, etc.

### (PB3923) GERMLINE NFKB1 VARIANT IS INDICATIVE OF INFERIOR OVERALL AND POST-RELAPSE SURVIVAL OUTCOMES IN PATIENTS WITH MANTLE CELL LYMPHOMA: ANALYSIS OF 459 CZECH PATIENTS REGISTRY COHORT

**Topic:** 21. Lymphoma biology & translational research

Jana Karolová*[1, 2], Jihyun Song[3], Kristina Forsterová[1], Marek Trneny#[1], Josef Prchal[1, 2, 3], Pavel Klener[1, 2]

*[1]General University Hospital, Charles University, First Dept. of Internal Medicine- Hematology, Prague, Czechia;[2]First Faculty of Medicine, Charles University, Institute of Pathological Physiology, Prague, Czechia; [3]Huntsman Cancer Institute, University of Utah, Hematology and hematologic malignancies, Salt Lake City, United States of America;*

### Background

Mantle cell lymphoma (MCL) is a heterogeneous, frequently relapsing B-cell lymphoma. Germline *NFKB1* variant can modulate inflammatory and hypoxia-inducible factor (HIF) signaling, potentially influencing MCL outcomes.

### Aims

To evaluate associations between germline *NFKB1* variant and clinical features and survival in MCL.

### Methods

We analyzed 459 patients with histologically confirmed MCL diagnosed between 2000 and 2024 at Charles University General Hospital (Prague) and recorded in the Czech non-Hodgkin lymphoma registry NiHiL (NCT03199066). The evolutionarily enriched *NFKB1* variant (rs230511), identified in Andeans, modulates inflammation and HIF signaling and is present in ~30% of Europeans (PMID: 39971917). Our MCL cohort was genotyped for the *NFKB1* variant as C/C, C/T, or T/T. Progression-free survival (PFS) and overall survival (OS) were measured from diagnosis; post-relapse endpoints were second PFS (PFS-2; from first relapse to progression) and second OS (OS-2; from first relapse to death). Survival was estimated by the Kaplan–Meier method and compared by the log-rank test. C-reactive protein (CRP) was measured across genotypes using nonparametric tests with Holm adjustment.

### Results

Baseline characteristics were balanced with a median age of 67 years and stage IV disease in 89.5% of cases. Genotype frequencies were C/C 217 (47.3%), C/T 210 (45.8%), and T/T 32 (7.0%), similar to those observed in healthy Europeans. Age did not differ across genotypes ($p$=0.411), and analysis of CRP at the time of diagnosis showed no statistically significant genotype associations. The MCL International Prognostic Index (MIPI) differed significantly, with higher median MIPI in C/T and T/T (both 6.3) vs C/C (6.1; $p$=0.038), suggesting an association of *NFKB1* heterozygosity/homozygosity with higher-risk biology. Frontline response rates and PFS were comparable; median PFS was 61.7 (C/C), 46.3 (C/T), and 44.3 months (T/T). OS differed by genotype ($p$=0.05): median OS 131.1 months (C/C) vs 84.3 (C/T; $p$=0.018) vs 73.1 (T/T; $p$=0.241). Post-relapse analyses (95 C/C, 84 C/T, 15 T/T) showed inferior outcomes for C/T and T/T (global $p$=0.0208): median PFS-2 26.9 months (C/C) vs 9.7 (C/T;$p$=0.018) vs 14.3 (T/T; $p$=0.662); median OS-2 54.4 months (C/C) vs 16.5 (C/T; $p$=0.007) vs 44.8 (T/T; $p$=0.511; global $p$=0.008, see Figure 1). The number of second or subsequent relapses did not differ significantly among the C/T, T/T, and C/C groups. However, disease-related deaths were 65 (31%) in C/T, 12 (37.5%) in T/T, and 55 (25.3%) in C/C patients. Results suggest that the poorer survival outcomes for C/T and T/T patients may be a consequence of a higher rate of disease-related deaths rather than an increased frequency of second or subsequent relapses. Evaluation of molecular mechanisms underlying these observations is in progress.

**Copyright Information:** (Online) ISSN: 2572-9241
©2026 The Author(s). HemaSphere is published by John Wiley & Sons Ltd on behalf of European Hematology Association. This is an open access Abstract Book distributed under the Attribution-NonCommercial-NoDerivs (CC BY-NC-ND), which allows third parties to download the articles and share them with others as long as they credit the author and the Abstract Book, but they cannot change the content in any way or use them commercially.
**Abstract Book Citations:** Authors, Title, HemaSphere, 2026;10:(S1):pages. The abstract book DOIs can be found on the “Author Information” page.
**Disclaimer:** Articles published in the journal HemaSphere exclusively reflect the opinions of the authors. The authors are responsible for all content in their abstracts including accuracy of the facts, statements, citing resources, etc.

### Summary/Conclusion

In this large registry-based MCL cohort, the *NFKB1* C/T and T/T genotypes were associated with inferior OS and markedly worse post-relapse survival despite similar frontline disease control. These findings suggest that germline *NFKB1* variation may contribute to relapse biology and treatment resistance, warranting validation in independent analyses adjusted for other prognostic factors and treatments.

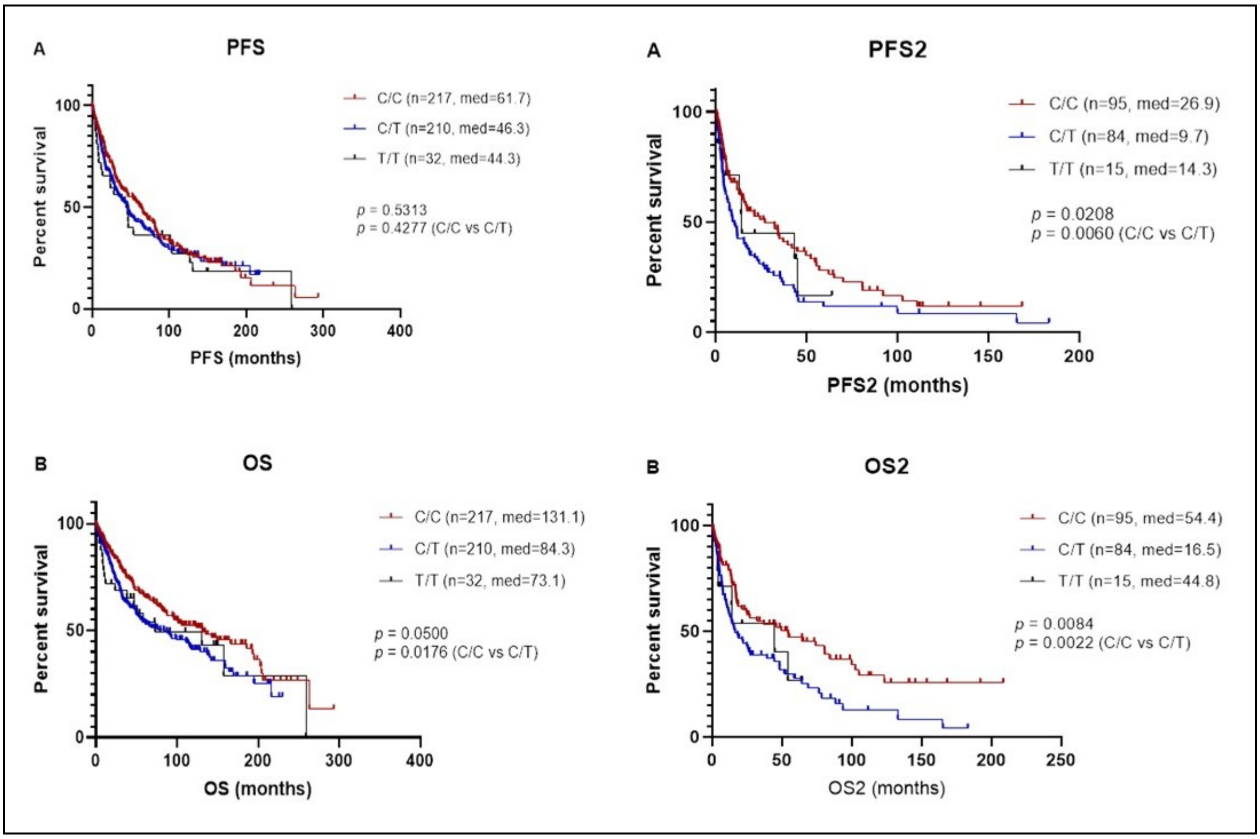

Figure 1: Progression-free survival (PFS), overall survival (OS), second progression-free survival (PFS2), and second overall survival (OS2) by treatment sequence. Kaplan–Meier curves for (A) PFS and (B) OS (months) comparing C/C (n=217, red), C/T (n=210, blue), and T/T (n=32, black). Median PFS: 61.7, 46.3, and 44.3 months; median OS: 131.1, 84.3, and 73.1 months, respectively. Log-rank p-values are shown (overall and C/C vs C/T). Second progression-free survival (PFS2) and second overall survival (OS2) by treatment sequence. Kaplan–Meier curves for (A) PFS2 and (B) OS2 (months) comparing C/C (n=95, red), C/T (n=84, blue), and T/T (n=15, black). Median PFS2: 26.9, 9.7, and 14.3 months; median OS2: 54.4, 16.5, and 44.8 months, respectively. Log-rank p-values are shown (overall and C/C vs C/T).

**Copyright Information:** (Online) ISSN: 2572-9241
©2026 The Author(s). HemaSphere is published by John Wiley & Sons Ltd on behalf of European Hematology Association. This is an open access Abstract Book distributed under the Attribution-NonCommercial-NoDerivs (CC BY-NC-ND), which allows third parties to download the articles and share them with others as long as they credit the author and the Abstract Book, but they cannot change the content in any way or use them commercially.
**Abstract Book Citations:** Authors, Title, HemaSphere, 2026;10:(S1):pages. The abstract book DOIs can be found on the "Author Information" page.
**Disclaimer:** Articles published in the journal HemaSphere exclusively reflect the opinions of the authors. The authors are responsible for all content in their abstracts including accuracy of the facts, statements, citing resources, etc.

### (PB3924) ELEVATED DOUBLE-NEGATIVE T CELLS AND ALTERED B-CELL MEMORY COMPARTMENTS AS IMMUNOPHENOTYPIC BIOMARKERS OF UNDERLYING INBORN ERRORS OF IMMUNITY IN YOUNG PATIENTS WITH LYMPHOPROLIFERATIVE DISORDERS

**Topic:** 21. Lymphoma biology & translational research

Eleonora Gambineri*[1], Martina Burattin[2], Cesare Sala[2], Daiana Flores[3], Maria Luisa Coniglio[3], Claudia Priolo[3], Marta Voarino[1], Agnese Roveta[1], Giorgio Costagliola[4], Elena Chiocca[3], Francesco Pegoraro[1], Annalisa Tondo[3]

*[1]University of Florence, Meyer Children's Hospital, IRCCS, Florence, Italy;[2]University of Florence, NEUROFARBA Department, Florence, Italy; [3]Meyer Children's Hospital, IRCCS, Florence, Italy; [4]Azienda Ospedaliero Universitaria Pisana, Pisa, Italy;*

### Background

Inborn errors of immunity (IEI) are increasingly recognized as drivers of lymphoproliferative disorders (LPD) in children and adolescents, yet immune dysregulation often manifests without classical infectious phenotypes, delaying diagnosis and appropriate management.

### Aims

We hypothesized that distinct immunophenotypic signatures, detectable by standard flow cytometry, could serve as accessible early biomarkers identifying IEI-associated LPD before genetic confirmation, enabling timely precision intervention.

### Methods

We prospectively enrolled 38 patients aged ≤20 years presenting with polyclonal LPD (PL, n=21) or malignant lymphoma (ML, n=17) persisting >6 months off therapy. All underwent extended flow cytometry immunophenotyping and genomic analysis (targeted panel and/or whole-exome sequencing) filtered against IUIS 2024 IEI gene lists. IEI diagnosis required pathogenic or likely pathogenic variants and/or ESID clinical criteria. Immunophenotypic parameters were compared with age-matched healthy controls using appropriate non-parametric testing.

### Results

Confirmed IEI were identified in 42% of patients (16/38), with a higher prevalence in PL (57%) than ML (24%). Pathogenic variants involved *FAS*, *PIK3CD*, *STAT3*, *NFKB1/2*, *PRKCD*, *IKZF1*, *SH2D1A*, and *TACI*. An additional 34% harbored variants of uncertain significance in IEI-associated or lymphoma-predisposing genes (*CTLA4*, *STAT5A*, *ATM*), yielding an overall diagnostic yield of 66%. Critically, the majority lacked classical infection-related immunodeficiency features.
Two immunophenotypic parameters robustly discriminated IEI-positive from IEI-negative patients and healthy controls: significantly elevated circulating double-negative T cells (DNT; $CD3^{+}CD4^{-}CD8^{-}TCR\alpha\beta^{+}$, >2% of $CD3^{+}$ lymphocytes; $p<0.01$) and expanded unswitched memory B cells ($CD27^{+}IgD^{+}IgM^{+}$; $p<0.001$). These signatures were consistent across multiple IEI genetic subtypes, persisted independently of lymphoma histology, and were detectable prior to genetic workup. Notably, all IEI identified in ML occurred exclusively in B-cell lymphomas (Burkitt, DLBCL, Hodgkin), underscoring the role of B-cell immune dysregulation in lymphomagenesis.

**Copyright Information:** (Online) ISSN: 2572-9241
©2026 The Author(s). HemaSphere is published by John Wiley & Sons Ltd on behalf of European Hematology Association. This is an open access Abstract Book distributed under the Attribution-NonCommercial-NoDerivs (CC BY-NC-ND), which allows third parties to download the articles and share them with others as long as they credit the author and the Abstract Book, but they cannot change the content in any way or use them commercially.
**Abstract Book Citations:** Authors, Title, HemaSphere, 2026;10:(S1):pages. The abstract book DOIs can be found on the “Author Information” page.
**Disclaimer:** Articles published in the journal HemaSphere exclusively reflect the opinions of the authors. The authors are responsible for all content in their abstracts including accuracy of the facts, statements, citing resources, etc.

### Summary/Conclusion

In young patients with LPD, elevated DNT cells and unswitched memory B-cell expansion constitute robust, flow cytometry-based screening biomarkers for underlying IEI identifiable before and independently of genetic results. With over 60% of patients carrying IEI-associated variants, often without overt immunodeficiency, these immunophenotypic signatures should prompt systematic genomic workup. Integration of this two-parameter screening approach into the diagnostic pathway of LPD in children and young adults could accelerate IEI diagnosis, guide gene-targeted therapies (sirolimus, abatacept), and transform surveillance strategies advocating for precision immunology as a standard of care in young patients with hematological malignancies and immune dysregulation.

**Copyright Information:** (Online) ISSN: 2572-9241
©2026 The Author(s). HemaSphere is published by John Wiley & Sons Ltd on behalf of European Hematology Association. This is an open access Abstract Book distributed under the Attribution-NonCommercial-NoDerivs (CC BY-NC-ND), which allows third parties to download the articles and share them with others as long as they credit the author and the Abstract Book, but they cannot change the content in any way or use them commercially.
**Abstract Book Citations:** Authors, Title, HemaSphere, 2026;10:(S1):pages. The abstract book DOIs can be found on the "Author Information" page.
**Disclaimer:** Articles published in the journal HemaSphere exclusively reflect the opinions of the authors. The authors are responsible for all content in their abstracts including accuracy of the facts, statements, citing resources, etc.

### (PB3925) ALK KINASE ACTIVATION USING PTPN1/PTPN2 DEGRADER-DRUGS AS TWO-PRONGED ANTI-LYMPHOMA STRATEGY IN ANAPLASTIC LARGE CELL LYMPHOMA

**Topic:** 21. Lymphoma biology & translational research

Nicola Mora*[1], Yunpeng Bai[2], Catello Giordano[1], Jinmin Miao[2], Aleksandra Fesiuk[1], Michaela Schlederer[1], Merjem Bećirović[3], Magdalena Kršić[4], Stefania Massari[1, 5], Sabine Lagger[5], Christine Damm-Welk[6], Jonatan Kendler[7], Zihan Qu[8], Jingmei Yu[2], Haoran Zhang[2], Ahmed Abou-Shanab[2], Zheng Zhang[9], Haibin Zhou[10], Yuzhi Wang[10], Lena Illert[4], Lukas Kenner[1, 5], Tony Tiganis[11], Weiguo Tao[2, 8, 9, 12], Shaomeng Wang[10], Zhong Zhang[2, 8, 12, 13], Olaf Merkel[1]

*[1]Department of Pathology, Medical University of Vienna, Vienna, Austria;[2]Borch Department of Medicinal Chemistry and Molecular Pharmacology, Purdue University, West Lafayette, Indiana, United States of America; [3]Competence Center for Periodontal Research, University Clinic of Dentistry, Medical University of Vienna, Vienna, Austria; [4]Institut für Allgemeine Pathologie und Pathologische Anatomie, TUM Universitätsklinikum Klinikum rechts der Isar, Munich, Germany; [5]Unit of Laboratory Animal Pathology, University of Veterinary Medicine Vienna, Vienna, Austria; [6]Department of Pediatric Hematology and Oncology, University Hospital Hamburg-Eppendorf, Hamburg, Germany; [7]Department of Biological Sciences and Pathobiology, Pharmacology and Toxicology, University of Veterinary Medicine Vienna, Vienna, Austria; [8]The James Tarpo Jr. and Margaret Tarpo Department of Chemistry, Purdue University, West Lafayette, Indiana, United States of America; [9]Department of Biochemistry, Purdue University, West Lafayette, Indiana, United States of America; [10]Department of Medicinal Chemistry, College of Pharmacy and Rogel Cancer Center, University of Michigan, Ann Arbor, Michigan, United States of America; [11]Monash Biomedicine Discovery Institute, Monash University Department of Biochemistry and Molecular Biology, Monash University, Melbourne, Australia; [12]Purdue University Institute for Cancer Research, West Lafayette, Indiana, United States of America; [13]Purdue University Institute for Drug Discovery, West Lafayette, Indiana, United States of America;*

### Background

Anaplastic large cell lymphoma (ALCL) is an aggressive CD30 T-cell lymphoma with around 50% of patients bearing the typical NPM-ALK fusion. The JAK/STAT signaling pathway is highly active in ALCL and STAT3 and STAT5 are described as tumor promoters. As second line of treatment after chemotherapy ALK inhibitors are now widely utilized, but quite a large subset of patients develop resistance. Potential new treatment are critical to overcome this resistance and inducing a complete remission.

### Methods

Transgenic ALCL mouse model with and without STAT1 abrogation; Immunohistochemistry on ALCL patient tissues; resazurin assay; Annexin V/PI staining using flow cytometry; PTPN1/2 phosphatase PROTAC double degrader DU-14; ALCL cells with and without CRISPR-mediated STAT1 knockout; Immuno-blotting; murine xenograft models of ALCL, ALK+; proteomic and phospho-proteomic; FACS analysis of T-cell activation; OPAL multiplex IHC

**Copyright Information:** (Online) ISSN: 2572-9241
©2026 The Author(s). HemaSphere is published by John Wiley & Sons Ltd on behalf of European Hematology Association. This is an open access Abstract Book distributed under the Attribution-NonCommercial-NoDerivs (CC BY-NC-ND), which allows third parties to download the articles and share them with others as long as they credit the author and the Abstract Book, but they cannot change the content in any way or use them commercially.
**Abstract Book Citations:** Authors, Title, HemaSphere, 2026;10:(S1):pages. The abstract book DOIs can be found on the "Author Information" page.
**Disclaimer:** Articles published in the journal HemaSphere exclusively reflect the opinions of the authors. The authors are responsible for all content in their abstracts including accuracy of the facts, statements, citing resources, etc.

### Results

Active JAK-STAT pathway is the main driver of ALCL. Recently a new concept of oncogenic over-stimulation as potential therapy avenue has been introduced. We observed that within the JAK-STAT pathway, protein tyrosine phosphatases (PTPs) are essential to maintain the balance in the oncogenic stimulation allowing new signal to restart the stimuli cascade. We analysed a public dataset of 1800 cell lines where they knocked-out every genes and observed a strong expression of PTPN1 and PTPN2 in all ALCL cells and a high sensitivity of ALK+, ALCL cells to the knock-out. We reproduced the effect of the knock-out with a double PROTAC degrader called DU-14 and with western blot we observed a strong upregulation of pNPM-ALK and pSTAT1. We later confirmed the high specificity of the degrader with proteomic and phospho-proteomic analysis. We furthermore showed DU-14 activity on resistant ALCL cells to ALK inhibitor. Meanwhile we performed IHC on patient tissues and observed high levels of STAT1 Tyr701 phosphorylation raising the question of its role in lymphomagenesis. To clarify this, we have created a STAT1 knockout in the established CD4-NPM-ALK mouse model. We saw strongly reduced overall survival in the STAT1-/- mice indicating that STAT1 is a tumor suppressor. Finally, in an immune-deficient murine xenograft ALCL, ALK+ model DU-14 treatment led to complete abrogation of tumor growth. Immunohistochemistry staining of the tumors showed PTPN1/2 degradation and activation of STAT1 Tyr701 phosphorylation. Furthermore, we tested DU-14 on immune-competent mice engrafted with murine ALK+, ALCL and we observed a full abolition of the tumors. We then, through FACS analysis of draining lymphnodes and multiplex IHC confirmed T-cell activation and T-cell infiltration in the tumors showing the ability of DU-14 to also induce immune-activation.

### Summary/Conclusion

This research may contribute to increase the attention on this oncogenic over-activation therapeutic approach. DU-14 has shown significant apoptosis induction in ALK+, ALCL cells through increase phosphorylation of pNPM-ALK and pSTAT1. In addition, DU-14 has shown on-tumor activity in NSG treated mice engrafted with human ALK+ ALCL cells and strong immune-activation in immune-competent mice engrafted with murine cells counterparts and this have contributed to prolonged remission in untreated mice for one year observation time period. Together this results support a novel and promising therapeutic avenue for clinical intervention in $ALK^{+}$ ALCL

**Copyright Information:** (Online) ISSN: 2572-9241
©2026 The Author(s). HemaSphere is published by John Wiley & Sons Ltd on behalf of European Hematology Association. This is an open access Abstract Book distributed under the Attribution-NonCommercial-NoDerivs (CC BY-NC-ND), which allows third parties to download the articles and share them with others as long as they credit the author and the Abstract Book, but they cannot change the content in any way or use them commercially.
**Abstract Book Citations:** Authors, Title, HemaSphere, 2026;10:(S1):pages. The abstract book DOIs can be found on the "Author Information" page.
**Disclaimer:** Articles published in the journal HemaSphere exclusively reflect the opinions of the authors. The authors are responsible for all content in their abstracts including accuracy of the facts, statements, citing resources, etc.

### (PB3926) COMBINATION OF GOLIDOCITINIB WITH CHIDAMIDE FOR PERIPHERAL T-CELL LYMPHOMA: POSSIBLE MECHANISMS AND THERAPEUTIC POTENTIAL IN INHIBITING TUMOR PROLIFERATION AND MODULATING THE IMMUNE MICROENVIRONMENT

**Topic:** 21. Lymphoma biology & translational research

Kaiyi Liu[1], Ruixin Zheng[1], Bing Pan[1], Li Li*[1]

[1]*The Second Hospital of Dalian Medical University, Dalian, China;*

### Background

Peripheral T-cell lymphoma (PTCL) is a rare and highly heterogeneous hematologic malignancy. Golidocitinib, a novel highly selective JAK1 inhibitor, has demonstrated promising anti-tumor potential against PTCL. The present study evaluated the direct cytotoxic effects of golidocitinib monotherapy and its combination with chidamide against PTCL, analyzed their anti-inflammatory and immunomodulatory effects on PTCL, and explored whether synergistic multi-mechanistic interactions could enhance anti-tumor efficacy. Subsequently, we performed an exploratory clinical evaluation of this combination strategy in a pilot cohort of four patients with PTCL.

### Aims

This study confirmed the pivotal role of golidocitinib in the treatment of PTCL and proposed a combination therapy strategy of golidocitinib and chidamide, providing a novel synergistic treatment approach for PTCL patients.

### Methods

Four classic PTCL cell lines were treated with golidocitinib and chidamide at indicated concentrations and time gradients, either as single agents or in combination, and the combination index (CI) was calculated. Meanwhile, the effects of golidocitinib alone and the combination regimen on the JAK-STAT3 signaling pathway were examined by Western blot analysis, and RNA sequencing was performed to analyze changes in cytokines and signaling pathways. Furthermore, a co-culture model of macrophages and tumor cells was established. Changes in cytokine levels in the supernatant, the number and apoptosis rate of tumor cells in the upper chamber, alterations in related signaling pathways, and the polarization status of macrophages in the lower chamber were detected. Subsequently, we evaluated the clinical efficacy of golidocitinib (150 mg once daily) combined with chidamide (20 mg twice weekly) in four patients with PTCL. The cohort included two chemo-free first-line treatment patients (1 PTCL-NOS, 1 AITL) and two fourth-line treatment patients (1 PTCL-NOS, 1 AITL).

### Results

Golidocitinib, alone and in combination, significantly inhibits the proliferation of various human PTCL cell lines. Moreover, the drug targets the JAK-STAT signaling pathway, downregulates proliferation-related pathways, and reduces the expression of key inflammatory cytokines. In the co-culture system, golidocitinib alone and in combination effectively reversed macrophage M2 polarization, markedly decreased the levels of core inflammatory cytokines, and exerted a synergistic pro-apoptotic effect on NK-92 cells. Building on these preclinical findings, we translated this combination regimen into clinical practice. Two chemo-free first-line treatment patients achieved partial responses (PR) after two cycles, while two fourth-line treatment patients attained complete remission (CR) following four cycles.

**Copyright Information:** (Online) ISSN: 2572-9241
©2026 The Author(s). HemaSphere is published by John Wiley & Sons Ltd on behalf of European Hematology Association. This is an open access Abstract Book distributed under the Attribution-NonCommercial-NoDerivs (CC BY-NC-ND), which allows third parties to download the articles and share them with others as long as they credit the author and the Abstract Book, but they cannot change the content in any way or use them commercially.
**Abstract Book Citations:** Authors, Title, HemaSphere, 2026;10:(S1):pages. The abstract book DOIs can be found on the “Author Information” page.
**Disclaimer:** Articles published in the journal HemaSphere exclusively reflect the opinions of the authors. The authors are responsible for all content in their abstracts including accuracy of the facts, statements, citing resources, etc.

### Summary/Conclusion

Golidocitinib alone and its combination with chidamide exert anti-tumor effects by inhibiting the JAK/STAT3 signaling pathway. The combination synergistically enhances anti-proliferative activity and suppresses pro-inflammatory signaling, promotes macrophage polarization, inhibits M2-driven immune escape, and remodels the tumor immune microenvironment. These findings are reflected in the clinical responses observed across all four types of peripheral T-cell lymphoma (PTCL) patients, including cases of refractory disease achieving complete remission. This laboratory-to-clinical consistency provides evidence supporting the combination therapy as a promising new treatment paradigm for PTCL.

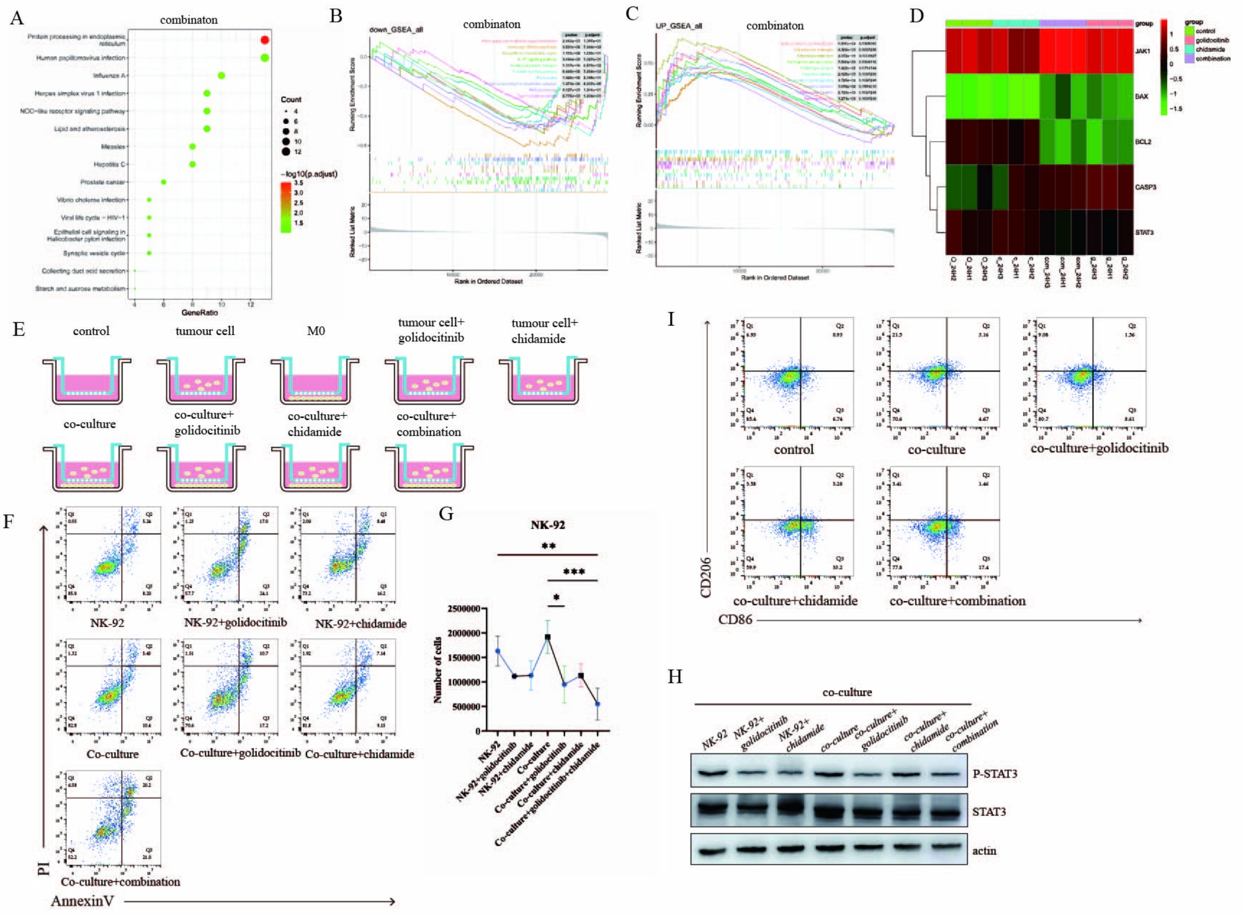

(A)Transcriptome sequencing was performed after 24 hours of combined treatment with golidocitinib and chidamide. KEGG , GSEA (B, C), and gene expression heatmap analyses (D) showed the enrichment of pathway-related genes. A co-culture model was established (E) and cells were treated with golidocitinib (25 μmol/L), chidamide (2.5 μmol/L) alone or in combination for 48 hours. Tumor cells in the upper chamber were collected and counted (G), apoptosis was analyzed by Annexin V-PI double staining (F), and the expression of P-STAT3 protein was detected by Western blot analysis (H). Collect macrophages from the lower chamber and determine the expression of macrophage surface markers by flow cytometry (I).

**Copyright Information:** (Online) ISSN: 2572-9241
©2026 The Author(s). HemaSphere is published by John Wiley & Sons Ltd on behalf of European Hematology Association. This is an open access Abstract Book distributed under the Attribution-NonCommercial-NoDerivs (CC BY-NC-ND), which allows third parties to download the articles and share them with others as long as they credit the author and the Abstract Book, but they cannot change the content in any way or use them commercially.
**Abstract Book Citations:** Authors, Title, HemaSphere, 2026;10:(S1):pages. The abstract book DOIs can be found on the "Author Information" page.
**Disclaimer:** Articles published in the journal HemaSphere exclusively reflect the opinions of the authors. The authors are responsible for all content in their abstracts including accuracy of the facts, statements, citing resources, etc.

### (PB3927) TARC & MDC DEPLETE PERIPHERAL ACTIVATED T CELLS AND T REGULATORY CELLS IN CLASSIC HODGKIN LYMPHOMA: A GHSG NIVAHL TRIAL ANALYSIS

**Topic:** 21. Lymphoma biology & translational research

Terézia Kolenová*[1], Paul J. Bröckelmann[2], Wouter Plattel[3], Maria A. Garcia-Marquez[4], Martin Thelen[4], Hans A. Schlösser[4], Arjan Diepstra[1], Lydia Visser[1]

*[1]University Medical Center Groningen, University of Groningen, Department of Pathology and Medical Biology, Groningen, The Netherlands; [2]University Hospital of Cologne, Department I of Internal Medicine and German Hodgkin Study Group (GHSG), Cologne, Germany; [3]University Medical Center Groningen, Department of Hematology, Groningen, The Netherlands; [4]University of Cologne, Faculty of Medicine and University Hospital Cologne, Center for Molecular Medicine Cologne, Cologne, Germany;*

### Background

The unique immune-cell-dominant tumor microenvironment (TME) of classic Hodgkin lymphoma (cHL) suggests a central role of the immune system in its pathogenesis. A relevant cHL biomarker, known to be produced at high levels by the malignant Hodgkin and Reed-Sternberg (HRS) cells, is the chemokine TARC. TARC is a high-affinity ligand for CCR4, expressed on T helper 2 (Th2) and T regulatory (Treg) cells, which are abundantly present in the cHL TME. In contrast, the percentages of Treg cells, T follicular helper cells, activated T cells, and other immune cells were decreased in the peripheral blood of treatment-naïve cHL patients compared to healthy controls.

### Aims

This study aims to define the relationship between chemokine levels and depleted peripheral immune cells.

### Methods

ELISA was used to measure chemokine levels in serum samples from 78 early-stage unfavorable cHL patients treated in the GHSG NIVAHL study and 18 healthy controls, with previously determined peripheral immune-cell counts by FACS. Patients and healthy controls provided informed consent. The chemokines MDC, CCL19, CXCL13, and CCL1, along with TARC, were correlated to the proportions of peripheral immune cells: T reg ($CD25^{high}FoxP3^{+}$), Tfh (CXCR5+), activated T cell (CD25+), naïve and central memory T cells (CD62L+), and activated B cells (CD86+). Spearman's rank correlation test was used for the analysis.

### Results

TARC was the most pronounced, but also MDC, CXCL13, and CCL19 levels were significantly higher in the serum of cHL patients than in healthy controls (p<0.0001), whereas CCL1 did not differ significantly. MDC has also been found to be produced by the HRS cells. TARC, MDC, and CXCL13 showed a significant decrease in the serum levels after treatment initiation (p<0.0001). At first restaging, when patients received 4 infusions of either nivolumab or nivolumab combined with 2 cycles of chemotherapy, there was no significant difference between the chemokine levels of the two treatment arms. Although most patients achieved a complete response at the end of treatment, at first restaging, TARC, CCL19, and CXCL13 showed significantly higher median levels in patients with a Deauville score (DS) >3 compared to patients classified based on the DS score as in complete response (score ≤3). At baseline, the strongest correlations were found between TARC and T regulatory cells (r=-0.536, p<0.0001) and CD25+T cells (r=-0.557, p<0.0001). MDC showed a weak negative correlation with T regulatory cells (r=-0.412, p<0.0001) and CD25+ T cells (r=-0.473, p<0.0001).

**Copyright Information:** (Online) ISSN: 2572-9241
©2026 The Author(s). HemaSphere is published by John Wiley & Sons Ltd on behalf of European Hematology Association. This is an open access Abstract Book distributed under the Attribution-NonCommercial-NoDerivs (CC BY-NC-ND), which allows third parties to download the articles and share them with others as long as they credit the author and the Abstract Book, but they cannot change the content in any way or use them commercially.
**Abstract Book Citations:** Authors, Title, HemaSphere, 2026;10:(S1):pages. The abstract book DOIs can be found on the "Author Information" page.
**Disclaimer:** Articles published in the journal HemaSphere exclusively reflect the opinions of the authors. The authors are responsible for all content in their abstracts including accuracy of the facts, statements, citing resources, etc.

**Summary/Conclusion**

Based on the correlation and physiological functions, we conclude that TARC and MDC actively recruit activated T cells and Treg cells from the periphery to the TME. By depleting the periphery and enriching TME, HRS cells can create an immune- suppressive environment, protecting the tumor cells and enabling their growth.

**Copyright Information:** (Online) ISSN: 2572-9241
©2026 The Author(s). HemaSphere is published by John Wiley & Sons Ltd on behalf of European Hematology Association. This is an open access Abstract Book distributed under the Attribution-NonCommercial-NoDerivs (CC BY-NC-ND), which allows third parties to download the articles and share them with others as long as they credit the author and the Abstract Book, but they cannot change the content in any way or use them commercially.
**Abstract Book Citations:** Authors, Title, HemaSphere, 2026;10:(S1):pages. The abstract book DOIs can be found on the “Author Information” page.
**Disclaimer:** Articles published in the journal HemaSphere exclusively reflect the opinions of the authors. The authors are responsible for all content in their abstracts including accuracy of the facts, statements, citing resources, etc.

### (PB3928) SELINEXOR IN COMBINATION WITH R-GDP FOR RELAPSED/REFRACTORY DIFFUSE LARGE B-CELL LYMPHOMA PATIENTS WITH TP53 ALTERATIONS: A PROSPECTIVE, SINGLE-ARM, PHASE II STUDY

**Topic:** 21. Lymphoma biology & translational research

Ying Fang*[1], Minxing Yue[1], Qing Shi[1], Muchen Zhang[1], Weili Zhao[1], Li Wang[1]

[1]*Shanghai Institute of Hematology, shanghai, China;*

### Background

*TP53* alterations are detected in approximately 20–25% of diffuse large B-cell lymphoma (DLBCL) cases and are associated with inferior outcomes and chemoresistance, particularly in the relapsed/refractory (R/R) setting. Effective treatment strategies for *TP53*-altered R/R DLBCL remain an unmet clinical need. We evaluated the clinical activity and explored the underlying molecular mechanisms of selinexor, a selective XPO1 inhibitor, combined with R-GDP (SR-GDP) in R/R DLBCL patients harboring *TP53* alterations.

### Aims

To evaluate the clinical efficacy and survival outcomes of selinexor combined with R-GDP (SR-GDP) in patients with *TP53*-altered R/R DLBCL and to investigate the underlying molecular mechanisms supporting this therapeutic strategy.

### Methods

47 R/R DLBCL patients with *TP53* alterations were enrolled at our center since November 2023 (27 with *TP53* mutation, 4 with *TP53* deletion, and 16 with both *TP53* mutation and deletion). The study is registered at ClinicalTrials.gov (NCT06062641). *In vitro* studies were conducted using the *TP53*-mutant DB B-cell lymphoma cell line. Cell cycle distribution was assessed by flow cytometry, DNA damage by immunofluorescence, and cytokine production by ELISA.

### Results

The median age was 55 years (range, 17–81), and 61.7% were male. At enrollment, 69.6% had stage III–IV disease, 78.7% had elevated LDH, and 23.4% had ≥2 extranodal sites. The best overall response rate was 78.7%, including a complete response rate of 25.5%. The 2-year progression-free survival (PFS) and overall survival (OS) rates were 65.2% (95% CI, 48.7–77.6%) and 67.5% (95% CI, 51.1–79.5%), respectively. Mechanically, selinexor demonstrated synergistic cytotoxicity when combined with cisplatin *in vitro*. Cisplatin alone induced S-phase arrest, whereas the addition of selinexor increased G0/G1-phase accumulation and reduced S-phase proportion, consistent with enhanced checkpoint activation. Combination treatment resulted in sustained γH2AX signaling, indicating persistent DNA double-strand breaks. Selinexor-treated cells also showed increased intracellular double-stranded DNA levels, accompanied by upregulation of interferon-β and interferon-γ, suggesting activation of innate immune signaling pathways.

### Summary/Conclusion

SR-GDP demonstrated encouraging clinical activity in R/R DLBCL patients with *TP53* alterations. Mechanistic findings indicate that selinexor enhances DNA damage persistence and may promote innate immune activation, providing a biological rationale for its use in *TP53*-aberrant DLBCL.

**Copyright Information:** (Online) ISSN: 2572-9241
©2026 The Author(s). HemaSphere is published by John Wiley & Sons Ltd on behalf of European Hematology Association. This is an open access Abstract Book distributed under the Attribution-NonCommercial-NoDerivs (CC BY-NC-ND), which allows third parties to download the articles and share them with others as long as they credit the author and the Abstract Book, but they cannot change the content in any way or use them commercially.
**Abstract Book Citations:** Authors, Title, HemaSphere, 2026;10:(S1):pages. The abstract book DOIs can be found on the "Author Information" page.
**Disclaimer:** Articles published in the journal HemaSphere exclusively reflect the opinions of the authors. The authors are responsible for all content in their abstracts including accuracy of the facts, statements, citing resources, etc.

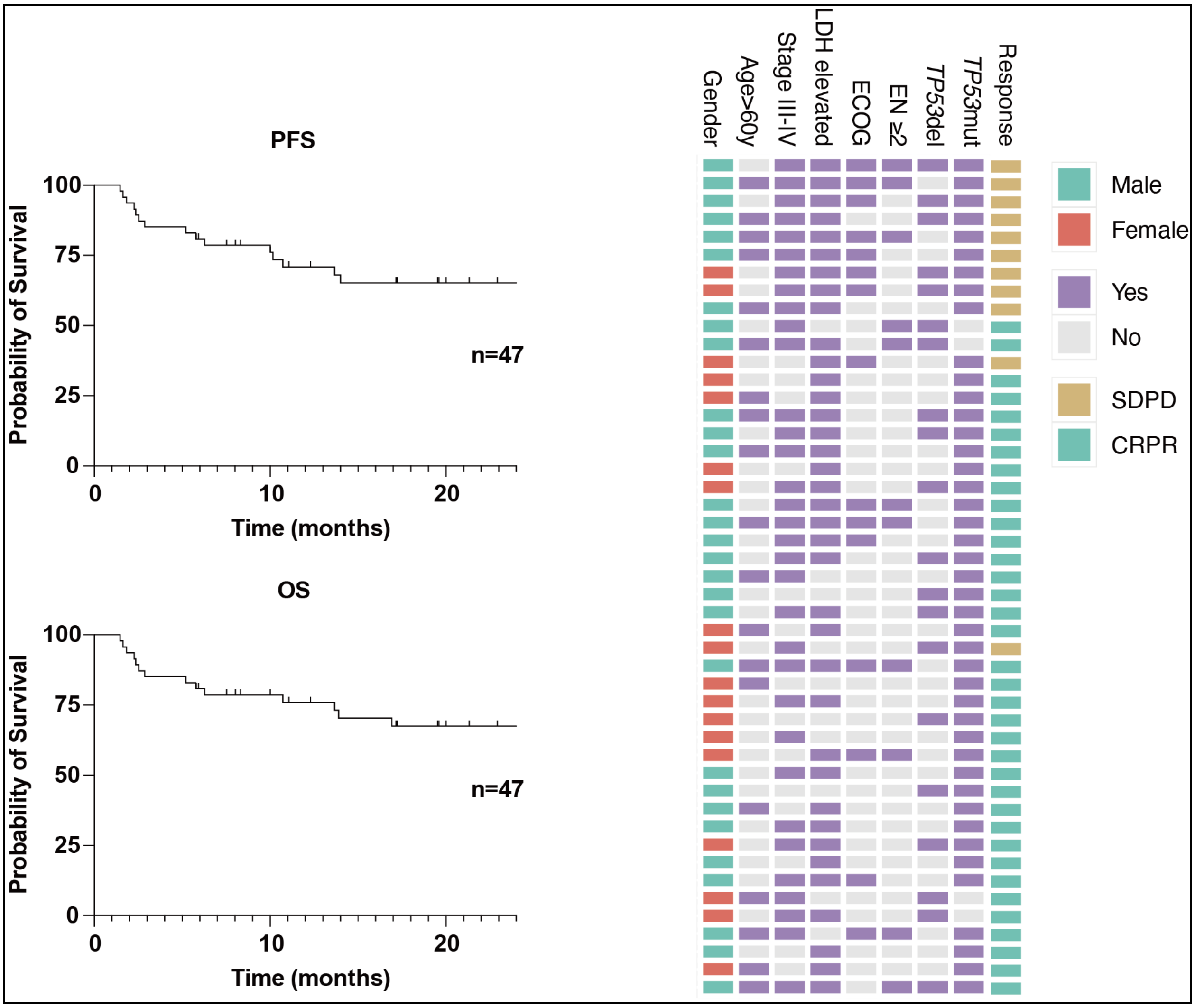

**Copyright Information:** (Online) ISSN: 2572-9241
©2026 The Author(s). HemaSphere is published by John Wiley & Sons Ltd on behalf of European Hematology Association. This is an open access Abstract Book distributed under the Attribution-NonCommercial-NoDerivs (CC BY-NC-ND), which allows third parties to download the articles and share them with others as long as they credit the author and the Abstract Book, but they cannot change the content in any way or use them commercially.
**Abstract Book Citations:** Authors, Title, HemaSphere, 2026;10:(S1):pages. The abstract book DOIs can be found on the "Author Information" page.
**Disclaimer:** Articles published in the journal HemaSphere exclusively reflect the opinions of the authors. The authors are responsible for all content in their abstracts including accuracy of the facts, statements, citing resources, etc.

### (PB3929) EMERGING ROLE OF SAMHD1 IN ANTI-TUMOR IMMUNE RESPONSES AND SENSITIVITY TO CHEMOTHERAPY IN CLASSICAL HODGKIN LYMPHOMA

**Topic:** 21. Lymphoma biology & translational research

Dora Trogrlic*[1], Eleni Psaroudaki[1], Ying Yang[1], Erophili Bougka[1], Christian Nordentoft[1], Nikolas Herold[2], Andreas Lundqvist[1], Georgios Rassidakis[1]

*[1]Karolinska Institutet, Oncology-Pathology, Stockholm, Sweden; [2]Karolinska Institutet, Department of Women's and Children's Health, Stockholm, Sweden;*

### Background

The tumor microenvironment (TME) plays a pivotal role in the pathogenesis of classical Hodgkin lymphoma (cHL) because of the multiple and complex interactions of neoplastic Hodgkin and Reed Sternberg (HRS) cells with the inflammatory cells, through numerous immune response mediators (i.e. cytokines, chemokines, interferons) secreted by both HRS and TME cells. SAMHD1, a dNTP hydrolase, is involved in anti-tumor immune responses through its function in DNA damage mechanisms and its biological association with the cGAS-STING anti-tumor response pathway. In a previous study, we showed that SAMHD1 expression is an independent adverse prognostic factor in cHL (Xagoraris et al, Br J Haemat, 2021), however, the mechanisms underlying its clinical significance are unknown to date.

### Aims

To investigate the biologic impact of SAMHD1 on anti-tumor immune responses and sensitivity to chemotherapy in cHL.

### Methods

The in vitro system included 6 cHL cell lines, in 3 of which (L1236, L428, L540) SAMHD1-deficient clones (KO) were generated using CRISPR-Cas9 gene editing. To further explore the biological role of SAMHD1, the effects of plasmid-mediated forced expression of SAMHD1 were analyzed in 2 cHL cell lines with low (L428) or undetectable (MDAV) endogenous SAMHD1 protein levels. The cHL cell lines were treated with a STING agonist, STING inhibitor (C-176), or doxorubicin. Expression and activation of proteins including critical components of the cGAS-STING pathway were analyzed by Western blot. Immune responses were evaluated using RT-qPCR for gene expression (mRNA level) of critical immunomodulators, targeted proteomics with Olink Target 48 Cytokine panel on culture supernates, as well as 51Chromium-based NK cell killing assays.

**Copyright Information:** (Online) ISSN: 2572-9241
©2026 The Author(s). HemaSphere is published by John Wiley & Sons Ltd on behalf of European Hematology Association. This is an open access Abstract Book distributed under the Attribution-NonCommercial-NoDerivs (CC BY-NC-ND), which allows third parties to download the articles and share them with others as long as they credit the author and the Abstract Book, but they cannot change the content in any way or use them commercially.
**Abstract Book Citations:** Authors, Title, HemaSphere, 2026;10:(S1):pages. The abstract book DOIs can be found on the “Author Information” page.
**Disclaimer:** Articles published in the journal HemaSphere exclusively reflect the opinions of the authors. The authors are responsible for all content in their abstracts including accuracy of the facts, statements, citing resources, etc.

**Results**

SAMHD1-deficient (KO) and -proficient (WT) cHL cells showed differential gene expression of critical modulators of antitumor immune responses among the cHL cell lines tested. The effects of SAMHD1 deficiency were associated with changes in the activation status of cGAS-STING pathway, including phosphorylation of TBK1, IRF3 and IRF7. SAMHD1 deficiency resulted in decreased secretion of IL6, CSF2, TNF, CCL2/7, and others in cell culture supernates using targeted proteomics. STING stimulation resulted in significantly increased IFNβ gene expression by HRS cell in SAMHD1-proficient but not in SAMHD1-deficient cHL cell lines, associated with activation of the cGAS-STING pathway. STING inhibition also resulted in differential changes in immunomodulatory cytokines. Forced expression of SAMHD1 resulted in upregulation of CXCL10 and -at a lesser degree- IFNβ gene expression (mRNA level) in cultured HRS cells associated with changes in cGAS-STING pathway. These effects were linked to lower levels of CXCL9, IL10, TNF, IL17a and CXCL8 in MDA-V cells following SAMHD1 forced expression as shown by proteomic profiling using OLINK platform. Treatment of SAMHD1-proficient cHL cells with doxorubicin resulted in lower cell viability as compared to SAMHD1-deficient cells in L1236 and L428 but not in L540. The effects on cell growth and viability were associated with changes in apoptosis-regulating proteins leading to Caspase –3 and –8 cleavage.

**Summary/Conclusion**

SAMHD1 expression affects the anti-tumor immune responses partially through the cGAS-STING pathway. The immunomodulatory functions of SAMHD1 may contribute to the TME shaping in cHL. In addition, SAMHD1 expression seems toconfer resistance to doxorubicin treatment, however, at a variable degree among cHL cell lines.

**Copyright Information:** (Online) ISSN: 2572-9241
©2026 The Author(s). HemaSphere is published by John Wiley & Sons Ltd on behalf of European Hematology Association. This is an open access Abstract Book distributed under the Attribution-NonCommercial-NoDerivs (CC BY-NC-ND), which allows third parties to download the articles and share them with others as long as they credit the author and the Abstract Book, but they cannot change the content in any way or use them commercially.
**Abstract Book Citations:** Authors, Title, HemaSphere, 2026;10:(S1):pages. The abstract book DOIs can be found on the “Author Information” page.
**Disclaimer:** Articles published in the journal HemaSphere exclusively reflect the opinions of the authors. The authors are responsible for all content in their abstracts including accuracy of the facts, statements, citing resources, etc.

### (PB3930) PROFILING CLONAL DYNAMICS AND T CELL STATES IN PATIENTS WITH B-CELL LYMPHOMA TREATED WITH COMMERCIAL CD20XCD3 T-CELL ENGAGERS

**Topic:** 21. Lymphoma biology & translational research

Gottfried von Keudell*[1], Ben Segev[1], Emma Logan[1], Jon Arnason[1], Michael Leukam[1], Matthew Weinstock[1], Hazal Toros[1], Isabel Saldarriaga[1], Jacalyn Rosenblatt[1], Ioannis Vlachos[1], David Avigan[1], Dimitra Karagkouni[1], Giulia Cheloni[1]

[1]*Beth Israel Deaconess Medical Center, Harvard Medical School, Boston, United States of America;*

### Background

T cell engagers (TCE) have revolutionized the treatment of B-cell non-Hodgkin lymphomas (NHL). However, a large proportion of patient do not respond or eventually relapse with poor prognosis. The biological factors underlying response and resistance remain incompletely defined. A central unanswered question is whether TCE-mediated CD3 engagement results merely in transient, TCR-agnostic bystander cytotoxicity or instead triggers antigen-specific T-cell immunity. We propose a two-phase immune model for TCE therapy: (i) an initial wave of TCR-independent bystander activation, leading to tumor cell lysis, antigen release, and inflammatory signaling, followed by (ii) a secondary TCR-dependent phase characterized by the expansion and persistence of tumor-specific effector-memory T cell clonotypes maintaining long-term tumor control.

### Aims

To define the temporal sequence of immune events induced by TCE, improve understanding of response and relapse

### Methods

Single-cell RNA and TCR sequencing (10x Genomics) was performed on PBMCs from NHL patients (3 LBCL, 1 FL) collected pre-TCE and one month post-treatment. Patients were stratified by response: CR (n=2), PR (n=1), and PD (n=1). Analyses focused on T cell states, clonal dynamics, and associations with clinical response.

### Results

Within LBCL patients, CR was characterized by higher proportions of activated and cytotoxic T cell subsets, including CD4 and CD8 cytotoxic lymphocytes (CTLs), IFNγ high, and highly proliferating CD8 T cell clusters, both pre- and post-TCE, compared to PR and PD. At the post-TCE timepoint, the patent in CR patient demonstrated a pronounced increase in CD4 and CD8 CTLs relative to pre-TCE. In contrast, PR showed more modest increases, primarily within the CD8 cytotoxic compartment, while PD patients displayed minimal changes across T cell subsets between pre- and post-TCE. Consistent with these shifts, TCR repertoire analysis revealed a marked increase in expanded clonotypes post-TCE in the CR patient, with only limited T cell clonal proliferation in PR and negligible changes in PD. The FL patient exhibited a distinct T cell composition dominated by CD4 T cell subsets, including naïve/central memory T cells, Tregs, and CD4 CTL/GZMK high cells; despite achieving CR, this patient relapsed three months post-TCE.

**Copyright Information:** (Online) ISSN: 2572-9241
©2026 The Author(s). HemaSphere is published by John Wiley & Sons Ltd on behalf of European Hematology Association. This is an open access Abstract Book distributed under the Attribution-NonCommercial-NoDerivs (CC BY-NC-ND), which allows third parties to download the articles and share them with others as long as they credit the author and the Abstract Book, but they cannot change the content in any way or use them commercially.
**Abstract Book Citations:** Authors, Title, HemaSphere, 2026;10:(S1):pages. The abstract book DOIs can be found on the "Author Information" page.
**Disclaimer:** Articles published in the journal HemaSphere exclusively reflect the opinions of the authors. The authors are responsible for all content in their abstracts including accuracy of the facts, statements, citing resources, etc.

**Summary/Conclusion**

Within LBCL patients, CR was characterized by higher proportions of activated and cytotoxic T cell subsets, both pre- and post-TCE, compared to PR and PD. At the post-TCE timepoint, the patent in CR patient demonstrated a pronounced increase in CD4 and CD8 CTLs relative to pre-TCE. In contrast, PR showed more modest increases, primarily within the CD8 cytotoxic compartment, while PD patients displayed minimal changes across T cell subsets between pre- and post-TCE. Consistent with these shifts, TCR repertoire analysis revealed a marked increase in expanded clonotypes post-TCE in the CR patient, with only limited T cell clonal proliferation in PR and negligible changes in PD. The FL patient exhibited a distinct T cell composition dominated by CD4 T cell subsets, i.e. Tregs; despite achieving CR, this patient relapsed three months post-TCE.These preliminary data in LBCL support our hypothesis indicating that clinical response to TCE is associated by expansion of cytotoxic T cell states and increased clonal proliferation indicative of a TCR-mediated T cell response. The findings in the FL patient point to potentially disease-specific T cell composition, emphasizing the need for further study.

**Copyright Information:** (Online) ISSN: 2572-9241
©2026 The Author(s). HemaSphere is published by John Wiley & Sons Ltd on behalf of European Hematology Association. This is an open access Abstract Book distributed under the Attribution-NonCommercial-NoDerivs (CC BY-NC-ND), which allows third parties to download the articles and share them with others as long as they credit the author and the Abstract Book, but they cannot change the content in any way or use them commercially.
**Abstract Book Citations:** Authors, Title, HemaSphere, 2026;10:(S1):pages. The abstract book DOIs can be found on the "Author Information" page.
**Disclaimer:** Articles published in the journal HemaSphere exclusively reflect the opinions of the authors. The authors are responsible for all content in their abstracts including accuracy of the facts, statements, citing resources, etc.

### (PB3931) TOWARDS A MOUSE MODEL FOR LYMPHOPROLIFERATION INDUCED BY IL4R MUTATION IN PMBL

**Topic:** 21. Lymphoma biology & translational research

Sarah Bailly*[1, 2, 3], Harsh K. V. Goyal[4], Elise Sepulchre[2], Gabriel Levy[1, 2, 5, 6], Emile Brandt[2], Alessandra Camboni[7, 8], Eric van den Neste[3], Vincent Camus[9], Stefan Constantinescu[1, 2, 5, 10]

*[1]Signal Transduction and Molecular Hematology, Ludwig Institute for Cancer Research, Brussels, Belgium;[2]Signal Transduction on Molecular Hematology, de Duve Institute, Université Catholique de Louvain, Brussels, Belgium; [3]Cliniques universitaires Saint-Luc, UCLouvain, Hematology, Brussels, Belgium; [4]Old Road Campus Research Building, Oxford University, Ludwig Institute for Cancer Research, Oxford, United Kingdom; [5]WELBIO, Walloon Excellence in Life Sciences and Biotechnology, Brussels, Belgium; [6]Cliniques universitaires Saint-Luc, Pediatric Hematology and Oncology, Brussels, Belgium;[7]Gynecology Research Unit, Institut de Recherche Expérimentale et Clinique, Université Catholique de Louvain, Brussels, Belgium; [8]Cliniques universitaires Saint-Luc, Anatomopathology, Brussels, Belgium; [9]Center Henri Becquerel, Department of Hematology and INSERM U1245, Rouen, France; [10]Nuffield Department of Medicine, Oxford University, Ludwig Institute for Cancer Research, Oxford, United Kingdom;*

### Background

Primary mediastinal B-cell lymphoma (PMBL) is characterized by constitutive activation of the JAK–STAT pathway. Recurrent IL4R mutations are a specific molecular hallmark of PMBL, but their functional relevance remains unclear. The transmembrane hotspot mutation IL4R I242N is the most frequently reported variant, but its biological consequences have not been fully elucidated.

### Aims

To define the mechanistic basis and in vivo impact of theIL4R I242N mutation in PMBL.

### Methods

We used complementary in vitro and in vivo approaches, including signaling and proliferation assays, JAK-deficient models, structural modeling, and a murine bone marrow transplantation model using retrovirally transduced bone marrow cells expressing IL4R I242N.

### Results

IL4R I242N induced robust ligand-independent STAT6 activation and cytokine-independent proliferation across multiple cellular systems. Sustained growth under cytokine deprivation and upregulation of STAT6 target genes confirmed functional activation. Mechanistically, the mutation promoted constitutive receptor assembly through enhanced transmembrane interactions, as supported by targeted alanine mutagenesis and NanoBRET analyses. Signaling strictly depended on canonical type II IL4 receptor components, involving the mutated IL4Rα itself, IL13Rα1, JAK1, and TYK2, while γc and JAK3 were dispensable. Pharmacologic JAK1 inhibition (itacitinib) selectively reduced mutant-driven proliferation, further supporting JAK1 dependency. In vivo, using a murine bone marrow transplantation model, IL4R I242N expression conferred a proliferative advantage to hematopoietic progenitors and resulted in thymic enlargement, splenomegaly, cervical lymphoid masses, expansion of peripheral $CD19^{+}CD23^{+}$ B-cell populations, and reduced survival. No overt lymphoma was observed. Histopathological analyses revealed diffuse non–B lymphoid infiltration of thymic, cervical tissues, and spleen, consistent with an early lymphoproliferative state and indicating that additional oncogenic events are required for full malignant transformation.

**Copyright Information:** (Online) ISSN: 2572-9241
©2026 The Author(s). HemaSphere is published by John Wiley & Sons Ltd on behalf of European Hematology Association. This is an open access Abstract Book distributed under the Attribution-NonCommercial-NoDerivs (CC BY-NC-ND), which allows third parties to download the articles and share them with others as long as they credit the author and the Abstract Book, but they cannot change the content in any way or use them commercially.
**Abstract Book Citations:** Authors, Title, HemaSphere, 2026;10:(S1):pages. The abstract book DOIs can be found on the "Author Information" page.
**Disclaimer:** Articles published in the journal HemaSphere exclusively reflect the opinions of the authors. The authors are responsible for all content in their abstracts including accuracy of the facts, statements, citing resources, etc.

**Summary/Conclusion**

In PMBL, IL4R I242N drives ligand-independent STAT6 activation through constitutive type II IL4 receptor signaling and confers a cytokine-independent proliferative advantage with functional dependence on JAK1. In a murine transplantation model, mutant IL4R induces an early lymphoproliferative phenotype involving thymic remodeling and peripheral B-cell expansion, without evidence of overt lymphoma. Together, these data identify IL4R I242N as an early driver of aberrant STAT6 signaling in PMBL. These results establish a novel system to investigate additional oncogenic events required for PMBL development.

**Copyright Information:** (Online) ISSN: 2572-9241
©2026 The Author(s). HemaSphere is published by John Wiley & Sons Ltd on behalf of European Hematology Association. This is an open access Abstract Book distributed under the Attribution-NonCommercial-NoDerivs (CC BY-NC-ND), which allows third parties to download the articles and share them with others as long as they credit the author and the Abstract Book, but they cannot change the content in any way or use them commercially.
**Abstract Book Citations:** Authors, Title, HemaSphere, 2026;10:(S1):pages. The abstract book DOIs can be found on the "Author Information" page.
**Disclaimer:** Articles published in the journal HemaSphere exclusively reflect the opinions of the authors. The authors are responsible for all content in their abstracts including accuracy of the facts, statements, citing resources, etc.

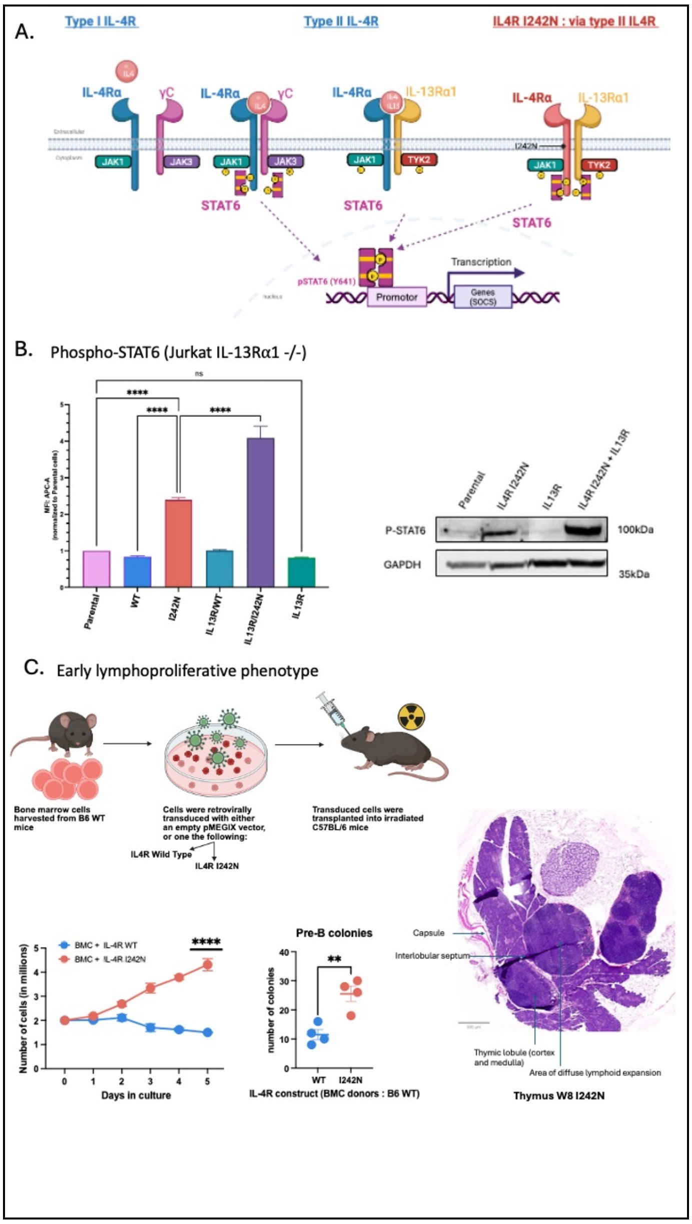

Figure (A) Schematic overview of IL-4R signaling. The PMBL-associated IL4R I242N mutation promotes ligand-independent STAT6 activation via the type II receptor. (B) In IL13Rα1-negative Jurkat cells, mutant IL4R increases phospho-STAT6, further enhanced by IL13Rα1 co-expression, supporting type II receptor dependence. Statistical analyses were performed using one-way ANOVA with Tukey's test. ****P < 0.0001; **P < 0.01; ns, not significant (n = 6). (C) In a murine transplantation model, IL4R I242N–expressing bone marrow cells show a cytokine-independent growth advantage and increased pre-B colonies. Statistical analyses included two-way ANOVA for growth curves (2 independent experiments, 5 mice per experiment) and unpaired t-test for colony assays (n = 4). ****P < 0.0001; **P < 0.01. Eight weeks post-transplantation, mice display early thymic lymphoid expansion without overt lymphoma.

**Copyright Information:** (Online) ISSN: 2572-9241
©2026 The Author(s). HemaSphere is published by John Wiley & Sons Ltd on behalf of European Hematology Association. This is an open access Abstract Book distributed under the Attribution-NonCommercial-NoDerivs (CC BY-NC-ND), which allows third parties to download the articles and share them with others as long as they credit the author and the Abstract Book, but they cannot change the content in any way or use them commercially.
**Abstract Book Citations:** Authors, Title, HemaSphere, 2026;10:(S1):pages. The abstract book DOIs can be found on the "Author Information" page.
**Disclaimer:** Articles published in the journal HemaSphere exclusively reflect the opinions of the authors. The authors are responsible for all content in their abstracts including accuracy of the facts, statements, citing resources, etc.

### (PB3932) MYC REARRANGEMENT IN PEDIATRIC NON-BURKITT MATURE B-CELL LYMPHOMA: PROGNOSTIC IMPACT AND ASSOCIATED CLINICAL AND MOLECULAR FEATURES

**Topic:** 21. Lymphoma biology & translational research

Yang Li[1], Ying Liu[2], Yanlong Duan[3], Pan Wu[4], Ling Jin[3], Xiaowen Zhai[5], Jianwen Zhou[6], Xiaomei Yang[7], Ansheng Liu[8], Xiaojun Yuan[9], Yunpeng Dai[10], Yueping Jia[11], Jian Wang[12], Rong Liu[13], Lingzhen Wang[14], Baoxi Zhang[15], Qinlong Zheng*[1], Yonghong Zhang[2]

*[1]Beijing GoBroad Boren Hospital, Molecular Laboratory Diagnostics, Beijing, China;[2]Beijing GoBroad Boren Hospital, Department of Pediatric Lymphoma, Beijing, China; [3]National Center for Children's Health, Beijing Children's Hospital, Capital Medical University, Department of Hematology/Oncology, Beijing, China; [4]Hunan Children's Hospital, Department of Hematology, Changsha, China; [5]Children's Hospital of Fudan University, National Center for Children's Health, Department of Hematology &Oncology, Shanghai, China; [6]Zhengzhou Children's Hospital, Department of Hematology & Oncology, Zhengzhou, China; [7]Shandong University Affiliated Hospital (Jinan Children's Hospital), Department of Pediatric Hematology/Oncology, Jinan, China; [8]Xi'an Children's Hospital, Department of Hematologic Oncology, Xi'an, China;[9]Xinhua Hospital, Shanghai Jiao Tong University School of Medicine, Department of Pediatric Hematology/Oncology, Shanghai, China; [10]Shandong Provincial Hospital Affiliated to Shandong University, Department of Pediatrics Hematology and Endocrinology, Jinan, China; [11]Peking University People's Hospital, Department of Pediatrics, Beijing, China; [12]Anhui Provincial Children's Hospital, Department of Hematology and Oncology, Hefei, China; [13]Capital institute of pediatric, Department of Hematology/Oncology, Beijing, China;[14]Affiliated Hospital of Qingdao University, Department of Pediatric Hematology and Oncology, Qingdao, China; [15]The Second Hospital of Hebei Medical University, Department of Pediatrics, Shijiazhuang, China;*

### Background

In adult mature B-cell lymphomas, *MYC* rearrangement has been demonstrated to be an adverse prognostic factor, particularly when accompanied by *BCL2* or *BCL6* rearrangement. In pediatric mature B-cell lymphomas, the presence of *MYC* rearrangement typically defines Burkitt lymphoma (BL). However, its prognostic significance in non-Burkitt subtypes—including diffuse large B-cell lymphoma (DLBCL) and high-grade B-cell lymphoma (HGBL)—has not been well established.

### Aims

This study aimed to evaluate the prognostic impact of *MYC* rearrangement in pediatric non-Burkitt mature B-cell lymphoma and to characterize the clinical and molecular genetic features of this subgroup, thereby informing risk stratification and individualized treatment strategies.

### Methods

This retrospective study enrolled 418 patients aged <18 years who were diagnosed with non-Burkitt mature B-cell lymphoma from the China Network of Childhood Lymphoma (CNCL) between 2017 and 2025. *MYC* rearrangement status was assessed by fluorescence in situ hybridization (FISH). Targeted next-generation sequencing (NGS) was performed on samples from 52 patients. The study endpoints were event-free survival (EFS) and overall survival (OS). Statistical analyses were performed to evaluated the association between *MYC* rearrangement and prognosis, and to compare clinical characteristics and mutation profiles between *MYC*-positive and *MYC*-negative groups.

**Copyright Information:** (Online) ISSN: 2572-9241
©2026 The Author(s). HemaSphere is published by John Wiley & Sons Ltd on behalf of European Hematology Association. This is an open access Abstract Book distributed under the Attribution-NonCommercial-NoDerivs (CC BY-NC-ND), which allows third parties to download the articles and share them with others as long as they credit the author and the Abstract Book, but they cannot change the content in any way or use them commercially.
**Abstract Book Citations:** Authors, Title, HemaSphere, 2026;10:(S1):pages. The abstract book DOIs can be found on the "Author Information" page.
**Disclaimer:** Articles published in the journal HemaSphere exclusively reflect the opinions of the authors. The authors are responsible for all content in their abstracts including accuracy of the facts, statements, citing resources, etc.

### Results

The median age was 10 years (range 1–18 years). DLBCL accounted for 188 cases (45.0%), HGBL for 143 cases (34.2%), follicular lymphoma (FL) for 30 cases (7.2%), primary mediastinal large B-cell lymphoma (PMBCL) for 11 cases (2.6%), and other subtypes for 46 cases (11.0%). Among the 340 patients with available FISH results, 89 (26.2%) were *MYC*-positive and 251 (73.8%) were *MYC*-negative. Survival analysis demonstrated that *MYC*-positive patients had significantly inferior EFS (log-rank $p = 0.0021$) and OS (log-rank $p = 0.014$) compared to *MYC*-negative patients (Fig. 1a).
Clinically, *MYC*-positive patients showed significantly higher frequencies of bone marrow (BM) involvement ($p < 0.001$), bulky disease ($p = 0.05$), and central nervous system (CNS) involvement ($p = 0.005$) compared to *MYC*-negative patients.
Among the 52 patients who underwent targeted NGS (19 MYC-positive, 33 MYC-negative), the most frequently mutated genes were *TP53* (31%), *ID3* (23%), *CCND3* (21%), *ARID1A* (17%), and *DDX3X* (15%, Fig. 1c). Mutations in *TP53*, *ID3*, *ARID1A*, and *DDX3X* were significantly more common in *MYC*-positive patients than in *MYC*-negative patients (all $p < 0.05$, Fig. 1d). Notably, mutations in epigenetic regulator genes—including *EZH2*, *KMT2C*, and *KMT2D*—were exclusively detected in *MYC*-negative patients (Fig.1d).

### Summary/Conclusion

This study demonstrates that *MYC* rearrangement is an adverse prognostic factor in Chinese children with non-Burkitt mature B-cell lymphoma. It is associated with aggressive clinical features and a distinct mutational profile characterized by enrichment of *TP53*, *ID3*, *ARID1A*, and *DDX3X* mutations. These findings highlight the potential of *MYC* rearrangement status as a biomarker for risk stratification and provide a basis for future studies investigating intensified or targeted therapies for this high-risk subgroup. (Yang Li and Ying Liu contributed equally, Qinlong Zheng and Yonghong Zhang contributed equally).

**Copyright Information:** (Online) ISSN: 2572-9241
©2026 The Author(s). HemaSphere is published by John Wiley & Sons Ltd on behalf of European Hematology Association. This is an open access Abstract Book distributed under the Attribution-NonCommercial-NoDerivs (CC BY-NC-ND), which allows third parties to download the articles and share them with others as long as they credit the author and the Abstract Book, but they cannot change the content in any way or use them commercially.
**Abstract Book Citations:** Authors, Title, HemaSphere, 2026;10:(S1):pages. The abstract book DOIs can be found on the "Author Information" page.
**Disclaimer:** Articles published in the journal HemaSphere exclusively reflect the opinions of the authors. The authors are responsible for all content in their abstracts including accuracy of the facts, statements, citing resources, etc.

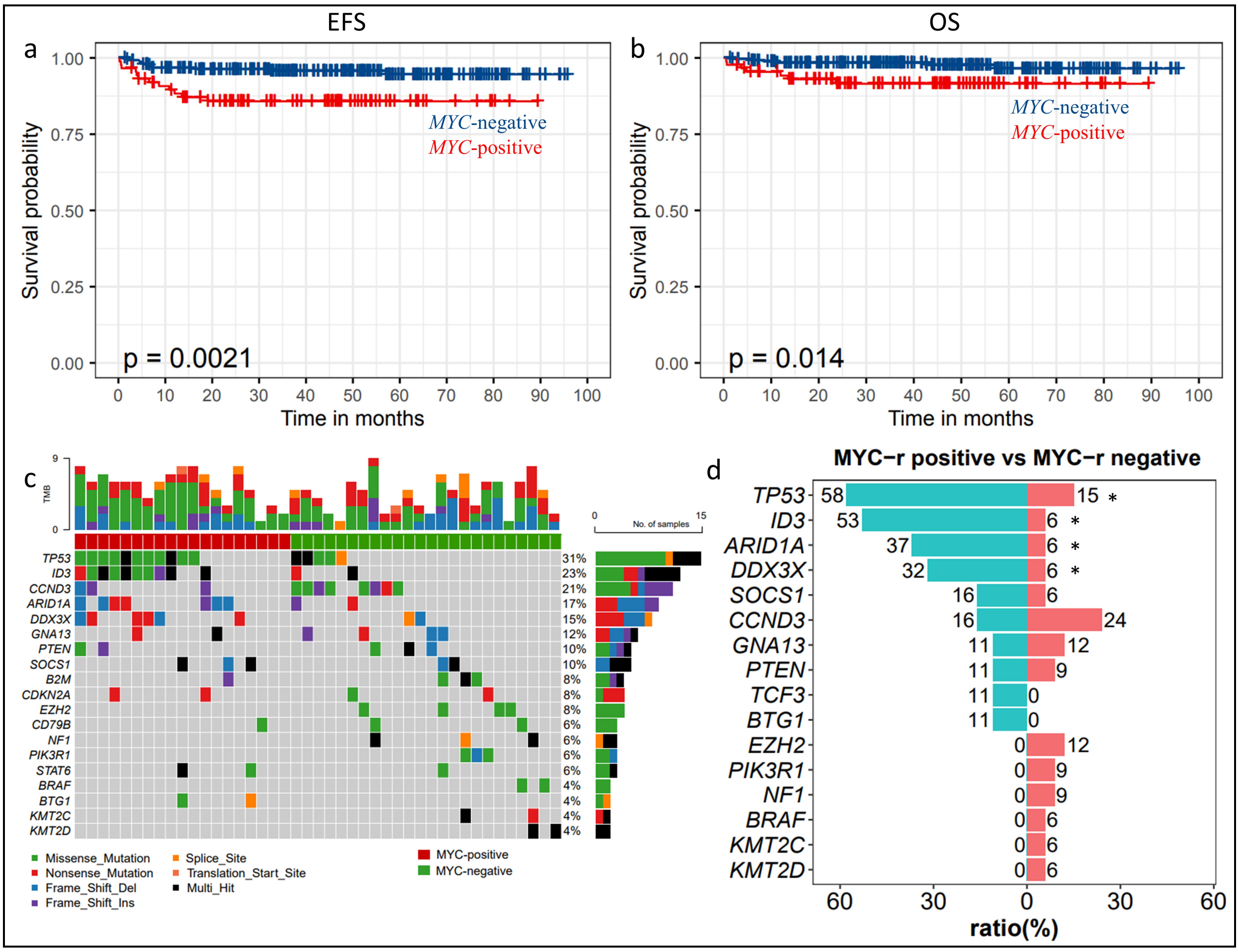

Figure 1. The prognostic impact of MYC rearrangement and its molecular mutation characteristics.

**Copyright Information:** (Online) ISSN: 2572-9241
©2026 The Author(s). HemaSphere is published by John Wiley & Sons Ltd on behalf of European Hematology Association. This is an open access Abstract Book distributed under the Attribution-NonCommercial-NoDerivs (CC BY-NC-ND), which allows third parties to download the articles and share them with others as long as they credit the author and the Abstract Book, but they cannot change the content in any way or use them commercially.
**Abstract Book Citations:** Authors, Title, HemaSphere, 2026;10:(S1):pages. The abstract book DOIs can be found on the "Author Information" page.
**Disclaimer:** Articles published in the journal HemaSphere exclusively reflect the opinions of the authors. The authors are responsible for all content in their abstracts including accuracy of the facts, statements, citing resources, etc.

### (PB3933) GENETICALLY FAITHFUL, TRANSCRIPTIONALLY TRANSFORMED: MICROENVIRONMENT-DRIVEN DIVERGENCE IN MATCHED LBCL PRECLINICAL MODELS

**Topic:** 21. Lymphoma biology & translational research

Dmitry Manakov*[1], Alexandra Dolnikova[1], Arnost Polak[1], Silvia Spanikova[1], Lucie Winkowska[2], Eva Pokorna[1], Vaclava Polivkova[3], Jitka Křížková[3], Pavel Semerak[3], Katerina MacHova Polakova[3], Magdalena Klanova[1, 4], Marek Trněný[4], Pavel Klener[1, 4]

*[1]Institute of Pathological Physiology, First Faculty of Medicine, Charles University, Prague, Czechia;[2]CLIP, Department of Pediatric Hematology/Oncology, Second Faculty of Medicine and University Hospital Motol and Homolka, Prague, Czechia; [3]Institute of Hematology and Blood Transfusion, Prague, Czechia;[4]First Department of Medicine-Hematology, General University Hospital Prague and First Faculty of Medicine, Charles University, Prague, Czechia;*

**Background**

Patient-derived xenografts (PDXs) and cell lines (PDCLs) of large B-cell lymphoma (LBCL) broadly retain primary tumor genetics, yet bulk transcriptomic profiling cannot resolve whether they preserve the intratumoral transcriptional heterogeneity that can drive clonal evolution and determine drug response.

**Aims**

To resolve, at single-cell resolution, transcriptional landscapes of matched primary tumor, PDX, PDCL, and PDCL-based xenografts (CDX) from LBCL patients, and to determine whether model-specific transcriptional programs arise from clonal selection or epigenetic adaptation to distinct tumor microenvironments (TME).

**Methods**

PDXs from pathology-confirmed LBCL were propagated in NSG mice; PDCLs were established from the same tumours in vitro. CDXs were established by xenotransplantation of PDCLs into mice. Viable cells were CD45+ sorted and profiled by 5′ scRNAseq with paired BCR/TCR V(D)J sequencing. scRNAseq QC included ambient RNA correction (decontX) and scDblFinder + V(D)J-based doublet removal. Samples were analyzed in Seurat. Cell types were assigned using canonical marker genes; BCR V(D)J, combined with light-chain restriction and clonotype analysis, identified malignant B cells. Samples were additionally profiled using whole exome sequencing (WES).

**Copyright Information:** (Online) ISSN: 2572-9241
©2026 The Author(s). HemaSphere is published by John Wiley & Sons Ltd on behalf of European Hematology Association. This is an open access Abstract Book distributed under the Attribution-NonCommercial-NoDerivs (CC BY-NC-ND), which allows third parties to download the articles and share them with others as long as they credit the author and the Abstract Book, but they cannot change the content in any way or use them commercially.
**Abstract Book Citations:** Authors, Title, HemaSphere, 2026;10:(S1):pages. The abstract book DOIs can be found on the “Author Information” page.
**Disclaimer:** Articles published in the journal HemaSphere exclusively reflect the opinions of the authors. The authors are responsible for all content in their abstracts including accuracy of the facts, statements, citing resources, etc.

**Results**

We profiled 46,609 cells from two primary LBCL samples (P062, ascites; P144, peripheral blood) and PDX, PDCL, and CDX samples derived from the primary samples (8 samples in total, Fig. 1A). B-cell receptor (BCR) light-chain restriction confirmed 85–100% malignant purity across all samples (Fig. 1B); TME cells of human origin were present in PT and PDX only. On joint UMAP, malignant cells clustered strictly by sample of origin. In P062 (Fig. 1C), the dominant “proliferative” PT state (blue, 56% of PT) propagated to all models (61–96%), while an “immunologically active” state with MHC I/II antigen presentation and IgM expression (green, 41% of PT) was entirely lost upon derivation of both PDX and PDCL. Besides the dominant “proliferative” state, the PDCL acquired a new “hypermetabolic” state (orange, 36%), subsequently inherited also by the CDX (32%). In P144 (Fig. 1D), 98% of PT cells occupied a single “G1 quiescent” state characterized by expression of KLF2 (green). PDX partially preserved the "G1 quiescent" state (9%) and acquired 3 new transcriptional states: a "tumor-proximal" state (violet, 29%), a "proliferative" state (brown, 15%), and a "xenograft-adapted" state (orange, 47%). In contrast, PDCL occupied 3 new transcriptional states: "hypermetabolic" (blue, 65%), "quiescent" (red, 23%), and "hyperproliferative" (purple, 11%). CDX converged on the "xenograft-adapted" state observed in PDX (98%). BTG1, ZFP36L2, and FCMR defined a conserved PT program lost in all models. Integration of matched WES confirmed a shared copy-number landscape across all models in both patients, with HVCN1 p.W45R showing no cluster-specific enrichment in P144, establishing transcriptional divergence as epigenetically rather than genetically driven.

**Summary/Conclusion**

We demonstrated that PDX and PDCL cells, despite sharing the same genomic landscape with the donor primary lymphoma cells, may assume different to highly different transcriptional states, which must be considered in the context of specific research objectives. Moreover, our data suggest that CDX represent the least relevant models compared to PDX and PDCL, and should be avoided in preclinical research, and replaced by PDX.

*Grant support AZV NU23-03-00172*

**Copyright Information:** (Online) ISSN: 2572-9241
©2026 The Author(s). HemaSphere is published by John Wiley & Sons Ltd on behalf of European Hematology Association. This is an open access Abstract Book distributed under the Attribution-NonCommercial-NoDerivs (CC BY-NC-ND), which allows third parties to download the articles and share them with others as long as they credit the author and the Abstract Book, but they cannot change the content in any way or use them commercially.
**Abstract Book Citations:** Authors, Title, HemaSphere, 2026;10:(S1):pages. The abstract book DOIs can be found on the “Author Information” page.
**Disclaimer:** Articles published in the journal HemaSphere exclusively reflect the opinions of the authors. The authors are responsible for all content in their abstracts including accuracy of the facts, statements, citing resources, etc.

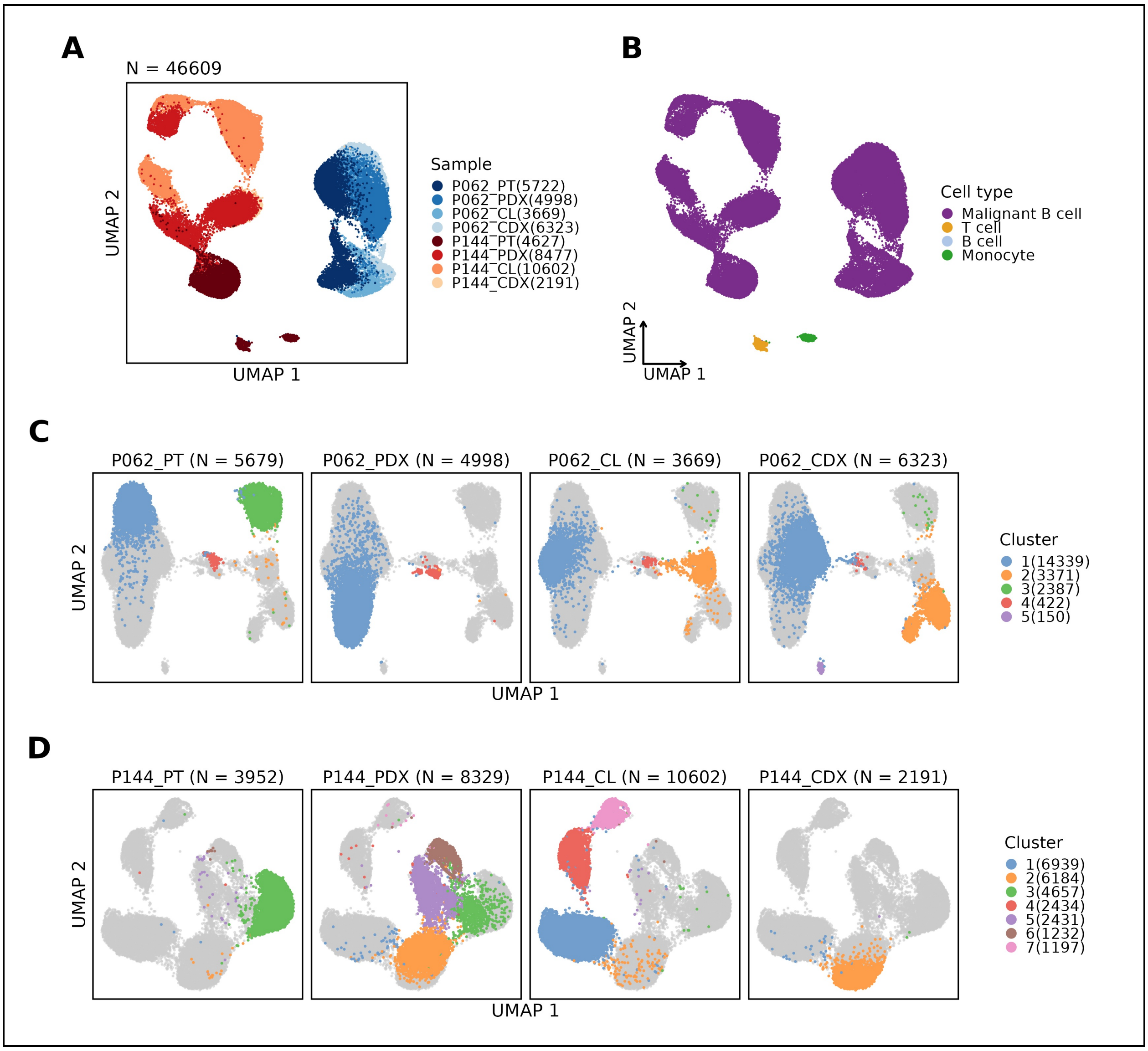

Figure 1. Single-cell transcriptional landscape of LBCL preclinical models. (A) Joint UMAP of 46,609 cells from two LBCL patients (P062, blues; P144, reds/oranges), coloured by sample of origin. Cell counts per sample in parentheses. (B) Same UMAP coloured by cell type; TME cells are restricted to PT and PDX. (C–D) UMAPs of malignant B cells from P062 (20,669 cells; C) and P144 (25,074 cells; D), each faceted by model (PT, PDX, PDCL, CDX). Cells are coloured by Leiden cluster (resolution 0.1 for P062, 5 clusters; resolution 0.3 for P144, 7 clusters); grey points represent cells from other facets shown for spatial reference.

**Copyright Information:** (Online) ISSN: 2572-9241
©2026 The Author(s). HemaSphere is published by John Wiley & Sons Ltd on behalf of European Hematology Association. This is an open access Abstract Book distributed under the Attribution-NonCommercial-NoDerivs (CC BY-NC-ND), which allows third parties to download the articles and share them with others as long as they credit the author and the Abstract Book, but they cannot change the content in any way or use them commercially.
**Abstract Book Citations:** Authors, Title, HemaSphere, 2026;10:(S1):pages. The abstract book DOIs can be found on the “Author Information” page.
**Disclaimer:** Articles published in the journal HemaSphere exclusively reflect the opinions of the authors. The authors are responsible for all content in their abstracts including accuracy of the facts, statements, citing resources, etc.

### (PB3934) HBB-CTCF-FEN1 AXIS MEDIATES PLATINUM RESISTANCE VIA ACTIVATING DNA DAMAGE REPAIR IN DLBCL

**Topic:** 21. Lymphoma biology & translational research

Xutao Guo*[1], Yunxia Zhang[1]

[1]*Nanfang Hospital, Southern Medical University, Department of Hematology, GUANGZHOU, China;*

### Background

Pola-R-CHP is the frontline standard for diffuse large B-cell lymphoma (DLBCL), with 21.5% of patients progressing/relapsing and 30%-40% in high-risk subgroups (MYC/BCL2 double-hit, TP53 mutation). Platinum-based salvage chemotherapy is the mainstay for relapsed/refractory DLBCL, yet platinum resistance is a critical bottleneck. DNA damage repair (DDR) hyperactivation drives platinum resistance, and Flap Endonuclease 1 (FEN1) is aberrantly overexpressed in platinum-resistant DLBCL, while its upstream transcriptional regulatory mechanism remains unclear.

### Aims

To identify the novel upstream regulatory axis of FEN1 in DLBCL, clarify its mechanism in mediating platinum resistance via DDR, and explore its potential as a prognostic biomarker and therapeutic target.

### Methods

HBB was screened as a differentially expressed gene in DLBCL via GEO datasets, with its prognostic value validated by tissue microarray. Its oncogenic role was confirmed by knockdown/overexpression assays in OCI-LY3 and U2932 cells. RNA-seq after HBB silencing identified DDR-related downstream genes, verified by qPCR and WB. Bioinformatics (STRING, hTFtarget, HumanTFDB) and Co-IP validated CTCF as the bridge between HBB and FEN1. Nuclear-cytoplasmic fractionation, ChIP-qPCR and dual-luciferase reporter assay clarified the HBB-CTCF-FEN1 axis mechanism. γ-H2AX foci imaging, comet assay and IC50 detection explored platinum resistance in vitro, with in vivo validation via nude mouse xenograft models.

### Results

HBB was highly expressed in DLBCL (FC>8, P<10^-20), an independent poor prognostic factor (HR=2.61, 95%CI:1.46-4.65), and correlated with pathological grade. HBB knockdown inhibited DLBCL proliferation/metastasis, while overexpression had opposite effects. HBB silencing downregulated DDR pathways (NES<-1.5), with FEN1 as the core target (FC=0.203, highest basal expression). CTCF bound HBB most strongly (score=352); HBB promoted CTCF nuclear translocation and FEN1 promoter binding to upregulate FEN1 transcription. HBB overexpression enhanced DDR capacity, reduced platinum-induced DNA damage and increased platinum resistance, while HBB/FEN1 silencing reversed resistance and sensitized cells to platinum. In vivo, HBB promoted tumor growth and platinum resistance via FEN1; targeting the HBB-CTCF-FEN1 axis enhanced platinum's anti-tumor efficacy, with reduced tumor volume/weight, elevated γ-H2AX and decreased FEN1 in tumor tissues.

**Copyright Information:** (Online) ISSN: 2572-9241
©2026 The Author(s). HemaSphere is published by John Wiley & Sons Ltd on behalf of European Hematology Association. This is an open access Abstract Book distributed under the Attribution-NonCommercial-NoDerivs (CC BY-NC-ND), which allows third parties to download the articles and share them with others as long as they credit the author and the Abstract Book, but they cannot change the content in any way or use them commercially.
**Abstract Book Citations:** Authors, Title, HemaSphere, 2026;10:(S1):pages. The abstract book DOIs can be found on the "Author Information" page.
**Disclaimer:** Articles published in the journal HemaSphere exclusively reflect the opinions of the authors. The authors are responsible for all content in their abstracts including accuracy of the facts, statements, citing resources, etc.

**Summary/Conclusion**

A novel HBB-CTCF-FEN1 transcriptional regulatory axis was identified in DLBCL. Aberrantly overexpressed HBB acts as a transcriptional cofactor, interacting with CTCF to upregulate FEN1, thereby enhancing DDR capacity and mediating platinum resistance. This axis uncovers a new molecular mechanism of platinum resistance in DLBCL; HBB may serve as a prognostic biomarker for predicting platinum-based chemotherapy efficacy. Targeting the HBB-CTCF-FEN1 axis holds great promise to reverse platinum resistance and improve the efficacy of platinum-based salvage chemotherapy, providing a novel therapeutic strategy for DLBCL.

**Copyright Information:** (Online) ISSN: 2572-9241
©2026 The Author(s). HemaSphere is published by John Wiley & Sons Ltd on behalf of European Hematology Association. This is an open access Abstract Book distributed under the Attribution-NonCommercial-NoDerivs (CC BY-NC-ND), which allows third parties to download the articles and share them with others as long as they credit the author and the Abstract Book, but they cannot change the content in any way or use them commercially.
**Abstract Book Citations:** Authors, Title, HemaSphere, 2026;10:(S1):pages. The abstract book DOIs can be found on the "Author Information" page.
**Disclaimer:** Articles published in the journal HemaSphere exclusively reflect the opinions of the authors. The authors are responsible for all content in their abstracts including accuracy of the facts, statements, citing resources, etc.

### (PB3935) DIVERGENT GENOMIC LANDSCAPES IN DLBCL: NOVEL NFKBIE ALTERATIONS AND TP53 DOMINANCE IN INDIAN PATIENTS CHALLENGE WESTERN FRAMEWORKS

**Topic:** 21. Lymphoma biology & translational research

Rahul Bhargav*[1], Shrinidhi Nathany[1], Moin Makda[2], Swarsat Kaushik Nath[1], Hrithik Bagga[1], Siddharth Sagar[1], Haristuti Verma[1], Anusha Swaminathan[1], Vikas Dua[1], Dinesh Bhurani[3], Ragesh Nair[4], Rayaz Ahmed[5]

*[1]Fortis Memorial Research Institute, Gurugram, India; [2]Netaji Subhas University of Technology, New Delhi, India; [3]Rajiv Gandhi Cancer Institute & Research Centre, New Delhi, India; [4]Meitra Hospital, Kozhikode, India; [5]Max Hospital, Saket, New Delhi, India;*

### Background

Diffuse large B-cell lymphoma (DLBCL) is the most common aggressive lymphoma worldwide, yet its genomic landscape in South Asian populations remains largely uncharacterized. Current molecular classification systems, including the MCD, BN2, EZB, and A53 subtypes and precision medicine strategies are derived almost exclusively from Western cohorts of predominantly European ancestry, raising fundamental questions about their universal applicability.

### Aims

The aim of this study was to characterize the genomic landscape of diffuse large B-cell lymphoma (DLBCL) in an Indian (South Asian) cohort through targeted next-generation sequencing and to perform a comparative analysis against large Western reference cohorts to evaluate the applicability of existing molecular classification systems and identify population-specific mutational patterns.

### Methods

We performed targeted next-generation sequencing on formalin-fixed, paraffin-embedded tissue from 44 Indian DLBCL patients (42 using the Ion AmpliSeq Liverpool Lymphoid Network Panel [60 genes]; 2 using the Genes2Me Lymphoma NGS Panel [75 genes]) and conducted an integrated four-cohort comparative analysis against three large Western reference cohorts: Memorial Sloan Kettering Cancer Center (MSK; N=363; IMPACT-HEME-400 panel), Duke University (N=1,001; 150-gene targeted panel), and The Cancer Genome Atlas (TCGA Pan-Cancer Atlas; N=48; whole-exome sequencing), comprising 1,456 patients in total. Gene-level mutation frequencies, variant functional classes, pathway-level mutation patterns, co-mutation relationships, hotspot distributions, aberrant somatic hypermutation (aSHM) target involvement, and survival associations were systematically compared across all cohorts using Fisher's exact test, Benjamini–Hochberg FDR correction, Kaplan–Meier analysis, and multivariate Cox proportional hazards regression.

### Results

Indian DLBCL exhibited a fundamentally distinct genomic architecture defined by five principal features. First, PIM1 — the canonical aSHM target and adverse prognostic marker — was markedly depleted (4.5% vs 19.1% Western; OR=0.20, $P=1.0\times10^{-2}$), anchoring a broader depletion of aSHM target mutations (36.4% vs 58.1%; OR=0.41, P=0.005). Second, truncating NFKBIE (IκBε) mutations were enriched >11-fold (6.8% vs 0.6%; OR=11.4, $P=4.7\times10^{-3}$), identifying an alternative NF-κB activation mechanism through inhibitor loss-of-function rather than MYD88 gain-of-function; NFKBIE mutation was associated with near-universal complete response in the Duke cohort (97.0% vs 82.5%; P=0.031). Third, TP53 was the most frequently mutated gene (27.3% vs 14.2%; OR=2.27, P=0.027), driving enrichment of the cell cycle/apoptosis pathway (61.4% vs 37.8%; P=0.002). Fourth, novel population-specific drivers EGR2 (OR=67.2, $P=2.6\times10^{-3}$) and NOTCH1 (OR=8.54, $P=9.1\times10^{-3}$) were identified, both virtually absent from >1,400 Western patients. Fifth, truncating mutations overall were enriched ~4-fold (40.9% vs 14.9%; OR=3.95, $P=5.9\times10^{-13}$), consistent with tumor suppressor–driven biology.

**Copyright Information:** (Online) ISSN: 2572-9241
©2026 The Author(s). HemaSphere is published by John Wiley & Sons Ltd on behalf of European Hematology Association. This is an open access Abstract Book distributed under the Attribution-NonCommercial-NoDerivs (CC BY-NC-ND), which allows third parties to download the articles and share them with others as long as they credit the author and the Abstract Book, but they cannot change the content in any way or use them commercially.
**Abstract Book Citations:** Authors, Title, HemaSphere, 2026;10:(S1):pages. The abstract book DOIs can be found on the "Author Information" page.
**Disclaimer:** Articles published in the journal HemaSphere exclusively reflect the opinions of the authors. The authors are responsible for all content in their abstracts including accuracy of the facts, statements, citing resources, etc.

**Summary/Conclusion**

Indian DLBCL harbors a genomically distinct mutational program characterized by tumor suppressor–driven biology, alternative NF-κB pathway wiring, depleted aSHM signatures, and novel population-specific driver genes. These findings challenge the universality of Western-derived molecular classification systems and have direct implications for precision medicine in South Asian populations. Prospective multi-ethnic validation is essential to refine population-specific classifiers.

**Copyright Information:** (Online) ISSN: 2572-9241
©2026 The Author(s). HemaSphere is published by John Wiley & Sons Ltd on behalf of European Hematology Association. This is an open access Abstract Book distributed under the Attribution-NonCommercial-NoDerivs (CC BY-NC-ND), which allows third parties to download the articles and share them with others as long as they credit the author and the Abstract Book, but they cannot change the content in any way or use them commercially.
**Abstract Book Citations:** Authors, Title, HemaSphere, 2026;10:(S1):pages. The abstract book DOIs can be found on the "Author Information" page.
**Disclaimer:** Articles published in the journal HemaSphere exclusively reflect the opinions of the authors. The authors are responsible for all content in their abstracts including accuracy of the facts, statements, citing resources, etc.

### (PB3936) THE INDOLEDIONE DERIVATIVE 211H SELECTIVELY TARGETS TP53 R175H-MUTANT AGGRESSIVE B-CELL LYMPHOMA VIA INDUCTION OF APOPTOSIS AND FERROPTOSIS

**Topic:** 21. Lymphoma biology & translational research

Wenhua Wang*[1], Jiayi Han[1], Xia Liu[1], Xinjie Bi[1], Xianhuo Wang[1], Huilai Zhang[1]

[1] *Tianjin Medical University Institute and Hospital, Tianjin, China;*

### Background

TP53 mutation is a well-established adverse prognostic factor in aggressive B-cell lymphomas, highlighting the clinical importance of developing novel therapeutic strategies for this patient population.

### Aims

Building on this background, the present study aims to: Systematically characterize the TP53 mutational spectrum in aggressive B-cell lymphoma and evaluate its association with patient prognosis; Screen a small-molecule compound library provided by the School of Pharmaceutical Sciences of Shandong University to identify candidate compounds that selectively eliminate TP53R175H-mutant aggressive B-cell lymphoma cells; Investigate the cytotoxic mechanisms and molecular pathways underlying the anti-tumor effects of the identified compound, in order to provide new therapeutic insights and a theoretical foundation for patients with TP53R175H-mutant aggressive B-cell lymphoma.

### Methods

We first analyzed the correlation between TP53 mutations and the prognosis as well as related clinical indicators in 2813 patients with aggressive B-cell lymphoma, based on genomic and clinical data. Using a CCK-8-based high-throughput screening of a small-molecule compound library provided by the School of Pharmaceutical Sciences, Shandong University, we identified compound 211H, an indoledione derivative, as a selective killer of cells harboring the TP53 R175H mutation. We further evaluated the effects of 211H in TP53 R175H-mutant aggressive B-cell lymphoma cell lines and an NSG mouse xenograft model, and explored the underlying mechanisms by which 211H exerts its selective cytotoxicity.

### Results

In our institutional cohort of patients with aggressive B-cell lymphoma, the TP53 mutation rate was 27.7% (n=149). Survival analysis revealed that overall TP53 mutation, all hotspot mutations, and individual hotspot mutations—including R175, G245, R248, and R273—were significantly associated with poor overall survival (OS).The novel indole-dione compound 211H selectively induces apoptosis and G2-phase cell cycle arrest in TP53R175H-mutant aggressive B-cell lymphoma cells in vitro. It also demonstrates potent antitumor activity and favorable biosafety in vivo. Further mechanistic investigation indicates that the cytotoxic effect of 211H on TP53R175H-mutant cells is not only mediated by apoptosis but also involves the induction of ferroptosis. The ferroptosis-inhibitory phenotype specifically occurs in TP53R175H-mutant cells. 211H directly binds to the deubiquitinase USP8, disrupts its interaction with SLC7A11, and thereby promotes K48-linked polyubiquitination and subsequent degradation of SLC7A11 protein, ultimately activating the ferroptosis pathway and leading to cell death.

**Copyright Information:** (Online) ISSN: 2572-9241
©2026 The Author(s). HemaSphere is published by John Wiley & Sons Ltd on behalf of European Hematology Association. This is an open access Abstract Book distributed under the Attribution-NonCommercial-NoDerivs (CC BY-NC-ND), which allows third parties to download the articles and share them with others as long as they credit the author and the Abstract Book, but they cannot change the content in any way or use them commercially.
**Abstract Book Citations:** Authors, Title, HemaSphere, 2026;10:(S1):pages. The abstract book DOIs can be found on the "Author Information" page.
**Disclaimer:** Articles published in the journal HemaSphere exclusively reflect the opinions of the authors. The authors are responsible for all content in their abstracts including accuracy of the facts, statements, citing resources, etc.

### Summary/Conclusion

As an indoledione derivative, 211H synergistically induces apoptosis and ferroptosis, leading to potent and selective elimination of TP53 R175H-mutant aggressive B-cell lymphoma. These findings provide a novel theoretical basis for the clinical treatment of this malignancy.

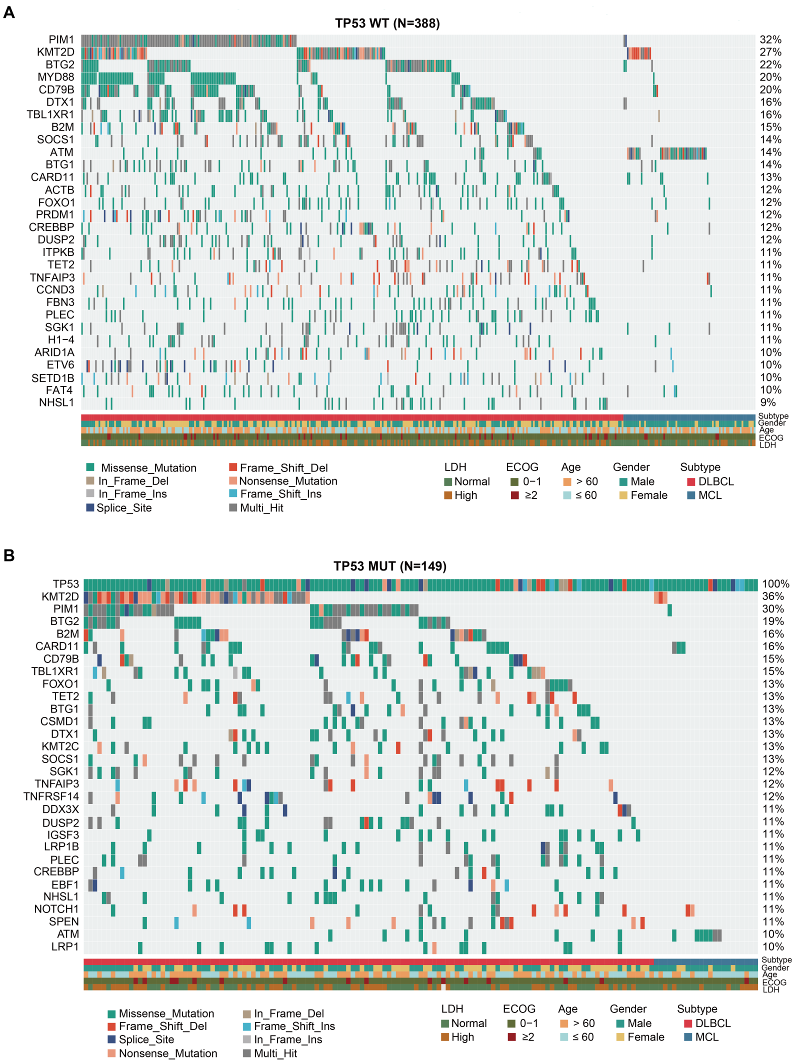

**Copyright Information:** (Online) ISSN: 2572-9241
©2026 The Author(s). HemaSphere is published by John Wiley & Sons Ltd on behalf of European Hematology Association. This is an open access Abstract Book distributed under the Attribution-NonCommercial-NoDerivs (CC BY-NC-ND), which allows third parties to download the articles and share them with others as long as they credit the author and the Abstract Book, but they cannot change the content in any way or use them commercially.
**Abstract Book Citations:** Authors, Title, HemaSphere, 2026;10:(S1):pages. The abstract book DOIs can be found on the "Author Information" page.
**Disclaimer:** Articles published in the journal HemaSphere exclusively reflect the opinions of the authors. The authors are responsible for all content in their abstracts including accuracy of the facts, statements, citing resources, etc.

### (PB3937) A PEPTIDE TARGETING OUTER MEMBRANE PROTEIN OF B-CELL LYMPHOMA EXHIBITS TARGETED THERAPY POTENTIAL AND APPLICATION

**Topic:** 21. Lymphoma biology & translational research

Qian Cheng[1], Ying Shen[1], Qian Wang[1], Wanying Chen[1], Qisi Lu[1], Kaiyu Sheng[1], Zhou Jin[1], Dandan Lin[1], Haiwen Huang*[1]

[1] *The First Affiliated Hospital Of Soochow University, Hematology, Suzhou, China;*

### Background

**Background:** B-cell lymphoma have significantly improved patient survival, however, approximately 30% of patients develop relapsed/refractory disease, which exhibits poor response to conventional therapies and unfavorable prognosis. To address this therapeutic gap, targeted therapy, aimed at enhancing treatment precision, holds promise for improving outcomes. This study employed phage display technology（PhD）to screen for peptides targeting B-cell surface proteins and investigate their biological functions in lymphoma cells. We developed a novel phage display-based peptide-drug conjugate (PDC) strategy that utilizes tumor-specific peptides to deliver cytotoxic payloads ( the pro-apoptotic KLA peptide), enabling precise tumor killing while minimizing off-target toxicity

### Aims

We developed a novel phage display-based peptide-drug conjugate (PDC) strategy that utilizes tumor-specific peptides to deliver cytotoxic payloads ( the pro-apoptotic KLA peptide), enabling precise tumor killing while minimizing off-target toxicity.

### Methods

**Methods:**
Through three rounds of biopanning against lymphoma cell lines using a 12-mer phage library. Seven phage monoclonal clones were isolated, sequenced, and analyzed to identify candidate peptides (C1 and C2). These were conjugated to cytotoxic peptide KLA to generate PDCs C1-KLA, C2-KLA, with scrambled-sequence NsG-KLA as control. Peptide specificity was validated via ELISA, flow cytometry, and Western blotting across multiple lymphoma cell lines. Functional assays were performed after conjugating C1 and C2 to the pro-apoptotic peptide KLA (C1-KLA/C2-KLA), with NsG-KL.

### Results

**Results:**
C1 exhibited nanomolar affinity for lymphoma cells, with >90% target engagement at 20 μM with negligible cross-reactivity to normal B-cells ($p<0.001$). Synthetic peptides and their NsG controls were generated for downstream experiments. Encouragingly, in vitro cytotoxicity, apoptosis, proliferation assays, C1-KLA demonstrated significant pro-apoptotic, anti-proliferative, and cell growth-inhibitory effects. Conjugating to the KLA, C1-KLA induced caspase-3-dependent apoptosis (3.4-fold increase vs control, $p<0.001$) and inhibited proliferation. In murine xenograft model established with subcutaneous injection of human B-cell lymphoma cells in nude mice, C1-KLA achieved objective responses in 100% of cases (at 120 mg/kg Q3D x 4 ), the tumor weight in the treatment group was significantly reduced compared to the control group ($p<0.001$), C1-KLA achieved tumor regression with no observable toxicity.

**Copyright Information:** (Online) ISSN: 2572-9241
©2026 The Author(s). HemaSphere is published by John Wiley & Sons Ltd on behalf of European Hematology Association. This is an open access Abstract Book distributed under the Attribution-NonCommercial-NoDerivs (CC BY-NC-ND), which allows third parties to download the articles and share them with others as long as they credit the author and the Abstract Book, but they cannot change the content in any way or use them commercially.
**Abstract Book Citations:** Authors, Title, HemaSphere, 2026;10:(S1):pages. The abstract book DOIs can be found on the “Author Information” page.
**Disclaimer:** Articles published in the journal HemaSphere exclusively reflect the opinions of the authors. The authors are responsible for all content in their abstracts including accuracy of the facts, statements, citing resources, etc.

**Summary/Conclusion**

**Conclusion:** Through comprehensive phage display screening and functional validation, we have identified C1 as a high-affinity targeting peptide that specifically recognizes a conserved epitope expressed in B-cell lymphoma cell lines. This PDC C1-KLA selectively targets a surface-exposed lymphoma-specific epitope, enabling tumor-restricted delivery of KLA. These findings highlight C1-KLA as a novel and highly effective targeted therapeutic molecule specifically directed against lymphoma cell surface proteins, addressing the critical need for precision therapies in relapsed/refractory cases. This study establishes C1-KLA as a promising therapeutic candidate for B-cell lymphoma, with potential applicability to other CD19-negative malignancies.This demonstrates broad applicability across hematologic malignancies expressing well-defined surface antigens, offering a streamlined and accelerated development pathway for precision-targeted immunotherapies.

**Copyright Information:** (Online) ISSN: 2572-9241
©2026 The Author(s). HemaSphere is published by John Wiley & Sons Ltd on behalf of European Hematology Association. This is an open access Abstract Book distributed under the Attribution-NonCommercial-NoDerivs (CC BY-NC-ND), which allows third parties to download the articles and share them with others as long as they credit the author and the Abstract Book, but they cannot change the content in any way or use them commercially.
**Abstract Book Citations:** Authors, Title, HemaSphere, 2026;10:(S1):pages. The abstract book DOIs can be found on the “Author Information” page.
**Disclaimer:** Articles published in the journal HemaSphere exclusively reflect the opinions of the authors. The authors are responsible for all content in their abstracts including accuracy of the facts, statements, citing resources, etc.

### (PB3938) DECIPHERING THE SPATIAL ARCHITECTURE OF LYMPHOMA

**Topic:** 21. Lymphoma biology & translational research

Maria Fanouria Chatziaslani*[1], Alvaro Lopez Malizia[1], Mustafa Al Sibai[1], Daniela Grases[1], Gerardo Ferrer[1], Laura Mondragon Martinez[1], Gaël Roué[1], Tomas Navarro[1], Eduard Porta[1], Manel Esteller[1]

*[1]Josep Carreras Leukaemia Research Institute, Barcelona, Spain;*

### Background

Lymphoma comprises a highly heterogeneous group of neoplasms arising from diverse immune cell precursors. Subtypes differ markedly in their localization, cell of origin, molecular profiles and clinical outcomes. Understanding the origins of the disease is of great scientific interest since lymphoma affects millions of people globally.

### Aims

Our main aim was to construct a Spatial Architecture Atlas of Lymphoma. This would set a ground rule of how the Lymphoma Microenvironment is structured in space and which are the key contributors to disease progression and growth. Our goal is to identify pan-lymphoma patterns that go beyond the clinical diagnosis of the subtype and/or the genomic landscape of the patient.

### Methods

We employed spatial transcriptomics using the 10X Genomics Visium platform on tissue samples from a cohort of 107 lymphoma patients and the 10X Genomics Xenium 5K platform for 21 representative patients of the cohort. Unlike bulk RNA-sequencing, spatial transcriptomics retains the tissue architecture, enabling high-resolution mapping of gene expression within the lymphoma microenvironment (LME). We leveraged the advantages of both technologies, with Visium providing whole-transcriptome coverage and Xenium providing sub-cellular resolution, creating a powerful dataset.

### Results

Our analysis highlights the heterogeneity of the LME composition and structure of cell types across -and within- subtypes. By quantifying genomics and phenotypic heterogeneity of the lymphoma cells, we show that the functional spatial landscape of the LME drives the phenotypic specialization of lymphoma clones and contributes ~4.5 times more than the genomic landscape of the patient. We identified spatially structured niches (neighborhoods) that constitute the functional landscape of the LME which revealed specialized activities. Spatially-aware unsupervised analysis of our cohort resulted in structured pan-lymphoma biological signatures. Finally, we identified interaction families and candidate therapeutic targets using computational approaches.

### Summary/Conclusion

Our findings demonstrate that the spatial architecture and function of the Lymphoma Microenvironment dictate the phenotype of lymphoma clones instead of the cellular composition or genomic landscape alone. This suggests that the “where” and “how” are superior to the “what”. Our study is a great example of the clinical utility and potential of Spatial Transcriptomics (ST) technologies. ST data could revolutionize patient stratification and therapeutic approaches by providing a granular view of the cancer battlefield that is the lymphoma micro-environment.

**Copyright Information:** (Online) ISSN: 2572-9241
©2026 The Author(s). HemaSphere is published by John Wiley & Sons Ltd on behalf of European Hematology Association. This is an open access Abstract Book distributed under the Attribution-NonCommercial-NoDerivs (CC BY-NC-ND), which allows third parties to download the articles and share them with others as long as they credit the author and the Abstract Book, but they cannot change the content in any way or use them commercially.
**Abstract Book Citations:** Authors, Title, HemaSphere, 2026;10:(S1):pages. The abstract book DOIs can be found on the “Author Information” page.
**Disclaimer:** Articles published in the journal HemaSphere exclusively reflect the opinions of the authors. The authors are responsible for all content in their abstracts including accuracy of the facts, statements, citing resources, etc.

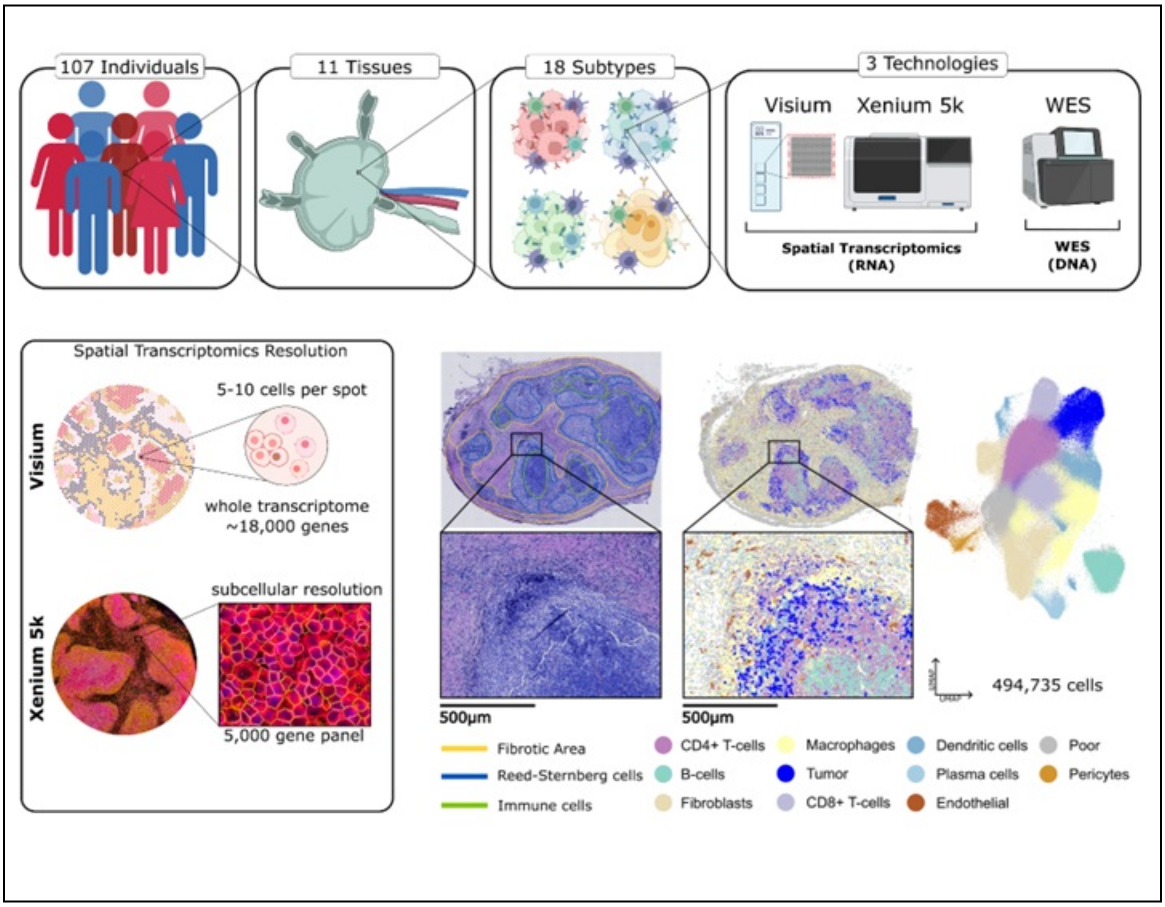

**Copyright Information:** (Online) ISSN: 2572-9241
©2026 The Author(s). HemaSphere is published by John Wiley & Sons Ltd on behalf of European Hematology Association. This is an open access Abstract Book distributed under the Attribution-NonCommercial-NoDerivs (CC BY-NC-ND), which allows third parties to download the articles and share them with others as long as they credit the author and the Abstract Book, but they cannot change the content in any way or use them commercially.
**Abstract Book Citations:** Authors, Title, HemaSphere, 2026;10:(S1):pages. The abstract book DOIs can be found on the "Author Information" page.
**Disclaimer:** Articles published in the journal HemaSphere exclusively reflect the opinions of the authors. The authors are responsible for all content in their abstracts including accuracy of the facts, statements, citing resources, etc.

### (PB3939) CHARACTERIZATION OF IMMUNE BIOMARKERS IN THE PERIPHERAL BLOOD IN CORRELATION WITH TOTAL METABOLIC TUMOR VOLUME IN CLASSICAL HODGKIN LYMPHOMA.

**Topic:** 21. Lymphoma biology & translational research

Zaid Mansur*[1, 2], Lotta Hansson[1, 2], Kia Heimersson[1], Bjorn Wahlin[2, 3], Tom Aadrian Mulder[1, 2], Alexandra Psychou[4], Carmen Cananau[4], Anna Kistner[4, 5], Marzia Palma[1, 2]

*[1]Department of Oncology-Pathology, Karolinska Institutet, Stockholm, Sweden;[2]Lymphoma Unit, Department of Hematology, Karolinska University Hospital, Stockholm, Sweden; [3]Department of Medicine, Huddinge, Karolinska Institutet, Stockholm, Sweden; [4]Department of Nuclear Medicine and Medical Physics, Karolinska University Hospital, Stockholm, Sweden; [5]Department of Molecular Medicine and Surgery, Karolinska Institutet, Stockholm, Sweden;*

### Background

In classical Hodgkin lymphoma (cHL), the malignant Hodgkin Reed-Sternberg cells orchestrate a complex lymphocyte predominant tumor microenvironment that sustains tumor survival. We previously described (*Mulder et al, Hemasphere 2022*) disease-related changes in circulating lymphocytes and plasma proteins in a consecutive cohort of patients (pts) with newly diagnosed cHL. We now expanded the study to correlate with metabolic tumor volume (MTV) assessed by $^{18}$F-FDG PET/CT, a quantitative imaging biomarker reported as strong independent predictor of outcome.

### Aims

To evaluate peripheral blood (PB) immune biomarkers in relation to tumor burden defined by MTV and to explore their association with clinical outcome.

### Methods

PB samples were obtained at baseline (BL) before start of primary treatment, at end of treatment (EoT), and at 6-12 months following EoT. PB mononuclear cells were analyzed using flow-cytometry (FACS) and analysis of plasma biomarkers was performed using a 92-plex Olink inflammatory panel. MTV from routine FDG-PET/CT scans was quantified by two independent radiologists with a standardized uptake value (SUV) threshold of > 2.5. Total lesion glycolysis (TLG) was calculated by multiplying MTV by the average SUV.

According to Swedish national guidelines, pts with advanced stage cHL (stages IIB-IV) who achieve PET-negativity after 2 chemotherapy cycles (PET2) are evaluated with conventional CT imaging at EoT. To enable correlation of immune parameters with EoT MTV, PET2-negative patients were classified as PET negative at EoT for analytical purposes.

**Copyright Information:** (Online) ISSN: 2572-9241
©2026 The Author(s). HemaSphere is published by John Wiley & Sons Ltd on behalf of European Hematology Association. This is an open access Abstract Book distributed under the Attribution-NonCommercial-NoDerivs (CC BY-NC-ND), which allows third parties to download the articles and share them with others as long as they credit the author and the Abstract Book, but they cannot change the content in any way or use them commercially.
**Abstract Book Citations:** Authors, Title, HemaSphere, 2026;10:(S1):pages. The abstract book DOIs can be found on the "Author Information" page.
**Disclaimer:** Articles published in the journal HemaSphere exclusively reflect the opinions of the authors. The authors are responsible for all content in their abstracts including accuracy of the facts, statements, citing resources, etc.

### Results

PB samples were obtained from a total of 103 pts. Median age at diagnosis was 36 (18-84) and 51 (49.5%) had advanced stage disease. So far, 51 pts with complete imaging and immune data were included in the present analysis, of whom 45% had advanced stage disease. MTV at baseline ($MTV_{BL}$) did not differ between pts with limited- and advanced stage disease (median 126 vs. 129 mL, p = 0.248). Among pts with an available PET at interim- and EoT (n=9), there was no difference in MTV between the two time points (p=0.0938). 18/48 pts had unmeasurable MTV following frontline therapy. $MTV_{BL}$ correlated significantly with the IPS risk factors ESR (r=0.45, p=0.001), hemoglobin (r = –0.32, p=0.024) and albumin (r = –0.37, p=0.007) but not with leukocyte or lymphocyte count. Stratification by median $MTV_{BL}$ (127 mL) showed no significant difference in 2-year event free survival between the groups of low and high MTV (95.8% vs 88.5%, p=0.366).
Higher baseline cytokine levels were associated with higher MTV, with the strongest positive associations observed for CXCL13, IL-10, CCL19, and TNFRSF14 (Fig. A). These cytokines were similarly associated with TLG, consistent with findings observed for MTV, and were significantly reduced following frontline therapy. None of the 4 cytokines, $MTV_{BL}$ or IPS factors predicted a state of unmeasurable MTV at EoT in a univariate logistic regression model. Correlation of lymphocyte subsets with $MTV_{BL}$ showed significant and strongest associations with activated regulatory- and checkpoint protein expressing T cells suggesting a tumor-mediated suppression of anti-tumoral immune response (Fig. B).

### Summary/Conclusion

A peripheral proinflammatory immune composition mirrored baseline tumor volume and metabolic burden but did not independently predict early metabolic response in this cohort. These findings suggest that circulating immune alterations primarily reflect disease extent rather than treatment sensitivity.

**Copyright Information:** (Online) ISSN: 2572-9241
©2026 The Author(s). HemaSphere is published by John Wiley & Sons Ltd on behalf of European Hematology Association. This is an open access Abstract Book distributed under the Attribution-NonCommercial-NoDerivs (CC BY-NC-ND), which allows third parties to download the articles and share them with others as long as they credit the author and the Abstract Book, but they cannot change the content in any way or use them commercially.
**Abstract Book Citations:** Authors, Title, HemaSphere, 2026;10:(S1):pages. The abstract book DOIs can be found on the “Author Information” page.
**Disclaimer:** Articles published in the journal HemaSphere exclusively reflect the opinions of the authors. The authors are responsible for all content in their abstracts including accuracy of the facts, statements, citing resources, etc.

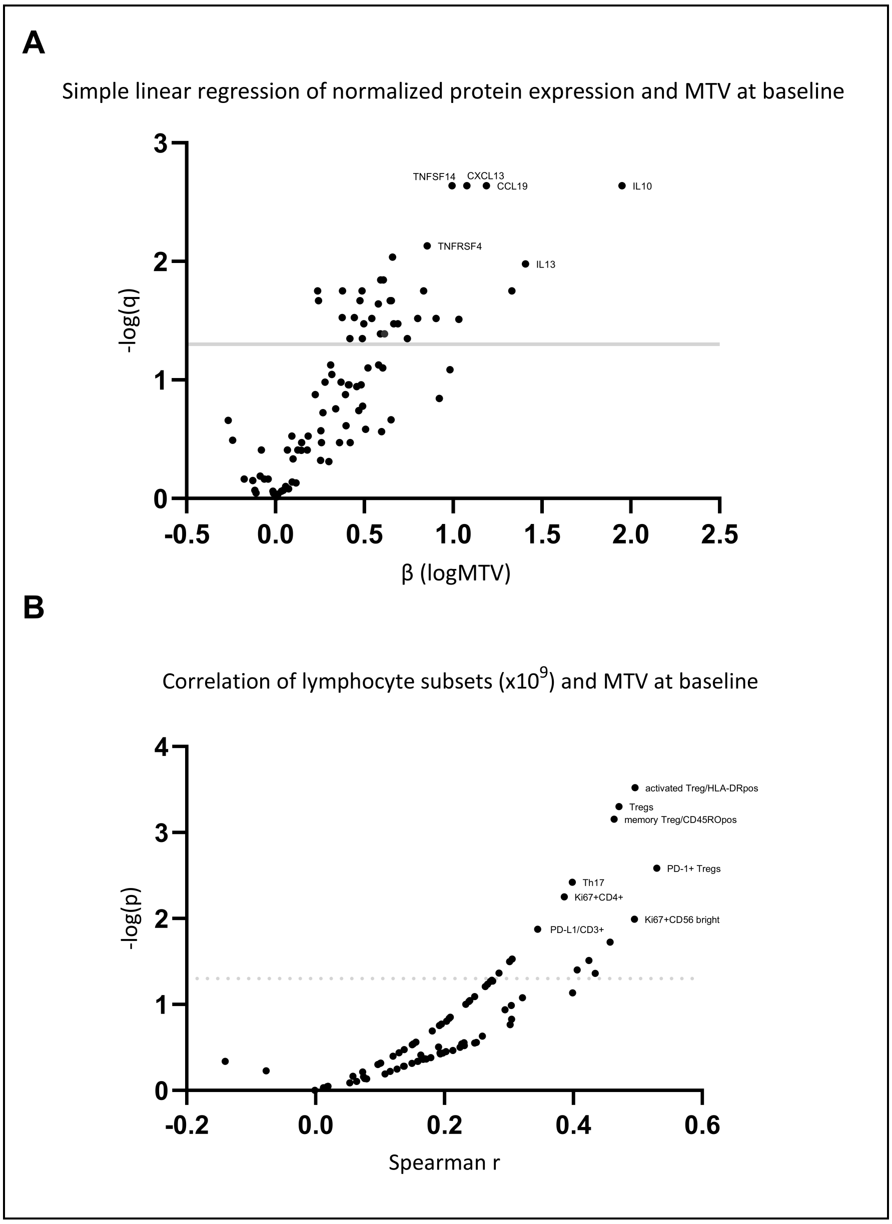

Copyright Information: (Online) ISSN: 2572-9241
©2026 The Author(s). HemaSphere is published by John Wiley & Sons Ltd on behalf of European Hematology Association. This is an open access Abstract Book distributed under the Attribution-NonCommercial-NoDerivs (CC BY-NC-ND), which allows third parties to download the articles and share them with others as long as they credit the author and the Abstract Book, but they cannot change the content in any way or use them commercially.
Abstract Book Citations: Authors, Title, HemaSphere, 2026;10:(S1):pages. The abstract book DOIs can be found on the "Author Information" page.
Disclaimer: Articles published in the journal HemaSphere exclusively reflect the opinions of the authors. The authors are responsible for all content in their abstracts including accuracy of the facts, statements, citing resources, etc.

### (PB3940) CD70-TARGETED IMMUNO-PET/CT FOR DETECTING HETEROGENEOUS CD70 EXPRESSION IN LYMPHOMAS: A PILOT CLINICAL STUDY

**Topic:** 21. Lymphoma biology & translational research

Xiaona Zheng[1], You Zhang[2], Jianchen Fang[3], Lianghua Li[2], Shuxian An[2], Wenzhi Jia[2], Jianjun Liu[2], Zhou Jiang[3], Zebing Liu[3], Weijun Wei[2], Tingting Liu*[1]

*[1]Renji Hospital, School of Medicine, Shanghai JiaoTong University, Department of Hematology, Shanghai, China;[2]Renji Hospital, School of Medicine, Shanghai JiaoTong University, Department of Nuclear Medicine, Shanghai, China; [3]Renji Hospital, School of Medicine, Shanghai JiaoTong University, Department of Pathology, Shanghai, China;*

### Background

CD70 represents a promising therapeutic target for lymphoma. The expression patterns of CD70 and the diagnostic value of CD70-targeted immuno-PET/CT in lymphomas remain unknown.

### Methods

We investigated CD70 expression and explored the diagnostic value of CD70-targeted immuno-PET/CT in 154 patients with lymphoma.

### Results

Analysis of 154 lymphoma specimens showed CD70 positivity in 97 patients (63%), with a trend toward higher expression in older patients ($P = 0.0014$) and a possible inverse relationship with levels of B-cell lymphoma 6 ($P = 0.001$). In an ongoing clinical trial (), 20 patients with lymphoma underwent CD70-targeted immuno-PET/CT. The mean $SUV_{max}$ of the CD70-targeted tracer was 3.4 ± 2.9 in 189 lymphoma lesions, significantly higher than that of the liver, spleen, and blood vessels ($P < 0.01$ for all). However, tracer uptake showed subtype-dependent variation, with diffuse large B-cell lymphoma and mucosa-associated lymphoid tissue lymphoma exhibiting the highest tracer uptake. No significant difference emerged between nodal and extranodal lesions ($P = 0.319$). The tracer uptake correlated well with CD70 expression ($r = 0.6655$, $P < 0.0001$). Moreover, CD70-targeted immuno-PET/CT showed noninferior diagnostic performance compared with [$^{18}$F]FDG across all evaluated categories, achieving detection rates of 100% for extranodal lesions and 97.7% for lymph node lesions and improving the detection of intracranial lesions. CD70-targeted immuno-PET/CT effectively monitored the therapeutic responses in 4 patients.

### Summary/Conclusion

CD70-targeted immuno-PET/CT effectively visualizes CD70 expression in patients with lymphoma, potentially serving as a valuable tool for improving CD70-targeted therapies.

**Copyright Information:** (Online) ISSN: 2572-9241
©2026 The Author(s). HemaSphere is published by John Wiley & Sons Ltd on behalf of European Hematology Association. This is an open access Abstract Book distributed under the Attribution-NonCommercial-NoDerivs (CC BY-NC-ND), which allows third parties to download the articles and share them with others as long as they credit the author and the Abstract Book, but they cannot change the content in any way or use them commercially.
**Abstract Book Citations:** Authors, Title, HemaSphere, 2026;10:(S1):pages. The abstract book DOIs can be found on the "Author Information" page.
**Disclaimer:** Articles published in the journal HemaSphere exclusively reflect the opinions of the authors. The authors are responsible for all content in their abstracts including accuracy of the facts, statements, citing resources, etc.

### (PB3941) LONG-READ SEQUENCING IMPROVES THE CYTOGENOMIC DIAGNOSIS OF NON-HODGKIN LYMPHOMA AND REVEALS ADDITIONAL INFORMATION

**Topic:** 21. Lymphoma biology & translational research

Amber Verhasselt*[1], Geneviève Ameye[2], Justine Vanhevel[2], Thomas Tousseyn[3, 4], Johanna Vets[4], Katrina Rack[2], Jonas Demeulemeester[5, 6], Lucienne Michaux[1, 2], Barbara Dewaele[2], Jolien De Bie[2]

*[1]KU Leuven, Department of Human Genetics, Leuven, Belgium;[2]UH Leuven, Center of Human Genetics, Leuven, Belgium;[3]UH Leuven, Department of Pathology, Leuven, Belgium; [4]KU Leuven, Department of Imaging & Pathology, Leuven, Belgium;[5]KU Leuven, Department of Oncology, Leuven, Belgium; [6]VIB-KU Leuven, Center for Cancer Biology, Leuven, Belgium;*

### Background

Non-Hodgkin Lymphoma (NHL) is a heterogeneous hematologic malignancy that arises from the clonal proliferation of lymphocytes. NHL subtypes are often characterized by specific structural variants (SV) and copy number alterations (CNA). The standard-of-care (SOC) genetic tests to detect these, chromosome banding analysis (CBA) and fluorescent in-situ hybridization (FISH), are labor-intensive and have limited resolution. Most notably, CBA relies on a viable cell culture and FISH is a targeted technique, requiring prior knowledge on the expected disease entity.
We recently demonstrated that Optical Genome Mapping (OGM) can overcome these limitations and improve diagnostic yields by robust detection of clinically relevant SV/CNA. However, its limited resolution (~500bp) means that breakpoint positions are approximate and single nucleotide variants (SNV) or short insertions/deletions (indels) are not detected. Nevertheless, identifying pathogenic small variants can guide clinical decision making in NHL. Long-read sequencing (LRS) not only provides a genome-wide view of SV/CNA with a higher sensitivity than SOC/OGM, but also allows profiling of small variants and DNA methylation in a single assay.

### Aims

We anticipate introducing LRS will further increase diagnostic yields and simplify workflows, but it also offers the opportunity to detect novel pathogenic variants underpinning aggressive lymphomas where current genetic knowledge is limited.

### Methods

To demonstrate the potential of LRS, we profiled a diverse retrospective cohort of 60 cases spanning a variety of NHL subtypes with ONT Nanopore or PacBio HiFi sequencing and performed a direct comparison to OGM and SOC for the detection of clinically relevant TIER1 and TIER2 genetic aberrations (Verhasselt *et al*, Mod Pathol 2026).

**Copyright Information:** (Online) ISSN: 2572-9241
©2026 The Author(s). HemaSphere is published by John Wiley & Sons Ltd on behalf of European Hematology Association. This is an open access Abstract Book distributed under the Attribution-NonCommercial-NoDerivs (CC BY-NC-ND), which allows third parties to download the articles and share them with others as long as they credit the author and the Abstract Book, but they cannot change the content in any way or use them commercially.
**Abstract Book Citations:** Authors, Title, HemaSphere, 2026;10:(S1):pages. The abstract book DOIs can be found on the “Author Information” page.
**Disclaimer:** Articles published in the journal HemaSphere exclusively reflect the opinions of the authors. The authors are responsible for all content in their abstracts including accuracy of the facts, statements, citing resources, etc.

**Results**

In this cohort, LRS-based variant calling detected 37/39 (95%) and 47/50 (94%) of TIER1 variants reported by SOC or OGM, respectively, as well as 28/33 (85%) and 69/75 (92%) of TIER2 variants.
SV missed by the LRS variant calling tools, were present in the raw data at low variant allele frequencies and could thus be rescued.
CNA that were missed by LRS, were often also missed by OGM and were present in subtypes with lower tumor percentages (e.g. angioimmunoblastic T-cell lymphoma), indicating both technologies require a certain amount of tumor infiltration for successful variant detection.
In contrast, 10 TIER1 and 43 TIER2 additional variants were detected only by OGM and LRS, while 6 TIER2 variants were only detected by LRS. Additionally, many SV in lymphoid-related or COSMIC genes were detected in this cohort, which require further investigation. LRS also improved breakpoint determination and resolved complex rearrangements.
Furthermore, mutational analysis uncovered 240 small variants (SNV and indels) with predicted high or moderate impact in genes associated with lymphoid malignancies. The top altered genes in this cohort were found to be *TP53* (11 cases), *KMT2D* (8 cases), *PTPRD* (7 cases), *TET2*, *ATM* and *SMARCA4* (all 6 cases), *ARID1A*, *CREBBP* and *IL7R* (all 5 cases).
Finally, when analyzing recurrent translocations, such as the *IGH::MYC* translocation in B-NHL, a difference in DNA methylation pattern between the normal and rearranged allele can be detected.

**Summary/Conclusion**

In conclusion, by allowing the simultaneous and high-resolution detection of SV, CNA, small variants and DNA methylation patterns, LRS increases the diagnostic yield and allows in-depth genomic characterization of NHL subtypes.

**Copyright Information:** (Online) ISSN: 2572-9241
©2026 The Author(s). HemaSphere is published by John Wiley & Sons Ltd on behalf of European Hematology Association. This is an open access Abstract Book distributed under the Attribution-NonCommercial-NoDerivs (CC BY-NC-ND), which allows third parties to download the articles and share them with others as long as they credit the author and the Abstract Book, but they cannot change the content in any way or use them commercially.
**Abstract Book Citations:** Authors, Title, HemaSphere, 2026;10:(S1):pages. The abstract book DOIs can be found on the “Author Information” page.
**Disclaimer:** Articles published in the journal HemaSphere exclusively reflect the opinions of the authors. The authors are responsible for all content in their abstracts including accuracy of the facts, statements, citing resources, etc.

### (PB3942) PROTEIN KINASE CK2 AS A THERAPEUTIC HUB IN MANTLE CELL LYMPHOMA: FROM INFLAMMATION TO DRUG RESISTANCE

**Topic:** 21. Lymphoma biology & translational research

Juairia Uddin Dekha*[1, 2, 3], Jessica Ceccato[1, 2], Amir Yami[1, 2, 3], Laura Quotti Tubi[1, 2], Iris Haxhiu[1, 2], Marco Fantuz[2], Nicolò Danesin[1], Greta Scapinello[1, 2], Marco Carraro[1], Andrea Visentin[1], Fabrizio Vianello[1, 2], Livio Trentin[1], Francesco Piazza[1, 2], Sabrina Manni[1, 2]

*[1]Hematology Unit, Department of Medicine, University of Padova, Padova, Italy;[2]Veneto Institute of Molecular Medicine (VIMM), Padova, Italy; [3]Department of Surgery, Oncology and Gastroenterology, Padova, Italy;*

### Background

Mantle Cell Lymphoma (MCL) is a largely incurable B-cell malignancy. Oncogenic signaling, epigenetic plasticity and tumor-associated pro-inflammatory programs contribute to disease progression. Protein kinase CK2 is constitutively active and overexpressed in MCL, in which it sustains tumor growth and survival. In MCL, growing evidence suggests that the Myc-driven oncogenic pathway may influence immune-suppressive inflammatory networks within the tumor microenvironment (TME). In other lymphoid and myeloid tumors CK2 has been shown to control c-Myc activity. Moreover, Bromodomain-containing protein 4 (BRD4), a member of the BET family of epigenetic regulators, controls pivotal determinants of lymphoid tumors, such as c-Myc and BCL2. BET inhibitors (BETi) have demonstrated promising preclinical activity in MCL; however, both intrinsic and acquired resistance limit their therapeutic efficacy. CK2 has been reported to phosphorylate BRD4 and regulate transcriptional and survival programs, suggesting that it may contribute to adaptive responses to BET inhibition.

### Aims

This study aimed to explore the role of CK2 in oncogenic transcription, inflammatory and immunomodulatory cytokine production, and resistance to BET inhibition in MCL.

### Methods

MCL cell lines were treated with CK2 inhibitors (CX-4945, SGC-CK2-1) or subjected to CK2 gene silencing alone and in combination with BETi (JQ-1, INCB054329). A JQ-1-resistant MCL cell line was generated through exposure to increasing concentrations of JQ-1 until acquired resistance. Cell proliferation, apoptosis, gene and protein expression were evaluated by MTT assays, flow cytometry RT-qPCR and immunoblot analysis. Cytokine and chemokine secretion was evaluated using a human cytokine array kit with validation through RT-qPCR. Publicly available transcriptomic datasets were analyzed to explore inflammatory signatures.

**Copyright Information:** (Online) ISSN: 2572-9241
©2026 The Author(s). HemaSphere is published by John Wiley & Sons Ltd on behalf of European Hematology Association. This is an open access Abstract Book distributed under the Attribution-NonCommercial-NoDerivs (CC BY-NC-ND), which allows third parties to download the articles and share them with others as long as they credit the author and the Abstract Book, but they cannot change the content in any way or use them commercially.
**Abstract Book Citations:** Authors, Title, HemaSphere, 2026;10:(S1):pages. The abstract book DOIs can be found on the "Author Information" page.
**Disclaimer:** Articles published in the journal HemaSphere exclusively reflect the opinions of the authors. The authors are responsible for all content in their abstracts including accuracy of the facts, statements, citing resources, etc.

**Results**

CK2 inhibition or silencing in combination with BETi significantly downregulated c-Myc expression in MCL cells. This effect was associated with reduced expression of two c-Myc targets with immunosuppressive function, IL-10 and PD-L1. Cytokine profiling revealed that CK2 promotes the production of pro-inflammatory (IL-1β, TNFα, OPN and MIF) as well as immunosuppressive cytokines (IL-10, CCL18 and HGF) mediators. Transcriptomic analysis of publicly available datasets identified upregulation of pro-inflammatory genes (IFNγ, CXCR4, TGFβ) in MCL patients B cells vs healthy individuals, increased CSNK2B in relapsed MCL patients vs newly diagnosed B cells and altered IL10 expression in bone marrow T cells from relapsed MCL patients as compared to newly diagnosed ones. Notably, BETi-resistant MCL cells displayed increased expression of CK2 subunits, together with upregulation of c-Myc and anti-apoptotic proteins. CK2 pharmacological inhibition reduced viability, induced apoptosis and restored sensitivity to BET inhibition, supporting its role in adaptive resistance.

**Summary/Conclusion**

These findings identify CK2 as a key driver of oncogenic transcription, inflammatory signaling, and adaptive resistance in MCL. CK2 inhibition disrupts tumor-promoting cytokine networks and restores sensitivity to BET inhibitors, supporting combination strategies aimed at enhancing therapeutic efficacy and overcoming drug resistance.

**Copyright Information:** (Online) ISSN: 2572-9241
©2026 The Author(s). HemaSphere is published by John Wiley & Sons Ltd on behalf of European Hematology Association. This is an open access Abstract Book distributed under the Attribution-NonCommercial-NoDerivs (CC BY-NC-ND), which allows third parties to download the articles and share them with others as long as they credit the author and the Abstract Book, but they cannot change the content in any way or use them commercially.
**Abstract Book Citations:** Authors, Title, HemaSphere, 2026;10:(S1):pages. The abstract book DOIs can be found on the "Author Information" page.
**Disclaimer:** Articles published in the journal HemaSphere exclusively reflect the opinions of the authors. The authors are responsible for all content in their abstracts including accuracy of the facts, statements, citing resources, etc.

### (PB3943) FASCIN AS A NOVEL POTENTIAL PROGNOSTIC BIOMARKER AND THERAPEUTIC TARGET IN T-CELL LYMPHOMA

**Topic:** 21. Lymphoma biology & translational research

María José Sánchez de Las Matas Garre[1], Dolores Gutiérrez-Meca Maestre*[2, 2], Andrea Sarabia-Pérez[3], Diogo Ribeiro[3], Ana Albaladejo-González[3], Asunción Beltrán Videla[1], Marta Romera Martinez[2], Fátima Póstigo-Corrales[1], María Dolores López-Abellán[1], Ana Tapia-Abellán[1, 4], Pablo Conesa-Zamora[1, 3], Ginés Luengo-Gil[1, 3], Ana María Hurtado López[3], Ana Belén Arroyo-Rodríguez[1, 3]

*[1]Servicio de Anatomía Patológica, Hospital General Universitario Santa Lucía, IMIB., Cartagena, Spain;[2]Servicio de Hematología, Hospital General Universitario Santa Lucía, Cartagena, Spain; [3]Universidad Católica San Antonio de Murcia (UCAM), Cartagena, Spain; [4]Grupo de Investigación en Inmunología, Departamento de Bioquímica y Biología Molecular B e Inmunología, Facultad de Medicina, Universidad de Murcia, Murcia, Spain;*

**Background**

T-cell lymphomas (TCL) are clinically heterogeneous malignancies with limited targeted options, underscoring the need for novel prognostic biomarkers and actionable therapeutic targets. Fascin (encoded by *FSCN1*) is an actin-bundling protein implicated in tumour cell migration, invasion, aggressiveness and chemotherapy resistance in several solid cancers, but remains poorly characterised in TCL. Fascin inhibition has shown anti-tumour activity in other models, supporting its evaluation in TCL.

**Aims**

To determine whether fascin associates with adverse outcome in T-cell lymphoma and represents a selective therapeutic vulnerability.

**Methods**

Fascin protein expression was assessed by immunohistochemistry (IHC) in FFPE diagnostic biopsies from 28 TCL patients, predominantly nodal TCL (mainly PTCL-NOS and AITL/TFH-type lymphomas), with occasional ALCL and T-lymphoblastic cases. Expression levels were correlated with overall survival (OS) using a predefined high/low cut-off. Public datasets (GSE94669, GSE250050) were analysed to compare *FSCN1* expression across lymphoma cell lines and to explore treatment resistance in T/NK lymphoma. Fascin protein levels in healthy donor T lymphocytes and selected T-cell neoplastic cell lines (Jurkat, T-ALL; SR-786, ALK+ ALCL) were confirmed by Western blot and IHC prior to functional viability assays. Cell viability was evaluated using an XTT assay following treatment with anti-fascin compounds (imipramine, a repurposed antidepressant, and NP-G2-044).

**Results**

Within our cohort, fascin expression was heterogeneous across TCL subtypes, without clear restriction to a specific histological entity. High fascin expression identified a subset of patients with inferior OS (HR=4.077; 95% CI 1.228-13.530; p=0.022). RNA-seq data from T/NK lymphoma suggested higher *FSCN1* expression in chemotherapy-resistant samples compared with treatment-sensitive cases and healthy donors (GSE250050). In 46 lymphoma cell lines (12 T-cell and 34 B-cell), *FSCN1* expression was significantly higher in T-NHL compared with B-NHL (GSE94669; p<0.001), suggesting preferential upregulation in T-cell malignancies. Finally, healthy T lymphocytes showed minimal/absent fascin expression compared with malignant T-cell lines. Pharmacological fascin inhibition selectively reduced viability of neoplastic T-cell lines while sparing healthy T cells: IC50 values for imipramine and NP-G2-044 were 13 µM and 17 µM in Jurkat (T-ALL), and 70 µM and 75 µM in SR-786 (ALK+ ALCL), respectively.

**Copyright Information:** (Online) ISSN: 2572-9241
©2026 The Author(s). HemaSphere is published by John Wiley & Sons Ltd on behalf of European Hematology Association. This is an open access Abstract Book distributed under the Attribution-NonCommercial-NoDerivs (CC BY-NC-ND), which allows third parties to download the articles and share them with others as long as they credit the author and the Abstract Book, but they cannot change the content in any way or use them commercially.
**Abstract Book Citations:** Authors, Title, HemaSphere, 2026;10:(S1):pages. The abstract book DOIs can be found on the "Author Information" page.
**Disclaimer:** Articles published in the journal HemaSphere exclusively reflect the opinions of the authors. The authors are responsible for all content in their abstracts including accuracy of the facts, statements, citing resources, etc.

**Summary/Conclusion**

Converging transcriptomic, clinical, and functional evidence indicates that fascin is enriched in T-cell malignancies, associates with adverse outcome, and represents a potential therapeutic vulnerability. These findings support further validation and preclinical development of fascin-targeted strategies in TCL.

*This work was supported by a grant from the FEHH-SEHH and by Ministerio de Ciencia e Innovation through the grant Ramón (Grant RYC2022-037702-I, funded by MCIU/AEI/10.13039/501100011033 and by the ESF+).*

**Copyright Information:** (Online) ISSN: 2572-9241
©2026 The Author(s). HemaSphere is published by John Wiley & Sons Ltd on behalf of European Hematology Association. This is an open access Abstract Book distributed under the Attribution-NonCommercial-NoDerivs (CC BY-NC-ND), which allows third parties to download the articles and share them with others as long as they credit the author and the Abstract Book, but they cannot change the content in any way or use them commercially.
**Abstract Book Citations:** Authors, Title, HemaSphere, 2026;10:(S1):pages. The abstract book DOIs can be found on the "Author Information" page.
**Disclaimer:** Articles published in the journal HemaSphere exclusively reflect the opinions of the authors. The authors are responsible for all content in their abstracts including accuracy of the facts, statements, citing resources, etc.

### (PB3944) SHIKONIN TRIGGERS FERRITINOPHAGY-DRIVEN FERROPTOSIS IN DIFFUSE LARGE B-CELL LYMPHOMA VIA THE C-MYC-ADPGK-AS1-EIF4G1/MT2A AXIS

**Topic:** 21. Lymphoma biology & translational research

Can Chen*[1], Pengfei Shi[1], Zhenzhen Chen[1], Shenxian Qian[1]

[1]*Hangzhou First People's Hospital, Hangzhou, China;*

### Background

Diffuse large B-cell lymphoma (DLBCL) is an aggressive malignancy and novel therapeutic strategies are needed, particularly for refractory disease. Ferritinophagy releases iron from ferritin and can fuel ferroptosis, an iron-dependent form of regulated cell death driven by lipid peroxidation. Shikonin, a natural compound derived from Lithospermum erythrorhizon, has antitumor activity, but its mechanistic basis in DLBCL is not fully defined.

### Aims

To determine whether shikonin induces ferritinophagy and ferroptosis in DLBCL and to define the underlying regulatory network involving lncRNA ADPGK-AS1.

### Methods

Shikonin cytotoxicity was assessed in DLBCL cells using MTT assays. Ferroptosis dependency was evaluated using the ferroptosis inhibitor Liproxstatin-1 (Lip-1). Lipid peroxidation and ferroptosis-related readouts were measured by flow cytometry and biochemical kits. Ferritinophagy and ferroptosis markers were quantified by Western blot, qPCR, and immunofluorescence. Mechanistic studies included gene knockdown and overexpression, dual-luciferase reporter assays, RNA pulldown, proteomics, RNA binding protein immunoprecipitation (RIP), RNA FISH, and ChIP. In vivo validation was performed in a xenograft model.

### Results

Shikonin suppressed DLBCL cell growth and induced an iron metabolism and oxidative stress phenotype consistent with ferritinophagy activation and ferroptosis. Co-treatment with Lip-1 partially rescued shikonin-induced cytotoxicity and ferroptosis-associated changes, supporting ferroptosis as a functional component of shikonin activity. The lncRNA ADPGK-AS1 emerged as a key regulator of ferroptosis sensitivity in DLBCL. ADPGK-AS1 acted as a protective factor against ferroptosis by post-transcriptionally regulating MT2A and interacting with eIF4G1 to control MT2A translation in OCI-LY3 cells. Shikonin downregulated c-Myc, which in turn reduced ADPGK-AS1 expression. Forced c-Myc expression reversed shikonin-mediated ADPGK-AS1 downregulation. Antitumor effects and pathway modulation were confirmed in xenograft experiments.

### Summary/Conclusion

Shikonin induces ferritinophagy and ferroptosis in DLBCL through disruption of intracellular iron homeostasis and enhanced oxidative stress. This process is modulated by a c-Myc-regulated lncRNA axis in which ADPGK-AS1, via eIF4G1-dependent control of MT2A translation, attenuates ferroptosis. These findings clarify shikonin's mechanism of action in DLBCL and identify ADPGK-AS1 and the eIF4G1/MT2A node as potential therapeutic targets.

**Copyright Information:** (Online) ISSN: 2572-9241
©2026 The Author(s). HemaSphere is published by John Wiley & Sons Ltd on behalf of European Hematology Association. This is an open access Abstract Book distributed under the Attribution-NonCommercial-NoDerivs (CC BY-NC-ND), which allows third parties to download the articles and share them with others as long as they credit the author and the Abstract Book, but they cannot change the content in any way or use them commercially.
**Abstract Book Citations:** Authors, Title, HemaSphere, 2026;10:(S1):pages. The abstract book DOIs can be found on the "Author Information" page.
**Disclaimer:** Articles published in the journal HemaSphere exclusively reflect the opinions of the authors. The authors are responsible for all content in their abstracts including accuracy of the facts, statements, citing resources, etc.

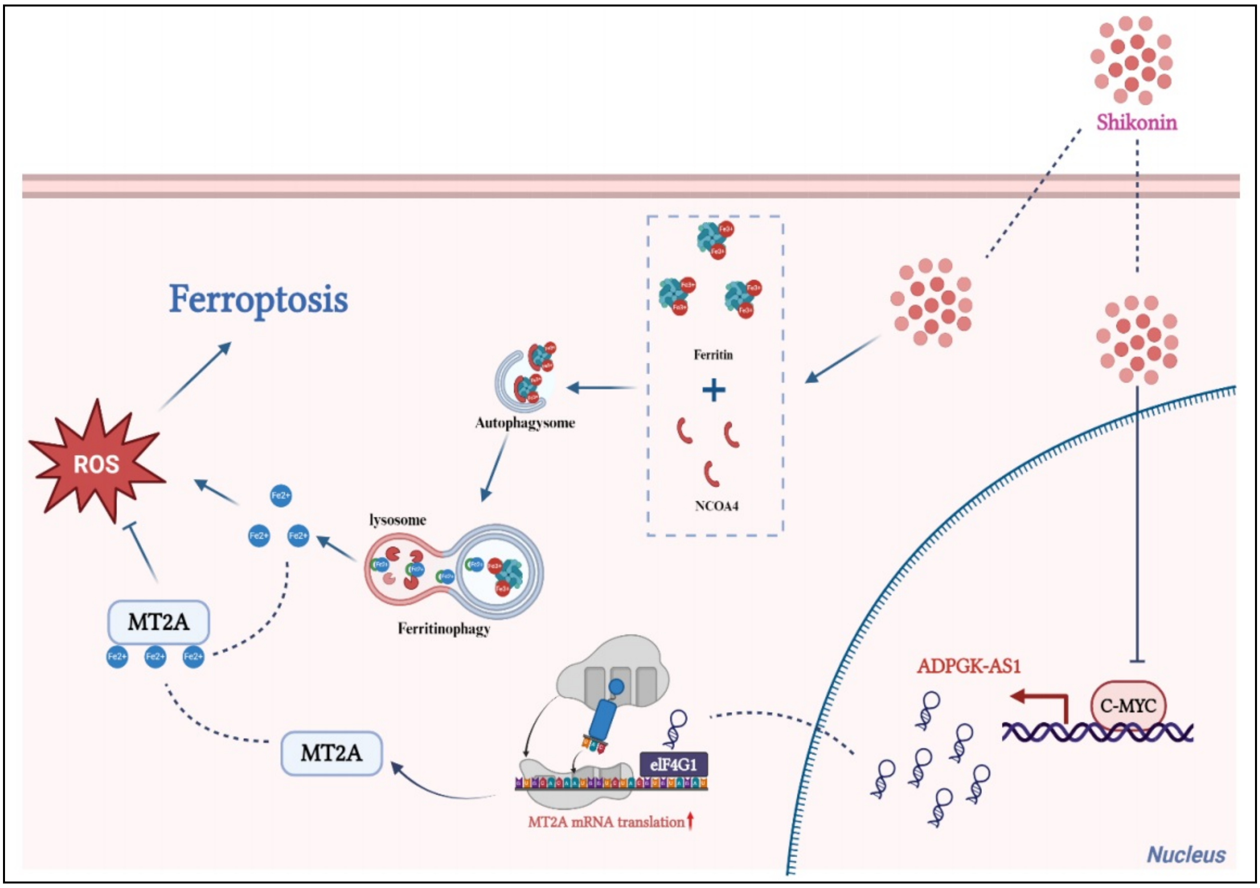

Proposed mechanism of shikonin-induced ferritinophagy and ferroptosis in diffuse large B-cell lymphoma. Shikonin promotes NCOA4-mediated ferritinophagy, in which ferritin is delivered to autophagosomes and subsequently degraded in lysosomes, resulting in increased release of ferrous iron ($Fe^{2+}$). The expanded labile iron pool enhances reactive oxygen species (ROS) accumulation and lipid peroxidation, thereby triggering ferroptotic cell death. In parallel, shikonin suppresses c-Myc, leading to downregulation of the long non-coding RNA ADPGK-AS1. ADPGK-AS1 interacts with eIF4G1 to facilitate MT2A mRNA translation. MT2A acts as an antioxidant and metal-binding protein that restrains iron-driven oxidative stress. Reduced ADPGK-AS1 expression decreases MT2A protein synthesis, amplifies ROS, and sensitizes DLBCL cells to ferroptosis. Solid arrows indicate activation or promotion. Blunt-ended lines indicate inhibition. Dashed lines indicate indirect regulation. Abbreviations: DLBCL, diffuse large B-cell lymphoma. NCOA4, nuclear receptor coactivator 4. ROS, reactive oxygen species. MT2A, metallothionein 2A. eIF4G1, eukaryotic translation initiation factor 4 gamma 1. lncRNA, long non-coding RNA.

**Copyright Information:** (Online) ISSN: 2572-9241
©2026 The Author(s). HemaSphere is published by John Wiley & Sons Ltd on behalf of European Hematology Association. This is an open access Abstract Book distributed under the Attribution-NonCommercial-NoDerivs (CC BY-NC-ND), which allows third parties to download the articles and share them with others as long as they credit the author and the Abstract Book, but they cannot change the content in any way or use them commercially.
**Abstract Book Citations:** Authors, Title, HemaSphere, 2026;10:(S1):pages. The abstract book DOIs can be found on the "Author Information" page.
**Disclaimer:** Articles published in the journal HemaSphere exclusively reflect the opinions of the authors. The authors are responsible for all content in their abstracts including accuracy of the facts, statements, citing resources, etc.

### (PB3946) PATIENT CELL EX VIVO DRUG PROFILING IDENTIFIES P53-LINKED VULNERABILITIES IN REFRACTORY LYMPHOID MALIGNANCIES

**Topic:** 21. Lymphoma biology & translational research

Arvid Cederlund*[1, 2], Nona Struyf[3, 4], Lucia Rico Pizarro[3, 4], Päivi Östling[3, 4], Kristina Sonnevi[1, 2], Björn Wahlin[1, 2], Tom Erkers[3, 4]

*[1]Karolinska Institute, Department of medicine huddinge, Stockholm, Sweden;[2]Karolinska University Hospital, ME Hematology, Stockholm, Sweden; [3]Karolinska Institute, Department of Oncology-Pathology, Stockholm, Sweden; [4]Science for life laboratory, Stockholm, Sweden;*

### Background

Aggressive lymphoid malignancies, ALL and lymphoma, exhibit poor prognosis when relapsed or refractory (R/R). Despite recent advances chemotherapy retains an important role for disease control and bridging therapy. However, inferior responses are common. Using functional precision-medicine approaches to tailor treatment might help to improve responses.

### Aims

To assess the feasibility of rapid-turnover functional drug testing on patient samples and explore patterns of drug sensitivity and resistance in R/R lymphoid malignancies.

### Methods

Malignant cell samples were obtained from adult patients with R/R aggressive lymphoma or ALL treated at Karolinska University Hospital. Malignant cells were isolated and underwent drug sensitivity and resistance testing (DSRT) using a library of 527 oncological drugs spanning 5 concentrations. DSRT incubation was for 24 hours following which cell viability was assessed using an ATP-based measurement and generating drug sensitivity scores (DSS). To control for general toxicity selective DSS (sDSS) were calculated by subtracting the DSS of healthy bone-marrow cells from the DSS of patient samples.

**Copyright Information:** (Online) ISSN: 2572-9241
©2026 The Author(s). HemaSphere is published by John Wiley & Sons Ltd on behalf of European Hematology Association. This is an open access Abstract Book distributed under the Attribution-NonCommercial-NoDerivs (CC BY-NC-ND), which allows third parties to download the articles and share them with others as long as they credit the author and the Abstract Book, but they cannot change the content in any way or use them commercially.
**Abstract Book Citations:** Authors, Title, HemaSphere, 2026;10:(S1):pages. The abstract book DOIs can be found on the "Author Information" page.
**Disclaimer:** Articles published in the journal HemaSphere exclusively reflect the opinions of the authors. The authors are responsible for all content in their abstracts including accuracy of the facts, statements, citing resources, etc.

### Results

Until 24 February 2026, 27 samples from 19 lymphoma and 7 ALL patients, with one ALL patient sampled twice, have been collected. Of 27 samples, five were excluded because of insufficient cell yields (n=3) or failed assay quality-control (n=2). Characteristics of patients are presented in Table 1.

| Characteristics | All included | Successfully analyzed |
|---|---|---|
| Age (mean, range) | 49.8 (18-74) | 50.7 (21-74) |
| Male (n, %) | 17 (63%) | 14 (64%) |
| B-cell lymphoma (n, %) | 13 (48%) | 10 (45%) |
| T-cell lymphoma (n, %) | 6 (22%) | 4 (18%) |
| Lymphoblastic disease (n, %) | 8 (30%)* | 8 (36%)* |
| Lines of therapy (median, range) | 4 (1-8) | 3.5 (1-8) |
| p53-aberrant (n, %) | 6 (22%) | 5 (23%) |

Table 1 *One ALL patient sampled twice.

*Ex vivo* drug sensitivity and resistance testing revealed low sDSS scores for commonly used chemotherapeutic agents including anthracyclines, platinum compounds and nucleoside analogues. Navitoclax, a BCL-2/BCL-xl inhibitor, demonstrated the most consistent sensitivity across samples. Venetoclax, a BCL-2 inhibitor, also demonstrated consistent sensitivity.

In this cohort of mixed lymphatic malignancies, few risk markers overlap. However, p53-aberrancy, defined as TP53 mutation, del(17p) or p53 overexpression, is a known adverse factor across diagnoses. Comparing p53-aberrant to p53 wild-type samples p53-aberrant samples demonstrated significantly higher sDSS for dasatinib (median sDSS 12.4 vs -3.2 SE=1.30) with every p53-aberrant sample demonstrating sensitivity regardless of diagnosis (DLBCL n = 2, T-LBL n = 1, MCL n = 1, Burkitt-like LBCL n = 1). Furthermore, we observed a trend towards increased sDSS for PI3K-inhibitors in p53-aberrant samples.

### Summary/Conclusion

We conclude that rapid turnover *ex vivo* DSRT is possible for ALL patients and most lymphoma patients with the main difficulty being acquiring an adequate malignant cell sample. Specific sensitivity to dasatinib in p53-aberrant samples has been reported in CLL but here it appears to transcend diagnosis and requires further investigation in lymphoid malignancies.

**Copyright Information:** (Online) ISSN: 2572-9241
©2026 The Author(s). HemaSphere is published by John Wiley & Sons Ltd on behalf of European Hematology Association. This is an open access Abstract Book distributed under the Attribution-NonCommercial-NoDerivs (CC BY-NC-ND), which allows third parties to download the articles and share them with others as long as they credit the author and the Abstract Book, but they cannot change the content in any way or use them commercially.
**Abstract Book Citations:** Authors, Title, HemaSphere, 2026;10:(S1):pages. The abstract book DOIs can be found on the "Author Information" page.
**Disclaimer:** Articles published in the journal HemaSphere exclusively reflect the opinions of the authors. The authors are responsible for all content in their abstracts including accuracy of the facts, statements, citing resources, etc.

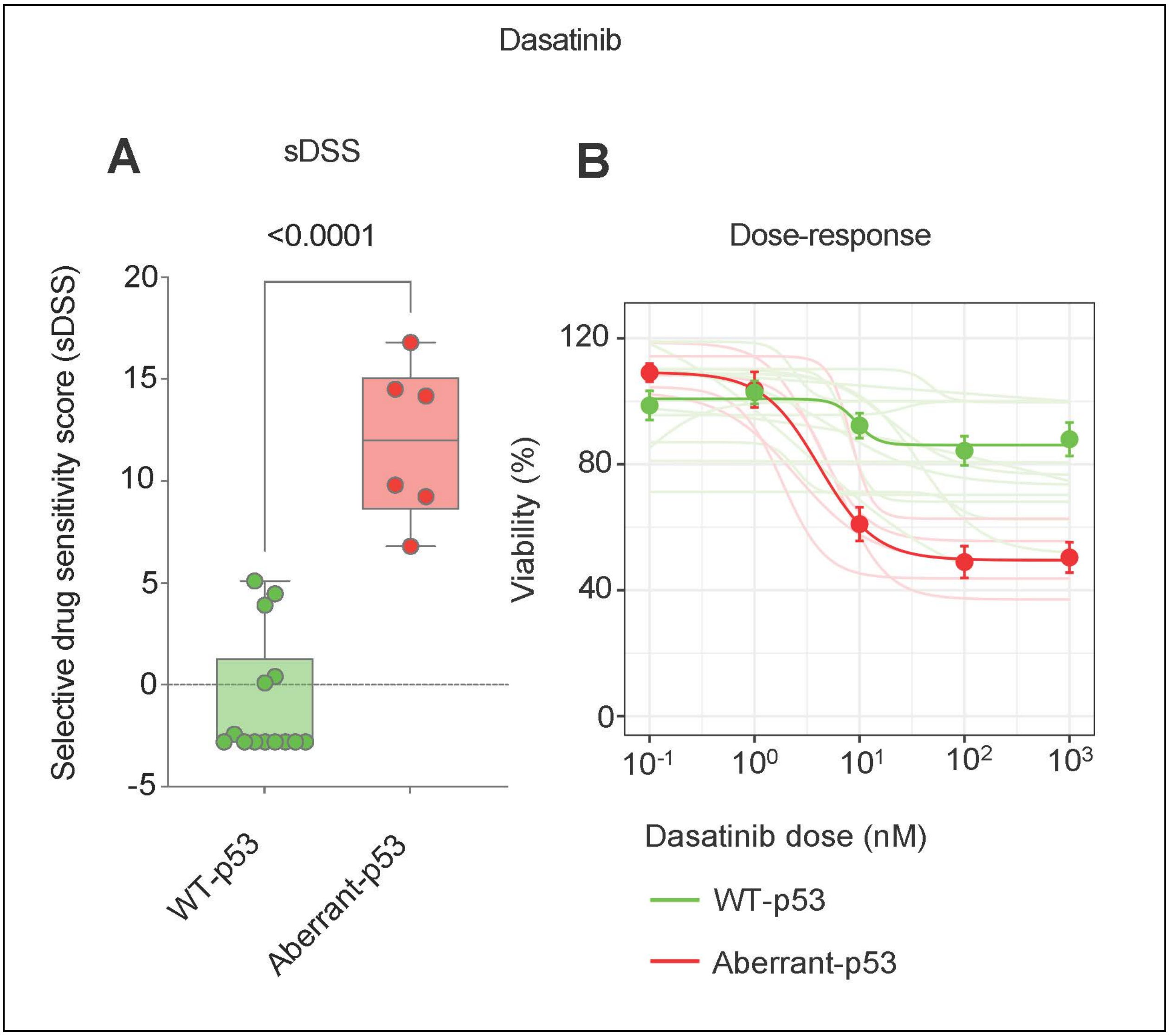

Figure 1 A: Box plot comparing selective drug sensitivity scores (sDSS) for dasatinib in p53-aberrant vs p53-wild type samples. B: Dose-response curves for dasatinib in p53-aberrant vs p53-wild type samples.

**Copyright Information:** (Online) ISSN: 2572-9241
©2026 The Author(s). HemaSphere is published by John Wiley & Sons Ltd on behalf of European Hematology Association. This is an open access Abstract Book distributed under the Attribution-NonCommercial-NoDerivs (CC BY-NC-ND), which allows third parties to download the articles and share them with others as long as they credit the author and the Abstract Book, but they cannot change the content in any way or use them commercially.
**Abstract Book Citations:** Authors, Title, HemaSphere, 2026;10:(S1):pages. The abstract book DOIs can be found on the "Author Information" page.
**Disclaimer:** Articles published in the journal HemaSphere exclusively reflect the opinions of the authors. The authors are responsible for all content in their abstracts including accuracy of the facts, statements, citing resources, etc.

### (PB3947) RED BLOOD CELL DISTRIBUTION WIDTH (RDW) ASSOCIATES WITH INFLAMMATORY COMPLICATIONS AFTER ANTI-CD19 CHIMERIC ANTIGEN RECEPTOR (CAR) T-CELL TREATMENT OF B-CELL LYMPHOMAS

**Topic:** 21. Lymphoma biology & translational research

Beatrice Berneck*[1, 2], David Reher[1, 2], Katharina Ruderich[1, 2], Georg-Nikolaus Franke[1, 2], Marco Herling[1, 2], Carmen Diana Herling[1, 2], Uwe Platzbecker[1, 2, 3], Klaus Metzeler[1, 2], Madlen Jentzsch[1, 2], Vladan Vucinic[1, 2, 4]

*[1]University Hospital Leipzig, Clinic and Policlinic for Hematology, Cell Therapy, Hemostaseology and Infectious Diseases, Leipzig, Germany; [2]Cancer Center Central Germany, Leipzig, Germany; [3]University Hospital Dresden, Dresden, Germany; [4]Fraunhofer Institute for Cellular Therapy and Immunology, Leipzig, Germany;*

### Background

Chimeric antigen receptor (CAR) T-cell therapy has revolutionized the treatment of patients (pts) with refractory or relapsed (r/r) B-cell lymphoma. However, CAR T-cell treatment can be associated with inflammatory complications, like cytokine release syndrome (CRS) and immune effector cell associated neurotoxicity syndrome (ICANS). Unfortunately, we still lack reliable and cost-effective biomarkers predicting efficacy or side-effects. The red blood cell distribution width (RDW) is part of automated routine output of blood cell counters. It describes the variation in size of red blood cells and has been studied as a marker of physiological stress and systemic inflammation.

### Aims

Here, we investigated the predictive value of RDW at lymphodepletion (LD) for CRS, ICANS, and treatment efficacy of CAR T cells as well as its association with laboratory-based clinical risk scores, and the development of clonal hematopoiesis (CH).

### Methods

This exploratory and retrospective study enrolled pts (≥ 18 years, n=90) with r/r large B-cell lymphoma (LBCL, n=78) or follicular lymphoma (FL, n=12) who received CAR T-cell therapy (tisagen-lecleucel [tisa-cel], n=38, axicabtagene ciloleucel [axi cel], n=48 und lisocabtagene maraleucel [liso-cel], n=4) from 02/2019 to 06/2025 at our center. CH was detected during post-CAR T follow up in 10/10 pts (CH+). We identified associations of RDW with several clinical scores by distance correlation (dCor) indicating non-independence and rank-based measures (Spearman's rho or Kendall's tau).

**Copyright Information:** (Online) ISSN: 2572-9241
©2026 The Author(s). HemaSphere is published by John Wiley & Sons Ltd on behalf of European Hematology Association. This is an open access Abstract Book distributed under the Attribution-NonCommercial-NoDerivs (CC BY-NC-ND), which allows third parties to download the articles and share them with others as long as they credit the author and the Abstract Book, but they cannot change the content in any way or use them commercially.
**Abstract Book Citations:** Authors, Title, HemaSphere, 2026;10:(S1):pages. The abstract book DOIs can be found on the "Author Information" page.
**Disclaimer:** Articles published in the journal HemaSphere exclusively reflect the opinions of the authors. The authors are responsible for all content in their abstracts including accuracy of the facts, statements, citing resources, etc.

**Results**

We found no evidence for differing RDW distributions at LD between LBCL and FL pts (median: 14.8 vs. 16.6; Wilcoxon-Mann-Whitney (WMW) p=0.57, estimated fold change (EFC): 0.97 [95% CI: 0.84-1.07]) and among CAR T products tisa-cel, axi-cel, and liso-cel (Kruskal-Wallis (KW) p=0.39, pairwise EFC: 0.90-1.10 [CIs range: 0.74-1.34]), as well as disease status at LD (medians for CR, PR, SD, PD: 14.5, 15.9, 14.7, 14.6 KW p=0.29, pairwise EFC: 0.92-1.09 [CIs range: 0.81-1.29]). Noteworthy, RDW weakly correlated with the CAR-HEMATOTOX score (dCor=0.12; Kendall's tau=0.27 [0.10-0.42], p=0.005) and the EASIX score (dCor=0.07; Spearman's rho=0.27 [0.05-0.46], p=0.013;). Pts with high CAR-HEMATOTOX score (≥2) tended to have higher RDW levels (WMW, p=0.048, EFC: 1.09 [1.00-1.18]). The ROC analysis determined an optimal cut-off at 17.5% for RDW, based on a comparison of the CRS vs. non-CRS group. Interestingly, lactate dehydrogenase, a marker of tumor burden, showed rather weak correlation with RDW (dCor=0.04; rho=0.23 [0.02-0.43], p=0.030), whereas hemoglobin showed a moderate negative correlation (dCor=0.23; rho=-0.52 [-0.66--0.34], p<0.0001). Higher RDW was associated with reduced PFS (median: 2.96 months) compared to low RDW pts (median: 9.72 months; log-rank (LR) p=0.013) and with shorter OS (LR p=0.04). CH+ pts without response to bridging showed higher RDW levels at apheresis (WMW p=0.017EFC: 1.37 [1.07-1.43]). Three of those 10 CH+ pts developed myeloid neoplasms during further follow-up.

**Summary/Conclusion**

Our analysis indicates an increased RDW has the potential to predict a higher risk of CRS, and associates with shorter PFS and OS, which would implicate to employ closer patient monitoring. The predictive role of RDW should be further explored in larger populations.

**Copyright Information:** (Online) ISSN: 2572-9241
©2026 The Author(s). HemaSphere is published by John Wiley & Sons Ltd on behalf of European Hematology Association. This is an open access Abstract Book distributed under the Attribution-NonCommercial-NoDerivs (CC BY-NC-ND), which allows third parties to download the articles and share them with others as long as they credit the author and the Abstract Book, but they cannot change the content in any way or use them commercially.
**Abstract Book Citations:** Authors, Title, HemaSphere, 2026;10:(S1):pages. The abstract book DOIs can be found on the "Author Information" page.
**Disclaimer:** Articles published in the journal HemaSphere exclusively reflect the opinions of the authors. The authors are responsible for all content in their abstracts including accuracy of the facts, statements, citing resources, etc.

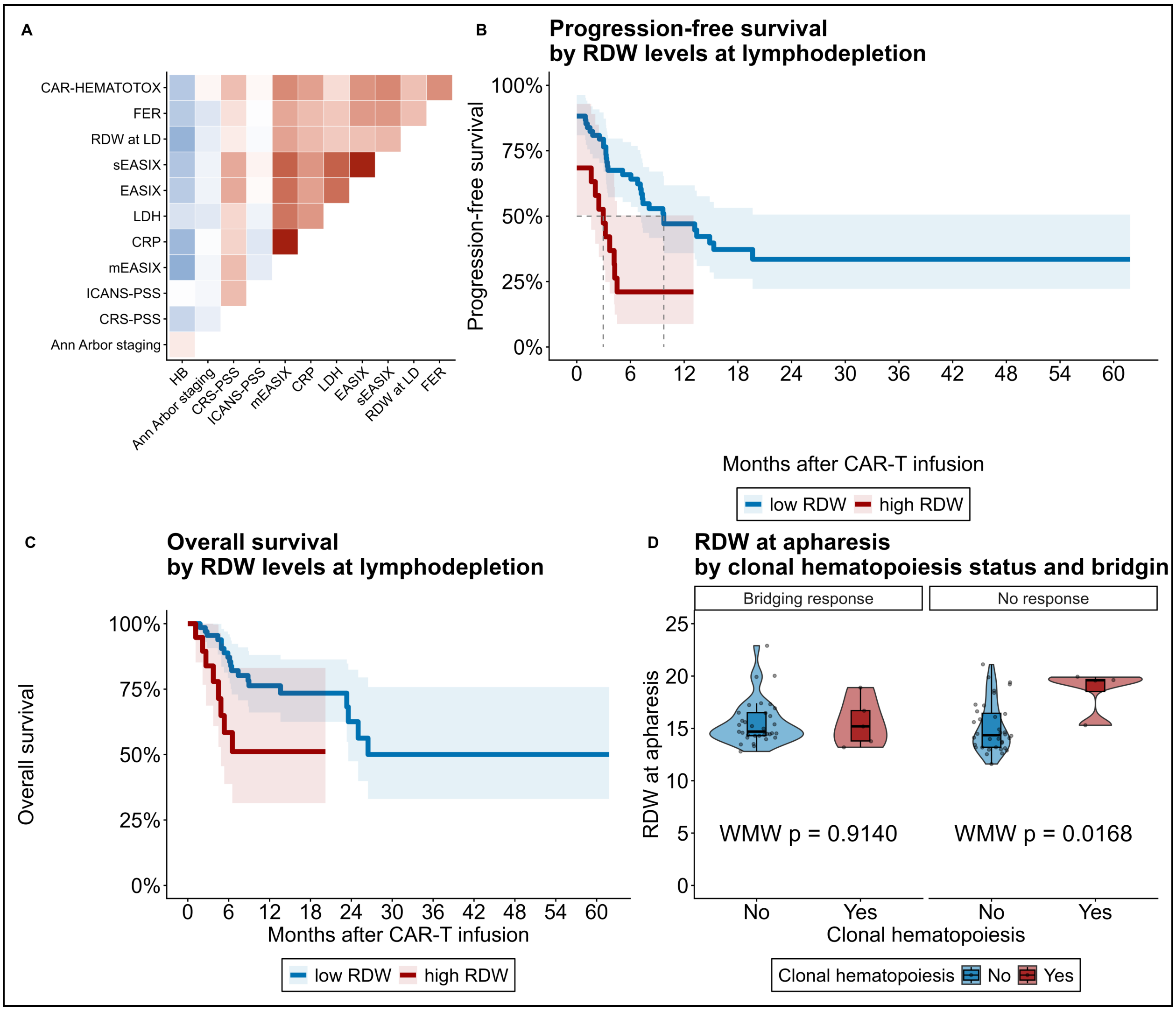

Fig.: A) Heatmap of correlations between RDW at LD and scores. B) PFS according to high vs. low RDW at LD. C) OS according to high vs. low RDW at LD. D) RDW at apheresis stratified by bridging response and CH status.

**Copyright Information:** (Online) ISSN: 2572-9241
©2026 The Author(s). HemaSphere is published by John Wiley & Sons Ltd on behalf of European Hematology Association. This is an open access Abstract Book distributed under the Attribution-NonCommercial-NoDerivs (CC BY-NC-ND), which allows third parties to download the articles and share them with others as long as they credit the author and the Abstract Book, but they cannot change the content in any way or use them commercially.
**Abstract Book Citations:** Authors, Title, HemaSphere, 2026;10:(S1):pages. The abstract book DOIs can be found on the "Author Information" page.
**Disclaimer:** Articles published in the journal HemaSphere exclusively reflect the opinions of the authors. The authors are responsible for all content in their abstracts including accuracy of the facts, statements, citing resources, etc.

### (PB3948) DLBCLASS-DEFINED MOLECULAR SUBSETS OF HEPATITIS C VIRUS-ASSOCIATED DIFFUSE LARGE B-CELL LYMPHOMAS

**Topic:** 21. Lymphoma biology & translational research

Michele Merli*[1], Silvia Zibellini[2], Roberta Sciarra[2], Marco Lucioni[3, 4], Roberta Riboni[4], Daniela Furlan[5], Francesca Magnoli[6], Benedetta Bianchi[7], Caterina Zerbi[2], Manuel Gotti[2], Virginia Valeria Ferretti[8], Chiara Varraso[2], Laura Libera[6], Alessandro Bosi[1], Cecilia Anna Fidanza[1], Alessandro Mazzacane[2], Martha Berliner[2], Giuseppe Neri[4], Andrea Ferrario[7], Ettore Rizzo[9], Federica De Paoli[9], Stefano La Rosa[6, 10], Marta Coscia[7, 11], Francesco Passamonti[1, 12], Marco Paulli[3, 4], Luca Arcaini[2, 3]

*[1]Fondazione IRCCS Ca' Granda, Ospedale Maggiore Policlinico, Hematology, Milan, Italy; [2]Fondazione IRCCS Policlinico San Matteo, Hematology, Pavia, Italy; [3]University of Pavia, Molecular Medicine, Pavia, Italy; [4]Fondazione IRCCS Policlinico San Matteo, Anatomic Pathology, Pavia, Italy; [5]University of Insubria, Anatomic Pathology, Varese, Italy; [6]Ospedale di Circolo e Fondazione Macchi - ASST Sette Laghi, Anatomic Pathology, Varese, Italy; [7]Ospedale di Circolo e Fondazione Macchi - ASST Sette Laghi, Hematology, Varese, Italy; [8]Fondazione IRCCS Policlinico San Matteo, Biostatistics and Clinical Trial Center, Pavia, Italy; [9]enGenome, Pavia, Italy; [10]University of Insubria, Medicine and Technology innoviation, Varese, Italy; [11]University of Insubria, Medicine and Surgery, Varese, Italy; [12]University of Milan, Oncology and Hemato-Oncology, Milan, Italy;*

### Background

The genetic landscape of hepatitis C virus (HCV)-associated diffuse large B-cell lymphoma (DLBCL) has recently been thoroughly characterized in a series of 54 patients, including FISH, gene-expression profiling with the Lymph2Cx assay, and next-generation sequencing (NGS) using a 184-gene panel. *LymphGen* cluster analysis classified 54% of cases into specific subsets, with BN2 being the most common subgroup (48% of classified tumors), indicating a preferential marginal zone origin (Sciarra et al, BJH 2024). Recently, a new neural network-based molecular classifier, DLB*class*, was shown to assign nearly all tumors to their respective clusters (C1-C5) (Chapuy et al, Blood 2025).

### Aims

We applied the DLB*class* classifier to our series of HCV-associated DLBCL to evaluate its classification performance compared to *LymphGen* in this setting and to explore the prognostic impact of different clusters.

### Methods

The web-based DLB*class* tool was tested using our series' NGS results to assign each case to a specific cluster with a confidence value. The high-accuracy threshold was set at >0.7. C1-C5 subgroups were compared with *LymphGen*-defined clusters and analyzed for overall survival (OS) and progression-free survival (PFS).

**Copyright Information:** (Online) ISSN: 2572-9241
©2026 The Author(s). HemaSphere is published by John Wiley & Sons Ltd on behalf of European Hematology Association. This is an open access Abstract Book distributed under the Attribution-NonCommercial-NoDerivs (CC BY-NC-ND), which allows third parties to download the articles and share them with others as long as they credit the author and the Abstract Book, but they cannot change the content in any way or use them commercially.
**Abstract Book Citations:** Authors, Title, HemaSphere, 2026;10:(S1):pages. The abstract book DOIs can be found on the "Author Information" page.
**Disclaimer:** Articles published in the journal HemaSphere exclusively reflect the opinions of the authors. The authors are responsible for all content in their abstracts including accuracy of the facts, statements, citing resources, etc.

### Results

All 54 tumors were assigned to a DLB*class* cluster (33, 61%, with confidence >0.7), specifically 25 (46%) to C1, 1 (2%) to C2, 8 (15%) to C3, 11 (20%) to C4, and 9 (17%) to C5. Among the more frequent clusters, 76% of C1, 63% of C3, 45% of C4, and 44% of C5 were classified with confidence >0.7. C5 DLBCLs were enriched in ABC (71%), while C3 was enriched in GCB tumors (63%). C1 and C4 showed a mixed distribution of cell-of-origin. C1 cases were enriched by the coexistence of a marginal zone lymphoma (MZL) component (32%), spleen (44%), and extranodal involvement (36%). C1 DLBCLs were characterized by BCL6 translocations (84%) and activating mutations in NOTCH2 (32%) and NF-κB pathways (56%), thus resembling the defining genetic alterations of MZL.
A direct comparison between the DLB*class* and *LymphGen* assignments showed that 40% of C1 DLBCLs were classified as BN2, 38% of C3 DLBCLs as EZB, 45% of C4 tumors as ST2, and only 33% of C5 DLBCLs as MCD. Conversely, of the 25 cases classified as “other” by *LymphGen*, 12 (48%) were assigned to C1, 1 to C2, 5 to C3, 3 to C4, and 4 to C5, with the only “ambiguous” case (BN2/N1) classified as C1 (Figure 1). Overall, despite a small number of cases, DLB*class* subgroups showed a borderline difference in 2-year OS (p=0.078), with better outcomes in C1 (76%) and C4 (68%) patients, and worse outcomes in C5 (48%) and C3 (33%) patients. Additionally, similar to the original HCV-negative DLB*class* series, among ABC-enriched tumors, 2-year PFS was better in C1 versus C5 patients (76% vs 33%, p=0.14), whereas in subtypes enriched in GCB tumors (C3 and C4), PFS was not significantly different (57% vs 64%, p=0.55). Finally, eradication of HCV using direct-acting antivirals (DAA) appeared to influence overall survival mainly in the C1 subgroup, with 10 treated patients exhibiting a 5-year OS of 86%, compared to 49% in the remaining ones (n=15, p=0.03).

### Summary/Conclusion

DLB*class* assigned all cases of HCV-associated DLBCL to a definitive cluster, classifying about 50% of these cases in the C1 group. This figure highlights a likely marginal zone origin and indicates a potential benefit from a molecularly targeted therapy approach (i.e. addition of BTK inhibitors) and from HCV eradication using DAA.

**Copyright Information:** (Online) ISSN: 2572-9241
©2026 The Author(s). HemaSphere is published by John Wiley & Sons Ltd on behalf of European Hematology Association. This is an open access Abstract Book distributed under the Attribution-NonCommercial-NoDerivs (CC BY-NC-ND), which allows third parties to download the articles and share them with others as long as they credit the author and the Abstract Book, but they cannot change the content in any way or use them commercially.
**Abstract Book Citations:** Authors, Title, HemaSphere, 2026;10:(S1):pages. The abstract book DOIs can be found on the “Author Information” page.
**Disclaimer:** Articles published in the journal HemaSphere exclusively reflect the opinions of the authors. The authors are responsible for all content in their abstracts including accuracy of the facts, statements, citing resources, etc.

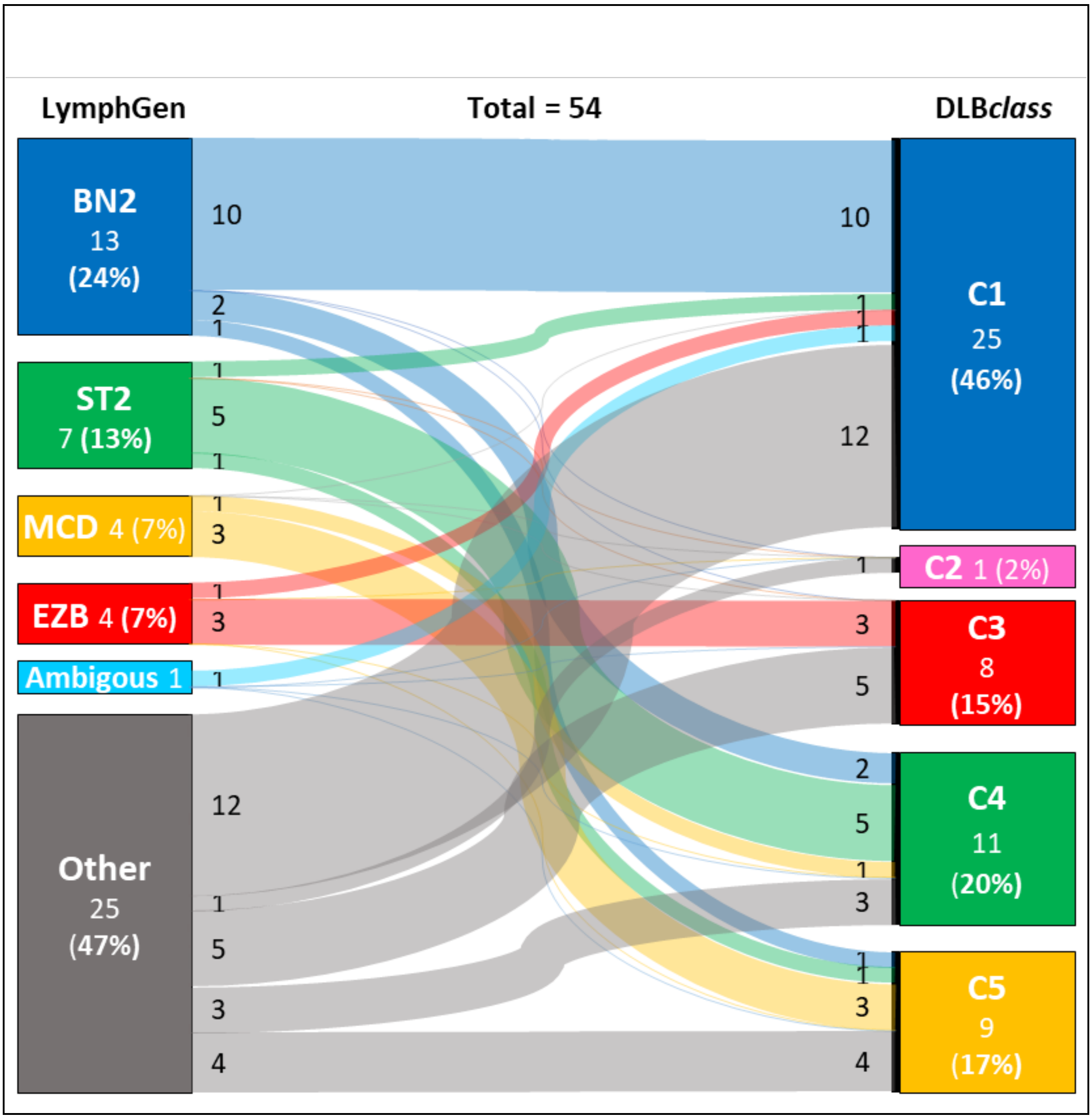

Figure 1. DLBclass and LymphGen classification of 54 patients with HCV-associated diffuse large B-cell lymphoma.

**Copyright Information:** (Online) ISSN: 2572-9241
©2026 The Author(s). HemaSphere is published by John Wiley & Sons Ltd on behalf of European Hematology Association. This is an open access Abstract Book distributed under the Attribution-NonCommercial-NoDerivs (CC BY-NC-ND), which allows third parties to download the articles and share them with others as long as they credit the author and the Abstract Book, but they cannot change the content in any way or use them commercially.
**Abstract Book Citations:** Authors, Title, HemaSphere, 2026;10:(S1):pages. The abstract book DOIs can be found on the "Author Information" page.
**Disclaimer:** Articles published in the journal HemaSphere exclusively reflect the opinions of the authors. The authors are responsible for all content in their abstracts including accuracy of the facts, statements, citing resources, etc.

### (PB3949) NF-KB–DRIVEN B-CELL LYMPHOMAS: DISTINCT INFLUENCE OF REL AND MYD88 ON THE CELLULAR TRAJECTORY LEADING TO B-CELL TRANSFORMATION

**Topic:** 21. Lymphoma biology & translational research

Dilara Sensoy*[1], Jean Feuillard[2], Christelle Vincent-Fabert[1], Nathalie Faumont[1]

*[1]Contrôle de la Réponse Immune B et Lymphoproliférations (CRIBL) UMR CNRS 7276 INSERM 1262 - Team 2MB2C, Haute-Vienne, Limoges, France; [2]Contrôle de la Réponse Immune B et Lymphoproliférations (CRIBL) UMR CNRS 7276 INSERM 1262 - Team 2MB2C, University Hospital Center and Faculty of Medicine of Limoges, Haute-Vienne, Limoges, France;*

### Background

B lymphocytes are particularly susceptible to malignant transformation, which is closely linked to the dynamic processes of the germinal center (GC) reaction and the integration of signals from the B-cell receptor (BCR) and the innate immune system. The BCR is a key activator of the canonical NF-κB pathway, playing a pivotal role in B-cell survival and clonal selection during peripheral B-cell development.

In diffuse large B-cell lymphomas (DLBCL), distinct genetic alterations converge towards the aberrant and sustained activation of NF-κB. Gains and amplifications of the *REL* gene locus, within the 2p15–16.1 region, are preferentially associated with germinal center B-cell–like DLBCLs (GCB-DLBCLs), whereas the MYD88.L265P mutation is predominantly found in post-GC lymphomas, including activated B-cell–like DLBCLs (ABC-DLBCLs) and Waldenström's macroglobulinemia.

### Aims

In light of this dichotomy, we hypothesized that the nature of the activated NF-κB module influences the cellular trajectory that drives B-cell transformation. This study aimed to investigate how the cooperation between BCR signalling and different NF-κB modules reshapes the signal hierarchy and determines the B-cell fate during and beyond the GC reaction.

### Methods

We used conditional mouse models that expressed REL-YFP fsf and MYD88.L252P-YFP fsf in B-cells via the Cre recombinase under the control of CD19 locus. Following antigenic immunization protocols with sheep red blood cells, B-cell populations are analyzed by flow cytometry to assess differentiation compartments and immunoglobulin isotype expression. Follicular architecture and GC organization are examined by immunofluorescence on spleen histological sections. fsf: floxed STOP cassette.

### Results

Analysis of B-cell differentiation trajectories revealed distinct reorganizations of B-cell compartments following immunization depending on the activated NF-κB module. Overexpression of *REL* is associated with an expansion of the follicular B-cell compartment and enhanced entry of B cells into GC. In contrast, constitutive MYD88 activation results in reduced GC participation and the accumulation of B-cells with an extrafollicular phenotype.

**Copyright Information:** (Online) ISSN: 2572-9241
©2026 The Author(s). HemaSphere is published by John Wiley & Sons Ltd on behalf of European Hematology Association. This is an open access Abstract Book distributed under the Attribution-NonCommercial-NoDerivs (CC BY-NC-ND), which allows third parties to download the articles and share them with others as long as they credit the author and the Abstract Book, but they cannot change the content in any way or use them commercially.
**Abstract Book Citations:** Authors, Title, HemaSphere, 2026;10:(S1):pages. The abstract book DOIs can be found on the "Author Information" page.
**Disclaimer:** Articles published in the journal HemaSphere exclusively reflect the opinions of the authors. The authors are responsible for all content in their abstracts including accuracy of the facts, statements, citing resources, etc.

**Summary/Conclusion**

These findings suggest that *REL* activation is closely linked to BCR signalling, promoting GC engagement and selection. Conversely, constitutive MYD88 activation appears to be partially independent of BCR signalling driving B-cells towards either extrafollicular differentiation through early cooperation with the BCR, or transient GC entry followed by premature exit via bypass of selection checkpoints.

Overall, the nature of the activated NF-κB module determines the temporal integration of BCR signals and critically influences B-cell fate and transformation trajectories.

**Copyright Information:** (Online) ISSN: 2572-9241
©2026 The Author(s). HemaSphere is published by John Wiley & Sons Ltd on behalf of European Hematology Association. This is an open access Abstract Book distributed under the Attribution-NonCommercial-NoDerivs (CC BY-NC-ND), which allows third parties to download the articles and share them with others as long as they credit the author and the Abstract Book, but they cannot change the content in any way or use them commercially.
**Abstract Book Citations:** Authors, Title, HemaSphere, 2026;10:(S1):pages. The abstract book DOIs can be found on the "Author Information" page.
**Disclaimer:** Articles published in the journal HemaSphere exclusively reflect the opinions of the authors. The authors are responsible for all content in their abstracts including accuracy of the facts, statements, citing resources, etc.

### (PB3950) DEEP 52-PARAMETER FLOW CYTOMETRY REVEALS PROFOUND PERIPHERAL T-CELL SENESCENCE, EXHAUSTION, AND DISTINCT MICROENVIRONMENTAL SIGNATURES IN RELAPSED/REFRACTORY AGGRESSIVE B-CELL LYMPHOMAS

**Topic:** 21. Lymphoma biology & translational research

Guoai Su[1], Ting Zhang[1], Yajing Zhang*[1]

[1]*Beijing GoBroad Boren Hospital, Beijing, China;*

### Background

The efficacy of advanced immunotherapies, including CAR-T cells and bispecific T-cell engagers, fundamentally depends on host immune fitness. Heavily pretreated patients with relapsed/refractory (R/R) aggressive B-cell lymphomas (primarily DLBCL) frequently fail autologous cellular therapies. While tumor-intrinsic escape mechanisms are well-documented, the high-resolution architecture of the systemic immune "microenvironment" remains insufficiently mapped. Understanding host immune dysfunction is pivotal for patient stratification and optimizing next-generation immuno-oncology sequences.

### Aims

To comprehensively profile the peripheral immune microenvironment in heavily pretreated R/R aggressive B-cell lymphoma patients using fine 52-parameter flow cytometry, aiming to identify predictive signatures of immune exhaustion, senescence, and treatment legacy.

### Methods

In an observational cohort study, from 2024.04 to 2026.02, peripheral blood from R/R aggressive B-cell lymphoma patients (N=42, modeled to include the representative 9 distinct phenotypes analyzed) failing ≥2 prior lines (including targeted/CAR-T therapies) was evaluated. A customized 52-color multiparametric flow cytometry panel was utilized. The panel comprehensively quantified T-cell memory subsets (Naive, Tcm, Tem, TEMRA), exhaustion markers (PD-1, TIM-3), Treg subsets, B-cell maturation, NK cell functional stages, and monocyte classifications.

### Results

Heavily pretreated R/R aggressive B-cell lymphoma patients harbor a structurally devastated and highly heterogeneous peripheral immune system. The predominant signature is irreversible T-cell senescence (massive TEMRA accumulation), profound naive T-cell loss, and high PD-1/TIM-3 exhaustion burden, presenting a severely hostile environment for autologous cellular therapies. High-dimensional peripheral immune phenotyping acts as a critical "liquid biopsy for immune fitness," emphasizing the urgent need for pre-treatment immune-reconditioning, use of allogeneic off-the-shelf platforms, or combination strategies with dual checkpoint blockades in this refractory population.

**Copyright Information:** (Online) ISSN: 2572-9241
©2026 The Author(s). HemaSphere is published by John Wiley & Sons Ltd on behalf of European Hematology Association. This is an open access Abstract Book distributed under the Attribution-NonCommercial-NoDerivs (CC BY-NC-ND), which allows third parties to download the articles and share them with others as long as they credit the author and the Abstract Book, but they cannot change the content in any way or use them commercially.
**Abstract Book Citations:** Authors, Title, HemaSphere, 2026;10:(S1):pages. The abstract book DOIs can be found on the "Author Information" page.
**Disclaimer:** Articles published in the journal HemaSphere exclusively reflect the opinions of the authors. The authors are responsible for all content in their abstracts including accuracy of the facts, statements, citing resources, etc.

**Summary/Conclusion**

Heavily pretreated R/R aggressive B-cell lymphoma patients harbor a structurally devastated and highly heterogeneous peripheral immune system. The predominant signature is irreversible T-cell senescence (massive TEMRA accumulation), profound naive T-cell loss, and high PD-1/TIM-3 exhaustion burden, presenting a severely hostile environment for autologous cellular therapies. High-dimensional peripheral immune phenotyping acts as a critical "liquid biopsy for immune fitness," emphasizing the urgent need for pre-treatment immune-reconditioning, use of allogeneic off-the-shelf platforms, or combination strategies with dual checkpoint blockades in this refractory population.

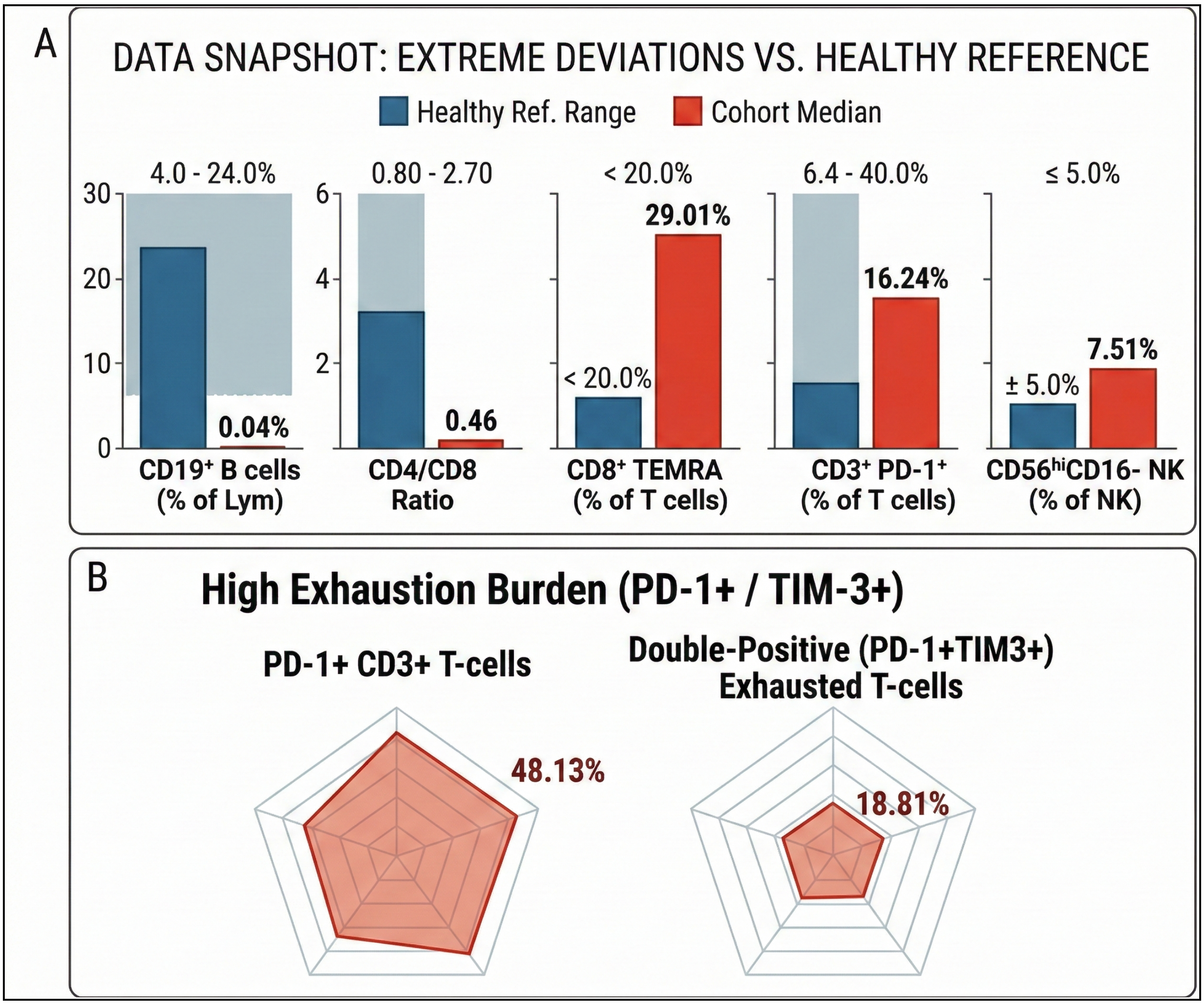

Profound structural deviation and terminal T-cell exhaustion signature in the peripheral immune microenvironment of R/R aggressive B-cell lymphomas.

**Copyright Information:** (Online) ISSN: 2572-9241
©2026 The Author(s). HemaSphere is published by John Wiley & Sons Ltd on behalf of European Hematology Association. This is an open access Abstract Book distributed under the Attribution-NonCommercial-NoDerivs (CC BY-NC-ND), which allows third parties to download the articles and share them with others as long as they credit the author and the Abstract Book, but they cannot change the content in any way or use them commercially.
**Abstract Book Citations:** Authors, Title, HemaSphere, 2026;10:(S1):pages. The abstract book DOIs can be found on the "Author Information" page.
**Disclaimer:** Articles published in the journal HemaSphere exclusively reflect the opinions of the authors. The authors are responsible for all content in their abstracts including accuracy of the facts, statements, citing resources, etc.

### (PB3951) CXCL8+ TUMOR-ASSOCIATED MACROPHAGES PROMOTE THE PROGRESSION OF EPSTEIN-BARR VIRUS-POSITIVE DIFFUSE LARGE B-CELL LYMPHOMA BY ANGIOGENESIS

**Topic:** 21. Lymphoma biology & translational research

Yan-Li Li*[1], Li Yan[2], Feng-Ya Nan[1], Hui-Ping Wang[2], Zhimin Zhai[2]

*[1]Department of Pathology, School of Basic Medical Sciences, Anhui Medical University, Hefei, Anhui, China;[2]Department of Hematology/Hematologic Diseases Research Center, The Second Affiliated Hospital of Anhui Medical University/Anhui Medical University, Hefei, Anhui, China;*

### Background

Epstein-Barr virus-positive (EBV+) diffuse large B-cell lymphoma (DLBCL) represents a rare aggressive B-cell lymphoma subtype characterized by an adverse clinical outcome with limited treatment options. Tumor-associated macrophages (TAMs) constitute the major innate immune population in the tumor immune microenvironment (TIME) where they facilitate crucial regulatory processes. Although recent limited evidence indicates that the TAMs (especially M2-type) are associated with the tumor progression of lymphoma, its role in EBV+ DLBCL remains poorly understood.

### Aims

This study aimed to identify and validate the role and function of CXCL8 + TAMs in the malignant progression of EBV+ DLBCL.

### Methods

We applied single-cell RNA sequencing (scRNA-seq) analyses of formalin-fixed and paraffin-embedded (FFPE) samples from two EBV+ DLBCL patients and two Epstein-Barr virus-negative (EBV-) DLBCL patients to explore the specific macrophage subpopulations in TIME and multiplex immunofuorescence staining used to verify its presence in EBV+ DLBCL samples. Macrophages (Macs) were differentiated from THP-1 cells cultured with phorbol 12-myristate 13-acetate (PMA). We detected CXCL8 expression and secretion in Macs by qPCR and ELISA, respectively, and constructed the CXCL8-knockdown (CXCL8-KD), CXCL8-overexpressing (CXCL8-OE), and control (CXCL8-NC and CXCL8-Sh-NC) in Macs by lentiviral transduction according to the manufacturer's protocol. The proliferation, migration, apoptosis, angiogenesis and signaling pathway were detected by CCK8 test, transwell assay, flow cytometry (FC), the tube-forming ability of human umbilical vein endothelial cells (HUVECs) by an inverted microscope and Western blot (WB) analysis, respectively in vitro. The subcutaneous cotransplantation models in BALB/c nude mice were used to verify the function of CXCL8+ Macs in the progression of EBV+ DLBCL.

### Results

The scRNA-seq results of FFPE samples revealed that Macs were heterogeneous and the significant differences in the TIME between EBV+ DLBCL and EBV- DLBCL. We interestly discovered and identifed the specific CXCL8+ TAMs, which were almost exclusively existing in EBV+ DLBCL samples and simultaneously expressed CD206 (M2-TAMs marker). CXCL8+ TAMs promote EBV+ DLBCL cell lines (Farage) cell proliferation, migration, anti-apoptosis ability and HUVECs angiogenesis in vitro. Mechanistically, we found that CXCL8+ TAMs promoted angiogenesis due to NF-kappa B signaling pathway activation. CXCL8 knockdown in TAMs or administration of CXCL8 neutralizing antibody was associated with decreased tumor growth, anti-angiogenesis and increased survival time in the xenograft model.

**Copyright Information:** (Online) ISSN: 2572-9241
©2026 The Author(s). HemaSphere is published by John Wiley & Sons Ltd on behalf of European Hematology Association. This is an open access Abstract Book distributed under the Attribution-NonCommercial-NoDerivs (CC BY-NC-ND), which allows third parties to download the articles and share them with others as long as they credit the author and the Abstract Book, but they cannot change the content in any way or use them commercially.
**Abstract Book Citations:** Authors, Title, HemaSphere, 2026;10:(S1):pages. The abstract book DOIs can be found on the "Author Information" page.
**Disclaimer:** Articles published in the journal HemaSphere exclusively reflect the opinions of the authors. The authors are responsible for all content in their abstracts including accuracy of the facts, statements, citing resources, etc.

**Summary/Conclusion**

Our study demonstrates that CXCL8+ TAMs plays an important role in the progression of EBV+ DLBCL by stimulating cell proliferation, migration, anti-apoptosis and angiogenesis. The inhibition of CXCL8 may, therefore, be a potential new target for anticancer therapy in EBV+ DLBCL.

**Copyright Information:** (Online) ISSN: 2572-9241
©2026 The Author(s). HemaSphere is published by John Wiley & Sons Ltd on behalf of European Hematology Association. This is an open access Abstract Book distributed under the Attribution-NonCommercial-NoDerivs (CC BY-NC-ND), which allows third parties to download the articles and share them with others as long as they credit the author and the Abstract Book, but they cannot change the content in any way or use them commercially.
**Abstract Book Citations:** Authors, Title, HemaSphere, 2026;10:(S1):pages. The abstract book DOIs can be found on the "Author Information" page.
**Disclaimer:** Articles published in the journal HemaSphere exclusively reflect the opinions of the authors. The authors are responsible for all content in their abstracts including accuracy of the facts, statements, citing resources, etc.

### (PB3952) EXOSOMAL BCYRN1 DRIVES IMMUNOSUPPRESSION IN NK/T-CELL LYMPHOMA BY TRIGGERING WNT/Β-CATENIN-MEDIATED M2 MACROPHAGE POLARIZATION

**Topic:** 21. Lymphoma biology & translational research

Lijie Zeng[1], Jia Guo[1], Liang Wang*[1]

[1]*Beijing Tongren Hospital, Capital Medical University, Beijing, China;*

### Background

Natural killer/T-cell lymphoma (NKTCL) is aggressive and frequently chemoresistant, partly due to an immunosuppressive tumor microenvironment (TME).

### Aims

Tumor-derived exosomes (TEXs) deliver lncRNAs that modulate TME dynamics; however, the role of exosomal lncRNA BCYRN1 in macrophage polarization and therapy resistance is unknown.

### Methods

*In vitro* and *in vivo* assays elucidated BCYRN1's functions and mechanisms in NKTCL progression (including RNA-seq, co-culture, flow cytometry, RIP, Western blot, ubiquitination assays, and xenograft models).

### Results

Serum exosomal BCYRN1 was significantly elevated in NKTCL patients. The NKTCL TME was enriched with M2-like tumor-associated macrophages (TAMs). NKTCL-derived TEXs delivered BCYRN1 to macrophages, inducing M2 polarization (increased CD206, IL-10, Arg1 and decreased CD80, IL-12, iNOS ) via activation of the Wnt/β-catenin pathway, specifically through Wnt1 upregulation. Mechanistically, BCYRN1 functioned as a modular scaffold, binding HNRNPK, HNRNPM, and PABPC1. HNRNPK and HNRNPM stabilized BCYRN1, while BCYRN1 enhanced PABPC1 stability via USP10-mediated deubiquitination. Stabilized PABPC1 bound and increased Wnt1 mRNA stability, amplifying Wnt/β-Catenin signaling. BCYRN1-induced M2 macrophages promoted tumor cell proliferation and conferred resistance to asparaginase and gemcitabine i*n vitro*, while accelerating tumor growth *in vivo.*

### Summary/Conclusion

NKTCL-derived exosomal lncRNA BCYRN1 orchestrates a scaffold-mediated signaling axis (HNRNPK/HNRNPM-BCYRN1-PABPC1/USP10-Wnt1) to drive macrophage M2 polarization. This BCYRN1-dependent immune reprogramming fosters chemoresistance and fuels NKTCL progression. Targeting this axis represents a promising strategy to overcome immune evasion and enhance therapeutic efficacy.

**Copyright Information:** (Online) ISSN: 2572-9241
©2026 The Author(s). HemaSphere is published by John Wiley & Sons Ltd on behalf of European Hematology Association. This is an open access Abstract Book distributed under the Attribution-NonCommercial-NoDerivs (CC BY-NC-ND), which allows third parties to download the articles and share them with others as long as they credit the author and the Abstract Book, but they cannot change the content in any way or use them commercially.
**Abstract Book Citations:** Authors, Title, HemaSphere, 2026;10:(S1):pages. The abstract book DOIs can be found on the "Author Information" page.
**Disclaimer:** Articles published in the journal HemaSphere exclusively reflect the opinions of the authors. The authors are responsible for all content in their abstracts including accuracy of the facts, statements, citing resources, etc.

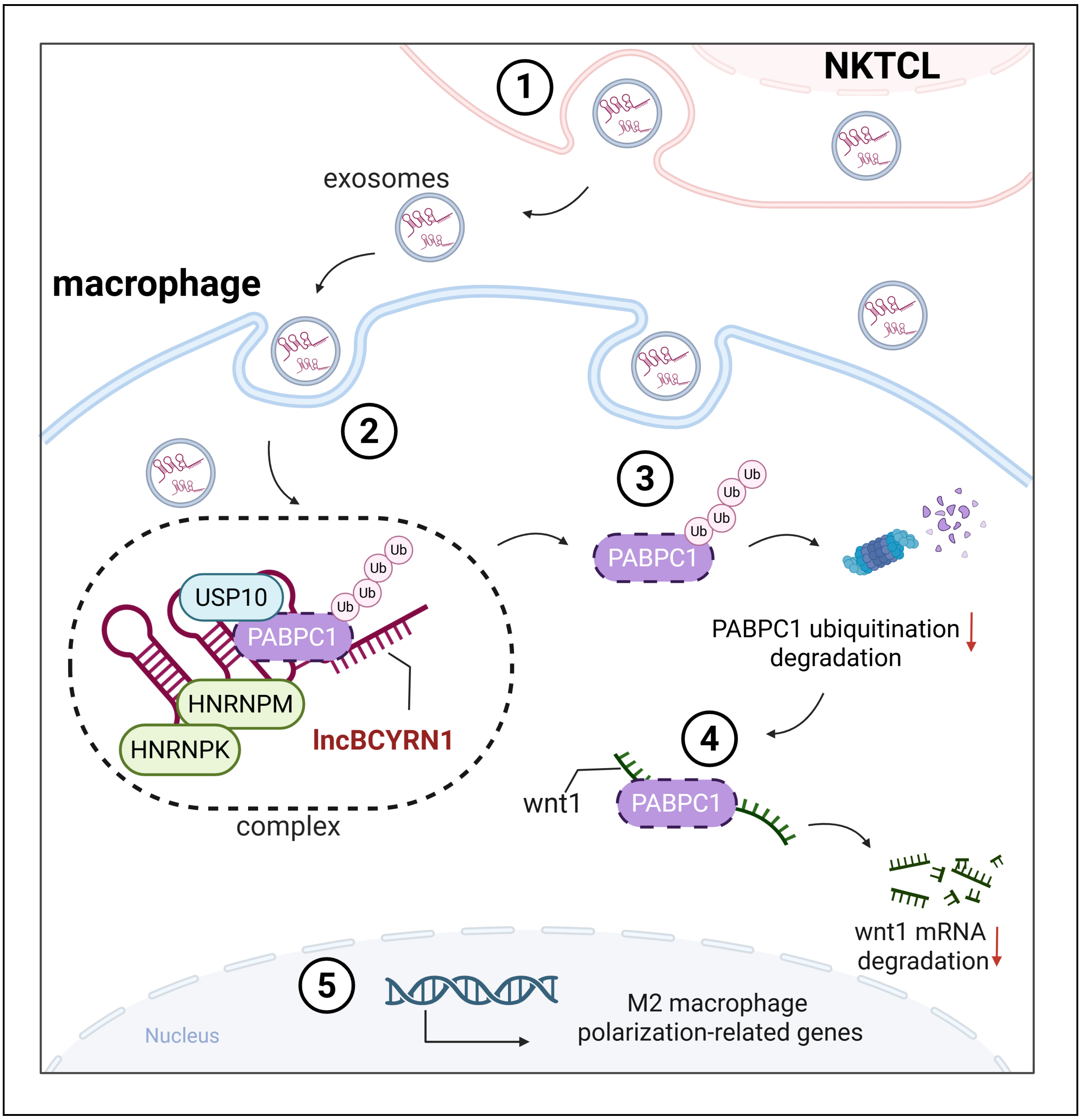

**Copyright Information:** (Online) ISSN: 2572-9241
©2026 The Author(s). HemaSphere is published by John Wiley & Sons Ltd on behalf of European Hematology Association. This is an open access Abstract Book distributed under the Attribution-NonCommercial-NoDerivs (CC BY-NC-ND), which allows third parties to download the articles and share them with others as long as they credit the author and the Abstract Book, but they cannot change the content in any way or use them commercially.
**Abstract Book Citations:** Authors, Title, HemaSphere, 2026;10:(S1):pages. The abstract book DOIs can be found on the "Author Information" page.
**Disclaimer:** Articles published in the journal HemaSphere exclusively reflect the opinions of the authors. The authors are responsible for all content in their abstracts including accuracy of the facts, statements, citing resources, etc.

### (PB3953) SINGLE-CELL RNA SEQUENCING ON FORMALIN-FIXED AND PARAFFIN-EMBEDDED (FFPE) TISSUE IDENTIFIED THE TUMOR MICROENVIRONMENT HETEROGENEITY IN EPSTEIN-BARR VIRUS-POSITIVE DIFFUSE LARGE B-CELL LYMPHOMA

**Topic:** 21. Lymphoma biology & translational research

Yan-Li Li*[1], Li Yan[2], Feng-Ya Nan[1], Hui-Ping Wang[2], Zhimin Zhai[2]

*[1]Department of Pathology, School of Basic Medical Sciences, Anhui Medical University, Hefei, Anhui, China;[2]Department of Hematology/Hematologic Diseases Research Center, The Second Affiliated Hospital of Anhui Medical University/Anhui Medical University, Hefei, Anhui, China;*

### Background

Epstein-Barr virus-positive (EBV+) diffuse large B-cell lymphoma (DLBCL) represents a rare aggressive B-cell lymphoma subtype characterized by an adverse clinical outcome with limited treatment options.The phenomenon potentially links to EBV infection-mediated remodeling of the tumor immune microenvironment (TIME) , but the single-cell RNA sequencing (scRNA-seq) on formalin-fixed and paraffin-embedded (FFPE) tissue to systematically map TIME heterogeneity in EBV+ DLBCL remains still poorly studied.

### Aims

This study aimed to characterize the TIME architecture of EBV+ DLBCL.

### Methods

For the Chromium Single Cell Gene Expression Flex (scFFPE-seq) workflow, 25μm FFPE curls were collected into a tube prior to serial sectioning for scFFPE-seq. These pooled 25μm curls (50 μm total) were treated as a single replicate. The samples were thawed and recounted. Single-cell library preparation was conducted following the manufacturer's protocol for the Chromium Fixed RNA Profiling Reagent Kits for Singleplexed Samples (CG000477 from 10× Genomics). The cells were loaded onto the Chromium X/iX to generate single-cell gel beads in emulsions (GEMs). Subsequently, the libraries were sequenced on a NovaSeq 6000 system (Illumina, San Diego, CA, USA) with an approximate 200 million reads per library.  Sequencing reads were aligned against reference transcriptome GRCh38 and unique molecule identifiers (UMIs) were quantified using Cell Ranger, version 7.1.0 (10× Genomics). Subsequent analyses were performed using R (version 4.3.2) and the Seurat package (version 5.0.2). Identification of cell types and subtypes by nonlinear dimensional reduction (t-SNE).

### Results

Using scRNA-seq on FFPE samples from two EBV+ DLBCL patients and two Epstein-Barr virus-negative (EBV-) DLBCL patients based on 50,934 cells, constructed a high-resolution panoramic map of the TIME, encompassing six distinct cell types. Differential analysis between the two groups revealed a decreased proportion of macrophage populations in EBV+ DLBCL, while the proportion of T/NK cells was significantly elevated. Further analysis of macrophage subpopulations revealed that CXCL8+PD-L1+CD206+ macrophage subtype was specifically present in EBV+ DLBCL. Functional enrichment analysis indicated that this subtype exhibited significant pro-angiogenic activity and possibly participated in immunosuppression, potentially representing a key mechanism underpinning the stronger aggressiveness of EBV+ DLBCL. Analysis of T cell subsets revealed that CD8+ T cells in EBV+ DLBCL predominantly enriched in the late functional stages, whereas CD8+ T cells in EBV- DLBCL were primarily enriched in early stages. This suggested EBV infection may promote tumor progression by inducing CD8+ T cell exhaustion. Additionally, functional enrichment analysis indicated that endothelial cells exhibited positive regulation of cell-cell adhesion and response to hypoxia in EBV+ DLBCL.

**Copyright Information:** (Online) ISSN: 2572-9241
©2026 The Author(s). HemaSphere is published by John Wiley & Sons Ltd on behalf of European Hematology Association. This is an open access Abstract Book distributed under the Attribution-NonCommercial-NoDerivs (CC BY-NC-ND), which allows third parties to download the articles and share them with others as long as they credit the author and the Abstract Book, but they cannot change the content in any way or use them commercially.
**Abstract Book Citations:** Authors, Title, HemaSphere, 2026;10:(S1):pages. The abstract book DOIs can be found on the "Author Information" page.
**Disclaimer:** Articles published in the journal HemaSphere exclusively reflect the opinions of the authors. The authors are responsible for all content in their abstracts including accuracy of the facts, statements, citing resources, etc.

**Summary/Conclusion**

This study provides the first evidence from FFPE samples of the TIME heterogeneity in EBV+ DLBCL, providing insights into the novel immunotherapy strategies for these patients.

**Copyright Information:** (Online) ISSN: 2572-9241
©2026 The Author(s). HemaSphere is published by John Wiley & Sons Ltd on behalf of European Hematology Association. This is an open access Abstract Book distributed under the Attribution-NonCommercial-NoDerivs (CC BY-NC-ND), which allows third parties to download the articles and share them with others as long as they credit the author and the Abstract Book, but they cannot change the content in any way or use them commercially.
**Abstract Book Citations:** Authors, Title, HemaSphere, 2026;10:(S1):pages. The abstract book DOIs can be found on the "Author Information" page.
**Disclaimer:** Articles published in the journal HemaSphere exclusively reflect the opinions of the authors. The authors are responsible for all content in their abstracts including accuracy of the facts, statements, citing resources, etc.

### (PB3954) DISCOVERY OF CDK9/EZH2 INHIBITORS TO ENHANCE ANTI-PROLIFERATIVE ACTIVITIES FOR THE TREATMENT OF DLBCL

**Topic:** 21. Lymphoma biology & translational research

Yixue Han[1], Lina Tian[2], Yanfen Fang[3], Xin Li[2], Jian Ding[3], Yi Chen[3], Youhong Hu*[4]

*[1]Anhui University of Chinese Medicine, Hefei, China;[2]Bohai Rim Advanced Research Institute for Drug Discovery,, Yantai, China; [3]Shanghai Institute of Materia Medica, Chinese Academy of Sciences, Shanghai, China;[4]Bohai Rim Advanced Research Institute for Drug Discovery/Shanghai Institute of Materia Medica, Shandong Laboratory of Yantai Drug Discovery, Yantai, China;*

### Background

CDK9 is frequently dysregulated in various malignancies, including hematological cancers and solid tumors. Its inhibition impedes Ser2 phosphorylation on the RNA polymerase II CTD, leading to reduced expression of short-lived oncoproteins such as c-Myc and Mcl-1. This mechanism has been widely observed across multiple cancer types, making CDK9 a promising therapeutic target. In diffuse large B-cell lymphoma (DLBCL), CDK9 inhibition downregulates Mcl-1 but concurrently upregulates H3K27me3 levels. EZH2 inhibitors can reverse this increase in H3K27me3, and the combination shows synergistic antitumor effects. This interplay highlights the therapeutic potential of dual CDK9/EZH2 inhibition. Then, we previously synthesized a series of dual inhibitors targeting CDK9 and EZH2 by linking CDK9 inhibitors (KI-ARV-03/SNS-032) and EZH2 inhibitor (C24), and found compound D16 exhibited potent anti-proliferative activities in DLBCL and various solid tumor cells.

### Aims

To further explore the generality of this approach and to leverage the CDK9-EZH2 synergy, we designed and synthesized a novel series of dual-target CDK9/EZH2 inhibitors to enhance the anti-proliferative effect of DLBCL.

### Methods

Synthesis of Compounds; CDK9 Kinase and EZH2 Assay; Cell Viability Assay; Western blotting analysis of **A3**, **A5**, **A6**, **A8**, and **A9** in U-2932 (EZH2 wild type; wt) and KARPAS-422 (EZH2 mutant; mut) cells. (RNA pol Ⅱ, p-pol Ⅱ S2, c-Myc, and Mcl-1 are marker proteins of CDK9.); Effect of **A9** on apoptosis and DNA damage.

### Results

We initially employed linkers of varying chain lengths to connect the self-discovered CDK9 inhibitor **Hu754** and **EPZ6438** pharmacophores based on **D16**. Compounds **A1**-**A4** were synthesized and evaluated, which revealed that the compound with a two-carbon linker exhibited potent dual CDK9/EZH2 inhibitory activity. Subsequently, we explored cyclic linkers, yielding compounds **A6**-**A8**, and further shortened the linker to a single carbon atom to obtain **A5** and **A9**. All synthesized compounds retained dual CDK9/EZH2 inhibitory activity, with **A9** displaying the most potent cellular activity.
We selected several representative compounds (**A3**, **A5**, **A6**, **A8**, and **A9**) to investigate their effects on the CDK9 signaling pathway in DLBCL cells by the western blotting. The results showed that in U2932 and KARPAS-422 cells, following treatment with compound **A3** or **A9** at a concentration of 100 nM, the expression levels of RNA pol Ⅱ, p-pol Ⅱ S2, c-Myc, and Mcl-1 were all significantly downregulated, with this effect being particularly pronounced in KARPAS-422 cells. These findings suggest that compound **A9** enhances the function of CDK9 by binding to EZH2.
**A9** significantly increased the apoptosis rate and induced DNA damage in EZH2 wild-type cells (U-2932) compared to single-target inhibitors. The reduced expression of the DNA double-strand break repair protein Rad51 and the increased expression of γ-H2AX.

**Copyright Information:** (Online) ISSN: 2572-9241
©2026 The Author(s). HemaSphere is published by John Wiley & Sons Ltd on behalf of European Hematology Association. This is an open access Abstract Book distributed under the Attribution-NonCommercial-NoDerivs (CC BY-NC-ND), which allows third parties to download the articles and share them with others as long as they credit the author and the Abstract Book, but they cannot change the content in any way or use them commercially.
**Abstract Book Citations:** Authors, Title, HemaSphere, 2026;10:(S1):pages. The abstract book DOIs can be found on the "Author Information" page.
**Disclaimer:** Articles published in the journal HemaSphere exclusively reflect the opinions of the authors. The authors are responsible for all content in their abstracts including accuracy of the facts, statements, citing resources, etc.

**Summary/Conclusion**

The inherent on-target toxicity of CDK9 inhibitors leads to a narrow therapeutic window and limited efficacy as a monotherapy, thus significantly hampering the development of such targeted agents. As a result, no CDK9 inhibitor has yet received marketing approval. In recent years, dual-target inhibitors directed against CDK9 have gradually become a research focus. This work not only validates the pharmacophore fusion approach for designing dual-target inhibitors but also identifies **A9** as a promising lead compound for further development toward CDK9/EZH2-driven malignancies.

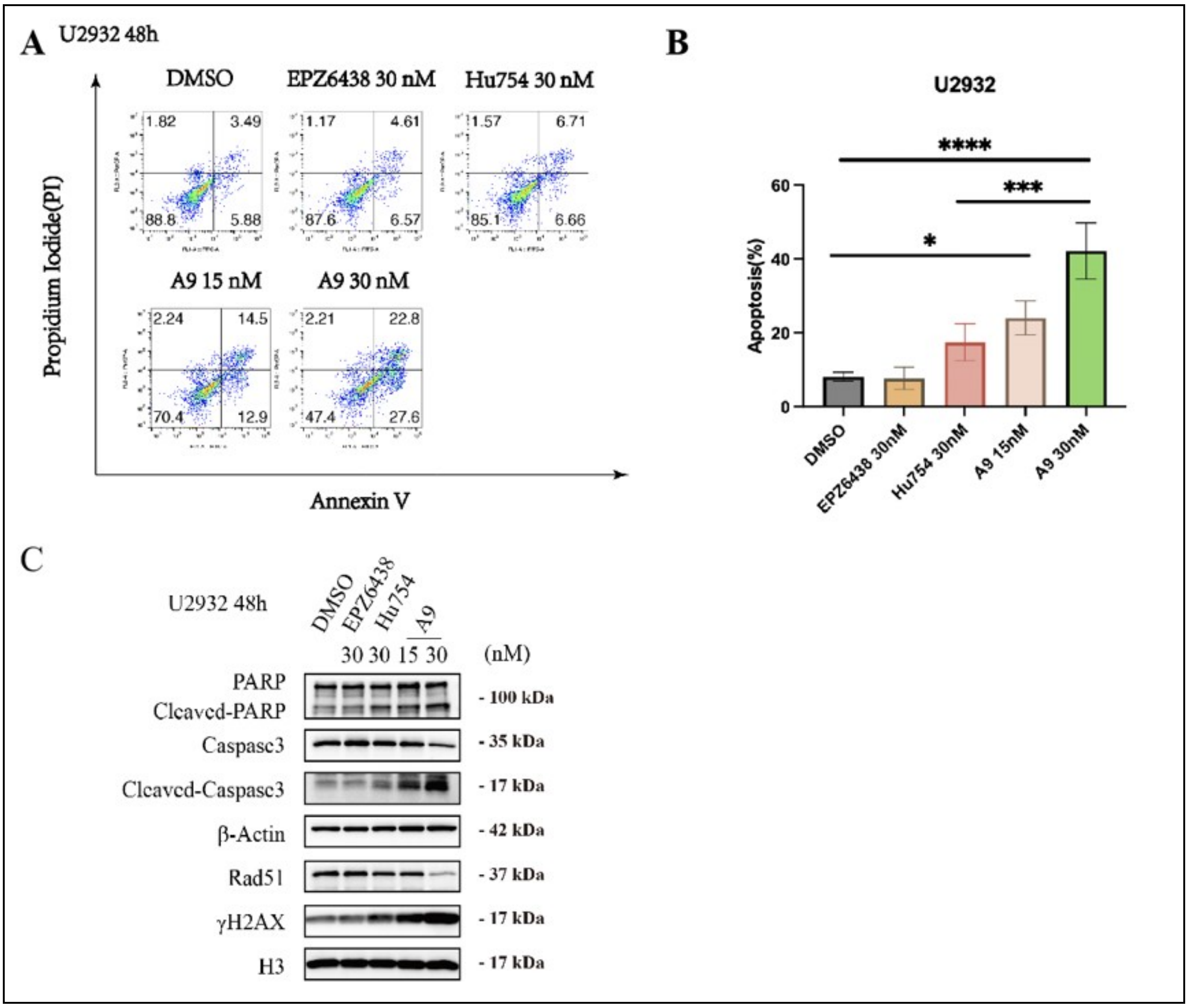

Effect of A9 on apoptosis and DNA damage. (A) U-2932 cells were treated with A9 for 48 hours, then collected and stained with Annexin V/PI for flow cytometry analysis. (B) Quantitative evaluation of apoptosis in U-2932 cells after 48 hours of A9 pretreatment. (C) Levels of apoptosis-related proteins in U-2932 cells following 48 hours of A9 pretreatment.

**Copyright Information:** (Online) ISSN: 2572-9241
©2026 The Author(s). HemaSphere is published by John Wiley & Sons Ltd on behalf of European Hematology Association. This is an open access Abstract Book distributed under the Attribution-NonCommercial-NoDerivs (CC BY-NC-ND), which allows third parties to download the articles and share them with others as long as they credit the author and the Abstract Book, but they cannot change the content in any way or use them commercially.
**Abstract Book Citations:** Authors, Title, HemaSphere, 2026;10:(S1):pages. The abstract book DOIs can be found on the "Author Information" page.
**Disclaimer:** Articles published in the journal HemaSphere exclusively reflect the opinions of the authors. The authors are responsible for all content in their abstracts including accuracy of the facts, statements, citing resources, etc.

### (PB3955) PERIPHERAL WHOLE BLOOD GENE EXPRESSION BIOMARKERS FOR DIFFERENTIATION OF TB ADENITIS, B-CELL LYMPHOMA, AND OTHER CAUSES OF PERIPHERAL LYMPHADENOPATHY

**Topic:** 21. Lymphoma biology & translational research

Nonhlanhla Mabuza*[1], Karryn Brown[1], Jenna Oosthuizen[1], Ahlisha Chetty[1], Denis Awany[1], Thomas Scriba[1], David Richardson[1], Simon Mendelsohn[1], Estelle Ramona Verburgh[1]

[1]*University of Cape Town, Cape Town, South Africa;*

### Background

Clinical features of tuberculous lymphadenitis (TBL) overlap with those of lymphoma, complicating diagnosis of lymphadenopathy in tuberculosis (TB)- and HIV-endemic settings. Current diagnostic approaches are often invasive, resource-intensive, and may delay appropriate treatment. Whole-blood gene expression biomarkers represent a promising non-invasive approach for disease differentiation.

### Aims

We aimed to evaluate the diagnostic utility of whole-blood gene expression biomarkers in distinguishing TBL from B-cell lymphoma.

### Methods

We conducted a cross-sectional study of 93 adults (≥18 years) referred for lymph node biopsy at Groote Schuur Hospital, Cape Town, between December 2022 and June 2025. Diagnostic categories—TBL, B-cell lymphoma, and other causes of lymphadenopathy—were assigned following review of clinical, cytology, histology, and microbiology records. Whole-blood RNA was collected in PAXgene tubes and cryopreserved. A TB-specific gene expression panel derived from published RNA-sequencing data was measured using microfluidic RT-qPCR on the BioMark platform.

### Results

Among 20 participants with TBL and 51 with B-cell lymphoma, 36 genes were identified as differentially expressed, of which 34 were upregulated in TBL relative to B-cell lymphoma. Two genes, *KCNH8* and *DNASE1L3*, demonstrated strong discriminatory performance with $\log_2$ fold changes of 2.1 (95%CI 1.4–3.0; adjusted p = $4.7\times10^{-4}$) and 2.0 (95%CI 1.3–2.8; adjusted p = $1.4\times10^{-4}$), respectively, and both achieving an AUC of 0.84 (95%CI 0.73–0.96). In contrast, only two genes were downregulated in TBL relative to B-cell lymphoma. *BMX* showed a $\log_2$ fold change of -1.0 (95%CI -1.6– -0.4; adjusted p = 0.03) with modest discrimination (AUC 0.71; 95%CI 0.59–0.83), while *BCL6* had a $\log_2$ fold change of -0.5 (95%CI -0.9– -0.1; adjusted p = 0.05) and AUC 0.68 (95%CI 0.55–0.82).

### Summary/Conclusion

These findings suggest that whole-blood gene expression profiling can potentially discriminate tuberculosis lymphadenitis from lymphoma, with further validation required in larger, diverse cohorts.

**Copyright Information:** (Online) ISSN: 2572-9241
©2026 The Author(s). HemaSphere is published by John Wiley & Sons Ltd on behalf of European Hematology Association. This is an open access Abstract Book distributed under the Attribution-NonCommercial-NoDerivs (CC BY-NC-ND), which allows third parties to download the articles and share them with others as long as they credit the author and the Abstract Book, but they cannot change the content in any way or use them commercially.
**Abstract Book Citations:** Authors, Title, HemaSphere, 2026;10:(S1):pages. The abstract book DOIs can be found on the "Author Information" page.
**Disclaimer:** Articles published in the journal HemaSphere exclusively reflect the opinions of the authors. The authors are responsible for all content in their abstracts including accuracy of the facts, statements, citing resources, etc.

### (PB3956) ATP5E LACTYLATION MEDIATED BY TP53 MUTATION PROMOTES PROGRESSION OF DIFFUSE LARGE B-CELL LYMPHOMA

**Topic:** 21. Lymphoma biology & translational research

Linquan Zhan*[1], Chenyang Jiang[1], Tiange Lu[1], Hua Wang[1], Xiangxiang Zhou[1]

[1]*Shandong Provincial Hospital Affiliated to Shandong First Medical University, Department of Hematology, Jinan, Shandong, China;*

### Background

TP53-mutant diffuse large B-cell lymphoma (DLBCL) is associated with poor prognosis and inferior therapeutic response. Lactylation (Kla) is a newly identified post-translational modification, playing critical roles in tumor progression. However, the extent and regulatory mechanisms of aberrant Kla in TP53-mutant DLBCL remain unclear.

### Aims

This study aims to elucidate how mutant TP53 (MUT-TP53) affects lactate production and lactylation in DLBCL and to pinpoint key lactylation targets connecting MUT-TP53 to mitochondrial changes and cancer progression.

### Methods

TP53 mutation spectrum in DLBCL was sourced from IARC TP53 database. RNA-seq identified transcriptional changes linked to TP53 alterations. Serum lactate levels were compared between DLBCL patients and healthy controls, with tissue Kla levels analyzed for correlations with progression-free survival (PFS) and overall survival (OS). Quantitative proteomics identified lactylated substrates, highlighting ATP5E Lys44 lactylation (K44la) as a potential modification. Statistical significance was determined using two-sided tests with $P<0.05$.

### Results

In DLBCL, TP53 mutations mainly occur in Exon 5, leading to varied TP53 protein expression compared to naive B-cells. TCGA data reveals high activity in oxidative phosphorylation pathways in TP53-mutated DLBCL patients. RNA-seq shows enhanced metabolism, cell cycle, and DNA replication in DLBCL. Knocking down mutant TP53 doesn't affect the cell cycle but reduces LDHA expression, lactate levels, and APR-246 sensitivity. Conversely, knocking down wild-type TP53 boosts cell proliferation and shortens the G0/G1 phase. APR-246 treatment decreases lactate production and induces cell cycle arrest.
We observed higher serum lactate levels and increased Kla in DLBCL tissues, correlating with poorer survival. DLBCL cell lines exhibited significant Kla. Sodium lactate enhanced Kla, whereas glycolysis or LDH inhibition reduced it. Proteomics identified lactylated proteins involved in cell cycle, mitochondrial function, transcription, and signaling, with ATP5E's K44 being a key Kla site. We investigated the role of ATP5E K44la in DLBCL, finding that ATP5E is overexpressed and further increased by LNa treatment, with K44la boosting its expression. Overexpressing WT ATP5E promoted cell growth, while the K44R mutation suppressed it. DEGs related to ATP5E WT and K44R were linked to DNA replication, mitochondrial function, cell cycle, and OXPHOS. WT cells had lower mtDNA, higher mitochondrial membrane potential, reduced mtROS, and increased ATP, whereas K44R reversed these. Mass spectrometry showed TP53 interacts with ATP5E. MUT-TP53 knockdown, but not WT-TP53, reduced Kla. mIHC confirmed TP53 and Kla co-localization in DLBCL. ATP5E overexpression strengthened its interaction with TP53, while K44 mutation weakened it. Functional tests revealed MUT-TP53 knockdown increased mtROS and decreased mitochondrial membrane potential, suggesting TP53 mutation raises Kla in DLBCL.

**Copyright Information:** (Online) ISSN: 2572-9241
©2026 The Author(s). HemaSphere is published by John Wiley & Sons Ltd on behalf of European Hematology Association. This is an open access Abstract Book distributed under the Attribution-NonCommercial-NoDerivs (CC BY-NC-ND), which allows third parties to download the articles and share them with others as long as they credit the author and the Abstract Book, but they cannot change the content in any way or use them commercially.
**Abstract Book Citations:** Authors, Title, HemaSphere, 2026;10:(S1):pages. The abstract book DOIs can be found on the "Author Information" page.
**Disclaimer:** Articles published in the journal HemaSphere exclusively reflect the opinions of the authors. The authors are responsible for all content in their abstracts including accuracy of the facts, statements, citing resources, etc.

**Summary/Conclusion**

MUT-TP53 creates a lactate-lactylation pathway in DLBCL by increasing LDHA/lactate and promoting ATP5E K44la, which stabilizes ATP5E, boosts mitochondrial remodeling, and supports tumor growth. This highlights lactate metabolism, lactylation, ATP5E K44la, and mutant-p53 as potential therapeutic targets.

**Copyright Information:** (Online) ISSN: 2572-9241
©2026 The Author(s). HemaSphere is published by John Wiley & Sons Ltd on behalf of European Hematology Association. This is an open access Abstract Book distributed under the Attribution-NonCommercial-NoDerivs (CC BY-NC-ND), which allows third parties to download the articles and share them with others as long as they credit the author and the Abstract Book, but they cannot change the content in any way or use them commercially.
**Abstract Book Citations:** Authors, Title, HemaSphere, 2026;10:(S1):pages. The abstract book DOIs can be found on the "Author Information" page.
**Disclaimer:** Articles published in the journal HemaSphere exclusively reflect the opinions of the authors. The authors are responsible for all content in their abstracts including accuracy of the facts, statements, citing resources, etc.

### (PB3957) COMPREHENSIVE GENOMIC PROFILING OF LYMPHOID MALIGNANCIES USING TARGETED PANELS IN A SOUTH ASIAN–ENRICHED INTERNATIONAL COHORT: MUTATIONAL LANDSCAPE, CLONAL ARCHITECTURE, AND THERAPEUTIC ACTIONABILITY

**Topic:** 21. Lymphoma biology & translational research

Rahul Bhargav*[1], Shrinidhi Nathany[1], Moin Makda[2], Swarsat Kaushik Nath[1], Hrithik Bagga[1], Siddharth Sagar[1], Haristuti Verma[1], Anusha Swaminathan[1], Vikas Dua[1], Dinesh Bhurani[3], Ragesh Nair[4], Rayaz Ahmed[5]

*[1]Fortis Memorial Research Institute, Gurugram, India; [2]Netaji Subhas University of Technology, New Delhi, India;[3]Rajiv Gandhi Cancer Institute & Research Centre, New Delhi, India; [4]Meitra Hospital, Kozhikode, India; [5]Max Hospital, Saket, New Delhi, India;*

### Background

Genomic profiling of lymphoid malignancies informs diagnosis, risk stratification, and therapy selection. However, data from South Asian populations remain critically underrepresented in global genomic databases, limiting the generalisability of molecular classification frameworks developed predominantly in Western cohorts. Targeted next-generation sequencing panels offer a scalable approach for molecular characterisation in resource-variable settings.

### Aims

To characterise the mutational landscape, clonal architecture, variant allele frequency (VAF) distribution, and therapeutic actionability across the full spectrum of lymphoid malignancies in a South Asian–enriched international cohort using the Ion AmpliSeq Liverpool Lymphoid Network 60-gene panel.

### Methods

We profiled 155 consecutive patients (103 male, 52 female; median age 59 years, range 4–88) with suspected or confirmed lymphoid neoplasms. Specimens included FFPE biopsies (n=81), peripheral blood (n=37), bone marrow aspirates (n=34), and other sample types (n=3). After quality-controlled deduplication of extraction artefacts, 353 unique somatic variants across 74 genes were analysed. Functional annotation included ACMG classification, AMP/ASCO/CAP tiering, LymphGen subtyping, clonal hierarchy inference, and therapeutic actionability assessment.

**Copyright Information:** (Online) ISSN: 2572-9241
©2026 The Author(s). HemaSphere is published by John Wiley & Sons Ltd on behalf of European Hematology Association. This is an open access Abstract Book distributed under the Attribution-NonCommercial-NoDerivs (CC BY-NC-ND), which allows third parties to download the articles and share them with others as long as they credit the author and the Abstract Book, but they cannot change the content in any way or use them commercially.
**Abstract Book Citations:** Authors, Title, HemaSphere, 2026;10:(S1):pages. The abstract book DOIs can be found on the "Author Information" page.
**Disclaimer:** Articles published in the journal HemaSphere exclusively reflect the opinions of the authors. The authors are responsible for all content in their abstracts including accuracy of the facts, statements, citing resources, etc.

**Results**

The cohort encompassed DLBCL (n=44), CLL/SLL (n=15), plasma cell neoplasms (n=14), T-cell lymphoma/leukaemia (n=15), follicular lymphoma (n=10), and 24 additional entities including MCL (n=4), cHL (n=4), MZL (n=4), LPL/WM (n=9), HCL (n=3), and others. The most frequently mutated genes were KMT2D (27 patients, 17.4%), TP53 (26, 16.8%), NOTCH1 (16, 10.3%), TET2 (14, 9.0%), and MYD88 (14, 9.0%). Median VAF was 26.0% (IQR 9.0–44.0%). Clonal hierarchy classification identified 257 clonal/founder events (median VAF 33.0%) and 86 subclonal events (median VAF 11.1%; Kruskal-Wallis $P=1.05\times10^{-15}$). MYD88 p.Leu252Pro (equivalent to p.L265P) was the single most recurrent hotspot (14 patients). Among the 44 DLBCL cases, A53-like (TP53-driven) biology was highly prevalent. Notably, the cohort showed a relative depletion of classical MCD features (MYD88/CD79B driven; 18.2%), with NF-κB activation frequently achieved via alternative mechanisms, such as truncating mutations in NFKBIE and CARD11 alterations, which bypass canonical proximal signaling. Epigenetic and chromatin remodelling was the most frequently deregulated pathway overall (56 patients, 36.1%), characterized by complex co-mutations involving KMT2D, CREBBP, EP300, and ARID1A. Crucially, investigational or approved targeted therapies were applicable to 76.1% of patients. This high actionability rate was primarily driven by Tier I/II variants conferring potential sensitivity to EZH2 inhibitors (e.g., EZH2 hotspot mutations, biallelic KMT2D loss), BTK inhibitors, and PARP inhibitors for DNA-damage response defective clones (e.g., ATM loss). Furthermore, the sequencing successfully identified highly trackable clonal mutations (e.g., TP53 R273C, MYD88 L265P) establishing a robust foundation for non-invasive ctDNA MRD monitoring.

**Summary/Conclusion**

Genomic profiling of this South Asian-enriched cohort reveals core similarities with Western data (KMT2D/TP53 predominance) alongside distinct biology, including depleted classical MCD signatures and alternative NF-κB activation in DLBCL. Finding actionable therapeutic targets and ctDNA MRD markers in >77% of patients highlights the clinical power of NGS for underrepresented populations.

**Copyright Information:** (Online) ISSN: 2572-9241
©2026 The Author(s). HemaSphere is published by John Wiley & Sons Ltd on behalf of European Hematology Association. This is an open access Abstract Book distributed under the Attribution-NonCommercial-NoDerivs (CC BY-NC-ND), which allows third parties to download the articles and share them with others as long as they credit the author and the Abstract Book, but they cannot change the content in any way or use them commercially.
**Abstract Book Citations:** Authors, Title, HemaSphere, 2026;10:(S1):pages. The abstract book DOIs can be found on the "Author Information" page.
**Disclaimer:** Articles published in the journal HemaSphere exclusively reflect the opinions of the authors. The authors are responsible for all content in their abstracts including accuracy of the facts, statements, citing resources, etc.

### (PB3958) ENHANCING RISK STRATIFICATION IN DIFFUSE LARGE B-CELL LYMPHOMA THROUGH ARTIFICIAL INTELLIGENCE: A SYSTEMATIC REVIEW

**Topic:** 21. Lymphoma biology & translational research

Dragos-Claudiu Popescu*[1], Mihnea-Alexandru Găman[2]

*[1] "Carol Davila" University of Medicine and Pharmacy, Faculty of Medicine, Bucharest, Romania;[2] Stefan S. Nicolau Institute of Virology, Department of Cellular and Molecular Pathology, Bucharest, Romania;*

**Background**

Diffuse large B-cell lymphoma (DLBCL) is a biologically heterogeneous malignancy with variable clinical outcomes. Current conventional prognostic tools, such as the International Prognostic Index (IPI), often lack sufficient precision at the individual patient level, prompting increasing interest in artificial intelligence (AI) for enhanced risk stratification.

**Aims**

To systematically map and evaluate the performance of AI and machine learning (ML) techniques in DLBCL outcome prediction and risk stratification.

**Methods**

Systematic literature review of manuscripts published through July 2025 and indexed in three reference electronic databases (PubMed/MEDLINE, Scopus, Cochrane Library) that evaluated the potential impact of AI in DLBCL prognosis. Inclusion criteria: i) original research articles; ii) application of AI/ML or deep learning techniques; iii) prediction of clinical outcomes, risk stratification, or prognostic modelling in DLBCL patients.

**Copyright Information:** (Online) ISSN: 2572-9241
©2026 The Author(s). HemaSphere is published by John Wiley & Sons Ltd on behalf of European Hematology Association. This is an open access Abstract Book distributed under the Attribution-NonCommercial-NoDerivs (CC BY-NC-ND), which allows third parties to download the articles and share them with others as long as they credit the author and the Abstract Book, but they cannot change the content in any way or use them commercially.
**Abstract Book Citations:** Authors, Title, HemaSphere, 2026;10:(S1):pages. The abstract book DOIs can be found on the "Author Information" page.
**Disclaimer:** Articles published in the journal HemaSphere exclusively reflect the opinions of the authors. The authors are responsible for all content in their abstracts including accuracy of the facts, statements, citing resources, etc.

### Results

The literature search yielded a total of 215 records, of which 35 were discarded for being duplicates. Of the remaining 180 manuscripts screened, 91 studies met the inclusion criteria for synthesis. The studies were categorized by input modality: PET/CT imaging (n=30), gene expression profiling (n=19), specific mutational signatures (n=18), clinical risk features (n=8), multi-omics integration (n=5), digital pathology (n=3), conventional histopathology automations (n=2), ctDNA (n=3), microRNA-based models (n=2), enhanced risk scores (n=8) and improved algorithms for CT imaging (n=1). The analysed studies frequently demonstrated superior predictive performance over standard IPI scores, with Area Under the Curve (AUC) or C-index values exceeding 0.80 in high-performing models. Multi-omics integration and ctDNA-based models showed particularly strong potential for clinical translation.

Radiomics and deep learning models applied to PET/CT achieved predictive performance metrics with AUC values ranging from 0.66 to 0.92, where combined clinical-radiomic signatures frequently exceeded 0.83 AUCs. Molecular-driven models utilising gene expression profiling and specific mutational signatures yielded superior stratification, with AUCs for immune-subtype classifiers reaching ~0.95 and survival prediction C-indices between 0.78–0.87. AI models employing clinical features or multi-omics integration displayed robust discrimination with C-indices of 0.70–0.89, consistently outperforming the standard IPI benchmark (~0.70).

| **Input data for the AI model** | **AUC / C-index Range** | **Performance vs IPI** |
|---|---|---|
| *Clinical Features* | 0.70 – 0.89 | Superior (IPI ~0.70) |
| *PET/CT Radiomics* | 0.66 – 0.92 | Superior |
| *Genomics/Transcriptomics* | 0.72 – 0.98 | Superior |
| *Multi-omics Integration* | 0.67 – 0.83 | Superior |

### Summary/Conclusion

AI/ML methods are increasingly used in DLBCL to improve prognostic accuracy by leveraging data types with diverse inputs. These approaches allow for enhanced stratification, superior to traditional indices (namely IPI), supporting the early identification of high-risk patients. Future investigations should focus on external validation and improvement of model interpretability to facilitate real-world workflow integration.

**Copyright Information:** (Online) ISSN: 2572-9241
©2026 The Author(s). HemaSphere is published by John Wiley & Sons Ltd on behalf of European Hematology Association. This is an open access Abstract Book distributed under the Attribution-NonCommercial-NoDerivs (CC BY-NC-ND), which allows third parties to download the articles and share them with others as long as they credit the author and the Abstract Book, but they cannot change the content in any way or use them commercially.
**Abstract Book Citations:** Authors, Title, HemaSphere, 2026;10:(S1):pages. The abstract book DOIs can be found on the “Author Information” page.
**Disclaimer:** Articles published in the journal HemaSphere exclusively reflect the opinions of the authors. The authors are responsible for all content in their abstracts including accuracy of the facts, statements, citing resources, etc.

### (PB3959) THE CIRCULATING IGG N-GLYCOME AS A NOVEL BIOMARKER OF INFLAMMATION AND PROGNOSIS IN DIFFUSE LARGE B-CELL LYMPHOMA

**Topic:** 21. Lymphoma biology & translational research

Vibor Milunovic*[1], Maja Pučić Baković[2], Frano Vučković[2], Ivana Duvnjak-Orešković[2], Karla Misura Jakobac[1], Marin Kursar[1], Inga Mandac Smoljanovic[1], Martina Bogeljic Patekar[1], Delfa Radić-Krišto[1], Slavko Gašparov[1]

[1]*Clinical Hospital Merkur, Zagreb, Croatia;* [2]*Laboratory for Glycobiology, Genos, Zagreb, Croatia;*

### Background

Diffuse large B-cell lymphoma (DLBCL) is characterized by pronounced biological and clinical heterogeneity. While clinical indices like the International Prognostic Index (IPI) are standard, they often fail to capture the complex host-immune interactions that drive disease progression. Immunoglobulin G (IgG) *N*-glycosylation is a critical modulator of humoral immunity and systemic inflammation, yet its role as a circulating biomarker in DLBCL remains largely unexplored.

### Aims

To characterize the plasma IgG *N*-glycome in treatment-naive DLBCL patients and evaluate its association with molecular subtypes (GCB vs. non-GCB), established clinical prognostic indices, and survival outcomes.

### Methods

Plasma samples were collected from 55 treatment-naive DLBCL patients and 54 age- and sex-matched healthy controls. IgG *N*-glycans were analyzed using capillary gel electrophoresis with laser-induced fluorescence (CGE-LIF), resolving 27 *N*-glycan peaks and six derived traits (e.g., galactosylation, sialylation, core-fucosylation). Subtypes were determined via the Hans algorithm. To identify differences between cases and controls and to evaluate associations with clinical prognostic indices, linear regression models were employed. Survival outcomes, specifically the probability of relapse and death, were assessed using the Cox proportional hazards model. In all regression analyses, age and sex were included as additional covariates to account for potential confounding. *P*-values were adjusted for multiple testing using the Benjamini-Hochberg False Discovery Rate (FDR) method, with an adjusted $P<0.05$ considered statistically significant.

### Results

DLBCL patients exhibited a significant pro-inflammatory glycan signature compared to controls, defined by increased agalactosylation (G0: effect=0.464, *P*=2.98E-02) and decreased digalactosylation (G2: effect=-0.441, *P*=3.79E-02) and sialylation (S: effect=-0.570, *P*=1.76E-02). Within molecular subtypes, GCB-DLBCL was uniquely associated with a higher abundance of afucosylated structures (A2G1: *P*=2.38E-02; A2BG2: *P*=1.28E-02), suggesting a potentially more active ADCC-mediated immune environment.
Significant correlations were found between decreased galactosylation and higher risk scores across IPI, R-IPI, and NCCN-IPI models. The most robust associations occurred with the inflammation-based ICPS, where high inflammatory burden strongly correlated with increased agalactosylated glycans (G0: effect=0.578, *P*=2.39E-04) and reduced digalactosylation (G2: effect=-0.670, *P*=2.93E-06). Prognostically, a decrease in the sialylated glycans (e.g., A2[6]G1S1: effect=-0.975, *P*=4.28E-03) and an increase in *N*-glycan structures containing bisecting N-acetylglucosamine (A2BG2, FA2[6]BG1) were independent predictors of a higher probability of relapse.

**Copyright Information:** (Online) ISSN: 2572-9241
©2026 The Author(s). HemaSphere is published by John Wiley & Sons Ltd on behalf of European Hematology Association. This is an open access Abstract Book distributed under the Attribution-NonCommercial-NoDerivs (CC BY-NC-ND), which allows third parties to download the articles and share them with others as long as they credit the author and the Abstract Book, but they cannot change the content in any way or use them commercially.
**Abstract Book Citations:** Authors, Title, HemaSphere, 2026;10:(S1):pages. The abstract book DOIs can be found on the "Author Information" page.
**Disclaimer:** Articles published in the journal HemaSphere exclusively reflect the opinions of the authors. The authors are responsible for all content in their abstracts including accuracy of the facts, statements, citing resources, etc.

**Summary/Conclusion**

Circulating IgG *N*-glycosylation may serve as a robust, minimally invasive biomarker of the systemic inflammatory state and disease severity in DLBCL. The distinct glycan signatures associated with molecular subtypes and clinical outcomes suggest that the IgG glycome could enhance current risk stratification and provide novel insights into the host-immune landscape of aggressive B-cell lymphomas.

**Copyright Information:** (Online) ISSN: 2572-9241
©2026 The Author(s). HemaSphere is published by John Wiley & Sons Ltd on behalf of European Hematology Association. This is an open access Abstract Book distributed under the Attribution-NonCommercial-NoDerivs (CC BY-NC-ND), which allows third parties to download the articles and share them with others as long as they credit the author and the Abstract Book, but they cannot change the content in any way or use them commercially.
**Abstract Book Citations:** Authors, Title, HemaSphere, 2026;10:(S1):pages. The abstract book DOIs can be found on the "Author Information" page.
**Disclaimer:** Articles published in the journal HemaSphere exclusively reflect the opinions of the authors. The authors are responsible for all content in their abstracts including accuracy of the facts, statements, citing resources, etc.

### (PB3960) IRF4 AND SERBP1 EXPRESSION ARE ASSOCIATED WITH POOR RESPONSES TO GUADECITABINE (SGI-110) THERAPY IN PERIPHERAL T CELL LYMPHOMA

**Topic:** 21. Lymphoma biology & translational research

Kiarash Behrouzfar*[1], Chathuri Abeyakoon[2], Mark Waltham[1], Pranav Dorwal[1, 3], Belina Maher[1, 2], Zahra Sabouri-Thompson[1], Kate Scull[1], Daniella Brasacchio[1], Robin Gasiorowski[4, 5], Jake Shortt[1, 2, 6], Paul Yeh[1, 2]

*[1]Monash University, Medicine, Clayton, Victoria, Australia;[2]Monash Health, Haematology, Clayton, Victoria, Australia;[3]Diagnostic Genomics and Anatomical Pathology Monash Health, Clayton, Vic, Australia;[4]Concord Repatriation General Hospital, Haematology, Concord, NSW, Australia; [5]University of Sydney, Sydney, NSW, Australia;[6]University of Melbourne, Sir Peter MacCallum Department of Oncology, Parkville, Victoria, Australia;*

### Background

Peripheral T cell lymphomas (PTCL) are aggressive non-Hodgkin lymphomas with a 5-year survival < 50%, and a lack of effective salvage therapies. Although hypomethylating agents (HMA) offer a potential novel treatment for PTCL, less than half of these patients respond to therapy. This highlights the necessity of developing predictive approach to inform selection of patients for HMA therapy.

### Aims

We aimed to identify gene expression profiles and candidate genes expressed in PTCL predicting response to HMA therapy.

### Methods

We performed RNA sequencing on Formalin Fixed Paraffin Embedded (FFPE) samples (n=11) that were previously collected from lymph node (n=9) and skin (n=2) of PTCL patients before being treated with Guadecitabine in single arm phase II clinical trial (ACTRN12618000028202). Patient characteristics distribution is outlined in the table 1. We leveraged differential gene expression analysis workflow in addition to Gene Set Enrichment Analysis (GSEA) to characterise gene expression profiles of samples from Guadecitabine-treated patients dichotomised by the best clinical response. Patients with Progressive Disease (PD) were classified as 'non-responders', while those with Stable Disease beyond 2 cycles (SD), Partial Response (PR) or Complete Response (CR) were classified as 'responders'. We applied the Leave-One-Out Cross-Validation (LOOCV) method and mirrorCheck to identify genes that are insensitive to outlier and reciprocal contrast effects.

**Copyright Information:** (Online) ISSN: 2572-9241
©2026 The Author(s). HemaSphere is published by John Wiley & Sons Ltd on behalf of European Hematology Association. This is an open access Abstract Book distributed under the Attribution-NonCommercial-NoDerivs (CC BY-NC-ND), which allows third parties to download the articles and share them with others as long as they credit the author and the Abstract Book, but they cannot change the content in any way or use them commercially.
**Abstract Book Citations:** Authors, Title, HemaSphere, 2026;10:(S1):pages. The abstract book DOIs can be found on the "Author Information" page.
**Disclaimer:** Articles published in the journal HemaSphere exclusively reflect the opinions of the authors. The authors are responsible for all content in their abstracts including accuracy of the facts, statements, citing resources, etc.

| Characteristic | Patients (n=11) |
|---|---|
| **Age, median [range]** | 71 [57-88] |
| **Gender, n (%)** | |
| Male | 9 (81%) |
| Female | 2 (19%) |
| **PTCL subtype, n (%)** | |
| AITL | 7 (63.6%) |
| PTCL-TFH | 2 (18.1%) |
| ALCL | 1 (9.09%) |
| PTCL-NOS | 1 (9.09%) |
| **Extent of disease at study entry, n (%)** | |
| Stage 3 | 4 (36%) |
| Stage 4 | 7 (64%) |

**Table 1. Pre-treatment patient characteristics.**

### Results

Our analysis revealed consistent and reliable expression of *IRF4* and *SERBP1* in non-responders versus responders to Guadecitabine (Figure 1. A & B). Moreover, GSEA demonstrated the greater enrichment of gene sets associated with cell cycle and cell proliferation in non-responders (Figure 1. C). The gene sets that are functionally related to methylation were also significantly enriched in non-responders compared to responders in this cohort.

**Copyright Information:** (Online) ISSN: 2572-9241
©2026 The Author(s). HemaSphere is published by John Wiley & Sons Ltd on behalf of European Hematology Association. This is an open access Abstract Book distributed under the Attribution-NonCommercial-NoDerivs (CC BY-NC-ND), which allows third parties to download the articles and share them with others as long as they credit the author and the Abstract Book, but they cannot change the content in any way or use them commercially.
**Abstract Book Citations:** Authors, Title, HemaSphere, 2026;10:(S1):pages. The abstract book DOIs can be found on the "Author Information" page.
**Disclaimer:** Articles published in the journal HemaSphere exclusively reflect the opinions of the authors. The authors are responsible for all content in their abstracts including accuracy of the facts, statements, citing resources, etc.

### Summary/Conclusion

We identified *IRF4* and *SERBP1* as candidate genes predicting resistance to HMA therapy in PTCL. Their elevated expression in non-responders, together with the enrichment of gene sets related to cell proliferation and methylation, suggests their potential role in lymphoma progression and HMA sensitivity. These findings warrant validation in larger independent cohorts and functional studies to determine whether *IRF4* and *SERBP1* may serve as predictive biomarkers for HMA resistance and to clarify their mechanistic contribution to PTCL pathobiology.

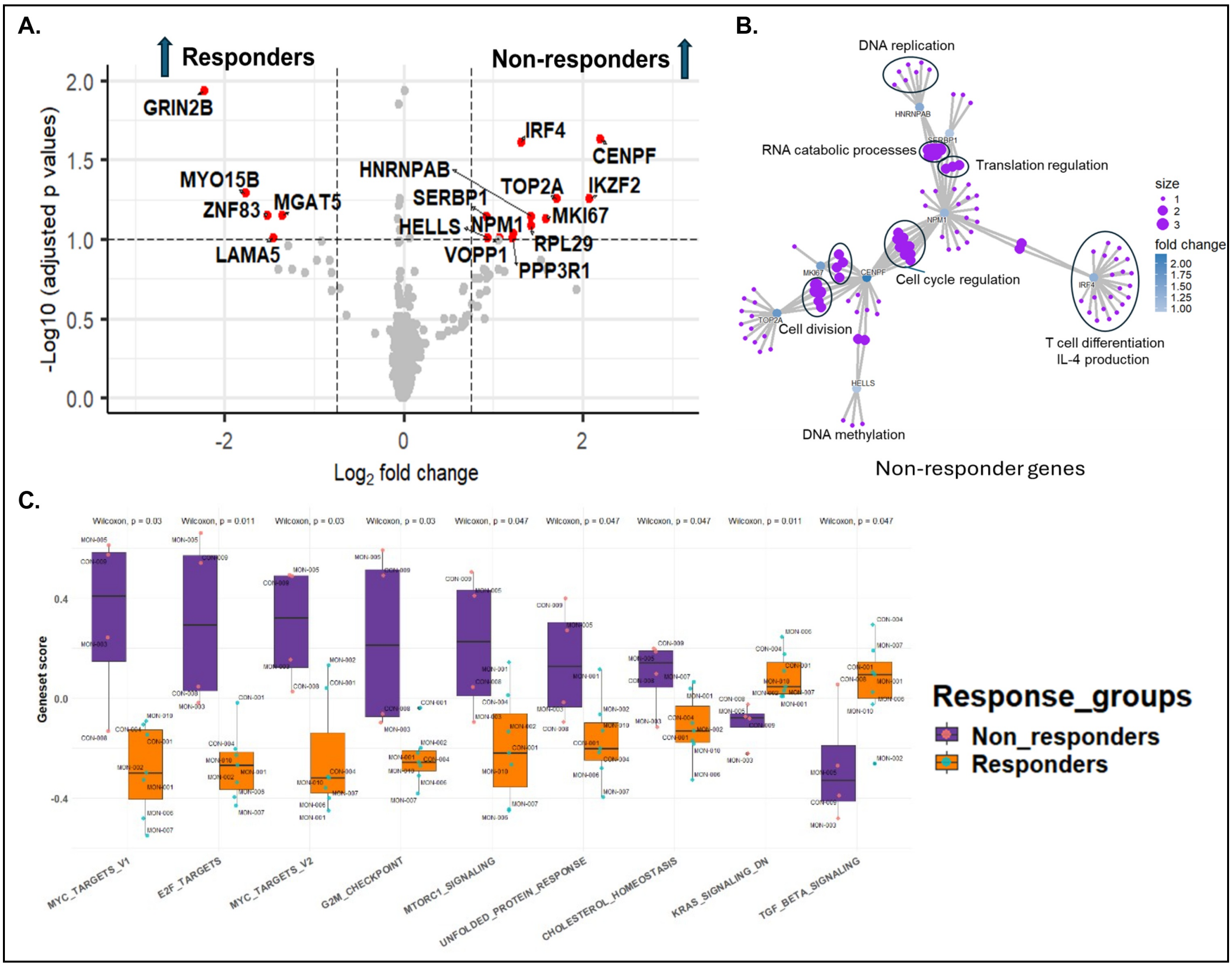

Figure 1. A. Volcano plot demonstrated genes differentially expressed between response groups (adjusted p value<0.1 and log2foldchange >0.75). B. Differentially expressed genes in non-responders that remained significant after multiple leave-one-out iterations. C. Boxplots show the enrichment scores of those HALLMARK gene sets that were significantly enriched in non-responders compared to responders.

**Copyright Information:** (Online) ISSN: 2572-9241
©2026 The Author(s). HemaSphere is published by John Wiley & Sons Ltd on behalf of European Hematology Association. This is an open access Abstract Book distributed under the Attribution-NonCommercial-NoDerivs (CC BY-NC-ND), which allows third parties to download the articles and share them with others as long as they credit the author and the Abstract Book, but they cannot change the content in any way or use them commercially.
**Abstract Book Citations:** Authors, Title, HemaSphere, 2026;10:(S1):pages. The abstract book DOIs can be found on the "Author Information" page.
**Disclaimer:** Articles published in the journal HemaSphere exclusively reflect the opinions of the authors. The authors are responsible for all content in their abstracts including accuracy of the facts, statements, citing resources, etc.

### (PB3961) THE AUTO/PARACRINE NETWORK OF INTERFERON-ALPHA MAINTAINS LATENCY OF EPSTEIN-BARR VIRUS IN B-CELL LYMPHOPROLIFERATIVE DISORDERS: A POTENTIAL THERAPEUTIC TARGET

**Topic:** 21. Lymphoma biology & translational research

Ryo Yanagiya*[1, 2], Hiroshi Ureshino[1, 2], Mai Fujita[1, 2], Moe Kawasaki[2], Keisuke Kidoguchi[1], Yuta Yamamoto[1, 2], Seiichi Kato[3], Shinya Kimura[1, 2]

*[1]Saga University, Division of Hematology and Oncology, Department of Internal Medicine, Saga, Japan;[2]Saga University, Department of Drug Discovery and Biomedical Sciences, Saga, Japan; [3]Faculty of Medicine, Saga University, Department of Pathology and Microbiology, Saga, Japan;*

### Background

Epstein-Barr virus (EBV) is a oncogenic virus that is latent in human memory B cells and reactivates in aging or immunocompromised condition, which results in lymphoproliferative disorders (EBV-LPD). The most common histological type of EBV-LPD is diffuse large B cell lymphoma (DLBCL). DLBCL type EBV-LPD without spontaneous regression shows poor prognosis so that a novel treatment strategy is requred.
EBV is known to have two forms of infection: "latent" (without viral genome replication) and "lytic" (occuring viral genome replication for producing progenies) infection. EBV should maintain latency for preventing apoptosis of host cells due to progeny virus production. Previous studies demonstrated that various drugs such as DNA hypomethylating agents induce lytic infection and subsequent apoptosis in Burkitt lymphoma-derived cell lines, an another type of EBV-associated lymphoma, indicating that forced induction of lytic infection is a potential treatment strategy against EBV-associated neoplasms. However, the approach to induce lytic infection in EBV-LPD remains unestablished.

### Aims

In this study, we aimed to elucidate the mechanisms maintaining viral latency in EBV-LPD using B-lymphoblastoid cell lines (LCLs), an experimental model of DLBCL type EBV-LPD, to establish an approach to induce lytic infection.

### Methods

An EBV with complete genome was obtained from Akata cell line. LCL was established by infecting healthy volunteer-derived B cells with EBV. The efficiency of inducing lytic infection by DNA hypomethylating agents (HMAs; Decitabine and its orally available prodrug OR-2100) was analyzed by qPCR measuring cellular EBV copies. Differentially expressed (DE) gene analyses and gene set enrichment analyses (GSEA) were performed using DESeq2 and clusterProfiler, respectively. Function of tumor-associated macrophages (TAMs) were analyzed using THP-1 cells activated by phorbol 12-myristate 13-acetate and exposed to culture medium of LCLs, as TAMs were required for EBV-LPD growth (*Blood*. 2018, etc.). For knockdown of interest genes, LCL was transduced with shRNA. For *in vivo* cell proliferation analysis, LCLs were intravenously inoculated into BRJ mice and tumor growth and survival periods were observed.

**Copyright Information:** (Online) ISSN: 2572-9241
©2026 The Author(s). HemaSphere is published by John Wiley & Sons Ltd on behalf of European Hematology Association. This is an open access Abstract Book distributed under the Attribution-NonCommercial-NoDerivs (CC BY-NC-ND), which allows third parties to download the articles and share them with others as long as they credit the author and the Abstract Book, but they cannot change the content in any way or use them commercially.
**Abstract Book Citations:** Authors, Title, HemaSphere, 2026;10:(S1):pages. The abstract book DOIs can be found on the “Author Information” page.
**Disclaimer:** Articles published in the journal HemaSphere exclusively reflect the opinions of the authors. The authors are responsible for all content in their abstracts including accuracy of the facts, statements, citing resources, etc.

**Results**

HMAs failed to induce lytic infection in LCL at the dose that caused lytic infection and subsequent apoptosis in Akata (100 nM, 72 hours), indicating that LCL has the unique manners to keep viral latency. GSEA of DE genes between Akata and LCL indicated that expression of both Interferon-alpha (IFN-α), a cytokine preventing viral reproduction, and its responsive genes were upregulated in LCL, suggesting that LCL autocrines IFN-α. In addition, TAMs also produce IFN-α by receiving IFN-α from LCLs via IRF3/7, indicating that TAMs amplify paratumoral IFN-α secretion using positive feedback machinary. These overactivation of IFN-α signaling was validated by analysing microarray data of EBV positive- and negative-DLBCL patients. Knockdown of *IFNA2* in LCL significantly induced lytic infection and exhibiting cytotoxicity against LCLs both *in vitro* and *in vivo* settings.

**Summary/Conclusion**

IFN-α is a well-known cytokine contributing to antiviral innate immunity. In this study, we successfully elucidated that EBV cleverly takes advantage of this antiviral effect of IFN-α to maintain their latency in LPD cells.
In conclusion, the auto/paracrine of IFN-α in EBV-LPD has a crucial role in maintaing viral latency, and functional blockade of IFN-α is a potential novel treatment modality.

**Copyright Information:** (Online) ISSN: 2572-9241
©2026 The Author(s). HemaSphere is published by John Wiley & Sons Ltd on behalf of European Hematology Association. This is an open access Abstract Book distributed under the Attribution-NonCommercial-NoDerivs (CC BY-NC-ND), which allows third parties to download the articles and share them with others as long as they credit the author and the Abstract Book, but they cannot change the content in any way or use them commercially.
**Abstract Book Citations:** Authors, Title, HemaSphere, 2026;10:(S1):pages. The abstract book DOIs can be found on the "Author Information" page.
**Disclaimer:** Articles published in the journal HemaSphere exclusively reflect the opinions of the authors. The authors are responsible for all content in their abstracts including accuracy of the facts, statements, citing resources, etc.

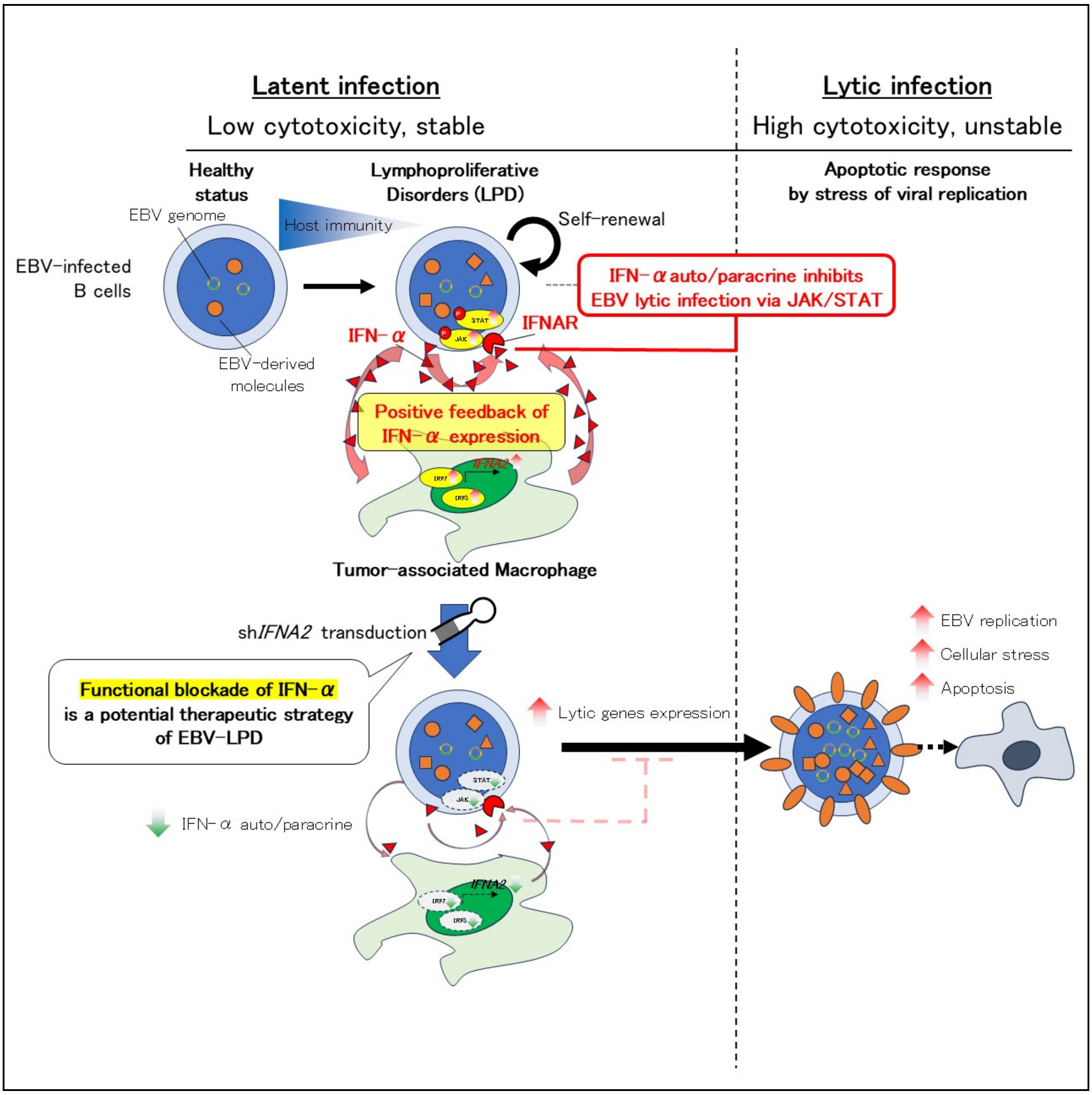

Copyright Information: (Online) ISSN: 2572-9241
©2026 The Author(s). HemaSphere is published by John Wiley & Sons Ltd on behalf of European Hematology Association. This is an open access Abstract Book distributed under the Attribution-NonCommercial-NoDerivs (CC BY-NC-ND), which allows third parties to download the articles and share them with others as long as they credit the author and the Abstract Book, but they cannot change the content in any way or use them commercially.
Abstract Book Citations: Authors, Title, HemaSphere, 2026;10:(S1):pages. The abstract book DOIs can be found on the "Author Information" page.
Disclaimer: Articles published in the journal HemaSphere exclusively reflect the opinions of the authors. The authors are responsible for all content in their abstracts including accuracy of the facts, statements, citing resources, etc.

### (PB3962) MECHANISM AND TARGETED INTERVENTION OF TARGETING PHGDH-MEDIATED ENDOGENOUS SERINE SYNTHESIS IN DOUBLE-HIT LYMPHOMA

**Topic:** 21. Lymphoma biology & translational research

Wenyu Shi*[1], Nan Chen[1], Hui Jin[2], Jianyong Li[2]

*[1]Affiliated Hospital of Nantong University, Nantong, China;[2]The First Affiliated Hospital of Nanjing Medical University, Nanjing, China;*

### Background

Amino acid metabolic reprogramming drives diffuse large B-cell lymphoma (DLBCL) progression, but remains poorly defined in double-hit lymphoma (DHL)—a highly aggressive DLBCL subtype with concurrent MYC and Bcl-2 translocations. Our preliminary integrated analysis of GEO datasets, untargeted metabolomics, and single-cell RNA sequencing (scRNA-seq) identified elevated serine metabolism as a key DHL metabolic abnormality, which may contribute to pathogenesis by perturbing cellular redox homeostasis and poor prognosis. Serine hydroxymethyltransferase 2 (SHMT2), a key mitochondrial serine catabolism enzyme, modulates redox homeostasis via regulating NAD(P)H/NAD(P)$^{+}$ dynamic conversion.

### Aims

Based on preliminary findings, this study aims to: (1) Define the metabolic reprogramming features, clinical correlations, and molecular basis of DHL's high serine demand; (2) Elucidate PHGDH-mediated endogenous serine synthesis' biological functions and upstream regulatory mechanisms in DHL progression; (3) Uncover how PHGDH-driven serine synthesis enhances SHMT2-mediated mitochondrial serine catabolism to suppress ferroptosis, and explore therapeutic strategies targeting the MYC/Bcl-2-PHGDH axis to induce ferroptosis.

### Methods

Clinical DHL and non-DHL DLBCL tumor/plasma samples underwent metabolomics to assess serine, redox changes, and clinical correlations; MYC/BCL2 translocations were confirmed by FISH. Serine-deprived/complete cultures evaluated endogenous synthesis. PHGDH knockdown/overexpression and inhibition defined function, while MYC knockdown plus BCL2 inhibition assessed regulation (ChIP-qPCR, RIP, luciferase). Multi-omics and functional assays examined ferroptosis and metabolism, and combination therapy was tested in vitro/in vivo. Co-IP, isotope tracing, imaging, and spatial metabolomics/IHC characterized PHGDH–SHMT2 interaction and related targets.

**Copyright Information:** (Online) ISSN: 2572-9241
©2026 The Author(s). HemaSphere is published by John Wiley & Sons Ltd on behalf of European Hematology Association. This is an open access Abstract Book distributed under the Attribution-NonCommercial-NoDerivs (CC BY-NC-ND), which allows third parties to download the articles and share them with others as long as they credit the author and the Abstract Book, but they cannot change the content in any way or use them commercially.
**Abstract Book Citations:** Authors, Title, HemaSphere, 2026;10:(S1):pages. The abstract book DOIs can be found on the "Author Information" page.
**Disclaimer:** Articles published in the journal HemaSphere exclusively reflect the opinions of the authors. The authors are responsible for all content in their abstracts including accuracy of the facts, statements, citing resources, etc.

### Results

(1) **Active serine metabolism:** GEO/scRNA-seq showed elevated serine metabolism and high PHGDH linked to poor prognosis; LC-MS/MS confirmed higher plasma serine with serine–methionine/redox enrichment in DHL.
(2) **PHGDH drives dependence:** Serine deprivation affected non-DHL more, while DHL upregulated PHGDH. PHGDH inhibition/knockdown reduced intracellular serine and preferentially suppressed DHL proliferation.
(3) **Induces ferroptosis:** PHGDH targeting triggered lipid peroxidation, mitochondrial damage, $Fe^{2+}$ accumulation, and reduced respiration, consistent with ferroptosis.
(4) **Via SHMT2:** SHMT2 correlated with PHGDH; PHGDH inhibition lowered SHMT2 and NADPH/$NADP^{+}$, with mitochondrial PHGDH–SHMT2 colocalization.
(5) **In vivo synergy:** NCT-503 outperformed serine deprivation, and NCT-503 + Erastin most effectively suppressed DHL xenografts without weight loss.

### Summary/Conclusion

DHL shows metabolic reprogramming driven by abnormal serine metabolism and high PHGDH, associated with poor prognosis. DHL depends on PHGDH-mediated endogenous serine synthesis. PHGDH promotes SHMT2-linked mitochondrial metabolism and NADPH production, suppressing ferroptosis. Targeting PHGDH induces ferroptosis, and combination with a ferroptosis inducer shows strong in vivo efficacy. The MYC/BCL2–PHGDH–SHMT2 axis represents a potential biomarker and precision therapeutic target in DHL.

**Copyright Information:** (Online) ISSN: 2572-9241
©2026 The Author(s). HemaSphere is published by John Wiley & Sons Ltd on behalf of European Hematology Association. This is an open access Abstract Book distributed under the Attribution-NonCommercial-NoDerivs (CC BY-NC-ND), which allows third parties to download the articles and share them with others as long as they credit the author and the Abstract Book, but they cannot change the content in any way or use them commercially.
**Abstract Book Citations:** Authors, Title, HemaSphere, 2026;10:(S1):pages. The abstract book DOIs can be found on the "Author Information" page.
**Disclaimer:** Articles published in the journal HemaSphere exclusively reflect the opinions of the authors. The authors are responsible for all content in their abstracts including accuracy of the facts, statements, citing resources, etc.

### (PB3963) THE DIAGNOSTIC ACCURACY OF 18F-FDG PET/CT IN HEMOPHAGOCYTIC LYMPHOHISTIOCYTOSIS: A DIAGNOSTIC META-ANALYSIS

**Topic:** 21. Lymphoma biology & translational research

Mahmoud Einieh*[1], Sara Hassan[1], Kristina Alaiyoud[1], Zeina Beqain[1], Jiaqi Liang[1], Anna Hornyák[2], Laszlo Pinczes[2]

*[1]University of Debrecen, Debrecen, Hungary; [2]University of Debrecen, Department of Internal Medicine, Debrecen, Hungary;*

### Background

Hemophagocytic lymphohistiocytosis (HLH) is a rare, life-threatening hyperinflammatory syndrome that is frequently secondary (sHLH) to infections, autoimmune diseases, or malignancies (Mal-HLH), most commonly lymphoma in adults. Diagnosis is often delayed due to overlapping clinical features with severe infection or advanced malignancy, and identification of an underlying lymphoma remains challenging when conventional imaging fails to localize occult disease. Whole-body 18F-FDG PET/CT, widely used in lymphoma staging, can detect both metabolically active neoplastic lesions and immune activation, suggesting potential utility in the diagnostic evaluation of HLH. However, the diagnostic performance of 18F-FDG PET/CT for detecting underlying malignancy in HLH has not been systematically evaluated.

### Aims

To assess the diagnostic accuracy of 18F-FDG PET/CT in detecting underlying malignancies, particularly lymphomas, in adults with suspected HLH.

### Methods

This systematic review and diagnostic meta-analysis included 12 observational studies evaluating adult patients with suspected sHLH undergoing 18F-FDG PET/CT. A comprehensive literature search was performed across five databases (PubMed, Embase, Cochrane, Web of Science, and CNKI). Diagnostic performance was assessed using pooled sensitivity, specificity, summary receiver operating characteristic (SROC) analysis, and area under the curve (AUC).

### Results

This diagnostic meta-analysis encompassed a total of 436 adult patients with suspected sHLH. The mean age was 47 years, and 52.5% of the patients were male. Pooled analysis demonstrated high overall diagnostic accuracy of 18F-FDG PET/CT with a sensitivity of 0.918 (95% CI: 0.818–0.965, Figure 1a) and specificity of 0.804 (95% CI: 0.657–0.897). SROC curve analysis confirmed excellent discriminative performance (AUC = 0.876) (Figure 1b). The subgroup analysis of Mal-HLH showed a sensitivity of 0.965 (95% CI: 0.870–0.991) and a specificity of 0.727 (95% CI: 0.159–0.974).

### Summary/Conclusion

18F-FDG PET/CT demonstrates robust and reliable diagnostic accuracy for detecting underlying malignancies in adult patients with HLH, supporting its use as a valuable diagnostic tool in this high-risk population.

**Copyright Information:** (Online) ISSN: 2572-9241
©2026 The Author(s). HemaSphere is published by John Wiley & Sons Ltd on behalf of European Hematology Association. This is an open access Abstract Book distributed under the Attribution-NonCommercial-NoDerivs (CC BY-NC-ND), which allows third parties to download the articles and share them with others as long as they credit the author and the Abstract Book, but they cannot change the content in any way or use them commercially.
**Abstract Book Citations:** Authors, Title, HemaSphere, 2026;10:(S1):pages. The abstract book DOIs can be found on the "Author Information" page.
**Disclaimer:** Articles published in the journal HemaSphere exclusively reflect the opinions of the authors. The authors are responsible for all content in their abstracts including accuracy of the facts, statements, citing resources, etc.

**Figure 1a. FDG-PET Sensitivity in HLH Forest Plot**

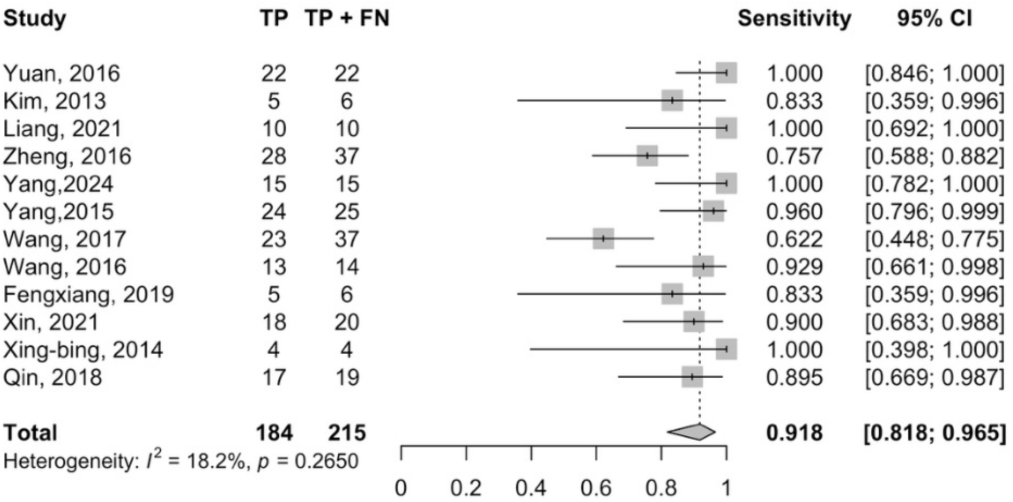

**Figure 1b. SROC Curve for FDG-PET in HLH**

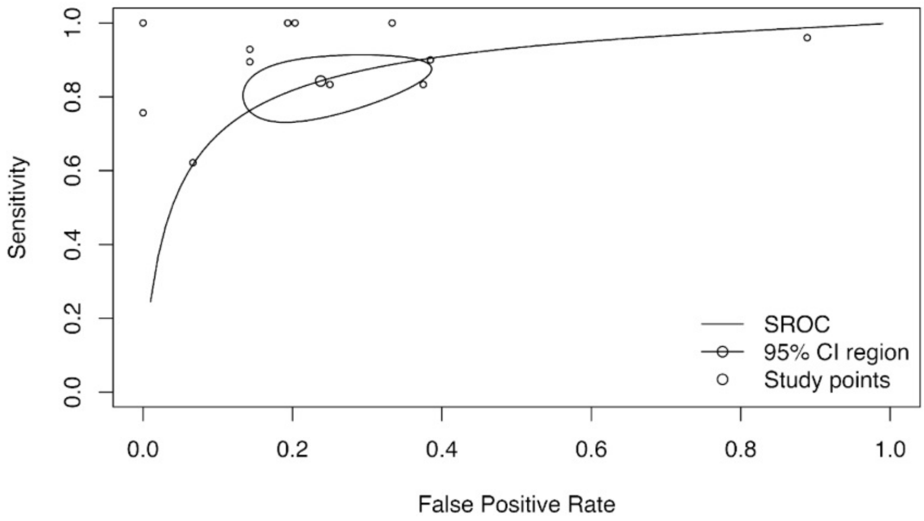

**Copyright Information:** (Online) ISSN: 2572-9241
©2026 The Author(s). HemaSphere is published by John Wiley & Sons Ltd on behalf of European Hematology Association. This is an open access Abstract Book distributed under the Attribution-NonCommercial-NoDerivs (CC BY-NC-ND), which allows third parties to download the articles and share them with others as long as they credit the author and the Abstract Book, but they cannot change the content in any way or use them commercially.
**Abstract Book Citations:** Authors, Title, HemaSphere, 2026;10:(S1):pages. The abstract book DOIs can be found on the "Author Information" page.
**Disclaimer:** Articles published in the journal HemaSphere exclusively reflect the opinions of the authors. The authors are responsible for all content in their abstracts including accuracy of the facts, statements, citing resources, etc.

### (PB3964) CIRCULATING IMMUNOMODULATORY CELL SUBSETS IN NEWLY DIAGNOSED DIFFUSE LARGE B-CELL LYMPHOMA: A MULTIPARAMETRIC FLOW CYTOMETRY STUDY

**Topic:** 21. Lymphoma biology & translational research

Aikaterini Aliki Keller[1], Athina Damianaki[1], Ioannis Moysis Skianis[1], Antigoni Linardaki[1], Maria Ximeri[2], Anastasia Komodromou[2], Christophoros Chontos[2], Panagiotis Verginis[1, 3], Christina Kalpadaki*[1]

*[1]Laboratory of Hematology, School of Medicine, University of Crete and University General Hospital of Heraklion, Heraklion, Greece; [2]Department of Hematology, School of Medicine, University of Crete and University General Hospital of Heraklion, Heraklion, Greece; [3]Laboratory of Immune Regulation and Tolerance, School of Medicine, University of Crete, Heraklion, Greece;*

### Background

The immune microenvironment plays a critical role in the pathogenesis and progression of diffuse large B-cell lymphoma (DLBCL). While tumor-infiltrating immune cells have been extensively studied, the prognostic and biological significance of circulating immunomodulatory cell subsets in DLBCL remains insufficiently characterized.

### Aims

To characterize peripheral blood cellular subpopulations with immunomodulatory properties in newly diagnosed DLBCL patients and to explore associations with clinical features and outcome.

### Methods

Peripheral blood samples from 18 newly diagnosed DLBCL patients and 14 age-matched healthy controls (HC) were analyzed. Peripheral blood mononuclear cells were isolated from fresh samples and examined using multiparametric flow cytometry with a 19-antibody panel (CD15, CD11b, CD14, CD19, CD206, CD33, CD45, HLA-DR, TIM-3, CD4, CD8, CD3, PD-1, PD-L1, CD38, CD25, CD16, CD127, OX-40). Total monocytes were defined as $CD11b^{+}CD33^{+}HLA\text{-}DR^{bright}$ cells and further classified into classical ($CD14^{+}CD16^{-}$), intermediate ($CD14^{+}CD16^{+}$), and non-classical ($CD14^{-}CD16^{+}$) subsets. Regulatory T cells (Tregs) were identified as $CD4^{+}CD25^{+}CD127^{-}$. Expression of OX-40, PD-1, and TIM-3 was evaluated across T-cell subsets, while PD-L1 and CD206 expression was assessed on monocyte populations. The study was conducted in accordance with the Declaration of Helsinki and approved by the Ethics Committee of the University Hospital of Crete.

### Results

The median age of patients was 65 years. According to Ann Arbor staging, 6 patients had stage IA, 7 had stage IIA, 2 had stage IIIB, 2 had stage IVA, and 1 had stage IVB disease. Compared with HC, DLBCL patients demonstrated a significant increase in circulating Tregs and $CD8^{+}$ T cells and a significant decrease in classical monocytes. OX-40 expression was significantly reduced across T-cell populations ($CD3^{+}$, $CD4^{+}$, $CD8^{+}$, and Tregs) in DLBCL patients compared with HC. PD-1 expression was significantly increased in $CD4^{+}$ T cells in DLBCL. TIM-3 expression did not differ between groups (Figure 1B). PD-L1 expression on classical and non-classical monocytes showed significantly higher levels in DLBCL patients (Figure 1A) No statistically significant associations were found between immunological parameters (Treg frequency, PD-1, OX-40, TIM-3 expression) and clinical characteristics, including age, stage, or IPI score.

**Copyright Information:** (Online) ISSN: 2572-9241
©2026 The Author(s). HemaSphere is published by John Wiley & Sons Ltd on behalf of European Hematology Association. This is an open access Abstract Book distributed under the Attribution-NonCommercial-NoDerivs (CC BY-NC-ND), which allows third parties to download the articles and share them with others as long as they credit the author and the Abstract Book, but they cannot change the content in any way or use them commercially.
**Abstract Book Citations:** Authors, Title, HemaSphere, 2026;10:(S1):pages. The abstract book DOIs can be found on the "Author Information" page.
**Disclaimer:** Articles published in the journal HemaSphere exclusively reflect the opinions of the authors. The authors are responsible for all content in their abstracts including accuracy of the facts, statements, citing resources, etc.

### Summary/Conclusion

This study demonstrates that newly diagnosed DLBCL is associated with systemic immune dysregulation characterized by expansion of circulating Tregs, reduced OX-40 expression across T-cell subsets, and increased PD-1 expression in CD4⁺ T cells, consistent with an exhausted or functionally impaired T-cell phenotype. Decreased classical monocyte frequencies and increased PD-L1 expression in DLBCL patients suggest a shift toward an immunosuppressive myeloid profile. These findings support the presence of peripheral immune checkpoint dysregulation in DLBCL and warrant further investigation in larger cohorts to clarify their prognostic and therapeutic implications.

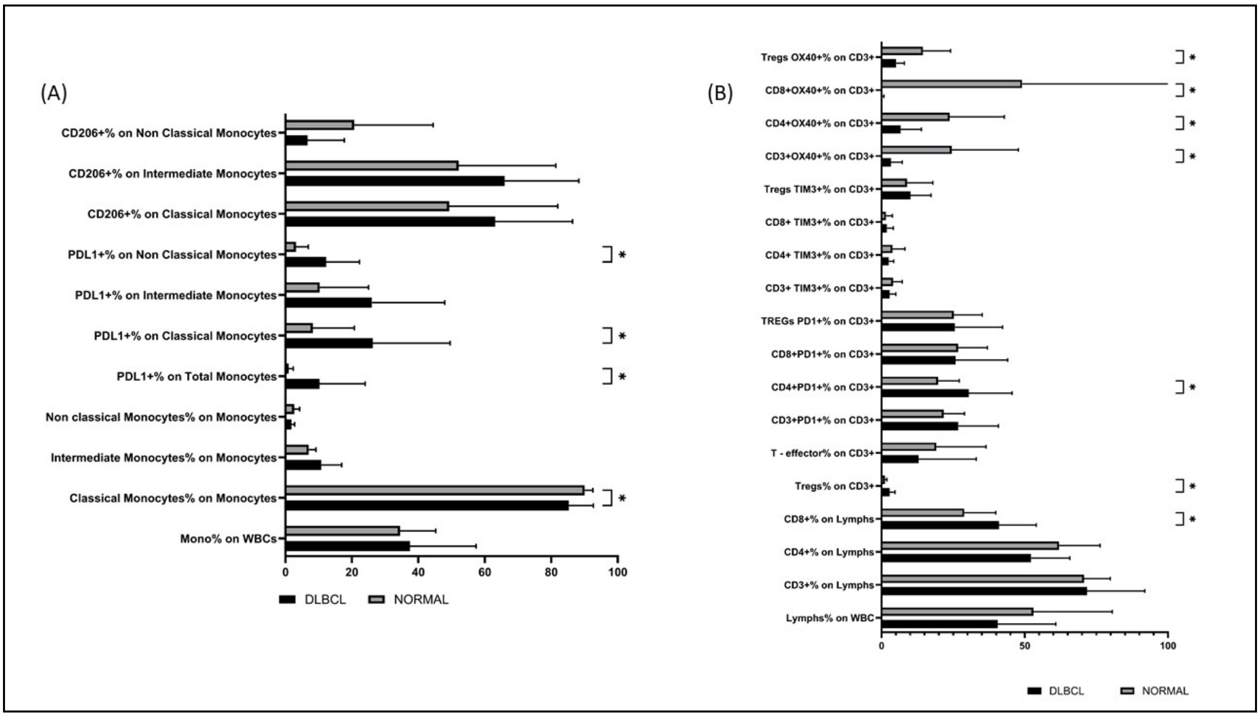

Figure 1: A) Comparisson of monocyte sub-populations between DLBCL patients and healthy controls. B) Comparisson of T cell sub-populations between DLBCL patients and helathy controls.

**Copyright Information:** (Online) ISSN: 2572-9241
©2026 The Author(s). HemaSphere is published by John Wiley & Sons Ltd on behalf of European Hematology Association. This is an open access Abstract Book distributed under the Attribution-NonCommercial-NoDerivs (CC BY-NC-ND), which allows third parties to download the articles and share them with others as long as they credit the author and the Abstract Book, but they cannot change the content in any way or use them commercially.
**Abstract Book Citations:** Authors, Title, HemaSphere, 2026;10:(S1):pages. The abstract book DOIs can be found on the "Author Information" page.
**Disclaimer:** Articles published in the journal HemaSphere exclusively reflect the opinions of the authors. The authors are responsible for all content in their abstracts including accuracy of the facts, statements, citing resources, etc.

### (PB3965) CAUSAL RELATIONSHIP BETWEEN PSMB1 AND FOLLICULAR LYMPHOMA RISK: INSIGHTS FROM MENDELIAN RANDOMIZATION AND POTENTIAL DRUG TARGETS

**Topic:** 21. Lymphoma biology & translational research

Li Huang[1], Ting Niu*[2]

*[1]Department of Hematology, The Affiliated Hospital of Southwest Medical University, Department of Hematology, Luzhou, Sichuan Province, China; [2]Department of Hematology, Institute of Hematology, West China Hospital, Sichuan University, Department of Hematology, Chengdu, Sichuan, China;*

### Background

Follicular lymphoma (FL) is a common type of non-Hodgkin lymphoma characterized by its indolent nature and complex genetic landscape.The immunochemotherapy regimen represented by anti-CD20 monoclonal antibody combined with chemotherapy has greatly improved the prognosis of patients . In particular, the introduction of rituximab maintenance therapy has significantly prolonged progression-free survival (PFS), making FL a manageable chronic disease . Despite the high initial treatment response rate, FL is still considered an incurable disease. Most patients will experience multiple relapses, and with the increase in the number of treatment lines, the disease-free survival period gradually shortens. Additionally, a subset of patients (approximately 20%) will experience disease progression (POD24) within 24 months after initial diagnosis, and these patients have an extremely poor prognosis, constituting a high-risk group in clinical practice. In recent years, with a deeper understanding of the tumor microenvironment and biological behaviors of FL, as well as the development of new targeted drugs and immunotherapy, the treatment of FL is gradually moving from pure immunochemotherapy towards more personalized and mechanism-based treatments,including immunomodulatory drugs, targeted agents, and immunotherapies like bispecific antibodies and CAR-T cells. In this context, the identification and validation of new, actionable drug targets are crucial for further improving patient outcomes.

### Aims

This study aims to identify potential drug targets for FL by integrating pharmacogenetic data and exploring the causal relationships between specific proteins and FL risk using Mendelian Randomization (MR) analysis.

### Methods

We utilized the GEO (Gene Expression Omnibus) database to obtain the FL dataset GSE32018, which includes samples from 23 FL patients and 7 control subjects. Drug target predictions were made using the SuperPred and SEA databases, resulting in 180 relevant target genes. We then extracted protein quantitative trait locus (pQTL) data from the deCODE database and performed two-sample MR analysis using the TwoSampleMR R package. Sensitivity analyses, including heterogeneity and pleiotropy tests, were conducted to validate the robustness of our findings. Additionally, Summary-data-based Mendelian Randomization (SMR) and color analyses were performed to assess the potential causal relationships and shared genetic signals.

### Results

Our MR analysis first identified a significant causal relationship between PSMB1 and FL, with PSMB1 levels showing a negative correlation with disease risk. Further sensitivity analyses validated the robustness of this association, while SMR and colocalization analyses excluded the presence of horizontal pleiotropy or genetic confounding. In addition to PSMB1, protein-protein interaction (PPI) network analysis identified 11 interacting proteins, and potential drug candidate molecules were screened through the DrugBank database. ROC(Receiver Operating Characteristic) curve analysis demonstrated that multiple proteins exhibited high diagnostic accuracy in distinguishing FL from control samples.

**Copyright Information:** (Online) ISSN: 2572-9241
©2026 The Author(s). HemaSphere is published by John Wiley & Sons Ltd on behalf of European Hematology Association. This is an open access Abstract Book distributed under the Attribution-NonCommercial-NoDerivs (CC BY-NC-ND), which allows third parties to download the articles and share them with others as long as they credit the author and the Abstract Book, but they cannot change the content in any way or use them commercially.
**Abstract Book Citations:** Authors, Title, HemaSphere, 2026;10:(S1):pages. The abstract book DOIs can be found on the "Author Information" page.
**Disclaimer:** Articles published in the journal HemaSphere exclusively reflect the opinions of the authors. The authors are responsible for all content in their abstracts including accuracy of the facts, statements, citing resources, etc.

**Summary/Conclusion**

This study provides compelling evidence for the role of PSMB1 as a potential drug target in FL, highlighting the importance of integrating genetic and proteomic data in drug discovery. Future research should focus on validating these findings in clinical settings and exploring the therapeutic implications of targeting PSMB1 in FL treatment.

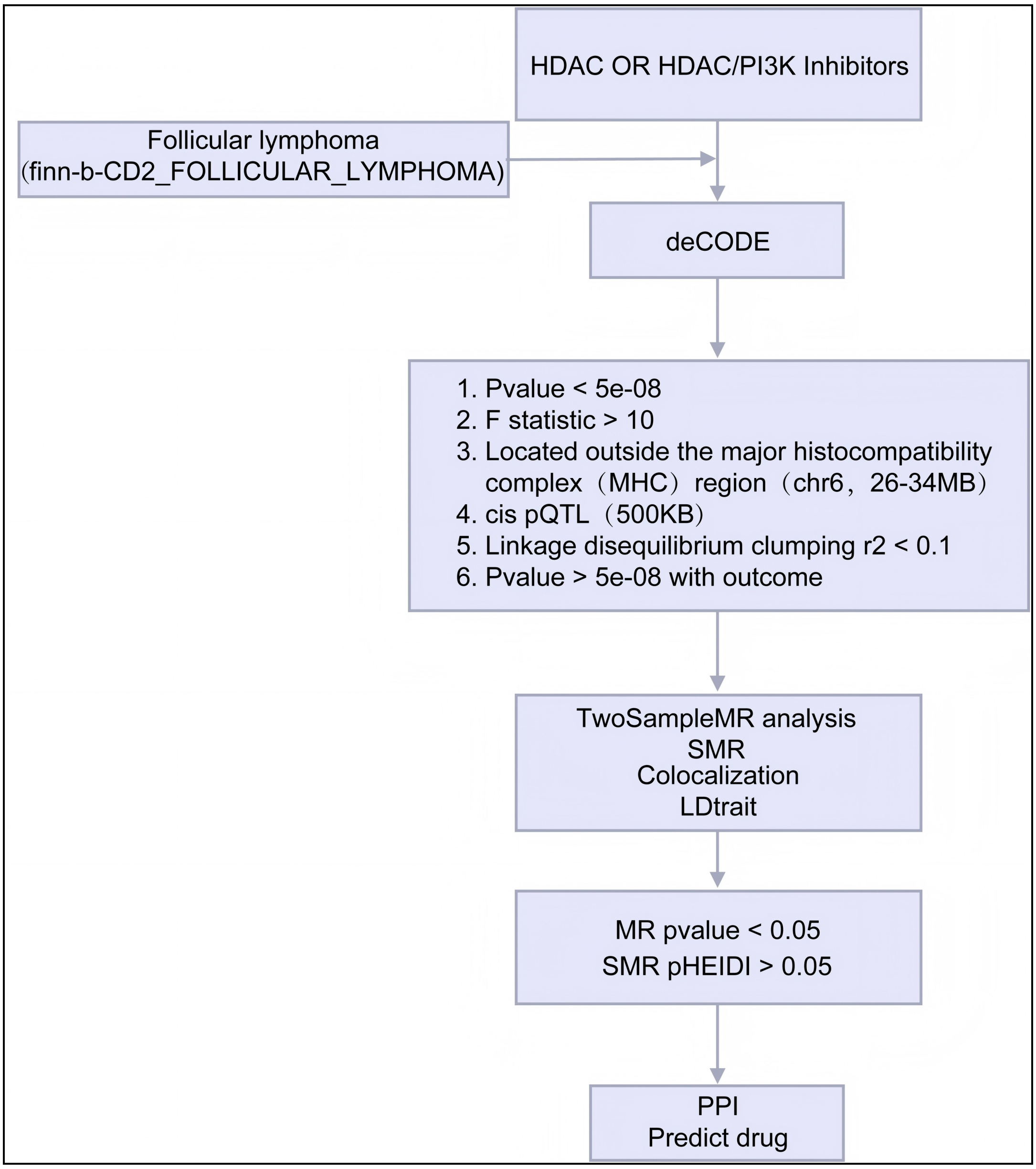

Figure 1 technology roadmap

**Copyright Information:** (Online) ISSN: 2572-9241
©2026 The Author(s). HemaSphere is published by John Wiley & Sons Ltd on behalf of European Hematology Association. This is an open access Abstract Book distributed under the Attribution-NonCommercial-NoDerivs (CC BY-NC-ND), which allows third parties to download the articles and share them with others as long as they credit the author and the Abstract Book, but they cannot change the content in any way or use them commercially.
**Abstract Book Citations:** Authors, Title, HemaSphere, 2026;10:(S1):pages. The abstract book DOIs can be found on the "Author Information" page.
**Disclaimer:** Articles published in the journal HemaSphere exclusively reflect the opinions of the authors. The authors are responsible for all content in their abstracts including accuracy of the facts, statements, citing resources, etc.

### (PB3966) IMMUNOREGULATORY CYTOKINE GENE POLYMORPHISMS AS DETERMINANTS OF DISEASE AGGRESSIVENESS IN B-CELL NON-HODGKIN LYMPHOMA: A CASE–CONTROL STUDY FROM SOUTHEASTERN EUROPE (SERBIA)

**Topic:** 21. Lymphoma biology & translational research

Milica Apostolovic Stojanovic*[1], Milica Radojkovic[1]

[1]*Clinical Hospital Center "Dr Dragisa Misovic - Dedinje", Department of Hematology, Belgrade, Serbia;*

### Background

Host genetic variability within immunoregulatory cytokine genes may modulate tumor microenvironment and influence disease biology in B-cell non-Hodgkin lymphoma (B-NHL). Data from
Southeastern Europe are scarce, and the prognostic relevance of cytokine promoter polymorphisms remains insufficiently defined.

### Aims

To investigate the prevalence and clinical impact of selected cytokine gene polymorphisms in Serbian patients with B-NHL and to evaluate their association with markers of aggressive disease and survival.

### Methods

This case–control study included 51 patients with histologically confirmed B-NHL and 100 age- and sex-matched healthy controls. Clinical parameters comprised Ann Arbor stage, B symptoms, extranodal involvement, IPI score, relapse, and overall survival (OS).
Laboratory variables included leukocyte count, hemoglobin, platelet count, LDH, albumin, fibrinogen, and ESR.
Genomic DNA was analyzed using PCR-RFLP for promoter polymorphisms: IL-6 G(−597)A, IL-10 T(−3575)A, TNF-α G(−308)A, and LT-α A(+252)G.
Associations were evaluated using $\chi^2$/Fisher's exact test and logistic regression (odds ratios [OR] with 95% confidence intervals [CI]).
Survival was assessed by Kaplan–Meier and Cox proportional hazards models. Statistical significance was defined as $p < 0.05$.

### Results

Genotype distributions in controls conformed to Hardy–Weinberg equilibrium. Carriers of the IL-6 −597A allele had an increased risk of thrombocytopenia (OR 2.3; 95% CI 1.1–4.8; p=0.028) and relapse (OR 2.1; 95% CI 1.0–4.5; p=0.041). The IL-10 −3575 TT genotype was significantly associated with elevated LDH (OR 3.4; 95% CI 1.6–7.2; p=0.001) and high/intermediate-high IPI (OR 2.7; 95% CI 1.2–5.9; p=0.014). In survival analysis, IL-10 TT carriers demonstrated inferior overall survival (median OS 38 vs 62 months; log-rank p=0.032). In multivariate Cox regression adjusting for IPI, IL-10 TT remained an independent predictor of shorter OS (HR 2.2; 95% CI 1.1–4.6; p=0.027). No significant associations were observed for TNF-α or LT-α polymorphisms.

### Summary/Conclusion

Summary/Conclusion:
In this Southeastern European cohort, IL-6 and IL-10 promoter polymorphisms were significantly associated with laboratory and clinical indicators of aggressive B-NHL. Importantly, the IL-10 −3575 TT genotype independently predicted inferior overall survival. In the era of CAR-T and other cellular immunotherapies, host cytokine gene polymorphisms may represent an additional layer of biological stratification deserving prospective evaluation. These findings support further validation in larger multicenter studies.

**Copyright Information:** (Online) ISSN: 2572-9241
©2026 The Author(s). HemaSphere is published by John Wiley & Sons Ltd on behalf of European Hematology Association. This is an open access Abstract Book distributed under the Attribution-NonCommercial-NoDerivs (CC BY-NC-ND), which allows third parties to download the articles and share them with others as long as they credit the author and the Abstract Book, but they cannot change the content in any way or use them commercially.
**Abstract Book Citations:** Authors, Title, HemaSphere, 2026;10:(S1):pages. The abstract book DOIs can be found on the "Author Information" page.
**Disclaimer:** Articles published in the journal HemaSphere exclusively reflect the opinions of the authors. The authors are responsible for all content in their abstracts including accuracy of the facts, statements, citing resources, etc.

### (PB3967) 3D EX VIVO PLATFORM FOR HIGH-THROUGHPUT IMMUNOTHERAPY SCREENING IN RELAPSED/REFRACTORY LYMPHOMA PATIENTS.

**Topic:** 21. Lymphoma biology & translational research

Fabien Gava[1], Paul Marcoux[1], Neus Serrat[2], Pauline Gravelle[1], Sonia Quertinmont[1], Marie Tosolini[1], Carla Faria[3], Juan García Valero[2], Christina Schniederjohann[4], Cathy Quelen[1], Fanny Bouquet[5], Catia Fonsecia[5], Sylvia Herter[6], Laura Codarri Deak[6], Ron McCord[7], Eugene Chiang[7], Peter-Martin Bruch[4], Loic Ysebaert[8], Patricia Perez Galan[2], Laurent Camille[9], Christine Bezombes*[1]

*[1]Research Center in Cancer de Toulouse, Toulouse, France; [2]Institut d'Investigacions Biomèdiques August Pi i Sunyer, Barcelona, Spain; [3]Oncology department UNIL-CHUV, Bernhard Gentner laboratory, Agora Research Center, Lausanne, Switzerland; [4]Medical Faculty and University Hospital Düsseldorf, Department of Hematology, Heidelberg, Germany; [5]F.Hoffmann-La Roche, Basel, Switzerland; [6]Roche Innovation Center Zürich, Schlieren, Schlieren, Switzerland; [7]Genentech, San Francisco, United States of America; [8]Department of Hematology, IUCT-O, Toulouse, France; [9]Institut Universitaire du Cancer de Toulouse - Oncopole, Dpt of pathology, Toulouse, France;*

### Background

Relapsed/refractory (R/R) B-cell lymphomas (B-NHL) remain incurable, with limited predictive biomarkers to guide immunotherapy. Preclinical platforms that faithfull preserve immune context and viability of patient samples are critical needed for relevant high-throughput drug testing.

### Aims

We aimed to generate a pioneering 3D *ex vivo* plaform for immunotherapy containing a comprehensive repository of 3D NHL avatars, fully annotated with immune-infiltration data to assess drug efficacy,  identify predictive response signatures and rapidly redirect non-responding patients toward more effective treatments, ultimately improving patient outcomes

### Methods

The Patient-Derived Lymphoma Spheroids (PDLS) were established in a scaffold- and matrix-free system from peripheral blood or lymph node biopsies of R/R follicular lymphoma, transformed follicular lymphoma or diffuse large B-cell lymphoma. Multiparametric flow cytometry were performed to evaluate the efficacy of ICP blockers, bispecific antibody and co-activator/co-stimulator molecules. scRNAsequencing and CODEX experiments completed the analyses.

### Results

- PDLS preserve viability, immune composition and exhaustion profile of original samples
- Integrated workflow for 3D imaging, drug testing, immune profiling, and B-cell depletion
- The PDLS platform serves as a predictive tool for personalized therapy guidance

**Copyright Information:** (Online) ISSN: 2572-9241
©2026 The Author(s). HemaSphere is published by John Wiley & Sons Ltd on behalf of European Hematology Association. This is an open access Abstract Book distributed under the Attribution-NonCommercial-NoDerivs (CC BY-NC-ND), which allows third parties to download the articles and share them with others as long as they credit the author and the Abstract Book, but they cannot change the content in any way or use them commercially.
**Abstract Book Citations:** Authors, Title, HemaSphere, 2026;10:(S1):pages. The abstract book DOIs can be found on the "Author Information" page.
**Disclaimer:** Articles published in the journal HemaSphere exclusively reflect the opinions of the authors. The authors are responsible for all content in their abstracts including accuracy of the facts, statements, citing resources, etc.

**Summary/Conclusion**

PDLS maintain the cellular composition, T-cell activation and immune escape profiles of the original tumors, supporting multiplexed screening of single-agent and combination therapies within clinically relevant timeframes. This scalable model, recapitulates immune–tumor cell interactions, provides rapid assessment of immunotherapy efficacy, and bridges preclinical research with clinical application in aggressive B-cell lymphomas.

**Copyright Information:** (Online) ISSN: 2572-9241
©2026 The Author(s). HemaSphere is published by John Wiley & Sons Ltd on behalf of European Hematology Association. This is an open access Abstract Book distributed under the Attribution-NonCommercial-NoDerivs (CC BY-NC-ND), which allows third parties to download the articles and share them with others as long as they credit the author and the Abstract Book, but they cannot change the content in any way or use them commercially.
**Abstract Book Citations:** Authors, Title, HemaSphere, 2026;10:(S1):pages. The abstract book DOIs can be found on the “Author Information” page.
**Disclaimer:** Articles published in the journal HemaSphere exclusively reflect the opinions of the authors. The authors are responsible for all content in their abstracts including accuracy of the facts, statements, citing resources, etc.

### (PB3968) CLONAL TRANSFORMATION OF COLD AGGLUTININ DISEASE TO DIFFUSE LARGE B-CELL LYMPHOMA

**Topic:** 21. Lymphoma biology & translational research

Agnieszka Malecka*[1, 2, 3], Henrik Tor Anderson Tvedt[1, 4], Signe Spetalen[2, 4], Gunhild Trøen[2], Sigbjorn Berentsen[5], Ludvig Munthe[3, 6], Geir E. Tjønnfjord[1, 4]

*[1]Oslo University Hospital, Department of Haematology, Oslo, Norway;[2]Oslo University Hospital, Department of Pathology, Oslo, Norway; [3]University of Oslo, PRIMA – Center for Precision Immunology, Oslo, Norway; [4]University of Oslo, Institute of Clinical Medicine, Oslo, Norway; [5]Haugesund Hospital, Helse Fonna Trust, Department of Research and Innovation, Haugesund, Norway; [6]Oslo University Hospital, Department of Immunology, Oslo, Norway;*

### Background

Cold Agglutinin Disease (CAD) is an autoimmune hemolytic anemia characterized by an indolent bone marrow B cell proliferation secreting monoclonal IgM autoantibodies targeting the I antigen on erythrocytes, leading to complement activation and hemolysis. It has been unclear whether this entity can progress to diffuse large B-cell lymphoma (DLBCL). Development of DLBCL has been described in patients with CAD, but the clonal relationship has not been addressed. A key feature of CAD is a clonotype with highly restricted immunoglobulin variable region usage, specifically *IGHV4-34,* allowing binding to the I antigen.

### Aims

Determine whether DLBCL in CAD patients represents true clonal transformation or an unrelated metachronous lymphoma. Additionally, we aimed to define molecular differences between transforming and non-transforming CAD cases.

### Methods

Here, we report on two CAD patients with DLBCL. Clonal relationship was assessed on fresh tissue using the LymphoTrack® Dx IGHV Leader Somatic Hypermutation Assay Kit A (Invivoscribe) on MiSeq™; FFPE samples were tested with the IdentiClone® IGH Gene Clonality Assay with ABI fluorescence due to fragmented DNA. Clonal matches were identified via CDR3 comparison using IMGT (www.imgt.org), and phylogeny was analyzed using www.phylogeny.fr. One case had prior profiling in a CAD cohort by next-generation sequencing, RNA-seq, and cytogenetic microarrays; original data were reanalyzed to compare the transformed case with untransformed cases. UMAP was performed in R (umap) and visualized in 2D with ggplot2.

**Copyright Information:** (Online) ISSN: 2572-9241
©2026 The Author(s). HemaSphere is published by John Wiley & Sons Ltd on behalf of European Hematology Association. This is an open access Abstract Book distributed under the Attribution-NonCommercial-NoDerivs (CC BY-NC-ND), which allows third parties to download the articles and share them with others as long as they credit the author and the Abstract Book, but they cannot change the content in any way or use them commercially.
**Abstract Book Citations:** Authors, Title, HemaSphere, 2026;10:(S1):pages. The abstract book DOIs can be found on the "Author Information" page.
**Disclaimer:** Articles published in the journal HemaSphere exclusively reflect the opinions of the authors. The authors are responsible for all content in their abstracts including accuracy of the facts, statements, citing resources, etc.

**Results**

We present the first fully documented cases of CAD undergoing clonal transformation to DLBCL, confirmed through PCR-based clonality testing and clinical data.
Approximately 2 years after CAD diagnosis and achieving partial remission following bendamustine–rituximab treatment, the first patient developed DLBCL. In-depth IGHV sequencing identified an identical clonotype across CAD and DLBCL with identical CDR3 and concordant IGHV mutations; and phylogenetic analysis of CAD- and DLBCL-derived sequences showed clustering of the shared clone, further supporting clonal relationship (Figure 1A, B). In addition, we reanalyzed pre-DLBCL genomic and transcriptomic data for this patient, which distinguished her from CAD cases that did not transform. Pre-transformation genomics revealed DLBCL-typical mutations (*KMT2D* nonsense, activating *CARD11*), numerous potentially oncogenic mutations, NF-κB pathway alterations, and a high burden of mutations in chromatin modification/organization, consistent with aggressive lymphoma-like features. Whole-exome sequencing at diagnosis showed a significantly higher mutation burden (Figure 1C). UMAP of RNA-seq (full transcriptome) placed this patient apart from healthy controls and other CAD cases (Figure 1D).
After CAD diagnosis at age 69, the second patient received multiple treatments with only partial, short-lived responses. Nine years later he developed gastric DLBCL; PCR clonality testing revealed that the gastric biopsy harboured the same clone as identified at age 72 (Figure 1E). Following successful DLBCL treatment, CAD recurred, and the same clone was again detectable in a bone marrow sample 8 years later.

**Summary/Conclusion**

This study demonstrates clonal transformation of CAD into DLBCL through the expansion of a minor, therapy-resistant subclone. Our findings emphasize the importance of molecular monitoring in CAD patients, particularly those exhibiting a high somatic mutation burden.

**Copyright Information:** (Online) ISSN: 2572-9241
©2026 The Author(s). HemaSphere is published by John Wiley & Sons Ltd on behalf of European Hematology Association. This is an open access Abstract Book distributed under the Attribution-NonCommercial-NoDerivs (CC BY-NC-ND), which allows third parties to download the articles and share them with others as long as they credit the author and the Abstract Book, but they cannot change the content in any way or use them commercially.
**Abstract Book Citations:** Authors, Title, HemaSphere, 2026;10:(S1):pages. The abstract book DOIs can be found on the “Author Information” page.
**Disclaimer:** Articles published in the journal HemaSphere exclusively reflect the opinions of the authors. The authors are responsible for all content in their abstracts including accuracy of the facts, statements, citing resources, etc.

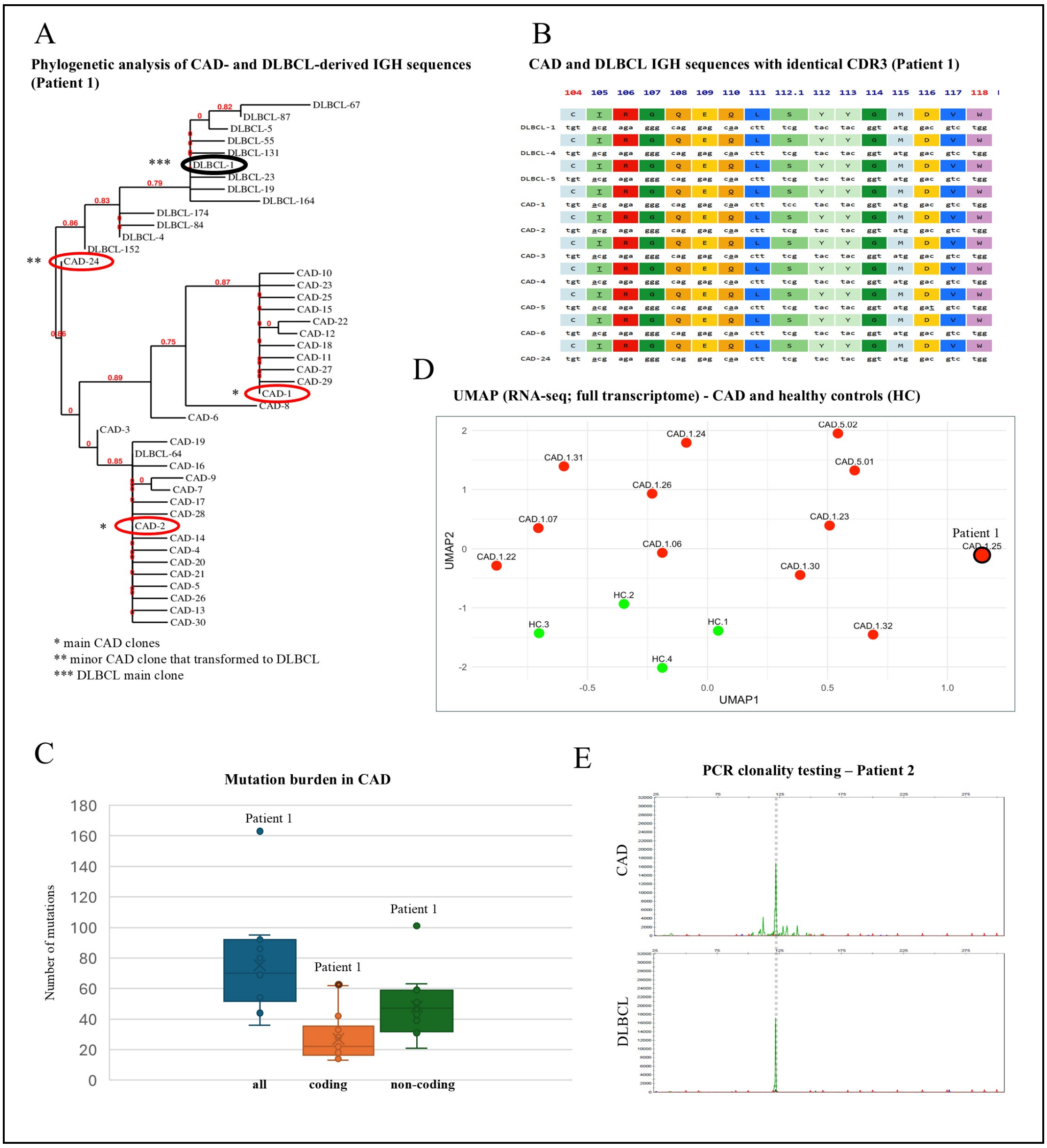

**Copyright Information:** (Online) ISSN: 2572-9241
©2026 The Author(s). HemaSphere is published by John Wiley & Sons Ltd on behalf of European Hematology Association. This is an open access Abstract Book distributed under the Attribution-NonCommercial-NoDerivs (CC BY-NC-ND), which allows third parties to download the articles and share them with others as long as they credit the author and the Abstract Book, but they cannot change the content in any way or use them commercially.
**Abstract Book Citations:** Authors, Title, HemaSphere, 2026;10:(S1):pages. The abstract book DOIs can be found on the "Author Information" page.
**Disclaimer:** Articles published in the journal HemaSphere exclusively reflect the opinions of the authors. The authors are responsible for all content in their abstracts including accuracy of the facts, statements, citing resources, etc.

### (PB3970) MDM2 INHIBITION INDUCES AN ADAPTIVE AKT–IDO1 AXIS THAT LIMITS ANTITUMOR EFFICACY IN DIFFUSE LARGE B-CELL LYMPHOMA

**Topic:** 21. Lymphoma biology & translational research

Xue Xu[1], Chengtao Sun*[2]

*[1]The Second Qilu Hospital of Shandong University, Department of Health Care/ Department of Geriatrics Gerontology, Jinan, China; [2]The First Affiliated Hospital of Shandong First Medical University & Shandong Provincial Qianfoshan Hospital, Shandong Lung Cancer Institute, Department of Oncology, Jinan, China;*

### Background

MDM2 inhibitors have emerged as promising targeted therapies for tumors retaining wild-type TP53, including subsets of diffuse large B-cell lymphoma (DLBCL). However, therapeutic responses to MDM2 inhibition are frequently transient, indicating the presence of adaptive resistance mechanisms. Indoleamine 2,3-dioxygenase 1 (IDO1), a critical immunometabolic enzyme that promotes immune tolerance, remains poorly characterized following MDM2-targeted therapy. This study aimed to investigate whether MDM2 inhibition activates an adaptive AKT–IDO1 signaling program that constrains antitumor efficacy in DLBCL.

### Aims

This study aimed to investigate whether MDM2 inhibition activates an adaptive AKT–IDO1 signaling axis that constrains antitumor efficacy in diffuse large B-cell lymphoma (DLBCL), and to evaluate the therapeutic potential of combined MDM2 and IDO1 blockade.

### Methods

DLBCL cell lines harboring wild-type TP53 were treated with the MDM2 inhibitor RG7388. AKT activation and IDO1 expression were evaluated by immunoblotting and quantitative PCR. Pharmacologic inhibition of AKT was employed to assess its role in RG7388-induced IDO1 upregulation. Functional responses were determined by cell proliferation and apoptosis assays. Combined treatment with RG7388 and the IDO1 inhibitor 1-L-MT was assessed in vitro. Public transcriptomic datasets (TCGA and GEO) were analyzed to examine associations among MDM2 expression, AKT pathway activation, and immunoregulatory gene signatures.

### Results

MDM2 inhibitor RG7388 elicited an early p53 response followed by a delayed but robust increase in AKT phosphorylation and IDO1 expression in TP53 wild-type DLBCL cells. Pharmacologic blockade of AKT effectively abrogated RG7388-induced IDO1 upregulation, supporting a mechanistic link between AKT activation and IDO1 induction. Analysis of public datasets revealed enrichment of PI3K–AKT and immunoregulatory pathways in MDM2-high DLBCL tumors. Combined treatment with an MDM2 inhibitor and an IDO1 inhibitor significantly enhanced antitumor activity compared with single-agent treatment, as evidenced by reduced tumor cell proliferation and increased apoptosis in vitro.

### Summary/Conclusion

These findings identify an adaptive AKT–IDO1 signaling axis as a previously unrecognized mechanism limiting the antitumor efficacy of MDM2 inhibition in DLBCL. This study provides a mechanistic rationale for combining MDM2 inhibitors with IDO1-targeted immunometabolic therapy to overcome adaptive immune tolerance. Targeting AKT–IDO1 crosstalk may represent a therapeutic vulnerability in DLBCL treated with MDM2-directed agents.

**Copyright Information:** (Online) ISSN: 2572-9241
©2026 The Author(s). HemaSphere is published by John Wiley & Sons Ltd on behalf of European Hematology Association. This is an open access Abstract Book distributed under the Attribution-NonCommercial-NoDerivs (CC BY-NC-ND), which allows third parties to download the articles and share them with others as long as they credit the author and the Abstract Book, but they cannot change the content in any way or use them commercially.
**Abstract Book Citations:** Authors, Title, HemaSphere, 2026;10:(S1):pages. The abstract book DOIs can be found on the "Author Information" page.
**Disclaimer:** Articles published in the journal HemaSphere exclusively reflect the opinions of the authors. The authors are responsible for all content in their abstracts including accuracy of the facts, statements, citing resources, etc.

### (PB3971) BONE MARROW NK-CELL PERCENTAGE AS A DETERMINANT OF THE CIRCULATING ADCC EFFECTOR POOL IN FOLLICULAR AND DIFFUSE LARGE B-CELL LYMPHOMA, WITH AND WITHOUT BONE MARROW INFILTRATION.

**Topic:** 21. Lymphoma biology & translational research

Ricardo Francisco Garcia Muñoz[1, 2], Sofia Rincon-Lopez*[1], Johelys Atencio-Matos[1], Olalla Pereda-Sainz[1], Javier Larreina-Perez[1], Andrea Campeny-Najara[1], Ada Esteban Figuerola[1], Montserrat Hernandez-Perez[1], Puy Garrastachu-Zumaran[3], Maria Velasco-Ruiz[4], Isabel Garcia-Bosque[5], Carmen Rodriguez-Lefler[5], Estefania Ruiz de Gaona[5], Jesus Feliu Sanchez[1], Alfonso Martin-Carnicero[6], Juan Cabello[2]

*[1]Universitary Hospital San Pedro, Hematology, Logroño, Spain;[2]CIBIR (Center for Biomedical Research of La Rioja, Logroño, Spain; [3]Universitary Hospital San Pedro, Nuclear Medicine, Logroño, Spain;[4]Universitary Hospital San Pedro, Radiology Department, Logroño, Spain; [5]Hospital Calahorra, Hematology, Calahorra, Spain;[6]Universitary Hospital San Pedro, Oncology, Logroño, Spain;*

### Background

The efficacy of anti-CD20 therapy depends on NK cell–mediated antibody-dependent cellular cytotoxicity (ADCC). The bone marrow is the primary site of NK-cell development; therefore, higher marrow NK-cell percentages should translate into greater effector availability in peripheral blood and tissues. We previously showed in DLBCL that ≥14% marrow NK cells predict superior disease control. Bone marrow infiltration—common in follicular lymphoma—may disrupt this NK-cell axis.

### Aims

To evaluate whether bone marrow NK-cell percentage reflects the size of the peripheral NK-cell effector pool, and to determine how marrow infiltration alters this relationship.

### Methods

Eighty-six newly diagnosed B-cell non-Hodgkin lymphoma patients (59 follicular lymphoma, 27 DLBCL) were assessed at diagnosis. NK-cell percentages in bone marrow and absolute NK counts in blood were quantified by flow cytometry. Bone marrow infiltration was determined by biopsy and/or flow cytometry. Correlation and linear regression analyses compared infiltrated vs. non-infiltrated marrow.

### Results

Bone marrow NK-cell percentage correlated with peripheral NK counts only in non-infiltrated marrow. In non-infiltrated patients (n=48), correlations were strong (Pearson $r = 0.63$, $p < 0.0001$; Spearman $\rho = 0.59$, $p < 0.0001$), with a regression slope of 18.9 NK/μL per 1% marrow NK ($R^2 = 0.40$). In infiltrated marrow (n=38), no association was observed (Pearson $r = 0.05$, $p = 0.77$; Spearman $\rho = 0.08$, $p = 0.64$), with a flat slope ($\beta = 2.5$ NK/μL per 1%, $R^2 = 0.003$). Correlations differed significantly (Fisher $Z = -2.42$, $p = 0.015$). Marrow NK percentages were lower in infiltrated vs. non-infiltrated patients (≈9% vs. ≈15%, $p < 0.001$), and peripheral NK counts were also reduced (median 145 vs. 215 NK/μL, $p = 0.02$).

**Copyright Information:** (Online) ISSN: 2572-9241
©2026 The Author(s). HemaSphere is published by John Wiley & Sons Ltd on behalf of European Hematology Association. This is an open access Abstract Book distributed under the Attribution-NonCommercial-NoDerivs (CC BY-NC-ND), which allows third parties to download the articles and share them with others as long as they credit the author and the Abstract Book, but they cannot change the content in any way or use them commercially.
**Abstract Book Citations:** Authors, Title, HemaSphere, 2026;10:(S1):pages. The abstract book DOIs can be found on the "Author Information" page.
**Disclaimer:** Articles published in the journal HemaSphere exclusively reflect the opinions of the authors. The authors are responsible for all content in their abstracts including accuracy of the facts, statements, citing resources, etc.

**Summary/Conclusion**

In non-infiltrated marrow, NK-cell percentage strongly predicts the size of the circulating effector pool, supporting a functional marrow-to-periphery NK-cell axis essential for ADCC. Bone marrow infiltration disrupts this axis, reducing NK availability and potentially limiting anti-CD20 effectiveness **(Figure 1)**. These findings support incorporating NK-cell assessment into risk stratification and immunotherapy planning in B-cell lymphomas.

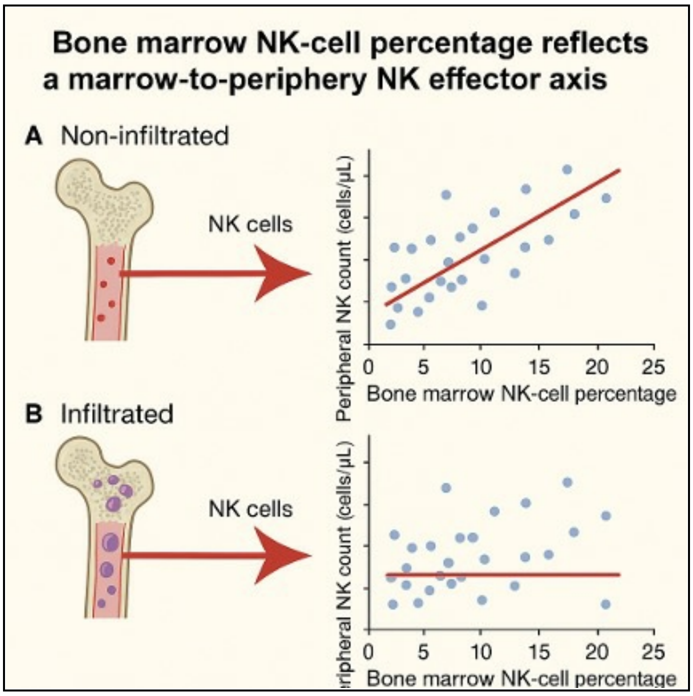

Figure 1: Bone marrow NK-cell percentage reflects a functional marrow-to-periphery NK effector axis in the absence of infiltration. (A) In non-infiltrated marrow, higher NK-cell percentages correlate with increased peripheral NK-cell counts, indicating preserved NK-cell output. (B) In infiltrated marrow, this correlation is lost, suggesting disruption of the marrow-to-periphery NK-cell axis and reduced circulating NK-cell availability.

**Copyright Information:** (Online) ISSN: 2572-9241
©2026 The Author(s). HemaSphere is published by John Wiley & Sons Ltd on behalf of European Hematology Association. This is an open access Abstract Book distributed under the Attribution-NonCommercial-NoDerivs (CC BY-NC-ND), which allows third parties to download the articles and share them with others as long as they credit the author and the Abstract Book, but they cannot change the content in any way or use them commercially.
**Abstract Book Citations:** Authors, Title, HemaSphere, 2026;10:(S1):pages. The abstract book DOIs can be found on the "Author Information" page.
**Disclaimer:** Articles published in the journal HemaSphere exclusively reflect the opinions of the authors. The authors are responsible for all content in their abstracts including accuracy of the facts, statements, citing resources, etc.

### (PB3972) SELINEXOR COMBINED WITH RADIATION THERAPY: A NOVEL STRATEGY FOR DOSE REDUCTION IN EXTRANODAL NK/T-CELL LYMPHOMA

**Topic:** 21. Lymphoma biology & translational research

Cheng-Ji Wang[1], Xindi Liu[1], Liang Wang*[1]

[1]*Beijing Tongren Hospital, Capital Medical University, Beijing, China;*

### Background

Extranodal NK/T-cell lymphoma (ENKTCL) is a highly aggressive malignancy with high prevalence in East Asia. Radiotherapy (RT) is essential for early-stage disease, but conventional doses (≥50 Gy) often cause severe toxicities including mucositis, dermatitis, and dysphagia, significantly compromising quality of life. Recent studies suggest that low-dose RT (<50 Gy) may achieve comparable survival outcomes in patients achieving complete response after chemotherapy, but the optimal strategy for dose de-escalation remains undefined. Inspired by recent findings that the XPO1 inhibitor KPT-330 (selinexor) enhances radiosensitivity in central nervous system lymphoma by impairing DNA damage repair through IRF3-BARD1-BRCA1 pathway inhibition.

### Aims

We hypothesized that selinexor could serve as a radiosensitizer in ENKTCL, enabling effective tumor control at reduced radiation doses.

### Methods

We utilized two established ENKTCL cell lines, SNK-6 and YT, to evaluate the synergistic effects of selinexor combined with ionizing radiation (IR). Cells were separately treated with selinexor at concentrations of 0 (control), 10 nM, 20 nM, and 40 nM, radiation doses of 0, 2 Gy, 4 Gy, and 8 Gy, or 10nM Selinexor+2Gy RT, 20nM Selinexor+4Gy RT, 40nM Selinexor+8Gy RT. Cell proliferation was assessed using the Cell Counting Kit-8 (CCK-8) assay at 24 hours post-treatment. Apoptosis was quantified by flow cytometry using Annexin V-FITC/PI double staining. Double-stranded RNA (dsRNA) levels in ENKTCL cells were detected by immunofluorescence.

### Results

Selinexor inhibited ENKTCL cell proliferation and induced apoptosis in a concentration-dependent manner, as did radiotherapy in a dose-dependent fashion—though radiotherapy alone demonstrated more potent effects. Notably, the combination of 10 nM selinexor with 2 Gy radiation achieved equivalent anti-proliferative and pro-apoptotic effects as 4 Gy radiation alone; similarly, 20 nM selinexor combined with 4 Gy radiation matched the cytotoxicity of 8 Gy alone. These findings indicate robust radiosensitization, suggesting potential for radiation dose reduction. Immunofluorescence analysis revealed that while selinexor monotherapy did not alter dsRNA levels, combination treatment significantly enhanced radiation-induced dsRNA accumulation in ENKTCL cells. Given that elevated dsRNA correlates with improved disease control, selinexor may function as a radiosensitizer through this mechanism, enabling effective dose de-escalation without compromising therapeutic efficacy.

**Copyright Information:** (Online) ISSN: 2572-9241
©2026 The Author(s). HemaSphere is published by John Wiley & Sons Ltd on behalf of European Hematology Association. This is an open access Abstract Book distributed under the Attribution-NonCommercial-NoDerivs (CC BY-NC-ND), which allows third parties to download the articles and share them with others as long as they credit the author and the Abstract Book, but they cannot change the content in any way or use them commercially.
**Abstract Book Citations:** Authors, Title, HemaSphere, 2026;10:(S1):pages. The abstract book DOIs can be found on the “Author Information” page.
**Disclaimer:** Articles published in the journal HemaSphere exclusively reflect the opinions of the authors. The authors are responsible for all content in their abstracts including accuracy of the facts, statements, citing resources, etc.

**Summary/Conclusion**

This preclinical study provides the first evidence that the XPO1 inhibitor selinexor acts as a potent radiosensitizer in ENKTCL cell lines. By inducing dsRNA accumulation in ENKTCL cells when combining Selinexor and RT, selinexor enables effective tumor cell killing at substantially reduced radiation doses. These findings support the clinical development of selinexor-RT combination therapy as a strategy to de-escalate radiation doses in early-stage ENKTCL, potentially reducing severe RT-related toxicities while maintaining therapeutic efficacy. Future studies will validate these findings in patient-derived xenograft models and explore the translational potential for clinical trials.

**Copyright Information:** (Online) ISSN: 2572-9241
©2026 The Author(s). HemaSphere is published by John Wiley & Sons Ltd on behalf of European Hematology Association. This is an open access Abstract Book distributed under the Attribution-NonCommercial-NoDerivs (CC BY-NC-ND), which allows third parties to download the articles and share them with others as long as they credit the author and the Abstract Book, but they cannot change the content in any way or use them commercially.
**Abstract Book Citations:** Authors, Title, HemaSphere, 2026;10:(S1):pages. The abstract book DOIs can be found on the “Author Information” page.
**Disclaimer:** Articles published in the journal HemaSphere exclusively reflect the opinions of the authors. The authors are responsible for all content in their abstracts including accuracy of the facts, statements, citing resources, etc.

### (PB3973) REAL-WORLD MUTATIONAL LANDSCAPE OF LYMPHOID MALIGNANCIES: CHARACTERIZATION OF A SINGLE-CENTER COHORT

**Topic:** 21. Lymphoma biology & translational research

Carmen Amorós*[1], Margarita Monzón[1], Mª José Lis[1], Karla Javier[1], Rosa Collado Nieto[1], Sonia Marti[1], Armando Mena Durán[1], Paula Sánchez[1], Deborah Pérez[1], Maria Monera[1], Carmen García-Mora[2], Esther Diez-Recio[3], Esther Roselló[4], Josefa Marco Buades[1], Mariam Ibáñez Company[1]

*[1]Hematology and Hemotherapy Department. Consorcio Hospital General Universitario de Valencia. Hematology and Hemotherapy Research Group. Fundación Investigación Hospital General de Valencia., Valencia, Spain; [2]Department of Radiation Oncology. Consorcio Hospital General Universitario de Valencia. Fundación de Investigación Hospital General Universitario de Valencia, VALENCIA, Spain; [3]Department of Dermatology. Consorcio Hospital General Universitario de Valencia. Fundación de Investigación Hospital General Universitario de Valencia, VALENCIA, Spain; [4]Department of Pathology. Consorcio Hospital General Universitario de Valencia. Fundación de Investigación Hospital General Universitario de Valencia, VALENCIA, Spain;*

### Background

The implementation of next-generation sequencing (NGS) in routine lymphoma diagnostics enables systematic identification of recurrent and clinically relevant genomic alterations. However, real-world molecular data derived from heterogeneous, consecutive cohorts remain limited.

### Aims

We aimed to describe the clinicopathological and mutational landscape of lymphoid neoplasms analyzed with targeted NGS in daily practice.

### Methods

We retrospectively analyzed 50 consecutive patients diagnosed with lymphoid neoplasms at a tertiary single center (2019–2025). Molecular profiling was performed using a custom lymphoma-targeted NGS panel (HealthinCode) covering the full coding regions of 62 recurrently mutated genes in B- and T-cell lymphomas. Sequencing achieved a mean UMI-collapsed depth >800× from DNA derived from peripheral blood, bone marrow, or FFPE tissue. Bioinformatic analysis was conducted with DataGenomics, and variants were classified according to current guideline-based criteria. Descriptive analyses were performed by lymphoma subtype and mutational status.

**Copyright Information:** (Online) ISSN: 2572-9241
©2026 The Author(s). HemaSphere is published by John Wiley & Sons Ltd on behalf of European Hematology Association. This is an open access Abstract Book distributed under the Attribution-NonCommercial-NoDerivs (CC BY-NC-ND), which allows third parties to download the articles and share them with others as long as they credit the author and the Abstract Book, but they cannot change the content in any way or use them commercially.
**Abstract Book Citations:** Authors, Title, HemaSphere, 2026;10:(S1):pages. The abstract book DOIs can be found on the “Author Information” page.
**Disclaimer:** Articles published in the journal HemaSphere exclusively reflect the opinions of the authors. The authors are responsible for all content in their abstracts including accuracy of the facts, statements, citing resources, etc.

### Results

Median age was 63 years (range 52–68); 70% were male. Diffuse large B-cell lymphoma (DLBCL, including EBV-positive, double-hit and high-grade B-cell lymphoma) represented 40% of cases, followed by follicular (20%), mantle cell (10%), Hodgkin (14%), indolent B-cell neoplasms (18%), and peripheral T-cell or other rare lymphomas (10%).
NGS detected at least one pathogenic/likely pathogenic mutation in 24 patients (48%), with a median of 2 altered genes/case (range 1–5). Most frequently affected pathways involved epigenetic regulation and immune/NF-κB or JAK/STAT signaling. Epigenetic modifiers (*KMT2D, CREBBP, EZH2, DNMT3A, TET2, ARID1A*) were mutated in 35–40% of cases: *KMT2D* and *CREBBP* (10% each), *EZH2* (8–10%), and *DNMT3A, TET2* or *ARID1A* (6–10% across subtypes). Alterations in NF-κB/BCR signaling (*TNFAIP3, CD79B, CARD11, B2M*) and JAK/STAT components (*SOCS1, STAT3, STAT6*) were observed in 25–30% overall; recurrent events included *TNFAIP3* (6–8%), *SOCS1* (4–6%), *STAT3/STAT6* (6–8% combined), and *B2M* (6%). *TP53* mutations occurred in 8–10%, enriched in aggressive B-cell lymphomas and often co-occurring with other alterations. No consistent association between FISH abnormalities and specific mutations was identified.
Follicular lymphoma showed the expected t(14;18) with recurrent *KMT2D, CREBBP, EZH2* and *TNFRSF14* mutations. DLBCL frequently harbored *TP53, ARID1A, TNFAIP3* and *CD58* alterations, sometimes with *MYC/BCL2/BCL6* rearrangements. *MYD88* (±*CXCR4*) was enriched in lymphoplasmacytic and marginal zone lymphomas, whereas *RHOA*-G17V, *IDH2* and *TET2* characterized angioimmunoblastic T-cell lymphoma. Among aggressive B-cell lymphomas, 60% achieved complete remission with R-CHOP–based regimens; patients harboring pathogenic mutations, particularly *TP53*-mutated and double-hit cytogenetics cases, showed an early relapse compared with NGS-negative cases (55% vs 75% CR rate). No clear differences were observed in indolent entities.

### Summary/Conclusion

In this real-world cohort, targeted NGS revealed a high prevalence of recurrent alterations affecting epigenetic modifiers and signaling pathways across lymphoma subtypes. The identification of high-risk genomic subsets underscores the clinical relevance of integrating NGS into routine diagnostics for biological risk stratification and potential therapeutic tailoring.

**Copyright Information:** (Online) ISSN: 2572-9241
©2026 The Author(s). HemaSphere is published by John Wiley & Sons Ltd on behalf of European Hematology Association. This is an open access Abstract Book distributed under the Attribution-NonCommercial-NoDerivs (CC BY-NC-ND), which allows third parties to download the articles and share them with others as long as they credit the author and the Abstract Book, but they cannot change the content in any way or use them commercially.
**Abstract Book Citations:** Authors, Title, HemaSphere, 2026;10:(S1):pages. The abstract book DOIs can be found on the “Author Information” page.
**Disclaimer:** Articles published in the journal HemaSphere exclusively reflect the opinions of the authors. The authors are responsible for all content in their abstracts including accuracy of the facts, statements, citing resources, etc.

### (PB3974) PRESERVED B-CELL IDENTITY WITH ANTIGEN SIGNALING SUPPRESSION IN LARGE B-CELL LYMPHOMA OF IMMUNE-PRIVILEGED SITES (LBCL-IPS): EVIDENCE FOR A PAX5–CR2 AXIS OF IMMUNE ADAPTATION

**Topic:** 21. Lymphoma biology & translational research

Sabrina Khan*[1], Ariz Akhter[2], Arisha Khan[3], Ramez Shaker[1], Tariq Roshan[2], Adnan Mansoor[2]

*[1]University College Cork, School of Medicine, Cork, Ireland; [2]University of Calgary and Alberta Precision Laboratories, Pathology & Laboratory Medicine, Calgary, Canada; [3]University of Alberta, School of Public Health, Edmonton, Canada;*

### Background

Large B-cell lymphoma arising in immune-privileged sites (LBCL-IPS), including the central nervous system (CNS) and testis, represents a biologically distinct subset of diffuse large B-cell lymphoma, not otherwise specified (DLBCL-NOS). They are characterized by an immune-restricted microenvironment with limited antigen exposure and reduced immune surveillance. These conditions impose selective pressures that influence tumor survival and signaling behavior. However, the transcriptional mechanisms enabling LBCL-IPS persistence under reduced antigen-driven stimulation remain unclear. PAX5 is a master transcription factor that preserves B-cell lineage identity and stabilizes B-cell programming, while CR2 (CD21) enhances antigen-dependent B-cell receptor signaling. We investigated whether coordinated alterations in PAX5 and CR2 expression show adaptive transcriptional programming in LBCL-IPS in comparison to DLBCL-NOS.

### Aims

To characterize differential expression of PAX5 and CR2 in LBCL-IPS compared with de novo DLBCL and evaluate their biological implications in immune-restricted tumor environments.

### Methods

Nanostring gene expression profiling was performed on diagnostic biopsies from LBCL-IPS (n=40), further classified by anatomical site (CNS and testis), and de novo DLBCL-NOS (n=40). Cell-of-origin classification was determined using the Lymph2CX assay, and only cases belonging to the activated B-cell (ABC) subtype were included. Median expression levels of PAX5 and CR2 were compared between groups, and $log_2$ fold change ($log_2FC$), p-values, and false discovery rate–adjusted q-values were calculated to assess statistical significance.

**Copyright Information:** (Online) ISSN: 2572-9241
©2026 The Author(s). HemaSphere is published by John Wiley & Sons Ltd on behalf of European Hematology Association. This is an open access Abstract Book distributed under the Attribution-NonCommercial-NoDerivs (CC BY-NC-ND), which allows third parties to download the articles and share them with others as long as they credit the author and the Abstract Book, but they cannot change the content in any way or use them commercially.
**Abstract Book Citations:** Authors, Title, HemaSphere, 2026;10:(S1):pages. The abstract book DOIs can be found on the "Author Information" page.
**Disclaimer:** Articles published in the journal HemaSphere exclusively reflect the opinions of the authors. The authors are responsible for all content in their abstracts including accuracy of the facts, statements, citing resources, etc.

### Results

PAX5 expression was significantly higher in LBCL-IPS (median 2966.62) compared to DLBCL-NOS (median 341.14; $log_2FC$ +2.53 and p<0.0001), indicating strong maintenance of B-cell lineage identity. PAX5 is a key transcription factor that stabilizes B-cell programming, supporting survival in the absence of external differentiation cues. In contrast, CR2 was downregulated in LBCL-IPS (median 126.37 vs 254.71 in de novo; $log_2FC$ –2.61 and p<0.01). CR2 encodes a co-receptor that enhances BCR signaling in response to antigen-bound complement. Its reduced expression suggests reduced reliance on antigen-driven activation. This aligns with the immune-restricted nature of the CNS and testis, where antigen presentation and immune surveillance are limited. Together, this pattern reflects a tumor phenotype characterized by lineage preservation (PAX5) and functional immune silencing (CR2).

Table 1.

| | DLBCL Median | LBC-IPS Median | Log2 Fold Change | p value | q value |
|---|---|---|---|---|---|
| **CR2** | 126.37 | 254.71 | -2.61 | 8.49E-03 | 0.01 |
| **PAX5** | 341.14 | 2966.62 | 2.53 | 7.48E-06 | 2.25E-05 |

$Log_2$ fold change ($log_2FC$) is calculated as LBCL-IPS relative to DLBCL-NOS. q values represent false discovery rate–adjusted p values.

### Summary/Conclusion

LBCL-IPS indicates a coordinated transcriptional program marked by sustained B-cell lineage identity through elevated PAX5 expression and reduced antigen-dependent signaling via CR2 downregulation. This pattern likely reflects tumor adaptation to immune-privileged environments and highlights a biologically distinct mechanism differentiating LBCL-IPS from de novo DLBCL. The PAX5–CR2 axis may represent a marker of immune adaptation and warrants further investigation for its potential role in disease biology and therapeutic targeting in immune-restricted lymphomas.

**Copyright Information:** (Online) ISSN: 2572-9241
©2026 The Author(s). HemaSphere is published by John Wiley & Sons Ltd on behalf of European Hematology Association. This is an open access Abstract Book distributed under the Attribution-NonCommercial-NoDerivs (CC BY-NC-ND), which allows third parties to download the articles and share them with others as long as they credit the author and the Abstract Book, but they cannot change the content in any way or use them commercially.
**Abstract Book Citations:** Authors, Title, HemaSphere, 2026;10:(S1):pages. The abstract book DOIs can be found on the "Author Information" page.
**Disclaimer:** Articles published in the journal HemaSphere exclusively reflect the opinions of the authors. The authors are responsible for all content in their abstracts including accuracy of the facts, statements, citing resources, etc.

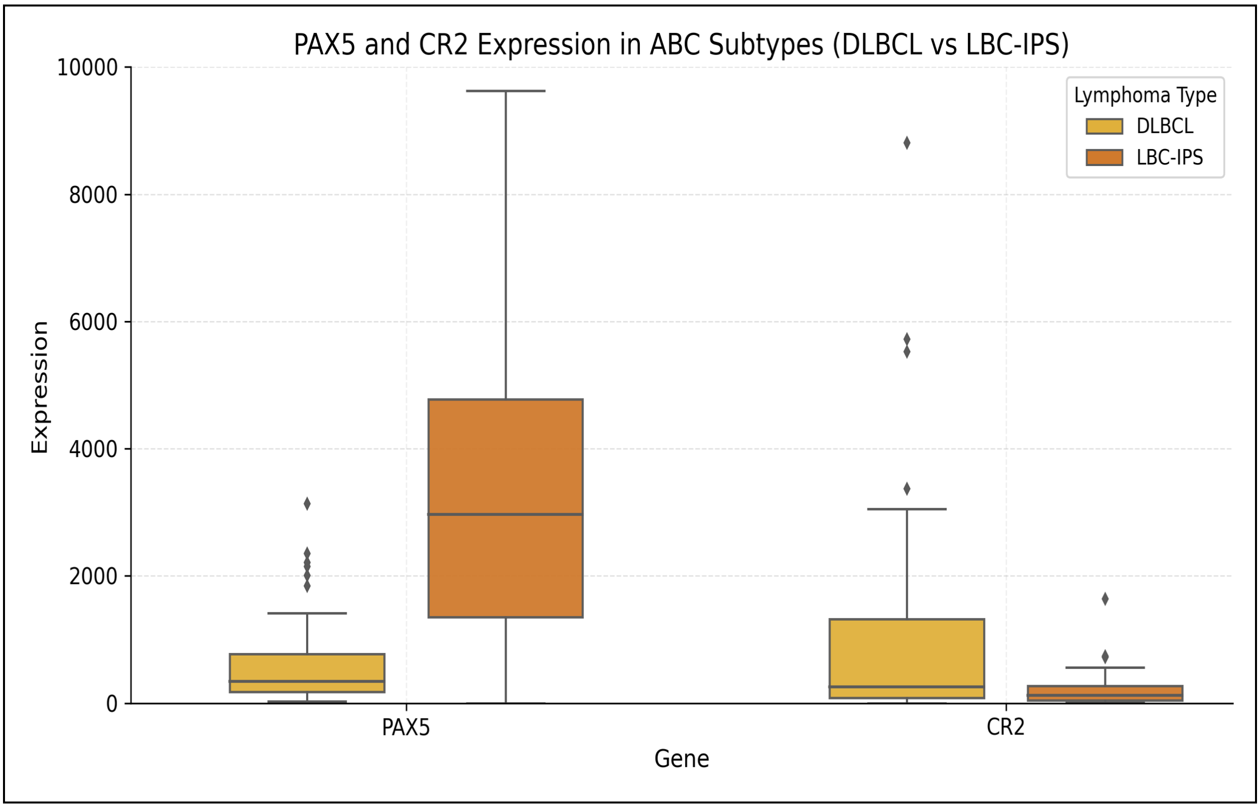

Boxplots of PAX5 and CR2 expression in ABC-subtype DLBCL and LBC-IPS (n=40 per group). Boxes represent interquartile range with median; whiskers and diamonds indicate range and outliers, respectively. NanoString gene expression data (ABC, R-ABC, C-ABC only).

**Copyright Information:** (Online) ISSN: 2572-9241
©2026 The Author(s). HemaSphere is published by John Wiley & Sons Ltd on behalf of European Hematology Association. This is an open access Abstract Book distributed under the Attribution-NonCommercial-NoDerivs (CC BY-NC-ND), which allows third parties to download the articles and share them with others as long as they credit the author and the Abstract Book, but they cannot change the content in any way or use them commercially.
**Abstract Book Citations:** Authors, Title, HemaSphere, 2026;10:(S1):pages. The abstract book DOIs can be found on the "Author Information" page.
**Disclaimer:** Articles published in the journal HemaSphere exclusively reflect the opinions of the authors. The authors are responsible for all content in their abstracts including accuracy of the facts, statements, citing resources, etc.

### (PB3975) CLINICAL AND PHARMACOGENOMIC DETERMINANTS OF DELAYED METHOTREXATE CLEARANCE IN ADULTS RECEIVING HIGH-DOSE METHOTREXATE

**Topic:** 21. Lymphoma biology & translational research

Yuwen Wang*[1], Hsing-Yu Lin[1], Tsung-Yu Pai[1], Chih-Fen Huang[2], Hsin-An Hou[3]

*[1]National Taiwan University Hospital, Department of Pharmacy, Taipei, Taiwan;[2]Graduate Institute of Clinical Pharmacy, College of Medicine, National Taiwan University, Taipei, Taiwan; [3]National Taiwan University Hospital, Division of Hematology, Department of Internal Medicine, Taipei, Taiwan;*

### Background

High-dose methotrexate (HD-MTX) is a key component in the treatment of acute lymphoblastic leukemia and aggressive lymphomas and required intensive inpatient monitoring to mitigate the risk of toxicities. Despite standardized supportive care, delayed MTX clearance remains a major contributor to MTX-related adverse effects. The hepatic uptake transporter OATP1B1, encoded by *SLCO1B1*, has been implicated in MTX disposition, yet prospective data in adult populations remain limited.

### Aims

To evaluate the impact of *SLCO1B1* polymorphism and clinical factors associated with delayed MTX clearance in adults receiving HD-MTX.

### Methods

Adult patients (≥20 years) with hematologic malignancies treated with HD-MTX (≥ 1000 mg/m²) between September 2022 and March 2024 were prospectively enrolled. Delayed clearance was defined as MTX levels >10 µM at 24 hours, >1 µM at 48 hours, or >0.1 µM at 72 hours. Acute kidney injury (AKI) was defined according to KDIGO criteria. Genotyping including *SLCO1B1, MTHFR C677T, and MTHFR A1298C was performed using a next-generation sequencing platform.* Logistic regression analyses were conducted to identify independent factors of delayed MTX clearance.

### Results

Forty-six patients were included, with a slight male predominance (N=27, 58.7%). The median age was 60.8 years and the mean baseline serum creatinine was 0.7±0.2 mg/dL. All patients received at least one cycle of HD-MTX with standardized supportive care, including adequate hydration and urine alkalization. The median administered dose was 4,250 mg/m², and no clinically significant drug–drug interactions were identified. Delayed MTX clearance occurred in 39.1% of patients and was associated with AKI, which developed in 28.3% of patients. Patients with delayed MTX clearance were significantly older than those without delayed clearance (P = 0.007). Variables with P <0.20, including age, baseline serum creatinine, MTX dose, and *SLCO1B1* function, were entered into a multivariate logistic regression model. Older age remained an independent predictor of delayed MTX clearance (OR 1.10, 95% CI 1.017–1.191, P = 0.017), while *SLCO1B1* function showed a borderline association (P = 0.051). In an exploratory subgroup of patients receiving intensified dosing (> 4000 mg/m²), patients with decreased/poor *SLCO1B1* function demonstrated higher 24-hour MTX levels than those with normal function (7.1±11.4 µM vs. 1.4±1.9 µM, P = 0.087). These findings suggest a potential dose-dependent pharmacogenomic effect, whereby transporter capacity may become rate-limiting under higher MTX exposure.

**Copyright Information:** (Online) ISSN: 2572-9241
©2026 The Author(s). HemaSphere is published by John Wiley & Sons Ltd on behalf of European Hematology Association. This is an open access Abstract Book distributed under the Attribution-NonCommercial-NoDerivs (CC BY-NC-ND), which allows third parties to download the articles and share them with others as long as they credit the author and the Abstract Book, but they cannot change the content in any way or use them commercially.
**Abstract Book Citations:** Authors, Title, HemaSphere, 2026;10:(S1):pages. The abstract book DOIs can be found on the “Author Information” page.
**Disclaimer:** Articles published in the journal HemaSphere exclusively reflect the opinions of the authors. The authors are responsible for all content in their abstracts including accuracy of the facts, statements, citing resources, etc.

**Summary/Conclusion**

In this single-center Taiwanese cohort, delayed MTX clearance and AKI remained common despite standardized supportive care, particularly among older patients. Although *SLCO1B1* genotype did not reach conventional statistical significance, the observed effect size and exposure patterns—especially under intensified dosing—support a potential pharmacogenomic influence on MTX disposition. These findings warrant validation in larger studies and further investigation of pharmacogenomic-guided strategies to optimize HD-MTX therapy.

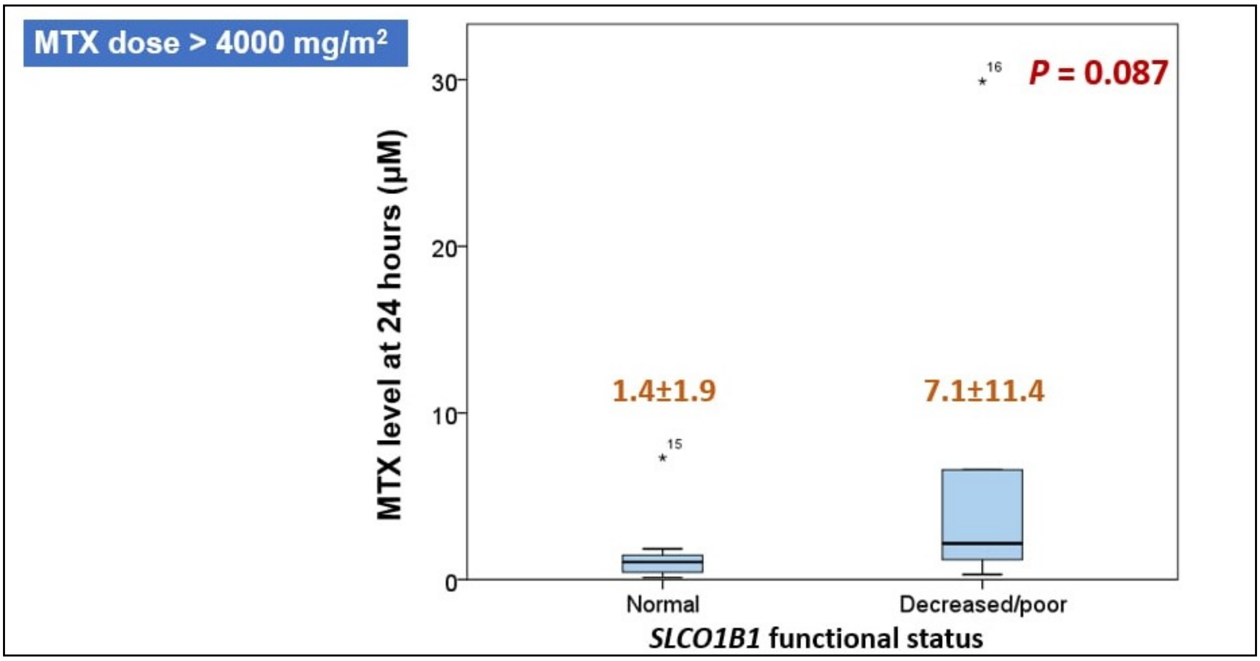

Distribution of MTX concentrations at 24 hours in patients receiving intensified dosing according to SLCO1B1 functional status

**Copyright Information:** (Online) ISSN: 2572-9241
©2026 The Author(s). HemaSphere is published by John Wiley & Sons Ltd on behalf of European Hematology Association. This is an open access Abstract Book distributed under the Attribution-NonCommercial-NoDerivs (CC BY-NC-ND), which allows third parties to download the articles and share them with others as long as they credit the author and the Abstract Book, but they cannot change the content in any way or use them commercially.
**Abstract Book Citations:** Authors, Title, HemaSphere, 2026;10:(S1):pages. The abstract book DOIs can be found on the "Author Information" page.
**Disclaimer:** Articles published in the journal HemaSphere exclusively reflect the opinions of the authors. The authors are responsible for all content in their abstracts including accuracy of the facts, statements, citing resources, etc.

### (PB3976) ALTERATIONS IN CELLULAR METABOLISM UNDERLIE VENETOCLAX RESISTANCE IN DLBCL

**Topic:** 21. Lymphoma biology & translational research

Daisy Tran[1], Evelyn Teh[2], Susanne Penny[2], Devenand Pinto[2], Samuel Saibil[1], John Kuruvilla[1], Rob Laister*[1]

*[1]Princess Margaret Cancer Centre, Toronto, Canada;[2]National Research Coucil of Canada, Halifax, Canada;*

### Background

Large B cell lymphoma is a heterogeneous lymphoid malignancy where the expression of anti-apoptotic protein BCL-2 is often dysregulated by chromosomal translocation and/or overexpression. Patients with BCL-2 dysregulation have worse prognosis under standard chemoimmunotherapy treatment regimens and represent a clinical unmet need. BCL-2 inhibitors such as venetoclax provide an opportunity to target BCL-2, however, resistance to BCL-2 inhibition has been observed. The goal of our study is to delineate the metabolic basis of venetoclax resistance in lymphoma, to better understand BCL-2 biology and to aid in identifying potential routes for the development of combination therapies.

### Aims

The aims of the this study are to understand how different aspects cellular metabolism are altered in venetoclax resistant lymphoma cells to better understand the effects of continued exposure to venetoclax and to potentially use this information as a basis for designing rationale drug combinations.

### Methods liquid-chromatorgraphy mass spectrometry, flow cytometry, electron microscopy, bioenergetic measurements, mouse xenograft models

### Results

We observed that venetoclax resistant (VR) cell lines have significantly altered bioenergetics as compared to venetoclax sensitive (VS) cells. The VR cells are more metabolically active at baseline and more flexible following stress. We also observed greater mitochondrial ATP production rates and, consequently, greater reactive oxygen species production. Using electron microscopy, we find that VR cells contain both fissed and fused mitochondria, whereas mitochondria in VS cells take on a more uniform morphology. In line with the observed mitochondrial phenotype, VR cells trend towards greater mitochondrial mass and mass spectrometry proteomics revealed upregulated pathways related to mitochondrial protein degradation, aerobic respiration, and mitochondrial translation. The VR cells appear to activate the pathways to induce mitchondrial biogenesis which is in keeping with the electron microscopy data which is indicative of mitochondrial turnover. VR SUDHL-4 lymphoma cells engraft readily into NSG mice where they result in an aggressive disease phenotype.

### Summary/Conclusion

VR lymphoma cells have significantly altered bioenergetic programs originating in part through increased expression of mitochondrial proteins related to oxidative phosphorylation.  We interpret these results to suggest that the mitochondria in VR cells, while operating at elevated levels of oxidative phosphorylation and producing elevated levels of ROS, activate additional pathways for the surveillance and removal of damaged or unfolded proteins. Changes in mitochondrial dynamics appear to be employed to recycle mitochondria that are damaged as a result of eleveted levels of oxidative phosphoryaltion.

**Copyright Information:** (Online) ISSN: 2572-9241
©2026 The Author(s). HemaSphere is published by John Wiley & Sons Ltd on behalf of European Hematology Association. This is an open access Abstract Book distributed under the Attribution-NonCommercial-NoDerivs (CC BY-NC-ND), which allows third parties to download the articles and share them with others as long as they credit the author and the Abstract Book, but they cannot change the content in any way or use them commercially.
**Abstract Book Citations:** Authors, Title, HemaSphere, 2026;10:(S1):pages. The abstract book DOIs can be found on the “Author Information” page.
**Disclaimer:** Articles published in the journal HemaSphere exclusively reflect the opinions of the authors. The authors are responsible for all content in their abstracts including accuracy of the facts, statements, citing resources, etc.

### (PB3977) IDENTIFICATION OF DRUGGABLE AND GUT MICROBIOTA-ASSOCIATED PROGNOSTIC GENES AND EXPLORATION OF THEIR REGULATORY MECHANISMS IN FOLLICULAR LYMPHOMA VIA BULK RNA-SEQ AND MENDELIAN RANDOMIZATION

**Topic:** 21. Lymphoma biology & translational research

Heng Li*[1], Hongling Peng[1]

[1] *The Second Xiangya Hospital of Central South University, Changsha, China;*

### Background

Follicular lymphoma (FL) is a common indolent non-Hodgkin lymphoma, characterized by high recurrence rates in patients. Current treatments only alleviate symptoms but fail to improve long-term prognosis. Moreover, the systematic identification of druggable genes (DGs) in FL remains insufficient, and the association mechanisms between gut microbiota (GM) and FL progression, treatment response as well as prognosis have not yet been elucidated.

### Aims

To identify DGs in FL and to elucidate the association mechanisms between gut microbiota (GM) and FL progression, treatment response as well as prognosis.

### Methods

Differentially expressed genes (DEGs) were intersected with DGs and gut microbiota-related genes (GRGs) to screen out candidate genes. Subsequently, Mendelian randomization (MR), univariate Cox regression, and random forest (RF) analyses were performed to identify prognostic genes. Furthermore, a prognostic model was established and validated. Gene set enrichment analysis (GSEA) was conducted for pathway enrichment, and immune cell infiltration patterns were evaluated. Additionally, potential drug prediction and molecular docking analyses were implemented for the identified prognostic genes.

### Results

Integration of DEGs with DGs and GRGs yielded 64 candidate genes. A total of 2 prognostic genes (CA4 and MMP9) were identified via univariate Cox regression, MR, and RF analysis. The prognostic model demonstrated reliable predictive efficacy in both training and validation analyses. GSEA results showed down-regulation of the lysosome pathway and up-regulation of the drug metabolism-cytochrome P450 pathway in the high-risk group. Immune microenvironment analysis revealed significantly elevated neutrophil scores in this group, with a significant positive correlation between CA4 and neutrophils. Furthermore, targeted drug prediction confirmed strong binding affinities between CA4 and trichlormethiazide, as well as between MMP9 and carboxylated glucosamine.

### Summary/Conclusion

Overall, CA4 and MMP9 were identified as druggable prognostic genes associated with gut microbiota in FL. This finding provides novel insights into the clinical diagnosis and targeted therapy of FL.

**Copyright Information:** (Online) ISSN: 2572-9241
©2026 The Author(s). HemaSphere is published by John Wiley & Sons Ltd on behalf of European Hematology Association. This is an open access Abstract Book distributed under the Attribution-NonCommercial-NoDerivs (CC BY-NC-ND), which allows third parties to download the articles and share them with others as long as they credit the author and the Abstract Book, but they cannot change the content in any way or use them commercially.
**Abstract Book Citations:** Authors, Title, HemaSphere, 2026;10:(S1):pages. The abstract book DOIs can be found on the "Author Information" page.
**Disclaimer:** Articles published in the journal HemaSphere exclusively reflect the opinions of the authors. The authors are responsible for all content in their abstracts including accuracy of the facts, statements, citing resources, etc.

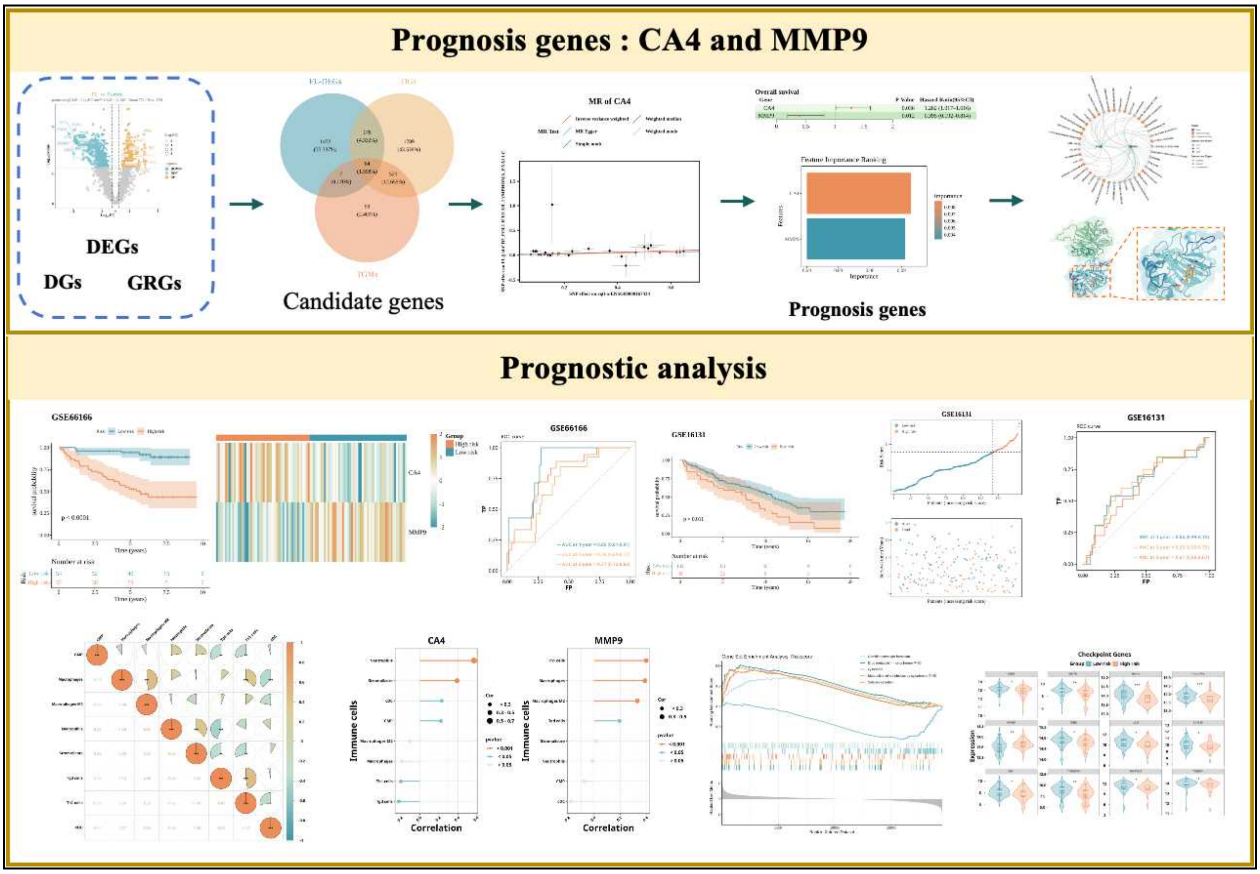

Graphic abstract of the study

**Copyright Information:** (Online) ISSN: 2572-9241
©2026 The Author(s). HemaSphere is published by John Wiley & Sons Ltd on behalf of European Hematology Association. This is an open access Abstract Book distributed under the Attribution-NonCommercial-NoDerivs (CC BY-NC-ND), which allows third parties to download the articles and share them with others as long as they credit the author and the Abstract Book, but they cannot change the content in any way or use them commercially.
**Abstract Book Citations:** Authors, Title, HemaSphere, 2026;10:(S1):pages. The abstract book DOIs can be found on the "Author Information" page.
**Disclaimer:** Articles published in the journal HemaSphere exclusively reflect the opinions of the authors. The authors are responsible for all content in their abstracts including accuracy of the facts, statements, citing resources, etc.

### (PB3980) A DOUBLE-EDGED SWORD: HOW THERAPY-INDUCED SENESCENCE DRIVES TUMOUR DISSEMINATION IN NON-HODGKIN LYMPHOMA

**Topic:** 21. Lymphoma biology & translational research

Basseem Radwan*[1], Cristina Guardia[1], Joan Balibrea[1], Elena Castillo[1], Mariia Sydorenko[1], David Olivares[1], Verónica Rodilla[1]

[1]*Josep Carreras Leukaemia Research Institute, Cancer Heterogeneity and Hierarchies, Badalona, Barcelona, Spain;*

### Background

Non-Hodgkin lymphoma (NHL) comprises a heterogeneous group of predominantly B-cell lymphoid malignancies with diverse clinical outcomes [1]. The standard first-line treatment, R-CHOP, relies on non-selective chemotherapeutics; cyclophosphamide, doxorubicin, and vincristine [2]. NHL remains a major global health burden due to frequent relapse and resistance[3], potentially driven by accumulation of therapy-associated senescent cells in different tissues.
Cellular senescence is defined by sustained cell cycle arrest and secretion of pro-inflammatory cytokines, chemokines, proteases, and growth factors, termed the senescence-associated secretory phenotype [4]. Evidence implicates senescence in hematologic malignancies, including acute myeloid leukaemia and multiple myeloma [5–7]. However, whether a therapy-induced senescence (TIS) microenvironment promotes NHL progression remains unclear. Detection of TIS is also challenging due to the lack of a definitive marker. Addressing this gap is essential for relapse prevention and early identification of at-risk patients.

### Methods

We combined single-cell RNA sequencing, proteomics, and functional *in vitro* and *in vivo* assays to profile senescence signatures and consequences in tissues frequently colonised by NHL cells. Blood samples were analysed for circulating senescence markers.

### Results

CHOP induced senescence in non-malignant liver, lung, and spleen cells in immunocompetent mouse models. It increased lymphoma metastasis, endothelial damage, and hepatic neutrophil recruitment. This neutrophil influx promoted lymphoma extravasation and dissemination, supporting their role in TIS-driven dissemination. This effect was reversed by the senolytic navitoclax, highlighting the potential for identifying patient who could benefit from combined R-CHOP and senolytic strategies.
Senescence-associated gene expression was elevated in blood from CHOP-treated animals. Raman microscopy enables label-free, high-sensitivity biochemical phenotyping. A Raman-based blood assay is under development to improve patient stratification and identify susceptibility to cumulative TIS.

### Summary/Conclusion

Our findings demonstrate that CHOP induces multi-organ senescence, vascular alterations, and neutrophil recruitment, contributing to increased metastatic burden. The circulating TIS signature in treated animal models highlights its potential as a minimally invasive biomarker for monitoring TIS as in NHL patients.

**Copyright Information:** (Online) ISSN: 2572-9241
©2026 The Author(s). HemaSphere is published by John Wiley & Sons Ltd on behalf of European Hematology Association. This is an open access Abstract Book distributed under the Attribution-NonCommercial-NoDerivs (CC BY-NC-ND), which allows third parties to download the articles and share them with others as long as they credit the author and the Abstract Book, but they cannot change the content in any way or use them commercially.
**Abstract Book Citations:** Authors, Title, HemaSphere, 2026;10:(S1):pages. The abstract book DOIs can be found on the “Author Information” page.
**Disclaimer:** Articles published in the journal HemaSphere exclusively reflect the opinions of the authors. The authors are responsible for all content in their abstracts including accuracy of the facts, statements, citing resources, etc.

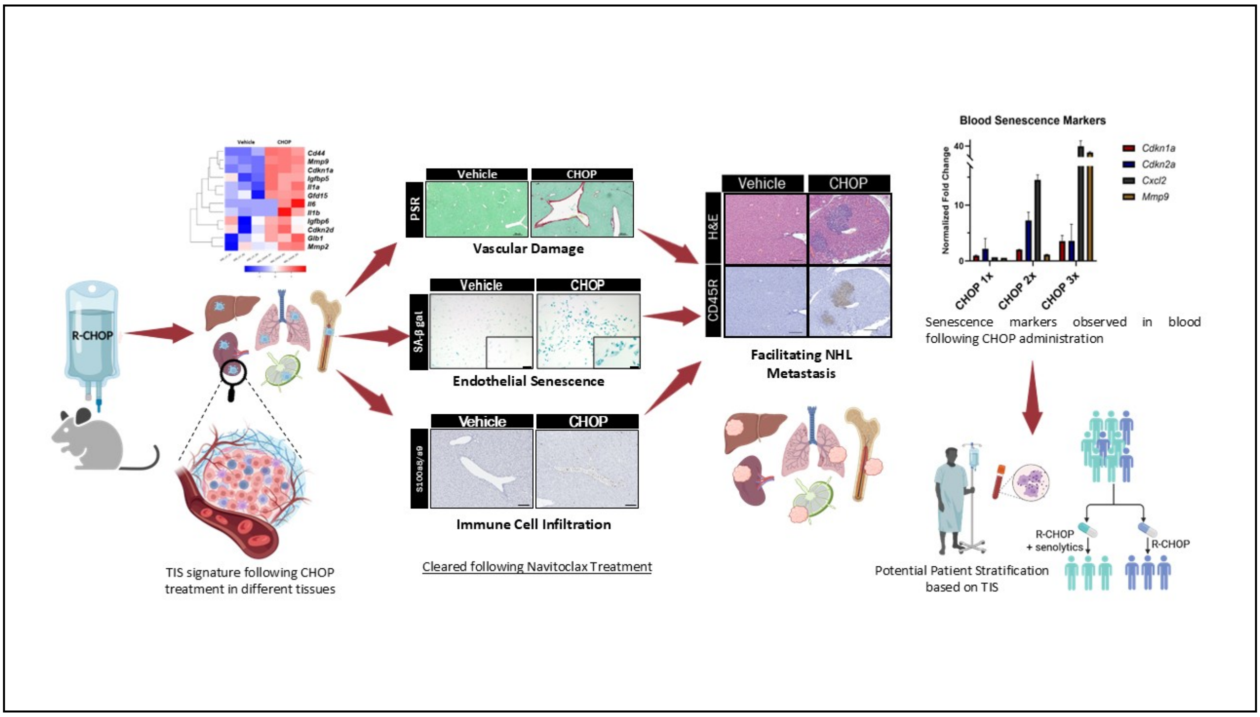

PSR: picrosirius red staining. SA-β-gal: Senescence-associated β-galactosidase staining.

**Copyright Information:** (Online) ISSN: 2572-9241
©2026 The Author(s). HemaSphere is published by John Wiley & Sons Ltd on behalf of European Hematology Association. This is an open access Abstract Book distributed under the Attribution-NonCommercial-NoDerivs (CC BY-NC-ND), which allows third parties to download the articles and share them with others as long as they credit the author and the Abstract Book, but they cannot change the content in any way or use them commercially.
**Abstract Book Citations:** Authors, Title, HemaSphere, 2026;10:(S1):pages. The abstract book DOIs can be found on the "Author Information" page.
**Disclaimer:** Articles published in the journal HemaSphere exclusively reflect the opinions of the authors. The authors are responsible for all content in their abstracts including accuracy of the facts, statements, citing resources, etc.

### (PB3981) IMPLEMENTATION OF TRBC-BASED T-CELL CLONALITY TESTING IN A RESOURCE-LIMITED EUROPEAN CENTER

**Topic:** 21. Lymphoma biology & translational research

Elsuarta Calliku*[1], Valentina Semanaj[1], Adela Perolla[1], Alma Cili[1], Teuta Curaj[1, 1], Polikron Pulluqi[1], Arben Ivanaj[1]

[1]*University Hospital Centre " Mother Teresa", Tiranë, Albania;*

### Background

The accurate identification of T-cell clonality is a fundamental tool for the diagnose of the mature T-cell lymphoproliferative disorders (T-LPD). Actual conventional assessment relies on the study of the molecular T-cell receptor (TCR) gene rearrangement, and this sometime may be time-consuming, costly, and not universally available. The use of Flow cytometric evaluation of TCR β constant region expression (TRBC1/TRBC2) has emerged recently as a real practical alternative, exploiting the mutually exclusive expression of TRBC1 or TRBC2 in the normal αβ T-cell populations, in order to identify the monoclonal expansions.

### Aims

Our aim was to to assess the feasibility and also the diagnostic performance of TRBC1/TRBC2 restriction analysis as a rapid surrogate marker of T-cell clonality when implemented within routine flow cytometric practice in a real-world hematology setting.

### Methods

We conducted a retrospective evaluation of thirteen consecutive patients referred in our Clinic of Hematology for immunophenotypic investigation of a suspected lymphoproliferative disorders from january 2024 to january 2026. The multiparameter flow cytometry panels incorporated also TRBC1 and TRBC2 antibodies within the standard lineage and maturation markers normally used. Clonality was defined by the dominant expression of either TRBC1 or TRBC2 (>85–90%) with the reciprocal suppression of the alternative constant region. Peripheral blood and bone marrow samples were accurately analyzed using a six-color Beckman Coulter DxFLEX flow cytometer and immunophenotypic characteristics of detected clones were recorded.

### Results

We have included in this study thirteen patients, predominantly representing an elderly population investigated for suspected mature T-cell neoplasia. TRBC restriction consistent with monoclonal T-cell proliferation was detected in all cases we have studied (13/13, 100%). The TRBC2-restricted clones predominated in 9/13 cases (69%), while the TRBC1 restriction was observed in 4/13 cases (31%). So, in all the evaluable cases TRBC restriction compatible with monoclonal T-cell proliferation was identified and our data demonstrated that TRBC2-restricted clones were more frequent than TRBC1-restricted clones. The clonal populations involved both CD4+ and CD8+ T-cell subsets and commonly exhibited immunophenotypic aberrancies, including antigen loss and atypical CD4/CD8 co-expression. The integration of TRBC analysis made possible the immediate detection of the clonality during primary flow cytometric assessment, demonstrating the need for initial molecular testing.

### Summary/Conclusion

Flow cytometric TRBC1/TRBC2 restriction represents a rapid, reproducible, and clinically applicable surrogate for T-cell clonality assessment. The Real-world implementation within routine hematology diagnostics facilitates in this way the early identification of mature T-cell lymphoproliferative disorders and supports the importance of the incorporation of TRBC analysis into standard immunophenotyping workflows, particularly in resource-constrained environments.

**Copyright Information:** (Online) ISSN: 2572-9241
©2026 The Author(s). HemaSphere is published by John Wiley & Sons Ltd on behalf of European Hematology Association. This is an open access Abstract Book distributed under the Attribution-NonCommercial-NoDerivs (CC BY-NC-ND), which allows third parties to download the articles and share them with others as long as they credit the author and the Abstract Book, but they cannot change the content in any way or use them commercially.
**Abstract Book Citations:** Authors, Title, HemaSphere, 2026;10:(S1):pages. The abstract book DOIs can be found on the “Author Information” page.
**Disclaimer:** Articles published in the journal HemaSphere exclusively reflect the opinions of the authors. The authors are responsible for all content in their abstracts including accuracy of the facts, statements, citing resources, etc.

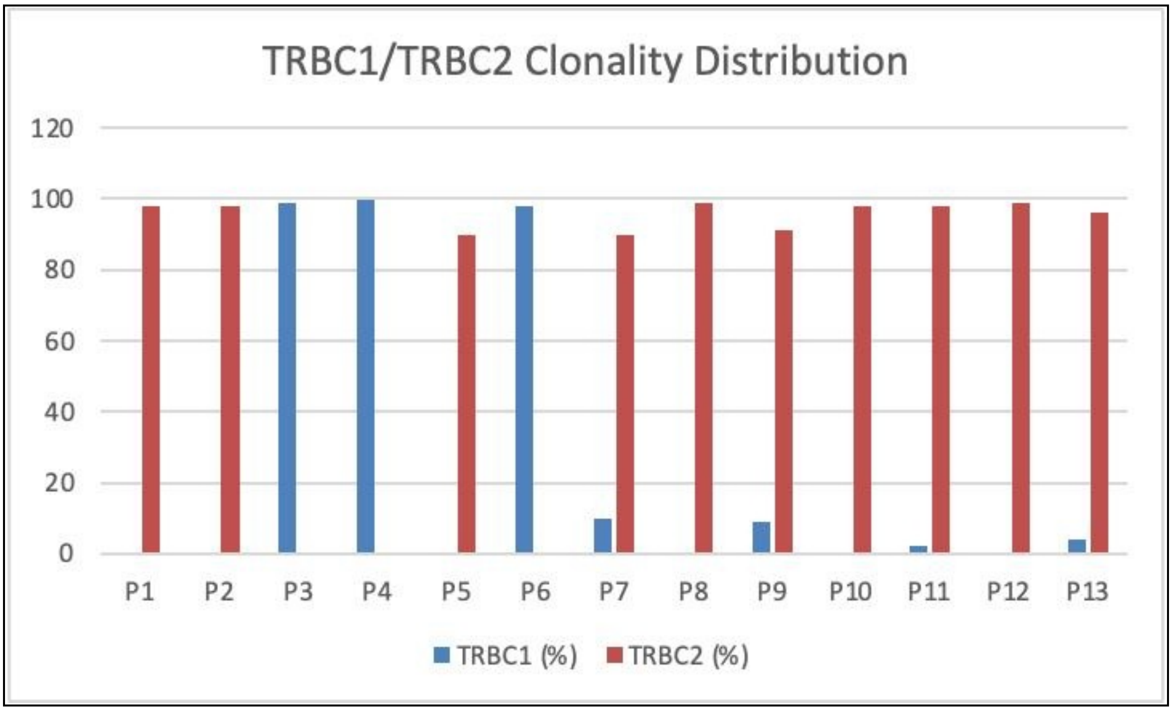

TRBC1/TRBC2 Clonality distribution

Copyright Information: (Online) ISSN: 2572-9241
©2026 The Author(s). HemaSphere is published by John Wiley & Sons Ltd on behalf of European Hematology Association. This is an open access Abstract Book distributed under the Attribution-NonCommercial-NoDerivs (CC BY-NC-ND), which allows third parties to download the articles and share them with others as long as they credit the author and the Abstract Book, but they cannot change the content in any way or use them commercially.
Abstract Book Citations: Authors, Title, HemaSphere, 2026;10:(S1):pages. The abstract book DOIs can be found on the "Author Information" page.
Disclaimer: Articles published in the journal HemaSphere exclusively reflect the opinions of the authors. The authors are responsible for all content in their abstracts including accuracy of the facts, statements, citing resources, etc.

### (PB3982) PROMISING OUTCOMES WITH DUAL EPIGENETIC THERAPY FOR OLDER PATIENTS (>70 YEARS) WITH NEWLY DIAGNOSED ANGIOIMMUNOBLASTIC T-CELL LYMPHOMA

**Topic:** 21. Lymphoma biology & translational research

Chao Hu[1], Chunmei Yang*[2], Hongyan Tong[3], Jie Jin[4]

*[1]The First Affiliated Hospital, Zhejiang University School of Medicine, Department of Hematology, Hangzhou, China;[2]the First Affiliated Hospital, Zhejiang University School of Medicine, Department of hematology, Hangzhou, China; [3]The First Affiliated Hospital, Zhejiang University School of Medicine, department of hematoloy, Hangzhou, China; [4]The First Affiliated Hospital Zhejiang University School of Medicine, department of hematology, Hangzhou, China;*

### Background

Previous studies have reported that the median onset age of angioimmunoblastic T-cell lymphoma (AITL) is approximately 65 years, with a 5-year overall survival (OS) rate of only 30%. Elderly patients aged over 70 years cannot tolerate conventional intensive chemotherapy and have poorer performance status, with a 5-year OS rate of less than 20%. Therefore, novel therapeutic strategies are urgently needed to improve the current situation. The abnormal epigenetic modifications in the development and progression of AITL have garnered considerable attention. Three recurring-mutation genes (TET2, DNMT3A and IDH2), involved in the regulation of DNA methylation, were high-frequency mutation rate in AITL patients. And, hypoacetylated histones are associated with closed the development of AITL. Previous studies demonstrated that azacitidine combined with chidamide was effective in patients with R/R AITL and had a favorable safety profile.

### Aims

Hence, this study aimed to investigate the efficacy and safety of chidamide combined with azacitidine in the treatment of newly diagnosed elderly AITL patients (>70 Years).

### Methods

We enrolled elderly patients (>70 Years) with newly diagnosed AITL treated with chidamide plus azacitidine in the Department of Hematology, the First Affiliated Hospital of Zhejiang University. Patients received 5-azacytidine at 75 mg/m2 subcutaneously for 5 consecutive days every 21 days and oral chidamide at 20 mg twice a week. The primary endpoint of the study is to explore the efficacy and safety.

### Results

Enrollment of patients into the study occurred from March 15, 2024, to July 15, 2025, and comprised a cohort of 7 patients. The median age was 71 years (range, 71-81), with 57.1% being female. All patients presented with advanced disease (stage III-IV). Three patients had a poor performance status (ECOG ≥2), and the median international prognostic index (IPI) was 3 at the time of diagnosis. Among the patients, 3(42.9%) had elevated lactate dehydrogenase and 4 (57.1%) had bone marrow involvement. The best ORR was 85.7% (6/7), with a complete remission rate (CR) of 42.9% and partial remission rate (PR) of 42.9%. Patients were monitored until either the date of death or the last follow-up date. The median duration of follow-up was 13.6 months. The median progression-free survival (PFS) and OS was not estimable. The 1 year PFS and OS rate was 85.7% and 100%, respectively. Treatment was generally well tolerated with expected side effects. Treatment-emergent grade 3 to 4 hematologic toxicities included neutropenia (57.1%), thrombocytopenia (57.1%) and anemia (42.9%), with febrile neutropenia (28.6%). Grade 3 to 4 nonhematologic toxicities included 42.9% fatigue and 28.6% diarrhea.

**Copyright Information:** (Online) ISSN: 2572-9241
©2026 The Author(s). HemaSphere is published by John Wiley & Sons Ltd on behalf of European Hematology Association. This is an open access Abstract Book distributed under the Attribution-NonCommercial-NoDerivs (CC BY-NC-ND), which allows third parties to download the articles and share them with others as long as they credit the author and the Abstract Book, but they cannot change the content in any way or use them commercially.
**Abstract Book Citations:** Authors, Title, HemaSphere, 2026;10:(S1):pages. The abstract book DOIs can be found on the “Author Information” page.
**Disclaimer:** Articles published in the journal HemaSphere exclusively reflect the opinions of the authors. The authors are responsible for all content in their abstracts including accuracy of the facts, statements, citing resources, etc.

**Summary/Conclusion**

Chidamide combined with azacitidine shows favorable efficacy, few adverse reactions and good tolerability in elderly patients (>70 Years) with AITL, which is worthy of further clinical promotion and application.

**Copyright Information:** (Online) ISSN: 2572-9241
©2026 The Author(s). HemaSphere is published by John Wiley & Sons Ltd on behalf of European Hematology Association. This is an open access Abstract Book distributed under the Attribution-NonCommercial-NoDerivs (CC BY-NC-ND), which allows third parties to download the articles and share them with others as long as they credit the author and the Abstract Book, but they cannot change the content in any way or use them commercially.
**Abstract Book Citations:** Authors, Title, HemaSphere, 2026;10:(S1):pages. The abstract book DOIs can be found on the "Author Information" page.
**Disclaimer:** Articles published in the journal HemaSphere exclusively reflect the opinions of the authors. The authors are responsible for all content in their abstracts including accuracy of the facts, statements, citing resources, etc.

### (PB3983) PROGNOSTIC IMPACT OF TP53 ABERRATION IN RICHTER TRANSFORMATION DURING THE ERA OF TARGETED AGENTS: A SINGLE-CENTER EXPERIENCE

**Topic:** 21. Lymphoma biology & translational research

Maria D'amato*[1], Claudia Barate'[2], Gabriele Bolognesi[1], Sara Galimberti[1]

*[1]Department of Clinical and Experimental Medicine, Haematology Unit, University of Pisa, Pisa, Italy;[2]Department of Hematology and Oncology, Hematology Unit, Niguarda Metropolitan Hospital, Pisa, Italy;*

### Background

Richter Transformation (RT) represents the clinico-pathological evolution of chronic lymphocytic leukemia (CLL) into a secondary, highly aggressive lymphoid malignancy, typically a diffuse large B-cell lymphoma (DLBCL). In the current landscape of hematology, RT remains one of the most significant therapeutic challenges, particularly for patients who have already been exposed to and failed modern targeted agents like BTK inhibitors or BCL-two antagonists. This comprehensive study aims to report on the clinical characteristics, complex molecular features, diverse treatment approaches, and survival outcomes of RT cases managed within ourac institution. A specific and primary focus was placed on the prognostic weight of **TP53 aberrations**, which include both gene mutations and deletions.

### Aims

The primary objective of this case series was to characterize the clinical and biological landscape of RT in a real-world institutional setting during the era of novel oral agents, evaluate the prevalence and prognostic significance of **TP53 aberrations** within this high-risk population, analyze the correlation between molecular profiles and the clinical response to various therapeutic strategies, including chemo-immunotherapy and targeted combinations, assess overall survival outcomes to identify the most critical biomarkers driving treatment failure and disease progression.

### Methods

We performed a retrospective review of **12** consecutive cases of RT diagnosed between **2012** and **2025**. All diagnoses were verified through histological and immunophenotypic examination. The analysis focused on patient demographics, previous CLL treatment history, RT histological variants, and genomic markers (including IGHV status, del17p, TP53 mutations, and complex karyotypes). Clinical response was graded according to the **2014** Lugano classification, and overall survival (OS) was calculated from the date of RT diagnosis.

### Results

The cohort had a median age of 66 years (range 56–86) at the time of transformation. Notably, 11 patients (91.7%) were pre-treated for CLL, with 7 having received BTK inhibitors. High-risk genetic profiles were prevalent: 5 patients (41.7%) harbored TP53 mutations, 4 (33.3%) showed del17p, and 3 (25%) presented a complex karyotype.
RT histology was DLBCL-type in 8 patients (66.7%), Hodgkin-like in 3 (25%), and mixed in 1 (8.3%). Induction strategies varied between chemo-immunotherapy (n=6), novel targeted combinations (n=4), and cellular products (n=2). The cohort achieved an overall response rate of 41.7%. However, TP53-aberrant patients faced significantly poorer prognoses, with a median OS of only 5.2 months, contrasted with 13.8 months in the wild-type group. All deaths or losses to follow-up occurred within the DLBCL subgroup carrying high-risk molecular markers.

**Copyright Information:** (Online) ISSN: 2572-9241
©2026 The Author(s). HemaSphere is published by John Wiley & Sons Ltd on behalf of European Hematology Association. This is an open access Abstract Book distributed under the Attribution-NonCommercial-NoDerivs (CC BY-NC-ND), which allows third parties to download the articles and share them with others as long as they credit the author and the Abstract Book, but they cannot change the content in any way or use them commercially.
**Abstract Book Citations:** Authors, Title, HemaSphere, 2026;10:(S1):pages. The abstract book DOIs can be found on the "Author Information" page.
**Disclaimer:** Articles published in the journal HemaSphere exclusively reflect the opinions of the authors. The authors are responsible for all content in their abstracts including accuracy of the facts, statements, citing resources, etc.

**Summary/Conclusion**

This real-life case series provides a vivid illustration of the aggressive nature of Richter Transformation in patients heavily pre-treated for CLL. Our data confirms that, even in the era of novel drugs, the presence of a **TP53 aberration** stands as the most formidable adverse prognostic factor. Despite the integration of targeted therapies, outcomes remain largely unsatisfactory, emphasizing the urgent need for early identification of high-risk cases and the development of innovative, non-chemotherapeutic strategies.

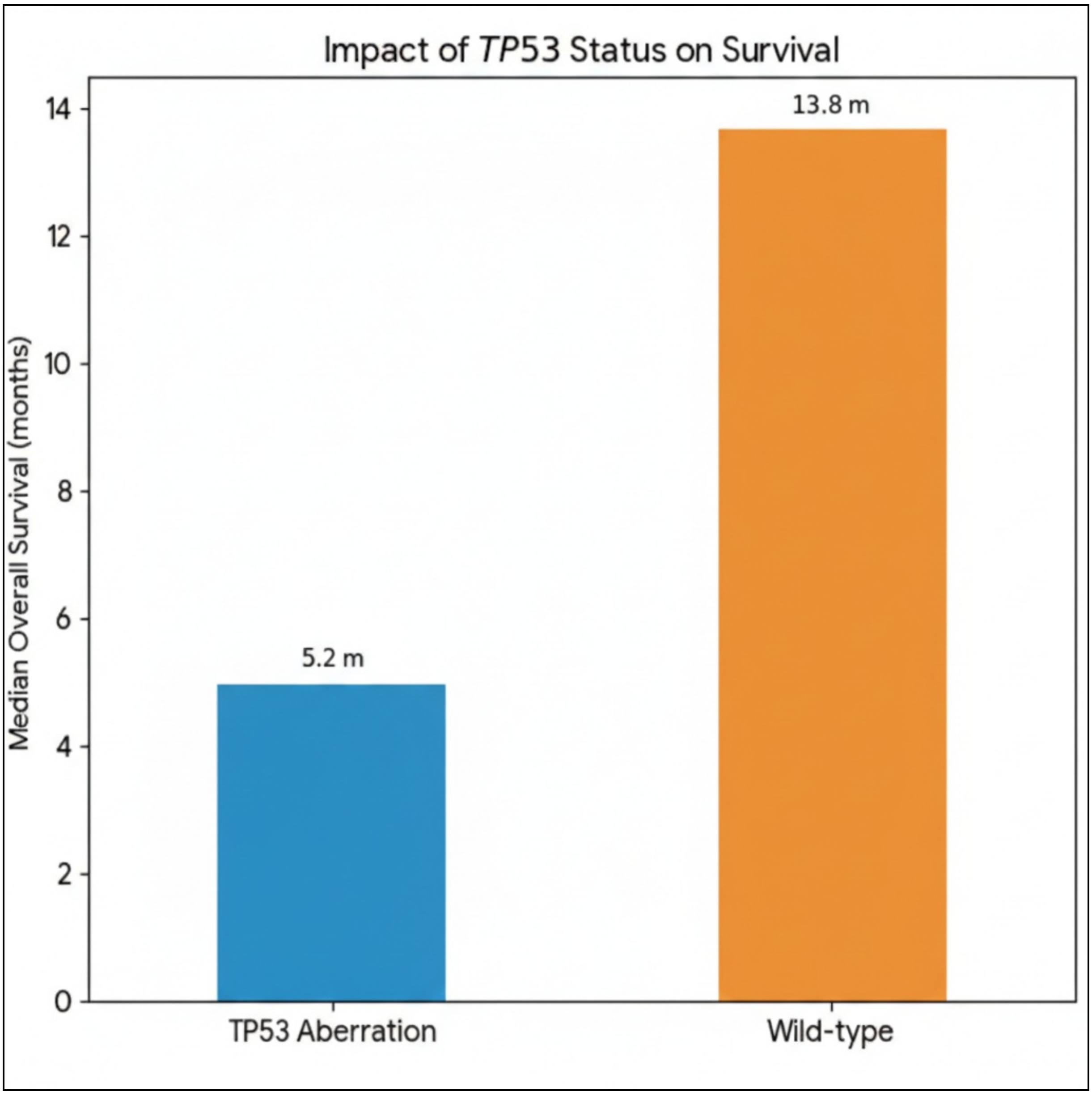

**Copyright Information:** (Online) ISSN: 2572-9241
©2026 The Author(s). HemaSphere is published by John Wiley & Sons Ltd on behalf of European Hematology Association. This is an open access Abstract Book distributed under the Attribution-NonCommercial-NoDerivs (CC BY-NC-ND), which allows third parties to download the articles and share them with others as long as they credit the author and the Abstract Book, but they cannot change the content in any way or use them commercially.
**Abstract Book Citations:** Authors, Title, HemaSphere, 2026;10:(S1):pages. The abstract book DOIs can be found on the "Author Information" page.
**Disclaimer:** Articles published in the journal HemaSphere exclusively reflect the opinions of the authors. The authors are responsible for all content in their abstracts including accuracy of the facts, statements, citing resources, etc.

### (PB3984) FGF DRIVES ENVIRONMENT MEDIATED MULTI-DRUG RESISTANCE IN B-CELL MALIGNANCIES

**Topic:** 21. Lymphoma biology & translational research

Matthew Timmins*[1, 2], Jingu Chen[1, 3], Andrew Moore[1, 4], Sophia Kogut[5], Seth Frietze[5], Ingo Ringshausen[1, 4]

*[1]Department of Haematology, Wellcome - MRC Cambridge Stem Cell Institute, Cambridge, United Kingdom;[2]Department of Haematology, Addenbrooke's Hospital, Cambridge University Hospital, Cambridge, United Kingdom; [3]Department of Biochemistry and Molecular Biology, West China School of Basic Medical Sciences & Forensic Medicine, Sichuan University, Chengdu, People's Republic of China., Sichuan, China; [4]Cancer Institute, University College London, London, United Kingdom; [5]Department of Biomedical and Health Sciences, University of Vermont, Vermont, Canada;*

### Background

Novel targeted therapies have substantially improved the prognosis of CLL and other B-cell lymphomas. However, a significant fraction of patients will relapse despite initial deep remissions. Studies have highlighted that the tumour microenvironment provides protective niches in which tumour cells receive survival signals promoting drug resistance. We have previously shown PKC-β to be a bone marrow stroma cell (BMSC) - autonomous signalling pathway critical for survival of malignant B cells (1,2). PKC-β dependent cell contact factors have been shown to regulate drug resistance, but the role of soluble-factors remains unknown.

### Results

Here we demonstrate novel mechanistic insights into stroma-mediated drug resistance in CLL and B-ALL. Stromal PKC-β activation regulates gene expression of distinct FGF ligands that promote a drug-resistant secretome. FGFR inhibition overcomes drug resistance to both targeted and non-targeted therapies. Mechanistically antagonising the putative receptors of FGF prevents therapy-induced ERK activation and BCL-xL stabilisation in CLL cells, substantially lowering their apoptotic threshold. Translationally, the significance of this pathway was demonstrated *in-vivo* through combination therapy of FGFR-inhibitors with anti-leukemic therapies, prolonging survival in EμTCL-1 diseased mice.

### Summary/Conclusion

Our data demonstrate PKC-β as a novel regulator of the secretome in bone marrow stroma cells and that targeting signalling cascades with small molecule inhibitors offers a novel therapeutic option to overcome multi-drug resistance in B-cell malignancies.

**Copyright Information:** (Online) ISSN: 2572-9241
©2026 The Author(s). HemaSphere is published by John Wiley & Sons Ltd on behalf of European Hematology Association. This is an open access Abstract Book distributed under the Attribution-NonCommercial-NoDerivs (CC BY-NC-ND), which allows third parties to download the articles and share them with others as long as they credit the author and the Abstract Book, but they cannot change the content in any way or use them commercially.
**Abstract Book Citations:** Authors, Title, HemaSphere, 2026;10:(S1):pages. The abstract book DOIs can be found on the "Author Information" page.
**Disclaimer:** Articles published in the journal HemaSphere exclusively reflect the opinions of the authors. The authors are responsible for all content in their abstracts including accuracy of the facts, statements, citing resources, etc.

### (PB3985) MECHANISM-GUIDED HDAC INHIBITION INDUCES COMPLETE RESPONSE IN PRIMARY REFRACTORY GATA3-POSITIVE PTCL-NOS: A BIOLOGICALLY INFORMED THERAPEUTIC STRATEGY

**Topic:** 21. Lymphoma biology & translational research

Alba Manasa Gonzalez Arias*[1], Sandra Garcia Lazaro[1], Lucia Rico Taverna[1], Alvaro Gonzalo Sanchez[1], Veronica Martinez Navarro[1], Irene Hervas Gonzalez[1], Maria Mar Diaz Goizueta[1], María Luisa Bengochea-Casado[1], Ramon Rodriguez Gonzalez[1], Margarita Berberana Fernandez DE Murias[1], Maria Jose Requena Rodriguez[1], Rosalia Riaza Grau[1]

*[1]HOSPITAL UNIVERSITARIO SEVERO OCHOA, HEMATHOLOGY AND HEMOTHERAPY, Madrid, Spain;*

### Background

Peripheral T-cell lymphoma, not otherwise specified (PTCL-NOS), particularly the GATA3 subtype, represents a critical unmet medical need. PTCL-GATA3 is characterized by high genomic instability and a Th2-like signature, leading to a dismal 5-year overall survival of only 19%. Literature indicates that GATA3+ patients frequently exhibit primary refractoriness to anthracycline-based induction (CHOP/CHOEP), with no significant survival benefit compared to supportive care. While allogeneic stem cell transplantation (allo-SCT) is the only reliable curative strategy in the relapsed/refractory (R/R) setting, patient-specific factors often preclude this option, highlighting the need for biologically informed therapeutic approaches.

### Aims

To report a rare case of primary refractory Stage IVA GATA3 PTCL-NOS in a young patient in whom mechanism-based precision therapy with the HDAC inhibitor Belinostat achieved a Complete Response (CR) after the failure of multiple standard salvage lines and formal exclusion from allo-SCT.

### Methods

A 34-year-old male with morbid obesity was diagnosed in June 2024 with Stage IVA GATA3-PTCL-NOS, presenting with massive splenomegaly (26 cm), splenic infarcts, and a high proliferative index (Ki67 60%). The patient demonstrated marked chemo-refractoriness to consecutive lines of treatment. First-line CHOEP-TIT resulted in primary progression after 6 cycles. Subsequent second-line DHAP was refractory and complicated by septic shock (Serratia marcescens) and acute renal failure. Third-line Pralatrexate had to be truncated before completing the first cycle due to intolerable Grade IV mucositis, severe cytopenias, and recurrent bacteremia. Despite his young age, the patient was formally rejected for allo-SCT by the referral center due to social characteristics and a high-risk clinical profile. Given the absence of curative options, fourth-line therapy with the HDAC inhibitor Belinostat was initiated in June 2025. Additional molecular studies are ongoing to explore potential predictive biomarkers of response to HDAC inhibition.

### Results

The patient achieved a Complete Response (CR) with a Deauville Score of 2 and a reduction of splenomegaly to 17 cm after 4 cycles of Belinostat. Following the 6th cycle, he underwent consolidation with Autologous Stem Cell Transplantation (ASCT) in November 2025. The post-transplant course was severe, involving abdominal septic shock and suspected intestinal ischemia, but the patient achieved full recovery. As of February 2026, he remains in morphometabolic stability (SUV max 2.2), is clinically asymptomatic, and has successfully returned to work.

**Copyright Information:** (Online) ISSN: 2572-9241
©2026 The Author(s). HemaSphere is published by John Wiley & Sons Ltd on behalf of European Hematology Association. This is an open access Abstract Book distributed under the Attribution-NonCommercial-NoDerivs (CC BY-NC-ND), which allows third parties to download the articles and share them with others as long as they credit the author and the Abstract Book, but they cannot change the content in any way or use them commercially.
**Abstract Book Citations:** Authors, Title, HemaSphere, 2026;10:(S1):pages. The abstract book DOIs can be found on the "Author Information" page.
**Disclaimer:** Articles published in the journal HemaSphere exclusively reflect the opinions of the authors. The authors are responsible for all content in their abstracts including accuracy of the facts, statements, citing resources, etc.

### Summary/Conclusion

This case underscores the limited efficacy of conventional cytotoxic chemotherapy in biologically defined high-risk GATA3-positive PTCL-NOS. It further supports the integration of molecular subtype information into therapeutic decision-making, suggesting that HDAC inhibition may provide a bridge to consolidation when allo-SCT is not an option. Although limited to a single case, this observation supports further investigation of HDAC inhibition in biologically defined GATA3-PTCL subsets. Our ongoing management strategy, in the event of relapse, includes reassessment of transplant eligibility and consideration of HDAC inhibition re-challenge based on prior response. Molecular studies are also in progress to explore potential biomarkers predicting response to HDAC inhibition in high-risk GATA3-positive PTCL-NOS.

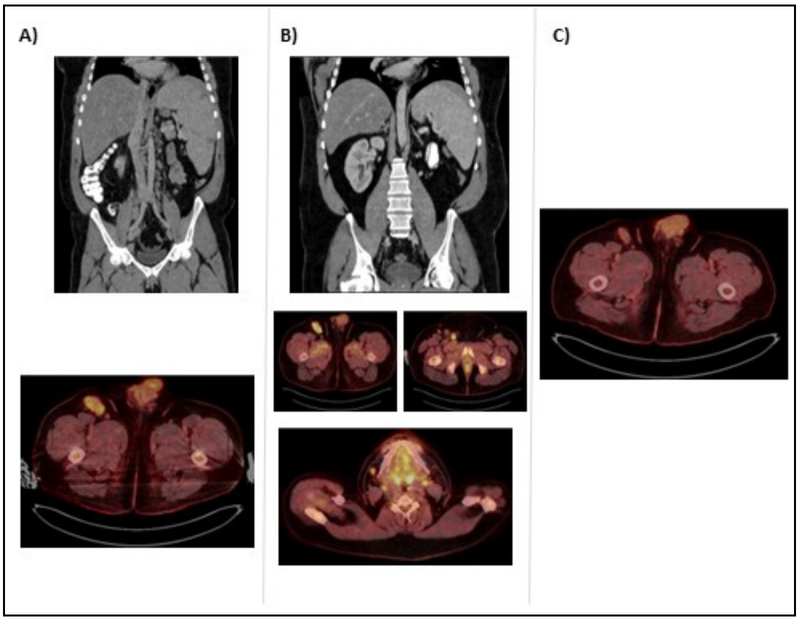

Sequential 18F-FDG PET/CT imaging illustrating clinical evolution. (A) Baseline (July 2024): Initial staging of Stage IVA GATA3 PTCL-NOS showing 21-cm splenomegaly and hypermetabolic right iliac and inguinal lymphadenopathy (SUV max 4.38). (B) Progression (November 2024): Disease progression after first-line CHOEP-TIT, demonstrating increased metabolic activity in right iliac (SUV max 5.83) and inguinal nodes (SUV max 7.0), alongside new bilateral laterocervical involvement (SUV max 5.12). (C) Complete Response (September 2025): Metabolic complete response after 4 cycles of 4th-line Belinostat, with reduction of splenomegaly to 17 cm and minimal residual right inguinal uptake (SUV max 1.87; Deauville Score 2).

**Copyright Information:** (Online) ISSN: 2572-9241
©2026 The Author(s). HemaSphere is published by John Wiley & Sons Ltd on behalf of European Hematology Association. This is an open access Abstract Book distributed under the Attribution-NonCommercial-NoDerivs (CC BY-NC-ND), which allows third parties to download the articles and share them with others as long as they credit the author and the Abstract Book, but they cannot change the content in any way or use them commercially.
**Abstract Book Citations:** Authors, Title, HemaSphere, 2026;10:(S1):pages. The abstract book DOIs can be found on the “Author Information” page.
**Disclaimer:** Articles published in the journal HemaSphere exclusively reflect the opinions of the authors. The authors are responsible for all content in their abstracts including accuracy of the facts, statements, citing resources, etc.

### (PB3986) EMERGENCE OF T-CELL LARGE GRANULAR LYMPHOCYTE EXPANSION DURING IMMUNE RECONSTITUTION AFTER TREATMENT OF SECOND RELAPSE OF HAIRY CELL LEUKEMIA

**Topic:** 21. Lymphoma biology & translational research

Abdellah Seghiri[1], Ikram Sadki[2], Soumaya Barkhoukh[2], Zahida Aqodad[1], Samia Sabri[1, 2], Houda Bachir[1], Siham Hamaz[1], Habiba Bennesser Alaoui*[1], Khalid Serraj Andaloussi[1]

*[1]Laboratory of Immuno-Hematology and Cell Therapy, Faculty of Medicine and Pharmacy of Oujda (FMPO), Mohammed First University, Oujda, Morocco., Internal Medicine, Mohammed VI University Hospital of Oujda, OUJDA PPAL, Morocco; [2]Laboratory of Immuno-Hematology and Cell Therapy, Faculty of Medicine and Pharmacy of Oujda (FMPO), Mohammed First University, Oujda, Morocco., OUJDA PPAL, Morocco;*

### Background

Hairy Cell Leukemia (HCL) is an indolent B-cell neoplasm highly responsive to purine analogs. Although durable remissions are common, relapses occur. Secondary lymphoproliferative expansions during immune reconstitution are uncommon and incompletely characterized. Among them, T-cell large granular lymphocytes (T-LGL) may emerge and pose diagnostic and therapeutic challenges.

### Aims

To describe the emergence of T-LGL expansion following treatment of a second HCL relapse and discuss its potential relationship with immune reconstitution.

### Results

A 58-year-old man with type 2 diabetes and prior ischemic strokes was diagnosed with HCL in 2018 and achieved complete remission after cladribine. In April 2025, he presented with second relapse characterized by thrombocytopenia ($60\times10^9$/L), neutropenia ($0.8\times10^9$/L), and minor residual B-cell population (~3%) expressing CD19+, CD20++, CD11c+, CD123+, CD25+, and CD103+. Bone marrow examination confirmed limited B-cell infiltration with reticulin fibrosis (MF1–2); BRAF mutation was positive.
He received cladribine combined with rituximab, achieving a good clinico-biological response with persistent grade 2–3 neutropenia. During follow-up, peripheral blood immunophenotyping revealed expansion of two T-LGL populations: CD4+ (57% of lymphocytes) and CD8+ (18%). B cells remained profoundly depleted (<0.01%), while NK cells were preserved. Bone marrow reassessment (November 2025) showed mild hypocellularity (MF1) without residual HCL. Molecular studies revealed the presence of a clonal activating STAT3 mutation.
Given the absence of symptoms or severe cytopenias, a watchful waiting strategy was adopted.

### Summary/Conclusion

T-LGL expansion may occur during immune reconstitution following B-cell–depleting therapy for relapsed HCL. Whether this represents reactive immune remodeling or early clonal evolution remains unclear. Systematic immunophenotypic and molecular monitoring is essential to distinguish benign post-treatment immune reconstitution from secondary lymphoproliferative disorders requiring intervention.
**Informed consent was obtained from the patient.**

**Copyright Information:** (Online) ISSN: 2572-9241
©2026 The Author(s). HemaSphere is published by John Wiley & Sons Ltd on behalf of European Hematology Association. This is an open access Abstract Book distributed under the Attribution-NonCommercial-NoDerivs (CC BY-NC-ND), which allows third parties to download the articles and share them with others as long as they credit the author and the Abstract Book, but they cannot change the content in any way or use them commercially.
**Abstract Book Citations:** Authors, Title, HemaSphere, 2026;10:(S1):pages. The abstract book DOIs can be found on the "Author Information" page.
**Disclaimer:** Articles published in the journal HemaSphere exclusively reflect the opinions of the authors. The authors are responsible for all content in their abstracts including accuracy of the facts, statements, citing resources, etc.

### (PB3987) THE NF-KAPPA B INDUCING KINASE INHIBITOR SMI1 PREVENTS EXPERIMENTAL GRAFT-VERSUS-HOST DISEASE IN HUMANIZED MICE

**Topic:** 22. Stem cell transplantation - Experimental

Charline Beguin*[1, 2], Oswin Kwan[1], Joan Somja[3], Sophie Dubois[1], Jo Caers[1, 2], Yves Beguin[1, 2], Gregory Ehx[1, 4], Emmanuel Dejardin[5], Caroline Ritacco[1], Frederic Baron[1, 2]

*[1]University of Liège, Hematology Research Unit, Groupe Interdisciplinaire de Génoprotéomique Appliquée (GIGA)-I³, Liège, Belgium; [2]University Hospital of Liège (CHU de Liège), Department of Medicine, Division of Hematology, Liège, Belgium; [3]University Hospital of Liège (CHU de Liège), Department of Anatomical and Cytological Pathology, Liège, Belgium;[4]Walloon ExceLlence (WEL) Research Institute, Walloon Excellence in Life Sciences and Biotechnology (WELBIO) Department, Wavre, Belgium; [5]University of Liège, Laboratory of Molecular Immunology and Signal Transduction, Groupe Interdisciplinaire de Génoprotéomique Appliquée (GIGA)-I³, Liège, Belgium;*

**Background**

Allogeneic stem cell transplantation remains a cornerstone of the treatment of some hematological malignancies, although it is still associated with a significant morbidity and mortality which are mainly related to graft-versus-host disease (GVHD). NF-kB signaling pathways play a determining role in the pathogenesis of the disease in part through OX40 receptor signaling via the NF-kappa B inducing kinase (NIK) in donor T-cells[1].

**Aims**

In order to determine whether NIK inhibition could become a therapeutic strategy against GVHD in humans, we assessed the ability of the NIK inhibitor SMI1[2] at preventing GVHD in a humanized mouse model combining xenogeneic and allogeneic GVHD[3].

**Methods**

NSG-HLA-A2-HHD mice were transplanted with human HLA-A2 negative peripheral blood mononuclear cells (PBMC) on day 0. In a first experiment, SMI1 was administered by oral gavage twice daily from day 1 to day 20. Blood samples were drawn on days 14, 20 and 28 and stained for spectral flow cytometry to assess immune reconstitution. Single cell RNA sequencing and TCR repertoire analyses were performed on human $CD3^+$ T-cells isolated from day 20 blood samples. In a second experiment, SMI1 was administered for 5 weeks with a tapering protocol. Blood samples were drawn on days 14, 20, 27, 35 and 42. In addition, 10 mice were sacrificed on day 21 to perform spectral flow cytometry and histology on organs.

**Copyright Information:** (Online) ISSN: 2572-9241
©2026 The Author(s). HemaSphere is published by John Wiley & Sons Ltd on behalf of European Hematology Association. This is an open access Abstract Book distributed under the Attribution-NonCommercial-NoDerivs (CC BY-NC-ND), which allows third parties to download the articles and share them with others as long as they credit the author and the Abstract Book, but they cannot change the content in any way or use them commercially.
**Abstract Book Citations:** Authors, Title, HemaSphere, 2026;10:(S1):pages. The abstract book DOIs can be found on the “Author Information” page.
**Disclaimer:** Articles published in the journal HemaSphere exclusively reflect the opinions of the authors. The authors are responsible for all content in their abstracts including accuracy of the facts, statements, citing resources, etc.

### Results

In the first experiment, treated mice harbored lower human CD45$^+$ chimerism, lower CD8$^+$ T-cells counts and a longer persistence of regulatory T-cells (Treg). Single cell RNA sequencing evidenced a lower proportion of effector/effector memory CD8$^+$ T-cells but a higher proportion of Treg and naive CD4$^+$ T-cells in treated mice. TCR sequencing showed that treated mice exhibited a broader TCR repertoire diversity with decreased clonality. Furthermore, genes involved in the cytotoxic function of T-cells, such as Granulysin, Granzyme B and Perforin 1, were downregulated in treated mice. GVHD scores were lower during treatment but a rebound effect was observed at treatment discontinuation and survival did not differ significantly between the two groups. In the second experiment, blood test results were consistent with our previous observations while, importantly, the slow tapering of the drug decreased the rebound effect. A significantly prolonged survival was observed in treated mice with 55% of treated mice surviving beyond day 50 versus no control mice. Finally, spectral flow cytometry and histological analyses confirmed lower T-cell infiltration and lower GVHD scores in GVHD target organs on day 21 after PBMC infusion.

### Summary/Conclusion

The NIK inhibitor SMI1 attenuated xenogeneic/allogeneic GVHD in a humanized mouse model.

References:

1. Murray SE, Polesso F, Rowe AM, et al. NF-κB-inducing kinase plays an essential T cell-intrinsic role in graft-versus-host disease and lethal autoimmunity in mice. *Journal of Clinical Investigation*. 2011;121(12):4775-4786. doi:10.1172/JCI44943
2. Brightbill HD, Suto E, Blaquiere N, et al. NF-κB inducing kinase is a therapeutic target for systemic lupus erythematosus. *Nat Commun*. 2018;9(1). doi:10.1038/s41467-017-02672-0
3. Ehx G, Somja J, Warnatz HJ, et al. Xenogeneic Graft-Versus-Host Disease in Humanized NSG and NSG-HLA-A2/HHD Mice. *Front Immunol*. 2018;9. doi:10.3389/fimmu.2018.01943

**Copyright Information:** (Online) ISSN: 2572-9241
©2026 The Author(s). HemaSphere is published by John Wiley & Sons Ltd on behalf of European Hematology Association. This is an open access Abstract Book distributed under the Attribution-NonCommercial-NoDerivs (CC BY-NC-ND), which allows third parties to download the articles and share them with others as long as they credit the author and the Abstract Book, but they cannot change the content in any way or use them commercially.
**Abstract Book Citations:** Authors, Title, HemaSphere, 2026;10:(S1):pages. The abstract book DOIs can be found on the "Author Information" page.
**Disclaimer:** Articles published in the journal HemaSphere exclusively reflect the opinions of the authors. The authors are responsible for all content in their abstracts including accuracy of the facts, statements, citing resources, etc.

### (PB3988) CSF3R/STAT3 SIGNALING ENHANCES ANTIGEN PRESENTATION IN MTECS AND PROMOTES CENTRAL TOLERANCE ESTABLISHMENT

**Topic:** 22. Stem cell transplantation - Experimental

Huidong Guo*[1], Xinya Jiang[1], Shuang Fan[1], Xiaojun Huang[1]

[1]*Peking University People's Hospital, Beijing, China;*

### Background

Central T cell tolerance is established in the thymic medulla and critically depends on medullary thymic epithelial cells (mTECs), particularly AIRE⁺ mTECs, which mediate the expression of tissue-restricted antigens (TRAs). Thymic injury resulting from hematopoietic stem cell transplantation (HSCT), irradiation, or aging severely impairs mTEC function and disrupts central tolerance. Despite this, effective strategies to restore thymic epithelial integrity remain limited. In our previous study, we elucidated the transcriptome atlas associated with successful restoration of immune homeostasis following allogeneic HSCT and observed that recipients of haploidentical HSCT exhibited elevated levels of thymic output. This suggests that the G-CSF/ATG-based "Beijing Protocol" may promote thymic regeneration. However, the underlying molecular mechanisms remain unclear.

### Aims

This study aims to determine whether granulocyte colony-stimulating factor (G-CSF) administered after transplantation regulates thymic regeneration and central tolerance, and to clarify the molecular mechanisms involved.

### Methods

Murine models of allogeneic HSCT, irradiation, and aging were employed to assess thymic injury and regeneration. G-CSF was administered systemically, and thymic architecture, cellularity, and T cell developmental subsets were analyzed by flow cytometry and histology. AIRE and TRA expression were evaluated by quantitative PCR and immunostaining. Functional restoration of central tolerance was assessed through analysis of thymocyte selection, peripheral T cell phenotype, and autoimmunity-related parameters.

### Results

Treatment with G-CSF significantly improved thymic cellularity and restored medullary architecture in models of transplantation, irradiation, and aging. Mechanistically, mTECs expressed the G-CSF receptor (CSF3R) and responded directly to G-CSF through STAT3 activation. This signaling pathway enhanced the expression of AIRE and upregulated the transcription of tissue-restricted antigens (TRAs) in mTECs, thereby facilitating efficient positive and negative selection of double-positive thymocytes. These improvements led to increased thymic output, a more balanced peripheral T cell compartment, and reduced signs of impaired central tolerance. Multiomics analysis further revealed a positive correlation between STAT3 activation and the expression of TRA-related transcription factors, suggesting that STAT3 may directly promote their activity.

### Summary/Conclusion

G-CSF acts as a previously unrecognized regulator of thymic epithelial regeneration by activating CSF3R/STAT3 signaling in mTECs. Through upregulation of AIRE and restoration of antigen presentation capacity, G-CSF facilitates re-establishment of central tolerance following thymic injury. These findings identify a translatable strategy to promote immune reconstitution and tolerance after HSCT and other thymus-damaging conditions.

**Copyright Information:** (Online) ISSN: 2572-9241
©2026 The Author(s). HemaSphere is published by John Wiley & Sons Ltd on behalf of European Hematology Association. This is an open access Abstract Book distributed under the Attribution-NonCommercial-NoDerivs (CC BY-NC-ND), which allows third parties to download the articles and share them with others as long as they credit the author and the Abstract Book, but they cannot change the content in any way or use them commercially.
**Abstract Book Citations:** Authors, Title, HemaSphere, 2026;10:(S1):pages. The abstract book DOIs can be found on the "Author Information" page.
**Disclaimer:** Articles published in the journal HemaSphere exclusively reflect the opinions of the authors. The authors are responsible for all content in their abstracts including accuracy of the facts, statements, citing resources, etc.

### (PB3989) ANTIBODY REPERTOIRE OF AN AML PATIENT UPON ALLOGENEIC HEMATOPOIETIC STEM CELL TRANSPLANTATION

**Topic:** 22. Stem cell transplantation - Experimental

Rivka de Jongh*[1, 2], Sabine de Ruiter[3], Etsuko Yasuda[4], Naobi Felix-Pantophlet[3], Haye Nijhuis[3], Rogers Nahui-Palomino[4], Gestur Vidarsson[1, 2], Mette Hazenberg[1, 5], Tim Beaumont[3, 4, 6]

*[1]Sanquin Blood Supply, Immunoglobulin Research, Amsterdam, The Netherlands; [2]Utrecht University, Utrecht Institute for Pharmaceutical Sciences and Bijvoet Center for Biomolecular Research, Department of Biomolecular Mass Spectrometry and Proteomics, Utrecht, The Netherlands; [3]Amsterdam University Medical Centre, Department of Medical Microbiology and Infection Prevention, Amsterdam, The Netherlands; [4]Amsterdam University Medical Centre, Experimental Immunology, Amsterdam, The Netherlands; [5]Amsterdam University Medical Centre, Department of Hematology, Amsterdam, The Netherlands; [6]Amsterdam University Medical Centre, Amsterdam Institute for Immunology and Infectious Diseases, Amsterdam, The Netherlands;*

### Background

Allogeneic hematopoietic progenitor cell transplantation (HCT) is effective against acute myeloid leukemia (AML) when a potent graft-versus-leukemia response is generated. While cytotoxic T-cells and NK cells have proven to be important effectors in tumor immunotherapy, the role of tumor specific B-cells and antibodies in graft-versus-leukemia immunity has been understudied. We identified antibodies directed against AML cells by studying the donor antibody response of elite responder AML patients. Target identification revealed CD43s and U5 snRNP200 as membrane expressed tumor antigens (Gillissen *et al.*, 2017, Blood advances & Gillissen *et al.*, 2018, Blood), suggesting that more information about the B-cell anti-tumor response can be revealed when studied in more detail. Detailed analyses of B-cell reconstitution following allogeneic HCT are, however, relatively scarce.

### Aims

Perform an in-depth analysis of the tumor-specific antibody and B-cell repertoire following allogeneic HCT and to compare this to the virus-specific B-cell and antibody repertoire, and to examine the outcome to the B-cell repertoire present in the original donor graft.

### Methods

PBMC were isolated from an elite responder who remained leukemia-free 2 years after FLAMSA-RIC allogeneic HCT for refractory AML. We cultured approximately 1200 to 1500 Bcl-6 and B-xL immortalized classical memory ($CD27^{+}IgD^{-}$) and non-classical memory ($CD27^{+}IgD^{+}$ and $CD27^{-}IgD^{-}$) $CD19^{+}$ B-cells at single cell level. Supernatants of these antibody secreting B-cells were tested by flow cytometry for binding to the AML cell lines THP1, OCI-AML3, MOLM13, U937, HL60, SH2 and K562, and binding to common viral peptides including CMV, influenza and RSV by Luminex. B-cell receptors will be sequenced to compare their characteristics, including VDJ gene usage and somatic hypermutations, to the B-cells in the donor transplant.

**Copyright Information:** (Online) ISSN: 2572-9241
©2026 The Author(s). HemaSphere is published by John Wiley & Sons Ltd on behalf of European Hematology Association. This is an open access Abstract Book distributed under the Attribution-NonCommercial-NoDerivs (CC BY-NC-ND), which allows third parties to download the articles and share them with others as long as they credit the author and the Abstract Book, but they cannot change the content in any way or use them commercially.
**Abstract Book Citations:** Authors, Title, HemaSphere, 2026;10:(S1):pages. The abstract book DOIs can be found on the "Author Information" page.
**Disclaimer:** Articles published in the journal HemaSphere exclusively reflect the opinions of the authors. The authors are responsible for all content in their abstracts including accuracy of the facts, statements, citing resources, etc.

### Results

We identified 70 THP1-binding clones in the classical memory B-cell pool and 84 THP1-binding clones in the non-classical memory population of the patient. Some of the antibodies also interacted with one or more of the other AML cell lines. Most AML-binding antibodies from the classical memory population were of the IgG1 isotype (88%), followed by 7% IgG3 and 1% IgM. In the non-classical memory populations, 65% of the AML-binding antibodies were IgG1, 11% IgG3 and 13% IgM. THP1-binding IgG1 antibodies of the patient had less somatic hypermutations (average classical memory B-cells: 10 and non-classical memory B-cells: 8,5) than matured antibodies in healthy adults (average: 26) (Burkovitz *et al*, 2013, FEBS Journal). These antibodies are not specific for the previously identified U5 snRNP200, so they will be further studied for antigen identification. The classical memory pool also contained RSV- (3) and Influenza- specific (15) B-cell clones, compared to 2 and 9 RSV- and Influenza-specific clones in the non-classical memory B-cell popoulations. Similar analysis of the donor transplant is underway.

### Summary/Conclusion

AML-directed antibodies are present in an elite responder AML patient 2 years after allogeneic HCT. Even at this late timepoint after allogeneic HCT, the antibody repertoire seems less mature than that of healthy people. With our unique in-depth comparison to the donor transplant, we can contribute to a better understanding of the biology of the B-cell reconstitution and the generation of AML specific humoral immunity after allogeneic HCT.

**Copyright Information:** (Online) ISSN: 2572-9241
©2026 The Author(s). HemaSphere is published by John Wiley & Sons Ltd on behalf of European Hematology Association. This is an open access Abstract Book distributed under the Attribution-NonCommercial-NoDerivs (CC BY-NC-ND), which allows third parties to download the articles and share them with others as long as they credit the author and the Abstract Book, but they cannot change the content in any way or use them commercially.
**Abstract Book Citations:** Authors, Title, HemaSphere, 2026;10:(S1):pages. The abstract book DOIs can be found on the “Author Information” page.
**Disclaimer:** Articles published in the journal HemaSphere exclusively reflect the opinions of the authors. The authors are responsible for all content in their abstracts including accuracy of the facts, statements, citing resources, etc.

### (PB3990) HEMATOPOIETIC RECONSTITUTION IN MICE WITH BONE MARROW ACUTE RADIATION SYNDROME FOLLOWING COMBINED TREATMENT OF STEM CELLS

**Topic:** 22. Stem cell transplantation - Experimental

Fang Zhou*[1], Xuan Liu[1], Yixi Hou[1]

[1] *The 960th Hospital of The People's Liberation Army (PLA) Joint Logistics Support Force, Jinan, China;*

### Background

By co-culturing hematopoietic stem cells (HSCs) and mesenchymal stem cells (MSCs) in a three-dimensional micro-scaffold, a biomimetic hematopoietic bone marrow microenvironment can be assembled. This approach, compared to HSC transplantation alone, significantly accelerated hematopoietic reconstitution and reduced the incidence of graft-versus-host disease (GVHD) in treating severe/very severe bone marrow acute radiation syndrome (ARS). Although both haploidentical HSCs combined with MSCs and umbilical cord blood HSCs combined with MSCs have demonstrated considerable therapeutic potential, a systematic comparison study in a controlled animal model is lacking.

### Aims

This study aims to comparatively evaluate the therapeutic efficacy of Haploidentical Hematopoietic Stem Cell Transplantation (Haplo-HSCT) Combined with Mesenchymal Stem Cells (MSCs) and Umbilical Cord Blood Hematopoietic Stem Cell Transplantation (UCBT) Combined with MSCs in Mice with Bone Marrow Acute Radiation Syndrome (ARS).

### Methods

CB6F1 mice, aged 8-10 weeks, were exposed to 8 Gy of $^{60}$Co γ-ray irradiation to establish a bone marrow-type ARS model. The mice were then randomly divided into three groups: a haploidentical hematopoietic stem cell combined with mesenchymal stem cell transplantation group (Haplo-MSC group), an umbilical cord blood hematopoietic stem cell combined with mesenchymal stem cell transplantation group (UCB-MSC group), and a saline group (blank control group). The survival rate, peripheral blood cell counts, pathological changes in target organs, colony formation of bone marrow cells, and graft-versus-host disease (GVHD) were observed.

### Results

All mice in the normal saline control group died. The 30-day survival rate was 65% in the Haplo-MSC group and 60% in the UCB-MSC group. The Log-rank test showed no significant difference in survival time between the two transplantation groups ($P = 0.561$). No significant differences were observed in WBC, PLT, RBC, and Hb counts between the Haplo-MSC group and UCB-MSC group 28 days after transplantation (P = 0.07, 0.597, 0.730, and 0.452, respectively). The pathological changes in bone marrow cells indicated that bone marrow failure occurred later in the UCB-MSC group than in the Haplo-MSC group, whereas the Haplo-MSC group exhibited faster engraftment of hematopoietic stem cells. Furthermore, the numbers of CFU-E, CFU-GM, and CFU-GEMM were significantly different between the two groups ($P < 0.05$).

### Summary/Conclusion

Both the Haplo-MSC and UCB-MSC regimens promoted hematopoietic engraftment and reconstitution in bone marrow-type ARS mice exposed to lethal-dose radiation. However, the onset of bone marrow failure occurred later in mice treated with the UCB-MSC regimen. In contrast, mice treated with the Haplo-MSC regimen exhibited faster engraftment.

**Copyright Information:** (Online) ISSN: 2572-9241
©2026 The Author(s). HemaSphere is published by John Wiley & Sons Ltd on behalf of European Hematology Association. This is an open access Abstract Book distributed under the Attribution-NonCommercial-NoDerivs (CC BY-NC-ND), which allows third parties to download the articles and share them with others as long as they credit the author and the Abstract Book, but they cannot change the content in any way or use them commercially.
**Abstract Book Citations:** Authors, Title, HemaSphere, 2026;10:(S1):pages. The abstract book DOIs can be found on the “Author Information” page.
**Disclaimer:** Articles published in the journal HemaSphere exclusively reflect the opinions of the authors. The authors are responsible for all content in their abstracts including accuracy of the facts, statements, citing resources, etc.

### (PB3991) THE INFLUENCE OF DIFFERENT COLLAGENASES ON THE ANALYSIS OF IMMUNE CELLS IN SALIVARY GLANDS OF MICE WITH CHRONIC GRAFT-VERSUS-HOST DISEASE

**Topic:** 22. Stem cell transplantation - Experimental

Ziyi Hu*[1], Qingxiao Song[1], Xi Zhang[1]

[1]*Medical Center of Hematology, Xinqiao Hospital of Army Medical University, Blood Ecology and Smart Cell Science Innovation Center, State Key Laboratory of Trauma and Chemical Poisoning, Chongqing Key Laboratory of Hematology and Microenvironment, Chongqing, China;*

### Background

The salivary gland is an target organ of cGVHD, however, mechanisms of salivary gland cGVHD is unclear.

### Aims

Therefore, optimizing the method to isolate immune cells from salivary gland is crucial.

### Methods

Establish the major MHC mismatch model, including the non-GVHD control group and the cGVHD group(n=15). On the 45$^{th}$ day after transplantation, salivary glands were collected(n=6~12), H&E and Masson's Trichrome staining were performed to assess the degree of injury and fibrosis. Flow cytometry analysis were performed to compare the immune cells population and phenotype between the groups without collagenase digestion, collagenaseⅡdigestion, and collagenase D digestion.

### Results

Compared to the non-GVHD group, mice in the cGVHD group showed weight loss□□101.2±4.19□% vs □66.77±11.72□%□P<0.0001□, hunched backs after transplantation□0±0 vs 2.11±0.59□P<0.0001□, the overall survival rate was significantly lower. The salivary glands of cGVHD mice showed acinar atrophy, cells infiltration□2±0 vs 3.67±0.52□P<0.0001□ and fibrosis□□4.11±0.87□% vs □14.54±4.05□%□P=0.0001□. Collagenase digestion significantly enhanced the yield and viability of mononuclear cells□P<0.05□, and among the tested protocols, digestion with collagenase II resulted in the greatest recovery of mononuclear cells□P<0.01□. However, collagenaseⅡdigestion significantly reduced the expression level of CD4 on T cells□□31.98±12.23□% vs □5.64±2.05□%□P=0.0052□, while collagenase D had no significant impact on CD4□□31.98±12.23□% vs □31.51±16.37□%□P=0.9973□. In the cGVHD group, the method containing collagenase D could recover more CD4$^+$ T cells, while the method containing collagenaseⅡcould recover more CD8$^+$ T cells□P<0.05□. In addition, the number of NK cells was significantly increased in both groups with collagenase□P<0.05□. In myeloid cells, collagenaseⅡdigestion significantly reduced the proportion of Ly6C$^{lo}$CD11b$^+$Ly6G$^+$ neutrophils□□0.34±0.2□% vs □0.05±0.01□%□P□0.05□, while collagenase D had no effect□□0.34±0.2□% vs □0.49±0.19□%□P=0.28□. In cGVHD tissues, the number of CD11b$^+$Ly6C$^+$Ly6G$^-$ monocytes rcovered with collagenase D digestion were significantly higher than those with collagenaseⅡdigestion□0.01±0 vs 0.06±0.05□P=0.0384□, while the number of macrophages rcovered with collagenaseⅡ digestion were significantly higher than those with collagenase D digestion□1.42±0.56 vs 0.57±0.36□P<0.0001□.

**Copyright Information:** (Online) ISSN: 2572-9241
©2026 The Author(s). HemaSphere is published by John Wiley & Sons Ltd on behalf of European Hematology Association. This is an open access Abstract Book distributed under the Attribution-NonCommercial-NoDerivs (CC BY-NC-ND), which allows third parties to download the articles and share them with others as long as they credit the author and the Abstract Book, but they cannot change the content in any way or use them commercially.
**Abstract Book Citations:** Authors, Title, HemaSphere, 2026;10:(S1):pages. The abstract book DOIs can be found on the "Author Information" page.
**Disclaimer:** Articles published in the journal HemaSphere exclusively reflect the opinions of the authors. The authors are responsible for all content in their abstracts including accuracy of the facts, statements, citing resources, etc.

**Summary/Conclusion**

This study compared the efficacy of digestion methods with different collagenase on the number and phenotype of immune cells in the salivary glands in the setting of allo-HSCT. The results suggested that the collagenase digestion significantly increased the number and viability of mononuclear cells. The method containing collagenaseⅡcould obtain the highest number of mononuclear cells and $CD8^{+}$ T cells, NK cells, and macrophages; the collagenase D was better for $CD4^{+}$ T cells, neutrophils, and monocytes.

**Copyright Information:** (Online) ISSN: 2572-9241
©2026 The Author(s). HemaSphere is published by John Wiley & Sons Ltd on behalf of European Hematology Association. This is an open access Abstract Book distributed under the Attribution-NonCommercial-NoDerivs (CC BY-NC-ND), which allows third parties to download the articles and share them with others as long as they credit the author and the Abstract Book, but they cannot change the content in any way or use them commercially.
**Abstract Book Citations:** Authors, Title, HemaSphere, 2026;10:(S1):pages. The abstract book DOIs can be found on the "Author Information" page.
**Disclaimer:** Articles published in the journal HemaSphere exclusively reflect the opinions of the authors. The authors are responsible for all content in their abstracts including accuracy of the facts, statements, citing resources, etc.

### (PB3992) DIAGNOSTIC VALUE OF PATHOGEN METAGENOMIC NEXT-GENERATION SEQUENCING (MNGS) FOR EARLY BLOODSTREAM INFECTIONS IN PEDIATRIC ALLOGENEIC HEMATOPOIETIC STEM CELL TRANSPLANTATION RECIPIENTS

**Topic:** 23. Stem cell transplantation - Clinical

Yang Wan*[1], Xiaolan Li[2], Lipeng Liu[3], Chen Xia[2], Aoli Zhang[3], Xiaoyan Zhang[3], Xiaoming Liu[2], Ren Yuanyuan[2], Ye Guo[3], Yumei Chen[2], Xiaofan Zhu[3], Wenyu Yang[2], Fang Liu[3]

*[1]Institute of Hematology & Blood Diseases Hospital, Chinese Academy of Medical Sciences & Peking Union Medical College, Pediatric Hematology and Oncology Center, Tianjin, China; [2]Institute of Hematology & Blood Diseases Hospital, Chinese Academy of Medical Sciences & Peking Union Medical College, TIANJIN, China; [3]Institute of Hematology & Blood Diseases Hospital, Chinese Academy of Medical Sciences & Peking Union Medical College, Tianjin, China;*

### Background

Early bloodstream infection (EBSI) remains a principal determinant of morbidity and mortality after paediatric allogeneic haematopoietic stem-cell transplantation (allo-HSCT). Metagenomic next-generation sequencing (mNGS) offers a culture-independent, high-throughput approach for comprehensive pathogen identification.

### Aims

We investigated the diagnostic performance of plasma mNGS for EBSI in children undergoing allo-HSCT.

### Methods

In this single-centre, retrospective cohort study, we consecutively enrolled 161 transplant episodes performed in 157 paediatric patients at the Pediatric Hematology and Oncology Center, Institute of Hematology, Chinese Academy of Medical Sciences, between January 2024 and April 2025. Blood cultures and plasma mNGS were systematically collected from conditioning initiation to day +30 post-transplant. Group comparisons employed Fisher's exact test.

**Copyright Information:** (Online) ISSN: 2572-9241
©2026 The Author(s). HemaSphere is published by John Wiley & Sons Ltd on behalf of European Hematology Association. This is an open access Abstract Book distributed under the Attribution-NonCommercial-NoDerivs (CC BY-NC-ND), which allows third parties to download the articles and share them with others as long as they credit the author and the Abstract Book, but they cannot change the content in any way or use them commercially.
**Abstract Book Citations:** Authors, Title, HemaSphere, 2026;10:(S1):pages. The abstract book DOIs can be found on the "Author Information" page.
**Disclaimer:** Articles published in the journal HemaSphere exclusively reflect the opinions of the authors. The authors are responsible for all content in their abstracts including accuracy of the facts, statements, citing resources, etc.

**Results**

Blood-culture-confirmed BSI was documented in 18 of 161 episodes (11.2%). Disease-specific incidence rates were: ALL 19.4 % (6/31), AML 10.5 % (4/38), acquired aplastic anaemia/other marrow failure 7.4 % (4/54), myelodysplastic syndrome 4.5 % (1/22) and other haematologic malignancies 25.0 % (3/12). Donor-specific BSI rates were 11.5 % (7/61) after haploidentical, 15.3 % (11/72) after unrelated cord-blood and 0 % (0/34) after matched-sibling transplantation. There were no statistically significant differences were found across disease subtypes (P = 0.31) or donor sources (P = 0.60).Predominant blood-culture isolates were Streptococcus mitis, Klebsiella pneumoniae and Escherichia coli.
Plasma mNGS was obtained in 39 episodes, yielding 22 positives (56.4 %). Among febrile episodes, blood culture was positive in 9/39 (23.1 %). mNGS identified bacterial infection in 8, viral infection in 6 and combined bacterial–viral infection in 8 cases. Leading bacteria were K. pneumoniae, Pseudomonas aeruginosa and S. mitis. Dominant viruses were HHV-6B, CMV and BK virus. Disease-stratified mNGS positivity was 100 % (5/5) for ALL, 75.0 % (6/8) for AML, 33.3 % (1/3) for MDS and 37.5 % (6/16) for severe aplastic anaemia. AML/ALL group exhibited significantly higher positivity than the combined MDS/SAA group (84.6 % vs 36.8 %, P = 0.017). Donor-stratified mNGS positivity was 52.1 % (12/23) after unrelated cord blood, 69.2 % (9/13) after haploidentical and 33.3 % (1/3) after matched-sibling transplantation (P > 0.05).
Eight mNGS-positive patients with neurological symptoms underwent cerebrospinal fluid mNGS; seven were positive, revealing S. mitis with HHV-6 (two), HHV-6 alone (two), S. mitis alone (one), P. aeruginosa (one) and K. pneumoniae (one). Two infection-related deaths occurred before day +30.

**Summary/Conclusion**

mNGS demonstrates high diagnostic value for early infections post-HSCT, particularly in AML/ALL patients, with superior detection of mixed and viral infections compared to blood culture. CNS involvement was common in mNGS-positive cases, highlighting the need for comprehensive pathogen screening in high-risk pediatric HSCT recipients.

**Copyright Information:** (Online) ISSN: 2572-9241
©2026 The Author(s). HemaSphere is published by John Wiley & Sons Ltd on behalf of European Hematology Association. This is an open access Abstract Book distributed under the Attribution-NonCommercial-NoDerivs (CC BY-NC-ND), which allows third parties to download the articles and share them with others as long as they credit the author and the Abstract Book, but they cannot change the content in any way or use them commercially.
**Abstract Book Citations:** Authors, Title, HemaSphere, 2026;10:(S1):pages. The abstract book DOIs can be found on the "Author Information" page.
**Disclaimer:** Articles published in the journal HemaSphere exclusively reflect the opinions of the authors. The authors are responsible for all content in their abstracts including accuracy of the facts, statements, citing resources, etc.

### (PB3993) THE PROGNOSTIC IMPACT OF CEBPA MUTATIONS IN ACUTE MYELOID LEUKEMIA PATIENTS UNDERGOING ALLOGENEIC HEMATOPOIETIC STEM CELL TRANSPLANTATION

**Topic:** 23. Stem cell transplantation - Clinical

Xianjing Zhang*[1], Dan Feng[1], Yigeng Cao[1], Mingyue Xu[1], Yuqing Liu[1], Junren Chen[1], Erlie Jiang[1]

*[1]Institute of Hematology & Blood Diseases Hospital, Chinese Academy of Medical Sciences & Peking Union Medical College, Tianjin, China;*

### Background

Acute myeloid leukemia (AML) is a heterogeneous hematological malignancy that frequently harbors genetic abnormalities. Myelodysplasia-related gene mutations and cytogenetic abnormalities in AML patients are strongly associated with clinical outcomes and play a pivotal role in guiding therapeutic strategies. The presence of mutations in the basic leucine zipper (bZIP) region of the CEBPA gene in patients with AML is defined as a favorable risk marker according to the guidelines. However, the value of allogeneic hematopoietic stem cell transplantation (allo-HSCT) in AML patients with CEBPA mutations remains to be clarified.

### Aims

We aimed to investigate the value and clinical impact of CEBPA mutations in patients undergoing allo-HSCT.

### Methods

We retrospectively screened a cohort of 173 patients with de novo AML who were treated between January, 2014 and March, 2025 at the institute of Hematology and Blood Diseases Hospital, Peking Union Medical College, and the Chinese Academy of Medical Sciences (IHCAMS). Furthermore, we enrolled 345 patients with newly diagnosed AML without CEBPA mutations, who received first allo-HSCT as the control group.

### Results

Patients carrying $CEBPA^{mut}$ had a significantly higher complete remission (CR) rate after first induction chemotherapy (80% vs. 68%, $P < 0.006$) and had a better 5-year PFS than $CEBPA^{wt}$ group (60.8% vs. 55.0%, $P = 0.016$). Failure to achieve remission after first induction chemotherapy as well as before transplantation, the presence of CEBPA mutation before allo-HSCT and the absence of maintenance therapy were proven to be statistically significant factors for worse survival (all $P < 0.001$). Surprisingly, survival analysis revealed that patients with nontypical in-frame bZIP mutations had a favorable outcome of PFS than typical bZIP mutations ($CEBPA^{\text{in-frame bZIP}}$ 59.9% vs. $CEBPA^{\text{no in-frame bZIP}}$ 63.3% vs. $CEBPA^{wt}$ 55.0%, $P = 0.045$). Interestingly, patients harboring both FLT3-ITD and CEBPA mutations had a significantly longer 5-year PFS than other groups ($CEBPA^{mut}$/$FLT3\text{-}ITD^{mut}$ 75.9% vs. $CEBPA^{mut}$/$FLT3\text{-}ITD^{wt}$ 57.3% vs. $CEBPA^{wt}$ 55.0%, $P = 0.02$). Conversely, the presence of GATA2 mutations (PFS: $CEBPA^{mut}$/$GATA2^{mut}$ 43.2% vs. $CEBPA^{mut}$/$GATA2^{wt}$ 63.8% vs. $CEBPA^{wt}$ 55.0%, $P = 0.04$) showed no benefit.

**Copyright Information:** (Online) ISSN: 2572-9241
©2026 The Author(s). HemaSphere is published by John Wiley & Sons Ltd on behalf of European Hematology Association. This is an open access Abstract Book distributed under the Attribution-NonCommercial-NoDerivs (CC BY-NC-ND), which allows third parties to download the articles and share them with others as long as they credit the author and the Abstract Book, but they cannot change the content in any way or use them commercially.
**Abstract Book Citations:** Authors, Title, HemaSphere, 2026;10:(S1):pages. The abstract book DOIs can be found on the "Author Information" page.
**Disclaimer:** Articles published in the journal HemaSphere exclusively reflect the opinions of the authors. The authors are responsible for all content in their abstracts including accuracy of the facts, statements, citing resources, etc.

### Summary/Conclusion

In summary, our findings suggest that the absence of CEBPA mutation, clearance of MRD in CR before transplantation and post-transplant maintenance therapy were associated with favorable survival. Moreover, patients harboring a non-typical in-frame shift in the bZIP region or CEBPA$^{mut}$ subjects together with FLT3-ITD co-mutation exhibited prolonged progression-free survival and benefited from allo-HSCT.

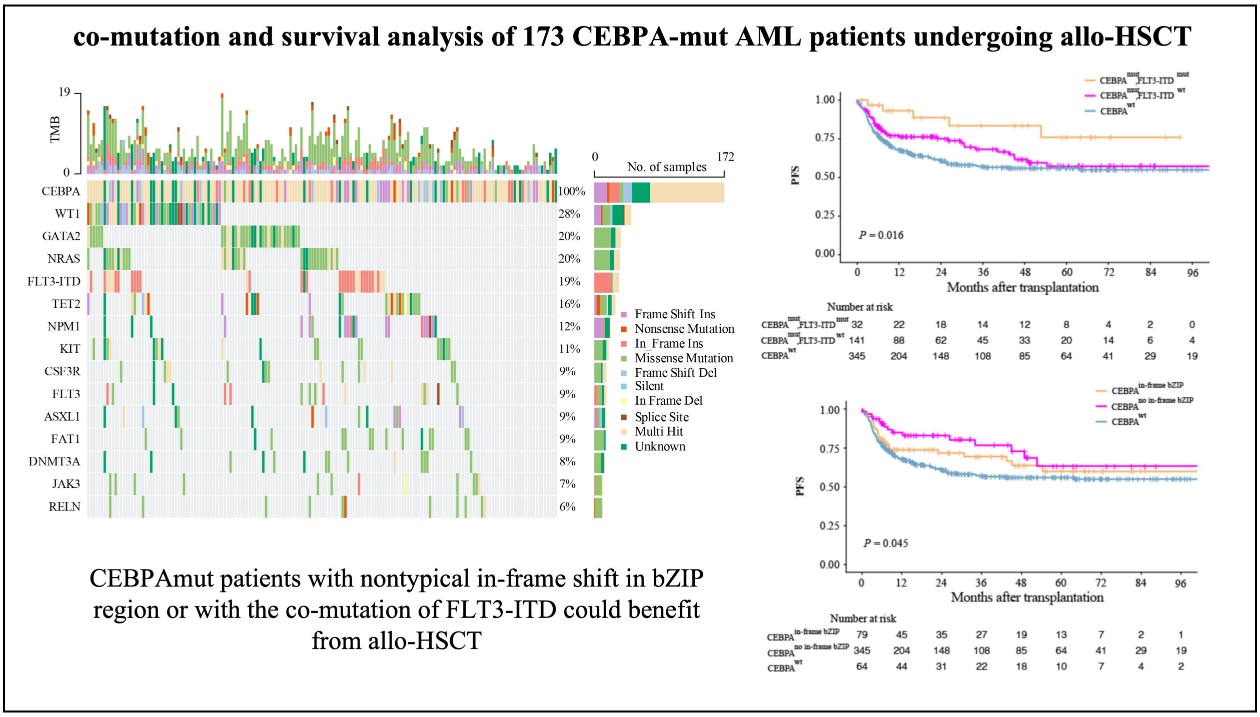

**Copyright Information:** (Online) ISSN: 2572-9241
©2026 The Author(s). HemaSphere is published by John Wiley & Sons Ltd on behalf of European Hematology Association. This is an open access Abstract Book distributed under the Attribution-NonCommercial-NoDerivs (CC BY-NC-ND), which allows third parties to download the articles and share them with others as long as they credit the author and the Abstract Book, but they cannot change the content in any way or use them commercially.
**Abstract Book Citations:** Authors, Title, HemaSphere, 2026;10:(S1):pages. The abstract book DOIs can be found on the "Author Information" page.
**Disclaimer:** Articles published in the journal HemaSphere exclusively reflect the opinions of the authors. The authors are responsible for all content in their abstracts including accuracy of the facts, statements, citing resources, etc.

### (PB3994) SINGLE-CENTER EXPERIENCE WITH AUTOLOGOUS TRANSPLANTATION OF NON-CRYOPRESERVED HEMATOPOIETIC STEM CELLS IN MULTIPLE MYELOMA: ANALYSIS OF 183 CASES

**Topic:** 23. Stem cell transplantation - Clinical

Salima Oukid*[1], Guezlane Sherifa[1], Fatima Lamraoui[1], Souad Taoussi[1], Hamida Brahimi[1], Bouchakor Moussa Yamina[1], Nabila Rekab[1], Khadoudj Mounira Benlabiod[1], Mohamed Mezroud[2], Ahmed Amine Ziani[1], Amina Rebouh[1], Ferdi Zineb[2], Bradai Mohamed[1]

*[1]Faculty of Medicine, University of Blida 1. Research Laboratory on Malignant Hemopathies and Hemoglobinopathies, Faculty of Medicine., Department of Hematology, EHS ELCC., Blida, Algeria; [2]Department of Hematology, EHS ELCC, Blida, Algeria;*

### Background

Treatment of eligible patients with Multiple Myeloma (MM) is based on therapeutic intensification followed by autologous hematopoietic stem cell transplantation. In the absence of cryopreservation facilities, we use fresh non-cryopreserved grafts in our practice.

### Aims

We report our experience regarding the feasibility of non-cryopreserved autologous hematopoietic stem cell transplantation in a cohort of 183 transplanted MM.

### Methods

From January 2019 to January 2026, we collected 183 cases of MM: 99 men and 84 women. The mean age was 54.3 years (33–70). Stage III predominated at 97% according to the Salmon and Durie classification, with renal involvement (B) in 16 cases (8.7%). According to the ISS classification: stage I: 47%, stage II: 23%, and stage III: 30%. Response evaluation at the end of induction therapy according to IMWG criteria showed: CR: 59 cases (32.2%), VGPR: 92 cases (50.3%), and PR: 32 cases (17.5%). Stem cell mobilization was achieved with G-CSF for 4 to 5 days, followed by graft collection, which was stored at 4°C. Intensification was based on Melphalan at 200 mg/m² on day -1 in cases of normal renal function, and 140 mg in cases of renal insufficiency. Evaluation was performed at day +100 post-transplant.

### Results

One apheresis session was performed in 135 patients (73.8%) and 2 sessions in 48 patients (26.2%); 3 cases of mobilization failure were recorded. The mean treated blood volume was 1.7 (0.6–3) over 230 procedures. The mean infused graft content was $7.7\times10^6$CD34+ cells/kg (1.57-30). The mean time to neutrophil recovery was 8.47 days (5-14). Regarding transfusion requirements: the mean number of platelet concentrates was 2.82 (1-17), and 1.8 (1-8) units of packed red blood cells were administered in 58 patients. No adverse events were noted during graft reinfusion. Mucositis was observed in 152 cases (84%), including 71 cases (46.7%) of grade III/IV. Day100 evaluation was performed in 141 patients: CR 37 cases (36 CR and 1 death during ASCT), VGPR 73 cases (41 converted to CR, 32 remained VGPR) and PR 31 cases (12 converted to CR, 16 to VGPR, and 3 remained PR). Outcome of 172 patients: 146 alive, 26 deceased. Relapse occurred in 48 out of 152 cases, with a mean time to relapse of 26.6 months (5–108).

**Copyright Information:** (Online) ISSN: 2572-9241
©2026 The Author(s). HemaSphere is published by John Wiley & Sons Ltd on behalf of European Hematology Association. This is an open access Abstract Book distributed under the Attribution-NonCommercial-NoDerivs (CC BY-NC-ND), which allows third parties to download the articles and share them with others as long as they credit the author and the Abstract Book, but they cannot change the content in any way or use them commercially.
**Abstract Book Citations:** Authors, Title, HemaSphere, 2026;10:(S1):pages. The abstract book DOIs can be found on the "Author Information" page.
**Disclaimer:** Articles published in the journal HemaSphere exclusively reflect the opinions of the authors. The authors are responsible for all content in their abstracts including accuracy of the facts, statements, citing resources, etc.

**Summary/Conclusion**

**Discussion**

Our data are consistent with those of the Urueta study, which analyzed 216 MM patients with a median age of 54 years (29–75). The median neutrophil recovery time was 14 days vs 7.9 days in our cohort. They reported 3 deaths vs. 1 in our study. The studies by Wanesson and Jaime support the effectiveness of non-cryopreserved stem cell transplantation, thus avoiding patient exposure to cryoprotectants such as dimethyl sulfoxide.

**Conclusion**

Fresh autologous hematopoietic stem cells are a viable alternative and secure to cryopreservative autologous transplantation in MM. This technique is less complex and less costly than cryopreservation, with comparable results. However, its limitations include the inability to store part of the graft for delayed transplantation, tandem transplantation, or a second transplant in case of relapse.

**Copyright Information:** (Online) ISSN: 2572-9241
©2026 The Author(s). HemaSphere is published by John Wiley & Sons Ltd on behalf of European Hematology Association. This is an open access Abstract Book distributed under the Attribution-NonCommercial-NoDerivs (CC BY-NC-ND), which allows third parties to download the articles and share them with others as long as they credit the author and the Abstract Book, but they cannot change the content in any way or use them commercially.
**Abstract Book Citations:** Authors, Title, HemaSphere, 2026;10:(S1):pages. The abstract book DOIs can be found on the "Author Information" page.
**Disclaimer:** Articles published in the journal HemaSphere exclusively reflect the opinions of the authors. The authors are responsible for all content in their abstracts including accuracy of the facts, statements, citing resources, etc.

### (PB3995) TBF VERSUS STANDARD CONDITIONING IN ALLOGENEIC HSCT: A SYSTEMATIC REVIEW AND META-ANALYSIS OF COMPARATIVE STUDIES FROM THE FIRST POOLED ANALYSIS OF THIOTEPA-BUSULFAN-FLUDARABINE OUTCOMES

**Topic:** 23. Stem cell transplantation - Clinical

Yashasvi Srivastava*[1], Shankar Biswas[1]

[1]*Ivano-Frankivsk National Medical University, Ivano-Frankivsk, Ukraine;*

### Background

Thiotepa-busulfan-fludarabine (TBF) has emerged as a widely adopted conditioning regimen for allogeneic hematopoietic stem cell transplantation (allo-HSCT), particularly in the haploidentical and cord blood settings. Several registry-based comparative studies have evaluated TBF against standard regimens including busulfan-cyclophosphamide (BuCy), busulfan-fludarabine (BuFlu), and treosulfan-fludarabine (TreoFlu). However, no pooled analysis has synthesized these data to determine the overall efficacy and safety profile of TBF relative to standard conditioning.

### Aims

To perform the first systematic review and meta-analysis comparing TBF with standard myeloablative conditioning regimens in adults undergoing allo-HSCT for hematologic malignancies.

### Methods

We systematically searched PubMed, Embase, and Scopus through February 2026. Comparative studies evaluating TBF versus BuCy, BuFlu, TreoFlu, or other standard regimens reporting at least one transplant outcome were eligible. Conference abstracts were included with no language restriction. Hazard ratios (HRs) with 95% confidence intervals (CIs) were pooled using random-effects models (REML) with Hartung-Knapp adjustment. Subgroup analyses were performed by comparator type and disease. Risk of bias was assessed using the Newcastle-Ottawa Scale (NOS). The protocol was registered on PROSPERO.

### Results

Nine studies (6,332 patients; 1,889 TBF, 4,443 comparator) were included. All were retrospective comparative studies with low risk of bias (NOS 7-9/9). TBF was associated with significantly lower relapse incidence (8 studies; HR 0.57, 95% CI 0.34-0.94; P=0.033; $I^2$=60.1%) but significantly higher non-relapse mortality (8 studies; HR 1.46, 95% CI 1.04-2.05; P=0.033; $I^2$=40.5%). These opposing effects resulted in no significant difference in overall survival (HR 0.91, 95% CI 0.61-1.35; P=0.58; $I^2$=70.0%), leukemia-free/disease-free survival (HR 0.92, 95% CI 0.61–1.40; P=0.66; $I^2$=64.3%), or GVHD-free relapse-free survival (HR 0.94, 95% CI 0.58–1.52; P=0.64). Rates of acute GVHD grade II-IV (HR 1.07, 95% CI 0.82–1.40; P=0.51; $I^2$=0%) and chronic GVHD (HR 0.71, 95% CI 0.42-1.19; P=0.13) were comparable. Subgroup analyses showed consistent anti-relapse benefit across comparators, with the NRM increase most pronounced against BuFlu. Leave-one-out and trim-and-fill analyses confirmed the robustness of findings. Egger's test showed no significant publication bias (P>0.10 for all outcomes).

### Summary/Conclusion

This first meta-analysis of TBF conditioning demonstrates a significant 43% reduction in relapse offset by 46% higher NRM, yielding comparable survival to standard regimens. These findings support risk-adapted use of TBF, favoring patients at high relapse risk who can tolerate increased transplant-related toxicity. Prospective randomized trials are warranted.

**Copyright Information:** (Online) ISSN: 2572-9241
©2026 The Author(s). HemaSphere is published by John Wiley & Sons Ltd on behalf of European Hematology Association. This is an open access Abstract Book distributed under the Attribution-NonCommercial-NoDerivs (CC BY-NC-ND), which allows third parties to download the articles and share them with others as long as they credit the author and the Abstract Book, but they cannot change the content in any way or use them commercially.
**Abstract Book Citations:** Authors, Title, HemaSphere, 2026;10:(S1):pages. The abstract book DOIs can be found on the "Author Information" page.
**Disclaimer:** Articles published in the journal HemaSphere exclusively reflect the opinions of the authors. The authors are responsible for all content in their abstracts including accuracy of the facts, statements, citing resources, etc.

### (PB3996) EFFICACY ANALYSIS OF DECITABINE IN MAINTENANCE THERAPY AFTER ALLOGENEIC HEMATOPOIETIC STEM CELL TRANSPLANTATION FOR ACUTE LEUKEMIA

**Topic:** 23. Stem cell transplantation - Clinical

Lihua Kong[1], Xiaolin Yu[1], Xiaochen Song[1], Lei Deng[1], Fang Zhou*[1]

[1] *The 960th Hospital of The People's Liberation Army (PLA) Joint Logistics Support Force, Jinnan, China;*

### Background

Acute leukemia is a group of highly heterogeneous hematological diseases, allogeneic hematopoietic stem cell transplantation (allo-HSCT) is an effective method for curing malignant blood diseases. However, disease recurrence after transplantation remains one of the important factors limiting long-term survival. There is an urgent need for effective maintenance treatment strategies that can both improve the long-term survival rate of patients with acute leukemia and exert anti-tumor effects. Decitabine induces DNA hypomethylation by inhibiting DNA methyltransferase, restores the expression of tumor suppressor genes, and thereby exerts immunomodulatory and anti-leukemic effects. In maintenance therapy after transplantation for acute leukemia, decitabine can improve long-term survival and enhance the efficacy of transplantation.

### Aims

To investigate the effect of decitabine as maintenance therapy after allo-HSCT in acute leukemia.

### Methods

A retrospective analysis was performed on the clinical data of patients with acute leukemia who underwent allo-HSCT at the 960th Hospital of the People's Liberation Army from January 1, 2023 to December 31, 2024. According to the inclusion and exclusion criteria, a total of 49 patients were enrolled in the study, including 37 patients in the experimental group who received decitabine maintenance therapy after allo-HSCT, and 12 patients in the control group who did not receive maintenance therapy after allo-HSCT. The disease-free survival (DFS) rate and overall survival (OS) rate were compared between the two groups.

### Results

The estimated 2-year OS was 94.4% (95% CI, 86.95%-100%) in the decitabine group and 61.4% (95% CI, 22.79%-100%) in the control group for an HR of 0.091 (95% CI, 0.016-0.512; $P = 0.007$)The estimated 2-year DFS was 78.4% (95% CI, 65.07%-91.73%) in the decitabine group and 54.5% (95% CI, 17.85%-91.15%) in the control group for an HR of 0.353 (95% CI, 0.114-1.089; $P = 0.07$)

### Summary/Conclusion

Maintenance therapy with decitabine after allo-HSCT for acute leukemia can improve DFS and OS.

**Copyright Information:** (Online) ISSN: 2572-9241
©2026 The Author(s). HemaSphere is published by John Wiley & Sons Ltd on behalf of European Hematology Association. This is an open access Abstract Book distributed under the Attribution-NonCommercial-NoDerivs (CC BY-NC-ND), which allows third parties to download the articles and share them with others as long as they credit the author and the Abstract Book, but they cannot change the content in any way or use them commercially.
**Abstract Book Citations:** Authors, Title, HemaSphere, 2026;10:(S1):pages. The abstract book DOIs can be found on the “Author Information” page.
**Disclaimer:** Articles published in the journal HemaSphere exclusively reflect the opinions of the authors. The authors are responsible for all content in their abstracts including accuracy of the facts, statements, citing resources, etc.

### (PB3997) SEQUENTIAL DECITABINE-BASED ALLOGENEIC STEM CELL TRANSPLANTATION IN PATIENTS WITH AML IN ACTIVE DISEASE

**Topic:** 23. Stem cell transplantation - Clinical

Maddalena Vittoria Ponti*[1, 2], Alessandro Criscimanna[1, 2], Greta Morello Pasin[1, 2], Lorenzo Lazzari[1], Sara Mastaglio[1], Maria Teresa Lupo Stanghellini[1, 2], Annalisa Ruggeri[1], Andrea Assanelli[1], Massimo Bernardi[1], Fabio Ciceri[1, 2], Luca Vago[1, 2]

*[1]IRCCS San Raffaele Scientific Institute, Hematology and Bone Marrow Transplantation Unit, Milano, Italy; [2]Vita-Salute San Raffaele University, Milano, Italy;*

### Background

Allo-HCT is primarily intended as a consolidation approach in the treatment of high-risk AML, and outcome of patients transplanted with active disease is traditionally dismal. However, emerging randomized data suggest that immediate HCT based on sequential conditioning may achieve comparable long-term results to attempting conventional salvage chemotherapy followed by HCT.
HMAs, including decitabine, are of particular interest in sequential approaches due to their immunomodulatory properties. Through DNA demethylation they enhance tumor-associated antigen expression, potentially strengthening the GvL response while providing disease control. A phase III trial in HR-MDS and sAML showed that adding decitabine to conditioning significantly reduced relapse and improved survival compared to standard conditioning, further supporting this approach.

### Aims

In this context, we report on a case series of patients with R/R AML treated with sequential HMA-venetoclax–based therapy followed by HCT, with the aim of evaluating feasibility and postHCT outcomes in this high-risk population.

### Methods

We conducted a retrospective analysis of 9 patients who underwent sequential allo-HCT for R/R AML in active disease at time of transplant.

### Results

Among our 9 patients, 6 were diagnosed with sAML, 4 had ELN adverse risk AML, 3 had adverse karyotype and mutations included TP53 (n=1), FLT3-ITD (n=2), and RUNX1 (n=2). The median number of prior lines of therapy was 2 (1-4), 7 patients underwent intensive chemotherapy, 6 had prior HMA exposure and 8 prior Venetoclax exposure. Two had received a prior allo-HCT.
Median bone marrow blast infiltration at the time sequential treatment was 10% (6-31%). Pre-conditioning regimen consisted of Decitabine in all patients, in 5 patients it was associated with Venetoclax based on prior exposure, in 1 Gilteritinib was also added, while in 1 Decitabine was associated with Daratumumab. Decitabine was started at a median of 13 (11-17) days before HCT, for a median of 5 days at 20 mg/mq.
All patients proceeded to allo-HCT, donor source were PBSC in 7 patients and cord blood in 2, donor was matched related in 1, unrelated in 6 and haploidentical in 2. Conditioning regimen was myeloablative in all patients. Neutrophil and platelet engraftment was achieved in all evaluable patients in a median of 24 and 36 days respectively, with kinetics comparable to our center’s historical cohort.
At the first post allo-HCT assessment (performed between day 30 and day 45) all 9 patients had achieved morphological complete remission. Four patients underwent maintenance therapy, mainly consisting of HMA +/- venetoclax. After a median follow-up of 5.5 months (1-20), 2 patients relapsed (day 167 and day 489) and two died in remission due to infectious complications (day 193 and 236). The remaining 5 patients are alive and in remission with a median FU of 3.5 months.

**Copyright Information:** (Online) ISSN: 2572-9241
©2026 The Author(s). HemaSphere is published by John Wiley & Sons Ltd on behalf of European Hematology Association. This is an open access Abstract Book distributed under the Attribution-NonCommercial-NoDerivs (CC BY-NC-ND), which allows third parties to download the articles and share them with others as long as they credit the author and the Abstract Book, but they cannot change the content in any way or use them commercially.
**Abstract Book Citations:** Authors, Title, HemaSphere, 2026;10:(S1):pages. The abstract book DOIs can be found on the “Author Information” page.
**Disclaimer:** Articles published in the journal HemaSphere exclusively reflect the opinions of the authors. The authors are responsible for all content in their abstracts including accuracy of the facts, statements, citing resources, etc.

**Summary/Conclusion**

In this small high-risk cohort, decitabine-based sequential conditioning enabled successful allo-HCT in patients with active disease, achieving post-transplant CR in all patients. These findings, to be confirmed in larger studies, suggest that decitabine-based preconditioning may help creating a therapeutic window for patients with very high-risk AML, allowing post-transplant maintenance approaches to synergize with the graft-versus-tumor effect.

**Copyright Information:** (Online) ISSN: 2572-9241
©2026 The Author(s). HemaSphere is published by John Wiley & Sons Ltd on behalf of European Hematology Association. This is an open access Abstract Book distributed under the Attribution-NonCommercial-NoDerivs (CC BY-NC-ND), which allows third parties to download the articles and share them with others as long as they credit the author and the Abstract Book, but they cannot change the content in any way or use them commercially.
**Abstract Book Citations:** Authors, Title, HemaSphere, 2026;10:(S1):pages. The abstract book DOIs can be found on the "Author Information" page.
**Disclaimer:** Articles published in the journal HemaSphere exclusively reflect the opinions of the authors. The authors are responsible for all content in their abstracts including accuracy of the facts, statements, citing resources, etc.

### (PB3998) TREOSULFAN-BASED REDUCED-INTENSITY CONDITIONING FOR ALLOGENEIC HCT IN OLDER AND HIGHLY COMORBID PATIENTS: A SINGLE-CENTER REAL-WORLD COHORT

**Topic:** 23. Stem cell transplantation - Clinical

MacArena Castillo Godoy*[1], Maria Orviz Garcia[1], Ana María Pilar González Rodríguez[1], Ana Julia Gonzalez Huerta[1], Soledad González Muñiz[1], Pilar Palomo[1], Joud Zanabili[1]

[1]*Hospital Universitario Central de Asturias, Oviedo, Spain;*

### Background

Allogeneic hematopoietic stem cell transplantation (alloHCT) is the only potential curative treatment for patients. Nevertheless, older or comorbid alloHCT recipients are at greater risk for several alloHCT-related complications. Treosulfan has emerged as an optimal part of prior HCT conditioning regimens in this patient population.

### Aims

Analyze the outcome of patients who underwent alloHCT with treosulfan-based conditioning for myeloid neoplasms.

### Methods

We retrospectively analyzed the outcome of adult pts with myeloid neoplasms who underwent alloHCT with treosulfan-based conditioning from a matched or mismatched donor from June 2023 to December 2025 at the Hospital Universitario Central Asturias. The conditioning consisted of a combination of treosulfan 10g/m2 /day on days -6 to -4, fludarabine, 30mg/m2 /day on days -6 to -2. Survival outcomes were estimated using Kaplan–Meier methods.

**Copyright Information:** (Online) ISSN: 2572-9241
©2026 The Author(s). HemaSphere is published by John Wiley & Sons Ltd on behalf of European Hematology Association. This is an open access Abstract Book distributed under the Attribution-NonCommercial-NoDerivs (CC BY-NC-ND), which allows third parties to download the articles and share them with others as long as they credit the author and the Abstract Book, but they cannot change the content in any way or use them commercially.
**Abstract Book Citations:** Authors, Title, HemaSphere, 2026;10:(S1):pages. The abstract book DOIs can be found on the "Author Information" page.
**Disclaimer:** Articles published in the journal HemaSphere exclusively reflect the opinions of the authors. The authors are responsible for all content in their abstracts including accuracy of the facts, statements, citing resources, etc.

**Results**

We included 34 patients, 23 (67.6%) were male with a median age of 68 years (range 48-75). Median follow-up was 573 days. Patient and transplant characteristics are listed in Table 1. Stem cell source was peripheral blood in all cases. The patients´ baseline diseases were: acute myeloblastic leukemia (41.2%), myelodysplastic syndrome (38.2%) and other myeloid neoplasms (20.6%). Nine from 14 AML patients (64.3%) had assigned as adverse genetic risk group at the 2022 ELN. Among 13 patients with MDS, 6 patients had scored as intermediate-1 IPSS-R, 6 as intermediate-2 and 1 as high-risk group.

The median time to neutrophil engraftment was 17.5 days (range 16–20.8), 18 days for platelet engraftment (range 14–22), significantly slower after haplo-HSCT (26 vs. 15 days; p=0.0001). Considering early toxicity, 11 of 34 patients (32-4%) suffered from mucositis (grade ≥2 in 14.7%) and 3 patients from SOS (8.8%). Eight of 34 patients (23.5%) developed acute GVHD (grade II-III, no one grade IV), and 23.5% (8 patients) had chronic GVHD of any grade (only 5 moderate-severe).

At day +100, 24 patients had achieved CR (70.6%), from whom 21 (87.5%) got MRD negative. Relapse occurred in 8 patients (23.5%) with a median of 77 days. Median OS was not reached and median PFS was 2.4 years; the 2-year OS was 67% and RFS was 59.9%. NRM was 14.7%. Main causes of death were disease relapse (4 patients), infections (1 patients), GVHD (2 patients) and other causes in remainder.

| | |
|---|---|
| **Fitness / Comorbidity** | |
| HCT-CI > 2, n (%) | 25 (73.5) |
| ECOG ≤ 2, n (%) | 33 (97.1) |
| **Diagnosis** | |
| AML, n (%) | 14 (41.2) |
| MDS, n (%) | 13 (38.2) |
| Other myeloid neoplasms, n (%) | 7 (20.6) |
| **Donor type** | |
| MRD, n (%) | 10 (29.4) |
| MUD 10/10, n (%) | 14 (41.2) |
| MMUD 9/10, n (%) | 2 (5.9) |
| Haploidentical, n (%) | 8 (23.5) |
| **GVHD prophylaxis** | |
| ATG + MTX + CNI, n (%) | 23 (67.6) |
| PTCy + MMF + CNI, n (%) | 9 (26.5) |
| Other, n (%) | 2 (5.9) |
| **CMV prophylaxis** | |
| Letermovir, n (%) | 14 (41.2) |
| **Pre-transplant comorbibidities and health conditions, %** | |
| Pulmonary comorbidity, n (%) | 9 (26.5) |
| Hepatic comorbidity, n (%) | 4 (11.8) |
| Neurologic comorbidity, n (%) | 1 (2.9) |
| Age > 60 with comorbidity, n (%) | 10 (29.4) |
| Age > 70, n (%) | 7 (20.6) |
| Second transplant, n (%) | 3 (8.8) |
| Additional secondary reason present, n (%) | 13 (38.2) |

**Copyright Information:** (Online) ISSN: 2572-9241
©2026 The Author(s). HemaSphere is published by John Wiley & Sons Ltd on behalf of European Hematology Association. This is an open access Abstract Book distributed under the Attribution-NonCommercial-NoDerivs (CC BY-NC-ND), which allows third parties to download the articles and share them with others as long as they credit the author and the Abstract Book, but they cannot change the content in any way or use them commercially.
**Abstract Book Citations:** Authors, Title, HemaSphere, 2026;10:(S1):pages. The abstract book DOIs can be found on the "Author Information" page.
**Disclaimer:** Articles published in the journal HemaSphere exclusively reflect the opinions of the authors. The authors are responsible for all content in their abstracts including accuracy of the facts, statements, citing resources, etc.

**Summary/Conclusion**

Our retrospective analysis suggests that a treosulfan-based RIC in an elderly and population with comorbidities is feasible, with an excellent engraftment rate and a limited early toxicity, GVHD and NRM. These data outline the patient profiles and practicaldrivers that most often led to treosulfan use in routine transplant care.

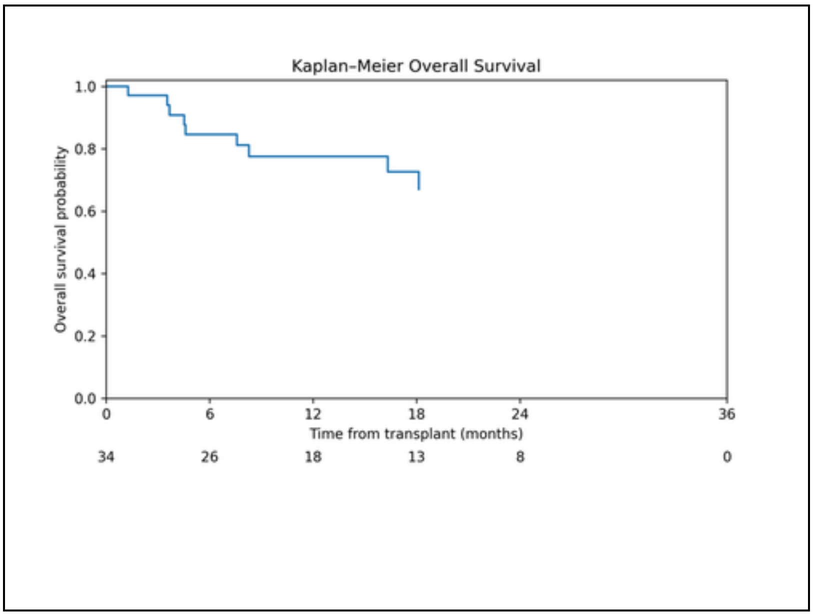

**Copyright Information:** (Online) ISSN: 2572-9241
©2026 The Author(s). HemaSphere is published by John Wiley & Sons Ltd on behalf of European Hematology Association. This is an open access Abstract Book distributed under the Attribution-NonCommercial-NoDerivs (CC BY-NC-ND), which allows third parties to download the articles and share them with others as long as they credit the author and the Abstract Book, but they cannot change the content in any way or use them commercially.
**Abstract Book Citations:** Authors, Title, HemaSphere, 2026;10:(S1):pages. The abstract book DOIs can be found on the "Author Information" page.
**Disclaimer:** Articles published in the journal HemaSphere exclusively reflect the opinions of the authors. The authors are responsible for all content in their abstracts including accuracy of the facts, statements, citing resources, etc.

### (PB3999) EARLY OUTCOMES OF LOW DOSE TBI PLUS THIOTEPA FLUDARABINE CYTARABINE CYCLOPHOSPHAMIDE (T-FACT) CONDITIONING FOR UMBILICAL CORD BLOOD TRANSPLANTATION

**Topic:** 23. Stem cell transplantation - Clinical

Can Chen*[1], Pengfei Shi[1], Shenxian Qian[1]

[1]*Hangzhou First People's Hospital, Hangzhou, China;*

**Background**

Umbilical cord blood transplantation (UCBT) expands donor availability for patients with acute myeloid leukemia (AML) and myelodysplastic syndromes (MDS). Delayed engraftment, graft-versus-host disease (GVHD), and relapse remain major challenges.

**Aims**

Umbilical cord blood transplantation (UCBT) expands donor availability for patients with acute myeloid leukemia (AML) and myelodysplastic syndromes (MDS). Delayed engraftment, graft-versus-host disease (GVHD), and relapse remain major challenges.

**Methods**

This prospective, single-center study was registered at the Chinese Clinical Trial Registry as ChiCTR2500108003. Consecutive adults with AML or MDS undergoing UCBT were included. Conditioning consisted of TBI 4 Gy on day -10, thiotepa 5 mg/kg on day -9, cytarabine total 10 g/m² and fludarabine total 180 mg/m² from day -8 to day -4, and cyclophosphamide 2 g/m² on days -3 and -2. GVHD prophylaxis was cyclosporine A plus mycophenolate mofetil. Outcomes included engraftment, GVHD, viral reactivation, relapse, 100-day non-relapse mortality (NRM), and Kaplan-Meier estimates of overall survival (OS), progression-free survival (PFS), and GVHD-free relapse-free survival (GRFS). Data cut-off was 27 Feb 2026.The study was approved by the Institutional Review Board of Hangzhou First People's Hospital and conducted in accordance with the Declaration of Helsinki. Written informed consent was obtained from all participants.

**Results**

Eleven patients were analyzed. Median age was 49 years (range 35 to 68). Eight patients had AML and three had MDS. High-risk disease was present in 10 of 11 patients (91%). Eight of 11 patients (73%) were MRD-positive before transplantation. Four of 11 patients (36%) had relapsed or refractory disease. Three of 11 patients (27%) underwent a second allogeneic transplant. Most patients received a single cord unit (9 of 11). Neutrophil and platelet engraftment occurred in all patients, with a median of 24 days (range 16 to 30) and 34 days (range 25 to 45), respectively. Early donor chimerism assessed by STR was 82.0% at day +8 (range 30.6 to 99.8, n=11) and 99.4% at day +15 (range 97.0 to 99.9, n=7). Grade II to IV acute GVHD occurred in 3 of 11 patients (27%). No chronic GVHD was observed. HHV-6 reactivation occurred in 8 of 11 patients (73%), with a median onset at day +17 (range +14 to +33). EBV viremia occurred in 1 of 11 patients (9%). No CMV viremia or CMV disease was documented. Three patients relapsed (27%). Two patients died (18%), both after relapse. The 100-day NRM was 0%. With a median follow-up of 249 days (range 110 to 461) among survivors, the estimated 1-year OS was 77.8% and the estimated 1-year PFS was 69.3%.

**Summary/Conclusion**

T-FACT conditioning for adult UCBT was feasible and resulted in universal engraftment with zero 100-day NRM in a predominantly high-risk, MRD-positive AML and MDS cohort. Early OS and PFS were encouraging. Longer follow-up is required to confirm durability and to obtain mature GRFS estimates.

**Copyright Information:** (Online) ISSN: 2572-9241
©2026 The Author(s). HemaSphere is published by John Wiley & Sons Ltd on behalf of European Hematology Association. This is an open access Abstract Book distributed under the Attribution-NonCommercial-NoDerivs (CC BY-NC-ND), which allows third parties to download the articles and share them with others as long as they credit the author and the Abstract Book, but they cannot change the content in any way or use them commercially.
**Abstract Book Citations:** Authors, Title, HemaSphere, 2026;10:(S1):pages. The abstract book DOIs can be found on the "Author Information" page.
**Disclaimer:** Articles published in the journal HemaSphere exclusively reflect the opinions of the authors. The authors are responsible for all content in their abstracts including accuracy of the facts, statements, citing resources, etc.

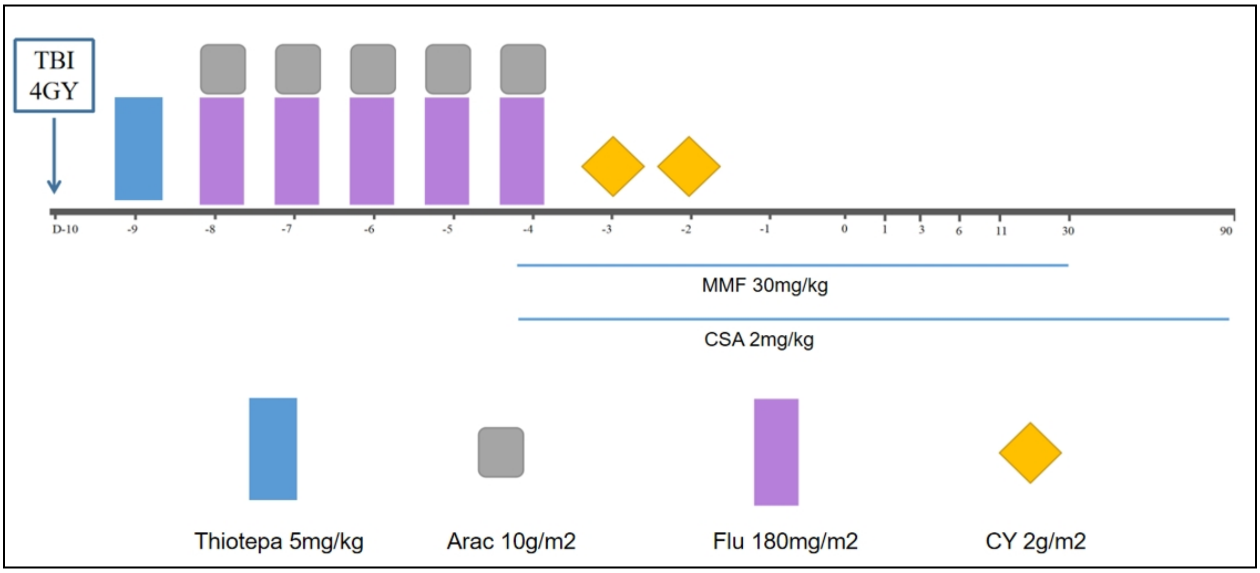

**Copyright Information:** (Online) ISSN: 2572-9241
©2026 The Author(s). HemaSphere is published by John Wiley & Sons Ltd on behalf of European Hematology Association. This is an open access Abstract Book distributed under the Attribution-NonCommercial-NoDerivs (CC BY-NC-ND), which allows third parties to download the articles and share them with others as long as they credit the author and the Abstract Book, but they cannot change the content in any way or use them commercially.
**Abstract Book Citations:** Authors, Title, HemaSphere, 2026;10:(S1):pages. The abstract book DOIs can be found on the "Author Information" page.
**Disclaimer:** Articles published in the journal HemaSphere exclusively reflect the opinions of the authors. The authors are responsible for all content in their abstracts including accuracy of the facts, statements, citing resources, etc.

### (PB4000) EMAPALUMAB IN TREATMENT-EXPERIENCED PRIMARY HLH: SAFETY, EFFICACY, AND TRANSPLANT OUTCOMES FROM THE OPEN-LABEL, SINGLE-ARM POST-AUTHORIZATION STUDY SOBI.EMAPALUMAB-104 IN CHINESE PATIENTS

**Topic:** 23. Stem cell transplantation - Clinical

Rui Zhang*[1], Xiaowen Zhai[2], Jianpei Fang[3], Andrew Danquah[4], Ben Peace[4], Per Hedlund[4], Andrew Ramsey[5], Rafael Krmar[4], Brian Jamieson[5]

*[1]Beijing Children's Hospital, Capital Medical University, National Center for Children's Health, Beijing, China;[2]Children's Hospital of Fudan University, Shanghai, China; [3]Sun Yat-sen Memorial Hospital, Sun Yat-sen University, Guangzhou City, China; [4]Sobi, Stockholm, Sweden; [5]Sobi, Inc., Morrisville, United States of America;*

### Background

Primary hemophagocytic lymphohistiocytosis (pHLH) is a life-threatening hyperinflammatory syndrome requiring rapid disease control and timely transition to hematopoietic stem cell transplant (HSCT). Emapalumab is a fully human monoclonal antibody that neutralizes interferon-γ (IFN-γ), a key cytokine in HLH pathogenesis. Data on treatment-experienced Chinese patients are limited.

### Aims

Assess safety, specifically permanent discontinuation of emapalumab due to emapalumab-related adverse events (AEs).

### Methods

Sobi.EMAPALUMAB-104 (NCT05744063) was an open-label, single-arm, multicenter, post-authorization study in China. Treatment-experienced pHLH patients received emapalumab twice weekly (first dose 1 mg/kg; stepwise increases up to 10 mg/kg were permitted) on background glucocorticoid (GC; ≥5 mg/m$^2$/day, dexamethasone equivalent, tapered or increased per investigator's judgement). Emapalumab treatment continued until conditioning for HSCT began or until HSCT was not deemed indicated. Analyses were descriptive on the All Treated set (receipt of ≥1 emapalumab infusion). Proportions were summarized with Clopper–Pearson 95% CIs.

### Results

Sixteen patients were screened; 13 were treated (All treated analysis set; **Table 1**).
No patient permanently discontinued emapalumab due to emapalumab-related AEs.
All patients experienced ≥1 treatment-emergent AE (TEAE); seven patients (53.8%) had TEAEs meeting SAE criteria (most commonly infections; 4/13 [30.8%]). Emapalumab-related TEAEs occurred in 9 (69.2%) and related serious TEAEs in 3 (23.1%), respiratory syncytial virus pneumonia, pneumonia and sepsis. One patient (7.7%) experienced emapalumab-related TEAEs that led to temporary interruption. No infusion reactions were experienced. Treatment-emergent laboratory abnormalities were frequent across hematology, biochemistry, coagulation, and sCD25, consistent with disease activity. Two deaths (15.4%) occurred prior to HSCT conditioning; both were attributed to disease progression and were not related to emaplaumab.
Overall response at Week 8/EOT was achieved by 10/13 (76.9%): complete response 5 (38.5%), partial response 4 (30.8%), HLH improvement 1 (7.7%). Two patients (15.4%) were non-responders and 1 (7.7%) had missing EOT data due to early withdrawal. GC tapering (≥50% reduction for ≥7 days) occurred in 6/13 (46.2%). Survival to start of HSCT conditioning was achieved by 11/13 (84.6%). HSCT was performed in 9/13 (69.2%); engraftment was achieved in 8/9 (88.9%), with a mean donor chimerism of 96.5% (range 73.1–100.0). No deaths occurred post-HSCT (survival post-HSCT, 100%). Overall survival rate at 12 months was 84.6%.

**Copyright Information:** (Online) ISSN: 2572-9241
©2026 The Author(s). HemaSphere is published by John Wiley & Sons Ltd on behalf of European Hematology Association. This is an open access Abstract Book distributed under the Attribution-NonCommercial-NoDerivs (CC BY-NC-ND), which allows third parties to download the articles and share them with others as long as they credit the author and the Abstract Book, but they cannot change the content in any way or use them commercially.
**Abstract Book Citations:** Authors, Title, HemaSphere, 2026;10:(S1):pages. The abstract book DOIs can be found on the "Author Information" page.
**Disclaimer:** Articles published in the journal HemaSphere exclusively reflect the opinions of the authors. The authors are responsible for all content in their abstracts including accuracy of the facts, statements, citing resources, etc.

**Summary/Conclusion**

In treatment-experienced Chinese patients with pHLH, emapalumab plus GC resulted in rapid, clinically meaningful disease control, substantial GC tapering, and successful transition to HSCT for most patients, with 100% post-HSCT survival. Safety was consistent with mechanism of action and disease context, infections were the most common AEs, and no permanent discontinuations were due to emapalumab-related AEs. These outcomes are broadly consistent with the pivotal NI-0501-04 primary analysis (Locatelli et al, 2020). They also support emapalumab as a well-tolerated and effective bridge to HSCT in this critically ill population of treatment-experienced Chinese patients with pHLH.

**Table 1: Demographics and Baseline Disease Features**

| | N=13 |
|---|---|
| **Age, years, median (range)** | 7 (3-33) |
| **Sex, male, n (%)** | 8 (61.5%) |
| **pHLH diagnosis n (%)** | |
| Confirmed pHLH | 9 (69.2%) |
| Suspected pHLH | 4 (30.8%) |
| **Disease features*, n (%)** | |
| Fever | 13 (100%) |
| Splenomegaly | 13 (100%) |
| Hypertriglyceridemia[a] and/or hypofibrinogenemia[b] | 9 (69.2%) |
| Cytopenias[c] | 8 (61.5%) |
| Hyperferritinemia[d] | 8 (61.5%) |

*Not mutually exclusive

[a]Fasting triglycerides ≥3 mmol/L or ≥265 mg/dL); [b]Hypofibrinogenemia (≤1.5 g/L); [c]Affecting 2 of 3 lineages in the peripheral blood (hemoglobin <90 g/L; platelets <100 x $10^9$/L; neutrophils <1 x $10^9$/L); [d]Ferritin ≥500 ug/L

pHLH, Primary hemophagocytic lymphohistiocytosis

Copyright Information: (Online) ISSN: 2572-9241
©2026 The Author(s). HemaSphere is published by John Wiley & Sons Ltd on behalf of European Hematology Association. This is an open access Abstract Book distributed under the Attribution-NonCommercial-NoDerivs (CC BY-NC-ND), which allows third parties to download the articles and share them with others as long as they credit the author and the Abstract Book, but they cannot change the content in any way or use them commercially.
Abstract Book Citations: Authors, Title, HemaSphere, 2026;10:(S1):pages. The abstract book DOIs can be found on the "Author Information" page.
Disclaimer: Articles published in the journal HemaSphere exclusively reflect the opinions of the authors. The authors are responsible for all content in their abstracts including accuracy of the facts, statements, citing resources, etc.

### (PB4001) EXPLORING THE EFFICACY AND SAFETY OF LOW-DOSE ATG COMBINED WITH LOW-DOSE PTCY IN PREVENTING GVHD AFTER ALLOGENEIC HEMATOPOIETIC STEM CELL TRANSPLANTATION

**Topic:** 23. Stem cell transplantation - Clinical

Li Jing Mao*[1]

[1]*Yantai Yuhuangding Hospital, Yantai, China;*

**Background**

In recent years, growing evidence has demonstrated the potential of anti-thymocyte globulin (ATG) combined with post-transplant cyclophosphamide (PTCy) as an effective strategy for graft-versus-host disease (GVHD) prophylaxis. This regimen appears to significantly reduce the incidence of both acute and chronic GVHD. However, the optimal dosing of ATG in combination with PTCy requires further investigation through well-designed clinical trials.

**Aims**

To retrospectively analyze the efficacy and safety of low-dose anti-thymocyte globulin (ATG) combined with low-dose post-transplant cyclophosphamide (PTCY) in preventing graft-versus-host disease (GVHD) after allogeneic hematopoietic stem cell transplantation (allo-HSCT).

**Methods**

Clinical data from 29 patients who underwent allo-HSCT at Yantai Yuhuangding Hospital between June 2022 and May 2025 were retrospectively analyzed (AML: 14 cases, MDS: 8 cases, ALL: 5 cases, MPAL: 2 cases; Haploidentical transplantation: 24 cases, Unrelated donor transplantation: 5 cases). The GVHD prophylaxis regimen consisted of low-dose ATG (2.5 mg/kg on days -2 and -1), fractionated low-dose PTCY (25 mg/kg on days +3 and +4), cyclosporine (2.5 mg/kg /day starting from day +5), and mycophenolate mofetil (0.5 g twice daily from day +5).

**Results**

The median age of the 29 patients was 48.5 years (range: 16–65), with 16 males and 13 females. The median follow-up was 561 days. The median time to neutrophil engraftment was 12 days (range: 9–21), and the median time to platelet engraftment was 13 days (range: 9–38). One patient experienced primary graft failure (graft rejection) and underwent a second transplantation with a different donor on day +37, achieving successful engraftment. At D+100⊠the cumulative incidence of grade I–IV acute GVHD (aGVHD) was 8.4%, the incidence of grade III–IV aGVHD was 3.8%⊠with only one case of grade IV intestinal aGVHD. The 1-year Chronic GVHD (cGVHD) incidence: 17.4% (moderate-to-severe cGVHD: 5.9%). At D+100⊠non-relapse mortality(NRM):7%, EBV reactivation⊠48.3%(plasma EBV DNA >1000 copies/mL :7.4%; EBV-PTLD: one case), CMV reactivation: 51.7%(CMV DNA >1000 copies/mL :27.6%). D+30,The incidence of bloodstream infection (BSI)  was 13.8%. The incidence of grade I–II hemorrhagic cystitis was 10.3%, with no grade III–IV cases. Grade I–II oral mucositis was observed in 31%, with no severe cases. No significant cardiac adverse events or grade ≥2 fluid overload (FO) were reported. The 1-year relapse rate was 17.2%. The 1-year overall survival (OS) and progression-free survival (PFS) rates were 77.3% and 62.5%. Among the 24 non-ALL patients, the 1-year OS and PFS were 80.9% and 70.3%. By the end of follow-up, six patients had died due to relapse (n=2), shock (n=1), cGVHD (n=1), TMA (n=1), and secondary graft failure (n=1).

**Copyright Information:** (Online) ISSN: 2572-9241
©2026 The Author(s). HemaSphere is published by John Wiley & Sons Ltd on behalf of European Hematology Association. This is an open access Abstract Book distributed under the Attribution-NonCommercial-NoDerivs (CC BY-NC-ND), which allows third parties to download the articles and share them with others as long as they credit the author and the Abstract Book, but they cannot change the content in any way or use them commercially.
**Abstract Book Citations:** Authors, Title, HemaSphere, 2026;10:(S1):pages. The abstract book DOIs can be found on the "Author Information" page.
**Disclaimer:** Articles published in the journal HemaSphere exclusively reflect the opinions of the authors. The authors are responsible for all content in their abstracts including accuracy of the facts, statements, citing resources, etc.

**Summary/Conclusion**

GVHD prophylaxis with low-dose ATG combined with fractionated low-dose PTCy in allogeneic hematopoietic stem cell transplantation demonstrated excellent tolerability, with a remarkably low incidence of grade III-IV GVHD and absence of cardiac adverse events,  However, due to the small sample size and short follow-up, further prospective, large-scale, multicenter randomized controlled trials are needed to validate the superiority of this regimen.

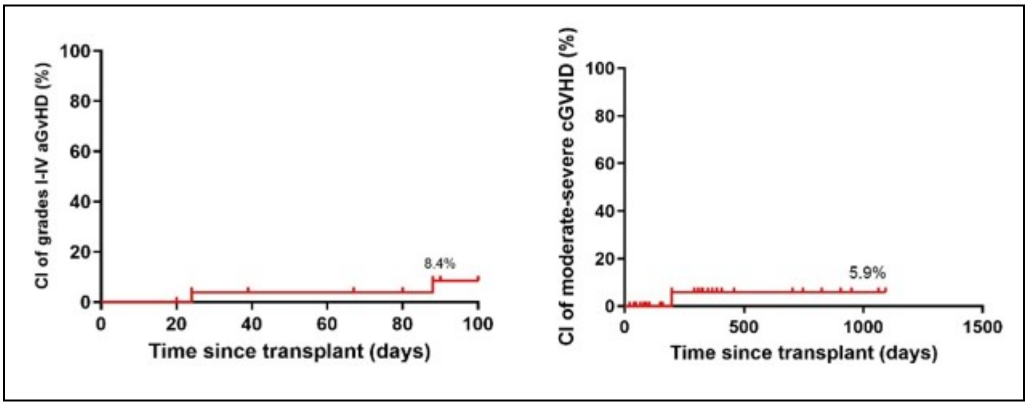

At D+100[illegible]the cumulative incidence of grade I–IV acute GVHD (aGVHD) was 8.4%. The 1-year Chronic GVHD (cGVHD) incidence: 17.4% (moderate-to-severe cGVHD: 5.9%).

**Copyright Information:** (Online) ISSN: 2572-9241
©2026 The Author(s). HemaSphere is published by John Wiley & Sons Ltd on behalf of European Hematology Association. This is an open access Abstract Book distributed under the Attribution-NonCommercial-NoDerivs (CC BY-NC-ND), which allows third parties to download the articles and share them with others as long as they credit the author and the Abstract Book, but they cannot change the content in any way or use them commercially.
**Abstract Book Citations:** Authors, Title, HemaSphere, 2026;10:(S1):pages. The abstract book DOIs can be found on the “Author Information” page.
**Disclaimer:** Articles published in the journal HemaSphere exclusively reflect the opinions of the authors. The authors are responsible for all content in their abstracts including accuracy of the facts, statements, citing resources, etc.

### (PB4002) HISTOLOGY-GUIDED DIAGNOSIS REFINES MANAGEMENT OF LIVER TOXICITY AFTER STEM CELL TRANSPLANTATION ALLOWING TARGETED TREATMENT

**Topic:** 23. Stem cell transplantation - Clinical

Alessandro Bruno*[1], Federica Pedica[1, 2], Andrea Municchi[1], Elisa Diral[1], Lorenzo Lazzari[1], Daniela Clerici[1], Acerbis Andrea[1], Raffaella Greco[1], Francesca Farina[1], Luca Vago[1, 2], Simona Piemontese[1], Sara Mastaglio[1], Fabio Giglio[1], Annalisa Ruggeri[1], Sarah Marktel[1], Consuelo Corti[1], Andrea Assanelli[1], Maurilio Ponzoni[1, 2], Fabio Ciceri[1, 2], Maria Teresa Lupo Stanghellini[1]

*[1]Ospedale San Raffaele, Milano, Italy; [2]Università Vita-Salute San Raffaele, Milano, Italy;*

### Background

Identifying the cause of hepatotoxicity following allogeneic transplantation (allo-HSCT) is essential for to ensure the most appropriate treatment is provided. The introduction of drugs with hepatotoxic potential, such as checkpoint (CPI) and tyrosine kinase inhibitors, has further complicated the diagnostic process in cases of post-transplant liver injury extremely complex. Moreover, hepatic graft-versus-host disease (hGvHD) is a common complication after allo-HSCT. Its presentation is often ambiguous, manifesting either as liver dysfunction with hyperbilirubinemia, elevated transaminases and/or progressive cholestasis. These features significantly overlap with other post-transplant hepatic conditions, including drug-induced liver injury (DILI), viral hepatitis, sinusoidal obstruction syndrome (SOS/VOD), sepsis, iron overload, metabolic conditions (MAFLD) and disease relapse, making the differential diagnosis challenging. In this context, liver biopsy plays an important role in the diagnostic work-up but, despite its diagnostic value, it may be challenging to perform due to potential clinical instability, comorbid conditions, thrombocytopenia, coagulation abnormalities and the urgency of starting treatment.

### Aims

The aim of our study is to confirm the pivotal role of early liver biopsy in patients with post allo-HSCT liver injury, allowing the initiation of targeted therapy while avoiding the potential toxicities associated with empirical treatment.

### Methods

We analyzed 52 patients who underwent allo-HSCT at our center and in whom a liver biopsy was performed to assess hepatic injury from 2011 to 2026. hGvHD was considered in the differential diagnosis in all cases. Through close collaboration with our Pathology Unit, we obtained timely histological results that, paired with clinical, laboratory and radiological findings, allowed prompt and accurate diagnosis.

### Results

Fifty-five percent of patients were male. Median age at allo-HSCT was 49 years. Sixty-seven percent of patients had a diagnosis of myeloid malignancy, while 27% of lymphoid malignancy. Donor types included matched related donors (21%), mismatched related donors (29%), unrelated donors (46%), and cord blood (4%). Median follow-up was 39 months. Only 36% of patients had bilirubin elevation, median peak ALT levels and median peak ALP levels before treatment initiation were 327 U/L and 215 U/L respectively. Thirty-eight percent of patients showed iron overload, as indicated by elevated transferrin saturation. Autoantibodies (ANA, AMA, ASMA, ANCA) were positive in 46% of cases. Twenty-three percent of patients had recent viral reactivation (11% CMV, 4% HHV6, 4% CMV+HHV6, 4% HBV), while 62% had concomitant potentially hepatotoxic drugs ongoing at time of liver damage. A diagnosis of GvHD for other systemic features was ultimately made in 83% of patients; however, in only 65% of cases hGvHD was histologically confirmed by liver biopsy. Other diagnosis included DILI (13%), CPI hepatitis (6%), SOS/VOD (4%), metabolic/iron overload (4%), viral hepatitis (2%), disease relapse (2%) or mixed etiologies (4%) (Figure 1).

**Copyright Information:** (Online) ISSN: 2572-9241
©2026 The Author(s). HemaSphere is published by John Wiley & Sons Ltd on behalf of European Hematology Association. This is an open access Abstract Book distributed under the Attribution-NonCommercial-NoDerivs (CC BY-NC-ND), which allows third parties to download the articles and share them with others as long as they credit the author and the Abstract Book, but they cannot change the content in any way or use them commercially.
**Abstract Book Citations:** Authors, Title, HemaSphere, 2026;10:(S1):pages. The abstract book DOIs can be found on the "Author Information" page.
**Disclaimer:** Articles published in the journal HemaSphere exclusively reflect the opinions of the authors. The authors are responsible for all content in their abstracts including accuracy of the facts, statements, citing resources, etc.

### Summary/Conclusion

Early liver biopsy proved to be a crucial tool in the management of liver toxicity facilitating timely and accurate diagnosis. Notably, it prevented unnecessary empirical therapy in approximately 35% of patients, thereby avoiding potential toxicities and allowing targeted treatment.

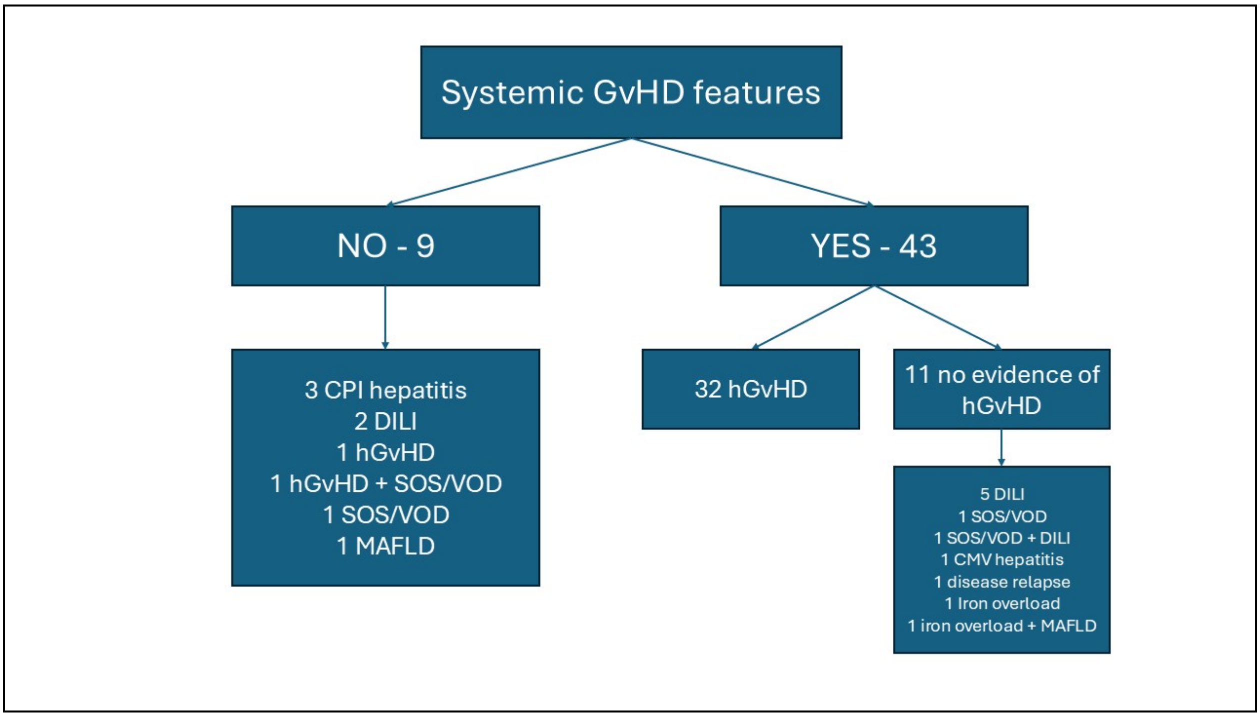

Figure 1. Histological diagnosis of patients who underwent liver biopsy.

**Copyright Information:** (Online) ISSN: 2572-9241
©2026 The Author(s). HemaSphere is published by John Wiley & Sons Ltd on behalf of European Hematology Association. This is an open access Abstract Book distributed under the Attribution-NonCommercial-NoDerivs (CC BY-NC-ND), which allows third parties to download the articles and share them with others as long as they credit the author and the Abstract Book, but they cannot change the content in any way or use them commercially.
**Abstract Book Citations:** Authors, Title, HemaSphere, 2026;10:(S1):pages. The abstract book DOIs can be found on the "Author Information" page.
**Disclaimer:** Articles published in the journal HemaSphere exclusively reflect the opinions of the authors. The authors are responsible for all content in their abstracts including accuracy of the facts, statements, citing resources, etc.

### (PB4003) THE IMPACT OF ATG/ALC RATIO ON OUTCOME IN ADULT PATIENTS UNDERWENT ALLOGENEIC STEM CELL TRANSPLANT FOR HEMATOLOGICAL MALIGNANCIES: A RETROSPECTIVE SINGLE CENTER ANALYSIS.

**Topic:** 23. Stem cell transplantation - Clinical

Giuseppe Sapienza[1], Corinne Spoto*[1], Roberto Bono[1], Cristina Rotolo[1], Luca Castagna[1]

[1]*Centro Trapianto di Midollo - A.O. Ospedali Riuniti Villa Sofia Cervello, PALERMO, Italy;*

### Background

Rabbit anti-thymocyte globulin (ATG) is widely used for graft-versus-host disease (GVHD) prophylaxis in matched related and unrelated donor allogeneic hematopoietic stem cell transplantation (allo-HSCT), with randomized trials showing reduced acute and chronic GVHD. However, in vivo exposure to ATG is variable, and optimal dosing remains difficult to define due to marked interpatient pharmacokinetic variability. Based on pediatric data linking ATG exposure to immune reconstitution and survival, pharmacokinetic models were extended to adults to enable exposure-adjusted dosing. In these models, baseline absolute lymphocyte count (ALC) is a key determinant of ATG clearance, supporting ALC-guided strategies. The concordance between weight-based dosing and model-suggested dosing in adult matched donor allo-HSCT remains largely unexplored

### Aims

We aimed to assess the concordance between administered weight-based ATG dosing and model-suggested ALC-adjusted dosing in adult matched donor allo-HSCT, and to descriptively evaluate clinical outcomes in patients classified as underdosed according to the model.

### Methods

We retrospectively analysed 120 adult patients who underwent allogeneic HSCT between 2010 and 2025 from matched unrelated (MUD) or matched related donors (MRD) receiving ATG-based GVHD prophylaxis. ATG dose was 5 mg/kg (day -2 and -1) for all patients. The median follow-up was 41 months (range, 2–189). For each patient, we calculated the model-suggested cumulative ATG dose based on baseline ALC and derived an ATG ratio defined as the administered cumulative dose divided by the theoretical ALC-based dose. Patients were classified according to ATG ratio (<1 vs ≥1).

### Results

Main patient characteristics are reported in the Table. The median ATG ratio was 0.77 (range, 0.32–1.42). Overall, 98 patients (82%) were classified as underdosed (ratio <1). The median administered cumulative dose was 398 mg compared with a model-suggested dose of 449 mg, corresponding to a median difference of −103.5 mg (range, −857 to +176).
In the overall cohort, OS was 66% at 1 year, with corresponding 100-day and 1y NRM of 17% and 23%, and relapse incidence of 6% and 21%. The CI of grade II–IV aGVHD was 15% at day 100, while 2y-cGVHD incidence was 15%. The CI of first viral infection was 10% at day 30, 20% at day 100, and 23% at day 180.
Among underdosed patients, grade II–IV aGVHD occurred in 14% at day 100, cGVHD in 11% and 14% at 1 and 2 years, respectively, and first viral infection in 11%, 21%, and 23% at day 30, day 100, and day 180.

**Copyright Information:** (Online) ISSN: 2572-9241
©2026 The Author(s). HemaSphere is published by John Wiley & Sons Ltd on behalf of European Hematology Association. This is an open access Abstract Book distributed under the Attribution-NonCommercial-NoDerivs (CC BY-NC-ND), which allows third parties to download the articles and share them with others as long as they credit the author and the Abstract Book, but they cannot change the content in any way or use them commercially.
**Abstract Book Citations:** Authors, Title, HemaSphere, 2026;10:(S1):pages. The abstract book DOIs can be found on the “Author Information” page.
**Disclaimer:** Articles published in the journal HemaSphere exclusively reflect the opinions of the authors. The authors are responsible for all content in their abstracts including accuracy of the facts, statements, citing resources, etc.

**Summary/Conclusion**

In this real-world matched donor allo-HSCT cohort, conventional weight-based ATG dosing showed a marked discrepancy compared with model-suggested ALC-adjusted dosing, with over 80% of patients classified as underdosed. Descriptive outcomes in the underdosed subgroup did not reveal substantial differences. Pharmacokinetic studies indicate that achieving optimal ATG exposure is critical for transplant outcomes and that ALC-based dosing improves target attainment. Given the immunomodulatory effect of ATG, CMV reactivation should be, partially, interpreted within the context of pre-letermovir practice. Larger multicentre prospective studies are required to validate ALC-guided dosing and better define the therapeutic window balancing GVHD, relapse, and infectious risk in matched donor allo-HSCT.

| TABLE 1 | |
|---|---|
| Patient age | 54 (15-74) |
| Diagnosis<br>AML<br>ALL<br>MDS<br>Others | <br>78 (66%)<br>20 (16)<br>20 (16)<br>2 (2%) |
| Disease status<br>CR1<br>CR2<br>CR3<br>Active | <br>73 (61)<br>24 (20)<br>1 (1)<br>22 (18) |
| Donor<br>MRD<br>MUD | <br>27 (22%)<br>93 (78%) |
| Source<br>HSC<br>BM | <br>111 (92%)<br>9 (8%) |
| Conditioning<br>MAC<br>RIC | <br>110 (92%)<br>10 (8%) |
| TCI<br>Low<br>Int<br>high | <br>11 (10%)<br>64 (53%)<br>45 (37%) |
| Median ALC at first day of ATG | 140 (0-3020) |
| Median Actual ATG dose | 398 (200-900) |
| Median Calculated ATG dose/Kg | 449 (400-1457) |

**Copyright Information:** (Online) ISSN: 2572-9241
©2026 The Author(s). HemaSphere is published by John Wiley & Sons Ltd on behalf of European Hematology Association. This is an open access Abstract Book distributed under the Attribution-NonCommercial-NoDerivs (CC BY-NC-ND), which allows third parties to download the articles and share them with others as long as they credit the author and the Abstract Book, but they cannot change the content in any way or use them commercially.
**Abstract Book Citations:** Authors, Title, HemaSphere, 2026;10:(S1):pages. The abstract book DOIs can be found on the “Author Information” page.
**Disclaimer:** Articles published in the journal HemaSphere exclusively reflect the opinions of the authors. The authors are responsible for all content in their abstracts including accuracy of the facts, statements, citing resources, etc.

### (PB4004) CMV-SEROPOSITIVE COLLATERAL DONORS SUPERIOR TO CMV-SERONEGATIVE DONORS TO IMPACT OUTCOMES OF ALLOGENEIC HEMATOPOIETIC STEM CELL TRANSPLANTATION

**Topic:** 23. Stem cell transplantation - Clinical

Jing Liu[1], Yuan-Yuan Zhang[1], Xiao-Dong Mo[1], Yuqian Sun[1], Meng Lv[1], Xiaosu Zhao[1], Yifei Cheng[1], Haixia Fu[1], Lanping Xu[1], Xiaohui Zhang[1], Xiao-Jun Huang[1], Yu Wang*[1]

[1]*Peking University People's Hospital & Peking University Institute of Hematology, Beijing, China;*

### Background

*When a serological matched donor for a CMV-seropositive recipient is unavailable, a CMV-seropositive collateral donor might be an alternative option. However, relative data from the Chinese population is currently limit.*

### Aims

How to make a donor selection between a CMV-seropositive collateral donor and a CMV-seronegative donor.

### Methods

This retrospective study collected data from patients with hematological malignancies who received allogeneic grafts from CMV-seropositive  collateral donors and CMV-seronegative donors at Peking University People's Hospital between January 2017 and March 2025 (excluding second allogeneic hematopoietic stem cell transplantation). The study compared CMV infection-related events, overall survival (OS), non-relapse mortality (NRM), and cumulative incidence of relapse (CIR) between the two groups.

**Copyright Information:** (Online) ISSN: 2572-9241
©2026 The Author(s). HemaSphere is published by John Wiley & Sons Ltd on behalf of European Hematology Association. This is an open access Abstract Book distributed under the Attribution-NonCommercial-NoDerivs (CC BY-NC-ND), which allows third parties to download the articles and share them with others as long as they credit the author and the Abstract Book, but they cannot change the content in any way or use them commercially.
**Abstract Book Citations:** Authors, Title, HemaSphere, 2026;10:(S1):pages. The abstract book DOIs can be found on the "Author Information" page.
**Disclaimer:** Articles published in the journal HemaSphere exclusively reflect the opinions of the authors. The authors are responsible for all content in their abstracts including accuracy of the facts, statements, citing resources, etc.

### Results

The median follow-up time was 885 days (Interquartile range, IQR: 350–1307 days). Without letermovir prophylaxis, the incidence of refractory CMV viremia (87.5% vs 50.0%�p = 0.007) of the CMV-seronegative donor group was significantly higher than that of the CMV-seropositive collateral donor group, and similarly the median clearance of initial CMV viremia of the CMV-seronegative donor group was longer�25 days vs 17 days�p = 0.004) compared to the CMV-seropositive collateral donor group. Letermovir prophylaxis had significantly reduced the incidence of refractory CMV viremia of the CMV-seronegative donor group (40.7% vs 87.5%�p = 0.003), but which of the CMV-seronegative donor group was still higher than that of the CMV-seropositive collateral donor group (40.7% vs 12.0%�p = 0.020). However, the 3-year OS rate, NRM rate, and CIR between the two groups were comparable. (see table 1)

CMV disease was an independent risk factor affecting OS (HR 3.0, 95%CI: 1.3–7.3, p = 0.014) and NRM (HR 3.1, 95%CI: 1.1–8.8, p = 0.035) of patients who received allogeneic hematopoietic stem cell transplantation. Logistic regression analysis identified refractory CMV viremia as an independent risk factor for CMV disease (OR 32.7, 95%CI: 3.1–342.6, p = 0.004). In addition, logistic regression analysis showed that CMV-seronegative donor without letermovir prophylaxis was an independent risk factor for refractory CMV viremia (p = 0.005).

Table 1 Transplant outcomes between CMV-seronegative donors and CMV-seropositive collateral donors in the pre-letermovir era and letermovir era

| | **the pre-letermovir era** | | | **letermovir era** | | |
|---|---|---|---|---|---|---|
| | CMV-seronegative donors,n = 16 | CMV-seropositive collateral donors, n = 58 | P value | CMV-seronegative donors,n = 27 | CMV-seropositive collateral donors,n = 25 | P value |
| Neutrophil engraftment time, median�95%CI� | +12(10–14) days | +14(13–14) days | 0.880 | +12(11–13) days | +14(13–15) days | 0.001 |
| Platelet engraftment time, median�95%CI� | +21(13–29) days | +16(13–19) days | 0.408 | +14(13–15) days | +19(17–21) days | 0.109 |
| Refractory CMV viremia, n(%) | 14(87.5%) | 29(50.0%) | 0.007 | 11(40.7%) | 3(12.0%) | 0.020 |
| CMV disease, n(%) | 4(25.0%) | 6(10.3%) | 0.129 | 2(7.4%) | 5(20.0%) | 0.184 |
| 3�OS�95%CI� | 75.0%�53.8%–96.2%� | 80.0%�68.6%–91.4%� | 0.479 | 88.3%�75.8%–100.0%� | 76.4%�58.0%–94.8%� | 0.622 |
| 3�NRM�95%CI� | 19.6%�0%–39.6%� | 9.8%�1.8%–17.8%� | 0.278 | 11.7%�0%–24.2%� | 20.1%�2.8%–37.4%� | 0.865 |
| 3�CIR�95%CI� | 6.7%�0%–19.2%� | 19.8%�8.0%–31.0%� | 0.291 | 11.8%�0%–24.3%� | 8.2%�0%–19.0%� | 0.629 |

### Summary/Conclusion

CMV-seronegative donors were associated with increased CMV-related events, and although improvements have been seen in the letermovir era, CMV-seropositive collateral donors have potential advantages in CMV infection control. However, overall survival was comparable between the two groups.

**Copyright Information:** (Online) ISSN: 2572-9241
©2026 The Author(s). HemaSphere is published by John Wiley & Sons Ltd on behalf of European Hematology Association. This is an open access Abstract Book distributed under the Attribution-NonCommercial-NoDerivs (CC BY-NC-ND), which allows third parties to download the articles and share them with others as long as they credit the author and the Abstract Book, but they cannot change the content in any way or use them commercially.
**Abstract Book Citations:** Authors, Title, HemaSphere, 2026;10:(S1):pages. The abstract book DOIs can be found on the “Author Information” page.
**Disclaimer:** Articles published in the journal HemaSphere exclusively reflect the opinions of the authors. The authors are responsible for all content in their abstracts including accuracy of the facts, statements, citing resources, etc.

### (PB4005) RISK FACTORS AND OUTCOMES OF PATIENTS WITH ACUTE MYELOID LEUKEMIA RELAPSING AFTER ALLOGENEIC HEMATOPOIETIC CELL TRANSPLANTATION. RESULTS FROM A SINGLE INSTITUTION.

**Topic:** 23. Stem cell transplantation - Clinical

Inés Zugasti*[1], Carlos Jimenez Vicente[1], Marta Moris[1], Victor Torrecillas[1], Albert Cortés[1], Sandra Castaño-Díez[1], Francesca Guijarro Tomas[1], Juan José Rodríguez-Sevilla[1], Monica Lopez-Guerra[1], Alexandra Martínez Roca[1], Beatriz Merchan[1], Adriana Cuartas[1], Marina Cornejo[1], Laia Guardia[1], Laura Rosiñol Dachs[1], Enric Carreras[1], Francesc Fernandez Aviles[1], Carmen Martínez[1], María Suárez-Lledó[1], Marina Diaz Beya[1], Montserrat Rovira Tarrats[1], Maria Queralt Salas[1], Jordi Esteve Reyner[1]

[1]*Hospital Clinic de Barcelona, Hematology, Barcelona, Spain;*

### Background

Relapse after allogeneic hematopoietic cell transplantation (alloHCT) remains the leading cause of death in AML, with reported 2-year survival below 20%. Recently, more sensitive measurable residual disease monitoring and the availability of targeted therapies have generated growing interest in the management of this setting.

### Aims

To evaluate outcomes and prognostic factors at the time of AML relapse after alloHCT with a specific focus on ELN2022 reassessment, relapse pattern, and treatment intensity

### Methods

We retrospectively analyzed adult AML patients relapsed after alloHCT between 2010-2024 at our center. ELN2022 and Refined ELN2022 risk classifications, including the Adverse-Plus category (complex karyotype, *MECOM* rearrangement, and/or *TP53* mutation or deletion; *Jiménez-Vicente et al. BCJ.2025*) were reassessed at relapse. Outcomes were measured from the time of relapse

**Copyright Information:** (Online) ISSN: 2572-9241
©2026 The Author(s). HemaSphere is published by John Wiley & Sons Ltd on behalf of European Hematology Association. This is an open access Abstract Book distributed under the Attribution-NonCommercial-NoDerivs (CC BY-NC-ND), which allows third parties to download the articles and share them with others as long as they credit the author and the Abstract Book, but they cannot change the content in any way or use them commercially.
**Abstract Book Citations:** Authors, Title, HemaSphere, 2026;10:(S1):pages. The abstract book DOIs can be found on the "Author Information" page.
**Disclaimer:** Articles published in the journal HemaSphere exclusively reflect the opinions of the authors. The authors are responsible for all content in their abstracts including accuracy of the facts, statements, citing resources, etc.

**Results**

Eighty-five patients were included (**Fig1A**). Relapse pattern was morphologic in 69 (81%), MRD-relapse in 10 (12%), and isolated extramedullary relapse in 6 (7%)
Sixty-one patients received salvage therapy. Among those with morphologic relapse (n=51), 20 received intensive chemotherapy, achieving responses (CR, Cri or MLFS) in 11 cases, whereas 31 received low-intensive approaches, resulting in disease control in only 4 cases (median OS from post-transplant relapse: 32.3 vs. 4.3m, respectively; p<0.0001). Of the 10 patients with MRD-relapse, 9 received pre-emptive treatment - HMA (2), VENAZA (4), FLT inhibitor (1), menin inhibitor (1), and exclusively DLI (1) - leading to remission in 6 patients. Overall, 20 patients underwent a second alloHCT and/or received DLI.
With a median follow-up of 17.5 months, 68 patients died. Median OS following relapse was 6.8m (95%CI, 4.1-8.7). Relapse pattern was strongly prognostic: morphologic relapse had significantly worse OS compared with those with MRD-relapse (n=10, median not reached vs. 4.6m; p<0.01) or isolated extramedullary (n=6, median not reached vs. 4.6 months; p<0.01). Early relapse (<6m post-alloHCT), ECOG >2, and age >60 years were also associated with inferior OS
ELN2022 and Refined ELN2022 classifications were reassessed at relapse. Reclassification occurred in 12 patients (14%), including 4 favorable-to-adverse, 5 intermediate-to-adverse, and 3 favorable-to-intermediate shifts. Among patients classified as ELN adverse risk, 38 of 56 (67%) were reclassified as ELNAdvPlus. According to ELN2022 risk (fav n=10, interm n=19, adv n=56), OS differed markedly between groups (p<0.0001), with median OS of 52.2m (95%CI, 46.9-NA), 8.7m (95%CI, 6.4-NA), and 4.2m (95%CI, 2.7-7.2), respectively. When assessing the Refined ELN2022 risk classification (**Fig1B**), no differences were observed in OS between Intermediate and Adverse groups (median 8.7 vs 8.1m; p=0.43), whereas AdvPlus patients had a significantly inferior OS compared with ELN Adverse patients (median 3.2 vs 8.1m; p=0.04)
In the multivariable analysis for OS, morphological relapse (HR 2.6, p=0.006), ECOG>2 (HR 2.5, p<0.01), age at relapse (>60y) (HR 1.8, p=0.043), and Refined ELN2022 criteria (HR 1.6, p=0.004) were significant predictors of inferior survival (**Fig1C**)

**Summary/Conclusion**

Post-transplant AML relapse remains associated with poor survival, particularly in patients with early or morphologic relapse and those with impaired ECOG. Refined ELN2022 reassessment at relapse identified an AdvPlus subgroup of patients with worse outcomes. Future studies are required to validate its applicability on this setting

**Copyright Information:** (Online) ISSN: 2572-9241
©2026 The Author(s). HemaSphere is published by John Wiley & Sons Ltd on behalf of European Hematology Association. This is an open access Abstract Book distributed under the Attribution-NonCommercial-NoDerivs (CC BY-NC-ND), which allows third parties to download the articles and share them with others as long as they credit the author and the Abstract Book, but they cannot change the content in any way or use them commercially.
**Abstract Book Citations:** Authors, Title, HemaSphere, 2026;10:(S1):pages. The abstract book DOIs can be found on the "Author Information" page.
**Disclaimer:** Articles published in the journal HemaSphere exclusively reflect the opinions of the authors. The authors are responsible for all content in their abstracts including accuracy of the facts, statements, citing resources, etc.

A

| ICC 2022 classification | N=85 (%) |
|---|---|
| Acute myeloid leukemia with *RUNX1::RUNX1T1* rearrangement | 1 (1.2) |
| Acute myeloid leukemia with *CBFB::MYH11* rearrangement | 6 (7.1) |
| Acute myeloid leukemia with *DEK::NUP214* rearrangement | 1 (1.2) |
| Acute myeloid leukemia with *BCR::ABL1* rearrangement | 1 (1.2) |
| Acute myeloid leukemia with *KMT2A* rearrangement | 6 (7.1) |
| Acute myeloid leukemia with *MECOM* rearrangement | 3 (3.5) |
| Acute myeloid leukemia with mutated *NPM1* | 11 (12.9) |
| Acute myeloid leukemia with *TP53* mutation | 9 (10.6) |
| Acute myeloid leukemia, myelodysplasia-related<br>*Cytogenetic changes*<br>*Myelodisplasia mutations* | 29 (34.1)<br>*18*<br>*11* |
| Acute myeloid leukemia, NOS | 14 (16.5) |
| Acute leukemia of ambiguous lineage, T/myeloid | 1 (1.2) |
| LMA/SMD with myelodysplasia-related mutations | 1 (1.2) |
| SMD/LMA con mut *TP53* | 2 (2.4) |

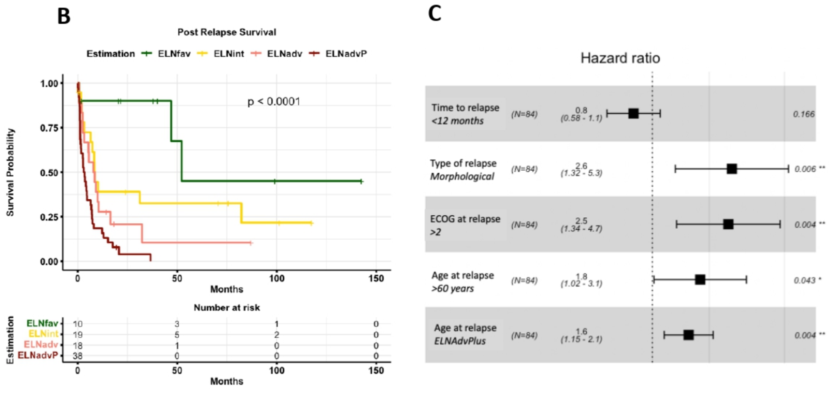

Figure 1. A. Baseline disease classification of the cohort according to the ICC 2022 criteria at diagnosis. B. Post-relapse overall survival stratified by Refined ELN 2022 risk. Median post-relapse OS was 52.2 months (95% CI, 46.9–NA) for ELN favorable, 8.7 months (95% CI, 6.4–NA) for ELN intermediate, 8.5 months (95% CI, 5.4–NA) for ELN adverse, and 3.2 months (95% CI, 1.6–6.8) for ADV+ patients (log-rank p < 0.0001). Median follow-up was 17.5 months. C. Multivariable Cox regression analysis for post-relapse overall survival (OS), including time to relapse, ECOG performance status, age at relapse, and ELN risk at diagnosis. Hazard ratios (HR) with 95% confidence intervals (CI) are shown.

**Copyright Information:** (Online) ISSN: 2572-9241
©2026 The Author(s). HemaSphere is published by John Wiley & Sons Ltd on behalf of European Hematology Association. This is an open access Abstract Book distributed under the Attribution-NonCommercial-NoDerivs (CC BY-NC-ND), which allows third parties to download the articles and share them with others as long as they credit the author and the Abstract Book, but they cannot change the content in any way or use them commercially.
**Abstract Book Citations:** Authors, Title, HemaSphere, 2026;10:(S1):pages. The abstract book DOIs can be found on the "Author Information" page.
**Disclaimer:** Articles published in the journal HemaSphere exclusively reflect the opinions of the authors. The authors are responsible for all content in their abstracts including accuracy of the facts, statements, citing resources, etc.

### (PB4006) MOBILIZATION OF PROGENITOR CELLS IN HEALTHY DONORS: IMPACT ON QUALITY OF LIFE AND ENDOTHELIAL ACTIVATION

**Topic:** 23. Stem cell transplantation - Clinical

Monica Molina-Martinez*[1], Paola Charry Espana[1], Maria Queralt Salas[2], Joan Cid[1], Elena Cahuancama[1], Teresa Jofre[1], María Suárez-Lledó[2], Francesc Fernandez Aviles[2], Carmen Martínez[2], Laura Rosiñol Dachs[2], Laia Guardia[2], Ana Belén Moreno-Castaño[3], Julia Martinez-Sanchez[3], Maribel Díaz-Ricart[3], Montserrat Rovira Tarrats[2], Miquel Lozano[1]

*[1]Hospital Clinic de Barcelona, Apheresis and Cellular Therapy Unit, Hemotherapy and Hemostasis Department (ICAMS), Barcelona, Spain; [2]Hospital Clinic de Barcelona, Hematopoietic Transplantation Unit, Hematology Department (ICAMS), Barcelona, Spain; [3]Hospital Clinic de Barcelona, Hemostasis and Erithropathology Laboratory (CDB), Barcelona, Spain;*

### Background

Allogenic hematopoietic cell transplantation is a key treatment strategy for hematological diseases. In recent years, bone marrow harvesting has been replaced by the collection of hematopoietic progenitor cells from peripheral blood after mobilization of healthy donors. In a steady-state mobilization, only cytokines, such as granulocyte-colony stimulating factor (G-CSF) are used. Most healthy donors who receive G-CSF complain about bone pain and dizziness, but the real impact of this procedure on quality of life (QoL) scores and endothelial activation markers has not been explored.

### Aims

This study evaluates the impact of the mobilization process on healthy related hematopoietic cell donors.

### Methods

We conducted a prospective observational study in all healthy donors mobilized at our institution between August/2024 and December/2025. Mobilization consisted of 5 µg/kg/12 hours of G-CSF during 4 consecutive days. On the 5th day we performed a CD34+ cell count and administered 0.24 mg/kg of plerixafor 6 hours before the apheresis when the CD34+ cell count was < 20,000 cells/mL. After providing written consent, donors completed the EQ-5D-3L questionnaire for evaluating QoL before initiating the mobilization and on day of the apheresis. Demographics were collected prospectively. Endothelial activation and stress index (EASIX) was calculated before and after G-CSF administration to indirectly evaluate endothelial activation. Paired comparisons were performed using McNemar and T-student tests.

**Copyright Information:** (Online) ISSN: 2572-9241
©2026 The Author(s). HemaSphere is published by John Wiley & Sons Ltd on behalf of European Hematology Association. This is an open access Abstract Book distributed under the Attribution-NonCommercial-NoDerivs (CC BY-NC-ND), which allows third parties to download the articles and share them with others as long as they credit the author and the Abstract Book, but they cannot change the content in any way or use them commercially.
**Abstract Book Citations:** Authors, Title, HemaSphere, 2026;10:(S1):pages. The abstract book DOIs can be found on the “Author Information” page.
**Disclaimer:** Articles published in the journal HemaSphere exclusively reflect the opinions of the authors. The authors are responsible for all content in their abstracts including accuracy of the facts, statements, citing resources, etc.

**Results**

Twenty-eight donors were included: 14 men and 14 women, with a median age of 46 years (SD +/- 19.52 years). Most donors had no comorbidities (75%), and 18% were active smokers. Fifteen (54%) donors needed an extra dose of G-CSF on the 5 th day before the apheresis and plerixafor was administered to 4 (14%) donors. Mean circulating CD34+ cell count increased from 2,161.9 ± 1,112.2 cells/mL at baseline to 112,703 ± 64,865 cells/mL after mobilization. The mean CD34+ cell collected was 10.83 ± 4.75 × $10^6$ cells/kg of recipient body weight.

As shown in Figure 1, the overall QoL score (scale 0-100%) decreased from 89.93 +/- 11.56 pre-mobilization to 85.39 +/- 12.98 post-mobilization (P= 0.081). While mobility, self-care and anxiety remained unchanged, differences were observed in daily activities and pain. Four donors (14%) reported difficulties in daily activities post-mobilization compared with 0 at baseline (p 0.125). Pain increased notably despite the anti-inflammatory medication received during the mobilization: 46% reported mild pain and 4% severe pain after mobilization versus 7% mild pain and no sever pain at baseline of (P < 0.001).

Lastly, a statistically significant increase in EASIX was observed following mobilization (0.55 +/- 0.23 vs 2.24 +/- 1.0 respectively, P < 0.001), suggesting endothelial activation.

**Summary/Conclusion**

Although peripheral blood stem cell donation is considered a secure procedure, mobilization with G-CSF had a measurable impact on donor well-being, particularly in terms of pain and daily activity limitations Furthermore, the observed increase in EASIX suggests transient endothelial activation. These findings highlight the importance of close clinical monitoring and supportive care strategies to safeguard the well-being of healthy donors.

| EQ-5D-3L | Pre-mobilization, n (%) | Post-mobilization, n (%) | *P value* |
|---|---|---|---|
| **Mobility** | | | 1 |
| No problem | 27 (96) | 27 (96) | |
| Some problems | 1 (4) | 1 (44) | |
| Cannot move | 0 | 0 | |
| **Self-care** | | | 1 |
| No problem | 28 (100) | 28 (100) | |
| Some problems | 0 | 0 | |
| Cannot do self-care | 0 | 0 | |
| **Daily activity** | | | 0.125 |
| No problem | 28 (100) | 24 (86) | |
| Some problems | 0 | 4 (14) | |
| Cannot do daily activity | 0 | 0 | |
| **Pain** | | | **<0.001** |
| No pain | 26 (93) | 14 (50) | |
| Mild | 2 (7) | 13 (46) | |
| Severe | 0 | 1 (4) | |
| **Anxiety** | | | 1 |
| No anxiety | 24 (86) | 24 (86) | |
| Mild | 4 (14) | 4 (14) | |
| Severe | 0 | 0 | |
| **Rage quality of life (0-100%)** | 89.93 +/- 11.56 | 85.39 +/- 12.98 | 0.081 |
| **EASIX** | 0.55 +/- 0.23 | 2.24 +/- 1.0 | **<0.001** |

**Copyright Information:** (Online) ISSN: 2572-9241
©2026 The Author(s). HemaSphere is published by John Wiley & Sons Ltd on behalf of European Hematology Association. This is an open access Abstract Book distributed under the Attribution-NonCommercial-NoDerivs (CC BY-NC-ND), which allows third parties to download the articles and share them with others as long as they credit the author and the Abstract Book, but they cannot change the content in any way or use them commercially.
**Abstract Book Citations:** Authors, Title, HemaSphere, 2026;10:(S1):pages. The abstract book DOIs can be found on the "Author Information" page.
**Disclaimer:** Articles published in the journal HemaSphere exclusively reflect the opinions of the authors. The authors are responsible for all content in their abstracts including accuracy of the facts, statements, citing resources, etc.

### (PB4007) IMPACT OF PULMONARY FUNCTION TESTING ON BRONCHIOLITIS OBLITERANS SYNDROME AND OUTCOME AFTER HEMATOPOIETIC STEM CELL TRANSPLANT

**Topic:** 23. Stem cell transplantation - Clinical

Antonia Ricchiuto[1], Eleonora Prete[1], Francesco Aji[1], Rosa De Francesco[1], Loredana Panico[1], Giuseppina Greco[1], Silvia Sibilla[1], Francesca Donatelli[1], Nicoletta Pizzileo[1], Domenico D'agostino[1], Guido Volpe[1], Vincenzo Pavone[1], Pasquale Barone[1], Anna Mele*[1]

[1]*Cardinale G.Panico Hospital, Hematology and Bone Marrow Transplant, TRICASE, Italy;*

### Background

Bronchiolitis obliterans syndrome (BOS) is a progressive small airway disease that can develop after hematopoietic stem cell transplantation (HSCT), often with poor therapeutic response and progression to respiratory failure. Diagnosis relies on pulmonary function testing (PFT).

### Aims

We investigated the role of PFT as a predictive marker in BOS and graft-versus-host disease (GvHD) after HSCT.

### Methods

We retrospectively analyzed allogeneic HSCT recipients from January 2018 to May 2025 at Cardinale G. Panico Hospital (Tricase, Italy) with available baseline PFT. BOS was defined using modified 2005 NIH criteria. Mid-expiratory flow at 75% of forced vital capacity (MEF75), 50% (MEF50), 25% (MEF25), and the mid-expiratory flow range between 75% and 25% (MEF75–25) ≤60% predicted were evaluated as additional markers of small airway obstruction and as potential predictors of BOS and GvHD. Univariate and multivariate logistic regression were performed using SPSS 20.0. Kaplan–Meier estimates evaluated time-to-event outcomes.

### Results

Among 105 included patients (median age 56 years), 60% had a past or current smoking history, and 6% had pre-transplant chronic obstructive pulmonary disease (COPD). Acute leukemia was the most common indication for HSCT. Overall, 69% of patients received a matched unrelated donor transplant (table 1). BOS was diagnosed in 7 (7%) patients, at a median of 297 days (243-344) after HSCT; all had evidence of chronic GVHD at another site. Patients' clinical features, hematologic disease, transplant-related variables, smoking history and pre-transplant COPD diagnosis were not significantly associated with BOS (table 1). We analyzed the role of pre-transplant PFTs in the development of BOS, acute and chronic GvHD. Pre-transplant MEF25 ≤60% was significantly more frequent in BOS patients (86% vs 47%, p=0.05, table 1). MEF75 ≤60% was associated with acute GvHD (19% vs 3%, p=0.004), while no PFT parameter correlated with chronic GvHD.

**Copyright Information:** (Online) ISSN: 2572-9241
©2026 The Author(s). HemaSphere is published by John Wiley & Sons Ltd on behalf of European Hematology Association. This is an open access Abstract Book distributed under the Attribution-NonCommercial-NoDerivs (CC BY-NC-ND), which allows third parties to download the articles and share them with others as long as they credit the author and the Abstract Book, but they cannot change the content in any way or use them commercially.
**Abstract Book Citations:** Authors, Title, HemaSphere, 2026;10:(S1):pages. The abstract book DOIs can be found on the "Author Information" page.
**Disclaimer:** Articles published in the journal HemaSphere exclusively reflect the opinions of the authors. The authors are responsible for all content in their abstracts including accuracy of the facts, statements, citing resources, etc.

| | Total (n = 105) | With BOS (n = 7, 7%) | Without BOS (n = 98, 93%) | P value |
|---|---|---|---|---|
| **Male patients, n (%)** | 64 (61) | 2 (29) | 62 (63) | 0,75 |
| **Age (yr), median (range)**<br>o > 65 yr, n (%) | 56 (17-71)<br>17 (16) | 55 (20-61)<br>0 | 56 (17-71)<br>17 (17) | 0,67 |
| **Smoking, n (%)**<br>o Current, n (%) | 56 (60)<br>14 (15) | 4 (57)<br>0 | 52 (60)<br>14 (16) | 0,89 |
| **Pre-HSCT COPD, n (%)** | 6 (6) | 0 | 6 (6) | 0,493 |
| **Acute leukemia, n (%)** | 61 (58) | 5 (71) | 56 (57) | |
| **Lymphoma, n (%)** | 22 (21) | 0 | 22 (22) | 0,366 |
| **Others, n (%)** | 22 (21) | 2 (29) | 20 (20) | |
| **Complete remission at HSCT, n (%)** | 65 (62) | 6 (86) | 59 (60) | 0,14 |
| **Matched-unrelated donor, n (%)** | 72 (69) | 2 (29) | 70 (71) | 0,45 |
| **Myeloablative Conditioning regimen, n (%)** | 63 (60) | 4 (57) | 59 (60) | 0,176 |
| **Antithymocyte globulin prophylaxis, n (%)** | 64 (61) | 5 (71) | 59 (60) | 0,56 |
| **FEV1 (%), median (range)**<br>**≤ 80 %, n (%)** | 103 (49-153)<br>9 (9) | 98 (75-112)<br>1 (14) | 103 (49-153)<br>8 (8) | 0,59 |
| **FEV1/FVC, median (range)**<br>**≤ 70 %, n (%)** | 80 (58-96)<br>5 (5) | 82 (75-89)<br>0 | 80 (58-95)<br>5 (5) | 0,53 |
| **MEF75 (%), median (range)**<br>**≤ 60 %, n (%)** | 100 (37-149)<br>6 (6) | 108 (91-125)<br>0 | 99 (37-145)<br>6 (6) | 0,43 |
| **MEF50 (%), median (range)**<br>**≤ 60 %, n (%)** | 85 (28-174)<br>14 (14) | 89 (64-98)<br>0 | 85 (28-174)<br>14 (15) | 0,27 |
| **MEF25 (%), median (range)**<br>**≤ 60 %, n (%)** | 61 (12-203)<br>51 (50) | 52 (48-103)<br>6 (86) | 63 (12-203)<br>45 (47) | 0,05 |
| **MEF75-25 (%), median (range)**<br>**≤ 60 %, n (%)** | 78 (21-168)<br>20 (20) | 74 (60-95)<br>1 (14) | 81 (21-168)<br>19 (20) | 0,71 |

### Summary/Conclusion

We identified novel pre-transplant PFT abnormalities associated with the development of BOS and acute GvHD. These findings may help identify a high-risk population and guide preventive or early therapeutic interventions.

**Copyright Information:** (Online) ISSN: 2572-9241
©2026 The Author(s). HemaSphere is published by John Wiley & Sons Ltd on behalf of European Hematology Association. This is an open access Abstract Book distributed under the Attribution-NonCommercial-NoDerivs (CC BY-NC-ND), which allows third parties to download the articles and share them with others as long as they credit the author and the Abstract Book, but they cannot change the content in any way or use them commercially.
**Abstract Book Citations:** Authors, Title, HemaSphere, 2026;10:(S1):pages. The abstract book DOIs can be found on the "Author Information" page.
**Disclaimer:** Articles published in the journal HemaSphere exclusively reflect the opinions of the authors. The authors are responsible for all content in their abstracts including accuracy of the facts, statements, citing resources, etc.

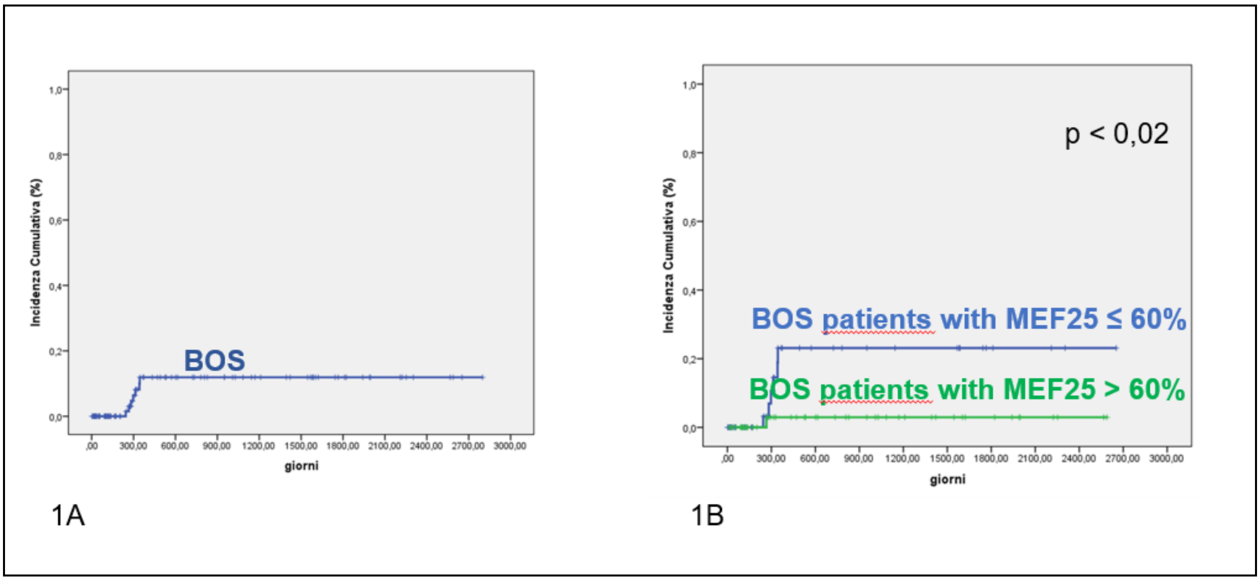

**Copyright Information:** (Online) ISSN: 2572-9241
©2026 The Author(s). HemaSphere is published by John Wiley & Sons Ltd on behalf of European Hematology Association. This is an open access Abstract Book distributed under the Attribution-NonCommercial-NoDerivs (CC BY-NC-ND), which allows third parties to download the articles and share them with others as long as they credit the author and the Abstract Book, but they cannot change the content in any way or use them commercially.
**Abstract Book Citations:** Authors, Title, HemaSphere, 2026;10:(S1):pages. The abstract book DOIs can be found on the “Author Information” page.
**Disclaimer:** Articles published in the journal HemaSphere exclusively reflect the opinions of the authors. The authors are responsible for all content in their abstracts including accuracy of the facts, statements, citing resources, etc.

### (PB4008) STUDY ON THE CORRELATION BETWEEN CCR5 EXPRESSION IN IMMUNE CELLS AND ACUTE GRAFT-VERSUS-HOST DISEASE AFTER ALLOGENEIC HEMATOPOIETIC STEM CELL TRANSPLANTATION

**Topic:** 23. Stem cell transplantation - Clinical

Heng Liu*[1], Tao Wu[1]

[1] *The 940th Hospital of Joint Logistics Support force of Chinese People's Liberation Army, hematology, Gansu, China;*

### Background

Allogeneic hematopoietic stem cell transplantation (allo-HSCT) serves as an effective therapeutic approach for curing various benign and malignant hematological disorders. However, post-transplant complications significantly impact treatment outcomes. Acute graft-versus-host disease (aGVHD), characterized by its high incidence and mortality rates, remains a major obstacle to the success of allo-HSCT. Helper T cell type 1 (Th1 cells) play a central role in promoting inflammatory responses during aGVHD, while CD8+ T cells, as the primary effector cells, cause damage to target organs by directly killing host cells and tissues. CCR5 and its ligands play an important role in the initiation and progression of aGVHD, particularly in the inflammatory responses of target organs such as the intestine, liver, and skin. The differential expression levels of CCR5 in various immune cells may influence the progression of aGVHD.

### Aims

To investigate the relationship between C-C chemokine receptor type 5 (CCR5) expression rates in immune cells and aGVHD in patients after allo-HSCT.

### Methods

Flow cytometry was used to detect CCR5 expression levels on peripheral blood Th1 cells, $CD8^{+}$T cells, Treg cells, and NK cells in 24 allo-HSCT patients, followed by statistical analysis.

### Results

Among the patients, 11 developed aGVHD (aGVHD group) and 13 did not (control group). Compared with the control group, the aGVHD group exhibited significantly higher CCR5 expression in Th1 cells (90.2% *vs* 57.9%, $P<0.01$). The CCR5 expression rate in $CD8^{+}$T cells was 85.9% ± 9.8% of the aGVHD group versus 62.5% ± 28.0% of the control group, showing a statistically significant difference ($P=0.013$). No significant differences were observed in CCR5 expression on Treg or NK cells between the two groups. After treatment, aGVHD patients showed significantly reduced CCR5 expression on Th1 cells compared to pre-treatment levels (46.8% ± 23.8% *vs* 82.1% ± 17.6%, $P<0.01$). Similarly, CCR5 expression in $CD8^{+}$T cells decreased significantly post-treatment (64.9% ± 25.1% *vs* 85.9% ± 11.4%, $P=0.035$). No significant changes occurred in CCR5 expression in Treg or NK cells.

### Summary/Conclusion

Upregulated CCR5 expression on Th1 and $CD8^{+}$T cells may promote the occurrence and progression of aGVHD after allo-HSCT.

**Copyright Information:** (Online) ISSN: 2572-9241
©2026 The Author(s). HemaSphere is published by John Wiley & Sons Ltd on behalf of European Hematology Association. This is an open access Abstract Book distributed under the Attribution-NonCommercial-NoDerivs (CC BY-NC-ND), which allows third parties to download the articles and share them with others as long as they credit the author and the Abstract Book, but they cannot change the content in any way or use them commercially.
**Abstract Book Citations:** Authors, Title, HemaSphere, 2026;10:(S1):pages. The abstract book DOIs can be found on the "Author Information" page.
**Disclaimer:** Articles published in the journal HemaSphere exclusively reflect the opinions of the authors. The authors are responsible for all content in their abstracts including accuracy of the facts, statements, citing resources, etc.

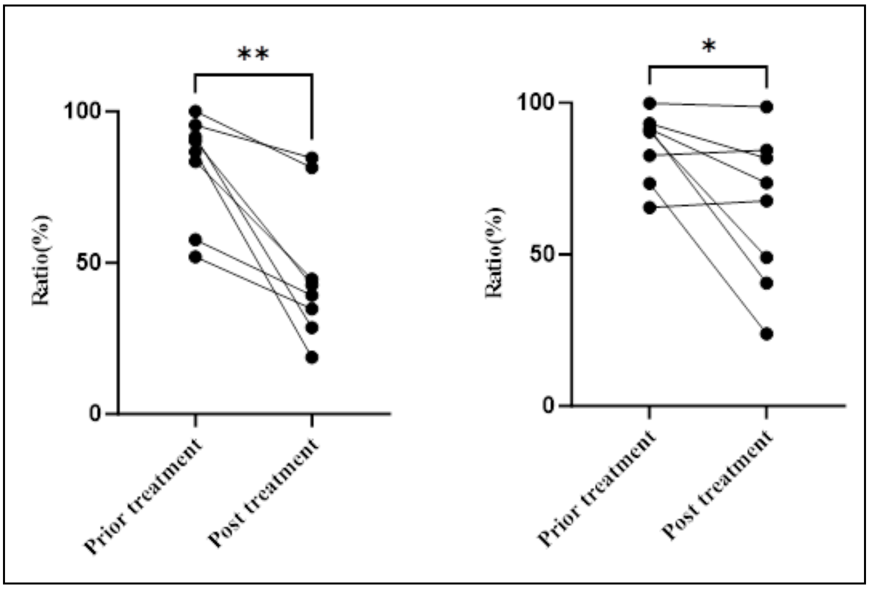

**Copyright Information:** (Online) ISSN: 2572-9241
©2026 The Author(s). HemaSphere is published by John Wiley & Sons Ltd on behalf of European Hematology Association. This is an open access Abstract Book distributed under the Attribution-NonCommercial-NoDerivs (CC BY-NC-ND), which allows third parties to download the articles and share them with others as long as they credit the author and the Abstract Book, but they cannot change the content in any way or use them commercially.
**Abstract Book Citations:** Authors, Title, HemaSphere, 2026;10:(S1):pages. The abstract book DOIs can be found on the "Author Information" page.
**Disclaimer:** Articles published in the journal HemaSphere exclusively reflect the opinions of the authors. The authors are responsible for all content in their abstracts including accuracy of the facts, statements, citing resources, etc.

### (PB4009) HIGH-DOSE MELPHALAN FOLLOWED BY DONOR STEM CELL INFUSION AS SALVAGE THERAPY FOR RELAPSED/REFRACTORY ACUTE LEUKEMIA AFTER ALLO-HSCT: A MULTICENTER, SINGLE-ARM, RETROSPECTIVE STUDY

**Topic:** 23. Stem cell transplantation - Clinical

Minglu Li*[1], Jingbo Wang[1], Rongmu Luo[1]

[1]*Aerospace Center Hospital, Hematology, Beijing, China;*

### Background

Relapsed/refractory acute leukemia (AL) after allogeneic hematopoietic stem cell transplantation (allo-HSCT) confers an extremely dismal prognosis, with no established standardized salvage regimen for this high-risk population. Immunosuppressant tapering and conventional chemotherapy provide only limited clinical efficacy for this patient group.

### Aims

This study aimed to comprehensively evaluate the efficacy and safety of high-dose melphalan followed by sequential donor stem cell infusion in patients with post-allo-HSCT relapsed/refractory AL.

### Methods

This was a multicenter, single-arm, retrospective study. A total of 37 consecutive patients with post-allo-HSCT relapsed/refractory AL, who had inadequate response to immunosuppressant tapering and/or prior chemotherapy, were enrolled. All patients received single-administration high-dose melphalan (140-200 mg/m², adjusted for organ function), followed by sequential donor peripheral blood stem cell infusion. The primary endpoint was overall response rate (ORR). Secondary endpoints included overall survival (OS), leukemia-free survival (LFS), 100-day treatment-related mortality (TRM), and adverse event profile. Survival outcomes were estimated via the Kaplan-Meier method, between-group differences were compared using the log-rank test, and prognostic factors were assessed using univariate and multivariate Cox proportional hazards regression models. A significance threshold of $P < 0.05$ was used for all statistical analyses.

### Results

At a median follow-up of 5.1 months , the ORR of the full cohort was 78.4% . The complete remission (CR) rate was 48.6% . All patients who achieved CR obtained 100% minimal residual disease negativity. The 100-day TRM was 2.7% . The median OS of the full cohort was 176 days, with a 6-month OS rate of 46.7% , 1-year OS rate of 22.0% , and 2-year OS rate of 17.6% . Patients who achieved remission had a significantly prolonged median OS of 196 days, compared with 90 days in non-remission patients (P=0.0017). The median LFS was 92 days, with a 6-month LFS rate of 25.1% , 1-year LFS rate of 16.7%, and 2-year LFS rate of 16.7%. The engraftment rates of white blood cells and platelets were 94.6% and 91.9%, with median engraftment times of 17.5 days and 17 days, respectively. The most common non-hematologic adverse events included infection (35.1%), thrombotic microangiopathy (21.6%), and acute graft-versus-host disease (18.9%), with grade II-IV acute graft-versus-host disease occurring in 2.7% of patients. The overall relapse rate was 59.5% , with a 1-year cumulative incidence of relapse of 56.8% , and disease relapse was the leading cause of death (58.3%). Univariate Cox regression analysis identified that higher CD34+ cell dose (P=0.005) and response achievement (P=0.004) were significant favorable factors for OS; higher CD34+ cell dose (P=0.027) was significantly associated with improved LFS. No prognostic risk factors for survival outcomes were identified in multivariate Cox regression analysis, which was likely attributed to the limited sample size of the cohort.

**Copyright Information:** (Online) ISSN: 2572-9241
©2026 The Author(s). HemaSphere is published by John Wiley & Sons Ltd on behalf of European Hematology Association. This is an open access Abstract Book distributed under the Attribution-NonCommercial-NoDerivs (CC BY-NC-ND), which allows third parties to download the articles and share them with others as long as they credit the author and the Abstract Book, but they cannot change the content in any way or use them commercially.
**Abstract Book Citations:** Authors, Title, HemaSphere, 2026;10:(S1):pages. The abstract book DOIs can be found on the "Author Information" page.
**Disclaimer:** Articles published in the journal HemaSphere exclusively reflect the opinions of the authors. The authors are responsible for all content in their abstracts including accuracy of the facts, statements, citing resources, etc.

**Summary/Conclusion**

For patients with post-allo-HSCT relapsed/refractory acute leukemia who failed conventional treatment, high-dose melphalan followed by sequential donor stem cell infusion is a highly effective and well-tolerated salvage regimen. This strategy can induce a high rate of secondary remission, create a critical therapeutic window for patients to receive second allo-HSCT or other consolidation therapies.

**Copyright Information:** (Online) ISSN: 2572-9241
©2026 The Author(s). HemaSphere is published by John Wiley & Sons Ltd on behalf of European Hematology Association. This is an open access Abstract Book distributed under the Attribution-NonCommercial-NoDerivs (CC BY-NC-ND), which allows third parties to download the articles and share them with others as long as they credit the author and the Abstract Book, but they cannot change the content in any way or use them commercially.
**Abstract Book Citations:** Authors, Title, HemaSphere, 2026;10:(S1):pages. The abstract book DOIs can be found on the "Author Information" page.
**Disclaimer:** Articles published in the journal HemaSphere exclusively reflect the opinions of the authors. The authors are responsible for all content in their abstracts including accuracy of the facts, statements, citing resources, etc.

### (PB4011) IMPACT OF PRE-TRANSPLANT HEMATOLOGIC RECOVERY STATUS ON THE PROGNOSIS OF ALLOGENEIC HEMATOPOIETIC STEM CELL TRANSPLANTATION IN PATIENTS WITH ACUTE MYELOID LEUKEMIA

**Topic:** 23. Stem cell transplantation - Clinical

Hailing Wang*[1], Yu Wang[1]

*[1]Peking University People's Hospital, Beijing, China;*

### Background

Allo-HSCT is a curative treatment for AML. While complete remission (CR) is the standard goal, many patients achieve morphologic remission without full hematologic recovery (CRi/CRh/MLFS) prior to transplant. Whether these states adversely affect prognosis by increasing relapse or transplant-related mortality remains controversial. In the era of measurable residual disease (MRD) monitoring, identifying the independent impact of hematologic recovery status is crucial for optimizing transplant timing and improving patient outcomes.

### Aims

To investigate the impact of different pre-transplant hematologic recovery statuses on the prognosis of patients with acute myeloid leukemia (AML) undergoing allogeneic hematopoietic stem cell transplantation (allo-HSCT).

### Methods

A retrospective analysis was conducted on 340 patients with AML (excluding acute promyelocytic leukemia) who received allo-HSCT between 2020 and 2021. Based on their pre-transplant status, patients were divided into a complete remission (CR) group (n=297) and an incomplete hematologic recovery group (n=43), which included CR with partial hematologic recovery (CRh), CR with incomplete hematologic recovery (CRi), and morphologic leukemia-free state (MLFS). Post-transplant overall survival (OS), disease-free survival (DFS), cumulative incidence of relapse (CIR), and non-relapse mortality (NRM) were compared between the two cohorts.

### Results

The median follow-up was 1559 days. There were no statistically significant differences between the CR group and the incomplete recovery group regarding the 3-year OS (85.7% vs. 85.8%, P=0.964), 3-year DFS (85.0% vs. 85.8%, P=0.952), 3-year CIR (7.35% vs. 4.76%, P=0.472), and NRM (6.96% vs. 9.47%, P=0.569). Multivariate Cox regression analysis demonstrated that after adjusting for confounding factors including age, disease risk stratification, and measurable residual disease (MRD) status, incomplete pre-transplant hematologic recovery was not an independent risk factor for OS (HR=1.021, 95% CI: 0.429-2.429, P=0.962).

### Summary/Conclusion

A pre-transplant status of CRh, CRi, or MLFS does not adversely affect the long-term survival of AML patients following allo-HSCT. For patients who are MRD-negative but experience delayed hematopoietic recovery after chemotherapy, early bridging to transplantation is feasible without unnecessarily waiting for complete hematologic recovery.

**Copyright Information:** (Online) ISSN: 2572-9241
©2026 The Author(s). HemaSphere is published by John Wiley & Sons Ltd on behalf of European Hematology Association. This is an open access Abstract Book distributed under the Attribution-NonCommercial-NoDerivs (CC BY-NC-ND), which allows third parties to download the articles and share them with others as long as they credit the author and the Abstract Book, but they cannot change the content in any way or use them commercially.
**Abstract Book Citations:** Authors, Title, HemaSphere, 2026;10:(S1):pages. The abstract book DOIs can be found on the "Author Information" page.
**Disclaimer:** Articles published in the journal HemaSphere exclusively reflect the opinions of the authors. The authors are responsible for all content in their abstracts including accuracy of the facts, statements, citing resources, etc.

### (PB4012) PROGNOSTIC RELEVANCE OF SPLEEN VOLUME AT ALLOGENEIC HEMATOPOIETIC STEM CELL TRANSPLANTATION IN PATIENTS WITH ACUTE MYELOID LEUKEMIA (AML)

**Topic:** 23. Stem cell transplantation - Clinical

Johanna Broermann*[1, 2], Clara Wall[1, 2], Jule Ussmann[1, 2], Jacob Jendro[1, 2], Lara Bischof[1, 2], Donata Backhaus[1, 2], Dominic Brauer[1, 2], Georg-Nikolaus Franke[1, 2], Vladan Vucinic[1, 2], Carmen Diana Herling[1, 2], Marco Herling[1, 2], Timm Denecke[3], Klaus Metzeler[1, 2], Hans-Jonas Meyer[3], Sebastian Schwind[1, 2], Madlen Jentzsch[1, 2]

*[1]University of Leipzig Medical Center, Department for Hematology, Cell Therapy, Hemostaseology and Infectious Diseases, Leipzig, Germany; [2]Cancer Center Central Germany, Leipzig, Germany; [3]University of Leipzig Medical Center, Department for Diagnostic and Interventional Radiology, Leipzig, Germany;*

### Background

An allogeneic hematopoietic stem cell transplantation (HSCT) is a potentially curative treatment for patients (pts) with AML. In myeloproliferative neoplasms (MPN), splenomegaly associates with graft rejection, delayed engraftment, higher cumulative incidence of relapse (CIR), and shorter overall survival (OS). In AML, the impact of spleen volume (SV) at HSCT is less clear, especially regarding modern risk factors as measurable residual disease (MRD) status and European LeukemiaNet (ELN) 2022 genetic risk.

### Aims

To evaluate the prognostic impact of SV on engraftment kinetics and outcome in patients with AML undergoing HSCT.

### Methods

We retrospectively analyzed 316 AML pts (median age 63, range 21-76 years) who underwent HSCT in first or second complete remission with or without blood count recovery (CRc, 78%) or without CRc (22%). Conditioning regimens were non-myeloablative (79%), or of reduced intensity (21%). Donors were matched sibling (11%), matched (66%) or mismatched unrelated (22%), or haploidentical (1%). ELN2022 risk at diagnosis was 19% favorable, 24% intermediate, and 57% adverse. In computed tomography scans performed up to 7 weeks before HSCT, SV was measured three-dimensionally. Pts-individual normal SV (NSV) was defined according to sex and height as published by Chow *et al.* (RSNA, 2016). In pts with spleen enlargement, the degree of elevated volume was calculated as percentage increase relative to the individual upper limit of normal (ULN). Using the median increase (43% of SV) as cutoff, pts with a SV above individual ULN were further categorized into a high (HSV), and very high (VHSV) group.

**Copyright Information:** (Online) ISSN: 2572-9241
©2026 The Author(s). HemaSphere is published by John Wiley & Sons Ltd on behalf of European Hematology Association. This is an open access Abstract Book distributed under the Attribution-NonCommercial-NoDerivs (CC BY-NC-ND), which allows third parties to download the articles and share them with others as long as they credit the author and the Abstract Book, but they cannot change the content in any way or use them commercially.
**Abstract Book Citations:** Authors, Title, HemaSphere, 2026;10:(S1):pages. The abstract book DOIs can be found on the "Author Information" page.
**Disclaimer:** Articles published in the journal HemaSphere exclusively reflect the opinions of the authors. The authors are responsible for all content in their abstracts including accuracy of the facts, statements, citing resources, etc.

**Results**

Men had larger absolute SV than women (median 309 ml *vs* 191 ml, *P*<.001), but when applying individual sex- and height-adapted values, there was no difference in the proportion of SV above ULN according to sex (*P*=.12). Pts with a SV above ULN were younger (*P*=.03), had fewer *de novo* AML (*P*=.02), more often *JAK2* mutations (*P*=.004), but no distinct ELN2022 risk (*P*=.21). They also less often had CRc at HSCT (*P*=.003) and - in pts in CRc – no different molecular MRD status prior to HSCT (*P*=.17).
While graft rejection was not more frequent in pts with a SV above ULN (*P*=.71), time to engraftment was significantly longer (platelets and leukocytes, both *P*<.001), with the longest delay observed in VHSV pts (Fig. 1A, B). In the entire cohort, a SV above ULN was associated with higher CIR (*P*=.02 Fig. 1C) and shorter OS (*P*=.02), but non-relapse mortality (NRM, *P*=.81) was similar across SV groups. Stratification into HSV and VHSV did not provide additional prognostic value.
In multivariate analyses, pts sex, ELN2022 risk, conditioning intensity, and remission status at HSCT associated with CIR, and remission status at HSCT and HCT-CI risk associated with OS, while SV did
not remain an independent prognostic factor. In subgroup analyses according to remission at HSCT, a SV above ULN associated with shorter OS in MRD-negative pts (*P*=.01), but not in MRD-positive pts (*P*=.70) or pts without CRc (*P*=.70). Similarly, no difference in OS according to SV was seen in ELN2022 favorable (*P*=.30), intermediate (*P*=.30), or adverse risk pts (*P*=.50).

**Summary/Conclusion**

In AML pts, SV above ULN was associated with delayed engraftment, higher CIR, and shorter OS. However, unlike in MPN, SV was not an independent risk factor for CIR and OS in multivariate analyses and subgroup analyses considering ELN2022 risk and molecular MRD at HSCT.

**Copyright Information:** (Online) ISSN: 2572-9241
©2026 The Author(s). HemaSphere is published by John Wiley & Sons Ltd on behalf of European Hematology Association. This is an open access Abstract Book distributed under the Attribution-NonCommercial-NoDerivs (CC BY-NC-ND), which allows third parties to download the articles and share them with others as long as they credit the author and the Abstract Book, but they cannot change the content in any way or use them commercially.
**Abstract Book Citations:** Authors, Title, HemaSphere, 2026;10:(S1):pages. The abstract book DOIs can be found on the "Author Information" page.
**Disclaimer:** Articles published in the journal HemaSphere exclusively reflect the opinions of the authors. The authors are responsible for all content in their abstracts including accuracy of the facts, statements, citing resources, etc.

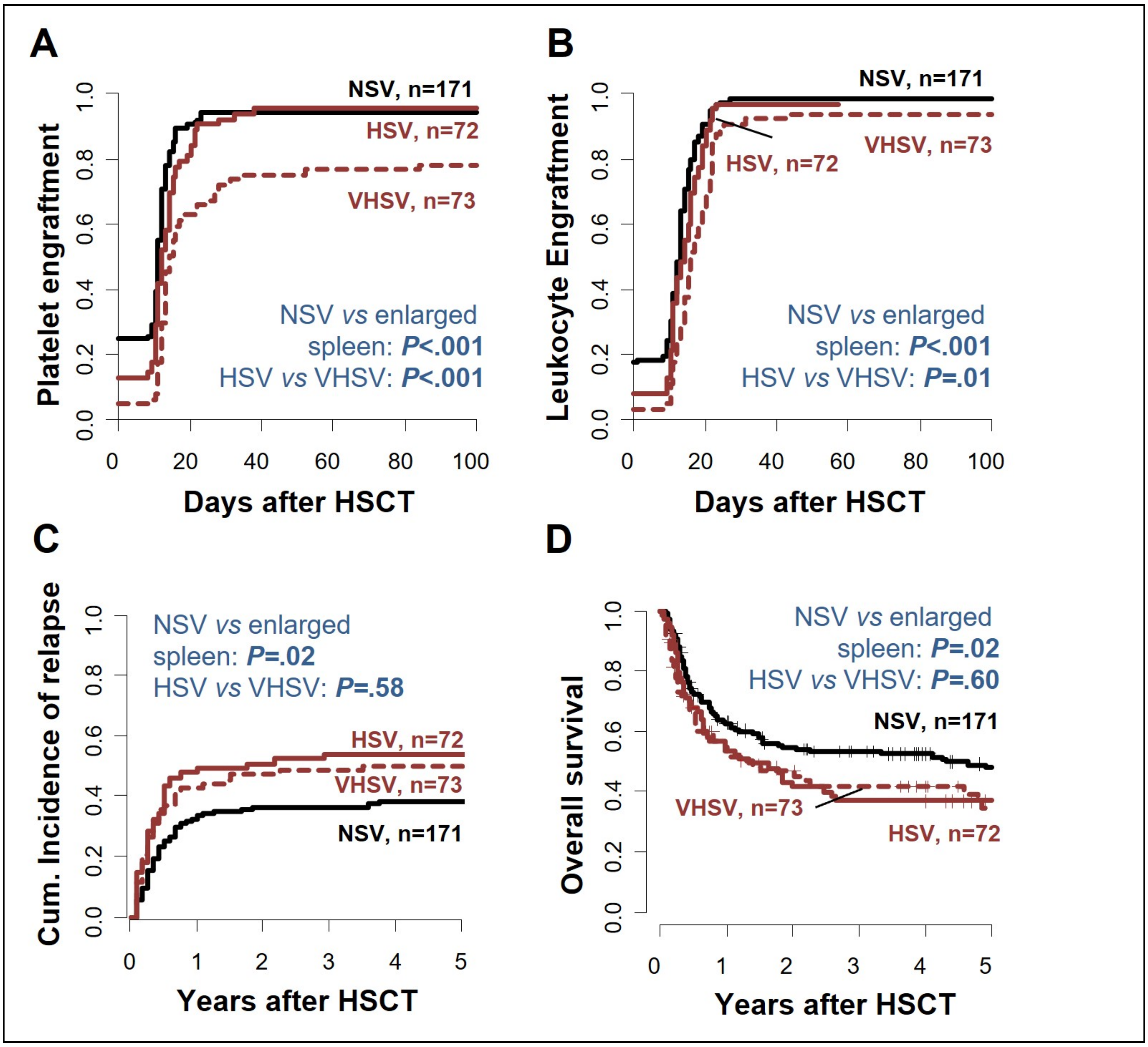

**Copyright Information:** (Online) ISSN: 2572-9241
©2026 The Author(s). HemaSphere is published by John Wiley & Sons Ltd on behalf of European Hematology Association. This is an open access Abstract Book distributed under the Attribution-NonCommercial-NoDerivs (CC BY-NC-ND), which allows third parties to download the articles and share them with others as long as they credit the author and the Abstract Book, but they cannot change the content in any way or use them commercially.
**Abstract Book Citations:** Authors, Title, HemaSphere, 2026;10:(S1):pages. The abstract book DOIs can be found on the "Author Information" page.
**Disclaimer:** Articles published in the journal HemaSphere exclusively reflect the opinions of the authors. The authors are responsible for all content in their abstracts including accuracy of the facts, statements, citing resources, etc.

### (PB4013) IMPACT OF DYSLIPIDEMIA ON ALLOGENEIC TRANSPLANTATION OUTCOMES AND CARDIOVASCULAR MORTALITY IN PATIENTS WITH ACUTE LEUKEMIAS IN THE POST-TRANSPLANT CYCLOPHOSPHAMIDE ERA WITH UP TO 9 YEARS FOLLOW-UP

**Topic:** 23. Stem cell transplantation - Clinical

Sema Secilmis[1], Burcu Aslan Candir[2], Uğur Hatipoğlu[2], Mert Seyhan[2], Bahar Uncu Ulu[2], Tugce Yiğenoglu[2], Dicle Iskender[2], Merih Kizil Cakar[1], Turgay Ulas*[2], Mehmet Sinan Dal[2], Fevzi Altuntas[2]

*[1]Etlik City Hospital, Department of Hematology and Bone Marrow Transplantation Center,, Ankara,, Türkiye;[2]University of Health Sciences, Ankara Oncology Training and Research Hospital, Ankara, Türkiye;*

### Background

Dyslipidemia contributes to endothelial dysfunction and cardiovascular morbidity. However, its impact on outcomes after allogeneic hematopoietic stem cell transplantation (allo-HSCT), particularly in the post-transplant cyclophosphamide (PTCy) era, remains unclear.

### Aims

To evaluate the association between pre-transplant non–high-density lipoprotein cholesterol (non–HDL-C) levels and transplant outcomes, including GVHD, survival, relapse, and cardiovascular mortality with up to 9 years, in acute leukemia patients receiving PTCy-based allo-HSCT.

### Methods

We retrospectively analyzed patients with acute leukemias undergoing matched sibling donor allo-HSCT with myeloablative or reduced-intensity conditioning and uniform GVHD prophylaxis consisting of PTCy (50 mg/kg ×2), cyclosporine, and anti-thymocyte lymphoglobulin. Patients were stratified according to non–HDL-C levels (<160 mg/dL vs ≥160 mg/dL).Risk stratification for non–HDL cholesterol is derived from the NCEP ATP III classification and the ESC/EAS guideline approach defining non–HDL-C targets as 30 mg/dL above LDL-C goals. Engraftment kinetics, veno-occlusive disease (VOD), cumulative incidence of acute and chronic GVHD, relapse, non-relapse mortality (NRM), GVHD-free/relapse-free survival (GRFS), overall survival (OS), CMV reactivation, and cardiovascular mortality were analyzed.

### Results

Neutrophil (median 14 vs 15 days) and platelet engraftment (median 13 vs 14 days) were comparable between groups (all p>0.05). The cumulative incidences of grade II–IV and III–IV acute GVHD at day +100 and moderate–severe chronic GVHD at 1 year did not differ significantly. One-year NRM, relapse-free survival, and CMV reactivation rates were similar across cohorts.
One-year GRFS was 66.6% in the non–HDL-C <160 mg/dL group and 52.0% in the ≥160 mg/dL group (HR 0.68, 95% CI 0.40–1.15; p=0.15). Median GRFS was 150 days (95% CI 120–330) vs 270 days (95% CI 154–not reached), respectively. With a median follow-up of 60 months (range 0.5–108), median OS was not reached in the <160 mg/dL group and was 67 months (95% CI 13–not reached) in the ≥160 mg/dL group (log-rank p=0.21). No cardiovascular death events occurred during follow-up (Figure).

**Copyright Information:** (Online) ISSN: 2572-9241
©2026 The Author(s). HemaSphere is published by John Wiley & Sons Ltd on behalf of European Hematology Association. This is an open access Abstract Book distributed under the Attribution-NonCommercial-NoDerivs (CC BY-NC-ND), which allows third parties to download the articles and share them with others as long as they credit the author and the Abstract Book, but they cannot change the content in any way or use them commercially.
**Abstract Book Citations:** Authors, Title, HemaSphere, 2026;10:(S1):pages. The abstract book DOIs can be found on the "Author Information" page.
**Disclaimer:** Articles published in the journal HemaSphere exclusively reflect the opinions of the authors. The authors are responsible for all content in their abstracts including accuracy of the facts, statements, citing resources, etc.

**Summary/Conclusion**

In this homogeneous matched sibling donor cohort receiving standardized PTCy-based GVHD prophylaxis, pre-transplant dyslipidemia defined by elevated non–HDL-C was not associated with adverse transplant outcomes or long-term 9-years cardiovascular mortality.

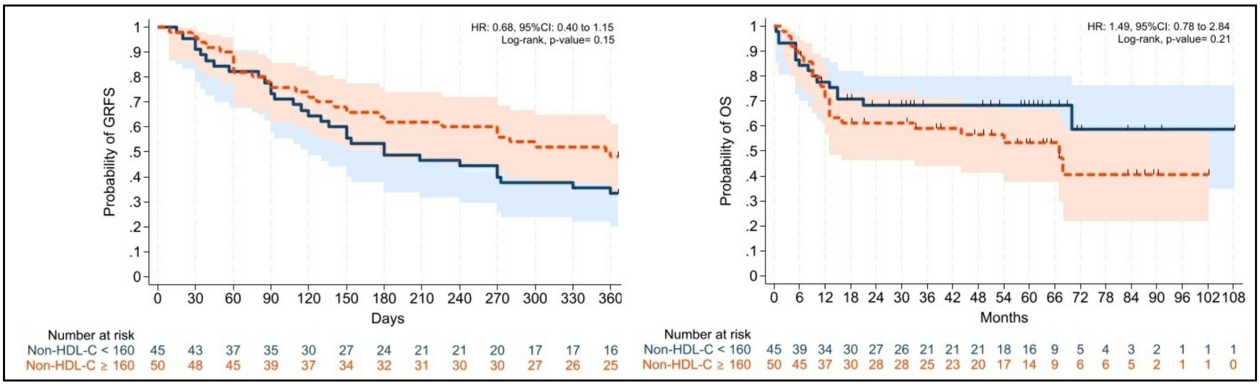

**Copyright Information:** (Online) ISSN: 2572-9241
©2026 The Author(s). HemaSphere is published by John Wiley & Sons Ltd on behalf of European Hematology Association. This is an open access Abstract Book distributed under the Attribution-NonCommercial-NoDerivs (CC BY-NC-ND), which allows third parties to download the articles and share them with others as long as they credit the author and the Abstract Book, but they cannot change the content in any way or use them commercially.
**Abstract Book Citations:** Authors, Title, HemaSphere, 2026;10:(S1):pages. The abstract book DOIs can be found on the "Author Information" page.
**Disclaimer:** Articles published in the journal HemaSphere exclusively reflect the opinions of the authors. The authors are responsible for all content in their abstracts including accuracy of the facts, statements, citing resources, etc.

### (PB4014) EARLY OUTCOMES OF ALLOGENEIC HAEMATOPOIETIC STEM CELL TRANSPLANTATION IN A MALAYSIAN PRIVATE QUATERNARY MEDICAL CENTRE: AN ONGOING COHORT STUDY

**Topic:** 23. Stem cell transplantation - Clinical

Kevin Tey[1], Naim Kamaruddin[1], Hong Huat Hoh[1], Kian Meng Chang[2], Kim Wah Ho[2], Siong Leng Hon[2], Andy Tang*[1, 2]

*[1]Sunway Medical Centre, Clinical Research Centre, Bandar Sunway, Malaysia;[2]Sunway Medical Centre, Haematology, Bandar Sunway, Malaysia;*

### Background

Allogeneic haematopoietic stem cell transplantation (allo-HSCT) is a potentially curative therapy for haematological disorders. Outcome data of allo-HSCT programmes in Malaysia remain limited. This study reports an interim analysis of transplant characteristics and early outcomes from an ongoing allo-HSCT programme in a private quaternary Malaysian hospital.

### Aims

This study aims to evaluate the transplant outcomes, focusing on overall survival, mortality, graft-versus-host (GVHD) incidence and severity, and infection rates, to improve clinical practice and contribute to the global understanding of HSCT efficacy and complications.

### Methods

This retrospective cohort study included 186 patients who underwent first allo-HSCT at Sunway Medical Centre between 2018 and 2025. Demographic characteristics, donor type, conditioning approach, and early post-transplant complications were analysed. Overall survival (OS) was estimated using the Kaplan-Meier, with patients censored at last known follow-up. As recruitment and follow-up are ongoing, survival estimates reflect available follow-up at the time of data cut-off.

### Results

Among the 186 allo-HSCT patients, 86% were Malaysians and 14% non-Malaysians. The median age at transplantation was 39 years (range: 18-70 years), with 53.2% were male. Acute leukaemia was the predominant indication (72.0%). Other indications included myelodysplastic syndrome/myeloproliferative neoplasm (11.3%), lymphoid neoplasm (9.1%), chronic leukaemia (4.3%), aplastic anaemia (2.7%), and other/rare hematologic neoplasms (0.5%). Most patients received transplants from haploidentical donors (53.8%), followed by matched-related donors (39.8%) and matched-unrelated donors (5.9%); ABO mismatch was present in 19.6% transplants. 9.1% of the patients had grade III-IV acute graft-versus-host disease (aGVHD), with the majority occurring in haploidentical transplants (60.6%, $p = 0.390$). Infections occurred in 76.9%. Disease relapse during available follow-up was 12.9%. At the time of analysis, the overall survival was 61.3% with a median follow up of 18 months. The primary cause of mortality was non-transplant-related causes (30.6%), followed by infection (26.4%), disease progresssion (23.6%) and GVHD (19.4%).

### Summary/Conclusion

This interim evaluation demonstrates acceptable early complication rates within our allo-HSCT programme in comparison to APBMT and EBMT registry report. This reflects real-world practice in a private quaternary Malaysian centre. Continued follow-up is required to determine long-term survival and relapse outcomes.

**Copyright Information:** (Online) ISSN: 2572-9241
©2026 The Author(s). HemaSphere is published by John Wiley & Sons Ltd on behalf of European Hematology Association. This is an open access Abstract Book distributed under the Attribution-NonCommercial-NoDerivs (CC BY-NC-ND), which allows third parties to download the articles and share them with others as long as they credit the author and the Abstract Book, but they cannot change the content in any way or use them commercially.
**Abstract Book Citations:** Authors, Title, HemaSphere, 2026;10:(S1):pages. The abstract book DOIs can be found on the "Author Information" page.
**Disclaimer:** Articles published in the journal HemaSphere exclusively reflect the opinions of the authors. The authors are responsible for all content in their abstracts including accuracy of the facts, statements, citing resources, etc.

### (PB4015) FEASIBILITY AND CLINICAL OUTCOMES OF PELVIC FLOOR THERAPY IN FEMALE ALLOGENEIC HCT RECIPIENTS WITH CHRONIC GENITAL GVHD

**Topic:** 23. Stem cell transplantation - Clinical

Portia Smallbone*[1], Nichole Samms[1], George Chen[1], Jin Im[1], Richard E. Champlin[1], Elizabeth J. Shpall[1], Amin Majid Alousi[1]

*[1]MD Anderson Cancer Center, Houston, United States of America;*

### Background

Chronic dyspareunia is common post allogeneic stem cell transplantation (HCT). Chronic genital graft-versus-host disease (vGVHD) is an underrecognized complication, associated with dyspareunia, vaginal stenosis, introital narrowing, and significant impairment of sexual and emotional quality of life. While medical management including corticosteroid therapy is standard, the role of dedicated pelvic floor therapy (PFT) in genital cGVHD remains poorly defined.

### Aims

To evaluate the feasibility and clinical outcomes of pelvic floor therapy in female HCT recipients, including those with vGVHD.

### Methods

We performed a retrospective chart review of adult female HCT recipients referred for pelvic therapy between 2018-2025. Clinical characteristics, NIH cGVHD staging criteria, therapy engagement, pain scores and functional outcomes were evaluated. PFT consisted of 40-60 minutes sessions including education, manual myofascial release, pelvic floor muscle exercises, and structured home dilator therapy.

### Results

29 patients post-HCT received pelvic therapy. Dyspareunia was the predominant presenting symptom (90%), with 50% reporting dryness and 20% reporting additional pain symptoms or non-vulvar symptoms. 10 patients had confirmed chronic vaginal GVHD, 13 demonstrated documented pelvic floor hypertonicity, while 6 were referred for incontinence.
Among patients with vGVHD, all patients had concurrent cGVHD at referral (median NIH score 6.5 (range; 1-9)). Multi-organ involvement was common, most frequently oral (60%), ocular (60%) and fascial (50%). Median age at HCT was 51 years (range 30–57), and median time from HCT to pelvic therapy initiation was 23.1 months (range; 6.1-115.7m). All but one patient received prophylaxis with post-transplant cyclophosphamide, mycophenolate and tacrolimus.
Most patients were receiving established GVHD-directed therapy at referral: 60% were on concurrent systemic immunosuppression (tacrolimus, ruxolitinib, or belumosudil), 60% were using topical corticosteroids, and 70% topical estrogen therapy. Two patients required escalation of systemic therapy (oral corticosteroids and ruxolitinib) for progression of extra-genital GVHD during follow-up. Four patients had clitoral phimosis or labial resorption, and three patients required surgical management (lysis of adhesions or reconstruction) during disease course. 30% were sexually active at time of referral, with 2 patients commencing sexual activity by completion of PFT.
Median number of PFT sessions completed was 2.5 (range 1-24), with 40% completing prolonged therapy (13-24 sessions). Baseline median pain score was 7/10 (range 2-10), improving to 3.5/10 at 6 months in patients with available follow-up. All patients completing prolonged therapy reported ≥50% subjective improvement, with reduction in median pain score.
Documented barriers to completion (n=8) included surgical intervention (n=3), geographical or logistical constraints (n=3) and pain limiting adherence (n=2); no specific barrier was documented in the remaining cases.

**Copyright Information:** (Online) ISSN: 2572-9241
©2026 The Author(s). HemaSphere is published by John Wiley & Sons Ltd on behalf of European Hematology Association. This is an open access Abstract Book distributed under the Attribution-NonCommercial-NoDerivs (CC BY-NC-ND), which allows third parties to download the articles and share them with others as long as they credit the author and the Abstract Book, but they cannot change the content in any way or use them commercially.
**Abstract Book Citations:** Authors, Title, HemaSphere, 2026;10:(S1):pages. The abstract book DOIs can be found on the “Author Information” page.
**Disclaimer:** Articles published in the journal HemaSphere exclusively reflect the opinions of the authors. The authors are responsible for all content in their abstracts including accuracy of the facts, statements, citing resources, etc.

**Summary/Conclusion**

Pelvic floor therapy is feasible in female HCT recipients, including those with chronic vGVHD. Clinically meaningful reductions in pain scores were observed, particularly among patients with prolonged engagement. These findings support integration of structured pelvic floor therapy into multidisciplinary management of vGVHD and warrant prospective evaluation.

**Copyright Information:** (Online) ISSN: 2572-9241
©2026 The Author(s). HemaSphere is published by John Wiley & Sons Ltd on behalf of European Hematology Association. This is an open access Abstract Book distributed under the Attribution-NonCommercial-NoDerivs (CC BY-NC-ND), which allows third parties to download the articles and share them with others as long as they credit the author and the Abstract Book, but they cannot change the content in any way or use them commercially.
**Abstract Book Citations:** Authors, Title, HemaSphere, 2026;10:(S1):pages. The abstract book DOIs can be found on the "Author Information" page.
**Disclaimer:** Articles published in the journal HemaSphere exclusively reflect the opinions of the authors. The authors are responsible for all content in their abstracts including accuracy of the facts, statements, citing resources, etc.

### (PB4016) ERAVACYCLINE IN ALLOGENEIC STEM CELL TRANSPLANT RECIPIENTS: A REAL-WORLD STUDY OF DRUG-DRUG INTERACTIONS WITH CYCLOSPORINE/TACROLIMUS AND ANTI-INFECTIVE EFFICACY

**Topic:** 23. Stem cell transplantation - Clinical

Jieya Luo*[1], Jingqi Li[1], Xinhui Zheng[1], Xin Chen[1], Erlie Jiang[1]

*[1]State Key Laboratory of Experimental Hematology, National Clinical Research Center for Blood Diseases, Haihe Laboratory of Cell Ecosystem, Institute of Hematology & Blood Diseases Hospital, Chinese Academy of Medical Sciences & Peking Union Medical College, Hematopoietic Stem Cell Transplantation Center, Tianjin, China;*

### Background

Calcineurin inhibitors (CNIs) are standard prophylaxis for acute graft-versus-host disease (aGVHD) after allogeneic hematopoietic stem cell transplantation (allo-HSCT). Given their narrow therapeutic window and variable bioavailability, rigorous therapeutic drug monitoring is mandatory, particularly during serious infections. Eravacycline, a novel synthetic tetracycline, shares the CYP3A4 metabolic pathway with CNIs, yet comprehensive data on their potential pharmacokinetic interactions are scarce.

### Aims

This study aimed to evaluate the drug-drug interactions between eravacycline and CNIs, as well as its anti-infective efficacy and safety in allo-HSCT.

### Methods

This single-center, retrospective study was conducted at the Institute of Hematology & Blood Diseases Hospital from August 2023 to December 2025, enrolling allo-HSCT recipients who received CNIs and eravacycline (50 mg q12h). CNI concentration-to-dose (C/D) ratios were monitored at a median interval of every 3 days before, during, and after eravacycline administration, with mean C/D ratios calculated for each phase. The Wilcoxon signed-rank test and LOWESS regression were used to analyze trends over time.

### Results

A total of 70 patients were included, comprising 48 receiving cyclosporine and 22 receiving tacrolimus. Eravacycline was initiated at a median of 10 (IQR 4–17.3) days post-transplant; 64.3% received therapy during neutropenia phase and 8.6% during aGVHD phase. The median treatment duration was 11 (IQR 7–15) days . Pulmonary (41.4%) and bloodstream (38.6%) infections predominated, with *Stenotrophomonas maltophilia* (38.6%) as the leading pathogen.
In the cyclosporine group, co-administration with eravacycline increased the C/D ratio by 13.2% (P=0.015), requiring a mean 10% dose reduction to maintain therapeutic concentrations. Within five half-lives after discontinuation, C/D ratios increased by an additional 9.9% (P<0.01), necessitating a further 11% dose reduction. In the tacrolimus group, C/D ratios increased by 52.3% during co-administration (P=0.011), prompting dose reductions of 33.3%–36.5%, with no significant change after discontinuation (P=0.691). These trends persisted in patients receiving concomitant azoles/ letermovir and in those with non-terminal status and grade 0–2 liver and kidney function. Patients with aGVHD exhibited higher and more variable C/D ratios. Despite increased exposure, the rate of supratherapeutic concentrations remained low (5.0 %).
After excluding patients with end-stage multi-organ failure, grade 3 adverse events occurred in 3.5% during treatment and 2.6% within five half-lives post-discontinuation. Clinically, the remission rate was 81.4%. The 28-day all-cause and infection-related mortality rates were 18.6% and 4.3%, respectively. One-year overall survival was 52.3% (95% CI 41.1%–66.7%).

**Copyright Information:** (Online) ISSN: 2572-9241
©2026 The Author(s). HemaSphere is published by John Wiley & Sons Ltd on behalf of European Hematology Association. This is an open access Abstract Book distributed under the Attribution-NonCommercial-NoDerivs (CC BY-NC-ND), which allows third parties to download the articles and share them with others as long as they credit the author and the Abstract Book, but they cannot change the content in any way or use them commercially.
**Abstract Book Citations:** Authors, Title, HemaSphere, 2026;10:(S1):pages. The abstract book DOIs can be found on the “Author Information” page.
**Disclaimer:** Articles published in the journal HemaSphere exclusively reflect the opinions of the authors. The authors are responsible for all content in their abstracts including accuracy of the facts, statements, citing resources, etc.

### Summary/Conclusion

This study provided the first clinical evidence of a pharmacokinetic interaction between eravacycline and CNIs in allo-HSCT recipients. Eravacycline increased CNI exposure during co-administration and induced a transient post-discontinuation elevation that resolved within approximately two weeks. Although the magnitude of the interaction was moderate, intensified therapeutic drug monitoring and stepwise dose adjustments were recommended during and shortly after eravacycline therapy, particularly with tacrolimus. In the post-transplant setting, eravacycline demonstrated robust anti-infective efficacy with an acceptable safety profile.

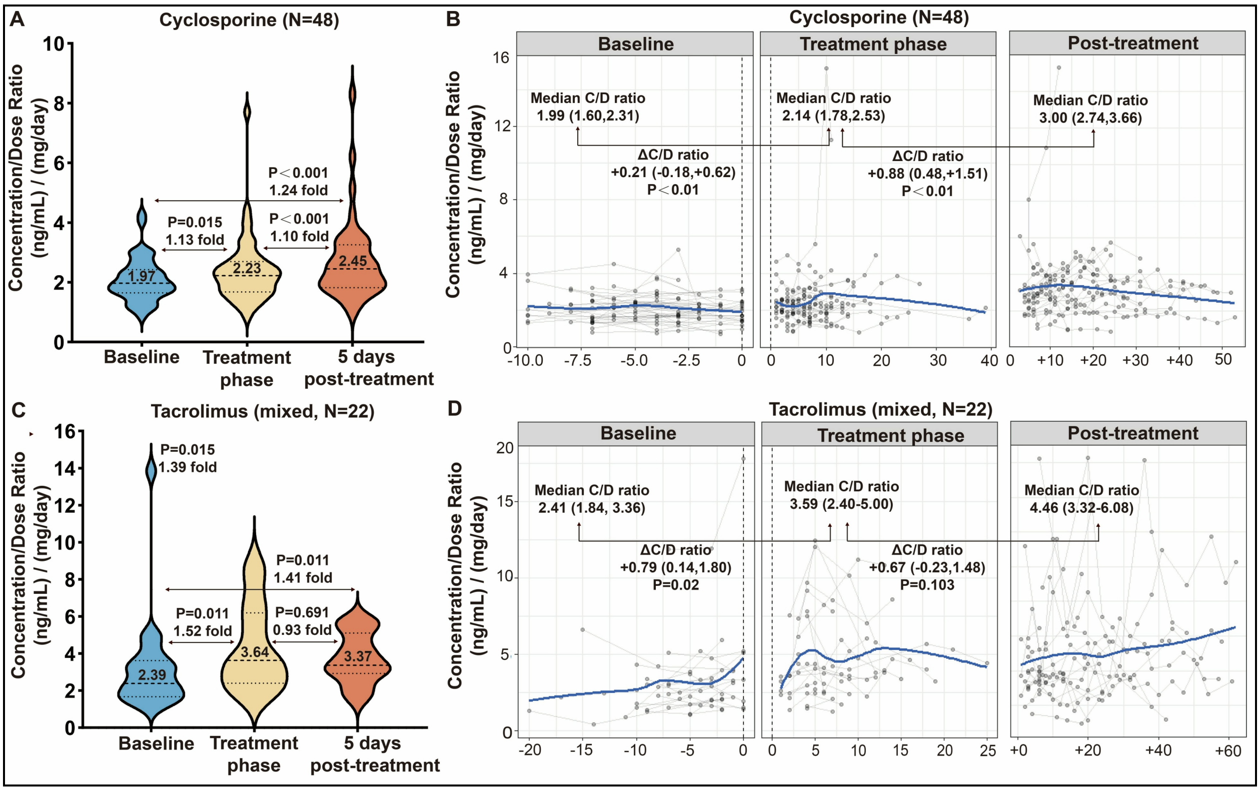

Changes in CNI concentration-to-dose (C/D) Before and After Eravacycline Coadministration

**Copyright Information:** (Online) ISSN: 2572-9241
©2026 The Author(s). HemaSphere is published by John Wiley & Sons Ltd on behalf of European Hematology Association. This is an open access Abstract Book distributed under the Attribution-NonCommercial-NoDerivs (CC BY-NC-ND), which allows third parties to download the articles and share them with others as long as they credit the author and the Abstract Book, but they cannot change the content in any way or use them commercially.
**Abstract Book Citations:** Authors, Title, HemaSphere, 2026;10:(S1):pages. The abstract book DOIs can be found on the "Author Information" page.
**Disclaimer:** Articles published in the journal HemaSphere exclusively reflect the opinions of the authors. The authors are responsible for all content in their abstracts including accuracy of the facts, statements, citing resources, etc.

### (PB4017) ANALYSIS OF CLINICAL FEATURES AND DRUG-RESISTANT INFECTIONS IN PATIENTS WITH EXTREMELY PROLONGED HOSPITAL STAYS FOLLOWING HAPLOIDENTICAL PERIPHERAL BLOOD STEM CELL TRANSPLANTATION

**Topic:** 23. Stem cell transplantation - Clinical

Yafang MA*[1], Jiejin Qian[1], Yanling Ren[1], Yu Xu[1], Shasha Wang[1], Chen Mei[1], Xianbo Huang[1], Hongyan Tong[1]

[1] *The First Affiliated Hospital, College of Medicine, Zhejiang University, Hangzhou, China;*

### Background

Extremely prolonged hospital stays (EPHS, >90 days within 180 days post-transplant) correlate with unfavorable prognosis in haploidentical peripheral blood stem cell transplantation (haplo-PBSCT), but there is limited clinical data on this issue, particularly regarding drug-resistant infections. As a mainstream modality for hematological diseases, haplo-PBSCT confers donor accessibility but increases the risk of acute graft-versus-host disease (aGVHD) and severe infections, which may exacerbate EPHS. Prolonged hospitalization further induces a vicious cycle of persistent immunosuppression and recurrent infections. The clinical features of EPHS, risk factors for drug-resistant infections, and the prognostic impact of key complications in this population remain uncharacterized, impeding effective clinical management.

### Aims

To explore the clinical risk factors, major post-transplant complications and survival outcomes of haplo-PBSCT patients with EPHS, clarify the risk factors for drug-resistant bacterial infections, and investigate the prognostic impact of invasive fungal disease (IFD) in this specific cohort.

### Methods

We conducted a single-center retrospective study at the Department of Hematology, the First Affiliated Hospital of Zhejiang University School of Medicine, enrolling 40 haplo-PBSCT patients with EPHS treated from December 2020 to December 2025.

### Results

The median length of stay (LOS) was 115.5 days (91–180 days), with 57.5% of patients hospitalized for 91–120 days and 52.5% admitted twice. The cohort had a median age of 49.5 years (range: 16–72 years), with 55.0% male, 65.0% in complete remission before transplantation, and 75.0% undergoing high-intensity conditioning regimens (transplant conditioning intensity, TCI: 4–6). Among them, 27.5% (11/40) developed drug-resistant bacterial infections, with bloodstream infections occurring mostly within 2 months post-transplant and non-bloodstream infections emerging after acute graft-versus-host disease (aGVHD). IFD was identified in 22.5% (9/40) patients, 8 of whom had concurrent aGVHD. IFD was a risk factor for inferior overall survival (OS) (1-year OS: 20.7% vs 64.1%, HR=0.136, 95%CI:0.028–0.654, P=0.013). Univariate analysis revealed that age ≥60 years (OR=4.600, 95%CI:1.038~20.381, P=0.045) and shorter time from diagnosis to HSCT (<3 months: 66.7% vs ≥6 months: <15%, P=0.011) increased the risk of drug-resistant infections, while higher TCI score was associated with lower infection risk (66.7% in TCI 1–2 vs 20.0% in TCI 4–6, P=0.049). Patients with drug-resistant infections had a significantly longer median LOS (131 days vs 107 days, Z=-2.046, P=0.041). Notably, infection was the most common cause of EPHS (90 episodes, 43.9%), and aGVHD was the most common cause of readmission (11 episodes, 21.6%).

**Copyright Information:** (Online) ISSN: 2572-9241
©2026 The Author(s). HemaSphere is published by John Wiley & Sons Ltd on behalf of European Hematology Association. This is an open access Abstract Book distributed under the Attribution-NonCommercial-NoDerivs (CC BY-NC-ND), which allows third parties to download the articles and share them with others as long as they credit the author and the Abstract Book, but they cannot change the content in any way or use them commercially.
**Abstract Book Citations:** Authors, Title, HemaSphere, 2026;10:(S1):pages. The abstract book DOIs can be found on the "Author Information" page.
**Disclaimer:** Articles published in the journal HemaSphere exclusively reflect the opinions of the authors. The authors are responsible for all content in their abstracts including accuracy of the facts, statements, citing resources, etc.

### Summary/Conclusion

In conclusion, EPHS patients after haplo-PBSCT have high incidences of drug-resistant infections and IFD, with IFD predicting poor survival. Age ≥60 years, short diagnosis-to-transplant time and low TCI are risk factors for drug-resistant infections, and infection is the core driver of prolonged hospitalization. Individualized TCI selection, early aGVHD management and targeted infection prevention may improve clinical outcomes of these patients.

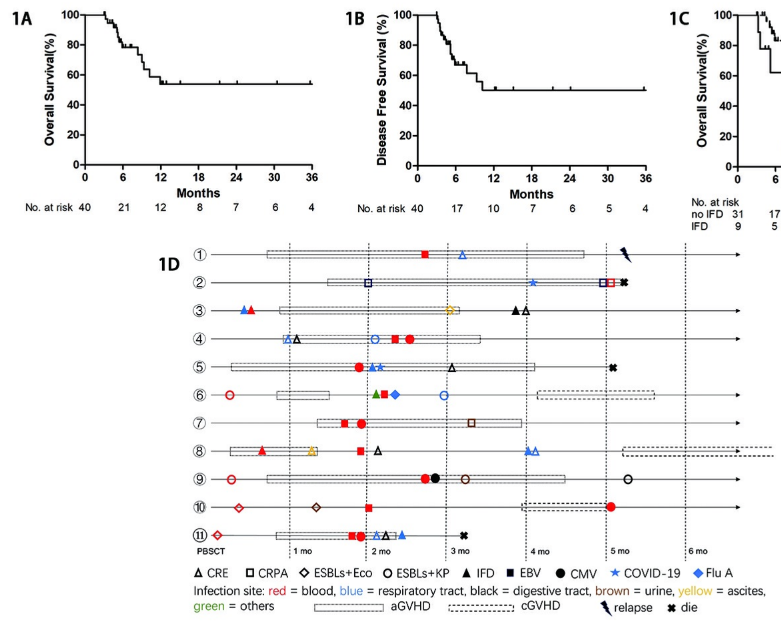

**Figure 1. Survival outcomes of the entire cohort and complication timelines in patients with multidrug-resistant bacterial infections post haplo-PBSCT**

1A. Kaplan-Meier curve for OS of the entire cohort; 1B. Kaplan-Meier curve for DFS of the entire cohort; 1C. OS stratified by IFD status, confirming IFD as a key factor for poor prognosis with significant OS reduction; 1D. 6-month post-transplant complication timelines in 11 patients with multidrug-resistant bacterial infections

Notes: 1 – 11 denote individual patient numbers, with each horizontal line representing the disease process of one patient. Complications are limited to GVHD, multidrug-resistant bacterial infections, as well as selected viral infections and invasive fungal diseases.

Abbreviations: mo = Month; PBSCT = Peripheral Blood Stem Cell Transplantation; GVHD = Graft-Versus-Host Disease; aGVHD = acute Graft-Versus-Host Disease; cGVHD = chronic Graft-Versus-Host Disease; CRE = Carbapenem-Resistant Enterobacterales; CRPA = Carbapenem-Resistant Pseudomonas aeruginosa; ESBLs = Extended-Spectrum Beta-Lactamases; Eco = Escherichia coli; KP = Klebsiella pneumoniae; IFD = Invasive Fungal Disease; EBV = Epstein-Barr Virus; CMV = Cytomegalovirus; Flu A = Influenza A Virus; OS = Overall survival; DFS = Disease-free survival.

**Copyright Information:** (Online) ISSN: 2572-9241
©2026 The Author(s). HemaSphere is published by John Wiley & Sons Ltd on behalf of European Hematology Association. This is an open access Abstract Book distributed under the Attribution-NonCommercial-NoDerivs (CC BY-NC-ND), which allows third parties to download the articles and share them with others as long as they credit the author and the Abstract Book, but they cannot change the content in any way or use them commercially.
**Abstract Book Citations:** Authors, Title, HemaSphere, 2026;10:(S1):pages. The abstract book DOIs can be found on the "Author Information" page.
**Disclaimer:** Articles published in the journal HemaSphere exclusively reflect the opinions of the authors. The authors are responsible for all content in their abstracts including accuracy of the facts, statements, citing resources, etc.

### (PB4018) INTERACTION BETWEEN IN VIVO T CELL DEPLETION AND PHARMACOKINETIC-BASED BUSULFAN CONDITIONING REGIMEN IN COMPLETE REMISSION ACUTE MYELOID LEUKEMIA (AML).

**Topic:** 23. Stem cell transplantation - Clinical

Luca Castagna*[1], Roberto Bono[1], Cristina Rotolo[1], Giuseppe Sapienza[1], Corinne Spoto[1], Carmelo Costanza[1], Sergio Indelicato[1], Caterina Patti[1], Antonino Mulè[1], Valeria Calafiore[1], Giulia Arrigo[1], Alessandra Santoro[1], Laura Di Noto[1]

*[1]AOR Villa Sofia Cervello, Palermo, Italy;*

### Background

Several disease-, patient- and procedure-related factors influence outcomes after allogenic hematopoietic stem cell transplantation (allo-SCT) in patients with acute myeloid leukemia (AML). Among procedure variables, the intensity of the conditioning regimens plays a pivotal role.

Busulfan (Bu), a widely used alkylating agent in conditioning regimens, is characterized by a narrow therapeutic index and marked inter-individual pharmacokinetic (PK) variability, potentially leading to under- or overexposure. Another key procedure-related determinant of transplant outcome is graft versus host prophylaxis (GVHD) regimen. In vivo T-cell depletion through serotherapy with anti-thymocyte globulin (ATG/ATLG) is widely adopted in matched unrelated donor (MUD) and HLA identical sibling transplants, significantly reducing the incidence of chronic GVHD.

### Aims

The aim of this study were to evaluate the interactions on outcomes between BU-based conditioning regimen with PK and inclusion of ATG as GVHD prophylaxis.

### Methods

Between January 2010 and August 2025, 111 patients satisfying the following inclusion criteria were included: complete remission (CR) AML, busulfan-based myeloablative conditioning regimen (MAC) with or without PK guided-dosing, matched related donor (MRD) or matched unrelated donor (MUD) and GVHD prophylaxis with or without ATG.

Patients received fixed-dose BU until October 2022; thereafter, BU dosing was adjusted to achieve a predefined target area-under-the-concentration-time curve (AUC).

PK assessment was performed after the first fixed dose of BU (100 mg/m2) and repeated after the 3rd dose. Two target exposure levels were predefined:  AUC16 for patients aged 65-70 years and/or with comorbidity index (HCT-CI) ≥3, and AUC20 for the remaining patients. GVHD prophylaxis for HLAid sibling transplantation was cyclosporine A (CSA) plus short course methotrexate (MTX). From 2021 onwards, patients undergoing transplantation from either matched related donor (MRD) or matched unrelated donor (MUD) received CSA+MTX+ATG, latter administered at a total dose 5 mg/kg on days -2 and -1

Patients were stratified into three cohorts according to exposure to BU-PK and in vivo T-cell depletion with ATG: (1) no PU-BK/no ATG; (2) ATG without BU-PK; (3) BU-PK and ATG. The median follow-up was 60 months (range 2-190).

**Copyright Information:** (Online) ISSN: 2572-9241
©2026 The Author(s). HemaSphere is published by John Wiley & Sons Ltd on behalf of European Hematology Association. This is an open access Abstract Book distributed under the Attribution-NonCommercial-NoDerivs (CC BY-NC-ND), which allows third parties to download the articles and share them with others as long as they credit the author and the Abstract Book, but they cannot change the content in any way or use them commercially.
**Abstract Book Citations:** Authors, Title, HemaSphere, 2026;10:(S1):pages. The abstract book DOIs can be found on the "Author Information" page.
**Disclaimer:** Articles published in the journal HemaSphere exclusively reflect the opinions of the authors. The authors are responsible for all content in their abstracts including accuracy of the facts, statements, citing resources, etc.

**Results**

In the overall population, the 2y OS, LFS, 100-day, 1y NRM, 1y cumulative incidence (CI) of relapse (CIR), and 1y GRFS were 62%, 58%, 9%, 17%, 25% and 58%, respectively. The 6-month CI of grade 2-4, 3-4 aGVHD, and 1y moderate-severe cGVHD were 28%, 9%, and 14%, respectively.
In Table we reported the outcomes according to cohorts. Overall, clinical outcomes were numerically better in the cohort BU-PK/ATG and moderate-severe cGVHD CI was statistically significant. Of note, 1y-GRFS in the BU-PK/ATG was 84%. These favorable results were not blunted by an increased risk of relapse.

| | No BU-PK/no ATG | ATG | BU-PK/ATG | pvalue |
|---|---|---|---|---|
| **OS @2y** | 62% | 56% | 68% | 0,58 |
| **LFS @2y** | 51% | 54% | 68% | 0,52 |
| **NRM @100 days** | 7% | 15% | 0 | 0,35 |
| **NRM @1y** | 18% | 24% | 0 | 0,35 |
| **CIR @1y** | 20% | 15% | 11% | 0,81 |
| **aGVHD 2-4** | 25% | 36% | 22% | 0,4 |
| **aGVHD 3-4** | 11% | 7% | 6% | 0,79 |
| **S/M cGVHD** | 34% | 0 | 6% | 0,005 |
| **GRFS @1y** | 42% | 63% | 84% | 0.08 |

**Summary/Conclusion**

These findings confirm the protective effect of ATG in reducing the incidence of moderate/severe cGVHD in CR AML patients. Moreover, the introduction of BU-PK associated to ATG appears to further improve the transplant tolerability leading to 1y GRFS of 84%. Notably, no cases of NRM were observed in BU-PK/ATG cohort.

**Copyright Information:** (Online) ISSN: 2572-9241
©2026 The Author(s). HemaSphere is published by John Wiley & Sons Ltd on behalf of European Hematology Association. This is an open access Abstract Book distributed under the Attribution-NonCommercial-NoDerivs (CC BY-NC-ND), which allows third parties to download the articles and share them with others as long as they credit the author and the Abstract Book, but they cannot change the content in any way or use them commercially.
**Abstract Book Citations:** Authors, Title, HemaSphere, 2026;10:(S1):pages. The abstract book DOIs can be found on the “Author Information” page.
**Disclaimer:** Articles published in the journal HemaSphere exclusively reflect the opinions of the authors. The authors are responsible for all content in their abstracts including accuracy of the facts, statements, citing resources, etc.

### (PB4019) ALTERNATIVE DONORS, PARTICULARLY HAPLOIDENTICAL DONORS, ARE SAFE AND EFFECTIVE FOR ELDERLY PATIENTS WITH ADVERSE-RISK AML: A MULTICENTER TROPHY STUDY

**Topic:** 23. Stem cell transplantation - Clinical

Wen-Xuan Huo*[1], Chuanhe Jiang[2], Congxiao Zhang[3], Zhen Shang[4], Miaomiao Zhao[5], Jinyun Lai[6], Yingjun Chang[1], Jiong Hu[2], Ling Wang[2], Yu Wang[1], Yang Yang[4], Wang Chun[7], Chuxian Zhao[7], Yi Yu[3], Linghui Xia[5], Hua Jin[6], Wei Shi[5], Yang Cao[4], Yanmin Zhao[3], Jia Chen[8], Xiaoxia Hu[2], Xiao-Dong Mo[1]

*[1]Peking University People's Hospital, Beijing, China; [2]State Key Laboratory of Medical Genomics, Shanghai Institute of Hematology, National Research Center for Translational Medicine, Shanghai Rui Jin Hospital, Shanghai Jiao Tong University School of Medicine, Shanghai, China; [3]Bone Marrow Transplantation Center, The First Affiliated Hospital, Zhejiang University School of Medicine, Hangzhou, China; [4]Department of Hematology, Tongji Hospital, Tongji Medical College, Huazhong University of Science and Technology, Wuhan, China; [5]Institute of Hematology, Union Hospital, Tongji Medical College, Huazhong University of Science and Technology, Wuhan, China; [6]Department of Hematology, Nanfang Hospital, Southern Medical University, Guanhgzhou, China; [7]Shanghai Zhaxin Traditional Chinese and Westen Medicine Hospital, Shanghai, China, Shanghai, China; [8]National Clinical Research Center for Hematologic Diseases, Jiangsu Institute of Hematology, The First Affiliated Hospital of Soochow University, Institute of Blood and Marrow Transplantation, Collaborative Innovation Center of Hematology, Soochow University, Suzhou, China;*

### Background

Acute myeloid leukemia (AML) is one of the most life-threatening hematologic malignancies in adults and allogeneic stem cell transplantation (allo-HSCT) has been recommended to be the standard consolidation therapy for adverse-risk AML. However, the incidence of AML increases with advancing age.Human leukocyte antigen (HLA) identical sibling donors (ISDs) are considered as the preferred option for allo-HSCT. However, for elderly AML patients, ISDs are often of advanced age or unsuitable for donation due to poor organ function, making alternative donors increasingly essential. Nevertheless, it remains unclear whether alternative donor, particularly the haploidentical related donor (HID) HSCT can achieve comparable clinical outcomes with ISD HSCT in elderly patients with adverse-risk AML.

### Aims

In this real-world study of the TROPHY group, we aimed to compare the clinical outcomes among ISD, HID and URD HSCT in elderly patients with adverse-risk AML, and we also aimed to compared the post-transplant outcomes between younger alternative donor, particularly the younger HID, and older ISD groups in patients receiving allo-HSCT at CR.

### Methods

This is a multicenter, retrospective designed study, based on the transplant database of TROPHY group from Peking University Institute of Hematology (PUIH), Wuhan Tongji Hospital, Shanghai Ruijin Hospital, Nanfang Hospital, Wuhan Union Hospital, and the First Affiliated Hospital of Zhejiang University. Consecutive AML patients receiving allo-HSCT from March 2017 and April 2025 were screened, and the eligibility criteria were as follows: (1) aged ≥ 55 years; (2) adverse-risk AML based on ELN 2022 criteria; and (3) achieved CR before allo-HSCT. The last follow-up was September 30, 2025.

**Copyright Information:** (Online) ISSN: 2572-9241
©2026 The Author(s). HemaSphere is published by John Wiley & Sons Ltd on behalf of European Hematology Association. This is an open access Abstract Book distributed under the Attribution-NonCommercial-NoDerivs (CC BY-NC-ND), which allows third parties to download the articles and share them with others as long as they credit the author and the Abstract Book, but they cannot change the content in any way or use them commercially.
**Abstract Book Citations:** Authors, Title, HemaSphere, 2026;10:(S1):pages. The abstract book DOIs can be found on the "Author Information" page.
**Disclaimer:** Articles published in the journal HemaSphere exclusively reflect the opinions of the authors. The authors are responsible for all content in their abstracts including accuracy of the facts, statements, citing resources, etc.

### Results

We compared the clinical outcomes among ISD, haploidentical related donor (HID), and unrelated donor (URD) HSCT in elderly patients with adverse-risk AML, and donors were categorized into younger (≤ 45years) and older (> 45 years) groups. The 2-year probabilities of relapse (22.4% *vs* 24.3% vs 15.5 %, *P* = 0.517), non-relapse mortality (15.6% *vs* 18.3% *vs* 20.8%, *P* = 0.848), leukemia-free survival (LFS, 62.0% *vs* 57.2% *vs* 63.6%, *P* = 0.786), and overall survival (OS, 68.6% *vs* 64.6% *vs* 73.1%, *P* = 0.688) after allo-HSCT were comparable among ISD, HID, and URD cohorts. In patients receiving allo-HSCT at complete remission, post-transplant outcomes of the older ISD group were generally comparable to those of the younger alternative donor and younger HID groups. The 2-year total cGVHD rate was higher in the older ISD group (80.0%) compared with the younger HID group (42.7%, *P* = 0.018) in patients aged 55–59 years.In multivariate analysis, *TP53* mutation was associated with a higher risk of relapse, a poorer LFS, and a worse OS. MRD positivity before HSCT was associated with a higher risk of relapse and a worse LFS.

### Summary/Conclusion

This study clarified the previously undefined role of alternative donors in a disease-specific cohort of elderly patients with adverse-risk AML. **We report for the first time that younger alternative donors, particularly younger HIDs, are a safe and viable option** for these patients.

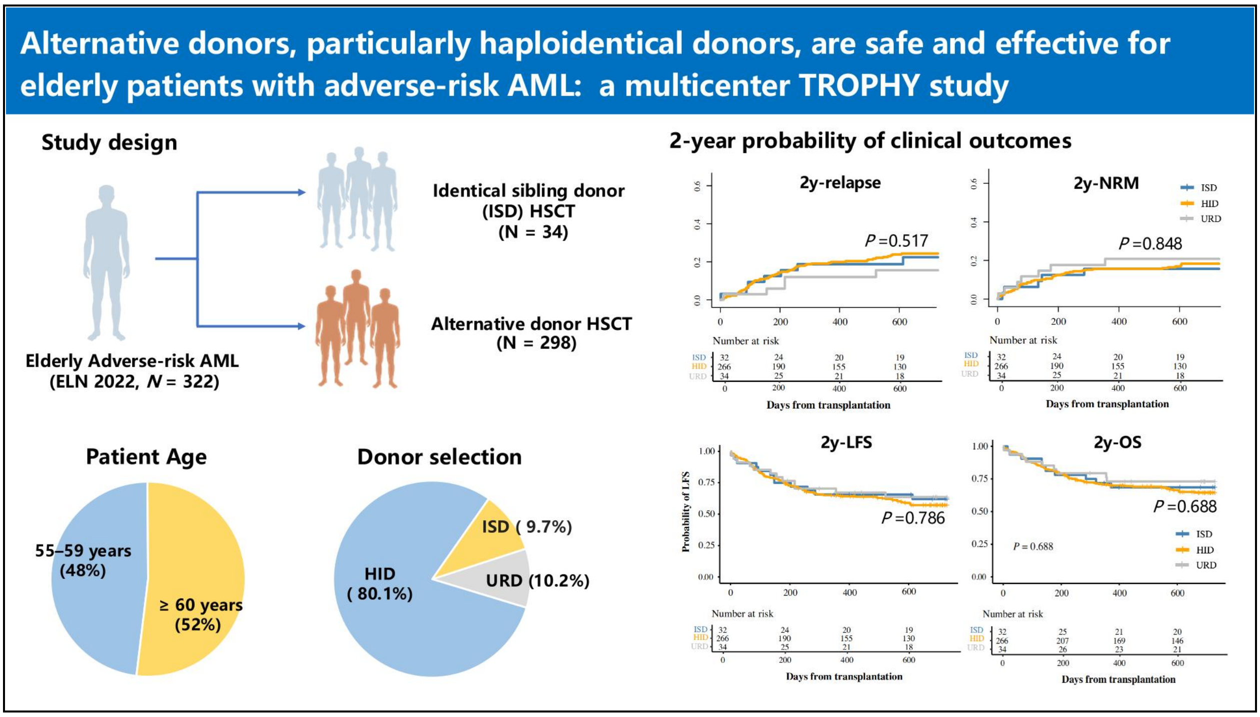

**Copyright Information:** (Online) ISSN: 2572-9241
©2026 The Author(s). HemaSphere is published by John Wiley & Sons Ltd on behalf of European Hematology Association. This is an open access Abstract Book distributed under the Attribution-NonCommercial-NoDerivs (CC BY-NC-ND), which allows third parties to download the articles and share them with others as long as they credit the author and the Abstract Book, but they cannot change the content in any way or use them commercially.
**Abstract Book Citations:** Authors, Title, HemaSphere, 2026;10:(S1):pages. The abstract book DOIs can be found on the "Author Information" page.
**Disclaimer:** Articles published in the journal HemaSphere exclusively reflect the opinions of the authors. The authors are responsible for all content in their abstracts including accuracy of the facts, statements, citing resources, etc.

### (PB4020) CHRONIC KIDNEY DISEASE PREDICTS DELAYED PLATELET ENGRAFTMENT, INCREASED TRANSFUSION REQUIREMENTS, AND HIGHER INFECTIOUS MORBIDITY FOLLOWING AUTOLOGOUS STEM CELL TRANSPLANTATION

**Topic:** 23. Stem cell transplantation - Clinical

Anamarija Vrkljan Vuk*[1], David Čičić[1], Matija Crnogorac[1], Ena Soric[1], Ozren Jakšić[1, 2], Ivan Durlen[1], Ivana Bedalov Crnkovic[1], Zeljko Prka[1]

*[1]University Hospital Dubrava, Zagreb, Croatia; [2]University of Zagreb School of Medicine, Zagreb, Croatia;*

### Background

Autologous hematopoietic stem cell transplantation (ASCT) is considered feasible in patients with chronic kidney disease (CKD); however, pre-existing renal impairment is associated with increased transplant-related morbidity. Uraemia-induced immune dysregulation impairs neutrophil, monocyte, and T-cell function, elevating infectious risk during post-conditioning neutropenia. Additionally, CKD-associated platelet dysfunction — driven by accumulation of uraemic toxins — may impede post-ASCT haematological reconstitution, while anaemia of chronic disease compounds post-transplant transfusion dependence.

### Aims

To evaluate post-ASCT neutrophil and platelet engraftment kinetics, transfusion requirements, and infectious complications in CKD versus non-CKD patients.

### Methods

Retrospective analysis of consecutive ASCT patients treated at our Centre during 2024–2025. Engraftment times, transfusion data, and infectious events were extracted from institutional electronic health records and compared between CKD and non-CKD patients. CKD was classified per KDIGO criteria. Between-group differences were assessed using Student's t-test and Chi-square test.

### Results

The cohort comprised 55 patients: 26 CKD and 29 non-CKD, with a median age of 68 years (IQR 40–75) and 60 years (IQR 27–63), respectively; 9 (34%) and 13 (44%) were female. All received ASCT for multiple myeloma (MM) or non-Hodgkin lymphoma (NHL); MM was significantly more frequent in the CKD group (20/26, 77% vs. 13/29, 44%; p=0.001). Four CKD patients (15%) were on chronic haemodialysis at the time of ASCT. Median eGFR pre-, peri-, and post-ASCT in the CKD group was 44.0–41.5–44.2 mL/min/1.73m² and remained stable (p=0.17). Median ANC engraftment time was comparable between groups: 10 days (IQR 9–16) in CKD versus 10 days (IQR 10–12) in non-CKD patients (p=0.1). In contrast, platelet recovery was significantly delayed in CKD patients: median 13.2 days (IQR 10–25) versus 12 days (IQR 9–18; p=0.008). CKD patients also required markedly greater transfusion support: irradiated RBC units totalled 73 versus 32 (p=0.01), and irradiated platelet units 602 versus 322 (p=0.01). The 4 haemodialysis-dependent CKD patients accounted for 24% of all irradiated RBC units administered to the CKD group. Infectious complications occurred significantly more frequently in CKD patients (23/26; 88%) than in non-CKD patients (19/29; 65%; p=0.02), most commonly as enterocolitis, bacteraemia, and urinary tract infections. One CKD patient died due to sepsis; no deaths occurred in the non-CKD group.

**Copyright Information:** (Online) ISSN: 2572-9241
©2026 The Author(s). HemaSphere is published by John Wiley & Sons Ltd on behalf of European Hematology Association. This is an open access Abstract Book distributed under the Attribution-NonCommercial-NoDerivs (CC BY-NC-ND), which allows third parties to download the articles and share them with others as long as they credit the author and the Abstract Book, but they cannot change the content in any way or use them commercially.
**Abstract Book Citations:** Authors, Title, HemaSphere, 2026;10:(S1):pages. The abstract book DOIs can be found on the "Author Information" page.
**Disclaimer:** Articles published in the journal HemaSphere exclusively reflect the opinions of the authors. The authors are responsible for all content in their abstracts including accuracy of the facts, statements, citing resources, etc.

**Summary/Conclusion**

While neutrophil engraftment was preserved, CKD patients demonstrated significantly delayed platelet reconstitution, higher irradiated RBC and platelet transfusion requirements, and a markedly increased rate of post-ASCT infectious complications compared to non-CKD patients. These findings highlight CKD as a distinct clinical phenotype requiring tailored post-transplant management, including heightened infectious vigilance with eGFR-guided antimicrobial dosing, and individualised transfusion and platelet-stimulating strategies.

**Copyright Information:** (Online) ISSN: 2572-9241
©2026 The Author(s). HemaSphere is published by John Wiley & Sons Ltd on behalf of European Hematology Association. This is an open access Abstract Book distributed under the Attribution-NonCommercial-NoDerivs (CC BY-NC-ND), which allows third parties to download the articles and share them with others as long as they credit the author and the Abstract Book, but they cannot change the content in any way or use them commercially.
**Abstract Book Citations:** Authors, Title, HemaSphere, 2026;10:(S1):pages. The abstract book DOIs can be found on the "Author Information" page.
**Disclaimer:** Articles published in the journal HemaSphere exclusively reflect the opinions of the authors. The authors are responsible for all content in their abstracts including accuracy of the facts, statements, citing resources, etc.

### (PB4021) DOUBLE-UNIT CORD BLOOD VERSUS HAPLOIDENTICAL DONOR TRANSPLANTATION: A RETROSPECTIVE SINGLE-CENTER ANALYSIS OF SURVIVAL OUTCOMES

**Topic:** 23. Stem cell transplantation - Clinical

Jiangbo Wan*[1], Wen-Hao Zhang[2]

*[1]Xinhua Hospital Affiliated to Shanghai Jiaotong University School of Medicine, Hematology, Shanghai, China;[2]Fudan University Shanghai Cancer Center, Shanghai, China;*

### Background

Hematopoietic stem cell transplantation (HSCT) is a cornerstone therapy for hematologic malignancies, with HLA-matched sibling donors (MSD) considered optimal—yet available to only [illegible]30% of patients. For the remainder, haploidentical donor (HID) and umbilical cord blood (UCB) serve as key alternatives. HID transplantation, facilitated by post-transplant cyclophosphamide (PTCy) and donor-specific antibody (DSA) management, offers near-universal donor availability and outcomes comparable to MSD. UCB transplantation requires less stringent HLA matching and also demonstrates similar survival to MSD, though limited cell dose—especially in adults—remains a challenge. Double-unit UCB (DUCB) has been adopted to overcome this limitation, yielding survival rates akin to HID. Despite their widespread use, direct comparisons between DUCB and HID regarding engraftment kinetics, survival, and complications are scarce.

### Aims

This study aims to evaluate and compare survival outcomes, engraftment, and complications associated with DUCB and HID transplantation at our center.

### Methods

This retrospective analysis included 97 patients with hematologic malignancies who underwent allo-HSCT at Xinhua Hospital, Shanghai Jiao Tong University School of Medicine, between August 2014 and May 2024. All of the patients had transplantation indications. Patients were assigned to unrelated DUCB transplantation (n = 40) or HID transplantation (n = 57) based on donor availability. A hierarchical allocation algorithm was implemented wherein HID transplantation was prioritized over DUCB transplantation when eligible donors were available for both modalities. Patient characteristics and transplantation outcomes were evaluated.

### Results

In this retrospective analysis of 97 patients with hematologic malignancies (2014–2024), transplantation outcomes were compared between haploidentical donors (HID, n=57) and double-unit umbilical cord blood (DUCB, n=40). HID demonstrated significantly faster neutrophil (14 vs 22.5 days, p<0.001) and platelet recovery (15 vs 44.5 days, p<0.001). Grade II-IV acute graft-versus-host-disease (GVHD) (16.8% vs 47.7%, p<0.001) and chronic GVHD (10.3% vs 28.6%, p=0.033) were lower with HID, though severe III-IV acute GVHD showed no difference. Five-year survival outcomes were comparable: overall survival (OS) (56.7% vs 48.9%, p=0.573), relapse-free survival (RFS) (76.7% vs 89.7%, p=0.210), GVHD-free relapse-free survival (GRFS) (48% vs 46.3%, p=0.410), and Non-relapse mortality (NRM) (35.4% vs 47%, p=0.188).

**Copyright Information:** (Online) ISSN: 2572-9241
©2026 The Author(s). HemaSphere is published by John Wiley & Sons Ltd on behalf of European Hematology Association. This is an open access Abstract Book distributed under the Attribution-NonCommercial-NoDerivs (CC BY-NC-ND), which allows third parties to download the articles and share them with others as long as they credit the author and the Abstract Book, but they cannot change the content in any way or use them commercially.
**Abstract Book Citations:** Authors, Title, HemaSphere, 2026;10:(S1):pages. The abstract book DOIs can be found on the "Author Information" page.
**Disclaimer:** Articles published in the journal HemaSphere exclusively reflect the opinions of the authors. The authors are responsible for all content in their abstracts including accuracy of the facts, statements, citing resources, etc.

**Summary/Conclusion**

For patients lacking HLA-matched sibling donors, both DUCB and HID transplantation represent viable alternatives, demonstrating no statistically significant differences in OS, RFS, or GRFS. However, HID transplantation is associated with faster hematopoietic recovery and lower incidence of GVHD, rendering it a more favorable option in clinical practice, particularly for patients at high risk of early transplantation-related complications.

**Copyright Information:** (Online) ISSN: 2572-9241
©2026 The Author(s). HemaSphere is published by John Wiley & Sons Ltd on behalf of European Hematology Association. This is an open access Abstract Book distributed under the Attribution-NonCommercial-NoDerivs (CC BY-NC-ND), which allows third parties to download the articles and share them with others as long as they credit the author and the Abstract Book, but they cannot change the content in any way or use them commercially.
**Abstract Book Citations:** Authors, Title, HemaSphere, 2026;10:(S1):pages. The abstract book DOIs can be found on the "Author Information" page.
**Disclaimer:** Articles published in the journal HemaSphere exclusively reflect the opinions of the authors. The authors are responsible for all content in their abstracts including accuracy of the facts, statements, citing resources, etc.

### (PB4022) EFFICACY AND SAFETY OF MARIBAVIR INTHE TREATMENT OF 10 PATIENTS WITH CMV END-ORGAN DISEASE AFTER ALLOGENEIC HEMATOPOIETIC STEM CELL TRANSPLANTATION

**Topic:** 23. Stem cell transplantation - Clinical

Zhicong Du[1], Fang Xu[2], Yun Wang[2], Baiqing Sun[2], Na Zhang[1], Chunqiong Xu[1], Shanshan Zhang[1], Jianli Wang[1], Juju Xu[1], Yue Lu*[1]

*[1]Beijing Lu Daopei Hospital, beijing, China;[2]Hebei Yanda Lu Daopei Hospital, langfang, China;*

### Background

Cytomegalovirus (CMV) end-organ disease is a severe complication after allogeneic hematopoietic stem cell transplantation (allo-HSCT). Traditional nucleoside antiviral agents as first-line treatments are limited in clinical use due to significant adverse effects. Maribavir has been increasingly used for the treatment of CMV infection in recent years. However, clinical efficacy and safety analyses in small cohorts of patients with post-HSCT CMV end-organ disease remain limited. This study analyzed the medical records of 10 patients to provide clinical evidence for clinical practice.

### Aims

To evaluate the efficacy and safety of maribavir in the treatment of CMV end-organ disease following allo-HSCT, and to provide clinical evidence for antiviral management in this population.

### Methods

We retrospectively analyzed the clinical data of 10 patients diagnosed with CMV end-organ disease after allo-HSCT and treated with maribavir between December 2023 and April 2025. The final follow-up date was October 31, 2025.

### Results

Of the 10 patients with CMV end-organ disease, 6 were male and 4 were female, with a median age of 36 years (range, 18–68 years). Pre-transplant CMV IgG serostatus: 9 cases were D+/R+ and 1 case was D-/R+. All 10 patients received letermovir (LET) prophylaxis from day +6 to day +100 post-transplantation. No CMV infection occurred within day +100, whereas 7 patients developed late-onset CMV viremia at a median of 153 days (range, 116–180 days) after transplantation. The median peak plasma CMV DNA load was 54,000 copies/mL (range, 1100–630,000 copies/mL), and the median duration of CMV viremia was 27 days (range, 8–52 days).  All 7 patients who had previously received conventional anti-CMV agents were then treated with maribavir.Among the 10 patients with CMV end-organ disease, 5 had CMV pneumonia and 5 had CMV gastroenteritis, occurring at a median of 151 days (range, 123–213 days) post-transplantation. Six patients had concurrent CMV viremia at the onset of CMV end-organ disease. Additional comorbidities included: 6 cases with concomitant overlapping GVHD/cGVHD, 4 cases with EBV-related diseases (2 EBV pneumonia, 2 EBV enteritis), 1 case with poor graft function, 1 case with capillary leak syndrome, and 1 case with severe bacterial infection. All patients treated with maribarvir when diagnosed with CMV end-organ diease. The median time to gastrointestinal symptom remission in 5 patients with CMV gastroenteritis was 19 days (range, 8–42 days). The median time to radiological resolution in 5 patients with CMV pneumonia was 4 weeks (range, 2–8 weeks). After maribavir discontinuation, CMV viremia recurred in 3 patients at a median of 81 days (range, 18–83 days) off therapy, with a median peak CMV DNA load of 1100 copies/mL (range, 770–3300 copies/mL). Sustained CMV DNA negativity was achieved in 2 patients after re-initiation of maribavir. One patient refused maribavir for personal reasons and progressed to CMV pneumonia 2 weeks after recurrent CMV viremia. One patient developed dysgeusia, which resolved after maribavir cessation. No worsening cytopenia, increased creatinine or other drug-related adverse events were observed. At the final follow-up, 9 patients alive,1 patient died due to severe pneumonia.

**Copyright Information:** (Online) ISSN: 2572-9241
©2026 The Author(s). HemaSphere is published by John Wiley & Sons Ltd on behalf of European Hematology Association. This is an open access Abstract Book distributed under the Attribution-NonCommercial-NoDerivs (CC BY-NC-ND), which allows third parties to download the articles and share them with others as long as they credit the author and the Abstract Book, but they cannot change the content in any way or use them commercially.
**Abstract Book Citations:** Authors, Title, HemaSphere, 2026;10:(S1):pages. The abstract book DOIs can be found on the “Author Information” page.
**Disclaimer:** Articles published in the journal HemaSphere exclusively reflect the opinions of the authors. The authors are responsible for all content in their abstracts including accuracy of the facts, statements, citing resources, etc.

### Summary/Conclusion

Maribavir shows favorable clinical efficacy in the treatment of CMV end-organ disease after allo-HSCT, with mild adverse effects and good tolerability. However, given the small sample size, further validation in large-scale prospective studies is warranted.

**Fingure1:Clinical characteristics of 10 patients with CMV end-organ disease after allo-HSCT treated with maribavir**

| Clinical characteristics | Number of patients, N=10 |
|---|---|
| **Sex** | |
| Male/Female | 6/4 |
| **Median age (years)** | 36 (18-68) |
| **Primary disease** | |
| AML/ALL/MDS/SAA-PNH | 7/1/1/1 |
| **HCT-CI score** | |
| ⩾2 points | 4（40%） |
| **Disease Risk Stratification** | |
| High-risk disease | 4（40%） |
| **Immunotherapy or targeted therapy before transplant** | 7（70%） |
| **Transplant type** | |
| Haploidentical/unrelated | 8/2 |
| **Donor/recipient CMV IgG serostatus** | |
| D−/R+ | 1（10%） |
| D+/R+ | 9（90%） |
| **Secondary allo-HSCT** | 1（10%） |
| **Conditioning regimen** | |
| Bu-based / TBI-based | 9/1 |
| **GVHD prophylaxis** | |
| CSA+MMF+sMTX | 9（90%） |
| FK506+MMF+sMTX | 1（10%） |
| **Type of ATG** | |
| Rabbit anti-T-cell immunoglobulin | 5（50%） |
| Rabbit anti-thymocyte globulin (rATG) | 5（50%） |
| **Letermovir prophylaxis** | 10 (day+6 to+100) |
| **aGVHD (grade III–IV)** | 4(40%) |
| **Median lymphocyte subsets within day +100 post-transplant (cells/μL)** | |
| CD3+/CD4+/CD8+ at day +30 | 47.5/11/22 |
| CD3+/CD4+/CD8+ at day +60 | 104/15/62 |
| CD3+/CD4+/CD8+ at day +100 | 121/66/109 |
| **CMV reactivation within day +100** | 0 |
| **Other viral reactivation within day +100** | |
| EBV viremia | 6（60%）(3 treated with rituximab) |
| BKV viremia | 2（20%） |
| **Post-transplant DLI** | 5（50%） |
| **High-dose steroids before CMV disease (prednisone ≥ 1 mg/kg)** | 4（40%） |

| | |
|---|---|
| **Late CMV viremia before CMV disease** | 7（70%） |
| Median onset of late CMV viremia (days) | 153（116-180） |
| Median peak CMV DNA load (copies/mL) | 54000(1100-630000) |
| Median duration of CMV viremia (days) | 27（8-52） |
| **Type of CMV end-organ disease** | |
| CMV pneumonia | 5（50%） |
| CMV enteritis | 4（40%） |
| CMV gastritis | 1（10%） |
| **Immunosuppression at CMV disease onset** | |
| CNI + ⩾2 agents | 4（40%） |
| **Median time to CMV disease after HSCT (days)** | 151（123-213） |
| **Median time from CMV viremia to CMV disease (days)** | 5（1-87） |
| **Median time from aGVHD to CMV disease (days)** | 101（40-164） |
| **Median NEU at CMV disease (×10⁹/L)** | 0.85（0.4-2） |
| **Median CD4+ count at CMV disease (cells/μL)** | 94（23-252） |
| **Coinfection with other viruses** | |
| EBV | 4（2 EBV pneumonia, 2 EBV enteritis） |
| **Comorbidities at CMV disease** | |
| Overlapping GVHD/cGVHD | 5/1（60%） |
| Poor graft function | 1（10%） |
| Capillary Leak Syndrome | 1（10%） |
| Severe bacterial infection | 3（30%） |
| **Maribavir initiation** | |
| First-line | 5（50%） |
| Second-line treatment | 5（50%） |
| **Median creatinine at maribavir start (μmol/L)** | 50.3（35.4-92） |
| **Median NEU at maribavir start (×10⁹/L)** | 0.85（0.4-2） |
| **Median duration of maribavir** | 4 weeks (2-8 weeks) |
| **Combined therapy with maribavir** | |
| Intravenous immunoglobulin (IVIG) | 10（100%） |
| Foscarnet | 2（20%） |
| **Maribavir-related adverse events** | |
| Dysgeusia | 1（10%） |
| **Late CMV viremia recurrence** | 3（30%） |
| **Median follow-up (days)** | 335 (182-810) |
| **Vital status** | |
| Alive | 9（90%） |
| Dead | 1（10%） |

Copyright Information: (Online) ISSN: 2572-9241
©2026 The Author(s). HemaSphere is published by John Wiley & Sons Ltd on behalf of European Hematology Association. This is an open access Abstract Book distributed under the Attribution-NonCommercial-NoDerivs (CC BY-NC-ND), which allows third parties to download the articles and share them with others as long as they credit the author and the Abstract Book, but they cannot change the content in any way or use them commercially.
Abstract Book Citations: Authors, Title, HemaSphere, 2026;10:(S1):pages. The abstract book DOIs can be found on the "Author Information" page.
Disclaimer: Articles published in the journal HemaSphere exclusively reflect the opinions of the authors. The authors are responsible for all content in their abstracts including accuracy of the facts, statements, citing resources, etc.

### (PB4023) CLINICOPATHOLOGIC FEATURES OF RENAL THROMBOTIC MICROANGIOPATHY AFTER ALLOGENEIC HSCT: ASSOCIATION WITH CHRONIC GVHD AND MACROPHAGE INFILTRATION

**Topic:** 23. Stem cell transplantation - Clinical

Atsushi Uehara*[1], Naoya Fukuda[1], Hajime Sakuma[1], Kazuki Nagao[1], Daichi Terunuma[1], Kosuke Shima[1], Masanori Toho[1], Kentaro Narita[1], Masami Takeuchi[1], Tomo Suzuki[1], Kosei Matsue[1]

[1]*Kameda Medical Center, Kamogawa, Japan;*

### Background

Renal dysfunction after allogeneic hematopoietic stem cell transplantation (allo-HSCT) is an important complication and an independent predictor of mortality. However, knowledge in this area remains limited because etiologies and clinical presentations are heterogeneous and kidney biopsies are rarely performed. In particular, although several reports have suggested an association between post–allo-HSCT renal dysfunction and thrombotic microangiopathy (TMA) and/or GvHD, the underlying mechanisms and pathologic findings remain poorly characterized.

### Aims

We aimed to characterize the renal histopathologic findings in post–allo-HSCT renal dysfunction and to investigate their association with GvHD, treatment response, and renal outcomes.

### Methods

We retrospectively analyzed patients who underwent allo-HSCT at Kameda Medical Center between January 2014 and February 2026 and subsequently received a kidney biopsy. Biopsy specimens were evaluated by light and electron microscopy and immunohistochemistry for CD3, CD4, CD8, CD19, TIA-1, CD68, and CD163.

### Results

A total of 169 patients underwent allo-HSCT during the study period, with 12 (7.1%) subsequently undergoing a kidney biopsy. Renal TMA was the most frequent pathologic diagnosis (n=6), and these cases were analyzed in detail. Other diagnoses included membranous nephropathy, adenovirus nephropathy, drug-induced tubulointerstitial nephritis, IgA nephropathy, focal segmental glomerulosclerosis, and nonspecific tubular injury (one case each).
Among the renal TMA cases, the median age at the time of kidney biopsy was 53 years (range, 34–67). The median time from allo-HSCT to biopsy was 300 days (range, 165–406). Indications for biopsy were progressive renal dysfunction (n=5) and persistent proteinuria (n=1). Donor sources included haploidentical peripheral blood in 4 cases and single-unit umbilical cord blood in 2 cases. The median eGFR at biopsy was 42.5 mL/min/1.73m$^2$ (range, 13.5–73.7). When evaluated using the TMA Harmonization Panel Consensus Recommended Diagnostic Criteria, only 1 of the 6 cases with renal TMA fulfilled the criteria. Four patients had a history of acute GvHD. At the time of kidney biopsy, 5 of the 6 patients had chronic GvHD, involving the skin, gastrointestinal tract, lungs, and liver.
Infiltration of CD3-positive cells into the tubulointerstitium was observed in all cases (**Figure A**), with glomerular infiltration also noted in 3 cases. In 3 cases, infiltration of foam cells was observed in the glomeruli and interstitium. These cells were positive for CD68 and CD163, exhibiting an M2 macrophage-like phenotype (**Figure B–D**).
Patients were managed with the discontinuation of tacrolimus and immunosuppressive therapy including prednisolone, mycophenolate mofetil, and ibrutinib. eGFR improved in one patient treated with prednisolone, whereas no improvement was observed in the remaining cases.

**Copyright Information:** (Online) ISSN: 2572-9241
©2026 The Author(s). HemaSphere is published by John Wiley & Sons Ltd on behalf of European Hematology Association. This is an open access Abstract Book distributed under the Attribution-NonCommercial-NoDerivs (CC BY-NC-ND), which allows third parties to download the articles and share them with others as long as they credit the author and the Abstract Book, but they cannot change the content in any way or use them commercially.
**Abstract Book Citations:** Authors, Title, HemaSphere, 2026;10:(S1):pages. The abstract book DOIs can be found on the “Author Information” page.
**Disclaimer:** Articles published in the journal HemaSphere exclusively reflect the opinions of the authors. The authors are responsible for all content in their abstracts including accuracy of the facts, statements, citing resources, etc.

### Summary/Conclusion

In our cohort with a high rate of kidney biopsy, renal TMA emerged as the predominant pathologic finding, even in the absence of systemic manifestations of TMA. The high frequency of T-cell infiltration in the interstitium and glomeruli, along with the high prevalence of concurrent chronic GvHD, suggests a potential association between renal TMA and GvHD. Furthermore, frequent macrophage infiltration may provide important clues to the pathogenesis and inform optimal treatment strategies.

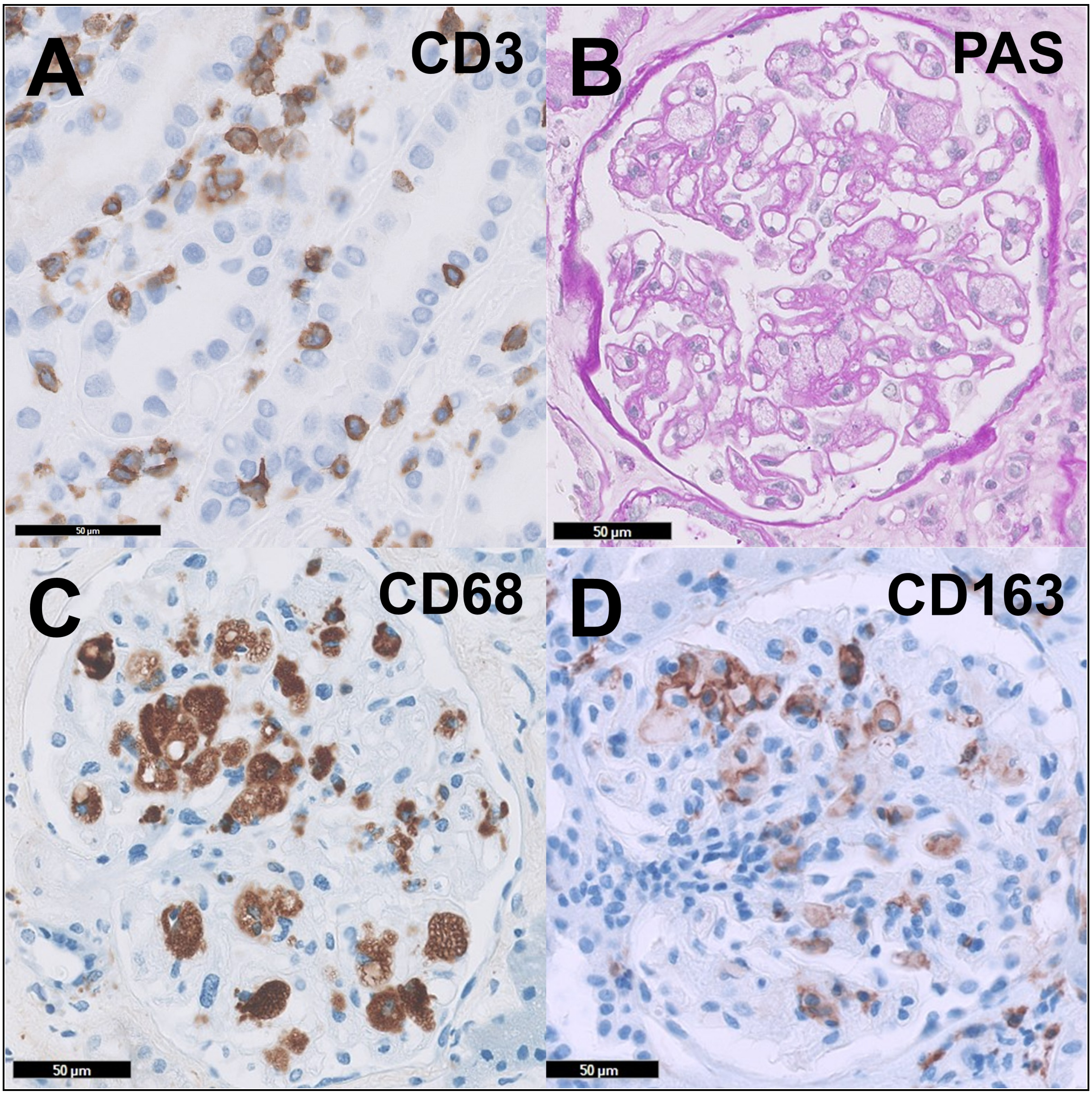

Copyright Information: (Online) ISSN: 2572-9241
©2026 The Author(s). HemaSphere is published by John Wiley & Sons Ltd on behalf of European Hematology Association. This is an open access Abstract Book distributed under the Attribution-NonCommercial-NoDerivs (CC BY-NC-ND), which allows third parties to download the articles and share them with others as long as they credit the author and the Abstract Book, but they cannot change the content in any way or use them commercially.
Abstract Book Citations: Authors, Title, HemaSphere, 2026;10:(S1):pages. The abstract book DOIs can be found on the “Author Information” page.
Disclaimer: Articles published in the journal HemaSphere exclusively reflect the opinions of the authors. The authors are responsible for all content in their abstracts including accuracy of the facts, statements, citing resources, etc.

### (PB4024) DONOR LYMPHOCYTE INFUSION AFTER ALLOGENEIC HEMATOPOIETIC STEM CELL TRANSPLANTATION IN THE POST-CYCLOPHOSPHAMIDE ERA: A SINGLE-CENTER EXPERIENCE.

**Topic:** 23. Stem cell transplantation - Clinical

Amagoia Ruiz Martin*[1], Melissa Torres[1], Leslie González-Pinedo[1], Maria Mar Perera Alvarez[1], Jorge Gayoso Cruz[1], Alejandro Morales Curbelo[1], Cynthia Acosta Fleitas[1], Luisa Guerra Domínguez[1], Carlos Rodriguez Medina[1], Maria Teresa Gomez Casares[1, 2]

*[1]Hospital Universitario de Gran Canaria Doctor Negrín, Hematology, Las Palmas de Gran Canaria, Spain;[2]Universidad de Las Palmas de Gran Canaria, Las Palmas de Gran Canaria, Spain;*

### Background

Relapse remains the principal cause of treatment failure after allogeneic hematopoietic stem cell transplantation (allo-HSCT). In the post-transplant cyclophosphamide (PTCy) era, donor lymphocyte infusion (DLI) represents a key strategy to enhance the graft-versus-leukemia (GVL) effect, although its efficacy and toxicity profile require further clarification. In our institutional transplant program, GVL potentiation is actively pursued through early tapering of immunosuppression and the use of DLI in selected high-risk patients, aiming to restore donor immune surveillance while balancing the risk of graft-versus-host disease (GVHD).

### Methods

We conducted a retrospective analysis of adult patients receiving DLI after allo-HSCT between January 2018 and December 2025 at a single institution. Indications were categorized as prophylactic, pre-emptive (measurable residual disease (MRD) positivity and/or mixed chimerism), or therapeutic (hematologic relapse). All patients received PTCy-based GVHD prophylaxis. We evaluated response, relapse, GVHD incidence, and survival outcomes.

### Results

From 2018 to 2025, a total of 22 patients with different hematological malignancies received DLI. The median age was 53.6 years and 54.5% were male. The different underlying diseases were: 16 Acute Myeloid Leukemia (73%), 4 Acute Lymphoid Leukemia (18%), 1 Myelodysplastic Syndrome (4.5%) and 1 T-cell Non-Hodgkin Lymphoma (4.5%). Patients received allo-HSCT from related HLA-identical (35%), haploidentical (45%) and unrelated donors (20%). With respect to disease status prior HSCT, 4 patients had persistent disease (18%), 18 complete response (CR), 11 of them presented negative minimal residual disease (MRD) and the other 7 had positive MRD (molecular, cytogenetics or immunophenotypic markers). 1 patient with MDS had not received any previous treatment. On day +30 post-alloHSCT, all presented CR, 8 of them complete chimerism (CC) and the other 14 mixed chimerism (MC). The median time to DLI indication was 203 days (95-570). The median DLI dose was 2 (1-4). In the treatment group, 75% received DLI in combination with chemotherapy (figure 1) and 1 in monotherapy. In the prophylaxis group, 25% received it in combination with Sorafenib and the other 75% in monotherapy. In the preemptive group, 45% received it in combination with Azacitidine and 55% in monotherapy.

Related to response after DLI over the 17 patients with myeloid neoplasm; in treatment group (n=2), all patients were refractory. In preemptive group (n=7): 1/7 achieved CR, negative MRD and CC, 4/7 had negative MRD and MC. In the prophylaxis group (high risk patients)( (n=8), 3/8 remained in CR, negative MDR and CC.

Therefore, 47% of all patients achieved CR with negative MRD and the other 53% presented refractoriness. GVHD rate was 65%. After a median time of follow-up of 20 months, 53% are in remission. 9 patients died, 7 of them due to relapse (77%) and the other 2 because of infections.

**Copyright Information:** (Online) ISSN: 2572-9241
©2026 The Author(s). HemaSphere is published by John Wiley & Sons Ltd on behalf of European Hematology Association. This is an open access Abstract Book distributed under the Attribution-NonCommercial-NoDerivs (CC BY-NC-ND), which allows third parties to download the articles and share them with others as long as they credit the author and the Abstract Book, but they cannot change the content in any way or use them commercially.
**Abstract Book Citations:** Authors, Title, HemaSphere, 2026;10:(S1):pages. The abstract book DOIs can be found on the "Author Information" page.
**Disclaimer:** Articles published in the journal HemaSphere exclusively reflect the opinions of the authors. The authors are responsible for all content in their abstracts including accuracy of the facts, statements, citing resources, etc.

### Summary/Conclusion

In our cohort, remission was achieved in approximately 40–50% of patients, suggesting that within the PTCy platform, DLI is feasible and immunologically active, particularly in the prophylactic and MRD-positive settings, with manageable toxicity. However, response durability was limited, especially in overt relapse settings, highlighting the need for improved immune monitoring and combinatorial strategies to optimize graft-versus-leukemia (GVL) effects while minimizing graft-versus-host disease (GVHD) risk.

**Figure 1. Transplant features and outcomes of patients receiving DLI.**

| Cases | Diagnosis | Donor type | Pre-DLI situation | DLI indication | DLI n | Max DLI dose* | Adjunctive Therapy | Response | GVHD | OS | Death cause |
|---|---|---|---|---|---|---|---|---|---|---|---|
| 1 | AML | HAPLO | CR | PROPHYLACTIC | 3 | 1x10^7 | - | CR | YES/a | 7y+6m | - |
| 2 | AML | HAPLO | CR | PROPHYLACTIC | 3 | 1x10^8 | - | CR | YES/a | 2y | RELAPSE |
| 3 | AML | HLA-id | CR | PROPHYLACTIC | 1 | 1x10^5 | - | CR | - | 4y+5m | - |
| 4 | AML | HAPLO | CR | PROPHYLACTIC | 3 | 1x10^7 | Sorafenib | CR | YES/c | 5y+3m | - |
| 5 | AML | DNE 10 | CR | PROPHYLACTIC | 1 | 1x10^6 | - | CR | - | 8m | - |
| 6 | AML | HLA-id | CR | PROPHYLACTIC | 4 | 1x10^7 | - | CR | - | 4y+10m | - |
| 7 | ALL | HLA-id | CR | PROPHYLACTIC | 3 | 1x10^7 | - | CR | - | 3y+4m | - |
| 8 | AML | HAPLO | CR | PROPHYLACTIC | 4 | 1x10^7 | Sorafenib | CR | - | 1y+9m | RELAPSE |
| 9 | AML | HAPLO | CR | PROPHYLACTIC | 3 | 1x10^7 | - | CR | YES/c | 4y+9m | - |
| 10 | AML | HAPLO | MC | PREEMPTIVE | 2 | 1x10^7 | Azacitidine | CR | YES/a | 8m | INFECTION |
| 11 | AML | HLA-id | MC | PREEMPTIVE | 1 | 1x10^6 | Azacitidine | CR | YES/a | 1y+6m | INFECTION |
| 12 | AML | HLA-id | MC | PREEMPTIVE | 2 | 1x10^7 | - | CR | YES/c | 5y+4m | RELAPSE |
| 13 | AML | HLA-id | MC | PREEMPTIVE | 1 | 1x10^6 | Azacitidine | - | - | 2m | INFECTION |
| 14 | AML | HAPLO | MC | PREEMPTIVE | 1 | 1x10^6 | - | CR | YES/c | 6m | - |
| 15 | AML | DNE 10 | MC | PREEMPTIVE | 1 | 1x10^6 | Azacitidine | CR | - | 2m | - |
| 16 | MDS | DNE 10 | MC | PREEMPTIVE | 4 | 5x10^7 | - | - | - | 3m | - |
| 17 | ALL | HAPLO | MC | PREEMPTIVE | 1 | 1x10^5 | - | RELAPSE | YES/a | 5m | RELAPSE |
| 18 | ALL | DNE 10 | MC | PREEMPTIVE | 3 | 1x10^7 | - | RELAPSE | - | 8m | - |
| 19 | AML | HLA-id | RELAPSE | TREATMENT | 2 | 1x10^7 | FLAG-IDA | RELAPSE | - | 4m | RELAPSE |
| 20 | AML | HLA-id | RELAPSE | TREATMENT | 1 | 1x10^6 | Azacitidine | RELAPSE | - | 2m | RELAPSE |
| 21 | t-NHL | HAPLO | RELAPSE | TREATMENT | 3 | 1x10^7 | - | CR | YES/a | 5y | - |
| 22 | ALL | HAPLO | RELAPSE | TREATMENT | 3 | 1x10^7 | Venetoclax + Bendamustine | CR | YES/a | 2y+6m | - |

AML, acute myeloid leukemia; ALL, acute lymphoblastic leukemia; MDS, myelodysplastic syndrome; MSD, matched sibling donor; Haplo, haploidentical HSCT; MUD 10/10, matched unrelated donor; YES/a, acute GVHD, YES/c, chronic GVHD; MC, mixed chimerism; CR, complete remission; PD, progressive disease; OS, overall survival; y+m, years and months; Max DLI dose, maximum DLI dose in $CD3^+$ cells/kg.

**Copyright Information:** (Online) ISSN: 2572-9241
©2026 The Author(s). HemaSphere is published by John Wiley & Sons Ltd on behalf of European Hematology Association. This is an open access Abstract Book distributed under the Attribution-NonCommercial-NoDerivs (CC BY-NC-ND), which allows third parties to download the articles and share them with others as long as they credit the author and the Abstract Book, but they cannot change the content in any way or use them commercially.
**Abstract Book Citations:** Authors, Title, HemaSphere, 2026;10:(S1):pages. The abstract book DOIs can be found on the "Author Information" page.
**Disclaimer:** Articles published in the journal HemaSphere exclusively reflect the opinions of the authors. The authors are responsible for all content in their abstracts including accuracy of the facts, statements, citing resources, etc.

### (PB4025) IMPACT OF PREEMPTIVE PCR MONITORING ON CYTOMEGALOVIRUS REACTIVATION OUTCOMES FOLLOWING ALLOGENEIC HSCT: A HIGH-SEROPREVALENCE SETTING EXPERIENCE

**Topic:** 23. Stem cell transplantation - Clinical

Rofaida Aroua[1], Meriem Abdennouri[1], Rania Rahmouni[1], Farida Harieche[1], Malek Benakli*[1]

[1]*Hematology and Bone Marrow Transplantation department, Pierre and Marie Curie Center, Algiers, Algeria;*

### Background

Cytomegalovirus (CMV) reactivation remains a significant complication following allogeneic hematopoietic stem cell transplantation (allo-HSCT), particularly in regions with near-universal CMV seroprevalence. We evaluated the effectiveness of a preemptive monitoring strategy on clinical outcomes in this specific epidemiological context.

### Methods

We conducted a retrospective analysis of 126 consecutive patients who underwent allo-HSCT at our center between January 2024 and December 2024. CMV viral load was monitored weekly using quantitative PCR (qPCR), with preemptive antiviral therapy initiated at a threshold of >1000 copies/mL. Survival analysis was performed using the Kaplan-Meier method, and prognostic factors were compared using the log-rank test.

### Results

The cohort included 126 patients (median age: 23 years; range: 3–67). CMV seroprevalence was 98%, with D+/R+ (Donor+/Recipient+) being the predominant pair. The crude incidence of CMV reactivation was 40.5% (n=51), while the cumulative incidence reached 77.4% at 18 months. The median time to first reactivation was 41 days (IQR: 35–55). Despite these high reactivation rates, CMV infection had no significant impact on overall survival (OS) (p=0.79). The 18-month OS for the entire cohort was 81.0%. Acute graft-versus-host disease (aGVHD) was identified as the only significant negative prognostic factor (p<0.001), reducing survival from 88% to 54%. A strong association was observed between CMV reactivation and aGVHD (60.6% vs. 39.4%). Notably, no cases of symptomatic CMV organ disease were documented.

### Summary/Conclusion

In settings with near-universal CMV seropositivity, weekly preemptive qPCR monitoring effectively neutralizes the survival impact of CMV reactivation despite its high incidence. This strategy successfully prevents progression to symptomatic CMV disease. While CMV management is optimized by this approach, acute GVHD remains the primary barrier to survival, necessitating further focus on its prevention and control.

**Copyright Information:** (Online) ISSN: 2572-9241
©2026 The Author(s). HemaSphere is published by John Wiley & Sons Ltd on behalf of European Hematology Association. This is an open access Abstract Book distributed under the Attribution-NonCommercial-NoDerivs (CC BY-NC-ND), which allows third parties to download the articles and share them with others as long as they credit the author and the Abstract Book, but they cannot change the content in any way or use them commercially.
**Abstract Book Citations:** Authors, Title, HemaSphere, 2026;10:(S1):pages. The abstract book DOIs can be found on the “Author Information” page.
**Disclaimer:** Articles published in the journal HemaSphere exclusively reflect the opinions of the authors. The authors are responsible for all content in their abstracts including accuracy of the facts, statements, citing resources, etc.

### (PB4026) ENGRAFTMENT SYNDROME IN AUTOLOGOUS HAEMATOPOIETIC STEM CELLTRANSPLANTATION: INCIDENCE, ASSOCIATED FACTORS AND CLINICAL IMPACT

**Topic:** 23. Stem cell transplantation - Clinical

Alberto Porro Camarero[1], Alejandro Lada Colunga[1], Marco Hernández Martín[1], Ana Pilar Gonzalez Rodríguez[1], Ana Julia Gonzalez Huerta[1], Pilar Palomo Moraleda[1], Joud Zanabili Al Sibai*[1], Soledad Gonzalez Rodríguez[1], Lucia Morais Brais[1], Angel Ramírez Payer[1], Carmen Loredo Álvarez[1], Laura Gonzalez Alvarez[1], Nicolas DE Lucas Torres[1], MacArena Castillo Godoy[1]

[1]*HOSPITAL UNIVERSITARIO CENTRAL DE ASTURIAS, oviedo, Spain;*

### Background

Engraftment syndrome (ES) is an inflammatory complication of autologous haematopoietic stem cell transplantation (ASCT) occurring around
neutrophil recovery and characterised by non-infectious fever and capillary leak manifestations

### Aims

To evaluate incidence, associated factors and clinical impact of engraftment syndrome in ASCT recipients.

### Methods

We performed a single-centre, retrospective study including 130 consecutive ASCT episodes performed between 2023 and 2025. Collected
variables included demographics, underlying disease, disease status, number of prior treatment lines, HCT-CI score, conditioning regimen and graft
characteristics (CD34+ ×10⁶/kg, infused volume and viability). Pre-ASCT laboratory parameters were C-reactive protein (CRP), lactate dehydrogenase,
creatinine and platelet count. Variables related to ES were collected according to the Maiolino criteria and included non-infectious fever (≥38°C without
microbiological documentation or response to antimicrobials within ±24 h of engraftment), weight gain >3%, maculopapular rash, non-infectious diarrhoea
and non-infectious pulmonary infiltrates. Additional data included CRP and procalcitonin at fever, time to neutrophil recovery, use or withdrawal of
filgrastim, steroid treatment, length of stay, ICU admission and day +100 mortality. Statistical analyses were performed using SPSS. Comparisons between
ES and non-ES episodes were performed using chi-square or Fisher's exact test for categorical variables and Mann–Whitney U test for continuous variables.

**Copyright Information:** (Online) ISSN: 2572-9241
©2026 The Author(s). HemaSphere is published by John Wiley & Sons Ltd on behalf of European Hematology Association. This is an open access Abstract Book distributed under the Attribution-NonCommercial-NoDerivs (CC BY-NC-ND), which allows third parties to download the articles and share them with others as long as they credit the author and the Abstract Book, but they cannot change the content in any way or use them commercially.
**Abstract Book Citations:** Authors, Title, HemaSphere, 2026;10:(S1):pages. The abstract book DOIs can be found on the "Author Information" page.
**Disclaimer:** Articles published in the journal HemaSphere exclusively reflect the opinions of the authors. The authors are responsible for all content in their abstracts including accuracy of the facts, statements, citing resources, etc.

**Results**

Median age at ASCT was 61 years (IQR 51–66), and 68% of episodes occurred in male patients. Underlying diseases were multiple myeloma
(50%), B-cell non-Hodgkin lymphoma (21%) and other diagnoses (T-cell non-Hodgkin lymphoma, Hodgkin lymphoma, primary CNS lymphoma, amyloidosis
[5 cases], plasma cell leukaemia, solid tumours and non-malignant diseases); amyloidosis showed a significantly higher incidence of ES compared with
other diagnoses (p≈0.03). Median HCT-CI was 2 (IQR 0–3). Median CD34+ dose was 3.98 $\times 10^6$/kg (IQR 3.00–5.27), infused volume 408 mL (IQR 288–532)
and graft viability 84% (IQR 76–89). Median length of stay was 16 days (IQR 14–19). Clinical ES was diagnosed in 22/130 episodes (16.9%), while 56/130
(43.1%) fulfilled Maiolino criteria. Compared with non-ES episodes, ES was associated with higher pre-ASCT creatinine (p≈0.01) and a trend towards higher
HCT-CI (p≈0.08); CD34+ dose (median 4.40 vs 3.93 $\times 10^6$/kg; p≈0.64) and infused volume (median 442 vs 404 mL; p≈0.29) were similar between groups.
ES typically developed around day +10 and was characterised by non-infectious fever (100%), weight gain >3% (~77%), non-infectious diarrhoea (~60%),
rash (~33%) and non-infectious pulmonary infiltrates (~25%). CRP at fever was higher in ES (p≈0.01), whereas procalcitonin was comparable. Length of
stay was longer in ES (median ~18 vs 16 days; p≈0.02), with no significant differences in ICU admission or day +100 mortality. Among episodes fulfilling
criteria but not clinically labelled as ES (35/130; 26.9% of the cohort), an alternative diagnosis was documented in 68.6% and 11.4% improved after
filgrastim withdrawal.

**Summary/Conclusion**

In our single-centre ASCT cohort (2023–2025), engraftment syndrome defined by the Maiolino criteria was a frequent complication associated
with increased inflammatory response and longer hospitalisation, without a clear impact on early mortality. Amyloidosis appeared to be associated with a
particularly high risk of ES.

**Copyright Information:** (Online) ISSN: 2572-9241
©2026 The Author(s). HemaSphere is published by John Wiley & Sons Ltd on behalf of European Hematology Association. This is an open access Abstract Book distributed under the Attribution-NonCommercial-NoDerivs (CC BY-NC-ND), which allows third parties to download the articles and share them with others as long as they credit the author and the Abstract Book, but they cannot change the content in any way or use them commercially.
**Abstract Book Citations:** Authors, Title, HemaSphere, 2026;10:(S1):pages. The abstract book DOIs can be found on the "Author Information" page.
**Disclaimer:** Articles published in the journal HemaSphere exclusively reflect the opinions of the authors. The authors are responsible for all content in their abstracts including accuracy of the facts, statements, citing resources, etc.

### (PB4027) THE APPLICATION OF VON WILLEBRAND FACTOR IN THE PRE-EMPTIVE TREATMENT OF TRANSPLANT-ASSOCIATED THROMBOTIC MICROANGIOPATHY:A REAL-WORLD EXPERIENCE FROM CHINESE SINGLE-CENTER

**Topic:** 23. Stem cell transplantation - Clinical

Chengwei Luo[1], Jianyu Weng*[1], Suijing Wu[1], Wei Ling[1], Xiaomei Chen[1], Lisi Huang[1]

*[1]Guangdong Provincial People´s Hospital, Department of Hematology, Guangzhou, Guangdong, China;*

### Background

Transplant-associated thrombotic microangiopathy (TA-TMA) is a fatal complication after allogeneic hematopoietic stem cell transplantation (allo-HSCT).Our previous report suggested Von Willebrand factor(vWF) is a useful predictor and prognostic measure for TA-TMA, which may help clinicians identify and manage this life-threatening disease earlier.The corresponding sensitivity of vWF(≥325%) in the diagnosis of TA-TMA was 86.96%, and the specificity of vWF (≥325%) in the diagnosis of TA-TMA was 67.27%.Patients with ≥325% vWF had a higher 2-year cumulative hazard of TA-TMA and a lower 2-year survi val compared with those with <325% vWF. However, This needs to be verified whether vWF can intervene the pre-emptive treatment of TA-TMA.

### Aims

This pilot study is to evaluate the efficacy of Von Willebrand Factor in the pre-emptive treatment of Transplant-Associated Thrombotic Microangiopathy

### Methods

In this retrospective single-center study, a total of 106 consecutive patients underwent allo-HSCT at Guang Dong Provincial People's Hospital between January 2019 and December 2021 were enrolled.Low-molecular-weight heparin combined with plasma exchange were given in the patients with ≥325% vWF.Those patients with <325% vWF were given watching and waiting.The purpose of this study is to evaluate the efficacy of Von Willebrand Factor in the pre-emptive treatment of Transplant-Associated Thrombotic Microangiopathy.

### Results

The overall incidence of TA-TMA in our patient population was 14.2% (15/106) at a median 55 days post-transplant.The median follow-up was 686 days (range: 11 to 1173 days).There were 55 patients with vWF≥325% and 51 patients with vWF<325%.After the pre-emptive treatment, Patients with ≥325% vWF had a lower 2-year hazard of TA-TMA(18% vs 53.1%; p<0.001) and a higher 2-year survival (85.5% vs 32.1%; p<0.001) compared with historical control group.After the pre-emptive treatment,Patients with TA-TMA had significantly higher 2-year survival compared with historical control group (80% vs 34.8%; p< 0.001). After the pre-emptive treatment,Patients with ≥325% vWF had a same 2-year survival (85.5% vs 84.3%; P =0.933) and hazard of TA-TMA (18% vs 9.8%; P=0.143) compared with those with <325% vWF(Figure 1 and 2)

### Summary/Conclusion

The preliminary results of this study showed Von Willebrand factor may be an effective intervention indicators, which can guide pre-emptive treatment of transplant-associated thrombotic microangiopathy. Whether Von Willebrand factor can be used as a biological marker for early treatment of TA-TMA needs to be confirmed by further randomized controlled trials.

**Copyright Information:** (Online) ISSN: 2572-9241
©2026 The Author(s). HemaSphere is published by John Wiley & Sons Ltd on behalf of European Hematology Association. This is an open access Abstract Book distributed under the Attribution-NonCommercial-NoDerivs (CC BY-NC-ND), which allows third parties to download the articles and share them with others as long as they credit the author and the Abstract Book, but they cannot change the content in any way or use them commercially.
**Abstract Book Citations:** Authors, Title, HemaSphere, 2026;10:(S1):pages. The abstract book DOIs can be found on the "Author Information" page.
**Disclaimer:** Articles published in the journal HemaSphere exclusively reflect the opinions of the authors. The authors are responsible for all content in their abstracts including accuracy of the facts, statements, citing resources, etc.

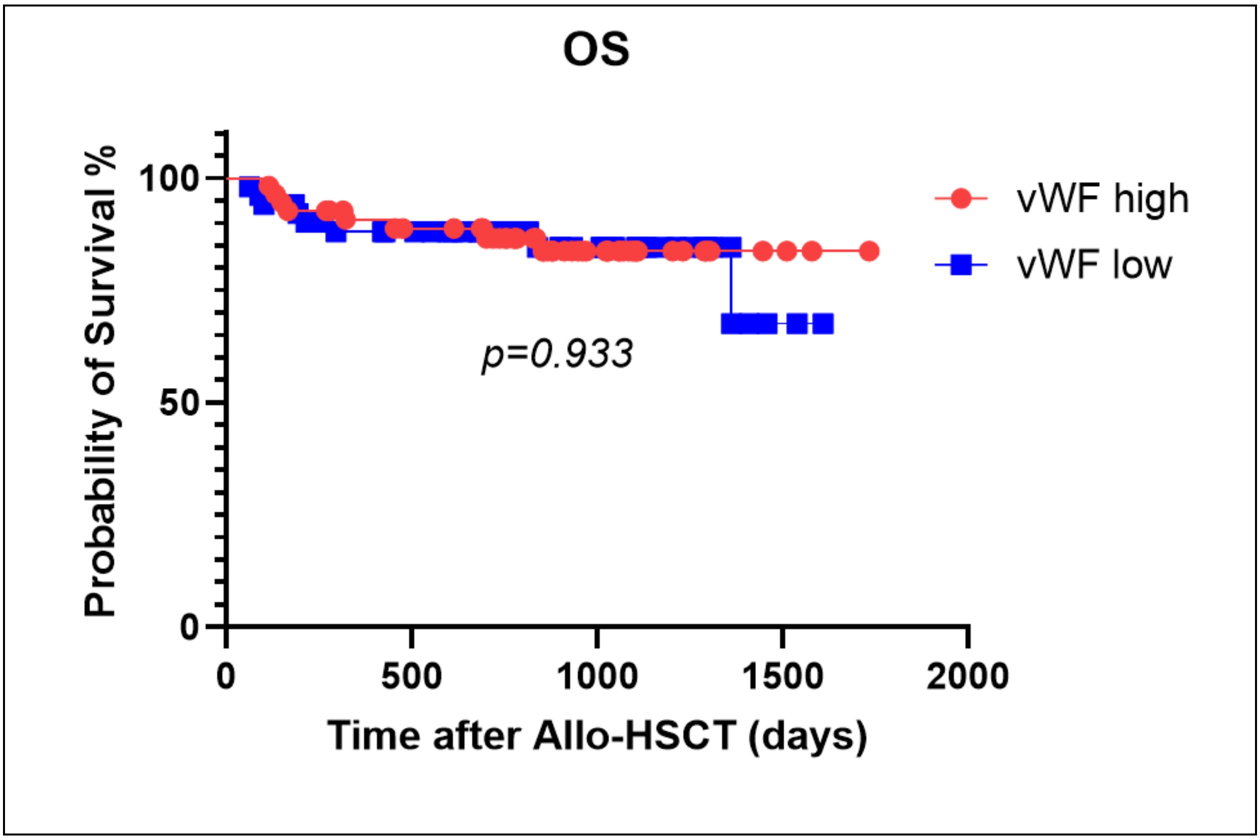

**Copyright Information:** (Online) ISSN: 2572-9241
©2026 The Author(s). HemaSphere is published by John Wiley & Sons Ltd on behalf of European Hematology Association. This is an open access Abstract Book distributed under the Attribution-NonCommercial-NoDerivs (CC BY-NC-ND), which allows third parties to download the articles and share them with others as long as they credit the author and the Abstract Book, but they cannot change the content in any way or use them commercially.
**Abstract Book Citations:** Authors, Title, HemaSphere, 2026;10:(S1):pages. The abstract book DOIs can be found on the "Author Information" page.
**Disclaimer:** Articles published in the journal HemaSphere exclusively reflect the opinions of the authors. The authors are responsible for all content in their abstracts including accuracy of the facts, statements, citing resources, etc.

### (PB4028) PSYCHIATRIC COMPLICATIONS AFTER HEMATOPOIETIC STEM CELL TRANSPLANTATION AND THEIR ASSOCIATION WITH EARLY CLINICAL OUTCOMES

**Topic:** 23. Stem cell transplantation - Clinical

Dana Taub*[1], Manuela Estrada-Schweineberg[2], Juliana Pardo[2], Maria Uchima-Vera[2], Felix Duarte[1], Elena Velasquez[1], Juliana Gomezese[3], Juliana Castro[1], Paula Perez[4], Andrea Pantoja[4], Laura Roa[4], Carmenza Camargo[2], Karen Milena Chaves Garcia[2], Claudia Agudelo-López[1, 2], Beatriz Wills[1, 2], Guillermo Enrique Quintero Vega[1, 2], Soraya Aparicio[1, 2]

*[1]Universidad de los Andes, Facultad de Medicina, Bogota, Colombia;[2]Fundación Santa Fe de Bogotá, Colombia, Colombia; [3]Universidad de la Sabana, Bogotá, Colombia; [4]Universidad del Bosque, Bogotá, Colombia;*

### Background

Hematopoietic stem cell transplantation (HSCT) induces profound inflammatory and psychological stress. Although acute psychiatric complications are common, their timing and association with early transplant-related outcomes remain insufficiently characterized in real-world cohorts.

### Aims

To characterize psychiatric complications within the first 100 days after HSCT and to evaluate their association with early transplant-related outcomes.

### Methods

We conducted a retrospective cohort study of adult patients undergoing autologous or allogeneic HSCT between 2022 and 2025 at a quaternary center. Psychiatric complications were defined as clinically documented psychiatric syndromes requiring formal psychiatric evaluation and/or psychotropic treatment. Events were categorized according to transplant phase. Primary outcome was neuropsychiatric complication; secondary outcomes included transplant-related toxicities and 100-day mortality. Multivariable logistic regression models were constructed adjusting for age, sex and transplant type.

**Copyright Information:** (Online) ISSN: 2572-9241
©2026 The Author(s). HemaSphere is published by John Wiley & Sons Ltd on behalf of European Hematology Association. This is an open access Abstract Book distributed under the Attribution-NonCommercial-NoDerivs (CC BY-NC-ND), which allows third parties to download the articles and share them with others as long as they credit the author and the Abstract Book, but they cannot change the content in any way or use them commercially.
**Abstract Book Citations:** Authors, Title, HemaSphere, 2026;10:(S1):pages. The abstract book DOIs can be found on the "Author Information" page.
**Disclaimer:** Articles published in the journal HemaSphere exclusively reflect the opinions of the authors. The authors are responsible for all content in their abstracts including accuracy of the facts, statements, citing resources, etc.

### Results

A total of 110 patients were included (median age 55.5 years; IQR 46–63; 51.8% female). 78.2% underwent autologous HSCT. Multiple myeloma was the most frequent diagnosis (44.5%), followed by aggressive lymphoma (30.9%) and acute myeloid leukemia (10%).

Psychiatric symptoms occurred in 69 patients (62.7%). Among these, 30 (43.5%) received a de novo psychiatric diagnosis. Adjustment disorder with anxious and/or depressive features (19 patients, 17.3%) was the predominant diagnosis, followed by depressive disorder (3.6%) and delirium (2.7%). Psychotropic treatment was initiated in 34 patients (31.9%). Symptoms most frequently emerged during mobilization (39.1%) and early/deep aplasia (31.4% combined).

In multivariable analysis adjusting for age and transplant type, neutropenia during the recovery (OR 2.90; 95% CI 1.01–8.36; p=0.048) and mucositis (OR 2.35; 95% CI 1.03–5.38; p=0.043) were independently associated with psychiatric complications. Nine patients (8.2%) died within 100 days. Overall psychiatric complications were not associated with mortality. However, adjustment disorder was independently associated with increased mortality after adjustment for age, sex, ans transplant type (OR 3.73 95% CI 1.10–12.59; p=0.034).

| Table 1. Psychiatric Symptoms and Timing After HSCT | |
|---|---|
| **Psychiatric Symptoms**<br>(Percentages exceed 100% because patients could present with more than one symptom) | |
| Anxious affect | 38.2% |
| Prevalent worry | 26.4% |
| Depressed mood | 19.1% |
| Sleep disturbances | 8.2% |
| Suicidal ideation | 0.9% |
| **Transplant Phase of Symptom Onset (n=69)** | |
| Pre-transplant (mobilization) | 39,1% |
| Conditioning | 18,8% |
| Early aplasia (≤ day +5) | 17,4% |
| Deep aplasia (day +5 to engraftment) | 14,5% |
| Engraftment phase | 2,9% |
| Early post-engraftment (until discharge) | 7,2% |

### Summary/Conclusion

Psychiatric complications are frequent during early HSCT and clustered around periods of heightened transplant-related toxicity, including neutropenia and mucositis. While overall psychiatric symptoms were not associated with 100-day mortality, adjustment disorder with depressive and/or anxious mood was independently associated with increased early mortality after adjustment for age, sex, and transplant type.  Although causality cannot be inferred, this finding identifies early psychiatric manifestations as potential clinical markers of transplant-related risk. Prospective studies incorporating structured neuropsychiatric assessment are warranted.

**Copyright Information:** (Online) ISSN: 2572-9241
©2026 The Author(s). HemaSphere is published by John Wiley & Sons Ltd on behalf of European Hematology Association. This is an open access Abstract Book distributed under the Attribution-NonCommercial-NoDerivs (CC BY-NC-ND), which allows third parties to download the articles and share them with others as long as they credit the author and the Abstract Book, but they cannot change the content in any way or use them commercially.
**Abstract Book Citations:** Authors, Title, HemaSphere, 2026;10:(S1):pages. The abstract book DOIs can be found on the "Author Information" page.
**Disclaimer:** Articles published in the journal HemaSphere exclusively reflect the opinions of the authors. The authors are responsible for all content in their abstracts including accuracy of the facts, statements, citing resources, etc.

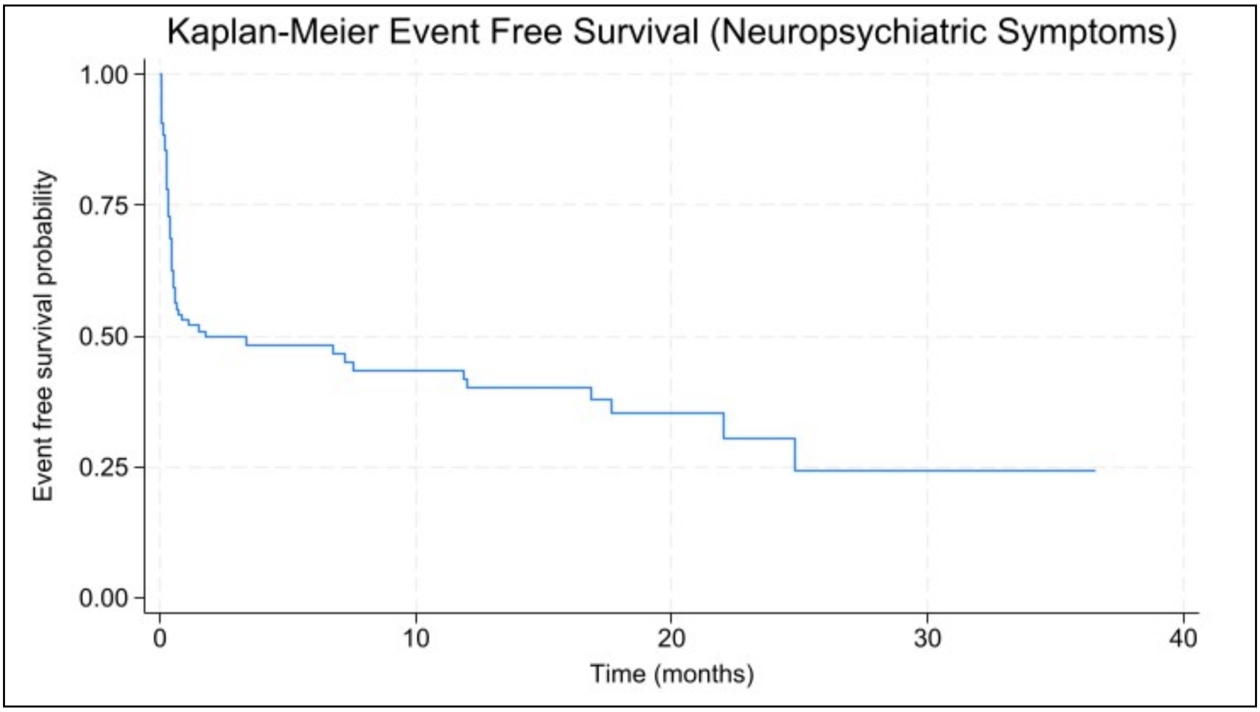

**Copyright Information:** (Online) ISSN: 2572-9241
©2026 The Author(s). HemaSphere is published by John Wiley & Sons Ltd on behalf of European Hematology Association. This is an open access Abstract Book distributed under the Attribution-NonCommercial-NoDerivs (CC BY-NC-ND), which allows third parties to download the articles and share them with others as long as they credit the author and the Abstract Book, but they cannot change the content in any way or use them commercially.
**Abstract Book Citations:** Authors, Title, HemaSphere, 2026;10:(S1):pages. The abstract book DOIs can be found on the "Author Information" page.
**Disclaimer:** Articles published in the journal HemaSphere exclusively reflect the opinions of the authors. The authors are responsible for all content in their abstracts including accuracy of the facts, statements, citing resources, etc.

### (PB4029) RESELECT2: A TRIAL EVALUATING ELTROMBOPAG AND RUXOLITINIB FOR THE TREATMENT OF POOR GRAFT FUNCTION FOLLOWING ALLOGENEIC STEM CELL TRANSPLANTATION AND RELAPSED/REFRACTORY SEVERE APLASTIC ANAEMIA

**Topic:** 23. Stem cell transplantation - Clinical

Daniel North[1, 2], Ashvind Prabahran*[1, 3, 4, 5], Syed Asif Ali Shah[1, 6], Digsu Koye[2], Sabine Braat[2], Jessica Klass[1], Alex Rivalland[1], Duncan Purtill[7], Safia Belbachir[7], Joanne Davis[1], Lynette Chee[1, 8], Rachel Koldej[1, 2], David Ritchie[1, 2, 8]

*[1]Royal Melbourne Hospital, Melbourne, Australia; [2]University of Melbourne, Melbourne, Australia; [3]Monash Health, Melbourne, Australia; [4]Austin Health, Melbourne, Australia; [5]Monash University, Melbourne, Australia; [6]St. Vincent's Hospital, Melbourne, Australia; [7]Fiona Stanley Hospital, Perth, Australia; [8]Peter MacCallum Cancer Centre, Melbourne, Australia;*

### Background

Poor graft function (PGF) following allogeneic stem cell transplantation (alloSCT) is characterised by persistent cytopenias despite full donor chimerism. PGF impacts 10-15% of alloSCT patients and is associated with a high burden of morbidity and mortality. PGF lacks an established standard of care, calling for the development of novel strategies. Prior studies have evaluated treatment of PGF with eltrombopag, a TPO mimetic used in the treatment of severe aplastic anaemia (SAA), with responses observed in some patients. Ruxolitinib is a JAK inhibitor that suppresses the production of cytokines in T and NK cells, used in the treatment of graft-vs-host disease (GVHD). While in the context of GVHD cytopenias have been associated with use of ruxolitinib, preclinical studies have demonstrated that ruxolitinib improves haematopoietic function in murine models of GVHD and immune-mediated bone marrow failure. RESELECT2 is an ongoing multicentre investigator-initiated single-arm clinical trial evaluating combination eltrombopag and ruxolitinib for the treatment of PGF following alloSCT and relapsed/refractory SAA. After recruitment of the first 10 patients, a protocol defined interim safety analysis was performed.

### Aims

To report the interim safety analysis from an ongoing study evaluating the use of combination eltrombopag and ruxolitinib for the treatment of PGF following alloSCT and relapsed/refractory SAA.

### Methods

Eligible participants include patients with PGF defined as ≥2 lineage cytopenias (Hb ≤ 80; plts ≤30 to D40-60, or ≤50 post D60; or neutropenia requiring GCSF support), and ≥95% donor chimerism. SAA patients refractory to 1$^{st}$ line immune suppression and ineligible for alloSCT are also included. Study treatment involves ruxolitinib 10mg BD and eltrombopag at a response adapted dose of 50-150mg daily, for 12 weeks. Primary endpoints include rate of complete response (CR) at 12 weeks defined as transfusion/GCSF independence with neutrophils >1.5x10$^9$/L and platelets >100x10$^9$/L and incidence of adverse events (AEs) on study. A total of 20 participants will be recruited, and outcomes will be compared with a historical cohort of patients from the primary study site who fulfil trial inclusion criteria.

**Copyright Information:** (Online) ISSN: 2572-9241
©2026 The Author(s). HemaSphere is published by John Wiley & Sons Ltd on behalf of European Hematology Association. This is an open access Abstract Book distributed under the Attribution-NonCommercial-NoDerivs (CC BY-NC-ND), which allows third parties to download the articles and share them with others as long as they credit the author and the Abstract Book, but they cannot change the content in any way or use them commercially.
**Abstract Book Citations:** Authors, Title, HemaSphere, 2026;10:(S1):pages. The abstract book DOIs can be found on the "Author Information" page.
**Disclaimer:** Articles published in the journal HemaSphere exclusively reflect the opinions of the authors. The authors are responsible for all content in their abstracts including accuracy of the facts, statements, citing resources, etc.

**Results**

Between 30/01/2025 and 30/01/2026 10 participants were recruited across 2 Australian sites. These included 9 patients with PGF, and 1 with SAA. After a mean exposure to trial therapy of 53 days, 15 grade 2-4 non-haematological AEs were experienced by 8 patients. These included 1 grade 3 osteoporotic fracture and 2 grade 3 CMV reactivations, with no grade 4 events. Haematological adverse events in the context of ruxolitinib were uncommon. One patient required dose reduction of ruxolitinib due to emergent grade 4 neutropenia, and one patient ceased ruxolitinib due to persistent pre-existing grade 4 thrombocytopenia. One patient experienced a transient reduction from grade 3 to grade 4 thrombocytopenia, which resolved with no dose modifications prior to normalisation of platelet count.

Four patients with PGF have completed 12 weeks of treatment and are evaluable for the primary endpoint. While CR at 12 weeks was not observed in any patients, 3 of 4 achieved independence from transfusion/GCSF support. No response was observed in the patient with SAA.

**Summary/Conclusion**

Interim results from the RESELECT2 trial demonstrate that combination eltrombopag and ruxolitinib has a favourable safety profile. Efficacy will be evaluated following trial completion.

**Copyright Information:** (Online) ISSN: 2572-9241
©2026 The Author(s). HemaSphere is published by John Wiley & Sons Ltd on behalf of European Hematology Association. This is an open access Abstract Book distributed under the Attribution-NonCommercial-NoDerivs (CC BY-NC-ND), which allows third parties to download the articles and share them with others as long as they credit the author and the Abstract Book, but they cannot change the content in any way or use them commercially.
**Abstract Book Citations:** Authors, Title, HemaSphere, 2026;10:(S1):pages. The abstract book DOIs can be found on the "Author Information" page.
**Disclaimer:** Articles published in the journal HemaSphere exclusively reflect the opinions of the authors. The authors are responsible for all content in their abstracts including accuracy of the facts, statements, citing resources, etc.

### (PB4030) EFFECTS OF COMPREHENSIVE NURSING CARE MODEL ON COMPLICATIONS AND QUALITY OF LIFE IN PATIENTS UNDERGOING HEMATOPOIETIC STEM CELL TRANSPLANTATION

**Topic:** 23. Stem cell transplantation - Clinical

Yuqing Liu*[1], Mingyue Xu[1], Yuqiu Liu[1], Xianjing Zhang[1], Erlie Jiang[1]

[1] *Institute of Hematology & Blood Diseases Hospital, Chinese Academy of Medical Sciences & Peking Union Medical College, Tianjin, China;*

### Background

Previous studies have highlighted the importance of comprehensive nursing care model in managing chronic diseases. however, the evidence of comprehensive nursing interventions specifically designed for the HSCT peri-transplantation period of hematopoietic stem cell transplantation (HSCT) remains limited.

### Aims

To investigate the application effect of comprehensive nursing care model in the peri-transplantation period of patients undergoing HSCT, and to provide practical evidence for improving patients' survival quality and complication prevention and control.

### Methods

A total of 80 patients who received HSCT from January 2022 to December 2024 in the IHCAMS (Institute of Hematology and Blood Diseases Hospital, Peking Union Medical College) were selected. A prospective controlled study design was adopted, dividing patients into a conventional nursing group and a comprehensive nursing care group. The comprehensive nursing care included pre-transplantation psychological assessment and health education, transplantation-period infection and bleeding risk stratified management, refined nursing during the immunosuppressive period, early identification and intervention of complications, and continuous nursing care after discharge. The incidence of infection, graft-versus-host disease (GVHD) and nursing compliance, and quality of life scores were compared between the two groups.

### Results

The incidence of infection and GVHD in the comprehensive nursing care group were significantly lower than those in the conventional nursing group ($P < 0.05$), while treatment compliance and quality of life scores were significantly improved ($P < 0.05$). The incidence of nursing risk events during the peri-transplantation period decreased significantly, and patients' psychological status and self-management ability were improved.

### Summary/Conclusion

Comprehensive nursing care can effectively reduce the incidence of complications in HSCT patients, and improve nursing quality and patients' quality of life, and has positive significance for improving transplantation success rate. It is worthy of promotion and application in clinical nursing practice.

**Copyright Information:** (Online) ISSN: 2572-9241
©2026 The Author(s). HemaSphere is published by John Wiley & Sons Ltd on behalf of European Hematology Association. This is an open access Abstract Book distributed under the Attribution-NonCommercial-NoDerivs (CC BY-NC-ND), which allows third parties to download the articles and share them with others as long as they credit the author and the Abstract Book, but they cannot change the content in any way or use them commercially.
**Abstract Book Citations:** Authors, Title, HemaSphere, 2026;10:(S1):pages. The abstract book DOIs can be found on the "Author Information" page.
**Disclaimer:** Articles published in the journal HemaSphere exclusively reflect the opinions of the authors. The authors are responsible for all content in their abstracts including accuracy of the facts, statements, citing resources, etc.

### (PB4031) ALLO-SCT WITH TREOSULFAN COMPARED WITH BU-BASED REGIMENS: A SINGLE CENTER RETROSPECTIVE REAL-WORLD EXPERIENCE IN PATIENTS WITH ACUTE LEUKEMIA OR MYELODISPLASTIC SYNDROME /MYELOFIBROSYS DISEASE

**Topic:** 23. Stem cell transplantation - Clinical

Anna Mele*[1], Eleonora Prete[1], Rosa De Francesco[1], Stefania Citiso[1], Francesco Aji[1], Guido Volpe[1], Francesca Anna Ciardo De Marti[1], Silvia Sibilla[1], Giuseppina Greco[1], Francesca Donatelli[1], Domenico D'agostino[1], Nicoletta Pizzileo[1], Maria Rosaria Morciano[1], Antonella Orlando[1], Vincenzo Pavone[1]

[1]*Cardinale G.Panico Hospital, Hematology and Bone Marrow Transplant, TRICASE, Italy;*

### Background

Allo-SCT is a potentially curative treatment for Acute Leukemia (AL) or myelodisplastic Syndromes (MDSs)/ Myelofibrosys disease (MFI). Treosulfan has myeloablative and immunosuppressive properties and has shown promise in allo-SCT with less TRM.

### Aims

We analyze real-world allo-SCT performed in our center between 2020 and 2025 in AL or MDS/MFI patients and treated with treosulfan (TReo) or Busulfan (BU)-based conditioning regimens

### Methods

For analysis purpose, patients were grouped in TReo-group (n=29; TReo30-Flu150=27 and TT10-TReo30-Flu=2) and Bu-group (n=38; Bu12,8-Cy120=7, Bu 8 or 12,8-Flu 160:8+5, TT10-Flu150-Bu9= 18). Baseline, allo-SCT characteristics, engraftment and safety profiles between TReo and BU-group were compared.

### Results

Overall, 67 patients with at least 3 months follow-up were analyzed. Patients' and transplants' characteristics were reported in table 1. We observed in TReo-group a greater proportion of elderly patients (median age 64 years vs 51, p<0,001), MDSs (45% vs 18%, p<0,05) or >=3 HCT score (24% vs 5%, p<0,05). GvHD prophylaxis more frequently included ATG in TReo-group (69% vs 39%, p<0,02) vs post CY (14% vs 46%, p<0,006). No differences in terms of CR at allo-sCT, donor type, median CD34+ infused was observed. Time to neutrophyl or platelet engraftment was similar in the two groups. The median time to neutrophyl recovery (ANC>1x10^9/L) was 16 days (range, 12-38) and 16 days (range, 9-55) after TReo and Bu-group, respectively. The median time to platelet engraftment (PLT>50x10^9/L) was 18 days (range, 4-60) in TReo vs 22 days (range, 11-150)in Bu-group, p NS. We observed almost shorter hospital stay in TReo-group (29 days,range 10-60 vs 33 days, range 18-90; p=0,06). Focusing on safety profiles, the incidence of febrile neutropenia (55% vs 86%, p<0,005) and grade III-IV mucosites (24% vs 62%, p<0,01) were lower in TReo-group. No differences in terms of onset acute GvHD (17% vs 34%), renal (10% vs 5%) or liver toxicitiy (38% vs 35%) and chronic GvHD (17% vs 15%) were observed.
Moreover, the 2y cumulative incidence of relapse (28% vs 37%, p NS), 2y Overall Survival (58% vs 48%, p NS) was similar in the TReo or Bu-group. The Kaplan-Meier estimates for 1y TRM were 12% (2 patients experienced septic shock) and 20% (4 septic shock, 1 graft failure and 1 MOF) the TReo and Bu-group, respectively; p=0,06.

**Copyright Information:** (Online) ISSN: 2572-9241
©2026 The Author(s). HemaSphere is published by John Wiley & Sons Ltd on behalf of European Hematology Association. This is an open access Abstract Book distributed under the Attribution-NonCommercial-NoDerivs (CC BY-NC-ND), which allows third parties to download the articles and share them with others as long as they credit the author and the Abstract Book, but they cannot change the content in any way or use them commercially.
**Abstract Book Citations:** Authors, Title, HemaSphere, 2026;10:(S1):pages. The abstract book DOIs can be found on the "Author Information" page.
**Disclaimer:** Articles published in the journal HemaSphere exclusively reflect the opinions of the authors. The authors are responsible for all content in their abstracts including accuracy of the facts, statements, citing resources, etc.

### Summary/Conclusion

Despite a higher number of high risk patients (elderly or >=3 HCT score) in TReo-group, the efficay and safety of treosulfan based regimen prior Allo-SCT in real-world setting was comparable with BU-group. Our data suggest lower incidence of febrile neutropenia and severe mucosites with almost shorter hospital stay.

Table 1: Baseline and transplant characteristics of enrolled patients

| | Treo-group 29 (43) | BU-group 38 (57) | p |
|---|---|---|---|
| Male, n (%) | 21 (72) | 21 (55) | 0,12 |
| Median age, range<br>> 55 years | 64 (44-71)<br>24 (83) | 51 (20-66)<br>15 (39) | <br>**0,001** |
| CR pre Allo-SCT, n (%) | 27 (93) | 34 (90) | 0,91 |
| Diagnosis, n (%)<br>AL<br>MDS or MFI | <br>16 (55)<br>13 (45) | <br>31 (82)<br>7 (18) | <br>**0,05** |
| >=3 HCT Score, n (%) | 7 (24) | 2 (5) | **0,02** |
| >=4 EBMT Score, n (%) | 22 (82) | 22 (63) | 0,21 |
| Donor type, n (%)<br>Sibling<br>MUD<br>MMUD<br>Haplo | <br>7 (21)<br>22 (76)<br>6 (21)<br>1 (3) | <br>5 (13)<br>26 (69)<br>13 (34)<br>7 (18) | 0,09 |
| PBSC Source, n (%) | 29 (100) | 35 (92) | 0,14 |
| Median CD34+ infused, range<br>>6x10^6/Kg | 5,88 (3-12,8)<br>14 (48) | 5,78 (1,37-18,9)<br>18 (47) | <br>0,94 |
| PT-CY prophylaxis, n (%) | 4 (14) | 16 (46) | **0,006** |
| ATG prophylaxis, n (%) | 20 (69) | 15 (39) | **0,02** |

Figure 1: Cumulative Incidence of Relapse (RI), Overall Survival (OS) and Transplant Related Mortality (TRM) after Treo or Bu allo-SCT.

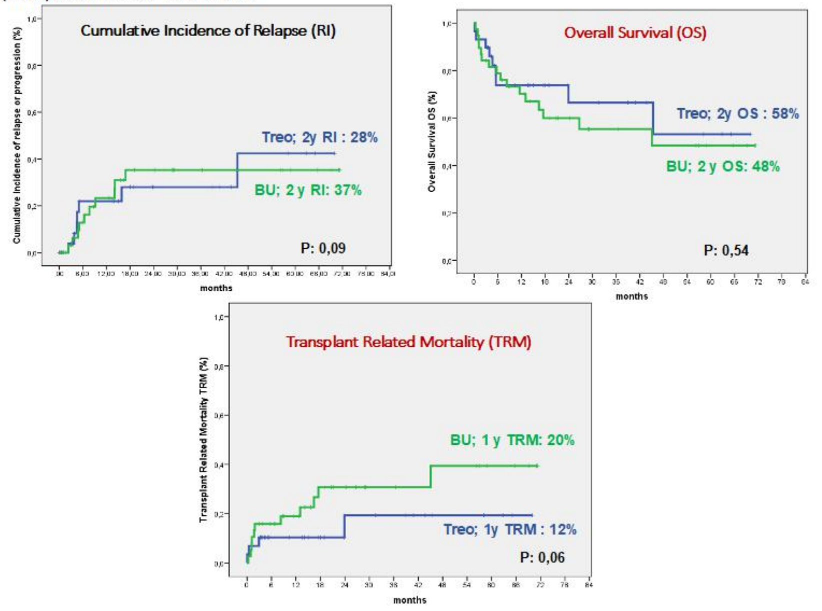

**Copyright Information:** (Online) ISSN: 2572-9241
©2026 The Author(s). HemaSphere is published by John Wiley & Sons Ltd on behalf of European Hematology Association. This is an open access Abstract Book distributed under the Attribution-NonCommercial-NoDerivs (CC BY-NC-ND), which allows third parties to download the articles and share them with others as long as they credit the author and the Abstract Book, but they cannot change the content in any way or use them commercially.
**Abstract Book Citations:** Authors, Title, HemaSphere, 2026;10:(S1):pages. The abstract book DOIs can be found on the "Author Information" page.
**Disclaimer:** Articles published in the journal HemaSphere exclusively reflect the opinions of the authors. The authors are responsible for all content in their abstracts including accuracy of the facts, statements, citing resources, etc.

### (PB4032) COMPARATIVE OUTCOMES OF HAPLOIDENTICAL AND MATCHED SIBLING DONOR TRANSPLANTS IN SEVERE APLASTIC ANEMIA

**Topic:** 23. Stem cell transplantation - Clinical

Fang Zhou*[1], Zhipeng Li[1], Xiaolin Yu[1], Xiaochen Song[1], Lei Deng[1], Fanjun Kong[1], Bo Xin[1]

[1]*The 960th Hospital of The People's Liberation Army (PLA) Joint Logistics Support Force, Jinan, China;*

### Background

In the absence of a matched sibling donor (MSD) for severe aplastic anemia (SAA) patients who have failed first-line immunosuppressive therapy (IST), haploidentical donor (HID) is a salvage treatment option.

### Aims

The aim of this research is to assess the difference between MSD and HID in SAA patients.

### Methods

Single-center retrospective study was performed on 94 SAA patients (45 from MSD; 49 from HID). The overall survival (OS) rate and failure-free survival (FFS) rate were evaluated and compared using Kaplan–Meier curves and log-rank tests. A competing risk regression model was used to evaluate neutrophil and platelet engraftment, acute graft-versus-host disease (aGVHD), chronic graft-versus-host disease (cGVHD), and transplantation-related mortality in which death was a competing event. Univariate and multivariate were assessed by Cox regression. Chimerism was evaluated at 1, 2, 3 months following transplantation.

### Results

After 3-months of transplantation, the objective response was 75% in the HID group versus 77.8% in the MSD group ($P$ = 0.802). The 28 days cumulative incidence of neutrophil engraftment was 91.8% versus 91.1% (Hazard Ratio [HR] 0.69, 95% CI, 0.46-1.04; $P$ = 0.075) in the HID and MSD. The 100 days cumulative incidence of platelet engraftment was 89.8% versus 91.1% (HR 0.7, 95% CI, 0.46-1.07; $P$ = 0.1) in the two groups. The estimated 5 years OS and FFS were 76.9% versus 86.7% ($P$ = 0.387), and 62.7% versus 80% ($P$ = 0.141) in the two groups. The cumulative incidence of aGVHD was 32.8% versus 13.4% (HR 2.91, 95% CI, 1.17-7.22; $P$ = 0.021) in the two groups. The cumulative incidence of cGVHD was 20.7% versus 2.4% (HR 8.27, 95% CI, 1.01-67.7; $P$ = 0.049) in the two groups. In multivariate analysis, aGVHD was associated with fludarabine-based conditioning regimen(vs. no, HR = 2.382, 95% CI 1.035-5.483, $P$ = 0.041) and graft source peripheral blood and bone marrow (vs. no, HR = 2.654, 95% CI 1.449-4.863, $P$ = 0.002). The MSD group had 21.05%, 44.44%, and 47.06% mixed chimerism at 1, 2, and 3 months after transplantation, respectively. MC was not detected in the HID group.

### Summary/Conclusion

Compared with MSD, HID demonstrated equivalent outcomes in response rate, hematopoietic recovery, OS, and FFS.

**Copyright Information:** (Online) ISSN: 2572-9241
©2026 The Author(s). HemaSphere is published by John Wiley & Sons Ltd on behalf of European Hematology Association. This is an open access Abstract Book distributed under the Attribution-NonCommercial-NoDerivs (CC BY-NC-ND), which allows third parties to download the articles and share them with others as long as they credit the author and the Abstract Book, but they cannot change the content in any way or use them commercially.
**Abstract Book Citations:** Authors, Title, HemaSphere, 2026;10:(S1):pages. The abstract book DOIs can be found on the "Author Information" page.
**Disclaimer:** Articles published in the journal HemaSphere exclusively reflect the opinions of the authors. The authors are responsible for all content in their abstracts including accuracy of the facts, statements, citing resources, etc.

### (PB4033) EFFECT OF COMPREHENSIVE NURSING MODEL ON COMPLICATIONS AND QUALITY OF LIFE IN PATIENTS UNDERGOING ALLOGENEIC HEMATOPOIETIC STEM CELL TRANSPLANTATION

**Topic:** 23. Stem cell transplantation - Clinical

Yuqing Liu*[1], Yuqiu Liu[1], Yi He[1], Wenjun Xie[1]

*[1]Institute of Hematology & Blood Diseases Hospital, Chinese Academy of Medical Sciences & Peking Union Medical College, Tian Jin, China;*

### Aims

This study aimed to explore the application effect of systematic nursing intervention in the peri-transplant period of patients undergoing hematopoietic stem cell transplantation (HSCT), and to provide practical evidence for improving patients' quality of life and preventing and controlling complications.

### Methods

A total of 80 patients who underwent HSCT from January 2022 to December 2024 were selected and divided into a routine nursing group and a comprehensive nursing intervention group by a prospective controlled study method. The comprehensive nursing measures included pre-transplant psychological assessment and health education, hierarchical management of infection and bleeding risks during transplantation, refined nursing in the immunosuppressive period, early identification and intervention of complications, and continuous nursing after discharge. The incidence of infection, incidence of graft-versus-host disease (GVHD), nursing compliance and quality of life scores were compared between the two groups.

### Results

The incidence of infection and GVHD in the comprehensive nursing intervention group were significantly lower than those in the routine nursing group (P⊠0.05), and the patients' treatment compliance and quality of life scores were significantly improved (P⊠0.05). The incidence of nursing risk events during the peri-transplant period decreased significantly, and the patients' psychological state and self-management ability were improved.

### Summary/Conclusion

Systematic and phased nursing intervention can effectively reduce the incidence of complications in HSCT patients, improve nursing quality and patients' quality of life, and play a positive role in improving the success rate of transplantation, which is worthy of popularization and application in clinical nursing practice.

**Copyright Information:** (Online) ISSN: 2572-9241
©2026 The Author(s). HemaSphere is published by John Wiley & Sons Ltd on behalf of European Hematology Association. This is an open access Abstract Book distributed under the Attribution-NonCommercial-NoDerivs (CC BY-NC-ND), which allows third parties to download the articles and share them with others as long as they credit the author and the Abstract Book, but they cannot change the content in any way or use them commercially.
**Abstract Book Citations:** Authors, Title, HemaSphere, 2026;10:(S1):pages. The abstract book DOIs can be found on the "Author Information" page.
**Disclaimer:** Articles published in the journal HemaSphere exclusively reflect the opinions of the authors. The authors are responsible for all content in their abstracts including accuracy of the facts, statements, citing resources, etc.

### (PB4034) LATENT CLASSES OF PSYCHOLOGICAL DISTRESS AND ITS ASSOCIATED FACTORS IN PATIENTS UNDERGOING HEMATOPOIETIC STEM CELL TRANSPLANTATION DURING THE PERI-TRANSPLANT PERIOD

**Topic:** 23. Stem cell transplantation - Clinical

Yuqiu Liu*[1], Yiming Gao[1], Xiaoyu Zhang[1], Yi He[1]

[1]*Institute of Hematology & Blood Diseases Hospital, Chinese Academy of Medical Sciences & Peking Union Medical College, Tian Jin, China;*

### Aims

This study aimed to analyze the latent classes of psychological distress and its associated factors in patients undergoing hematopoietic stem cell transplantation (HSCT) during the peri-transplant period, so as to provide empirical references for improving the mental health of this population.

### Methods

A convenient sampling method was adopted to select patients receiving primary HSCT in a specialized hematological hospital in Tianjin from January to December 2024 as the research subjects. A questionnaire survey was conducted among the participants using the General Information Questionnaire, Depression Anxiety Stress Scales, Connor-Davidson Resilience Scale-10 and Family APGAR Index. Latent profile analysis was used to identify the latent classes of patients' psychological distress, and Logistic regression analysis was applied to explore the associated factors of these latent classes.

### Results

A total of 223 patients were finally included in the study, and their psychological distress was classified into four latent classes, which were named as the distress type (33.2%), anxiety type (26.5%), depression type (17.9%) and adaptation type (22.4%) according to their characteristics. There were statistically significant differences in age, economic status, psychological resilience scores and family care scores among patients with different latent classes of psychological distress (P[illegible]0.05).

### Summary/Conclusion

Psychological distress in HSCT patients during the peri-transplant period is heterogeneous with distinct latent classes. Medical staff should timely assess the psychological distress of inpatients, focus on patients with distress, anxiety and depression types, and implement targeted interventions based on their characteristics to reduce psychological distress and promote mental health.

**Copyright Information:** (Online) ISSN: 2572-9241
©2026 The Author(s). HemaSphere is published by John Wiley & Sons Ltd on behalf of European Hematology Association. This is an open access Abstract Book distributed under the Attribution-NonCommercial-NoDerivs (CC BY-NC-ND), which allows third parties to download the articles and share them with others as long as they credit the author and the Abstract Book, but they cannot change the content in any way or use them commercially.
**Abstract Book Citations:** Authors, Title, HemaSphere, 2026;10:(S1):pages. The abstract book DOIs can be found on the "Author Information" page.
**Disclaimer:** Articles published in the journal HemaSphere exclusively reflect the opinions of the authors. The authors are responsible for all content in their abstracts including accuracy of the facts, statements, citing resources, etc.

### (PB4035) LETERMOVIR FOR CYTOMEGALOVIRUS PREVENTION IN RELAPSED/REFRACTORY ACUTE LEUKEMIA AND LYMPHOMA RECEIVING SEQUENTIAL CD7 CAR-T CELL THERAPY BRIDGING ALLOGENEIC HEMATOPOIETIC STEM CELL TRANSPLANTATION

**Topic:** 23. Stem cell transplantation - Clinical

Linwei Shi*[1], Aiming Pang[1]

[1]*Institute of Hematology & Blood Diseases Hospital, Chinese Academy of Medical Sciences & Peking Union Medical College, Institute of Hematology & Blood Diseases Hospital, Tianjin, China;*

### Background

To evaluate the real-world safety and efficiency of letermovir in preventing cytomegalovirus infection in patients undergoing sequential CD7 CAR-T cell therapy bridging allogeneic hematopoietic stem cell transplantation for relapsed/refractory acute leukemia and lymphoma.

### Methods

This single-center, retrospective, observational study enrolled patients with relapsed/refractory acute leukemia and lymphoma who received sequential CD7 CAR-T cell therapy bridging allo-HSCT at the Institute of Hematology & Blood Diseases Hospital, Chinese Academy of Medical Sciences[illegible]1st June, 2024- 1st May, 2025[illegible]. All enrolled patients were scheduled to receive letermovir for CMV prophylaxis.Prophylaxis was initiated within one week after transplantation and continued until day 100 post-allo-HSCT. The primary endpoint was the breakthrough CMV infection rate.

### Results

A total of 9 patients were enrolled, including 5 with T-lymphoblastic leukemia/lymphoma, 3 with T-cell acute lymphoblastic leukemia (T-ALL), and 1 with mixed-phenotype acute leukemia. Eight patients received autologous CAR-T cell therapy, while 1 received allogeneic CAR-T cells. The median time from diagnosis to CAR-T cell infusion was 119 days (83-285 days), and the median time from CAR-T cell infusion to hematopoietic stem and progenitor cell (HSPC) infusion was 57 days (41-74 days). The median follow-up time from CAR-T cell infusion was 5.6 months (2.5-11.9 months), and the median overall survival (OS) for all patients was 10.5 months (6.6-16.6 months).
All 9 patients who received CD7 CAR-T cell therapy bridging allo-HSCT started letermovir prophylaxis within one week after transplantation initiation **(Figure 1)**. The median duration of letermovir prophylaxis was 100 days (19-100 days). During prophylaxis, 3 patients (33.3%) required intensive care unit admission due to severe infections. One patient (11.1%) experienced a breakthrough CMV infection on day 14 post-transplantation, with an initial CMV DNA load of 914 copies/mL; the infection resolved after 2 days of adjunctive ganciclovir therapy, with subsequent CMV DNA conversion to negative. Five patients completed letermovir prophylaxis until day 100 post-transplant, and none of these patients developed CMV infection from drug discontinuation until the end of follow-up. However, 3 patients (33.3%) developed Epstein-Barr virus (EBV) infection during letermovir prophylaxis, including 1 case of refractory EBV infection. The median time to EBV viremia onset was 60 days post-transplantation, with a mean duration of 27.0 days. Four patients died due to infections post-transplantation; notably, 3 of these 4 patients had EBV viremia, with 1 death directly attributed to EBV infection and the others to severe infections. Letermovir prophylaxis demonstrated a favorable safety profile, with no serious adverse events related to the drug observed.

**Copyright Information:** (Online) ISSN: 2572-9241
©2026 The Author(s). HemaSphere is published by John Wiley & Sons Ltd on behalf of European Hematology Association. This is an open access Abstract Book distributed under the Attribution-NonCommercial-NoDerivs (CC BY-NC-ND), which allows third parties to download the articles and share them with others as long as they credit the author and the Abstract Book, but they cannot change the content in any way or use them commercially.
**Abstract Book Citations:** Authors, Title, HemaSphere, 2026;10:(S1):pages. The abstract book DOIs can be found on the “Author Information” page.
**Disclaimer:** Articles published in the journal HemaSphere exclusively reflect the opinions of the authors. The authors are responsible for all content in their abstracts including accuracy of the facts, statements, citing resources, etc.

### Summary/Conclusion

In a real-world setting, letermovir demonstrated significant efficacy in preventing CMV infection in patients with acute leukemia and lymphoma undergoing sequential CD7 CAR-T cell therapy bridging allo-HSCT. The high risk of CMV reactivation post-CAR-T therapy has been confirmed in multiple studies. The CMV infection rate observed in this study is significantly lower than previously reported rates for CD7 CAR-T bridging transplantation. Further large-scale cohorts will hold greater promise to verify letermovir for CMV prophylaxis in high-risk populations undergoing CD7 CAR-T bridging allo-HSCT.

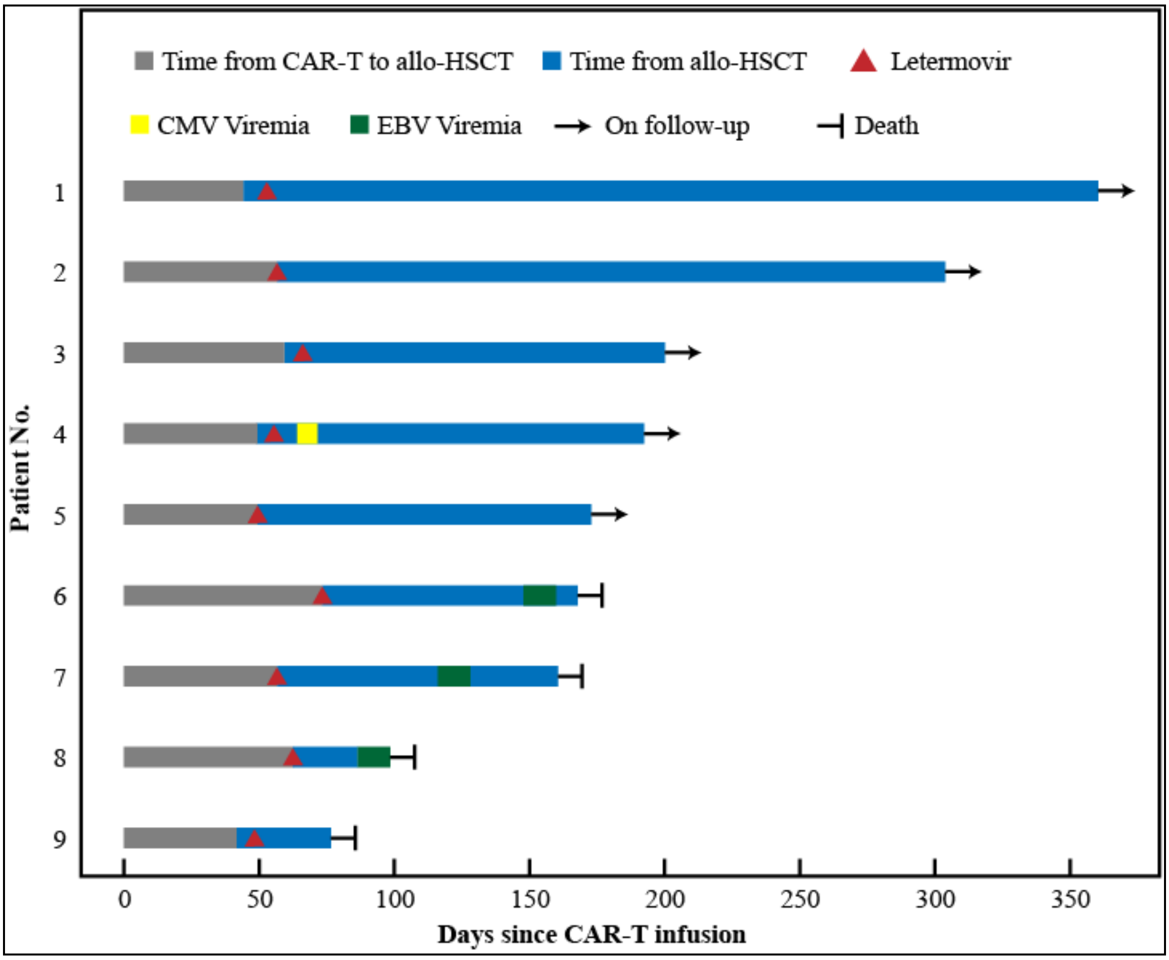

All 9 patients who received CD7 CAR-T cell therapy bridging allo HSCT started letermovir prophylaxis within one week after transplantation initiation

Copyright Information: (Online) ISSN: 2572-9241
©2026 The Author(s). HemaSphere is published by John Wiley & Sons Ltd on behalf of European Hematology Association. This is an open access Abstract Book distributed under the Attribution-NonCommercial-NoDerivs (CC BY-NC-ND), which allows third parties to download the articles and share them with others as long as they credit the author and the Abstract Book, but they cannot change the content in any way or use them commercially.
Abstract Book Citations: Authors, Title, HemaSphere, 2026;10:(S1):pages. The abstract book DOIs can be found on the “Author Information” page.
Disclaimer: Articles published in the journal HemaSphere exclusively reflect the opinions of the authors. The authors are responsible for all content in their abstracts including accuracy of the facts, statements, citing resources, etc.

### (PB4036) DEVELOPMENT AND APPLICATION EVALUATION OF AN OCCUPATIONAL STRESS REGULATION SYSTEM FOR NURSES IN HEMATOPOIETIC STEM CELL TRANSPLANTATION UNITS BASED ON THE NJSS

**Topic:** 23. Stem cell transplantation - Clinical

Xiaoxu Ren*[1], Ruihong Liu[1], Siyang Zhai[1], Zhu Lan[1], Meijing Liu[1], Jinshaung Geng[1], Wenjun Xie[1, 1]

*[1]State Key Laboratory of Experimental Hematology, National Clinical Research Center for Blood Diseases, Institute of Hematology & Blood Diseases Hospital, Chinese Academy of Medical Sciences & Peking Union Medical College, tianjin, China;*

### Background

The transplant unit (i.e., laminar airflow ward) represents a highly specialized clinical setting dedicated to the completion of hematopoietic stem cell transplantation (HSCT) for patients with hematological diseases. Throughout the transplantation process, patients are consistently in critical condition, requiring medical staff to maintain sustained high concentration. This prolonged intense focus, coupled with psychological tension and limited external communication, imposes significant occupational stress on nurses. Persistent stress may lead to physiological disorders and impairments, including illness and mortality. Our team's investigation revealed that 77.8% of nurses in this unit were experiencing occupational stress.

### Aims

Based on the assessment results of the Nurse Job Stress Scale (NJSS), an occupational stress regulation system for nurses in the hematopoietic stem cell transplant unit was developed, and its effects on alleviating nurse stress, improving mental health, and enhancing nursing quality were evaluated.

### Methods

A pre-post comparative design was employed. In March 2024, a baseline survey was conducted among all nurses (n=50) in the transplant unit using the NJSS and the Chinese version of the Perceived Stress Scale (CPSS) to identify primary stressors. Based on the SMART principle, a comprehensive intervention program was developed covering five dimensions: workflow optimization, professional competence enhancement, time management, psychological support, and team culture cultivation. The intervention was implemented from June 2024 to May 2025. Post-intervention evaluation was conducted using the CPSS, the Nurse Burnout Scale (NBS), the Subjective Well-being Scale, and a patient satisfaction questionnaire.

### Results

After the intervention, nurses' CPSS scores significantly decreased from (38.7 ± 5.2) at baseline to (28.61 ± 4.8) ($P < 0.01$); the Nurse Burnout Scale (NBS) scores decreased from (110.7 ± 5.3) to (81.3 ± 4.3); subjective well-being scores increased from (69.4 ± 9.1) to (75.6 ± 8.3) ($P < 0.05$); and patient satisfaction during hospitalization increased from 90% before the intervention to 99.6% ($P < 0.05$).

### Summary/Conclusion

The occupational stress regulation system developed based on the NJSS assessment effectively reduced perceived stress and burnout among nurses in the transplant unit, enhanced their subjective well-being, and indirectly contributed to improved patient satisfaction. With its clear structure and strong practicality, this system provides a valuable reference for occupational health management among nurses in similar specialized units.

**Copyright Information:** (Online) ISSN: 2572-9241
©2026 The Author(s). HemaSphere is published by John Wiley & Sons Ltd on behalf of European Hematology Association. This is an open access Abstract Book distributed under the Attribution-NonCommercial-NoDerivs (CC BY-NC-ND), which allows third parties to download the articles and share them with others as long as they credit the author and the Abstract Book, but they cannot change the content in any way or use them commercially.
**Abstract Book Citations:** Authors, Title, HemaSphere, 2026;10:(S1):pages. The abstract book DOIs can be found on the "Author Information" page.
**Disclaimer:** Articles published in the journal HemaSphere exclusively reflect the opinions of the authors. The authors are responsible for all content in their abstracts including accuracy of the facts, statements, citing resources, etc.

### (PB4037) RUXOLITINIB COMBINED WITH LOW-DOSE STEROIDS FOR GRADE II ACUTE GRAFT-VERSUS-HOST DISEASE: A SINGLE-CENTER, SINGLE-ARM, PHASE II CLINICAL TRIAL

**Topic:** 23. Stem cell transplantation - Clinical

Dai-Hong Liu*[1], Hanlin Xue[1]

[1]*Chinese PLA General Hospita, State Key Laboratory of Experimental Hematology, Beijing, China;*

### Background

The application of allogeneic hematopoietic stem cell transplantation (allo-HSCT) has become increasingly widespread. However, acute graft-versus-host disease (aGVHD) remains a major cause of morbidity and mortality, significantly impacting transplant outcomes.Steroids (1-2 mg/kg/day) as first-line therapy for aGVHD are frequently associated with significant infectious and metabolic complications and often lead to steroid-refractory aGVHD. Previous work by our team showed that ruxolitinib combined with 1-2 mg/kg/day steroids could significantly improve the 28-day objective response rate (ORR) in steroid-refractory aGVHD.

### Aims

This study aimed to evaluate the efficacy and safety of low-dose steroids (0.5 mg/kg/day) combined with ruxolitinib as initial therapy for aGVHD.

### Methods

From January 2025 to December 2025, 15 newly diagnosed patients with grade II aGVHD were enrolled in this single-arm study. All patients received a regimen of 0.5 mg/kg/day steroids plus ruxolitinib.

### Results

The objective response rates (ORR) on day 7 and day 28 were 93.33% (95% CI: 69.8–99.7%) and 80.0% (95% CI: 51.8–95.7%), respectively. The complete response (CR) rate at day 56 was 66.67% (95% CI: 38.4–88.1%). The 180-day overall survival (OS) was 90.90% (95% CI:67.6-98.5%), non-relapse mortality (NRM) was 9.09% (95% CI:1.5-32.3%), and failure-free survival (FFS) was 43.75% (95% CI: 19.8–70.1%). The main adverse events within 30 days after enrollment included electrolyte disturbances (73.33%), EBV reactivation (73.33%), and anemia (66.67%),all of which were managed effectively and resolved with appropriate interventions.

### Summary/Conclusion

The regimen of 0.5 mg/kg/day steroids combined with ruxolitinib demonstrated promising efficacy and a manageable safety profile in patients with grade II aGVHD. Further patient enrollment is ongoing. This approach may reduce corticosteroid exposure and associated toxicity, and warrants further validation in randomized controlled trials.

**Copyright Information:** (Online) ISSN: 2572-9241
©2026 The Author(s). HemaSphere is published by John Wiley & Sons Ltd on behalf of European Hematology Association. This is an open access Abstract Book distributed under the Attribution-NonCommercial-NoDerivs (CC BY-NC-ND), which allows third parties to download the articles and share them with others as long as they credit the author and the Abstract Book, but they cannot change the content in any way or use them commercially.
**Abstract Book Citations:** Authors, Title, HemaSphere, 2026;10:(S1):pages. The abstract book DOIs can be found on the "Author Information" page.
**Disclaimer:** Articles published in the journal HemaSphere exclusively reflect the opinions of the authors. The authors are responsible for all content in their abstracts including accuracy of the facts, statements, citing resources, etc.

### (PB4038) A REAL-WORLD, MULTI-CENTER, RETROSPECTIVE STUDY OF AUTOLOGOUS HEMATOPOIETIC STEM CELL TRANSPLANTATION IN 33 HIV-ASSOCIATED LYMPHOMA IN CHINA (CALL-006 STUDY)

**Topic:** 23. Stem cell transplantation - Clinical

Chaoyu Wang*[1], Jun Liu[1], Haiyan Min[2], Juanjuan Chen[3], Weiping Liu[4], Linjun Zhao[5], Hongyun Xing[6], Cong Wang[7], Yao Liu[1]

*[1]Chongqing university cancer hospital, Chong, China; [2]Yunnan Provincial Infectious Diseases Hospital, Kunming, China; [3]Nanfang Hospital, Southern Medical University, Guangzhou, China;[4]Peking University Cancer Hospital, Beijing, China; [5]Peking University International Hospital, Beijing, China; [6]Affiliated Hospital of Southwest Medical University, Sichuan, China;[7]Gansu Wuwei Cancer Hospital, Gansu, China;*

### Background

In the era of combination antiretroviral therapy (cART), patients with HIV-associated lymphoma have become more tolerant to higher-intensity chemotherapy, enhancing the feasibility of autologous hematopoietic stem cell transplantation (ASCT). Nevertheless, clinical data on ASCT in this patient population remain limited.

### Aims

In this study, we retrospectively analyzed the clinical characteristics and outcomes of 33 patients with HIV-associated lymphoma who underwent ASCT across eight centers, with the aim of providing further evidence regarding the feasibility and efficacy of ASCT in this setting.

### Methods

We performed a retrospective multi-center analysis of the clinical characteristics and outcomes of 33 patients with HIV-associated lymphoma who underwent ASCT at eight centers.

### Results

Among the 33 patients, 81.82% underwent ASCT as first-line consolidation therapy, while 18.18% received it as salvage therapy. All patients successfully collected sufficient $CD34^+$ cells following G-CSF mobilization. The single-collection success rate was higher in those receiving ASCT as first-line consolidation (51.9%) compared with the salvage group (33.3%). Neutrophil engraftment occurred at a median of 11 days (range: 8–24 days), and platelet engraftment at a median of 15 days (range: 8–41 days). The 100-days post-transplantation mortality rate was 3% (1/33), with one patient in the salvage therapy group dying from disease progression. Median survival was significantly longer in the CR group at the first post-transplant efficacy assessment (81 months) than in the PR group (26.8 months) (p=0.003). Patients with $CD4^+$ T cell counts ≥ 200/μL at lymphoma diagnosis also exhibited better median survival (44.5 months) than those with counts<200/μL(19.1 months)(p=0.030).

### Summary/Conclusion

Our findings demonstrate that ASCT is feasible for patients with HIV-associated lymphoma in the cART era, and that its administration as first-line consolidation therapy following CR is associated with improved patient outcomes.

**Copyright Information:** (Online) ISSN: 2572-9241
©2026 The Author(s). HemaSphere is published by John Wiley & Sons Ltd on behalf of European Hematology Association. This is an open access Abstract Book distributed under the Attribution-NonCommercial-NoDerivs (CC BY-NC-ND), which allows third parties to download the articles and share them with others as long as they credit the author and the Abstract Book, but they cannot change the content in any way or use them commercially.
**Abstract Book Citations:** Authors, Title, HemaSphere, 2026;10:(S1):pages. The abstract book DOIs can be found on the "Author Information" page.
**Disclaimer:** Articles published in the journal HemaSphere exclusively reflect the opinions of the authors. The authors are responsible for all content in their abstracts including accuracy of the facts, statements, citing resources, etc.

### (PB4039) THE INFLUENCE OF CMV STATUS OF DONOR-RECIPIENT PAIRS ON THE DEVELOPMENT OF ACUTE GRAFT-VERSUS-HOST DISEASE

**Topic:** 23. Stem cell transplantation - Clinical

Nechay Oksana*[1], Dziuba Alena[1], Morozova Oksana[1], Strongin Yuri[1], Tatsiana Talako[1], Alena Hlaz[1], Amvrosieva Tamara[2], Natalya Milanovich[1], Uss Anatoly[1]

*[1]Minsk Scientific and Practical Center for Surgery, Transplantology and Hematology, Minsk, Belarus;[2]Research Institute of Hygiene, Toxicology, Epidemiology, Virology, and Microbiology, Minsk, Belarus;*

### Background

Current research highlights a complex immunological relationship between cytomegalovirus (CMV) infection and graft-versus-host disease (GVHD): viral reactivation can trigger T-cell alloreactivity, while GVHD therapy (one or more lines of immunosuppression) creates conditions for uncontrolled viral replication. Despite advances in prophylactic antiviral therapy, the prognostic significance of pretransplant CMV status (donor-recipient serodiscordance) in relation to the risk of developing acute and chronic GVHD remains a subject of debate.

### Aims

To evaluate the impact of CMV status of donor-recipient pairs on the development of acute graft-versus-host disease in recipients of allogeneic hematopoietic stem cell transplantation (alloHSCT).

### Methods

A single-center retrospective study included analysis of data from 70 patients who underwent alloHSCT from 2018 to 2023 at the Minsk Scientific and Practical Center for Surgery, Transplantology and Hematology, Belarus. The study included 44 men and 26 women, median age 31 (18-59) years. Patients were transplanted with the following nosologies: acute myeloid leukemia - 31 patients (44.2%), acute lymphoblastic leukemia - 18 patients (25.7%), aplastic anemia - 7 (10.0%), chronic myelogenous leukemia 5 (7.1%), myelodysplastic syndrome - 3 (4.3%), mixed cell leukemia - 2 (2.9%), primary myelofibrosis - 2 (2.9%).

### Results

In this study, 32 (45.7%) transplantations were performed from related donors and 38 (54.3%) from unrelated donors. The source of the graft was bone marrow in 21 (30.0%) cases, and peripheral stem cells in 49 (70.0%). 23 (32.9%) donor-recipient pairs were seronegative for CMV (IgG -). Seronegative donors were selected for 17 (24.3%) CMV-seropositive recipients, and seropositive donors were selected for 26 (37.1%) seropositive recipients. Acute GVHD developed in 34 (48.5%) recipients of all alloHSCT. Among recipients without acute GVHD, donor and recipient serodiscordance for CMV was observed in 6 (24.0%) recipients, and seroconcordance for CMV among recipients without acute GVHD was determined in 15 (60.0%) recipients ($p = 0.014$).

### Summary/Conclusion

The study results confirm the significant role of pre-transplant CMV status in predicting immunological complications. Seroconcordance between donor and recipient was found to be a favorable prognostic factor, significantly reducing the risk of developing acute GVHD ($p = 0.014$).

**Copyright Information:** (Online) ISSN: 2572-9241
©2026 The Author(s). HemaSphere is published by John Wiley & Sons Ltd on behalf of European Hematology Association. This is an open access Abstract Book distributed under the Attribution-NonCommercial-NoDerivs (CC BY-NC-ND), which allows third parties to download the articles and share them with others as long as they credit the author and the Abstract Book, but they cannot change the content in any way or use them commercially.
**Abstract Book Citations:** Authors, Title, HemaSphere, 2026;10:(S1):pages. The abstract book DOIs can be found on the “Author Information” page.
**Disclaimer:** Articles published in the journal HemaSphere exclusively reflect the opinions of the authors. The authors are responsible for all content in their abstracts including accuracy of the facts, statements, citing resources, etc.

### (PB4040) EARLY ST2 AND REG3A KINETICS PREDICT ADVERSE OUTCOMES FOLLOWING MYELOABLATIVE ALLOGENEIC STEM CELL TRANSPLANTATION

**Topic:** 23. Stem cell transplantation - Clinical

Syed Asif Ali Shah*[1, 2], Fatima Saeed[1, 2], Mandy Ludford Menting[1, 2], Jessica Klass[1], Joanne Davis[1], Rachel Koldej[1, 2], David Ritchie[1, 2, 3]

*[1]Royal Melbourne Hospital, Melbourne, Australia; [2]University of Melbourne, Melbourne, Australia; [3]Peter MacCallum Cancer, Melbourne, Australia;*

### Background

Conditioning-related tissue injury is a critical initiating event in the pathogenesis of acute graft-versus-host disease (aGVHD) following allogeneic hematopoietic cell transplantation (HCT). Two biomarkers ST2 (suppression of tumorigenicity 2) and REG3a (regenerating islet-derived protein 3 alpha), are markers of endothelial injury and gastrointestinal epithelial injury respectively and have been used to predict patient outcome from GVHD. We hypothesised that changes in these biomarkers immediately following the completion of conditioning may reflect the degree of conditioning-induced tissue damage and may therefore predict the onset of GVHD or treatment related mortality (TRM).

### Aims

To evaluate whether early fold changes in ST2 and REG3α predict adverse outcomes following HCT and to assess differences according to conditioning intensity.

### Methods

We performed a retrospective analysis of patients enrolled in a prospective institutional biomarker study, with serial blood sampling performed following informed consent in patients undergoing allogeneic stem cell transplantation. ST2 and REG3α levels in serum were measured pre-conditioning, on day 0 and on day 14 HCT by ELISA, and fold change was calculated.
Patients were stratified by conditioning intensity into myeloablative conditioning (MAC) and reduced-toxicity conditioning (FluMel). The MAC cohort uniformly received a busulfan–cyclophosphamide (BuCy)-based regimen, with anti-thymocyte globulin (ATG) added in unrelated donor transplants.
The primary endpoint was adverse outcome, defined as a composite of grade 3–4 acute GVHD, ICU admission, or multi-organ failure.
Receiver operating characteristic (ROC) analysis was performed to assess predictive performance. A combined biomarker model was constructed defining low risk (no elevation in either biomarker) and high risk (elevation in ≥1 biomarker).
Biomarker elevation for ST2 was defined using ROC-derived optimal cutoff values. Given the lack of a clear ROC-defined threshold for REG3α, elevation was defined using the cohort median. Categorical outcomes between groups were compared using Fisher's exact test.

**Copyright Information:** (Online) ISSN: 2572-9241
©2026 The Author(s). HemaSphere is published by John Wiley & Sons Ltd on behalf of European Hematology Association. This is an open access Abstract Book distributed under the Attribution-NonCommercial-NoDerivs (CC BY-NC-ND), which allows third parties to download the articles and share them with others as long as they credit the author and the Abstract Book, but they cannot change the content in any way or use them commercially.
**Abstract Book Citations:** Authors, Title, HemaSphere, 2026;10:(S1):pages. The abstract book DOIs can be found on the "Author Information" page.
**Disclaimer:** Articles published in the journal HemaSphere exclusively reflect the opinions of the authors. The authors are responsible for all content in their abstracts including accuracy of the facts, statements, citing resources, etc.

**Results**

In the MAC cohort (n=28), day 0 ST2 fold change over pre-conditioning demonstrated predictive ability for adverse outcomes (AUC 0.72) and grade 3–4 GVHD (AUC 0.77).
Using the combined biomarker model, low-risk patients had a lower incidence of adverse outcomes compared to high-risk patients (14% vs 47%, p = 0.18), demonstrating a trend toward improved risk stratification. REG3α showed a similar directional trend and contributed to risk stratification.
In the FluMel cohort (n=61), ROC analysis did not demonstrate significant predictive discrimination. However, in exploratory analysis, a higher incidence of adverse outcomes was observed in the high-risk group compared to low-risk patients (42.5% vs 21%, p = 0.21), demonstrating a similar directional trend.

**Summary/Conclusion**

Early post-transplant increases in ST2 are associated with clinically significant adverse outcomes following myeloablative transplantation. A simplified combined biomarker model incorporating ST2 and REG3α identifies a low-risk population.
While predictive performance was attenuated in reduced-toxicity conditioning, a consistent directional trend was observed, suggesting that biomarker analysis may provide an opportunity to intervene prior to the onset of adverse outcomes. Prospective validation in larger cohorts is warranted.

**Copyright Information:** (Online) ISSN: 2572-9241
©2026 The Author(s). HemaSphere is published by John Wiley & Sons Ltd on behalf of European Hematology Association. This is an open access Abstract Book distributed under the Attribution-NonCommercial-NoDerivs (CC BY-NC-ND), which allows third parties to download the articles and share them with others as long as they credit the author and the Abstract Book, but they cannot change the content in any way or use them commercially.
**Abstract Book Citations:** Authors, Title, HemaSphere, 2026;10:(S1):pages. The abstract book DOIs can be found on the "Author Information" page.
**Disclaimer:** Articles published in the journal HemaSphere exclusively reflect the opinions of the authors. The authors are responsible for all content in their abstracts including accuracy of the facts, statements, citing resources, etc.

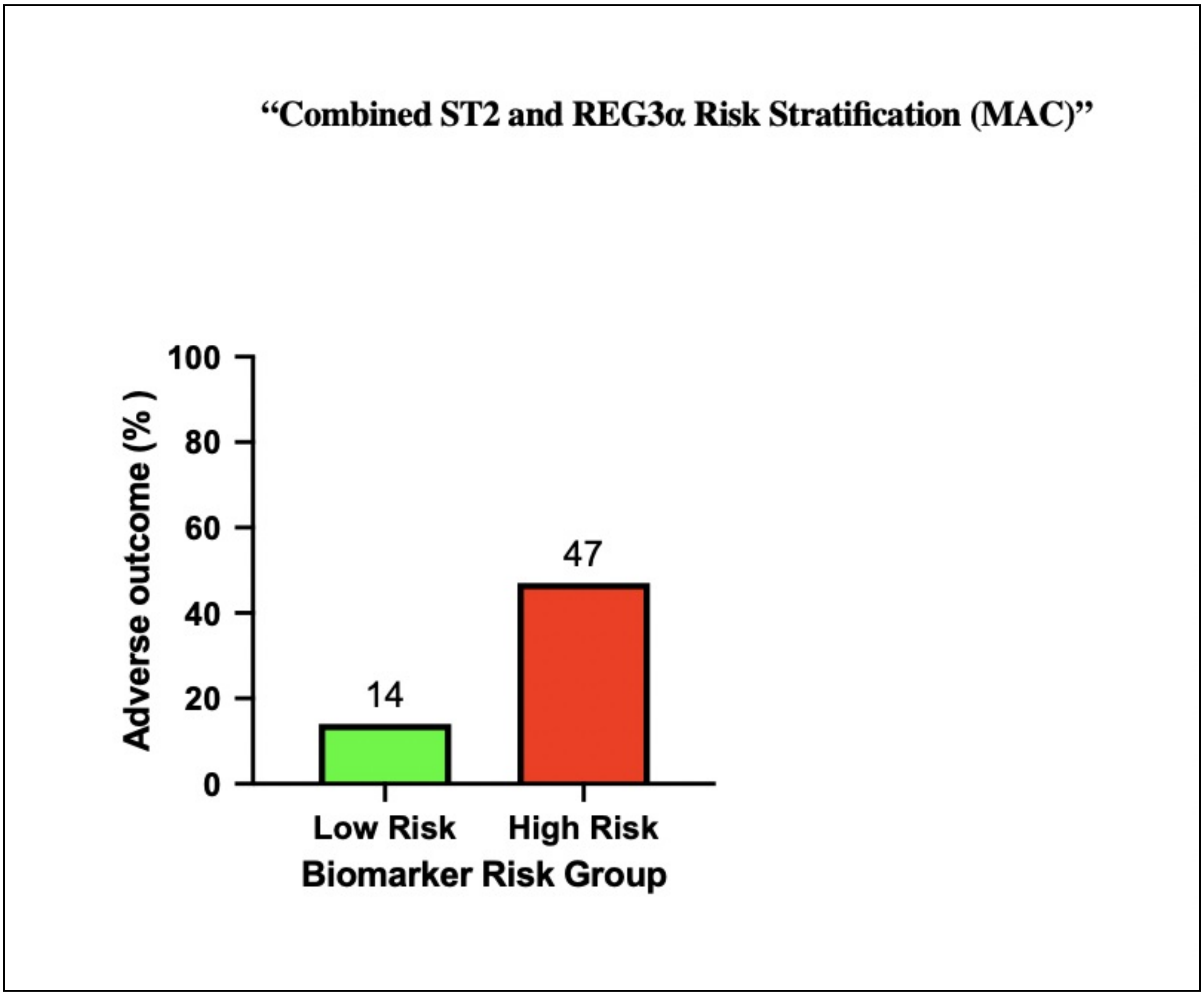

Figure 1: Combined ST2 and REG3α biomarker-based risk stratification in the myeloablative conditioning cohort. Patients were categorized as low risk (no elevation in either biomarker) and high risk (elevation in ≥1 biomarker). The high-risk group demonstrated a higher incidence of adverse outcomes (47%) compared to the low-risk group (14%).

**Copyright Information:** (Online) ISSN: 2572-9241
©2026 The Author(s). HemaSphere is published by John Wiley & Sons Ltd on behalf of European Hematology Association. This is an open access Abstract Book distributed under the Attribution-NonCommercial-NoDerivs (CC BY-NC-ND), which allows third parties to download the articles and share them with others as long as they credit the author and the Abstract Book, but they cannot change the content in any way or use them commercially.
**Abstract Book Citations:** Authors, Title, HemaSphere, 2026;10:(S1):pages. The abstract book DOIs can be found on the "Author Information" page.
**Disclaimer:** Articles published in the journal HemaSphere exclusively reflect the opinions of the authors. The authors are responsible for all content in their abstracts including accuracy of the facts, statements, citing resources, etc.

### (PB4041) LEUCOGEN COMBINED WITH G-CSF FOR THE TREATMENT OF MYELOSUPPRESSION AFTER AUTO-HSCT: A SINGLE-CENTER, RANDOMIZED CONTROLLED TRIAL

**Topic:** 23. Stem cell transplantation - Clinical

Na Zhang*[1], Qian Dong[1], Cuicui Peng[1], Dongfeng Zeng[1]

[1]*Daping Hospital, Army Medical University, Department of Hematology, Chongqing, China;*

### Background

Prior to autologous hematopoietic stem cell transplantation (auto-HSCT), patients receive high-dose conditioning chemotherapy, which is followed by a period of myelosuppression. During this myelosuppressive phase, neutropenia and thrombocytopenia are the most common manifestations. Febrile neutropenia in patients undergoing auto-HSCT is associated with an increased risk of sepsis and mortality. Granulocyte colony-stimulating factor (G-CSF) is commonly used to accelerate neutrophil recovery. Leucogen is approved in China for the prevention and treatment of leukopenia and thrombocytopenia.Studies have confirmed that Leucogen is effective in both the prevention and treatment of leukopenia.

### Aims

To verify whether leucogen can shorten the time to neutrophil and platelet engraftment.

### Methods

In this randomized controlled study, we compared the efficacy and safety of Leucogen (60 mg orally three times daily), with or without G-CSF, in patients undergoing auto-HSCT. The trial was registered with the Chinese Clinical Trial Registry (ChiCTR2500103184). Patients in Group A received Leucogen (60 mg orally three times daily) starting on Day 1 post-transplant, with G-CSF (10 μg/kg) added on Day 5. Patients in Group B received G-CSF (10 μg/kg) alone starting on Day 5 post-transplant. The primary outcomes were the times to neutrophil and platelet engraftment, as well as the safety profile of the treatment regimens.

### Results

A total of 21 patients were enrolled from April 2025, including 9 females and 12 males, with an age range of 21 to 70 years (mean age, 53.95±10.94 years). In Group A, the median time to neutrophil engraftment was 11.33±1.50 days, and the median time to platelet engraftment was 13.00±2.63 days. In Group B, the median time to neutrophil engraftment was 13.44±1.51 days, and the median time to platelet engraftment was 17.78±3.70 days. No treatment-related adverse events were observed. The differences between the two groups were statistically significant ($P < 0.05$).

### Summary/Conclusion

Leucogen significantly reduced the time to neutrophil and platelet engraftment in patients undergoing auto-HSCT, with no drug-related adverse reactions observed.

**Copyright Information:** (Online) ISSN: 2572-9241
©2026 The Author(s). HemaSphere is published by John Wiley & Sons Ltd on behalf of European Hematology Association. This is an open access Abstract Book distributed under the Attribution-NonCommercial-NoDerivs (CC BY-NC-ND), which allows third parties to download the articles and share them with others as long as they credit the author and the Abstract Book, but they cannot change the content in any way or use them commercially.
**Abstract Book Citations:** Authors, Title, HemaSphere, 2026;10:(S1):pages. The abstract book DOIs can be found on the “Author Information” page.
**Disclaimer:** Articles published in the journal HemaSphere exclusively reflect the opinions of the authors. The authors are responsible for all content in their abstracts including accuracy of the facts, statements, citing resources, etc.

### (PB4042) ALEMTUZUMAB AS A SUBSTITUTE FOR ATG IN THE FBM (FLUDARABINE, BCNU, MELPHALAN) CONDITIONING REGIMEN: COMPARABLE OUTCOMES IN PATIENTS WITH HIGH HCT-CI AND AGE > 60, BUT INFERIOR OUTCOMES IN AML

**Topic:** 23. Stem cell transplantation - Clinical

Merle Weniger[1], Natalie Schub[1], Thomas Spanberger[1, 2], Thomas Valerius[1], Martin Gramatzki[1], Andreas Guenther*[1, 3]

[1]*Christian-Albrechts University of Kiel, Division of Stem Cell Transplantation and Immunotherapy, Department of Medicine II, Kiel, Germany;* [2]*Klinikum Ottakring, 1.Med. Abteilung – Zentrum für Onkologie und Hämatologie mit Ambulanz und Palliativstation, Vienna, Austria;* [3]*Practice Hematology & Oncology, Koblenz, Germany;*

### Background

In recent years, conditioning regimens with reduced or intermediate intensity have been developed to extend the curative approach of allogeneic stem cell transplantation to elderly and medically unfit patients. The combination of fludarabine, BCNU, and melphalan (FBM) has demonstrated efficacy and good tolerability in acute myeloid leukemia and other malignancies. A particular point of interest is the kind of immunosuppression accompanying the FBM conditioning regimen.

### Aims

At our center two approaches to T-cell depletion were used: the monoclonal CD52 antibody alemtuzumab or rabbit anti–T-lymphocyte globulin (ATG) (Neovii Biotech, Gräfelfing, Germany). This retrospective single-center study analyzes the long-term outcome associated with both approaches.

### Methods

All patients who underwent allogeneic hematopoietic stem cell transplantation (HSCT) at our center between 2010 and 2016 and received FBM as the conditioning regimen were included in the study. Of the 132 patients, at the physician's discretion 92 were treated with ATG while 40 received alemtuzumab. According to institutional standards, patients transplanted from a 9/10 or 10/10 HLA matched unrelated donor (MUD) were considered to receive ATG at a dose of 40 mg/kg, whereas in matched related donors (MRD) the dose was reduced to 30 mg/kg. In contrast, alemtuzumab was administered at a total dose of 30 mg for MUD transplants and 20 mg for MRD transplants. To assess the acute toxicity of the conditioning regimen, clinical symptoms were recorded according to the Common Terminology Criteria for Adverse Events (CTCAE). Endpoints for long-term outcomes included overall survival, relapse rate, chronic graft-versus-host disease (cGvHD), and cause of death. The median follow-up was 1,483 days.

### Results

The median age was comparable between the two groups (alemtuzumab: 60 years; ATG: 58 years). However, patients treated with alemtuzumab had a significantly higher comorbidity burden compared with the ATG cohort (median HCT-CI: 3 vs. 1). Notably, acute tolerability was better with alemtuzumab than with ATG, and the rate of acute GvHD was significantly lower (44% vs. 68%, $p = 0.01$). Overall survival was statistically superior in the ATG group ($p = 0.04$). However, analyzed by disease category this difference was observed only in patients with AML. Importantly, the higher mortality rate among AML patients treated with alemtuzumab was attributable to both increased disease-related and treatment-related mortality. No statistically significant survival benefit of ATG was observed in patients older than 60 years or in those with a high HCT-CI.

**Copyright Information:** (Online) ISSN: 2572-9241
©2026 The Author(s). HemaSphere is published by John Wiley & Sons Ltd on behalf of European Hematology Association. This is an open access Abstract Book distributed under the Attribution-NonCommercial-NoDerivs (CC BY-NC-ND), which allows third parties to download the articles and share them with others as long as they credit the author and the Abstract Book, but they cannot change the content in any way or use them commercially.
**Abstract Book Citations:** Authors, Title, HemaSphere, 2026;10:(S1):pages. The abstract book DOIs can be found on the "Author Information" page.
**Disclaimer:** Articles published in the journal HemaSphere exclusively reflect the opinions of the authors. The authors are responsible for all content in their abstracts including accuracy of the facts, statements, citing resources, etc.

**Summary/Conclusion**

In this single-center study, the FBM conditioning regimen demonstrated strong efficacy and good tolerability when used with both ATG as well as alemtuzumab. Alemtuzumab provided improved control of acute GvHD. Alemtuzumab appears to be particularly useful as substitute for ATG in elderly patients and those with significant comorbidities. However, alemtuzumab should be used with caution in AML patients, where ATG may be of benefit, potentially through improved disease control.

**Copyright Information:** (Online) ISSN: 2572-9241
©2026 The Author(s). HemaSphere is published by John Wiley & Sons Ltd on behalf of European Hematology Association. This is an open access Abstract Book distributed under the Attribution-NonCommercial-NoDerivs (CC BY-NC-ND), which allows third parties to download the articles and share them with others as long as they credit the author and the Abstract Book, but they cannot change the content in any way or use them commercially.
**Abstract Book Citations:** Authors, Title, HemaSphere, 2026;10:(S1):pages. The abstract book DOIs can be found on the "Author Information" page.
**Disclaimer:** Articles published in the journal HemaSphere exclusively reflect the opinions of the authors. The authors are responsible for all content in their abstracts including accuracy of the facts, statements, citing resources, etc.

### (PB4043) FEASIBILITY AND SAFETY OF REDUCED-DOSE POST-TRANSPLANT CYCLOPHOSPHAMIDE COMBINED WITH RABBIT ATG FOR GRAFT-VERSUS-HOST DISEASE PROPHYLAXIS

**Topic:** 23. Stem cell transplantation - Clinical

Swapnil Tripathi*[1], Himani Gupta[1], Vaisshnavi Goel[1], Narendra Agrawal[1]

[1] *Rajiv Gandhi Cancer Institute and Research Centre, Hemato-oncology and BMTU, New Delhi, India;*

### Background

Graft-versus-host disease (GvHD) remains a major cause of morbidity and mortality after allogeneic hematopoietic stem cell transplantation (allo-HSCT). Post-transplant cyclophosphamide (PTCy) has improved GvHD control compared with conventional calcineurin inhibitor (CNI) plus methotrexate regimens. However, the standard dose (50 mg/kg/day ×2) has been associated with infectious complications, hemorrhagic cystitis, cardiotoxicity, and delayed immune reconstitution. Dose-reduction strategies aim to minimize toxicity while maintaining efficacy. Data on low-dose PTCy combined with rabbit anti-thymocyte globulin (rATG) remain limited.

### Aims

To evaluate the feasibility, safety, and efficacy of low-dose PTCy (25 mg/kg/day ×2) combined with rATG and cyclosporine for GvHD prophylaxis in allo-HSCT recipients.

### Methods

We conducted a single-center retrospective case series of 15 consecutive allo-HSCT recipients. Diagnoses included acute leukemias (n=12), lymphoma (n=1), aplastic anemia (n=1), and familial HLH (n=1). Thirteen patients received myeloablative conditioning and two received reduced-intensity conditioning.
GvHD prophylaxis consisted of PTCy 25 mg/kg on days +3 and +4 (total 50 mg/kg), rATG 2.5 mg/kg total divided over days −2 and −1, and oral cyclosporine from day −1 (target trough 200–300 ng/mL; tapered from day +60 and stopped by day +90).
Mycophenolate mofetil was added from day +5 to +35 in haploidentical transplants. Primary endpoints included acute and chronic GvHD. Secondary endpoints included engraftment, CMV reactivation, organ toxicity, relapse, and survival.

### Results

Median age was 25 years (range 17–55). Median CD34+ dose was $6.2\times10^6$/kg. Median neutrophil and platelet engraftment occurred at 13 and 12 days, respectively. Acute GvHD occurred in 2 patients (13%)—both grade II (one gastrointestinal, one skin). No grade III–IV cases were observed. Chronic GvHD developed in 3 patients (20%), all mild (skin n=2, mucosal n=1). Overall GvHD incidence was 27%. CMV reactivation occurred in 7 patients (47%), including one case of CMV colitis; all responded to intravenous ganciclovir. One patient who received letermovir prophylaxis remained CMV-free through day +120 despite steroid exposure. Transient mild tremors were observed in 3 patients following total body irradiation–based conditioning and resolved spontaneously. No hemorrhagic cystitis, cardiotoxicity, graft dysfunction, relapse, or death occurred. At a median follow-up of 156 days, overall survival and event-free survival were 100%.

**Copyright Information:** (Online) ISSN: 2572-9241
©2026 The Author(s). HemaSphere is published by John Wiley & Sons Ltd on behalf of European Hematology Association. This is an open access Abstract Book distributed under the Attribution-NonCommercial-NoDerivs (CC BY-NC-ND), which allows third parties to download the articles and share them with others as long as they credit the author and the Abstract Book, but they cannot change the content in any way or use them commercially.
**Abstract Book Citations:** Authors, Title, HemaSphere, 2026;10:(S1):pages. The abstract book DOIs can be found on the "Author Information" page.
**Disclaimer:** Articles published in the journal HemaSphere exclusively reflect the opinions of the authors. The authors are responsible for all content in their abstracts including accuracy of the facts, statements, citing resources, etc.

**Summary/Conclusion**

Low-dose PTCy (25 mg/kg ×2) combined with low-dose rATG and cyclosporine provided excellent GvHD control with rapid engraftment and minimal organ toxicity in this small cohort. However, CMV reactivation was frequent, likely reflecting augmented T-cell depletion from combined ATG and PTCy. Routine antiviral prophylaxis should be strongly considered in this setting. Larger prospective studies are warranted to validate these findings and clarify the necessity of ATG when using reduced-dose PTCy.

**Copyright Information:** (Online) ISSN: 2572-9241
©2026 The Author(s). HemaSphere is published by John Wiley & Sons Ltd on behalf of European Hematology Association. This is an open access Abstract Book distributed under the Attribution-NonCommercial-NoDerivs (CC BY-NC-ND), which allows third parties to download the articles and share them with others as long as they credit the author and the Abstract Book, but they cannot change the content in any way or use them commercially.
**Abstract Book Citations:** Authors, Title, HemaSphere, 2026;10:(S1):pages. The abstract book DOIs can be found on the "Author Information" page.
**Disclaimer:** Articles published in the journal HemaSphere exclusively reflect the opinions of the authors. The authors are responsible for all content in their abstracts including accuracy of the facts, statements, citing resources, etc.

### (PB4044) FAILURE RATES OF HAEMATOPOIETIC STEM CELL MOBILISATION IN RELAPSED-REFRACTORY HODGKIN LYMPHOMA. A SINGLE CENTRE EXPERIENCE AT GROOTE SCHUUR HOSPITAL

**Topic:** 23. Stem cell transplantation - Clinical

David Richardson*[1], Thomas Muza[1], Karryn Brown[1], Jenna Oosthuizen[1], Estelle Ramona Verburgh[1]

*[1]University of Cape Town and Groote Schuur Hospital, Department of Medicine, Cape Town, South Africa;*

### Background

Hodgkin lymphoma (HL) is one of the most treatable cancers, with most patients achieving durable remission with first-line therapy. However, up to 30% of patients develop relapsed or refractory disease (rrHL) requiring salvage chemotherapy followed by autologous stem cell transplantation (ASCT). Successful ASCT depends on adequate peripheral blood CD34+ stem cell (PBCD34) mobilization, but reported mobilization failure rates in lymphoma patients vary widely, ranging from 5-40%. In low-income regions, plerixafor access is limited and only became available at our center in June 2021. In this context, identifying patients at risk of poor mobilization may optimize the use of limited plerixafor stock and improve transplant rates. However, ASCT data from homogeneous rrHL cohorts in developing nations and human immunodeficiency virus (HIV)-endemic regions remain limited.

### Aims

To determine PBCD34 mobilization failure rates in rrHL patients eligible for ASCT at Groote Schuur Hospital between 2013 and 2023 and to identify factors associated with poor mobilization pre- and post-the introduction of plerixafor.

### Methods

A retrospective cohort study was conducted of rrHL patients undergoing stem cell mobilization for planned ASCT at a single tertiary centre. Mobilization failure was defined as inability to collect $\geq 2 \times 10^6$ CD34+ cells/kg. Clinical characteristics, treatment exposures, mobilisation kinetics, and outcomes were analysed, with multivariable analyses used to identify predictors of failure.

### Results

Of 72 patients, 82% successfully mobilized at first harvest, 91% of whom proceeded to transplant. In contrast, only 31% of patients with first harvest mobilization failure ultimately achieved harvest. Successful stem cell harvest in a single collection was associated with day-1 PBCD34 cell counts, but no clinical or pathological features were significant in univariate regression. Receiver operator characteristic curves showed that day-1 PBCD34 counts <6.9 cells/µL predicted mobilization failure (area under the curve 0.855, sensitivity 92%, specificity 68%). In patients with reduced baseline PBCD34 counts, administration of plerixafor was associated with improved PBCD34 mobilisation and once plerixafor was available, no harvest failures occurred. Compared to no transplant, ASCT was associated with a substantial survival advantage (12-month survival 96% vs. 75%; 5-year survival 71% vs. 19%). Mobilization outcomes in virally suppressed, optimised HIV-positive patients were comparable to HIV-negative cohorts.

### Summary/Conclusion

Stem cell mobilisation capacity is a key determinant of ASCT eligibility and thus survival in rrHL. Early PBCD34 cell count thresholds provide practical tools for predicting mobilisation failure and guiding selective, pre-emptive plerixafor use in resource-constrained settings. The homogeneous nature of this cohort strengthens the validity of these findings and supports the development of rrHL-specific mobilisation strategies applicable to low- and middle-income environments.

**Copyright Information:** (Online) ISSN: 2572-9241
©2026 The Author(s). HemaSphere is published by John Wiley & Sons Ltd on behalf of European Hematology Association. This is an open access Abstract Book distributed under the Attribution-NonCommercial-NoDerivs (CC BY-NC-ND), which allows third parties to download the articles and share them with others as long as they credit the author and the Abstract Book, but they cannot change the content in any way or use them commercially.
**Abstract Book Citations:** Authors, Title, HemaSphere, 2026;10:(S1):pages. The abstract book DOIs can be found on the “Author Information” page.
**Disclaimer:** Articles published in the journal HemaSphere exclusively reflect the opinions of the authors. The authors are responsible for all content in their abstracts including accuracy of the facts, statements, citing resources, etc.

**Figure 1. Overcoming mobilization failure and improving disease outcomes through selective plerixafor administration**

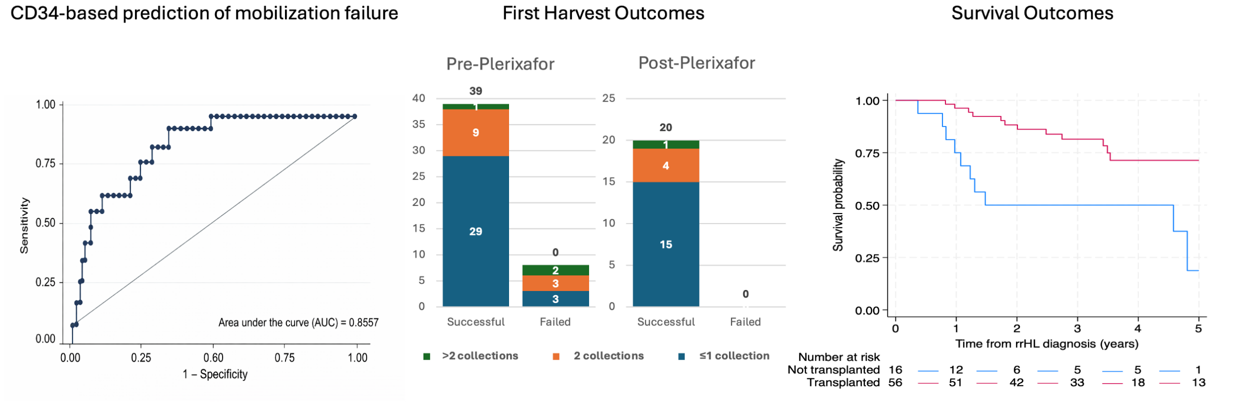

**A) Receiver operator characteristics curve indicated preharvest CD34 threshold of 6.9 cells/ul had 92% sensitivity and 68% specificity for failed harvest. This allows accurate prediction of need for Plerixafor**

**B) Implementation of selective, pre-emptive plerixafor use eliminated harvest failure after its introduction in June 2021**

**C) Kaplan-Meier curve showing patients undergoing ASCT achieved markedly improved 1- and 5-year overall survival (96% [86-99] vs 75% [46-90]; and 71% [54-83] vs 19% [1-52], respectively).**

**Copyright Information:** (Online) ISSN: 2572-9241
©2026 The Author(s). HemaSphere is published by John Wiley & Sons Ltd on behalf of European Hematology Association. This is an open access Abstract Book distributed under the Attribution-NonCommercial-NoDerivs (CC BY-NC-ND), which allows third parties to download the articles and share them with others as long as they credit the author and the Abstract Book, but they cannot change the content in any way or use them commercially.
**Abstract Book Citations:** Authors, Title, HemaSphere, 2026;10:(S1):pages. The abstract book DOIs can be found on the “Author Information” page.
**Disclaimer:** Articles published in the journal HemaSphere exclusively reflect the opinions of the authors. The authors are responsible for all content in their abstracts including accuracy of the facts, statements, citing resources, etc.

### (PB4045) POST-TRANSPLANT CYCLOPHOSPHAMIDE PLUS LOW-DOSE ATG: EFFECTIVE GVHD PROPHYLAXIS IN HAPLOIDENTICAL HEMATOPOIETIC STEM CELL TRANSPLANTATION

**Topic:** 23. Stem cell transplantation - Clinical

Xiaojuan An[1], Yu Tao[2], Yun MA[1], Hu Lina[3], Guoqiang Li[3], Peng Ke[3], Chun Feng[3], Huijun Li[3], Jun Wang[1], Jinhui He[1], Jing Huang[1], Miaoling Qiu[1], Xiaoling Qiu[1], Patrick Chu[1], Jiong Hu[4], Hua Yang[1], Jihao Zhou*[3]

*[1]The University of Hongkong-Shenzhen Hospital, Department of Hematology, Shenzhen, China;[2]The First Affiliated Hospital,School of Medicine,Southern University of Science and technology, Department of Hematology, Shenzhen, China; [3]Shenzhen People's Hospital, Department of Hematology, Shenzhen, China;[4]Shanghai Institute of Hematology, Blood and Marrow Transplantation Center, Collaborative Innovation Center of Hematology, Department of Hematology, Ruijin Hospital, Shanghai Jiao Tong University School of Medicine, Shanghai, China;*

### Background

While post-transplant cyclophosphamide (PTCy) is commonly used in Haploidentical hematopoietic stem cell transplantation (Haplo-HSCT) as Graft-Versus-Host Disease(GVHD) Prophylaxis, the incidence of moderate-to-severe GVHD remains substantial.Preclinical and clinical data suggest synergistic effects between PTCy and ATG. PTCy in conjunction with ATG appears as a potentially effective GVHD prevention strategy in Haplo-HCT.

### Aims

In this study, we adopted a novel strategy combining medium-dose PTCy (80 mg/kg) with low-dose, delayed ATG (2.5 mg/kg), administered after neutrophil engraftment, aiming to evaluate the safety and efficacy of this regimen for GVHD prophylaxis in Haplo-HSCT.

### Methods

We retrospectively analyzed 102 patients with hematologic malignancies who underwent first-time Haplo-HSCT at Shenzhen People's Hospital and The University of Hong Kong-Shenzhen Hospital. Patients were divided into two groups: PTCy-ATG (n=28) and ATG-alone (n=74). All received myeloablative conditioning. GVHD prophylaxis in the PTCy-ATG group consisted of PTCy (40mg/kg/day on days +3 and +4), low-dose ATG (2.5mg/kg on day +15 or later, with neutrophil-engraftment), mycophenolate mofetil, and cyclosporine. The ATG-alone group received pre-transplant ATG (7.5-10mg/kg) plus cyclosporine, mycophenolate mofetil, and short-term methotrexate. Endpoints included engraftment, GVHD, infections, survival, relapse, and immune reconstitution.

### Results

Patient characteristics were balanced between groups. Neutrophil recovery was delayed in the PTCy-ATG group (median 16 vs. 14 days, p=0.027). The PTCy-ATG group had significantly lower incidence of grade I–IV acute GVHD (18.2% vs. 47.9%, p=0.008) and a trend toward reduced grade II–IV acute GVHD (14.6% vs. 34.2%, p=0.051). Chronic GVHD rates were comparable. The PTCy-ATG group also exhibited lower CMV reactivation (21.8% vs. 50.0%), EBV reactivation (7.1% vs. 14.9%), bloodstream infections (3.6% vs. 23%, p<0.05), and hemorrhagic cystitis (7.1% vs. 32.4%, p<0.05). No significant differences were observed in sinusoidal obstruction syndrome, thrombotic microangiopathy, 3-year overall survival (68.8% vs. 68.0%), disease-free survival (68.8% vs. 67.2%), or GVHD-free relapse-free survival (57.1% vs. 56.76%). No relapses occurred in the PTCy-ATG group versus 9.5% in the ATG-alone group, though non-relapse mortality was numerically higher (31.2% vs. 23.3%). Immune reconstitution was similar between groups.

**Copyright Information:** (Online) ISSN: 2572-9241
©2026 The Author(s). HemaSphere is published by John Wiley & Sons Ltd on behalf of European Hematology Association. This is an open access Abstract Book distributed under the Attribution-NonCommercial-NoDerivs (CC BY-NC-ND), which allows third parties to download the articles and share them with others as long as they credit the author and the Abstract Book, but they cannot change the content in any way or use them commercially.
**Abstract Book Citations:** Authors, Title, HemaSphere, 2026;10:(S1):pages. The abstract book DOIs can be found on the “Author Information” page.
**Disclaimer:** Articles published in the journal HemaSphere exclusively reflect the opinions of the authors. The authors are responsible for all content in their abstracts including accuracy of the facts, statements, citing resources, etc.

### Summary/Conclusion

Medium-dose PTCy combined with low-dose, delayed ATG is an effective and safe GVHD prophylaxis regimen in Haplo-HSCT, associated with significant reductions in acute GVHD, viral reactivation, and key transplant-related complications, without compromising survival outcomes. The differential profile in relapse and non-relapse mortality warrants further investigation.

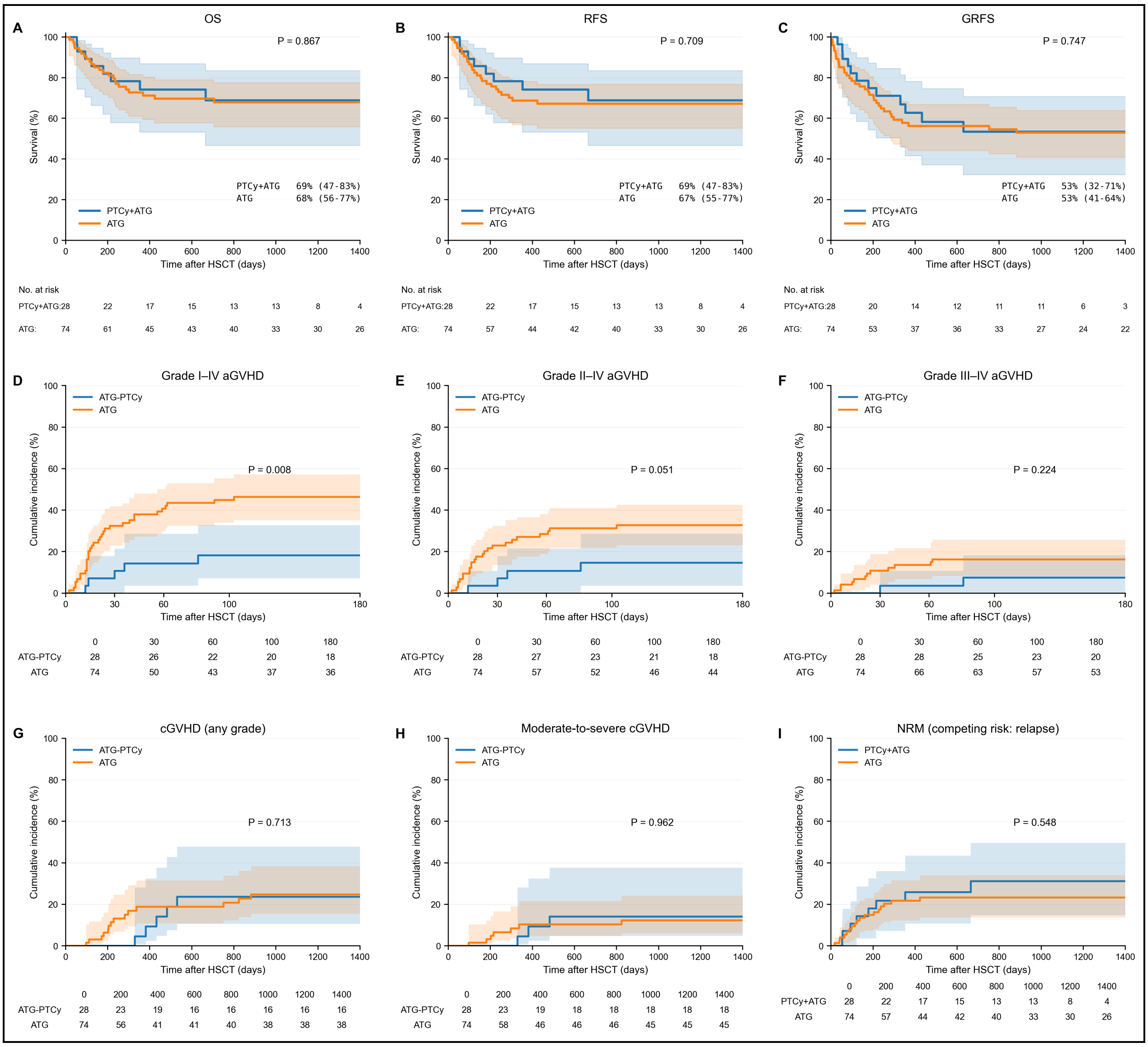

**Copyright Information:** (Online) ISSN: 2572-9241
©2026 The Author(s). HemaSphere is published by John Wiley & Sons Ltd on behalf of European Hematology Association. This is an open access Abstract Book distributed under the Attribution-NonCommercial-NoDerivs (CC BY-NC-ND), which allows third parties to download the articles and share them with others as long as they credit the author and the Abstract Book, but they cannot change the content in any way or use them commercially.
**Abstract Book Citations:** Authors, Title, HemaSphere, 2026;10:(S1):pages. The abstract book DOIs can be found on the "Author Information" page.
**Disclaimer:** Articles published in the journal HemaSphere exclusively reflect the opinions of the authors. The authors are responsible for all content in their abstracts including accuracy of the facts, statements, citing resources, etc.

### (PB4046) RETROSPECTIVE STUDY OF SINUSOIDAL OBSTRUCTION SYNDROME IN PEDIATRIC PATIENTS WITH PRE-TRANSPLANT INOTUZUMAB OZOGAMICIN :A SINGLE CENTER STUDY FROM CHINA

**Topic:** 23. Stem cell transplantation - Clinical

Chen Zhou*[1], Xiaodong Wang[1], Chengjuan Luo[1], Xia Qin[1], Uet Yu[1], Xiaohang Huang[1], Yuchen Lin[1], Xinan Wang[1], Manpin Zhang[1], Aiyun Song[1], Jingyu Gao[1], Qi Luo[1], Jing Chen[1]

*[1]Shanghai Children's Medical Center,Shanghai Jiao Tong University School of Medicine, Blood and Marrow Transplantation Center, Shanghai, China;*

### Background

Inotuzumab ozogamicin (InO) is an anti-CD22 antibody conjugated to calicheamicin that is effective against CD22- positive B acute lymphoblastic leukemia (B-ALL) giving chances and hopes for remission to patients with relapsed or refractory (r/r) B-ALL.However, it is associated with increased risk of sinusoidal obstruction syndrome (SOS) ,which is potentially a life-threatening complication that can be observed after allogeneic hematopoietic stem cell transplantation (HSCT) .

### Aims

We studied patients who received an anti-CD22-calicheamicin conjugate (inotuzumab ozogamicin) to gain insight into mechanisms of sinusoidal injury and to find potential contributing risk factors.

### Methods

**Study population and conditioning regimen**

We conducted a retrospective analysis of patients treated in Shanghai Children's Medical Center that received Ino before HSCT from 2023 to 2025. Conditioning regimens studied included myeloablative (MAC) or reduced-intensity (RIC) conditioning.MAC regimen includes total body irradiation (TBI) 12Gy or busulfan plus cyclophosphamide 120mg/kg and antithymocyte globulin 7.5mg/kg. RIC regimen includes two categories, the one is TBI 2-3Gy plus fludarabine,melphalan,cetirizine and cyclophosphamide ,the other one is busulfan plus fludarabine,melphalan and cyclophosphamide.

**Diagnosis of SOS**

the European Society of Bone Marrow Transplant (EBMT) has established new clinical diagnostic criteria for SOS in pediatric patients in 2018 and our center confirm the diagnosis of SOS according to this new criteria.

**SOS prophylaxis and treatment**

Ursodiol and alprostadil were routinely administered for SOS prophylaxis for all patients undergoing HSCT at our center. Defibrotide was used for patients diagnosis of severe SOS, and for the mild SOS supportive treatment was the most important.

**Statistical analysis**

Baseline characteristics were reported descriptively. Fisher's exact test was used to assess the contingency tables and the Wilcoxon rank sum test was performed to examine the difference on medians. The significance of prognostic factors was tested using the logistic regression analysis. Survival estimates were calculated using the Kaplan-Meier method and the comparison was performed by Log-rank test. Statistical significance was set at the $P < 0.05$ level; all P values were two-sided. Statistical analyses were performed using SPSS 22.0

**Copyright Information:** (Online) ISSN: 2572-9241
©2026 The Author(s). HemaSphere is published by John Wiley & Sons Ltd on behalf of European Hematology Association. This is an open access Abstract Book distributed under the Attribution-NonCommercial-NoDerivs (CC BY-NC-ND), which allows third parties to download the articles and share them with others as long as they credit the author and the Abstract Book, but they cannot change the content in any way or use them commercially.
**Abstract Book Citations:** Authors, Title, HemaSphere, 2026;10:(S1):pages. The abstract book DOIs can be found on the "Author Information" page.
**Disclaimer:** Articles published in the journal HemaSphere exclusively reflect the opinions of the authors. The authors are responsible for all content in their abstracts including accuracy of the facts, statements, citing resources, etc.

**Results**

Between January 2023 and December 2025, we identified 30 patients who received Ino before HSCT⊠all of our patients received just one cycle of Ino therapy.5 patients(16.7%) developed SOS after HSCT and one of them was graded as very severe SOS.We analyzed risk factors reported contributing to SOS in HSCT with prior use of Ino, including the interval from the last administration of Ino to HSCT⊠< 40days or >40days⊠, Disease Status⊠CR1⊠CR2 or > CR2⊠ ⊠conditioning regimen⊠MAC or RIC⊠, Dual alkylators for conditioning and GVHD prophylaxis. Interval time < 40days is the only significant facor both in univariate analysis(*P*=0.015) and multivariate analysis(*P*=0.002).

**Summary/Conclusion**

Inotuzumab has proven to effective in R/R ALL but SOS may require more attention in patients with prior use of Ino⊠and increasing the interval time (preferably more than 40 days) from the last administration of Ino to HSCT may reduce the occurrence of this fatal complications.

**Copyright Information:** (Online) ISSN: 2572-9241
©2026 The Author(s). HemaSphere is published by John Wiley & Sons Ltd on behalf of European Hematology Association. This is an open access Abstract Book distributed under the Attribution-NonCommercial-NoDerivs (CC BY-NC-ND), which allows third parties to download the articles and share them with others as long as they credit the author and the Abstract Book, but they cannot change the content in any way or use them commercially.
**Abstract Book Citations:** Authors, Title, HemaSphere, 2026;10:(S1):pages. The abstract book DOIs can be found on the “Author Information” page.
**Disclaimer:** Articles published in the journal HemaSphere exclusively reflect the opinions of the authors. The authors are responsible for all content in their abstracts including accuracy of the facts, statements, citing resources, etc.

### (PB4047) ANALYSIS OF INFLUENCING FACTORS OF SLEEP QUALITY AND NURSING INTERVENTIONS IN PATIENTS UNDERGOING HEMATOPOIETIC STEM CELL TRANSPLANTATION

**Topic:** 23. Stem cell transplantation - Clinical

Yushuai Guo*[1], Yuqiu Liu[1], Wenjun Xie[1], Yi He[1]

[1]*Institute of Hematology & Blood Diseases Hospital, Chinese Academy of Medical Sciences & Peking Union Medical College, Tian Jin, China;*

### Aims

To investigate the current status of sleep disorders and related influencing factors in patients undergoing hematopoietic stem cell transplantation (HSCT) during hospitalization, construct a scientific, systematic and operable nursing intervention program, and provide theoretical basis and clinical practice reference for improving patients' sleep quality, relieving physical and mental pain, enhancing treatment compliance, and promoting post-transplant rehabilitation and long-term quality of life.

### Methods

A total of 92 patients who received HSCT in the laminar flow ward of our hospital from June to December 2024 were selected as the research subjects. General information questionnaire, Pittsburgh Sleep Quality Index (PSQI), Self-Rating Anxiety Scale (SAS), Self-Rating Depression Scale (SDS) and self-designed sleep influencing factor scale were used for investigation. The incidence of sleep disorders was counted, and univariate and multivariate Logistic regression analyses were performed. Nursing interventions were implemented for patients with sleep disorders.

### Results

Among the 92 patients, 78 suffered from sleep disorders, with an incidence rate of 84.78%. The main manifestations included difficulty falling asleep, frequent nocturnal awakenings, light sleep, early awakening and daytime sleepiness. Anxiety and depression, physical pain and discomfort, environmental noise and light interference, nocturnal therapeutic operations, adverse drug reactions, and circadian rhythm disturbance were identified as independent risk factors for sleep disorders (P[illegible]0.05). After the implementation of comprehensive nursing interventions, the scores of PSQI, SAS and SDS in patients decreased significantly compared with those before intervention, and the sleep quality and emotional state were improved remarkably.

### Summary/Conclusion

The incidence of sleep disorders is high in HSCT patients, which is jointly affected by multiple factors. Multidimensional, individualized and whole-course comprehensive nursing interventions can effectively improve sleep quality, alleviate negative emotions, reduce the risk of complications, and have important clinical significance for promoting patients' rehabilitation.

**Copyright Information:** (Online) ISSN: 2572-9241
©2026 The Author(s). HemaSphere is published by John Wiley & Sons Ltd on behalf of European Hematology Association. This is an open access Abstract Book distributed under the Attribution-NonCommercial-NoDerivs (CC BY-NC-ND), which allows third parties to download the articles and share them with others as long as they credit the author and the Abstract Book, but they cannot change the content in any way or use them commercially.
**Abstract Book Citations:** Authors, Title, HemaSphere, 2026;10:(S1):pages. The abstract book DOIs can be found on the "Author Information" page.
**Disclaimer:** Articles published in the journal HemaSphere exclusively reflect the opinions of the authors. The authors are responsible for all content in their abstracts including accuracy of the facts, statements, citing resources, etc.

### (PB4048) HERPESVIRUS INFECTION IN PATIENTS EARLY AFTER ALLOGENEIC HEMATOPOIETIC STEM CELL TRANSPLANTATION

**Topic:** 23. Stem cell transplantation - Clinical

Nechay Oksana*[1], Dziuba Alena[1], Morozova Oksana[1], Strongin Yuri[1], Tatsiana Talako[1], Alena Hlaz[1], Amvrosieva Tamara[2], Natalya Milanovich[1], Uss Anatoly[1]

*[1]Minsk Scientific and Practical Center for Surgery, Transplantology and Hematology, Minsk, Belarus;[2]Research Institute of Hygiene, Toxicology, Epidemiology, Virology, and Microbiology, Minsk, Belarus;*

### Background

Infectious complications are one of the main causes of mortality after allogeneic hematopoietic stem cell transplantation (alloHSCT). The etiology of infectious complications varies at different times after allogeneic hematopoietic stem cell transplantation. While bacterial infections play a leading role during the cytopenia period, viral pathogens become more prevalent after hematopoiesis is established.

### Aims

To determine the frequency of development of herpesvirus infections in allo-HSCT recipients, activation time and clinical manifestations.

### Methods

The study included 70 participants: 35 donors and 35 alloHSCT recipients: 18 with acute leukemia, 4 with chronic myeloid leukemia, 3 with myelodysplastic syndrome, 6 with aplastic anemia, 2 with Hodgkin's lymphoma, and 2 with primary myelofibrosis.12 patients underwent alloHSCT from a related donor, 23 from an unrelated donor (14 - HLA 1010 compatible, 7 - 9/10 compatible), and 2 patients underwent haploidentical alloHSCT (9/10 and 6/10). All donors underwent serological blood testing for cytomegalovirus (CMV), Epstein-Barr virus (EBV), and human herpesvirus-6 (HHV-6) prior to stimulation, as well as polymerase chain reaction (PCR) testing of blood and urine for CMV, EBV, and HHV-6.
All patients underwent serological blood testing for CMV, EBV, and HHV-6 prior to conditioning, and on days +7, +14, +30, +60, +90, and +120 after allo-HSCT. If potential signs of herpesvirus infections were detected, blood and urine were tested using PCR.

### Results

All allo-HSCT recipients (100%) were CMV seropositive (IgG). Among donors, CMV seropositivity was 80.0% (28 of 35). Seropositivity among related donors was 90.0% (n=11), while among unrelated donors, it was 73.9% (n=14). Of the 35 allo-HSCT recipients, 7 (20.0%) experienced clinically significant CMV reactivation: median day +22 (range +14 to +170). The main manifestations were skin manifestations, low-grade fever, and hemorrhagic cystitis. Of these patients, 5 (71.4%) had seronegative donors (CMV IgG-). Isolated manifestations of EBV were recorded in 2 patients (5.7%) at days +18 and +120, with skin lesions and myalgia. HHV-6 was detected in 6 patients (17.1%) after alloHSCT: median at day +85 (range +14 to +365). Main manifestations: hemorrhagic cystitis, secondary graft hypofunction. Combined reactivation of CMV and HHV-6 was recorded in 4 patients (11.4%): median +68 days (from +14 to +150) with manifestations such as acute kidney injury and hemorrhagic cystitis. Activation of all studied viruses was detected in 3 patients (8.6%) with a median +57 days (from +14 to +150), accompanied by profound thrombocytopenia, febrile fever in the absence of verified bacterial infection. In one patient, activation of all herpesviruses was detected against the background of a four-component regimen for the treatment of acute GVHD

**Copyright Information:** (Online) ISSN: 2572-9241
©2026 The Author(s). HemaSphere is published by John Wiley & Sons Ltd on behalf of European Hematology Association. This is an open access Abstract Book distributed under the Attribution-NonCommercial-NoDerivs (CC BY-NC-ND), which allows third parties to download the articles and share them with others as long as they credit the author and the Abstract Book, but they cannot change the content in any way or use them commercially.
**Abstract Book Citations:** Authors, Title, HemaSphere, 2026;10:(S1):pages. The abstract book DOIs can be found on the "Author Information" page.
**Disclaimer:** Articles published in the journal HemaSphere exclusively reflect the opinions of the authors. The authors are responsible for all content in their abstracts including accuracy of the facts, statements, citing resources, etc.

**Summary/Conclusion**

The manifestations of herpesvirus infections after allo-HSCT are varied, appear at different times, and require regular monitoring and treatment. Particular attention is required in CMV IgG-seropositive recipients with a CMV IgG-seronegative donor; among these recipients, CMV reactivation was 71.4%.

**Copyright Information:** (Online) ISSN: 2572-9241
©2026 The Author(s). HemaSphere is published by John Wiley & Sons Ltd on behalf of European Hematology Association. This is an open access Abstract Book distributed under the Attribution-NonCommercial-NoDerivs (CC BY-NC-ND), which allows third parties to download the articles and share them with others as long as they credit the author and the Abstract Book, but they cannot change the content in any way or use them commercially.
**Abstract Book Citations:** Authors, Title, HemaSphere, 2026;10:(S1):pages. The abstract book DOIs can be found on the "Author Information" page.
**Disclaimer:** Articles published in the journal HemaSphere exclusively reflect the opinions of the authors. The authors are responsible for all content in their abstracts including accuracy of the facts, statements, citing resources, etc.

### (PB4049) ALLOGENEIC TRANSPLANTATION FOR CMML-DERIVED AML: IMPACT OF ANTECEDENT CMML DISEASE FEATURES ON POST-TRANSPLANT OUTCOMES

**Topic:** 23. Stem cell transplantation - Clinical

Xiaohong Liu[1, 1], Yue Lu[1], Xingyu Cao[1], Deyan Liu*[1]

[1]*Beijing Lu Daopei Hospital, Beijing, China;*

### Background

Chronic myelomonocytic leukemia (CMML) is a biologically heterogeneous myeloid neoplasm with approximately 20% of patients progressing to secondary acute myeloid leukemia (s-AML), particularly CMML-2, which is associated with rapid leukemic evolution and dismal survival. Allogeneic hematopoietic stem cell transplantation (allo-HSCT) remains the only curative option, but post-transplant outcomes in CMML-derived AML remain poorly defined owing to the rarity of this biologically distinct and highly selected population.

### Aims

To evaluate transplant outcomes and prognostic determinants in patients undergoing allo-HSCT following CMML transformation to AML.

### Methods

We retrospectively analyzed 15 patients with CMML-derived AML who underwent allo-HSCT between January 2015 and May 2025 at two transplant centers. Overall survival (OS) and progression-free survival (PFS) were estimated using the Kaplan–Meier method. Follow-up was censored on January 1, 2026.

### Results

Among 15 patients, 11 (73.3%) had CMML-2 and 4 (26.7%) CMML-1. Median age was 50 years (range, 28–63), including 9 males (60.0%) and 6 females (40.0%). Haploidentical HSCT was performed in 12 patients (80%) and matched unrelated donor HSCT in 3 (20%). Median time from CMML diagnosis to AML transformation was 3.03 months (0.23–65.3). Patients received a median of 2 chemotherapy cycles during the CMML phase (1–10) and 2 after AML transformation (0–10). Complex karyotype was observed in 20% and non-complex abnormalities in 33.3%. Twelve patients harbored ≥3 gene mutations, including *TP53 (n=2), ASXL1 (n=6), FLT3-ITD (n=3), RUNX1 (n=4), SRSF2 (n=3), SF3B1 (n=2), and BCOR (n=1).*
Before HSCT, MRD-negative CR was achieved in 4 patients (26.7%), MRD-positive CR in 5 (33.3%) with median bone marrow blasts of 10.34% (0.03–40.4), and 6 (40%) remained in non-remission with median blasts of 19.5% (13.5–42). Median infused doses were $8.29\times10^8$/kg (4.11–17.36) for mononuclear cells and $4.86\times10^6$/kg (2.37–11.01) for $CD34^+$ cells. All patients achieved engraftment, with neutrophil and platelet recovery at medians of 14 (10–20) and 17 (12–100) days.
CMML-1 showed slower leukemic evolution than CMML-2 (8.37 vs 3.03 months) and higher pre-HSCT CR rates (100% vs 45.5%). After a median follow-up of 7.3 months (2.03–43.23), 1-year OS and PFS were 43.8% (95% CI, 23.9–80.2%) and 44.4% (24.6–80.5%), respectively, with 100-day grade III–IV acute GVHD of 26.67% (95% CI, 3.36–49.97%). CMML-1 patients (n=4) had superior outcomes compared with CMML-2 (n=11), with 1-year OS/PFS of 75.0% (95% CI, 42.6–100) versus 36.4% (95% CI, 16.6–79.5). Abnormal or complex karyotypes (n=8) were associated with inferior survival versus normal karyotype (n=7) (37.5% vs 57.1%). Pre-transplant CR (n=9) yielded OS 48.6% and PFS 50.0%, compared with 33.3% OS/PFS in NR patients (n=6), although differences were not statistically significant (OS P=0.064; PFS P=0.106), showing consistent adverse trends.

**Copyright Information:** (Online) ISSN: 2572-9241
©2026 The Author(s). HemaSphere is published by John Wiley & Sons Ltd on behalf of European Hematology Association. This is an open access Abstract Book distributed under the Attribution-NonCommercial-NoDerivs (CC BY-NC-ND), which allows third parties to download the articles and share them with others as long as they credit the author and the Abstract Book, but they cannot change the content in any way or use them commercially.
**Abstract Book Citations:** Authors, Title, HemaSphere, 2026;10:(S1):pages. The abstract book DOIs can be found on the “Author Information” page.
**Disclaimer:** Articles published in the journal HemaSphere exclusively reflect the opinions of the authors. The authors are responsible for all content in their abstracts including accuracy of the facts, statements, citing resources, etc.

**Summary/Conclusion**

Allo-HSCT provides meaningful survival for CMML-derived AML despite high-risk disease characteristics. Patients evolving from CMML-1 demonstrated more favorable transplant outcomes. Early transplant referral and achieving remission before HSCT may improve outcomes.

**Copyright Information:** (Online) ISSN: 2572-9241
©2026 The Author(s). HemaSphere is published by John Wiley & Sons Ltd on behalf of European Hematology Association. This is an open access Abstract Book distributed under the Attribution-NonCommercial-NoDerivs (CC BY-NC-ND), which allows third parties to download the articles and share them with others as long as they credit the author and the Abstract Book, but they cannot change the content in any way or use them commercially.
**Abstract Book Citations:** Authors, Title, HemaSphere, 2026;10:(S1):pages. The abstract book DOIs can be found on the "Author Information" page.
**Disclaimer:** Articles published in the journal HemaSphere exclusively reflect the opinions of the authors. The authors are responsible for all content in their abstracts including accuracy of the facts, statements, citing resources, etc.

### (PB4050) QUANTITATIVE EBV LOAD AND RELAPSE-FREE SURVIVAL AFTER ALLOGENEIC HEMATOPOIETIC STEM CELL TRANSPLANTATION

**Topic:** 23. Stem cell transplantation - Clinical

Jiaqi Tan*[1], Weng Jianyu[1], Chengwei Luo[1], Suijing Wu[1], Wei Ling[1], Xiaomei Chen[1], Lisi Huang[1], Xin Du[1]

[1]*Guangdong Provincial People´s Hospital (Guangdong Academy of Medical Sciences), Southern Medical University, Department of Hematology, Guangzhou, China;*

### Background

EBV reactivation is a common complication post-allogeneic hematopoietic stem cell transplantation (allo-HSCT), but its prognostic impact remains controversial. This study integrated single-center clinical data and meta-analysis to clarify the association between EBV viral load and post-transplant outcomes, aiming to guide clinical monitoring and management.

### Aims

This study aimed to 1) identify risk factors for EBV reactivation post-allo-HSCT; 2) explore the correlation between EBV viral load stratification and relapse-free survival (RFS)/overall survival (OS); 3) verify the prognostic value of EBV infection via meta-analysis to guide individualized clinical monitoring and management.

### Methods

A retrospective single-center analysis included 157 adult patients who underwent allo-HSCT between 2019–2021. EBV load was monitored via quantitative real-time PCR, with reactivation (EBV-R) defined as ≥500 copies/mL. Patients were stratified into four groups by EBV load: non-EBV-R (0–500 copies/mL), low-load (500–7,482 copies/mL), intermediate-load (7,482–39,350 copies/mL), and high-load (>39,350 copies/mL). For the meta-analysis, 11 eligible studies (2,459 cases) were identified via PubMed, and pooled odds ratios (OR) for relapse-free survival (RFS) and overall survival (OS) were calculated using Review Manager 5.

### Results

EBV-R occurred in 71.3% (112/157) of patients at a median 48.5 days post-transplant. Independent risk factors for EBV-R included advanced age ($p$=0.008), antithymocyte globulin (ATG) use ($p$<0.0001), HLA mismatch ($p$=0.0005), myeloid tumors ($p$=0.0053), and CMV co-infection ($p$=0.0126). Stratified survival analysis showed the intermediate EBV-load group had significantly better RFS than non-EBV-R/low-load groups (2-year RFS: 67.9% vs. 28.8%, $p$=0.025) and numerically superior OS (2-year OS: 71.4% vs. 38.4%, $p$=0.107). High EBV-load patients had comparable RFS/OS to non-EBV-R/low-load groups. Meta-analysis (after excluding heterogeneous studies) confirmed EBV infection was associated with increased RFS (OR=1.26, 95%CI=1.02–1.55, $I^2$=27%) without compromising OS (OR=1.01, 95%CI=0.80–1.59, $I^2$=49%). Nine patients (8.04%) with EBV-R developed post-transplant lymphoproliferative disorder (PTLD), all with high EBV loads (>34,800 copies/mL).

### Summary/Conclusion

Moderate EBV reactivation (7,482–39,350 copies/mL) correlates with improved RFS post-allo-HSCT, potentially via immune-stimulatory effects. High EBV loads predict PTLD but do not worsen survival with appropriate intervention. These findings support individualized EBV monitoring and risk-adapted management strategies in transplant recipients.

**Copyright Information:** (Online) ISSN: 2572-9241
©2026 The Author(s). HemaSphere is published by John Wiley & Sons Ltd on behalf of European Hematology Association. This is an open access Abstract Book distributed under the Attribution-NonCommercial-NoDerivs (CC BY-NC-ND), which allows third parties to download the articles and share them with others as long as they credit the author and the Abstract Book, but they cannot change the content in any way or use them commercially.
**Abstract Book Citations:** Authors, Title, HemaSphere, 2026;10:(S1):pages. The abstract book DOIs can be found on the "Author Information" page.
**Disclaimer:** Articles published in the journal HemaSphere exclusively reflect the opinions of the authors. The authors are responsible for all content in their abstracts including accuracy of the facts, statements, citing resources, etc.

### (PB4051) DOES RENAL FUNCTION MATTER? CHRONIC KIDNEY DISEASE DEFINES A SEPARATE CLINICAL PHENOTYPE IN AUTOLOGOUS HEMATOPOIETIC STEM CELL TRANSPLANTATION

**Topic:** 23. Stem cell transplantation - Clinical

Anamarija Vrkljan Vuk*[1, 1], Matija Crnogorac[1], David Čičić[1], Ena Soric[1], Ozren Jakšić[1, 2], Ivan Durlen[1], Ivana Bedalov Crnkovic[1], Zeljko Prka[1]

*[1]University Hospital Dubrava, Zagreb, Croatia; [2]University of Zagreb School of Medicine, Zagreb, Croatia;*

### Background

Chronic kidney disease (CKD) is increasing in global prevalence. Given its pathophysiological characteristics, CKD may significantly affect clinical course, transplant-related toxicity, and outcomes in patients undergoing autologous hematopoietic stem cell transplantation (ASCT), potentially representing a distinct clinical phenotype requiring individualized management.

### Aims

To assess the prevalence and clinicopathological characteristics of CKD in ASCT patients, and to evaluate the impact of pre-existing CKD on renal function trajectories and transplant-associated nephrotoxicity.

### Methods

Retrospective analysis of consecutive ASCT patients treated at our Center during 2024–2025. Clinical data, comorbidities, and serial renal function parameters were extracted from institutional electronic health records and compared between CKD and non-CKD patients. CKD was classified per KDIGO criteria. Persistent renal impairment was defined as failure to recover renal function within 3 months post-ASCT, determined in collaboration with dedicated nephrologists. Between-group differences were assessed using Student's t-test and Chi-square test.

### Results

The analysis included 26 CKD and 29 non-CKD patients, with median ages of 68 (IQR 40–75) and 60 years (IQR 27–63); 9 (34%) and 13 (44%) were female. All received ASCT for multiple myeloma (MM) or non-Hodgkin lymphoma (NHL). MM was more prevalent in the CKD group (20/26, 77% vs. 13/29, 44%; p=0.001). No significant differences were found in type 2 diabetes (17% vs. 23%; p=0.5), arterial hypertension (80% vs. 65%; p=0.2), or cardiovascular disease (24% vs. 23%; p=0.9) — classical CKD risk factors. Four CKD patients (15%) were on chronic haemodialysis at ASCT. Acute kidney injury (AKI) occurred in 7 CKD patients (27%); 2 required continuous renal replacement therapy, 6 recovered, and 1 died. No AKI occurred in the non-CKD group. In the CKD cohort, SCr (median: 129–142.5–147.5 µmol/L) and eGFR (44.0–41.5–44.2 mL/min/1.73m²) remained stable throughout (p=0.15; p=0.17). In non-CKD patients, renal function was preserved overall (p=0.17), with a non-significant transient SCr rise (p=0.07) and a significant transient eGFR decline peri-ASCT (p=0.04).

### Summary/Conclusion

CKD in ASCT patients appears driven primarily by the underlying haematological malignancy — notably MM — rather than classical risk factors. Despite high-dose conditioning, CKD patients maintained stable renal function post-ASCT, and most who developed AKI recovered. These findings support CKD as a distinct clinical phenotype in ASCT, warranting tailored conditioning, nephroprotective strategies, and structured renal follow-up. Cohort expansion is planned to further characterize long-term outcomes.

**Copyright Information:** (Online) ISSN: 2572-9241
©2026 The Author(s). HemaSphere is published by John Wiley & Sons Ltd on behalf of European Hematology Association. This is an open access Abstract Book distributed under the Attribution-NonCommercial-NoDerivs (CC BY-NC-ND), which allows third parties to download the articles and share them with others as long as they credit the author and the Abstract Book, but they cannot change the content in any way or use them commercially.
**Abstract Book Citations:** Authors, Title, HemaSphere, 2026;10:(S1):pages. The abstract book DOIs can be found on the "Author Information" page.
**Disclaimer:** Articles published in the journal HemaSphere exclusively reflect the opinions of the authors. The authors are responsible for all content in their abstracts including accuracy of the facts, statements, citing resources, etc.

### (PB4052) ASSOCIATIONS OF APPENDICULAR SKELETAL MUSCLE MASS INDEX WITH CLINICAL INDICATORS IN PATIENTS DURING HEMATOPOIETIC RECONSTITUTION AFTER ALLOGENEIC HEMATOPOIETIC STEM CELL TRANSPLANTATION

**Topic:** 23. Stem cell transplantation - Clinical

Fangfang Wu*[1], Li Wang[1], Xiaomao Zheng[1], Lu He[1], Quanxi Tang[1], Peiyan Kong[1], Lei Gao[1], Xin Du[1], Xi Zhang[1]

[1] *The Second Affiliated Hospital of Army Medical University, Hematology Medical Center, Chongqing, China;*

### Background

Hematopoietic reconstitution is a critical period for patients undergoing allogeneic hematopoietic stem cell transplantation (allo-HSCT). Malnutrition, inflammation, and decreased skeletal muscle mass are common during this phase and closely associated with clinical outcomes. Appendicular skeletal muscle mass index (ASMI) is a key indicator of nutritional status and physical function, but its related factors remain unclear in allo-HSCT recipients during hematopoietic reconstitution.

### Aims

To investigate the factors associated with the appendicular skeletal muscle mass index in patients during the hematopoietic reconstitution phase after allogeneic hematopoietic stem cell transplantation, so as to provide a basis for clinical nutritional support, functional assessment and rehabilitation interventions.

### Methods

A total of 198 allo-HSCT patients in hematopoietic reconstitution period were enrolled in this cross-sectional study. General demographic and clinical data were collected, including age, gender, education level, primary diagnosis, disease course, and body mass index (BMI). Grip strength, serum albumin, pre-albumin, white blood cell count, hemoglobin, platelet count, and neutrophil-to-lymphocyte ratio (NLR) were measured. Linear regression analysis was performed to explore the correlations between ASMI and the above indicators.

### Results

Linear regression analysis demonstrated that ASMI was significantly correlated with grip strength ß=0.294,95%CI(0.03,0.07),t=5.054,p<0.01,serum albumin ß= 0.294,95%CI(0.016,0.113), t=2.682,p<0.01, NLR ß=0.268,95%CI(0.002-0.022),t=2.478,p<0.05, and age ß=0.24,95%CI(0.001,0.025),t=2.104,p<0.05. Specifically, higher grip strength and serum albumin levels were positively associated with ASMI, while elevated NLR and older age were negatively correlated with ASMI. No significant correlations were observed between ASMI and other general or laboratory indicators in the regression model.

### Summary/Conclusion

In allo-HSCT patients during hematopoietic reconstitution, ASMI is closely related to grip strength, nutritional status, systemic inflammatory response, and age. Early assessment of muscle mass, nutritional intervention and anti-inflammatory management may help improve skeletal muscle status and physical function in this population.

**Copyright Information:** (Online) ISSN: 2572-9241
©2026 The Author(s). HemaSphere is published by John Wiley & Sons Ltd on behalf of European Hematology Association. This is an open access Abstract Book distributed under the Attribution-NonCommercial-NoDerivs (CC BY-NC-ND), which allows third parties to download the articles and share them with others as long as they credit the author and the Abstract Book, but they cannot change the content in any way or use them commercially.
**Abstract Book Citations:** Authors, Title, HemaSphere, 2026;10:(S1):pages. The abstract book DOIs can be found on the “Author Information” page.
**Disclaimer:** Articles published in the journal HemaSphere exclusively reflect the opinions of the authors. The authors are responsible for all content in their abstracts including accuracy of the facts, statements, citing resources, etc.

### (PB4053) CONTINUOUS COTRIMOXAZOLE PROPHYLAXIS DOES NOT AFFECT ENGRAFTMENT IN PATIENTS UNDERGOING ALLOGENEIC STEM CELL TRANSPLANTATION

**Topic:** 23. Stem cell transplantation - Clinical

Benedikt Färber[1], Jan Räder[1], Leopold Alexander Schawe[1], Jan Vorwerk[1], Jan Morf[1], Helen Sievert[1], Andrea Feindel[1], Niklas Gebauer[1], Nikolaschristiancornelius Bubnoff[1], Friederike Wortmann[1], Lorenz Oelschlaeger*[1]

[1]*University Cancer Center Schleswig-Holstein, Hematology and Oncology, Lübeck, Germany;*

### Background

Allogeneic hematopoietic stem cell transplantation (allo-HSCT) is associated with a high risk of infectious complications in the post-transplant period. Trimethoprim-sulfamethoxazole (Cotrimoxazole) is often used as standard prophylaxis against Pneumocystis jirovecii (PJP) but its use is often delayed in the early post-transplant setting because of reported myelotoxicity and possibly impaired engraftment.

### Aims

To evaluate the impact of continuous cotrimoxazole prophylaxis after allo-HSCT on leukocyte and platelet engraftment.

### Methods

We retrospectively analyzed patients who underwent allo-HSCT at our center between 2022 and 2025 and compared engraftment outcomes between patients receiving continuous versus interrupted cotrimoxazole prophylaxis, re-initiated after engraftment. Exposure patterns reflected routine institutional prophylaxis practice during the study period. Cotrimoxazole prophylaxis was administered orally at a dose of 960mg three times weekly. Leukocyte engraftment was defined as leukocyte count ≥1,000/µl and platelet engraftment as a platelet count ≥20,000/µl on three consecutive days without transfusion dependency. Categorical variables were compared using Fisher´s exact text and continuous variables using the nonparametric Mann-Whitney U- Test. Time-to-event analyses were performed using the Kaplan-Meier method.

### Results

A total of 92 patients were included of whom 29 received continuous cotrimoxazole (cCotrim) and 63 received interrupted cotrimoxazole (iCotrim). Male patients were more frequent in the cCotrim group (79% vs. 60%). Underlying diseases included acute myeloid leukemia (52% vs. 61%), myelodysplastic syndromes (7% vs. 16%), and myeloproliferative neoplasms (21% vs. 10%) in the cCotrim and iCotrim groups, respectively. Immunosuppresive regimens did not include methotrexate but involved anti-thymocyte globulin (cCotrim: 71%, iCotrim: 83%) or post-transplant cyclophosphamide (cCotrim: 29%, iCotrim: 17%). Baseline characteristics were largely comparable between groups with no significant differences in age (mean 60 vs. 62 years, p = 0.60), sex distribution (p = 0.10), or the transplanted CD34+ donor stem cell dose per kilogram body weight (median 7.3 × $10^6$/kg vs. 6.05 × $10^6$/kg, p = 0.08), or donor type (79% matched unrelated donors in both groups). ECOG performance status was better in the cCotrim group (median 0, range 0-1 vs. median 1, range 0-2, p=0.00).  Only five patients in the iCotrim group had an ECOG score of 2.

Median time to leukocyte engraftment was 17 days (95% CI 16–20) in the cCotrim group and 18 days (95% CI 15–20) in the iCotrim group (p= 0.10). Median time to thrombocyte engraftment was 15 days for cCotrim (95% CI: 13–25) and 18 days for iCotrim (95% CI: 16–23; p= 0.27). No breakthrough PJP infection occurred. Thirty-day mortality did not differ between the groups (7.9% vs. 6.7%, p= 0.99)

**Copyright Information:** (Online) ISSN: 2572-9241
©2026 The Author(s). HemaSphere is published by John Wiley & Sons Ltd on behalf of European Hematology Association. This is an open access Abstract Book distributed under the Attribution-NonCommercial-NoDerivs (CC BY-NC-ND), which allows third parties to download the articles and share them with others as long as they credit the author and the Abstract Book, but they cannot change the content in any way or use them commercially.
**Abstract Book Citations:** Authors, Title, HemaSphere, 2026;10:(S1):pages. The abstract book DOIs can be found on the "Author Information" page.
**Disclaimer:** Articles published in the journal HemaSphere exclusively reflect the opinions of the authors. The authors are responsible for all content in their abstracts including accuracy of the facts, statements, citing resources, etc.

**Summary/Conclusion**

Continuous cotrimoxazole prophylaxis after allo-HSCT was not associated with delayed leukocyte or platelet engraftment compared with interrupted prophylaxis. Despite concerns regarding myelotoxicity, continuous administration appeared safe with respect to engraftment and was not associated with breakthrough Pneumocystis jirovecii infections, supporting its feasibility in the early post-transplant period.

**Copyright Information:** (Online) ISSN: 2572-9241
©2026 The Author(s). HemaSphere is published by John Wiley & Sons Ltd on behalf of European Hematology Association. This is an open access Abstract Book distributed under the Attribution-NonCommercial-NoDerivs (CC BY-NC-ND), which allows third parties to download the articles and share them with others as long as they credit the author and the Abstract Book, but they cannot change the content in any way or use them commercially.
**Abstract Book Citations:** Authors, Title, HemaSphere, 2026;10:(S1):pages. The abstract book DOIs can be found on the "Author Information" page.
**Disclaimer:** Articles published in the journal HemaSphere exclusively reflect the opinions of the authors. The authors are responsible for all content in their abstracts including accuracy of the facts, statements, citing resources, etc.

### (PB4054) A QUALITATIVE STUDY ON THE CARE NEEDS OF PATIENTS FROM THE PERSPECTIVES OF PATIENTS, CAREGIVERS AND HEALTHCARE PROVIDERS

**Topic:** 23. Stem cell transplantation - Clinical

Yeyan He*[1]

[1]*Xinqiao Hospital, Medical Center of Hematology, Chong Qin, China;*

### Background

Patients who undergo hematopoietic stem cell transplantation require long-term care. However, most existing studies focus on a single perspective. Nevertheless, there are often differences in the understanding of needs among patients, caregivers, and healthcare providers, which can lead to a lack of coordinated care. To establish a collaborative care mechanism, this study intends to integrate the perspectives of all three parties and deeply explore their care needs.

### Aims

Understanding the perspectives of "patients - caregivers - healthcare providers", and deeply exploring the care needs of patients after hematopoietic stem cell transplantation, will provide a basis for constructing a comprehensive and personalized continuous care plan.

### Methods

Using the phenomenological research method, from June to December 2025, 120 patients in the transplant ward, 116 patient caregivers, 26 doctors and 30 nurses were selected as the research subjects for semi-structured interviews. The Colaizzi phenomenological data seven-step analysis method was used to extract the themes.

### Results

Four core demand themes have been extracted: physiological function reconstruction, psychological and social adaptation, information support, and family care capacity. Patients focus on symptom management and reintegration into society; caregivers emphasize caregiving skills and psychological support; healthcare providers pay attention to professional guidance and collaborative support.

### Summary/Conclusion

From the perspectives of patients, caregivers and healthcare providers, the care needs of patients after hematopoietic stem cell transplantation involve multiple aspects. The care for patients needs to integrate various demands and establish a "synergistic and complementary" mechanism. In the future, through the development of multimodal integrated health education, the design of individualized follow-up paths, the promotion of the "guardian-support" family model and the "medical-social-economic" integrated support network, full-cycle personalized care can be achieved.

**Copyright Information:** (Online) ISSN: 2572-9241
©2026 The Author(s). HemaSphere is published by John Wiley & Sons Ltd on behalf of European Hematology Association. This is an open access Abstract Book distributed under the Attribution-NonCommercial-NoDerivs (CC BY-NC-ND), which allows third parties to download the articles and share them with others as long as they credit the author and the Abstract Book, but they cannot change the content in any way or use them commercially.
**Abstract Book Citations:** Authors, Title, HemaSphere, 2026;10:(S1):pages. The abstract book DOIs can be found on the "Author Information" page.
**Disclaimer:** Articles published in the journal HemaSphere exclusively reflect the opinions of the authors. The authors are responsible for all content in their abstracts including accuracy of the facts, statements, citing resources, etc.

### (PB4055) FIRST-LINE MARIBAVIR TREATMENT FOR PATIENTS WITH CMV INFECTION INTOLERANT TO CONVENTIONAL ANTIVIRAL DRUGS

**Topic:** 23. Stem cell transplantation - Clinical

Andrej Pešić[1], Nevena Bešević[1], Milena Todorovic Balint*[1, 2]

*[1]Clinic for Hematology, University Clinical Center of Serbia, Bone Marrow Transplant Unit, Belgrade, Serbia;[2]Faculty of Medicine, University of Belgrade, Belgrade, Serbia;*

### Background

Maribavir is an anti-viral drug approved for the treatment of resistant or refractory cytomegalovirus (CMV) infection after allogeneic stem cell transplantation. Recently, ECIL-10 guidelines recommended its consideration as a first-line treatment for patients with neutropenia (BI) or renal function impairment (BII).

### Aims

Our objective was to describe the efficacy and safety of maribavir as a first-line antiviral agent in patients with CMV infection intolerant to conventional antiviral therapies.

### Methods

We retrospectively analyzed clinical characteristics and outcomes of patients with CMV infection or disease treated with maribavir as a first-line antiviral therapy at the University Clinical Center of Serbia between July 2022 and January 2026. Descriptive statistical analyses were performed.

**Copyright Information:** (Online) ISSN: 2572-9241
©2026 The Author(s). HemaSphere is published by John Wiley & Sons Ltd on behalf of European Hematology Association. This is an open access Abstract Book distributed under the Attribution-NonCommercial-NoDerivs (CC BY-NC-ND), which allows third parties to download the articles and share them with others as long as they credit the author and the Abstract Book, but they cannot change the content in any way or use them commercially.
**Abstract Book Citations:** Authors, Title, HemaSphere, 2026;10:(S1):pages. The abstract book DOIs can be found on the "Author Information" page.
**Disclaimer:** Articles published in the journal HemaSphere exclusively reflect the opinions of the authors. The authors are responsible for all content in their abstracts including accuracy of the facts, statements, citing resources, etc.

**Results**

A total of 12 patients were eligible for the study. The median follow-up from maribavir administration was 21 months (range 3-39). Nine patients (75%) received a transplant from matched
unrelated donor, while one patient each received a transplant from a matched related donor, a mismatched unrelated donor, and a haploidentical donor. All but one patient with a haploidentical
donor received anti-T-lymphocyte globulin before the transplant. Myeloablative conditioning was used in seven patients (58%). Eight patients (67%) had a CMV seronegative donor. All of the patients received CMV prophylaxis with letermovir for 100 days and only one patient had a breakthrough infection while still receiving it. CMV infection or disease were diagnosed after a mean of 126 days after transplantation (range 90-168). Three cases (25%) were diagnosed with CMV disease, all of them had CMV colitis. All patients received maribavir monotherapy in a standard dose (400mg twice daily) for a duration of eight weeks, with no cases of premature discontinuation of the drug. Before the administration of maribavir, grade I-IV neutropenia was present in eleven (92%) patients, while grade I-III renal impairment was present in seven (58%) patients. The median peak blood CMV copy number was 16145 copies/mL (range 1305-1864500). The infection resolved in all cases during maribavir treatment, and viremia clearance was achieved after a median of 18.5 days (range 11-31). Four reinfections were documented after discontinuation of the drug, occurring at a median of 20 days (range 17-79). Patients who did not experience CMV reinfection had a viremia clearance in a median of 16.5 days, compared to 28 days in those developing reinfection. Three patients (25%) needed a hospitalization for the initial CMV episode, while additional two cases of hospitalization were needed for the treatment of reinfection. Among four patients with CMV reinfection, one achieved successful treatment with valganciclovir and another with maribavir retreatment. The remaining two patients did not respond to multiple lines of therapy, including valganciclovir, foscarnet, cidofovir, and maribavir. During treatment with maribavir, two patients (17%) had a grade II dysgeusia, while only one case of neutropenia worsening was documented. Seven (58%) out of twelve patients are alive, with two deaths related to CMV, two to relapse and one to acute graft-versus-host disease. Drug resistance testing by next-generation sequencing was not performed.

**Summary/Conclusion**

Maribavir is an effective and safe treatment option for CMV infection in patients with neutropenia and/or renal impairment after allogeneic stem cell transplantation.

**Copyright Information:** (Online) ISSN: 2572-9241
©2026 The Author(s). HemaSphere is published by John Wiley & Sons Ltd on behalf of European Hematology Association. This is an open access Abstract Book distributed under the Attribution-NonCommercial-NoDerivs (CC BY-NC-ND), which allows third parties to download the articles and share them with others as long as they credit the author and the Abstract Book, but they cannot change the content in any way or use them commercially.
**Abstract Book Citations:** Authors, Title, HemaSphere, 2026;10:(S1):pages. The abstract book DOIs can be found on the “Author Information” page.
**Disclaimer:** Articles published in the journal HemaSphere exclusively reflect the opinions of the authors. The authors are responsible for all content in their abstracts including accuracy of the facts, statements, citing resources, etc.

### (PB4056) AUTOLOGOUS STEM CELL TRANSPLANTATION IN PATIENTS OVER 65 WITH LYMPHOMA AND MULTIPLE MYELOMA: FEASIBILITY AND OUTCOMES – A MOROCCAN EXPERIENCE

**Topic:** 23. Stem cell transplantation - Clinical

Zineb Guessous*[1], Maryame Ahnach[1]

[1]*University Mohammed VI of Health Sciences, Casablanca, Morocco;*

### Background

Autologous hematopoietic stem cell transplantation (ASCT) is a central component of the therapeutic strategy for several hematologic malignancies, including lymphomas and multiple myeloma. While ASCT is a standard first-line therapy for eligible younger patients, its use in individuals over 65 years remains debated due to a higher prevalence of comorbidities and reduced tolerance to intensive conditioning regimens.

### Methods

This study was conducted from September 2024 to June 2025 and included patients over 65 years of age with lymphoma or multiple myeloma who underwent autologous hematopoietic stem cell transplantation (ASCT). Analyzed parameters included comorbidities according to the Sorror HCT-CI score, graft cellularity, tolerance to the conditioning regimen, and post-transplant complications.

### Results

Our study included a cohort of 19 patients who underwent autologous hematopoietic stem cell transplantation (ASCT), including 14 with multiple myeloma and 5 with lymphoma, comprising 11 women and 8 men (female-to-male ratio 1.4; male-to-female 0.8). The median age at transplant was 68.6 years (range 65–74). Comorbidity assessment using the Sorror HCT-CI score showed low risk (score 0) in most patients, and intermediate risk (score 1–2) in 7 patients; no patient had a score ≥3.
Diffuse large B-cell lymphoma was the most frequent lymphoma subtype (3 cases), followed by one mantle cell lymphoma and one Hodgkin lymphoma, all in complete remission at the time of transplant. Among multiple myeloma patients, 9 had very good partial response (VGPR) and 5 were in complete remission (CR) before ASCT. Induction therapy included bortezomib plus cyclophosphamide in 6 patients, bortezomib plus lenalidomide in 6 patients, and bortezomib plus thalidomide in 2 patients.
Stem cell mobilization was performed with G-CSF alone for 5 days in 5 patients and G-CSF combined with plerixafor in 14 patients. Double apheresis was required in 4 patients, while 2 patients experienced mobilization failure. The mean graft cellularity was 5.47 × $10^6$ CD34+ cells/kg (range 2.8–11.6 × $10^6$).
Reduced-dose alkylating agent conditioning was applied in 12 patients. Acute toxicities were primarily gastrointestinal, observed in 10 patients, with 6 requiring parenteral nutrition. Febrile episodes occurred in 50% of patients, with Gram-negative bacteremia documented in 38%. No transplant-related mortality was reported. Neutrophil recovery occurred at a median of 11 days (range 8–14)

### Summary/Conclusion

Autologous hematopoietic stem cell transplantation (ASCT) is an effective and well-tolerated therapeutic option in patients over 65 years of age. Chronological age is no longer an absolute contraindication, but careful assessment of comorbidities using validated scores such as the HCT-CI, along with a tailored geriatric approach, is essential. Conditioning regimen adjustments and optimization of supportive care allow broader access to ASCT in older adults, even within the challenges of the Moroccan healthcare context.

**Copyright Information:** (Online) ISSN: 2572-9241
©2026 The Author(s). HemaSphere is published by John Wiley & Sons Ltd on behalf of European Hematology Association. This is an open access Abstract Book distributed under the Attribution-NonCommercial-NoDerivs (CC BY-NC-ND), which allows third parties to download the articles and share them with others as long as they credit the author and the Abstract Book, but they cannot change the content in any way or use them commercially.
**Abstract Book Citations:** Authors, Title, HemaSphere, 2026;10:(S1):pages. The abstract book DOIs can be found on the "Author Information" page.
**Disclaimer:** Articles published in the journal HemaSphere exclusively reflect the opinions of the authors. The authors are responsible for all content in their abstracts including accuracy of the facts, statements, citing resources, etc.

### (PB4057) POST TRANSPLANT LYMPHOPROLIFERATIVE DISEASE AFTER MISMATCHED ALLOGENEIC TRANSPLANT IN PATIENTS RECEIVING POST-TRANSPLANT CYCLOPHOSPHAMIDE, CNI AND ABATACEPT AS GRAFT VERSUS HOST DISEASE PROPHYLAXIS

**Topic:** 23. Stem cell transplantation - Clinical

Guillermo Ramos Moreno*[1], Laura Pardo Gambarte[2], Jose María Sánchez-Pina[1], Javier Cornago Navascués[2], Adolfo Jesús Sáez Marín[1], Laura Solán[2], Magdalena Corona[1], M Nieves Lopez[1], Maria-Luisa Palacios-Berraquero[1], Pilar Martínez Sánchez[1], Daniel Gil-Alós[1], Joaquín Martinez-Lopez[1], José Luis López-Lorenzo[2], Maria Calbacho[1]

*[1]Hospital 12 de Octubre, Madrid, Spain;[2]Fundación Jiménez Díaz, Madrid, Spain;*

### Background

In patients lacking matched donors for allogeneic stem-cell transplant (Allo-SCT), the use of mismatched unrelated or haploidentical donors is a growing option. The use of Abatacept has been studied as a means for prophylaxis of graft versus host disease (GVHD) in these cases with promising results. However, the added immunosuppressive effect could lead to a higher risk of viral reactivation.

### Aims

To describe five cases of post-transplant lymphoproliferative disorder (PTLD) after GVHD prophylaxis with post-transplant cyclophosphamide (PTCY), Abatacept and calcineurin inhibitors (CNI) occurring at two academic hospitals in Madrid.

### Methods

We retrospectively analysed 5 cases of PTLD in patients receiving allo-SCT in which Abatacept was used as GVHD prophylaxis. 4 patients were treated at 12 de Octubre University Hospital and 1 patient was treated at Fundación Jiménez Díaz University Hospital. Clinical and laboratory data were extracted from institutional electronic records.

### Results

Patient baseline characteristics are summarized in Table 1. All patients received PTCY at 50mg/kg for two consecutive days (+3,+4), Cyclosporin A, Mycophenolate Mofetil and Abatacept at a dose of 10mg/kg for a total of 4 doses. One patient (Patient 3) received anti-thymocyte globulin (ATG) as part of the conditioning regimen. This patient also received PTLD prophylaxis with Rituximab.
All patients received treatment for PTLD with rituximab, administered weekly for four doses, reaching complete remission (CR) in 3 cases, with partial response in one case (Patient 3) and progression in another (Patient 4). Patient 3 received one cycle of R-CHOP and patient 4 received 4 additional doses of Rituximab, both achieving CR. With a current median follow-up of 187 days after diagnosis of PTLD, all patients are in CR and alive.

### Summary/Conclusion

Our report suggests some shared characteristics between the cases. Four out of five cases had involvement of the gastrointestinal tract, and in two cases it was the only site involved, both of which presented with relatively low Epstein-Barr virus (EBV) viremia. Patient 1 had a rising EBV viremia detectable already at day 67, with a slow increase until diagnosis at day +167 after allo-SCT. This could point towards a more indolent form of PTLD.
All cases were preceded by treatment for GVHD with corticosteroids and ruxolitinib (except 1 patient). Immunosuppression is a driver of PTLD, so regular monitoring of EBV viremia, in the setting of GVHD, is crucial. With our current sample size, it is not possible to know if the addition of Abatacept has played a role in the development of PTLD, but the findings warrant caution and

**Copyright Information:** (Online) ISSN: 2572-9241
©2026 The Author(s). HemaSphere is published by John Wiley & Sons Ltd on behalf of European Hematology Association. This is an open access Abstract Book distributed under the Attribution-NonCommercial-NoDerivs (CC BY-NC-ND), which allows third parties to download the articles and share them with others as long as they credit the author and the Abstract Book, but they cannot change the content in any way or use them commercially.
**Abstract Book Citations:** Authors, Title, HemaSphere, 2026;10:(S1):pages. The abstract book DOIs can be found on the “Author Information” page.
**Disclaimer:** Articles published in the journal HemaSphere exclusively reflect the opinions of the authors. The authors are responsible for all content in their abstracts including accuracy of the facts, statements, citing resources, etc.

additional studies.

**Table 1: Patient Characteristics.**

| | Patient 1 | Patient 2 | Patient 3 | Patient 4 | Patient 5 |
|---|---|---|---|---|---|
| **Age at PTLD diagnosis (yr)** | 17 | 27 | 27 | 63 | 39 |
| **Patient sex (M/F)** | M | M | F | F | M |
| **Underlying disase** | AML | CTCL | AA | AML | MPAL |
| **HLA match** | Haplo | Haplo | MMURD | Haplo | Haplo |
| **Conditioning intensity** | MAC | MAC | RIC | MAC | MAC |
| **D/R EBV status** | +/+ | +/- | +/+ | +/+ | +/+ |
| **D/R CMV status** | +/+ | +/+ | +/+ | +/+ | +/+ |
| **Time to PTLD diagnosis (days post transplant)** | 164 | 200 | 91 | 152 | 149 |
| **Staging at diagnosis (Ann-Arbor)** | IV | IV | I | IV | I |
| **Involved organs** | Colonic, liver, osseus and nodal | Isolated gastric | Base of tongue, gastric, nodal, osseus | Nodal involvement at both sides of the diaphragm | Isolated pharyngeal lesion |
| **Treatment recieved** | Rituximab | Rituximab | Rituximab and R-CHOP | Rituximab | Rituximab |

AML: Acute Myeloid Leukemia MMURD: Mismatched unrelated donor MAC: Myeloablative conditioning, CTCL: Cutaneous T Cell Lymphoma. Haplo: Haploidentical. MPAL: Mixed phenotype acute leukaemia.

**Copyright Information:** (Online) ISSN: 2572-9241
©2026 The Author(s). HemaSphere is published by John Wiley & Sons Ltd on behalf of European Hematology Association. This is an open access Abstract Book distributed under the Attribution-NonCommercial-NoDerivs (CC BY-NC-ND), which allows third parties to download the articles and share them with others as long as they credit the author and the Abstract Book, but they cannot change the content in any way or use them commercially.
**Abstract Book Citations:** Authors, Title, HemaSphere, 2026;10:(S1):pages. The abstract book DOIs can be found on the "Author Information" page.
**Disclaimer:** Articles published in the journal HemaSphere exclusively reflect the opinions of the authors. The authors are responsible for all content in their abstracts including accuracy of the facts, statements, citing resources, etc.

### (PB4058) TANDEM HIGH-DOSE CHEMOTHERAPY AND AUTOLOGOUS STEM CELL TRANSPLANTATION FOR RELAPSED AND REFRACTORY GERM CELL TUMORS: A SINGLE-CENTER EXPERIENCE

**Topic:** 23. Stem cell transplantation - Clinical

Kameliya Milcheva*[1], Krasen Venkov[1], Ivan Tonev[1], Milcho Mincheff[1], Andriyana Bankova[1], Georgi Mihaylov[1]

*[1]Specialized Hospital for Active Treatment of Hematological Diseases, Sofia, Bulgaria;*

### Background

Germ cell tumors (GCTs) are the most prevalent solid malignancies among young men aged 15–45 years. While the treatment of GCTs is highly effective, the management of relapsed or refractory (R/R) disease remains controversial. Retrospective evidence, suggests that HDCT/ASCT offers superior long-term survival, especially when integrated as an early salvage strategy. Furthermore, tandem HDCT cycles are designed to maximize dose intensity while minimizing cumulative organ toxicity.

### Aims

We present a single-center clinical experience using SWENOTECA IV tandem protocol to assess its safety profile and therapeutic outcomes in a high-risk patient group with R/R GCTs in Bulgaria.

### Methods

This is a retrospective, single-center study, including nine male patients with R/R GCTs referred to our HSCT center by their treating oncologist between 2019 and 2022.The therapeutic strategy followed the SWENOTECA IV protocol. The primary endpoints were OS and PFS. Survival probabilities were estimated using the Kaplan-Meier method. Statistical analysis was performed using Python (version 3.10) utilizing the lifelines library for survival modeling.

**Copyright Information:** (Online) ISSN: 2572-9241
©2026 The Author(s). HemaSphere is published by John Wiley & Sons Ltd on behalf of European Hematology Association. This is an open access Abstract Book distributed under the Attribution-NonCommercial-NoDerivs (CC BY-NC-ND), which allows third parties to download the articles and share them with others as long as they credit the author and the Abstract Book, but they cannot change the content in any way or use them commercially.
**Abstract Book Citations:** Authors, Title, HemaSphere, 2026;10:(S1):pages. The abstract book DOIs can be found on the “Author Information” page.
**Disclaimer:** Articles published in the journal HemaSphere exclusively reflect the opinions of the authors. The authors are responsible for all content in their abstracts including accuracy of the facts, statements, citing resources, etc.

### Results

Patient characteristics are summarized in Table 1. The median age of the patients was 32.6 years (range 23–41). At initial diagnosis, 66.7% of patients presented with metastatic disease (T2N2M1).Stem cell mobilization was successful in all patients with a mean CD34+ yield of 8.38 × $10^6$/kg.With a median follow-up of 39.3 months, the median OS was 8.4 months and the median PFS was 5.9 months. The ORR post-ASCT was 33.3%. Kaplan-Meier analysis (Fig. 1A , 1B) demonstrated a 1-year OS and PFS of both 44.4%. The survival curve reached a stable plateau after the initial 18-month high-risk window, suggesting that patients, who remain progression-free at this time point achieve durable long-term remission.Hematopoietic recovery was comparable after both ASCT. The cumulative safety profile showed that regimen-related toxicities were predictable and successfully managed, despite the intensive nature of the regimen. Febrile neutropenia occurred in 88.9% of cases but was successfully resolved with standard antibiotic treatment protocols. Grade 3–4 nephrotoxicity was observed in only one patient, requiring temporary hemodialysis, there was no treatment-related mortality.

Table 1. Treatment characteristics and patient outcones

| **Characteristic** | **Patients, n (%)** |
|---|---|
| **First-Line Therapy** | |
| BEP (Bleomycin, Etoposide, Cisplatin) | 5 (55.5%) |
| PEI (Cisplatin, Etoposide, Ifosfamide) | 4 (44.5%) |
| **Response** to First-Line Therapy | |
| Objective Response (CR + PR) | 2 (22.3%) |
| Stable/Progressive Disease (SD + PD) | 7 (77.7%) |
| **Second-Line (Salvage) Therapy** | |
| TIP (Paclitaxel, Ifosfamide, Cisplatin) | 8 (88.8%) |
| PEI (Cisplatin, Etoposide, Ifosfamide) | 1 (11.1%) |
| **Response Before HSCT (after salvage)** | |
| Objective Response (CR + PR) | 5 (55.5%) |
| Stable Disease (SD) | 3 (33.3%) |
| Progressive Disease (PD) | 1 (11.1%) |
| **Post-ASCT Response** | |
| Complete Response (CR) | 3 (33.3%) |
| Progressive Disease (PD) | 5 (55.5%) |
| Not Evaluated (NA) | 1 (11.1%) |

### Summary/Conclusion

Tandem HDCT followed by ASCT is an intensive but safe salvage therapy for patients with R/R GSTs. The procedure is characterized by predictable toxicities and excellent hematopoietic recovery. While long-term survival is lower in highly refractory cases, the achievement of a survival plateau confirms the curative potential of this treatment. Future results from the TIGER trial will further refine whether intensification to three cycles of HDCT and ASCT should become the new international standard.

**Copyright Information:** (Online) ISSN: 2572-9241
©2026 The Author(s). HemaSphere is published by John Wiley & Sons Ltd on behalf of European Hematology Association. This is an open access Abstract Book distributed under the Attribution-NonCommercial-NoDerivs (CC BY-NC-ND), which allows third parties to download the articles and share them with others as long as they credit the author and the Abstract Book, but they cannot change the content in any way or use them commercially.
**Abstract Book Citations:** Authors, Title, HemaSphere, 2026;10:(S1):pages. The abstract book DOIs can be found on the "Author Information" page.
**Disclaimer:** Articles published in the journal HemaSphere exclusively reflect the opinions of the authors. The authors are responsible for all content in their abstracts including accuracy of the facts, statements, citing resources, etc.

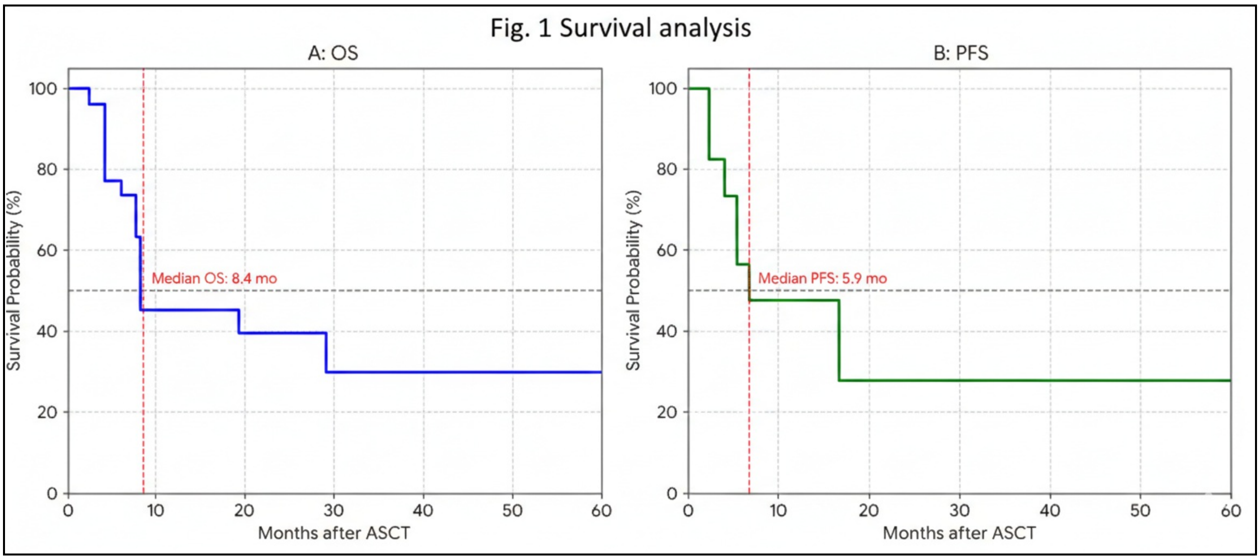

**Copyright Information:** (Online) ISSN: 2572-9241
©2026 The Author(s). HemaSphere is published by John Wiley & Sons Ltd on behalf of European Hematology Association. This is an open access Abstract Book distributed under the Attribution-NonCommercial-NoDerivs (CC BY-NC-ND), which allows third parties to download the articles and share them with others as long as they credit the author and the Abstract Book, but they cannot change the content in any way or use them commercially.
**Abstract Book Citations:** Authors, Title, HemaSphere, 2026;10:(S1):pages. The abstract book DOIs can be found on the "Author Information" page.
**Disclaimer:** Articles published in the journal HemaSphere exclusively reflect the opinions of the authors. The authors are responsible for all content in their abstracts including accuracy of the facts, statements, citing resources, etc.

### (PB4059) BARRIERS TO ACCESS AND TIME TO ALLOGENEIC HEMATOPOIETIC STEM CELL TRANSPLANTATION IN ACUTE MYELOID LEUKEMIA: A COHORT FROM NORTHERN BRAZIL

**Topic:** 23. Stem cell transplantation - Clinical

Thiago Carneiro*[1], Vanessa Ribeiro[1], Rosana Silva[1], Lucyana Leão[1], Jamila Tavares[1], Camila Reis[1], João Rodrigues[2], Luiza Souza[2], Joao Carlos Saraiva F[1]

*[1]Hospital Ophir Loyola / UEPA, Belém, Brazil; [2]Universidade do Estado do Pará, Belém, Brazil;*

### Background

Allogeneic hematopoietic stem cell transplantation (allo-HSCT) remains the only potentially curative strategy for a substantial proportion of adults with high-risk acute myeloid leukemia (AML). Despite advances in donor availability, access to allo-HSCT remains profoundly unequal in low- and middle-income countries (LMICs), particularly in geographically remote regions. In the Brazilian Amazon, the absence of local allo-HSCT infrastructure may result in prolonged delays and attrition along the transplant pathway.

### Aims

To evaluate access to allo-HSCT, time intervals along the transplant pathway, and reasons for failure to reach transplantation among adult patients with AML treated at a public referral center in Northern Brazil.

### Methods

We conducted a retrospective observational cohort study including adult patients diagnosed with AML between 2020 and 2024 at a tertiary public hospital in the Brazilian Amazon. Clinical, demographic, and transplant-related data were extracted from medical records. Allo-HSCT indication, performance, and time intervals from diagnosis to transplant indication and from indication to transplant were analyzed. Reasons for non-performance of allo-HSCT were systematically categorized. Analyses were descriptive.

### Results

Ninety-eight patients were included (median age 52 years; 56% female). Allo-HSCT was formally indicated in 46.9% of patients; however, only 14.3% ultimately underwent transplantation. Among transplanted patients, the median time from diagnosis to transplant indication was 105 days. Median time from indication to allo-HSCT was 238 days for related donors and 408 days for unrelated donors. Failure to proceed to transplantation was most frequently due to death prior to allo-HSCT (34.5%), clinical or social contraindications (26.2%), and referral to exclusive palliative care (23.8%). Disease relapse and patient withdrawal were less common causes. Overall, substantial attrition occurred between transplant indication and procedure performance.

### Summary/Conclusion

In this Amazonian LMIC setting, access to allo-HSCT for AML is severely constrained by prolonged waiting times and structural barriers rather than by lack of formal transplant indication. Delays between indication and transplantation are associated with high rates of pre-transplant mortality and transition to palliative care, resulting in loss of access to potentially curative therapy. These findings underscore the urgent need to streamline referral pathways and expand regional transplant capacity to reduce inequities and improve survival in underserved populations.

**Copyright Information:** (Online) ISSN: 2572-9241
©2026 The Author(s). HemaSphere is published by John Wiley & Sons Ltd on behalf of European Hematology Association. This is an open access Abstract Book distributed under the Attribution-NonCommercial-NoDerivs (CC BY-NC-ND), which allows third parties to download the articles and share them with others as long as they credit the author and the Abstract Book, but they cannot change the content in any way or use them commercially.
**Abstract Book Citations:** Authors, Title, HemaSphere, 2026;10:(S1):pages. The abstract book DOIs can be found on the "Author Information" page.
**Disclaimer:** Articles published in the journal HemaSphere exclusively reflect the opinions of the authors. The authors are responsible for all content in their abstracts including accuracy of the facts, statements, citing resources, etc.

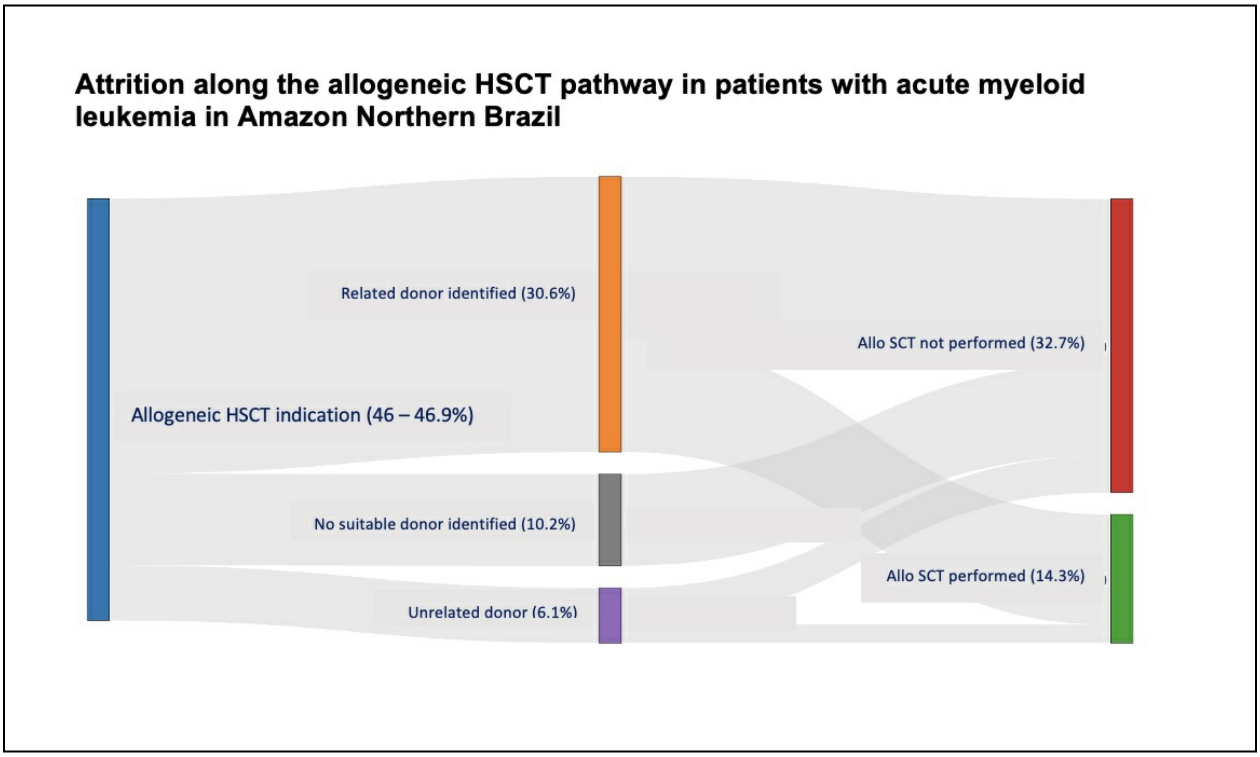

**Copyright Information:** (Online) ISSN: 2572-9241
©2026 The Author(s). HemaSphere is published by John Wiley & Sons Ltd on behalf of European Hematology Association. This is an open access Abstract Book distributed under the Attribution-NonCommercial-NoDerivs (CC BY-NC-ND), which allows third parties to download the articles and share them with others as long as they credit the author and the Abstract Book, but they cannot change the content in any way or use them commercially.
**Abstract Book Citations:** Authors, Title, HemaSphere, 2026;10:(S1):pages. The abstract book DOIs can be found on the "Author Information" page.
**Disclaimer:** Articles published in the journal HemaSphere exclusively reflect the opinions of the authors. The authors are responsible for all content in their abstracts including accuracy of the facts, statements, citing resources, etc.

### (PB4060) OMISSION OF METHOTREXATE WAS NOT ASSOCIATED WITH WORSE CLINICAL OUTCOMES IN PATIENTS HAVING ALLOGENEIC HAEMATOPOIETIC STEM CELL TRANSPLANT

**Topic:** 23. Stem cell transplantation - Clinical

Kasys Meira Gervatauskas*[1, 2, 3], Brijesh Gautama[2], Thales Dalessandro[3], Caroline Besley[2]

*[1]IDOR - D'Or Institute of Research and Education, São Paulo, Brazil; [2]University Hospitals Bristol and Weston, Haematology and Oncology, Bristol, United Kingdom; [3]Hospital das Clinicas da Faculdade de Medicina da Universidade de São Paulo, Hematologia, São Paulo, Brazil;*

### Background

Allogeneic Haematopoietic Stem Cell Therapy (HSCT) is a crucial treatment for a variety of condition, bearing, however, severe side effects, such as the Graft Versus Host Disease (GVHD). The use of post-transplant methotrexate (ptMTX) and calcineurin inhibitors is regarded as the most common prophylactic regimen.
The dose and days of methotrexate given to this end are not uniform between the different services. In clinical practice, the omission of doses of methotrexate has been a recurrent issue, and there are some retrospective studies that have addressed the possible impacts of these omissions. The results of these studies are, however, controversial

### Aims

To this date, there are no randomized clinical trials (RCTs) done to assess the omission of post-transplant methotrexate at the usual 45mg/m² dose.  This study aimed to assess the impact of such omission in clinical outcomes.

### Methods

This is a single centre, retrospective analysis of all the patients that underwent HSCT between 2019 and 2023 using post-transplant methotrexate. All patients were more than 18 years old. The main regimen of ptMTX is 15mg/m² on day +1 and 10mg/m² in days +3, +6 and +11 (45mg/m² in total). Subjects all received a Myeloablative Conditioning (MAC). Donors would either be fully matched siblings or unrelated donors (MSD and MUD, respectively).
Patients were divided into the omission (<45mg/m²) or no omission groups. Overall survival (OS), relapse rates, non-relapse mortality (NRM) and rates of cGVHD were estimated using Kaplan-Meier method and the Log-rank method for univariate analysis. Death was the main competing risk for cGVHD.
Hematopoietic Cell Transplantation-Comorbidity Index (HCT-CI) and Disease Risk according to previously established criteria  were assessed as baseline characteristics and variables in the studied population. We chose to use the age cutoff of 50 years based on previous data that used a similar threshold
All the statistical calculations were made using IBM SPSS Statistics 29.0.1.0.

### Results

40 patients were included in the final analisys. All but one patient used CsA. 19(47.5%) had omissions in the methotrexate doses (omission group-OG). There were no significant differences in baseline characteristics..
Kaplan-Meier OS curves for the OG and the non-OG were similar in the OG (51.3% OG vs 65.3% non-OG at 4 years; p 0.319) at three years. There were no differences between groups in cGVHD (42% OG vs 47% non-OG; HR 0.95 CI 0.37-2.5, p 0.95). Methotrexate omission had a non-statistically, albeit significant, association in the aGVHD rates (52% OG vs 42% non-OG; RR 1.8 CI 0.53-6.6, p 0.3). Severe aGVHD (more than grade II) occurred in 36% of the patients that had omissions in the ptMTX in comparison to 19% on the other group, (RR 2.4; CI 0.59-10.3 p 0.2).

**Copyright Information:** (Online) ISSN: 2572-9241
©2026 The Author(s). HemaSphere is published by John Wiley & Sons Ltd on behalf of European Hematology Association. This is an open access Abstract Book distributed under the Attribution-NonCommercial-NoDerivs (CC BY-NC-ND), which allows third parties to download the articles and share them with others as long as they credit the author and the Abstract Book, but they cannot change the content in any way or use them commercially.
**Abstract Book Citations:** Authors, Title, HemaSphere, 2026;10:(S1):pages. The abstract book DOIs can be found on the "Author Information" page.
**Disclaimer:** Articles published in the journal HemaSphere exclusively reflect the opinions of the authors. The authors are responsible for all content in their abstracts including accuracy of the facts, statements, citing resources, etc.

### Summary/Conclusion

This retrospective analysis contributes to our current understanding of GVHD prophylaxis and ptMTX management. The lack of day 11 dose of methotrexate appears not to have interfered with GVHD rates and survival in our patients. This finding is in line with some of the previous studies on the same subject, adding to the conflicting data regarding day 11 omission.
We could notice a striking difference of the OS and NRM between groups at 4 years, favouring the non-OG, although not statistically significant. This finding is likely due to the confusional bias of patients clinically unwell having their ptMTX omitted. Graft-Versus-Host Disease rates were similar between groups.

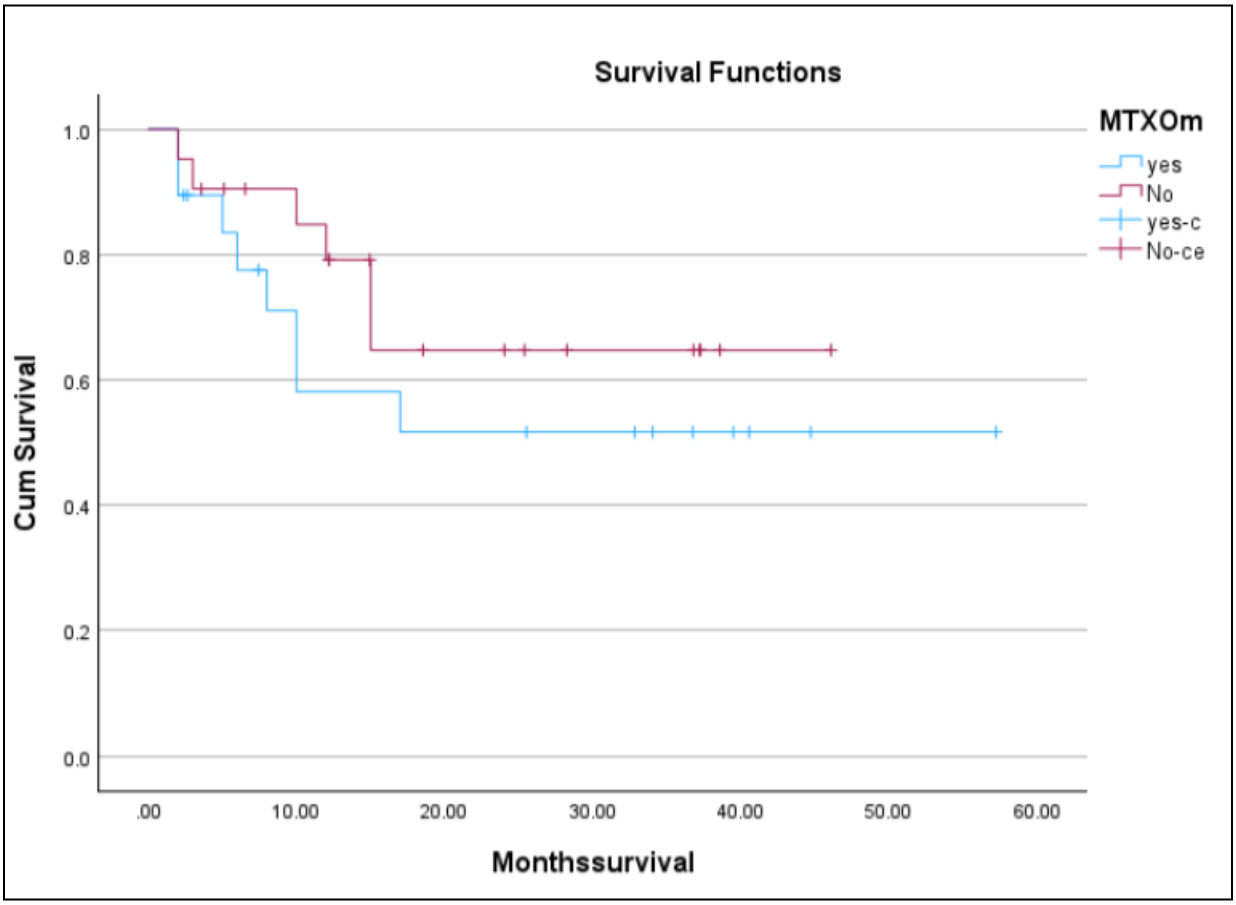

OS in the different groups (OG vs non-OG)

**Copyright Information:** (Online) ISSN: 2572-9241
©2026 The Author(s). HemaSphere is published by John Wiley & Sons Ltd on behalf of European Hematology Association. This is an open access Abstract Book distributed under the Attribution-NonCommercial-NoDerivs (CC BY-NC-ND), which allows third parties to download the articles and share them with others as long as they credit the author and the Abstract Book, but they cannot change the content in any way or use them commercially.
**Abstract Book Citations:** Authors, Title, HemaSphere, 2026;10:(S1):pages. The abstract book DOIs can be found on the "Author Information" page.
**Disclaimer:** Articles published in the journal HemaSphere exclusively reflect the opinions of the authors. The authors are responsible for all content in their abstracts including accuracy of the facts, statements, citing resources, etc.

### (PB4061) SAFETY MANAGEMENT OF ORAL MEDICATIONS FOR HEMATOPOIETIC STEM CELL TRANSPLANTATION PATIENTS

**Topic:** 23. Stem cell transplantation - Clinical

Li Zeng*[1], Xin Du[1]

[1]*Xinqiao Hospital of Army Medical University, Chong, China;*

### Background

Patients undergoing hematopoietic stem cell transplantation (HSCT) often require a large number of oral medications with complex administration methods, which increases the risk of medication errors and compromises treatment quality.

### Aims

To evaluate the application effect of patient participation in medication safety management on reducing medication errors during the hospital-to-home transition period for HSCT patients.

### Methods

A total of 36 HSCT patients admitted to our department from June 2024 to December 2025 were randomly divided into a control group (n=18) and an observation group (n=18). The control group received routine care, while the observation group received additional medication safety management. From the day of transplantation, patients were involved in the oral medication regimen according to the prescribed plan. The patient participation protocol included: ① Careful participation: The responsible nurse explained the functions, dosing times, and requirements of oral medications, emphasizing the importance of adherence to medical advice; ② Informed participation: The nurse inquired about the patient's understanding of medication knowledge and any additional questions, recorded their concerns, and provided timely responses; ③ Decision-making participation: The nurse taught patients to observe adverse reactions after medication, the correct method for missed doses or vomiting, and to communicate any discomfort to healthcare providers promptly. On the day of discharge, the patient or primary caregiver was asked to repeat the dosage and administration method. Follow-up visits were conducted at 7 days, 1 month, and 2 months post-discharge to assess medication accuracy and correct any errors. The incidence of medication errors and medication adherence were compared between the two groups.

### Results

The incidence of medication deviation in the observation group was significantly lower than that in the control group ($P<0.01$), and the medication adherence in the observation group was higher than that in the control group ($P<0.01$).

### Summary/Conclusion

Patient participation in medication safety management can effectively reduce medication deviations during the hospital-to-home transition period for hematopoietic stem cell transplantation patients, improve their medication adherence, thereby enhancing nursing quality and improving treatment outcomes.

**Copyright Information:** (Online) ISSN: 2572-9241
©2026 The Author(s). HemaSphere is published by John Wiley & Sons Ltd on behalf of European Hematology Association. This is an open access Abstract Book distributed under the Attribution-NonCommercial-NoDerivs (CC BY-NC-ND), which allows third parties to download the articles and share them with others as long as they credit the author and the Abstract Book, but they cannot change the content in any way or use them commercially.
**Abstract Book Citations:** Authors, Title, HemaSphere, 2026;10:(S1):pages. The abstract book DOIs can be found on the “Author Information” page.
**Disclaimer:** Articles published in the journal HemaSphere exclusively reflect the opinions of the authors. The authors are responsible for all content in their abstracts including accuracy of the facts, statements, citing resources, etc.

### (PB4062) REAL-WORLD EFFICACY AND SAFETY OF MARIBAVIR VERSUS CONVENTIONAL THERAPY FOR CMV INFECTION WITH GANCICLOVIR TREATMENT FAILURE AFTER HEMATOPOIETIC STEM CELL TRANSPLANTATION

**Topic:** 23. Stem cell transplantation - Clinical

Suhyun Park*[1], Sami Yang[1], Jiyoung Choi[1], Hyewon Han[1]

[1]*Asan Medical Center, Department of Pharmacy, Seoul, South Korea;*

### Background

Ganciclovir (GCV) remains the standard first-line therapy for cytomegalovirus (CMV) infection in hematopoietic stem cell transplantation (HSCT) recipients; however, its use is frequently limited by treatment failure due to antiviral resistance and dose-limiting toxicities, particularly myelosuppression. Maribavir (MBV), a novel antiviral agent that selectively inhibits UL97 kinase via a distinct mechanism, has emerged as an alternative for refractory or GCV-intolerant CMV infection. After reimbursement approval in April 2024, MBV has been increasingly used in clinical practice in South Korea; however, real-world data remain limited.

### Aims

This study aimed to evaluate the real-world efficacy and safety of maribavir compared with conventional therapy in patients with ganciclovir treatment failure.

### Methods

A retrospective study was conducted among adult hematopoietic stem cell transplant recipients with ganciclovir-resistant, refractory, or intolerant CMV infection at Asan Medical Center between January 2023 and July 2025. Among these patients, 17 patients who continued GCV therapy or were switched to foscarnet (FOS) were classified as the conventional therapy group, while 12 patients who were switched from GCV to MBV comprised the MBV group. The primary outcome was the CMV clearance rate. Secondary outcomes included the virologic response at week 2, time to CMV clearance, and the incidence of adverse drug reactions. Statistical analyses were performed using Fisher's exact test, Kaplan–Meier analysis, and the log-rank test.

### Results

The CMV clearance rate was significantly higher in the MBV group than in the conventional therapy group (66.7% [8/12] vs. 23.5% [4/17]; odds ratio [OR], 6.50; 95% confidence interval [CI], 1.26–33.58; $p = 0.029$). The virologic response rate at week 2 was numerically higher in the MBV group (91.7% [11/12]) compared with the conventional therapy group (58.8% [10/17]), although this difference did not reach statistical significance (OR, 7.70; 95% CI, 0.80–74.10; $p = 0.093$). The median time to CMV clearance was 33.0 days (95% CI, 9.2–56.8) in the MBV group, whereas the median could not be estimated in the conventional therapy group because more than half of the patients did not achieve CMV clearance (log-rank test, $p = 0.013$). In addition, treatment discontinuation due to grade ≥4 hematologic toxicity occurred in 29.4% of patients receiving conventional therapy, whereas no such events were observed with MBV.

### Summary/Conclusion

In patients with ganciclovir treatment failure, maribavir demonstrated higher CMV clearance rates, more rapid virologic responses, and a lower incidence of hematologic toxicity compared with conventional therapy. Despite limitations inherent to its retrospective, single-center design and small sample size, this study provides real-world evidence supporting maribavir as an effective and safer salvage option for CMV infection after HSCT.

**Copyright Information:** (Online) ISSN: 2572-9241
©2026 The Author(s). HemaSphere is published by John Wiley & Sons Ltd on behalf of European Hematology Association. This is an open access Abstract Book distributed under the Attribution-NonCommercial-NoDerivs (CC BY-NC-ND), which allows third parties to download the articles and share them with others as long as they credit the author and the Abstract Book, but they cannot change the content in any way or use them commercially.
**Abstract Book Citations:** Authors, Title, HemaSphere, 2026;10:(S1):pages. The abstract book DOIs can be found on the "Author Information" page.
**Disclaimer:** Articles published in the journal HemaSphere exclusively reflect the opinions of the authors. The authors are responsible for all content in their abstracts including accuracy of the facts, statements, citing resources, etc.

### (PB4063) STEM CELL SUPPORT AS A SALVAGE THERAPY FOR PROLONGED SEVERE HEMATOLOGICAL TOXICITY FOLLOWING CAR-T CELL THERAPY

**Topic:** 23. Stem cell transplantation - Clinical

Changming Dong*[1], Mengyu Ruan[1], Wenjie Xiong[1], Dehui Zou[1]

[1]*Institute of Hematology and Blood Diseases Hospital, Chinese Academy of Medical Sciences and Peking Union Medical College, Tianjin, China;*

### Background

Although CAR-T cell therapy has revolutionized clinical outcomes for patients with relapsed/refractory hematologic malignancies, its application is associated with distinct adverse effects. In addition to cytokine release syndrome (CRS) and immune effector cell-associated neurotoxicity syndrome (ICANS), CAR-T therapy frequently induces hematological toxicity. Notably, prolonged and severe impaired hematopoietic recovery constitutes a major cause of secondary infections, bleeding events, and increased non-relapse mortality. Given that CD34+ stem cells have been successfully utilized to treat graft failure or poor graft function following allogeneic and autologous stem cell transplantation, stem cell infusion (without conditioning regimens) may represent an effective salvage approach for growth factor-refractory and transfusion-dependent CAR-T-associated cytopenias. However, its role in CAR-T-related hematological toxicity remains unclear, and relevant clinical experience is limited.

### Aims

To evaluate the safety and efficacy of autologous hematopoietic stem cell (CD34+) and/or mesenchymal stem cell (MSC) infusion as a salvage therapy for prolonged severe hematological toxicity following chimeric antigen receptor T-cell (CAR-T) therapy.

### Methods

This study retrospectively analyzed 25 patients who received stem cell infusions for prolonged severe cytopenia (defined as an absolute neutrophil count $<0.5\times10^9$/L and/or platelet count $<20\times10^9$/L on day 28 post-infusion) following CAR-T therapy between November 2023 and October 2025. The cohort included 11 patients with diffuse large B-cell lymphoma (treated with CD19 CAR-T) and 14 patients with multiple myeloma (treated with BCMA CAR-T). The primary outcomes were engraftment and hematopoietic reconstitution (time to engraftment, cumulative incidence of engraftment) and survival outcomes.

### Results

Among the 18 patients with poor neutrophil engraftment, 17 (94.4%) achieved successful engraftment, with a median time to engraftment of 11 days and a 30-day cumulative incidence of engraftment of 88.9%. Of the 24 patients with poor platelet engraftment, 21 (87.5%) achieved successful engraftment, with a median time to engraftment of 16.5 days and a 30-day cumulative incidence of engraftment of 75.0%. Neutrophil, platelet, and hemoglobin levels on day 30 and day 90 post-stem cell infusion were significantly improved compared to baseline levels pre-infusion ($P < 0.001$). Subgroup analysis revealed no statistically significant differences in engraftment time or cumulative incidence of engraftment among patients receiving different stem cell types ($CD34^+$ stem cells, MSCs, or combined infusion) (all $P > 0.05$). At the 12-month follow-up, the best overall response rate (ORR) was 88.0%, the complete remission (CR) rate was 80%, and the 1-year progression-free survival and overall survival rates were both 78.4%.

**Copyright Information:** (Online) ISSN: 2572-9241
©2026 The Author(s). HemaSphere is published by John Wiley & Sons Ltd on behalf of European Hematology Association. This is an open access Abstract Book distributed under the Attribution-NonCommercial-NoDerivs (CC BY-NC-ND), which allows third parties to download the articles and share them with others as long as they credit the author and the Abstract Book, but they cannot change the content in any way or use them commercially.
**Abstract Book Citations:** Authors, Title, HemaSphere, 2026;10:(S1):pages. The abstract book DOIs can be found on the “Author Information” page.
**Disclaimer:** Articles published in the journal HemaSphere exclusively reflect the opinions of the authors. The authors are responsible for all content in their abstracts including accuracy of the facts, statements, citing resources, etc.

**Summary/Conclusion**

Stem cell infusion represents a safe and effective salvage treatment strategy for patients experiencing prolonged severe cytopenia after CAR-T therapy. It facilitates rapid and durable hematopoietic reconstitution without compromising the long-term anti-tumor efficacy of CAR-T cells. Notably, MSCs demonstrated comparable efficacy to $CD34^+$ stem cells in promoting hematopoietic recovery, offering a more accessible alternative treatment option for patients lacking pre-cryopreserved autologous stem cells.

**Copyright Information:** (Online) ISSN: 2572-9241
©2026 The Author(s). HemaSphere is published by John Wiley & Sons Ltd on behalf of European Hematology Association. This is an open access Abstract Book distributed under the Attribution-NonCommercial-NoDerivs (CC BY-NC-ND), which allows third parties to download the articles and share them with others as long as they credit the author and the Abstract Book, but they cannot change the content in any way or use them commercially.
**Abstract Book Citations:** Authors, Title, HemaSphere, 2026;10:(S1):pages. The abstract book DOIs can be found on the "Author Information" page.
**Disclaimer:** Articles published in the journal HemaSphere exclusively reflect the opinions of the authors. The authors are responsible for all content in their abstracts including accuracy of the facts, statements, citing resources, etc.

### (PB4064) COMPREHENSIVE NURSING MANAGEMENT DURING THE APPLICATION OF MESENCHYMAL STEM CELL IN THE TREATMENT OF ACUTE INTESTINAL GRAFT VERSUS HOST DISEASE AFTER ALLOGENEIC HEMATOPOIETIC STEM CELL TRANSPLANTATION

**Topic:** 23. Stem cell transplantation - Clinical

Mingyue Xu*[1], Xianjing Zhang[1], Guiying Liu[1], Yuqing Liu[1], Yigeng Cao[1], Erlie Jiang[1]

[1]*Institute of Hematology & Blood Diseases Hospital, Chinese Academy of Medical Sciences & Peking Union Medical College, Tianjin, China;*

### Background

Currently, mesenchymal stem cells (MSC) are the primary cellular therapeutic agent used to treat acute gastrointestinal graft versus host disease (aGI-GVHD) after allogeneic hematopoietic stem cell transplantation (allo-HSCT). However, MSC infusions can trigger adverse events and provoke emotional distress in patients.

### Aims

This study examined the clinical impact of comprehensive nursing management throughout the entire MSC treatment trajectory.

### Methods

We enrolled 30 patients who received MSC therapy for aGI-GVHD between October, 2024 and November, 2025 in the IHCAMS (Institute of Hematology and Blood Diseases Hospital, Peking Union Medical College). We established a “screening–infusion–follow-up” Comprehensive nursing system for these patients. We developed a comprehensive nursing system that started with baseline assessment, consent disclosure and the preparation of a ready environment before clinical trial. During infusion, we adhered strictly to the clinical protocol and kept a real-time watch for any adverse reactions. Moreover, we gave patients clear and upfront education about possible reactions they might experience during the infusion. After infusion, we closely monitored for complications, provided step-by-step rehabilitation guidance and scheduled regular follow-ups. Furthermore, we ensured that clinical data was recorded accurately and reported promptly.

### Results

After comprehensive nursing management, patient compliance reached 100%. Eight subjects developed post-infusion fever that resolved promptly with supportive care. No cases of CRS, nausea, vomiting, rash or any other adverse events were observed. MSC-related reactions were infrequent and managed immediately. The completeness of the patients’ clinical-trial data achieved 100 %. In addition, intestinal function of patients recovered well.

### Summary/Conclusion

Comprehensive nursing management throughout MSC therapy effectively reduced infusion-related risks and improved the safety for patients with aGI-GVHD. Our nursing strategy established a standardized template for future cell-infusion protocols in clinical trials.

**Copyright Information:** (Online) ISSN: 2572-9241
©2026 The Author(s). HemaSphere is published by John Wiley & Sons Ltd on behalf of European Hematology Association. This is an open access Abstract Book distributed under the Attribution-NonCommercial-NoDerivs (CC BY-NC-ND), which allows third parties to download the articles and share them with others as long as they credit the author and the Abstract Book, but they cannot change the content in any way or use them commercially.
**Abstract Book Citations:** Authors, Title, HemaSphere, 2026;10:(S1):pages. The abstract book DOIs can be found on the “Author Information” page.
**Disclaimer:** Articles published in the journal HemaSphere exclusively reflect the opinions of the authors. The authors are responsible for all content in their abstracts including accuracy of the facts, statements, citing resources, etc.

### (PB4065) LOW-DOSE BLINATUMOMAB FOR DESENSITIZATION OF POSITIVE DONOR-SPECIFIC ANTI-HLA ANTIBODY (DSA) BEFORE ALLOGENIC STEM CELL TRANSPLANTATION

**Topic:** 23. Stem cell transplantation - Clinical

Chunlei Lu[1], Lina Feng[1], Yu Sun[1], Mei Wang[1], Qi Gao[1], Yuejun Li[1], Yu Wang[1], Fang Liu*[1]

[1]*Shanghai New Daopei Hospital, Shanghai, China;*

### Background

Donor-specific anti-HLA antibodies (DSAs) are antibodies in the recipient directed against donor class I/II HLA antigens. The presence of DSA is known to be a major obstacle in HLA mismatched allogenic stem cell transplantation (allo-SCT) due to the increased risk of engraftment failure. So far there're no established methods for DSA desensitization, some study reported rituximab be useful by depleting B cells, however, it's time-consuming and easy to rebound due to the existence of antibody producing plasma cells, especially for those with high antibody titers.

CD19 is expressed not only on B cells but also on plasma cells. Blinatumomab is a bispecific antibody construct that engages T cells with target cells by binding CD3 and CD19, leading to destruction of $CD19^+$ B cells and plasma cells consequently reducing antibodies produced by the cells.

### Aims

We report two cases who underwent allo-HCT after the clearance of high-titer DSA using 2 weeks of low dose blinatumomab, and successfully achieved engraftment.

### Methods

Allo-HCT candidates in our hospital were screened for DSA before transplantation by using the LUMINEX technique. The DSA value were expressed in mean fluorescence intensity (MFI). Case 1 is a 38-year-old female diagnosed with Myelodysplastic syndrome. The MFI of DSA was 16,747 before treatment. After four doses of weekly Rituximab at 375mg/$m^2$, the DSA MFI keeps arising to 37,318. Case 2 is a 30-year-old female diagnosed with very severe aplastic anemia. Before treatment, the MFI of HLA class I and class II DSA titer was as high as 78,788 and 207,137 respectively. Both patients received treatment of blinatumomab at 9ug/d for 14 days. The DSA was monitored during the treatment.

### Results

After the treatment with blinatumomab, DSA of both patients gradually decreased, the change of the MIF is shown in the figure. Case1 received an unrelated donor transplantation 6 weeks later, case2 received a double cord blood transplant 10 weeks later after two plasma exchanges, both patients successfully restored hematopoiesis with a complete donor chimerism.

### Summary/Conclusion

Our study shows that low dose blinatumomab can effectively decrease DSA before allo-SCT. Further and larger scale studies are needed to better explore the best methods of successful desensitization.

**Copyright Information:** (Online) ISSN: 2572-9241
©2026 The Author(s). HemaSphere is published by John Wiley & Sons Ltd on behalf of European Hematology Association. This is an open access Abstract Book distributed under the Attribution-NonCommercial-NoDerivs (CC BY-NC-ND), which allows third parties to download the articles and share them with others as long as they credit the author and the Abstract Book, but they cannot change the content in any way or use them commercially.
**Abstract Book Citations:** Authors, Title, HemaSphere, 2026;10:(S1):pages. The abstract book DOIs can be found on the "Author Information" page.
**Disclaimer:** Articles published in the journal HemaSphere exclusively reflect the opinions of the authors. The authors are responsible for all content in their abstracts including accuracy of the facts, statements, citing resources, etc.

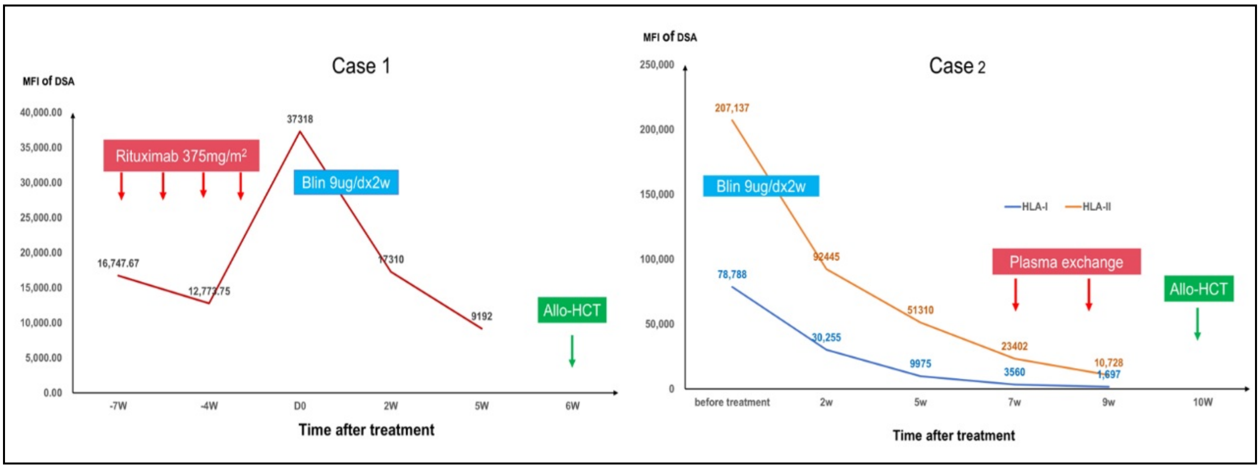

The change of DSA after treatment

**Copyright Information:** (Online) ISSN: 2572-9241
©2026 The Author(s). HemaSphere is published by John Wiley & Sons Ltd on behalf of European Hematology Association. This is an open access Abstract Book distributed under the Attribution-NonCommercial-NoDerivs (CC BY-NC-ND), which allows third parties to download the articles and share them with others as long as they credit the author and the Abstract Book, but they cannot change the content in any way or use them commercially.
**Abstract Book Citations:** Authors, Title, HemaSphere, 2026;10:(S1):pages. The abstract book DOIs can be found on the "Author Information" page.
**Disclaimer:** Articles published in the journal HemaSphere exclusively reflect the opinions of the authors. The authors are responsible for all content in their abstracts including accuracy of the facts, statements, citing resources, etc.

### (PB4066) VITAMIN D MONITORING AND REPLACEMENT IN HAEMATOPOIETIC STEM CELL TRANSPLANTATION: A CLINICAL AUDIT OF PRACTICE AGAINST REGIONAL GUIDELINES

**Topic:** 23. Stem cell transplantation - Clinical

Maeve O'shea*[1], Victoria Potter[1], Michelle Kenyon[1]

[1]*Kings College Hospital, London, United Kingdom;*

### Background

Vitamin D deficiency is highly prevalent among haematopoietic stem cell transplantation (HSCT) recipients, driven by prolonged hospitalisation, limited sunlight exposure and treatment-related factors. Increasing evidence suggests that suboptimal vitamin D status is not only common but clinically significant, being linked with poorer bone health, delayed immune recovery and a greater risk and severity of Graft versus Host Disease (GvHD) (Mancin et al., 2024). As a modifiable factor, vitamin D represents an important yet under recognised aspect of supportive transplant care. The South East London (SEL) Clinical Guideline for the Management of Vitamin D Deficiency and Insufficiency in Adults (2003) recommends routine screening and treatment using defined loading and maintenance regimens.

### Aims

To evaluate local vitamin D monitoring and replacement practices in HSCT patients against regional guideline standards and identify areas for improvement.

### Methods

A retrospective review was undertaken of all patients who underwent HSCT between October 2023 and October 2024 at King's College Hospital. Of 255 patients, 10 who died within six months post-transplant were excluded, leaving 245 evaluable cases (151 autologous, 94 allogeneic). Patient age ranged from 17 to 75 years (mean 55.2). Data were extracted from the electronic patient record (EPIC) at three timepoints: pre-transplant, three months and six months post-transplant. Vitamin D status was classified as deficient (<25 nmol/L), insufficient (25–50 nmol/L) or sufficient (>50 nmol/L). Prescribing of loading or maintenance vitamin D was compared with SEL guideline recommendations.

### Results

Vitamin D testing occurred in 18.3% of patients pre-transplant, 15.6% at three months and 16.0% at six months, substantially below the ≥90% target for high-risk populations. Among those tested, 62.8% were deficient or insufficient pre-transplant, 55.2% at three months and 45.0% at six months. Of the total cohort, 50.6% received any vitamin D prescription, but prescribing was largely empirical and often unrelated to measured status.

Among 20 patients with confirmed deficiency, only 9 (45%) received an appropriate loading dose. Of 36 patients with insufficiency, 15 (42%) received maintenance supplementation. Allogeneic recipients were marginally more likely to be tested (20.2% vs 17.2% autologous) but were less consistently treated at six months, when none of 3 deficient patients received a loading dose.

**Table 1. Vitamin D testing and Deficiency Rates by Timepoint**

| Timepoint | Patients tested n (%) | Deficient n (%) | Insufficient n (%) |
|---|---|---|---|
| Pre-transplant | 45 (18.3) | 10 (23.3) | 17 (39.5) |
| 3 months | 38 (15.6) | 5 (10.5) | 17 (44.7) |
| 6 months | 39 (16.0) | 5 (12.5) | 13 (32.5) |

**Copyright Information:** (Online) ISSN: 2572-9241
©2026 The Author(s). HemaSphere is published by John Wiley & Sons Ltd on behalf of European Hematology Association. This is an open access Abstract Book distributed under the Attribution-NonCommercial-NoDerivs (CC BY-NC-ND), which allows third parties to download the articles and share them with others as long as they credit the author and the Abstract Book, but they cannot change the content in any way or use them commercially.
**Abstract Book Citations:** Authors, Title, HemaSphere, 2026;10:(S1):pages. The abstract book DOIs can be found on the "Author Information" page.
**Disclaimer:** Articles published in the journal HemaSphere exclusively reflect the opinions of the authors. The authors are responsible for all content in their abstracts including accuracy of the facts, statements, citing resources, etc.

**Summary/Conclusion**

Vitamin D monitoring and replacement in HSCT patients at our centre are inconsistent and substantially below guideline recommendations. Testing occurred in fewer than one in five patients, and only 45% of those with deficiency received the recommended loading regimen. These results reflect international variation in practice (Ros-Soto et al., 2019; Kenny et al., 2019) and highlight a need for systematic improvement.

Following presentation of these findings to the haematology consultant group, work is underway to introduce automatic vitamin D testing at defined HSCT milestones and implement a standard operating procedure for supplementation. A re-audit is planned within 12 months to evaluate improvement in compliance and patient outcomes.

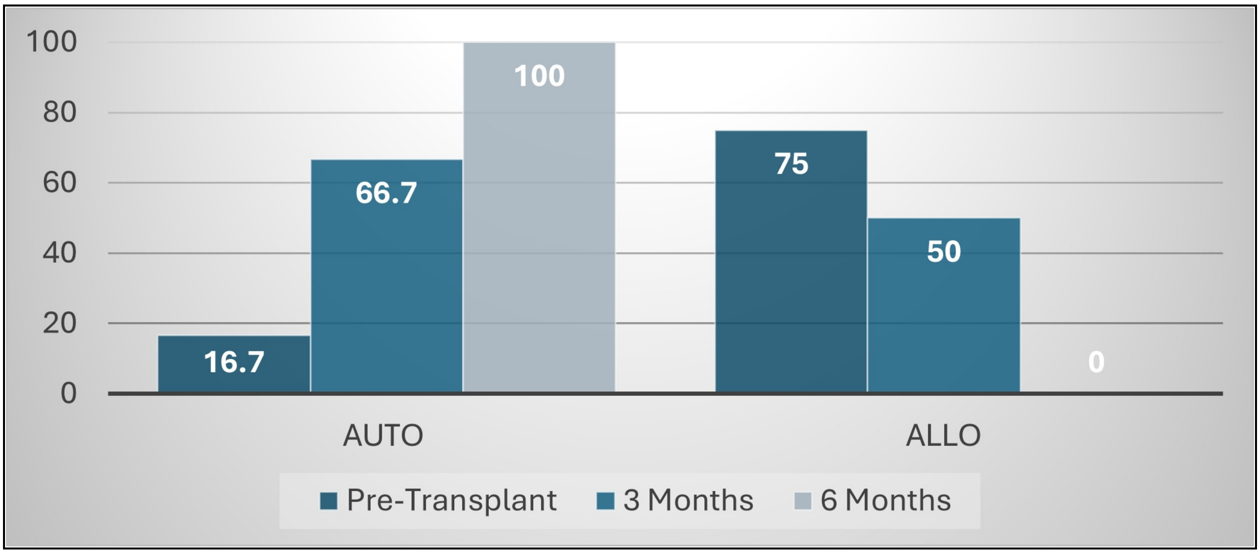

**Copyright Information:** (Online) ISSN: 2572-9241
©2026 The Author(s). HemaSphere is published by John Wiley & Sons Ltd on behalf of European Hematology Association. This is an open access Abstract Book distributed under the Attribution-NonCommercial-NoDerivs (CC BY-NC-ND), which allows third parties to download the articles and share them with others as long as they credit the author and the Abstract Book, but they cannot change the content in any way or use them commercially.
**Abstract Book Citations:** Authors, Title, HemaSphere, 2026;10:(S1):pages. The abstract book DOIs can be found on the "Author Information" page.
**Disclaimer:** Articles published in the journal HemaSphere exclusively reflect the opinions of the authors. The authors are responsible for all content in their abstracts including accuracy of the facts, statements, citing resources, etc.

### (PB4067) ALLOGENEIC HEMATOPOIETIC STEM CELL TRANSPLANTATION IN THE ADULT POPULATION WITH SEVER APLASTIC ANEMIA OF THE REPUBLIC OF BELARUS

**Topic:** 23. Stem cell transplantation - Clinical

Nechay Oksana*[1], Dziuba Alena[1], Morozova Oksana[1], Strongin Yuri[1], Tatsiana Talako[1], Natallia Kokhnovich[1], Natallia Vazhnik[1], Viktoryia Hrynevich[1], Viktirya Smolnikova[1], Natalya Milanovich[1], Uss Anatoly[1]

*[1]Minsk Scientific and Practical Center for Surgery, Transplantology and Hematology, Minsk, Belarus;*

### Background

Allogeneic hematopoietic stem cell transplantation (allo-HSCT) is the most effective treatment for severe aplastic anemia (SAA) and is considered the treatment of choice for young patients with an HLA-identical related donor. In recent years, the approach to allogeneic hematopoietic stem cell transplantation in the Republic of Belarus has undergone changes.

### Aims

To evaluate the results of allo-HSCT performed from 2014 to 2024 at the State Institution “Minsk Scientific and Practical Center for Surgery, Transplantology and Hematology”.

### Methods

We analyzed 24 cases of allo-HSCT for SAA. The study included 9 men and 15 women, median age 27 (21-30) years. In 8 patients (33.3%), an association of SAA with paroxysmal nocturnal hemoglobinuria (PNH) was found with a median PNH clone of 9.0% (2.5%-13.8%). The conditioning regimen used was cyclophosphamide (25 mg/kg), fludarabine phosphate (25 mg/kg), and antithymocyte immunoglobulin (25 mg/kg). In 2014, two patients underwent a conditioning regimen without the use of fludarabine phosphate. Prevention of acute graft-versus-host disease (GVHD) was performed with methylprednisolone (0.5 mg/kg) in combination with cyclosporine A (3 mg/kg/day for related HSCT, n=11) or with cyclosporine A (3 mg/kg/day) and mycophenolate mofetil (2 g/day for unrelated HSCT, n=13). The sources of hematopoietic stem cells were stimulated peripheral blood stem cells (PBSCs, n=13) and bone marrow (BM, n=11). The median cellularity by the number of CD34+ cells was 1.3 (0.9-1.8)x106/kg for BM (related HSCT), and 9.9 (7.8-10.3)x106/kg for PBSCs (unrelated HSCT). Most often, donors were men (n=18; 75%).

### Results

In allo-HSCT performed from 2014 to 2020 (n=12), the source of HSC was bone marrow in most cases (n=8; 66.7%), and allo-HSCT from related donors predominated (n=8; 66.7%). Among allo-HSCT in the period 2021 to 2024 (n=12), an increase in the number of transplants with PBSC as a source of HSCs (n=9; 75%) and an increase in the number of transplants from unrelated donors (n=9; 75%) were noted. ABO-incompatible allo-HSCT was performed between 2014 and 2020 in 3 cases: major - 1 and minor - 2, in the period 2021 to 2024. – in 4 cases: major – 1, minor – 3. All allo-HSCT in 2020 were performed from fully HLA-compatible donors; among transplants from 2021 to 2024, 2 allo-HSCT were performed from unrelated donors with partial HLA incompatibility. With the accumulation of clinical experience in managing patients with SAA/PNH, an increase in the size of the PNH clone, for which allo-HSCT was successfully performed, is observed: from 2014 to 2020, the size of the PNH clone ranged from 2.5% to 16% (n=4), from 2021 to 2024 – from 2.1% to 70.4% (n=4). Three-year overall survival of patients (n=15) was 80%, five-year overall survival (n=12) – 75%; Transplant mortality is 8.3%. The causes of death were sepsis and systemic fungal infection. Graft failure developed in 6 patients (25%): 3 had primary graft failure (1 had graft failure, 2 had restoration of native hematopoiesis), 2 had secondary graft hypofunction, and 1 had mixed chimerism.

**Copyright Information:** (Online) ISSN: 2572-9241
©2026 The Author(s). HemaSphere is published by John Wiley & Sons Ltd on behalf of European Hematology Association. This is an open access Abstract Book distributed under the Attribution-NonCommercial-NoDerivs (CC BY-NC-ND), which allows third parties to download the articles and share them with others as long as they credit the author and the Abstract Book, but they cannot change the content in any way or use them commercially.
**Abstract Book Citations:** Authors, Title, HemaSphere, 2026;10:(S1):pages. The abstract book DOIs can be found on the “Author Information” page.
**Disclaimer:** Articles published in the journal HemaSphere exclusively reflect the opinions of the authors. The authors are responsible for all content in their abstracts including accuracy of the facts, statements, citing resources, etc.

**Summary/Conclusion**

Over the past four years, there has been an increase in the number of allo-HSCTs from unrelated donors using PBSCs as the source of HSCs. Allo-HSCT is an effective treatment for patients with SAA, including SAA/PNH with varying PNH clone sizes. To reduce the incidence of post-transplant complications, it is necessary to assess potential risk factors for their development at all stages, including the allo-HSCT planning stage.

**Copyright Information:** (Online) ISSN: 2572-9241
©2026 The Author(s). HemaSphere is published by John Wiley & Sons Ltd on behalf of European Hematology Association. This is an open access Abstract Book distributed under the Attribution-NonCommercial-NoDerivs (CC BY-NC-ND), which allows third parties to download the articles and share them with others as long as they credit the author and the Abstract Book, but they cannot change the content in any way or use them commercially.
**Abstract Book Citations:** Authors, Title, HemaSphere, 2026;10:(S1):pages. The abstract book DOIs can be found on the “Author Information” page.
**Disclaimer:** Articles published in the journal HemaSphere exclusively reflect the opinions of the authors. The authors are responsible for all content in their abstracts including accuracy of the facts, statements, citing resources, etc.

### (PB4068) REAL-WORLD OUTCOMES OF AUTOLOGOUS STEM CELL TRANSPLANTATION USING BEACE CONDITIONING IN RELAPSED OR REFRACTORY HODGKIN LYMPHOMA

**Topic:** 23. Stem cell transplantation - Clinical

Thiago Carneiro*[1], Rosana Silva[1], Lucyana Leão[1], Camila Reis[1], Débora Do Vale[1], Joao Carlos Saraiva F[1]

[1]*Hospital Ophir Loyola / UEPA, Belém, Brazil;*

### Background

High-dose chemotherapy followed by autologous hematopoietic stem cell transplantation (auto-HSCT) remains a cornerstone of curative therapy for relapsed or refractory Hodgkin lymphoma (HL). The BEAM regimen is widely used; however, recurrent shortages of key agents such as carmustine and melphalan have limited its availability in routine practice. As a result, alternative conditioning regimens have been adopted in real-world settings. BeACE, combining bendamustine, cytarabine, etoposide, and cyclophosphamide, represents a pragmatic alternative, although real-world data on its feasibility, toxicity, and outcomes remain scarce.

### Aims

To evaluate the safety, feasibility, and early clinical outcomes of auto-HSCT using BeACE conditioning in patients with relapsed or refractory HL treated at a public tertiary referral center.

### Methods

We conducted a retrospective, observational cohort study based on systematic review of medical records from Ophir Loyola Hospital, a public tertiary referral center for hematologic malignancies located in Belém, Brazil, in the Brazilian Amazon region. Consecutive adult patients with relapsed or refractory HL who underwent auto-HSCT with BeACE conditioning were included. Data were abstracted from medical records.

The BeACE regimen consisted of bendamustine 200 mg/m² on day −6; etoposide 266 mg/m² daily on days −6 to −4; cytarabine 2,000 mg/m² daily on days −5 and −4; and cyclophosphamide 1,800 mg/m² daily on days −3 to −1, with mesna uroprotection. Disease status prior to transplantation was classified as complete remission (CR) or partial remission (PR). Relapse was considered only when a documented relapse date was recorded. Toxicity, engraftment, and outcomes were analyzed descriptively.

### Results

Between December 2023 and October 2025, 11 patients with relapsed or refractory HL underwent auto-HSCT using BeACE conditioning. Median age was 32 years (range, 27–55), and 63.6% were male. Prior to transplantation, 8 patients (72.7%) were in CR and 3 (27.3%) in PR. Salvage therapy before HSCT included pembrolizumab-based regimens in 3 patients (27.3%) and brentuximab vedotin in 1 patient (9.1%); the remaining 7 patients (63.6%) achieved disease control with chemotherapy alone.

The median infused $CD34^+$ cell dose was 3.7 $\times 10^6$/kg (range, 2.5–6.0). Median time to neutrophil engraftment was 10 days (range, 9–11). Febrile neutropenia occurred in 72.7% of patients. Mucositis, including all cases presenting with diarrhea, was observed in 36.4%. One patient (9.1%) developed a documented bloodstream infection, and one experienced catheter-associated thrombosis. No patients required intensive care unit support, and no transplant-related mortality was observed by day +100.

After a median follow-up of approximately 17 months (range, 3.7–25.5), one patient (9.1%) experienced documented relapse, and one patient (9.1%) died. At last follow-up, 81.8% of patients were alive and relapse-free.

**Copyright Information:** (Online) ISSN: 2572-9241
©2026 The Author(s). HemaSphere is published by John Wiley & Sons Ltd on behalf of European Hematology Association. This is an open access Abstract Book distributed under the Attribution-NonCommercial-NoDerivs (CC BY-NC-ND), which allows third parties to download the articles and share them with others as long as they credit the author and the Abstract Book, but they cannot change the content in any way or use them commercially.
**Abstract Book Citations:** Authors, Title, HemaSphere, 2026;10:(S1):pages. The abstract book DOIs can be found on the "Author Information" page.
**Disclaimer:** Articles published in the journal HemaSphere exclusively reflect the opinions of the authors. The authors are responsible for all content in their abstracts including accuracy of the facts, statements, citing resources, etc.

**Summary/Conclusion**

In this real-world cohort, auto-HSCT using BeACE conditioning demonstrated rapid hematopoietic recovery, manageable toxicity, and absence of early transplant-related mortality in patients with relapsed or refractory HL. Favorable early disease control was observed both in patients bridged with immunotherapy or antibody–drug conjugates and in those rescued with chemotherapy alone. These findings support BeACE as a feasible and safe conditioning regimen for patients with relapsed or refractory HL

**Table. Baseline characteristics, prior therapy, transplant features, and outcomes**

| Variable | Result (N = 11) |
|---|---|
| **Age,** years, median (range) | 32 (27–55) |
| **Male sex,** n (%) | 7 (63.6) |
| **Disease status pre-HSCT,** n (%) | |
| • Complete remission (CR) | 8 (72.7) |
| • Partial remission (PR) | 3 (27.3) |
| **Prior salvage therapy**, n (%) | |
| • Pembrolizumab-based | 3 (27.3) |
| • Brentuximab vedotin | 1 (9.1) |
| • Chemotherapy alone | 7 (63.6) |
| **$CD34^+$ cells infused** ($\times 10^6$/kg), median (range) | 3.7 (2.5–6.0) |
| **Neutrophil engraftment** (days), median (range) | 10 (9–11) |
| **Febrile neutropenia,** n (%) | 8 (72.7) |
| **Mucositis**, n (%) | 4 (36.4) |
| **Documented bloodstream infection,** n (%) | 1 (9.1) |
| **Catheter-associated thrombosis,** n (%) | 1 (9.1) |
| **ICU admission**, n (%) | 0 (0) |
| **Transplant-related mortality (≤ day +100)** | 0 |
| **Relapse,** n (%) | 1 (9.1) |
| **Death** (any cause), n (%) | 1 (9.1) |
| **Alive and relapse-free at last follow-up,** n (%) | 9 (81.8) |
| **Median follow-up,** months (range) | 17 (3.7–25.5) |

**Copyright Information:** (Online) ISSN: 2572-9241
©2026 The Author(s). HemaSphere is published by John Wiley & Sons Ltd on behalf of European Hematology Association. This is an open access Abstract Book distributed under the Attribution-NonCommercial-NoDerivs (CC BY-NC-ND), which allows third parties to download the articles and share them with others as long as they credit the author and the Abstract Book, but they cannot change the content in any way or use them commercially.
**Abstract Book Citations:** Authors, Title, HemaSphere, 2026;10:(S1):pages. The abstract book DOIs can be found on the "Author Information" page.
**Disclaimer:** Articles published in the journal HemaSphere exclusively reflect the opinions of the authors. The authors are responsible for all content in their abstracts including accuracy of the facts, statements, citing resources, etc.

### (PB4069) CLINICAL IMPACT OF POOLED BUFFY COAT–DERIVED GRANULOCYTE TRANSFUSION SUPPORT IN NEUTROPENIC PATIENTS UNDERGOING HEMATOPOIETIC STEM CELL TRANSPLANTATION

**Topic:** 23. Stem cell transplantation - Clinical

Nikhil Nagpal[1], Ashish Jain[2], Diksha Kumari[2], Deepali Chauhan[2], Arjun Kachhwaha[1], Daljit Kaur[2], Gita Negi[2], Uttam Kumar Nath*[1]

*[1]All India Institute of Medical Sciences, Rishikesh, Department of Medical Oncology Hematology, Rishikesh, India,[2]All India Institute of Medical Sciences, Rishikesh, Rishikesh, India;*

### Background

Profound neutropenia following hematopoietic stem cell transplantation (HSCT) represents a critical window of vulnerability to severe infection and sepsis. Although apheresis-derived granulocyte concentrates are recommended for adjunctive therapy, they remain inaccessible in many low- and middle-income settings. Pooled buffy coat–derived granulocyte transfusions are frequently used as a pragmatic alternative; however, data regarding the effective granulocyte dose delivered and associated clinical outcomes are limited.

### Aims

To evaluate the granulocyte dose achieved with pooled buffy coat transfusions and to assess hematologic response, infection control, and safety in neutropenic HSCT recipients with sepsis.

### Methods

We performed a retrospective analysis of five consecutive HSCT recipients (three allogeneic, two autologous) who developed microbiologically documented infections during profound neutropenia and received pooled buffy coat–derived granulocyte support. Granulocyte yield per transfusion, peri-transfusion total leukocyte count (TLC), microbiologic clearance, fever trends, and adverse events were analyzed across transfusion episodes.

### Results

All patients had infections caused by multidrug-resistant organisms, including *Stenotrophomonas maltophilia*, MDR *Escherichia coli*, *Staphylococcus epidermidis*, *Enterococcus faecium*, *Klebsiella pneumoniae*, and enteric pathogens detected on multiplex stool panels. Granulocyte yield per transfusion ranged from **0.24–0.58 × $10^{10}$ cells**, with a **mean delivered dose of 4.07 × $10^{9}$ granulocytes**.
The mean TLC increased from **45/µL pre-transfusion to 63/µL post-transfusion** (mean increment +18/µL; $p = 0.24$). Despite modest laboratory increments, most transfusion episodes were followed by **defervescence within 24–48 hours** and progressive clinical stabilization. Follow-up cultures became negative, indicating infection control.
Granulocyte support was continued until neutrophil engraftment, occurring between **Day +9 and Day +14** in evaluable patients. No transfusion-related adverse reactions were observed.

**Copyright Information:** (Online) ISSN: 2572-9241
©2026 The Author(s). HemaSphere is published by John Wiley & Sons Ltd on behalf of European Hematology Association. This is an open access Abstract Book distributed under the Attribution-NonCommercial-NoDerivs (CC BY-NC-ND), which allows third parties to download the articles and share them with others as long as they credit the author and the Abstract Book, but they cannot change the content in any way or use them commercially.
**Abstract Book Citations:** Authors, Title, HemaSphere, 2026;10:(S1):pages. The abstract book DOIs can be found on the “Author Information” page.
**Disclaimer:** Articles published in the journal HemaSphere exclusively reflect the opinions of the authors. The authors are responsible for all content in their abstracts including accuracy of the facts, statements, citing resources, etc.

**Summary/Conclusion**

Pooled buffy coat–derived granulocyte transfusions provided a **safe and clinically meaningful adjunctive therapy** for managing severe infections during post-transplant neutropenia, even with lower measurable granulocyte doses than conventional apheresis products. The observed discordance between limited leukocyte increments and clear clinical improvement suggests functional antimicrobial benefit. This strategy may represent a **feasible alternative in resource-constrained transplant programs**, warranting prospective evaluation to define optimal dosing and indications.

**Copyright Information:** (Online) ISSN: 2572-9241
©2026 The Author(s). HemaSphere is published by John Wiley & Sons Ltd on behalf of European Hematology Association. This is an open access Abstract Book distributed under the Attribution-NonCommercial-NoDerivs (CC BY-NC-ND), which allows third parties to download the articles and share them with others as long as they credit the author and the Abstract Book, but they cannot change the content in any way or use them commercially.
**Abstract Book Citations:** Authors, Title, HemaSphere, 2026;10:(S1):pages. The abstract book DOIs can be found on the “Author Information” page.
**Disclaimer:** Articles published in the journal HemaSphere exclusively reflect the opinions of the authors. The authors are responsible for all content in their abstracts including accuracy of the facts, statements, citing resources, etc.

### (PB4070) SECOND HEMATOPOIETIC STEM CELL TRANSPLANTATION WITH A LOW-DOSE TBI–CONTAINING CONDITIONING REGIMEN AFTER GRAFT FAILURE IN Β-THALASSEMIA MAJOR: EXPERIENCE FROM THREE PEDIATRIC CASES

**Topic:** 23. Stem cell transplantation - Clinical

Seda Aras Gürcan*[1], Sema Aylan Gelen[1], Damet Bağışov[1, 1, 1], Shahad Al Amoudi[1], Koray Yalçın[1]

[1]*Acıbadem Üniversitesi Hastanesi, pediatric hematology, altunizade -istanbul, Türkiye;*

### Background

Allogeneic hematopoietic stem cell transplantation (HSCT) remains the only curative treatment for transfusion-dependent β-thalassemia major. However, primary or secondary graft failure represents a significant clinical challenge, particularly in patients with substantial iron overload and immunological risk factors. Data regarding optimal conditioning regimens for salvage HSCT that provide adequate immunosuppression while minimizing toxicity are limited.

### Aims

We report the clinical characteristics, iron overload profiles, and post-transplant outcomes of three pediatric patients with β-thalassemia major who underwent a second HSCT using a treosulfan-based conditioning regimen incorporating low-dose total body irradiation (TBI).

### Methods

Three pediatric patients with transfusion-dependent β-thalassemia major who developed graft failure after their first allogeneic HSCT were retrospectively evaluated. Pre-transplant iron burden was assessed using serum ferritin levels along with cardiac and hepatic T2* magnetic resonance imaging (MRI). Immunological risk assessment included panel reactive antibody (PRA) testing and donor-specific antibody (DSA) analysis.
For the second HSCT, all patients received a conditioning regimen consisting of fludarabine (30 mg/m²/day), treosulfan (14 g/m²/day), thiotepa (10 mg/kg), and low-dose TBI (200 cGy). Peripheral blood stem cells were used as the graft source, obtained from a matched sibling donor in two patients and a 10/10 HLA-matched unrelated donor in one patient. Patients were monitored for engraftment, donor chimerism, transplant-related toxicities, and graft-versus-host disease (GVHD).

### Results

Following the first HSCT, all patients developed progressive loss of donor chimerism and recurrence of transfusion dependence. Before the second HSCT, ferritin levels ranged from 2,800 to 4,300 ng/mL, and cardiac and hepatic T2* MRI demonstrated moderate-to-severe iron deposition.
After the second HSCT, neutrophil engraftment occurred between days +14 and +17, and platelet engraftment between days +14 and +21. Early complete donor chimerism (100%) was achieved in all three patients. The median follow-up after the second transplant was 5 months (range, 1.5–7 months). During follow-up, no graft loss, severe acute GVHD, or significant organ toxicity was observed. One patient developed CMV reactivation, which was successfully managed with antiviral therapy. At last follow-up, all patients remain transfusion independent.

**Copyright Information:** (Online) ISSN: 2572-9241
©2026 The Author(s). HemaSphere is published by John Wiley & Sons Ltd on behalf of European Hematology Association. This is an open access Abstract Book distributed under the Attribution-NonCommercial-NoDerivs (CC BY-NC-ND), which allows third parties to download the articles and share them with others as long as they credit the author and the Abstract Book, but they cannot change the content in any way or use them commercially.
**Abstract Book Citations:** Authors, Title, HemaSphere, 2026;10:(S1):pages. The abstract book DOIs can be found on the “Author Information” page.
**Disclaimer:** Articles published in the journal HemaSphere exclusively reflect the opinions of the authors. The authors are responsible for all content in their abstracts including accuracy of the facts, statements, citing resources, etc.

**Summary/Conclusion**

In pediatric patients with β-thalassemia major and a history of graft failure with significant iron overload, a treosulfan–fludarabine–thiotepa–based conditioning regimen incorporating low-dose TBI appears to be an effective salvage transplantation strategy, resulting in successful engraftment, sustained donor chimerism, and acceptable early post-transplant outcomes.

**Copyright Information:** (Online) ISSN: 2572-9241
©2026 The Author(s). HemaSphere is published by John Wiley & Sons Ltd on behalf of European Hematology Association. This is an open access Abstract Book distributed under the Attribution-NonCommercial-NoDerivs (CC BY-NC-ND), which allows third parties to download the articles and share them with others as long as they credit the author and the Abstract Book, but they cannot change the content in any way or use them commercially.
**Abstract Book Citations:** Authors, Title, HemaSphere, 2026;10:(S1):pages. The abstract book DOIs can be found on the “Author Information” page.
**Disclaimer:** Articles published in the journal HemaSphere exclusively reflect the opinions of the authors. The authors are responsible for all content in their abstracts including accuracy of the facts, statements, citing resources, etc.

### (PB4071) EQUIVALENT EFFICACY BUT GREATER EFFICIENCY: PEGFILGRASTIM VERSUS FILGRASTIM FOR STEM CELL MOBILIZATION IN MULTIPLE MYELOMA

**Topic:** 23. Stem cell transplantation - Clinical

Yanlin Zhang[1], Xin-Yi Qin[1], Chun Cao[1], Zhi-Ming Luo[1], Ying Zhang[1], Han-Qing Zeng[1], Jian-Chuan Deng[1], Yun Luo*[1]

[1] *The Second Affiliated Hospital of Chongqing Medical University, Department of Hematology, Chongqing, China;*

### Background

Autologous stem cell transplantation (ASCT) is standard for eligible multiple myeloma (MM) patients, requiring efficient stem cell mobilization. Pegfilgrastim (PEG), a long-acting G-CSF, offers pharmacokinetic advantages over daily filgrastim (FIL), but real-world comparative data in MM are limited.

### Aims

To compare the efficacy, safety, and procedural efficiency of PEG versus FIL for stem cell mobilization in MM patients undergoing ASCT.

### Methods

We conducted a retrospective single-center study of 102 MM patients (PEG=49, FIL=53). Endpoints included $CD34^+$ cell yield, mobilization success ($\geq 2\times10^6$/kg), optimal yield ($\geq 5\times10^6$/kg), mobilization duration, apheresis sessions, engraftment times, and safety. Subgroup analysis by cyclophosphamide use and propensity score matching (PSM) were performed.

### Results

Baseline characteristics were balanced except for higher cyclophosphamide use in the PEG group (69.4% vs. 34.0%, p=0.001). Median $CD34^+$ yields (3.90 vs. 4.99 $\times10^6$/kg, p=0.096), success rates (91.7% vs. 94.2%), and optimal rates (35.4% vs. 50.0%) did not differ significantly. FIL yielded higher total mononuclear cells (p=0.0003). In the cyclophosphamide subgroup, PEG shortened mobilization duration (median 10 vs. 15 days, p<0.001) and reduced apheresis sessions (p=0.017). Engraftment times and safety profiles were similar between groups.

### Summary/Conclusion

PEG and FIL show comparable stem cell mobilization efficacy in MM patients. PEG significantly shortens mobilization time and reduces apheresis sessions when combined with cyclophosphamide, supporting its use as a logistically efficient alternative in chemo-mobilization protocols.

**Copyright Information:** (Online) ISSN: 2572-9241
©2026 The Author(s). HemaSphere is published by John Wiley & Sons Ltd on behalf of European Hematology Association. This is an open access Abstract Book distributed under the Attribution-NonCommercial-NoDerivs (CC BY-NC-ND), which allows third parties to download the articles and share them with others as long as they credit the author and the Abstract Book, but they cannot change the content in any way or use them commercially.
**Abstract Book Citations:** Authors, Title, HemaSphere, 2026;10:(S1):pages. The abstract book DOIs can be found on the "Author Information" page.
**Disclaimer:** Articles published in the journal HemaSphere exclusively reflect the opinions of the authors. The authors are responsible for all content in their abstracts including accuracy of the facts, statements, citing resources, etc.

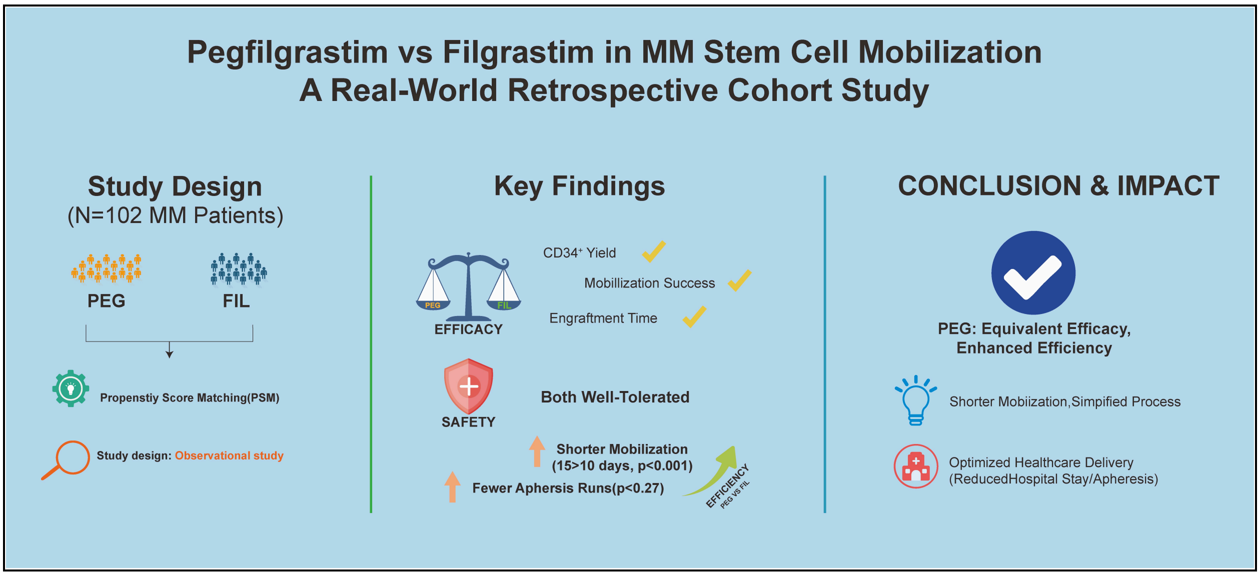

**Copyright Information:** (Online) ISSN: 2572-9241
©2026 The Author(s). HemaSphere is published by John Wiley & Sons Ltd on behalf of European Hematology Association. This is an open access Abstract Book distributed under the Attribution-NonCommercial-NoDerivs (CC BY-NC-ND), which allows third parties to download the articles and share them with others as long as they credit the author and the Abstract Book, but they cannot change the content in any way or use them commercially.
**Abstract Book Citations:** Authors, Title, HemaSphere, 2026;10:(S1):pages. The abstract book DOIs can be found on the “Author Information” page.
**Disclaimer:** Articles published in the journal HemaSphere exclusively reflect the opinions of the authors. The authors are responsible for all content in their abstracts including accuracy of the facts, statements, citing resources, etc.

### (PB4072) PERI-TRANSPLANT NURSING CARE FOR A PATIENT WITH ACUTE MYELOID LEUKEMIA COMPLICATED WITH HEMOPHILIA A UNDERGOING ALLOGENEIC HEMATOPOIETIC STEM CELL TRANSPLANTATION

**Topic:** 23. Stem cell transplantation - Clinical

Yuqiu Liu*[1], Lixue Zhang[1], Yiming Gao[1], Wenjun Xie[1]

[1]*Institute of Hematology & Blood Diseases Hospital, Chinese Academy of Medical Sciences & Peking Union Medical College, Tian Jin, China;*

### Aims

This study aimed to summarize the key points of peri-transplant nursing care for a patient with acute myeloid leukemia (AML) complicated with hemophilia A undergoing allogeneic hematopoietic stem cell transplantation (allo-HSCT), and provide clinical references for the transplant nursing of patients with such rare comorbidities.

### Methods

Refined peri-transplant nursing care was implemented for a 23-year-old male patient with AML complicated with hemophilia A admitted to our hospital in August 2025. Centering on the core issue of high bleeding risk, comprehensive bleeding risk prevention and control measures were adopted, including precise supplementation of coagulation factors, dual monitoring of overt and occult bleeding, safety management and personalized health education. Intelligent all-dimensional condition monitoring was supplemented with real-time monitoring via camera, electrocardiogram and personal digital assistant (PDA). Standardized nursing care for complications was carried out, with closed-loop management such as mucosal care and phased rehabilitation training for infections, disuse syndrome and other complications. Meanwhile, targeted psychological care was provided, and the patient's anxiety was alleviated combined with health education to improve treatment compliance.

### Results

No severe bleeding events such as intracranial hemorrhage or disuse syndrome occurred in the patient during the peri-transplant period. Complications including fever, diarrhea, grade I oral ulcer and hypokalemia were relieved within 3-5 days after symptomatic nursing, and the serum potassium level returned to normal. The patient successfully passed the myelosuppression period on the 15th day after transplantation, achieved granulocyte engraftment and hematopoietic reconstitution, and met the discharge criteria from the transplant unit. During the 1-month follow-up after discharge, the patient had good joint function without bleeding or infection events.

### Summary/Conclusion

The core of transplant nursing for AML patients complicated with hemophilia A is to address the dual coagulation disorders caused by the superposition of coagulation factor deficiency and thrombocytopenia. A comprehensive nursing plan including comprehensive bleeding prevention and control, intelligent condition monitoring, standardized complication nursing and targeted psychological care can effectively reduce the risks of bleeding and complications during the peri-transplant period, ensure the smooth completion of transplantation for patients, and provide effective practical experience for the clinical nursing of such rare cases.

**Copyright Information:** (Online) ISSN: 2572-9241
©2026 The Author(s). HemaSphere is published by John Wiley & Sons Ltd on behalf of European Hematology Association. This is an open access Abstract Book distributed under the Attribution-NonCommercial-NoDerivs (CC BY-NC-ND), which allows third parties to download the articles and share them with others as long as they credit the author and the Abstract Book, but they cannot change the content in any way or use them commercially.
**Abstract Book Citations:** Authors, Title, HemaSphere, 2026;10:(S1):pages. The abstract book DOIs can be found on the "Author Information" page.
**Disclaimer:** Articles published in the journal HemaSphere exclusively reflect the opinions of the authors. The authors are responsible for all content in their abstracts including accuracy of the facts, statements, citing resources, etc.

### (PB4073) EARLY TREATMENT WITH BELUMOSUDIL IMPROVES THE CLINICAL OUTCOME OF CGVHD

**Topic:** 23. Stem cell transplantation - Clinical

Akihisa Hino*[1], Tomoaki Ueda[1], Shinsuke Kusakabe[1], Hidenori Kasahara[1], Keigo Akuta[1], Hiroyuki Takamori[1], Jiro Fujita[1], Kentaro Fukushima[1], Naoki Hosen[1, 2, 3]

*[1]The University of Osaka Graduate School of Medicine, Hematology and Oncology, Suita, Japan;[2]The University of Osaka, World Premier International Immunology Frontier Research Centre, Suita, Japan; [3]The University of Osaka, Integrated Frontier Research for Medical Science Division, Institute for Open and Transdisciplinary Research Initiatives (OTRI), Suita, Japan;*

### Background

Chronic graft-versus-host disease (cGVHD) is a refractory disease, causing tissue inflammation and fibrosis in various organs. In recent years, the advent of novel therapeutic agents has precipitated substantial developments in the management of cGVHD. Belumosudil is one of the novel therapeutic agents for cGVHD. Belumosudil promotes downregulation of inflammatory cytokines and improves fibrosis by inhibiting Rho-associated coiled-coil containing protein kinase 2 (ROCK2). Belumosudil is approved in many countries as the third-line treatment for cGVHD. This drug is permitted for use in earlier lines in a few countries, including Japan.

### Aims

We consider that the early inhibition of ROCK2 prevents fibrosis and leads to favorable outcomes in cGVHD. However, there is no clear evidence regarding the impact of prior treatment history or treatment timing on the therapeutic efficacy of Belumosudil. We are permitted to use Belumosudil earlier than many other countries.  Based on this background, we examined the impact of early Belumosudil use on cGVHD treatment.

### Methods

We retrospectively analyzed 22 patients who received Belumosudil for cGVHD at The University of Osaka Hospital from May 2024 to December 2025. The median duration of cGVHD was 228 days (range: 11-5370 days). There were 17 cases that received early treatment with Belumosudil within one year after cGVHD onset. Thirteen cases received Belumosudil as second-line treatment after steroid therapy. Nine cases received this drug as third-line or beyond treatment. Baseline organ dysfunction was present in the following organs: Eye (n=3), Skin (n=7), Lung (n=4), Gastrointestinal tract (n=5), Mouth (n=3), Liver (n=3), Joint (n=1), Serositis (n=3). Baseline assessment and treatment efficacy evaluation of cGVHD in each organ were performed based on the 2014 National Institutes of Health Consensus Criteria. Treatment efficacy was evaluated at both the short-term (1–3 months) and long-term (6–12 months) time points.

### Results

Belumosudil significantly improved cGVHD scores in each organ within 3 months after treatment ($p<0.0001$). Further improvement in the cGVHD score was observed only in the gastrointestinal tract at 6 months and beyond after treatment. Both the early treatment (within 1 year of cGVHD onset) and the late treatment (1 year or later after cGVHD onset) significantly improved cGVHD scores (Figure1 A, B). In particular, the early treatment produced a greater improvement in cGVHD scores compared to the late treatment ($p=0.009$) (Figure1 C). This result demonstrated the effectiveness of early treatment for Belumosudil. No significant difference in treatment efficacy was observed between second-line treatment and third-line or beyond treatment. In organ-specific comparisons, treatment efficacy was low for the lungs in both early and late treatment groups. Complete remission was achieved for serositis in both early and late treatment groups.

**Copyright Information:** (Online) ISSN: 2572-9241
©2026 The Author(s). HemaSphere is published by John Wiley & Sons Ltd on behalf of European Hematology Association. This is an open access Abstract Book distributed under the Attribution-NonCommercial-NoDerivs (CC BY-NC-ND), which allows third parties to download the articles and share them with others as long as they credit the author and the Abstract Book, but they cannot change the content in any way or use them commercially.
**Abstract Book Citations:** Authors, Title, HemaSphere, 2026;10:(S1):pages. The abstract book DOIs can be found on the "Author Information" page.
**Disclaimer:** Articles published in the journal HemaSphere exclusively reflect the opinions of the authors. The authors are responsible for all content in their abstracts including accuracy of the facts, statements, citing resources, etc.

### Summary/Conclusion

Initiating Belumosudil within one year of cGVHD onset significantly improved cGVHD outcomes. However, the choice of second-line treatment versus third-line or beyond treatment did not affect the results. Regardless of prior treatment, early initiation of Belumosudil after cGVHD onset was shown to deliver effective results.

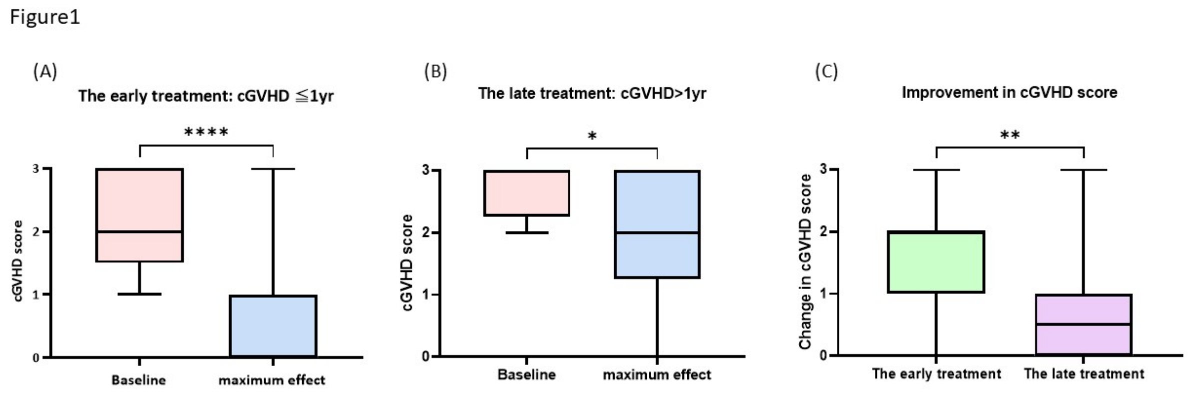

**Copyright Information:** (Online) ISSN: 2572-9241
©2026 The Author(s). HemaSphere is published by John Wiley & Sons Ltd on behalf of European Hematology Association. This is an open access Abstract Book distributed under the Attribution-NonCommercial-NoDerivs (CC BY-NC-ND), which allows third parties to download the articles and share them with others as long as they credit the author and the Abstract Book, but they cannot change the content in any way or use them commercially.
**Abstract Book Citations:** Authors, Title, HemaSphere, 2026;10:(S1):pages. The abstract book DOIs can be found on the "Author Information" page.
**Disclaimer:** Articles published in the journal HemaSphere exclusively reflect the opinions of the authors. The authors are responsible for all content in their abstracts including accuracy of the facts, statements, citing resources, etc.

### (PB4074) EFFICACY AND SAFETY OF AUTOLOGOUS HEMATOPOIETIC STEM CELL TRANSPLANTATION IN THE TREATMENT OF PEDIATRIC ATYPICAL TERATOID/RHABDOID TUMOR: A RETROSPECTIVE STUDY

**Topic:** 23. Stem cell transplantation - Clinical

Jianming Fang*[1], Han Gong[1], Ran Zheng[1], Wei Zhou[1], Hongyan Liu[1]

*[1]Beijing Jingdu Children's Hospital, Beijing, China;*

### Background

Atypical teratoid/rhabdoid tumor (AT/RT) is a rare, highly malignant central nervous system embryonal tumor that predominantly affects infants and young children. In recent years, despite the clinical application of comprehensive treatment modalities such as surgery, chemotherapy, and radiotherapy, the survival rate of patients remains unsatisfactory, with a long-term survival rate of 15-20% . With the application of autologous hematopoietic stem cell transplantation (auto-HSCT), the survival rate has slightly improved compared with previous years. However, due to the frequent rapid recurrence or progression of the disease, the overall treatment still faces enormous challenges. This study retrospectively analyzed the clinical data of 4 children with AT/RT admitted to Beijing Jingdu Children's Hospital from July 2020 to January 2025, summarized the therapeutic effect of autologous peripheral blood stem cell transplantation (auto-HSCT), and further explored the clinical value of this treatment regimen combined with literature review.

### Aims

To investigate the efficacy and safety of autologous hematopoietic stem cell transplantation (Auto-HSCT) in the treatment of childhood atypical teratoid/rhabdoid tumor (AT/RT).

### Methods

A retrospective analysis was performed on the clinical data of 4 children with AT/RT admitted to Beijing Kyoto Children's Hospital from July 2020 to January 2025. All patients received the thiotepa + carboplatin + etoposide (TCE) conditioning regimen. The conditioning-related toxicity was evaluated, and hematopoietic reconstitution, complications and prognosis were observed

### Results

The median transplant age of the 4 children was 3 years and 3 months, and all achieved successful autologous stem cell collection. Grade Ⅳ myelosuppression occurred in all patients after conditioning, with no severe organ dysfunction. Neutrophil engraftment was achieved on days +10 to +12 post-transplant, and all transplantations were successful. One case recurred and died 2 months after transplantation, and the other 3 cases remained in disease-free survival. The median overall survival of the 4 children was 71 months, with a median post-transplant survival of 18 months

### Summary/Conclusion

Auto-HSCT combined with the TCE conditioning regimen for childhood AT/RT has controllable adverse reactions and favorable efficacy, which can prolong the survival time of children and provide the possibility of achieving long-term disease-free survival.

**Copyright Information:** (Online) ISSN: 2572-9241
©2026 The Author(s). HemaSphere is published by John Wiley & Sons Ltd on behalf of European Hematology Association. This is an open access Abstract Book distributed under the Attribution-NonCommercial-NoDerivs (CC BY-NC-ND), which allows third parties to download the articles and share them with others as long as they credit the author and the Abstract Book, but they cannot change the content in any way or use them commercially.
**Abstract Book Citations:** Authors, Title, HemaSphere, 2026;10:(S1):pages. The abstract book DOIs can be found on the "Author Information" page.
**Disclaimer:** Articles published in the journal HemaSphere exclusively reflect the opinions of the authors. The authors are responsible for all content in their abstracts including accuracy of the facts, statements, citing resources, etc.

### (PB4075) EFFECT OF IMPROVED WEARING METHOD OF PORTABLE ELECTROCARDIOGRAM MONITOR IN PATIENTS UNDERGOING HEMATOPOIETIC STEM CELL TRANSPLANTATION DURING TREATMENT IN ISOLATION CHAMBER

**Topic:** 23. Stem cell transplantation - Clinical

Xinrong Cui*[1], Yuqiu Liu[1], Wenjun Xie[1], Yi He[1]

[1]*Institute of Hematology & Blood Diseases Hospital, Chinese Academy of Medical Sciences & Peking Union Medical College, Tian Jin, China;*

### Aims

To explore the application effect of the improved wearing method of portable electrocardiogram (ECG) monitor in patients undergoing hematopoietic stem cell transplantation (HSCT) during treatment in the isolation chamber, and to analyze its safety, effectiveness and influence on patients' comfort level.

### Methods

A total of 80 patients receiving treatment in the isolation chamber at the HSCT Center of our hospital from January 2025 to December 2025 were selected and randomly divided into the control group and the observation group with 40 cases in each group by the random number table method. The control group adopted traditional ECG monitor electrodes and conventional pasting method, while the observation group used the improved wearing method with hypoallergenic flexible electrode patches fixed by sterile transparent dressings. The incidence of electrode-related skin complications (including contact dermatitis, skin damage and infection), electrode displacement/shedding rate, incidence of analysis difficulties caused by signal artifacts were compared between the two groups. Meanwhile, the subjective comfort level of patients was evaluated by the comfort scale.

### Results

The incidence of electrode-related skin complications in the observation group was 7.5%, which was significantly lower than 35.0% in the control group ($P<0.01$). The electrode displacement/shedding rate and the incidence of signal artifacts in the observation group were 5.0% and 12.5% respectively, both significantly lower than 22.5% and 32.5% in the control group ($P<0.05$). The results of comfort scale scoring showed that the scores of pain, pruritus and overall comfort in the observation group were significantly better than those in the control group ($P<0.01$).

### Summary/Conclusion

Patients undergoing HSCT in the isolation chamber have fragile skin and high risk of infection. The improved wearing method of portable ECG monitor can significantly reduce the risks of skin damage and infection, effectively ensure the continuity and accuracy of ECG signal monitoring, and greatly improve the comfort level of patients during treatment. It is a safe and efficient monitoring scheme suitable for this group of patients.

**Copyright Information:** (Online) ISSN: 2572-9241
©2026 The Author(s). HemaSphere is published by John Wiley & Sons Ltd on behalf of European Hematology Association. This is an open access Abstract Book distributed under the Attribution-NonCommercial-NoDerivs (CC BY-NC-ND), which allows third parties to download the articles and share them with others as long as they credit the author and the Abstract Book, but they cannot change the content in any way or use them commercially.
**Abstract Book Citations:** Authors, Title, HemaSphere, 2026;10:(S1):pages. The abstract book DOIs can be found on the "Author Information" page.
**Disclaimer:** Articles published in the journal HemaSphere exclusively reflect the opinions of the authors. The authors are responsible for all content in their abstracts including accuracy of the facts, statements, citing resources, etc.

### (PB4076) A JOURNEY MAP STUDY OF THE PSYCHOLOGICAL EXPERIENCE OF PATIENTS UNDERGOING HEMATOPOIETIC STEM CELL TRANSPLANTATION

**Topic:** 23. Stem cell transplantation - Clinical

Qiuhui Zhang*[1], Jiongtong Fan[1], Junying Han[1]

[1] *Institute of Hematology & Blood Diseases Hospital, Chinese Academy of Medical Sciences & Peking Union Medical College, Transplantation Center, tianjin, China;*

### Background

Hematopoietic stem cell transplantation (HSCT) is an effective means of treating various blood diseases. With the advancement of transplantation technology and the extension of patient survival, the psychological experience and quality of life of patients are increasingly receiving attention. However, the process of hematopoietic stem cell therapy is complex, requiring high-dose radiation and chemotherapy, long-term protective isolation, and the threat of various complications. The psychological experience of patients is a complex, dynamic, and environmentally influenced issue. Although research has confirmed the prevalence and harm of psychological distress, there is still a lack of systematic and in-depth research on how patients cope with these psychological challenges throughout the entire transplantation process. Journey map is a tool and method based on the concept of "patient-centered", which visualizes the interaction between patients and various elements in the medical system. It presents their psychological experience trajectories, key touchpoints, and emotional fluctuations at different stages in a time series, providing empirical evidence for the development of staged and precise psychological support interventions in clinical practice.

### Aims

To dynamically identify the psychological experiences and needs of patients undergoing HSCT at different stages of their illness trajectory using a journey map approach, thereby providing a basis for optimizing psychological management and developing targeted nursing strategies.

### Methods

A descriptive qualitative study was conducted. From November to December 2025, semi-structured interviews were performed with 15 patients who had undergone HSCT and were attending follow-up appointments at a tertiary hospital in Tianjin, China. Interview data were managed using NVivo 15.0 software, and thematic analysis was performed using Colaizzi's seven-step method. A journey map was subsequently developed based on the findings.

### Results sing the four phases of the HSCT patient pathway—decision-making period, waiting period for transplantation, peri-transplantation period, and recovery period—as the temporal axis, 40 core themes were extracted across three dimensions: tasks, emotions, and pain points. The findings revealed a complete psychological evolution process in patients, progressing from decisional dilemmas and uncertainty-related anxiety during the waiting period, through traumatic experiences in the peri-transplantation period, to identity reconstruction and post-traumatic growth during the recovery period. A psychological experience journey map for HSCT patients was constructed based on these findings.

**Copyright Information:** (Online) ISSN: 2572-9241
©2026 The Author(s). HemaSphere is published by John Wiley & Sons Ltd on behalf of European Hematology Association. This is an open access Abstract Book distributed under the Attribution-NonCommercial-NoDerivs (CC BY-NC-ND), which allows third parties to download the articles and share them with others as long as they credit the author and the Abstract Book, but they cannot change the content in any way or use them commercially.
**Abstract Book Citations:** Authors, Title, HemaSphere, 2026;10:(S1):pages. The abstract book DOIs can be found on the "Author Information" page.
**Disclaimer:** Articles published in the journal HemaSphere exclusively reflect the opinions of the authors. The authors are responsible for all content in their abstracts including accuracy of the facts, statements, citing resources, etc.

**Summary/Conclusion**

The disease management journey for HSCT patients is prolonged, and their psychological experiences are characterized by being[illegible], dynamic, and complex. In the future, healthcare professionals should accurately deliver dynamic and personalized psychological support targeting patients' core pain points and emotional needs at different phases. This approach can help patients effectively cope with the psychological challenges of transplantation, promote psychological adaptation and post-traumatic growth, and ultimately enhance their overall quality of life.

**Copyright Information:** (Online) ISSN: 2572-9241
©2026 The Author(s). HemaSphere is published by John Wiley & Sons Ltd on behalf of European Hematology Association. This is an open access Abstract Book distributed under the Attribution-NonCommercial-NoDerivs (CC BY-NC-ND), which allows third parties to download the articles and share them with others as long as they credit the author and the Abstract Book, but they cannot change the content in any way or use them commercially.
**Abstract Book Citations:** Authors, Title, HemaSphere, 2026;10:(S1):pages. The abstract book DOIs can be found on the "Author Information" page.
**Disclaimer:** Articles published in the journal HemaSphere exclusively reflect the opinions of the authors. The authors are responsible for all content in their abstracts including accuracy of the facts, statements, citing resources, etc.

### (PB4077) REPEATED ALLOGENEIC HEMATOPOIETIC STEM CELL TRANSPLANTATION IN A PATIENT WITH AML (CLINICAL OBSERVATION)

**Topic:** 23. Stem cell transplantation - Clinical

Alena Hlaz*[1], Volha Mishkova[2], Natalia Kovshik[1], Ihar Iskrou[1], Uss Anatoly[1]

*[1]Minsk Scientific and Practical Center for Surgery, Transplantology and Hematology, Minsk, Belarus;[2]Belarusian Research Center for Pediatric Oncology, Hematology, and Immunology, Minsk, Belarus;*

### Background

Allogeneic hematopoietic stem cell transplantation (allo-HSCT) remains the standard curative approach for the majority of hematologic malignancies. Disease relapse and graft failure represent the leading causes of allo-HSCT failure. In selected cases, repeat allo-HSCT may be considered as a therapeutic option; however, the optimal donor selection strategy in this setting remains unclear.

### Aims

To evaluate the impact of donor selection on the outcomes of repeat allo-HSCT.

### Methods

A 37-year-old patient was diagnosed with acute myeloid leukemia (FAB M2). Following induction polychemotherapy, first complete remission with negative minimal residual disease (MRD–) was achieved. Given the patient's young age and high risk of relapse, allo-HSCT was indicated.
Due to the absence of a fully matched related donor, transplantation was performed from an unrelated 9/10 HLA-mismatched donor (female), ABO-compatible and CMV-IgG negative. The graft contained 4.83 × $10^6$ $CD34^+$ cells/kg. Conditioning consisted of busulfan and fludarabine (BuFlu).
On day +30 post-transplant, no evidence of disease relapse was detected; however, hematopoietic recovery did not occur, consistent with primary graft failure. In the absence of alternative donors within the country, a second allo-HSCT from the same donor was performed 7 weeks later, using bone marrow as the stem cell source. The $CD34^+$ cell dose was 0.86 × $10^6$/kg. Conditioning consisted of fludarabine and cyclophosphamide (FluCy).
Hematopoietic recovery was again absent by day +30 after the second transplant. Consequently, a haploidentical allo-HSCT from the patient's mother (HLA 5/10 match) was performed 7 weeks later, using FluCy conditioning. By day +30 after the third transplant, engraftment was achieved; however, primary graft hypofunction developed. The patient remains transfusion-dependent 160 days after the third allo-HSCT.

### Results

Neither a change in stem cell source nor donor substitution guaranteed successful engraftment in this case, particularly when repeat allo-HSCT was performed due to primary graft failure.

**Copyright Information:** (Online) ISSN: 2572-9241
©2026 The Author(s). HemaSphere is published by John Wiley & Sons Ltd on behalf of European Hematology Association. This is an open access Abstract Book distributed under the Attribution-NonCommercial-NoDerivs (CC BY-NC-ND), which allows third parties to download the articles and share them with others as long as they credit the author and the Abstract Book, but they cannot change the content in any way or use them commercially.
**Abstract Book Citations:** Authors, Title, HemaSphere, 2026;10:(S1):pages. The abstract book DOIs can be found on the "Author Information" page.
**Disclaimer:** Articles published in the journal HemaSphere exclusively reflect the opinions of the authors. The authors are responsible for all content in their abstracts including accuracy of the facts, statements, citing resources, etc.

**Summary/Conclusion**

Donor selection plays a critical role in repeat allo-HSCT and should be carefully re-evaluated after graft failure. However, in the setting of primary engraftment failure requiring urgent retransplantation, clinical urgency, limited donor availability, and patient condition may outweigh theoretical benefits of donor change. This case highlights the complexity of decision-making in repeat allo-HSCT and underscores the need for individualized strategies, as modification of donor or stem cell source alone may be insufficient to ensure successful engraftment.

**Copyright Information:** (Online) ISSN: 2572-9241
©2026 The Author(s). HemaSphere is published by John Wiley & Sons Ltd on behalf of European Hematology Association. This is an open access Abstract Book distributed under the Attribution-NonCommercial-NoDerivs (CC BY-NC-ND), which allows third parties to download the articles and share them with others as long as they credit the author and the Abstract Book, but they cannot change the content in any way or use them commercially.
**Abstract Book Citations:** Authors, Title, HemaSphere, 2026;10:(S1):pages. The abstract book DOIs can be found on the “Author Information” page.
**Disclaimer:** Articles published in the journal HemaSphere exclusively reflect the opinions of the authors. The authors are responsible for all content in their abstracts including accuracy of the facts, statements, citing resources, etc.

### (PB4078) THE PROGNOSTIC IMPACT OF HAPLOIDENTICAL PERIPHERAL HEMATOPOIETIC STEM CELL TRANSPLANTATION COMBINED WITH CORD BLOOD INFUSION ON PATIENTS WITH ACUTE LEUKEMIA

**Topic:** 23. Stem cell transplantation - Clinical

Pan Pan*[1], Aiming Pang[1], Erlie Jiang[1]

*[1]Institute of Hematology & Blood Diseases Hospital, Chinese Academy of Medical Sciences & Peking Union Medical College, Tianjin, China;*

### Background

Currently, studies have explored the combined transplantation of haploidentical hematopoietic stem cells and umbilical cord blood for the treatment of hematologic malignancies such as acute myeloid leukemia, aiming to achieve synergistic effects that promote hematopoietic engraftment, reduce the incidence of graft-versus-host disease, and decrease the risk of disease relapse. Preliminary clinical observations indicate that this combined transplantation strategy has shown promising therapeutic efficacy.

### Aims

To investigate the efficacy and prognostic factors of haploidentical peripheral hematopoietic stem cell transplantation combined with third-party umbilical cord blood in patients with acute leukemia.

### Methods

Clinical data from 47 acute leukemia patients who underwent haploidentical peripheral hematopoietic stem cell transplantation combined with third-party umbilical cord blood at the Institute of Hematology, Chinese Academy of Medical Sciences, between January 2021 and June 2023, were retrospectively analyzed.

### Results

Among the 47 patients, 31 were male and 16 were female, with a median age of 38(range: 9-60) years. Underlying diseases included acute myeloid leukemia(32 cases), acute lymphoblastic leukemia(13 cases), and mixed-phenotype acute leukemia(2 cases). A total of 17 patients(36.17%) had extramedullary infiltration and/or central nervous system leukemia. The median time from diagnosis to transplantation was 7.33(range: 4.8-52.8) months. At the time of transplantation, 21.3%(10/47) of patients were in refractory or relapsed status, and 42.55%(20/47) were molecularly positive. All patients achieved successful neutrophil engraftment (median time: 14 days) and platelet engraftment (median time: 17 days). The cumulative incidence of grade Ⅱ-Ⅳ acute GVHD at 100 days post-transplantation was 31.5%, and the cumulative incidence of moderate-to-severe chronic GVHD at 3 years was 21.8%. After a median follow-up of 35.93 months, the 1-year and 3-year overall survival rates were 78.5% and 67.9%, respectively. The 3-year progression-free survival rate was 54.6%, and the 3-year cumulative relapse rate was 27.2%. Subgroup analysis showed that patients who achieved complete remission within 2 chemotherapy cycles and had no relapse or extramedullary involvement had a 3-year overall survival rate of 87.0%. Multivariate analysis identified relapsed/refractory status(*P*=0.049) and the presence of extramedullary and/or central nervous system involvement(*P*=0.025) as independent adverse prognostic factors for overall survival.

**Copyright Information:** (Online) ISSN: 2572-9241
©2026 The Author(s). HemaSphere is published by John Wiley & Sons Ltd on behalf of European Hematology Association. This is an open access Abstract Book distributed under the Attribution-NonCommercial-NoDerivs (CC BY-NC-ND), which allows third parties to download the articles and share them with others as long as they credit the author and the Abstract Book, but they cannot change the content in any way or use them commercially.
**Abstract Book Citations:** Authors, Title, HemaSphere, 2026;10:(S1):pages. The abstract book DOIs can be found on the “Author Information” page.
**Disclaimer:** Articles published in the journal HemaSphere exclusively reflect the opinions of the authors. The authors are responsible for all content in their abstracts including accuracy of the facts, statements, citing resources, etc.

**Summary/Conclusion**

Haploidentical peripheral hematopoietic stem cell transplantation combined with third-party umbilical cord blood can achieve stable engraftment in acute leukemia patients without significantly increasing the risk of GVHD. This strategy may improve transplantation outcomes, particularly for non-refractory/relapsed acute leukemia patients without extramedullary/central nervous system involvement.

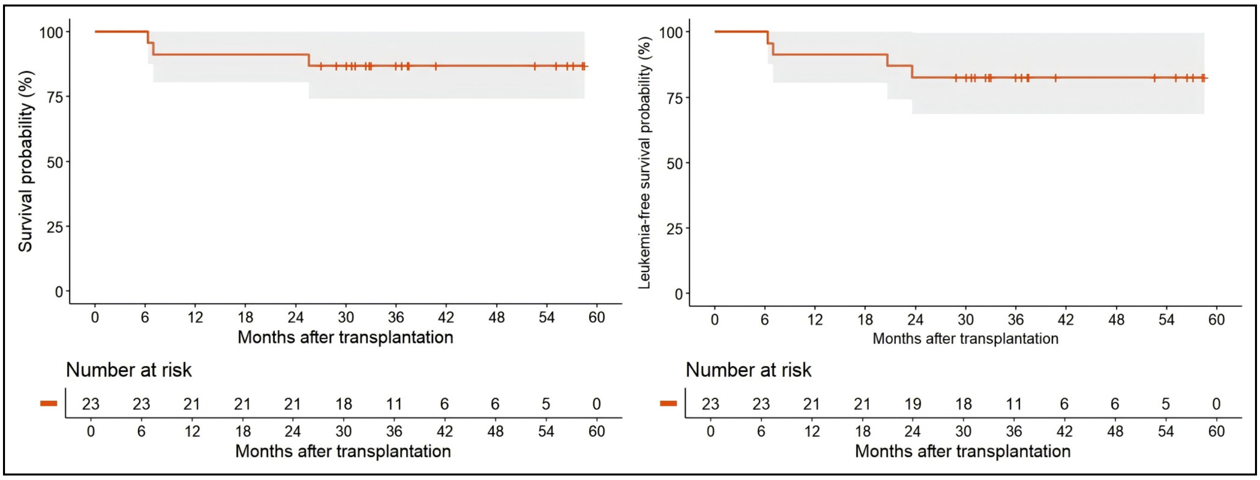

OS and DFS of patients who achieved complete remission within 2 chemotherapy cycles and had no relapse or extramedullary involvement

**Copyright Information:** (Online) ISSN: 2572-9241
©2026 The Author(s). HemaSphere is published by John Wiley & Sons Ltd on behalf of European Hematology Association. This is an open access Abstract Book distributed under the Attribution-NonCommercial-NoDerivs (CC BY-NC-ND), which allows third parties to download the articles and share them with others as long as they credit the author and the Abstract Book, but they cannot change the content in any way or use them commercially.
**Abstract Book Citations:** Authors, Title, HemaSphere, 2026;10:(S1):pages. The abstract book DOIs can be found on the "Author Information" page.
**Disclaimer:** Articles published in the journal HemaSphere exclusively reflect the opinions of the authors. The authors are responsible for all content in their abstracts including accuracy of the facts, statements, citing resources, etc.

### (PB4079) CLINICAL OUTCOMES IN TP53-MUTATED CLONAL HEMATOPOIESIS: A SINGLE-CENTER RETROSPECTIVE STUDY

**Topic:** 24. Hematopoiesis, stem cells and microenvironment

Kelly Chien[1], Jacob Herstein[2], Manuel Maroun[1], Guillermo Montalban-Bravo[1], Koichi Takahashi[1], Danielle Hammond[1], Nicholas Short[1], Guillermo Garcia-Manero[1], Courtney Dinardo[1], Sanam Loghavi*[3]

*[1]The University of Texas MD Anderson Cancer Center, Leukemia, Houston, United States of America;[2]The University of Texas Health Science Center at Houston, Internal Medicine, Houston, United States of America; [3]The University of Texas MD Anderson Cancer Center, Hematopathology, Houston, United States of America;*

### Background

Clonal hematopoiesis (CH), including CH of indeterminate potential (CHIP) and clonal cytopenia of undetermined significance (CCUS), is defined by myeloid neoplasm (MN)-associated somatic mutations in the absence of known hematologic malignancy and may precede MNs, such as myelodysplastic syndromes (MDS) and acute myeloid leukemia (AML). *TP53*-mutated (*TP53*m) CH has been assumed to be high risk, but clinical outcomes are poorly characterized.

### Aims

We aim to evaluate the clinicopathologic characteristics and outcomes of patients with *TP53*m CH.

### Methods

We retrospectively assessed patients with *TP53m* CH seen at a tertiary cancer center. Inclusion required pathogenic or likely pathogenic *TP53* mutations of presumed hematopoietic origin identified on bone marrow, peripheral blood, or plasma next-generation sequencing.

**Copyright Information:** (Online) ISSN: 2572-9241
©2026 The Author(s). HemaSphere is published by John Wiley & Sons Ltd on behalf of European Hematology Association. This is an open access Abstract Book distributed under the Attribution-NonCommercial-NoDerivs (CC BY-NC-ND), which allows third parties to download the articles and share them with others as long as they credit the author and the Abstract Book, but they cannot change the content in any way or use them commercially.
**Abstract Book Citations:** Authors, Title, HemaSphere, 2026;10:(S1):pages. The abstract book DOIs can be found on the "Author Information" page.
**Disclaimer:** Articles published in the journal HemaSphere exclusively reflect the opinions of the authors. The authors are responsible for all content in their abstracts including accuracy of the facts, statements, citing resources, etc.

**Results**

62 patients with *TP53*m CH were included; 26 (42%) CHIP and 36 (58%) CCUS. Median age was 68 years (range, 23–81) with 30 male patients (48%). History of tobacco, alcohol, and illicit drug use was noted in 30 (48%), 31 (50%), and 7 (11%) patients, respectively. Common comorbidities included cardiovascular disease (n=51, 82%), diabetes (n=17, 27%), and rheumatologic disorders (n=13, 21%). 53 patients (85%) had a history of non-myeloid malignancy, with 41 (77%) receiving prior cytotoxic chemotherapy, 20 radiation (32%), and 13 (21%) stem cell transplant and/or cellular therapy. By CH Risk Score (CHRS), 4 (7%), 38 (61%), and 20 (32%) patients were considered low-, intermediate-, and high-risk of myeloid transformation. The 36 CCUS patients were classified as 10 (28%) low, 9 (25%) intermediate, and 17 (47%) high risk patients by the Clonal Cytopenia Risk Score (CCRS). Median *TP53* VAF was 5% (range, 1–54), and 30 patients (46%) had additional somatic mutations. 12 patients (19%) were *TP53* double-hit. *TP53* immunohistochemistry on bone marrow specimens is pending.

Median time from prior malignancy to CH detection was 42.3 months (range, 0.2–365.0). With a median follow-up time of 60.1 months (95% confidence interval [CI], 43.0–70.5), median overall survival (mOS) from CH detection was 67.5 months (95% CI, 59.0–not estimable [NE]). A total of 15 patients (24%) progressed to MNs (12 MDS [80%], 3 AML [20%]) with a median time to transformation of 20.2 months (95% CI: 6.8–53.9). mOS from time of MN diagnosis was 20.1 months (95% CI, 7.3–NE) for MDS and 0.3 months (95% CI, 0.3–NE) for AML patients. Patients who transformed to MNs were more likely to have had tobacco (p=0.026) or illicit drug use (p=0.031), higher comorbidity burdens (p=0.031), rheumatologic disorders (p=0.016), previous stem cell transplant/cellular therapy (p=0.042), and CCUS (p=0.035). No differences were observed by *TP53* VAF. Of those who transformed, 7 (47%) were intermediate- and 8 (53%) high-risk by CHRS. CCRS showed low, intermediate, and high risk in 4 (33%), 1 (9%), and 7 (58%) of the 12 *TP53*m CCUS patients who progressed to MNs, respectively.

**Summary/Conclusion**

*TP53*m CH is associated with significant comorbidity burdens and oncologic history. Existing risk stratification tools poorly predict myeloid transformation in these patients. Further clinicopathologic characterization of *TP53*m CH is warranted.

**Copyright Information:** (Online) ISSN: 2572-9241
©2026 The Author(s). HemaSphere is published by John Wiley & Sons Ltd on behalf of European Hematology Association. This is an open access Abstract Book distributed under the Attribution-NonCommercial-NoDerivs (CC BY-NC-ND), which allows third parties to download the articles and share them with others as long as they credit the author and the Abstract Book, but they cannot change the content in any way or use them commercially.
**Abstract Book Citations:** Authors, Title, HemaSphere, 2026;10:(S1):pages. The abstract book DOIs can be found on the “Author Information” page.
**Disclaimer:** Articles published in the journal HemaSphere exclusively reflect the opinions of the authors. The authors are responsible for all content in their abstracts including accuracy of the facts, statements, citing resources, etc.

### (PB4080) CHANGES IN EXTRACELLULAR MATRIX PROTEINS IN MULTIPOTENT MESENCHYMAL STROMAL CELLS IN ACUTE LEUKEMIA

**Topic:** 24. Hematopoiesis, stem cells and microenvironment

Nina Drize[1], Nataliya Petinati*[1], Aleksandra Sadovskaya[1], Igor Smirnov[1], Olga Pobegyts[1], Georgiy Arapidi[1], Anastasya Vasilieva[1], Olga Aleshina[1], Elena Parovichnikova[1]

[1]*National Medical Research Center for Hematology, Moscow, Russia;*

### Background

More and more data indicate the role of changes in the extracellular matrix in leukemia pathogenesis. Many cells are involved in the formation of the matrix, including mesenchymal stem cells and their progeny, multipotent mesenchymal stromal cells (MSCs). In patients with acute leukemia (AL), the properties of MSCs and their ability to maintain normal hematopoiesis are altered, and their ability to form and maintain extracellular matrix (ECM) remains obscure.

### Aims

To analyze changes in ECM proteins in MSCs of patients at AL onset and during treatment.

### Methods

MSCs were derived from bone marrow samples of 8 healthy donors and 18 AL patients at onset, in remission, and after allogeneic hemopoietic stem cell transplantation (HSCT), and cultured for 3 passages. Proteomic analysis was performed with an Orbitrap Q Exactive HF-X mass spectrometer and Scaffold 5 software. For functional enrichment analysis we selected significantly ($p<0.05$) differing proteins with |log Fold Change|>2.

**Copyright Information:** (Online) ISSN: 2572-9241
©2026 The Author(s). HemaSphere is published by John Wiley & Sons Ltd on behalf of European Hematology Association. This is an open access Abstract Book distributed under the Attribution-NonCommercial-NoDerivs (CC BY-NC-ND), which allows third parties to download the articles and share them with others as long as they credit the author and the Abstract Book, but they cannot change the content in any way or use them commercially.
**Abstract Book Citations:** Authors, Title, HemaSphere, 2026;10:(S1):pages. The abstract book DOIs can be found on the "Author Information" page.
**Disclaimer:** Articles published in the journal HemaSphere exclusively reflect the opinions of the authors. The authors are responsible for all content in their abstracts including accuracy of the facts, statements, citing resources, etc.

### Results

A total of 81 ECM proteins were identified in MSCs. In the proteome of MSCs of the patients at AL onset 19 proteins were decreased compared to donor MSCs (Table). These are primarily proteins that organize collagen fibrils, including various types of collagen, fibronectin and fibulin. Thus, deterioration of ECM, a crucial niche component, occurs. In remission, levels of several of those proteins increased compared to the onset, while some remained reduced compared to donors' cells. After HSCT, compared to remission, MSCs show an increased level of proteins involved in integrin and BMP signaling pathways (HTRA1, FSTL1). Compared to donors, MSCs after HSCT have a decreased level of proteins related to collagen synthesis and metabolism.
COL1A1, COL1A2 and COL5A1 were downregulated in MSCs of all patients' groups compared to donors. COL6A1 and COL6A2 decreased at the onset, but normalized in remission, and COL6A3 was increased in remission and after HSCT compared to onset and donor cells. ECM-shaping enzymes such as LOXL2, P4HA1 and P4HA2 were also decreased at the onset. LOXL2 levels normalized in remission and even exceeded donor MSCs' after HSCT, while P4HA1 and P4HA2 remained downregulated in MSCs of all patients' groups.
Table. ECM proteins differentially present in MSCs

| Comparison groups | Proteins |
|---|---|
| O-MSCs<D-MSCs | FBLN1,BLN2,FN1,MFGE8,EMILIN1,HSPG2,LOXL2,P4HA1,P4HA2,COL1A1, COL1A2,COL5A1,COL6A1,COL6A2,COL11A1,COL12A1 |
| O-MSCs<R-MSCs | FBLN1,FN1,LAMA4,HSPG2,LOXL2,TGFBI,EMILIN1,TIMP3,COL6A1,COL6A2, COL6A3 |
| O-MSCs<HSCT-MSCs | FBLN1,FN1,ITGB1,LOXL2,HSPG2,HTRA1,POSTN,TGFBI,EMILIN1,MFGE8,TNC, COL1A2,COL5A1,COL5A2,COL6A1,COL6A2,COL6A3 |
| R-MSCs<D-MSCs | POSTN,LAMB2,P4HA1,P4HA2,COL1A1,COL1A2,COL5A1,COL11A1 |
| R-MSCs>D-MSCs | FBLN2,TIMP3,SEMA7A,DCN,LAMA4,LAMB1,LAMC1,IGFBP3,COL6A3 |
| HSCT-MSCs<D-MSCs | SPARC,MFGE8,LAMB2,SERPINH1,P4HA1,P4HA2,COL1A1,COL1A2,COL5A1 |
| HSCT-MSCs>D-MSCs | LOXL2,FSTL1,IGFBP3,LAMB1,LAMC1,SERPINE1,POSTN,TNC,ITGB1,THBS1, TGFBI,HTRA1,COL6A2,COL6A3 |
| HSCT-MSCs>R-MSCs | ITGB1,P4HA2,POSTN,FSTL1,HTRA1,HSPG2,LOXL2,FN1 |

D-MSCs – donors' MSCs
O-MSCs – MSCs of patients at the AL onset
R-MSCs – MSCs of patients in remission
HSCT-MSCs – MSCs of patients after allo-HSCT

### Summary/Conclusion

Changes in the level of ECM proteins in MSCs of AL patients can indicate disturbed regulation of hematopoiesis processes. In AL, upon achieving remission and after allo-HSCT, the balance of ECM is not fully redressed.

Copyright Information: (Online) ISSN: 2572-9241
©2026 The Author(s). HemaSphere is published by John Wiley & Sons Ltd on behalf of European Hematology Association. This is an open access Abstract Book distributed under the Attribution-NonCommercial-NoDerivs (CC BY-NC-ND), which allows third parties to download the articles and share them with others as long as they credit the author and the Abstract Book, but they cannot change the content in any way or use them commercially.
Abstract Book Citations: Authors, Title, HemaSphere, 2026;10:(S1):pages. The abstract book DOIs can be found on the "Author Information" page.
Disclaimer: Articles published in the journal HemaSphere exclusively reflect the opinions of the authors. The authors are responsible for all content in their abstracts including accuracy of the facts, statements, citing resources, etc.

### (PB4081) CLONAL HEMATOPOIESIS IN CAR-T CELL PREPARATIONS OF PATIENTS TREATED WITH ANTI-CD19 OR ANTI-BCMA CAR-T CELLS

**Topic:** 24. Hematopoiesis, stem cells and microenvironment

Lea Naomi Eder*[1], Julia Thomson[1], Laura Horn[1], Lisa Schäfer[1], Elzbieta Brzuszkiewicz[1], Christina Ganster[1], Detlef Haase[1], Gerald Wulf[1]

[1] *University Medical Center Göttingen, Clinic of Hematology and Medical Oncology, Göttingen, Germany;*

### Background

Clonal hematopoiesis (CH) is a frequent phenomenon in hematopoietic precursor cells of elderly patients, associated with an increased risk of progression to myelodysplastic neoplasms (MDS) or acute myeloid leukemia (AML). Some CH mutations are also associated with previous chemo- or radiotherapy and increased risks for therapy-associated hematopoietic neoplasms. CAR-T cell therapy is an important therapy option for patients with refractory or relapsed aggressive B-cell lymphoma and multiple myeloma. Patients receiving this therapy have often been pretreated with several treatment lines including immunochemotherapy. It is well known that these patients have a higher prevalence of clonal hematopoiesis but evidence for the impact of CH on CAR-T treatment outcomes are sparse, except for mutations of the tumor suppressor gene TP53.

### Aims

We addressed the clinical impact of CH on functionality of CAR-T-cells by analyzing CH in CAR-T cell preparations with respect to treatment response and CAR-T associated side effects such as cytokine releasing syndrome (CRS) and immune effector-cell associated neurotoxicity syndrome (ICANS).

### Methods

Following IRB approval residual material after infusion set flushing of the CAR-T preparations was collected from 43 patients and analyzed by next generation sequencing (NGS) using a gene panel including the most frequently affected genes in CH. Clinical data were extracted from the clinical CAR-T program documentation.

### Results

43 patients were included in this study, 15 patients with multiple myeloma, 19 patients with diffuse large cellular B-cell lymphoma, 6 patients with mantle cell lymphoma and 3 patients with indolent B-cell lymphoma. All patients were treated with EMA approved anti-CD19 (28) or anti-BCMA CAR-T cell products (15).
We found mutations in 14/43 (33%) CD3+ cell preparations. The most frequently affected genes were DNMT3A, BCOR and TET2. We also found mutations in PPM1D, CBL, JAK2, CUX1 and EZH2. The median VAF was 5% (range 1-93%). In five patients corresponding NGS data from bone marrow (BM) or CD34+ peripheral blood cells (PB) before CAR-T cell therapy were available. In three patients mutations of TP53 were present in PB or BM, but could not be detected in the CAR-T cells (level of detection 1% VAF). The cells of two patients had mutations in BCOR and DNMT3A, which both were also found in the corresponding CAR-T cell preparations. We found no associations of CH with disease, treatment response, overall survival, incidence of post CAR-T cell cytopenia, ICANS or CRS.

**Copyright Information:** (Online) ISSN: 2572-9241
©2026 The Author(s). HemaSphere is published by John Wiley & Sons Ltd on behalf of European Hematology Association. This is an open access Abstract Book distributed under the Attribution-NonCommercial-NoDerivs (CC BY-NC-ND), which allows third parties to download the articles and share them with others as long as they credit the author and the Abstract Book, but they cannot change the content in any way or use them commercially.
**Abstract Book Citations:** Authors, Title, HemaSphere, 2026;10:(S1):pages. The abstract book DOIs can be found on the "Author Information" page.
**Disclaimer:** Articles published in the journal HemaSphere exclusively reflect the opinions of the authors. The authors are responsible for all content in their abstracts including accuracy of the facts, statements, citing resources, etc.

**Summary/Conclusion**

CH is a phenomenon not only associated with hematopoietic precursor cells and myeloid diseases. Our study shows that CH may also appear in lymphatic cells. In order to learn more about the influence of these mutations regarding adverse events, therapy response or overall survival, data from larger cohorts with extended follow ups appear warranted.

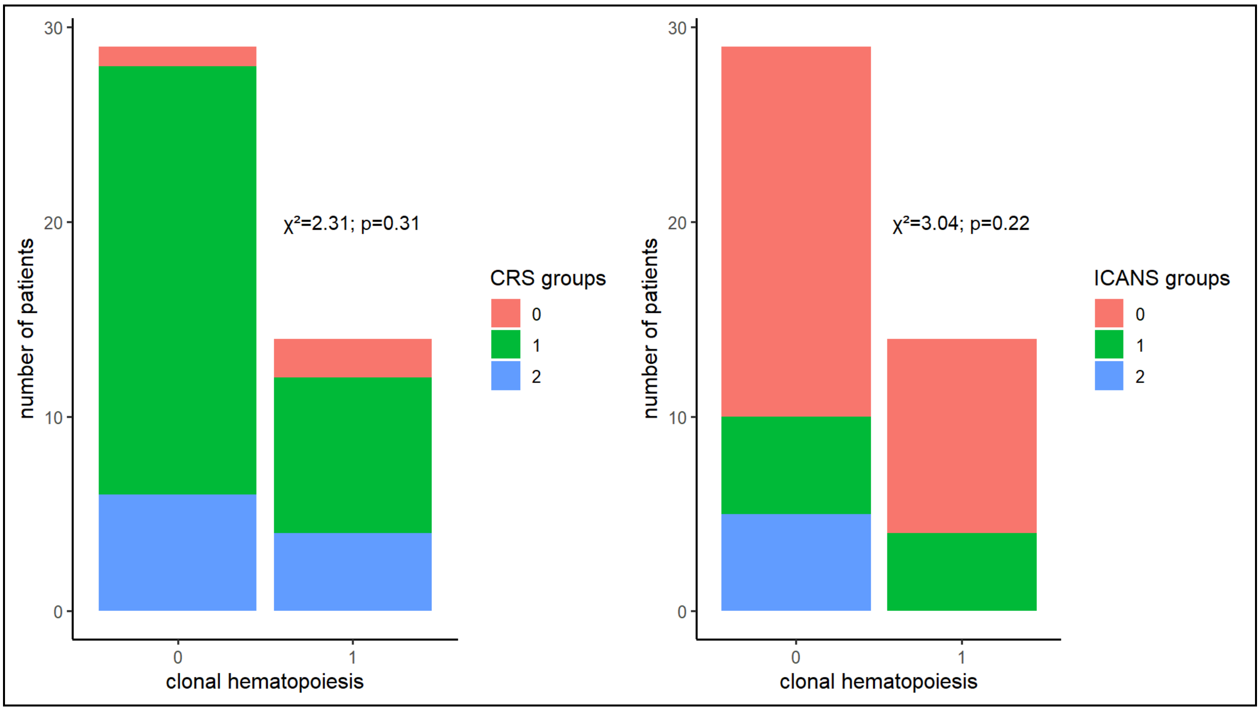

**Copyright Information:** (Online) ISSN: 2572-9241
©2026 The Author(s). HemaSphere is published by John Wiley & Sons Ltd on behalf of European Hematology Association. This is an open access Abstract Book distributed under the Attribution-NonCommercial-NoDerivs (CC BY-NC-ND), which allows third parties to download the articles and share them with others as long as they credit the author and the Abstract Book, but they cannot change the content in any way or use them commercially.
**Abstract Book Citations:** Authors, Title, HemaSphere, 2026;10:(S1):pages. The abstract book DOIs can be found on the "Author Information" page.
**Disclaimer:** Articles published in the journal HemaSphere exclusively reflect the opinions of the authors. The authors are responsible for all content in their abstracts including accuracy of the facts, statements, citing resources, etc.

### (PB4082) DISEASE-SPECIFIC BIOLOGICAL CLUSTERING OF SECOND PRIMARY MALIGNANCIES IN HEMATOLOGY PATIENTS: EVIDENCE FROM A LARGE TERTIARY COHORT

**Topic:** 24. Hematopoiesis, stem cells and microenvironment

Metban Mastanzade*[1], Ulviyya Hasanzade[1], Nidanur Eryiğit[2], Meryem Merve Ören Çelik[3], Miraç Yılmaz[2], Simge Erdem[1], Mustafa Murat Özbalak[1], İpek Yönal Hindilerden[1], Mustafa Nuri Yenerel[1], Sevgi Kalayoglu Besisik[1]

*[1]Istanbul University, Istanbul Faculty of Medicine, Department of Internal Medicine, Division of Hematology, Istanbul, Türkiye; [2]Istanbul University, Istanbul Faculty of Medicine, Department of Internal Medicine, Istanbul, Türkiye;[3]Istanbul University, Istanbul Faculty of Medicine, Department of Public Health, Istanbul, Türkiye;*

### Background

Improved survival in hematologic malignancies has led to increasing recognition of second primary cancers. Whether these malignancies reflect background population cancer risk or arise from shared biological mechanisms remains uncertain.

### Aims

To evaluate the epidemiologic distribution, demographic profile, primary disease spectrum, and temporal characteristics of dual malignancies in a large tertiary hematology cohort and to explore evidence of disease-specific biological clustering.

### Methods

We retrospectively analyzed 3,864 consecutive patients with hematologic malignancies. Dual malignancy was defined as two histologically distinct primary cancers diagnosed synchronously (≤6 months) or metachronously (>6 months). Continuous variables are presented as median (range), and categorical variables as n (%). Observed secondary malignancy patterns were compared with Turkey 2020 national cancer statistics. Primary and secondary cancer distributions are summarized in Tables 1 and 2.

### Results

Dual malignancies were identified in 313 patients (8.1%). The median age was 65 years (range, 20–94), with 157 males (50.2%) and 156 females (49.8%).
As shown in Table 1, plasma cell disorders constituted the largest primary disease category (33.5%), followed by aggressive and indolent B-cell lymphomas, Hodgkin lymphoma, chronic lymphocytic leukemia, and myeloid neoplasms, including myeloproliferative and myelodysplastic syndromes.
According to Table 2, hematologic malignancies accounted for 31.9% of second cancers, representing the dominant secondary category. Among solid tumors, papillary thyroid carcinoma was most frequent (10.5%), followed by breast cancer (8.6%), lung adenocarcinoma (8.3%), colon cancer (7.7%), and prostate cancer (7.3%).
Most dual malignancies were metachronous (76.7%), while 23.3% were synchronous. Hematologic–hematologic associations demonstrated longer latency compared with hematologic–solid combinations (median 74 vs 61 months), with intervals extending up to 26 years.
Compared with national cancer statistics, hematologic second malignancies were markedly overrepresented relative to their expected population proportion (8–10%). Papillary thyroid carcinoma also appeared disproportionately frequent. In contrast, the frequencies of lung, breast, prostate and bladder cancers were broadly comparable to national trends.

**Copyright Information:** (Online) ISSN: 2572-9241
©2026 The Author(s). HemaSphere is published by John Wiley & Sons Ltd on behalf of European Hematology Association. This is an open access Abstract Book distributed under the Attribution-NonCommercial-NoDerivs (CC BY-NC-ND), which allows third parties to download the articles and share them with others as long as they credit the author and the Abstract Book, but they cannot change the content in any way or use them commercially.
**Abstract Book Citations:** Authors, Title, HemaSphere, 2026;10:(S1):pages. The abstract book DOIs can be found on the "Author Information" page.
**Disclaimer:** Articles published in the journal HemaSphere exclusively reflect the opinions of the authors. The authors are responsible for all content in their abstracts including accuracy of the facts, statements, citing resources, etc.

**Summary/Conclusion**

Dual malignancies in hematology patients exhibit a distinct epidemiologic signature rather than a uniform increase across cancer types. The selective enrichment of hematologic second malignancies and papillary thyroid carcinoma, together with prolonged latency in hematologic–hematologic combinations, suggests the contribution of shared biological susceptibility, clonal predisposition, treatment-related genomic instability, and immune dysregulation.
In addition, increased surveillance intensity and cross-sectional imaging during long-term follow-up may partially contribute to the higher detection of certain tumors, particularly papillary thyroid carcinoma.
These findings indicate that secondary cancer development in hematology populations likely reflects an interplay between biological vulnerability and surveillance-related detection, underscoring the need for mechanism-informed and risk-adapted long-term monitoring strategies.

**Copyright Information:** (Online) ISSN: 2572-9241
©2026 The Author(s). HemaSphere is published by John Wiley & Sons Ltd on behalf of European Hematology Association. This is an open access Abstract Book distributed under the Attribution-NonCommercial-NoDerivs (CC BY-NC-ND), which allows third parties to download the articles and share them with others as long as they credit the author and the Abstract Book, but they cannot change the content in any way or use them commercially.
**Abstract Book Citations:** Authors, Title, HemaSphere, 2026;10:(S1):pages. The abstract book DOIs can be found on the “Author Information” page.
**Disclaimer:** Articles published in the journal HemaSphere exclusively reflect the opinions of the authors. The authors are responsible for all content in their abstracts including accuracy of the facts, statements, citing resources, etc.

| Table1: Primary Disease Category | Specific Diagnosis | n | % |
|---|---|---|---|
| **Plasma Cell Disorders** | Multiple Myeloma (MM) | 69 | 22.0 |
| | MGUS | 36 | 11.5 |
| **Aggressive B-cell Lymphomas** | Diffuse Large B-Cell Lymphoma / aggressive NHL | 39 | 12.5 |
| **Indolent B-cell Lymphomas** | Indolent NHL | 14 | 4.5 |
| | Follicular Lymphoma (FL) | 6 | 1.9 |
| **Hodgkin Lymphoma** | | 32 | 10.2 |
| **Chronic Lymphocytic Leukemia** | CLL | 24 | 7.7 |
| **Myeloproliferative Neoplasms (MPN)** | Polycythemia vera / ET / PMF | 38 | 12.1 |
| **Chronic Myeloid Leukemia** | CML | 16 | 5.1 |
| **Myelodysplastic Syndromes** | MDS | 27 | 8.6 |
| **Acute Leukemias** | AML | 5 | 1.6 |
| | ALL | 2 | 0.6 |
| **T-cell Neoplasms** | T-LGL | 4 | 1.3 |
| | PTCL | 1 | 0.3 |
| **Total** | | **313** | **100** |

| Table 2: Secondary Malignancy | n (%) |
|---|---|
| **Hematologic malignancies** | **100 (31.9)** |
| • **MGUS** | 28 (8.9) |
| • **Myeloproliferative neoplasms** | 13 (4.2) |
| • **Myelodysplastic syndrome** | 11 (3.5) |
| • **Aggressive lymphoma** | 11 (3.5) |
| • **Other hematologic neoplasms** | 37 (11.8) |
| **Papillary thyroid carcinoma** | 33 (10.5) |
| **Breast cancer** | 27 (8.6) |
| **Lung adenocarcinoma** | 26 (8.3) |
| **Colon cancer** | 24 (7.7) |
| **Prostate cancer** | 23 (7.3) |
| **Squamous cell carcinoma** | 15 (4.8) |
| **Basal cell carcinoma** | 10 (3.2) |
| **Head and neck cancer** | 10 (3.2) |
| **Renal cell carcinoma** | 8 (2.6) |
| **Bladder cancer** | 8 (2.6) |
| **Gastric cancer** | 7 (2.2) |
| **Gynecologic malignancies** | 6 (1.9) |
| **Total** | **313 (100)** |

Figure 1. Distribution of primary hematologic diseases and secondary malignancies in the dual malignancy cohort (n=313).

**Copyright Information:** (Online) ISSN: 2572-9241
©2026 The Author(s). HemaSphere is published by John Wiley & Sons Ltd on behalf of European Hematology Association. This is an open access Abstract Book distributed under the Attribution-NonCommercial-NoDerivs (CC BY-NC-ND), which allows third parties to download the articles and share them with others as long as they credit the author and the Abstract Book, but they cannot change the content in any way or use them commercially.
**Abstract Book Citations:** Authors, Title, HemaSphere, 2026;10:(S1):pages. The abstract book DOIs can be found on the "Author Information" page.
**Disclaimer:** Articles published in the journal HemaSphere exclusively reflect the opinions of the authors. The authors are responsible for all content in their abstracts including accuracy of the facts, statements, citing resources, etc.

### (PB4083) PRE-TRANSPLANT IMMUNE AND INFLAMMATORY STATUS PREDICT DELAYED HEMATOPOIETIC RECONSTITUTION AFTER AUTOLOGOUS STEM CELL TRANSPLANTATION IN LYMPHOMA

**Topic:** 24. Hematopoiesis, stem cells and microenvironment

Huaibin Zhang*[1], Ran Longrong[2], Xi Quan[1], Zhiming Luo[1], Nan Zhang[1], Jian-Chuan Deng[1]

*[1]The Second Affiliated Hospital of Chongqing Medical University, Department of Hematology, Chongqing, China;[2]Chongqing University Cancer Hospital, Chongqing, China;*

### Background

Factors associated with delayed hematopoietic reconstitution following autologous hematopoietic stem cell transplantation (ASCT) in lymphoma remain incompletely understood. The impact of pre-transplant circulating immune cell subsets and inflammatory cytokine profiles on delayed engraftment has been poorly investigated.

### Aims

To analyze the incidence of delayed hematopoietic reconstitution in 111 lymphoma patients undergoing ASCT from multiple centers, and to evaluate the effects of pre-transplant circulating immune cells, inflammatory cytokines, and clinical characteristics on delayed engraftment.

### Methods

A total of 111 lymphoma patients who underwent ASCT between July 2022 and December 2024 at Chongqing University Cancer Hospital and the Second Affiliated Hospital of Chongqing Medical University were enrolled. Pre-transplant clinical data, peripheral circulating immune cell subsets, and inflammatory cytokines were collected. Associations between these parameters and delayed hematopoietic reconstitution were analyzed. Survival follow-up was performed to explore the relationship between circulating immune cell profiles and long-term outcomes.

### Results

Median time to neutrophil engraftment was 10 days (range 8–39 days), and median time to platelet engraftment was 12 days (range 5–39 days). Patients were stratified into non-delayed and delayed groups based on thresholds of neutrophil engraftment >11 days and platelet engraftment >13 days. The delayed group showed significantly higher levels of activated CD4+CD25+ T cells and regulatory T cells. Levels of IL-2R and IL-6 were also higher in the delayed group, though without statistical significance. Older age, worse nutritional status, higher LDH, higher β2-microglobulin, and lower pre-transplant hemoglobin were independently associated with delayed engraftment (all $P<0.05$).

### Summary/Conclusion

In this multicenter study of 111 lymphoma patients treated with ASCT, delayed hematopoietic reconstitution was linked to elevated pre-transplant activated CD4+CD25+ T cells and regulatory T cells. Although IL-2R and IL-6 did not reach statistical significance, a trend toward higher levels was observed in the delayed engraftment group. Older age, poor nutritional status, elevated LDH, β2-microglobulin, and low hemoglobin were significant clinical risk factors. Pre-transplant immune activation and inflammatory status, combined with baseline clinical features, may serve as clinically useful predictors for delayed hematopoietic reconstitution after ASCT in lymphoma.

**Copyright Information:** (Online) ISSN: 2572-9241
©2026 The Author(s). HemaSphere is published by John Wiley & Sons Ltd on behalf of European Hematology Association. This is an open access Abstract Book distributed under the Attribution-NonCommercial-NoDerivs (CC BY-NC-ND), which allows third parties to download the articles and share them with others as long as they credit the author and the Abstract Book, but they cannot change the content in any way or use them commercially.
**Abstract Book Citations:** Authors, Title, HemaSphere, 2026;10:(S1):pages. The abstract book DOIs can be found on the "Author Information" page.
**Disclaimer:** Articles published in the journal HemaSphere exclusively reflect the opinions of the authors. The authors are responsible for all content in their abstracts including accuracy of the facts, statements, citing resources, etc.

### (PB4084) COMPARATIVE EVALUATION OF PRIMARY B-CELL CULTIVATION SYSTEMS

**Topic:** 24. Hematopoiesis, stem cells and microenvironment

Hulda Haraldsdottir*[1, 2, 3], Vidthdyan Veeravakaran[1, 2, 3], Helle Høholt[1, 3], Hanne Due[1, 2, 3], Karen Dybkær[1, 2, 3]

*[1]Aalborg University Hospital, Department of Hematology, Aalborg, Denmark;[2]Aalborg University, Department of Clinical Medicine, Gistrup, Denmark; [3]Aalborg University Hospital, Clinical Cancer Research Center, Aalborg, Denmark;*

### Background

Primary human B-cells from peripheral blood are essential for investigating both physiological B-cell mechanisms and the molecular processes underlying malignant transformation. However, sustaining primary B-cells *in vitro* remains challenging due to their dependence on regulated microenvironment signals controlling survival, activation, and differentiation. In contrast to immortalized cell lines, primary B-cells rapidly undergo apoptosis without such stimuli. To overcome this limitation, several *in vitro* systems have been developed, including feeder-cell-based co-cultures and cytokine stimulation protocols. These approaches vary considerably in composition, complexity, cost, and in the cellular programs they elicit.

### Aims

Thorough characterization and comparative evaluation of the primary B-cell culture systems are essential to guide selection of systems tailored to specific research objectives. Therefore, we aim to systematically compare two complementary approaches, primary B-cell monoculture and feeder-cell-based co-culture, focusing on expansion dynamics, differentiation potential and applicability for functional studies.

### Methods

The two complementary human primary B-cell culture systems were evaluated under five stimulating conditions: 1) unstimulated 2) CD40 ligand addition, 3) CD40 ligand + IL-4, 4) CD40 ligand + IL-21, and 5) CD40 ligand + IL-4 + IL-21. Expansion rates and immunophenotypes were determined for each condition, and analysis are ongoing of genetic clonotyping of the B-cell receptor, profiling of differentiation specific transcription factors, and B-cell transfection efficiencies.

### Results

Addition of IL-4 and/or IL-21 was essential for sustained expansion of primary B-cells in both culture systems over 7 days. The highest expansion was observed in monoculture with combined CD40 ligand, IL-4, and IL-21 cytokine stimulation, increasing cell numbers from 0.5 million to 47 million within 14 days, providing foundation for high-throughput experiments. The feeder-cell-based co-culture system resulted in higher degrees of differentiation diversity assessed by immunophenotyping throughout the cultivation time compared to monoculture, including increased proportions of activated naïve B-cells and plasmablasts.

### Summary/Conclusion

Both primary B-cell culture systems are robust platforms for functional studies. The simpler monoculture enables rapid large-scale expansion, whereas the feeder-cell-based co-culture promotes enhanced immunophenotypic differentiation. These findings provide a framework for selecting culture models according to experimental objectives, including high-throughput applications and functional studies.

**Copyright Information:** (Online) ISSN: 2572-9241
©2026 The Author(s). HemaSphere is published by John Wiley & Sons Ltd on behalf of European Hematology Association. This is an open access Abstract Book distributed under the Attribution-NonCommercial-NoDerivs (CC BY-NC-ND), which allows third parties to download the articles and share them with others as long as they credit the author and the Abstract Book, but they cannot change the content in any way or use them commercially.
**Abstract Book Citations:** Authors, Title, HemaSphere, 2026;10:(S1):pages. The abstract book DOIs can be found on the "Author Information" page.
**Disclaimer:** Articles published in the journal HemaSphere exclusively reflect the opinions of the authors. The authors are responsible for all content in their abstracts including accuracy of the facts, statements, citing resources, etc.

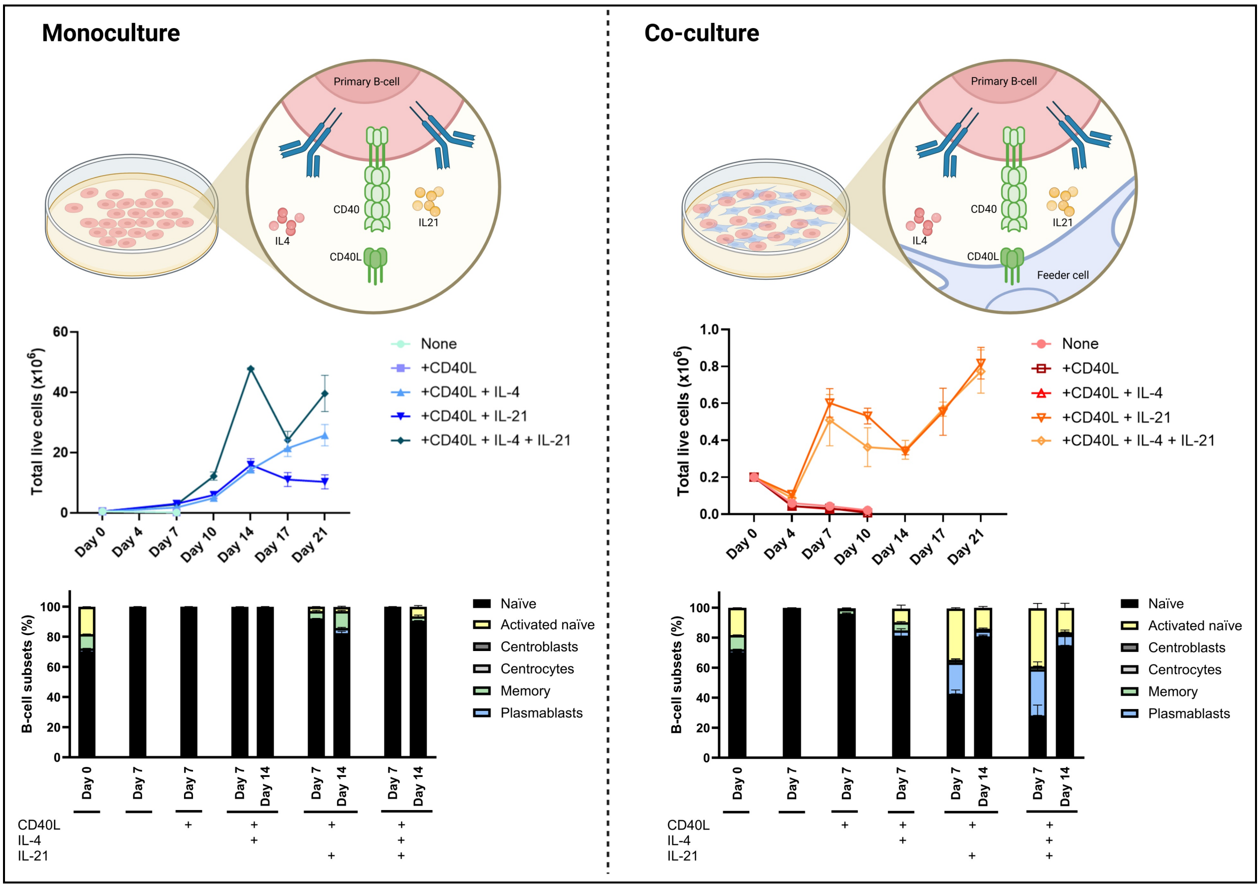

**Copyright Information:** (Online) ISSN: 2572-9241
©2026 The Author(s). HemaSphere is published by John Wiley & Sons Ltd on behalf of European Hematology Association. This is an open access Abstract Book distributed under the Attribution-NonCommercial-NoDerivs (CC BY-NC-ND), which allows third parties to download the articles and share them with others as long as they credit the author and the Abstract Book, but they cannot change the content in any way or use them commercially.
**Abstract Book Citations:** Authors, Title, HemaSphere, 2026;10:(S1):pages. The abstract book DOIs can be found on the "Author Information" page.
**Disclaimer:** Articles published in the journal HemaSphere exclusively reflect the opinions of the authors. The authors are responsible for all content in their abstracts including accuracy of the facts, statements, citing resources, etc.

### (PB4086) EFFICACY ANALYSIS OF SWITCHING FROM C5 TO FACTOR B INHIBITORS IN PATIENTS WITH PAROXYSMAL NOCTURNAL HEMOGLOBINURIA: A REAL-WORLD STUDY

**Topic:** 24. Hematopoiesis, stem cells and microenvironment

Liyan Li[1, 2, 3], Yao Zhang[1, 2, 3], Yuanli Li[1, 2, 3], Hui Liu[1, 2, 3], Zhaoyun Liu[1, 2, 3], Rong Fu*[1, 2, 3]

*[1]Department of Hematology, Tianjin Medical University General Hospital, 154 Anshan Street, Heping District, Tian Jin, China; [2]Tianjin Key Laboratory of Bone Marrow Failure and Malignant Hemopoietic Clone Control, Tian Jin, China;[3]Tianjin Institute of Hematology, Tian Jin, China;*

### Background

Paroxysmal nocturnal hemoglobinuria (PNH) is a rare acquired clonal hematopoietic stem cell disorder characterized primarily by intravascular hemolysis, bone marrow failure, and a high risk of thrombosis. C5 complement inhibitors have ushered in a new era of targeted therapy for PNH and significantly improved patient survival. However, they do not effectively control extravascular hemolysis. Intravenous administration and frequent dosing challenges to patient compliance. Factor B inhibitors, which target the alternative complement pathway, block complement activation at a more proximal level, providing a novel therapeutic strategy to address these unmet clinical needs.

### Aims

To evaluate the efficacy and adverse events of switching to factor B inhibitors in real-world PNH patients who had suboptimal responses to C5 complement inhibitors.

### Methods

A retrospective analysis was conducted on 15 real-world PNH patients with inadequate response to C5 complement inhibitors who were switched to factor B inhibitors. Based on clinical decision-making, 7 patients were switched to iptacopan and 8 patients to HRS-5965. Red blood cell count (RBC), hemoglobin (Hb), lactate dehydrogenase (LDH), bilirubin, reticulocytes, free hemoglobin, and adverse events were assessed before switching and at 4, 8, and 12 weeks after switching.

### Results

After switching to factor B inhibitors, RBC and Hb levels gradually increased over time. Compared with baseline, Hb increased from 82±19.67g/L to 122.4±11.99g/L, and RBC increased from $2.38\pm0.77\times10^{12}$/L to $3.87\pm0.93\times10^{12}$/L; both differences were statistically significant ($P<0.0001$), indicating marked improvement in anemia. LDH levels decreased from 460.66±508.06U/L to 196.9±36.8U/L ($P=0.0182$). Total bilirubin decreased from 41.46±24.43U/L to 15.63±6.76U/L ($P=0.0024$), indirect bilirubin from 31.58±23.59U/L to 9.21±4.35U/L ($P=0.0003$), and reticulocytes from 249.7±95.32U/L to 113.29±35.94U/L ($P=0.0001$). All differences were statistically significant, suggesting a significant improvement in hemolysis. No significant changes were observed in white blood cell (WBC) or platelet (PLT) counts before and after switching (WBC: $3.47\pm1.19\times10^9$/L to $3.24\pm1.09\times10^9$/L, $P=0.8402$; PLT: $121.86\pm54.2\times10^9$/L to $106.21\pm47.87\times10^9$/L, $P=0.1320$). Some patients experienced adverse events commonly associated with complement inhibitors, including infections, cytopenia, and liver function abnormalities. These events were generally manageable, and no serious adverse events occurred.

**Copyright Information:** (Online) ISSN: 2572-9241
©2026 The Author(s). HemaSphere is published by John Wiley & Sons Ltd on behalf of European Hematology Association. This is an open access Abstract Book distributed under the Attribution-NonCommercial-NoDerivs (CC BY-NC-ND), which allows third parties to download the articles and share them with others as long as they credit the author and the Abstract Book, but they cannot change the content in any way or use them commercially.
**Abstract Book Citations:** Authors, Title, HemaSphere, 2026;10:(S1):pages. The abstract book DOIs can be found on the "Author Information" page.
**Disclaimer:** Articles published in the journal HemaSphere exclusively reflect the opinions of the authors. The authors are responsible for all content in their abstracts including accuracy of the facts, statements, citing resources, etc.

**Summary/Conclusion**

Real-world data indicate that switching from C5 complement inhibitors to factor B inhibitors in PNH patients can effectively control hemolysis and improve hematologic parameters. Factor B inhibitors show promising potential in individualized treatment strategies for PNH. Larger sample sizes and longer-term follow-up are required to further validate their efficacy and safety.

**Copyright Information:** (Online) ISSN: 2572-9241
©2026 The Author(s). HemaSphere is published by John Wiley & Sons Ltd on behalf of European Hematology Association. This is an open access Abstract Book distributed under the Attribution-NonCommercial-NoDerivs (CC BY-NC-ND), which allows third parties to download the articles and share them with others as long as they credit the author and the Abstract Book, but they cannot change the content in any way or use them commercially.
**Abstract Book Citations:** Authors, Title, HemaSphere, 2026;10:(S1):pages. The abstract book DOIs can be found on the "Author Information" page.
**Disclaimer:** Articles published in the journal HemaSphere exclusively reflect the opinions of the authors. The authors are responsible for all content in their abstracts including accuracy of the facts, statements, citing resources, etc.

### (PB4087) ASSOCIATION OF CLONAL HEMATOPOIESIS WITH INDETERMINATE POTENTIAL (CHIP) WITH OUTCOME OF CHECKPOINT BLOCKADE IMMUNOTHERAPY IN MALIGNANT MELANOMA

**Topic:** 24. Hematopoiesis, stem cells and microenvironment

Philipp Faustmann*[1], German Demidov[2], Stephan Ossowski[2], Olga Kelemen[2], Andrea Forschner[3], Öner Öznur[4, 5], Michael Bitzer[4, 5], Claudia Lengerke[1, 6], Christopher Schroeder[2], Malte Roerden[1]

*[1]Internal Medicine II, University Hospital Tuebingen, Tuebingen, Germany; [2]Institute for Medical Genetics and Applied Genomics, University Hospital Tuebingen, Tuebingen, Germany; [3]Department of Dermatology, University Hospital Tuebingen, Tuebingen, Germany; [4]Internal Medicine I, University Hospital Tuebingen, Tuebingen, Germany; [5]Center for Personalized Medicine, University Hospital Tuebingen, Tuebingen, Germany; [6]German Cancer Consortium (DKTK), partner side Tuebingen, a partnership between DKFZ and University Hospital Tuebingen, Tuebingen, Germany;*

### Background

Clonal hematopoiesis of indeterminate potential (CHIP) is defined as the occurrence of myeloid gene mutations in the absence of hematopoietic disease or cytopenias with the variant allele frequency greater than 2%. Frequently affected genes include *TET2*, *ASXL1*, and *DNMT3A.*
The clinical significance of CHIP in solid tumor patients remains poorly characterized. At the same time, data show that CHIP mutations occur at significantly higher frequencies in patients with solid tumors compared to healthy individuals.
Solid tumors pose unique challenges for immunotherapy approaches due to their complex, immunosuppressive tumor microenvironment (TME). This immunosuppression is multifactorial and comprises physical barriers to immune cell infiltration, hypoxic conditions that impair effector function, and recruitment of immunosuppressive cells. CHIP-derived clones likewise infiltrate the TME and become an integral part of this complex immunological landscape, potentially modulating anti-tumor immune responses. Limited data have recently shown an association between CHIP mutations and prognosis in patients with solid tumors treated with immune checkpoint inhibitors (ICI). Furthermore, it has been demonstrated that data collected during routine diagnostic procedures, such as blood counts, can help predict the outcome of ICI treatment.

### Aims

The objective of this study was to investigate the prevalence of CHIP in melanoma patients and its impact on the outcomes of ICI treatment.

### Methods

A dataset was obtained from patients whose cases were reviewed by the local molecular tumor board between January 1, 2021, and December 31, 2024. The objective of the dataset was to screen for the diagnosis of melanoma. All melanoma patients treated with ICI were selected for analysis. In preparation for the meeting of the molecular tumor board, tumor and blood samples from all patients were subject to panel sequencing. The presence of CHIP-associated mutations was searched for in the patients' blood, and a comparison was made between the overall survival (OS) and progression-free survival (PFS) of patients with relevant mutations and those without relevant mutations. A multivariate Cox analysis was performed to study the influence of CHIP and clinical data, collected within four weeks before the start of ICI treatment, on the two endpoints.

**Copyright Information:** (Online) ISSN: 2572-9241
©2026 The Author(s). HemaSphere is published by John Wiley & Sons Ltd on behalf of European Hematology Association. This is an open access Abstract Book distributed under the Attribution-NonCommercial-NoDerivs (CC BY-NC-ND), which allows third parties to download the articles and share them with others as long as they credit the author and the Abstract Book, but they cannot change the content in any way or use them commercially.
**Abstract Book Citations:** Authors, Title, HemaSphere, 2026;10:(S1):pages. The abstract book DOIs can be found on the "Author Information" page.
**Disclaimer:** Articles published in the journal HemaSphere exclusively reflect the opinions of the authors. The authors are responsible for all content in their abstracts including accuracy of the facts, statements, citing resources, etc.

### Results

A total of 20 with and 179 melanoma patients without CHIP-associated mutations were identified. The median age of patients with and without CHIP was 67 and 60, respectively. 45% of CHIP patients were female and 55% were male. The median OS for patients with CHIP and without CHIP was 17.5 months and 20 months, respectively. The median PFS for patients with and without CHIP was 6 months and 5 months, respectively. No statistically significant difference was observed for both endpoints in univariate analysis. But cox multivariate regression revealed a significantly worse OS for CHIP-positive patients but no difference in PFS. Furthermore, higher hemoglobin levels were associated with a better OS. In univaraite analysis higher ECOG and age were associated with worse OS, but not in multivariate analysis.

### Summary/Conclusion

In our malignant melanoma patient cohort, presence of CHIP was associated with worse OS for ICI treatment, in line with previous studies that reported a trend toward inferior outcomes for patients with CHIP. Nevertheless, further research is necessary to determine the impact of CHIP on the outcome of ICI treatment.

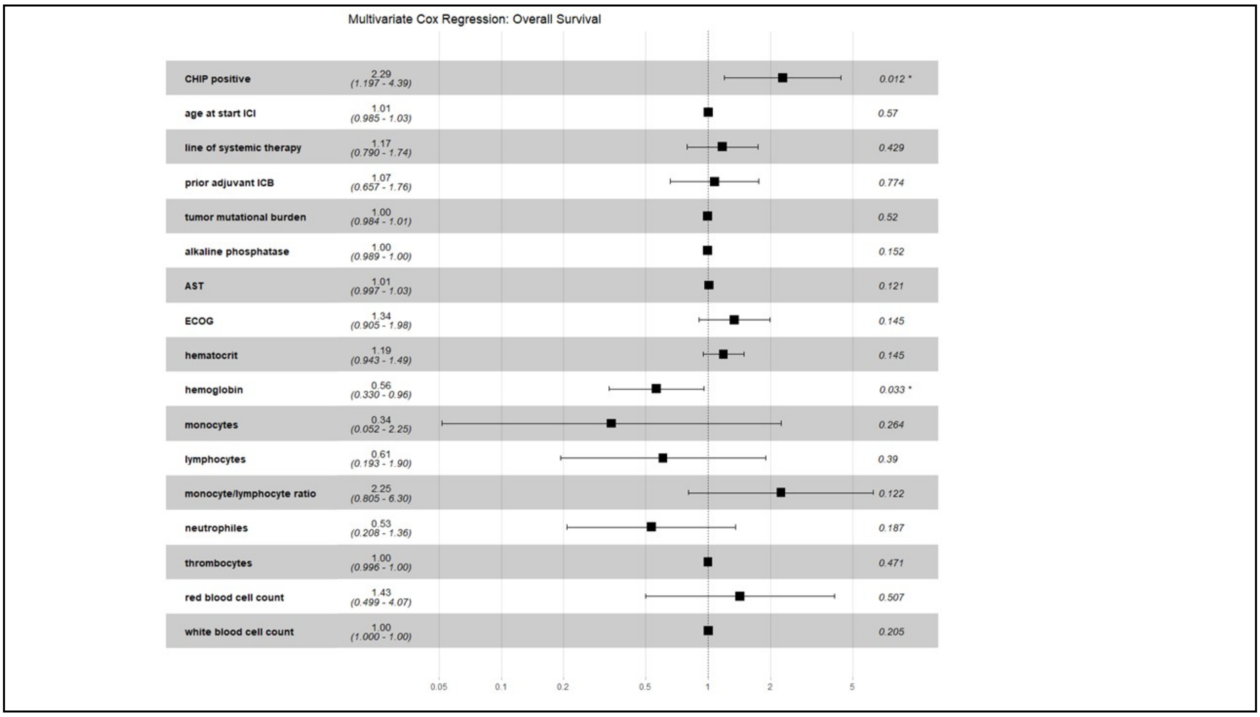

**Copyright Information:** (Online) ISSN: 2572-9241
©2026 The Author(s). HemaSphere is published by John Wiley & Sons Ltd on behalf of European Hematology Association. This is an open access Abstract Book distributed under the Attribution-NonCommercial-NoDerivs (CC BY-NC-ND), which allows third parties to download the articles and share them with others as long as they credit the author and the Abstract Book, but they cannot change the content in any way or use them commercially.
**Abstract Book Citations:** Authors, Title, HemaSphere, 2026;10:(S1):pages. The abstract book DOIs can be found on the "Author Information" page.
**Disclaimer:** Articles published in the journal HemaSphere exclusively reflect the opinions of the authors. The authors are responsible for all content in their abstracts including accuracy of the facts, statements, citing resources, etc.

### (PB4088) TRANSCRIPTOME SEQUENCING–BASED STUDY OF THE MOLECULAR MECHANISMS UNDERLYING ABERRANT ERYTHROID DIFFERENTIATION IN PAROXYSMAL NOCTURNAL HEMOGLOBINURIA

**Topic:** 24. Hematopoiesis, stem cells and microenvironment

Liyan Li*[1], Zewei Chen[1], Hui Liu[1], Zhaoyun Liu[1], Rong Fu[1]

[1] *Tianjin Medical University General Hospital, Tianjin, China;*

### Background

Paroxysmal nocturnal hemoglobinuria (PNH) is a clonal hematopoietic disorder caused by acquired PIGA mutations. Beyond complement-mediated hemolysis, inflammatory cues from the marrow microenvironment and aberrant intracellular signaling are implicated in persistent anemia and restricted erythropoiesis. We previously identified sustained downregulation of TRAF family member-associated NF-κB activator (TANK) in PNH-related erythroid cells and showed that TANK overexpression partially rescues erythroid differentiation defects in a PIGA-knockout background. However, the downstream molecular network and key signaling nodes regulated by TANK remain unclear.

### Aims

To define transcriptomic changes induced by TANK overexpression in a PIGA-deficient erythroid model and to validate key signaling axes suggested by pathway analyses, focusing on MAPK and TNFα/NF-κB.

### Methods

A PIGA-knockout (KO-PIGA) K562 cell line was used as an in vitro PNH model. Cells were transduced with lentiviral empty vector (OE-NC) or TANK overexpression construct (OE-TANK), then induced toward erythroid differentiation for 4 days. Bulk RNA sequencing was performed (Illumina NovaSeq 6000, PE150). Differentially expressed genes (DEGs) were identified using DESeq2. GO and KEGG enrichment analyses and GSEA (ClusterProfiler) were conducted. Key pathway activity was validated by Western blot for phosphorylated and total p38 and p65.

### Results

RNA-seq revealed marked transcriptomic remodeling in OE-TANK vs OE-NC, with 446 DEGs (28 upregulated, 418 downregulated). GO enrichment highlighted immune and signaling-related terms including immune response (GO:0006954, FDR=0.0066), integral component of plasma membrane (GO:0005887, FDR=0.0066), and transmembrane signaling receptor activity (GO:0004888, FDR=0.0107). KEGG analysis showed significant enrichment of the MAPK signaling pathway ($P=6.86\times10^{-5}$, FDR=0.0087). GSEA suggested a negative enrichment trend for HALLMARK_TNFA_SIGNALING_VIA_NFKB (NES=−1.790, P=0.0877, FDR=0.0737). Consistent with these findings, Western blot demonstrated increased p38 activation in OE-TANK cells: p-p38/p38 = 1.065±0.279 vs 0.717±0.139 in OE-NC (P=0.0211). In contrast, NF-κB activation was reduced: p-p65/p65 = 0.550±0.140 vs 1.303±0.783 in OE-NC (P=0.0127).

**Copyright Information:** (Online) ISSN: 2572-9241
©2026 The Author(s). HemaSphere is published by John Wiley & Sons Ltd on behalf of European Hematology Association. This is an open access Abstract Book distributed under the Attribution-NonCommercial-NoDerivs (CC BY-NC-ND), which allows third parties to download the articles and share them with others as long as they credit the author and the Abstract Book, but they cannot change the content in any way or use them commercially.
**Abstract Book Citations:** Authors, Title, HemaSphere, 2026;10:(S1):pages. The abstract book DOIs can be found on the "Author Information" page.
**Disclaimer:** Articles published in the journal HemaSphere exclusively reflect the opinions of the authors. The authors are responsible for all content in their abstracts including accuracy of the facts, statements, citing resources, etc.

**Summary/Conclusion**

In a PIGA-deficient erythroid model, TANK overexpression induces broad transcriptional changes dominated by downregulated inflammatory and receptor/signaling programs, with pathway analyses pointing to MAPK and TNFα/NF-κB axes. Protein-level validation confirms enhanced p38 MAPK phosphorylation and suppressed p65 phosphorylation, supporting a model in which TANK promotes erythroid differentiation by rebalancing pro-differentiation MAPK signaling and dampening inflammatory NF-κB activity. These results provide mechanistic evidence that TANK may act as a critical signaling modulator contributing to erythroid dysfunction in PNH.

**Copyright Information:** (Online) ISSN: 2572-9241
©2026 The Author(s). HemaSphere is published by John Wiley & Sons Ltd on behalf of European Hematology Association. This is an open access Abstract Book distributed under the Attribution-NonCommercial-NoDerivs (CC BY-NC-ND), which allows third parties to download the articles and share them with others as long as they credit the author and the Abstract Book, but they cannot change the content in any way or use them commercially.
**Abstract Book Citations:** Authors, Title, HemaSphere, 2026;10:(S1):pages. The abstract book DOIs can be found on the "Author Information" page.
**Disclaimer:** Articles published in the journal HemaSphere exclusively reflect the opinions of the authors. The authors are responsible for all content in their abstracts including accuracy of the facts, statements, citing resources, etc.

### (PB4089) HIGH BASELINE PREVALENCE OF CLONAL HEMATOPOIESIS MUTATIONS IN CANCER PATIENTS: A SINGLE-INSTITUTION RETROSPECTIVE ANALYSIS

**Topic:** 24. Hematopoiesis, stem cells and microenvironment

Monika Kulasekaran*[1], Chelsie MacKanos[1]

[1]*University of Missouri, Columbia, United States of America;*

### Background

Clonal hematopoiesis (CH) mutations are frequently observed in cancer patients and are associated with increased risks of therapy-related complications, cardiovascular events, and secondary hematologic malignancies. While CH prevalence in the general population is typically 10–20% in older adults, emerging evidence suggests substantially higher rates at baseline in individuals with solid tumors—potentially reflecting shared risk factors or disease-related clonal selection. This single-institution retrospective study aimed to quantify the baseline prevalence of CH mutations in a real-world cohort of cancer patients from the University of Missouri, USA.

### Methods

We conducted a retrospective study of 300 consecutive adult patients with solid malignancies who underwent next-generation sequencing (NGS) at our center between 2020 and 2022. Baseline CH was defined as mutations detected prior to chemotherapy exposure. Demographic data, cancer type, smoking history, and vital status were collected from electronic medical records. Prevalence was calculated overall and stratified by age, sex, smoking status, and cancer group. Survival was assessed using Kaplan-Meier estimates.

### Results

The cohort was dominated by gastrointestinal (36%) and thoracic (35%) cancers, with a median age at diagnosis of 64 years. Baseline CH mutations were identified in approximately 30% of patients—markedly higher than expected in age-matched non-cancer populations. Prevalence was significantly greater in patients aged ≥60 years (29–30% vs 21% in <60 years; p<0.05) and showed non-significant trends toward elevation in smokers and thoracic malignancies. The most frequently mutated genes were DNMT3A and TET2. Baseline CH status was not significantly associated with overall survival (log-rank p=0.55).

### Summary/Conclusion

This single-institution retrospective analysis from 2020–2022 demonstrates a high baseline prevalence of CH mutations (≈30%) in cancer patients, exceeding rates reported in the general population and supporting the hypothesis of enhanced clonal expansion in malignancy. DNMT3A and TET2 were the dominant drivers. Prospective multicenter studies are warranted to clarify the mechanistic links, prognostic impact, and therapeutic implications of baseline CH in solid tumors.

**Copyright Information:** (Online) ISSN: 2572-9241
©2026 The Author(s). HemaSphere is published by John Wiley & Sons Ltd on behalf of European Hematology Association. This is an open access Abstract Book distributed under the Attribution-NonCommercial-NoDerivs (CC BY-NC-ND), which allows third parties to download the articles and share them with others as long as they credit the author and the Abstract Book, but they cannot change the content in any way or use them commercially.
**Abstract Book Citations:** Authors, Title, HemaSphere, 2026;10:(S1):pages. The abstract book DOIs can be found on the “Author Information” page.
**Disclaimer:** Articles published in the journal HemaSphere exclusively reflect the opinions of the authors. The authors are responsible for all content in their abstracts including accuracy of the facts, statements, citing resources, etc.

### (PB4090) ANGIOGENIC PLASTICITY OF VASCULAR ENDOTHELIUM IN THE TUMOR MICROENVIRONMENT OF ACUTE LEUKEMIA AND TRANSFORMING GROWTH FACTOR BETA 1

**Topic:** 24. Hematopoiesis, stem cells and microenvironment

Vira Barilka*[1], Volodymyr Matlan[2, 2], Olha Shalay[1], Sofiya Prymak[1], Vasyl Novak[1]

*[1]State Institution "Institute of Blood Pathology and Transfusion Medicine of National Academy of Medical Science of Ukraine", Laboratory of Immunology and Genetics of Blood Neoplasms, Lviv, Ukraine; [2]«State Non-Profit Enterprise «Danylo Halytsky Lviv National Medical University», Faculty of Postgraduate Studies, Lviv, Ukraine;*

### Background

The problem of angiogenesis in the tumor microenvironment of acute leukemias (AL) remains unresolved. This is due to the property of leukemic cells to acquire the phenotype of endothelial cells (mimicry), integrate into the vascular wall and induce pathological angioplasty. On the other hand, leukemic cells are a powerful source of secretion of the proangiogenic, growth-regulatory and procoagulant cytokine, transforming growth factor beta 1 (TGFβ1), due to which they are able to induce angiogenesis from pre-existing capillaries and carry out transendothelial migration of leukemia to target organs. However, the ultimate role of TGFβ1 in angiogenesis in the tumor microenvironment of AL is not well understood.

### Aims

To determine the proliferative activity of vascular endothelium (VE) after exposure to plasma or 24-hour supernatants of peripheral blood blasts (Sp) of patients with Al (pts). The obtained results compared with the effect of endogenous TGFβ1 (enTGFβ1) in experimental samples and with the effect of recombinant human TGFβ1 (rh TGFβ1; Gibco, UK) in vitro.

### Methods

The study included 10 pts with primary untreated AL (5 pts with AML and 5 pts with ALL in adult) . The median age was 68 years. The number of blasts in peripheral blood was 58,62 ± 10,53%. The control group consisted of 15 blood donors.The concentrations of enTGFβ1 in plasma and Sp were determined by biological methods using the CCL 64 line. VE was obtained according to E. A. Jaffe, 1973. VE proliferation was determined after treatment of VE monolayers with plasma or 24-hour Sp of blasts cells of AL pts. To compare the effects of enTGFβ1, VE was incubated with different doses of rh TGFβ1 (range from 0.5 to 15 ng/mL). Treated VE were incubated for 72 hours, after which the cells were labeled with 3H-thymidine and the proliferation index (PI) was calculated. VE cells that were not treated before the study were used as controls.

### Results

The concentration of enTGFβ1 in plasma of AL pts was higher than in healthy donors ($p<0.001$), while the concentration of enTGFβ1 in Sp blasts was significantly lower than in healthy individuals. Plasma AL pts showed a weaker effect on VE proliferation (IP = 1,24 ± 0,18) than Sp blasts (IP = 1,73 ± 0,34; $p < 0,05$). It should be noted that the content of enTGFβ1 in Sp blasts was twice as high as in plasma AL pts ($p<0.05$) and inversely correlated with the number of leukemic cells (r = - 0,40). Low doses of rhTGFβ1 (0.5 - 1.9 ng/ml) strongly stimulated the growth of VE (IP = 2,5 ± 0,43; r = - 0,9). Under these conditions, the largest number of round vessel-like structures was detected in the field of view of the inverted microscope. With increasing concentrations of rhTGFβ1 from 2.0 to 4.8 ng/ml, the VE was in a quiescent state (PI = 1,4 ± 0,19). Even higher doses of rhTGFβ1 (4,2 – 11,06 ng/ml) caused a cytostatic effect of VE (PI = 0,76 ± 0,2).

**Copyright Information:** (Online) ISSN: 2572-9241
©2026 The Author(s). HemaSphere is published by John Wiley & Sons Ltd on behalf of European Hematology Association. This is an open access Abstract Book distributed under the Attribution-NonCommercial-NoDerivs (CC BY-NC-ND), which allows third parties to download the articles and share them with others as long as they credit the author and the Abstract Book, but they cannot change the content in any way or use them commercially.
**Abstract Book Citations:** Authors, Title, HemaSphere, 2026;10:(S1):pages. The abstract book DOIs can be found on the "Author Information" page.
**Disclaimer:** Articles published in the journal HemaSphere exclusively reflect the opinions of the authors. The authors are responsible for all content in their abstracts including accuracy of the facts, statements, citing resources, etc.

**Summary/Conclusion**

Obtained data indicated that plasma as well as Sp of blasts cell fom AL pts stimulated proliferative activity of endothelial cells in vitro. Leukemic cells from AL pts were a potent source of enTGFβ1 secretion, which was able to accumulate and modulate the angiogenic properties of VE and may pathogenetically influence on the course of AL. This may suggest that angiogenic plasticity under influence of enTGFβ1 may be important in modifying the tumor microenvironment in AL and indicate important targets in the treatment of AL.

**Copyright Information:** (Online) ISSN: 2572-9241
©2026 The Author(s). HemaSphere is published by John Wiley & Sons Ltd on behalf of European Hematology Association. This is an open access Abstract Book distributed under the Attribution-NonCommercial-NoDerivs (CC BY-NC-ND), which allows third parties to download the articles and share them with others as long as they credit the author and the Abstract Book, but they cannot change the content in any way or use them commercially.
**Abstract Book Citations:** Authors, Title, HemaSphere, 2026;10:(S1):pages. The abstract book DOIs can be found on the "Author Information" page.
**Disclaimer:** Articles published in the journal HemaSphere exclusively reflect the opinions of the authors. The authors are responsible for all content in their abstracts including accuracy of the facts, statements, citing resources, etc.

### (PB4091) ALTERATIONS IN MITOCHONDRIAL PROTEINS WITHIN MULTIPOTENT MESENCHYMAL STROMAL CELLS AS ACUTE LEUKEMIA ADVANCES

**Topic:** 24. Hematopoiesis, stem cells and microenvironment

Nataliya Petinati*[1], Aleksandra Sadovskaya[1], Nina Drize[1], Igor Smirnov[1], Valeria Surimova[1, 1], Olga Pobegyts[1], Georgiy Arapidi[1], Maria Lagarkova[1], Anastasya Vasilieva[1], Olga Aleshina[2], Elena Parovichnikova[1]

*[1]National Medical Research Center for Hematology, Moscow, Russia;[2]Federal Scientific and Clinical Center of Physicochemical Medicine named after Academician Yu. M. Lopukhin of the Federal Medical and Biological Agency, Moscow, Russia;*

### Background

Acute leukemia (AL) significantly affects multipotent mesenchymal stromal cells (MSCs). As the disease advances, MSCs' normal functions and their capacity to support hematopoiesis diminish. MSCs’ mitochondrial (MT) biogenesis and metabolism are disrupted.

### Aims

To analyze changes in MT proteins in MSCs of patients at AL onset and during treatment.

### Methods

MSCs were derived from bone marrow samples of 8 healthy donors and 18 AL patients at onset, in remission, and after allogeneic hemopoietic stem cell transplantation (HSCT), and cultured for 3 passages. Proteomic analysis was performed with an Orbitrap Q Exactive HF-X mass spectrometer and Scaffold 5 software. For functional enrichment analysis we selected significantly ($p<0.05$) differing proteins with |log Fold Change|>2. 302 mitochondrial proteins were analyzed

**Copyright Information:** (Online) ISSN: 2572-9241
©2026 The Author(s). HemaSphere is published by John Wiley & Sons Ltd on behalf of European Hematology Association. This is an open access Abstract Book distributed under the Attribution-NonCommercial-NoDerivs (CC BY-NC-ND), which allows third parties to download the articles and share them with others as long as they credit the author and the Abstract Book, but they cannot change the content in any way or use them commercially.
**Abstract Book Citations:** Authors, Title, HemaSphere, 2026;10:(S1):pages. The abstract book DOIs can be found on the “Author Information” page.
**Disclaimer:** Articles published in the journal HemaSphere exclusively reflect the opinions of the authors. The authors are responsible for all content in their abstracts including accuracy of the facts, statements, citing resources, etc.

**Results**

At AL onset, patients' MSCs had elevated levels of ATPase and respiratory chain complex IV subunits compared to donor cells. In remission and post-HSCT, ATPase subunits, including ATPase inhibitor ATP5IF1, decreased, while respiratory chain complex I subunits and cytochrome c increased compared to donors.
C1QBP increased at the onset and in remission but normalized after HSCT. It regulates many processes, including respiration and MT fusion. MFF and FIS1, essential for MT fission, decreased in remission, and FIS1 did not normalize after HSCT. It implies that MT in patients' MSCs are predisposed to fusion over fission.
MT ribosomal proteins (MRPs) content changed. At the onset, two small subunit MRPs decreased. In remission and after HSCT, while some MRPs decreased, others increased. Notable is increase in MRPL56 (or LACTB) which also regulates MT phospholipid metabolism.
Levels of transmembrane transporters were altered. In remission, TIMM50, inner membrane transporter of proteins, increased and SLC25A12, aspartate/glutamate antiporter, decreased. After HSCT, SCL25A22 (glutamate/H+ symporter) and MTCH2 were upregulated while protein channel TOMM40 was downregulated compared to donors. MTCH2 controls membrane permeability and plays a role in apoptosis.
Proteins that increased after HSCT compared to pre-HSCT relate to lipid metabolism.
Proteins associated with drug metabolism were upregulated in remission. This can be due to the treatment and indicates atypical MT metabolism.
Table. Mitochondrial proteins differentially present in MSCs

| Compared groups | Proteins |
|---|---|
| O-MSCs>D-MSCs | NDUFB8,COX5B,CYP24A1,C1QBP,ATP5F1D,ATP5F1A,SUCLG2 |
| O-MSCs<D-MSCs | MRPS5,MRPS36,COX7A2L,DLST,ATP5PO,PPT1 |
| R-MSCs>D-MSCs | MRPS9,MRPS28,MRPS27,MRPL9,MRPL18,MRPL34,MRPL50,MRPL55,LACTB,SUCLG2,C1QBP,NUDT9,MAP2K1,MAP2K3,MAP2K4,NDUFS3,CPT1A,TAB1,FAHD1,CMC1,CYC1,COX5B,IDH3B,ACAD9 |
| R-MSCs<D-MSCs | ATP5ME,ATP5IF1,AK2,AK4,MTRES1,MRPS7,MRPS10,MRPS14,MRPL3,COX7A2L,FIS1 |
| O-MSCs>R-MSCs | ATP5ME,ATP5F1D,SLC25A12,MFF |
| O-MSCs<R-MSCs | MRPL11,MRPL38,MRPL50,MRPL55,MAP2K1,MAP2K3,DECR1,ACAT1,SLIRP,TIMM50,PDHX,PPT1 |
| HSCT-MSCs>D-MSCs | MRPS5,MRPS9,MRPS22,MRPS30,MRPS31,LACTB,HADHB,MAVS,MAP2K1,SLC25A22,PPA2,ACAT1,PPT1,NDUFA10,MTCH2,GLS,NDUFS6,SUCLG2,FAHD1,CYC1,PDHX |
| HSCT-MSCs<D-MSCs | NDUFV2,YARS2,ATP5ME,ATP5IF1,ATP5F1A,FIS1,COX7A2L,PRDX5,MRPL27,TOMM40 |
| HSCT-MSCs>R-MSCs | PPT1,ACAT1,AK4,GLS |
| HSCT-MSCs<R-MSCs | MRPL34,MRPL46,C1QBP,NUMA1,ATP5IF1 |

D-MSCs – donors' MSCs
O-MSCs – MSCs of patients at the AL onset
R-MSCs – MSCs of patients in remission
HSCT-MSCs – MSCs of patients after allo-HSCT

**Summary/Conclusion**

Significant changes in MT proteins are observed. Such events contribute to metabolic dysfunctions and compromise MSCs activity

**Copyright Information:** (Online) ISSN: 2572-9241
©2026 The Author(s). HemaSphere is published by John Wiley & Sons Ltd on behalf of European Hematology Association. This is an open access Abstract Book distributed under the Attribution-NonCommercial-NoDerivs (CC BY-NC-ND), which allows third parties to download the articles and share them with others as long as they credit the author and the Abstract Book, but they cannot change the content in any way or use them commercially.
**Abstract Book Citations:** Authors, Title, HemaSphere, 2026;10:(S1):pages. The abstract book DOIs can be found on the "Author Information" page.
**Disclaimer:** Articles published in the journal HemaSphere exclusively reflect the opinions of the authors. The authors are responsible for all content in their abstracts including accuracy of the facts, statements, citing resources, etc.

### (PB4092) CHRONIC NEUTROPENIA IN CHILDREN: A SINGLE-CENTER EXPERIENCE

**Topic:** 24. Hematopoiesis, stem cells and microenvironment

Jelena Roganovic*[1, 2]

*[1]Children's Hospital Zagreb, Department for Pediatric Oncology and Hematology, Zagreb, Croatia;[2]Faculty of Biomedicine and Drug Development, University of Rijeka, Rijeka, Croatia;*

### Background

Neutropenia is a frequent laboratory finding in children and a common reason for referral to pediatric hematologists. While acute neutropenia is most often caused by infections (primarily post-viral), chronic neutropenia encompasses a broad spectrum of underlying conditions. Several expert groups have published guidelines for the accurate diagnosis, follow-up, and management of childhood neutropenia – particularly chronic forms – however, until recently, routine clinical practice remained heterogeneous, driven primarily by the physician's experience and local protocols rather than by standardized, evidence-based recommendations. This study aimed to present institutional experience with the diagnostic work-up, management strategies, and follow-up of children with chronic neutropenia.

### Aims

This study aims to examine the etiology, clinical course, interventions, and outcomes of children with chronic neutropenia admitted to a tertiary care pediatric hospital in Croatia.

### Methods

Fifty-seven children with neutropenia who were consecutively admitted to the day hospital or inpatient unit at the Division of Hematology and Oncology, Department of Pediatrics, Clinical Hospital Center Rijeka, Croatia, between January 1, 2013, and December 31, 2021, were enrolled. Patients with chemotherapy-induced febrile neutropenia were excluded from the study. Of the 57 subjects, children with acute neutropenia (ANC recovered to ≥1500/µL within three months) were further excluded. Additionally, two patients with chronic neutropenia were lost to follow-up. After these exclusions, data from 48 children (23 males and 25 females), all of Caucasian origin, were included in the final analysis. The median age at diagnosis was 7.75 months (IQR 5.125–11.75). Thirteen (27%) patients were younger than 6 months, 30 (63%) were between 6 months and 5 years of age, and 5 (10%) were older than 5 years of age. These age categories were defined based on recognized differences in the etiology and clinical course of pediatric neutropenia according to age at onset, rather than on developmental pediatric classifications.

### Results

The median absolute neutrophil count (ANC) at presentation was 487/µL (IQR 198.5–837.5), and 52% of the patients had severe neutropenia. Autoimmune neutropenia (AIN) was diagnosed in 22 cases, chronic idiopathic neutropenia (CIN) in 25 cases, and congenital neutropenia in one case. Antineutrophil antibodies were detected in 44% of the children tested. During follow-up, 23% received granulocyte-colony stimulating factor (G-CSF) and 21% received antibiotics. The median follow-up duration was 21 months (IQR 12–32.75), during which 83% achieved spontaneous remission, with a median time to remission of 13.5 months. Lower ANC at diagnosis was associated with more frequent rehospitalizations, longer time to remission and longer follow-up. No significant differences were found between AIN and CIN in terms of age, ANC at diagnosis, time to referral, or time to remission, although AIN cases were followed for longer.

**Copyright Information:** (Online) ISSN: 2572-9241
©2026 The Author(s). HemaSphere is published by John Wiley & Sons Ltd on behalf of European Hematology Association. This is an open access Abstract Book distributed under the Attribution-NonCommercial-NoDerivs (CC BY-NC-ND), which allows third parties to download the articles and share them with others as long as they credit the author and the Abstract Book, but they cannot change the content in any way or use them commercially.
**Abstract Book Citations:** Authors, Title, HemaSphere, 2026;10:(S1):pages. The abstract book DOIs can be found on the "Author Information" page.
**Disclaimer:** Articles published in the journal HemaSphere exclusively reflect the opinions of the authors. The authors are responsible for all content in their abstracts including accuracy of the facts, statements, citing resources, etc.

**Summary/Conclusion**

Pediatric chronic isolated neutropenia, including AIN and CIN, generally follows a mild clinical course with a low incidence of severe infections. Most children achieve spontaneous remission within one year. Comprehensive genetic testing is essential in congenital cases. Adherence to European guidelines supports standardized diagnosis, follow-up, and management, thereby improving patient care.

**Copyright Information:** (Online) ISSN: 2572-9241
©2026 The Author(s). HemaSphere is published by John Wiley & Sons Ltd on behalf of European Hematology Association. This is an open access Abstract Book distributed under the Attribution-NonCommercial-NoDerivs (CC BY-NC-ND), which allows third parties to download the articles and share them with others as long as they credit the author and the Abstract Book, but they cannot change the content in any way or use them commercially.
**Abstract Book Citations:** Authors, Title, HemaSphere, 2026;10:(S1):pages. The abstract book DOIs can be found on the "Author Information" page.
**Disclaimer:** Articles published in the journal HemaSphere exclusively reflect the opinions of the authors. The authors are responsible for all content in their abstracts including accuracy of the facts, statements, citing resources, etc.

### (PB4093) CYTOKINE RELEASE SYNDROME STATE ANTECEDENT TO MACROPHAGE ACTIVATION SYNDROME-HEMOPHAGOCYTIC LYMPHOHISTIOCYTOSIS SECONDARY TO SYSTEMIC JUVENILE IDIOPATHIC ARTHRITIS.

**Topic:** 24. Hematopoiesis, stem cells and microenvironment

Zein Sheikh*[1], Roaa Aljohani[2], Nagah Elmusharaf[3], Amal Albeihany[3], Mohammed Almakadi[3]

*[1]Prince Mohammed Bin Abdulaziz Hospital, Ministry of National Guard Health Affairs, Internal Medicine, Madinah, Saudi Arabia; [2]College of Medicine, Taibah University, Madinah, Saudi Arabia;[3]King Faisal Specialist Hospital & Research Centre, Madinah, Saudi Arabia;*

### Background

Hemophagocytic lymphohistiocytosis (HLH) is a serious hyperinflammatory syndrome with potentially grave prognosis if not suspected timely due to secondary multi-organ failure. Mutually and irrespective of the subtype, HLH is generally characterized by persistent fever, cytopenias, hyperferritinemia, and hepatosplenomegaly.

### Aims

To describe an atypical clinical presentation of HLH in an otherwise healthy 14-year-old girl.

### Methods

Our patient presented with a 4-week history of quotidian fever, evanescent rash, lymphadenopathy, and arthritis involving medium and large joints. Initial laboratory testing revealed leukocytosis with neutrophilia (19.2 $\times 10^9$/L) normal hemoglobin and platelet counts, elevated aspartate aminotransferase (73 U/L), lactate dehydrogenase (LDH) (637 U/L), C-reactive protein (43.5 mg/L), erythrocyte sedimentation rate (76 mm/h), and ferritin of 1,675 µg/L, rising to 10,000 µg/L. Peripheral blood smear was unremarkable. Infectious and autoimmune workup was negative. Imaging showed no hepatosplenomegaly.
Few days later, she acutely deteriorated with hypotension and respiratory failure, requiring intensive care unit admission. Ferritin escalated to 46,759 µg/L, LDH to 1,793 U/L, and leukocytosis to 35.79 $\times 10^9$/L. Notably, triglycerides, fibrinogen, and platelet counts remained within normal ranges at that stage.

### Results

Despite not fulfilling any criteria of HLH subcategories, the significant clinical deterioration prompted immediate treatment for presumed evolving macrophage activation syndrome (MAS) secondary to systemic juvenile idiopathic arthritis (sJIA) using high-dose intravenous pulse methylprednisolone with anakinra. Within 24 hours, ferritin exceeded 100,000 µg/L, LDH rose above 1,800 U/L, triglycerides increased to 6.39 mmol/L, and leukocytosis progressed to 41 $\times 10^9$/L. Intravenous immunoglobulin and cyclosporine were subsequently added.
Over the following 48 hours, fever defervesced, and hemodynamic stability was achieved. Thrombocytopenia and hypofibrinogenemia then developed, which was only transient, consistent with evolving MAS. She demonstrated an excellent clinical and laboratory response to therapy and was discharged home on tapering oral corticosteroids, anakinra, and cyclosporine.

**Copyright Information:** (Online) ISSN: 2572-9241
©2026 The Author(s). HemaSphere is published by John Wiley & Sons Ltd on behalf of European Hematology Association. This is an open access Abstract Book distributed under the Attribution-NonCommercial-NoDerivs (CC BY-NC-ND), which allows third parties to download the articles and share them with others as long as they credit the author and the Abstract Book, but they cannot change the content in any way or use them commercially.
**Abstract Book Citations:** Authors, Title, HemaSphere, 2026;10:(S1):pages. The abstract book DOIs can be found on the “Author Information” page.
**Disclaimer:** Articles published in the journal HemaSphere exclusively reflect the opinions of the authors. The authors are responsible for all content in their abstracts including accuracy of the facts, statements, citing resources, etc.

**Summary/Conclusion**

Our case highlights few challenges. It is conceivable to say that the current HLH criteria address an overwhelming inflammatory state that has evolved to prevent secondary end-organ damage. Our patient suffered a dramatic clinical deterioration due to HLH that was "dissociative" from HLH criteria necessitating prompt intervention. As majority of the HLH literature is driven by pediatric and adult studies, the literature is scarce in adolescents highlighting a potential unmet need. Hyperferritinemia can be elevated in patients with HLH due to other contributing conditions like sJIA in our patient. However, hyperferritinemia exceeding 10,000 μg/L has a sensitivity and specificity for HLH exceeding 90%. Ferritin levels measurement is cost-effective and clinically informative in cases of unexplained fever, especially that dedicated cytokines assays require specialized facilities and have longer turnaround time. The striking hyperferritinemia was the "whistleblower" for the evolving MAS-HLH due to sJIA.
We are unaware of any published similar case in the literature. Our case presents a unique aspect of the HLH spectrum reminiscent to what is usually observed when cytokines are rapidly liberated by means of CAR T-cell or BiTE therapy.

**Copyright Information:** (Online) ISSN: 2572-9241
©2026 The Author(s). HemaSphere is published by John Wiley & Sons Ltd on behalf of European Hematology Association. This is an open access Abstract Book distributed under the Attribution-NonCommercial-NoDerivs (CC BY-NC-ND), which allows third parties to download the articles and share them with others as long as they credit the author and the Abstract Book, but they cannot change the content in any way or use them commercially.
**Abstract Book Citations:** Authors, Title, HemaSphere, 2026;10:(S1):pages. The abstract book DOIs can be found on the "Author Information" page.
**Disclaimer:** Articles published in the journal HemaSphere exclusively reflect the opinions of the authors. The authors are responsible for all content in their abstracts including accuracy of the facts, statements, citing resources, etc.

### (PB4094) THE HEMATOPOIESIS OF RADIX ASTRAGALUS POLYSACCHARIDE RAP IN ANAEMIC MICE THROUGH REGULATING THE DIFFERENTIATION OF HEMATOPOIETIC STEM CELLS

**Topic:** 24. Hematopoiesis, stem cells and microenvironment

Jing Li*[1]

[1]*Hong Kong Baptist University, School of Chinese Medicine, Hong Kong, Hong Kong;*

### Background

The World Health Organisation estimates that approximately a quarter of the population worldwide suffers from anaemia. However, first-line clinical therapies cannot meet the needs of patients with different types of anaemia, especially those with haemorrhagic and aplastic anaemia. Although Radix Astragali, one of the most well-known Chinese medicinal herbs, has been used clinically for thousands of years to nourish the blood, its development remains limited by unclear key ingredients and mechanisms. Radix Astragali Polysaccharides (RAP) are considered the most important component for their diverse therapeutic activities, including immunomodulatory, anti-cancer, anti-viral, fatigue resistance, and senescence-delaying effects, yet haematopoietic effects and mechanisms are rarely reported.

### Aims

Therefore, it is essential to evaluate the erythropoietic effects of RAP in haemorrhagic and aplastic anaemia in vitro and in vivo.

### Methods

Hemorrhagic Anaemic mice model were established through loosing 300μL blood, and aquired Aplastic animia mice were injected Epirubicin via tail vein. The hemogram of blood were tested by hemocytometer. The spleen index was used to evaluate the extramedullary haematopoiesis. Differentiation of hamatopoietic stem and progenitor cells were identified by flow cytometry. Cytokine levels were studied by Elisa. Triple Quadrupole Liquid Chromatography-Mass Spectrometry confirmed the delivery of RAP into bone marrow.

### Results

In this study, RAP was found to significantly increase red blood cells, haemoglobin, and haematocrit in anaemic mice, with concomitant evidence of extramedullary haematopoiesis in the spleen decreased. Furthermore, RAP treatment was observed to increase reticulocytes, different types of erythroblasts, and promote the differentiation of haematopoietic stem cells (HSCs) towards short-term HSC-multipotent progenitor 2-common myeloid progenitor-megakaryocyte erythrocyte progenitor. In addition, this erythropoietic effect of RAP was confirmed in HSCs isolated from mice. Moreover, Triple Quadrupole Liquid Chromatography-Mass Spectrometry identified RAP in bone marrow interstitial fluid.

### Summary/Conclusion

In conclusion, our findings reveal that RAP is the main component of Radix Astragali that exerts erythropoietic effects by accelerating the differentiation of HSCs into erythroblasts, highlighting its potential to alleviate anaemia.

**Copyright Information:** (Online) ISSN: 2572-9241
©2026 The Author(s). HemaSphere is published by John Wiley & Sons Ltd on behalf of European Hematology Association. This is an open access Abstract Book distributed under the Attribution-NonCommercial-NoDerivs (CC BY-NC-ND), which allows third parties to download the articles and share them with others as long as they credit the author and the Abstract Book, but they cannot change the content in any way or use them commercially.
**Abstract Book Citations:** Authors, Title, HemaSphere, 2026;10:(S1):pages. The abstract book DOIs can be found on the "Author Information" page.
**Disclaimer:** Articles published in the journal HemaSphere exclusively reflect the opinions of the authors. The authors are responsible for all content in their abstracts including accuracy of the facts, statements, citing resources, etc.

### (PB4095) SEVERE METHOTREXATE-INDUCED PANCYTOPENIA FOLLOWED BY EXTREME FILGRASTIM-ASSOCIATED HYPERLEUKOCYTOSIS IN SUBACUTE CUTANEOUS LUPUS ERYTHEMATOSUS

**Topic:** 24. Hematopoiesis, stem cells and microenvironment

Teuta Dedej*[1], Resmije Tozharaku[2], Adela Perolla[2], Arben Ivanaj[2], Alma Cili[2]

[1]*mother teresa hospital, laboratory, Tirana, Albania;* [2]*Hematology department, Tirana, Albania;*

### Background

Methotrexate (MTX) is a cornerstone therapy for many immune-mediated disorders. Although commonly well-tolerated at low doses, severe myelosuppression including pancytopenia can occur, particularly in the presence of risk factors such as renal dysfunction and drug interactions. Granulocyte-colony stimulating factor (filgrastim) is widely used to treat MTX-induced neutropenia, but extreme leukocyte responses may lead to hyperleukocytosis and potential leukostasis.

### Methods

### Case overview

A 39-year-old woman with a 4-year history of subacute cutaneous lupus erythematosus, treated with MTX (7.5 mg/week; cumulative dose 530 mg), hydroxychloroquine, folic acid, and glucocorticoids, presented for routine follow-up. Three months earlier, CBC showed mild macrocytic anemia and leukopenia. At presentation she complained of fatigue and oral sores; labs revealed severe pancytopenia with marked neutropenia and thrombocytopenia. Bone marrow aspiration showed hypocellularity with megaloblastic changes.

**Management**

MTX was discontinued. Supportive care included:

- **Folinic acid rescue**
- **Blood and platelet transfusions**
- **Empiric antibiotics and antifungals**
- **Methylprednisolone**
- **Filgrastim 5 µg/kg/day for 5 days**

Hematologic parameters improved over time; however, the WBC count rose dramatically after filgrastim cessation, peaking at 115,560/mm³ without clinical leukostasis.

**Copyright Information:** (Online) ISSN: 2572-9241
©2026 The Author(s). HemaSphere is published by John Wiley & Sons Ltd on behalf of European Hematology Association. This is an open access Abstract Book distributed under the Attribution-NonCommercial-NoDerivs (CC BY-NC-ND), which allows third parties to download the articles and share them with others as long as they credit the author and the Abstract Book, but they cannot change the content in any way or use them commercially.
**Abstract Book Citations:** Authors, Title, HemaSphere, 2026;10:(S1):pages. The abstract book DOIs can be found on the “Author Information” page.
**Disclaimer:** Articles published in the journal HemaSphere exclusively reflect the opinions of the authors. The authors are responsible for all content in their abstracts including accuracy of the facts, statements, citing resources, etc.

### Results

### Results

### Table 1: Laboratory Findings Over Time

| **Parameter (units)** | **3 Months** | **Admission** | **Nadir** | **Peak After Filgrastim** | **Week - 8 8 -Week follow UP** |
|---|---|---|---|---|---|
| Hemoglobin (g/dL) | 11.7 | 8.8 | 6.4 | 10.5 | 13.0 |
| MCV (fL) | 103.2 | 97.7 | - | - | - |
| WBC (/mm³) | 3,910 | 1200 | 770 | 115560 | 11300 |
| ANC (/mm³) | - | 570 | 270 | - | - |
| Platelets (/mm³) | 182,000 | 37000 | 16000 | 377000 | 162000 |
| Creatinine (mg/dL) | - | 1.58 | - | - | 1.31 |

### Table 2. Identified Risk Factors for MTX Toxicity

| Risk Factors | Present |
|---|---|
| Renal impairment | Yes |
| Macrocytosis | yes |
| Oral myositis’ | Yes |
| Hydroxychloroquine interaction | Yes |
| Long term MTX exposure | Yes |
| Folate deficiency | Suspected |

**Copyright Information:** (Online) ISSN: 2572-9241
©2026 The Author(s). HemaSphere is published by John Wiley & Sons Ltd on behalf of European Hematology Association. This is an open access Abstract Book distributed under the Attribution-NonCommercial-NoDerivs (CC BY-NC-ND), which allows third parties to download the articles and share them with others as long as they credit the author and the Abstract Book, but they cannot change the content in any way or use them commercially.
**Abstract Book Citations:** Authors, Title, HemaSphere, 2026;10:(S1):pages. The abstract book DOIs can be found on the “Author Information” page.
**Disclaimer:** Articles published in the journal HemaSphere exclusively reflect the opinions of the authors. The authors are responsible for all content in their abstracts including accuracy of the facts, statements, citing resources, etc.

**Summary/Conclusion**

**Discussion**
MTX-induced pancytopenia can be unpredictable and severe. Risk factors in this case included impaired renal function, macrocytosis, mucositis, and concomitant hydroxychloroquine therapy affecting MTX clearance. Leukocyte rebound after G-CSF can reach extreme levels (>100,000/mm³), a phenomenon reported in a minority of patients but one that carries risk.
**Conclusion**

- **MTX can cause severe pancytopenia even at low doses**
- **CBC monitoring is essential for early detection**
- **Filgrastim-associated hyperleukocytosis, though rare, requires vigilant follow-up**

**References**

- Lim AY, Gaffney K, Scott DG. Methotrexate-induced pancytopenia: Serious and under-reported? *Rheumatology*. 2005;44:1051–1055.
- Gonzalez-Ibarra F, et al. Methotrexate Induced Pancytopenia. *Case Rep Rheumatol*. 2014.
- Gutierrez-Ureña S, et al. Pancytopenia secondary to MTX therapy in rheumatoid arthritis. *Arthritis Rheum*. 1996;39:272–276.
- FDA Prescribing Information: Filgrastim.

**Copyright Information:** (Online) ISSN: 2572-9241
©2026 The Author(s). HemaSphere is published by John Wiley & Sons Ltd on behalf of European Hematology Association. This is an open access Abstract Book distributed under the Attribution-NonCommercial-NoDerivs (CC BY-NC-ND), which allows third parties to download the articles and share them with others as long as they credit the author and the Abstract Book, but they cannot change the content in any way or use them commercially.
**Abstract Book Citations:** Authors, Title, HemaSphere, 2026;10:(S1):pages. The abstract book DOIs can be found on the "Author Information" page.
**Disclaimer:** Articles published in the journal HemaSphere exclusively reflect the opinions of the authors. The authors are responsible for all content in their abstracts including accuracy of the facts, statements, citing resources, etc.

### (PB4096) HEMATOGON-LIKE APPEARANCE IN FLOW CYTOMETRY EVALUATION: CAUTION

**Topic:** 24. Hematopoiesis, stem cells and microenvironment

İrfan Yavaşoğlu*[1], Sencer Küçük[1], Yusufcan Aygül[1], Ayse Hilal Eroglu Kucukdiler[1], Atakan Turgutkaya[1], Ali Zahit Bolaman[1]

[1]*Aydın Adnan Menderes University, Hematology, Aydın, Türkiye;*

### Aims

Hematogons are known as lymphoid-appearing cells that are important, particularly in the evaluation of acute lymphoblastic leukaemia (ALL), but whose structure is still debated. We attempted to examine the hematogon-like appearance we observed in flow cytometry practice.

### Methods

For our study, we retrospectively collected samples from 44 patients (22 male, 22 female) aged 22–84 years who underwent flow cytometry (Beckman Coulter Navious 3L10C, 10 colours, 3 lasers) for various reasons.

### Results

For our study, we retrospectively collected samples from 44 patients (22 male, 22 female) aged 22–84 years who underwent flow cytometry (Beckman Coulter Navious 3L10C, 10 colours, 3 lasers) for various reasons. None of the patients had a diagnosis of ALL. Nineteen of them had NHL (18 B-cell, 1 T-cell), 6 of them had anaemia (MDS, haemolytic anaemia), 6 of them had Hodgkin's lymphoma (HL), 4 of them had CLL, 4 of them had AML, and 5 of them had myeloma. All samples were from the initial diagnosis. There was no bone marrow involvement in lymphoma patients. The areas shown in the image contained 0.2-4.4% cells. There was strong CD 19 staining. CD 45, TDT, CD38, CD 34, and CD10 were evaluated in these areas.

### Summary/Conclusion

In conclusion, it is important to evaluate the haematogone-like cell structure in the presence of this appearance in flow cytometry evaluation. CD45, TdT, CD38, CD34, CD10, CD22, CD43, CD20, cIgM+, and sIg+ should be performed to determine haematogones and their stages. Disease involvement should be kept in mind. It is unclear whether it indicates a disease-specific prognosis.
Image: CD45-SS appearance in the bone marrow of a patient with MDS (blue marked area)

**Copyright Information:** (Online) ISSN: 2572-9241
©2026 The Author(s). HemaSphere is published by John Wiley & Sons Ltd on behalf of European Hematology Association. This is an open access Abstract Book distributed under the Attribution-NonCommercial-NoDerivs (CC BY-NC-ND), which allows third parties to download the articles and share them with others as long as they credit the author and the Abstract Book, but they cannot change the content in any way or use them commercially.
**Abstract Book Citations:** Authors, Title, HemaSphere, 2026;10:(S1):pages. The abstract book DOIs can be found on the “Author Information” page.
**Disclaimer:** Articles published in the journal HemaSphere exclusively reflect the opinions of the authors. The authors are responsible for all content in their abstracts including accuracy of the facts, statements, citing resources, etc.

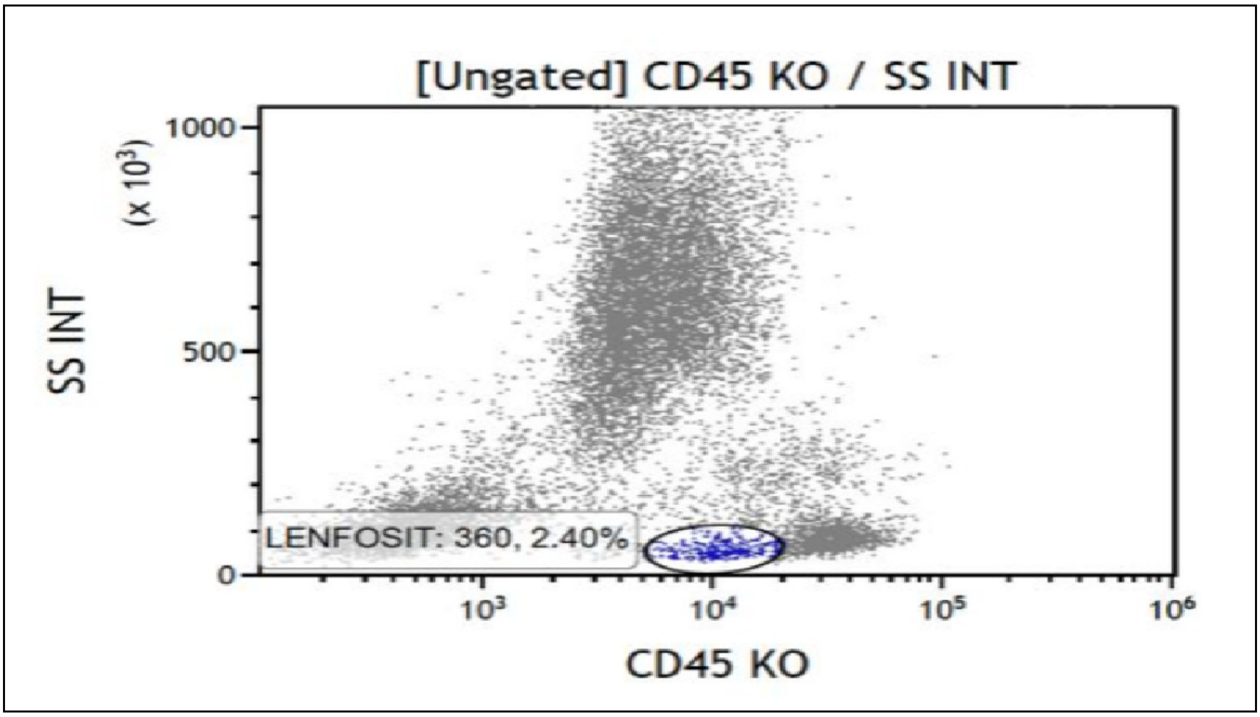

**Copyright Information:** (Online) ISSN: 2572-9241
©2026 The Author(s). HemaSphere is published by John Wiley & Sons Ltd on behalf of European Hematology Association. This is an open access Abstract Book distributed under the Attribution-NonCommercial-NoDerivs (CC BY-NC-ND), which allows third parties to download the articles and share them with others as long as they credit the author and the Abstract Book, but they cannot change the content in any way or use them commercially.
**Abstract Book Citations:** Authors, Title, HemaSphere, 2026;10:(S1):pages. The abstract book DOIs can be found on the "Author Information" page.
**Disclaimer:** Articles published in the journal HemaSphere exclusively reflect the opinions of the authors. The authors are responsible for all content in their abstracts including accuracy of the facts, statements, citing resources, etc.

### (PB2536) FROM BRAF V600E SCREENING TO EXTENDED NGS: ACTIONABLE MAPK ALTERATIONS AND TARGETED THERAPY IN ADULT HISTIOCYTOSIS

**Topic:** 25. Gene therapy, cellular immunotherapy and vaccination - Biology & translational research

Ramón Diez-Feijóo*[1, 2, 3], Clara Nadal Pellise[2], Concepción Fernández-Rodriguez[1, 4], Clara Montané[2], Thalia Guiulfo Knell[2], Lucía Gómez-Pérez[1, 2], Sara García Ávila[1, 2, 3], Maria Alicia Senin Magan[1, 2], Patricia Vélez[1, 2], Cristina Baca[2], Eva Gimeno[1, 2, 3], Ivonne Vázquez[4], Luis Colomo[4], Blanca Sanchez-Gonzalez[1, 2, 3], Beatriz Bellosillo[1, 4], Christelle Ferrà[1, 2, 3]

*[1]Group of Applied Clinical Research in Hematology, Cancer Research Program, Barcelona, Spain;[2]Department of Hematology, Hospital del Mar, Barcelona, Spain; [3]Pompeu Fabra University, Barcelona, Spain; [4]Department of Pathology, Hospital del Mar, Barcelona, Spain;*

### Background

Histiocytic neoplasms, including Langerhans cell histiocytosis (LCH) and non-Langerhans cell histiocytosis (non-LCH), are rare disorders with heterogeneous clinical behavior. Activating alterations of the MAPK signaling pathway represent a key pathogenic mechanism and have opened the door to targeted therapeutic approaches. However, the integration of molecular findings into real-world treatment strategies remains poorly described.

### Aims

To characterize the genetic landscape of LCH and non-LCH and to evaluate treatment strategies, with particular emphasis on molecularly-guided targeted treatments in a single center.

### Methods

We retrospectively reviewed adult patients diagnosed with histiocytic disorders at Hospital del Mar in Barcelona, between 2021 and 2025. Collected data included clinical presentation, organ involvement, histopathological diagnosis, molecular alterations, treatments and response. *BRAF* p.(V600E) was first assessed by allele-specific testing in all cases;*BRAF* p.(V600E)–negative cases were subsequently profiled using a targeted 45-gene NGS panel enriched for MAPK/ERK and PI3K–AKT–mTOR alterations (*MAP2K1/2, KRAS/NRAS, ARAF, RAF1, PIK3CA, PTEN, AKT1–3, MTOR*) and key RTKs (*EGFR/ERBB2-4, FGFR1–4, PDGFRA*).

**Copyright Information:** (Online) ISSN: 2572-9241
©2026 The Author(s). HemaSphere is published by John Wiley & Sons Ltd on behalf of European Hematology Association. This is an open access Abstract Book distributed under the Attribution-NonCommercial-NoDerivs (CC BY-NC-ND), which allows third parties to download the articles and share them with others as long as they credit the author and the Abstract Book, but they cannot change the content in any way or use them commercially.
**Abstract Book Citations:** Authors, Title, HemaSphere, 2026;10:(S1):pages. The abstract book DOIs can be found on the "Author Information" page.
**Disclaimer:** Articles published in the journal HemaSphere exclusively reflect the opinions of the authors. The authors are responsible for all content in their abstracts including accuracy of the facts, statements, citing resources, etc.

**Results**

Five patients were included, with a median age at diagnosis of 60 years (range 37–60); four were female. Three patients were diagnosed with LCH and two with non-LCH, including Erdheim–Chester disease (ECD) and Rosai–Dorfman disease (RDD). Clinical presentation varied according to the organs involved. Notably, diabetes insipidus was the initial manifestation in two cases (one LCH and one ECD), prompting evaluation for pituitary involvement. Overall, disease sites included the pituitary region (2/5), bone (2/5), soft tissues/skin (2/5), lung (1/5), liver (1/5), and breast (1/5). In LCH (n=3), *MAP2K1* alterations were identified in 2/3 cases, both harboring the recurrent p.(E102_I103del), while the remaining case was not evaluable due to insufficient material. Among non-LCH, *BRAF* p.(V600E) was detected in the ECD case, and *KRAS* p.(K117N) in the RDD case.
First-line treatment consisted of cytarabine for 12 cycles in the 3 LCH cases, achieving metabolic complete response (mCR) in two patients and partial response (PR) in one. In the ECD case, vemurafenib (a *BRAF* inhibitor) 480 mg twice daily was used as first line, leading to a very good partial response after 3 months. In the RDD case, an initial watch-and-wait strategy was adopted due to localized disease. Only one LCH patient (*MAP2K* mutated) relapsed and was treated in second line with trametinib (MEK/MAPK-pathway inhibitor) 2 mg daily; the dose was reduced to 0.5 mg daily due to severe toxicity (bilateral pleural effusion). The patient maintained partial response with complete toxicity resolution. After a median follow-up of 14 months (range 6–56), all patients were alive at last follow-up.

**Summary/Conclusion**

In this single-center series, histiocytic neoplasms showed a consistent enrichment of MAPK-pathway alterations (*MAP2K1*, *BRAF*, *KRAS*) across LCH and non-LCH, supporting systematic molecular profiling beyond *BRAF* p.(V600E) screening. Clinical presentation was heterogeneous, with diabetes insipidus as a sentinel symptom in many cases. Cytarabine achieved high response rates, and molecularly guided targeted therapy (BRAF/MEK inhibition) also provided meaningful disease control, supporting the use of genotype-driven treatment strategies in clinical practice.

**Copyright Information:** (Online) ISSN: 2572-9241
©2026 The Author(s). HemaSphere is published by John Wiley & Sons Ltd on behalf of European Hematology Association. This is an open access Abstract Book distributed under the Attribution-NonCommercial-NoDerivs (CC BY-NC-ND), which allows third parties to download the articles and share them with others as long as they credit the author and the Abstract Book, but they cannot change the content in any way or use them commercially.
**Abstract Book Citations:** Authors, Title, HemaSphere, 2026;10:(S1):pages. The abstract book DOIs can be found on the “Author Information” page.
**Disclaimer:** Articles published in the journal HemaSphere exclusively reflect the opinions of the authors. The authors are responsible for all content in their abstracts including accuracy of the facts, statements, citing resources, etc.

| Age at Dx | Sex | Disease type | Diagnostic sample | Clinical presentation | Organs involved | Molecular alteration | 1st-line treatment | Response | Relapse | 2nd-line treatment | Dose adjustment | Response (2L) | PFS (months) | OS (months) |
|---|---|---|---|---|---|---|---|---|---|---|---|---|---|---|
| 60 | F | LCH | Bone marrow (aspirate/biopsy) | Diabetes insipidus and Subcutaneous mass | Pituitary, bone, soft tissues | *MAP2K1* c.304_309del, p.(Glu102_Ile103del) 10.5% VAF | Cytarabine ×12 cycles | mCR | No | | | | 51 | 51 |
| 60 | F | LCH | Bone biopsy | Temporal mass | Bone | *MAP2K1* c.303_308del, p.(Glu102_Ile103del) 27% VAF | Cytarabine ×12 cycles | PR | Yes | Trametinib 2 mg/day | Yes, reduced to 0.5 mg/day | PR | 15 | 53 |
| 60 | M | LCH | Liver biopsy | Asthenia and myalgias | Lung, liver | Insufficient material | Cytarabine ×12 cycles | mCR | No | | | | 6 | 6 |
| 53 | F | non-LCH (ECD) | Bone biopsy | Diabetes insipidus, imbalance impairment, myalgias, fever | Pituitary, bone, skin | *BRAF* p.(Val600Glu) | Vemurafenib 480 mg twice daily | PR | No | | No | | 7 | 7 |
| 37 | F | non-LCH (RDD) | Breast biopsy | Breast mass | breast, thoracic lymph nodes | *KRAS* c.351A>T p.(Lys117Asn) 5.5% VAF | Watch-and-wait | - | | | | | 14 | 14 |

F, female; M, male, LCH, Langerhans cell histiocytosis; non-LCH, non–Langerhans cell histiocytosis; ECD, Erdheim–Chester disease; RDD, Rosai–Dorfman disease; VAF, variant allele frequency; mCR, metabolic complete response; PR, partial response; PET/CT, positron emission tomography/computed tomography.

**Copyright Information:** (Online) ISSN: 2572-9241
©2026 The Author(s). HemaSphere is published by John Wiley & Sons Ltd on behalf of European Hematology Association. This is an open access Abstract Book distributed under the Attribution-NonCommercial-NoDerivs (CC BY-NC-ND), which allows third parties to download the articles and share them with others as long as they credit the author and the Abstract Book, but they cannot change the content in any way or use them commercially.
**Abstract Book Citations:** Authors, Title, HemaSphere, 2026;10:(S1):pages. The abstract book DOIs can be found on the "Author Information" page.
**Disclaimer:** Articles published in the journal HemaSphere exclusively reflect the opinions of the authors. The authors are responsible for all content in their abstracts including accuracy of the facts, statements, citing resources, etc.

### (PB2537) LINKING IMMUNOTHERAPY AND CONGESTIVE HEART FAILURE: A PHARMACOVIGILANCE ANALYSIS

**Topic:** 25. Gene therapy, cellular immunotherapy and vaccination - Biology & translational research

Aditya Lakkamsani*[1], Nikhita Vemulapalli[1], Ronak Patel[1], Suhaib Hyder[1], Viktoriya Bikeyeva[1], Eli Ehrenpreis[1]

[1]*Advocate Lutheran General Hospital, Internal Medicine, Park Ridge, United States of America;*

### Background

Immune checkpoint inhibitors (ICIs) have greatly advanced the field of oncology- providing novel options for advanced disease and improving outcomes. They have also introduced new and varied side effects- specifically cardiovascular. Congestive heart failure (CHF) is a very common medical condition, with high mortality, that has been reported as an immunotherapy-associated side effect.

### Aims

The aim of this study is to determine if commonly used immunotherapy regimens have significant signaling of CHF using the U.S. Food and Drug Administration Adverse Event Reporting System (FAERS) database, a voluntary reporting system of adverse drug reactions (ADRs).

### Methods

The immunotherapies studied include PD-1 inhibitors (pembrolizumab, nivolumab, cemiplimab), PD-L1 inhibitors (atezolizumab, durvalumab, avelumab), CTLA-4 inhibitors (ipilimumab, tremelimumab), and combination therapies (nivolumab/ipilimumab, durvalumab/tremelimumab). 314,620 total ADRs were noted in the FAERS system for the aforementioned therapies between September 10, 2012 and January 15, 2026; with 644 total reports of CHF. The Reporting Odds Ratio (ROR) was the metric used to determine if signaling was present. ROR= (positive reports with drug/negative reports with drug)/(positive reports without drug/negative reports without drug). A ROR >1 is considered a positive signal for an ADR. 95% confidence intervals (CI), and p-values were calculated to assess the strength and significance of association between each therapy and CHF reporting. P-values were significant (≤0.05), unless stated otherwise.

**Copyright Information:** (Online) ISSN: 2572-9241
©2026 The Author(s). HemaSphere is published by John Wiley & Sons Ltd on behalf of European Hematology Association. This is an open access Abstract Book distributed under the Attribution-NonCommercial-NoDerivs (CC BY-NC-ND), which allows third parties to download the articles and share them with others as long as they credit the author and the Abstract Book, but they cannot change the content in any way or use them commercially.
**Abstract Book Citations:** Authors, Title, HemaSphere, 2026;10:(S1):pages. The abstract book DOIs can be found on the "Author Information" page.
**Disclaimer:** Articles published in the journal HemaSphere exclusively reflect the opinions of the authors. The authors are responsible for all content in their abstracts including accuracy of the facts, statements, citing resources, etc.

**Results**

Nivolumab/ipilimumab (ROR 4.52, 95% CI 3.83-5.33) and durvalumab/tremelimumab (ROR 17.9, 95% CI 14.27-22.25) combinations showed disproportionately higher signaling. PD-1 inhibitors pembrolizumab (ROR 0.67, 95% CI 0.56-0.80) and nivolumab (ROR 0.64, 95% CI 0.53-0.77) showed lower signaling. Cemiplimab had no CHF reports. PD-L1 inhibitors atezolizumab (ROR 0.66, 95% CI 0.49-0.88) and durvalumab (ROR 0.54, 95% CI 0.34-0.86) showed lower signaling. Avelumab did not show significant signaling. The CTLA-4 inhibitor ipilimumab (ROR 0.52, 95% CI 0.38-0.70) showed lower signaling, while tremelimumab did not show significant signaling. Class differences were noted with lower signaling in PD-1 inhibitors (ROR 0.65, 95% CI 0.57-0.74), PD-L1 inhibitors (ROR 0.61, 95% CI 0.48-0.78), and CTLA-4 inhibitors (ROR 0.52, 95% CI 0.39-0.70) while combination therapies (ROR 5.89, 95% CI 5.14-6.75) had higher overall signaling.
(See Table 1)

Table 1:

| **Therapy** | **Reports** | **ROR** | **95% CI** | **p-value** |
|---|---|---|---|---|
| Pembrolizumab | 127 | 0.67 | 0.56-0.80 | 0.0001 |
| Nivolumab | 119 | 0.64 | 0.53-0.77 | 0.0001 |
| Cemiplimab | 0 | 0 | 0 | 0 |
| Atezolizumab | 47 | 0.66 | 0.49-0.88 | 0.0049 |
| Durvalumab | 18 | 0.54 | 0.34-0.86 | 0.0094 |
| Avelumab | 4 | 0.51 | 0.19-1.37 | 0.1823 |
| Ipilimumab | 43 | 0.52 | 0.38-0.70 | 0.0001 |
| Tremelimumab | 4 | 0.57 | 0.21-1.53 | 0.2661 |
| Nivolumab/ipilimumab | 198 | 4.52 | 3.83-5.33 | 0.0001 |
| Durvalumab/tremelimumab | 84 | 17.9 | 14.27-22.45 | 0.0001 |
| **Class** | **Reports** | **ROR** | **95% CI** | **p-value** |
| PD-1 | 246 | 0.65 | 0.57-0.74 | 0.0001 |
| PD-L1 | 69 | 0.61 | 0.48-0.78 | 0.0001 |
| CTLA-4 | 47 | 0.52 | 0.39-0.70 | 0.0001 |
| Combination | 282 | 5.89 | 5.14-6.75 | 0.0001 |

**Copyright Information:** (Online) ISSN: 2572-9241
©2026 The Author(s). HemaSphere is published by John Wiley & Sons Ltd on behalf of European Hematology Association. This is an open access Abstract Book distributed under the Attribution-NonCommercial-NoDerivs (CC BY-NC-ND), which allows third parties to download the articles and share them with others as long as they credit the author and the Abstract Book, but they cannot change the content in any way or use them commercially.
**Abstract Book Citations:** Authors, Title, HemaSphere, 2026;10:(S1):pages. The abstract book DOIs can be found on the “Author Information” page.
**Disclaimer:** Articles published in the journal HemaSphere exclusively reflect the opinions of the authors. The authors are responsible for all content in their abstracts including accuracy of the facts, statements, citing resources, etc.

**Summary/Conclusion**

Both class and individual differences were noted by the data- with monotherapies showing overall reduced signaling. Combination ICI regimens, on the other hand, demonstrated significantly higher CHF signaling, most notably durvalumab/tremelimumab. These findings suggest that more extensive cardiac screening and monitoring may be indicated in patients starting combination ICI therapy and that further study is warranted.

**Copyright Information:** (Online) ISSN: 2572-9241
©2026 The Author(s). HemaSphere is published by John Wiley & Sons Ltd on behalf of European Hematology Association. This is an open access Abstract Book distributed under the Attribution-NonCommercial-NoDerivs (CC BY-NC-ND), which allows third parties to download the articles and share them with others as long as they credit the author and the Abstract Book, but they cannot change the content in any way or use them commercially.
**Abstract Book Citations:** Authors, Title, HemaSphere, 2026;10:(S1):pages. The abstract book DOIs can be found on the “Author Information” page.
**Disclaimer:** Articles published in the journal HemaSphere exclusively reflect the opinions of the authors. The authors are responsible for all content in their abstracts including accuracy of the facts, statements, citing resources, etc.

### (PB4097) DEVELOPMENT OF A CETUXIMAB MEDITOPE-BASED SAFETY MODULE FOR AN ANTI-BCMA CAR-T CELL PRODUCT

**Topic:** 25. Gene therapy, cellular immunotherapy and vaccination - Biology & translational research

Ekaterina Fefelova*[1], Nataliia Ivanova[1], Daniil Chistyakov[1], Dmitry Dianov[1], Tatiana Nenasheva[1], Yana Serdyuk[1], Olga Maryakhina[1], Apollinaria Bogoliubova[1]

[1]*National Medical Research Center for Hematology, Moscow, Russia;*

### Background

Anti-BCMA CAR-T cell therapy has demonstrated remarkable success in treating relapsed/refractory multiple myeloma (r/r MM), but managing severe toxicities such as cytokine release syndrome (CRS) or secondary CAR+ malignancies remains a critical challenge. Designing structural elements for targeted CAR-T cell depletion *in vivo* using clinically available antibodies represents a compelling strategy to enhance the safety profile of CAR-T therapies.

### Aims

To engineer a cetuximab meditope-based safety module to enhance anti-BCMA CAR-T therapy management

### Methods

We generated two second-generation anti-BCMA CAR constructs with differences in antigen-recognition domains: one with a conventional scFv and another with three cetuximab meditopes within the scFv sequence. CAR-T cell products were produced via lentiviral transduction of CD3+ T cells isolated from healthy donors. The potency of both CAR-T products was rigorously compared *in vitro*. We evaluated transduction efficacy, expansion ratio and cytotoxic activity against a BCMA-expressing Jurkat cell line. Next, a long-term co-culture assay was performed to evaluate the dynamics of T cell phenotype changes as well as expression of exhaustion markers (PD-1, TIM3, LAG3) and IFNγ production during repeated antigen exposure. Finally, the efficacy of CAR+ T cells depletion was assessed in an antibody-dependent cellular cytotoxicity (ADCC) assay upon cetuximab administration.

### Results

Our data demonstrate that cetuximab meditope utilization does not compromise the core functionality of anti-BCMA CAR-T cell products. Meditope-incorporating anti-BCMA CAR-T cells exhibited expansion rates and transduction efficacy comparable to canonical ones. Their potency such as cytotoxic activity as well as IFNγ production were fully preserved throughout long-term co-culture assay. Notably, meditope-incorporating CAR-T cells displayed a favourable immunophenotype profile, showing a trend toward a reduced frequency of terminal effector cells (Eff) during repeated antigen exposure. Additionally, we confirmed effective NK cell-mediated depletion of CAR-T cells in the presence of cetuximab.

### Summary/Conclusion

We have successfully developed a novel anti-BCMA CAR-T cell product featuring a meditope-incorporating scFv which exhibits a favorable functional profile and can be effectively depleted in the presence of cetuximab. This approach offers a practical safety solution for managing serious adverse events, such as severe CRS or CAR+ secondary malignancies.

**Copyright Information:** (Online) ISSN: 2572-9241
©2026 The Author(s). HemaSphere is published by John Wiley & Sons Ltd on behalf of European Hematology Association. This is an open access Abstract Book distributed under the Attribution-NonCommercial-NoDerivs (CC BY-NC-ND), which allows third parties to download the articles and share them with others as long as they credit the author and the Abstract Book, but they cannot change the content in any way or use them commercially.
**Abstract Book Citations:** Authors, Title, HemaSphere, 2026;10:(S1):pages. The abstract book DOIs can be found on the "Author Information" page.
**Disclaimer:** Articles published in the journal HemaSphere exclusively reflect the opinions of the authors. The authors are responsible for all content in their abstracts including accuracy of the facts, statements, citing resources, etc.

### (PB4098) FUNCTIONAL EVALUATION OF CAR-T CELLS WITH LOW-AFFINITY ANTI-CD19 RECEPTOR: ACTIVITY, CYTOTOXICITY AND MODULATION BY IBRUTINIB

**Topic:** 25. Gene therapy, cellular immunotherapy and vaccination - Biology & translational research

Anna Serdyuk*[1], Dmitry Dianov[1], Ekaterina Fefelova[1], Nataliia Ivanova[1], Tatiana Nenasheva[1], Konstantin Baranov[2], Apollinaria Bogoliubova[1]

*[1]National Medical and Research Center for Hematology, Moscow, Russia;[2]Institute of Molecular and Cellular Biology SB RAS, Novosibirsk, Russia;*

### Background

The majority of approved anti-CD19 CAR-T therapies utilize the FMC63-derived scFv as an antigen-recognition domain. However, the affinity of the antigen-recognition domain is a critical parameter influencing CAR signaling, tonic activity and subsequent propensity toward T-cell exhaustion. Low-affinity scFv may offer a unique functional profile, which may significantly affect therapy efficacy and safety. Moreover, pharmacological agents such as ibrutinib could potentially attenuate tonic signaling and preserve CAR-T cell functional fitness, thereby improving overall product quality.

### Aims

To investigate the functional properties of CAR-T cells engineered with a  novel low-affinity anti-CD19 scFv (XS6) and to evaluate the potential of ibrutinib to mitigate the tonic signaling and to improve the CAR-T product quality.

### Methods

We compared the affinity and kinetic parameters of canonical (FMC63) and low-affinity (XS6) scFvs using Bio-Layer Interferometry. Using GoldenGate assembly we generated six second-generation CAR constructs combining two antigen-recognition domains (FMC63/XS6) with three costimulatory domains (CD28/OX40/4-1BB). Then we assessed the rate of CAR-mediated activation using CAR-transduced Jurkat E6.1 triple-reporter cell lines. To assess phenotype and cytotoxic function, CAR-T products were generated from healthy donor CD3+ T cells, and cytotoxicity and proliferation were examined in short- (2-day) and long-term (12-day) assays against CD19+ target cells. Finally we assessed the ibrutinib effect by adding it during activation or expansion steps of manufacturing and further characterisation of immunophenotype and cytotoxic properties of CAR-T products.

### Results

XS6 displayed substantially lower affinity to CD19 compared with FMC63 (KD 2,39x10-6 M and 2,17x10-8 M, respectively), along with distinct association/dissociation rates. Moreover, XS6 CAR-T cells exhibited higher activation levels than FMC63 CAR-T cells regardless of the costimulatory domain, and comparable phenotypic characteristics. This translated to superior cytotoxic activity and IFNγ production of XS6 CAR-T cells within short-term and long-term cytotoxicity assays. Exposure to ibrutinib significantly reduced activation in both FMC63 and XS6 CAR-T cells, together with decreased expression of exhaustion markers (LAG3, PD1) and activation markers (CD25, Ki67), indicating successful attenuation of tonic signalling and improved CAR-T product quality.

### Summary/Conclusion

We present a novel low-affinity anti-CD19 CAR (XS6), that drives potent T cell activation and robust cytotoxicity. We demonstrate that ibrutinib may effectively mitigate the excessive activation and reduce the expression of exhaustion markers, offering a promising strategy to improve the safety and functional fitness of CAR-T products.

**Copyright Information:** (Online) ISSN: 2572-9241
©2026 The Author(s). HemaSphere is published by John Wiley & Sons Ltd on behalf of European Hematology Association. This is an open access Abstract Book distributed under the Attribution-NonCommercial-NoDerivs (CC BY-NC-ND), which allows third parties to download the articles and share them with others as long as they credit the author and the Abstract Book, but they cannot change the content in any way or use them commercially.
**Abstract Book Citations:** Authors, Title, HemaSphere, 2026;10:(S1):pages. The abstract book DOIs can be found on the "Author Information" page.
**Disclaimer:** Articles published in the journal HemaSphere exclusively reflect the opinions of the authors. The authors are responsible for all content in their abstracts including accuracy of the facts, statements, citing resources, etc.

### (PB4099) TARGETING ALTERNATIVE B-CELL ANTIGENS TO OVERCOME CD19 CAR-T RESISTANCE IN PRECLINICAL MODELS OF ALL AND LYMPHOMA

**Topic:** 25. Gene therapy, cellular immunotherapy and vaccination - Biology & translational research

Agata Braniewska*[1], Mikołaj Jacyno[1, 2], Magdalena Winiarska[1], Marta Krawczyk[1, 2]

*[1]Mossakowski Medical Research Institute, Polish Academy Of Sciences, Department of Immunology, Warsaw, Poland;[2]Doctoral School of Translational Medicine, Mossakowski Medical Research Institute, Polish Academy of Sciences, Centre of Postgraduate Medical Education, Warsaw, Poland;*

### Background

T cells modified to express chimeric antigen receptors (CARs) directed against the B-cell surface protein CD19 (CD19 CAR-T) are now emerging as a standard treatment option for individuals with relapsed or refractory non-Hodgkin lymphoma (NHL) or acute lymphoblastic leukemia (ALL). Nevertheless, the overall effectiveness of this therapy is substantially limited by acquired resistance that occurs in the course of the treatment. One of the predominant mechanisms underlying relapse is antigen escape, characterized by loss or downregulation of CD19 expression on malignant cells.
Consequently, considerable efforts are being directed toward the identification and development of alternative therapeutic strategies targeting other B-cell–associated antigens to overcome resistance and improve outcomes in patients relapsing after CD19-directed CAR-T therapy.

### Aims

The aim of this study is to evaluate alternative CAR-T cell strategies in preclinical models of CD19 CAR-T–resistant B-cell lymphoma and B-ALL in order to identify effective salvage strategies for the treatment of relapse following CD19-directed CAR-T therapy.

### Methods

We used in-house preclinical *in vitro* models of CD19 CAR-T–resistant B-cell lymphoma (Raji) and B-ALL (Nalm-6) exposed to either 4-1BB– or CD28–FMC63–based CD19 CAR-T cells generated in our laboratory. Resistant cell lines were screened by flow cytometry for the expression of various surface markers, including CD20, CD22, and CD37. Corresponding CAR-T cells were produced by retroviral transduction of primary T cells isolated from buffy coats of healthy donors. CAR-T constructs were provided by Sébastien Wälchli from the Department of Cellular Therapy, Translational Research Unit, Oslo University Hospital. Cytotoxicity, degranulation, and cytokine production by CAR-T cells were evaluated using flow cytometry–based assays.

### Results

We observed that all tested alternative CAR-T targets (CD20, CD22, CD37) exhibited cytotoxic activity against CD19 CAR-T–resistant Raji and Nalm-6 cells. Interestingly, the efficacy of CAR-T treatment did not always strictly correlate with the expression of the target antigen. For example, irrespective of CD37 expression, CD37 CAR-T cells were less potent in killing Nalm-6 cells previously exposed to 4-1BB–based CD19 CAR-T compared with those exposed to CD28–based CD19 CAR-T cells. Our results suggest that mechanisms other than antigen loss can contribute to the resistance of tumor cells exposed to 4-1BB–based CD19 CAR-T.

**Copyright Information:** (Online) ISSN: 2572-9241
©2026 The Author(s). HemaSphere is published by John Wiley & Sons Ltd on behalf of European Hematology Association. This is an open access Abstract Book distributed under the Attribution-NonCommercial-NoDerivs (CC BY-NC-ND), which allows third parties to download the articles and share them with others as long as they credit the author and the Abstract Book, but they cannot change the content in any way or use them commercially.
**Abstract Book Citations:** Authors, Title, HemaSphere, 2026;10:(S1):pages. The abstract book DOIs can be found on the “Author Information” page.
**Disclaimer:** Articles published in the journal HemaSphere exclusively reflect the opinions of the authors. The authors are responsible for all content in their abstracts including accuracy of the facts, statements, citing resources, etc.

**Summary/Conclusion**

Targeting CD20, CD22, or CD37 via CAR-T cells is a promising therapeutic option for patients with B-cell malignancies resistant to CD19 CAR-T. However, more detailed studies on intrinsic vulnerabilities of tumor cells to CAR-T cells are warranted to better design salvage therapies for relapsing patients.

**Copyright Information:** (Online) ISSN: 2572-9241
©2026 The Author(s). HemaSphere is published by John Wiley & Sons Ltd on behalf of European Hematology Association. This is an open access Abstract Book distributed under the Attribution-NonCommercial-NoDerivs (CC BY-NC-ND), which allows third parties to download the articles and share them with others as long as they credit the author and the Abstract Book, but they cannot change the content in any way or use them commercially.
**Abstract Book Citations:** Authors, Title, HemaSphere, 2026;10:(S1):pages. The abstract book DOIs can be found on the “Author Information” page.
**Disclaimer:** Articles published in the journal HemaSphere exclusively reflect the opinions of the authors. The authors are responsible for all content in their abstracts including accuracy of the facts, statements, citing resources, etc.

### (PB4100) THE EFFECT OF TYROSINE KINASE INHIBITORS ON THE FUNCTIONAL AND PHENOTYPIC CHARACTERISTICS OF ANTI-CD19 CAR-T CELL PRODUCT

**Topic:** 25. Gene therapy, cellular immunotherapy and vaccination - Biology & translational research

Valeriia Grabareva*[1], Ekaterina Fefelova[1], Tatiana Nenasheva[1], Nataliia Ivanova[1], Olga Aleshina (Gavrilina)[1], Apollinaria Bogoliubova[1]

[1]*National Medical Research Center for Hematology, Laboratory of Translational Immunology, Moscow, Russia;*

### Background

Patients with Philadelphia chromosome–positive (Ph+) B-cell acute lymphoblastic leukemia (B-ALL) or lymphoid blast crisis of chronic myeloid leukemia (CML) receive continuous tyrosine kinase inhibitor (TKI) therapy prior to being considered for CAR-T cell treatment. Despite limited experimental evidence, TKI washout period is commonly required prior to leukapheresis and CAR-T cell manufacturing [1].
TKIs may also been used during CAR-T production due to their ability to modulate T-cell proliferation and differentiation. In patients with diffuse large B-cell lymphoma (DLBCL), leukapheresis material and CAR-T products are often enriched with poorly functional terminally differentiated effector T cells, which is associated with reduced therapeutic efficacy. By non-specifically inhibiting T-cell signalling pathways, TKIs may promote the manufacturing of CAR-T products with a favourable phenotypic composition.

### Aims

To evaluate the impact of TKI exposure on the functional and phenotypic characteristics of anti-CD19 CAR-T cell products.

### Methods

The study was conducted in two parts. First, we enrolled seven patients (4 B-ALL, 3 CML) receiving continuous TKI therapy with either dasatinib or ponatinib. Blood samples were collected before (Cmin) and 2 hours after (Cmax) daily TKI administration. T lymphocytes were used to manufacture second-generation anti-CD19 CAR-T cells (CD28-CD3ζ). We assessed transduction efficiency, expansion, cytotoxicity against CD19+ NALM6 cells, and immunophenotype at key manufacturing stages. In a second experimental series, T cells from six DLBCL patients were used to generate CAR-T cells under a modified protocol, with ponatinib (10, 20, or 200 nM) added during the expansion step. The resulting products were characterized for expansion, transduction, and phenotypic changes.

### Results

Our findings indicate that T cells obtained during ongoing TKI therapy retained full capacity for activation, transduction, and expansion with no manufacturing failures. The final cellular products were enriched in naïve and central memory T-cell subsets and displayed robust *in vitro* cytotoxicity against CD19+ target cells, suggesting that prior TKI exposure does not compromise CAR-T product quality. The addition of ponatinib during transduction of T-cells from DLBCL patients induced a dose-dependent reduction in expansion but increased the proportion of naïve CAR-T cells, accompanied by a trend toward a reduced CD4/CD8 ratio.

**Copyright Information:** (Online) ISSN: 2572-9241
©2026 The Author(s). HemaSphere is published by John Wiley & Sons Ltd on behalf of European Hematology Association. This is an open access Abstract Book distributed under the Attribution-NonCommercial-NoDerivs (CC BY-NC-ND), which allows third parties to download the articles and share them with others as long as they credit the author and the Abstract Book, but they cannot change the content in any way or use them commercially.
**Abstract Book Citations:** Authors, Title, HemaSphere, 2026;10:(S1):pages. The abstract book DOIs can be found on the "Author Information" page.
**Disclaimer:** Articles published in the journal HemaSphere exclusively reflect the opinions of the authors. The authors are responsible for all content in their abstracts including accuracy of the facts, statements, citing resources, etc.

**Summary/Conclusion**

Our results challenge the current requirements for TKI washout prior to leukapheresis, demonstration that prior TKI therapy does not negatively affect activation, transduction, or cytotoxicity of anti-CD19 CAR-T cells. Moreover, incorporating TKIs into manufacturing process may improve the phenotypic composition of CAR-T cell products resulting the better quality of the final cell product.

**Copyright Information:** (Online) ISSN: 2572-9241
©2026 The Author(s). HemaSphere is published by John Wiley & Sons Ltd on behalf of European Hematology Association. This is an open access Abstract Book distributed under the Attribution-NonCommercial-NoDerivs (CC BY-NC-ND), which allows third parties to download the articles and share them with others as long as they credit the author and the Abstract Book, but they cannot change the content in any way or use them commercially.
**Abstract Book Citations:** Authors, Title, HemaSphere, 2026;10:(S1):pages. The abstract book DOIs can be found on the "Author Information" page.
**Disclaimer:** Articles published in the journal HemaSphere exclusively reflect the opinions of the authors. The authors are responsible for all content in their abstracts including accuracy of the facts, statements, citing resources, etc.

### (PB4101) EX VIVO EFFECTS OF EXTRACORPOREAL PHOTOPHERESIS ON PATIENT-DERIVED MONONUCLEAR CELLS (MNC) FOLLOWING CAR T-CELL THERAPY

**Topic:** 25. Gene therapy, cellular immunotherapy and vaccination - Biology & translational research

Paula Renauld*[1, 2], Sabine Seiffert[3], Victoria Menger[1, 2], Maik Friedrich[3], Anja Grahnert[3], David Reher[1, 2], Enrica Bach[1, 2], Beatrice Berneck[1, 2], Carmen Diana Herling[1, 2], Georg-Nikolaus Franke[1, 2], Madlen Jentzsch[1, 2], Klaus Metzeler[1, 2], Ulrike Koehl[3, 4], Andreas Boldt[3], Ulrich Sack[3], Uwe Platzbecker[1, 2, 5], Marco Herling[1, 2], Janine Kirchberg[1, 2], Vladan Vucinic[1, 2, 4]

*[1]University Leipzig, Department for Hematology, Cellular Therapy, Hemostaseology and Infectious Diseases, Leipzig, Germany; [2]Comprehensive Cancer Center Central Germany, Leipzig, Germany; [3]Institute for Clinical Immunology, Leipzig, Germany; [4]Fraunhofer Institute for Celltherapy and Immunology, Leipzig, Germany; [5]Technical University Dresden, Dresden, Germany;*

### Background

Chimeric Antigen Receptor (CAR) T-cell therapy has revolutionized the treatment of B-cell non-Hodgkin lymphoma (NHL) but remains limited by severe inflammatory side-effects, including cytokine release syndrome (CRS) and immune effector cell-associated neurotoxicity syndrome (ICANS). Sustained CAR-T cell expansion has also been linked to delayed on-target off-tumor late toxicities (e.g., non-ICANS neurotoxicity). Current management relies on IL-6 blockade and corticosteroids, which may increase non-relapse morbidity and infectious complications. Strategies capable of mitigating excessive immune activation without compromising antitumor function are therefore needed. Extracorporeal photopheresis (ECP) represents a potential immunomodulatory approach in this context.

### Aims

To investigate whether ECP has selective immunomodulatory in-vitro effects on patient-derived MNCs following CAR T-cell therapy by modulating proliferative and inflammatory responses while preserving cytotoxic function.

### Methods

Twenty-four patients undergoing CD19-directed CAR T-cell therapy were included (median age 65.5 years; 75% had DLBCL). Patients received axicabtagene ciloleucel (N=19), tisagenlecleucel (N=3), or lisocabtagene maraleucel (N=2) following fludarabine/cyclophosphamide lymphodepletion. Peripheral blood was collected a median of +14 days post-infusion; additional samples for cytokine analyses were obtained pre-lymphodepletion and on day +34. Ex vivo ECP was performed using 8-methoxypsoralen (340 ng/mL) and UVA irradiation (2 J/cm$^2$); untreated paired aliquots from the same patients served as controls. Mononuclear cells were cultured ≥48 hours prior to analysis. T-cell proliferative responses were assessed under CD3/CD28 stimulation by VPD450 dilution (n=3) and phosphorylated STAT5 (pSTAT5; N=7). Cytotoxicity was evaluated in 24-hour co-culture assays with CD19$^+$ Raji cells (E:T 6.7:1; N=7). T-cell subset composition was analyzed longitudinally (0-72 h; N=15). All data were acquired by flow cytometry (FACSLyric™, BD Biosciences). Pro-inflammatory, anti-inflammatory, activation-associated, antiviral cytokines and chemokine CCL2 were quantified by multiplex bead-based immunoassay.

Copyright Information: (Online) ISSN: 2572-9241
©2026 The Author(s). HemaSphere is published by John Wiley & Sons Ltd on behalf of European Hematology Association. This is an open access Abstract Book distributed under the Attribution-NonCommercial-NoDerivs (CC BY-NC-ND), which allows third parties to download the articles and share them with others as long as they credit the author and the Abstract Book, but they cannot change the content in any way or use them commercially.
**Abstract Book Citations:** Authors, Title, HemaSphere, 2026;10:(S1):pages. The abstract book DOIs can be found on the “Author Information” page.
**Disclaimer:** Articles published in the journal HemaSphere exclusively reflect the opinions of the authors. The authors are responsible for all content in their abstracts including accuracy of the facts, statements, citing resources, etc.

### Results

After a median follow-up of 7.06 months [95% CI: 3.35-10.6], median progression-free survival was 8.97 months, and median overall survival was not reached (12 months overall survival estimated as 84.7% with 4 patients still at risk and 2 observed events). 11 (46%) patients developed CRS and 5 (21%) ICANS. Across paired samples, ECP exposure was associated with an estimated 45.3-fold reduction of in-vitro proliferation (Wilcoxon signed rank (WSR) p=0.002, 95% CI: 24.0 to 113.6-fold reduction). We found no evidence for differences in CD4$^{+}$/CD8$^{+}$/CAR$^{+}$/Treg composition or cytokine profiles; preliminary analyses suggest lower production of inflammatory cytokines (CCL2, IL-6, IL-1beta, TNF-alpha) and the growth factor IL-2 after ECP treatment at d+34 post-infusion. Short-term cytotoxic activity was comparable between conditions (WSR p= 0.94, estimated fold change: 0.28 [-3.21-4.66]).

### Summary/Conclusion

ECP exposure was associated with attenuated proliferative signaling and expansion without detectable impairment of immediate cytotoxic function. These findings suggest that modulation of proliferative dynamics may represent a targetable axis to mitigate expansion-associated toxicities while preserving antitumor activity. Clinical data are required to determine the translational relevance of ECP in this setting.

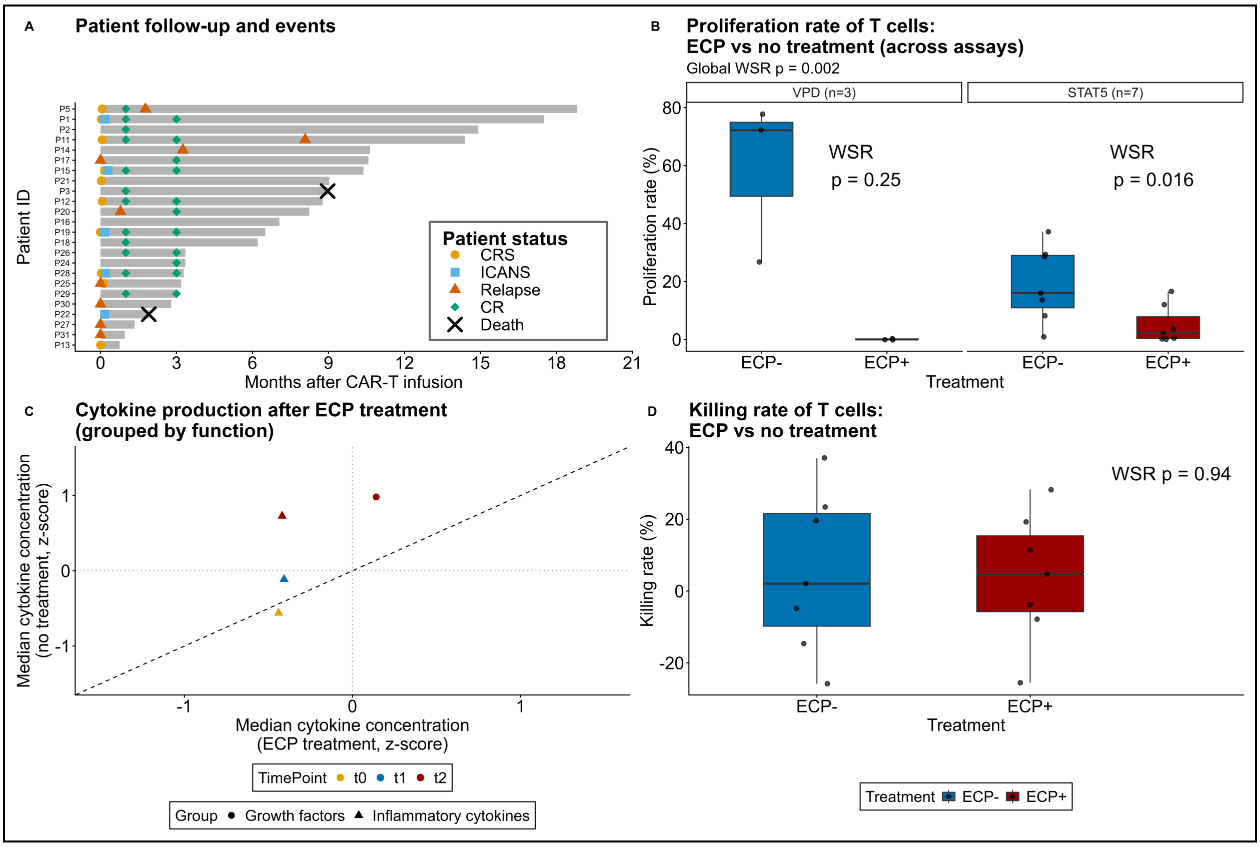

**Copyright Information:** (Online) ISSN: 2572-9241
©2026 The Author(s). HemaSphere is published by John Wiley & Sons Ltd on behalf of European Hematology Association. This is an open access Abstract Book distributed under the Attribution-NonCommercial-NoDerivs (CC BY-NC-ND), which allows third parties to download the articles and share them with others as long as they credit the author and the Abstract Book, but they cannot change the content in any way or use them commercially.
**Abstract Book Citations:** Authors, Title, HemaSphere, 2026;10:(S1):pages. The abstract book DOIs can be found on the “Author Information” page.
**Disclaimer:** Articles published in the journal HemaSphere exclusively reflect the opinions of the authors. The authors are responsible for all content in their abstracts including accuracy of the facts, statements, citing resources, etc.

### (PB4102) METABOLIC REMODELLING UNDER THE STRESS OF EX VIVO CULTURE OF HUMAN HAEMATOPOIETIC STEM CELLS

**Topic:** 25. Gene therapy, cellular immunotherapy and vaccination - Biology & translational research

Wenjuan MA*[1, 2], Maya Lopez[1, 2], Angelica Varesi[3, 4], Helen Demollin[5, 6], Maria-Eleni Lalioti[5, 6], Tomoya Isobe[1, 2], Yuhui Zhou[1, 2], Yurui Sun[1, 2], Hugo Bastos[1, 2], Yuran Wang[1, 2], Carys Johnson[1, 2], Berthold Gottgens[1, 2], Stephanie Xie[4, 7], John Dick[3, 4], Nina Cabezas[5, 6], Elisa Laurenti[1, 2]

*[1]Cambridge Stem Cell Institute, University of Cambridge, Cambridge, United Kingdom; [2]Department of Haematology, University of Cambridge, Cambridge, United Kingdom; [3]Department of Molecular Genetics, University of Toronto, Toronto, Canada; [4]Princess Margaret Cancer Centre, University Health Network, Toronto, Canada;[5]Max-Planck-Institute of Immunobiology and Epigenetics, Freiburg, Germany; [6]Laboratory of Stem Cell Biology and Ageing, Department of Health Sciences and Technology, Swiss Federal Institute of Technology (ETH Zürich), Zürich, Switzerland; [7]Department of Medical Biophysics, University of Toronto, Toronto, Canada;*

### Background

HSC-based Gene Therapy (GT) fundamentally requires *ex vivo* culture of hematopoietic stem cells (HSCs). However, a major clinical challenge for HSC GT arises from the loss of HSC function during *ex vivo* culture, posing a high risk of delayed or failed blood recovery in patients. Recent work from our laboratory has demonstrated that more than 66% of functional HSCs are lost within the early adaptation to *ex vivo* culture (approximately 24 hours) with significant transcriptional remodelling in genes linked to metabolism (Johnson et al, 2024).

### Aims

Metabolic switches are known key regulators of HSC function *in vivo,* but if and how they globally contribute to early loss of human HSCs function during *ex vivo* culture is less well understood.

### Methods

Here we have combined scRNAseq and low-input metabolomics to generate a kinetically resolved predictive model of metabolomic switches occurring during the first division of human HSC *ex vivo* in GT culture conditions and upon induction of differentiation.

### Results

We identify that metabolic shifts occur dynamically over 72 hours of culture but are most pronounced in the first 24 hours and are independent of cell cycle progression. Flux through one carbon metabolism was dynamically changed in the first 24 hours of culture. Pharmacological inhibition of key one-carbon metabolism enzymes changed HSC and progenitor survival, cell cycle and regenerative capacity *ex vivo.*

### Summary/Conclusion

Collectively, our data contribute to a better understanding of the interplay between stem cell stress, metabolism, and regeneration during clinically relevant human HSC culture, and will aid in the identification of new preclinical strategies to improve HSC function *ex vivo.*

**Copyright Information:** (Online) ISSN: 2572-9241
©2026 The Author(s). HemaSphere is published by John Wiley & Sons Ltd on behalf of European Hematology Association. This is an open access Abstract Book distributed under the Attribution-NonCommercial-NoDerivs (CC BY-NC-ND), which allows third parties to download the articles and share them with others as long as they credit the author and the Abstract Book, but they cannot change the content in any way or use them commercially.
**Abstract Book Citations:** Authors, Title, HemaSphere, 2026;10:(S1):pages. The abstract book DOIs can be found on the "Author Information" page.
**Disclaimer:** Articles published in the journal HemaSphere exclusively reflect the opinions of the authors. The authors are responsible for all content in their abstracts including accuracy of the facts, statements, citing resources, etc.

### (PB4103) CD19 MUTATIONAL LANDSCAPE OF TUMOR CELLS RESISTANT TO CD19-4-1BB CAR-T THERAPY

**Topic:** 25. Gene therapy, cellular immunotherapy and vaccination - Biology & translational research

Marta Krawczyk*[1, 2, 3], Klaudyna Fidyt[2, 4], Narcis Fernandez-Fuentes[4], Monika Pepek[2, 5], Ana Gutiérrez-Fernández[6, 7], Pablo Bousquets-Muñoz[6, 7], Jacinta Davis[8], Andrei Thomas-Tikhonenko[8, 9], Xose S. Puente[6, 7], Magdalena Winiarska[1, 2]

*[1]Mossakowski Medical Research Institute, Polish Academy of Sciences, Department of Immunology, Warsaw, Poland;[2]Medical University of Warsaw, Department of Immunology, Warsaw, Poland; [3]Doctoral School of Translational Medicine, Mossakowski Medical Research Institute, Polish Academy of Sciences, Centre of Postgraduate Medical Education, Warsaw, Poland; [4]Josep Carreras Leukaemia Research Institute, Stem cell biology, developmental leukemia and immunotherapy group, Barcelona, Spain; [5]University of Rzeszow, Department of Hematology, Faculty of Medicine, Warsaw, Poland;[6]Universidad de Oviedo, Departamento de Bioquímica y Biología Molecular, Instituto Universitario de Oncología (IUOPA), Oviedo, Spain; [7]Centro de Investigación Biomédica en Red de Cáncer (CIBERONC), Madrid, Spain; [8]Children's Hospital of Philadelphia, Division of Cancer Pathobiology, Philadelphia, United States of America; [9]University of Pennsylvania, Department of Pathology & Laboratory Medicine, Philadelphia, United States of America;*

### Background

CD19 CAR-T cell therapy has revolutionized the treatment of relapsed/refractory (r/r) B-cell malignancies. Approved CAR-T products use 4-1BB or CD28 as signaling platforms. Although CD19 loss is a major cause of resistance, the effect of the CAR costimulatory domain on *CD19* genetic changes in tumor cells is unclear. Here, using in vitro resistance models, we directly compare 4-1BB and CD28 CARs in the context of *CD19* mutation–driven resistance.

### Aims

Evaluation of *CD19* gene changes in tumor cells resistant to CD19 CAR-T therapy.

### Methods

In vitro resistance models were obtained by repeatedly (for 19 cycles) exposing Nalm-6 and Raji cells to CD19(FMC63) CAR-T cells with either 4-1BB or CD28 costimulatory domain. Models were characterized via cytotoxicity assays, flow cytometry (FC), and RT-qPCR. RNA next-generation sequencing (RNAseq), Oxford Nanopore Technologies long-read sequencing (ONT-LR-seq), limited dilution cloning, and amplicon-based NGS were used to identify *CD19* genetic changes.

### Results

The results show that CD19-4-1BB CAR-T cells induced complete resistance of tumor B cells, whereas CD19-CD28 CAR-T cells only partially reduced tumor sensitivity. Epitope-specific antibody analysis confirmed total CD19/FMC63-specific loss with 4-1BB CAR-T and moderate reduction with CD28 CAR-T cells. Transcriptomic analysis revealed that resistance in 4-1BB-exposed cells, but not in CD28-treated cells, was driven by *CD19* mutations, loss of heterozygosity (LOH), and alternative splicing (intron 2/6 retention and exon 2 skipping). RNAseq identified a high-VAF frameshift mutation (in exon 3) with LOH in the Raji-4-1BB model, and frameshift (in exon 2) plus missense (R163H) mutations in Nalm-6-4-1BB. Using the limited dilution cloning method, we confirmed that both mutations in Nalm-6-4-1BB cells are heterozygous and affect both alleles of the *CD19* gene. Amplicon-based ultra-deep NGS revealed that both mutations in the Nalm-6-4-1BB model were detectable at ~2% VAF after 14 cycles and dominated the population after 16 cycles. Additionally, we observed that all discovered mutations disrupted the FMC63 epitope and impaired CD19 detection by FC and immunohistochemistry. Moreover, *CD19* aberrations identified in our in vitro models were also reported in r/r B-ALL and lymphoma patients relapsed following CD19-4-1BB CAR-T therapy.

**Copyright Information:** (Online) ISSN: 2572-9241
©2026 The Author(s). HemaSphere is published by John Wiley & Sons Ltd on behalf of European Hematology Association. This is an open access Abstract Book distributed under the Attribution-NonCommercial-NoDerivs (CC BY-NC-ND), which allows third parties to download the articles and share them with others as long as they credit the author and the Abstract Book, but they cannot change the content in any way or use them commercially.
**Abstract Book Citations:** Authors, Title, HemaSphere, 2026;10:(S1):pages. The abstract book DOIs can be found on the "Author Information" page.
**Disclaimer:** Articles published in the journal HemaSphere exclusively reflect the opinions of the authors. The authors are responsible for all content in their abstracts including accuracy of the facts, statements, citing resources, etc.

**Summary/Conclusion**

4-1BB-based CD19 CAR-T therapy drives antigen escape dependent on *CD19* genetic changes. Initially undetectable *CD19* mutations rapidly dominate the tumor population and impair proper FMC63 epitope detection. In clinical settings, CD19-mutation-mediated acquired resistance after 4-1BB-CD19 CAR-T cells cannot be properly predicted.
*Funding: National Science Centre 2020/39/O/NZ6/01434, 2023/49/N/NZ7/03096, 2023/50/A/NZ6/00423; Polish National Agency for Academic Exchange NAWA BPI/PST/2024/1/00127/DEC/1.*

**Copyright Information:** (Online) ISSN: 2572-9241
©2026 The Author(s). HemaSphere is published by John Wiley & Sons Ltd on behalf of European Hematology Association. This is an open access Abstract Book distributed under the Attribution-NonCommercial-NoDerivs (CC BY-NC-ND), which allows third parties to download the articles and share them with others as long as they credit the author and the Abstract Book, but they cannot change the content in any way or use them commercially.
**Abstract Book Citations:** Authors, Title, HemaSphere, 2026;10:(S1):pages. The abstract book DOIs can be found on the "Author Information" page.
**Disclaimer:** Articles published in the journal HemaSphere exclusively reflect the opinions of the authors. The authors are responsible for all content in their abstracts including accuracy of the facts, statements, citing resources, etc.

### (PB4104) BISPECIFIC T-CELL ENGAGERS AS SALVAGE THERAPY FOR RELAPSED/REFRACTORY B-CELL-DERIVED MALIGNANCIES AFTER CD19 CAR-T THERAPY

**Topic:** 25. Gene therapy, cellular immunotherapy and vaccination - Biology & translational research

Mikołaj Jacyno*[1, 2], Agata Braniewska[1], Emilia Bednarska[1], Magdalena Drużyńska[1], Magdalena Winiarska[1, 3], Marta Krawczyk[1, 2]

*[1]Department of Immunology, Mossakowski Medical Research Institute, Polish Academy of Sciences, Warsaw, Poland;[2]Doctoral School of Translational Medicine, Mossakowski Medical Research Institute, Polish Academy of Sciences, Centre of Postgraduate Medical Education, Warsaw, Poland; [3]Department of Immunology, Medical University of Warsaw, Warsaw, Poland;*

### Background

CD19 CAR-T cells are the new standard in the treatment of relapsed/refractory (R/R) B cell malignancies. However, a significant proportion of patients show resistance to this therapy. One of the most common resistance mechanisms involves mutations in the CD19 gene that disrupts the epitope recognized by CAR-T cells. Bispecific T-cell Engagers (BiTEs) are novel immunotherapeutic approach to treat R/R B cell malignancies. BiTEs can simultaneously bind tumor-associated antigen and CD3 on T cell, which results in formation of immunological synapse and T-cell activation. Currently there are four FDA-approved BiTEs with indication to treat B cell malignancies: CD3-CD19 (Blinatumomab), CD3-CD20 (Mosunetuzumab, Glofitamab and Epcoritamab).

### Aims

The aim is to investigate whether BiTEs can act as a salvage therapy after CD19 CAR-T failure. Additionally, we attempted to better understand the mechanisms driving the resistance to BiTEs.

### Methods

To assess the cytotoxic effects of BiTEs on CD19 CAR-T resistant cell lines, tumor target cells were incubated with stimulated T cells at different effectors to targets ratio (E:T) and with either Blinatumomab or Epcoritamab at different concentrations. T cells were stimulated by seeding freshly isolated Peripheral Blood Mononuclear Cells (PBMCs) onto anti-CD3/anti-CD28-antibody-coated plate with addition of Interleukin-2. Flow cytometry was employed to monitor CD19 and CD20 expression. In order to generate *in vitro* models resistant to Blinatumomab and Epcoritamab, RS4;11 and Raji cell lines were repeatedly co-cultured with stimulated T cells and either Blinatumomab or Epcoritamab.

### Results

Obtained data shows that Raji cells resistant to CD19 CAR-T after repeated exposure to CD19-4-1BB CAR-T cells (Raji 4-1BB) or after repeated exposure to CD19-CD28 CAR-T cells (Raji CD28) are less sensitive to Blinatumomab treatment (Figure A). Simultaneously, Nalm-6 4-1BB cells, are less sensitive (Figure B), while Nalm-6 CD28 retain their susceptibility to Blinatumomab treatment. Interestingly, Raji cells resistant to CD19 CAR-T cells remain sensitive to Epcoritamab (Figure C). So far generated model resistant to BiTEs, shows modulation of CD19 and CD20 already after 5 passages.

**Copyright Information:** (Online) ISSN: 2572-9241
©2026 The Author(s). HemaSphere is published by John Wiley & Sons Ltd on behalf of European Hematology Association. This is an open access Abstract Book distributed under the Attribution-NonCommercial-NoDerivs (CC BY-NC-ND), which allows third parties to download the articles and share them with others as long as they credit the author and the Abstract Book, but they cannot change the content in any way or use them commercially.
**Abstract Book Citations:** Authors, Title, HemaSphere, 2026;10:(S1):pages. The abstract book DOIs can be found on the "Author Information" page.
**Disclaimer:** Articles published in the journal HemaSphere exclusively reflect the opinions of the authors. The authors are responsible for all content in their abstracts including accuracy of the facts, statements, citing resources, etc.

### Summary/Conclusion

Our results provide strong evidence that BiTEs can act as a salvage therapy after failure of CD19 CAR-T cell treatment. As observed in Nalm-6 cell line, disruption of FMC63 epitope does not severely impact the effectives of Blinatumomab, despite shared target antigen. Lymphoma cells resistant to CD19 CAR-T remain less sensitive to Blinatumomab, but they are still sensitive to CD20-targeting Epcoritamab. Additionally, B-cell malignancies may develop resistance to BiTEs, rendering our resistance model particularly valuable for investigating underlying mechanisms.

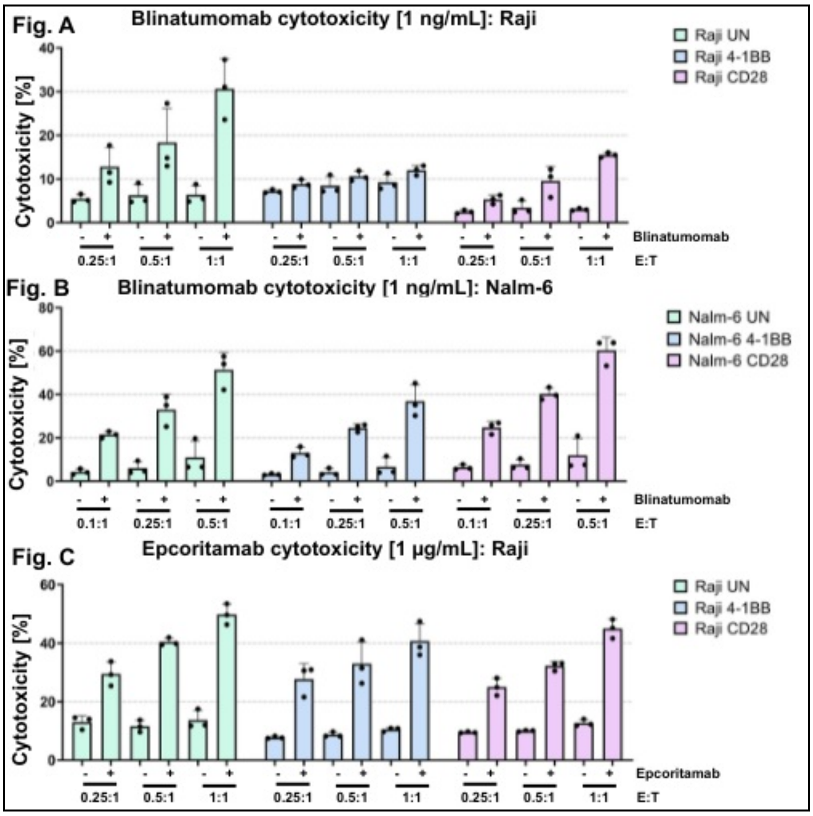

**Copyright Information:** (Online) ISSN: 2572-9241
©2026 The Author(s). HemaSphere is published by John Wiley & Sons Ltd on behalf of European Hematology Association. This is an open access Abstract Book distributed under the Attribution-NonCommercial-NoDerivs (CC BY-NC-ND), which allows third parties to download the articles and share them with others as long as they credit the author and the Abstract Book, but they cannot change the content in any way or use them commercially.
**Abstract Book Citations:** Authors, Title, HemaSphere, 2026;10:(S1):pages. The abstract book DOIs can be found on the "Author Information" page.
**Disclaimer:** Articles published in the journal HemaSphere exclusively reflect the opinions of the authors. The authors are responsible for all content in their abstracts including accuracy of the facts, statements, citing resources, etc.

### (PB4106) PRELIMINARY OBSERVATION OF GROWTH AND PUBERTAL DEVELOPMENT FOLLOWING CAR-T CELL THERAPY FOR SYSTEMIC LUPUS ERYTHEMATOSUS CHILDREN.

**Topic:** 26. Gene therapy, cellular immunotherapy and vaccination - Clinical

Jing Pan*[1, 2], Wenjing Zhang[2], Yanhong Huang[2, 3], Siyu Liu[2], Sha Li[2], Zelin Wang[2], Chen Chen[4]

*[1]State Key Laboratory of Experimental Hematology, National Clinical Research Center for Blood Diseases, Haihe Laboratory of Cell Ecosystem, Institute of Hematology & Blood Diseases Hospital, Chinese Academy of Medical College, Sciences & Peking Union Medical College, Beijing, China; [2]Beijing Gobroad Hospital, Beijing, China; [3]Beijing Jishuitan Hospital, 31 Xinjiekou East Street, Xicheng District, Beijing, China; [4]Gobroad Research Center, No. 58, Haiqu Park, Distr. Pudong, Shanghai, China;*

### Background

Prolonged glucocorticoid and immunosuppressive therapy in systemic lupus erythematosus (SLE) children can cause a range of unique adverse effects, including impaired linear growth and disrupted pubertal development. Chimeric antigen receptor T-cell (CAR-T) therapy has achieved initial efficacy in the treatment of lupus, potentially allowing for rapid discontinuation of chronic immunosuppression. However, studies involving pediatric patients and examining the impact of CAR-T therapy on their growth and developmental outcomes remain scarce.

### Aims

To evaluate the safety and efficacy of CAR-T therapy in r/r SLE, while observing its impact on children's growth and pubertal development.

### Methods

Pediatric r/r SLE patients (>age 10 years old) were allowed to enrolled in our clinical trial (NCT06947460), besides monitoring safety (primarily evaluated through dose-limiting toxicity) and the efficacy (SLEDAI, LLDAS, SRI-4) of CAR-T cell therapy for r/r SLE children, improvement of growth and pubertal development was monitored. Baseline height was measured in all patients before CAR-T cell infusion, followed by regular assessments at 3-month intervals post-treatment according to a predefined follow-up plan, assessed by the criteria of the Standard for Height Level Classification among Children and Adolescents Aged 7–18 Years (2018 Version). Pubertal development was also assessed using Tanner staging.

### Results

Between Jun 12 and Sep 20, 2025, four r/r SLE children were enrolled. All patients stopped glucocorticoids and immunosuppressants pre-infusion and remained drug-free. No DLTs occurred within 28 days after infusion. All patients experienced grade 1 CRS; 25% had grade 2 hematologic toxicity. No neurotoxicity was observed and all toxicities resolved with supportive care. As of 20 February 2026, all 4 patients completed efficacy assessments at months 1, 3, and 6. Remission rates were 75%/75%/75% (DORIS), 75%/100%/75% (LLDAS), and 75%/75%/75% (SRI-4). One patient had a grade 3 viral infection and fully recovered after hospitalization. SLEDAI and PGA scores decreased by 2–6 and 0.5–2 points, respectively. From drug discontinuation to 6 months after CAR-T infusion, all patients demonstrated catch-up growth. Compared with 2018 height references, 50% patients exhibited reduced height prior to treatment discontinuation: the 12-year-old girl (144 cm) was approximately 3 cm below the expected height (147 cm), and the 11-year-old girl (133 cm) was about 1 cm below the expected height (134 cm). The remaining patients had heights within the normal range. Height gains ranged from +1 cm to +5 cm. All patients exhibited secondary sexual characteristics within the normal Tanner stages, indicating that they had not missed the normal window for pubertal development.

**Copyright Information:** (Online) ISSN: 2572-9241
©2026 The Author(s). HemaSphere is published by John Wiley & Sons Ltd on behalf of European Hematology Association. This is an open access Abstract Book distributed under the Attribution-NonCommercial-NoDerivs (CC BY-NC-ND), which allows third parties to download the articles and share them with others as long as they credit the author and the Abstract Book, but they cannot change the content in any way or use them commercially.
**Abstract Book Citations:** Authors, Title, HemaSphere, 2026;10:(S1):pages. The abstract book DOIs can be found on the "Author Information" page.
**Disclaimer:** Articles published in the journal HemaSphere exclusively reflect the opinions of the authors. The authors are responsible for all content in their abstracts including accuracy of the facts, statements, citing resources, etc.

**Summary/Conclusion**

CAR-T cell therapy shows favorable safety and efficacy profile for r/r SLE children. Building upon this foundation, our data further suggest that this therapy may mitigate growth and development toxicity associated with conventional immunosuppressive therapy, offering a novel therapeutic strategy with distinct advantages for pediatric SLE patients.

**Copyright Information:** (Online) ISSN: 2572-9241
©2026 The Author(s). HemaSphere is published by John Wiley & Sons Ltd on behalf of European Hematology Association. This is an open access Abstract Book distributed under the Attribution-NonCommercial-NoDerivs (CC BY-NC-ND), which allows third parties to download the articles and share them with others as long as they credit the author and the Abstract Book, but they cannot change the content in any way or use them commercially.
**Abstract Book Citations:** Authors, Title, HemaSphere, 2026;10:(S1):pages. The abstract book DOIs can be found on the "Author Information" page.
**Disclaimer:** Articles published in the journal HemaSphere exclusively reflect the opinions of the authors. The authors are responsible for all content in their abstracts including accuracy of the facts, statements, citing resources, etc.

### (PB4107) COAGULOPATHY IN CAR T CELL–TREATED PATIENTS WITH LYMPHOPROLIFERATIVE DISORDERS: A RETROSPECTIVE, SINGLE-CENTER OBSERVATIONAL STUDY

**Topic:** 26. Gene therapy, cellular immunotherapy and vaccination - Clinical

Ignazio Santo*[1], Riccardo Morganti[2], Giulia Leoni[3], Gaspare Tancredi[3], Sara Galimberti[3], Enrico Orciuolo[4]

[1]*Azienda USL Toscana Nord Ovest, HEMATOLOGY, Lido di Camaiore, Italy;*[2]*Hematology Division, AOUP, Pisa, Italy, Pisa, Italy;* [3]*University of Pisa, HEMATOLOGY, Pisa, Italy;*[4]*Azienda Ospedaliera Universitaria Pisana, HEMATOLOGY, Pisa, Italy;*

### Background

CAR-T therapy improves outcomes in relapsed/refractory lymphoid malignancies but can cause coagulopathy. Massive cytokine release leads to endothelial injury, platelet activation, excess thrombin, and reduced fibrinolysis, inducing an initial hypercoagulable state.
Clinically, it causes bleeding, thrombosis, and sometimes DIC (Disseminated Intravascular Coagulopathy), with frequent laboratory abnormalities.

### Aims

To evaluate, in a retrospective single-center cohort of patients with lymphoproliferative disorders treated with CAR-T cells, the incidence of post-infusion thrombotic and hemorrhagic events (within 60 days of discharge) and changes in major coagulation parameters.
Additionally, we analyzed cases of overt DIC and identified predictive risk factors using univariate analysis.

### Methods

For each patient treated with CAR T therapy, laboratory, epidemiological, and clinical data were collected.
Laboratory data included coagulation parameters (prothrombin activity, PT, aPTT, fibrinogen, D-dimer, ATIII, Hb, and PLT count), whose temporal variations were assessed at four timepoints: pre- and post-lymphodepletion (t0, t1), acute phase (t2) and subacute phase (t3); the parameters were stratified for each type of CAR-T product used.
Epidemiological variables included: age and sex.
Clinical data included: diagnosis, line of therapy, type of CAR-T, International Prognostic Index (IPI) at diagnosis, Ann Arbor (A.A.) stage, bulky disease, history of DVT/PE, bridging therapy, CAR-HEMATOTOX score, vein-to-vein time, occurrence and grade of CRS and ICANS, use of tocilizumab, IL-6 levels, and CAR-T cell expansion kinetics.
Statistical analysis used descriptive statistics, repeated-measures ANOVA, and SPSS ($p < 0.05$).

**Copyright Information:** (Online) ISSN: 2572-9241
©2026 The Author(s). HemaSphere is published by John Wiley & Sons Ltd on behalf of European Hematology Association. This is an open access Abstract Book distributed under the Attribution-NonCommercial-NoDerivs (CC BY-NC-ND), which allows third parties to download the articles and share them with others as long as they credit the author and the Abstract Book, but they cannot change the content in any way or use them commercially.
**Abstract Book Citations:** Authors, Title, HemaSphere, 2026;10:(S1):pages. The abstract book DOIs can be found on the “Author Information” page.
**Disclaimer:** Articles published in the journal HemaSphere exclusively reflect the opinions of the authors. The authors are responsible for all content in their abstracts including accuracy of the facts, statements, citing resources, etc.

### Results

Between November 2020 and August 2025, a total of 72 patients with lymphoproliferative disorders treated with CAR-T therapy were included.
Severe hemorrhagic events were rare (1 out of 72 pt, 1.3%), whereas thrombotic events occurred in 4.1% of cases (3 out of 72 pt). Analysis of coagulation parameters showed significant temporal variations in PT, fibrinogen, prothrombin activity, ATIII, PLT, and Hb while aPTT did not demonstrate significant changes. D-dimer was significant only in patients treated with brexucabtagene autoleucel.
Overt DIC was observed in 5.5% of patients (4 out of 72 pt), with additional subclinical cases (3 out of 72 pt, 4.1%). The Overall incidence of coagulation abnormalities was 9.6%. Mortality rate associated with overt DIC was 1 out of 4 pt (25%).
Factors associated with overt-DIC included age, diagnosis, bulky disease, CAR-HEMATOTOX score, vein-to-vein time, severity of CRS/ICANS, IL-6 serum levels, and CAR-T cell expansion ($p<0.05$).
No significant association with DIC was observed for IPI, A.A. stage, presence of CRS, or prior thrombotic history. Bridging therapy and tocilizumab use, although not significantly associated, showed a trend toward potential significance.

### Summary/Conclusion

Thrombotic events often occur after discharge, indicating a need for prolonged anticoagulant prophylaxis. Hemostasis shows a biphasic pattern: early inflammation with high fibrinogen and PT changes, followed by consumption with low fibrinogen and ATIII. Overt DIC (5.5%) carries high mortality. Risk factors include young age, Brexu-cel use, bulky disease, high CAR-HEMATOTOX score, short vein-to-vein time, severe CRS/ICANS, high IL-6 serum level, and strong CAR-T expansion.
Future approaches should target inflammation and endothelial protection.

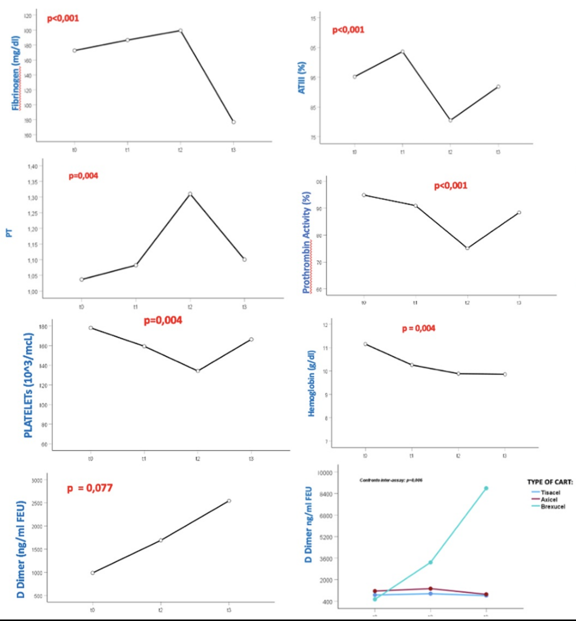

| DATA | Overt DIC | | p-value |
|---|---|---|---|
| | NO | YES | |
| Age at treatment | 60 (10) | 45,5 (15) | 0,007 |
| Gender | | | 0,453 |
| M | 38 | 3 | |
| F | 30 | 1 | |
| Diagnosis | | | <0,001 |
| DLBCL | 53 | 2 | |
| Mant | 6 | 0 | |
| Foll | 6 | 0 | |
| LLAB | 1 | 2 | |
| PMBCL | 2 | 0 | |
| Line of therapy | 2,6 (0,49) | 2,25 (0,50) | 0,169 |
| Type of CAR-T | | | 0,080 |
| Tisacel | 16 | 0 | |
| Axicel | 44 | 2 | |
| Brexucel | 8 | 2 | |
| IPI at diagnosis | | | 0,244 |
| Low | 26 | 1 | |
| Intermediate | 18 | 0 | |
| High | 24 | 3 | |
| A.A stage | | | 0,444 |
| I | 2 | 0 | |
| II | 8 | 1 | |
| III | 13 | 0 | |
| IV | 44 | 1 | |
| Bulky Disease | | | 0,003 |
| no | 49 | 0 | |
| si | 19 | 4 | |
| History of DVT/PE | | | 0,289 |
| no | 62 | 3 | |
| si | 6 | 1 | |

| DATA | Overt DIC | | p-value |
|---|---|---|---|
| | NO | YES | |
| BRIDGE THERAPY TO CAR-T | | | 0,082 |
| NO | 30 | 0 | |
| SI | 38 | 4 | |
| CARHEMATOTOX | | | <0,001 |
| Low Risk | 52 | 0 | |
| High Risk | 16 | 4 | |
| VEIN TO VEIN TIME | 45,5 (48,5) | 30,5 (6,03) | 0,028 |
| CRS | | | 0,357 |
| NO | 12 | 0 | |
| SI | 56 | 4 | |
| CRS GRADE | 1,10 (0,74) | 2 (0,82) | 0,021 |
| ICANS | | | 0,013 |
| NO | 54 | 1 | |
| SI | 14 | 3 | |
| ICANS GRADE | 0,44 (0,92) | 2 (1,41) | 0,002 |
| TOCILIZUMAB USE | | | 0,074 |
| NO | 31 | 0 | |
| SI | 37 | 4 | |
| IL 6 MAX PEAK | 84 (105) | 367 (241) | <0,001 |
| PEAK DAY MAXIMUM IL 6 | 6,35 (3,76) | 6,67 (3,06) | 0,885 |
| CAR-T EXPANSION PEAK MAX | 19,22 (15,35) | 59,46 (37,76) | 0,01 |
| PEAK DAY CAR-T MAX EPANSION | 7,85 (1,41) | 8,5 (2,12) | 0,572 |

FIRST IMAGE: Coagulation parameters with temporal variation; SECOND IMAGE: overt DIC and predictive risk factors using univariate analysis.

Copyright Information: (Online) ISSN: 2572-9241
©2026 The Author(s). HemaSphere is published by John Wiley & Sons Ltd on behalf of European Hematology Association. This is an open access Abstract Book distributed under the Attribution-NonCommercial-NoDerivs (CC BY-NC-ND), which allows third parties to download the articles and share them with others as long as they credit the author and the Abstract Book, but they cannot change the content in any way or use them commercially.
Abstract Book Citations: Authors, Title, HemaSphere, 2026;10:(S1):pages. The abstract book DOIs can be found on the "Author Information" page.
Disclaimer: Articles published in the journal HemaSphere exclusively reflect the opinions of the authors. The authors are responsible for all content in their abstracts including accuracy of the facts, statements, citing resources, etc.

### (PB4108) COMPARISON OF NEUTROPENIA-DRIVEN VERSUS PROPHYLACTIC ADMINISTRATION OF GRANULOCYTE COLONY-STIMULATING FACTOR AFTER LYMPHODEPLETION IN 4-1BB-COSTIMULATED CD19 CAR-T THERAPY

**Topic:** 26. Gene therapy, cellular immunotherapy and vaccination - Clinical

Hui Liu*[1], Xiaoxia Wang[2], Wen Lei[1], Panpan Chen[1], Yuan Li[3], Wenbin Qian[1]

*[1]the Second Affiliated Hospital, College of Medicine, Zhejiang University, Department of Hematology, Hangzhou, China; [2]Dongyang Hospital Affiliated to Wenzhou Medical University, Dongyang, China;[3]The Affiliated Hospital of Jiaxing University, Jiaxing, China;*

### Background

After receiving CAR-T therapy, most patients may experience neutropenia of varying duration, including long-term hematologic toxicity that can persist for several months, due to multiple factors such as lymphodepleting chemotherapy, prior multi-line treatments, and inflammatory responses triggered by CAR-T therapy. In clinical practice, granulocyte colony-stimulating factor (G-CSF) is commonly administered to patients to accelerate neutrophil recovery, or given prophylactically to prevent or alleviate neutropenia.

### Aims

Given the lack of a clear framework for integrating pegylated G-CSF into the CD19 CAR-T therapy protocol for B-cell lymphoma, we conducted a retrospective study in patients with B-cell lymphoma who received 4-1BB-costimulated CD19 CAR-T therapy, try to analyze the efficacy and safety of pegylated G-CSF administration, with the goal of generating evidence to inform the comprehensive management of CAR-T therapy.

### Methods

We enrolled R/R B-cell lymphoma patients who received 4-1BB-costimulated CD19 CAR-T Therapy, both from clinical trials or with commercial approvals, heretofore since June 2017 to December 2023, Patients with bone marrow involvement were excluded from the study. For patients who received neutropenia-driven administration, G-CSF 5 μg/kg was given in duration of grade Ⅳ neutropenia with absolute neutrophil count (ANC) < 500 cells/μl. For patients who received prophylactic administration, a single dose of pegylated G-CSF 100 μg/kg was given after lymphodepletion at day -2.

**Copyright Information:** (Online) ISSN: 2572-9241
©2026 The Author(s). HemaSphere is published by John Wiley & Sons Ltd on behalf of European Hematology Association. This is an open access Abstract Book distributed under the Attribution-NonCommercial-NoDerivs (CC BY-NC-ND), which allows third parties to download the articles and share them with others as long as they credit the author and the Abstract Book, but they cannot change the content in any way or use them commercially.
**Abstract Book Citations:** Authors, Title, HemaSphere, 2026;10:(S1):pages. The abstract book DOIs can be found on the “Author Information” page.
**Disclaimer:** Articles published in the journal HemaSphere exclusively reflect the opinions of the authors. The authors are responsible for all content in their abstracts including accuracy of the facts, statements, citing resources, etc.

**Results**

30 patients (53.6%) exposed to neutropenia-driven administration of G-CSF, and 26 patients (46.4%) patients received prophylactic pegylated G-CSF treatment for neutropenia. In the neutropenia-driven group, the ORR was 56.7% (17/30), with 13 CR (43.3%) and 4 PR. In contrast, the prophylactic group had an ORR of 69.2% (18/26) (*P*=0.322; RR=1.222, 95% CI: 0.815-1.829), comprising 12 CR (46.2%) (*P*=0.828; RR=1.065, 95% CI: 0.594-1.907) and 6 PR. Rates of stable and progressive disease were comparable between the groups. In the neutropenia-driven group, the median duration of neutropenia following lymphodepletion was 8 days (range, 0-11 days), versus 5 days (range, 0-11 days) in the prophylaxis group, A significant difference was observed between the two groups (*P*=0.0015). Patients in the neutropenia-driven group ($0.2\times10^9$/L, range experienced a more profound neutropenia, as evidenced by a significantly lower median nadir ANC compared to the prophylaxis group ($0.2\times10^9$/L vs. $0.3\times10^9$/L, *P*=0.0058). No statistically significant differences were observed between the two groups in either the overall incidence of CRS (*P*=0.198; RR=0.875, 95% CI: 0.733-1.043) or the incidence of grade ≥3 CRS (*P*=1.000; RR=0.577, 95% CI: 0.055-6.073). The difference did not reach statistical significance in either the overall incidence of ICANS (*P*=0.352; RR=0.641, 95% CI: 0.241-1.705) or the incidence of grade ≥3 ICANS (*P*=1.000; RR=0.769, 95% CI: 0.139-4.262).  No statistically significant difference was observed between the two groups in OS and PFS.

**Summary/Conclusion**

Prophylactic pegylated G-CSF administration in patients with B-cell lymphoma who received 4-1BB-costimulated CD19 CAR-T therapy can effectively shortens neutropenia duration with no elevated risks of CRS or ICANS, representing a safe clinical strategy.

**Copyright Information:** (Online) ISSN: 2572-9241
©2026 The Author(s). HemaSphere is published by John Wiley & Sons Ltd on behalf of European Hematology Association. This is an open access Abstract Book distributed under the Attribution-NonCommercial-NoDerivs (CC BY-NC-ND), which allows third parties to download the articles and share them with others as long as they credit the author and the Abstract Book, but they cannot change the content in any way or use them commercially.
**Abstract Book Citations:** Authors, Title, HemaSphere, 2026;10:(S1):pages. The abstract book DOIs can be found on the "Author Information" page.
**Disclaimer:** Articles published in the journal HemaSphere exclusively reflect the opinions of the authors. The authors are responsible for all content in their abstracts including accuracy of the facts, statements, citing resources, etc.

### (PB4110) RECOMBINANT HUMAN THROMBOPOIETIN FACILITATES PLATELET RECOVERY IN B-CELL MALIGNANCY PATIENTS TREATED WITH CAR-T THERAPY

**Topic:** 26. Gene therapy, cellular immunotherapy and vaccination - Clinical

Xiaoqing Jiang*[1], Liqiong Liu[1], Zhengyi Jia[1], Yuan Ye[1], Xiaoxuan Liu[1], Xiaofan Chen[1], Jizi Liu[1], Zhi Guo[1]

[1]*Affiliated Nanshan Hospital of Shenzhen University, Department of Hematology, Shenzhen, China;*

### Background

Immune effector cell-associated thrombocytopenia is a common complication of chimeric antigen receptor T-cell (CAR-T) therapy, carrying high risks for severe or fatal outcomes. Data onclinical utility of recombinant human thrombopoietin (rhTPO) for thrombocytopenia in patients with B-cell malignancies receiving CAR-T therapy remains limited.
Methods：This study aimed to investigate the clinical effectiveness and safety of rhTPO administered to CAR-T-treated patients with thrombocytopenia over a 90-day observation period (Days 0–90).

### Aims

This study aimed to investigate the clinical effectiveness and safety of rhTPO administered to CAR-T-treated patients with thrombocytopenia over a 90-day observation period (Days 0–90).

### Methods

This retrospective study included 21 B-cell malignancy patients with post-CAR-T thrombocytopenia (<50×10⁹/L) who received rhTPO from August 2023 to December 2025. With Day 0 designated as the initiation of rhTPO therapy (baseline), longitudinal trends in platelet counts and transfusion burden were assessed during the 90-day observation period (Days 0–90).

### Results

All patients (100%, 21/21) developed Grade 3–4 thrombocytopenia (platelet count ≤50×10⁹/L), with 76.2% (16/21) having severe thrombocytopenia (≤20×10⁹/L). The median platelet count at baseline (Day 0) was 47.00×10⁹/L (IQR 35.5–62), which decreased to 33.50×10⁹/L (IQR 22.00–45.50) by Day 7 (P=0.038). Subsequently, platelet counts recovered significantly, reaching 69.00×10⁹/L (IQR 30.25–156.50) at Day 28 (P=0.035), 97.00×10⁹/L (IQR 54.50–134.50) at Day 60 (P=0.003), and stabilized at 116.00×10⁹/L (IQR 45.50–133.50) by Day 90 (P=0.009).
Regarding transfusion requirements, the transfusion rate was 61.9% during the first week (Days 0–7), remained at 57.1% during Days 8–14, decreased to 38.1% during Days 15–21, and dropped to 23.8% during Days 29–60. Notably, 47.6% of patients (10/21) achieved complete transfusion independence after Day 28.
In terms of safety, no rhTPO-related thrombotic events or Grade 3–4 non-hematologic adverse events were observed throughout the study period.

### Summary/Conclusion

Our findings indicate that despite an obligatory lag phase marked by an early platelet nadir, rhTPO effectively reversed severe CAR-T-associated thrombocytopenia achieving significant recovery and transfusion independence from Day 28 onwards. Consequently, we recommend ensure adequate treatment duration and provide intensified transfusion support during the first two weeks.

**Copyright Information:** (Online) ISSN: 2572-9241
©2026 The Author(s). HemaSphere is published by John Wiley & Sons Ltd on behalf of European Hematology Association. This is an open access Abstract Book distributed under the Attribution-NonCommercial-NoDerivs (CC BY-NC-ND), which allows third parties to download the articles and share them with others as long as they credit the author and the Abstract Book, but they cannot change the content in any way or use them commercially.
**Abstract Book Citations:** Authors, Title, HemaSphere, 2026;10:(S1):pages. The abstract book DOIs can be found on the “Author Information” page.
**Disclaimer:** Articles published in the journal HemaSphere exclusively reflect the opinions of the authors. The authors are responsible for all content in their abstracts including accuracy of the facts, statements, citing resources, etc.

### (PB4111) OUTPATIENT-AT-HOME (OATH) RAMP-UP OF BISPECIFIC ANTIBODIES FOR LYMPHOMA AND MYELOMA: A REAL-WORLD SINGLE-CENTER EXPERIENCE

**Topic:** 26. Gene therapy, cellular immunotherapy and vaccination - Clinical

Beatriz Merchan Muñoz*[1], Alexandra Martínez Roca[1], Cristina Gallego[1], Nuria Ballestar[1], Estel Güell[1], Irene Martínez-Mesa[1], Natalia Elena Gomez[1], Esther Carcelero[1], Inés Monge[1], Laura Magnano Mayer[1], Armando Lopez Guillermo[1], Natalia Tovar[1], Laura Rosiñol Dachs[1], Carlos Fernández de Larrea[1], Francesc Fernandez Aviles[1]

*[1]Hospital Clínic Barcelona, Barcelona, Spain;*

### Background

Bispecific antibodies (BsAbs) have demonstrated significant clinical activity in hematologic malignancies. Step-up dosing (ramp-up) is required to mitigate cytokine release syndrome (CRS) and immune effector cell–associated neurotoxicity syndrome (ICANS); it has traditionally required inpatient monitoring. Real-world data on structured outpatient strategies for BsAb ramp-up remain scarce.

### Aims

To evaluate the feasibility and safety of BsAb ramp-up using an Outpatient-At-Home (OATH) model.

### Methods

A real-world observational study was conducted including consecutive adult patients undergoing BsAb ramp-up within an OATH program for lymphoma and myeloma. BsAbs included mosunetuzumab, epcoritamab, elranatamab, teclistamab and talquetamab.

Eligibility was based on predefined clinical and social . Patients were required to remain within 30 minutes of the hospital and have 24-hour caregiver availability; temporary accommodation or professional caregivers were provided when needed. Patients and caregivers received structured education, including symptom recognition, caregiver-performed immune effector cell encephalopathy (ICE) scoring, and an adverse-event action plan.

The first step-up dose was administered in a day-care unit, while subsequent step-up and full doses were administered at home. Prophylactic oral dexamethasone (10 mg) was administered 24 hours after each step-up dose; one patient with multiple myeloma also received prophylactic tocilizumab due to bulky disease. Monitoring included twice-daily telephone assessments during the 48 hours after each step-up dose and laboratory testing every 72 hours, with immediate access to hospital care ward if needed.

### Results

Between January 2025 and February 2026, 20 patients underwent BsAb ramp-up under the OATH model (Table 1). Median age was 59 years (range, 31–84), with a median of 3 prior treatment lines (range, 1–7). Prior autologous HCT and CAR T-cell therapy were reported in 35% and 50% of patients, respectively.

Median at-home follow-up was 17 days (range, 11–31). CRS occurred in 7 patients (35%), including 6 grade 1 and 1 grade 2 events, with a median duration of 1 day (range, 1–2). No grade ≥3 CRS or ICANS were observed. Disease flare occurred in 2 patients (10%).

Overall, 3 patients (15%) required hospital admission, due to grade 2 CRS (n=1), rapid disease progression with fever (n=1), and disease flare with acute kidney injury (n=1). No ICU admissions or treatment-related deaths occurred.

**Copyright Information:** (Online) ISSN: 2572-9241
©2026 The Author(s). HemaSphere is published by John Wiley & Sons Ltd on behalf of European Hematology Association. This is an open access Abstract Book distributed under the Attribution-NonCommercial-NoDerivs (CC BY-NC-ND), which allows third parties to download the articles and share them with others as long as they credit the author and the Abstract Book, but they cannot change the content in any way or use them commercially.
**Abstract Book Citations:** Authors, Title, HemaSphere, 2026;10:(S1):pages. The abstract book DOIs can be found on the “Author Information” page.
**Disclaimer:** Articles published in the journal HemaSphere exclusively reflect the opinions of the authors. The authors are responsible for all content in their abstracts including accuracy of the facts, statements, citing resources, etc.

Tocilizumab was only administered in 3 patients (15%). Two patients with grade 1 CRS were managed entirely within the OATH setting. All remaining adverse events were managed with protocol-mandated dexamethasone, without inpatient care.

Table 1: Baseline Characteristics

| **Baseline Characteristics** | **n=20** |
|---|---|
| **Age,** median (range), years | 59 (31-84) |
| **Sex,** n (%) | |
| Female | 4 (20) |
| Male | 16 (80) |
| **Haematologic malignancy,** n (%) | |
| FL | 8 (40) |
| DBLCL | 4 (20) |
| MM | 8 (40) |
| **Ann Arbor stage III–IV** (lymphoma), n (%) | 12 (100) |
| **Extramedullary disease** (MM), n (%) | 4 (50) |
| **LDH,** median (range), U/L | 181 (136–523) |
| **≥3 prior therapy lines, n (%)** | 13 (65) |
| **Prior autologous HCT,** n (%) | 7 (35) |
| **Prior CART-cell therapy,** n (%) | 10 (50) |
| **Bispecific antibody,** n (%) | |
| Mosunetuzumab | 6 (30) |
| Epcoritamab | 6 (30) |
| Elranatamab | 2 (10) |
| Teclistamab | 1 (5) |
| Talquetamab | 5 (25) |

**Copyright Information:** (Online) ISSN: 2572-9241
©2026 The Author(s). HemaSphere is published by John Wiley & Sons Ltd on behalf of European Hematology Association. This is an open access Abstract Book distributed under the Attribution-NonCommercial-NoDerivs (CC BY-NC-ND), which allows third parties to download the articles and share them with others as long as they credit the author and the Abstract Book, but they cannot change the content in any way or use them commercially.
**Abstract Book Citations:** Authors, Title, HemaSphere, 2026;10:(S1):pages. The abstract book DOIs can be found on the “Author Information” page.
**Disclaimer:** Articles published in the journal HemaSphere exclusively reflect the opinions of the authors. The authors are responsible for all content in their abstracts including accuracy of the facts, statements, citing resources, etc.

**Summary/Conclusion**

BsAb ramp-up using an OATH model is feasible and safe in selected patients, allowing most adverse events to be managed without inpatient care and resulting in low rate of hospital admission.

**Copyright Information:** (Online) ISSN: 2572-9241
©2026 The Author(s). HemaSphere is published by John Wiley & Sons Ltd on behalf of European Hematology Association. This is an open access Abstract Book distributed under the Attribution-NonCommercial-NoDerivs (CC BY-NC-ND), which allows third parties to download the articles and share them with others as long as they credit the author and the Abstract Book, but they cannot change the content in any way or use them commercially.
**Abstract Book Citations:** Authors, Title, HemaSphere, 2026;10:(S1):pages. The abstract book DOIs can be found on the "Author Information" page.
**Disclaimer:** Articles published in the journal HemaSphere exclusively reflect the opinions of the authors. The authors are responsible for all content in their abstracts including accuracy of the facts, statements, citing resources, etc.

### (PB4112) HBF-INDUCING GENE THERAPIES FOR THE Β-HEMOGLOBINOPATHIES: IMPLICATIONS FOR FUTURE PREGNANCIES

**Topic:** 26. Gene therapy, cellular immunotherapy and vaccination - Clinical

Mathilde Gavillet*[1], Hélène Legardeur[1], Cecile Adam[1], Pradip Dashraath[2], David Baud[1], Raffaele Renella[1]

*[1]Lausanne University Hospital, Obstetrics, Lausanne, Switzerland; [2]National University Hospital, Department of Obstetrics and Gynecology, Division of Maternal-Fetal Medicine, Singapore, Singapore;*

### Background

The most recent somatic gene therapies strategies for β-hemoglobinopathies - sickle cell disease and β-thalassemia – aim at stable re-expression of high level of fetal hemoglobin (HbF) are transforming the contemporary therapeutic landscape. HbF, expressed during in utero life and physiologically silenced after birth, has a higher affinity for oxygen allowing for transplacental exchange of oxygen. Its persistence at significant levels in maternal circulation could impair fetal oxygenation during subsequent pregnancies.

### Aims

To anticipate the obstetric risks in this emerging population, we synthesize evidence on pregnancy outcomes in women with hereditary persistence of fetal hemoglobin (HPFH)—a naturally occurring model of elevated HbF—to apprehend potential maternal and fetal impacts in individuals having undergone gene therapy for β-hemoglobinopathies.

### Methods

Unpublished data issued from our local patient's registry were combined with a review of the literature. Four different PubMed database search were executed with the following key words: "Pregnancy" AND "hereditary persistence fetal hemoglobin", "Pregnancy Outcome" AND "Fetal Hemoglobin", "Pregnancy " AND "delta-beta thalassemia", "Pregnancy" AND "Hb E-beta thalassemia" on December 10th 2024.

### Results

Evidence is very limited with 61 pregnancies reported from 40 individuals: 22 pregnancies in persons with sickle cell disease (qualitative β-globin defects) and 39 with thalassemia syndromes (quantitative β-globin defects), with one third of the later (13/39) having HbF≥50%. Our data analysis confirms that moderately elevated HbF level (10–30%) reduces both maternal and fetal complications in sickle cell disease. However, pregnancy outcomes in individuals with thalassemia syndrome and HbF levels exceeding 50%, as observed after gene editing in β-thalassemia, show concerningly high rate of fetal morbidity. Half of neonates (6/13) suffered severe intrauterine fetal growth restriction (<3rd percentile), and an additional 8.3% (2/13) were between the 3rd and 10th centiles. For these patients, elevated HbF in maternal blood might blunt the maternal-fetal oxygen affinity gradient and contribute to placental insufficiency and negative fetal impact. Based on these elements, we outline pragmatic adaptation of the current recommendations for antenatal monitoring in pregnancies with supraphysiological level of maternal HbF.

**Copyright Information:** (Online) ISSN: 2572-9241
©2026 The Author(s). HemaSphere is published by John Wiley & Sons Ltd on behalf of European Hematology Association. This is an open access Abstract Book distributed under the Attribution-NonCommercial-NoDerivs (CC BY-NC-ND), which allows third parties to download the articles and share them with others as long as they credit the author and the Abstract Book, but they cannot change the content in any way or use them commercially.
**Abstract Book Citations:** Authors, Title, HemaSphere, 2026;10:(S1):pages. The abstract book DOIs can be found on the "Author Information" page.
**Disclaimer:** Articles published in the journal HemaSphere exclusively reflect the opinions of the authors. The authors are responsible for all content in their abstracts including accuracy of the facts, statements, citing resources, etc.

**Summary/Conclusion**

As gene therapies aim for a cure and are offered to teenagers and young adults, the medical community should be able to care for these patients' post-therapy life which, for number of them, will likely include the wish to bear children. These patients deserve to be informed of the challenges ahead and cared for to the best of our abilities. The current quality of data is too limited and the authors advocate for a renewed effort in identifying patients with HPFH, the best available surrogate, and report pregnancy outcome in this collective. Multidisciplinary guidelines will then need to be developed to ensure maternal safety and optimize perinatal outcomes within this evolving therapeutic landscape.

**Copyright Information:** (Online) ISSN: 2572-9241
©2026 The Author(s). HemaSphere is published by John Wiley & Sons Ltd on behalf of European Hematology Association. This is an open access Abstract Book distributed under the Attribution-NonCommercial-NoDerivs (CC BY-NC-ND), which allows third parties to download the articles and share them with others as long as they credit the author and the Abstract Book, but they cannot change the content in any way or use them commercially.
**Abstract Book Citations:** Authors, Title, HemaSphere, 2026;10:(S1):pages. The abstract book DOIs can be found on the "Author Information" page.
**Disclaimer:** Articles published in the journal HemaSphere exclusively reflect the opinions of the authors. The authors are responsible for all content in their abstracts including accuracy of the facts, statements, citing resources, etc.

### (PB4114) THE RATIO OF NAIVE AND STEM CELL MEMORY T CELLS TO TERMINAL EFFECTOR T CELLS PREDICTS THE POTENCY OF EPCORITAMAB-MEDIATED CYTOTOXICITY IN B-CELL NEOPLASMS

**Topic:** 26. Gene therapy, cellular immunotherapy and vaccination - Clinical

Sheng-Hsuan Chien*[1, 2, 3], Yi-Ling Li[1], Li-Chun Shin[1], Fu-Ru Li[1], Chia Jen Liu[1, 3], Chih-Cheng Chen*[2, 4]

*[1]Taipei Veterans General Hospital, Division of Transfusion, Department of Medicine, Taipei, Taiwan;[2]National Yang Ming Chiao Tung University, Institute of Clinical Medicine, Taipei, Taiwan; [3]National Yang Ming Chiao Tung University, Faculty of Medicine, Taipei, Taiwan; [4]Taipei Veterans General Hospital, Department of Medical Oncology, Taipei, Taiwan;*

### Background

The clinical efficacy of CD3xCD20 bispecific antibodies (bsAbs), such as epcoritamab, is heavily dependent on the quality of the host's endogenous T-cell compartment. T-cell populations are categorized into functional subsets: naïve (TN), stem cell memory (TSCM), central memory (TCM), effector memory (TEM), and terminal effector (Teff) T cells.

### Aims

We hypothesized that a higher proportion of "younger" T-cell phenotypes (TN and TSCM) relative to terminal effector cells (Teff) would correlate with enhanced bispecific antibody-mediated anti-tumor activity.

### Methods

T-cell immunophenotypes from 10 patients (N=10) were characterized via flow cytometry using CCR7 and CD45RA surface markers (Figure A.). Functional cytotoxicity was assessed using an *in vitro* co-culture system where patient-derived T cells were incubated with CFSE-labeled Raji cells at an Effector-to-Target (E:T) ratio of 2:1. Co-culture cells were treated with 20 ng/mL of epcoritamab for 3 hours or medium only for control (Figure B). Cytotoxicity was calculated as:100% *(% of CFSE in control -% of CFSE in epcoritamab)/ % of CFSE in control. Linear regression was employed to analyze the correlation between the (TN +T SCM / Teff ratio (X-axis) and the percentage of cytotoxicity (Y-axis).

### Results

Our analysis revealed a significant positive correlation between the (TN + TSCM) / T eff ratio and epcoritamab-mediated T-cell cytotoxicity. Linear regression yielded an $R^2$ of 0.6081 and a P value = 0.0078 (Figure C.), indicating that approximately 60% of the variance in cytotoxic response is explained by the baseline T-cell differentiation state. Patients with a higher frequency of early-lineage T-cell subsets demonstrated markedly more potent tumor-cell lysis, while a shift toward terminal differentiation was associated with diminished therapeutic efficacy.

### Summary/Conclusion

These findings suggest that pre-treatment T-cell immunophenotyping, specifically the ratio of TN and TSCM to Teff cells, serves as a significant predictive biomarker for epcoritamab-mediated cytotoxicity. Monitoring these subsets may assist in personalizing bispecific antibody therapy and identifying patients most likely to achieve a robust clinical response.

**Copyright Information:** (Online) ISSN: 2572-9241
©2026 The Author(s). HemaSphere is published by John Wiley & Sons Ltd on behalf of European Hematology Association. This is an open access Abstract Book distributed under the Attribution-NonCommercial-NoDerivs (CC BY-NC-ND), which allows third parties to download the articles and share them with others as long as they credit the author and the Abstract Book, but they cannot change the content in any way or use them commercially.
**Abstract Book Citations:** Authors, Title, HemaSphere, 2026;10:(S1):pages. The abstract book DOIs can be found on the “Author Information” page.
**Disclaimer:** Articles published in the journal HemaSphere exclusively reflect the opinions of the authors. The authors are responsible for all content in their abstracts including accuracy of the facts, statements, citing resources, etc.

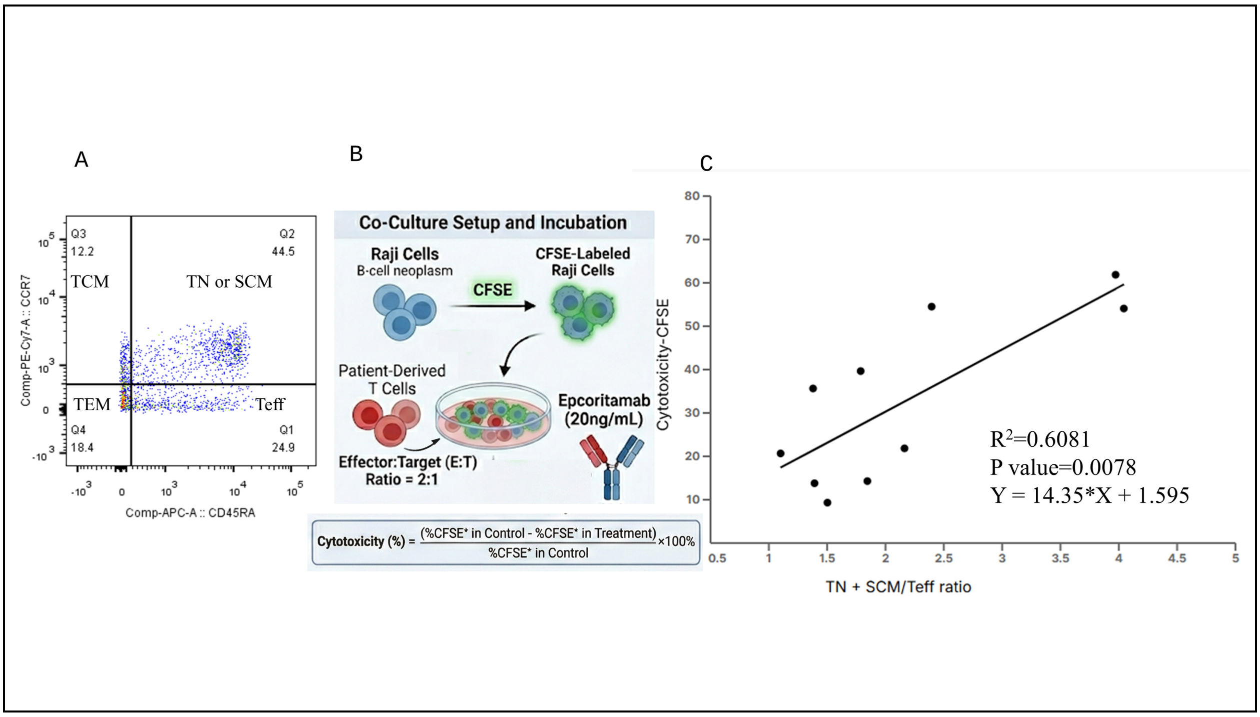

(A) Representative flow cytometry plot illustrating the gating strategy for T-cell subsets based on CCR7 and CD45RA expression. (B) Schematic representation of the in vitro co-culture workflow. (C) Linear regression analysis demonstrating the relationship between the (TN+TSCM)/Teff ratio and epcoritamab-mediated T-cell cytotoxicity.

**Copyright Information:** (Online) ISSN: 2572-9241
©2026 The Author(s). HemaSphere is published by John Wiley & Sons Ltd on behalf of European Hematology Association. This is an open access Abstract Book distributed under the Attribution-NonCommercial-NoDerivs (CC BY-NC-ND), which allows third parties to download the articles and share them with others as long as they credit the author and the Abstract Book, but they cannot change the content in any way or use them commercially.
**Abstract Book Citations:** Authors, Title, HemaSphere, 2026;10:(S1):pages. The abstract book DOIs can be found on the "Author Information" page.
**Disclaimer:** Articles published in the journal HemaSphere exclusively reflect the opinions of the authors. The authors are responsible for all content in their abstracts including accuracy of the facts, statements, citing resources, etc.

### (PB4115) RESULTS OF THE ANTI-CD19+ CAR-T THERAPY FOR R/R B-ALL IN CHILDREN AND YOUNG ADULTS IN THE REPUBLIC OF BELARUS

**Topic:** 26. Gene therapy, cellular immunotherapy and vaccination - Clinical

Inna Praliaskouskaya*[1], Volha Karas[1], Alexander Meleshko[1], Dzmitry Lutskovich[1], Natallia Klimkovich[2], Maryia Keraz[1]

*[1]Belarusian Research Center for Pediatric Oncology, Hematology, and Immunology, Minsk, Belarus;[2]Belarusian State Medical Univercity, Minsk, Belarus;*

### Background

Chimeric antigen receptor T-cell (CAR-T) therapy is a groundbreaking treatment for cancer that has shown significant promise in patients with relapsed/refractory acute lymphoblastic leukemia (R/R B-ALL). This abstract presents single-center results of anti-CD19 CAR-T therapy using locally manufactured CD19 CAR-T cells

### Methods

Nineteen patients (pts) with R/R B-ALL underwent anti-CD19 CAR-T therapy at the Belarusian Research Center for Pediatric Oncology, Hematology, and Immunology from 2020 to 2025. The median age at the time of CAR-T infusion was 11,2 years (min 3,7; max 30,3). Patients received the median amount of 2 (min 1; max 5) lines of treatment before CAR-T therapy. Eleven patients didn't have morphological remission, 4 pts had positive minimal residual disease (MRD+) measured by flow cytometry and/or RT-PCR, and 4 pts were MRD negative (-). The CAR construct consisted of mFMC63 single-chain variable fragment, IgG4 hinge, CD28 transmembrane domain, 4-1BB, and CD3ζ cytoplasmic signaling domain. A truncated form of the epidermal growth factor receptor served as a surface marker for tracking CAR-T cells. Lymphocytes were collected via leukapheresis followed by density centrifugation. The CAR-T products were manually produced from the separated CD4 and CD8 T cells, then were activated using CD3/CD28 Dynabeads and transduced with a lentiviral vector. CD4 and CD8 T cells were cultured separately in a medium containing IL-7, IL-15, and IL-21 and mixed before infusion in a ratio close to 1:1. After lymphodepletion, a single dose of CAR-T cells with a median dosage of 0,9 mln (0,1 – 2,75) cells/kg was infused.

### Results

The median peak CAR-T expansion in the blood reached 44.5 (1,683 – 7845,7) cells/µL on day 10 after infusion. Three out of 19 pts died before the initial evaluation of the therapy. Of the remaining 16 pts, the overall response rate (ORR) was 87,5% (14/16 pts). MRD negative remission was confirmed in 11 out of 14 responded pts (78,5%). Subsequently, 8 out of 14 pts underwent allogeneic hematopoietic stem cell transplantation. Regarding treatment toxicity, 5 out of 19 pts (26,3%) experienced CRS grade ≥3, including one patient with CRS grade 5. Median time to the start of CRS was 6 days (min 1; max 13). ICANS grade ≥3 was observed in 5 out of 19 pts (26,3%) including grade 5 in 3 pts. Median time to the start of ICANS was 8 days (min 4; max 10). Early ICAHT was registered in 10 out of 19 pts (52,6%). Grade ≥3 infection experienced 7 out of 19 pts (36,8%), including grade 5 in 2 pts. We didn't find statistically significant correlation between any grade CRS, ICANS, early ICAHT and tumor burden, CD4/CD8 CAR-T cell ratio, amount of previous lines of treatment. One-year overall survival (OS) rate for altogether MRD+ and MRD negative pts was significantly higher (75%±15,3%) compared to pts without morphological remission (36,4%±14,5%) (p=0,038), indicating that tumor burden has negative effect on the results of CAR-T therapy. The three-year OS rate was 52,6% ± 11.5%, and 3-year event-free survival rate was 28,1% ± 11.1%.

**Copyright Information:** (Online) ISSN: 2572-9241
©2026 The Author(s). HemaSphere is published by John Wiley & Sons Ltd on behalf of European Hematology Association. This is an open access Abstract Book distributed under the Attribution-NonCommercial-NoDerivs (CC BY-NC-ND), which allows third parties to download the articles and share them with others as long as they credit the author and the Abstract Book, but they cannot change the content in any way or use them commercially.
**Abstract Book Citations:** Authors, Title, HemaSphere, 2026;10:(S1):pages. The abstract book DOIs can be found on the "Author Information" page.
**Disclaimer:** Articles published in the journal HemaSphere exclusively reflect the opinions of the authors. The authors are responsible for all content in their abstracts including accuracy of the facts, statements, citing resources, etc.

**Summary/Conclusion**

CAR-T therapy is very effective in achieving complete remission in patients with R/R B-ALL, still remaining a bridge to further alloHSCT. The associated toxicity is manageable with timely intervention. Our results align well with real-world data, highlighting the significant role of CAR-T therapy in improving outcomes for patients with R/R B-ALL.

**Copyright Information:** (Online) ISSN: 2572-9241
©2026 The Author(s). HemaSphere is published by John Wiley & Sons Ltd on behalf of European Hematology Association. This is an open access Abstract Book distributed under the Attribution-NonCommercial-NoDerivs (CC BY-NC-ND), which allows third parties to download the articles and share them with others as long as they credit the author and the Abstract Book, but they cannot change the content in any way or use them commercially.
**Abstract Book Citations:** Authors, Title, HemaSphere, 2026;10:(S1):pages. The abstract book DOIs can be found on the "Author Information" page.
**Disclaimer:** Articles published in the journal HemaSphere exclusively reflect the opinions of the authors. The authors are responsible for all content in their abstracts including accuracy of the facts, statements, citing resources, etc.

### (PB4116) OUTCOMES AND DURATION OF HOSPITALISATION IN PATIENTS RECEIVING CAR-T CELL THERAPY FOR B-CELL LYMPHOMA: A SINGLE CENTRE REVIEW

**Topic:** 26. Gene therapy, cellular immunotherapy and vaccination - Clinical

Sara Stefani*[1], Chao Jiang[1], Lisanne Schoutens[1], Andrew Morrow[1], Joanna Han[1], Natalia Czub[1], Kirsten Rendall[1], Kushani Ediriwickrema[1], Graham Collins[1], Katerina Panopoulou[1]

[1]*Oxford University Hospitals, Oxford, United Kingdom;*

### Background

Studies show that a shorter brain-to-vein (B2V) and vein-to-vein (V2V) time might improve outcomes before CD-19 directed CAR-T infusion, however the effects on longer term disease control are not clear. Most UK centres mandate hospitalisation for at least 14 days following CAR-T infusion, however others have trialled shorter inpatient stays, or even ambulatory CAR-T treatment.

### Aims

To understand how B2V/V2V time and pre-infusion disease status influence CAR-T outcomes, and to explore variables affecting duration of hospitalisation in patients receiving CAR-T.

### Methods

Patients who received CAR-T for DLBCL and PMBCL at Oxford University Hospitals between June 2023 and December 2025 were included. Data on demographics, bridging, complications, and outcomes, were retrospectively collected via electronic patient records. B2V was defined as days between decision to proceed with CAR-T and infusion, and V2V as days between apheresis and infusion. Descriptive statistics were utilised to describe the population, complication rates, and outcomes. Independent variables were analysed by independent sample T-test and one-way ANOVA test; survival was estimated via the Kaplan Meier method.

### Results

49 patients met the inclusion criteria. 54% were male; median age was 61 (IQR 53-72). 94% received CAR-T for DLBCL (7% with CNS relapse), and 6% for PMBCL. 43% received CAR-T as second line therapy, and 57% as third line. The most common bridging regimens were radiotherapy (29%), RGDP (27%), and Pola-BR (20%); 68% received 1 cycle, 26% 2, and 6% 3 cycles. Median B2V and V2V times were 50 and 38 days respectively. Prior to CAR-T infusion, 18% were in complete metabolic response (CMR), 55% partial metabolic response (PMR), and 27% progressive disease (PD). 94% received Axi-cel, and 6% Liso-cel. 92% developed CRS, and 51% ICANS. The median interval between CAR-T cell infusion and being fit for discharge was 14 days (IQR 13-22); 31% were fit to be discharged before day 14. Factors influencing length of the inpatient stay included ICU admission ($p=0.025$) and viral infections ($p<0.05$).
Median follow-up was 13 months. Of 45 patients who had a PET scan at 3-months post CAR-T, 67% were in CMR, 20% PMR, and 13% PD; 3-month mortality was 7%. Disease status pre-CAR-T infusion strongly correlated with response at 3 months; 78% of patients who were in CMR, and 72% of those in PMR at infusion achieved CMR at 3 months, compared to 45% of those who had PD ($p=0.001$). One-year overall survival was 59%. Response at 3 months predicted longer term survival ($p<0.001$), with 87% of those in CMR at 3 months still in remission at 1-year follow up.
There was no significant association between B2V or V2V time and outcome at 3 months. Median B2V time was 50 days for those in CMR, 49 days for those in PMR, and 48 days for patients with PD. Median V2V was 37 days for those in CMR, 38 days for those in PMR, and 35 for those with PD.

**Copyright Information:** (Online) ISSN: 2572-9241
©2026 The Author(s). HemaSphere is published by John Wiley & Sons Ltd on behalf of European Hematology Association. This is an open access Abstract Book distributed under the Attribution-NonCommercial-NoDerivs (CC BY-NC-ND), which allows third parties to download the articles and share them with others as long as they credit the author and the Abstract Book, but they cannot change the content in any way or use them commercially.
**Abstract Book Citations:** Authors, Title, HemaSphere, 2026;10:(S1):pages. The abstract book DOIs can be found on the “Author Information” page.
**Disclaimer:** Articles published in the journal HemaSphere exclusively reflect the opinions of the authors. The authors are responsible for all content in their abstracts including accuracy of the facts, statements, citing resources, etc.

**Summary/Conclusion**

While logistical delays (B2V/V2V) are a focus of service delivery, they did not significantly impact post-infusion outcomes in this cohort. Instead, disease status on the day of infusion—largely driven by bridging efficacy—is the critical predictor of long-term remission. Our findings also suggest that nearly one-third of patients could be managed via ambulatory pathways, with ICU admission and viral complications being the primary barriers to early discharge. These data support the use of intensive bridging (including RT and novel agents) to achieve pre-infusion disease control.

**Copyright Information:** (Online) ISSN: 2572-9241
©2026 The Author(s). HemaSphere is published by John Wiley & Sons Ltd on behalf of European Hematology Association. This is an open access Abstract Book distributed under the Attribution-NonCommercial-NoDerivs (CC BY-NC-ND), which allows third parties to download the articles and share them with others as long as they credit the author and the Abstract Book, but they cannot change the content in any way or use them commercially.
**Abstract Book Citations:** Authors, Title, HemaSphere, 2026;10:(S1):pages. The abstract book DOIs can be found on the “Author Information” page.
**Disclaimer:** Articles published in the journal HemaSphere exclusively reflect the opinions of the authors. The authors are responsible for all content in their abstracts including accuracy of the facts, statements, citing resources, etc.

### (PB4117) FULLY OUTPATIENT ADMINISTRATION OF BLINATUMOMAB AS CONSOLIDATION IN ADULT B-CELL PRECURSOR ACUTE LYMPHOBLASTIC LEUKEMIA: A REAL-WORLD EXPERIENCE

**Topic:** 26. Gene therapy, cellular immunotherapy and vaccination - Clinical

Beatriz Merchan Muñoz*[1], Alexandra Martínez Roca[1], Nuria Ballestar[1], Cristina Gallego[1], Anna Estanyol[1], Montserrat Carreño[1], Susana Segura[1], Laura Villa[1], Esther Carcelero[1], Inés Monge[1], David López Sisamon[1], Iñaki Nos[1], Marina Diaz Beya[1], Nuria Martinez-Cibrian[1], Jordi Esteve Reyner[1], Francesc Fernandez Aviles[1]

[1]*Hospital Clínic Barcelona, Barcelona, Spain;*

### Background

The addition of Blinatumomab, a CD19×CD3 bispecific T-cell engager (BiTE), to consolidation chemotherapy has improved overall survival in Ph negative adult B-cell precursor acute lymphoblastic leukemia (Ph- BCP-ALL). Therefore, it has increasingly been incorporated into first-line treatment schedules. Owing to the risks of cytokine release syndrome (CRS) and neurotoxicity, initial inpatient administration is recommended. Evidence on fully outpatient delivery supported by structured home-care programs remains limited.

### Aims

To evaluate the feasibility and safety of fully outpatient blinatumomab consolidation in adult patients with Ph- BCP-ALL in first complete morphological remission (CR1).

### Methods

We performed a real-world observational study including consecutive adult newly diagnosed with Ph- BCP-ALL in CR1 who received blinatumomab consolidation within a fully outpatient program between August 2025 and March 2026.
Blinatumomab was administered as a continuous intravenous infusion for 28 days at a constant rate (2.5 mL/h) via a programmable, lockable infusion pump with 96-hour infusion bags. Treatment was initiated in the outpatient day-care unit with short-term observation; in the absence of early toxicity, infusion continued at home. All subsequent care, pump changes, and monitoring occurred at the patient's home.
A specialized home-care team conducted structured follow-up including telephone assessments every 12 hours during the first 14 days of each cycle to evaluate symptoms, vital signs, infusion device function, and Immune Effector Cell Encephalopathy score. From day 14 onward, monitoring frequency was individualized according to clinical stability. In-person nursing visits with blood tests were carried out every 96 hours for pump replacement. CRS and neurotoxicity were graded according to ASTCT criteria

**Copyright Information:** (Online) ISSN: 2572-9241
©2026 The Author(s). HemaSphere is published by John Wiley & Sons Ltd on behalf of European Hematology Association. This is an open access Abstract Book distributed under the Attribution-NonCommercial-NoDerivs (CC BY-NC-ND), which allows third parties to download the articles and share them with others as long as they credit the author and the Abstract Book, but they cannot change the content in any way or use them commercially.
**Abstract Book Citations:** Authors, Title, HemaSphere, 2026;10:(S1):pages. The abstract book DOIs can be found on the "Author Information" page.
**Disclaimer:** Articles published in the journal HemaSphere exclusively reflect the opinions of the authors. The authors are responsible for all content in their abstracts including accuracy of the facts, statements, citing resources, etc.

**Results**

Ten fully outpatient blinatumomab consolidation cycles were administered in 6 patients (6 completed one cycle; 4 completed two cycles). Median age was 45.5 years (range 22–76). Median duration of at-home follow-up was 28 days (range 28–33).
CRS occurred in 3 patients (30%), all grade 1, with a median onset on day +3 of cycle 1 (range day +2 to +4). All events resolved after administration of additional dexamethasone, without hospital admission. No grade ≥2 CRS or ICANS events were observed.
Non-CRS adverse events included grade 2 transaminitis (n=2), one deep vein thrombosis, and two infectious complications (one upper respiratory tract infection due to respiratory syncytial virus and one catheter-related *Pseudomonas aeruginosa* infection). The catheter-related infection resolved after catheter removal and antibiotic therapy, requiring a temporary 3-day interruption of blinatumomab but no hospitalization.
Infusion device–related issues prompted 8 additional outpatient visits, due to pump occlusion alarms, blood reflux, or accidental port-a-cath disconnection. All were managed without hospital admission. No ICU admissions or treatment-related deaths occurred.

**Summary/Conclusion**

Fully outpatient blinatumomab consolidation for adult patients with Ph- BCP-ALL in first remission is feasible and safe within a structured home-care program, with low toxicity, no hospital admissions, and effective management of adverse events and device-related issues in the outpatient setting.

**Copyright Information:** (Online) ISSN: 2572-9241
©2026 The Author(s). HemaSphere is published by John Wiley & Sons Ltd on behalf of European Hematology Association. This is an open access Abstract Book distributed under the Attribution-NonCommercial-NoDerivs (CC BY-NC-ND), which allows third parties to download the articles and share them with others as long as they credit the author and the Abstract Book, but they cannot change the content in any way or use them commercially.
**Abstract Book Citations:** Authors, Title, HemaSphere, 2026;10:(S1):pages. The abstract book DOIs can be found on the “Author Information” page.
**Disclaimer:** Articles published in the journal HemaSphere exclusively reflect the opinions of the authors. The authors are responsible for all content in their abstracts including accuracy of the facts, statements, citing resources, etc.

### (PB4118) REAL-WORLD INCIDENCE AND MANAGEMENT OF CRS AND ICANS FOLLOWING CD3×CD20 BISPECIFIC ANTIBODY THERAPY IN B-CELL LYMPHOMAS

**Topic:** 26. Gene therapy, cellular immunotherapy and vaccination - Clinical

Youssef (Joe) Samaha*[1], Roshan Asrani[2], Alexis Ludy[2], Noa Rippel[2], Theodora Anagnostou[1]

*[1]Icahn School of Medicine at Mount Sinai, New York, United States of America;[2]Icahn School of Medicine at Mount Sinai, Hematology/Oncology, New York, United States of America;*

### Background

CD3xCD20 bispecific antibodies (BsAbs) are used for relapsed/refractory B-cell lymphomas, but cytokine release syndrome (CRS) and immune effector cell–associated neurotoxicity syndrome (ICANS) remain clinically relevant toxicities. Real-world management varies without standardized pathways.

### Aims

To characterize incidence, severity, timing, and management of CRS and ICANS following CD3xCD20 BsAbs, and to evaluate ICU utilization and CRS/ICANS-related mortality as markers of toxicity severity.

### Methods

We performed a retrospective, single-center review of adults with lymphoma treated with CD3xCD20 BsAbs at Mount Sinai Hospital (Jan 2021–Dec 2024). CRS and ICANS were graded per ASTCT criteria. Exploratory comparisons used Fisher's exact test. CRS/ICANS-related mortality was defined as death primarily attributable to CRS or ICANS on chart review.

### Results

Among 63 patients, 35 received glofitamab, 25 epcoritamab, and 5 mosunetuzumab. Median age was 65 years (IQR 57–73). Aggressive B-cell lymphomas comprised 43/63 (68.3%), indolent lymphomas 13/63 (20.6%), and other entities 7/63 (11.1%). CRS occurred in 35/63 (55.6%): grade 1 in 18 (28.6%), grade 2 in 13 (20.6%), and grade 3 in 4 (6.3%); no grade 4–5 events occurred. Grade >=2 CRS occurred in 17/63 (27.0%). By agent, CRS occurred in 19/35 (54.3%) with glofitamab, 15/25 (60.0%) with epcoritamab, and 1/5 (20.0%) with mosunetuzumab; grade >=2 CRS occurred in 11/35 (31.4%), 6/25 (24.0%), and 0/5 (0%), respectively.

All CRS and ICANS events occurred early (median 1–3 days; range 1–7). Grade 1 CRS (n=18) was managed with supportive care alone in 10 (55.6%), tocilizumab in 6 (33.3%), and corticosteroids in 2 (11.1%). Grade 2 CRS (n=13) was treated with tocilizumab and corticosteroids in 10 (76.9%) each, including combination therapy in 9 (69.2%); supportive care alone was used in 2 (15.4%). All grade 3 CRS (n=4) received tocilizumab and corticosteroids.

ICANS occurred in 6/63 (9.5%): grade 1 in 1 (1.6%), grade 3 in 4 (6.3%), and grade 4 in 1 (1.6%); grade >=3 ICANS occurred in 5/63 (7.9%). All ICANS cases occurred with concurrent CRS. Incidence was 5/35 (14.3%) with glofitamab, 1/25 (4.0%) with epcoritamab, and 0/5 (0%) with mosunetuzumab. All ICANS cases received corticosteroids; 5/6 (83.3%) received anakinra.

Nine of 63 patients (14.3%) required ICU admission (glofitamab 7/35 [20.0%], epcoritamab 2/25 [8.0%], mosunetuzumab 0/5 [0%]). Differences in grade >=2 CRS and grade >=3 ICANS across agents were not statistically significant (all p>0.05). CRS resolved in 32/35 patients (91.4%). CRS/ICANS-related mortality occurred in 3/35 patients with CRS (8.6%), all with aggressive B-cell lymphomas treated with glofitamab and severe CRS/ICANS.

**Copyright Information:** (Online) ISSN: 2572-9241
©2026 The Author(s). HemaSphere is published by John Wiley & Sons Ltd on behalf of European Hematology Association. This is an open access Abstract Book distributed under the Attribution-NonCommercial-NoDerivs (CC BY-NC-ND), which allows third parties to download the articles and share them with others as long as they credit the author and the Abstract Book, but they cannot change the content in any way or use them commercially.
**Abstract Book Citations:** Authors, Title, HemaSphere, 2026;10:(S1):pages. The abstract book DOIs can be found on the "Author Information" page.
**Disclaimer:** Articles published in the journal HemaSphere exclusively reflect the opinions of the authors. The authors are responsible for all content in their abstracts including accuracy of the facts, statements, citing resources, etc.

### Summary/Conclusion

CD3xCD20 BsAbs were associated with predominantly low-grade CRS and 9.5% ICANS, with all events occurring within the first week. Grade >=2 CRS, grade >=3 ICANS, ICU utilization, and CRS/ICANS-related mortality identified a clinically significant subset requiring escalation. Given early onset kinetics, centers should prioritize proactive monitoring during treatment initiation and standardized, severity-based management pathways.

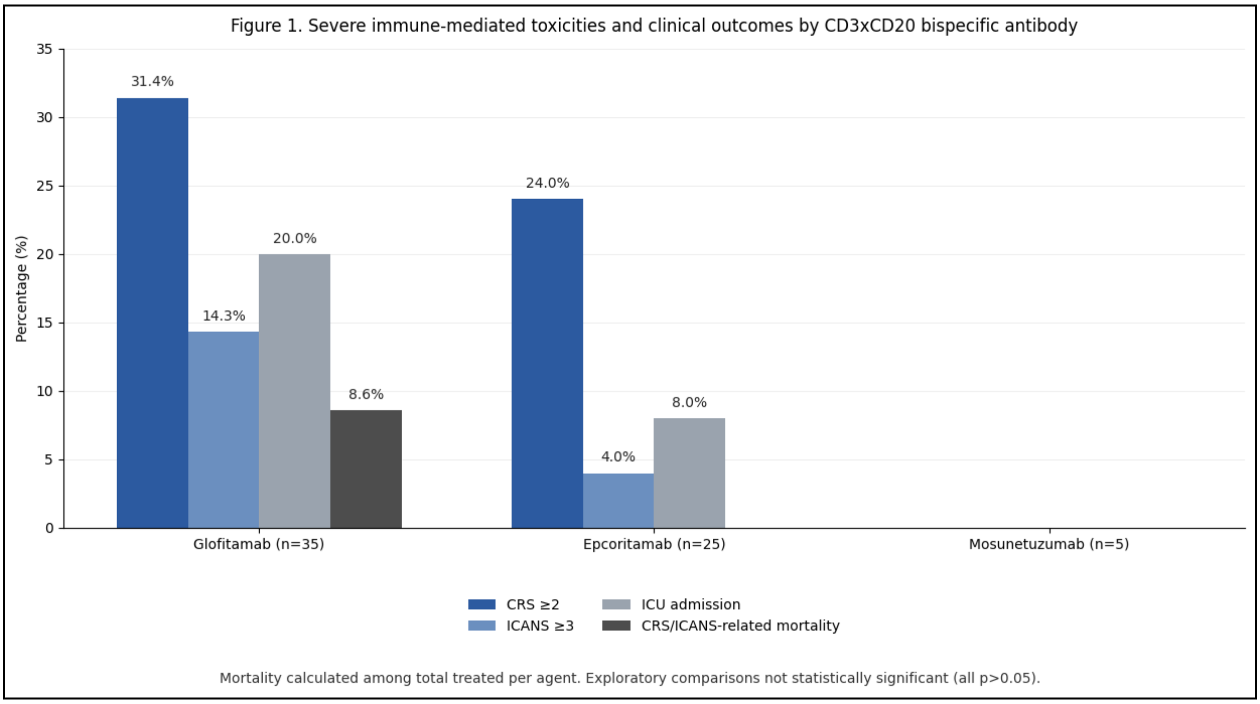

Mortality calculated among total treated per agent. CRS graded per ASTCT criteria; CRS endpoint shown is grade ≥2. ICANS graded per ASTCT criteria; ICANS endpoint shown is grade ≥3. ICU admission reflects any toxicity-related ICU-level care. Exploratory comparisons between agents were not statistically significant (all $p>0.05$).

**Copyright Information:** (Online) ISSN: 2572-9241
©2026 The Author(s). HemaSphere is published by John Wiley & Sons Ltd on behalf of European Hematology Association. This is an open access Abstract Book distributed under the Attribution-NonCommercial-NoDerivs (CC BY-NC-ND), which allows third parties to download the articles and share them with others as long as they credit the author and the Abstract Book, but they cannot change the content in any way or use them commercially.
**Abstract Book Citations:** Authors, Title, HemaSphere, 2026;10:(S1):pages. The abstract book DOIs can be found on the "Author Information" page.
**Disclaimer:** Articles published in the journal HemaSphere exclusively reflect the opinions of the authors. The authors are responsible for all content in their abstracts including accuracy of the facts, statements, citing resources, etc.

### (PB4119) OBSERVATIONAL STUDY OF LOCALLY PRODUCED CD19-TARGETED CAR-T CELL THERAPY FOR PATIENTS WITH RELAPSED/REFRACTORY DIFFUSE LARGE B CELL LYMPHOMA

**Topic:** 26. Gene therapy, cellular immunotherapy and vaccination - Clinical

Kevin Tey*[1], Joyin Lee[1], Naim Kamaruddin[1], Hong Huat Hoh[1], Dhakshyani Kalai Chelvan[1], Kian Meng Chang[2], Chieh-Lee Wong[2], Andy Tang[2], Siong Leng Hon[2], Henning Loo[2], Kim Wah Ho[2]

*[1]Sunway Medical Centre, Clinical Research Centre, Bandar Sunway, Malaysia;[2]Sunway Medical Centre, Haematology, Bandar Sunway, Malaysia;*

### Background

Chimeric antigen receptor T-cell (CAR-T) therapy targeting CD19 has emerged as a ground-breaking treatment for relapsed or refractory diffuse large B-cell lymphoma (RR-DLBCL), demonstrating remarkable efficacy in clinical trials globally. However, its implementation in Malaysia faces unique challenges, including high costs, limited infrastructure, and the limited clinical data on locally produced CD19-targeted CAR-T.

### Aims

The present study aims to evaluate the safety and efficacy of locally produced CD19 CAR-T therapy in RR-DLBCL patients.

### Methods

This observational study examined RR-DLBCL patients at Sunway Medical Centre who underwent CD19 CAR-T therapy through compassionate use after failing two or more lines of treatment. Informed consent was obtained from the patients before extracting their demographic data, clinical presentations, treatment parameters, and outcomes from medical records. Statistical analysis was subsequently conducted to elucidate the frequencies, measures of central tendencies, duration-to-event, as well as treatment indicators and outcomes through inferential T-test.

### Results

A total of 19 patients with RR-DLBCL had undergone CD19 CAR-T therapy. A near proportionate ratio of male to female patients (0.9:1) was observed, with a majority of patients being of Chinese ethnicity (78.9%) and aged between 25 to 71 years (median: 55 years) at CD19 CAR-T infusion. The median number of treatments received prior to CD19 CAR-T therapy was three (range: 2 to 5). CD19 CAR-T therapy was administered between 6 to 91 months (median: 16 months) following initial diagnosis. Most patients received one infusion of CD19 CAR-T with a mean dose of $2.96 \times 10^6$ and mean cell viability of 86.1%. Twelve patients experienced grade I-II cytokine release syndrome (CRS) between day 7 to 9 post-infusion, while two patients presented with grade III-IV CRS. The most common symptom exhibited by patients with CRS was high fever (71.4%). Four patients developed immune effector cell-associated neurotoxicity syndrome (ICANS) in a median of 10.5 days post-infusion, with the most common symptom exhibited being tremors (66.0%). Subsequently, four patients experienced infection with a median of 3 days (range:1-13 days). Additionally, three patients developed hemophagocytic lymphohistiocytosis within 4 to 34 days post infusion. Complete response was achieved in 31.6% patients, with 21.1% patients showing partial response, 10.5% with stable disease, and 36.8% patients exhibiting disease progression. Patients with >3 lines of treatment prior to CD19 CAR-T infusion had a significantly higher risk (75.0%) of ICANS ($p = 0.013$) compared to patients with ≤3 lines of treatment. Patients receiving CAR-T dose with $\leq 3 \times 10^6$ cells/kg had a slightly better disease response (72.7%, $p = 0.053$) and overall survival (72.7%, $p < 0.001$) compared to patients receiving $>3 \times 10^6$ cells/kg (disease response: 40%; overall survival: 20%).

**Copyright Information:** (Online) ISSN: 2572-9241
©2026 The Author(s). HemaSphere is published by John Wiley & Sons Ltd on behalf of European Hematology Association. This is an open access Abstract Book distributed under the Attribution-NonCommercial-NoDerivs (CC BY-NC-ND), which allows third parties to download the articles and share them with others as long as they credit the author and the Abstract Book, but they cannot change the content in any way or use them commercially.
**Abstract Book Citations:** Authors, Title, HemaSphere, 2026;10:(S1):pages. The abstract book DOIs can be found on the “Author Information” page.
**Disclaimer:** Articles published in the journal HemaSphere exclusively reflect the opinions of the authors. The authors are responsible for all content in their abstracts including accuracy of the facts, statements, citing resources, etc.

**Summary/Conclusion**

In this small observational study, CD19 CAR-T therapy was observed to be relatively safe with the low incidence of high-grade (III/IV) CRS and ICANS in patients with RR-DLBCL. However, further monitoring of cytokine levels post-infusion is recommended, especially for patients experiencing a concurrent infective episode. Additionally, long-term monitoring of circulatory CD19 CAR-T cells post-infusion would provide a more comprehensive overview of the efficacy and longevity of CD19 CAR-T cell therapy for RR-DLBCL, particularly in patients with disease progression.

**Copyright Information:** (Online) ISSN: 2572-9241
©2026 The Author(s). HemaSphere is published by John Wiley & Sons Ltd on behalf of European Hematology Association. This is an open access Abstract Book distributed under the Attribution-NonCommercial-NoDerivs (CC BY-NC-ND), which allows third parties to download the articles and share them with others as long as they credit the author and the Abstract Book, but they cannot change the content in any way or use them commercially.
**Abstract Book Citations:** Authors, Title, HemaSphere, 2026;10:(S1):pages. The abstract book DOIs can be found on the "Author Information" page.
**Disclaimer:** Articles published in the journal HemaSphere exclusively reflect the opinions of the authors. The authors are responsible for all content in their abstracts including accuracy of the facts, statements, citing resources, etc.

### (PB4120) IMMUNE EFFECTOR CELL (IEC)–ASSOCIATED HEMOPHAGOCYTIC LYMPHOHISTIOCYTOSIS–LIKE SYNDROME (IEC-HS) IN PATIENTS WITH HEMATOLOGIC MALIGNANCIES: A SYSTEMATIC REVIEW AND META-ANALYSIS

**Topic:** 26. Gene therapy, cellular immunotherapy and vaccination - Clinical

Leen Haj Saleh[1], Marrita Rabadi[1], Aadhila Manthiri[2], Ayman Dalol[1], Usra Elshaikh[3], Abdulrahman F. Al-Mashdali[4], Mohammed Mohammed[4], Shehab Fareed*[4]

*[1]Qatar University, College of Medicine, Doha, Qatar; [2]Qatar University, College of Pharmacy, Doha, Qatar; [3]Qatar University, Department of Public Health, College of Health Sciences, Doha, Qatar; [4]Hamad Medical Corporation, Hematology Department, National Center for Cancer Care and Research, Doha, Qatar;*

### Background

Chimeric antigen receptor (CAR) T-cell therapy has transformed hematologic malignancy treatment, achieving high remission rates in relapsed/refractory disease. However, it can trigger immune effector cell–associated HLH-like syndrome (IEC-HS), a severe hyperinflammatory toxicity distinct from classical HLH.

### Aims

We conducted a systematic review and meta-analysis to estimate pooled incidence, characterize outcomes, evaluate management strategies, and identify risk factors for IEC-HS following CAR-T therapy.

### Methods

A comprehensive literature search of PubMed, Scopus, and Embase was performed to identify relevant studies. Data on incidence, clinical and laboratory features, management, and survival outcomes were extracted. Pooled incidence estimates were calculated using a random-effects model with Freeman–Tukey double arcsine transformation. Heterogeneity was assessed using the $I^2$ statistic, and publication bias evaluated with Egger's regression test.

### Results

Out of 692 records, 35 studies including 8,573 CAR-T recipients were analyzed, of whom 396 developed IEC-HS/HLH. 18 studies were eligible for meta-analysis. The pooled incidence of IEC-HS was 10.0% (95% CI: 7.0–14.0; $I^2$ = 92.4%). Mortality among affected patients was substantial, with HLH-related deaths reported despite immunosuppressive therapy.

### Summary/Conclusion

Our meta-analysis shows that IEC-HS occurs in a notable proportion of patients after CAR T-cell therapy and is associated with significant morbidity. These findings emphasize the need for standardized diagnostic criteria and validated management strategies to improve recognition and outcomes.

**Copyright Information:** (Online) ISSN: 2572-9241
©2026 The Author(s). HemaSphere is published by John Wiley & Sons Ltd on behalf of European Hematology Association. This is an open access Abstract Book distributed under the Attribution-NonCommercial-NoDerivs (CC BY-NC-ND), which allows third parties to download the articles and share them with others as long as they credit the author and the Abstract Book, but they cannot change the content in any way or use them commercially.
**Abstract Book Citations:** Authors, Title, HemaSphere, 2026;10:(S1):pages. The abstract book DOIs can be found on the "Author Information" page.
**Disclaimer:** Articles published in the journal HemaSphere exclusively reflect the opinions of the authors. The authors are responsible for all content in their abstracts including accuracy of the facts, statements, citing resources, etc.

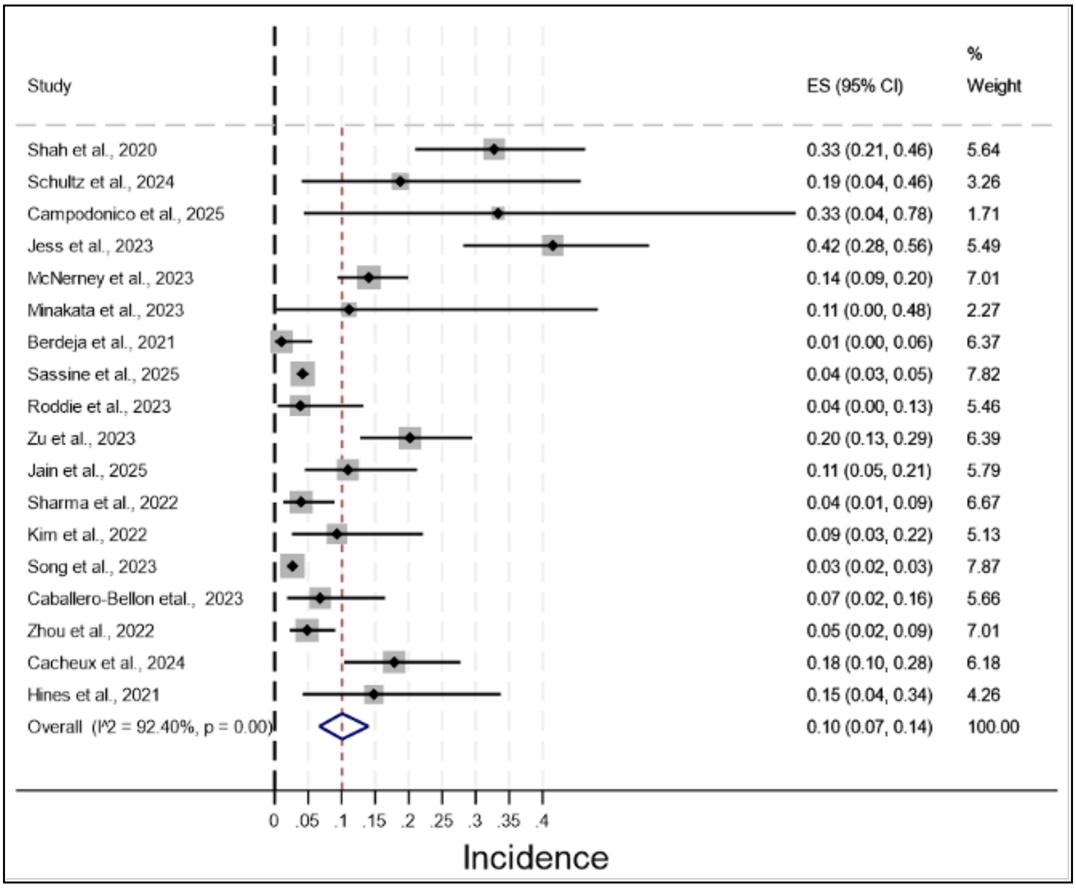

Meta-analysis of HLH Pooled Cumulative Incidence

**Copyright Information:** (Online) ISSN: 2572-9241
©2026 The Author(s). HemaSphere is published by John Wiley & Sons Ltd on behalf of European Hematology Association. This is an open access Abstract Book distributed under the Attribution-NonCommercial-NoDerivs (CC BY-NC-ND), which allows third parties to download the articles and share them with others as long as they credit the author and the Abstract Book, but they cannot change the content in any way or use them commercially.
**Abstract Book Citations:** Authors, Title, HemaSphere, 2026;10:(S1):pages. The abstract book DOIs can be found on the "Author Information" page.
**Disclaimer:** Articles published in the journal HemaSphere exclusively reflect the opinions of the authors. The authors are responsible for all content in their abstracts including accuracy of the facts, statements, citing resources, etc.

### (PB4122) ASSOCIATION BETWEEN INFUSION TIME AND OUTCOMES OF ANTI-CD19 CAR-T CELL THERAPY

**Topic:** 26. Gene therapy, cellular immunotherapy and vaccination - Clinical

Nicole-Ann Lim*[1], Yeow Kheong Woon[1], Kye Ling Wong[1], Francesca Lorraine Wei Inng Lim[1], Shin Yeu Ong[1], Chandramouli Nagarajan[1], Xiu Hue Lee[1], Jing Yuan Tan[1], William Hwang[1], Jeffrey Quek[1], Than Hein[1], Yeh Ching Linn[1], Tertius Tuy[1], Chieh Hwee Ang[1], Aloysius Ho[1], Yunxin Chen[1], Lawrence Cheng Kiat Ng[1]

*[1]Singapore General Hospital, Haematology, Singapore, Singapore;*

### Background

Circadian rhythm is a biologic cycle of approximately 24hours that regulates many physiologic and behavioural functions in living beings, that include immune system, cellular metabolism and proliferation. Preclinical work indicates that antitumor T-cell immunity oscillates across the circadian cycle, reflected by phase-specific differences in CD8+ tumor infiltration and function. Recent studies suggested that time of infusion potentially affect the effectiveness of immunotherapy and CAR-T cell therapy in cancer treatment.

### Aims

To evaluate the association of time of CAR-T infusion with clinical efficacy and safety outcome in Singapore – an equatorial country with minimal seasonal circadian variation.

### Methods

We retrospectively analyse data of patients underwent CD19 CAR-T therapy for aggressive large B-cell lymphoma from the Singapore General Hospital CAR-T registry (2020-2025).

**Copyright Information:** (Online) ISSN: 2572-9241
©2026 The Author(s). HemaSphere is published by John Wiley & Sons Ltd on behalf of European Hematology Association. This is an open access Abstract Book distributed under the Attribution-NonCommercial-NoDerivs (CC BY-NC-ND), which allows third parties to download the articles and share them with others as long as they credit the author and the Abstract Book, but they cannot change the content in any way or use them commercially.
**Abstract Book Citations:** Authors, Title, HemaSphere, 2026;10:(S1):pages. The abstract book DOIs can be found on the “Author Information” page.
**Disclaimer:** Articles published in the journal HemaSphere exclusively reflect the opinions of the authors. The authors are responsible for all content in their abstracts including accuracy of the facts, statements, citing resources, etc.

### Results

Total 39 patients were included in the analysis. Based on a median daily infusion time of 1330 HR, patients were categorized into an early infusion cohort (early infusers; 0800–1355 HR; n=19) and a late infusion cohort (late infusers; after 1400 HR; n=20).
Baseline characteristics were similar between the two cohorts with respect to age, sex, ECOG performance status, and histological subtypes. Yescarta was used in 21% and 40% of patients in the early and late infusion cohorts, respectively (p=0.20). Late infusers had a higher median number of prior lines of therapy compared with early infusers (3 vs 2), resulting in a greater proportion of patients with >2 prior lines of therapy in the late infusion cohort (70% vs 37%, p=0.04). At the time of CAR-T infusion, 42% of early infusers were in CR/PR compared with 30% of late infusers (p=0.43). Numerically more early infusers had elevated LDH levels pre-infusion compared with late infusers, 53% vs 35% (p=0.26). (Table 1).
At a median follow-up of 13 months, a trend toward a higher CR rate was observed in early infusers compared with late infusers (63% vs 45%, p=0.26). This was associated with superior 1-year PFS in the early infusion cohort (67% vs 53%, p=0.03). One-year OS was similar between early and late infusers (75% vs 82%, p=0.39). Trend towards more frequent ≥grade 3 CAR-T–related toxicities were observed in early infusers compared with late infusers, including CRS (16% vs 0%, p=0.13), ICANS (21% vs 5%, p=0.84), and infections (47% vs 20%, p=0.26). Non-relapse mortality was 5% in both cohorts. Notably, on multivariate analysis adjusted for early vs late infusion time, elevated vs normal LDH and 1-2 vs >2 prior line of therapies, none retained prognostic significance.

**Table 1. Baseline characteristic (n=39)**

| | | Early Infusion (8am-2pm) n=19 | % | Late Infusion (after 2pm) N=20 | % | p-value |
|---|---|---|---|---|---|---|
| Median age | | 61 (37-76) | | 58 (20-80) | | |
| Gender | Male | 9 | 47 | 10 | 50 | 0.87 |
| | Female | 10 | 53 | 10 | 50 | |
| ECOG | 0-1 | 16 | 84 | 19 | 95 | 0.27 |
| | 2-3 | 3 | 16 | 1 | 5 | |
| Diseases | DLBCL | 11 | 58 | 11 | 55 | 0.85 |
| | tFL | 8 | 42 | 8 | 50 | |
| | PMBL | 0 | 0 | 1 | 5 | |
| Median line prior therapies | | 2 (1-5) | | 3 (1-8) | | |
| | 1-2 | 12 | 63 | 6 | 30 | 0.04* |
| | >2 | 7 | 37 | 14 | 70 | |
| Prior ASCT | | 3 | 16 | 5 | 25 | 0.48 |
| Disease status at CAR-T infusion | CR/PR | 8 | 42 | 6 | 30 | 0.43 |
| | SD/PD | 11 | 58 | 14 | 70 | |
| CD19 CAR-T product | Yescarta | 4 | 21 | 8 | 40 | 0.20 |
| | Kymriah/41BB academic | 15 | 79 | 12 | 60 | |
| Elevated LDH at pre-infusion | | 10/19 | 53 | 7/20 | 35 | 0.26 |

### Summary/Conclusion

Earlier CAR-T infusion time may be associated with improved PFS despite higher pre-infusion LDH levels and increased rates of grade ≥3 toxicities. However, these findings should be interpreted with caution due to imbalances in the number of prior therapies between cohorts and the small sample size.

**Copyright Information:** (Online) ISSN: 2572-9241
©2026 The Author(s). HemaSphere is published by John Wiley & Sons Ltd on behalf of European Hematology Association. This is an open access Abstract Book distributed under the Attribution-NonCommercial-NoDerivs (CC BY-NC-ND), which allows third parties to download the articles and share them with others as long as they credit the author and the Abstract Book, but they cannot change the content in any way or use them commercially.
**Abstract Book Citations:** Authors, Title, HemaSphere, 2026;10:(S1):pages. The abstract book DOIs can be found on the "Author Information" page.
**Disclaimer:** Articles published in the journal HemaSphere exclusively reflect the opinions of the authors. The authors are responsible for all content in their abstracts including accuracy of the facts, statements, citing resources, etc.

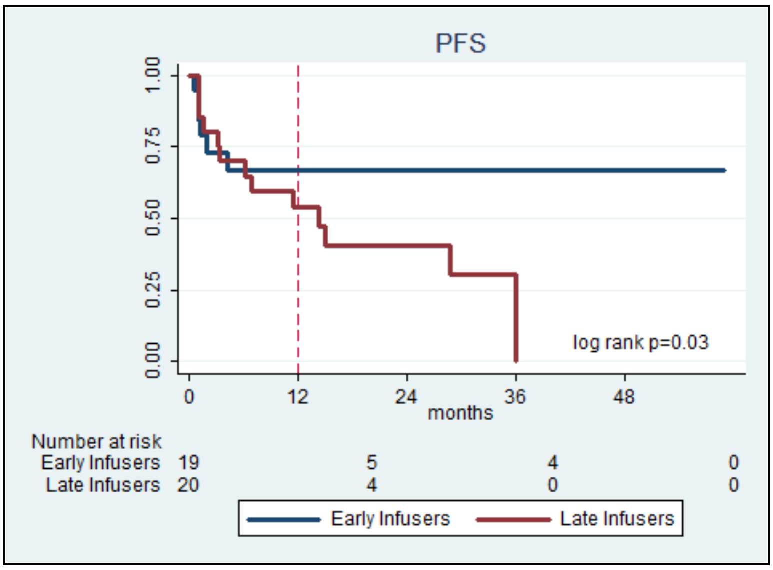

**Copyright Information:** (Online) ISSN: 2572-9241
©2026 The Author(s). HemaSphere is published by John Wiley & Sons Ltd on behalf of European Hematology Association. This is an open access Abstract Book distributed under the Attribution-NonCommercial-NoDerivs (CC BY-NC-ND), which allows third parties to download the articles and share them with others as long as they credit the author and the Abstract Book, but they cannot change the content in any way or use them commercially.
**Abstract Book Citations:** Authors, Title, HemaSphere, 2026;10:(S1):pages. The abstract book DOIs can be found on the "Author Information" page.
**Disclaimer:** Articles published in the journal HemaSphere exclusively reflect the opinions of the authors. The authors are responsible for all content in their abstracts including accuracy of the facts, statements, citing resources, etc.

### (PB4123) YUPINGFENG FOR THE MANAGEMENT OF PERSISTENT THROMBOCYTOPENIA FOLLOWING IMMUNOTHERAPIES: A CASE SERIES

**Topic:** 26. Gene therapy, cellular immunotherapy and vaccination - Clinical

Xuzhao Zhang*[1], Qian Luo[1], Huawei Jiang[1], Xi Qiu[1], Zhaoxing Wu[1], Liansheng Huang[1], Yun Liang[1], Wenbin Qian[1], Yang Xu[1]

[1] *The Second Affiliated Hospital of Zhejiang University School of Medicine, Hangzhou, China;*

### Background

T-cell engaging immunotherapies, including chimeric antigen receptor (CAR)-T cells and bispecific antibodies, have transformed the management of relapsed/refractory hematologic malignancies . Despite their remarkable efficacy, these treatments are frequently complicated by prolonged cytopenias, with thrombocytopenia posing a major clinical challenge due to the associated bleeding risk and potential treatment delays . This condition, termed immune effector cell-associated hematotoxicity (ICAHT), is now understood to involve complex immune-mediated mechanisms that extend beyond the transient myelosuppression caused by lymphodepleting chemotherapy. A recent comparative analysis has critically demonstrated that thrombopoietin receptor agonists (TPO-RAs), the primary pharmacologic intervention, provide no significant benefit over supportive care alone in this setting, highlighting a critical therapeutic gap.

### Aims

Yupingfeng (YPF) is a classic traditional Chinese medicine formula composed of *Astragalus membranaceus*, *Atractylodes macrocephala*, and *Saposhnikovia divaricata*. Modern pharmacological studies have revealed that YPF and its constituent compounds possess potent immunomodulatory properties, including the regulation of T-cell subsets and the suppression of pro-inflammatory cytokine production. Given this profile, we hypothesized that YPF might address the immune dysregulation underpinning ICAHT. Herein, we report a series of five patients in whom YPF was associated with the resolution of persistent or early-onset thrombocytopenia following CAR-T or bispecific antibody therapy.

### Methods

We describe five patients with relapsed/refractory hematologic malignancies who developed substantial thrombocytopenia after BCMA- or CD19-targeted immunotherapy.

### Results

Three initial patients developed persistent thrombocytopenia refractory to TPO-RAs (hetrombopag or romiplostim). In these refractory cases, administration of the traditional Chinese medicine Yupingfeng (YPF) was associated with rapid platelet recovery within 10-20 days, which persisted even after YPF discontinuation. Based on this consistent response, two subsequent patients received YPF at thrombocytopenia onset, resulting in prompt platelet normalization without progression to prolonged thrombocytopenia. Critically, antitumor efficacy from immunotherapy was maintained in all patients, and no YPF-related adverse events were observed.

### Summary/Conclusion

This series provides the first clinical evidence that Yupingfeng may be a highly effective and well-tolerated therapeutic option for managing immunotherapy-associated thrombocytopenia, particularly in TPO-RA refractory cases, while its early application shows potential for preventing persistent cytopenia development. These compelling findings warrant formal prospective investigation.

**Copyright Information:** (Online) ISSN: 2572-9241
©2026 The Author(s). HemaSphere is published by John Wiley & Sons Ltd on behalf of European Hematology Association. This is an open access Abstract Book distributed under the Attribution-NonCommercial-NoDerivs (CC BY-NC-ND), which allows third parties to download the articles and share them with others as long as they credit the author and the Abstract Book, but they cannot change the content in any way or use them commercially.
**Abstract Book Citations:** Authors, Title, HemaSphere, 2026;10:(S1):pages. The abstract book DOIs can be found on the "Author Information" page.
**Disclaimer:** Articles published in the journal HemaSphere exclusively reflect the opinions of the authors. The authors are responsible for all content in their abstracts including accuracy of the facts, statements, citing resources, etc.

### (PB4124) EXPERIENCE OF ANTI-CD19 CAR-T THERAPY IN RUSSIA FOR ADULT PATIENTS WITH REFRACTORY/RELAPSED B-CELL ACUTE LYMPHOBLASTIC LEUKEMIA AND B-CELL NON-HODGKIN'S LYMPHOMAS.

**Topic:** 26. Gene therapy, cellular immunotherapy and vaccination - Clinical

Olga Aleshina (Gavrilina)*[1], Ekaterina Kotova[1], Galstyan Gennady[1], Michail Maschan[1], Evgeniy Zvonkov[1], Nelli Gabeeva[1], Daria Koroleva[1], Yana Mangasarova[1], Runisa Abdurashidova[1], Karina Akezheva[1], Apollinaria Bogoliubova[1], Natalia Ivanova[1], Yana Serdyuk[1], Dinara Barakova[1], Maxim Telyashov[1], Dmitriy Pershin[2], Alexey Kazachenok[2], Yakov Muzalevsky[2], Larisa Kuzmina[1], Vera Troitskaya[1], Elena Parovichnikova[1]

*[1]National Medical research center for Hematology, Moscow, Russia;[2]Dmitriy Rogachev National Medical Research Center of Pediatric Hematology, Moscow, Russia;*

### Background

The long-term outcome for relapsed and refractory B-cell acute lymphoblastic leukemia (r/r B-ALL) and relapsed and refractory B-cell lymphomas (B-NHL) is extremely poor, with 3-year overall survival rates below 20-30%. Anti-CD19 CAR T-cell therapy is a novel treatment approach for this cohort of patients.

### Aims

To evaluate the outcomes and toxicity of anti-CD19 CAR T-cell therapy in 15 adult patients with r/r B-ALL and B-NHL who received therapy under a hospital exemption.

### Methods

From 2020 to 2024, anti-CD19 CAR-T cell therapy was administered to eight adult patients with r/r B-ALL and 7 adult patients with B-NHL (1 – mantle cell lymphoma, 1 - follicular lymphoma, 5 – DLBCL) by NRCH-CAR-T-2020 pilot study (approved by the local ethics committee). All patients had no alternative effective and safe treatment options. After signing an informed consent, all patients underwent lymphodepletion with fludarabine and cyclophosphamide on days -5 to -2 of therapy, CRS prophylaxis with tocilizumab and anti-CD19 CAR-T cell infusion on day 0. The efficacy and safety of CAR-T therapy were assessed in November 2025.

### Results

Infusions of locally produced (academic) anti-CD19 CAR-T lymphocytes were performed in 8 adult (age 19–37 years, median 28 years) patients with r/r B-ALL (5 with early relapse, 2 with MRD persistence and 1 with primary refractory disease) and in 7 adult (age 21–69 years, median 42 years) patients with r/r B-NHL (2 with early relapse, 1 with MRD persistence and 4 with primary refractory disease). All patients received 1 to 6 (median 3) lines of chemotherapy and/or immunotherapy before CAR-T therapy. Thirteen patients received anti-CD19 CAR-T, and 2 patients with B-ALL received anti-CD19/CD22 CAR-T. The median of infused CAR-T lymphocytes was $1.5 \times 10^6$ CAR+ cells/kg (range: 0.15 to $4 \times 10^6$). All patients (100%) achieved an overall response to therapy (complete remission or complete remission without recovery of hemogram parameters) on day 28 after CAR-T administration. Immune complications were noted in 6 of 15 patients (40%): CRS — in 33% of cases (CRS 1–3 (50%), CRS 2–2 (33%), CRS 3–1 (17%) patient), ICANS grade 3— in 1 patient (6,6%). There were no fatal outcomes from complications of CAR-T therapy. The median follow-up period for patients was 16 (2–48) months. Six of 15 patients (40%) died from relapse and progression; all of them developed a relapse with a median of 2 months after therapy. Nine patients (60%) are under observation. Three of the five patients with ALL in complete remission with a median of 3 months after CAR-T therapy underwent allo-HSCT. The 12-months' overall survival rate was 60%. Median OS is not reached and exceeds 7 months.

**Copyright Information:** (Online) ISSN: 2572-9241
©2026 The Author(s). HemaSphere is published by John Wiley & Sons Ltd on behalf of European Hematology Association. This is an open access Abstract Book distributed under the Attribution-NonCommercial-NoDerivs (CC BY-NC-ND), which allows third parties to download the articles and share them with others as long as they credit the author and the Abstract Book, but they cannot change the content in any way or use them commercially.
**Abstract Book Citations:** Authors, Title, HemaSphere, 2026;10:(S1):pages. The abstract book DOIs can be found on the "Author Information" page.
**Disclaimer:** Articles published in the journal HemaSphere exclusively reflect the opinions of the authors. The authors are responsible for all content in their abstracts including accuracy of the facts, statements, citing resources, etc.

**Summary/Conclusion**

CAR-T therapy is an effective approach in a cohort of patients with r/r B-ALL and B-NHL malignancies, who have little chance of achieving favorable long-term results with other therapies. Presented are the first results of CAR-T therapy in adult patients with r/r B-ALL and B-NHL. Access to CAR-T therapy in Russia is currently expanding, and new data will be presented in the near future.

**Copyright Information:** (Online) ISSN: 2572-9241
©2026 The Author(s). HemaSphere is published by John Wiley & Sons Ltd on behalf of European Hematology Association. This is an open access Abstract Book distributed under the Attribution-NonCommercial-NoDerivs (CC BY-NC-ND), which allows third parties to download the articles and share them with others as long as they credit the author and the Abstract Book, but they cannot change the content in any way or use them commercially.
**Abstract Book Citations:** Authors, Title, HemaSphere, 2026;10:(S1):pages. The abstract book DOIs can be found on the “Author Information” page.
**Disclaimer:** Articles published in the journal HemaSphere exclusively reflect the opinions of the authors. The authors are responsible for all content in their abstracts including accuracy of the facts, statements, citing resources, etc.

### (PB4125) INFLAMMATORY MARKER KINETICS AND THEIR ASSOCIATION WITH CRS AND ICANS IN A REAL-WORLD CAR-T COHORT

**Topic:** 26. Gene therapy, cellular immunotherapy and vaccination - Clinical

Maryam Hajiabbasi*[1, 1], Steven C. Tsai[1]

[1]*David Geffen School of Medicine, University of California, Department of Hematology/Oncology, Los Angeles, United States of America;*

### Background

Chimeric antigen receptor T-cell (CAR-T) therapy is an established treatment for relapsed or refractory hematologic malignancies with improved clinical outcomes. Cytokine release syndrome (CRS) and immune effector cell–associated neurotoxicity syndrome (ICANS) remain common toxicities. We evaluated real-world inflammatory markers and their association with these toxicities.

### Aims

To evaluate the incidence and timing of CRS, ICANS, and delayed neurotoxicity and their associations with baseline and dynamic inflammatory markers in a real-world CAR-T cohort.

### Methods

In this retrospective single-center cohort of 93 patients treated with CAR-T, CRS and ICANS were graded per ASTCT criteria; delayed neurotoxicity was defined as events >14 days post-infusion. Inflammatory markers (CRP, ferritin, LDH) were collected at baseline, peak, and day 14. Logistic regression (log2-transformed markers) evaluated associations with toxicity, adjusting for product. Correlation between marker peak timing and CRS onset was analyzed.

**Copyright Information:** (Online) ISSN: 2572-9241
©2026 The Author(s). HemaSphere is published by John Wiley & Sons Ltd on behalf of European Hematology Association. This is an open access Abstract Book distributed under the Attribution-NonCommercial-NoDerivs (CC BY-NC-ND), which allows third parties to download the articles and share them with others as long as they credit the author and the Abstract Book, but they cannot change the content in any way or use them commercially.
**Abstract Book Citations:** Authors, Title, HemaSphere, 2026;10:(S1):pages. The abstract book DOIs can be found on the "Author Information" page.
**Disclaimer:** Articles published in the journal HemaSphere exclusively reflect the opinions of the authors. The authors are responsible for all content in their abstracts including accuracy of the facts, statements, citing resources, etc.

**Results**

Ninety-three patients received CAR-T (Carvykti n=48; Yescarta n=17; Breyanzi n=16; Aucatzyl n=5; Tecartus n=4; others n=3). CRS occurred in 64 (68.8%) and ICANS in 22 (23.7%). CRS was highest with Yescarta (94%; median onset 2 days), followed by Carvykti (66.7%; 7 days) and Breyanzi (62.5%; 5 days). ICANS was most frequent with Yescarta (64.7%; 5 days), then Breyanzi (25%; 5 days) and Carvykti (10.4%; 6 days). Delayed non-ICANS neurotoxicity occurred in 8 patients (8.6%), most commonly with Carvykti (4/48).
Baseline CRP was not associated with CRS (OR 0.86, 95% CI 0.34–2.19, p=0.76), nor was baseline ferritin (OR 1.47, 95% CI 0.56–3.85, p=0.43). However, higher baseline ferritin was associated with CRS grade ≥2 (OR 1.41 per doubling, 95% CI 1.00–1.99, p=0.050); median ferritin was higher in grade ≥2 vs 0–1 CRS (930 vs 345, p=0.027). Baseline LD showed a borderline association (OR 2.46, 95% CI 0.88–6.88, p=0.087).
Higher baseline ferritin was associated with ICANS (OR 1.56 per doubling, 95% CI 1.15–2.12, p=0.004), remaining significant after adjustment for therapy (adjusted OR 1.54, 95% CI 1.06–2.22, p=0.023). Baseline CRP was not associated with ICANS.
CRP peak day was strongly correlated with CRS onset (Spearman ρ=0.74, p<0.001), peaking 1 day after onset (IQR 0–1). Ferritin peak day also correlated with onset and peaked 3 days after CRS. The lag between CRP and ferritin peaks was significant (median difference 3 days, p<0.001).
Early CRS (≤3 days) was associated with ICANS in unadjusted analysis (OR 8.36, 95% CI 2.53–27.64, p=0.001) but not after adjustment for therapy (OR 2.91, p=0.17).
CAR-T product type was the strongest predictor of delayed neurotoxicity, with higher odds observed for Carvykti. Peak ferritin (OR 1.43, p=0.044) and day 14 ferritin (OR 1.52, p=0.024) were associated in univariable analysis but not after adjustment. CRP and ALC were not associated. Findings should be interpreted with caution given the small number of events.

**Summary/Conclusion**

In this real-world cohort, CRS and ICANS incidence varied by CAR-T product. Baseline ferritin was independently associated with ICANS and with severe CRS. CRP closely tracked the timing of CRS onset, peaking one day after onset, whereas ferritin peaked later. Delayed non-ICANS neurotoxicity was uncommon and was most strongly associated with CAR-T product type; Sustained ferritin elevation was associated with delayed non-ICANS neurotoxicity in univariable analysis.

**Copyright Information:** (Online) ISSN: 2572-9241
©2026 The Author(s). HemaSphere is published by John Wiley & Sons Ltd on behalf of European Hematology Association. This is an open access Abstract Book distributed under the Attribution-NonCommercial-NoDerivs (CC BY-NC-ND), which allows third parties to download the articles and share them with others as long as they credit the author and the Abstract Book, but they cannot change the content in any way or use them commercially.
**Abstract Book Citations:** Authors, Title, HemaSphere, 2026;10:(S1):pages. The abstract book DOIs can be found on the "Author Information" page.
**Disclaimer:** Articles published in the journal HemaSphere exclusively reflect the opinions of the authors. The authors are responsible for all content in their abstracts including accuracy of the facts, statements, citing resources, etc.

### (PB4126) SHOULD LIVER DISEASE LIMIT GENE THERAPY IN HEMOPHILIA B?

**Topic:** 26. Gene therapy, cellular immunotherapy and vaccination - Clinical

Krishna Gundabolu*[1], Alex Nester[1], Marcel Devetten[1], Nathalie Khoury[2]

*[1]University of Nebraska Medical Center, Internal Medicine - Division of Hematology Oncology, Omaha, United States of America; [2]University of Nebraska Medical Center, Internal Medicine - Division of Gastroenterology and Hepatology, Omaha, United States of America;*

### Background

Gene therapy in Hemophilia had provided a curative treatment opportunity of this debilitating congenital bleeding disorder otherwise. Etranacogen Dezaparvovec (ETRA) is an adeno-associated virus (AAV) based factor IX gene therapy using recombiant AAV5 capsid contaning human factor IX Padua (R338L) varaint. Factor IX Padua, faactor IX specific activity is sustained at a higher levels compared to wild type factor IX. It is approved in the USA for moderate- severe hemophilia B. These therapies need good liver function so that transduction of the vector and expression of factor IX protein is not impaired. Patients with past history of Hepatitis B or C were not excluded in the phase 2b or phase 3 HOPE-B trial but advanced liver fibrosis defined by FibroScan $^{TM}$ score of > or equal to 9 kPa were excluded in the HOPE-B trial. Limited data is avaiable on the utility of ETRA in patients with advanced liver disease.

### Aims

We outline the sustained efficacy of ETRA with stable factor IX activity post infusion with acceptable post infusion course in a patient with chronic liver disease from past hepatitis C and advance fibrosis.

### Methods

A 41 year old man with history of severe congenital hemophilia B without inhibitor, transfuion acquired chronic hepatits C and chronic liver disease was considered for ETRA therapy. An ultrasound of the abdomen 8 years before the infusion showed heterogenous and nodular liver parenchyma suggestive of liver cirrhosis. He later received hepatitis C directed antiviral therapy and acheived a complete remission from it. Due to obesity and intermittent mild elevation of ALT (1-1.5 times upper limit of normal-ULN) a diagnosis of coexisting metabolic dysfunciton-associated steatotic liver disease (MASLD) was made and patient was recommend to lose weight and control his type II diabetes mellitus. Prior to gene therapy he was on recombinant factor IX, Fc fusion protein for prophylaxis and continued to suffer joint bleeds with an annualized bleed rate of 4-6. During the evaluation for ETRA, we consulted hepatology who performed a fibroscan which showed Metavir Fibrosis 3 - intermediate probability for fibrosis and cirrhosis with a score of 10.2 kPa. Liver fibrosis markers measured on his blood using FIBROSpect II index was 41 suggestive of Metavir Fibrosis 0-1 indicating good liver function.

### Results

Five weeks after the infusion of ETRA, he had mild elevation of ALT to 2 times ULN indicating immune mediate hepatotoxicity which was managed with prednisone for 6 weeks with no recurrence. His factor IX levels are outlined below. No joint bleeds were reported following the ETRA infusion.

| Timeline | Before infusion | 1 month | 2 months | 3 months | 4 months | 7 months |
|---|---|---|---|---|---|---|
| Factor IX level % | 32 (on prophylaxis) | 45 | 68 | 52 | 52 | 53 |

**Copyright Information:** (Online) ISSN: 2572-9241
©2026 The Author(s). HemaSphere is published by John Wiley & Sons Ltd on behalf of European Hematology Association. This is an open access Abstract Book distributed under the Attribution-NonCommercial-NoDerivs (CC BY-NC-ND), which allows third parties to download the articles and share them with others as long as they credit the author and the Abstract Book, but they cannot change the content in any way or use them commercially.
**Abstract Book Citations:** Authors, Title, HemaSphere, 2026;10:(S1):pages. The abstract book DOIs can be found on the "Author Information" page.
**Disclaimer:** Articles published in the journal HemaSphere exclusively reflect the opinions of the authors. The authors are responsible for all content in their abstracts including accuracy of the facts, statements, citing resources, etc.

**Summary/Conclusion**

Patients with severe hemophilia B with chronic liver disease could be considered for a curative gene therapy with ETRA when the liver disease is considered compensated inspite of moderately advanced fibrosis by FibroScan. A multidiciplinary care coordination with hepatology colleagues is crucial to not exclude patients from this therapy when the liver function is considered adequate. Liver disease not associated from prior chroic hepatitis B or C, due to MASLD should be managed with proper counseling of patients about lifestyle measures and weight loss before gene therapy for improved outcomes following ETRA.

**Copyright Information:** (Online) ISSN: 2572-9241
©2026 The Author(s). HemaSphere is published by John Wiley & Sons Ltd on behalf of European Hematology Association. This is an open access Abstract Book distributed under the Attribution-NonCommercial-NoDerivs (CC BY-NC-ND), which allows third parties to download the articles and share them with others as long as they credit the author and the Abstract Book, but they cannot change the content in any way or use them commercially.
**Abstract Book Citations:** Authors, Title, HemaSphere, 2026;10:(S1):pages. The abstract book DOIs can be found on the “Author Information” page.
**Disclaimer:** Articles published in the journal HemaSphere exclusively reflect the opinions of the authors. The authors are responsible for all content in their abstracts including accuracy of the facts, statements, citing resources, etc.

### (PB4127) OBSERVATIONAL STUDY PROTOCOL FOR EARLY DIFFERENTIATION OF CYTOKINE RELEASE SYNDROME AND SEPSIS IN CAR-T RECIPIENTS WITH HAEMATOLOGICAL MALIGNANCIES USING THE IL-6/PCT RATIO: THE DRACARYS STUDY

**Topic:** 26. Gene therapy, cellular immunotherapy and vaccination - Clinical

Anas Ibraheem*[1], Melanie Dalby[2, 3]

*[1]The London Clinic, Oncology, London, United Kingdom; [2]King's College Hospital NHS Foundation Trust, Haematology, London, United Kingdom; [3]King's College London School of Cancer & Pharmaceutical Sciences, London, United Kingdom;*

### Background

Chimeric Antigen Receptor T-cell (CAR-T) therapy has revolutionised treatment for haematological malignancies, demonstrating remarkable efficacy in B-cell leukaemias, lymphomas, and multiple myeloma. However, severe toxicities—particularly Cytokine Release Syndrome (CRS) and sepsis—present significant clinical challenges. Both conditions share overlapping features, including fever, hypotension, and multi-organ dysfunction, making timely and accurate differentiation essential. CRS is driven by excessive cytokine release, predominantly IL-6, and is treated with IL-6 receptor blockade (tocilizumab) and corticosteroids. Sepsis, by contrast, results from a dysregulated immune response to infection and requires antibiotics, as well as supportive care. Due to diagnostic uncertainty, clinicians often treat both conditions empirically. This can lead to inappropriate therapies—immunosuppressives may worsen sepsis, while antibiotics in CRS contribute to antimicrobial resistance and unnecessary healthcare burden. Existing biomarkers, such as IFN-ϒ and IL-1β, have shown potential but are limited by cost, availability, and the lack of rapid bedside implementation. There is a pressing need for a clinically accessible and reliable biomarker to distinguish CRS from sepsis in CAR-T patients.

### Aims

This study assesses the IL-6/procalcitonin (PCT) ratio as a diagnostic biomarker to distinguish cytokine release syndrome from sepsis in patients with haematological malignancies receiving CAR-T therapy, with a primary focus on relapsed/refractory B-cell lymphomas. Using a prospective observational approach, it aims to derive data-driven diagnostic cut-offs, describe biomarker dynamics during the first 14 days post–CAR-T infusion, validate findings in other CAR-T indications, and explore associations with short-term clinical outcomes to support real-time clinical decision-making.

### Methods

We hypothesise that the IL-6/procalcitonin (PCT) ratio will improve diagnostic accuracy. IL-6 is elevated in both conditions, while PCT is more specific to bacterial infection. However, PCT alone may be unreliable in immunocompromised patients, such as those receiving CAR-T therapy. The IL-6/PCT ratio is expected to reduce inter-individual variability and address limitations inherent to each marker when used alone. In this multi-centre, observational, prospective study, we will evaluate the IL-6/PCT ratio in CAR-T patients who develop fever within 14 days of infusion. Consecutive eligible patients will be enrolled before lymphodepletion. The sampling timeline for biomarker measurement is summarised in Table 1. The primary analysis will focus on relapsed/refractory B-cell lymphomas, with a prespecified expansion/validation across other CAR-T indications. Blinded clinical adjudication (two independent clinicians, third tie-breaker) will serve as the standard of reference. We will assess diagnostic performance using Receiver Operating Characteristic (ROC) analysis to determine sensitivity, specificity, and optimal cutoffs.

### Results

Protocol is under implementation; data collection pending, and no results available yet.

**Copyright Information:** (Online) ISSN: 2572-9241
©2026 The Author(s). HemaSphere is published by John Wiley & Sons Ltd on behalf of European Hematology Association. This is an open access Abstract Book distributed under the Attribution-NonCommercial-NoDerivs (CC BY-NC-ND), which allows third parties to download the articles and share them with others as long as they credit the author and the Abstract Book, but they cannot change the content in any way or use them commercially.
**Abstract Book Citations:** Authors, Title, HemaSphere, 2026;10:(S1):pages. The abstract book DOIs can be found on the "Author Information" page.
**Disclaimer:** Articles published in the journal HemaSphere exclusively reflect the opinions of the authors. The authors are responsible for all content in their abstracts including accuracy of the facts, statements, citing resources, etc.

**Summary/Conclusion**

This study, titled *DRACARYS* (Differentiating Reactions—CRS versus sepsis—After CAR-Ts), is expected to determine whether the IL-6/PCT ratio can support early differentiation between CRS and sepsis in CAR-T recipients. It aims to enhance diagnostic precision, guide timely and appropriate treatment, and reduce both clinical complications and unnecessary healthcare expenditures.

| Day | Event | Biomarker collection | Notes |
|---|---|---|---|
| D0 | CAR-T infusion (baseline) | Yes (once) | Pre-infusion baseline biomarkers. |
| D1–D14 | Post-infusion monitoring | Once daily[1] | Routine collection; timing recorded. |
| Tocilizumab/ steroids (any day) | Before administration | Yes (extra sample)[2] | Sample drawn just before immunosuppressive therapy; Draw immediately before the dose. |
| Event-triggered (any day) | Clinical deterioration | Yes (at discretion)[3] | If new/worsening features suggest CRS or sepsis. |

[1]If a sample is collected within 2 hours of the scheduled routine draw (either ≤2 h before or ≤2 h after), do not perform an additional routine sample. Count that sample as the daily draw and proceed with the next scheduled time-point. Applies equally to event-triggered and pre-immunomodulator samples; pre-dose timing takes precedence when both apply.
[2]Ideally, take the sample before rescue immunomodulation; do not delay urgent care.
[3]Do not add an extra interim sample during a continuous febrile period. Take an additional sample only if the patient has been afebrile ≥12 h, or immediately before a planned immunomodulator dose. If another tocilizumab/steroid dose is scheduled within 6–8 h, defer to the next pre-dose sample.

**Copyright Information:** (Online) ISSN: 2572-9241
©2026 The Author(s). HemaSphere is published by John Wiley & Sons Ltd on behalf of European Hematology Association. This is an open access Abstract Book distributed under the Attribution-NonCommercial-NoDerivs (CC BY-NC-ND), which allows third parties to download the articles and share them with others as long as they credit the author and the Abstract Book, but they cannot change the content in any way or use them commercially.
**Abstract Book Citations:** Authors, Title, HemaSphere, 2026;10:(S1):pages. The abstract book DOIs can be found on the "Author Information" page.
**Disclaimer:** Articles published in the journal HemaSphere exclusively reflect the opinions of the authors. The authors are responsible for all content in their abstracts including accuracy of the facts, statements, citing resources, etc.

### (PB4128) BILATERAL CYTOMEGALOVIRUS RETINITIS FOLLOWING BCMA-DIRECTED CAR-T CELL THERAPY IN MULTIPLE MYELOMA

**Topic:** 26. Gene therapy, cellular immunotherapy and vaccination - Clinical

Yanhan Zhou[1], Yiyi Li[1], Juntao Zhang[1], Ying Lu[1], Peipei Ye*[1]

[1]*Affiliated People's Hospital of Ningbo University, Ningbo, China;*

### Background

CMV retinitis is a severe sight-threatening opportunistic infection in immunocompromised populations. BCMA-directed CAR-T therapy is an effective treatment for relapsed/refractory MM, but it induces profound immunosuppression, increasing the risk of CMV reactivation and end-organ involvement. CMV retinitis after BCMA CAR-T therapy is rare, especially bilateral cases. Asian populations have a high CMV seropositivity rate , an independent risk factor for post-CAR-T CMV infection. This complication is underrecognized clinically, with high misdiagnosis rates and management strategies extrapolated from hematopoietic stem cell transplantation.

### Aims

To report a rare case of bilateral CMV retinitis in an MM patient after BCMA CAR-T therapy, analyze its risk factors, diagnosis, treatment and prognosis, and propose practical clinical monitoring and treatment strategies for this complication.

### Methods

A retrospective analysis was conducted on the clinical data of a 70-year-old female MM patient with type 2 diabetes who developed bilateral CMV retinitis after BCMA CAR-T therapy, including medical history, chemotherapy, CAR-T treatment, immunosuppressant use, CMV monitoring, ophthalmic symptom onset, misdiagnosis process, diagnosis and treatment. Relevant literature on post-CAR-T CMV retinitis was also reviewed and summarized.

### Results

The patient had multiple high-risk factors: multiple lines of chemotherapy, tocilizumab, infliximab and high-dose corticosteroids (cumulative prednisolone 780 mg) for CAR-T-related adverse events, and type 2 diabetes. CMV viremia reactivated 3 months post-CAR-T infusion with a viral load below the intervention threshold, without anti-CMV treatment. She developed bilateral blurred vision in July 2025, was misdiagnosed with glaucoma locally, and was confirmed to have bilateral CMV retinitis in our hospital in August 2025 via aqueous humor PCR (right eye CMV-DNA: $2.24\times10^5$ copies/mL; left eye: 1710 copies/mL), with undetectable systemic CMV-DNA. Initial intravenous ganciclovir was switched to foscarnet due to thrombocytopenia, combined with weekly bilateral intravitreal ganciclovir injections. CMV-DNA remained positive after 4 weeks of initial treatment, so injections were extended for 3 weeks (7 total). Re-evaluation revealed improved visual acuity, resolved retinal lesions and negative aqueous humor CMV-DNA. No recurrence was noted by December 2025, and the patient's MM was well-controlled post-CAR-T.

**Copyright Information:** (Online) ISSN: 2572-9241
©2026 The Author(s). HemaSphere is published by John Wiley & Sons Ltd on behalf of European Hematology Association. This is an open access Abstract Book distributed under the Attribution-NonCommercial-NoDerivs (CC BY-NC-ND), which allows third parties to download the articles and share them with others as long as they credit the author and the Abstract Book, but they cannot change the content in any way or use them commercially.
**Abstract Book Citations:** Authors, Title, HemaSphere, 2026;10:(S1):pages. The abstract book DOIs can be found on the "Author Information" page.
**Disclaimer:** Articles published in the journal HemaSphere exclusively reflect the opinions of the authors. The authors are responsible for all content in their abstracts including accuracy of the facts, statements, citing resources, etc.

### Summary/Conclusion

Bilateral CMV retinitis after BCMA CAR-T therapy for MM is a rare severe complication, associated with multiple immunosuppressive risk factors, and delayed diagnosis is mainly caused by early misdiagnosis. Aqueous humor puncture and PCR are key for early diagnosis, avoiding disease progression to surgical intervention. Individualized systemic plus local antiviral therapy achieves good efficacy, and persistent negative aqueous humor CMV-DNA is a safe withdrawal indicator. For high-risk patients, comprehensive immune monitoring (lymphocyte subsets for 3-12 months), weekly CMV testing within 3-6 months, and baseline dilated funduscopic examination plus regular ophthalmic follow-up within 3-12 months after CAR-T are recommended. Hematologists should raise awareness of this complication and advise patients to seek experienced ophthalmic care promptly for ocular symptoms. Prophylactic antiviral therapy is not recommended due to the low incidence of this complication.

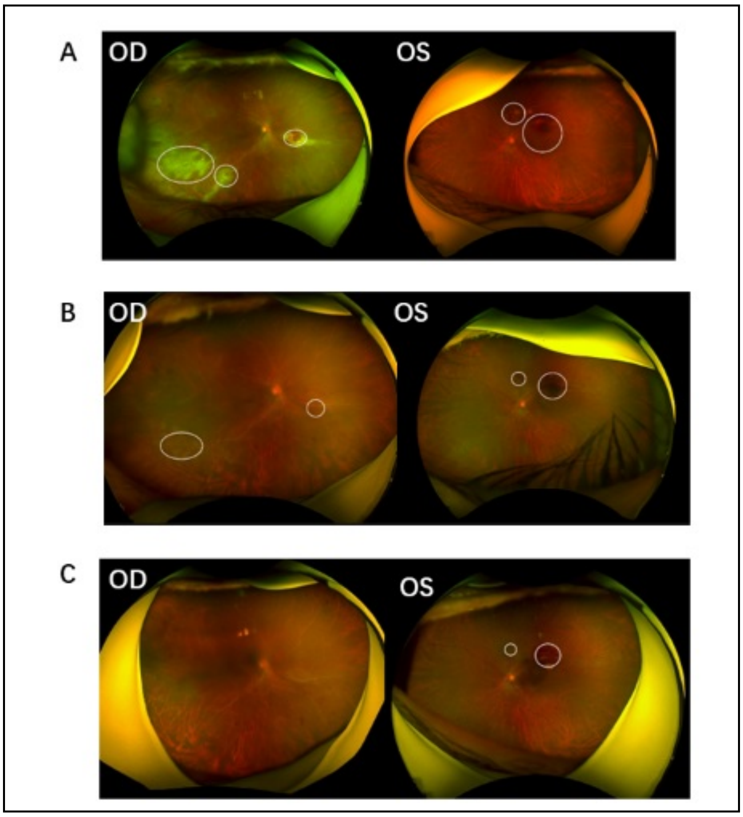

**Copyright Information:** (Online) ISSN: 2572-9241
©2026 The Author(s). HemaSphere is published by John Wiley & Sons Ltd on behalf of European Hematology Association. This is an open access Abstract Book distributed under the Attribution-NonCommercial-NoDerivs (CC BY-NC-ND), which allows third parties to download the articles and share them with others as long as they credit the author and the Abstract Book, but they cannot change the content in any way or use them commercially.
**Abstract Book Citations:** Authors, Title, HemaSphere, 2026;10:(S1):pages. The abstract book DOIs can be found on the "Author Information" page.
**Disclaimer:** Articles published in the journal HemaSphere exclusively reflect the opinions of the authors. The authors are responsible for all content in their abstracts including accuracy of the facts, statements, citing resources, etc.

### (PB4129) EXTENDED IMMUNE PROFILING REVEALS DISTINCT IMMUNE ARCHITECTURE IN POST-HSCT RECONSTITUTION AND PRE-CAR-T SURVEILLANCE: A REAL-WORLD FLOW CYTOMETRY STUDY

**Topic:** 26. Gene therapy, cellular immunotherapy and vaccination - Clinical

Aastha Gupta*[1], Rahul Bhargav[1], Anusha Swaminathan[1], Nikita Choudhary[1], Vikas Dua[1], Kanika Verma[1], Moinul Haq[2]

*[1]Fortis memorial research institute, Gurugram, India; [2]Agilus Diagnostics, Gurugram, India;*

### Background

Advanced cellular therapies such as hematopoietic stem cell transplantation (HSCT) and chimeric antigen receptor T-cell (CAR-T) have significantly improved relapse-free and overall survival in patients with hematological malignancies. However,these interventions impose substantial immune stress on recipients, necessitating a delicate balance between effective anti-tumor immunity and prevention of complications like relapse, graft-versus-host disease and reactivation of infections.
Extended immune profiling with multiparameter flow cytometry is increasingly employed to assess immune competence in patients undergoing HSCT. Likewise, baseline immune surveillance profiling prior to CAR-T therapy may help identify determinants of CAR-T persistence, relapse risk and the potential need for post-therapy vaccination strategies. Despite this, real-world data describing compartment-specific immune reconstitution post HSCT and baseline immune architecture in pre-CAR-T patients remain limited in developing-world settings, particularly with respect to maturation subsets within the B-cell and T-cell compartments.

### Aims

1. To describe compartment-specific immune recovery across early (≤100 days), intermediate (101–365 days), and late (>365 days) post-HSCT timepoints.
2. To define baseline immune surveillance profiles in patients undergoing pre-CAR-T evaluation using extended immune phenotyping.

### Methods

We retrospectively analysed 40 consecutive immune profiling samples tested using flow cytometry immune reconstitution panel. This panel extensively looked at absolute counts and percentages for lymphocytes, T-cell maturation subsets (CD4 and CD8 naïve, central/transitional memory, effector memory), B-cell maturation subsets (total B cells, pre-germinal center cells, switched memory B cells), NK cells, neutrophils and monocytes. Results are presented as medians. Samples were categorized as post-HSCT (n=12), pre-CAR-T evaluation (n=8), or immune evaluation for other indications (n=18). Two patients were post-CART.
Post-HSCT among each time point were 4 samples each.

**Copyright Information:** (Online) ISSN: 2572-9241
©2026 The Author(s). HemaSphere is published by John Wiley & Sons Ltd on behalf of European Hematology Association. This is an open access Abstract Book distributed under the Attribution-NonCommercial-NoDerivs (CC BY-NC-ND), which allows third parties to download the articles and share them with others as long as they credit the author and the Abstract Book, but they cannot change the content in any way or use them commercially.
**Abstract Book Citations:** Authors, Title, HemaSphere, 2026;10:(S1):pages. The abstract book DOIs can be found on the “Author Information” page.
**Disclaimer:** Articles published in the journal HemaSphere exclusively reflect the opinions of the authors. The authors are responsible for all content in their abstracts including accuracy of the facts, statements, citing resources, etc.

**Results**

- Post-HSCT immune recovery was strongly time-dependent. Early post-HSCT samples demonstrated profound lymphopenia with near-absent B cells (median 1.45/µL), CD4 T cells (median 32.5/µL), NK cells (median 4.1/µL) and undetectable CD4 naïve T cells and switched memory B cells.
- Intermediate post-HSCT samples showed marked quantitative recovery of lymphocytes and T-cell subsets with persistent low naïve pools. An early recovery of total B-cells (495/ µL) was noted in contrast to the published literature.
- Late post-HSCT samples demonstrated qualitative maturation with recovery of CD4 naïve T cells(median 134/µL),CD8 naïve T cells (median 75.6/µL) and switched memory B cells (median 72.3/µL).
- Pre-CAR-T patients exhibited a distinct immune surveillance profile characterized by low lymphocyte counts(median 982/µL), marked B-cell depletion (median 26.4/µL), severely reduced naïve T-cell compartments and relative preservation of NK cells.

**Summary/Conclusion**

Extended flow cytometric immune profiling demonstrates compartment-specific and maturation-dependent immune recovery after HSCT, with early profound adaptive immune deficiency followed by effector expansion and later qualitative maturation of naïve and memory compartments.
Pre-CAR-T patients exhibited a baseline immune profile characterized by severe B-cell depletion, reduced central memory reserves, and effector-skewed T-cell architecture, supporting "post-therapy exhausted immune system" pattern.

**Copyright Information:** (Online) ISSN: 2572-9241
©2026 The Author(s). HemaSphere is published by John Wiley & Sons Ltd on behalf of European Hematology Association. This is an open access Abstract Book distributed under the Attribution-NonCommercial-NoDerivs (CC BY-NC-ND), which allows third parties to download the articles and share them with others as long as they credit the author and the Abstract Book, but they cannot change the content in any way or use them commercially.
**Abstract Book Citations:** Authors, Title, HemaSphere, 2026;10:(S1):pages. The abstract book DOIs can be found on the "Author Information" page.
**Disclaimer:** Articles published in the journal HemaSphere exclusively reflect the opinions of the authors. The authors are responsible for all content in their abstracts including accuracy of the facts, statements, citing resources, etc.

### (PB4130) COMPLEMENT BLOCKADE REDUCES CHRONIC INFLAMMATION, HEMOLYSIS AND ORGAN DAMAGE IN A SICKLE CELL MOUSE MODEL

**Topic:** 27. Sickle cell disease

Thiago MacIel*[1], Nabiha Sbeih[1], Rachel Rignault[1], Geoffrey Cheminet[1], Isabelle Chevalme[1], Slimane Allali[2], Camille Brochier[3], Anna Kiialainen[4], Tovo David[4], Olivier Hermine[5]

*[1]Institut Imagine INSERM UMR1163, Laboratory of Cellular and Molecular Mechanisms of Hematological Disorders and Therapeutical Implications, Paris, France; [2]Hôpital Necker-Enfants Malades, Department of General Pediatrics and Pediatric Infectious Diseases, Sickle Cell Center, Paris, France; [3]Institut Roche, Boulogne-Billancourt, France; [4]F. Hoffmann-La Roche Ltd, Roche Pharma Research & Early Development, Basel, Switzerland; [5]Hôpital Necker-Enfants Malades, Department of Hematology, Paris, France;*

### Background

Complement alternative pathway (AP) activation contributes to intravascular hemolysis, endothelial dysfunction and progressive organ damage in sickle cell disease (SCD). We previously showed that complement inhibition reduces hemolysis in Townes HbSS mice. However, the long-term impact of proximal (factor B) versus terminal (C5) blockade, alone or combined, on chronic inflammation and multi-organ injury in chronic SCD remains unclear.

### Aims

To determine whether inhibition of terminal complement (anti-C5 antibody, BB5.1), alternative pathway activation (factor B antisense oligonucleotide, ASO fB), or their combination reduces hemolysis, endothelial activation, and organ injury in a chronic sickle cell disease (SCD) mouse model.

### Methods

Townes HbSS mice (8 weeks) received weekly ASO fB (100 mg/kg), twice-weekly anti-C5 (BB5.1, 30 mg/kg), or both, for 12 weeks. HbAA and isotype/ASO controls were included. Plasma complement components (factor B, C3b, C5a, sC5b9), red blood cell (RBC) C3/C3b opsonization and hemolysis markers (LDH, plasma hemoglobin, bilirubin) were assessed. Endothelial/adhesion markers (VCAM-1, ICAM-1, VE-cadherin, selectins, CD44), thrombo-inflammatory markers (TAT, D-dimer), histologic injury scores (liver, lung, kidney), and tissue RNA sequencing (liver, lung, kidney) were analyzed.

**Copyright Information:** (Online) ISSN: 2572-9241
©2026 The Author(s). HemaSphere is published by John Wiley & Sons Ltd on behalf of European Hematology Association. This is an open access Abstract Book distributed under the Attribution-NonCommercial-NoDerivs (CC BY-NC-ND), which allows third parties to download the articles and share them with others as long as they credit the author and the Abstract Book, but they cannot change the content in any way or use them commercially.
**Abstract Book Citations:** Authors, Title, HemaSphere, 2026;10:(S1):pages. The abstract book DOIs can be found on the "Author Information" page.
**Disclaimer:** Articles published in the journal HemaSphere exclusively reflect the opinions of the authors. The authors are responsible for all content in their abstracts including accuracy of the facts, statements, citing resources, etc.

### Results

ASO fB significantly reduced plasma factor B and C3b levels and markedly decreased RBC C3/C3b opsonization. All treatments reduced C5a and sC5b9 levels. Both monotherapies and the combination significantly reduced hemolysis.
HbSS mice exhibited progressive endothelial activation and elevated thrombin–antithrombin (TAT) complexes. Complement inhibition, particularly ASO fB and the combination therapy, reduced multiple endothelial and adhesion markers (VE-cadherin, ICAM-1, P-selectin, and E-selectin) as well as TAT levels, consistent with attenuation of vascular inflammation.
Histologically, HbSS mice developed age-dependent organ damage, most prominently in the liver, lung, and kidney. Complement blockade reduced hepatic and pulmonary injury scores, with the combination therapy demonstrating the broadest protective effect. Renal injury markers (KIM-1 and NGAL) were significantly reduced across treatment groups.
Bulk RNA sequencing revealed marked enrichment of inflammatory and immune activation pathways in HbSS liver, lung, and kidney compared with HbAA controls. ASO fB attenuated transcriptional programs associated with complement activation, leukocyte adhesion, chemotaxis, and hemostasis, whereas BB5.1 dampened key inflammatory and proliferative signaling pathways, including IL-6 and MAPK cascades. Combination therapy induced broader anti-inflammatory transcriptional reprogramming, reducing immune activation and vascular inflammatory interactions.

### Summary/Conclusion

Complement activation drives hemolysis, endothelial dysfunction, and chronic multi-organ inflammatory injury in this SCD model. Alternative pathway inhibition via factor B targeting effectively suppressed complement amplification and vascular activation, while C5 blockade modulated downstream effector and inflammatory signaling pathways. The complementary mechanisms observed with dual inhibition translated into broader protection against vascular inflammation and organ damage. These findings support further investigation of complement-directed strategies, including combined alternative pathway and C5 inhibition, to mitigate chronic vascular and organ injury in SCD.

**Copyright Information:** (Online) ISSN: 2572-9241
©2026 The Author(s). HemaSphere is published by John Wiley & Sons Ltd on behalf of European Hematology Association. This is an open access Abstract Book distributed under the Attribution-NonCommercial-NoDerivs (CC BY-NC-ND), which allows third parties to download the articles and share them with others as long as they credit the author and the Abstract Book, but they cannot change the content in any way or use them commercially.
**Abstract Book Citations:** Authors, Title, HemaSphere, 2026;10:(S1):pages. The abstract book DOIs can be found on the "Author Information" page.
**Disclaimer:** Articles published in the journal HemaSphere exclusively reflect the opinions of the authors. The authors are responsible for all content in their abstracts including accuracy of the facts, statements, citing resources, etc.

### (PB4131) URICA: A NON-INVASIVE URINARY CARBONIC ANHYDRASE 1 ASSAY FOR DETECTION OF INTRAVASCULAR HAEMOLYSIS - MULTICENTRE VALIDATION AND POINT-OF-CARE DEVELOPMENT

**Topic:** 27. Sickle cell disease

Sanja Brolih*[1]

[1]*University of Oxford, Department of Physiology, Anatomy, and Genetics, Oxford, United Kingdom;*

### Background

Intravascular haemolysis accompanies diverse hematologic disorders, yet conventional blood-based markers ( plasma-free haemoglobin (PFH), lactate dehydrogenase (LDH), bilirubin, and haptoglobin) are variably sensitive, nonspecific, and unsuitable for point-of-care use. Carbonic anhydrase 1 (CA1), an abundant erythrocyte cytosolic enzyme, is released during red cell destruction and detectable in urine, offering a mechanistically grounded, non-invasive biomarker.

### Aims

This study aims to evaluate urinary CA1 as a marker of intravascular haemolysis across inherited anaemias and sickle-cell disease, and to present results from inpatient and outpatient cohorts at Milton Keynes University Hospital (MKUH) NHS Foundation Trust and Oxford University Hospitals (OUH) NHS Foundation Trust.

### Methods

Urine CA1 and haemoglobin (Hb) were quantified using enzyme-linked immunosorbent assay and immunoblotting. CA1:Hb stoichiometry was applied to distinguish intravascular haemolysis from urogenital blood contamination and associations with PFH, LDH, bilirubin, Hb, C-reactive protein (CRP), and clinical variables were assessed. To enable rapid and cost-effective detection, a lateral flow device was developed and analytically validated.

### Results

Amongst inherited anaemias, urinary CA1 levels were highest in sickle cell disease. CA1 concentrations positively correlated with serum LDH and inversely correlated with blood Hb, supporting biological specificity for haemolysis. Reference urine samples were predominantly CA1-negative/Hb-negative, further confirming CA1 as an independent marker of intravascular haemolysis. Most notably, the lateral flow device demonstrated excellent sensitivity and specificity for urinary CA1 detection.

### Summary/Conclusion

Urinary CA1 is a sensitive, mechanistically informed biomarker of intravascular haemolysis, particularly in sickle cell disease. Its urine-based detection overcomes limitations of conventional blood assays and enables non-invasive monitoring across inpatient and outpatient settings. The development of a validated lateral flow platform supports rapid, low-cost, point-of-care and potential home-based testing, with broad applicability in both high-resource and global health settings where laboratory access is limited.

**Copyright Information:** (Online) ISSN: 2572-9241
©2026 The Author(s). HemaSphere is published by John Wiley & Sons Ltd on behalf of European Hematology Association. This is an open access Abstract Book distributed under the Attribution-NonCommercial-NoDerivs (CC BY-NC-ND), which allows third parties to download the articles and share them with others as long as they credit the author and the Abstract Book, but they cannot change the content in any way or use them commercially.
**Abstract Book Citations:** Authors, Title, HemaSphere, 2026;10:(S1):pages. The abstract book DOIs can be found on the “Author Information” page.
**Disclaimer:** Articles published in the journal HemaSphere exclusively reflect the opinions of the authors. The authors are responsible for all content in their abstracts including accuracy of the facts, statements, citing resources, etc.

### (PB4132) CAUSES, IMPACTS, AND MANAGEMENT OF SICKLE CELL PATIENTS WITH ELEVATED FERRITIN

**Topic:** 27. Sickle cell disease

James Lamb*[1], Anisa Vrapi[2], Vivian Garcia[2], Ruth Jolley[2], Raeesa Auturally[2], Martin Besser[2]

*[1]School of Clinical Medicine, University of Cambridge, Cambridge, United Kingdom;[2]Addenbrooke's Hospital, Department of Haematology, Cambridge, United Kingdom;*

### Background

Elevated serum ferritin in sickle cell disease (SCD) can reflect transfusional iron overload (IOL) but also inflammation and liver disease. Whilst SCD-related haemolysis causes urinary iron loss, repeated transfusions lead to iron accumulation; partially mitigated by iron-neutral exchange methods. IOL in SCD primarily affects the liver, leading to fibrosis and cirrhosis; cardiac and endocrine complications are less common. As guidance largely extrapolates from thalassemia IOL, SCD specific evidence is needed. UK guidance emphasises ferritin monitoring, MRI liver iron concentration (LIC) assessment and timely chelation initiation.

### Aims

To assess the prevalence, causes, monitoring and management of elevated serum ferritin in adult SCD.

### Methods

A retrospective audit of electronic patient records at Addenbrookes Hospital. Inclusion criteria were >16 years old, confirmed SCD and most recent ferritin >500µg/L. Data collected was transfusional history, ferritin monitoring, MRI LIC and cardiac MRI use, chelation, and IOL-related pathology. Practice was benchmarked against UK guidance.

### Results

Of 144 SCD patients, 133 (92.4%) had ferritin measured (mean 247 ug/L; median 111ug/L; range 7–3095). The index group comprised 16 patients (12.0%) with ferritin >500 ug/L, of whom 7 (5.3%) were >1000ug/L. Most were female (75%) with a mean age of 32 years (17-51). 8 patients were regularly transfused (6 exchange, 2 top-up), 5 were sporadically transfused (1 exchange, 3 top-up, 1 mixed), and 3 were not transfused. Probable causes were transfusional IOL (n=10), acute inflammation (n=2), viral hepatitis (n=1), rheumatological disease (n=1), and unclear in 2. Monitoring was inconsistent. Of 4 patients on >3-monthly top-up transfusions, 1 had regular ferritin monitoring and 1 had MRI LIC. Conversely, of 7 on exchange with rising ferritin, 5 had regular ferritin monitoring and all had MRI LIC. Cardiac MRI was overused; 1 met criteria (LIC >15 mg/g), while 5 were scanned without indication. Regarding IOL impact, 7 patients had abnormal LFTs (excluding bilirubin), 4 had liver disease, no cardiomyopathy was identified, 3 had endocrine pathology, and 2 had siderophile infections (salmonella). Attribution to IOL versus SCD is unclear due to overlapping pathology. Of 11 patients meeting chelation criteria, 6 were chelated. Drug choice was primarily deferasirox.

**Copyright Information:** (Online) ISSN: 2572-9241
©2026 The Author(s). HemaSphere is published by John Wiley & Sons Ltd on behalf of European Hematology Association. This is an open access Abstract Book distributed under the Attribution-NonCommercial-NoDerivs (CC BY-NC-ND), which allows third parties to download the articles and share them with others as long as they credit the author and the Abstract Book, but they cannot change the content in any way or use them commercially.
**Abstract Book Citations:** Authors, Title, HemaSphere, 2026;10:(S1):pages. The abstract book DOIs can be found on the "Author Information" page.
**Disclaimer:** Articles published in the journal HemaSphere exclusively reflect the opinions of the authors. The authors are responsible for all content in their abstracts including accuracy of the facts, statements, citing resources, etc.

**Summary/Conclusion**

IOL in SCD can be obscured by overlapping inflammatory and iron-related ferritin elevation, risking under-recognition. In this cohort, transfusional exposure was the main driver of raised ferritin. Further, assessing clinical impact requires disentangling relative contributions of SCD and IOL to pathology. This complexity is reflected in inconsistencies in monitoring with underutilisation of MRI LIC and overuse of cardiac MRI. Thorough transfusion histories and ferritin monitoring are essential. Secondly, this study shows exchange methods do not fully mitigate IOL; notably, post-hoc analysis found many of these patients had delayed apheresis appointments. This may reflect transfused blood, ordinarily removed at the following apheresis session, being processed by the reticular endothelial system. Overall, these findings highlight the need for rigorous longitudinal iron monitoring and adherence to exchange regimes to manage IOL in adult SCD.

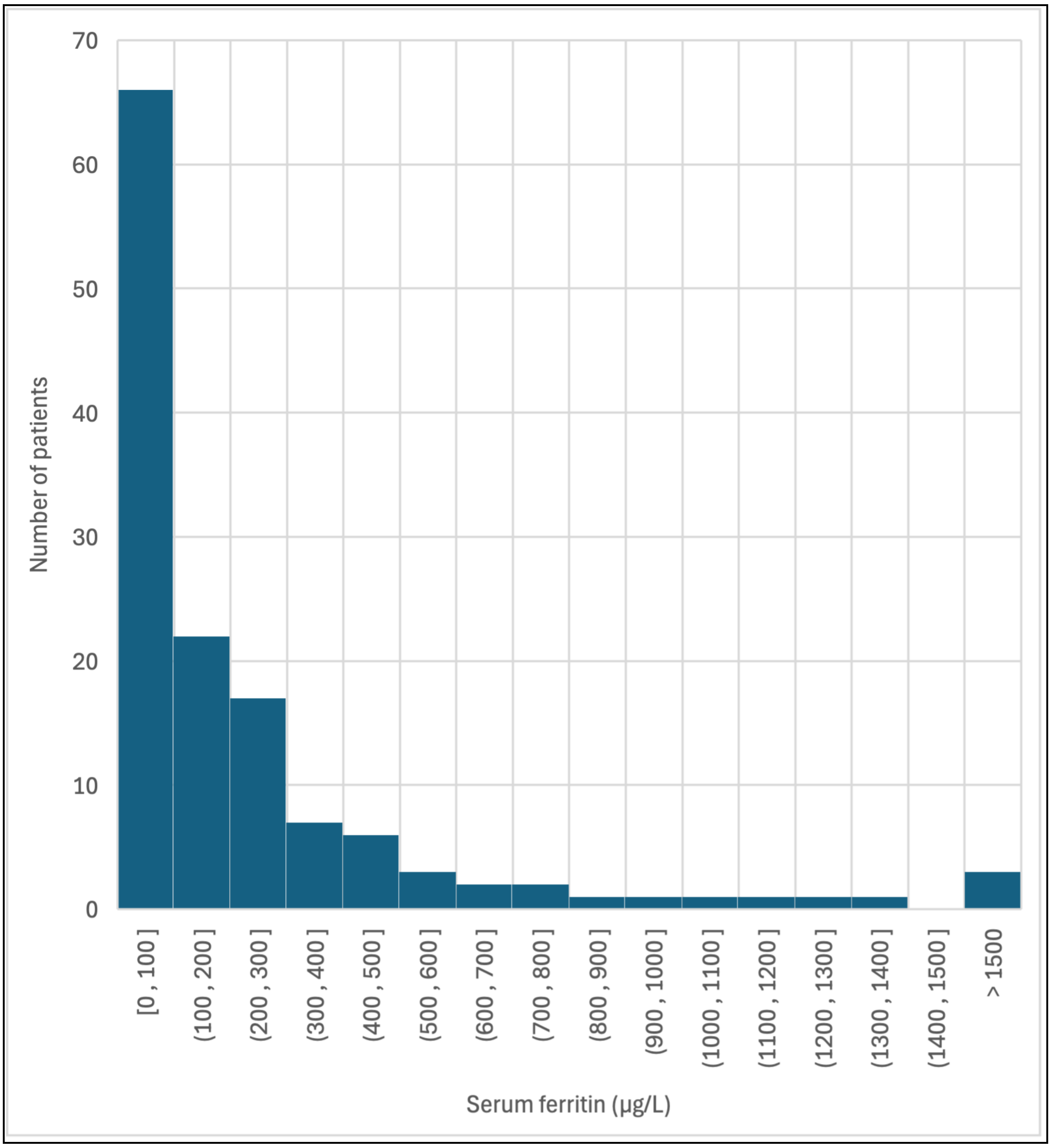

**Copyright Information:** (Online) ISSN: 2572-9241
©2026 The Author(s). HemaSphere is published by John Wiley & Sons Ltd on behalf of European Hematology Association. This is an open access Abstract Book distributed under the Attribution-NonCommercial-NoDerivs (CC BY-NC-ND), which allows third parties to download the articles and share them with others as long as they credit the author and the Abstract Book, but they cannot change the content in any way or use them commercially.
**Abstract Book Citations:** Authors, Title, HemaSphere, 2026;10:(S1):pages. The abstract book DOIs can be found on the "Author Information" page.
**Disclaimer:** Articles published in the journal HemaSphere exclusively reflect the opinions of the authors. The authors are responsible for all content in their abstracts including accuracy of the facts, statements, citing resources, etc.

### (PB4133) CHARACTERIZATION OF PATIENTS WITH SICKLE CELL DISEASE IN SPAIN: AN 8-YEAR SPAN STUDY

**Topic:** 27. Sickle cell disease

Maria Ordoñez Garcia*[1], Ane Brieva Lopez[1], Rafael Garcia Carretero[2], Mariana Cerrato Salas[1], Pavel Manuel Acevedo Pérez[1], Juan Sastre Ortega[1], Maria Angeles Andreu Costa[1]

*[1]Hospital Universitario de Móstoles, Madrid, Spain;[2]Hospital Universitario Severo Ochoa, Madrid, Spain;*

### Background

Sickle cell disease (SCD) is an inherited structural hemoglobinopathy with an increasing prevalence in Spain due to migratory patterns. Despite the increasing relevance of this condition, comprehensive long-term studies utilizing large-scale national databases are still needed to fully understand the demographic and clinical landscape of SCD in Spain.

### Aims

This study aims to characterize the population of patients diagnosed with various forms of sickle cell disease—including Hb-SS, Hb-C, and sickle cell beta-thalassemia (both $\beta^0$ and $\beta^+$)—hospitalized in Spain over an eight-year period.

### Methods

We conducted an observational, descriptive, nationwide study using the Minimum Basic Data Set (MBDS-H), a centralized registry covering approximately 95–97% of Spanish hospital discharges. Patients with ICD-10-CM codes for SCD (D57.x) were identified between January 1, 2016, and December 31, 2023. Demographic variables, clinical complications, and in-hospital mortality were analyzed.

**Copyright Information:** (Online) ISSN: 2572-9241
©2026 The Author(s). HemaSphere is published by John Wiley & Sons Ltd on behalf of European Hematology Association. This is an open access Abstract Book distributed under the Attribution-NonCommercial-NoDerivs (CC BY-NC-ND), which allows third parties to download the articles and share them with others as long as they credit the author and the Abstract Book, but they cannot change the content in any way or use them commercially.
**Abstract Book Citations:** Authors, Title, HemaSphere, 2026;10:(S1):pages. The abstract book DOIs can be found on the "Author Information" page.
**Disclaimer:** Articles published in the journal HemaSphere exclusively reflect the opinions of the authors. The authors are responsible for all content in their abstracts including accuracy of the facts, statements, citing resources, etc.

### Results

A total of 9,905 hospitalizations were recorded, corresponding to 1,285 unique patients. The median age of unique patients was 16 years (IQR: 7, 27), with a predominant pediatric and young adult distribution. The HbSS phenotype was the most common (27%, n=991), while sickle cell thalassemia $\beta^0$ and $\beta^+$ were rare (each <0.1%, n=2). Hospitalizations showed an upward trend, rising from approximately 1,000 in 2016 to over 1,500 in 2023, with a notable decrease in 2020 during the COVID-19 pandemic. The most frequent complication was vaso-occlusive crisis (30% of admissions). In-hospital mortality was low at 0.6%.

Table 1. Baseline Demographic and Clinical Characteristics of Patients with Sickle Cell Disease

| **Characteristic** | **N = 1,285**[1] |
|---|---|
| **Age** | 16 (7, 27) |
| **Sex (women)** | 608 (47%) |
| **HIV infection** | 3 (0.2%) |
| **Hematopoietic stem-cell transplantation** | 32 (2.5%) |
| **Allelic phenotype of sickle cell disease** | |
| SS | 991 (27%) |
| SC | 292 (8.1%) |
| S$\beta^o$ | 2 (<0.1%) |
| S$\beta^+$ | 2 (<0.1%) |

[1]Median (IQR) or Frequency (%)

### Summary/Conclusion

SCD hospitalizations in Spain are increasing and primarily affect younger populations. Despite a high recurrence of admissions (average 7.7 per patient), the low mortality rate suggests effective hospital management within the Spanish National Health System. These findings emphasize the need for continued epidemiological monitoring and specialized care resources.

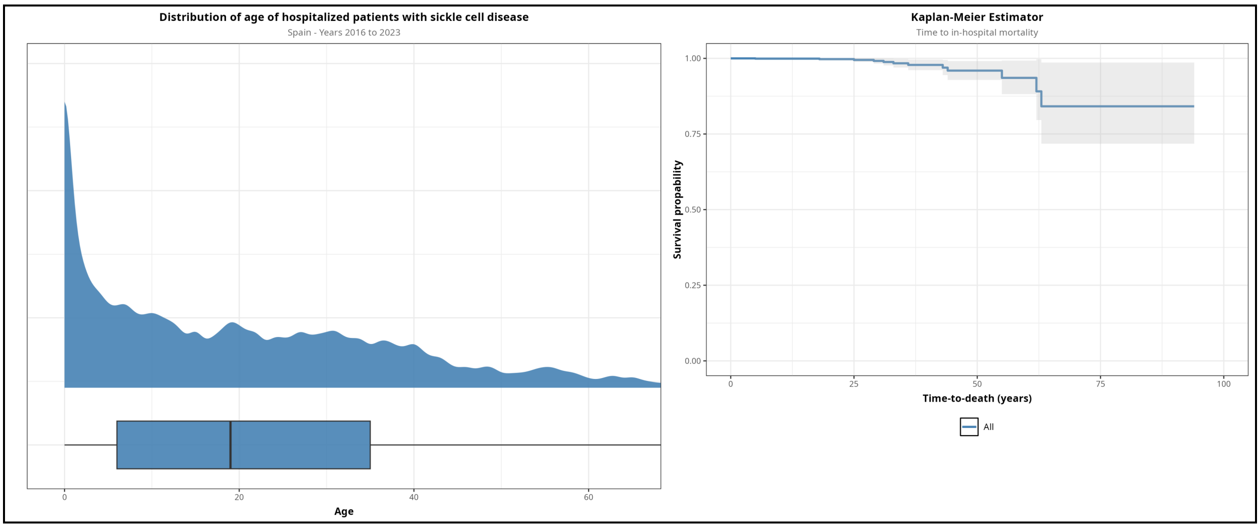

Figure 1. Age distribution of hospitalized patients with sickle cell disease and Kaplan-Meier survival analysis.

**Copyright Information:** (Online) ISSN: 2572-9241
©2026 The Author(s). HemaSphere is published by John Wiley & Sons Ltd on behalf of European Hematology Association. This is an open access Abstract Book distributed under the Attribution-NonCommercial-NoDerivs (CC BY-NC-ND), which allows third parties to download the articles and share them with others as long as they credit the author and the Abstract Book, but they cannot change the content in any way or use them commercially.
**Abstract Book Citations:** Authors, Title, HemaSphere, 2026;10:(S1):pages. The abstract book DOIs can be found on the "Author Information" page.
**Disclaimer:** Articles published in the journal HemaSphere exclusively reflect the opinions of the authors. The authors are responsible for all content in their abstracts including accuracy of the facts, statements, citing resources, etc.

### (PB4134) CORRELATION BETWEEN FETAL HEMOGLOBIN AND CLINICAL OUTCOMES IN SICKLE CELL DISEASE: A MODEL-BASED META-ANALYSIS

**Topic:** 27. Sickle cell disease

Bahru Habtemariam*[1], Jackson Rutecki[1], Arthur Lo[1], Alexis Krolick[1], Katherine L. Ender[1], Amy Simon[1], Sunita Goyal[1], Srikanth Ambati[1]

*[1]Beam Therapeutics Inc., Cambridge, MA, United States of America;*

### Background

Elevated fetal hemoglobin (HbF) ameliorates clinical manifestations of sickle cell disease (SCD). Sustained, pancellular HbF expression into adulthood confers non-sickling and anti-sickling effects. Clinically, HbF levels of 10–20% are associated with improved survival in individuals with SCD, while levels approaching or exceeding 30–40%, as observed in patients with hereditary persistence of HbF, are linked to a markedly attenuated disease course. Although HbF induction is a well-established therapeutic strategy in SCD, a comprehensive quantitative relationship between HbF levels and clinical outcomes has not been defined.

### Aims

This model-based meta-analysis aimed to define the quantitative relationship between HbF levels and clinical outcomes, as well as markers of anemia and hemolysis, in SCD.

### Methods

A literature search was conducted using PubMed and Google Scholar with the term 'Impact of HbF on Sickle Cell Disease Manifestations'. Articles were screened for relevance; data were digitized from published plots or simulated from summary tables and then fit to models to determine the relationship between HbF and outcomes. Synthetic populations consisting of 10,000 patients were constructed from population- and individual-level statistics of 12 references. Non-normal data were normalized with log and logit transformations in regression. Poisson regression was applied to represent vaso-occlusive crises (VOCs) in response to HbF, while logistic regression was used to correlate HbF with retinopathy, stroke, and mortality. Linear regression was used to correlate HbF with total hemoglobin (Hb) and reticulocytes. Emax models were used to correlate HbF with bilirubin, lactate dehydrogenase (LDH), and arginase I.

### Results

In a synthetic population, annual VOCs followed a Poisson distribution, and historical data suggested that ~90% of patients would be VOC free for 12 months (VF12) if HbF reached ~40% (Figure 1A). Annual VOC frequency decreased from 2.5/year at 0% HbF to <0.5/year at 20% HbF, corresponding to an estimated 8% reduction in VOC rate per 1% HbF increase (Figure 1B). In a separate synthetic population, ~20% HbF prevented most retinopathy cases, while in synthetic patients who experienced a stroke, 30% HbF reduced recurrent stroke risk to near zero over 10 years. Modeling hydroxyurea (HU) treatment data predicted improved survival versus controls, with HbF levels as low as 1 g/dL associated with ~85% 10-year survival, and survival increased with higher HbF.

Increased HbF was associated with improved markers of anemia and hemolysis, accompanied by proportional increases in total Hb. High HbF was strongly associated with reticulocyte reductions, with model-predicted reticulocyte values falling to normal levels (<100,000 cells/$mm^3$) at ~35% HbF. Elevated HbF also showed strong non-linear correlations with bilirubin, LDH, and arginase I reductions to normal or near normal levels.

**Copyright Information:** (Online) ISSN: 2572-9241
©2026 The Author(s). HemaSphere is published by John Wiley & Sons Ltd on behalf of European Hematology Association. This is an open access Abstract Book distributed under the Attribution-NonCommercial-NoDerivs (CC BY-NC-ND), which allows third parties to download the articles and share them with others as long as they credit the author and the Abstract Book, but they cannot change the content in any way or use them commercially.
**Abstract Book Citations:** Authors, Title, HemaSphere, 2026;10:(S1):pages. The abstract book DOIs can be found on the "Author Information" page.
**Disclaimer:** Articles published in the journal HemaSphere exclusively reflect the opinions of the authors. The authors are responsible for all content in their abstracts including accuracy of the facts, statements, citing resources, etc.

**Summary/Conclusion**

Meta-analysis of published data shows that HbF is strongly correlated with key clinical outcomes in SCD, including VOCs, retinopathy, stroke, and mortality, as well as laboratory parameters of anemia and hemolysis. The non-linear relationships observed in the models suggest that increased HbF is associated with clinically meaningful improvements across these parameters, and that this correlation is continuous, plateauing at HbF of >60%. These findings support HbF as a useful pharmacodynamic endpoint that is a predictor of clinical benefit for evaluating HbF-inducing therapies in SCD.

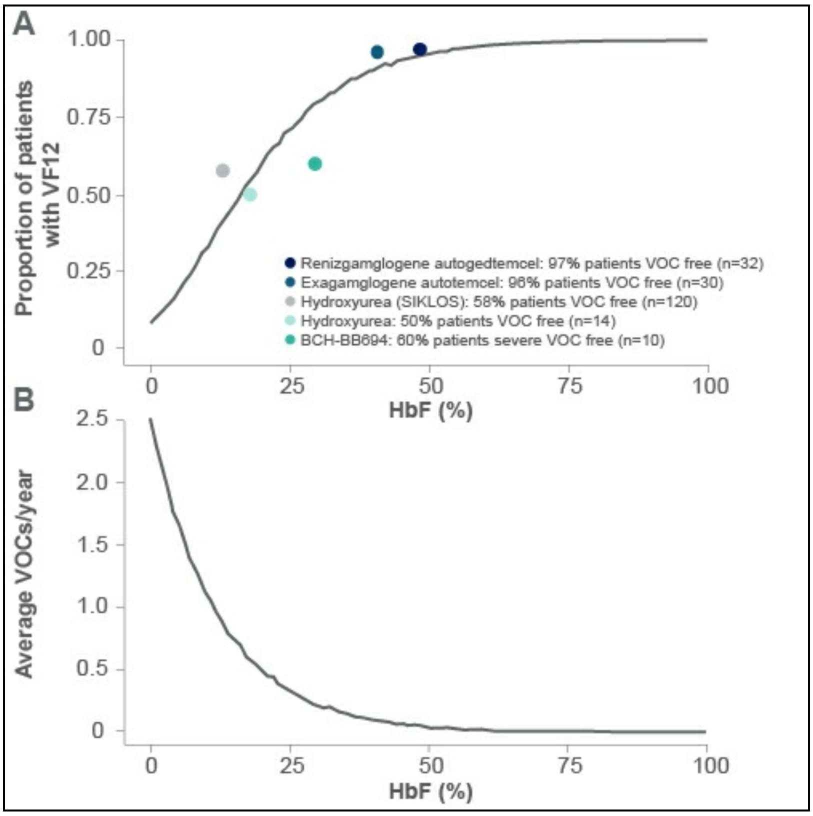

**Copyright Information:** (Online) ISSN: 2572-9241
©2026 The Author(s). HemaSphere is published by John Wiley & Sons Ltd on behalf of European Hematology Association. This is an open access Abstract Book distributed under the Attribution-NonCommercial-NoDerivs (CC BY-NC-ND), which allows third parties to download the articles and share them with others as long as they credit the author and the Abstract Book, but they cannot change the content in any way or use them commercially.
**Abstract Book Citations:** Authors, Title, HemaSphere, 2026;10:(S1):pages. The abstract book DOIs can be found on the “Author Information” page.
**Disclaimer:** Articles published in the journal HemaSphere exclusively reflect the opinions of the authors. The authors are responsible for all content in their abstracts including accuracy of the facts, statements, citing resources, etc.

### (PB4135) AN EVALUATION OF THE ACCURACY, ACCESSIBILITY, AND CLINICAL RELIABILITY OF A SPECIALIZED AI TOOL FOR SICKLE CELL PATIENT EDUCATION ON WEB AND SOCIAL MEDIA PLATFORMS

**Topic:** 27. Sickle cell disease

Zacharie Liman-Tinguiri*[1, 2], Lewis Thomas[1, 3], Vincent Malcor Deydier De Pierrefeu[2, 4], Colombe Chedal-Anglay[2, 4], Fatima Bensiradj[2, 4], Pablo Bartolucci[2, 4], Frédéric Galactéros[2, 4]

*[1]SCKIN Sickle Cell Knowledge and Information Network, New York, United States of America;[2]RED Network (Erythrocyte Disorders & Sickle Cell Disease Network), Créteil, France; [3]The SickleVerse, London, United Kingdom; [4]Henri Mondor University Hospital, AP-HP, Créteil, France;*

### Background

Most sickle cell patients live in middle and low income countries where access to medical expertise or information is scarce. Consequently patients turn to universally accessible social media and messaging platforms to get answers to their questions. To address the need for high quality information, we developed a specialized AI tool, grounded exclusively in reliable sources of knowledge, driven by system prompts prioritizing clinical reliability, patient accessibility, with clear safety guardrails.

### Aims

To evaluate the accuracy, accessibility, and clinical reliability of a specialized AI agent for Sickle Cell Disease education compared to various general-purpose large language models (LLMs). A secondary objective is to assess the efficacy of our specialized retrieval methods and determine how underlying model characteristics—specifically parameter size, open-weight architecture, and market popularity—affect clinical performance.

### Methods

We are utilizing a curated dataset of clinical queries, alongside expert-validated reference answers, developed by researchers from the Réseau d'Érythropoïèse et de Drépanocytose (RED). This standardized prompt set is being administered to our specialized RAG-enabled agent (SickleCellPedia) and five general-purpose large language models, encompassing both proprietary and open-weight architectures (ChatGPT, Gemini, Anthropic, DeepSeek, and Mistral). The generated outputs will be compiled and subjected to a rigorous, independent review by a panel of medical professionals. Evaluators will be strictly blinded to the source model of each response. Using the RED expert answers as the baseline, the panel will score the outputs across three predefined dimensions: accuracy (absence of clinical errors or hallucinations), accessibility (plain-language readability for non-scientists), and clinical reliability (alignment with expert medical consensus).

### Results

Data collection and blinded expert evaluations are currently ongoing. The final analysis will quantify the performance discrepancies between the specialized RAG architecture and general-purpose LLMs. We will present comparative metrics on how underlying model characteristics—specifically parameter size, open-weight availability, and market popularity—impact clinical safety and readability scores.

**Copyright Information:** (Online) ISSN: 2572-9241
©2026 The Author(s). HemaSphere is published by John Wiley & Sons Ltd on behalf of European Hematology Association. This is an open access Abstract Book distributed under the Attribution-NonCommercial-NoDerivs (CC BY-NC-ND), which allows third parties to download the articles and share them with others as long as they credit the author and the Abstract Book, but they cannot change the content in any way or use them commercially.
**Abstract Book Citations:** Authors, Title, HemaSphere, 2026;10:(S1):pages. The abstract book DOIs can be found on the "Author Information" page.
**Disclaimer:** Articles published in the journal HemaSphere exclusively reflect the opinions of the authors. The authors are responsible for all content in their abstracts including accuracy of the facts, statements, citing resources, etc.

### Summary/Conclusion

As patients increasingly utilize web and social media platforms for health information, validating the safety of AI tools is critical. The findings from this evaluation will provide crucial evidence on whether RAG-enabled agents, fortified with specialized prompts and safety guardrails, offer a safer and more accessible alternative to generic LLMs. Furthermore, our results will elucidate the tradeoffs between clinical safety and accessibility based on model size, and establish the comparative performance of open-parameter versus closed proprietary models. Ultimately, these insights will guide the robust development of future AI applications, ensuring they can safely and effectively serve the Sickle Cell Disease community and other specialized patient populations.

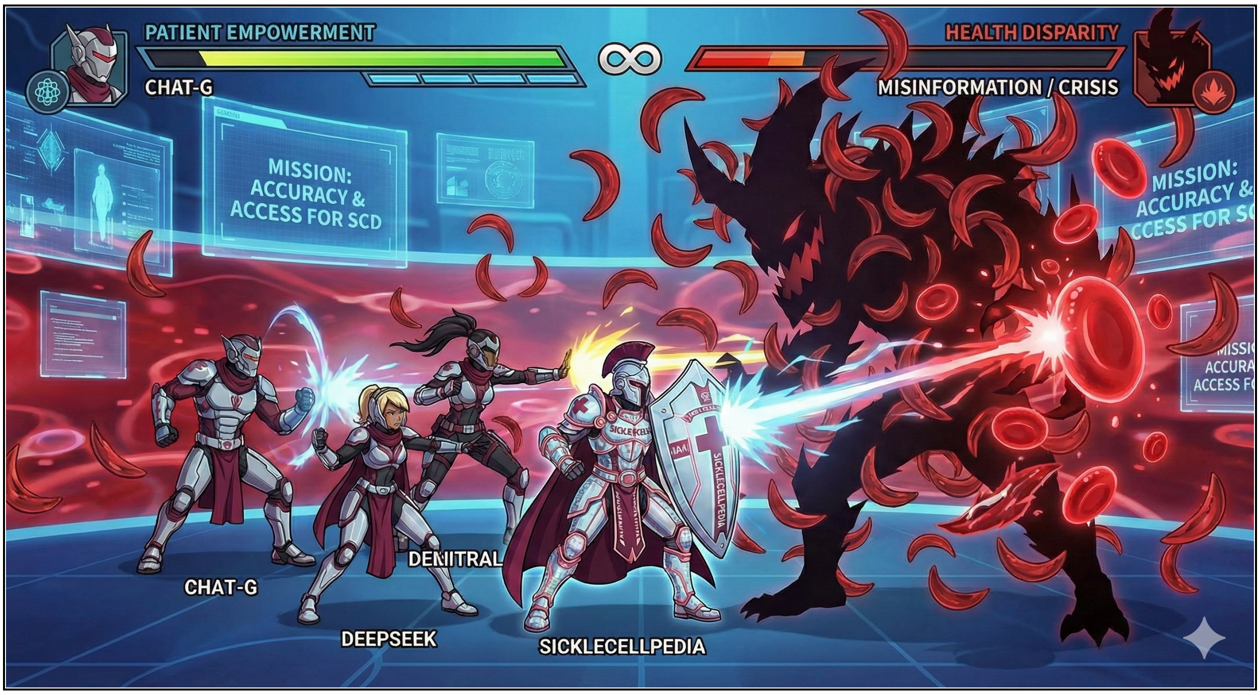

Generated by Nano Banana

**Copyright Information:** (Online) ISSN: 2572-9241
©2026 The Author(s). HemaSphere is published by John Wiley & Sons Ltd on behalf of European Hematology Association. This is an open access Abstract Book distributed under the Attribution-NonCommercial-NoDerivs (CC BY-NC-ND), which allows third parties to download the articles and share them with others as long as they credit the author and the Abstract Book, but they cannot change the content in any way or use them commercially.
**Abstract Book Citations:** Authors, Title, HemaSphere, 2026;10:(S1):pages. The abstract book DOIs can be found on the "Author Information" page.
**Disclaimer:** Articles published in the journal HemaSphere exclusively reflect the opinions of the authors. The authors are responsible for all content in their abstracts including accuracy of the facts, statements, citing resources, etc.

### (PB4136) PATTERN AND OUTCOMES OF SICKLE CELL DISEASE IN SOUTH SUDAN: INSIGHTS FROM HIGH-VOLUME CLINICAL PRACTICE

**Topic:** 27. Sickle cell disease

Buga Kenyi*[1]

[1]*Juba Teaching Hospital, Internal Medicine, Juba, South Sudan;*

### Background

Background
Sickle cell disease (SCD) is a leading cause of morbidity and mortality in sub-Saharan Africa, but data from fragile, post-conflict health systems like South Sudan remain scarce. Al Sabah Children's Hospital in Juba serves as a major referral center, managing high volumes of pediatric SCD cases in a resource-limited setting.

### Aims

Aims
To describe the clinical patterns, hematologic characteristics, and short-term outcomes among patients with sickle cell disease attending a high-volume pediatric center in South Sudan.

### Methods

Methods
We conducted a prospective observational study of 100 consecutive patients with confirmed sickle cell disease (HbSS) at Al Sabah Children's Hospital, Juba, over 3 months. Data included demographics, complete blood count parameters, frequency of vaso-occlusive crises (VOC), infectious complications, blood transfusion requirements, hospital admissions, and acute complications. Analysis was descriptive.

### Results

Results
Median age was 13 years (interquartile range not reported), with slight male predominance. Chronic anemia was universal (mean steady-state hemoglobin 7.9 g/dL), and one-third of patients had levels below 7 g/dL. Most patients experienced ≥1 VOC during the study period. Blood transfusions were required in 40% (mainly for acute anemia and crisis-related complications). Infectious episodes—predominantly malaria and respiratory infections—occurred in nearly one-third of patients and often triggered crises. Acute chest syndrome and stroke were uncommon but significant; no in-hospital mortality occurred. Key challenges included inconsistent blood supply, lack of hydroxyurea, and absence of structured preventive care.

### Summary/Conclusion

Conclusion
At Al Sabah Children's Hospital, SCD manifests with severe chronic anemia, frequent VOCs, and high transfusion needs, exacerbated by resource constraints. These findings underscore the need for improved transfusion services, disease-modifying therapies, and preventive programs in South Sudan, providing baseline data from a high-burden, underreported context.

**Copyright Information:** (Online) ISSN: 2572-9241
©2026 The Author(s). HemaSphere is published by John Wiley & Sons Ltd on behalf of European Hematology Association. This is an open access Abstract Book distributed under the Attribution-NonCommercial-NoDerivs (CC BY-NC-ND), which allows third parties to download the articles and share them with others as long as they credit the author and the Abstract Book, but they cannot change the content in any way or use them commercially.
**Abstract Book Citations:** Authors, Title, HemaSphere, 2026;10:(S1):pages. The abstract book DOIs can be found on the "Author Information" page.
**Disclaimer:** Articles published in the journal HemaSphere exclusively reflect the opinions of the authors. The authors are responsible for all content in their abstracts including accuracy of the facts, statements, citing resources, etc.

### (PB4137) METHODS TO MODEL LONGITUDINAL BIOMARKERS AND SICKLE CELL DISEASE PROGRESSION USING OBSERVATIONAL DATA FROM ENGLAND

**Topic:** 27. Sickle cell disease

Giovanna Barcelos*[1], Anna Filonenko[2], Sara Carvalho[3], Lili Jiang[4], Jennifer Davidson[5], Reto Wirz[1], Caoimhe Rice[5]

*[1]Pfizer AG, Zürich, Switzerland; [2]Pfizer, Berlin, Germany; [3]Thermo Fisher Scientific, Lisbon, Portugal; [4]Pfizer Inc, New York, United States of America; [5]Thermo Fisher Scientific, London, United Kingdom;*

### Background

In sickle cell disease (SCD), hemoglobin (Hb) and other markers of hemolysis (e.g., lactate dehydrogenase, bilirubin, haptoglobin) fluctuate due to ongoing hemolysis and transfusion. In observational data, these biomarkers are recorded at irregular and widely spaced time points. Therefore, summarization using simple metrics (e.g., point estimates or mean values) may not capture longitudinal patterns or account for time-varying confounding. Analytical strategy should account for the structural limitations of observational biomarkers and clinical outcomes data as imperfect proxies for the complex course of SCD progression.

### Aims

To explore analytical approaches for characterizing longitudinal relationship between lab biomarkers and the onset of complications in SCD.

### Methods

For the purpose of this research, we used hemoglobin as our biomarker lab data, and arrhythmia was the complication used to illustrate the disease progression. 3,846 patients who met the criteria for SCD with ≥1 Hb reading were identified from linked Clinical Practice Research Datalink (CPRD)-Hospital Episode Statistics (HES) data during the study period of 01/04/2007 to 31/03/2019 and patients were indexed on their first Hb reading within this period. We then performed an assessment of the available Hb readings followed by time series analyses to understand patterns in Hb over time and those with SCD who developed arrhythmia, and those who did not.
We explored the relevance of components of Hb including period mean Hb, proportion of time spent without severe anemia (Hb <8g/dl), and Hb variance in univariable Cox proportional hazards (CPH) regression. We evaluated associations between Hb and incident arrhythmia using three approaches: (1) standard CPH regression with explanatory variables period mean Hb and proportion of time without severe anemia with death treated as a competing risk. (2) weighed CPH regression with time-varying Hb and covariates; and (3) joint models combining a linear mixed-effects model for Hb trajectory with a survival model. Models were adjusted for demographic and clinical covariates.

**Copyright Information:** (Online) ISSN: 2572-9241
©2026 The Author(s). HemaSphere is published by John Wiley & Sons Ltd on behalf of European Hematology Association. This is an open access Abstract Book distributed under the Attribution-NonCommercial-NoDerivs (CC BY-NC-ND), which allows third parties to download the articles and share them with others as long as they credit the author and the Abstract Book, but they cannot change the content in any way or use them commercially.
**Abstract Book Citations:** Authors, Title, HemaSphere, 2026;10:(S1):pages. The abstract book DOIs can be found on the “Author Information” page.
**Disclaimer:** Articles published in the journal HemaSphere exclusively reflect the opinions of the authors. The authors are responsible for all content in their abstracts including accuracy of the facts, statements, citing resources, etc.

**Results**

A total of 22,431 Hb readings were observed in the cohort, with a median of 3 (IQR: 2 to 7) readings per person over a median follow up of 4.6 years with a median of 9.6 months between readings. Mean Hb for the cohort during follow up was 11g/dl. 17.4% of patients had ≥1 reading of <8g/dl and patients spent a mean of 6.7 months with severe anemia. 12.7% of patients had≥1 transfusion.

Hazard ratios differed substantially by modeling approach (Table), with statistical associations observed in standard and weighted Cox models but a null association in joint models.

Table. Comparison of different modeling results

| Condition | Patients at risk (number of events) | CPH | Weighted CPH | Joint models |
|---|---|---|---|---|
| Exposure | | Period mean Hb | Time-updating Hb | Longitudinal Hb trajectory |
| Arrhythmia, Hazard ratio (95% confidence interval) | 3709 (314) | 0.90 (0.86-0.95) | 0.79 (0.67-0.92) | 1.01 (0.96-1.06) |

**Summary/Conclusion**

Hazard ratios for SCD complications varied across statistical models. The analyses were substantially limited by missing, sparse, and potentially informative testing and values of biomarker. Future research should prioritize systematic lab data collection and further methodological development to model multiple longitudinal biomarker trajectories (Hb and hemolysis) and their associations with SCD progression.

**Copyright Information:** (Online) ISSN: 2572-9241
©2026 The Author(s). HemaSphere is published by John Wiley & Sons Ltd on behalf of European Hematology Association. This is an open access Abstract Book distributed under the Attribution-NonCommercial-NoDerivs (CC BY-NC-ND), which allows third parties to download the articles and share them with others as long as they credit the author and the Abstract Book, but they cannot change the content in any way or use them commercially.
**Abstract Book Citations:** Authors, Title, HemaSphere, 2026;10:(S1):pages. The abstract book DOIs can be found on the “Author Information” page.
**Disclaimer:** Articles published in the journal HemaSphere exclusively reflect the opinions of the authors. The authors are responsible for all content in their abstracts including accuracy of the facts, statements, citing resources, etc.

### (PB4138) AUDIT OF COMPLIANCE WITH THE BRITISH SOCIETY FOR HAEMATOLOGY GUIDELINE ON THE MANAGEMENT OF SICKLE CELL DISEASE IN PREGNANCY AND RELATED OUTCOMES: A SINGLE TERTIARY CENTRE RETROSPECTIVE STUDY

**Topic:** 27. Sickle cell disease

Elena Capbun*[1], Omer Elchanan[1], Stanley MacMurray[1], Alice Yau[1], Bernadette Jenner[2], Charlotte Patient[2], Catherine Barlow[2], Laura Kessack[3], James Bamber[3], Megan Jones[3], Tamsin Pool[3], Vivian Garcia[4], Ruth Jolley[4], Martin Besser[4]

*[1]University of Cambridge, Clinical School of Medicine, Cambridge, United Kingdom;[2]Cambridge University Hospitals NHS Foundation Trust, Department of Obstetrics, Cambridge, United Kingdom; [3]Cambridge University Hospitals NHS Foundation Trust, Department of Anaesthetics, Cambridge, United Kingdom; [4]Cambridge University Hospitals NHS Foundation Trust, Department of Haematology, Cambridge, United Kingdom;*

### Background

Sickle cell disease (SCD) in pregnancy poses significant maternal and fetal risks, requiring specialised multidisciplinary team (MDT) management.

### Aims

To (1) assess adherence to national standards across antenatal,intrapartum and post-partum care, and (2) describe maternal and neonatal outcomes in pregnancies complicated by SCD.

### Methods

We retrospectively audited pregnancies referred to Addenbrooke's Hospital, Cambridge, UK (May 2022 – May 2025). Electronic records were reviewed using the British Society for Haematology (BSH) proforma and National Institute of Health and Care Excellence (NICE) guidelines to evaluate compliance and pregnancy outcomes.

**Copyright Information:** (Online) ISSN: 2572-9241
©2026 The Author(s). HemaSphere is published by John Wiley & Sons Ltd on behalf of European Hematology Association. This is an open access Abstract Book distributed under the Attribution-NonCommercial-NoDerivs (CC BY-NC-ND), which allows third parties to download the articles and share them with others as long as they credit the author and the Abstract Book, but they cannot change the content in any way or use them commercially.
**Abstract Book Citations:** Authors, Title, HemaSphere, 2026;10:(S1):pages. The abstract book DOIs can be found on the "Author Information" page.
**Disclaimer:** Articles published in the journal HemaSphere exclusively reflect the opinions of the authors. The authors are responsible for all content in their abstracts including accuracy of the facts, statements, citing resources, etc.

### Results

Twenty-eight pregnancies were included (60.7% HbSC, 39.3% HbSS) with a median maternal age at booking of 31.5 (IQR 29.0 – 36.3) years. The cohort included two pregnancy losses and four terminations of pregnancy. 35.7% had baseline SCD-related comorbidities (sickle retinopathy (n = 4), mild cardiac decompensation (n = 2), venous thrombosis (n = 2)).
All patients were managed within a joint obstetric-haematology MDT. The median gestation at haematology referral was 15.0 (IQR 10.8 – 28.0, range 6.0 – 35) weeks. All were offered pre-partum aspirin (two declined) and low-molecular-weight heparin (one declined pre-partum, one non-compliant post-partum). Folic acid was offered in 96.4% of cases. Pre-partum and post-partum prophylactic antibiotics were offered in 89.3% and 84.6% of cases, respectively. Although 96.3% of women underwent screening, partner screening was offered in only 88.5% of cases and counselling for identified carriers was lower at 66.7%. All local deliveries had a delivery and anaesthetics plan, senior and MDT involvement and fetal monitoring. Intra-partum analgesia was used in 95.2%, with one patient declining pain relief. Of note, contraceptive counselling was documented in only 46.4%, with only one of these being given comprehensive advice prior to discharge.
Maternal sickle crises occurred in 35.7% (19 crises total, 16 requiring admission: 3 acute chest) at a median gestation of 32.4 (IQR 23.5 – 37.0) weeks. A total of 8 patients were transfused throughout pregnancy (4 planned exchanges, 2 planned top-ups and 3 unplanned top-ups post-delivery), receiving a median of 2 (IQR 1 – 3.5) transfusions at a median interval of 7 (IQR 6 – 8) weeks and 27.4 (IQR 18.1 – 37) weeks' gestation. Pre-eclampsia occurred in 9.5% and postpartum haemorrhage in 19.0%, with no HDU admissions or deaths.
Live birth rate was 78.6% with a median gestation age at delivery of 38.6 (IQR 38.2 – 39.3) weeks. Eleven pregnancies were delivered by Caesarean birth (10 emergency) and ten via vaginal delivery (2 instrumental). Preterm delivery <37 weeks occurred only in twins (n = 1). Only one case of small-gestational age and restricted intra-uterine growth were recorded. Four neonates required NICU admission.

### Summary/Conclusion

This audit identified high overall adherence to BSH guidelines for pharmacological prophylaxis and intrapartum care within a tertiary MDT setting. The involvement of a joint obstetric-haematology MDT remains a cornerstone of care, likely contributing to the favourable neonatal outcomes and the absence of maternal mortality or HDU admissions observed in this cohort. Significant gaps persist in early antenatal referral, partner screening and counselling and post-partum contraceptive advice. Service redesign may focus on standardising documentation and patient advice to ensure holistic compliance.

**Copyright Information:** (Online) ISSN: 2572-9241
©2026 The Author(s). HemaSphere is published by John Wiley & Sons Ltd on behalf of European Hematology Association. This is an open access Abstract Book distributed under the Attribution-NonCommercial-NoDerivs (CC BY-NC-ND), which allows third parties to download the articles and share them with others as long as they credit the author and the Abstract Book, but they cannot change the content in any way or use them commercially.
**Abstract Book Citations:** Authors, Title, HemaSphere, 2026;10:(S1):pages. The abstract book DOIs can be found on the "Author Information" page.
**Disclaimer:** Articles published in the journal HemaSphere exclusively reflect the opinions of the authors. The authors are responsible for all content in their abstracts including accuracy of the facts, statements, citing resources, etc.

### (PB4139) PATHCRISP-SICKLEDETECT: A NEXT GENERATION TOOL FOR SICKLE CELL SCREENING

**Topic:** 27. Sickle cell disease

Utkarsh Nikhade*[1], Annes Siji[1], Salim Shaikh[1], Mandar Naik[1], Kanikah Mehndiratta[1], Kruthika Bs[1], Vandana Hegde[1], Vaijayanti Gupta[1], Arvind Venkatesan[1], Vijay Chandru[1], Reety Arora[1]

[1]*CRISPRBITS Private Limited, Bengaluru, India;*

### Background

Sickle cell disease (SCD) is a blood disorder characterised by sickle-shaped RBCs. This abnormality of RBCs leads to critical complications like severe vaso-occlusive crises, anaemia, acute chest syndrome and stroke requiring urgent medical attention to prevent organ damage. It is a genetic condition caused by a point mutation in the seventh codon of the beta-globin gene, leading to the substitution of glutamic acid to valine.
One allele or carrier status confers resistance to malaria, but two alleles lead to a diseased condition. Africa and India have the highest burden of sickle cell disease in the world. 50% to 80% of diseased cases die before age 5 in low-income and resource-deficient countries. So, the first step towards reducing this load is widespread screening to identify and control the magnification of disease-causing recessive sickle allele in a population.

### Aims

Development of a CRISPR-based point-of-care diagnostic solution capable of swiftly and accurately detecting the sickle cell allele.

### Methods

We developed a *PathCrisp-SickleDetect* assay, a rapid SNP-detection system based on CRISPR-Cas12b, capable of detecting disease and carrier status. We designed two specific single-guide RNAs, each capable of detecting the presence or absence of rs334. The Cas12b RNP complex recognises the target DNA for sickle cell. This recognition then triggers trans-cleavage of the surrounding ssDNA reporter. This resulting fluorescence can distinguish wild-type, homozygous (SCD), and heterozygous (carrier status) genotypes. The assay delivers results in under 2 hours with minimal equipment, and the PathCrisp software for automated genotype calling analyses the data.

### Results

We have validated our *PathCrisp-SickleDetect* on 144 patient samples obtained from various sample matrices, including whole blood, dried blood spots, buccal swabs, and prenatal samples and demonstrated 100% concordance with Sanger sequencing. The nucleotide-specific interaction between the single-guide RNA and the target DNA provides the assay with unprecedented accuracy. The lyophilised reagents enable room-temperature storage and field deployment.

### Summary/Conclusion

*PathCrisp-SickleDetect* offers a cost-effective, robust, and sensitive point-of-care diagnostic solution that bridges the gap between laboratory diagnostics and primary healthcare, enabling SCD diagnosis in the general population and newborns. Lyophilisation simplified the workflow and added an extra layer of reagent stability, making the kit suitable for diverse clinical settings.

**Copyright Information:** (Online) ISSN: 2572-9241
©2026 The Author(s). HemaSphere is published by John Wiley & Sons Ltd on behalf of European Hematology Association. This is an open access Abstract Book distributed under the Attribution-NonCommercial-NoDerivs (CC BY-NC-ND), which allows third parties to download the articles and share them with others as long as they credit the author and the Abstract Book, but they cannot change the content in any way or use them commercially.
**Abstract Book Citations:** Authors, Title, HemaSphere, 2026;10:(S1):pages. The abstract book DOIs can be found on the “Author Information” page.
**Disclaimer:** Articles published in the journal HemaSphere exclusively reflect the opinions of the authors. The authors are responsible for all content in their abstracts including accuracy of the facts, statements, citing resources, etc.

### (PB4140) EFFECT OF THE ANTI-SICKLING DRUG VOXELOTOR ON INFLAMMATORY AND ANGIOGENIC PATHWAYS IN ADULT PATIENTS WITH SICKLE CELL DISEASE

**Topic:** 27. Sickle cell disease

Sanjay Thakoerdin*[1], Kadère Konté[2], Maud Zwolsman[1, 3], Lydian De Ligt[1, 3], Bart Biemond[1, 3], Erfan Nur[1, 3]

*[1]Amsterdam UMC, Hematology, Amsterdam, The Netherlands; [2]Amsterdam Umc, Amsterdam, The Netherlands; [3]Sanquin, Amsterdam, The Netherlands;*

### Background

In sickle cell disease (SCD), chronic intravascular hemolysis and ischemia-reperfusion injury due to recurrent microvascular occlusion result in oxidative stress, chronic inflammation, and hypoxia-induced angiogenesis. We recently showed that plasma values of inflammatory and angiogenic proteins are increased in patients with SCD. Voxelotor is a sickle hemoglobin (HbS) polymerization inhibitor that reduces hemolysis, resulting in increased hemoglobin (Hb) levels. However, the effect of this reduction in hemolysis on the angiogenic and inflammatory pathways in SCD remains unclear.

### Aims

This study aimed to evaluate the effect of voxelotor on plasma values of angiogenic and inflammatory proteins in adult patients with the severe SCD genotypes (HbSS and HbS$\beta^0$-thalassemia).

### Methods

Stored paired plasma samples from the single-center, prospective, open-label COVERAGE study (Amsterdam UMC) were analyzed. Twenty preselected proteins were quantified using Proximity Extension Assay. Values were analyzed on the log2 scale; within-patient differences were assessed using paired t-tests. Results are reported as geometric means per time point and geometric mean ratios (GMRs) with 95% confidence intervals. Multiple testing was controlled using Benjamini–Hochberg false discovery rate. Correlations with Hb, leukocyte count, and HbS/HbF percentages were assessed.

### Results

A total of 13 patients (age 36±9 (mean±SD) years; 69% female) were included in this study. After 3 months, Voxelotor increased Hb by 2.3±1.1 g/dL and significantly reduced hemolysis markers (reticulocytes, LDH, bilirubin). Angiogenesis/hypoxia-related proteins decreased after therapy: ANGPT2 (GMR 0.75, 95% CI 0.64–0.89), EGLN1 (0.61, 0.49–0.76), EPO (0.66, 0.44–0.99), and VEGFA (0.74, 0.57–0.94). Soluble G-CSFR also decreased (0.86, 0.79–0.92). At baseline, proteins across angiogenesis, inflammation and neutrophil activation correlated with laboratory parameters reflecting hemolysis ($r \geq 0.5$, $p < 0.05$), including inverse associations of Hb with VEGFA ($r = -0.58$) and IL-1β ($r = -0.72$).

### Summary/Conclusion

In adults with severe SCD, voxelotor therapy was associated with attenuation of hypoxia/angiogenesis response. These findings support a link between chronic intravascular hemolysis and neoangiogenic activation in SCD. Although voxelotor was withdrawn from global markets in September 2024 due to emerging safety concerns, next-generation HbS polymerization inhibitors such as osivelotor remain in clinical development. Therefore, the results of this study contribute to a greater understanding of the effect of this class of polymerization inhibitors on hypoxia response, angiogenesis and inflammation.

**Copyright Information:** (Online) ISSN: 2572-9241
©2026 The Author(s). HemaSphere is published by John Wiley & Sons Ltd on behalf of European Hematology Association. This is an open access Abstract Book distributed under the Attribution-NonCommercial-NoDerivs (CC BY-NC-ND), which allows third parties to download the articles and share them with others as long as they credit the author and the Abstract Book, but they cannot change the content in any way or use them commercially.
**Abstract Book Citations:** Authors, Title, HemaSphere, 2026;10:(S1):pages. The abstract book DOIs can be found on the "Author Information" page.
**Disclaimer:** Articles published in the journal HemaSphere exclusively reflect the opinions of the authors. The authors are responsible for all content in their abstracts including accuracy of the facts, statements, citing resources, etc.

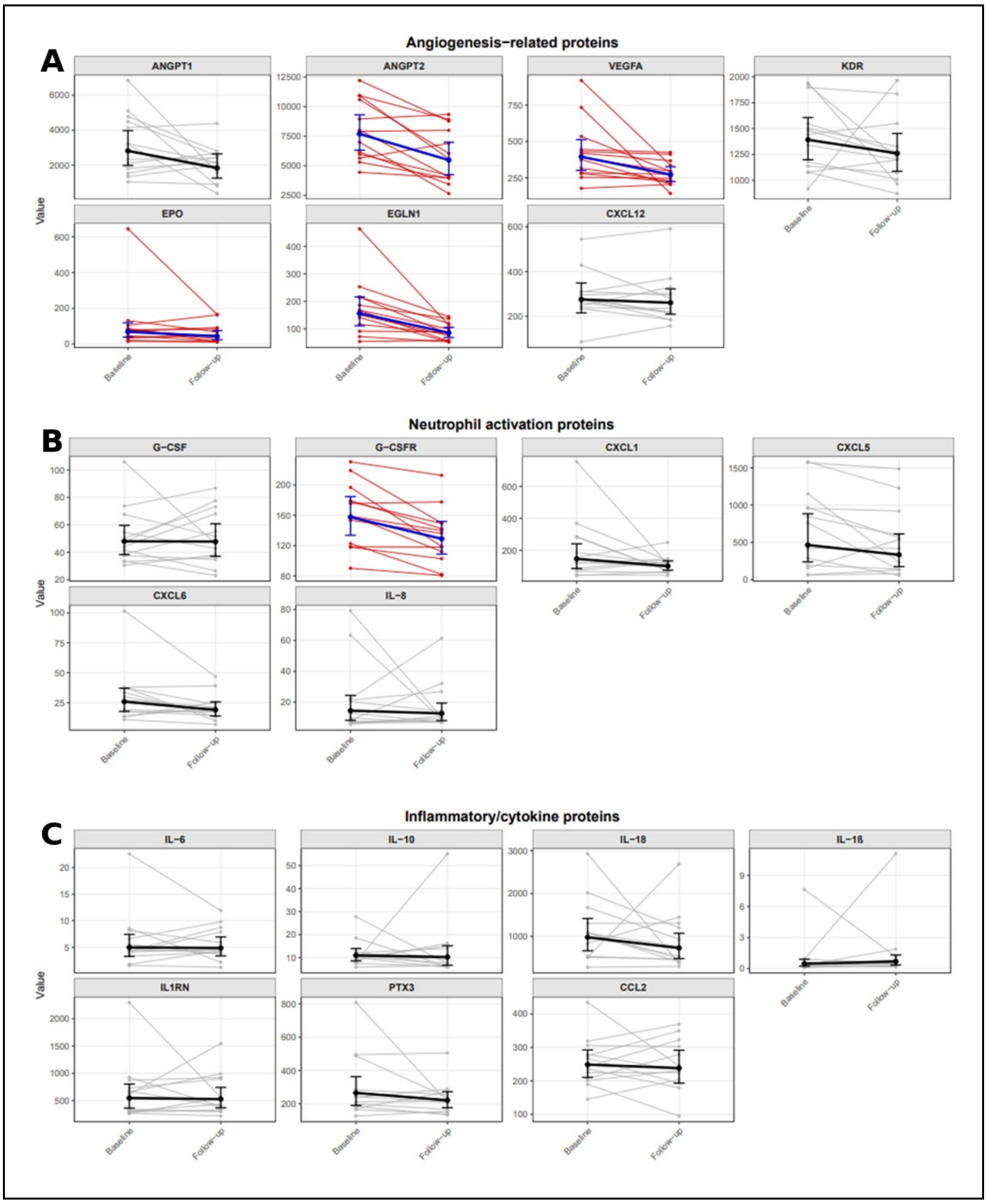

Figure 1A:Longitudinal individual plasma protein markers related to angiogenesis and response to hypoxia at baseline and after 3 months of therapy with voxelotor. Figure 1B: Longitudinal individual neutrophil activation plasma protein markers at baseline and after 3 months of therapy with voxelotor. Figure 1C: Longitudinal individual inflammatory/cytokine plasma protein markers at baseline and after 3 months of therapy with voxelotor. Black lines show geometric mean after log2 transformation and back transformation with 95% CI. Red trajectories indicate significant biomarker changes after false discovery rate (FDR) correction with blue lines showing the geometric mean with 95% CI.

**Copyright Information:** (Online) ISSN: 2572-9241
©2026 The Author(s). HemaSphere is published by John Wiley & Sons Ltd on behalf of European Hematology Association. This is an open access Abstract Book distributed under the Attribution-NonCommercial-NoDerivs (CC BY-NC-ND), which allows third parties to download the articles and share them with others as long as they credit the author and the Abstract Book, but they cannot change the content in any way or use them commercially.
**Abstract Book Citations:** Authors, Title, HemaSphere, 2026;10:(S1):pages. The abstract book DOIs can be found on the "Author Information" page.
**Disclaimer:** Articles published in the journal HemaSphere exclusively reflect the opinions of the authors. The authors are responsible for all content in their abstracts including accuracy of the facts, statements, citing resources, etc.

### (PB4141) PARVOVIRUS B19-ASSOCIATED PAIN CRISES IN PEDIATRIC PATIENTS WITH HBSC SICKLE CELL DISEASE: CLINICAL CHARACTERISTICS AND COMPLICATIONS

**Topic:** 27. Sickle cell disease

Matthias Bleeke*[1], Sabine Ebert[1], Beate Ebert[1]

[1] *University Medical Center Hamburg-Eppendorf, Dept. of Pediatric Hematology and Oncology, Hamburg, Germany;*

### Background

Infections with parvovirus B19 (PB19) lead to aplastic crisis in patients with sickle cell disease. However, in patients with HbSC sickle cell disease, acute anemia is less severe than in HbSS genotype, but acute pain is a common symptom of PB19 infection. In addition, patients with HbSC disease have an increased risk of critical complications such as bone marrow necrosis and fat embolism syndrome in the context of PB19 infection.

### Aims

This study aims to describe differences in the clinical course of pain crises in pediatric patients with HbSC disease with and without acute PB19 infection.

### Methods

We retrospectively analyzed data from our institution on acute pain crises requiring inpatient treatment in children and adolescents with HbSC sickle cell disease between 2013 and 2025. Data on clinical symptoms, laboratory parameters, treatment and complications were compared between patients with and without evidence of concomitant PB19 infection ($PB19^+$, $PB19^-$). Groups were compared using chi-square, Fisher's exact and Mann-Whitney tests.

### Results

A total of 38 acute pain crises were included, 11 of which had evidence of acute PB19 infection with a median PB19 viral load of 9.8 x $10^6$ cop/mL. All patients presented with pain, but there were marked differences regarding the pain location with a clear predominance of back pain in $PB19^+$ (90.9%), while limb pain was most common in $PB19^-$ (85.2%).

Pain therapy with opioids was administered in 16/27 (59.3%) of the $PB19^-$ group, but in 11/11 (100%) of the $PB19^+$ group ($p = 0.016$). Among patients who received pain treatment with opioids, the median duration of opioid treatment was significantly longer in $PB19^+$ with 9 days than in $PB19^-$ with 3 days ($p < 0.001$). The total length of inpatient treatment was significantly longer for $PB19^+$ patients, with a median of 10 days, than for PB19- patients, with a median of 3 days ($p < 0.001$).

$PB19^+$ patients required oxygen therapy more frequently (81.1 % vs. 3.7 %, $p < 0.001$). Major complications occurred in three $PB19^+$ patients: respiratory failure requiring invasive ventilation occurred in two $PB19^+$ patients, one $PB19^+$ patient suffered transient hemiparesis in the course of the infection.

At admission, hemoglobin and reticulocytes were significantly lower in $PB19^+$ than $PB19^-$, consistent with aplastic crisis, while there were no significant differences in initial LDH and CRP. However, a significant increase of LDH and CRP was observed in $PB19^+$ over the course of inpatient treatment with median peak levels for LDH being 1474 vs. 412 U/L ($p < 0.001$) and for CRP being 162 vs. 15 mg/L ($p < 0.001$). All three $PB19^+$ patients with major complications showed an LDH > 2000 U/L on day 2 or 3 of inpatient treatment, respectively.

**Copyright Information:** (Online) ISSN: 2572-9241
©2026 The Author(s). HemaSphere is published by John Wiley & Sons Ltd on behalf of European Hematology Association. This is an open access Abstract Book distributed under the Attribution-NonCommercial-NoDerivs (CC BY-NC-ND), which allows third parties to download the articles and share them with others as long as they credit the author and the Abstract Book, but they cannot change the content in any way or use them commercially.
**Abstract Book Citations:** Authors, Title, HemaSphere, 2026;10:(S1):pages. The abstract book DOIs can be found on the "Author Information" page.
**Disclaimer:** Articles published in the journal HemaSphere exclusively reflect the opinions of the authors. The authors are responsible for all content in their abstracts including accuracy of the facts, statements, citing resources, etc.

**Summary/Conclusion**

Pain crises in pediatric HbSC patients showed significant differences in terms of clinical presentation, treatment requirements, and laboratory parameter trajectories, depending on an association with acute PB19 infections. The combination of severe back pain and elevated LDH and CRP levels may indicate bone marrow necrosis as a known complication of PB19 infection in non-HbSS sickle cell patients. Patients with severe respiratory and neurological complications showed LDH levels > 2000 U/L from day 2 of inpatient treatment. Therefore, LDH progression could provide an indication of a severe course of PB19 infection in HbSC patients, and could help to determine the need for therapeutic measures, such as blood exchange transfusion.

**Copyright Information:** (Online) ISSN: 2572-9241
©2026 The Author(s). HemaSphere is published by John Wiley & Sons Ltd on behalf of European Hematology Association. This is an open access Abstract Book distributed under the Attribution-NonCommercial-NoDerivs (CC BY-NC-ND), which allows third parties to download the articles and share them with others as long as they credit the author and the Abstract Book, but they cannot change the content in any way or use them commercially.
**Abstract Book Citations:** Authors, Title, HemaSphere, 2026;10:(S1):pages. The abstract book DOIs can be found on the “Author Information” page.
**Disclaimer:** Articles published in the journal HemaSphere exclusively reflect the opinions of the authors. The authors are responsible for all content in their abstracts including accuracy of the facts, statements, citing resources, etc.

### (PB4142) EXCHANGE VS SIMPLE TRANSFUSION FOR CHRONIC SICKLE CELL DISEASE: A SYSTEMATIC REVIEW AND META-ANALYSIS

**Topic:** 27. Sickle cell disease

Yashasvi Srivastava*[1], Shankar Biswas[2]

*[1]Ivano-Frankivsk National Medical University, Ivano-Frankivsk, Ukraine; [2]Ivano-Frankivsk National Medical University, Internal Medicine, Ivano-Frankivsk, Ukraine;*

### Background

Chronic transfusion is essential in SCD management for stroke prevention and organ protection. Transfusions can be delivered as simple (top-up) or exchange (automated/manual). ASH 2020 guidelines acknowledged uncertainty about the optimal modality for chronic therapy (1). Existing meta-analyses compared automated versus manual exchange (2), but none have quantitatively compared exchange versus simple transfusion for chronic non-perioperative SCD management.

### Aims

To compare exchange versus simple transfusion for chronic SCD management regarding iron overload, alloimmunization, blood utilization, and transfusion reactions.

### Methods

PubMed, Embase, and Scopus were searched through February 2026 without language restrictions, including conference abstracts. Comparative studies (≥10 patients) of exchange versus simple transfusion in SCD patients on chronic non-perioperative transfusion were included. Alloimmunization was pooled as incidence rate ratio (IRR) using random-effects meta-analysis. Other outcomes were synthesized narratively. Risk of bias was assessed via Newcastle-Ottawa Scale (NOS); certainty via GRADE.

### Results

Five observational studies (n=399; 1994–2022) from USA, France, and UK were included. NOS scores: 6–8/9.
*Alloimmunization (2 studies; 23,735 units):* Exchange had significantly lower per-unit alloimmunization (IRR=0.19; 95%CI: 0.12–0.31; p<0.0001; $I^2$=0%). Patient-level prevalence was not different (OR=1.47; 0.76–2.83), reflecting higher unit exposure with exchange.
*Iron overload (2 studies):* In the SIT trial secondary analysis (n=83), 12-month ferritin was markedly lower with automated exchange (median 355 ng/mL) versus simple transfusion (1801 ng/mL; p<0.001). Adjusted analysis confirmed 958 ng/mL greater ferritin increase with simple transfusion (95%CI: 574–1342; p<0.001) (3). In a paired analysis (n=7), erythrocytapheresis reduced annual iron load by 87% (p<0.01) (4).
*Blood utilization (3 studies):* Exchange required 23–73% more donor blood per year and 2–11-fold more units per patient (5–7).
*Transfusion reactions (2 studies):* No hemolytic reactions in exchange arms (0/71) versus 5 events in simple/conventional arms (5/162).
*VOC (1 study):* A conference abstract reported significant VOC reduction switching from manual to automated exchange but no difference versus simple transfusion.
All outcomes: very low to low certainty (GRADE).

**Copyright Information:** (Online) ISSN: 2572-9241
©2026 The Author(s). HemaSphere is published by John Wiley & Sons Ltd on behalf of European Hematology Association. This is an open access Abstract Book distributed under the Attribution-NonCommercial-NoDerivs (CC BY-NC-ND), which allows third parties to download the articles and share them with others as long as they credit the author and the Abstract Book, but they cannot change the content in any way or use them commercially.
**Abstract Book Citations:** Authors, Title, HemaSphere, 2026;10:(S1):pages. The abstract book DOIs can be found on the "Author Information" page.
**Disclaimer:** Articles published in the journal HemaSphere exclusively reflect the opinions of the authors. The authors are responsible for all content in their abstracts including accuracy of the facts, statements, citing resources, etc.

### Summary/Conclusion

This first meta-analysis of exchange versus simple transfusion for chronic SCD management shows exchange is associated with significantly lower per-unit alloimmunization and substantially less iron overload, at the cost of higher blood consumption. The absence of RCTs limits certainty. Randomized trials comparing these modalities are needed.

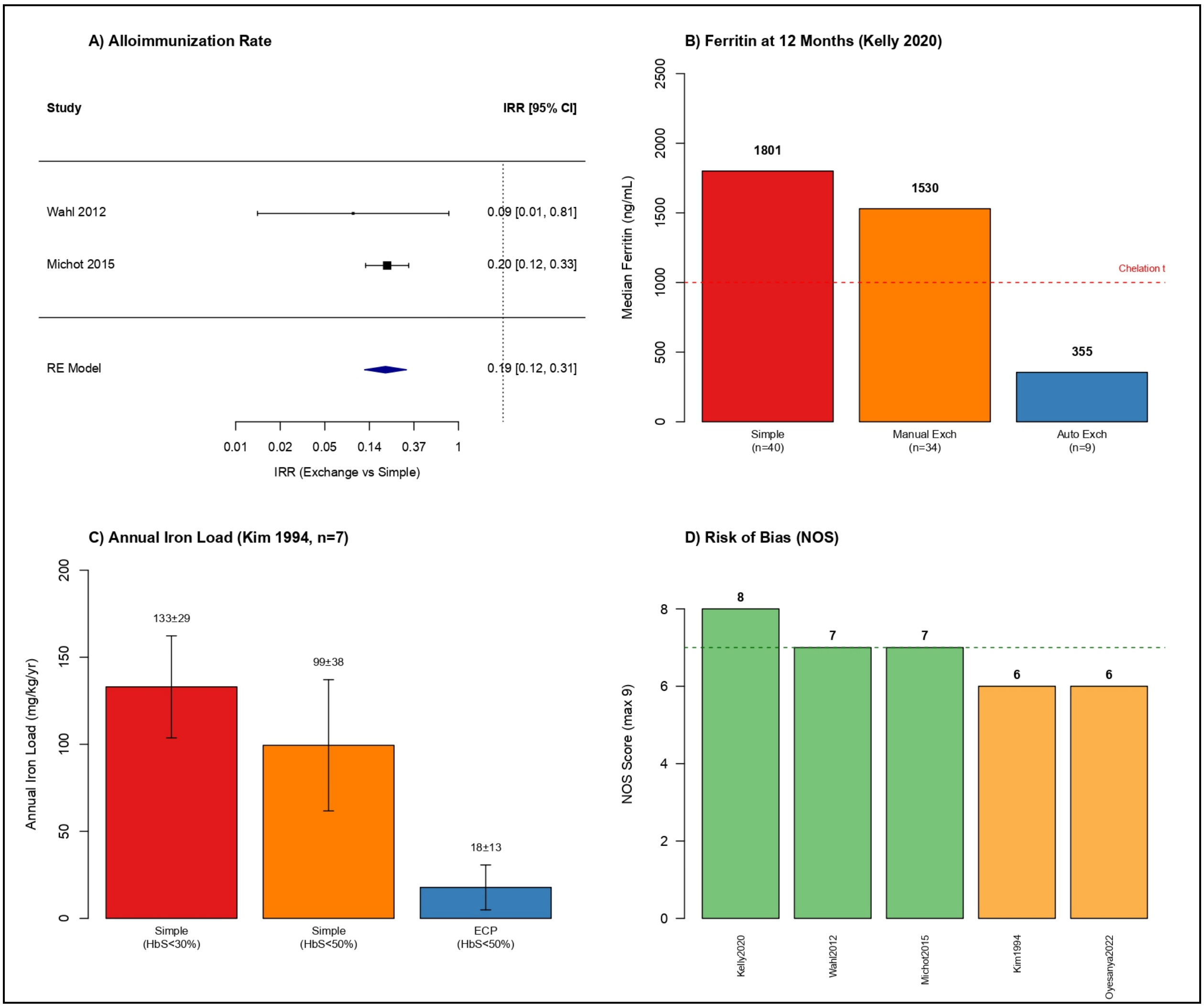

4 Panel Fig

**Copyright Information:** (Online) ISSN: 2572-9241
©2026 The Author(s). HemaSphere is published by John Wiley & Sons Ltd on behalf of European Hematology Association. This is an open access Abstract Book distributed under the Attribution-NonCommercial-NoDerivs (CC BY-NC-ND), which allows third parties to download the articles and share them with others as long as they credit the author and the Abstract Book, but they cannot change the content in any way or use them commercially.
**Abstract Book Citations:** Authors, Title, HemaSphere, 2026;10:(S1):pages. The abstract book DOIs can be found on the "Author Information" page.
**Disclaimer:** Articles published in the journal HemaSphere exclusively reflect the opinions of the authors. The authors are responsible for all content in their abstracts including accuracy of the facts, statements, citing resources, etc.

### (PB4143) LONG-TERM FOLLOW-UP OF HYDROXYUREA IN SEVERE SICKLE CELL DISEASE TREATED WITH HYDROXYUREA :A RETROSPECTIVE STUDY

**Topic:** 27. Sickle cell disease

Bradai Mohamed*[1], Hamida Brahimi[1], Ahmed Amine Ziani[1], Fatima Lamraoui[1], Souad Taoussi[1], Yamina Bouchakour Moussa[1, 1], Salima Oukid[1], Khadoudj Mounira Benlabiod[1], Nabila Rekab[1]

*[1]EHS ELCC, Blida, Algeria, Hematology department, Blida, Algeria;*

### Background

Hydroxyurea (HU) represents the standard disease-modifying therapy for preventing acute complications in severe sickle cell disease (SCD). However, limited data are available regarding its very long-term impact on chronic degenerative complications, growth, fertility, and leukemogenic risk.

### Aims

To evaluate the long-term clinical and biological efficacy and safety of hydroxyurea in patients with severe sickle cell disease (SCD) followed for more than a decade, focusing on the occurrence of major complications, chronic visceral damage, mortality, and long-term effects on growth, fertility, and the risk of myelodysplasia.

### Methods

This retrospective monocentric study (2001–2025) included 47 patients (25 SS, 21 Sβ-thalassemia, 1 SC). The median age at HU initiation was 11 years [ 5–33], with a male-to-female ratio of 0.9.
Indications for HU included recurrent severe vaso-occlusive crises (VOC) (>3/year, n=28), acute chest syndrome (ACS, n=11), severe symptomatic anemia (n=6), and poly-alloimmunization (n=2). The starting dose was 15 mg/kg/day, adjusted to a mean maintenance dose of 20 ± 3 mg/kg/day.

### Results

After a median follow-up of 14 years [10–24], the annual frequency of VOC/ACS decreased by 84%, with a 72% reduction in transfusion requirements. The mean hemoglobin gain was +1.8 g/dL [0.8–3.2].
Chronic complications (renal, cardiac, pulmonary) were rare. Two new cases of femoral head avascular necrosis and one spontaneously resolving hemorrhagic stroke were observed. No leg ulcers or new splenectomies were reported. Growth and pubertal development were satisfactory.
Eleven pregnancies occurred in four female patients : 7 pregnancies followed temporary discontinuation of hydroxyurea (HU) in two women with homozygous sickle cell disease (HbSS; G3P3 and G2P2), resulting in seven healthy children without complications. ; 4 additional pregnancies were maintained under HU therapy, which was introduced during the third trimester as an alternative to transfusion; all resulted in healthy infants without complications.
Moreover, 4 pregnancies were reported in partners of male patients receiving HU.
Overall tolerance and treatment adherence were satisfactory.
Six deaths occurred during follow-up (acute severe ACS, n=2; multiorgan failure in one patient treated with high-dose corticosteroids for COVID-19 infection, n=1; undetermined causes, n=2; acute leukemia, n=1). The case of acute leukemia was diagnosed after two years of hydroxyurea exposure, without an established causal relationship.

**Copyright Information:** (Online) ISSN: 2572-9241
©2026 The Author(s). HemaSphere is published by John Wiley & Sons Ltd on behalf of European Hematology Association. This is an open access Abstract Book distributed under the Attribution-NonCommercial-NoDerivs (CC BY-NC-ND), which allows third parties to download the articles and share them with others as long as they credit the author and the Abstract Book, but they cannot change the content in any way or use them commercially.
**Abstract Book Citations:** Authors, Title, HemaSphere, 2026;10:(S1):pages. The abstract book DOIs can be found on the “Author Information” page.
**Disclaimer:** Articles published in the journal HemaSphere exclusively reflect the opinions of the authors. The authors are responsible for all content in their abstracts including accuracy of the facts, statements, citing resources, etc.

**Summary/Conclusion**

Hydroxyurea demonstrates sustained efficacy and good long-term tolerance in severe sickle cell disease. When initiated early, it significantly reduces acute morbidity, delays chronic complications, and preserves growth and fertility, without a clear signal of increased leukemogenic risk. These findings support early and long-term use of hydroxyurea in childhood and adulthood.

**Copyright Information:** (Online) ISSN: 2572-9241
©2026 The Author(s). HemaSphere is published by John Wiley & Sons Ltd on behalf of European Hematology Association. This is an open access Abstract Book distributed under the Attribution-NonCommercial-NoDerivs (CC BY-NC-ND), which allows third parties to download the articles and share them with others as long as they credit the author and the Abstract Book, but they cannot change the content in any way or use them commercially.
**Abstract Book Citations:** Authors, Title, HemaSphere, 2026;10:(S1):pages. The abstract book DOIs can be found on the "Author Information" page.
**Disclaimer:** Articles published in the journal HemaSphere exclusively reflect the opinions of the authors. The authors are responsible for all content in their abstracts including accuracy of the facts, statements, citing resources, etc.

### (PB4144) EXTENDED ERYTHROCYTE GENOTYPE AND TRANSFUSION COMPLICATIONS IN SICKLE CELL DISEASE: PRELIMINARY ANALYSIS FROM A REAL-WORLD COHORT IN ITALY

**Topic:** 27. Sickle cell disease

Claudia Sorgato*[1], Tiziana Tison[2], Alessandro Lanti[2], Mirco D'agnolo[1], Maria Paola Boaro[3], Giulia Reggiani[1, 3], Raffaella Colombatti[1, 3]

*[1]Department of Woman's and Child's Health, University of Padova, Padova, Italy;[2]Immunotransfusion Unit, Azienda Ospedale-Università di Padova, Padova, Italy; [3]Pediatric Hematology-Oncology Unit, Department of Woman's and Child's Health, Azienda Ospedale-Università di Padova, Padova, Italy;*

### Background

Sickle Cell Disease (SCD) is the most prevalent hemoglobinopathy worldwide. In Countries where SCD is rare but increasing due to population movements, transfusion management poses significant challenges. The primary risk is alloimmunization, often leading to Delayed Hemolytic Transfusion Reactions (DHTRs), driven by the antigenic mismatch between predominantly Caucasian donors and recipients of African descent. This study represents a report of extended erythrocyte genotypes in Italy and their correlation with transfusion complications in a pediatric SCD cohort.

### Aims

The aims were to evaluate AB0/Rh/Kell and extended genotypes distribution across ethnic groups, assess alloimmunization frequency and clinical outcomes and explore correlations between genotypes and clinical-laboratory variables.

### Methods

Patients with SCD followed by the Pediatric Onco-hematology and the Immunotransfusion Unit of the AOPD (Azienda Ospedaliera di Padova) from 2007 to 2024 were retrospectively selected. Inclusion criteria required a confirmed SCD mutation (HbS/HbS, HbS/HbC, HbS/βthal+, HbS/βthal0) and available extended erythrocyte genotyping. Data were integrated from the "Sickle Cell Disease Database" and the "EmoNet" system. Statistical analysis was performed using GraphPad Prism (v. 8.4.0), with significance set at $p<0,05$.

### Results

75/168 patients (42 M, 33F), most of them <12 years old and diagnosed with SCD mainly between 0 and 3 years, were eligible at time of study. 68% of them were of sub-Saharan origin, while 12.8% came from Southern Europe. The most frequent genotype was HbS/HbS (87%) and the most frequent Rh phenotype was "ccDee" (54.7%), characteristic of African populations but rare in Caucasians. Extended genotyping showed that Jkb, Fya, Fyb and S antigens were significantly less expressed in patients of sub-Saharan origin compared to Caucasians ($p<0,05$). Even if 49/75 (65%) patients received RBC transfusions (15/75 on chronic regimens, *Figure 1A*), the alloimmunization rate (persistently positive Indirect Antiglobulin Test, *Figure 1B*) was only 9% (7/75), with anti-S, anti-Fyb, and anti-CW being the most frequent antibodies (*Figure 1C*). Two patients (one Caucasian, one African) experienced severe recurrent DHTRs; notably, both lacked common Caucasian antigens (Fya, Fyb, Jkb, S) (*Figure 1D*) and developed the corresponding antibodies. No significant correlations were found between specific minor antigens and laboratory variables or hospitalization rates.

**Copyright Information:** (Online) ISSN: 2572-9241
©2026 The Author(s). HemaSphere is published by John Wiley & Sons Ltd on behalf of European Hematology Association. This is an open access Abstract Book distributed under the Attribution-NonCommercial-NoDerivs (CC BY-NC-ND), which allows third parties to download the articles and share them with others as long as they credit the author and the Abstract Book, but they cannot change the content in any way or use them commercially.
**Abstract Book Citations:** Authors, Title, HemaSphere, 2026;10:(S1):pages. The abstract book DOIs can be found on the "Author Information" page.
**Disclaimer:** Articles published in the journal HemaSphere exclusively reflect the opinions of the authors. The authors are responsible for all content in their abstracts including accuracy of the facts, statements, citing resources, etc.

**Summary/Conclusion**

This study provides Italian data on the impact of extended erythrocyte genotyping in preventing transfusion-related complications in SCD. Our findings reveal an alloimmunization rate lower than reported in international literature, likely due to the proactive use of extended genotyping in our center—a practice not yet uniformly standardized across Europe or Italy. However, the significant "antigenic gap" between the Italian donor pool and SCD recipients remains a critical issue. These results underscore the urgent need to: improve the implementation of universal extended genotyping for all SCD patients and donors in our center and in Italy; develop strategies to recruit non-Caucasian donors to improve matching; implement a national registry including transfusion-specific genotypes; collaboration within European Reference Networks (ERN) to standardize matching protocols and develop alternative therapies.

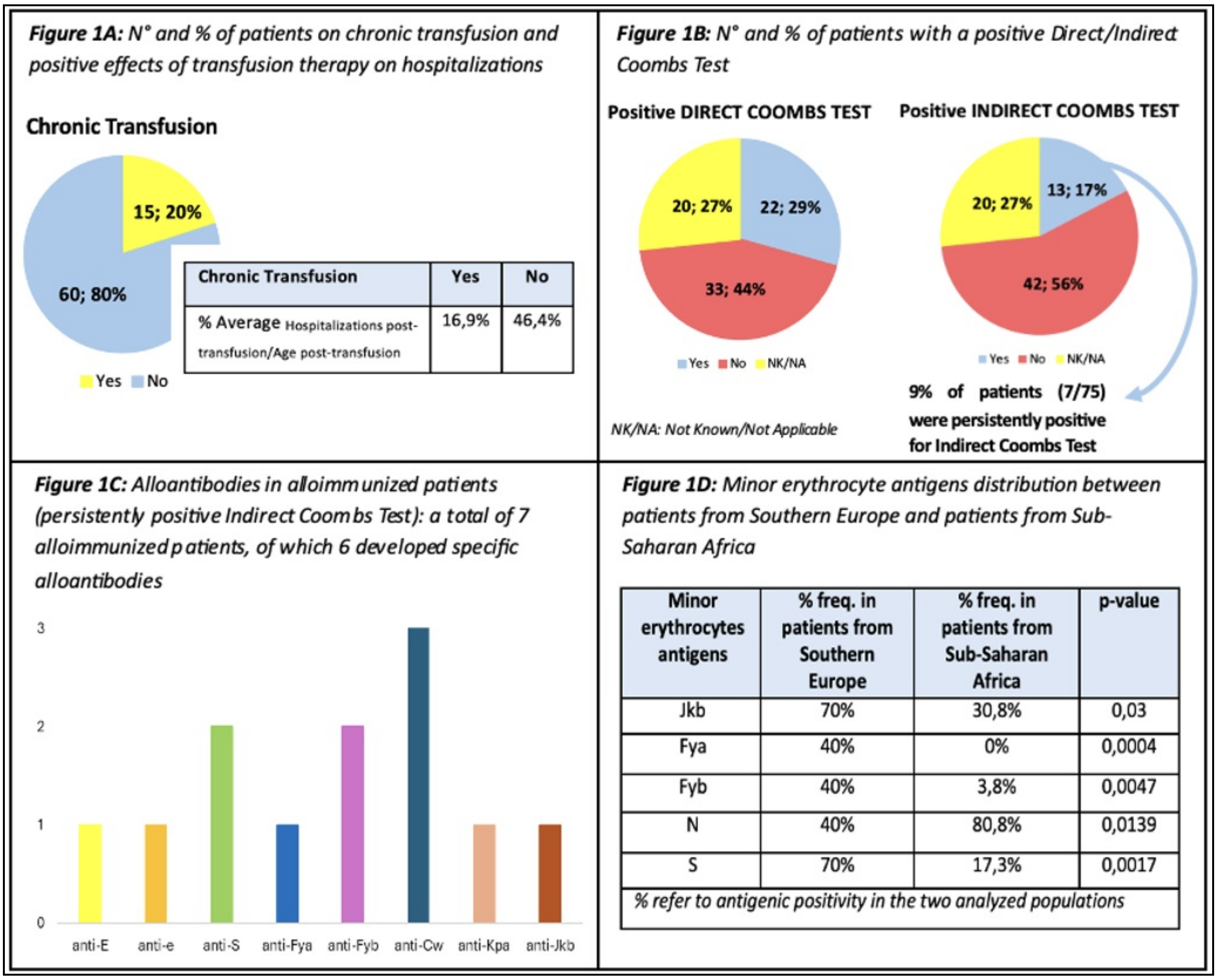

**Copyright Information:** (Online) ISSN: 2572-9241
©2026 The Author(s). HemaSphere is published by John Wiley & Sons Ltd on behalf of European Hematology Association. This is an open access Abstract Book distributed under the Attribution-NonCommercial-NoDerivs (CC BY-NC-ND), which allows third parties to download the articles and share them with others as long as they credit the author and the Abstract Book, but they cannot change the content in any way or use them commercially.
**Abstract Book Citations:** Authors, Title, HemaSphere, 2026;10:(S1):pages. The abstract book DOIs can be found on the "Author Information" page.
**Disclaimer:** Articles published in the journal HemaSphere exclusively reflect the opinions of the authors. The authors are responsible for all content in their abstracts including accuracy of the facts, statements, citing resources, etc.

### (PB4145) RECOGNIZING THE BURDEN OF HEMOGLOBINOPATHIES: THE IMPACT OF ACCREDITED MEDICAL EDUCATION

**Topic:** 27. Sickle cell disease

S Christy Rohani-Montez*[1], Sanneke Koekkoek[1], Tiphaine Barras[1]

[1]*Medscape Education Global, London, United Kingdom;*

### Background

The burden of disease for patients with transfusion-dependent beta-thalassemia (TDT) or sickle cell disease (SCD) and their caregivers is enormous, particularly with regards to the treatment burden for TDT and vaso-occlusive crises (VOCs) for SCD; and the disease-associated complications for both. Poor awareness of this burden in the healthcare system has resulted in substandard care.

### Aims

To address these clinical gaps, we developed a CME-accredited online educational activity featuring a discussion between 2 expert faculty.

### Methods

Physicians participated in a 30-minute video-based, online CME activity led by 2 experts with synchronized slides (https://www.medscape.org/viewarticle/1000286). The effects of the education were assessed using a 3-question, repeated pairs, pre-assessment/post-assessment study design. One question assessed confidence. Pre- to post-assessment differences were evaluated using the McNemar's test, with $P \leq 05$ being significant. The activity launched in 3/2024 and data were collected through 12/2025.

### Results

Overall significant improvements were seen after participation for both haematologists (correct response rate of 86% at pre-assessment vs 93% at post-assessment; P<.001, Cohen's d= 0.49, N=90), and paediatricians (correct response rate of 67% at pre-assessment vs 81% at post-assessment; P<.001, Cohen's d= 0.75, N=189). Specifically, significant improvements were observed in clinicians' awareness of acute chest syndrome as a primary cause of death in SCD with VOC complications, in identifying liver fibrosis as a common sequelae of iron overload in TDT, and recognition of gene therapy as a recently approved therapy for both. After participating, 26% of haematologists and 43% of paediatricians had measurable improved confidence related to understanding the patient burden associated with hemoglobinopathies. In addition, 45% of haematologists and 40% of paediatricians said they intend to discuss the possibility of gene therapy with patients who are eligible; 24% of haematologists and of haematologists and 40% of paediatricians 32% of paediatricians said they would recommend SCD or TDT patient advocacy groups to patients more often; and 28% of both specialties said they intend to ask their patients about their disease burden.

### Summary/Conclusion

This study demonstrates the success of online expert-led video-based education in improving knowledge of the burden of hemoglobinopathies and awareness of new therapeutic approaches. This may lead to higher prioritisation in managing the large patient burden common for so many patients.

**Copyright Information:** (Online) ISSN: 2572-9241
©2026 The Author(s). HemaSphere is published by John Wiley & Sons Ltd on behalf of European Hematology Association. This is an open access Abstract Book distributed under the Attribution-NonCommercial-NoDerivs (CC BY-NC-ND), which allows third parties to download the articles and share them with others as long as they credit the author and the Abstract Book, but they cannot change the content in any way or use them commercially.
**Abstract Book Citations:** Authors, Title, HemaSphere, 2026;10:(S1):pages. The abstract book DOIs can be found on the "Author Information" page.
**Disclaimer:** Articles published in the journal HemaSphere exclusively reflect the opinions of the authors. The authors are responsible for all content in their abstracts including accuracy of the facts, statements, citing resources, etc.

### (PB4146) SETTING UP PRIORITIES FOR RESEARCH ON SICKLE CELL AND GENOMICS: A COMMUNITY-LED PRIORITY SETTING PARTNERSHIP

**Topic:** 27. Sickle cell disease

Rutendo Muzambi[1], Rashae Peart[2], John James[2], Suzannah Kinsella[3], Frédéric B. Piel*[1]

*[1]Imperial College London, School of Public Health, London, United Kingdom;[2]Sickle Cell Society, London, United Kingdom;* *[3]James Lind Alliance, London, United Kingdom;*

### Background

The phenotypic heterogeneity experienced by people living with sickle cell (SC) present many challenges for the management of this condition. Although some biomarkers of severity have been identified, recent advances in genomics research offer huge potential towards a better understanding of the complexity of SC and for personalised medicine.

### Aims

The aim of this partnership between the James Lind Alliance, Genomics England and the UK Sickle Cell Society was to coproduce with patients, carers and healthcare professional a process to identify and prioritise unanswered research questions for genomics research that matter most to people with SC and those supporting their care.

### Methods

A Priority Setting Partnership follows a standard methodology based on a series of successive steps driven by input from community surveys and workshops. Through these steps, we gathered diverse lived experiences and clinical perspectives from participants, and identified priorities for research on genomics and SC, while accounting for the strength of existing evidence.

**Copyright Information:** (Online) ISSN: 2572-9241
©2026 The Author(s). HemaSphere is published by John Wiley & Sons Ltd on behalf of European Hematology Association. This is an open access Abstract Book distributed under the Attribution-NonCommercial-NoDerivs (CC BY-NC-ND), which allows third parties to download the articles and share them with others as long as they credit the author and the Abstract Book, but they cannot change the content in any way or use them commercially.
**Abstract Book Citations:** Authors, Title, HemaSphere, 2026;10:(S1):pages. The abstract book DOIs can be found on the “Author Information” page.
**Disclaimer:** Articles published in the journal HemaSphere exclusively reflect the opinions of the authors. The authors are responsible for all content in their abstracts including accuracy of the facts, statements, citing resources, etc.

### Results

The process resulted in a consensus top 37 list of research priorities for SC and genomics. The top 10 priorities are presented in *Table 1*. These priorities span key areas where genomic research could have high impact, including community interests in identifying new effective therapies to prolong life; using genomics to predict, prevent, or reduce severe complications; enabling stratification of treatment strategies through genomics; improving acute and chronic pain management; understanding genetic factors in treatment response; and clarifying the genetic mechanisms associated with differences in SC crisis frequency. Other priorities include reducing adverse reactions to blood transfusions and translating genomic advances into personalised care approaches.

**Table**: Top priorities for genomics and sickle cell (SC) research

| | |
|---|---|
| 1 | How can genomics identify new effective therapies for SC that can prolong life or cure SC? |
| 2 | Can genomics and genomic treatment predict, prevent or reduce severe SC complications, such as organ damage, stroke and bone pain? |
| 3 | How can genomics help to decide the best treatments based on a person with SC's genetic profile? |
| 4 | How can genomics improve acute and chronic pain management strategies for people with SC? |
| 5 | Can genomics be used to better understand and avoid adverse reactions to blood transfusions? |
| 6 | What is the role of genomics in improving the management of complications and outcomes of SC in older adults? |
| 7 | How effective is genomic treatment in reducing SC milder complications that negatively impact day-to-day life such as pains, fatigue and susceptibility to infection? |
| 8 | How can clinicians translate genomics advances into personalised tailored treatments for SC care? |
| 9 | What are the genetic factors that explain differences in responses to SC medications? |
| 10 | What are the genetic factors and mechanisms linked to the triggering and frequency of a SC crisis? |

### Summary/Conclusion

By embedding community experience in the research agenda, these priorities provide a roadmap for scientists and funding bodies to align genomic research with real-world needs. The ultimate goal is to improve clinical outcomes and quality of life for people with SC. This process underscores the importance of inclusive, patient-centred research priority setting in advancing equitable genomic science. Although this work focused on the United Kingdom, the priorities identified have worldwide relevance.

**Copyright Information:** (Online) ISSN: 2572-9241
©2026 The Author(s). HemaSphere is published by John Wiley & Sons Ltd on behalf of European Hematology Association. This is an open access Abstract Book distributed under the Attribution-NonCommercial-NoDerivs (CC BY-NC-ND), which allows third parties to download the articles and share them with others as long as they credit the author and the Abstract Book, but they cannot change the content in any way or use them commercially.
**Abstract Book Citations:** Authors, Title, HemaSphere, 2026;10:(S1):pages. The abstract book DOIs can be found on the "Author Information" page.
**Disclaimer:** Articles published in the journal HemaSphere exclusively reflect the opinions of the authors. The authors are responsible for all content in their abstracts including accuracy of the facts, statements, citing resources, etc.

### (PB4147) EVALUATION OF KNOWLEDGE AND PREVALENCE OF PRIAPISM AND ERECTILE DYSFUNCTION AMONGST SICKLE CELL DISEASE PATIENTS IN A LARGE TERTIARY CENTRE

**Topic:** 27. Sickle cell disease

Noor Al-Zubaidi*[1], Hilesa Asiedu[1], Chevone Chambers-Russell[1], Rachel Kesse-Adu[1], Kate Gardner[1], Dale Seviar[1]

*[1]Guy's and St Thomas' NHS Foundation Trust, London, United Kingdom;*

### Background

Sickle cell disease (SCD) is an inherited condition caused by a genetic mutation that leads to the production of HbS. Under stressful conditions HbS polymerises leading to sickle shaped red cells. This can lead to anaemia and vaso-occlusion presenting with pain, organ damage and increased susceptibility to infections. Priapism is a urological emergency that patients with SCD can present with. It is a painful, sustained unwanted penile erection. Priapism can have significant impact on patient's sexual, reproductive, mental health and can lead to erectile dysfunction. The prevalence of priapism in SCD is varied in literature, ranging 30-90%.The standard of care for SCD patients in the UK is to offer a comprehensive annual review (AR) in a specialist centre to provide holistic review of psychosocial health, ensure consistent comprehensive care. One of the aims of AR is ensuring patient education on their condition. At our centre, a large specialist haemoglobinopathy team in a tertiary hospital in London we manage care of 1199 patients with SCD (755 with HbSS, 331 with HbSC, 32 with HbS beta-plus thalassaemia and 16 with HbS beta-zero thalassaemia). In response to previously noted low levels of understanding and awareness amongst male patients of priapism we developed a compilation of educational tools in 2022.

### Aims

1.Explore the understanding, knowledge and prevalence of priapism amongst male sickle cell patients attending clinic. 2.Review the assessment of priapism during annual review for SCD patients by healthcare professionals

### Methods

An anonymous self-administered questionnaire was distributed to male patients with SCD attending OP clinic. The questionnaire included demographic information about age, a clinical question about their sickle cell genotype. Questions to assess individuals' knowledge and experience of priapism. In addition, participants were asked if priapism affected their sleep, daily activities and sexual activity. Erectile dysfunction (ED) was assessed using the International Index of Erectile Function Score (IIEF). We also undertook a retrospective medical record review of the AR visits that male patients had over a month in 2025 to check healthcare professionals' assessment of priapism.

### Results

48 male patients filled out the questionnaire. The median age range of the participants was 26-35 years of age. 60% reported having HbSS, 21% HbSC while 14% did not respond to the genotype question. 81% of participants were aware of priapism. A significant number of patients (45%) had experienced priapism. Of those who had experienced priapism, 31% experienced it once, 18% frequently and 9% very frequently. 27% of those experiencing priapism reported a significant episode leading to a hospital visit. 48% of participants stated they would like more information on priapism. 35 patients filled the IIEF score for ED; 60% had no ED, 11% had mild ED, 21% mild-moderate and moderate ED and 1 patient reported severe ED. 17 male patients had an annual review in the SCD clinic over 1 month in 2025. The median age of patients was 36-45 years. Only 35% of patients were assessed for priapism.

**Copyright Information:** (Online) ISSN: 2572-9241
©2026 The Author(s). HemaSphere is published by John Wiley & Sons Ltd on behalf of European Hematology Association. This is an open access Abstract Book distributed under the Attribution-NonCommercial-NoDerivs (CC BY-NC-ND), which allows third parties to download the articles and share them with others as long as they credit the author and the Abstract Book, but they cannot change the content in any way or use them commercially.
**Abstract Book Citations:** Authors, Title, HemaSphere, 2026;10:(S1):pages. The abstract book DOIs can be found on the "Author Information" page.
**Disclaimer:** Articles published in the journal HemaSphere exclusively reflect the opinions of the authors. The authors are responsible for all content in their abstracts including accuracy of the facts, statements, citing resources, etc.

**Summary/Conclusion**

Prior to developing educational material about priapism in 2022 awareness of priapism in our cohort was less than 50%. These tools have had measurable impact however this audit shows a need to improve education regarding priapism to males with SCD. This can be improved by addressing it in clinic, and making priapism discussion mandatory at AR visit of male patients.

**Copyright Information:** (Online) ISSN: 2572-9241
©2026 The Author(s). HemaSphere is published by John Wiley & Sons Ltd on behalf of European Hematology Association. This is an open access Abstract Book distributed under the Attribution-NonCommercial-NoDerivs (CC BY-NC-ND), which allows third parties to download the articles and share them with others as long as they credit the author and the Abstract Book, but they cannot change the content in any way or use them commercially.
**Abstract Book Citations:** Authors, Title, HemaSphere, 2026;10:(S1):pages. The abstract book DOIs can be found on the "Author Information" page.
**Disclaimer:** Articles published in the journal HemaSphere exclusively reflect the opinions of the authors. The authors are responsible for all content in their abstracts including accuracy of the facts, statements, citing resources, etc.

### (PB4148) PATIENT JOURNEY AND UNMET NEEDS IN SICKLE CELL DISEASE: RESULTS FROM A MIXED-METHODS REGIONAL STUDY IN CUKUROVA REGION, TURKIYE

**Topic:** 27. Sickle cell disease

Kubra Karaman[1], Nurgul Yonyul*[2], Hatice Ilgen Sasmaz[2], Ali Bülent Antmen[2]

*[1]Novo Nordisk, Medical Affairs Department, Istanbul, Türkiye;[2]Acıbadem Adana Hospital, Pediatric Hematology and Oncology Department and Bone Marrow Transplantation Unit, Adana, Türkiye;*

### Background

Sickle cell disease (SCD) is a lifelong hereditary hemoglobinopathy associated with recurrent vaso-occlusive crises, organ complications, and impaired quality of life. Despite advances in supportive care, many patients continue to experience frequent pain episodes, limited access to comprehensive care, and psychosocial challenges. Understanding the real-world patient journey is essential for identifying unmet needs and improving disease management strategies.

### Methods

A mixed-methods study was conducted to evaluate the experiences, treatment patterns, and unmet needs of individuals with SCD. Quantitative data were collected through a structured 25-minute survey administered to 51 patients or caregivers from the provinces of Adana, Hatay, and Mersin between November and December 2024. Qualitative insights were obtained via in-depth, semi-structured interviews with three patients or caregivers in February 2025. The study explored disease history, pain experiences, healthcare utilization, treatment patterns, psychosocial impact, and expectations from new therapies.

**Copyright Information:** (Online) ISSN: 2572-9241
©2026 The Author(s). HemaSphere is published by John Wiley & Sons Ltd on behalf of European Hematology Association. This is an open access Abstract Book distributed under the Attribution-NonCommercial-NoDerivs (CC BY-NC-ND), which allows third parties to download the articles and share them with others as long as they credit the author and the Abstract Book, but they cannot change the content in any way or use them commercially.
**Abstract Book Citations:** Authors, Title, HemaSphere, 2026;10:(S1):pages. The abstract book DOIs can be found on the "Author Information" page.
**Disclaimer:** Articles published in the journal HemaSphere exclusively reflect the opinions of the authors. The authors are responsible for all content in their abstracts including accuracy of the facts, statements, citing resources, etc.

### Results

Fourteen patients (28%) were between 6–13 years of age, 8 patients (15%) were between 14–17 years, and 29 patients (57%) were older than 18 years. Twenty six of of the patients enrolled in the study were female and twenty five were male.
The average duration since diagnosis was approximately 18 years, reflecting the lifelong burden of the disease. Pain crises emerged as the most significant clinical challenge. Two out of three patients reported experiencing vaso-occlusive crises every two to three months or more frequently. Hospital utilization was high, with 92% of patients reporting hospitalization due to painful crises, and more than half describing their inpatient pain as severe.

Most patients (69%) were receiving pharmacologic treatment, with approximately one-third reporting hydroxyurea use or transfusion therapy. The most commonly reported positive treatment outcome was reduction in pain, followed by decreased crisis frequency and improved ability to perform daily activities. However, treatment-related challenges included frequent hospitalizations, psychological distress, and financial burden.

The social impact of SCD was substantial. Patients reported decreased school or work performance, frequent healthcare visits, and reduced participation in social activities. Only 10% of patients had received psychological support despite reported anxiety, depression, and social stigma. Although nearly all patients had received information from physicians, only 61% considered themselves knowledgeable about the disease and treatment options.

Regarding future therapies, 43% of patients expressed hope in new treatment options, with primary expectations including reduced crisis frequency, pain control, and disease stabilization.

### Summary/Conclusion

SCD imposes a substantial clinical, psychological, and socioeconomic burden on patients and families. Frequent pain crises, high hospitalization rates, limited disease knowledge, and inadequate psychosocial support remain major unmet needs. Interventions focusing on improved pain management, patient education, multidisciplinary care models, and enhanced access to innovative therapies may significantly improve outcomes and quality of life for individuals with SCD.

**Copyright Information:** (Online) ISSN: 2572-9241
©2026 The Author(s). HemaSphere is published by John Wiley & Sons Ltd on behalf of European Hematology Association. This is an open access Abstract Book distributed under the Attribution-NonCommercial-NoDerivs (CC BY-NC-ND), which allows third parties to download the articles and share them with others as long as they credit the author and the Abstract Book, but they cannot change the content in any way or use them commercially.
**Abstract Book Citations:** Authors, Title, HemaSphere, 2026;10:(S1):pages. The abstract book DOIs can be found on the "Author Information" page.
**Disclaimer:** Articles published in the journal HemaSphere exclusively reflect the opinions of the authors. The authors are responsible for all content in their abstracts including accuracy of the facts, statements, citing resources, etc.

### (PB4149) PROFILING PATIENTS WITH SICKLE CELL DISEASE REQUIRING ECULIZUMAB AS FIRST LINE TREATMENT FOR DELAYED HEMOLYTIC TRANSFUSION REACTION

**Topic:** 27. Sickle cell disease

Simone Villaboni*[1], Filippo Mazzi[1], Simone Cesaro[2], Elisa Bonetti[2], Nadi Mirra[3], Valeria Maria Pinto[4], Annalisa Peli[5], Jacopo Ceolan[1], Francesca Begali[1], Roberto Bertazzo[1], Lucia De Franceschi[1]

*[1]Policlinico Rossi Borgo Roma, Azienda Ospedaliera Univesitaria Integrata di Verona, Malattie Rare Immunoematologiche Benigne, Verona, Italy; [2]Ospedale Borgo Trento, Oncoematologia pediatrica, Verona, Italy; [3]IRCCS Ca' Granda Ospedale Maggiore Policlinico Foundation, Paediatric Haematology, Paediatric Day Hospital, Milano, Italy; [4]Dipartimento di Ematologia e Terapie Cellulari, Ospedale policlinico san martino, Genova, Genova, Italy; [5]A.S.S.T Spedali Civili di Brescia, Unità di Ematologia, Brescia, Italy;*

### Background

Delayed hemolytic transfusion reaction (DHTR) represents a serious and life-threatening complication in patients with sickle cell disease (SCD). A key role of chronic unresolved inflammation has been reported to contribute to over-activation of innate and adaptive immune response, increasing the biocomplexity of SCD clinical manifestation. Up to now growing evidence supports the participation of overactivated alternative complement pathways in DHTR in patients with SCD.

### Aims

Description of clinical and laboratory trends of clinical cases, in order to profile patients with sickle cell anemia who require eculizumab as first-line treatment for delayed hemolytic transfusion reaction

### Methods

This is a multicenter retrospective case series of 5 SCD patients who developed DHTR with severe organ manifestations between 2022 and 2025 treated with Eculizumab before transfusion. DHTR was diagnosed based on occurrence of acute hemolysis within 20 days post-transfusion, decreased hemoglobin, presence of hemoglobinuria, detection of new alloantibodies.

### Results

Median time to DHTR onset was 7 days (range 0–12). Hb nadirs ranged 3.9–6.7 g/dL (mean drop 4.3 g/dL); LDH peaked at 1,173–2,881 IU/L. Reticulocytopenia was always present (nadirs 22–201 $\times 10^9$/L) as well as hemoglobinuria. Ferritin rose up to 1426–6966 µg/L and CRP to 19-289 mg/L. New alloantibodies (anti-e, anti-M, anti-S, anti-$Jk^a$) were detected in 60%; DAT IgG+C3d positivity in 40%. Intensive care unit admission was required in 100% due to acute respiratory failure related to acute chest syndrome (40%), acute kidney failure (20%), or neurological events from fat embolism (20%). All patients presented general symptoms of sickle cell related vaso-occlusive crisis such as severe pain at hospital admission. Patients were fist treated with corticosteroids (80%), intravenous immunoglobulins (60%), plasma exchange (60%), and erythropoietin (80%). At 2-3 days after hospital admission, despite the ongoing treatment, patients showed worsened of organ sufferance, and worsened anemia, potentially requiring transfusion to prevent multi-organ failure. Patients were treated with eculizumab (600-900 mg/patient once per week after the first administration) and transfused within 1-2 hours with matched red cells (total 2-5 units per patient). This strategy allowed to increase and maintain Hb (1.5–2.4 g/dL increment) without exacerbation of hemolysis and rapid reduction of hemoglobinuria. Mortality was 0%; complete recovery occurred in 60%.

**Copyright Information:** (Online) ISSN: 2572-9241
©2026 The Author(s). HemaSphere is published by John Wiley & Sons Ltd on behalf of European Hematology Association. This is an open access Abstract Book distributed under the Attribution-NonCommercial-NoDerivs (CC BY-NC-ND), which allows third parties to download the articles and share them with others as long as they credit the author and the Abstract Book, but they cannot change the content in any way or use them commercially.
**Abstract Book Citations:** Authors, Title, HemaSphere, 2026;10:(S1):pages. The abstract book DOIs can be found on the "Author Information" page.
**Disclaimer:** Articles published in the journal HemaSphere exclusively reflect the opinions of the authors. The authors are responsible for all content in their abstracts including accuracy of the facts, statements, citing resources, etc.

**Summary/Conclusion**

SCD patients with severe DHTR and organ complications might be safely transfused immediately after eculizumab administration. Our data help in profiling SCD patients with DHTR who might benefit from eculizumab infusion as first line treatment. Systematic multi-parameter charting may serve as a practical clinical tool for real-time monitoring and treatment decision-making in future DHTR episodes.

**Copyright Information:** (Online) ISSN: 2572-9241
©2026 The Author(s). HemaSphere is published by John Wiley & Sons Ltd on behalf of European Hematology Association. This is an open access Abstract Book distributed under the Attribution-NonCommercial-NoDerivs (CC BY-NC-ND), which allows third parties to download the articles and share them with others as long as they credit the author and the Abstract Book, but they cannot change the content in any way or use them commercially.
**Abstract Book Citations:** Authors, Title, HemaSphere, 2026;10:(S1):pages. The abstract book DOIs can be found on the "Author Information" page.
**Disclaimer:** Articles published in the journal HemaSphere exclusively reflect the opinions of the authors. The authors are responsible for all content in their abstracts including accuracy of the facts, statements, citing resources, etc.

### (PB4150) BEYOND CRISIS COUNTS: REAL-WORLD MULTIDIMENSIONAL BURDEN IN SICKLE CELL DISEASE SUPPORTS URGENT NEED FOR TRANSFORMATIVE THERAPIES

**Topic:** 27. Sickle cell disease

Sarah Hendry[1], Sam Young*[1]

[1]*Spherix Global Insights, Exton, United States of America;*

### Background

Annual vaso-occlusive crisis (VOC) frequency is frequently used to define “severe” sickle cell disease (SCD), often at ≥5 VOCs per year. However, emerging natural history data demonstrate wide dispersion in crisis burden, including patients experiencing double-digit events annually (Piel et al., N Engl J Med, 2017). As gene therapies enter clinical practice, accurate identification of high-burden patients becomes critical.

### Aims

To quantify contemporary clinical, humanistic, and socioeconomic burden in SCD and assess implications for treatment escalation and gene therapy candidacy.

### Methods

Patient Chart Dynamix™: SCD 2026 evaluated 133 HbSS charts from 86 U.S. hematologist/oncologists. Patient Voice Dynamix™: SCD 2025 surveyed 33 U.S. adults with SCD. Outcomes included VOC frequency, transfusion burden, hospitalization, medication intensity, quality of life, emotional impact, and financial toxicity.

### Results

Physician-reported mean annual VOCs were 5.2. Fifty-four percent received regular transfusions and patients were prescribed a mean 5.7 medications. Despite intensive management, 63% were described as only “moderately controlled”. Patients reported a markedly higher mean of 9.5 VOCs annually, with 58% experiencing ≥5 VOCs. Seventy percent required hospitalization for their most recent VOC, averaging 6.6 inpatient days. Symptom burden was pervasive: fatigue (94%), chronic pain (85%), acute pain (73%). Emotional distress and financial toxicity were substantial, with 64% reporting medical debt. Importantly, high transfusion burden, hospitalization, and multidimensional morbidity extended beyond patients narrowly meeting a ≥5 VOC definition of severity.

### Summary/Conclusion

Contemporary SCD burden remains profound despite polypharmacy and transfusion support. Crisis-based thresholds underestimate total disease impact and risk excluding highly burdened patients from consideration for transformative therapies. These findings support multidimensional criteria – including transfusion dependence, hospitalization, and quality-of-life impairment – to inform timely escalation to gene therapy and other disease-modifying interventions.

**Copyright Information:** (Online) ISSN: 2572-9241
©2026 The Author(s). HemaSphere is published by John Wiley & Sons Ltd on behalf of European Hematology Association. This is an open access Abstract Book distributed under the Attribution-NonCommercial-NoDerivs (CC BY-NC-ND), which allows third parties to download the articles and share them with others as long as they credit the author and the Abstract Book, but they cannot change the content in any way or use them commercially.
**Abstract Book Citations:** Authors, Title, HemaSphere, 2026;10:(S1):pages. The abstract book DOIs can be found on the “Author Information” page.
**Disclaimer:** Articles published in the journal HemaSphere exclusively reflect the opinions of the authors. The authors are responsible for all content in their abstracts including accuracy of the facts, statements, citing resources, etc.

### (PB4151) EXPLORATORY EVALUATION OF LIPID AND PLATELET PARAMETERS FOLLOWING GLP-1 RECEPTOR AGONIST EXPOSURE IN ADULTS WITH SICKLE CELL DISEASE: A PILOT RETROSPECTIVE ANALYSIS

**Topic:** 27. Sickle cell disease

Burak Bahar*[1]

[1]*Brown University, Pathology and Laboratory Medicine, Providence, United States of America;*

### Background

Sickle cell disease (SCD) is characterized by chronic hemolysis, endothelial dysfunction, inflammation, and altered lipid metabolism. Individuals with SCD frequently exhibit lower baseline low-density lipoprotein (LDL) cholesterol compared with non-SCD populations. Platelet activation and thrombocytosis also contribute to SCD-associated vasculopathy.

### Aims

Glucagon-like peptide-1 receptor agonists (GLP-1 RAs) improve metabolic and inflammatory parameters in general populations; however, their hematologic and metabolic effects in SCD have not been described. We conducted a pilot study to explore lipid and platelet trends following GLP-1 exposure in adults with SCD.

### Methods

We performed a retrospective analysis of adults with HbS-confirmed SCD receiving GLP-1 RA therapy identified through an Epic Caboodle data warehouse. Lipid (LDL, HDL, triglycerides) and platelet measurements were extracted relative to GLP-1 initiation. Pre-exposure values were defined as laboratory measurements obtained 180 days prior to therapy start; post-exposure values were defined as values obtained within 180 days of therapy initiation. Eighteen unique patients contributed LDL and HDL data, 19 contributed triglyceride data, and 21 contributed platelet data. Due to limited availability of paired lipid measurements, lipid comparisons were performed primarily at the measurement level using two-sided Mann–Whitney U tests. Platelet analyses included paired patient-level comparisons.

### Results

Among 18 patients contributing LDL data (12 pre and 10 post measurements), mean LDL decreased from 98.2 mg/dL pre-exposure to 79.4 mg/dL post-exposure (−18.8 mg/dL), demonstrating a nonsignificant trend toward lower post-exposure LDL ($p=0.093$). Triglycerides decreased from 155.7 mg/dL to 133.0 mg/dL (−22.7 mg/dL; $p=0.449$) among 19 patients. HDL remained stable (42.8 mg/dL pre vs 43.7 mg/dL post; $p=0.923$). Among 21 patients with platelet data (12 with paired measurements), no significant change in platelet count was observed following GLP-1 exposure (paired median change −9 $\times 10^3/\mu L$; Wilcoxon $p=0.34$). No signal of reactive thrombocytosis was detected at the measurement level ($p=0.75$).

### Summary/Conclusion

In this pilot SCD cohort, GLP-1 RA exposure was associated with directional reductions in LDL cholesterol and triglycerides at the measurement level without evidence of increased platelet counts. Although statistical significance was not achieved and paired lipid data were limited, these preliminary findings suggest metabolic responsiveness without hematologic destabilization in adults with SCD. Larger, prospectively designed studies incorporating standardized longitudinal sampling and integration of hemolysis and inflammatory biomarkers are warranted to clarify potential vascular implications.

**Copyright Information:** (Online) ISSN: 2572-9241
©2026 The Author(s). HemaSphere is published by John Wiley & Sons Ltd on behalf of European Hematology Association. This is an open access Abstract Book distributed under the Attribution-NonCommercial-NoDerivs (CC BY-NC-ND), which allows third parties to download the articles and share them with others as long as they credit the author and the Abstract Book, but they cannot change the content in any way or use them commercially.
**Abstract Book Citations:** Authors, Title, HemaSphere, 2026;10:(S1):pages. The abstract book DOIs can be found on the “Author Information” page.
**Disclaimer:** Articles published in the journal HemaSphere exclusively reflect the opinions of the authors. The authors are responsible for all content in their abstracts including accuracy of the facts, statements, citing resources, etc.

### (PB4152) TRIAL IN PROGRESS: DREAM – A PHASE 3, RANDOMIZED, PLACEBO-CONTROLLED, DOUBLE-BLIND STUDY EVALUATING OSIVELOTOR IN SICKLE CELL DISEASE

**Topic:** 27. Sickle cell disease

Modupe Idowu*[1], Santosh Saraf[2], Perla Eleftheriou[3], Gurpreet Brar[4], Tamara Bratt[4], Geoffrey Allen[5]

*[1]University of Texas Health McGovern Medical School, Houston, TX, United States of America;[2]University of Illinois Chicago, Chicago, IL, United States of America; [3]University College London Hospital, London, United Kingdom; [4]Pfizer Inc, Groton, CT, United States of America; [5]Pfizer Inc, Collegeville, PA, United States of America;*

### Background

Sickle cell disease (SCD) is characterized by hemolytic anemia, vaso-occlusion, and progressive end organ damage. The most prominent complication is vaso-occlusive crisis (VOC), an acutely painful episode that often requires treatment at a medical facility. Osivelotor, an investigational agent being evaluated as a potential treatment for SCD, is a second-generation hemoglobin S (HbS) polymerization inhibitor that increases Hb–oxygen affinity and stabilizes Hb in the oxygenated form. Compared to voxelotor, the first-generation HbS polymerization inhibitor, osivelotor has a distinct chemical structure leading to differences in Hb binding (stoichiometry), longer half-life, and improved pharmacokinetic profile. In a 12-week, phase 2 clinical trial and a long-term open-label extension study, osivelotor improved Hb and was well tolerated in patients with SCD, while the rate of VOCs was decreased compared with the rate during 12 months prior to treatment.

### Aims

To describe the phase 3 randomized, double-blind, placebo-controlled portion of the ongoing osivelotor DREAM study.

### Methods

DREAM is a combined phase 2/3 clinical trial of osivelotor in SCD (NCT05431088). The phase 3 portion is a randomized, double-blind, placebo-controlled study evaluating the efficacy and safety of osivelotor versus placebo in adults and adolescents with SCD. After informed consent and a 4-week screening period, patients enter a 48-week double-blind treatment period, followed by either a 12-week follow-up or an open-label extension (**Figure**). For adults (aged ≥18 years), key eligibility criteria are SCD with HbSS or HbS$\beta^0$ genotype, screening Hb 5.5–10.5 g/dL, and ≥2 to ≤10 VOCs in the prior 12 months. Stable hydroxyurea and/or L-glutamine are permitted. Adolescents (aged 12 to <18 years) will be enrolled after safety and tolerability data for adult participants have been reviewed. Based on the findings of increased deaths and VOCs in 2 post-marketing trials conducted largely in sub-Saharan Africa with voxelotor, participants in or having recently relocated from these regions will be excluded per agreement with the US Food and Drug Administration. In total, approximately 332 participants will be randomized 1:1 to placebo or osivelotor, administered orally. Adult participants will receive a loading dose for 7 days (osivelotor 300 mg once daily) followed by maintenance doses through Week 48 (150 mg once daily); the dose for adolescent participants will be selected after review of further data for adults.

### Results

Primary endpoints include Hb response (>1 g/dL increase from baseline) at Week 48 and annualized rate of VOCs through end of Week 48. To provide the most robust evaluation, VOCs will be adjudicated by a blinded independent expert panel. An independent unblinded data monitoring committee is reviewing safety data every 3 months.

**Copyright Information:** (Online) ISSN: 2572-9241
©2026 The Author(s). HemaSphere is published by John Wiley & Sons Ltd on behalf of European Hematology Association. This is an open access Abstract Book distributed under the Attribution-NonCommercial-NoDerivs (CC BY-NC-ND), which allows third parties to download the articles and share them with others as long as they credit the author and the Abstract Book, but they cannot change the content in any way or use them commercially.
**Abstract Book Citations:** Authors, Title, HemaSphere, 2026;10:(S1):pages. The abstract book DOIs can be found on the "Author Information" page.
**Disclaimer:** Articles published in the journal HemaSphere exclusively reflect the opinions of the authors. The authors are responsible for all content in their abstracts including accuracy of the facts, statements, citing resources, etc.

**Summary/Conclusion**

Data from the DREAM trial will help researchers better understand the potential efficacy and safety of osivelotor in participants with SCD.

**Figure:** Osivelotor DREAM trial: phase 3 study design

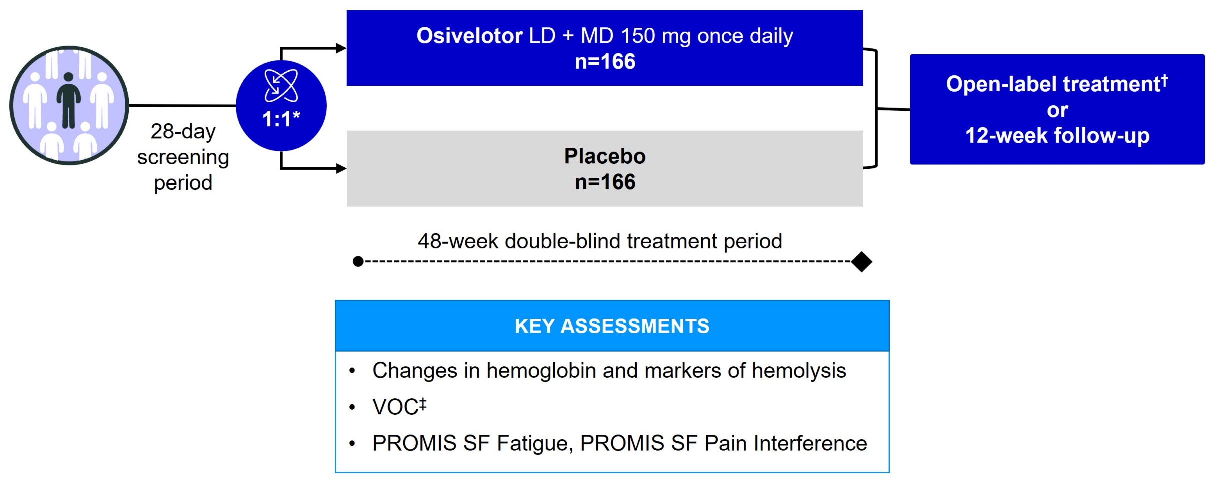

*Randomization will be stratified by hydroxyurea use (yes or no), geographic region (USA, high-income countries other than the USA, or low-to-middle income countries), and VOCs in the prior 12 months (2–4 or 5–10). †The open-label period could continue for up to 2 years after the last participant completes the 48-week double-blind period. ‡For analyses, VOC is defined as an acute episode of pain that has no medically determined cause other than a vaso-occlusive event, results in a visit to a medical facility (hospitalization, emergency department, urgent care center, outpatient clinic, or infusion center), and requires parenteral narcotic agents, parenteral NSAIDs, or an increase in oral narcotics. LD, loading dose; MD, maintenance dose; NSAID, nonsteroidal anti-inflammatory drug; PROMIS, Patient-Reported Outcomes Measurement Information System; SF, Short Form; USA, United States of America; VOC, vaso-occlusive crisis.

**Copyright Information:** (Online) ISSN: 2572-9241
©2026 The Author(s). HemaSphere is published by John Wiley & Sons Ltd on behalf of European Hematology Association. This is an open access Abstract Book distributed under the Attribution-NonCommercial-NoDerivs (CC BY-NC-ND), which allows third parties to download the articles and share them with others as long as they credit the author and the Abstract Book, but they cannot change the content in any way or use them commercially.
**Abstract Book Citations:** Authors, Title, HemaSphere, 2026;10:(S1):pages. The abstract book DOIs can be found on the “Author Information” page.
**Disclaimer:** Articles published in the journal HemaSphere exclusively reflect the opinions of the authors. The authors are responsible for all content in their abstracts including accuracy of the facts, statements, citing resources, etc.

### (PB4153) TRAVEL MEDICINE FOR SICKLE CELL DISEASE

**Topic:** 27. Sickle cell disease

Abdulrahman Nasiri*[1], Manal Alshammari[2], Hamad Alghethber[3], Reem Alkharras[3]

*[1]Imam Mohammad Ibn Saud Islamic University, College of Medicine, Riyadh, Saudi Arabia;[2]King Salman Specialist Hospital, Department of Hematology, Hail, Saudi Arabia; [3]Security Forces Hospital, Department of Medicine, Riyadh, Saudi Arabia;*

### Background

Sickle cell disease (SCD) affects over 20 million people worldwide, yet travel-related health considerations remain poorly understood. Travel exposes patients to hypoxia, dehydration, and infection, which can trigger red blood cell sickling and precipitate vaso-occlusive crises . Furthermore, recent data suggest sickle cell trait (SCT) may not be a benign condition under specific environmental stresses, such as high altitude.

### Aims

To systematically review existing research on travel medicine considerations, complications, and management strategies for individuals with SCD and SCT.

### Methods

A scoping review was conducted adhering to PRISMA-ScR guidelines. Five major databases were searched from inception to December 2024 for human studies involving SCD or SCT and travel-related content. Two independent reviewers screened records and extracted data regarding study characteristics, population demographics, and clinical outcomes.

### Results

Eighteen studies encompassing over 2,000 participants met the inclusion criteria. Commercial aviation appears relatively safe for most patients, with a 1.4% complication rate reported in the largest flight series. Conversely, high-altitude exposure carries substantial risks, with crisis rates reaching up to 66% at 6,320 feet for certain genotypes. Notably, chronic high-altitude exposure in SCT carriers is associated with significantly increased risks of chronic kidney disease, pulmonary embolism, and pregnancy complications . In pediatric travelers, infectious diseases, particularly malaria and digestive septicemia in endemic regions, represent the primary threat.

### Summary/Conclusion ravel medicine for SCD has evolved from restrictive policies to individualized risk assessment. While commercial aviation safety is well-documented, high-altitude exposure and endemic infections require rigorous pre-travel evaluation. Significant research gaps remain, particularly the lack of randomized controlled trials, pediatric-specific guidelines, and standardized risk assessment tools.

**Copyright Information:** (Online) ISSN: 2572-9241
©2026 The Author(s). HemaSphere is published by John Wiley & Sons Ltd on behalf of European Hematology Association. This is an open access Abstract Book distributed under the Attribution-NonCommercial-NoDerivs (CC BY-NC-ND), which allows third parties to download the articles and share them with others as long as they credit the author and the Abstract Book, but they cannot change the content in any way or use them commercially.
**Abstract Book Citations:** Authors, Title, HemaSphere, 2026;10:(S1):pages. The abstract book DOIs can be found on the "Author Information" page.
**Disclaimer:** Articles published in the journal HemaSphere exclusively reflect the opinions of the authors. The authors are responsible for all content in their abstracts including accuracy of the facts, statements, citing resources, etc.

### (PB4154) BIOCULTURAL HYBRIDIZATION OF HEMATOLOGICAL PROTOCOLS: A TRANS-CONTINENTAL MODEL RESOLVING THE ADHERENCE PARADOX IN SICKLE CELL MANAGEMENT

**Topic:** 27. Sickle cell disease

Emmanuel Busera*[1]

[1]*Afro Asia Institute of Strategic Studies, Medical Anthropology, Nairobi, Kenya;*

### Background

In the Afro-Asian corridor, Sickle Cell Disease (SCD) management faces a "Paradox of Underperformance" where Western clinical standards often conflict with Traditional Healing Systems (THS). While protocols prioritize hydroxyurea and transfusion, a policy gap exists regarding the 68% of patients who concurrently utilize THS. This research investigates the "hybridization" of care to resolve the disconnect between institutional models and regional patient belief systems.

### Aims

The primary objective of this research is to resolve the "Paradox of Underperformance" in Sickle Cell Disease (SCD) management within the Afro-Asian corridor by bridging the gap between clinical hematology and medical anthropology.

The study specifically aims to:

Investigate the "Hybridization" of Care: To analyze the socio-clinical disconnect between institutional medical models and regional patient belief systems, particularly for the 68% of the SCD population that concurrently utilizes Traditional Healing Systems (THS).

Evaluate Treatment Abandonment Drivers: To identify the specific anthropological and systemic factors that contribute to the 42% treatment abandonment rate observed in exclusionary Western clinical models.

Validate the "Co-Creation" Framework: To assess the efficacy of a dual-track care model where THS is acknowledged for psychosocial support and pain management, while Western clinical protocols maintain strict control over infection prophylaxis, hydroxyurea administration, and transfusion.

Develop a Policy Roadmap: To propose a "Co-Governance" framework that aligns national health acts and mental health legislation in West Africa and South Asia with EHA international clinical standards to improve long-term patient stability and protocol adherence.

### Methods

A multi-sited ethnographic and clinical review was conducted across health clusters in West Africa and South Asia ($n=1,250$). The design integrated legal analysis of national acts with 200 qualitative depth interviews and 1,050 longitudinal patient observations. Informed consent was obtained for all participants, with data meticulously coded for transparency.

**Copyright Information:** (Online) ISSN: 2572-9241
©2026 The Author(s). HemaSphere is published by John Wiley & Sons Ltd on behalf of European Hematology Association. This is an open access Abstract Book distributed under the Attribution-NonCommercial-NoDerivs (CC BY-NC-ND), which allows third parties to download the articles and share them with others as long as they credit the author and the Abstract Book, but they cannot change the content in any way or use them commercially.
**Abstract Book Citations:** Authors, Title, HemaSphere, 2026;10:(S1):pages. The abstract book DOIs can be found on the "Author Information" page.
**Disclaimer:** Articles published in the journal HemaSphere exclusively reflect the opinions of the authors. The authors are responsible for all content in their abstracts including accuracy of the facts, statements, citing resources, etc.

**Results**

Data-driven analysis reveals that exclusionary Western models led to a 42% treatment abandonment rate within 24 months. Conversely, facilities implementing a "Co-Creation" model—where THS is acknowledged for psychosocial pain management while Western protocols handle prophylaxis—demonstrated a 26% increase in protocol adherence. "Biocultural Hybridization" significantly advances patient retention in high-burden regions.

**Summary/Conclusion**

Results support a "Co-Governance" framework for SCD that aligns African and Asian mental health legislation with EHA clinical standards. Anthropological integration is essential for overcoming the paradox of underperformance, providing a policy-relevant roadmap for improving long-term hematological outcomes.

**Copyright Information:** (Online) ISSN: 2572-9241
©2026 The Author(s). HemaSphere is published by John Wiley & Sons Ltd on behalf of European Hematology Association. This is an open access Abstract Book distributed under the Attribution-NonCommercial-NoDerivs (CC BY-NC-ND), which allows third parties to download the articles and share them with others as long as they credit the author and the Abstract Book, but they cannot change the content in any way or use them commercially.
**Abstract Book Citations:** Authors, Title, HemaSphere, 2026;10:(S1):pages. The abstract book DOIs can be found on the “Author Information” page.
**Disclaimer:** Articles published in the journal HemaSphere exclusively reflect the opinions of the authors. The authors are responsible for all content in their abstracts including accuracy of the facts, statements, citing resources, etc.

### (PB4155) EFFICACY AND SAFETY OF PYRUVATE KINASE-R ACTIVATORS IN SICKLE CELL DISEASE: A SYSTEMATIC REVIEW AND META-ANALYSIS

**Topic:** 27. Sickle cell disease

Yashasvi Srivastava*[1], Shankar Biswas[1], Urooj Iftekhar[2], Dileep Raja Kuraku[3, 3]

*[1]Ivano-Frankivsk National Medical University, Internal Medicine, Ivano-Frankivsk, Ukraine; [2]Tbilisi State Medical University, Tbilisi, Georgia; [3]National Pirogov Memorial Medical University, Vinnytsya, Ukraine;*

### Background

Sickle cell disease (SCD) affects ~7.7 million people worldwide, with ~300,000 births annually. For decades, hydroxyurea stood essentially alone as a disease-modifying oral therapy. Three agents approved 2017–2019 briefly widened options, but crizanlizumab lost European approval in August 2023 (STAND trial: no VOC benefit), and voxelotor was withdrawn globally in September 2024 due to a fatal events imbalance the effectively resetting the field. Pyruvate kinase-R (PKR) activators address the upstream red cell pathology: by raising erythrocyte ATP and lowering 2,3-DPG they simultaneously improve red cell survival and reduce HbS polymerisation. Mitapivat (RISE UP) and etavopivat (HIBISCUS) have both completed Phase 2 RCTs in SCD, but their evidence has never been pooled.

### Aims

To provide the first systematic review and meta-analysis of PKR activators in SCD across all available controlled trials.

### Methods

PubMed, EMBASE, Cochrane Central, and ClinicalTrials.gov were searched through December 2025. Controlled trials of any PKR activator versus placebo in SCD patients were eligible. Four studies qualified: two Phase 2 RCTs (RISE UP, HIBISCUS) as primary evidence; two Phase 1 studies (Xu 2022, Saraf 2024) for sensitivity analyses. Primary outcomes: Hb response (≥1.0 g/dL rise from baseline), Hb change from baseline (WMD), annualised VOC rate ratio, and serious adverse events (SAE). DerSimonian-Laird random-effects models were primary; fixed-effects and combined-dose models were pre-specified sensitivities. Risk of bias was assessed using RoB 2.0.

### Results

RISE UP randomised 79 patients (mitapivat 50/100 mg BID vs. placebo; 12 weeks) and HIBISCUS enrolled 60 patients (etavopivat 200/400 mg QD vs. placebo; 52 weeks). Baseline Hb was 8.4–8.8 g/dL across both trials, with 65–81% of participants receiving concomitant hydroxyurea. PKR activators produced a statistically significant and clinically meaningful improvement in Hb from baseline in RISE UP, with a pooled WMD of +1.07 g/dL (95% CI 0.73–1.41; p<0.001; $I^2$=0%). VOC rate was substantially reduced in HIBISCUS, with a per-protocol rate ratio of 0.38 (95% CI 0.21–0.69; p=0.001; $I^2$=0%), representing a 62% reduction versus placebo. Hb response rates were markedly higher with active treatment in both trials (RISE UP: 46–50% vs. 3.7%; HIBISCUS: 25–38% vs. 10.5%), though scale differences between trials precluded cross-study risk ratio pooling. VOC sensitivity analysis in the ITT population yielded a rate ratio of 0.55 (95% CI 0.30–0.99), confirming robustness. SAE risk was not significantly elevated with either agent (risk ratio 1.23; 95% CI 0.61–2.50; p=0.56; $I^2$=0%), and all sensitivity models were directionally consistent.

**Copyright Information:** (Online) ISSN: 2572-9241
©2026 The Author(s). HemaSphere is published by John Wiley & Sons Ltd on behalf of European Hematology Association. This is an open access Abstract Book distributed under the Attribution-NonCommercial-NoDerivs (CC BY-NC-ND), which allows third parties to download the articles and share them with others as long as they credit the author and the Abstract Book, but they cannot change the content in any way or use them commercially.
**Abstract Book Citations:** Authors, Title, HemaSphere, 2026;10:(S1):pages. The abstract book DOIs can be found on the "Author Information" page.
**Disclaimer:** Articles published in the journal HemaSphere exclusively reflect the opinions of the authors. The authors are responsible for all content in their abstracts including accuracy of the facts, statements, citing resources, etc.

### Summary/Conclusion

This first meta-analysis of PKR activators in SCD shows consistent haemoglobin improvement and a 62% reduction in VOC rate, with no safety signal. RISE UP Phase 3 topline data (November 2025: Hb response 40.6% vs. 2.9%, p<0.0001) corroborate these findings. With two SCD therapies recently lost from the market, PKR activation, orally administered, well tolerated, and mechanistically rational, stands as the most credible emerging treatment option for patients facing this disease with too few choices.

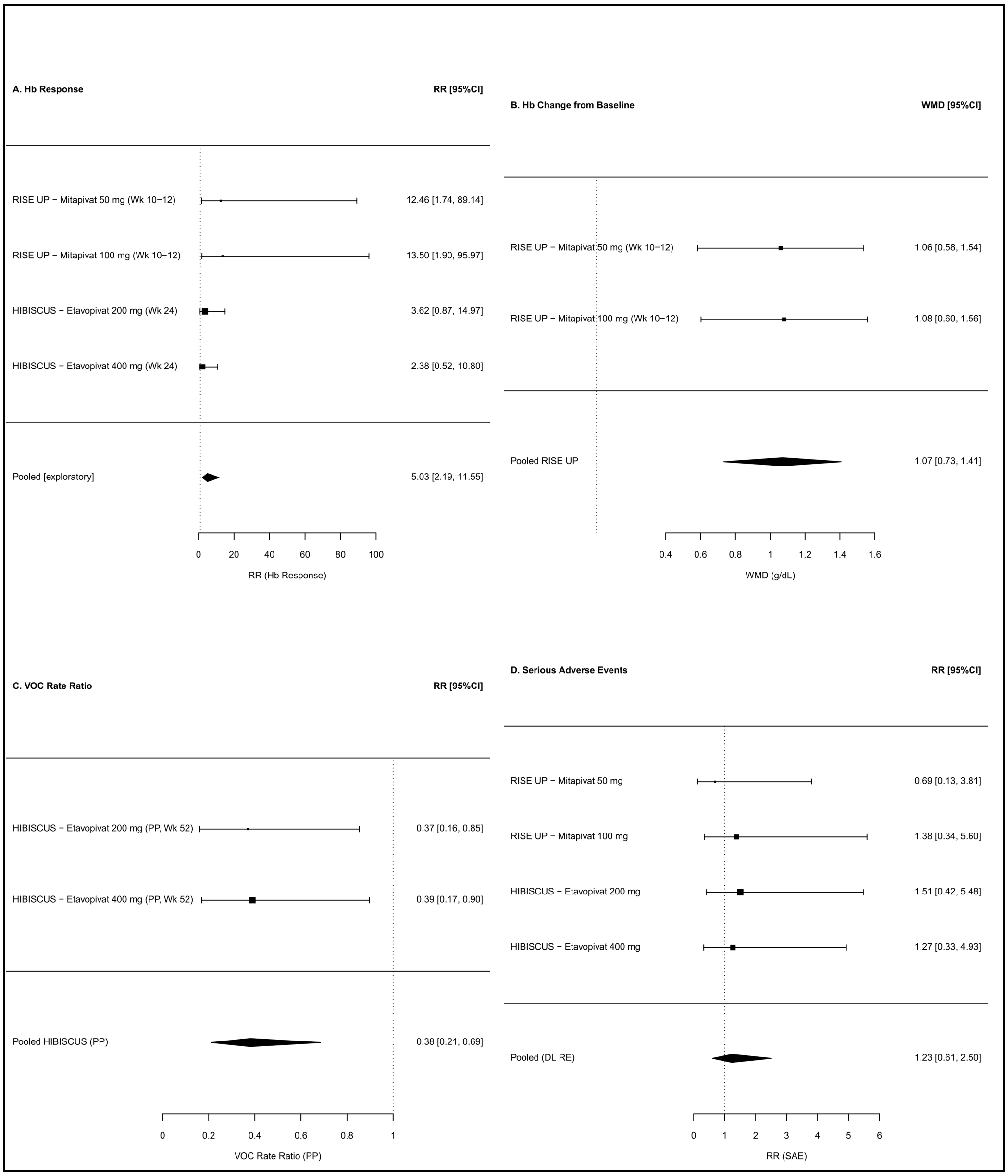

Abbreviations: CI, confidence interval; DL RE, DerSimonian-Laird random-effects model; Hb, haemoglobin; PP, per-protocol; PKR, pyruvate kinase-R; RR, risk ratio; SAE, serious adverse event; SCD, sickle cell disease; VOC, vaso-occlusive crisis; WMD, weighted mean difference; Wk, week. Panel A (Hb response) pooled estimate is exploratory given cross-trial heterogeneity in response definitions and timepoints; Panel C VOC rate ratio reflects HIBISCUS per-protocol population only, as RISE UP was not powered for VOC; Panel D shows no statistically significant excess SAE risk with either agent across all dose arms.

**Copyright Information:** (Online) ISSN: 2572-9241
©2026 The Author(s). HemaSphere is published by John Wiley & Sons Ltd on behalf of European Hematology Association. This is an open access Abstract Book distributed under the Attribution-NonCommercial-NoDerivs (CC BY-NC-ND), which allows third parties to download the articles and share them with others as long as they credit the author and the Abstract Book, but they cannot change the content in any way or use them commercially.
**Abstract Book Citations:** Authors, Title, HemaSphere, 2026;10:(S1):pages. The abstract book DOIs can be found on the "Author Information" page.
**Disclaimer:** Articles published in the journal HemaSphere exclusively reflect the opinions of the authors. The authors are responsible for all content in their abstracts including accuracy of the facts, statements, citing resources, etc.

### (PB4156) FACTORS ASSOCIATED WITH RECURRENCE OF VENOUS THROMBOEMBOLISM IN SICKLE CELL DISEASE: A RETROSPECTIVE ANALYSIS

**Topic:** 27. Sickle cell disease

Husna Thalib[1], Ammarah Tayyebah[1], Abdul Haseeb*[2]

*[1]Batterjee Medical College, Jeddah, Saudi Arabia;[2]Shifa College of Medicine, Islamabad, Pakistan;*

**Background**

Sickle Cell Disease (SCD) patients have an elevated risk of venous thromboembolism (VTE). Identifying factors that predict VTE recurrence could improve patient management, but data in this area are limited.

**Aims**

To investigate clinical and hematologic factors linked to VTE recurrence in patients with SCD.

**Methods**

We retrospectively analyzed medical records of 42 SCD patients diagnosed with a first VTE event between 2017 and 2023 at a single tertiary hospital. Clinical data (age, gender, hydroxyurea use) and baseline hematologic parameters (hemoglobin, reticulocyte percentage, platelet count) at initial VTE diagnosis were collected. Patients were followed for a median of 2.5 years to detect VTE recurrence. Cox proportional hazards regression assessed predictors of recurrence.

**Results**

Among the 42 patients, 11 (26%) developed recurrent VTE. Higher reticulocyte percentage at baseline was significantly associated with recurrence (Hazard Ratio [HR] 1.15 per 1% increase; 95% Confidence Interval [CI]: 1.03–1.29; p=0.01). Patients not receiving hydroxyurea had a higher likelihood of recurrence (HR 2.4; 95% CI: 1.1–5.2; p=0.03). Female gender was not significantly associated with recurrence. Hemoglobin and platelet counts showed no significant effect.

**Summary/Conclusion**

This small cohort study suggests that elevated reticulocyte counts, and lack of hydroxyurea treatment are linked to higher VTE recurrence risk in SCD patients. Larger prospective studies are needed to confirm these findings and guide management.

**Copyright Information:** (Online) ISSN: 2572-9241
©2026 The Author(s). HemaSphere is published by John Wiley & Sons Ltd on behalf of European Hematology Association. This is an open access Abstract Book distributed under the Attribution-NonCommercial-NoDerivs (CC BY-NC-ND), which allows third parties to download the articles and share them with others as long as they credit the author and the Abstract Book, but they cannot change the content in any way or use them commercially.
**Abstract Book Citations:** Authors, Title, HemaSphere, 2026;10:(S1):pages. The abstract book DOIs can be found on the “Author Information” page.
**Disclaimer:** Articles published in the journal HemaSphere exclusively reflect the opinions of the authors. The authors are responsible for all content in their abstracts including accuracy of the facts, statements, citing resources, etc.

### (PB4157) A STUDY OF THE COMPLICATIONS IN SICKLE CELL DISEASE PATIENTS AND THE INFLUENCE OF PRIMARY MODIFIERS ON THESE COMPLICATIONS: A SINGLE CENTRE EXPERIENCE FROM A TERITIARY CARE CENTRE OF EASTERN INDIA

**Topic:** 27. Sickle cell disease

Rajat Mohapatra*[1], Priyanka Samal[1], Soudamini Mahapatra[1], Anchal Tandon[1]

*[1]IMS AND SUM HOSPITAL, CLINICAL HEMATOLOGY, BHUBANESWAR, India;*

### Background

In India the prevalence of Sickle cell disease is estimated to be 4.3% and in Odisha it is 9.1%. The most important clinical manifestation of Sickle Cell Disease is pain which occurs due to tissue ischemia and infarction. Repeated episodes of ischemia-reperfusion injury and the effects of hemolysis result in chronic end organ damage.Improvement in management of complications has increased survival of newborns to adulthood. Ninety percent of strokes can be prevented by Trans-cranial Doppler screening. There is high prevalence of Vitamin D deficiency, osteopenia and osteoporosis in Sickle Cell Disease patients which can cause increased episodes of vaso-occlusive crisis. Ocular consequences of Sickle Cell Disease include retinopathy. The kidney is compromised early in life with both glomerular and tubular damage in individuals with Sickle cell Disease. Evaluating the complications of Sickle Cell Disease would enable us to take proper steps in delaying end organ damage.

### Aims

To study the spectrum of complications in sickle cell disease patients and the impact of primary modifiers (HbF and alpha globin mutation) on these complications.

### Methods

A prospective study was conducted on Sickle Cell Disease patients who presented to our Department between June 2024 to June 2025.

**Copyright Information:** (Online) ISSN: 2572-9241
©2026 The Author(s). HemaSphere is published by John Wiley & Sons Ltd on behalf of European Hematology Association. This is an open access Abstract Book distributed under the Attribution-NonCommercial-NoDerivs (CC BY-NC-ND), which allows third parties to download the articles and share them with others as long as they credit the author and the Abstract Book, but they cannot change the content in any way or use them commercially.
**Abstract Book Citations:** Authors, Title, HemaSphere, 2026;10:(S1):pages. The abstract book DOIs can be found on the “Author Information” page.
**Disclaimer:** Articles published in the journal HemaSphere exclusively reflect the opinions of the authors. The authors are responsible for all content in their abstracts including accuracy of the facts, statements, citing resources, etc.

### Results

60 diagnosed cases of Sickle Cell Disease were included in the study. 68.3% cases were males and 31.7% cases were females. The median age at presentation was 21.5 years (8-54 years). 90% cases were HbSS and 10% were Hb Beta S type. The median hemoglobin was 8.4 gm/dl, with a median HbS% being 79.55%. The median Retic count was 3.6%. Alpha Thalassemia mutation study could only be done in 50% of the patients due to financial issues. 71% of the patients didn't have any mutation, 23% of the patients had alpha 3.7 mutation, 4% had alpha 4.3 mutation and 2% had p.Gly23= mutation. The most common complication noted was Bone Mineral Disease (43.3%) followed by Acute Chest Syndrome (28.3%), Hepatopathy (25%) and a series of other complications were also seen. Osteopenia was noted in 40% and osteoporosis in 3% of our patients. The Total bilirubin, direct bilirubin, SGOT and SGPT, significantly correlated with Sickle Cell Hepatopathy. The Urine Albumin Creatinine ratio also significantly correlated with Sickle Cell Nephropathy. 8% of our patients had Pulmonary Arterial Hypertension.

| COMPLICATIONS | EFFECT OF ALPHA THALASSEMIA MUTATION |
|---|---|
| Bone Mineral Disease | Equivocal |
| Renal Function, Albuminuria | Reduces risk |
| TRJ Velocity | Reduces risk |
| Acute Chest Syndrome | Reduces risk |
| Cholelithiasis | Reduces risk |
| Splenic infarct | Reduces risk |

| COMPLICATIONS | EFFECT OF FETAL HEMOGLOBIN |
|---|---|
| Bone Mineral Disease | Equivocal |
| Renal function, Albuminuria | Equivocal |
| TRJ Velocity | Protective effect |
| Acute Chest Syndrome | Equivocal |
| Splenic infarct | Equivocal |
| Cholelithiasis | Protective effect |

### Summary/Conclusion

Sickle Cell disease patients can have a wide variety of complications and be associated with genetic modifiers. In our study, Elevated fetal hemoglobin level showed protective effect for Tricuspid regurgitant velocity and cholelithiasis (although not statistically significant). Sickle Cell Nephropathy, Tricuspid Regurgitant velocity, Cholelithiasis, Splenic infarct and Acute Chest Syndrome were seen to have reduced risk with Alpha Thalassemia gene mutation (although not statistically significant). So, it is important to have idea about the primary genetic modifiers and their impact on these complications.

**Copyright Information:** (Online) ISSN: 2572-9241
©2026 The Author(s). HemaSphere is published by John Wiley & Sons Ltd on behalf of European Hematology Association. This is an open access Abstract Book distributed under the Attribution-NonCommercial-NoDerivs (CC BY-NC-ND), which allows third parties to download the articles and share them with others as long as they credit the author and the Abstract Book, but they cannot change the content in any way or use them commercially.
**Abstract Book Citations:** Authors, Title, HemaSphere, 2026;10:(S1):pages. The abstract book DOIs can be found on the "Author Information" page.
**Disclaimer:** Articles published in the journal HemaSphere exclusively reflect the opinions of the authors. The authors are responsible for all content in their abstracts including accuracy of the facts, statements, citing resources, etc.

### (PB4158) SEMEN PARAMETERS AND ELEVATED SPERM DNA FRAGMENTATION IN MEN WITH SICKLE CELL DISEASE: FINDINGS AND PERSPECTIVES

**Topic:** 27. Sickle cell disease

Anna Paloma Martins Rocha Ribeiro*[1], José de Bessa Júnior[1], Jean Carlos Contreras[1], Caroline Silva[1], Marcelo Peixoto[1], Rodolfo MacEdo Cruz[1], Lidiane de Jesus[1], Claudiana Bomfim de Almeida[1], Juliana Miranda[1], Carlos Augusto Molina[2], Eduardo Miranda[3], Ângela Guimarães[1]

*[1]University State of Feira de Santana, Health, Feira de Santana, Brazil;[2]Hospital das Clínicas of the Faculty of Medicine of Ribeirão Preto, University of São Paulo, Health, Ribeirão Preto, Brazil; [3]Unichristus University Center, Health, Fortaleza, Brazil;*

### Background

Adult men with sickle cell disease may present with normal semen analysis, although infertility is frequently reported in this population, attributed both to the disease itself and to treatments.In this study 37 patients from a referral center were evaluated by semen analysis and sperm DNA fragmentation.Despite mostly normal seminal parameters, the median SDNAIF was 28.0%, evidencing high fragmentation and wide variation in DNA integrity. Barriers such as religious issues and discomfort with the examination limited participation. This is the first study to investigate sperm DNA fragmentationin men with sickle cell disease, highlighting the importance of longitudinal research to clarify the impact of the condition on fertility and guide reproductive management.

### Aims

Describe seminal parameters and sperm DNA fragmentation in men with sickle cell disease.

### Methods

Men aged 18 or older, diagnosed with sickle cell disease, responded to a structured interview about sociodemographic variables (age, sex, self-declared race/color, and number of children) and clinical variables (type of hemoglobinopathy, use of hydroxyurea, occurrence of priapism, and fertility history).
Subsequently, participants were instructed to undergo hormonal laboratory tests and provide a semen sample, which was properly frozen and sent for sperm DNA fragmentation analysis by flow cytometry. The complete semen analysis was used as the primary method, being considered the gold standard for evaluating male fertility. The procedures and criteria established by the World Health Organization (WHO, 2021) were adopted to ensure the standardization and clinical interpretation of the results.
The cutoff point for IFDNA evaluation was 20%.

### Results

37 patients completed the interview. Of these, 24 provided semen samples for analysis,13 did not participate in this step.sSix refused the test for religious reasons (due to the prohibition of masturbation) and seven cited discomfort.Semen analysis revealed significant variability among participants. The median values of seminal parameters were within the normal range, despite the variability presente.DNA Fragmentation Index showed a median of 28.0%, indicating high levels of fragmentation and considerable heterogeneity in sperm DNA integrity among participants (Figure 1).

**Copyright Information:** (Online) ISSN: 2572-9241
©2026 The Author(s). HemaSphere is published by John Wiley & Sons Ltd on behalf of European Hematology Association. This is an open access Abstract Book distributed under the Attribution-NonCommercial-NoDerivs (CC BY-NC-ND), which allows third parties to download the articles and share them with others as long as they credit the author and the Abstract Book, but they cannot change the content in any way or use them commercially.
**Abstract Book Citations:** Authors, Title, HemaSphere, 2026;10:(S1):pages. The abstract book DOIs can be found on the “Author Information” page.
**Disclaimer:** Articles published in the journal HemaSphere exclusively reflect the opinions of the authors. The authors are responsible for all content in their abstracts including accuracy of the facts, statements, citing resources, etc.

### Summary/Conclusion

The data showed sperm concentration and motility within normal limits, although isolated alterations in morphology were observed according to rigorous criteria. Despite the disease and its treatments potentially affecting spermatogenesis, the overall semen quality was relatively preserved.

The main finding was the high prevalence of sperm DNA fragmentation, a condition associated with a lower probability of natural conception, recurrent spontaneous abortions, and worse results in assisted reproduction. The observed median was 28%, above the cutoff point frequently associated with subfertility.Fragmentation can be influenced by several factors; however, there are no consistent descriptions in the literature of this alteration specifically in men with sickle cell disease, which reinforces the relevance of the findings.

Even with strategies to expand access to testing, a significant portion of men were not evaluated, suggesting important barriers to reproductive care. The results reinforce the need for innovative strategies to expand access to fertility research and ensure that men with sickle cell disease can plan their reproductive lives in an informed and supported manner.

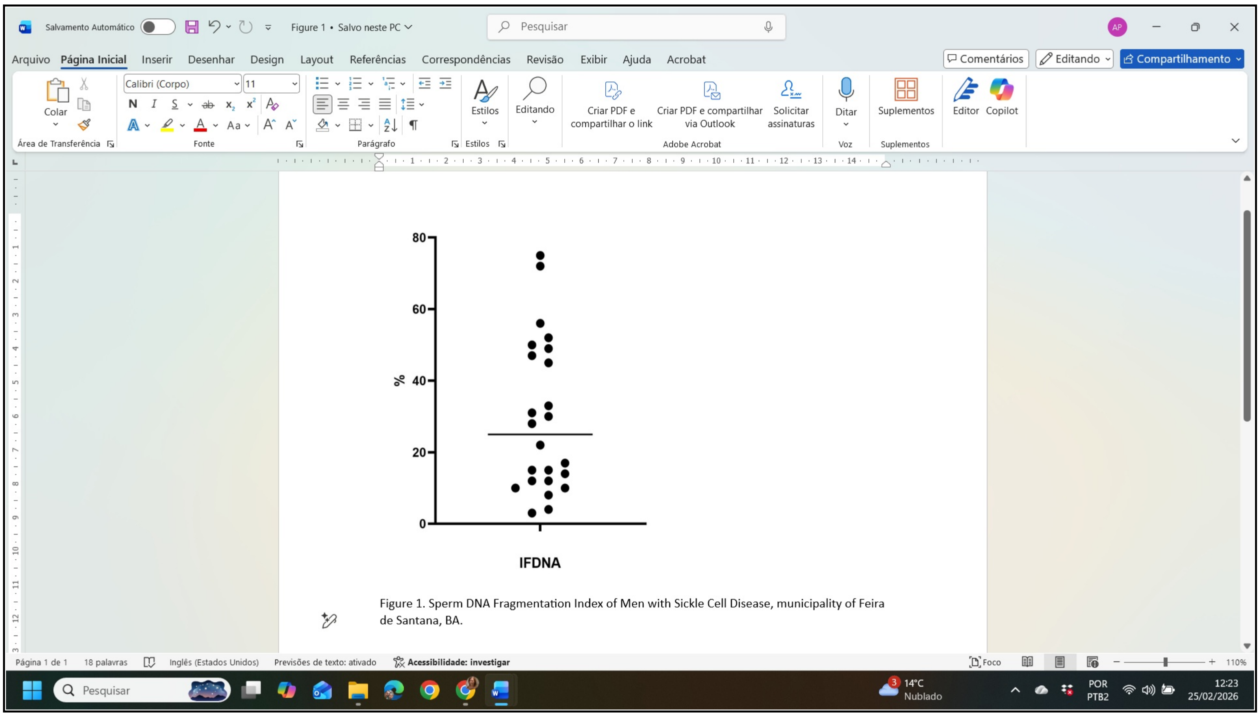

**Copyright Information:** (Online) ISSN: 2572-9241
©2026 The Author(s). HemaSphere is published by John Wiley & Sons Ltd on behalf of European Hematology Association. This is an open access Abstract Book distributed under the Attribution-NonCommercial-NoDerivs (CC BY-NC-ND), which allows third parties to download the articles and share them with others as long as they credit the author and the Abstract Book, but they cannot change the content in any way or use them commercially.
**Abstract Book Citations:** Authors, Title, HemaSphere, 2026;10:(S1):pages. The abstract book DOIs can be found on the “Author Information” page.
**Disclaimer:** Articles published in the journal HemaSphere exclusively reflect the opinions of the authors. The authors are responsible for all content in their abstracts including accuracy of the facts, statements, citing resources, etc.

### (PB4159) UNUSUAL MUSCULOSKELETAL COMPLICATIONS IN SICKLE CELL TRAIT: A CASE REPORT OF BONE INFARCTS, MARROW NECROSIS, AND MYONECROSIS

**Topic:** 27. Sickle cell disease

Saloni Talreja*[1], Naman Lodha[1], Durga Meena[1], Pyrus Bhellum[2], Reshu Lohiya[2], Ritik Bajaj[1], Gopal Bohra[1], Pawan Garg[3]

*[1]AIIMS Jodhpur, General Medicine, Jodhpur, India;[2]AIIMS Jodhpur, General medicine, Jodhpur, India;[3]AIIMS Jodhpur, Diagnostic and Interventional Radiology, Jodhpur, India;*

### Background

Sickle cell trait (SCT) affects nearly 300 million individuals worldwide and is traditionally regarded as a benign carrier state. However, under stress conditions such as hypoxia, dehydration, acidosis, or corticosteroid exposure, transient erythrocyte sickling may occur, leading to vaso-occlusive complications. Musculoskeletal manifestations including avascular necrosis (AVN) and bone infarcts are rare in SCT, while myonecrosis with sterile abscess formation is exceedingly uncommon.

### Aims

To describe a rare presentation of SCT manifesting as multifocal bone infarcts, marrow necrosis, bilateral AVN, and myonecrosis with sterile collections following acute systemic stress.

### Methods

We report a 38-year-old male construction worker with no prior comorbidities who developed severe bilateral hip pain after recovery from acute diarrheal illness complicated by hypoxia, pancytopenia, viral encephalitis, and high-dose corticosteroid therapy. Clinical, laboratory, radiological, histopathological, and hemoglobin electrophoresis findings were analyzed.

### Results

Imaging revealed bilateral femoral head AVN, multifocal vertebral and sacral bone infarcts, marrow necrosis, and intramuscular/intermuscular fluid collections in the iliopsoas and gluteal muscles. Despite surgical drainage, all bacterial, fungal, and mycobacterial cultures were sterile. Bone marrow biopsy demonstrated extensive necrosis with reactive changes. High-performance liquid chromatography confirmed SCT (HbA 62.5%, HbS 26%, HbF <1%).Whole blood PCR sequencing mutation analysis showed mutation at 6th codon (A>T) in heterozygous form further confirming the diagnosis .The collections were attributed to vaso-occlusive myonecrosis with sterile abscess formation. The patient was managed conservatively with analgesia, physiotherapy, hydroxyurea, and L-glutamine, with planned bilateral hip replacement.

### Summary/Conclusion

This case highlights that SCT, though usually benign, may under significant physiological stress manifest with severe vaso-occlusive complications resembling sickle cell disease. Recognition of sterile myonecrosis and marrow infarction in SCT is critical to avoid unnecessary surgical interventions and prolonged antimicrobial therapy. Clinicians should maintain high suspicion for ischemic complications in SCT patients presenting with unexplained musculoskeletal pain and sterile collections.

**Copyright Information:** (Online) ISSN: 2572-9241
©2026 The Author(s). HemaSphere is published by John Wiley & Sons Ltd on behalf of European Hematology Association. This is an open access Abstract Book distributed under the Attribution-NonCommercial-NoDerivs (CC BY-NC-ND), which allows third parties to download the articles and share them with others as long as they credit the author and the Abstract Book, but they cannot change the content in any way or use them commercially.
**Abstract Book Citations:** Authors, Title, HemaSphere, 2026;10:(S1):pages. The abstract book DOIs can be found on the “Author Information” page.
**Disclaimer:** Articles published in the journal HemaSphere exclusively reflect the opinions of the authors. The authors are responsible for all content in their abstracts including accuracy of the facts, statements, citing resources, etc.

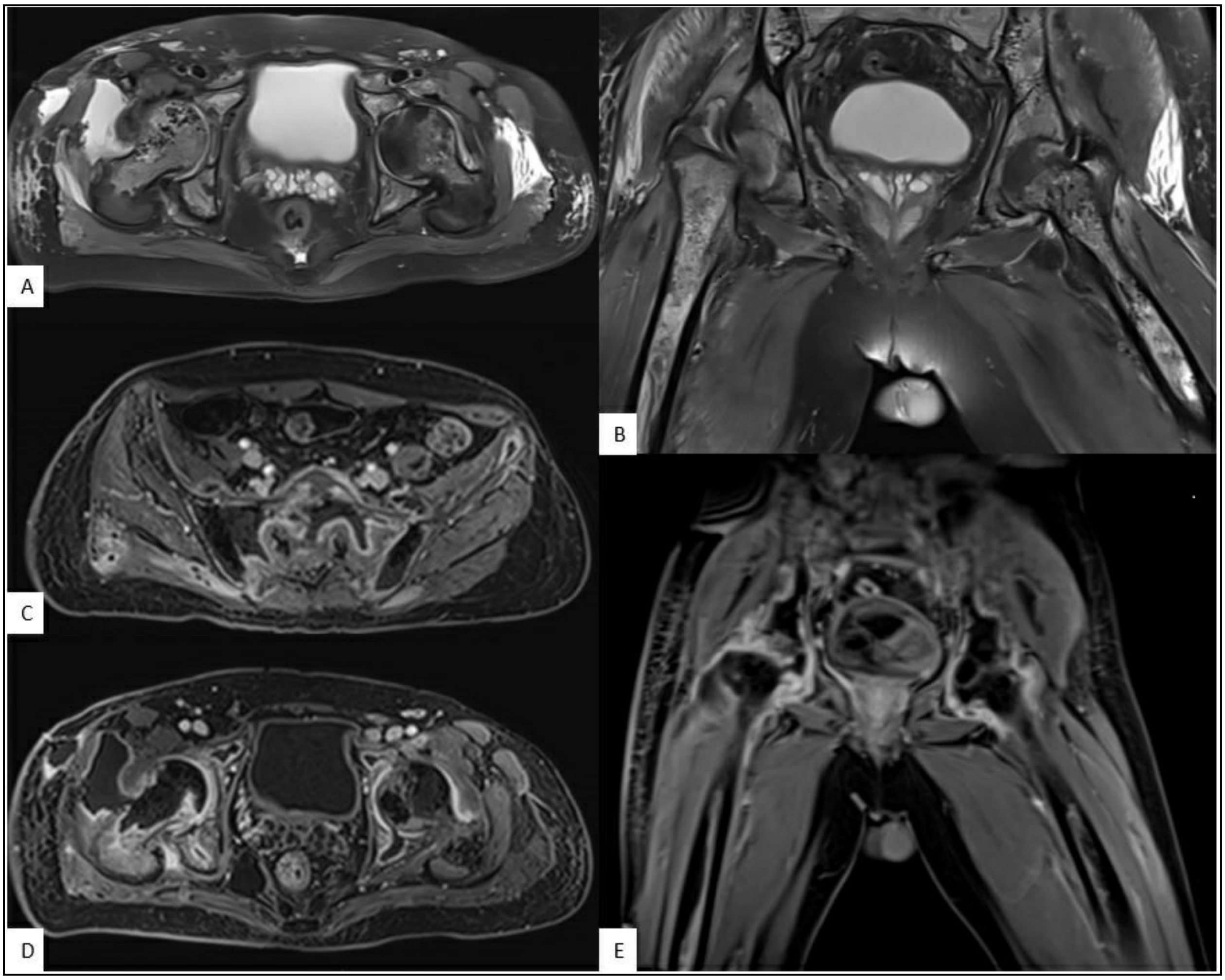

Figure 1: MRI proton density (PD) fat-saturated axial (A), coronal (B), post-contrast T1 W axial (C, D), and coronal (E) images at the level of the hip joint and pelvis, showing multifocal geographical areas of altered marrow signal intensity with peripheral post-contrast enhancement involving bilateral proximal femoral epiphysis, pelvic bones, and sacrum, suggestive of bone infarct and avascular necrosis. Diffuse enhancing synovial thickening is seen involving bilateral hip joints. Peripherally enhancing collection seen involving the right anterior thigh region.

**Copyright Information:** (Online) ISSN: 2572-9241
©2026 The Author(s). HemaSphere is published by John Wiley & Sons Ltd on behalf of European Hematology Association. This is an open access Abstract Book distributed under the Attribution-NonCommercial-NoDerivs (CC BY-NC-ND), which allows third parties to download the articles and share them with others as long as they credit the author and the Abstract Book, but they cannot change the content in any way or use them commercially.
**Abstract Book Citations:** Authors, Title, HemaSphere, 2026;10:(S1):pages. The abstract book DOIs can be found on the “Author Information” page.
**Disclaimer:** Articles published in the journal HemaSphere exclusively reflect the opinions of the authors. The authors are responsible for all content in their abstracts including accuracy of the facts, statements, citing resources, etc.

### (PB4160) ENERGIZEKIDS: MITAPIVAT IN PEDIATRIC PATIENTS WITH NON–TRANSFUSION-DEPENDENT ALPHA- OR ΒETA-THALASSEMIA

**Topic:** 28. Thalassemias

Kathryn Dickerson*[1], Yesim Aydinok[2], Maria Domenica Cappellini[3], Thomas D. Coates[4, 5], Antonis Kattamis[6], Janet L. Kwiatkowski[7, 8], Raffaella Origa[9], Sujit Sheth[10], Vip Viprakasit[11], Jayme Dahlin[12], Jeremie H. Estepp[12], Hui Shao[12], Olivia Roginski[12], Nicole R. Zane[12], Ali T. Taher[13]

*[1]Division of Pediatric Hematology and Oncology, Department of Pediatrics, UT Southwestern Medical Center, Dallas, United States of America; [2]Department of Pediatric Hematology, Ege University Faculty of Medicine, Izmir, Türkiye;[3]Fondazione IRCCS Ca' Granda Policlinico Hospital, University of Milan, Milan, Italy; [4]Department of Pediatrics, University of Southern California Keck School of Medicine, Los Angeles, United States of America; [5]Hematology Section, Cancer and Blood Disease Institute, Children's Hospital Los Angeles, Los Angeles, United States of America; [6]Thalassemia Unit, First Department of Pediatrics, National and Kapodistrian University of Athens, Athens, Greece; [7]Division of Hematology, Children's Hospital of Philadelphia, Philadelphia, United States of America; [8]Department of Pediatrics, Perelman School of Medicine of the University of Pennsylvania, Philadelphia, United States of America; [9]Department of Medical Sciences and Public Health, University of Cagliari, SC Microcitemie e Anemie Rare, ARNAS Brotzu, Cagliari, Italy; [10]Division of Hematology and Oncology, Department of Pediatrics, Weill Cornell Medicine, New York, United States of America; [11]Department of Pediatrics & Thalassemia Center, Faculty of Medicine, Siriraj Research Hospital, Mahidol University, Bangkok, Thailand; [12]Agios Pharmaceuticals, Inc., Cambridge, United States of America; [13]Division of Hematology and Oncology, Department of Internal Medicine, American University of Beirut Medical Center, Beirut, Lebanon;*

### Background

Children with non–transfusion-dependent (NTD) thalassemia do not require regular blood transfusions but remain at risk for iron overload, multiple complications including organ damage, and impaired quality of life Mitapivat, a first-in-class oral allosteric activator of pyruvate kinase, including the red blood cell (RBC)-specific (PKR) and PKM2 isoforms, is approved to be used in adults with α- or β-thalassemia, regardless of transfusion needs, in the US and Saudi Arabia.

### Aims

To describe the design of the ENERGIZEKids study, which will evaluate the efficacy, pharmacokinetics, and safety of mitapivat in children with NTD α- or β-thalassemia.

**Copyright Information:** (Online) ISSN: 2572-9241
©2026 The Author(s). HemaSphere is published by John Wiley & Sons Ltd on behalf of European Hematology Association. This is an open access Abstract Book distributed under the Attribution-NonCommercial-NoDerivs (CC BY-NC-ND), which allows third parties to download the articles and share them with others as long as they credit the author and the Abstract Book, but they cannot change the content in any way or use them commercially.
**Abstract Book Citations:** Authors, Title, HemaSphere, 2026;10:(S1):pages. The abstract book DOIs can be found on the “Author Information” page.
**Disclaimer:** Articles published in the journal HemaSphere exclusively reflect the opinions of the authors. The authors are responsible for all content in their abstracts including accuracy of the facts, statements, citing resources, etc.

**Methods**

ENERGIZEKids is a global, phase 3, randomized, double-blind, placebo-controlled trial. Eligible patients aged 1 to <18 years will enroll in staggered age cohorts: 12 to <18 years (cohort 1, n ≥12), 6 to <12 years (cohort 2, n ≥12), and 1 to <6 years (cohort 3; n ≥9 [≥2 children aged <3 years]). In a 24-week double-blind period (DBP), patients will be randomized 2:1 to receive mitapivat (100 mg, 75 mg, or 50 mg) or placebo twice daily (BID) orally (**Figure**). Doses will be assigned based on age and body weight to approximate exposure at the approved dose of mitapivat 100 mg BID for adults with thalassemia. The key inclusion criteria are weight ≥7 kg; documented α- or β-thalassemia diagnosis; hemoglobin (Hb) concentration ≤10.0 g/dL (average of ≥2 measurements ≥7 days apart); non–transfusion dependent, defined as ≤5 transfusion episodes within 24 weeks before randomization, no RBC transfusions within 8 weeks before providing informed consent/assent, and no RBC transfusion during screening; and, for patients taking hydroxyurea, a stable dose for ≥16 weeks before randomization. Key exclusion criteria include homozygous or heterozygous sickle Hb or HbC, prior receipt of gene therapy or bone marrow or stem cell transplantation, and concomitant treatment with or receipt within 18 weeks before randomization of luspatercept or hematopoietic stimulating agents. The primary endpoint is Hb response, defined as a ≥1.0-g/dL increase from baseline in average Hb concentration from Week 12 through Week 24. Secondary endpoints include other measures of Hb improvement, hemolysis, erythropoiesis, iron metabolism, and quality of life; pharmacokinetics; and safety. The Hb response analysis will use a Bayesian logistic regression model with Hb response status (yes/no) as the dependent variable and treatment arm as the independent variable. Patients who complete the DBP can enroll in the open-label extension and receive mitapivat for up to another 144 weeks (**Figure**).

**Results**

Recruitment is planned for approximately 18 sites in 10 countries.

**Summary/Conclusion**

The ENERGIZEKids trial will evaluate the efficacy, pharmacokinetics, and safety of mitapivat in children with NTD α- or β-thalassemia. As there are no approved therapies for NTD α- or β-thalassemia in children, mitapivat is a potential disease-modifying treatment to address a significant unmet need.

**Copyright Information:** (Online) ISSN: 2572-9241
©2026 The Author(s). HemaSphere is published by John Wiley & Sons Ltd on behalf of European Hematology Association. This is an open access Abstract Book distributed under the Attribution-NonCommercial-NoDerivs (CC BY-NC-ND), which allows third parties to download the articles and share them with others as long as they credit the author and the Abstract Book, but they cannot change the content in any way or use them commercially.
**Abstract Book Citations:** Authors, Title, HemaSphere, 2026;10:(S1):pages. The abstract book DOIs can be found on the “Author Information” page.
**Disclaimer:** Articles published in the journal HemaSphere exclusively reflect the opinions of the authors. The authors are responsible for all content in their abstracts including accuracy of the facts, statements, citing resources, etc.

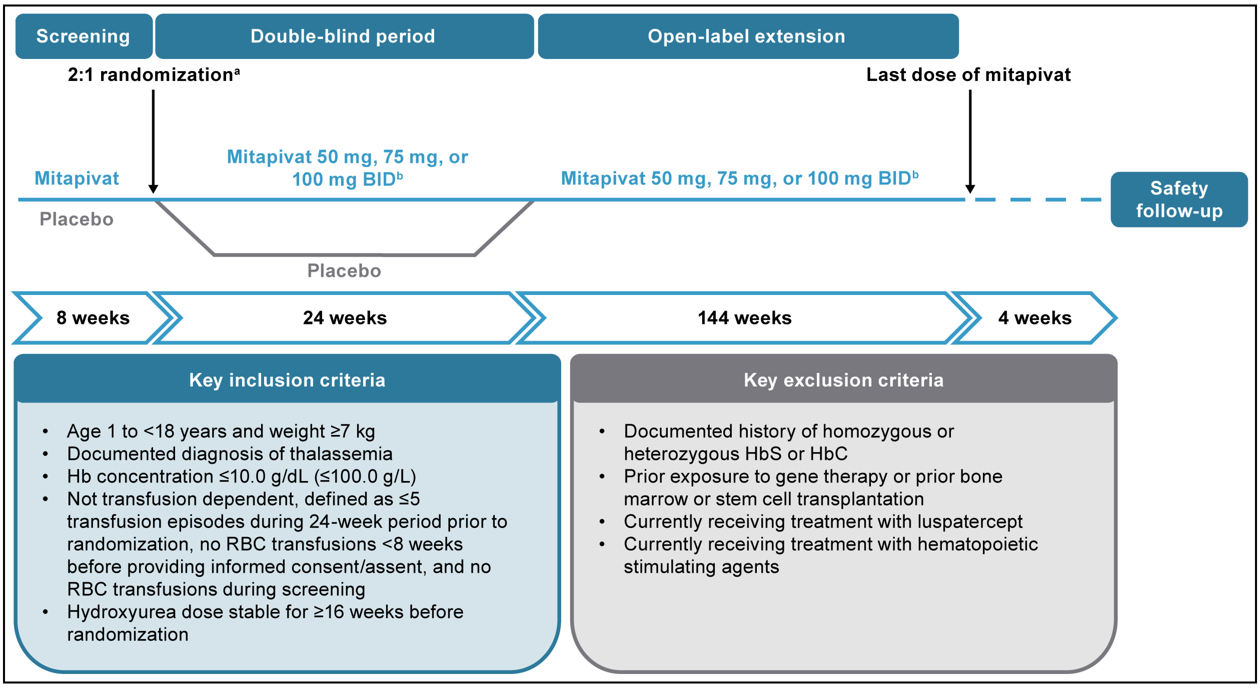

Figure. ENERGIZEKids study design. Footnote a: Randomization will be stratified by age: 12 to <18 years, 6 to <12 years, 1 to <6 years. Footnote b: Mitapivat dose will be assigned based on age and body weight to achieve systemic exposure comparable to the recommended adult clinical dose of 100 mg BID. BID, twice daily; Hb, hemoglobin; HbC, hemoglobin C; HbS, sickle hemoglobin; RBC, red blood cell.

Copyright Information: (Online) ISSN: 2572-9241
©2026 The Author(s). HemaSphere is published by John Wiley & Sons Ltd on behalf of European Hematology Association. This is an open access Abstract Book distributed under the Attribution-NonCommercial-NoDerivs (CC BY-NC-ND), which allows third parties to download the articles and share them with others as long as they credit the author and the Abstract Book, but they cannot change the content in any way or use them commercially.
Abstract Book Citations: Authors, Title, HemaSphere, 2026;10:(S1):pages. The abstract book DOIs can be found on the "Author Information" page.
Disclaimer: Articles published in the journal HemaSphere exclusively reflect the opinions of the authors. The authors are responsible for all content in their abstracts including accuracy of the facts, statements, citing resources, etc.

### (PB4161) INVESTIGATION OF FACTORS ASSOCIATED WITH THE DEVELOPMENT OF NEPHROLITHIASIS AND RENAL CYSTS IN PATIENTS WITH TRANSFUSION-DEPENDENT Β-THALASSEMIA: A RETROSPECTIVE DESCRIPTIVE STUDY

**Topic:** 28. Thalassemias

Theodora Maria Venou*[1], Esther Mbonde Mouangue[1], Athina Fufula[1], Katerina Pentidou[1], Ioanna Christodoulou[1], Efthymia Vlachaki[1]

*[1]Adult Thalassemia Unit, 2nd Department of Internal Medicine, Hippokration Geneneral Hospital, Thessaloniki, Greece;*

### Background

Blood transfusions and safe iron chelation are the cornerstones of treatment and a necessary condition for improving survival in patients with transfusion-dependent β-thalassemia. However, new complications such as malignancy development, cardiac arrhythmias, endocrine disorders, and renal dysfunction are emerging.

### Aims

The aim of this study was to investigate factors associated with the occurrence of nephrolithiasis and renal cysts in patients with transfusion-dependent β-thalassemia.

### Methods

A total of 74 patients who receive regular transfusions with pre-transfusion Hb >9.5–10 g/dL were retrospectively analyzed. Of these, 18 presented nephrolithiasis (Group A: mean age at diagnosis 38.8 ± 14.0 years), 19 presented renal cysts (Group B: 40.8 ± 13.5 years), and 40 constituted the control group (Group C: 42.0 ± 13.0). Data were collected from patient records, including age at diagnosis, serum ferritin, serum uric acid levels, bone mineral density measurements by Dual-energy X-ray Absorptiometry (DEXA), type of iron chelation therapy and serum creatinine levels. Three patients had both stones and cysts simultaneously. Estimated glomerular filtration rate (eGFR) was calculated using the CKD-EPI formula. Statistical analysis was performed using SPSS 29.0.1.

### Results

Group A showed significantly higher serum creatinine levels (0.90 ± 0.17 mg/dL vs 0.80 ± 0.14, $p = 0.0012$) and significantly lower eGFR (82.6 ± 16.5 vs 97.4 ± 17.6 ml/min/1.73m², $p = 0.0038$) compared with the control group. No statistically significant difference was found regarding age (38.8 ± 14.0 vs 42.0 ± 13.0 years, $p = 0.33$) or uric acid levels (4.75 [4.33–6.00] vs 4.70 [2.98–5.62] mg/dL, $p = 0.20$), hemoglobin, or ferritin. Similarly, in Group B no statistically significant differences were observed in age (40.8 ± 13.5 years), serum creatinine (0.80 ± 0.14 mg/dL), eGFR (93.1 ± 21.9 ml/min/1.73m²), or uric acid (4.85 [4.20–6.30] mg/dL) compared with the control group ($p > 0.05$). Notably, both nephrolithiasis and renal cysts were significantly associated with the presence of bone metabolism disorders ($p < 0.001$). No statistically significant difference was found between Groups A and B versus controls ($p = 0.118$ and $p = 0.533$ respectively) regarding splenectomy status. In Group A, most patients (10/18, 55.6%) received Deferasirox, without a statistically significant difference compared with Group C, whereas a statistically significant association was found between type of iron chelation therapy and occurrence of cysts ($p < 0.0001$).

### Summary/Conclusion

Nephrolithiasis was associated with deterioration of renal function, whereas the presence of stones and cysts was linked to coexisting disorders of bone metabolism. Systematic monitoring of renal and metabolic status is considered essential in the management of patients with transfusion-dependent β-thalassemia.

**Copyright Information:** (Online) ISSN: 2572-9241
©2026 The Author(s). HemaSphere is published by John Wiley & Sons Ltd on behalf of European Hematology Association. This is an open access Abstract Book distributed under the Attribution-NonCommercial-NoDerivs (CC BY-NC-ND), which allows third parties to download the articles and share them with others as long as they credit the author and the Abstract Book, but they cannot change the content in any way or use them commercially.
**Abstract Book Citations:** Authors, Title, HemaSphere, 2026;10:(S1):pages. The abstract book DOIs can be found on the "Author Information" page.
**Disclaimer:** Articles published in the journal HemaSphere exclusively reflect the opinions of the authors. The authors are responsible for all content in their abstracts including accuracy of the facts, statements, citing resources, etc.

### (PB4162) ENERGIZEKIDS-T: MITAPIVAT IN PEDIATRIC PATIENTS WITH TRANSFUSION-DEPENDENT ALPHA- OR BETA-THALASSEMIA

**Topic:** 28. Thalassemias

Janet L. Kwiatkowski*[1, 2], Yesim Aydinok[3], Thomas D. Coates[4, 5], Kathryn Dickerson[6], Antonis Kattamis[7], Raffaella Origa[8], Sujit Sheth[9], Ali T. Taher[10], Vip Viprakasit[11], Jeremie H. Estepp[12], Jayme Dahlin[12], Lauren Czapla[12], Shihao Shen[12], Nicole R. Zane[12], Maria Domenica Cappellini[13]

*[1]Division of Hematology, Children's Hospital of Philadelphia, Philadelphia, United States of America;[2]Department of Pediatrics, Perelman School of Medicine of the University of Pennsylvania, Philadelphia, United States of America; [3]Department of Pediatric Hematology, Ege University Faculty of Medicine, Izmir, Türkiye; [4]Hematology Section, Cancer and Blood Disease Institute, Children's Hospital Los Angeles, Los Angeles, United States of America; [5]Department of Pediatrics, University of Southern California Keck School of Medicine, Los Angeles, United States of America; [6]Division of Pediatric Hematology and Oncology, Department of Pediatrics, UT Southwestern Medical Center, Dallas, United States of America; [7]Thalassemia Unit, First Department of Pediatrics, National and Kapodistrian University of Athens, Athens, Greece; [8]Department of Medical Sciences and Public Health, University of Cagliari, SC Microcitemie e Anemie Rare, ARNAS Brotzu, Cagliari, Italy; [9]Division of Hematology and Oncology, Department of Pediatrics, Weill Cornell Medicine, New York, United States of America; [10]Division of Hematology and Oncology, Department of Internal Medicine, American University of Beirut Medical Center, Beirut, Lebanon; [11]Department of Pediatrics & Thalassemia Center, Faculty of Medicine, Siriraj Research Hospital, Mahidol University, Bangkok, Thailand; [12]Agios Pharmaceuticals, Inc., Cambridge, United States of America; [13]Fondazione IRCCS Ca' Granda Policlinico Hospital, University of Milan, Milan, Italy;*

### Background

Children with transfusion-dependent (TD) thalassemia require lifelong, regular red blood cell (RBC) transfusions for survival and may experience serious complications related to both the disease itself, and to transfusion therapy including transfusion reactions, iron overload, alloimmunization, and high treatment burden. Mitapivat, a first-in-class oral allosteric activator of pyruvate kinase, including the RBC-specific (PKR) and PKM2 isoforms, is approved to be used in adults with α- or β-thalassemia, regardless of transfusion needs, in the US and Saudi Arabia.

### Aims

To describe the design of the ENERGIZEKids-T study, which will evaluate the efficacy, pharmacokinetics, and safety of mitapivat in pediatric patients with TD α- or β-thalassemia.

**Copyright Information:** (Online) ISSN: 2572-9241
©2026 The Author(s). HemaSphere is published by John Wiley & Sons Ltd on behalf of European Hematology Association. This is an open access Abstract Book distributed under the Attribution-NonCommercial-NoDerivs (CC BY-NC-ND), which allows third parties to download the articles and share them with others as long as they credit the author and the Abstract Book, but they cannot change the content in any way or use them commercially.
**Abstract Book Citations:** Authors, Title, HemaSphere, 2026;10:(S1):pages. The abstract book DOIs can be found on the "Author Information" page.
**Disclaimer:** Articles published in the journal HemaSphere exclusively reflect the opinions of the authors. The authors are responsible for all content in their abstracts including accuracy of the facts, statements, citing resources, etc.

**Methods**

ENERGIZEKids-T is a global, phase 3, randomized, double-blind, placebo-controlled trial. Eligible patients aged 1 to <18 years will enroll in staggered age cohorts: 12 to <18 years (cohort 1, n ≥12), 6 to <12 years (cohort 2, n ≥12), and 1 to <6 years (cohort 3; n ≥9 [≥2 children aged <3 years]). In a 48-week double-blind period (DBP), patients will be randomized 2:1 to mitapivat (100 mg, 75 mg, or 50 mg) or placebo twice daily (BID) orally (**Figure**). Doses will be assigned based on age and body weight to approximate exposure at the approved dose of mitapivat 100 mg BID for adults with thalassemia. The key inclusion criteria are weight ≥7 kg, documented α- or β-thalassemia diagnosis, transfusion dependence (6-20 transfusion episodes in the 24 weeks before randomization, with a ≤6-week transfusion-free period), and for patients taking hydroxyurea, a stable dose for ≥16 weeks before randomization. Key exclusion criteria include homozygous or heterozygous sickle hemoglobin (Hb) or HbC, prior receipt of gene therapy or bone marrow or stem cell transplantation, and concomitant treatment with or receipt ≥36 weeks before randomization of luspatercept or hematopoietic stimulating agents. The primary endpoint is transfusion reduction response (TRR), defined as a ≥50% reduction in transfused RBC volume (weight-normalized) in any consecutive 12-week period through Week 48 vs historical volume. Secondary endpoints include additional measures of transfusion burden, iron metabolism/overload, pharmacokinetics, and safety. The transfusion reduction analysis will use a Bayesian logistic regression model with TRR status (yes/no) as the dependent variable and treatment as the independent variable. Patients who complete the DBP can enroll in the open-label extension and receive mitapivat for up to another 144 weeks (**Figure**).

**Results**

Recruitment is planned for approximately 18 sites in 10 countries.

**Summary/Conclusion**

The ENERGIZEKids-T trial will evaluate the efficacy, pharmacokinetics, and safety of mitapivat in children with TD α- or β-thalassemia. As there are no approved therapies for children with α-thalassemia and limited options for β-thalassemia, mitapivat is a potential disease-modifying treatment to address a significant unmet need.

**Copyright Information:** (Online) ISSN: 2572-9241
©2026 The Author(s). HemaSphere is published by John Wiley & Sons Ltd on behalf of European Hematology Association. This is an open access Abstract Book distributed under the Attribution-NonCommercial-NoDerivs (CC BY-NC-ND), which allows third parties to download the articles and share them with others as long as they credit the author and the Abstract Book, but they cannot change the content in any way or use them commercially.
**Abstract Book Citations:** Authors, Title, HemaSphere, 2026;10:(S1):pages. The abstract book DOIs can be found on the “Author Information” page.
**Disclaimer:** Articles published in the journal HemaSphere exclusively reflect the opinions of the authors. The authors are responsible for all content in their abstracts including accuracy of the facts, statements, citing resources, etc.

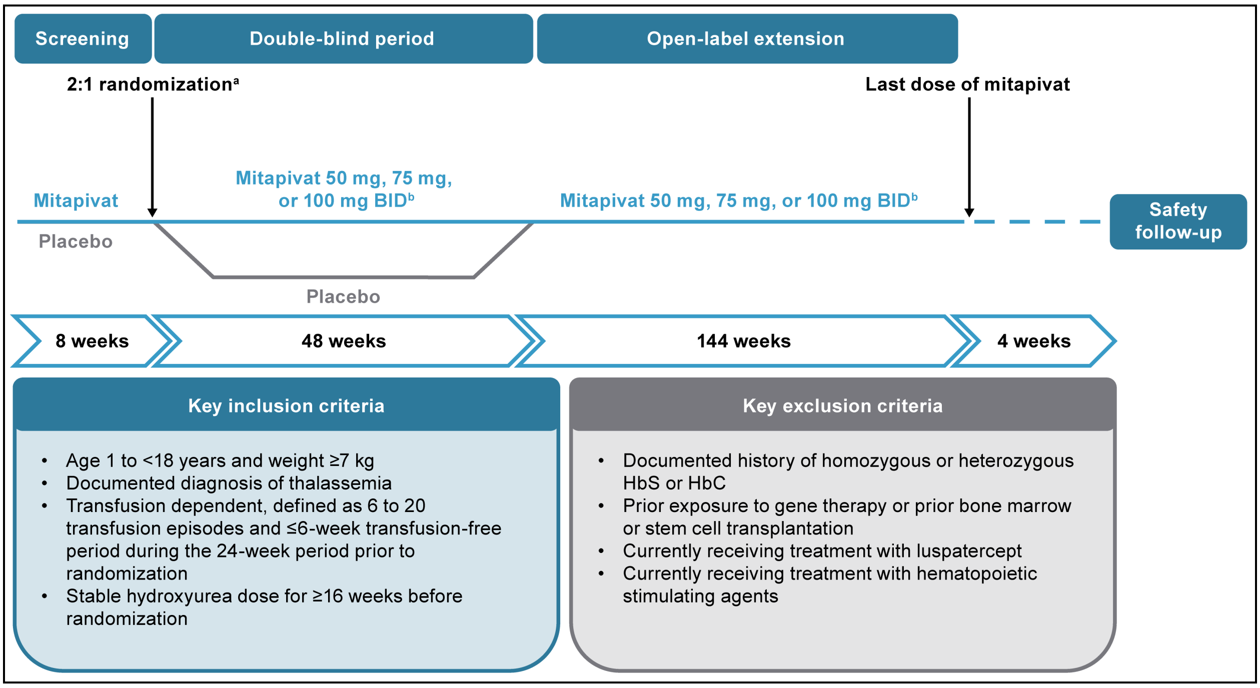

Figure. ENERGIZEKids-T study design. Footnote a: Randomization will be stratified by age: 12 to <18 years, 6 to <12 years, 1 to <6 years. Footnote b: Mitapivat dose will be assigned based on age and body weight to achieve systemic exposure comparable to the recommended adult clinical dose of 100 mg BID. BID, twice daily; HbC, hemoglobin C; HbS, sickle hemoglobin.

**Copyright Information:** (Online) ISSN: 2572-9241
©2026 The Author(s). HemaSphere is published by John Wiley & Sons Ltd on behalf of European Hematology Association. This is an open access Abstract Book distributed under the Attribution-NonCommercial-NoDerivs (CC BY-NC-ND), which allows third parties to download the articles and share them with others as long as they credit the author and the Abstract Book, but they cannot change the content in any way or use them commercially.
**Abstract Book Citations:** Authors, Title, HemaSphere, 2026;10:(S1):pages. The abstract book DOIs can be found on the "Author Information" page.
**Disclaimer:** Articles published in the journal HemaSphere exclusively reflect the opinions of the authors. The authors are responsible for all content in their abstracts including accuracy of the facts, statements, citing resources, etc.

### (PB4163) CLINICAL SIGNIFICANCE AND PREDICTORS OF PROLONGED ACTIVATED PARTIAL THROMBOPLASTIN TIME IN ADULTS WITH TRANSFUSION-DEPENDENT THALASSEMIA: A SINGLE-CENTER STUDY

**Topic:** 28. Thalassemias

Mariam Goubran*[1], Hayley Merkeley[1]

[1]*University of British Columbia, Division of Hematology, Vancouver, Canada;*

### Background

Transfusion-dependent thalassemias (TDT) are severe inherited hemoglobinopathies characterized by ineffective erythropoiesis and lifelong dependence on regular red blood cell transfusions. Patients with TDT are recognized to have a hypercoagulable phenotype and increased thrombotic risk. Prolongation of activated partial thromboplastin time (aPTT) has been reported in pediatric TDT cohorts; however, its etiology and clinical significance remain poorly understood, particularly in adults.

### Aims

To characterize the prevalence and clinical significance of prolonged aPTT in adults with TDT and to identify laboratory predictors associated with aPTT prolongation.

### Methods

We conducted a single-center observational study of adult patients with TDT followed through the Adult Red Cell Disorder Program at St. Paul's Hospital. Longitudinal data collected over a 5-year period included aPTT, ferritin, lactate dehydrogenase (LDH), reticulocyte count, and liver iron concentration (LIC) measured by MRI. Logistic regression and non-parametric testing were used to assess associations between laboratory variables and aPTT prolongation. Subgroup analyses were performed in patients with both normal and prolonged aPTT measurements over time. Receiver operating characteristic (ROC) curve analyses were used to assess the discriminatory performance of LIC and aPTT. Institutional ethics approval was obtained.

### Results

Forty-four patients were included, of whom 8 (18%) demonstrated prolonged aPTT at their most recent assessment. LIC was significantly higher in patients with prolonged aPTT compared with those with normal aPTT (median 7.7 vs. 2.9 mg/g, $p = 0.007$) and was independently associated with aPTT prolongation on logistic regression. ROC analysis demonstrated excellent discrimination of prolonged aPTT by LIC (AUC 0.807), with an optimal LIC threshold of >4.3 mg/g. In a subset of patients with paired normal and prolonged aPTT measurements, transition to prolonged aPTT was associated with a significant increase in LIC ($p = 0.024$).

### Summary/Conclusion

Prolonged aPTT is observed in a subset of adults with transfusion-dependent thalassemia and is strongly associated with increased liver iron concentration. These findings suggest a potential link between iron overload and coagulation abnormalities in TDT and support further evaluation of prolonged aPTT in this population. Importantly, the observed relationship raises the possibility that aPTT, a widely available and low-cost test, could serve as a surrogate marker to identify patients at risk of clinically significant iron overload in resource-limited settings where access to MRI-based LIC assessment is constrained. Future studies should explore the role of aPTT abnormalities in non–transfusion-dependent thalassemia and other iron overload states.

**Copyright Information:** (Online) ISSN: 2572-9241
©2026 The Author(s). HemaSphere is published by John Wiley & Sons Ltd on behalf of European Hematology Association. This is an open access Abstract Book distributed under the Attribution-NonCommercial-NoDerivs (CC BY-NC-ND), which allows third parties to download the articles and share them with others as long as they credit the author and the Abstract Book, but they cannot change the content in any way or use them commercially.
**Abstract Book Citations:** Authors, Title, HemaSphere, 2026;10:(S1):pages. The abstract book DOIs can be found on the "Author Information" page.
**Disclaimer:** Articles published in the journal HemaSphere exclusively reflect the opinions of the authors. The authors are responsible for all content in their abstracts including accuracy of the facts, statements, citing resources, etc.

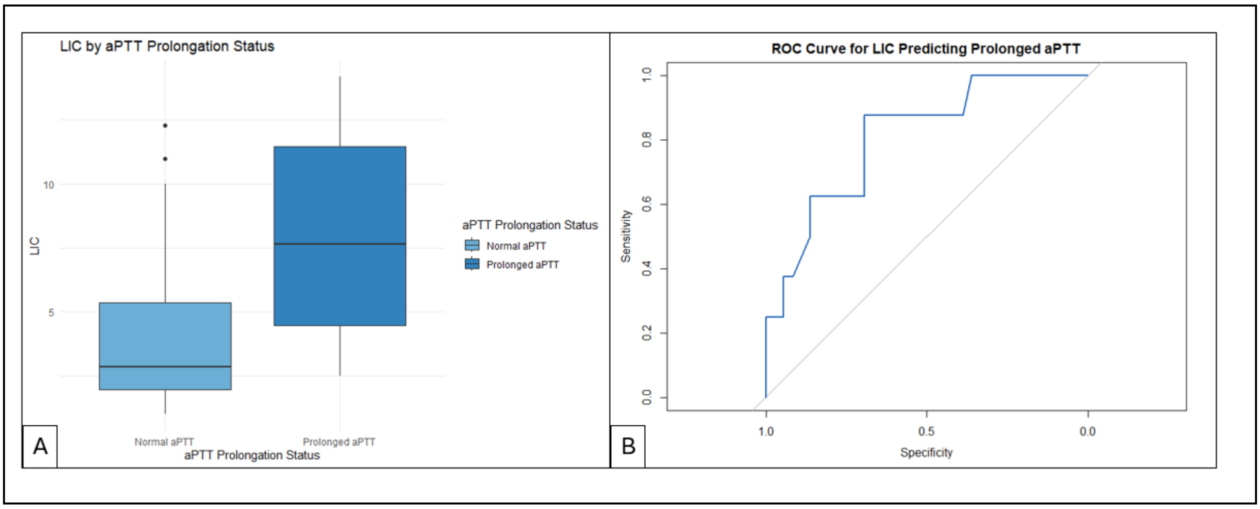

A) LIC is significantly higher in patients with prolonged aPTT (median=7.7, IQR 4.5-11.5) vs patients with normal aPTT (median=2.9, IQR 2.0-5.4) with a p-value of 0.007. B) ROC curve analysis suggests a threshold LIC of >4.3mg/g with specificity of 0.875 and sensitivity 0.694 with an area under the curve of 0.807 suggesting excellent ability of LIC to discriminate between patients with normal vs. prolonged aPTT.

**Copyright Information:** (Online) ISSN: 2572-9241
©2026 The Author(s). HemaSphere is published by John Wiley & Sons Ltd on behalf of European Hematology Association. This is an open access Abstract Book distributed under the Attribution-NonCommercial-NoDerivs (CC BY-NC-ND), which allows third parties to download the articles and share them with others as long as they credit the author and the Abstract Book, but they cannot change the content in any way or use them commercially.
**Abstract Book Citations:** Authors, Title, HemaSphere, 2026;10:(S1):pages. The abstract book DOIs can be found on the “Author Information” page.
**Disclaimer:** Articles published in the journal HemaSphere exclusively reflect the opinions of the authors. The authors are responsible for all content in their abstracts including accuracy of the facts, statements, citing resources, etc.

### (PB4164) MCS DUPLICATION AND INVERSION IN THE Α-GLOBIN REGULATORY REGION: PEDIGREE ANALYSIS OF AN Α-THALASSEMIA HBH DISEASE FAMILY

**Topic:** 28. Thalassemias

Weijia Yang*[1], Shiping Chen[2], Li Wang[1, 2], Susu Zhao[1], Qingping Kang[1], Min Dou[1]

[1]*Guilin People's Hospital, Department of Eugenics and Genetics, Guilin, China;*[2]*BGI Genomics Co., Ltd., Shenzhen, China;*

### Background

Alpha-thalassemia is one of the most common inherited disorders worldwide. Approximately 5%–20% of the global population carries one or more α-thalassemia mutations. Normal individuals possess two pairs of α-globin genes (αα/αα). In patients with α-thalassemia, deletions may remove one or both genes on a chromosome, such as the -$\alpha^{3.7}$ and -$\alpha^{4.2}$ deletions in $\alpha^+$-thalassemia, and the --SEA and --MED deletions in $\alpha^0$-thalassemia. Inactivation of three α-globin genes leads to excessive β-globin chain production and formation of $\beta_4$ tetramers (HbH). HbH disease is characterized by variable hemolysis and marked clinical heterogeneity. Homozygous $\alpha^0$-thalassemia (--/--) causes $\gamma_4$ tetramer formation, known as Hb Bart's hydrops fetalis syndrome, which usually results in intrauterine or early neonatal death. These α-globin variants lie within the α-globin gene cluster and can be detected by routine thalassemia genetic testing.
However, rare mutations outside the α-globin cluster are often undetectable by conventional assays. The MCS-R regulatory elements are critical for α-globin synthesis. The human α-globin gene cluster is located at 16p13.3, with the arrangement 5'-ζ2-ψζ1-ψα2-ψα1-α2-α1-θ1-3'. Four highly conserved multispecies conserved sequences (MCS-R1 to MCS-R4) lie 30–70 kb upstream, corresponding to erythroid-specific DNase I hypersensitive sites HS-48, HS-40, HS-33, and HS-10, which mediate long-range regulation of downstream gene expression. Deletion of the major regulatory element MCS-R2 (HS-40) severely reduces α-globin expression. Heterozygous HS-40 deletion alone causes $\alpha^0$-thalassemia, and its co-inheritance with --SEA leads to Hb Bart's hydrops fetalis.
In recent years, a few cases of α-thalassemia caused by MCS-R duplication have been reported. Here, we describe a pedigree in which the proband was identified by long-read whole-genome sequencing to carry a complex rearrangement involving inversion and duplication of the α-globin regulatory MCS-R region (chr16:137,807–202,853) combined with heterozygous --SEA deletion, resulting in an HbH disease phenotype.

### Aims

We identified a novel complex inversion-duplication involving MCS regulatory elements as a rare cause of HbH disease, and long-read sequencing is crucial for resolving genotype-phenotype discrepancies in α-thalassemia.

### Methods

A pediatric patient with inconsistent genotype-phenotype was initially genotyped as --SEA/αα by routine testing. Targeted capture sequencing suggested a duplication in the NPRL3 region upstream of the α-globin cluster, but short-read sequencing could not define its size or structural arrangement. Long-read whole-genome sequencing (LR-WGS) and PCR validation were performed.

### Results

LR-WGS identified an inversion at chr16:102812–199333, accompanied by two inverted duplications (74464 bp and 23114 bp, total 96478 bp). The right breakpoint of this complex inversion-duplication (INV+DUP) lay between NPRL3 and HBZ. The variant was confirmed in the proband's mother and maternal grandmother. The final genotype was --SEA/(αα)NC_000016.10:g.102711_176193dup(inv);g.176209_199348dup, consistent with HbH disease.

**Copyright Information:** (Online) ISSN: 2572-9241
©2026 The Author(s). HemaSphere is published by John Wiley & Sons Ltd on behalf of European Hematology Association. This is an open access Abstract Book distributed under the Attribution-NonCommercial-NoDerivs (CC BY-NC-ND), which allows third parties to download the articles and share them with others as long as they credit the author and the Abstract Book, but they cannot change the content in any way or use them commercially.
**Abstract Book Citations:** Authors, Title, HemaSphere, 2026;10:(S1):pages. The abstract book DOIs can be found on the "Author Information" page.
**Disclaimer:** Articles published in the journal HemaSphere exclusively reflect the opinions of the authors. The authors are responsible for all content in their abstracts including accuracy of the facts, statements, citing resources, etc.

### Summary/Conclusion

The α-globin regulatory region (HBA-LCR) overlaps with NPRL3. The novel duplication increases the physical distance between HS-40 and the α2/α1 genes, likely disrupting long-range regulation and reducing α-globin expression, leading to α⁺-thalassemia.

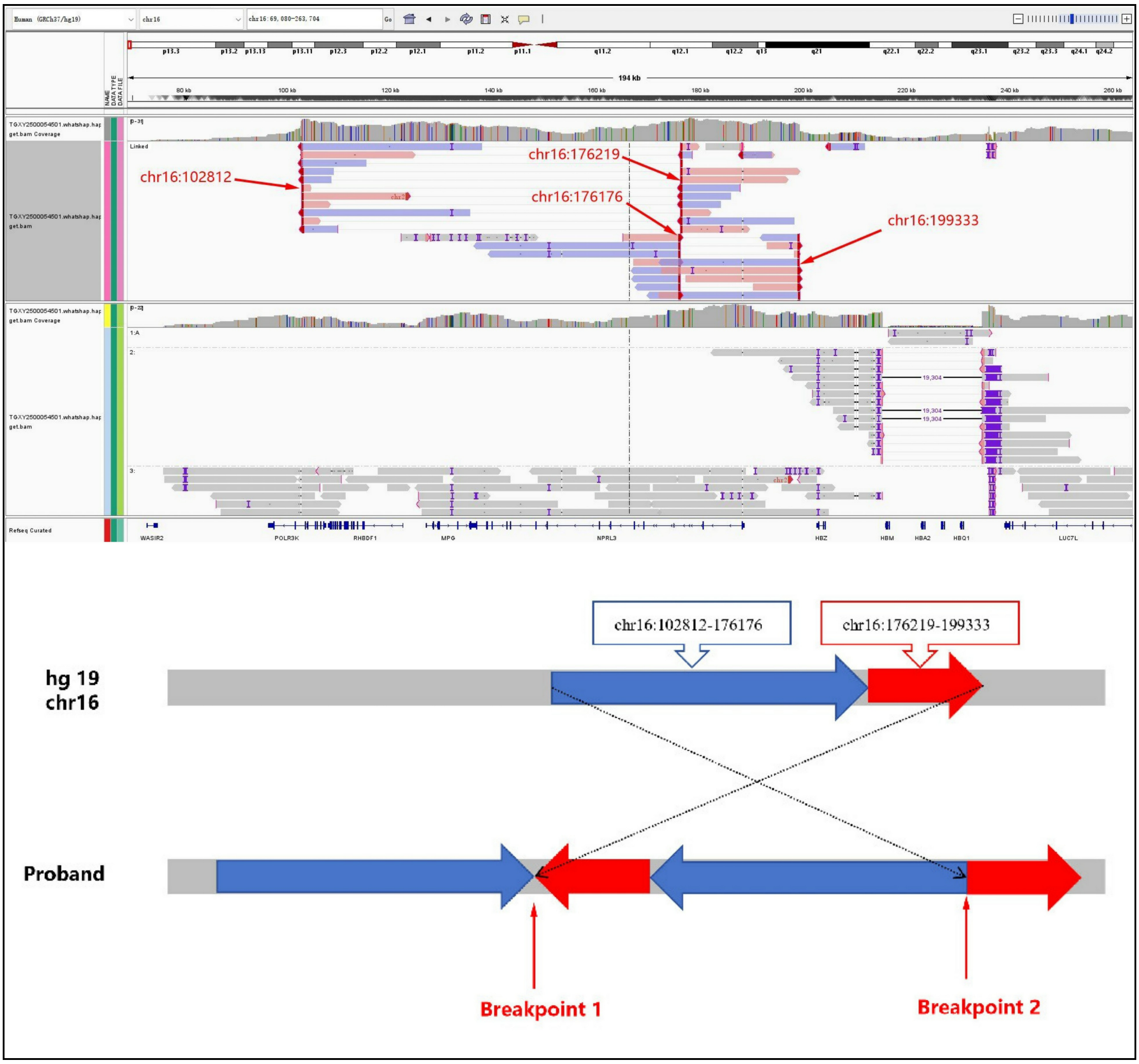

**Copyright Information:** (Online) ISSN: 2572-9241
©2026 The Author(s). HemaSphere is published by John Wiley & Sons Ltd on behalf of European Hematology Association. This is an open access Abstract Book distributed under the Attribution-NonCommercial-NoDerivs (CC BY-NC-ND), which allows third parties to download the articles and share them with others as long as they credit the author and the Abstract Book, but they cannot change the content in any way or use them commercially.
**Abstract Book Citations:** Authors, Title, HemaSphere, 2026;10:(S1):pages. The abstract book DOIs can be found on the "Author Information" page.
**Disclaimer:** Articles published in the journal HemaSphere exclusively reflect the opinions of the authors. The authors are responsible for all content in their abstracts including accuracy of the facts, statements, citing resources, etc.

### (PB4165) THE ROLE OF NOVEL RETICULOCYTE INDICES IN DISTINGUISHING BETA-THALASSEMIA TRAIT FROM IRON DEFICIENCY ANEMIA

**Topic:** 28. Thalassemias

Ikbel Ghachem*[1], Mohamed Yassine Kaabar[2], Eya Thabet[3], Hamida Khémiri Guerbouj[4], Wafa Skouri[4], Haifa Tounsi[4], Asma Bachali[1]

*[1]University of Tunis El Manar, Faculty of Medicine of Tunis, Laboratory of Biology, Tahar Maamouri Hospital, Nabeul, Tunisia; [2]University of Monastir, Faculty of Pharmacy of Monastir, Laboratory of Biology, Tahar Maamouri Hospital,, Nabeul, Tunisia; [3]Laboratory of Biology, Taher Maamouri Hospital, Nabeul, Tunisia;[4]University of Tunis El Manar, Faculty of medicine of Tunis, Internal medicine Department, Taher Maamouri Hospital, Nabeul, Tunisia;*

### Background

Microcytic, hypochromic anemia is a common diagnostic challenge, with iron deficiency anemia (IDA) and beta-thalassemia trait (BT) being the two most frequent causes. Accurate differentiation is clinically essential, as the treatment pathways for these conditions are entirely distinct. Recently, interest has grown in using reticulocyte parameters, such as the reticulocyte hemoglobin equivalent (RET-He), the hemoglobin content difference between reticulocytes and mature cells (Delta-CH), and the microcytosis/hypochromia (MH) ratio, as rapid, accessible tools for this differential diagnosis.

### Aims

To assess the diagnostic accuracy of RET-He, Delta-CH, and the MH ratio in differentiating IDA from BT within a local patient population.

### Methods

This prospective, single-center study enrolled 71 patients with confirmed IDA, 31 patients with BT, and 84 healthy controls. All participants underwent a complete blood count and reticulocyte analysis on the Mindray® BC6200 hematology analyzer. Additionally, ferritin and C-reactive protein levels were measured using the COBAS6000 platform. Anemia was defined according to WHO criteria (hemoglobin <13 g/dL in men, <12 g/dL in women), with IDA specifically characterized by ferritin <30 ng/mL in the absence of elevated inflammatory markers. Receiver operating characteristic (ROC) curve analysis was employed to determine the diagnostic performance of each parameter, including the area under the curve (AUC), sensitivity, specificity, and predictive values.

**Copyright Information:** (Online) ISSN: 2572-9241
©2026 The Author(s). HemaSphere is published by John Wiley & Sons Ltd on behalf of European Hematology Association. This is an open access Abstract Book distributed under the Attribution-NonCommercial-NoDerivs (CC BY-NC-ND), which allows third parties to download the articles and share them with others as long as they credit the author and the Abstract Book, but they cannot change the content in any way or use them commercially.
**Abstract Book Citations:** Authors, Title, HemaSphere, 2026;10:(S1):pages. The abstract book DOIs can be found on the "Author Information" page.
**Disclaimer:** Articles published in the journal HemaSphere exclusively reflect the opinions of the authors. The authors are responsible for all content in their abstracts including accuracy of the facts, statements, citing resources, etc.

**Results**

The mean RET-He was significantly lower in the IDA cohort (21.5 ± 2.6 pg) compared to the BT (19.18 ± 1.16 pg) and healthy groups (27.15 ± 1.3 pg). The median Delta-CH values showed no significant difference between the IDA (0.7 [-0.02 to 1.7]), BT (0.5 [0.2 to 1.1]), and healthy groups (0.7 [-0.2 to 1.2]) (p=0.54).
The median percentages of microcytic (Micro%) and hypochromic (Hypo%) red blood cells were significantly higher (p<0.001) in the BT group (61.1% [48.3-61.6] and 57.5% [52.1-66.7], respectively) compared to the IDA group (24.7% [16.5-36.1] and 32.5% [18.6-49.8], respectively) and the healthy group (1.45% [0.9-2.5] and 0.9% [0.5-1.6]).
Consequently, the derived MH ratio was significantly (p<0.001) lower in the IDA group (0.7 [0.7-0.8]) compared to the BT group (1.02 [0.9-1.1]) and the healthy group (1.57 [1.2-1.8]).
ROC analysis confirmed the poor discriminatory power of RET-He (AUC: 0.197; 95% CI: 0.11–0.28; p<0.001) and Delta-CH (AUC: 0.45; 95% CI: 0.34–0.57; p=0.38). The ROC curves for Micro% and Hypo% showed an AUC of 0.93 (95% CI: 0.88–0.98) and 0.83 (95% CI: 0.75–0.9); p<0.0001, respectively. The MH ratio demonstrated an AUC of 0.84 (95% CI: 0.72–0.88; p<0.0001) (Figure1).
A Micro% cut-off above 39.3% confirmed BT, achieving 96.6% sensitivity, 83.1% specificity, a 70.7% positive predictive value (PPV), and a 98.3% negative predictive value (NPV), with an overall accuracy of 96.6%.
A Hypo% threshold above 52.65% confirmed BT, achieving 76.6% sensitivity, 83.1% specificity, a 65.7% PPV, and an 89.3% NPV, with an overall accuracy of 76.6%.
An MH ratio cut-off of 0.85 confirmed the diagnosis of BT, yielding 86% sensitivity, 77.4% specificity, and a 93.2% NPV, with an overall accuracy of 86.6%.

**Summary/Conclusion**

The MH ratio is a simple, rapid, and powerful biomarker that could be integrated into the routine workup of microcytic anemia to guide appropriate clinical management.

**Copyright Information:** (Online) ISSN: 2572-9241
©2026 The Author(s). HemaSphere is published by John Wiley & Sons Ltd on behalf of European Hematology Association. This is an open access Abstract Book distributed under the Attribution-NonCommercial-NoDerivs (CC BY-NC-ND), which allows third parties to download the articles and share them with others as long as they credit the author and the Abstract Book, but they cannot change the content in any way or use them commercially.
**Abstract Book Citations:** Authors, Title, HemaSphere, 2026;10:(S1):pages. The abstract book DOIs can be found on the "Author Information" page.
**Disclaimer:** Articles published in the journal HemaSphere exclusively reflect the opinions of the authors. The authors are responsible for all content in their abstracts including accuracy of the facts, statements, citing resources, etc.

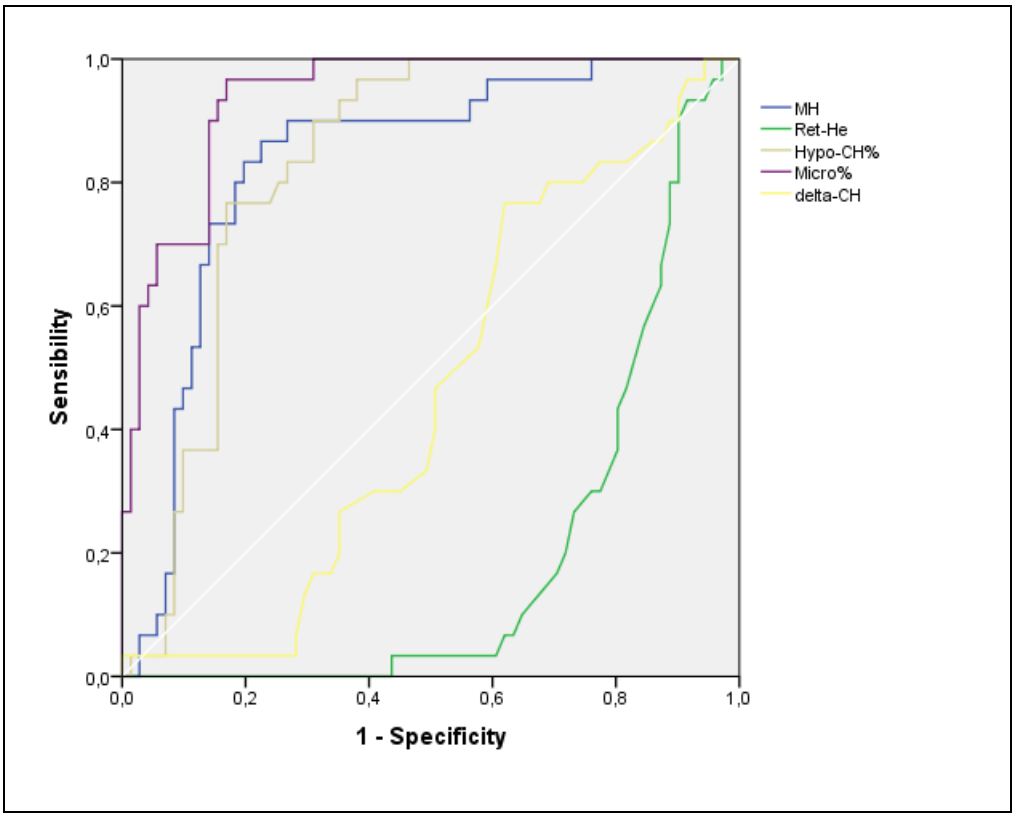

ROC curve analysis of RET-He, Delta-CH, Micro%, Hypo%, and the MH ratio for distinguishing beta-thalassemia trait from iron deficiency anemia

**Copyright Information:** (Online) ISSN: 2572-9241
©2026 The Author(s). HemaSphere is published by John Wiley & Sons Ltd on behalf of European Hematology Association. This is an open access Abstract Book distributed under the Attribution-NonCommercial-NoDerivs (CC BY-NC-ND), which allows third parties to download the articles and share them with others as long as they credit the author and the Abstract Book, but they cannot change the content in any way or use them commercially.
**Abstract Book Citations:** Authors, Title, HemaSphere, 2026;10:(S1):pages. The abstract book DOIs can be found on the "Author Information" page.
**Disclaimer:** Articles published in the journal HemaSphere exclusively reflect the opinions of the authors. The authors are responsible for all content in their abstracts including accuracy of the facts, statements, citing resources, etc.

### (PB4166) HYDROXYUREA IN REAL-WORLD SICKLE CELL DISEASE CARE: THREE-CENTER GREEK COHORT SHOWS FEWER PAIN CRISES AND HOSPITALIZATIONS, INCLUDING TRANSFUSED PATIENTS

**Topic:** 28. Thalassemias

Sofia Delicou[1], Aikaterini Poulaki*[2], Maria Moraki[1], Despina Papadopoulou[3], Petroula Krestinidi[4], Aikaterini Chatzouli[4], Lazaros Karaloizos[4], Aikaterini Xydaki[1], Despoina Pantelidou[3], Michail Diamantidis[4]

*[1]Hippokrateio General Hospital, Thalassemia and Sickle Cell Unit, Expertise Center of Hemoglobinopathies and Their Complications, Athens, Greece; [2]National and Kapodistrian University of Athens, 2nd Dept of Internal Medicine, Athens, Greece; [3]AHEPA University Hospital, Thalassemia Unit, 1st Department of Internal Medicine, Thessaloniki, Greece; [4]General Hospital of Larissa, Thalassemia and Sickle Cell Disease Unit, Department of Hematology, Larissa, Greece;*

### Background

Hydroxyurea (HU) reduces vaso-occlusive morbidity and improves hematologic markers in sickle cell disease (SCD), despite its' well-known bone marrow myelosuppression. Real-world multicenter data from Greece, including transfusion subgroups, are limited.

### Aims

To evaluate HU impact on (i) pain crises/year and hospitalizations/year (pre-HU vs on-HU) and (ii) hematologic parameters (baseline vs last: Hb, HbS, HbF, MCV). Secondarily, to assess whether response differs by transfusion status and to describe HU dose, duration, and adherence.

### Methods

A retrospective multicenter study from three Greek SCD centers including 161 patients was conducted. Age was calculated using 2025 as reference year. HU exposure included ~~start~~ the year of HU initiation, the current/interruption year (HU duration), initial and maximum total HU dose (mg/day), and adherence (%). Within-patient comparisons ~~used~~ the Wilcoxon signed-rank test (two-sided) was used. Patients were classified as transfused if transfusions/year >0 during follow-up.

**Copyright Information:** (Online) ISSN: 2572-9241
©2026 The Author(s). HemaSphere is published by John Wiley & Sons Ltd on behalf of European Hematology Association. This is an open access Abstract Book distributed under the Attribution-NonCommercial-NoDerivs (CC BY-NC-ND), which allows third parties to download the articles and share them with others as long as they credit the author and the Abstract Book, but they cannot change the content in any way or use them commercially.
**Abstract Book Citations:** Authors, Title, HemaSphere, 2026;10:(S1):pages. The abstract book DOIs can be found on the "Author Information" page.
**Disclaimer:** Articles published in the journal HemaSphere exclusively reflect the opinions of the authors. The authors are responsible for all content in their abstracts including accuracy of the facts, statements, citing resources, etc.

### Results

Median age was 54 years (IQR 45.8-61.0); 104 female, 56 male (1 missing). Genotypes: S/β n=137, S/S n=21, S/β/ααα n=2 (1 missing). Median HU duration was 13 years (IQR 8-17). Adherence was high (median 100%, IQR 95-100). Initial HU dose was 500 mg/day (IQR 500-775, range 250-1500) and maximum HU dose 1000 mg/day (IQR 750-1000, range 250-1500).

Pain crises/year decreased from median 3 (IQR 1-4) pre-HU to 1 (IQR 0-2) on-HU (p<0.001). Hospitalizations/year decreased from 1 (IQR 0-2) to 0 (IQR 0-1) (p<0.001). Although HbF and MCV increased (both p<0.001), while HbS decreased (p<0.001); Hb showed no significant change (p=0.354).

Transfusion subgroup analysis was the following: non-transfused n=110, transfused n=46 (missing transfusion data n=5). Both subgroups showed significant reductions in pain crises and hospitalizations (non-transfused: both p<0.001; transfused: pain p=0.000006, hospitalizations p=0.000061) and significant increases in HbF and MCV (non-transfused: both p<0.001; transfused: HbF p=0.000284, MCV p=0.006588). Change-score comparisons between transfused vs non-transfused were not significant (all p>0.05).

| Outcome category | Outcome | Pre/Baseline Median (IQR) | On-HU/Last Median (IQR) | p-value |
|---|---|---|---|---|
| Clinical | Pain crises/year | 3 (1-4) | 1 (0-2) | <0.001 |
| Clinical | Hospitalizations/year | 1 (0-2) | 0 (0-1) | <0.001 |
| Hematologic | Hb (g/dL) | 9.15 (8.0-10.2) | 9.40 (8.2-10.1) | 0.354 |
| Hematologic | HbS (%) | 72.1 (66.0-78.0) | 64.4 (50.4-71.4) | <0.001 |
| Hematologic | HbF (%) | 8.3 (4.1-14.1) | 13.4 (8.3-21.8) | <0.001 |
| Hematologic | MCV (fL) | 75.9 (69.5-87.7) | 84.3 (78.0-92.0) | <0.001 |

### Summary/Conclusion

In a three-center Greek real-world SCD cohort with long HU exposure, high adherence, and titration up to ~1000 mg/day were observed. HU was associated with clinically meaningful reductions in pain crises and hospitalizations and expected hematologic responses (↑HbF, ↑MCV, ↓HbS). Benefits were consistent in transfused and non-transfused patients, without evidence of differential response by transfusion status.

**Copyright Information:** (Online) ISSN: 2572-9241
©2026 The Author(s). HemaSphere is published by John Wiley & Sons Ltd on behalf of European Hematology Association. This is an open access Abstract Book distributed under the Attribution-NonCommercial-NoDerivs (CC BY-NC-ND), which allows third parties to download the articles and share them with others as long as they credit the author and the Abstract Book, but they cannot change the content in any way or use them commercially.
**Abstract Book Citations:** Authors, Title, HemaSphere, 2026;10:(S1):pages. The abstract book DOIs can be found on the “Author Information” page.
**Disclaimer:** Articles published in the journal HemaSphere exclusively reflect the opinions of the authors. The authors are responsible for all content in their abstracts including accuracy of the facts, statements, citing resources, etc.

### (PB4167) TOO LATE TO DIAGNOSE: PATTERNS OF DELAY IN ADULT HAEMOGLOBINOPATHIES

**Topic:** 28. Thalassemias

Aikaterini Poulaki*[1], Aikaterini Xydaki[2], Maria Moraki[2], Maria Kozanitou[2], Sofia Papagianni[2], Iliana Mani[2], Christos Savvidis[2], Elena Papatheodorou[2], Sabrina Goga[2], Georgia-Ioanna Kokkineli[2], Argiri Vlachopoulou[2], Ioannis Ilias[2], Constantina Angeli[2], Ioannis Koskinas[2], Sophia Delicou[2]

*[1]National and Kapodistrian University of Athens, 2nd Dept of Internal Medicine, Athens, Greece;[2]Hippokrateio General Hospital, Thalassemia and Sickle Cell Unit, Expertise Center of Hemoglobinopathies and Their Complications, Athens, Greece;*

### Background

Hemoglobinopathies are inherited diseases caused primarily by mutations affecting the globin genes. Being themselves the most frequent genetic pathogenies in humans, it is of no surprise that hemoglobinopathy heterozygotes consist of up to 15% of the population in areas with historically endemic malaria. Among the various clinical syndromes that exist, thalassemias and sickle cell disease (SCD) are by far the most common. Owing to the vigorous newborn screening and targeted prenatal counseling along with the severe clinical phenotype of nearly all homozygous states, most cases of hemoglobinopathies are readily diagnosed in infancy or early childhood. Being therefore diagnosed in adulthood is rare, at least in high income countries, and almost always follows a painstaking and yearlong process of diagnostic inadequacy, unresolved symptoms, healthcare costs and affected QoL.

### Aims

To describe the clinical phenotypes of late diagnosed hemoglobinopathies. To raise awareness of this possibly distinct and polymorphous clinical entity. To gain insight into the genetic variability of the disease.

### Methods

We retrospectively studied 19 adult patients that were diagnosed with haemoglobinopathies at the expertise center of hemoglobinopathies of a tertiary Greek hospital during the last decade. Median age at diagnosis was 39.0 years (IQR 31.0 - 53.5); 5 patients (26.3%) experienced very late diagnosis (≥54 years). Laboratory evaluation, clinical phenotype as well HBB and HBA1/2 genotyping are hereby presented (Table 1).

### Results

Combined α/β genotypes, as compound heterozygosity, comprised 63.2% of all cases. 3 out of 19 patients were compound SCD-β+ heterozygotes. Key laboratory values at diagnosis were as follows: median Hgb 9.7 g/dL (IQR 9.0-10.9), MCV 63.6 fL (61.8- 67.6), RDW 19.5% (16.6-21.8), sFerritin 201.0 (89.6–283.9) while 2 out of 19 patients had already had clinically apparent, MRI evaluated, hemochromatosis. Frequently missed red flags included hematinic factor supplementation refractory microcytic anemia (63.2%), splenomegaly (21.1%), early onset cholelithiasis (15%), inexplicable bone pain and/or arthralgias (16%). 2 out of 19 patients had transfusion dependent anemia of unknown cause, 1 patient had already received erythropoiesis stimulating agents (ESA) to correct the anemia. Patients had already consulted a median of 2 specialties before diagnosis, always including an internist while for 4 of them their diagnostic process initiated in childhood. All patients had abnormal Hb electrophoresis at first consultation. See also the Table.

**Copyright Information:** (Online) ISSN: 2572-9241
©2026 The Author(s). HemaSphere is published by John Wiley & Sons Ltd on behalf of European Hematology Association. This is an open access Abstract Book distributed under the Attribution-NonCommercial-NoDerivs (CC BY-NC-ND), which allows third parties to download the articles and share them with others as long as they credit the author and the Abstract Book, but they cannot change the content in any way or use them commercially.
**Abstract Book Citations:** Authors, Title, HemaSphere, 2026;10:(S1):pages. The abstract book DOIs can be found on the "Author Information" page.
**Disclaimer:** Articles published in the journal HemaSphere exclusively reflect the opinions of the authors. The authors are responsible for all content in their abstracts including accuracy of the facts, statements, citing resources, etc.

**Summary/Conclusion**

In this cohort of 19 adults with late-diagnosed haemoglobinopathies (median age at diagnosis 39.0 years; 26.3% ≥54 years), diagnostic delay followed a reproducible pattern: persistent iron-refractory microcytosis/anemia (63.2%) was repeatedly managed as hematinic deficiency, while multisystem clues (e.g., splenomegaly, early cholelithiasis, bone pain/arthralgias) were under-recognized. All patients had abnormal Hb electrophoresis at first specialist consultation, indicating missed opportunities for earlier recognition. Routine Hb fractionation/HPLC and prompt genotyping, should be considered in adults with iron-refractory microcytosis, particularly when systemic features coexist, to avoid inappropriate therapies and prevent complications such as iron overload.

| Patiet ID | Age at Diagnosis | Phenotype | Gene1 | Gene2 | Gene3 |
|---|---|---|---|---|---|
| P1 | 39 | Intermedia | HBB: c.-151C>T | HBB: c.92+1G>A | Triple a (anti3.7) |
| P2 | 68 | Intermedia | HBB: c.92+1G>A | Triple a (anti3.7) | |
| P3 | 33 | Intermedia | HBB: c.92+1G>A | Triple a (anti3.7) | |
| P4 | 44 | HBH disease (mild) | -(a)20.5 | -a3.7 | |
| P5 | 74 | HBH disease (mild) | -(a)20.5 | -a3.7 | |
| P6 | 30 | SCD | HBB: c.20A>T | Sicilian δβ | |
| P7 | 28 | Intermedia | HBB: c.92+1G>A | Triple a (anti4.2) | |
| P8 | 32 | Intermedia | HBB: c.92+1G>A | Triple a (anti3.7) | |
| P9 | 32 | Intermedia | HBB: c.92+1G>A | HBA2: c.231G>A | |
| P10 | 75 | SCD | HBB: c.20A>T | HBB: c.118C>T | -a3.7 |
| P11 | 52 | Intermedia | HBB: c.92+1G>A | Triple a (anti3.7) | |
| P12 | 29 | Intermedia | HBB: c.92+6T>C | IVS1-n6/polyA | |
| P13 | 24 | SCD | HBB: c.20A>T | C.388G>C (HBB Crete) | |
| P14 | 29 | Intermedia | HBB: c.92+1G>A | HBB: c. *96T>C | |
| P15 | 45 | Intermedia | HBB c.9321 G>A | Triple a (anti3.7) | |
| P16 | 44 | Intermedia | HBB: c.118C>T | Triple a (anti3.7) | |
| P17 | 57 | Intermedia | HBB: c.92+1G>A | Triple a (anti4.2) | |
| P18 | 34 | Intermedia | HBB: c.92+1G>A | Triple a (anti3.7) | |
| P19 | 55 | Intermedia | Sicilian δβ | HBA2: c.*+94A>G (aPolyAa/aa) | |

**Copyright Information:** (Online) ISSN: 2572-9241
©2026 The Author(s). HemaSphere is published by John Wiley & Sons Ltd on behalf of European Hematology Association. This is an open access Abstract Book distributed under the Attribution-NonCommercial-NoDerivs (CC BY-NC-ND), which allows third parties to download the articles and share them with others as long as they credit the author and the Abstract Book, but they cannot change the content in any way or use them commercially.
**Abstract Book Citations:** Authors, Title, HemaSphere, 2026;10:(S1):pages. The abstract book DOIs can be found on the "Author Information" page.
**Disclaimer:** Articles published in the journal HemaSphere exclusively reflect the opinions of the authors. The authors are responsible for all content in their abstracts including accuracy of the facts, statements, citing resources, etc.

### (PB4168) DISTRIBUTION AND CLINICAL CORRELATES OF HEPATIC FAT IN REGULARLY TRANSFUSED NON-TRANSFUSION-DEPENDENT THALASSEMIA PATIENTS

**Topic:** 28. Thalassemias

Antonella Meloni*[1], Laura Pistoia[1], Vincenzo Positano[1], Angelica Barone[2], Marilena Serra[3], Valentina Carrai[4], Piera Giovangrossi[5], Paola Maria Grazia Sanna[6], Maria Grazia Roberti[7], Michela Zerbini[8], Massimiliano Missere[9], Andrea Barison[1]

*[1]Fondazione G. Monasterio CNR-Regione Toscana, Pisa, Italy; [2]Azienda Ospedaliero-Universitaria di Parma, Parma, Italy; [3]Ospedale "V. Fazzi", Lecce, Italy; [4]Azienda Ospedaliero - Universitaria Careggi, Firenze, Italy; [5]Ospedale S. M. Goretti, Latina, Italy; [6]Azienda Ospedaliero-Universitaria di Sassari, Sassari, Italy; [7]Azienda Ospedaliero-Universitaria OO.RR. Foggia, Foggia, Italy; [8]Ospedale del Delta, Lagosanto (FE), Italy; [9]Responsible Research Hospital, Campobasso, Italy;*

### Background

Patients with non-transfusion-dependent thalassemia (NTDT) who initiate regular transfusion therapy (RT-NTDT) later in life present a unique clinical phenotype, characterized by the interplay between chronic anemia and secondary transfusional iron loading. While iron-mediated organ damage is well-documented, the prevalence and clinical significance of liver steatosis (LS) in this population remain poorly defined.

### Aims

This multicenter, cross-sectional study aimed to quantify hepatic fat fraction (FF) using magnetic resonance imaging (MRI) in RT-NTDT patients and to evaluate its associations with demographic, clinical, and biochemical parameters, tissue iron overload, and glucose metabolism alterations.

### Methods

Ninety-nine RT-NTDT patients (61.6% female; mean age 47.13±13.66years) from the Extension-Myocardial Iron Overload in Thalassemia (E-MIOT) Network underwent standardized MRI for the assessment of hepatic, pancreatic, and cardiac iron, as well as hepatic FF. All patients were receiving regular transfusions (>4 units/year) and chelation therapy. Results were compared with 301 patients with transfusion-dependent thalassemia (TDT). Glucose metabolism was evaluated via the oral glucose tolerance test (OGTT).

### Results

Despite comparable liver iron concentration (LIC) levels, RT-NTDT patients exhibited a significantly lower hepatic FF than TDT patients (1.29±2.67% vs. 5.01±7.69%; $p<0.0001$).
In the RT-NTDT cohort, hepatic FF did not correlate with sex, age, splenectomy status, or body mass index. Furthermore, no significant differences in FF were observed based on hepatitis C virus status. While FF showed no correlation with serum ferritin, triglycerides, or cholesterol levels, it was significantly associated with the ALT/AST ratio ($R=0.334$; $p=0.011$). No correlations were found between hepatic FF and MRI-derived LIC, pancreatic $R2^*$, or cardiac $R2^*$ values.
Disturbances in glucose metabolism were present in 23.5% of patients. While LIC was similar between patients with normal and impaired glucose tolerance, the presence of glucose metabolism alterations was associated with significantly higher hepatic FF (2.99±3.92% vs. 0.94±2.22%; $p=0.009$) (Figure 1A) and global pancreatic $R2^*$ (89.69±68.19 Hz vs. 65.97±51.85 Hz; $p=0.026$).
ROC analysis demonstrated that a hepatic FF>0.82% predicted an abnormal OGTT with 55.0% sensitivity and 80.0% specificity (AUC=0.69). Similarly, a pancreatic $R2^*$>38.66 Hz predicted an abnormal OGTT with 95.0% sensitivity and 40.6% specificity (AUC=0.67). There was no difference in discriminatory power between these two parameters ($p=0.779$) (Figure 1B).

**Copyright Information:** (Online) ISSN: 2572-9241
©2026 The Author(s). HemaSphere is published by John Wiley & Sons Ltd on behalf of European Hematology Association. This is an open access Abstract Book distributed under the Attribution-NonCommercial-NoDerivs (CC BY-NC-ND), which allows third parties to download the articles and share them with others as long as they credit the author and the Abstract Book, but they cannot change the content in any way or use them commercially.
**Abstract Book Citations:** Authors, Title, HemaSphere, 2026;10:(S1):pages. The abstract book DOIs can be found on the "Author Information" page.
**Disclaimer:** Articles published in the journal HemaSphere exclusively reflect the opinions of the authors. The authors are responsible for all content in their abstracts including accuracy of the facts, statements, citing resources, etc.

### Summary/Conclusion

In RT-NTDT patients, hepatic fat accumulation is generally low but serves as a significant surrogate marker of metabolic dysfunction. Our findings suggest a synergistic role for hepatic steatosis and pancreatic iron in the pathogenesis of dysglycemia. Even minimal increases in hepatic FF may reflect early insulin resistance, suggesting that MRI-based fat quantification can complement pancreatic R2* for a more comprehensive metabolic risk assessment.

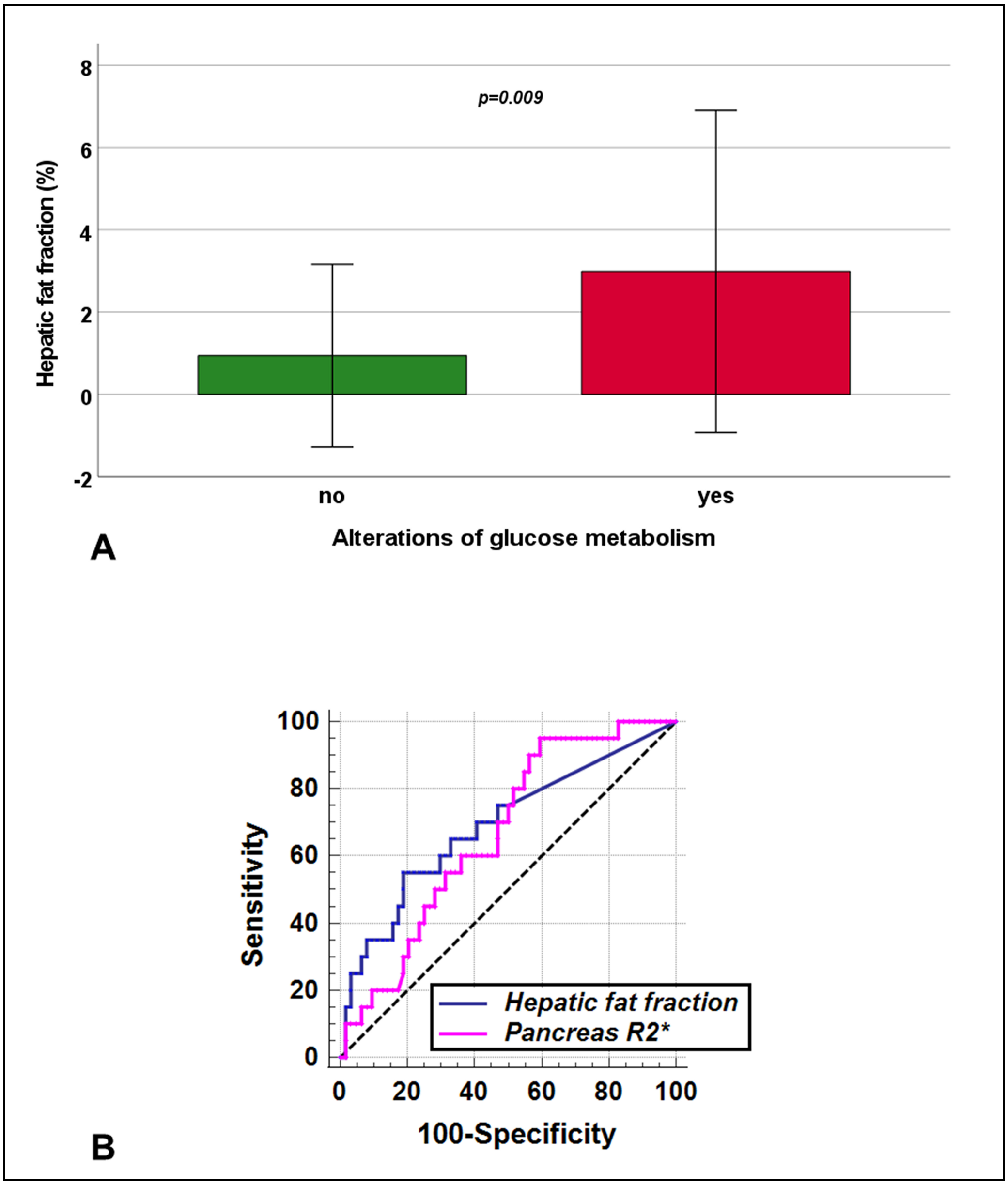

Figure 1. A) Comparison of hepatic fat fraction between RT-NTDT patients without and with alterations of glucose metabolism. Bar graphs show mean ± standard deviation. B) ROC curve analysis of hepatic fat fraction (blue) and global pancreas R2* (fuchsia pink) to identify an altered glucose metabolism in RT-NTDT patients.

**Copyright Information:** (Online) ISSN: 2572-9241
©2026 The Author(s). HemaSphere is published by John Wiley & Sons Ltd on behalf of European Hematology Association. This is an open access Abstract Book distributed under the Attribution-NonCommercial-NoDerivs (CC BY-NC-ND), which allows third parties to download the articles and share them with others as long as they credit the author and the Abstract Book, but they cannot change the content in any way or use them commercially.
**Abstract Book Citations:** Authors, Title, HemaSphere, 2026;10:(S1):pages. The abstract book DOIs can be found on the "Author Information" page.
**Disclaimer:** Articles published in the journal HemaSphere exclusively reflect the opinions of the authors. The authors are responsible for all content in their abstracts including accuracy of the facts, statements, citing resources, etc.

### (PB4169) HEMOGLOBIN VARIANTS DETECTED IN A TERTIARY HOSPITAL IN ARGENTINA: A 5YEAR EXPERIENCE

**Topic:** 28. Thalassemias

Debora Magali Assum[1], Catherine Vitali[1], José Amigone[1], Maria Laura Herrera Andino[2], Carlos Hollmann[2], Victoria Rojana[2], Luciana Guanchiale[2], Monica Freiberg[1], Santiago De Loredo[3], Ana Lisa Basquiera[2], Raul Capra[1]

*[1]Hospital Universitario de Córdoba, Laboratory, Córdoba, Argentina;[2]Hospital Universitario de Córdoba, Hematology service, Córdoba, Argentina; [3]Hospital Universitario de Córdoba, Diabetes, Córdoba, Argentina;*

### Background

Hemoglobinopathies are the most common monogenic disorders globally. More than a thousand hemoglobin (Hb) variants have been described, and their correct identification is essential due to their clinical implications and their potential to interfere with the diagnosis of other conditions, including diabetes mellitus. Accurate diagnosis requires an integrated strategy that combines clinical information with laboratory findings.

### Aims

The objective of this study was to analyze the Hb variants identified at the Hospital Privado Universitario de Córdoba over a 5year period and, based on this experience and current evidence, propose a diagnostic and clinical management approach for hematologists.

### Methods

This observational and descriptive study included whole blood samples collected between 2021 and 2025 in EDTA for hemoglobin evaluation. Samples were obtained either from patients undergoing routine hematological assessment due to suspicion of a hemoglobin variant, or from individuals in whom HbA1c measurement revealed an abnormal electrophoretic pattern. Hb variant analysis was performed using capillary electrophoresis with Capillarys 2 and 3 Octa systems (Sebia) and Phoresis v9.2.0 software.

### Results

A total of 842 patients were analyzed, of whom 253 (30%) presented altered profiles (Figure1A). The distribution of variants was as follows (Figure 1B): 60.4% showed elevated Hb A2 compatible with thalassemia, and among these, 30.7% also exhibited increased fetal Hb. Hb S was identified in 22.1% of patients, isolated Hb F in 9.5%, Hb C in 3.6%, Hb E in 2.4%, and Hb D in 1.2%. In 0.8% of cases, unidentified variants migrating in zone 11 were detected, representing candidates for genetic confirmation. Based on this series, a diagnostic and clinical pathway was proposed: i) Initial confirmation and characterization should include close collaboration between clinicians and laboratory staff, combined with a complete hematologic evaluation and a targeted clinical history addressing family background, ethnicity, anemia, transfusion history, and chronic disease; ii) Risk classification should differentiate asymptomatic carriers, who typically require education, preventive recommendations, and genetic counseling, from clinically significant forms that warrant referral to specialized hematology consultation; iii) Patientcentered interventions should include counseling for patients and families, incorporation into a hospital registry, and coordinated communication with other medical specialties to ensure adequate followup and address reproductive implications and chronic complications. To operationalize this workflow, an interdisciplinary group dedicated to hemoglobinopathies was established at our institution in 2026.

**Copyright Information:** (Online) ISSN: 2572-9241
©2026 The Author(s). HemaSphere is published by John Wiley & Sons Ltd on behalf of European Hematology Association. This is an open access Abstract Book distributed under the Attribution-NonCommercial-NoDerivs (CC BY-NC-ND), which allows third parties to download the articles and share them with others as long as they credit the author and the Abstract Book, but they cannot change the content in any way or use them commercially.
**Abstract Book Citations:** Authors, Title, HemaSphere, 2026;10:(S1):pages. The abstract book DOIs can be found on the "Author Information" page.
**Disclaimer:** Articles published in the journal HemaSphere exclusively reflect the opinions of the authors. The authors are responsible for all content in their abstracts including accuracy of the facts, statements, citing resources, etc.

**Summary/Conclusion**

In conclusion, Hb variants are frequent in clinical practice, and although many patients are asymptomatic, early identification is essential. The hematologist should serve as the central coordinator of the diagnostic and therapeutic process, ensuring diagnostic accuracy, effective communication, risk stratification, and the implementation of a structured action plan that ranges from prevention and genetic counseling to the management of longterm complications. A standardized institutional protocol such as the one proposed is crucial to guarantee equitable, highquality care for all individuals with hemoglobinopathies in our healthcare setting.

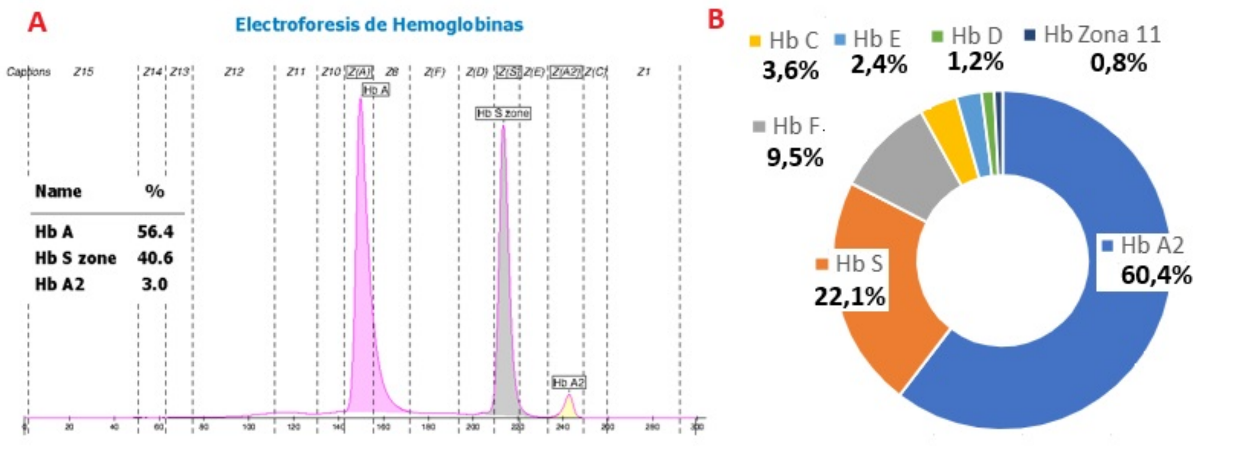

**A** Hemoglobin electrophoresis with HbA/S pattern. **B** Graph representing the observed distribution.

**Copyright Information:** (Online) ISSN: 2572-9241
©2026 The Author(s). HemaSphere is published by John Wiley & Sons Ltd on behalf of European Hematology Association. This is an open access Abstract Book distributed under the Attribution-NonCommercial-NoDerivs (CC BY-NC-ND), which allows third parties to download the articles and share them with others as long as they credit the author and the Abstract Book, but they cannot change the content in any way or use them commercially.
**Abstract Book Citations:** Authors, Title, HemaSphere, 2026;10:(S1):pages. The abstract book DOIs can be found on the "Author Information" page.
**Disclaimer:** Articles published in the journal HemaSphere exclusively reflect the opinions of the authors. The authors are responsible for all content in their abstracts including accuracy of the facts, statements, citing resources, etc.

### (PB4170) INTEGRATED HEMATOLOGICAL AND GENETIC ANALYSIS IDENTIFIES DELTA-BETA THALASSEMIA IN THE HUNGARIAN POPULATION

**Topic:** 28. Thalassemias

Agnes Mosolygo Lukacs*[1], Zoltan Mezei[1], Anita Bartha Tatar[1], Lilla Szabo[1], Janos Kappelmayer[1], Anikó Ujfalusi[2]

*[1]Unviversity of Debrecen, Faculty of Medicine, Deartment of Laboratory Medicine, Debrecen, Hungary;[2]Unviversity of Debrecen, Faculty of Medicine, Department of Medical Genetics, Debrecen, Hungary;*

### Background

Beta-thalassemia is an inherited hemoglobin disorder characterized by reduced or absent beta-globin chain synthesis, resulting in variable clinical phenotypes. It is primarily caused by point mutations in the beta-globin gene (*HBB*). However, approximately 5% of beta-hemoglobinopathies result from large, heterogeneous deletions affecting the human beta-globin gene cluster. Among these, delta-beta thalassemias represent a distinct subgroup characterized by deletions involving both the delta- and beta-globin genes, leading to compensatory elevation of fetal hemoglobin (HbF) levels. The clinical and hematological presentation of delta-beta thalassemia may overlap with classical beta-thalassemia trait, which can complicate differential diagnosis without comprehensive molecular analysis.

### Aims

Our aim was the comprehensive hematological and molecular genetic characterization of beta-thalassemia cases, with a focus on identifying rare deletion variants within the beta-globin gene cluster that may not be detected by standard sequencing methods.

### Methods

Laboratory investigation of beta-thalassemia included measurement of red blood cell indices using an automated hematology analyzer (HGB, HCT, MCV, MCHC). Quantification of hemoglobin fractions, including hemoglobin F (HbF) and hemoglobin $A_2$ ($HbA_2$), was performed by high-performance liquid chromatography (HPLC). Genetic analysis of the beta-globin gene (*HBB*) was carried out by Sanger sequencing. Cases with negative Sanger sequencing results were further analyzed by multiplex ligation-dependent probe amplification (MLPA) targeting the beta-globin gene cluster to detect copy number variations (CNVs).

### Results

Based on hematological and chromatographic findings, genetic testing was performed in 315 patients between 2022 and 2025. Pathogenic variants in the HBB gene were identified by sequencing in 255 cases (79%). Among the sequencing-negative cases (n = 60), MLPA was performed in 23 patients to detect CNVs. Large deletions involving the beta-globin gene cluster were identified in 18 of these cases: seven were Lepore-type deletions, while eleven were Sicilian-type deletions consistent with delta-beta thalassemia. Sicilian-type cases showed elevated HbF levels and normal $HbA_2$ values. Hematological parameters differed between the two subtypes: decreased MCV and HCT were characteristic of the Sicilian type, whereas normal values were observed in the Lepore type.

### Summary/Conclusion

The applied diagnostic algorithm, integrating comprehensive hematological and genetic findings, enables reliable differential diagnosis and allows detection of both point mutations and large deletions in the *HBB* gene as well as across the entire beta-globin gene cluster, thereby providing clinically relevant information for the evaluation of thalassemias.

**Copyright Information:** (Online) ISSN: 2572-9241
©2026 The Author(s). HemaSphere is published by John Wiley & Sons Ltd on behalf of European Hematology Association. This is an open access Abstract Book distributed under the Attribution-NonCommercial-NoDerivs (CC BY-NC-ND), which allows third parties to download the articles and share them with others as long as they credit the author and the Abstract Book, but they cannot change the content in any way or use them commercially.
**Abstract Book Citations:** Authors, Title, HemaSphere, 2026;10:(S1):pages. The abstract book DOIs can be found on the "Author Information" page.
**Disclaimer:** Articles published in the journal HemaSphere exclusively reflect the opinions of the authors. The authors are responsible for all content in their abstracts including accuracy of the facts, statements, citing resources, etc.

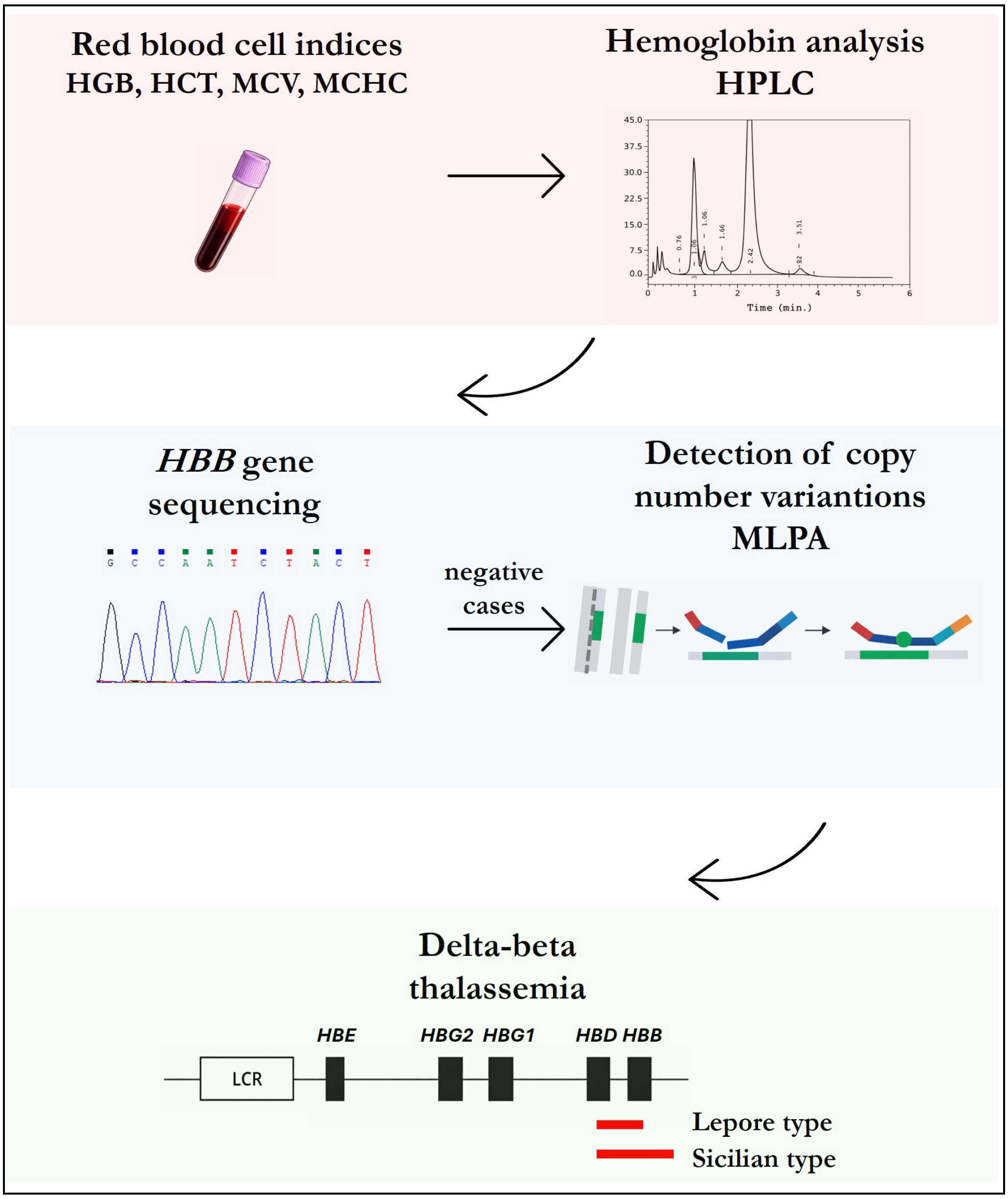

Diagnostic workflow of delta–beta thalassemia. Evaluation begins with red blood cell indices and hemoglobin analysis by HPLC, followed by HBB gene sequencing and MLPA for copy number variation analysis in negative cases, enabling identification of Lepore and Sicilian types.

**Copyright Information:** (Online) ISSN: 2572-9241
©2026 The Author(s). HemaSphere is published by John Wiley & Sons Ltd on behalf of European Hematology Association. This is an open access Abstract Book distributed under the Attribution-NonCommercial-NoDerivs (CC BY-NC-ND), which allows third parties to download the articles and share them with others as long as they credit the author and the Abstract Book, but they cannot change the content in any way or use them commercially.
**Abstract Book Citations:** Authors, Title, HemaSphere, 2026;10:(S1):pages. The abstract book DOIs can be found on the "Author Information" page.
**Disclaimer:** Articles published in the journal HemaSphere exclusively reflect the opinions of the authors. The authors are responsible for all content in their abstracts including accuracy of the facts, statements, citing resources, etc.

### (PB4171) INTERACTIVE CASE-BASED MEDICAL EDUCATION SIGNIFICANTLY IMPROVES COMPETENCE IN MANAGING HEMOGLOBINOPATHIES ACROSS THE LIFECYCLE

**Topic:** 28. Thalassemias

S Christy Rohani-Montez*[1], Sanneke Koekkoek[1], Tiphaine Barras[1], Rachel Kesse-Adu[2]

*[1]Medscape Education Global, London, United Kingdom;[2]Guy's and St Thomas' NHS Foundation Trust, London, United Kingdom;*

### Background

Because of the rarity of beta-thalassemia (TDT) and sickle cell disease (SCD) in Europe, many physicians are not familiar with these patients' needs across the disease course and at different stages of life.

### Aims

To improve best practices in managing patients with hemoglobinopathies across the lifecycle, we developed a CME-accredited interactive online case-based educational activity featuring 1 TDT case and 1 SCD case.

### Methods

Physicians worked through each case, answering questions along the way with evidence-based feedback regarding correct responses (https://www.medscape.org/viewarticle/across-lifecycle-patients-hemoglobinopathies-using-our-best-2023a1000x77/1). The education effects were assessed using a 3-question, repeated pairs, pre-assessment/post-assessment study design. One question assessed confidence. Differences from pre- to post-assessment were evaluated using McNemar's test. The activity launched in 02/2024 and data were collected through 12/2025.

### Results

1,417 physicians participated in the education, with 180 completing both cases and all pre- and post-assessment questions. Significant improvements were seen, with 63% of haematologists having correct responses at pre-assessment, improving to 87% post case completion (P<.001). The corresponding figures for paediatricians were 51% pre-assessment vs 76% at post-assessment (P<.001). Specifically, significant improvements were observed in recommending transfusion, assessing nonadherence to chelation therapy, and recommending gene therapy at various stages of life. When this cohort was split into those who managed <7 vs ≥7 patients, higher baseline knowledge and improvements were seen amongst those with the higher patient loads (figure).
After participating, 46% of haematologists and 56% of paediatricians had measurable improved confidence related to implementing appropriate treatment approaches for patients with hemoglobinopathies. Again, when the cohort was split by patient load, a higher confidence both at baseline and post-education was seen for those managing >7 patients (figure).

### Summary/Conclusion

This study demonstrates the success of simple online, case-based education in improving competence and implementation of appropriate interventions for patients with hemoglobinopathies. This could result in patients achieving their life stage-specific goals earlier and in overall improved outcomes.

**Copyright Information:** (Online) ISSN: 2572-9241
©2026 The Author(s). HemaSphere is published by John Wiley & Sons Ltd on behalf of European Hematology Association. This is an open access Abstract Book distributed under the Attribution-NonCommercial-NoDerivs (CC BY-NC-ND), which allows third parties to download the articles and share them with others as long as they credit the author and the Abstract Book, but they cannot change the content in any way or use them commercially.
**Abstract Book Citations:** Authors, Title, HemaSphere, 2026;10:(S1):pages. The abstract book DOIs can be found on the "Author Information" page.
**Disclaimer:** Articles published in the journal HemaSphere exclusively reflect the opinions of the authors. The authors are responsible for all content in their abstracts including accuracy of the facts, statements, citing resources, etc.

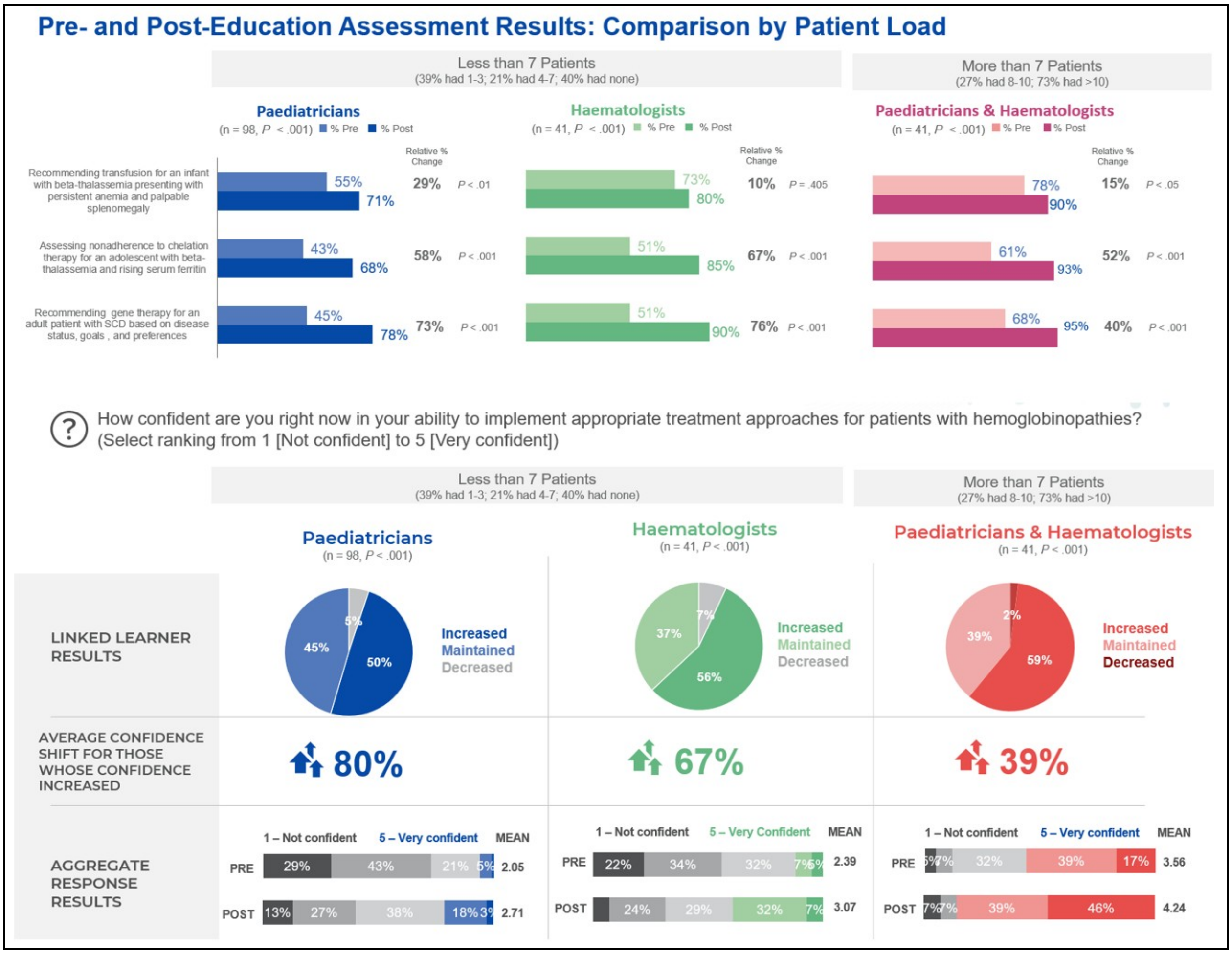

Figure

**Copyright Information:** (Online) ISSN: 2572-9241
©2026 The Author(s). HemaSphere is published by John Wiley & Sons Ltd on behalf of European Hematology Association. This is an open access Abstract Book distributed under the Attribution-NonCommercial-NoDerivs (CC BY-NC-ND), which allows third parties to download the articles and share them with others as long as they credit the author and the Abstract Book, but they cannot change the content in any way or use them commercially.
**Abstract Book Citations:** Authors, Title, HemaSphere, 2026;10:(S1):pages. The abstract book DOIs can be found on the “Author Information” page.
**Disclaimer:** Articles published in the journal HemaSphere exclusively reflect the opinions of the authors. The authors are responsible for all content in their abstracts including accuracy of the facts, statements, citing resources, etc.

### (PB4172) PHENOTYPIC AND GENOTYPIC ANALYSIS OF TWO RARE CASES OF --SEA/-Α⁴·³ HBH DISEASE RUNNING TITLE: ANALYSIS OF --SEA/-Α⁴·³ HBH DISEASE

**Topic:** 28. Thalassemias

Weijia Yang*[1], Xiaoyan Huang[2], Shiping Chen[2], Qingping Kang[1]

[1]Guilin People's Hospital, Department of Eugenics and Genetics, Guilin, China;[2]BGI Genomics Co., Ltd., Shenzhen, China;

### Background

Thalassemia is a group of hereditary autosomal recessive disorders characterized by defective synthesis of hemoglobin (Hb).The main types include α- and β-thalassemia,  Globally, 5%–20% of the population carries one or more α-thalassemia variants. Prevalence varies by region, being highest in tropical and subtropical countries, particularly in Southeast Asia and the Mediterranean region [1]. The Guangxi Zhuang Autonomous Region in China is a high-prevalence area for thalassemia, with a carrier rate as high as 20%–25% [2]. Zeng D et al. reported using multiplex ligation-dependent probe amplification (MLPA), Sanger sequencing, and single-molecule real-time sequencing to identify rare thalassemia genes. Among 55,281 samples in the Guilin area of Guangxi, 16,442 cases of thalassemia were confirmed (detection rate 29.74%), with detection rates for α-, β-, and composite α+β thalassemia being 18.57%, 9.99%, and 1.18%, respectively [3].
Currently, there are over 130 known α-thalassemia variant types[4]. Common thalassemia gene testing covers 90%–95% of variant types in the Chinese population, with a detection rate of 91.7% via common thalassemia gene testing. However, approximately 8.3% of rare variants remain missed due to technical limitations [5].
Based on the number of α-globin gene deletions, it can be classified into silent carrier, trait, HbH disease, and Hb Bart's hydrops fetalis. HbH disease is typically caused by the deletion or dysfunction of three α-genes, with clinical manifestations ranging from asymptomatic to transfusion-dependent anemia.

This study reports two unrelated adult males, common thalassemia testing yielded a result of α-thalassemia ($--^{SEA}/\alpha\alpha$) and β-thalassemia ($\beta^N/\beta^N$). Due to the inconsistency between the genotype and clinical phenotype, long-read sequencing was performed using the CycloneSEQ platform (single-molecule nanopore sequencing). They were ultimately confirmed to have rare deletional HbH disease: $--^{SEA}/-\alpha^{4.3}$.

### Aims

To report two cases of hemoglobin H (HbH) disease where the phenotype was inconsistent with routine genetic testing results, explore their true genotypes and clinical characteristics, and evaluate the application value of single-molecule nanopore sequencing (CycloneSEQ platform) in detecting rare thalassemia variants.

### Methods

Common thalassemia gene testing was performed on two adult male patients who presented with different complications. The results showed $--^{SEA}/\alpha\alpha$, which was significantly inconsistent with their hematological parameters. Subsequently, long-read sequencing of the α-globin gene cluster was conducted using the CycloneSEQ platform (single-molecule nanopore sequencing technology), and specific primers were designed based on the results for PCR verification.

**Copyright Information:** (Online) ISSN: 2572-9241
©2026 The Author(s). HemaSphere is published by John Wiley & Sons Ltd on behalf of European Hematology Association. This is an open access Abstract Book distributed under the Attribution-NonCommercial-NoDerivs (CC BY-NC-ND), which allows third parties to download the articles and share them with others as long as they credit the author and the Abstract Book, but they cannot change the content in any way or use them commercially.
**Abstract Book Citations:** Authors, Title, HemaSphere, 2026;10:(S1):pages. The abstract book DOIs can be found on the "Author Information" page.
**Disclaimer:** Articles published in the journal HemaSphere exclusively reflect the opinions of the authors. The authors are responsible for all content in their abstracts including accuracy of the facts, statements, citing resources, etc.

**Results**

The two patients were ultimately diagnosed with rare deletional HbH disease with the genotype $--^{SEA}/-\alpha^{4.3}$ (NC_000016.10:g.176935-181274DEL). The deletion range includes the α1 gene and is accompanied by an X-Y-Z segment recombination, which is reported here for the first time. Both patients exhibited varying degrees of anemia and complications, suggesting that the same genotype can manifest significant phenotypic heterogeneity.

**Summary/Conclusion**

The $-\alpha^{4.3}$ deletion is a newly discovered rare deletion type that cannot be detected by routine testing methods. Single-molecule nanopore sequencing technology effectively identifies rare variants, helping to improve the level of precise diagnosis of thalassemia.

**Copyright Information:** (Online) ISSN: 2572-9241
©2026 The Author(s). HemaSphere is published by John Wiley & Sons Ltd on behalf of European Hematology Association. This is an open access Abstract Book distributed under the Attribution-NonCommercial-NoDerivs (CC BY-NC-ND), which allows third parties to download the articles and share them with others as long as they credit the author and the Abstract Book, but they cannot change the content in any way or use them commercially.
**Abstract Book Citations:** Authors, Title, HemaSphere, 2026;10:(S1):pages. The abstract book DOIs can be found on the “Author Information” page.
**Disclaimer:** Articles published in the journal HemaSphere exclusively reflect the opinions of the authors. The authors are responsible for all content in their abstracts including accuracy of the facts, statements, citing resources, etc.

Figure5: Schematic diagram of the α-globin gene cluster (showing the -α4.3 deletion and X-Y-Z segment recombination)

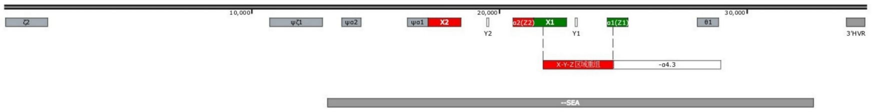

Figure 6: Agarose gel electrophoresis image of targeted amplification products

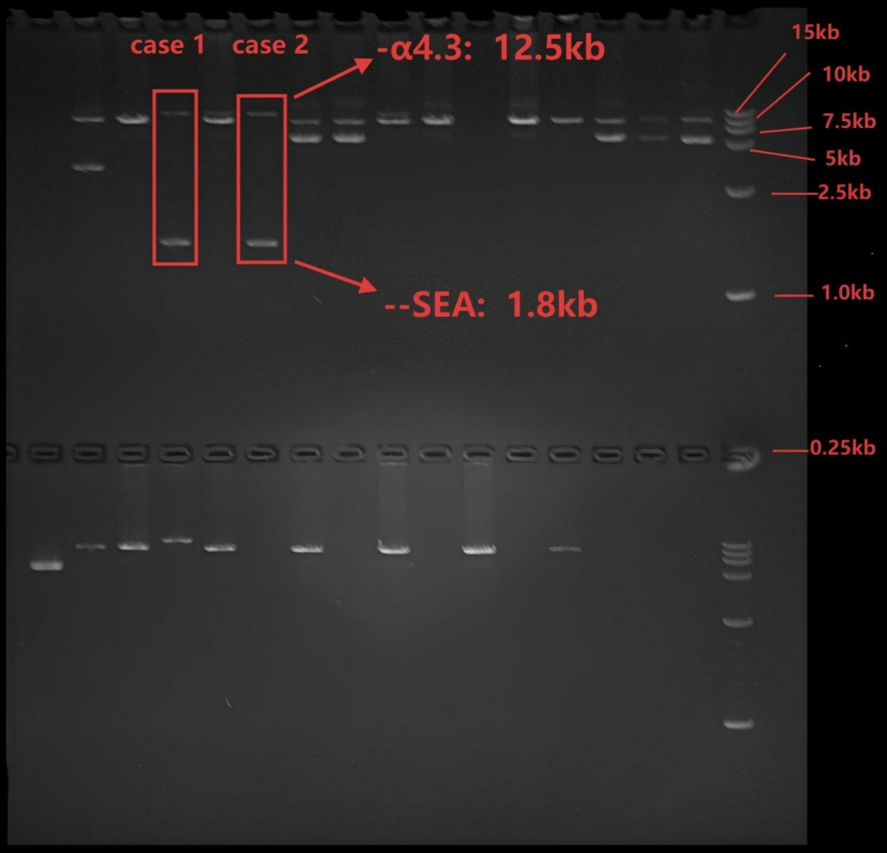

**Copyright Information:** (Online) ISSN: 2572-9241
©2026 The Author(s). HemaSphere is published by John Wiley & Sons Ltd on behalf of European Hematology Association. This is an open access Abstract Book distributed under the Attribution-NonCommercial-NoDerivs (CC BY-NC-ND), which allows third parties to download the articles and share them with others as long as they credit the author and the Abstract Book, but they cannot change the content in any way or use them commercially.
**Abstract Book Citations:** Authors, Title, HemaSphere, 2026;10:(S1):pages. The abstract book DOIs can be found on the "Author Information" page.
**Disclaimer:** Articles published in the journal HemaSphere exclusively reflect the opinions of the authors. The authors are responsible for all content in their abstracts including accuracy of the facts, statements, citing resources, etc.

### (PB4173) LIPID PROFILE IN TRANSFUSION DEPENDENT THALASSEMIA PEDIATRIC PATIENTS

**Topic:** 28. Thalassemias

Alkistis Adramerina*[1], Maria Pinelopi Tritou[1], Elpida Emmanouilidou-Fotoulaki[1], Labib Tarazi[2], Aikaterini Teli[1], Marina Economou[1]

*[1]Aristotle University of Thessaloniki, Thessaloniki, Greece; [2]Tomografia AE, Medical Center, Thessaloniki, Greece;*

### Background

Dyslipidemia is recognized in patients with transfusion-dependent thalassemia (TDT), although the pathophysiological mechanisms remain incompletely understood. Hypocholesterolemia has been attributed to increased erythropoietic activity and hepatic dysfunction secondary to iron overload. Similarly, hypertriglyceridemia has been associated with impaired extrahepatic lipolytic activity, resulting from increased oxidative stress induced by iron overload.

### Aims

To assess the lipid profile in pediatric patients with TDT and to evaluate possible associations with disease characteristics.

### Methods

Medical records of pediatric patients with TDT followed at a single referral center were reviewed. Collected data included demographics, age at initiation of transfusion therapy, chelation treatment, lipid profile parameters (total cholesterol [TC], LDL, HDL, apolipoprotein A1, apolipoprotein B, triglycerides, lipoprotein(a)), pre-transfusion hemoglobin levels, serum ferritin, liver iron concentration (LIC), and cardiac iron assessed by MRI T2*.

### Results

Fifteen patients were included (male:female ratio 1.1:1), with a mean age of 12 years (4–18) and mean BMI 18.8kg/m$^2$. Median age at initiation of transfusion was 23months and mean pre-transfusion Hb level was 9.3g/dL (8.7–10). As for chelation therapy, 7(46.7%) patients were on deferasirox, 4(26.7%) on deferiprone and the rest 4(26.7%) on deferioxamine.
Regarding iron overload, the median ferritin level was 875ng/mL (IQR 733). Mild and moderate liver hemosiderosis were documented in 7(46.7%) and 3(20%) patients, respectively, based on LIC measurements (mean LIC 5.1 Fe mg/g dw). No pathological findings were detected on cardiac MRI T2* (mean cardiac MRI T2* 34.8msec).
Lipid abnormalities were observed in 13 patients(86.7%), all of whom had abnormalities in at least two parameters measured. Decreased HDL was present in all affected patients, decreased TC in 11(78.6%), hypertriglyceridemia in 5(33.3%), decreased Apo A1 in 4(26.7%), and increased Apo B in 1(6.7%). No abnormalities were noted in LDL or Lp(a) levels.
Statistical analysis showed no significant correlation between lipid profile abnormalities and markers of iron overload (ferritin, LIC, cardiac T2*), pre-transfusion Hb, or chelation therapy.  Notably, hypocholesterolemia was significantly associated with higher body mass index (BMI) ($p = 0.015$), while normal Apo A1 levels were associated with younger age at initiation of transfusion therapy ($p = 0.06$).

**Copyright Information:** (Online) ISSN: 2572-9241
©2026 The Author(s). HemaSphere is published by John Wiley & Sons Ltd on behalf of European Hematology Association. This is an open access Abstract Book distributed under the Attribution-NonCommercial-NoDerivs (CC BY-NC-ND), which allows third parties to download the articles and share them with others as long as they credit the author and the Abstract Book, but they cannot change the content in any way or use them commercially.
**Abstract Book Citations:** Authors, Title, HemaSphere, 2026;10:(S1):pages. The abstract book DOIs can be found on the "Author Information" page.
**Disclaimer:** Articles published in the journal HemaSphere exclusively reflect the opinions of the authors. The authors are responsible for all content in their abstracts including accuracy of the facts, statements, citing resources, etc.

**Summary/Conclusion**

Previous pediatric studies have reported lipid profile abnormalities in TDT patients, however in the context of significant iron overload (ferritin >2000 ng/mL). The present report confirms the presence of dyslipidemia in children with TDT, albeit, in the absence of severe iron overload.
Further investigation is required to clarify the underlying mechanisms and determine the clinical significance, which may have implications for long-term cardiovascular risk and require management strategies.

**Copyright Information:** (Online) ISSN: 2572-9241
©2026 The Author(s). HemaSphere is published by John Wiley & Sons Ltd on behalf of European Hematology Association. This is an open access Abstract Book distributed under the Attribution-NonCommercial-NoDerivs (CC BY-NC-ND), which allows third parties to download the articles and share them with others as long as they credit the author and the Abstract Book, but they cannot change the content in any way or use them commercially.
**Abstract Book Citations:** Authors, Title, HemaSphere, 2026;10:(S1):pages. The abstract book DOIs can be found on the "Author Information" page.
**Disclaimer:** Articles published in the journal HemaSphere exclusively reflect the opinions of the authors. The authors are responsible for all content in their abstracts including accuracy of the facts, statements, citing resources, etc.

### (PB4175) Β-THALASSEMIA IN KAZAKHSTAN: MOLECULAR CHARACTERIZATION OF HBB MUTATIONS IN PATIENTS FROM CENTRAL AND NORTHERN REGIONS OUTSIDE THE THALASSEMIA BELT

**Topic:** 28. Thalassemias

Meruyert Satayeva*[1], Anton Klodzinsky[1], Aliya Baimurzina[1]

[1]*Hematology Center LLP, Hematology, Astana, Kazakhstan;*

### Background

Beta-thalassemia is endemic to the “thalassemia belt” (Mediterranean, Middle East, South/Southeast Asia). Kazakhstan, in Central Asia, is not traditionally considered part of this belt, yet clinically significant cases are increasingly recognized in central and northern regions. However, molecular data from these regions remain scarce, and diagnostic pathways are not standardized.

### Aims

To describe HBB variants detected in a consecutive referral-based cohort evaluated for suspected β-thalassemia in central/northern Kazakhstan and to guide development of a tiered diagnostic approach.

### Methods

We enrolled 37 consecutive referred patients with microcytic, hypochromic anemia in whom iron deficiency was excluded; a Mentzer index <13 was used as a screening enrichment criterion. HBB coding regions and exon–intron boundaries were analyzed by Sanger sequencing. Variants were interpreted using ACMG/AMP criteria. This approach does not detect large deletions/duplications (copy-number variants) or complex rearrangements and may miss regulatory lesions outside the sequenced regions.

### Results

Pathogenic/likely pathogenic HBB variants were identified in 17/37 patients (45.9%), comprising 13 distinct mutations, indicating high allelic heterogeneity within this referred series. The most frequent variants were IVS2-1G>A (c.315+1G>A, $\beta^0$; n=4, 23.5%) and CD8(-AA) (c.25_26delAA, $\beta^0$; n=3, 17.6%). $\beta^0$ alleles predominated (12/17, 70.6%), consistent with enrichment for clinically significant phenotypes in a referral cohort. Additional variants included IVS2-654 (c.316-197C>T, $\beta^+$), IVS1-1G>A ($\beta^0$), IVS1-2T>C ($\beta^+$), IVS1-110G>A ($\beta^+$), IVS1-130G>C, CD8/9(+G) ($\beta^0$), codon 17 (c.52A>T, $\beta^0$), codon 39 (c.118C>T, $\beta^0$), c.135del ($\beta^0$), and a CAP+20(C>T)/IVS2-745C>G (c.316-106C>G) combination in cis. No pathogenic/likely pathogenic HBB variant was identified in 20/37 (54.1%), suggesting non-HBB etiologies (e.g., α-thalassemia or other causes of microcytosis), undetectable HBB alterations, or both.

### Summary/Conclusion

This small, consecutive, referral-based series provides initial data on HBB variants observed among patients evaluated for microcytosis after iron deficiency exclusion in central/northern Kazakhstan and highlights substantial allelic heterogeneity. Given the proportion of molecularly unconfirmed cases and the limits of Sanger sequencing, a stepwise pathway incorporating deletion/duplication testing and α-globin and/or broader hemoglobinopathy panels is warranted. Larger studies with phenotypic correlation and population-based sampling are needed to define regional prevalence and genotype–phenotype relationships.

**Copyright Information:** (Online) ISSN: 2572-9241
©2026 The Author(s). HemaSphere is published by John Wiley & Sons Ltd on behalf of European Hematology Association. This is an open access Abstract Book distributed under the Attribution-NonCommercial-NoDerivs (CC BY-NC-ND), which allows third parties to download the articles and share them with others as long as they credit the author and the Abstract Book, but they cannot change the content in any way or use them commercially.
**Abstract Book Citations:** Authors, Title, HemaSphere, 2026;10:(S1):pages. The abstract book DOIs can be found on the “Author Information” page.
**Disclaimer:** Articles published in the journal HemaSphere exclusively reflect the opinions of the authors. The authors are responsible for all content in their abstracts including accuracy of the facts, statements, citing resources, etc.

### (PB4176) NATIONAL MAPPING OF BETA-THALASSEMIA TRAIT IN TUNISIA: EVALUATION OF ERYTHROCYTE INDICES AND IMPACT OF THRESHOLD ADAPTATION

**Topic:** 28. Thalassemias

Malek Haouari[1], Mohamed Yassine Kaabar[2], Sonia Chouaieb[3], Ikbel Ghachem*[4], Asma Bachali[4]

*[1]Laboratory of Biology, Taher Maamouri Hospital, Nabeul, Tunisia;[2]University of Monastir, Faculty of Pharmacy of Monastir, Laboratory of Biology, Tahar Maamouri Hospital,, Nabeul, Tunisia; [3]University of Monastir, Faculty of Pharmacy of Monastir, Laboratory of Biology, Habib Thamer Hospital, Tunis, Tunisia; [4]University of Tunis El Manar, Faculty of Medicine of Tunis, Laboratory of Biology, Tahar Maamouri Hospital, Nabeul, Tunisia;*

### Background

Beta-thalassemia is the most frequent hemoglobinopathy in Tunisia, with a historically estimated prevalence of 2.21%. This monogenic disorder, caused by defective β-globin chain synthesis, represents a major public health concern due to its clinical, social, and economic implications. Persistent high rates of consanguinity and internal migration sustain significant epidemiological pressure, particularly in inland regions. Given the high cost and limited accessibility of confirmatory tests such as hemoglobin electrophoresis and molecular analysis, erythrocyte indices derived from automated complete blood counts offer a simple, accessible, and cost-effective screening alternative.

### Aims

This study aimed to (i) generate an updated national map of beta-thalassemia trait (BTT) prevalence, (ii) assess the diagnostic performance of six commonly used erythrocyte indices, and (iii) evaluate the impact of population-specific threshold adaptation on screening sensitivity.

### Methods

A retrospective multicenter study was conducted in September 2023, including data from 26 hospitals across Tunisia. Six erythrocyte indices Mentzer, Ehsani, Shine & Lal, Srivastava, Ricerca, and RDWI, were calculated for each subject. Two sets of thresholds were compared: conventional international thresholds and thresholds adapted to the Tunisian population. Inter-index agreement was measured using Cohen's Kappa coefficient, and regional prevalence patterns were analyzed.

### Results

Data from major referral hospitals, revealed a national BTT prevalence of 3.7% with adapted thresholds compared to 2.5% with conventional thresholds. Mentzer, Ehsani, and Srivastava indices demonstrated the highest diagnostic reliability (Kappa > 0.80), whereas RDWI and Ricerca indices performed less effectively. Geographic mapping confirmed high endemicity in the Northwest and Central-West regions (Figure 1). Hardy–Weinberg calculations indicate a theoretical beta-thalassemia major frequency of approximately 0.035%, emphasizing the importance of systematic and early screening.

### Summary/Conclusion

Adapting erythrocyte index thresholds to the Tunisian population significantly improves screening sensitivity. Integration into automated laboratory systems can enable early triage, targeted genetic counseling, and optimized prenatal diagnosis. These findings also support the potential development of machine-learning algorithms to create population-specific predictive models, thereby enhancing the effectiveness of national prevention and public health strategies.

**Copyright Information:** (Online) ISSN: 2572-9241
©2026 The Author(s). HemaSphere is published by John Wiley & Sons Ltd on behalf of European Hematology Association. This is an open access Abstract Book distributed under the Attribution-NonCommercial-NoDerivs (CC BY-NC-ND), which allows third parties to download the articles and share them with others as long as they credit the author and the Abstract Book, but they cannot change the content in any way or use them commercially.
**Abstract Book Citations:** Authors, Title, HemaSphere, 2026;10:(S1):pages. The abstract book DOIs can be found on the "Author Information" page.
**Disclaimer:** Articles published in the journal HemaSphere exclusively reflect the opinions of the authors. The authors are responsible for all content in their abstracts including accuracy of the facts, statements, citing resources, etc.

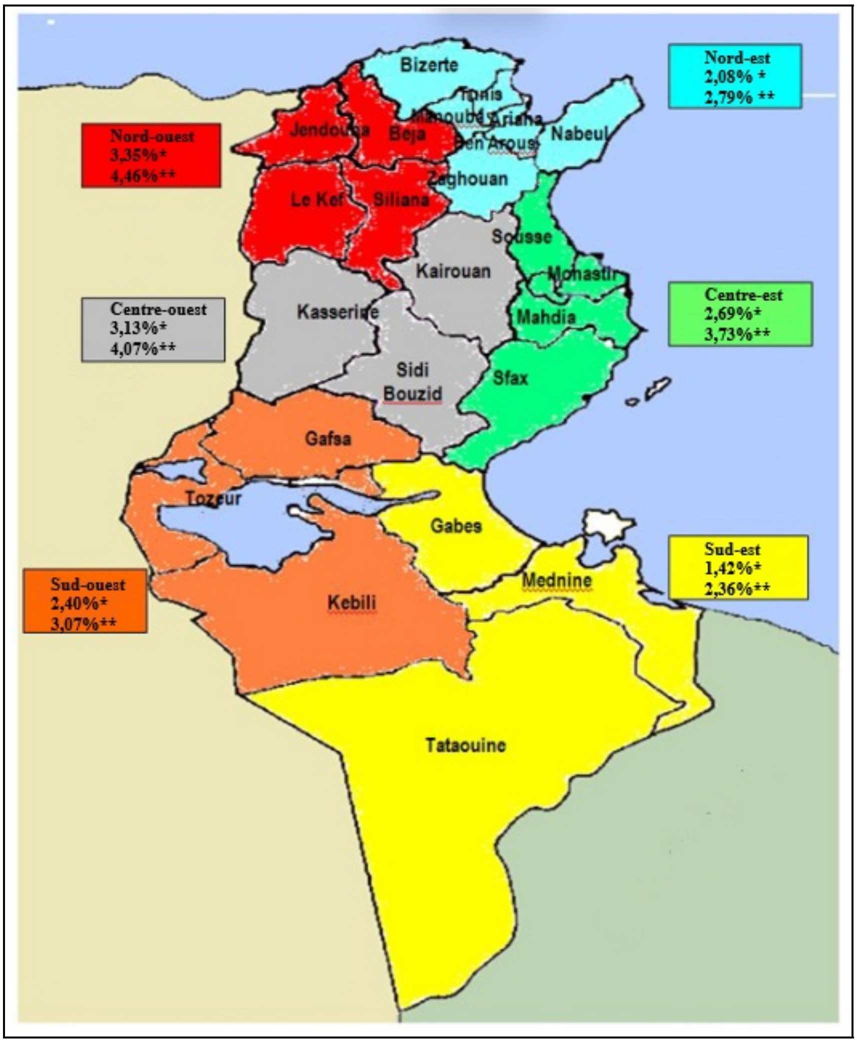

Figure 1 : Geographic mapping of Beta-Thalassemia Trait in Tunisia

Copyright Information: (Online) ISSN: 2572-9241
©2026 The Author(s). HemaSphere is published by John Wiley & Sons Ltd on behalf of European Hematology Association. This is an open access Abstract Book distributed under the Attribution-NonCommercial-NoDerivs (CC BY-NC-ND), which allows third parties to download the articles and share them with others as long as they credit the author and the Abstract Book, but they cannot change the content in any way or use them commercially.
Abstract Book Citations: Authors, Title, HemaSphere, 2026;10:(S1):pages. The abstract book DOIs can be found on the "Author Information" page.
Disclaimer: Articles published in the journal HemaSphere exclusively reflect the opinions of the authors. The authors are responsible for all content in their abstracts including accuracy of the facts, statements, citing resources, etc.

### (PB4177) AN INNOVATIVE APPROACH FOR THE EVALUATION OF RESPONSE TO LUSPATERCEPT IN TRANSFUSION-DEPENDENT BETA-THALASSEMIA

**Topic:** 28. Thalassemias

Daniele Lello Panzieri*[1, 2], Natalia Scaramellini[3, 4], Simona Leoni[3, 4], Ali T. Taher[5], Maria Cappellini[4], Irene Motta[3, 4]

*[1]Università degli Studi di Milano, Milano, Italy;[2]King's College London, Red Cell Haematology, School of Cancer and Pharmaceutical Sciences, Comprehensive Cancer Centre, London, United Kingdom; [3]Università Degli Studi Di Milano, Department of Clinical Sciences and Community Health, Dipartimento di Eccellenza 2023-2027, University of Milan, Milan, Italy, Milano, Italy; [4]Fondazione IRCCS Ca' Granda Ospedale Maggiore Policlinico, SC Medicina ad Indirizzo Metabolico, Milan, Italy; [5]American University of Beirut Medical Center, Division of Hematology and Oncology, Department of Internal Medicine, Beirut, Lebanon;*

### Background

Transfusion-dependent β-thalassemia is the most severe form of β-thalassemia, in which regular blood transfusions are needed to survive. The therapeutic landscape for this disease is in constant evolution, from a transfusion-only practice to a new-drug era. Luspatercept is actually approved for the treatment of anemia in both transfusion-dependent thalassemia (TDT) and non transfusion-dependent thalassemia.

### Aims

To propose a new approach for the evaluation of response to luspatercept in TDT patients.

### Methods

Careful review of the literature and implementation of a mathematical rule, with a new way for evaluating the response.

**Copyright Information:** (Online) ISSN: 2572-9241
©2026 The Author(s). HemaSphere is published by John Wiley & Sons Ltd on behalf of European Hematology Association. This is an open access Abstract Book distributed under the Attribution-NonCommercial-NoDerivs (CC BY-NC-ND), which allows third parties to download the articles and share them with others as long as they credit the author and the Abstract Book, but they cannot change the content in any way or use them commercially.
**Abstract Book Citations:** Authors, Title, HemaSphere, 2026;10:(S1):pages. The abstract book DOIs can be found on the "Author Information" page.
**Disclaimer:** Articles published in the journal HemaSphere exclusively reflect the opinions of the authors. The authors are responsible for all content in their abstracts including accuracy of the facts, statements, citing resources, etc.

**Results**

The definition of hematological response to luspatercept is a reduction in transfusion burden of at least 33% in any 12-week interval. This definition comes from the phase 3 clinical trial and was then adopted by both FDA and EMA and is currently used in common clinical practice.

The “33% reduction rule” from the phase 3 BELIEVE trial was based upon the expected resulting reduction of Liver Iron Concentration (LIC) following the reduction of transfusion burden. Indeed, secondary iron overload in transfusion-dependent thalassemia is a direct consequence of transfusions, containing approximately 200mg of iron for each blood bag. Knowing the number of blood bags transfused in a period and the expected reduction in transfusion burden allow to estimating the reduction of LIC, using the reverse Angelucci’s formula. Angelucci’s formula states a direct proportionality between body iron stores and LIC: total body iron = 10.6 x LIC. When applying this mathematical formula to our context, total body iron can be changed to iron overload, therefore defining how much that precise iron loading can impact the LIC. Knowing the LIC target reduction, this formula can generate the reduction of iron overload needed to achieve the endpoint.

Since iron overload is nowadays one of the greatest burdens of disease in transfusion-dependent thalassemia, evaluating the effectiveness of therapy with luspatercept in terms of expected reduction of LIC, makes much sense. This approach can therefore lead to the individualization of the threshold for response, based on each patient's needs.

In more practical terms, an LIC reduction of at least 3 mg/g dw in one year has been established as clinically meaningful by a board of experts and used as an endpoint in past relevant clinical trials. This threshold of 3 mg/g dw can be raised or lowered following the different clinical needs of each patient.

We can provide step-by-step calculations to obtain the individualized transfusion burden reduction (Figure 1).

This new approach can tailor the evaluation of response to luspatercept based on the iron overload of each patient and can also help in better defining those patients who will benefit from a longer-term treatment from those who should quickly discontinue the therapy.

It must be underlined that the patient's response may fluctuate over time. Moreover, the relationship between iron intake and iron overload is not always direct, as stated by Angelucci's formula, since it can be influenced by many factors, including adherence to iron chelation therapy and the effects of luspatercept itself.

**Summary/Conclusion**

In real-world experience, the evaluation of response to luspatercept poses many challenges and can be difficult to tailor to each patient’s needs. This new method for the evaluation of response in TDT patients, based on iron overload, introduces an innovative patient-centered approach.

**Copyright Information:** (Online) ISSN: 2572-9241
©2026 The Author(s). HemaSphere is published by John Wiley & Sons Ltd on behalf of European Hematology Association. This is an open access Abstract Book distributed under the Attribution-NonCommercial-NoDerivs (CC BY-NC-ND), which allows third parties to download the articles and share them with others as long as they credit the author and the Abstract Book, but they cannot change the content in any way or use them commercially.
**Abstract Book Citations:** Authors, Title, HemaSphere, 2026;10:(S1):pages. The abstract book DOIs can be found on the “Author Information” page.
**Disclaimer:** Articles published in the journal HemaSphere exclusively reflect the opinions of the authors. The authors are responsible for all content in their abstracts including accuracy of the facts, statements, citing resources, etc.

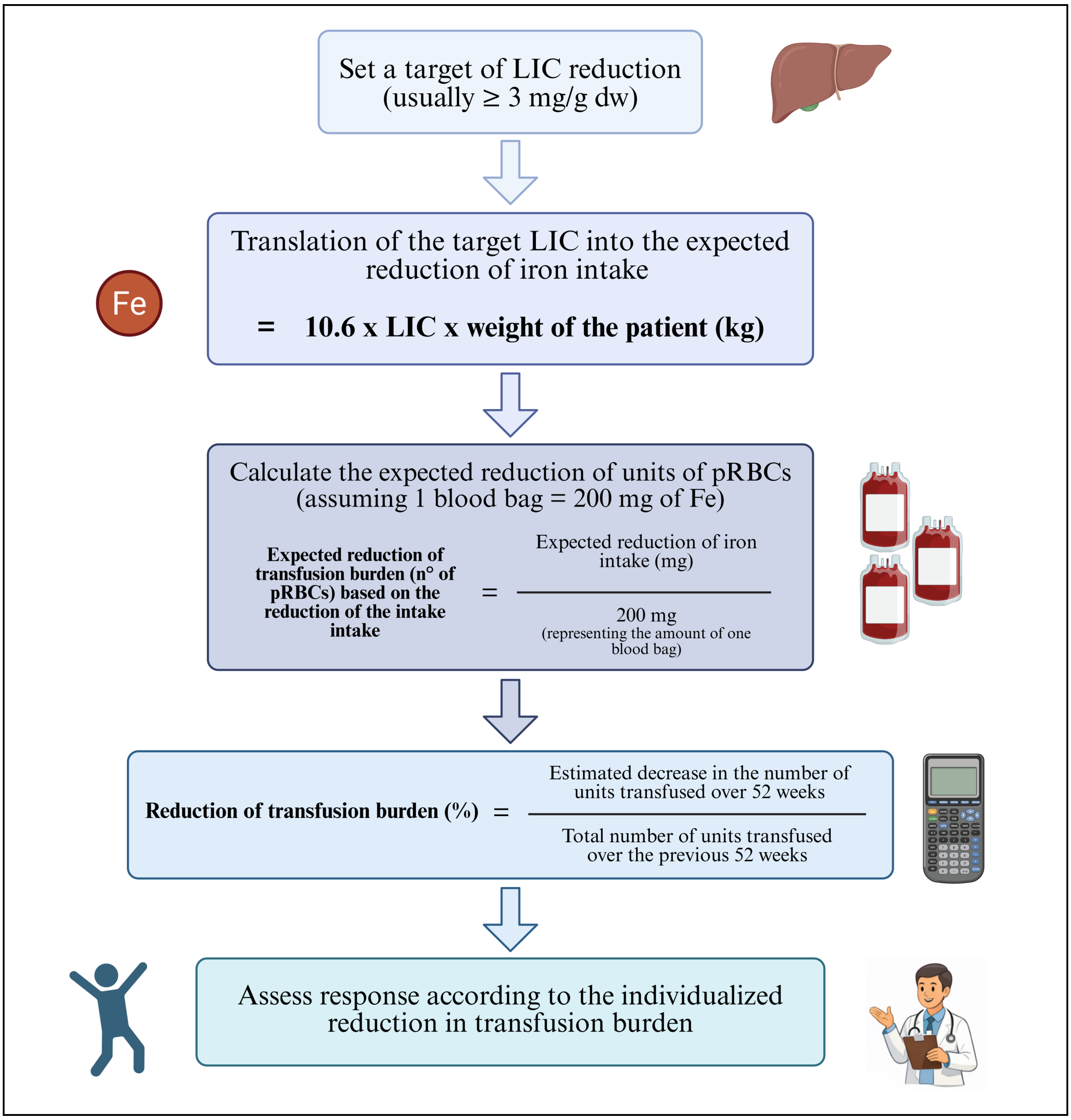

Figure 1. Individualized approach for the evaluation of response to luspatercept in TDT patients, based on expected reduction in the LIC consequent to the reduction of the transfusion burden.

Copyright Information: (Online) ISSN: 2572-9241
©2026 The Author(s). HemaSphere is published by John Wiley & Sons Ltd on behalf of European Hematology Association. This is an open access Abstract Book distributed under the Attribution-NonCommercial-NoDerivs (CC BY-NC-ND), which allows third parties to download the articles and share them with others as long as they credit the author and the Abstract Book, but they cannot change the content in any way or use them commercially.
Abstract Book Citations: Authors, Title, HemaSphere, 2026;10:(S1):pages. The abstract book DOIs can be found on the "Author Information" page.
Disclaimer: Articles published in the journal HemaSphere exclusively reflect the opinions of the authors. The authors are responsible for all content in their abstracts including accuracy of the facts, statements, citing resources, etc.

### (PB4178) THE SILENT EPIDEMIC: CHRONIC PAIN ETIOLOGY AND MANAGEMENT STRATEGIES IN TRANSFUSION-DEPENDENT THALASSEMIA

**Topic:** 28. Thalassemias

Abdulrahman Nasiri*[1], Mostafa Mohammed Saleh[2], Manal Alshammari[3], Ali Alahmari[2], Reem Alkharras[4], Fahed Almhareb[2]

*[1]Imam Mohammad Ibn Saud Islamic University, College of Medicine, Riyadh, Saudi Arabia;[2]King Faisal Specialist Hospital & Research Centre, Department of Hematology, Riyadh, Saudi Arabia; [3]King Salman Specialist Hospital, Department of Hematology, Hail, Saudi Arabia; [4]Security Forces Hospital, Department of Medicine, Riyadh, Saudi Arabia;*

### Background

Advances in hypertransfusion and iron chelation have fundamentally shifted the survival trajectory of transfusion-dependent thalassemia (TDT), transforming it into a chronic condition. However, this longevity has exposed a pervasive and under-addressed morbidity: chronic pain. Despite profoundly impairing health-related quality of life, pain in TDT remains poorly characterized, lacking disease-specific guidelines, and is frequently neglected in standard hematological protocols.

### Aims

To systematically map the epidemiological burden, multifactorial pathogenesis, and therapeutic interventions for chronic pain in TDT, and to propose a novel, disease-specific analgesic framework.

### Methods

We conducted a comprehensive scoping review across PubMed/MEDLINE, EMBASE, Scopus, and the Cochrane Library for studies published from database inception to January 2026. Included were original research, systematic reviews, and clinical guidelines evaluating chronic pain in TDT. Two independent reviewers performed the screening, data extraction, and synthesis across three core domains: epidemiological burden, etiological factors, and management strategies.

### Results

Of 1,247 identified citations, 78 studies met the inclusion criteria. Contemporary 2025 data reveal a high prevalence of chronic pain ranging from 32% to 55% in adult cohorts, with over 90% of affected patients reporting moderate-to-severe intensity. The pathogenesis is distinctly multifactorial, driven primarily by thalassemia-associated osteoporosis (affecting 50-65% of adults), deferiprone-associated arthropathy (5-15%), extramedullary hematopoiesis, and iron-overload-induced endocrinopathies. Management is complicated by treatment-related vulnerabilities; while bisphosphonates demonstrate robust efficacy for skeletal pain, the use of standard NSAIDs and opioids is severely constrained by prevalent iron-overload-induced hepatic and renal comorbidities.

### Summary/Conclusion

Chronic pain is a dominant, core morbidity of the contemporary TDT phenotype. To address the fragmented and empirical nature of current treatments, we propose a TDT-specific analgesic framework that integrates metabolic optimization, targeted anti-resorptive therapy, and cautious pharmacotherapy. Dedicated randomized controlled trials evaluating targeted analgesic strategies and the formal integration of validated pain assessment tools are urgently required to match survival gains with quality of life improvements.

**Copyright Information:** (Online) ISSN: 2572-9241
©2026 The Author(s). HemaSphere is published by John Wiley & Sons Ltd on behalf of European Hematology Association. This is an open access Abstract Book distributed under the Attribution-NonCommercial-NoDerivs (CC BY-NC-ND), which allows third parties to download the articles and share them with others as long as they credit the author and the Abstract Book, but they cannot change the content in any way or use them commercially.
**Abstract Book Citations:** Authors, Title, HemaSphere, 2026;10:(S1):pages. The abstract book DOIs can be found on the "Author Information" page.
**Disclaimer:** Articles published in the journal HemaSphere exclusively reflect the opinions of the authors. The authors are responsible for all content in their abstracts including accuracy of the facts, statements, citing resources, etc.

### (PB4179) A READILY AVAILABLE MARKER FOR A TARGETED THERAPY: NRBC/WBC RATIO IN LUSPATERCEPT-TREATED BETA-THALASSEMIA AND MYELODYSPLASTIC SYNDROME

**Topic:** 28. Thalassemias

Adela-Sara Ionescu*[1, 2], Viola Maria Popov[1], Mihaela-Sabrina Herdea[1, 2], Gratiela-Elena Dobre[1], Ana-Maria Petraru[1], Florina Oana Gianina Pătrînoiu[1], Oana Dragoi Cozmaciuc[1], Geanina Ofiteru[1], Adriana Francu[1, 2], Mihaela Andreescu[1, 2]

*[1]Colentina Clinical Hospital, Hematology I, Bucharest, Romania;[2]Titu Maiorescu University, Faculty of Medicine, Bucharest, Romania;*

### Background

Ineffective erythropoiesis is central to both beta-thalassemia (BT) and myelodysplastic syndromes (MDS), despite distinct pathophysiology. Luspatercept enhances late-stage erythroid maturation via modulation of the TGF-β/SMAD pathway.
While biomarkers such as erythropoietin, GDF-15, and soluble transferrin receptor have been explored, accessible dynamic markers remain insufficiently characterized. The nucleated red blood cell to white blood cell ratio (NRBC/WBC), derived from routine complete blood counts, may represent a low-cost marker of stress erythropoiesis.

### Aims

To evaluate the NRBC/WBC as a potential dynamic marker of erythropoietic activity and biological changes under luspatercept therapy in BT and MDS, beyond hemoglobin and transfusion burden as conventional endpoints.

### Methods

This retrospective observational study included 21 patients (9 BT- major, intermedia and minor phenotypes, 12 MDS- ring and non-ring sideroblast types) assessed at baseline and at 1, 2, 3, and 6 months after luspatercept initiation. All patients received luspatercept at a standardized dose.
Studied laboratory parameters included hemoglobin, complete blood count with differential, neutrophil-to-lymphocyte ratio (NLR), platelet-to-lymphocyte ratio, NRBC/WBC, red blood cell distribution width, mean corpuscular volume, lactate dehydrogenase (LDH), ferritin, and transfusion burden (defined as red blood cell units per 4 weeks, consistent with monthly follow-up assessments).
Repeated measures correlation assessed intra-individual associations between NRBC/WBC and studied laboratory parameters.
Spearman correlation was applied to explore associations with IPSS-R and IPSS-M scores in MDS. Kruskal-Wallis tests compared median NRBC/WBC ratios across groups, and receiver operating characteristic (ROC) curve analysis evaluated discriminatory performance. A p-value <0.05 was considered statistically significant.
The study was approved by the local ethics committee.

### Results

In BT, NRBC/WBC showed a significant inverse longitudinal association with absolute lymphocyte count (r=-0.46, p=0.004), without significant correlations with other hematologic parameters, LDH, ferritin, or transfusion burden.
In the MDS, NRBC/WBC demonstrated significant inverse associations with LDH (r=-0.39, p=0.005), neutrophil count (r=-0.43, p=0.001), and NLR (r=-0.30, p=0.03), without significant correlations with other hematologic parameters, ferritin, or transfusion burden, nor with IPSS-R or IPSS-M scores.
Overall, associations were modest (r=-0.30 to -0.46, 9-21% shared variance).
Kruskal-Wallis and ROC analyses showed a trend toward NRBC/WBC improvement that did not reach statistical significance (p=0,406 in both cohorts) (image1)

**Copyright Information:** (Online) ISSN: 2572-9241
©2026 The Author(s). HemaSphere is published by John Wiley & Sons Ltd on behalf of European Hematology Association. This is an open access Abstract Book distributed under the Attribution-NonCommercial-NoDerivs (CC BY-NC-ND), which allows third parties to download the articles and share them with others as long as they credit the author and the Abstract Book, but they cannot change the content in any way or use them commercially.
**Abstract Book Citations:** Authors, Title, HemaSphere, 2026;10:(S1):pages. The abstract book DOIs can be found on the “Author Information” page.
**Disclaimer:** Articles published in the journal HemaSphere exclusively reflect the opinions of the authors. The authors are responsible for all content in their abstracts including accuracy of the facts, statements, citing resources, etc.

### Summary/Conclusion

NRBC/WBC demonstrated modest but significant longitudinal associations with selected laboratory parameters under luspatercept therapy, without correlation to hemoglobin or transfusion burden. This may reflect luspatercept's late-stage mechanism and the confounding effect of transfusional support.
NRBC/WBC may capture dynamic shifts in erythropoietic activity occurring alongside immune and inflammatory changes.
Given the exploratory design, multiple comparisions, and small, biologically heterogeneous cohorts, these findings require cautious interpretation and validation in larger, disease-specific, prospective studies and over an extended follow-up period.

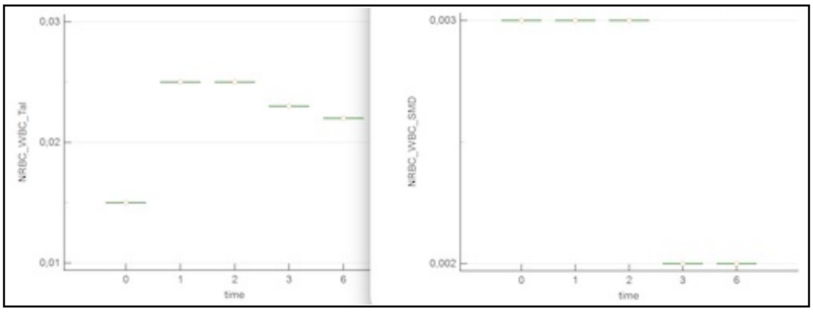

Image 1- Trend improvement in NRBC/WBC.

**Copyright Information:** (Online) ISSN: 2572-9241
©2026 The Author(s). HemaSphere is published by John Wiley & Sons Ltd on behalf of European Hematology Association. This is an open access Abstract Book distributed under the Attribution-NonCommercial-NoDerivs (CC BY-NC-ND), which allows third parties to download the articles and share them with others as long as they credit the author and the Abstract Book, but they cannot change the content in any way or use them commercially.
**Abstract Book Citations:** Authors, Title, HemaSphere, 2026;10:(S1):pages. The abstract book DOIs can be found on the "Author Information" page.
**Disclaimer:** Articles published in the journal HemaSphere exclusively reflect the opinions of the authors. The authors are responsible for all content in their abstracts including accuracy of the facts, statements, citing resources, etc.

### (PB4180) COMPARATIVE ANALYSIS OF HEALTHCARE AND NON-HEALTHCARE THALASSEMIA CONTENT ON TIKTOK: QUALITY, ENGAGEMENT, AND MISINFORMATION PATTERNS

**Topic:** 28. Thalassemias

Ayesha Farooqui*[1, 2], Martin Castillo[3], David Dulaimy[4], Rayyan Haqqani[5], Marwah Farooqui[3]

*[1]agios, Cambridge, United States of America; [2]MCPHS, Brookline, United States of America; [3]universtiy of illinois chicago, Chicago, United States of America; [4]university of rhode island, providence, United States of America;[5]Waubonsie High School, naperville, United States of America;*

### Background

Thalassemia is an inherited hemoglobin disorder caused by mutations in the α- or β-globin genes, resulting in chronic anemia of variable severity.[1] It is most prevalent in the Middle East, South and Southeast Asia, and the Mediterranean, with rising prevalence in the United States and Europe due to migration.[2] Clinical presentation ranges from asymptomatic carriers to transfusion-dependent disease complicated by iron overload and multi-organ morbidity. Management includes transfusion with iron chelation, disease-modifying therapies, stem cell transplantation, and emerging gene therapy.[1] Barriers to care are influenced by social determinants of health, making patient education essential for optimizing outcomes.[3] Social media platforms such as TikTok increasingly serve as sources of health information and peer support.

### Aims

This study evaluates the quality, engagement, and treatment portrayal of thalassemia-related TikTok content, comparing healthcare and non-healthcare authors while assessing misinformation, language distribution, and access-related themes.

### Methods

A cross-sectional content analysis of TikTok was performed using the hashtags #thalassemia, #betathalassemia, and #alphathalassemia. The first 30 videos from each hashtag were analyzed (n=90). Videos were categorized by author type (healthcare vs non-healthcare). Informational quality was assessed using mDISCERN total score and Global Quality Score (GQS). Engagement was measured by number of likes. Mann–Whitney U testing (MATLAB® R2025b) compared quality and engagement between author groups overall and within each hashtag. Descriptive analyses characterized content type, treatment representation, misinformation, access-related themes, and language distribution. Videos focused on sickle cell disease were excluded.

### Results

Of 90 videos, 62% were created by non-healthcare individuals. Healthcare posts demonstrated higher GQS across all hashtags ($p \le 0.0117$) and higher mDISCERN scores within #betathalassemia ($p=0.0299$). Engagement differed only within #alphathalassemia, where non-healthcare posts had more likes ($p=0.0209$). Overall, 22% of videos contained misinformation, occurring more frequently among non-healthcare authors (29% vs 6%), particularly within #betathalassemia and #alphathalassemia. Descriptively, #thalassemia content was primarily educational (66.7%) with minimal treatment discussion. #betathalassemia emphasized transfusion (60%), transplant (30%), and advanced therapies including gene therapy (23.3%). In contrast, #alphathalassemia more frequently referenced iron-related therapies (23.3%) and access barriers (23.3%), with limited discussion of advanced treatments.

### Summary/Conclusion

Thalassemia-related content on TikTok varies by author type and disease subtype. Healthcare-authored posts demonstrate higher

**Copyright Information:** (Online) ISSN: 2572-9241
©2026 The Author(s). HemaSphere is published by John Wiley & Sons Ltd on behalf of European Hematology Association. This is an open access Abstract Book distributed under the Attribution-NonCommercial-NoDerivs (CC BY-NC-ND), which allows third parties to download the articles and share them with others as long as they credit the author and the Abstract Book, but they cannot change the content in any way or use them commercially.
**Abstract Book Citations:** Authors, Title, HemaSphere, 2026;10:(S1):pages. The abstract book DOIs can be found on the "Author Information" page.
**Disclaimer:** Articles published in the journal HemaSphere exclusively reflect the opinions of the authors. The authors are responsible for all content in their abstracts including accuracy of the facts, statements, citing resources, etc.

informational quality, whereas non-healthcare posts account for greater engagement and higher rates of misinformation. Treatment narratives differ across hashtags, reflecting disease severity and common management patterns. As content visibility may be influenced by platform algorithms, findings should be interpreted cautiously. Partnerships with healthcare organizations and community advisory groups, such as the Thalassaemia International Federation, may improve digital accuracy, engagement, and patient education.

| | |
|---|---|
| **Thalassemia** | |
| **Comparison** | **P-value** |
| mDISCERN | 0.4081 |
| GQS | 0.0117 |
| Likes | 0.0923 |
| **Beta Thal** | |
| **Comparison** | **P-value** |
| mDISCERN | 0.0299 |
| GQS | 0.0068 |
| Likes | 0.1813 |
| **Alpha Thal** | |
| **Comparison** | **P-value** |
| mDISCERN | 0.4961 |
| GQS | 8.14E-04 |
| Likes | 0.0209 |

**Copyright Information:** (Online) ISSN: 2572-9241
©2026 The Author(s). HemaSphere is published by John Wiley & Sons Ltd on behalf of European Hematology Association. This is an open access Abstract Book distributed under the Attribution-NonCommercial-NoDerivs (CC BY-NC-ND), which allows third parties to download the articles and share them with others as long as they credit the author and the Abstract Book, but they cannot change the content in any way or use them commercially.
**Abstract Book Citations:** Authors, Title, HemaSphere, 2026;10:(S1):pages. The abstract book DOIs can be found on the "Author Information" page.
**Disclaimer:** Articles published in the journal HemaSphere exclusively reflect the opinions of the authors. The authors are responsible for all content in their abstracts including accuracy of the facts, statements, citing resources, etc.

### (PB4181) NON-INVASIVE PRENATAL DIAGNOSIS OF HAEMOGLOBINOPATHIES: FROM CONCEPT TO CLINICAL REALITY

**Topic:** 28. Thalassemias

Irene Leonidou*[1]

[1] *King's College Hospital, Fetal Medicine, London, United Kingdom;*

**Background**

Haemoglobinopathies are the most prevalent monogenic disorders worldwide and are a major cause of childhood morbidity and mortality. Preventive strategies such as carrier screening and prenatal diagnosis have reduced disease incidence in regions with established programmes. However, definitive prenatal diagnosis currently depends on invasive procedures, including chorionic villus sampling and amniocentesis, which carry a small but significant risk of pregnancy loss.

**Aims**

This narrative review aims to explore the genetic basis and global burden of haemoglobinopathies, outline current screening strategies and invasive diagnostic approaches and critically evaluate the emerging role of non-invasive prenatal diagnosis (NIPD) for sickle cell disease and thalassaemias.

**Methods**

The review synthesises published literature and clinical trial evidence on NIPD methodologies for haemoglobinopathies. Key approaches examined include relative haplotype dosage and relative mutation dosage, alongside discussion of their technical principles, performance, and practical considerations.

**Results**

Multiple NIPD trials demonstrate promising diagnostic accuracy and the potential for earlier, safer prenatal diagnosis without procedure-related miscarriage risk. However, challenges remain, including variable fetal fraction, technical complexity, cost, and limitations in accuracy. Significant global inequities persist in access to screening and diagnostic services, particularly in regions with the highest disease burden.

**Summary/Conclusion**

NIPD represents a potentially transformative alternative to invasive prenatal diagnosis for haemoglobinopathies, with encouraging results from emerging methodologies. Despite this promise, technical, economic and equity-related barriers currently limit widespread clinical implementation. Although NIPD could improve acceptability and uptake of prenatal diagnosis for haemoglobinopathies, further methodological refinement, large-scale validation, and careful implementation are required before routine clinical adoption can be achieved.

**Copyright Information:** (Online) ISSN: 2572-9241
©2026 The Author(s). HemaSphere is published by John Wiley & Sons Ltd on behalf of European Hematology Association. This is an open access Abstract Book distributed under the Attribution-NonCommercial-NoDerivs (CC BY-NC-ND), which allows third parties to download the articles and share them with others as long as they credit the author and the Abstract Book, but they cannot change the content in any way or use them commercially.
**Abstract Book Citations:** Authors, Title, HemaSphere, 2026;10:(S1):pages. The abstract book DOIs can be found on the "Author Information" page.
**Disclaimer:** Articles published in the journal HemaSphere exclusively reflect the opinions of the authors. The authors are responsible for all content in their abstracts including accuracy of the facts, statements, citing resources, etc.

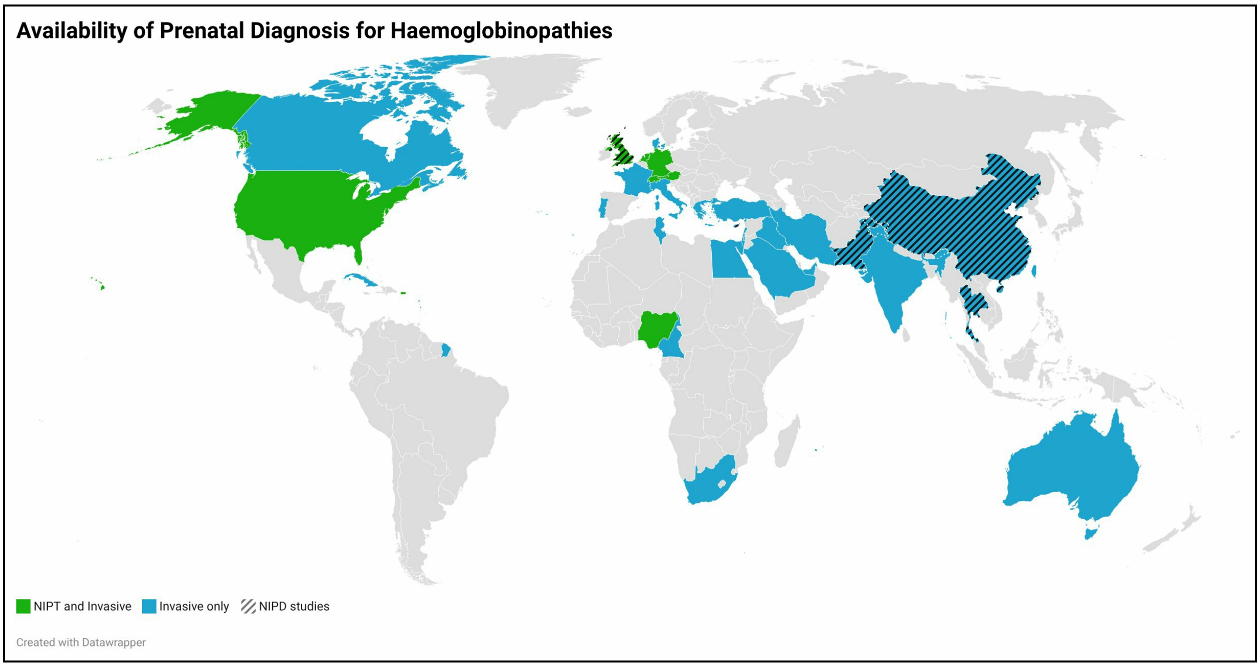

Global availability of prenatal diagnostic approaches for haemoglobinopathies. Countries coloured blue represent availability of invasive prenatal diagnosis alone and those coloured green represent both invasive and commercial NIPT methods. Countries with shading indicate where NIPD studies have been reported. Countries included were identified through a keyword search of English language literature.

**Copyright Information:** (Online) ISSN: 2572-9241
©2026 The Author(s). HemaSphere is published by John Wiley & Sons Ltd on behalf of European Hematology Association. This is an open access Abstract Book distributed under the Attribution-NonCommercial-NoDerivs (CC BY-NC-ND), which allows third parties to download the articles and share them with others as long as they credit the author and the Abstract Book, but they cannot change the content in any way or use them commercially.
**Abstract Book Citations:** Authors, Title, HemaSphere, 2026;10:(S1):pages. The abstract book DOIs can be found on the "Author Information" page.
**Disclaimer:** Articles published in the journal HemaSphere exclusively reflect the opinions of the authors. The authors are responsible for all content in their abstracts including accuracy of the facts, statements, citing resources, etc.

### (PB4182) BEYOND DIAGNOSIS: A SYSTEMATIC REVIEW OF ARTIFICIAL INTELLIGENCE AND DEEP LEARNING IN MONITORING IRON OVERLOAD AND ORGAN TOXICITY IN BETA-THALASSEMIA

**Topic:** 28. Thalassemias

Abdulrahman Nasiri*[1], Mostafa Mohammed Saleh[2], Manal Alshammari[3], Ali Alahmari[2], Reem Alkharras[4], Fahed Almhareb[2]

*[1]Imam Mohammad Ibn Saud Islamic University, College of Medicine, Riyadh, Saudi Arabia;[2]King Faisal Specialist Hospital & Research Centre, Department of Hematology, Riyadh, Saudi Arabia; [3]King Salman Specialist Hospital, Department of Hematology, Hail, Saudi Arabia; [4]Security Forces Hospital, Department of Medicine, Riyadh, Saudi Arabia;*

### Background

In the management of beta-thalassemia major (B-TM), mitigating the consequences of transfusional iron overload is paramount to preventing organ toxicity and reducing mortality. Conventional monitoring methods, including serum ferritin and T2* magnetic resonance imaging (MRI), possess well-documented limitations in precision and prognostic capability. Artificial intelligence (AI) and deep learning (DL) are emerging as powerful analytical tools to enhance the accuracy and predictive power of iron overload assessment.

### Aims

To systematically evaluate the role, performance, and clinical applicability of AI and DL models in the assessment of iron overload and associated organ toxicity in patients with beta-thalassemia.

### Methods

This systematic review was conducted in accordance with the Preferred Reporting Items for Systematic Reviews and Meta-Analyses (PRISMA) 2020 guidelines. A comprehensive search of PubMed, Scopus, and Web of Science was performed for studies published between January 1, 2019, and January 6, 2026. Studies were included if they were original research applying AI or DL models to quantify iron overload, predict organ damage, or stratify risk in patients with beta-thalassemia and included a performance evaluation. The Prediction model Risk Of Bias ASsessment Tool (PROBAST) was used to formally assess the risk of bias and applicability.

### Results

The literature search yielded 482 records. After a rigorous screening process, 8 studies were included in the final qualitative synthesis. These studies demonstrated the application of AI/DL across three critical domains: (1) automated, rapid quantification of hepatic iron from MRI R2*/T2* data using physics-informed deep learning; (2) automated cardiac MRI segmentation and classification of myocardial iron overload using U-Net-based architectures; and (3) prediction of low bone mass using machine learning classifiers on clinical data. The formal risk of bias assessment revealed that a majority of the included studies were at high risk of bias, primarily due to the use of retrospective designs and a lack of robust external validation.

### Summary/Conclusion

AI and DL models demonstrate considerable potential to transform the monitoring of iron overload and organ toxicity in beta-thalassemia. However, the translation of these models into routine clinical practice is currently impeded by significant methodological limitations. Future research must prioritize rigorous, multi-center prospective studies, strict adherence to reporting guidelines such as PROBAST-AI, and the development of explainable AI to build clinical trust and ensure patient safety.

**Copyright Information:** (Online) ISSN: 2572-9241
©2026 The Author(s). HemaSphere is published by John Wiley & Sons Ltd on behalf of European Hematology Association. This is an open access Abstract Book distributed under the Attribution-NonCommercial-NoDerivs (CC BY-NC-ND), which allows third parties to download the articles and share them with others as long as they credit the author and the Abstract Book, but they cannot change the content in any way or use them commercially.
**Abstract Book Citations:** Authors, Title, HemaSphere, 2026;10:(S1):pages. The abstract book DOIs can be found on the "Author Information" page.
**Disclaimer:** Articles published in the journal HemaSphere exclusively reflect the opinions of the authors. The authors are responsible for all content in their abstracts including accuracy of the facts, statements, citing resources, etc.

### (PB4183) GLUCOSE-6-PHOSPHATE ISOMERASE DEFICIENCY: AN UPDATED COHORT OF 14 PATIENTS HIGHLIGHTING DIAGNOSTIC CHALLENGES AND AN EXPANDED MUTATIONAL SPECTRUM

**Topic:** 29. Enzymopathies, membranopathies and other red blood cell disorders

Guzel Rakhimova*[1], Zhanna Kuzminova[1], Svetlana Kapranova[1], Marina Lokhmatova[1], Nataliya Smetanina[1]

*[1]Dmitry Rogachev National Medical Research Center Of Pediatric Hematology, Oncology And Immunology, Moscow, Russia;*

### Background

Glucose-6-phosphate isomerase (GPI) deficiency is a rare autosomal recessive cause of hereditary nonspherocytic hemolytic anemia (HNSHA). It is frequently underdiagnosed due to phenotypic overlap with other inherited hemolytic disorders and limited access to molecular diagnostic testing.

### Aims

To characterize the clinical presentation, management outcomes, and mutational spectrum in a pediatric cohort with genetically confirmed GPI deficiency.

### Methods

A single-center retrospective analysis was performed in patients younger than 18 years diagnosed with GPI deficiency between 2018 and 2025. Diagnosis was confirmed using NGS–based gene panels or whole-genome sequencing. Clinical, laboratory, and genetic data were extracted from medical records. This updated cohort includes one additional patient initially misdiagnosed with congenital dyserythropoietic anemia type 1 (CDA-1).

### Results

Fourteen patients from 13 families were analyzed (11 males; median diagnosis age: 3.5 years). All presented with hemolytic anemia of variable severity (median Hb 69.5 g/L, range 40–94), reticulocytosis (median 15.7%), and indirect hyperbilirubinemia (median TBil 88.3 µmol/L). Neurological involvement, including epilepsy, tetraparesis, and psychomotor delay, was observed in two siblings (14%). While consistent with GPI deficiency (neuroleukin function), additional variants of uncertain significance (VUS) in CREBBP and WASHC5 were identified in these patients; segregation analysis is ongoing. Splenectomy was performed in three patients (21%), resulting in reduced transfusion requirements but persistent hemolysis. One patient developed portal vein thrombosis following splenectomy. Genetic analysis identified 14 distinct GPI variants, including 8 novel mutations. Functional enzyme assay confirmed the pathogenicity of novel variants, demonstrating 14.6% residual enzymatic activity. The newly included case, initially labeled as CDA-1, highlights NGS utility in differentiating GPI deficiency from phenotypically similar disorders.
***Table 1.*** *Key cohort characteristics (N=14).*

**Copyright Information:** (Online) ISSN: 2572-9241
©2026 The Author(s). HemaSphere is published by John Wiley & Sons Ltd on behalf of European Hematology Association. This is an open access Abstract Book distributed under the Attribution-NonCommercial-NoDerivs (CC BY-NC-ND), which allows third parties to download the articles and share them with others as long as they credit the author and the Abstract Book, but they cannot change the content in any way or use them commercially.
**Abstract Book Citations:** Authors, Title, HemaSphere, 2026;10:(S1):pages. The abstract book DOIs can be found on the "Author Information" page.
**Disclaimer:** Articles published in the journal HemaSphere exclusively reflect the opinions of the authors. The authors are responsible for all content in their abstracts including accuracy of the facts, statements, citing resources, etc.

| Parameter | Value |
|---|---|
| Age at diagnosis, years (median, range) | 3.5 (0.3–16.5) |
| Male sex, n (%) | 11 (79%) |
| Hemoglobin at crisis, g/L (median, range) | 69.5 (40–94) |
| Reticulocytes, % (median, range) | 15.7 (2.4–26.8) |
| Total bilirubin, µmol/L (median, range) | 88.3 (22.9–192) |
| Neurological involvement, n (%) | 2 (14%) |
| Splenectomy performed, n (%) | 3 (21%) |
| Distinct GPI variants identified | 14 total (8 novel*) |
| Most frequent GPI variant | c.1039C>T p.(Arg347Cys), 35% |

**Novel variants were supported by in silico prediction; functional enzyme assay demonstrated 14.6% residual activity. Additional variants of uncertain significance (VUS) in CREBBP and WASHC5 were identified in siblings with neurological involvement; clinical significance is under investigation.*

### Summary/Conclusion

GPI deficiency demonstrates marked clinical and genetic heterogeneity. Our real-world data underscore the critical role of NGS in the diagnostic evaluation of unexplained hemolytic anemia, particularly in cases with overlapping phenotypes such as CDA. We report an expanded spectrum of pathogenic GPI variants supported by functional validation and highlight the need for individualized management, including cautious consideration of splenectomy. Early genetic confirmation may reduce diagnostic delay and optimize patient care. Comprehensive genomic profiling may also identify additional variants influencing disease phenotype.

**Copyright Information:** (Online) ISSN: 2572-9241
©2026 The Author(s). HemaSphere is published by John Wiley & Sons Ltd on behalf of European Hematology Association. This is an open access Abstract Book distributed under the Attribution-NonCommercial-NoDerivs (CC BY-NC-ND), which allows third parties to download the articles and share them with others as long as they credit the author and the Abstract Book, but they cannot change the content in any way or use them commercially.
**Abstract Book Citations:** Authors, Title, HemaSphere, 2026;10:(S1):pages. The abstract book DOIs can be found on the "Author Information" page.
**Disclaimer:** Articles published in the journal HemaSphere exclusively reflect the opinions of the authors. The authors are responsible for all content in their abstracts including accuracy of the facts, statements, citing resources, etc.

### (PB4184) COMBINATION RITUXIMAB AND MMF IN PEDIATRIC AIHA: A THERAPEUTIC ADVANTAGE.

**Topic:** 29. Enzymopathies, membranopathies and other red blood cell disorders

Zhanna Kuzminova*[1]

[1]*Dmitry Rogachev National Medical Research Center Of Pediatric Hematology, Oncology and Immunology, Moscow, Russia;*

### Background

Autoimmune hemolytic anemia (AIHA) has a high risk of relapse. In cases refractory to standard therapy (corticosteroids) and in recurrent cases, patients require immunosuppressive therapy to achieve remission.
Rituximab has been widely used for the treatment of refractory AIHA, with a high efficacy rate of up to 79%. Mycophenolate mofetil (MMF) reduces T- and B-lymphocyte proliferation and also demonstrates good efficacy in immune cytopenias (80%). However, as the response to MMF occurs gradually, more aggressive therapy is usually required in the acute phase of hemolysis.

### Aims

To evaluate the efficacy of combination therapy with rituximab and MMF in pediatric AIHA.

### Methods

A retrospective analysis of treatment efficacy and remission duration in patients with AIHA who received rituximab and MMF as monotherapy, or as combination therapy.

### Results

The analyzed data were divided into three groups for comparison:
Group 1 – 35 patients who received rituximab monotherapy at a dose of 375 mg/m² for a course of 2-7 administrations. Two patients experienced adverse reactions to the administrations (allergic reactions) that prevented further therapy.
A total of 26/35 patients (74.3%) responded to rituximab, and 17 patients (65%) maintained remission with a median duration of 9 months (range, 1 month to 11 years).
Group 2 – 22 patients who received MMF monotherapy at a dose of 30-50 mg/kg/day. One patient experienced toxicity in the form of colitis; however, this did not require treatment discontinuation.
A total of 17/22 patients (77.3%) responded to therapy, and 16/17 (94%) maintained remission with a median duration of 10 months (range, 1 month to 6 years). Two patients maintained remission after discontinuing MMF therapy (for 2.5 and 5 years).
Group 3 – 27 patients who received combination therapy of rituximab 375 mg/m² (2-6 injections) and MMF at a dose of 25-50 mg/kg/day. All 27 patients (100%) responded to therapy, and 26/27 patients (96%) maintained remission, with a median duration of 18 months (range, 1 month to 5 years). One patient experienced toxicity from MMF therapy. Five patients maintain remission after completion of therapy, with a duration ranging from 1 month to 3 years.
There was no statistically significant difference in response rates between groups 1 and 2 ($p>0.05$), whereas significantly higher response rates were observed in group 3 compared to groups 1 and 2 ($p<0.001$). Furthermore, a longer remission duration was observed in group 3 compared to groups 1 and 2 ($p<0.001$).

### Summary/Conclusion

Combination immunosuppressive therapy with rituximab and MMF is a highly effective treatment for AIHA and allows for the achievement of long-term remission.

**Copyright Information:** (Online) ISSN: 2572-9241
©2026 The Author(s). HemaSphere is published by John Wiley & Sons Ltd on behalf of European Hematology Association. This is an open access Abstract Book distributed under the Attribution-NonCommercial-NoDerivs (CC BY-NC-ND), which allows third parties to download the articles and share them with others as long as they credit the author and the Abstract Book, but they cannot change the content in any way or use them commercially.
**Abstract Book Citations:** Authors, Title, HemaSphere, 2026;10:(S1):pages. The abstract book DOIs can be found on the “Author Information” page.
**Disclaimer:** Articles published in the journal HemaSphere exclusively reflect the opinions of the authors. The authors are responsible for all content in their abstracts including accuracy of the facts, statements, citing resources, etc.

### (PB4185) ENDOCRINE COMPLICATIONS IN PATIENTS WITH INEFFECTIVE ERYTHROPOIESIS: A SINGLE-CENTER EXPERIENCE

**Topic:** 29. Enzymopathies, membranopathies and other red blood cell disorders

Gamze Gürsoy*[1], Ugur Unluturk[2], Şule Ünal Cangül[1]

*[1]Hacettepe University Faculty of Medicine, Department of Pediatric Hematology, Ankara, Türkiye;[2]Hacettepe University Faculty of Medicine, Department of Internal Diseases Endocrinology Subdivision, Ankara, Türkiye;*

### Background

Congenital dyserythropoietic anemias (CDA) and pyruvate kinase deficiency (PKD) are rare inherited anemias characterized by ineffective erythropoiesis. Both transfusion-dependent (TD) and non-transfusion-dependent (NTD) patients are at risk for iron overload and related endocrine complications. While endocrine disorders are well-described in transfusion-dependent conditions such as thalassemia, data regarding endocrine complications in CDA and PKD, particularly in NTD patients, remain limited.

### Aims

We aimed to evaluate the prevalence and spectrum of endocrine complications in patients with CDA and PKD

### Methods

A total of 28 patients with genetically diagnosed CDA or PKD were evaluated. Endocrine assessment included anthropometric measurements, metabolic, thyroid, growth, adrenal, gonadal, and bone parameters.Iron status was assessed using serum ferritin and hepatic and cardiac iron concentration with T2*MRI. Endocrine complications were analysed in relation to age, transfusion status, iron overload, and disease characteristics.

### Results

Among the 28 patients (PKD: n = 17, CDA: n = 11), the cohort included 14 males and 14 females. The mean age was 18.3 ± 11.3 years (range: 5–49).Twenty patients (71%) were classified as non–transfusion dependent (NTD), whereas all transfusion-dependent (TD) patients were receiving iron chelation therapy. The mean serum ferritin level among the patients was 710 µg/L. Among 13 patients with hepatic T2* assessment, 5 exhibited severe iron accumulation, 3 moderate, 1 mild, and 4 had no detectable hepatic iron accumulation, while none of the patients had cardiac iron overload.
Among the patients, 24 (85%) had at least one endocrinological complication. Eight patients had one endocrine disorder, seven had two endocrine disorders, and nine had three or more concurrent endocrinological complications. Vitamin D deficiency was the most common finding (n:15,53.6%), followed by insulin resistance/prediabetes or diabetes mellitus (n:8,28%), short stature (n:7,25%), subclinical hypothyroidism (n:6,21%), osteopenia (n:4,14%), hyperprolactinemia (n:3, 10%). Other rare endocrine problems included hypogonadotropic hypogonadism, hyperparathyroidism, pituitary microadenoma, obesity, steroid-induced adrenal insufficiency, and subclinical adrenal insufficiency.
The presence of endocrine disorders was not significantly associated with age, gender, PKD-CDA group, transfusion dependency, ferritin levels, or T2* MRI findings. However, TD patients demonstrated a higher endocrinopathy rate compared to NTD patients although the difference was not statistically significant (%100 vs %80, p:0,2). When vitamin D deficiency, which is highly prevalent in the general population, is excluded from the analysis of endocrine disorders; prevalence was 87% in TD patients and %65 in NTD's (p:0,2).

**Copyright Information:** (Online) ISSN: 2572-9241
©2026 The Author(s). HemaSphere is published by John Wiley & Sons Ltd on behalf of European Hematology Association. This is an open access Abstract Book distributed under the Attribution-NonCommercial-NoDerivs (CC BY-NC-ND), which allows third parties to download the articles and share them with others as long as they credit the author and the Abstract Book, but they cannot change the content in any way or use them commercially.
**Abstract Book Citations:** Authors, Title, HemaSphere, 2026;10:(S1):pages. The abstract book DOIs can be found on the "Author Information" page.
**Disclaimer:** Articles published in the journal HemaSphere exclusively reflect the opinions of the authors. The authors are responsible for all content in their abstracts including accuracy of the facts, statements, citing resources, etc.

### Summary/Conclusion

In the literature, there is very limited information on endocrinopathies in patients with CDA and PKD. Endocrine disturbances are common and well-recognized in TD thalassemia patients, while they are also observed in NTD thalassemia, albeit at a lower prevalence. In our study group, we observed that endocrine complications were not associated with the transfusion status of the patient. These findings suggest that endocrine dysfunction may not be solely related to iron overload but may also reflect intrinsic disease-related mechanisms. Therefore, early endocrinologic screening and multidisciplinary follow-up are essential to prevent long-term morbidity in this patient population.

**Copyright Information:** (Online) ISSN: 2572-9241
©2026 The Author(s). HemaSphere is published by John Wiley & Sons Ltd on behalf of European Hematology Association. This is an open access Abstract Book distributed under the Attribution-NonCommercial-NoDerivs (CC BY-NC-ND), which allows third parties to download the articles and share them with others as long as they credit the author and the Abstract Book, but they cannot change the content in any way or use them commercially.
**Abstract Book Citations:** Authors, Title, HemaSphere, 2026;10:(S1):pages. The abstract book DOIs can be found on the "Author Information" page.
**Disclaimer:** Articles published in the journal HemaSphere exclusively reflect the opinions of the authors. The authors are responsible for all content in their abstracts including accuracy of the facts, statements, citing resources, etc.

### (PB4186) SIROLIMUS AS AN EFFECTIVE TREATMENT IN CHILDREN WITH CHRONIC/REFRACTORY PRIMARY AUTOIMMUNE HEMOLYTIC ANEMIA

**Topic:** 29. Enzymopathies, membranopathies and other red blood cell disorders

Maurizio Miano*[1], Valentina Savino[1], Riccardo Morini[1], Gianluca Dell'orso[1], Damiano Lemmi[1], Eugenia Mariani[1], Maria Carla Giarratana[1], Luca Arcuri[1], Francesca Fioredda[1], Elena Palmisani[1], Carlo Dufour[1]

*[1]IRCCS Istituto Giannina Gaslini, Dept. of Pediatric Haematology/oncology, Genoa, Italy;*

### Background

Autoimmune hemolytic anemia (AIHA) is a very rare disorder that may follow a severe life-threatening course. Therapeutic management beyond first-line corticosteroids remains challenging for patients who relapse or become steroid-dependent. The use of immunosuppressors have demonstrated encouraging results, especially in children. Sirolimus is a mTOR inhibitor which has shown efficacy in patients wityh autoimmune lymphoproliferative syndrome (ALPS) and Evans syndrome (ES). However, data on its use in primary AIHA in the absence of these conditions are very limited.

### Aims

To retrospectively evaluate the efficacy and safety of sirolimus as maintenance therapy in pediatric patients with steroid-dependent or steroid-refractory primary AIHA and pure red cell aplasia (PRCA).

### Methods

We conducted a retrospective single-center study including children with chronic or refractory primary AIHA/PRCA treated with sirolimus. Patients with multilineage cytopenia, ALPS or other underlying congenital disorders were excluded. Sirolimus was administered at a starting dose of 2 mg/m², with subsequent dose adjustments based on blood levels. Treatment response was defined as complete (CR) in the presence of age-adjusted hemoglobin normalisation and absence of clinical evidence of hemolysis, or partial (PR) in the case of a sustained haemoglobin increase ≥2 g/dL from baseline with reduced transfusion requirements, despite persistent minor hemolysis.

### Results

Fifteen children (median age 4.3 years, range 0.1-16) with AIHA received sirolimus for refractory or steroid-dependent disease. In three patients AIHA was also associated with pure cell anemia. Median hemoglobin at diagnosis was 5.6 g/dL (range 2.1–11.3 g/dL). All patients received corticosteroids, with or without immunoglobulins, as first-line therapy. Sirolimus was started after a median of 341 days from disease onset (range 4–3562 days) as second-line (n=4), third-line (n=5), fourth-line (n=3), or later-line therapy (n=2); the remaining patient received sirolimus during the acute phase. All patients responded to treatment: 13 (87%) achieved CR and 2 (13%) achieved PR. Median time to response was 44 days (range 16–244 days), with a median sirolimus trough level at response of 7.1 ng/mL (range 4.54–13.67 ng/mL). Six patients relapsed during treatment (3) or after discontinuation (3), which resolved after dose adjustment or drug reintroduction. Sirolimus was well tolerated, and no patient discontinued therapy because of adverse events. Median follow-up was 6.4 years (range 2–23 years).

**Copyright Information:** (Online) ISSN: 2572-9241
©2026 The Author(s). HemaSphere is published by John Wiley & Sons Ltd on behalf of European Hematology Association. This is an open access Abstract Book distributed under the Attribution-NonCommercial-NoDerivs (CC BY-NC-ND), which allows third parties to download the articles and share them with others as long as they credit the author and the Abstract Book, but they cannot change the content in any way or use them commercially.
**Abstract Book Citations:** Authors, Title, HemaSphere, 2026;10:(S1):pages. The abstract book DOIs can be found on the “Author Information” page.
**Disclaimer:** Articles published in the journal HemaSphere exclusively reflect the opinions of the authors. The authors are responsible for all content in their abstracts including accuracy of the facts, statements, citing resources, etc.

**Summary/Conclusion**

These findings support the use of sirolimus in children with primary AIHA or PRCA, particularly in cases of steroid-dependent or refractory disease. The high response rate, durable efficacy, and favorable safety profile suggest that sirolimus represents a valuable steroid-sparing therapeutic option. Prospective studies are warranted to further define its role in treatment algorithms for these rare conditions.

**Copyright Information:** (Online) ISSN: 2572-9241
©2026 The Author(s). HemaSphere is published by John Wiley & Sons Ltd on behalf of European Hematology Association. This is an open access Abstract Book distributed under the Attribution-NonCommercial-NoDerivs (CC BY-NC-ND), which allows third parties to download the articles and share them with others as long as they credit the author and the Abstract Book, but they cannot change the content in any way or use them commercially.
**Abstract Book Citations:** Authors, Title, HemaSphere, 2026;10:(S1):pages. The abstract book DOIs can be found on the "Author Information" page.
**Disclaimer:** Articles published in the journal HemaSphere exclusively reflect the opinions of the authors. The authors are responsible for all content in their abstracts including accuracy of the facts, statements, citing resources, etc.

### (PB4187) IGA DRIVEN AUTOIMMUNE HEMOLYTIC ANEMIA: A MONOCENTRIC ITALIAN REPORT

**Topic:** 29. Enzymopathies, membranopathies and other red blood cell disorders

Elisa Tarasconi*[1, 2], Nicoletta Revelli[1], Giacinto Luca Pedone[1, 3], Wilma Barcellini[1], Bruno Fattizzo[1, 4]

*[1]Fondazione IRCCS Ca' Granda Ospedale Maggiore Policlinico, Milan, Italy; [2]SC Medicina Generale II, Ospedale San Paolo, ASST Santi Paolo E Carlo, Università Degli Studi Di Milano, Milan, Italy; [3]Department of Pathophysiology and Transplantation, Università degli Studi di Milano, Milan, Italy; [4]Department of Oncology and Onco-Hematology, University of Milan, Milan, Italy;*

### Background

Atypical autoimmune hemolytic anemias (AIHA) include those negative at the direct antiglobulin test (DAT), as well as the ones driven by IgA autoantibodies. The latter are rare with limited evidence on clinical severity and management beyond first-line steroids.

### Aims

To better understand clinical presentation, severity and management of IgA-mediated AIHA.

### Methods

We retrospectively evaluated patients with IgA-positive AIHA, confirmed by monospecific DAT, followed at a tertiary hematologic center in Milan, Italy, since May 1978.

### Results

13 patients with IgA positive AIHA were identified among 409 AIHA cases followed at our center. Table 1 summarizes clinical, laboratory, and therapeutic characteristics of this cohort. The median age was 60 years (range 33–86), with a female predominance (9/13; 69.2%). Reported signs/symptoms included fatigue (3/8, 27.5%), hepatomegaly (2/13; 15.4%), splenomegaly (1/13; 7.7%), and jaundice (1/6; 16.7%). Mean hemoglobin was 7.9 g/dL (range 5–10.5), mean total bilirubin was 4.42 mg/dL (1.2–14.29), and LDH levels 648.9 U/L (270–1105). All patients tested positive for IgA and 9/13 (69.2%) had dual positivity for IgA and IgG.
All patients (12/12; 100%) received corticosteroids frontline. Rituximab and IVIg were used in 4/12 (33.3%) and 2/12 (16.7%) patients respectively as second- or third-line treatment, and transfusions were required in 3/13 patients (23.1%). One patient received bortezomib as fourth-line therapy achieving partial response, then relapsed and received a second cycle of bortezomib, mycophenolate mofetil, and plasma exchange with no response. Complement inhibition was considered due to C4 consumption, though not yet obtained off label.
Median follow-up was 36 months (range 3–108). 7/12 patients (63.6%) experienced a relapse, 2 patients (2/12, 16.7%) developed G2 infections, and 2 (2/12, 16.7%) showed thrombotic events. No death was reported.

**Table 1.** Patient characteristics, treatments and adverse events

**Copyright Information:** (Online) ISSN: 2572-9241
©2026 The Author(s). HemaSphere is published by John Wiley & Sons Ltd on behalf of European Hematology Association. This is an open access Abstract Book distributed under the Attribution-NonCommercial-NoDerivs (CC BY-NC-ND), which allows third parties to download the articles and share them with others as long as they credit the author and the Abstract Book, but they cannot change the content in any way or use them commercially.
**Abstract Book Citations:** Authors, Title, HemaSphere, 2026;10:(S1):pages. The abstract book DOIs can be found on the "Author Information" page.
**Disclaimer:** Articles published in the journal HemaSphere exclusively reflect the opinions of the authors. The authors are responsible for all content in their abstracts including accuracy of the facts, statements, citing resources, etc.

| | |
|---|---|
| Patients, n | 13 |
| Median age, y (range) | 60 (33 - 86) |
| Female, n/N (%) | 9/13 (69.2) |
| Clinical manifestations<br>Fatigue, n/N (%)<br>Dyspnea, n/N (%)<br>Chest pain, n/N (%)<br>Abdominal pain, n/N (%)<br>Hepatomegaly, n/N (%)<br>Splenomegaly, n/N (%)<br>Jaundice, n/N (%) | <br>3/8 (37.5)<br>1/8 (12.5)<br>2/8 (25)<br>1/8 (12.5)<br>2/13 (15.4)<br>1/13 (7.7)<br>1/6 (16.7) |
| Laboratory values<br>Hb (g/dL), mean (range)*<br>Platelets (x109/L), mean (range)°<br>Bilirubin (mg/dL), mean (range)§<br>LDH (U/L), mean (range)◇ | <br>7.93 (5.0 - 10.6)<br>334.7 (200 - 567)<br>4.42 (1.20 - 14.29)<br>648.9 (270 - 1105) |
| DAT<br>IgA, n/N (%)<br>IgA + IgG, n/N (%)<br>IgA + IgG + IgM, n/N (%)<br>Complement activation, n/N (%) | <br>13/13 (100)<br>9/13 (69.2)<br>1/13 (7.7)<br>2/13 (15.4) |
| Treatments<br>Steroids, n/N (%)<br>IVIg, n/N (%)<br>Rituximab, n/N (%)<br>Splenectomy, n/N (%)<br>Plasmaferesi, n/N (%)<br>Transfusions, n/N (%) | <br>12/12 (100)<br>2/12 (16.7)<br>4/12 (33.3)<br>0/11 (0)<br>0/11 (0)<br>3/13 (23.1) |
| Follow-up (months), median (range) ∫ | 36 (3 - 108) |
| Relapse, n/N (%) | 7/11 (63.6) |
| Adverse events, n/N (%)<br>Death, n/N (%)<br>Infections, n/N (%)<br>Thrombosis, n/N (%) | 4/12 (33.3)<br>0/12 (0)<br>2/12 (16.7)<br>2/12 (16.7) |

### Summary/Conclusion

IgA-mediated AIHA is a rare and heterogeneous subtype of AIHA characterised by frequent dual antibody positivity and suboptimal corticosteroids response. Despite the generally favorable prognosis, relapses and complications remain common, highlighting the need for careful management. The exclusion of atypical forms of AIHA from clinical trials remains an issue in the advanced line setting.

**Copyright Information:** (Online) ISSN: 2572-9241
©2026 The Author(s). HemaSphere is published by John Wiley & Sons Ltd on behalf of European Hematology Association. This is an open access Abstract Book distributed under the Attribution-NonCommercial-NoDerivs (CC BY-NC-ND), which allows third parties to download the articles and share them with others as long as they credit the author and the Abstract Book, but they cannot change the content in any way or use them commercially.
**Abstract Book Citations:** Authors, Title, HemaSphere, 2026;10:(S1):pages. The abstract book DOIs can be found on the "Author Information" page.
**Disclaimer:** Articles published in the journal HemaSphere exclusively reflect the opinions of the authors. The authors are responsible for all content in their abstracts including accuracy of the facts, statements, citing resources, etc.

### (PB4188) THE TWO-STEP MODEL OF RED BLOOD CELL DEATH: IDENTIFICATION OF DISTINCT PROCOAGULANT SUBPOPULATIONS AND THEIR PHYSIOLOGICAL RELEVANCE

**Topic:** 29. Enzymopathies, membranopathies and other red blood cell disorders

Ivan Chabin*[1], Nadezhda Podoplelova[1, 2], Kirill Butov[1, 3], Ekaterina Koltosa[1, 2], Sergei Obydennyi[1, 2], Nataliya Smetanina[1], Mikhail Panteleev[1, 2, 4]

*[1]Dmitry Rogachev National Medical Research Center Of Pediatric Hematology, Oncology and Immunology, Moscow, Russia; [2]Federal scientific budgetary institution Center for theoretical problems of physico-chemical pharmacology of the Russian Academy of Sciences, Moscow, Russia; [3]Federal State Autonomous Educational Institution of Higher Education «N.I. Pirogov Russian National Research Medical University» of the Ministry of Health of the Russian Federation, Moscow, Russia; [4]Federal State Budget Educational Institution of Higher Education M.V.Lomonosov Moscow State University, Moscow, Russia;*

### Background

Red blood cell (RBC) elimination is traditionally attributed to senescence or haemolysis; however, a programmed cell death pathway, termed eryptosis, was proposed twenty years ago as an analogue to apoptosis. Phosphatidylserine (PS) exposure is considered a hallmark of eryptosis, yet it is also associated with physiological senescence. Consequently, the precise boundaries between these processes remain poorly defined, likely due to limitations in detection methods. While lactadherin is a highly sensitive PS probe that mediates RBC clearance in vivo, most experimental studies still rely on annexin V, which requires high PS density for binding. This reliance on annexin V may explain why the prevalence and role of PS-exposing RBCs remain a subject of ongoing debate. In this study, we employed both PS probes to resolve these problems and characterize the stages of the RBC death process, and analyzed RBCs from the whole blood.

### Aims

To characterize the dynamics of PS exposure using high-sensitivity fluorescent probes and procoagulant activity of PS-exposing RBCs.

### Methods

RBCs and platelets were isolated from healthy donors and patients with hereditary RBC disorders (informed consent obtained). RBCs were stimulated with A23187 (calcium ionophore), or PMA (PKC activator), or $H_2O_2$ (oxidative stress) and analyzed via flow cytometry and confocal microscopy using annexin V-AF647 and recombinant $C_2$-fragment of lactadherin conjugated with mNeonGreen. The use of home-made recombinant $C_2$-lactadherin enabled dual-staining with annexin V for subpopulation characterization. Procoagulant activity was quantified by assembly the intrinsic tenase complex (FVIIIa/FIXa), followed by a chromogenic assay to measure Factor Xa generation.

**Copyright Information:** (Online) ISSN: 2572-9241
©2026 The Author(s). HemaSphere is published by John Wiley & Sons Ltd on behalf of European Hematology Association. This is an open access Abstract Book distributed under the Attribution-NonCommercial-NoDerivs (CC BY-NC-ND), which allows third parties to download the articles and share them with others as long as they credit the author and the Abstract Book, but they cannot change the content in any way or use them commercially.
**Abstract Book Citations:** Authors, Title, HemaSphere, 2026;10:(S1):pages. The abstract book DOIs can be found on the “Author Information” page.
**Disclaimer:** Articles published in the journal HemaSphere exclusively reflect the opinions of the authors. The authors are responsible for all content in their abstracts including accuracy of the facts, statements, citing resources, etc.

### Results

We identified two distinct PS-exposing RBC subpopulations upon stimulation: a lactadherin-positive, annexin V-negative (Ldh+AnnV-) state and a double-positive (Ldh+AnnV+) state (Fig. 1). Within subsequent storage after stimulation the smooth transit from Ldh+AnnV- to Ldh+AnnV+ state was observed. Confocal imaging revealed non-uniform, punctate lactadherin binding, suggesting PS microclustering prior to global membrane scrambling.
Functionally, Ldh+/AnnV- RBC exhibited a "slightly procoagulant" phenotype (intrinsic tenase activity ~50% of procoagulant platelet), whereas Ldh+AnnV+ cell were highly procoagulant (~10-fold higher than procoagulant platelet). Notably, only the Ldh+AnnV- subpopulation was detectable in healthy circulation (0.05–0.1% of RBCs). In patients with hereditary spherocytosis and pyruvate kinase deficiency, Ldh+ subpopulation of RBCs was significantly elevated (0,27 and 0,46%, respectively), correlating with disease severity.

### Summary/Conclusion

Our data suggest a revised two-step model of RBC death. RBCs primarily transition into a mildly procoagulant (Lact+/AnnV-) state, which is sufficient for physiological clearance. Progression to the highly procoagulant AnnV+ state occurs rarely under normal conditions but is accelerated in erythropathies, potentially contributing to thrombotic risk.

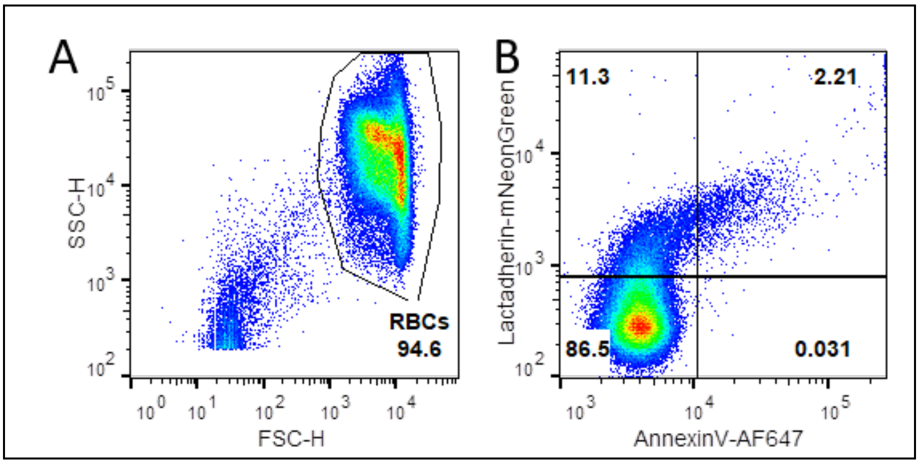

Figure 1. Two subpopulation of PS-exposing RBCs after A23187 stimulation. (A) FSC/SSC plot with RBCs gating is present, (B) Lactadherin/Annexin V plot with gating for subpopulation is presented. Absence of Ldh-AnnV+ subpopulation is observed.

**Copyright Information:** (Online) ISSN: 2572-9241
©2026 The Author(s). HemaSphere is published by John Wiley & Sons Ltd on behalf of European Hematology Association. This is an open access Abstract Book distributed under the Attribution-NonCommercial-NoDerivs (CC BY-NC-ND), which allows third parties to download the articles and share them with others as long as they credit the author and the Abstract Book, but they cannot change the content in any way or use them commercially.
**Abstract Book Citations:** Authors, Title, HemaSphere, 2026;10:(S1):pages. The abstract book DOIs can be found on the "Author Information" page.
**Disclaimer:** Articles published in the journal HemaSphere exclusively reflect the opinions of the authors. The authors are responsible for all content in their abstracts including accuracy of the facts, statements, citing resources, etc.

### (PB4189) THE EFFICACY OF DARATUMUMAB IN COLD AGGLUTININ DISEASE PATIENTS REFRACTORY TO PRIOR B-CELL OR COMPLEMENT-DIRECTED THERAPIES

**Topic:** 29. Enzymopathies, membranopathies and other red blood cell disorders

Nicole Japzon*[1, 2], Hannah George[1, 2], Maria Gabriel[1], Jindriska Lindsay[1, 3], Charalampia Kyriakou[1], Shirley D'sa[1], Jahanzaib Khwaja[1]

*[1]University College London Hospitals Trust, Haematology, London, United Kingdom;[2]The University of Queensland Faculty of Medicine, Brisbane, Australia; [3]East Kent Hospitals University NHS Foundation Trust, Kent, United Kingdom;*

### Background

Cold agglutinin disease (CAD) is a rare clonal autoimmune haemolytic anaemia characterised by IgM antibodies that bind to red cell antigens optimally at 4°C. CAD can result in clinically significant anaemia and debilitating circulatory symptoms. Daratumumab is a monoclonal antibody targeting CD38 and has been used in patients with refractory CAD.

### Aims

This study aimed to retrospectively evaluate the efficacy of plasma-cell depletion using daratumumab in a cohort of patients with refractory CAD after prior B-cell or complement-directed therapies.

### Methods

Patients treated with daratumumab for CAD following tertiary centre review between 2019-2025 were evaluated. Baseline characteristics, prior therapies and outcomes were collected. Response was defined as either normalisation of haemoglobin (Hb) or a sustained increase in Hb >20 g/L on at least two consecutive measurements, together with improvement in circulatory symptoms where present.

**Copyright Information:** (Online) ISSN: 2572-9241
©2026 The Author(s). HemaSphere is published by John Wiley & Sons Ltd on behalf of European Hematology Association. This is an open access Abstract Book distributed under the Attribution-NonCommercial-NoDerivs (CC BY-NC-ND), which allows third parties to download the articles and share them with others as long as they credit the author and the Abstract Book, but they cannot change the content in any way or use them commercially.
**Abstract Book Citations:** Authors, Title, HemaSphere, 2026;10:(S1):pages. The abstract book DOIs can be found on the "Author Information" page.
**Disclaimer:** Articles published in the journal HemaSphere exclusively reflect the opinions of the authors. The authors are responsible for all content in their abstracts including accuracy of the facts, statements, citing resources, etc.

**Results**

Seven patients were identified (4 male, 3 female): 6/7 primary CAD, 1/7 secondary to Waldenstrom macroglobulinaemia. At CAD diagnosis, median age was 62 (range 48-69) years, 6 had a detectable serum monoclonal IgM paraprotein and 1 IgG. 4/7 had a concurrent cryoglobulinemia (n=3 type I and n=1 type III). Median bone marrow trephine clonal infiltration was 7.5% (range 0-12.5) and 1/7 had MYD88 mutation detected. Median prior lines of therapy were 2 (range 1-4): 6/7 rituximab monotherapy; 3/7 chemoimmunotherapy (R-bendamustine, R-CHOP, chlorambucil, VCD, lenalidomide); 2/7 completement inhibitor. Median time from CAD diagnosis to daratumumab initiation was 105 months (1-192).

Indication for daratumumab was haemolytic anaemia (3/7), circulatory symptoms (1/7) and a combination of both (3/7). Circulatory symptoms were grade 3 (n=1 gangrene) and grade 2 (n=3 Raynaud-like symptoms affecting daily activities). The median number of monthly cycles administered was 3 (range 1–68). Treatment was interrupted in one patient due to incidental hepatitis E infection and response was regained upon treatment resumption. No treatment-related adverse events were observed in the remaining patients.

Clinical responses were observed in 4 patients (57%): haemolytic alone in 1/3, circulatory symptoms alone in 1/1 and 2/3 demonstrated both. Improvement in circulatory symptoms was seen in 75% (all of whom had grade 2 severity) whilst the non-responder had grade 3. Median duration of therapy was 3 months (range 1–76). After 6 months, median Hb improved to 147 g/L and monoclonal paraprotein reduced (Table 1).

At a median follow-up of 41 months (range: 4-80) after daratumumab initiation, median overall survival was not reached. Two deaths occurred due to severe haemolysis and relapsed lymphoma, respectively. Three remain on maintenance therapy for >36 months.

**Summary/Conclusion**

Treatment with daratumumab resulted in overall response in 57% of patients in this refractory cohort. Circulatory response seen in 75% and treatment was well tolerated. This highlights the potential importance of plasma-cell directed therapies in immune-driven disorders.

| | Beginning of treatment | After treatment |
|---|---|---|
| Haemoglobin g/L | 73 (60-150) | 147 (118-149) |
| Bilirubin µmol/L | 30.5 (8-101) | 20 (10-33) |
| Monoclonal protein, g/L | 8 (0-42) | 3 (0-45) |
| Serum kappa free light chain, mg/L | 24.8 (24.8-321) | 25.1 (16.5-41.4) |
| Serum lambda free light chain, mg/L | 11.1 (7.83-33) | 9.0 (5.4-11.5) |
| Serum free kappa: lambda Light Chain Ratio | 2.01 (1.05-41) | 2.8 (1.5-7.67) |

Table 1. Key parameters at baseline and 3-6 months after treatment

**Copyright Information:** (Online) ISSN: 2572-9241
©2026 The Author(s). HemaSphere is published by John Wiley & Sons Ltd on behalf of European Hematology Association. This is an open access Abstract Book distributed under the Attribution-NonCommercial-NoDerivs (CC BY-NC-ND), which allows third parties to download the articles and share them with others as long as they credit the author and the Abstract Book, but they cannot change the content in any way or use them commercially.
**Abstract Book Citations:** Authors, Title, HemaSphere, 2026;10:(S1):pages. The abstract book DOIs can be found on the "Author Information" page.
**Disclaimer:** Articles published in the journal HemaSphere exclusively reflect the opinions of the authors. The authors are responsible for all content in their abstracts including accuracy of the facts, statements, citing resources, etc.

### (PB4190) BEYOND MORPHOLOGY: RED BLOOD CELL FUNCTION, OXIDATIVE STRESS, AND IRON REGULATION IN HEREDITARY ELLIPTOCYTOSIS

**Topic:** 29. Enzymopathies, membranopathies and other red blood cell disorders

Vasiliki Chouliara*[1, 2], Polyxeni Delaporta[1, 2], Alkmini Anastasiadi[3], Aimilia Mantzou[4], Stavros Glentis[1, 2], Ilona Binenbaum[1, 2], Evangelos Premetis[4], Marianna Tzanoudaki[4], Konstantinos Bistas[1, 2], Elena Chatzikalil[1, 2], Efthalia-Faidra Agiomavriti-Stefanopoulou[1, 2], Ioannis Kereres[3], Ioannis Papassotiriou[4], Vasileios Tzounakas[3], Antonis Kattamis[1, 2]

*[1]Division of Pediatric Hematology-Oncology, First Department of Pediatrics, National and Kapodistrian University of Athens, Athens, Greece; [2]"Aghia Sophia" Children's Hospital, ERN-EuroBloodNet Center, Athens, Greece;[3]University of Patras, School of Medicine, Department of Biochemistry, Patras, Greece; [4]First Department of Pediatrics, National and Kapodistrian University of Athens, School of Medicine, Athens, Greece;*

### Background

Hereditary elliptocytosis (HE) is an inherited Red Blood Cell (RBC) membrane disorder caused by the disruption of the horizontal linkages of RBC cytoskeleton which results in impairing membrane deformability and in RBCs becoming elliptical. While most patients (pts) with common HE are either asymptomatic or have mild symptoms, they may still experience hemolytic crises especially during infancy or infections. Data regarding erythropoiesis and oxidative stress in HE-pts are limited.

### Aims

To investigate markers of erythropoiesis, angiogenesis and iron metabolism in HE-pts and to explore biochemical properties and functionality of HE-RBCs including membrane stability, redox balance and proteostasis capacity

### Methods

HE-pts followed at our unit and who had signed informed consent were evaluated. All pts underwent genetic analysis via targeted-Next Generation Sequencing (MiSeq, Ilumina) and/or Sanger Sequencing. Clinical and laboratory data were collected. Erythropoietin (EPO), soluble transferrin receptors (sTfR), Hepcidin (Hep-25), Placental Growth Factor (PlGF), and soluble fms-like Tyrosine Kinase 1 (sFlt-1) at steady phase were measured by means of immunoenzymatic assays. RBC characteristics including lysis, oxidative burden, proteasome activity, malondialdehyde (MDA) were assessed by spectrophoto/fluorometry and flow cytometry and compared to healthy controls. Values of $p<0.05$ were considered significant.

**Copyright Information:** (Online) ISSN: 2572-9241
©2026 The Author(s). HemaSphere is published by John Wiley & Sons Ltd on behalf of European Hematology Association. This is an open access Abstract Book distributed under the Attribution-NonCommercial-NoDerivs (CC BY-NC-ND), which allows third parties to download the articles and share them with others as long as they credit the author and the Abstract Book, but they cannot change the content in any way or use them commercially.
**Abstract Book Citations:** Authors, Title, HemaSphere, 2026;10:(S1):pages. The abstract book DOIs can be found on the "Author Information" page.
**Disclaimer:** Articles published in the journal HemaSphere exclusively reflect the opinions of the authors. The authors are responsible for all content in their abstracts including accuracy of the facts, statements, citing resources, etc.

### Results

7 HE-pts (2 males) of median age: 10.8 years, range:4.2-41, belonging to 2 families were included. 3 pts experienced neonatal jaundice and 2 pts hemolytic crisis requiring hospitalizations. Genetic analysis revealed a Pathogenic/Likely Pathogenic variant in *EPB41* gene (*NM_001376013.1:c.C1522T:p.Arg508Ter*) for family A (5 members) and *SPTA1* gene *(NM_003126.4:c.460_462dup:p.Leu155dup*) for family B (2 members). The Eosin-5′-maleimide binding test was either normal or inconclusive as 3 pts had reduced Mean Fluorescence Intensity Ratio and 1 pt showed a reduced footprint. In HE-pts the PlGF levels were significantly lower compared to the reported levels for apparently healthy individuals (p=0.015) while sFLT-1 levels did not differ significantly (p=0.578), resulting in a higher ratio of the two proteins sFLT-1/PlGF, suggestive of a switch towards the antiangiogenic condition. Hep-25 levels were significantly lower than in controls (p=0.019). STfR levels were elevated (p=0.015) while EPO values were within normal range. HE-RBCs presented increased hemolysis (measured as free hemoglobin, p=0.017) compared to controls. Intrinsic reactive oxygen species (ROS) did not differ significantly from controls, while diamide-induced ROS production and MDA levels were elevated (p=0.002). Chymotrypsin-like proteasome activity was reduced in the membrane (p=0.011) but increased in the cytosol (p=0.003). Further analysis revealed higher hemoglobin oxidation either as hemichromes (p=0.011) or as methemoglobin (p=0.024). Laboratory parameters are summarized in Figure 1.

### Summary/Conclusion

This exploratory analysis provides preliminary results of biochemical differences concerning both proteins and lipids in HE-pts warranting further investigation. We found that despite the mild clinical phenotype, there is significant lipid peroxidation of the membrane bilayer, protein and hemoglobin oxidation and membrane destabilization. The consequences of these alterations in determining the clinical course of HE and the development of complications need to be evaluated in larger studies.

| LABORATORY PARAMETERS | MEDIAN | RANGE |
|---|---|---|
| Free Hb (mg/dl) | 17.72 | 10.28-68.38 |
| Intrinsic ROS (MFI/mg of protein) | 1,315.66 | 1,152.20-1,652.07 |
| Diamide-induced ROS (MFI/mg of protein) | 1,470.37 | 1,308.53-3,008.02 |
| Intracellular Ca2+ (MFI/mg of protein) | 15,291.35 | 13,350.35-17,021.72 |
| Hemichromes (nM/mg) | 5.82 | 3.74-6.55 |
| Methemoglobin (%) | 0.71 | 0.37-0.96 |
| Cytosol: Chymotrypsin-Like Proteasome Activity (MFI/mg of protein) | 80,405.48 | 69,804.73-100,428.39 |
| Membrane: Chymotrypsin-Like Proteasome Activity (MFI/mg of protein) | 322,645.41 | 305,992.74-387,857.16 |
| Malondialdehyde (mM/ml RBC) | 58.42 | 47.97-71.28 |
| Hb Z-Score: Measured Hb-Mean Hb(Population)/Sd(Population | (-2) | (-2.8) - (-0.5) |
| Erythropoietin (mIU /ml) | 10.3 | 6.32-14 |
| Hepcidin-25 (ng/ml) | 35.05 | 27.6-65.8 |
| STfR (mg/L) | 1.98 | 1.29-3.3 |
| PlGF (pg/ml) | 6.75 | 3.53-11.3 |
| sFlt-1 (pg/ml) | 392 | 354-484 |
| LDH (U/L) | 217 | 123-331 |
| Ferritin (μg/L) | 29 | 14-108 |

Figure 1: Laboratory parameters of HE-patients

Copyright Information: (Online) ISSN: 2572-9241
©2026 The Author(s). HemaSphere is published by John Wiley & Sons Ltd on behalf of European Hematology Association. This is an open access Abstract Book distributed under the Attribution-NonCommercial-NoDerivs (CC BY-NC-ND), which allows third parties to download the articles and share them with others as long as they credit the author and the Abstract Book, but they cannot change the content in any way or use them commercially.
**Abstract Book Citations:** Authors, Title, HemaSphere, 2026;10:(S1):pages. The abstract book DOIs can be found on the “Author Information” page.
**Disclaimer:** Articles published in the journal HemaSphere exclusively reflect the opinions of the authors. The authors are responsible for all content in their abstracts including accuracy of the facts, statements, citing resources, etc.

### (PB4191) EARLY COMPLETE RESPONSE (CR60) IDENTIFIES A DISTINCT PROGNOSTIC SUBGROUP IN FIRST LINE STEROID-TREATED WARM AUTOIMMUNE HEMOLYTIC ANEMIA(WAIHA)

**Topic:** 29. Enzymopathies, membranopathies and other red blood cell disorders

Mattia D'antiga*[1], Anna Giordano[1], Andrea Serafin[1], Francesco Angotzi[1], Alessandro Cellini[1], Arianna Bevilacqua[1], Nicolò Danesin[1], Giovanni Leone[1], Francesco Piazza[1], Cinzia Vio[2], Alessandro Lanti[2], Livio Trentin[1], Fabrizio Vianello[1], Andrea Visentin[1]

*[1]University of Padua, Hematology Unit, Department of Medicine, Padova, Italy;[2]Azienda ospedale università Padova, Immunotransfusion Unit, Department of Medicine, Padua, Italy;*

### Background

Steroids remains the standard first-line therapy for warm Autoimmune Hemolytic Anemia (wAIHA), yet relapse is frequent. Guidelines differ on optimal full-dose duration and tapering, and whether treatment duration or early response kinetics better predict long-term outcome remains unclear.

### Aims

Evaluate the impact of full dose steroid duration on relapse-free survival (RFS) and to determine whether early hemolytic response identifies patients at differential relapse risk.

### Methods

We retrospectively analyzed consecutive wAIHA patients treated with first-line steroids alone between 2017 and February 2026. Inclusion required positive direct antiglobulin test (DAT); cold agglutinin disease was excluded.
Complete response was defined as hemoglobin > 120 g/L after 21 day of steroid treatment with normalization of bilirubin, LDH, haptoglobin and retyculocytes.
A 60-day landmark was selected to reflect early post-induction clinical assessment (CR60 = CR within 60 days from onset). The primary endpoint was RFS. Time-to-event outcomes were analyzed using Kaplan-Meier and Cox regression. Predictors of CR60 were assessed by logistic regression.

### Results

Among 124 identified cases, 47 were evaluable. Median age was 75 years (56-80); 60% were females and 62% had secondary wAIHA.  Forty-seven percent were DAT IgG without C3d.
Median full-dose prednisone duration was 21.5 days.  64% were steroid-responsive.
After a median follow-up of 25 months (95%CI 20-40), median RFS was 8 months (95%CI 2.5-20). Overall, 65% (30/46) patients achieved CR (median time to CR: 61 days (95% CI 40-114).
Full-dose duration (>21 days), DAT profile and secondary disease were not associated with RFS. Achievement of CR significantly reduced relapse risk (HR 0.29, 95%CI 0.13–0.63, p=0.002). Using the predefined landmark, CR60 identified distinct prognostic groups: 24-months RFS was 56% in CR60 vs 35% in non-CR60 patients (Figure 1, HR 0.32 95%CI 0.10-0.96, p= 0.042).
Early reticulocyte normalization (before day 60) strongly predicted CR60 (OR 39.0, 95% CI 6.3–769, p=0.001) and CR at any time (OR 5.67, 95%CI 1.33-24.11, p=0.019) whereas haptoglobin kinetics were not associated.

**Copyright Information:** (Online) ISSN: 2572-9241
©2026 The Author(s). HemaSphere is published by John Wiley & Sons Ltd on behalf of European Hematology Association. This is an open access Abstract Book distributed under the Attribution-NonCommercial-NoDerivs (CC BY-NC-ND), which allows third parties to download the articles and share them with others as long as they credit the author and the Abstract Book, but they cannot change the content in any way or use them commercially.
**Abstract Book Citations:** Authors, Title, HemaSphere, 2026;10:(S1):pages. The abstract book DOIs can be found on the "Author Information" page.
**Disclaimer:** Articles published in the journal HemaSphere exclusively reflect the opinions of the authors. The authors are responsible for all content in their abstracts including accuracy of the facts, statements, citing resources, etc.

**Summary/Conclusion**

Full-dose steroid duration did not influence relapse-free survival, nor did baseline characteristics. These findings question duration-driven strategies and suggest that prolonged exposure does not substantially modify disease trajectory.
In contrast, early response kinetics were prognostically relevant. CR achievement significantly reduced relapse risk, and the 60-day landmark identified patients with distinct long-term outcomes. Early reticulocyte normalization further supports the biological relevance of timely marrow recovery as a marker of effective disease control.
While limited by sample size and retrospective design, the magnitude and consistency of these associations suggest that early depth and timing response may better reflect disease biology than treatment duration. These data shift the focus from "how long steroids are given" to "how rapidly and completely hemolysis is controlled", particularly in an elderly population vulnerable to steroid toxicity.
Early complete response within 60 days identifies patients with significantly lower relapse risk. A 60-day CR landmark may serve as a clinically actionable endpoint to support risk-adapted management and identify patients who could benefit from earlier therapeutic escalation Prospective validation is warranted.

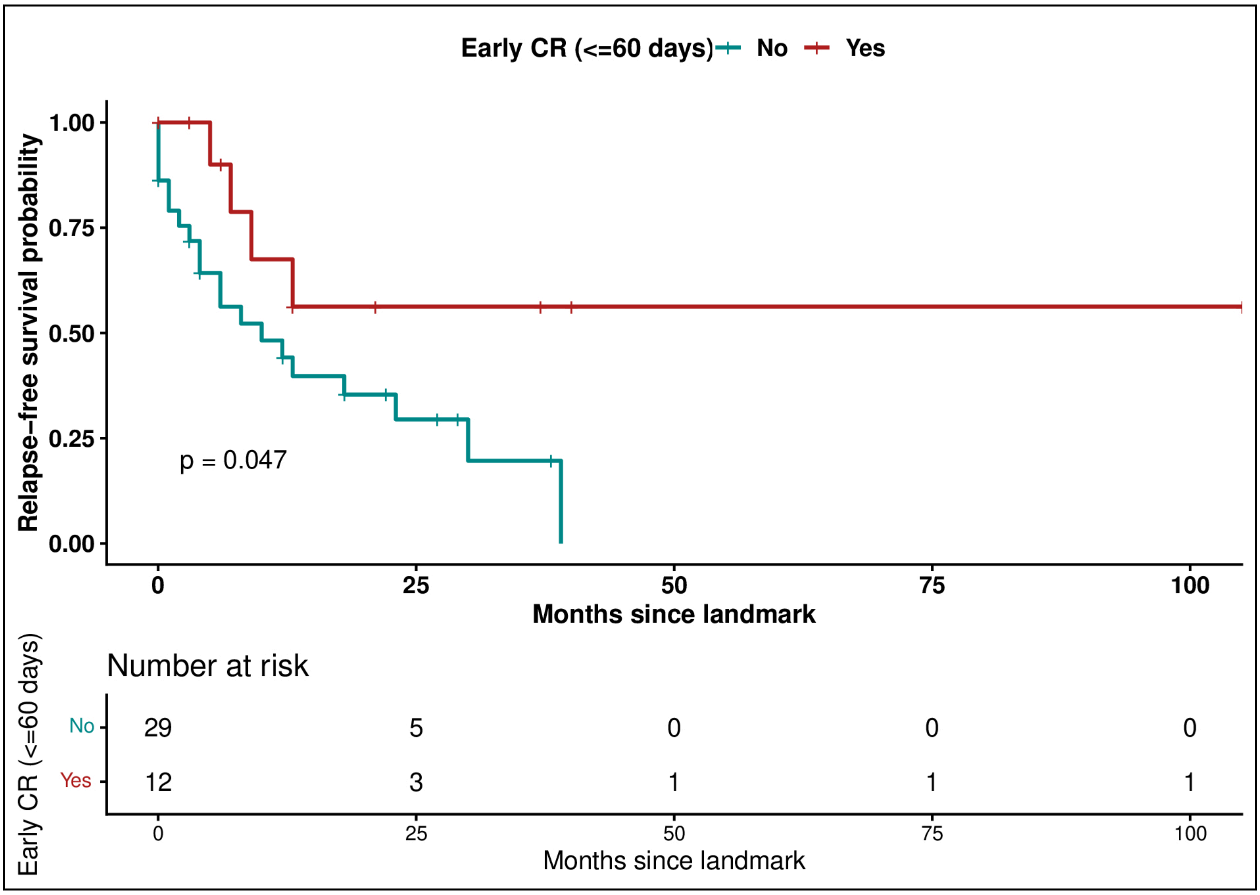

Figure 1

**Copyright Information:** (Online) ISSN: 2572-9241
©2026 The Author(s). HemaSphere is published by John Wiley & Sons Ltd on behalf of European Hematology Association. This is an open access Abstract Book distributed under the Attribution-NonCommercial-NoDerivs (CC BY-NC-ND), which allows third parties to download the articles and share them with others as long as they credit the author and the Abstract Book, but they cannot change the content in any way or use them commercially.
**Abstract Book Citations:** Authors, Title, HemaSphere, 2026;10:(S1):pages. The abstract book DOIs can be found on the "Author Information" page.
**Disclaimer:** Articles published in the journal HemaSphere exclusively reflect the opinions of the authors. The authors are responsible for all content in their abstracts including accuracy of the facts, statements, citing resources, etc.

### (PB4193) SAFETY OF LONG-TERM RILIPRUBART (SAR445088) TREATMENT IN COLD AGGLUTININ DISEASE: AN OPEN-LABEL STUDY IN RILIPRUBART-NAIVE AND PREVIOUSLY EXPOSED PARTICIPANTS

**Topic:** 29. Enzymopathies, membranopathies and other red blood cell disorders

Alexander Röth*[1], Wilma Barcellini[2], Shirley D'sa[3], Laure Malinge[4], Marek Wardecki[5], Shashi Bhushan[6]

*[1]Department of Hematology and Stem Cell Transplantation, West German Cancer Center, University Hospital Essen, University of Duisburg-Essen, Essen, Germany; [2]Fondazione IRCCS Ca' Granda Ospedale Maggiore Policlinico, Milan, Italy; [3]UCLH Centre for Waldenström's Macroglobulinaemia and Related Conditions, University College London Hospitals NHS Foundation Trust, London, United Kingdom; [4]Aixial (for Sanofi), Boulogne-Billancourt, France; [5]Sanofi, Warsaw, Poland; [6]Sanofi, Cambridge, Massachusetts, United States of America;*

### Background

Cold agglutinin disease (CAD) is a rare autoimmune hemolytic anemia caused by IgM-induced complement pathway activation. Inhibition of complement protein complex C1 has emerged as a viable treatment option for CAD. Riliprubart (SAR445088), a second-generation classical complement inhibitor, is a humanized monoclonal antibody that selectively inhibits the activated form of C1s.

### Aims

To investigate the long-term safety, tolerability, pharmacokinetics (PK), and pharmacodynamics (PD) of riliprubart in adult participants with CAD, who were previously exposed or naive to riliprubart.

### Methods

Eligible participants were enrolled into a Phase 1b, multicenter, open-label, nonrandomized extension study (NCT04802057). In Part 1, participants received riliprubart 30 mg/kg intravenous (IV) dose on Day 1 and 600 mg subcutaneously (SC) every 4 weeks (Q4W) from Day 29. In Part 2, participants continuing from Part 1 and participants with CAD who were naive to riliprubart received riliprubart 3.5 g IV every 3 months plus an additional riliprubart 3.5 g IV dose administered on Day 29. The primary endpoint was long-term safety and tolerability of riliprubart. Secondary endpoints included markers of anemia (hemoglobin) and complement mediated hemolysis (complement activity [CH50]). PK/PD analysis was performed for an earlier cut-off date (Chow et al 2025). The study was prematurely terminated by the Sponsor due to a business decision.

**Copyright Information:** (Online) ISSN: 2572-9241
©2026 The Author(s). HemaSphere is published by John Wiley & Sons Ltd on behalf of European Hematology Association. This is an open access Abstract Book distributed under the Attribution-NonCommercial-NoDerivs (CC BY-NC-ND), which allows third parties to download the articles and share them with others as long as they credit the author and the Abstract Book, but they cannot change the content in any way or use them commercially.
**Abstract Book Citations:** Authors, Title, HemaSphere, 2026;10:(S1):pages. The abstract book DOIs can be found on the "Author Information" page.
**Disclaimer:** Articles published in the journal HemaSphere exclusively reflect the opinions of the authors. The authors are responsible for all content in their abstracts including accuracy of the facts, statements, citing resources, etc.

**Results**

Study database lock date was 27 March 2025. Six of ten participants, who were enrolled and exposed to study intervention in either Part 1 (9 participants) or Part 2 (9 participants: 8 participants who rolled over from Part 1 and 1 naive participant) continued in the study until the premature termination of the study.
Cumulative duration of riliprubart exposure was 27.2 participant years (16.4 in Part 1 and 10.9 in Part 2). Median duration of exposure through both study parts was 39.3 (range: 9 to 46) months. TEAEs at system organ class level included general disorders and administration site conditions (n=7 [70%]), infections and infestations (n=6 [60%]), vascular disorders (n=5 [40%]), gastrointestinal disorders, and nervous system disorders (each reported in 4 [40%] participants). No deaths reported. Three TEAEs led to permanent discontinuation of riliprubart: Waldenström's macroglobulinemia (n=1), cyanosis (n=1), and elevated antinuclear antibody (n=1).
Except for worsening of hypertension (n=2) and increased antinuclear antibody titer (AE of special interest; n=1), no changes were observed in laboratory evaluations, vital signs, or electrocardiogram parameters. There were no reports of either pre-existing or treatment-emergent anti-drug antibodies.
Sustained control of hemolysis and anemia with quarterly IV dosing of riliprubart was evident for up to week 84 in Part 2. Mean and median hemoglobin level was maintained around 12 g/dL during Part 2. Except for one measurement at Part 1 W84, CH50 levels declined to below the lower limit of quantitation (10 IU/mL) after the first administration of riliprubart in both Parts 1 and 2.

**Summary/Conclusion**

In adult participants with CAD, long-term treatment with riliprubart (two dosing regimens in SC or IV) was generally safe and well tolerated, regardless of prior exposure, with few TEAEs reported as related to the study treatment. These findings provide valuable safety and efficacy data supporting the inhibition of complement C1s with riliprubart as a therapeutic approach in CAD.

**Copyright Information:** (Online) ISSN: 2572-9241
©2026 The Author(s). HemaSphere is published by John Wiley & Sons Ltd on behalf of European Hematology Association. This is an open access Abstract Book distributed under the Attribution-NonCommercial-NoDerivs (CC BY-NC-ND), which allows third parties to download the articles and share them with others as long as they credit the author and the Abstract Book, but they cannot change the content in any way or use them commercially.
**Abstract Book Citations:** Authors, Title, HemaSphere, 2026;10:(S1):pages. The abstract book DOIs can be found on the “Author Information” page.
**Disclaimer:** Articles published in the journal HemaSphere exclusively reflect the opinions of the authors. The authors are responsible for all content in their abstracts including accuracy of the facts, statements, citing resources, etc.

**Table 1. Adverse event profile in the safety population**

| | Part 1 (N=9) | Part 2 (N=9)* | All (N=10) |
|---|---|---|---|
| Any TEAE, n (%) | 8 (88.9) | 8 (88.9) | 9 (90.0) |
| Any severe TEAE, n (%) | 3 (33.3) | 4 (44.4) | 5 (50.0) |
| Any treatment emergent SAE, n (%) | 3 (33.3) | 1 (11.1) | 3 (30.0) |
| Any TEAEs occurring within 24 hours of infusion (i.e. same day or day after the infusion), n (%) | 1 (11.1) | 4 (44.4) | 5 (50.0) |
| Any TEAE leading to permanent study intervention discontinuation, n (%) | 0 | 3 (33.3) | 3 (30.0) |
| Any TEAE leading to permanent study discontinuation, n (%) | 0 | 0 | 3 (30.0) |
| Any treatment emergent AESI, n (%) | 0 | 1 (11.1) | 1 (10.0) |
| Any TEAE related to IMP, n (%) | 5 (55.6) | 4 (44.4) | 7 (70.0) |

TEAE, treatment emergent adverse event, SAE: serious adverse event, AESI: adverse event of special interest
An adverse event is considered severe if the grading is ≥3
N = Number of participants treated within each group, n (%) = number and % of participants with at least one TEAE in each Category
*Included 8 participants who rolled over from Part 1 and 1 naïve participant

Copyright Information: (Online) ISSN: 2572-9241
©2026 The Author(s). HemaSphere is published by John Wiley & Sons Ltd on behalf of European Hematology Association. This is an open access Abstract Book distributed under the Attribution-NonCommercial-NoDerivs (CC BY-NC-ND), which allows third parties to download the articles and share them with others as long as they credit the author and the Abstract Book, but they cannot change the content in any way or use them commercially.
Abstract Book Citations: Authors, Title, HemaSphere, 2026;10:(S1):pages. The abstract book DOIs can be found on the "Author Information" page.
Disclaimer: Articles published in the journal HemaSphere exclusively reflect the opinions of the authors. The authors are responsible for all content in their abstracts including accuracy of the facts, statements, citing resources, etc.

### (PB4194) GENOTYPE–PHENOTYPE CORRELATIONS OF HEREDITARY SPHEROCYTOSIS IN THE RUSSIAN FEDERATION: A POPULATION-BASED REGISTRY STUDY

**Topic:** 29. Enzymopathies, membranopathies and other red blood cell disorders

Mariia Luniakova[1], Svetlana Mann[2], Natalia Sokolova[3], Ivan Fisyun[4], Elena Raykina[5], Nataliya Smetanina*[6]

*[1]Ryazan State Medical University, Clinic of hematology, oncology and immunology, Ryazan, Russia;[2]Dmitry Rogachev National Medical Research Center Of Pediatric Hematology, Oncology and Immunology, Moscow, Russia; [3]Children's City Multidisciplinary Clinical Specialized Center of High Medical Technologies, Saint Petersburg, Russia; [4]Kruglaya Scientific-clinical multidisciplinary center for medical care for mothers and children, Orel, Russia; [5]Dmitry Rogachev National Research Center of Pediatric Hematology, Oncology, and Immunology, Moscow, Russia; [6]Dmitry Rogachev National Research Center of Pediatric Hematology, Oncology and Immunology, Moscow, Russia;*

### Background

Hereditary spherocytosis (HS) is the most common inherited hemolytic anemia in populations of Northern European descent, with an estimated prevalence of 1:2,000–5,000. HS results from defects in erythrocyte membrane and cytoskeletal proteins encoded primarily by *ANK1* (ankyrin-1), *SPTB1* (beta-spectrin), *SLC4A1* (band 3), *SPTA1* (alpha-spectrin) and *EPB42* (protein 4.2). The molecular spectrum of HS varies across populations; however, large-scale genetic data from the Russian Federation remain scarce.

### Aims

To characterize the molecular-genetic landscape of HS in Russian patients and to analyze genotype–phenotype correlations based on a national population-based registry.

### Methods

A population-based registry of pediatric patients with clinically confirmed HS was established across the Russian Federation. Demographic, clinical, and laboratory data were collected systematically. Molecular-genetic analysis targeting *ANK1, SPTB1, SLC4A1, SPTA1* and *EPB42* was performed in 241 of 485 registered patients using NGS targeted panel or whole genome sequencing. Detected variants were classified according to ACMG/AMP guidelines.

### Results

The registry enrolled 485 patients with confirmed HS from 86 centers across Russia. The distribution of causative mutations across genes was as follows: *SPTB1* — 91 unique variants (45,3 % of all mutations); *ANK1* — 69 variants (34,3 %); *SLC4A1* — 26 variants (12,9 %); *SPTA1* — 15 variants (7,5%). In total, 201 distinct variants were detected, of which 22 were recurrent (found in ≥2 unrelated patients). The most frequent recurrent mutations included c.4267C>T in *SPTB1* (n=7), c.44339-99C>T in *SPTA1* (n=6) and c.3754C>T, c.4306C>T, c.4462C>T in *ANK1* (n=3). Gene-map analysis demonstrated clustering of variants in specific functional domains. 58,3% of identified variants were novel, not previously reported in ClinVar or HGMD. Genotype–phenotype analysis revealed that *SLC4A1* mutations were associated with a milder clinical phenotype: over 55% of patients carrying *SLC4A1* variants presented with mild disease, and no severe HS cases were observed in this group. In contrast, patients harboring *SPTB1*, *ANK1*, or *SPTA1* mutations predominantly presented with moderate disease. The proportion of mild cases did not exceed 40%, while severe forms were observed in no more than 20% of patients, with mild, moderate, and severe phenotypes distributed in comparable proportions across these three genotype groups.

**Copyright Information:** (Online) ISSN: 2572-9241
©2026 The Author(s). HemaSphere is published by John Wiley & Sons Ltd on behalf of European Hematology Association. This is an open access Abstract Book distributed under the Attribution-NonCommercial-NoDerivs (CC BY-NC-ND), which allows third parties to download the articles and share them with others as long as they credit the author and the Abstract Book, but they cannot change the content in any way or use them commercially.
**Abstract Book Citations:** Authors, Title, HemaSphere, 2026;10:(S1):pages. The abstract book DOIs can be found on the "Author Information" page.
**Disclaimer:** Articles published in the journal HemaSphere exclusively reflect the opinions of the authors. The authors are responsible for all content in their abstracts including accuracy of the facts, statements, citing resources, etc.

**Summary/Conclusion**

This study represents the largest genotype–phenotype analysis of hereditary spherocytosis in the pediatric population of the Russian Federation to date. In the majority of Russian patients, HS was associated with defects in beta-spectrin and ankyrin, while 58.3% of identified variants were novel, indicating a distinctive mutational landscape in the Russian population. A clear genotype–phenotype correlation was established for *SLC4A1*: band 3 protein deficiency was exclusively associated with mild and, less frequently, moderate disease. The population-based registry proved to be an effective tool for systematic molecular-genetic analysis and identification of genotype–phenotype correlations in patients with HS in the Russian Federation.

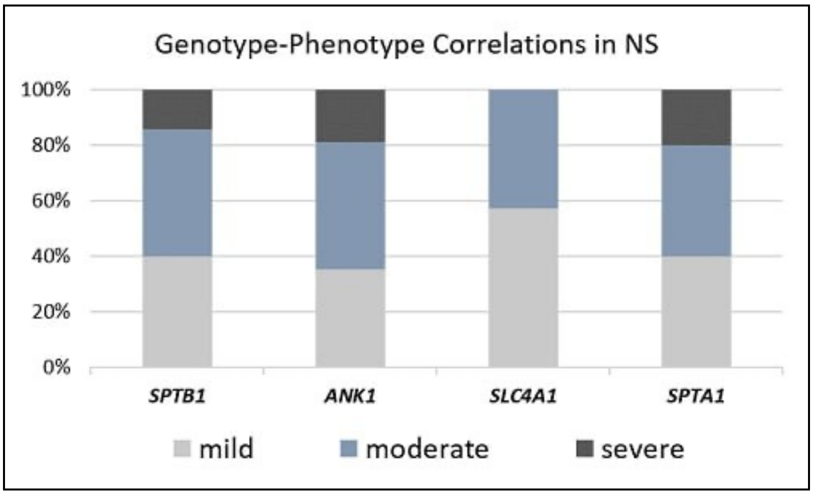

**Copyright Information:** (Online) ISSN: 2572-9241
©2026 The Author(s). HemaSphere is published by John Wiley & Sons Ltd on behalf of European Hematology Association. This is an open access Abstract Book distributed under the Attribution-NonCommercial-NoDerivs (CC BY-NC-ND), which allows third parties to download the articles and share them with others as long as they credit the author and the Abstract Book, but they cannot change the content in any way or use them commercially.
**Abstract Book Citations:** Authors, Title, HemaSphere, 2026;10:(S1):pages. The abstract book DOIs can be found on the "Author Information" page.
**Disclaimer:** Articles published in the journal HemaSphere exclusively reflect the opinions of the authors. The authors are responsible for all content in their abstracts including accuracy of the facts, statements, citing resources, etc.

### (PB4195) GAMGERTAMIG (OM336), A NOVEL B CELL MATURATION ANTIGEN (BCMA) X CD3 T CELL ENGAGER ANTIBODY, AMELIORATES DISEASE IN PATIENTS WITH WARM OR COLD AUTOIMMUNE HEMOLYTIC ANEMIA (AIHA)

**Topic:** 29. Enzymopathies, membranopathies and other red blood cell disorders

Philip Choi[1], Danny Hsu[2], Charlie Zhou*[3], Neelufar Mozaffarian[3]

*[1]Canberra Health Services, Haematology, Garran, Australia;[2]Liverpool Hospital and University of New South Wales, New South Wales, Australia; [3]Ouro Medicines, San Francisco, United States of America;*

### Background

Autoimmune hemolytic anemia (AIHA), including warm (wAIHA) and cold subtypes (cold agglutinin disease [CAD]), is an autoantibody driven disease with high unmet need. Current therapies include immunosuppressive and/or cytotoxic drugs, often associated with adverse effects and incomplete or non-durable efficacy. Long-lived plasma cells are critical producers of autoantibodies in AIHA but are not specifically targeted by current therapies. Gamgertamig (OM336) is an investigational, humanized, T cell engager (TCE) antibody targeting B cell maturation antigen (BCMA), leading to potent depletion of plasma and memory B cells, with the goal of "resetting" the immune system. >60 patients with highly active autoimmune diseases have received gagmertamig to date. Prior compassionate use of gamgertamig in patients with wAIHA has demonstrated compelling results [Zhang et al, NEJM, 2025].

### Aims

To report the initial experience for gamgertamig in patients with AIHA in a clinical trial setting, including the first report of a patient with CAD treated with a BCMA-targeting TCE.

**Copyright Information:** (Online) ISSN: 2572-9241
©2026 The Author(s). HemaSphere is published by John Wiley & Sons Ltd on behalf of European Hematology Association. This is an open access Abstract Book distributed under the Attribution-NonCommercial-NoDerivs (CC BY-NC-ND), which allows third parties to download the articles and share them with others as long as they credit the author and the Abstract Book, but they cannot change the content in any way or use them commercially.
**Abstract Book Citations:** Authors, Title, HemaSphere, 2026;10:(S1):pages. The abstract book DOIs can be found on the "Author Information" page.
**Disclaimer:** Articles published in the journal HemaSphere exclusively reflect the opinions of the authors. The authors are responsible for all content in their abstracts including accuracy of the facts, statements, citing resources, etc.

### Methods

NCT07083960 is an open-label, Phase 1b, multiple ascending dose, multinational study to assess the safety and tolerability of gamgertamig in adults with active autoimmune cytopenias (e.g., ITP, wAIHA, CAD or mixed forms). Eligible patients receive gamgertamig as a fractionated dose for a total of up to four subcutaneous injections. Three patients with AIHA have been enrolled, including one patient with CAD and two patients with wAIHA (Table 1).

**Table 1. Baseline Demographics**

| Patient # | Diagnosis | Age, years | Sex | Prior medications for AIHA | Medications for AIHA at enrolment | Baseline Hb (g/dL) | Gamgertamig regimen |
|---|---|---|---|---|---|---|---|
| 1 | CAD (PMHx of RA & CLL) | 76 | F | Zanubrutinib (CLL), Rituximab, Darbepoietin, Adalimumab (RA), Methotrexate (RA) | Zanubrutinib (CLL) | 8.6 | High dose |
| 2 | wAIHA | 44 | F | Prednisolone, Rituximab, Mycophenolate, Povetacicept | Prednisolone, Folic Acid | 6.8 | Mid dose |
| 3 | wAIHA | 71 | M | Prednisolone, Rituximab, Mycophenolate | Prednisolone, Mycophenolate | 9.9 | High dose |

Copyright Information: (Online) ISSN: 2572-9241
©2026 The Author(s). HemaSphere is published by John Wiley & Sons Ltd on behalf of European Hematology Association. This is an open access Abstract Book distributed under the Attribution-NonCommercial-NoDerivs (CC BY-NC-ND), which allows third parties to download the articles and share them with others as long as they credit the author and the Abstract Book, but they cannot change the content in any way or use them commercially.
Abstract Book Citations: Authors, Title, HemaSphere, 2026;10:(S1):pages. The abstract book DOIs can be found on the "Author Information" page.
Disclaimer: Articles published in the journal HemaSphere exclusively reflect the opinions of the authors. The authors are responsible for all content in their abstracts including accuracy of the facts, statements, citing resources, etc.

**Results**

Administration of gamgertamig has been completed in two patients. Gamgertamig was well-tolerated, with no cytokine release syndrome (CRS), immune effector cell-associated neurotoxicity syndrome (ICANS), infections, or serious adverse events.
At baseline for the patient with CAD, hemoglobin (Hb) was 8.6 g/dL which continued to decline, requiring one unit of packed red cells on Day 12 which resulted in a temporary improvement in Hb (9.2 g/dL on Day 15). After completion of gamgertamig, Hb improved to 10.6 g/dL on Day 81 with associated declines in lactate dehydrogenase (LDH), indirect bilirubin and increase in haptoglobin.
In the patient with AIHA, the Hb at baseline was 6.8 g/dL which increased to 8.5 g/dL by Day 15. LDH and reticulocytes were both significantly reduced by Day 15.
Preliminary PK were consistent with data from other gamgertamig trials. The patients will continue to be followed to Week 52 to assess ongoing clinical response.

**Summary/Conclusion**

This is the first clinical report of gamgertamig in patients with AIHA in a clinical trial setting. Preliminary efficacy data suggest improvements in anemia and biomarkers of hemolysis following a short course of gamgertamig; short-term safety and PK were favorable. These data warrant additional clinical investigation, with longer-term follow-up to assess the durability of response.

**Copyright Information:** (Online) ISSN: 2572-9241
©2026 The Author(s). HemaSphere is published by John Wiley & Sons Ltd on behalf of European Hematology Association. This is an open access Abstract Book distributed under the Attribution-NonCommercial-NoDerivs (CC BY-NC-ND), which allows third parties to download the articles and share them with others as long as they credit the author and the Abstract Book, but they cannot change the content in any way or use them commercially.
**Abstract Book Citations:** Authors, Title, HemaSphere, 2026;10:(S1):pages. The abstract book DOIs can be found on the "Author Information" page.
**Disclaimer:** Articles published in the journal HemaSphere exclusively reflect the opinions of the authors. The authors are responsible for all content in their abstracts including accuracy of the facts, statements, citing resources, etc.

### (PB4196) UPDATED HEMATOLOGIC GENOTYPE–PHENOTYPE ASSOCIATION IN GAUCHER DISEASE: A RETROSPECTIVE ANALYSIS OF LABORATORY PROFILES IN DIFFERENT GBA VARIANTS

**Topic:** 29. Enzymopathies, membranopathies and other red blood cell disorders

Estefania Rossetti[1], Silvia Eandi Eberle[1], Fernando Aguirre[1], Andrea Barrios[1], Jaqueline Vanesa Avalos Gomez*[1]

[1]*Hospital de Pediatría Dr. Juan P. Garrahan, Hematology, Buenos Aires, Argentina;*

### Background

Gaucher disease (GD) exhibits heterogeneous hematologic manifestations that may reflect underlying *GBA* variants. Cytopenias, particularly neutropenia and thrombocytopenia, are central features, yet their distribution across specific genotypes remains insufficiently characterized.

### Aims

To evaluate hematological involvement in patients with gaucher disease at diagnosis, and to analyze genotype-phenotype correlation.

### Methods

Observational, analytical, retrospective study, conducted in patients from a single center in Argentina with a confirmed diagnosis (by enzymatic testing and/or molecular diagnosis) of Gaucher disease, who initiated disease-specific therapy.
Variables analyzed included: hematological parameters (Hb-hemoglobin, WBC-white blood count, platelet count), and genetic variants detected (*GBA* gene mutations).
Genotype comparisons (N370S/RecNcil vs N370S/L444P vs L444P/?; ≥2 cases/group) were performed with the program MedCal.
Significance was set at α=0.05.

### Results

A total of 52 patients with a diagnosis of gaucher disease were analyzed. The Female/Male ratio was 1.1/1, age at diagnosis was 4.9 years (IQR 0.6-14.9). Median Hb levels was 10.4gr/dL (IQR 3.6-14.8), median WBC 6.05* 10^9/L (IQR 2.3-11), and median platelet count 102* 10^9/L (IQR 15.6-380).
Twenty-nine patients had complete hematologic and genetic data and were therefore included in the genotype-phenotype analysis.
Median levels of Hb, WBC and platelet count between different variants are described in table 1. Thrombocytopenia (<150*10^9/L) was present in 68.9% (20/29). The most frequent genotypes were N370S/RecNcil (n=8) and N370S/L444P (n=7).
Genotypes with ≥2 cases were included for comparative analysis: N370S/RecNcil (n=8), N370S/L444P (n=7), and L444P/? (n=3).
Differences in WBC and between genotypes N370S/RecNcil and N370S/L444P were not statistically significant (*p=0.66*), however there were statistically significant differences between N370S/RecNcil and L444P/? *(p=0.05*) and N370S/L444P with L444P/? (*p=0.008*). Similar findings were observed with absolute neutrophil count. Hb and platelet levels did not show statistically significant differences between genotypes.

**Copyright Information:** (Online) ISSN: 2572-9241
©2026 The Author(s). HemaSphere is published by John Wiley & Sons Ltd on behalf of European Hematology Association. This is an open access Abstract Book distributed under the Attribution-NonCommercial-NoDerivs (CC BY-NC-ND), which allows third parties to download the articles and share them with others as long as they credit the author and the Abstract Book, but they cannot change the content in any way or use them commercially.
**Abstract Book Citations:** Authors, Title, HemaSphere, 2026;10:(S1):pages. The abstract book DOIs can be found on the "Author Information" page.
**Disclaimer:** Articles published in the journal HemaSphere exclusively reflect the opinions of the authors. The authors are responsible for all content in their abstracts including accuracy of the facts, statements, citing resources, etc.

**Summary/Conclusion**

In this single-center cohort of patients with gaucher disease, cytopenias were common at diagnosis, with thrombocytopenia being the most frequent hematologic abnormality. While hemoglobin and platelet levels did not significantly differ across genotypes, white blood cell and absolute neutrophil counts showed significant variation, particularly in patients carrying the L444P/? genotype. These findings suggest a potential genotype–phenotype correlation, with certain *GBA* variants possibly associated with more pronounced leukocyte involvement at presentation.
Although limited by the retrospective design and small subgroup sizes, this study contributes to the understanding of hematologic heterogeneity in gaucher disease and highlights the importance of integrating genetic data into the initial clinical evaluation. Larger, multicenter studies are warranted to further clarify the impact of specific *GBA* variants on hematologic expression and disease severity.

**Copyright Information:** (Online) ISSN: 2572-9241
©2026 The Author(s). HemaSphere is published by John Wiley & Sons Ltd on behalf of European Hematology Association. This is an open access Abstract Book distributed under the Attribution-NonCommercial-NoDerivs (CC BY-NC-ND), which allows third parties to download the articles and share them with others as long as they credit the author and the Abstract Book, but they cannot change the content in any way or use them commercially.
**Abstract Book Citations:** Authors, Title, HemaSphere, 2026;10:(S1):pages. The abstract book DOIs can be found on the "Author Information" page.
**Disclaimer:** Articles published in the journal HemaSphere exclusively reflect the opinions of the authors. The authors are responsible for all content in their abstracts including accuracy of the facts, statements, citing resources, etc.

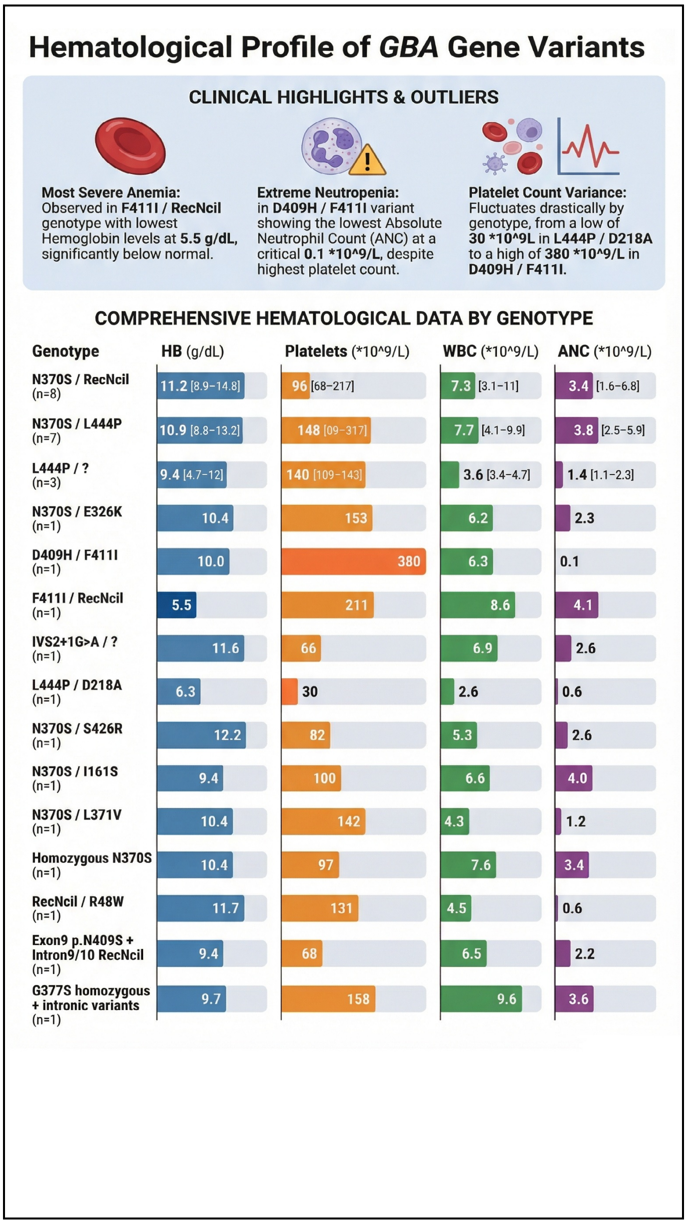

**Copyright Information:** (Online) ISSN: 2572-9241
©2026 The Author(s). HemaSphere is published by John Wiley & Sons Ltd on behalf of European Hematology Association. This is an open access Abstract Book distributed under the Attribution-NonCommercial-NoDerivs (CC BY-NC-ND), which allows third parties to download the articles and share them with others as long as they credit the author and the Abstract Book, but they cannot change the content in any way or use them commercially.
**Abstract Book Citations:** Authors, Title, HemaSphere, 2026;10:(S1):pages. The abstract book DOIs can be found on the "Author Information" page.
**Disclaimer:** Articles published in the journal HemaSphere exclusively reflect the opinions of the authors. The authors are responsible for all content in their abstracts including accuracy of the facts, statements, citing resources, etc.

### (PB4197) ORELABRUTINIB FOR THE TREATMENT OF REFRACTORY/RELAPSED AUTOIMMUNE HAEMOLYTIC ANAEMIA/EVANS SYNDROME: RESULTS FROM AN OPEN-LABEL, PHASE 2 TRIAL

**Topic:** 29. Enzymopathies, membranopathies and other red blood cell disorders

Hui Liu[1], Kai Ding[1], Wei Zhang[1], Mengting Che[1], Limin Xing[1], Yihao Wang[1], Weiwei Qi[1], Qing Shao[1], Jin Chen[1], Huaquan Wang[1], Jia Song[1], Lijuan Li[1], Zonghong Shao[1], Rong Fu*[1]

[1]*Tianjin Medical University General Hospital, Hematology, Tianjin, China;*

### Summary/Conclusion

Currently, there is no effective treatment for refractory/relapsed (R/R) autoimmune haemolytic anemia (AIHA), associated with poor quality of life. Bruton tyrosine kinase (BTK) inhibitors have begun to be used in some autoimmune diseases. We initiated the clinical trial of Orelabrutinib treatment on R/R AIHA/Evans Syndrome, which has completed the enrollment of 20 patients (ChiCTR2100045594). All the patients will receive 100mg qd of orelabrutinib treatment for 12 weeks, and be followed up for 48 weeks. Followed up until June 2025, 16 patients completed the treatment of 12 weeks, 1 patient was lost to follow-up in the second month of treatment, 1 patients withdrew due to ineffective treatment, and 2 patient withdrew due to severe skin bleeding and infection. After 12 weeks of treatment, ORR rate was 80% (16/20), CR rate was 45% (9/20). The average hemoglobin increased from 80.5g/L at baseline to 121.5g/L at week 12, with a more significant increase in wAIHA patients. Fifteen patients have completed a one-year follow-up, including 8 cases of CR, 2 case of PR, and 5 cases of relapse. We also observed a significant decrease in the proportion of $CD19^{+}$B lymphocytes and $CD5^{+}CD19^{+}$B cell subsets after treatment. For the safety, we observed mild infections (upper respiratory tract infection, urinary tract infection, pneumonia, etc.), skin bruising, conjunctival hemorrhage, bone marrow suppression, and elevated glutamic-pyruvic transaminase or glutamic oxaloacetic transaminase levels. Only one patient stopped treatment due to skin bruising. Orelabrutinib is expected to become a new second-line treatment for R/R AIHA/Evans syndrome

**Copyright Information:** (Online) ISSN: 2572-9241
©2026 The Author(s). HemaSphere is published by John Wiley & Sons Ltd on behalf of European Hematology Association. This is an open access Abstract Book distributed under the Attribution-NonCommercial-NoDerivs (CC BY-NC-ND), which allows third parties to download the articles and share them with others as long as they credit the author and the Abstract Book, but they cannot change the content in any way or use them commercially.
**Abstract Book Citations:** Authors, Title, HemaSphere, 2026;10:(S1):pages. The abstract book DOIs can be found on the “Author Information” page.
**Disclaimer:** Articles published in the journal HemaSphere exclusively reflect the opinions of the authors. The authors are responsible for all content in their abstracts including accuracy of the facts, statements, citing resources, etc.

### (PB4198) EMERGENCY DEPARTMENT UTILIZATION RATES FOR TRANSITIONS OF CARE PATIENTS WHO ESTABLISHED ADULT HEMATOLOGIC CARE AT SCD CENTER IN TEXAS IN 2023

**Topic:** 29. Enzymopathies, membranopathies and other red blood cell disorders

Alexandra Sova[1], Amanda Gregg*[1], Modupe Idowu[1]

[1]*University of Texas at Houston, Houston, United States of America;*

### Background

Sickle cell disease affects 1 in 350 non-Hispanic Black newborns in the United States For a subgroup of this population, adults aged 20-24 years, there is a 2.4 fold increase in mortality as patients transition into adulthood and away from pediatric care. Research has demonstrated that the transition to adulthood is a challenging time for SCD patients as they establish care as adults. In 2023, the Texas published data on ED visits by patients with SCD and reported 19,109 ED visits by individuals with SCD and that 13.2% of visits were made by 18-24yo. We examined ED utilization rates among transition patients defined as 18-25yo who established care at the Adult Sickle Cell center in 2023 to the statewide report.

### Aims

Given the increased mortality rate seen in this population, we aim to learn how best to advocate resources for them which requires state and federal funding. By comparing local SCD population ED utilization rates to national and other state averages, we can identify care gaps that contribute to higher ED utilization rates.

### Methods

We performed a retrospective chart review of 49 patients with sickle cell between the ages of 18-25yo at a comprehensive adult sickle cell center in Houston, Texas in 2023. We looked at their ED visits up to 2 years after establishing care at the SCC in 2023, as reported in the EMR. After determining the average ED utilization rate for our population, we compared it to known ED utilization rates of various populations to determine utilization rate significance. The populations considered were the general TX population, national sickle cell population based on Medicaid reporting, and state specific sickle cell populations, specifically CA and NC.

### Results

Based on the 49 patients that established care, the mean ED utilization for 2 years after establishing care was 4.53 (SD 4.34) visits per person. The majority of patients (32%) had no ED visits, while the next majority (26%) had 11 or more ED visits. Of the patients with 11 or more ED visits, 69% of the patients were of the HBSS genotype, 15% were of SC, 7% sickle beta plus thalassemia, and 7% sickle beta zero thalassemia. The average ED utilization rate for the general Texas population was 0.49 visits per person based on CDC reporting, and ED utilization was significantly higher in our population as expected. The national SCD population has an average ED utilization rate of 2.96, and our utilization rate was significant in comparison ($p<0.05$) based on t-test calculations. In CA, based on a study from 2005-2014 for the SCD population, the average ED utilization rate was 2.1, and our utilization rate was significant in comparison ($p<0.001$) based on t-test calculations. In NC, based on a study from 2013-2019, the average ED utilization rate was 3.6, and our utilization rate was not significant in comparison.

**Copyright Information:** (Online) ISSN: 2572-9241
©2026 The Author(s). HemaSphere is published by John Wiley & Sons Ltd on behalf of European Hematology Association. This is an open access Abstract Book distributed under the Attribution-NonCommercial-NoDerivs (CC BY-NC-ND), which allows third parties to download the articles and share them with others as long as they credit the author and the Abstract Book, but they cannot change the content in any way or use them commercially.
**Abstract Book Citations:** Authors, Title, HemaSphere, 2026;10:(S1):pages. The abstract book DOIs can be found on the "Author Information" page.
**Disclaimer:** Articles published in the journal HemaSphere exclusively reflect the opinions of the authors. The authors are responsible for all content in their abstracts including accuracy of the facts, statements, citing resources, etc.

**Summary/Conclusion**

When comparing TX ED visits with CA, TX has a higher utilization rate than CA but was on par with NC. In CA, the ED average was 2.71, in comparison to our sample mean of 4.53. Additionally, CA published ED utilization rates by age range in 2018, with the 20-29 age group having the highest utilization rate at 3.64 ED visits per person. Similar to other studies, patients with hgb SS disease are the most likely to go to the ED for care as seen in our patient population with 69% of patients having 11+ ED visits. We plan to continue investigating the possible reasons why transitions of care patients utilize the ED more frequently and furthermore may increase mortality.

Figure 1.

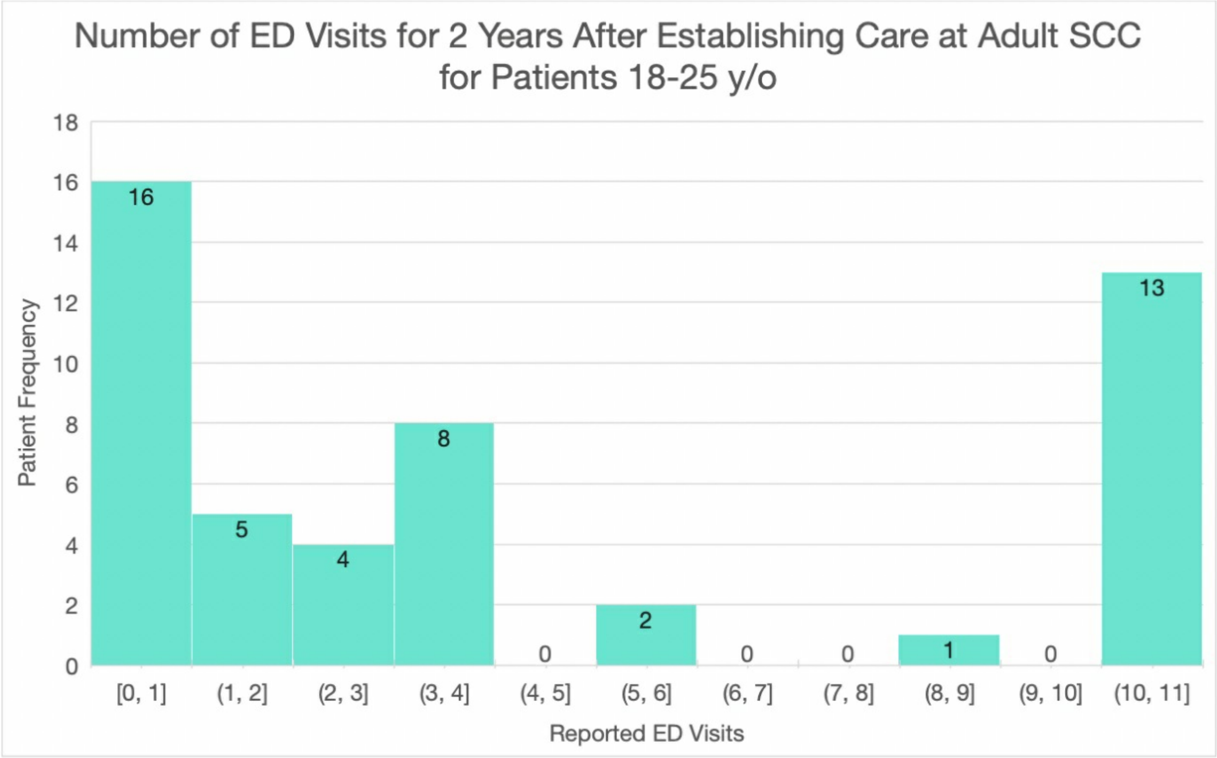

**Copyright Information:** (Online) ISSN: 2572-9241
©2026 The Author(s). HemaSphere is published by John Wiley & Sons Ltd on behalf of European Hematology Association. This is an open access Abstract Book distributed under the Attribution-NonCommercial-NoDerivs (CC BY-NC-ND), which allows third parties to download the articles and share them with others as long as they credit the author and the Abstract Book, but they cannot change the content in any way or use them commercially.
**Abstract Book Citations:** Authors, Title, HemaSphere, 2026;10:(S1):pages. The abstract book DOIs can be found on the "Author Information" page.
**Disclaimer:** Articles published in the journal HemaSphere exclusively reflect the opinions of the authors. The authors are responsible for all content in their abstracts including accuracy of the facts, statements, citing resources, etc.

### (PB4199) UNEXPECTED RESULTS IN NGS TESTING PERFORMED IN 33 PATIENTS DIAGNOSED WITH G6PD DEFICIENCY

**Topic:** 29. Enzymopathies, membranopathies and other red blood cell disorders

Marta M. Carracedo*[1], Miguel Gómez[2], Dylan Rudolf[1], Eduardo Jose Salido Fierrez[3], María Sánchez-Villalobos[3], Monica Lopez-Duarte[4], Cristina Alburquerque-Prieto[4], Elena Cela[5], Jorge Huerta[5], Cristina Seri[6], Ml Martin[7], María Irene Nuevo López[8], Ml Bengoechea[9], C Vicente[10], Silvia de la Iglesia[11], J Solano[12], L Garcia Alonso[13], Isabel Vicuña Andrés[14], Jorge Labrador[15], Julián Sevilla[16], Y Mozo[17], V Recasens[18], S Villanueva-Medina[19], Paloma Ropero[1], Fernando Ataulfo González Fernández[2], Ana María Villegas Martínez[2], Celina Benavente[2], Jorge M. Nieto[1]

*[1]Instituto de Investigación Sanitaria del Hospital Clínico San Carlos, Madrid, Spain;[2]Hospital Clinico San Carlos, Madrid, Spain; [3]Hospital Virgen de la Arrixaca, Murcia, Spain;[4]Hospital de Valdecilla, Santander, Spain;[5]Hospital Gregorio Marañón, Madrid, Spain; [6]Hospital Gómez Ulla, Madrid, Spain; [7]Hospital de Salamanca, Salamanca, Spain;[8]Hospital de Guadalajara, Guadalajara, Spain; [9]Hospital Severo Ochoa, Madrid, Spain; [10]BR Salud, Madrid, Spain; [11]Hospital Puerta de Hierro, Madrid, Spain; [12]Hospital Nuestra Señora del Prado, Madrid, Spain; [13]Hospital de Getafe, Madrid, Spain; [14]Hospital La Princesa, Madrid, Spain; [15]Hospital de Burgos, Madrid, Spain; [16]Hospital Niño Jesús, Madrid, Spain; [17]Hospital La Paz, Madrid, Spain; [18]Hospital Miguel Servet, Madrid, Spain; [19]Hospital Infanta Leonor, Madrid, Spain;*

### Background

Glucose-6-phosphate dehydrogenase (G6PD) deficiency is a recessive X-linked enzymatic disorder that causes hemolytic anemia outbreaks as it affects cellular defense mechanisms against oxidative stress. When suspected, an enzymatic test can be performed, having good results in males, but not always in females. Genetic study can help determine the genotype of these patients.

### Aims

To perform a G6PD variant description in our cohort of patients diagnosed with G6PD deficiency.

### Methods

We retrospectively studied 35 patients that were finally diagnosed with G6PD deficiency by NGS analysis. This study includes 18 Spanish hospitals. The patients had different clinical suspicions (23 G6PD deficiencies, 7 unexplained hemolytic anemia, 2 elliptocytosis/pyropoikilocytosis, 1 spherocytosis, 1 stomatocytosis and 1 methemoglobinemia). The pathogenicity of the variants found was analyzed using the ACMG criteria.

### Results

59 pathogenic(P)/likely pathogenic(LP) variants were found in the cohort, including 12 unique G6PD variants, as well as 4 variants in different anemia related genes (SPTA1, SEC23B, SLC2A1). Two of the found variants have not been previously described, summarized in Image 1. 18 patients presented a pathogenic G6PD haplotype (13 G6PD A-, 3 G6PD Santamaria and 2 G6PD A-(968)).
A 34% diagnosis change was found in our study. In our cohort, 23 men and 12 women were included, with diagnosis change ratios of 26% versus 50% in the female patients.
Three patients with a double pathology were detected, including 1 cryohydrocytosis, 1 pyropoikilocytosis (SPTA1 variant plus the Low-expression allele, Lyon (LELY)), and 1 spherocytosis, (SPTA1 variant plus LELY). A DCA type II carrier was also found.
32 patients with hemolytic anemia outbreaks were detected. Chronic hemolytic anemia was detected only in 1 patient, who also presented pyropoikilocytosis. In the two remaining patients, clinical information was not available.

**Copyright Information:** (Online) ISSN: 2572-9241
©2026 The Author(s). HemaSphere is published by John Wiley & Sons Ltd on behalf of European Hematology Association. This is an open access Abstract Book distributed under the Attribution-NonCommercial-NoDerivs (CC BY-NC-ND), which allows third parties to download the articles and share them with others as long as they credit the author and the Abstract Book, but they cannot change the content in any way or use them commercially.
**Abstract Book Citations:** Authors, Title, HemaSphere, 2026;10:(S1):pages. The abstract book DOIs can be found on the "Author Information" page.
**Disclaimer:** Articles published in the journal HemaSphere exclusively reflect the opinions of the authors. The authors are responsible for all content in their abstracts including accuracy of the facts, statements, citing resources, etc.

**Summary/Conclusion**

In our environment, men are more affected than women (2:1). We consider that female patients can be underdiagnosed as the G6PD gene is located in the X chromosome. This implies that, as they have a heterozygous genotype, their phenotype will be altered due to the random inactivation of this chromosome, making them mosaics for the pathology. This fact can produce a wide phenotype range in females between not presenting any symptoms, to suffer serious anemia outbreaks. This way, females with mild phenotype tend to be not diagnosed. This implies that the ratio between male and female could be more equal.
Chronic hemolytic anemia is rarely presented in this pathology, at least given the genetic variations found in our environment. This way, the chronic hemolytic anemia found in one patient could be caused by the doble pathology (pyropoikilocytosis) co-occurring in them.
One in three patients suffered a diagnosis change in our cohort. This states the relevance of the genetic study of patients with hemolytic anemia. This becomes more evident in female patients, as the enzymatic testing can result in false negative, due to the random inactivation of the X chromosome.

| Gene | Variant | Location | ACMG classification |
|---|---|---|---|
| G6PD (NM_001360016.2) | c.262_264del (Phe88del) | Chr X exon 4 | LP (PP4, PM2, PM4) |
| SLC2A1 (NM_006516.4) | c.953C>T (Thr318Met) | Chr 1 exon 7 | P (PM1, PP2, PM2, PP3) |

*Image 1. Novel genetic variants in G6PD and SLC2A1 genes. The table presents the new variants found, the gene they were found in, its location, and its ACMG classification: Pathogenic (P) or Likely pathogenic (LP). PM1: Non-truncating non-synonymous variant is located in a mutational hot spot and/or critical and well-established functional domain. PP2: Missense variant in a gene with low rate of benign missense mutations and for which missense mutation is a common mechanism of a disease. PM2: Extremely low frequency in gnomAD population databases. PP3: For a missense or a splicing region variant, computational prediction tools unanimously support a deleterious effect on the gene. PP4: Patient's phenotype or family history is highly specific for a disease with a single genetic etiology. PM4: Protein length changes resulting from in-frame deletions/insertions in a non-repeat region or a stop-loss variant.*

**Copyright Information:** (Online) ISSN: 2572-9241
©2026 The Author(s). HemaSphere is published by John Wiley & Sons Ltd on behalf of European Hematology Association. This is an open access Abstract Book distributed under the Attribution-NonCommercial-NoDerivs (CC BY-NC-ND), which allows third parties to download the articles and share them with others as long as they credit the author and the Abstract Book, but they cannot change the content in any way or use them commercially.
**Abstract Book Citations:** Authors, Title, HemaSphere, 2026;10:(S1):pages. The abstract book DOIs can be found on the "Author Information" page.
**Disclaimer:** Articles published in the journal HemaSphere exclusively reflect the opinions of the authors. The authors are responsible for all content in their abstracts including accuracy of the facts, statements, citing resources, etc.

### (PB4200) ANTICOAGULATION STRATEGIES IN PAROXYSMAL NOCTURNAL HEMOGLOBINURIA: A SYSTEMATIC REVIEW

**Topic:** 29. Enzymopathies, membranopathies and other red blood cell disorders

Bianca Constantin*[1, 2], Mariam Shenouda[3], Alla Iansavitchene[4], Ian Chin-Yee[4, 5], Alejandro Lazo-Langner[4, 5], Cyrus Hsia[4], Benjamin Chin-Yee[4, 5]

[1]*Fundeni Clinical Institute, Hematology, Bucharest, Romania;* [2]*Carol Davila University of Medicine and Pharmacy, Bucharest, Romania;* [3]*University of Toronto, Internal Medicine, Toronto, Canada;* [4]*London Health Sciences Centre, London, Canada;* [5]*Western University, London, Canada;*

**Background**

Paroxysmal nocturnal hemoglobinuria (PNH) is a rare clonal hematopoietic disorder characterized by complement-mediated hemolysis, bone marrow failure, and high risk of thrombosis and associated mortality. Although complement inhibitors reduce thrombotic incidence, optimal anticoagulation strategies have not been clearly established across clinical contexts, including primary prophylaxis, secondary prevention, anticoagulant selection, and management during high-risk situations such as pregnancy or breakthrough hemolysis.

**Aims**

To evaluate anticoagulant strategies and clinical outcomes in patients with PNH in various clinical settings.

**Methods**

A systematic review was conducted according to a preregistered protocol (PROSPERO CRD420251101985). Two reviewers independently performed screening, data extraction, and risk-of-bias assessment. Due to clinical and methodological heterogeneity, results were synthesized descriptively.

**Copyright Information:** (Online) ISSN: 2572-9241
©2026 The Author(s). HemaSphere is published by John Wiley & Sons Ltd on behalf of European Hematology Association. This is an open access Abstract Book distributed under the Attribution-NonCommercial-NoDerivs (CC BY-NC-ND), which allows third parties to download the articles and share them with others as long as they credit the author and the Abstract Book, but they cannot change the content in any way or use them commercially.
**Abstract Book Citations:** Authors, Title, HemaSphere, 2026;10:(S1):pages. The abstract book DOIs can be found on the “Author Information” page.
**Disclaimer:** Articles published in the journal HemaSphere exclusively reflect the opinions of the authors. The authors are responsible for all content in their abstracts including accuracy of the facts, statements, citing resources, etc.

### Results

Database searches identified 1,354 records; after title and abstract screening, 488 full texts were reviewed. 16 studies met inclusion criteria evaluating primary thromboprophylaxis, 19 studies addressed anticoagulation for secondary prevention following prior thrombosis, 4 studies evaluated outcomes according to anticoagulant class and dosing strategies, and 18 studies examined anticoagulation management in high-risk clinical scenarios.

**Primary thromboprophylaxis:** 16 evaluated primary thromboprophylaxis and consisted predominantly of retrospective cohorts and registry analyses with highly heterogeneous populations. Direct comparative evidence was limited. In the pre-complement inhibitor era, one cohort demonstrated a substantial reduction in thrombotic events among patients with large PNH clones receiving vitamin K antagonist prophylaxis compared with untreated patients with high long-term thrombotic risk, at the cost of infrequent but clinically significant bleeding. In contemporary cohorts, prophylaxis with vitamin K antagonists, heparins, or direct oral anticoagulants were reported; however, most studies lacked comparator groups and rarely stratified outcomes according to complement inhibitor exposure.

**Secondary prevention:** 19 studies addressed anticoagulation following prior thrombosis. Most patients received long-term anticoagulation in combination with complement inhibition, with low recurrence rates reported in contemporary cohorts. 3 studies described discontinuation of anticoagulation after initiation of complement inhibitors with low thrombotic recurrence.

**High-risk clinical scenarios:** 18 studies evaluated anticoagulation in high-risk settings, predominantly pregnancy. Across 131 reported pregnancies in women with PNH, anticoagulation was administered in over 80% of cases, most commonly prophylactic or intermediate-dose LMWH. In the complement inhibitor era, thrombotic events during pregnancy or postpartum were rare, bleeding complications were predominantly non-major, and maternal mortality was reported only in isolated cases, with generally favorable fetal outcomes.

### Summary/Conclusion

Anticoagulation remains an important component in management of PNH; however, evidence is heterogeneous and major gaps remain regarding patient selection, duration of therapy, and anticoagulant choice. Future registry-based studies which systematically document anticoagulation use, thrombotic and bleeding outcomes may help to clarify optimal anticoagulation strategies in PNH across clinical contexts.

**Copyright Information:** (Online) ISSN: 2572-9241
©2026 The Author(s). HemaSphere is published by John Wiley & Sons Ltd on behalf of European Hematology Association. This is an open access Abstract Book distributed under the Attribution-NonCommercial-NoDerivs (CC BY-NC-ND), which allows third parties to download the articles and share them with others as long as they credit the author and the Abstract Book, but they cannot change the content in any way or use them commercially.
**Abstract Book Citations:** Authors, Title, HemaSphere, 2026;10:(S1):pages. The abstract book DOIs can be found on the "Author Information" page.
**Disclaimer:** Articles published in the journal HemaSphere exclusively reflect the opinions of the authors. The authors are responsible for all content in their abstracts including accuracy of the facts, statements, citing resources, etc.

### (PB4201) EPIDEMIOLOGY AND NATURAL HISTORY OF WARM AUTOIMMUNE HEMOLYTIC ANEMIA (WAIHA): INSIGHTS FROM LABORATORY TEST RESULTS IN ONTARIO, CANADA

**Topic:** 29. Enzymopathies, membranopathies and other red blood cell disorders

Jennifer Leung[1], Benjamin Chin-Yee*[2], Hasan Ghaffar[3], Fernando Camacho[4], Bernie Sattin[5], Christine Cserti[3], Cyrus Hsia[2]

*[1]Queen's Univeristy, Kingston, Canada; [2]Western Univeristy, London, Canada; [3]University of Toronto, Toronto, Canada; [4]Damos Inc, Toronto, Canada; [5]Johnson & Johnson, Toronto, Canada;*

### Background

Warm autoimmune hemolytic anemia (wAIHA) is a rare immune-mediated hemolytic disorder mediated by warm-reactive IgG autoantibodies. Its symptoms are serious and often life-threatening, frequently leading to recurrent red blood cell transfusions, hospitalizations, and increased mortality. In depth knowledge is scarce, and often grouped with other AIHA's. Improving our understanding of the epidemiology and natural history of wAIHA is essential to deepen our comprehension of the disease and improve patient outcomes.

### Aims

To describe the incidence and prevalence of wAIHA in Ontario, Canada, as well as the disease course.

### Methods

The OPTUM EHR database was used to validate lab-test-based definitions that could identify wAIHA cases in a previously validated health-claims-based "gold" standard; these conditions were further refined based on clinician input. The database of a large community laboratory network serving Ontario was then retrospectively analyzed to identify adults (>=18 years old) who met these lab conditions between January 1, 2016 and December 31, 2023. The results were used to estimate the incidence and prevalence of wAIHA using a Bayesian approach. To evaluate disease course, active clinical disease was defined as ≥1 hemoglobin test result <100 g/L in a calendar year.

### Results

Multiple lab-test-based case definitions were validated against an ICD-10-based health claims definition (Table 1). wAIHA-LAB1 (Hb < 100 g/L and positive DAT) had a low sensitivity (0.356) which improved when restricted to association with a hematologist or oncologist (0.776). All definitions have high specificity. The Bayesian model for wAIHA-LAB1 estimated a mean 389 annual incident cases, corresponding to a standardized incidence of 2.3/100,000 people (95% credibility interval = 0.11-4.74 per 100,000), and a prevalence of 11.5 - 16.1 cases/100,000. This was stable over the time period examined. Across all lab-test-based definitions, the dataset showed a female predominance (63-70%), with the majority (55-64%) of cases in patients ≥65. In the first follow up year, 62% had ongoing disease, decreasing to 35% in the 6th year of follow up (without accounting for mortality or loss of follow up). Case volumes were generally evenly distributed across the province.

**Copyright Information:** (Online) ISSN: 2572-9241
©2026 The Author(s). HemaSphere is published by John Wiley & Sons Ltd on behalf of European Hematology Association. This is an open access Abstract Book distributed under the Attribution-NonCommercial-NoDerivs (CC BY-NC-ND), which allows third parties to download the articles and share them with others as long as they credit the author and the Abstract Book, but they cannot change the content in any way or use them commercially.
**Abstract Book Citations:** Authors, Title, HemaSphere, 2026;10:(S1):pages. The abstract book DOIs can be found on the "Author Information" page.
**Disclaimer:** Articles published in the journal HemaSphere exclusively reflect the opinions of the authors. The authors are responsible for all content in their abstracts including accuracy of the facts, statements, citing resources, etc.

**Summary/Conclusion**

We report the first estimate of the incidence and prevalence of wAIHA across Canada's most populous province, using a novel lab-test-based approach. Epidemiology, and clinical course were consistent across multiple lab-test-based definitions of wAIHA. In keeping with prior estimates, we observed a female predominance and an increasing incidence and prevalence with advancing age. Our lab-test-based case definitions offers a scalable approach for population-level surveillance and could support the development of public health monitoring tools, including real-time detection dashboards. The persistence of active disease, evidenced by prolonged anemia in a substantial proportion of patients over 6-years of follow up, highlights a significant unmet need in the management of wAIHA.

Table 1. Sensitivity and specificity for lab-test-based wAIHA definitions for Bayesian model

| | **Sensitivity** | | | **Specificity** | |
|---|---|---|---|---|---|
| **Case** | lower bound | Estimate | Upper bound | Low | Mean |
| **wAIHA-ICD vs. wAIHA-LAB1** | 0.30 | 0.356200 | 0.40 | 0.99 | 0.99987 |
| **wAIHA-ICD vs. wAIHA-LAB2** | 0.55 | 0.608760 | 0.65 | 0.99 | 0.99343 |
| **wAIHA-ICD-HO vs. wAIHA-LAB1-HO** | 0.72 | 0.775862 | 0.90 | 0.99 | 0.99990 |

wAIHA-ICD: 2 or more wAIHA ICD-10 code entries (D59.11) between OCT/01/2020 and JUL/31/2024, and no AIHA diagnostic code (D59.1) from OCT/01/2015 -SEP/30/2020

wAIHA-ICD-HO: 2 or more wAIHA ICD-10 code entries **by a hematologist or oncologist** (D59.11) between OCT/01/2020 and JUL/31/2024, and no AIHA diagnostic code (D59.1) from OCT/01/2015 -SEP/30/2020

wAIHA-LAB1: Positive DAT test result between OCT/01/2020 – JUL/31/2024, and no AIHA code (D59.1) from OCT/01/2015-SEP/30/2020, and Hb < 10 within 4 weeks of DAT result.

wAIHA-LAB2: Hb < 10 between OCT/01/2020 – JUL/31/2024, no AIHA code (D59.1) from OCT/01/2015-SEP/30/2020, and within 4 weeks of the Hb result, at least one of: high reticulocyte, high indirect bilirubin, low haptoglobin, high lactate dehydrogenase and/or positive DAT

wAIHA-LAB1: Positive DAT test result between OCT/01/2020 – JUL/31/2024, and no AIHA code (D59.1) from OCT/01/2015-SEP/30/2020, and Hb < 10 within 4 weeks of DAT result. All tests ordered by a hematologist or oncologist

Table 1

**Copyright Information:** (Online) ISSN: 2572-9241
©2026 The Author(s). HemaSphere is published by John Wiley & Sons Ltd on behalf of European Hematology Association. This is an open access Abstract Book distributed under the Attribution-NonCommercial-NoDerivs (CC BY-NC-ND), which allows third parties to download the articles and share them with others as long as they credit the author and the Abstract Book, but they cannot change the content in any way or use them commercially.
**Abstract Book Citations:** Authors, Title, HemaSphere, 2026;10:(S1):pages. The abstract book DOIs can be found on the "Author Information" page.
**Disclaimer:** Articles published in the journal HemaSphere exclusively reflect the opinions of the authors. The authors are responsible for all content in their abstracts including accuracy of the facts, statements, citing resources, etc.

### (PB4202) DIFFERENT DISEASES, DIFFERENT RESPONSES: TREATMENT OUTCOMES IN COLD AGGLUTININ DISEASE VS SYNDROME

**Topic:** 29. Enzymopathies, membranopathies and other red blood cell disorders

Uros Markovic*[1], Sebastiano Salvatore Pergolizzi[2], Gaetano Giuffrida[1], Dario Leotta[1, 3], Gabriella Santuccio[1, 3], Annalisa Chiarenza[1], Amalia Figuera[1], Lara Gullo[1], Stephanie Grasso[1], Dario Frenna[4], Sara Celauro[4], Francesco Di Raimondo[1, 3], Alessandra Romano[1, 3]

*[1]Hematology and Bone Marrow Transplatation Unit, A.O.U. Policlinico "G. Rodolico - S.Marco", Catania, Italy; [2]Transfusion Medicine Unit, A.O.U. Policlinico "G. Rodolico - S.Marco", Catania, Italy; [3]Post-Graduation School of Haematology, University of Catania, Catania, Italy; [4]University of Catania, Catania, Italy;*

### Background

Cold autoimmune hemolytic anemia (cAIHA) is caused by IgM autoantibody-based and complement-mediated both intra- and extravascular hemolysis when exposed to cold temperatures. It is divided in cold agglutinin disease (CAD), generally chronic and idiopathic or caused by indolent lymphoproliferative B cell infiltration, and cold agglutinin syndrome (CAS) secondary to infections, autoimmune disorders or paraneoplastic that has acute and self-limiting presentation.

### Aims

Evaluate differences in treatment response and remission rates between CAD and CAS patients using different therapies.

### Methods

Thirty-three patients were diagnosed with cAIHA between January 2020 and May 2025 with positive direct antiglobulin test (DAT) and cold agglutinin titer of at least 1:64 independently from concomitant IgG presence. They received immunosuppressive therapy at a median age of 63 years due to moderate/severe hemolytic anemia, and were evaluated retrospectively after a median follow-up (FUP) of 18.5 months. Nineteen patients (58%) with monoclonal B cell bone marrow infiltration were diagnosed as CAD, while 14 of them (42%) had secondary causes and were defined as CAS.

**Copyright Information:** (Online) ISSN: 2572-9241
©2026 The Author(s). HemaSphere is published by John Wiley & Sons Ltd on behalf of European Hematology Association. This is an open access Abstract Book distributed under the Attribution-NonCommercial-NoDerivs (CC BY-NC-ND), which allows third parties to download the articles and share them with others as long as they credit the author and the Abstract Book, but they cannot change the content in any way or use them commercially.
**Abstract Book Citations:** Authors, Title, HemaSphere, 2026;10:(S1):pages. The abstract book DOIs can be found on the "Author Information" page.
**Disclaimer:** Articles published in the journal HemaSphere exclusively reflect the opinions of the authors. The authors are responsible for all content in their abstracts including accuracy of the facts, statements, citing resources, etc.

**Results**

CAS patients were slightly older (65 vs 61 years respectively), had mixed AIHA with cold IgM prevalence more frequently (64% vs 32%, p=0.085, Odds ratio (OR) 0.26, 95% CI 0.06-1.10) and lower median Hb level (5 vs 8.5 g/dL), while higher agglutinin titer was present in CAD group (1:1024 vs 1:512, Fisher's exact test p=0.015). Mixed AIHA and agglutinin titer levels were confirmed as statistically significant using binary logistic regression for multivariate analysis (p=0.03 each).
In the CAD subgroup after a median FUP of 31 months out of 14 patients that received first-line corticosteroids (CCS) alone, response was achieved in 60%, although only two of them (14%) remained in remission following treatment discontinuation. Fourteen CAS patients were treated with CCS alone, 85% responded, and in eight (58%) remission was maintained after median FUP of 11 months, evidencing higher remission probability (Odds ratio (OR) 0.12, 95% CI 0.02-0.79, Fisher's exact test p=0.04) (Fig. 1).
Rituximab led to durable remission in both 88% CAD patients (14/16) and 50% CAS (3/6) independently from treatment line.
Although CAS patients had lower relapse incidence following remission (9 vs 41%), there was no statistical significance probably due to shorter follow-up period.
Median overall survival (OS) for the study cohort was not reached using Kaplan-Meier analysis, while relapse-free survival (RFS) was 37 months, with no impact in terms of survival based on CAD status. As for the separate survival analysis, CAD patients had a better trend of OS if Hb>8 g/dL (p=0.052, HR 0.16, 95% CI 0.03-1.02), while younger age (<65 years) was predictive of both superior OS (p=0.03) and positive trend of RFS (p=0.055) in the CAS group.
Ten patients (30%) had subsequent relapse after a median period of 10 months, while twelve patients passed away during follow-up at a median age of 74 years.

**Summary/Conclusion**

Even though cAIHA patients are generally treated with rituximab, it is not always possible, due to delayed DAT results. Furthermore, CCS can be of aid in case of mixed antibodies with cold agglutinin prevalence. In our experience CAS patients are more likely to achieve remission following CCS alone, probably due to concomitant IgG presence, without impacting survival outcomes, although rituximab remains the first choice.

**Copyright Information:** (Online) ISSN: 2572-9241
©2026 The Author(s). HemaSphere is published by John Wiley & Sons Ltd on behalf of European Hematology Association. This is an open access Abstract Book distributed under the Attribution-NonCommercial-NoDerivs (CC BY-NC-ND), which allows third parties to download the articles and share them with others as long as they credit the author and the Abstract Book, but they cannot change the content in any way or use them commercially.
**Abstract Book Citations:** Authors, Title, HemaSphere, 2026;10:(S1):pages. The abstract book DOIs can be found on the "Author Information" page.
**Disclaimer:** Articles published in the journal HemaSphere exclusively reflect the opinions of the authors. The authors are responsible for all content in their abstracts including accuracy of the facts, statements, citing resources, etc.

**Figure 1: Corticosteroid-remission rate in CAD versus CAS patients**

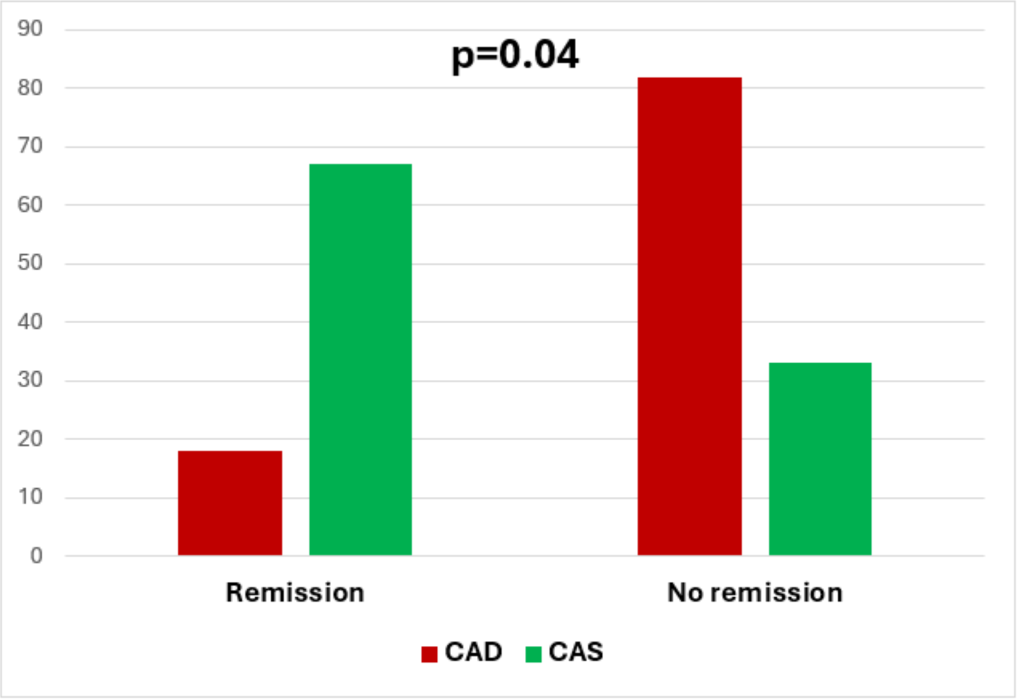

Abbreviations: CAD – Cold agglutinin disease; CAS – Cold agglutinin syndrome.

**Copyright Information:** (Online) ISSN: 2572-9241
©2026 The Author(s). HemaSphere is published by John Wiley & Sons Ltd on behalf of European Hematology Association. This is an open access Abstract Book distributed under the Attribution-NonCommercial-NoDerivs (CC BY-NC-ND), which allows third parties to download the articles and share them with others as long as they credit the author and the Abstract Book, but they cannot change the content in any way or use them commercially.
**Abstract Book Citations:** Authors, Title, HemaSphere, 2026;10:(S1):pages. The abstract book DOIs can be found on the "Author Information" page.
**Disclaimer:** Articles published in the journal HemaSphere exclusively reflect the opinions of the authors. The authors are responsible for all content in their abstracts including accuracy of the facts, statements, citing resources, etc.

### (PB4203) HEREDITARY STOMATOCYTOSIS IN CHILDREN OF THE RUSSIAN FEDERATION

**Topic:** 29. Enzymopathies, membranopathies and other red blood cell disorders

Vladislav Klyukhin*[1], Svetlana Mann[1], Elena Raykina[1], Nataliya Smetanina[1]

[1]*Dmitry Rogachev National Research Center of Pediatric Hematology, Oncology, and Immunology, Moscow, Russia;*

### Background

Hereditary stomatocytosis (HSts) is a group of rare hereditary anemia characterized by impaired electrolyte transport, leading to changes in membrane integrity and the appearance of stomatocytes in the peripheral blood. The majority of these anemias are transmitted in an autosomal dominant pattern and are linked to pathogenic variants, predominantly in the *RHAG*, *KCNN4*, and *PIEZO1* genes, and to a lesser extent in *ABCG5*, *ABCG8*, *SLC2A1*, and *ABCB6*. The clinical and laboratory manifestations of such patients are characterized by macrocytic hemolytic anemia of varying severity, reticulocytosis, and splenomegaly.

### Aims

To characterize the clinical and genetic features of HSts in a Russian pediatric cohort.

### Methods

We analyzed 12 patients with a confirmed diagnosis of HSts of whom 10 were male, and 2 were female. The mean age was 13.7 years (range: 1–53 years). The DNA sequencing was performed on the DNBSEQ-T7 platform (MGI, China) using paired-end reading (150x2) with an average depth of 38x and 98% coverage at a minimum reading depth of 10x. The MGIEasy FS PCR-Free DNA Library Prep Set (MGI, China) was utilized for sample preparation.

### Results

Ten patients were diagnosed with dehydrated stomatocytosis (DHS), and two with overhydrated stomatocytosis (OHS). Severe symptoms were observed in 3 of 12 patients, while 9 of 12 had mild clinical manifestations. In 8 patients diagnosed with HSt, the pathological variant was identified in the *PIEZO1* gene, while in the remaining four patients, it was identified in the *KCNN4* and *RHAG* genes, with 2 patients per gene. Missense variants in the *KCNN4* and *RHAG* genes were predominantly located in exons (exons 2, 6, and 7), and one splice variant was identified in intron 3 of the *RHAG* gene **(c.492+1G>T),** which is novel and has not been previously described. The present study found that all mutations in the *PIEZO1* gene occurred within exons. Of particular note is the identification of four new variants: three missense variants — **c.7285C>T, p.(Pro2429Ser); c.3284A>C, p.(Asn1095Thr); c.3033G>A, p.(Met1011Ile),** as well as a large duplication of 26 kilobases.

Laboratory tests revealed mild anemia in all groups. In the *PIEZO1* group (n = 8), the median Hb was 120 g/L (105-153), MCV was 86 fl (75–94), and mean corpuscular hemoglobin concentration (MCHC) was 368 g/L (353–382). Furthermore, high reticulocytosis was observed, with a median value of 0.235 (0.08–0.44). In the *KCNN4* group (n = 2), Hb level was 102–105 g/L, and the MCHC was 341–345 g/L. In the *RHAG* group (n = 2), the most significant variability in MCHC was observed (310–361 g/L), as well as a pronounced tendency toward macrocytosis (increase in MCV to 109.7). LDH activity in the subgroups exhibited no significant variation, while ferritin levels fluctuated between 31 and 1200 µg/L. The most pronounced distribution of ferritin levels (ranging from 34.7 to 1200) was observed in patients with a mutation in *PIEZO1*, indicative of the extent of iron overload in this cohort.

**Copyright Information:** (Online) ISSN: 2572-9241
©2026 The Author(s). HemaSphere is published by John Wiley & Sons Ltd on behalf of European Hematology Association. This is an open access Abstract Book distributed under the Attribution-NonCommercial-NoDerivs (CC BY-NC-ND), which allows third parties to download the articles and share them with others as long as they credit the author and the Abstract Book, but they cannot change the content in any way or use them commercially.
**Abstract Book Citations:** Authors, Title, HemaSphere, 2026;10:(S1):pages. The abstract book DOIs can be found on the “Author Information” page.
**Disclaimer:** Articles published in the journal HemaSphere exclusively reflect the opinions of the authors. The authors are responsible for all content in their abstracts including accuracy of the facts, statements, citing resources, etc.

**Summary/Conclusion**

This study offers the inaugural comprehensive clinical and genetic characterization of pediatric patients diagnosed with hereditary stomatocytosis within the Russian Federation. The identification of four new variants, as well as one large rearrangement, underscores the molecular-genetic heterogeneity of the studied population and underscores the necessity of employing next-generation sequencing testing in conjunction with other methods of genetic verification to reach a definitive diagnosis.

| Gene | HGVS (coding) | HGVS (protein) | Exon/intron | Variant type | Disease /Phenotype |
|---|---|---|---|---|---|
| *RHAG* | c.492+1G>T | - | Intron 3 | Splice-site | OHS/mild |
| *PIEZO1* | c.7285C>T | p.(Pro2429Ser) | Exon 50 | Missense | DHS/moderate |
| *PIEZO1* | c.3284A>C | p.(Asn1095Thr) | Exon 23 | Missense | DHS/moderate |
| *PIEZO1* | c.3033G>A | p.(Met1011Ile) | Exon 22 | Missense | DHS/mild |
| *PIEZO1* | - | p.(?) | - | Duplication | DHS/mild |

**Copyright Information:** (Online) ISSN: 2572-9241
©2026 The Author(s). HemaSphere is published by John Wiley & Sons Ltd on behalf of European Hematology Association. This is an open access Abstract Book distributed under the Attribution-NonCommercial-NoDerivs (CC BY-NC-ND), which allows third parties to download the articles and share them with others as long as they credit the author and the Abstract Book, but they cannot change the content in any way or use them commercially.
**Abstract Book Citations:** Authors, Title, HemaSphere, 2026;10:(S1):pages. The abstract book DOIs can be found on the "Author Information" page.
**Disclaimer:** Articles published in the journal HemaSphere exclusively reflect the opinions of the authors. The authors are responsible for all content in their abstracts including accuracy of the facts, statements, citing resources, etc.

### (PB4204) SPLEEN TYROSINE KINASE AND BRUTON'S TYROSINE KINASE INHIBITORS FOR THE MANAGEMENT OF WARM AUTOIMMUNE HEMOLYTIC ANEMIA: A SYSTEMATIC REVIEW AND SINGLE-ARM META-ANALYSIS

**Topic:** 29. Enzymopathies, membranopathies and other red blood cell disorders

Mehak Dang*[1], Aashray Raj[2], Harkirat Kaur Sandhu[3], Abdrabo Gamal Motawea[4], Hosana Candreva[5], Sahithi Mani Chandana Paramkusam[6], Mahta Moradi[7], Fahad Alabbas[8]

*[1]JSS Medical College, Mysore, India;[2]Government Medical College, Bhavnagar, India;[3]Punjab institute of medical science, Jalandhar, India; [4]Faculty of Medicine, Menoufia University, menoufia, Egypt;[5]Department of Medicine, Rio de Janeiro State University, Department of Medicine, Rio de Janeiro, Brazil; [6]Katuri Medical College and Hospital, Guntur, India;[7]Department of Medicine, Iran University of Medical Sciences, Tehran, Iran; [8]Prince Sultan Military Medical City, Riyadh, Saudi Arabia;*

### Background

Autoimmune hemolytic anemia (AIHA) is a heterogeneous immune-mediated disorder characterized by autoantibody-driven red blood cell destruction. In warm AIHA (wAIHA), IgG-mediated, Fcγ receptor–dependent erythrophagocytosis predominates. For several years, corticosteroids, immunosuppressants, and splenectomy have been the mainstay of treatment. Although corticosteroids remain first-line therapy, relapse and steroid dependence are frequent, and optimal second-line strategies remain undefined. Spleen tyrosine kinase (SYK) and Bruton's tyrosine kinase (BTK), key mediators of Fcγ receptor signaling, represent rational therapeutic targets whose inhibition may attenuate immune-mediated hemolysis; however, their efficacy and safety have not been systematically evaluated.

### Aims

To evaluate the clinical efficacy and safety of SYK and BTK inhibitors in adult patients with wAIHA.

### Methods

A systematic literature search was performed across PubMed, Embase, and Cochrane Library from inception to January 2026. This systematic review and meta-analysis followed the Cochrane Handbook for Systematic Reviews of Interventions and PRISMA guidelines. The protocol was prospectively registered in PROSPERO (CRD420261297871). Inclusion criteria comprised randomized controlled trials (RCTs) and observational studies that evaluated adult patients with wAIHA receiving either SYK or BTK inhibitors. The primary outcome assessed was Overall hemoglobin response, defined as Hgb >10 g/dL with a change of ≥2 g/dL from baseline, whereas durable hemoglobin response, need for rescue treatment, and safety profile were the secondary outcomes. Proportions were pooled using a random-effects model with logit transformation and reported with 95% confidence intervals (CI). Statistical analyses were performed using R (version 4.5.2). Heterogeneity was assessed using $I^2$ statistics.

**Copyright Information:** (Online) ISSN: 2572-9241
©2026 The Author(s). HemaSphere is published by John Wiley & Sons Ltd on behalf of European Hematology Association. This is an open access Abstract Book distributed under the Attribution-NonCommercial-NoDerivs (CC BY-NC-ND), which allows third parties to download the articles and share them with others as long as they credit the author and the Abstract Book, but they cannot change the content in any way or use them commercially.
**Abstract Book Citations:** Authors, Title, HemaSphere, 2026;10:(S1):pages. The abstract book DOIs can be found on the "Author Information" page.
**Disclaimer:** Articles published in the journal HemaSphere exclusively reflect the opinions of the authors. The authors are responsible for all content in their abstracts including accuracy of the facts, statements, citing resources, etc.

### Results

Six studies comprising 137 patients were included in the analysis. Four studies with 105 patients evaluated SYK inhibitors (including two phase II trials, one phase III trial, and one retrospective study). Two studies with 32 patients evaluated BTK inhibitors (one phase II study and one retrospective study).
The pooled analysis of SYK inhibitors demonstrated an overall hemoglobin response of 54.37% (95% CI 44.71–63.71; $I^2$ = 16.8%). A durable hemoglobin response was achieved in 40.51% (95% CI 30.29–51.62; $I^2$ = 0%). Rescue treatment was required in 23.55% (95% CI 12.18–40.62; $I^2$ = 70.8%). The incidence of treatment-emergent adverse events (TEAEs) was 50.12% (95% CI 32.58–67.63; $I^2$ = 64.1%), while serious adverse events occurred in 36.96%. The most commonly reported adverse events included hypertension (22.62%) and gastrointestinal symptoms, such as nausea (14.13%), abdominal pain (14.89%), and diarrhea (32.39%).
The pooled analysis of BTK inhibitors showed an overall hemoglobin response of 62.50% (95% CI: 44.90–77.32; $I^2$ = 0%), and the incidence of TEAEs was 40.63% (95% CI: 25.26–58.08; $I^2$ = 0%).

### Summary/Conclusion

Meta-analysis of SYK and BTK inhibitors demonstrate moderate efficacy with an acceptable safety profile in adult patients with wAIHA and may represent promising steroid-sparing targeted therapies. However, the evidence is limited by small sample sizes, single-arm study designs, and short follow-up duration. Larger randomized controlled trials with longer follow-up are required to establish comparative efficacy, durability of response, and long-term safety.

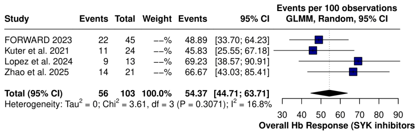

Figure 1a

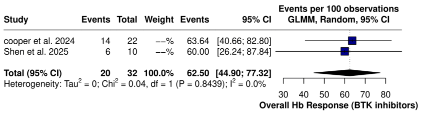

Figure 1b

Figure 1a demonstrates overall hemoglobin response in SYK inhibitors. Figure 1b demonstrates overall hemoglobin response in BTK inhibitors.

Copyright Information: (Online) ISSN: 2572-9241
©2026 The Author(s). HemaSphere is published by John Wiley & Sons Ltd on behalf of European Hematology Association. This is an open access Abstract Book distributed under the Attribution-NonCommercial-NoDerivs (CC BY-NC-ND), which allows third parties to download the articles and share them with others as long as they credit the author and the Abstract Book, but they cannot change the content in any way or use them commercially.
Abstract Book Citations: Authors, Title, HemaSphere, 2026;10:(S1):pages. The abstract book DOIs can be found on the "Author Information" page.
Disclaimer: Articles published in the journal HemaSphere exclusively reflect the opinions of the authors. The authors are responsible for all content in their abstracts including accuracy of the facts, statements, citing resources, etc.

### (PB4205) MACROCYTOSIS AND HYPERHOMOCYSTEINEMIA AS CLUES TO CHRONIC NITROUS OXIDE USE: A SINGLE-CENTER CASE SERIES

**Topic:** 29. Enzymopathies, membranopathies and other red blood cell disorders

Mateo Barba Cavigiolo*[1], Ignacio Lopera-Rodríguez[2], Marta Baranda Arias[3], Jesús Francisco Rodríguez Fernández[4], Raquel Valencia Martín[5], Amparo Fernández Simón[4], Ángela María Núñez Jaldón[4], Salvador Payán-Pernía[6]

*[1]Department of Hematology, Hospital Universitario Virgen del Rocío, Sevilla, Spain;[2]Department of Neurology, Hospital Universitario Virgen del Rocío, Sevilla, Spain; [3]Department of Mental Health, Hospital Universitario Virgen del Rocío, Sevilla, Spain; [4]Department of Emergency Medicine, Hospital Universitario Virgen del Rocío, Sevilla, Spain;[5]Department of Preventive Medicine and Public Health, Hospital Universitario Virgen del Rocío, Sevilla, Spain; [6]Department of Hematology, Hospital Universitario Virgen del Rocío and Instituto de Biomedicina de Sevilla (IBiS)-CSIC, Universidad de Sevilla, Sevilla, Spain;*

### Background

Recreational use of nitrous oxide ($N_2O$), commonly known as laughing gas, is increasing in Europe. It is inexpensive, easy to obtain and use, now available in high-volume cartridges, and widely perceived as safe. Its toxicity is mainly related to functional vitamin B12 deficiency. Through oxidation of the cobalt atom, $N_2O$ inactivates methylcobalamin and inhibits methionine synthase. This impairs the production of methionine and tetrahydrofolate, which are required for myelin and DNA synthesis, respectively, leading to secondary homocysteine elevation. Progressive depletion of cobalamin stores and, secondarily, of adenosylcobalamin (AdoCbl), may also inhibit methylmalonyl-CoA mutase.

### Aims

To raise awareness of the hematological and non-hematological manifestations of chronic $N_2O$ use by describing the epidemiological, clinical, and laboratory features of a patient series.

### Methods

Retrospective analysis of patients attended between October 2024 and February 2026 at a tertiary hospital in southern Spain with manifestations associated with chronic $N_2O$ use. A multidisciplinary working group involving Hematology, Neurology, Mental Health, Emergency Medicine, and Preventive Medicine/Public Health was established for case identification and management.

**Copyright Information:** (Online) ISSN: 2572-9241
©2026 The Author(s). HemaSphere is published by John Wiley & Sons Ltd on behalf of European Hematology Association. This is an open access Abstract Book distributed under the Attribution-NonCommercial-NoDerivs (CC BY-NC-ND), which allows third parties to download the articles and share them with others as long as they credit the author and the Abstract Book, but they cannot change the content in any way or use them commercially.
**Abstract Book Citations:** Authors, Title, HemaSphere, 2026;10:(S1):pages. The abstract book DOIs can be found on the “Author Information” page.
**Disclaimer:** Articles published in the journal HemaSphere exclusively reflect the opinions of the authors. The authors are responsible for all content in their abstracts including accuracy of the facts, statements, citing resources, etc.

### Results

Ten patients were included (Table 1). All required hospital admission. In three patients, the main presenting complaint was behavioral disturbance without neurological manifestations; in the remaining patients, neurological symptoms predominated (ascending acroparesthesias in all, gait ataxia in 6/7, and acute urinary retention in 3). Two patients were unable to walk independently at discharge. Only one patient had macrocytic anemia, whereas macrocytosis was present in 6/10. Marked homocysteine elevation was observed; methylmalonic acid was not assessed. No predisposing cause for vitamin B12 deficiency was identified. All patients were treated with intramuscular cyanocobalamin, usually daily or on alternate days, although hydroxocobalamin is the formulation of choice in the current protocol due to its faster intracellular uptake.

Table 1: Epidemiological, clinical, and laboratory characteristics of the patients.

| Variable | Result |
|---|---|
| Male sex | 8 (80%) |
| Age at presentation (years) | 26.9 ± 4.2 |
| Other drug use (excluding alcohol/tobacco) | 8 (80%) |
| History of thrombosis | 0 (0%) |
| Frequency of use – Daily | 6 (60%) |
| Frequency of use – Weekends | 4 (40%) |
| Months of use prior to presentation (n=8) | 18.9 ± 14.3 |
| Oral ulcers | 1 (10%) |
| Fatigue | 1 (10%) |
| Glossitis | 1 (10%) |
| Hemoglobin (g/dL) | 13.69 ± 1.54 |
| MCV (fL) | 99.36 ± 4.52 |
| Platelets (×10⁹/L) | 292.6 ± 58.1 |
| Neutrophils (×10⁹/L) | 4.05 ± 0.97 |
| Vitamin B12 (pg/mL) (187 – 883) | 265.3 ± 83.8 |
| Folate (ng/mL) (2.9 - 20.0) | 9.51 ± 11.77 |
| Homocysteine (mg/L) (0.68 - 2.16) | 11.86 ± 7.68 |

Continuous variables are presented as means ± standard deviations, and categorical variables as n (%).

### Summary/Conclusion

Macrocytosis without anemia in young patients presenting with psychiatric symptoms, paresthesias, gait ataxia, or thrombosis (although not observed in our series) should prompt measurement of homocysteine and methylmalonic acid and raise suspicion of chronic nitrous oxide use.

**Copyright Information:** (Online) ISSN: 2572-9241
©2026 The Author(s). HemaSphere is published by John Wiley & Sons Ltd on behalf of European Hematology Association. This is an open access Abstract Book distributed under the Attribution-NonCommercial-NoDerivs (CC BY-NC-ND), which allows third parties to download the articles and share them with others as long as they credit the author and the Abstract Book, but they cannot change the content in any way or use them commercially.
**Abstract Book Citations:** Authors, Title, HemaSphere, 2026;10:(S1):pages. The abstract book DOIs can be found on the "Author Information" page.
**Disclaimer:** Articles published in the journal HemaSphere exclusively reflect the opinions of the authors. The authors are responsible for all content in their abstracts including accuracy of the facts, statements, citing resources, etc.

### (PB4206) CASE REPORTS OF PEGCETACOPLAN TREATMENT IN PAROXYSMAL NOCTURNAL HEMOGLOBINURIA (PNH) - A SYSTEMATIC REVIEW

**Topic:** 29. Enzymopathies, membranopathies and other red blood cell disorders

Koo Wilson[1], Zalmai Hakimi*[1], Vikte Lionikaite[2], Fiston Vuvu[3], Barbara Czech[1]

*[1]Sobi, Stockholm, Sweden; [2]Oxford Pharmagenesis, Oxford, United Kingdom; [3]Apellis Pharmaceuticals, Waltham, MA, United States of America;*

### Background

Case reports are a valuable complement to clinical evidence from large-scale studies. The in-depth reporting can provide insight into patient disposition, clinical setting, treatment approaches and outcomes, especially in complex situations which make patients otherwise ineligible for clinical trials. Pegcetacoplan is a targeted, proximal complement 3 (C3) and C3b inhibitor for paroxysmal nocturnal hemoglobinuria (PNH), and was the first approved proximal inhibitor monotherapy in the treatment of adult patients with PNH who have haemolytic anaemia. Since the availability of pegcetacoplan in 2021, an increasing number of case reports are being published on real-world outcomes observed across a diverse range of patients.

### Aims

This systematic literature review (SLR) aims to summarize clinical characteristics, prior treatment history, haematologic and patient-reported outcomes, and safety reported in adult patients with PNH treated with pegcetacoplan.

### Methods

The SLR (registered on PROSPERO CRD420251043506) followed 2020 Preferred Reporting Items for Systematic reviews and Meta-Analyses (PRISMA) guidelines. Searches included publications, abstracts and presentations of pegcetacoplan case reports (to January 2026) in English, in adults (age ≥18 years) with PNH treated with pegcetacoplan.

### Results

Across the 19 case reports identified, patient ages ranged widely (24–79), with PNH managed with pegcetacoplan in patients with different clinical profiles, including associated aplastic anaemia, surgery or pregnancy. All but one case reported on patients who were switched from treatment with a C5 inhibitor to pegcetacoplan, and typically due to extravascular haemolysis. After pegcetacoplan initiation, reports note safety and improved outcomes across haematological markers (including haemoglobin levels, absolute reticulocyte count, or lactate dehydrogenase) and reduction of or independence from red-blood cell transfusions. Health-related quality of life (HRQoL) measures were reported in 3 out of 19 case reports, noting improvements in fatigue, dyspnea, daily activity, and overall health-related HRQoL.

### Summary/Conclusion

The clinical value of pegcetacoplan is backed by substantial evidence from clinical trials and large real-world studies, showing regulation of both intravascular and extravascular haemolysis, and overall improved PNH disease control. The published case reports summarized here highlight the efficacy and safety of pegcetacoplan in adult patients with PNH, showing beneficial haematologic response across a broad range of real-world clinical scenarios. Findings should be interpreted in the context of publication bias and small sample size, but overall support the effectiveness and safety of pegcetacoplan in patients with PNH.

**Copyright Information:** (Online) ISSN: 2572-9241
©2026 The Author(s). HemaSphere is published by John Wiley & Sons Ltd on behalf of European Hematology Association. This is an open access Abstract Book distributed under the Attribution-NonCommercial-NoDerivs (CC BY-NC-ND), which allows third parties to download the articles and share them with others as long as they credit the author and the Abstract Book, but they cannot change the content in any way or use them commercially.
**Abstract Book Citations:** Authors, Title, HemaSphere, 2026;10:(S1):pages. The abstract book DOIs can be found on the "Author Information" page.
**Disclaimer:** Articles published in the journal HemaSphere exclusively reflect the opinions of the authors. The authors are responsible for all content in their abstracts including accuracy of the facts, statements, citing resources, etc.

### (PB4207) WHEN HIGH FERRITIN MISLEADS: A HOMOZYGOUS CP GENE MUTATION REVEALING ACERULOPLASMINEMIA

**Topic:** 29. Enzymopathies, membranopathies and other red blood cell disorders

Sevil Sadri*[1]

[1] *istanbul training and research hospital, hematology, istanbul, Türkiye;*

### Background

Systemic and cerebral iron accumulation as a result of ceruloplasmin insufficiency is the hallmark of aceruloplasminemia, an uncommon autosomal recessive condition of iron metabolism. Microcytic anemia, hyperferritinemia, diabetes mellitus, and increasing neurological symptoms are the usual presentations. In order to avoid irreparable organ damage, especially neurological problems, early identification is essential.

### Aims

To demonstrate the significance of taking into account this uncommon condition in the differential diagnosis of iron metabolism problems by presenting a genetically verified case of aceruloplasminemia that was assessed for microcytic anemia and raised ferritin levels.

### Methods

A 46-year-old male patient with type 1 diabetes mellitus was assessed for cognitive deterioration, chronic hyperferritinemia, and microcytic anemia. Laboratory evaluations encompassed a complete blood count, iron metrics, and metabolic analysis. Genetic study was conducted utilizing polymerase chain reaction (PCR)-based single-gene mutation analysis focused on the CP gene (NM_000096.4).

### Results

The patient had high ferritin levels and microcytic anemia without any signs of inflammation or other iron overload. A homozygous CP gene variant c.1192_1196del was found through genetic testing. Consanguinity between the patient and his spouse was shown via family history. His daughter had the same homozygous mutation, according to screening of family relatives. At diagnosis, no other organ involvement was found. The patient was started on iron chelation therapy with deferasirox, and a long-term clinical follow-up was planned.

### Summary/Conclusion

Patients with microcytic anemia and hyperferritinemia should be suspected of having aceruloplasminemia, especially if they also have diabetes mellitus and consanguinity. Timely beginning of chelation therapy and surveillance to prevent worsening neurological and systemic consequences is made possible by early genetic diagnosis.

**Copyright Information:** (Online) ISSN: 2572-9241
©2026 The Author(s). HemaSphere is published by John Wiley & Sons Ltd on behalf of European Hematology Association. This is an open access Abstract Book distributed under the Attribution-NonCommercial-NoDerivs (CC BY-NC-ND), which allows third parties to download the articles and share them with others as long as they credit the author and the Abstract Book, but they cannot change the content in any way or use them commercially.
**Abstract Book Citations:** Authors, Title, HemaSphere, 2026;10:(S1):pages. The abstract book DOIs can be found on the “Author Information” page.
**Disclaimer:** Articles published in the journal HemaSphere exclusively reflect the opinions of the authors. The authors are responsible for all content in their abstracts including accuracy of the facts, statements, citing resources, etc.

### (PB4208) PRACTICE VARIATION SURVEY ON INVESTIGATIONS AND MANAGEMENT OF EYTHROCYTOSIS: A CANADIAN PERSPECTIVE

**Topic:** 29. Enzymopathies, membranopathies and other red blood cell disorders

Romel Abou-Akl[1], Chai Phua[1], Benjamin Chin-Yee[1], Cyrus Hsia[1], Jenny Ho*[2]

*[1]Schulich School of Medicine & Dentistry, Western University, London, Canada;[2]Schulich School of Medicine & Dentistry, Western University, Division of Hematology, Department of Medicine, London, Canada;*

### Background

The causes of erythrocytosis are diverse and work up can thus be extensive. Currently, there are no published consensus recommendations on how to approach the investigation of erythrocytosis. Consequently, the diagnostic approach will vary between physicians and range from limited to comprehensive. Furthermore, management of secondary erythrocytosis is likely variable due to limited evidence-based guidance.

### Aims

Our study aimed to survey Canadian specialists to determine how they approached the investigation of erythrocytosis and management of secondary erythrocytosis.

### Methods

We developed a survey that could be electronically completed in 10 minutes. Participants included hematologists, medical oncologists, and internists from across Canada. Our survey which was constructed in REDCAP, included 37 clinical questions and was distributed through email. We addressed diagnostic investigations and queried how frequently investigations were performed. Respondents were provided with a spectrum of response options as follows: "Routinely", "If initial testing is inconclusive", "In the correct clinical context", "Never", "Never, but would consider if available to me", and "Other". We queried an extensive list of investigations (n=30) related to basic and specialized laboratory blood testing, molecular testing, bone marrow testing, imaging studies, and other investigations outside these categories. We addressed clinical management of secondary erythrocytosis and asked specific management questions (n=7) and provided response options that included: "Routinely", "Never", and "In specific clinical context". Survey data was analyzed using descriptive analysis to identify trends, as well as, differences between specific groups of practitioners. The data was analyzed descriptively to identify overall practice patterns and variations between practitioner groups. Practice variation was further quantified using normalized Shannon entropy.

### Results

We sent out 453 emails and a total of 78 survey responses (17.2% response rate) were received. Findings demonstrated significant variability in both the diagnosis of erythrocytosis and management of secondary erythrocytosis. Approximately half the (48.6%) clinical questions achieved ≥ 50% agreement in clinical decision among respondents, and only 9/37 (24.3%) exceeded 70% consensus. A majority of clinical practices (>50%) demonstrated high normalized Shannon entropy, confirming significant dispersion in practice patterns. Additionally, differences were observed between hematologists and general internists, particularly regarding the use of advanced investigations such as molecular testing. Routine JAK2 V617F testing was reported by 74% of hematologists compared to 46% of general internists (OR 3.27, 95% CI 0.99–10.8; Fisher's exact p=0.094), suggesting a trend toward higher routine testing among hematologists that did not reach statistical significance.

**Copyright Information:** (Online) ISSN: 2572-9241
©2026 The Author(s). HemaSphere is published by John Wiley & Sons Ltd on behalf of European Hematology Association. This is an open access Abstract Book distributed under the Attribution-NonCommercial-NoDerivs (CC BY-NC-ND), which allows third parties to download the articles and share them with others as long as they credit the author and the Abstract Book, but they cannot change the content in any way or use them commercially.
**Abstract Book Citations:** Authors, Title, HemaSphere, 2026;10:(S1):pages. The abstract book DOIs can be found on the "Author Information" page.
**Disclaimer:** Articles published in the journal HemaSphere exclusively reflect the opinions of the authors. The authors are responsible for all content in their abstracts including accuracy of the facts, statements, citing resources, etc.

**Summary/Conclusion**

This study provides evidence of significant practice variation in the evaluation of erythrocytosis and the management of secondary erythrocytosis across Canadian specialists. Given the smaller sample size, the analysis was exploratory and intended to characterize patterns of practice rather than establish definitive group differences. The findings highlight gaps in consensus and underscore the need for larger, adequately powered studies and the development of evidence-informed guidelines to standardize approaches to the diagnosis and management of erythrocytosis.

**Copyright Information:** (Online) ISSN: 2572-9241
©2026 The Author(s). HemaSphere is published by John Wiley & Sons Ltd on behalf of European Hematology Association. This is an open access Abstract Book distributed under the Attribution-NonCommercial-NoDerivs (CC BY-NC-ND), which allows third parties to download the articles and share them with others as long as they credit the author and the Abstract Book, but they cannot change the content in any way or use them commercially.
**Abstract Book Citations:** Authors, Title, HemaSphere, 2026;10:(S1):pages. The abstract book DOIs can be found on the "Author Information" page.
**Disclaimer:** Articles published in the journal HemaSphere exclusively reflect the opinions of the authors. The authors are responsible for all content in their abstracts including accuracy of the facts, statements, citing resources, etc.

### (PB4209) HB TAK CAUSES ERYTHROCYTOSIS, AN ELEVATED IRON LEVEL AND CHILBLAINS IN COLD EXPOSURE

**Topic:** 29. Enzymopathies, membranopathies and other red blood cell disorders

Stefanie Gröpper*[1], Aristoteles Giagounidis[1], Sabine Haase[2], Dorothee Schoensteiner[3], Holger Cario[4]

*[1]Marienhospital Düsseldorf, Clinic for oncology, hematology and palliative care, Duesseldorf, Germany;[2]Marienhospital Duesseldorf, Spezialisiertes hämatologisches Labor, Duesseldorf, Germany; [3]University Hospital Ulm, Zentrale Einrichtungen klinische Chemie, Ulm, Germany; [4]University Hospital Ulm, Department of pediatric, Ulm, Germany;*

### Background

Hb Tak is a rare, high-affinity Hb variant, found in southeast asia and caused by an insertion of 2 nucleotides (AC) between codons 146 and 147 in the HBB gene (HBB:c.441_442insAC). This leads to an elongated beta chain with impaired T-state formation with subsequent increased oxygen affinity, a high p50 and mild instability. In heterozygosity, Hb Tak usually is associated with variant percentage of 30-40% in capillary electrophoresis. Heterozygous patients present asymptomatic erythrocytosis with Hb values between 130-170 g/l.
HbE (HBB:c.Glu26Lys) is one of the most common mutations worldwide, with the highest prevalence in the Asia-Pacific region. The prevalence is up to 50% carrier rate. Hb E is unstable an tends to hemolysis in oxidative stress. The p50 value is normal to slightly reduced.
Only two reports of compound heterozygous Hb Tak/Hb E are available in literature. Both describe an asymptomatic erythrocytosis without other symptoms or iron metabolism disorders.

### Aims

This report shows that in individual patients Hb Tak may lead to symptomatic chilblains on hands and feet and impaired iron metabolism due to vasoconstriction and reduced oxygen release when exposed to cold.

### Methods

These are the first two cases of heterocygous Hb Tak in Germany. Comprehensive hematological and molecular genetic testing was performed. Phlebotomy was initiated for symptomatic chilblains caused by increased viscosity.

### Results

Two women (aged 34 and 31), one with heterozygosity for Hb Tak and one with compound heterozygosity for Hb Tak/Hb E respectively, show symptomatic chilblains on their extremities during the winter months in Europe. In the heterozygous only patient, the Hb Tak percentage is 34% with Hb of 134 g/l, and a serum ferritin of 134 ng/ml. The chilblains appears rarely and less severe. The 31-year-old compound heterozygous Hb Tak/Hb E patient has 61% Hb Tak, 33.5% Hb E, a Hb of 178 g/l, a p50 of 12 mmHg and a serum ferritin level of 300 ng/ml without signs of hemolysis. Chilblains occur nearly every winter on hands and feet.

### Summary/Conclusion

Due to the instability of HbE, the amount of Hb Tak in our compound heterozygous patient is higher than in heterozygosity for Hb Tak. This results in erythrocytosis because of the high oxygen affinity. Chilblains may conditioned by oxygen deficiency situations like vasoconstriction in cold. But even heterozygous Hb Tak carriers can exhibit hyperviscosity symptoms in cold conditions due to the resulting erythrocytosis. Phlebotomy may reduce viscosity and optimize iron balance in these patients to prevent the occurrence of chilblains upon exposure to cold in this rare, high-affinity Hb variant.

**Copyright Information:** (Online) ISSN: 2572-9241
©2026 The Author(s). HemaSphere is published by John Wiley & Sons Ltd on behalf of European Hematology Association. This is an open access Abstract Book distributed under the Attribution-NonCommercial-NoDerivs (CC BY-NC-ND), which allows third parties to download the articles and share them with others as long as they credit the author and the Abstract Book, but they cannot change the content in any way or use them commercially.
**Abstract Book Citations:** Authors, Title, HemaSphere, 2026;10:(S1):pages. The abstract book DOIs can be found on the “Author Information” page.
**Disclaimer:** Articles published in the journal HemaSphere exclusively reflect the opinions of the authors. The authors are responsible for all content in their abstracts including accuracy of the facts, statements, citing resources, etc.

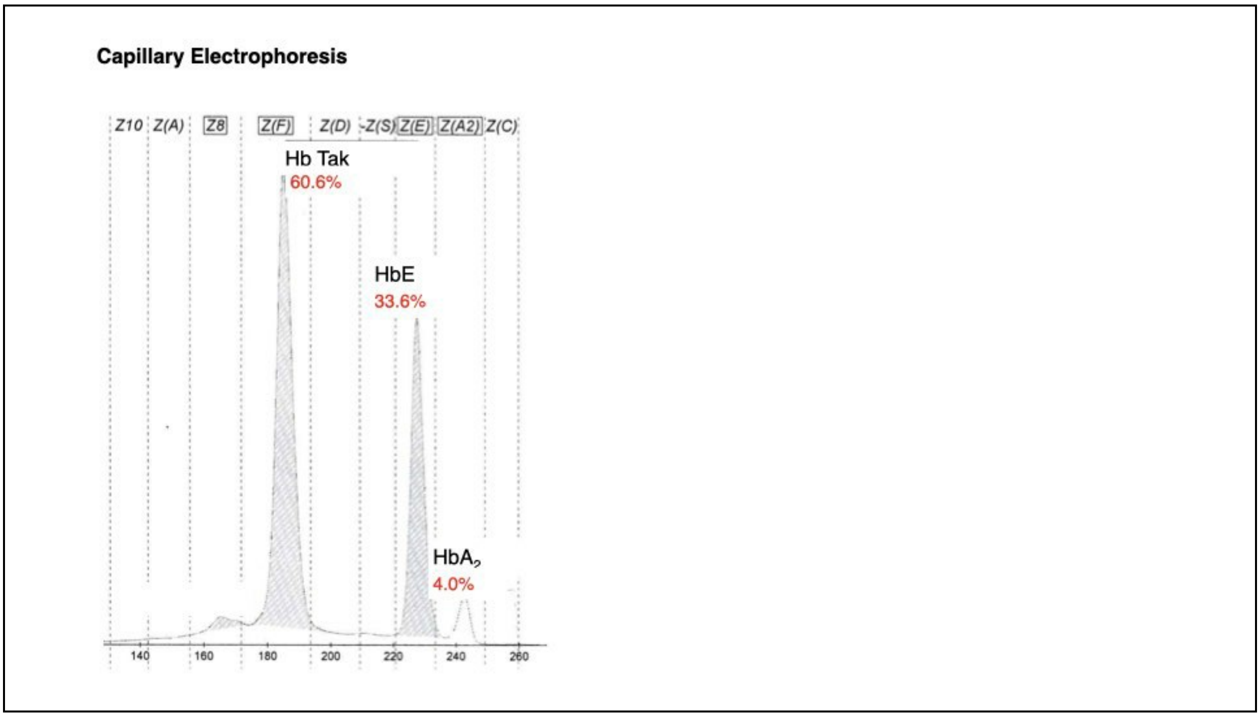

Capillary electrophoresis in the compound heterozygous Hb Tak/Hb E patient

**Copyright Information:** (Online) ISSN: 2572-9241
©2026 The Author(s). HemaSphere is published by John Wiley & Sons Ltd on behalf of European Hematology Association. This is an open access Abstract Book distributed under the Attribution-NonCommercial-NoDerivs (CC BY-NC-ND), which allows third parties to download the articles and share them with others as long as they credit the author and the Abstract Book, but they cannot change the content in any way or use them commercially.
**Abstract Book Citations:** Authors, Title, HemaSphere, 2026;10:(S1):pages. The abstract book DOIs can be found on the "Author Information" page.
**Disclaimer:** Articles published in the journal HemaSphere exclusively reflect the opinions of the authors. The authors are responsible for all content in their abstracts including accuracy of the facts, statements, citing resources, etc.

### (PB4210) BEYOND HEMATOLOGIC CLUES: PROTEINURIA REVEALING IMERSLUND–GRASBECK SYNDROME

**Topic:** 29. Enzymopathies, membranopathies and other red blood cell disorders

Çağrı Coşkun*[1], Bora Gülhan[2], Selin Aytaç[1]

*[1]Hacettepe University Faculty of Medicine, Pediatric Hematology, Ankara, Türkiye;[2]Hacettepe University Faculty of Medicine, Pediatric Nephrology, Ankara, Türkiye;*

### Background

Imerslund-Gräsbeck syndrome (IGS) is a rare autosomal recessive cause of hereditary vitamin B12 deficiency resulting from selective intestinal malabsorption. It is classically characterized by megaloblastic anemia and proteinuria. However, hematological findings may be absent in some patients, leading to delayed diagnosis.

### Aims

This case report aims to highlight the clinical, biochemical, and genetic features of Imerslund–Gräsbeck syndrome in a child with persistent vitamin B12 deficiency and proteinuria, and to discuss the implications for diagnosis and lifelong parenteral therapy.

### Results

A 12-year-old boy was referred for fatigue and recurrent vitamin B12 deficiency despite adequate nutritional intake. He had a 5-year history of intermittent proteinuria. The family history revealed parental consanguinity, and none of the relatives had previously been diagnosed with vitamin B12 deficiency or evaluated for proteinuria. Physical and neurological examinations were unremarkable. Laboratory evaluation revealed anemia with low hemoglobin (10.6 g/dL) and elevated mean corpuscular volume (93 fL), accompanied by markedly reduced serum vitamin B12 levels (<137 pg/mL). The homocysteine level was 7 µmol/L (reference range 0–15), while both serum and urinary methylmalonic acid levels were elevated. Peripheral smear demonstrated hypersegmented neutrophils without overt macrocytosis. Persistent proteinuria (spot urine protein/creatinine ratio: 0.95) was detected, while renal imaging was normal.
Given the coexistence of unexplained vitamin B12 deficiency and proteinuria, IGS was suspected. Genetic analysis revealed a homozygous pathogenic variant in the AMN gene (c.1161dupG; p.Arg388Glufs) and a heterozygous variant in the CUBN gene. This mutation was also found in the patient's parents. The parents were carriers of the mutation in the AMN gene (c.1161dupG/p. Arg388Glufs). Parenteral hydroxocobalamin therapy resulted in normalization of vitamin B12 levels, whereas proteinuria persisted.

### Summary/Conclusion

This case highlights that inherited causes such as Imerslund–Gräsbeck syndrome should be considered in pediatric patients presenting with macrocytic anemia and recurrent or treatment-resistant vitamin B12 deficiency, particularly when there is a family history of consanguinity and isolated proteinuria. Early genetic diagnosis facilitates appropriate lifelong parenteral therapy and careful monitoring of hematologic and renal parameters.

**Copyright Information:** (Online) ISSN: 2572-9241
©2026 The Author(s). HemaSphere is published by John Wiley & Sons Ltd on behalf of European Hematology Association. This is an open access Abstract Book distributed under the Attribution-NonCommercial-NoDerivs (CC BY-NC-ND), which allows third parties to download the articles and share them with others as long as they credit the author and the Abstract Book, but they cannot change the content in any way or use them commercially.
**Abstract Book Citations:** Authors, Title, HemaSphere, 2026;10:(S1):pages. The abstract book DOIs can be found on the “Author Information” page.
**Disclaimer:** Articles published in the journal HemaSphere exclusively reflect the opinions of the authors. The authors are responsible for all content in their abstracts including accuracy of the facts, statements, citing resources, etc.

**Table 1. Laboratory Findings of the Patient at Presentation**

| Parameter | Result | Reference Range |
|---|---|---|
| Hemoglobin | 10.6 g/dL | 12–16 |
| MCV | 93 fL | 77-91 |
| RDW | 14.8 % | 11.6–17 |
| Leukocyte count | 11.3 × $10^9/mm^3$ | 4–11 |
| Platelet count | 403 × $10^9/mm^3$ | 150–450 |
| Reticulocyte | 0.7 % | 0.5–2 |
| LDH | 254 U/L | 110–295 |
| Total bilirubin | 0.39 mg/dL | 0.3–1.2 |
| Direct bilirubin | 0.13 mg/dL | 0–0.2 |
| AST | 79 U/L | 0–50 |
| ALT | 42 U/L | 0–38 |
| Serum iron | 44 µg/dL | 20–140 |
| TIBC | 375 µg/dL | 228–428 |
| Ferritin | 27.2 ng/mL | 20–336 |
| Folate | 7.3 ng/mL | 3.1–19.9 |
| Vitamin B12 | <137 pg/mL | 126–590 |
| Homocysteine | 7 µmol/L | 0–15 |
| Urine protein | 1+ | Negative |
| Spot urine protein/creatinine ratio | 0.95 | <0.2 |
| 24-hour urine protein | 16.5 $mg/m^2/day$ | <4 $mg/m^2/day$ |

**Copyright Information:** (Online) ISSN: 2572-9241
©2026 The Author(s). HemaSphere is published by John Wiley & Sons Ltd on behalf of European Hematology Association. This is an open access Abstract Book distributed under the Attribution-NonCommercial-NoDerivs (CC BY-NC-ND), which allows third parties to download the articles and share them with others as long as they credit the author and the Abstract Book, but they cannot change the content in any way or use them commercially.
**Abstract Book Citations:** Authors, Title, HemaSphere, 2026;10:(S1):pages. The abstract book DOIs can be found on the "Author Information" page.
**Disclaimer:** Articles published in the journal HemaSphere exclusively reflect the opinions of the authors. The authors are responsible for all content in their abstracts including accuracy of the facts, statements, citing resources, etc.

### (PB4211) TRIPLE-HIT GENETICS: ATP11C, UGT1A1, AND HFE DRIVE REFRACTORY HYPERBILIRUBINEMIA WITH COMPENSATED HEMOLYSIS

**Topic:** 29. Enzymopathies, membranopathies and other red blood cell disorders

Mariam Ibáñez Company*[1], Carlos Alventosa-Mateu[2], Margarita Monzón[1], José Cervera[3], Karla Javier[1], Rosa Collado Nieto[1], Alejandro Fernandez[2], Mercedes Latorre[2], Inmaculada Castelló[2], Beatriz Sorribes[1], Juan José Uriquijo[2], Josefa Marco Buades[1], Mª José Lis[1]

*[1]Hematology and Hemotherapy Department. Consorcio Hospital General Universitario de Valencia. Hematology and Hemotherapy Research Group. Fundación Investigación Hospital General de Valencia., Valencia, Spain; [2]Hepatology Unit. Digestive Diseases Department. Consorcio Hospital General Universitario de Valencia., Valencia, Spain; [3]Genetics Unit. Hospital Universitario y Politécnico La Fe. Valencia. Hematology Research Group, Instituto de Investigación Sanitaria La Fe, Valencia. Centro de Investigación Biomédica en Red de Cáncer (CIBERONC), Madrid., Valencia, Spain;*

### Background

Persistent hyperbilirubinemia in adults is frequently attributed to Gilbert syndrome (GS); however, disproportionately elevated bilirubin levels or associated metabolic abnormalities should prompt evaluation for additional genetic contributors.

**Copyright Information:** (Online) ISSN: 2572-9241
©2026 The Author(s). HemaSphere is published by John Wiley & Sons Ltd on behalf of European Hematology Association. This is an open access Abstract Book distributed under the Attribution-NonCommercial-NoDerivs (CC BY-NC-ND), which allows third parties to download the articles and share them with others as long as they credit the author and the Abstract Book, but they cannot change the content in any way or use them commercially.
**Abstract Book Citations:** Authors, Title, HemaSphere, 2026;10:(S1):pages. The abstract book DOIs can be found on the “Author Information” page.
**Disclaimer:** Articles published in the journal HemaSphere exclusively reflect the opinions of the authors. The authors are responsible for all content in their abstracts including accuracy of the facts, statements, citing resources, etc.

### Results

We report a 45-year-old male with chronic jaundice and a prior GS diagnosis (heterozygous *UGT1A1**28). Serum total bilirubin was persistently elevated (5 mg/dL) (Table1). A defining feature was refractory systemic iron overload (elevated transferrin saturation) despite regular phlebotomy. Although the patient carried the *HFE*-H63D variant, this genotype alone does not fully explain the degree of iron dysregulation. Liver MRI and elastography were unremarkable, excluding fibrosis or significant macroscopic iron deposition; mild splenomegaly was present.
Clinical exome sequencing identified a hemizygous truncating variant in *ATP11C* (c.1788_1789del;p.Val598Serfs*9). A second pathogenic loss-of-function variant in *UGT1A1* (c.300del;p.Phe100Leufs*10) established compound heterozygosity and markedly reduced bilirubin conjugation capacity.
*ATP11C* encodes the major erythrocyte phospholipid flippase responsible for maintaining membrane lipid asymmetry and is linked to congenital hemolytic anemia. Its deficiency leads to phosphatidylserine externalization, promoting premature splenic clearance of red blood cells. Hematological analysis confirmed compensated hemolysis, evidenced by normal hemoglobin levels alongside increased reticulocytes and reduced haptoglobin. Experimental models suggest that *ATP11C* is also required for appropriate basolateral localization of hepatic uptake transporters. Loss of function may therefore impair bilirubin uptake, partially resembling mechanisms described in Rotor syndrome.
This phenotype represents a genetic triad in which chronic compensated hemolysis converges with dual hepatic dysfunction: impaired uptake via ATP11C-dependent transporters and reduced conjugation from UGT1A1. This synergistic "triple-hit" mechanism likely accounts for the refractory biochemical profile.

**Table 1:** Relevant biochemical and hematological parameters for the patient

| Laboratory parameter | Value | Reference range | Unit |
|---|---|---|---|
| Total bilirubin | **5.5** | 0.3-1.2 | mg/dL |
| Unconjugated bilirubin | **4.65** | 3.2-4.3 | mg/dL |
| Conjugated bilirubin | **0.7** | < 0.2 | mg/dL |
| AST | 23 | 3-50 | U/L |
| ALT | 22 | 3-50 | U/L |
| Alkaline phosphatase | 60 | 30-120 | U/L |
| GGT | 15 | 8-55 | U/L |
| Hemoglobine | **14.1** | 13.5-18 | g/dL |
| Mean corpuscular volume | 101 | 78-100 | fL |
| Ferritin | 61 | 20-250 | µg/L |
| Serum iron | **227** | 70-180 | µg /dL |
| Transferrin saturation | **75.2** | 25-50 | % |
| Haptoglobin | **37** | 40-270 | mg/dL |
| Direct Coombs test | Negative | Negative | |
| Reticulocytes | **2.63** | 0.5-1.86 | % |

### Summary/Conclusion

In conclusion, this case expands the clinical spectrum of *ATP11C*-associated disease and highlights the importance of extended genetic evaluation in patients with atypical or disproportionate hyperbilirubinemia. Given that progressive liver damage, cirrhosis, and hepatocellular carcinoma have been described in association with this genetic defect, close multidisciplinary surveillance is mandatory. Further studies are required to investigate if acquired genetic events mostly linked to myeloid malignancies could impact the refractory iron overload, while providing deeper insights into the patients' long-term hematopoietic compensatory mechanisms.

**Copyright Information:** (Online) ISSN: 2572-9241
©2026 The Author(s). HemaSphere is published by John Wiley & Sons Ltd on behalf of European Hematology Association. This is an open access Abstract Book distributed under the Attribution-NonCommercial-NoDerivs (CC BY-NC-ND), which allows third parties to download the articles and share them with others as long as they credit the author and the Abstract Book, but they cannot change the content in any way or use them commercially.
**Abstract Book Citations:** Authors, Title, HemaSphere, 2026;10:(S1):pages. The abstract book DOIs can be found on the "Author Information" page.
**Disclaimer:** Articles published in the journal HemaSphere exclusively reflect the opinions of the authors. The authors are responsible for all content in their abstracts including accuracy of the facts, statements, citing resources, etc.

### (PB4212) THROMBOTIC THROMBOCYTOPENIC PURPURA AND AUTOIMMUNE CYTOPENIAS ASSOCIATED WITH NEWLY DIAGNOSED SYSTEMIC LUPUS ERYTHEMATOSUS

**Topic:** 29. Enzymopathies, membranopathies and other red blood cell disorders

Teresa Botin Lopez*[1], Sophie Vanderputten[1], Mark Gurney[1], Bernadette Lynch[1], Liam Smyth[2], Amjad Hayat[1], Mark Comerford[1]

[1]*Galway University Hospital, Galway, Ireland;* [2]*St Vincent's University Hospital, Dublin, Ireland;*

### Background

There are only a few case reports in the literature of Systemic Lupus Erythematosus (SLE) and thrombotic thrombocytopenic purpura (TTP) presenting simultaneously.

TTP is exceptionally rare in SLE patients with an incidence of <0.5%[(1)].
TTP is one type of thrombotic microangiopathy (TMA) characterized by the combination of microangiopathic hemolytic anemia, severe thrombocytopenia, and organ impairment.
The loss of the activity of ADAMTS-13, an enzyme able to cleave ultra-large von Willebrand factor (vWF) multimers, is the major cause of TTP pathogenesis.
The autoantibodies in SLE patients can also cause endothelial damage and aberrant fibrinolysis, inciting the development of TTP.
TTP complicated with SLE often has a sudden onset, rapid progression, severe clinical course, and poor prognosis.

### Aims

This case describes a 31y/o Brazilian female who presented to a community hospital in the west of Ireland with abdominal pain and weight loss. One month prior she had experienced polyarthralgias, mainly in her hands, requiring daily NSAIDs, and a malar rash on her forehead whenever she was exposed to sunlight (figure 1b).
On admission she was anaemic (Hb 9,5 g/dL) and thrombocytopaenic (47 G/L) with a raised LDH. A blood film showed 7-8 fragments/field with occasional spherocytes (figure 1a). No blast cells were seen. Her direct Coombs test was positive. PLASMIC score was 5. ADAMST13 was found to be <1% and a diagnosis of Thrombotic Thrombocytopenic Purpura (TTP) was made. She was given plasma, IV Methylprednisolone and transferred to a large tertiary centre for Plasma Exchange (PLEX).
She was admitted to HDU with severe abdominal pain and vomiting. A CT mesenteric angiogram showed cholitis and small bowel obstruction but no signs of ischemia. An NGT was initially inserted and surgery was not necessary.
She received 11 rounds of PLEX and continued IV methylprednisolone in the HDU setting before being switched to a tapering dose of oral prednisolone. She also received weekly 375 mg/m2 rituximab. Caplacizumab was not available. Following completion of her PLEX her platelets were still low (80 G/L) and began to drop again despite normal levels of ADAMS 13.
At this time, autoimmune panel returned a positive ANA, speckled pattern with 1:1280 titre. Her ENA was also positive for anti-smith and anti-RNP, with a weak positive Anti-Ro antibody so rheumatology consult was requested.

### Methods

Given her symptoms of polyarthralgia, malar rash on sun exposure and weight loss, along with her anaemia, thrombocytopaenia, and positive antibodies, her combined EULAR/ACR criteria score for SLE was 32, conferring a diagnosis of SLE, possibly leading on to the severe haematological manifestation of TTP along with autoimmune cytopenias.
The patient was then started on hydroxychloroquine allowing a progressive recovery of the platelet count.

**Copyright Information:** (Online) ISSN: 2572-9241
©2026 The Author(s). HemaSphere is published by John Wiley & Sons Ltd on behalf of European Hematology Association. This is an open access Abstract Book distributed under the Attribution-NonCommercial-NoDerivs (CC BY-NC-ND), which allows third parties to download the articles and share them with others as long as they credit the author and the Abstract Book, but they cannot change the content in any way or use them commercially.
**Abstract Book Citations:** Authors, Title, HemaSphere, 2026;10:(S1):pages. The abstract book DOIs can be found on the "Author Information" page.
**Disclaimer:** Articles published in the journal HemaSphere exclusively reflect the opinions of the authors. The authors are responsible for all content in their abstracts including accuracy of the facts, statements, citing resources, etc.

### Results

A systematic literature review suggests that SLE and TTP co-exist, with 51 articles identified reporting 87 patients who were diagnosed with both TTP and SLE.

SLE was diagnosed prior to TTP in 53 (61%), subsequent to TTP in 11 (13%) and simultaneously with TTP in 23 (26%) patients[2].

### Summary/Conclusion

1. Kadam, A.et al. (2024). Systemic Lupus Erythematosus (SLE) and Thrombotic Thrombocytopenic Purpura (TTP) Presenting as Recurrent Strokes: A Case Report. *Cureus*, [online] 16(5)
2. Hunt, L et al. (2004). Thrombotic Thrombocytopenic Purpura (TTP) and Systemic Lupus Erythematosus (SLE): Distinct but Potentially Overlapping Syndromes. *Blood*, 104(11), pp.858–858.

**Copyright Information:** (Online) ISSN: 2572-9241
©2026 The Author(s). HemaSphere is published by John Wiley & Sons Ltd on behalf of European Hematology Association. This is an open access Abstract Book distributed under the Attribution-NonCommercial-NoDerivs (CC BY-NC-ND), which allows third parties to download the articles and share them with others as long as they credit the author and the Abstract Book, but they cannot change the content in any way or use them commercially.
**Abstract Book Citations:** Authors, Title, HemaSphere, 2026;10:(S1):pages. The abstract book DOIs can be found on the “Author Information” page.
**Disclaimer:** Articles published in the journal HemaSphere exclusively reflect the opinions of the authors. The authors are responsible for all content in their abstracts including accuracy of the facts, statements, citing resources, etc.

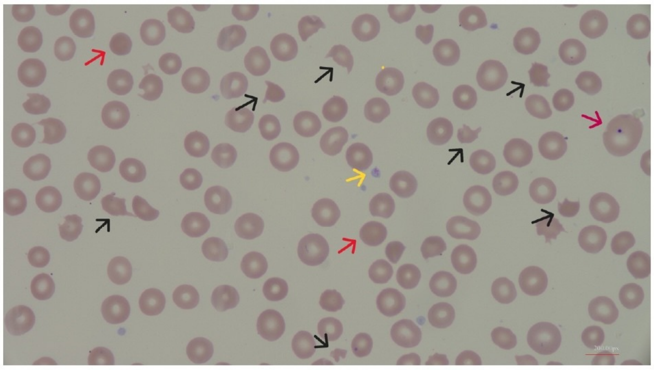

Figure 1a. Blood film review showed a real thrombocytopenia (yellow arrow), more than 8-9 red cell fragments/field (black arrows), occasional spherocytes (red arrows) and Howell-Jolly bodies (pink arrow).

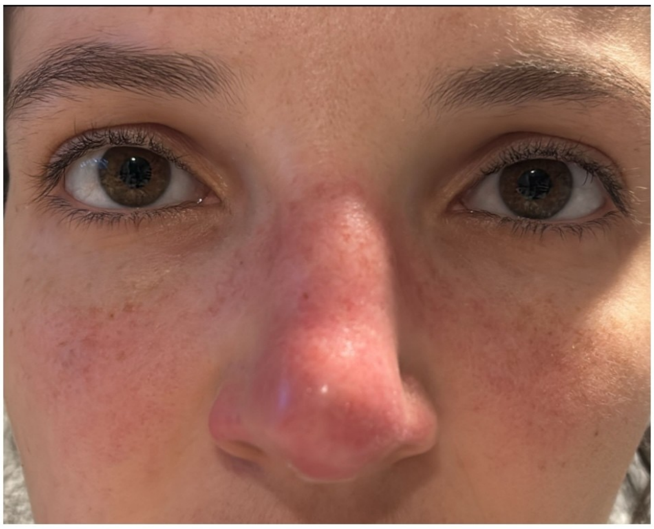

Figure 1b. Erythematous rash across the cheeks and the bridge of the nose "malar rash"

**Copyright Information:** (Online) ISSN: 2572-9241
©2026 The Author(s). HemaSphere is published by John Wiley & Sons Ltd on behalf of European Hematology Association. This is an open access Abstract Book distributed under the Attribution-NonCommercial-NoDerivs (CC BY-NC-ND), which allows third parties to download the articles and share them with others as long as they credit the author and the Abstract Book, but they cannot change the content in any way or use them commercially.
**Abstract Book Citations:** Authors, Title, HemaSphere, 2026;10:(S1):pages. The abstract book DOIs can be found on the "Author Information" page.
**Disclaimer:** Articles published in the journal HemaSphere exclusively reflect the opinions of the authors. The authors are responsible for all content in their abstracts including accuracy of the facts, statements, citing resources, etc.

### (PB4213) MEDICAL EDUCATION IN HOLISTIC PATIENT-CENTRED CARE FOR PAROXYSMAL NOCTURNAL HEMOGLOBINURIA IDENTIFIES SPECIALTY-SPECIFIC GAPS

**Topic:** 29. Enzymopathies, membranopathies and other red blood cell disorders

S Christy Rohani-Montez*[1], Keisha Peters[1], Tiphaine Barras[1]

[1]*Medscape Education Global, London, United Kingdom;*

### Background

The relatively recent availability of a variety of therapies for paroxysmal nocturnal hemoglobinuria (PNH) has significantly improved outcomes for this rare condition. Choosing the right therapeutic approach for the right patient to enhance quality of life is now possible but many clinicians lack the necessary knowledge and competence to optimize holistic care.

### Aims

We sought to improve physician knowledge regarding patient preferences and how to incorporate this information into clinical decisions by providing expert-led education.

### Methods

Physicians participated in an online CME activity divided into 4 segments delivered by different speakers including a patient, with synchronized slides (https://www.medscape.org/viewarticle/1002332). The education effects were assessed using a 3-question, repeated pairs, pre-assessment/post-assessment study design. One question assessed confidence. Differences from pre- to post-assessment were evaluated using the McNemar's test with $P \leq .05$ as significant. The activity launched in 03/25 and data were collected through 12/25.

### Results

38 haematologists and 479 nurses completed all pre- and post-assessment questions. Significant improvements for both specialties were seen regarding knowing how to manage a patient with clinically significant fatigue and poor adherence to PNH therapy, with 47% of haematologists but only 10% of nurses answering correctly at baseline, improving post-education to 66% ($P<.05$) and 16% respectively ($P<.001$). With regards to patient preference, more haematologists than nurses correctly identified lower infusion frequency (versus time to administer or financial reasons) as being a primary preference for patients with 66% of haematologists answering this question correctly at baseline, increasing to 71% post education (ns). The corresponding figures for nurses were 31% at baseline and 43% post education ($P<.001$).
After completing the activity, 64% of haematologists and 32% of nurses had measurable improved confidence related to implementing a patient-centred approach to select treatments that fit the needs and lifestyle of patients with PNH.

### Summary/Conclusion

This study shows stark differences between haematologists and nurses in their knowledge and competence in considering PNH patient preferences and needs when determining a management approach. Although significant improvements were achieved via this online education for both audiences, the need for further education particularly for nurses stands out.

**Copyright Information:** (Online) ISSN: 2572-9241
©2026 The Author(s). HemaSphere is published by John Wiley & Sons Ltd on behalf of European Hematology Association. This is an open access Abstract Book distributed under the Attribution-NonCommercial-NoDerivs (CC BY-NC-ND), which allows third parties to download the articles and share them with others as long as they credit the author and the Abstract Book, but they cannot change the content in any way or use them commercially.
**Abstract Book Citations:** Authors, Title, HemaSphere, 2026;10:(S1):pages. The abstract book DOIs can be found on the "Author Information" page.
**Disclaimer:** Articles published in the journal HemaSphere exclusively reflect the opinions of the authors. The authors are responsible for all content in their abstracts including accuracy of the facts, statements, citing resources, etc.

### (PB4214) CASTLEMAN DISEASE-RARE CAUSE OF COLD AGGLUTININ SYNDROME

**Topic:** 29. Enzymopathies, membranopathies and other red blood cell disorders

Angéla Csirmaz[1], Ferenc Magyari[1], Arpád Illés[1], Boglarka Csilla Brugos*[1]

[1]*University of Debrecen, Internal Medicine Division of Hematology, Debrecen, Hungary;*

### Background

Castleman disease is a rare, atypical lymphoproliferative disease with systemic symptoms, the etiology of which is not precisely known, and it has both unicentric and multicentric forms. Multicentric Castleman disease can be further classified into HHV-8-associated and idiopathic forms, which mainly affect immunosuppressed patients. The diagnosis is often delayed because the symptoms are similar to those of many malignant lymphoproliferative diseases, autoimmune and autoinflammatory diseases, and infections, leading to diagnostic difficulties. Autoimmune hemolytic anemia (AIHA) can be cold or warm type, primary, or secondary, and can be caused by various pathologies. The association of cold AIHA, also known as cold agglutinin syndrome, with CD is rare in the literature.

### Aims

The 45-year-old man was examined in pulmonology in 2020 due to chronic cough; chest CT confirmed mediastinal lymphadenomegaly. His axillary lymph node core biopsy confirmed reactive abnormalities, and histology obtained during mediastinoscopy in 2022 was also non-pathological. In March 2024, he was referred to our hematology department due to hemolytic anemia and lymphadenomegaly. Laboratory tests indicated eosinophilia, hematuria, and proteinuria, and blood group serology confirmed cold-type AIHA, cold agglutinin titer 1:64, and hypocomplementemia was confirmed. Based on this, we concluded that he had cold agglutinin syndrome, and immunoserology, bone marrow biopsy, and lymph node biopsy were performed to investigate the underlying condition. Myeloid NGS did not confirm any genetic abnormalities. Corticosteroids were initially started due to hemolysis. A surgical biopsy was performed on the lymph nodes with increased activity on PET/CT, and histological examination revealed two rare conditions: Rosai Dorfman disease and hyaline-vascular CD, based on sinus histiocytosis. Emergency surgery was performed in thoracic surgery to remove the lung lobe indicated by PET/CT and lymphadenectomy. In addition to corticosteroid treatment, we started antiCD20 treatment (rituximab) due to severe hemolysis, and he received 6 cycles. Histological examination of the lung finally confirmed plasma cell CD, and therefore, anti-IL-6 and siltuximab treatment was started in February 2025. His blood count stabilized. He has received 15 cycles so far, and a control chest CT confirmed regression.

### Summary/Conclusion

The association between cold-type AIHA and CD is rare, and in our case we would like to highlight the diagnostic challengesof cold agglutinin syndrome.

**Copyright Information:** (Online) ISSN: 2572-9241
©2026 The Author(s). HemaSphere is published by John Wiley & Sons Ltd on behalf of European Hematology Association. This is an open access Abstract Book distributed under the Attribution-NonCommercial-NoDerivs (CC BY-NC-ND), which allows third parties to download the articles and share them with others as long as they credit the author and the Abstract Book, but they cannot change the content in any way or use them commercially.
**Abstract Book Citations:** Authors, Title, HemaSphere, 2026;10:(S1):pages. The abstract book DOIs can be found on the “Author Information” page.
**Disclaimer:** Articles published in the journal HemaSphere exclusively reflect the opinions of the authors. The authors are responsible for all content in their abstracts including accuracy of the facts, statements, citing resources, etc.

### (PB4215) "UNMASKING ANEMIA IN PEDIATRIC CKD: IS HEPCIDIN A MORE RELIABLE INDICATOR THAN FERRITIN AND TSAT?"

**Topic:** 30. Iron metabolism, deficiency and overload

Ahmed Almenshawy*[1], Amr Fathy[1], Mai Korkor[1], Rana Shaat[1]

[1]*Mansoura University faculty of medicine, Clinical Pathology, Elmansoura, Egypt;*

### Background

Hepcidin is a key regulator of iron homeostasis. It inhibits intestinal iron absorption and iron release from macrophages by binding to the iron exporter ferroportin which results in reducing circulating iron availability [1]. In chronic kidney disease (CKD), hepcidin metabolism is profoundly affected due to reduced renal clearance, chronic inflammation, and iron imbalance, leading to impaired erythropoiesis and the development of anemia [2,3]. Pediatric non-dialysis CKD as chronic disease patients are particularly vulnerable to hepcidin-mediated iron restriction, which can worsen anemia and complicate management [4]. While conventional iron markers such as ferritin and transferrin saturation (TSAT) are commonly used, they are influenced by inflammation and may not accurately reflect functional iron deficiency [5]. Therefore, hepcidin has been proposed as a potentially more sensitive biomarker for assessing iron status and anemia in this population [2,5].

### Aims

The aim was to evaluate the relationship between serum hepcidin levels and anemia indices in children with non-dialysis CKD, and to assess whether hepcidin provides better clinical insight compared to ferritin and TSAT.

### Methods

We conducted a cross-sectional study including 40 pediatric CKD patients and 40 controls. Demographic, hematological, and iron profile data were collected.

### Results

Serum Hepcidin levels were significantly elevated in children group with chronic kidney disease (CKD) compared to healthy controls group (median 262.2 vs. 219.9 ng/mL, P= 0.033). Hepcidin level has showed strong positive correlations with serum ferritin ($r = 0.566$, $P < 0.001$) and parathyroid hormone ($r = 0.565$, $p < 0.001$), while it was inversely correlated with glomerular filtration rate ($r = -0.497$, $p = 0.001$), hemoglobin ($r = -0.496$, $p = 0.001$), iron ($r = -0.467$, $p = 0.002$), total iron-binding capacity ($r = -0.446$, $p = 0.004$), and vitamin D ($r = -0.478$, $p = 0.002$). Receiver operating characteristic analysis revealed a modest discriminative value of hepcidin for differentiating CKD from controls (AUC = 0.638, $p = 0.033$, cut-off = 210.7 ng/mL, 72.5% sensitivity, 50% specificity), indicating it is associated with but not superior to ferritin or transferrin saturation in assessing iron status.

### Summary/Conclusion

Hepcidin elevation in pediatric CKD reflects disrupted iron homeostasis, inflammation, and declining renal function. Although correlated with traditional iron markers, its diagnostic performance does not exceed that of ferritin or transferrin saturation. However, given its close relationship with renal function, vitamin D, and parathyroid activity, hepcidin may serve as an additional biomarker reflecting the combined effects of iron dysregulation, anemia, and secondary hyperparathyroidism in CKD-related anemia

**Copyright Information:** (Online) ISSN: 2572-9241
©2026 The Author(s). HemaSphere is published by John Wiley & Sons Ltd on behalf of European Hematology Association. This is an open access Abstract Book distributed under the Attribution-NonCommercial-NoDerivs (CC BY-NC-ND), which allows third parties to download the articles and share them with others as long as they credit the author and the Abstract Book, but they cannot change the content in any way or use them commercially.
**Abstract Book Citations:** Authors, Title, HemaSphere, 2026;10:(S1):pages. The abstract book DOIs can be found on the "Author Information" page.
**Disclaimer:** Articles published in the journal HemaSphere exclusively reflect the opinions of the authors. The authors are responsible for all content in their abstracts including accuracy of the facts, statements, citing resources, etc.

| | AUC | *P* | 95% CI | Cut off | Sensitivity (%) | Specificity (%) |
|---|---|---|---|---|---|---|
| **Hepcidin** | 0.638 | 0.033 | 0.516-0.760 | 210.7 | 72.5 | 50.0 |

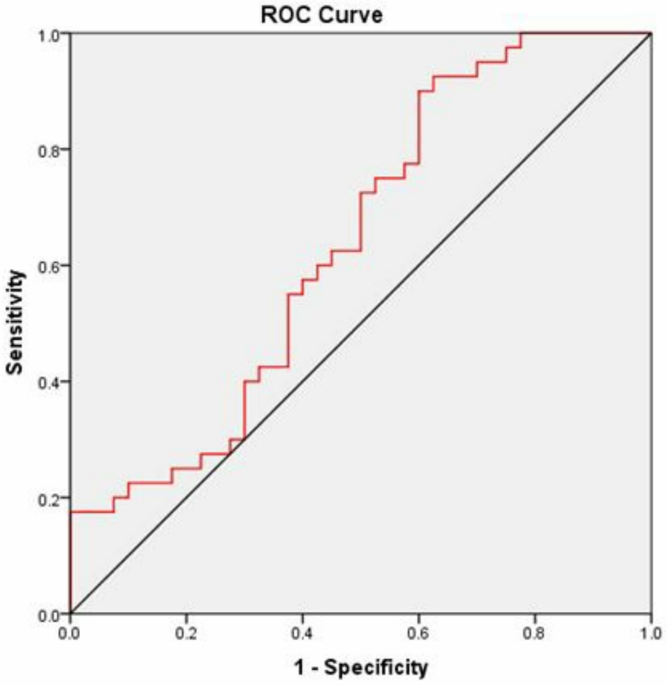

**Copyright Information:** (Online) ISSN: 2572-9241
©2026 The Author(s). HemaSphere is published by John Wiley & Sons Ltd on behalf of European Hematology Association. This is an open access Abstract Book distributed under the Attribution-NonCommercial-NoDerivs (CC BY-NC-ND), which allows third parties to download the articles and share them with others as long as they credit the author and the Abstract Book, but they cannot change the content in any way or use them commercially.
**Abstract Book Citations:** Authors, Title, HemaSphere, 2026;10:(S1):pages. The abstract book DOIs can be found on the "Author Information" page.
**Disclaimer:** Articles published in the journal HemaSphere exclusively reflect the opinions of the authors. The authors are responsible for all content in their abstracts including accuracy of the facts, statements, citing resources, etc.

### (PB4216) THE ‘IRON GAMES’ OF AGING: DIVERGENT CLONAL EVOLUTION IN ELDERLY PATIENTS WITH UNEXPLAINED ORAL IRON-REFRACTORY ANEMIA (UOIRA)

**Topic:** 30. Iron metabolism, deficiency and overload

Liri Seraj*[1]

[1]*Memorial Regional Hospital, Fier, Albania;*

### Background

Unexplained oral iron-refractory anemia (UOIRA) in older adults, defined as anemia unresponsive to adequate oral iron when inflammatory markers are normal and common causes excluded, presents a significant diagnostic challenge. We distinguish UOIRA from genetic IRIDA (TMPRSS6-related) and propose it may represent **Iron-Refractory Anemia of Undetermined Significance (IRAUS),** a pre-clonal or clonal hematopoietic state analogous to MGUS or CHIP, capable of evolving into overt myeloid neoplasia. The clinical trajectory of UOIRA patients following intravenous (IV) iron repletion has not been well characterized.

### Aims

To describe the divergent hematological outcomes in elderly patients with UOIRA following IV iron therapy and to propose IRAUS as a conceptual framework linking this presentation to underlying clonal hematopoiesis.

### Methods

Retrospective analysis of eleven patients (ages 66–85) with iron deficiency anemia unresponsive to oral iron (failure to achieve ≥1 g/dL hemoglobin rise after 4–6 weeks with confirmed compliance). All had negative investigations for occult GI bleeding (upper and lower endoscopy), malabsorption (celiac serology, duodenal biopsy), and chronic inflammation (normal CRP, ESR, ferritin pattern consistent with true iron deficiency). Each received IV iron therapy (iron sucrose or ferric carboxymaltose) and was followed longitudinally with serial CBC and iron studies. Bone marrow evaluation and mutation analysis were performed when clinically indicated.

### Results

Three distinct clinical trajectories emerged following IV iron administration:
**Group 1 — Progression to MDS (n=7, 64%):** Despite normalization of iron parameters (ferritin 150–800 ng/mL post-treatment), anemia persisted or worsened. Bone marrow biopsies confirmed MDS: multilineage dysplasia (n=4), excess blasts-1 (n=2), single lineage dysplasia (n=1). Time to diagnosis: 3–18 months.
**Group 2 — Unmasking of PV (n=2, 18%):** JAK2 V617F-positive patients converted from apparent iron deficiency anemia to overt erythrocytosis (Hct >49%) within 8–12 weeks of iron repletion. Both had retrospective findings suggestive of masked MPN: elevated RBC count despite low Hb, low serum erythropoietin, and mild splenomegaly.
**Group 3 — Suspected MPN under investigation (n=1, 9%):** Progressive splenomegaly following iron therapy. JAK2 V617F, CALR, and MPL negative; bone marrow biopsy and extended myeloid panel pending.
**Group 4 — Sustained response (n=1, 9%):** Durable normalization of hemoglobin and ferritin without evidence of clonal disease after 24 months of follow-up.

**Copyright Information:** (Online) ISSN: 2572-9241
©2026 The Author(s). HemaSphere is published by John Wiley & Sons Ltd on behalf of European Hematology Association. This is an open access Abstract Book distributed under the Attribution-NonCommercial-NoDerivs (CC BY-NC-ND), which allows third parties to download the articles and share them with others as long as they credit the author and the Abstract Book, but they cannot change the content in any way or use them commercially.
**Abstract Book Citations:** Authors, Title, HemaSphere, 2026;10:(S1):pages. The abstract book DOIs can be found on the “Author Information” page.
**Disclaimer:** Articles published in the journal HemaSphere exclusively reflect the opinions of the authors. The authors are responsible for all content in their abstracts including accuracy of the facts, statements, citing resources, etc.

**Summary/Conclusion**

In this series, 91% (10/11) of elderly UOIRA patients demonstrated evidence of clonal myeloid disease, supporting our proposal that UOIRA represents **IRAUS,** a clonal-adjacent state warranting systematic screening. IV iron functions as an unmasking intervention rather than a disease-modifying factor: in JAK2+ clones, correcting iron deficiency reveals masked PV; in pre-MDS states, persistent anemia despite iron repletion signals ineffective erythropoiesis. We recommend comprehensive hematological evaluation, including molecular profiling where available, prior to or concurrent with IV iron therapy in elderly UOIRA patients. IRAUS demands the same clinical vigilance as MGUS. Limitations include retrospective design, small sample size, and lack of baseline molecular profiling. Prospective studies with systematic pre-treatment evaluation are needed.

**Copyright Information:** (Online) ISSN: 2572-9241
©2026 The Author(s). HemaSphere is published by John Wiley & Sons Ltd on behalf of European Hematology Association. This is an open access Abstract Book distributed under the Attribution-NonCommercial-NoDerivs (CC BY-NC-ND), which allows third parties to download the articles and share them with others as long as they credit the author and the Abstract Book, but they cannot change the content in any way or use them commercially.
**Abstract Book Citations:** Authors, Title, HemaSphere, 2026;10:(S1):pages. The abstract book DOIs can be found on the "Author Information" page.
**Disclaimer:** Articles published in the journal HemaSphere exclusively reflect the opinions of the authors. The authors are responsible for all content in their abstracts including accuracy of the facts, statements, citing resources, etc.

### (PB4217) TOXICITY ASPECTS OF PRIMARY IRON OVERLOAD ON RED BLOOD CELLS: PRELIMINARY RESULTS

**Topic:** 30. Iron metabolism, deficiency and overload

Elena Chatzikalil*[1, 2], Alkmini Anastasiadi[3], Polyxeni Delaporta[1, 2], Stavros Glentis[1, 2], Ilona Binenbaum[1, 2], Konstantinos Liontakis[3], Vasiliki Chouliara[1, 2], Konstantinos Bistas[1, 2], Vasiliki Maravgaki[1, 2], Vangelis Mitros[1, 2], Efthalia-Faidra Agiomavriti-Stefanopoulou[1, 2], Dimitra Kyriakopoulou[1, 2], Ioannis Papassotiriou[1, 2], Vasileios Tzounakas[3], Antonis Kattamis[1, 2]

*[1]Thalassemia Unit, First Department of Pediatrics, National and Kapodistrian University of Athens, Athens, Greece;[2]"Aghia Sophia" Children's Hospital, ERN-EuroBloodNet Center, Athens, Greece; [3]University of Patras, School of Medicine, Department of Biochemistry, Patras, Greece;*

### Background

Despite the limited number of studies on artificially iron loaded cell models focusing on changes in red blood cells-RBCs, evidence on the influence of excess iron on patient-derived RBCs is still lacking.

### Aims

To provide a quantitative analysis of the toxic influence of primary iron overload on RBCs parameters of freshly drawn patient samples. Secondly, to investigate whether persistent hyperferritinemia-HF without iron overload is distinctly related with toxicity on RBCs.

### Methods

6 patients-pts with hereditary HF: 4/6 with primary iron overload (Hereditary Hemochromatosis-HH type 1: 2 pts, HH type 2A: 2 pts) and 2/6 without primary iron overload (Hereditary Hyperferritinemia Cataract Syndrome-HHCT), were included. Pts underwent hematology and iron (serum ferritin-SF, iron, transferrin saturation-TSAT) blood tests, and Magnetic Resonance Imaging-MRI when indicated, for evaluating iron accumulation on liver and heart. Genetic analysis was performed by targeted Next Generation Sequencing-NGS (39 iron-related genes) and Sanger sequencing for confirming identified mutations. Pure RBCs were assessed for their integrity (propensity to lysis), redox equilibrium (ROS production), hemichromes and methemoglobin-MetHb formation, and proteasomal activity using spectro-/fluoro-metric approaches against healthy controls. RStudio v.4.4.1 was used for statistical analysis. Values of $p < 0.05$ were considered statistically significant.

### Results

Iron and TSAT were increased in HH pts, and within normal ranges in HHCS pts. SF was highly increased in both groups. MRI on HH pts showed mild to severe iron accumulation in the liver and normal iron load in the heart. Erythrocytic analysis revealed significant variations for both groups (HH, HHCS). For HH pts, propensity to proteotoxic lysis (p<0.05), and membrane's hemichromes (p=0.001) were significantly increased *vs* controls. Accordingly, MetHb presented an increased tendency (p=0.09). Proteasome activity was significantly elevated in the cytosol (p<0.05) but decreased in the membrane (p<0.001). No significant differences were observed comparing RBCs of HHCS pts *vs* controls, but large-to-huge effect sizes (Hedges' g) were observed for free hemoglobin (g=3.75) and mechanical lysis (g=1.71), suggesting potentially meaningful biological differences. When compared to HH pts, HHCS RBCs' propensity to lysis, phenyl-hydrazine induced ROS generation and membrane hemichromes were significantly decreased (p<0.05). Increased proteasomal degradation in RBCs membrane was observed *vs* HH pts, while when inhibiting proteasomal activity, their RBCs were significantly more resistant to lysis *vs* HH pts. Values are presented in Figure 1.

**Copyright Information:** (Online) ISSN: 2572-9241
©2026 The Author(s). HemaSphere is published by John Wiley & Sons Ltd on behalf of European Hematology Association. This is an open access Abstract Book distributed under the Attribution-NonCommercial-NoDerivs (CC BY-NC-ND), which allows third parties to download the articles and share them with others as long as they credit the author and the Abstract Book, but they cannot change the content in any way or use them commercially.
**Abstract Book Citations:** Authors, Title, HemaSphere, 2026;10:(S1):pages. The abstract book DOIs can be found on the "Author Information" page.
**Disclaimer:** Articles published in the journal HemaSphere exclusively reflect the opinions of the authors. The authors are responsible for all content in their abstracts including accuracy of the facts, statements, citing resources, etc.

**Summary/Conclusion**

Considering that increased ROS directly cause cellular toxicity and exacerbate RBC lysis, and MetHb participates in systemic iron absorption, RBC-related results reflect HH excess iron load and iron-related organ damage. HF without iron overload is associated with reduced hemoglobin derivatives (MetHb) and better response to oxidative stimuli *vs* HH pts, as anticipated considering the normal iron load and the absence of systemic oxidative toxicity in HHCS. We also proved ferritin L-subunit variants' (HHCS pts) increased susceptibility to proteasomal degradation compared to iron-bound ferritin. We suggest RBCs as a key aspect of primary iron overload toxicity, further providing insights for the distinct oxidative patterns of high serum ferritin. We are planning to extensively validate our results in larger cohorts.

| | Pt 1 (HH type 1, p.C282Y/ p.C282Y) | Pt 2 (HH type 1, p.C282Y/ p.C282Y) | Pt 3 (HH type 2A, p.G320V/ p.G320V) | Pt 4 (HH type 2A, p.A256G/ p.Y286C) | Mean (HH) | SD (HH) | Pt 5 (HHCS) | Pt 6 (HHCS) | Mean (HHCS) | SD (HHCS) |
|---|---|---|---|---|---|---|---|---|---|---|
| Serum iron panel | | | | | | | | | | |
| Iron (gr/dl) | 163 | 209 | 431 | 273 | 269 | 117.1 | 30 | 121 | 75.5 | 64.3 |
| Ferritin (ng/ml) | 316 | 412 | 286 | 1820 | 708.5 | 742.9 | 2070 | 2000 | 2035 | 49.5 |
| TSAT (%) | 54 | 59 | 89 | 87 | 72.3 | 18.3 | 11.5 | 16.5 | 14 | 3.5 |
| MRI results | | | | | | | | | | |
| LIC (mg Fe/g.d.w.) | 19 | 16 | 7.3 | 8.9 | 12.8 | 5.6 | | | | |
| T2* (msec) | 28.5 | 30.7 | 37.6 | 34.9 | 32.9 | 4.1 | | | | |
| RBC analysis results | | | | | | | | | | |
| Mechanical lysis (%) | 0.351 | 0.603 | 0.288 | 0.839 | 1.2 | 0.2 | 13.9 | 11.1 | 0.5 | 0.3 |
| Proteotoxic lysis (mg/dl) | 102.8 | 131.3 | 127.1 | 114.9 | 119.1 | 12.8 | 57.7 | 75.4 | 66.5 | 12.5 |
| Phenyl-hydrazine induced ROS (MFI/mg) | 38141 | 33989 | 35913 | 34478 | 35630 | 1862 | 26377 | 29171 | 27774 | 1976 |
| Hemichromes (nM/mg) | 6.3 | 7.9 | 6.2 | 6 | 6.6 | 0.9 | 4.9 | 4.4 | 4.7 | 0.4 |
| Methemoglobin (%) | 0.9 | 0.4 | 0.4 | 0.6 | 0.6 | 0.2 | 0.4 | 0.3 | 0.3 | 0.1 |
| Cytosol: Chymotrypsin-like proteasome activity (MFI/mg of protein) | 119983 | 84504 | 84050 | 76149 | 91171 | 19587 | 73463 | 54515 | 63989 | 13398 |
| Cytosol: Trypsin-like proteasome activity (MFI/mg of protein) | 154516 | 119999 | 86175 | 98078 | 114692 | 30019 | 86720 | 67730 | 77225 | 13428 |
| Cytosol: Caspase-like proteasome activity (MFI/mg of protein) | 97129 | 72071 | 72297 | 78519 | 80004 | 11801 | 80657 | 59176 | 69916 | 15189 |
| Membrane: Chymotrypsin-like proteasome activity (MFI/mg of protein) | 36871 | 10796 | 435153 | 289198 | 193004 | 204505 | 437453 | 380806 | 409130 | 40055 |
| Membrane: Trypsin-like proteasome activity (MFI/mg of protein) | 53526 | 51912 | 491003 | 403327 | 249942 | 230530 | 447005 | 419237 | 433121 | 19635 |
| Membrane: Caspase-like proteasome activity (MFI/mg of protein) | 119534 | 151874 | 427908 | 362495 | 265453 | 152754 | 372496 | 400549 | 386522 | 19836 |

**Figure 1.** Presentation of the variables assessed by serum iron assays, MRI, and spectrometry, for each patient. Mean values significantly increased ($p < 0.05$) compared to controls are highlighted in red color. HH patients are characterized by excess systemic iron overload, as indicated by TSAT and LIC measurements. HHCS, despite their increased serum ferritin levels, present a normal iron load. Significant differences were observed in RBCs' propensity to lysis, ROS, membrane hemichromes and proteasome activity in HH patients. HHCS pts presented decreased RBCs' membrane lysis, ROS, and membrane hemichromes compared to HH patients, whereas an elevated proteasomal degradation in RBC membrane was demonstrated. [HH: hereditary hemochromatosis; HHCS: hereditary hyperferritinemia-cataract syndrome; LIC: liver iron concentration; Pt: patient; TSAT: transferrin saturation]

**Copyright Information:** (Online) ISSN: 2572-9241
©2026 The Author(s). HemaSphere is published by John Wiley & Sons Ltd on behalf of European Hematology Association. This is an open access Abstract Book distributed under the Attribution-NonCommercial-NoDerivs (CC BY-NC-ND), which allows third parties to download the articles and share them with others as long as they credit the author and the Abstract Book, but they cannot change the content in any way or use them commercially.
**Abstract Book Citations:** Authors, Title, HemaSphere, 2026;10:(S1):pages. The abstract book DOIs can be found on the "Author Information" page.
**Disclaimer:** Articles published in the journal HemaSphere exclusively reflect the opinions of the authors. The authors are responsible for all content in their abstracts including accuracy of the facts, statements, citing resources, etc.

### (PB4218) EVALUATING THE DISEASE MODIFYING EFFECT OF TFR2 VARIANTS IN HEMOGLOBINOPATHIES

**Topic:** 30. Iron metabolism, deficiency and overload

Immacolata Andolfo[1, 2], Rosa Manca[3], Silverio Perrotta[3], Raffaella Origa[4, 5], Alessia Perrotta[1, 3], Antonella Nostroso[1, 2], Roberta Russo[1, 2], Giada Giuliani[6, 7], Antonella Nai[8, 9], Lucia De Franceschi*[6, 7]

*[1]University of Naples Federico II, Department of Molecular Medicine and Medical Biotechnology, Naples, Italy;[2]CEINGE Biotecnologie Avanzate Franco Salvatore, Naples, Italy; [3]University of Campania "L. Vanvitelli", Naples, Italy; [4]University of Cagliari, Department of Medical Sciences and Public Health, Cagliari, Italy; [5]Ospedale Pediatrico Microcitemico, ARNAS G.Brotzu, SC Microcitemie e Anemie Rare, Cagliari, Italy; [6]University of Verona, Department of Engineering for Innovative Medicine, Verona, Italy; [7]Azienda Ospedaliera Integrata of Verona, Verona, Italy; [8]IRCCS Ospedale San Raffaele, Regulation of Iron Metabolism Unit, Division of Genetics and Cellular Biology, Milan, Italy; [9]Vita-Salute San Raffaele University, Milan, Italy;*

### Background

Transferrin receptor 2 (TFR2) in an iron-sensor predominantly expressed in the liver, where it contributes to the transcriptional activation of the iron-regulatory hormone hepcidin, and in erythroid cells, where it attenuates sensitivity to erythropoietin (EPO), partnering with its receptor. Consistent with this dual function, studies in murine models have shown that hepatic *Tfr2* deficiency causes iron-overload, whereas loss of *Tfr2* in the hematopoietic compartment enhances erythropoiesis in wild-type mice and ameliorates anemia in murine models of β-thalassemia.
While the role of TFR2 in systemic iron regulation is well established also in humans, where inactivating mutations cause hereditary hemochromatosis, its function in human erythropoiesis and its involvement in human erythroid disorders remain poorly characterized. Notably, several genome-wide association studies have identified *TFR2*-single nucleotide polymorphisms (SNPs) associated with increased red blood cell count in healthy individuals, supporting a conserved role for TFR2 in erythroid regulation.

### Aims

Here, we aim to investigate the relevance of TFR2 in human erythropoiesis and its potential role as a disease modifier in hemoglobinopathies.

### Methods

To investigate the relevance of TFR2 in human hemoglobinopathies, we will apply a custom 125-gene next-generation sequencing panel for hereditary red blood cells and iron metabolism disorders (Nostroso A, et al. Hemasphere 2026) to patients with non-transfusion-dependent ß-thalassemia (NTDT) and sickle cell disease (SCD). About 200 adult patients were enrolled, excluding individuals under chronic transfusion regimen, with current or recent (within 6 months) iron chelation therapy, or with coexisting hematologic or iron disorders. This approach will enable comprehensive sequencing of *TFR2*, together with other key genes involved in iron homeostasis and erythropoiesis (including *HAMP, HFE, HJV, TF, TMPRSS6, EPO,* and *EPOR*), to correlate the presence of coding variants with clinical parameters such as anemia severity, iron-overload, circulating erythroblasts, reticulocyte counts, and lactate dehydrogenase levels. These analyses will clarify whether common or rare variants in *TFR2 gene* influence patient's phenotype.
In parallel, to functionally validate the impact of *TFR2* loss of function on human thalassemic or SCD erythropoiesis, CD34+ cells will be isolated from NTDT and SCD patients without *TFR2* mutations (n=10/genotype) and subjected to CRISPR-Cas9-mediated *TFR2* inactivation. Edited and control cells will be compared for viability, morphology, proliferation, maturation profile, enucleation efficiency, oxidative status, and hemichrome formation.

**Copyright Information:** (Online) ISSN: 2572-9241
©2026 The Author(s). HemaSphere is published by John Wiley & Sons Ltd on behalf of European Hematology Association. This is an open access Abstract Book distributed under the Attribution-NonCommercial-NoDerivs (CC BY-NC-ND), which allows third parties to download the articles and share them with others as long as they credit the author and the Abstract Book, but they cannot change the content in any way or use them commercially.
**Abstract Book Citations:** Authors, Title, HemaSphere, 2026;10:(S1):pages. The abstract book DOIs can be found on the "Author Information" page.
**Disclaimer:** Articles published in the journal HemaSphere exclusively reflect the opinions of the authors. The authors are responsible for all content in their abstracts including accuracy of the facts, statements, citing resources, etc.

### Results

We expect the first results of the study will be ready for presentation at the meeting.

### Summary/Conclusion

Overall, these studies will define the role of TFR2 as a regulator of human erythropoiesis and assess its potential as a therapeutic target for hemoglobinopathies, as demonstrated in murine models. In addition, this approach may uncover novel genetic modifiers of disease severity in β-thalassemic and sickle cell disease.

Work supported by European Union-Next Generation EU_PNRR M6/C2 PNRR-POC-2023-12378393 grant (CUP: C43C24000430007).

**Copyright Information:** (Online) ISSN: 2572-9241
©2026 The Author(s). HemaSphere is published by John Wiley & Sons Ltd on behalf of European Hematology Association. This is an open access Abstract Book distributed under the Attribution-NonCommercial-NoDerivs (CC BY-NC-ND), which allows third parties to download the articles and share them with others as long as they credit the author and the Abstract Book, but they cannot change the content in any way or use them commercially.
**Abstract Book Citations:** Authors, Title, HemaSphere, 2026;10:(S1):pages. The abstract book DOIs can be found on the "Author Information" page.
**Disclaimer:** Articles published in the journal HemaSphere exclusively reflect the opinions of the authors. The authors are responsible for all content in their abstracts including accuracy of the facts, statements, citing resources, etc.

### (PB4219) AGE-DEPENDENT ATTENUATION OF ORAL IRON REPLETION IN IRON DEFICIENCY: ELDERLY PATIENTS AS A WINDOW INTO UNEXPLAINED ORAL IRON-REFRACTORY ANEMIA (UOIRA)

**Topic:** 30. Iron metabolism, deficiency and overload

Liri Seraj*[1], Rialda Myrtaj[2], Arlind Seraj[3], Evriola Dede[2], Rudina Degjoni[2], Aureta Bruci[2]

*[1]Memorial Regional Hospital, Internal Medicine/Hematology, Fier, Albania; [2]Memorial Regional Hospital, Fier, Albania; [3]Pharma Flow qms, Copenhagen, Denmark;*

### Background

Oral iron remains first-line therapy for iron deficiency (ID) and iron deficiency anemia (IDA) globally. However, a subset of patients fails to respond adequately, a phenomenon increasingly recognized as unexplained oral iron-refractory anemia (UOIRA). Clinical experience suggests elderly patients are disproportionately affected, yet prospective age-stratified data on oral iron responsiveness remain scarce, particularly from resource-limited settings where oral iron is often the only accessible treatment.

### Aims

To evaluate age-dependent differences in oral iron repletion and to characterize the elderly non-responder phenotype in a prospective cohort of ID/IDA patients.

### Methods

We prospectively enrolled 530 patients with oral iron-refractory ID/IDA at Memorial Hospital of Fier, Albania (2023-2025). All patients had treatable causes of refractoriness identified and addressed before restarting oral iron supplementation. Hematologic parameters (HGB, RBC, MCV, MCH) and serum ferritin were measured at baseline and follow-up. Of 200 patients with paired data, 122 had documented age and were stratified into young (<40, n=23), middle-aged (40-64, n=52), and elderly (>=65, n=47). Response was defined as any HGB increase; normalization as HGB >=12 g/dL.

### Results

Mean age was 55.8 years (range 16-89). A stepwise age-dependent attenuation of iron repletion response was observed. HGB improved across all groups, but the magnitude declined with age: young +0.74 g/dL, middle-aged +0.57 g/dL, elderly +0.40 g/dL. Response rates followed the same gradient: 69.6%, 73.1%, and 57.4%, respectively. Among anemic patients (HGB <12), the disparity was more pronounced: HGB normalization was achieved in 50.0% of young, 59.1% of middle-aged, but only 25.0% of elderly patients. Elderly non-responders (42.6% of the elderly cohort) exhibited a distinct phenotype: paradoxically higher baseline ferritin (75.6 vs 57.2 ng/mL in responders) and higher baseline HGB (12.2 vs 10.8 g/dL), suggesting functional iron restriction rather than absolute deficiency. Baseline ferritin was significantly higher in the elderly group overall (median 41.2 vs 13.1 ng/mL in young, p<0.01), consistent with inflammation-mediated iron sequestration. Elderly patients also had higher mean CRP (2.07 vs 0.85 mg/L in middle-aged).

**Copyright Information:** (Online) ISSN: 2572-9241
©2026 The Author(s). HemaSphere is published by John Wiley & Sons Ltd on behalf of European Hematology Association. This is an open access Abstract Book distributed under the Attribution-NonCommercial-NoDerivs (CC BY-NC-ND), which allows third parties to download the articles and share them with others as long as they credit the author and the Abstract Book, but they cannot change the content in any way or use them commercially.
**Abstract Book Citations:** Authors, Title, HemaSphere, 2026;10:(S1):pages. The abstract book DOIs can be found on the "Author Information" page.
**Disclaimer:** Articles published in the journal HemaSphere exclusively reflect the opinions of the authors. The authors are responsible for all content in their abstracts including accuracy of the facts, statements, citing resources, etc.

**Summary/Conclusion**

Oral iron repletion response attenuates progressively with age, with elderly patients showing the lowest response and normalization rates. The elderly non-responder phenotype, characterized by higher ferritin and inflammatory markers despite persistent anemia, may represent early or subclinical UOIRA. These findings support systematic investigation of elderly patients with unexplained oral iron-refractory anemia for underlying functional iron restriction, chronic inflammation, or occult clonal hematopoiesis. Prospective studies incorporating hepcidin and molecular profiling are warranted.

**Copyright Information:** (Online) ISSN: 2572-9241
©2026 The Author(s). HemaSphere is published by John Wiley & Sons Ltd on behalf of European Hematology Association. This is an open access Abstract Book distributed under the Attribution-NonCommercial-NoDerivs (CC BY-NC-ND), which allows third parties to download the articles and share them with others as long as they credit the author and the Abstract Book, but they cannot change the content in any way or use them commercially.
**Abstract Book Citations:** Authors, Title, HemaSphere, 2026;10:(S1):pages. The abstract book DOIs can be found on the "Author Information" page.
**Disclaimer:** Articles published in the journal HemaSphere exclusively reflect the opinions of the authors. The authors are responsible for all content in their abstracts including accuracy of the facts, statements, citing resources, etc.

### (PB4220) IMPACT OF ORAL IRON SUPPLEMENTATION ON FATIGUE AND PHYSICAL PERFORMANCE IN YOUNG WOMEN WITH IRON DEFICIENCY ANEMIA

**Topic:** 30. Iron metabolism, deficiency and overload

Ines Mezghani*[1], Yesmine Fourati[1], Mohamed Achraf Harrabi[2], Rahma Ayed[3], Mouna Turki[4], Sonia Salhi[5], Fatma Ayadi[3]

*[1]Laboratory of Biochemistry, Hedi Chaker University Hospital, Sfax, Tunisia;[2]Research Laboratory Education, Motricité, Sport et Santé, EM2S, LR19JS01, High Institute of Sport and Physical Education of Sfax, sfax, Tunisia; [3]Laboratory of Biochemistry, Habib Bourguiba University Hospital, sfax, Tunisia; [4]Laboratory of Biochemistry, Hedi Chaker University Hospital, sfax, Tunisia; [5]Sports Performance Optimization (LR09SEP01), National Center of Medecine and Science in Sports (CNMSS), sfax, Tunisia;*

### Background

Iron deficiency anemia (IDA) is the most prevalent nutritional deficiency worldwide, disproportionately affecting women of reproductive age. It is commonly associated with reduced physical performance and increased fatigue. While iron supplementation is known to restore hematological parameters, its effects on muscle function in young women remain poorly documented, justifying the need for further clinical investigation.

### Aims

Evaluate the effects of oral ferrous sulfate supplementation on physical performance including aerobic fitness, muscle strength and endurance in young women of reproductive age diagnosed with iron deficiency anemia.

### Methods

This is a prospective clinical trial conducted at the Leather and Footwear Vocational Training Center of Sfax over a period of 8 weeks. Young women with IDA were recruited.Each participant received 494.5 mg ferrous sulfate per day for 8 weeks.
Physical performance was evaluated through three complementary assessments: the 20m shuttle test for aerobic fitness, the Killy test for static lower limb muscle endurance, and an isometric dynamometer for maximal quadriceps strength, conducted at baseline and following the 8-week supplementation period.
Fatigue was assessed using the French version of the Multidimensional Fatigue Inventory (MFI). It is a valid and reliable self-questionnaire for women with anemia.It includes 5 items covering general fatigue, physical fatigue, mental fatigue,reduced activity,and motivation.
Statistical analyses were conducted using IBM SPSS® statistics software version 25.

### Results sixteen women with iron deficiency anemia, aged 18 to 30 years, were enrolled in the present study.
The mean age was 21.69 ± 1.92 years and the mean body mass index (BMI) was 21.32 ± 2.88 kg/m².
After 8 weeks of ferrous sulfate supplementation(160 mg elemental iron/day), significant improvements were observed in hematological parameters, with Hb concentration increasing by 2.18 g/dL ($P < 0.001$) and serum ferritin by 2.19 µg/L ($P < 0.001$).
Lower limb muscle endurance also increased significantly ($P = 0.006$).
All five dimensions of fatigue measured by the MFI-20 improved significantly, with reductions in general fatigue ($P < 0.001$), physical fatigue ($P < 0.001$), mental fatigue ($P < 0.001$), reduced activity ($P < 0.001$) and reduced motivation ($P = 0.006$). In contrast, neither maximal isometric quadriceps strength ($P = 0.86$) nor aerobic fitness ($P = 0.008$) reached statistical significance after the supplementation period.

**Copyright Information:** (Online) ISSN: 2572-9241
©2026 The Author(s). HemaSphere is published by John Wiley & Sons Ltd on behalf of European Hematology Association. This is an open access Abstract Book distributed under the Attribution-NonCommercial-NoDerivs (CC BY-NC-ND), which allows third parties to download the articles and share them with others as long as they credit the author and the Abstract Book, but they cannot change the content in any way or use them commercially.
**Abstract Book Citations:** Authors, Title, HemaSphere, 2026;10:(S1):pages. The abstract book DOIs can be found on the "Author Information" page.
**Disclaimer:** Articles published in the journal HemaSphere exclusively reflect the opinions of the authors. The authors are responsible for all content in their abstracts including accuracy of the facts, statements, citing resources, etc.

**Summary/Conclusion**

Eight weeks of oral ferrous sulfate supplementation restored iron status and improved muscle endurance and all dimensions of fatigue in young women with IDA. However, aerobic fitness and maximal muscle strength remained unchanged,suggesting that iron repletion alone may be insufficient to fully restore physical performance.

**Copyright Information:** (Online) ISSN: 2572-9241
©2026 The Author(s). HemaSphere is published by John Wiley & Sons Ltd on behalf of European Hematology Association. This is an open access Abstract Book distributed under the Attribution-NonCommercial-NoDerivs (CC BY-NC-ND), which allows third parties to download the articles and share them with others as long as they credit the author and the Abstract Book, but they cannot change the content in any way or use them commercially.
**Abstract Book Citations:** Authors, Title, HemaSphere, 2026;10:(S1):pages. The abstract book DOIs can be found on the "Author Information" page.
**Disclaimer:** Articles published in the journal HemaSphere exclusively reflect the opinions of the authors. The authors are responsible for all content in their abstracts including accuracy of the facts, statements, citing resources, etc.

### (PB4221) INFLAMMATION-RELATED HEMATOLOGIC AND IRON-AXIS HETEROGENEITY ON ADMISSION IN COMBAT CASUALTIES WITH PROLONGED LIMB ISCHEMIA DURING THE RUSSIA-UKRAINE WAR

**Topic:** 30. Iron metabolism, deficiency and overload

Pavlo Dubovetskyi*[1], Svitlana Husieva[2], Ian Goncharov[1], Vitalii Orlov[1]

*[1]National Military Medical Clinical Center "Main Military Clinical Hospital", Clinic of hematology, Ukraine,[2]Ukrainian Military Medical Academy, Department of Military Therapy, Kyiv, Ukraine;*

### Background

During the ongoing Russia–Ukraine war, combat-related prolonged limb ischemia develops in a highly heterogeneous clinical context, including variable hemorrhage burden, tourniquet load, time-to-tertiary care, pre-transfer transfusion exposure, and extent of surgery (amputation vs fasciotomy). These factors may substantially influence admission hematologic indices and iron parameters. In this setting, anemia is expected to be multifactorial, combining inflammatory iron restriction with post-hemorrhagic and iron-deficiency components.

### Aims

To assess associations between inflammatory markers and admission hematologic and iron parameters in combat casualties with prolonged limb ischemia.

### Methods

Retrospective observational analysis of 84 combat casualties treated at a tertiary military center between 2022 and 2024 during the Russia–Ukraine war. All analyses were performed on admission samples to minimize treatment-related confounding. Spearman's correlation analysis was performed. Variables included erythrocyte count (RBC), hemoglobin level, red cell distribution width (RDW), platelet count, absolute monocyte count, C-reactive protein (CRP), ferritin, and serum iron.

### Results

Hemoglobin level ranged 55-128 g/L; RBC 1.16-4.29×10^12/L; RDW 12.0-18.3%; platelet count 28-504 × 10^9/L; absolute monocyte count 0.09-1.82×10^9/L; CRP 16.3-401.1 mg/L; ferritin 221.5-1676.0 µg/L; and serum iron 0.9-10.8 µmol/L. Spearman correlation analysis demonstrated weak-to-moderate statistically significant associations ($p<0.05$ for reported correlations). Ferritin showed inverse correlations with erythroid parameters: erythrocyte count-ferritin ($\rho=-0.33$) and hemoglobin level-ferritin ($\rho=-0.35$). CRP demonstrated an inverse association with serum iron ($\rho=-0.24$), consistent with inflammation-associated hypoferremia. Ferritin demonstrated a weak positive association with serum iron ($\rho=0.20$), indicating limited interpretability of ferritin as a surrogate of iron availability under wartime confounding. Platelet count correlated positively with CRP ($\rho=0.19$) and with ferritin ($\rho=0.29$). The strongest association was observed between ferritin and absolute monocyte count ($\rho=0.56$), consistent with inflammation-related innate immune activation.

**Copyright Information:** (Online) ISSN: 2572-9241
©2026 The Author(s). HemaSphere is published by John Wiley & Sons Ltd on behalf of European Hematology Association. This is an open access Abstract Book distributed under the Attribution-NonCommercial-NoDerivs (CC BY-NC-ND), which allows third parties to download the articles and share them with others as long as they credit the author and the Abstract Book, but they cannot change the content in any way or use them commercially.
**Abstract Book Citations:** Authors, Title, HemaSphere, 2026;10:(S1):pages. The abstract book DOIs can be found on the "Author Information" page.
**Disclaimer:** Articles published in the journal HemaSphere exclusively reflect the opinions of the authors. The authors are responsible for all content in their abstracts including accuracy of the facts, statements, citing resources, etc.

**Summary/Conclusion**

In combat-related prolonged limb ischemia during the Russia–Ukraine war, admission inflammatory markers were associated with multi-lineage hematologic and iron-axis perturbations. The inverse associations of ferritin with erythroid parameters, together with CRP-associated hypoferremia and ferritin-monocyte correlation, support a clinically relevant inflammation-hematology axis. However, the magnitude of correlations indicates that ferritin cannot be interpreted as a standalone surrogate of iron availability in this heterogeneous wartime population. These findings are compatible with combined-genesis anemia and justify prospective validation with standardized iron panels (including transferrin saturation and hepcidin) to inform iron management in inflammatory trauma settings.

**Copyright Information:** (Online) ISSN: 2572-9241
©2026 The Author(s). HemaSphere is published by John Wiley & Sons Ltd on behalf of European Hematology Association. This is an open access Abstract Book distributed under the Attribution-NonCommercial-NoDerivs (CC BY-NC-ND), which allows third parties to download the articles and share them with others as long as they credit the author and the Abstract Book, but they cannot change the content in any way or use them commercially.
**Abstract Book Citations:** Authors, Title, HemaSphere, 2026;10:(S1):pages. The abstract book DOIs can be found on the "Author Information" page.
**Disclaimer:** Articles published in the journal HemaSphere exclusively reflect the opinions of the authors. The authors are responsible for all content in their abstracts including accuracy of the facts, statements, citing resources, etc.

### (PB4222) PANCYTOPENIA REVEALING SEVERE IRON DEFICIENCY ANEMIA: AN UNUSUAL PRESENTATION

**Topic:** 30. Iron metabolism, deficiency and overload

Rihab Ben Sghaier*[1], Mariem Chaabane[1], Wafa Chenbah[1], Ghassen Saidi[1], Haifa Regaieg[1], Bouteraa Walid[1], Monia Zaier[1], Kmira Zahra[1], Nesrine Ben Sayed[1], Yosra Ben Youssef[1]

[1]*Farhat Hached University Hospital, Clinical Hematology Department, Sousse, Tunisia;*

### Background

Iron deficiency anemia (IDA) is the most common cause of anemia worldwide. It is classically associated with reactive thrombocytosis. Leukopenia and thrombocytopenia are uncommon findings, and pancytopenia is exceptionally rare. When present, it usually raises suspicion of bone marrow failure, hematologic malignancy, infection, or autoimmune disease.

### Aims

To report a rare case of severe iron deficiency anemia presenting as pancytopenia and to highlight the importance of excluding other etiologies before retaining this diagnosis.

### Methods

Data were retrospectively collected from the patient’s medical records, including clinical findings, laboratory investigations, bone marrow examination, treatment, and follow-up.

**Copyright Information:** (Online) ISSN: 2572-9241
©2026 The Author(s). HemaSphere is published by John Wiley & Sons Ltd on behalf of European Hematology Association. This is an open access Abstract Book distributed under the Attribution-NonCommercial-NoDerivs (CC BY-NC-ND), which allows third parties to download the articles and share them with others as long as they credit the author and the Abstract Book, but they cannot change the content in any way or use them commercially.
**Abstract Book Citations:** Authors, Title, HemaSphere, 2026;10:(S1):pages. The abstract book DOIs can be found on the “Author Information” page.
**Disclaimer:** Articles published in the journal HemaSphere exclusively reflect the opinions of the authors. The authors are responsible for all content in their abstracts including accuracy of the facts, statements, citing resources, etc.

### Results

A 43-year-old woman presented with progressive pallor and asthenia evolving over several months. Physical examination revealed marked pallor (WHO performance status 2) without hemorrhagic manifestations, infection, lymphadenopathy, splenomegaly, or hepatomegaly.
Initial laboratory evaluation showed pancytopenia with severe microcytic anemia: hemoglobin 3.2 g/dL, MCV 56 fL, MCH 16.4 pg, WBC 2,300/mm³ (ANC 1,500/mm³), and platelets 69,000/mm³. RDW was 21%. Peripheral blood smear showed no blasts or dysplastic features. Biochemistry revealed no hemolysis.
Etiological workup demonstrated normal vitamin B12 (275 pg/mL) and folate levels (5.21 ng/mL). Ferritin was profoundly decreased at 2.17 ng/mL. Viral serologies and autoimmune screening were negative.
Given the unexpected association of iron deficiency with pancytopenia, bone marrow biopsy was performed to rule out marrow failure or infiltration. It revealed a normocellular marrow with preserved maturation and no malignant infiltration.
The patient received two units of packed red blood cells followed by intravenous iron therapy for four weeks, then oral iron supplementation. A progressive correction of all hematologic parameters was observed.
At four months follow-up, hemoglobin increased to 13.2 g/dL, WBC to 6,400/mm³, platelets to 119,000/mm³, and ferritin to 104 ng/mL. All three hematopoietic lineages recovered after iron repletion, confirming iron deficiency as the underlying cause (Table1).

| | 06/09/25 | 09/09/25 | 16/09/25 | 30/09/25 | 28/10/25 | 27/01/26 |
|---|---|---|---|---|---|---|
| WBC/mm$^3$ | 2300 | 2800 | 4300 | 4000 | 4900 | 6400 |
| ANC/mm$^3$ | 1500 | 1800 | 3000 | 2700 | 3600 | 4600 |
| HB (g/dL) | 3.2 | 5 | 8.4 | 7.6 | 10.6 | 13.2 |
| MCV (fl) | 56 | 56.8 | 65 | 74 | 76 | 86 |
| MCHC ( pg) | 16 | 16.4 | 20 | | 23.2 | 29.7 |
| Platelets/mm$^3$ | 69000 | 70000 | 122000 | 120000 | 146000 | 119000 |
| Ferritin level ng/mL | | 2.14 | | | | 104 |
| Treatment | | Transfusion of 2 concentrates of erythocytes | Iron iv | Oral Iron | Oral Iron | Oral Iron |

### Summary/Conclusion

Although iron deficiency anemia is typically associated with thrombocytosis, it can exceptionally present as pancytopenia. This diagnosis should only be considered after careful exclusion of bone marrow failure syndromes, hematologic malignancies, infections, and autoimmune disorders. Recognition of this rare presentation is important to avoid unnecessary aggressive investigations and to ensure appropriate treatment.

**Copyright Information:** (Online) ISSN: 2572-9241
©2026 The Author(s). HemaSphere is published by John Wiley & Sons Ltd on behalf of European Hematology Association. This is an open access Abstract Book distributed under the Attribution-NonCommercial-NoDerivs (CC BY-NC-ND), which allows third parties to download the articles and share them with others as long as they credit the author and the Abstract Book, but they cannot change the content in any way or use them commercially.
**Abstract Book Citations:** Authors, Title, HemaSphere, 2026;10:(S1):pages. The abstract book DOIs can be found on the “Author Information” page.
**Disclaimer:** Articles published in the journal HemaSphere exclusively reflect the opinions of the authors. The authors are responsible for all content in their abstracts including accuracy of the facts, statements, citing resources, etc.

### (PB4223) ORAL DEFERASIROX FOR IRON OVERLOAD IN HEREDITARY HEMOCHROMATOSIS WITH LIVER CIRRHOSIS;A CASE REPORT ON LONG TERM SAFETY AND EFFICACY

**Topic:** 30. Iron metabolism, deficiency and overload

Chithrangani Batugedara*[1], Charlotte Bradbury[2]

*[1]University Hospital Bristol and Weston NHS Foundation Trust, Haematology, Bristol, United Kingdom;[2]University of Bristol, Faculty of Health Sciences, Bristol, United Kingdom;*

### Background

Hereditary hemochromatosis( HH), most commonly due to HFE C282Y homozygosity, leads to progressive iron overload and organ damage particularly affecting liver, heart and endocrine glands.
Serum ferritin levels >1000 µg/L signify the risk of significant cardiac and hepatic iron overload.  Notably up to 25% of the HH-related liver cirrhosis patients are at risk of developing hepatocellular carcinoma.
Phlebotomy remains the mainstay of treatment; however patients with anaemia, cytopaenias or cirrhosis may not tolerate venesection. The European Association for the Study of the Liver (EASL) guidelines advise caution or avoidance of deferasirox in patients with advanced liver disease, due to the potential of hepatic and renal toxicity. Data on its use in cirrhotic HH management remain limited.

### Aims

To describe long term safety an efficacy of low dose oral deferasirox in an elderly HH patient with cirrhosis who was unable to tolerate conventional phlebotomy

### Methods

A detailed clinical review of laboratory parameters, MRI T2* findings , treatment course and adverse event monitoring was performed over 2.5 year treatment period.

**Copyright Information:** (Online) ISSN: 2572-9241
©2026 The Author(s). HemaSphere is published by John Wiley & Sons Ltd on behalf of European Hematology Association. This is an open access Abstract Book distributed under the Attribution-NonCommercial-NoDerivs (CC BY-NC-ND), which allows third parties to download the articles and share them with others as long as they credit the author and the Abstract Book, but they cannot change the content in any way or use them commercially.
**Abstract Book Citations:** Authors, Title, HemaSphere, 2026;10:(S1):pages. The abstract book DOIs can be found on the "Author Information" page.
**Disclaimer:** Articles published in the journal HemaSphere exclusively reflect the opinions of the authors. The authors are responsible for all content in their abstracts including accuracy of the facts, statements, citing resources, etc.

**Results**

A 77-year-old woman with C282Y homozygous HH presented with severe iron overload ( ferritin >2000 µg/L, transferrin saturation 100%)and Fibroscan confirmed fibrosis (22.6 kPa), consistent with cirrhosis.
Initial biweekly 250 mL phlebotomy led to progressive anaemia ( Hb~100-116g/L) with minimal improvement in iron burden.
MRI T2* demonstrated very severe hepatic siderosis (T2* 1.79ms) without cardiac involvement.
Following multidisciplinary review, low dose oral deferasirox was initiated at 7 mg/kg (450 mg daily) despite the standard contradictions in cirrhosis and CKD stage 3. Laboratory monitoring ( with FBC, Liver and Renal functions, and Urine protein: creatinine ratio) was performed weekly for the first month and monthly thereafter. Low volume phlebotomy continued in parallel with a reduced frequency.
Treatment was well tolerated apart from manageable diarrhoea. Renal and hepatic parameters remained stable, with dose adjustments between 360-450mg/day for tolerability.
Ferritin declined to <1000µg/L by 10months and normalised to 388µg/L after 18 months, permitting cessation of venesection.
Deferasirox was tapered and discontinued after about 2.5 years. Follow up MRI T2* demonstrated marked reduction in hepatic iron (14.2ms). No auditory or ophthalmology toxicity occured.
The patient remains clinically stable with no active treatment for more than a year with a Hb of 116g/L, ferritin 62µg/L, preserved renal functions and improved quality of life.

**References**

1. Hoffbrand AV, Higgs DR, Keeling DM, Mehta AB. *Postgraduate Haematology*, 7th ed. Wiley-Blackwell; 2016.
2. European Association for the Study of the Liver. EASL clinical practice guidelines for HFE hemochromatosis. *J Hepatol.* 2022.
3. British Committee for Standards in Haematology. Diagnosis and therapy of genetic haemochromatosis: Review and 2017 update.
4. SPC *Exjade (deferasirox),* electronic Medicines Compendium (eMC).

5. Ahley C, Dunleavy A. *The Renal Drug Handbook*, 5th ed. CRC Press;2018

**Summary/Conclusion**

This case adds to the limited literature supporting deferasirox in HH patients with cirrhosis. While routine use in this population is not recommended, deferasirox can be a viable option in selected patients with intolerance to phlebotomy, with multidisciplinary oversight. Careful patient selection, low-dose initiation, and rigorous monitoring are essential to minimise risk and optimise outcomes.

**Copyright Information:** (Online) ISSN: 2572-9241
©2026 The Author(s). HemaSphere is published by John Wiley & Sons Ltd on behalf of European Hematology Association. This is an open access Abstract Book distributed under the Attribution-NonCommercial-NoDerivs (CC BY-NC-ND), which allows third parties to download the articles and share them with others as long as they credit the author and the Abstract Book, but they cannot change the content in any way or use them commercially.
**Abstract Book Citations:** Authors, Title, HemaSphere, 2026;10:(S1):pages. The abstract book DOIs can be found on the "Author Information" page.
**Disclaimer:** Articles published in the journal HemaSphere exclusively reflect the opinions of the authors. The authors are responsible for all content in their abstracts including accuracy of the facts, statements, citing resources, etc.

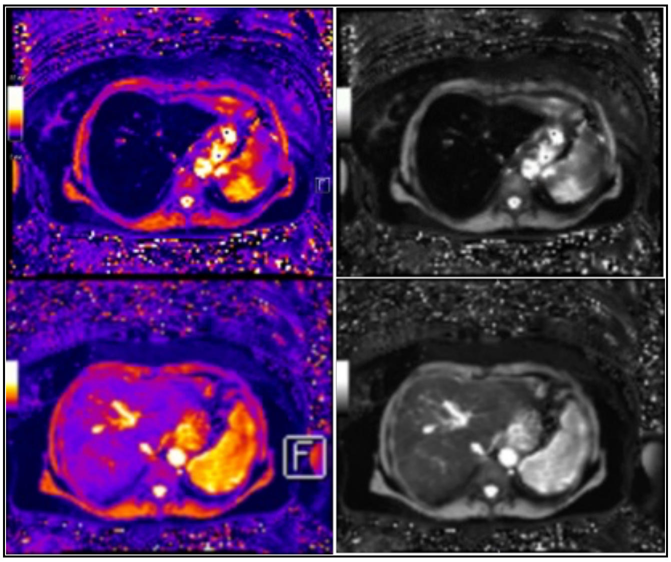

Figure 1-Liver T2* MRI Top 2 - December 2021 Bottom 2 - January 2024

**Copyright Information:** (Online) ISSN: 2572-9241
©2026 The Author(s). HemaSphere is published by John Wiley & Sons Ltd on behalf of European Hematology Association. This is an open access Abstract Book distributed under the Attribution-NonCommercial-NoDerivs (CC BY-NC-ND), which allows third parties to download the articles and share them with others as long as they credit the author and the Abstract Book, but they cannot change the content in any way or use them commercially.
**Abstract Book Citations:** Authors, Title, HemaSphere, 2026;10:(S1):pages. The abstract book DOIs can be found on the "Author Information" page.
**Disclaimer:** Articles published in the journal HemaSphere exclusively reflect the opinions of the authors. The authors are responsible for all content in their abstracts including accuracy of the facts, statements, citing resources, etc.

### (PB4224) HEREDITARY HYPERFERRITINEMIA–CATARACT SYNDROME WITH MUSCULOSKELETAL MANIFESTATIONS

**Topic:** 30. Iron metabolism, deficiency and overload

Faisal Alghamdi[1], Noura Alsaid*[1], Mawaddah Sabr[2], Sayed Amin[2]

*[1]King Salman medical city, Medina, Saudi Arabia;[2]Uhod hospital, Medina, Saudi Arabia;*

### Background

Hereditary hyperferritinemia–cataract syndrome (HHCS) is a rare autosomal-dominant disorder caused by pathogenic variants in the iron-responsive element (IRE) of the L-ferritin gene (FTL). It is characterized by isolated hyperferritinemia without systemic iron overload and early-onset cataracts.

### Aims

To report a pediatric case of isolated hyperferritinemia associated with familial early-onset cataracts and to confirm the molecular diagnosis.

### Methods

This is a case report with targeted genomic evaluation. Clinical history, family pedigree, ophthalmologic examination, comprehensive iron studies, inflammatory markers and whole-exome sequencing were reviewed.

### Results

An 11-year-old girl was evaluated during anemia screening for recurrent bilateral lower limb pain. Laboratory investigations revealed isolated hyperferritinemia (serum ferritin 2000 ng/mL) with normal hemoglobin indices, transferrin and iron studies, and no inflammatory or hepatic abnormalities. Retrospective history identified progressive bilateral visual impairment and slit-lamp examination demonstrated posterior subcapsular cataracts. Family history revealed bilateral cataracts across four successive generations. Whole-exome sequencing identified a heterozygous c.-168G>A variant in the 5′-UTR IRE of FTL, previously reported as pathogenic variant associated with HHCS. The combination of markedly elevated ferritin, normal iron indices, early bilateral cataracts, and autosomal-dominant inheritance confirmed the diagnosis. To our knowledge, this is the first molecularly confirmed HHCS case reported from the Middle East and the first described with an association of lower limb pain.

### Summary/Conclusion

This case highlights the importance of recognizing HHCS to avoid misdiagnosis and inappropriate treatment. Molecular confirmation, multidisciplinary collaboration, and genetic counseling are crucial for optimal care. Increased awareness among pediatricians, hematologists and ophthalmologists will improve diagnostic accuracy and patient outcomes.

**Copyright Information:** (Online) ISSN: 2572-9241
©2026 The Author(s). HemaSphere is published by John Wiley & Sons Ltd on behalf of European Hematology Association. This is an open access Abstract Book distributed under the Attribution-NonCommercial-NoDerivs (CC BY-NC-ND), which allows third parties to download the articles and share them with others as long as they credit the author and the Abstract Book, but they cannot change the content in any way or use them commercially.
**Abstract Book Citations:** Authors, Title, HemaSphere, 2026;10:(S1):pages. The abstract book DOIs can be found on the “Author Information” page.
**Disclaimer:** Articles published in the journal HemaSphere exclusively reflect the opinions of the authors. The authors are responsible for all content in their abstracts including accuracy of the facts, statements, citing resources, etc.

### (PB4225) PREDICTORS OF DISEASE-RELATED COMPLICATIONS IN AUTOIMMUNE HEMOLYTIC ANEMIAS: A SINGLE CENTER ANALYSIS

**Topic:** 31. Infections in hematology (incl. supportive care/therapy)

Bruno Fattizzo*[1], Federica Catania[2], Giacinto Pedone[1], Marta Bortolotti[1], Riccardo Colombo[1], Francesco Passamonti[1], Wilma Barcellini[1, 1]

*[1]Fondazione IRCCS Ca' Granda Ospedale Maggiore Policlinico, Milan, Italy;[2]University of Turin, Turin, Italy;*

**Background**

Autoimmune hemolytic anemias (AIHAs) are heterogeneous disorders characterized by immune-mediated hemolysis and variable clinical severity. Beyond anemia-related morbidity, patients are exposed to infectious, thrombotic, and skeletal complications that may significantly affect long-term outcomes. Robust predictors of event-free survival (EFS) and validated prognostic tools are still lacking.

**Aims**

To evaluate EFS for infections, thrombosis, osteoporosis, and mortality in a monocentric retrospective AIHA cohort, to estimate cumulative incidence of major complications, and to identify independent predictors for the development of prognostic scores.

**Methods**

We retrospectively analyzed 279 consecutive AIHA patients followed since 2010 until 2025 at a referral center in Milan, Italy. Median age was 62 years (8–94), 55.2% female. AIHA subtypes were warm IgG (42.7%), warm IgG+C (16.5%), cold (27.6%), mixed (6.8%), and atypical (6.5%). Median baseline hemoglobin was 7.3 g/dL and LDH 509 U/L. Median number of therapy lines was 2 (0–11). Treatments included steroids/IVIG (85.7%), rituximab (54.5%), erythropoietin (12.9%), immunosuppressants (9.0%), targeted therapies (13.3%), chemoimmunotherapy (2.2%), plasma exchange (0.7%), and splenectomy (2.9%). Cumulative incidence functions and Cox regression models were used. Multivariate models were internally weighted to derive prognostic scores; discrimination was assessed by C-index.

**Results**

After a median follow-up of 49 months (0–510), thrombosis occurred in 18.6% (median 3 months) and infections in 32.6% (median 14 months) (Figure 1). Thrombosis showed an early increase, reaching ~20% at 12 months and ~30% at 5 years, then plateauing. Infection incidence rose progressively, reaching ~25% at 12 months, ~40% at 5 years, and ~70% at long-term follow-up. Osteoporosis occurred in 16.4%, vertebral fractures in 5.2%. Overall mortality was 4.3%. In univariate analysis for infection, male sex, older age, elevated leukocyte parameters, warm AIHA, and steroids/IVIG exposure were associated with higher risk, while cold AIHA and selected rituximab/targeted therapy exposures were protective. In multivariate analysis for infection, independent predictors were: age (HR 1.02 per year, p<0.001), steroids/IVIG exposure (HR 2.91, p=0.026), low neutrophils (<5000/ µL;HR 1.74, p=0.027), low lymphocytes (<3560/µL; HR 1.75, p=0.040), and high LDH (>863 U/L), which was protective (HR 0.47, p=0.018). Rituximab (HR 0.69, p=0.101) and higher baseline hemoglobin (HR 0.68, p=0.125) showed protective trends. The infection model demonstrated good discrimination (C-index 0.700). For thrombosis, univariate analysis identified high neutrophil count as the main predictor. In multivariate analysis, high neutrophils remained independently associated with thrombosis (HR 2.91, p=0.010), while other variables were not significant. The thrombosis model showed acceptable discrimination (C-index 0.676).

**Copyright Information:** (Online) ISSN: 2572-9241
©2026 The Author(s). HemaSphere is published by John Wiley & Sons Ltd on behalf of European Hematology Association. This is an open access Abstract Book distributed under the Attribution-NonCommercial-NoDerivs (CC BY-NC-ND), which allows third parties to download the articles and share them with others as long as they credit the author and the Abstract Book, but they cannot change the content in any way or use them commercially.
**Abstract Book Citations:** Authors, Title, HemaSphere, 2026;10:(S1):pages. The abstract book DOIs can be found on the “Author Information” page.
**Disclaimer:** Articles published in the journal HemaSphere exclusively reflect the opinions of the authors. The authors are responsible for all content in their abstracts including accuracy of the facts, statements, citing resources, etc.

**Summary/Conclusion**

AIHA is associated with substantial long-term morbidity, characterized by early thrombotic risk and progressively increasing infectious burden. Age, steroid exposure, and lymphopenia independently predict infection, whereas neutrophilia possibly related to chronic autoinflammation was associated with thrombotic risk. A multivariate-derived prognostic scores, based on these readily available parameters, will possibly improve risk stratification and guide individualized surveillance and preventive strategies.

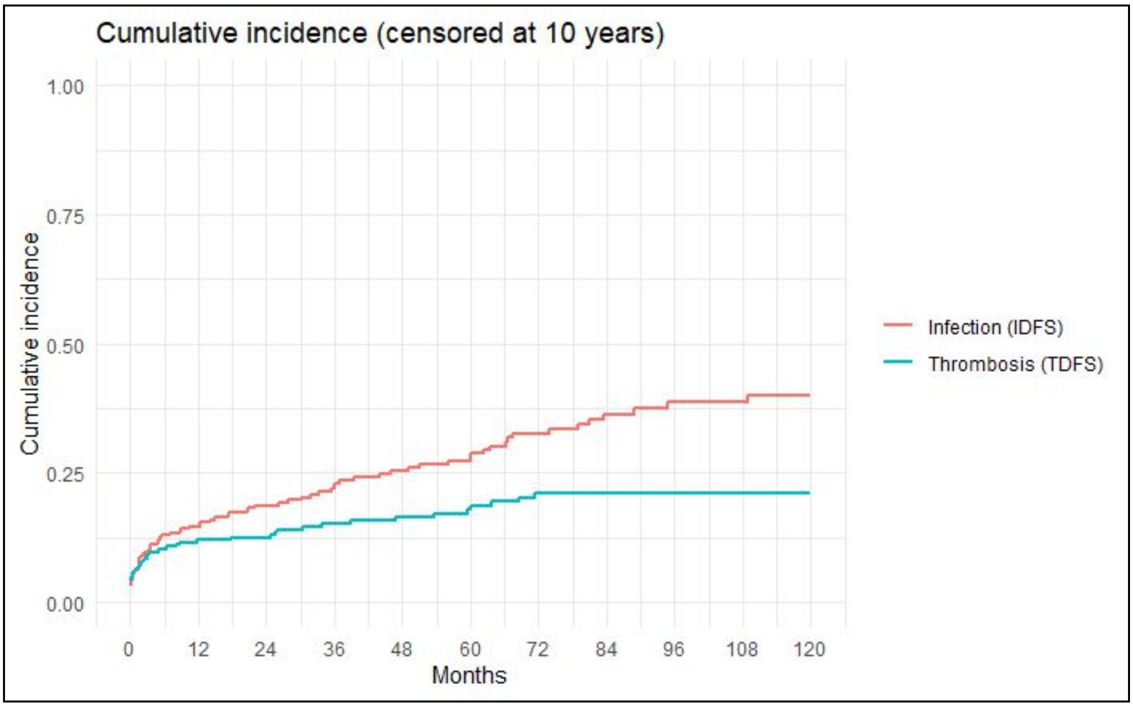

Figure 1: cumulative incidence of thrombosis and infections in autoimmune hemolytic anemias patients.

Copyright Information: (Online) ISSN: 2572-9241
©2026 The Author(s). HemaSphere is published by John Wiley & Sons Ltd on behalf of European Hematology Association. This is an open access Abstract Book distributed under the Attribution-NonCommercial-NoDerivs (CC BY-NC-ND), which allows third parties to download the articles and share them with others as long as they credit the author and the Abstract Book, but they cannot change the content in any way or use them commercially.
**Abstract Book Citations:** Authors, Title, HemaSphere, 2026;10:(S1):pages. The abstract book DOIs can be found on the "Author Information" page.
**Disclaimer:** Articles published in the journal HemaSphere exclusively reflect the opinions of the authors. The authors are responsible for all content in their abstracts including accuracy of the facts, statements, citing resources, etc.

### (PB4226) PROGNOSTIC ANALYSIS OF FUNGAL BLOODSTREAM INFECTIONS IN HEMATOLOGIC DISEASES BASED ON NGS

**Topic:** 31. Infections in hematology (incl. supportive care/therapy)

Yingying MA[1], Ying Chen[1], Qin Wen[1], Xi Zhang[1], Cheng Zhang*[1]

[1]*Medical Center of Hematology, Institute of Science Innovation for Blood Ecology and Intelligent Cells, Xinqiao Hospital of Army Medical University, Chongqing, China;*

### Background

Patients with hematologic diseases, particularly hematologic malignancies, often undergo intensive chemotherapy or allogeneic hematopoietic stem cell transplantation (allo-HSCT), which compromises immune function. The possibility of neutropenia accompanied by fever exceeds 80%, thereby increasing the risk of pathogen infection and the use of antimicrobial therapy. Fungal bloodstream infection is a rare type of infection in patients with hematological diseases. Antifungal drug therapy is usually used when empirical coverage of bacterial treatment is ineffective. However, once fungal bloodstream infection occurs, the prognosis of patients is extremely poor.

### Aims

This study aims to evaluate the prognostic analysis of fungal bloodstream infection treatment in hematological diseases based on next-generation sequencing (NGS).

### Methods

Hematological patients with fever who underwent mNGS examination at six blood disease centers nationwide from January 2020 to May 2023 were included. Univariate and multivariate logistic regression models were employed to analyze the risk factors for bloodstream infections in hematological disorders, and to evaluate the associations between empirical antifungal coverage against Mucorales, distinct antifungal therapeutic regimens, and long-term survival rates.

### Results

A total of 205 hematological patients with fever were enrolled in this retrospective cohort study. The median follow-up time was 116 days (range, 0–1,650 days), and the median age was 45 years (range, 2–76 years); 61% of the patients were male. Observing the outcomes and survival of patients at 1, 3, and 6 months, the median survival time of all patients during the 6-month observation period was 119 days (0-180 days). Univariate and Cox regression analyses showed that gender, site of infection, mucormycosis, concomitant bacterial infections, gastrointestinal complications, and cardiac dysfunction were the main risk factors affecting patients' survival time within 6 months, as shown in Fig1. Further subgroup analysis was conducted on patients with mucormycosis infection, comparing empirical antifungal coverage against Mucor prior to mNGS detection and distinct therapeutic regimens. The results indicated that empirical coverage against Mucor may improve patient prognosis [hazard ratio (HR) 1.533, 95% confidence interval (CI) 0.712–3.298; P = 0.275]. Additionally, the therapeutic efficacy varied among different antifungal regimens, with superior outcomes observed in patients receiving amphotericin B monotherapy or combination therapy with amphotericin B plus isavuconazole/posaconazole.

**Copyright Information:** (Online) ISSN: 2572-9241
©2026 The Author(s). HemaSphere is published by John Wiley & Sons Ltd on behalf of European Hematology Association. This is an open access Abstract Book distributed under the Attribution-NonCommercial-NoDerivs (CC BY-NC-ND), which allows third parties to download the articles and share them with others as long as they credit the author and the Abstract Book, but they cannot change the content in any way or use them commercially.
**Abstract Book Citations:** Authors, Title, HemaSphere, 2026;10:(S1):pages. The abstract book DOIs can be found on the "Author Information" page.
**Disclaimer:** Articles published in the journal HemaSphere exclusively reflect the opinions of the authors. The authors are responsible for all content in their abstracts including accuracy of the facts, statements, citing resources, etc.

**Summary/Conclusion**

Gender, age, mucormycosis, concurrent bacterial infections, gastrointestinal bleeding, and cardiac dysfunction are risk factors affecting the survival time of patients with fungal bloodstream infections. Empirical treatment with antifungal agents can improve patient prognosis, with amphotericin B or a combination of amphotericin B and esoconazole/posaconazole showing better efficacy in treating fungal infections compared to other regimens.

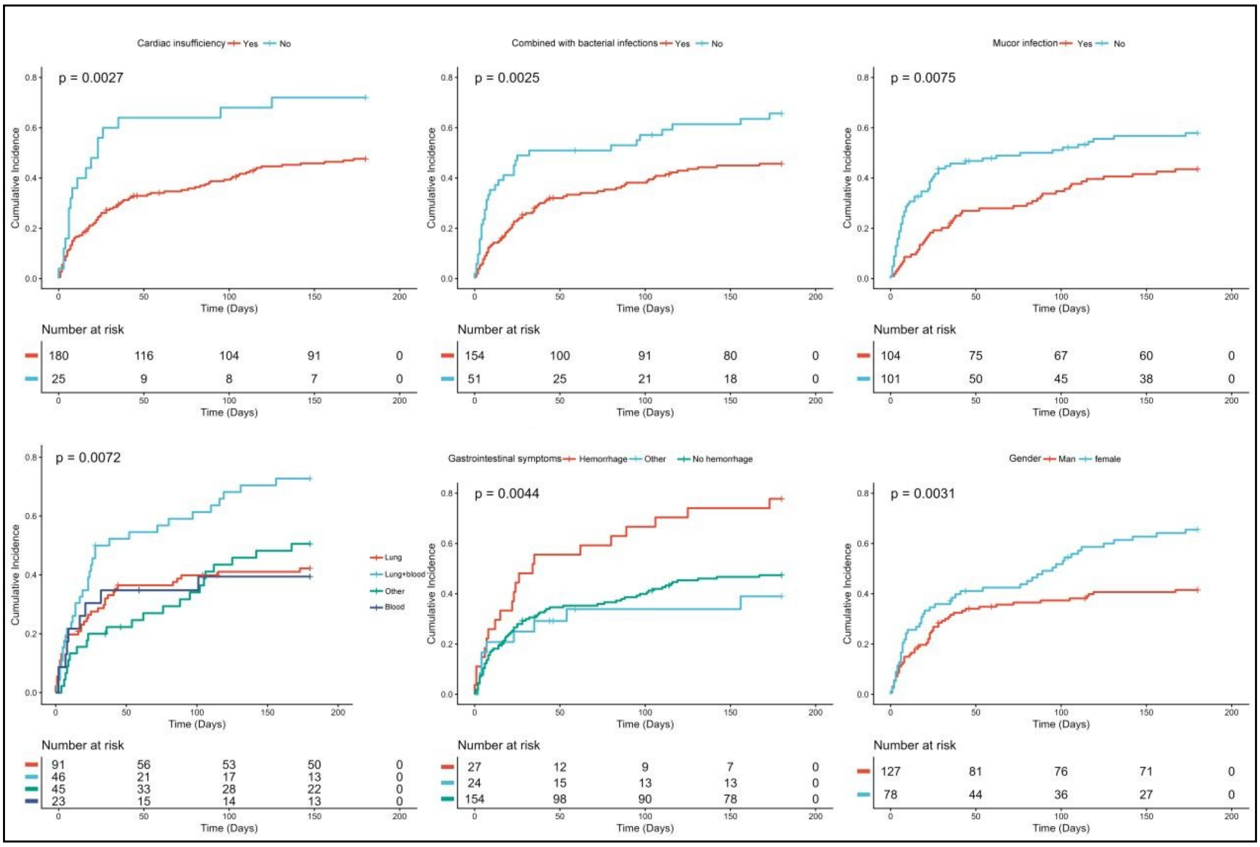

Fig. 1. Analysis of the impact of cardiac insufficiency, concomitant bacterial infection, Mucorales infection, site of infection, gastrointestinal complications, and sex on prognosis in hematological patients with fever within 6 months

**Copyright Information:** (Online) ISSN: 2572-9241
©2026 The Author(s). HemaSphere is published by John Wiley & Sons Ltd on behalf of European Hematology Association. This is an open access Abstract Book distributed under the Attribution-NonCommercial-NoDerivs (CC BY-NC-ND), which allows third parties to download the articles and share them with others as long as they credit the author and the Abstract Book, but they cannot change the content in any way or use them commercially.
**Abstract Book Citations:** Authors, Title, HemaSphere, 2026;10:(S1):pages. The abstract book DOIs can be found on the "Author Information" page.
**Disclaimer:** Articles published in the journal HemaSphere exclusively reflect the opinions of the authors. The authors are responsible for all content in their abstracts including accuracy of the facts, statements, citing resources, etc.

### (PB4227) EFFECTIVENESS OF IMMUNOGLOBULIN-REPLACEMENT THERAPY IN PATIENTS WITH NON-HODGKIN LYMPHOMA PRESENTING HYPOGAMMAGLOBULINEMIA

**Topic:** 31. Infections in hematology (incl. supportive care/therapy)

Antoine Azar*[1], Jiani Zhou[2], Elisabeth Calderón-Gómez[3], James Jordan[2], David Gomez[3], Igor Rossa-Ramm[3], Elisabet Viayna[3]

*[1]Johns Hopkins University School of Medicine, Division of Allergy and Clinical Immunology, Baltimore, United States of America; [2]Grifols Shared Services North America (SSNA), Global HEOR & RWE, Durham, United States of America;[3]Grifols, Sant Cugat del Valles, Spain;*

### Background

Hypogammaglobulinemia (HGG) is a common complication in patients with non-Hodgkin lymphoma (NHL), which can be exacerbated by anticancer immunosuppressive therapies used for the management of the disease. HGG increases the risk of infection and associated morbidity and mortality, potentially mitigated by immunoglobulin-replacement therapy (IgRT).

### Aims

To assess the effect of 10% Human Intravenous Immunoglobulin Caprylate-Chromatography-Purified (IGIV-C10%) on the odds of severe infections and infections of any kind in NHL patients with HGG.

### Methods

A retrospective real-world evidence (RWE) study was conducted using data from IQVIA PharMetrics Plus, a large U.S. health plan claims database. The study included adult NHL patients who developed HGG (ICD-10-CM 80.1, 80.3, 80.9) and initiated treatment with IGIV-C10% between Jul 2018, and Jun 2024. Index date was defined as the first IGIV-C10% administration. Patients were required to have 6-month continuous enrollment before and after index and have received at least 4 doses of IGIV-C10% within the 6 months post-index. Patients with primary immunodeficiency or other conditions that may have led to the use of IgRT at any time in patient records were excluded (Figure 1). The primary outcome was the proportion of patients with a severe infection, defined by the presence of a record meeting one of the following criteria: 1) hospitalization with a primary admission code for infection; or 2) use of injectable antimicrobials; or 3) serious infections code meeting the FDA Sentinel criteria. The proportion of patients with severe infections and infections of any kind during the 6-month post-index (exposed) was compared to that during the 6-month pre-index (unexposed). Generalized estimating equations (GEE) based logistic models were used to estimate odds ratio (OR) and 95% confidence intervals (CIs), adjusting for changes to the anticancer therapy and seasonality.

### Results

A total of 130 NHL patients were included. Mean (SD) age was 62.9 (12.0) years, and 56.2% (N=73) were female. Pre-index, B-cell monoclonal antibodies, kinase inhibitors, and alkylating agents constituted the most common treatments, representing 25.4%, 11.5%, and 10.8%, respectively. In the 6 months post-index, most common treatments were B-cell monoclonal antibodies (15.4%), kinase inhibitors (11.5%), and immunomodulatory imide drugs (IMiDs) (5.4%). Additionally, 60.8% and 44.6% of patients received corticosteroids during the pre- and post-index period, respectively. On average, patients received 5.8 (1.3) doses of IGIV-C10%, with a mean (SD) time of 4.6 (1.1) weeks between doses. IGIV-C10% was associated with an adjusted 46% and 64% lower risk of severe infections (OR: 0.54, 95% CI: 0.31, 0.94, $p<0.05$) and infections of any kind (OR: 0.36, 95% CI: 0.17, 0.74, $p<0.01$), respectively.

**Copyright Information:** (Online) ISSN: 2572-9241
©2026 The Author(s). HemaSphere is published by John Wiley & Sons Ltd on behalf of European Hematology Association. This is an open access Abstract Book distributed under the Attribution-NonCommercial-NoDerivs (CC BY-NC-ND), which allows third parties to download the articles and share them with others as long as they credit the author and the Abstract Book, but they cannot change the content in any way or use them commercially.
**Abstract Book Citations:** Authors, Title, HemaSphere, 2026;10:(S1):pages. The abstract book DOIs can be found on the "Author Information" page.
**Disclaimer:** Articles published in the journal HemaSphere exclusively reflect the opinions of the authors. The authors are responsible for all content in their abstracts including accuracy of the facts, statements, citing resources, etc.

### Summary/Conclusion

Regular use of IGIV-C10% (at least 4 doses over 6 months) was associated with significantly lower likelihood of developing both severe infections and infections of any kind among NHL patients presenting with HGG.

**Figure 1. Study design**

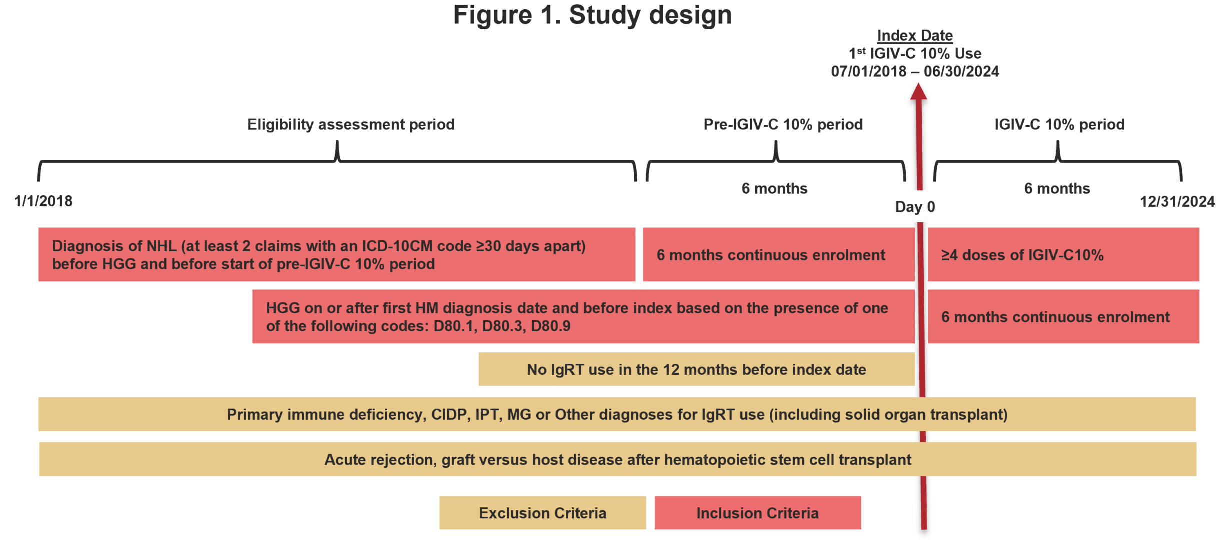

CIDP: Chronic Inflammatory Demyelinating Polyneuropathy; HGG: hypogammaglobulinemia; IGIV-C10%: 10% Human Intravenous Immunoglobulin Caprylate-Chromatography-Purified; IgRT: Immunoglobulin Replacement Therapy; ITP: Immune Thrombocytopenia; MG: Myasthenia Gravis; NHL: Non-Hodgkin lymphoma

**Copyright Information:** (Online) ISSN: 2572-9241
©2026 The Author(s). HemaSphere is published by John Wiley & Sons Ltd on behalf of European Hematology Association. This is an open access Abstract Book distributed under the Attribution-NonCommercial-NoDerivs (CC BY-NC-ND), which allows third parties to download the articles and share them with others as long as they credit the author and the Abstract Book, but they cannot change the content in any way or use them commercially.
**Abstract Book Citations:** Authors, Title, HemaSphere, 2026;10:(S1):pages. The abstract book DOIs can be found on the “Author Information” page.
**Disclaimer:** Articles published in the journal HemaSphere exclusively reflect the opinions of the authors. The authors are responsible for all content in their abstracts including accuracy of the facts, statements, citing resources, etc.

### (PB4228) ANALYSIS OF ADVERSE DRUG REACTIONS AND CMV INFECTIONS DURING LETERMOVIR THERAPY: A FAERS DATABASE STUDY

**Topic:** 31. Infections in hematology (incl. supportive care/therapy)

Zhangjie Chen*[1], Sisi Zhen[1], Xin Chen[1], Sizhou Feng[1]

[1]*Institute of Hematology & Blood Diseases Hospital, Chinese Academy of Medical Sciences & Peking Union Medical College, Tianjin, China;*

### Background

Letermovir is an antiviral agent approved for CMV infection prophylaxis in transplant recipients. This study aimed to evaluate adverse drug reactions (ADRs) during letermovir treatment.

### Aims

We aim to explore ADRs associated with letermovir treatment using FAERS database.

### Methods

Data from October 2017 to September 2024 were extracted from FAERS database. Disproportionality analysis was performed to detect adverse events potentially associated with letermovir. Time-to-onset analysis was conducted, logistic and cox regression were performed to identify risk factors.

### Results

The most frequently reported ADR was infections and infestations at SOC level, CMV infection at HLT level and CMV infection reactivation at PT level. Novel safety signals included cardiac disorder (cardiac arrest, heart failure), gastrointestinal/hepatobiliary disorder (colitis, hematochezia, hepatic failure), renal failure, various CMV-related events and transplant-related complications. The median time to onset for overall ADRs was 25 days (Figure A), and 33 days for events associated with CMV infections (Figure B), with both showing significantly delayed onset time for intravenous formulations compared to tablets (Figure C, D). Older age was identified as a protective factor against CMV infections, while geographic disparities revealed increased risks in Europe and decreased risks in North America.

### Summary/Conclusion

Severe cardiac, hepatic and renal complications were found to be associated with letermovir. CMV breakthrough infections remain a concern, and leukemia relapse risk requires further investigations.

**Copyright Information:** (Online) ISSN: 2572-9241
©2026 The Author(s). HemaSphere is published by John Wiley & Sons Ltd on behalf of European Hematology Association. This is an open access Abstract Book distributed under the Attribution-NonCommercial-NoDerivs (CC BY-NC-ND), which allows third parties to download the articles and share them with others as long as they credit the author and the Abstract Book, but they cannot change the content in any way or use them commercially.
**Abstract Book Citations:** Authors, Title, HemaSphere, 2026;10:(S1):pages. The abstract book DOIs can be found on the "Author Information" page.
**Disclaimer:** Articles published in the journal HemaSphere exclusively reflect the opinions of the authors. The authors are responsible for all content in their abstracts including accuracy of the facts, statements, citing resources, etc.

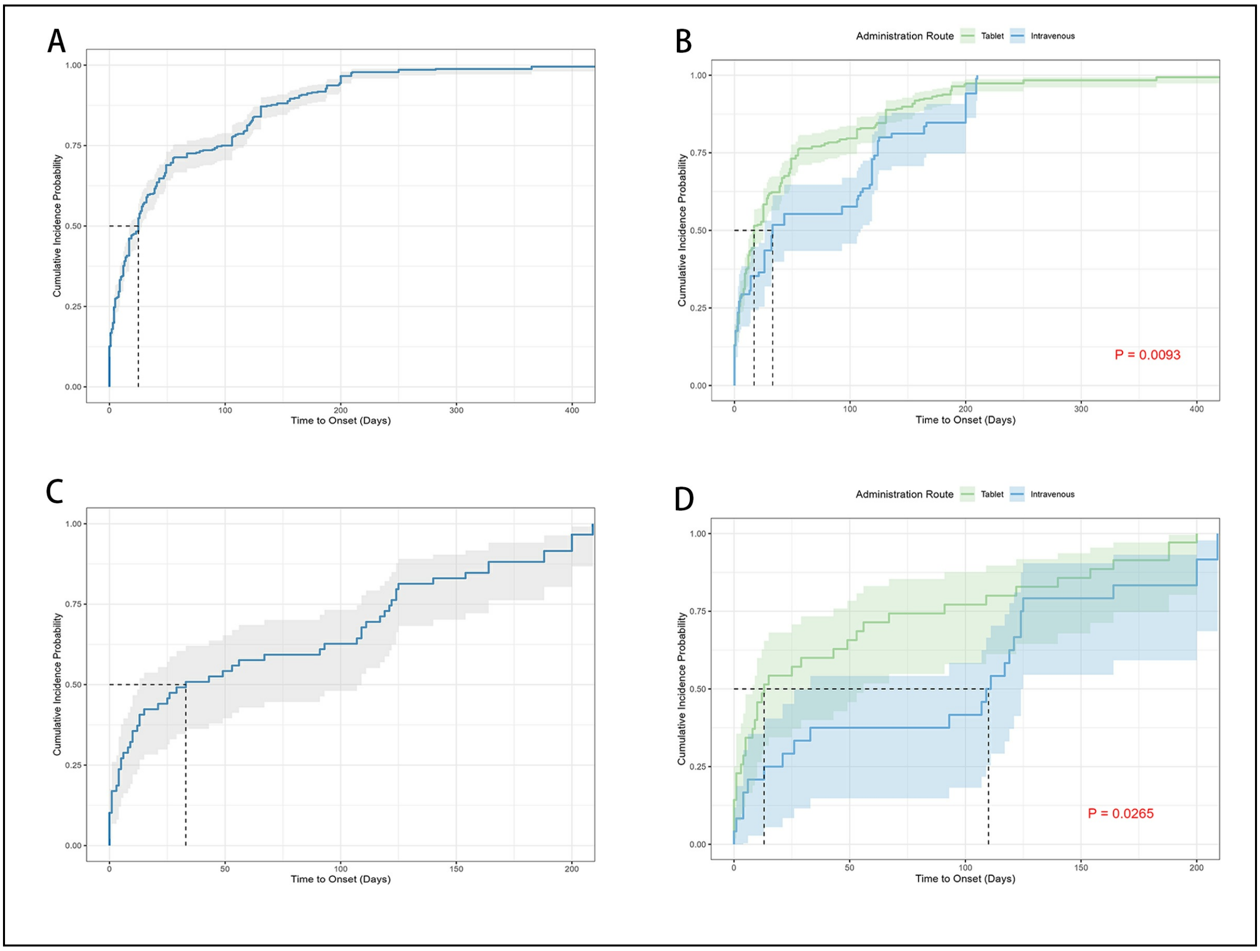

Cumulative incidence probability of ADRs associated with letermovir. Panels (A) and (C) showed cumulative incidence probability of overall ADRs (A) and CMV infections (C) during letermovir treatment. Panels (B) and (D) illustrated stratification by administration route (tablet vs. intravenous) for overall ADRs (B) and CMV infections (D) during letermovir treatment. ADRs: adverse drug events.

**Copyright Information:** (Online) ISSN: 2572-9241
©2026 The Author(s). HemaSphere is published by John Wiley & Sons Ltd on behalf of European Hematology Association. This is an open access Abstract Book distributed under the Attribution-NonCommercial-NoDerivs (CC BY-NC-ND), which allows third parties to download the articles and share them with others as long as they credit the author and the Abstract Book, but they cannot change the content in any way or use them commercially.
**Abstract Book Citations:** Authors, Title, HemaSphere, 2026;10:(S1):pages. The abstract book DOIs can be found on the "Author Information" page.
**Disclaimer:** Articles published in the journal HemaSphere exclusively reflect the opinions of the authors. The authors are responsible for all content in their abstracts including accuracy of the facts, statements, citing resources, etc.

### (PB4229) REAL-WORLD EXPERIENCE OF SULBACTAM–DURLOBACTAM IN HEMATOLOGIC MALIGNANCY PATIENTS WITH SUSPECTED CARBAPENEM-RESISTANT ACINETOBACTER BAUMANNII INFECTION

**Topic:** 31. Infections in hematology (incl. supportive care/therapy)

Chang Zhang[1], Xin Li*[1]

[1] *The Third Xiangya Hospital of Central South University, Hematology, Changsha, China;*

### Background

Carbapenem-resistant *Acinetobacter baumannii* (CRAB) remains a major cause of hospital-acquired infection with high mortality and limited therapeutic options. Patients with hematologic malignancies are particularly vulnerable due to intensive chemotherapy, prolonged neutropenia, hematopoietic stem cell transplantation (HSCT), and repeated exposure to broad-spectrum antibiotics. Sulbactam–durlobactam is a recently approved β-lactam/β-lactamase inhibitor combination targeting *A. baumannii*. Although phase III data have demonstrated its efficacy and improved safety compared with polymyxins, real-world experience remains limited, and hematologic malignancy patients have been underrepresented in available studies.

### Aims

To describe the real-world clinical outcomes and safety of sulbactam–durlobactam in heavily pretreated hematologic malignancy patients treated for suspected CRAB infection.

### Methods

We retrospectively analyzed consecutive hematologic malignancy patients who received sulbactam–durlobactam between April 2025 and February 2026 at a tertiary hematology center. Inclusion required microbiological evidence of *A. baumannii* by next-generation sequencing (NGS) or conventional culture in the setting of strong clinical suspicion of CRAB infection. Clinical improvement within 7±2 days (resolution of fever and improvement of infection-related signs), 28-day all-cause mortality, and renal safety were assessed.

### Results

A total of 10 patients were included. Median age was 44.5 years (range 16–73). 5 had prior allogeneic HSCT and 5 had neutropenia at infection onset. Pulmonary infection was the predominant presentation. At the time of sulbactam–durlobactam initiation, therapeutic options were considered limited in all patients. 9/10 had received prior carbapenem therapy without adequate response, and 4/10 had previously received polymyxin-based therapy due to treatment failure or toxicity, representing a salvage setting. Clinical improvement within 7±2 days was observed in 9/10 (90%) patients. The 28-day all-cause mortality was 1/10 (10%). Microbiological diagnosis was primarily supported by NGS, whereas conventional cultures were positive in only 1/10 (10%). No acute kidney injury or new renal replacement therapy occurred during treatment. During 90-day follow-up, no confirmed recurrence of *A. baumannii* infection was documented.

### Summary/Conclusion

In this small real-world cohort of heavily pretreated hematologic malignancy patients with limited therapeutic options, sulbactam–durlobactam demonstrated encouraging early clinical responses with acceptable safety. These findings provide preliminary clinical evidence supporting its use in this underrepresented high-risk population and highlight the need for further evaluation.

**Copyright Information:** (Online) ISSN: 2572-9241
©2026 The Author(s). HemaSphere is published by John Wiley & Sons Ltd on behalf of European Hematology Association. This is an open access Abstract Book distributed under the Attribution-NonCommercial-NoDerivs (CC BY-NC-ND), which allows third parties to download the articles and share them with others as long as they credit the author and the Abstract Book, but they cannot change the content in any way or use them commercially.
**Abstract Book Citations:** Authors, Title, HemaSphere, 2026;10:(S1):pages. The abstract book DOIs can be found on the “Author Information” page.
**Disclaimer:** Articles published in the journal HemaSphere exclusively reflect the opinions of the authors. The authors are responsible for all content in their abstracts including accuracy of the facts, statements, citing resources, etc.

### (PB4230) CARBAPENEM-SPARING STRATEGIES IN TREATING SECOND FEBRILE EPISODE IN ONCOHEMATOLOGIC PATIENTS: A MONOCENTRIC RETROSPECTIVE OBSERVATIONAL STUDY

**Topic:** 31. Infections in hematology (incl. supportive care/therapy)

Lorenzo Celona*[1], Marco Cerrano[1], Stefano D'ardia[1], Chiara Frairia[1], Valentina Giai[1], Elisa Santambrogio[1], Irene Urbino[1], Eleonora Boscaro[1], Federica Catania[1], Giulia Berutto[1], Bianca Patarnello[1], Chiara Maria Dellacasa[1], Carolina Secreto[1], Davide Stella[1], Camilla Gariazzo[1], Roberto Freilone[1], Alessandro Busca[1], Ernesta Audisio[1]

*[1]AOU Città della Salute e della Scienza di Torino, SC Hematology, Turin, Italy;*

### Background

In oncohematologic patients undergoing chemotherapy or cellular therapies, carbapenems have traditionally been considered the reference antibiotic treatment for severe Gram-negative infections, particularly in neutropenic patients. However, their use is associated with an increased risk of multiresistant bacteria selection, leading to severe hospital infections.
Carbapenem-sparing strategies aim to optimize antimicrobial therapy by reducing selective pressure without compromising outcomes. ECIL-10 guidelines for febrile neutropenia in leukemia highlight the need for a risk-stratified and personalized approach for antimicrobial therapy that also considers local resistance epidemiology.

### Aims

This study is intended to analyze the feasibility of carbapenem-sparing strategies for the empirical or targeted therapy of infections in hospitalized oncohematologic patients.

### Methods

We conducted a retrospective observational study including all hospitalizations of oncohematologic patients in the Hematology Department of “Città della Scienza e della Salute” Hospital in Turin, Italy during 2024.

**Copyright Information:** (Online) ISSN: 2572-9241
©2026 The Author(s). HemaSphere is published by John Wiley & Sons Ltd on behalf of European Hematology Association. This is an open access Abstract Book distributed under the Attribution-NonCommercial-NoDerivs (CC BY-NC-ND), which allows third parties to download the articles and share them with others as long as they credit the author and the Abstract Book, but they cannot change the content in any way or use them commercially.
**Abstract Book Citations:** Authors, Title, HemaSphere, 2026;10:(S1):pages. The abstract book DOIs can be found on the “Author Information” page.
**Disclaimer:** Articles published in the journal HemaSphere exclusively reflect the opinions of the authors. The authors are responsible for all content in their abstracts including accuracy of the facts, statements, citing resources, etc.

**Results**

A total of 364 hospitalizations were analyzed, 229 males and 135 females, with a mean age of 58 years. The median length of hospitalization was 30 days.
The reasons for hospitalization were intensive chemotherapy in 123 cases (33.8%), semi-intensive chemotherapy in 111 cases (30.5%), 14 (3.8%) autologous hematopoietic stem cell transplantation (HSCT), 31 (8.5%) allogeneic HSCT, 28 (7.6%) immunotherapies, bispecific antibodies or CAR-T cells, and finally 56 (15,3%) patients hospitalized for complications.
No patients received antibiotic prophylaxis.
Infectious complications requiring antibiotic therapy occurred in 229 hospitalizations (63%). Rectal screening for multidrug-resistant organisms was performed in all patients: ESBL-positive bacteria were detected in 51 patients (14%) and KPC-producing bacteria in 12 patients (3.3%).
The cumulative incidence was: 20.9 % for bacteremia, 15% for clinically documented infections and 23.6% for fever of unknown origin (FUO).
Carbapenems were used as first-line therapy for the first febrile episode in 21 patients (9%), mainly in critical conditions, with a median treatment duration of 8.4 days.
A second febrile episode occurred in 86 hospitalizations. In 61 cases (70%), a carbapenem was administered, with a median duration of 7 days. The ratio between median carbapenem therapy duration and median length of hospitalization was 0.39 for first-line therapy and 0.31 for second-line therapy.
Carbapenem use was quantified using days of therapy (DOT). A total of 498 carbapenem DOT were administered for second febrile episodes. Notably, 87 DOT (17.5% of total second-line carbapenem use) were prescribed in patients with a final diagnosis of FUO, without hemodynamic instability or documented resistant colonization.

**Summary/Conclusion**

This retrospective observational study highlights and objectively quantifies the inappropriate overuse of carbapenems in the treatment of second febrile episodes. Specifically, 17.5% of second-line carbapenem consumption could potentially have been avoided according to ECIL-10 guidelines.
An optimized strategy reserving carbapenems for hemodynamically unstable patients or those with documented colonization by resistant pathogens may help reduce the spread of multidrug-resistant bacteria without compromising patient safety, especially in a context with a low rate of multidrug-resistant organisms colonizations.

**Copyright Information:** (Online) ISSN: 2572-9241
©2026 The Author(s). HemaSphere is published by John Wiley & Sons Ltd on behalf of European Hematology Association. This is an open access Abstract Book distributed under the Attribution-NonCommercial-NoDerivs (CC BY-NC-ND), which allows third parties to download the articles and share them with others as long as they credit the author and the Abstract Book, but they cannot change the content in any way or use them commercially.
**Abstract Book Citations:** Authors, Title, HemaSphere, 2026;10:(S1):pages. The abstract book DOIs can be found on the “Author Information” page.
**Disclaimer:** Articles published in the journal HemaSphere exclusively reflect the opinions of the authors. The authors are responsible for all content in their abstracts including accuracy of the facts, statements, citing resources, etc.

### (PB4231) EFFICACY OF EARLY LOW DOSE OF RITUXIMAB IN PROPHYLAXIS OF EBV REACTIVATION AND EBV RELATED POST-TRANSPLANT LYMPHOPROLIFERATIVE DISORDER AFTER ALLOGENEIC HEMATOPOIETIC CELL TRANSPLANTATION

**Topic:** 31. Infections in hematology (incl. supportive care/therapy)

Fang Liu*[1], Gele Tong[2], Lina Feng[1], Chunlei Lu[1], Yu Sun[1], Mei Wang[1], Qi Gao[1], Yuejun Li[1], Yu Wang[1]

[1]*Shanghai New Daopei Hospital, Shanghai, China;*[2]*Hebei Yanda Lu Daopei Hospital, Langfang, China;*

### Background

The incidence of Epstein-Barr virus (EBV) reactivation is reported to be increased after allogeneic hematopoietic cell transplantation(allo-HCT) in letermovir era in several studies, ranging from 20% to 58%. EBV related post-transplant lymphoproliferative disorder (EBV-PTLD) is a life-threatening complication and a major cause of non-relapse mortality. Prophylaxis of EBV reactivation is needed. The majority of EBV-PTLD are due to proliferation of B-cells after allo-HCT. Early clearance of B cells may eliminate the proliferation sites of EBV, reducing the incidence of EBV reactivation and mortality of EBV-PTLD.

### Aims

To explore the efficacy of early low dose of Rituximab after allo-HCT on the incidence of EBV viremia and EBV-related PTLD.

### Methods

From Feb 2024 to Dec 2025, a total of 124 patients with hematological disease underwent allo-HCT at Shanghai new dao-pei Hospital. All patients were EBV-specific IgG positive before HCT. All patients received ATG based conditioning, and letermovir for CMV prophylaxis. All patients were treated with Rituximab 200mg on the 5th day after stem cell reinfusion (the dose for children was adjusted according to their weight), a $2^{nd}$ dose was used in 10 patients 3 weeks later due to prolonged severe immunosuppression.

EBV screening is performed once a week by q-PCR and more frequent if EBV DNA positive. Recurrent EBV viremia is diagnosed by detection of EBV-DNA in plasma. PTLD was screened in patients with EBV viremia by lymph node ultrasound and biopsy if needed. The incidence of EBV viremia and PTLD and treatment-related toxicity was retrospectively analyzed, and the incidence of GVHD, CMV reactivation, and the survival status was observed.

**Copyright Information:** (Online) ISSN: 2572-9241
©2026 The Author(s). HemaSphere is published by John Wiley & Sons Ltd on behalf of European Hematology Association. This is an open access Abstract Book distributed under the Attribution-NonCommercial-NoDerivs (CC BY-NC-ND), which allows third parties to download the articles and share them with others as long as they credit the author and the Abstract Book, but they cannot change the content in any way or use them commercially.
**Abstract Book Citations:** Authors, Title, HemaSphere, 2026;10:(S1):pages. The abstract book DOIs can be found on the “Author Information” page.
**Disclaimer:** Articles published in the journal HemaSphere exclusively reflect the opinions of the authors. The authors are responsible for all content in their abstracts including accuracy of the facts, statements, citing resources, etc.

### Results

The median age of the patients was 31 years (range, 2-67). 49 pts were females and 75 were males. Diagnosis was AML/MDS in 34 pts, ALL/LBL in 85 pts and severe aplastic anemia in 5 pts. The stem cell donors were from haploidentical donors in 69 pts, unrelated donors in 39 pts, matched sibling donors in 14 pts, and cord blood in 2 pts.

The median follow-up was 177 days (60[illegible]388 days). Of the 107 pts who follow up above 100days, only 3 (2.4%) developed early EBV viremia in 100 days, and 3 [illegible]2.4%[illegible]developed late EBV viremia after 100 days. 4 pts was recovered after the treatment of rituximab.1 patient progressed to PTLD and died; one died of EBV related HLH. There were no severe adverse effect or aggravated infection events during the medication process. Cumulative incidences (CI) of grade II-IV and grade III-IV aGVHD day +100 were 12% and 5.6% respectively. Incidence of CMV reactivation was 14.5%. Till last follow up, most of the patients presented with B-cell deficiency and hypoimmunoglobulinemia, requiring IVIg replacement therapy. 117(94.3%) patients were still alive with disease free, 7 patients died, with the causes being one case of aGVHD, one case of PTLD, one case of recurrence of the original disease, three cases of TA-TMA, and one case of organ failure.

### Summary/Conclusion

Our study shows that early low dose of rituximab after allo-HCT can significantly reduce incidence of EBV viremia and the risk of EBV-related PTLD, without severe adverse effect despite delayed humoral immune reestablishment. It will provide an effective prophylactic strategy for patients at high risk of EBV and reduce transplant-related mortality after allo-HCT.

**Copyright Information:** (Online) ISSN: 2572-9241
©2026 The Author(s). HemaSphere is published by John Wiley & Sons Ltd on behalf of European Hematology Association. This is an open access Abstract Book distributed under the Attribution-NonCommercial-NoDerivs (CC BY-NC-ND), which allows third parties to download the articles and share them with others as long as they credit the author and the Abstract Book, but they cannot change the content in any way or use them commercially.
**Abstract Book Citations:** Authors, Title, HemaSphere, 2026;10:(S1):pages. The abstract book DOIs can be found on the "Author Information" page.
**Disclaimer:** Articles published in the journal HemaSphere exclusively reflect the opinions of the authors. The authors are responsible for all content in their abstracts including accuracy of the facts, statements, citing resources, etc.

### (PB4232) INFECTIOUS COMPLICATIONS IN PATIENTS WITH ACUTE MYELOID LEUKEMIA TREATED WITH AZACITIDINE-VENETOCLAX COMBINATION – RESULTS OF VENINFAZA 3.0 STUDY BY POLISH ADULT LEUKEMIA GROUP

**Topic:** 31. Infections in hematology (incl. supportive care/therapy)

Joanna Drozd-Sokolowska*[1], Karol Lis[1], Elzbieta Sienkiewicz[2], Oktawia Sosnia[3], Katarzyna Budziszewska[3], Anna Kulikowska de Nałęcz[4], Edyta Subocz[5], Agnieszka Podgórska[5], Małgorzata Gajewska[6], Joanna Rolbiecka[7], Łukasz Bołkun[8], Renata Guzicka-Kazimierczak[9], Anna Kopinska[10], Jagoda Rytel[1], Jadwiga Dwilewicz-Trojaczek[1], Agnieszka Wierzbowska[11], Lidia Gil[12], Grzegorz Basak[1], Krzysztof Madry[1]

*[1]Medical University of Warsaw, Warsaw, Poland;[2]Warsaw University of Technology, Department of Mathematics and Information Science, Warsaw, Poland; [3]Institute of Hematology and Transfusion Medicine, Warsaw, Poland;[4]Department of Hematology, Hemato-Oncology and Internal Diseases, Opole, Opole, Poland; [5]Clinical Hospital of the Ministry of Internal Affairs and Administration with the Warmia-Mazury Oncology Centre in Olsztyn, Olsztyn, Poland; [6]Military Institute of Medicine-National Research Institute, Warsaw, Poland; [7]Medical University of Gdansk, Gdansk, Poland; [8]Medical University of Bialystok, Bialystok, Poland; [9]Pomeranian Medical University, Szczecin, Poland; [10]Medical University of Silesia, Katowice, Poland; [11]Medical University of Lodz, Lodz, Poland; [12]Poznan University of Medical Sciences, Poznan, Poland;*

### Background

Azacitidine combined with venetoclax (AZA–VEN) is the standard treatment for acute myeloid leukemia (AML) in treatment-naïve (TN) patients ineligible for intensive chemotherapy. Under specific circumstances it may also be used in relapsed/refractory (RR) setting. Despite its wide use, data on infectious complications of AZA–VEN treatment coming from real-world setting is limited.

### Aims

To evaluate the frequency and types of infectious complications occurring during the first three cycles of AZA–VEN in TN and RR AML

### Methods

A retrospective multicenter analysis of patients treated with AZA–VEN in Polish hematology centers, aimed at evaluating the incidence and spectrum of infectious complications occurring during the first three treatment cycles.

**Copyright Information:** (Online) ISSN: 2572-9241
©2026 The Author(s). HemaSphere is published by John Wiley & Sons Ltd on behalf of European Hematology Association. This is an open access Abstract Book distributed under the Attribution-NonCommercial-NoDerivs (CC BY-NC-ND), which allows third parties to download the articles and share them with others as long as they credit the author and the Abstract Book, but they cannot change the content in any way or use them commercially.
**Abstract Book Citations:** Authors, Title, HemaSphere, 2026;10:(S1):pages. The abstract book DOIs can be found on the "Author Information" page.
**Disclaimer:** Articles published in the journal HemaSphere exclusively reflect the opinions of the authors. The authors are responsible for all content in their abstracts including accuracy of the facts, statements, citing resources, etc.

**Results**

Among 149 patients included in the study, 77 (51.7%) were male, the median age at AZA-VEN initiation was 72 years (range, 24-92). 123 (82.6%) patients received AZA-VEN for TN, 26 (17.4%) for RR AML. In the analyzed cohort 36 (24.3%) suffered from diabetes mellitus, 13 (8.7%) from chronic obstructive pulmonary disease, and 16 (10.7%) from heart failure. Forty (26.8%) patients did have a history of other malignancy. Thirty (21.1%) patients had gastrointestinal colonization with multidrug-resistant bacteria. During the first cycle the majority of patients was scheduled for any anti-microbial prophylaxis (78.5%), most frequently posaconazole (37, 24.8%), levofloxacin (18, 12.1%), acyclovir (79, 53%), and trimethoprim/sulfamethoxazole (45, 30.2%). The total number of patients receiving prophylaxis increased with the increasing number of cycles.
124 episodes grade 3 or higher developed in 85 (57.0%) patients, 71 during the first, 37 during the second, and 16 during the third cycle. Concerning clinical diagnosis, most frequently patients developed pneumonia (45 episodes; 31 during cycle 1), neutropenic fever (27 episodes; 18 during cycle 1), sepsis (32 episodes; 12 during cycle 1) with 14 patients experiencing septic shock, and urinary tract infection (13 episodes; 7 during cycle 1). *Clostridioides difficile* infection was diagnosed in 7 (4.7%) patients. The etiology of infections was bacterial in 79 cases, viral in 18, with most cases attributable to SARS-CoV2 infection (12), and fungal in 14. Eighteen patients (12.1%) died because of infections. Antimicrobial prophylaxis in Cycle 1 did not translate into a significant change in the incidence of infectious complications (p-values in the range of 0.6-1.0).

**Summary/Conclusion**

Infections were a frequent complication of AZA-VEN treatment. Most episodes occurred during the first cycle. Pneumonia, neutropenic fever and sepsis were the most frequent infectious complications. Bacterial etiology was the most prevalent, nevertheless fungal infections constituted >10% of the diagnosed episodes. No uniform mode of prophylaxis was identified in the analyzed population. Antimicrobial prophylaxis in Cycle 1 did not translate into lower incidence of infectious complications.

**Copyright Information:** (Online) ISSN: 2572-9241
©2026 The Author(s). HemaSphere is published by John Wiley & Sons Ltd on behalf of European Hematology Association. This is an open access Abstract Book distributed under the Attribution-NonCommercial-NoDerivs (CC BY-NC-ND), which allows third parties to download the articles and share them with others as long as they credit the author and the Abstract Book, but they cannot change the content in any way or use them commercially.
**Abstract Book Citations:** Authors, Title, HemaSphere, 2026;10:(S1):pages. The abstract book DOIs can be found on the "Author Information" page.
**Disclaimer:** Articles published in the journal HemaSphere exclusively reflect the opinions of the authors. The authors are responsible for all content in their abstracts including accuracy of the facts, statements, citing resources, etc.

### (PB4233) SELINEXOR PLUS R-EPOCH AS FIRST-LINE THERAPY FOR HIV-ASSOCIATED DIFFUSE LARGE B-CELL LYMPHOMA: A PROOF-OF-CONCEPT STUDY (CALL-003)

**Topic:** 31. Infections in hematology (incl. supportive care/therapy)

Chaoyu Wang*[1], Yixing Zhou[1], Chensi Zeng[1], Bingling Guo[1], Yao Liu[1]

[1]*Chongqing university cancer hospital, Chong, China;*

### Background

Selinexor is an oral selective inhibitor of nuclear export compound that blocks exportin 1 (XPO1), which is the principal nuclear export receptor that frequently overexpressed in DLBCL and is associated with inferior clinical outcomes . Preclinical studies indicated that selinexor exhibits anti-lymphoma activity and synergizes with R-CHOP-based chemoimmunotherapy by enhancing apoptosis, suppressing oncogene translation, and up-regulating CD20 expression. A previous single-arm trial reported durable responses of selinexor monotherapy in heavily-pretreated DLBCL, and a phase I trial demonstrated encouraging tolerability and response of selinexor combined with R-CHOP in frontline treatment for HIV-negative patients with non-Hodgkin lymphoma. Beyond its antitumor activity, XPO1 inhibition has been shown to impede HIV replication through nuclear retention of Rev and other HIV regulatory proteins, suggesting an intrinsic antiviral benefit.

### Aims

This dual mechanism raises the intriguing possibility that selinexor-based regimens could simultaneously control lymphoma and suppress HIV. However, the evidence of integrating selinexor into standard chemoimmunotherapy for HIV-associated DLBCL is lacking. Building on this rationale, we conducted the first investigation of selinexor plus R-EPOCH (XR-EPOCH) administered concurrently with cART in patients with HIV-associated DLBCL.

### Methods

We conducted a proof-of-concept study evaluating selinexor in combination with R-EPOCH in patients with previously untreated HIV-associated DLBCL. The trial was approved by the Institutional Review Board of our hospital (CZLS2023028-A), performed in accordance with the Declaration of Helsinki, and registered at the Chinese Clinical Trial Registry (ChiCTR2300069941). All participants provided written informed consent before enrollment.

### Results

Eligible patients received ≥ 4 cycles of selinexor at a dose of 60 mg orally on days 1, 8 and 15 of each 21-day cycle in combination with standard R-EPOCH regimen, and selinexor maintenance therapy at 60 mg orally once weekly. The primary endpoint was complete response. Among 10 enrolled patients, the primary endpoint of complete response was achieved by 80% of patients, with an overall response rate of 100%. At a median follow-up of 22 months, median progression-free and overall survival were not reached, with a 1-year survival rate of 90.0% (95% confidence interval, 78.4 to 95.3) for both. The antitumor effects occurred in parallel with and meaningful immune reconstitution with 7 patients achieved CD4-positive T-cell level >200 cells/μL, and effective suppression of HIV viremia with 8 patients achieved undetectable HIV RNA levels and 2 showed near-complete viral suppression while on concomitant antiretroviral therapy. No hepatitis B virus or Epstein-Barr virus reactivation occurred. The most common grade 3-4 treatment-emergence adverse events were hematologic toxicities, primarily neutropenia (80%) and thrombocytopenia (40%).

**Copyright Information:** (Online) ISSN: 2572-9241
©2026 The Author(s). HemaSphere is published by John Wiley & Sons Ltd on behalf of European Hematology Association. This is an open access Abstract Book distributed under the Attribution-NonCommercial-NoDerivs (CC BY-NC-ND), which allows third parties to download the articles and share them with others as long as they credit the author and the Abstract Book, but they cannot change the content in any way or use them commercially.
**Abstract Book Citations:** Authors, Title, HemaSphere, 2026;10:(S1):pages. The abstract book DOIs can be found on the "Author Information" page.
**Disclaimer:** Articles published in the journal HemaSphere exclusively reflect the opinions of the authors. The authors are responsible for all content in their abstracts including accuracy of the facts, statements, citing resources, etc.

**Summary/Conclusion**

XR-EPOCH demonstrated promising anti-tumor activity and tolerability, suggesting its potential as a therapeutic strategy that kills two birds with one stone.

**Copyright Information:** (Online) ISSN: 2572-9241
©2026 The Author(s). HemaSphere is published by John Wiley & Sons Ltd on behalf of European Hematology Association. This is an open access Abstract Book distributed under the Attribution-NonCommercial-NoDerivs (CC BY-NC-ND), which allows third parties to download the articles and share them with others as long as they credit the author and the Abstract Book, but they cannot change the content in any way or use them commercially.
**Abstract Book Citations:** Authors, Title, HemaSphere, 2026;10:(S1):pages. The abstract book DOIs can be found on the "Author Information" page.
**Disclaimer:** Articles published in the journal HemaSphere exclusively reflect the opinions of the authors. The authors are responsible for all content in their abstracts including accuracy of the facts, statements, citing resources, etc.

### (PB4234) CLINICAL ANALYSIS OF 17 CASES OF HUMAN PARVOVIRUS B19 INFECTION FOLLOWING CHEMOTHERAPY IN ADULT LEUKEMIA PATIENTS

**Topic:** 31. Infections in hematology (incl. supportive care/therapy)

Lingrui Li*[1]

*[1]State Key Laboratory of Experimental Hematology, National Clinical Research Center for Blood Diseases, Haihe Laboratory of Cell Ecosystem, Institute of Hematology & Blood Diseases Hospital, Chinese Academy of Medical Sciences & Peking Union Medical College, Tianjin, China;*

### Background

Human parvovirus B19 (B19V) infection is an uncommon but clinically significant complication in patients with hematologic malignancies undergoing chemotherapy. Due to immunosuppression, such patients are at increased risk for persistent anemia and delayed hematologic recovery, yet clinical recognition of B19V infection remains limited.

### Aims

To investigate the clinical characteristics, treatment approaches, and outcomes of B19V infection following chemotherapy in adult patients with leukemia.

### Methods

This retrospective study included patients admitted to the Leukemia Diagnosis and Treatment Center of the Institute of Hematology and Blood Diseases Hospital, Chinese Academy of Medical Sciences, between June 2022 and July 2025. Eligible cases were defined as newly diagnosed adult patients with leukemia who received chemotherapy and were subsequently suspected of having B19V infection based on metagenomic next-generation sequencing (mNGS) of peripheral venous blood. A total of 17 patients with B19V infection were finally confirmed by polymerase chain reaction (PCR) testing and were enrolled in the analysis.

### Results

Based on the IVIG treatment dose and the patients' response to the treatment, the patients were divided into three groups. Group A was named the suboptimal response group, consisting of five patients. These patients were all in the granulocytopenic period or the bone marrow suppression period after chemotherapy when they were infected, and all received IVIG injection treatment after infection. However, the improvement in HGB was limited or lacked sustainability, and the transfusion dependence was not significantly reversed, suggesting that they were IVIG low-response/non-responders. The median dose intensity of IVIG used in this group was 6.88 g/day, and the median total dose was 25 g. The average ΔHGB in the suboptimal response group was -5.33 g/L. Most of the patients in this group had severe infection symptoms and were complicated with bacterial infections. Group C was named the significant benefit group, consisting of seven patients. These patients were mostly in the granulocytopenic recovery period or the granulocytopenic period when they were infected. After receiving an adequate dose of IVIG during the infection-related window, HGB showed a quantifiable increase, accompanied by a relief of transfusion dependence, indicating a clear IVIG response. The median dose intensity of IVIG used in this group was 10.13 g/day, and the median total dose was 48.75 g. The average ΔHGB in the significant benefit group was +29.33 g/L. Group B was named the self-limiting/support treatment group, consisting of five patients. This group mainly received blood transfusion support or observation strategies, lacking IVIG intervention or quantifiable response evidence. The clinical course was more consistent with self-limitation or dominated by the underlying disease course. Patients in this group had significantly higher transfusion dependence than the other two groups, with progressive decline in HB, which could lead to forced postponement of further chemotherapy courses.

**Copyright Information:** (Online) ISSN: 2572-9241
©2026 The Author(s). HemaSphere is published by John Wiley & Sons Ltd on behalf of European Hematology Association. This is an open access Abstract Book distributed under the Attribution-NonCommercial-NoDerivs (CC BY-NC-ND), which allows third parties to download the articles and share them with others as long as they credit the author and the Abstract Book, but they cannot change the content in any way or use them commercially.
**Abstract Book Citations:** Authors, Title, HemaSphere, 2026;10:(S1):pages. The abstract book DOIs can be found on the "Author Information" page.
**Disclaimer:** Articles published in the journal HemaSphere exclusively reflect the opinions of the authors. The authors are responsible for all content in their abstracts including accuracy of the facts, statements, citing resources, etc.

### Summary/Conclusion

The possibility of B19V infection is often overlooked. When there is a progressive decline in hemoglobin levels accompanied by moderate or higher fever, B19V infection should be suspected. This viral infection lacks specific treatment methods. Symptomatic treatment with immunoglobulin and blood transfusion generally yields better therapeutic effects.

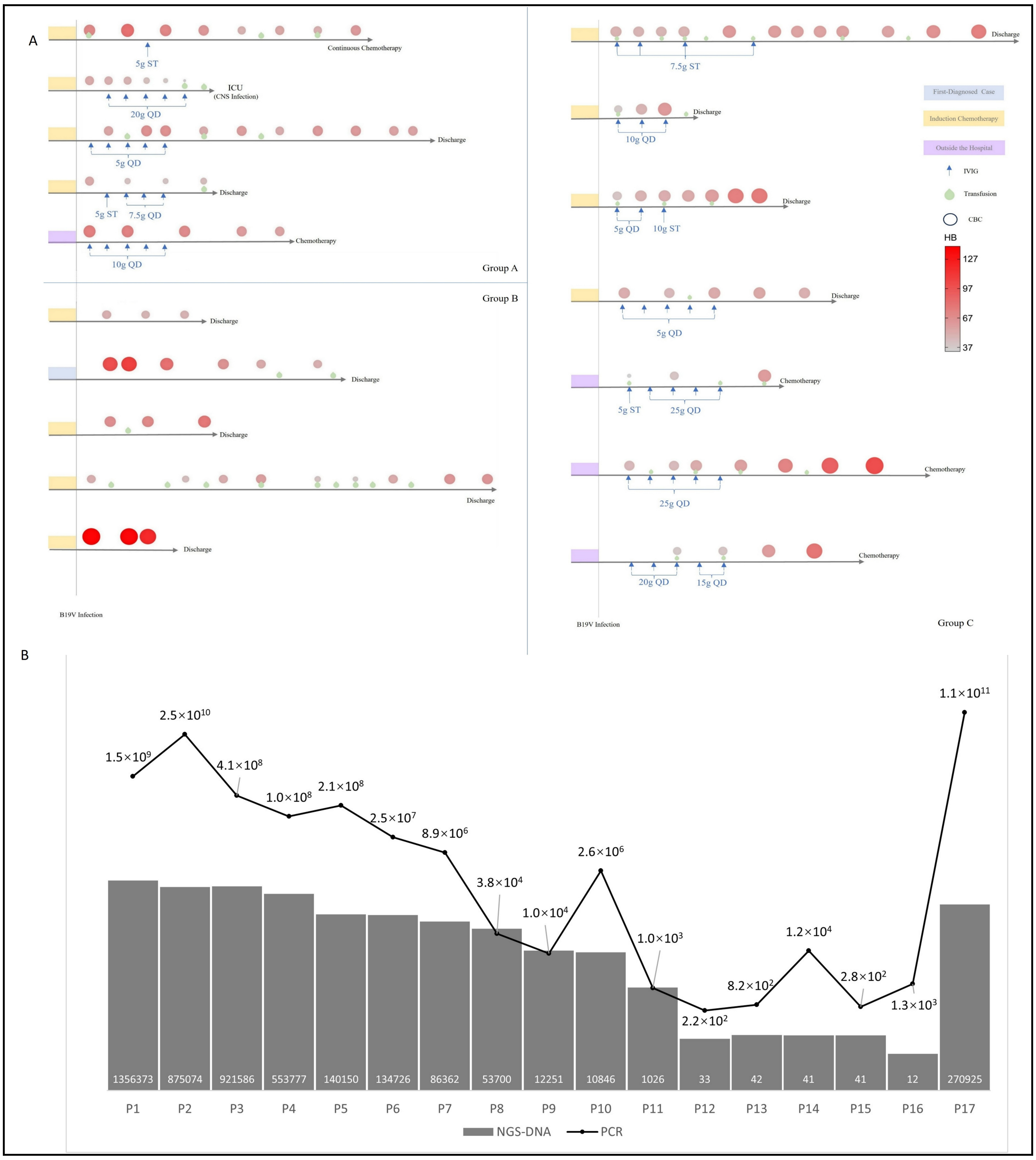

(A)Patient treatment timeline(B)Comparison of mNGS and PCR test results: The gray column chart represents the number of mNGS sequences for each sample (P1-P17), while the black line graph shows the corresponding PCR quantitative results (expressed in copy numbers) for each sample. The overall trend indicates that the results of the two detection methods are generally positively correlated. When the PCR copy number is high, the number of sequences obtained by NGS detection is also relatively large.

**Copyright Information:** (Online) ISSN: 2572-9241
©2026 The Author(s). HemaSphere is published by John Wiley & Sons Ltd on behalf of European Hematology Association. This is an open access Abstract Book distributed under the Attribution-NonCommercial-NoDerivs (CC BY-NC-ND), which allows third parties to download the articles and share them with others as long as they credit the author and the Abstract Book, but they cannot change the content in any way or use them commercially.
**Abstract Book Citations:** Authors, Title, HemaSphere, 2026;10:(S1):pages. The abstract book DOIs can be found on the "Author Information" page.
**Disclaimer:** Articles published in the journal HemaSphere exclusively reflect the opinions of the authors. The authors are responsible for all content in their abstracts including accuracy of the facts, statements, citing resources, etc.

### (PB4235) CLINICAL CHARACTERISTIC AND OUTCOME OF HUMAN HERPESVIRUS 6 ENCEPHALITIS AFTER PEDIATRIC ALLOGENEIC HEMATOPOIETIC STEM CELL TRANSPLANTATION

**Topic:** 31. Infections in hematology (incl. supportive care/therapy)

Leran Li*[1], Ye Guo[1]

[1] *Institute of Hematology & Blood Diseases Hospital, Chinese Academy of Medical Sciences, Tianjin, China;*

### Background

Human herpesvirus 6 (HHV-6) encephalitis is a severe and potentially fatal complication following allogeneic hematopoietic stem cell transplantation (allo-HSCT), particularly in pediatric patients during the early post-HSCT period. Comprehensive data integrating clinical manifestation, immune status, pathogen spectrum, and long-term outcome in children remain scarce.

### Aims

To characterize the clinical manifestation, immunological feature, pathogen spectrum, risk factor, and outcome of HHV-6 encephalitis in pediatric patients after allo-HSCT.

### Methods

We enrolled pediatric HHV-6 encephalitis patients following allo-HSCT between November 2019 and September 2025 at the Institute of Hematology & Blood Diseases Hospital, Chinese Academy of Medical Sciences, then conducted a 1:3 nested case-control cohort study based on HSCT time. Clinical characteristics, immune reconstitution parameters, laboratory and neuroimaging findings, metagenomic next-generation sequencing (mNGS) results, treatment strategies, and outcomes were analyzed. Logistic regression was used to identify risk factors, and survival outcomes were assessed using Kaplan–Meier analysis.

### Results

HHV-6 encephalitis occurred in 14 of 303 patients (4.6%), with a median onset of 22 days after HSCT. The most common clinical manifestation was seizures, observed in 50.0 % of cases; fever and altered mental status each occurred in 42.9 % of cases. Younger age was independently associated with HHV-6 encephalitis (OR 0.75, 95% CI 0.61–0.92, P = 0.007). All patients exhibited profound lymphopenia at onset, with markedly reduced $CD4^+$ and $CD8^+$ T-cell counts. Elevated inflammatory markers, including interleukin-6, interleukin-10, and lactate dehydrogenase, were observed in all cases. Hyponatremia was present in 78.6% of patients, and neuroimaging abnormalities were detected in 57.1%, predominantly as multifocal cerebral lesions.
Cerebrospinal fluid mNGS detected HHV-6 sequences in all patients, with a median read count of 223, while conventional HHV-6 PCR was positive in only 58.3% of tested cases. Intracranial mixed infections were identified in 64.3% of patients, most commonly Epstein–Barr virus and cytomegalovirus. Following antiviral therapy with foscarnet and/or ganciclovir combined with intravenous immunoglobulin, 71.4% of patients showed clinical improvement, while 4 patients died. The 3-year overall survival (OS) was lower in the encephalitis group compared with controls (68.6% vs 87.6%, P = 0.086), and the 3-year relapse-free survival (RFS) was significantly inferior (55.6% vs 83.0%, P = 0.0107).

**Copyright Information:** (Online) ISSN: 2572-9241
©2026 The Author(s). HemaSphere is published by John Wiley & Sons Ltd on behalf of European Hematology Association. This is an open access Abstract Book distributed under the Attribution-NonCommercial-NoDerivs (CC BY-NC-ND), which allows third parties to download the articles and share them with others as long as they credit the author and the Abstract Book, but they cannot change the content in any way or use them commercially.
**Abstract Book Citations:** Authors, Title, HemaSphere, 2026;10:(S1):pages. The abstract book DOIs can be found on the "Author Information" page.
**Disclaimer:** Articles published in the journal HemaSphere exclusively reflect the opinions of the authors. The authors are responsible for all content in their abstracts including accuracy of the facts, statements, citing resources, etc.

**Summary/Conclusion**

HHV-6 encephalitis predominantly occurs early after pediatric allo-HSCT and is characterized by severe neurological symptoms, profound cellular immunosuppression, and frequent intracranial mixed infections. mNGS demonstrates superior sensitivity over conventional PCR for HHV-6 detection and provides critical information on co-pathogens. HHV-6 encephalitis is associated with significantly inferior RFS, underscoring the need for intensified early surveillance, immune monitoring, and comprehensive pathogen assessment in high-risk pediatric patients.

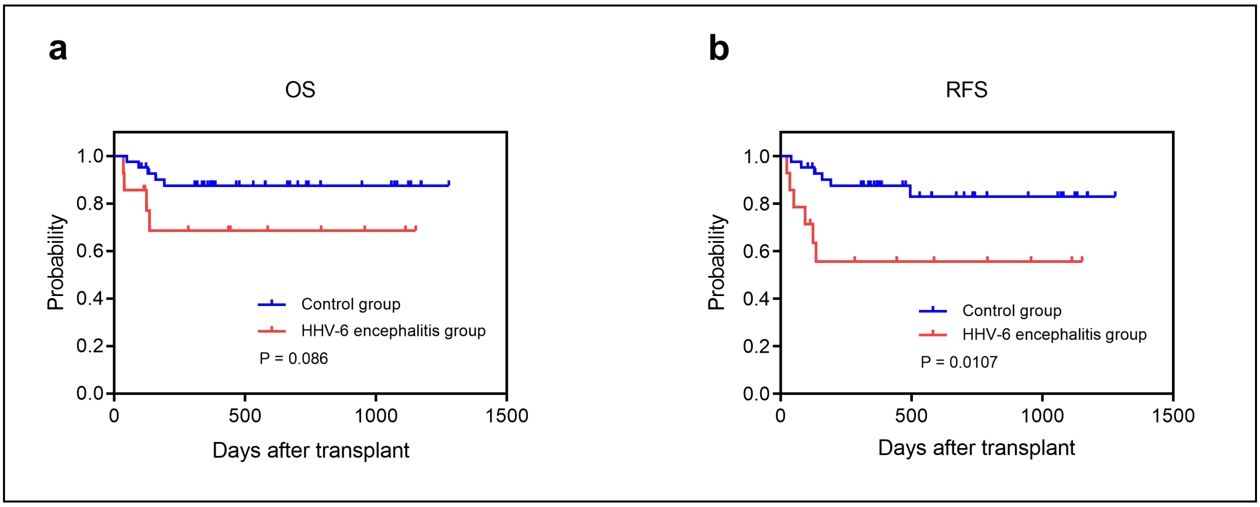

OS and RFS by Kaplan-Meier method for HHV-6 encephalitis patients (n = 14) and control (n = 42) patients.

**Copyright Information:** (Online) ISSN: 2572-9241
©2026 The Author(s). HemaSphere is published by John Wiley & Sons Ltd on behalf of European Hematology Association. This is an open access Abstract Book distributed under the Attribution-NonCommercial-NoDerivs (CC BY-NC-ND), which allows third parties to download the articles and share them with others as long as they credit the author and the Abstract Book, but they cannot change the content in any way or use them commercially.
**Abstract Book Citations:** Authors, Title, HemaSphere, 2026;10:(S1):pages. The abstract book DOIs can be found on the "Author Information" page.
**Disclaimer:** Articles published in the journal HemaSphere exclusively reflect the opinions of the authors. The authors are responsible for all content in their abstracts including accuracy of the facts, statements, citing resources, etc.

### (PB4236) EFFICACY OF IVIG REPLACEMENT THERAPY FOR INFECTION PROPHYLAXIS IN PATIENTS WITH LYMPHOPROLIFERATIVE DISEASES FOLLOWING AUTO-HSCT

**Topic:** 31. Infections in hematology (incl. supportive care/therapy)

Olga Gerasimovich*[1], Ihar Iskrou[2]

*[1]State Institution "Minsk Scientific and Practical Center for Surgery, Transplantology and Hematology", Department of Hematology and Bone Marrow Transplantation No. 1, Minsk, Belarus; [2]The Institute for Advanced Training and Retraining of Healthcare Personnel at the Belarusian State Medical University, Minsk, Belarus;*

### Background

Infectious complications (IC) are the leading cause of mortality after autologous hematopoietic stem cell transplantation (auto-HSCT) in patients with lymphoproliferative diseases (LPD). The development of hypogammaglobulinemia—a significant risk factor for these complications—depends on the specific LPD entity and the nature of prior therapy, including immunochemotherapy and radiation.

### Aims

The aim of this study was to evaluate the effect of immunoglobulin replacement therapy on the frequency and severity of IC in patients with LPD undergoing auto-HSCT.

### Methods

Data from 92 patients with LPD after auto-HSCT were analyzed: multiple myeloma (MM) — 49 (53.3%), Hodgkin lymphoma (HL) — 23 (25%), and non-Hodgkin lymphoma (NHL) — 20 (21.7%). The median age was 47.1 [35.7; 59.3] years, with a male-to-female ratio of 1.9:1. Infectious complications were graded according to the CTCAE version 5.0. Within the study framework, intravenous immunoglobulin was administered at immunomodulatory doses: 0.2 g/kg for febrile neutropenia and up to 0.4 g/kg for infectious complications during post-transplant cytopenia. The observation period covered 3 months after auto-HSCT.
Analysis of the obtained results and assessment of pharmacoeconomic efficacy. Statistical analysis was performed using R-statistics v4.1.1, odds ratio estimation and Fisher's exact test; the number needed to treat (NNT).

### Results

The baseline IgG level ranged from 3.71 g/L to 11.14 g/L (median 7.35 g/L). Hypogammaglobulinemia was diagnosed in 9 (18.3%) patients with MM, 2 (8.7%) with HL, and 7 (35%) with NHL. Statistical analysis revealed that hypogammaglobulinemia was detected significantly more often in NHL patients compared to the HL group (OR 7.1; 95% CI 1.091–79.5; Fisher's exact test p=0.029) and the MM group (OR 2.7; 95% CI 1.165–56.9; Fisher's exact test p=0.047).
Eighteen (19.6%) patients had moderate or severe hypogammaglobulinemia and they received immunoglobulin replacement therapy at a starting dose of 0.2 g/kg during the preparation stage for conditioning. During the cytopenic phase following auto-HSCT, episodes of febrile neutropenia were recorded in 77 patients. All of these patients also received a prophylactic dose of intravenous immunoglobulin. Among the 92 patients, 33 developed 38 infectious episodes, including 19 microbiologically confirmed bloodstream infections. All of these patients received a single therapeutic dose of intravenous immunoglobulin (IVIG) at 0.4 g/kg of body weight. Administration of replacement therapy prior to conditioning in patients with hypogammaglobulinemia was associated with a 2.17-fold increase in the odds of a favorable outcome (absence of bloodstream infection) (95% CI 1.67–2.99; p=0.027). The number needed to treat (NNT) to prevent one infectious episode was 3 (p=0.007). No lethal outcomes were recorded during the three-month observation period.

**Copyright Information:** (Online) ISSN: 2572-9241
©2026 The Author(s). HemaSphere is published by John Wiley & Sons Ltd on behalf of European Hematology Association. This is an open access Abstract Book distributed under the Attribution-NonCommercial-NoDerivs (CC BY-NC-ND), which allows third parties to download the articles and share them with others as long as they credit the author and the Abstract Book, but they cannot change the content in any way or use them commercially.
**Abstract Book Citations:** Authors, Title, HemaSphere, 2026;10:(S1):pages. The abstract book DOIs can be found on the "Author Information" page.
**Disclaimer:** Articles published in the journal HemaSphere exclusively reflect the opinions of the authors. The authors are responsible for all content in their abstracts including accuracy of the facts, statements, citing resources, etc.

**Summary/Conclusion**

**Monitoring IgG levels and correcting hypogammaglobulinemia** in patients with LPD, particularly those with NHL and prior anti-CD20 monoclonal antibody therapy, are advisable before auto-HSCT.
**Prophylactic administration** of immunoglobulin preparations reduces the risk of infectious complications, which impacts quality of life and event-free survival rates.
**The pharmacoeconomic effectiveness of IVIG** in patients with LPD is justified by cost savings due to direct medical costs (hospitalization costs, examinations for severe IC, and the use of reserve antibacterial and antifungal agents).

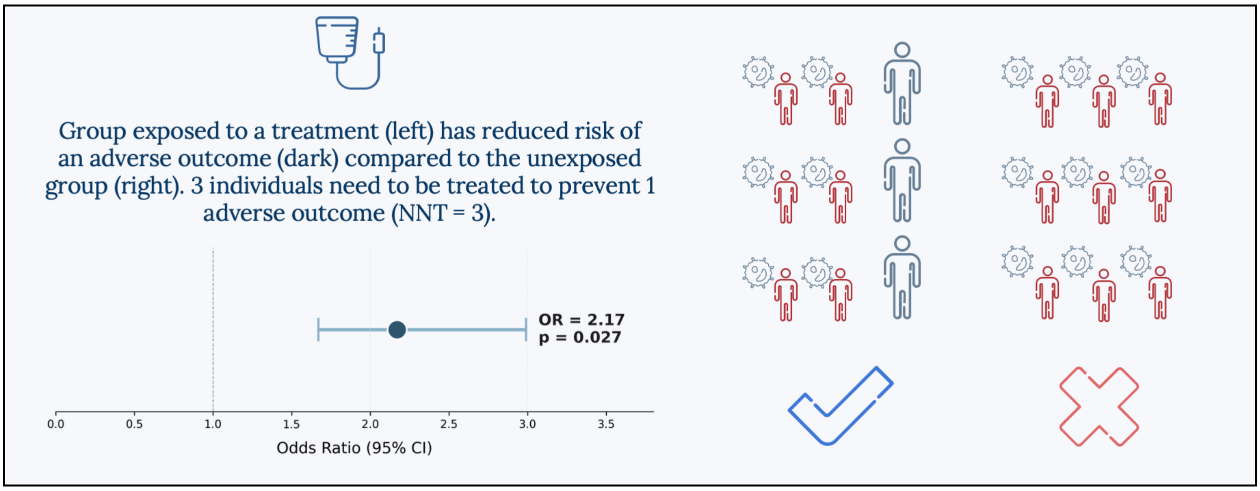

Favorable Outcome with Pre-HSCT IgG Therapy (Absence of BSI)

**Copyright Information:** (Online) ISSN: 2572-9241
©2026 The Author(s). HemaSphere is published by John Wiley & Sons Ltd on behalf of European Hematology Association. This is an open access Abstract Book distributed under the Attribution-NonCommercial-NoDerivs (CC BY-NC-ND), which allows third parties to download the articles and share them with others as long as they credit the author and the Abstract Book, but they cannot change the content in any way or use them commercially.
**Abstract Book Citations:** Authors, Title, HemaSphere, 2026;10:(S1):pages. The abstract book DOIs can be found on the "Author Information" page.
**Disclaimer:** Articles published in the journal HemaSphere exclusively reflect the opinions of the authors. The authors are responsible for all content in their abstracts including accuracy of the facts, statements, citing resources, etc.

### (PB4237) AN EHA-SWG INFECTIONS IN HEMATOLOGY SURVEY FOR INFECTIOUS DISEASES TRAINING IN HEMATOLOGY TRAINEES: A CALL FOR ACTION

**Topic:** 31. Infections in hematology (incl. supportive care/therapy)

Paschalis Evangelidis*[1], Eleni Gavriilaki[1], Nikolaos Kotsiou[1], Ioanna Sakellari[2], Oliver A. Cornely[3], Livio Pagano[4], Jon Salmanton-García[3]

*[1]Aristotle University of Thessaloniki, Second Propedeutic Department of Internal Medicine, Thessaloniki, Greece; [2]G. Papanicolaou Hospital, Hematology Department - BMT Unit, Thessaloniki, Greece; [3]University of Cologne, Cologne, Germany; [4]Universita Cattolica del Sacro Cuore, Rome, Italy;*

### Background

Infectious diseases (ID) constitute a significant cause of mortality and morbidity in patients with hematological malignancies. Therefore, training hematology residents and fellows in ID prevention, prophylaxis, and management is crucial. However, no study to date has evaluated the current landscape of ID training-education that hematology residents and fellows receive worldwide.

### Aims

Understanding the importance of providing quality ID care in hematology patients and recognizing the aforementioned literature gap, we decided to conduct the current study, aiming to evaluate the current status of ID training, clinical exposure, and self-reported confidence among hematology residents and fellows, investigating also their perspectives for improvement of the education provided.

### Methods

This is a cross-sectional, web-based survey study. Eligible participants were hematology residents/fellows, whereas responses from non-trainees were excluded during data cleaning. The questionnaire included: 7 questions regarding the demographic/employment data of participants, 7 concerning exposure to ID cases in clinical practice, 5 about the content and education methods in ID used in their department, 5 on their self-reported confidence in the management of infectious complications, and 4 examining their satisfaction with current ID training and potential future perspectives. The survey was distributed to hematology residents/ fellows via the newsletter of Young-EHA. Data collection took place between July 2025 and February 2026.

### Results

Eighty-five hematology residents/fellows completed the survey (Table 1 and Figure 1). Regarding their clinical exposure to ID cases, 57.6% reported their department had a standardized protocol for infection prevention and control, whereas an organized antimicrobial stewardship (AMS) program was available for 25.9% of the participants. Only 7.1% of the study respondents did not have access to ID specialists. Concerning educational activities and ID education components, training on AMS was available for 27.1%, and ID lectures/seminars for 42.4%, whereas ID case-based discussions were noted by 68.2%, interdisciplinary ID rounds by 52.9%, and simulation-based ID training only for 8.2%. As far as their self-confidence in the management of ID, 47.1% mentioned that they were confident in managing infectious complications, 61.2%, 40%, and 34.1% considered themselves confident in choosing empirical antibacterial, antifungal, and antiviral regimens, respectively. The majority of them (35.3%) were found to be moderately satisfied with the ID training in their institution. As key training gaps, the following were identified: insufficient focus on AMS (49.4%), lack of training in infection prevention and control (41.2%), lack of multidisciplinary collaboration experience (32.9%), and gaps in laboratory interpretation and microbiology (36.5%). Structured ID modules were the most frequently suggested improvement (70.6%), followed by AMS training (55.3%) and simulation-based education (49.4%). Finally, case-based learning was the preferred educational format (76.5%), with interdisciplinary clinical rounds (56.5%) and clinical ID electives (54.1%) next.

**Copyright Information:** (Online) ISSN: 2572-9241
©2026 The Author(s). HemaSphere is published by John Wiley & Sons Ltd on behalf of European Hematology Association. This is an open access Abstract Book distributed under the Attribution-NonCommercial-NoDerivs (CC BY-NC-ND), which allows third parties to download the articles and share them with others as long as they credit the author and the Abstract Book, but they cannot change the content in any way or use them commercially.
**Abstract Book Citations:** Authors, Title, HemaSphere, 2026;10:(S1):pages. The abstract book DOIs can be found on the "Author Information" page.
**Disclaimer:** Articles published in the journal HemaSphere exclusively reflect the opinions of the authors. The authors are responsible for all content in their abstracts including accuracy of the facts, statements, citing resources, etc.

### Summary/Conclusion

Our findings highlight substantial heterogeneity and unmet educational needs in ID training among hematology trainees, underlining the necessity for structured curricula, AMS integration, and interdisciplinary learning strategies.

**Figure 1. Geographic distribution of participants (N=85)**

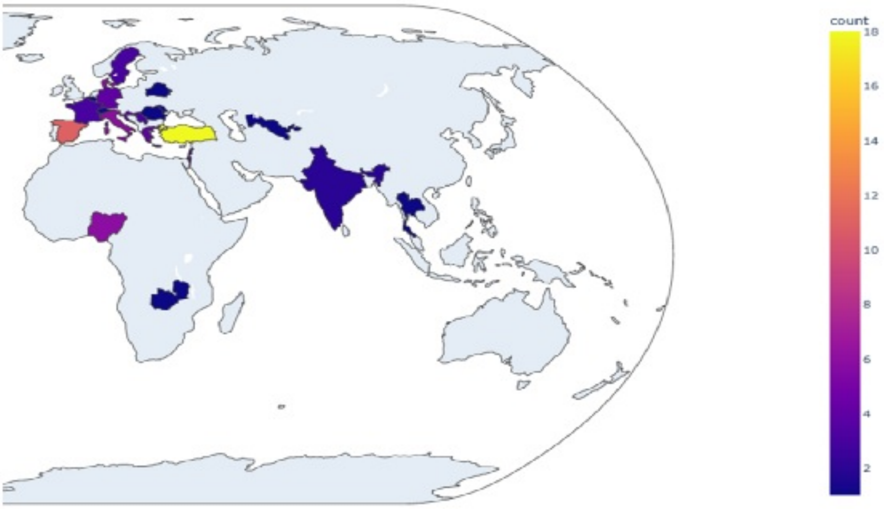

**Table 1. Participant characteristics**

| | |
|---|---|
| Median age (IQR 25–75) | 31 (29–34) |
| Median residency/fellowship year (IQR 25–75) | 3 (2–3) of 4 (3–5) |
| Affiliated institution, n (%) | |
| Secondary hospital | 1 (1.2) |
| Tertiary hospital | 15 (17.6) |
| University hospital | 69 (81.2) |
| HSCT/CAR-T center, n (%) | 63 (74.1) |
| Patient population, n (%) | |
| Adult | 69 (81.2) |
| Pediatric | 10 (11.8) |
| Both | 6 (7.1) |

Abbreviations: CAR-T: chimeric antigen receptor T-cell; HSCT: hematopoietic stem cell transplantation; IQR: interquartile range.

**Copyright Information:** (Online) ISSN: 2572-9241
©2026 The Author(s). HemaSphere is published by John Wiley & Sons Ltd on behalf of European Hematology Association. This is an open access Abstract Book distributed under the Attribution-NonCommercial-NoDerivs (CC BY-NC-ND), which allows third parties to download the articles and share them with others as long as they credit the author and the Abstract Book, but they cannot change the content in any way or use them commercially.
**Abstract Book Citations:** Authors, Title, HemaSphere, 2026;10:(S1):pages. The abstract book DOIs can be found on the "Author Information" page.
**Disclaimer:** Articles published in the journal HemaSphere exclusively reflect the opinions of the authors. The authors are responsible for all content in their abstracts including accuracy of the facts, statements, citing resources, etc.

### (PB4238) THE BIDIRECTIONAL RELATIONSHIP BETWEEN IMMUNOSUPPRESSION AND PNEUMOCYSTIS JIROVECII PNEUMONIA IN B CELL LYMPHOMA PATIENTS TREATED WITH RITUXIMAB: A RETROSPECTIVE STUDY FROM CHINA

**Topic:** 31. Infections in hematology (incl. supportive care/therapy)

Chaoyu Wang*[1], Xiping Liang[1], Yao Liu[1]

[1]*Chongqing university cancer hospital, Chong, China;*

**Background**

Pneumocystis jirovecii pneumonia (PJP) is a life-threatening infection in HIV patients, and has emerged as a critical complication of high morbidity in patients with malignancies. However, there is a scarcity of data on this association in lymphoma.

**Aims**

Moreover, existing evidence is largely derived from small cohort studies or case series, no specific risk factors or immune function of PJP have been presented. The aim of this study was to estimate the risk factors and immune function for PCP infection in B-cell lymphoma with treatment of RTX.

**Methods**

The medical records of lymphoma who received rituximab between Jan 2019 and Jan 2023 were retrospectively evaluated. Clinical feature and immune function were determined with and without PJP.

**Results**

There were 989 patients receiving rituximab, from which 22 patients diagnosed with PJP. The median patient age was 54.3 years. We observed that advanced disease stage ($p=0.032$), poorer performance status ($p=0.044$), patients receiving rituximab as first line, and significantly immunosuppression including decreased lymphocyte ($p=0.032$), CD4 ($p=0.042$) and CD4/CD8 ($p=0.035$) are more likely to have PJP. Patients diagnosed with PJP exhibited abnormalities on computed tomography (CT) as patchy opacities and ground-glass opacities. Common presentations of fever and dyspnea were observed, with a subset of patients requiring ventilator support. Following PJP infection, a revealed inflammatory (CRP, TNF-α, IL-6, LDH,) along with a decline in CD4+ T-cell and lymphocytes was observed ($P<0.05$). Administration of 3–4 cycles of rituximab ($p=0.044$), poor performance status ($p=0.038$), and impaired immune function ($p<0.05$) were identified as significant risk factors associated with the development of PJP.

**Summary/Conclusion**

PJP was associated with more advanced disease, poorer performance status, and impaired immunity. PJP infection induces a pronounced inflammatory and sever immunosuppression. In clinical practice, patients with rituximab treatment presenting with fever, dyspnea and severe immunosuppression should raise clinical suspicion of PJP infection.

**Copyright Information:** (Online) ISSN: 2572-9241
©2026 The Author(s). HemaSphere is published by John Wiley & Sons Ltd on behalf of European Hematology Association. This is an open access Abstract Book distributed under the Attribution-NonCommercial-NoDerivs (CC BY-NC-ND), which allows third parties to download the articles and share them with others as long as they credit the author and the Abstract Book, but they cannot change the content in any way or use them commercially.
**Abstract Book Citations:** Authors, Title, HemaSphere, 2026;10:(S1):pages. The abstract book DOIs can be found on the "Author Information" page.
**Disclaimer:** Articles published in the journal HemaSphere exclusively reflect the opinions of the authors. The authors are responsible for all content in their abstracts including accuracy of the facts, statements, citing resources, etc.

### (PB4239) ASSOCIATION OF HEPATITIS B AND C VIRUS INFECTIONS WITH MAJOR HEMATOLOGIC MALIGNANCIES IN AZERBAIJAN: A POPULATION-BASED CASE–CONTROL STUDY

**Topic:** 31. Infections in hematology (incl. supportive care/therapy)

Gunel Aliyeva*[1], Chingiz Asadov[1], Aytan Shirinova[1], Naila Ismail[1]

[1]*National Center Hematology and Transfuziology, Baku, Azerbaijan;*

**Background**

Chronic hepatitis B virus (HBV) and hepatitis C virus (HCV) infections have been linked to B-cell lymphoid neoplasms, but data from regions with intermediate endemicity, such as Azerbaijan, are limited.

**Aims**

To assess the association between chronic HBV and HCV infections and major hematologic malignancies in adults in Azerbaijan.

**Methods**

We performed a retrospective observational study including 797 adults diagnosed with hematologic malignancies (non-Hodgkin lymphoma [NHL], acute lymphoblastic leukemia [ALL], chronic lymphocytic leukemia [CLL], multiple myeloma, acute myeloid leukemia, myeloproliferative neoplasms) at the National Center of Hematology and Transfusiology (2018–2023). Diagnoses followed WHO criteria. HBV and HCV status at diagnosis was determined serologically (HBsAg, anti-HCV). An external control group comprised 38,789 adults from a national sero-epidemiological survey. Odds ratios (ORs) with 95% confidence intervals (CIs) were estimated from 2×2 tables and multivariable logistic regression adjusted for age and sex.

**Results**

Among 797 patients, the most frequent diagnoses were ALL (32.0%), AML (24.2%), CLL (15.4%), and NHL (7.8%). HCV seropositivity was higher in NHL than in controls (12.9% vs 3.0%), corresponding to an OR of 4.8 (95% CI, 2.3–9.7; P<0.001). HBV infection was also associated with NHL (6.5% vs 2.6%; OR, 2.6; 95% CI, 1.2–5.6; P=0.046). For ALL, HCV seropositivity was 7.5% vs 3.0% in controls (OR, 2.5; 95% CI, 1.6–4.0; P<0.001), with no significant association for HBV. Both viruses were associated with CLL (HBV: 6.5% vs 2.6%; OR, 2.6; 95% CI, 1.6–4.3; P=0.009; HCV: 6.5% vs 3.0%; OR, 2.2; 95% CI, 1.3–3.8; P=0.025). No significant associations were observed for multiple myeloma, AML, or myeloproliferative neoplasms. Virus–malignancy associations were confined to B-cell lymphoid neoplasms.

**Summary/Conclusion**

In this Azerbaijani cohort, chronic HBV and HCV infections were independently associated with an increased risk of NHL, ALL, and CLL, with the strongest effect observed for HCV and NHL. These findings support routine HBV/HCV screening and guideline-based management in patients with B-cell hematologic malignancies in regions with intermediate endemicity, and highlight the potential contribution of hepatitis virus control to blood cancer prevention strategies. A schematic figure summarizing proposed mechanisms of HBV/HCV-driven B-cell lymphomagenesis, integrating CD81-mediated HCV signaling and HBV DNA integration–induced genomic instability (Figue 1).

**Copyright Information:** (Online) ISSN: 2572-9241
©2026 The Author(s). HemaSphere is published by John Wiley & Sons Ltd on behalf of European Hematology Association. This is an open access Abstract Book distributed under the Attribution-NonCommercial-NoDerivs (CC BY-NC-ND), which allows third parties to download the articles and share them with others as long as they credit the author and the Abstract Book, but they cannot change the content in any way or use them commercially.
**Abstract Book Citations:** Authors, Title, HemaSphere, 2026;10:(S1):pages. The abstract book DOIs can be found on the “Author Information” page.
**Disclaimer:** Articles published in the journal HemaSphere exclusively reflect the opinions of the authors. The authors are responsible for all content in their abstracts including accuracy of the facts, statements, citing resources, etc.

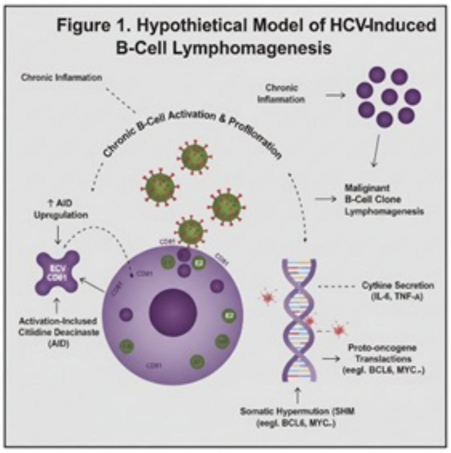

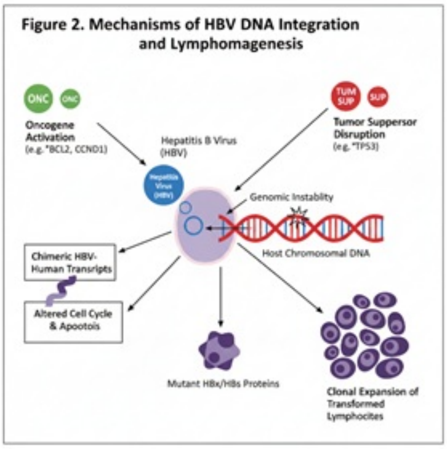

Figure legend

**Schematic overview of how chronic HCV and HBV infections may promote B‑cell lymphomagenesis**. Left panel: HCV envelope glycoprotein E2 engages the CD81 receptor on B cells, activating MAPK and NF‑κB signaling pathways, upregulating activation-induced cytidine deaminase (AID), and inducing off‑target somatic mutations in oncogenes (e.g., BCL6, MYC) that drive clonal B‑cell expansion. Right panel: HBV DNA integrates into host chromosomal DNA in lymphoid cells, leading to activation of proto‑oncogenes, disruption of tumor suppressor genes, production of truncated HBx/HBs proteins with altered transactivation activity, and formation of viral–host chimeric transcripts, collectively contributing to genomic instability and B‑cell malignant transformation.

**Copyright Information:** (Online) ISSN: 2572-9241
©2026 The Author(s). HemaSphere is published by John Wiley & Sons Ltd on behalf of European Hematology Association. This is an open access Abstract Book distributed under the Attribution-NonCommercial-NoDerivs (CC BY-NC-ND), which allows third parties to download the articles and share them with others as long as they credit the author and the Abstract Book, but they cannot change the content in any way or use them commercially.
**Abstract Book Citations:** Authors, Title, HemaSphere, 2026;10:(S1):pages. The abstract book DOIs can be found on the “Author Information” page.
**Disclaimer:** Articles published in the journal HemaSphere exclusively reflect the opinions of the authors. The authors are responsible for all content in their abstracts including accuracy of the facts, statements, citing resources, etc.

### (PB4240) REAL-WORLD USAGE OF HYALURONIDASE-FACILITATED SUBCUTANEOUS IMMUNOGLOBULIN 10% IN PATIENTS WITH MULTIPLE MYELOMA DIAGNOSED WITH SECONDARY IMMUNODEFICIENCY: AN INTERIM ANALYSIS

**Topic:** 31. Infections in hematology (incl. supportive care/therapy)

Efstathios Kastritis*[1], Silvia Sánchez-Ramón[2], Agoston Szabo[3], Bogusław MacHaliński[4], Albert Pérez Montaña[5], Jie Chen[6], Barbara McCoy[7], Marta Kamieniak[6], Stefan Wietek[7]

*[1]National and Kapodistrian University of Athens, Department of Clinical Therapeutics, Athens, Greece;[2]Complutense University of Madrid, Department of Clinical Immunology, Institute of Laboratory Medicine, Hospital Clínico San Carlos, Madrid, Spain; [3]Rigshospitalet, University of Copenhagen, Department of Haematology, Copenhagen, Denmark;[4]Pomeranian Medical University, Department of Hematology and Transplantology, Szczecin, Poland; [5]Hospital Universitari Son Espases, Hematology Department, Palma de Mallorca, Spain; [6]Takeda Development Center Americas, Inc., Cambridge, MA, United States of America; [7]Baxalta Innovations GmbH, a Takeda company, Vienna, Austria;*

### Background

Patients with multiple myeloma (MM) are susceptible to secondary immunodeficiency (SID) owing to both their underlying disease and/or immunosuppressive therapy. HyMMy (TAK-771-5006; NCT05879757) is an ongoing, prospective, non-interventional, observational study of hyaluronidase-facilitated subcutaneous immunoglobulin (fSCIG) 10% in adult patients with MM and SID across 25 centers in Europe.

### Aims

This interim analysis evaluated fSCIG 10% use and associated outcomes at 3-month follow up.

### Methods

Enrolled patients had a diagnosis of MM and met criteria for SID, defined as severe, recurrent or persistent infection(s) despite appropriate anti-infective treatment, and proven specific antibody failure or a serum IgG trough level of < 4 g/L (excluding paraprotein). All patients provided informed consent. The primary objective was to assess real-world infusion parameters of fSCIG 10%. Secondary objectives included healthcare resource utilization, MM disease status and adverse events (AEs). Statistical analysis was descriptive.

**Copyright Information:** (Online) ISSN: 2572-9241
©2026 The Author(s). HemaSphere is published by John Wiley & Sons Ltd on behalf of European Hematology Association. This is an open access Abstract Book distributed under the Attribution-NonCommercial-NoDerivs (CC BY-NC-ND), which allows third parties to download the articles and share them with others as long as they credit the author and the Abstract Book, but they cannot change the content in any way or use them commercially.
**Abstract Book Citations:** Authors, Title, HemaSphere, 2026;10:(S1):pages. The abstract book DOIs can be found on the "Author Information" page.
**Disclaimer:** Articles published in the journal HemaSphere exclusively reflect the opinions of the authors. The authors are responsible for all content in their abstracts including accuracy of the facts, statements, citing resources, etc.

**Results**

As of May 5, 2025, 67 patients were enrolled; of these, 43 had completed 3 months of follow up. The mean (standard deviation [SD]) age was 64.9 (11.26) years and 53.7% were female. At baseline (enrollment), patients with MM had bone lesions (82.4%), anemia (49.0%), renal insufficiency (27.5%) or hypercalcemia (13.7%), and patients received steroids (61.2%), anti-plasma cell monoclonal antibodies (49.3%), immunomodulatory drugs (46.3%), proteasome inhibitors (28.4%) and bispecific antibodies (14.9%). The median (interquartile range [IQR]) time from MM to SID diagnosis was 30 (8–67) months and the time from SID diagnosis to fSCIG 10% initiation was 2 (0–6) months. At baseline, 41.6% (57/137) of fSCIG 10% infusions were received every 4 weeks; this increased to 82.2% (97/118) of infusions at 3 months. The mean (SD) dose was 0.36 (0.225) g/kg at baseline and 0.37 (0.319) g/kg at 3 months. At baseline and at 3 months, median (IQR) total infusion volume was 200 (100–250) mL and 300 (210–400) mL, respectively; infusion rate was 133.3 (100–250) mL/h and 210.5 (165–224) mL/h; maximum infusion rate was 240 (240–250) mL/h and 300 (245–300) mL/h; infusion duration was 80 (60–120) minutes and 95 (80–110) minutes; and number of infusion sites was 2 (1–3) and 2.5 (2–3). At baseline and at 3 months, 91.1% (123/135) and 92.2% (129/119) of infusions were in the abdomen, respectively. At baseline, 90.5% (124/137) of infusions occurred in the hospital and 8.8% (12/137) at home; at 3 months, 66.1% (78/118) occurred in hospital and 33.9% (40/118) at home. No infusions were discontinued, slowed or interrupted. Four patients discontinued treatment owing to lack of effectiveness (n = 1) or because they enrolled in another study (n = 3). At 3 months, all treatment-related AEs were mild (1.5%) to moderate (3.0%) in severity, with no serious treatment-related AEs reported (Table). One patient died (2.2%) from an infection unrelated to treatment.

**Summary/Conclusion**

This interim analysis shows the feasibility of using fSCIG 10% as a treatment option for patients with MM and SID in real-world setting, potentially offering the flexibility of home infusions with a favorable safety profile. Final study data from up to 12 months of follow-up are needed to confirm these findings.

This abstract was previously submitted to the European Myeloma Network Meeting, April 2026. Study/writing funder: Takeda Development Center Americas, Inc./Takeda Pharmaceuticals International AG.

**Copyright Information:** (Online) ISSN: 2572-9241
©2026 The Author(s). HemaSphere is published by John Wiley & Sons Ltd on behalf of European Hematology Association. This is an open access Abstract Book distributed under the Attribution-NonCommercial-NoDerivs (CC BY-NC-ND), which allows third parties to download the articles and share them with others as long as they credit the author and the Abstract Book, but they cannot change the content in any way or use them commercially.
**Abstract Book Citations:** Authors, Title, HemaSphere, 2026;10:(S1):pages. The abstract book DOIs can be found on the "Author Information" page.
**Disclaimer:** Articles published in the journal HemaSphere exclusively reflect the opinions of the authors. The authors are responsible for all content in their abstracts including accuracy of the facts, statements, citing resources, etc.

| Table. Any treatment-related AEs reported at 3 months (N = 67)[a] | | | | |
|---|---|---|---|---|
| **System organ class** | **Preferred term** | **Grade** | **Patients, n (%)** | **Events, n** |
| Gastrointestinal disorders | Diarrhea | 2 | 1 (1.5) | 1 |
| Infections and infestations | Upper respiratory tract infection | 2 | 1 (1.5) | 1 |
| Skin and subcutaneous tissue disorders | Pruritus | 1 | 1 (1.5) | 1 |

[a]Treatment-related AEs included those considered by the investigator as related or possibly related to treatment. AE terms were reported according to MedDRA version 27.0. The severity of AEs was classified according to CTCAE criteria as follows: 1, mild; 2, moderate, 3, severe; 4, life-threatening; 5, death.

AE, adverse event; CTCAE, Common Terminology Criteria for Adverse Events; MeDRA, Medical Dictionary for Regulatory Activities.

Copyright Information: (Online) ISSN: 2572-9241
©2026 The Author(s). HemaSphere is published by John Wiley & Sons Ltd on behalf of European Hematology Association. This is an open access Abstract Book distributed under the Attribution-NonCommercial-NoDerivs (CC BY-NC-ND), which allows third parties to download the articles and share them with others as long as they credit the author and the Abstract Book, but they cannot change the content in any way or use them commercially.
Abstract Book Citations: Authors, Title, HemaSphere, 2026;10:(S1):pages. The abstract book DOIs can be found on the "Author Information" page.
Disclaimer: Articles published in the journal HemaSphere exclusively reflect the opinions of the authors. The authors are responsible for all content in their abstracts including accuracy of the facts, statements, citing resources, etc.

### (PB4243) SCRUB TYPHUS-ASSOCIATED HEMOPHAGOCYTIC LYMPHOHISTIOCYTOSIS IN CHILDREN: CLINICAL FEATURES AND THE DIAGNOSTIC UTILITY OF METAGENOMIC NEXT-GENERATION SEQUENCING

**Topic:** 31. Infections in hematology (incl. supportive care/therapy)

Qihong Lin*[1], Shilin Liu[1]

[1]*Shenzhen Children's Hospital, SHEN ZHEN, China;*

**Background**

Scrub typhus, caused by Orientia tsutsugamushi, can trigger severe secondary hemophagocytic lymphohistiocytosis (HLH), a life-threatening hyperinflammatory syndrome. Early identification of the trigger is critical for guiding therapy, yet diagnosing scrub typhus in this setting remains challenging.

**Aims**

To characterize the clinical and laboratory features of pediatric scrub typhus-associated HLH and evaluate the diagnostic utility of metagenomic next-generation sequencing (mNGS).

**Methods**

A retrospective analysis was conducted on 12 children diagnosed with scrub typhus-associated HLH between July 2023 and July 2025. HLH diagnosis followed HLH-2004 criteria, and scrub typhus was confirmed via mNGS of peripheral blood and/or cerebrospinal fluid (CSF). Clinical presentation, laboratory parameters, treatment, and outcomes were reviewed.

**Results**

The median age was 7.5 years. All patients presented with prolonged fever (median duration: 9 days). Common signs included lymphadenopathy (100%), eschar (66.7%), hepatomegaly (58.3%), and splenomegaly (66.7%). Notably, 33.3% (4/12) were initially misdiagnosed with Kawasaki disease. All patients fulfilled HLH criteria, exhibiting marked hyperferritinemia (median: 5,355 ng/mL) and elevated soluble CD25 (median: 5,172 U/mL). mNGS from peripheral blood confirmed O. tsutsugamushi in all cases. CSF mNGS was positive in 4 out of 5 tested patients, accompanied by elevated cytokines (IL-6, IL-10, IFN-γ), indicating neuroinflammation. Severe complications included pulmonary hemorrhage (n=3) and shock (n=3). All patients survived following targeted antibiotic therapy (doxycycline/azithromycin) combined with immunomodulators (corticosteroids, IVIG, and/or ruxolitinib).

**Summary/Conclusion**

Scrub typhus is an important cause of secondary HLH in children from endemic regions. mNGS proves to be a rapid and sensitive tool for etiological diagnosis, enabling timely and appropriate management. The findings support the use of mNGS, including CSF analysis in severe cases, to identify central nervous system involvement. Awareness of potential complications like pulmonary hemorrhage is crucial in managing these critically ill patients.

**Copyright Information:** (Online) ISSN: 2572-9241
©2026 The Author(s). HemaSphere is published by John Wiley & Sons Ltd on behalf of European Hematology Association. This is an open access Abstract Book distributed under the Attribution-NonCommercial-NoDerivs (CC BY-NC-ND), which allows third parties to download the articles and share them with others as long as they credit the author and the Abstract Book, but they cannot change the content in any way or use them commercially.
**Abstract Book Citations:** Authors, Title, HemaSphere, 2026;10:(S1):pages. The abstract book DOIs can be found on the “Author Information” page.
**Disclaimer:** Articles published in the journal HemaSphere exclusively reflect the opinions of the authors. The authors are responsible for all content in their abstracts including accuracy of the facts, statements, citing resources, etc.

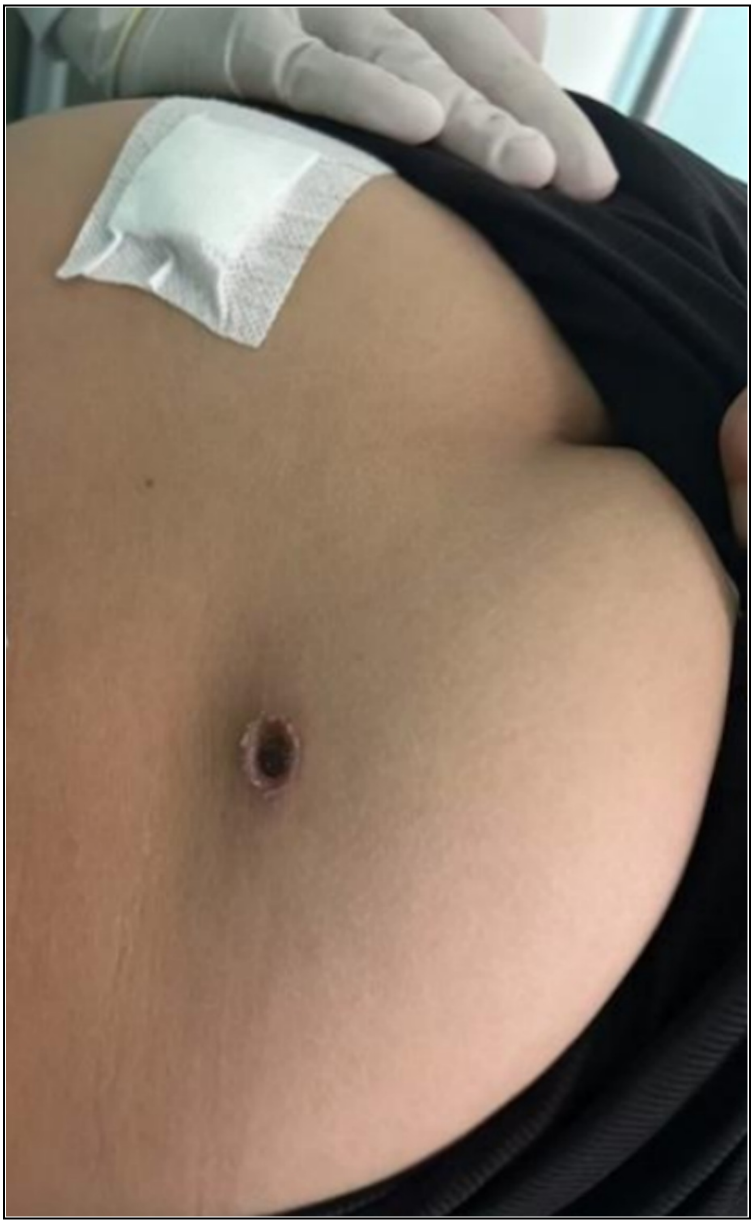

**Copyright Information:** (Online) ISSN: 2572-9241
©2026 The Author(s). HemaSphere is published by John Wiley & Sons Ltd on behalf of European Hematology Association. This is an open access Abstract Book distributed under the Attribution-NonCommercial-NoDerivs (CC BY-NC-ND), which allows third parties to download the articles and share them with others as long as they credit the author and the Abstract Book, but they cannot change the content in any way or use them commercially.
**Abstract Book Citations:** Authors, Title, HemaSphere, 2026;10:(S1):pages. The abstract book DOIs can be found on the "Author Information" page.
**Disclaimer:** Articles published in the journal HemaSphere exclusively reflect the opinions of the authors. The authors are responsible for all content in their abstracts including accuracy of the facts, statements, citing resources, etc.

### (PB4244) THE APPLICATION OF LETERMOVIR FOR POST - HAPLOIDENTICAL TRANSPLANTATION PREVENTION IN CHILDREN WITH ACUTE LEUKEMIA DID NOT ELEVATE THE INCIDENCE OF LATE - ONSET CYTOMEGALOVIRUS INFECTION

**Topic:** 31. Infections in hematology (incl. supportive care/therapy)

Huili Zhu[1], Zhijie Wei*[1]

[1]*Hebei Yanda Lu Daopei Hospital, Langfang, China;*

### Background

Letermovir prophylaxis was initiated on day +1 after haploidentical transplantation in children with acute leukemia. Literature reports that the incidence of CMV infection within 100 days after transplantation was reduced to 26.09%. However, other studies have shown that letermovir prophylaxis may delay CMV-specific immune reconstitution, leading to an increased incidence of late-onset CMV infection from 100 days to 360 days after transplantation, reaching up to 63.00%.

### Aims

This study aimed to analyze the incidence of late-onset CMV infection and non-relapse mortality from 100 days to 360 days after transplantation in children with acute leukemia who received letermovir prophylaxis for 100 days.

### Methods

Thirty children with acute leukemia who underwent haploidentical transplantation at Hebei Yanda Lu Daopei Hospital from October 1, 2022 to October 1, 2025 were included. All patients received myeloablative conditioning regimens, including 21 cases based on busulfan (Bu) and 9 cases based on total body irradiation (TBI). The sources of stem cells were G-CSF-mobilized bone marrow + peripheral blood stem cells in 27 cases, G-CSF-mobilized peripheral blood stem cells in 3 cases, On Day 3, the reinfusion of unrelated cord blood was carried out.The median time to initiate letermovir prophylaxis was +1 day, and the median duration of letermovir prophylaxis was 100 days. The median follow-up time was 20.98 months.

### Results

Among the 30 patients with acute leukemia, the median age at diagnosis was 14.86 years, including 12 cases of acute lymphoblastic leukemia, 16 cases of acute myeloid leukemia, and 2 cases of acute mixed-lineage leukemia. The median number of MNCs reinfused was $18.29\times10^{8}$/kg, and the median number of CD34+ cells was $14.15\times10^{6}$/kg. Letermovir prophylaxis was initiated on day +1 after transplantation, and the median duration of letermovir prophylaxis was 100 days. All patients achieved engraftment of white blood cells, and all achieved engraftment of platelets.The incidence of CMV infection within 100 days after transplantation was 23.30%, the incidence within 1 year was 36.7%, and the incidence from 100 days to 360 days after transplantation was 13.30% (lower than the 63.00% reported in previous literature). The incidence of EBV infection within 100 days after transplantation was 18.10%, and there was no EBV infection from 100 days to 360 days after transplantation. During the follow-up period, one patient died of intracranial fungal infection. Four patients relapsed, including one with hematological relapse + extramedullary relapse (orbit) at +106 days, one with molecular relapse at +127 days after transplantation, one with hematological relapse at +312 days after transplantation, and one with hematological relapse at +80 days after transplantation. The first two patients achieved complete remission after treatment, and the other two are currently under treatment. The estimated 4-year overall survival was 85.70%, and the estimated 4-year disease-free survival (DFS) was 79.54%, with an estimated 4-year non-relapse mortality of 13.70%.

**Copyright Information:** (Online) ISSN: 2572-9241
©2026 The Author(s). HemaSphere is published by John Wiley & Sons Ltd on behalf of European Hematology Association. This is an open access Abstract Book distributed under the Attribution-NonCommercial-NoDerivs (CC BY-NC-ND), which allows third parties to download the articles and share them with others as long as they credit the author and the Abstract Book, but they cannot change the content in any way or use them commercially.
**Abstract Book Citations:** Authors, Title, HemaSphere, 2026;10:(S1):pages. The abstract book DOIs can be found on the "Author Information" page.
**Disclaimer:** Articles published in the journal HemaSphere exclusively reflect the opinions of the authors. The authors are responsible for all content in their abstracts including accuracy of the facts, statements, citing resources, etc.

**Summary/Conclusion**

Letermovir prophylaxis for 100 days initiated on day +1 after haploidentical transplantation in children with acute leukemia ,led to a reduction in the incidence of CMV within 100 days after transplantation. Moreover, it did not increase the incidence of late - onset CMV from 100 days to 360 days after transplantation, nor did it reduce the disease - free survival rate after transplantation.

**Copyright Information:** (Online) ISSN: 2572-9241
©2026 The Author(s). HemaSphere is published by John Wiley & Sons Ltd on behalf of European Hematology Association. This is an open access Abstract Book distributed under the Attribution-NonCommercial-NoDerivs (CC BY-NC-ND), which allows third parties to download the articles and share them with others as long as they credit the author and the Abstract Book, but they cannot change the content in any way or use them commercially.
**Abstract Book Citations:** Authors, Title, HemaSphere, 2026;10:(S1):pages. The abstract book DOIs can be found on the "Author Information" page.
**Disclaimer:** Articles published in the journal HemaSphere exclusively reflect the opinions of the authors. The authors are responsible for all content in their abstracts including accuracy of the facts, statements, citing resources, etc.

### (PB4245) THE VALUE OF PRESEPSIN IN FEBRILE NEUTROPENIA IN NON-HODGKIN LYMPHOMAS

**Topic:** 31. Infections in hematology (incl. supportive care/therapy)

Karla Misura Jakobac*[1], Vibor Milunovic[1], Vesna Kusec[2, 3], Pero Hrabac[3], Marko Martinovic[1, 4], Slobodanka Ostojic Kolonic[1, 3], Delfa Radic-Kristo[1, 3], Gordana Pavlisa[3, 5]

*[1]University hospital Merkur, Zagreb, Croatia;[2]Department of Innovative Diagnostics, Children's Hospital Srebrnjak, Zagreb, Croatia; [3]School of Medicine, University of Zagreb, Zagreb, Croatia;[4]School of Medicine, Catholic University of Croatia, Zagreb, Croatia; [5]Department for Respiratory Diseases Jordanovac, University Hospital Centre Zagreb, Zagreb, Croatia;*

### Background

Febrile neutropenia (FN) is a serious, potentially fatal complication of therapy in patients with non-Hodgkin lymphoma (NHL). Early and accurate differentiation between infectious and noninfectious causes of FN remains challenging despite existing diagnostic methods, highlighting the urgent need for more reliable diagnostic tools.

### Aims

The aim was to evaluate the significance of presepsin (PSP), a biomarker of the innate immune response, as an early marker for differentiating infectious from noninfectious causes of FN and to assess its prognostic role in predicting clinical course and risk of mortality.

### Methods

A prospective clinical study enrolled 52 participants: 34 patients with febrile neutropenia stratified by microbiological/clinical evidence of infection and 18 neutropenic patients as controls. Demographic and clinical characteristics were recorded, and laboratory parameters were measured on day 1 for all participants and repeated on day 3 in the FN cohort; treatment trajectories and clinical outcomes were documented. Comparative analyses evaluated intergroup differences, variable correlations, and associations with survival using appropriate inferential statistics.

### Results

Baseline presepsin concentrations were significantly higher in the group of patients with FN and proven infection compared with FN patients without proven infection ($p = 0.024$). Baseline PSP and procalcitonin (PCT) concentrations were significantly higher in Gram-negative bacterial infections ($p < 0.001$ for PSP, $p = 0.034$ for PCT), suggesting a possible role in predicting bacterial etiology. Elevated baseline PSP concentrations correlated significantly with mortality; each 1 ng/mL increase in PSP concentration was associated with a 4% increase in risk of death ($p < 0.002$). Conventional biochemical inflammatory markers, CRP and PCT, did not demonstrate the same level of sensitivity or specificity for early differentiation of infectious etiologies and monitoring therapeutic response.

### Summary/Conclusion

PSP proved to be a useful diagnostic and prognostic marker in patients with NHL and FN. Elevated baseline PSP concentrations were associated with confirmed infectious events, worse clinical outcomes, and higher mortality, making PSP a negative prognostic factor in this specific cohort of hematologic patients with treatment-related complications.

**Copyright Information:** (Online) ISSN: 2572-9241
©2026 The Author(s). HemaSphere is published by John Wiley & Sons Ltd on behalf of European Hematology Association. This is an open access Abstract Book distributed under the Attribution-NonCommercial-NoDerivs (CC BY-NC-ND), which allows third parties to download the articles and share them with others as long as they credit the author and the Abstract Book, but they cannot change the content in any way or use them commercially.
**Abstract Book Citations:** Authors, Title, HemaSphere, 2026;10:(S1):pages. The abstract book DOIs can be found on the "Author Information" page.
**Disclaimer:** Articles published in the journal HemaSphere exclusively reflect the opinions of the authors. The authors are responsible for all content in their abstracts including accuracy of the facts, statements, citing resources, etc.

### (PB4246) EVALUATION AND FOLLOW-UP RESULTS OF 154 CHILDREN WITH NEUTROPENIA

**Topic:** 31. Infections in hematology (incl. supportive care/therapy)

Aysegul Unuvar*[1], Seyma Nur Ozkul Safi[2], Gulcan Erbas[1], Osman Kuleli[1], Deniz Tugcu[1], Serap Karaman[1]

*[1]Istanbul University, Istanbul School of Medicine, Division of Pediatric Hematology&Oncology, Istanbul, Türkiye;[2]Istanbul University, Istanbul School of Medicine, Department of Pediatrics, Istanbul, Türkiye;*

### Background

Neutropenia, defined as a decrease in the absolute neutrophil count (ANC) below the lower limit of the normal range for the age and ethnic origin. It is a common cause of referral to pediatric hematologists.

### Aims

Our aim was to evaluate the medical history, clinical and laboratory findings, treatment features, and outcomes of children with neutropenia who were referred to our outpatient clinic.

### Methods

Out of 291 children who were referred with neutropenia between May 2016 and June 2021, 154 children who were regularly followed-up for at least 4 years or until recovery, were included in this study.

### Results

The median age at the first diagnosis of neutropenia was 8 months (range: 20 days-194 months), of whom 92 (59.8%) were male. A history of parental consanguinity was identified in 28 children (n=28), and 12 children had a family history of neutropenia. Fifty four percent of children (n=83) had a history of infection at the time of diagnosis, and 43.8% (n=39) of these children were diagnosed with infection-related neutropenia at the follow-up. Among children with regular medication use (n=19), a total of 4 children receiving sodium valproate, levetiracetam, and zidovudin (n=2) were diagnosed with drug-induced neutropenia.The mean ANC was 645.3±374.34/mm$^3$ (range: 0 - 1490 /mm$^3$; mild neutropenia: 8%, moderate: 37%, severe: 55%). Leukopenia was detected in 48% of children, and lymphopenia in 7.1%. Anemia was detected in 23%, and all these children were diagnosed with iron deficiency anemia (The children with anemias other than iron deficiency anemia or thrombocytopenia were not included in this study). Genetic analysis was performed on 60 children who were initially suspected of having congenital neutropenia or who had chronic neutropenia, and the gene mutations were detected in only 8 patients (7 congenital neutropenias;HAX1 (n=3), ELANE (n=2), G6PC3 (n=1), SBDS (n=1), and one with metabolic disease; VPS16 mutation).All children were classified according to The European Guidelines on Diagnosis and Management of Neutropenia in Adults and Children of EHA and the EuNet-INNOCHRON COST Action in 2023. Among the cases; 49.4% were diagnosed with acquired primary or idiopathic neutropenia, 25.3% with infection-related, 4.5% with congenital neutropenias, 4.5% with immunodeficiency-associated, 2.6% with drug-induced neutropenia, 1.3% due to vitamin B12 deficiency, 0.6% with metabolic disease-associated, and 0.6% with neutropenia secondary to autoimmune disease. The etiology of neutropenia could not be determined in 17 cases (11%). Of 154 children, 20 patients received G-CSF therapy, and all of them responded. During the follow-up period; 38% (n=59) required hospitalization, while 1.9% (n=3) in the pediatric ICU. Neutropenia resolved in 85% (n=131), while 15% continued to have neutropenia. The median time to recovery from neutropenia was 9 months for the entire group (range: 1 month-64 months). Infection-related neutropenia resolved at a median of one month, acquired primary or idiopathic neutropenia at 19 months, and nutrition or drug-related neutropenia at 2 months. No significant relationship was found between the duration of neutropenia and history of prematurity, sex, or iron deficiency anemia. No deaths occurred due to neutropenia.

**Copyright Information:** (Online) ISSN: 2572-9241
©2026 The Author(s). HemaSphere is published by John Wiley & Sons Ltd on behalf of European Hematology Association. This is an open access Abstract Book distributed under the Attribution-NonCommercial-NoDerivs (CC BY-NC-ND), which allows third parties to download the articles and share them with others as long as they credit the author and the Abstract Book, but they cannot change the content in any way or use them commercially.
**Abstract Book Citations:** Authors, Title, HemaSphere, 2026;10:(S1):pages. The abstract book DOIs can be found on the "Author Information" page.
**Disclaimer:** Articles published in the journal HemaSphere exclusively reflect the opinions of the authors. The authors are responsible for all content in their abstracts including accuracy of the facts, statements, citing resources, etc.

**Summary/Conclusion**

In conclusion; our findings highlight the etiology, complications, and outcomes of 154 children with neutropenia. The recovery times of neutropenia, determined according to etiology, may provide valuable contribution to the literature.

**Copyright Information:** (Online) ISSN: 2572-9241
©2026 The Author(s). HemaSphere is published by John Wiley & Sons Ltd on behalf of European Hematology Association. This is an open access Abstract Book distributed under the Attribution-NonCommercial-NoDerivs (CC BY-NC-ND), which allows third parties to download the articles and share them with others as long as they credit the author and the Abstract Book, but they cannot change the content in any way or use them commercially.
**Abstract Book Citations:** Authors, Title, HemaSphere, 2026;10:(S1):pages. The abstract book DOIs can be found on the "Author Information" page.
**Disclaimer:** Articles published in the journal HemaSphere exclusively reflect the opinions of the authors. The authors are responsible for all content in their abstracts including accuracy of the facts, statements, citing resources, etc.

### (PB4247) A RANDOMIZED PHASE 3 STUDY: EFFICACY, SAFETY AND PK OF SUBCUTANEOUS IMMUNOGLOBULIN TO PREVENT INFECTIONS IN PATIENTS WITH HEMATOLOGICAL MALIGNANCIES AND HYPOGAMMAGLOBULINEMIA. THE EXCELL STUDY.

**Topic:** 31. Infections in hematology (incl. supportive care/therapy)

Ciprian Tomuleasa*[1], Luke M. Pittman[2], Dominik Chraniuk[3], Dubravka Zupanic Krmek[4], Lavinika Atanaskovic[5], Marina Acosta Enslen[6], Michelle McCrory[7], David Vilardell[8], Sandra Fernandez[8], Elisabeth Calderón-Gómez[8], Enrikas Vainorius[6], Alejhandra Lei[6]

*[1]Iuliu Hatieganu University of Medicine and Pharmacy, Cluj-Napoca, Romania;[2]Walter Reed Medical Center, Bethesda, United States of America; [3]MICS Medical Center Torun, Torun, Poland;[4]Clinical Hospital Sveti Duh, Zagreb, Croatia;[5]Military Medical Academy, Clinic for Hematology, Belgrade, Serbia; [6]Grifols, Scientific Innovation Office, Reseatch Triangle Park, United States of America; [7]Grifols, Scientific Innovation Office, Dublin, Ireland;[8]Grifols, Scientific Innovation Office, Barcelona, Spain;*

### Background

Secondary immunodeficiencies are common in hematological malignancies, including B-cell chronic lymphocytic leukemia (CLL), multiple myeloma (MM) and non-Hodgkin lymphoma (NHL). Disease- and treatment-related hypogammaglobulinemia (HGG) predisposes patients to serious or recurrent infections. Intravenous immunoglobulin replacement therapy reduces serious bacterial infections; however, evidence from large, controlled trials of subcutaneous immunoglobulin (SCIg) in this population remains limited.

### Aims

To evaluate the efficacy, safety, and pharmacokinetics (PK) of SCIg 20% over 1 year to reduce major bacterial infections in adults with B-cell CLL, MM, or NHL with HGG and recurrent or severe infections.

### Methods

EXCELL is a randomized, multicenter, parallel-group, double-blind, placebo-controlled phase 3 clinical trial. Eligible patients are adults with B-cell CLL, MM or NHL; serum IgG <5 g/L; and either ≥1 severe infection (≥ grade 3) or recurrent bacterial/viral infections (≥ 3) within 12 months before screening. Patients are randomized 1:1 to SCIg 20% plus standard medical treatment (SMT) or placebo plus SMT. SCIg 20% includes a loading dose of 150 mg/kg/day on days 1-5, followed by 300 mg/kg every 2 weeks (weeks 3-51), with a 30-day follow-up. SMT comprises active and supportive therapies required for the underlying malignancy (Figure 1). Written informed consent is obtained before any study-specific procedures.

### Results

Enrollment is ongoing at 61 locations across North America and Europe. Endpoints: The primary endpoint is the annualized rate of major bacterial infections. Secondary endpoints include time to
first major bacterial infection; time to first non-major bacterial infection and to first infection of any type; proportion of patients with ≥1 major bacterial infection and with ≥1 bacterial infection; annualized rate of all bacterial infections; antibiotic exposure (days); number and duration of hospitalizations for any infection and major bacterial infection; annualized rate of all infections (serious and non-serious); and validated infections. PK analysis will be performed in a subset of patients. Exploratory endpoints include change from baseline in quality of life (EQ-5D-5L; SF-20) and treatment satisfaction (TSQM). Safety endpoints include adverse events, vital signs, physical examinations, and laboratory assessments.

**Copyright Information:** (Online) ISSN: 2572-9241
©2026 The Author(s). HemaSphere is published by John Wiley & Sons Ltd on behalf of European Hematology Association. This is an open access Abstract Book distributed under the Attribution-NonCommercial-NoDerivs (CC BY-NC-ND), which allows third parties to download the articles and share them with others as long as they credit the author and the Abstract Book, but they cannot change the content in any way or use them commercially.
**Abstract Book Citations:** Authors, Title, HemaSphere, 2026;10:(S1):pages. The abstract book DOIs can be found on the "Author Information" page.
**Disclaimer:** Articles published in the journal HemaSphere exclusively reflect the opinions of the authors. The authors are responsible for all content in their abstracts including accuracy of the facts, statements, citing resources, etc.

**Summary/Conclusion**

EXCELL should provide pivotal evidence on the efficacy, safety and PK of biweekly SCIg 20% to reduce major bacterial infections in adults with B-cell CLL, MM, or NHL and HGG (NCT05645107;
EudraCT 2022-502193-16-00).

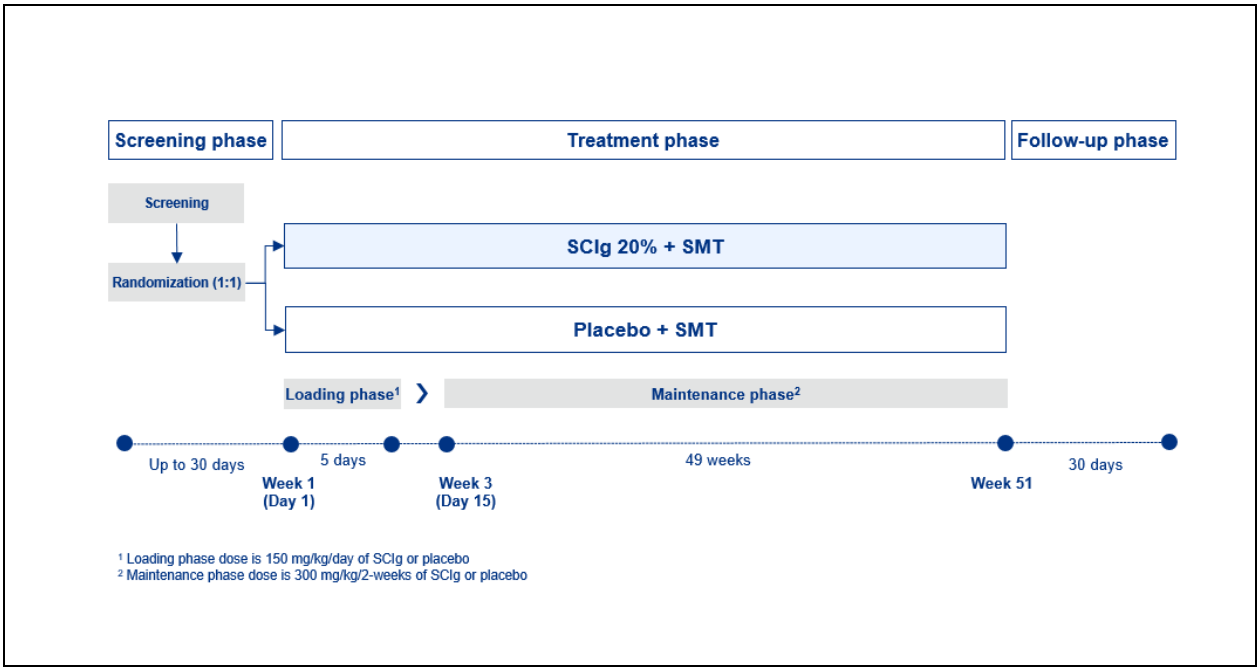

Figure 1. EXCELL study schema. Abbreviations: SCIg, subcutaneous immunoglobulin; SMT, standard medical treatment.

**Copyright Information:** (Online) ISSN: 2572-9241
©2026 The Author(s). HemaSphere is published by John Wiley & Sons Ltd on behalf of European Hematology Association. This is an open access Abstract Book distributed under the Attribution-NonCommercial-NoDerivs (CC BY-NC-ND), which allows third parties to download the articles and share them with others as long as they credit the author and the Abstract Book, but they cannot change the content in any way or use them commercially.
**Abstract Book Citations:** Authors, Title, HemaSphere, 2026;10:(S1):pages. The abstract book DOIs can be found on the "Author Information" page.
**Disclaimer:** Articles published in the journal HemaSphere exclusively reflect the opinions of the authors. The authors are responsible for all content in their abstracts including accuracy of the facts, statements, citing resources, etc.

### (PB4248) REAL-WORLD SAFETY OUTCOMES OF BTK INHIBITORS IN CHRONIC LYMPHOCYTIC LEUKEMIA: TEN-YEAR EXPERIENCE FROM A TERTIARY REFERRAL CENTER

**Topic:** 31. Infections in hematology (incl. supportive care/therapy)

Santiago Lizarazo[1], Manuela Estrada-Schweineberg[2], Dana Taub[1], Juliana Castro*[1], Angel Maestre[2], Laura Ortiz[2], Juan Jaimes[1], Felix Duarte[1], Maria Uchima-Vera[2], Paula Perez[3], Andrea Pantoja[3], Laura Roa[3], Laura Guevera[1], Carmenza Camargo[2], Karen Milena Chaves Garcia[2], Claudia Agudelo-López[1, 2], Guillermo Enrique Quintero Vega[1, 2], Beatriz Wills[1, 2]

*[1]Universidad de los Andes, Facultad de Medicina, Bogota, Colombia;[2]Fundación Santa Fe de Bogotá, Colombia, Colombia;* *[3]Universidad del Bosque, Bogotá, Colombia;*

### Background

Bruton's tyrosine kinase inhibitors (BTKi) have transformed chronic lymphocytic leukemia (CLL) management. However, immune modulation and off-target effects may increase susceptibility to infections, cardiovascular events, bleeding and secondary malignancies. While safety data from clinical trials are well established, real-world evidence, particularly from Latin American cohorts, remains scarce.

### Aims

To describe the incidence, spectrum, timing, and clinical impact of major adverse events in patients with CLL treated with BTKi in routine clinical practice.

### Methods

We conducted a retrospective cohort study including consecutive adults with CLL who initiated BTKi therapy between 2015 and 2025 at a quaternary referral center in Bogotá, Colombia. Clinically significant adverse events included severe infections, cardiovascular events, major bleeding, and secondary malignancies. Time-to-event outcomes were estimated using Kaplan–Meier methods. Analyses were descriptive.

### Results

Thirty patients were included (median age 62.5 years; 50% male). All had adequate ECOG performance status. Baseline infection risk factors included hypogammaglobulinemia (20%), prior malignancy (16.7%), and age >70 years (13.3%). Ibrutinib was administered in 93.3% of patients and acalabrutinib in 6.7%; 56.7% received BTKi as first-line therapy.
During follow-up, 60% of patients experienced at least one adverse event. Infections were the most frequent toxicity, occurring in 43.3%. The respiratory tract was the predominant site (75%). Antimicrobial prophylaxis was administered in the majority of patients, and 13.3% required immunoglobulin replacement during therapy. Cumulative incidence of infection was 11.1% at 3 months and 23.8% at 6 and 12 months. The first infection occurred 14 days after initiation, with most events developing within the first 6 months. The infection rate was 2.38 episodes per 100 patient-months, and median infection-free survival was not reached.
Among patients with infections, 37.5% required hospitalization and 18.8% intensive care admission. Infection-related mortality was 6.7%. Infectious complications led to permanent BTKi discontinuation in 13.3% and temporary interruption in 10%. No baseline clinical factors were significantly associated with infection risk in exploratory analyses.
Cardiovascular events occurred in 6.7% of patients, minor bleeding in 3.3%, and no major hemorrhagic events were observed. Secondary malignancies developed in 10% of patients, predominantly hematologic.

**Copyright Information:** (Online) ISSN: 2572-9241
©2026 The Author(s). HemaSphere is published by John Wiley & Sons Ltd on behalf of European Hematology Association. This is an open access Abstract Book distributed under the Attribution-NonCommercial-NoDerivs (CC BY-NC-ND), which allows third parties to download the articles and share them with others as long as they credit the author and the Abstract Book, but they cannot change the content in any way or use them commercially.
**Abstract Book Citations:** Authors, Title, HemaSphere, 2026;10:(S1):pages. The abstract book DOIs can be found on the "Author Information" page.
**Disclaimer:** Articles published in the journal HemaSphere exclusively reflect the opinions of the authors. The authors are responsible for all content in their abstracts including accuracy of the facts, statements, citing resources, etc.

**Summary/Conclusion**

In this real-world Latin American cohort, adverse events during BTKi therapy were frequent and clinically meaningful. Infections were the predominant toxicity, occurring early and resulting in substantial rates of hospitalization, intensive care admission, and treatment modification. Cardiovascular and malignant complications were less common but clinically relevant. These findings underscore the burden of infectious complications during BTKi therapy and highlight the need for close monitoring, particularly during the initial months of treatment.

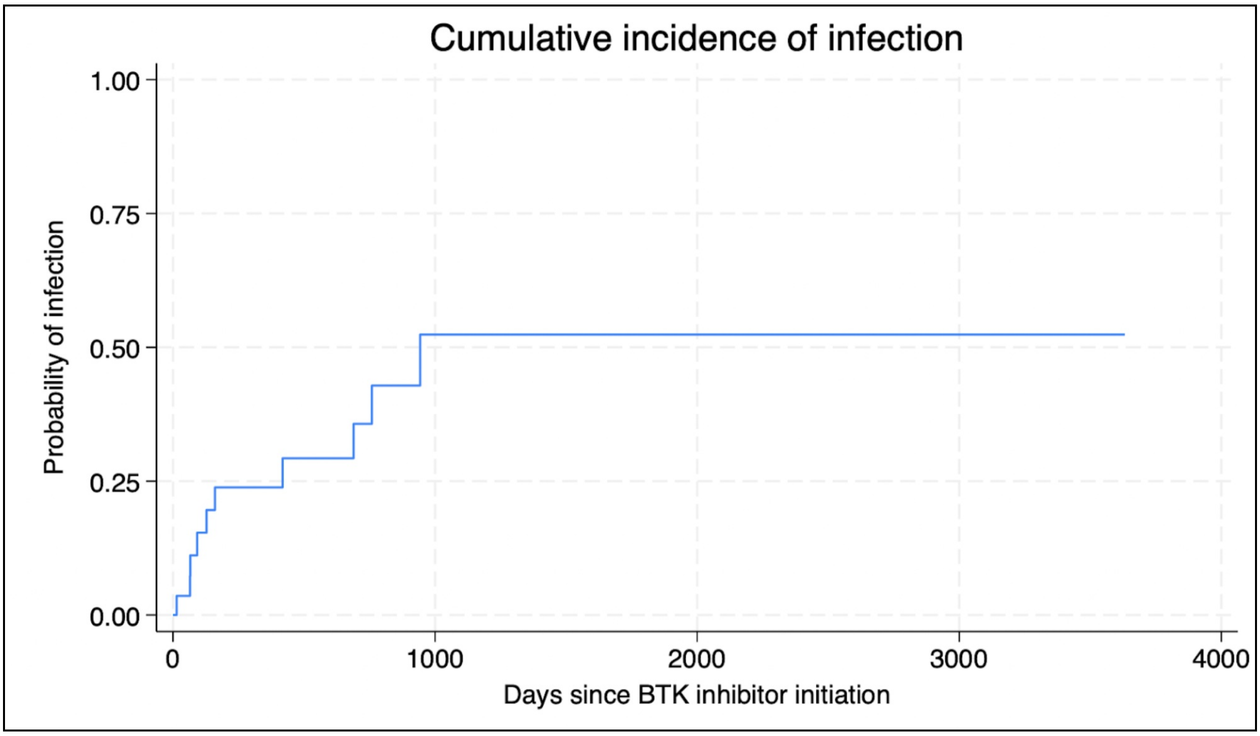

Kaplan–Meier estimate of time to first infection during BTKi therapy.

**Copyright Information:** (Online) ISSN: 2572-9241
©2026 The Author(s). HemaSphere is published by John Wiley & Sons Ltd on behalf of European Hematology Association. This is an open access Abstract Book distributed under the Attribution-NonCommercial-NoDerivs (CC BY-NC-ND), which allows third parties to download the articles and share them with others as long as they credit the author and the Abstract Book, but they cannot change the content in any way or use them commercially.
**Abstract Book Citations:** Authors, Title, HemaSphere, 2026;10:(S1):pages. The abstract book DOIs can be found on the "Author Information" page.
**Disclaimer:** Articles published in the journal HemaSphere exclusively reflect the opinions of the authors. The authors are responsible for all content in their abstracts including accuracy of the facts, statements, citing resources, etc.

### (PB4249) INFECTION-RELATED TREATMENT INTERRUPTIONS AND IMPACT ON SURVIVAL OUTCOMES IN HEMATOLOGIC MALIGNANCIES

**Topic:** 31. Infections in hematology (incl. supportive care/therapy)

Batyrkhan Kuatov*[1], Yi-Chun Lin[2], Lei Ni[2], Louise McCrink[2], Sorrel Wolowacz[2], Melike Inkaya Taskin[3]

*[1]CSL, Bern, Switzerland; [2]RTI Health Solutions, Manchester, United Kingdom; [3]CSL, Istanbul, Türkiye;*

### Background

Patients with hematologic malignancies (HM) and secondary immunodeficiency (SID) are at high risk of serious and recurrent infections, which are a substantial cause of morbidity and mortality across HM types. Further, infections often cause cancer treatment interruptions and delays, which can compromise therapeutic efficacy and therefore impact survival, health-related quality of life (HRQoL), and healthcare resource utilization. Though infection-related complications are clinically meaningful in this population, many are potentially preventable through optimized supportive care.

### Aims

This targeted literature review (TLR) aimed to identify and summarize available evidence on infection-related cancer treatment interruptions and their impact on disease progression, survival outcomes, HRQoL, and costs in patients with HM and SID.

### Methods

Articles published between 2016 and 2025 were identified using electronic medical literature databases (MEDLINE, Embase, Cochrane Library), desktop internet searches (Google Scholar, PubMed), and by reviewing citations from reference lists of key articles (snowball approach). Search terms included combinations of free text, Medical Subject Headings or Emtree terms.
Literature was screened across multiple myeloma (MM), chronic lymphocytic leukemia (CLL), non-Hodgkin lymphoma (NHL), and broader HMs.
Results were screened for relevance with priority given to high-quality sources (eg, systematic reviews, large observational studies).
Key top-level data extracted included parameter by indication, population (eg, SID/hypogammaglobulinemia; HM subtype), type of infection, intervention/drug class, study type, and country.

### Results

Overall, 1,093 potentially relevant studies were identified (databases n=1,033; internet n=40; reference lists n=20). SID status was reported in few studies (n=7), therefore broader studies where an SID status may be inferred were included for a total of 57 studies for data extraction. Notably, data from SID-specific studies were limited to percentages of infection-related treatment interruptions and infection-attributable deaths.
Of the 57 total studies, 14 were in broad HMs, 13 in MM, 12 in CLL, and 12 in NHL. In total, 33 studies reported data on probability of, or reason for, treatment interruption, with 29 reporting interruptions due to infection. Seventeen studies reported hazard ratios or survival curves following treatment interruption, but most (n=15) did not report infection-specific data. Limited data on duration of treatment delay or impact on subsequent treatment were found for HMs, and no data specific to MM, CLL, or NHL was available. Infection-attributable death was reported in 22 studies, which included both broad HMs and specific subtypes. No infection-specific data on cancer treatment modification following interruption were found.
Data on the economic and HRQoL impacts of treatment interruptions were limited: 4 studies reported on broad interruption-related costs and 2 reported on utilities during interruption and recovery.

**Copyright Information:** (Online) ISSN: 2572-9241
©2026 The Author(s). HemaSphere is published by John Wiley & Sons Ltd on behalf of European Hematology Association. This is an open access Abstract Book distributed under the Attribution-NonCommercial-NoDerivs (CC BY-NC-ND), which allows third parties to download the articles and share them with others as long as they credit the author and the Abstract Book, but they cannot change the content in any way or use them commercially.
**Abstract Book Citations:** Authors, Title, HemaSphere, 2026;10:(S1):pages. The abstract book DOIs can be found on the “Author Information” page.
**Disclaimer:** Articles published in the journal HemaSphere exclusively reflect the opinions of the authors. The authors are responsible for all content in their abstracts including accuracy of the facts, statements, citing resources, etc.

### Summary/Conclusion

Existing studies reveal emerging evidence pointing to infection-related treatment interruptions in patients with HM, but data are limited, warranting further research. Investigations into infection-associated treatment interruptions in patients with HM and confirmed SID are especially critical, as this growing population is characterized by a markedly increased susceptibility to infections.

**Heat map of number of studies identified overall, with infection-related treatment interruption data, and with SID patients**

| Category | Outcome | MM | CLL | NHL | Other HM* | Other cancer |
|---|---|---|---|---|---|---|
| Treatment interruption metrics | Probability of treatment interruption | n=6 total (Infection data n=5) | n=6 total (Infection data n=5; (SID-specific n=2) | n=6 total (Infection data n=4) | n=5 total (Infection data n=5) | n=3 total (Infection data n=3; SID-specific n=1) |
| Treatment interruption metrics | Reasons for treatment interruption | n=3 total (Infection data n=2) | n=5 total (Infection data n=4) | | n=1 total (Infection data n=1) | n=1 total (Infection data n=1) |
| Treatment interruption metrics | Duration of delay | | | | n=1 total (Infection data n=1) | n=2 total (Infection data n=2) |
| Treatment interruption metrics | Dose reduction | | n=1 total | | | |
| Treatment interruption metrics | Relative dose intensity | n=2 total | n=1 total | n=2 total | | |
| Treatment interruption metrics | Missed treatment cycles | | | | | |
| Treatment interruption metrics | Time to next treatment | n=2 total | n=1 total | | | |
| Treatment interruption metrics | Impact on subsequent treatment | | n=1 total | | n=2 total (Infection data n=1) | n=2 total (Infection data n=2) |
| Impact of interruption on: Disease progression and survival | Progression-free survival/overall survival/hazard ratio/curves/other | n=2 total | n=3 total | n=6 total (Infection data n=1) | n=5 total (Infection data n=1) | n=1 total |
| Impact of interruption on: Disease progression and survival | Infection-attributable death | n=5 total (Infection data n=5; SID-specific n=1) | n=5 total (Infection data n=5; SID-specific n=2) | n=4 total (Infection data n=4; SID-specific n=1) | n=6 total (Infection data n=6; SID-specific n=1) | n=2 total (Infection data n=2; SID-specific n=1) |
| Impact of interruption on: Costs and health-related quality of life | Cost | | | | n=3 total | n=1 total |
| Impact of interruption on: Costs and health-related quality of life | Utility | | n=2 total | | | |

### of studies with infection data: 5+, 4, 3, 2, 1, 0

*Included patients with other HMs or HM not specific to a single indication.

CLL, chronic lymphocytic leukemia; HM, hematologic malignancy; MM, multiple myeloma; NHL, non-Hodgkin lymphoma; SID, secondary immunodeficiency

**Copyright Information:** (Online) ISSN: 2572-9241
©2026 The Author(s). HemaSphere is published by John Wiley & Sons Ltd on behalf of European Hematology Association. This is an open access Abstract Book distributed under the Attribution-NonCommercial-NoDerivs (CC BY-NC-ND), which allows third parties to download the articles and share them with others as long as they credit the author and the Abstract Book, but they cannot change the content in any way or use them commercially.
**Abstract Book Citations:** Authors, Title, HemaSphere, 2026;10:(S1):pages. The abstract book DOIs can be found on the "Author Information" page.
**Disclaimer:** Articles published in the journal HemaSphere exclusively reflect the opinions of the authors. The authors are responsible for all content in their abstracts including accuracy of the facts, statements, citing resources, etc.

### (PB4250) PROBIOTIC CULTURES OF MICROORGANISMS – AS A COMPONENT OF THE PREVENTION OF INFECTIOUS AND INFLAMMATORY COMPLICATIONS IN PATIENTS WITH LEUKEMIA

**Topic:** 31. Infections in hematology (incl. supportive care/therapy)

Alla Rybalska*[1], Nadiya Goryainova[1], Elena Melnik[1], Nadia Skachkova[1], Tetiana Kalynychenko[1], Svetlana Serhutina[1]

[1]*National Scientific Center for Radiation Medicine, Hematology and Oncology, Kyiv, Ukraine;*

### Background

The human microbiota is extremely sensitive to the negative effects of various factors, in particular, to the effects of chemotherapeutic drugs used in the treatment of leukemia patients. The consequences of long courses of chemotherapy treatment are the risk of developing infectious-inflammatory complications and secondary immunosuppression. Probiotics can be one of the means of correcting these pathological conditions.

### Aims

Prevention of the development of infectious and inflammatory complications in patients with oncohematological pathology by normalizing the composition of the microbiota of biotopes.

### Methods

Microbiological. immunological, ELISA.

### Results

In the dynamics of probiotic therapy, the quantitative and species composition of the microflora of the upper respiratory tract (URT) biotopes of 32 patients with leukemia was determined, including: 15 patients with CLL, 7 - CML, 9 - AML, and 1 - ALL. The age of the patients ranged from 40 to 87 years. A two-component probiotic with the composition of Lactobacillus acidophilus (LA-5), Bifidobacterium Lactis (BB-12) - $2x10^9$ CFU was used by 16 patients. A multicomponent probiotic preparation containing Lactobacillus plantarum CETC7484, Lactobacillus plantarum CETC7485, Pediococcus acidilactici CETC748 - $3x10^9$ CFU was consumed by 16 people. Probiotics were prescribed according to the following scheme: 1 capsule per day for 4 weeks. Informed consent was obtained from all patients to participate in the study. The exclusion criteria for patients from the study were: neutropenia, presence of infectious and inflammatory complications, and relapse phase of the disease. Microbiological and immunological studies were conducted before and after the end of taking the drugs. According to the results of the study, 320 strains of bacterial cultures, yeast-like and mycelial fungi were isolated and identified from the URT of leukemia patients. A positive effect of probiotic cultures on the microbiocenosis of the URT of patients with leukemia was established, which was manifested by the elimination of probable factors of infectious and inflammatory complications in 58.0% of people - when using a bicomponent preparation and in 50.0% of patients - when using a multicomponent preparation. The positive effect was also confirmed by a decrease in the titers of potential pathogens to the limits of physiological values in 56.1% and 40.0% of patients, respectively. Under the influence of probiotics, the ability of circulating staphylococci to produce certain pathogenic enzymes was lost: fibrinolysin, plasmacoagulase (9.6-19.2% of strains). It was found that the use of a bicomponent probiotic contributed to an increase in the index of the final phase of phagocytosis of neutrophil granulocytes in the peripheral blood of patients (76.9±2.3% - before taking the probiotic, 86.0±2.3% - after taking the probiotic, $p < 0.05$). The levels of C-reactive protein (10.14±2.32 mg/l) and interleukin-6 (0 pg/ml) in the blood serum of patients after taking probiotic cultures indicated the absence of an acute phase of the inflammatory reaction in the body.

**Copyright Information:** (Online) ISSN: 2572-9241
©2026 The Author(s). HemaSphere is published by John Wiley & Sons Ltd on behalf of European Hematology Association. This is an open access Abstract Book distributed under the Attribution-NonCommercial-NoDerivs (CC BY-NC-ND), which allows third parties to download the articles and share them with others as long as they credit the author and the Abstract Book, but they cannot change the content in any way or use them commercially.
**Abstract Book Citations:** Authors, Title, HemaSphere, 2026;10:(S1):pages. The abstract book DOIs can be found on the “Author Information” page.
**Disclaimer:** Articles published in the journal HemaSphere exclusively reflect the opinions of the authors. The authors are responsible for all content in their abstracts including accuracy of the facts, statements, citing resources, etc.

**Summary/Conclusion**

The antagonistic nature of the relationship between the studied representatives of opportunistic microorganisms and probiotic strains contributes to the restoration of the composition of the microbiota of patients, the elimination of opportunistic pathogens and the reduction of the risk of developing infectious and inflammatory complications.

**Copyright Information:** (Online) ISSN: 2572-9241
©2026 The Author(s). HemaSphere is published by John Wiley & Sons Ltd on behalf of European Hematology Association. This is an open access Abstract Book distributed under the Attribution-NonCommercial-NoDerivs (CC BY-NC-ND), which allows third parties to download the articles and share them with others as long as they credit the author and the Abstract Book, but they cannot change the content in any way or use them commercially.
**Abstract Book Citations:** Authors, Title, HemaSphere, 2026;10:(S1):pages. The abstract book DOIs can be found on the "Author Information" page.
**Disclaimer:** Articles published in the journal HemaSphere exclusively reflect the opinions of the authors. The authors are responsible for all content in their abstracts including accuracy of the facts, statements, citing resources, etc.

### (PB4251) RISK FACTORS OF REFRACTORY CLINICALLY SIGNIFICANT CMV INFECTION IN HEMATOPOIETIC STEM CELL TRANSPLANTATION PATIENTS

**Topic:** 31. Infections in hematology (incl. supportive care/therapy)

Jen Pei Huang*[1], Hsueng Mei Liu[2], Chiu-Mei Yeh[3], Chun-Kuang Tsai[3], Chia Jen Liu[2, 3]

*[1]Taipei Veterans General Hospital, Department of Oncology, Taipei, Taiwan;[2]Taipei Veterans General Hospital, Department of Medicine, Division of Transfusion Medicine, Taipei, Taiwan; [3]Taipei Veterans General Hospital, Department of Medicine, Division of Hematology, Taipei, Taiwan;*

### Background

Clinically significant cytomegalovirus infection (CS-CMVi) is a significant challenge in patients undergoing allogeneic hematopoietic stem cell transplantation (HSCT). Although evidence supports prophylactic strategies, limited studies have addressed risk factors for refractory CS-CMVi.

### Aims

We aimed to identify the risk factors for refractory CMV infection and to clarify its association with early mortality in this population.

### Methods

We retrospectively analyzed patients with hematologic diseases who underwent allogeneic HSCT at Taipei Veterans General Hospital, Taiwan, between January 1, 2008, and August 31, 2023. Refractory CS-CMVi was defined as persistent CMV viremia or a higher peak viral load after 14 days of treatment. The primary endpoint was the occurrence of refractory CS-CMVi within 1 year after HSCT.

### Results

A total of 628 patients who underwent allogeneic HSCT were enrolled. Twenty patients (3.18%) developed refractory CS-CMVi. The one-year cumulative incidence of refractory CS-CMVi was 3.8%. In multivariate analysis, antithymocyte globulin (ATG) use (95% CI 1.02–9.11) and engraftment failure (95% CI 1.31–8.96) were significant risk factors for refractory CS-CMVi. Refractory CS-CMVi was also identified as an independent one-year mortality risk factor after allogeneic HSCT (95% CI 1.41–12.48).

### Summary/Conclusion

ATG use and engraftment failure were risk factors for refractory CS-CMVi in allogeneic HSCT, and refractory CS-CMVi was an independent predictor for mortality within one year after transplantation.

**Copyright Information:** (Online) ISSN: 2572-9241
©2026 The Author(s). HemaSphere is published by John Wiley & Sons Ltd on behalf of European Hematology Association. This is an open access Abstract Book distributed under the Attribution-NonCommercial-NoDerivs (CC BY-NC-ND), which allows third parties to download the articles and share them with others as long as they credit the author and the Abstract Book, but they cannot change the content in any way or use them commercially.
**Abstract Book Citations:** Authors, Title, HemaSphere, 2026;10:(S1):pages. The abstract book DOIs can be found on the "Author Information" page.
**Disclaimer:** Articles published in the journal HemaSphere exclusively reflect the opinions of the authors. The authors are responsible for all content in their abstracts including accuracy of the facts, statements, citing resources, etc.

### (PB4252) PRE-TRANSPLANT COLONIZATION WITH CARBAPENEMASE-PRODUCING ENTEROBACTERIACEAE AND CLINICAL OUTCOMES IN HSCT: A REAL-WORLD COHORT STUDY

**Topic:** 31. Infections in hematology (incl. supportive care/therapy)

Andrea Pantoja*[1], Paula Perez[1], Juliana Pardo[2], Manuela Estrada-Schweineberg[2], Maria Uchima-Vera[2], Felix Duarte[3], Elena Velasquez[3], Laura Roa[1], Carmenza Camargo[2], Karen Milena Chaves Garcia[2], Beatriz Wills[2, 3], Claudia Agudelo-López[2, 3], Guillermo Enrique Quintero Vega[2, 3]

*[1]Universidad del Bosque, Bogotá, Colombia;[2]Fundación Santa Fe de Bogotá, Colombia, Colombia;[3]Universidad de los Andes, Facultad de Medicina, Bogota, Colombia;*

### Background

Carbapenemase-producing Enterobacteriaceae (CPE) represent a major global public health threat due to limited therapeutic options and high mortality. Their clinical impact in hematopoietic stem cell transplantation (HSCT) recipients, particularly in Latin American real-world settings, remains insufficiently characterized.

### Aims

To evaluate whether pre-transplant CPE colonization is associated with increased hospitalization burden and reduced survival among adult HSCT recipients.

### Methods

We conducted a retrospective cohort study of adult HSCT recipients with pre-transplant CPE rectal screening between 2022 and 2025 at a Latin American quaternary transplant center. Patients were classified as CPE-colonized or non-colonized. Primary outcomes were length of hospital stay (LOS) and overall survival. Categorical variables were compared using chi-square or Fisher's exact tests, and continuous variables using the Mann–Whitney U test. The association between colonization and bacteremia was assessed using Fisher's exact test due to the small number of events. To evaluate the independent association between colonization and LOS, a median (quantile) regression model was performed adjusting for transplant type. Logistic regression was used to estimate odds ratios (OR) and 95% confidence intervals (CI) for the association between colonization and mortality.

### Results

Sixty-three patients were included. The median age was 53 years (IQR 41-64), and 54% were female. The most frequent indication for HSCT was multiple myeloma (41.2%), followed by acute lymphoblastic leukemia and mantle cell lymphoma (9.5% each). Autologous transplantation was predominant (76.1%). Median follow-up was 12.7 months (95% CI 7.9–15.6)
Pre-transplant CPE colonization was identified in 9 patients (14.3%). Baseline characteristics did not significantly differ between colonized and non-colonized patients. Neutropenia occurred in 84.1% of patients, mucositis in 55.6%, and bacteremia in 9.5%. Overall median LOS was 25 days (IQR 21–31).
CPE colonization was not associated with documented CPE bacteremia during hospitalization (11.1% vs 9.4%; p=1.00). In median regression analysis adjusted for transplant type, colonization was independently associated with an 8-day increase in median hospital stay (95% CI 0.47–15.53; p=0.038). CPE positivity was associated with increased odds of mortality (OR 0.28, 95% CI 0.06–1.25; p=0.095), although this did not reach statistical significance.

**Copyright Information:** (Online) ISSN: 2572-9241
©2026 The Author(s). HemaSphere is published by John Wiley & Sons Ltd on behalf of European Hematology Association. This is an open access Abstract Book distributed under the Attribution-NonCommercial-NoDerivs (CC BY-NC-ND), which allows third parties to download the articles and share them with others as long as they credit the author and the Abstract Book, but they cannot change the content in any way or use them commercially.
**Abstract Book Citations:** Authors, Title, HemaSphere, 2026;10:(S1):pages. The abstract book DOIs can be found on the "Author Information" page.
**Disclaimer:** Articles published in the journal HemaSphere exclusively reflect the opinions of the authors. The authors are responsible for all content in their abstracts including accuracy of the facts, statements, citing resources, etc.

**Summary/Conclusion**

In this real-world cohort, baseline CPE colonization was not associated with documented CPE bacteremia during transplantation hospitalization. However, after adjustment for transplant type, colonized patients experienced a significantly prolonged hospital stay, with an 8-day increase in median length of stay. Although the association with mortality did not reach statistical significance, the observed effect size suggests potential clinical relevance. These findings indicate that CPE carriage may function less as a predictor of infection and more as a marker of baseline vulnerability, prior healthcare exposure, or antibiotic pressure. Larger cohorts are needed to clarify its prognostic implications in transplant populations.

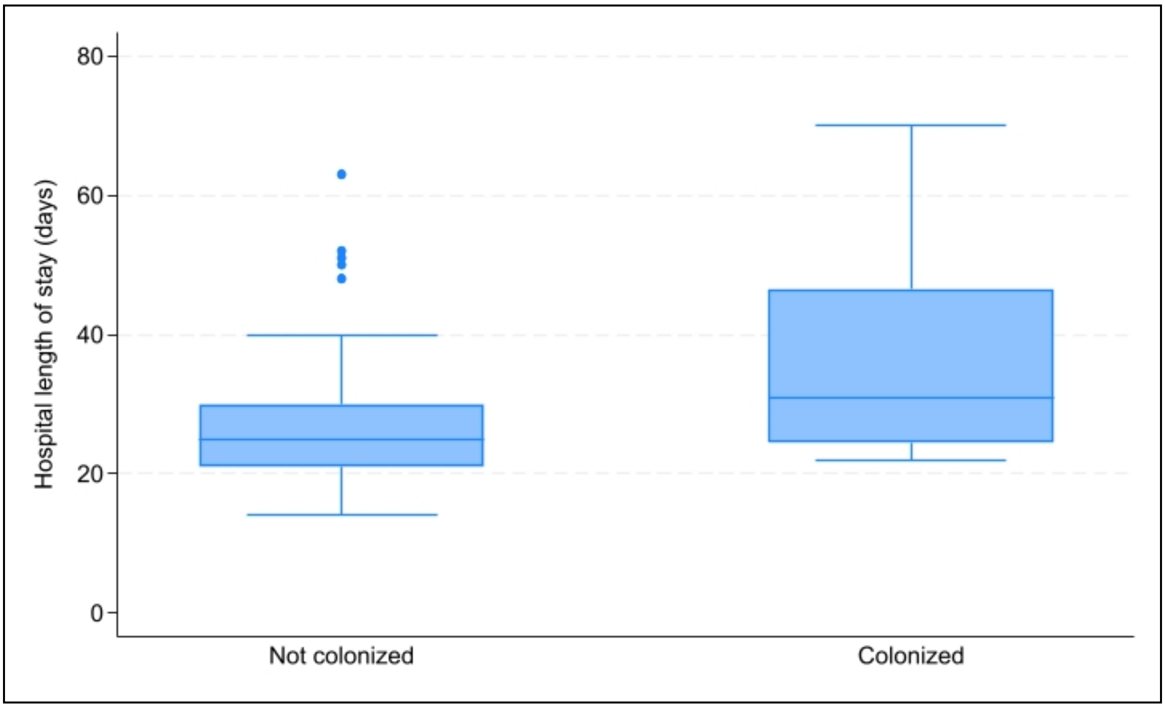

**Copyright Information:** (Online) ISSN: 2572-9241
©2026 The Author(s). HemaSphere is published by John Wiley & Sons Ltd on behalf of European Hematology Association. This is an open access Abstract Book distributed under the Attribution-NonCommercial-NoDerivs (CC BY-NC-ND), which allows third parties to download the articles and share them with others as long as they credit the author and the Abstract Book, but they cannot change the content in any way or use them commercially.
**Abstract Book Citations:** Authors, Title, HemaSphere, 2026;10:(S1):pages. The abstract book DOIs can be found on the "Author Information" page.
**Disclaimer:** Articles published in the journal HemaSphere exclusively reflect the opinions of the authors. The authors are responsible for all content in their abstracts including accuracy of the facts, statements, citing resources, etc.

### (PB4253) MULTIDRUG-RESISTANT ACINETOBACTER SPP. BLOODSTREAM INFECTIONS IN HEMATOLOGIC PATIENTS: CLINICAL CHARACTERISTICS, GENOMIC FEATURES, AND ERAVACYCLINE-BASED COMBINATION THERAPY

**Topic:** 31. Infections in hematology (incl. supportive care/therapy)

Wenjing Guo*[1], Jia Li[1], Qingsong Lin[1], Sizhou Feng[1]

*[1]State Key Laboratory of Experimental Hematology, National Clinical Research Center for Blood Diseases, Haihe Laboratory of Cell Ecosystem, Institute of Hematology & Blood Diseases Hospital, Chinese Academy of Medical Sciences & Peking Union Medical College, Tianjin, China;*

### Background

Multidrug-resistant (MDR) *Acinetobacter* spp. bloodstream infection (BSI) is concerning in hematologic patients, but integrated clinical and genomic analyses are scarce.

### Methods

We retrospectively analyzed 62 hematologic patients with *Acinetobacter* spp. BSI over 13 years, including 30 MDR and 32 non-MDR cases. Whole-genome sequence (WGS) was conducted on 23 MDR isolates. Checkerboard assays evaluated the *in vitro* activity of eravacycline combined with sulbactam, meropenem, cefoperazone-sulbactam, polymyxin B, and levofloxacin.

### Results

Female (66.67% vs. 34.38%, *P*=0.022) and adult (≥14 years; 80.00% vs. 53.12%, *P*=0.049) patients were more common in the MDR group. Prior carbapenem exposure, prolonged neutropenia, and respiratory tract colonization were associated with MDR-BSI. The majority of MDR cases did not receive appropriate empirical therapy within 24 hours (40.00% vs. 90.62%, *P*<0.001). The overall 30-day mortality was 38.71%, significantly higher in MDR than non-MDR patients (53.33% vs. 25.00%, *P*=0.043). Among MDR subgroup, 30-day non-survivors had more profound cytopenias and higher inflammatory markers at BSI onset (*P*<0.05). WGS revealed diverse sequence types and capsular loci across the isolates, including 14 non-*A.baumannii* and 9 *A.baumannii* strains. Among 20 carbapenemase-positive isolates, $bla_{\text{OXA-23}}$ (n=9) predominated in *A.baumannii*, while $bla_{\text{OXA-58}}$ (n=7) and $bla_{\text{NDM-1}}$ (n=7) were restricted to non-*A.baumannii* isolates. Eravacycline demonstrated potent *in vitro* activity ($MIC_{50/90}$=0.125/0.5 mg/L) and showed synergy with sulbactam (52.17%), meropenem (43.48%), and cefoperazone-sulbactam (39.13%), while combinations with polymyxin B (86.96%) and levofloxacin (69.57%) were primarily additive.

### Summary/Conclusion

Eravacycline exhibited strong intrinsic activity and favorable synergy with sulbactam, supporting its potential role in treating MDR *Acinetobacter* spp. infections in this high-risk population.

**Copyright Information:** (Online) ISSN: 2572-9241
©2026 The Author(s). HemaSphere is published by John Wiley & Sons Ltd on behalf of European Hematology Association. This is an open access Abstract Book distributed under the Attribution-NonCommercial-NoDerivs (CC BY-NC-ND), which allows third parties to download the articles and share them with others as long as they credit the author and the Abstract Book, but they cannot change the content in any way or use them commercially.
**Abstract Book Citations:** Authors, Title, HemaSphere, 2026;10:(S1):pages. The abstract book DOIs can be found on the "Author Information" page.
**Disclaimer:** Articles published in the journal HemaSphere exclusively reflect the opinions of the authors. The authors are responsible for all content in their abstracts including accuracy of the facts, statements, citing resources, etc.

### (PB4254) IMPACT OF CARBAPENEM-RESISTANT ENTEROBACTERALES COLONIZATION IN HEMATOLOGICAL PATIENTS: A SINGLE-CENTER EXPERIENCE

**Topic:** 31. Infections in hematology (incl. supportive care/therapy)

Daniela Clerici*[1], Flavia Badalucco Ciotta[2], Maria Helena Parrinello[1], Raffaele Dell' Acqua[2], Vincenzo Spagnuolo[2], Enzo Scifo[3], Francesca Farina[1], Marco Ripa[2], Raffaella Greco[1], Consuelo Corti[1], Silvia Carletti[3], Fabio Ciceri[1], Antonella Castagna[2]

*[1]IRCCS San Raffaele Scientific Institute, Hematology and Bone Marrow Transplant Unit, Milan, Italy; [2]IRCCS San Raffaele Scientific Institute, Department of Infectious Diseases, Milan, Italy; [3]IRCCS San Raffaele Scientific Institute, Laboratory of Microbiology and Virology, Milan, Italy;*

### Background

Antimicrobial resistance represents an open issue in the contest of public health. In hematological setting, infections caused by multi drug resistant (MDR) pathogen still constitute a significant cause of mortality despite the development of novel antimicrobial drug in recent years. Antimicrobial surveillance serves as a useful tool to guide antimicrobial therapy in patients with a high infectious risk.

### Aims

We aimed to evaluate the impact of Carbapenem-resistant *Enterobacterales* (CRE) colonization on the development of infections sustained by the colonizing pathogen in hematological patients.

### Methods

We retrospectively collected data from adult patients admitted to the Hematology Department of IRCCS San Raffaele Scientific Institute (Milan, Italy) who were colonized by CRE between January 2021 and March 2024. Active surveillance was performed through regular assessment of colonization using rectal swab at admission and weekly thereafter. We collected data regarding infection complications.

**Copyright Information:** (Online) ISSN: 2572-9241
©2026 The Author(s). HemaSphere is published by John Wiley & Sons Ltd on behalf of European Hematology Association. This is an open access Abstract Book distributed under the Attribution-NonCommercial-NoDerivs (CC BY-NC-ND), which allows third parties to download the articles and share them with others as long as they credit the author and the Abstract Book, but they cannot change the content in any way or use them commercially.
**Abstract Book Citations:** Authors, Title, HemaSphere, 2026;10:(S1):pages. The abstract book DOIs can be found on the “Author Information” page.
**Disclaimer:** Articles published in the journal HemaSphere exclusively reflect the opinions of the authors. The authors are responsible for all content in their abstracts including accuracy of the facts, statements, citing resources, etc.

**Results**

Overall, 32 patients were enrolled. The majority of them were affected by acute leukemia (62.5%). The most recent hematological therapy was allogeneic stem cell transplantation (SCT) in the majority of cases (62.5%), followed by chemotherapy (18.8%), autologous SCT (9.4%) and CAR T-cell therapy (9.4%). The first positive surveillance swab was registered at median time of 11 days (range 3-18) from the last hematological therapy.
Regarding the colonizing pathogen, *Klebsiella pneumoniae* (43.8%) was the most prevalent isolate, followed by *Escherichia coli* (28.1%), *Citrobacter freundii* (12.5%), *Enterobacter cloacae* (12.5%) and *Klebsiella oxytoca* (3.1%). The predominant resistance mechanism was VIM (68.8%), followed by NDM (21.8%) and KPC (6.3%). One *Klebsiella pneumoniae* isolate (3.1%) harbored both KPC and VIM resistance mechanism.
Overall, 24/32 (75%) patients developed febrile neutropenia with a microbiologically documented infection, caused by bacterial pathogens in 15 out of 24 cases.
Overall, 20/32 (62.5%) patients received antimicrobial therapy active against the colonizing pathogen at the onset of neutropenic fever, mainly ceftazidime-avibactam plus aztreonam.
Only 5/32 (15.6%) patients developed infection sustained by the colonizing pathogen. All patients were affected by high risk haematological disease, 3 patients had previously received salvage chemotherapy and 2 allogeneic SCT. All infections were sustained by *Klebsiella pneumoniae* (3 NDM-producing, 1 VIM-producing, 1 KPC-producing). The onset of infection was at median time of 22 days (0-36) from the first detection of colonization. The type of infection included 2 central line-associated bloodstream infections (CLABSI), 2 bloodstream infections and 1 nosocomial pneumonia. All 5 patients received antimicrobial therapy against the colonizing pathogen. No patients developed septic shock, and no patient died from infection-related complications.

**Summary/Conclusion**

Our data confirm the relevance of CRE as emerging pathogens in hematological patients. An active surveillance program with colonization screening represents a useful tool for guiding early targeted antimicrobial therapy, resulting in a low mortality rate in this high risk setting. Antimicrobial stewardship programs are instrumental in ensuring appropriate patient selection for antimicrobial therapy, thereby mitigating the emergence of MDR pathogens.

**Copyright Information:** (Online) ISSN: 2572-9241
©2026 The Author(s). HemaSphere is published by John Wiley & Sons Ltd on behalf of European Hematology Association. This is an open access Abstract Book distributed under the Attribution-NonCommercial-NoDerivs (CC BY-NC-ND), which allows third parties to download the articles and share them with others as long as they credit the author and the Abstract Book, but they cannot change the content in any way or use them commercially.
**Abstract Book Citations:** Authors, Title, HemaSphere, 2026;10:(S1):pages. The abstract book DOIs can be found on the “Author Information” page.
**Disclaimer:** Articles published in the journal HemaSphere exclusively reflect the opinions of the authors. The authors are responsible for all content in their abstracts including accuracy of the facts, statements, citing resources, etc.

### (PB4255) INFECTIOUS COMPLICATIONS AND CLINICAL OUTCOMES DURING HIGH-DOSE CYTARABINE CONSOLIDATION WITHOUT ROUTINE ANTIMICROBIAL PROPHYLAXIS IN ACUTE MYELOID LEUKEMIA: A SINGLE-CENTER RETROSPECTIVE STUDY

**Topic:** 31. Infections in hematology (incl. supportive care/therapy)

Tugcan Alp Kirkizlar*[1], Hakki Onur Kirkizlar[1], Ahmet Balci[1], Guray Aygun[1], Ahmet Muzaffer Demir[1]

[1]*Trakya University, Hematology, Edirne, Türkiye;*

### Background

Most studies investigating the role of antibacterial prophylaxis in acute myeloid leukemia (AML) have focused predominantly on patients undergoing induction chemotherapy rather than consolidation therapy. However, there is a limited body of evidence specifically evaluating the risk of infection during high-dose cytarabine (HiDAC). consolidation.

### Aims

We aimed to evaluate the frequency of febrile neutropenia (FN), the distribution of infections, and clinical outcomes in patients receiving a HiDAC regimen in which routine anti-infective prophylaxis was not administered.

### Methods

In this retrospective single-center cohort study, 26 pts treated with HiDACconsolidation regimen and 58 cycles of treatment were analyzed. While routine anti-infective prophylaxis was not administered, prophylactic strategies were determined on the basis of patient-specific needs arising from prior treatment cycles.

### Results

The median age was 49y and 53.8% of the pts were male. 65.5% of the pts were AML defining genetic abnormalities according to WHO classification. 76.9% of the pts were in morphological CR prior HiDAC consolidation. The median number of HiDAC cycle was 2 and the rate of FN was 92.3% in cohort. In all treatment cycles, the median day of severe neutropenia was 12 days. The presence of FN was 91.4% and the median day of FN was 16th day of the cycle. 46.5% of FN episodes were FUO.
The overall mortality rate was 38.5% (10 pts), of which 30% (3 pts) was attributable to infection-related causes.
Infection prophylaxis was required in 40 cycles. Antibacterial prophylaxis was administered in 8 cycles, antiviral prophylaxis in 11 cycles, and antifungal prophylaxis in 35 cycles, based on patient-specific needs. G-CSF was used in 16 cycles, one of which was not complicated by FN. 24 episodes occurred during the 1st cycle, 17 during the s2nd cycle, 11 during the 3rd cycle and one during the 4th cycle. There was no statistically significant difference in the incidence of FN episodes among cycles (p:0.705).
Bacterial growth was observed in 18 cycles with positive urine cultures in 6 cycles and positive blood cultures in 15 cycles.*E. coli* was the most frequently isolated pathogen in both sources. 94.2% of the FN episodes were grade 1. Fungal infections were observed in 14 cycles and were classified as ‘possible’. Fungal infections occured in 35.7%, 28.6% and 35.7% of the 1st, 2nd and 3rd HiDAC cycles, respectively.
FN was observed in 92.2% of patients in CR and 85.7% of those in PR prior to HiDAC, with no significant difference between groups (p:0.487).
The mean overall survival was 1,691.4 days (95% CI, 1,224.77–2,158.03). FN was observed in 93.4% of patients, and all deaths occurred among those who experienced FN, with no significant difference between groups (p:0.238).

**Copyright Information:** (Online) ISSN: 2572-9241
©2026 The Author(s). HemaSphere is published by John Wiley & Sons Ltd on behalf of European Hematology Association. This is an open access Abstract Book distributed under the Attribution-NonCommercial-NoDerivs (CC BY-NC-ND), which allows third parties to download the articles and share them with others as long as they credit the author and the Abstract Book, but they cannot change the content in any way or use them commercially.
**Abstract Book Citations:** Authors, Title, HemaSphere, 2026;10:(S1):pages. The abstract book DOIs can be found on the “Author Information” page.
**Disclaimer:** Articles published in the journal HemaSphere exclusively reflect the opinions of the authors. The authors are responsible for all content in their abstracts including accuracy of the facts, statements, citing resources, etc.

### Summary/Conclusion

In our cohort from a center not routinely using antimicrobial prophylaxis, FN occurred at a very high rate during HiDAC consolidation. Although infection-related mortality was relatively limited, infectious complications were frequent across all treatment cycles, with no significant difference in incidence between cycles or remission status prior to consolidation.
These findings suggest that patients receiving HiDAC consolidation remain at substantial risk for infectious events, underscoring the need for careful monitoring and individualized prophylactic strategies.

Table 1. Features of 26 patients treated with HiDAC consolidation

| Parameters | Values |
|---|---|
| Age | Median: 49 years (20-62) |
| Gender | Female: 46.2%, Male: 53.8% |
| CCI | Median: 3 (2-5) |
| Bone marrow blast % at diagnosis | Median: 70% (20-90) |
| AML classification | AML MDS related: 15.4%<br>AML defining genetic abnormalities: 65.5%<br>AML, defined by differentiation: 11.5%<br>AML, MPN transformation: 3.8%<br>Myeloid sarcoma: 3.8% |
| Disease status prior HIDAC | CR: 76.9%, PR :23.1% |
| @ first cycle, Hb (g/dL) | Median: 9.7 (7.5-14.1) |
| @ first cycle, Neut (/mm³) | Median: 3250 (850-10 220) |
| @ first cycle, PLT (/mm³) | Median: 209 000 (40 000-670 000) |
| @ first cycle, CRP (mg/L) | Median: 7 (1-76) |
| @ first cycle, GFR (ml/m²) | Median: 111 (40-146) |
| @ first cycle, Albumin (g/dL) | Median: 3.75 (2.7-4.5) |
| @ first cycle, duration of severe neutopenia (days) | Median: 11 (6-17) |
| Presence of FN | 92.3% |
| Mortality rate | 38.5% |
| Infection related mortality | 30% |
| Follow-up time (days) | Median: 626 (62-2661) |

Table 2. Features of 58 HiDAC cycles

| Parameters | Values |
|---|---|
| Distribution of HIDAC cycles | 1: 44.8%/ 2: 31%/ 3: 22.4%/ 4: 1.7% |
| Duration of severe neutropenia (days) | Median: 12 (4-35) |
| The use of G-CSF | 27.6% |
| The presence of FN | 91.4% |
| The median day of FN episode | 16 |
| The origin of FN episode | FUO: 46.5%<br>Bacteremia: 36.2%<br>Respiratory: 24.1%<br>Soft tissue: 15.5%<br>Urinary: 10.3%<br>Gastrointestinal: 5.2%<br>Thrombophlebitis: 6.9% |
| The grade of infection: | Gr 3: 94.2%/ G 4: 1.7%/ G5: 5.8% |
| Fungal infection rate | 26.4% |
| The median length of hospital stay | 25 (8-43) |

Table 1 and 2

Copyright Information: (Online) ISSN: 2572-9241
©2026 The Author(s). HemaSphere is published by John Wiley & Sons Ltd on behalf of European Hematology Association. This is an open access Abstract Book distributed under the Attribution-NonCommercial-NoDerivs (CC BY-NC-ND), which allows third parties to download the articles and share them with others as long as they credit the author and the Abstract Book, but they cannot change the content in any way or use them commercially.
Abstract Book Citations: Authors, Title, HemaSphere, 2026;10:(S1):pages. The abstract book DOIs can be found on the "Author Information" page.
Disclaimer: Articles published in the journal HemaSphere exclusively reflect the opinions of the authors. The authors are responsible for all content in their abstracts including accuracy of the facts, statements, citing resources, etc.

### (PB4257) STRUCTURE OF PATHOGENS CAUSING BLOODSTREAM INFECTIONS IN PATIENTS WITH ACUTE LEUKEMIA

**Topic:** 31. Infections in hematology (incl. supportive care/therapy)

Veranika Staina*[1], Iryna Lendzina[1], Ihar Iskrou[2]

[1]*State Institution «Minsk Scientific and Practical Center for Surgery, Transplantology and Hematology», Minsk, Belarus;*
[2]*Educational Institution «Belarusian state medical University», Minsk, Belarus;*

### Background

Patients with acute leukemia  face a high risk of infectious complications resulting from both secondary immunodeficiency caused by the disease itself and immunosuppression following chemotherapy. Most treatment protocols for AML and ALL have a myelosuppressive effect, with an expected period of severe neutropenia exceeding 7 days, which is the most significant risk factor for developing infectious complications. One of the major clinical challenges in managing febrile neutropenia is overcoming carbapenem resistance, as recent data indicate a growing prevalence of carbapenem-resistant gram-negative bacteria in bloodstream infections among patients with myelotoxic agranulocytosis.

### Aims

To analyze the frequency and structure of pathogens of bloodstream infections in patients with acute leukemia during myelotoxic agranulocytosis after chemotherapy, and to assess the level of carbapenem-resistance of the most significant pathogens.

### Methods

Venous blood samples collected for bacteriological culture from patients with febrile neutropenia treated at the Hematology Department No. 3 of the Minsk Scientific and Practical Center for Surgery, Transplantology, and Hematology in 2025 were analyzed. Microorganisms were identified using a Microflex LT/SH mass spectrometer, microorganism identification and antibiotic susceptibility testing were performed using a VITEK 2 Compact 60 bacteriological analyzer. The interpretation of minimum inhibitory concentrations was based on EUCAST standards.

### Results

A total of 211 blood samples were assessed, from which 28 pathogens were isolated. The rate of positive blood cultures was 13.3%. The majority of bloodstream infections were caused by Gram-negative bacteria (67.9%), mainly from the Enterobacteriaceae family (60.7%). Among these, *Kl. pneumoniae* accounted for 25% and *E. coli* for 35.7%. Non-fermenting bacteria were represented by isolated cases of *P. aeruginosa* (n=1) and *A. baumannii* (n=1). Among Gram-positive microorganisms, coagulase-negative staphylococci were predominant (66.7%), which may indicate contamination of venous blood samples with opportunistic pathogens from the skin. *Enterococcus* spp. were identified in 22.2% of cases, and a single case of *S. aureus* bacteremia was detected. Analysis of antimicrobial susceptibility of the key Gram-negative pathogens showed marked resistance among*K. pneumoniae* isolates, with 57.1% demonstrating carbapenem resistance, while all *E. coli* isolates remained fully susceptible to carbapenems. Empirical antibacterial therapy followed the standard operating procedure "Initial Rational Antibacterial Therapy for Febrile Neutropenia in Hematology Patients in Hospitals." For patients with acute leukemia during induction, consolidation, or after chemotherapy courses associated with severe neutropenia expected to last more than 10 days, protected β-lactam antibiotics were recommended as first-line empirical treatment. Application of this regimen resulted in a 30-day overall survival rate of 78.6%.

**Copyright Information:** (Online) ISSN: 2572-9241
©2026 The Author(s). HemaSphere is published by John Wiley & Sons Ltd on behalf of European Hematology Association. This is an open access Abstract Book distributed under the Attribution-NonCommercial-NoDerivs (CC BY-NC-ND), which allows third parties to download the articles and share them with others as long as they credit the author and the Abstract Book, but they cannot change the content in any way or use them commercially.
**Abstract Book Citations:** Authors, Title, HemaSphere, 2026;10:(S1):pages. The abstract book DOIs can be found on the "Author Information" page.
**Disclaimer:** Articles published in the journal HemaSphere exclusively reflect the opinions of the authors. The authors are responsible for all content in their abstracts including accuracy of the facts, statements, citing resources, etc.

**Summary/Conclusion**

According to a scientific study conducted under similar conditions from 2022 to 2024, the carbapenem resistance rate of Kl. pneumoniae was 85.2%. In this study, lower rates of carbapenem resistance were observed in Gram-negative bacteria (Fig. 1). The introduction of an empirical antibacterial therapy regimen using protected β-lactam antibiotics allows for increased treatment efficacy and the widespread use of carbapenems, which is important for combating the development of antibiotic resistance.

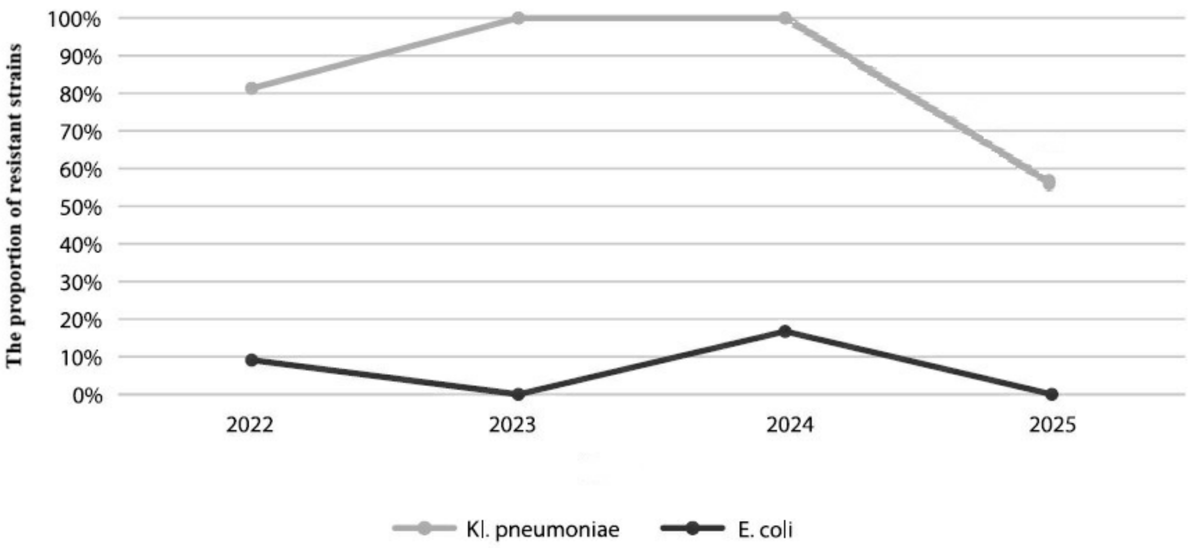

**Fig. 1. Dynamics of carbapenem resistance of the most significant gram-negative microorganisms (Kl. pneumoniae and E. coli)**

**Copyright Information:** (Online) ISSN: 2572-9241
©2026 The Author(s). HemaSphere is published by John Wiley & Sons Ltd on behalf of European Hematology Association. This is an open access Abstract Book distributed under the Attribution-NonCommercial-NoDerivs (CC BY-NC-ND), which allows third parties to download the articles and share them with others as long as they credit the author and the Abstract Book, but they cannot change the content in any way or use them commercially.
**Abstract Book Citations:** Authors, Title, HemaSphere, 2026;10:(S1):pages. The abstract book DOIs can be found on the "Author Information" page.
**Disclaimer:** Articles published in the journal HemaSphere exclusively reflect the opinions of the authors. The authors are responsible for all content in their abstracts including accuracy of the facts, statements, citing resources, etc.

### (PB4258) REAL-WORLD EFFECTIVENESS OF IMMUNOGLOBULIN-REPLACEMENT THERAPY IN MITIGATING INFECTION RISK AMONG PATIENTS WITH NON-HODGKIN LYMPHOMA, MULTIPLE MYELOMA, AND CHRONIC LYMPHOCYTIC LEUKEMIA

**Topic:** 31. Infections in hematology (incl. supportive care/therapy)

Antoine Azar*[1], Elisabeth Calderón-Gómez[2], Jerrod Nelms[3], Jiani Zhou[3], James Jordan[3], Igor Rossa-Ramm[2], Elisabet Viayna[2]

*[1]Johns Hopkins University School of Medicine, Division of Allergy and Clinical Immunology, Baltimore, United States of America; [2]Grifols, Sant Cugat del Valles, Spain; [3]Grifols Shared Services North America (SSNA), Global HEOR & RWE, Durham, United States of America;*

### Background

Patients with hematological malignancies such as non-Hodgkin lymphoma (NHL), multiple myeloma (MM) or chronic lymphocytic leukemia (CLL) are at a high risk of hypogammaglobulinemia (HGG), which is associated with an increased risk of infections that may preclude the use of certain anti-cancer therapies, hindering patient care. Immunoglobulin-replacement therapy (IgRT) reduces the risk of infections associated with HGG. However, given the introduction of novel, highly immunosuppressive anti-cancer therapies and the evolving treatment paradigm, up-to-date real-world evidence (RWE) assessing the effectiveness of IgRT is warranted.

### Aims

To assess the effectiveness of 10% Human Intravenous Immunoglobulin Caprylate-Chromatography-Purified (IGIV-C10%) in patients with NHL, MM or CLL and HGG.

### Methods

A retrospective RWE study including patients with NHL, MM, or CLL was conducted using TriNetX Dataworks, a data source consisting of electronic heath records from over 100 million patients in the US. Patients were required to have HGG (IgG levels<700 mg/dL or ICD-10-CM codes 80.1, 80.3, or 80.9) following the diagnosis of NHL, MM or CLL, and to be subsequently treated with at least four doses of IGIV-C10% over the 6-month follow-up period. Index date was defined as the first IGIV-C10% dose between Jun-2021 and Jun-2025. Patients treated with IgRT within 12 months prior to index or who had a record for primary immunodeficiency or other conditions requiring use of immunoglobulin any time prior to index were excluded (Figure 1). Effectiveness of IGIV-C10% was assessed by comparing the proportion of patients experiencing severe infections or infections of any kind during the 6-month post-IGIV-C10% vs. the 6-month pre-IGIV-C10% periods using generalized estimating equations logistic models. Distribution of patients by IgG levels across the pre- and post-index periods was also assessed.

**Copyright Information:** (Online) ISSN: 2572-9241
©2026 The Author(s). HemaSphere is published by John Wiley & Sons Ltd on behalf of European Hematology Association. This is an open access Abstract Book distributed under the Attribution-NonCommercial-NoDerivs (CC BY-NC-ND), which allows third parties to download the articles and share them with others as long as they credit the author and the Abstract Book, but they cannot change the content in any way or use them commercially.
**Abstract Book Citations:** Authors, Title, HemaSphere, 2026;10:(S1):pages. The abstract book DOIs can be found on the “Author Information” page.
**Disclaimer:** Articles published in the journal HemaSphere exclusively reflect the opinions of the authors. The authors are responsible for all content in their abstracts including accuracy of the facts, statements, citing resources, etc.

### Results

A total of 277 patients were included: 87, 118 and 72 patients diagnosed with NHL, MM, and CLL, respectively. Mean (SD) age was 69.6 (10.0) years, and 55.2% were male. Age and sex distribution was similar across malignancies. Most common anti-cancer therapies used among the overall population were B-cell monoclonal antibodies both during the pre- (35.4%) and post-IGIV-C10% periods (27.4%). During the pre-IGIV-C10% period, 12.6%, 5.1% and 4.2% of NHL, MM, and CLL patients, respectively, were treated with a Chimeric Antigen Receptor T-cell therapy; and bispecific antibodies were used in 5.7% and 4.6% of NHL patients and 15.3% and 17.8% of MM patients pre- and post-IGIV-C10%, respectively, and 1.4% of CLL patients pre-IGIV-C10%. Patients received 5.0 (2.0) doses of IGIV-C10% over 6 months. Proportion of patients with a severe infection (19.9% vs. 10.1%) or an infection of any kind (70.8% vs. 54.5%) were 57% (OR: 0.43, 95% CI: 0.27, 0.68, p<0.001) and 66% (OR: 0.44, 95% CI: 0.32, 0.60, p<0.001) lower, respectively, during the post-IGIV-C10% period. Patients presented lower IgG levels during the pre-IGIV-C10% period, with median levels increasing from 278.5 to 504.0 mg/dL (p<0.001). Correspondingly, the proportion of patients with IgG<400 mg/dL decreased from 93.6% to 82.9%, and those achieving IgG>700 mg/dL increased from 47.4% to 78.7% (p<0.001).

### Summary/Conclusion

Regular use of IGIV-C10% was associated with lower odds of severe infections and infections of any kind, as well as lower proportion of patients with HGG, which is essential to ensure optimal patient care.

**Figure 1.** Study design

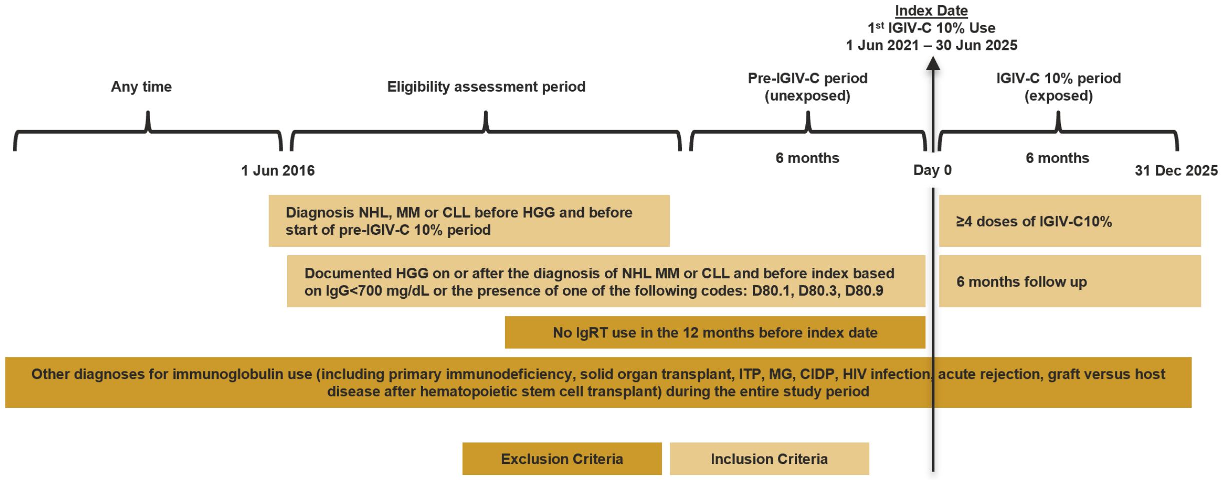

CIDP: Chronic Inflammatory Demyelinating Polyneuropathy; CLL: Chronic Lymphocytic Leukemia; HGG: Hypogammaglobulinemia; HIV: Human Immunodeficiency Virus; IGIV-C10%: 10% Human Intravenous Immunoglobulin Caprylate-Chromatography-Purified; IgRT: Immunoglobulin-Replacement Therapy; ITP: Immune Thrombocytopenia; MG: Myasthenia Gravis; MM: Multiple Myeloma, NHL: Non-Hodgkin Lymphoma

**Copyright Information:** (Online) ISSN: 2572-9241
©2026 The Author(s). HemaSphere is published by John Wiley & Sons Ltd on behalf of European Hematology Association. This is an open access Abstract Book distributed under the Attribution-NonCommercial-NoDerivs (CC BY-NC-ND), which allows third parties to download the articles and share them with others as long as they credit the author and the Abstract Book, but they cannot change the content in any way or use them commercially.
**Abstract Book Citations:** Authors, Title, HemaSphere, 2026;10:(S1):pages. The abstract book DOIs can be found on the "Author Information" page.
**Disclaimer:** Articles published in the journal HemaSphere exclusively reflect the opinions of the authors. The authors are responsible for all content in their abstracts including accuracy of the facts, statements, citing resources, etc.

### (PB4259) ANALYSIS OF BACTERIAL COLONIZATION OF INFUSION CONNECTORS IN GENERAL WARDS FOR PATIENTS WITH HEMATOLOGICAL MALIGNANCIES AND EXPLORATION OF COUNTERMEASURES

**Topic:** 31. Infections in hematology (incl. supportive care/therapy)

Chuqian Xiao*[1]

[1]*Xinqiao Hospital, Army Medical University, Department of Hematology, chongqing, China;*

### Background

Catheter-related bloodstream infection is a common healthcare-associated infection in patients with hematological malignancies, with bacterial colonization of infusion connectors as a key inducing factor. Patients with hematological malignancies are in a state of immunodeficiency with significantly increased infection risk, while non-standard disinfection of infusion connectors in clinical practice leads to poor prevention and control of bacterial colonization. Relevant research on the actual status of bacterial colonization of infusion connectors in such patients in general wards is still insufficient, which necessitates in-depth investigation to provide evidence for clinical infection control.

### Aims

To investigate the bacterial colonization status and disinfection/decontamination effect of infusion connectors in general wards for patients with hematological malignancies, and to provide clinical references for the safety management of intravenous infusion in such patients.

### Methods

A total of 59 indwelling infusion connectors of patients with hematological malignancies were selected as the control group; 52 disinfected infusion connectors during the same period were enrolled as Observation Group A, and the nurses' disinfection duration and methods for the connectors were recorded. Targeted training on the disinfection methods of infusion connectors was conducted for medical staff, after which 50 disinfected infusion connectors were selected as Observation Group B. All infusion connectors were sampled and sent for bacterial culture.

### Results

Bacteria were detected in 41 connectors in the control group, with a detection rate of 69.5%. Among them, 30 connectors had one strain of bacteria detected each, 10 connectors had two strains each, and 1 connector had three strains. In Observation Group A, bacteria were detected in 8 connectors (detection rate: 15.3%); in Observation Group B, bacteria were detected in 4 connectors (detection rate: 8.0%). The difference in the bacterial culture detection rate before and after the training was statistically significant ($\chi^2$=84.418, P<0.001).

### Summary/Conclusion

Infusion connection devices are highly susceptible to contamination, and effective disinfection can significantly reduce bacterial colonization. Patients with hematological malignancies have impaired immune function and are thus at a high risk of infection. Medical staff should recognize the risks of infusion-related infections, standardize infusion operations, strengthen the management of infusion devices, and further explore more effective disinfection methods.

**Copyright Information:** (Online) ISSN: 2572-9241
©2026 The Author(s). HemaSphere is published by John Wiley & Sons Ltd on behalf of European Hematology Association. This is an open access Abstract Book distributed under the Attribution-NonCommercial-NoDerivs (CC BY-NC-ND), which allows third parties to download the articles and share them with others as long as they credit the author and the Abstract Book, but they cannot change the content in any way or use them commercially.
**Abstract Book Citations:** Authors, Title, HemaSphere, 2026;10:(S1):pages. The abstract book DOIs can be found on the "Author Information" page.
**Disclaimer:** Articles published in the journal HemaSphere exclusively reflect the opinions of the authors. The authors are responsible for all content in their abstracts including accuracy of the facts, statements, citing resources, etc.

### (PB4260) METROLOGICAL CHARACTERISTICS OF THE METHOD FOR DETERMINING THE CONTENT OF ANTI-D ANTIBODIES BASED ON FLOW CYTOFLUOROMETRY

**Topic:** 31. Infections in hematology (incl. supportive care/therapy)

Natalia Vildanova*[1], Ekaterina Konovalova[1], Elena Kormshchikova[1], Natalia Isaeva[1], Anastasia Zagarskikh[1]

[1]*FSFSI KRIHandBT FMBA of Russia, Kirov, Russia;*

### Background

The determination of anti-D antibodies by flow cytofluorometry is based on the specific binding of anti-D IgG toRhD-positive red blood cells, which is detected by the level of luminescence caused by fluorochrome-labeled secondary anti-IgGantibodies. The State Pharmacopoeia of the Russia regulates the use of this method for quality control of the potency of humananti-D immunoglobulin. However there is no data on the use of flow cytofluorometry for testing the plasma donations of anti-D donors.

### Aims

To carry out a metrological characterization of the method for determining the content of anti-D antibodies based on flow cytofluorometry.

### Methods

The Human anti-D immunoglobulin andImmunorho Kedrion, samples of plasma with antibody titers were studied 1:128, 1:1024, 1:4096, installed in the reaction of indirecthemagglutination in gel, and the international standard of anti-Dimmunoglobulin (NIBSC code: 16/332). To construct the calibration curve, five double dilutions of a standard of anti-Dantibodies were prepared with a certified value of 35.2 (35.2 –58.2) IU/ml in indirect hemagglutination and 36.4 ± 11.6 IU/ml inmethods of ELISA and flow cytofluorimetry, starting from 1.8 IU/ml. Suspensions of humanerythrocytes with phenotypes 0R1R1 and 0rr from a set of reagents ID-DiaCell I-II-III and secondary antibodies Anti-HumanIgG (Fc-specific)-FITC were used in the work. Erythrocytes were analyzed using a FACS Canto$^{TM}$ II, and MFI was measured at a wavelength of 488nm.

**Copyright Information:** (Online) ISSN: 2572-9241
©2026 The Author(s). HemaSphere is published by John Wiley & Sons Ltd on behalf of European Hematology Association. This is an open access Abstract Book distributed under the Attribution-NonCommercial-NoDerivs (CC BY-NC-ND), which allows third parties to download the articles and share them with others as long as they credit the author and the Abstract Book, but they cannot change the content in any way or use them commercially.
**Abstract Book Citations:** Authors, Title, HemaSphere, 2026;10:(S1):pages. The abstract book DOIs can be found on the "Author Information" page.
**Disclaimer:** Articles published in the journal HemaSphere exclusively reflect the opinions of the authors. The authors are responsible for all content in their abstracts including accuracy of the facts, statements, citing resources, etc.

**Results**

The specificity of the determination of anti-D antibodieswas confirmed by a low MFI (less than 37 units) in blank andcontrol samples consisting of a mixture of human erythrocyteswith phenotypes 0R1R1, 0rr, phosphate buffer solution and the studied samples.

The analytical range of the methods was established from 0.16IU/ml to 1.80 IU/ml, which corresponded to the MFI range from50 to 1000 cont. units. In this range, the average coefficient of variation when testing each sample in two repetitions was 8.3%(95% CI: 6.9% – 9.6%), the maximum value was 29.5% Thecalibration dependences were characterized by high coefficients of determination $R^2 > 0.95$ (0.9967; 95% CI: 0.9945 – 0.9989). The angular coefficient was 1.31 (95% CI: 1.26 – 1.37), the free termof the equation was 2.60 (95% CI: 2.46 – 2.74). The coefficient of variation of the linear regression parameters – 4.9% and 5.7% –indicated the stability of the calibration dependencies.

When testing the international standard, the concentration of anti-D antibodies was 313.2 ± 39.7 IU/ml, the specified range includeda certified value of 297 IU/ml, which confirms the correctness of the method. The range of offset of the determination resultsrelative to the certified value did not exceed 17.1%.

The content of anti-D IgG in the foreign anti-D immunoglobulinwas 970.0 ± 109.5 IU/ml, in the preparation of domesticproduction – 501.1 ± 67.5 IU/ml. The potency of plasma is 19.3 ±2.6 IU/ml (with an anti-D antibody titer of 1:4096), 8.6 ± 0.7IU/ml (with an anti-D antibody titer of 1:1024) and 0.4 ± 0.1IU/ml (with an titer of 1:128). The average coefficient of variationof the potency for the samples of the same name was 13.1% (95%CI: 8.2% – 19.4%). At the same time, the maximum coefficient of variation of 19.4% was obtained when testing the plasma with an anti-D antibodies concentration of 0.4 IU/ml, close to the lowervalue of the analytical range.

**Summary/Conclusion**

The flow cytofluorometry suitable forevaluating the potency of the plasma donations of anti-D donors and anti-D immunoglobulin.

**Copyright Information:** (Online) ISSN: 2572-9241
©2026 The Author(s). HemaSphere is published by John Wiley & Sons Ltd on behalf of European Hematology Association. This is an open access Abstract Book distributed under the Attribution-NonCommercial-NoDerivs (CC BY-NC-ND), which allows third parties to download the articles and share them with others as long as they credit the author and the Abstract Book, but they cannot change the content in any way or use them commercially.
**Abstract Book Citations:** Authors, Title, HemaSphere, 2026;10:(S1):pages. The abstract book DOIs can be found on the “Author Information” page.
**Disclaimer:** Articles published in the journal HemaSphere exclusively reflect the opinions of the authors. The authors are responsible for all content in their abstracts including accuracy of the facts, statements, citing resources, etc.

### (PB4261) CLINICAL AND IMMUNOLOGICAL CHARACTERISTICS OF COVID-19 INFECTION AND REINFECTION IN HOSPITALIZED PATIENTS WITH LYMPHOMA

**Topic:** 31. Infections in hematology (incl. supportive care/therapy)

Yijie Li[1, 2], Jiaojiao Zhang*[1, 3, 4], Jian-qing Mi[1, 3, 4], Min Zhou[1, 2]

*[1]Ruijin Hospital Affiliated to Shanghai Jiao Tong University School of Medicine, Shanghai, China;[2]Department of Pulmonary and Critical Care Medicine, Ruijin Hospital , Shanghai Jiao Tong University School of Medicine, Institute of Respiratory Diseases Shanghai Jiao Tong University School of Medicine, Shanghai, China; [3]Shanghai Institute of Hematology, State Key Laboratory of Medical Genomics, National Research Center for Translational Medicine at Shanghai, Ruijin Hospital, Shanghai jiao Tong University School of Medicine, Shanghai, China; [4]Shanghai Key Laboratory of Gene Editing and Cell-based Immunotherapy for Hematological Diseases, Ruijin Hospital, Shanghai jiao Tong University School of Medicine, Shanghai, China;*

### Background

Lymphoma patients are at substantially elevated risk for COVID-19 and its complications owing to disease-related and treatment-induced immunosuppression(chemotherapy, targeted agents, and immunosuppressants). Despite the high clinical burden, data on COVID-19 reinfection patterns and immunological determinants of reinfection risk in this population remain limited.

### Aims

To characterize the clinical and immunological features of COVID-19 infection and reinfection in hospitalized lymphoma patients, to describe the pathogen spectrum of recurrent pulmonary infections, and to identify hematologic and cellular immune biomarkers associated with SARS-CoV-2 reinfection risk.

### Methods

We retrospectively analyzed 278 lymphoma patients with confirmed COVID-19 admitted to Ruijin Hospital, Shanghai Jiao Tong University School of Medicine, from November 2022 to November 2023. Patients were followed up for recurrent pulmonary infections, with the follow-up period extending until May 2025 and they were grouped into non-recurrent (n=206) and recurrent pulmonary infection cohorts (n=72). Pathogen profiles (throat swab, sputum culture,bronchoalveolar lavage fluid, next-generation sequencing), hematologic indices (WBC, Neutrophil count, NLR), inflammatory markers (CRP, PCT, IL-6), and cellular immune parameters (CD3+, CD4+, CD8+ T cells; immunoglobulins) were compared between groups using Mann-Whitney U test or chi-square test, with P<0.05 as the significance threshold. Among recurrent cases, patients were further stratified by SARS-CoV-2 reinfection status.

### Results

The cohort comprised 59.35% males (age range 17–92 years). Severe/critical COVID- 19 occurred in 20.08% (49/244). The overall case fatality rate was 5.76% (16/278); patients aged ≥60 years accounted for 87.5% of deaths. Co-infection with other pulmonary pathogens at baseline was detected in 40.29% (112/278). Recurrent pulmonary infection occurred in 25.90% (72/278). Pathogen analysis from 50 recurrent cases identified 88 isolates (>30species); viruses predominated (46.59%), with SARS-CoV-2 the most frequent single pathogen (30.55%, 22/72), followed by Gram-negative bacteria (37.5%) and fungi (12.5%). The recurrent infection group had significantly lower WBC, neutrophil count, and NLR than the non-recurrent group (all P<0.05). Among patients with SARS-CoV-2 reinfection (n=22) vs. those without(n=50), NLR was significantly lower (P=0.032). CD4+
T cell levels were markedly elevated in the SARS-CoV-2 reinfection group (443.23±303.41 vs. 258.65±253.57 cells/μL; P=0.021), while immunoglobulin levels showed no significant intergroup differences.

**Copyright Information:** (Online) ISSN: 2572-9241
©2026 The Author(s). HemaSphere is published by John Wiley & Sons Ltd on behalf of European Hematology Association. This is an open access Abstract Book distributed under the Attribution-NonCommercial-NoDerivs (CC BY-NC-ND), which allows third parties to download the articles and share them with others as long as they credit the author and the Abstract Book, but they cannot change the content in any way or use them commercially.
**Abstract Book Citations:** Authors, Title, HemaSphere, 2026;10:(S1):pages. The abstract book DOIs can be found on the “Author Information” page.
**Disclaimer:** Articles published in the journal HemaSphere exclusively reflect the opinions of the authors. The authors are responsible for all content in their abstracts including accuracy of the facts, statements, citing resources, etc.

### Summary/Conclusion

Lymphoma patients with COVID-19 carry a substantially elevated risk of recurrent pulmonary infection and SARS-CoV-2 reinfection, with mortality far exceeding that of the general COVID-19 population. SARS-CoV-2 remains the predominant recurrent pathogen,consistent with viral persistence or reinfection in immunocompromised hosts. Reduced NLR, and elevated CD4+ T cell levels may serve as practical biomarkers for early identification of high-risk individuals. Intensified pathogen surveillance, dynamic immune monitoring, and prompt antiviral intervention are critical to reducing severe disease and improving outcomes in this vulnerable population.

Table1:Comparison Table of laboratory Test Indicators for recurrent Pulmonary infection (n=278)

| | | Non-recurrent (n=206) | Recurrent pulmonary infection (n=72) | $X^2$ Z | P |
|---|---|---|---|---|---|
| WBC (×10*9/L) | | 6.23 ± 13.81 | 4.89 ± 4.89 | −2.128 | 0.033 |
| Hb (g/L) | | 113.65 ± 28.21 | 111.89 ± 27.81 | −0.588 | 0.557 |
| RBC (×10*9/L) | | 3.95 ± 2.83 | 4.11 ± 4.62 | −1.408 | 0.159 |
| CRP (g/L) | | 32.37 ± 56.49 | 33.86 ± 49.00 | −0.102 | 0.919 |
| NE (×10*9/L) | | 3.55 ± 3.33 | 2.92 ± 3.43 | −2.307 | 0.021 |
| Lym (×10*9/L) | | 1.10 ± 1.24 | 1.89 ± 5.24 | −0.42 | 0.674 |
| NLR | < 0.7 | 12 (6.1%) | 10 (14.3%) | 6.144 | 0.046 |
| PCT (ng/L) | 0.7−3 | 136 (69.0%) | 49 (70%) | −1.824 | 0.068 |
| | > 3 | 49 (24.9%) | 11 (15.7%) | | |
| | | 1.94 ± 14.91 | 0.44 ± 1.25 | | |
| IL-6 (pg/L) | | 34.37 ± 62.43 | 33.20 ± 57.26 | −0.918 | 0.359 |

Table2:Statistical table of Overall Pathogenic bacteria Classification for Recurrent infections

| Bacteria | Proportion (%) | Fungus | Proportion (%) | Virus | Proportion (%) | Else | Proportion (%) |
|---|---|---|---|---|---|---|---|
| G- | | | | | | | |
| Klebsiella pneumoniae | 7 (7.95%) | Candida albicans | 4 | SARS-CoV-2 | 22 (25%) | Chlamydia | 1 |
| Stenotrophomonas maltophilia | 4 (4.55%) | Aspergillus | 3 | Epstein-Barr virus | 5 (5.68%) | Mycoplasma pneumoniae | 2 |
| Baumanii | 3 (3.41%) | Pneumocystis carinii | 1 | Cytomegalovirus | 4 (4.55%) | | |
| Escherichia coli | 3 (3.41%) | Cryptococcus | 1 | Torque teno virus | 3 (3.41%) | | |
| Pseudomonas aeruginosa | 2 | Else | 2 | Respiratory syncytial virus | 2 | | |
| Serratia | 2 | | | Influenza a virus | 3 | | |
| Limonobacter brucellosis | 1 | | | Sendai virus | 1 | | |
| Moraxella catarrhalis | 1 | | | Human rhinovirus | 1 | | |
| Haemophilus influenzae | 1 | | | | | | |
| New onion Burkholderia | 1 | | | | | | |
| G+ | | | | | | | |
| Staphylococcus epidermidis | 1 | | | | | | |
| Streptococcus granulosus | 1 | | | | | | |
| Staphylococcus haemolyticus | 1 | | | | | | |
| Enterococcus faecium | 5 (5.68%) | | | | | | |

Copyright Information: (Online) ISSN: 2572-9241
©2026 The Author(s). HemaSphere is published by John Wiley & Sons Ltd on behalf of European Hematology Association. This is an open access Abstract Book distributed under the Attribution-NonCommercial-NoDerivs (CC BY-NC-ND), which allows third parties to download the articles and share them with others as long as they credit the author and the Abstract Book, but they cannot change the content in any way or use them commercially.
Abstract Book Citations: Authors, Title, HemaSphere, 2026;10:(S1):pages. The abstract book DOIs can be found on the “Author Information” page.
Disclaimer: Articles published in the journal HemaSphere exclusively reflect the opinions of the authors. The authors are responsible for all content in their abstracts including accuracy of the facts, statements, citing resources, etc.

### (PB4262) THE APPLICATION OF SEMI-RECUMBENT POSITION COMBINED WITH PARTIAL WITHDRAWAL OF GUIDEWIRE IN THE PREVENTION OF INTERNAL JUGULAR VEIN MALPOSITION OF PICC

**Topic:** 31. Infections in hematology (incl. supportive care/therapy)

Yang Peng*[1]

[1] *The Second Affiliated Hospital of Army Medical University, Hematology Medical Center, ChongQing, China;*

### Background

Peripherally Inserted Central Catheter (PICC) refers to a catheter inserted into the central vein via peripheral veins such as the basilic vein, median cubital vein, and cephalic vein. It is widely used in clinical practice. However, catheter malposition is prone to occur during catheterization. Foreign studies report an incidence rate of 7.87% to 35%, with internal jugular vein malposition being the most common. Domestic literature reports an incidence rate of 3.3% to 12.3% for internal jugular vein malposition. Besides disease and anatomical factors, the vast majority of internal jugular vein malpositions are caused by improper body position or ineffective neck flexion, which fails to effectively compress the internal jugular vein.

### Aims

At present, head deviation and finger compression are commonly used in clinical practice to block the entrance of the internal jugular vein (2–3 cm above the clavicle, lateral to the sternocleidomastoid muscle), thereby preventing malposition into the internal jugular vein.
However, these methods have certain limitations in actual operation.
For emaciated patients, those with poor cooperation, limited neck movement, disturbance of consciousness, or tracheotomy, improper application of head deviation or finger compression may fail to effectively block the internal jugular vein entrance.

### Methods

We adopted the semi-recumbent position combined with partial guidewire withdrawal to prevent PICC malposition into the internal jugular vein. Specifically, during PICC insertion, when the catheter was advanced to 15 cm, the guidewire was withdrawn by 2–3 cm, and the catheter was slowly advanced while elevating the head of the bed to 30°–45°.

After withdrawing the guidewire by 2–3 cm, the unsupported segment of the distal catheter reached 4–5 cm (with the distal 2 cm of the PICC being guidewire-free). This rendered the distal catheter soft and floating within the subclavian vein, facilitating its entry into the superior vena cava along the blood flow. The semi-recumbent position reduced the pressure in the superior vena cava and increased venous return. Under gravity, the soft-tipped PICC catheter smoothly entered the superior vena cava, thereby reducing the risk of internal jugular vein malposition. This technique does not require patient head rotation and allows the neck to remain naturally relaxed.

### Results

This helps improve patient comfort and is particularly suitable for patients with head and neck disorders.

### Summary/Conclusion

The method has been applied clinically in several cases with favorable outcomes.

**Copyright Information:** (Online) ISSN: 2572-9241
©2026 The Author(s). HemaSphere is published by John Wiley & Sons Ltd on behalf of European Hematology Association. This is an open access Abstract Book distributed under the Attribution-NonCommercial-NoDerivs (CC BY-NC-ND), which allows third parties to download the articles and share them with others as long as they credit the author and the Abstract Book, but they cannot change the content in any way or use them commercially.
**Abstract Book Citations:** Authors, Title, HemaSphere, 2026;10:(S1):pages. The abstract book DOIs can be found on the "Author Information" page.
**Disclaimer:** Articles published in the journal HemaSphere exclusively reflect the opinions of the authors. The authors are responsible for all content in their abstracts including accuracy of the facts, statements, citing resources, etc.

### (PB4263) UTILITY OF CHEST X RAY IN FEBRILE NEUTROPENIA

**Topic:** 31. Infections in hematology (incl. supportive care/therapy)

Khalid Shedeed[1], Rita Zeka*[1]

[1]*Queen Elizabeth Hospital, University Hospital Birmingham, Haematology, Birmingham, United Kingdom;*

### Background

Neutropenic fever is an expected occurrence in haematology patients undergoing stem cell transplant or CAR-T therapy. Chest X ray has traditionally been used as a screening test to exclude pneumonia.

### Aims

This study aimed to examine the utility of this method in the diagnosis and treatment of febrile neutropenia. As well as examining the total imaging the patients had in the same admission.

### Methods

We conducted a single centre retrospective and prospective cohort study over a 6-month period at a bone marrow transplant unit in a tertiary care hospital in Birmingham, United Kingdom. We examined 62 adult patients with febrile neutropenia undergoing autologous or allogeneic stem cell transplant or CAR-T therapy. 74% of patients were examined retrospectively and 26% prospectively. The patients were categorised based on occurrence of respiratory symptoms at the time chest X ray was performed. We assessed the frequency of abnormal chest X ray or CT results, as well as examining if this had therapeutic implications on management.

### Results

Chest X ray was performed in 23 patients (group 1) as initial investigation and CT chest in 8 patients (group 2) as initial investigation. 18 patients (78.2%) having chest X ray and 6 patients (75%) having CT chest did not have respiratory symptoms or oxygen requirement. In Group 1, 8 patients had respiratory symptoms of which 1 had positive imaging findings, 4 of these went on to have CT chest as a follow up investigation. In Group 2, 2 patients had respiratory symptoms of which 1 had positive findings on the CT. Out of the 62 patients 12.9% were diagnosed with chest infection based on imaging findings, 62.5% of these patients were asymptomatic.
The total number of chest X rays performed was 46 (average 0.74), total number of CT performed was 60 (average 0.96). The total number of imaging for all patients was 106 (average 2.26).

### Summary/Conclusion

We found no cases where chest X ray results had therapeutic implications in management of febrile neutropenia, therefore making it safe to be excluded in absence of respiratory symptoms.

**Copyright Information:** (Online) ISSN: 2572-9241
©2026 The Author(s). HemaSphere is published by John Wiley & Sons Ltd on behalf of European Hematology Association. This is an open access Abstract Book distributed under the Attribution-NonCommercial-NoDerivs (CC BY-NC-ND), which allows third parties to download the articles and share them with others as long as they credit the author and the Abstract Book, but they cannot change the content in any way or use them commercially.
**Abstract Book Citations:** Authors, Title, HemaSphere, 2026;10:(S1):pages. The abstract book DOIs can be found on the "Author Information" page.
**Disclaimer:** Articles published in the journal HemaSphere exclusively reflect the opinions of the authors. The authors are responsible for all content in their abstracts including accuracy of the facts, statements, citing resources, etc.

### (PB4264) IMPACT OF ANTIMICROBIAL-RESISTANT INFECTIONS ON OUTCOMES AND LONG-TERM HEALTHCARE UTILIZATION IN HEMATOLOGIC MALIGNANCIES

**Topic:** 31. Infections in hematology (incl. supportive care/therapy)

Batyrkhan Kuatov*[1], Yi-Chun Lin[2], Lei Ni[2], Louise McCrink[2], Sorrel Wolowacz[2], Melike Inkaya Taskin[3]

*[1]CSL, Bern, Switzerland; [2]RTI Health Solutions, Manchester, United Kingdom; [3]CSL, Istanbul, Türkiye;*

### Background

Patients with hematologic malignancies (HM) and secondary immune deficiency (SID) are at high risk of serious and recurrent infections, which are a key driver of morbidity and mortality in HM. The global rise of antimicrobial resistance (AMR) is a particular concern in SID, where management of infections includes repeated courses of prophylactic or therapeutic antibiotics; however, the impact of AMR on clinical outcomes and healthcare-resource utilization (HCRU) in patients with SID and HM is poorly characterized.

### Aims

This targeted literature review aimed to summarize available evidence on AMR infections in patients with HM and SID, focusing on prevalence, incidence, severity, and associated mortality, utility and HCRU (hospitalizations, costs).

### Methods

Articles published between 2016 and 2025 were identified using electronic medical literature databases (MEDLINE, Embase, Cochrane Library), desktop internet searches (Google Scholar, PubMed), and reviewing reference lists of key studies. Pre-specified search terms included combinations of free text, Medical Subject Headings, and Emtree terms. Publications were screened for HM subtype and confirmed/probable SID/hypogammaglobulinemia (HGG); high-quality sources (eg, systematic reviews and large observational studies) were prioritized. Data extracted from relevant publications included: clinical and HCRU parameters; population (SID in multiple myeloma [MM], chronic lymphocytic leukemia [CLL], non-Hodgkin lymphoma [NHL], other cancer); infection type; intervention details; AMR status; study type; country.

### Results

Overall, 147 records were identified for screening (databases: n= 56; internet: n=76; reference lists: n=15). SID populations were reported in only 3 studies; therefore, studies reporting on AMR infections in patients with HM or other cancers were also included, as such infections are likely to be associated with SID due to underlying disease and treatment-related immunosuppression.
After screening, 41 publications were selected for data extraction (n=3 confirmed SID/HGG; n=38 probable SID). Four studies included patients with MM (n=0 reported CLL or NHL specifically), 34 with other HM, and 3 with other cancers.
In total, 20 and 22 studies, respectively, reported prevalence and mortality data for AMR infections. Few studies reported other outcomes: incidence (n=4) and severity (n=2) of AMR infection; intensive care unit (ICU) stay (n=1); hospitalization length-of-stay (n=4); impact of AMR on infection treatment effectiveness (n=4); impact of prophylaxis on AMR infection (n=1).
No studies reported utility or costs associated with AMR infections in confirmed or probable SID populations.
While 24 studies reported infection prophylaxis regimen, the impact of infection prophylaxis on AMR infection (n=1) and the impact of AMR on infection treatment efficacy (n=1) was scarcely reported. Of note, a meta-analysis that included an SID population showed the relative risk of developing an AMR infection was 2.05 (95% confidence interval, 1.88–2.23) with, vs. without, antibiotic prophylaxis.

**Copyright Information:** (Online) ISSN: 2572-9241
©2026 The Author(s). HemaSphere is published by John Wiley & Sons Ltd on behalf of European Hematology Association. This is an open access Abstract Book distributed under the Attribution-NonCommercial-NoDerivs (CC BY-NC-ND), which allows third parties to download the articles and share them with others as long as they credit the author and the Abstract Book, but they cannot change the content in any way or use them commercially.
**Abstract Book Citations:** Authors, Title, HemaSphere, 2026;10:(S1):pages. The abstract book DOIs can be found on the "Author Information" page.
**Disclaimer:** Articles published in the journal HemaSphere exclusively reflect the opinions of the authors. The authors are responsible for all content in their abstracts including accuracy of the facts, statements, citing resources, etc.

### Summary/Conclusion

Data on the impact of AMR infections in patients with HM and SID are sparse, but existing data provide preliminary evidence of an increased AMR risk with antibiotic prophylaxis in these patients. Our findings highlight key evidence limitations, underscoring the need for further research to better understand outcomes and healthcare consequences in this vulnerable and expanding patient population.

**Heat map of number of AMR studies identified per variable of interest by cancer type**

| | | MM | CLL | NHL | Other HM* | Other cancer |
|---|---|---|---|---|---|---|
| Clinical outcomes | Prevalence | n=2 (SID-specific n=1) | | | n=17 (SID-specific n=1) | n=2 |
| | Incidence | | | | n=3 | n=1 |
| | Severity | | | | n=2 | |
| | Hospitalisation | | | | | |
| | ICU stay | | | | | n=1 |
| | Hospitalisation length of stay | | | | n=4 | |
| | ICU length of stay | | | | | |
| | Readmission | | | | | |
| | AMR infection-attributable mortality | n=2 | | | n=20 | n=2 |
| | Impact of AMR on infection treatment efficacy | | | | n=4 | |
| | Impact of infection prophylaxis on AMR | | | | n=1 (SID-specific n=1) | |
| Costs and HRQoL | Utility | | | | | |
| | Duration of utility loss | | | | | |
| | Cost | | | | | |

5+ studies
4 studies
3 studies
2 studies
1 study
0 studies

*Included patients with other HMs or HM not specific to a single indication.

AMR, antimicrobial resistance; CLL, chronic lymphocytic leukemia; HM, hematologic malignancy; HRQoL, health-related quality of life; ICU, intensive care unit; MM, multiple myeloma; NHL, non-Hodgkin lymphoma; SID, secondary immunodeficiency

**Copyright Information:** (Online) ISSN: 2572-9241
©2026 The Author(s). HemaSphere is published by John Wiley & Sons Ltd on behalf of European Hematology Association. This is an open access Abstract Book distributed under the Attribution-NonCommercial-NoDerivs (CC BY-NC-ND), which allows third parties to download the articles and share them with others as long as they credit the author and the Abstract Book, but they cannot change the content in any way or use them commercially.
**Abstract Book Citations:** Authors, Title, HemaSphere, 2026;10:(S1):pages. The abstract book DOIs can be found on the "Author Information" page.
**Disclaimer:** Articles published in the journal HemaSphere exclusively reflect the opinions of the authors. The authors are responsible for all content in their abstracts including accuracy of the facts, statements, citing resources, etc.

### (PB4265) CANDIDEMIA IN HEMATOLOGIC MALIGNANCIES : A SINGLE-CENTER EXPERIENCE

**Topic:** 31. Infections in hematology (incl. supportive care/therapy)

Nour Siala*[1], Maha Charfi[1], Fakhfekh Yousra[1], Faten Kallel[1], Nahed Khemakhem[2], Imen Frikha[1], Imen Ben Amor[1], Rahma Mallek[1], Ines Turki[1], Asma Koubaa[1], Hanen Charfi[1], Aicha Ben Kahla[1], Moez Medhaffar[1], Ali Ayadi[2], Moez Elloumi[1]

[1]*Hematology department Hedi Chaker Hospital, Sfax, Tunisia;* [2]*Parasitology laboratory Habib Bourguiba hospital, Sfax, Tunisia;*

### Background

Invasive fungal infections (IFIs) are a significant complication in oncology and hematology. Immunosuppression in patients with hematologic malignancies promotes the growth of infectious agents, primarily from the Candida and Aspergillus genera. Systemic candidiasis can be either Candida bloodstream infection (candidemia) or deep-seated candidal infection involving visceral organs. Candida yeasts disseminate hematogenously to various organs, adhere to tissues, and lead to significant therapeutic challenges.

### Aims

This study aimed to describe the epidemiological, clinical, microbiological, and therapeutic characteristics of candidemia in our department.

### Methods

We conducted a retrospective study including patients with hematologic malignancies who developed candidemia during treatment and were followed in the Hematology Department of Hedi Chaker Hospital between 2020 and 2024. Investigations for secondary localization were systematically performed on all patients with candidemia, including fundoscopic examination, transthoracic echocardiography, and thoraco-abdomino-pelvic computed tomography.

### Results

A total of 24 patients were identified, with a median age of 36 years (range: 1–57 years). Seventy-five percent had acute myeloid leukemia, while 17% had acute lymphoblastic leukemia. Eight percent of cases involved relapsed hematologic malignancies. Four patients were receiving voriconazole for pulmonary aspergillosis at the time of candidemia. Regarding risk factors for candidiasis, 88% of the patients had profound neutropenia, which lasted a median of 17 days (range: 10–45 days), and all were receiving broad-spectrum antibiotic therapy. Eight patients (33%) had a central venous catheter at the time of infection. Candida tropicalis was the most frequently identified species (62%), followed by C. parapsilosis, C. glabrata, and C. albicans (8% each). Deep-seated candidiasis was documented in 11 patients (46%), with pulmonary (73%) and hepatic (64%) being the most frequent localizations. All patients received antifungal therapy within four days of receiving the candidemia diagnosis. A complete response was achieved in 10 patients (42%). Five patients (21%) had died by the last follow-up.

**Copyright Information:** (Online) ISSN: 2572-9241
©2026 The Author(s). HemaSphere is published by John Wiley & Sons Ltd on behalf of European Hematology Association. This is an open access Abstract Book distributed under the Attribution-NonCommercial-NoDerivs (CC BY-NC-ND), which allows third parties to download the articles and share them with others as long as they credit the author and the Abstract Book, but they cannot change the content in any way or use them commercially.
**Abstract Book Citations:** Authors, Title, HemaSphere, 2026;10:(S1):pages. The abstract book DOIs can be found on the "Author Information" page.
**Disclaimer:** Articles published in the journal HemaSphere exclusively reflect the opinions of the authors. The authors are responsible for all content in their abstracts including accuracy of the facts, statements, citing resources, etc.

**Summary/Conclusion**

Candidiasis is the most common invasive fungal infection (IFI), accounting for about 80% of cases. Strategies that reduce the duration of neutropenia through growth factor administration and limit exposure to broad-spectrum antibiotics in neutropenic patients may decrease the incidence of these infections.
In our study, Candida tropicalis was the predominant species, consistent with published data reporting it as one of the most frequently documented in systemic candidiasis. The observed mortality rate of 21% aligns with previously reported rates ranging from 20% to 40% in this high-risk population. Recognizing risk factors associated with IFIs early on may facilitate prompt diagnosis and the initiation of antifungal therapy, thereby improving survival outcomes.

**Copyright Information:** (Online) ISSN: 2572-9241
©2026 The Author(s). HemaSphere is published by John Wiley & Sons Ltd on behalf of European Hematology Association. This is an open access Abstract Book distributed under the Attribution-NonCommercial-NoDerivs (CC BY-NC-ND), which allows third parties to download the articles and share them with others as long as they credit the author and the Abstract Book, but they cannot change the content in any way or use them commercially.
**Abstract Book Citations:** Authors, Title, HemaSphere, 2026;10:(S1):pages. The abstract book DOIs can be found on the “Author Information” page.
**Disclaimer:** Articles published in the journal HemaSphere exclusively reflect the opinions of the authors. The authors are responsible for all content in their abstracts including accuracy of the facts, statements, citing resources, etc.

### (PB4266) CANDIDEMIA GUIDELINE ADHERENCE VIA MOBILE APP – DEVELOPMENT AND DATA COLLECTION

**Topic:** 31. Infections in hematology (incl. supportive care/therapy)

Oliver A. Cornely[1, 2, 3, 4], Nico Bekaan*[1, 2], Rosanne Sprute[1, 2, 3]

*[1]University of Cologne, Faculty of Medicine and University Hospital Cologne, Institute of Translational Research, Cologne Excellence Cluster on Cellular Stress Responses in Aging-Associated Diseases (CECAD), Cologne, Germany; [2]University of Cologne, Faculty of Medicine and University Hospital Cologne, Department I of Internal Medicine, Excellence Center for Medical Mycology (ECMM), Cologne, Germany; [3]German Centre for Infection Research (DZIF), Partner Site Bonn-Cologne, Cologne, Germany; [4]University of Cologne, Faculty of Medicine and University Hospital Cologne, Clinical Trials Centre Cologne (ZKS Köln), Cologne, Germany;*

### Background

Adherence to evidence-based guidelines improves outcomes in patients with candidemia. The European Confederation of Medical Mycology Quality (EQUAL) Candida Score provides a standardized method to assess compliance with candidemia guidelines. Previous studies have shown that higher EQUAL score values are associated with improved survival rates.

### Aims

To develop and implement a mobile application that integrates the EQUAL Candida Score, enabling clinicians to rapidly assess guideline adherence and to facilitate international data collection for quality monitoring and research.

### Methods

A mobile application was developed to implement the EQUAL Candida Score. Cross-platform compatibility for iOS and Android was achieved using the Flutter framework, which enables native performance on both operating systems from a single codebase.
Server-side infrastructure was implemented using PHP and MySQL to handle data transmission and storage. To support multilingual distribution, an international team of experts was brought together using software Tolgee for collaborative translations.
The application interface guides clinicians through a structured checklist for diagnosis, treatment and follow-up. Inputs are evaluated on the results page, which also presents the scorecard information and provides links to references.
Users are asked to confirm whether a real and completed case is being documented before submission.

### Results

The first stable version of the app was released in November 2023 via Google Play and the Apple App Store. Until 25$^{th}$ February 2026 2009 cases were transmitted including 718 real cases according to self-classification and after cleaning.
The real cases were primarily submitted from Germany (369 cases), followed by Brazil (42), Turkey (35), the Philippines (22), Argentina (18) and other 54 countries. This distribution can be explained by local spread, as well as international sharing through the involvement of translators and at conferences. 425 users reported real cases, with 22% submitting multiple reports. On average it took 44 seconds to go through the checklist.

### Summary/Conclusion

The EQUAL Candida Score app enables rapid and systematic evaluation of guideline adherence for candidemia management.
Its cross-platform design and multilingual capability support widespread clinical use and international research collaboration.
Ongoing development will focus on data-driven insights to further optimize guideline implementation and patient outcomes.

**Copyright Information:** (Online) ISSN: 2572-9241
©2026 The Author(s). HemaSphere is published by John Wiley & Sons Ltd on behalf of European Hematology Association. This is an open access Abstract Book distributed under the Attribution-NonCommercial-NoDerivs (CC BY-NC-ND), which allows third parties to download the articles and share them with others as long as they credit the author and the Abstract Book, but they cannot change the content in any way or use them commercially.
**Abstract Book Citations:** Authors, Title, HemaSphere, 2026;10:(S1):pages. The abstract book DOIs can be found on the “Author Information” page.
**Disclaimer:** Articles published in the journal HemaSphere exclusively reflect the opinions of the authors. The authors are responsible for all content in their abstracts including accuracy of the facts, statements, citing resources, etc.

### (PB4267) BETIBEGLOGENE AUTOTEMCEL STEM CELL COLLECTION IN THREE Β-THALASSEMIA PATIENTS

**Topic:** 32. Transfusion medicine

Lefan Zhuang*[1], Deisen Lauro[1], Allen Lin[1], Jennifer Woo[2], Ryan Jackson[1], Shirong Wang[1], Shan Yuan[1]

*[1]City of Hope, Duarte, United States of America;[2]City of Hope, Irvine, United States of America;*

### Background

Betibeglogene autotemcel (beti-cel) is an autologous gene-therapy product requiring robust mobilization and collection of CD34⁺ hematopoietic stem cells. Ensuring efficient apheresis yield is critical for successful product manufacturing.

### Aims

We report three real-world cases of beti-cel collections performed in two patients with E-β thalassemia and one with β thalassemia major, highlighting mobilization approaches, collection characteristics, and CD34⁺ cell yields.

### Methods

Data were reviewed from three individuals: a 6-year-old male with E-β thalassemia, a 28-year-old female with β-thalassemia major, and a 31-year-old female with E-β thalassemia. All underwent mobilization with daily G-CSF starting 4 days prior to first collection day through the last collection day. Plerixafor was given 4.5 to 6 hours prior to the start of each collection. Apheresis was performed on consecutive collection days using Spectra Optia (software version 12.1) and ACD-A anticoagulant ratio of 10:1.

### Results

All three patients successfully completed mobilization and collection without reported adverse events. The 6-year-old male demonstrated the highest yield, with a mean total CD34⁺ collection of 54.4 $\times 10^6$/kg over three days, of which 45.6 $\times 10^6$/kg was sent (manufacturing incomplete at time of writing). The 28-year-old female collected 48.9 $\times 10^6$/kg over two days, with 35.3 $\times 10^6$/kg sent for manufacturing, and a manufactured beti-cel product of 7.85 $\times 10^6$/kg (22.2% of sent dose). One month post infusion this patient continues to be RBC transfusion dependent. The 31-year-old female achieved a total collection of 35.2 $\times 10^6$/kg over three days, with 30.0 $\times 10^6$/kg sent for manufacturing, and a manufactured beti-cel product of 7.85 $\times 10^6$/kg (26.1% of sent dose).

### Summary/Conclusion

Across three diverse patients, mobilization using G-CSF with plerixafor produced good CD34⁺ yields for beti-cel manufacturing. These findings support the reliability of standard mobilization and apheresis protocols for robust gene-therapy collections in β-thalassemia major and E-β thalassemia patients.

**Copyright Information:** (Online) ISSN: 2572-9241
©2026 The Author(s). HemaSphere is published by John Wiley & Sons Ltd on behalf of European Hematology Association. This is an open access Abstract Book distributed under the Attribution-NonCommercial-NoDerivs (CC BY-NC-ND), which allows third parties to download the articles and share them with others as long as they credit the author and the Abstract Book, but they cannot change the content in any way or use them commercially.
**Abstract Book Citations:** Authors, Title, HemaSphere, 2026;10:(S1):pages. The abstract book DOIs can be found on the "Author Information" page.
**Disclaimer:** Articles published in the journal HemaSphere exclusively reflect the opinions of the authors. The authors are responsible for all content in their abstracts including accuracy of the facts, statements, citing resources, etc.

### (PB4268) IMPLEMENTING PATIENT BLOOD MANAGEMENT (PBM): REVIEW OF TRANSFUSION PRACTICE IN THE GYNECOLOGY SERVICE

**Topic:** 32. Transfusion medicine

Maria Teresa Calderón López*[1], María Piñero Montes[1], Gonzalo del Campo Balguerías[2], Pablo Jiménez Montero[1], Julia García Espeso[1], Cristina Serí Merino[1], Jesús Viñas Soler[1], Edna Margarita Sandoval Barreto[1], Maria Jose Otero Martinez-Fornes[1], Jose Rogelio Díaz Valdés[1]

*[1]Hospital Central De La Defensa Gómez Ulla, Madrid, Spain;[2]Hospital Universitario 12 De Octubre, Madrid, Spain;*

### Background

Red blood cell transfusion is a common practice in surgical departments. When performed on women of childbearing age, it is especially relevant due to the possibility of developing erythrocyte alloantibodies that can cause hemolytic disease of the fetus and newborn (HFDN). For this reason, every effort is made to transfuse these women with Rh and Kell-compatible red blood cells. A primary objective is to avoid unnecessary transfusions if iron supplementation can be sufficient.

### Aims

To analyze the indications for packed red blood cell (PRBC) transfusions in the Gynecology Department at our Center, a 520-bed university hospital, to implement improvement measures as part of the Patient Blood Management (PBM) project.

### Methods

We retrospectively analyzed red blood cell transfusions requested by the Gynecology Department of our hospital from January to December last year. We reviewed the requests, complete blood counts, and pre-transfusion iron studies of the patients and consulted the minimum necessary data from their medical records to complete this analysis. Data collection was carried out using the Blood Bank's software, and the patients health records.

### Results

In a whole year, the Gynecology Department requested transfusions of 64 units of packed red blood cells (pRBC) for 29 patients. Two of these 29 patients were excluded from the analysis due to advanced age and anemia of chronic disease. The data of the remaining 27 patients were analyzed. The data are presented in the tables:

| **Study population** | |
|---|---|
| Number of patients | 27 |
| Median age of patients | 35 years old (18-53) |
| Median number of pRBC transfused per patient | 2 (1-5) |

**Copyright Information:** (Online) ISSN: 2572-9241
©2026 The Author(s). HemaSphere is published by John Wiley & Sons Ltd on behalf of European Hematology Association. This is an open access Abstract Book distributed under the Attribution-NonCommercial-NoDerivs (CC BY-NC-ND), which allows third parties to download the articles and share them with others as long as they credit the author and the Abstract Book, but they cannot change the content in any way or use them commercially.
**Abstract Book Citations:** Authors, Title, HemaSphere, 2026;10:(S1):pages. The abstract book DOIs can be found on the "Author Information" page.
**Disclaimer:** Articles published in the journal HemaSphere exclusively reflect the opinions of the authors. The authors are responsible for all content in their abstracts including accuracy of the facts, statements, citing resources, etc.

| | |
|---|---|
| Total pRBC transfused | 61 |
| Number of patients receiving more than 2 pRBC | 6 |
| | |
| **Laboratory data** | |
| **Pre-transfusion** | |
| Hb (g/dl) | 7 (4.2 – 8.8) median and range<br>Hb ≥7 in 14 patients (52%)<br>Hb ≥8 in 3 patients (11%) |
| VCM (fl) | 83 (58 - 98) median and range |
| Ferritin (only requested in 8 patients) (ng/ml) | 10 (2-31) median and range<br>(normal values 13-150) |
| Pregnant women with ferritin<20 in 3rd trimester blood work | 3/8 (37%) |
| Pregnant women with Hb<11 in 3rd trimester blood work | 2/8 (25%) |
| **Post-transfusion** | |
| Hb | 9.1 g/dl (6.8-11.9) median and range |

| **Etiology of anemia** | | **Patients** |
|---|---|---|
| OBGyn (11) | Vaginal delivery | 3 |
| | Cesarean section | 4 |

**Copyright Information:** (Online) ISSN: 2572-9241
©2026 The Author(s). HemaSphere is published by John Wiley & Sons Ltd on behalf of European Hematology Association. This is an open access Abstract Book distributed under the Attribution-NonCommercial-NoDerivs (CC BY-NC-ND), which allows third parties to download the articles and share them with others as long as they credit the author and the Abstract Book, but they cannot change the content in any way or use them commercially.
**Abstract Book Citations:** Authors, Title, HemaSphere, 2026;10:(S1):pages. The abstract book DOIs can be found on the "Author Information" page.
**Disclaimer:** Articles published in the journal HemaSphere exclusively reflect the opinions of the authors. The authors are responsible for all content in their abstracts including accuracy of the facts, statements, citing resources, etc.

| | | |
|---|---|---|
| | Abortion | 1 |
| | Ectopic pregnancy | 3 |
| Uterin fibroids | | 12 |
| Gynecologic oncology surgery | | 3 |
| Benign gynecologic surgery (abscess) | | 1 |
| Total | | 27 |

In the group of pregnant women who received red blood cell transfusions at the end of the pregnancy, marked iron deficiency was present in more than one-third of them; also, in those who received transfusions for metrorrhagia due to uterine fibroids. A review of medical records revealed that the vast majority were receiving oral iron therapy, but this support was insufficient, ineffective, or not monitored after its initiation to allow for a switch to intravenous iron if oral iron was not tolerated or absorbed. It is noteworthy that ferritin levels were not measured in many patients; only serum iron levels were measured.

### Summary/Conclusion

In almost half of the patients (13/27), red blood cell transfusions could have been avoided, at least in part, with proper treatment and monitoring of iron deficiency anemia. It´s very important given the risk of alloimmunization and HFDN with transfusions in women of childbearing age. These results underscore the importance of implementing patient blood management strategies, emphasizing early diagnosis and treatment of iron deficiency anemia to minimize unnecessary transfusions and improve both maternal and fetal outcomes.

**Copyright Information:** (Online) ISSN: 2572-9241
©2026 The Author(s). HemaSphere is published by John Wiley & Sons Ltd on behalf of European Hematology Association. This is an open access Abstract Book distributed under the Attribution-NonCommercial-NoDerivs (CC BY-NC-ND), which allows third parties to download the articles and share them with others as long as they credit the author and the Abstract Book, but they cannot change the content in any way or use them commercially.
**Abstract Book Citations:** Authors, Title, HemaSphere, 2026;10:(S1):pages. The abstract book DOIs can be found on the "Author Information" page.
**Disclaimer:** Articles published in the journal HemaSphere exclusively reflect the opinions of the authors. The authors are responsible for all content in their abstracts including accuracy of the facts, statements, citing resources, etc.

### (PB4269) ARE FRESH FROZEN PLASMA TRANSFUSIONS ORDERED APPROPRIATELY FOR THE PATIENTS IN THE EMERGENCY MEDICINE DEPARTMENT? A PRACTICE AUDIT IN A TERTIARY CARE TEACHING HOSPITAL

**Topic:** 32. Transfusion medicine

Darija Bogdanic*[1], Mirela Raos[1, 2], Fini Plenkovic[1], Ines Bojanic[1, 2]

*[1]University Hospital Centre Zagreb, ZAGREB, Croatia;[2]University of Zagreb, School of Medicine, ZAGREB, Croatia;*

**Background**

Fresh frozen plasma (FFP) can be a life saving threatment option, but also a high-risk one because of transfusion reactions. Per guidlines indications are limited, but the number of ordered units in the Emergency Medicine Department (EMD) suggests otherwise.

**Aims**

The aim was to assess the ordering and utilisation of FFP in EMD of a tertiary care teaching hospital, and evaluate the appropriateness of its use.

**Methods**

A retrospective analysis of FFP requests for adult patients in EMD between January 2025 and December 2025  was conducted. Data were collected manually using the hospital and transfusion service information systems. The completeness of the request form completion was analysed, as well as the appropriateness of ordering FFP, which was assessed according to the patient's diagnosis, indications, and subsequent treatment.

**Copyright Information:** (Online) ISSN: 2572-9241
©2026 The Author(s). HemaSphere is published by John Wiley & Sons Ltd on behalf of European Hematology Association. This is an open access Abstract Book distributed under the Attribution-NonCommercial-NoDerivs (CC BY-NC-ND), which allows third parties to download the articles and share them with others as long as they credit the author and the Abstract Book, but they cannot change the content in any way or use them commercially.
**Abstract Book Citations:** Authors, Title, HemaSphere, 2026;10:(S1):pages. The abstract book DOIs can be found on the "Author Information" page.
**Disclaimer:** Articles published in the journal HemaSphere exclusively reflect the opinions of the authors. The authors are responsible for all content in their abstracts including accuracy of the facts, statements, citing resources, etc.

**Results**

During one year, out of 5167 units transfused in our hospital, 333 (6.4%) FFP units were ordered in EMD for 107 (10.3%) patients out of 1036 FFP transfused patients. The patients' median age was 69.3 years (range 36.7-90.8 years), and 78 (72.9%) were male.
There were 110 requests for FFP transfusions, 71 (64.5%), 55 (50%), and 54 (49%) requisition forms had no diagnosis, clinical indication and laboratory data, respectively; 22 (20%) had some other laboratory value, but no PT or INR noted.
Out of issued units, 250 (75.1%) were intended for 68 (63.6%) hospitalised surgical patients, 55 (16.5%) units for 23 (21.5%) patients set for nonsurgical wards admission and 28 (8.4%) for 16 (14.9%) non-hospitalised patients.
Out of 68 surgical patients, 5 (4.6%) were admitted through EMD for an allocated liver transplantation, 27 (25.2%) for aortic dissection or aneurysm rupture, 13 (12.1%) for abdominal surgery, and 11 (10.3%) to cardiac or vascular wards.
Regarding 39 (36.4%) nonsurgical and non-hospitalised patients, they were treated mostly for bleeding: 15 (14%) gastrointestinal, 3 (2.8%) respiratory, 1 (0.93%) ophthalmologic bleeding and hematuria 3 (2.8%). Other reasons were preprocedural, surgical threatment not requiring hospitalisation, infections, angioedema and unknown in one (0.93%) patient.
Ten (9%) requests were for 1 FFP unit; 8 for patients on anticoagulant therapy, of which 6 had bleeding (hematuria, abdominal wall, rectal, and thoracic bleedings), and 2 were to be operated on (acute abdomen, hip fracture). One patient had angioedema, and one had FV deficiency.
There were 47 (42.7%) requests for 2 FFP units, meaning that more than half had suboptimal doses of FFP ordered. Out of them, 16 went to surgical wards, 14 to internal medicine wards, of whom 8 had bleeding. The rest of the units were requested for unadmitted patients, of whom one was for unknown reasons.
Patients who had three or more units ordered were mostly set for hospital admission and operation, and those requests were written for that purpose and later transfused during the surgery. In 4 cases, however, patients had gastrointestinal bleeding and were hospitalised at internal medicine wards.
Although in EMD the request for emergency transfusion of AB blood type FFP could be expected, in our centre, only 5 units were issued to one patient with aortic dissection.

**Summary/Conclusion**

Current FFP ordering and utilisation at our EMD have been suboptimal, and better strategies for monitoring of indications and dosing are needed, as inappropriate use raises important questions about rationality, cost, and risk implications.

**Copyright Information:** (Online) ISSN: 2572-9241
©2026 The Author(s). HemaSphere is published by John Wiley & Sons Ltd on behalf of European Hematology Association. This is an open access Abstract Book distributed under the Attribution-NonCommercial-NoDerivs (CC BY-NC-ND), which allows third parties to download the articles and share them with others as long as they credit the author and the Abstract Book, but they cannot change the content in any way or use them commercially.
**Abstract Book Citations:** Authors, Title, HemaSphere, 2026;10:(S1):pages. The abstract book DOIs can be found on the "Author Information" page.
**Disclaimer:** Articles published in the journal HemaSphere exclusively reflect the opinions of the authors. The authors are responsible for all content in their abstracts including accuracy of the facts, statements, citing resources, etc.

### (PB4270) EVALUATION OF RED BLOOD CELL TRANSFUSION PRACTICES: A SINGLE-CENTER AUDIT FROM TUNISIA

**Topic:** 32. Transfusion medicine

Ikbel Ghachem*[1], Mohamed Yassine Kaabar[2], Aya Kahlaoui[3], Selma Ben Cheick[4], Lamia Sghair[5], Asma Bachali[1]

*[1]University of Tunis El Manar, Faculty of Medicine of Tunis, Laboratory of Biology, Tahar Maamouri Hospital, Nabeul, Tunisia; [2]University of Monastir, Faculty of Pharmacy of Monastir, Tahar Maamouri Hospital, Nabeul, Tunisia;[3]Faculty of medicine of Tunis, NABEUL, Tunisia; [4]Laboratory of Biology, Taher Maamouri Hospital, NABEUL, Tunisia;[5]Laboratory of Biology, Taher Maamouri Hospital, Nabeul, Tunisia;*

### Background

Red blood cell (RBC) transfusion is a vital intervention for managing anemia, yet inappropriate prescribing poses patient safety risks and strains blood bank resources. Adherence to established guidelines is critical for optimizing transfusion practices.

### Aims

This study aimed to assess the conformity of RBC prescription practices with the current French National Authority for Health (HAS) recommendations and to identify factors associated with non-compliant prescribing.

### Methods

We conducted a retrospective, descriptive analysis of all RBC orders processed at the Tahar Maamouri University Hospital (Nabeul, Tunisia) over a one-year period. Prescriptions were evaluated against a structured algorithm based on HAS guidelines. Key assessment criteria included: pre-transfusion hemoglobin (Hb) level, patient's clinical context (including tolerance of anemia and comorbidities), and the appropriateness of the quantity and specifications (e.g., phenotyping) of RBC units ordered.

### Results

A total of 500 transfusion episodes were analyzed. Overall, 85% of prescriptions were found to be compliant with guidelines. The remaining 15% of non-compliant prescriptions originated predominantly from the general surgery department. The primary reasons for non-compliance were an inadequate evaluation of the patient's clinical tolerance to anemia (51%) and failure to adhere to the recommended Hb threshold (49%). While compliant in principle, 33% of appropriate prescriptions called for an excessive number of RBC units, a pattern significantly associated with pre-transfusion Hb levels ≥ 8 g/dL. Critical safety omissions were also noted, with requests for phenotyped RBCs absent for 21% of multi-transfused patients and 35% of women of childbearing age. In compliant prescriptions that were transfused, 18% of the units administered were deemed excessive. Multivariate analysis identified that prescribing based solely on the pre-transfusion Hb level, without considering clinical context, increased the risk of a non-compliant prescription nearly five-fold (OR=4.9; 95% CI [2.9-5.2]; p<0.001). Conversely, the presence of documented comorbidities was a protective factor against non-compliance (OR=0.3; 95% CI [0.1-0.6]; p=0.003).

### Summary/Conclusion

This audit reveals that while a majority of RBC transfusions align with guidelines, significant areas for improvement exist, particularly in surgical departments. Key interventions, including targeted educational programs and the integration of digital decision-support tools, are needed to address the over-prescription of units and to enhance compliance with special transfusion requirements for at-risk patient populations.

**Copyright Information:** (Online) ISSN: 2572-9241
©2026 The Author(s). HemaSphere is published by John Wiley & Sons Ltd on behalf of European Hematology Association. This is an open access Abstract Book distributed under the Attribution-NonCommercial-NoDerivs (CC BY-NC-ND), which allows third parties to download the articles and share them with others as long as they credit the author and the Abstract Book, but they cannot change the content in any way or use them commercially.
**Abstract Book Citations:** Authors, Title, HemaSphere, 2026;10:(S1):pages. The abstract book DOIs can be found on the "Author Information" page.
**Disclaimer:** Articles published in the journal HemaSphere exclusively reflect the opinions of the authors. The authors are responsible for all content in their abstracts including accuracy of the facts, statements, citing resources, etc.

### (PB4271) SEVERE THYROTOXICOSIS MANAGED WITH THERAPEUTIC PLASMA EXCHANGE: REGIONAL EVIDENCE FOR STRENGTHENING ASFA GUIDELINES

**Topic:** 32. Transfusion medicine

Bala Bhasker Poluru Mranikrinda*[1]

[1] *hamad medical corporation, doha, Qatar;*

**Background**

Thyrotoxicosis is a serious endocrine emergency with mortality reaching 25%. Crises may be triggered by infection, trauma, emergency surgery, or abrupt cessation of antithyroid medications. Therapeutic plasma exchange (TPE) is recommended when antithyroid drugs cannot be used due to toxicity, are ineffective, or when rapid clinical control is required.

**Aims**

The aim of this study is to evaluate the clinical effectiveness, safety, and perioperative outcomes of TPE in patients with severe thyrotoxicosis requiring surgical intervention.

**Methods**

A retrospective review was conducted on all patients who received TPE before thyroidectomy at Hamad Medical Corporation (August 2022–August 2025). Demographic, clinical, laboratory, TPE-related, and perioperative data were analyzed.

**Results**

Fourteen patients were included (57% female; mean age 42 years). Average reduction of free T4 was 58.4% (18.8% to 75.6%) and average reduction of free T3 was 64.3% (8.8% to 89.8%). Indications included poor response to therapy (n=9), agranulocytosis (n=3), hepatotoxicity (n=1), urgent orthopedic surgery (n=1), Hernia surgery (n=1), neurologic surgery (n=1) and cardiac (n=5) or neurologic (n=2) complications. Thirty-six TPE sessions were performed (mean 2.5); two were aborted due to flow issues and two had mild allergic reactions. Eleven patients underwent total thyroidectomy, and one each underwent surgeries for Glioblastoma, Inguinal hernia and intertrochanteric fracture hip. No perioperative thyroid storm occurred despite some persistent biochemical hyperthyroidism.

**Summary/Conclusion**

TPE is a safe and effective adjunct for severe thyrotoxicosis, particularly in patient's intolerant of antithyroid drugs, unresponsive to medical therapy, or requiring urgent surgery. This cohort, one of the largest from the Middle East, supports consideration for upgrading the ASFA recommendation for TPE in thyrotoxicosis from Category II, Grade 2C to a higher evidence grade, potentially Category II, Grade 1A.

**Copyright Information:** (Online) ISSN: 2572-9241
©2026 The Author(s). HemaSphere is published by John Wiley & Sons Ltd on behalf of European Hematology Association. This is an open access Abstract Book distributed under the Attribution-NonCommercial-NoDerivs (CC BY-NC-ND), which allows third parties to download the articles and share them with others as long as they credit the author and the Abstract Book, but they cannot change the content in any way or use them commercially.
**Abstract Book Citations:** Authors, Title, HemaSphere, 2026;10:(S1):pages. The abstract book DOIs can be found on the "Author Information" page.
**Disclaimer:** Articles published in the journal HemaSphere exclusively reflect the opinions of the authors. The authors are responsible for all content in their abstracts including accuracy of the facts, statements, citing resources, etc.

### (PB4272) TURKEY'S FIRST "GOLDEN BLOOD" CASE: A REGULATOR-TYPE RHNULL PHENOTYPE

**Topic:** 32. Transfusion medicine

Vahap Okan*[1], Mustafa Tekin[2, 2, 2]

*[1]Department of Internal Medicine, Division of Hematology, Faculty of Medicine, Gaziantep University, Gaziantep, Türkiye, gaziantep, Türkiye; [2]Blood Bank, Gaziantep University Şahinbey Hospital, Gaziantep, Türkiye, gaziantep, Türkiye;*

### Background

A 30-year-old female (N.S., born June 12, 1995) was referred at 31 weeks of her third pregnancy for routine antenatal antibody screening. Her medical history included two previous deliveries (2017 and 2022), after which she received six units of packed red blood cells without documented adverse reactions.
Initial serologic testing identified her as blood group A Rh-negative. However, extended Rh phenotyping demonstrated complete absence of D, C, c, E, and e antigens. The indirect antiglobulin test was positive, whereas the direct antiglobulin test was negative.
Antibody identification using an 11-cell panel revealed strong (3+ AHG) pan-reactivity, consistent with anti-total Rh (anti-Rh29).
Hematologic evaluation demonstrated chronic compensated hemolytic anemia: hemoglobin levels 8.9–11.4 g/dL, RBC count 2.24–3.4 $\times10^6$/μL, elevated reticulocytes (7.79–9%), increased bilirubin, and markedly decreased haptoglobin (0.01–0.05 g/L). Peripheral smear showed numerous spherocytes (30–40%), stomatocytes, and polychromasia.
Molecular genetic analysis revealed a homozygous c.1067+1G>A splice-site mutation in the RHAG gene (RHAG*01N.07 allele), confirming regulator-type Rhnull phenotype.

### Results

The Rhnull phenotype arises through two mechanisms:

1. **Regulator type**, caused by mutations in the RHAG gene
2. **Amorph type**, resulting from defects in both RHD and RHCE genes

In regulator-type Rhnull, mutations in RHAG impair expression of Rh-associated glycoprotein (RhAG), which is essential for membrane assembly and expression of Rh proteins. Without functional RhAG, Rh antigens fail to be expressed on the erythrocyte surface.
Deficiency of the Rh complex compromises membrane structural stability and alters cation permeability, leading to chronic hemolysis. Clinically, patients typically exhibit mild to moderate compensated hemolytic anemia but may require transfusion during physiological stress such as pregnancy.
Transfusion management is particularly challenging because anti-Rh29 reacts with all Rh-positive RBCs. Compatible donors must also be Rhnull, making international rare donor registries essential. In pregnancy, maternal anti-total Rh antibodies may cross the placenta and cause hemolytic disease of the fetus and newborn (HDFN), necessitating close surveillance and multidisciplinary management.
This case emphasizes the importance of advanced serologic and molecular diagnostics in atypical Rh typing. Standard Rh typing alone may fail to detect this phenotype.

**Copyright Information:** (Online) ISSN: 2572-9241
©2026 The Author(s). HemaSphere is published by John Wiley & Sons Ltd on behalf of European Hematology Association. This is an open access Abstract Book distributed under the Attribution-NonCommercial-NoDerivs (CC BY-NC-ND), which allows third parties to download the articles and share them with others as long as they credit the author and the Abstract Book, but they cannot change the content in any way or use them commercially.
**Abstract Book Citations:** Authors, Title, HemaSphere, 2026;10:(S1):pages. The abstract book DOIs can be found on the "Author Information" page.
**Disclaimer:** Articles published in the journal HemaSphere exclusively reflect the opinions of the authors. The authors are responsible for all content in their abstracts including accuracy of the facts, statements, citing resources, etc.

**Summary/Conclusion**

We report the first genetically confirmed regulator-type Rhnull phenotype in Türkiye, established by identification of a homozygous RHAG splice-site mutation (c.1067+1G>A; RHAG*01N.07). Recognition of such rare phenotypes is essential for patient safety, transfusion planning, and obstetric management. Enrollment in rare donor registries and strategic blood management are critical components of care.

**Copyright Information:** (Online) ISSN: 2572-9241
©2026 The Author(s). HemaSphere is published by John Wiley & Sons Ltd on behalf of European Hematology Association. This is an open access Abstract Book distributed under the Attribution-NonCommercial-NoDerivs (CC BY-NC-ND), which allows third parties to download the articles and share them with others as long as they credit the author and the Abstract Book, but they cannot change the content in any way or use them commercially.
**Abstract Book Citations:** Authors, Title, HemaSphere, 2026;10:(S1):pages. The abstract book DOIs can be found on the "Author Information" page.
**Disclaimer:** Articles published in the journal HemaSphere exclusively reflect the opinions of the authors. The authors are responsible for all content in their abstracts including accuracy of the facts, statements, citing resources, etc.

### (PB4273) IMPROVING PLATELET TRANSFUSION PRACTICES IN AN ONCOLOGY CENTER THROUGH A QUALITY IMPROVEMENT (QI) INITIATIVE

**Topic:** 32. Transfusion medicine

Shruti Prem Sudha[1], Taif Najeebi*[1], Nabil Abdelfattah[1], Fatima Sajjad[1], Ali Alsharif[1], Aly Rashed[1], Uzma Zaidi[1], Mohammed Abouelela[1], Lateefa Daraj[1], Latifa Alkaabi[1], Manu Venkatesan[2], Suha Hejres[2], Yasmeen Humran[2], Cigdem Ozturk[1], Noora Butti[1], Rehab Ibrahim[2], Tarkan Yetisyigit[1], Shiv Rana[1], Mahmoud Elsobeiey[1], Ahmed Shammaa[1]

*[1]Bahrain Oncology Center, Muharraq, Bahrain; [2]King Hamad University Hospital, Muharraq, Bahrain;*

### Background

Platelet transfusions are a common supportive therapy in oncology, however inappropriate use increases healthcare costs, and leads to shortage of this resource for other patients.

### Aims

This quality improvement (QI) project aimed to audit platelet transfusion practices in terms of adherence to guidelines, and to implement interventions to optimize adherence.

### Methods

A retrospective audit of platelet transfusions over a 6-month period was done in a tertiary oncology center. Data collected included transfusion indications and thresholds, WHO grade of bleeding, post-transfusion increments, transfusion reactions, and deaths from bleeding. Adherence was measured against institutional policy and AABB standards. Department-wise trends in platelet utilization were analyzed. The audit results were shared with the clinical and QI teams, areas for improvement identified, and interventions introduced to encourage adherence.

**Copyright Information:** (Online) ISSN: 2572-9241
©2026 The Author(s). HemaSphere is published by John Wiley & Sons Ltd on behalf of European Hematology Association. This is an open access Abstract Book distributed under the Attribution-NonCommercial-NoDerivs (CC BY-NC-ND), which allows third parties to download the articles and share them with others as long as they credit the author and the Abstract Book, but they cannot change the content in any way or use them commercially.
**Abstract Book Citations:** Authors, Title, HemaSphere, 2026;10:(S1):pages. The abstract book DOIs can be found on the "Author Information" page.
**Disclaimer:** Articles published in the journal HemaSphere exclusively reflect the opinions of the authors. The authors are responsible for all content in their abstracts including accuracy of the facts, statements, citing resources, etc.

**Results**

The baseline audit screened 670 transfusion episodes, the most common indications were prophylaxis for thrombocytopenia (74%), and treatment for active bleeding (26%). Platelet thresholds for prophylactic transfusion observed were: $10x10^9$/L in 28 %, $20x10^9$/L in 56 %, and in 16 % transfusions were for platelet counts more than $20x10^9$/L for non-standard indications. In the therapeutic transfusion group, the majority of transfusions (57%) were for minor grades (WHO grade 1 or 2) bleeding. Effective platelet count increments post-transfusion were seen in 66 %. Ninety-nine percent of therapeutic transfusions resulted in resolution of bleeding (45% needed more than 1 unit to control bleeding), while two patients died of bleeding. There was one documented transfusion reaction. There were 30 inappropriate requests for more than one apheresis unit per transfusion, found to be due to ordering error on the part of the junior physician.
Overall 60% transfusions were not adherent to guidelines. The principal reasons for non-adherence were use of inappropriate thresholds for prophylactic transfusion without clinical justification (in 77%), followed by use of transfusion for minor grades of bleeding (in 21%). Monthly and department-wise trends showed consistent high rates of non-adherence.
The QI interventions introduced after the audit included changes to the electronic blood bank order mandating physician justification for prophylactic thresholds used, documentation of WHO grade of bleeding for therapeutic transfusions, and need for clarification of requests of more than one apheresis unit. Blood bank staff were trained to flag potentially inappropriate requests, and ask for more information in such cases. Educational sessions for junior and senior doctors were conducted department-wise to reinforce adherence to guidelines. Platelet transfusion threshold cards were circulated, and displayed in areas of easy visibility in resident rooms. A re-audit is planned 3 months later to assess the efficacy of these interventions in improving adherence to guidelines.

**Summary/Conclusion**

Auditing appropriateness of platelet utilization led to identifying areas for improvement in the use of platelet transfusions in oncology patients. A large proportion of platelet transfusions were inappropriate, suggesting the need for improved awareness, and stricter enforcement of transfusion policies among physicians. Multi-faceted interventions like education and EMR-based prompts were introduced, and ongoing staff training and regular audits will be maintained to promote rational use of blood products.

**Copyright Information:** (Online) ISSN: 2572-9241
©2026 The Author(s). HemaSphere is published by John Wiley & Sons Ltd on behalf of European Hematology Association. This is an open access Abstract Book distributed under the Attribution-NonCommercial-NoDerivs (CC BY-NC-ND), which allows third parties to download the articles and share them with others as long as they credit the author and the Abstract Book, but they cannot change the content in any way or use them commercially.
**Abstract Book Citations:** Authors, Title, HemaSphere, 2026;10:(S1):pages. The abstract book DOIs can be found on the “Author Information” page.
**Disclaimer:** Articles published in the journal HemaSphere exclusively reflect the opinions of the authors. The authors are responsible for all content in their abstracts including accuracy of the facts, statements, citing resources, etc.

### (PB4275) REAL-WORLD PRACTICE OF THERAPEUTIC APHERESIS HEMATOLOGIST-SUPERVISED: A TWO-YEAR DESCRIPTIVE STUDY IN A COLOMBIAN TERTIARY CENTER

**Topic:** 32. Transfusion medicine

Silvia Rey-Beltrán[1], Liliana Landazabal Camargo[2], María Luna-González[3, 4], Angela Maria Peña Castellanos[2, 3], Luis Antonio Salazar[2, 3], Manuel Rosales Acevedo[2, 3], Daniel Santamaria Uribe[2], Claudia Sossa-Melo*[1, 2]

*[1]Universidad Autónoma de Bucaramanga, Santander, Bucaramanga, Colombia;[2]Fundación Oftalmológica de Santander - FOSCAL, Floridablanca, Colombia; [3]Programa para el Tratamiento de Enfermedades Hemato-oncológicas de Santander-PROTEHOS, Santander, Floridablanca, Colombia; [4]Universidad Autónoma de Bucaramanga-UNAB, Facultad de Ciencias de la Salud, Santander, Bucaramanga, Colombia;*

### Background

Therapeutic apheresis (TA) is an extracorporeal modality utilized as a primary or adjunctive treatment across a broad spectrum of diseases. Its clinical application is standardized by the American Society for Apheresis (ASFA), whose 2023 guidelines categorize evidence-based indications to guide optimal patient care.

### Aims

To characterize the clinical practices and procedural outcomes of TA using the Spectra Optia System at a tertiary center in Santander, Colombia.

### Methods

We conducted a secondary analysis of medical records for all TA procedures Hematologist-supervised, performed between 2024 and 2026. Central venous catheters were used in 100% of cases per institutional protocol. Collected data included demographics, clinical indications, procedural characteristics, and adverse events (AEs). AEs were classified by severity (mild, moderate, severe) and analyzed according to procedure type.

### Results

A total of 245 TA sessions were performed on 47 patients, 29 females (61,7%), mean age 49.66 ± 17.59 years.  Procedure types included therapeutic plasma exchange (TPE; 91.49%), leukocytapheresis (6.38%), and thrombocytapheresis (2.13%). The mean number of sessions was 5.53 (range 4–7) for TPE, 2 (range 1–3) for leukocytapheresis, and 1 for thrombocytapheresis. Primary indications for TPE were neuromyelitis optica spectrum disorder (32.56%), multiple sclerosis (13.95%), and Guillain-Barré syndrome (11.63%). All leukocytapheresis and thrombocytapheresis procedures were indicated for hyperleukocytosis (100%) and thrombocytosis (100%), respectively. AEs occurred in 71.02% of all procedures. These were predominantly moderate (62.04%; requiring supportive medication, primarily due to hypocalcemia, hypofibrinogenemia, and prolonged PT/aPTT) or mild (8.1%; dyspnea, urticaria, anemia, thrombocytopenia). Severe AEs, specifically transfusion-associated circulatory overload (TACO) and hypotension, were rare (0.82%) (Figure 1). Notably, no procedure-related mortalities occurred.

### Summary/Conclusion

Hematologist-supervised TA at our center is a safe and effective intervention. High adherence to the evidence-based 2023 ASFA guidelines ensures optimal patient management and minimizes the risk of severe adverse events.

**Copyright Information:** (Online) ISSN: 2572-9241
©2026 The Author(s). HemaSphere is published by John Wiley & Sons Ltd on behalf of European Hematology Association. This is an open access Abstract Book distributed under the Attribution-NonCommercial-NoDerivs (CC BY-NC-ND), which allows third parties to download the articles and share them with others as long as they credit the author and the Abstract Book, but they cannot change the content in any way or use them commercially.
**Abstract Book Citations:** Authors, Title, HemaSphere, 2026;10:(S1):pages. The abstract book DOIs can be found on the "Author Information" page.
**Disclaimer:** Articles published in the journal HemaSphere exclusively reflect the opinions of the authors. The authors are responsible for all content in their abstracts including accuracy of the facts, statements, citing resources, etc.

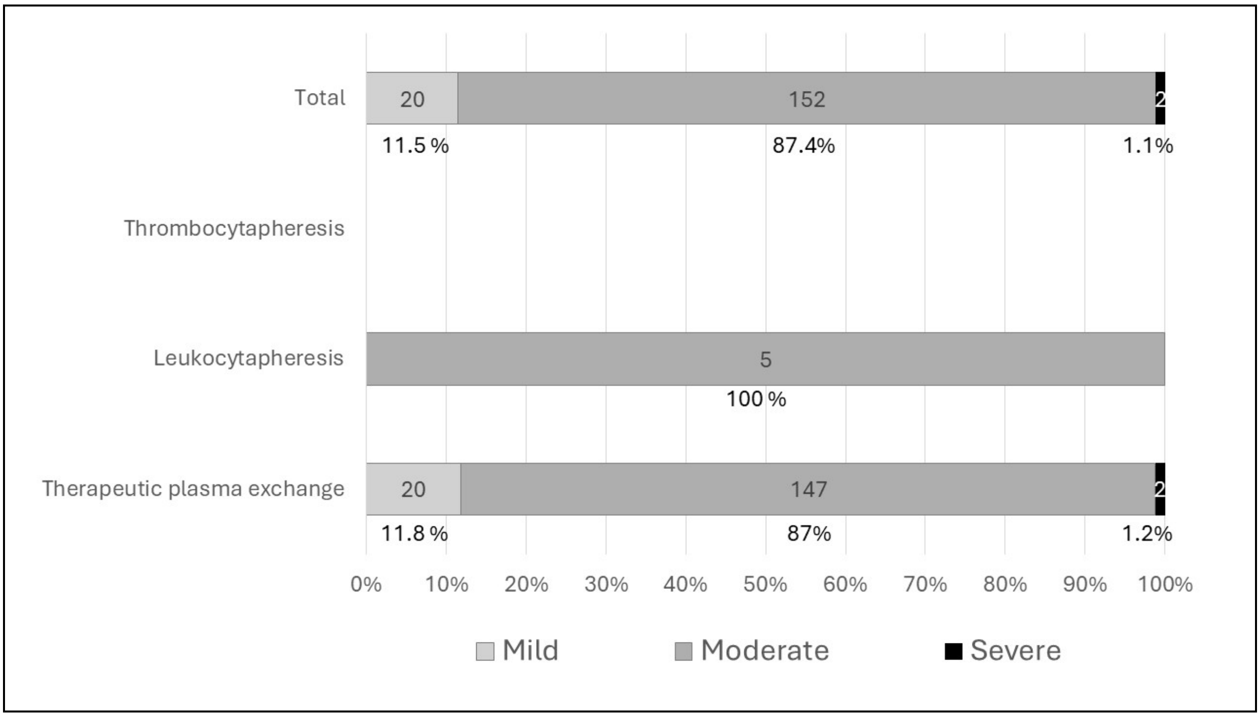

Adverse Events by Apheresis Modality

**Copyright Information:** (Online) ISSN: 2572-9241
©2026 The Author(s). HemaSphere is published by John Wiley & Sons Ltd on behalf of European Hematology Association. This is an open access Abstract Book distributed under the Attribution-NonCommercial-NoDerivs (CC BY-NC-ND), which allows third parties to download the articles and share them with others as long as they credit the author and the Abstract Book, but they cannot change the content in any way or use them commercially.
**Abstract Book Citations:** Authors, Title, HemaSphere, 2026;10:(S1):pages. The abstract book DOIs can be found on the "Author Information" page.
**Disclaimer:** Articles published in the journal HemaSphere exclusively reflect the opinions of the authors. The authors are responsible for all content in their abstracts including accuracy of the facts, statements, citing resources, etc.

### (PB4276) DECENTRALIZED BLOOD COMPONENT PREPARATION IN A RESOURCE-LIMITED HOSPITAL: INTERRUPTED TIME-SERIES ANALYSIS OF IMPLEMENTATION, DISRUPTION AND RECOVERY

**Topic:** 32. Transfusion medicine

Juliet Deche*[1], Betty Chepkaitany[1]

[1]*MOI COUNTY AND REFERRAL HOSPITAL VOI, LABORATORY DEPARTMENT, VOI, Kenya;*

### Background

Limited access to diverse blood components constrains transfusion practice in many resource-limited settings. In August 2024, a hospital-based satellite transfusion unit installed component preparation technology, enabling local production of platelet concentrates and cryoprecipitate in addition to existing products.

### Aims

To evaluate the impact of decentralized component preparation on transfusion utilization patterns and assess service resilience under real-world disruptions.

### Methods

A retrospective interrupted time-series analysis was conducted using transfusion data from January 2024 to December 2025. Seven months pre-implementation were compared with 17 months post-implementation. Monthly utilization trends were analyzed, including periods affected by industrial action (September 2024) and blood bag shortages (May 2025).

### Results

A total of 674 units were transfused pre-implementation which translated to (96 units/month) and 1534 units post-implementation (128 units/month), representing a 33% increase in monthly transfusion activity.
Pre-implementation distribution: packed red cells (98.2%), whole blood (1.2%), fresh frozen plasma (0.6%), platelets (0%).
Post implementation distribution: Packed red cells (94.1%), whole blood (2.0%), fresh frozen plasma (2.1%), platelets (1.9%).
Fresh frozen plasma utilization increased in more than threefold proportionally. Platelet concentrates were introduced for the first time following the equipment installation accounting for 29 transfusions in that period. Industrial action in september 2024 reduced total transfusions to 12 units. Blood bag shortages in May 2025 resulted in temporary suspension of component separation and increased whole blood use; however, platelet production resumed thereafter with no further shortages. Despite these disruptions, expanded component access was sustained overall.
Component preparation was associated with a 33% increase in monthly transfusion activity and a shift from 1.8% to 5.9% non-red cell component utilization.

| VARIABLE | PRE-IMPLEMENTATION (7 MONTHS) | POST-IMPLEMENTATION (17 MONTHS) |
|---|---|---|
| TOTAL UNITS TRANSFUSED | 674 | 1534 |
| PACKED RED CELLS | 662 (98.2%) | 1443 (94.1%) |
| WHOLE BLOOD | 8 (1.2%) | 30 (2.0%) |
| FRESH FROZEN PLASMA | 4 (0.6%) | 32 (2.1%) |
| PLATELET CONCENTRATES | 0 (0%) | 29 (1.9%) |
| MEAN UNITS PER MONTH | 96 | 128 |
| NON-RED CELL COMPONENTS | (1.8%) | (5.9%) |

**Copyright Information:** (Online) ISSN: 2572-9241
©2026 The Author(s). HemaSphere is published by John Wiley & Sons Ltd on behalf of European Hematology Association. This is an open access Abstract Book distributed under the Attribution-NonCommercial-NoDerivs (CC BY-NC-ND), which allows third parties to download the articles and share them with others as long as they credit the author and the Abstract Book, but they cannot change the content in any way or use them commercially.
**Abstract Book Citations:** Authors, Title, HemaSphere, 2026;10:(S1):pages. The abstract book DOIs can be found on the "Author Information" page.
**Disclaimer:** Articles published in the journal HemaSphere exclusively reflect the opinions of the authors. The authors are responsible for all content in their abstracts including accuracy of the facts, statements, citing resources, etc.

**Summary/Conclusion**

Decentralized component preparation significantly increased transfusion capacity, product diversity and expanded access to platelet therapy in a resource-limited hospital. Although workforce and supply chain disruptions temporarily affected service delivery, recovery was achieved and sustained. Strengthening transfusion services in low middle income (LMIC) settings requires both technological investment and system resilience.

**Copyright Information:** (Online) ISSN: 2572-9241
©2026 The Author(s). HemaSphere is published by John Wiley & Sons Ltd on behalf of European Hematology Association. This is an open access Abstract Book distributed under the Attribution-NonCommercial-NoDerivs (CC BY-NC-ND), which allows third parties to download the articles and share them with others as long as they credit the author and the Abstract Book, but they cannot change the content in any way or use them commercially.
**Abstract Book Citations:** Authors, Title, HemaSphere, 2026;10:(S1):pages. The abstract book DOIs can be found on the "Author Information" page.
**Disclaimer:** Articles published in the journal HemaSphere exclusively reflect the opinions of the authors. The authors are responsible for all content in their abstracts including accuracy of the facts, statements, citing resources, etc.

### (PB4277) COMPARISON OF THE COAGULATION POTENTIAL OF FREEZE-DRIED PLASMA DISSOLVED IN SALINE AND STERILE WATER

**Topic:** 32. Transfusion medicine

Svetlana Ziganshina[1], Ekaterina Konovalova[1], Elena Rosina[1], Sergey Ignatiev[1], Aleksey Lyanguzov[1], Elena Kormshchikova*[1]

[1]*Kirov Research Institute of Hematology and Blood Transfusion under the Federal Medical Biological Agence, Kirov, Russia;*

### Background

The use of freeze-dried plasma (FDP) makes it possible to increase the availability of transfusion therapy and the efficiency of medical care for victims with acute massive blood loss in extreme conditions. The blood component is obtained by lyophilization, which is the drying of plasma from a frozen state under vacuum. The use of such technology makes it possible to preserve the properties of biological material. Nevertheless, the lyophilization process is accompanied by phase transitions (crystallization, sublimation) and an increase pH. Before transfusion, the FDP is dissolved in a normal saline (NS), which increases the osmolarity. These factors can significantly affect the hemostatic properties of the blood component. During quality control of FDP the use of NS with adjusted pH 2.0, tris-buffered saline with pH 7.9 and sterile water during sample preparation leads to an increase in factor VIII activity compared to the use of NS. Of these solvents, sterile water may be an alternative to NS for the rehydration of freeze-dried plasma prior to transfusion.

### Aims

Comparison of the coagulation potential of freeze-dried plasma dissolved in saline and sterile water.

### Methods

Materials and methods. Plasma lyophilization was carried out in containers Plazmapak (RU No. RZN 2025/24964, Profit Farm LLC) using a laboratory lyophilization unit ULL-4.5M (NPP LioTekh LLC, Russia). The residual moisture content of the obtained samples was assessed by vacuum drying of an accurate sample. Freeze-dried plasma samples weighing 1.5 g were rehydrated in 17.3 ml of the preparation "Sodium chloride, 0.9% infusion solution" (n = 42). Total protein was measured using the colorimetric method according to the instructions for use of the reagent kit (OLVEX DIAGNOSTIKUM LLC, Russia). The activity of factor VIII was determined using the STA Compact MAX automatic hemostasis analyzer (Diagnostica STAGO, France) and compatible reagent kits.

### Results

Lyophilization of the plasma was performed on the ULL-4.5M unit. It has a two–way power supply to the material: conductive – due to heating of the shelf, and radiation - from screens located above the material. This made it possible to intensify the process of drying the blood component. The production of freeze-dried plasma involved filling 300 ml plasma into containers Plasmapack, shock freezing of the blood component and holding at a temperature of minus (80 ± 2) 0C for at least 12 hours, loading the containers into a freeze-drying chamber pre-cooled to a temperature of minus (40 ± 5) 0C and holding for at least 1 hour, gradual heating of the blood component up to a shelf temperature of minus 20 0C at a rate of 10 0C / hour and from minus 20 0C to 30 0C at a rate of 5 0C / hour at a process pressure of 8-10 Pa. When the set pressure was exceeded, the heat supply to the material stopped automatically. For uniform drying of the freeze-dried plasma, the heating of the screens was adjusted in the range (70 ± 15) % of the set maximum power. The total drying time was 40 - 46 hours. The samples obtained met the requirements of regulatory documentation: residual moisture — less than 2%, total protein — more than 50 g/L, factor VIII activity — at least 0.5 IU/ml, sterility.

**Copyright Information:** (Online) ISSN: 2572-9241
©2026 The Author(s). HemaSphere is published by John Wiley & Sons Ltd on behalf of European Hematology Association. This is an open access Abstract Book distributed under the Attribution-NonCommercial-NoDerivs (CC BY-NC-ND), which allows third parties to download the articles and share them with others as long as they credit the author and the Abstract Book, but they cannot change the content in any way or use them commercially.
**Abstract Book Citations:** Authors, Title, HemaSphere, 2026;10:(S1):pages. The abstract book DOIs can be found on the "Author Information" page.
**Disclaimer:** Articles published in the journal HemaSphere exclusively reflect the opinions of the authors. The authors are responsible for all content in their abstracts including accuracy of the facts, statements, citing resources, etc.

**Summary/Conclusion**

Freeze-dried plasma was obtained in polymer containers using membrane drying technology. The use of durable, lightweight and compact lyophilization containers has made it possible to obtain a high-quality blood component for the correction of coagulopathy in massive bleeding.

**Copyright Information:** (Online) ISSN: 2572-9241
©2026 The Author(s). HemaSphere is published by John Wiley & Sons Ltd on behalf of European Hematology Association. This is an open access Abstract Book distributed under the Attribution-NonCommercial-NoDerivs (CC BY-NC-ND), which allows third parties to download the articles and share them with others as long as they credit the author and the Abstract Book, but they cannot change the content in any way or use them commercially.
**Abstract Book Citations:** Authors, Title, HemaSphere, 2026;10:(S1):pages. The abstract book DOIs can be found on the "Author Information" page.
**Disclaimer:** Articles published in the journal HemaSphere exclusively reflect the opinions of the authors. The authors are responsible for all content in their abstracts including accuracy of the facts, statements, citing resources, etc.

### (PB4278) BOMBAY PHENOTYPE DIAGNOSED DURING PREGNANCY: CASE REPORT

**Topic:** 32. Transfusion medicine

Peterson Gontijo*[1, 2], Marcia T Delamain[1], Glaciano Ribeiro[1]

*[1]Ciencias Medicas de Minas Gerais, Hematology, Belo Horizonte, Brazil;[2]Grupo Pulsa, Hematology, Belo Horizonte, Brazil;*

### Background

The Bombay phenotype is characterized by the absence of ABO blood antigen expression on erythrocytes. Patients with this blood pattern have mutations in recessive alleles of the FUT1 gene, which encodes the expression of the fructosyltransferase enzyme, responsible for converting common precursors into the H antigen. As a consequence, red blood cells do not express molecules such as A, B, and H antigens, and anti-H antibodies are formed, leading to incompatibility of dissimilar phenotypes in situations where blood transfusion is necessary. Due to its rarity, the literature on the prevalence of this phenotype is scarce, with most publications relating to epidemiological data from India and the Asian continent, where there is a higher incidence of this alteration.

### Aims

To report a case of Bombay phenotype in a young woman from Belo Horizonte. Method: A case report was conducted, with retrospective data collection through the analysis of medical record data provided and authorized by the patient. In addition, research was conducted in relevant databases such as PubMed and BVS on the topic.

### Results

This is a case of a 25-year-old female patient, previously with an O+ phenotype, who at 6 months of gestation presented a positive indirect Coombs test. The initial investigation consisted of several laboratory tests and ultrasounds to monitor peak systolic velocity in order to rule out fetal anemia. Near the end of the pregnancy, during blood collection for a possible transfusion, the patient's sample was sent to Hemominas for further investigation. The ABO/RhD phenotyping results revealed the "Oh" (Bombay)/Positive phenotype, confirming the presence of the Bombay phenotype. The direct Coombs test was negative, while the irregular antibody screening (PAI) was positive, identifying an irregular anti-H antibody. Despite the maternal diagnosis, the fetus did not present any risks or complications related to blood incompatibility. The patient's complete phenotyping demonstrated: ABO: Oh, RhD: Positive, C: Positive, c: Positive, E: Positive, e: Positive, K(Kell): Negative, k(Celano): Positive, Kpb: Positive, Jka: Positive, Jkb: Positive, M: Positive, N: Negative, S: Negative, s: Positive, Lea: Positive, Leb: Negative, Fya: Positive, Fyb: Positive, Lub: Positive. The child was tested shortly after birth and confirmed to be A+, not presenting the Bombay phenotype.

### Summary/Conclusion

Identifying this rare condition is crucial for ensuring transfusion safety and for family genetic counseling. Collaboration between prenatal services and specialized blood banks is essential for the proper management of these cases, preventing complications and guaranteeing maternal and fetal health. Furthermore, scientific publications related to this pregnancy-associated phenotype are of great importance for building and expanding a database, which is currently insufficient.

**Copyright Information:** (Online) ISSN: 2572-9241
©2026 The Author(s). HemaSphere is published by John Wiley & Sons Ltd on behalf of European Hematology Association. This is an open access Abstract Book distributed under the Attribution-NonCommercial-NoDerivs (CC BY-NC-ND), which allows third parties to download the articles and share them with others as long as they credit the author and the Abstract Book, but they cannot change the content in any way or use them commercially.
**Abstract Book Citations:** Authors, Title, HemaSphere, 2026;10:(S1):pages. The abstract book DOIs can be found on the “Author Information” page.
**Disclaimer:** Articles published in the journal HemaSphere exclusively reflect the opinions of the authors. The authors are responsible for all content in their abstracts including accuracy of the facts, statements, citing resources, etc.

### (PB4279) PRODUCTION OF FREEZE-DRIED PLASMA IN POLIMER CONTAINERS USING MEMBRANE DRYING TECHNOLOGY

**Topic:** 32. Transfusion medicine

Svetlana Ziganshina[1], Ekaterina Konovalova[2], Elena Rosina[2], Sergey Ignatiev[2], Aleksey Lyanguzov[2], Elena Kormshchikova*[2]

*[1]Kirov Research Institute of Hematology and Blood Transfusion under the Federal Medical Biological Agence, Laboratory of blood preparations, Kirov, Russia; [2]Kirov Research Institute of Hematology and Blood Transfusion under the Federal Medical Biological Agence, Kirov, Russia;*

### Background

Bleeding and related complications are responsible for more than 25 % of injury-related deaths. It is known that about 40% of deaths due to blood loss in peacetime and about 90 % in combat can be prevented with a timely transfusion care. In conditions of limited opportunities for using fresh frozen plasma in emergency situations, the use of freeze-dried plasma has significant advantages. Today, the most promising approach is to produce this blood component in polymer containers using membrane technology, which ensures plasma drying in a closed system while maintaining sterility.

### Aims

To obtain freeze-dried plasma in polymer containers using membrane drying technology.

### Methods

Plasma lyophilization was carried out in containers Plazmapak (RU No. RZN 2025/24964, Profit Farm LLC) using a laboratory lyophilization unit ULL-4.5M (NPP LioTekh LLC, Russia). The residual moisture content of the obtained samples was assessed by vacuum drying of an accurate sample. Freeze-dried plasma samples weighing 1.5 gwere rehydrated in 17.3 ml of the preparation "Sodium chloride,0.9% infusion solution" (n = 42). Total protein was measuredusing the colorimetric method according to the instructions foruse of the reagent kit (OLVEX DIAGNOSTIKUM LLC,Russia). The activity of factor VIII was determined using the STA Compact MAX automatic hemostasis analyzer(Diagnostica STAGO, France) and compatible reagent kits.

### Results ophilization of the plasma was performed onthe ULL-4.5M unit. It has a two–way power supply to the material: conductive – due to heating of the shelf, and radiation -from screens located above the material. This made it possibleto intensify the process of drying the blood component. The production of freeze-dried plasma involved filling 300 mlplasma into containers Plasmapack, shock freezing of the blood component and holding at a temperature of minus (80 ± 2) $^{0}$Cfor at least 12 hours, loading the containers into a freeze-drying chamber pre-cooled to a temperature of minus (40 ± 5) $^{0}$C andholding for at least 1 hour, gradual heating of the blood component up to a shelf temperature of minus 20 $^{0}$C at a rate of 10 $^{0}$C / hour and from minus 20 $^{0}$C to 30 $^{0}$C at a rate of 5 $^{0}$C /hour at a process pressure of 8-10 Pa. When the set pressure was exceeded, the heat supply to the material stopped automatically. For uniform drying of the freeze-dried plasma, the heating of the screens was adjusted in the range (70 ± 15) % of the setmaximum power. The total drying time was 40 - 46 hours. The samples obtained met the requirements of regulatorydocumentation: residual moisture — less than 2%, total protein— more than 50 g/L, factor VIII activity — at least 0.5 IU/ml,sterility.

**Copyright Information:** (Online) ISSN: 2572-9241
©2026 The Author(s). HemaSphere is published by John Wiley & Sons Ltd on behalf of European Hematology Association. This is an open access Abstract Book distributed under the Attribution-NonCommercial-NoDerivs (CC BY-NC-ND), which allows third parties to download the articles and share them with others as long as they credit the author and the Abstract Book, but they cannot change the content in any way or use them commercially.
**Abstract Book Citations:** Authors, Title, HemaSphere, 2026;10:(S1):pages. The abstract book DOIs can be found on the "Author Information" page.
**Disclaimer:** Articles published in the journal HemaSphere exclusively reflect the opinions of the authors. The authors are responsible for all content in their abstracts including accuracy of the facts, statements, citing resources, etc.

**Summary/Conclusion**

Freeze-dried plasma was obtained in polymercontainers using membrane drying technology. The use of durable, lightweight and compact lyophilization containers has made it possible to obtain a high-quality blood component forthe correction of coagulopathy in massive bleeding.

**Copyright Information:** (Online) ISSN: 2572-9241
©2026 The Author(s). HemaSphere is published by John Wiley & Sons Ltd on behalf of European Hematology Association. This is an open access Abstract Book distributed under the Attribution-NonCommercial-NoDerivs (CC BY-NC-ND), which allows third parties to download the articles and share them with others as long as they credit the author and the Abstract Book, but they cannot change the content in any way or use them commercially.
**Abstract Book Citations:** Authors, Title, HemaSphere, 2026;10:(S1):pages. The abstract book DOIs can be found on the "Author Information" page.
**Disclaimer:** Articles published in the journal HemaSphere exclusively reflect the opinions of the authors. The authors are responsible for all content in their abstracts including accuracy of the facts, statements, citing resources, etc.

### (PB4280) MANAGEMENT CHALLENGES IN PEDIATRIC REFRACTORY AUTOIMMUNE HEMOLYTIC ANEMIA: A CASE REPORT OF THERAPEUTIC DEADLOCK ,AND SEVERE TRANSFUSION REACTION

**Topic:** 32. Transfusion medicine

Imene Larous*[1], Mouna Messasset[2]

*[1]centre hospitalo-universitaire Setif, Laboratoire du pole pédiatrique, SETIF, Algeria;[2]Centre hospitalo-universiatire Setif, pole pédiatrique, setif, Algeria;*

### Background

Autoimmune hemolytic anemia (AIHA) rarely affects children, with a frequency of 8 cases per million. It is characterized by premature destruction of red blood cells, which can be life-threatening. Depending on the antibody involved, a distinction can be made between warm antibody AIHA, which is the most common (60% to 70% of cases), and cold antibody AIHA, in which hemolysis is triggered by exposure to cold, with an optimum temperature of 4°C.

### Aims

To highlight the complex management challenges of severe, refractory pediatric autoimmune hemolytic anemia (AIHA) by presenting a case complicated by a severe transfusion reaction and subsequent therapeutic impasse, emphasizing the critical need for rigorous transfusion protocols and further therapeutic options.

### Methods

A girl from southeastern Algeria was diagnosed with AIHA at the age of 8 months. The condition was revealed following persistent asthenia, mucocutaneous pallor, and jaundice. Laboratory tests revealed severe anemia with hemoglobin at 3 g/dL; normocytic, normochromic, regenerative. Despite first-line treatment with corticosteroids and immunoglobulins, the patient required a splenectomy at the age of 5. Since then, the patient has been dependent on occasional blood transfusions with iso-group, iso-Rhesus, and phenotyped red blood cell concentrates.

### Results

In August 2025, at the age of 12, the patient underwent an emergency transfusion at a public hospital in southern Algeria. The patient received two units of untyped O-negative red blood cells. In this child, who had received multiple transfusions, this triggered a severe acute hemolytic transfusion reaction, with signs of acute hemolysis: chills, fever, and headaches. This condition was complicated by acute renal failure. The direct Coombs test was positive with anti-IgG globulin. The antibody detected was anti-Kell specificity associated with anti-D. The results of the investigation were not communicated, as the patient was transferred to the pediatric department of the Setif University Hospital for better care. Today, due to the lack of improvement in transfusion efficacy in this patient, despite a count of 37°C of transfused red blood cells, and the impossibility of using rituximab due to the presence of a bronchiolitis epidemic and the splenectomy complicating her hospitalization, the medical team is faced with a relative therapeutic impasse.

### Summary/Conclusion

This clinical case, beyond its rarity, is a powerful reminder of the need for close collaboration between peripheral centers and specialized services, the imperative need to comply with phenotyping rules in polytransfused patients, and the constant search for new therapeutic strategies for these severe and complex forms of AHAI in children.

**Copyright Information:** (Online) ISSN: 2572-9241
©2026 The Author(s). HemaSphere is published by John Wiley & Sons Ltd on behalf of European Hematology Association. This is an open access Abstract Book distributed under the Attribution-NonCommercial-NoDerivs (CC BY-NC-ND), which allows third parties to download the articles and share them with others as long as they credit the author and the Abstract Book, but they cannot change the content in any way or use them commercially.
**Abstract Book Citations:** Authors, Title, HemaSphere, 2026;10:(S1):pages. The abstract book DOIs can be found on the "Author Information" page.
**Disclaimer:** Articles published in the journal HemaSphere exclusively reflect the opinions of the authors. The authors are responsible for all content in their abstracts including accuracy of the facts, statements, citing resources, etc.

### (PB4281) BEYOND IGM: SELECTIVE DETECTION OF CLINICALLY RELEVANT IGG ANTI-ABO ANTIBODIES IN PERIPARTUM WOMEN.

**Topic:** 32. Transfusion medicine

Katarzyna Popko*[1], Anna Stelmaszczyk-Emmel[1], Małgorzata Skrzypczak-Pamięta[2], Olga Kowal[2], Wioletta Zarlak[2], Grażyna Szlaga[2], Sabina Sobolewska[1], Urszula Demkow[1]

*[1]Medical University of Warsaw, Department of Laboratory Medicine and Clinical Immunology of Developmental Age, Warsaw, Poland; [2]Children's Hospital of Medical University of Warsaw, Laboratory of Blood Serology, Warsaw, Poland;*

### Background

The ABO blood group system represents the most clinically significant blood group in transfusion medicine and transplantation. It is unique among blood group systems due to the presence of naturally occurring alloantibodies directed against A and/or B antigens absent from the individual's own erythrocytes. Although IgM antibodies predominate, IgG anti-A and anti-B antibodies are also of considerable clinical relevance. IgG antibodies against ABO antigens may occur independently of prior alloimmunization and can cross the placenta. Consequently, they may contribute to the pathogenesis of hemolytic disease of the fetus and newborn (HDFN), particularly in group O mothers. ABO incompatibility is observed in 20–25% of pregnancies; however, clinically significant HDFN develops less frequently. The underlying mechanism involves maternal IgG binding to A and/or B antigens on fetal red blood cells, resulting in extravascular hemolysis and neonatal hyperbilirubinemia.

### Aims

The aim of the study was to develop a method for neutralizing naturally occurring IgM antibodies through adsorption with reagent red blood cells, thereby enabling the detection of immune IgG antibodies, and to assess their prevalence in peripartum women.

### Methods

The identification of IgG antibodies in the presence of naturally occurring IgM immunoglobulins represents a diagnostic challenge. Currently employed IgM neutralization methods are suboptimal and frequently result in serum protein denaturation, thereby precluding subsequent identification of IgG antibodies. In this study, a protocol for IgM neutralization was developed based on adsorption with reagent red blood cells under refrigerated incubation conditions. Residual IgG antibodies were subsequently detected using a column agglutination technique (IAT). A total of 138 women with blood groups O, A, and B were included. The study population comprised 44 (31.88%) group O, 54 (39.13%) group A, and 40 (28.99%) group B samples. In all cases, ABO typing, IgM neutralization, and detection of IgG anti-A and/or anti-B antibodies were performed.

### Results

The identification of IgG antibodies in the presence of naturally occurring IgM immunoglobulins represents a diagnostic challenge. Currently employed IgM neutralization methods are suboptimal and frequently result in serum protein denaturation, thereby precluding subsequent identification of IgG antibodies. In this study, a protocol for IgM neutralization was developed based on adsorption with reagent red blood cells under refrigerated incubation conditions. Residual IgG antibodies were subsequently detected using a column agglutination technique (IAT). A total of 138 women with blood groups O, A, and B were included (group AB was excluded due to the physiological absence of anti-A and anti-B antibodies). The study population comprised 44 (31.88%) group O, 54 (39.13%) group A, and 40 (28.99%) group B samples. In all cases, ABO typing, IgM neutralization, and detection of IgG anti-A and/or anti-B antibodies were performed.

**Copyright Information:** (Online) ISSN: 2572-9241
©2026 The Author(s). HemaSphere is published by John Wiley & Sons Ltd on behalf of European Hematology Association. This is an open access Abstract Book distributed under the Attribution-NonCommercial-NoDerivs (CC BY-NC-ND), which allows third parties to download the articles and share them with others as long as they credit the author and the Abstract Book, but they cannot change the content in any way or use them commercially.
**Abstract Book Citations:** Authors, Title, HemaSphere, 2026;10:(S1):pages. The abstract book DOIs can be found on the "Author Information" page.
**Disclaimer:** Articles published in the journal HemaSphere exclusively reflect the opinions of the authors. The authors are responsible for all content in their abstracts including accuracy of the facts, statements, citing resources, etc.

**Summary/Conclusion**

The study demonstrates that adsorption of IgM antibodies using ABO-incompatible red blood cells may serve as an alternative diagnostic approach for the detection of IgG antibodies within the ABO system. The occurrence of warm IgG anti-ABO antibodies is strongly associated with ABO blood group phenotype, with group O individuals significantly more likely to produce IgG anti-A and/or anti-B antibodies than individuals with groups A or B.

**Copyright Information:** (Online) ISSN: 2572-9241
©2026 The Author(s). HemaSphere is published by John Wiley & Sons Ltd on behalf of European Hematology Association. This is an open access Abstract Book distributed under the Attribution-NonCommercial-NoDerivs (CC BY-NC-ND), which allows third parties to download the articles and share them with others as long as they credit the author and the Abstract Book, but they cannot change the content in any way or use them commercially.
**Abstract Book Citations:** Authors, Title, HemaSphere, 2026;10:(S1):pages. The abstract book DOIs can be found on the "Author Information" page.
**Disclaimer:** Articles published in the journal HemaSphere exclusively reflect the opinions of the authors. The authors are responsible for all content in their abstracts including accuracy of the facts, statements, citing resources, etc.

### (PB4572) IMMUNOGLOBULIN-ASSOCIATED TRANSFUSION-RELATED ACUTE LUNG INJURY & TRANSFUSION-ASSOCIATED CIRCULATORY OVERLOAD OVERLAP IN REFRACTORY IMMUNE THROMBOCYTOPENIA

**Topic:** 32. Transfusion medicine

Milica Apostolovic Stojanovic*[1], Aleksandar Stojanovic[2], Milica Radojkovic[3]

[1]*Clinical Hospital Center "Dr Dragisa Misovic - Dedinje", Department of Hematology, Belgrade, Serbia;*[2]*Medical faculty, University of Belgrade, Serbia, Belgrade, Serbia;* [3]*Clinical Hospital Center "Dr Dragisa Misovic - Dedinje", Beograd (Voždovac) 11010, Serbia;*

### Background

Transfusion-related acute lung injury (TRALI) is one of the most serious complications of blood transfusion and has been rarely documented to occur after IVIG infusion. Transfusion-related acute lung injury (TRALI) and transfusion-associated circulatory overload (TACO) are major causes of acute respiratory compromise after blood product exposure. Increasing evidence suggests these entities may represent overlapping pathophysiologic processes rather than mutually exclusive syndromes.
Intravenous immunoglobulin (IVIG), widely used in refractory immune thrombocytopenia (ITP), exerts immunomodulatory effects through Fcγ-receptor (FcγR) interactions. FcγR-mediated neutrophil activation and complement amplification may contribute to endothelial injury and capillary leak, providing a plausible mechanistic link to acute lung injury.

### Aims

We report a biomarker-supported TRALI/TACO overlap phenotype following IVIG in refractory ITP, highlighting diagnostic and mechanistic implications.

### Methods

Clinical presentation, laboratory parameters, arterial blood gases, radiologic findings, and echocardiographic assessment were analyzed in a patient who developed acute respiratory deterioration during IVIG infusion. Infectious causes were excluded. Natriuretic peptides and structured cardiac evaluation were used to assess hydrostatic contribution.

### Results

A 76-year-old woman with refractory ITP on chronic corticosteroid therapy received IVIG (1 g/kg) prior to colonoscopy for anemia secondary to rectorrhagia (hemoglobin 85 g/L). Three hours after completed infusion , she developed abrupt dyspnea, hypertensive crisis (200/100 mmHg), and hypoxemia ($PaO_2$ 68 mmHg). Chest radiography demonstrated bilateral pulmonary infiltrates without cardiomegaly. NT-proBNP was markedly elevated (5,600 ng/L; reference <165 ng/L). Electrocardiography was normal. Transthoracic echocardiography showed preserved left ventricular systolic function (LVEF 55%), TAPSE 22 mm, estimated systolic pulmonary artery pressure 35 mmHg, and no structural abnormalities. Renal function was preserved and IgA deficiency excluded.
Mechanical ventilation was not required. Rapid clinical improvement occurred within 48 hours, with radiologic resolution by 72 hours. Platelet count normalized within two days.
The abrupt onset of bilateral infiltrates and hypoxemia without overt cardiac dysfunction supported immune-mediated lung injury consistent with TRALI. However, marked natriuretic peptide elevation, severe hypertension, and favorable response to diuretics suggested concomitant hydrostatic stress, indicating a TRALI/TACO overlap phenotype.
FcγR-driven immune activation in refractory ITP may represent a priming factor for endothelial vulnerability.

### Summary/Conclusion

In refractory ITP, IVIG may precipitate an acute lung injury spectrum integrating Fcγ-mediated inflammatory mechanisms and hydrostatic stress.
TRALI and TACO should be conceptualized as intersecting syndromes in hematology patients.
Incorporating natriuretic peptides and structured cardiac assessment into IVIG-related adverse event algorithms may improve diagnostic precision and guide individualized management.

**Copyright Information:** (Online) ISSN: 2572-9241
©2026 The Author(s). HemaSphere is published by John Wiley & Sons Ltd on behalf of European Hematology Association. This is an open access Abstract Book distributed under the Attribution-NonCommercial-NoDerivs (CC BY-NC-ND), which allows third parties to download the articles and share them with others as long as they credit the author and the Abstract Book, but they cannot change the content in any way or use them commercially.
**Abstract Book Citations:** Authors, Title, HemaSphere, 2026;10:(S1):pages. The abstract book DOIs can be found on the "Author Information" page.
**Disclaimer:** Articles published in the journal HemaSphere exclusively reflect the opinions of the authors. The authors are responsible for all content in their abstracts including accuracy of the facts, statements, citing resources, etc.

### (PB4282) UPFRONT SINGLE AGENT ROMIPLOSTIM IN PRIMARY ACUTE ITP IS AN EFFECTIVE OPTION. ANALYSIS OF REAL-WORLD DATA OF THE UK ADULT ITP-REGISTRY DURING THE COVID PANDEMIC

**Topic:** 33. Platelet disorders

Frederick Chen*[1, 2], Haroon Miah[1, 2], David Allsup[3], Tina Biss[4], Joannes Hermans[5], Benjamin Gray[6], Giorgia Saccullo[7], Jamie Wilson[8], Chetan Patalappa[9], Jason Coppell[10], Raymond Dang[11], Emma Drasar[12], Ana Ortuno-Cabrero[13], Gillian Evans[14], Kate Foley[15], Adam Forbes[16], Louise Fryearson[17], Mari Kilner[18], Stephen Jones[19], Vickie McDonald[20], Maria Mushkbar[21], Roderick Oakes[22], Michael Desborough[23], Sue Pavord[23], Susan Robinson[20], Drew Provan[1, 24]

*[1]Barts Health NHS Trust, Clinical Haematology Royal London Hospital, London, United Kingdom;[2]Queen Mary University, Blizard Institute, London, United Kingdom; [3]Hull York Medical School, University of Hull, Hull, United Kingdom;[4]THE NEWCASTLE UPON TYNE HOSPITALS NHS FOUNDATION TRUST, Newcastle, United Kingdom; [5]Nottingham University Hospitals NHS Trust, Nottingham, United Kingdom; [6]University Hospitals Dorset NHS Foundation Trust, Poole, United Kingdom;[7]SHEFFIELD TEACHING HOSPITALS NHS FOUNDATION TRUST, Sheffield, United Kingdom; [8]UNIVERSITY HOSPITALS SUSSEX NHS FOUNDATION TRUST, Haematology, Chichester, United Kingdom; [9]Bolton NHS Foundation Trust, Haematology, Bolton, United Kingdom; [10]ROYAL DEVON UNIVERSITY HEALTHCARE NHS FOUNDATION TRUST, Haematology, Exeter, United Kingdom; [11]SOUTH TEES HOSPITALS NHS FOUNDATION TRUST, Middlesbrough, United Kingdom; [12]Whittington Health NHS Trust, London, United Kingdom; [13]Whittington Health NHS Trust, Haematology, London, United Kingdom; [14]EAST KENT HOSPITALS UNIVERSITY NHS FOUNDATION TRUST, Canterbury, United Kingdom; [15]YORK AND SCARBOROUGH TEACHING HOSPITALS NHS FOUNDATION TRUST), York, United Kingdom; [16]Royal Cornwall Hospitals NHS Trust, Truro, United Kingdom;[17]South Warwickshire NHS Foundation Trust, Warwick, United Kingdom; [18]North Tyneside General Hospital, Northumbria NHST, North Tyneside, United Kingdom; [19]SHERWOOD FOREST HOSPITALS NHS FOUNDATION TRUST, Nottinghamshire, United Kingdom; [20]Guy's and St Thomas' NHS Foundation Trust, London, United Kingdom;[21]University Hospitals Coventry and Warwickshire NHS Foundation Trust, Haematology, Coventry, United Kingdom; [22]North Cumbria Integrated Care NHS Foundation Trust, Carlisle, United Kingdom; [23]Oxford University Hospitals NHS Foundation Trust, Oxford, United Kingdom;[24]Queen Mary University, London, United Kingdom;*

### Background

TPO-RA agonists are increasingly used as first line therapy in acute ITP patients refractory to, intolerant of, or dependant on corticosteroids. We analysed the real-world data obtained during the COVID-19 pandemic, when UK haematologists had access to universal use of TPO-RA for upfront treatment of ITP to preserve immune responses to COVID infection and immunisation.

### Aims

To assess the efficacy of upfront romiplostim in a cohort of ITP patients treated without bias for severe disease.

### Methods

From the UK Adult-ITP Registry (Research Ethics: 07/H0718/57) all patients who received upfront single-agent romiplostim, and romiplostim following initial steroids +/- intravenous immunoglobulins (IVIg) within 30 days of diagnosis were identified from February 2020 to February 2023 during the COVID pandemic. R-software was used for statistical analysis, including Kaplan-Meier plots and t-tests.

**Copyright Information:** (Online) ISSN: 2572-9241
©2026 The Author(s). HemaSphere is published by John Wiley & Sons Ltd on behalf of European Hematology Association. This is an open access Abstract Book distributed under the Attribution-NonCommercial-NoDerivs (CC BY-NC-ND), which allows third parties to download the articles and share them with others as long as they credit the author and the Abstract Book, but they cannot change the content in any way or use them commercially.
**Abstract Book Citations:** Authors, Title, HemaSphere, 2026;10:(S1):pages. The abstract book DOIs can be found on the "Author Information" page.
**Disclaimer:** Articles published in the journal HemaSphere exclusively reflect the opinions of the authors. The authors are responsible for all content in their abstracts including accuracy of the facts, statements, citing resources, etc.

### Results

Thirty-four patients were treated with romiplostim (median age: 54.5 years; 18 (53%) male). Seventeen received single-agent romiplostim and 17 received initial corticosteroids+/-IVIg, followed by romiplostim. The romiplostim dose ranged from 1-3μg/Kg/week. The lowest median platelet counts prior to starting romiplostim was $11x10^{9}$/L (IQR:3-23) in the single-agent group, and $3x10^{9}$/L (IQR:1-10.7), (p=0.028) in the steroid+/-ivIg-romiplostim group, indicating a more severe disease. The median time-to-response to platelets of $30x10^{9}$/L and $50x10^{9}$/L for the whole cohort was 7.5 days (IQR:5.5-15) and 12 days (IQR:7-21) respectively. By 100 days, 94% of patients achieved platelets of $30\times10^{9}$/L, and 91% achieved $50\times10^{9}$/L. In the single-agent romiplostim group, the median time-to-response for achieving these thresholds was 9 (IQR:7-16) and 13.5 days (IQR:7-21) respectively, and for the steroid-romiplostim group, both thresholds were achieved within 7 days (IQR:5-14). The difference in response between the two groups was not statistically significant (p= 0.78;0.76). The median time-to-failure (platelets<30 or switch to a new treatment) among 32 responders was 609 days. There was a trend towards lower failure amongst the patients who had corticosteroids+/-IVIg and romiplostim, compared to those on single-agent romiplostim, but this was not statistically significant. Fourteen of 32 responders (44%) had a sustained response. Ten of 14 responders (71%) were off romiplostim for a median of 818.5 days at the latest follow-up.

### Summary/Conclusion

Our real-world data showed good responses of acute ITP to upfront romiplostim both as a single-agent and following initial steroids+/IVIg within 30 days of diagnosis. The responses observed were similar to the outcome data of the pivotal romiplostim trials for chronic ITP. The difference in platelet counts before starting romiplostim between the two groups suggests that there may be a case for stratified first line use of single-agent romiplostim in acute ITP, where patients with higher platelet counts may be stratified to single-agent romiplostim and those with lower platelet counts to combination therapy of romiplostim and steroids. However, larger studies are required to confirm this.

**Copyright Information:** (Online) ISSN: 2572-9241
©2026 The Author(s). HemaSphere is published by John Wiley & Sons Ltd on behalf of European Hematology Association. This is an open access Abstract Book distributed under the Attribution-NonCommercial-NoDerivs (CC BY-NC-ND), which allows third parties to download the articles and share them with others as long as they credit the author and the Abstract Book, but they cannot change the content in any way or use them commercially.
**Abstract Book Citations:** Authors, Title, HemaSphere, 2026;10:(S1):pages. The abstract book DOIs can be found on the “Author Information” page.
**Disclaimer:** Articles published in the journal HemaSphere exclusively reflect the opinions of the authors. The authors are responsible for all content in their abstracts including accuracy of the facts, statements, citing resources, etc.

### (PB4283) EXPLORATION OF THE TREATMENT AND ECONOMIC EVALUATION OF HETROMBOPAG PLUS RECOMBINANT HUMAN THROMBOPOIETIN FOR CHEMOTHERAPY-INDUCED THROMBOCYTOPENIA IN LYMPHOMA

**Topic:** 33. Platelet disorders

Xueyi Pan*[1], Chaolun Chen[1], Qinlan Qiu[1], Shimei Yang[1], Mingjie Li[1], Zebing Guan[1], Lanlan Zhou[1], Wenbin Zeng[1], Hong Wang[1], Qiuhua Zhu[1], Yanan Liu[1], Yanmin Gao[1], Shiqiu Qiu[1], Huan Li[1], Yuemei Yuan[1], Hanling Zhang[1], Guanqiao Ruan[1]

[1]*The First Affiliated Hospital of Guangdong Pharmaceutical University, Hematology, Guangzhou, China;*

### Background

Chemotherapy-induced thrombocytopenia (CTIT), especially grade 3/4 CTIT, is a frequent and clinically significant complication in patients with lymphoma, often leading to bleeding risk, platelet transfusion, and chemotherapy delay. Recombinant human thrombopoietin (rhTPO) is commonly used, but its high cost and injection burden limit long-term use. Hetrombopag, an oral thrombopoietin receptor agonist, may provide a cost-effective alternative.

### Aims

To evaluate the effectiveness, safety, and cost-effectiveness of hetrombopag, alone or in combination with rhTPO, compared with eltrombopag- or rhTPO-based strategies in lymphoma patients with grade 3/4 CTIT.

### Methods

This retrospective study included adult lymphoma patients who developed grade 3/4 CTIT and were treated at a single tertiary hematology center between December 2023 and July 2025. Patients received hetrombopag (7.5 mg once daily), eltrombopag (75 mg once daily), rhTPO (300 U/kg once daily), or a combination of a TPO receptor agonist (hetrombopag or eltrombopag) with rhTPO. Clinical outcomes included platelet response, nadir platelet count, time to platelet recovery (≥50, ≥75, and ≥100×10⁹/L), bleeding events, and platelet transfusion frequency. A cost-effectiveness analysis was conducted. Th1/Th2 cytokines and regulatory T cells (Treg) were analyzed in a subset of patients to explore immune dysregulation associated with CTIT.

### Results

A total of 269 patients with lymphoma and grade 3/4 CTIT were included: hetrombopag+rhTPO (n=39), hetrombopag monotherapy (- n=24), rhTPO monotherapy (n=70), eltrombopag+rhTPO (n=23), eltrombopag monotherapy (n=60), and controls without thrombopoietic agents (n=53). Baseline platelet counts were comparable across groups. All thrombopoietic treatments significantly increased platelet counts and resulted in higher nadir platelet levels compared with controls (overall P<0.001). Hetrombopag+rhTPO achieved the fastest platelet recovery, with mean time to platelet counts ≥50, ≥75, and ≥100×10⁹/L of 4.0, 5.2, and 6.6 days, respectively, which was shorter than hetrombopag monotherapy (5.4, 7.3, and 9.3 days) and rhTPO monotherapy (5.6, 7.3, and 9.5 days). Hetrombopag monotherapy demonstrated platelet recovery comparable to rhTPO. Bleeding events and platelet transfusion rates were significantly lower in all thrombopoietic treatment groups than in controls. Notably, hetrombopag monotherapy achieved transfusion rates comparable to hetrombopag plus rhTPO, while both were superior to rhTPO alone. In the cost analysis, hetrombopag monotherapy showed the lowest total treatment cost, and hetrombopag-based strategies were more cost-effective than rhTPO. Among 88 patients with immune profiling, Treg, IL-6, and IL-10 levels significantly decreased after platelet recovery to ≥100×10⁹/L.

**Copyright Information:** (Online) ISSN: 2572-9241
©2026 The Author(s). HemaSphere is published by John Wiley & Sons Ltd on behalf of European Hematology Association. This is an open access Abstract Book distributed under the Attribution-NonCommercial-NoDerivs (CC BY-NC-ND), which allows third parties to download the articles and share them with others as long as they credit the author and the Abstract Book, but they cannot change the content in any way or use them commercially.
**Abstract Book Citations:** Authors, Title, HemaSphere, 2026;10:(S1):pages. The abstract book DOIs can be found on the "Author Information" page.
**Disclaimer:** Articles published in the journal HemaSphere exclusively reflect the opinions of the authors. The authors are responsible for all content in their abstracts including accuracy of the facts, statements, citing resources, etc.

**Summary/Conclusion**

In patients with lymphoma and grade 3/4 CTIT, hetrombopag initiated at 7.5 mg provides platelet recovery comparable to rhTPO with superior economic advantages. Combination therapy with rhTPO further accelerates platelet recovery.

**Copyright Information:** (Online) ISSN: 2572-9241
©2026 The Author(s). HemaSphere is published by John Wiley & Sons Ltd on behalf of European Hematology Association. This is an open access Abstract Book distributed under the Attribution-NonCommercial-NoDerivs (CC BY-NC-ND), which allows third parties to download the articles and share them with others as long as they credit the author and the Abstract Book, but they cannot change the content in any way or use them commercially.
**Abstract Book Citations:** Authors, Title, HemaSphere, 2026;10:(S1):pages. The abstract book DOIs can be found on the "Author Information" page.
**Disclaimer:** Articles published in the journal HemaSphere exclusively reflect the opinions of the authors. The authors are responsible for all content in their abstracts including accuracy of the facts, statements, citing resources, etc.

### (PB4284) LONG TERM FOLLOW-UP (≥10 YEARS) OF SPLENECTOMIZED PATIENTS FOR IMMUNE THROMBOCYTOPENIA : RESULTS FROM A SINGLE-CENTER COHORT OF 85 PATIENTS

**Topic:** 33. Platelet disorders

Hamida Brahimi*[1], Nabila Rekab[1], Khadoudj Mounira Benlabiod[1], Fatima Lamraoui[1], Souad Taoussi[1], Salima Oukid[1], Yamina Bouchakour Moussa[1], Mohamed Mezroud[1], Bradai Mohamed[1]

*[1]EHS ELCC, Blida, Algeria, Hematology department, Blida, Algeria;*

### Background

Splenectomy is a major therapeutic option in immune thrombocytopenia (ITP) as second-line treatment, particularly in resource-limited settings where access to thrombopoietin receptor agonists (TPO-RAs) is restricted.. However, very long-term follow-up data (>10 years) are scarce, particularly in resource-limited settings where splenectomy retains a key role.

### Aims

To evaluate long-term hematological efficacy, overall survival, and late complications in splenectomized ITP patients with ≥10 years of follow-up.

### Methods

Single-center retrospective study including 85 out of 193 patients splenectomized for ITP between 1993 and 2014, with ≥10 years of follow-up. The cohort comprised 63 women and 22 men, median age 29 years (5-65) at splenectomy. Procedures were performed by laparoscopy (n=37) or laparotomy (n=48). Overall survival (OS) and relapse-free survival (RFS) were calculated using the Kaplan-Meier method.

**Copyright Information:** (Online) ISSN: 2572-9241
©2026 The Author(s). HemaSphere is published by John Wiley & Sons Ltd on behalf of European Hematology Association. This is an open access Abstract Book distributed under the Attribution-NonCommercial-NoDerivs (CC BY-NC-ND), which allows third parties to download the articles and share them with others as long as they credit the author and the Abstract Book, but they cannot change the content in any way or use them commercially.
**Abstract Book Citations:** Authors, Title, HemaSphere, 2026;10:(S1):pages. The abstract book DOIs can be found on the "Author Information" page.
**Disclaimer:** Articles published in the journal HemaSphere exclusively reflect the opinions of the authors. The authors are responsible for all content in their abstracts including accuracy of the facts, statements, citing resources, etc.

**Results**

85 patients total, none having received rituximab pre-splenectomy. Overall response rate was 92.5% (79/85), with 90% complete and 10% partial responses among responders. 6% were primary non-responders.
After a median follow-up of 219 months (18.3 years), response durability declined over time: 63.3% of initial responders maintained treatment-free response at 10 years, falling to 27.5% at 25 years. Relapses occurred in 29/79 responders (36.7%), with a mean time to relapse of 86 months (range 3–232), including 7 late relapses beyond the 10-year mark — underscoring the need for lifelong follow-up even after apparent sustained remission.
Among the 33 patients with primary failure or relapse, second-line treatments included corticosteroids (17), rituximab (13), and romiplostim (6) — reflecting a real-world, heterogeneous rescue approach.
Excellent long-term survival: 100% at 15 years, 94.6% at 20 years, and 87.3% at 25 years. The 4 deaths (mean delay 245 months) were all unrelated to splenectomy or ITP per se.
Complications were uncommon. Infections were the most notable concern — 7 episodes in 6 patients with 2 hospitalizations — though the overall burden appears low given the follow-up duration. Other events included one patient with recurrent thromboembolism and 9 patients developing autoimmune or hematological diseases, consistent with the known background risk in this population. No procedure-related mortality was recorded.

**Summary/Conclusion**

This study reports the longest follow-up (≥18 years) of a cohort of splenectomized ITP patients. Splenectomy provides durable remission in nearly two-thirds of patients at 10 years, with excellent long-term vital prognosis and a low rate of very late complications. The unprecedented observation of late relapses >10 years warrants prolonged vigilance. In resource-limited settings, splenectomy remains a reference therapeutic option. Further analyses are needed to identify predictive factors for durable response and late complications.

**Copyright Information:** (Online) ISSN: 2572-9241
©2026 The Author(s). HemaSphere is published by John Wiley & Sons Ltd on behalf of European Hematology Association. This is an open access Abstract Book distributed under the Attribution-NonCommercial-NoDerivs (CC BY-NC-ND), which allows third parties to download the articles and share them with others as long as they credit the author and the Abstract Book, but they cannot change the content in any way or use them commercially.
**Abstract Book Citations:** Authors, Title, HemaSphere, 2026;10:(S1):pages. The abstract book DOIs can be found on the “Author Information” page.
**Disclaimer:** Articles published in the journal HemaSphere exclusively reflect the opinions of the authors. The authors are responsible for all content in their abstracts including accuracy of the facts, statements, citing resources, etc.

### (PB4285) LONG-TERM MATERNAL AND NEONATAL OUTCOMES OF IMMUNE THROMBOCYTOPENIA DURING PREGNANCY: A 20-YEAR MONOCENTRIC COHORT STUDY

**Topic:** 33. Platelet disorders

Hamida Brahimi*[1], Khadoudj Mounira Benlabiod[1], Nabila Rekab[1], Souad Taoussi[1], Fatima Lamraoui[1], Salima Oukid[1], Yamina Bouchakour Moussa[1], Guezlane Sherifa[1], Bradai Mohamed[1]

*[1]EHS ELCC, Blida, Algeria, Hematology department, Blida, Algeria;*

**Background**

Immune thrombocytopenia (ITP) during pregnancy represents a high-risk condition due to the potential worsening of thrombocytopenia and the occurrence of maternal and fetal complications. Real-world data with long-term follow-up remain limited.

**Aims**

To analyze the clinical course, therapeutic management, mode of delivery, and maternal–fetal outcomes in patients with primary ITP during pregnancy.

**Methods**

This retrospective monocentric study was conducted between 2005 and 2025 and included 69 patients corresponding to 111 pregnancies (including one twin pregnancy). Clinical, biological, therapeutic, and obstetrical parameters were analyzed.

**Results**

Among the 111 pregnancies, 5 (4.5%) were prematurely terminated (4 spontaneous abortions and 1 therapeutic abortion unrelated to ITP).
The 106 ongoing pregnancies resulted in 107 newborns (one twin pregnancy), including 101 live births, 4 stillbirths, and 2 early neonatal deaths related to congenital malformations.
The mean age at ITP diagnosis was 25 years (range 3–40) and 30 years (range 19–40) at the time of pregnancy, with a median ITP-to-pregnancy interval of 62 months (range 1–293). Twenty-two patients (32%) had undergone splenectomy.
A worsening of thrombocytopenia was observed in 40 pregnancies (36%; 40/111), occurring in 50% of cases during the first trimester. Specific treatment was required in 27 pregnancies (24%; 27/111), primarily consisting of corticosteroids, and in two cases intravenous immunoglobulins (IVIG).
Peripartum hematologic preparation was performed in 32 pregnancies (29%; 32/111), including corticosteroid boluses in 14 cases, IVIG in 11 cases, and platelet transfusions in 7 cases.
Regarding maternal complications, 3 postpartum hemorrhages were recorded (2.8%; 3/106 deliveries) and 8 cases of postpartum worsening thrombocytopenia (7.5%; 8/106).
The mode of delivery was as follows: 85 vaginal deliveries (80.2%; 85/106), 21 cesarean sections (19.8%; 21/106), mainly for obstetrical indications.
Neonatal thrombocytopenia was observed in 11 newborns (10.3%; 11/107), with a mean platelet count of 22.8 G/L (range 2–53), including 7 severe cases (<50 G/L). Eight newborns (7.5%; 8/107) required specific treatment, mainly with IVIG.

**Copyright Information:** (Online) ISSN: 2572-9241
©2026 The Author(s). HemaSphere is published by John Wiley & Sons Ltd on behalf of European Hematology Association. This is an open access Abstract Book distributed under the Attribution-NonCommercial-NoDerivs (CC BY-NC-ND), which allows third parties to download the articles and share them with others as long as they credit the author and the Abstract Book, but they cannot change the content in any way or use them commercially.
**Abstract Book Citations:** Authors, Title, HemaSphere, 2026;10:(S1):pages. The abstract book DOIs can be found on the “Author Information” page.
**Disclaimer:** Articles published in the journal HemaSphere exclusively reflect the opinions of the authors. The authors are responsible for all content in their abstracts including accuracy of the facts, statements, citing resources, etc.

**Summary/Conclusion**

This monocentric cohort with long-term follow-up confirms that ITP may worsen early during pregnancy, requiring treatment in approximately one-quarter of cases. Maternal hemorrhagic complications remain rare, whereas neonatal thrombocytopenia affects about 10% of newborns. These findings confirm that the mode of delivery is predominantly vaginal and determined by obstetrical indications. They emphasize the importance of structured multidisciplinary follow-up involving obstetricians, hematologists, neonatologists, and intensivists.

**Copyright Information:** (Online) ISSN: 2572-9241
©2026 The Author(s). HemaSphere is published by John Wiley & Sons Ltd on behalf of European Hematology Association. This is an open access Abstract Book distributed under the Attribution-NonCommercial-NoDerivs (CC BY-NC-ND), which allows third parties to download the articles and share them with others as long as they credit the author and the Abstract Book, but they cannot change the content in any way or use them commercially.
**Abstract Book Citations:** Authors, Title, HemaSphere, 2026;10:(S1):pages. The abstract book DOIs can be found on the "Author Information" page.
**Disclaimer:** Articles published in the journal HemaSphere exclusively reflect the opinions of the authors. The authors are responsible for all content in their abstracts including accuracy of the facts, statements, citing resources, etc.

### (PB4286) SERUM VITAMIN D LEVELS IN PEDIATRIC PATIENTS WITH NEWLY DIAGNOSED AND CHRONIC IMMUNE THROMBOCYTOPENIA

**Topic:** 33. Platelet disorders

Emine Turkkan*[1], Elber Ulutas[2]

*[1]University of Health Sciences, Hamidiye Faculty of Medicine, Prof dr Cemil Tascioglu City Hospital, Pediatrics, Pediatric Hematology-Oncology, Istanbul, Türkiye; [2]Prof dr Cemil Tascioglu City Hospital, Pediatrics, Istanbul, Türkiye;*

### Background

In recent years, the immunomodulatory role of vitamin D has been increasingly elucidated. Vitamin D modulates immune and inflammatory responses and levels of serum 25-hydroxyvitamin D (25-OHD) have been found to be significantly lower in patients with several autoimmune diseases than in the healthy population.

### Aims

This study aimed to determine serum 25-OHD levels in pediatric patients diagnosed with immune thrombocytopenic purpura (ITP) and to evaluate the relationship between serum 25-OHD levels and disease severity, clinical features, and laboratory findings.

### Methods

This prospective case-control study was conducted at a research and training hospital in Istanbul, Turkey. The study was approved by ethics committee of the hospital. After informed consent was obtained, 101 pediatric patients diagnosed with ITP and 101 age- and sex-matched healthy controls were included. Demographic data, serum 25-OHD levels, and laboratory parameters were recorded. Serum 25-OHD level was measured using a commercially available chemiluminescent immunoassay (UniCel DxI Immunoassay Systems). Disease severity was assessed using the modified Buchanan bleeding score and Statistical analyses were performed using Number Cruncher Statistical System 2020 (Kaysville, Utah, USA); appropriate parametric and non-parametric tests were applied according to the distribution of variables.

### Results

A total of 202 children (20 acute, 81 chronic ITP and 101 healthy control) were included (mean age: 10.34±4.66 years; 52.2% were female). The mean serum 25-OHD level of the entire sample was 17.97±9.2 ng/mL, Serum 25-OHD levels were significantly lower in the chronic ITP group compared to the control group (15,66±8,94 vs 18,67±8,25 ng/mL, $p<0.05$), whereas no significant difference was observed in the acute ITP group compared to the control (23,68±11,86 vs 18,67±8,25 ng/mL, $p>0.05$). Serum 25-OHD levels were significantly higher in males than in females among patients with total ITP group ($p<0.05$). Bleeding scores were significantly higher in the acute ITP group compared to the chronic group ($p<0.01$). Correlation analyses revealed a negative correlation between serum 25-OHD levels and age, indicating that vitamin D levels decreased with increasing age ($p<0.001$).

### Summary/Conclusion

Vitamin D deficiency was found to be common among children with ITP, and low serum 25-OHD levels were particularly evident in chronic cases. The significant association between serum vitamin D levels and chronic ITP suggests a possible modulatory role of vitamin D in the disease pathogenesis and development of chronicity. Furthermore, the correlation of vitamin D levels with demographic parameters such as age and sex indicates that assessing vitamin D status may be clinically useful in the follow-up of pediatric ITP patients.

**Copyright Information:** (Online) ISSN: 2572-9241
©2026 The Author(s). HemaSphere is published by John Wiley & Sons Ltd on behalf of European Hematology Association. This is an open access Abstract Book distributed under the Attribution-NonCommercial-NoDerivs (CC BY-NC-ND), which allows third parties to download the articles and share them with others as long as they credit the author and the Abstract Book, but they cannot change the content in any way or use them commercially.
**Abstract Book Citations:** Authors, Title, HemaSphere, 2026;10:(S1):pages. The abstract book DOIs can be found on the "Author Information" page.
**Disclaimer:** Articles published in the journal HemaSphere exclusively reflect the opinions of the authors. The authors are responsible for all content in their abstracts including accuracy of the facts, statements, citing resources, etc.

### (PB4287) PREDICTING FATIGUE IN PRIMARY IMMUNE THROMBOCYTOPENIA: A MACHINE LEARNING ANALYSIS USING TRANSFORMER-BASED NEURAL NETWORKS

**Topic:** 33. Platelet disorders

Justin Oosterlee*[1], Tim Dreier[1], Dino Mehic[1], Michael Fillitz[2], Cihan Ay[1], Ingrid Pabinger[1]

*[1]Medical University of Vienna, Department of Internal Medicine I, Division of Haematology and Haemostaseology, Vienna, Austria; [2]Hanusch Hospital, 3rd Department for Internal Medicine, Vienna, Austria;*

### Background

Primary immune thrombocytopenia (ITP) is a bleeding disorder caused by autoimmune-mediated destruction and impaired production of platelets. Fatigue is a common symptom and significantly impacts patients' quality of life, yet its cause and factors associated with it are poorly understood.

### Aims

To identify factors associated with fatigue in primary ITP.

### Methods

Patients from the Vienna ITP Registry, a prospective cohort study for adult primary ITP, were assessed for fatigue using the FACIT-Fatigue questionnaire. All patients with available fatigue data were included.
The primary analysis employed TabPFN[1] v2.5[2], a transformer-based neural network model designed for small tabular datasets, using all available clinical and laboratory parameters (n=103). Features with >20% missing data were excluded. Sensitivity analyses were performed to assess robustness by (i) restricting features to clinical parameters alone (n=19), laboratory parameters alone (n=84), or a hypothesis-driven subset of 8 predictors with strong clinical priors for fatigue (haemoglobin, ferritin, transferrin saturation, platelet count, vitamin D, age, sex, bleeding score, current ITP treatment), and (ii) substituting elastic net penalised regression (with k-nearest neighbour imputation for missing values) as an alternative modelling approach across all feature sets. All models were evaluated using 5-fold, 10-times repeated cross-validation with shared fold assignments to ensure direct comparability. Performance was assessed by the out-of-fold root mean squared error (RMSE) and the coefficient of determination $R^2$ with bootstrapped 95% confidence intervals.

### Results

183 patients (106 female) with a median FACIT-Fatigue score of 43 were analysed. Patient characteristics are listed in Table 1.
In the primary analysis, TabPFN with all 103 features achieved an RMSE of 11.61 (95% confidence interval [CI] 10.41–12.78) and $R^2$ of 0.015 (95% CI −0.054-0.066), explaining less than 2% of variance in fatigue scores. Sensitivity analyses yielded consistent results: restricting to clinical ($R^2$ 0.004), laboratory ($R^2$ 0.009), or hypothesis-driven features ($R^2$ 0.079) did not meaningfully improve prediction. Elastic net regression performed comparably on reduced feature sets (clinical characteristics $R^2$ 0.002 and hypothesis-driven selection $R^2$ 0.065) but showed significant overfitting on the full laboratory ($R^2$ −0.169) and combined sets ($R^2$ −0.199). No model-feature set combination achieved clinically meaningful fatigue prediction (Figure 1).

**Copyright Information:** (Online) ISSN: 2572-9241
©2026 The Author(s). HemaSphere is published by John Wiley & Sons Ltd on behalf of European Hematology Association. This is an open access Abstract Book distributed under the Attribution-NonCommercial-NoDerivs (CC BY-NC-ND), which allows third parties to download the articles and share them with others as long as they credit the author and the Abstract Book, but they cannot change the content in any way or use them commercially.
**Abstract Book Citations:** Authors, Title, HemaSphere, 2026;10:(S1):pages. The abstract book DOIs can be found on the "Author Information" page.
**Disclaimer:** Articles published in the journal HemaSphere exclusively reflect the opinions of the authors. The authors are responsible for all content in their abstracts including accuracy of the facts, statements, citing resources, etc.

| Characteristic | N = 183[1] |
|---|---|
| Female | 106 (58%) |
| Age (years) | 48 (33, 62) |
| FACIT-Fatigue score | 43 (32, 49) |
| Platelet count (x$10^9$/L) | 66 (37, 101) |
| Disease duration (months) | 28 (0, 143) |
| Newly Diagnosed | 66 (36%) |
| Persistent | 15 (8.2%) |
| Chronic | 102 (56%) |
| Splenectomy | 11 (6%) |
| Current treatment | 73 (40%) |
| Past treatment lines | 1 (0, 3) |

[1]n (%); Median (Q1, Q3)

### Summary/Conclusion

Despite leveraging a highly characterised ITP cohort and employing TabPFN v2.5, a transformer-based architecture specifically designed to handle small-sample data, fatigue in ITP could not be meaningfully predicted using standard clinical and laboratory data. The inability of both neural networks and penalised regression models to find features associated with fatigue highlight the heterogeneity of ITP and underscores that ITP-related fatigue as a complex and multifactorial symptom, which may transcend routine laboratory parameters. Due to its complexity, further research with larger sample sizes is needed to elucidate the mechanisms of ITP-related fatigue.

**Copyright Information:** (Online) ISSN: 2572-9241
©2026 The Author(s). HemaSphere is published by John Wiley & Sons Ltd on behalf of European Hematology Association. This is an open access Abstract Book distributed under the Attribution-NonCommercial-NoDerivs (CC BY-NC-ND), which allows third parties to download the articles and share them with others as long as they credit the author and the Abstract Book, but they cannot change the content in any way or use them commercially.
**Abstract Book Citations:** Authors, Title, HemaSphere, 2026;10:(S1):pages. The abstract book DOIs can be found on the "Author Information" page.
**Disclaimer:** Articles published in the journal HemaSphere exclusively reflect the opinions of the authors. The authors are responsible for all content in their abstracts including accuracy of the facts, statements, citing resources, etc.

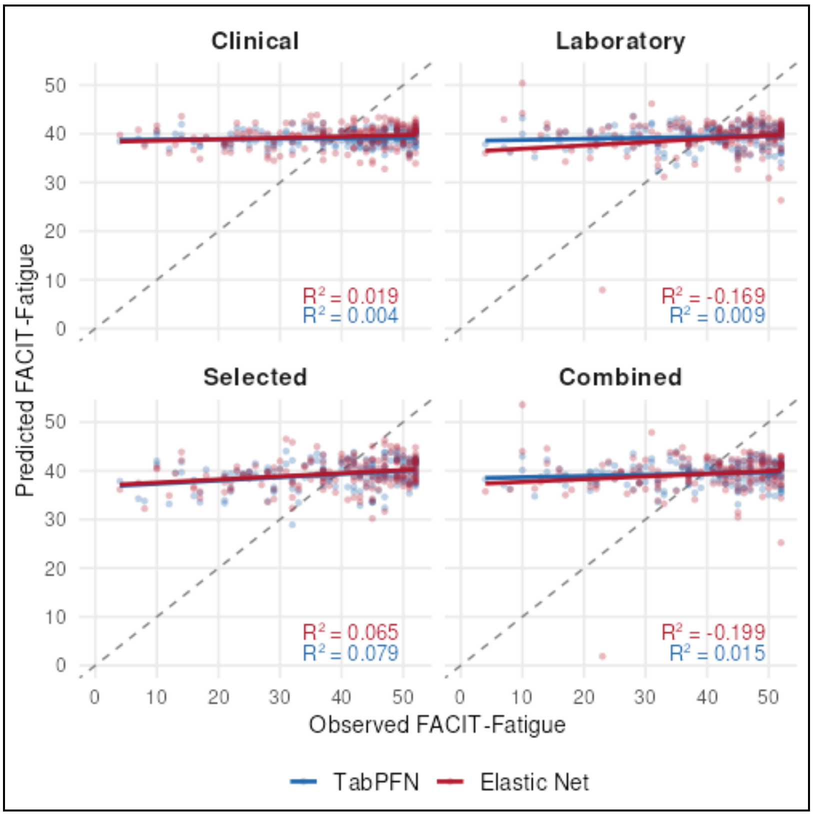

Figure 1. Predicted versus observed FACIT-Fatigue scores from out-of-fold cross-validation for TabPFN and elastic net regression across four feature set configurations. Dashed lines indicate perfect prediction. $R^2$ values are shown per model and panel.

**Copyright Information:** (Online) ISSN: 2572-9241
©2026 The Author(s). HemaSphere is published by John Wiley & Sons Ltd on behalf of European Hematology Association. This is an open access Abstract Book distributed under the Attribution-NonCommercial-NoDerivs (CC BY-NC-ND), which allows third parties to download the articles and share them with others as long as they credit the author and the Abstract Book, but they cannot change the content in any way or use them commercially.
**Abstract Book Citations:** Authors, Title, HemaSphere, 2026;10:(S1):pages. The abstract book DOIs can be found on the "Author Information" page.
**Disclaimer:** Articles published in the journal HemaSphere exclusively reflect the opinions of the authors. The authors are responsible for all content in their abstracts including accuracy of the facts, statements, citing resources, etc.

### (PB4288) A PATIENT-REPORTED MEASURE OF DISEASE CONTROL IN IMMUNE THROMBOCYTOPENIA (ITP): DEVELOPMENT AND CONTENT VALIDITY OF THE ITP CONTROL TOOL (ITP-CT) FOR ROUTINE CLINICAL CARE

**Topic:** 33. Platelet disorders

Waleed Ghanima*[1], Sandhya Panch[2], Hanny Al-Samkari[3], Danielle Boyle[4], Caroline Kruse[5], Barbara Lovrencic[6], Mervyn Morgan[7], Pablo Manuel Bianculli[8], Matias Cordoba[9], Ramiya Kumar[10], Owen Cooper[11], Matthew Reaney[12], Maria Belen Rodriguez[9], Nichola Cooper[13]

*[1]Østfold Hospital Trust, Grålum, Norway, and Institute of Clinical Medicine, University of Oslo, Oslo, Norway;[2]University of Washington/Fred Hutchinson Cancer Center, Seattle, WA, United States of America; [3]Harvard Medical School/Massachusetts General Hospital, Boston, MA, United States of America; [4]International ITP Alliance, ITP Australia and New Zealand, Cairns, Australia; [5]International ITP Alliance, Platelet Disorder Support Association, Cleveland, Ohio, United States of America; [6]International ITP Alliance, Associazione Italiana Porpora Immune Trombocitopenica, Caprino Veronese, VR, Italy;[7]International ITP Alliance, ITP Support Association, Bolnhurst, United Kingdom; [8]Sanofi, Buenos Aires, Argentina; [9]Sanofi, Cambridge, MA, United States of America; [10]IQVIA, Bangalore, India; [11]IQVIA, London, United Kingdom; [12]IQVIA, Reading, United Kingdom; [13]Imperial College London, London, United Kingdom;*

### Background

In immune thrombocytopenia (ITP), evaluation of platelet counts and bleeding remain central to disease monitoring. However, patients' lived experiences encompass additional aspects of disease that are often under-recognized by clinicians. There is no standardized measure to assess the multifaceted nature of patient-perceived disease control. To address this gap, we have begun the development of the ITP control tool (ITP-CT), a patient-reported outcome measure that supports the evaluation of disease control. In stage I of the ITP-CT development, discussions between ITP clinicians and ITP patient representatives identified key determinants of ITP disease control, resulting in an 8-item draft ITP-CT questionnaire for evaluation with a 4-week recall period.

### Aims

We report stage II of the ITP-CT development, which aims to assess the relevance, understandability, and usability of the ITP-CT by patients.

### Methods

18 adults with ITP were recruited to take part in a 1:1 90-minute interview. Interviews were conducted in 4 rounds (each with 3 to 7 participants) and included a concept elicitation (CE) phase to access patient perspective of symptoms and impacts of ITP control, and a cognitive debriefing (CD) phase to access comprehension, relevance, and usability of the draft ITP-CT. Between rounds, interview data and feedback were reviewed to inform revisions to item wording, examples, response options, and instructions in the draft ITP-CT before use in subsequent rounds. Following all interview rounds, the ITP-CT was finalized.

**Copyright Information:** (Online) ISSN: 2572-9241
©2026 The Author(s). HemaSphere is published by John Wiley & Sons Ltd on behalf of European Hematology Association. This is an open access Abstract Book distributed under the Attribution-NonCommercial-NoDerivs (CC BY-NC-ND), which allows third parties to download the articles and share them with others as long as they credit the author and the Abstract Book, but they cannot change the content in any way or use them commercially.
**Abstract Book Citations:** Authors, Title, HemaSphere, 2026;10:(S1):pages. The abstract book DOIs can be found on the "Author Information" page.
**Disclaimer:** Articles published in the journal HemaSphere exclusively reflect the opinions of the authors. The authors are responsible for all content in their abstracts including accuracy of the facts, statements, citing resources, etc.

### Results

Patients were 72% female, had a median age of 49.5 years (age range: 26 to 76), and had a median time since diagnosis of 12.0 years (range: 0.3-61.2). Patients represented 11 US states and varied in education, employment, platelet counts (16,000-297,000 platelets/µL), and treatment status (72% receiving treatment), reflecting diverse patient experiences. During the CE phase, patients identified 41 unique concepts relevant to ITP control. Of the variables identified in stage I, the most common included bruising (100%), fatigue (89%), low platelet count (89%), need to be cautious (67%), impact on diet (61%), hospitalizations (61%), interaction with friends and family (56%), and brain fog (44%) (Figure). Most participants associated control with stability/predictability in platelet count and normalcy in lifestyle, while some emphasized reduced treatment burden, fewer symptoms, and sense of security. ITP "out of control" was often associated with uncertainty and disruption, such as unexpected platelet drops, fatigue, urgent medical attention, and frequent symptoms. In the CD phase, all participants (100%) reported that the instructions for completing the ITP-CT were easy to follow, items were relevant and clear and found the recall period appropriate. Most patients (88.2%) found the tool helpful in preparing for visits with health care providers. Patient feedback in rounds 1-3 led to improvements, such as clearer item definitions, providing concrete examples of symptoms and adjusting response options to reduce overlap and ambiguity. No further changes were made after round 4.

### Summary/Conclusion

The ITP-CT is a concise and multidimensional tool that was developed involving ITP clinicians and patients and validated through ITP patient interviews to enable patient-centered management and shared decision-making.

Figure. Participant feedback on top signs, symptoms and impacts related to ITP disease control.

| ITP control tool item mapping | Signs, symptoms, impact related to ITP control | Percentage of patient mentions during concept elicitation (n=18) |
|---|---|---|
| **Item 1:** Skin bleeding or bleeding elsewhere | Bruising | 100% |
| | Petechiae | 72% |
| | Gum bleeds | 44% |
| | Nose bleeds | 44% |
| | Skin bleeding | 39% |
| | Blood blisters | 22% |
| | Menstrual bleeds | 22% |
| **Item 2:** Physical fatigue | Fatigue | 89% |
| **Item 3:** Brain fog and/or difficulties concentrating | Brain fog | 44% |
| **Item 4:** Concerns about platelet counts | Low platelet count | 89% |
| | Concerns about platelet count | 22% |
| **Item 5:** Feeling worried, anxious, sad, or depressed | Worried/Anxious | 28% |
| | Mental stress | 50% |
| | Frustration | 44% |
| **Item 6:** Impact on day to day activities or habits | Need to be cautious | 67% |
| | Impact on diet | 61% |
| | Impact on sports/exercise | 56% |
| | Impact on daily tasks/routine | 56% |
| | Requires more time to care | 39% |
| | Impact on work | 39% |
| | Impact on hobbies | 33% |
| | Impact to medical care | 33% |
| **Item 7:** Impact on interactions with family, friends and/or partner | Interaction with friends and family | 56% |
| **Item 8:** Type of care needed from health care provider | Hospitalizations/health care needs | 61% |

**Copyright Information:** (Online) ISSN: 2572-9241
©2026 The Author(s). HemaSphere is published by John Wiley & Sons Ltd on behalf of European Hematology Association. This is an open access Abstract Book distributed under the Attribution-NonCommercial-NoDerivs (CC BY-NC-ND), which allows third parties to download the articles and share them with others as long as they credit the author and the Abstract Book, but they cannot change the content in any way or use them commercially.
**Abstract Book Citations:** Authors, Title, HemaSphere, 2026;10:(S1):pages. The abstract book DOIs can be found on the "Author Information" page.
**Disclaimer:** Articles published in the journal HemaSphere exclusively reflect the opinions of the authors. The authors are responsible for all content in their abstracts including accuracy of the facts, statements, citing resources, etc.

### (PB4289) A NOVEL GERMLINE GP1BA VARIANT RESPONSIBLE FOR MONOALLELIC BERNARD-SOULIER SYNDROME IN A MULTI-GENERATIONAL FAMILY WITH INHERITED MACROTHROMBOCYTOPENIA

**Topic:** 33. Platelet disorders

Carlo Zaninetti*[1], Luise Porges[1], Natascha Klinkel[2], Eva Klopocki[3, 4], Carmen Freyer[1], Stefanie Spiegler[5, 6], Thomas Thiele[1], Andreas Greinacher[1]

*[1]Institute for Transfusion Medicine, University Medicine Greifswald., Greifswald, Germany;[2]Department of Pediatric Hematology and Oncology, University Hospital Giessen, Giessen, Germany; [3]Institute of Human Genetics, University of Würzburg, Würzburg, Germany; [4]Institute for Clinical Genetics and Genomic Medicine, University Hospital Würzburg., Würzburg, Germany;[5]Institute of Physics, University of Greifswald, Greifswald, Germany., Greifswald, Germany; [6]German Centre for Cardiovascular Research (DZHK), partner site Greifswald., Greifswald, Germany;*

### Background

Classical Bernard-Soulier syndrome (BSS) is a rare autosomal recessive platelet disorder caused by biallelic variants in GP1BA, GP1BB or GP9, encoding the subunits of the platelet glycoprotein (GP)Ib/IX/V complex, the receptor for von Willebrand factor. Patients typically present with mucocutaneous bleeding, moderate to severe macrothrombocytopenia, markedly reduced GPIb/IX/V expression and a pronounced platelet adhesion defect. In contrast, monoallelic variants in GP1BA or GP1BB give rise to a dominantly inherited macrothrombocytopenia with only partially reduced GPIb/IX/V expression and, at most, mild platelet dysfunction. Although bleeding symptoms are usually mild, correct recognition of monoallelic BSS is crucial to avoid misdiagnosis, particularly as immune thrombocytopenia. Here, we describe an individual from a large family with monoallelic BSS due to a previously unreported GP1BA variant.

### Aims

To delineate the clinical, laboratory and genetic features of an autosomal dominant inherited thrombocytopenia in a large pedigree.

### Methods

Platelet evaluation comprised full blood count, flow cytometry, light microscopy, standard and confocal immunofluorescence microscopy on the peripheral blood smears. Genetic analysis was performed by next-generation sequencing, and segregation of the candidate variant within the family was confirmed by Sanger sequencing.

### Results

The proband, a 15-year-old Caucasian male, was referred for workup of an incidentally detected thrombocytopenia. He exhibited mild thrombocytopenia (mean platelet count 139 × $10^9$/L) and a minimal bleeding history (ISTH BAT score 1). Light microscopy showed platelet macrocytosis with occasional giant platelets. Immunofluorescence and flow cytometry demonstrated a partial reduction of GPIbα and GPIbβ expression on platelets. In view of these findings and a family history consistent with autosomal dominant thrombocytopenia, monoallelic BSS was suspected. NGS revealed a heterozygous GP1BA exon 2 variant (NM_000173.7): c.455T>C, p.(Leu152Pro), which is absent from gnomAD and other major population databases. The variant fully segregated with thrombocytopenia over three generations. It replaces a leucine with a proline within a leucine-rich repeat domain and is predicted to impair folding and stability of the GPIb/IX/V complex. According to current criteria, the variant was classified as likely pathogenic.

**Copyright Information:** (Online) ISSN: 2572-9241
©2026 The Author(s). HemaSphere is published by John Wiley & Sons Ltd on behalf of European Hematology Association. This is an open access Abstract Book distributed under the Attribution-NonCommercial-NoDerivs (CC BY-NC-ND), which allows third parties to download the articles and share them with others as long as they credit the author and the Abstract Book, but they cannot change the content in any way or use them commercially.
**Abstract Book Citations:** Authors, Title, HemaSphere, 2026;10:(S1):pages. The abstract book DOIs can be found on the "Author Information" page.
**Disclaimer:** Articles published in the journal HemaSphere exclusively reflect the opinions of the authors. The authors are responsible for all content in their abstracts including accuracy of the facts, statements, citing resources, etc.

**Summary/Conclusion**

This novel *GP1BA* variant expands the mutational spectrum of monoallelic BSS, a relatively frequent cause of inherited macrothrombocytopenia in Europe. This case underlines the diagnostic impact of careful family history and detailed platelet morphology assessment as cost-effective and informative tools in the identification of hereditary platelet disorders.

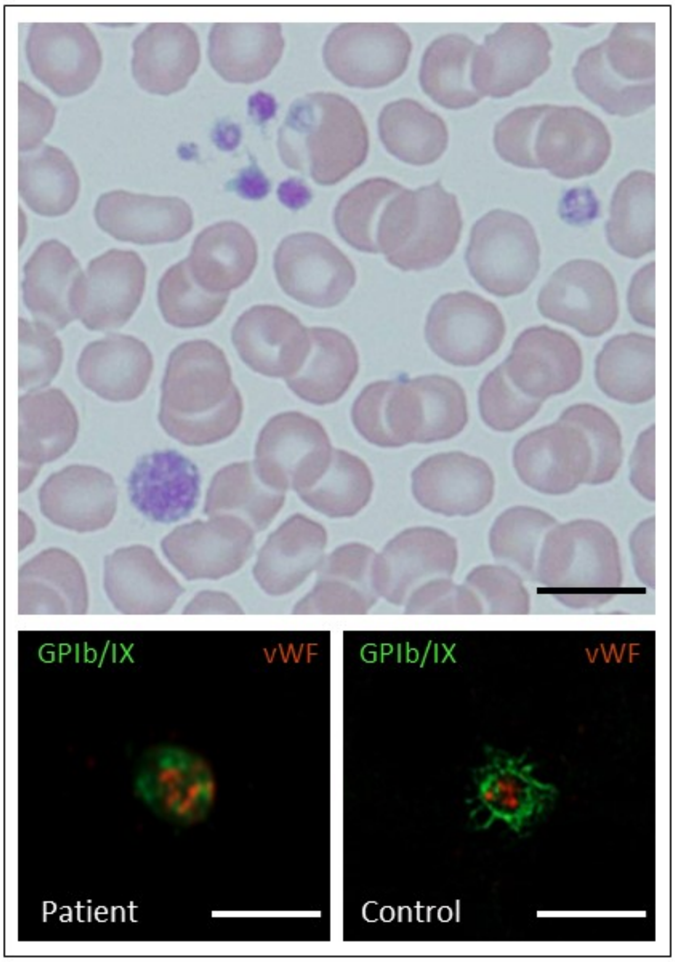

Figure caption. Heterogeneous platelet population with enlarged and giant platelets on light microscopy (top) and reduced GPIb/IX surface expression compared with a healthy control on confocal immunofluorescence microscopy (bottom; bottom; counterstained for von Willebrand factor, vWF). Scale bars, 10µm.

**Copyright Information:** (Online) ISSN: 2572-9241
©2026 The Author(s). HemaSphere is published by John Wiley & Sons Ltd on behalf of European Hematology Association. This is an open access Abstract Book distributed under the Attribution-NonCommercial-NoDerivs (CC BY-NC-ND), which allows third parties to download the articles and share them with others as long as they credit the author and the Abstract Book, but they cannot change the content in any way or use them commercially.
**Abstract Book Citations:** Authors, Title, HemaSphere, 2026;10:(S1):pages. The abstract book DOIs can be found on the "Author Information" page.
**Disclaimer:** Articles published in the journal HemaSphere exclusively reflect the opinions of the authors. The authors are responsible for all content in their abstracts including accuracy of the facts, statements, citing resources, etc.

### (PB4290) REAL-WORLD STUDY OF HETROMBOPAG FOR CHEMOTHERAPY-INDUCED THROMBOCYTOPENIA IN CANCER PATIENTS

**Topic:** 33. Platelet disorders

Yu Gao[1], Li Zuo[1], Meixing Wang[1], Ning Zhang[2], Wenqing Rong[3], Guangru Xu[4], Xiaodong Zhu[5], Yuedi Dai*[1]

*[1]Minhang Branch, Fudan University Shanghai Cancer Center, Department of Medical Oncology, Shanghai, China;[2]Jinshan Hosoital of Fudan University, Department of Pharmacology, Shanghai, China; [3]Shanghai Seventh People's Hospital, Department of Medical Oncology, Shanghai, China; [4]Shanghai Pudong New Area People's Hospital, Department of Medical Oncology, Shanghai, China; [5]Fudan University Shanghai Cancer Center, Department of Medical Oncology, Shanghai, China;*

**Background**

Chemotherapy-induced thrombocytopenia (CIT) is one of the most common chemotherapy-related hematologic toxicities. It increases bleeding risk, prolongs hospital stays, raises healthcare costs, and can be fatal in severe cases. Hetrombopag is a novel oral non-peptide thrombopoietin receptor agonist (TPO-RA) that effectively improves patient outcomes in CIT while demonstrating favorable safety and tolerability. However, despite supporting clinical evidence, real-world application data across different tumor types and baseline platelet levels remain scarce, particularly lacking large-scale, multicenter practice evidence.

**Aims**

This prospective, multicenter real-world study aims to evaluate the efficacy and safety of hetrombopag in treating CIT in a real-world setting.

**Methods**

Patients with CIT (platelet count <75 × 10⁹/L) receiving hetrombopag treatment were enrolled from five hospitals. The primary endpoint was the proportion achieving a platelet count (PLT) ≥75 × 10⁹/L within two weeks. Secondary endpoints included the proportion achieving an increase of at least 50 × 10⁹/L from baseline or PLT ≥100 × 10⁹/L within two weeks. All participants provided written informed consent.

**Results**

A total of 75 patients were included in the analysis. The median age was 64 years (range 24–81 years); 33.3% (25/75) had gastric cancer, 22.7% (17/75) had colorectal cancer, 12.0% (9/75) had ovarian cancer, and 6.7% (5/75) had lung cancer. The median baseline platelet count was 56 × 10⁹/L (range 8–71 × 10⁹/L). Among 75 evaluable patients, the median duration of treatment with hetrombopag was 6 days (range 3–36 days), with a median starting dose of 5 mg (range 2.5–7.5 mg). 82.7% (62/75) initiated hetrombopag at 5 mg QD without dose adjustment. Overall, 77.3% (58/75; 95% CI, 65.9–85.9%) achieved the primary endpoint. Among patients with baseline PLT levels of 50 × 10⁹/L ≤ PLT ≤ 75 × 10⁹/L, 25 × 10⁹/L ≤ PLT ≤ 50 × 10⁹/L, and PLT < 25 × 10⁹/L, the proportions achieving the study endpoint were 85.7% (42/49), 53.3% (8/15), and 72.7% (8/11), respectively. Among gastric and colorectal cancer patients, the proportions reaching the primary endpoint were 88.0% (22/25) and 82.4% (14/17), respectively. 46.7% (35/75; 95% CI, 35.2–58.5%) of patients achieved the secondary endpoint, with a high proportion of 81.8% (9/11) among patients with baseline PLT <25×10⁹/L. Adverse events were generally mild; 24.0% (18/75) of patients experienced treatment-emergent adverse events (TEAEs), with no Grade ≥3 TEAEs, no drug-related adverse events, and no fatal TEAEs during the study period.

**Copyright Information:** (Online) ISSN: 2572-9241
©2026 The Author(s). HemaSphere is published by John Wiley & Sons Ltd on behalf of European Hematology Association. This is an open access Abstract Book distributed under the Attribution-NonCommercial-NoDerivs (CC BY-NC-ND), which allows third parties to download the articles and share them with others as long as they credit the author and the Abstract Book, but they cannot change the content in any way or use them commercially.
**Abstract Book Citations:** Authors, Title, HemaSphere, 2026;10:(S1):pages. The abstract book DOIs can be found on the "Author Information" page.
**Disclaimer:** Articles published in the journal HemaSphere exclusively reflect the opinions of the authors. The authors are responsible for all content in their abstracts including accuracy of the facts, statements, citing resources, etc.

**Summary/Conclusion**

This study provides evidence for the efficacy and safety of hetrombopag in treating CIT in a real-world setting. Hetrombopag may offer a novel therapeutic strategy for CIT.

**Copyright Information:** (Online) ISSN: 2572-9241
©2026 The Author(s). HemaSphere is published by John Wiley & Sons Ltd on behalf of European Hematology Association. This is an open access Abstract Book distributed under the Attribution-NonCommercial-NoDerivs (CC BY-NC-ND), which allows third parties to download the articles and share them with others as long as they credit the author and the Abstract Book, but they cannot change the content in any way or use them commercially.
**Abstract Book Citations:** Authors, Title, HemaSphere, 2026;10:(S1):pages. The abstract book DOIs can be found on the "Author Information" page.
**Disclaimer:** Articles published in the journal HemaSphere exclusively reflect the opinions of the authors. The authors are responsible for all content in their abstracts including accuracy of the facts, statements, citing resources, etc.

### (PB4291) EFFICACY AND SAFETY OF HETROMBOPAG COMBINED WITH PREDNISONE AS FIRST-LINE THERAPY FOR PRIMARY IMMUNE THROMBOCYTOPENIA (ITP)

**Topic:** 33. Platelet disorders

Xifeng Dong*[1], Weinan Lin[1], Ting Wang[1], Shaoxue Ding[1], Qing Shao[1], Rong Fu[1]

[1] *Tianjin Medical University General Hospital, Tianjin, China;*

### Background

Primary immune thrombocytopenia (ITP) is an acquired autoimmune hemorrhagic disease characterized by continuous thrombocytopenia and an increased risk of bleeding. Prednisone is the standard first-line treatment for primary ITP. However, its clinical application is often limited by short-term relapse, glucocorticoid dependence, and adverse effects associated with prolonged steroid use.

### Aims

This study aims to evaluate the efficacy and safety of combining hetrombopag with prednisone in reducing relapse rates and minimizing glucocorticoid exposure duration in primary ITP patients.

### Methods

This is a single-arm, single-center, and exploratory phase II clinical study, aiming to evaluate the efficacy and safety of hetrombopag combined with prednisone as first-line therapy. Adult patients with clearly diagnosed ITP whose platelet counts of < $30\times10^9$/L or platelet counts of < $50\times10^9$/L accompanied by significant bleeding (bleeding score ≥ 2 points), were eligible for the study. Eligible patients will receive an 8-week treatment. The treatment plan is as follows: Prednisone, 1 mg/kg/d, with a maximum dose of 80 mg/d, administered in divided doses or as a single dose. Once the treatment takes effect, the dosage should be reduced as soon as possible and discontinued within 6 to 8 weeks. Patients who show no response to prednisone within 2 weeks should have the drug reduced and discontinued promptly. Hetrombopag should be administered within 7 days after the initiation of glucocorticoid treatment, with an initial dose of 5 mg/d, orally. The maximum adjusted dose is 7.5 mg. For patients whose platelet count remains < $50\times10^9$/L after 2 weeks of treatment, the dose of hetrombopag should be increased to 7.5 mg/d. The primary endpoint was the proportion of patients who remained effective for 26 weeks after treatment.

### Results

As of December 31, 2025, 49 patients were enrolled, among whom 63.3% (31/49) were female. The newly diagnosed ITP accounted for 73.5%, while the others were chronic and persistent ITP. The median age of these patients was 58.0 y (range, 19-79). The median platelet count at baseline was $11\times10^9$/L (range, 1-39). Overall, the proportion of patients who remained effective for 26 weeks after treatment was 84.1% (37/44). 91.7% (44/48) of patients had response after 8 weeks of treatment. Response was defined as the platelet count of at least $30\times10^9$/L (the platelet counts should be tested at least twice consecutively, with an interval of at least 7 days), at least doubling of the baseline count, and absence of bleeding. 54.2% (26/48) of patients had complete response. Complete response was defined as the platelet count of at least $100\times10^9$/L (the platelet counts should be tested at least twice consecutively, with an interval of at least 7 days), at least doubling of the baseline count, and absence of bleeding. The median time required for response is 5 days (1-43). No related thrombotic events or hepatotoxicity were observed.

**Copyright Information:** (Online) ISSN: 2572-9241
©2026 The Author(s). HemaSphere is published by John Wiley & Sons Ltd on behalf of European Hematology Association. This is an open access Abstract Book distributed under the Attribution-NonCommercial-NoDerivs (CC BY-NC-ND), which allows third parties to download the articles and share them with others as long as they credit the author and the Abstract Book, but they cannot change the content in any way or use them commercially.
**Abstract Book Citations:** Authors, Title, HemaSphere, 2026;10:(S1):pages. The abstract book DOIs can be found on the "Author Information" page.
**Disclaimer:** Articles published in the journal HemaSphere exclusively reflect the opinions of the authors. The authors are responsible for all content in their abstracts including accuracy of the facts, statements, citing resources, etc.

**Summary/Conclusion**

The combination of hetrombopag and prednisone demonstrated rapid, durable efficacy in primary ITP patients. This regimen significantly reduced glucocorticoid exposure in both dose and duration, whileeffectively lowering bleeding risks. These findings suggest that this combination therapy could serve as apromising first-line treatment strategy. Further randomized controlled trials are warranted to validate long-term outcomes.

**Copyright Information:** (Online) ISSN: 2572-9241
©2026 The Author(s). HemaSphere is published by John Wiley & Sons Ltd on behalf of European Hematology Association. This is an open access Abstract Book distributed under the Attribution-NonCommercial-NoDerivs (CC BY-NC-ND), which allows third parties to download the articles and share them with others as long as they credit the author and the Abstract Book, but they cannot change the content in any way or use them commercially.
**Abstract Book Citations:** Authors, Title, HemaSphere, 2026;10:(S1):pages. The abstract book DOIs can be found on the "Author Information" page.
**Disclaimer:** Articles published in the journal HemaSphere exclusively reflect the opinions of the authors. The authors are responsible for all content in their abstracts including accuracy of the facts, statements, citing resources, etc.

### (PB4292) LONG-TERM OUTCOMES OF THROMBOPOIETIN RECEPTOR AGONISTS IN CHILDREN WITH IMMUNE THROMBOCYTOPENIA, WITH SPECIAL REFERENCE TO THE OFF-LABEL USE OF AVATROMBOPAG

**Topic:** 33. Platelet disorders

Aikaterini Michalopoulou[1], Stefanos Saslis*[1], Athina Dettoraki[1], Nefeli Papageorgiou[1], Helen Karelioti[1], Sotiria Thymianou[1], Zoey Kapsimali[1], Eleni Pergantou[1]

*[1] "Aghia Sophia" Children's Hospital, Hemophilia Centre/Hemostasis and Thrombosis Unit, Athens, Greece;*

### Background

Immune thrombocytopenia (ITP) is an autoimmune disorder characterized by isolated thrombocytopenia (platelet count <100 $\times 10^9$/L) and increased bleeding risk. Although 70%-80% of pediatric patients with ITP experience remission, approximately 20%-30% will develop longer-lasting disease, defined as persistent thrombocytopenia lasting ≥ 3 months but <12 months (pITP) or chronic thrombocytopenia lasting ≥12 months (cITP). Thrombopoietin-Receptor Agonists (TPO-RAs), i.e., eltrombopag and romiplostim are increasingly used in pediatric patients, usually as a second-line therapy in ITP management.

### Aims

To evaluate the long-term outcome of TPO-RAs use in children with ITP, with special reference to the off-label use of avatrombopag, a newer oral TPO-RA, in refractory cases.

### Methods

This is a retrospective observational study conducted in a single pediatric reference centre for bleeding disorders in Greece. It included patients aged 1-18 years with ITP who received TPO-RAs ( eltrombopag, romiplostim or avatrombopag) over a 6-year period (2019-2025). Clinical evaluation, hematology and liver function tests (in eltrombopag use) were performed regularly and doses were adjusted according to platelet counts.

### Results

Twenty-seven patients with ITP (11 girls,16 boys) were treated with TPO-RAs for a median period of 20 (range: 2-80) months. Median age at ITP diagnosis was 5.5 (range: 1-16) years of age and median time from diagnosis to TPO-RA initiation was 15 (range: 1-156) months. Baseline platelet count at diagnosis was 8,000 (range: 1,000-42,000)/$mm^3$. Twenty-three patients received eltrombopag ( pITP: 8 and cITP :15) and four patients with cITP were treated sequentially with both eltrombopag and romiplostim. Bone marrow evaluation prior to drug initiation was performed in all but two cases. In 81% of patients there was a satisfying and durable response, with a median time of 14 (range: 7-60) days to platelet count increase. Additional therapies during treatment were needed in 40% of patients, while eltrombopag/romiplostim were discontinued in 10% due to remission and in 1.5% of patients due to lack of response. It is worth mentioning that eltrombopag was also off-labelly prescribed in a girl with inherited thrombocytopenia (MYH-9 related disorder) to increase platelet count before invasive procedures with good, but transient response. Among the 19 patients with cITP, two adolescents failed to respond to multiple therapies (including IVIG, corticosteroids, eltrombopag/romiplostim and rituximab) and presented with bleeding diathesis, so it was decided to try a newer TPO-RA, avatrombopag (approved for cITP treatment in adults). After approval from national authorities, both patients received avatrombopag with very good platelet count response after 15 days and without any adverse events. After a sustained and durable response, the first patient (15.5 year-old girl) discontinued the drug after 8 months, achieving remission, while the second patient (15 year-old boy) is still taking avatrombopag for 14 months, preserving good platelet counts > 50.000/$mm^3$.

**Copyright Information:** (Online) ISSN: 2572-9241
©2026 The Author(s). HemaSphere is published by John Wiley & Sons Ltd on behalf of European Hematology Association. This is an open access Abstract Book distributed under the Attribution-NonCommercial-NoDerivs (CC BY-NC-ND), which allows third parties to download the articles and share them with others as long as they credit the author and the Abstract Book, but they cannot change the content in any way or use them commercially.
**Abstract Book Citations:** Authors, Title, HemaSphere, 2026;10:(S1):pages. The abstract book DOIs can be found on the "Author Information" page.
**Disclaimer:** Articles published in the journal HemaSphere exclusively reflect the opinions of the authors. The authors are responsible for all content in their abstracts including accuracy of the facts, statements, citing resources, etc.

**Summary/Conclusion**

In our study, TPO-RAs were effective with favorable safety profile for treating pediatric ITP demonstrating good long-term outcome. In two refractory cases, with significant impairment of quality of life and high risk of severe bleeding, the newer TPO-RA avatrombopag proved an effective and well-tolerated alternative therapeutic option.

**Copyright Information:** (Online) ISSN: 2572-9241
©2026 The Author(s). HemaSphere is published by John Wiley & Sons Ltd on behalf of European Hematology Association. This is an open access Abstract Book distributed under the Attribution-NonCommercial-NoDerivs (CC BY-NC-ND), which allows third parties to download the articles and share them with others as long as they credit the author and the Abstract Book, but they cannot change the content in any way or use them commercially.
**Abstract Book Citations:** Authors, Title, HemaSphere, 2026;10:(S1):pages. The abstract book DOIs can be found on the "Author Information" page.
**Disclaimer:** Articles published in the journal HemaSphere exclusively reflect the opinions of the authors. The authors are responsible for all content in their abstracts including accuracy of the facts, statements, citing resources, etc.

### (PB4293) A PROSPECTIVE, SINGLE-ARM STUDY OF ROMIPLOSTIM N01 FOR PLATELET ENGRAFTMENT AFTER AUTOLOGOUS STEM CELL TRANSPLANTATION IN MULTIPLE MYELOMA

**Topic:** 33. Platelet disorders

Min Ruan[1], Haitao Xu[2], Fengbo Jin[3], Xijun Zhu[4], Fengxiang Jin[5], Junjie Zhou[1], Jian Hong[1], Zhangbiao Long[1], Jifei Dai[1], Xinglin Liang[1], Qingsheng Li[1], Jian Ge*[1]

*[1]The First Affiliated Hospital of Anhui Medical University, Hefei, China;[2]Anqing municipal hospital, Anqing, China;[3]The First Affiliated Hospital of Anhui Medical University North District, Hefei, China; [4]Xuancheng People's Hospital, The affiliated Xuancheng Hospital of Wannan Medical College, Xuancheng, China; [5]Tongling People's Hospital, Tongling, China;*

### Background

Delayed or failed platelet engraftment is a common complication after autologous stem cell transplantation (ASCT) in patients with multiple myeloma (MM), leading to increased bleeding risk, transfusion dependency, and reduced quality of life. A standardized treatment to promote platelet recovery is currently lacking. Romiplostim N01 is a novel, long-acting thrombopoietin receptor agonist (TPO-RA) with demonstrated efficacy in primary immune thrombocytopenia and chemotherapy-induced thrombocytopenia. However, its role in post-ASCT platelet engraftment has not been fully evaluated in prospective studies.

### Aims

This study aims to evaluate the efficacy and safety of romiplostim N01 in promoting platelet engraftment in MM patients following ASCT.

### Methods

This is a prospective, single-arm, multicenter, exploratory clinical study. We plan to enroll 100 eligible patients with newly diagnosed MM. All patients will receive subcutaneous romiplostim N01 (3 μg/kg, once a week) starting on day +1 post-ASCT, for up to 6 weeks. The primary endpoint is the time to platelet engraftment, defined as the first day of a platelet count ≥ $20\times10^9$/L sustained for ≥ 7 consecutive days without transfusion support. Secondary endpoints include time to platelet recovery, total volume of platelet transfusions and the incidence of adverse events (AEs).

### Results

As of January 2026, 20 patients with multiple myeloma were enrolled in the study, including 13 males and 7 females. The median age was 56 years (range, 30–70), and all patients (100%) were classified as DS stage IIIA. All patients received melphalan conditioning prior to autologous stem cell transplantation, followed by administration of romiplostim N01 starting on day +1 post-transplantation. The median number of romiplostim N01 administrations was 3 (range, 2–4). During treatment, the platelet count reached its nadir after day +7 following autologous stem cell transplantation with the value of 14 (range, 6–67). The median time to platelet engraftment was day +13 (range, 9–20). On day +21, platelet engraftment was achieved in all patients (100%), and platelet counts recovered to ≥ $50\times10^9$/L for the first time in 13 patients (65%). All patients (100%) received platelet transfusion, with a mean total platelet transfusion requirement of 3.9 apheresis platelet units (95% CI, 2.73–5.07). No adverse events related to romiplostim N01 were observed during the treatment period.

**Copyright Information:** (Online) ISSN: 2572-9241
©2026 The Author(s). HemaSphere is published by John Wiley & Sons Ltd on behalf of European Hematology Association. This is an open access Abstract Book distributed under the Attribution-NonCommercial-NoDerivs (CC BY-NC-ND), which allows third parties to download the articles and share them with others as long as they credit the author and the Abstract Book, but they cannot change the content in any way or use them commercially.
**Abstract Book Citations:** Authors, Title, HemaSphere, 2026;10:(S1):pages. The abstract book DOIs can be found on the "Author Information" page.
**Disclaimer:** Articles published in the journal HemaSphere exclusively reflect the opinions of the authors. The authors are responsible for all content in their abstracts including accuracy of the facts, statements, citing resources, etc.

**Summary/Conclusion**

This study will provide prospective evidence on the efficacy and safety of romiplostim N01 for promoting platelet engraftment in MM patients post-ASCT. The preliminary findings are expected to support its potential as a treatment option to reduce post-transplant bleeding complications and platelet transfusion dependency, thereby improving patient outcomes.

**Copyright Information:** (Online) ISSN: 2572-9241
©2026 The Author(s). HemaSphere is published by John Wiley & Sons Ltd on behalf of European Hematology Association. This is an open access Abstract Book distributed under the Attribution-NonCommercial-NoDerivs (CC BY-NC-ND), which allows third parties to download the articles and share them with others as long as they credit the author and the Abstract Book, but they cannot change the content in any way or use them commercially.
**Abstract Book Citations:** Authors, Title, HemaSphere, 2026;10:(S1):pages. The abstract book DOIs can be found on the "Author Information" page.
**Disclaimer:** Articles published in the journal HemaSphere exclusively reflect the opinions of the authors. The authors are responsible for all content in their abstracts including accuracy of the facts, statements, citing resources, etc.

### (PB4294) BCMA-DIRECTED T-CELL-ENGAGER INDUCES SUSTAINED REMISSION IN REFRACTORY IMMUNE THROMBOCYTOPENIA

**Topic:** 33. Platelet disorders

Michael Korenkov*[1], Maximilian Zuleeg[2], Julian Liebaert[1], Vincent Fregona[3], Sebastian Serve[1], Julia Jesse[1], David Böckle[1], Stephan Bohl[1], Frederik Damm[1], Lars Bullinger[1], Ulrich Keller[1], Adrian Schwarzer[2], Frederik Albach[1], Thomas Thiele[2], Simon Haas[3], Jan Krönke[2], Marie Luise Hütter-Krönke[1]

*[1]Charité, Department of Hematology, Oncology and Cancer Immunology, Berlin, Germany;[2]University Medicine Greifswald, Internal Medicine C, Hematology, Oncology, Stem Cell Transplantation and Palliative Care, Greifswald, Germany; [3]Max Delbrück Center for Molecular Medicine in the Helmholtz Association, Berlin Institute for Medical Systems Biology,, Berlin, Germany;*

### Background

Immune thrombocytopenia (ITP) is an acquired autoimmune disorder characterized by immune-mediated platelet destruction and impaired immune tolerance, leading to increased bleeding risk. Multidrug-refractory ITP remains a major clinical challenge with limited durable treatment options, underscoring the need to explore novel strategies that address the underlying autoimmune pathology. In this context T-cell engaging therapies have been successfully applied in the treatment of several autoimmune diseases.

### Aims

To evaluate the safety and therapeutic potential of a bispecific BCMA-directed T-cell engager therapy in two patients with heavily pretreated ITP.

### Methods

Two patients with severe ITP that were refractory to multiple agents were selected from two hospitals to receive off label treatment with a fixed duration of the BCMA-directed T-cell-engager teclistamab. Clinical and laboratory data were collected to assess safety and hematologic response. Additionally, high dimensional spectral flow cytometry of bone marrow and peripheral blood has been performed in one patient.

### Results

Both patients (one male, one female) had received multiple lines of therapy, including corticosteroids, immunoglobulins, at least two different thrombopoetin-receptor-agonists, fostamatinib, immunosuppressive agents and rituximab without achieving a durable response. Fixed-duration teclistamab was well tolerated with only one patient experiencing grade 1 cytokine release syndrome and manageable transient neutropenia observed in both patients. The patients received different dosages: Patient 1 received 2 step-ups and four full doses; Patient 2 received 2 step-ups and one full dose. Platelet counts increased within days, exceeding 50/nl by days 3 and 9, respectively, and normalized within two weeks, allowing discontinuation of all ITP therapies. The first patient had Evans syndrome with mild concomitant autoimmune hemolytic anemia. Treatment led to sustained remission of both conditions, with normalization of platelet counts, as well as hemoglobin increase and conversion to a negative direct antiglobulin test. High-dimensional spectral flow cytometry in patient 1 showed complete plasma cell depletion in the bone marrow and a shift toward activated T-cell phenotypes with a reduction of naïve T cells in peripheral blood and bone marrow after teclistamab. Both patients are in treatment free remission for 5.5 and 4.5 months, respectively, and have a greatly increased quality of life.

**Copyright Information:** (Online) ISSN: 2572-9241
©2026 The Author(s). HemaSphere is published by John Wiley & Sons Ltd on behalf of European Hematology Association. This is an open access Abstract Book distributed under the Attribution-NonCommercial-NoDerivs (CC BY-NC-ND), which allows third parties to download the articles and share them with others as long as they credit the author and the Abstract Book, but they cannot change the content in any way or use them commercially.
**Abstract Book Citations:** Authors, Title, HemaSphere, 2026;10:(S1):pages. The abstract book DOIs can be found on the “Author Information” page.
**Disclaimer:** Articles published in the journal HemaSphere exclusively reflect the opinions of the authors. The authors are responsible for all content in their abstracts including accuracy of the facts, statements, citing resources, etc.

**Summary/Conclusion**

The unexpected rapid response observed within days of teclistamab therapy despite the immunoglobulin half-life of >3 weeks can be explained by the swift and complete plasma-cell eradication by teclistamab and fast consumption of anti-platelet antibodies, which rapidly shifts the balance between autoantibodies and available platelets towards platelets. Bone marrow investigations imply that sole B cell depletion by fostamatinib or rituximab while sparing plasma cells was not sufficient for inducing response in our patients. Instead, depletion of plasma cells by teclistamab was associated with rapid platelet response implying that auto reactive plasma cells are primarily driving ITP. The successful and well-tolerated treatment outcomes in these two patients provide a rationale for prospective clinical studies to systematically assess the safety, therapeutic efficacy, and optimal dosing of BCMA-directed T-cell–engaging therapies in ITP and AIHA.

**Copyright Information:** (Online) ISSN: 2572-9241
©2026 The Author(s). HemaSphere is published by John Wiley & Sons Ltd on behalf of European Hematology Association. This is an open access Abstract Book distributed under the Attribution-NonCommercial-NoDerivs (CC BY-NC-ND), which allows third parties to download the articles and share them with others as long as they credit the author and the Abstract Book, but they cannot change the content in any way or use them commercially.
**Abstract Book Citations:** Authors, Title, HemaSphere, 2026;10:(S1):pages. The abstract book DOIs can be found on the “Author Information” page.
**Disclaimer:** Articles published in the journal HemaSphere exclusively reflect the opinions of the authors. The authors are responsible for all content in their abstracts including accuracy of the facts, statements, citing resources, etc.

### (PB4295) HEALTH-RELATED QUALITY OF LIFE (HRQOL) AMONG PATIENTS WITH IMMUNE THROMBOCYTOPENIA (ITP) TREATED WITH FOSTAMATINIB IN THE FORTE STUDY

**Topic:** 33. Platelet disorders

Amber Afzal[1], Robert Kribs[2], Sumit Sawhney[3], Mohit Narang*[4], Robert Numerof[5], Francois Di Trapani[5], Aaron Sheppard[5], Bing Miu[5], Ruchika Goel[6]

*[1]Washington University School of Medicine, Department of Medicine, St. Louis, United States of America;[2]University of Kansas Health System, Hematologic Malignancies and Cellular Therapeutics, Westwood, United States of America; [3]HCA East Florida, Department of Internal Medicine, Fort Lauderdale, United States of America; [4]Maryland Oncology Hematology, US Oncology Research, Columbia, United States of America; [5]Rigel Pharmaceuticals, Inc., South San Francisco, United States of America; [6]Simmons Cancer Institute at SIU School of Medicine, Departments of Internal Medicine and Pediatrics, Springfield, United States of America;*

### Background

Primary ITP is an autoimmune disorder causing increased bleeding risk from immune-mediated platelet destruction. Patients with ITP experience substantial HRQoL impairment, most notably fatigue similar to other autoimmune diseases, but few studies report HRQoL outcomes with ITP-directed therapy. We investigated the effect of fostamatinib, a spleen tyrosine kinase inhibitor currently approved by the FDA and EMA for adults with previously treated ITP, on HRQoL.

### Aims

To examine HRQoL in adults with ITP enrolled in the FORTE study (NCT04904276) as measured by the 10-domain ITP Patient Assessment Questionnaire (ITP-PAQ).

### Methods

FORTE was a prospective, single-arm study of fostamatinib as second-line therapy conducted at 14 sites in the US. Consenting adults with ITP and insufficient response to corticosteroids were followed for up to 12 mo. HRQoL outcomes were assessed at baseline and during follow-up at the treating physician's discretion. Patients were included in the analysis if they had a baseline assessment and ≥1 postbaseline ITP-PAQ assessment after receiving ≥1 dose of fostamatinib. Follow-up assessments were grouped by study weeks (1-8, 19-28, and 36-54). Results were calculated as raw mean ITP-PAQ domain scores and least-squares mean (LSM) changes from baseline and were compared with the corresponding minimally important difference (MID) for clinically relevant improvement in each domain. LSM changes from baseline were calculated using a linear mixed-effects model adjusted for baseline score and grouped study week.

### Results

Among 15 enrolled patients who received fostamatinib, 10 patients (6 women) had ≥1 postbaseline ITP-PAQ assessment, 9 of whom had a postbaseline platelet count improvement >50×$10^9$/L. Among the evaluable patients, scores for all ITP-PAQ domains, except Women's Reproductive Health, increased over time (**Table**). Improvements in mean ITP-PAQ scores exceeded the MID at 36-54 wk in 7 of 10 domains, including Overall QoL. Statistically significant ($P$<0.05) LSM improvements from baseline at 36-54 wk were observed for Overall QoL, Activity, Bother-Physical Health, Fatigue/Sleep, Fear, Social Activity, and Work domains. For the ITP-PAQ Item 10 (physical fatigue) at 36-54 wk (n=5), mean change from baseline (SE) was 40.0 (18.7) points, and a significant LSM improvement from baseline score was observed (38.9; $P$=0.02).

**Copyright Information:** (Online) ISSN: 2572-9241
©2026 The Author(s). HemaSphere is published by John Wiley & Sons Ltd on behalf of European Hematology Association. This is an open access Abstract Book distributed under the Attribution-NonCommercial-NoDerivs (CC BY-NC-ND), which allows third parties to download the articles and share them with others as long as they credit the author and the Abstract Book, but they cannot change the content in any way or use them commercially.
**Abstract Book Citations:** Authors, Title, HemaSphere, 2026;10:(S1):pages. The abstract book DOIs can be found on the "Author Information" page.
**Disclaimer:** Articles published in the journal HemaSphere exclusively reflect the opinions of the authors. The authors are responsible for all content in their abstracts including accuracy of the facts, statements, citing resources, etc.

**Summary/Conclusion**

Fostamatinib use in the FORTE study was associated with a meaningful improvement in HRQoL assessed by ITP-PAQ within 1-8 wk of initiating treatment, and the greatest magnitude of improvement was noted at 36-54 wk. Specifically, clinically meaningful improvements in fatigue-related measures were observed at 36-54 wk of treatment. Limitations of this analysis include small sample size and lack of a comparator group.

**Table: Mean (SE) Change From Baseline in ITP-PAQ Scores**

| **Domain** | **MID** | **1-8 wk (n=9)** | **19-28 wk (n=4)** | **36-54 wk (n=5)** |
|---|---|---|---|---|
| Overall QoL | 8-12 | **14.1 (8.2)** | **37.5 (21.6)** | **52.0 (15.8)** |
| Activity | 10-15 | **16.7 (13.2)** | **37.5 (22.2)** | **57.5 (22.2)** |
| Bother-Physical Health | 8-12 | **14.5 (8.1)** | **43.1 (8.4)** | **52.8 (17.9)** |
| Social Activity | 8-12 | 9.7 (6.4) | **29.7 (12.1)** | **45.0 (20.5)** |
| Psychological Health | 8-12 | 5.0 (9.8) | **22.5 (20.5)** | **28.0 (13.4)** |
| Symptoms | 8-12 | -1.4 (5.6) | **13.8 (15.3)** | **22.5 (16.0)** |
| Fatigue/Sleep | 10-15 | 3.0 (6.3) | 6.8 (11.1) | **27.5 (15.3)** |
| Fear | ND | -0.6 (6.5) | 30.0 (12.1) | 43.0 (14.5) |
| Work | ND | 7.2 (5.2) | 15.0 (10.6) | 25.0 (8.8) |
| Women's Reproductive Health | 8-12 | (n=5)<br>-5.8 (8.1) | (n=3)<br>**18.1 (14.1)** | (n=2)<br>0 (0) |

Bold values exceed the MID. ND, not defined.

**Copyright Information:** (Online) ISSN: 2572-9241
©2026 The Author(s). HemaSphere is published by John Wiley & Sons Ltd on behalf of European Hematology Association. This is an open access Abstract Book distributed under the Attribution-NonCommercial-NoDerivs (CC BY-NC-ND), which allows third parties to download the articles and share them with others as long as they credit the author and the Abstract Book, but they cannot change the content in any way or use them commercially.
**Abstract Book Citations:** Authors, Title, HemaSphere, 2026;10:(S1):pages. The abstract book DOIs can be found on the "Author Information" page.
**Disclaimer:** Articles published in the journal HemaSphere exclusively reflect the opinions of the authors. The authors are responsible for all content in their abstracts including accuracy of the facts, statements, citing resources, etc.

### (PB4296) THE RESPONSE TO GLUCOCORTICOIDS AS FIRST-LINE TREATMENT FOR ITP PATIENTS IS INFERIOR IN THOSE AGED ≥56 YEARS COMPARED TO YOUNGER PATIENTS

**Topic:** 33. Platelet disorders

Wanting Feng*[1], Yuanyuan Zhu[2], Weijia Huang[1, 2, 2], Ninghong Zhao[2], Jie Sun[1, 2]

*[1]Zhejiang University School of Medicine, Hangzhou, China;[2]The First Affiliated Hospital, Zhejiang University School of Medicine, Hangzhou, China;*

### Background

Immune thrombocytopenia (ITP) is an acquired immune-mediated disorder characterized by isolated thrombocytopenia, affecting individuals of all ages. Currently, glucocorticoids remain the first-line therapy for patients of any age. However, elderly patients are more susceptible to the adverse effects of glucocorticoid therapy. In the era of precision medicine, with the availability of newer agents such as TPO-RAs, whether glucocorticoid treatment should be stratified by age warrants further investigation.

### Aims

To compare the efficacy, relapse risk, and safety of glucocorticoid monotherapy in newly diagnosed adult ITP patients across different age groups, in order to provide evidence supporting personalized clinical management of ITP.

### Methods

This retrospective analysis was conducted at The First Affiliated Hospital of Zhejiang University School of Medicine. Patients diagnosed with ITP according to the American Society of Hematology 2019 guidelines were divided into two groups based on age: the 18–55 years group and the ≥56 years group. All patients received glucocorticoids (prednisone 1 mg/kg/day or dexamethasone 10 mg/day). After response was achieved, the dose was tapered as soon as possible and discontinued within 6–8 weeks. The primary endpoint was the relapse rate at 6 months after treatment; secondary endpoints were the overall response rate and incidence of adverse reactions. Informed consent was obtained from all patients in accordance with the Declaration of Helsinki.

### Results

From January 2020 to June 2025, a total of 4,598 ITP patients were enrolled, of whom 1,723 had a follow-up duration exceeding 6 months. Among these, 105 patients received first-line treatment with glucocorticoids alone, including 65 patients aged 18–55 years and 40 patients aged ≥56 years. No statistically significant differences were observed between the two groups in baseline characteristics such as median platelet count at onset and proportion of severe cases (both $p > 0.05$). The 6-month relapse rate was significantly higher in the ≥56 age group (52.5% vs. 32.31%, $p = 0.041$). There was no significant difference in the initial overall response rate between the two groups (89.2% vs. 92.5%, $p > 0.05$). The ≥56 age group showed a slightly higher incidence of dizziness, hyperglycemia, Cushing's syndrome, and gastrointestinal bleeding, but these differences were not statistically significant.

### Summary/Conclusion

Glucocorticoids demonstrate comparable initial efficacy and adverse effects across different age groups; however, patients≥56 years have a higher risk of relapse. These findings suggest that glucocorticoid monotherapy should be avoided as first-line treatment in this age group. Further studies are needed to determine which therapeutic agent or combination of agents is most appropriate for ITP patients in this population.

**Copyright Information:** (Online) ISSN: 2572-9241
©2026 The Author(s). HemaSphere is published by John Wiley & Sons Ltd on behalf of European Hematology Association. This is an open access Abstract Book distributed under the Attribution-NonCommercial-NoDerivs (CC BY-NC-ND), which allows third parties to download the articles and share them with others as long as they credit the author and the Abstract Book, but they cannot change the content in any way or use them commercially.
**Abstract Book Citations:** Authors, Title, HemaSphere, 2026;10:(S1):pages. The abstract book DOIs can be found on the “Author Information” page.
**Disclaimer:** Articles published in the journal HemaSphere exclusively reflect the opinions of the authors. The authors are responsible for all content in their abstracts including accuracy of the facts, statements, citing resources, etc.

### (PB4297) EFFICACY AND SAFETY OF FULL-DOSE VERSUS NON-FULL-DOSE DEXAMETHASONE IN IMMUNE THROMBOCYTOPENIA

**Topic:** 33. Platelet disorders

Aoxue Tan*[1], Yimei Feng[1], Xi Zhang[1]

[1]*Xinqiao Hospital, Army Medical University, Chongqing, China;*

### Background

Glucocorticoids are the cornerstone of emergency and first-line treatment for primary immune thrombocytopenia (ITP). High-dose dexamethasone (40 mg/day for 4 days) is commonly used in clinical practice; however, patients frequently report significant systemic discomfort during treatment. Current therapeutic goals in ITP have expanded from merely achieving a safe platelet count to also improving patients' quality of life.

### Aims

This study aimed to compare the efficacy and safety of full-dose versus non-full-dose dexamethasone in ITP, in order to identify an optimal dosing strategy that maintains platelet response while reducing steroid-related adverse effects.

### Methods

ITP patients requiring emergency or first-line treatment were enrolled and assigned according to treatment regimen. The full-dose group (Group A, n=13) received oral dexamethasone 40 mg/day for 4 consecutive days. The non-full-dose group (Group B, n=14) received oral dexamethasone 20 mg/day (n=6) or 30 mg/day (n=8) for 4 days. Patients were preferentially assigned to the non-full-dose group if they were aged >60 years, had comorbidities such as hypertension or diabetes, had baseline platelet counts ≥20×10⁹/L, and showed no significant bleeding manifestations.
Steroid-related adverse effects were assessed using a self-designed quality-of-life questionnaire based on glucocorticoid-related toxicities involving eight major organ systems. The questionnaire included 20 items (e.g., insomnia, dizziness, abdominal pain, palpitations, skin rash), with severity scores recorded. Platelet counts, clinical response, and adverse-effect scores were compared between groups.

### Results

A total of 27 patients were included (Group A: 13; Group B: 14), comprising 9 males and 18 females, with a median age of 37 years (range, 16–64). The median disease duration was 5 months (range, 2–163) in Group A and 15 months (range, 3–295) in Group B. The mean baseline platelet count was 16.5×10⁹/L in Group A and 23.5×10⁹/L in Group B. One week after treatment, mean platelet counts increased to 127.8×10⁹/L in Group A and 117.0×10⁹/L in Group B, with no significant difference (P=0.586). At 4 weeks, mean platelet counts were 70.3×10⁹/L and 57.7×10⁹/L, respectively (P=0.581).
At week 1, the overall response rate was 85% in Group A (CR 31%, PR 54%) and 83% in Group B (CR 50%, PR 33%). At week 4, response rates were 87% (CR 25%, PR 63%) and 82% (CR 18%, PR 64%), respectively.
The mean adverse-effect score was significantly higher in Group A than in Group B (23.9±5.5 vs. 20.7±4.7, P=0.040), indicating fewer steroid-related adverse effects in the non-full-dose group, while platelet responses were comparable.

**Copyright Information:** (Online) ISSN: 2572-9241
©2026 The Author(s). HemaSphere is published by John Wiley & Sons Ltd on behalf of European Hematology Association. This is an open access Abstract Book distributed under the Attribution-NonCommercial-NoDerivs (CC BY-NC-ND), which allows third parties to download the articles and share them with others as long as they credit the author and the Abstract Book, but they cannot change the content in any way or use them commercially.
**Abstract Book Citations:** Authors, Title, HemaSphere, 2026;10:(S1):pages. The abstract book DOIs can be found on the "Author Information" page.
**Disclaimer:** Articles published in the journal HemaSphere exclusively reflect the opinions of the authors. The authors are responsible for all content in their abstracts including accuracy of the facts, statements, citing resources, etc.

**Summary/Conclusion**

Non-full-dose dexamethasone achieves short-term efficacy comparable to full-dose dexamethasone in patients with ITP, while significantly reducing steroid-related adverse effects and improving quality of life. Reduced-dose dexamethasone may therefore be a preferred option for selected patients, particularly the elderly and those with comorbidities.

**Copyright Information:** (Online) ISSN: 2572-9241
©2026 The Author(s). HemaSphere is published by John Wiley & Sons Ltd on behalf of European Hematology Association. This is an open access Abstract Book distributed under the Attribution-NonCommercial-NoDerivs (CC BY-NC-ND), which allows third parties to download the articles and share them with others as long as they credit the author and the Abstract Book, but they cannot change the content in any way or use them commercially.
**Abstract Book Citations:** Authors, Title, HemaSphere, 2026;10:(S1):pages. The abstract book DOIs can be found on the "Author Information" page.
**Disclaimer:** Articles published in the journal HemaSphere exclusively reflect the opinions of the authors. The authors are responsible for all content in their abstracts including accuracy of the facts, statements, citing resources, etc.

### (PB4298) ROMIPLOSTIM N01 AS SWITCH THERAPY IN CANCER THERAPY-RELATED THROMBOCYTOPENIA: A REAL-WORLD STUDY

**Topic:** 33. Platelet disorders

Yingzhe Ju*[1, 1], Tingting Cui[2], Miao Chen[3, 3]

*[1]Beijing No.6 Hospital, Department of Hematology, Beijing, China;[2]Beijing No.1 hospital, Department of Hematology, Beijing, China;[3]Peking Union Medical College Hospital, hematology, Beijing, China;*

### Background

CTIT (~30% solid tumors) causes chemo modifications impairing outcomes; current treatments limited. Romiplostim yields 81-93% response in CTIT but evidence mainly Western. Romiplostim N01 (Chinese biosimilar) lacks local real-world data. Xu et al reported 91.3% ORR (N=46) but heterogeneous pretreatments, no failure criteria/stratification. CTIT etiologies (aplasia, immune) may affect response, yet stratification lacking. Whether TPO-RA-refractory Chinese patients benefit from switching to Romiplostim N01 is unknown. This study evaluates its short-term efficacy/safety in this population, focusing on etiological subgroups & impact on subsequent anti-cancer treatment completion.

### Aims

Real-world data on romiplostim biosimilars in cancer therapy-related thrombocytopenia (CTIT) remain limited, particularly regarding switch therapy and etiological stratification in the Chinese population. This exploratory study aims to preliminarily investigate the short-term efficacy and safety of Romiplostim N01 (Ruilisheng®, Qilu Pharmaceutical), a thrombopoietin receptor agonist (TPO-RA) biosimilar, as a switch therapy in Chinese CTIT patients with refractory or inadequate response to prior TPO-RAs.

### Methods

Patients with CTIT who received Romiplostim N01 at two centers between January 2024 and June 2025 were enrolled. All patients had prior exposure to at least one TPO-RA and were switched to Romiplostim N01 due to treatment failure or inadequate response, with clearly defined response criteria. The primary outcomes were overall response rate (ORR) and complete response rate (CRR) within 4 weeks. Stratified analyses were performed based on thrombocytopenia etiology (aplastic anemia [AA], immune thrombocytopenia [ITP], and other CTIT). Logistic regression was used to identify predictors of CR.

### Results

A total of 51 patients (median age 59 years; 64.7% female) were included. Within 4 weeks, ORR was 88.2% and CRR was 68.6%. Among patients switching due to failure of prior TPO-RA therapy (78.4%), ORR and CRR were 87.5% and 65.0%, respectively. Etiological stratification revealed variable responses: the AA subgroup (n=9) achieved 88.9% ORR but only 55.6% CRR; the ITP subgroup (n=6) achieved 100% ORR; and the other CTIT subgroup (n=36) achieved 86.1% ORR and 69.4% CRR. In multivariate analysis, male sex was identified as an independent predictor of CR (OR = 0.064, 95% CI: 0.007–0.617, P = 0.017). Dose escalation after initial non-response led to response in 6/9 (66.7%) patients. Adverse events were mild and self-limited, with no thrombotic events. Among 42 patients with an indication for continued anti-cancer therapy, 88.1% successfully completed subsequent treatment.Although no statistically significant dose-response relationship was observed (P for trend = 0.369), dose escalation was performed in patients with no response to initial low-dose therapy, and 66.7% of these patients still achieved a response. Only one patient discontinued treatment due to adverse events, and no thrombotic events occurred. Among 42 patients with an indication for continued anti-cancer therapy, 88.1% successfully completed subsequent treatment.

**Copyright Information:** (Online) ISSN: 2572-9241
©2026 The Author(s). HemaSphere is published by John Wiley & Sons Ltd on behalf of European Hematology Association. This is an open access Abstract Book distributed under the Attribution-NonCommercial-NoDerivs (CC BY-NC-ND), which allows third parties to download the articles and share them with others as long as they credit the author and the Abstract Book, but they cannot change the content in any way or use them commercially.
**Abstract Book Citations:** Authors, Title, HemaSphere, 2026;10:(S1):pages. The abstract book DOIs can be found on the "Author Information" page.
**Disclaimer:** Articles published in the journal HemaSphere exclusively reflect the opinions of the authors. The authors are responsible for all content in their abstracts including accuracy of the facts, statements, citing resources, etc.

**Summary/Conclusion**

Romiplostim N01 is an effective and safe short-term option for Chinese CTIT patients with inadequate response to prior TPO-RAs. The differential responses across etiological subgroups highlight the importance of etiology-guided treatment strategies. Long-term safety requires validation in future prospective studies.

**Copyright Information:** (Online) ISSN: 2572-9241
©2026 The Author(s). HemaSphere is published by John Wiley & Sons Ltd on behalf of European Hematology Association. This is an open access Abstract Book distributed under the Attribution-NonCommercial-NoDerivs (CC BY-NC-ND), which allows third parties to download the articles and share them with others as long as they credit the author and the Abstract Book, but they cannot change the content in any way or use them commercially.
**Abstract Book Citations:** Authors, Title, HemaSphere, 2026;10:(S1):pages. The abstract book DOIs can be found on the "Author Information" page.
**Disclaimer:** Articles published in the journal HemaSphere exclusively reflect the opinions of the authors. The authors are responsible for all content in their abstracts including accuracy of the facts, statements, citing resources, etc.

### (PB4299) MOLECULAR DIAGNOSIS IS VITAL TO THE CONGENITAL THROMBOTIC THROMBOCYTOPENIC PURPURA IN CHILDREN: THE FIRST CASE OF C-TTP IN UKRAINE WITH NOVEL MUTATIONS

**Topic:** 33. Platelet disorders

Halyna Moroz*[1], Sergii Dyma[2], Natalia Karpenko[2], Natalia Derbenova[2], Tetiana Kramar[3], Olena Galchinska[4], Natalia Trofimova[3], Olga Medvedieva[3], Kateryna Kiblitska[3]

*[1]Shupyk National Healthcare University of Ukraine, therapy, family therapy, hematology and transfusiology, Kyiv, Ukraine;[2]Kyiv Regional Oncology Center, Department of pediatric hematology/oncology, Kyiv, Ukraine; [3]National specialized children's hospital "Okhmatdyt", Kyiv, Ukraine; [4]Kyiv Regional Oncology Center, Kyiv, Ukraine;*

### Background

Congenital thrombotic thrombocytopenic purpura (cTTP; Upshaw-Schulman syndrome) is an ultra-rare hereditary thrombotic microangiopathy caused by biallelic pathogenic variants in ADAMTS13. The condition is characterized by thrombocytopenia, microangiopathic hemolytic anemia and ischemic organ injury. Congenital TTP is often not recognized at the first occurrence of disease manifestation, e.g. in the newborn period or during childhood. The phenotype of cTTP is highly variable depending on molecular genetic alterations. To date, more than 450 causative mutations spread throughout all ADAMTS13 protein domains have been reported globally. ADAMTS13 assays, and the possibility of sequencing the ADAMTS13 gene allows the diagnosis of congenital TTP to be quickly and efficiently confirmed and development of an optimized management strategy.

### Methods

We present the first case of cTTP in Ukraine, confirmed by molecular genetic methods of research with mutations in the ADAMTS13 gene that have not yet been described.

### Results

We present a case of a 7 year old girl with no know family history of hematological disorders. She presented with episodes of thrombocytopenia, petechiae after acute viral infection since 1 year old. At presentation to our department the patient was documented to have thrombocytopenia (8x109/l), anemia. The diagnosis of immune thrombocytopenia was initially reported and treated with immunoglobulin, prednisolone, dexamethasone, rituximab without positive effect. Monitoring of laboratory parameters revealed moderate bilirubinemia, increased lactate dehydrogenase, decreased haptoglobin levels, reticulocytosis, and a negative direct Coombs test. TTP was then suspected; the patient was treated with 20ml/kg of fresh –frozen plasma, which promptly resulted in total recovery of thrombocytes (225x109/l).
The patient was found to have severe ADAMTS13 deficiency (3.2%) without inhibitor. NGS testing identified a novel compound heterozygous variants c.1244+1G>A (p.?), c.3608T>C(p.11e1203Thr) in gene ADAMTS13 (NM139025.5), absent from population databases and classified as not described. She remains stable on prophylactic fresh frozen plasma infusions every 3-4 weeks

### Summary/Conclusion

Biochemical and genetic confirmation enables timely plasma therapy and counseling. Novel ADAMTS13 variants continue to expand the mutational spectrum and underscore the variability of genotype-phenotype correlations.

**Copyright Information:** (Online) ISSN: 2572-9241
©2026 The Author(s). HemaSphere is published by John Wiley & Sons Ltd on behalf of European Hematology Association. This is an open access Abstract Book distributed under the Attribution-NonCommercial-NoDerivs (CC BY-NC-ND), which allows third parties to download the articles and share them with others as long as they credit the author and the Abstract Book, but they cannot change the content in any way or use them commercially.
**Abstract Book Citations:** Authors, Title, HemaSphere, 2026;10:(S1):pages. The abstract book DOIs can be found on the "Author Information" page.
**Disclaimer:** Articles published in the journal HemaSphere exclusively reflect the opinions of the authors. The authors are responsible for all content in their abstracts including accuracy of the facts, statements, citing resources, etc.

### (PB4300) PLATELET ACTIVATION PROFILES IN IRON DEFICIENCY

**Topic:** 33. Platelet disorders

Ioannis Moysis Skianis*[1], Athina Damianaki[1], Paraskevi Spatharaki[1], Myrto Kallimorou[1], Vasiliki Gkalea[1, 2], Christina Kalpadaki[1]

*[1]Laboratory of Hematology, School of Medicine, University of Crete and University General Hospital of Heraklion, Heraklion, Greece; [2]Laboratory of Hematology, Alexandra General Hospital, Athens, Greece;*

### Background

Iron deficiency (ID) and iron deficiency anemia (IDA) are global health burdens frequently associated with reactive thrombocytosis. While the reversal of thrombocytosis with iron replacement is well documented, data on the functional state—specifically the activation status—of platelets in ID remain limited.

### Aims

To evaluate platelet activation profiles in premenopausal women with iron deficiency compared with healthy individuals.

### Methods

This study enrolled female patients with confirmed iron deficiency (ferritin < 30 ng/mL), with or without thrombocytosis. Patients with active bleeding or those using anticoagulants/antiplatelet agents and medications affecting hemostasis within 10 days of sampling were excluded. Whole blood was collected in sodium citrate from healthy controls [N=18] and patients [N=11]. Platelet function was assessed via light transmission aggregometry (LTA) and flow cytometry (Beckman Coulter Navios EX) per ISTH guidelines. We quantified surface expression of the activated state of platelet integrin αIIbβ3 (PAC-1), P-selectin (CD62P), an alpha granule membrane-integrated protein, and CD63, a marker of dense granule function, on platelets identified using forward scatter, side scatter, and CD41 expression. The samples were analyzed at rest (not stimulated, NS), after stimulation with ADP (10 µM), and after stimulation with the thrombin receptor-activating peptide (TRAP, 50 µM). Light transmission aggregometry (LTA) was used to assess platelet reactivity following activation with collagen (5 µg/ml), ADP (5 µM), epinephrine (15 µM), ristocetin (1,2 mg/ml), and arachidonic acid (1,5 U/ml). The study was conducted in accordance with the Declaration of Helsinki and approved by the Ethics Committee of the University Hospital of Crete.

### Results

Mean hemoglobin levels in patients with ID and healthy subjects were 9,7 g/dl and 13,7 g/dl, respectively (p-value = <0.01). The mean platelet count in patients with ID and healthy subjects was 287 K/µL and 331 K/µL, respectively (p-value = 0,14). Patients with iron deficiency exhibited increased spontaneous expression of CD62P, CD63, and PAC-1 in resting platelets compared to healthy controls. After ADP activation, platelets from iron-deficient patients showed decreased CD62P expression. Following TRAP activation, no significant differences were observed between healthy subjects and patients (Figure 1). Moreover, no differences in the maximal platelet activation amplitude were observed after the addition of all tested agonists in patients with iron deficiency compared with healthy subjects in the LTA studies.

**Copyright Information:** (Online) ISSN: 2572-9241
©2026 The Author(s). HemaSphere is published by John Wiley & Sons Ltd on behalf of European Hematology Association. This is an open access Abstract Book distributed under the Attribution-NonCommercial-NoDerivs (CC BY-NC-ND), which allows third parties to download the articles and share them with others as long as they credit the author and the Abstract Book, but they cannot change the content in any way or use them commercially.
**Abstract Book Citations:** Authors, Title, HemaSphere, 2026;10:(S1):pages. The abstract book DOIs can be found on the "Author Information" page.
**Disclaimer:** Articles published in the journal HemaSphere exclusively reflect the opinions of the authors. The authors are responsible for all content in their abstracts including accuracy of the facts, statements, citing resources, etc.

**Summary/Conclusion**

Our observations demonstrate that platelets from patients with iron deficiency exhibit increased activation at rest. Interestingly, after ADP excitation, the non-stimulated baseline activation does not correlate with an increased expression of activation markers, indicating a mild “exhaustion” phenotype. Ex vivo stimulation with a strong agonist reveals no differences in platelet reactivity. The above remarks suggest a subclinical platelet activation in patients with iron deficiency that does not correlate with an increased thrombotic risk, although larger cohorts and clinical studies are warranted.

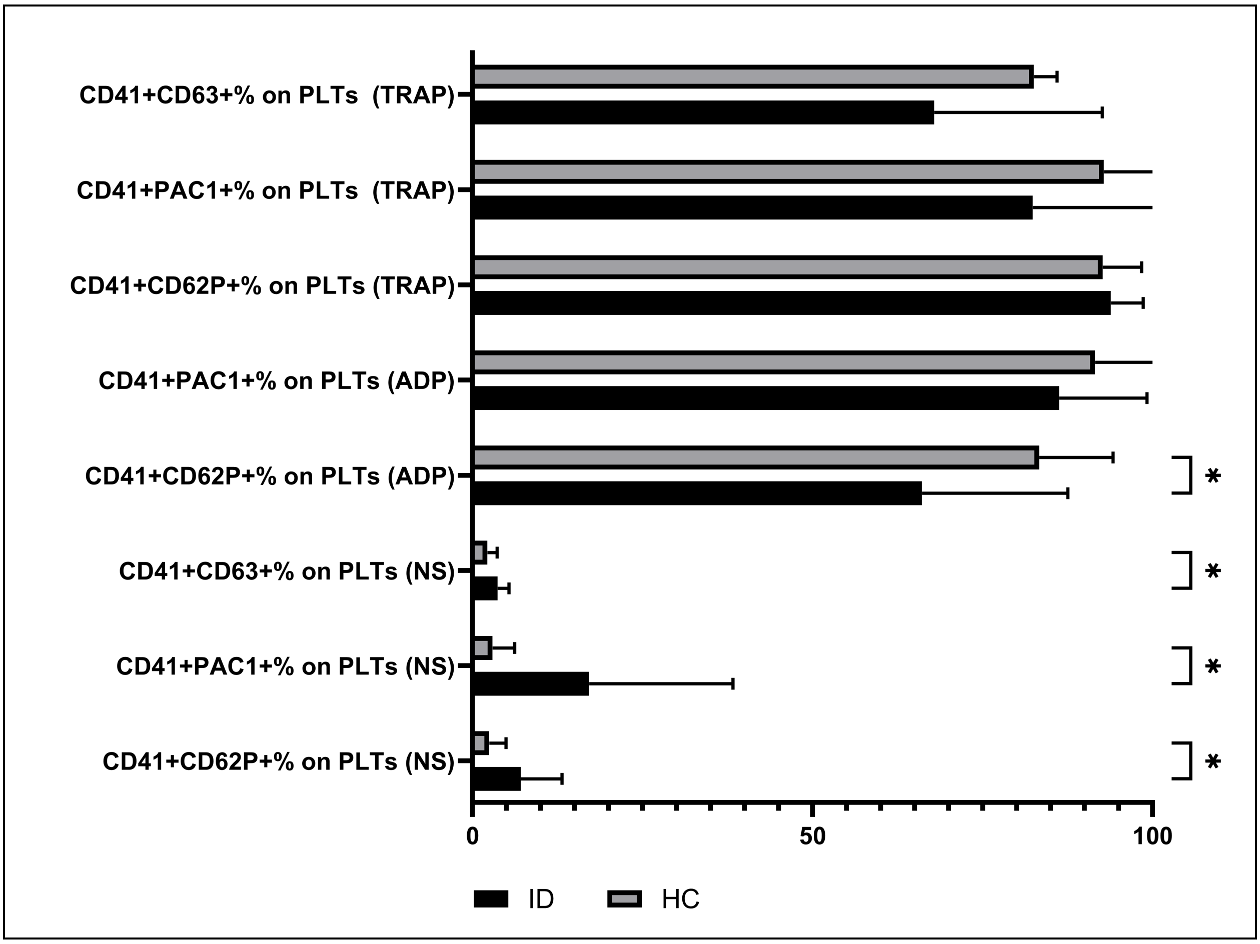

Figure 1: Comparisson of the expression of CD62P, PAC1 and CD63 on platelets of patients with irod deficiency (ID) and healthy controls (HC).

**Copyright Information:** (Online) ISSN: 2572-9241
©2026 The Author(s). HemaSphere is published by John Wiley & Sons Ltd on behalf of European Hematology Association. This is an open access Abstract Book distributed under the Attribution-NonCommercial-NoDerivs (CC BY-NC-ND), which allows third parties to download the articles and share them with others as long as they credit the author and the Abstract Book, but they cannot change the content in any way or use them commercially.
**Abstract Book Citations:** Authors, Title, HemaSphere, 2026;10:(S1):pages. The abstract book DOIs can be found on the “Author Information” page.
**Disclaimer:** Articles published in the journal HemaSphere exclusively reflect the opinions of the authors. The authors are responsible for all content in their abstracts including accuracy of the facts, statements, citing resources, etc.

### (PB4301) EVALUATING THE PERFORMANCE OF THE ANYSIS-200 AND T-TAS01 COMPARED TO CONVENTIONAL PLATELET FUNCTION TESTING

**Topic:** 33. Platelet disorders

Nienke Meskes*[1, 2], Dave Hellenbrand[2], Erik Beckers[1], Floor Heubel-Moenen[1], Yvonne Henskens[2]

*[1]Maastricht University Medical Center (MUMC+), Hematology, Internal Medicine, Maastricht, The Netherlands; [2]Maastricht University Medical Center (MUMC+), Central Diagnostic Laboratory, Maastricht, The Netherlands;*

### Background

The assessment of platelet function is essential for evaluating congenital and acquired platelet function defects, but remains challenging due to the limited sensitivity and specificity of current laboratory tests.

### Aims

This study evaluates the performance of the newly developed screening tests Anysis-200 and Total Thrombus Analyzing System (T-TAS01) in comparison with conventional assays for the laboratory diagnosis of platelet function disorders (PFD).

### Methods

Data from the Predictors of Bleeding in Adult Hemostasis Patients (ProBe-AHP) study were assessed. All patients referred to the MUMC+ for evaluation of a bleeding tendency were enrolled and underwent a comprehensive hemostasis work-up including PFA-200, Anysis-200, T-TAS01, light transmission aggregometry (LTA) and lumiaggregometry for ATP release testing. LTA with ATP release served as the reference standard for diagnosing platelet function disorders, defined as an abnormal response to at least two LTA agonists and/or one ATP-release agonist.

### Results

In 35 patients evaluated for bleeding tendency, platelet function was assessed using all methods. The Anysis-200 showed 65% agreement with the PFA-200 for the ADP and 41% for the EPI cartridge, while T-TAS01 and PFA-200 showed 69% and 41% agreement for ADP and EPI, respectively. Compared with the LTA and/or ATP release results, Anysis-200 yielded NPVs/PPVs of 79%/47% (EPI) and 65%/40% (ADP), with sensitivities/specificities of 52%/75% (EPI) and 71%/33% (ADP). For T-TAS01, the NPV, PPV, sensitivity and specificity were 71%, 57%, 83% and 40%, respectively.

### Summary/Conclusion

Anysis-200 and T-TAS01 show moderate agreement with conventional testing and acceptable NPV, but limited PPV and specificity. They may support screening for platelet function disorders, but cannot replace reference diagnostics.

**Copyright Information:** (Online) ISSN: 2572-9241
©2026 The Author(s). HemaSphere is published by John Wiley & Sons Ltd on behalf of European Hematology Association. This is an open access Abstract Book distributed under the Attribution-NonCommercial-NoDerivs (CC BY-NC-ND), which allows third parties to download the articles and share them with others as long as they credit the author and the Abstract Book, but they cannot change the content in any way or use them commercially.
**Abstract Book Citations:** Authors, Title, HemaSphere, 2026;10:(S1):pages. The abstract book DOIs can be found on the "Author Information" page.
**Disclaimer:** Articles published in the journal HemaSphere exclusively reflect the opinions of the authors. The authors are responsible for all content in their abstracts including accuracy of the facts, statements, citing resources, etc.

### (PB4302) REAL-WORLD EFFICACY AND SAFETY OF THE MTOR INHIBITOR SIROLIMUS IN ELDERLY PATIENTS WITH IMMUNE THROMBOCYTOPENIA

**Topic:** 33. Platelet disorders

Aoxue Tan*[1], Yimei Feng[1], Xiaoqi Wang[1], Jia Liu[1], Chen Li[1], Xi Zhang[1]

[1]*Xinqiao Hospital, Army Medical University, Chongqing, China;*

**Background**

With the acceleration of global population aging, the incidence of immune thrombocytopenia (ITP) in elderly patients is increasing. Due to age-related physiological decline, multiple comorbidities, and polypharmacy, treatment of elderly ITP patients poses particular challenges. Identifying therapeutic strategies that balance efficacy and safety remains an important clinical issue. Our previous study demonstrated that sirolimus is an effective and low-toxicity treatment for ITP (Feng YM et al., 2020). Therefore, this study aimed to evaluate the real-world efficacy and safety of the mTOR inhibitor sirolimus in elderly patients with ITP.

**Aims**

To assess the efficacy and safety of sirolimus in the treatment of elderly patients with ITP in real-world clinical practice.

**Methods**

Sirolimus was initiated at a dose of 2 mg once daily and adjusted according to trough blood concentrations to maintain levels between 5–12 ng/mL. After achieving stable efficacy, the dose was tapered by 0.5 mg every month until discontinuation. Treatment responses and adverse events were retrospectively analyzed.

**Results**

A total of 29 elderly patients were enrolled, including 10 males and 19 females. The median age was 67 years (range, 60–85), and the median disease duration was 19 months (range, 1–144). Common comorbidities included chronic obstructive pulmonary disease (n=1), Parkinson's disease (n=1), cerebral infarction (n=2), hypertension (n=5), diabetes mellitus (n=6), hepatitis B infection (n=1), Sjögren's syndrome (n=3), thyroid dysfunction (n=4), gastric ulcer (n=1), and rheumatoid arthritis (n=1). The median duration of sirolimus treatment was 3.5 months (range, 1–75). The median time to initial response was 21 days (range, 7–91). The overall response rates (ORR) at 1, 2, 3, and 6 months were 68.0% (CR 16% + PR 52%), 82% (CR 18% + PR 64%), 82% (CR 14% + PR 68%), and 75% (CR 30% + PR 45%), respectively. The mean platelet count increased from 28 × $10^9$/L at baseline to 63 × $10^9$/L, 67 × $10^9$/L, 75 × $10^9$/L, and 80 × $10^9$/L at 1, 2, 3, and 6 months after treatment, respectively. The most common adverse events were grade 1–2 oral ulcers (3 cases, 10%), grade 1 lower limb edema (3 cases, 10%), grade 1 hyperlipidemia (2 cases, 7%), grade 1 rash (1 case, 3%), and grade 2 diarrhea (1 case, 3%). All adverse events were manageable with symptomatic treatment and did not result in treatment discontinuation.

**Summary/Conclusion**

Sirolimus is an effective and well-tolerated treatment option for elderly patients with ITP in real-world practice, demonstrating favorable efficacy with a low incidence of manageable adverse events.

**Copyright Information:** (Online) ISSN: 2572-9241
©2026 The Author(s). HemaSphere is published by John Wiley & Sons Ltd on behalf of European Hematology Association. This is an open access Abstract Book distributed under the Attribution-NonCommercial-NoDerivs (CC BY-NC-ND), which allows third parties to download the articles and share them with others as long as they credit the author and the Abstract Book, but they cannot change the content in any way or use them commercially.
**Abstract Book Citations:** Authors, Title, HemaSphere, 2026;10:(S1):pages. The abstract book DOIs can be found on the "Author Information" page.
**Disclaimer:** Articles published in the journal HemaSphere exclusively reflect the opinions of the authors. The authors are responsible for all content in their abstracts including accuracy of the facts, statements, citing resources, etc.

### (PB4303) DESIGN OF A RANDOMIZED, DOUBLE-BLIND, PLACEBO-CONTROLLED, PHASE 2/3 STUDY WITH AN OPEN-LABEL EXTENSION TO EVALUATE EFGARTIGIMOD IV IN PEDIATRIC PARTICIPANTS WITH IMMUNE THROMBOCYTOPENIA (ADVANCE JR)

**Topic:** 33. Platelet disorders

John Grainger*[1, 2], David Diaz Perez[3], Anca Colita[4], Thais Murciano Carillo[5], Nathalie Aladjidi[6], Janki Brahmbhatt[7], Lan Lan[7], Filip Matthijssens[7], Thomas Kühne[8], Nichola Cooper[9]

*[1]Royal Manchester Children's Hospital, Manchester, United Kingdom;[2]University of Manchester, Faculty of Medicine, Manchester, United Kingdom; [3]Hospital Materno-Infantil Universitario Gregorio Marañón, Madrid, Spain;[4]Institutul Clinic Fundeni, Bucharest, Romania; [5]Hospital Universitario Vall d'Hebron, Barcelona, Spain;[6]Centre Hospitalier Universitaire de Bordeaux, Bordeaux, France; [7]argenx, Ghent, Belgium; [8]University Children's Hospital, Basel, Switzerland; [9]Imperial College London, London, United Kingdom;*

### Background

Primary immune thrombocytopenia (ITP) is an autoimmune disorder mediated predominantly by platelet autoantibodies, resulting in thrombocytopenia, bleeding, and decreased quality of life. Despite currently available treatments, there is still an unmet need in the treatment of primary ITP in pediatric patients as clinical data in this population are limited. Efgartigimod, a human IgG1-derived Fc fragment engineered to prevent recycling of IgG, reduces IgG levels, including pathogenic IgG autoantibodies. Efgartigimod has demonstrated adequate safety and efficacy in adults with primary ITP, supporting its evaluation in pediatric patients.

### Aims

ADVANCE Jr (NCT07194850) aims to confirm the appropriate dose of efgartigimod intravenous (IV) for patients with chronic ITP aged 12 to <18 years. It will also evaluate the pharmacokinetics (PK), pharmacodynamics (PD), safety, tolerability, immunogenicity, and activity of efgartigimod IV.

### Methods

ADVANCE Jr is a multicenter (across Europe, UK, and Japan), randomized, double-blind, parallel-arm, placebo-controlled study. The study population will include ~24 pediatric participants with chronic ITP. Participants will have a documented diagnosis of primary ITP (>12 months), a baseline mean platelet count $<30\times10^9$/L, and will have previously received and responded to ≥1 prior ITP therapy (corticosteroids, IVIg, or anti-D immunoglobulin) but subsequently had an insufficient response. Following screening, participants will be randomized 2:1 and enter a 24-week double-blind treatment period (DBTP), where they will receive efgartigimod IV 12 mg/kg or placebo IV once weekly for 4 weeks, then weekly or once every other week based on platelet levels. The DBTP will be followed by 2 sequential, open-label treatment periods lasting up to 52 weeks each, where all participants will receive efgartigimod weekly or every other week based on platelet levels. Stable doses of 1 prespecified concomitant ITP therapy will be permitted, but initiation of new ITP treatments or dose escalation beyond baseline will not be allowed during the study. Patients with a complete response (platelet counts of $\geq100\times10^9$/L for ≥24 consecutive weeks in the open-label treatment period and an absence of bleeding during that time) are permitted to temporarily discontinue treatment. An 8-week safety follow-up period will follow the last efgartigimod dose.

**Copyright Information:** (Online) ISSN: 2572-9241
©2026 The Author(s). HemaSphere is published by John Wiley & Sons Ltd on behalf of European Hematology Association. This is an open access Abstract Book distributed under the Attribution-NonCommercial-NoDerivs (CC BY-NC-ND), which allows third parties to download the articles and share them with others as long as they credit the author and the Abstract Book, but they cannot change the content in any way or use them commercially.
**Abstract Book Citations:** Authors, Title, HemaSphere, 2026;10:(S1):pages. The abstract book DOIs can be found on the "Author Information" page.
**Disclaimer:** Articles published in the journal HemaSphere exclusively reflect the opinions of the authors. The authors are responsible for all content in their abstracts including accuracy of the facts, statements, citing resources, etc.

### Results

The primary endpoint is a compartmental, model-driven analysis evaluating age- and size-related effects on clearance and volume of distribution of efgartigimod, along with changes from baseline in total IgG levels over time, to determine the appropriate dose. Secondary endpoints include PK/PD effects assessed by serum concentrations and total IgG levels over time; safety and tolerability assessed by the incidence and severity of adverse events; efficacy assessed by sustained platelet responses, disease control, platelet count change from baseline, and bleeding severity; immunogenicity; and the effects of efgartigimod IV on clinical outcome assessments using the EQ-5D-5L, KIT Child Self-Report, and pedsFACIT-F. Measurement of immature platelet fraction over time is an exploratory endpoint.

### Summary/Conclusion

ADVANCE Jr will confirm an appropriate dose of efgartigimod IV in pediatric patients with chronic ITP and will provide model-predicted evidence of treatment response. Recruitment is ongoing.

**Copyright Information:** (Online) ISSN: 2572-9241
©2026 The Author(s). HemaSphere is published by John Wiley & Sons Ltd on behalf of European Hematology Association. This is an open access Abstract Book distributed under the Attribution-NonCommercial-NoDerivs (CC BY-NC-ND), which allows third parties to download the articles and share them with others as long as they credit the author and the Abstract Book, but they cannot change the content in any way or use them commercially.
**Abstract Book Citations:** Authors, Title, HemaSphere, 2026;10:(S1):pages. The abstract book DOIs can be found on the “Author Information” page.
**Disclaimer:** Articles published in the journal HemaSphere exclusively reflect the opinions of the authors. The authors are responsible for all content in their abstracts including accuracy of the facts, statements, citing resources, etc.

### (PB4304) EFFICACY AND SAFETY OF HETROMBOPAG IN CHILDREN WITH PRIMARY IMMUNE THROMBOCYTOPENIA: A LONGITUDINAL PROSPECTIVE COHORT STUDY

**Topic:** 33. Platelet disorders

Aixue Liu*[1], Ting Sun[2], Lei Zhang[2]

[1]*Tianjin Medical University, Tianjing, China;* [2]*National Clinical Research Center for Blood Diseases, Tianjing, China;*

### Background

Hetrombopag, an oral thrombopoietin receptor agonist (TPO-RA), has been approved to increase platelet counts and reduce bleeding risks in adults with immune thrombocytopenia (ITP). Therefore, the efficacy and safety of hetrombopag in pediatric ITP remain unestablished.

### Aims

We conducted a prospective clinical study (ClinicalTrials.gov number, NCT06107582) to assess the efficacy and safety of hetrombopag for children with primary ITP.

### Methods

Children aged ≥6 to <18 years with a confirmed diagnosis of primary ITP and a baseline platelet count ≤30×$10^9$/L were enrolled and treated with hetrombopag at an initial dose of 5 mg or 7.5 mg once daily for up to 24 weeks. Doses were adjusted based on the platelet count. The primary efficacy endpoint was defined as the proportion of patients achieving a platelet count of ≥50×$10^9$/L within 4 weeks without rescue therapy (confirmed by two consecutive measurements at least 7 days apart).

### Results

A total of 11 children with ITP received hetrombopag treatment. During the 24 weeks treatment period, 7 (63.6%, 95% CI: 31.1 – 88.1) patients achieved the primary endpoint at week 4. Among these patients, 2 patients who had previous treatment failure with eltrombopag or a relapse after eltrombopag treatment also reached the primary efficacy. all the patients achieved platelet count ≥50 × $10^9$/L at least once. The median time to the first platelet count ≥50 × $10^9$/L was 2.3 weeks (range: 1 – 7). At week 24, a total of 72.7% the patients (95% CI: 39.0 – 93.9) achieved overall response (OR), with a median cumulative response duration of 22 weeks (range: 2.0 – 23.0).The proportion of patients with WHO bleeding scale grades 1–4 decreased from 54.5% at baseline to 9% at week 24, with only grade 1 bleeding events reported during follow-up. Among the 5 (45.5%) patients using combination medications at study entry, all successfully tapered or discontinued concomitant medications by week 24. During treatment with hetrombopag, rescue therapy was required in only 2 (18.1%) patients. The transaminase elevation reported in 1 (9.1%) patient, which resolved with medical management. No death or serious adverse events were reported.

### Summary/Conclusion

Hetrombopag showed efficacy in elevating and sustaining platelet counts for children with ITP, and has a favorable safety profile in the pediatric population. Notably,
hetrombopag can also be an effective therapy for children who are unresponsive or relapse with eltrombopag.

**Copyright Information:** (Online) ISSN: 2572-9241
©2026 The Author(s). HemaSphere is published by John Wiley & Sons Ltd on behalf of European Hematology Association. This is an open access Abstract Book distributed under the Attribution-NonCommercial-NoDerivs (CC BY-NC-ND), which allows third parties to download the articles and share them with others as long as they credit the author and the Abstract Book, but they cannot change the content in any way or use them commercially.
**Abstract Book Citations:** Authors, Title, HemaSphere, 2026;10:(S1):pages. The abstract book DOIs can be found on the "Author Information" page.
**Disclaimer:** Articles published in the journal HemaSphere exclusively reflect the opinions of the authors. The authors are responsible for all content in their abstracts including accuracy of the facts, statements, citing resources, etc.

### (PB4305) SPLENECTOMY VS ELTROMBOPAG: A RETROSPECTIVE STUDY IN IMMUNE THROMBOCYTOPENIA

**Topic:** 33. Platelet disorders

Mustafa Berkay Tastekin*[1], Umit Yavuz Malkan[2]

*[1]Hacettepe University, Internal Medicine, Ankara, Türkiye; [2]Hacettepe University, Internal Medicine, Hematology, Ankara, Türkiye;*

### Background

Immune thrombocytopenia (ITP) is an autoimmune hematological disorder characterized by isolated thrombocytopenia. Corticosteroids and intravenous immunoglobulin (IVIG) are the most frequently used first-line treatments. For second-line therapy, splenectomy and eltrombopag are the most common clinical options.

### Aims

In our single-center retrospective study, we aimed to contribute to the management of ITP by comparing the efficacy and side effects of splenectomy versus eltrombopag as second-line treatments.

### Methods

Our study involved a retrospective archive review of patients diagnosed with ITP at our center from January 1980 to 2025, to provide a comprehensive analysis of the patient group

### Results

A total of 94 patients who either underwent splenectomy or received eltrombopag as a second-line therapy for ITP were analyzed. Of the patients, 63 (%67) were female. The mean age at diagnosis was 41.7 (±18.3) years. The median follow-up period after treatment was 36.0 months (10.3–72.8 IQR). In eight patients, both treatment modalities were administered at different times, ensuring that their periods of action did not overlap. Therefore a total of 102 treatment modalities were administered, consisting of 68 (66.7%) splenectomies and 34 (33.3%) eltrombopag treatments. Response criteria were categorized as follows: complete response (platelets >100x10³/µL), partial response (50–100x10³/µL), and non-response (<50x10³/µL). Of the 68 splenectomy cases, complete response, partial response, and no response were observed in 62 (91.2%), 4 (5.9%), and 2 (2.9%) patients, respectively. In the eltrombopag group, 28 (82.4%) patients achieved a complete response and 6 (17.6%) showed a partial response, while no patients were found to be non-responsive. Although it did not reach statistical significance (p=0.09), a clinically relevant difference was observed between the two treatments. When comparing platelet counts one month after treatment, the values were found to be significantly higher in the splenectomy group (p<0.001). At the three-month follow-up, platelet counts remained significantly higher in favor of splenectomy (p=0.003). When comparing complications, the splenectomy group presented with bleeding in 4 (5.9%) cases and thrombosis in 3 (4.4%) cases (2 portal-splenic and 1 femoral vein thrombosis). Regarding the eltrombopag group, complications included 2 (5.9%) cases of bleeding and 2 (5.9%) cases of thrombosis, specifically one portal vein thrombosis and one pulmonary thromboembolism. Thrombosis was identified on days 60 and 11 after starting eltrombopag therapy, respectively. No life-threatening bleeding events were observed in either group. No significant difference was found between the two treatment groups in terms of side effects.

**Copyright Information:** (Online) ISSN: 2572-9241
©2026 The Author(s). HemaSphere is published by John Wiley & Sons Ltd on behalf of European Hematology Association. This is an open access Abstract Book distributed under the Attribution-NonCommercial-NoDerivs (CC BY-NC-ND), which allows third parties to download the articles and share them with others as long as they credit the author and the Abstract Book, but they cannot change the content in any way or use them commercially.
**Abstract Book Citations:** Authors, Title, HemaSphere, 2026;10:(S1):pages. The abstract book DOIs can be found on the “Author Information” page.
**Disclaimer:** Articles published in the journal HemaSphere exclusively reflect the opinions of the authors. The authors are responsible for all content in their abstracts including accuracy of the facts, statements, citing resources, etc.

**Summary/Conclusion**

Our study involved a comparative analysis of second-line therapy treatments, focusing on their efficacy and safety profiles. Although it did not reach statistical significance, our study observed a higher rate of complete response in the splenectomy group, which may be considered clinically significant. Platelet counts were significantly higher in the splenectomy group at both the first and third months post-treatment. No statistically significant difference was observed between the groups concerning bleeding and thrombotic complications. In conclusion, splenectomy may be preferred over eltrombopag as the second line treatment in immune thrombocytopenia since the superior response and similar complication rates.

**Copyright Information:** (Online) ISSN: 2572-9241
©2026 The Author(s). HemaSphere is published by John Wiley & Sons Ltd on behalf of European Hematology Association. This is an open access Abstract Book distributed under the Attribution-NonCommercial-NoDerivs (CC BY-NC-ND), which allows third parties to download the articles and share them with others as long as they credit the author and the Abstract Book, but they cannot change the content in any way or use them commercially.
**Abstract Book Citations:** Authors, Title, HemaSphere, 2026;10:(S1):pages. The abstract book DOIs can be found on the “Author Information” page.
**Disclaimer:** Articles published in the journal HemaSphere exclusively reflect the opinions of the authors. The authors are responsible for all content in their abstracts including accuracy of the facts, statements, citing resources, etc.

### (PB4306) BEYOND THE PLATELET: GRAY PLATELET SYNDROME MISDIAGNOSED AS REFRACTORY ITP IN A PATIENT WITH RECURRENT PULMONARY INFECTIONS

**Topic:** 33. Platelet disorders

Lucy González Villarroel*[1]

[1] *Regional Hospital 1° de Octubre ISSSTE, Hematology/Internal Medicine, Mexico City, Mexico;*

### Background

Gray Platelet Syndrome (GPS) is a rare inherited macrothrombocytopenia caused by NBEAL2 mutations, resulting in absent platelet alpha-granules. These granules are critical reservoirs for hemostatic and inflammatory proteins including von Willebrand factor and fibrinogen. GPS represents a true multi-systemic disorder: defective protein packaging causes granule content leakage into bone marrow, driving progressive myelofibrosis, while emerging evidence links alpha-granule deficiency to impaired innate immunity and increased infection susceptibility. Its rarity and overlapping features with Primary Immune Thrombocytopenia (ITP) frequently result in misdiagnosis and inappropriate immunosuppressive therapy.

### Aims

To report a case of Gray Platelet Syndrome initially misdiagnosed as refractory ITP and to highlight the clinical importance of recognizing the atypical infectious phenotype associated with NBEAL2 deficiency to prevent futile treatments.

### Methods

A 62-year-old male presented with long-standing thrombocytopenia and a striking history of recurrent pulmonary infections, including multiple pneumonia episodes and pulmonary tuberculosis. Initially diagnosed with primary ITP, he received sequential treatment escalation: corticosteroids, rituximab, splenectomy, and finally eltrombopag. Despite aggressive immunosuppression, he remained refractory with persistent hemorrhagic manifestations (epistaxis, extensive hematomas) and persistently elevated mean platelet volume (MPV). The atypical treatment resistance and unusual infectious phenotype prompted comprehensive diagnostic re-evaluation: peripheral blood smear, flow cytometry (CD62P/CD61, CD41a/CD42b, and CD41a/CD61 analysis), platelet aggregometry, and bone marrow biopsy.

### Results

The diagnostic re-evaluation revealed:

- **Peripheral blood smear:** Large, pale, characteristic "gray" platelets.
- **Flow cytometry:** Analysis revealed intrinsic structural defects and abnormal activation patterns consistent with alpha-granule deficiency.
- **Platelet aggregometry:** Severe functional impairment with epinephrine and ADP, incompatible with isolated ITP.
- **Bone marrow biopsy:** Megakaryocytic hyperplasia with dysplastic features and early myelofibrosis.

Integration of refractory macrothrombocytopenia, profound platelet dysfunction, and recurrent respiratory infections established the diagnosis of Gray Platelet Syndrome.

**Copyright Information:** (Online) ISSN: 2572-9241
©2026 The Author(s). HemaSphere is published by John Wiley & Sons Ltd on behalf of European Hematology Association. This is an open access Abstract Book distributed under the Attribution-NonCommercial-NoDerivs (CC BY-NC-ND), which allows third parties to download the articles and share them with others as long as they credit the author and the Abstract Book, but they cannot change the content in any way or use them commercially.
**Abstract Book Citations:** Authors, Title, HemaSphere, 2026;10:(S1):pages. The abstract book DOIs can be found on the "Author Information" page.
**Disclaimer:** Articles published in the journal HemaSphere exclusively reflect the opinions of the authors. The authors are responsible for all content in their abstracts including accuracy of the facts, statements, citing resources, etc.

**Summary/Conclusion**

This case exemplifies a critical diagnostic pitfall: inherited platelet disorders masquerading as "refractory ITP." GPS should be considered when elevated MPV accompanies treatment resistance, particularly with atypical comorbidities. The patient's recurrent pneumonia and tuberculosis illuminate the underrecognized immunological dimension of NBEAL2 deficiency, suggesting alpha-granules play previously unappreciated roles in pulmonary immune surveillance. Timely GPS diagnosis prevents futile—and potentially harmful—immunosuppression and splenectomy (which paradoxically increased this patient's infection risk), while enabling appropriate monitoring for progressive myelofibrosis. This case advocates for incorporating platelet function testing and genetic evaluation into diagnostic algorithms for presumed refractory ITP, especially when clinical phenotypes extend beyond isolated thrombocytopenia.

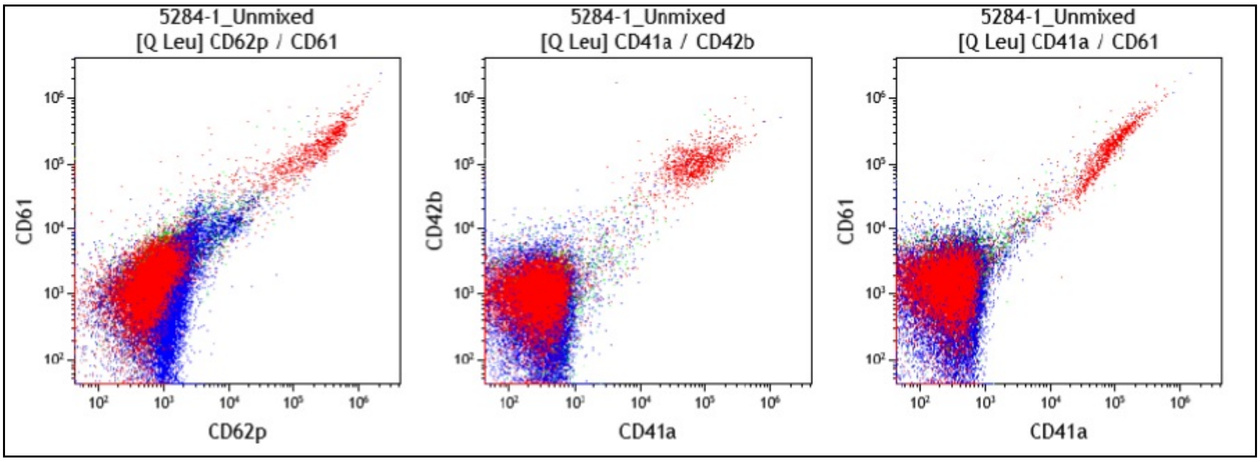

**Copyright Information:** (Online) ISSN: 2572-9241
©2026 The Author(s). HemaSphere is published by John Wiley & Sons Ltd on behalf of European Hematology Association. This is an open access Abstract Book distributed under the Attribution-NonCommercial-NoDerivs (CC BY-NC-ND), which allows third parties to download the articles and share them with others as long as they credit the author and the Abstract Book, but they cannot change the content in any way or use them commercially.
**Abstract Book Citations:** Authors, Title, HemaSphere, 2026;10:(S1):pages. The abstract book DOIs can be found on the "Author Information" page.
**Disclaimer:** Articles published in the journal HemaSphere exclusively reflect the opinions of the authors. The authors are responsible for all content in their abstracts including accuracy of the facts, statements, citing resources, etc.

### (PB4307) WHEN REFRACTORY "ITP" IS NOT ITP: DIAGNOSTIC DELAY IN ACQUIRED AMEGAKARYOCYTIC THROMBOCYTOPENIA AND CURATIVE HAPLOIDENTICAL TRANSPLANTATION

**Topic:** 33. Platelet disorders

Serkan Guven*[1]

[1]*Çanakkala Mehmet Akif Ersoy State Hospital, Hematology, Çanakkale, Türkiye;*

### Background

Acquired amegakaryocytic thrombocytopenia (AAT) is an uncommon but potentially life-threatening cause of isolated thrombocytopenia characterized by selective bone marrow megakaryocyte depletion. Its clinical presentation often mimics Immune thrombocytopenia (ITP), leading to misdiagnosis, ineffective immunosuppression, and delayed curative intervention.

### Aims

To highlight a critical diagnostic pitfall in refractory thrombocytopenia and demonstrate the curative potential of haploidentical allogeneic hematopoietic stem cell transplantation (allo-HSCT) in AAT.

### Methods

A detailed clinical, morphological, cytogenetic, and molecular evaluation of a young patient initially treated as ITP was performed.

### Results

A 21-year-old female presented with severe thrombocytopenia ($12 \times 10^3/\mu L$) and mucocutaneous bleeding. She was managed as ITP with corticosteroids, IVIG, and eltrombopag for approximately two months. However, platelet counts failed to improve, and she became transfusion-dependent, requiring multiple platelet and red blood cell transfusions.
Bone marrow examination revealed 40% cellularity with marked megakaryocytic depletion and relative erythroid hyperplasia, without dysplasia or fibrosis. The absence of compensatory megakaryocytic proliferation was incompatible with ITP and established the diagnosis of AAT. Cytogenetics, FISH, and a 141-gene myeloid NGS panel excluded clonal evolution.
Given refractory disease and ongoing transfusion dependency, haploidentical allo-HSCT from her father was performed on 02.05.2024. Engraftment was successful, and at 6 months post-transplant, the patient achieved complete hematologic remission with sustained platelet recovery. Mild cutaneous graft-versus-host disease was effectively controlled.

### Summary/Conclusion

Refractory thrombocytopenia labeled as ITP should prompt early bone marrow evaluation to exclude AAT. Diagnostic delay may increase morbidity and transfusion burden. Haploidentical allo-HSCT can provide durable remission and should be considered in refractory cases without matched donors.

**Copyright Information:** (Online) ISSN: 2572-9241
©2026 The Author(s). HemaSphere is published by John Wiley & Sons Ltd on behalf of European Hematology Association. This is an open access Abstract Book distributed under the Attribution-NonCommercial-NoDerivs (CC BY-NC-ND), which allows third parties to download the articles and share them with others as long as they credit the author and the Abstract Book, but they cannot change the content in any way or use them commercially.
**Abstract Book Citations:** Authors, Title, HemaSphere, 2026;10:(S1):pages. The abstract book DOIs can be found on the "Author Information" page.
**Disclaimer:** Articles published in the journal HemaSphere exclusively reflect the opinions of the authors. The authors are responsible for all content in their abstracts including accuracy of the facts, statements, citing resources, etc.

### (PB4308) AUTOLOGOUS STEM CELL TRANSPLANTATION IN MULTIREFRACTORY IMMUNE THROMBOCYTOPENIA: A CASE REPORT

**Topic:** 33. Platelet disorders

Vahap Okan*[1], Nuray Gül Açar[1], Buğra Sağlam[2], Ali Eser[3]

*[1]gaziantep üniversitesi, gaziantep, Türkiye; [2]medical point hospital, hematoloji, gaziantep, Türkiye; [3]medical point hospital, hematoloji, GAZİANTEP, Türkiye;*

**Background**

Immune thrombocytopenia (ITP) is typically manageable with corticosteroids, immunomodulatory agents, and thrombopoietin receptor agonists. Nevertheless, a minority of patients develop refractory disease requiring alternative therapeutic strategies. Hematopoietic stem cell transplantation (HSCT) has rarely been used in this setting and is considered a last-line option for highly selected patients.

**Aims**

To present a rare case of multirefractory ITP successfully treated with autologous stem cell transplantation.

**Methods**

Clinical features, laboratory findings, and treatment responses of a 36-year-old female patient with refractory ITP were retrospectively evaluated.

**Results**

The patient presented with menorrhagia, mucocutaneous bleeding, and severe thrombocytopenia. Secondary causes were excluded. Initial corticosteroid therapy produced only transient response followed by relapse. Sequential treatments including intravenous immunoglobulin, eltrombopag, rituximab, romiplostim, cyclosporine, azathioprine, vincristine, and daratumumab failed to induce sustained remission.
Bone marrow examination showed increased megakaryocytes without fibrosis or blast excess. Splenectomy performed in 2023 achieved temporary remission lasting approximately ten months before relapse. Due to persistent treatment resistance, the patient underwent autologous HSCT in May 2025.
No transplantation-related complications occurred, and platelet counts normalized following transplantation, indicating complete hematologic response.

**Summary/Conclusion**

Approximately 5–10% of ITP cases follow a refractory course. HSCT may restore immune tolerance and induce durable remission, but should be reserved for carefully selected patients who have exhausted conventional therapies.
**This case highlights that autologous stem cell transplantation may represent a viable rescue strategy in carefully selected patients with multirefractory ITP when all standard therapies have failed.**

**Copyright Information:** (Online) ISSN: 2572-9241
©2026 The Author(s). HemaSphere is published by John Wiley & Sons Ltd on behalf of European Hematology Association. This is an open access Abstract Book distributed under the Attribution-NonCommercial-NoDerivs (CC BY-NC-ND), which allows third parties to download the articles and share them with others as long as they credit the author and the Abstract Book, but they cannot change the content in any way or use them commercially.
**Abstract Book Citations:** Authors, Title, HemaSphere, 2026;10:(S1):pages. The abstract book DOIs can be found on the "Author Information" page.
**Disclaimer:** Articles published in the journal HemaSphere exclusively reflect the opinions of the authors. The authors are responsible for all content in their abstracts including accuracy of the facts, statements, citing resources, etc.

### (PB4309) DURABLE REMISSION ACHIEVED WITH OBINUTUZUMAB IN A PEDIATRIC PATIENT WITH CHRONIC REFRACTORY ITP AND HYPERSENSITIVITY TO RITUXIMAB AND ROMIPLOSTIM

**Topic:** 33. Platelet disorders

Ahmad Al-Huniti*[1]

[1]*Mayo Clinic, Rochester, United States of America;*

### Background

Chronic refractory immune thrombocytopenia (ITP) in pediatric patients poses substantial therapeutic challenges, particularly when standard second-line agents are ineffective or contraindicated. Obinutuzumab, a glycoengineered type II anti-CD20 antibody, has demonstrated efficacy in adult patients with refractory ITP and related autoimmune cytopenias; however, its use in pediatric ITP has not been previously reported.

### Aims

This report aims to detail the clinical course, therapeutic intervention, and outcomes in the first documented pediatric case of chronic refractory ITP treated with obinutuzumab following severe hypersensitivity reactions to both rituximab and romiplostim.

### Methods

A retrospective chart review was conducted to collect medical history, treatment details, and clinical response. Remission of ITP was defined as a platelet count exceeding 100 × $10^9$/L for a minimum of three months in the absence of ITP-directed therapy.

### Results

A 16-year-old female was diagnosed with chronic ITP in 2017. Initial transient responses to corticosteroids and intravenous immunoglobulin were followed by a poor response to eltrombopag (2018–2019). Rituximab induced remission in 2019; however, a second course in 2021 was discontinued after the second dose due to anaphylaxis. Romiplostim was initiated but discontinued following a severe allergic rash. Subsequent treatment with eltrombopag 75 mg daily, mycophenolate 1000 mg daily, and hydroxychloroquine 400 mg daily failed to increase platelet counts above 10 × $10^9$/L. In March 2024, the patient was admitted to intensive care with a life-threatening hemorrhage secondary to severe epistaxis, resulting in hypotension and shock. Given the prior response to anti-CD20 therapy, a single obinutuzumab infusion (1000 mg, as per the NCT06094881 clinical trial) was administered in April 2024. Platelet counts increased from below 10 × $10^9$/L to approximately 400 × $10^9$/L within weeks, stabilizing at 150–300 × $10^9$/L. By eight months, eltrombopag and mycophenolate were discontinued; hydroxychloroquine was continued as an immunomodulator in collaboration with rheumatology due to positive ANA and mild antiphospholipid antibody positivity. Remission criteria were met and sustained through 18 months of follow-up. No obinutuzumab-related adverse events or bleeding recurrence were observed.

**Copyright Information:** (Online) ISSN: 2572-9241
©2026 The Author(s). HemaSphere is published by John Wiley & Sons Ltd on behalf of European Hematology Association. This is an open access Abstract Book distributed under the Attribution-NonCommercial-NoDerivs (CC BY-NC-ND), which allows third parties to download the articles and share them with others as long as they credit the author and the Abstract Book, but they cannot change the content in any way or use them commercially.
**Abstract Book Citations:** Authors, Title, HemaSphere, 2026;10:(S1):pages. The abstract book DOIs can be found on the "Author Information" page.
**Disclaimer:** Articles published in the journal HemaSphere exclusively reflect the opinions of the authors. The authors are responsible for all content in their abstracts including accuracy of the facts, statements, citing resources, etc.

**Summary/Conclusion**

Obinutuzumab induced rapid and durable remission in this first documented pediatric ITP case, allowing discontinuation of all ITP-directed therapies. These findings suggest that obinutuzumab may be considered for selected patients with severe allergic reactions to rituximab. Prospective studies are necessary to further define the safety and efficacy of obinutuzumab in pediatric ITP and to establish its role within treatment algorithms.

**Copyright Information:** (Online) ISSN: 2572-9241
©2026 The Author(s). HemaSphere is published by John Wiley & Sons Ltd on behalf of European Hematology Association. This is an open access Abstract Book distributed under the Attribution-NonCommercial-NoDerivs (CC BY-NC-ND), which allows third parties to download the articles and share them with others as long as they credit the author and the Abstract Book, but they cannot change the content in any way or use them commercially.
**Abstract Book Citations:** Authors, Title, HemaSphere, 2026;10:(S1):pages. The abstract book DOIs can be found on the "Author Information" page.
**Disclaimer:** Articles published in the journal HemaSphere exclusively reflect the opinions of the authors. The authors are responsible for all content in their abstracts including accuracy of the facts, statements, citing resources, etc.

### (PB4310) TREATMENT-FREE REMISSION AFTER HETROMBOPAG DISCONTINUATION IN PATIENTS WITH CHRONIC IMMUNE THROMBOCYTOPENIA IN CHINA: AN OBSERVATIONAL RETROSPECTIVE ANALYSIS IN REALWORLD CLINICAL PRACTICE

**Topic:** 33. Platelet disorders

Yimei Feng*[1], Jia Liu[1], Aoxue Tan[1], Chen Li[1], Xiaoqi Wang[1], Xi Zhang[1]

[1]*Xinqiao Hospital, Army Medical University, Chongqing, China;*

### Background

Chronic immune thrombocytopenia (ITP) is associated with prolonged disease, recurrent bleeding, and long-term treatment burden. Thrombopoietin receptor agonists (TPO-RAs) stimulate megakaryocytes and are important for chronic ITP management. Hetrombopag(HET), an oral non-peptide TPO-RA, is widely used in China, but data on platelet response and treatment-free remission (TFR) after discontinuation are limited.

### Aims

To systematically evaluate the feasibility of HET discontinuation and the durability of treatment-free remission in patients with chronic ITP who achieved a stable complete response (CR).

### Methods

Patients with chronic ITP who achieved an effective response to HET monotherapy and maintained CR (platelet count ≥100 ×10⁹/L) for more than 3 months were retrospectively enrolled. HET was tapered gradually, with dose reductions of 25–50% every 2 weeks following an alternate-day reduction strategy (reducing the dose on even days first, followed by odd days) until complete discontinuation. If platelet counts could not be maintained above 100 ×10⁹/L during dose reduction, the dose was escalated to the previous two-step effective level.

### Results

A total of 21 patients with chronic ITP (4M/17F; median age 59y, range 18–77) with relapsed/refractory disease and a median of 3 prior therapies were included. Median disease duration was 27 months (12–154). Initial HET dose was 5.0 mg/day (2.5–7.5), baseline platelet count 26 (3–30)×10⁹/L. Median time to CR was 31 days (6–60), and median CR duration during treatment was 21 months (3–35).
Mean platelet count at discontinuation was 182×10⁹/L; post-discontinuation, mean platelets were 104, 131, and 132×10⁹/L at weeks 1, 2, and 4. All patients maintained ≥30×10⁹/L at week 1, 90% at week 2; two patients had <30×10⁹/L at week 4 and resumed HET. Platelets ≥100×10⁹/L were maintained in 47.6%, 33.3%, and 33.3% at weeks 1, 2, and 4; at 2 months 33.3% (6/21), and at 3 months 28.6% (6/21) maintained CR. These six had mean HET exposure of 820 days and mean CR duration of 730 days. During 3-month follow-up, 15 patients resumed therapy due to platelet falling below 100 ×10⁹/L.
The relatively low TFR rate may be attributable to advanced age, long disease duration, and multiple prior lines of therapy, which could lead to rapid loss of platelet response after discontinuation. In addition, patients' anxiety and concern regarding platelet counts below 100 ×10⁹/L may have contributed to early retreatment. Longer duration of HET treatment were associated with a higher likelihood of successful discontinuation.

**Copyright Information:** (Online) ISSN: 2572-9241
©2026 The Author(s). HemaSphere is published by John Wiley & Sons Ltd on behalf of European Hematology Association. This is an open access Abstract Book distributed under the Attribution-NonCommercial-NoDerivs (CC BY-NC-ND), which allows third parties to download the articles and share them with others as long as they credit the author and the Abstract Book, but they cannot change the content in any way or use them commercially.
**Abstract Book Citations:** Authors, Title, HemaSphere, 2026;10:(S1):pages. The abstract book DOIs can be found on the "Author Information" page.
**Disclaimer:** Articles published in the journal HemaSphere exclusively reflect the opinions of the authors. The authors are responsible for all content in their abstracts including accuracy of the facts, statements, citing resources, etc.

| Variables | Value |
|---|---|
| Patients, n | 21 |
| Median age, years (range) | 59 (18–77) |
| Female, n (%) | 17 (81.0%) |
| Median disease duration, months (range) | 27 (12–154) |
| Relapsed/refractory ITP, n (%) | 21 (100%) |
| Median prior treatment regimens, n (range) | 3 (2–6) |
| Median time to CR, days (range) | 31 (6–60) |
| Mean platelet counts at discontinuation, $\times 10^9$/L | 182 |
| Platelet $\geq 100\times 10^9$/L at 1 months (TFR), n (%) | 7 (33.3%) |
| Platelet $\geq 100\times 10^9$/L at 2 months (TFR), n (%) | 7 (33.3%) |
| Platelet $\geq 100\times 10^9$/L at 3 months (TFR), n (%) | 6 (28.6%) |

### Summary/Conclusion

This is the first real-world study in China evaluating treatment-free remission after Hetrombopag discontinuation in patients with chronic ITP. For patients with chronic relapsed/refractory ITP who achieve a stable platelet response after treatment with Hetrombopag following multiple lines of therapy, long-term discontinuation is not recommended. Even if treatment discontinuation is considered, the maximum duration of drug withdrawal should not exceed three months.

**Copyright Information:** (Online) ISSN: 2572-9241
©2026 The Author(s). HemaSphere is published by John Wiley & Sons Ltd on behalf of European Hematology Association. This is an open access Abstract Book distributed under the Attribution-NonCommercial-NoDerivs (CC BY-NC-ND), which allows third parties to download the articles and share them with others as long as they credit the author and the Abstract Book, but they cannot change the content in any way or use them commercially.
**Abstract Book Citations:** Authors, Title, HemaSphere, 2026;10:(S1):pages. The abstract book DOIs can be found on the “Author Information” page.
**Disclaimer:** Articles published in the journal HemaSphere exclusively reflect the opinions of the authors. The authors are responsible for all content in their abstracts including accuracy of the facts, statements, citing resources, etc.

### (PB4311) IMMUNE THROMBOCYTOPENIA IN ADULTS: AN 8.5-YEAR RETROSPECTIVE SINGLE-CENTER STUDY

**Topic:** 33. Platelet disorders

Imane EL Johri*[1], Ikram Sadki[1], Zahida Aqodad[1], Samia Sabri[1], Houda Bachir[1], Siham Hamaz[1], Habiba Bennesser Alaoui[1], Khalid Serraj Andaloussi[1]

*[1]Laboratory of Immuno-Hematology and Cell Therapy, Faculty of Medicine and Pharmacy of Oujda (FMPO), Mohammed First University, Oujda, Morocco., Internal medicine, Oujda, Morocco;*

### Background

Primary immune thrombocytopenia (ITP) is a rare autoimmune disorder characterized by isolated thrombocytopenia (<100,000/mm³) in the absence of an identifiable cause. The diagnosis is established by excluding other causes of thrombocytopenia. IT may present as an acute form, mainly in children and often post-infectious, or as a chronic form affecting adults, considered primary in approximately 80% of cases.

### Aims

To analyze the epidemiological profile, clinical presentation, paraclinical findings, and therapeutic management of adult patients with immune thrombocytopenia in Morocco (Africa), providing insights into disease patterns, treatment responses, and outcomes.

### Methods

We conducted a retrospective descriptive study including 62 adult patients followed for immune thrombocytopenia in the Department of Internal Medicine at Mohammed VI University Hospital, Oujda, over an 8.5-year period from January 2016 to July 2024. Epidemiological, clinical, paraclinical, and therapeutic data were collected and analyzed.

### Results

The mean age was 43 years, with a female predominance (74%), resulting in a female-to-male ratio of 2.87. Hemorrhagic manifestations were observed in 84% of patients, predominantly mucocutaneous bleeding (81.25%), with severe bleeding occurring in 27% of cases. Primary ITP accounted for 71% of cases, while secondary forms represented 29%. Treatment was administered to 96.8% of patients. Corticosteroids were used in 96.8% of cases, intravenous immunoglobulins in 30.64%, vincristine in 32.25%, rituximab in 22.85%, thrombopoietin receptor agonists in 27.41%, splenectomy in 27.41%, and azathioprine in 1.6%. The highest response rates were observed after splenectomy, with a 100% initial response rate and a sustained remission rate of 52.94%.

### Summary/Conclusion

Immune thrombocytopenia predominantly affects adult women and is frequently associated with hemorrhagic manifestations. Corticosteroids remain the first-line therapy, while splenectomy provides the highest long-term remission rates in selected patients.

**Copyright Information:** (Online) ISSN: 2572-9241
©2026 The Author(s). HemaSphere is published by John Wiley & Sons Ltd on behalf of European Hematology Association. This is an open access Abstract Book distributed under the Attribution-NonCommercial-NoDerivs (CC BY-NC-ND), which allows third parties to download the articles and share them with others as long as they credit the author and the Abstract Book, but they cannot change the content in any way or use them commercially.
**Abstract Book Citations:** Authors, Title, HemaSphere, 2026;10:(S1):pages. The abstract book DOIs can be found on the "Author Information" page.
**Disclaimer:** Articles published in the journal HemaSphere exclusively reflect the opinions of the authors. The authors are responsible for all content in their abstracts including accuracy of the facts, statements, citing resources, etc.

### (PB4312) FIRST-DOSE OXALIPLATIN–INDUCED IMMUNE THROMBOCYTOPENIA WITHOUT HYPERSENSITIVITY: A DIAGNOSTIC PITFALL

**Topic:** 33. Platelet disorders

Muhiddin Er[1], Muzaffer Uğraklı[1], Taha Ulutan Kars*[2]

*[1]University of Health Science, Konya City Hospital, Medical Oncology, Konya, Türkiye;[2]University of Health Science, Konya City Hospital, Hematology, Konya, Türkiye;*

**Background**

Oxaliplatin is a cornerstone chemotherapeutic agent in gastrointestinal malignancies and is most commonly associated with mild-to-moderate thrombocytopenia due to myelosuppression. In rare cases, oxaliplatin may cause immune-mediated thrombocytopenia, typically after multiple treatment cycles and frequently accompanied by hypersensitivity reactions. Early-onset presentations without allergic features are uncommon and may be misinterpreted as chemotherapy-related myelosuppression, leading to delayed diagnosis and potentially serious complications.

**Aims**

To highlight an atypical presentation of oxaliplatin-induced immune thrombocytopenia (OIIT) occurring after the first dose and in the absence of hypersensitivity reactions, and to emphasize key clinical features that help distinguish immune-mediated thrombocytopenia from chemotherapy-induced myelosuppression.

**Methods**

We report a detailed clinical and laboratory evaluation of a patient who developed severe isolated thrombocytopenia following first-dose oxaliplatin. Alternative causes of thrombocytopenia, including bone marrow suppression, thrombotic microangiopathy, splenic sequestration, infection, and drug-related non-immune mechanisms, were systematically excluded. Temporal association, transfusion response, corticosteroid treatment effect, and causality assessment using the Naranjo Adverse Drug Reaction Probability Scale were analyzed.

**Results**

A 68-year-old man with locally advanced rectal cancer received oxaliplatin as part of a CAPOX regimen following neoadjuvant chemoradiotherapy. Baseline platelet count was 228,000/µL. On day 21 after the first oxaliplatin dose, platelet count abruptly decreased to 30,000/µL and further declined to 15,000/µL within two days, with preserved hemoglobin, leukocyte counts, renal function, and no evidence of hemolysis. No hypersensitivity or infusion-related reactions were observed. Platelet transfusion resulted in no increment, indicating transfusion refractoriness. High-dose dexamethasone therapy led to rapid and sustained platelet recovery, reaching 212,000/µL. Oxaliplatin was permanently discontinued, and no recurrent hematologic abnormalities occurred. The Naranjo score was 8, supporting a probable causal relationship.

**Summary/Conclusion**

This case demonstrates that OITT may occur after a single exposure and without hypersensitivity manifestations. Abrupt isolated thrombocytopenia, lack of response to platelet transfusion, and rapid corticosteroid-induced recovery are key features suggestive of an immune-mediated mechanism. Awareness of early-onset OIIT is essential to avoid misdiagnosis as myelosuppression and to ensure prompt, potentially life-saving management.

**Copyright Information:** (Online) ISSN: 2572-9241
©2026 The Author(s). HemaSphere is published by John Wiley & Sons Ltd on behalf of European Hematology Association. This is an open access Abstract Book distributed under the Attribution-NonCommercial-NoDerivs (CC BY-NC-ND), which allows third parties to download the articles and share them with others as long as they credit the author and the Abstract Book, but they cannot change the content in any way or use them commercially.
**Abstract Book Citations:** Authors, Title, HemaSphere, 2026;10:(S1):pages. The abstract book DOIs can be found on the “Author Information” page.
**Disclaimer:** Articles published in the journal HemaSphere exclusively reflect the opinions of the authors. The authors are responsible for all content in their abstracts including accuracy of the facts, statements, citing resources, etc.

### (PB4313) RECURRENT IMMUNE THROMBOCYTOPENIA AFTER SPLENECTOMY: WHY SEARCHING FOR AN ACCESSORY SPLEEN MATTERS

**Topic:** 33. Platelet disorders

Ikram Sadki*[1], Soumaya Barkhoukh[1], Abdellah Seghiri[1], Houda Bachir[1], Siham Hamaz[1], Habiba Bennesser Alaoui[1], Khalid Serraj Andaloussi[1]

*[1]Immuno-Hematology and Cellular Therapy Laboratory. Faculty of Medicine and Pharmacy of Oujda (FMPO ). University of Mohamed First of Oujda. Morocco, Department of Internal Medicine, Mohammed VI University Hospital of Oujda, Oujda, Morocco;*

### Background

Immune thrombocytopenia (ITP) is an acquired autoimmune disorder characterized by immune-mediated platelet destruction and impaired platelet production. Splenectomy remains an effective second-line therapeutic option for patients with chronic or refractory disease, achieving durable remission in a substantial proportion of cases. However, disease relapse after splenectomy occurs in up to 20–30% of patients, representing a significant clinical challenge. One of the most important yet frequently overlooked causes of post-splenectomy relapse is the presence of an accessory spleen. We report a teaching case of ITP relapse 7 years after splenectomy due to the presence of an accessory spleen

### Aims

Highlighting the importance of searching for an accessory spleen in cases of relapse of immune thrombocytopenia after splenectomy

### Methods

**Case Presentation:** A 38-year-old woman with a history of immune thrombocytopenia diagnosed in 2016 underwent splenectomy in 2018, achieving complete and sustained remission. Seven years later, she was admitted for severe isolated thrombocytopenia (12,000/mm$^3$) discovered during preoperative assessment. Clinical examination was unremarkable without bleeding manifestations. Extensive evaluation excluded secondary causes of thrombocytopenia. Bone marrow examination supported peripheral platelet destruction. Abdominal imaging revealed an accessory spleen, confirmed by computed tomography. The patient received corticosteroid therapy with a favorable hematologic response.

### Results

Discussion : Accessory spleens are ectopic splenic tissues that retain normal splenic structure and function. They are identified in 10–30% of the general population and in up to 50% of splenectomized patients investigated for recurrent ITP. Failure to identify accessory splenic tissue represents a well-recognized cause of post-splenectomy relapse. Moreover, prevention of splenic tissue spillage during laparoscopic splenectomy is essential, as dissemination of splenic fragments may result in splenosis or functional accessory spleen formation. Accessory spleens may preserve the immune microenvironment responsible for platelet destruction, thereby contributing to disease persistence or relapse after splenectomy.

**Copyright Information:** (Online) ISSN: 2572-9241
©2026 The Author(s). HemaSphere is published by John Wiley & Sons Ltd on behalf of European Hematology Association. This is an open access Abstract Book distributed under the Attribution-NonCommercial-NoDerivs (CC BY-NC-ND), which allows third parties to download the articles and share them with others as long as they credit the author and the Abstract Book, but they cannot change the content in any way or use them commercially.
**Abstract Book Citations:** Authors, Title, HemaSphere, 2026;10:(S1):pages. The abstract book DOIs can be found on the "Author Information" page.
**Disclaimer:** Articles published in the journal HemaSphere exclusively reflect the opinions of the authors. The authors are responsible for all content in their abstracts including accuracy of the facts, statements, citing resources, etc.

**Summary/Conclusion**

Recurrent immune thrombocytopenia following splenectomy should systematically raise suspicion for accessory splenic tissue, particularly in patients with late relapse after an excellent initial response, as post-splenectomy recurrence may represent a potentially curable condition rather than true treatment failure.

**Copyright Information:** (Online) ISSN: 2572-9241
©2026 The Author(s). HemaSphere is published by John Wiley & Sons Ltd on behalf of European Hematology Association. This is an open access Abstract Book distributed under the Attribution-NonCommercial-NoDerivs (CC BY-NC-ND), which allows third parties to download the articles and share them with others as long as they credit the author and the Abstract Book, but they cannot change the content in any way or use them commercially.
**Abstract Book Citations:** Authors, Title, HemaSphere, 2026;10:(S1):pages. The abstract book DOIs can be found on the "Author Information" page.
**Disclaimer:** Articles published in the journal HemaSphere exclusively reflect the opinions of the authors. The authors are responsible for all content in their abstracts including accuracy of the facts, statements, citing resources, etc.

### (PB4314) EFFICACY AND SAFETY OF PIRTOBRUTINIB IN ADULTS WITH IMMUNE THROMBOCYTOPENIA: A PHASE 1/2 DOSE-FINDING STUDY IN PROGRESS

**Topic:** 33. Platelet disorders

Waleed Ghanima*[1, 2], Catherine Broome[3], Tomás José González-López[4], Ananya Guntur[5], Chunxiao Wang[5], Sasha Amdur[5], James Pauff[5]

*[1]Department of Research and Haemato-Oncology, Østfold Hospital Trust, Grålum, Norway;[2]Institute of Clinical Medicine, University of Oslo, Oslo, Norway; [3]MedStar Georgetown Cancer Institute at MedStar Georgetown University Hospital, Washington, United States of America; [4]Department of Hematology, Hospital Universitario de Burgos, Burgos, Spain;[5]Eli Lilly and Company, Clinical Research, Indianapolis, IN, United States of America;*

### Background

Immune thrombocytopenia (ITP) is an autoimmune disorder characterized by reduced platelet counts and increased risk of bleeding. Pirtobrutinib, a highly selective, non-covalent, reversible Bruton tyrosine kinase (BTK) inhibitor, may offer advantages for patients with ITP due to its optimized pharmacokinetic properties, once-daily dosing, and specificity for BTK. Preclinical ITP models show pirtobrutinib effectively reduces B-cell activity and autoantibody production.

### Aims

This phase 1/2 study aims to optimize the dosing of pirtobrutinib in adults pre-treated for primary ITP.

### Methods

This study consists of 2 parts: phase 1 dose escalation and phase 2 dose optimization. Phase 1 is an open-label portion where dose escalation will be driven by the BOIN method.~ 9-18 participants will be enrolled into sequential dose levels, starting at the lowest dose of oral pirtobrutinib, once daily for 12 weeks. The primary objective is to assess the safety and tolerability of pirtobrutinib and select doses for phase 2. Secondary objectives will assess the efficacy by platelet response rate, the extent of disease control, and describe the PK of pirtobrutinib. Phase 2 is a randomized double-blind dose optimization design where ~ 10 participants will be randomized to evaluate ≥2 dose levels of pirtobrutinib versus placebo. Patients will be treated for 12 weeks to assess the stability of response. The primary objective is to assess the efficacy of pirtobrutinib versus placebo, by stable platelet response rate (platelet count ≥50 k/µL on ≥ 4 of 6 consecutive biweekly visits between weeks 14-24, without rescue therapy and prohibited concomitant medications affecting efficacy). Secondary objectives will evaluate the safety, PK, and additional efficacy endpoints of pirtobrutinib. Patients aged ≥18 with confirmed diagnosis of primary ITP for ≥3 months prior to enrollment, and history of response to ≥1 prior line of therapy are eligible. Exclusion criteria include treatment other than corticosteroids or TPO-RA for ITP within 2 weeks of visit 1, use of anticoagulants or antiplatelet therapy, history of any thrombotic or embolic event within 12 months before screening, hematologic malignancy, and significant cardiovascular disease.

### Results

This study is a Trial in Progress, and enrollment is ongoing.

### Summary/Conclusion

This study is a Trial in Progress, and enrollment is ongoing.

**Copyright Information:** (Online) ISSN: 2572-9241
©2026 The Author(s). HemaSphere is published by John Wiley & Sons Ltd on behalf of European Hematology Association. This is an open access Abstract Book distributed under the Attribution-NonCommercial-NoDerivs (CC BY-NC-ND), which allows third parties to download the articles and share them with others as long as they credit the author and the Abstract Book, but they cannot change the content in any way or use them commercially.
**Abstract Book Citations:** Authors, Title, HemaSphere, 2026;10:(S1):pages. The abstract book DOIs can be found on the "Author Information" page.
**Disclaimer:** Articles published in the journal HemaSphere exclusively reflect the opinions of the authors. The authors are responsible for all content in their abstracts including accuracy of the facts, statements, citing resources, etc.

### (PB4315) TARGETING LONG-LIVED PLASMA CELLS FOR THE TREATMENT OF ACQUIRED COAGULATION DISORDERS

**Topic:** 33. Platelet disorders

Xinya Peng*[1], Li Zhou[1]

[1] *The Second Hospital of Anhui Medical University, Hefei, China;*

### Background

Long-lived plasma cells (LLPCs), with their distinct immunophenotype of CD38$^{++}$/CD138$^{+}$, are key immune cells responsible for the continuous secretion of antibodies. They play a crucial role in the chronicity and high recurrence rate of acquired immune-mediated thrombocytopenias, such as Immune Thrombocytopenia (ITP), and are central to the pathogenesis of acquired coagulation disorders like acquired hemophilia A (AHA).These cells reside in specialized niches, primarily in the bone marrow, where they continuously produce pathogenic autoantibodies without antigenic stimulation. In ITP, they generate antiplatelet autoantibodies that mediate platelet destruction or impair production; in AHA, they produce neutralizing antibodies against coagulation factor VIII (FVIII), inhibiting its function and leading to spontaneous or severe post-traumatic bleeding that can be life-threatening.

### Aims

This characteristic renders traditional B-cell-targeted therapies, such as Rituximab, less effective, as Rituximab primarily targets CD20$^{+}$ B cells and fails to eliminate CD20-negative LLPCs, a major factor contributing to relapse and treatment resistance in AHA. Thus, direct targeting of LLPCs has emerged as a promising innovative therapeutic approach for both conditions.

### Methods

To investigate CD38$^{++}$ long-lived plasma cell (LLPC)-targeted strategies in antibody-mediated acquired coagulation/cytopenic disorders (ITP, AHA), we searched CNKI and PubMed using keywords such as LLPCs, ITP, and AHA, retrieving approximately 90 articles. Included studies covered almost four therapies(CD38mAb, proteasome inhibitors, immunosuppressants, CAR-T), mostly small-sample prospective, phase II or case reports, with limited large RCTs. Overall safety and tolerability were favorable.

### Results

Monoclonal antibodies against CD38, like Daratumumab, effectively deplete CD38$^{++}$ LLPCs through ADCC and CDC, with clinical trials in ITP showing high response rates and good tolerability. In AHA, combining CD38 antibodies with conventional immunosuppressants has potential in removing anti-FVIII antibody-secreting LLPCs, rapidly reducing inhibitor levels and offering new treatment options for refractory cases. Proteasome inhibitors, such as Bortezomib, disrupt critical LLPC survival signals to induce apoptosis and reduce autoantibody secretion, proving effective in refractory ITP and serving as rescue therapy for recurrent or refractory AHA when combined with Rituximab.

### Summary/Conclusion

CD38-targeted CAR-T cell therapy enables precise elimination of LLPCs but faces off-target toxicity due to low CD38 expression on normal immune cells, limiting its clinical translation. Larger-scale clinical trials are urgently needed to optimize regimens, verify CAR-T's safety and efficacy, and explore LLPC-immune molecule interactions to provide more precise and personalized therapeutic strategies.

**Copyright Information:** (Online) ISSN: 2572-9241
©2026 The Author(s). HemaSphere is published by John Wiley & Sons Ltd on behalf of European Hematology Association. This is an open access Abstract Book distributed under the Attribution-NonCommercial-NoDerivs (CC BY-NC-ND), which allows third parties to download the articles and share them with others as long as they credit the author and the Abstract Book, but they cannot change the content in any way or use them commercially.
**Abstract Book Citations:** Authors, Title, HemaSphere, 2026;10:(S1):pages. The abstract book DOIs can be found on the "Author Information" page.
**Disclaimer:** Articles published in the journal HemaSphere exclusively reflect the opinions of the authors. The authors are responsible for all content in their abstracts including accuracy of the facts, statements, citing resources, etc.

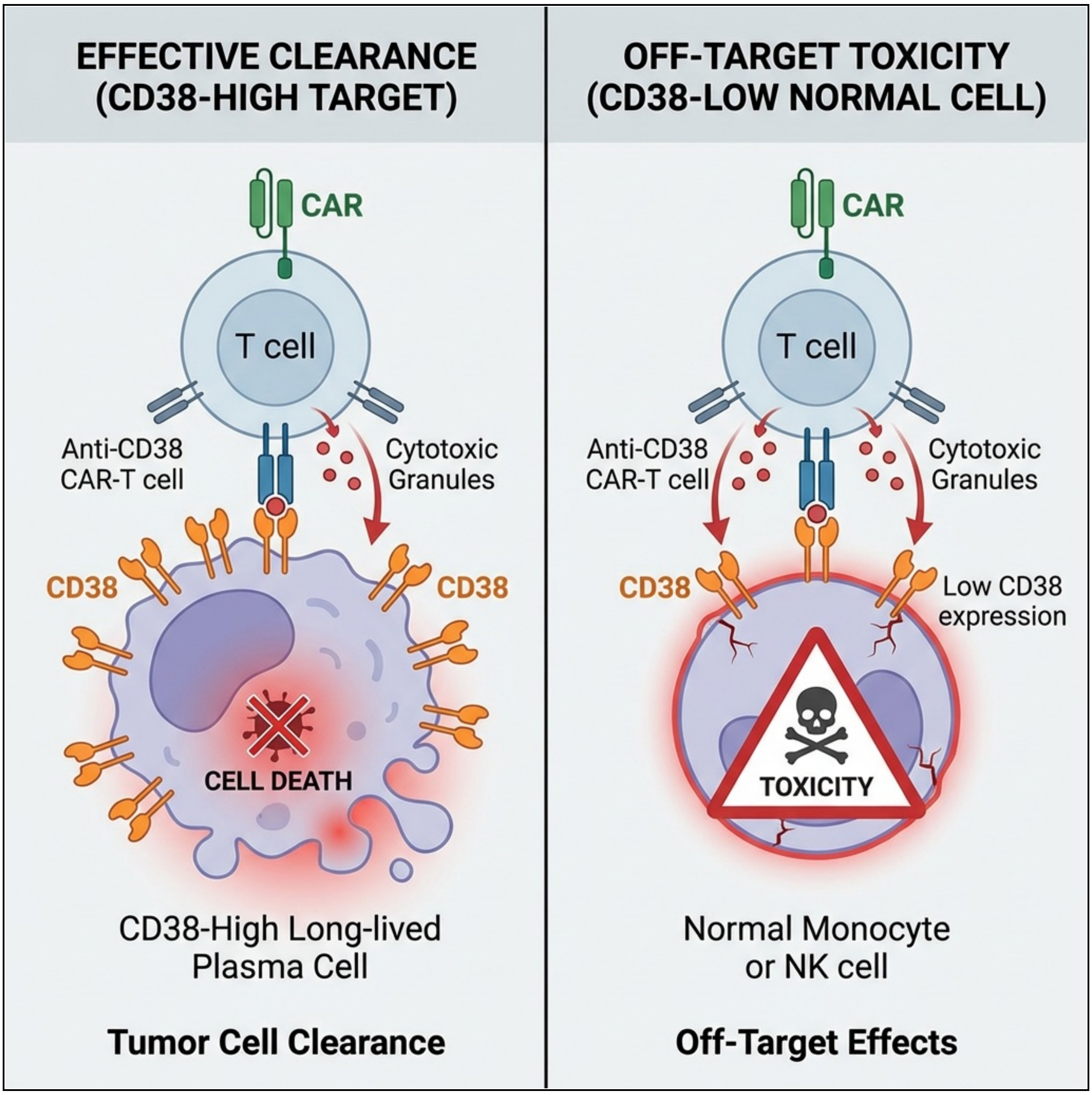

This images was generated by AI.

**Copyright Information:** (Online) ISSN: 2572-9241
©2026 The Author(s). HemaSphere is published by John Wiley & Sons Ltd on behalf of European Hematology Association. This is an open access Abstract Book distributed under the Attribution-NonCommercial-NoDerivs (CC BY-NC-ND), which allows third parties to download the articles and share them with others as long as they credit the author and the Abstract Book, but they cannot change the content in any way or use them commercially.
**Abstract Book Citations:** Authors, Title, HemaSphere, 2026;10:(S1):pages. The abstract book DOIs can be found on the "Author Information" page.
**Disclaimer:** Articles published in the journal HemaSphere exclusively reflect the opinions of the authors. The authors are responsible for all content in their abstracts including accuracy of the facts, statements, citing resources, etc.

### (PB4316) CLINICAL FEATURES OF EVANS SYNDROME: A SINGLE-CENTER CASE SERIES

**Topic:** 33. Platelet disorders

Mustafa Berkay Tastekin*[1], Umit Yavuz Malkan[2]

[1]*Hacettepe University, Internal Medicine, Ankara, Türkiye;* [2]*Hacettepe University, Internal Medicine, Hematology, Ankara, Türkiye;*

### Background

Evans syndrome (ES) is a form of multiple autoimmune cytopenia defined by the coexistence of AIHA and ITP, with the infrequent addition of autoimmune neutropenia. Splenectomy may benefit both entities by reducing the level of autoantibodies. Additionally, treatments such as rituximab, thrombopoietin receptor agonists, and immunosuppressive agents can be utilized in the management of the disease

### Aims

In our retrospective case series, we aimed to contribute to the literature by examining the clinical characteristics of patients diagnosed with Evans syndrome (ES).

### Methods

Data from patients diagnosed with ES between 1980 and 2025 were reviewed retrospectively in our single-center study.

### Results

A total of 14 patients diagnosed with Evans syndrome (ES) who presented to our center were included in the study. Eight patients (57.1%) were female. The median age of the patients was 40.0 (24.7–61.2) years, while the median age at diagnosis was 30.0 (14.0–52.5) years. The median follow-up period was 72.0 (20.4–110.9) months. At the time of diagnosis, the median laboratory values were as follows: hemoglobin 12.1 (11.2–15.5) g/dL, MCV 79.0 (74.5–85.9) fL, platelet count 19 (6–77) $\times 10^3$/µL, MPV 10.1 (8.5–11.4) fL, leukocyte count 7.9 (4.4–9.0) $\times 10^3$/µL, neutrophil count 4.7 (2.6–6.0) $\times 10^3$/µL, and LDH 187.5 (234.0–334.5) U/L. In terms of disease onset, 42.9% (n=6) of patients initially presented with ITP, 35.7% (n=5) with simultaneous AIHA and ITP, and 21.4% (n=3) with AIHA alone. Regarding first-line treatments, four patients each (28.6%) received prednisolone and a combination of prednisolone-IVIG, while one patient (7.1%) received high-dose prednisolone and another (7.1%) received IVIG monotherapy. Three patients (21.4%) directly proceeded to second-line therapy, while one patient (7.1%) received other types of treatment. Regarding platelet recovery, 54.5% (n=6) of patients who received treatment achieved a complete response. Second-line therapy was administered to eleven patients. The second-line consisted of splenectomy in 6 patients (54.5%), eltrombopag in 2 (18.2%), and rituximab-based combination regimens in 3 (27.3%). A complete response was observed in 50% (n=7) of the patients; however, an identical percentage experienced a relapse. At the final follow-up, the median laboratory values were as follows: hemoglobin 13.7 (12.8–15.6) g/dL, MCV 87.4 (83.2–91.9) fL, platelet count 133 (43–351) $\times 10^3$/µL, and MPV 10.7 (9.4–12.0) fL. A statistically significant difference was found between the parameters at the time of diagnosis and the final follow-up, with p-values of 0.041 for hemoglobin, 0.035 for MCV, and 0.026 for platelet counts. Complications included portal vein thrombosis in one patient; notably, no life-threatening bleeding was observed, with the exception of one case of mucocutaneous hemorrhage. There were no deaths among the patient cohort during follow-up.

**Copyright Information:** (Online) ISSN: 2572-9241
©2026 The Author(s). HemaSphere is published by John Wiley & Sons Ltd on behalf of European Hematology Association. This is an open access Abstract Book distributed under the Attribution-NonCommercial-NoDerivs (CC BY-NC-ND), which allows third parties to download the articles and share them with others as long as they credit the author and the Abstract Book, but they cannot change the content in any way or use them commercially.
**Abstract Book Citations:** Authors, Title, HemaSphere, 2026;10:(S1):pages. The abstract book DOIs can be found on the “Author Information” page.
**Disclaimer:** Articles published in the journal HemaSphere exclusively reflect the opinions of the authors. The authors are responsible for all content in their abstracts including accuracy of the facts, statements, citing resources, etc.

**Summary/Conclusion**

Despite high initial response rates, Evans syndrome is characterized by a high frequency of relapses. Consistent with the literature, despite high recurrence rates, the significant improvement in hematological parameters at the final visit underscores the therapeutic impact, regardless of subsequent relapses. Furthermore, no major complications occurred during the follow-up period. In our study, we aimed to contribute to the literature by presenting the clinical characteristics of patients diagnosed with Evans syndrome (ES).

**Copyright Information:** (Online) ISSN: 2572-9241
©2026 The Author(s). HemaSphere is published by John Wiley & Sons Ltd on behalf of European Hematology Association. This is an open access Abstract Book distributed under the Attribution-NonCommercial-NoDerivs (CC BY-NC-ND), which allows third parties to download the articles and share them with others as long as they credit the author and the Abstract Book, but they cannot change the content in any way or use them commercially.
**Abstract Book Citations:** Authors, Title, HemaSphere, 2026;10:(S1):pages. The abstract book DOIs can be found on the "Author Information" page.
**Disclaimer:** Articles published in the journal HemaSphere exclusively reflect the opinions of the authors. The authors are responsible for all content in their abstracts including accuracy of the facts, statements, citing resources, etc.

### (PB4317) MANAGEMENT AND THERAPEUTIC OUTCOMES OF PATIENTS WITH THROMBOTIC THROMBOCYTOPENIC PURPURA: A RETROSPECTIVE STUDY

**Topic:** 33. Platelet disorders

Marina Leon Gamazo*[1], Alfredo Gómez Lacuey[1], Ignacio De la Fuente Graciani[1], Pilar Fuertes[1], Scarlet Barrios Rodriguez[1], María Jesús Peñarrubia Ponce[1]

*[1]Hospital Clinico Universitario Valladolid, Valladolid, Spain;*

### Background

Thrombotic Thrombocytopenic Purpura (TTP) is a rare, life-threatening hematologic disorder characterized by the classic triad of thrombocytopenia, microangiopathic hemolytic anemia, and organ dysfunction—primarily neurological and renal. The annual incidence is estimated at 4–6 cases per million, corresponding to approximately 200–300 new cases per year in our country.
Common clinical presentations include fatigue, petechiae, and ecchymosis, along with transient neurological deficits such as headache, confusion, or altered mental status. Due to its clinical variability and rarity, diagnosis is often delayed. This delay is critical; untreated TTP carries a mortality rate exceeding 90%, whereas early intervention with therapeutic plasma exchange (TPE) reduces mortality to below 20%.
Diagnostic confirmation requires measuring ADAMTS13 activity levels and excluding other thrombotic microangiopathies.

### Aims

To describe baseline laboratory parameters, including thrombocytopenia, hemolytic anemia, and ADAMTS13 activity levels.
To review administered treatments and evaluate the subsequent therapeutic response.

### Methods

A retrospective descriptive study was conducted on a case series of patients diagnosed with thrombotic thrombocytopenic purpura (TTP) between 2021 and 2025.

### Results

Five patients were diagnosed over the five-year period. The primary clinical presentation was asthenia, accompanied by petechiae, bleeding, and neurological symptoms, which were present in 80% of cases. Due to the severity of neurological manifestations, 40% of patients required direct admission to the Intensive Care Unit.
Upon diagnostic suspicion, all patients initiated triple therapy consisting of corticosteroids, therapeutic plasma exchange, and rituximab. Treatment was initially discontinued in three patients after reaching the target platelet count; however, these cases exhibited refractoriness, necessitating treatment reintroduction and the addition of caplacizumab.
Following the addition of caplacizumab, patients showed clinical improvement with normalization of ADAMTS13 levels. One patient treated with caplacizumab experienced heavy menorrhagia, requiring transfusion support and administration of coagulation factors.

### Summary/Conclusion

TTP remains a critical condition requiring early diagnosis. The addition of caplacizumab facilitates faster platelet recovery, reduces the risk of relapse, and decreases disease-associated mortality. However, caution is advised, as blocking von Willebrand factor may increase the risk of bleeding complications. Early integration of caplacizumab into the treatment regimen significantly reduces mortality and shortens the duration of hospitalization.

**Copyright Information:** (Online) ISSN: 2572-9241
©2026 The Author(s). HemaSphere is published by John Wiley & Sons Ltd on behalf of European Hematology Association. This is an open access Abstract Book distributed under the Attribution-NonCommercial-NoDerivs (CC BY-NC-ND), which allows third parties to download the articles and share them with others as long as they credit the author and the Abstract Book, but they cannot change the content in any way or use them commercially.
**Abstract Book Citations:** Authors, Title, HemaSphere, 2026;10:(S1):pages. The abstract book DOIs can be found on the “Author Information” page.
**Disclaimer:** Articles published in the journal HemaSphere exclusively reflect the opinions of the authors. The authors are responsible for all content in their abstracts including accuracy of the facts, statements, citing resources, etc.

### (PB4318) FIRST-LINE TREATMENT OF IMMUNE THROMBOCYTOPENIA: PREDNISOLONE VERSUS PREDNISOLONE PLUS IVIG

**Topic:** 33. Platelet disorders

Mustafa Berkay Tastekin*[1], Umit Yavuz Malkan[2]

*[1]Hacettepe University, Internal Medicine, Ankara, Türkiye; [2]Hacettepe University, Internal Medicine, Hematology, Ankara, Türkiye;*

### Background

Immune thrombocytopenia (ITP) is an acquired autoimmune condition where a reduced platelet count results in an increased risk of bleeding. In the treatment of ITP, corticosteroids, intravenous immunoglobulin (IVIG), or a combination of both can be utilized as first-line therapy to prevent complications such as bleeding at the time of diagnosis

### Aims

In our research, the two most utilized first-line treatment regimens for ITP were compared in terms of efficacy and side effects. Thus, we aimed to enhance the existing body of literature with real-world clinical evidence

### Methods

Data from patients diagnosed with ITP between 1980 and 2025 were reviewed retrospectively in our single-center study.

### Results

The study included 100 patients treated with either prednisolone monotherapy or a combination of prednisolone and IVIG at our center. Of the patients included, 62 (62%) were female. The median age and median age at diagnosis were 45.0 (33.0–63.0) and 33.5 (21.2–54.0) years, respectively. The median follow-up duration was 42.8 (11.6–86.7) months. Prednisolone was administered to 59 (59%) patients, whereas 41 (41%) patients received methylprednisolone plus IVIG. Response criteria were categorized as follows: complete response (platelets >100x10³/µL), partial (50–100x10³/µL), and non-response (<50x10³/µL). Complete, partial, and non-response rates were 62.7%, 27.1%, and 10.2% in the prednisolone group, compared to 68.3%, 12.2%, and 19.5% in the prednisolone-IVIG group, respectively. No significant difference was found between the two groups (p=0.12). The median platelet counts at one month post-treatment were 125 (63–169) x10³/µL and 193 (94–225) x10³/µL for the prednisolone and prednisolone-IVIG groups, respectively. No statistically significant difference was observed between the groups regarding first-month platelet counts (p=0.28). In the third-month follow-up, the prednisolone and prednisolone-IVIG groups demonstrated median platelet counts of 111 (36–196) x10³/µL and 163 (81–302) x10³/µL, respectively and significant difference was observed between the groups (p=0.03). The median time to treatment response was 11 (7–27) days in the prednisolone group, compared to 5 (3–15) days in the prednisolone-IVIG group. A statistically significant difference was observed regarding the time to response (p=0.004), indicating that the response time was significantly shorter in the prednisolone-IVIG group. In responders, the relapse rate was 81.5% (n=44) for the prednisolone group compared to 75% (n=27) for the prednisolone-IVIG group. No statistically significant difference was found between the groups regarding relapse rates (p=0.46). No significant difference was observed regarding the side effect profiles of both treatment arms (p=0.19).

**Copyright Information:** (Online) ISSN: 2572-9241
©2026 The Author(s). HemaSphere is published by John Wiley & Sons Ltd on behalf of European Hematology Association. This is an open access Abstract Book distributed under the Attribution-NonCommercial-NoDerivs (CC BY-NC-ND), which allows third parties to download the articles and share them with others as long as they credit the author and the Abstract Book, but they cannot change the content in any way or use them commercially.
**Abstract Book Citations:** Authors, Title, HemaSphere, 2026;10:(S1):pages. The abstract book DOIs can be found on the "Author Information" page.
**Disclaimer:** Articles published in the journal HemaSphere exclusively reflect the opinions of the authors. The authors are responsible for all content in their abstracts including accuracy of the facts, statements, citing resources, etc.

**Summary/Conclusion**

Although no significant differences were observed between the two treatment arms in terms of therapeutic response, platelet counts at the first month, or disease relapse, the addition of IVIG to prednisolone therapy was found to significantly shorten the time to achieve a response. However, due to the higher cost of IVIG therapy, its use is more rational when reserved for life-threatening situations requiring a rapid increase in platelet counts or for selected patients where there are significant concerns regarding steroid-related side effects. In conclusion, addition of IVIG only shortens the time to achieve a response not the treatment response rates.

**Copyright Information:** (Online) ISSN: 2572-9241
©2026 The Author(s). HemaSphere is published by John Wiley & Sons Ltd on behalf of European Hematology Association. This is an open access Abstract Book distributed under the Attribution-NonCommercial-NoDerivs (CC BY-NC-ND), which allows third parties to download the articles and share them with others as long as they credit the author and the Abstract Book, but they cannot change the content in any way or use them commercially.
**Abstract Book Citations:** Authors, Title, HemaSphere, 2026;10:(S1):pages. The abstract book DOIs can be found on the "Author Information" page.
**Disclaimer:** Articles published in the journal HemaSphere exclusively reflect the opinions of the authors. The authors are responsible for all content in their abstracts including accuracy of the facts, statements, citing resources, etc.

### (PB4319) SUCCESSFUL TREATMENT OF A PATIENT WITH CONGENITAL THROMBOTIC THROMBOCYTOPENIC PURPURA (TTP) WITH RECOMBINANT ADAMTS13 - A CASE REPORT

**Topic:** 33. Platelet disorders

Marijo Vodanović*[1], Desiree Coen Herak[2], Ana Boban[3], Marija Miloš[4], Marino Narancic[5], Radovan Radonić[6], Ena Ranković[7], Ana Zelic Kerep[7], Drazen Pulanic[3, 3]

*[1]University Hospital Center Zagreb, University of Applied Health Sciences, Department of Internal Medicine, Division of Hematology, Zagreb, Croatia; [2]University Hospital Center Zagreb, Department of Laboratory Diagnostics, Faculty of Pharmacy and Biochemistry, University of Zagreb, Zagreb, Croatia; [3]University Hospital Center Zagreb, Medical School, University of Zagreb, Department of Internal Medicine, Division of Hematology, Zagreb, Croatia; [4]University Hospital Center Zagreb, Department of Laboratory Diagnostics, Zagreb, Croatia; [5]University Hospital Center Zagreb, Faculty of Pharmacy, University of Mostar, Bosnia and Herzegovina, Department of Internal Medicine, Division of Hematology, Zagreb, Croatia; [6]University Hospital Center Zagreb, Medical School, University of Zagreb, Department of Internal Medicine, Division of Intensive Care Unit, Zagreb, Croatia; [7]University Hospital Center Zagreb, Department of Internal Medicine, Division of Hematology, Zagreb, Croatia;*

### Background

Congenital TTP is a rare blood disease with a prevalence of 0.5 to 2 cases per million. It is caused by biallelic mutations in the gene ADAMTS13 and lead to a severy ADAMTS13 deficiency. Platelets bind to ultralarge vWF multimers, resulting in end-organ damage, consumptive thrombocytopenia and microangiopathic hemolytic anemia.

### Aims

The aim is to present the first experience of treatment with recombinant ADAMTS13 in a Croatian patient with congenital TTP who had previously end-organ damage (chronic renal failure, hemolytic anemia and neurological disorders).

### Methods

A 38 year-old female patient was diagnosed for cTTP at 3 years of age (ADAMTS13 < 1%). She had two pregnancies, in 2010. and 2012, and both ended earlier in the 35th and 36th gestational week, terminated by Ceasarian section. Patient was admitted to hospital >20 times due to exacerbations of cTTP, mostly in the ICU, due to the renal failure, hemolytic anemia, neurological disorders and thrombocytopenia. In acute episodes she was treated with hemodialysis, plasma exchange, steroids and FFP. Exacerbations were triggered by heavier menstrual bleeding, respiratory or gastrontestinal infections. A Patient was regularly treated with FFP 500 ml from the same donor every 2 weeks, because had an allergic reaction to FFP (urticaria, itching, periorbital edema), fatigue, and permanently dull headache, confusion and foggy memory, renal failure (creatinine 120 - 180 µmol/L). The last cTTP exacerbation appeared in February 2024, caused by RSV infection and pneumonia (platelet count $6x10^9$/L, creatinine 305 µmol/L, LDH 1295 U/L, Hemoglobin 9.7 g/dL).

### Results

The donation request for rADAMTS13 was sent in April 2024. Treatment was started on 2nd August 2024, at a dose of 40 IU/kg., with regular admission every two weeks. The maximum ADAMTS13 activity after rADAMTS13 treatment was 86% of normal activity and 8 % with FFP) with through activity of 2 - 3 % after 2 weeks. Treatment was well tolerated, without allergic reaction or other side effects. Symptoms such as headache, fatigue, confusion and foggy memory disappeared, renal parameters normalized with normal blood count. A patient has not experienced TTP events with normal laboratory tests in 19 -months follow up.

**Copyright Information:** (Online) ISSN: 2572-9241
©2026 The Author(s). HemaSphere is published by John Wiley & Sons Ltd on behalf of European Hematology Association. This is an open access Abstract Book distributed under the Attribution-NonCommercial-NoDerivs (CC BY-NC-ND), which allows third parties to download the articles and share them with others as long as they credit the author and the Abstract Book, but they cannot change the content in any way or use them commercially.
**Abstract Book Citations:** Authors, Title, HemaSphere, 2026;10:(S1):pages. The abstract book DOIs can be found on the "Author Information" page.
**Disclaimer:** Articles published in the journal HemaSphere exclusively reflect the opinions of the authors. The authors are responsible for all content in their abstracts including accuracy of the facts, statements, citing resources, etc.

**Summary/Conclusion**

This is the first Croatian experience with recombinant ADAMTS13, which is very effective and safe for patiens with cTTP. It restores normal hemostasis, prevents TTP relapses, with higher efficacy compared to standard therapy.

**Copyright Information:** (Online) ISSN: 2572-9241
©2026 The Author(s). HemaSphere is published by John Wiley & Sons Ltd on behalf of European Hematology Association. This is an open access Abstract Book distributed under the Attribution-NonCommercial-NoDerivs (CC BY-NC-ND), which allows third parties to download the articles and share them with others as long as they credit the author and the Abstract Book, but they cannot change the content in any way or use them commercially.
**Abstract Book Citations:** Authors, Title, HemaSphere, 2026;10:(S1):pages. The abstract book DOIs can be found on the "Author Information" page.
**Disclaimer:** Articles published in the journal HemaSphere exclusively reflect the opinions of the authors. The authors are responsible for all content in their abstracts including accuracy of the facts, statements, citing resources, etc.

### (PB4320) COVID-19 ASSOCIATED THROMBOTIC THROMBOCYTOPENIC PURPURA – THE USE OF REFLECTIVE TESTS

**Topic:** 33. Platelet disorders

Athanasia Agorasti*[1]

[1]*General Hospital of Xanthi, Haematology Laboratory, Xanthi, Greece;*

### Background

Coronavirus disease-2019 (COVID-19) caused by SARS-CoV-2 present with several immune manifestations, such as immune-mediated thrombotic thrombocytopenia. Acquired form of Thrombotic Thrombocytopenic Purpura (TTP) is caused by the development of inhibitory IgG autoantibody against the ADAMTS13 metalloprotease. TTP following SARS-CoV-2 infection has been reported in the literature. The International Organization for Standardization (ISO) 15189:2022 for Medical Laboratories places a strong emphasis on clinical interpretation and, if required, advisory services.

### Aims

The aim of this study is to report the laboratory workflow for thrombocytopenia investigation in an outpatient with COVID-19 following the suitable guidelines and in order to meet ISO 15189:2022 requirements.

### Methods

The core laboratory received blood samples drawn from a 44-year-old woman, who was referred to the Emergency Department. according to the clinical information she was suffering from COVID-19 (diagnosed as outpatient) and nose bleeding. Full blood count, coagulation tests, and routine biochemistry tests were requested.

### Results

Full blood count on Sysmex XN-1000™ analyzer revealed anemia (Hb 9 g/dL) and thrombocytopenia (PLT 5 x$10^9$/L), total white blood cells count of 6,55 x$10^9$/L, an absolute neutrophil count of 4,85 x$10^9$/L. Prothrombin time, activated partial thromboplastin time and fibrinogen levels were normal. Renal and liver function tests were abnormal (urea 76 mg/dL, creatinine 1,39 mg/dL, aspartate transaminase-AST 93 U/L, alanine transaminase-ALT 39 U/L). The blood smear examination on CellaVision® DC-1 (an intelligent digital hematology microscopy) revealed many schistocytes (>5%), polychromasia (8%) and thrombocytopenia (PLT 4,7 x$10^9$/L). As TTP, α life-threatening thrombotic microangiopathy, was suspected, we proceeded to reflective tests for hemolysis investigation: reticulocytes automated measurement (RET 4,5%), total bilirubin 2,89 mg/dL with an indirect bilirubin of 1,86 mg/Dl, serum lactate dehydrogenase levels of 1871 U/L, and undetectable haptoglobin levels (0,0 mg/dL). All results suggesting microangiopathic hemolysis. Special tests ADAMTS13 activity testing and ADAMTS13 antibody testing were not available.

### Summary/Conclusion

Thrombotic Thrombocytopenic Purpura should be suspected in COVID-19 patients who present with anemia and thrombocytopenia. In such life-threatening emergency conditions and in the context of ISO 15189:2022 requirements, the laboratory staff should execute all reflective tests added to existing requests in order to support the diagnosis as soon as possible.

**Copyright Information:** (Online) ISSN: 2572-9241
©2026 The Author(s). HemaSphere is published by John Wiley & Sons Ltd on behalf of European Hematology Association. This is an open access Abstract Book distributed under the Attribution-NonCommercial-NoDerivs (CC BY-NC-ND), which allows third parties to download the articles and share them with others as long as they credit the author and the Abstract Book, but they cannot change the content in any way or use them commercially.
**Abstract Book Citations:** Authors, Title, HemaSphere, 2026;10:(S1):pages. The abstract book DOIs can be found on the “Author Information” page.
**Disclaimer:** Articles published in the journal HemaSphere exclusively reflect the opinions of the authors. The authors are responsible for all content in their abstracts including accuracy of the facts, statements, citing resources, etc.

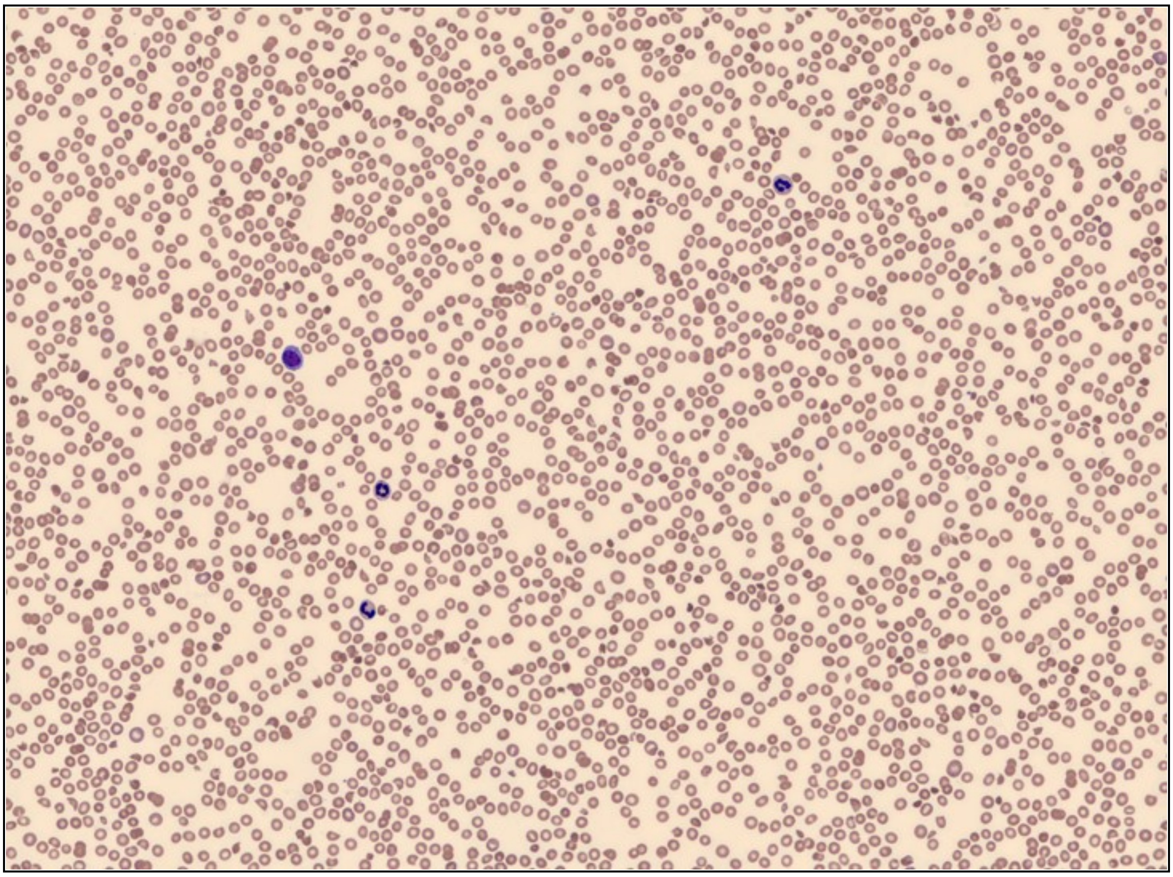

**Copyright Information:** (Online) ISSN: 2572-9241
©2026 The Author(s). HemaSphere is published by John Wiley & Sons Ltd on behalf of European Hematology Association. This is an open access Abstract Book distributed under the Attribution-NonCommercial-NoDerivs (CC BY-NC-ND), which allows third parties to download the articles and share them with others as long as they credit the author and the Abstract Book, but they cannot change the content in any way or use them commercially.
**Abstract Book Citations:** Authors, Title, HemaSphere, 2026;10:(S1):pages. The abstract book DOIs can be found on the "Author Information" page.
**Disclaimer:** Articles published in the journal HemaSphere exclusively reflect the opinions of the authors. The authors are responsible for all content in their abstracts including accuracy of the facts, statements, citing resources, etc.

### (PB4321) SEVERE VITAMIN B12 DEFICIENCY PRESENTING AS PSEUDO-THROMBOTIC MICROANGIOPATHY MIMICKING TTP

**Topic:** 33. Platelet disorders

Nour Gaaloul*[1], Guermazi Monia[1], Firas Cherif[1], Wafa Chenbah[1], Haifa Regaieg[1], Kmira Zahra[1], Nesrine Ben Sayed[1], Monia Zaier[2], Bouteraa Walid[2], Yosra Ben Youssef[3]

*[1]Farhat Hached University Hospital Sousse, Hematology Department, Sousse, Tunisia;[2]Farhat Hached university hospital, Hematology Department, sousse, Tunisia; [3]Farhat Hached University Hospital Sousse, Hematology Department, sousse, Tunisia;*

### Background

Immune-mediated thrombotic thrombocytopenic purpura (TTP) is a hematologic emergency requiring immediate plasma exchange. Rarely, severe vitamin B12 deficiency may present as pseudo–thrombotic microangiopathy (pseudo-TMA), creating a major diagnostic pitfall.

### Aims

To highlight the importance of recognizing vitamin B12 deficiency in patients presenting with a TTP-like syndrome

### Methods

We report the case of a 68-year-old man admitted with acute confusion and functional decline. Clinical, hematological, and biochemical data were analyzed, including PLASMIC score and ADAMTS13 activity.

### Results

Initial evaluation revealed severe pancytopenia (hemoglobin 1.6 g/dL, platelets $8\times10^9$/L), schistocytosis (5–6%), elevated LDH, indirect hyperbilirubinemia, mild renal impairment, fever, and neurological symptoms, fulfilling the classical TTP pentad. The PLASMIC score was 5, prompting urgent plasma exchange and corticosteroids.
After two sessions, the absence of hematological improvement raised suspicion of an alternative diagnosis. Repeat peripheral smear showed hypersegmented neutrophils and profound reticulocytopenia. ADAMTS13 activity subsequently returned within the normal range. Serum vitamin B12 level was severely decreased (<83 pg/mL).
Plasma exchange was discontinued and parenteral vitamin B12 was initiated, leading to rapid and sustained hematologic recovery within one week.

### Summary/Conclusion

Severe vitamin B12 deficiency can closely mimic TTP and expose patients to unnecessary plasma exchange. Normal ADAMTS13 activity, profound anemia with reticulocytopenia, and hypersegmented neutrophils are key diagnostic clues. Early recognition is essential to avoid invasive therapy and to initiate prompt vitamin replacement.

**Copyright Information:** (Online) ISSN: 2572-9241
©2026 The Author(s). HemaSphere is published by John Wiley & Sons Ltd on behalf of European Hematology Association. This is an open access Abstract Book distributed under the Attribution-NonCommercial-NoDerivs (CC BY-NC-ND), which allows third parties to download the articles and share them with others as long as they credit the author and the Abstract Book, but they cannot change the content in any way or use them commercially.
**Abstract Book Citations:** Authors, Title, HemaSphere, 2026;10:(S1):pages. The abstract book DOIs can be found on the “Author Information” page.
**Disclaimer:** Articles published in the journal HemaSphere exclusively reflect the opinions of the authors. The authors are responsible for all content in their abstracts including accuracy of the facts, statements, citing resources, etc.

### (PB4322) QUALITY OF LIFE AND JOINT HEALTH AFTER 5 YEARS OF EMICIZUMAB PROPHYLAXIS IN ADULTS WITH HAEMOPHILIA A AND ARTHROPATHY

**Topic:** 34. Bleeding disorders (congenital and acquired)

Karla Rener*[1, 2], Domen Plut[2, 3], Petra Dovč[4], Irena Preloznik Zupan[1, 2]

*[1]University Medical Centre Ljubljana, Department of Hematology, Ljubljana, Slovenia;[2]Faculty of Medicine, University of Ljubljana, Ljubljana, Slovenia; [3]University Medical Centre Ljubljana, Institute of Radiology, Ljubljana, Slovenia; [4]University Medical Centre Ljubljana, Orthopedic Clinic, Ljubljana, Slovenia;*

### Background

Severe haemophilia A (HA) complicated by haemophilic arthropathy substantially impairs health-related quality of life (HRQoL). Although emicizumab has demonstrated safety and efficacy in clinical trials, long-term real-world data in adults without factor VIII (FVIII) inhibitors and with established arthropathy remain scarce.

### Aims

To evaluate the long term impact of emicizumab prophylaxis on HRQoL and joint health over 5 years in adults with HA without FVIII inhibitors and with haemophilic arthropathy treated at the Slovenian Comprehensive Haemophilia Treatment Centre.

### Methods

This prospective observational study included all eligible adults with HA who initiated emicizumab prophylaxis between February 2020 and July 2021. HRQoL and joint health were assessed at baseline, at 1 year, and at 5 years using the Short Form-36 version 2 (SF-36v2) questionnaire and the mean HEAD-US score per joint, respectively. Differences across time points were analysed using the Friedman test, with post-hoc Wilcoxon signed-rank tests for pairwise comparisons.

### Results

Seventeen patients were evaluable at baseline; the median age was 54 years (range 31–65), 16 (94.1%) had severe HA, 13 (76.5%) had previously received FVIII prophylaxis, and 12 (70.6%) had one or more prosthetic joints. Median follow-up for the entire cohort was 5.2 years (range 1.1–5.8 years). Two patients (11.8%) discontinued emicizumab: one after a myocardial infarction with bleeding during dual antiplatelet therapy (1.1 years) and one after intracranial bleeding requiring prolonged FVIII treatment (1.2 years). Fifteen patients completed 5-year follow-up.
The median annual bleeding rate decreased from 5 (range 0–40) at baseline to 1 (range 0–4) at year 1 and remained low at year 5 (1, range 0–5) . Significant improvements were observed in Physical Functioning ($p=0.017$), Social Functioning ($p=0.014$), General Health ($p=0.011$), Bodily Pain ($p=0.009$), and the Physical Component Summary score ($p=0.031$). Improvements occurred within the first year and were sustained through year 5, with further improvement noted only in Bodily Pain. Other SF-36v2 domains and the Mental Component Summary did not show significant changes. HEAD-US scores remained stable over time ($p=0.46$).

### Summary/Conclusion

In this long-term real-world cohort of adults with HA and established haemophilic arthropathy, emicizumab prophylaxis was associated with sustained improvement or stability of HRQoL, durable bleeding control, and stable joint health over 5 years. These findings support the long-term use of emicizumab in adults with advanced haemophilic arthropathy in routine clinical practice.

**Copyright Information:** (Online) ISSN: 2572-9241
©2026 The Author(s). HemaSphere is published by John Wiley & Sons Ltd on behalf of European Hematology Association. This is an open access Abstract Book distributed under the Attribution-NonCommercial-NoDerivs (CC BY-NC-ND), which allows third parties to download the articles and share them with others as long as they credit the author and the Abstract Book, but they cannot change the content in any way or use them commercially.
**Abstract Book Citations:** Authors, Title, HemaSphere, 2026;10:(S1):pages. The abstract book DOIs can be found on the "Author Information" page.
**Disclaimer:** Articles published in the journal HemaSphere exclusively reflect the opinions of the authors. The authors are responsible for all content in their abstracts including accuracy of the facts, statements, citing resources, etc.

### (PB4323) EVALUATING THE CONFORMITY TO ASH 2019 GUIDELINES IN PEDIATRIC ITP MANAGEMENT: CLINICAL AND ECONOMIC OUTCOMES AT A SAUDI ARABIAN TERTIARY CARE CENTER.

**Topic:** 34. Bleeding disorders (congenital and acquired)

Rafif Alahmadi*[1], Faisal Alghamdi[2], Ahad Almutairi[1], Ghaida Almohammadi[1], Asem Alamri[2], Ahmed Mousa[2]

*[1]King Salman bin Abdulaziz Medical City, General pediatric, Madinah, Saudi Arabia;[2]King Salman bin Abdulaziz Medical City, Pediatric Hematology/ Oncology, Madinah, Saudi Arabia;*

### Background

Immune thrombocytopenia (ITP) is a common hematologic disorder in pediatric patients. While the 2019 American Society of Hematology (ASH) guidelines recommend observation for children with non-severe bleeding, clinical practice often varies. This study evaluates the management patterns, outcomes, and economic impact of adherence to ASH guidelines in a tertiary center in Saudi Arabia.

### Aims

To assess adherence to ASH 2019 in the management of newly diagnosed ITP, identify predictors of inappropriate therapy, and evaluate associated clinical outcomes and economic impact.

### Methods

A retrospective cohort study was conducted at King Salman bin Abdulaziz Medical City (KSAMCS), including 73 pediatric patients aged 6 months – 14 years with newly diagnosed ITP over 5 5-year period ending in 2025. Demographic, clinical, laboratory, management, outcome, and cost data were extracted from electronic medical records. Management was classified as either appropriate or inappropriate based on the 2019 ASH recommendations. Statistical analyses included chi-square/Fisher's exact test and Mann-Whitney tests. Univariate and multivariate logistic regression to identify predictors of treatment initiation and chronic ITP. Significance at p<0.05.

### Results

The mean age was 4.99 years. Treatment was initiated in 68.5% of cases (57.5% IVIG, 21.9% corticosteroids), while 31.5% underwent observation. Inappropriate therapy—pharmacologic treatment for Grade 0–1 bleeding—occurred in 61.6%. Inappropriate therapy was significantly associated with emergency room (ER) admission (p=0.003), presence of bleeding symptoms (p<0.001), and lower initial platelet counts (p=0.002). While inappropriate therapy (active treatment in mild cases) yielded higher complete remission rates at 72 hours (42.2% vs. 15.4%; p=0.006), it did not significantly improve long-term remission rates at 3 months. Crucially, appropriate therapy was associated with significantly lower median hospitalization costs ($697.55 vs. $2,284.34; p=0.001) and fewer ER revisits within one month (p=0.043).

**Copyright Information:** (Online) ISSN: 2572-9241
©2026 The Author(s). HemaSphere is published by John Wiley & Sons Ltd on behalf of European Hematology Association. This is an open access Abstract Book distributed under the Attribution-NonCommercial-NoDerivs (CC BY-NC-ND), which allows third parties to download the articles and share them with others as long as they credit the author and the Abstract Book, but they cannot change the content in any way or use them commercially.
**Abstract Book Citations:** Authors, Title, HemaSphere, 2026;10:(S1):pages. The abstract book DOIs can be found on the "Author Information" page.
**Disclaimer:** Articles published in the journal HemaSphere exclusively reflect the opinions of the authors. The authors are responsible for all content in their abstracts including accuracy of the facts, statements, citing resources, etc.

**Summary/Conclusion**

A key finding of this study is a persistent tendency toward overtreatment in children with minimal or no bleeding, despite clear ASH 2019 recommendations favoring observation. Much of this overtreatment appears driven by clinician anxiety surrounding severe thrombocytopenia, especially platelet counts <10 × $10^9$/L, even though evidence shows that bleeding risk correlates more strongly with clinical symptoms than with platelet numbers alone.
Our results demonstrate that overtreatment offers no long-term clinical advantage. Moreover, unnecessary pharmacologic therapy exposes children to avoidable risks, including adverse effects of IVIG and steroids. These findings emphasize the importance of reinforcing guideline-based care.

This abstract was previously presented at the ASPHO 2026 Annual Conference and published in Pediatric Blood & Cancer as part of the ASPHO 2026 Conference Abstract Supplement (Wiley Periodicals LLC).

**Figure 5: Timeline curves for platelets at different time points comparing treatment over time**

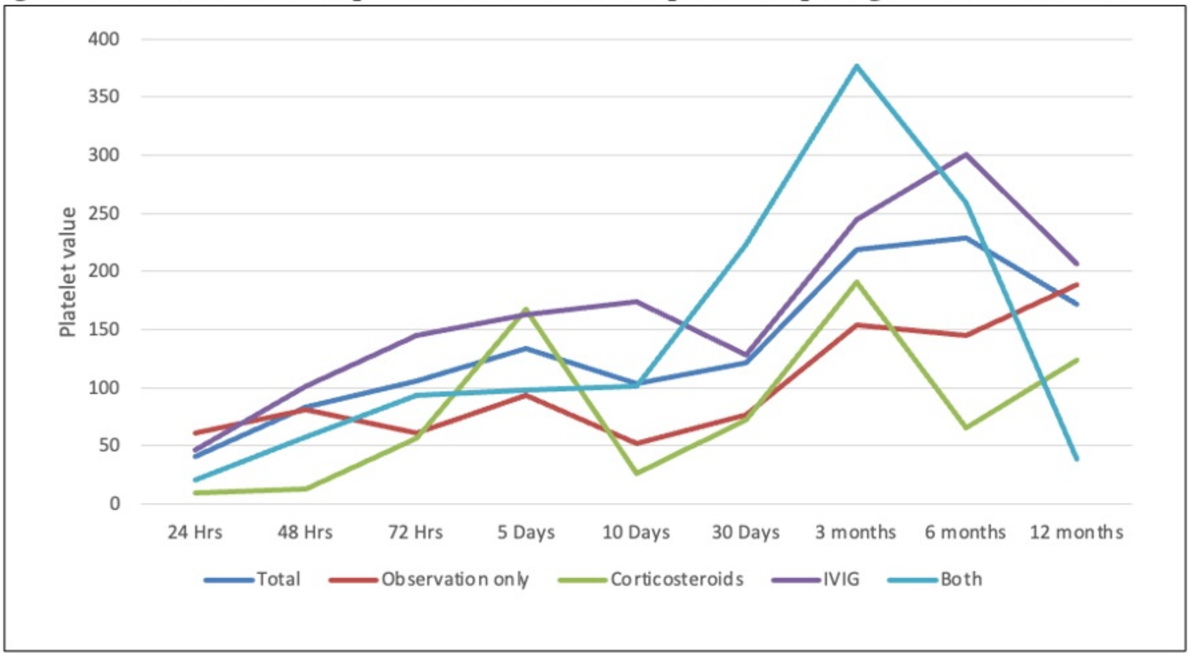

**Copyright Information:** (Online) ISSN: 2572-9241
©2026 The Author(s). HemaSphere is published by John Wiley & Sons Ltd on behalf of European Hematology Association. This is an open access Abstract Book distributed under the Attribution-NonCommercial-NoDerivs (CC BY-NC-ND), which allows third parties to download the articles and share them with others as long as they credit the author and the Abstract Book, but they cannot change the content in any way or use them commercially.
**Abstract Book Citations:** Authors, Title, HemaSphere, 2026;10:(S1):pages. The abstract book DOIs can be found on the "Author Information" page.
**Disclaimer:** Articles published in the journal HemaSphere exclusively reflect the opinions of the authors. The authors are responsible for all content in their abstracts including accuracy of the facts, statements, citing resources, etc.

### (PB4324) REPURPOSED ALK INHIBITORS IMPAIR PLASMA CELL SECRETORY FUNCTION THROUGH LTK INHIBITION

**Topic:** 34. Bleeding disorders (congenital and acquired)

Thea Kristin Våtsveen*[1], Valgerdur Bjørnsdottir[1], Elias Tjarnhage[2], John Imbery[2], Katrine Lund[1], Peter Hofgaard[2], Ankush Sharma[1], Waleed Ghanima[3], Pål Andre Holme[4], Hoa T. T. Tran[5], Britt Nakken[2], Hesso Farhan[6], Ludvig Munthe[1]

*[1]Precision immunotherapy alliance (PRIMA) Institute of Clinical Medicine, University of Oslo, Department of immunology, Oslo, Norway; [2]Department of clinical medicine, Department of immunology, Oslo, Norway; [3]Østfold Hospital Trust, Department of Medicine, Hematology-Oncology and research, Østfold Hospital Trust, Kalnes,, Kalnes, Norway; [4]Oslo University hospital, Department of Haematology, Oslo, Norway; [5]Akershus University Hospital, Department of Haematology, Lørenskog, Norway; [6]Medical University of Innsbruck, Institute of Patophysiology, Innsbruck, Austria;*

### Background

Plasma cells are highly secretory cells producing large amounts of immunoglobulins and is therefor dependent on endoplasmic reticulum (ER) proteostasis to maintain secretory capacity. Disruption of ER secretory pathways can induce unfolded protein response (UPR), ER stress and apoptosis. Leukocyte tyrosine kinase (LTK) is the first known ER-resident receptor tyrosine kinase, and responds to secretory cargo load and regulates ER secretory capacity. LTK has a sharing ~80% kinase-domain similarity to anaplastic lymphoma kinase (ALK), raising the possibility that clinically available ALK inhibitors might inhibit LTK and thereby perturb plasma cell secretion. Given that autoantibody-secreting plasma cells drive diseases such as immune thrombocytopenia (ITP), ITP provides a clinical context in which targeting plasma cell secretion could be therapeutically relevant

### Aims

To determine whether inhibition of LTK via repurposed ALK inhibitors disrupts plasma cell secretory function in a non-cancerous setting.

### Methods

LTK expression across B-cell maturation and its correlation with immunoglobulin and ER-gene programs were analyzed from transcriptomic datasets. CD19+ cells from healthy donors and ITP patient PBMCs were differentiated to plasmablasts/plasma cells using ImmunoCult. LTK subcellular localization was examined by immunofluorescence with Sec31A. ALK inhibitors; ceritinib, entrectinib, brigatinib, crizotinib, alectinib and lorlatinib were tested for effects on viability (CellTiter-Glo), plasma cell frequency (IRF4+/BLIMP1+ by flow cytometry), intracellular light-chain retention (flow cytometry), and secreted IgG (ELISA). ER-stress induction was assessed by CHOP and BiP expression (western blot). An in vivo xenograft with human PBMCs evaluated entrectinib effects on human bone marrow plasma cells.

### Results

LTK expression increases through B-cell maturation, peaks in bone marrow plasma cells, and significantly correlates with IgH and ER-secretory genes. LTK co-localized with ER-marker Sec31A. In plasma cells the IC50s of the inhibitors are in the low micromolar range (0.9–3.6 µM) in both healthy-donor and ITP patient-derived plasma cells. Ceritinib and entrectinib decreased viable plasma cell frequency and produced rapid intracellular retention of light chains with concomitant reduction in secreted IgG. Treatment upregulated CHOP and BiP, consistent with UPR activation and ER stress preceding apoptosis. In vivo, entrectinib markedly reduced human plasma cell frequency in bone marrow ($p = 0.0014$). Drug effect concordance and lack of expression of common off-targets in B-cells support an LTK-mediated mechanism.

**Copyright Information:** (Online) ISSN: 2572-9241
©2026 The Author(s). HemaSphere is published by John Wiley & Sons Ltd on behalf of European Hematology Association. This is an open access Abstract Book distributed under the Attribution-NonCommercial-NoDerivs (CC BY-NC-ND), which allows third parties to download the articles and share them with others as long as they credit the author and the Abstract Book, but they cannot change the content in any way or use them commercially.
**Abstract Book Citations:** Authors, Title, HemaSphere, 2026;10:(S1):pages. The abstract book DOIs can be found on the "Author Information" page.
**Disclaimer:** Articles published in the journal HemaSphere exclusively reflect the opinions of the authors. The authors are responsible for all content in their abstracts including accuracy of the facts, statements, citing resources, etc.

### Summary/Conclusion

LTK is a central regulator of plasma cell secretory homeostasis. Pharmacologic inhibition using repurposed ALK inhibitors impairs antibody secretion, triggers ER-stress/UPR and reduces plasma cell survival both in vitro and in vivo. These findings highlight LTK inhibition as a strategy to directly target pathological secretory plasma cells; ITP serves as a clinically relevant model for applications where eliminating autoantibody-secreting plasma cells may be beneficial. Further work should refine LTK-selective inhibitors and elucidate precise molecular mechanisms.

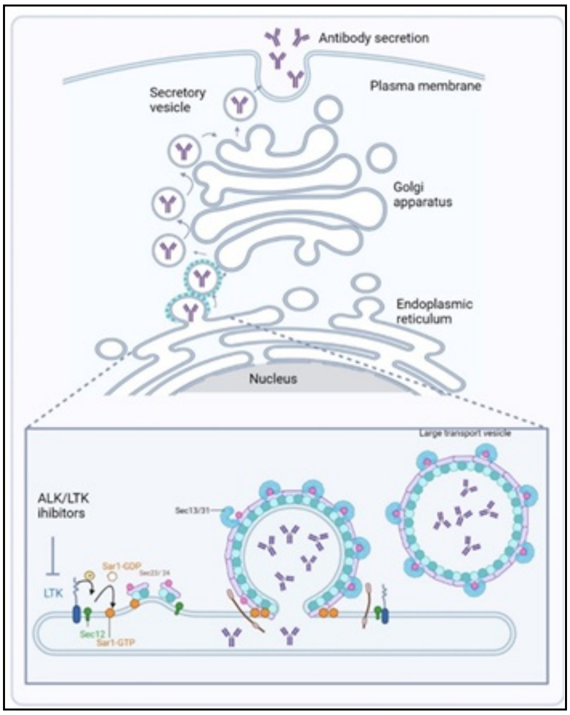

Figure 1. Illustration showing LTKs involvement in secretion from the ER. Figure created with Biorender

**Copyright Information:** (Online) ISSN: 2572-9241
©2026 The Author(s). HemaSphere is published by John Wiley & Sons Ltd on behalf of European Hematology Association. This is an open access Abstract Book distributed under the Attribution-NonCommercial-NoDerivs (CC BY-NC-ND), which allows third parties to download the articles and share them with others as long as they credit the author and the Abstract Book, but they cannot change the content in any way or use them commercially.
**Abstract Book Citations:** Authors, Title, HemaSphere, 2026;10:(S1):pages. The abstract book DOIs can be found on the "Author Information" page.
**Disclaimer:** Articles published in the journal HemaSphere exclusively reflect the opinions of the authors. The authors are responsible for all content in their abstracts including accuracy of the facts, statements, citing resources, etc.

### (PB4325) HEALTHCARE UTILIZATION AND TREATMENT PROFILE IN AUTOIMMUNE HEMOLYTIC ANEMIA IN COLOMBIA: A REAL-WORLD STUDY

**Topic:** 34. Bleeding disorders (congenital and acquired)

Renato Watanabe de Oliveira[1], Ligia Coelho[1], Gabriela Kanevsky*[2], Thais Zamboni Berra[3], Gustavo Pires Matheus[3], Claudia Casas[4, 5]

*[1]Johnson & Johnson Brasil, São Paulo, Brazil; [2]Johnson & Johnson Argentina, Buenos Aires, Argentina; [3]IQVIA, São Paulo, Brazil; [4]Fundación Universitaria de Ciencias de la Salud, Facultad de Medicina, Bogota, Colombia,[5]Hospital de San José, Servicio de Hematología, Bogota, Colombia;*

### Background

Autoimmune hemolytic anemia (AIHA) is a rare immune-mediated disorder that can lead to severe, life-threatening complications, yet no specific approved therapies exist. In Colombia, the lack of real-world evidence on AIHA management limits clinical and policy decision-making.

### Aims

To describe the resource use and treatment profile of patients with primary AIHA submitted to systemic second or subsequent treatment in Colombia, between 2018 and 2021.

### Methods

This claims-based study used the RIPS database (*Registros Individuales de Prestación de Servicios de Salud*), encompassing public and private healthcare settings. Adults (≥18 years) with an initial AIHA-related claim (ICD-10 D59.1; ≥1 inpatient or ≥2 outpatient claims) between 2018–2021 and at least 12 months of follow-up were included, while drug-induced AIHA (D59.0) or evidence of secondary AIHA were excluded. Treatments assessed included immunosuppressants and rescue therapies (IVIg, blood transfusion, plasmapheresis); corticosteroid use and splenectomy could not be evaluated. Demographics, treatment patterns, and hospitalizations were summarized descriptively for the primary AIHA patients, with rates reported per patient-year.

### Results

We identified 1,871 patients with AIHA, including 904 (48.3%) with primary and 967 (51.7%) with secondary disease; only patients with primary AIHA were included in the following analyses. Among primary AIHA patients, the mean age was 50.9 ± 20.1 years, 67.9% were female, and most resided in Bogotá (17.6%), Antioquia (14.3%), and Valle del Cauca (10.2%). Approximately 15.7% (n: 142) of patients received at least one second-line or rescue treatment, with 66.2% of them using immunosuppressants (azathioprine: 59.6%; rituximab: 45.7%); 33.8% were treated only with rescue therapies in the period. Among primary AIHA patients, rescue therapies were required by 6.4%; of them, with rescue therapy use rate of 0.2 (95%CI: 0.1 – 0.3) PPY; among these patients, 94.8% received blood transfusion. Hospitalizations occurred in 29.2% of patients (median stay 4 days), and 20.1% of these required ICU care (median 1 day), corresponding to a hospitalization rate of 1 (95%CI 0.7 – 1.4) event PPY.

**Copyright Information:** (Online) ISSN: 2572-9241
©2026 The Author(s). HemaSphere is published by John Wiley & Sons Ltd on behalf of European Hematology Association. This is an open access Abstract Book distributed under the Attribution-NonCommercial-NoDerivs (CC BY-NC-ND), which allows third parties to download the articles and share them with others as long as they credit the author and the Abstract Book, but they cannot change the content in any way or use them commercially.
**Abstract Book Citations:** Authors, Title, HemaSphere, 2026;10:(S1):pages. The abstract book DOIs can be found on the “Author Information” page.
**Disclaimer:** Articles published in the journal HemaSphere exclusively reflect the opinions of the authors. The authors are responsible for all content in their abstracts including accuracy of the facts, statements, citing resources, etc.

**Summary/Conclusion**

Primary AIHA imposes a notable clinical burden, with a group of patients requiring immunosuppressants, recurrent need for acute rescue interventions, and significant hospitalization and ICU rates. These findings highlight persistent gaps in long-term disease control and the need for more effective management strategies and new therapeutic options, though interpretation should consider the inherent limitations of claims-based data.

**Copyright Information:** (Online) ISSN: 2572-9241
©2026 The Author(s). HemaSphere is published by John Wiley & Sons Ltd on behalf of European Hematology Association. This is an open access Abstract Book distributed under the Attribution-NonCommercial-NoDerivs (CC BY-NC-ND), which allows third parties to download the articles and share them with others as long as they credit the author and the Abstract Book, but they cannot change the content in any way or use them commercially.
**Abstract Book Citations:** Authors, Title, HemaSphere, 2026;10:(S1):pages. The abstract book DOIs can be found on the "Author Information" page.
**Disclaimer:** Articles published in the journal HemaSphere exclusively reflect the opinions of the authors. The authors are responsible for all content in their abstracts including accuracy of the facts, statements, citing resources, etc.

### (PB4326) HEALTH-RELATED QUALITY OF LIFE OUTCOMES AND HEALTHCARE RESOURCE UTILISATION IN PATIENTS WITH COLD AGGLUTININ DISEASE (CAD) FROM THE SECOND INTERIM ANALYSIS OF THE CADENCE REGISTRY

**Topic:** 34. Bleeding disorders (congenital and acquired)

Francis Shupo*[1], Mounia Belouche[1], Yuxian Chen[2], Pascale Dequen-O'byrne[2], Louise Linsell[2], Keith R Abrams[2]

*[1]Recordati UK Ltd., Hemel Hempstead, United Kingdom;[2]Visible Analytics Ltd., Oxford, United Kingdom;*

### Background

Cold agglutinin disease (CAD) is a rare autoimmune hemolytic anemia characterized as a low-grade lymphoproliferative disorder of the bone marrow wherein autoantibodies bind to erythrocytes at temperatures ≤37°C, leading to complement-mediated hemolysis. CAD is associated with a substantial disease burden, including severe, debilitating fatigue and transfusion dependence which negatively impact health-related quality of life (HRQoL). CAD is also associated with an increased risk of thromboembolic events and mortality. CADENCE (Cold Agglutinin Disease Real-World Evidence Registry) is the first large, multinational, prospective registry study of adult CAD patients, designed to improve understanding of the natural history of the disease, treatment patterns, HRQoL outcomes, and healthcare resource utilisation (HCRU). The registry also includes patients treated with sutimlimab, a humanized monoclonal antibody that selectively inhibits the classical complement pathway by inactivating C1s.

### Aims

To investigate the impact of sutimlimab, the only approved therapy for CAD, on HRQoL outcomes and HCRU, compared to standard of care (SOC) in the real-world setting.

### Methods

HRQoL measures included the SF-36v2® physical and mental health component summary scores (PCS and MCS), and the FACIT-Fatigue Scale, collected at baseline, 6 and 12 months. Mixed-effect repeated measures models (MMRM) were fitted, including treatment group (sutimlimab vs. SOC), visit, treatment-by-visit interaction and prespecified baseline characteristics (age, sex, geographic region, and time from diagnosis to enrolment). Treatment was modelled as a time-varying covariate to account for treatment switching during follow-up. Incident data on hospitalisations, outpatient and emergency room visits were also collected and analysed using Poisson regression.

### Results

The analysis included 309 CAD patients with a median follow-up of 156 days (range: 1, 331), of which 35 patients were receiving sutimlimab at baseline. The remaining patients (SOC group) were receiving other drugs at baseline (n=94), treatment-naïve (n=127) or not currently treated (n=53). Five patients switched to sutimlimab during the study period (two were receiving other CAD treatments, and three no treatment at baseline). The overall least squares (LS) mean difference in SF-36 PCS across visits in patients receiving sutimlimab compared to SOC was 0.89 (95% CI: –2.47, 4.26), and 3.05 (95% CI: –0.53, 6.63) for SF-36 MCS. The FACIT-Fatigue subscale score was also numerically improved in patients receiving sutimlimab vs. SOC, with overall LS mean difference 3.22 (95% CI: –0.71, 7.16). Among patients receiving sutimlimab at baseline (n=35), 59 healthcare encounters were recorded during follow-up (none related to transfusion). In the SOC group (n=274), 193 healthcare encounters were reported (nine related to transfusion). Model-based incident rates are presented for each type of visit in the Figure; there were no statistically significant differences between groups.

**Copyright Information:** (Online) ISSN: 2572-9241
©2026 The Author(s). HemaSphere is published by John Wiley & Sons Ltd on behalf of European Hematology Association. This is an open access Abstract Book distributed under the Attribution-NonCommercial-NoDerivs (CC BY-NC-ND), which allows third parties to download the articles and share them with others as long as they credit the author and the Abstract Book, but they cannot change the content in any way or use them commercially.
**Abstract Book Citations:** Authors, Title, HemaSphere, 2026;10:(S1):pages. The abstract book DOIs can be found on the “Author Information” page.
**Disclaimer:** Articles published in the journal HemaSphere exclusively reflect the opinions of the authors. The authors are responsible for all content in their abstracts including accuracy of the facts, statements, citing resources, etc.

**Summary/Conclusion**

These findings provide real-world evidence supportive of the early real-world HRQoL and HCRU outcomes for sutimlimab, although interpretation is limited by the short duration of follow-up, and the small number of patients and events. Further planned analyses will inform the understanding of the long-term HRQoL and HCRU benefits of sutimlimab outside of the controlled clinical trial setting and its value in CAD patient management.

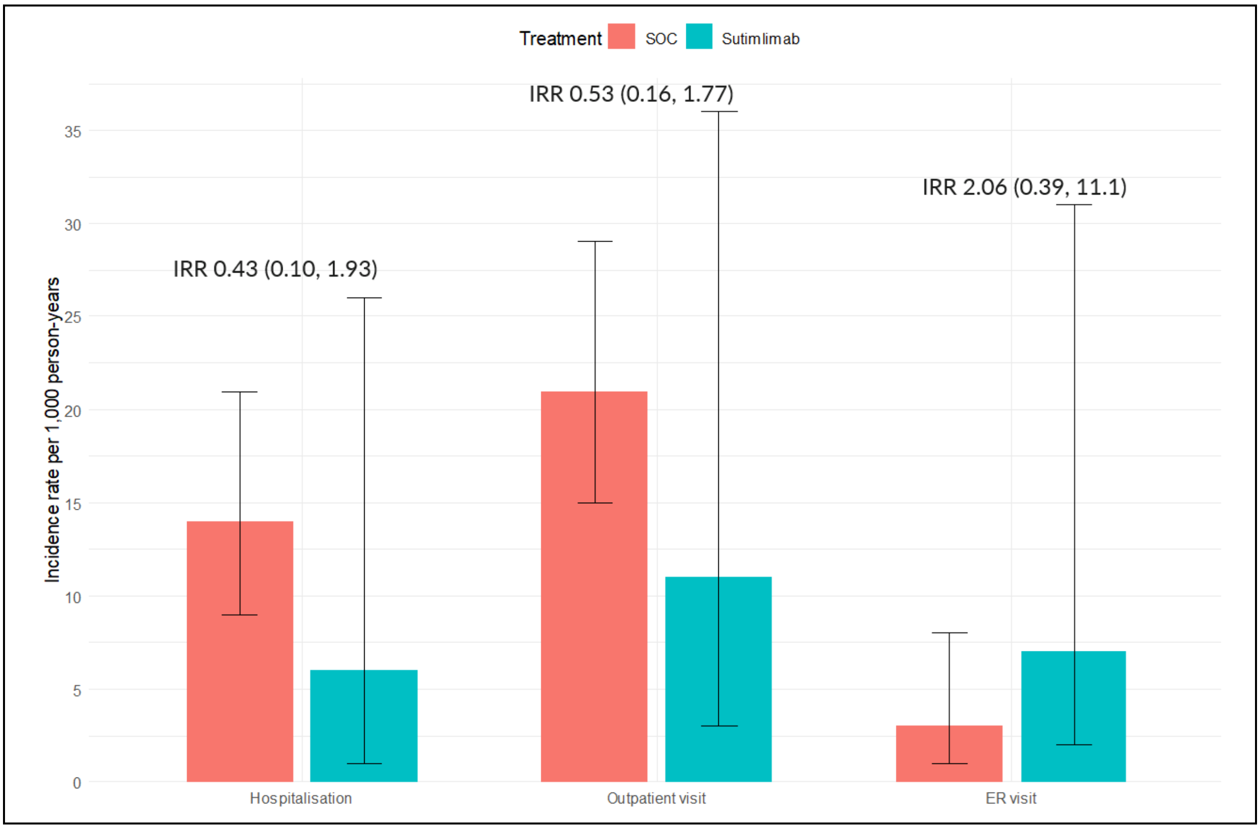

Figure: Model-based incidence rates and incidence rate ratios (IRR) for hospitalisation, outpatient visits, and emergency room (ER) visits by treatment group

**Copyright Information:** (Online) ISSN: 2572-9241
©2026 The Author(s). HemaSphere is published by John Wiley & Sons Ltd on behalf of European Hematology Association. This is an open access Abstract Book distributed under the Attribution-NonCommercial-NoDerivs (CC BY-NC-ND), which allows third parties to download the articles and share them with others as long as they credit the author and the Abstract Book, but they cannot change the content in any way or use them commercially.
**Abstract Book Citations:** Authors, Title, HemaSphere, 2026;10:(S1):pages. The abstract book DOIs can be found on the “Author Information” page.
**Disclaimer:** Articles published in the journal HemaSphere exclusively reflect the opinions of the authors. The authors are responsible for all content in their abstracts including accuracy of the facts, statements, citing resources, etc.

### (PB4328) REAL-WORLD TREATMENT PATTERNS AND CLINICAL OUTCOMES OF PATIENTS WITH PRIMARY AUTOIMMUNE HEMOLYTIC ANEMIA IN BRAZIL: A CLAIMS DATABASE STUDY

**Topic:** 34. Bleeding disorders (congenital and acquired)

Renato Watanabe de Oliveira[1], Ligia Coelho[1], Gabriela Kanevsky*[2], Thais Zamboni Berra[3], Gustavo Pires Matheus[3], Renato Sampaio Tavares[4]

*[1]Johnson & Johnson Brasil, São Paulo, Brazil; [2]Johnson & Johnson Argentina, Buenos Aires, Argentina; [3]IQVIA, São Paulo, Brazil; [4]Universidade Federal de Goiás, Faculdade de Medicina, Goiania, Brazil;*

### Background

Autoimmune hemolytic anemia (AIHA) is a rare disorder marked by immune-driven red blood cell destruction, in which a group of patients experience severe, potentially fatal disease requiring transfusions or intensive care. In Brazil, the scarcity of real-world evidence on AIHA management hampers informed clinical and policy decision-making.

### Aims

To describe the treatment profile of patients with primary AIHA submitted to second or subsequent-line treatment in the Brazilian public healthcare system, between 2013 and 2023.

### Methods

This retrospective observational study used claims data from DATASUS, which represents 75% of Brazilian population. We included adult patients (≥18 years old), with their first AIHA claim (ICD-10: D59.1; at least one inpatient or two outpatient claims) between 2013 and 2023, and with ≥12 months of follow-up. Patients with secondary or drug-induced AIHA (ICD-10 D59.0) were excluded. Treatments assessed included immunosuppressants, splenectomy or rescue therapies (IVIg, blood transfusion or plasmapheresis); corticosteroids are not available in the database. Demographics, treatment and hospitalizations were described using descriptive statistics, with rates calculated by number of events per patient-year (PPY).

### Results

A total of 2,893 patients with primary AIHA were identified. Patients had a mean age of 47.8 ± 19.2 years, were predominantly female (67.2%), and were mainly concentrated in the states of São Paulo (24.9%), Rio Grande do Sul (8.9%) and Minas Gerais (7.5%). Multimorbidity was frequent (mean 2.6 comorbidities), most commonly idiopathic thrombocytopenic purpura (2.1%) and septicemia (1.9%). A total of 1355 (46.8%) patients received systemic therapy; of them, 8.0% used immunosuppressants (cyclosporine: 69.7%; cyclophosphamide: 33.0%). Splenectomy was performed in 14.4% of total patients, 99.5% of them prior to immunosuppressant therapy. Of the total patients, 43.5%% required at least one rescue treatment in the period; of them, mainly blood transfusion (79.8%, mean use 1.2) and IVIg (20.8%; mean 4.3 administrations), with a rescue therapy rate of 2.3 (95%CI 2.0 – 2.6) uses PPY. Around 84% of all patients required at least one hospitalization in the period (median stay 7 days), corresponding to a hospitalization rate of 3 (95%CI 2.6 – 3.3) events PPY. Of all hospitalizations, 10.3% of them required ICU admission (median 10 days).

**Copyright Information:** (Online) ISSN: 2572-9241
©2026 The Author(s). HemaSphere is published by John Wiley & Sons Ltd on behalf of European Hematology Association. This is an open access Abstract Book distributed under the Attribution-NonCommercial-NoDerivs (CC BY-NC-ND), which allows third parties to download the articles and share them with others as long as they credit the author and the Abstract Book, but they cannot change the content in any way or use them commercially.
**Abstract Book Citations:** Authors, Title, HemaSphere, 2026;10:(S1):pages. The abstract book DOIs can be found on the "Author Information" page.
**Disclaimer:** Articles published in the journal HemaSphere exclusively reflect the opinions of the authors. The authors are responsible for all content in their abstracts including accuracy of the facts, statements, citing resources, etc.

**Summary/Conclusion**

Primary AIHA is associated with substantial clinical burden and intensive healthcare utilization, reflected in high dependence on rescue therapies and frequent hospitalizations. Our results suggest the need for new therapeutic strategies and treatment options for long-term disease control.

**Copyright Information:** (Online) ISSN: 2572-9241
©2026 The Author(s). HemaSphere is published by John Wiley & Sons Ltd on behalf of European Hematology Association. This is an open access Abstract Book distributed under the Attribution-NonCommercial-NoDerivs (CC BY-NC-ND), which allows third parties to download the articles and share them with others as long as they credit the author and the Abstract Book, but they cannot change the content in any way or use them commercially.
**Abstract Book Citations:** Authors, Title, HemaSphere, 2026;10:(S1):pages. The abstract book DOIs can be found on the "Author Information" page.
**Disclaimer:** Articles published in the journal HemaSphere exclusively reflect the opinions of the authors. The authors are responsible for all content in their abstracts including accuracy of the facts, statements, citing resources, etc.

### (PB4329) IMPACT OF EMICIZUMAB PROPHYLAXIS ON HEALTH-RELATED QUALITY OF LIFE IN SEVERE HEMOPHILIA A: LONG-TERM REAL-WORLD SINGLE-CENTER EXPERIENCE

**Topic:** 34. Bleeding disorders (congenital and acquired)

Ermelinda Longo*[1, 2], Uros Markovic[1, 3], Chiara Sorbello[1], Stephanie Grasso[1, 3], Antonino Giuffrida[4], Rita Chiaramonte[5], Alberto Corsaro[3], Salvatore La Penta[1], Gaetano Giuffrida[1, 3]

*[1]Hematology and Bone Marrow Transplatation Unit, A.O.U. Policlinico "G. Rodolico - S.Marco", Catania, Italy; [2]Post-Graduation School of Haematology, University of Catania, Catania, Italy; [3]Rare Hematological Diseases Unit, AOU Policlinico "G.Rodolico-S.Marco", Catania, Italy; [4]Radiology Department of AO "San Marco", A.O.U. Policlinico "G. Rodolico - San Marco", Catania, Italy., Catania, Italy; [5]Disability and Rehabilitation Unit, Department of Rehabilitation, Azienda Sanitaria Provinciale (ASP) of Catania, Catania, Italy;*

### Background

Emicizumab (EMI) is a bispecific monoclonal antibody that restores activated factor VIII activity independently from inhibitors. It has reshaped prophylactic management in Hemophilia A by providing sustained bleeding control through subcutaneous administration. Beyond bleeding reduction, its real-world impact on health-related quality of life (HRQoL) remains a key clinical outcome.

### Aims

Assess long-term impact of EMI prophylaxis on HRQoL and clinical outcomes in patients with severe Hemophilia A treated in routine practice.

### Methods

We conducted a retrospective single-center study including 16 male patients (median age 10.7 years, range 2.1–59.7, seven pediatric ones); five of them with inhibitors and receiving on-demand bypassing agents prior to EMI. Nine patients already had a median of four target joints (range 2-6). HRQoL was evaluated using the EQ-5D-5L before (pre-) and after (post-) EMI initiation. Clinical endpoints included annualized bleeding rate (ABR), treated ABR, hemarthrosis, Hemophilia Joint Health Score (HJHS), HEAD-US, and visual analogue scale (VAS). Pre- and post-EMI comparisons were performed using Wilcoxon signed-rank tests.

**Copyright Information:** (Online) ISSN: 2572-9241
©2026 The Author(s). HemaSphere is published by John Wiley & Sons Ltd on behalf of European Hematology Association. This is an open access Abstract Book distributed under the Attribution-NonCommercial-NoDerivs (CC BY-NC-ND), which allows third parties to download the articles and share them with others as long as they credit the author and the Abstract Book, but they cannot change the content in any way or use them commercially.
**Abstract Book Citations:** Authors, Title, HemaSphere, 2026;10:(S1):pages. The abstract book DOIs can be found on the "Author Information" page.
**Disclaimer:** Articles published in the journal HemaSphere exclusively reflect the opinions of the authors. The authors are responsible for all content in their abstracts including accuracy of the facts, statements, citing resources, etc.

**Results**

After a median follow-up of 53.5 months both total and treated ABR showed a numerical reduction in course of EMI compared to baseline, median 2 and 1 vs 0 episodes each respectively, with seven patients (44%) having zero bleeds. Annual hemarthrosis episodes declined significantly, respectively mean pre-EMI 1.1 episodes versus 0 post-EMI (p=0.046), with only two patients with hemarthrosis event in course of EMI. HEAD-US remained negative in six patients, improved in three patients (mean difference 11), and remained stable in another three due to permanent hemophilic arthropathy. Furthermore, HJHS showed a positive trend of improvement in seven patients with altered baseline values, with median pre- and post-EMI values of 35 and 22 respectively (p=0.058).
Out of 12 patients that had altered baseline HRQoL (median EQ-5D-5L value pre-EMI 10.5), six had complete normalization (50%), while in three the score improved (median value post-EMI 6) with a significant overall impact (p=0.002). Improvements were consistent across multiple domains, with the greatest benefit observed in usual activities (median pre-EMI 2.5, p=0.003), with enhanced ability to perform daily tasks, as well as mobility (median pre-EMI 2, p=0.014). Pain was evaluated using both VAS (median pre-EMI score 62.5) and EQ-5D-DL for pain (median pre-EMI 3) with significant reduction in both tests (p=0.003 and p=0.0067). Anxiety/depression also improved (p=0.014), while self-care showed a borderline positive trend (p=0.059). Individual median post-EMI EQ-5D-5L values were 1 for all categories. Positive overall HRQoL impact of EMI was confirmed even in a limited sample of five patients with inhibitors across all EQ-5D-5L score (p=0.04). They also suffered less from hemarthrosis (p=0.07) in course of EMI, and had less pain (VAS p=0.04).

**Summary/Conclusion**

In this long-term real-world cohort, emicizumab prophylaxis was associated with sustained improvements in patient-reported outcomes and functional status, particularly enhancing patients' ability to perform daily activities and pain reduction, alongside improvent in bleeding events, specifically hemarthrosis. These findings highlight the multidimensional benefit of EMI in routine practice, especially in inhibitor-positive patients, supporting its central role in modern Hemophilia A management.

**Copyright Information:** (Online) ISSN: 2572-9241
©2026 The Author(s). HemaSphere is published by John Wiley & Sons Ltd on behalf of European Hematology Association. This is an open access Abstract Book distributed under the Attribution-NonCommercial-NoDerivs (CC BY-NC-ND), which allows third parties to download the articles and share them with others as long as they credit the author and the Abstract Book, but they cannot change the content in any way or use them commercially.
**Abstract Book Citations:** Authors, Title, HemaSphere, 2026;10:(S1):pages. The abstract book DOIs can be found on the "Author Information" page.
**Disclaimer:** Articles published in the journal HemaSphere exclusively reflect the opinions of the authors. The authors are responsible for all content in their abstracts including accuracy of the facts, statements, citing resources, etc.

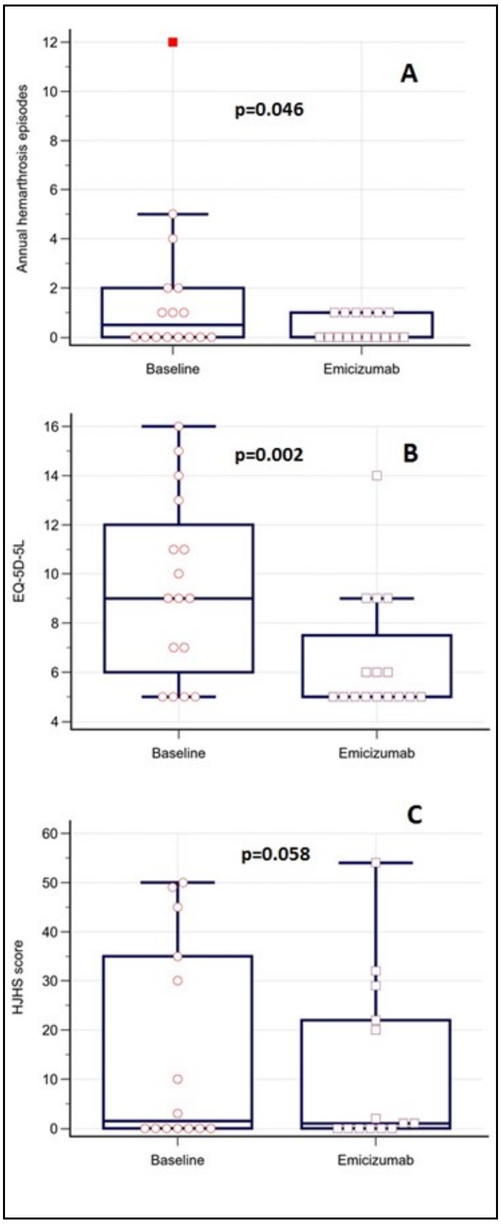

Figure 1. Paired dot plot analysis of annual hemarthrosis episodes (A), QoL score (EQ-5D-5L) (B) and HJHS (C) prior to and in course of emicizumab using Wilcoxon signed-rank test

**Copyright Information:** (Online) ISSN: 2572-9241
©2026 The Author(s). HemaSphere is published by John Wiley & Sons Ltd on behalf of European Hematology Association. This is an open access Abstract Book distributed under the Attribution-NonCommercial-NoDerivs (CC BY-NC-ND), which allows third parties to download the articles and share them with others as long as they credit the author and the Abstract Book, but they cannot change the content in any way or use them commercially.
**Abstract Book Citations:** Authors, Title, HemaSphere, 2026;10:(S1):pages. The abstract book DOIs can be found on the "Author Information" page.
**Disclaimer:** Articles published in the journal HemaSphere exclusively reflect the opinions of the authors. The authors are responsible for all content in their abstracts including accuracy of the facts, statements, citing resources, etc.

### (PB4330) MANAGEMENT OF PATIENTS WITH FACTOR VII DEFICIENCY IN SURGERY: A SINGLE-CENTER EXPERIENCE.

**Topic:** 34. Bleeding disorders (congenital and acquired)

Bolivar Luis Diaz Jordan*[1, 2]

*[1]Hospital General de Valdepeñas, Hematology, Valdepeñas, Spain;[2]Sociedad Castellano-Manchega de Hematología y Hemoterapia (SCMHH), Valdepeñas, Spain;*

### Background

Factor VII deficiency is a rare hemostasis disorder that commonly causes postoperative bleeding complications (depending on the level of intervention). Treatment with recombinant activated factor VII (rFVIIa) is the gold standard for this indication, although some adverse effects (especially thrombotic) are not uncommon and have been described in the literature.

### Aims

The aim of this study is to present our experience with ten scheduled major surgical procedures in patients with factor VII deficiency at a regional hospital.

### Methods

This retrospective, descriptive, single-center study compiles our experience with ten major surgical procedures in patients with factor VII deficiency at a Spanish regional hospital during the period 2020–2025. Preoperative, perioperative, and postoperative data, as well as demographic data, treatment, and side effects, were analyzed.

### Results

Ten surgical procedures were successfully performed, and no complications were observed in the nine patients with congenital FVII deficiency. The median FVII activity was 17.0% (range: 4.1–30.0%). Three tonsillectomies, three complex dental extractions, three orthopedic procedures, and one partial colectomy secondary to colon adenocarcinoma were performed. The median length of hospital stay was 8.5 days (range: 0–15 days). rFVIIa was administered in all procedures except for one dental extraction, which was performed using only antifibrinolytics without replacement. Fresh frozen plasma or packed red blood cells were not required in any case. No hemorrhagic or thrombogenic complications were observed.

### Summary/Conclusion

Patients with congenital FVII deficiency requiring surgery can be treated effectively and safely with rFVIIa and/or antifibrinolytics, depending on the procedure. rFVIIa was well tolerated, maintained effective hemostasis, and showed a good clinical prognosis after major surgery.

**Copyright Information:** (Online) ISSN: 2572-9241
©2026 The Author(s). HemaSphere is published by John Wiley & Sons Ltd on behalf of European Hematology Association. This is an open access Abstract Book distributed under the Attribution-NonCommercial-NoDerivs (CC BY-NC-ND), which allows third parties to download the articles and share them with others as long as they credit the author and the Abstract Book, but they cannot change the content in any way or use them commercially.
**Abstract Book Citations:** Authors, Title, HemaSphere, 2026;10:(S1):pages. The abstract book DOIs can be found on the "Author Information" page.
**Disclaimer:** Articles published in the journal HemaSphere exclusively reflect the opinions of the authors. The authors are responsible for all content in their abstracts including accuracy of the facts, statements, citing resources, etc.

### (PB4331) EARLY COAGULATION RECOVERY AFTER THERAPEUTIC LOW MOLECULAR WEIGHT HEPARIN IN TERM PREGNANCY CHALLENGES THE 24-HOUR DOGMA FOR NEURAXIAL ANESTHESIA

**Topic:** 34. Bleeding disorders (congenital and acquired)

Hélène Legardeur[1], Alexia Cuenoud[1], Maxime Zermatten[1], Alessandro Aliota[1], Francesco Grandoni[1], Lorenzo Alberio[1], Patrick Schoettker[1], David Baud[1], Mathilde Gavillet*[1]

[1]*Lausanne University Hospital, Obstetrics, Lausanne, Switzerland;*

### Background

Therapeutic anticoagulation during pregnancy poses a major challenge for peripartum management. Current guidelines require a 24-hour delay after the last full-dose low-molecular-weight heparin (LMWH) injection for safe epidural anesthesia placement. These recommendations apply to the general population and obstetric patients indistinctively, without considering the pregnancy-induced procoagulant context. Allowing earlier neuraxial anesthesia, the gold-standard analgesia during labor, would reduce maternal and fetal morbidity associated with general anesthesia and labor induction, thereby limiting immobilization during the highest-risk thromboembolic period. Anti-Xa activity is widely used to monitor LMWH treatment but is limited to pharmacodynamics assessment. Global coagulation assays (e.g. *ex vivo* thrombin generation assays) are of particular interest, as they integrate the overall coagulation potential.

### Aims

We aimed to characterize the evolution of overall coagulation balance following the last therapeutic dose of LMWH in term pregnant patients.

### Methods

This observational study included 26 full-term pregnant patients, receiving therapeutic dose LMWH. They were compared to 27 non-anticoagulated full-term pregnant patients, as well as non-anticoagulated non pregnant age- and gender-matched controls. Serial blood samples were collected at 4-hour intervals up to 24 hours after the last LMWH dose in the LMWH group; and once (at admission) for the controls. Standard coagulation tests, anti-Xa activity, surrogate markers of *in vivo* thrombin generation (prothrombin fragments 1 and 2, thrombin-antithrombin complexes, and D-dimers), and *ex vivo* thrombin generation were assessed.

### Results

Baseline demographic and routine coagulation tests were similar between groups. Anti-Xa activity was maximal 4 hours after injection, then declined progressively. Measurable residual anti-Xa activity persisted in 15/26 participants at 24 hours. Surrogate markers of *in vivo* thrombin formation were above the reference range in both pregnant groups throughout all time points. Ex vivo thrombin generation parameters similarly demonstrated substantial procoagulant context both in anticoagulated and non-anticoagulated pregnant individuals. The time-course shows an impact of LMWH that is maximal between 4 and 8 hours after injection with key parameters returning to values comparable to non-anticoagulated age- and gender-matched controls, i.e. values within or above the normal range, as soon as 12 hours post injection.

**Copyright Information:** (Online) ISSN: 2572-9241
©2026 The Author(s). HemaSphere is published by John Wiley & Sons Ltd on behalf of European Hematology Association. This is an open access Abstract Book distributed under the Attribution-NonCommercial-NoDerivs (CC BY-NC-ND), which allows third parties to download the articles and share them with others as long as they credit the author and the Abstract Book, but they cannot change the content in any way or use them commercially.
**Abstract Book Citations:** Authors, Title, HemaSphere, 2026;10:(S1):pages. The abstract book DOIs can be found on the “Author Information” page.
**Disclaimer:** Articles published in the journal HemaSphere exclusively reflect the opinions of the authors. The authors are responsible for all content in their abstracts including accuracy of the facts, statements, citing resources, etc.

### Summary/Conclusion

Pregnancy amplifies procoagulant drive and substantially blunts the biological impact of anticoagulation with LMWH. Using a comprehensive array of coagulation tests, we showed that in most full-term pregnant individuals, global coagulation potential is fully restored within 12 hours after the last LMWH injection. The data also show moderate correlation of anti-Xa activity and individual coagulation potential; with a 0.4 UI/ml cut-off seeming safe to extrapolate restored coagulation potential in this specific patient subgroup. The present study, albeit limited in size, supports that term pregnancy allows for and should prompt the reconsideration of the 24-hour interval, in favor of an individualized management plan, rather than the current practice based on a uniform "safety delay". These findings provide an opportunity for multidisciplinary discussion to improve the quality of care of high-risk pregnant persons.

**Figure 1:**

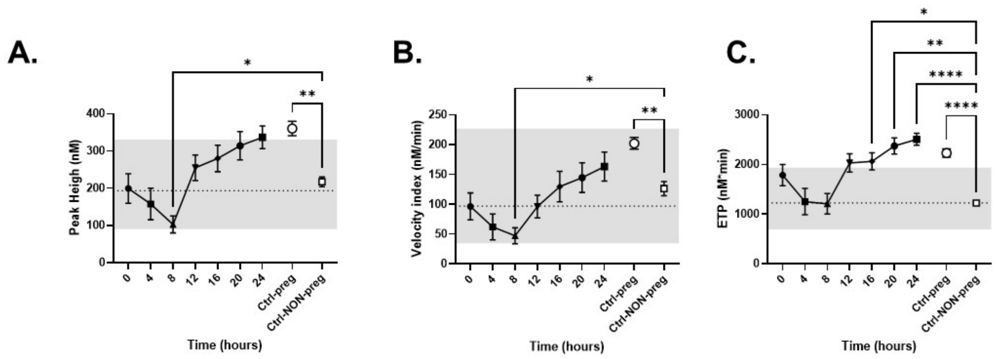

**Figure 1: *ex vivo* Thrombin generation parameters evolution over 24h since the last LMWH injection (t=0). A:** Peak Height, **B:** Velocity index, **C:** Endogenous Thrombin Potential (ETP). Values are represented as mean ± SEM for anticoagulated pregnant patients (black forms) and non-anticoagulated pregnant patients (open circle) and age- and gender matched controls (open square). The reference range is represented as grey area. Statistically significant comparisons with Ctrl-NON-preg are represented: * P <0.05, ** P< 0.01, *** P<0.001, **** P<0.0001.

**Copyright Information:** (Online) ISSN: 2572-9241
©2026 The Author(s). HemaSphere is published by John Wiley & Sons Ltd on behalf of European Hematology Association. This is an open access Abstract Book distributed under the Attribution-NonCommercial-NoDerivs (CC BY-NC-ND), which allows third parties to download the articles and share them with others as long as they credit the author and the Abstract Book, but they cannot change the content in any way or use them commercially.
**Abstract Book Citations:** Authors, Title, HemaSphere, 2026;10:(S1):pages. The abstract book DOIs can be found on the "Author Information" page.
**Disclaimer:** Articles published in the journal HemaSphere exclusively reflect the opinions of the authors. The authors are responsible for all content in their abstracts including accuracy of the facts, statements, citing resources, etc.

### (PB4332) CLINICAL OUTCOMES AND TREATMENT REQUIREMENTS IN PATIENTS WITH VON WILLEBRAND DISEASE: A SINGLE CENTER EXPERIENCE

**Topic:** 34. Bleeding disorders (congenital and acquired)

Gamze Gürsoy*[1], Cem Kurtoğlu[1], Nihan Avcu Oral[1], Çağrı Coşkun[1], Tekin Aksu[1], Selin Aytaç[1]

[1]*Hacettepe University Faculty of Medicine, Department of Pediatric Hematology, Ankara, Türkiye;*

### Background

Von Willebrand disease (VWD) is the most common inherited bleeding disorder.The diagnosis of VWD is established based on clinical presentation, coagulation test analysis, and family history. Treatment and prophylaxis strategies are individualized according to bleeding phenotype and VWD subtype.

### Aims

We aim to evaluate the clinical features and management of patients with VWD followed at our center.

### Methods

This study included patients diagnosed with vWD who were followed at the Pediatric Hematology Department of Hacettepe University Faculty of Medicine between 2020 and 2025 (N = 80). The diagnosis of vWD was established based on activated partial thromboplastin time, VWF antigen, ristocetin cofactor activity, bleeding time, factor VIII levels, along with family and bleeding history. Spontaneous bleeding episodes, trauma- and surgery-related bleeding, treatments administered, laboratory parameters, and imaging findings were retrospectively analyzed

### Results

Among the 80 patients, 33 (41.3%) were female and 47 (58.7%) were male. Fifty-six patients (70%) were classified as Type 1, 12 (15%) as Type 2, and 12 (15%) as Type 3. The mean age at diagnosis was 5.6 ± 4.4 years (range: 1 month–17 years). Patients with Type 3 VWD were diagnosed at a significantly younger age (2.2 ± 2.7 years). A positive family history of bleeding was present in 57 patients (71%), and parental consanguinity was identified in 28 patients (42%).The most common reason for referral was spontaneous bleeding, followed by family history of bleeding, easy bruising, prolonged bleeding after surgery or trauma, and incidental laboratory abnormalities. Spontaneous bleeding was observed in the majority of patients (n = 62), most commonly epistaxis, oropharyngeal bleeding, and ecchymosis. A smaller subset experienced hemarthrosis and gastrointestinal bleeding. Prophylactic plasma-derived VWF/FVIII (pd[VWF/FVIII]) concentrate was administered in patients with severe bleeding phenotype, including 2 patients with Type 1 and 12 patients with Type 3 VWD. Abnormal uterine bleeding (AUB) was common among female patients; 18 patients received antifibrinolytics, desmopressin, or hormonal therapy. In Type 3 patients with AUB and in patients with severe bleeding (Types 1 and 2), pd(VWF/FVIII) concentrate was administered. A total of 67 minor and major invasive procedures were evaluated. The relationship between procedure type, VWD subtype, bleeding history, and perioperative prophylaxis was analyzed. Prophylactic approaches—including desmopressin, antifibrinolytics, fresh frozen plasma, and pd(VWF/FVIII) concentrate—were observed to significantly improve procedural safety.

**Copyright Information:** (Online) ISSN: 2572-9241
©2026 The Author(s). HemaSphere is published by John Wiley & Sons Ltd on behalf of European Hematology Association. This is an open access Abstract Book distributed under the Attribution-NonCommercial-NoDerivs (CC BY-NC-ND), which allows third parties to download the articles and share them with others as long as they credit the author and the Abstract Book, but they cannot change the content in any way or use them commercially.
**Abstract Book Citations:** Authors, Title, HemaSphere, 2026;10:(S1):pages. The abstract book DOIs can be found on the “Author Information” page.
**Disclaimer:** Articles published in the journal HemaSphere exclusively reflect the opinions of the authors. The authors are responsible for all content in their abstracts including accuracy of the facts, statements, citing resources, etc.

**Summary/Conclusion**

Although VWD is one of the most common inherited bleeding disorders, diagnosis may be challenging in complex cases despite comprehensive laboratory evaluation. Genetic confirmation may not always be feasible and diagnosis may rely on clinic and treatment response. Type 3 VWD patients exhibited a severe bleeding phenotype in our study, we suggest that prophylactic management reduces bleeding frequency and related complications in these patients. Different prophylactic strategies are applied according to spontaneous bleeding, trauma-related events, and perioperative settings across VWD subtypes. With these individualized approaches, effective bleeding control was achieved in the majority of patients. Patient- and phenotype-based individualized prophylaxis strategies play a fundamental role in the safe follow-up and effective management of VWD.

**Copyright Information:** (Online) ISSN: 2572-9241
©2026 The Author(s). HemaSphere is published by John Wiley & Sons Ltd on behalf of European Hematology Association. This is an open access Abstract Book distributed under the Attribution-NonCommercial-NoDerivs (CC BY-NC-ND), which allows third parties to download the articles and share them with others as long as they credit the author and the Abstract Book, but they cannot change the content in any way or use them commercially.
**Abstract Book Citations:** Authors, Title, HemaSphere, 2026;10:(S1):pages. The abstract book DOIs can be found on the “Author Information” page.
**Disclaimer:** Articles published in the journal HemaSphere exclusively reflect the opinions of the authors. The authors are responsible for all content in their abstracts including accuracy of the facts, statements, citing resources, etc.

### (PB4333) EVOLVING MANAGEMENT OF CONGENITAL THROMBOTIC THROMBOCYTOPENIC PURPURA WITH RECOMBINANT ADAMTS13

**Topic:** 34. Bleeding disorders (congenital and acquired)

Nikhita Vemulapalli*[1], Rafia Ali[1], Aditya Lakkamsani[1], Taha Alrifai[2]

*[1]Advocate Lutheran General, Internal Medicine, Park Ridge, United States of America;[2]Advocate Lutheran General, Hematology/Oncology, Park Ridge, United States of America;*

### Background

Upshaw–Schulman syndrome, also known as congenital thrombotic thrombocytopenic purpura (cTTP), is a rare autosomal recessive thrombotic microangiopathy caused by inherited deficiency of ADAMTS13. This leads to uncontrolled platelet aggregation and microvascular thrombosis which is life threatening with reported mortality rates of 10–20% despite treatment. Historically, treatment of cTTP has relied on fresh frozen plasma (FFP) to replace ADAMTS13 activity. However, 2025 TTP treatment guidelines have reshaped management, emphasizing recombinant ADAMTS13 as an alternative to FFP. Despite this change, there are limited published real-world data regarding its use in management of cTTP.

### Aims

To provide emerging evidence of recombinant ADAMTS13 as an alternative to FFP in management of congenital thrombotic thrombocytopenic purpura (cTTP).

### Methods

This case follows a woman in her mid-thirties with a childhood diagnosis of cTTP. She has required prophylactic FFP transfusions every 2–3 weeks, with inability to extend beyond 4 weeks without acute exacerbations. Despite adherence to this regimen, she still has experienced recurrent hospitalizations for acute exacerbations, typically precipitated by missed FFP transfusions or viral illness. She has additionally undergone termination of two pregnancies due to life threatening complications despite increased frequency and volume of FFP during pregnancy. Given her ongoing morbidity, and incomplete disease control with plasma-based therapy, she was approved for recombinant ADAMTS13 therapy in 2024. While still needing this therapy every 2 weeks, the patient has since avoided emergent transfusions and hospitalizations, especially in the setting of inflammatory triggers.

### Results

This case highlights several aspects of cTTP management. It underscores the limitations of plasma-based therapy, particularly in patients with frequent triggers and pregnancy-related exacerbations. While FFP provides functional ADAMTS13, there is significant variability of enzyme bioavailability. This case also illustrates the disproportionate disease burden faced by women with cTTP, with pregnancy representing a uniquely high-risk and refractory state. The patient's stability following initiation of recombinant ADAMTS13 supports emerging evidence that targeted enzyme replacement may offer improved disease control, and enhanced quality of life especially in high risk states such as pregnancy.

### Summary/Conclusion

Our case highlights the efficacy of recombinant ADAMTS13 as prophylactic therapy. Future studies should focus on optimizing dosing strategies, particularly during high-risk states such as pregnancy, to better define maternal and fetal safety profiles, as well as to compare long-term disease control with FFP-based prophylaxis.

**Copyright Information:** (Online) ISSN: 2572-9241
©2026 The Author(s). HemaSphere is published by John Wiley & Sons Ltd on behalf of European Hematology Association. This is an open access Abstract Book distributed under the Attribution-NonCommercial-NoDerivs (CC BY-NC-ND), which allows third parties to download the articles and share them with others as long as they credit the author and the Abstract Book, but they cannot change the content in any way or use them commercially.
**Abstract Book Citations:** Authors, Title, HemaSphere, 2026;10:(S1):pages. The abstract book DOIs can be found on the "Author Information" page.
**Disclaimer:** Articles published in the journal HemaSphere exclusively reflect the opinions of the authors. The authors are responsible for all content in their abstracts including accuracy of the facts, statements, citing resources, etc.

### (PB4334) THERAPY ADJUSTMENT IN PATIENTS WITH COAGULATION DISORDERS AND MYELOPROLIFERATIVE NEOPLASMS

**Topic:** 34. Bleeding disorders (congenital and acquired)

Mercedes Gasior Kabat*[1], Maria Isabel Rivas Pollmar[1], María -Teresa Álvarez Román[1], Elena Monzón Manzano[1], Paula Acuña Butta[1], Monica Martin Salces[1], Ana Mendoza Martinez[1], Matías Facal Giuliani[1], Patricia Gonzalez Marugan[1], Víctor Jiménez-Yuste[1], Nora Butta[1]

[1]*Hospital Universitario La Paz, Madrid, Spain;*

**Background**

Philadelphia-negative Myeloproliferative Neoplasms (MPNs, Polycythemia Vera (PV), Essential thrombocythemia (ET) and Primary Myelofibrosis (PMF).) are clonal disorders affecting the stem-cell. Treatment is based on anti-platelet agents and cytoreduction. Main causes of morbidity/mortality are thrombotic/hemorrhagic complications, so it´s important to adjust thrombotic prophylaxis in patients with coagulation disorders.

**Aims**

The objective of this work it´s to raise awareness on the importance of recognizing bleeding disorders in patients with MPNs.

**Methods**

Platelet characteristics and functional assessment using flow cytometry and aspirin was stopped 7-10 days prior sample collection. VWD screening using chemoluminescence to determine VW molecule and chromogenic FVIII levels. FVII and FXI levels were determined with coagulative test. In 2 patients T-TAS® (Total Thrombus formation analysis System) was also performed.

**Results**

We present 13 patients diagnosed with a bleeding disorder and MPNs: 7 PV and 6 ET. Median age 48 years (24-71). Eight patients received cytoreductive treatment: hydroxycarbamide (6) and anagrelide (2) and 9 received low dose aspirin 100 mg daily.
Six patients studied due to bleeding complications, easy bruising in 4, epistaxis and gum bleeding in 1 and severe hemorrhage after thyroid-surgery in other that needed platelet transfusion. Patient with easy bruising diagnosed with mild VWD type 1, 2 with significant bruising and 1 with surgical bleeding were diagnosed with mild/moderate thrombopathy.
Two patients without bleeding diagnosed with mild Factor VII deficiency, one in pre-surgery assessment, the other in familiar screening. One PV with mild FXI deficiency due to altered coagulation assays. One patient with adquired Von Willebrand (VW) disease and a trauma-related muscular hematoma requiring VW factor treatment and surgery.
Due to coagulation disorder diagnosis and bleeding symptons, aspirin dose was decreased in 4 patients, and stopped in another 3.

**Summary/Conclusion**

Although preventing thrombosis in MPNs patients is a treatment goal, bleeding complications could also be frequent and severe. Mild hemorrhagic events, can be related to antiplatelets agents, for moderate/severe bleeding it´s important to exclude factor deficiencies/platelet disorders in MPNs or to have it in mind to adjust thrombotic prophylaxis.
In NMPs patients could be important to rule out bleeding disorders to adjust thromboprophylaxis and to avoid surgical complications.

**Copyright Information:** (Online) ISSN: 2572-9241
©2026 The Author(s). HemaSphere is published by John Wiley & Sons Ltd on behalf of European Hematology Association. This is an open access Abstract Book distributed under the Attribution-NonCommercial-NoDerivs (CC BY-NC-ND), which allows third parties to download the articles and share them with others as long as they credit the author and the Abstract Book, but they cannot change the content in any way or use them commercially.
**Abstract Book Citations:** Authors, Title, HemaSphere, 2026;10:(S1):pages. The abstract book DOIs can be found on the “Author Information” page.
**Disclaimer:** Articles published in the journal HemaSphere exclusively reflect the opinions of the authors. The authors are responsible for all content in their abstracts including accuracy of the facts, statements, citing resources, etc.

### (PB4335) MORTALITY AND HOSPITALIZATION IN AUTOIMMUNE HEMOLYTIC ANEMIA: A 14-YEAR POPULATION-BASED STUDY IN BRAZIL (2010-2023)

**Topic:** 34. Bleeding disorders (congenital and acquired)

Renato Watanabe de Oliveira[1], Ligia Coelho[1], Gabriela Kanevsky*[2], Caroline Castro[3], Julio Cesar Barbour[3], Renato Sampaio Tavares[4]

*[1]Johnson & Johnson Brasil, São Paulo, Brazil; [2]Johnson & Johnson Argentina, Buenos Aires, Argentina; [3]Precision Data, São Paulo, Brazil; [4]Universidade Federal de Goiás, Faculdade de Medicina, Goiania, Brazil;*

### Background

Autoimmune hemolytic anemia (AIHA) is a rare life-threatening disorder characterized by antibody-mediated red blood cell destruction. Population-based epidemiological data remain scarce globally, with no comprehensive studies from Latin America, limiting understanding of the disease burden, healthcare impact, and regional disparities.

### Aims

To estimate the cumulative and annual mortality and hospitalization rates attributed to AIHA in Brazil from 2010-2023, and to describe the demographic, clinical, and regional patterns of the disease.

### Methods

This retrospective ecological study used Brazilian national mortality (SIM) and hospitalization (SIH) databases. We included all deaths and hospitalizations (age ≥20 years) with ICD-10 code D59.1 as primary or secondary cause from 2010-2023. Standardized rates per 100,000 inhabitants used the 2016 mid-period population for cumulative rates and the population of each year for annual rates. The annual percent change (APC) was calculated using linear regression to assess temporal trends. Years of life lost (YLL) and years of potential life lost (YPLL) were estimated using the WHO Global Health Estimates standard life table.

### Results

Over 14 years, 737 deaths related to AIHA were recorded, resulting in a cumulative mortality rate of 0.52 per 100,000 individuals. The annual mortality rates were stable over the years varying from 0.03 per 100,000 in 2010 to 0.04 per 100,000 in 2023, with an APC of +1.58% (95% CI: -0.21 to 3.40; p=0.084). Mortality rates were higher in females compared to males, at 0.63 versus 0.40 per 100,000, respectively. Mortality also increased with age, from 0.35 per 100,000 in the 20-29 age group to 3.22 per 100,000 in those aged 80 and above. The states of Roraima (2.5), Acre (1.2) and Tocantins (1.0) presented the highest cumulative mortality rates per 100,000 inhabitants. AIHA-related deaths resulted in an estimated 26,659 YLL over the study period, with women accounting for a disproportionate share of the total burden (16,260 YLL) compared with men (10,400 YLL). YPLL totaled 14,526 years, indicating a substantial impact of premature mortality. A total of 3,215 patients accounted for 6,404 hospitalizations, with a cumulative rate of 2.27 per 100,000. Annual hospitalization rates were also stable over the years, varying between 0.22 per 100,000 in 2010 and 0.19 per 100,000 in 2023, with an APC of –0.77% (95% CI: –2.12 to 0.59; p=0.246). Hospitalization rates increased with age, from 4.57 per 100,000 in the 20-29 age group to 7.25 per 100,000 in those aged 80 and above. The states of Roraima 19.7), Rondonia (12.7) and Distrito Federal (11.2) had the highest cumulative hospitalization rate per 100,000. Among all hospitalizations, 80.82% were related to emergency visits and 9.6% to splenectomy. Blood transfusions were administered in 33.5% of hospitalizations.

**Copyright Information:** (Online) ISSN: 2572-9241
©2026 The Author(s). HemaSphere is published by John Wiley & Sons Ltd on behalf of European Hematology Association. This is an open access Abstract Book distributed under the Attribution-NonCommercial-NoDerivs (CC BY-NC-ND), which allows third parties to download the articles and share them with others as long as they credit the author and the Abstract Book, but they cannot change the content in any way or use them commercially.
**Abstract Book Citations:** Authors, Title, HemaSphere, 2026;10:(S1):pages. The abstract book DOIs can be found on the "Author Information" page.
**Disclaimer:** Articles published in the journal HemaSphere exclusively reflect the opinions of the authors. The authors are responsible for all content in their abstracts including accuracy of the facts, statements, citing resources, etc.

**Summary/Conclusion**

despite stable mortality and hospitalization rates over the last 14 years, AIHA is a disease with significant morbidity and mortality, associated with premature death, affecting women and older adults more frequently. The high reliance on emergency care and transfusions, together with regional rates disparities, suggests possible gaps in the standardized management of these patients. The results reinforce the need for improved therapeutic strategies and equitable access to specialized hematological care.

**Copyright Information:** (Online) ISSN: 2572-9241
©2026 The Author(s). HemaSphere is published by John Wiley & Sons Ltd on behalf of European Hematology Association. This is an open access Abstract Book distributed under the Attribution-NonCommercial-NoDerivs (CC BY-NC-ND), which allows third parties to download the articles and share them with others as long as they credit the author and the Abstract Book, but they cannot change the content in any way or use them commercially.
**Abstract Book Citations:** Authors, Title, HemaSphere, 2026;10:(S1):pages. The abstract book DOIs can be found on the “Author Information” page.
**Disclaimer:** Articles published in the journal HemaSphere exclusively reflect the opinions of the authors. The authors are responsible for all content in their abstracts including accuracy of the facts, statements, citing resources, etc.

### (PB4336) CURRENT CONCEPTS IN CHILDHOOD HEMOPHILIA

**Topic:** 34. Bleeding disorders (congenital and acquired)

Vladimir Iacomi*[1], Dorina Agachi[1], Ilinca Motelica[1], Olga Malai[1], Galina Esanu[1], Valentin Turea[1]

[1]*State University of Medicine and Pharmacy "Nicolae Testemitanu", Paediatrics, Chisinau, Moldova;*

### Background

Haemophilia is considered a major public health concern among rare diseases. Recent data shows more than 800000 people worldwide live with a form of haemophilia, but less than half have a confirmed diagnosis, highlighting a significant problem, especially in resource-limited countries. International partnerships and targeted funding for rare diseases have broadened access to genetic testing and inhibitor assessment. The lack of a national network of specialized centers and the need for strong epidemiological surveillance, enabled the creation of the National Registry for Hemophilia Patients.

### Aims

Assessment of the clinical manifestations, biochemical markers, and the genetic profile in hemophilia A and B in children from the Republic of Moldova. Development of a National Registry for the purpose of improvement of paraclinical diagnosis and development of personalized therapeutic regimen adapted to the type and severity of the disease.

### Methods

The research was held at the Pediatric Hematology Clinic of the State University of Medicine and Pharmacy „Nicolae Testemitanu”. The sample included 90 children aged 0–18 years, diagnosed with hemophilia and monitored in the clinic during 2018-2023. Participants underwent a comprehensive examination, which included standard clinical assessment and laboratory investigations to determine coagulation parameters, quantitative measurements of coagulation factors VIII and IX, inhibitor titers, and analyses to identify the spectrum of genetic mutations.

### Results

In 36.7% of cases (95% CI [26.71–46.62]), hemophilia was classified as sporadic, meaning that no known family history of the disease existed. The data showed that the number of affected joints ranged from 1 to 4, with multiple joint involvement: 24.4% (95% CI [15.57–33.32]) simultaneous 4 joints involvement; 19.8% (95% CI [0–0.00]) 3 joints involvement; and 23.3% (95% CI [14.60–32.07]) 2 joints involvement. In hemophilia B, the ankles were affected significantly less frequently compared to hemophilia A ($\chi^2$ = 7.04, df = 2, p = 0.032), suggesting a phenotypic feature of this form. Across the entire cohort, regardless of hemophilia type, the severe form predominated in 48.9% of cases (95% CI [38.56–59.22]). The most frequently identified mutation was of the missense type, found in 17.8% of cases (95% CI [9.88–25.68]). This mutation was observed in 18.5% of Hemophilia A cases and in 11.1% of Hemophilia B cases. The Inv Intr 22 mutation was recorded in 14.8% of cases (95% CI [6.31–20.36]), exclusively in Hemophilia A. In Hemophilia A, 4 patients (4.9%) (95% CI [0.19% - 8.70%]) had detectable inhibitors indicating a low incidence of this phenomenon. No cases of inhibitors were observed in Hemophilia B. Upon completion, the “Electronic Registry for Hemophilia Patients in the Republic of Moldova” was implemented as a national platform for the data collection, monitoring, and management of hemophilia patients.

**Copyright Information:** (Online) ISSN: 2572-9241
©2026 The Author(s). HemaSphere is published by John Wiley & Sons Ltd on behalf of European Hematology Association. This is an open access Abstract Book distributed under the Attribution-NonCommercial-NoDerivs (CC BY-NC-ND), which allows third parties to download the articles and share them with others as long as they credit the author and the Abstract Book, but they cannot change the content in any way or use them commercially.
**Abstract Book Citations:** Authors, Title, HemaSphere, 2026;10:(S1):pages. The abstract book DOIs can be found on the “Author Information” page.
**Disclaimer:** Articles published in the journal HemaSphere exclusively reflect the opinions of the authors. The authors are responsible for all content in their abstracts including accuracy of the facts, statements, citing resources, etc.

**Summary/Conclusion**

Genetic analysis highlighted the predominant mutations which were associated with moderate and severe forms of the disease. Inhibitor testing revealed the importance of their periodic monitoring to adjust treatment and prevent therapeutic failures due to immune resistance. The implementation of the national registry **facilitates** the collection of patient data and continuous monitoring of therapeutic efficacy.

**Copyright Information:** (Online) ISSN: 2572-9241
©2026 The Author(s). HemaSphere is published by John Wiley & Sons Ltd on behalf of European Hematology Association. This is an open access Abstract Book distributed under the Attribution-NonCommercial-NoDerivs (CC BY-NC-ND), which allows third parties to download the articles and share them with others as long as they credit the author and the Abstract Book, but they cannot change the content in any way or use them commercially.
**Abstract Book Citations:** Authors, Title, HemaSphere, 2026;10:(S1):pages. The abstract book DOIs can be found on the "Author Information" page.
**Disclaimer:** Articles published in the journal HemaSphere exclusively reflect the opinions of the authors. The authors are responsible for all content in their abstracts including accuracy of the facts, statements, citing resources, etc.

### (PB4337) CORRELATION BETWEEN ANTI-PLATELET AUTO ANTIBODIES, CLINICAL SEVERITY, AND TREATMENT RESPONSE IN PEDIATRIC IMMUNETHROMBOCYTOPENIC PURPURA (ITP)

**Topic:** 34. Bleeding disorders (congenital and acquired)

Imene Larous*[1]

[1]*centre hospitalo-universitaire Setif, Laboratoire du pole pédiatrique, SETIF, Algeria;*

### Background

Immune Thrombocytopenia (ITP) is characterized by thrombocytopenia partially attributable to the presence of anti-platelet autoantibodies, which induce platelet destruction by mononuclear phagocytes. These antibodies most frequently target antigens expressed on platelet membrane glycoproteins, often IgG antibodies directed against the glycoprotein complex GPIIb/IIIa and/or the GPIb/IX complex. Although ITP is a common cause of thrombocytopenia in childhood, its diagnosis remains one of exclusion. It is established in cases of persistent isolated thrombocytopenia in a previously healthy child, with no other etiology suspected based on medical history, clinical examination, complete blood count, and blood smear analysis, even in the absence of a definitive confirmatory laboratory test.

### Aims

It will evaluate the potential role of anti-platelet autoantibodies carracterisation as a tool for risk stratification and personalized treatment in children with ITP.

### Methods

The detection of antiplatelet antibodies has provided increasingly reliable results, and the severity of ITP as well as the response to treatment may be related to the specific nature of the involved autoantibodies. In a study using the indirect MAIPA technique, screening for antiplatelet antibodies was performed on samples from 10 patients aged 5 to 12 years, followed in the pediatric department of Setif University Hospital for ITP.

### Results

The findings revealed anti-GPIIb/IIIa antibodies in 40% of patients, anti-GPIb/IX in 60%, both antibodies in 30%, and anti-GPIa/IIa antibodies in 60% of cases. Variations in response to corticosteroids, intravenous immunoglobulins, as well as the severity of ITP, were observed depending on the type of antibody detected.

### Summary/Conclusion

The identification of specific antibody profiles may assist in determining, from the initial diagnosis, whether a patient is at risk of developing an acute or chronic form of the disease. This could aid in selecting
the most appropriate treatment for these patients.

**Copyright Information:** (Online) ISSN: 2572-9241
©2026 The Author(s). HemaSphere is published by John Wiley & Sons Ltd on behalf of European Hematology Association. This is an open access Abstract Book distributed under the Attribution-NonCommercial-NoDerivs (CC BY-NC-ND), which allows third parties to download the articles and share them with others as long as they credit the author and the Abstract Book, but they cannot change the content in any way or use them commercially.
**Abstract Book Citations:** Authors, Title, HemaSphere, 2026;10:(S1):pages. The abstract book DOIs can be found on the "Author Information" page.
**Disclaimer:** Articles published in the journal HemaSphere exclusively reflect the opinions of the authors. The authors are responsible for all content in their abstracts including accuracy of the facts, statements, citing resources, etc.

### (PB4339) SPECTRUM AND CLINICAL IMPACT OF THROMBOCYTOPENIA MANAGED IN A TERTIARY OBSTETRIC HAEMATOLOGY CENTRE: AN 18-MONTH COHORT STUDY.

**Topic:** 34. Bleeding disorders (congenital and acquired)

Alanna Allen*[1], Maeve Crowley[1]

[1]*CORK UNIVERSITY HOSPITAL, CORK, Ireland;*

### Background

Thrombocytopenia complicates up to 11% of pregnancies and poses challenges for delivery planning, neuraxial anaesthesia and haemorrhage management. Real world data describing the clinical impact of maternal thrombocytopenia remains limited.

### Aims

To define the aetiological spectrum of maternal thrombocytopenia in a tertiary centre and quantify its impact on peripartum management, delivery planning, and maternal-neonatal outcomes.

### Methods

Retrospective cohort study of women referred to the obstetric haematology service with thrombocytopenia over 18 months in a tertiary (~6500 deliveries/year) obstetric centre.

### Results

Thirty one women (33 pregnancies including two twin pregnancies) were included. Median maternal age was 33 years. Thirty five percent of women were nulliparous. Median booking platelet count was 132 $\times 10^9$/L and median nadir was 96 $\times 10^9$/L. The diagnostic distribution and associated treatment burden are summarised in Table 1. Final diagnoses were gestational thrombocytopenia (GT) in 19/31 (61%), immune thrombocytopenia (ITP) in 11/31 (35%) and B12 deficiency in 1/31 (3%). A significant proportion 22/31 (71%) of women had a prior history of thrombocytopenia, predominantly ITP or gestational thrombocytopenia.
Eight women (26%) required active treatment, predominantly within the ITP cohort. Corticosteroids were administered in 8 cases, IVIG in 3, and one patient required platelet transfusion. Among treated patients, 5/8 (63%) achieved a delivery platelet count >80 $\times 10^9$/L, supporting effective peripartum optimisation in the majority.  Neuraxial anaesthesia was requested in 27 pregnancies and achieved in 22 (81%); five cases were withheld due to thrombocytopenia. Mode of delivery was vaginal in 14 (45%) and Caesarean section in 16 (52%). Major PPH (>1000 mL) occurred in 3/31 (10%). Red cell transfusion was required in these patients. Tranexamic acid was administered in 23% of cases. Two intrauterine deaths occurred in women with GT, neither was attributable to thrombocytopenia. No cases of severe neonatal thrombocytopenia (<100 $\times 10^9$/L) were observed.
Table 1. Aetiology and Treatment Requirement in Maternal Thrombocytopenia (n=31)

| Diagnosis | N (%) | Required Active Treatment n (%) |
|---|---|---|
| Gestational thrombocytopenia | 19 (61%) | 0 (0%) |
| Immune thrombocytopenia | 11 (35%) | 8 (73%) |
| B12 deficiency | 1 (3%) | 0 (0%) |
| Total | 31 (100%) | 8 (26%) |

**Copyright Information:** (Online) ISSN: 2572-9241
©2026 The Author(s). HemaSphere is published by John Wiley & Sons Ltd on behalf of European Hematology Association. This is an open access Abstract Book distributed under the Attribution-NonCommercial-NoDerivs (CC BY-NC-ND), which allows third parties to download the articles and share them with others as long as they credit the author and the Abstract Book, but they cannot change the content in any way or use them commercially.
**Abstract Book Citations:** Authors, Title, HemaSphere, 2026;10:(S1):pages. The abstract book DOIs can be found on the "Author Information" page.
**Disclaimer:** Articles published in the journal HemaSphere exclusively reflect the opinions of the authors. The authors are responsible for all content in their abstracts including accuracy of the facts, statements, citing resources, etc.

**Summary/Conclusion**

Compared with national data from the Irish Maternity Indicator System (IMIS) released in 2024, our cohort demonstrated higher Caesarean section rates (52% vs 40.6%) and greater use of general anaesthesia for Caesarean section (12.5% vs 4.75%). Although gestational thrombocytopenia accounted for most cases over one-quarter required active intervention, largely driven by ITP. Fortunately there were no cases of severe neonatal thrombocytopenia observed. Specialist obstetric haematology involvement guided anaesthetic access, transfusion utilisation and delivery planning. This data supports development of risk adapted pathways to optimise resource allocation while safeguarding maternal and neonatal outcomes.

**Copyright Information:** (Online) ISSN: 2572-9241
©2026 The Author(s). HemaSphere is published by John Wiley & Sons Ltd on behalf of European Hematology Association. This is an open access Abstract Book distributed under the Attribution-NonCommercial-NoDerivs (CC BY-NC-ND), which allows third parties to download the articles and share them with others as long as they credit the author and the Abstract Book, but they cannot change the content in any way or use them commercially.
**Abstract Book Citations:** Authors, Title, HemaSphere, 2026;10:(S1):pages. The abstract book DOIs can be found on the "Author Information" page.
**Disclaimer:** Articles published in the journal HemaSphere exclusively reflect the opinions of the authors. The authors are responsible for all content in their abstracts including accuracy of the facts, statements, citing resources, etc.

### (PB4340) CLINICAL BURDEN OF HEMOPHILIA IN ABU DHABI, UNITED ARAB EMIRATES (UAE): REAL-WORLD INSIGHTS FROM THE MALAFFI PLATFORM

**Topic:** 34. Bleeding disorders (congenital and acquired)

Muna Alsafi*[1], Alia Aldhaheri[1], Subhashini Ganesan[2], Flavia Cantarutti[2], Hannah Wilson-Nortey[2], Islam Eltantawy[2], Thekra Mohamed[1], Shahrukh Hashmi[1], Asma Al Mannaie[1]

*[1]Department of Health, Abu Dhabi, United Arab Emirates;[2]Insights Research Organization and Solutions (IROS), Abu Dhabi, United Arab Emirates;*

### Background

Hemophilia A and B are the most prevalent severe hereditary bleeding disorders, resulting from genetic defects affecting clotting factors VIII and IX. These disorders cause extended or excessive bleeding, even after minor injuries. In the United Arab Emirates, the standard approach for severe cases involves preventive replacement therapy with clotting factor concentrates, initiated early to avoid complications. Although this treatment strategy improves patient outcomes and reduces hospital admissions, it remains expensive and requires frequent intravenous administration, creating ongoing clinical and financial obstacles.

### Aims

The purpose of this study is to assess the clinical burden of hemophilia and the use of healthcare resources in Abu Dhabi, UAE.

### Methods

Malaffi, Abu Dhabi's health information exchange platform, securely connects patient medical records among healthcare providers throughout the emirate. This study retrospectively reviewed Malaffi health records for patients with hemophilia A (ICD10: D66) or B (ICD10: D67) who received treatment from January 1, 2021, to April 14, 2023, focusing on demographics, healthcare usage, and clinical features.

### Results

Out of 146 patients identified, 83% (n=121) had hemophilia A. The majority of those with hemophilia A were male (>91%) and 65% (n=95) of all hemophilia patients were of Arab ethnicity. Pediatric patients made up 65% (n=95), with an average age of 6.3 ± 5.6 years, while adults comprised 35% (n=51), with a mean age of 30.4 ± 10.1 years. Pain and swelling were the predominant symptoms across both groups. High rates of comorbidities were noted, especially joint/arthritic conditions and anemia.

Table 1: **Hemophilia-related symptoms**

| Symptoms | Pediatric patients | Adult patients | Total |
|---|---|---|---|
| **Total no. of patients, n (%)** | **95 (65.1)** | **51 (34.9)** | **146 (100)** |
| Pain and swelling | 73 (76.8) | 45 (88.2) | 118 (80.8) |
| Abrasions, contusions, ecchymosis | 50 (52.6) | 23 (45.1) | 73 (50) |
| Hemostatic symptoms | 42 (44.2) | 19 (37.3) | 61 (41.8) |
| Nausea and vomiting | 39 (41.1) | 21 (41.2) | 60 (41.1) |
| Fatigue and weakness | 33 (34.7) | 22 (43.1) | 55 (37.7) |
| Headache | 23 (24.2) | 22 (43.1) | 45 (30.8) |
| Other joint and soft tissue-related symptoms | 24 (25.3) | 15 (29.4) | 39 (26.7) |
| Others | 15 (15.8) | 17 (33.3) | 32 (21.9) |

**Copyright Information:** (Online) ISSN: 2572-9241
©2026 The Author(s). HemaSphere is published by John Wiley & Sons Ltd on behalf of European Hematology Association. This is an open access Abstract Book distributed under the Attribution-NonCommercial-NoDerivs (CC BY-NC-ND), which allows third parties to download the articles and share them with others as long as they credit the author and the Abstract Book, but they cannot change the content in any way or use them commercially.
**Abstract Book Citations:** Authors, Title, HemaSphere, 2026;10:(S1):pages. The abstract book DOIs can be found on the "Author Information" page.
**Disclaimer:** Articles published in the journal HemaSphere exclusively reflect the opinions of the authors. The authors are responsible for all content in their abstracts including accuracy of the facts, statements, citing resources, etc.

**Summary/Conclusion**

This is the first study in the UAE to examine state (emirate)-level data on hemophilia, revealing a considerable clinical and healthcare burden in Abu Dhabi. The findings highlight significant healthcare utilization required to manage hemophilia, such as frequent visits to healthcare providers.

**Acknowledgements**
This study is conducted in collaboration with the Department of Health Abu Dhabi which granted researchers remote and secure data access to de-identified datasets. Views and opinions expressed in the text are those of the authors and do not necessarily reflect the official policy or position of the Department of Health or other affiliated institutions.

**Copyright Information:** (Online) ISSN: 2572-9241
©2026 The Author(s). HemaSphere is published by John Wiley & Sons Ltd on behalf of European Hematology Association. This is an open access Abstract Book distributed under the Attribution-NonCommercial-NoDerivs (CC BY-NC-ND), which allows third parties to download the articles and share them with others as long as they credit the author and the Abstract Book, but they cannot change the content in any way or use them commercially.
**Abstract Book Citations:** Authors, Title, HemaSphere, 2026;10:(S1):pages. The abstract book DOIs can be found on the "Author Information" page.
**Disclaimer:** Articles published in the journal HemaSphere exclusively reflect the opinions of the authors. The authors are responsible for all content in their abstracts including accuracy of the facts, statements, citing resources, etc.

### (PB4341) ACQUIRED HEMOPHILIA IN INDIA: A SINGLE CENTER CASE SERIES

**Topic:** 34. Bleeding disorders (congenital and acquired)

Kartikey Saini*[1], Himil Parikh[2], Vinu Balraam[3], Jasmita Dass[1], Mukul Aggarwal[1], Rishi Dhawan[1], Richa Chauhan[1], Ganesh Kumar Viswanathan[1], Tulika Seth[1], Manoranjan Mahapatra[1]

*[1]ALL INDIA INSTITUTE OF MEDICAL SCIENCES NEW DELHI, HEMATOLOGY, NEW DELHI, India;[2]HOC VEDANTA, AHMEDABAD, India; [3]Command Hospital, Panchkula, India;*

### Background

Acquired Hemophilia A (AHA) is a rare but serious autoimmune bleeding disorder (about 1.5 cases per million people yearly) with autoantibodies against factor VIII, leading to severe bleeding—typically in older adults or women after childbirth. This retrospective study evaluated clinical features, treatment responses, and outcomes in 10 AHA patients and the challenges faced in diagnosis, clinical feautres, management of the disease in resource limitied conditions.

### Aims

Assess titer-stratified IST outcomes and prognostic factors influencing CR, relapse,complications and mortality in AHA in resource constraint setting

### Methods

We reviewed 10 consecutive AHA cases (2019-2025) meeting criteria: acute bleeding/prolonged aPTT uncorrectable on mixing, FVIII <50%, and positive Bethesda inhibitor assay. Data included demographics, bleeding sites, labs (aPTT, FVIII, inhibitors), IST (steroids ± cyclophosphamide/rituximab), and outcomes (remission, relapse, mortality).

### Results

Median age was 64 years (range 28-84; 70% male); 30% had associations (postpartum, RA, HIV). All had soft tissue hematomas (100%), mucosal bleeding (30%), hemarthrosis (20%). Median aPTT 79s, FVIII <1% (80%), inhibitor 180 BU/mL (80% high-titer). 1 Patient had pseudoinhibitors to FIX in addition to FVIII. IST: steroids monotherapy (20%), combinations (80%).Byspassing agents and Emicizumab used in 1/10 patient. Complete remission: 60% (median 3 months); partial/clinical improvement: 30%; relapse: 20%; mortality: 10% (sepsis): 10% had haemmorhagic cystitis secondary to cummuative cyclophosphamide exposure. High-titer cases had delayed responses.

### Summary/Conclusion

This cohort shows high high-titer prevalence and diagnostic delays exacerbating severity, with 60% CR comparable to registries via titer-guided IST. Early diagnosis, bypassing agents, and aggressive high-titer IST are key; infections limit survival. These findings inform AHA management in India

**Copyright Information:** (Online) ISSN: 2572-9241
©2026 The Author(s). HemaSphere is published by John Wiley & Sons Ltd on behalf of European Hematology Association. This is an open access Abstract Book distributed under the Attribution-NonCommercial-NoDerivs (CC BY-NC-ND), which allows third parties to download the articles and share them with others as long as they credit the author and the Abstract Book, but they cannot change the content in any way or use them commercially.
**Abstract Book Citations:** Authors, Title, HemaSphere, 2026;10:(S1):pages. The abstract book DOIs can be found on the "Author Information" page.
**Disclaimer:** Articles published in the journal HemaSphere exclusively reflect the opinions of the authors. The authors are responsible for all content in their abstracts including accuracy of the facts, statements, citing resources, etc.

### (PB4342) HEMORRHAGIC PHENOTYPE AND FACTOR LEVELS IN VON WILLEBRAND DISEASE: IMPLICATIONS FOR SURGICAL SAFETY

**Topic:** 34. Bleeding disorders (congenital and acquired)

Mónica Fernández Pérez*[1], Carlos Pison[1], Angel Pereda[1]

[1]*Hospital Universitario Araba, Vitoria, Spain;*

### Background

Von Willebrand Disease (vWD) is the most common hereditary coagulopathy, characterized by a quantitative deficit (partial (type 1) or total (type 3)) or qualitative deficit (type 2) of the von Willebrand factor. Patients with vWD have a substantial bleeding risk, usually proportional to the degree of von Willebrand factor deficiency and factor VIII deficiency. Clinical manifestations often involve mucocutaneous bleeding. Treatment focuses on controlling or preventing bleeding by restoring primary hemostasis.

### Aims

The primary objective is to analyze the incidence and clinical-hemorrhagic correlation among patients with different types of vWD. Secondary objectives include analyzing various treatment types used in procedures and complications arising from them.

### Methods

This is a retrospective, single-center study at the University Hospital of Araba (HUA). We analyzed von Willebrand factor requests in our hospital from 1/01/2024 to 31/12/2025. Data, including gender, age, type of vWD, family history, clinic, ISTH-BAT, originating department, von Willebrand factor activity and antigen, treatment for surgery or procedures, and associated complications, were collected from medical records.

### Results

A total of 410 von Willebrand factor samples were analyzed at HUA, with 63.80% being women and 36.20% men. The incidence of vWD was 6% (25 patients), with 60% being women and 40% men. Types included 68% type 1, 12% type 2A, 4% type 2B, 8% type 2M, and 8% type 3. 84% had known family history of vWD. Initial symptoms included epistaxis (36%), metrorrhagia (20%), significant spontaneous hematomas (12%), gingival bleeding (4%), postpartum bleeding (8%), and surgery-related bleeding (4%). Regarding ISTH-BAT, 52% had a score over 3. Hematology accounted for 60% of requests, followed by gynecology (24%), and others (4% each). The average von Willebrand factor activity was 35%, and the antigen level was 43%. Patients with type 2M and 3 showed lower factor levels with more hemorrhagic symptoms postpartum and after surgery.
Forty percent of patients underwent surgeries or procedures, including dental extractions (16%), abdominal surgery (12%), orthopedic surgery (8%), and thoracic surgery (4%). During interventions, tranexamic acid was used in 16%, desmopressin in 12%, FVIII concentrate in 4%, iron therapy in 4%, and plasma in 4%. No patients developed complications postoperatively (TABLE 1).

### Summary/Conclusion

Our study confirms that vWD carries a substantial bleeding risk, with the severity of bleeding proportional to the degree of von Willebrand factor deficiency, as observed in the literature. With appropriate therapy and preparation, proper hemostasis can be ensured for invasive procedures, with risks being individualized at all times.

**Copyright Information:** (Online) ISSN: 2572-9241
©2026 The Author(s). HemaSphere is published by John Wiley & Sons Ltd on behalf of European Hematology Association. This is an open access Abstract Book distributed under the Attribution-NonCommercial-NoDerivs (CC BY-NC-ND), which allows third parties to download the articles and share them with others as long as they credit the author and the Abstract Book, but they cannot change the content in any way or use them commercially.
**Abstract Book Citations:** Authors, Title, HemaSphere, 2026;10:(S1):pages. The abstract book DOIs can be found on the "Author Information" page.
**Disclaimer:** Articles published in the journal HemaSphere exclusively reflect the opinions of the authors. The authors are responsible for all content in their abstracts including accuracy of the facts, statements, citing resources, etc.

| | |
|---|---|
| Summary of Patient Characteristics: | Total Patients: 25 |
| Gender | Female: 60% (15)<br>Male: 40% (10) |
| Average Age: | 34.84 years |
| Distribution of Von Willebrand Disease Types: | Type 1: 68% (17)<br>Type 2:<br>2A: 12% (3)<br>2B: 4% (1)<br>2M: 8% (2)<br>2N: 0<br>Type 3: 8% (2) |
| Family History: | Yes: 72% (18)<br>No: 28% (7) |
| Clinical Symptoms: | Epistaxis: 36% (9)<br>Metrorrhagia: 20% (5)<br>Significant Hematomas: 12% (3)<br>Gingival Bleeding: 4% (1)<br>Postpartum Bleeding: 8% (2)<br>Surgery-Related Bleeding: 4% (1)<br>No Hemorrhagic Symptoms: 16% (4) |
| ISTH-BAT Score Distribution: | 0: 16% (4)<br>1: 16% (4)<br>2: 16% (4)<br>3: 32% (8)<br>4: 8% (2)<br>5: 12% (3) |
| Von Willebrand Factor Activity and Antigen Levels: | Average Von Willebrand Factor Activity: 35%<br>Average Von Willebrand Factor Antigen Level: 43% |
| Type-specific Distribution of Von Willebrand Factor Activity: | Type 1: 38%<br>Type 2:<br>2A: 21%<br>2B: 20%<br>2M: 17%<br>Type 3: 14% |
| Type-specific Distribution of Von Willebrand Factor Antigen Level: | Type 1: 54%<br>Type 2:<br>2A: 49%<br>2B: 20.4%<br>2M: 46%<br>Type 3: 5% |
| Surgical Intervention: | Yes: 40% (10)<br>No: 60% (15) |
| Types of Surgeries: | Dental Extraction: 16% (4)<br>Abdominal Surgery: 12% (3)<br>Traumatological Surgery: 8% (2)<br>Thoracic Surgery: 4% (1) |
| Treatment During Surgery: | No Treatment: 60% (15)<br>Tranexamic Acid: 16% (4)<br>Desmopressin: 12% (3)<br>FVIII Concentrate: 4% (1)<br>Iron Therapy: 4% (1)<br>Plasma: 4% (1) |

TABLE 1: Characteristic of the patients

**Copyright Information:** (Online) ISSN: 2572-9241
©2026 The Author(s). HemaSphere is published by John Wiley & Sons Ltd on behalf of European Hematology Association. This is an open access Abstract Book distributed under the Attribution-NonCommercial-NoDerivs (CC BY-NC-ND), which allows third parties to download the articles and share them with others as long as they credit the author and the Abstract Book, but they cannot change the content in any way or use them commercially.
**Abstract Book Citations:** Authors, Title, HemaSphere, 2026;10:(S1):pages. The abstract book DOIs can be found on the "Author Information" page.
**Disclaimer:** Articles published in the journal HemaSphere exclusively reflect the opinions of the authors. The authors are responsible for all content in their abstracts including accuracy of the facts, statements, citing resources, etc.

### (PB4343) IMMUNE MEDIATED ACQUIRED VITAMIN K DEPENDENT COAGULATION FACTOR DEFICIENCY -A REPORT OF TWO CASES

**Topic:** 34. Bleeding disorders (congenital and acquired)

Alfadil Haroon*[1], Anwar Alkhadir[1], Hazzaa Alzahrani[1]

*[1]Cancer Center of Excellence, King Faisal Specialist Hospital and Research Center, Adult Hematology, stem cell transplant and cellular therapy section, Riyad, Saudi Arabia;*

### Background

Acquired vitamin K-dependent coagulation factor deficiency (VKCFD) is a rare cause of coagulopathy characterized by prolonged PT and elevated INR without anticoagulant use. It caused by malabsorption, liver disease, malignancy, autoimmune disorders, or drugs. Prompt recognition and management are essential for correction and prevention of hemorrhage.

### Aims

To describe two clinically cases of acquired VKCFD, highlight diagnostic challenges, and illustrate therapeutic strategies required for refractory or relapsing coagulopathy.

### Methods

Two patients with severe coagulopathy, bleeding, and markedly elevated INR with reduced VKCFs were evaluated; clinical, laboratory, imaging and treatment. Data were reviewed to assess etiology and therapeutic effectiveness.

**Copyright Information:** (Online) ISSN: 2572-9241
©2026 The Author(s). HemaSphere is published by John Wiley & Sons Ltd on behalf of European Hematology Association. This is an open access Abstract Book distributed under the Attribution-NonCommercial-NoDerivs (CC BY-NC-ND), which allows third parties to download the articles and share them with others as long as they credit the author and the Abstract Book, but they cannot change the content in any way or use them commercially.
**Abstract Book Citations:** Authors, Title, HemaSphere, 2026;10:(S1):pages. The abstract book DOIs can be found on the “Author Information” page.
**Disclaimer:** Articles published in the journal HemaSphere exclusively reflect the opinions of the authors. The authors are responsible for all content in their abstracts including accuracy of the facts, statements, citing resources, etc.

**Results**

Case 1: A 27-year-old woman with a history of idiopathic intracranial hypertension, recurrent early pregnancy loss, prior preeclampsia, depression, and suspected coagulopathy was admitted for evaluation of bilateral adnexal hemorrhagic lesions. She presented with right-lower abdominal pain and heavy menstrual bleeding with clots. She denied anticoagulant use. Examination showed mild tachycardia but hemodynamic stability, without mucocutaneous or joint bleeding. Laboratory tests revealed hemoglobin 8.7 g/dL, platelets 382 $\times 10^9$/L INR 1.8 rising to 2.7 and high PT and aPTT. There was no evidence of autoimmune-mediated coagulopathy; antiphospholipid and connective tissue disease panels were negative .Pelvic imaging showed bilateral ovarian hemorrhagic cystic lesions without active extravasation.Brain CT scan showed features of increased intracranial pressure without acute bleeding. The working diagnosis was acquired VKCFD coagulopathy. The patient received intravenous vitamin K resulting in a transient improvement in INR followed by recurrence. Subsequent initiation of prednisone and mycophenolate mofetil led to gradual stabilization of both INR and hemoglobin levels.
Case 2: A 19-year-old woman presented two years after sleeve gastrectomy with a two-month history of heavy menstrual bleeding and ecchymoses in March 2023. Laboratory evaluation showed INR >10 and markedly prolonged PT and aPTT. Factors II, VII, IX, and X were markedly reduced, while other coagulations factors were normal. She had no anticoagulant exposure or liver disease. Findings were consistent with acquired VKCFD likely due to postoperative malabsorption.

Despite parenteral and oral vitamin K with intermittent prothrombin complex concentrate (PCC), her INR fluctuated between >10 and 1.4 for months. Immunosuppressive therapy with prednisone and mycophenolate mofetil initiated in November 2023 normalized coagulation suggesting a possible immune-mediated component. In April 2024 when she was off Immunosuppressive, she relapsed with INR >10 and developed a spontaneous anterior mediastinal hematoma, successfully treated with PCC and vitamin K. Steroids were tapered and mycophenolate stopped. Since mid-2025 INR has remained stable (0.9–1.2) without bleeding.

**Summary/Conclusion**

These two cases highlight that acquired VKCFD may have an immune-mediated component beyond nutritional deficiency. Refractory coagulopathy unresponsive to vitamin K alone should prompt consideration of immunosuppressive therapy. The sustained response to prednisone and mycophenolate mofetil underscores the need for greater clinical awareness of immune dysregulation as an underrecognized driver of acquired VKCFD.

**Copyright Information:** (Online) ISSN: 2572-9241
©2026 The Author(s). HemaSphere is published by John Wiley & Sons Ltd on behalf of European Hematology Association. This is an open access Abstract Book distributed under the Attribution-NonCommercial-NoDerivs (CC BY-NC-ND), which allows third parties to download the articles and share them with others as long as they credit the author and the Abstract Book, but they cannot change the content in any way or use them commercially.
**Abstract Book Citations:** Authors, Title, HemaSphere, 2026;10:(S1):pages. The abstract book DOIs can be found on the "Author Information" page.
**Disclaimer:** Articles published in the journal HemaSphere exclusively reflect the opinions of the authors. The authors are responsible for all content in their abstracts including accuracy of the facts, statements, citing resources, etc.

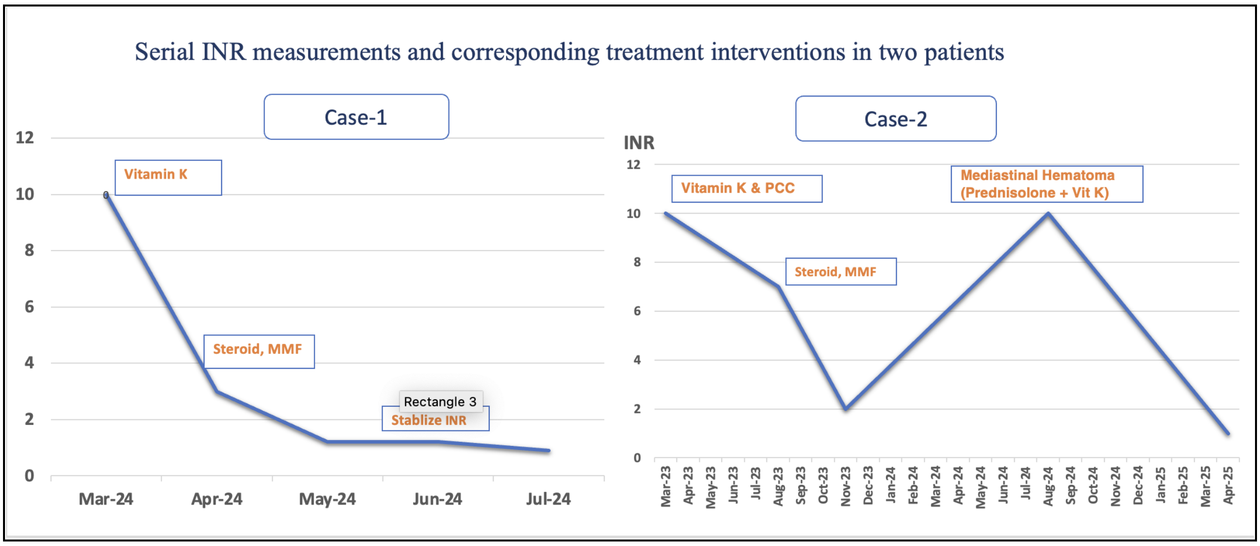

**Copyright Information:** (Online) ISSN: 2572-9241
©2026 The Author(s). HemaSphere is published by John Wiley & Sons Ltd on behalf of European Hematology Association. This is an open access Abstract Book distributed under the Attribution-NonCommercial-NoDerivs (CC BY-NC-ND), which allows third parties to download the articles and share them with others as long as they credit the author and the Abstract Book, but they cannot change the content in any way or use them commercially.
**Abstract Book Citations:** Authors, Title, HemaSphere, 2026;10:(S1):pages. The abstract book DOIs can be found on the "Author Information" page.
**Disclaimer:** Articles published in the journal HemaSphere exclusively reflect the opinions of the authors. The authors are responsible for all content in their abstracts including accuracy of the facts, statements, citing resources, etc.

### (PB4344) SUCCESSFUL SLEEVE GASTRECTOMY IN A MORBIDLY OBESE PATIENT WITH CONGENITAL HEMOPHILIA B: CHALLENGES AND LESSONS LEARNED

**Topic:** 34. Bleeding disorders (congenital and acquired)

Alfadil Haroon*[1], Arwa Almuwallad[1], Hazzaa Alzahrani[1]

[1]*Cancer Center of Excellence, King Faisal Specialist Hospital and Research Center, Adult Hematology, stem cell transplant and cellular therapy section, Riyad, Saudi Arabia;*

### Background

Hemophilia B, a congenital deficiency of factor IX, leads to a bleeding that complicates surgical procedures. Morbid obesity in hemophilic patients accelerates joint damage and reduces mobility. Bariatric surgery,is an effective weight reduction strategy, but requires meticulous perioperative planning in hemophilia to prevent hemorrhagic complications.

### Aims

To report successful perioperative management of laparoscopic sleeve gastrectomy in a morbidly obese adolescent with severe congenital Hemophilia B.

### Methods

Clinical data, per and postoperative factor IX replacement protocols, laboratory monitoring, and postoperative outcomes were reviewed and analyzed from institutional case chart.

### Results

Case presentation:
A 19-year-old male with severe congenital Hemophilia B (baseline factor IX activity: 0.01 IU/mL) presented with morbid obesity (weight: 169 kg, BMI: 50.5 kg/m²) and progressive hemophilic arthropathy secondary to recurrent hemarthroses. He had no documented inhibitor history and was managed with on-demand recombinant factor IX infusions rather than regular prophylaxis. Given worsening mobility, anticipated long-term disability and failed conservative weight management, laparoscopic sleeve gastrectomy was deemed necessary.
Preoperative multidisciplinary planning involved hematology and surgical teams to establish a structured perioperative factor IX replacement protocol targeting activity levels of 70–100%. On the day of surgery, recombinant factor IX 16,000 IU (100 IU/kg) was administered intravenously 30 minutes preoperatively and continued twice daily. Doses were subsequently tapered over five days: 12,000 IU BID on days 2–3, 10,000 IU BID on day 4, and 8,000 IU BID on day 5, with further adjustment from day 6 onward based on clinical status and measured factor IX activity levels. Tranexamic acid 1,500 mg IV every eight hours was administered concurrently from days 1 through 5 as adjunctive antifibrinolytic therapy. Daily monitoring of factor IX activity, hemoglobin, coagulation profile, and clinical bleeding signs was performed throughout the perioperative period.

The surgical procedure was completed without intraoperative complications. Factor IX activity remained within the target range throughout. Postoperative recovery was uneventful, and the patient was discharged on day 5. At follow-up, body weight decreased dramatically from 169 kg to 83 kg, with marked improvement in joint mobility and hemophilic arthropathy, demonstrating the broader therapeutic benefit of bariatric intervention in this complex patient population.

**Copyright Information:** (Online) ISSN: 2572-9241
©2026 The Author(s). HemaSphere is published by John Wiley & Sons Ltd on behalf of European Hematology Association. This is an open access Abstract Book distributed under the Attribution-NonCommercial-NoDerivs (CC BY-NC-ND), which allows third parties to download the articles and share them with others as long as they credit the author and the Abstract Book, but they cannot change the content in any way or use them commercially.
**Abstract Book Citations:** Authors, Title, HemaSphere, 2026;10:(S1):pages. The abstract book DOIs can be found on the “Author Information” page.
**Disclaimer:** Articles published in the journal HemaSphere exclusively reflect the opinions of the authors. The authors are responsible for all content in their abstracts including accuracy of the facts, statements, citing resources, etc.

### Summary/Conclusion

This case demonstrates that laparoscopic sleeve gastrectomy can be performed safely in patients with severe Hemophilia B when supported by a structured, weight-based perioperative factor IX replacement protocol and close hematological monitoring. A multidisciplinary approach is essential to minimize bleeding risk while optimizing surgical outcomes. Beyond weight reduction, bariatric surgery offers significant musculoskeletal benefit in hemophilic patients, potentially reducing long-term joint morbidity and improving overall quality of life and reduced factor consumption. This case adds to the limited literature and underscores the need for pharmacokinetic studies to optimize factor dosing in obese individuals with hemophilia B.

Table 1. Per and posoperative factor IX replacement regimen

| Day | Clinical Context | FIX Dose (IU/kg) | Dose (IU) | Frequency | Adjunctive Therapy |
|---|---|---|---|---|---|
| 0 (Surgery Day) | Pre- and intra-operative coverage | 100 | 16,000 | Every 12 h | Tranexamic acid 1,500 mg IV q8h (Day 1–5) |
| 1 | Immediate post-op | 71 | 12,000 | Every 12 h | Continued tranexamic acid |
| 2 | Early post-op | 71 | 12,000 | Every 12 h | Continued tranexamic acid |
| 3 | Early stabilization | 59 | 10,000 | Every 12 h | Continued tranexamic acid |
| 4 | Dose tapering | 59 | 10,000 | Every 12 h | Continued tranexamic acid |
| 5 | Further taper | 47 | 8,000 | Every 12 h | Last day of tranexamic acid |
| 6 onward | Tailored based on FIX levels and clinical stability | Adjusted to maintain FIX > 50 IU/dL | Individualized | Per lab guidance | None |

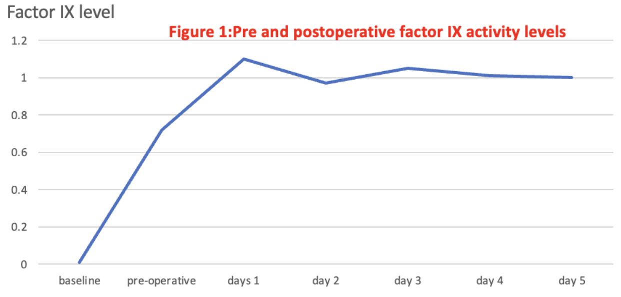

Figure 1:Pre and postoperative factor IX activity levels

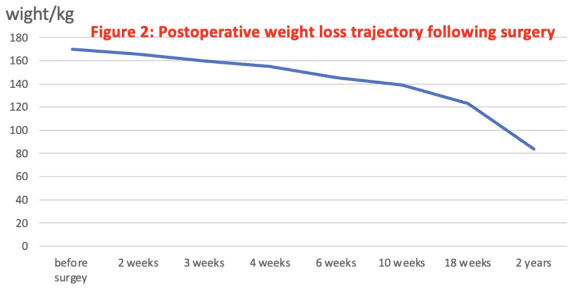

Figure 2: Postoperative weight loss trajectory following surgery

**Copyright Information:** (Online) ISSN: 2572-9241
©2026 The Author(s). HemaSphere is published by John Wiley & Sons Ltd on behalf of European Hematology Association. This is an open access Abstract Book distributed under the Attribution-NonCommercial-NoDerivs (CC BY-NC-ND), which allows third parties to download the articles and share them with others as long as they credit the author and the Abstract Book, but they cannot change the content in any way or use them commercially.
**Abstract Book Citations:** Authors, Title, HemaSphere, 2026;10:(S1):pages. The abstract book DOIs can be found on the "Author Information" page.
**Disclaimer:** Articles published in the journal HemaSphere exclusively reflect the opinions of the authors. The authors are responsible for all content in their abstracts including accuracy of the facts, statements, citing resources, etc.

### (PB4345) SUCCESSFUL PERIOPERATIVE MANAGEMENT OF ACQUIRED HEMOPHILIA B IN A PREGNANT PATIENT REFUSING BLOOD TRANSFUSION DUE TO RELIGIOUS BELIEFS

**Topic:** 34. Bleeding disorders (congenital and acquired)

Rogermar Villanueva*[1]

[1]*University of the Philippines- Philippine General Hospital, Internal Medicine- Division of Hematology, Manila, Philippines;*

**Background**

Acquired hemophilia B (AHB) is an ultra-rare, potentially life-threatening bleeding disorder, with pregnancy-associated cases and transfusion-free management scarcely documented worldwide. This report describes the perioperative management of a 33-year-old pregnant Filipino woman with AHB and concomitant iron deficiency anemia who categorically refused allogeneic blood products on religious grounds.

**Aims**

To describe the perioperative, transfusion-free management of pregnancy-complicated AHB using patient blood management strategies in a resource-limited setting.

**Methods**

This report describes a single patient, using data obtained retrospectively.

**Results**

Comprehensive work-up revealed a variably prolonged activated partial thromboplastin time, normal factor VIII and von Willebrand factor levels, and a high-titer factor IX inhibitor (10 Bethesda units), confirming acquired hemophilia B. Preoperative optimization followed patient blood management principles, including intravenous iron, subcutaneous erythropoietin, cessation of aspirin and low molecular weight heparin at appropriate gestational intervals, and formulation of a bloodless cesarean section plan with multidisciplinary input from hematology, obstetrics, anesthesia, allergy-immunology, neurology, and psychiatry.
At term, the patient underwent urgent repeat cesarean section with bilateral tubal ligation under spinal anesthesia, using tranexamic acid, meticulous surgical technique, and vigilant hemodynamic monitoring; estimated blood loss was 400 mL, and no blood products, factor concentrates, or bypassing agents were administered. The postoperative course was uneventful, with stable hemoglobin, absence of hemorrhagic or thrombotic complications, and healthy maternal and neonatal discharge.

**Summary/Conclusion**

This case demonstrates that, even in a resource-limited setting, pregnancy-complicated AHB can be managed safely without transfusion through early recognition, structured multidisciplinary planning, and rigorous application of blood conservation strategies, and it adds novel data to the sparse global literature on bloodless management of acquired hemophilia B in pregnancy.

**Copyright Information:** (Online) ISSN: 2572-9241
©2026 The Author(s). HemaSphere is published by John Wiley & Sons Ltd on behalf of European Hematology Association. This is an open access Abstract Book distributed under the Attribution-NonCommercial-NoDerivs (CC BY-NC-ND), which allows third parties to download the articles and share them with others as long as they credit the author and the Abstract Book, but they cannot change the content in any way or use them commercially.
**Abstract Book Citations:** Authors, Title, HemaSphere, 2026;10:(S1):pages. The abstract book DOIs can be found on the "Author Information" page.
**Disclaimer:** Articles published in the journal HemaSphere exclusively reflect the opinions of the authors. The authors are responsible for all content in their abstracts including accuracy of the facts, statements, citing resources, etc.

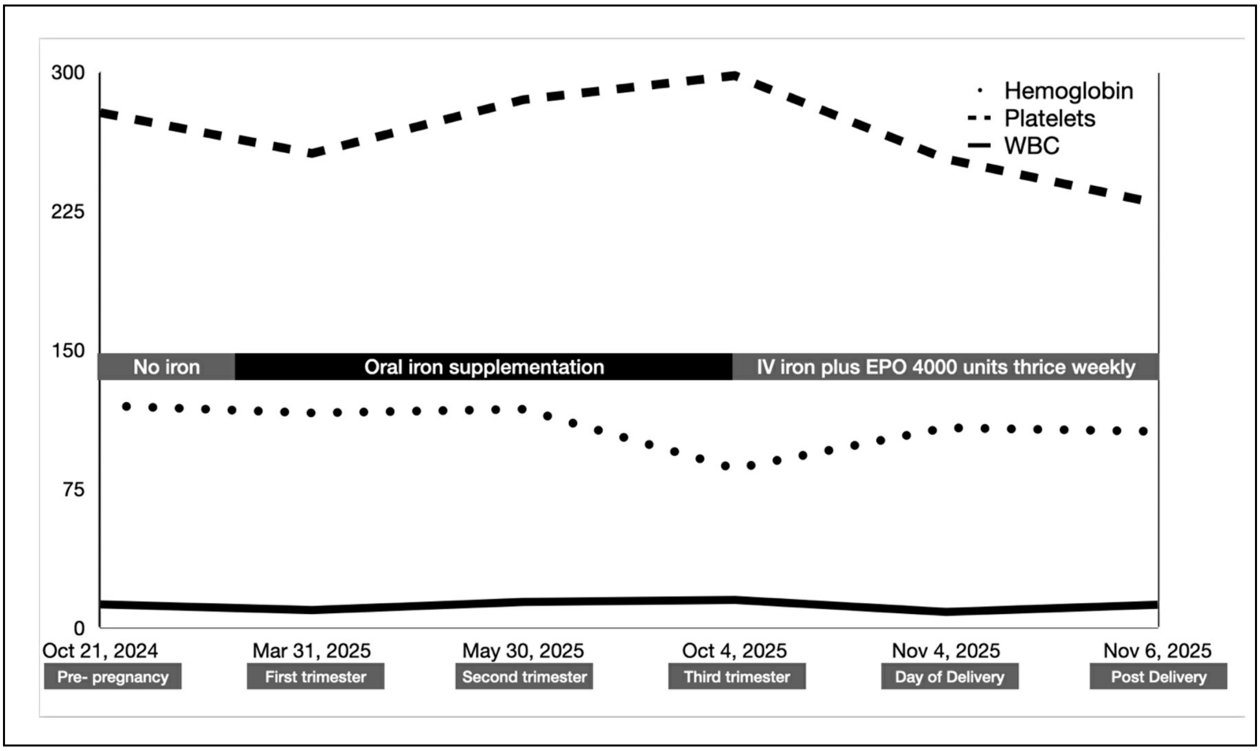

**Copyright Information:** (Online) ISSN: 2572-9241
©2026 The Author(s). HemaSphere is published by John Wiley & Sons Ltd on behalf of European Hematology Association. This is an open access Abstract Book distributed under the Attribution-NonCommercial-NoDerivs (CC BY-NC-ND), which allows third parties to download the articles and share them with others as long as they credit the author and the Abstract Book, but they cannot change the content in any way or use them commercially.
**Abstract Book Citations:** Authors, Title, HemaSphere, 2026;10:(S1):pages. The abstract book DOIs can be found on the "Author Information" page.
**Disclaimer:** Articles published in the journal HemaSphere exclusively reflect the opinions of the authors. The authors are responsible for all content in their abstracts including accuracy of the facts, statements, citing resources, etc.

### (PB4346) ACQUIRED VITAMIN K–DEPENDENT COAGULATION FACTOR DEFICIENCY OF UNKNOWN ETIOLOGY: A DIAGNOSTIC CHALLENGE

**Topic:** 34. Bleeding disorders (congenital and acquired)

Rubab Ali*[1], Asma Munir[1], Arsala Rashid[1], Alssa Alvi[2], Quratulain Ghalib[1], Fauzia Tabassum[1]

*[1]Services Institute of Medical Sciences/ Services Hospital, Hematology, Lahore, Pakistan;[2]Al Aleem Medical College, Lahore, Pakistan;*

### Background

Acquired deficiency of vitamin K–dependent coagulation factors (II, VII, IX, X) is most commonly secondary to malnutrition, malabsorption, hepatobiliary disease, antibiotic exposure, or vitamin K antagonists (warfarin, etc.). In contrast, idiopathic presentations in healthy young adults are very rare. Such cases pose a diagnostic dilemma, often mimicking congenital factor deficiencies, hepatic dysfunction or acquired inhibitors. Recognition is crucial because the condition is reversible yet potentially catastrophic if overlooked.

### Aims

To describe a rare case of idiopathic acquired VKCFD in a young adult female presenting with significant bleeding, and to highlight a structured diagnostic approach to prevent misdiagnosis and unnecessary interventions.

### Methods

A 21-year-old healthy female presented with sudden onset of painless hematuria and menorrhagia accompanied by spontaneous bruising over lower limbs. There was no history of trauma, drug intake (any anticoagulants or antibiotics), dietary restriction, liver disease, gastrointestinal symptoms, or family history of bleeding diathesis.
On examination, she had mild pallor and multiple bruises over lower limbs, no lymphadenopathy or organomegaly. Laboratory investigations revealed mild anemia, normal platelet count, and markedly prolonged PT and aPTT. Liver and renal function tests were within reference ranges.
Mixing studies demonstrated complete correction of PT and aPTT with normal and aged plasma, excluding circulating inhibitors. Factor assays showed markedly reduced activity of factors II, VII, IX, and X, with preserved factor V and VIII. Imaging and evaluation for occult liver or biliary pathology were unremarkable. No secondary cause could be identified despite comprehensive evaluation and detailed history taking.
Patient initially received parenteral vitamin K (10 mg) but reported a severe adverse reaction. Therapy was promptly shifted to oral vitamin K which was well tolerated. PT and aPTT normalized within 48 hours and hematuria resolved within 72 hours of treatment. She was discharged on oral vitamin K but was lost on follow-up; and presented two months later with left lower limb swelling and tenderness. Coagulation parameters were again markedly deranged. Upon inquiry, she stated non-compliance to advised treatment. Oral vitamin K was restarted along with FFP infusion to achieve rapid normalization of parameters and avoid intramuscular hematoma and subsequent contracture formation.

**Copyright Information:** (Online) ISSN: 2572-9241
©2026 The Author(s). HemaSphere is published by John Wiley & Sons Ltd on behalf of European Hematology Association. This is an open access Abstract Book distributed under the Attribution-NonCommercial-NoDerivs (CC BY-NC-ND), which allows third parties to download the articles and share them with others as long as they credit the author and the Abstract Book, but they cannot change the content in any way or use them commercially.
**Abstract Book Citations:** Authors, Title, HemaSphere, 2026;10:(S1):pages. The abstract book DOIs can be found on the “Author Information” page.
**Disclaimer:** Articles published in the journal HemaSphere exclusively reflect the opinions of the authors. The authors are responsible for all content in their abstracts including accuracy of the facts, statements, citing resources, etc.

**Results**

Following therapy, coagulation parameters normalized within 24-48 hours with complete clinical resolution of bleeding manifestations. Patient was discharged on oral vitamin K supplements and remains symptom free to date without recurrence. The clinical course presenting as new adult-onset bleeding tendency without prior personal or family history or known precipitating factors; was consistent with Idiopathic Acquired VKCFD; supported by mixing studies, specific factor assays and a rapid therapeutic response to vitamin K therapy.

**Summary/Conclusion**

Idiopathic acquired VKCFD is an exceptionally rare but reversible cause of significant bleeding in young adults which underlines the importance of including VKCFD in the differential diagnosis of combined pathway prolongation. This case highlights that VKCFD may arise spontaneously despite no identifiable risk factor; and understanding this can facilitate correct diagnosis, avoid misclassification as congenital coagulopathy and overtreatment; thus improving patient outcomes.

**Copyright Information:** (Online) ISSN: 2572-9241
©2026 The Author(s). HemaSphere is published by John Wiley & Sons Ltd on behalf of European Hematology Association. This is an open access Abstract Book distributed under the Attribution-NonCommercial-NoDerivs (CC BY-NC-ND), which allows third parties to download the articles and share them with others as long as they credit the author and the Abstract Book, but they cannot change the content in any way or use them commercially.
**Abstract Book Citations:** Authors, Title, HemaSphere, 2026;10:(S1):pages. The abstract book DOIs can be found on the "Author Information" page.
**Disclaimer:** Articles published in the journal HemaSphere exclusively reflect the opinions of the authors. The authors are responsible for all content in their abstracts including accuracy of the facts, statements, citing resources, etc.

### (PB4347) REFRACTORY IMMUNE THROMBOCYTOPENIA IN A CASE WITH SPONDYLOENCHONDRODYSPLASIA TREATED SUCCESSFULLY WITH RİTUXİMAB

**Topic:** 34. Bleeding disorders (congenital and acquired)

Yeter Düzenli Kar*[1], Ugur Mete[1], Fatma Aslan[2], Numan Katmer[1], Melike Sezgİn Evİm[1], Adalet Meral Güneş[1]

*[1]Bursa Uludag University, Pediatric Hematology, Bursa, Türkiye;[2]Erzurum City Hospital, Pediatric Hematology, Erzurum, Türkiye;*

### Background

Spondyloenchondrodysplasia (SPENCD) is a type of interferonopathy caused by biallelic mutations in ACP5 gene. It is characterized by metaphyseal lesions, neurological impairment and immune dysregulation associated with lupus-like features.

### Aims

In this article, we reported a 21-year-old male with SPENCD, presenting with recurrent thrombocytopenia episodes since he was 7 years old and refractory autoimmune hemolytic anemia at he was 11 years old.

### Methods

The pathophysiology and clinical presentation of SPENCDs were discussed with our patients findings and in light of the literature.

### Results

A 21-year-old male patient was first referred to our hospital when he was seven years old to investigate the etiology of thrombocytopenia. It was learned that he had a history of hypoxic birth and was being followed up by pediatric neurology and neurosurgery due to neuromotor developmental delay. His family history showed that his mother and father were second-degree relatives. In his physical examination, his weight was 16 kg (<3 percentile), height was 105 cm (<3 percentile), he could not walk or speak, and he had mental retardation. He had contractures in his hand and foot joints, and there was no organomegaly. He was unresponsive to intravenous immunoglobulin (IVIG) and responsive to steroids. The patient, who developed refractory autoimmune hemolytic anemia and splenomegaly at the age of 11, was diagnosed with Evans Syndrome and used steroids for 1.5 years. Direct chest X-rays taken at the age of 10 showed platyspondyly, no scoliosis at this age. At 16 years old minimal thoracolumbar scoliosis is relevant. Scoliosis progresses at 21 years old age. (Figure 1). Cranial magnetic resonance and computerized tomography at the age of 10 showed calcifications in the bilateral basal ganglia, thalamus and subcortical white matter of the cerebral hemisphere. Steroids were given for a short period of time as long as treatment was indicated. There was no response to eltrombopag and rompilastim treatments. In the patient's screening for collagen tissue diseases, ANA: 1 positive (1/1000), anti-Ds-DNA: 42 (0-20), C4: 0.28 g/L, C3c: 1.24 g/L, anticardiolipin screening and lupus anticoagulant were negative. In lymphocyte subsets, CD19: 0.8%, CD20: 0.7%, CD4/CD8: 0.1 and simultaneous peripheral lymphocyte count:1000/mm$^3$. In the whole exome sequencing (WES) analysis showed a homozygous c.155A > C (p.K52T) mutation was detected in the ACP5 gene, and the patient was diagnosed with SPENCD. Since the steroid response decreased after subdural bleeding when he was 21 years old, respectively mycophenolate mofetil, sirolumus, ruxolitinib were started and was not response obtained. Due to the patient's persistent low platelet count and history of frequent bleeding, rituximab 375 mg/m2 was administered for 4 weeks. The patient's platelet count exceeded 100,000/mm3 from the 3rd week onwards, and the patient did not require further treatment.

**Copyright Information:** (Online) ISSN: 2572-9241
©2026 The Author(s). HemaSphere is published by John Wiley & Sons Ltd on behalf of European Hematology Association. This is an open access Abstract Book distributed under the Attribution-NonCommercial-NoDerivs (CC BY-NC-ND), which allows third parties to download the articles and share them with others as long as they credit the author and the Abstract Book, but they cannot change the content in any way or use them commercially.
**Abstract Book Citations:** Authors, Title, HemaSphere, 2026;10:(S1):pages. The abstract book DOIs can be found on the "Author Information" page.
**Disclaimer:** Articles published in the journal HemaSphere exclusively reflect the opinions of the authors. The authors are responsible for all content in their abstracts including accuracy of the facts, statements, citing resources, etc.

**Summary/Conclusion**

The diagnosis of SPENCD should be kept in mind in patients with persistent thrombocytopenia, mental retardation and skeletal deformities since early childhood, especially if there are widespread cranial calcifications. Although these patients respond to steroids, long-term use may cause severe side effects. Rituximab may be the first-line biological agent of choice for autoimmune cytopenia of SPENCD, even with low peripheral blood CD20 levels.

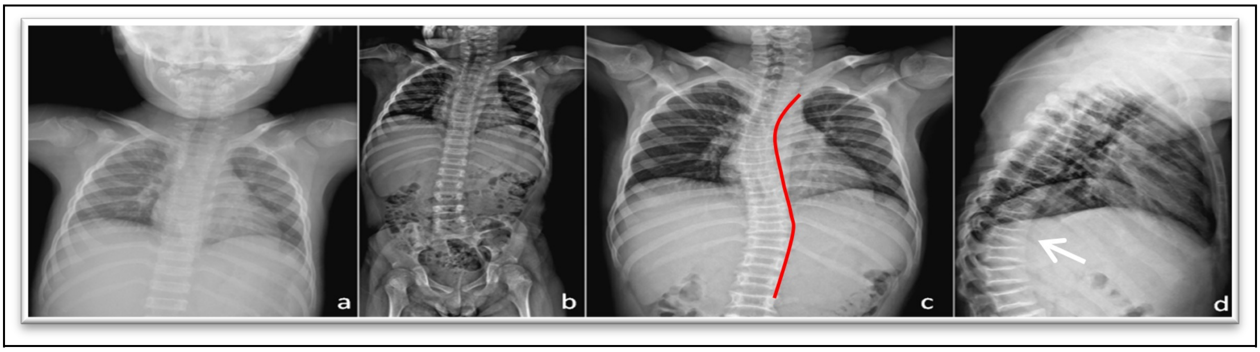

Figure 1. Direct chest X rays of the patient in 10 years (a), 16 years(b) and 21 (c,d) years old. The patient have platyspondyly (arrow in d) starting from 10 years old age, no scoliosis at this age. At 16 years old (b) minimal thoracolumber scoliosis is relevant. Scoliosis progresses at 21 years old age(red curved line).

**Copyright Information:** (Online) ISSN: 2572-9241
©2026 The Author(s). HemaSphere is published by John Wiley & Sons Ltd on behalf of European Hematology Association. This is an open access Abstract Book distributed under the Attribution-NonCommercial-NoDerivs (CC BY-NC-ND), which allows third parties to download the articles and share them with others as long as they credit the author and the Abstract Book, but they cannot change the content in any way or use them commercially.
**Abstract Book Citations:** Authors, Title, HemaSphere, 2026;10:(S1):pages. The abstract book DOIs can be found on the "Author Information" page.
**Disclaimer:** Articles published in the journal HemaSphere exclusively reflect the opinions of the authors. The authors are responsible for all content in their abstracts including accuracy of the facts, statements, citing resources, etc.

### (PB4348) POSTPARTUM ACQUIRED HEMOPHILIA A TREATED WITH RITUXIMAB

**Topic:** 34. Bleeding disorders (congenital and acquired)

Milica Apostolovic Stojanovic*[1], Aleksandar Stojanovic[2], Milica Radojkovic[1]

*[1]Clinical Hospital Center "Dr Dragisa Misovic - Dedinje", Department of Hematology, Belgrade, Serbia;[2]Medical faculty, University of Belgrade, Serbia, Belgrade, Serbia;*

### Background

Acquired hemophilia A (AHA) is a rare autoimmune bleeding disorder caused by inhibitory autoantibodies against factor VIII (FVIII). Postpartum AHA represents a distinct subset, accounting for approximately 7-21% of AHA cases and typically presents with skin or soft-tissue bleeding in patients without a prior bleeding history.

### Aims

To present a clinically relevant case of postpartum AHA successfully treated with off-label rituximab and to highlight diagnostic and therapeutic considerations.

### Methods

We describe a previously healthy 40-year-old woman who presented four months postpartum with spontaneous bruising and soft-tissue bleeding. Diagnostic evaluation included coagulation testing, mixing study, FVIII activity measurement, inhibitor quantification, and exclusion of secondary causes of AHA.

### Results

The patient developed hematomas of the right forearm, palm and an intramuscular thigh hematoma confirmed by ultrasound. Laboratory findings showed isolated prolongation of activated partial thromboplastin time (aPTT) with partial correction on mixing study, FVIII activity of 0.84%, and a FVIII inhibitor titer >2 Bethesda units/mL. Antinuclear antibodies, complement C3/C4, and ferritin levels were within normal ranges. Immunosuppressive therapy with corticosteroids combined with off-label rituximab was initiated. After four cycles of rituximab, bleeding resolved, the FVIII inhibitor was eradicated, FVIII activity normalized, and complete clinical remission was achieved.

### Summary/Conclusion

Postpartum AHA should be suspected in women with new-onset bleeding and isolated aPTT prolongation after delivery. Early recognition, exclusion of alternative causes of acquired coagulopathy, and prompt immunosuppressive therapy are essential. This case supports rituximab as an effective off-label option for inhibitor eradication and remission induction in postpartum AHA.

**Copyright Information:** (Online) ISSN: 2572-9241
©2026 The Author(s). HemaSphere is published by John Wiley & Sons Ltd on behalf of European Hematology Association. This is an open access Abstract Book distributed under the Attribution-NonCommercial-NoDerivs (CC BY-NC-ND), which allows third parties to download the articles and share them with others as long as they credit the author and the Abstract Book, but they cannot change the content in any way or use them commercially.
**Abstract Book Citations:** Authors, Title, HemaSphere, 2026;10:(S1):pages. The abstract book DOIs can be found on the "Author Information" page.
**Disclaimer:** Articles published in the journal HemaSphere exclusively reflect the opinions of the authors. The authors are responsible for all content in their abstracts including accuracy of the facts, statements, citing resources, etc.

### (PB4349) MIXING TEST APTT: A LOW-COST, RAPID SCREENING TEST FOR PROLONGED APTT THAT MAY BE IMMEDIATELY DIAGNOSTIC FOR ACQUIRED HEMORRHAGIC OR THROMBOTIC DISORDERS

**Topic:** 34. Bleeding disorders (congenital and acquired)

Sofia K. Chaniotaki[1], Anna Kalogianni[1], Eirini Manoli[1], Ioulia Chaliori*[1], Nikolaos Tsagarakis[1], Iris Koutri[2], Vasilios Bolanos[2], Georgios Papadopoulos[3], Andria Yiaskouri[1], Maria Siarkou[1], Eleni Bortzari[1], Dimitrios Liakopoulos[1], Athanasios Alexopoulos[1], Evanthia Farmaki[1], Christina Karela[4], Stavros Kalafatis[1], Stefanos Papadimitriou[1], Theodoros Marinakis[1], Efrosyni Nomikou[2], Veroniki Komninaka[1]

*[1]General Hospital "G. Gennimatas", Hematology Laboratory, Athens, Greece; [2]General Hospital "Hippokration", Blood Bank and Hemophilia Unit, Athens, Greece; [3]General Hospital "G. Gennimatas", Department of Urology, Athens, Greece; [4]General Hospital "G. Gennimatas", Department of Immunology, Athens, Greece;*

### Background

Mixing tests are able to identify factor deficiencies or inhibitors, and are also useful for the detection of lupus anticoagulant (LA). The mixing test–APTT (MT-APTT) is performed in cases of unexplained prolonged activated partial thromboplastin time (APTT). It consists of mixing the patient's plasma with normal plasma in a 1:1 ratio and measuring APTT immediately and after 2-hour incubation at 37°C, as certain inhibitors are time- and temperature-dependent [e.g., factor VIII- inhibitor (FVIII-inh) / acquired hemophilia A (AHA)]. There are two possible outcomes: mixing tests that present post-incubation correction tends to suggest the presence of factor deficiencies, where as those that are not corrected suggest the presence of "inhibitors". AHA is a rare, underreported hemorrhagic disease in non-hemophiliacs (1 case/$10^6$ persons/year). It causes sudden and severe skin and mucosal bleeding that can be life-threatening. The disease occurs more often in the elderly, it is usually idiopathic (50%) or secondary to other autoimmune diseases, malignancies, inflammatory disorders, postpartum or infectious triggers. Therapy aims to control the bleeding with hemostatic agents and the eradication of the inhibitor. Conversely LA represents the most enigmatic antibody population in patients with antiphospholipid syndrome and represents a paradox that is still unsolved: this class of antibody causes a phospholipid-dependent prolongation of the clotting time but is associated with an increased risk of thrombosis and pregnancy morbidity.

### Aims

To describe two cases in which the diagnosis was established through MT-APTT.

### Methods

Coagulation assays, coagulation factor measurements, and MT-APTT were performed on ACL-TOP 750 analyzers (IL/Werfen).

**Copyright Information:** (Online) ISSN: 2572-9241
©2026 The Author(s). HemaSphere is published by John Wiley & Sons Ltd on behalf of European Hematology Association. This is an open access Abstract Book distributed under the Attribution-NonCommercial-NoDerivs (CC BY-NC-ND), which allows third parties to download the articles and share them with others as long as they credit the author and the Abstract Book, but they cannot change the content in any way or use them commercially.
**Abstract Book Citations:** Authors, Title, HemaSphere, 2026;10:(S1):pages. The abstract book DOIs can be found on the "Author Information" page.
**Disclaimer:** Articles published in the journal HemaSphere exclusively reflect the opinions of the authors. The authors are responsible for all content in their abstracts including accuracy of the facts, statements, citing resources, etc.

### Results

<u>Case 1:</u> A 71-year-old woman with no prior bleeding history and no anticoagulation therapy presented with extensive ecchymoses (Figure 1) lasting for four months, progressively worsening, and prolonged APTT (46 sec). She was referred to our hospital for further investigation. Laboratory evaluation revealed isolated progressive APTT prolongation (57.1 sec). MT-APTT results were: Immediate APTTmix = 34.2 sec (correction), APTTmix after incubation = 47.2 sec (no correction). These findings suggested the presence of an acquired coagulation factor inhibitor, confirmed by FVIII-activity measurement of 1.6% (reference range: 50–150%).
<u>Case 2:</u> A 59-year-old man presented to the Urology Emergency department with symptoms of acute prostatitis. Prolonged APTT (81.9 sec) was detected and persisted throughout hospitalization, without anticoagulant use. MT-APTT results were: Immediate APTTmix = 61.9 sec (no correction), APTTmix after incubation = 70.5 sec (no correction). These findings suggested the presence of LA, which was confirmed using the dilute Russell's viper venom time (DRVVT) assay. The patient was found to have triple-positive antiphospholipid antibodies and was ultimately diagnosed with SLE.

### Summary/Conclusion

Isolated prolongation of APTT raises suspicion of either a coagulation factor deficiency or the presence of an inhibitor (such as LA or an acquired factor inhibitor, e.g. FVIII-inh) and requires further investigation. MT-APTT, a rapid and cost-effective method, may aid diagnostic evaluation and support appropriate patient management. Close collaboration between clinicians and laboratory physicians remains essential for timely diagnosis and optimal treatment.

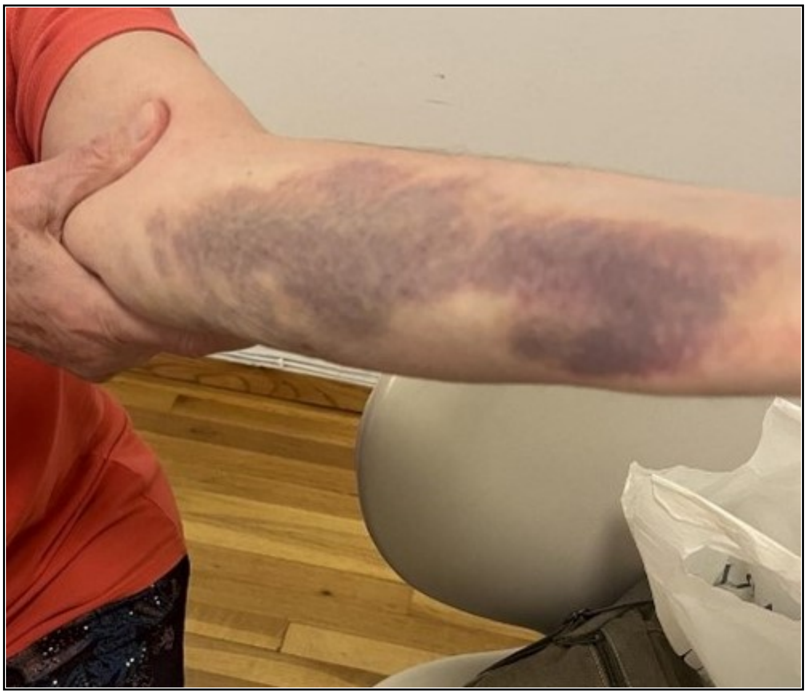

Figure 1: Case 1, extensive ecchymoses.

**Copyright Information:** (Online) ISSN: 2572-9241
©2026 The Author(s). HemaSphere is published by John Wiley & Sons Ltd on behalf of European Hematology Association. This is an open access Abstract Book distributed under the Attribution-NonCommercial-NoDerivs (CC BY-NC-ND), which allows third parties to download the articles and share them with others as long as they credit the author and the Abstract Book, but they cannot change the content in any way or use them commercially.
**Abstract Book Citations:** Authors, Title, HemaSphere, 2026;10:(S1):pages. The abstract book DOIs can be found on the "Author Information" page.
**Disclaimer:** Articles published in the journal HemaSphere exclusively reflect the opinions of the authors. The authors are responsible for all content in their abstracts including accuracy of the facts, statements, citing resources, etc.

### (PB4350) POPULATION PHARMACOKINETICS MODELING OF KOVALTRY BY REAL WORLD DATA: FREE INDIVIDUALIZED MEDICATION GUIDANCE FOR HEMOPHILIA PATIENTS IN CHINA

**Topic:** 34. Bleeding disorders (congenital and acquired)

Xiaoxu Wang[1], Lingling Chen[1], Tiantian Wang[2], Wenjuan Miao[1], Jixin Tian[1], Wei Liu[1], Lei Zhang[1], Jie Wang[2], Yan Cheng[2], Yunhai Fang[2], Renchi Yang[1], Feng Xue[1], Ping Zhang*[1]

*[1]State Key Laboratory of Experimental Hematology, National Clinical Research Center for Blood Diseases, Haihe Laboratory of Cell Ecosystem, Institute of Hematology & Blood Diseases Hospital, Chinese Academy of Medical Sciences & Peking Union Medical College, Tianjin, China; [2]Shandong Blood Center, Shandong Hemophilia Treatment Center, Jinan, China;*

### Background

Standard weight-based prophylaxis treatment is not safe or effective for all patients due to extreme interindividual variability in FVIII pharmacokinetics (PK), which complicates maintaining FVIII coagulating activity above the targeted level. Free personalized medication support is essential from both economic and safety perspectives in China. However, the PK-tailored individualized prevention is challenging due to the complex calculations and numerous blood samples required by the traditional PK methods. The population pharmacokinetic (PopPK) approach can reduce the number of samples for PK parameter to support precision medication for patients. Kovaltry, as one of the most widely used medications for hemophilia patients in China, urgently needs a PopPK model suitable for Chinese patients.

### Aims

The purpose is to develop a PopPK model using real world data in Chinese quantifying the influence between and within patients. Integrating the model into the Hemophilia Treatment Center Collaboration Network of China (HTCCNC) to support individualized treatment for a larger population of Chinese hemophiliacs to improve quality of life.

### Methods

31 Chinese patients' clinical routine prophylactic data were collected and used to develop the model. Phoenix NLME 8.3 was used to develop the PopPK model and quantify the effect of potential covariates on the PK parameters, including demographics, von Willebrand factor antigens (vWF:Ag) and blood groups. Meanwhile, external validation was performed using sparse data from 6 real-world prophylactic patients.

### Results

Kovaltry conformed with a 2-compartment model in Chinese HA patients. WT and vWF:Ag significantly affected the clearance (CL) of the central compartment (V), while fat-free mass (FFM) affected the distribution volume of peripheral compartment (V2) and V. Our final model demonstrated sufficient reproducibility for evaluating PK parameters via internal and external validation. Through comparison, our model's predicted PK parameters for 6 real-world prophylactic patients are close to those of WAPPS. The model enables accurate prediction of individual responses from sparse samples, supporting personalized prophylaxis adjustment in real-world clinical settings and being the first real-world data model integrated into HTCCNC.

### Summary/Conclusion

This is the first real-world data predictive model of Kovaltry integrated into HTCCNC. It enables PK-guided prophylaxis, improving patient outcomes and costs for a larger population of Chinese hemophiliacs.

**Copyright Information:** (Online) ISSN: 2572-9241
©2026 The Author(s). HemaSphere is published by John Wiley & Sons Ltd on behalf of European Hematology Association. This is an open access Abstract Book distributed under the Attribution-NonCommercial-NoDerivs (CC BY-NC-ND), which allows third parties to download the articles and share them with others as long as they credit the author and the Abstract Book, but they cannot change the content in any way or use them commercially.
**Abstract Book Citations:** Authors, Title, HemaSphere, 2026;10:(S1):pages. The abstract book DOIs can be found on the "Author Information" page.
**Disclaimer:** Articles published in the journal HemaSphere exclusively reflect the opinions of the authors. The authors are responsible for all content in their abstracts including accuracy of the facts, statements, citing resources, etc.

### (PB4351) ACQUIRED FACTOR XIII DEFICIENCY: A CASE SERIES ON DIAGNOSIS IN PATIENTS WITH UNEXPLAINED BLEEDING AND NORMAL COAGULATION PROFILES.

**Topic:** 34. Bleeding disorders (congenital and acquired)

Alfredo Gómez Lacuey*[1], Alicia Wendy Vega Harwood[1], Marina Leon Gamazo[1], Pilar Fuertes[1], Susana Herraez Rodriguez[1, 1], Scarlet Barrios Rodriguez[1], Maria Jose Cebeira[1], Carolina Bombin Canal[1]

[1]*HOSPITAL CLINICO VALLADOLID, VALLADOLID, Spain;*

### Background

Acquired factor XIII (FXIII) deficiency is a rare coagulation disorder that may occur in previously healthy patients with no personal or family history of bleeding. It is characterized by a functional reduction of FXIII, which plays a crucial role in fibrin clot stabilization. This explains why conventional coagulation tests are usually normal. The most common cause is the development of inhibitory autoantibodies, although increased consumption or underlying diseases may also be involved. Clinically, it presents with spontaneous or disproportionate bleeding, which can be severe, making early recognition essential. three cases were identified over one year among 300,000 inhabitants

### Aims

- Clinical Characterization: To evaluate the patterns and severity of hemorrhagic manifestations at the time of clinical presentation.
- Biochemical Correlation: To correlate laboratory findings—specifically FXIII activity levels—with the clinical phenotype and response to therapy

### Methods

A descriptive observational study was focused on three patients with no personal or familial history of hemostatic disorders who presented with unexpected, severe bleeding.

### Results

Three previously healthy patients developed excessive bleeding following a triggering event (major surgery or childbirth), despite normal conventional coagulation tests. In all cases, measurement of FXIII levels led to the diagnosis of acquired FXIII deficiency.
The first case was a postpartum woman who developed excessive bleeding and widespread hematomas, with a significant drop in hemoglobin levels. Despite a normal coagulation profile, she required plasma transfusion and antifibrinolytic treatment with tranexamic acid (Amchafibrin). FXIII levels were 57%.
The second case involved a woman with a history of thrombosis who was a candidate for kidney transplantation. She presented with preoperative bleeding that did not improve after administration of tranexamic acid or prothrombin complex concentrate. Clinical improvement was observed after initiation of fresh frozen plasma, and FXIII levels were 47%.
The third case was a patient who underwent coronary bypass surgery and subsequently developed persistent surgical wound bleeding with anemia and normal coagulation tests. Treatment with plasma controlled the bleeding. FXIII levels were markedly reduced at 12%.

**Copyright Information:** (Online) ISSN: 2572-9241
©2026 The Author(s). HemaSphere is published by John Wiley & Sons Ltd on behalf of European Hematology Association. This is an open access Abstract Book distributed under the Attribution-NonCommercial-NoDerivs (CC BY-NC-ND), which allows third parties to download the articles and share them with others as long as they credit the author and the Abstract Book, but they cannot change the content in any way or use them commercially.
**Abstract Book Citations:** Authors, Title, HemaSphere, 2026;10:(S1):pages. The abstract book DOIs can be found on the "Author Information" page.
**Disclaimer:** Articles published in the journal HemaSphere exclusively reflect the opinions of the authors. The authors are responsible for all content in their abstracts including accuracy of the facts, statements, citing resources, etc.

**Summary/Conclusion**

Acquired FXIII deficiency is a "diagnostic chameleon" that must be suspected in any patient exhibiting disproportionate bleeding in the presence of normal routine coagulation profiles.

- Early Detection: Quantitative FXIII activity assays should be prioritized over the older, less sensitive "clot solubility tests."
- Therapeutic Strategy: Replacement therapy via Fresh Frozen Plasma (FFP), Cryoprecipitate is essential to restore clot integrity.
- Prognostic Impact: Swift recognition prevents the administration of unnecessary procoagulants and reduces the morbidity associated with persistent, unexplained surgical hemorrhage.

**Copyright Information:** (Online) ISSN: 2572-9241
©2026 The Author(s). HemaSphere is published by John Wiley & Sons Ltd on behalf of European Hematology Association. This is an open access Abstract Book distributed under the Attribution-NonCommercial-NoDerivs (CC BY-NC-ND), which allows third parties to download the articles and share them with others as long as they credit the author and the Abstract Book, but they cannot change the content in any way or use them commercially.
**Abstract Book Citations:** Authors, Title, HemaSphere, 2026;10:(S1):pages. The abstract book DOIs can be found on the “Author Information” page.
**Disclaimer:** Articles published in the journal HemaSphere exclusively reflect the opinions of the authors. The authors are responsible for all content in their abstracts including accuracy of the facts, statements, citing resources, etc.

### (PB4352) SPINAL ANAESTHESIA IN ELDERLY PATIENTS WITH HIP FRACTURES ON APIXABAN THERAPY: A RETROSPECTIVE STUDY

**Topic:** 34. Bleeding disorders (congenital and acquired)

Eirini Papakitsou*[1], Eleftherios Papakitsos[2], George Papakitsos[3, 3]

*[1]Aristotle University Thessaloniki, Greece, Thessaloniki, Greece; [2]Aristotle University Thessaloniki, Thessaloniki, Greece; [3]general hospital arta, greece, Arta, Greece;*

### Background

This study reviews surgeries involving elderly patients with hip fractures on apixaban who underwent spinal anaesthesia (SA) within 72 hours of their last dose.

### Aims

The study aims to assess the potential safety of using SA for hip fracture surgery by reviewing consecutive cases at our hospital, involving patients with confirmed positive plasma concentrations of the anticoagulant.

### Methods

We performed a retrospective review of medical records of patients, who underwent hip fracture surgery under SA, while on apixaban therapy from January 2021, to January 2026. 65 patients were included in the study. SA was selected for all cases after a careful evaluation of the risks and benefits, considering both the patient’s clinical conditions and their preferences

### Results

There are several limitations. Its retrospective design and small sample size limit the generalizability of the findings. The use of neuraxial anaesthesia in patients receiving apixaban raises concerns about the risk of neuraxial bleeding. Daily evaluations were conducted throughout their hospitalization to assess for signs of spinal haematoma or persistent neurological deficits. Despite patients being on anticoagulation, no neurological complications occurred

### Summary/Conclusion

SA may be safely performed and practical in cases where the benefits of timely surgery outweigh the potential risks in select patients on apixaban, even when the drug has been discontinued for <72 hours. However, further research is crucial to optimize the management of anticoagulated patients requiring neuraxial anaesthesia

**Copyright Information:** (Online) ISSN: 2572-9241
©2026 The Author(s). HemaSphere is published by John Wiley & Sons Ltd on behalf of European Hematology Association. This is an open access Abstract Book distributed under the Attribution-NonCommercial-NoDerivs (CC BY-NC-ND), which allows third parties to download the articles and share them with others as long as they credit the author and the Abstract Book, but they cannot change the content in any way or use them commercially.
**Abstract Book Citations:** Authors, Title, HemaSphere, 2026;10:(S1):pages. The abstract book DOIs can be found on the “Author Information” page.
**Disclaimer:** Articles published in the journal HemaSphere exclusively reflect the opinions of the authors. The authors are responsible for all content in their abstracts including accuracy of the facts, statements, citing resources, etc.

### (PB4354) ASSESSMENT OF KNOWLEDGE ON HEMOSTASIS AMONG PHYSICIANS IN TRAINING: A DIAGNOSTIC STUDY

**Topic:** 34. Bleeding disorders (congenital and acquired)

Clara Leal Voltarelli*[1], Maria Eduarda Jabuonski Antosz[1], Marcia Olandoski[2], Bruna Olandoski Erbano[3], Jerusa Karina Miqueloto[4]

*[1]Hospital Universitário Cajuru, Curitiba, Brazil; [2]PUCPR - Pontifícia Universidade Católica do Paraná, Curitiba, Brazil; [3]CHC UFPR, Curitiba, Brazil; [4]Oncoclínicas, Curitiba, Brazil;*

**Background**

Hemostasis is a fundamental physiological process for bleeding control and safe clinical practice across multiple medical specialties. Despite its clinical relevance, previous studies suggest that physicians and medical students frequently demonstrate insufficient knowledge of coagulation mechanisms and laboratory test interpretation, which may result in diagnostic errors, inappropriate test ordering, and suboptimal patient management.

**Aims**

To assess the level of knowledge regarding hemostasis and coagulation among physicians and medical students at different stages of medical training, and to compare performance between these groups in order to identify potential educational gaps.

**Methods**

This cross-sectional, descriptive, and analytical study was conducted at a university hospital in southern Brazil between July and October 2025. A validated questionnaire composed of five multiple-choice and five open-ended questions addressing theoretical and practical aspects of hemostasis and coagulation testing was applied. A total of 192 participants were stratified into five groups: physicians, specialists, residents, Student 1 (medical students up to the ninth semester), and Student 2 (medical students from the tenth semester onward). Scores were converted to a 0–100 scale. Statistical analyses were performed using SPSS version 30.0, applying Chi-square tests, ANOVA, Kruskal-Wallis test, and Dunn post-hoc analysis, with $p < 0.05$ considered statistically significant.

**Copyright Information:** (Online) ISSN: 2572-9241
©2026 The Author(s). HemaSphere is published by John Wiley & Sons Ltd on behalf of European Hematology Association. This is an open access Abstract Book distributed under the Attribution-NonCommercial-NoDerivs (CC BY-NC-ND), which allows third parties to download the articles and share them with others as long as they credit the author and the Abstract Book, but they cannot change the content in any way or use them commercially.
**Abstract Book Citations:** Authors, Title, HemaSphere, 2026;10:(S1):pages. The abstract book DOIs can be found on the "Author Information" page.
**Disclaimer:** Articles published in the journal HemaSphere exclusively reflect the opinions of the authors. The authors are responsible for all content in their abstracts including accuracy of the facts, statements, citing resources, etc.

### Results

Significant differences were observed among groups regarding hemostasis knowledge scores ($p < 0.001$). Residents achieved the highest mean score (50.7 ± 15.8), followed by specialists (46.2 ± 14.0), whereas physicians demonstrated the lowest performance (28.4 ± 14.3). Student 1 (up to the ninth semester) and Student 2 (from the tenth semester onward) presented similar intermediate scores, with no statistically significant difference between these groups. Knowledge score distributions according to level of medical training are presented in **Table 1** and illustrated in **Figure 1.**

Table 1

Descriptive statistics of hemostasis knowledge scores by level of medical training

| Group | | Score | | | | | P value* |
|---|---|---|---|---|---|---|---|
| | n | Mean score | Standard deviation | Median | Minimum | Maximum | |
| Resident | 44 | 50.7 | 15.8 | 50 | 14 | 82 | |
| Especialist | 32 | 46.2 | 14 | 47.5 | 14 | 75 | |
| Student 2 | 36 | 39.6 | 15.7 | 39 | 9 | 82 | <0.001 |
| Student 1 | 46 | 38.0 | 11.4 | 37 | 17 | 70 | |
| Physician | 34 | 28.4 | 14.3 | 28 | 5 | 72 | |

* Kruskal–Wallis non-parametric test; $p < 0.05$ considered statistically significant.

Data are presented as mean ± standard deviation. Student 1: up to the ninth semester; Student 2: from the tenth semester onward.

### Summary/Conclusion

Relevant knowledge gaps in hemostasis were identified across all stages of medical training, with particularly low performance among physicians after graduation. These findings highlight the need for continuous medical education strategies and curricular improvements that reinforce the integration of theoretical knowledge and clinical application of coagulation principles to enhance diagnostic accuracy and patient safety.

**Copyright Information:** (Online) ISSN: 2572-9241
©2026 The Author(s). HemaSphere is published by John Wiley & Sons Ltd on behalf of European Hematology Association. This is an open access Abstract Book distributed under the Attribution-NonCommercial-NoDerivs (CC BY-NC-ND), which allows third parties to download the articles and share them with others as long as they credit the author and the Abstract Book, but they cannot change the content in any way or use them commercially.
**Abstract Book Citations:** Authors, Title, HemaSphere, 2026;10:(S1):pages. The abstract book DOIs can be found on the “Author Information” page.
**Disclaimer:** Articles published in the journal HemaSphere exclusively reflect the opinions of the authors. The authors are responsible for all content in their abstracts including accuracy of the facts, statements, citing resources, etc.

**Figure 1**
Distribution of hemostasis knowledge scores according to level of medical training

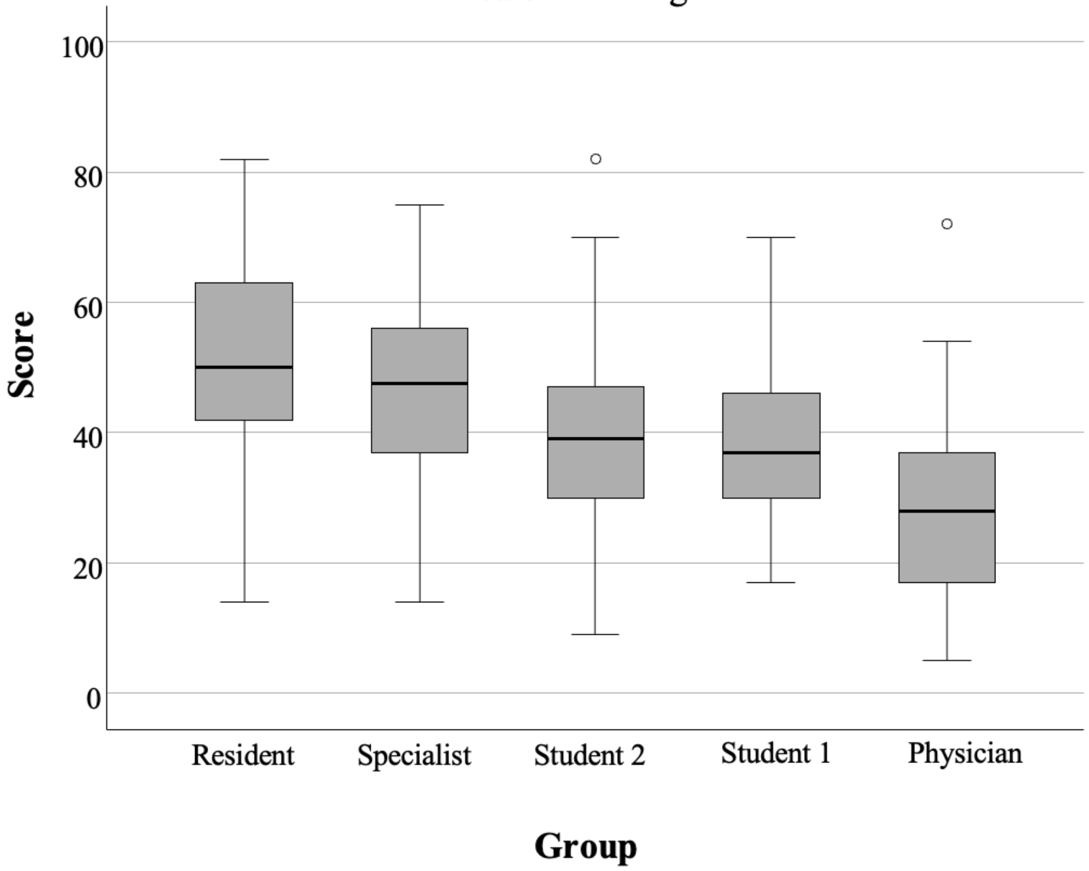

Boxplot showing median values, interquartile range, and minimum and maximum scores. Kruskal–Wallis test, $p < 0.001$.

**Copyright Information:** (Online) ISSN: 2572-9241
©2026 The Author(s). HemaSphere is published by John Wiley & Sons Ltd on behalf of European Hematology Association. This is an open access Abstract Book distributed under the Attribution-NonCommercial-NoDerivs (CC BY-NC-ND), which allows third parties to download the articles and share them with others as long as they credit the author and the Abstract Book, but they cannot change the content in any way or use them commercially.
**Abstract Book Citations:** Authors, Title, HemaSphere, 2026;10:(S1):pages. The abstract book DOIs can be found on the "Author Information" page.
**Disclaimer:** Articles published in the journal HemaSphere exclusively reflect the opinions of the authors. The authors are responsible for all content in their abstracts including accuracy of the facts, statements, citing resources, etc.

### (PB4355) ACQUIRED FACTOR VII DEFICIENCY ASSOCIATED WITH AGGRESSIVE T-CELL LYMPHOMA: A CASE REPORT

**Topic:** 34. Bleeding disorders (congenital and acquired)

Oumeyma Touati[1], Roua Hsasna[1], Emna Azza[1], Ben Othmen Aya[1], Yosr Ben Abdennabi[1], Marwa Bahri[1], Lamia Issaoui*[1]

[1]*Aziza Othmana Hospital, Tunis, Tunisia;*

### Background

Isolated acquired factor VII deficiency (aFVIID) is very rare. The majority of reported cases are secondary to infection or neoplasia. Malignant blood disorders associated with this deficiency are uncommon and there is little information available in the literature. In particular, few cases of aFVIID associated with aggressive lymphoma have been reported, hence the interest of our work.

### Results

We report the case of a 14-year-old male patient who was referred to our department for multiple lymphadenopathies and deterioration in general health. History of the patient reported no personal or family history of illness. Initial examination revealed cervical lymphadenopathies, with no signs of infection or hemorrhagic syndrome. Preoperative coagulation tests revealed an isolated decrease in prothrombin time (PT) to 59%, activated partial thromboplastin time was normal, and fibrinogen was elevated at 6.9 g/L. This decrease in PT was verified in a second sample. Coagulation factors testing was done : FII was at 106%, FV was at 88%, FX was at 134%, and FVII was at 45%. This decrease in FVII was confirmed in a second sample. A biopsy of a cervical lymph node was performed without hemorrhagic complications. Diagnosis was ALK-positive anaplastic large cell T-cell lymphoma. The patient was classified as Ann Arbor stage IV. The patient received first-line chemotherapy (CHOEP protocol). The coagulation tests performed after the first course of chemotherapy showed normalization of PT and FVII levels to 79% and 72%, respectively, without the use of specific replacement therapy.
Given the absence of a personal or family history of coagulation factor deficiency and the normalization of FVII levels after chemotherapy, the diagnosis of isolated aFVIID was retained.

### Summary/Conclusion

This case illustrates a rare association between aggressive lymphoma and aFVIID. Several hypotheses have been proposed of the pathophysiological mechanism of this deficiency, and they are likely to be intertwined. Rapid correction of the coagulation abnormality after chemotherapy may be a rare biological marker of tumor activity. Our case highlights the importance of systematic screening for coagulation disorders in aggressive lymphomas.

**Copyright Information:** (Online) ISSN: 2572-9241
©2026 The Author(s). HemaSphere is published by John Wiley & Sons Ltd on behalf of European Hematology Association. This is an open access Abstract Book distributed under the Attribution-NonCommercial-NoDerivs (CC BY-NC-ND), which allows third parties to download the articles and share them with others as long as they credit the author and the Abstract Book, but they cannot change the content in any way or use them commercially.
**Abstract Book Citations:** Authors, Title, HemaSphere, 2026;10:(S1):pages. The abstract book DOIs can be found on the "Author Information" page.
**Disclaimer:** Articles published in the journal HemaSphere exclusively reflect the opinions of the authors. The authors are responsible for all content in their abstracts including accuracy of the facts, statements, citing resources, etc.

### (PB4356) PATIENT-REPORTED BLEEDING BURDEN AND QUALITY OF LIFE IN HEMATOLOGIC DISORDERS: A SYSTEMATIC REVIEW

**Topic:** 34. Bleeding disorders (congenital and acquired)

Stephanie Quon*[1], Katherine Zheng[1], Sarah Miller[1]

[1]*University of British Columbia, Vancouver, Canada;*

### Background

Bleeding symptoms are common across hematologic disorders and may substantially affect quality of life, daily functioning, and treatment adherence. Despite their clinical importance, patient-reported bleeding burden and its impact on quality of life are inconsistently assessed in hematology research and practice.

### Aims

To systematically review patient-reported bleeding burden and its association with quality of life in adults with hematologic disorders.

### Methods

We searched MEDLINE, Embase, CINAHL, and PsycINFO from inception through June 2025 for studies reporting patient-reported bleeding outcomes in adults with inherited or acquired bleeding disorders, hematologic malignancies, or bone marrow failure syndromes. Outcomes included bleeding severity scores, symptom burden, and validated quality-of-life measures (e.g., EQ-5D, SF-36). Two reviewers independently screened studies and extracted data. Risk of bias was assessed using tools appropriate to study design.

### Results

Thirty-one studies involving 9,284 participants were included. Most focused on inherited bleeding disorders, particularly hemophilia and von Willebrand disease, with fewer studies addressing malignancy-associated or acquired cytopenias. Across conditions, greater patient-reported bleeding burden was consistently associated with worse quality-of-life outcomes, particularly in physical functioning, pain, vitality, and work or school productivity. In adults with hemophilia A, increasing annual bleeding frequency was associated with progressively lower EQ-5D-5L scores (approximately 0.92 with no recent bleeds versus 0.56 with five or more bleeds per year), alongside marked increases in work productivity loss. In von Willebrand disease, quality of life was consistently reduced compared with population norms, with greater impairment among individuals with severe bleeding phenotypes, joint bleeding, or heavy menstrual bleeding. Women with inherited bleeding disorders experiencing menorrhagia reported disproportionately lower quality-of-life scores. Considerable heterogeneity existed in bleeding assessment tools and outcome reporting.

### Summary/Conclusion

Patient-reported bleeding burden is associated with clinically meaningful impairments in quality of life across hematologic disorders. Standardized patient-reported bleeding and quality-of-life measures are needed to better inform clinical care, supportive interventions, and future research.

**Copyright Information:** (Online) ISSN: 2572-9241
©2026 The Author(s). HemaSphere is published by John Wiley & Sons Ltd on behalf of European Hematology Association. This is an open access Abstract Book distributed under the Attribution-NonCommercial-NoDerivs (CC BY-NC-ND), which allows third parties to download the articles and share them with others as long as they credit the author and the Abstract Book, but they cannot change the content in any way or use them commercially.
**Abstract Book Citations:** Authors, Title, HemaSphere, 2026;10:(S1):pages. The abstract book DOIs can be found on the “Author Information” page.
**Disclaimer:** Articles published in the journal HemaSphere exclusively reflect the opinions of the authors. The authors are responsible for all content in their abstracts including accuracy of the facts, statements, citing resources, etc.

### (PB4357) ADULT CIRCUMCISION PROCEDURE WITH FIBRIN GLUE IN A PATIENT WITH SEVERE HEMOPHILIA A

**Topic:** 34. Bleeding disorders (congenital and acquired)

Nil Güler*[1], Zafer Aybek[2], Kaan Kavaklı[3]

*[1]Pamukkale University, Faculty of Medicine, Hematology, Denizli, Türkiye;[2]Pamukkale University, Faculty of Medicine, Urology, Denizli, Türkiye; [3]Ege University Faculty of Medicine, Department of Pediatrics and Hematology, İzmir, Türkiye;*

### Background

Circumcision is a big step in some cultures and countries to become a man and accepting to adult life. In addition, circumcision is practiced in some religious such as Islam and Judaism as part of religion culture. Patients with hemophilia mostly don't circumcised because of bleeding risk.

### Methods

A 42-year-old male with severe hemophilia was applied for elective circumcision. He had been diagnosed at 10 months of age with baseline Factor VIII level <1%. After two radioisotope synovectomy procedures of the left knee, patient was under Factor VIII prophylaxis.
At admission, actual Factor VIII level: 2.2%, inhibitor levels <0.4 BU/mL. Factor 8 level was >1% due to ongoing prophylaxis (1500 IU recombinant Factor VIII, three times weekly).
Protocol was planned as: The patient weighed 70kg.
Preoperatively, 3500 IU Factor VIII was administered one hour before general anesthesia.
Patient's prescription was prepared for post of factor levels 80%. However, the target was revised to maintain levels at 100% at during the three post-operative days. Because the prescription had been prepared for an 80% target, 1500 IU doses were administered at 10-hour intervals for three days instead of 12-hour intervals.
An addition factor VIIII dose was administered 30 minutes prior to removal of the gauze dressing at 48 hours' post -surgery. The patient was discharged at 72nd hours postoperatively without any bleeding complication during whole period.
After discharge, factor VIII was given 2000 U/daily for another 4 days. Eighth days of surgery, Factor 8 prophylaxis was restarted as before (1500 U thrice weekly).
Due to logistic limitations, pre and post postoperative factor VIII levels couldn't have checked after factor replacement. However, pre and post- operative APTT levels were within normal limits.
Tranexamic acid was initiated one day prior to surgery and continued one week postoperatively at a dose of 20mg/kg/day divided into three doses.
Diazepam 5mg/daily was prescribed for one week to reduce the risk of erection related bleeding. Due to anxiety about possible bleeding, the patient continued diazepam for an additional weak after discharge.
Surgical technic: The patient was placed in the supine position under general anesthesia. The prepuce was retracted to release any existing adhesions between the foreskin and the glans penis and excised circumferentially using standard surgical technique. Meticulous hemostasis was achieved using monopolar cautery (set at 45W) ensuring no active bleeding from the dorsal vessels or frenular artery. The mucosal and skin edges were approximated and sutured using absorbable sutures in an interrupted fashion. Fibrin sealant (Tisseel) was applied circumferentially over the subcutaneous tissue and mucosal edges. The wound was dressed with antibiotic ointment, followed by the application of a Coban (self-adherent wrap) dressing for light compression and hemostasis.

**Copyright Information:** (Online) ISSN: 2572-9241
©2026 The Author(s). HemaSphere is published by John Wiley & Sons Ltd on behalf of European Hematology Association. This is an open access Abstract Book distributed under the Attribution-NonCommercial-NoDerivs (CC BY-NC-ND), which allows third parties to download the articles and share them with others as long as they credit the author and the Abstract Book, but they cannot change the content in any way or use them commercially.
**Abstract Book Citations:** Authors, Title, HemaSphere, 2026;10:(S1):pages. The abstract book DOIs can be found on the "Author Information" page.
**Disclaimer:** Articles published in the journal HemaSphere exclusively reflect the opinions of the authors. The authors are responsible for all content in their abstracts including accuracy of the facts, statements, citing resources, etc.

**Summary/Conclusion**

Circumcision can be performed safely with careful organization and protocols in Hemophila patents. In our center, we followed the 'İzmir Protocol', which aims to minimize total factor consumption with help of fibrin glue. Our Factor 8 usage amount for circumcision were higher as 335.71 U/kg compare to (102-600 U/Kg) İzmir Protocol. The reasons may be this circumcision were it was our center's first experience and potential bleeding risk associated with erectile function in adult man compare to young boys leaded us to be more conservative.

**Copyright Information:** (Online) ISSN: 2572-9241
©2026 The Author(s). HemaSphere is published by John Wiley & Sons Ltd on behalf of European Hematology Association. This is an open access Abstract Book distributed under the Attribution-NonCommercial-NoDerivs (CC BY-NC-ND), which allows third parties to download the articles and share them with others as long as they credit the author and the Abstract Book, but they cannot change the content in any way or use them commercially.
**Abstract Book Citations:** Authors, Title, HemaSphere, 2026;10:(S1):pages. The abstract book DOIs can be found on the "Author Information" page.
**Disclaimer:** Articles published in the journal HemaSphere exclusively reflect the opinions of the authors. The authors are responsible for all content in their abstracts including accuracy of the facts, statements, citing resources, etc.

### (PB4358) PATIENT WITH IMMUNE THROMBOCYTOPENIC PURPURA AGED 78 YEARS INDUCED BY COVID-19 VACCINE AFTER SECOND DOSE: A CASE REPORT

**Topic:** 34. Bleeding disorders (congenital and acquired)

Wenhui Liu*[1], Tao Wu[1], Yang Liu[1], Qiuyue Wu[1]

[1] *The 940th Hospital of Joint Logistics Support force of Chinese People's Liberation Army, hematology, Gansu, China;*

### Background

Since December 2019, COVID-19 caused by SARS-CoV-2 has spread widely worldwide, seriously threatening human life and health. Because there are no specific medicine against novel coronaviruses, COVID-19 vaccines become the most important preventive measure to control the COVID-19 pandemic. At present, there are several COVID-19 vaccines under development[1]: inactivated vaccine, live attenuated vaccine, recombinant subunit vaccine, adenoviral vector vaccine, deoxyribonucleic acid (DNA) vaccines and ribonucleic acid (RNA) vaccines. Three vaccines have been approved for marketing in China, which are inactivated vaccines, recombinant subunit vaccines and adenoviral vector vaccines. With massive vaccination and subsequent follow-up, vaccine-related adverse reactions have emerged in clinical practice. In this article, a case of immune thrombocytopenic purpura (ITP) induced by COVID-19 vaccine (Vero cells) is reported.

### Aims

COVID-19 has become a global pandemic, but treatment options remain limited. Up to now, vaccination is the main strategy to prevent transmission and reduce disease severity. In the initial clinical trials of vaccines, no significant adverse events other than rare allergic reactions were reported. However, with follow-up observations after massive vaccination, ITP induced by COVID-19 vaccines has attracted the attention of investigators. The aim of the present study was to raise the awareness of medical staff regarding this disease and to increase the vigilance of the general public. At the same time, the present study also provided an effective method to manage this type of adverse reaction to the COVID-19 vaccine.

### Methods

We selected a 78-year-old elderly female who presented with "oral bleeding for 2 days and scattered bleeding spots on the extremities for 1 day" after vaccination with the COVID-19 vaccine (Vero Cells) as the study subject. And blood routine analysis indicated a white blood cell count of $6.27\times10^{9}$/L(Reference Interval: 4-$10\times10^{9}$/L), hemoglobin levels of 144 g/L(Reference Interval: 110-150g/L) and platelet count (PLT) of $1\times10^{9}$/L(Reference Interval: 100-$300\times10^{9}$/L). Bone marrow cytomorphology showed thrombocytopenia, while no platelet-producing megakaryocytes were observed.

### Results

The patient was diagnosed with ITP and given symptomatic and supportive treatment, such as prednisone acetate 1 mg/kg, recombinant human thrombopoietin, intravenous injection of human immunoglobulin 0.4 g/kg and prevention of bleeding.

**Copyright Information:** (Online) ISSN: 2572-9241
©2026 The Author(s). HemaSphere is published by John Wiley & Sons Ltd on behalf of European Hematology Association. This is an open access Abstract Book distributed under the Attribution-NonCommercial-NoDerivs (CC BY-NC-ND), which allows third parties to download the articles and share them with others as long as they credit the author and the Abstract Book, but they cannot change the content in any way or use them commercially.
**Abstract Book Citations:** Authors, Title, HemaSphere, 2026;10:(S1):pages. The abstract book DOIs can be found on the "Author Information" page.
**Disclaimer:** Articles published in the journal HemaSphere exclusively reflect the opinions of the authors. The authors are responsible for all content in their abstracts including accuracy of the facts, statements, citing resources, etc.

**Summary/Conclusion**

At one week after the treatment was started, the patient's PLT began to increase and 9 days later, it returned to normal levels. The patient tolerated the treatment and is currently on maintenance therapy with the oral thrombopoietin receptor agonist Hytrombopag as an outpatient. Patient was followed up 4 months after discharge, the PLT was still >$100\times10^9$/L.

**Copyright Information:** (Online) ISSN: 2572-9241
©2026 The Author(s). HemaSphere is published by John Wiley & Sons Ltd on behalf of European Hematology Association. This is an open access Abstract Book distributed under the Attribution-NonCommercial-NoDerivs (CC BY-NC-ND), which allows third parties to download the articles and share them with others as long as they credit the author and the Abstract Book, but they cannot change the content in any way or use them commercially.
**Abstract Book Citations:** Authors, Title, HemaSphere, 2026;10:(S1):pages. The abstract book DOIs can be found on the "Author Information" page.
**Disclaimer:** Articles published in the journal HemaSphere exclusively reflect the opinions of the authors. The authors are responsible for all content in their abstracts including accuracy of the facts, statements, citing resources, etc.

### (PB323) JAK2 V617F AND THROMBOTIC RISK INDEPENDENT OF HEMOGLOBIN: POLYCYTHEMIA VERA VS SECONDARY ERYTHROCYTOSIS IN MEN

**Topic:** 35. Thrombosis and vascular biology

Motaz Almahmood*[1], Mohammad Abu-Tineh[2], Dalia Othman[1], Hatem Ahmed[1], Yuenu Wu[1], Mohammad Alazzeh[1], Jinming Song[3], Le Wang[1]

*[1]Tower Health Phoenixville Hospital, Phoenixville, United States of America;[2]Tower Health Reading Hospital, Reading, United States of America; [3]Moffitt Cancer Center, Tampa, United States of America;*

**Background**

Polycythemia vera (PV) is associated with increased thrombotic risk, and current guidelines recommend phlebotomy to maintain hematocrit 45%. However, whether thrombotic risk is driven primarily by elevated hemoglobin or by JAK2 V617F-mediated prothrombotic biology remains unclear. We compared thrombotic outcomes between JAK2+ PV and secondary erythrocytosis at equivalent hemoglobin levels in men.

**Aims**

To compare thrombotic outcomes in men with polycythemia vera and secondary erythrocytosis at similar hemoglobin levels, in order to assess whether thrombotic risk is more closely associated with JAK2 V617F–related disease biology than with erythrocytosis alone.

**Methods**

Retrospective cohort study using TriNetX, a federated network of 67 US healthcare organizations. We identified men aged 18-80 with hemoglobin ≥16.5 g/dL, comparing JAK2 V617F-positive patients (identified by CPT 81270) to those with secondary erythrocytosis (COPD, obstructive sleep apnea, smoking history, pulmonary hypertension, or renal malignancy). Secondary erythrocytosis patients with JAK2 positivity, PV diagnosis, testosterone, or erythropoietin use were excluded. Dehydration within 7 days of hemoglobin measurement was excluded in both cohorts. Propensity score matching balanced cohorts on hemoglobin, hematocrit, BMI, LDL cholesterol, HbA1c, platelets, leukocytes, hypertension, prior MI, and atrial fibrillation. Outcomes included composite thrombosis (MI, stroke/TIA, arterial embolism, VTE, PE, splanchnic thrombosis), arterial events, and venous events.

**Results**

After matching, 19,356 patients per cohort were analyzed (mean follow-up: 1,244 vs 1,395 days). Mean hemoglobin was 17.0 g/dL (PV) vs 16.4 g/dL (secondary). Composite thrombosis risk was lower in PV (10.2% vs 11.3%; OR 0.89, 95% CI 0.83–0.96; p=0.003). Arterial events were similarly lower in PV (7.5% vs 8.4%; OR 0.89, 95% CI 0.82–0.96; p=0.003). However, venous thrombosis showed increased hazard in PV (HR 1.13, 95% CI 1.03–1.25; p=0.013) despite similar absolute risk (4.9% vs 4.8%).

**Summary/Conclusion**

At equivalent hemoglobin levels, JAK2+ PV is not associated with increased arterial thrombotic risk compared to secondary erythrocytosis. However, PV demonstrates increased venous thrombotic hazard, suggesting JAK2-mediated mechanisms may specifically drive venous thrombosis independent of hemoglobin. These findings warrant prospective validation and may have implications for risk stratification in erythrocytosis.

**Copyright Information:** (Online) ISSN: 2572-9241
©2026 The Author(s). HemaSphere is published by John Wiley & Sons Ltd on behalf of European Hematology Association. This is an open access Abstract Book distributed under the Attribution-NonCommercial-NoDerivs (CC BY-NC-ND), which allows third parties to download the articles and share them with others as long as they credit the author and the Abstract Book, but they cannot change the content in any way or use them commercially.
**Abstract Book Citations:** Authors, Title, HemaSphere, 2026;10:(S1):pages. The abstract book DOIs can be found on the "Author Information" page.
**Disclaimer:** Articles published in the journal HemaSphere exclusively reflect the opinions of the authors. The authors are responsible for all content in their abstracts including accuracy of the facts, statements, citing resources, etc.

### (PB4359) THE ENDOTHELIAL ACTIVATION AND STRESS INDEX (EASIX) PREDICTS CLINICAL PROGRESSION TO ANTIPHOSPHOLIPID SYNDROME IN ANTIPHOSPHOLIPID ANTIBODY CARRIERS

**Topic:** 35. Thrombosis and vascular biology

Rodrigo Cantera Estefanía*[1], Rafael Gálvez[2], Jose Flores García[3], Héctor Cruz Barquín[3], Maria Oviedo Madrid[3], María Abando Casuso[3], Raquel García Ruiz[3], Marina Herrero López[3], Irene Gorostidi Álvarez[3], Ligia Gabrie Rodríguez[2], Marcos López Hoyos[4], Juan José Domínguez-García[3], María Luisa González Ponte[3], Belén González-Mesones Galán[3], Gala Aglaia Méndez Navarro[3], Jose Luis Hernández Hernández[5], Victor Manuel Martínez Taboada[2], Lucrecia Yáñez[3]

*[1]Hospital Universitario de Navarra, Hematology and Hemotherapy, Pamplona, Spain;[2]Hospital Universitario Marqués de Valdecilla, Rheumatology, Santander, Spain; [3]Hospital Universitario Marqués de Valdecilla, Hematology and Hemotherapy, Santander, Spain; [4]Hospital Universitario Marqués de Valdecilla, Immunology, Santander, Spain;[5]Hospital Universitario Marqués de Valdecilla, Internal Medicine, Santander, Spain;*

### Background

The endothelial activation and stress index (EASIX) is a validated biomarker of endothelial dysfunction in cellular therapies such as allogenic stem cell transplantation and CAR-T cell therapies. As endothelial injury is a hallmark of antiphospholipid syndrome (APS), EASIX could help to identify aPL-positive patients at high risk of complications.

### Aims

To evaluate whether EASIX and derived scores can predict ACR/EULAR domains (D) 1-3 (arterial thromboembolism (ATE), venous thromboembolism (VTE) and microvascular manifestations) among asymptomatic aPL carriers.

### Methods

We conducted a retrospective cohort study including aPL-positive individuals identified from January 2018 to March 2024 without clinical ACR/EULAR D1-4 at serologic confirmation. Baseline EASIX and derived scores were recorded at analytic confirmation. Association and discriminative ability for D1-3 were examined by logistic regression and ROC curve analyses. Multivariable models were built when AUC values exceeded 0.7.

### Results

A total of 985 patients were included (table 1). No significant differences in EASIX-based indices were observed according to autoimmune disease (AD) or prior antithrombotic treatment. Among aPL carriers with AD (n=282), EASIX demonstrated good discrimination D2 (AUC=0.850). Model 1, designed to predict ATE in this subgroup, was less discriminative than EASIX alone (AUC=0.827). Sensitivity analysis of raw EASIX at Youden-optimized cutoff (0.965) showed a high negative-predictive-value (0.994) with low positive-predictive-value (0.115, image 1a). In the entire cohort (n=985), $Log_2$EASIX was associated with D3 (OR 1.826; $p<0.001$), showing good discriminative performance (AUC=0.815). Model 2 improved prediction of microvascular thrombosis in all aPL carriers (AUC=0.887; sensitivity=0.889, specificity= 0.760, image 1b).
Table 1. Demographic characteristics.

**Copyright Information:** (Online) ISSN: 2572-9241
©2026 The Author(s). HemaSphere is published by John Wiley & Sons Ltd on behalf of European Hematology Association. This is an open access Abstract Book distributed under the Attribution-NonCommercial-NoDerivs (CC BY-NC-ND), which allows third parties to download the articles and share them with others as long as they credit the author and the Abstract Book, but they cannot change the content in any way or use them commercially.
**Abstract Book Citations:** Authors, Title, HemaSphere, 2026;10:(S1):pages. The abstract book DOIs can be found on the "Author Information" page.
**Disclaimer:** Articles published in the journal HemaSphere exclusively reflect the opinions of the authors. The authors are responsible for all content in their abstracts including accuracy of the facts, statements, citing resources, etc.

| | | aPL carriers N=985 | |
|---|---|---|---|
| | | N | % |
| Median age [IQR], years | | 53.0 [41–65] | |
| Women | | 686 | 69.6 |
| **CV risk factors** | HTN<180/110 mmHg | 345 | 35.0 |
| | HTN≥180/110 mmHg | 13 | 1.3 |
| | Chronic renal insufficiency | 83 | 8.4 |
| | DM (organ damage or long-term) | 39 | 4.0 |
| | DM (no organ damage + short-term) | 76 | 7.8 |
| | Active smoking | 227 | 23.0 |
| | Obesity | 143 | 14.5 |
| | HC≥310 mg/dL | 8 | 0.8 |
| | HC<310 mg/dL | 352 | 35.7 |
| **VTE risk factors** | Surgerywith general anesthesia | 69 | 7.0 |
| | Surgery<30 mins | 26 | 2.6 |
| | Mayor trauma with fractures | 17 | 1.7 |
| | Cancer | 47 | 4.8 |
| | Hormonal treatment | 49 | 5.0 |
| | Central venous catheter | 16 | 1.6 |
| | Severe infection | 64 | 6.5 |
| | Hospitalization | 140 | 14.2 |
| | Hospitalization (confined to bed) | 57 | 5.8 |
| | Hypercoagulability | 52 | 5.3 |
| | Autoimmune disease | 282 | 28.6 |
| | Pregnancy | 97 | 9.8 |
| **Clinical events** | VTE (D1) | 45 | 4.6 |
| | ATE (D2) | 40 | 4.1 |
| | Microvascular thrombosis (D3) | 20 | 2.0 |

DM: diabetes mellitus. HC: Hypercholesterolemia. HTN: arterial hypertension. LA: lupus anticoagulant.

**Table 2.** Predictive models.

**Copyright Information:** (Online) ISSN: 2572-9241
©2026 The Author(s). HemaSphere is published by John Wiley & Sons Ltd on behalf of European Hematology Association. This is an open access Abstract Book distributed under the Attribution-NonCommercial-NoDerivs (CC BY-NC-ND), which allows third parties to download the articles and share them with others as long as they credit the author and the Abstract Book, but they cannot change the content in any way or use them commercially.
**Abstract Book Citations:** Authors, Title, HemaSphere, 2026;10:(S1):pages. The abstract book DOIs can be found on the "Author Information" page.
**Disclaimer:** Articles published in the journal HemaSphere exclusively reflect the opinions of the authors. The authors are responsible for all content in their abstracts including accuracy of the facts, statements, citing resources, etc.

| Model | Dependent variable | Population | Variables included | B | p | OR | CI95% Inferior | Superior |
|---|---|---|---|---|---|---|---|---|
| 1 | D2 (ATE) | aPL carriers with AD | EASIX | 0.255 | **0.007** | 1.291 | 1.072 | 1.555 |
| | | | Age | 0.003 | 0.909 | 1.003 | 0.957 | 1.051 |
| | | | Women | 1.190 | 0.321 | 3.286 | 0.313 | 34.498 |
| | | | Laboratory domain score | 0.230 | **0.049** | 1.259 | 1.001 | 1.583 |
| 2 | D3 (microvascular thrombosis) | aPL carriers | EASIX | 0.025 | 0.520 | 1.025 | 0.950 | 1.107 |
| | | | Age | 0.015 | 0.420 | 1.016 | 0.978 | 1.054 |
| | | | Women | -0.231 | 0.662 | 0.794 | 0.282 | 2.233 |
| | | | HTN>180/110 mmHg | 1.798 | 0.072 | 6.038 | 0.851 | 42.861 |
| | | | Chronic renal insufficiency | 2.799 | **<0.0001** | 16.436 | 5.448 | 49.589 |
| | | | Obesity | -1.457 | 0.179 | 0.233 | 0.028 | 1.946 |
| | | | Cancer | 1.401 | 0.066 | 4.060 | 0.911 | 18.100 |

### Summary/Conclusion

EASIX and related indices may reflect endothelial activation and injury in aPL carriers, providing a shared pathophysiological basis between APS and hematologic malignancies that supports their potential role as predictors of thrombotic complications.

**Copyright Information:** (Online) ISSN: 2572-9241
©2026 The Author(s). HemaSphere is published by John Wiley & Sons Ltd on behalf of European Hematology Association. This is an open access Abstract Book distributed under the Attribution-NonCommercial-NoDerivs (CC BY-NC-ND), which allows third parties to download the articles and share them with others as long as they credit the author and the Abstract Book, but they cannot change the content in any way or use them commercially.
**Abstract Book Citations:** Authors, Title, HemaSphere, 2026;10:(S1):pages. The abstract book DOIs can be found on the "Author Information" page.
**Disclaimer:** Articles published in the journal HemaSphere exclusively reflect the opinions of the authors. The authors are responsible for all content in their abstracts including accuracy of the facts, statements, citing resources, etc.

**Figure 1a.** Sensitivity analysis with ROC curve for EASIX and Model-1 (arterial thrombosis prediction in aPL carriers with autoimmune disease).

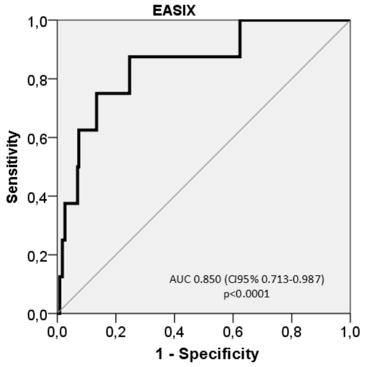

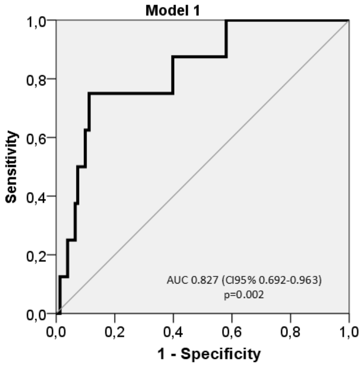

| | Max Youden | Priorize S | Priorize E |
|---|---|---|---|
| Cutoff | 0.965 | 0.590 | 18.200 |
| Youden | 0.628 | 0.377 | -0.004 |
| S | 0.875 | 1.000 | 0.000 |
| E | 0.753 | 0.377 | 0.996 |
| PPV | 0.115 | 0.056 | 0.000 |
| NPV | 0.994 | 1.000 | 0.960 |

| | Max Youden | Priorize S | Priorize E |
|---|---|---|---|
| Cutoff | 0.552 | 0.138 | 0.434 |
| Youden | 0.637 | 0.420 | -0.004 |
| S | 0.750 | 1.000 | 0.004 |
| E | 0.113 | 0.420 | 0.996 |
| PPV | 0.091 | 0.170 | 0.103 |
| NPV | 0.792 | 1.000 | 0.894 |

**Figure 1b** Sensitivity analysis with ROC curve for EASIX and Model-2 (microvascular thrombosis prediction in aPL carriers).

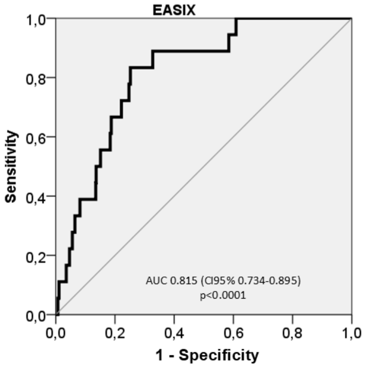

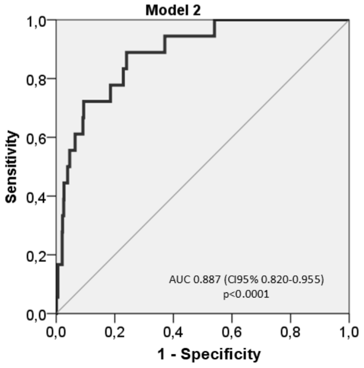

| | Max Youden | Priorize S | Priorize E |
|---|---|---|---|
| Cutoff | 0.970 | 0.580 | 10.730 |
| Youden | 0.581 | 0.391 | 0.048 |
| S | 0.833 | 1.000 | 0.056 |
| E | 0.748 | 0.391 | 0.993 |
| PPV | 0.650 | 0.033 | 0.125 |
| NPV | 0.996 | 1.000 | 0.981 |

| | Max Youden | Priorize S | Priorize E |
|---|---|---|---|
| Cutoff | 0.011 | 0.008 | 0.524 |
| Youden | 0.649 | 0.460 | 0.054 |
| S | 0.889 | 1.000 | 0.056 |
| E | 0.760 | 0.460 | 0.999 |
| PPV | 0.071 | 0.037 | 0.537 |
| NPV | 0.997 | 1.000 | 0.981 |

*AUC: area under the curve. CI: confidence interval. E: specificity.NPV: negative predictive value. PPV: positive predictive value. S: Sensitivity.*

**Copyright Information:** (Online) ISSN: 2572-9241
©2026 The Author(s). HemaSphere is published by John Wiley & Sons Ltd on behalf of European Hematology Association. This is an open access Abstract Book distributed under the Attribution-NonCommercial-NoDerivs (CC BY-NC-ND), which allows third parties to download the articles and share them with others as long as they credit the author and the Abstract Book, but they cannot change the content in any way or use them commercially.
**Abstract Book Citations:** Authors, Title, HemaSphere, 2026;10:(S1):pages. The abstract book DOIs can be found on the "Author Information" page.
**Disclaimer:** Articles published in the journal HemaSphere exclusively reflect the opinions of the authors. The authors are responsible for all content in their abstracts including accuracy of the facts, statements, citing resources, etc.

### (PB4360) DIAPS EVOLUTION AMONG ANTIPHOSPHOLIPID ANTIBODY CARRIERS: IMPACT OF ACR/EULAR LABORATORY DOMAINS, ASSOCIATED DISEASES AND PRIMARY THROMBOPROPHYLAXIS

**Topic:** 35. Thrombosis and vascular biology

Rodrigo Cantera Estefanía*[1], Rafael Gálvez[2], Maria Oviedo Madrid[3], María Abando Casuso[3], Héctor Cruz Barquín[3], Raquel García Ruiz[3], Marina Herrero López[3], Jose Flores García[3], Irene Gorostidi Álvarez[3], Ariadna García Ascacíbar[4], Ligia Gabrie Rodríguez[2], Lucrecia Yáñez[3], Marcos López Hoyos[5], Juan José Domínguez-García[3], María Luisa González Ponte[3], Belén González-Mesones Galán[3], Gala Aglaia Méndez Navarro[3], Jose Luis Hernández Hernández[6], Victor Manuel Martínez Taboada[2]

*[1]Hospital Universitario de Navarra, Hematology and Hemotherapy, Pamplona, Spain;[2]Hospital Universitario Marqués de Valdecilla, Rheumatology, Santander, Spain; [3]Hospital Universitario Marqués de Valdecilla, Hematology and Hemotherapy, Santander, Spain; [4]Hospital Universitario Araba, Hematology and Hemotherapy, Vitoria, Spain; [5]Hospital Universitario Marqués de Valdecilla, Immunology, Santander, Spain; [6]Hospital Universitario Marqués de Valdecilla, Internal Medicine, Santander, Spain;*

### Background

The Damage Index for Antiphospholipid Syndrome (DIAPS) is a validated tool to quantify cumulative organ damage in APS, but the pattern of damage accumulation in antiphospholipid antibody (aPL) carriers remains poorly defined. In addition, its longitudinal evolution is unclear, as most studies rely on cross-sectional cut-off points rather than time-dependent statistical approaches.

### Aims

To describe organ damage progression in aPL carriers using DIAPS and to assess the impact of serological profile, associated autoimmune disease (AD), and primary thromboprophylaxis. Additionally, to evaluate whether DIAPS at diagnosis predicts clinical APS development and survival.

### Methods

We performed a retrospective observational study including aPL carriers identified between January 2018 and March 2024 who fulfilled 2023 ACR/EULAR laboratory domains 7 or 8 and had no clinical APS domains 1–4 at serological diagnosis. DIAPS was assessed at baseline and biennially up to 10 years. Longitudinal DIAPS changes were analysed using linear mixed-effects models (LMM). Progression to clinical APS was evaluated with logistic regression. Overall survival (OS) and event-free survival (EFS, defined as absence of domains 1-4 ACR/EULAR) were analysed using Kaplan–Meier, log-rank, and age- and sex-adjusted Cox models.

**Copyright Information:** (Online) ISSN: 2572-9241
©2026 The Author(s). HemaSphere is published by John Wiley & Sons Ltd on behalf of European Hematology Association. This is an open access Abstract Book distributed under the Attribution-NonCommercial-NoDerivs (CC BY-NC-ND), which allows third parties to download the articles and share them with others as long as they credit the author and the Abstract Book, but they cannot change the content in any way or use them commercially.
**Abstract Book Citations:** Authors, Title, HemaSphere, 2026;10:(S1):pages. The abstract book DOIs can be found on the "Author Information" page.
**Disclaimer:** Articles published in the journal HemaSphere exclusively reflect the opinions of the authors. The authors are responsible for all content in their abstracts including accuracy of the facts, statements, citing resources, etc.

### Results

A total of 985 aPL carriers were included (69.6% women), with a median age of 53 years and a median follow-up of 2.84 years. DIAPS positivity increased from baseline to the end of follow-up (21.3% to 30.9%).
In the LMM, mean DIAPS scores were slightly higher in patients with persistent lupus anticoagulant (LA) compared with those without (0.429 vs 0.350), with a steeper increase in DIAPS over time (time*persistent LA interaction: F=2.103, p=0.041; Image 1a).
AD was linked to faster DIAPS progression (time*AD interaction: F=4.532, p<0.001; Image 1b). Primary thromboprophylaxis did not modify DIAPS progression (F=1.357, p=0.220).
In a multivariable logistic regression model adjusted by sex and age, higher baseline DIAPS scores were associated with increased risk of venous (OR 1.587, p=0.006), arterial (OR 1.896, p<0.001), and microvascular thrombosis (OR 2.737, p<0.001) during follow-up. DIAPS score was not associated with obstetric morbidity (OR 0.754, p=0.612).
Regarding OS and EFS, Patients with a DIAPS score ≥1 at diagnosis exhibited worse EFS in both univariate and multivariable analysis (table 1, Image 1c, 1d).

**Table 1.** Impact of DIAPS in survival.

| Primary endpoint | Statistical Analysis | Variables | p | Media | CI 95.0% for Media | | HR | CI 95.0% for HR | |
|---|---|---|---|---|---|---|---|---|---|
| | | | | | Inferior | Superior | | Inferior | Superior |
| OS | Kaplan-Meier + Log rank* | DIAPS at diagnosis (0 points) | 0.000 | 31.634 | 27.760 | 35.508 | - | - | - |
| | | DIAPS at diagnosis (≥1 points) | | 20.093 | 16.452 | 23.735 | - | - | - |
| | Cox regression** | Age (years) | 0.000 | - | - | - | 1.078 | 1.056 | 1.102 |
| | | Women | 0.026 | - | - | - | 0.525 | 0.297 | 0.927 |
| | | DIAPS at diagnosis | 0.004 | - | - | - | 1.504 | 1.142 | 1.981 |
| EFS | Kaplan-Meier + Log rank* | DIAPS at diagnosis (0 points) | 0.001 | 25.868 | 23.683 | 28.054 | - | - | - |
| | | DIAPS at diagnosis (≥1 points) | | 19.694 | 15.709 | 23.679 | - | - | - |
| | Cox regression** | Age (years) | 0.000 | - | - | - | 1.036 | 1.019 | 1.054 |
| | | Women | 0.476 | - | - | - | 0.828 | 0.493 | 1.391 |
| | | DIAPS at diagnosis | 0.003 | - | - | - | 1.470 | 1.139 | 1.896 |

*DIAPS was introduced as a dichotomous variable to perform Kaplan-Meier's graphics.
**DIAPS was introduced as a quantitative variable.

### Summary/Conclusion

Among aPL carriers, persistent LA and associated AD are linked to faster accumulation of organ damage measured by DIAPS. Baseline DIAPS at diagnosis identifies individuals at higher risk of future thrombotic manifestations and lower OS and EFS.

**Copyright Information:** (Online) ISSN: 2572-9241
©2026 The Author(s). HemaSphere is published by John Wiley & Sons Ltd on behalf of European Hematology Association. This is an open access Abstract Book distributed under the Attribution-NonCommercial-NoDerivs (CC BY-NC-ND), which allows third parties to download the articles and share them with others as long as they credit the author and the Abstract Book, but they cannot change the content in any way or use them commercially.
**Abstract Book Citations:** Authors, Title, HemaSphere, 2026;10:(S1):pages. The abstract book DOIs can be found on the "Author Information" page.
**Disclaimer:** Articles published in the journal HemaSphere exclusively reflect the opinions of the authors. The authors are responsible for all content in their abstracts including accuracy of the facts, statements, citing resources, etc.

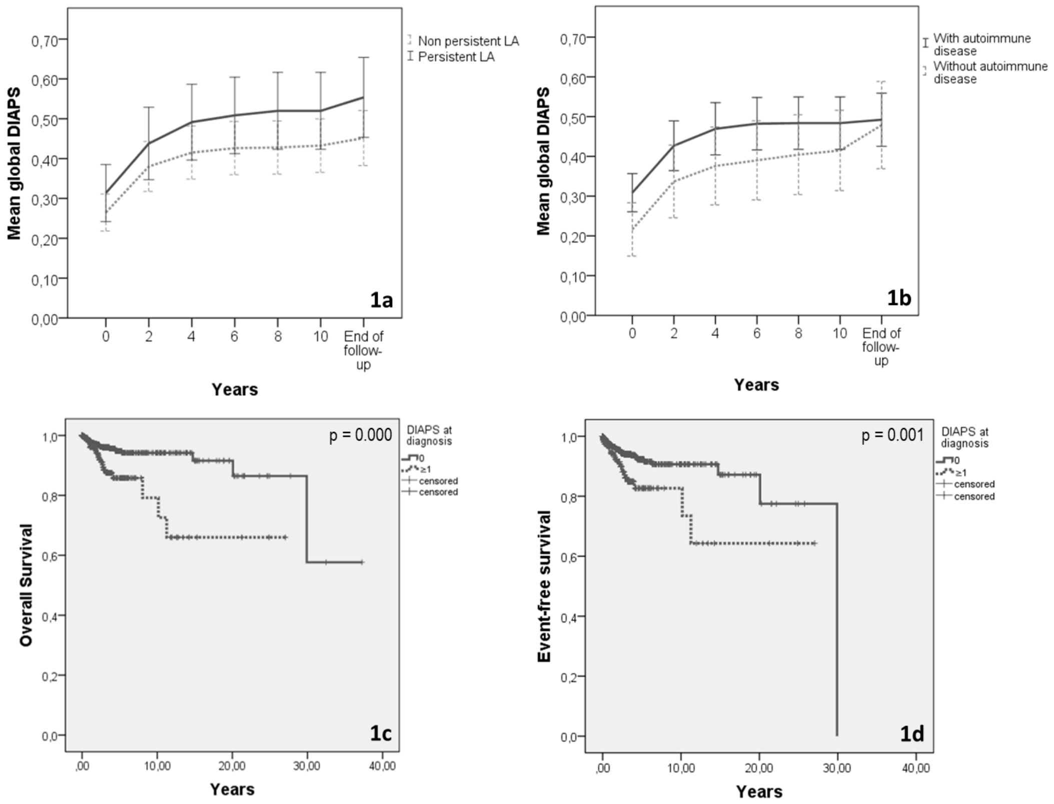

Image 1a. Mean Global DIAPS index evolution in time among persistent and non-persistent lupus anticoagulant.
Image 1b. Mean Global DIAPS index evolution in time among aPL carriers with and without autoimmune disease.
Image 1c. Kaplan–Meier univariate analysis of overall survival stratified by baseline DIAPS score.
Image 1d. Kaplan–Meier univariate analysis of event-free survival stratified by baseline DIAPS score.

Copyright Information: (Online) ISSN: 2572-9241
©2026 The Author(s). HemaSphere is published by John Wiley & Sons Ltd on behalf of European Hematology Association. This is an open access Abstract Book distributed under the Attribution-NonCommercial-NoDerivs (CC BY-NC-ND), which allows third parties to download the articles and share them with others as long as they credit the author and the Abstract Book, but they cannot change the content in any way or use them commercially.
Abstract Book Citations: Authors, Title, HemaSphere, 2026;10:(S1):pages. The abstract book DOIs can be found on the “Author Information” page.
Disclaimer: Articles published in the journal HemaSphere exclusively reflect the opinions of the authors. The authors are responsible for all content in their abstracts including accuracy of the facts, statements, citing resources, etc.

### (PB4361) CIRCULATING ENDOTHELIAL CELLS IN PATIENTS WITH POST-THROMBOTIC SYNDROME

**Topic:** 35. Thrombosis and vascular biology

Natalia Silina*[1], Olga Golovina[1], Natalia Korsakova[1], Olesia Matvienko[1], Kirill Komissarov[1], Vitaly Soldatenkov[1], Irina Krobinets[1]

*[1]Russian Research Institute of Hematology and Transfusiology, Saint Petersburg, Russia;*

**Background**

Post-thrombotic syndrome (PTS) is caused by organic damage to the deep veins of the lower extremities due to thrombosis, which is based on pathological changes in the vascular wall affecting endothelial cells (EC), which can lead to impaired endothelial function and, as a result, the development of recurrent thrombosis. Structural and functional changes in the endothelium in PTS can lead to disruption of the intercellular and cell-matrix interactions of the EC, their separation and release into the bloodstream – desquamation of the endothelium. An increase in the number of circulating endothelial cells (CEC) in peripheral blood can serve as a marker of damage and a pronounced degree of endothelial dysfunction (ED) and, as a result, procoagulant disorders that may cause thrombosis.

**Aim.** To evaluate the level of circulating endothelial cells as the marker of endothelial dysfunction in patients with post-thrombotic syndrome.

**Methods.** 52 patients with PTS (20 males and 32 females, median age 56 years) were examined. CEC was determined by flow cytofluorometry using monoclonal antibodies. Combinations of CD 31+ CD 146+ (desquamated endotheliocytes) and CD 45- CD 146+ (circulating mature EC) were used to detect CEC. The results are given as the number of cells per $10^4$ leukocytes. The control group consisted of 35 healthy individuals. The Statistica 12.0 was used. The median (Me) and the interquartile range (Q1-Q3) were determined. Mann-Whitney U–test was used to compare independent groups. The critical level of statistical significance was assumed to be 0.05.

**Results**. An increase in the number of desquamated endotheliocytes in patients compared to the healthy individuals (9.0; 6.4-14.0 cells $\times 10^4$ leukocytes versus 4.0; 3.0-5.0 cells $\times 10^4$ leukocytes, respectively; p=0.0000) and a high number of circulating mature EC compared to the control group (3.0; 1.6-5.2 cells $\times 10^4$ leukocytes versus 1.0; 1.0-2.0 cells $\times 10^4$ leukocytes, respectively; p=0.0000) confirms the presence of ED in patients with PTS.

**Conclusion.** A significant increase in the number of circulating endothelial cells in patients with PTS indicates a pronounced process of desquamation and structural and functional disorders of the endothelium – endothelial dysfunction, which may indicate an increased risk of thromboembolic complications.

**Copyright Information:** (Online) ISSN: 2572-9241
©2026 The Author(s). HemaSphere is published by John Wiley & Sons Ltd on behalf of European Hematology Association. This is an open access Abstract Book distributed under the Attribution-NonCommercial-NoDerivs (CC BY-NC-ND), which allows third parties to download the articles and share them with others as long as they credit the author and the Abstract Book, but they cannot change the content in any way or use them commercially.
**Abstract Book Citations:** Authors, Title, HemaSphere, 2026;10:(S1):pages. The abstract book DOIs can be found on the "Author Information" page.
**Disclaimer:** Articles published in the journal HemaSphere exclusively reflect the opinions of the authors. The authors are responsible for all content in their abstracts including accuracy of the facts, statements, citing resources, etc.

### (PB4362) USING ANTICOAGULATION IN THE MANAGEMENT OF LEFT VENTRICULAR THROMBOSIS: DATA REVIEW FROM A TERTIARY CARDIOLOGY CENTRE

**Topic:** 35. Thrombosis and vascular biology

Aisling Eves*[1], Jonathon Foggin[1]

*[1]Newcastle Upon Tyne Hospitals, Newcastle upon Tyne, United Kingdom;*

### Background

Left ventricular (LV) thrombus remains a common consequence of myocardial infarction however limited data around the appropriate anticoagulation strategy. The only approved anticoagulation is vitamin K antagonists, including Warfarin (1). However, previous studies have demonstrated that off label use of direct oral anticoagulants (DOACs) is becoming progressively more common (1).

### Aims

This study reviewed the current approach to anticoagulation of LV thrombus at a tertiary cardiology centre with the aim of determining whether DOACs is as efficacious as Warfarin with reduced complications.

### Methods

All patients with transthoracic echo scans carried out between 01/01/2024 - 25/05/2025 at Newcastle Upon Tyne Hospitals Trust with 'LV summary contains thrombus' were reviewed. 66 patients were included.
Electronic Patient Records were used to determine whether the patient had been anticoagulated prior to identification of LV thrombus and if so, the anticoagulant. Also, what anticoagulant (if any) was commenced on diagnosis and its duration (in weeks). Any thromboembolic complication or bleeding whilst on anticoagulation following diagnosis of LV thrombus was recorded. These were defined as any thromboembolism or bleeding noted in the inpatient or outpatient record. Outpatient records included clinic letters and the use of GP records.

### Results

The most commonly used anticoagulant in management of LV thrombus was DOACs (47%), followed by Warfarin (33%). The remaining patients received alternative anticoagulants, except a minority of patients who were not anticoagulated due to palliation. 3% of patients on DOACs experienced a bleeding complication, compared to 22% of patients anticoagulated on Warfarin.
There were no thromboembolic complications noted in any of the patient population.

**Copyright Information:** (Online) ISSN: 2572-9241
©2026 The Author(s). HemaSphere is published by John Wiley & Sons Ltd on behalf of European Hematology Association. This is an open access Abstract Book distributed under the Attribution-NonCommercial-NoDerivs (CC BY-NC-ND), which allows third parties to download the articles and share them with others as long as they credit the author and the Abstract Book, but they cannot change the content in any way or use them commercially.
**Abstract Book Citations:** Authors, Title, HemaSphere, 2026;10:(S1):pages. The abstract book DOIs can be found on the "Author Information" page.
**Disclaimer:** Articles published in the journal HemaSphere exclusively reflect the opinions of the authors. The authors are responsible for all content in their abstracts including accuracy of the facts, statements, citing resources, etc.

**Summary/Conclusion**

Limitations
Given factors such as geographical distribution and patients dying after commencing anti-coagulation, there was variation in access to medical records between patients. It is possible that some milder complications were not recorded. Some patients may have experienced complications but not presented or presented out of region. The patient sample only included those diagnosed from echocardiography data.
Conclusion and Recommendations
Our data found that bleeding complications were lower in patients anti-coagulated on DOACs compared to warfarin. This has also been found in previous studies (1).There was no difference in thromboembolic complications.
Whilst this study suggests that licensing DOACs for management of LV thrombus is appropriate, our sample size was small, and we would recommend a randomised control double blind trial prior to changes in prescribing guidelines. We predict that the use of DOACs would result in a cost saving and a reduction in hospital bed demand by avoiding the administration and follow up required in warfarinisation.

**Copyright Information:** (Online) ISSN: 2572-9241
©2026 The Author(s). HemaSphere is published by John Wiley & Sons Ltd on behalf of European Hematology Association. This is an open access Abstract Book distributed under the Attribution-NonCommercial-NoDerivs (CC BY-NC-ND), which allows third parties to download the articles and share them with others as long as they credit the author and the Abstract Book, but they cannot change the content in any way or use them commercially.
**Abstract Book Citations:** Authors, Title, HemaSphere, 2026;10:(S1):pages. The abstract book DOIs can be found on the “Author Information” page.
**Disclaimer:** Articles published in the journal HemaSphere exclusively reflect the opinions of the authors. The authors are responsible for all content in their abstracts including accuracy of the facts, statements, citing resources, etc.

### (PB4363) INFLAMMATORY BIOMARKERS' ROLE IN IMMUNE THROMBOTIC THROMBOCYTOPENIC PURPURA (ITTP)

**Topic:** 35. Thrombosis and vascular biology

Francesca Schieppati*[1, 2], Silvia Bolognini[1, 2], Chiara Ticozzi[1], Oana Cristina Cretu[1], Patricia Gomez Rosas[1, 3], Mariella D'adda[4], Valeria Cancelli[4], Riccardo Panebianco[4], Chiara Ambaglio[1], Elisa Galimberti[1], Luca Barcella[1], Anna Falanga[1, 2], Marina Marchetti[1, 2]

*[1]Hospital Papa Giovanni XXIII, Bergamo, Italy; [2]University of Milano-Bicocca, Milan, Italy; [3]Maastricht University Medical Center, Maastricht, The Netherlands; [4]Hospital Spedali Civili di Brescia, Brescia, Italy;*

### Background iTTP is a rare, life-threatening thrombotic microangiopathy caused by an immune-mediated severe ADAMTS13 deficiency. Current treatment with therapeutic plasma exchange (TPE), immunosuppression (IS), and caplacizumab has dramatically reduced mortality to <10%, although the severity of each acute episode remains unpredictable. While ADAMTS13 deficiency is central to pathogenesis, systemic inflammation and NETosis can contribute to disease severity.

### Aims

As part of a larger observational study on iTTP patients, we aim to investigate the role of biomarkers of NETosis (citrullinated H3 histone, H3Cit) and inflammation (tumor necrosis factor-alpha, TNF-α, and interleukin-6, IL-6) in acute iTTP and during treatment.

### Methods

Six patients (67% female; median age: 57 years, range 20–79) newly diagnosed with acute iTTP at the Hospitals of Bergamo and Brescia between Feb 2024 and Apr 2025 were evaluated. Plasma samples were collected at baseline (T0), and on days 6 (T6), 9 (T9), and 15 (T15) following diagnosis, during treatment. ADAMTS13 activity, anti-ADAMTS13 antibodies and H3Cit were assessed by ELISA. TNF-α and IL-6 levels were quantified by multiplex assay. The analyses were performed at the Hemostasis and Thrombosis Center of Bergamo Hospital. Clinical data and treatments were recorded. Statistical analyses were conducted using SPSS v21.0 and R v4.2.2.

### Results

At onset, 5/6 patients presented neurological symptoms of different severity. HIV infection, myelodysplastic syndrome (MDS), and autoimmune disorders were the most significant comorbidities. All patients received daily TPE, IS (including rituximab), and caplacizumab. Hematological remission was achieved in a median time of 3 days, except for the patient with MDS who reached a stable platelet count >20 $\times 10^9$/L by day 9. Platelets' rise correlated with LDH reduction (p=0.001). Median time to ADAMTS13 >20% was 8 days; from T0 to T15, ADAMTS13 activity increased (1–43%; p=0.03), and antibody titers declined (50 to 5 U/mL). During treatment, a significant decrease in H3Cit (p=0.0018) and TNF-α (p=0.015) plasma levels was observed over the different time points, while no significant changes were seen in IL6 levels. Specifically, H3Cit levels markedly decreased from T0 (5.90 ng/mL) to T6 (1.32)(p=0.017), T9 (0.55)(p=0.009), and T15 (0.69)(p=0.016). TNF-α also significantly declined from baseline to T9 (14.83 vs 4.90 pg/mL, p=0.035). Furthermore, a significant positive correlation was found between TNF-α levels and anti-ADAMTS13 antibodies (r=0.783; p<0.001). Interestingly, patients who received caplacizumab within 2 days of diagnosis (n=3) had significantly lower post-treatment TNF-α levels (4.6 vs 7.2, p=0.009) and showed a trend toward lower IL-6 levels (2.4 vs 7.3) compared to those who initiated treatment later (n=3).

**Copyright Information:** (Online) ISSN: 2572-9241
©2026 The Author(s). HemaSphere is published by John Wiley & Sons Ltd on behalf of European Hematology Association. This is an open access Abstract Book distributed under the Attribution-NonCommercial-NoDerivs (CC BY-NC-ND), which allows third parties to download the articles and share them with others as long as they credit the author and the Abstract Book, but they cannot change the content in any way or use them commercially.
**Abstract Book Citations:** Authors, Title, HemaSphere, 2026;10:(S1):pages. The abstract book DOIs can be found on the "Author Information" page.
**Disclaimer:** Articles published in the journal HemaSphere exclusively reflect the opinions of the authors. The authors are responsible for all content in their abstracts including accuracy of the facts, statements, citing resources, etc.

**Summary/Conclusion**

During iTTP treatment, a significant reduction in H3Cit and TNF-α was observed in parallel with hematologic and biochemical recovery, pointing to a role of these biomarkers as indicators of NET-driven inflammation and immune activation. The positive correlation between TNF-α and anti-ADAMTS13 antibodies further supports the link between inflammation and the autoimmune process. Additionally, this preliminary observation suggests that early initiation of caplacizumab may be associated with reduced inflammation. Despite the small sample size, these results highlight the potential for using NETosis and inflammation markers to monitor disease activity and optimize therapeutic strategies in iTTP.

**Copyright Information:** (Online) ISSN: 2572-9241
©2026 The Author(s). HemaSphere is published by John Wiley & Sons Ltd on behalf of European Hematology Association. This is an open access Abstract Book distributed under the Attribution-NonCommercial-NoDerivs (CC BY-NC-ND), which allows third parties to download the articles and share them with others as long as they credit the author and the Abstract Book, but they cannot change the content in any way or use them commercially.
**Abstract Book Citations:** Authors, Title, HemaSphere, 2026;10:(S1):pages. The abstract book DOIs can be found on the “Author Information” page.
**Disclaimer:** Articles published in the journal HemaSphere exclusively reflect the opinions of the authors. The authors are responsible for all content in their abstracts including accuracy of the facts, statements, citing resources, etc.

### (PB4364) CLINICAL MANAGEMENT OF CANCER-ASSOCIATED VENOUS THROMBOEMBOLISM IN DENMARK: A DESCRIPTIVE STUDY OF PRACTICE PATTERNS IN THE PAST DECADE

**Topic:** 35. Thrombosis and vascular biology

Anders K. Nielsen*[1], Helena Wisby[1], Peter Nielsen[2], Thure Overvad[1]

*[1]Aalborg University Hospital, Clinical Pharmacology, Aalborg, Denmark;[2]Aalborg University Hospital, Clinical Medicine, Gistrup, Denmark;*

### Background

Venous thromboembolism (VTE), consisting of deep vein thrombosis and pulmonary embolism, is a common and serious complication in patients with cancer. Dedicated guidelines for cancer-associated VTE exist, but some recommendations are vague and guidelines are not always followed in everyday clinical practice. Since 2022, direct oral anticoagulants (DOACs) have been recommended as first-line treatment for most patients with cancer in Denmark. However, the clinical uptake of these recommendations remains unexplored.

### Aims

To investigate routine clinical management of cancer-associated VTE in the North Denmark Region prior to (2017-2021) compared with after (2023-2025) the nationwide recommendation of DOACs as first-line treatment.

### Methods

We identified randomly selected patients with haematological or oncological cancer who were registered with an ICD-10 code for VTE in the hospital discharge registry. Only patients with imaging-confirmed VTE were included. Electronic medical records were manually reviewed and information about VTE type, incidental vs. non-incidental VTE, choice of anticoagulant, planned treatment duration, renal function, and platelet count (laboratory values only for the contemporary cohort) was entered into a secure web-based database (REDCap®). The study was based on data from two projects registered at Aalborg University Hospital (K2022-002 and K2025-147).

**Copyright Information:** (Online) ISSN: 2572-9241
©2026 The Author(s). HemaSphere is published by John Wiley & Sons Ltd on behalf of European Hematology Association. This is an open access Abstract Book distributed under the Attribution-NonCommercial-NoDerivs (CC BY-NC-ND), which allows third parties to download the articles and share them with others as long as they credit the author and the Abstract Book, but they cannot change the content in any way or use them commercially.
**Abstract Book Citations:** Authors, Title, HemaSphere, 2026;10:(S1):pages. The abstract book DOIs can be found on the "Author Information" page.
**Disclaimer:** Articles published in the journal HemaSphere exclusively reflect the opinions of the authors. The authors are responsible for all content in their abstracts including accuracy of the facts, statements, citing resources, etc.

### Results

Overall, we included 414 patients with confirmed cancer-associated VTE, of which 37% had haematological cancer. In the overall population, all patients were treated with anticoagulation, including the 12% with incidental VTE. A clear increase in the use of DOACs after the new recommendation was evident both among patients with haematological (41% to 77%) and oncological cancer (25% to 71%). Every patient treated with a DOAC had acceptable renal function and platelet count. Of the available DOACs, only apixaban and rivaroxaban were used. Low-molecular-weight heparin remained the drug of choice in most other patients.
Further descriptive characteristics from the two periods are shown in the Table.

**Table: Baseline characteristics and anticoagulant treatment in patients with cancer and VTE before and after the recommendation of DOACs as first line agents**

| Characteristics | 2017-2021 cohort (n = 221) | 2023-2025 cohort (n = 193) |
|---|---|---|
| **Underlying malignancy** | | |
| Haematology | 50.2% | 22.3%* |
| Oncology | 49.8% | 77.7% |
| **Type of VTE** | | |
| Pulmonary embolism | 62.9% | 61.7% |
| Deep vein thrombosis | 37.1% | 38.3% |
| **Anticoagulant Treatment** | | |
| DOAC | 33.0% | 72.5% |
| Low-molecular-weight heparin | 63.8% | 26.9% |
| Vitamin K antagonist | 3.2% | 0.5% |
| **DOAC choice** | | |
| rivaroxaban | 29.9% | 65.8% |
| apixaban | 3.2% | 6.7% |
| **Planned treatment duration** | | |
| Lifelong anticoagulation | 29.4% | 30.1% |
| Six months | 16.3% | 21.8% |
| Three months | 8.6% | 8.8% |
| Other duration | 4.5% | 6.2% |
| Not specified | 41.2% | 33.2% |
| Treatment duration specified (overall) | 58.8% | 66.8% |
| **Renal function (eGFR)** | | |
| < 30 ml/min | | 1.6% |
| 30–50 ml/min | | 10.9% |
| > 50 ml/min | | 87.6% |
| **Platelet count** | | |
| < 30 $x10^9/l$ | | 0,5% |
| > 50 $x10^9/l$ | | 99,5% |

* Only 43 patients with haematological cancer were registered with a VTE diagnosis in the 2023-2025 period, therefore the relatively smaller sample size.

Copyright Information: (Online) ISSN: 2572-9241
©2026 The Author(s). HemaSphere is published by John Wiley & Sons Ltd on behalf of European Hematology Association. This is an open access Abstract Book distributed under the Attribution-NonCommercial-NoDerivs (CC BY-NC-ND), which allows third parties to download the articles and share them with others as long as they credit the author and the Abstract Book, but they cannot change the content in any way or use them commercially.
Abstract Book Citations: Authors, Title, HemaSphere, 2026;10:(S1):pages. The abstract book DOIs can be found on the “Author Information” page.
Disclaimer: Articles published in the journal HemaSphere exclusively reflect the opinions of the authors. The authors are responsible for all content in their abstracts including accuracy of the facts, statements, citing resources, etc.

**Summary/Conclusion**

All patients with cancer-associated VTE, both symptomatic and incidental, were treated with anticoagulation. In line with guideline changes, a substantial increase in the use of DOACs was evident. Planned treatment duration was variable and remained unspecified in approximately 1/3 of VTE cases.

**Copyright Information:** (Online) ISSN: 2572-9241
©2026 The Author(s). HemaSphere is published by John Wiley & Sons Ltd on behalf of European Hematology Association. This is an open access Abstract Book distributed under the Attribution-NonCommercial-NoDerivs (CC BY-NC-ND), which allows third parties to download the articles and share them with others as long as they credit the author and the Abstract Book, but they cannot change the content in any way or use them commercially.
**Abstract Book Citations:** Authors, Title, HemaSphere, 2026;10:(S1):pages. The abstract book DOIs can be found on the “Author Information” page.
**Disclaimer:** Articles published in the journal HemaSphere exclusively reflect the opinions of the authors. The authors are responsible for all content in their abstracts including accuracy of the facts, statements, citing resources, etc.

### (PB4365) EXTENDED ANTICOAGULATION WITH DOACS IN PATIENTS AFFECTED BY HEMATOLOGIC MALIGNANCIES AND VENOUS THROMBOEMBOLISM: DOES LOW DOSE FIT ALL?

**Topic:** 35. Thrombosis and vascular biology

Mario Biglietto*[1], Silvia Sorella[1], Luca Vincenzo Cappelli[1], Bianca Maria Mandelli[1], Deborah Kasmi[1], Erminia Baldacci[1], Antonio Chistolini[1]

[1]*Sapienza, University of Rome, Department of Translational and Precision Medicine, Rome, Italy;*

### Background

Data on the extended treatment of venous thromboembolism (VTE) in the oncohematologic patients is lacking.

### Aims

The aim of our monocentric retrospective analysis was to evaluate the efficacy and safety in secondary prophylaxis of two different Direct Oral Anticoagulants (DOACs) treatment strategies (full-dose/low-dose) in onco-hematologic patients, based on the persistence of complete venous occlusion.

### Methods

We included adult oncohematologic patients with objectively confirmed VTE and at least 12 months of follow-up from the start of DOAC treatment. During the acute-phase of VTE treatment (first 3-6 months), the patients were treated with therapeutic dose of anticoagulants. In the extended therapy, the patients were treated with full-dose DOAC in case of persistent complete thrombosis and with low-dose DOAC in case of partial or complete vein recanalization and active cancer. Follow-up visits were scheduled every three months and included monitoring for bleeding (B-AEs) and thrombotic adverse events (T-AEs). Imaging with doppler ultrasound or computed tomography was performed every six months as per institutional policy, or earlier if recurrence was suspected. B-AEs were classified according to the ISTH criteria. Associations between anticoagulant dose and outcomes were analyzed with Cox proportional hazards models using time-varying exposure according to the Andersen–Gill formulation. Statistical significance was defined as $p < 0.05$.

### Results

Patients' characteristics and AEs are summarized in Table 1. In the time-varying Cox model adjusted for age and sex, low-dose therapy was associated with a lower, but not statistically significant, bleeding risk compared with full-dose therapy (HR 0.25, 95% CI 0.05–1.22, p=0.08). In the time-varying Cox model adjusted for age and sex, low-dose therapy was associated with a lower but not statistically significant hazard of VTE recurrence (HR 0.52, 95% CI 0.12–2.19, p=0.37).

### Summary/Conclusion

Our analysis suggests that a "low dose fits all" approach may be inappropriate for this high-risk population. Onco-hematologic patients with persistent complete VTE may benefit from continued full-dose anticoagulation, with the option to de-escalate to reduced-dose therapy once recanalization occurs. Our findings should be interpreted with caution for the limitation of this study including its retrospective design, small sample, single-center setting, lack of randomization.

**Copyright Information:** (Online) ISSN: 2572-9241
©2026 The Author(s). HemaSphere is published by John Wiley & Sons Ltd on behalf of European Hematology Association. This is an open access Abstract Book distributed under the Attribution-NonCommercial-NoDerivs (CC BY-NC-ND), which allows third parties to download the articles and share them with others as long as they credit the author and the Abstract Book, but they cannot change the content in any way or use them commercially.
**Abstract Book Citations:** Authors, Title, HemaSphere, 2026;10:(S1):pages. The abstract book DOIs can be found on the "Author Information" page.
**Disclaimer:** Articles published in the journal HemaSphere exclusively reflect the opinions of the authors. The authors are responsible for all content in their abstracts including accuracy of the facts, statements, citing resources, etc.

**Table 1.** Patients' characteristics and adverse events.

| **Patients' characteristics** | **N (%)*** |
|---|---|
| **Sex** | |
| Male | 78 (52.7) |
| Female | 70 (47.3) |
| **Age (years at thrombosis) – Median (IQR)** | 65.3 (18.9-90.9) |
| **Type of VTE** | |
| Typical site | 96 (64.9) |
| Atypical site | 47 (31.7) |
| PICC-related | 5 (3.4) |
| **Onco-hematologic disease** | |
| MPN | 61 (41.2) |
| Lymphoma | 55 (37.2) |
| MM | 17 (11.5) |
| PNH ± Aplastic anemia | 8 (5.4) |
| AL | 4 (2.7) |
| MDS | 3 (2) |
| **Acute treatment of VTE** | |
| DOACs | 119 (80.5) |
| Apixaban | 59 (39.9) |
| Edoxaban | 38 (25.7) |
| Rivaroxaban | 22 (14.9) |
| VKA | 29 (19.5) |
| **DOACs dosage during extended treatment of VTE** | |
| Full dose DOACs | 29 (19.5) |
| Full dose DOACs -> low dose DOACs[§] | 97 (65.4) |
| Low dose DOACs | 22 (15.1) |
| **Duration of extended treatment of VTE (months) – Median (IQR)** | 35.3 (12.8-119.4) |
| **Duration of full dose DOACs (months) during extended treatment of VTE° – Median (IQR)** | 13.4 (0.1-125.4) |
| **Time from full dose to low dose DOACs (months) during extended treatment of VTE° – Median (IQR)** | 12.2 (0.5-100.1) |
| **Duration of low dose DOACs (months) during extended treatment of VTE°– Median (IQR)** | 24.2 (1.3-109.4) |
| **Bleeding adverse events (full dose DOACs vs low dose DOACs) – Rate per 100 patient-years** | |
| Major | 0.7 vs 0.4 |
| CRNM | 9.2 vs 0.4 |
| **Thrombotic adverse events - Rate per 100 patient-years** | |
| During full dose DOACs | 4.3 |
| During low dose DOACs | 1.3 |

IQR: interquartile range, VTE: venous thromboembolism, PICC: peripherally inserted central catheter, MPN: myeloproliferative neoplasm, MM: multiple myeloma, PNH: paroxysmal nocturnal hemoglobinuria, AL: acute leukemia, MDS: myelodysplastic neoplasm, LMWH: low molecular weight heparin, DOACs: direct oral anticoagulants, VKA: vitamin K antagonists, CRNM: clinically relevant non major bleedings.
*If not otherwise specified.
[§]Patients were switched to low dose after partial or complete vein recanalization and persistence of active cancer.
°If applicable.

Copyright Information: (Online) ISSN: 2572-9241
©2026 The Author(s). HemaSphere is published by John Wiley & Sons Ltd on behalf of European Hematology Association. This is an open access Abstract Book distributed under the Attribution-NonCommercial-NoDerivs (CC BY-NC-ND), which allows third parties to download the articles and share them with others as long as they credit the author and the Abstract Book, but they cannot change the content in any way or use them commercially.
Abstract Book Citations: Authors, Title, HemaSphere, 2026;10:(S1):pages. The abstract book DOIs can be found on the "Author Information" page.
Disclaimer: Articles published in the journal HemaSphere exclusively reflect the opinions of the authors. The authors are responsible for all content in their abstracts including accuracy of the facts, statements, citing resources, etc.

### (PB4366) T744C POLYMORPHISM OF THE P2Y12 PLATELET RECEPTOR : INFLUENCE ON THE VARIABILITY OF BIOLOGICAL RESPONSE TO CLOPIDOGREL IN CORONARY PATIENTS

**Topic:** 35. Thrombosis and vascular biology

Rania EL Aissi*[1]

[1]*Fattouma Bourguiba Hospital, Laboratory of hematology, Monastir, Tunisia;*

### Background

Variability in platelet response to clopidogrel is multifactorial, involving both genetic and non-genetic determinants that may lead to reduced therapeutic efficacy.

### Aims

This study aims to assess the impact of the T744C polymorphism of the P2Y12 receptor on platelet response to clopidogrel in patients with coronary artery disease.

### Methods

This is a cross-sectional study led on patients with acute coronary syndrome treated with clopidogrel (75 mg/day for at least seven days). Platelet reactivity was assessed by the VerifyNow® P2Y12 test and high on treatment platelet reactivity was defined by a PRU ≥ 208. The genotyping of the T744C polymorphism of the P2Y12 receptor was performed by PCR- RFLP.

### Results

The study involved 150 coronary patients (135 men and 15 women) with a mean age of 58 ± 10 years. The VerifyNow®P2Y12 test showed that 33 % were resistant to clopidogrel. The genetic study showed that the T744C genotypes were TT 78%, CT 21.3%, and CC 0.7%, respectively. Analysis of the results of this study showed no statistically significant association between the presence of the P2Y12 (T744C) polymorphism and biological resistance to clopidogrel in coronary patients (OR=0.837; 95% CI [0.363–1.929]; p=0.676).

### Summary/Conclusion

At the conclusion of this study, no significant association was identified between the P2Y12 (T744C) polymorphism and biological non-responsiveness to clopidogrel.

**Copyright Information:** (Online) ISSN: 2572-9241
©2026 The Author(s). HemaSphere is published by John Wiley & Sons Ltd on behalf of European Hematology Association. This is an open access Abstract Book distributed under the Attribution-NonCommercial-NoDerivs (CC BY-NC-ND), which allows third parties to download the articles and share them with others as long as they credit the author and the Abstract Book, but they cannot change the content in any way or use them commercially.
**Abstract Book Citations:** Authors, Title, HemaSphere, 2026;10:(S1):pages. The abstract book DOIs can be found on the “Author Information” page.
**Disclaimer:** Articles published in the journal HemaSphere exclusively reflect the opinions of the authors. The authors are responsible for all content in their abstracts including accuracy of the facts, statements, citing resources, etc.

### (PB4367) IMPROVING VENOUS THROMBOEMBOLISM PROPHYLAXIS IN PANCREATIC ADENOCARCINOMA

**Topic:** 35. Thrombosis and vascular biology

Anika Noor*[1], Tisha Singhal[1]

*[1]King's College London, London, United Kingdom;*

### Background

Pancreatic adenocarcinoma carries a high risk of venous thromboembolism (VTE), approximately seven-fold higher than other adenocarcinomas. Drivers include procoagulant tumour biology, advanced disease and early systemic chemotherapy. In a cohort of 243 patients, 24% developed VTE, half within two months of diagnosis. Primary thromboprophylaxis reduces VTE from 31% to 8% (number needed to treat ≈4). Pancreatic cancer is classified high-risk within the Khorana score and guidelines recommend routine prophylaxis for eligible patients receiving systemic anticancer therapy. Despite this, implementation remains inconsistent.

### Aims

To evaluate baseline prophylaxis prescribing in ambulatory pancreatic adenocarcinoma and assess whether sequential clinician- and system-level interventions improve prescribing and communication within an acute oncology service.

### Methods

A two-cycle Plan–Do–Study–Act quality improvement project was undertaken between September 2025 and February 2026.

Cycle 1 (September–December 2025) identified clinician-level barriers. A baseline survey (n=14) included registrars (64%), consultants (29%) and acute oncology nurses (7%). Only 36% routinely offered prophylaxis, 29% never offered it and familiarity with guidance was limited (7% very familiar; 29% unaware). Barriers included uncertainty regarding eligibility (64%), lack of guidance awareness (~70%) and bleeding concerns (43%). These informed a targeted teaching session in November 2025. Post-intervention audit assessed prescribing and documentation.

Cycle 2 (December 2025–February 2026) addressed system-level barriers after minimal improvement with education alone. As 79% reported they would prescribe more with an electronic prompt and 93% requested clearer guidance, a standardised electronic smartphrase was embedded in the electronic patient record. Prospective audit collection continued.

### Results

Cycle 1 baseline audit (September–December 2025) showed inconsistent prescribing. Of 30 patients, 10 were on therapeutic anticoagulation and excluded. Pre-teaching (September–October; n=14): no eligible patients received prophylaxis. Post-teaching (November–December; n=16): one patient received prophylactic anticoagulation.

Cycle 2 demonstrated improvement. January 2026 (n=11): three patients on therapeutic anticoagulation; among eligible patients, six (55%) received prophylaxis and five did not (one planned after PICC insertion). One patient developed pulmonary embolism shortly after starting prophylactic apixaban.

February 2026 (n=5): none on therapeutic anticoagulation; three of five eligible patients (60%) received prophylaxis and two did not (one pending initiation, one not yet eligible pending systemic therapy). Overall uptake January–February was 56% compared with 0–6% in cycle 1. Mortality limited follow-up. Limitations include single-centre design and small sample size.

**Copyright Information:** (Online) ISSN: 2572-9241
©2026 The Author(s). HemaSphere is published by John Wiley & Sons Ltd on behalf of European Hematology Association. This is an open access Abstract Book distributed under the Attribution-NonCommercial-NoDerivs (CC BY-NC-ND), which allows third parties to download the articles and share them with others as long as they credit the author and the Abstract Book, but they cannot change the content in any way or use them commercially.
**Abstract Book Citations:** Authors, Title, HemaSphere, 2026;10:(S1):pages. The abstract book DOIs can be found on the "Author Information" page.
**Disclaimer:** Articles published in the journal HemaSphere exclusively reflect the opinions of the authors. The authors are responsible for all content in their abstracts including accuracy of the facts, statements, citing resources, etc.

**Summary/Conclusion**

Education alone was insufficient to improve VTE prophylaxis. Persistent gaps prompted a system-level intervention. Targeted education plus embedded electronic support improved prescribing and offers a scalable model to improve safety and consistency in high-risk oncology. However, findings are limited by single-centre design, small sample size, and restricted follow-up due to mortality.

These authors contributed equally and share first authorship.

**Copyright Information:** (Online) ISSN: 2572-9241
©2026 The Author(s). HemaSphere is published by John Wiley & Sons Ltd on behalf of European Hematology Association. This is an open access Abstract Book distributed under the Attribution-NonCommercial-NoDerivs (CC BY-NC-ND), which allows third parties to download the articles and share them with others as long as they credit the author and the Abstract Book, but they cannot change the content in any way or use them commercially.
**Abstract Book Citations:** Authors, Title, HemaSphere, 2026;10:(S1):pages. The abstract book DOIs can be found on the "Author Information" page.
**Disclaimer:** Articles published in the journal HemaSphere exclusively reflect the opinions of the authors. The authors are responsible for all content in their abstracts including accuracy of the facts, statements, citing resources, etc.

### (PB4368) EFFICACY AND SAFETY OF DIRECT ANTICOAGULANTS (DAOCS) COMPARED TO LOW MOLECULAR WEIGHT HEPARIN (LMWH) IN HEMATOLOGICAL MALIGNANCIES

**Topic:** 35. Thrombosis and vascular biology

Khalid Aladwani*[1], Hazzaa Alzahrani[2], Abdullah Alzahrani[1], Salman Almutairi[1], Abdulaziz Asiri[1]

*[1]King Faisal Specialist & Research Centre, Pharmacy Care Division, Riyadh, Saudi Arabia;[2]King Faisal Specialist & Research Centre, Riyadh, Saudi Arabia;*

### Background

Venous thromboembolism (VTE) is a commonly encountered complication in cancer patients particularly in patients with hematological malignancies. However, patients with hematological malignancies are underrepresented in most of the studies.

### Aims

The aim of this study is to evaluate the efficacy and safety of DOACs compared to enoxaparin in patients with hematological malignancies.

### Methods

A retrospective study revising database from January 2024 – March 2025. Including hematologic patients on DOACS or LMWH. Primary outcome is the probability of recurrent thrombosis within 6 months of VTE or occurnce of thrombosis within 6 months from time of malignancy diagnosis. Secondary outcomes are major bleeding and clinically relevant nonmajor bleeding

### Results

A total of 122 patients were included, of whom 66 received DOACs and 49 received enoxaparin. The primary indication for anticoagulation was treatment of thrombosis in 84 patients. Eight recurrent thrombosis events were documented, 5 among patients treated with enoxaparin and 3 among those treated with DOACs. In addition, there were 2 bleeding events (one major and minor bleeding) between patients treated with enoxaparin. There was no significant increase risk of recurrent thrombosis in patient treated with DOACs compared to patients treated with enoxaparin, (P value = 0.1825).

### Summary/Conclusion

In patients with hematological malignancies, the use of DOACs demonstrated efficacy and safety that appeared comparable to enoxaparin for both the treatment and prophylaxis of VTE. These results suggest DOACs may consider a viable option for this population. Further large, prospective studies are warranted to validate the efficacy and safety of DOACs in hematological malignancies.

**Copyright Information:** (Online) ISSN: 2572-9241
©2026 The Author(s). HemaSphere is published by John Wiley & Sons Ltd on behalf of European Hematology Association. This is an open access Abstract Book distributed under the Attribution-NonCommercial-NoDerivs (CC BY-NC-ND), which allows third parties to download the articles and share them with others as long as they credit the author and the Abstract Book, but they cannot change the content in any way or use them commercially.
**Abstract Book Citations:** Authors, Title, HemaSphere, 2026;10:(S1):pages. The abstract book DOIs can be found on the "Author Information" page.
**Disclaimer:** Articles published in the journal HemaSphere exclusively reflect the opinions of the authors. The authors are responsible for all content in their abstracts including accuracy of the facts, statements, citing resources, etc.

### (PB4369) ENDOTHELIAL DYSFUNCTION IS ASSOCIATED WITH INCREASED PROCOAGULANT MICROPARTICLE ACTIVITY IN PATIENTS WITH VARICOSE VEINS OF THE LOWER LIMBS

**Topic:** 35. Thrombosis and vascular biology

Natalia Korsakova*[1], Olesia Matvienko[1], Olga Golovina[1], Kirill Komissarov[1], Vyacheslav Burakov[1], Vitaly Soldatenkov[1], Natalia Silina[1]

*[1]Russian Research Institute of Hematology and Transfusiology, Saint Petersburg, Russia;*

### Background

Varicose veins (VVs) of the lower limbs are accompanied by chronic venous insufficiency and thromboembolic complications development. Important factors in the VVs pathogenesis include Inflammatory changes in the vascular wall associated with endothelial dysfunction (ED) and activation of blood cells with the formation of microparticles (MPs) with procoagulant potential. Early detection of ED and procoagulant changes in VVs can serve for timely prevention of thrombosis.

### Aims

The aim of the study was to evaluate ED and the procoagulant potential of blood plasma caused by MPs in patients with VVs.

### Methods

The study included 24 patients with VVs of the lower limbs. To detect ED, the level of von Willebrand factor (vWF) was evaluated with the immunoturbidimetric method. The procoagulant activity of MPs was determined using the calibrated automated thrombinography method using the "MP-reagent" (phospholipids 4 µmol), which allows to assess of the contribution of tissue factor (TF) exposed on MPs. Endogenous thrombin potential (ETP), peak thrombin concentration (Peak), and thrombin formation rate were determined. The control group consisted of 30 healthy individuals. The results were presented as the median and interquartile range, and Mann-Whitney and Spearman rank tests were used. $P<0.05$ was considered statistically significant.

### Results

In patients with VVs the level of vWF was significantly higher than in healthy individuals: 134.4 (113.0-186.3)% versus 107.0 (95.3-133.5)%, p=0.001. The ETP, Peak, and rate of thrombin formation in patients also significantly exceeded the control values: 906.8 (687.1-1024.1) nmol*min versus 566.5 (462.3-708.0) nmol*min, p=0.001; 76.7 (50.4-89.7) nmol versus 46.3 (32.9-67.2) nmol, p=0.009 and 14.1 (7.7-15.9) nmol/min versus 7.3 (5.2-12.6) nmol/min, p=0.018. The level of vWF correlated with ETP and Peak (R=0.514 and R=0.457, respectively, $p<0.05$).

### Summary/Conclusion

An increase in the level of vWF in patients with VVs indicates the development of ED. High levels and rate of thrombin generation under the influence of the "MP-reagent" in patients indicate an enhanced procoagulant potential, depending on the MPs of blood plasma. The detected correlation confirms the relationship of ED with the increase of procoagulant activity of MPs in VVs. Identification and correction of endothelial disturbances accompanied by activation of blood cells, expression of TF and formation of MPs can contribute to the prevention of prothrombotic conditions in VVs.

**Copyright Information:** (Online) ISSN: 2572-9241
©2026 The Author(s). HemaSphere is published by John Wiley & Sons Ltd on behalf of European Hematology Association. This is an open access Abstract Book distributed under the Attribution-NonCommercial-NoDerivs (CC BY-NC-ND), which allows third parties to download the articles and share them with others as long as they credit the author and the Abstract Book, but they cannot change the content in any way or use them commercially.
**Abstract Book Citations:** Authors, Title, HemaSphere, 2026;10:(S1):pages. The abstract book DOIs can be found on the "Author Information" page.
**Disclaimer:** Articles published in the journal HemaSphere exclusively reflect the opinions of the authors. The authors are responsible for all content in their abstracts including accuracy of the facts, statements, citing resources, etc.

### (PB4370) INCIDENCE, RECURRENCE, AND RISK FACTORS OF VENOUS THROMBOEMBOLIC EVENTS IN PATIENTS WITH PANCREATIC CANCER IN OMAN: A RETROSPECTIVE STUDY

**Topic:** 35. Thrombosis and vascular biology

Asma Al Adawi*[1], Khalid Nasser Alhashmi[2], Turkiya Al Harrasi[2]

*[1]Oman Medical Specialty Board, Hematopathology, Muscat, Oman; [2]Oman Medical Specialty Board, Muscat, Oman;*

### Background

Venous thromboembolism (VTE) is a leading cause of morbidity and mortality in cancer patients, with pancreatic cancer being among the most prothrombotic malignancies. Pancreatic cancer patients are classified as having intermediate or high Khorana scores, reflecting a substantial risk of VTE. Despite clinical guidelines advocating prophylaxis for high-risk patients, in Oman, prophylaxis is primarily limited to inpatient settings, using parenteral anticoagulants during hospital admissions.

### Aims

This study aims to evaluate the incidence and recurrence of VTE in pancreatic cancer patients and the risk factors of VTE, assesses the impact of prophylaxis on VTE prevention, and emphasizes the potential benefits of integrating direct oral anticoagulants into routine care.

### Methods

A retrospective cohort study was conducted at a tertiary care center in Oman, analyzing pancreatic cancer cases from 2017 to 2019. VTE incidence, recurrence, risk factors, and the impact of inpatient thromboprophylaxis were evaluated. Statistical analyses were performed using SPSS, with $p < 0.05$ considered significant. Ethical approval was obtained, and data were anonymized

### Results

A total of 55 patients with pancreatic cancer were included. The cumulative incidence of venous thromboembolism (VTE) was 29.1%, with 37.5% of affected patients experiencing recurrence. Patients who received chemotherapy and those with metastases demonstrated a higher incidence of VTE compared to others. In patient thromboprophylaxis (unfractionated heparin and low-molecular-weight heparin) was used in 40 patients (72.6%) and was associated with lower VTE rates than no prophylaxis (17.5% vs. 60%, $p = 0.002$). Bleeding complications associated with anticoagulant use were rare, occurring in only two patients (7.3%). Overall mortality was high (81.8%) and did not differ significantly between patients with and without VTE.

### Summary/Conclusion

These findings highlight the importance of VTE prevention in pancreatic cancer patients. Extending prophylaxis to include outpatient care and introducing direct oral anticoagulants, could reduce the risk of VTE.

**Copyright Information:** (Online) ISSN: 2572-9241
©2026 The Author(s). HemaSphere is published by John Wiley & Sons Ltd on behalf of European Hematology Association. This is an open access Abstract Book distributed under the Attribution-NonCommercial-NoDerivs (CC BY-NC-ND), which allows third parties to download the articles and share them with others as long as they credit the author and the Abstract Book, but they cannot change the content in any way or use them commercially.
**Abstract Book Citations:** Authors, Title, HemaSphere, 2026;10:(S1):pages. The abstract book DOIs can be found on the "Author Information" page.
**Disclaimer:** Articles published in the journal HemaSphere exclusively reflect the opinions of the authors. The authors are responsible for all content in their abstracts including accuracy of the facts, statements, citing resources, etc.

### (PB4371) PERICATHETER AND SYSTEMIC THROMBOSIS DURING CAR-T LYMPHOCYTAPHERESIS: INCIDENCE AND CLINICAL CORRELATES

**Topic:** 35. Thrombosis and vascular biology

Ilaria Pansini*[1, 2], Eugenio Galli[1, 2], Alessandro Corrente[2], Nicola Piccirillo[1, 2], Marcello Viscovo[1, 2], Stefan Hohaus[1, 2], Patrizia Chiusolo[1, 2], Federica Sorà[1, 2], Simona Sica[1, 2]

*[1]Fondazione Policlinico Universitario A. Gemelli IRCCS, Rome, Italy, Department of Laboratory and Hematological Sciences, Rome, Italy; [2]Università Cattolica del Sacro Cuore, Rome, Italy, Hematology Section, Department of Radiological and Hematological Sciences, Rome, Italy;*

### Background

Pericatheter thrombosis (PCT) is a recognised complication of large-bore venous catheters used for cell collection. An incidence of local thrombotic occlusion of approximately 1.8% has been described in literature for Mahurkar catheters used in peripheral blood stem cell collection. Despite these devices being widely used in chimeric antigen receptor t (CAR-T) cell manufacturing, the incidence and determinants of PCT in this setting are still unclear. This study provides a real-world evaluation of PCT in patients undergoing lymphocytapheresis for CAR-T therapy.

### Aims

The aim is to characterise thrombotic events and explore potentially relevant clinical or procedural factors.

### Methods

A retrospective, single-centre analysis was conducted on 158 adults evaluated for CAR-T therapy between September 2019 and October 2025. Of these patients, 62 who underwent lymphocytapheresis using a Mahurkar catheter were included in the analysis. The underlying diseases were large B-cell lymphoma (79.0%), mantle cell lymphoma (9.7%) and B-cell acute lymphoblastic leukaemia (11.3%). Clinical, laboratory, and procedural variables were collected at the time of catheter placement. Two predefined analytical strategies were employed: (1) comparison of patients with pericatheter thrombosis (PCT) versus all other patients (with or without thrombosis at distant sites); and (2) comparison of patients with any thrombotic event (PCT or distant thrombosis) versus those with no thrombosis. Continuous variables (age, platelet count, haemoglobin, white blood cell count, fibrinogen and D-dimer) were analysed using a Student's t-test, while categorical variables (sex, disease status, lactate dehydrogenase -LDH- level above or below the laboratory limit, Khorana score, anticoagulation, catheter site and procedural difficulty) were analysed using a chi-squared test or Fisher's exact test, as appropriate.

### Results

PCT occurred in 5 out of 62 patients (8%). An additional 8 patients (13%) developed thrombosis at non-catheter sites, for a total of 13 thrombotic events (21%). No continuous variable was associated with PCT, although platelet count showed a borderline trend ($p = 0.061$). Procedural difficulty was more frequent in PCT cases (66% vs. 18%), though this was not statistically significant after Yates's correction. Sex, LDH, Khorana score, anticoagulation, disease status, catheter site and other laboratory parameters showed no association with PCT. In the overall thrombosis analysis, no clinical or laboratory variable reached statistical significance, with platelet count again demonstrating a borderline trend ($p = 0.098$).

**Copyright Information:** (Online) ISSN: 2572-9241
©2026 The Author(s). HemaSphere is published by John Wiley & Sons Ltd on behalf of European Hematology Association. This is an open access Abstract Book distributed under the Attribution-NonCommercial-NoDerivs (CC BY-NC-ND), which allows third parties to download the articles and share them with others as long as they credit the author and the Abstract Book, but they cannot change the content in any way or use them commercially.
**Abstract Book Citations:** Authors, Title, HemaSphere, 2026;10:(S1):pages. The abstract book DOIs can be found on the “Author Information” page.
**Disclaimer:** Articles published in the journal HemaSphere exclusively reflect the opinions of the authors. The authors are responsible for all content in their abstracts including accuracy of the facts, statements, citing resources, etc.

**Summary/Conclusion**

The main finding of this study was that PCT during lymphocytapheresis for CAR-T manufacturing was more frequent than had been reported in historical series of Mahurkar catheters used for peripheral blood stem cell collection. Within the analysed population, no baseline clinical or laboratory variable was found to be associated with an increased risk of thrombosis. This suggests that CAR-T candidates undergoing lymphocytapheresis have a relatively homogeneous thrombotic risk profile. These observations highlight the need for comparative analyses with patients undergoing haematopoietic stem cell apheresis to determine whether the higher incidence observed reflects factors related to the procedure, the disease, or the population.

**Copyright Information:** (Online) ISSN: 2572-9241
©2026 The Author(s). HemaSphere is published by John Wiley & Sons Ltd on behalf of European Hematology Association. This is an open access Abstract Book distributed under the Attribution-NonCommercial-NoDerivs (CC BY-NC-ND), which allows third parties to download the articles and share them with others as long as they credit the author and the Abstract Book, but they cannot change the content in any way or use them commercially.
**Abstract Book Citations:** Authors, Title, HemaSphere, 2026;10:(S1):pages. The abstract book DOIs can be found on the "Author Information" page.
**Disclaimer:** Articles published in the journal HemaSphere exclusively reflect the opinions of the authors. The authors are responsible for all content in their abstracts including accuracy of the facts, statements, citing resources, etc.

### (PB4372) THROMBOEMBOLISM BEYOND MYELOPROLIFERATIVE NEOPLASMS: REAL-WORLD CHARACTERISTICS AND ANTICOAGULATION OUTCOMES IN NON-MYELOPROLIFERATIVE HEMATOLOGIC MALIGNANCIES

**Topic:** 35. Thrombosis and vascular biology

Otman Abdelmoula*[1], Maha EL Amani[1], Imane Chajai[1], Souad Fennan[1], Zineb Afroukh[1], Othman Doghmi[1], Amal Terfai[1], Elmaachi Nora[1], Naoufal Benlachgar[1], Soukaina Haidouri[1], Zoubida Tazi Mezalek[1]

[1]*Ibn Sina University Hospital Center, RABAT, Morocco;*

### Background

Thromboembolic events (TE) are a major source of morbidity in patients with hematologic malignancies. Beyond well-established risks in myeloproliferative neoplasms, TE also occur across a broad spectrum of non-myeloproliferative disorders. They may reflect intrinsic cancer-related prothrombotic mechanisms and/or "opportunistic" triggers such as infection, central venous catheter (CVC), immobilization, or treatment-related factors. Real-world data on TE phenotype, management, and bleeding complications in this heterogeneous setting remain limited.

### Aims

To describe the characteristics of TE in patients with non-myeloproliferative hematologic malignancies, explore associated clinical factors, and evaluate anticoagulant strategies.

### Methods

Retrospective observational study conducted in the clinical hematology department of Ibn Sina University Hospital (January 2022–December 2025). Adult patients with non-myeloproliferative hematologic malignancies complicated by TE were included. Collected data covered demographics, malignancy type/status and bulky disease, contextual factors (CVC, infection, immobilization), TE localization, anticoagulation (initial agent and switches), thrombotic recurrence, and bleeding complications.

### Results

A total of 61 patients were included. The median age at hematologic diagnosis was 53.5 years (range 10–85), with a balanced sex distribution (51.7% female). The cohort reflected a wide spectrum of hematologic diseases. The most frequent entities were diffuse large B-cell lymphoma (36.7%), Hodgkin lymphoma (13.3%), and multiple myeloma (11.7%). Other diagnoses included primary mediastinal B-cell lymphoma, adult T-cell leukemia/lymphoma, follicular lymphoma, acute B-lymphoblastic leukemia, chronic lymphocytic leukemia, mantle cell lymphoma, plasma cell leukemia, and rarer lymphoproliferative/histiocytic disorders such as Rosai–Dorfman disease and lymphomatoid granulomatosis.
TE were predominantly venous (98.3%). Pulmonary embolism accounted for approximately 30% of events and was often associated with deep vein thrombosis, particularly at femoral sites, while cervico-thoracic thromboses were also observed. TE occurred predominantly at the time of initial disease presentation (78.3%) and were frequently early events (<3 months) (67.8%). Concomitant potential triggers were common, including active infection (26.7%), immobilization (21.7%), and bulky disease (25%), suggesting a multifactorial prothrombotic context.
Initial anticoagulation consisted of low-molecular-weight heparin (LMWH) in 58.3% of patients and direct oral anticoagulants (DOACs; apixaban or rivaroxaban) in 41.6%. Thrombotic recurrence was rare (1.7%). Bleeding complications occurred in 16.7% of patients; bleeding events were not more frequent among patients treated with DOACs.

**Copyright Information:** (Online) ISSN: 2572-9241
©2026 The Author(s). HemaSphere is published by John Wiley & Sons Ltd on behalf of European Hematology Association. This is an open access Abstract Book distributed under the Attribution-NonCommercial-NoDerivs (CC BY-NC-ND), which allows third parties to download the articles and share them with others as long as they credit the author and the Abstract Book, but they cannot change the content in any way or use them commercially.
**Abstract Book Citations:** Authors, Title, HemaSphere, 2026;10:(S1):pages. The abstract book DOIs can be found on the "Author Information" page.
**Disclaimer:** Articles published in the journal HemaSphere exclusively reflect the opinions of the authors. The authors are responsible for all content in their abstracts including accuracy of the facts, statements, citing resources, etc.

**Summary/Conclusion**

In this real-world cohort of non-myeloproliferative hematologic malignancies, TE were mostly venous, early, and clustered around the initial disease phase, supporting a strong cancer-associated prothrombotic background. The frequent presence of contextual triggers (CVC, infection, immobilization, bulky disease) suggests that TE often results from an interplay between intrinsic risk and opportunistic factors. While LMWH remained the most common initial anticoagulant, DOACs were frequently used with apparently comparable outcomes. The non-negligible bleeding rate highlights the need for individualized risk assessment and careful monitoring in this population.

**Copyright Information:** (Online) ISSN: 2572-9241
©2026 The Author(s). HemaSphere is published by John Wiley & Sons Ltd on behalf of European Hematology Association. This is an open access Abstract Book distributed under the Attribution-NonCommercial-NoDerivs (CC BY-NC-ND), which allows third parties to download the articles and share them with others as long as they credit the author and the Abstract Book, but they cannot change the content in any way or use them commercially.
**Abstract Book Citations:** Authors, Title, HemaSphere, 2026;10:(S1):pages. The abstract book DOIs can be found on the "Author Information" page.
**Disclaimer:** Articles published in the journal HemaSphere exclusively reflect the opinions of the authors. The authors are responsible for all content in their abstracts including accuracy of the facts, statements, citing resources, etc.

### (PB4373) IS A "HARD STOP" ON ELECTRONIC CLINICAL NOTES ENOUGH TO IMPROVE ADHERENCE TO VENOUS THROMBOEMBOLISM NICE GUIDELINES ?

**Topic:** 35. Thrombosis and vascular biology

Silvia Lovato*[1], Ida Atti[1], Najah Hussein[1], Ektaa Modha[1]

[1]*Harefield Hospital, Guy's and St Thomas' NHS Foundation Trust, Haematology, Harefield, United Kingdom;*

### Background

VTE risk assessment is mandatory in UK for every patient admitted to any hospital according to the National Institute for Health and Care Excellence (NICE), national guidelines.tHEY states that all admitted patients must be assessed for VTE and bleeding risk on admission, with pharmacological prophylaxis initiated possibly within 14 hours when indicated. In 2024 only 84% of patients were evaluated within 24 hours down from 98% of previous years and in medical patients, only 14% had LWMH prescribed within 14 hours from admission,.One of the changes that may have caused this was a transition to a new electronic note system that did not have, like the previous one, a hard-stop system where doctors could not proceed with other tasks until VTE risk assessment was completed. Following these results, we introduced again a hard stop system to see if this could help improving compliance.

### Aims

The first aim of the improvement project was to assess if the introduction of the hard stop system improved the compare the compliance with NICE guidelines in completion of VTE assessment. The second was to evaluate if the number of medical patients receiving thromboprophylaxis within 14 hours increased.

### Methods

We compiled a list of patients admitted in medical and surgical wards from about patients, admitted in the period 17-25/09/25 from electronic notes to determine if the VTE assessments were done in a timely manner and if the LWMH was prescribed within 14 hours in medical patients.

### Results

We audited 139 admissions (74 medical, 65 surgical) audited, of those 97% (135/139) had VTE assessments done at some point during their admission, 99% of medical patients (73/74) and 91% (59/65) of surgical patients. Only 78% (108/139) ), 93% (69/74) of the medical patient and 60% (39/65) of surgical patients had VTE assessed within 24 hours of admission. The decision about thromboprophylaxis was documented only in 22% (31/138) of the forms, despite that patients received phrophylaxis when indicated, even the 4 patient where there was no evidence of VTE assesment.
Out of 74 medical patients 17 were already on some anticoagulant, so they did not qualify for thromboprophylaxis. Of the remaining patients in 10 starting prophilaxis was contraindicated because they had an invasive procedure booked and in 1 there was ongoing bleeding. Ot the remaining 46 only 56% (26/46) started the prophylaxis within 14 hours. In one case the reason documented was patient refusal, but in most cases there was not a clearly documented reason.

**Copyright Information:** (Online) ISSN: 2572-9241
©2026 The Author(s). HemaSphere is published by John Wiley & Sons Ltd on behalf of European Hematology Association. This is an open access Abstract Book distributed under the Attribution-NonCommercial-NoDerivs (CC BY-NC-ND), which allows third parties to download the articles and share them with others as long as they credit the author and the Abstract Book, but they cannot change the content in any way or use them commercially.
**Abstract Book Citations:** Authors, Title, HemaSphere, 2026;10:(S1):pages. The abstract book DOIs can be found on the "Author Information" page.
**Disclaimer:** Articles published in the journal HemaSphere exclusively reflect the opinions of the authors. The authors are responsible for all content in their abstracts including accuracy of the facts, statements, citing resources, etc.

**Summary/Conclusion**

The VTE guidelines compliance improved since the introduction of the hard stop from 84% to 97%.  Although in theory doctors cannot proceed with other tasks until VTE risk assessment is completed, there is obviously a way to get around this hard stop.  For surgical patients most likely doctors deferred the assessment after the surgery. The percentage of patients who started prophylaxis within 14 hours improved significantly from 14% to 56%, but the lack of cleare documentation may understimate this. Whit these new results we identified areas of improvement, mainly on timing of VTE assessment in surgical patients and incomplete of clear documentation of the clinical reasoning process, we can identify the department most affected and work in collaboration with them to further improve the compliance to the guideline and patient safety.

**Copyright Information:** (Online) ISSN: 2572-9241
©2026 The Author(s). HemaSphere is published by John Wiley & Sons Ltd on behalf of European Hematology Association. This is an open access Abstract Book distributed under the Attribution-NonCommercial-NoDerivs (CC BY-NC-ND), which allows third parties to download the articles and share them with others as long as they credit the author and the Abstract Book, but they cannot change the content in any way or use them commercially.
**Abstract Book Citations:** Authors, Title, HemaSphere, 2026;10:(S1):pages. The abstract book DOIs can be found on the "Author Information" page.
**Disclaimer:** Articles published in the journal HemaSphere exclusively reflect the opinions of the authors. The authors are responsible for all content in their abstracts including accuracy of the facts, statements, citing resources, etc.

### (PB4374) PROGNOSTIC ROLE OF FIBRINOGEN IN PATIENTS WITH NEUTROPENIC SEPSIS

**Topic:** 35. Thrombosis and vascular biology

Gülçin Çelebi*[1], İlkçe Akgün Kurtulmuş[1], Şükriye Miray Kılınçer Bozgül[1], Ajda Gunes[1], Devrim Bozkurt[1]

[1]*EGE UNIVERSITY, INTERNAL MEDICINE, IZMIR, Türkiye;*

### Background

Sepsis remains a major cause of morbidity and mortality in patients with hematologic malignancies who develop neutropenia. This study aimed to evaluate the associations between demographic, clinical, and laboratory parameters and mortality in neutropenic patients with hematologic malignancies during sepsis.

### Aims

The aim of the study is to investigate the roll of fibrinogene in neutropenic sepsis.

### Methods

The medical records of 105 neutropenic patients with hematologic malignancies diagnosed with sepsis in the Intensive Care Unit of Ege University between 2019 and 2024 were retrospectively reviewed. Demographic characteristics, comorbidities, sepsis focus, laboratory findings, organ dysfunction, appropriate antibiotic use, and
30-day mortality were recorded. Parameters associated with mortality were analyzed using chi-square, Fisher's exact, Student's t-test, and Mann–Whitney U test.

### Results

Of the 105 patients included, 67 (63.8%) were male and 38 (36.2%) were female, with a mean age of51.2±18.6 years. Comorbidities were present in 74.3% of patients, most commonly hypertension (26.7%). The most frequent focus of sepsis was pulmonary infections (44.8%). At ICU admission, 69.5% required vasopressors, and 57.1% developed respiratory failure during follow-up. Gram-negative bacteria were the most common pathogens (48.6%), and bacteremia was detected in 52.3%. The 30-day mortality rate was 70.2%. Mortality was significantly associated with low albumin (p=0.027), low systolic blood pressure (SBP, p=0.013), high heart rate (p=0.016), higher SOFA score (p<0.001), prolonged aPTT (p=0.024), low fibrinogen (p<0.001), elevated creatinine (p=0.008), low uric acid (p=0.005), elevated CRP (p=0.005), lower neutrophil count (p=0.002), thrombocytopenia (p<0.001), and elevated lactate (p<0.001). In multivariate analysis, only low fibrinogen level remained an independent risk factor for mortality, with an odds ratio of 0.994 (95% CI [0.988–1.000]).

### Summary/Conclusion

Sepsis in neutropenic patients with hematologic malignancies is associated with high mortality rates. Low fibrinogen, thrombocytopenia, high lactate, and elevated SOFA score were strongly associated with mortality. Among these, fibrinogen was identified as the sole independent protective factor. Our study demonstrated that each unit increase in fibrinogen reduced mortality risk by approximately 0.6%. Markedly decreased fibrinogen levels during sepsis may reflect both the severity of systemic inflammation and the development of consumptive coagulopathy. The finding that fibrinogen remained the only independent predictor highlights its potential as a valuable biomarker for early mortality prediction in clinical practice. Routine monitoring of fibrinogen in neutropenic patients with hematologic malignancies may facilitate early risk stratification and individualized treatment strategies. Furthermore, prospective studies are warranted to explore the potential of fibrinogen as a therapeutic target.

**Copyright Information:** (Online) ISSN: 2572-9241
©2026 The Author(s). HemaSphere is published by John Wiley & Sons Ltd on behalf of European Hematology Association. This is an open access Abstract Book distributed under the Attribution-NonCommercial-NoDerivs (CC BY-NC-ND), which allows third parties to download the articles and share them with others as long as they credit the author and the Abstract Book, but they cannot change the content in any way or use them commercially.
**Abstract Book Citations:** Authors, Title, HemaSphere, 2026;10:(S1):pages. The abstract book DOIs can be found on the "Author Information" page.
**Disclaimer:** Articles published in the journal HemaSphere exclusively reflect the opinions of the authors. The authors are responsible for all content in their abstracts including accuracy of the facts, statements, citing resources, etc.

### (PB4375) PSYCHOLOGICAL BURDEN OF CANCER-ASSOCIATED THROMBOSIS: IMPACT ON QUALITY OF LIFE, ADHERENCE AND INTEGRATED HEMATOLOGY CARE

**Topic:** 35. Thrombosis and vascular biology

Pedro Henrique Fernandes Do Carmo Las Casas*[1, 2], Grigorios Gerotziafas[1, 3, 4], Maria Jose Serna Muñoz[5], Yiannis Mitrou[6], Marina Marchetti[7], Alfonso Tafur[8], Victoria Bitsadze[3], Darko Antic[9], Corinne Frere[10, 11], Alexander Makatsariya[3], Laurent Garderet[12], Cihan Ay[13], Nikita Lomakin[14], Zoubida Tazi Mezalek[15], Mariella Catalano[16], Isabelle Mahe[17, 18], Joseph Gligorov[19], Patrick Van Dreden[1, 20], Jawed Fareed[4], Anna Falanga[7]

*[1]Saint Antoine University Hospital Assistance Publique Hôpitaux de Paris (AP-HP), Sorbonne University, INSERM UMR_S_938, CRSA, Research Department "Cancer, Vessels, Biology and Therapeutics" (CaVITE), Research Group "Cancer-Angiogenesis-Thrombosis and Haemostasis," Thrombosis Center, University Institute of Cancerology (UIC),, Paris, France; [2]Federal University of Minas Gerais, Faculty of Medicine, Belo Horizonte, Brazil; [3]The I.M. Sechenov First Moscow State Medical University (Sechenov University), Thrombosis Center, Department of Obstetrics, Gynecology and Perinatal Medicine,, Moscow, Russia; [4]Loyola University Stritch School of Medicine, Maywood, United States of America;[5]University Hospital Morales Meseguer, Hematology Department,, Murcia, Spain; [6]National and Kapodistrian University of Athens,, Department of Phylosophy, Athens, Greece; [7]Hospital Papa Giovanni XXIII, Department of Immunohematology and Transfusion Medicine, Bergamo, Italy;[8]Pritzker School of Medicine, University of Chicago, Vascular Medicine, Chigaco, United States of America; [9]University of Belgrade, Faculty of Medicine, Belgrade, Serbia; [10]Sorbonne Université, Department of Hematology, Pitié-Salpêtrière Hospital, Assistance Publique-Hôpitaux de Paris,, Paris, France; [11]INSERM UMRS-1166 Institute of Cardiometabolism and Nutrition, Paris, France; [12]Hôpital Universitaire Pitié-Salpêtrière, Assistance Publique Hôpitaux de Paris (AP-HP), Service Hématologie, Paris, France; [13]Medical University of Vienna, Department of Internal Medicine I, Clinical Division of Haematology and Haemostaseology,, Vienna, Austria; [14]Central Clinical Hospital of Presidential Administration, Cardiology Division, Moscow, Russia; [15]Centre Hospitalo-Universitaire Ibn Sina, Université Mohammed V de Rabat, Department of Internal Medicine, Hématologie Clinique, Rabat, Morocco; [16]University of Milan-L Sacco Hospital, Inter-University Research Center on Vascular Diseases,, Milan, Italy; [17]Hôpital Louis Mourier, Assistance Publique-Hôpitaux de Paris (AP-HP), Service de Médecine Interne,, Colombes, France; [18]Université Paris Cité, Paris, France; [19]Tenon Hospital, Assistance Publique-Hôpitaux de Paris (AP-HP), Sorbonne University, Medical Oncology Department, Paris, France; [20]Diagnostica Stago, Department of Clinical Research, Gennevilliers, France;*

### Background

Cancer-associated thrombosis (CAT) affects 5–20% of patients with malignancy and represents the second leading cause of death after cancer progression. Although its clinical impact is well established, the psychological burden associated with CAT remains insufficiently integrated into hematology practice. Venous thromboembolism (VTE), particularly pulmonary embolism (PE), may constitute a life-threatening experience that intensifies fear of death, anxiety about cancer recurrence, and existential distress, with potential long-term effects on health-related quality of life (HRQoL) and treatment engagement.

### Aims

This narrative review aims to evaluate the psychological impact of CAT on cancer patients, assess its effects on HRQoL, and identify gaps in patient education, adherence, and integrated psycho-oncological care. It also examines the influence of socioeconomic disparities and proposes a patient-centered model that integrates psychological support into CAT management.

**Copyright Information:** (Online) ISSN: 2572-9241
©2026 The Author(s). HemaSphere is published by John Wiley & Sons Ltd on behalf of European Hematology Association. This is an open access Abstract Book distributed under the Attribution-NonCommercial-NoDerivs (CC BY-NC-ND), which allows third parties to download the articles and share them with others as long as they credit the author and the Abstract Book, but they cannot change the content in any way or use them commercially.
**Abstract Book Citations:** Authors, Title, HemaSphere, 2026;10:(S1):pages. The abstract book DOIs can be found on the "Author Information" page.
**Disclaimer:** Articles published in the journal HemaSphere exclusively reflect the opinions of the authors. The authors are responsible for all content in their abstracts including accuracy of the facts, statements, citing resources, etc.

**Methods**

We conducted a narrative review of clinical cohorts, qualitative and quantitative studies, and mixed-methods research addressing CAT, psychological outcomes, HRQoL, and anticoagulation management. Evidence from longitudinal cohorts, including the PELICAN program, was analyzed together with randomized trials comparing low-molecular-weight heparin (LMWH) and direct oral anticoagulants (DOACs). Data extraction focused on prevalence and persistence of existential distress, anxiety, fear of recurrence, HRQoL impairment across domains, treatment-related burden, adherence patterns, and the reported impact of psychosocial interventions. Socioeconomic determinants and disparities in access to psycho-oncological support were examined when available.

**Results**

Up to 46% of advanced cancer patients experience clinically significant existential distress, often alongside depression and anxiety. CAT amplifies pre-existing distress and triggers additional existential anxiety. Symptomatic VTE, particularly PE, can be life-changing, leading to lasting psychological sequelae. Acute CAT adversely affects HRQoL, impairing physical, role, and social functioning, particularly in patients with recurrent events, disseminated disease, or poor performance status. The use of parenteral LMWH increases treatment-related stress, while DOACs improve convenience and perceived safety. Higher levels of emotional distress correlate with better anticoagulation adherence. Patients frequently report feeling insufficiently informed on CAT risk and management. Socioeconomic disadvantage is associated with higher distress and lower access to psycho-oncological support. Structured interventions such as cognitive behavioral therapy, meaning-centered therapy, CALM, psycho-dynamics therapy, and supportive counseling effectively reduce fear and existential distress and improve HRQoL.

**Summary/Conclusion**

CAT imposes a dual clinical and psychological burden in patients with cancer. Systematic psychological assessment, structured patient education, and integration of psycho-oncological support into hematology care pathways are supported by available evidence and may improve HRQoL and therapeutic adherence. Addressing socioeconomic vulnerability is essential to ensure equitable and patient-centered thrombosis management.

**Copyright Information:** (Online) ISSN: 2572-9241
©2026 The Author(s). HemaSphere is published by John Wiley & Sons Ltd on behalf of European Hematology Association. This is an open access Abstract Book distributed under the Attribution-NonCommercial-NoDerivs (CC BY-NC-ND), which allows third parties to download the articles and share them with others as long as they credit the author and the Abstract Book, but they cannot change the content in any way or use them commercially.
**Abstract Book Citations:** Authors, Title, HemaSphere, 2026;10:(S1):pages. The abstract book DOIs can be found on the “Author Information” page.
**Disclaimer:** Articles published in the journal HemaSphere exclusively reflect the opinions of the authors. The authors are responsible for all content in their abstracts including accuracy of the facts, statements, citing resources, etc.

### (PB4376) INCIDENCE AND RISK FACTORS FOR PORTAL VEIN THROMBOSIS AFTER SPLENECTOMY: A -20 YEAR SINGLE CENTER EXPERIENCE

**Topic:** 35. Thrombosis and vascular biology

Mahmoud Ayesh (Haj Yousef)*[1]

[1]*Jordan University of Science and Technology, King Abdullah University Hospital, Internal Medicine, Irbed, Jordan;*

### Background

Portal vein thrombosis (PVT) is a recognized but uncommon complication after splenectomy. Limited data exist on its incidence and risk factors.

### Aims

To find the incidence and risk factors for portal vein thrombosis in adult patients  after splenectomy

### Methods

We performed a retrospective review of medical records for all patients who underwent splenectomy at king Abdullah University hospital from September 2002 to December 2022 (n = 195). Incidence, timing, presenting features, operative approach (open vs laparoscopic) and potential risk factors for PVT were analyzed.

### Results

Portal vein thrombosis (PVT)occurred in 11 of 195 patients (5.6%). There was no significant difference in PVT incidence between open and laparoscopic splenectomy. Pain was the most frequent presenting symptom. Most PVT cases were diagnosed within the first postoperative month. The principal underlying diagnoses associated with PVT were autoimmune hemolytic anemia and lymphoma.

### Summary/Conclusion

Portal vein thrombosis (PVT) occurred in 11 of 195 patients (5.6%). There was no significant difference in PVT incidence between open and laparoscopic splenectomy. Pain was the most frequent presenting symptom. Most PVT cases were diagnosed within the first postoperative month. The principal underlying diagnoses associated with PVT were autoimmune hemolytic anemia and lymphoma.

**Copyright Information:** (Online) ISSN: 2572-9241
©2026 The Author(s). HemaSphere is published by John Wiley & Sons Ltd on behalf of European Hematology Association. This is an open access Abstract Book distributed under the Attribution-NonCommercial-NoDerivs (CC BY-NC-ND), which allows third parties to download the articles and share them with others as long as they credit the author and the Abstract Book, but they cannot change the content in any way or use them commercially.
**Abstract Book Citations:** Authors, Title, HemaSphere, 2026;10:(S1):pages. The abstract book DOIs can be found on the "Author Information" page.
**Disclaimer:** Articles published in the journal HemaSphere exclusively reflect the opinions of the authors. The authors are responsible for all content in their abstracts including accuracy of the facts, statements, citing resources, etc.

### (PB4377) CHARACTERIZATION AND TREATMENT OF VENOUS THROMBOTIC EVENTS IN PATIENTS WITH VEXAS SYNDROME: A SINGLE CENTRE EXPERIENCE

**Topic:** 35. Thrombosis and vascular biology

Chiara Ambaglio*[1], Francesca Schieppati[1], Elisa Galimberti[1], Marco Frigeni[2], Marina Marchetti[1], Luca Barcella[1], Anna Falanga[3]

*[1]Immunohematology and Transfusion Medicine Unit, ASST Papa Giovanni XXIII, Bergamo, Italy; [2]Hematology and Bone Marrow Transplant Unit, ASST Papa Giovanni XXIII, Bergamo, Italy; [3]School of Medicine and Surgery, University of Milano-Bicocca, Milano, Italy;*

### Background

VEXAS syndrome is an autoinflammatory condition first described in 2020. Reported venous thromboembolism (VTE) incidence is from 35 to 57%. Little is known about the best anticoagulant treatment duration, intensity, and the risk of recurrence.

### Aims

To describe the incidence of VTE and characteristics of venous thrombosis in patients with VEXAS syndrome followed at our Institution.

### Methods

All the patients had the pathogenic UBA1 somatic mutation and clinical features of VEXAS syndrome. We analyzed data on superficial vein thrombosis (SVT) and VTE defined as pulmonary embolism (PE) and/or deep vein thrombosis (DVT).

### Results

To date, seven male patients have been followed at our Institution. One patient deceased little after the diagnosis of VEXAS and had no thrombotic events; two patients are in complete remission after bone marrow transplantion and have no history of VTE or SVT. The other 4 experienced venous thrombotic events. Case 1. Age 69, in 2017 DVT of the right internal jugular and subclavian veins occurred, associated with a piriform sinus abscess; warfarin was started with INR target 2.5 (range 2-3). Then he had recurrent pleuritis and diagnosis of Horton arteritis. In 2018 during warfarin with INR in therapeutic range, bilateral SVT of the lower limbs and DVT of right popliteal vein occurred; INR target was increased to 3 (range 2.5-3.5). In august 2021, due to myelodisplastic syndrome appearance, the diagnosis of VEXAS syndrome was placed. In 2022 bone marrow transplantation was successfully performed, then anticoagulation was stopped without recurrence. Case 2. Age 74, diagnosis of VEXAS made in 2023 due to persistent eosinophilia, macrocytic anemia, dermatosis. Steroids, hydroxychloroquine and erithropoietin were started for progressive anemia. In 2024 due to left popliteal and femoral vein DVT, apixaban 5 mg bid was started. Six months  later, the affected veins were patent. Case 3. Age 82, in 2024 the diagnosis of VEXAS syndrome was done due to fevers, macrocytic anemia, IgM-k MGUS, pulmonary involvement; tocilizumab and steroids were started. Three months later he developed bilateral SVT and DVT (right superficial femoral vein; left popliteal vein). All veins were patent six months after starting anticoagulants. Full dose apixaban is still ongoing. Case 4. Age 76, in July 2024 he had a first SVT of the left leg, treated with Fondaparinux 2.5 mg od for 60 days; in December 2024 a second SVT of the right arm was treated with Fondaparinux 2.5 mg od until he had a third SVT of the left arm and was switched to Rivaroxaban 10 mg od. In April 2025 during regular anticoagulant another left SVT and a first DVT (popliteal, superficial and common femoral veins) occurred, so Dabigatran 150 mg bid was started. Four months later, only the common femoral vein was still thrombosed. Concomitant interstitial pneumonia and macrocytosis were found: VEXAS syndrome was diagnosed in September 2025.

**Copyright Information:** (Online) ISSN: 2572-9241
©2026 The Author(s). HemaSphere is published by John Wiley & Sons Ltd on behalf of European Hematology Association. This is an open access Abstract Book distributed under the Attribution-NonCommercial-NoDerivs (CC BY-NC-ND), which allows third parties to download the articles and share them with others as long as they credit the author and the Abstract Book, but they cannot change the content in any way or use them commercially.
**Abstract Book Citations:** Authors, Title, HemaSphere, 2026;10:(S1):pages. The abstract book DOIs can be found on the “Author Information” page.
**Disclaimer:** Articles published in the journal HemaSphere exclusively reflect the opinions of the authors. The authors are responsible for all content in their abstracts including accuracy of the facts, statements, citing resources, etc.

**Summary/Conclusion**

VEXAS-associated VTE incidence at our center is 57%, in line with current literature. Our cases' clinical history highlights different presentations of thrombotic events at different stages of disease. Recanalization of DVT can be achieved within months but risk of recurrence remains. The anticoagulant or anti-inflammatory therapy alone may not be sufficient to protect from thrombosis, possibly their association may be more efficacious. Alternative treatments for long-term indications are to be investigated.

**Copyright Information:** (Online) ISSN: 2572-9241
©2026 The Author(s). HemaSphere is published by John Wiley & Sons Ltd on behalf of European Hematology Association. This is an open access Abstract Book distributed under the Attribution-NonCommercial-NoDerivs (CC BY-NC-ND), which allows third parties to download the articles and share them with others as long as they credit the author and the Abstract Book, but they cannot change the content in any way or use them commercially.
**Abstract Book Citations:** Authors, Title, HemaSphere, 2026;10:(S1):pages. The abstract book DOIs can be found on the "Author Information" page.
**Disclaimer:** Articles published in the journal HemaSphere exclusively reflect the opinions of the authors. The authors are responsible for all content in their abstracts including accuracy of the facts, statements, citing resources, etc.

### (PB4378) RELATIONSHIP OF PLASMA DABIGATRAN CONCENTRATION WITH RENAL FUNCTION AND COAGULATION SCREENING TEST RESULTS

**Topic:** 35. Thrombosis and vascular biology

Marija Miloš*[1, 2, 3], Irena Matošević[2], Anamarija Bogić[1], Vlasta Cigula Kurajica[1], Ana Boban[4, 5], Marijo Vodanović[3, 4], Desiree Coen Herak[1, 3, 6]

*[1]Department of Laboratory Diagnostics, University Hospital Centre Zagreb, Zagreb, Croatia;[2]Faculty of Pharmacy, University of Mostar, Mostar, Bosnia and Herzegovina; [3]University of Applied Health Sciences, Zagreb, Croatia;[4]Department of Internal Medicine, University Hospital Centre Zagreb, Zagreb, Croatia; [5]School of Medicine, University of Zagreb, Zagreb, Croatia; [6]Faculty of Pharmacy and Biochemistry, University of Zagreb, Zagreb, Croatia;*

### Background

Dabigatran is a direct oral thrombin inhibitor whose elimination is predominantly mediated by the kidneys. Since impaired renal function may lead to drug accumulation and to an increased bleeding risk, therapeutic drug monitoring of dabigatran becomes clinically important.

### Aims

To determine trough dabigatran plasma concentrations in patients with different stages of renal function impairement and to examine the relationship of obtained concentrations with the biochemical parameters for renal function estimation, and screening coagulation tests.

### Methods

The study included 60 citrated plasma samples from 33 patients on dabigatran therapy. Dabigatran concentration was determined using a calibrated *in-house* diluted thrombin time method (dTT) with Test-Thrombin reagent on the automated coagulation analyzer Sysmex CS-5100 (Siemens, Germany). Coagulation screening tests, including prothrombin time (Innovin), activated partial thromboplastin time (Actin FS), thrombin time (Test Thrombin), and fibrinogen (Dade Thrombin), were measured on the same analyzer (Siemens). Biochemical parameters for renal function estimation (urea, creatinine, and estimated glomerular filtration rate (eGFR)) were measured on the Alinity CC biochemical analyzer with original Abbott reagents (Abbott Laboratories, USA).

### Results

Patients with impaired renal function had higher trough plasma dabigatran concentrations compared to those with preserved renal function (Figure 1). Correlation of dabigatran plasma concentrations with coagulation and biochemical parameters, depending on the dose used, is presented in Table 1. Statistically significant correlation with all investigated biochemical and coagulation parameters were found in the group *All doses.* A very similar pattern was obtained in the group *110 mg twice daily.* Correlation coefficients differ significantly in other specific groups of patients with respect to the administered drug dose, probably because of an extremely small number of subjects in these groups of patients.

**Copyright Information:** (Online) ISSN: 2572-9241
©2026 The Author(s). HemaSphere is published by John Wiley & Sons Ltd on behalf of European Hematology Association. This is an open access Abstract Book distributed under the Attribution-NonCommercial-NoDerivs (CC BY-NC-ND), which allows third parties to download the articles and share them with others as long as they credit the author and the Abstract Book, but they cannot change the content in any way or use them commercially.
**Abstract Book Citations:** Authors, Title, HemaSphere, 2026;10:(S1):pages. The abstract book DOIs can be found on the "Author Information" page.
**Disclaimer:** Articles published in the journal HemaSphere exclusively reflect the opinions of the authors. The authors are responsible for all content in their abstracts including accuracy of the facts, statements, citing resources, etc.

### Summary/Conclusion

Trough plasma dabigatran concentrations and coagulation screening test results vary widely across the patient population, depending on renal function and probably other patient characteristics, suggesting the implementation of plasma dabigatran concentration measurement together with the eGFR estimation in certain clinical situations such as renal failure, for safer and more effective use of dabigatran.

Table 1. Correlation coefficients between trough dabigatran plasma concentrations and coagulation parameters: prothrombin time (PT), activated partial thromboplastin time (aPTT), thrombin time (TT), and fibrinogen (Fib) and biochemical parameters for renal function estimation: urea, creatinine, and estimated glomerular filtration rate (eGFR)

| | **Dabigatran dose** | **PT r (P)** | **aPTT r (P)** | **TT r (P)** | **Fib r (P)** | **Urea r (P)** | **Creatinine r (P)** | **eGFR r (P)** |
|---|---|---|---|---|---|---|---|---|
| **Trough dabigatran plasma concentration (ng/mL)** | **All doses** | **-0.499 (0.001) N=39** | **0.681 (<0.001) N=42** | **0.922 (<0.001) N=25** | **0.564 (0.001) N=34** | **0.361 (0.016) N=44** | **0.399 (0.007) N=44** | **-0.546 (<0.001) N=44** |
| | **75 mg twice daily** | -0.700 (0.188) N=5 | 0.800 (0.200) N=4 | **0.975 (0.005) N=5** | 0.949 (0.051) N=4 | 0.721 (0.068) N=7 | 0.750 (0.052) N=7 | **-0.955 (0.001) N=7** |
| | **110 mg twice daily** | **-0.509 (0.022) N=20** | **0.619 (0.001) N=26** | **0.907 (<0.001) N=14** | **0.604 (0.005) N=20** | **0.501 (0.015) N=23** | 0.394 (0.063) N=23 | **-0.611 (0.002) N=23** |
| | **150 twice daily** | -0.247 (0.395) N=14 | **0.750 (0.005) N=12** | **0.900 (0.037) N=5** | 0.509 (0.133) N=10 | 0.205 (0.481) N=14 | -0,046 (0.875) N=14 | -0.137 (0.642) N=14 |

r - Spearman rank correlation

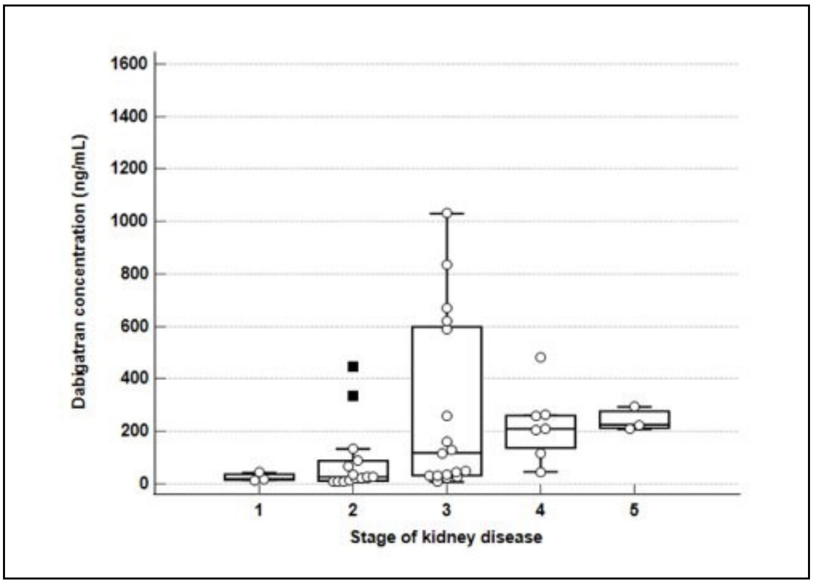

Figure 1. Trough dabigatran plasma concentrations in patients with different stages of kidney disease

**Copyright Information:** (Online) ISSN: 2572-9241
©2026 The Author(s). HemaSphere is published by John Wiley & Sons Ltd on behalf of European Hematology Association. This is an open access Abstract Book distributed under the Attribution-NonCommercial-NoDerivs (CC BY-NC-ND), which allows third parties to download the articles and share them with others as long as they credit the author and the Abstract Book, but they cannot change the content in any way or use them commercially.
**Abstract Book Citations:** Authors, Title, HemaSphere, 2026;10:(S1):pages. The abstract book DOIs can be found on the "Author Information" page.
**Disclaimer:** Articles published in the journal HemaSphere exclusively reflect the opinions of the authors. The authors are responsible for all content in their abstracts including accuracy of the facts, statements, citing resources, etc.

### (PB4379) HAEMOSTATIC DYSFUNCTION MIRRORS RENAL PATHOLOGY IN LUPUS NEPHRITIS: ROLE OF ANTITHROMBIN III

**Topic:** 35. Thrombosis and vascular biology

Ahmed Almenshawy*[1], Alaa Elsawi[1], Shaimaa Shimaa Abd El-Galeel[1], Dalia Younes[1], Mostafa Abdelsalam[1], Ahmed Abd Elwahab[1], Eman Nagy[1]

[1]*Mansoura University faculty of medicine, Clinical Pathology, Elmansoura, Egypt;*

### Background

Thrombo-inflammatory mechanisms play a pivotal role in the pathogenesis of systemic lupus erythematosus (SLE), particularly in lupus nephritis (LN), one of its most severe organ manifestations. Alterations in natural anticoagulants such as protein C (PC), protein S (PS), and antithrombin III (AT-III), as well as enhanced fibrinolysis reflected by elevated D-dimer, contribute to the hypercoagulable milieu frequently observed in SLE. Interleukin-6 (IL-6), a key inflammatory cytokine, is implicated in promoting endothelial injury and may interact with coagulation cascades. However, the clinical and pathological relevance of these haemostatic disturbances in LN remains insufficiently characterized.

### Aims

This study aimed to assess coagulation abnormalities and IL-6 levels in SLE patients, exploring their associations with renal involvement and disease activity.

### Methods

A cross-sectional observational study was conducted on 50 SLE patients: 28 with biopsy-proven LN and 22 with non-renal SLE. Demographic, clinical, and laboratory data were recorded. Disease activity was assessed using the SLE Disease Activity Index-2000 (SLEDAI-2K). Seventeen LN patients underwent renal biopsy, and histopathological activity and chronicity indices were evaluated according to ISN/RPS criteria. Plasma levels of PC, PS, AT-III, D-dimer, and IL-6 were measured by standard immunoassays and compared across subgroups. Correlations between coagulation parameters, complement components, and histologic indices were analyzed.

### Results

SLEDAI scores were comparable between LN and non-LN patients ($p = 0.424$). Overall, PC, PS, AT-III, D-dimer, and IL-6 levels did not differ significantly between groups. However, AT-III levels were significantly lower in active LN cases compared to inactive ones (93 [60–126] mg/dL vs. 131 [130–132] mg/dL; $p = 0.019$). AT-III showed a strong negative correlation with renal histopathologic activity index ($r = -0.649$, $p = 0.009$) and positive correlations with complement C3 ($r = 0.502$, $p = 0.004$) and C4 ($r = 0.484$, $p = 0.007$). IL-6 did not correlate significantly with haemostatic markers or SLEDAI score.

### Summary/Conclusion

Reduced AT-III levels closely parallel renal inflammatory activity and complement depletion in lupus nephritis, underscoring its value as a potential biochemical marker of disease activity. Haemostatic dysfunction in LN appears to reflect renal pathology rather than systemic disease burden. Integrating AT-III evaluation into clinical assessment may enhance early recognition of renal flares and refine therapeutic monitoring in SLE patients.

**Copyright Information:** (Online) ISSN: 2572-9241
©2026 The Author(s). HemaSphere is published by John Wiley & Sons Ltd on behalf of European Hematology Association. This is an open access Abstract Book distributed under the Attribution-NonCommercial-NoDerivs (CC BY-NC-ND), which allows third parties to download the articles and share them with others as long as they credit the author and the Abstract Book, but they cannot change the content in any way or use them commercially.
**Abstract Book Citations:** Authors, Title, HemaSphere, 2026;10:(S1):pages. The abstract book DOIs can be found on the "Author Information" page.
**Disclaimer:** Articles published in the journal HemaSphere exclusively reflect the opinions of the authors. The authors are responsible for all content in their abstracts including accuracy of the facts, statements, citing resources, etc.

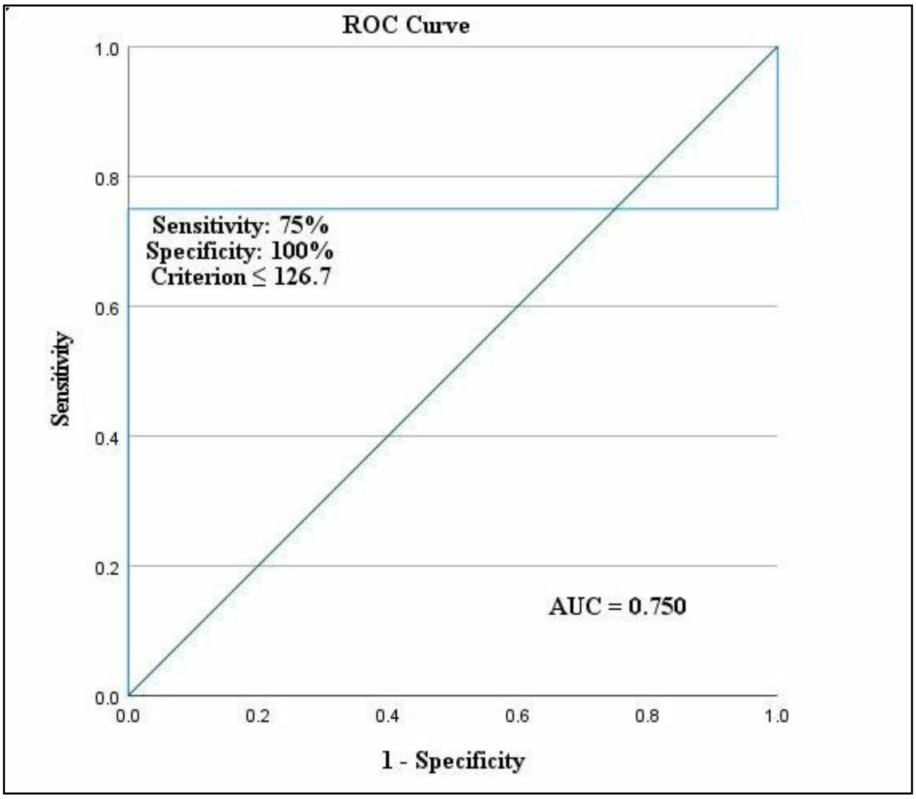

**Copyright Information:** (Online) ISSN: 2572-9241
©2026 The Author(s). HemaSphere is published by John Wiley & Sons Ltd on behalf of European Hematology Association. This is an open access Abstract Book distributed under the Attribution-NonCommercial-NoDerivs (CC BY-NC-ND), which allows third parties to download the articles and share them with others as long as they credit the author and the Abstract Book, but they cannot change the content in any way or use them commercially.
**Abstract Book Citations:** Authors, Title, HemaSphere, 2026;10:(S1):pages. The abstract book DOIs can be found on the "Author Information" page.
**Disclaimer:** Articles published in the journal HemaSphere exclusively reflect the opinions of the authors. The authors are responsible for all content in their abstracts including accuracy of the facts, statements, citing resources, etc.

### (PB4380) LIVING DONOR LIVER TRANSPLANTATION FOR CONGENITAL PLASMINOGEN DEFICIENCY: A NOVEL THERAPEUTIC APPROACH

**Topic:** 35. Thrombosis and vascular biology

Abdulrahman Nasiri*[1], Nawaf Aldawsari[2], Markus Boehnert[3], Bandar Aljudaibi[4], Khalid Bzeizi[4], Hazzaa Alzahrani[2]

*[1]Imam Mohammad Ibn Saud Islamic University, College of Medicine, Riyadh, Saudi Arabia;[2]King Faisal Specialist Hospital & Research Centre, Department of Hematology, Riyadh, Saudi Arabia; [3]King Faisal Specialist Hospital & Research Centre, ABDOMINAL TRANSPLANT & HEPATOBILIARY SURGERY CENTRE, Riyadh, Saudi Arabia; [4]King Faisal Specialist Hospital & Research Centre, LIVER & SMALL BOWEL HEALTH CENTRE, Riyadh, Saudi Arabia;*

### Background

Congenital plasminogen deficiency is an ultra-rare autosomal recessive disorder characterized by impaired fibrinolysis and ligneous disease, leading to progressive multi-organ complications and significant morbidity. Current treatment relies on lifelong plasminogen replacement therapy, which is associated with high cost, treatment burden, and limited availability. Because the liver is the primary site of plasminogen synthesis, liver transplantation represents a potential definitive therapeutic strategy by correcting the underlying synthetic defect.

### Aims

To evaluate the biochemical and early clinical outcomes of living donor liver transplantation as a therapeutic intervention for severe congenital plasminogen deficiency.

### Methods

We report a 22-year-old woman with genetically confirmed homozygous plasminogen deficiency and severe progressive disease despite replacement therapy. She developed ligneous conjunctivitis, hydrocephalus requiring multiple ventriculoperitoneal shunt revisions, and progressive neurological impairment. Baseline plasminogen level was 18% (reference range 80–120%). The patient underwent living donor liver transplantation using a right lobe graft from her twin sister on April 28, 2025. Post-transplant outcomes were assessed by serial plasminogen measurements, clinical monitoring, and evaluation of disease progression.

### Results

Liver transplantation was technically successful with no major perioperative complications. Plasminogen levels increased from 18% pre-transplant to 79% by postoperative day 12, reached 91–101% at one month, and remained stable within the normal range (84–93%) at three-month follow-up. No new ligneous lesions developed after transplantation. Laboratory parameters normalized, and donor recovery was uneventful. Neurological deficits remained unchanged, reflecting pre-existing irreversible damage rather than transplant failure.

### Summary/Conclusion

Living donor liver transplantation achieved rapid and sustained normalization of plasminogen levels, providing proof-of-concept evidence that transplantation can correct the underlying biochemical defect in congenital plasminogen deficiency. This approach may represent a potential curative strategy in selected patients with severe disease. Longer follow-up and additional cases are required to define long-term efficacy, clinical benefit, and optimal patient selection.

**Copyright Information:** (Online) ISSN: 2572-9241
©2026 The Author(s). HemaSphere is published by John Wiley & Sons Ltd on behalf of European Hematology Association. This is an open access Abstract Book distributed under the Attribution-NonCommercial-NoDerivs (CC BY-NC-ND), which allows third parties to download the articles and share them with others as long as they credit the author and the Abstract Book, but they cannot change the content in any way or use them commercially.
**Abstract Book Citations:** Authors, Title, HemaSphere, 2026;10:(S1):pages. The abstract book DOIs can be found on the "Author Information" page.
**Disclaimer:** Articles published in the journal HemaSphere exclusively reflect the opinions of the authors. The authors are responsible for all content in their abstracts including accuracy of the facts, statements, citing resources, etc.

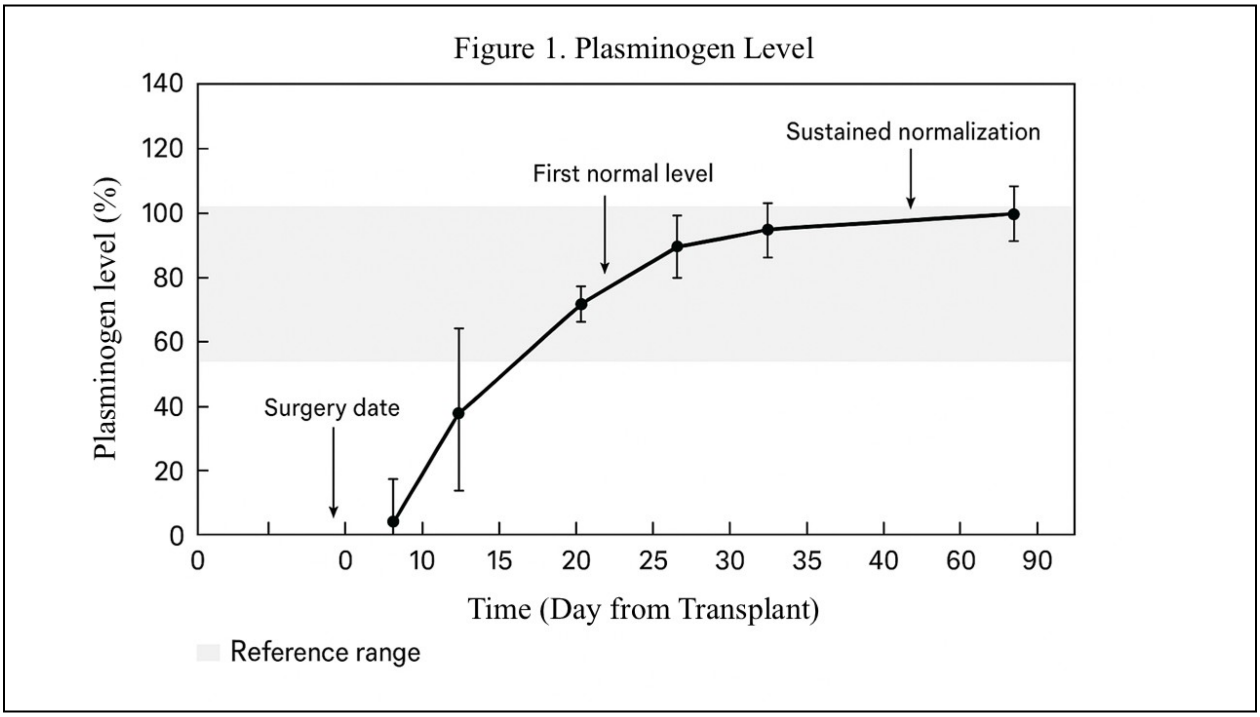

**Copyright Information:** (Online) ISSN: 2572-9241
©2026 The Author(s). HemaSphere is published by John Wiley & Sons Ltd on behalf of European Hematology Association. This is an open access Abstract Book distributed under the Attribution-NonCommercial-NoDerivs (CC BY-NC-ND), which allows third parties to download the articles and share them with others as long as they credit the author and the Abstract Book, but they cannot change the content in any way or use them commercially.
**Abstract Book Citations:** Authors, Title, HemaSphere, 2026;10:(S1):pages. The abstract book DOIs can be found on the "Author Information" page.
**Disclaimer:** Articles published in the journal HemaSphere exclusively reflect the opinions of the authors. The authors are responsible for all content in their abstracts including accuracy of the facts, statements, citing resources, etc.

### (PB4381) HEMATOLOGIC AND HEMOLYSIS-RELATED LABORATORY PROFILE IN PATIENTS WITH MECHANICAL HEART VALVES RECEIVING CHRONIC VITAMIN K ANTAGONIST THERAPY

**Topic:** 35. Thrombosis and vascular biology

Maximilian-George Zaides*[1, 2], Elisabeta Badila[2, 3], Viola Maria Popov[2], Mihaela Andreescu[1, 2]

*[1]Titu Maiorescu University, General Medicine, Bucharest, Romania;[2]Colentina Clinical Hospital, Hematology I, Bucharest, Romania;[3]Carol Davila University of Medicine and Pharmacy, Bucharest, Romania;*

### Background

Mechanical heart valves (MHV) require lifelong vitamin K antagonist (VKA) therapy and expose circulating blood elements to chronic shear stress, potentially leading to sustained coagulation activation and low-grade intravascular hemolysis. While INR monitoring guides anticoagulation intensity, the broader quantitative hematologic and hemolysis-related laboratory profile of these patients remains insufficiently characterized.

### Aims

To provide a quantitative descriptive assessment of coagulation, inflammatory, and hemolysis-related laboratory parameters in patients with MHV receiving chronic VKA therapy and to evaluate laboratory variation according to valve position.

### Methods

We conducted a retrospective observational study including 53 adult patients with mechanical heart valves treated with acenocoumarol. The valve distribution was: isolated aortic (n=23; 43%), isolated mitral (n=15; 28%), and double mechanical valves (n=15; 28%).
Laboratory variables included hemoglobin, platelet count, INR, prothrombin time, activated partial thromboplastin time, fibrinogen, D-dimers, CRP, ESR, NT-proBNP, and total, direct, and indirect bilirubin.For analyses of variables data we used nonparametric tests due low groups of patients (Mann Whithey test, p<0,05 was considered statistical significant). Continuous variables are presented as median (IQR), and analyses were performed using available-case data. Comparisons across valve positions (aortic, mitral, double) were conducted using non-parametric testing. Due to the very small number of patients receiving adjunctive antiplatelet therapy (n=4), treatment-group comparisons were considered exploratory and non-inferential.

### Results

In the overall cohort, INR (n=47) was 2.53 [1.80–3.36], consistent with therapeutic anticoagulation. Hemoglobin (n=48) was 12.15 g/dL [11.08–13.60], with values in the lower-normal range in a substantial proportion of patients. D-dimers (n=8) were 0.41 mg/L [0.27–0.64], and indirect bilirubin (n=4) was 1.02 mg/dL [0.16–1.87].
No statistically significant differences were observed between aortic, mitral, and double valve groups for INR (p=0.44), hemoglobin (p=0.84), D-dimers (p=0.23), or indirect bilirubin (p=0.47). Comparisons between VKA-only (n=49) and combined therapy (n=4) patients did not demonstrate significant laboratory differences; however, the extremely small antiplatelet subgroup severely limits statistical power and precludes meaningful comparative conclusions.
Dedicated intravascular hemolysis markers, including lactate dehydrogenase (LDH) and haptoglobin, were not systematically available.

**Copyright Information:** (Online) ISSN: 2572-9241
©2026 The Author(s). HemaSphere is published by John Wiley & Sons Ltd on behalf of European Hematology Association. This is an open access Abstract Book distributed under the Attribution-NonCommercial-NoDerivs (CC BY-NC-ND), which allows third parties to download the articles and share them with others as long as they credit the author and the Abstract Book, but they cannot change the content in any way or use them commercially.
**Abstract Book Citations:** Authors, Title, HemaSphere, 2026;10:(S1):pages. The abstract book DOIs can be found on the "Author Information" page.
**Disclaimer:** Articles published in the journal HemaSphere exclusively reflect the opinions of the authors. The authors are responsible for all content in their abstracts including accuracy of the facts, statements, citing resources, etc.

**Summary/Conclusion**

Patients with mechanical heart valves receiving chronic VKA therapy demonstrate stable therapeutic anticoagulation with hemoglobin values in the lower normal range and variable fibrinolysis markers. In this cohort, valve position was not associated with significant laboratory variation. Interpretation of potential low-grade hemolysis is limited by incomplete availability of dedicated hemolysis markers, particularly LDH and haptoglobin. Larger prospective studies incorporating standardized hemolysis panels and longitudinal assessment are warranted to better define the hematologic signature of mechanical valve carriers.

**Copyright Information:** (Online) ISSN: 2572-9241
©2026 The Author(s). HemaSphere is published by John Wiley & Sons Ltd on behalf of European Hematology Association. This is an open access Abstract Book distributed under the Attribution-NonCommercial-NoDerivs (CC BY-NC-ND), which allows third parties to download the articles and share them with others as long as they credit the author and the Abstract Book, but they cannot change the content in any way or use them commercially.
**Abstract Book Citations:** Authors, Title, HemaSphere, 2026;10:(S1):pages. The abstract book DOIs can be found on the “Author Information” page.
**Disclaimer:** Articles published in the journal HemaSphere exclusively reflect the opinions of the authors. The authors are responsible for all content in their abstracts including accuracy of the facts, statements, citing resources, etc.

### (PB4382) "IMPACT OF LOW-DOSE ASPIRIN ON VENOUS THROMBOEMBOLISM RECURRENCE AFTER ANTICOAGULATION IN CANCER PATIENTS: A RETROSPECTIVE COHORT ANALYSIS"

**Topic:** 35. Thrombosis and vascular biology

Shreya Singh Beniwal*[1], Raghabendra Kumar Mahato[2], Amerendra Kumar Mahato[3], Shankar Biswas[4], Manju Ramakrishnan[5], Kishankumar Patel[6]

*[1]Lady Hardinge Medical College, New Delhi, India;[2]Gandaki Medical College Teaching Hospital and Research Center, Pokhara, Nepal; [3]Nobel Medical College Teaching Hospital, Biratnagar, Nepal;[4]Ivano-Frankivsk National Medical University, Ivano-Frankivsk, Ukraine; [5]Emory University, Rollins School of Public Health Institution, Atlanta, United States of America;[6]David Tvildiani Medical University, Tbilisi, India;*

### Background

Venous thromboembolism (VTE) frequently occurs in cancer patients and represents a major cause of morbidity and mortality in this population.Despite growing recognition of the significane of aspirin research into its role in preventing recurrence among cancer patients post anticoagulation remains relatively underexplored.

### Aims our study aims to evaluate the efficacy and safety of low-dose aspirin in reducing VTE recurrence following completion of therapeutic anticoagulation in cancer patients.

### Methods

We performed a systematic literature search using PubMed, Scopus to identify existing data on low dose Aspirin on VTE.Based on identified gaps we conducted a retrospective analysis of 312 cancer patients with prior VTE who completed at least six months of therapeutic anticoagulation and achieved stable disease or remission. Patients were categorized into two cohorts: those receiving low-dose aspirin (81 mg daily) for 12 months post-anticoagulation (n=156) and those receiving no antithrombotic therapy (n=156). The primary endpoint was recurrent VTE within one year, while secondary endpoints included major bleeding (per ISTH criteria) and all-cause mortality. Kaplan-Meier estimates and Cox regression models evaluated outcomes, with statistical significance defined as $p<0.05$.

### Results

A total of 312 patients were analyzed. Recurrent VTE occurred in 8.3% of patients receiving low-dose aspirin compared to 14.7% in the control group (HR 0.52; 95% CI: 0.28–0.96; p = 0.04), indicating a statistically significant reduction in recurrence with aspirin. Rates of major bleeding were low and similar between groups (2.5% vs. 1.9%; p = 0.62). All-cause mortality showed no significant difference (p = 0.48). Importantly, no aspirin-related treatment discontinuations were reported, reinforcing the safety profile of this approach.

**Copyright Information:** (Online) ISSN: 2572-9241
©2026 The Author(s). HemaSphere is published by John Wiley & Sons Ltd on behalf of European Hematology Association. This is an open access Abstract Book distributed under the Attribution-NonCommercial-NoDerivs (CC BY-NC-ND), which allows third parties to download the articles and share them with others as long as they credit the author and the Abstract Book, but they cannot change the content in any way or use them commercially.
**Abstract Book Citations:** Authors, Title, HemaSphere, 2026;10:(S1):pages. The abstract book DOIs can be found on the "Author Information" page.
**Disclaimer:** Articles published in the journal HemaSphere exclusively reflect the opinions of the authors. The authors are responsible for all content in their abstracts including accuracy of the facts, statements, citing resources, etc.

**Summary/Conclusion**

In summary this research has provided evidence that low-dose aspirin significantly decreases the risk of recurrent VTE in cancer patients following completion of anticoagulation, without elevating major bleeding risk.our findings provide real-world support for aspirin as a practical and safe secondary prevention strategy.

**Copyright Information:** (Online) ISSN: 2572-9241
©2026 The Author(s). HemaSphere is published by John Wiley & Sons Ltd on behalf of European Hematology Association. This is an open access Abstract Book distributed under the Attribution-NonCommercial-NoDerivs (CC BY-NC-ND), which allows third parties to download the articles and share them with others as long as they credit the author and the Abstract Book, but they cannot change the content in any way or use them commercially.
**Abstract Book Citations:** Authors, Title, HemaSphere, 2026;10:(S1):pages. The abstract book DOIs can be found on the "Author Information" page.
**Disclaimer:** Articles published in the journal HemaSphere exclusively reflect the opinions of the authors. The authors are responsible for all content in their abstracts including accuracy of the facts, statements, citing resources, etc.

### (PB4383) REAL-WORLD MONOCENTRIC RETROSPECTIVE STUDY ON CANCER ASSOCIATED THROMBOSIS

**Topic:** 35. Thrombosis and vascular biology

Bruna Daraia*[1], Pamela Pinto[1], Anna Maria Anzelmo[1], Angela Cardascia[1], Davide Renna[1], Letizia Laera[1], Nicola Sasso[1], Vittoria Musci[1], Stella D'oronzo[1, 2], Francesco Gaudio[1, 2], Giammarco Surico[1]

*[1]Ecclesiastical Entity Regional General Hospital "F.Miulli", Oncology and Onco-Hematology Unit, Acquaviva delle Fonti, Italy; [2]LUM "Degennaro" University, Department of Medicine and Surgery, Casamassima (BA), Italy;*

### Background

Cancer-associated thrombosis (CAT) is a significant complication contributing to increased morbidity and mortality in cancer patients. The incidence of thromboembolic events varies according to several factors, including tumor type, treatment regimen, and patient demographics.

### Aims

This study aims to investigate the incidence of thrombosis in cancer patients at the "F. Miulli" General Regional Hospital, focusing on the impact of age, tumor histotype, and oncological therapies on thrombotic risk.

### Methods

This retrospective, monocentric study analyzed clinical data from 2,905 cancer patients treated between 2010 and 2025. Key variables, including age, cancer type, occurrence of thrombosis (at diagnosis or during follow-up), and oncological therapies (chemotherapy and hormonal treatment), were collected from electronic health records. Descriptive, bivariate, and multivariate analyses were performed, including logistic regression and Random Forest modeling, to identify risk factors for thrombosis.

### Results

The study found a significant association between older age and the incidence of thrombosis ($p < 0.05$). Pancreatic cancer, lung adenocarcinoma, and colon cancer were most frequently associated with thrombotic events. Chemotherapy and hormonal therapies were identified as significant contributors to thrombosis risk, with multivariate logistic regression confirming their role ($p = 0.002$). The Random Forest model highlighted pancreatic cancer as the most predictive factor for thrombosis.

### Summary/Conclusion

This study demonstrates that age, tumor histotype, and oncological therapies are key determinants of thrombosis risk in cancer patients. Personalized thrombosis prevention strategies, particularly for elderly patients and those with high-risk cancers, such as pancreatic and lung adenocarcinomas, should be considered to improve patient outcomes. Future prospective studies and trials are necessary to validate these findings and explore targeted thrombosis prevention strategies.

**Copyright Information:** (Online) ISSN: 2572-9241
©2026 The Author(s). HemaSphere is published by John Wiley & Sons Ltd on behalf of European Hematology Association. This is an open access Abstract Book distributed under the Attribution-NonCommercial-NoDerivs (CC BY-NC-ND), which allows third parties to download the articles and share them with others as long as they credit the author and the Abstract Book, but they cannot change the content in any way or use them commercially.
**Abstract Book Citations:** Authors, Title, HemaSphere, 2026;10:(S1):pages. The abstract book DOIs can be found on the "Author Information" page.
**Disclaimer:** Articles published in the journal HemaSphere exclusively reflect the opinions of the authors. The authors are responsible for all content in their abstracts including accuracy of the facts, statements, citing resources, etc.

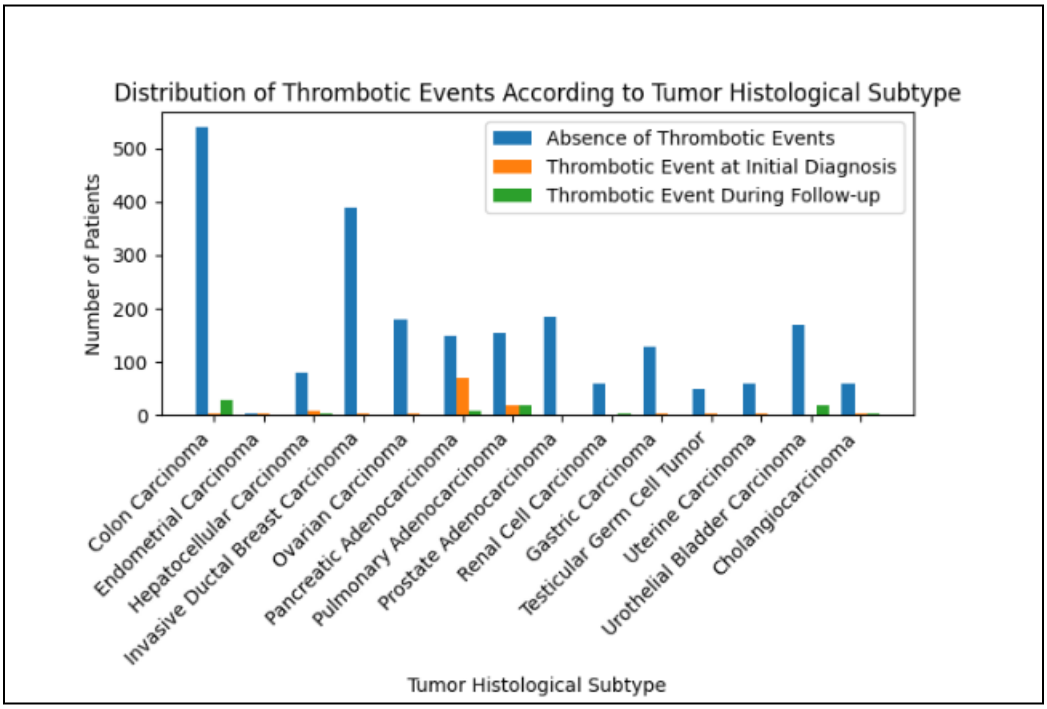

**Copyright Information:** (Online) ISSN: 2572-9241
©2026 The Author(s). HemaSphere is published by John Wiley & Sons Ltd on behalf of European Hematology Association. This is an open access Abstract Book distributed under the Attribution-NonCommercial-NoDerivs (CC BY-NC-ND), which allows third parties to download the articles and share them with others as long as they credit the author and the Abstract Book, but they cannot change the content in any way or use them commercially.
**Abstract Book Citations:** Authors, Title, HemaSphere, 2026;10:(S1):pages. The abstract book DOIs can be found on the "Author Information" page.
**Disclaimer:** Articles published in the journal HemaSphere exclusively reflect the opinions of the authors. The authors are responsible for all content in their abstracts including accuracy of the facts, statements, citing resources, etc.

### (PB4384) AN UNUSUAL PRESENTATION OF DELAYED ONSET HEPARIN INDUCED THROMBOCYTOPENIA POST FEMORAL-TIBIAL BYPASS USING HEPARIN BONDED GRAFT (PROPATEN): A SCOTTISH CENTRE EXPERIENCE FOR SHARED LEARNING

**Topic:** 35. Thrombosis and vascular biology

Wei Lynn Khor*[1, 1], Erica Tsoi[1, 1], Andrew Cross[1], Pareskevi (Vivian) Untiveros[1]

[1]*Aberdeen Royal Infirmary, Department of Haematology, Aberdeen, United Kingdom;*

### Background

Delayed Onset HIT is a subtype of autoimmune HIT and is defined by a decrease in platelet count occurring at least 5 days after heparin discontinuation, extending up to 3 weeks post-therapy. Its mechanism is attributed to heparin independent platelet activating mechanism, as heparin with its short half-life would typically be cleared from patient's system.

The PROPATEN® vascular graft is a heparin-bonded expanded polytetrafluoroethylene (ePTFE) conduit commonly used in peripheral arterial bypass when autologous vein is unavailable. Its luminal surface is covalently bonded with heparin to reduce thrombogenicity and improve patency. Heparin-bonded grafts have been associated with HIT and previous history of HIT is a contraindication.

Understanding the manifestation of delayed-onset HIT is essential in centres using heparin-bonded grafts for lower-limb revascularisation. Awareness of heparin-independent platelet activation, temporal dissociation of systemic heparin exposure and thrombocytopenia ; and the risks of heparin-bonded vascular grafts are essential in preventing delayed diagnosis and optimising management.

This background frames the context for our Scottish centre's experience with an unusual and severe presentation of delayed-onset HIT following femoral–tibial bypass using a heparin-bonded Propaten graft.

### Aims

To illustrate the real-world diagnostic and therapeutic challenges posed by delayed-onset HIT in the presence of graft-associated heparin exposure.

### Methods

Clinical, laboratory, and imaging data were reviewed retrospectively to describe a case of delayed-onset heparin-induced thrombocytopenia following vascular surgery

### Results

The patient developed abrupt, severe thrombocytopenia 13 days after initial heparin exposure, with platelet counts falling from normal to $<5 \times 10^9$/L within 48 hours. HIT ELISA testing returned strongly positive with a high optical density (OD=3.2), confirming immune-mediated HIT consistent with delayed onset. Despite cessation of systemic heparin and initiation of Argatroban, platelet recovery did not occur. Persistent thrombocytopenia was observed, with ongoing exposure from a heparin-bonded (Propaten) graft considered a potential contributing factor. The clinical course was complicated by graft thrombosis, myocardial injury, and progressive deterioration, culminating in death.

**Copyright Information:** (Online) ISSN: 2572-9241
©2026 The Author(s). HemaSphere is published by John Wiley & Sons Ltd on behalf of European Hematology Association. This is an open access Abstract Book distributed under the Attribution-NonCommercial-NoDerivs (CC BY-NC-ND), which allows third parties to download the articles and share them with others as long as they credit the author and the Abstract Book, but they cannot change the content in any way or use them commercially.
**Abstract Book Citations:** Authors, Title, HemaSphere, 2026;10:(S1):pages. The abstract book DOIs can be found on the "Author Information" page.
**Disclaimer:** Articles published in the journal HemaSphere exclusively reflect the opinions of the authors. The authors are responsible for all content in their abstracts including accuracy of the facts, statements, citing resources, etc.

**Summary/Conclusion**

This case demonstrates delayed-onset heparin-induced thrombocytopenia, confirmed by a strongly positive, high-optical-density HIT ELISA, in a complex post-operative vascular patient. Diagnosis was challenging due to multiple competing causes of thrombocytopenia and the condition progressed despite cessation of systemic heparin and initiation of argatroban. In refractory or rapidly progressive delayed-onset HIT, immunomodulatory therapies such as intravenous immunoglobulin and plasma exchange may be considered to reduce circulating pathogenic antibodies. Where a heparin-bonded vascular graft is present, ongoing antigen exposure should be considered as a potential perpetuating factor; although causality cannot be definitively established, case reports describe platelet recovery following graft explantation or re-lining with non-heparin-coated material.Early recognition of delayed-onset HIT and awareness of potential hidden sources of heparin exposure are essential to guide timely multidisciplinary management and improve outcomes.

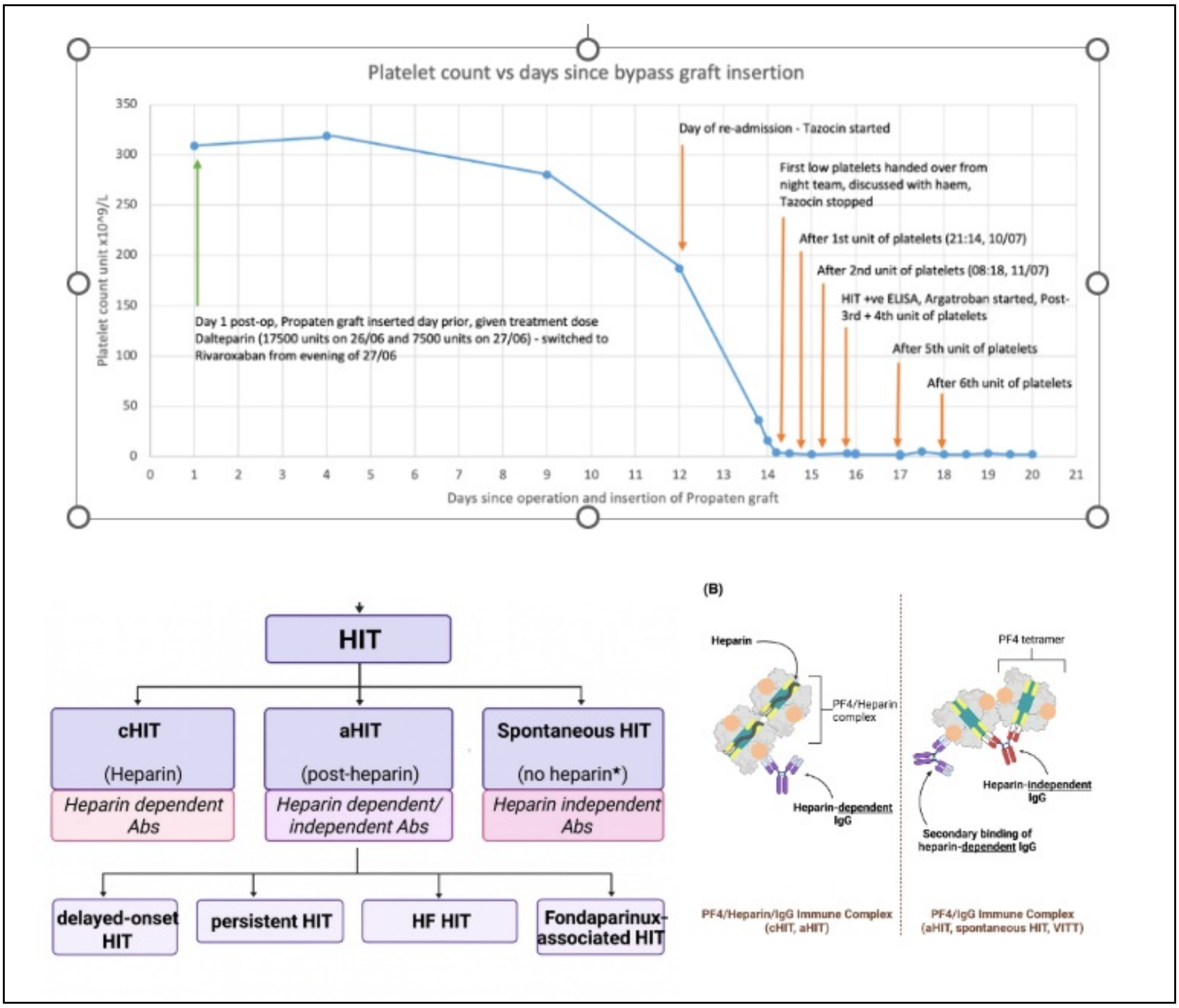

Graph: Platelet Count trend in relation to Propaten graft insertion Flow Chart: Delayed Onset HIT as a subcategory of Autoimmune HIT PIC: Heparin independent IgG ab

**Copyright Information:** (Online) ISSN: 2572-9241
©2026 The Author(s). HemaSphere is published by John Wiley & Sons Ltd on behalf of European Hematology Association. This is an open access Abstract Book distributed under the Attribution-NonCommercial-NoDerivs (CC BY-NC-ND), which allows third parties to download the articles and share them with others as long as they credit the author and the Abstract Book, but they cannot change the content in any way or use them commercially.
**Abstract Book Citations:** Authors, Title, HemaSphere, 2026;10:(S1):pages. The abstract book DOIs can be found on the "Author Information" page.
**Disclaimer:** Articles published in the journal HemaSphere exclusively reflect the opinions of the authors. The authors are responsible for all content in their abstracts including accuracy of the facts, statements, citing resources, etc.

### (PB4385) THROMBOTIC EVENTS DURING THE EARLY POST- AUTOLOGOUS STEM CELL TRANSPLANTATION (ASCT) PERIOD IN PATIENTS WITH MULTIPLE MYELOMA: THE EXPERIENCE OF A TERTIARY CENTER IN GREECE

**Topic:** 35. Thrombosis and vascular biology

Konstantina Toutoudaki*[1], Konstantinos Karamouzis[1], Eleni Korompoki[1], Evangelos Eleutherakis-Papaiakovou[1], Efstathios Kastritis[1], Evangelos Terpos[1], Maria Gavriatopoulou[1], Meletios A. Dimopoulos[1]

*[1]Medical School, National and Kapodistrian University of Athens, Department of Therapeutics, Plasma cell dyscrasia Unit, General Hospital Alexandra, Athens, Greece;*

### Background

Thrombosis during the peri-transplant period is a well described complication of autologous stem cell transplantation (ASCT) in patients with multiple myeloma (MM). However, diagnosis and management of thrombosis during the early post-transplant period is challenging, given the coexistence of thrombotic and haemorrhagic risks. Specific risk factors for thrombosis as well as type and duration of anticoagulation treatment remain elusive.

### Aims

Herein, the purpose of our study was to assess potential risk factors for thrombotic events during the early post-ASCT period in MM patients and provide further insight into anticoagulation management.

### Methods

We conducted a retrospective analysis on patients with MM undergoing ASCT since 2020, comparing clinical and laboratory parameters between transplanted MM patients diagnosed with at least one thrombotic event and transplanted age and MM subtype matched subjects without any thrombotic event. Data were obtained from patient's charts. All patients underwent ASCT as part of first line treatment.

**Copyright Information:** (Online) ISSN: 2572-9241
©2026 The Author(s). HemaSphere is published by John Wiley & Sons Ltd on behalf of European Hematology Association. This is an open access Abstract Book distributed under the Attribution-NonCommercial-NoDerivs (CC BY-NC-ND), which allows third parties to download the articles and share them with others as long as they credit the author and the Abstract Book, but they cannot change the content in any way or use them commercially.
**Abstract Book Citations:** Authors, Title, HemaSphere, 2026;10:(S1):pages. The abstract book DOIs can be found on the "Author Information" page.
**Disclaimer:** Articles published in the journal HemaSphere exclusively reflect the opinions of the authors. The authors are responsible for all content in their abstracts including accuracy of the facts, statements, citing resources, etc.

**Results**

We collected data by 20 patients, 11 of which presented with thrombosis during the early post-ASCT period, and we matched them with 9 transplanted patients without thrombotic complications during ASCT. Sex, clonal subtype, age, BMI and induction regimen were similar between the two subgroups. Site of thrombosis was mainly CVC-related, located either in the jugular vein (n=10), or the femoral vein (n=1), while a non CVC-related case of deep venous thrombosis (DVT) in the popliteal vein was reported (n=1). Three cases of CVC-related jugular vein thrombosis were complicated by sinus venous thrombosis. Thrombosis occurred at a mean of 16.9 days after transplantation. Median platelet count at the time of thrombosis detection by ultrasound or computed tomography was 94,600/mcl (Range: 30-238,000/mcl). The most common thrombosis related symptoms were low-grade fever (n= 7), site erythema/edema (n=6), and pain (n=5).

Most patients in both groups were treated with bortezomib, lenalidomide and dexamethasone, with or without daratumumab as induction therapy, while high-dose melphalan was the myeloablative regimen in all ASCT. Patients who experienced thrombosis had numerically increased baseline d-dimers at the time of the admission for ASCT, compared to those without thrombotic complications (median: 0.58 vs 1.01 mg/L, mean: 1.9 vs 0.9 p: 0.02). CRP during the peri-thrombotic period was significantly higher in patients with thrombosis (median: 52 vs 17.5, p: 0.03). Bacteremia, especially by staphylococcal species was associated with higher thrombotic risk (p: 0.04). Thrombosis was associated with longer hospitalisation (p:0.01).

Patients were treated while hospitalized with low molecular weight heparin switching to direct oral anticoagulants (DOACs) in 10 subjects after discharge, and fondaparinux for 1 patient. Treatment duration ranged from 3 months to life-long. Intracranial hemorrhage occurred in 1 case of CVC-related thrombosis, while on aticoagulation. No related deaths were reported.

**Summary/Conclusion**

Early post-ASCT period is considered as highly thrombogenic in patients with MM. Baseline increased d-dimers, high serum CRP levels and staphylococcal infections should raise suspicion for thrombotic events in transplanted patients with relevant symptoms. Prospective data on anticoagulation management in this vulnerable population are required.

**Copyright Information:** (Online) ISSN: 2572-9241
©2026 The Author(s). HemaSphere is published by John Wiley & Sons Ltd on behalf of European Hematology Association. This is an open access Abstract Book distributed under the Attribution-NonCommercial-NoDerivs (CC BY-NC-ND), which allows third parties to download the articles and share them with others as long as they credit the author and the Abstract Book, but they cannot change the content in any way or use them commercially.
**Abstract Book Citations:** Authors, Title, HemaSphere, 2026;10:(S1):pages. The abstract book DOIs can be found on the "Author Information" page.
**Disclaimer:** Articles published in the journal HemaSphere exclusively reflect the opinions of the authors. The authors are responsible for all content in their abstracts including accuracy of the facts, statements, citing resources, etc.

### (PB4386) COMPARATIVE STUDY OF THE PREVALENCE OF MTHFR GENETIC POLYMORPHISM IN PATIENTS WITH MPNS AND HAEMOPHILIA

**Topic:** 35. Thrombosis and vascular biology

Nataliia Shurko*[1], Myroslav Voroniak[1], Zvenyslava Maslyak[1], Sofiia Hudzij[1], Maryana Kokoruz[1], Svitlana Milyashkevych[1], Valeriia Krasivska[1], Oleksandra Stasyshyn[1]

*[1]SI "Institute of Blood Pathology and Transfusion Medicine NAMS of Ukraine", Lviv, Ukraine;*

### Background

The human organism is a multifunctional, interconnected system with compensatory mechanisms designed to eliminate physiological disorders. This is well illustrated by genetic mutations, some of which predispose to clotting, while others predispose to bleeding. Among the main factors contributing to blood clot formation in myeloproliferative neoplasms (MPNs) are hyperactive blood cells, endothelial dysfunction, inflammation, and genetic mutations (especially the JAK2 mutation). In Hemophilia, mutations in the FVIII or FIX genes are responsible for bleeding.
Mutations in the MTHFR gene are common variations that affect folate metabolism, potentially increasing the risk of thrombosis in MPNs or compensating for low FVIII/FIX levels, resulting in more efficient thrombin generation and attenuation of clinical symptoms.

### Aims to investigate and compare the prevalence of MTHFR mutations in patients with MPNs and Hemophilia, as they may affect the coagulation process.

### Methods

DNA extraction and isolation were performed using the “DNA purification from blood and body fluids” (GeneAll ExgeneTM kit, Turkey). Mutations were analyzed using allele-specific polymerase chain reaction (PCR) (Genmark GeneMAP™ kits, Turkey). Amplification was performed on a CFX 96 Real-Time System (Bio-Rad, USA).

**Copyright Information:** (Online) ISSN: 2572-9241
©2026 The Author(s). HemaSphere is published by John Wiley & Sons Ltd on behalf of European Hematology Association. This is an open access Abstract Book distributed under the Attribution-NonCommercial-NoDerivs (CC BY-NC-ND), which allows third parties to download the articles and share them with others as long as they credit the author and the Abstract Book, but they cannot change the content in any way or use them commercially.
**Abstract Book Citations:** Authors, Title, HemaSphere, 2026;10:(S1):pages. The abstract book DOIs can be found on the “Author Information” page.
**Disclaimer:** Articles published in the journal HemaSphere exclusively reflect the opinions of the authors. The authors are responsible for all content in their abstracts including accuracy of the facts, statements, citing resources, etc.

### Results

Under our supervision, there were 114 patients: MPNs (56) and Hemophilia A/B (58). Mutations in JAK2 V617F, CALR, MPL, and MTHFR were analyzed. The results of the studies are presented in Table 1.

**Table 1.** Frequency of mutation detection in patients with different nosologies

| № | Study group | MTFR C/T | | | MTFR A/C | | |
|---|---|---|---|---|---|---|---|
| | | Homozygotes, % | Heterozygotes, % | Norma, % | Homozygotes, % | Heterozygotes, % | Norma, % |
| 1. | MPNs JAK2 V617F + | 10.00 | 40.00 | 50.00 | 10.00 | 40.00 | 50.00 |
| 2. | MPNs Calr+ | 0 | 14.29 | 85.71 | 14.29 | 71.43 | 14.28 |
| 3. | MPNs MPL+ | 0 | 25.00 | 75.00 | 25.00 | 25.00 | 50.00 |
| 4. | MPNs Tripel negative | 0 | 42.86 | 57.14 | 14.29 | 57.14 | 28.57 |
| 5. | MPNs (totale) | 3.57 | 32.14 | 64.29 | 14.29 | 50.00 | 35.71 |
| 6. | Hemophilia | 10.34 | 65.52 | 24.14 | 10.34 | 55.17 | 34.48 |

Our studies demonstrate that in patients with a tendency to bleed (Hemophilia), the frequency of detection of the MTHFR C/T mutation is significantly higher (Homozygotes 2.9 times, Heterozygotes 2.03 times) than in patients with MPNs, in whom thrombohemorrhagic clinical symptoms are the main complication and a leading cause of death. A study of the frequency of detection of the MTHFR A/C mutation did not reveal such significant differences (0.72 and 1.1 times, respectively).

### Summary/Conclusion

Mutations in the MTHFR gene are common variations that affect folate metabolism, potentially increasing the risk of thrombosis. Our studies have confirmed the assumption that compensatory mechanisms are involved in the human organism: people with blood clotting disorders have a higher frequency of these mutations, and as a result, clinical symptoms are further reduced.

**Copyright Information:** (Online) ISSN: 2572-9241
©2026 The Author(s). HemaSphere is published by John Wiley & Sons Ltd on behalf of European Hematology Association. This is an open access Abstract Book distributed under the Attribution-NonCommercial-NoDerivs (CC BY-NC-ND), which allows third parties to download the articles and share them with others as long as they credit the author and the Abstract Book, but they cannot change the content in any way or use them commercially.
**Abstract Book Citations:** Authors, Title, HemaSphere, 2026;10:(S1):pages. The abstract book DOIs can be found on the “Author Information” page.
**Disclaimer:** Articles published in the journal HemaSphere exclusively reflect the opinions of the authors. The authors are responsible for all content in their abstracts including accuracy of the facts, statements, citing resources, etc.

### (PB4387) TIME IN THERAPEUTIC RANGE AFTER A CLINICAL EVENT: BASELINE STATUS AND LONGITUDINAL CHANGES

**Topic:** 35. Thrombosis and vascular biology

Mónica Fernández Pérez*[1], Carlos Pison[1], Angel Pereda[1]

[1]*Hospital Universitario Araba, Vitoria, Spain;*

### Background

Vitamin K antagonists are the anticoagulation therapy available for the primary and secondary prevention of venous and arterial thrombosis. The strict control of anticoagulant therapy through the INR reduces the risk of thrombosis or bleeding. INR control is suboptimal when time in therapeutic range (TTR) is < 60%.

### Aims

The main objective is to observe the differences in the TTR, after the presence of a thrombotic or hemorrhagic event

### Methods

First, a database of 291 patients was created. The patients started anticoagulation from November 2024 to April 2025 and were followed up for one year. Secondly, the patients data were collected: age, gender, reason for anticoagulation, presence of hemorrhagic or thrombotic event and TTR. Third, the TTR of anticoagulated patients was observed according to gender, age, reason for anticoagulation and event.

### Results

In this study, the 57.7% were men and 42.3% women. The mean age was 74.97 years. The main reasons for anticoagulation were Atrial Fibrillation (AF) in 61.51%, Deep Vein Thrombosis (DVT) in 14.43%, Pulmonary Thromboembolism (PTE) in 11% and 13.06% for other reasons. The 92,17% of the patients who started anticoagulation due to AF, were older than 60 years. AF and PTE predominated in men, however, DVT in women.
The 52.2% had a suboptimal TTR. The 48.6% of patients with AF and 40.6% with PTE had a TTR < 60, in opposite to patients with DVT, which was 66.66%. Patients < 60 years and women had worse TTR.
During the year of follow-up, 12.71% of the patients presented a thrombotic event and 24.39% a hemorrhagic event. After a thrombotic event, 91.66% presented a TTR >60 and after a hemorrhagic event, 81.70%.

### Summary/Conclusion

The factors associated with a low TTR are: female sex, DVT and age less than 60 years. After a hemorrhagic or thrombotic event, anticoagulated patients improve the TTR.

**Copyright Information:** (Online) ISSN: 2572-9241
©2026 The Author(s). HemaSphere is published by John Wiley & Sons Ltd on behalf of European Hematology Association. This is an open access Abstract Book distributed under the Attribution-NonCommercial-NoDerivs (CC BY-NC-ND), which allows third parties to download the articles and share them with others as long as they credit the author and the Abstract Book, but they cannot change the content in any way or use them commercially.
**Abstract Book Citations:** Authors, Title, HemaSphere, 2026;10:(S1):pages. The abstract book DOIs can be found on the "Author Information" page.
**Disclaimer:** Articles published in the journal HemaSphere exclusively reflect the opinions of the authors. The authors are responsible for all content in their abstracts including accuracy of the facts, statements, citing resources, etc.

| THROMBOTIC EVENT | % EVENTS |
|---|---|
| Deep Vain Thrombosis (DVT) | 6,18% (18) |
| Stroke | 3,43% (10) |
| Transient ischemic attack | 1,37% (4) |
| Pulmonary thromboembolism (PTE) | 0,68% (2) |
| Cerebral Thrombosis | 0,34% (1) |
| Splenic infarction | 0,34% (1) |
| Acute myocardial infarction | 0,34% (1) |
| NO | 87,29% (254) |
| TOTAL | 100% (291) |
| HEMORRHAGIC EVENT | % EVENTS |
| Hematuria | 5,49% (16) |
| Bruises | 4,12% (12) |
| Lower gastrointestinal bleeding | 3,78% (11) |
| Subarachnoid hemorrhage | 2,06% (6) |
| Rectorrhagia | 1,71% (5) |
| Subconjunctival hemorrhage | 1,03% (3) |
| Hemoptysis | 1,03% (3) |
| Gingivorrhagia | 0,68% (2) |
| Epistaxis | 0,68% (2) |
| Upper gastrointestinal bleeding | 0,68% (2) |
| Otorrhagia | 0,34% (1) |
| Oropharyngeal bleeding | 0,34% (1) |
| Hemorrhoids | 0,34% (1) |
| Acute anemia | 0,34% (1) |
| Metrorrhagia | 0,34% (1) |
| Lower gastrointestinal bleeding + Bruises | 0,34% (1) |
| Chronic anemia | 0,34% (1) |
| Hemarthrosis | 0,34% (1) |
| Vaginal Bleeding | 0,34% (1) |
| NO | 75,61% (220) |
| TOTAL | 100% (291) |

TABLE 1: Number and type of thromboembolic and hemorrhagic events with VKA that patients had during the year of follow-up

**Copyright Information:** (Online) ISSN: 2572-9241
©2026 The Author(s). HemaSphere is published by John Wiley & Sons Ltd on behalf of European Hematology Association. This is an open access Abstract Book distributed under the Attribution-NonCommercial-NoDerivs (CC BY-NC-ND), which allows third parties to download the articles and share them with others as long as they credit the author and the Abstract Book, but they cannot change the content in any way or use them commercially.
**Abstract Book Citations:** Authors, Title, HemaSphere, 2026;10:(S1):pages. The abstract book DOIs can be found on the "Author Information" page.
**Disclaimer:** Articles published in the journal HemaSphere exclusively reflect the opinions of the authors. The authors are responsible for all content in their abstracts including accuracy of the facts, statements, citing resources, etc.

### (PB4388) CLINICAL AND SEROLOGICAL CHARACTERIZATION OF ANTIPHOSPHOLIPID SYNDROME IN MEN: A SINGLE-CENTER STUDY

**Topic:** 35. Thrombosis and vascular biology

Lucy González Villarroel*[1]

[1]*Regional Hospital 1° de Octubre ISSSTE, Hematology/Internal Medicine, Mexico City, Mexico;*

### Background

Antiphospholipid Syndrome (APS) is predominantly diagnosed in women; however, the male phenotype often presents a more complex diagnostic challenge. While typically associated with a prothrombotic state, APS in men can also manifest with severe hematological cytopenias and a dense overlap with other systemic autoimmune diseases. The presence of positive Antinuclear Antibodies (ANA), anti-dsDNA, and ANCA suggests a broad loss of self-tolerance, potentially indicating a "lupus-like" syndrome or evolution toward systemic lupus erythematosus (SLE) that requires strategies beyond standard anticoagulation.

### Aims

To analyze the clinical manifestations and serological profiles of male patients with APS, emphasizing the diversity between thrombotic and hematological presentations and the role of adjunctive immunosuppression.

### Methods

We conducted a retrospective, single-center observational study of all newly diagnosed APS cases over an 8-month period (June 2025 to January 2026). Out of 25 new diagnoses, 19 (76%) were women and 6 (24%) were men. We evaluated the 6 male patients (median age 54 years) regarding their clinical history (thrombosis vs. cytopenias), smoking status, and extended serological markers (LA, aCL, aβ2GPI, ANA, anti-DNA, ANCA, Complement).

### Results

- **Clinical Profile:** 66.7% (4/6) of male patients presented with severe thrombotic events (PE, DVT, or Stroke). Notably, 33.3% (2/6) presented with non-thrombotic phenotypes: one patient with refractory pancytopenia and another 52-year-old patient with isolated thrombocytopenia without prior thrombosis. Smoking remained a prevalent comorbidity in the thrombotic subgroup.
- **Serology:** High-risk profiles were dominant. 100% of patients were positive for Lupus Anticoagulant. We observed extreme titers of aCL IgG (up to 199.9 U/mL) and aβ2GPI IgG. Immunological overlap was significant: 80% (4/6 tested) were ANA positive (titers 1:80–1:160). The hematological case with pancytopenia exhibited a more aggressive profile with positive anti-DNA (51.3 IU/mL) and C-ANCA (34.8 U/mL), while the sixth patient did not undergo SLE or vasculitis profiling.
- **Management:** 83.3% (5/6) of patients are managed with Vitamin K Antagonists (acenocoumarol). The 52-year-old patient with isolated thrombocytopenia did not receive anticoagulation.
- **Immunosuppression:** 83.3% of the cohort (5/6) received immunosuppression: 4 patients with azathioprine to control systemic autoimmunity and "aPL-vasculopathy," and the patient with isolated thrombocytopenia managed exclusively with prednisone.

**Copyright Information:** (Online) ISSN: 2572-9241
©2026 The Author(s). HemaSphere is published by John Wiley & Sons Ltd on behalf of European Hematology Association. This is an open access Abstract Book distributed under the Attribution-NonCommercial-NoDerivs (CC BY-NC-ND), which allows third parties to download the articles and share them with others as long as they credit the author and the Abstract Book, but they cannot change the content in any way or use them commercially.
**Abstract Book Citations:** Authors, Title, HemaSphere, 2026;10:(S1):pages. The abstract book DOIs can be found on the "Author Information" page.
**Disclaimer:** Articles published in the journal HemaSphere exclusively reflect the opinions of the authors. The authors are responsible for all content in their abstracts including accuracy of the facts, statements, citing resources, etc.

**Summary/Conclusion**

Male APS patients represent a high-risk subgroup with significant serological complexity. Our study highlights that while thrombosis is common, a significant subset of male patients presents with cytopenias (pancytopenia or isolated thrombocytopenia) without thrombotic events. Early integration of immunosuppression (azathioprine or prednisone) alongside or instead of anticoagulation—depending on the phenotype—is crucial to manage hematological manifestations and improve long-term outcomes in this diverse male cohort.

**Copyright Information:** (Online) ISSN: 2572-9241
©2026 The Author(s). HemaSphere is published by John Wiley & Sons Ltd on behalf of European Hematology Association. This is an open access Abstract Book distributed under the Attribution-NonCommercial-NoDerivs (CC BY-NC-ND), which allows third parties to download the articles and share them with others as long as they credit the author and the Abstract Book, but they cannot change the content in any way or use them commercially.
**Abstract Book Citations:** Authors, Title, HemaSphere, 2026;10:(S1):pages. The abstract book DOIs can be found on the "Author Information" page.
**Disclaimer:** Articles published in the journal HemaSphere exclusively reflect the opinions of the authors. The authors are responsible for all content in their abstracts including accuracy of the facts, statements, citing resources, etc.

### (PB4389) REAL WORLD DATA REGARDING CLINICAL AND LABORATORY CHARACTERISTICS OF 34 PATIENTS DIAGNOSED AND TREATED WITH VENOUS THROMBOSIS: IS THERE A RELEVANT SCORE?

**Topic:** 35. Thrombosis and vascular biology

Vasiliki Gougoula*[1], Michail Diamantidis[2], Michail Makris[1], Eleftheria Ztriva[1], Michail Giannakakis[1], Vasileios Patriarcheas[1], Marina Psoma[1], Stavroula Bountola[1], Vasileios Perifanis[1], Christos Savopoulos[1], Georgia Kaiafa[1]

*[1]Aristotle University, First Propaedeutic Department of Internal Medicine, Thessaloniki, Greece;[2]Thalassemia and Sickle Cell Disease Unit, Department of Hematology, Larissa, Greece;*

### Background

It is well established that non-O blood types (A, B, AB) are genetic risk factors for thrombosis with a 2- to 3-fold higher risk of venous thromboembolism (VTE), compared to type O. However, real-world data from Greek patients with VTE regarding clinical manifestations and laboratory characteristics, including the incidence of blood types, are limited.

### Aims

To describe primarily the clinical and laboratory characteristics of a cohort of Greek patients who were diagnosed and treated for VTE. Secondarily, to identify a possible score for predisposing factors leading to venous thrombosis, possibly also defining the severity and the anatomic determination of the thrombotic event.

### Methods

This retrospective study included 34 patients diagnosed and treated with VTE and one with left ventricular heart arterial thrombosis from 01/02/2025 to 01/02/2026. Statistical Analysis was performed with SPSS 21.0. Chi-square test, Pearson correlation coefficient, Mann Whitney U test plus the Shapiro-Wilk normality test were used.
Moreover, we incorporated factors like thrombophilia, factor VIII, von Willebrand factor (vWF) antigen, arterial hypertension, coronary heart disease, diabetes, dyslipidemia, atrial fibrillation, aortic valve disease, smoking, contraceptives, along with the history of neoplasms and vascular stroke to form a SCORE for VTE.

**Copyright Information:** (Online) ISSN: 2572-9241
©2026 The Author(s). HemaSphere is published by John Wiley & Sons Ltd on behalf of European Hematology Association. This is an open access Abstract Book distributed under the Attribution-NonCommercial-NoDerivs (CC BY-NC-ND), which allows third parties to download the articles and share them with others as long as they credit the author and the Abstract Book, but they cannot change the content in any way or use them commercially.
**Abstract Book Citations:** Authors, Title, HemaSphere, 2026;10:(S1):pages. The abstract book DOIs can be found on the "Author Information" page.
**Disclaimer:** Articles published in the journal HemaSphere exclusively reflect the opinions of the authors. The authors are responsible for all content in their abstracts including accuracy of the facts, statements, citing resources, etc.

**Results**

Mean age was 63.8 years (range 26-86) with 14 female and 21 male patients. One of them died, 33 patients are alive until today and data for 1 patient were unavailable at the recent follow-up. The blood types were as follows: O: 6 (17.1%), A: 20 (57.1%), B: 8 (22.9%), AB: 1 (2.9%), in contrast to the higher prevalence of the O blood group to the Greek population (44%). Interestingly, all 5 male patients with neoplasms (Hodgkin lymphoma, thyroid cancer, pancreatic cancer, IPMN, keratoacanthoma) and VTE had A blood type, while the only female (NHL) was of the O blood type.
Regarding the anatomical site of VTE, 19 patients were diagnosed with pulmonary embolism (PE), 8 had concomitant VTE in 2 different body areas, 3 had renal vein thrombosis (all males of A blood group), 2 suffered from portal vein thrombosis, whereas the remaining 2 had respectively greater saphenous vein and deep vein thrombosis.
All 8 patients with thrombosis in the abdomen (renal vein: 3, portal vein: 2, mesenteric vein: 1, inferior vena cava: 1, anterior and posterior tibial veins reaching the abdominal aorta: 1) were male with no females ($p=0.012<0.05$).
In the patients with PE, the Pulmonary Embolism Severity Index (PESI) Score increases with age ($p=0.026<0.05$), while it decreases when the number of platelets increases ($p=0.015<0.05$).
Finally, we proposed a SCORE for VTE, described in the Methods section, which differs between patients with VTE of the abdomen (Mean Score: 3.375 with Std. Deviation 1.4, Median Score: 3 with Range 1-6) compared with the non-abdominal VTE, in whom it is lower (Mean Score: 1.815 with Std. Deviation 1.545, Median Score: 1 with Range 0-5), ($p=0.02<0.05$, Mann Whitney).

**Summary/Conclusion**

We described the clinical and laboratory characteristics in a Greek real-world cohort with VTE. Moreover, we identified and propose for the first time a clinically meaningful SCORE for VTE, significantly higher for VTE of the abdomen, incorporating a variety of factors, aiming to highlight predisposing factors leading to VTE, possibly also predicting the severity and the anatomic site of the thrombotic event.

**Copyright Information:** (Online) ISSN: 2572-9241
©2026 The Author(s). HemaSphere is published by John Wiley & Sons Ltd on behalf of European Hematology Association. This is an open access Abstract Book distributed under the Attribution-NonCommercial-NoDerivs (CC BY-NC-ND), which allows third parties to download the articles and share them with others as long as they credit the author and the Abstract Book, but they cannot change the content in any way or use them commercially.
**Abstract Book Citations:** Authors, Title, HemaSphere, 2026;10:(S1):pages. The abstract book DOIs can be found on the "Author Information" page.
**Disclaimer:** Articles published in the journal HemaSphere exclusively reflect the opinions of the authors. The authors are responsible for all content in their abstracts including accuracy of the facts, statements, citing resources, etc.

### (PB4390) STABILITY OF THROMBOPHILIA SCREENING PARAMETERS: IMPACT OF TIME AND TEMPERATURE ON DIAGNOSTIC RELIABILITY

**Topic:** 35. Thrombosis and vascular biology

Selma Ben Cheick[1], Mohamed Yassine Kaabar[2], Malek Haouari[1], Ikbel Ghachem*[3], Hamida Khémiri Guerbouj[4], Roua Mankai[4], Zeineb Alaya[4], Wafa Skouri[4], Asma Bachali[3]

*[1]Faculty of pharmacy of Monastir, Nabeul, Tunisia;[2]University of Monastir, Faculty of Pharmacy of Monastir, Laboratory of Biology, Tahar Maamouri Hospital,, Nabeul, Tunisia; [3]University of Tunis El Manar, Faculty of Medicine of Tunis, Laboratory of Biology, Tahar Maamouri Hospital, Nabeul, Tunisia; [4]University of Tunis El Manar, Faculty of medicine of Tunis, Internal medicine Department, Taher Maamouri Hospital, Nabeul, Tunisia;*

### Background

Thrombophilia screenings are often requested in clinical settings (emergency or outpatient) and can be sent to the laboratory at any time. This creates logistical challenges involving storage at Room Temperature (RT) or +4°C overnight or during weekends.

### Aims

We evaluated the stability of Protein C (PC), Protein S (PS), Antithrombin (AT), and Lupus Anticoagulant (LA) assays (DRVVT, SCT) under these constraints.

### Methods

Between February 15th and 25th, 2026, blood samples from 10 randomly selected controls were analyzed at Taher Maamouri Hospital (Nabeul) using the ACL TOP (Instrumentation Laboratory). Stability was assessed from baseline ($H_0$) at 24h (n=6) and 48h (n=4) at both RT and +4°C. Variations were compared against a 15% clinical acceptability limit.

### Results

- PC & AT: Showed excellent stability at 24h (medians <10%), but PC degraded significantly at 48h at +4°C (median variation: 25%).
  • Protein S: While the median variation at 24h/+4°C was low (2%), the interquartile range (IQR) widened significantly (1.5%–20.4%) compared to RT (6.1%–14.0%), indicating unpredictable individual instability at +4°C. At 48h, PS was unstable under all conditions (medians >20%).
  • Lupus Anticoagulant: SCT was highly unstable at all time points (medians: 42% to 92%). DRVVT showed marked degradation at 24h at +4°C (22%) and at 48h at RT (24%).

### Summary/Conclusion

RT storage is preferable for up to 24h for PC, PS, and AT. Cold storage at +4°C triggers early, erratic instability in PS and LA assays. Specifically, SCT is unsuitable for delayed testing, emphasizing the need for immediate processing or specialized freezing protocols.

**Copyright Information:** (Online) ISSN: 2572-9241
©2026 The Author(s). HemaSphere is published by John Wiley & Sons Ltd on behalf of European Hematology Association. This is an open access Abstract Book distributed under the Attribution-NonCommercial-NoDerivs (CC BY-NC-ND), which allows third parties to download the articles and share them with others as long as they credit the author and the Abstract Book, but they cannot change the content in any way or use them commercially.
**Abstract Book Citations:** Authors, Title, HemaSphere, 2026;10:(S1):pages. The abstract book DOIs can be found on the “Author Information” page.
**Disclaimer:** Articles published in the journal HemaSphere exclusively reflect the opinions of the authors. The authors are responsible for all content in their abstracts including accuracy of the facts, statements, citing resources, etc.

### (PB4391) DETERMINATION OF THE OPTIMAL CUT-OFF VALUE OF APCR RATIO TO PREDICT THE PRESENCE OF FACTOR V LEIDEN MUTATION

**Topic:** 35. Thrombosis and vascular biology

Nouha Jaballah*[1], Salma Riahi[1], Eya Morjane[1], Yosra Dhaha[1], Donia Mbarki[1], Amina Bouatay[1]

[1]*Sahloul Hospital, Laboratory of hematology and Blood Bank, Sousse, Tunisia;*

### Background

The factor V Leiden mutation is the main hereditary risk factor for thrombophilia. Testing is indicated following an unexplained thromboembolic event, in the presence of a family history of thrombosis, or in specific obstetric complications. Initial screening is based on a functional test, activated protein C resistance (APCR), which assesses the capacity of activated protein C to inactivate activated factor V (Va).

### Aims

The objective of our study is to determine the optimal cut-off value for the APCR ratio to predict the presence of the factor V Leiden mutation.

### Methods

A retrospective, descriptive study was conducted, including all requests for APCR testing received at our laboratory. The Factor V Leiden (G1691A) mutation was detected using polymerase chain reaction–restriction fragment length polymorphism (PCR–RFLP) analysis, and APCR was measured via a chronometric assay on the ACL TOP analyzer. Standard preanalytical conditions were followed, particularly regarding the absence of drug interference. The presence of APCR was defined by a normalized ratio < 2.14, a threshold established according to the manufacturer's recommendations and based on the mean value obtained from 30 healthy control subjects.

### Results

A total of 62 specimens were analyzed. The sex ratio (M/F) was 0.51, and the mean age of patients was 36.6 ± 15.44 years (range: 8 months to 71 years). The most common clinical indications for APCR testing were peripheral venous thrombosis (25.8% of patients) and ischemic stroke (19.4%). APCR testing was positive in 35.5% of patients (n=22), and the factor V Leiden mutation was identified in 17.7% of patients (n=11). Of these, nine were heterozygous and two were homozygous for the mutation. Given the limited number of homozygous cases, the cut-off value was determined only for heterozygous mutations. The optimal APCR ratio cut-off for predicting the presence of the factor V Leiden mutation was 1.79 with a sensitivity of 90.9% and a specificity of 41%.

### Summary/Conclusion

Our results confirm the utility of the APCR test as an effective first-line screen to rule out the mutation. In cases of a positive result, subsequent confirmation by molecular analysis is essential.

**Copyright Information:** (Online) ISSN: 2572-9241
©2026 The Author(s). HemaSphere is published by John Wiley & Sons Ltd on behalf of European Hematology Association. This is an open access Abstract Book distributed under the Attribution-NonCommercial-NoDerivs (CC BY-NC-ND), which allows third parties to download the articles and share them with others as long as they credit the author and the Abstract Book, but they cannot change the content in any way or use them commercially.
**Abstract Book Citations:** Authors, Title, HemaSphere, 2026;10:(S1):pages. The abstract book DOIs can be found on the "Author Information" page.
**Disclaimer:** Articles published in the journal HemaSphere exclusively reflect the opinions of the authors. The authors are responsible for all content in their abstracts including accuracy of the facts, statements, citing resources, etc.

### (PB4392) RARE GENETIC VARIANTS AFFECTING FACTOR VIII AND PLATELET FUNCTION IN RECURRENT IDIOPATHIC VENOUS THROMBOEMBOLISM

**Topic:** 35. Thrombosis and vascular biology

Martina Mia Mitic*[1], Dusan Usjak[1], Sofija Dunjic Manevski[1], Dusica Basaric[2], Mirjana Kovac[2, 3], Marija Cumbo Stikic[1], Branko Tomic[1], Valentina Djordjevic[1]

*[1]Institute of molecular genetics and genetic engineering, University of Belgrade, Human Molecular Genetics & Genomics, Belgrade, Serbia; [2]Blood Transfusion Institute of Serbia, Hemostasis Department, Belgrade, Serbia;[3]Faculty of Medicine, University of Belgrade, Belgrade, Serbia;*

### Background

A substantial proportion of venous thromboembolism (VTE) remains idiopathic. Elevated factor VIII (FVIII) and altered platelet activation are increasingly associated with thrombotic risk. Regulation of FVIII plasma levels is well recognized to involve *LRP1*-mediated clearance. In addition, important roles of *SVEP1* and *LRP5* in platelet activation have been recently described. Rare variants in these genes were identified in preliminary whole-exome sequencing analysis of recurrent idiopathic VTE cohort, with one patient carrying a combination of three variants in LRP1, SVEP1, and LRP5 genes.

### Aims

To investigate the functional impact of rare variants in *LRP1* (p.Arg2978Trp), *SVEP1* (p.Ile1513Thr), and *LRP5* (p.Ala465Thr) on coagulation and platelet function.

### Methods

Functional analyses were performed using platelet poor plasma and involved overall haemostatic potential (OHP) assay, and measurements of FVIII:c and von Willebrand factor collagen-binding (VWF:CB) activity, as well as platelet factor 4 (PF4) and VWF antigen levels.

### Results

The analysis revealed significantly increased overall haemostatic ($p < 0.005$) and coagulation potentials ($p < 0.001$), with mildly but significantly reduced fibrinolysis potential ($p < 0.021$), compared with pooled normal plasma (PNP). Data were normally distributed and analyzed using the Student's t-test. Additionally, elevated levels of FVIII:c (299%), VWF:CB (230%), and VWF:Ag (200%) were observed, along with PF4:Ag level that was 1.58-fold higher than in PNP.

### Summary/Conclusion

The obtained results suggest that rare variants in the *LRP1*, *SVEP1*, and *LRP5* genes may promote a prothrombotic state underlying recurrent idiopathic VTE by disrupting FVIII–VWF homeostasis and enhancing platelet activation. However, further studies are needed to confirm these findings.

**Copyright Information:** (Online) ISSN: 2572-9241
©2026 The Author(s). HemaSphere is published by John Wiley & Sons Ltd on behalf of European Hematology Association. This is an open access Abstract Book distributed under the Attribution-NonCommercial-NoDerivs (CC BY-NC-ND), which allows third parties to download the articles and share them with others as long as they credit the author and the Abstract Book, but they cannot change the content in any way or use them commercially.
**Abstract Book Citations:** Authors, Title, HemaSphere, 2026;10:(S1):pages. The abstract book DOIs can be found on the "Author Information" page.
**Disclaimer:** Articles published in the journal HemaSphere exclusively reflect the opinions of the authors. The authors are responsible for all content in their abstracts including accuracy of the facts, statements, citing resources, etc.

### (PB4393) THROMBOEMBOLIC EVENTS IN PATIENTS WITH MALIGNANT HEMATOLOGIC DISORDERS: A RETROSPECTIVE STUDY OF 38 CASES

**Topic:** 35. Thrombosis and vascular biology

Wafa Chenbah*[1], Oumeyma Touati[1], Nadia Sassi[1], Rim Ferhi[1], Rihab Ben Sghaier[1], Wided Cherif[1], Guermazi Monia[1], Haifa Regaieg[1], Bouteraa Walid[1], Kmira Zahra[1], Monia Zaier[1], Nesrine Ben Sayed[1], Yosra Ben Youssef[1]

[1]*Hôpital Farhat Hached, hematolgy, Sousse, Tunisia;*

### Background

Malignant hematologic disorders represent a major risk factor for thromboembolic complications due to the hypercoagulable state induced by the disease itself and the adverse effects of certain antineoplastic agents. The occurrence of venous or arterial thrombotic events in these patients worsens prognosis by increasing morbidity and mortality.

### Methods

We conducted a retrospective study including 38 patients with malignant hematologic disorders complicated by thromboembolic events.

### Results

The mean age at diagnosis was 30 years (range: 4–75), with a sex ratio of 1.2. Identified cardiovascular risk factors included diabetes in one patient, smoking in two patients, and oral contraceptive use in two female patients.
The distribution of hematologic malignancies was as follows: 14 acute lymphoblastic leukemias, 5 non-Hodgkin lymphomas, 6 Hodgkin lymphomas, 4 acute myeloid leukemias, 4 multiple myelomas, and 5 myeloproliferative neoplasms.
Thrombotic events occurred during chemotherapy in 30 patients, before initiation of chemotherapy in 7 patients, and during disease progression without treatment in one patient.
Thromboembolic events were venous in 34 cases and arterial in 4 cases. Among venous events, there were 2 superficial thromboses and 32 deep vein thromboses, including 6 pulmonary embolisms, 12 cerebral venous thromboses, 5 superior vena cava thromboses, 6 internal jugular vein thromboses, and 3 lower-limb deep vein thromboses. Arterial events consisted of 4 ischemic strokes.
Thirteen patients had received L-asparaginase therapy. Six patients were on antiplatelet therapy for preventive purposes. None had a central venous catheter. The mean platelet count at the time of thrombosis was 120,000/mm³.
Thrombophilia screening was not performed in 13 patients, was negative in 24 patients, and revealed activated protein C resistance in one patient.
All patients received initial treatment with low-molecular-weight heparin, followed by oral anticoagulation in four patients. The duration of anticoagulation ranged from 3 to 6 months.
Thrombotic outcomes were favorable in 37 cases, with vascular recanalization achieved. One patient died as a result of ischemic stroke.
From a hematologic standpoint, 20 patients achieved complete remission, 5 died from their hematologic malignancy, 1 patient died from an arterial thrombotic event, and 12 patients remain on specific treatment.

**Copyright Information:** (Online) ISSN: 2572-9241
©2026 The Author(s). HemaSphere is published by John Wiley & Sons Ltd on behalf of European Hematology Association. This is an open access Abstract Book distributed under the Attribution-NonCommercial-NoDerivs (CC BY-NC-ND), which allows third parties to download the articles and share them with others as long as they credit the author and the Abstract Book, but they cannot change the content in any way or use them commercially.
**Abstract Book Citations:** Authors, Title, HemaSphere, 2026;10:(S1):pages. The abstract book DOIs can be found on the “Author Information” page.
**Disclaimer:** Articles published in the journal HemaSphere exclusively reflect the opinions of the authors. The authors are responsible for all content in their abstracts including accuracy of the facts, statements, citing resources, etc.

**Summary/Conclusion**

The occurrence of venous or arterial thromboembolic events in patients with malignant hematologic disorders represents an adverse prognostic factor. This study highlights the importance of systematic identification of thrombotic risk factors in these patients to optimize prevention and management strategies

**Copyright Information:** (Online) ISSN: 2572-9241
©2026 The Author(s). HemaSphere is published by John Wiley & Sons Ltd on behalf of European Hematology Association. This is an open access Abstract Book distributed under the Attribution-NonCommercial-NoDerivs (CC BY-NC-ND), which allows third parties to download the articles and share them with others as long as they credit the author and the Abstract Book, but they cannot change the content in any way or use them commercially.
**Abstract Book Citations:** Authors, Title, HemaSphere, 2026;10:(S1):pages. The abstract book DOIs can be found on the "Author Information" page.
**Disclaimer:** Articles published in the journal HemaSphere exclusively reflect the opinions of the authors. The authors are responsible for all content in their abstracts including accuracy of the facts, statements, citing resources, etc.

### (PB4394) MANAGEMENT OF GIANT CORONARY ARTERY ANEURYSMS AND THROMBOSIS SECONDARY TO KAWASAKI DISEASE: A FOUR-CASE SERIES

**Topic:** 35. Thrombosis and vascular biology

Çağrı Coşkun*[1], Tekin Aksu[1], Ebru Aypar[2], Seza Özen[3], Selin Aytaç[1]

*[1]Hacettepe University Faculty of Medicine, Pediatric Hematology, Ankara, Türkiye;[2]Hacettepe University Faculty of Medicine, Pediatric Cardiology, Ankara, Türkiye; [3]Hacettepe University Faculty of Medicine, pediatric rheumatology, Ankara, Türkiye;*

### Background

Kawasaki disease (KD) is the most common systemic vasculitis of childhood. Coronary artery involvement represents its most serious complication. Giant coronary artery aneurysms (CAA) carry a substantial risk of thrombosis and acute coronary syndrome. We present four cases with giant CAA and thrombotic complications, focusing on antithrombotic management and imaging strategies.

### Methods

Four patients diagnosed with KD and subsequently found to have a
coronary artery aneurysm and thrombosis were retrospectively evaluated. Clinical and imaging findings were analyzed along with anti-inflammatory and antithrombotic treatment strategies.

### Results

Four patients exhibited coronary involvement. Giant aneurysms were present in three cases, with thrombosis occurring predominantly within these segments. Antithrombotic therapy comprised acetylsalicylic acid (ASA), clopidogrel, low-molecular-weight heparin (LMWH), and warfarin, with dual or triple regimens required in patients with giant aneurysms.
Case 1 was diagnosed at 4 months of age with a medium-sized saccular LMCA aneurysm detected on echocardiography (echo) at diagnosis. Dual antiplatelet therapy with ASA and clopidogrel was initiated. Follow-up echo, performed approximately 3 weeks later, revealed LMCA diameters of 4.6 mm (Z score +8.8) and 4.8 mm (Z score +9.8), prompting discontinuation of clopidogrel and addition of LMWH to ASA. At 3-month follow-up, the LMCA diameter decreased to 3.7 mm (Z score +6). As there was no indication for anticoagulation, long-term treatment with ASA and clopidogrel was initiated.
Case 2 was found to have aneurysms in the RCA (12 mm) and LAD (7 mm), with thrombi in the RCA (6 mm) and LAD (1.5 mm), detected one month after diagnosis. PFA closure time was prolonged, but no ASA resistance was detected. Despite dual antiplatelet therapy, thrombus persisted, prompting transition from LMWH to warfarin, which subsequently reduced the thrombus size.
Case 3 was diagnosed with giant aneurysms in the right and left coronary arteries on initial echo. ASA, clopidogrel, and LMWH were initiated. Follow-up echo and cardiac CTA performed two months after diagnosis showed an increase in aneurysm size and thrombus within the aneurysms. Warfarin was then initiated. LMWH and clopidogrel were discontinued. However, an effective INR value could not be achieved. Treatment continued with LMWH, ASA, and clopidogrel (dual antiplatelet and single anticoagulant therapy). Clopidogrel was discontinued 3 days before the angiography procedure. The LMWH dose was skipped one day before the procedure.
Case 4 was started on ASA due to a coronary artery aneurysm. Toe necrosis was detected one week after diagnosis during hospitalization, prompting Doppler ultrasonography, which demonstrated the absence of flow in the dorsalis pedis artery. Heparin infusion was initiated, and treatment was switched to LMWH 10 days later.

**Copyright Information:** (Online) ISSN: 2572-9241
©2026 The Author(s). HemaSphere is published by John Wiley & Sons Ltd on behalf of European Hematology Association. This is an open access Abstract Book distributed under the Attribution-NonCommercial-NoDerivs (CC BY-NC-ND), which allows third parties to download the articles and share them with others as long as they credit the author and the Abstract Book, but they cannot change the content in any way or use them commercially.
**Abstract Book Citations:** Authors, Title, HemaSphere, 2026;10:(S1):pages. The abstract book DOIs can be found on the “Author Information” page.
**Disclaimer:** Articles published in the journal HemaSphere exclusively reflect the opinions of the authors. The authors are responsible for all content in their abstracts including accuracy of the facts, statements, citing resources, etc.

### Summary/Conclusion

In Kawasaki disease patients with giant coronary artery aneurysms, individualized antithrombotic therapy and close follow-up are critical to reduce thrombotic risk and adverse outcomes. Maintaining the INR within the therapeutic range, preferably toward the upper limit, and assessing antiplatelet responsiveness when indicated may help prevent treatment failure. While echo is essential for routine monitoring, coronary CTA enables comprehensive evaluation of aneurysm extent and complications. These findings support a multidisciplinary, patient-specific approach to long-term management.

**Table 1.** Summary of Four Cases with Giant Coronary Aneurysm and Thrombosis in Kawasaki Disease

| Age at Diagnosis | Sex | Coronary Artery Findings | Thrombosis | Treatment | Current Status |
|---|---|---|---|---|---|
| 4 months | M | LMCA saccular aneurysm, diffuse dilation | None | ASA + Clopidogrel → ASA + LMWH → ASA + Clopidogrel | Regression from a giant to a medium-sized aneurysm |
| 5 years | F | Giant aneurysm (LMCA, RCA) | LMCA and RCA thrombosis | ASA + Clopidogrel → ASA + LMWH → ASA + Warfarin | Persistent giant aneurysm, regressing thrombus |
| 5 years | M | Giant aneurysms in the right and left coronary arteries | Intra-aneurysmal thrombus | ASA + Clopidogrel + LMWH | Ongoing follow-up under triple therapy |
| 8 years | F | Diffuse dilation, aneurysm | Peripheral arterial thrombosis | ASA → Heparin → LMWH | Clinically stable |

**ASA:** Acetylsalicylic acid

**LMWH:** Low molecular weight heparin

**LMCA:** Left main coronary artery

**RCA:** Right coronary artery

**Copyright Information:** (Online) ISSN: 2572-9241
©2026 The Author(s). HemaSphere is published by John Wiley & Sons Ltd on behalf of European Hematology Association. This is an open access Abstract Book distributed under the Attribution-NonCommercial-NoDerivs (CC BY-NC-ND), which allows third parties to download the articles and share them with others as long as they credit the author and the Abstract Book, but they cannot change the content in any way or use them commercially.
**Abstract Book Citations:** Authors, Title, HemaSphere, 2026;10:(S1):pages. The abstract book DOIs can be found on the "Author Information" page.
**Disclaimer:** Articles published in the journal HemaSphere exclusively reflect the opinions of the authors. The authors are responsible for all content in their abstracts including accuracy of the facts, statements, citing resources, etc.

### (PB4395) SECONDARY THROMBOTIC THROMBOCYTOPENIC PURPURA IN STEROID-REFRACTORY THROMBOCYTOPENIA DURING A SEVERE SYSTEMIC LUPUS ERYTHEMATOSUS FLARE: A CASE REPORT

**Topic:** 35. Thrombosis and vascular biology

Neil Gabriel Encina*[1]

[1]*National Kidney & Transplant Institute, Internal Medicine, Quezon City, Philippines;*

### Background

Hematologic manifestations are common in Systemic Lupus Erythematosus (SLE), but the emergence of Secondary Thrombotic Thrombocytopenic Purpura (TTP) represents a rare, life-threatening complication. This report aims to describe the diagnostic challenge and therapeutic management of a patient presenting with steroid-refractory thrombocytopenia during a severe SLE flare, ultimately identified as secondary TTP.

### Methods

A 40-year-old female presented with fever, bicytopenia (hemoglobin 8.9 g/dL, platelets 61 x 10^9/L), and renal impairment (creatinine 2.28 mg/dL). Initial symptoms included a cough treated inadequately, prompting initiation of piperacillin-tazobactam for pneumonia-related sepsis. With an initial SLEDAI of 29, she was diagnosed with a severe SLE flare. Sensorial changes were initially attributed to uremia, necessitating hemodialysis. A diagnostic dilemma arose when her profound thrombocytopenia remained refractory (35-36 x 10^9/L) despite three days of pulse methylprednisolone. A high PLASMIC score of 6 and peripheral blood smear revealing schistocytes prompted initiation of therapeutic plasma exchange (TPE) while awaiting ADAMTS13 results. Subsequent testing confirmed an activity of 6.98%.

### Results

TPE replaced one plasma volume with fresh frozen plasma per session. This successfully restored platelet counts to 106 x 10^9/L. However, a therapeutic dilemma emerged as TPE and steroids induced profound iatrogenic secondary immunodeficiency presenting with a lymphocyte count 2%, facilitating complicated pyelonephritis from Candida albicans. Despite hematologic recovery, the patient succumbed to this opportunistic infection.

### Summary/Conclusion

Secondary TTP should be strongly suspected in SLE patients with thrombocytopenia that is refractory to conventional steroid therapy. Early recognition of the TMA pentad and prompt initiation of plasma exchange are critical to improving survival outcomes in this high-mortality subset of SLE patients. In resource-limited settings, initiating TPE based on clinical suspicion is vital, though it necessitates balancing intervention with the risk of fatal opportunistic infections.

**Copyright Information:** (Online) ISSN: 2572-9241
©2026 The Author(s). HemaSphere is published by John Wiley & Sons Ltd on behalf of European Hematology Association. This is an open access Abstract Book distributed under the Attribution-NonCommercial-NoDerivs (CC BY-NC-ND), which allows third parties to download the articles and share them with others as long as they credit the author and the Abstract Book, but they cannot change the content in any way or use them commercially.
**Abstract Book Citations:** Authors, Title, HemaSphere, 2026;10:(S1):pages. The abstract book DOIs can be found on the “Author Information” page.
**Disclaimer:** Articles published in the journal HemaSphere exclusively reflect the opinions of the authors. The authors are responsible for all content in their abstracts including accuracy of the facts, statements, citing resources, etc.

### (PB4396) SEVERE PEDIATRIC BEHCET DISEASE WITH IVC AND PORTAL VEIN THROMBOSIS

**Topic:** 35. Thrombosis and vascular biology

Ayşe Şimşek*[1], Müşerref Kasap Cüceoğlu[2]

*[1]Konya City Hospital, Pediatric Hematology and Oncology, Konya, Türkiye;[2]Konya City Hospital, Pediatric Rheumotology, Konya, Türkiye;*

### Background

Behçet's disease is a relap-remitting vasculitis with variable clinical manifestations, including mucocutaneous, ocular, neurological, and vascular involvement. Vascular Behçet Disease, particularly involving large veins such as the inferior vena cava and hepatic veins, is a rare but severe complication, often associated with increased morbidity and mortality.

### Results

A 14-year-old girl first presented with recurrent oral and genital aphthous ulcers and was diagnosed with **Behçet's disease (BD)** based on clinical findings and HLA-B51 positivity. Colchicine was initiated. Two years later, she developed persistent headaches and was diagnosed with **neuro-Behçet's disease with pseudotumor cerebri**, treated with corticosteroids and acetazolamide. Shortly afterward, she developed **inferior vena cava (IVC) thrombosis**, and cyclophosphamide, infliximab, and anticoagulation were started.
Due to poor adherence, she discontinued treatment for four months and was readmitted with headache, vomiting, visual blurring, and elevated inflammatory markers. Laboratory tests showed leukocytosis, high ESR/CRP, thrombocytosis followed by thrombocytopenia, coagulopathy, low natural anticoagulant levels, and abnormal liver enzymes. CSF studies were negative for infection. Immunosuppressive therapy was restarted.
She later presented with severe abdominal pain; imaging revealed **extensive thrombosis** involving the IVC, iliac, and hepatic veins with ascites. A heterozygous **MEFV M694V** mutation was detected. Despite aggressive immunosuppressive therapy, her condition deteriorated, and she died six weeks later.

### Summary/Conclusion

This case underscores the aggressive vascular and neurological course BD may take in pediatric patients, particularly in the setting of poor treatment adherence. The presence of a heterozygous M694V mutation in the MEFV gene also raises the possibility of a coexisting autoinflammatory disorder, such as familial Mediterranean fever (FMF), which may have contributed to the severity and treatment resistance. This outcome highlights the need for early recognition of severe disease phenotypes, timely initiation of aggressive therapy, and, crucially, patient adherence to long-term treatment protocols.

**Copyright Information:** (Online) ISSN: 2572-9241
©2026 The Author(s). HemaSphere is published by John Wiley & Sons Ltd on behalf of European Hematology Association. This is an open access Abstract Book distributed under the Attribution-NonCommercial-NoDerivs (CC BY-NC-ND), which allows third parties to download the articles and share them with others as long as they credit the author and the Abstract Book, but they cannot change the content in any way or use them commercially.
**Abstract Book Citations:** Authors, Title, HemaSphere, 2026;10:(S1):pages. The abstract book DOIs can be found on the "Author Information" page.
**Disclaimer:** Articles published in the journal HemaSphere exclusively reflect the opinions of the authors. The authors are responsible for all content in their abstracts including accuracy of the facts, statements, citing resources, etc.

### (PB4397) SPONTANEOUS LOBAR INTRACEREBRAL HEMORRHAGE REVEALING COMBINED INHERITED THROMBOPHILIA AND INCIDENTAL PULMONARY EMBOLISM: A THERAPEUTIC PARADOX

**Topic:** 35. Thrombosis and vascular biology

Maryam Arefi Sanaei*[1], Mario Folgueral Pérez[1, 2], Laura Fueyo Ramírez[1, 3]

*[1]Sacyl Hospital Santos Reyes, HEMATOLOGIA Y HEMOTERAPIA, Aranda de Duero, Spain;[2]Hospital, Jefe de sección de Radiodiagnóstico, Aranda de Duero, Burgos, Spain; [3]Hospital, Laboratorio, Aranda de Duero, Burgos, Spain;*

### Background

A 41-year-old female with no cardiovascular risk factors presented to the Emergency Department with a 15-day history of predominantly nocturnal headache (hemicranial, radiating to the right shoulder) and associated with nausea and occasional vomiting. In the days prior to admission, she developed fluctuating right-sided weakness and reported deviation of the mouth to the left.
She denied any history of trauma, head injury, recent infection, weight loss, recurrent epistaxis or easy bruising. Family history was negative for bleeding disorders (two healthy sisters; both parents alive without bleeding history). She reported stress and worked as a home care assistant.

### Aims

To report a case of spontaneous intracerebral hemorrhage (ICH) as the initial manifestation of combined inherited thrombophilia.

### Methods

We retrospectively reviewed the clinical, radiological, and hematological data of a 41-year-old woman admitted with non-traumatic ICH. Comprehensive vascular imaging and thrombophilia testing were performed.

### Results

In the Emergency Department, CBC, coagulation, biochemistry and brain CT scan were performed. In the Brain CT: Extensive right fronto-parietal intraparenchymal hemorrhage with significant surrounding edema and 4 mm contralateral midline shift. 24h after ICU admission, she developed neurological deterioration characterized by increased somnolence and poor command following. A repeat CT scan showed a right frontal intraparenchymal hematoma without significant changes compared to the previous study and no evidence of cerebral herniation.
Lab. tests showed microcytic iron-deficiency anemia. Fibrinogen, platelet function assay (PFA), prothrombin time (PT), von Willebrand factor antigen (VWF Ag), and D-dimer, tumor markers were within the normal range.
ECG & Cerebral angiography was normal.
One week later, body CT (to rule out underlying disease) revealed: Acute bilateral peripheral pulmonary embolism (incidental finding), Small cystic pancreatic lesion, Hepatic steatosis. Eco-doppler: no thrombosis.
THROMBOPHILIA evaluation: Performed >6 months after the hemorrhagic event: Heterozygous prothrombin G20210A mutation, Protein C deficiency (41%), Antithrombin deficiency (41%), factor VIII (287%), Hyperhomocysteinemia (55 µmol/L), Factor V Leiden mutation: Neg, antiphospholipid antibodies: Neg.

**Copyright Information:** (Online) ISSN: 2572-9241
©2026 The Author(s). HemaSphere is published by John Wiley & Sons Ltd on behalf of European Hematology Association. This is an open access Abstract Book distributed under the Attribution-NonCommercial-NoDerivs (CC BY-NC-ND), which allows third parties to download the articles and share them with others as long as they credit the author and the Abstract Book, but they cannot change the content in any way or use them commercially.
**Abstract Book Citations:** Authors, Title, HemaSphere, 2026;10:(S1):pages. The abstract book DOIs can be found on the “Author Information” page.
**Disclaimer:** Articles published in the journal HemaSphere exclusively reflect the opinions of the authors. The authors are responsible for all content in their abstracts including accuracy of the facts, statements, citing resources, etc.

### Summary/Conclusion

Inherited thrombophilia is predominantly associated with venous thromboembolism (VTE), while spontaneous intracerebral hemorrhage (ICH) in young adults is uncommon and typically secondary to structural or vascular abnormalities.
This case illustrates an unusual presentation of severe combined inherited thrombophilia manifesting as hemorrhagic stroke rather than thrombosis. The incidental pulmonary embolism created a significant therapeutic dilemma, requiring carefully timed anticoagulation.
After incidental detection of bilateral PE, prophylactic-dose low-molecular-weight heparin (enoxaparin) was started, although was contraindicated due to acute ICH. The patient tolerated treatment well without new hemorrhagic events. Resolved microcytic anemia after oral iron therapy; Helicobacter pylori infection treated.
Follow-up after 17 months post-ICH, the patient remains clinically stable, no new neurological deficit and no recurrent ICH or VTE.. The second angiography remained normal. She Currently anticoagulated with acenocoumarol (Sintrom®), INR: 2-2.8
Individualized risk assessment is essential to balance thrombotic and hemorrhagic risks in complex cases such as this.

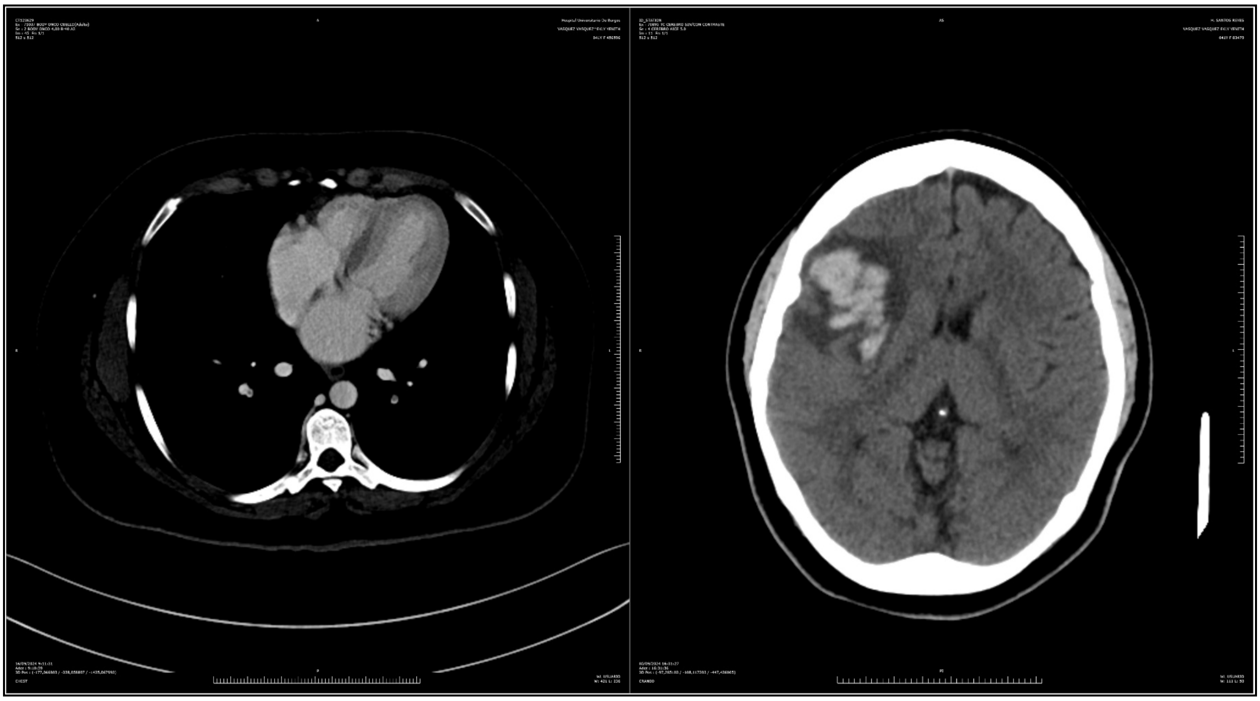

Right Image: intracerebral hemorrhage. Left image: bilateral PE

**Copyright Information:** (Online) ISSN: 2572-9241
©2026 The Author(s). HemaSphere is published by John Wiley & Sons Ltd on behalf of European Hematology Association. This is an open access Abstract Book distributed under the Attribution-NonCommercial-NoDerivs (CC BY-NC-ND), which allows third parties to download the articles and share them with others as long as they credit the author and the Abstract Book, but they cannot change the content in any way or use them commercially.
**Abstract Book Citations:** Authors, Title, HemaSphere, 2026;10:(S1):pages. The abstract book DOIs can be found on the "Author Information" page.
**Disclaimer:** Articles published in the journal HemaSphere exclusively reflect the opinions of the authors. The authors are responsible for all content in their abstracts including accuracy of the facts, statements, citing resources, etc.

### (PB4398) MULTIPLE-HIT PROTHROMBOTIC PHENOTYPE: PERSISTENT IGM ANTIPHOSPHOLIPID SYNDROME COMBINED WITH INHERITED PROTEIN S/C DEFICIENCY AND CHRONIC INTESTINAL INFLAMMATION LEADING TO RECURRENT VENOUS THROMBOSIS

**Topic:** 35. Thrombosis and vascular biology

Maryam Arefi Sanaei*[1]

[1]*Sacyl Hospital Santos Reyes, HEMATOLOGIA Y HEMOTERAPIA, Aranda de Duero, Spain;*

### Background

Venous thromboembolism (VTE) is frequently driven by single risk factors; however, overlapping inherited, autoimmune, and inflammatory conditions can create a cumulative “multi-hit” prothrombotic state. Antiphospholipid syndrome (APS) with persistent IgM positivity, when combined with inherited deficiencies of natural anticoagulants and chronic inflammatory triggers, represents a high-risk phenotype that challenges conventional management

### Aims

To illustrate a rare, complex multi-hit thrombotic phenotype integrating persistent IgM APS, inherited protein S/C deficiency, and chronic intestinal inflammation, highlighting its clinical and therapeutic implications

### Methods

We describe a 51-year-old female, active smoker (BMI 31.9 kg/m²) with prior gastric bypass (2011) and collagenous colitis refractory to budesonide, presenting with recurrent unprovoked VTE (three DVTs 2016–2017, one superficial venous thrombosis 2019) and labile INR on acenocoumarol. Chronic watery diarrhea (>10/day, fecal calprotectin 1000 µg/g) contributed to anticoagulation instability.
Comprehensive thrombophilia and autoimmune workup included repeated measurement of antiphospholipid antibodies and lupus anticoagulant.

### Results

Laboratory evaluation revealed protein S deficiency (62%), protein C deficiency (69%), persistent anticardiolipin IgM (82.4 U/mL), anti-β2 glycoprotein I IgM (54.7 U/mL), and lupus anticoagulant positive in two determinations, fulfilling APS criteria. IgG isotypes were negative. Fibrinogen was elevated (470 mg/dL) and homocysteine mildly increased (14.4 µmol/L); Factor V Leiden and prothrombin G20210A mutations were excluded.
The patient demonstrates recurrent thrombosis despite anticoagulation, highlighting a synergistic interplay of inherited deficiencies, autoimmune activation, and chronic intestinal inflammation, which together amplify thrombotic risk and destabilize vitamin K antagonist therapy.

### Summary/Conclusion

This case exemplifies a cumulative multi-hit prothrombotic phenotype where persistent IgM APS, inherited protein S/C deficiency, and chronic inflammatory triggers converge to drive recurrent VTE. Recognition of such overlapping mechanisms is critical for individualized anticoagulation strategies and multidisciplinary management. The case emphasizes that persistent IgM APS is clinically relevant when coupled with additional thrombotic risk factors, and that chronic inflammatory states can exacerbate coagulation dysregulation, posing challenges for conventional therapy.

**Copyright Information:** (Online) ISSN: 2572-9241
©2026 The Author(s). HemaSphere is published by John Wiley & Sons Ltd on behalf of European Hematology Association. This is an open access Abstract Book distributed under the Attribution-NonCommercial-NoDerivs (CC BY-NC-ND), which allows third parties to download the articles and share them with others as long as they credit the author and the Abstract Book, but they cannot change the content in any way or use them commercially.
**Abstract Book Citations:** Authors, Title, HemaSphere, 2026;10:(S1):pages. The abstract book DOIs can be found on the “Author Information” page.
**Disclaimer:** Articles published in the journal HemaSphere exclusively reflect the opinions of the authors. The authors are responsible for all content in their abstracts including accuracy of the facts, statements, citing resources, etc.

### (PB4399) A CASE REPORT AND LITERATURE REVIEW OF RENAL INJURY ASSOCIATED WITH CRYSTALLINE CRYOGLOBULINEMIA

**Topic:** 35. Thrombosis and vascular biology

Yang Liu*[1], Jin Lu[1]

[1]*Peking University People's Hospital, Hematology, Beijing, China;*

### Background

Cryocrystalglobulinemia is a rare subtype of monoclonal gammopathy-associated crystalline nephropathy, which is characterized by the deposition of crystalline monoclonal globulins in blood vessels and tissues, leading to vascular occlusion and multiple organ damage. Renal injury is one of the most common and severe clinical manifestations of the disease, and it is often manifested as acute kidney injury (AKI) with rapid progression, even leading to irreversible renal failure. Due to the low incidence of the disease and the lack of specific clinical manifestations, it is easy to be misdiagnosed and missed in clinical practice. In this study, we report a case of severe renal injury associated with cryocrystalglobulinemia complicated with multiple organ damage, so as to provide reference for clinical practice.

### Aims

To explore the clinical manifestations, pathological features, diagnosis and treatment of renal injury associated with cryocrystalglobulinemia, and to improve the clinical recognition of this rare disease.

### Methods

A 57-year-old male patient with renal injury induced by cryocrystalglobulinemia admitted to our hospital in April 2025 was retrospectively analyzed, and the relevant literature was reviewed.

### Results

The patient presented with purpuric rash aggravated by cold, acral necrosis, acute renal failure and multiple organ damage. Laboratory examinations showed positive IgG kappa in serum immunofixation electrophoresis, intravascular hemolysis, hypocomplementemia and elevated sC5b-9. Bone marrow immunophenotyping detected 0.17% kappa-positive clonal B cells after constant temperature transportation of specimens. Renal puncture biopsy confirmed crystalline cryoglobulinemic nephropathy (IgG2 kappa type, amyloid type) complicated with renal cortical necrosis, with crystalline pseudothrombi deposition in glomerular capillaries and typical Congo red staining positive. The patient was treated with CD20 monoclonal antibody combined with bortezomib and dexamethasone, and the hematological and pulmonary lesions improved, but renal function did not recover. The patient eventually died of multiple organ failure due to duodenal perforation.

### Summary/Conclusion

Cryocrystalglobulinemia is a rare disease with diverse clinical manifestations and severe multi-organ damage. Early cryoglobulin detection, biopsy of involved organs and accurate identification of bone marrow clonality are crucial for diagnosis. Targeted clonal therapy can improve the prognosis of partial patients, and comprehensive systemic evaluation is needed during treatment to avoid serious complications.

**Copyright Information:** (Online) ISSN: 2572-9241
©2026 The Author(s). HemaSphere is published by John Wiley & Sons Ltd on behalf of European Hematology Association. This is an open access Abstract Book distributed under the Attribution-NonCommercial-NoDerivs (CC BY-NC-ND), which allows third parties to download the articles and share them with others as long as they credit the author and the Abstract Book, but they cannot change the content in any way or use them commercially.
**Abstract Book Citations:** Authors, Title, HemaSphere, 2026;10:(S1):pages. The abstract book DOIs can be found on the "Author Information" page.
**Disclaimer:** Articles published in the journal HemaSphere exclusively reflect the opinions of the authors. The authors are responsible for all content in their abstracts including accuracy of the facts, statements, citing resources, etc.

### (PB4400) PEDIATRIC CEREBRAL VENOUS SINUS THROMBOSIS AS A RARE VASCULAR DISORDER IN INFANT: A FIRST CASE REPORT IN CAMBODIA

**Topic:** 35. Thrombosis and vascular biology

Chin Soey*[1]

[1]*National Pediatric Hospital, Pediatric Hemato-immunology and Medicine transfusion, phnompenh, Cambodia;*

### Background

### Introduction

Cerebral venous sinus thrombosis (CVST) in infants is a rare vascular disorder that presents with nonspecific symptoms leading to a delay in diagnosis and treatment. The estimated annual incidence is up to 7 cases per million among children. To our knowledge, no case was previously reported in Cambodia; therefore, we report our first case to share with our colleagues.

### Aims

Cerebral venous sinus thrombosis (CVST) in children is a rare and life-threatening cerebrovascular condition, and with increased clinical awareness and improved neuroradiological techniques, the disease can now be diagnosed more frequently and at earlier stages. The incidence of childhood CVST occurs between 0.4 and 0.7 children per 100,000 children per year [1]. The incidence is higher among neonates (30–50%) compared to other pediatric age groups. Neonates, in general, have a greater risk of poor outcomes, while children show a better outcome with full recovery [2]

### Methods

A 2-year-old boy of Cambodian origin presented to the department of pediatric hematology and immunology with 2-day history of fever, 3 episodes of partial seizures expressing right hand tonic and jerking without vomiting. *Past medical history* was not remarkable. His *physical examination* on arrival revealed a non-toxic appearance, fully alert, Glasgow coma score 15/15, low grade fever 38ºC, with normal vital signs and no signs of neurological impairment or meningeal involvement were detected.

### Results

Cerebral venous sinus thrombosis (CVST) in infants is a rare vascular disorder that presents with nonspecific symptoms leading to a delay in diagnosis and treatment. The estimated annual incidence is up to 7 cases per million among children[6]. Clinically, patients are presented with variable symptoms ranging from headache to seizure and coma in severe cases. Visual impairment due to an increase in intracranial pressure, and generalized neurology deficits are reported to be more common in children[7]. In our case, the clinical manifestations were fever, headache, vomiting associated with partial seizures expressing right hand tonic and jerking which were nonspecific symptoms.

**Copyright Information:** (Online) ISSN: 2572-9241
©2026 The Author(s). HemaSphere is published by John Wiley & Sons Ltd on behalf of European Hematology Association. This is an open access Abstract Book distributed under the Attribution-NonCommercial-NoDerivs (CC BY-NC-ND), which allows third parties to download the articles and share them with others as long as they credit the author and the Abstract Book, but they cannot change the content in any way or use them commercially.
**Abstract Book Citations:** Authors, Title, HemaSphere, 2026;10:(S1):pages. The abstract book DOIs can be found on the "Author Information" page.
**Disclaimer:** Articles published in the journal HemaSphere exclusively reflect the opinions of the authors. The authors are responsible for all content in their abstracts including accuracy of the facts, statements, citing resources, etc.

### Summary/Conclusion

CVST is a rare phenomenon with an ambiguous clinical presentation which include but are not limited to seizures, vomiting, hemiparesis, and the impact on cranial nerves creating a challenge for physicians to diagnose, especially in the limited setting of investigative facilities like Cambodia. This current case gives insight into how early detection and treatment resulted in a good outcome for the patient.

**Pediatric cerebral venous sinus thrombosis as a rare vascular disorder in infant: case report**

*Chin Soey[1*], Chean Sophâl[1]*

[1]Department of Pediatric Hematology and Immunology
National Pediatric Hospital, Phnom Penh, Cambodia

**ABSTRACT**

**Introduction**

Cerebral venous sinus thrombosis (CVST) in infants is a rare vascular disorder that presents with nonspecific symptoms leading to a delay in diagnosis and treatment. The estimated annual incidence is up to 7 cases per million among children. To our knowledge, no case was previously reported in Cambodia; therefore, we report our first case to share with our colleagues.

**Case Presentation**

A 2-year-old boy of Cambodian origin presented with a 2-day history of fever, 3 episodes of partial seizures expressing right hand tonic and jerking without vomiting. Past medical history was not remarkable. On physical examination, non-toxic appearance and fully alert with normal neurologic examination were observed with low grade fever 38ºC. Initial blood analyses showed a high level of D-dimer to 1735 mg/L, a hemoglobin level of 11.1 g/dL and an increased platelet count of 737 $x10^9$/L, white blood cell count of 20×10⁹/L, neutrophils of 12 $x10^9$/L and lymphocytes of 6 $x10^9$/L. APTT, PT, and INR were 41.4, 11 seconds, and 0.84, respectively. Cerebral CT scan with contrast shown as 5 mm CVST. This child was treated with oral Warfarin (direct oral anticoagulant), and the progression over the past 6-month monitoring showed normal neurological development and normal milestones for age whereas laboratory profiles showed varying levels of D-dimer.

**Conclusions**

CVST is a rare phenomenon with an ambiguous clinical presentation, creating a challenge for physicians to diagnose, especially in the limited setting of investigative facilities like Cambodia. This case gives insight into one of the many presentations in hopes of early detection and a good outcome.

**Keywords:** neurology, pediatric seizure, cerebral venous sinus thrombosis, CVST

**Copyright Information:** (Online) ISSN: 2572-9241
©2026 The Author(s). HemaSphere is published by John Wiley & Sons Ltd on behalf of European Hematology Association. This is an open access Abstract Book distributed under the Attribution-NonCommercial-NoDerivs (CC BY-NC-ND), which allows third parties to download the articles and share them with others as long as they credit the author and the Abstract Book, but they cannot change the content in any way or use them commercially.
**Abstract Book Citations:** Authors, Title, HemaSphere, 2026;10:(S1):pages. The abstract book DOIs can be found on the "Author Information" page.
**Disclaimer:** Articles published in the journal HemaSphere exclusively reflect the opinions of the authors. The authors are responsible for all content in their abstracts including accuracy of the facts, statements, citing resources, etc.

### (PB4401) IS LUPUS ANTICOAGULANT TESTING RELEVANT IN PATIENTS WITH AUTOIMMUNE HAEMOLYTIC ANAEMIA ?

**Topic:** 35. Thrombosis and vascular biology

Enna Maria Lourenco*[1], Nirmal Thomas[1], Anurag Pandey[1, 1], Amit Jakhi[1], Dr V.P Antia[1]

[1]*BREACH CANDY HOSPITAL TRUST, HAEMATOLOGY, MUMBAI, India;*

### Background

Lupus anticoagulant [also known as Lupus antibody or LA OR LAC or Lupus inhibitors] is an immunoglobin binds to phospholipids and proteins associated with the cell membrane. LA is a silent antibody that harms our own body instead of protecting it. Lupus anticoagulant is one of three malfunctioning antibodies [Lupus anticoagulant [LA] , anticardiolipin [ACA] and beta 2 Glycoprotein1 called antiphospholipid antibodies.LAC increase risk of blood clots [thrombosis] in veins or arteries. Autoimmune hemolytic anaemia [AIHA] is an autoimmune disease where there is a fall in red blood cells because of antibody-mediated red blood cell destruction.There are two categories of AIHA i.e warm and cold. In children with AIHA, some can be life-threating and immediate medical care is required..

### Aims

Patients having autoimmune hemolytic anemia especially suspected Venous Thromboembolism [VTE] , patients with increased aPTT tested for Lupus antibody [LA] to rule out underlying diseases in confirming or refuting the diagnosis which help to investigate unexplained clotting events and patients with increased aPTT.

### Methods

Study done at tertiary hospital, Mumbai, India. Blood sample tested for LA with blood test such as dilute Russell's viper venom time [ DRVVT ] and activated partial thromboplastin time [aPTT ]admitted with different clinical history and some also had suspected VTE disease .. The samples were tested on Stago compact Max 2 or Max 3.within 3 hrs.
The study of LA was carried out by using STA-Staclot DRVV Screen and STA Staclot DRVV Confirm and Sta control LA1 + 2 used on Stago Compact Max Instrument using Clot based assay. APTT automate used for APTT…Coag control N & P were used.
**Inclusion criteria:**Patients having AIHA with less Haemoglobin ,peripheral smear showing spherocytes [in warm AIHA ],auto agglutination[ in cold AIHA ],positive direct anticoagulant testing [DAT ] , suspected VTE disease and patients with increased aPTT
**Rejection Criteria**: Haemolysed or underfilled or overfilled or patient on blood thinners
**Blood Collected**: Blood samples collected in Sodium Citrate anticoagulant, 3.2 % [ratio 9:1] vacutainers.

**Copyright Information:** (Online) ISSN: 2572-9241
©2026 The Author(s). HemaSphere is published by John Wiley & Sons Ltd on behalf of European Hematology Association. This is an open access Abstract Book distributed under the Attribution-NonCommercial-NoDerivs (CC BY-NC-ND), which allows third parties to download the articles and share them with others as long as they credit the author and the Abstract Book, but they cannot change the content in any way or use them commercially.
**Abstract Book Citations:** Authors, Title, HemaSphere, 2026;10:(S1):pages. The abstract book DOIs can be found on the "Author Information" page.
**Disclaimer:** Articles published in the journal HemaSphere exclusively reflect the opinions of the authors. The authors are responsible for all content in their abstracts including accuracy of the facts, statements, citing resources, etc.

### Results

| **N:134** | LA | DRVVT confirm ratio | Results |
|---|---|---|---|
| **Control[Normal ] [n=15 ] 11.20 %,** | Negative | <1.20 | LA Negative |
| Patients with AIHA with underlying VTE [ n=15] 11.20 % | Positive | >1.20 | LA Positive<br>Mixing studies and confirmatory test positive |
| Patients with LA+ACP with AIHA [n=4 ] 3.0 % | Positive | >1.20 | LA Positive<br>Mixing studies and confirmatory test positive |
| ACA WITH AIHA [n=3 ] 2.24 % | LA Negative | <1.20 | LA Negative but ACA Positive |
| VTE without AIHA [n=6] 4.48 % | LA Positive | >1.20 | LA Positive<br>Mixing studies and confirmatory test positive |
| ACA without AIHA [n=5 ] 3.70 % | LA Negative | <1.20 | LA Negative but ACA Positive |
| Patients on anticoagulant [n=09 ]6.72 % | LA Positive | | LA False Positive<br>Patients on blood thinners. |
| Patients with other underlying history [n=77 ] 57.46 % | LA Negative | <1.20 | LA Negative |

### Summary/Conclusion

Presence of Lupus anticoagulant in autoimmune haemolytic anaemia [AIHA] in patients is significant, as it indicate a higher risk of developing VTE a potentially serious complication

Detection of Lupus anticoagulant in patient with AIHA helps in appropriate preventative measures. And also role of prophylactic anticoagulation. AIHA Patients with severe low anaemia <7.0 g/dl are at high risk of VTE.

**Key message**: Lupus anticoagulant testing helps in identifying patients at increased risk of venous thromboembolism [VTE] a major complication of Autoimmune haemolytic anaemia [AIHA

**Copyright Information:** (Online) ISSN: 2572-9241
©2026 The Author(s). HemaSphere is published by John Wiley & Sons Ltd on behalf of European Hematology Association. This is an open access Abstract Book distributed under the Attribution-NonCommercial-NoDerivs (CC BY-NC-ND), which allows third parties to download the articles and share them with others as long as they credit the author and the Abstract Book, but they cannot change the content in any way or use them commercially.
**Abstract Book Citations:** Authors, Title, HemaSphere, 2026;10:(S1):pages. The abstract book DOIs can be found on the "Author Information" page.
**Disclaimer:** Articles published in the journal HemaSphere exclusively reflect the opinions of the authors. The authors are responsible for all content in their abstracts including accuracy of the facts, statements, citing resources, etc.

### (PB4402) ISOLATED FACTOR XII DEFICIENCY PRESENTING WITH MUCOCUTANEOUS BLEEDING IN AN EARLY ADOLESCENT: A DIAGNOSTIC PARADOX

**Topic:** 35. Thrombosis and vascular biology

Adnan Khan*[1], Saifudeen A[2], Aamir Shamas[1], Suhail S[1], Ashikh Sheriff[1]

*[1]KIMS Health, Internal Medicine, Trivandrum, India;[2]KIMS Health, Benign Hematology, Trivandrum, India;*

### Background

Factor XII (FXII) deficiency is a rare inherited coagulation disorder characterized by markedly prolonged activated partial thromboplastin time (aPTT) without an associated bleeding phenotype. It is most often detected incidentally during coagulation screening. The presence of mucocutaneous bleeding in patients with FXII deficiency presents a diagnostic challenge and necessitates evaluation for alternative or coexisting causes.

### Aims

To describe an early adolescent girl with mucocutaneous bleeding found to have isolated FXII deficiency and to highlight the diagnostic approach in cases with discordance between laboratory abnormalities and clinical presentation.

### Methods

An 11-year-old girl with hypothyroidism on levothyroxine presented with recurrent left-sided epistaxis for two weeks and increased menstrual frequency for five months. ENT examination including nasal endoscopy and gynecologic evaluation with pelvic ultrasonography were unremarkable. Coagulation screening demonstrated prolonged prothrombin time (16.6 s), prolonged aPTT (35.3 s), and INR of 1.25. Extended coagulation studies included assays for Factors VIII, IX, XI, XII, and VII, along with von Willebrand factor (vWF) antigen and ristocetin cofactor activity.

### Results

Isolated FXII deficiency was identified with FXII activity of 38%, while all other intrinsic and extrinsic pathway factors and vWF parameters were within reference ranges. Despite the classical non-hemorrhagic nature of FXII deficiency, the patient had mucocutaneous bleeding and menstrual irregularity. Genetic testing to confirm hereditary deficiency was advised but was not performed due to financial constraints.

### Summary/Conclusion

Factor XII (FXII) deficiency is a rare inherited coagulation abnormality that typically causes a prolonged activated partial thromboplastin time without clinical bleeding. We report an 11-year-old girl presenting with recurrent epistaxis and increased menstrual frequency who was found to have isolated FXII deficiency during evaluation for suspected coagulopathy. ENT and gynecologic assessments were normal. Coagulation studies showed prolonged PT and aPTT, and factor assays revealed reduced FXII activity (38%) with normal levels of other coagulation factors and von Willebrand parameters. Although FXII deficiency is classically non-hemorrhagic, the patient had mucocutaneous bleeding, creating a diagnostic paradox. This case highlights the importance of correlating laboratory abnormalities with clinical findings and of investigating alternative causes of bleeding when prolonged aPTT is identified.

**Copyright Information:** (Online) ISSN: 2572-9241
©2026 The Author(s). HemaSphere is published by John Wiley & Sons Ltd on behalf of European Hematology Association. This is an open access Abstract Book distributed under the Attribution-NonCommercial-NoDerivs (CC BY-NC-ND), which allows third parties to download the articles and share them with others as long as they credit the author and the Abstract Book, but they cannot change the content in any way or use them commercially.
**Abstract Book Citations:** Authors, Title, HemaSphere, 2026;10:(S1):pages. The abstract book DOIs can be found on the "Author Information" page.
**Disclaimer:** Articles published in the journal HemaSphere exclusively reflect the opinions of the authors. The authors are responsible for all content in their abstracts including accuracy of the facts, statements, citing resources, etc.

| Parameter | Value |
|---|---|
| PT (s) | 16.6 |
| aPTT (s) | 35.3 |
| INR | 1.25 |
| Factor XII (%) | 38.0 |

**Copyright Information:** (Online) ISSN: 2572-9241
©2026 The Author(s). HemaSphere is published by John Wiley & Sons Ltd on behalf of European Hematology Association. This is an open access Abstract Book distributed under the Attribution-NonCommercial-NoDerivs (CC BY-NC-ND), which allows third parties to download the articles and share them with others as long as they credit the author and the Abstract Book, but they cannot change the content in any way or use them commercially.
**Abstract Book Citations:** Authors, Title, HemaSphere, 2026;10:(S1):pages. The abstract book DOIs can be found on the "Author Information" page.
**Disclaimer:** Articles published in the journal HemaSphere exclusively reflect the opinions of the authors. The authors are responsible for all content in their abstracts including accuracy of the facts, statements, citing resources, etc.

### (PB4403) MANAGING ONCOLOGY PATIENTS WITH ACKR1/DARC-ASSOCIATED NEUTROPENIA (ADAN): MULTI-CENTRE UK SURVEY

**Topic:** 36. Quality of life, ethics, supportive and palliative care

Lekshmy Balakrishnan[1], Haider Al-Qayyim[1], Joseph Henley[1], Hilesa Asiedu[1], Nita Prasannan[1], Alexandros Georgiou[1], Debra Josephs[1], Elias Pintus[1], Priya Sriskandarajah*[1]

[1]*Guy's and St Thomas' Hospital, London, United Kingdom;*

### Background

ACKR1/DARC-associated neutropenia (ADAN), also known as Duffy-null associated neutrophil count (DANC), is a common inherited variant affecting individuals of African and Middle Eastern ancestry characterised by chronically lower absolute neutrophil counts (ANC). Current ANC reference ranges do not account for ADAN, frequently leading to unnecessary investigation and disruption to cancer treatment pathways. Limited awareness among oncology clinicians and lack of clear guidance may contribute to treatment delays, dose reductions, inappropriate growth factor use, and exclusion from clinical trials.

### Aims

Assess awareness and knowledge of ADAN among oncology professionals across London and evaluate its perceived impact on chemotherapy decision-making.

### Methods

An electronic survey comprising three sections (participant background, scenario-based knowledge assessment, and knowledge of Duffy status and its clinical significance) was distributed to medical and clinical oncology professionals across five Southeast London hospitals. Two case-based scenarios evaluated management decisions for patients of African ancestry with low ANC prior to or during chemotherapy. Awareness of Duffy testing availability and perceived barriers to its implementation was also assessed.

### Results

44 oncology professionals responded to the survey; 64% worked in medical oncology and 34% in clinical oncology. Majority were consultants (61%) and based in tertiary or academic centres (88%). Gastrointestinal, lung, breast, and prostate oncology were the most represented subspecialties.
In a pre-chemotherapy scenario involving stage IV gastric cancer with chronic neutropenia, 68% said they would seek urgent haematology referral prior to treatment initiation, 7% would consider alternative therapy, and 21% would administer G-CSF.
In a second scenario involving a patient due to start cycle 2 of chemotherapy for lung cancer with longstanding asymptomatic neutropenia, 50% respondents said they would refer to haematology, 41% would administer G-CSF, 14% would delay treatment, and 9% would dose-reduce chemotherapy. Only 20% of respondents would proceed with full dosing, considering the neutropenia clinically insignificant.
Majority of respondents (52%) were unaware of ADAN. Of those who were aware of ADAN (48%), only 61% correctly identified that ADAN is associated with lower ANC without increased infection risk. Nearly all respondents (98%) did not know if Duffy-null testing was available through their local pathology service, and the most frequently reported barrier to testing was lack of awareness of this variant (80%). Majority (59%) also stated that isolated neutropenia from ADAN would impact chemotherapy delivery as well as dosing decisions. Importantly, 41% felt ADAN would influence decision to enrol patients into clinical trials, a theme picked up in the free-text responses.

**Copyright Information:** (Online) ISSN: 2572-9241
©2026 The Author(s). HemaSphere is published by John Wiley & Sons Ltd on behalf of European Hematology Association. This is an open access Abstract Book distributed under the Attribution-NonCommercial-NoDerivs (CC BY-NC-ND), which allows third parties to download the articles and share them with others as long as they credit the author and the Abstract Book, but they cannot change the content in any way or use them commercially.
**Abstract Book Citations:** Authors, Title, HemaSphere, 2026;10:(S1):pages. The abstract book DOIs can be found on the “Author Information” page.
**Disclaimer:** Articles published in the journal HemaSphere exclusively reflect the opinions of the authors. The authors are responsible for all content in their abstracts including accuracy of the facts, statements, citing resources, etc.

**Summary/Conclusion**

This survey demonstrates substantial knowledge gaps regarding ADAN among oncology professionals in London tertiary centres. Clinical uncertainty commonly results in haematology referral, treatment delay pending review, inappropriate growth factor use, or dose modification in patients with likely benign neutropenia. Limited awareness of Duffy testing further compounds this issue. Overall, although our sample size is small, these results highlight a need for standardised guidance and targeted educational initiatives to reduce inequities, prevent avoidable treatment delays, and optimise cancer care for patients of African ancestry.

**Copyright Information:** (Online) ISSN: 2572-9241
©2026 The Author(s). HemaSphere is published by John Wiley & Sons Ltd on behalf of European Hematology Association. This is an open access Abstract Book distributed under the Attribution-NonCommercial-NoDerivs (CC BY-NC-ND), which allows third parties to download the articles and share them with others as long as they credit the author and the Abstract Book, but they cannot change the content in any way or use them commercially.
**Abstract Book Citations:** Authors, Title, HemaSphere, 2026;10:(S1):pages. The abstract book DOIs can be found on the "Author Information" page.
**Disclaimer:** Articles published in the journal HemaSphere exclusively reflect the opinions of the authors. The authors are responsible for all content in their abstracts including accuracy of the facts, statements, citing resources, etc.

### (PB4404) QUALITY OF LIFE, PATIENT-REPORTED EXPECTATIONS AND EXPERIENCE OF THE FIRST 55 CAR-T TREATED PATIENTS AT OUR HOSPITAL

**Topic:** 36. Quality of life, ethics, supportive and palliative care

Annegret Meyer-Hari*[1], Martin Fehr[1], Gian-Andrea Lehmann[2], Matthias Volden[1, 3], Jutta Thierbach[3], Thomas Lehmann[1]

*[1]HOCH, St. Gallen Cantonal Hospital, Oncology-Hematology, St. Gallen, Switzerland;[2]Kantonsschule Trogen, Biology, Trogen, Switzerland; [3]Swiss Red Cross Blood Donation Foundation, Eastern Switzerland, Celltherapy, St. Gallen, Switzerland;*

### Background

Chimeric Antigen Receptor T-cell (CAR-T) therapy is a major breakthrough for treating relapsed or refractory blood cancers, offering durable remissions and improved survival. While evidence supports its efficacy and safety, less is known about its impact on quality of life (QoL) and how patient's expectations compare to their real experiences after treatment.

### Aims

To evaluate the outcome of CAR-T treated patients with respect to QoL, patient's expectations and patient-reported experience.

### Methods

The study was a retrospective observational analysis including all patients who received CAR-T cell therapy at our center between 2021 and the end of September 2025. QoL was assessed using the EORTC QLQ-C30 instrument, supplemented by a structured questionnaire evaluating patient expectations and experiences. Only patients with general consent are included and a declaration of consent was obtained for this survey.

### Results

The cohort included 55 patients; in 3 cases, cells were collected but not retransfused. Patient characteristics of the 52 evaluable patients were as follows: 22 female, 30 male; median age at retransfusion: 66.5 years; median follow-up: 333 days; completed questionnaires: 27 (response rate 66%). Underlying diseases were: Multiple myeloma (MM)(n=15), diffuse large B-cell lymphoma (DLBCL)(n=30), mantle cell lymphoma (MCL)(n=6), and B-acute lymphoblastic leukemia (B-ALL)(n=1). At data cut-off, 41 patients (78.8%) were alive, 11 deaths occurred (7 DLBCL, 2 MCL, 2 MM). Of the 41 patients alive, 34 did not receive subsequent treatment (20 DLBCL, 4 MCL, 9 MM, 1 B-ALL), while the remaining did (3 DLBCL, 4 MM). The following comparison highlights functional and symptom scores between the EORTC general population reference group and the CAR-T treated cohort (see table below). Higher functional scores indicate better perceived functioning, while higher symptom scores reflect greater symptom burden.

**Copyright Information:** (Online) ISSN: 2572-9241
©2026 The Author(s). HemaSphere is published by John Wiley & Sons Ltd on behalf of European Hematology Association. This is an open access Abstract Book distributed under the Attribution-NonCommercial-NoDerivs (CC BY-NC-ND), which allows third parties to download the articles and share them with others as long as they credit the author and the Abstract Book, but they cannot change the content in any way or use them commercially.
**Abstract Book Citations:** Authors, Title, HemaSphere, 2026;10:(S1):pages. The abstract book DOIs can be found on the "Author Information" page.
**Disclaimer:** Articles published in the journal HemaSphere exclusively reflect the opinions of the authors. The authors are responsible for all content in their abstracts including accuracy of the facts, statements, citing resources, etc.

| Function domains | General population (mean±SD) | CAR-T cohort (mean±SD) | Interpretation |
|---|---|---|---|
| Global health | 71 (22) | 70 (22) | No difference |
| Physical function | 90 (16) | 84 (16) | Slightly reduced in CAR-T cohort |
| Role function | 85 (25) | 72 (29) | Reduced in CAR-T cohort |
| Emotional function | 76 (23) | 79 (17) | Marginally better in CAR-T cohort |
| Cognitive function | 86 (20) | 87 (14) | No difference |
| Social function | 88 (23) | 66 (31) | Reduced in CAR-T cohort |
| **Symptom** | | | |
| Fatigue | 24 (24) | 36 (26) | Increased in CAR-T cohort |
| Pain | 21 (28) | 20 (26) | No difference |
| Insomnia | 22 (30) | 21 (26) | No difference |
| Financial difficulties | 10 (23) | 20 (28) | Higher in CAR-T cohort |

Patients rated the preparatory phase including information about the planned therapy, support from the treatment team and psychological preparation very positive (9±0.8). During and after hospital stay, patients felt very well cared for (9.3±1.3 and 8.8±1.6, respectively). Fear of relapse and safety in the first weeks at home were differently rated from 3-10 and 2-10, respectively. Before treatment, all patients were sure, that the CAR-T therapy was the right decision (8-10) while after treatment 24 out of 27 would definitely choose CAR-T therapy again (see figure 1).

### Summary/Conclusion

This retrospective observational study analyzed all patients who received CAR-T cell therapy at our hospital before the end of September 2025. Compared with the general population, the CAR-T cohort shows preserved global health, cognitive functioning and emotional resilience despite reduced physical, role, and social functioning. Fatigue and financial concerns are more pronounced, while pain and insomnia remain similar to normative data.

**Copyright Information:** (Online) ISSN: 2572-9241
©2026 The Author(s). HemaSphere is published by John Wiley & Sons Ltd on behalf of European Hematology Association. This is an open access Abstract Book distributed under the Attribution-NonCommercial-NoDerivs (CC BY-NC-ND), which allows third parties to download the articles and share them with others as long as they credit the author and the Abstract Book, but they cannot change the content in any way or use them commercially.
**Abstract Book Citations:** Authors, Title, HemaSphere, 2026;10:(S1):pages. The abstract book DOIs can be found on the “Author Information” page.
**Disclaimer:** Articles published in the journal HemaSphere exclusively reflect the opinions of the authors. The authors are responsible for all content in their abstracts including accuracy of the facts, statements, citing resources, etc.

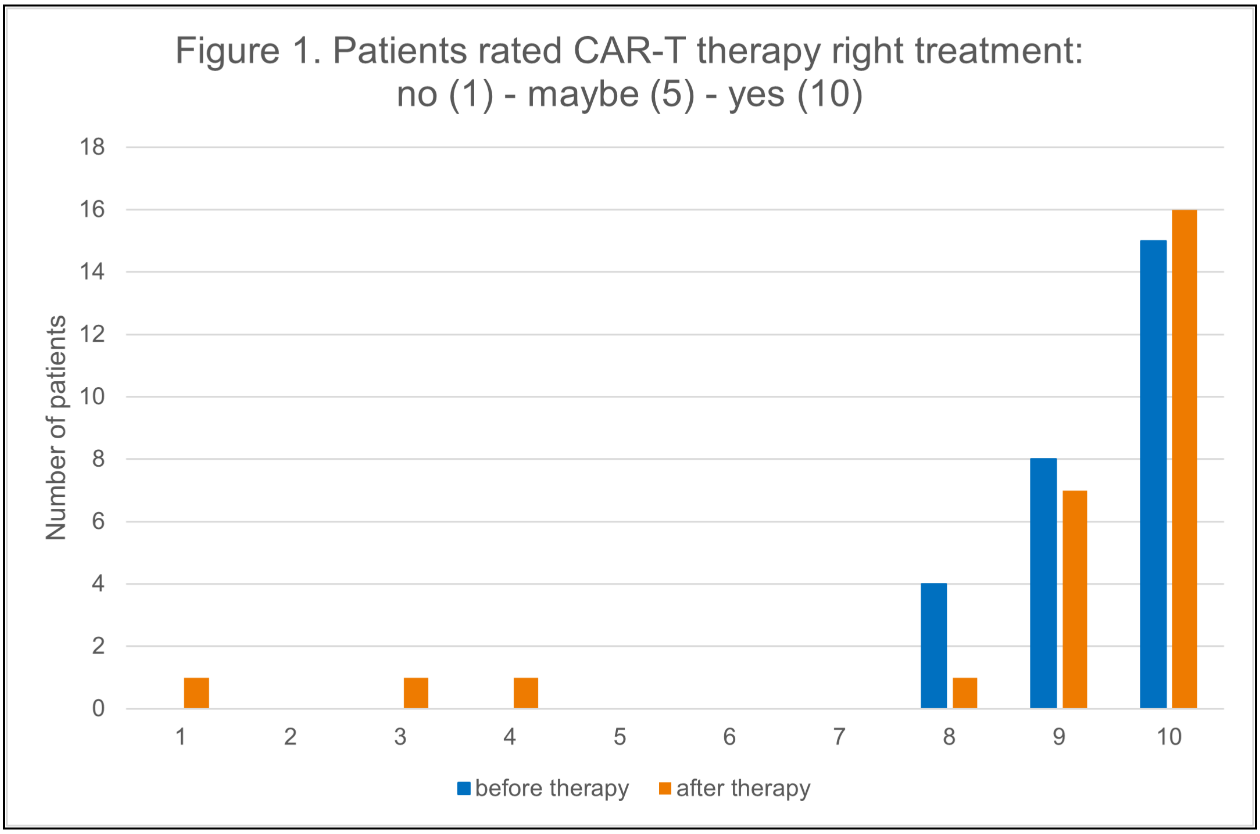

**Copyright Information:** (Online) ISSN: 2572-9241
©2026 The Author(s). HemaSphere is published by John Wiley & Sons Ltd on behalf of European Hematology Association. This is an open access Abstract Book distributed under the Attribution-NonCommercial-NoDerivs (CC BY-NC-ND), which allows third parties to download the articles and share them with others as long as they credit the author and the Abstract Book, but they cannot change the content in any way or use them commercially.
**Abstract Book Citations:** Authors, Title, HemaSphere, 2026;10:(S1):pages. The abstract book DOIs can be found on the "Author Information" page.
**Disclaimer:** Articles published in the journal HemaSphere exclusively reflect the opinions of the authors. The authors are responsible for all content in their abstracts including accuracy of the facts, statements, citing resources, etc.

### (PB4405) UNMET NEEDS AFTER AZACITIDINE FAILURE: REAL-WORLD OUTCOMES AND END-OF-LIFE MANAGEMENT IN MDS AND CMML

**Topic:** 36. Quality of life, ethics, supportive and palliative care

Adriana Cuartas*[1], Inés Zugasti[1], Sandra Castaño-Díez[2, 3], Ferran Balbastre-Ubeda[4], Francisco Jose Marrero[5], Laura Rama Sanchez[6], Carlos Jimenez Vicente[1, 3], Marina Cornejo[1], Albert Cortés[1, 3], Marta Moris[1], Paula Sastre[1], Eduardo Francisco Aguilar[1], Monica Lopez-Guerra[2, 3], Francesca Guijarro Tomas[2, 3], Alexandra Martínez Roca[1], Juan José Rodríguez-Sevilla[1, 3], Aina Cardús[3], Angela Llop[2, 3], Maria Carmen Barrera[7], Manoli Chicote[7], Teresa Gabarro[7], Esther Carcelero[8], Inés Monge[8], David López Sisamon[8], Marta Gomez Nuñez[6], Helena Pomares[5], Montserrat Arnan Sangerman[5], Andres Jerez[4], Albert Tuca[7], Jordi Esteve Reyner[1, 3], Deborath Moreno[7], Marina Diaz Beya[1, 3]

*[1]Hospital Clinic de Barcelona, Hematology, Barcelona, Spain;[2]Hospital Clinic de Barcelona, Hematopathology, Barcelona, Spain; [3]Instituto de Investigaciones Biomédicas August Pi i Sunyer (IDIBAPS), Barcelona, Spain;[4]Vall d'Hebron Institute of Oncology (VHIO), Vall d'Hebron hospital, Barcelona, Spain; [5]Institut Català d'Oncologia-Institut d'Investigació Biomèdica de Bellvitge (IDIBELL), L'Hospitalet de Llobregat, Barcelona, Spain, Barcelona, Spain; [6]Consorcio Corporación Sanitaria Parc Taulí de Sabadell, Hematology, Barcelona, Spain; [7]Hospital Clinic de Barcelona, Palliative care unit, Barcelona, Spain;[8]Hospital Clinic de Barcelona, Pharmacy, Barcelona, Spain;*

### Background

Azacitidine failure (AZA-f) in patients with myelodysplastic syndromes (MDS) and chronic myelomonocytic leukemia (CMML) remains a challenge due to the unfavourable prognosis and the lack of therapeutic options. Moreover, end-of-life management in these patients is not well established.

### Aims

To describe the clinical and biological features, prognosis, treatment options, and end-of-life management of AZA-f patients.

### Methods

We conducted a multicentre retrospective analysis of a cohort of MDS and CMML patients with AZA failure who were not eligible for bone marrow transplantation between 2016 and 2025. High-risk MDS/CMML (MDS/CMML-HR) was defined as IPSS-R > 3.5 or CPSS intermediate-2 or higher. Median follow-up was calculated using the reverse Kaplan–Meier method.

**Copyright Information:** (Online) ISSN: 2572-9241
©2026 The Author(s). HemaSphere is published by John Wiley & Sons Ltd on behalf of European Hematology Association. This is an open access Abstract Book distributed under the Attribution-NonCommercial-NoDerivs (CC BY-NC-ND), which allows third parties to download the articles and share them with others as long as they credit the author and the Abstract Book, but they cannot change the content in any way or use them commercially.
**Abstract Book Citations:** Authors, Title, HemaSphere, 2026;10:(S1):pages. The abstract book DOIs can be found on the "Author Information" page.
**Disclaimer:** Articles published in the journal HemaSphere exclusively reflect the opinions of the authors. The authors are responsible for all content in their abstracts including accuracy of the facts, statements, citing resources, etc.

**Results**

A total of 153 patients were included (**Figure 1A**). With a median follow-up of 64 months, overall survival (OS) after AZA-f was 7.5 months (95% CI, 6.2–11.8). For high-risk MDS/CMML, OS was 6.2 months (95% CI, 5.1–8.4), whereas low-risk patients had a median OS of 45.5 months (95% CI, 18.1–not reached).

At the time of AZA-f, only 88 patients (57.5%) were re-evaluated. Molecular tests were performed in only 37 cases (24%). At this time point, the presence of >10% blasts was associated with lower overall survival (5.9 months [95% CI, 4.5–8.6] vs 12.5 months [95% CI, 6.1–61.6]; p = 0.004).

Only 38 patients (24.8%) received a subsequent line of treatment after AZA-f, 17 of whom (44.7%) were enrolled in a clinical trial. The overall response rate (ORR) was 47.36% (cCR 31.5%, n = 12), with a median duration of response of 8.6 months (95% CI, 5.8–10). The most frequent second-line therapy was venetoclax plus hypomethylating agents (n = 13), with an ORR of 46.15% (cCR 38.5%). Patients who received treatment after AZA-f had significantly longer overall survival compared with those who received no further therapy (14.5 months [95% CI, 9.8–36] vs 6.3 months [95% CI, 4.8–9.6]; p = 0.002).

Palliative care support was initiated in 88 patients (57.5%), but referral occurred at the time of AZA-f in only 21 (13.7%). The median time to palliative care activation after AZA-f was 3.2 months (95% CI, 1.97–5.51) for patients who did not receive subsequent treatment versus 9.2 months (95% CI, 4.6–25.30) for those who did (p= 0.0044). Clinical follow-up was mainly performed by primary care support units (63.6%). Most patients referred to palliative care units were transfusion-dependent (78.4% for red blood cell units and 20.5% for platelet units). Median OS after palliative care activation was 37 days (95% CI, 25–57). Among deceased patients, those followed by support units were significantly less likely to die in hospital (42% vs 73%; p = 0.03). **Figure 1B** summarizes end-of-life management.

**Summary/Conclusion**

In high-risk MDS/CMML patients after AZA failure, therapeutic options are limited and prognosis remains poor. High-risk disease before AZA treatment and the presence of >10% blasts at AZA-f were identified as poor prognostic factors. A high proportion of these patients required red blood cell transfusion support once enrolled in Home Care Program and Support Teams Unit, a situation likely different from other oncologic scenarios managed by palliative care teams. Given the unfavourable outcomes, it is essential to facilitate access to second-line therapies, which may help improve survival, as well as to ensure the early integration of palliative care to optimize the management of these patients.

**Copyright Information:** (Online) ISSN: 2572-9241
©2026 The Author(s). HemaSphere is published by John Wiley & Sons Ltd on behalf of European Hematology Association. This is an open access Abstract Book distributed under the Attribution-NonCommercial-NoDerivs (CC BY-NC-ND), which allows third parties to download the articles and share them with others as long as they credit the author and the Abstract Book, but they cannot change the content in any way or use them commercially.
**Abstract Book Citations:** Authors, Title, HemaSphere, 2026;10:(S1):pages. The abstract book DOIs can be found on the "Author Information" page.
**Disclaimer:** Articles published in the journal HemaSphere exclusively reflect the opinions of the authors. The authors are responsible for all content in their abstracts including accuracy of the facts, statements, citing resources, etc.

Figure 1A.

| **Baseline characteristics at AZA initiation** | ***n*=153** |
|---|---|
| Age, years, median (range) | 77 (54-91) |
| Sex (male), n (%) | 108 (70.58) |
| ***IPSS-R,/ CPSS n (%)*** | ***n=136 (89)/ n=17 (11)*** |
| Lowrisk | 19 (14)/10 (58.8) |
| High risk | 117 (86)/ 7 (41.2) |
| **Overall response rate to AZA, n (%)** | 79 (51.6) |
| Median number of AZA cycles (range) | 8 (1-84) |
| **Characteristics at AZA-f** | ***n* = 88** |
| Relapse AML | 37 |
| Relapse MDS/CMML | 46 |
| Number of blasts, median (range) | 8% (1-19%) |
| ***IPSS-R, n / CPSS, n*** | ***n=42/ n=9*** |
| Lowrisk | 4 (9.5) / 4 (45) |
| High risk | 38 (90.5) / 5 (55) |
| ***2nd line therapy, n (%)*** | ***n = 38 (24.8)*** |
| Venetoclax-azacitidine/decitabine | 13 (33.3) |
| Clinicaltrial<br>Targeted theraphies (inhibitor *IDH*1/2 or FLT3) | 17 (44.7)<br>3 (17.6) |
| Chemotherapy regimens | 2 (6) |
| Lenalidomide | 3 (8) |
| ***ORR 2nd line therapy, n (%)*** | 18 (47.3) |

**Figure 1B: Flowchart end-of-life management**

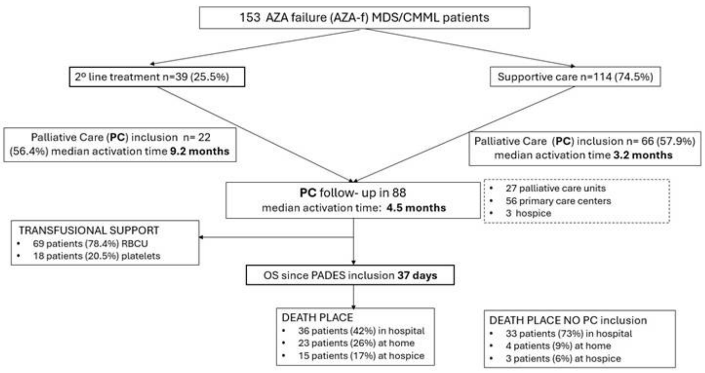

Figure 1. Figure 1A: Characteristics of the cohort. Figure 1B: Flowchart end-of-life management

**Copyright Information:** (Online) ISSN: 2572-9241
©2026 The Author(s). HemaSphere is published by John Wiley & Sons Ltd on behalf of European Hematology Association. This is an open access Abstract Book distributed under the Attribution-NonCommercial-NoDerivs (CC BY-NC-ND), which allows third parties to download the articles and share them with others as long as they credit the author and the Abstract Book, but they cannot change the content in any way or use them commercially.
**Abstract Book Citations:** Authors, Title, HemaSphere, 2026;10:(S1):pages. The abstract book DOIs can be found on the "Author Information" page.
**Disclaimer:** Articles published in the journal HemaSphere exclusively reflect the opinions of the authors. The authors are responsible for all content in their abstracts including accuracy of the facts, statements, citing resources, etc.

### (PB4406) SUPPORTIVE CARE NEEDS IN PATIENTS WITH LYMPHOMA

**Topic:** 36. Quality of life, ethics, supportive and palliative care

Mengyang Di*[1, 2], Chaitra Ujjani[1, 2], Stephen Smith[1, 2], Mazyar Shadman[1], Ryan Lynch[1, 2], Paul Martin[1, 2], Brian Till[1], Jennifer Huang[1], Kerry McMillen[1], Ajay Gopal[1, 2]

*[1]Fred Hutchinson Cancer Center, Seattle, United States of America;[2]University of Washington, Seattle, United States of America;*

### Background

Lymphoma is a heterogeneous group of malignancies and patients with different subtypes may exhibit varied supportive care needs. Recognizing these needs can help tailor supportive care services and improve patient outcomes. Since data on supportive care needs by lymphoma subtypes are lacking, we comprehensively examined these in our analysis.

### Methods

Our center has launched a supportive care screening program where all cancer patients are asked to complete a self-administered questionnaire at referral. The questionnaire covers 11 domains (please see domain details in **Figure**). For this analysis, we selected adult patients with lymphoma who were referred to our center 1/1/2023-7/31/2025 and completed the questionnaire. We analyzed descriptively the baseline characteristics and supportive care needs for any domain and for each domain individually. We examined the difference in supportive care need for each domain and for any domain, respectively, across lymphoma subtypes, using chi-square or Fisher's exact tests. Lymphoma subtypes were categorized by aggressive B (BCL) and T cell lymphoma (TCL), Hodgkin (HL), and indolent BCL and TCL. We also performed multivariable logistic regression to examine the associations between lymphoma subtypes and supportive care need for any domain, reporting odds ratio (OR) with 95% confidence interval (CI). Covariates included age, sex, race, and ethnicity.

**Copyright Information:** (Online) ISSN: 2572-9241
©2026 The Author(s). HemaSphere is published by John Wiley & Sons Ltd on behalf of European Hematology Association. This is an open access Abstract Book distributed under the Attribution-NonCommercial-NoDerivs (CC BY-NC-ND), which allows third parties to download the articles and share them with others as long as they credit the author and the Abstract Book, but they cannot change the content in any way or use them commercially.
**Abstract Book Citations:** Authors, Title, HemaSphere, 2026;10:(S1):pages. The abstract book DOIs can be found on the "Author Information" page.
**Disclaimer:** Articles published in the journal HemaSphere exclusively reflect the opinions of the authors. The authors are responsible for all content in their abstracts including accuracy of the facts, statements, citing resources, etc.

### Results

We included 663 patients (**Table**); 69%-79% patients with aggressive lymphoma (aggressive BCL and TCL, and HL, **Figure**) required any supportive care. In the univariable analysis across lymphoma subtypes (**Figure**), social work and nutrition needs were higher in patients with aggressive lymphoma. Physical therapy and palliative care needs were higher among those with aggressive BCL and TCL. Spiritual care need was particularly higher in patients with aggressive TCL, possibly driven by prognosis and non-White population (the latter often associated with higher spiritual care need). Whereas fertility care need was predominantly higher in those with HL, likely due to younger age. The supportive care need in any domain was significantly higher in patients with aggressive lymphoma. In the multivariable analysis, increased need of any supportive care was seen in patients with aggressive BCL (OR: 2.45, 95% CI: 1.67-3.61) and TCL (OR: 3.21, 95% CI: 1.22-8.43), compared with those with indolent BCL (reference). The need was similar in HL and indolent TCL, compared with the reference group. Age was the only other covariant associated with any supportive care need.

**Table. Baseline characteristics of the lymphoma population**

| | Aggressive BCL (N=235) | Aggressive TCL (N=29) | Hodgkin (N=55) | Indolent BCL (N=306) | Indolent TCL (N=38) | Overall (N=663) |
|---|---|---|---|---|---|---|
| Age, years, median (IQR) | 65 (54, 72) | 68 (54, 71) | 37 (28, 55) | 67 (57, 73) | 67 (53, 76) | 65 (54, 72) |
| Sex, male, n (%) | 143 (61) | 19 (66) | 30 (55) | 171 (56) | 24 (63) | 387 (58) |
| Race, White, n (%) | 200 (85) | 19 (66) | 44 (80) | 272 (89) | 33 (87) | 568 (86) |
| Ethnicity, non-Hispanic, n (%) | 214 (91) | 27 (93) | 48 (87) | 274 (90) | 32 (84) | 595 (90) |

### Summary/Conclusion

Our study showed a high need for supportive care in aggressive lymphoma, particularly aggressive TCL. Different supportive care needs across lymphoma subtypes were generally consistent with disease prognosis and demographic distributions. Our findings underscore the need of routine supportive care screening in aggressive lymphoma and potentially tailoring the screening tools for different subtypes.

**Copyright Information:** (Online) ISSN: 2572-9241
©2026 The Author(s). HemaSphere is published by John Wiley & Sons Ltd on behalf of European Hematology Association. This is an open access Abstract Book distributed under the Attribution-NonCommercial-NoDerivs (CC BY-NC-ND), which allows third parties to download the articles and share them with others as long as they credit the author and the Abstract Book, but they cannot change the content in any way or use them commercially.
**Abstract Book Citations:** Authors, Title, HemaSphere, 2026;10:(S1):pages. The abstract book DOIs can be found on the “Author Information” page.
**Disclaimer:** Articles published in the journal HemaSphere exclusively reflect the opinions of the authors. The authors are responsible for all content in their abstracts including accuracy of the facts, statements, citing resources, etc.

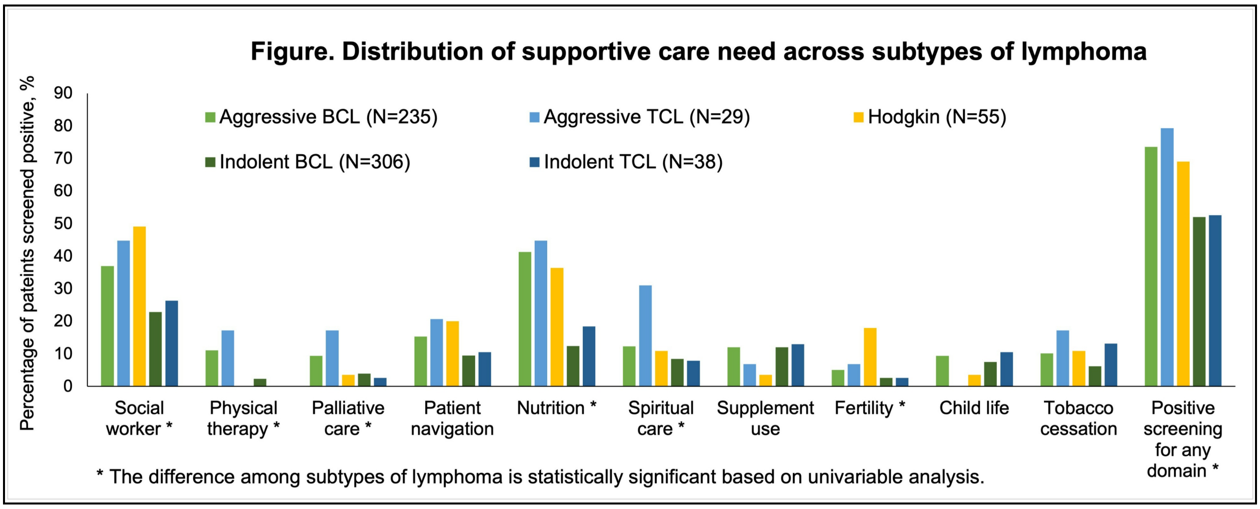

**Copyright Information:** (Online) ISSN: 2572-9241
©2026 The Author(s). HemaSphere is published by John Wiley & Sons Ltd on behalf of European Hematology Association. This is an open access Abstract Book distributed under the Attribution-NonCommercial-NoDerivs (CC BY-NC-ND), which allows third parties to download the articles and share them with others as long as they credit the author and the Abstract Book, but they cannot change the content in any way or use them commercially.
**Abstract Book Citations:** Authors, Title, HemaSphere, 2026;10:(S1):pages. The abstract book DOIs can be found on the "Author Information" page.
**Disclaimer:** Articles published in the journal HemaSphere exclusively reflect the opinions of the authors. The authors are responsible for all content in their abstracts including accuracy of the facts, statements, citing resources, etc.

### (PB4407) DEVELOPMENT OF THE ICMDS-PRO SCORE: AN INTERNATIONAL SURVEY OF PATIENT PRIORITIES ACROSS RISK GROUPS

**Topic:** 36. Quality of life, ethics, supportive and palliative care

Fabio Efficace*[1], Amer M. Zeidan[2], Gregory Abel[3], Esther N. Oliva[4], Daniela Krepper[1, 5], Lea Harrison[6], Alice Houk[7], Adel Naeem Abdullah Al-Obaidi[8], Katharina Götze[9], Ulrich Germing[10], Moshe Mittelman[11], Carmelo Gurnari[12, 13], Massimo Breccia[14], Adriano Venditti[13], Yu-Hung Wang[15], Francesca Tartaglia[1], Micaela Quarchioni[16], Fernando Barroso[17], Pierre Fenaux[18], Mikkael A. Sekeres[19], Rena Buckstein[20]

*[1]Italian Group for Adult Hematologic Diseases (GIMEMA), Data Center and Health Outcomes Research Unit, Rome, Italy, Rome, Italy; [2]Yale School of Medicine and Yale Comprehensive Cancer Center, Yale University, Section of Hematology, Department of Internal Medicine, New Haven, United States of America; [3]Dana-Farber Cancer Institute, Boston, MA; Harvard Medical School, Boston, United States of America; [4]4. Hematology Department, London North West University Healthcare NHS Trust, London, United Kingdom; [5]University Hospital of Psychiatry II, Medical University of Innsbruck, Innsbruck, Austria;[6]MDS Foundation Inc., New York, United States of America; [7]Aplastic Anemia & MDS International Foundation, Rockville, United States of America; [8]Adult Nursing, College of Nursing, University of AL Esraa, Iraq; Hematology and Bone Marrow Transplant Center, Medical City Complex, Ministry of Health, Baghdad, Iraq; [9]Department of Medicine III, Technical University of Munich School of Medicine and Health, Munich, Germany; [10]Clinic for Haematology, Oncology and Clinical Immunology, Düsseldorf University Hospital, Heinrich Heine University, Düsseldorf, Germany; [11]Department of Hematology, Tel-Aviv Sourasky Medical Center; Tel-Aviv University, School of Medicine, Tel-Aviv, Israel; [12]Translational Hematology & Oncology Research, Cleveland Clinic, Cleveland, United States of America; [13]Hematology Department of Biomedicine and Prevention, University of Rome "Tor Vergata", Rome, Italy; [14]Department of Translational and Precision Medicine, Hematology Sapienza University, Rome, Italy; [15]Department of Hematological Oncology, National Taiwan University Cancer Center, Taipei, Taiwan; [16]Hematology and Bone Marrow Transplant Unit, British Hospital of Buenos Aires, Buenos Aires, Argentina; [17]Department of Hematology, Hospital Universitario Walter Cantídio, Universidade Federal do Ceara, Fortaleza, Brazil; [18]Département (DMU) d'hématologie et immunologie, APHP Nord Service d'hématologie seniors Hôpital St Louis/université de Paris, Paris, France; [19]Sylvester Comprehensive Cancer Center, University of Miami, Miami, United States of America; [20]Department of Medicine, University of Toronto; Odette Cancer Centre, Sunnybrook Health Sciences Centre; Sunnybrook Research Institute, Toronto, Canada;*

### Background

In the rapidly evolving therapeutic landscape of myelodysplastic syndromes (MDS) the assessment of patient-reported outcomes (PROs) has become critical for informing benefit-risk evaluation of novel therapies in clinical trials.

### Aims

To develop a brief, harmonized, trial-ready PRO tool (icMDS PRO score) to support standardized assessment in clinical research and to identify and prioritize outcomes considered most important by patients with MDS across risk groups.

**Copyright Information:** (Online) ISSN: 2572-9241
©2026 The Author(s). HemaSphere is published by John Wiley & Sons Ltd on behalf of European Hematology Association. This is an open access Abstract Book distributed under the Attribution-NonCommercial-NoDerivs (CC BY-NC-ND), which allows third parties to download the articles and share them with others as long as they credit the author and the Abstract Book, but they cannot change the content in any way or use them commercially.
**Abstract Book Citations:** Authors, Title, HemaSphere, 2026;10:(S1):pages. The abstract book DOIs can be found on the "Author Information" page.
**Disclaimer:** Articles published in the journal HemaSphere exclusively reflect the opinions of the authors. The authors are responsible for all content in their abstracts including accuracy of the facts, statements, citing resources, etc.

### Methods

An issue list was developed through a systematic review of PRO measures used in MDS and refined with clinician input. Twenty-three issues were identified, covering symptoms, functional impairment, social impact, and treatment burden, including time spent on healthcare. Patients were asked to rate each issue on a 9-point Likert scale and to suggest additional items. An anonymized online survey collected sociodemographic and clinical data (self-assessed MDS risk group [lower vs higher], treatment status, comorbidities, disease duration). Treatment goals: quality of life (QoL), life expectancy, reduced transfusion need, and ease of administration were rated on a 5-point Likert scale. The survey was disseminated with the support of patient organizations, including the MDS Foundation and the Aplastic Anemia and MDS International Foundation (AAMDSIF), as well as professional networks. Ratings were summarized using means (SD) and compared between risk groups using the Wilcoxon rank-sum test. Analyses were conducted using all available data, with item-specific sample sizes varying due to missing responses.

### Results

A total of 348 patients from 17 countries provided informed consent to complete the survey. Most respondents were aged ≥60 years (82%), including 19% aged ≥80 years; 51% were male. Among 207 patients (59.5%) with evaluable information on disease risk, 64.3% had lower-risk and 35.7% higher-risk disease. Thirty-one percent had lived with MDS for >5 years; 54% were receiving active therapy, and 71.1% reported ≥1 comorbidity.
The highest-ranked issues were fatigue (mean 5.5, SD 2.9), physical fitness (mean 5.2, SD 2.9), impact on daily life (mean 5.0, SD 3.0), time spent on healthcare (mean 4.9, SD 2.7), and impact on social life (mean 4.3, SD 2.9), underscoring the central role of functional limitation and healthcare-related time burden in patients' lived experience.
Priorities differed significantly between lower- and higher-risk groups for 18 of 23 issues ($p<0.05$), demonstrating heterogeneity across risk categories. The largest differences were observed for time spent on healthcare ($\Delta=2.4$, $p<0.001$), impact on social life ($\Delta=2.4$, $p<0.001$), and change in caregiving ability ($\Delta=2.3$, $p<0.001$), all rated as more important by patients with higher-risk disease.
Importantly, improved QoL was rated as the most important treatment benefit (mean 4.5, SD 1.1), exceeding life expectancy (mean 4.2, SD 1.3) and reduction in transfusion need, highlighting a potential misalignment between patient priorities and traditional survival-dominated endpoint hierarchies.

### Summary/Conclusion

These findings provide empirical evidence on patient priorities across MDS risk groups and support the development of harmonized, trial-ready brief PRO tools. The observed heterogeneity between lower- and higher-risk disease suggests that risk-adapted versions of the icMDS-PRO score may further enhance alignment with disease-specific needs in future trials. Prospective psychometric validation is planned.

**Copyright Information:** (Online) ISSN: 2572-9241
©2026 The Author(s). HemaSphere is published by John Wiley & Sons Ltd on behalf of European Hematology Association. This is an open access Abstract Book distributed under the Attribution-NonCommercial-NoDerivs (CC BY-NC-ND), which allows third parties to download the articles and share them with others as long as they credit the author and the Abstract Book, but they cannot change the content in any way or use them commercially.
**Abstract Book Citations:** Authors, Title, HemaSphere, 2026;10:(S1):pages. The abstract book DOIs can be found on the "Author Information" page.
**Disclaimer:** Articles published in the journal HemaSphere exclusively reflect the opinions of the authors. The authors are responsible for all content in their abstracts including accuracy of the facts, statements, citing resources, etc.

### (PB4408) THE EFFECT OF A MINDFULNESS-BASED CANCER REHABILITATION PROGRAM BASED ON THE LIVERPOOL MINDFULNESS MODEL IN PATIENTS WITH MALIGNANT HEMATOLOGIC DISEASES: A QUASI-EXPERIMENTAL STUDY

**Topic:** 36. Quality of life, ethics, supportive and palliative care

Rongrong Wang[1], Jie Liu*[1], Jianlan Ren[1], Wei Zhang[1]

[1] *Tianjin Medical University General Hospital, Tianjin, China;*

### Background

Mindfulness-based cancer recovery training serves as an effective non-pharmacological complementary and alternative medicine intervention for improving negative emotions and treatment adherence among cancer patients, with widespread application in breast cancer patients. However, compared to solid tumor patients, those with malignant hematologic diseases face more intensive, prolonged, and toxic treatments due to the high heterogeneity of disease trajectories and prognoses. Furthermore, the timing, content, and outcomes of MBCR implementation among hematologic malignancy patients in China vary significantly. There is no standardized protocol for MBCR in this population, existing protocols lack detailed implementation guidelines, and the qualifications of intervention providers are inconsistent. Some clinicians, lacking a robust scientific foundation, adopt a “one-size-fits-all” approach when applying MBCR across different patient groups.

### Aims

To develop a mindfulness-based cancer rehabilitation training program for patients with malignant hematological diseases using the Liverpool Mindfulness Model as a theoretical framework, and to explore its effects on anxiety, depression, and insomnia in these patients, thereby providing practical methods for improving their physical and psychological symptoms.

### Methods

Using convenience sampling, patients with malignant hematological diseases who regularly visited a tertiary-level general hospital in Tianjin from May to December 2025 were selected as study subjects. Subjects enrolled from May to August 2025 formed the control group, receiving conventional psychological support measures including addressing patients' disease knowledge needs and verbal reassurance. Participants enrolled from September to December 2025 formed the experimental group, receiving a 6-week mindfulness-based cancer rehabilitation training program developed based on the Liverpool Mindfulness Model. Patients were assessed using the Generalized Anxiety Disorder-7 (GAD-7), Patient Health Questionnaire-9 (PHQ-9), and Insomnia Severity Index（ISI） at three time points: within one week of admission (T0), 24 hours after intervention completion (T1), and 30 days post-intervention (T2). Differences in scores between the two patient groups were compared. A generalized estimating equations (GEE) model was employed to examine the effects of time, group, and their interaction.

### Results

A total of 35 patients in the intervention group and 37 in the control group participated in and completed the study. At baseline, the intervention and control groups showed statistically significant differences in age ($P<0.001$), educational attainment ($P=0.013$), and disease diagnosis ($P<0.001$). After adjusting for these covariates, the intervention group showed significant improvements in anxiety, depression, and insomnia compared to the control group (e.g., $\beta$ value for anxiety within 24 hours after the 6-week intervention = -2.700, 95% confidence interval: -3.972 to -1.429, $p<0.001$; $\beta$ value at 1 month post-intervention = -3.309, 95% CI: -1.52 to -5.018, $p<0.001$).

**Copyright Information:** (Online) ISSN: 2572-9241
©2026 The Author(s). HemaSphere is published by John Wiley & Sons Ltd on behalf of European Hematology Association. This is an open access Abstract Book distributed under the Attribution-NonCommercial-NoDerivs (CC BY-NC-ND), which allows third parties to download the articles and share them with others as long as they credit the author and the Abstract Book, but they cannot change the content in any way or use them commercially.
**Abstract Book Citations:** Authors, Title, HemaSphere, 2026;10:(S1):pages. The abstract book DOIs can be found on the “Author Information” page.
**Disclaimer:** Articles published in the journal HemaSphere exclusively reflect the opinions of the authors. The authors are responsible for all content in their abstracts including accuracy of the facts, statements, citing resources, etc.

### Summary/Conclusion

The mindfulness-based cancer rehabilitation training program for malignant hematologic diseases, developed based on the Liverpool Mindfulness Model, significantly improves patients' anxiety, depression, and insomnia.

**The comparison of outcome variables by generalized estimating equation modeling.**

| Outcome | Time | Control group | Intervention group | Group effect | Time effect | Time*Group effect |
|---|---|---|---|---|---|---|
| | | M (P25, P75) | M (P25, P75) | β (95%CI), P | β (95%CI), P | β (95%CI), P |
| Anxiety | T0 | 7.00 (5.00, 10.00) | 5.00 (5.00, 7.00) | -1.551(−3.257,0.154), 0.075 | | |
| | T1 | 7.00 (5.00, 8.00) | 3.00 (2.00, 4.00) | | −0.757(−1.790,0.277), 0.069 | −2.700(−3.972, −1.429), <0.001 |
| | T2 | 5.00 (3.50, 7.00) | 2.00 (0, 2.00) | | −1.405(−2.921,0.110), 0.151 | −3.309(−5.018, −1.599), <0.001 |
| Depression | T0 | 7.00 (5.00, 11.00) | 7.00 (6.00, 9.00) | −0.320(−2.275,1.635), 0.748 | | |
| | T1 | 9.00 (6.00, 11.00) | 4.00 (3.00, 5.00) | | 0.135(−1.406,1.676), 0.864 | −3.964(−5.722, −2.205), <0.001 |
| | T2 | 8.00 (5.00, 11.50) | 3.00 (2.00, 3.00) | | −0.108 (−1.967,1.751), 0.909 | −5.292(−7.385, −3.199), <0.001 |
| Insomnia | T0 | 8.00 (5.00, 13.00) | 7.00 (5.00, 10.00) | −0.728(−3.407,1.951), 0.594 | | |
| | T1 | 9.00 (5.50, 12.50) | 4.00 (3.00, 6.00) | | −0.108(−1.889,2.105), 0.916 | −3.508(−5.713, −1.303), 0.002 |
| | T2 | 8.00 (5.00, 10.00) | 3.00 (1.00, 4.00) | | −1.216(−3.228,0.796), 0.236 | −3.898(−6.246, −1.550), 0.001 |

**Copyright Information:** (Online) ISSN: 2572-9241
©2026 The Author(s). HemaSphere is published by John Wiley & Sons Ltd on behalf of European Hematology Association. This is an open access Abstract Book distributed under the Attribution-NonCommercial-NoDerivs (CC BY-NC-ND), which allows third parties to download the articles and share them with others as long as they credit the author and the Abstract Book, but they cannot change the content in any way or use them commercially.
**Abstract Book Citations:** Authors, Title, HemaSphere, 2026;10:(S1):pages. The abstract book DOIs can be found on the "Author Information" page.
**Disclaimer:** Articles published in the journal HemaSphere exclusively reflect the opinions of the authors. The authors are responsible for all content in their abstracts including accuracy of the facts, statements, citing resources, etc.

### (PB4409) TEMPORAL RELATIONSHIP BETWEEN CARDIAC TROPONIN I ELEVATION AND ECHOCARDIOGRAPHIC DETECTION OF CANCER THERAPY–RELATED CARDIAC DYSFUNCTION IN ANTHRACYCLINE-TREATED NON-HODGKIN LYMPHOMA

**Topic:** 36. Quality of life, ethics, supportive and palliative care

Ryotaro Niwa*[1], Naru Tomigaki[1], Akimasa Sakamoto[1], Isamu Harima[1], Hiroaki Akiyama[1], Ikumi Takagi[1], Yoko Koduki[1], Yoshiharu Miyata[2], Nobuko Iwata[1], Seiji Kakiuchi[1]

*[1]Yodogawa Christian Hospital, Hematology, Osaka, Japan; [2]Kobe University Graduate School of Medicine, Department of AI and Digital Health Science, Hyogo, Japan;*

-->
### Background

Cancer therapy–related cardiac dysfunction (CTRCD) is an important complication of anthracycline therapy in patients with non-Hodgkin lymphoma (NHL). Contemporary cardio-oncology guidelines recommend surveillance with cardiac biomarkers and echocardiography including global longitudinal strain (GLS); however, frequent echocardiographic monitoring can be difficult to implement in routine hematology practice because of limited imaging resources and competing clinical priorities. Cardiac troponin I (TnI) reflects myocardial injury and may precede overt ventricular dysfunction, yet the temporal relationship between TnI elevation and echocardiographic detection of CTRCD in real-world hematology care remains incompletely characterized.

-->
### Aims

To characterize the temporal relationship between TnI elevation and echocardiographic detection of CTRCD in anthracycline-treated NHL patients in routine clinical practice.

-->
### Methods

We conducted a retrospective analysis of consecutive adult patients with NHL who received doxorubicin-containing chemotherapy between April 2023 and October 2025 at a single center. Patients with baseline left ventricular ejection fraction (LVEF) <50% or significant structural heart disease were excluded. High-sensitivity cardiac TnI was routinely measured before each chemotherapy cycle during treatment using a commercially available high-sensitivity immunoassay (upper limit of normal 25.0 ng/L). Additional post-treatment TnI measurements were included when available. Echocardiography including LVEF and GLS was performed at baseline and post-treatment, with additional imaging as clinically indicated. CTRCD was defined as a ≥10% decline in LVEF to <50% and/or a ≥15% relative reduction in GLS.

was low in 10 patients (19.6%), moderate in 30 (58.8%), and high in 11 (21.6%). Mean baseline LVEF and GLS were 64.9 ± 4.2% and −18.7 ± 2.2%, respectively. TnI elevation occurred in 28 patients (54.9%) at a median of chemotherapy cycle 5 (interquartile range [IQR] 4–6). Among TnI-positive patients, CTRCD was identified in 14 (50.0%). In contrast, among the 23 patients without TnI elevation, only one developed CTRCD (4.3%). No patient without TnI elevation developed symptomatic heart failure during a median follow-up of 14 months (IQR 7.0–21.5). Overall, CTRCD developed in 15 patients (29.4%). Among the 14 TnI-positive CTRCD cases, echocardiographic abnormalities were detected at the same treatment cycle as the first TnI elevation in 10 patients, 1 month later in 2 patients, 2 months later in 1 patient, and 3 months

**Copyright Information:** (Online) ISSN: 2572-9241
©2026 The Author(s). HemaSphere is published by John Wiley & Sons Ltd on behalf of European Hematology Association. This is an open access Abstract Book distributed under the Attribution-NonCommercial-NoDerivs (CC BY-NC-ND), which allows third parties to download the articles and share them with others as long as they credit the author and the Abstract Book, but they cannot change the content in any way or use them commercially.
**Abstract Book Citations:** Authors, Title, HemaSphere, 2026;10:(S1):pages. The abstract book DOIs can be found on the "Author Information" page.
**Disclaimer:** Articles published in the journal HemaSphere exclusively reflect the opinions of the authors. The authors are responsible for all content in their abstracts including accuracy of the facts, statements, citing resources, etc.

later in 1 patient. The median interval between the first TnI rise and CTRCD detection was 0 months (IQR 0–1). The median cumulative doxorubicin dose at CTRCD detection was 230 mg/m².

-->
**Results**

A total of 51 patients were included (median age 70 years; 23 males). Baseline Heart Failure Association-International Cardio-Oncology Society (HFA-ICOS) risk score was low in 10 patients (19.6%), moderate in 30 (58.8%), and high in 11 (21.6%). Mean baseline LVEF and GLS were 64.9 ± 4.2% and −18.7 ± 2.2%, respectively. TnI elevation occurred in 28 patients (54.9%) at a median of chemotherapy cycle 5 (interquartile range [IQR] 4–6). Among TnI-positive patients, CTRCD was identified in 14 (50.0%). In contrast, among the 23 patients without TnI elevation, only one developed CTRCD (4.3%). No patient without TnI elevation developed symptomatic heart failure during a median follow-up of 14 months (IQR 7.0–21.5). Overall, CTRCD developed in 15 patients (29.4%). Among the 14 TnI-positive CTRCD cases, echocardiographic abnormalities were detected at the same treatment cycle as the first TnI elevation in 10 patients, 1 month later in 2 patients, 2 months later in 1 patient, and 3 months later in 1 patient. The median interval between the first TnI rise and CTRCD detection was 0 months (IQR 0–1). The median cumulative doxorubicin dose at CTRCD detection was 230 mg/m².

-->
**Summary/Conclusion**

In this real-world cohort of anthracycline-treated NHL patients, TnI elevation showed a close temporal association with echocardiographic detection of CTRCD, with abnormalities most frequently identified at the same treatment cycle or shortly thereafter. These findings suggest that TnI elevation is typically detected concurrently with, or shortly before, imaging-detectable cardiac dysfunction rather than substantially preceding it. Prospective studies are warranted to further define the clinical implications of this temporal relationship.

**Copyright Information:** (Online) ISSN: 2572-9241
©2026 The Author(s). HemaSphere is published by John Wiley & Sons Ltd on behalf of European Hematology Association. This is an open access Abstract Book distributed under the Attribution-NonCommercial-NoDerivs (CC BY-NC-ND), which allows third parties to download the articles and share them with others as long as they credit the author and the Abstract Book, but they cannot change the content in any way or use them commercially.
**Abstract Book Citations:** Authors, Title, HemaSphere, 2026;10:(S1):pages. The abstract book DOIs can be found on the "Author Information" page.
**Disclaimer:** Articles published in the journal HemaSphere exclusively reflect the opinions of the authors. The authors are responsible for all content in their abstracts including accuracy of the facts, statements, citing resources, etc.

**Figure 1.** Temporal relationship between cardiac troponin I (TnI) elevation and echocardiographic detection of cancer therapy–related cardiac dysfunction (CTRCD).

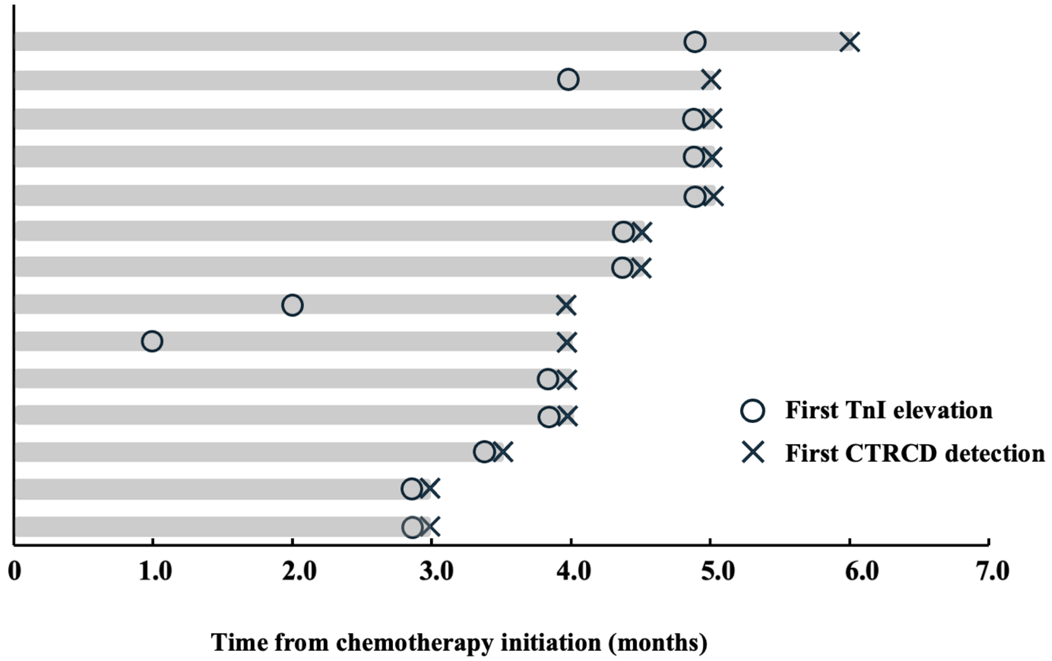

**Copyright Information:** (Online) ISSN: 2572-9241
©2026 The Author(s). HemaSphere is published by John Wiley & Sons Ltd on behalf of European Hematology Association. This is an open access Abstract Book distributed under the Attribution-NonCommercial-NoDerivs (CC BY-NC-ND), which allows third parties to download the articles and share them with others as long as they credit the author and the Abstract Book, but they cannot change the content in any way or use them commercially.
**Abstract Book Citations:** Authors, Title, HemaSphere, 2026;10:(S1):pages. The abstract book DOIs can be found on the "Author Information" page.
**Disclaimer:** Articles published in the journal HemaSphere exclusively reflect the opinions of the authors. The authors are responsible for all content in their abstracts including accuracy of the facts, statements, citing resources, etc.

### (PB4410) DEVELOPMENT OF A MULTIVARIATE REGRESSION-BASED PREDICTIVE MODEL FOR OPTIMAL INTERNAL JUGULAR VEIN CATHETER INSERTION DEPTH INPATIENTS WITH HEMATOLOGICAL DISEASES

**Topic:** 36. Quality of life, ethics, supportive and palliative care

Jing Zhao*[1, 2], Jianli Zhang[1, 2], Hongyan Shen[1, 2], Juanjuan Liu[1, 2], Xiaoyu Zhou[1, 2], Liwei Xu[1, 2], Yibo Wu[1, 3, 4, 5], Aiyun Jin[1, 2]

*[1]Bone Marrow Transplantation Center, the First Affiliated Hospital, Zhejiang University School of Medicine, Hangzhou, China;[2]the First Affiliated Hospital, Zhejiang University School of Medicine, Department of Nursing, Hangzhou, China; [3]Liangzhu Laboratory, Hangzhou, China; [4]Institute of Hematology, Zhejiang University, Hangzhou, China;[5]Zhejiang Province Engineering Research Center for Stem Cell and Immunity Therapy,, Hangzhou, China;*

### Background

The internal jugular vein (IJV) catheter is a commonly used central venous access device for patients with hematological diseases undergoing chemotherapy or hematopoietic stem cell transplantation. Accurate IJV catheter tip positioning is crucial for its safe and effective use. While intracavitary electrocardiography (IC-ECG) facilitates real-time catheter tip localization by detecting characteristic P-wave changes, this technique does not account for individual anatomical variations associated with age, sex, height, and weight. As a result, a clinically relevant discrepancy often remains between the depth achieved under IC-ECG guidance and the radiologically confirmed optimal position. Currently, no validated method exists for real-time prediction of optimal IJV catheter insertion depth during the procedure.

### Aims

To develop and validate an individualized predictive model for optimal right IJV catheter insertion depth by integrating patient-specific variables—sex, age, height, and weight—with IC-ECG guidance, thereby enabling real-time, precise catheter placement.

### Methods

Building upon existing IC-ECG-guided placement protocols, we retrospectively analyzed clinical data from hematology patients who underwent right IJV catheterization using IC-ECG at the First Affiliated Hospital, Zhejiang University School of Medicine, between August 2023 and January 2025. Univariate and multivariate regression analyses were conducted to identify determinants of optimal catheter depth and to construct a predictive model. The model was subsequently validated in a prospective cohort.

### Results

A total of 363 patients were included in the model development cohort (52.3% male; n=190). The most common diagnoses were acute myeloid leukemia (41.3%) and acute lymphoblastic leukemia (20.9%). The median catheter insertion depth was 16 cm (range: 11–22 cm). Despite IC-ECG guidance, post-procedural chest X-ray revealed a median deviation from the optimal depth of −0.6 cm (range: −4.8 to +3.7 cm), confirming persistent placement inaccuracy.
The optimal IJV insertion depth prediction model, incorporating age, height, weight, sex, and IC-ECG measured depth, was defined as:
$Y = 7.8421 - 0.0167 \times \text{age} + 0.1156 \times \text{sex (male=1, female=0)} + 0.0253 \times \text{height} + 0.0079 \times \text{weight} + 0.6923 \times \text{IC-ECG measured depth}$.
The model demonstrated excellent fit ($R^2 = 0.872$, $F = 484.12$, $P < 0.001$).
In a prospective validation cohort of 59 patients (50.8% male), real-time application of the model during IC-ECG-guided placement significantly improved accuracy, reducing the median deviation to 0 cm (range: −1 to +1 cm; $P < 0.05$ compared to the derivation cohort).

**Copyright Information:** (Online) ISSN: 2572-9241
©2026 The Author(s). HemaSphere is published by John Wiley & Sons Ltd on behalf of European Hematology Association. This is an open access Abstract Book distributed under the Attribution-NonCommercial-NoDerivs (CC BY-NC-ND), which allows third parties to download the articles and share them with others as long as they credit the author and the Abstract Book, but they cannot change the content in any way or use them commercially.
**Abstract Book Citations:** Authors, Title, HemaSphere, 2026;10:(S1):pages. The abstract book DOIs can be found on the "Author Information" page.
**Disclaimer:** Articles published in the journal HemaSphere exclusively reflect the opinions of the authors. The authors are responsible for all content in their abstracts including accuracy of the facts, statements, citing resources, etc.

**Summary/Conclusion**

An individualized predictive model integrating IC-ECG guidance with key patient characteristics enables real-time, accurate determination of optimal IJV catheter insertion depth. This integrative approach enhances placement precision, may lower complication risks, and supports safe and effective infusion therapy, thereby providing a scientific foundation for precision care in venous access management.

**Copyright Information:** (Online) ISSN: 2572-9241
©2026 The Author(s). HemaSphere is published by John Wiley & Sons Ltd on behalf of European Hematology Association. This is an open access Abstract Book distributed under the Attribution-NonCommercial-NoDerivs (CC BY-NC-ND), which allows third parties to download the articles and share them with others as long as they credit the author and the Abstract Book, but they cannot change the content in any way or use them commercially.
**Abstract Book Citations:** Authors, Title, HemaSphere, 2026;10:(S1):pages. The abstract book DOIs can be found on the "Author Information" page.
**Disclaimer:** Articles published in the journal HemaSphere exclusively reflect the opinions of the authors. The authors are responsible for all content in their abstracts including accuracy of the facts, statements, citing resources, etc.

### (PB4411) NOT ALL CYTOPENIAS ARE EQUAL: THE WEEKEND EFFECT IS ASSOCIATED WITH INCREASED IN-HOSPITAL MORTALITY IN PATIENTS ADMITTED WITH SEVERE THROMBOCYTOPENIA

**Topic:** 36. Quality of life, ethics, supportive and palliative care

Libi Feigin[1], Eugene Feigin[2], Yotam Bronstein*[3]

*[1]Internal Medicine Division, Internal Medicine Division, Tel Aviv, Israel; [2]Tel Aviv Sourasky Medical Center, Tel Aviv, Israel; [3]Tel Aviv Sourasky Medical Center, Hematology, Tel Aviv, Israel;*

### Background

The “weekend effect” describes worse outcomes among patients admitted on weekends, but evidence in internal medicine, and specifically among patients admitted with cytopenias is scarce. We examined whether weekend admission is associated with higher in-hospital mortality among patients admitted primarily for anemia, neutropenia, or thrombocytopenia.

### Aims

To assess the association between weekend admission and all-cause in-hospital mortality in internal medicine admissions with cytopenia as the primary diagnosis, and to determine whether effects differ by cytopenia subtype.

### Methods

Retrospective cohort study of adult admissions to internal medicine wards at Tel Aviv Sourasky Medical Center (2015–2023). Included admissions with anemia, neutropenia (including febrile neutropenia), or thrombocytopenia recorded as the primary admission diagnosis. Admissions were classified as weekday (Sunday–Thursday) or weekend (Friday–Saturday). The primary outcome was in-hospital mortality. Weekend vs weekday outcomes were compared using univariate tests; multivariable logistic regression adjusted for age, sex, and Charlson Comorbidity Index (CCI).

**Copyright Information:** (Online) ISSN: 2572-9241
©2026 The Author(s). HemaSphere is published by John Wiley & Sons Ltd on behalf of European Hematology Association. This is an open access Abstract Book distributed under the Attribution-NonCommercial-NoDerivs (CC BY-NC-ND), which allows third parties to download the articles and share them with others as long as they credit the author and the Abstract Book, but they cannot change the content in any way or use them commercially.
**Abstract Book Citations:** Authors, Title, HemaSphere, 2026;10:(S1):pages. The abstract book DOIs can be found on the “Author Information” page.
**Disclaimer:** Articles published in the journal HemaSphere exclusively reflect the opinions of the authors. The authors are responsible for all content in their abstracts including accuracy of the facts, statements, citing resources, etc.

### Results

Overall, 4,939 admissions were included: 4,101 (83.0%) weekday and 838 (17.0%) weekend. Anemia comprised 3,541 (71.7%) admissions (2,975 weekday; 566 weekend), with no differences in age, sex, or CCI by admission timing. Median hemoglobin was slightly higher on weekends (7.7 [IQR 6.7–8.9] g/dL) than weekdays (7.5 [IQR 6.5–8.7] g/dL; p=0.004). In-hospital mortality did not differ (6.9% weekend vs 5.0% weekday; p=0.08).
Neutropenia comprised 1,118 (22.6%) admissions (900 weekday; 218 weekend), including 834/1,118 (74.6%) febrile neutropenia; baseline age/sex/CCI were similar between groups. Median absolute neutrophil count was identical (0.1 [IQR 0–0.4] $\times 10^9$/L weekday vs 0.1 [IQR 0–0.4] $\times 10^9$/L weekend; p=0.3). Mortality was comparable overall (5.6% weekday vs 6.0% weekend; p=0.90) and in febrile neutropenia (4.9% weekday vs 6.0% weekend; p=0.6).
Thrombocytopenia comprised 280 (5.7%) admissions (226 weekday; 54 weekend), with no significant differences in baseline features or CCI. Platelet counts were similar (median 19 [IQR 9–43] $\times 10^9$/L weekday vs 22 [IQR 12–62] $\times 10^9$/L weekend; p=0.2) and did not differ by outcome (survivors 20 [IQR 10–49] vs non-survivors 20 [IQR 11–51] $\times 10^9$/L; p=0.7). In contrast to anemia/neutropenia, thrombocytopenia showed higher weekend mortality: 5.8% (13/226) weekday vs 18.5% (10/54) weekend (p=0.005); After adjustment, weekend admission in patients with thrombocytopenia remained strongly associated with increased in-hospital mortality (aOR 3.91, 95% CI 1.51–10.14; p=0.005).
Non-survivors were older (81.8 [IQR 69.6–85.9] vs 67.8 [IQR 47.1–79.1] years; p=0.0005) and had higher CCI (7 [IQR 6–8.5] vs 4 [IQR 1–6]; p<0.0001). Causes of death were similar by timing: sepsis predominated (weekday 7/13; weekend 7/10), with fewer deaths from malignancy progression (weekday 4/13; weekend 1/10) and major bleeding (weekday 2/13; weekend 2/10).

### Summary/Conclusion

No weekend effect was observed for admissions due to anemia or neutropenia. A clear weekend effect was confined to severe thrombocytopenia, with markedly higher in-hospital mortality despite similar baseline features. These findings highlight a vulnerable subgroup requiring tailored weekend considerations and support targeted pathways (early senior review, rapid evaluation, timely platelet support/specialist input, and proactive escalation for sepsis or bleeding).

**Copyright Information:** (Online) ISSN: 2572-9241
©2026 The Author(s). HemaSphere is published by John Wiley & Sons Ltd on behalf of European Hematology Association. This is an open access Abstract Book distributed under the Attribution-NonCommercial-NoDerivs (CC BY-NC-ND), which allows third parties to download the articles and share them with others as long as they credit the author and the Abstract Book, but they cannot change the content in any way or use them commercially.
**Abstract Book Citations:** Authors, Title, HemaSphere, 2026;10:(S1):pages. The abstract book DOIs can be found on the “Author Information” page.
**Disclaimer:** Articles published in the journal HemaSphere exclusively reflect the opinions of the authors. The authors are responsible for all content in their abstracts including accuracy of the facts, statements, citing resources, etc.

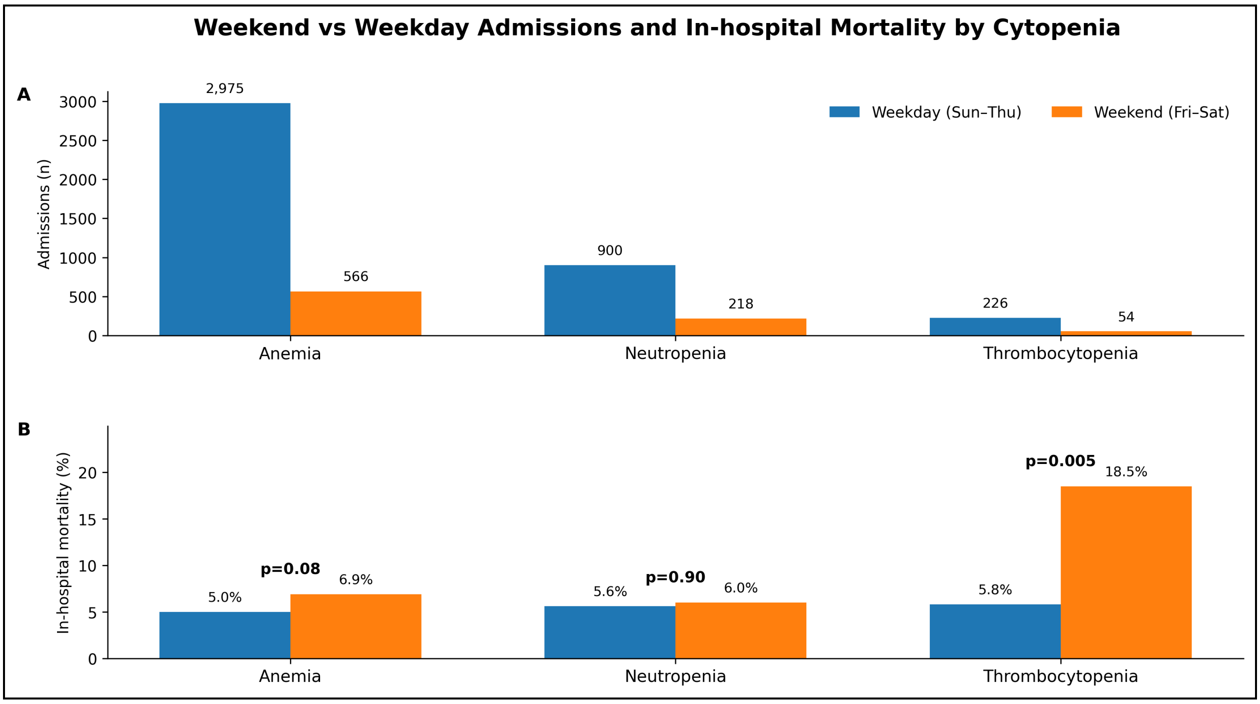

**Copyright Information:** (Online) ISSN: 2572-9241
©2026 The Author(s). HemaSphere is published by John Wiley & Sons Ltd on behalf of European Hematology Association. This is an open access Abstract Book distributed under the Attribution-NonCommercial-NoDerivs (CC BY-NC-ND), which allows third parties to download the articles and share them with others as long as they credit the author and the Abstract Book, but they cannot change the content in any way or use them commercially.
**Abstract Book Citations:** Authors, Title, HemaSphere, 2026;10:(S1):pages. The abstract book DOIs can be found on the "Author Information" page.
**Disclaimer:** Articles published in the journal HemaSphere exclusively reflect the opinions of the authors. The authors are responsible for all content in their abstracts including accuracy of the facts, statements, citing resources, etc.

### (PB4412) REDESIGNING A CANCER SUPPORTIVE CARE PROGRAM TO EMBED EQUITABLE PERSON-CENTRED CARE

**Topic:** 36. Quality of life, ethics, supportive and palliative care

Tricia Wright*[1, 2], Daniela Klarica[1], Bianca Cirone[1], Joanne Tan[1, 2], Olivia Cook[1, 2], Andrew Spencer[1, 2]

[1]*Alfred Health, Cancer Services, Melbourne, Australia;* [2]*Monash University, Medicine, Melbourne, Australia;*

### Background

The benefits of quality Cancer Supportive Care (CSC) are well established[1]. Health service structures drive efficacy models of supportive care within disease or targeted treatment groups. Whilst high standard supportive care (SC) outcomes are achieved this framework reduces the opportunity for a shared understanding and equitable access to best person-centred supportive care across all cancers. We describe how we redesigned our Supportive Care Program (SCP) for long-term survivors of stem cell transplant (SCT) to shift away from strict protocols toward patient-centred, equitable approach for all individuals with a cancer diagnosis in our metropolitan health service in Melbourne, Australia.

### Aims

To redesign our SCT long term follow up program using consumer interviews to inform the elements of an equitable supportive care program
To analyse 12-month readmission from SCT data to demonstrate the impact of the new approach

### Methods

The response to the interview question, '*What is the optimal supportive care clinic for you*?' from our previous study conducted in 2022 were analysed[2] The SCP redesign was informed from interview responses. Readmission data was collected for all individuals attending the revamped SCP clinic for a period of 12-months from discharge from an autologous or allogeneic SCT acute admission.

### Results

Of the 81 interview participants, 78 (96%) reported a preference for the SCP clinic to be more accessible through the continuum of their cancer experience. The figure summarises the final redesign of the Cancer SCP (CSCP). A multidisciplinary CSCP governance group was established to produce an organisation wide CSCP policy; create an ethics endorsed CSCP clinical database, oversee the clinical and research activity and establish engagement with consumer groups to contribute to ongoing co-design of the program. The CSCP clinic was refocussed to review patients at endorsed timepoints through diagnosis and treatment for dedicated SC interventions. The redesigned CSCP clinic provided SC to 155 individuals following discharge from autologous and allogeneic SCT. Of these individuals 73 (47%) required at least one readmission with a total of 133 readmissions across the group. Reasons for readmission included 40 (30%) respiratory viruses, 22 (16.5%) fever for investigation, 28 (21.1%) bacterial infection and 44 (33%) other medical or transplant related complication.

**Copyright Information:** (Online) ISSN: 2572-9241
©2026 The Author(s). HemaSphere is published by John Wiley & Sons Ltd on behalf of European Hematology Association. This is an open access Abstract Book distributed under the Attribution-NonCommercial-NoDerivs (CC BY-NC-ND), which allows third parties to download the articles and share them with others as long as they credit the author and the Abstract Book, but they cannot change the content in any way or use them commercially.
**Abstract Book Citations:** Authors, Title, HemaSphere, 2026;10:(S1):pages. The abstract book DOIs can be found on the "Author Information" page.
**Disclaimer:** Articles published in the journal HemaSphere exclusively reflect the opinions of the authors. The authors are responsible for all content in their abstracts including accuracy of the facts, statements, citing resources, etc.

**Summary/Conclusion**

Shifting the focus to a person-centred CSCP requires more than a measurement of individual unmet needs. Success requires embedding the principles of care within organisation governance to drive adaptive clinical systems to support consumer endorsed change. The impact of our established CSCP is demonstrated in highlighting readmission rates and causes across programs and diseases. This has led to a project proposal grounded in human factors theory[3] considering consumer, health care worker and organisation factors targeted at opportunities to reduce readmission rates, with an initial focus on improvements in the prevention of respiratory viral diseases in our vulnerable populations. This model seamlessly extends to empowering patients with any cancer diagnosis as active partners in their own health journey.

1. Scotté, F.,etal (2023). https://doi.org/10.3390/cancers15153860
2. Wright T et al, (2023) https://doi.org/10.1038/s41409-023-02055-8
3. Edwards III GF et al (2023) https://doi: 10.3389/frhs.2022.981450

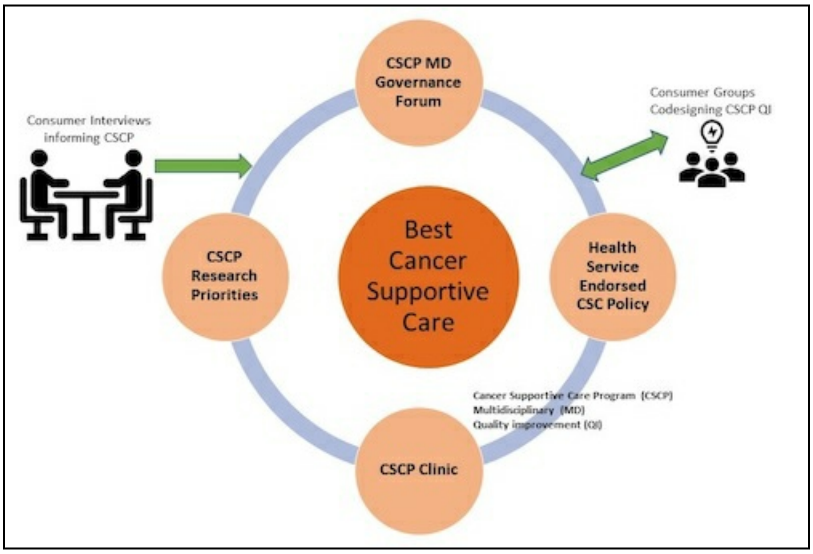

Figure CSCP components

**Copyright Information:** (Online) ISSN: 2572-9241
©2026 The Author(s). HemaSphere is published by John Wiley & Sons Ltd on behalf of European Hematology Association. This is an open access Abstract Book distributed under the Attribution-NonCommercial-NoDerivs (CC BY-NC-ND), which allows third parties to download the articles and share them with others as long as they credit the author and the Abstract Book, but they cannot change the content in any way or use them commercially.
**Abstract Book Citations:** Authors, Title, HemaSphere, 2026;10:(S1):pages. The abstract book DOIs can be found on the "Author Information" page.
**Disclaimer:** Articles published in the journal HemaSphere exclusively reflect the opinions of the authors. The authors are responsible for all content in their abstracts including accuracy of the facts, statements, citing resources, etc.

### (PB4413) TIMING IS EVERYTHING: SYNCHRONOUS ONSET AND EARLY SECOND MALIGNANCY AS KEY SURVIVAL DETERMINANTS IN MULTIPLE PRIMARY MALIGNANCIES - A REAL-WORLD COHORT

**Topic:** 36. Quality of life, ethics, supportive and palliative care

Alexandra-Diana Malenda*[1, 2], Daniela-Andreea Șerbu[1, 2], Elena Pirvu[1, 2], Diana Grasu[1, 2], Maria Bacanu[1, 2], Viola Maria Popov[1], Mihaela Andreescu[1, 2]

*[1]Colentina Clinical Hospital, Hematology I, Bucharest, Romania;[2]Titu Maiorescu University, Faculty of Medicine, Bucharest, Romania;*

### Background

Patients developing multiple primary malignancies (MPM) represent a clinically heterogeneous population with poorly defined prognostic determinants across the full spectrum of solid and hematologic malignancies.

### Aims

To identify predictors of overall survival (OS) in patients with MPM, focusing on timing and biological relationship between sequential malignancies.

### Methods

Retrospective single-center study (Jan 2020–Jan 2026). Inclusion criteria: age ≥18, ≥2 primary malignancies (solid/hematologic). Exclusion criteria: disease transformation/insufficient data. N=120. Synchronous MPM: within 6 months; metachronous: >6 months. OS was measured from first malignancy (M1) diagnosis. Kaplan–Meier, Cox regression (univariable/multivariable), chi-square, McNemar, Mann–Whitney U tests were performed (Jamovi v2.7.18).

### Results

The cohort included 67 males (56%) and 53 females (44%); median age at M1 was 61 years (18–89) and at second malignancy (M2) 68.5 years (22–90). Most patients had exactly 2 malignancies (98/120, 82%), while 18% had ≥3, including 2 cases with 5 and 6 malignancies. M1 was hematologic in 54% and solid in 46%; M2 was hematologic in 63% and solid in 37% (McNemar $\chi^2$=1.36; p=0.24). The most frequent M1 types were B-cell neoplasms (29%), breast (11%), Hodgkin lymphoma (9%); M2 most commonly comprised B-cell neoplasms (28%) and myeloid neoplasms (21%), reflecting a shift toward myeloid disease. High comorbidity (Charlson Comorbidity Index [CCI] ≥3) was present in 73% at M1 and 91% at M2, with median CCI rising from 3 to 6 (n=63, McNemar $\chi^2$=11.0, p<0.001). After a 91 months median follow-up, 45/120 patients (37.5%) had died; median OS was 220 months (95% CI 145–NR), with 1-, 3-, and 5-year OS of 93%, 86%, and 84%. The M1–M2 interval was the strongest survival predictor: patients with interval <2 years (n=44) had median OS of 90 months vs. 285 months for ≥2 years (n=76; HR 0.20; 95% CI 0.10–0.39; p<0.001), confirmed in sensitivity analysis restricted to exactly 2-malignancy patients (n=98; HR 0.18; 95% CI 0.09–0.38; p<0.001). Synchronous M2 (n=17, 14%) was associated with markedly inferior OS versus metachronous M2 (n=103, 86%; median 91 vs. 220 months; 5-year OS 64% vs. 88%; HR 3.61; 95% CI 1.63–8.00; p=0.002), strengthened on sensitivity analysis (HR 4.02; 95% CI 1.71–9.43; p=0.001). Male sex was associated with shorter OS (median 149 vs. 322 months; HR 1.93; 95% CI 1.01–3.69; p=0.048), though this did not reach significance in the 2-malignancy cohort (HR 1.99; p=0.064). Short M1–M2 intervals were more frequent in males (45% vs. 26%; $\chi^2$p=0.038). In multivariable Cox analysis (n=63, 22 events), higher CCI at M1 was the only independent predictor of inferior OS (HR 1.41/point; 95% CI 1.02–1.96; p=0.039); synchronous M2 showed a non-significant trend (HR 2.75; 95% CI 0.88–8.62; p=0.082). Patients with/without CCI data were comparable in age/sex, but those with CCI data had shorter M1–M2 intervals (p≈0.02). OS did not differ by M1 category (p=0.67) or sequencing pattern (p=0.58).

**Copyright Information:** (Online) ISSN: 2572-9241
©2026 The Author(s). HemaSphere is published by John Wiley & Sons Ltd on behalf of European Hematology Association. This is an open access Abstract Book distributed under the Attribution-NonCommercial-NoDerivs (CC BY-NC-ND), which allows third parties to download the articles and share them with others as long as they credit the author and the Abstract Book, but they cannot change the content in any way or use them commercially.
**Abstract Book Citations:** Authors, Title, HemaSphere, 2026;10:(S1):pages. The abstract book DOIs can be found on the "Author Information" page.
**Disclaimer:** Articles published in the journal HemaSphere exclusively reflect the opinions of the authors. The authors are responsible for all content in their abstracts including accuracy of the facts, statements, citing resources, etc.

**Summary/Conclusion**

A short M1–M2 interval (<2 years) and synchronous M2 were the strongest univariable predictors of inferior OS, both robust on sensitivity analysis. Higher CCI at M1 was the sole independent multivariable predictor. These findings support intensified surveillance for patients with M2 within 2 years of M1 and highlight comorbidity assessment as key to clinical decisions. Limitations: retrospective single-center design, small synchronous subgroup, incomplete CCI data, residual confounding.

**Copyright Information:** (Online) ISSN: 2572-9241
©2026 The Author(s). HemaSphere is published by John Wiley & Sons Ltd on behalf of European Hematology Association. This is an open access Abstract Book distributed under the Attribution-NonCommercial-NoDerivs (CC BY-NC-ND), which allows third parties to download the articles and share them with others as long as they credit the author and the Abstract Book, but they cannot change the content in any way or use them commercially.
**Abstract Book Citations:** Authors, Title, HemaSphere, 2026;10:(S1):pages. The abstract book DOIs can be found on the "Author Information" page.
**Disclaimer:** Articles published in the journal HemaSphere exclusively reflect the opinions of the authors. The authors are responsible for all content in their abstracts including accuracy of the facts, statements, citing resources, etc.

### (PB4414) REAL WORLD USE OF ERYTHROPOIESIS STIMULATING AGENTS IN PATIENTS RECEIVING MYELOSUPPRESSIVE CHEMOTHERAPY IN EUROPE

**Topic:** 36. Quality of life, ethics, supportive and palliative care

Vibeke Vergote[1], Adrian Alegre Amor[2], Amanda Ly[3], Scott Stryker[4], Leanne Goldstein[4], Sarah McGill[4], Amy Kimball*[4]

*[1]Universitair Ziekenhuis Leuven, Department of Haematology, Leuven, Belgium;[2]Hospital Universitario de La Princesa, Madrid, Spain; [3]Amgen Ltd, Uxbridge, London, United Kingdom; [4]Amgen Inc., Thousand Oaks, California, United States of America;*

### Background

In 2008, a safety review of clinical studies identified increased risk of mortality and/or tumour progression in cancer patients treated for chemotherapy-induced anaemia (CIA) when haemoglobin (Hb) levels were >12 g/dL at initiation of erythropoiesis stimulating agents (ESAs; Tonia T. et al, Cochrane Database Syst Rev. 2012;12(12):CD003407), prompting authorities to establish thresholds and targets for ESA use. The EU label for darbepoetin alfa recommends treatment for CIA when Hb levels are ≤10 g/dL with a target not exceeding 12 g/dL. In a phase 3 randomised trial, with baseline Hb ≤11 g/dL, use of darbepoetin alfa to a 12 g/dL Hb target was noninferior to placebo for overall survival and progression-free survival and superior to placebo for red blood cell transfusion or Hb ≤8 g/dL in patients with advanced-stage non-small cell lung carcinoma receiving myelosuppressive chemotherapy (Gascón P. et al, J Thorac Oncol. 2020;15(2):190-202). However, it remained to be confirmed if real-world use of ESAs conformed to labelling and trial experience.

### Aims

To describe real-world use of ESAs in patients receiving myelosuppressive chemotherapy and determine whether ESAs were prescribed in accordance with EU labelling.

### Methods

This retrospective observational study enrolled patients with non-myeloid malignancies treated with myelosuppressive chemotherapy between 06 January 2015 and 12 September 2024 across clinical sites in 6 countries. Patients were followed up from ESA treatment initiation (index date) until 9 weeks post-index date. Data were abstracted from medical records, pathology and pharmacy databases. Key variables for the descriptive analyses included: Hb levels at baseline and at weeks 3, 4, 6 and 9, demographic and clinical characteristics, including recent red blood cell (RBC) transfusions. Baseline Hb levels were categorised using thresholds of ≤10 g/dL (EU label) and ≤11 g/dL (Gascón P. et al, J Thorac Oncol. 2020;15(2):190-202). During follow-up, rapid Hb rises of ≥2 g/dL within any 4-week interval were analysed.

### Results

Data from 762 patients across 28 sites in France,Spain, Belgium, Italy, the United Kingdom and Germany were analysed. Most patients (97.8%) had baseline Hb ≤11 g/dL and 83.9% had Hb ≤10 g/dL. Characteristics are presented in Table 1. ESA use decreased steadily from 68.9% at week 3 to 24.9% at week 9; most (93.4%) patients had no dose modifications. During follow-up, the majority of patients had Hb values within the target range (> 10 g/dL to ≤12 g/dL) or below the target range (≤10 g/dL): 93.5% (471/504) among those with at least one Hb measure at week 3 and 82.9% (213/257) among those with at least one Hb measure at week 9. A ≥2 g/dL Hb rise during any 4-week interval among those whose follow-up did not end prior to day 63 was observed in 38.0% (274/721) of patients; of these 274, 3.3% (n=9) had a subsequent ESA dose reduction. In a subset of patients with baseline Hb >10 g/L and continued ESA prescription (n=31), 6 (19.4%) had a ≥2 g/dL Hb rise, ultimately representing 0.8% of all patients treated with ESAs in this study.

**Copyright Information:** (Online) ISSN: 2572-9241
©2026 The Author(s). HemaSphere is published by John Wiley & Sons Ltd on behalf of European Hematology Association. This is an open access Abstract Book distributed under the Attribution-NonCommercial-NoDerivs (CC BY-NC-ND), which allows third parties to download the articles and share them with others as long as they credit the author and the Abstract Book, but they cannot change the content in any way or use them commercially.
**Abstract Book Citations:** Authors, Title, HemaSphere, 2026;10:(S1):pages. The abstract book DOIs can be found on the "Author Information" page.
**Disclaimer:** Articles published in the journal HemaSphere exclusively reflect the opinions of the authors. The authors are responsible for all content in their abstracts including accuracy of the facts, statements, citing resources, etc.

### Summary/Conclusion

In this real-world study, ESA use showed high adherence to the recommended Hb initiation thresholds and targets. Findings support appropriate prescribing practice and good Hb response to ESAs in patients with myelosuppressive chemotherapy.

Table 1. Patient Demographic and Clinical Characteristics at Index Date° (N=762)

| Characteristic | Total (N = 762) |
|---|---|
| **Demographics** | |
| **Country, n (%)** | |
| Belgium | 169 (22.2) |
| France | 235 (30.85) |
| Germany | 6 (0.8) |
| Italy | 88 (11.55) |
| Spain | 232 (30.4) |
| United Kingdom | 32 (4.2) |
| **Age at index date, n (%)** | |
| 18–65 years | 333 (43.7) |
| 66–75 years | 265 (34.8) |
| 76–85 years | 150 (19.7) |
| >85 years | 14 (1.8) |
| **Sex, n (%)** | |
| Male | 398 (52.2) |
| Female | 364 (47.8) |
| **Clinical characteristics** | |
| **Primary malignancy by body system, n (%)** | |
| Haematologic | 369 (48.4) |
| *Lymphoma* | *283 (37.1)* |
| *Multiple myeloma* | *86 (11.3)* |
| Respiratory | 177 (23.2) |
| Gastrointestinal | 134 (17.6) |
| Reproductive | 55 (7.2) |
| Other / non-specified | 27 (3.5) |
| **Disease stage, n (%)** | |
| Metastatic | 358 (47.0) |
| Non-metastatic | 351 (46.1) |
| Not available | 53 (7.0) |
| **ESA type, n (%)** | |
| Long-acting | 399 (52.4) |
| Short-acting | 363 (47.6) |
| **RBC transfusions prior to index date (Day −93 to Day 0)** | |
| Patients with ≥1 RBC transfusion, n | 194 |
| Count of RBC transfusions | |
| Mean (SD) | 2.7 (2.79) |
| Median (Q1–Q3) | 2.0 (1.0–3.0) |
| Min–Max | 1–20 |
| Number of RBC units transfused, n | 193 |
| Mean (SD) | 3.9 (3.98) |
| Median (Q1–Q3) | 2.0 (2.0–4.0) |
| Min–Max | 1–28 |

ESA, erythropoiesis stimulating agent; RBC, red blood cells. Long-acting ESA: darbepoetin alfa; short-acting ESA: epoetin alfa, beta, theta, and zeta. SD (standard deviation). °Index date (Day 0) was the first recorded ESA prescription date.

**Copyright Information:** (Online) ISSN: 2572-9241
©2026 The Author(s). HemaSphere is published by John Wiley & Sons Ltd on behalf of European Hematology Association. This is an open access Abstract Book distributed under the Attribution-NonCommercial-NoDerivs (CC BY-NC-ND), which allows third parties to download the articles and share them with others as long as they credit the author and the Abstract Book, but they cannot change the content in any way or use them commercially.
**Abstract Book Citations:** Authors, Title, HemaSphere, 2026;10:(S1):pages. The abstract book DOIs can be found on the "Author Information" page.
**Disclaimer:** Articles published in the journal HemaSphere exclusively reflect the opinions of the authors. The authors are responsible for all content in their abstracts including accuracy of the facts, statements, citing resources, etc.

### (PB4415) ERYTHROPOIETIC PROTOPORPHYRIA LIFE IMPACT AND GENETIC HEALTH TRAJECTORY (EPP LIGHT) STUDY: A CROSS-SECTIONAL SURVEY OF ADULTS AND ADOLESCENTS IN EUROPE

**Topic:** 36. Quality of life, ethics, supportive and palliative care

Hetanshi Naik*[1], Kristen Wheeden[2], Susan Mathias[3], Chelsea Norregaard[4], Adaeze Q. Amaefule[4], Melanie Chin[4], Manisha Balwani[5]

*[1]Stanford University School of Medicine, Stanford, United States of America; [2]United Porphyrias Association, Bethesda, United States of America; [3]Health Outcomes Solutions, Winter Park, United States of America; [4]Disc Medicine, Watertown, United States of America; [5]Icahn School of Medicine at Mount Sinai, New York, United States of America;*

### Background

Erythropoietic protoporphyria and X-linked protoporphyria (collectively referred to as EPP) are rare genetic disorders of heme biosynthesis that lead to protoporphyrin IX (PPIX) accumulation in the blood. Individuals present with extreme photosensitivity, including severe skin pain due to sunlight exposure.

### Aims

To describe the impact and burden in terms of health-related quality of life, symptoms, and healthcare resource utilization in patients with EPP in Europe.

### Methods

Participants aged ≥12 years with a diagnosis of EPP from France (n=33), Germany (n=21), Italy (n=15), Spain (n=10), or UK (n=22) who provided consent completed a one-time online survey containing original items (EPP Impact Questionnaire [EPIQ], Sunlight Exposure Diary) and validated measures (PROMIS). The study received Ethics approval from each country.

**Copyright Information:** (Online) ISSN: 2572-9241
©2026 The Author(s). HemaSphere is published by John Wiley & Sons Ltd on behalf of European Hematology Association. This is an open access Abstract Book distributed under the Attribution-NonCommercial-NoDerivs (CC BY-NC-ND), which allows third parties to download the articles and share them with others as long as they credit the author and the Abstract Book, but they cannot change the content in any way or use them commercially.
**Abstract Book Citations:** Authors, Title, HemaSphere, 2026;10:(S1):pages. The abstract book DOIs can be found on the "Author Information" page.
**Disclaimer:** Articles published in the journal HemaSphere exclusively reflect the opinions of the authors. The authors are responsible for all content in their abstracts including accuracy of the facts, statements, citing resources, etc.

**Results**

In Europe, 91 adults (mean age=46 yrs) and 10 adolescents (mean age=14 yrs) completed the survey; 67% were female. Time to EPP diagnosis was ≥5 yrs (51%) and ≤4 years for 49%. Before diagnosis, the most commonly consulted clinician in France, Germany, Italy, and Spain was a dermatologist (80-91%), followed by a general practitioner/internist (40-86%). In the UK, a general practitioner/internist (82%) was most common, followed by a dermatologist (59%). Prior to EPP diagnosis, Germans reported the most physician visits (3.10±1.45), and those in the UK the fewest (2.09±1.44). A dermatologist most frequently made the EPP diagnosis (60%; range: 39% France-76% UK and Germany).

The most common prodromal (early warning) symptoms were sensitivity to heat/cold (84%), sensitivity to touch (83%), warmth (78%), burning (70%), itching (69%), and pain (68%). Most participants (73%) tolerated ≤30 minutes of sun exposure before experiencing prodromal symptoms (67-81% across countries). Between 79 and 90% experienced prodromal symptoms ≥1 time in the past 3 months, and 57-70% reported prodromal symptoms affected daily activities "quite a bit" or "very much." Other than in the UK (30%), a majority of participants (52-71%) "always" experienced prodromal symptoms before phototoxic reactions. Participants "often" or "always" experienced pain from indirect sunlight (53%), with pain from phototoxic reactions occurring within 30 minutes in indirect (58%) and direct (69%) sunlight. On a scale from 0 (no pain) to 10 (worst pain imaginable), the mean pain rating was 7.6±1.8. Phototoxic reactions affected daily activities "very much" (58%), and participants reported symptom improvement began after 2.3±1.4 days, with complete resolution taking 5.0±2.5 days. A majority (83%) reported that having more time in the sun without pain would be more meaningful than having fewer phototoxic reactions.

At least "some difficulty" in doing chores such as yardwork, gardening, or washing a car and going for a walk, run, or bike ride for ≥15 mins when sunny outside was reported by 61% of participants. At least "a little difficulty" in being able to run errands for ≥15 mins when sunny outside was reported by 67% of participants.

**Summary/Conclusion**

In Europe, EPP limits sun exposure tolerance and restricts participation in daily activities, resulting in negative impacts on physical and emotional functioning.

**Emotional Functioning Results**

| Emotion | France | Germany | Italy | Spain | UK |
|---|---|---|---|---|---|
| % Depressed/sad | 49 | 76 | 60 | 60 | 73 |
| % Anxious | 52 | 57 | 47 | 50 | 68 |
| % Isolated | 73 | 91 | 93 | 50 | 82 |
| % Frustrated | 94 | 68 | 73 | 90 | 100 |
| % Lonely | 73 | 81 | 67 | 60 | 64 |

**Copyright Information:** (Online) ISSN: 2572-9241
©2026 The Author(s). HemaSphere is published by John Wiley & Sons Ltd on behalf of European Hematology Association. This is an open access Abstract Book distributed under the Attribution-NonCommercial-NoDerivs (CC BY-NC-ND), which allows third parties to download the articles and share them with others as long as they credit the author and the Abstract Book, but they cannot change the content in any way or use them commercially.
**Abstract Book Citations:** Authors, Title, HemaSphere, 2026;10:(S1):pages. The abstract book DOIs can be found on the "Author Information" page.
**Disclaimer:** Articles published in the journal HemaSphere exclusively reflect the opinions of the authors. The authors are responsible for all content in their abstracts including accuracy of the facts, statements, citing resources, etc.

### (PB4416) FEASIBILITY AND ACCEPTABILITY OF HOME-BASED VIDEO RECORDING OF ORAL CHEMOTHERAPY ADMINISTRATION BY CAREGIVERS OF CHILDREN WITH ACUTE LYMPHOBLASTIC LEUKEMIA

**Topic:** 36. Quality of life, ethics, supportive and palliative care

Kara Kelly*[1], Paula Vincent[2], Hital Patel[2], Sharon Castellino[3], Diana Fridlyand[3], Gregory Fabiano[4], Karen Lutfey Spencer[5], Susan Lavalley[2], Elisa Rodriguez[2], Leonard Epstein[6], Mark Prince[7], Ebonee Harris[3], Elizabeth Bouchard[2]

*[1]Roswell Park Comprehensive Cancer Center; University at Buffalo Jacobs School of Medicine and Biomedical Sciences, Buffalo, United States of America; [2]Roswell Park Comprehensive Cancer Center, Buffalo, United States of America; [3]Emory University, Aflac Cancer and Blood Disorders Center, Children's Healthcare of Atlanta, Atlanta, United States of America; [4]Florida International University, Miamia, United States of America; [5]University of Colorado Denver, Denver, United States of America; [6]University at Buffalo Jacobs School of Medicine and Biomedical Sciences, Buffalo, United States of America; [7]Keck School of Medicine, University of Southern California, Los Angeles, United States of America;*

### Background

Adherence to oral chemotherapy is a critical concern in children with Acute Lymphoblastic Leukemia (ALL), as nonadherence to ≥10% of mercaptopurine (MP) chemotherapy doses is associated with a 4-fold risk of relapse. Parenting-related skills around medication administration impact risk for nonadherence. As advances in pediatric cancer care have shifted responsibility for care delivery to home-based settings, understanding the dynamics of caregiving at home has grown in importance. Caregiver reports of medication adherence are often unreliable. Direct observation offers a methodological approach to collect objective data that has increased reliability for home-based behaviors; yet inviting researchers into patient homes has raised issues of infectious exposure health risks, as well as logistical and privacy concerns by caregivers of children with ALL. Caregiver recording of medication administration using a study camera at home is an approach for obtaining direct observation data and has not previously been studied in children with ALL.

### Aims

To assess the feasibility and acceptability of direct observation using caregiver recordings of home-based MP administration in children with ALL.

### Methods

Caregivers of children ages 3 – 9 years diagnosed with ALL on home-based MP were recruited. English and/or Spanish speaking caregivers used a study camera to record one episode of MP administration (or attempt to administer) at home. Caregivers were instructed to begin recording 5 minutes prior, and to stop recording after child ingestion of MP. Feasibility was examined through rates of study enrollment and video submission. Acceptability was assessed using caregiver ratings of ease of camera use and burden of recording at home.

**Copyright Information:** (Online) ISSN: 2572-9241
©2026 The Author(s). HemaSphere is published by John Wiley & Sons Ltd on behalf of European Hematology Association. This is an open access Abstract Book distributed under the Attribution-NonCommercial-NoDerivs (CC BY-NC-ND), which allows third parties to download the articles and share them with others as long as they credit the author and the Abstract Book, but they cannot change the content in any way or use them commercially.
**Abstract Book Citations:** Authors, Title, HemaSphere, 2026;10:(S1):pages. The abstract book DOIs can be found on the "Author Information" page.
**Disclaimer:** Articles published in the journal HemaSphere exclusively reflect the opinions of the authors. The authors are responsible for all content in their abstracts including accuracy of the facts, statements, citing resources, etc.

### Results

Of 54 eligible families, 43 (80%) enrolled, exceeding the expected enrollment of >75%. Caregivers were primarily mothers (82%). Mean age was 35 years. 68% were white, 18% were black. 18% were Hispanic ethnicity. 45% had a high school diploma or less education. 40% were employed full-time. Among the children, 48% were female and mean age was 5 years. Most were publicly insured; 55% public only, whereas 38% received both public and private insurance coverage. Mean time from ALL diagnosis was 16 months (*SD*=7.03). 40 (93%) submitted a home video, exceeding *a priori* benchmarks of feasibility of ≥ 80% completion of a home video recording. 36 videos were in English, 3 were in English and Spanish, and 1 Spanish only. Median duration of videos was 67 seconds (range 15 seconds - 32 minutes 53 seconds). 31 (77%) rated the medication session as very typical. Only 3 (8%) rated the session as atypical. Caregivers deemed home recording methodology acceptable (4.25; *SD*=1.03, range 1-5) and low burden (4.77; *SD*=.58m, range 2-5). Videos captured objective data on where the families stored the oral MP, how oral MP is prepared, how the medication is administered in the real-world setting, and whether the MP was actually ingested.

### Summary/Conclusion

Caregiver-recorded video observation of oral chemotherapy administration in a diverse cohort of young children with ALL is a highly acceptable and feasible method for informing an understanding of family dynamics and the impact on medication adherence. This study demonstrates the potential utility of this method to understand other home-based behaviors and to practically inform the development of interventions to improve oral chemotherapy adherence rates.

**Copyright Information:** (Online) ISSN: 2572-9241
©2026 The Author(s). HemaSphere is published by John Wiley & Sons Ltd on behalf of European Hematology Association. This is an open access Abstract Book distributed under the Attribution-NonCommercial-NoDerivs (CC BY-NC-ND), which allows third parties to download the articles and share them with others as long as they credit the author and the Abstract Book, but they cannot change the content in any way or use them commercially.
**Abstract Book Citations:** Authors, Title, HemaSphere, 2026;10:(S1):pages. The abstract book DOIs can be found on the "Author Information" page.
**Disclaimer:** Articles published in the journal HemaSphere exclusively reflect the opinions of the authors. The authors are responsible for all content in their abstracts including accuracy of the facts, statements, citing resources, etc.

### (PB4417) THE PATIENT EXPERIENCE OF FATIGUE IN INDIVIDUALS WITH SICKLE CELL DISEASE, THALASSEMIA, AND PYRUVATE KINASE DEFICIENCY

**Topic:** 36. Quality of life, ethics, supportive and palliative care

Biree Andemariam*[1], Ninamaria Badalamenti[2], Rae Blaylark[3], Audra Boscoe[4], Lily Cannon[5], Ralph Colasanti[6], Daniel Ford[7], Rachael F. Grace[8], Holly John[4], Charles Jonassaint[9], Sarah Knight[7], Kevin H. M. Kuo[10, 11], Laurice Levine[12], Clarisse Lobo[13], Jake MacEy[7], Parija Patel[4], Tamara Schryver[14], Nirmish Shah[15], Sujit Sheth[16], Cassandra Trimnell[17], Alejandra Watson[18], Jill Welle[19], Teonna Woolford[20], Raffaella Colombatti[21]

*[1]New England Sickle Cell Institute, University of Connecticut Health, Farmington, United States of America;[2]Independent Contributor, Chicago, United States of America; [3]Sickle Cell Foundation of Minnesota, Minneapolis, United States of America; [4]Agios Pharmaceuticals, Inc., Cambridge, MA, United States of America;[5]Thalassaemia International Federation, Nicosia, Cyprus; [6]Cooley's Anemia Foundation, New York, United States of America;[7]Clarivate, London, United Kingdom;[8]Dana-Farber/Boston Children's Cancer and Blood Disorders Center, Boston, United States of America;[9]Emory University, Department of Hematology and Medical Oncology, Atlanta, United States of America; [10]Division of Hematology, Department of Medicine, University of Toronto, Toronto, ON, Canada; [11]Scarborough Health Network Research Institute, Scarborough, Canada; [12]Independent Contributor, Petaluma, United States of America; [13]Instituto Estadual de Hematologia Arthur de Siqueira Cavalcanti (HEMORIO), Department of Clinical Research, Rio de Janeiro, Brazil; [14]Independent Contributor, Minneapolis, MN, United States of America; [15]Duke University, Division of Hematology, Durham, United States of America;[16]Weill Cornell Medicine, Division of Hematology Oncology, Department of Pediatrics,, New York, United States of America; [17]Sickle Cell 101, San Francisco, United States of America; [18]Pyruvate Kinase Deficiency International Alliance (PKDIA), Wolverine Lake, United States of America; [19]Independent Contributor, Minnesota, United States of America;[20]Sickle Cell Reproductive Health Education Directive, Owing Mills, United States of America; [21]University of Padova, Department of Woman's and Child's Health, Padova, Italy;*

### Background

Hereditary hemolytic anemias (HHAs), including sickle cell disease (SCD), thalassemia, and pyruvate kinase (PK) deficiency, are rare inherited hematologic disorders that impair function of red blood cells. A multi-stakeholder initiative on unmet research needs in SCD, thalassemia, and PK deficiency identified physical and emotional fatigue as key factors affecting patient health-related quality of life that require greater understanding.

### Aims

Explore the patient experience of physical, mental, and emotional fatigue across SCD, thalassemia, and PK deficiency.

### Methods

Semi-structured, one-on-one interviews were conducted with participants aged ≥18 years from the USA, with a clinician-confirmed diagnosis of SCD, thalassemia, or PK deficiency. Transcripts were analyzed in ATLAS.ti using content analysis for concept elicitation. Quantitative descriptive statistics summarized demographic and clinical characteristics. Ranking and saliency analyses identified the most bothersome types and impacts of fatigue.

**Copyright Information:** (Online) ISSN: 2572-9241
©2026 The Author(s). HemaSphere is published by John Wiley & Sons Ltd on behalf of European Hematology Association. This is an open access Abstract Book distributed under the Attribution-NonCommercial-NoDerivs (CC BY-NC-ND), which allows third parties to download the articles and share them with others as long as they credit the author and the Abstract Book, but they cannot change the content in any way or use them commercially.
**Abstract Book Citations:** Authors, Title, HemaSphere, 2026;10:(S1):pages. The abstract book DOIs can be found on the "Author Information" page.
**Disclaimer:** Articles published in the journal HemaSphere exclusively reflect the opinions of the authors. The authors are responsible for all content in their abstracts including accuracy of the facts, statements, citing resources, etc.

**Results**

A total of 58 participants (n=20 SCD; n=20 thalassemia; n=18 PK deficiency) had a median (range) age of 42.5 years (19–74) and were mostly female (n=46 [79%]). Median (range) number of years since HHA diagnosis was 41.5 (0–60), and participants received a median (range) of 3.5 (0–44) blood transfusions in the past 12 months. Physical fatigue was reported as a bodily experience of "low energy" or feeling "heavy/weak". Mental fatigue was described as cognitive impairment resulting in "brain fog" or feeling "drained", leading to difficulty initiating, engaging with, and focusing on tasks. Emotional fatigue was reported as feeling depressed or irritable due to physical/mental fatigue, and the emotional toll of being unable to function like individuals without fatigue. Participants' experiences of physical, mental, and emotional fatigue were broadly consistent across the different HHAs. Across the three HHAs, physical fatigue was ranked the most bothersome type of fatigue. Impacts of fatigue were negative and pervasive: among the 58 participants, impacts on physical functioning (n=56), daily activities (eg, chores, self-care; n=55), work/study (n=54), sleep (n=52), cognition (eg, memory, concentration; n=51), relationships and social life (n=50), emotions (eg, depression, anxiety; n=46), and hobbies (n=41) were reported. Participants ranked cognitive problems and physical functioning as the most bothersome impacts of fatigue (Figure). Fatigue triggers included physical exertion, exercise, illness, inadequate rest, and work or caregiving responsibilities. Resting and pacing activities were the most frequently reported management strategies. Most participants had discussed fatigue with their physicians. These interactions varied from positive, to neutral, to negative; negative experiences included not feeling listened to, or feeling like the physician did not take action to address the fatigue. Most participants would be open to discussing fatigue with their physician in the future.

**Summary/Conclusion**

Fatigue is a complex, debilitating symptom experienced by patients with SCD, thalassemia, and PK deficiency. While physical fatigue was identified as the most bothersome type, mental and emotional fatigue contributed substantially to patients' overall fatigue burden. This study highlights the critical importance of raising awareness of the various manifestations and broad impacts of fatigue to better inform patient care across HHAs.

**Copyright Information:** (Online) ISSN: 2572-9241
©2026 The Author(s). HemaSphere is published by John Wiley & Sons Ltd on behalf of European Hematology Association. This is an open access Abstract Book distributed under the Attribution-NonCommercial-NoDerivs (CC BY-NC-ND), which allows third parties to download the articles and share them with others as long as they credit the author and the Abstract Book, but they cannot change the content in any way or use them commercially.
**Abstract Book Citations:** Authors, Title, HemaSphere, 2026;10:(S1):pages. The abstract book DOIs can be found on the "Author Information" page.
**Disclaimer:** Articles published in the journal HemaSphere exclusively reflect the opinions of the authors. The authors are responsible for all content in their abstracts including accuracy of the facts, statements, citing resources, etc.

**Figure.** Most bothersome impacts of fatigue across hereditary hemolytic anemias (N=57*)

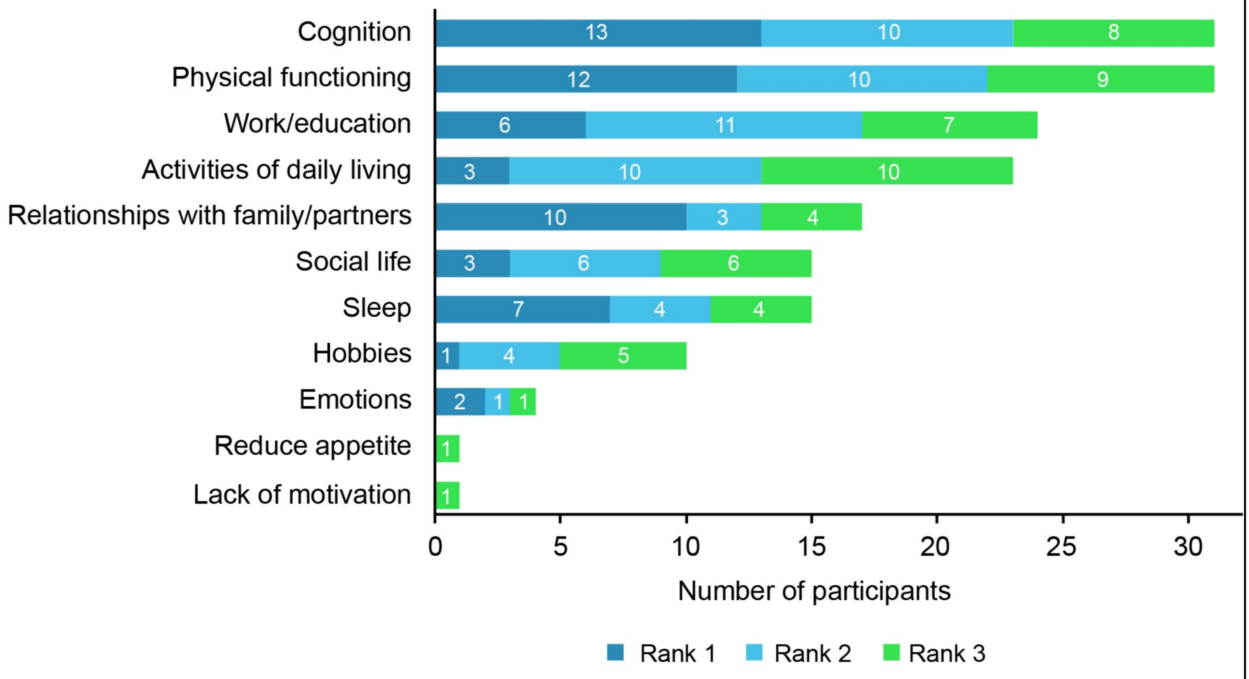

*One participant with thalassemia did not complete this ranking exercise.

**Copyright Information:** (Online) ISSN: 2572-9241
©2026 The Author(s). HemaSphere is published by John Wiley & Sons Ltd on behalf of European Hematology Association. This is an open access Abstract Book distributed under the Attribution-NonCommercial-NoDerivs (CC BY-NC-ND), which allows third parties to download the articles and share them with others as long as they credit the author and the Abstract Book, but they cannot change the content in any way or use them commercially.
**Abstract Book Citations:** Authors, Title, HemaSphere, 2026;10:(S1):pages. The abstract book DOIs can be found on the “Author Information” page.
**Disclaimer:** Articles published in the journal HemaSphere exclusively reflect the opinions of the authors. The authors are responsible for all content in their abstracts including accuracy of the facts, statements, citing resources, etc.

### (PB4418) STRESS COPING THEORY-BASED NURSING PROGRAM ENHANCES CAREGIVING OUTCOMES IN ACUTE LEUKEMIA

**Topic:** 36. Quality of life, ethics, supportive and palliative care

Hong Cheng*[1]

[1] *The First Affiliated Hospital, Zhejiang University ,School of Medicine, Department of Nursing, [illegible], China;*

### Background

Caregivers of acute leukemia (AL) patients face heavy physical and psychological burdens, yet few stress coping theory-based interventions address their positive experiences and coping needs. We aimed to explore the effects of a nursing intervention based on stress coping theory on primary caregivers of AL patients.

### Aims

Based on this theoretical foundation, this study applied stress coping theory to develop a primary caregiving intervention program for the main caregivers of patients with AL. We aim to enhance caregivers' positive aspects of caregiving, strengthen their perceived social support, and reduce caregiver burden and maladaptive coping strategies.

### Methods

Eighty primary caregivers of AL patients hospitalized between October 2023 and December 2024 were retrospectively enrolled and assigned to an intervention group (n = 40) and control group (n = 40). The intervention group received a 5-week stress coping theory-based nursing program in addition to routine care, while the control group received routine care only. Caregiver outcomes were assessed at baseline and one week post-intervention using the Positive Aspects of Caregiving Scale (PAC), Social Support Rating Scale (SSRS), Simplified Coping Style Questionnaire (SCSQ), and Zarit Burden Inventory (ZBI).

### Results

At baseline, there were no significant differences between the intervention and control groups in PAC, SSRS, SCSQ, or ZBI scores (all $P > 0.05$). After the 5-week intervention, the intervention group showed significantly higher PAC and SSRS scores and lower SCSQ negative coping and ZBI scores compared with the control group ($P < 0.05$). Within the intervention group, PAC, SSRS scores increased, and SCSQ negative coping and ZBI scores decreased significantly from baseline ($P < 0.05$).

### Summary/Conclusion

Nursing interventions based on stress coping theory effectively enhanced positive caregiving experiences and perceived social support while reducing caregiving burden and negative coping among primary caregivers of AL patients.

**Copyright Information:** (Online) ISSN: 2572-9241
©2026 The Author(s). HemaSphere is published by John Wiley & Sons Ltd on behalf of European Hematology Association. This is an open access Abstract Book distributed under the Attribution-NonCommercial-NoDerivs (CC BY-NC-ND), which allows third parties to download the articles and share them with others as long as they credit the author and the Abstract Book, but they cannot change the content in any way or use them commercially.
**Abstract Book Citations:** Authors, Title, HemaSphere, 2026;10:(S1):pages. The abstract book DOIs can be found on the "Author Information" page.
**Disclaimer:** Articles published in the journal HemaSphere exclusively reflect the opinions of the authors. The authors are responsible for all content in their abstracts including accuracy of the facts, statements, citing resources, etc.

### (PB4419) AUDIO SUMMARIES OF CONSULTATIONS AS AN 'EXTRA SET OF EARS': A QUALITATIVE INTERVIEW STUDY WITH PATIENTS WITH SICKLE CELL DISEASE AND THALASSEMIA

**Topic:** 36. Quality of life, ethics, supportive and palliative care

Lisbeth Birkelund*[1, 2], Henrik Frederiksen[1, 3], Dennis Lund Hansen[1, 3], Dorthe Susanne Nielsen[1, 4]

*[1]University of Southern Denmark, Department of Clinical Research, Odense M, Denmark;[2]Odense University Hospital, Odense C, Denmark; [3]Odense University Hospital, Department of Hematology, Odense C, Denmark;[4]Odense University Hospital, Department of Geriatric Medicine, Odense C, Denmark;*

### Background

Sickle cell disease and thalassemia are hereditary blood disorders characterized by abnormal hemoglobin, leading to chronic anemia and organ damage. Consequently, patients with these conditions often face complex treatment trajectories, including frequent hospital visits. As the diseases originate from malaria-endemic regions, they primarily affect individuals whose ethnic origins trace back to these areas and thus mainly affect ethnic minority populations in Denmark. While all patients must navigate and act on large amounts of complex medical information, language barriers may pose additional challenges in understanding and applying information provided during clinical consultations. In this context, audio-supported tools have been proposed to help patients recall information and support them in navigating treatment.

### Aims

This study explores how patients with sickle cell disease or thalassemia experience receiving audio summaries of hematology consultations, with the aim of understanding their perceived usefulness.

### Methods

The study is carried out in Denmark and uses a qualitative, phenomenological-hermeneutic approach. Following informed consent, semi-structured interviews were conducted with eight patients with sickle cell disease, thalassemia, or both. Two interviews included family caregivers, resulting in a total of ten informants. Participants had diverse ethnic backgrounds and varying levels of Danish proficiency; some were born in Denmark and fluent in Danish, while others had arrived as immigrants or refugees. According to participants' preferences, two interviews were mediated by a professional interpreter, one by a Danish-speaking family caregiver, and one was conducted in English. Transcripts are thematically analyzed, inspired by Paul Ricoeur's theory of interpretation.

### Results

Preliminary analysis points to one overarching theme: audio summaries functioning as an "extra set of ears" in constrained everyday lives, reflecting participants' experiences of support in recalling and understanding information. Patients described living with pain, fatigue, and limitations, affecting both themselves and their families. Notably, fatigue and cognitive constraints were experienced not only by patients but also by a family caregiver, who described the effort involved in receiving and recalling information from clinical consultations. In this context, audio summaries were perceived as helpful in supporting both patients' and caregivers' memory and understanding. Although not all participants had used them, they emphasized that audio summaries provide reassurance and can even strengthen connections with healthcare providers, contributing to a sense of being afforded more time. Factors influencing engagement with audio summaries included language barriers and patients' physical state during consultations.

**Copyright Information:** (Online) ISSN: 2572-9241
©2026 The Author(s). HemaSphere is published by John Wiley & Sons Ltd on behalf of European Hematology Association. This is an open access Abstract Book distributed under the Attribution-NonCommercial-NoDerivs (CC BY-NC-ND), which allows third parties to download the articles and share them with others as long as they credit the author and the Abstract Book, but they cannot change the content in any way or use them commercially.
**Abstract Book Citations:** Authors, Title, HemaSphere, 2026;10:(S1):pages. The abstract book DOIs can be found on the "Author Information" page.
**Disclaimer:** Articles published in the journal HemaSphere exclusively reflect the opinions of the authors. The authors are responsible for all content in their abstracts including accuracy of the facts, statements, citing resources, etc.

**Summary/Conclusion**

Although preliminary, the findings suggest that audio summaries can serve as a small yet effective communicative intervention, supporting patients in navigating complex treatment trajectories while also reducing caregiver burden associated with attending consultations. Their potential is not limited to sickle cell disease and thalassemia but may also extend to other serious hematological conditions. Audio summaries may also be particularly valuable in clinical areas such as geriatrics and oncology, where patients face cognitive or treatment-related challenges, as well as in consultations affected by language barriers more generally.

**Copyright Information:** (Online) ISSN: 2572-9241
©2026 The Author(s). HemaSphere is published by John Wiley & Sons Ltd on behalf of European Hematology Association. This is an open access Abstract Book distributed under the Attribution-NonCommercial-NoDerivs (CC BY-NC-ND), which allows third parties to download the articles and share them with others as long as they credit the author and the Abstract Book, but they cannot change the content in any way or use them commercially.
**Abstract Book Citations:** Authors, Title, HemaSphere, 2026;10:(S1):pages. The abstract book DOIs can be found on the “Author Information” page.
**Disclaimer:** Articles published in the journal HemaSphere exclusively reflect the opinions of the authors. The authors are responsible for all content in their abstracts including accuracy of the facts, statements, citing resources, etc.

### (PB4420) INVESTIGATING FINANCIAL HARDSHIPS IN PATIENTS WITH VEXAS. REAL-WORLD EVIDENCE FROM A MULTICENTER OBSERVATIONAL STUDY BY THE GIMEMA

**Topic:** 36. Quality of life, ethics, supportive and palliative care

Fabio Efficace*[1], Elisa Diral[2], Daniela Krepper[1, 3], Corrado Campochiaro[4], Fabrizio Pane[5], Giorgia Battipaglia[5], Erika Morsia[6], Jaqueline Ferrari[7], Marco Frigeni[8], Isabella Capodanno[9], Gabriele Todisco[10], Cinzia Sissa[11], Bruno Fattizzo[12], Cristina Papayannidis[13], Paola Guglielmelli[14], Chiara Cattaneo[15], Elena Crisà[16], Arianna Savi[17], Alfonso Fiumarella[18], Davide Firinu[19], Francesca Tartaglia[1], Laura Cicconi[20], Antonio Pierini[21], Paola Triggianese[22], Paola Fazi[1], Maria Teresa Voso[22], Carmelo Gurnari[22]

*[1]Italian Group for Adult Hematologic Diseases (GIMEMA), Data Center and Health Outcomes Research Unit, Rome, Italy, Rome, Italy; [2]Unit of Hematology and Bone Marrow Transplant, IRCCS San Raffaele Scientific Institute, Milan, Italy; [3]University Hospital of Psychiatry II, Medical University of Innsbruck, Innsbruck, Austria; [4]Unit of Immunology, Rheumatology, Allergy and Rare Diseases, IRCCS San Raffaele Hospital, Vita-Salute San Raffaele University, Milan, Italy; [5]Department of Clinical Medicine and Surgery, University Federico II, Hematology, Naples, Italy; [6]Hematology Unit, Department of Clinical and Molecular Sciences, DISCLIMO, Università Politecnica delle Marche, Ancona, Italy; [7]Division of Molecular Hematology and Precision Medicine, Fondazione IRCCS Policlinico San Matteo, Pavia, Italy; [8]Department of Oncology and Hematology, ASST Papa Giovanni XXIII, SC Ematologia, Bergamo, Italy; [9]SOC Ematologia Azienda USL-IRCSS di Reggio Emilia, Reggio Emilia, Italy; [10]Department of Oncology and Hematology, Humanitas Cancer Center, IRCCS Humanitas Research Hospital, Rozzano, Italy; [11]Section of Hematology, Department of Medicine, University of Verona, Verona, Italy; [12]Department of Oncology and Haemato-Oncology, University of Milan, Milan, Italy; [13]IRCCS Azienda Ospedaliero-Universitaria di Bologna, Istituto di Ematologia "Seràgnoli", Bologna, Italy; [14]Department of Experimental and Clinical Medicine, Center for Research and Innovation of Myeloproliferative Neoplasms (CRIMM), AOU Careggi, University of Florence, Florence, Italy; [15]Divisione di Ematologia, ASST-Spedali Civili di Brescia, Brescia, Italy; [16]Candiolo Cancer Institute, FPO-IRCCS, Candiolo, Turin, Italy; [17]Department of Clinical and Biological Sciences, University of Turin, Orbassano, Turin, Italy; [18]UOC ematologia ospedale A. tortora di Pagani -ASL di Salerno, Salerno, Italy; [19]Department of Medical Sciences and Public Health, University of Cagliari, Cagliari, Italy;[20]Department of Hematology, Polo Universitario Pontino, S.M. Goretti Hospital, Latina, Italy; [21]Institute of Hematology, Centro Ricerche Emato-Oncologiche, Ospedale S. Maria della Misericordia, University of Perugia, Perugia, Italy; [22]Department of Biomedicine and Prevention, University of Rome Tor Vergata, Rome, Italy;*

### Background

VEXAS (vacuoles, E1 enzyme, X-linked, autoinflammatory, somatic) is a newly recognized autoinflammatory syndrome characterized by severe multisystem morbidity. While its clinical impact is increasingly understood, information on the burden of financial hardships experienced by these patients is lacking.

### Aims

The primary objective of this analysis is to assess financial toxicity (FT) reported by patients with VEXAS and investigate its determinants. A secondary objective was to explore the relationship between FT and the health-related quality of life (HRQoL) profile of these patients.

**Copyright Information:** (Online) ISSN: 2572-9241
©2026 The Author(s). HemaSphere is published by John Wiley & Sons Ltd on behalf of European Hematology Association. This is an open access Abstract Book distributed under the Attribution-NonCommercial-NoDerivs (CC BY-NC-ND), which allows third parties to download the articles and share them with others as long as they credit the author and the Abstract Book, but they cannot change the content in any way or use them commercially.
**Abstract Book Citations:** Authors, Title, HemaSphere, 2026;10:(S1):pages. The abstract book DOIs can be found on the "Author Information" page.
**Disclaimer:** Articles published in the journal HemaSphere exclusively reflect the opinions of the authors. The authors are responsible for all content in their abstracts including accuracy of the facts, statements, citing resources, etc.

### Methods

This analysis is based on 59 patients diagnosed with VEXAS enrolled in a multicenter observational study by the Italian Group for Adult Hematologic Diseases (GIMEMA). Adult patients with a confirmed diagnosis of VEXAS, defined by a pathogenic mutation of the *UBA1* gene and consistent clinical manifestation, were included. Participants completed a predefined set of validated patient-reported outcome (PRO) measures, including the Patient-Reported Outcome for Fighting Financial Toxicity (PROFFIT) questionnaire. The PROFFIT score ranges from 0 to 100, with higher scores indicating greater FT. Potential determinants of FT analyzed for their association with the PROFFIT score included key aspects such as medical expenses, transport, and support from medical staff. HRQoL was assessed with the EORTC QLQ-C30, and HRQoL profiles were compared between patients with lower and higher FT, dichotomized by the median PROFFIT score.

### Results

Patients had a median age of 74 years (IQR 68.0-77.5), and the majority (69.5%) had at least an intermediate level of education. At the time of evaluation, 78% patients were receiving treatment for VEXAS, and 72.9% had concomitant diseases.
The mean PROFFIT score was 24.3 (SD 23.9), and no statistically significant differences were observed across sociodemographic or clinical subgroups. A high proportion of patients reported out-of-pocket payments for private medical consultations (77.6%) and additional medications or supplements (71.9%). However, these medical expenses were not associated with higher FT. In contrast, transport costs were associated with FT: PROFFIT scores increased progressively with greater distance from the treatment center (median 9.5 vs. 42.9; p = 0.051), and a considerable amount of travel expenses emerged as the only statistically significant determinant of financial toxicity (median 9.5 vs. 35.7; p = 0.001). The level of financial burden was also corroborated by the financial difficulties item from the EORTC QLQ-C30, indicating that 28.8% of patients reported financial difficulties. Exploratory analysis also indicated a statistically significant association between higher FT and lower global quality of life (p=0.009). Differences in the HRQoL profile by FT were more evident across functional outcomes rather than symptom aspects.

### Summary/Conclusion

Our findings indicate that FT is an important aspect to consider in patients with VEXAS and suggest that this is primarily driven by the perceived burden of travel-related expenses in a healthcare system of universal health care largely financed by government through a Beveridge model. Our results also suggest that patients with higher FT may report a lower HRQoL profile. Overall, these data underscore the need to systematically assess and address financial burden as part of comprehensive patient-centered care, even in the context of solidaristic health systems.

**Copyright Information:** (Online) ISSN: 2572-9241
©2026 The Author(s). HemaSphere is published by John Wiley & Sons Ltd on behalf of European Hematology Association. This is an open access Abstract Book distributed under the Attribution-NonCommercial-NoDerivs (CC BY-NC-ND), which allows third parties to download the articles and share them with others as long as they credit the author and the Abstract Book, but they cannot change the content in any way or use them commercially.
**Abstract Book Citations:** Authors, Title, HemaSphere, 2026;10:(S1):pages. The abstract book DOIs can be found on the "Author Information" page.
**Disclaimer:** Articles published in the journal HemaSphere exclusively reflect the opinions of the authors. The authors are responsible for all content in their abstracts including accuracy of the facts, statements, citing resources, etc.

### (PB4421) EFFICACY AND SAFETY OF HETROMBOPAG FOR SECONDARY PREVENTION OF LYMPHOMA TREATMENT-INDUCED THROMBOCYTOPENIA: AN EXPLORATORY CLINICAL STUDY

**Topic:** 36. Quality of life, ethics, supportive and palliative care

Ping Li[1], Hai-Chen Wei[1], Hui Wang[1], Ke Lu[1], Zengjun Li*[1]

*[1]Shandong Cancer Hospital and Institute, Shandong First Medical University and Shandong Academy of Medical Sciences, Shandong, China;*

### Background

Chemotherapy-induced thrombocytopenia (CIT) increases the risk of bleeding complications and the need for platelet transfusion and may lead to dose reductions or discontinuation of anti-tumor therapy. Thrombopoietin receptor agonists (TPO-RAs) stimulate platelet production by activating the thrombopoietin receptor.
Hetrombopag, an orally administered, small-molecule synthetic TPO-RA, has demonstrated promising efficacy in the treatment of CIT, with an acceptable safety profile. However, prospective evidence in patients with lymphoma remains lacking.

### Aims

This study aimed to evaluate the efficacy and safety of hetrombopag for the secondary prevention of CIT in patients with lymphoma, providing guidance for its clinical use.

### Methods

The present study was a multicenter, single-arm, exploratory study. This study was registered at chictr.org.cn (ChiCTR 2400094077). All informed consents were obtained from all participants before study entry.

Patients who had a platelet count < $50\times10^9$/L due to chemotherapy in the previous cycle and recovered to≥$100\times10^9$/L before the current chemotherapy cycle were enrolled. Eligible patients received hetrombopag (7.5mg, orally, daily) 24-48 hours after the current chemotherapy cycle, in a 14-day cycle for 2 or 3 cycles. After the last dose of hetrombopag, patients entered a 28-day safety follow-up period.

The primary endpoint was the occurrence rate of platelet count < $50\times10^9$/L during the first cycle of secondary prevention. Secondary endpoints included the duration of platelet count < $50 \times 10^9$/L during the first cycle; incidence of platelet count < $25 \times 10^9$/L during the first cycle; proportion of patients with treatment delay ≥ 4 days, dose reduction ≥ 15%, or treatment discontinuation due to thrombocytopenia; proportion of patients requiring platelet rescue therapy during the first cycle; and safety profile.

**Copyright Information:** (Online) ISSN: 2572-9241
©2026 The Author(s). HemaSphere is published by John Wiley & Sons Ltd on behalf of European Hematology Association. This is an open access Abstract Book distributed under the Attribution-NonCommercial-NoDerivs (CC BY-NC-ND), which allows third parties to download the articles and share them with others as long as they credit the author and the Abstract Book, but they cannot change the content in any way or use them commercially.
**Abstract Book Citations:** Authors, Title, HemaSphere, 2026;10:(S1):pages. The abstract book DOIs can be found on the "Author Information" page.
**Disclaimer:** Articles published in the journal HemaSphere exclusively reflect the opinions of the authors. The authors are responsible for all content in their abstracts including accuracy of the facts, statements, citing resources, etc.

**Results**

Between January, 2025 and November 2025, a total of 20 patients with lymphoma were included. The median age was 58 years (range: 27-85 years). Twelve patients had diffuse large B cell lymphoma, 5 had peripheral T-cell lymphoma, and 3 had follicular lymphoma or mantle cell lymphoma. The median baseline platelet count was $185\times10^9$/L (range: $113\text{-}286\times10^9$/L).
In the first cycle of secondary prevention, 5% (1/20) of patient had a platelet count < $50\times10^9$/L, and 60% (15/20) had a platelet count $\geq100\times10^9$/L. In the second cycle, 9% (1/11) of patients had a platelet count < $50\times10^9$/L, and 64% (7/11) had a platelet count $\geq100\times10^9$/L. None of the patients had a platelet count [illegible]$25\times10^9$/L during the whole treatment period. Two patients delayed and reduced the treatment dose due to CIT. Four patients received platelet transfusion. Regarding safety, one patient experienced grade 3 anemia, one had a grade 3 febrile neutropenia, one had a grade 4 pulmonary infection, all of which were deemed unrelated to hetrombopag treatment.

**Summary/Conclusion**

The preliminary results of secondary prevention with hetrombopag for CIT showed potential efficacy and an acceptable safety profile in patients with lymphoma. Further studies with larger sample size are warrant to validate these findings.

**Copyright Information:** (Online) ISSN: 2572-9241
©2026 The Author(s). HemaSphere is published by John Wiley & Sons Ltd on behalf of European Hematology Association. This is an open access Abstract Book distributed under the Attribution-NonCommercial-NoDerivs (CC BY-NC-ND), which allows third parties to download the articles and share them with others as long as they credit the author and the Abstract Book, but they cannot change the content in any way or use them commercially.
**Abstract Book Citations:** Authors, Title, HemaSphere, 2026;10:(S1):pages. The abstract book DOIs can be found on the "Author Information" page.
**Disclaimer:** Articles published in the journal HemaSphere exclusively reflect the opinions of the authors. The authors are responsible for all content in their abstracts including accuracy of the facts, statements, citing resources, etc.

### (PB4422) MEASUREMENT PROPERTIES OF PATIENT-REPORTED OUTCOME MEASURES IN MYELOPROLIFERATIVE NEOPLASMS: A COSMIN SYSTEMATIC REVIEW

**Topic:** 36. Quality of life, ethics, supportive and palliative care

Luise Stach von Goltzheim*[1], Sam Salek[2], Tatyana Ionova[3], Edward Laane[4], Esther N. Oliva[5], Nicole Skoetz[1], Ina Monsef[1], Julia Schroer[1], Mario Csenar[1]

*[1]Institute of Public Health, Faculty of Medicine and University Hospital Cologne, University of Cologne, Cologne, Germany; [2]School of Health, Medicine and Life Sciences, University of Hertfordshire, Hatfield, United Kingdom; [3]Quality of Life Monitoring Unit, Saint Petersburg State University Hospital, Saint Petersburg, Russia; [4]Hematology-Oncology Clinic, University of Tartu, Tartu, Estonia; [5]Hematology Department, London North West University Healthcare NHS Trust, London, United Kingdom;*

myelofibrosis (MF), essential thrombocythemia (ET), and polycythemia vera (PV), that are distinguished by clonal proliferation of myeloid cells and an increased risk in thrombotic events. The chronic nature of these diseases results in patients experiencing prolonged impact of disease and symptoms associated with disease and/or treatment. Therefore, systematic assessment and targeted enhancement of patient-reported outcomes in Ph- MPN patients is of particular significance. -->

**Background**

Philadelphia chromosome-negative (Ph-) myeloproliferative neoplasms (MPN) represent a group of hematological malignancies, namely myelofibrosis (MF), essential thrombocythemia (ET), and polycythemia vera (PV), that are distinguished by clonal proliferation of myeloid cells and an increased risk in thrombotic events. The chronic nature of these diseases results in patients experiencing prolonged impact of disease and symptoms associated with disease and/or treatment. Therefore, systematic assessment and targeted enhancement of patient-reported outcomes in Ph- MPN patients is of particular significance.

**Aims**

To assess and evaluate the validity in terms of measurement properties (MPs) of patient-reported outcome measures (PROMs) in Ph-MPN.

**Methods**

The present systematic review (PROSPERO: CRD42023447408) was conducted in accordance with the COSMIN-Manual for systematic reviews of PROMs (v2.0). A systematic search for validation studies of PROMs that are identified for use in Ph- MPN was conducted in the MEDLINE and EMBASE databases up to April 2025. Following screening of the search results and the inclusion of relevant validation studies, these studies were utilized to examine MPs and interpretability of the PROMs. Furthermore, the methodological quality, including risk of bias, of the validation studies was assessed. The results for each PROM were subsequently summarized. Finally, the certainty of the evidence was determined using a Grading of Recommendations, Assessment, Development and Evaluation (GRADE) approach adapted from COSMIN.

**Copyright Information:** (Online) ISSN: 2572-9241
©2026 The Author(s). HemaSphere is published by John Wiley & Sons Ltd on behalf of European Hematology Association. This is an open access Abstract Book distributed under the Attribution-NonCommercial-NoDerivs (CC BY-NC-ND), which allows third parties to download the articles and share them with others as long as they credit the author and the Abstract Book, but they cannot change the content in any way or use them commercially.
**Abstract Book Citations:** Authors, Title, HemaSphere, 2026;10:(S1):pages. The abstract book DOIs can be found on the "Author Information" page.
**Disclaimer:** Articles published in the journal HemaSphere exclusively reflect the opinions of the authors. The authors are responsible for all content in their abstracts including accuracy of the facts, statements, citing resources, etc.

**Results**

The systematic search identified a total of 52 reports on validation studies of twelve PROMs (including different versions of a tool) used in MF, ET, PV or Ph- MPN overall. At the PROM level, it was found that no reliable conclusions could be drawn for five PROMs (MFSAF, modified MFSAF, SC-MPN, EORTC QLQ-C30, and FACT-An) because the MPs examined in the validation studies were rated as indeterminate. For a further six PROMs (MFSAF v2.0, MPN-SAF, MPN-SAF TSS, modified MPN-SAF TSS, MPN-SD, EQ-5D), only one or two MPs could be evaluated, while the remaining MPs were either not examined or also classified as indeterminate. MFSAF v4.0 was subjected to the most comprehensive analysis. The instrument demonstrated sufficient content validity in terms of comprehensiveness, structural validity and internal consistency with low to high evidence certainty. However, content validity, reliability, construct validity, and responsiveness were found to be inconsistent, due to the underlying studies yielding divergent conclusions. At the level of the MPs, it was determined that cross-cultural validity, measurement error, and criterion validity had not been adequately examined in any validation study. Considering only conclusive assessments, internal consistency was assessed for one PROM, responsiveness for two, content validity, structural validity, and reliability for three, and construct validity for four PROMs.

**Summary/Conclusion**

MPs of PROMs utilized in Ph- MPN have been validated to varying degrees and the existence of multiple versions per instrument complicates their application. Despite observed correlations between PROMs, the extent to which results obtained with one instrument or version can be transferred to another remains unclear. Our results underscore the necessity for additional, more exhaustive validation studies to ensure the appropriate application and interpretation of PROMs in Ph- MPN.

**Copyright Information:** (Online) ISSN: 2572-9241
©2026 The Author(s). HemaSphere is published by John Wiley & Sons Ltd on behalf of European Hematology Association. This is an open access Abstract Book distributed under the Attribution-NonCommercial-NoDerivs (CC BY-NC-ND), which allows third parties to download the articles and share them with others as long as they credit the author and the Abstract Book, but they cannot change the content in any way or use them commercially.
**Abstract Book Citations:** Authors, Title, HemaSphere, 2026;10:(S1):pages. The abstract book DOIs can be found on the “Author Information” page.
**Disclaimer:** Articles published in the journal HemaSphere exclusively reflect the opinions of the authors. The authors are responsible for all content in their abstracts including accuracy of the facts, statements, citing resources, etc.

### (PB4423) COMPARISON OF IVOSIDENIB- AND VENETOCLAX-BASED TREATMENT SEQUENCING AND OUTCOMES IN PATIENTS WITH MIDH1 ACUTE MYELOID LEUKEMIA: A REAL-WORLD STUDY

**Topic:** 36. Quality of life, ethics, supportive and palliative care

Vanessa Kennedy*[1], Tarun Bhagnani[2], Weilong Li[2], Assaf Vestin[2], Anne Angiolillo[2], Debasish Mazumder[3], Utkarsh Parmar[3], Amer M. Zeidan[4]

*[1]Stanford University, Palo Alto, United States of America; [2]Servier Pharmaceuticals, Boston, United States of America; [3]Putnam Associates, Gurgaon, India; [4]Yale University, New Haven, United States of America;*

### Background

Ivosidenib (IVO)- and venetoclax (VEN)-based treatment regimens are widely used in patients with isocitrate dehydrogenase-mutated (mIDH1) acute myeloid leukemia (AML); however, evidence guiding optimal sequencing of these agents is limited.

### Aims

To compare real-world outcomes between two treatment sequences in mIDH1 AML: IVO followed by VEN (IVO→VEN) vs VEN followed by IVO (VEN→IVO).

### Methods

This retrospective cohort study used closed claims data from the Komodo Research Dataset, which includes longitudinal medical and pharmacy claims for >330 million US patients. Adult patients (≥18 years) with newly diagnosed AML between January 2018 and June 2025, with ≥180 days of continuous medical and pharmacy coverage prior to diagnosis, no prior leukemia history, and exposure to both IVO- and VEN-based regimens, were included. Outcomes included duration of treatment (DOT), time to next treatment (TTNT), frequency of red cell and platelet transfusions, healthcare resource utilization (i.e., hospitalizations, intensive care unit [ICU] admissions, emergency room [ER] visits), frequency of stem cell transplantation (SCT), and hematologic adverse events (HAEs). The first occurrence of either agent was designated as the index treatment and the treatment immediately following as subsequent treatment. Outcomes for both cohorts were assessed in index and subsequent treatment phases. Continuous variables were compared using t-test/Mann-Whitney test and categorical variables using Fisher's exact test. TTNT was assessed using Kaplan–Meier methods.

### Results

Of 130 eligible patients, 25 received IVO→VEN and 105 received VEN→IVO. Median age was 68 and 71 years, respectively; median Charlson Comorbidity Index was 1.0 in both cohorts. Median follow-up from index diagnosis was 22 and 18 months, respectively. During the index phase, DOT (median: 4.7 vs 3.0 months, $p$=0.0285; mean 6.9 vs 4.2 months, $p$=0.0328) and TTNT (median: 5.5 vs 3.9 months; $p$=0.0367) were longer for IVO than for VEN. Compared with VEN→IVO, the IVO→VEN cohort had a higher proportion of transfusion-independent patients during the index phase (52% vs 25.7%; $p$=0.0155) and lower per patient per month (PPPM) number of transfusions (0.57 vs 1.11; $p$<0.0001).
IVO→VEN had numerically lower proportion of AML-related hospitalization in index phase (40.0% vs 60.0%), lower PPPM number of hospitalizations (0.27 vs 1.37; $p$<0.0001), lower ICU admission rates (0% vs 4.8%), and numerically lower ER visit rates (8% vs 24.8%) than VEN→IVO. HAEs assessed within 3 months of treatment initiation were numerically lower for IVO. The proportion of patients proceeding to SCT was similar: 24.0% and 19.0% for IVO→VEN and VEN→IVO, respectively.
Similar patterns were observed in the subsequent phase, with IVO associated with longer DOT and fewer transfusions, hospitalizations, ICU and ER visits, and HAEs than VEN (Table).

**Copyright Information:** (Online) ISSN: 2572-9241
©2026 The Author(s). HemaSphere is published by John Wiley & Sons Ltd on behalf of European Hematology Association. This is an open access Abstract Book distributed under the Attribution-NonCommercial-NoDerivs (CC BY-NC-ND), which allows third parties to download the articles and share them with others as long as they credit the author and the Abstract Book, but they cannot change the content in any way or use them commercially.
**Abstract Book Citations:** Authors, Title, HemaSphere, 2026;10:(S1):pages. The abstract book DOIs can be found on the "Author Information" page.
**Disclaimer:** Articles published in the journal HemaSphere exclusively reflect the opinions of the authors. The authors are responsible for all content in their abstracts including accuracy of the facts, statements, citing resources, etc.

### Summary/Conclusion

In the real-world treatment of mIDH1 AML, IVO treatment phase was associated with longer treatment duration, greater transfusion independence – a surrogate for improved quality of life, lower intensity of acute care utilization, and a more favorable hematologic safety profile relative to VEN phase, irrespective of treatment line. Treatment sequence did not impact SCT rates. These findings support earlier integration of IVO within treatment sequencing to enhance treatment durability while reducing treatment-associated toxicity and acute care burden in patients with mIDH1 AML.

**Table.** Outcomes with IVO and VEN in Index and Subsequent Phases

| | Index treatment phase | | | Subsequent treatment phase | | |
|---|---|---|---|---|---|---|
| | *IVO→VEN (N=25) | *VEN→IVO (N=105) | *p*-value | IVO→*VEN (N=25) | VEN→*IVO (N=105) | *p*-value |
| **DOT, median (IQR)** | 4.7 (2.5-9.1) | 3.0 (2.1-5.2) | 0.0285 | 2.8 (2.6-4.7) | 3.8 (2.1-5.7) | 0.7840 |
| **DOT, mean (SD)** | 6.9 (5.7) | 4.2 (3.7) | 0.0328 | 4.0 (2.6) | 6.0 (8.5) | 0.0398 |
| **≥1 transfusions, N (%)** | 12 (48) | 78 (74.3) | 0.0155 | 18 (72) | 69 (65.7) | 0.6407 |
| PPPM (SE) transfusions | 0.57 | 1.11 | <0.0001 | 1.32 | 1.01 | <0.0059 |
| **≥1 AML-related hospitalization, N (%)** | 10 (40.0%) | 63 (60.0%) | 0.0776 | 17 (68.0%) | 42 (40.0%) | 0.0142 |
| PPPM (SE) Hospitalizations | 0.27 (0.23) | 1.37 (0.05) | <0.0001 | 1.14 (0.12) | 0.96 (0.06) | 0.2077 |
| Mean (SE) LOS per hospitalization, days | 14.53 (0.27) | 3.81 (0.06) | <0.0001 | 2.65 (0.12) | 2.44 (0.07) | 0.5368 |
| **≥1 ICU admissions, N (%)** | 0 (0.0%) | 5 (4.8%) | NE | 1 (4.0%) | 6 (5.7%) | >0.9999 |
| PPPM (SE) ICU admissions | 0 (0.0) | 0.35 (0.41) | NE | 0.17 (1.00) | 0.54 (0.29) | 0.1943 |
| Mean (SE) LOS per ICU admission, days | 0 (0.0) | 3.17 (0.35) | NE | 1.00 (0.87) | 3.17 (0.25) | 0.2823 |
| **≥1 ER visit, N (%)** | 2 (8.0%) | 26 (24.8%) | 0.1019 | 5 (20.0%) | 15 (14.3%) | 0.5381 |
| PPPM (SE) ER visits | 0.37 (0.45) | 0.24 (0.17) | 0.4081 | 0.35 (0.35) | 0.24 (0.22) | 0.4068 |
| **Safety outcomes, N (%)** | | | | | | |
| Neutropenia | 4 (16.0) | 21 (20.0) | 0.7826 | 7 (28.0) | 18 (17.1) | 0.259 |
| Febrile neutropenia | 11 (44.0) | 53 (50.5) | 0.6681 | 11 (44.0) | 37 (35.2) | 0.4907 |
| Thrombocytopenia | 9 (36.0) | 52 (49.5) | 0.2685 | 13 (52.0) | 42 (40.0) | 0.3679 |
| Anemia | 11 (44.0) | 57 (54.3) | 0.3811 | 13 (52.0) | 42 (40.0) | 0.3679 |

*Indicates IVO or VEN treatment phase. AML, acute myeloid leukemia; DOT, duration of treatment; ER, emergency room; ICU, intensive care unit; IQR, interquartile range; IVO, ivosidenib; LOS, length of stay; NE, not estimated; PPPM, per patient per month; SE, standard error; VEN, venetoclax.

**Copyright Information:** (Online) ISSN: 2572-9241
©2026 The Author(s). HemaSphere is published by John Wiley & Sons Ltd on behalf of European Hematology Association. This is an open access Abstract Book distributed under the Attribution-NonCommercial-NoDerivs (CC BY-NC-ND), which allows third parties to download the articles and share them with others as long as they credit the author and the Abstract Book, but they cannot change the content in any way or use them commercially.
**Abstract Book Citations:** Authors, Title, HemaSphere, 2026;10:(S1):pages. The abstract book DOIs can be found on the "Author Information" page.
**Disclaimer:** Articles published in the journal HemaSphere exclusively reflect the opinions of the authors. The authors are responsible for all content in their abstracts including accuracy of the facts, statements, citing resources, etc.

### (PB4424) FROM KNOWLEDGE TO SAFETY: A MULTIMODAL SIMULATION APPROACH IN CLINICAL HEMATOLOGY

**Topic:** 36. Quality of life, ethics, supportive and palliative care

Oumayma Hari*[1], Imane El Boutahiri[1], Siham Belkadi[1], Safae Regragui[1]

*[1]faculty of medecin, clinical hematology, tangier, Morocco;*

### Background

Medical errors account for an estimated 400,000 deaths annually, with at least half considered preventable, highlighting the urgent need for effective educational strategies to improve patient safety. In onco-hematological, patients are particularly vulnerable due to immunosuppression, treatment-related toxicity, and the complexity of their care pathways. In this high-risk setting, adverse outcomes may be exacerbated by lapses in aseptic practices, delayed recognition of clinical deterioration, or suboptimal management of emergencies such as febrile neutropenia.
Simulation-based learning (SBL) and "Room of Errors/Room of Horrors" (ROE/ROH) exercises have demonstrated educational benefits across several medical disciplines, particularly in critical care and surgical specialties. However, despite the complexity and fragility of hematology patients, the integration of structured simulation-based training in clinical hematology remains limited and insufficiently explored.

### Aims

Assess the impact of SBL on knowledge acquisition related to febrile neutropenia (FN).
Evaluate safety hazard detection using a hematology-specific Room of Errors exercise.
Explore associations between professional status, years of experience, and performance outcomes.

### Methods

The **SBL workshop** consisted of two clinical simulation scenarios focusing on febrile neutropenia (FN) (pediatric an adult patients). Participants completed an 8-item questionnaire before the simulation and at 3-month follow-up. The assessment addressed key aspects of FN management, including clinical semiology, diagnostic reasoning, therapeutic management, and timeliness of intervention. Paired comparisons were performed using the Wilcoxon and McNemar tests, and item difficulty indices were calculated.
The **Room of Errors (ROE) exercise** involved physicians and nurses inspecting a simulated hospital room representing a patient in post-allogeneic hematopoietic stem cell transplantation aplasia. Participants were asked to identify predefined safety hazards related to diet, aseptic precautions, and patient identification. Group comparisons were conducted using the Kruskal–Wallis test, and correlations with years of professional experience were assessed using Spearman's coefficient.

### Results

A total of 107 healthcare professionals participated (approximately 50% physicians and 50% nurses) from Morocco and Tunisia, with a mean professional experience of 4.6 years.
SBL led to a significant and sustained improvement in overall knowledge scores and across all domains (semiology, clinical reasoning, management; all $p < 0.001$). Greater relative improvements were observed for items with higher baseline difficulty indices ($p = 0.001$), suggesting enhanced acquisition of complex decision-making skills.
In the ROE exercise, physicians identified significantly more errors overall and specifically in aseptic precautions compared to nurses ($p < 0.008$ and $p < 0.006$, respectively). No significant differences were observed for diet-related or additional errors. Importantly, patient identification errors were infrequently detected across all professional groups.

**Copyright Information:** (Online) ISSN: 2572-9241
©2026 The Author(s). HemaSphere is published by John Wiley & Sons Ltd on behalf of European Hematology Association. This is an open access Abstract Book distributed under the Attribution-NonCommercial-NoDerivs (CC BY-NC-ND), which allows third parties to download the articles and share them with others as long as they credit the author and the Abstract Book, but they cannot change the content in any way or use them commercially.
**Abstract Book Citations:** Authors, Title, HemaSphere, 2026;10:(S1):pages. The abstract book DOIs can be found on the "Author Information" page.
**Disclaimer:** Articles published in the journal HemaSphere exclusively reflect the opinions of the authors. The authors are responsible for all content in their abstracts including accuracy of the facts, statements, citing resources, etc.

**Summary/Conclusion**

A multimodal simulation strategy combining SBL and a Room of Errors exercise significantly improves knowledge retention and uncovers critical patient safety gaps in clinical hematology. These findings support the integration of structured simulation-based training into hematology education programs to enhance clinical decision-making and strengthen a culture of safety in high-risk settings.

**Copyright Information:** (Online) ISSN: 2572-9241
©2026 The Author(s). HemaSphere is published by John Wiley & Sons Ltd on behalf of European Hematology Association. This is an open access Abstract Book distributed under the Attribution-NonCommercial-NoDerivs (CC BY-NC-ND), which allows third parties to download the articles and share them with others as long as they credit the author and the Abstract Book, but they cannot change the content in any way or use them commercially.
**Abstract Book Citations:** Authors, Title, HemaSphere, 2026;10:(S1):pages. The abstract book DOIs can be found on the "Author Information" page.
**Disclaimer:** Articles published in the journal HemaSphere exclusively reflect the opinions of the authors. The authors are responsible for all content in their abstracts including accuracy of the facts, statements, citing resources, etc.

### (PB4425) SYMPTOM BURDEN AND QUALITY OF LIFE IN CHINESE PATIENTS WITH CHRONIC GRAFT-VERSUS-HOST DISEASE: A MULTICENTER CROSS-SECTIONAL STUDY

**Topic:** 36. Quality of life, ethics, supportive and palliative care

Xiaoqi Wang*[1], Yi Luo[2], Xiaoli Liang[1], Shiqing Huang[1], Pengcheng He[3], Xiaoyu Zhu[4], Yajing Xu[5], Rongrong Liu[6], Yan Jinsong[7], Erlie Jiang[8], Dingming Wan[9], Sanbin Wang[10], Li Liu[11], Linghui Xia[12], Yicheng Zhang[13], Jianchuan Deng[14], Jun Peng[15], Linhui Wang[16], Xiiaolin Yi[17], Xiaohui Zhang[18], Xi Zhang[1]

*[1]Department of Hematology, The Second Affiliated Hospital, Army Medical University, chongqing, China;[2]Bone Marrow Transplantation Center, The First Affiliated Hospital, Zhejiang University School of Medicine, hangzhou, China; [3]Department of Hematology, The First Affiliated Hospital of Xi'an Jiaotong University, Xi'an, China; [4]Department of Hematology, The First Affiliated Hospital of USTC, hefei, China; [5]Department of Hematology, Xiangya Hospital, Central South University, changsha, China; [6]Department of Hematology, The First Affiliated Hospital of Guangxi Medical University, nanning, China;[7]Department of Hematology, The Second Hospital of Dalian Medical University, dalian, China; [8]State Key Laboratory of Experimental Hematology, National Clinical Research Center for Blood Diseases, Haihe Laboratory of Cell Ecosystem, Institute of Hematology & Blood Diseases Hospital, Chinese Academy of Medical Sciences, tianjin, China; [9]Department of Hematology, The First Affiliated Hospital of Zhengzhou University, zhengzhou, China; [10]Department of Hematology, 920th Hospital of Joint Logistics Support Force, kunming, China; [11]Department of Hematology, Tangdu Hospital, Air Force Medical University, Xi'an, China;[12]Institute of Hematology, Union Hospital, Tongji Medical College, Huazhong University of Science and Technology, wuhan, China; [13]Department of Hematology, Tongji Hospital, Tongji Medical College, Huazhong University of Science and Technology, wuhan, China; [14]Department of Hematology, The Second Affiliated Hospital of Chongqing Medical University, chongqing, China; [15]Department of Hematology, Qilu Hospital of Shandong University, jinan, China;[16]Department of Hematology, Guizhou Provincial People's Hospital, guiyang, China; [17]Department of Hematology, 923rd Hospital of Joint Logistics Support Force, nanning, China; [18]Peking University People's Hospital, Peking University Institute of Hematology, National Clinical Research Center for Hematologic Disease, Beijing, China;*

### Background

Chronic graft-versus-host disease (cGVHD) remains a major late complication after allogeneic hematopoietic stem cell transplantation (allo-HSCT), significantly impairing patients' long-term quality of life (QoL). However, comprehensive data on symptom burden and its specific impact on QoL in Chinese cGVHD patients are limited.

### Aims

This multicenter study aimed to systematically evaluate symptom burden and health-related QoL in Chinese cGVHD patients, and to identify key symptom drivers associated with QoL impairment.

### Methods

A multicenter cross-sectional study was conducted from February to August 2025 across 18 transplant centers in China, enrolling 579 cGVHD patients. Symptom burden was assessed using the Lee Symptom Scale (LSS). QoL was evaluated using the SF-36, FACT-BMT, and EQ-5D-5L instruments. Spearman correlation analysis was performed to examine associations between symptom burden and QoL measures.

**Copyright Information:** (Online) ISSN: 2572-9241
©2026 The Author(s). HemaSphere is published by John Wiley & Sons Ltd on behalf of European Hematology Association. This is an open access Abstract Book distributed under the Attribution-NonCommercial-NoDerivs (CC BY-NC-ND), which allows third parties to download the articles and share them with others as long as they credit the author and the Abstract Book, but they cannot change the content in any way or use them commercially.
**Abstract Book Citations:** Authors, Title, HemaSphere, 2026;10:(S1):pages. The abstract book DOIs can be found on the "Author Information" page.
**Disclaimer:** Articles published in the journal HemaSphere exclusively reflect the opinions of the authors. The authors are responsible for all content in their abstracts including accuracy of the facts, statements, citing resources, etc.

### Results

The mean LSS total score was 14.49±10.65, with the highest burden observed in energy (17.72±15.43) and eye/oral (16.88±17.13) domains. QoL assessment revealed substantial physical impairment (SF-36 Physical Component Summary: 39.06±9.02). LSS total scores were significantly correlated with SF-36 PCS (ρ=-0.514), FACT-BMT total (ρ=-0.589), and EQ-5D utility scores (ρ=-0.668) (all P<0.001). Among symptom domains, musculoskeletal symptoms showed the strongest negative correlation with SF-36 PCS (ρ=-0.494) and EQ-5D utility (ρ=-0.628). Psychological/emotional (ρ=-0.598) and energy (ρ=-0.499) symptoms were most strongly associated with FACT-BMT total scores (all P<0.001).

### Summary/Conclusion

Chinese cGVHD patients experience substantial symptom burden and impaired QoL, with strong correlations between them. Musculoskeletal symptoms, fatigue, and psychological distress emerge as the predominant drivers of QoL deterioration. These findings underscore the need for targeted interventions addressing these key symptom clusters in clinical management strategies.

**Copyright Information:** (Online) ISSN: 2572-9241
©2026 The Author(s). HemaSphere is published by John Wiley & Sons Ltd on behalf of European Hematology Association. This is an open access Abstract Book distributed under the Attribution-NonCommercial-NoDerivs (CC BY-NC-ND), which allows third parties to download the articles and share them with others as long as they credit the author and the Abstract Book, but they cannot change the content in any way or use them commercially.
**Abstract Book Citations:** Authors, Title, HemaSphere, 2026;10:(S1):pages. The abstract book DOIs can be found on the "Author Information" page.
**Disclaimer:** Articles published in the journal HemaSphere exclusively reflect the opinions of the authors. The authors are responsible for all content in their abstracts including accuracy of the facts, statements, citing resources, etc.

### (PB4426) BODY COMPOSITION ANALYSIS BY ELECTRICAL BIOIMPEDANCE AS A PROGNOSTIC FACTOR FOR SURVIVAL IN PATIENTS WITH HEMATOLOGICAL MALIGNANCIES.

**Topic:** 36. Quality of life, ethics, supportive and palliative care

Victor Urbalejo*[1], Dana Aline Perez Camargo[1], Omar Eduardo Fernandez Vargas[1], Gabriela Bravo Alvarez[1], Erika Thalia Copca Mendoza[1], Evelin Valeria Moreno Vargas[1], Kenia Guadalupe Martinez Muñoz[1], Francisco Ramirez Vargas[1], Karla Andrea Gómez Pereyra[1]

[1]*Instituto Nacional de Cancerología, Hematologia, Ciudad de México, Mexico;*

### Background

The prognostic role of body composition analysis (BCA) and phase angle (PhA) in adult patients with Hematologic Malignancies (HM) remains unclear. This study evaluates the impact of body composition parameters and PhA on overall survival (OS) and disease-free survival (DFS) in HM patients receiving intensive chemotherapy.
Recent research suggests a link between nutritional factors and treatment response in patients with HM, yet current risk assessment tools don't incorporate them. Malnutrition can lead to chemotherapy intolerance and poorer OS, while a high body mass index (BMI) is associated with greater toxicities and worse outcomes. However, these findings vary, and BMI's utility is limited by its slow response to dietary changes and inability to distinguish between fat and fat-free (lean) body mass.
In the context of cancer BCA, particularly PhA, has emerged as a promising prognostic marker, including in the acute myeloid leukemia, however, evidence of its utility in patients with HM is limited. Given the need for more precise methods to assess the impact of nutrition on HM outcomes, this study set out to explore the prognostic value of BCA and PhA in adult patients with HM.

### Aims

Describe the body composition analysis and phase angle in adult patients with Hematologic Malignancies and overall survival

### Methods

This is a single center retrospective analysis from tertiary care center from Mexico, that included patients who were older than 16 years old with a diagnosed HM, who received first line therapy from January 2019 to November 2023. The primary objective of this study was to assess the prognostic impact of BCA and PhA measurements on clinically relevant outcomes, including OS and DFS.

### Results

We included 492 patients with HM, 104 patients with multiple myeloma, 213 patients with Non-Hodgkin Lymphoma, 95 patients with Hodgkin Lymphoma, 55 patients with acute myeloid leukemia and 25 patients with chronic myeloid leukemia. The average age at diagnosis was of 48 (16-87) years. The majority (43%) belonged to the NHL, and 52.4% were female. The study cohort exhibited a categorization based on BMI, revealed that 191 (38.8%) patients were within the normal weight range, 150 (30.4%) were classified as overweight, 124 (25.2%) had obesity, while 27 (5%) patient fell into the underweight category. Serum albumin values indicated that 217 (44.1%) patients were hypoalbuminemic, with albumin levels below 4 g/dL. Following BCA, individuals exhibited a mean LMI of 17.2±2.6% and a FMI of 8.7 kg/m$^2$. The mean PhA was 4.6 degrees, with 329 (66.8%) patients demonstrating a PhA value under 4 degrees. Statistical analysis reveals a significant difference in patients with a phase angle less than 4 ($p=<0.0001$)

**Copyright Information:** (Online) ISSN: 2572-9241
©2026 The Author(s). HemaSphere is published by John Wiley & Sons Ltd on behalf of European Hematology Association. This is an open access Abstract Book distributed under the Attribution-NonCommercial-NoDerivs (CC BY-NC-ND), which allows third parties to download the articles and share them with others as long as they credit the author and the Abstract Book, but they cannot change the content in any way or use them commercially.
**Abstract Book Citations:** Authors, Title, HemaSphere, 2026;10:(S1):pages. The abstract book DOIs can be found on the "Author Information" page.
**Disclaimer:** Articles published in the journal HemaSphere exclusively reflect the opinions of the authors. The authors are responsible for all content in their abstracts including accuracy of the facts, statements, citing resources, etc.

### Summary/Conclusion

The present study demonstrates the prognostic value of body composition parameters in adult HM, particularly PhA as a robust predictor of survival. While BMI alone was not associated with OS, lower PhA correlated with poorer outcomes.
These results suggest that incorporating PhA and nutritional assessments into routine clinical practice could enhance risk stratification and inform supportive care strategies in HM management. Further research is needed to prospectively validate these findings, and to determine whether and which nutritional interventions targeting body composition could improve clinical outcomes.

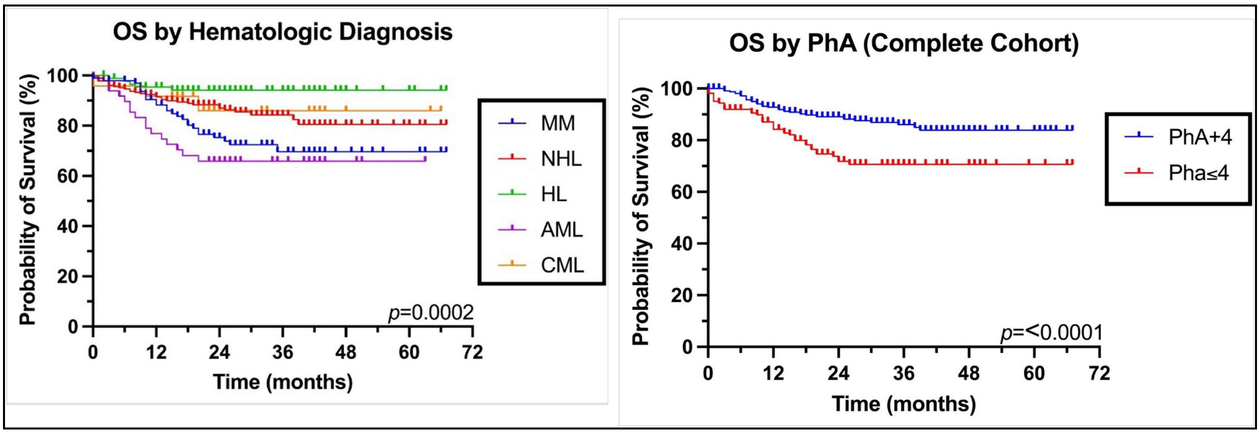

Copyright Information: (Online) ISSN: 2572-9241
©2026 The Author(s). HemaSphere is published by John Wiley & Sons Ltd on behalf of European Hematology Association. This is an open access Abstract Book distributed under the Attribution-NonCommercial-NoDerivs (CC BY-NC-ND), which allows third parties to download the articles and share them with others as long as they credit the author and the Abstract Book, but they cannot change the content in any way or use them commercially.
Abstract Book Citations: Authors, Title, HemaSphere, 2026;10:(S1):pages. The abstract book DOIs can be found on the "Author Information" page.
Disclaimer: Articles published in the journal HemaSphere exclusively reflect the opinions of the authors. The authors are responsible for all content in their abstracts including accuracy of the facts, statements, citing resources, etc.

### (PB4427) BARRIERS AND FACILITATORS OF ACCESS TO PSYCHOSOCIAL SUPPORT FOR PEOPLE LIVING WITH BLOOD CANCERS AND THEIR INFORMAL CAREGIVERS: A SCOPING REVIEW

**Topic:** 36. Quality of life, ethics, supportive and palliative care

Erica Fortune*[1], Meghan Gutierrez[2], Samantha Nier[3], Simon Purcell[4], Veronika Rysinova[5], Jhulia Salviano[6], Sally Snow[5], Maja Isabelle Strecker[4], Jack Watkins[4]

*[1]Cancer Support Community, Washington, United States of America;[2]Lymphoma Research Foundation, New York, United States of America; [3]Acute Leukemia Advocates Network, Bern, Switzerland; [4]Pfizer Inc., New York, United States of America;[5]Studio Health, London, United Kingdom; [6]Myeloma Patients Europe, Brussels, Belgium;*

### Background

Hematological malignancies are associated with significant psychological distress and disruption to social, work and family life. Psychosocial services and structured interventions can mitigate these effects, yet access remains inconsistent across geographies, health systems, disease types, and sociodemographic groups.

### Aims

To map and synthesize the evidence on barriers and facilitators of access to formal psychosocial support for people with hematological malignancies and their caregivers.

### Methods

This scoping review followed Joanna Briggs Institute methodology. Studies were included if they focused on adults with hematological malignancies and/or their caregivers (Population); involved psychosocial services or interventions delivered by trained professionals or organizations (Concept); and conducted in any care setting or country (Context). Searches were conducted in MEDLINE (Ovid), APA PsycInfo (Ovid), trial registries and preprint platforms. Eligible sources (2015–2025, English language) included quantitative, qualitative, mixed-methods, and review studies. Data were synthesized narratively to identify themes relating to access to psychosocial support. Patient organization representatives contributed to study design and interpretation.

### Results

5,272 records were screened, with 435 included in the narrative synthesis. Five themes were identified:**1) Service design and delivery**. Access was enhanced when services were appropriately timed and integrated into care pathways, tailored to individual and social context, and responsive to evolving needs across the disease course. Barriers arose when services were *ad hoc*, under-resourced, or deprioritized in relation to biomedical care. **2) Healthcare professionals' capacity and skills.** Specialist training, culturally competent, trauma-informed approaches, staffing continuity, and clearly delineated shared-care pathways facilitated access. Limited training or confidence in initiating psychosocial discussions, and inconsistent multidisciplinary coordination constrained access. **3) Awareness and communication of support needs**. Use of appropriate screening tools, proactive signposting, and clear, accessible information facilitated access. Patients' distress was frequently normalized, and caregivers were often not routinely screened or formally integrated into care pathways. **4) Practical, technological and financial factors.** Online delivery improved reach for rural and mobility-restricted populations but introduced barriers related to digital literacy, connectivity and privacy. Fatigue, sensory/mobility needs, childcare and financial constraints on patients and caregivers limited access. **5) Emotional, motivational and cultural factors.** Culturally sensitive framing of psychosocial care, and focus on trustbuilding and emotional safety facilitated access. Normalization of cancer-related distress, stigma around mental health and palliative care, and discomfort in group settings were access barriers.

**Copyright Information:** (Online) ISSN: 2572-9241
©2026 The Author(s). HemaSphere is published by John Wiley & Sons Ltd on behalf of European Hematology Association. This is an open access Abstract Book distributed under the Attribution-NonCommercial-NoDerivs (CC BY-NC-ND), which allows third parties to download the articles and share them with others as long as they credit the author and the Abstract Book, but they cannot change the content in any way or use them commercially.
**Abstract Book Citations:** Authors, Title, HemaSphere, 2026;10:(S1):pages. The abstract book DOIs can be found on the "Author Information" page.
**Disclaimer:** Articles published in the journal HemaSphere exclusively reflect the opinions of the authors. The authors are responsible for all content in their abstracts including accuracy of the facts, statements, citing resources, etc.

**Summary/Conclusion**

Psychosocial support in hematological malignancies remains inconsistently integrated into routine care, often delivered reactively, reliant on individual clinician initiative, and rarely inclusive of caregivers. Persistent gaps in systematic screening, workforce training, and formalised care pathways highlight the need for proactive, caregiver-inclusive models that address modifiable service- and system-level barriers to psychosocial care across the full illness trajectory.

**Copyright Information:** (Online) ISSN: 2572-9241
©2026 The Author(s). HemaSphere is published by John Wiley & Sons Ltd on behalf of European Hematology Association. This is an open access Abstract Book distributed under the Attribution-NonCommercial-NoDerivs (CC BY-NC-ND), which allows third parties to download the articles and share them with others as long as they credit the author and the Abstract Book, but they cannot change the content in any way or use them commercially.
**Abstract Book Citations:** Authors, Title, HemaSphere, 2026;10:(S1):pages. The abstract book DOIs can be found on the "Author Information" page.
**Disclaimer:** Articles published in the journal HemaSphere exclusively reflect the opinions of the authors. The authors are responsible for all content in their abstracts including accuracy of the facts, statements, citing resources, etc.

### (PB4428) QUALITY OF LIFE AFTER ALLOGENEIC HEMATOPOIETIC STEM CELL TRANSPLANTATION IN ADULT PATIENTS WITH HEMATOLOGICAL MALIGNANCIES: A SINGLE-CENTER RETROSPECTIVE ANALYSIS FROM SLOVAKIA

**Topic:** 36. Quality of life, ethics, supportive and palliative care

Iveta Oravcová*[1, 2], Eva Mikuskova[1], Andrej Vranovsky[1, 2], Silvia Cingelova[1], Miriam Ladická[1], Alica Slobodova[1], Radka Vasickova[1], Denis Urban[1], Rebeka Granakova[1], Lubos Drgona[1, 2], Zuzana Rusináková[1, 2]

*[1]National Cancer Institute, Department of Oncohematology, Bratislava, Slovakia;[2]Faculty of Medicine Comenius University, Oncohematology Clinic, Oncohematology Department, Bratislava, Slovakia;*

### Background

Allogeneic hematopoietic stem cell transplantation (alloSCT) as intensive therapeutic modality carries toxicity associated with significant short-term and long-term morbidity and mortality, and it also markedly affects physical, emotional, social, and functional aspects of recipients' quality of life (QOL).

### Aims

The aim of this retrospective cross-sectional study was to evaluate QOL in adult patients following alloSCT.

### Methods

We assessed QOL using standardized Functional Assessment of Cancer Therapy-Bone Marrow Transplant Scale (FACT-BMT) questionnaires completed by 74 patients with median age of 44 (22-62) years and collected at the National Cancer Institute from May 2022 till the end of November 2022, **Table 1**. Diagnosis were AML/ALL, MDS/MPN, HL, NHL, CLL, CML, multiple myeloma. Patients could also answer several additional questions regarding QOL. The clinical study was approved by Ethical Committee of National Cancer Institute in April 2022 with the number QOL01/2022/a.

### Results

Univariate analysis showed that patients who were more than 3 years post-alloSCT had significantly better functional well-being (FWB; p = 0.004) and significantly higher scores in all indices (FACT-TOI (p = 0.025), FACT-G (p = 0.026), and FACT-BMT (p = 0.019)), which assess both general and disease-specific quality of life, compared with patients who were less than 1-year post-alloSCT, **Image 1**. Multivariate analysis confirmed that patients more than 3 years post-alloSCT had a significantly higher probability of favorable outcomes across the social (SWB; p=0,039), emotional (EWB; p=0,007), functional (FWB; p=0,015), and BMTS (p=0,038) domains, as well as higher scores on the FACT-G (p=0,008) and FACT-BMT (p=0,001) indices, compared with patients less than 1-year post-alloSCT.
The most common negative changes associated with alloSCT reported by the patients were: 1.decreased energy, fatigue, and weakness; 2. loss of employment; 3. social isolation and inability to meet with close relatives and friends; 4. changes in taste, reduced appetite; sexual dysfunction (loss of libido); 5. limitation in sports activities and travel.
The most reported positive changes were: 1. lifesaving, re-evaluation of life priorities; 2. strengthening of family relationships and more time spent with family; 3. greater enjoyment of life; 4. more time devoted to self-care; 5.new friendships.
**Table 1:** Characteristics of the cohort

**Copyright Information:** (Online) ISSN: 2572-9241
©2026 The Author(s). HemaSphere is published by John Wiley & Sons Ltd on behalf of European Hematology Association. This is an open access Abstract Book distributed under the Attribution-NonCommercial-NoDerivs (CC BY-NC-ND), which allows third parties to download the articles and share them with others as long as they credit the author and the Abstract Book, but they cannot change the content in any way or use them commercially.
**Abstract Book Citations:** Authors, Title, HemaSphere, 2026;10:(S1):pages. The abstract book DOIs can be found on the "Author Information" page.
**Disclaimer:** Articles published in the journal HemaSphere exclusively reflect the opinions of the authors. The authors are responsible for all content in their abstracts including accuracy of the facts, statements, citing resources, etc.

| characteristic | N (%) |
|---|---|
| **Gender**<br>man<br>woman | <br>39 (52,7)<br>35 (47,3) |
| **Age at alloSCT, years**<br>median<br>18-39<br>≥ 40 | <br>44 (22-62)<br>29 (39,2)<br>45 (60,8) |
| **Time from alloSCT to questionnaire completing, years**<br>< 1<br>≥ 1– 3<br>≥ 3 | <br>19 (25,7)<br>10 (13,5)<br>45 (60,8) |
| **Donor type**<br>MSD<br>MUD<br>Haploidentical | <br>31 (41,9)<br>37 (50)<br>6 (8,1) |
| **Conditioning regimen**<br>MAC<br>RIC+NMA | <br>29 (39,2)<br>45 (60,8) |
| **TBI**<br>yes<br>no | <br>24 (32,4)<br>50 (67,6%) |
| **KR before alloSCT**<br>yes<br>no | <br>51 (68,9)<br>23 (31,1) |

**Copyright Information:** (Online) ISSN: 2572-9241
©2026 The Author(s). HemaSphere is published by John Wiley & Sons Ltd on behalf of European Hematology Association. This is an open access Abstract Book distributed under the Attribution-NonCommercial-NoDerivs (CC BY-NC-ND), which allows third parties to download the articles and share them with others as long as they credit the author and the Abstract Book, but they cannot change the content in any way or use them commercially.
**Abstract Book Citations:** Authors, Title, HemaSphere, 2026;10:(S1):pages. The abstract book DOIs can be found on the "Author Information" page.
**Disclaimer:** Articles published in the journal HemaSphere exclusively reflect the opinions of the authors. The authors are responsible for all content in their abstracts including accuracy of the facts, statements, citing resources, etc.

### Summary/Conclusion

This clinical study demonstrated a significant decline in QOL in the first year following alloSCT, with a progressive improvement in QOL after the third year post-transplantation. Given that alloSCT affects all domains of QOL, multidisciplinary care should be provided throughout the peri-transplant period and during long-term follow-up. Adequate patient education, psychological and social support for both patients and their families, and active communication among members of the multidisciplinary team contribute substantially to better treatment management, improved adherence, and, ultimately, improved quality of life.

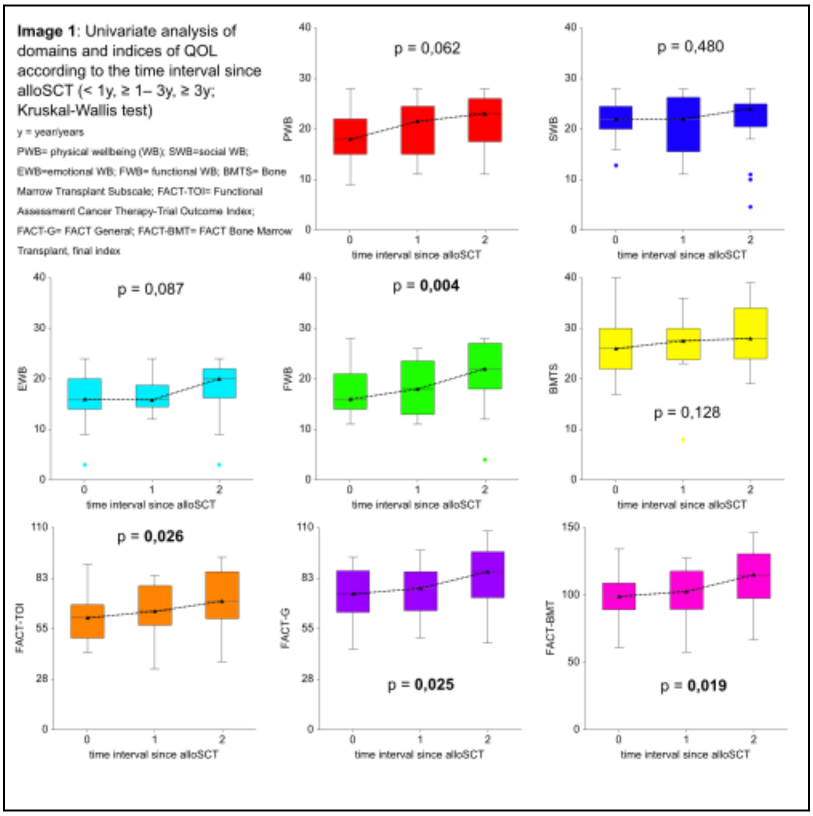

**Image 1**: Univariate analysis of domains and indices of QOL according to the time interval since alloSCT (< 1y, ≥ 1– 3y, ≥ 3y; Kruskal-Wallis test)
y = year/years
PWB= physical wellbeing (WB); SWB=social WB; EWB=emotional WB; FWB= functional WB; BMTS= Bone Marrow Transplant Subscale; FACT-TOI= Functional Assessment Cancer Therapy-Trial Outcome Index; FACT-G= FACT General; FACT-BMT= FACT Bone Marrow Transplant, final index

**Copyright Information:** (Online) ISSN: 2572-9241
©2026 The Author(s). HemaSphere is published by John Wiley & Sons Ltd on behalf of European Hematology Association. This is an open access Abstract Book distributed under the Attribution-NonCommercial-NoDerivs (CC BY-NC-ND), which allows third parties to download the articles and share them with others as long as they credit the author and the Abstract Book, but they cannot change the content in any way or use them commercially.
**Abstract Book Citations:** Authors, Title, HemaSphere, 2026;10:(S1):pages. The abstract book DOIs can be found on the "Author Information" page.
**Disclaimer:** Articles published in the journal HemaSphere exclusively reflect the opinions of the authors. The authors are responsible for all content in their abstracts including accuracy of the facts, statements, citing resources, etc.

### (PB4429) IMPACT OF PERIPHERAL NEUROPATHIC SYMPTOMS ON QUALITY OF LIFE IN PATIENTS WITH SMOLDERING MULTIPLE MYELOMA

**Topic:** 36. Quality of life, ethics, supportive and palliative care

Eileen Kurre*[1], Mareike Hampel[1], Kaya Miah[2], Sophia Schmidt[1], Annika Werly[1], Carsten Müller-Tidow[1], Marc-Steffen Raab[1], Hartmut Goldschmidt[1, 3], Elias K. Mai[1]

*[1]Heidelberg University Hospital and Medical Faculty Heidelberg, Heidelberg Myeloma Center, Department of Internal Medicine V, Heidelberg, Germany; [2]German Cancer Research Center (DKFZ) Heidelberg, Division of Biostatistics, Heidelberg, Germany; [3]National Center for Tumor Diseases (NCT) Heidelberg, Heidelberg, Germany;*

### Background

Smoldering multiple myeloma (SMM) is considered an asymptomatic malignant disease with a high risk of progression to multiple myeloma (MM) requiring treatment. In addition to the classic manifestations of the disease, patients may also develop peripheral neuropathies, which can significantly impact their quality of life (QoL). Currently, there are few studies that address neuropathic symptoms in the context of SMM.

### Aims

The aim of this study was to investigate the influence of peripheral neuropathies on the health-related (HR)-QoL of treatment-naive SMM patients.

### Methods

A monocentric, prospective, non-interventional, observational study without randomization was conducted. Data was collected using the validated EORTC questionnaires QoL-C30, QoL-MY20 and QoL-CIPN, as well as a data collection form. Evaluation was based on official scoring manuals, descriptive statistics as well as univariate and multivariable linear regression analyses. Non-parametric statistical tests, including the Mann–Whitney U test, were performed to investigate differences between the groups (i.e., patients with and without neurological symptoms; PN vs. non-PN).

### Results

A total of 127 patients participated in the study between Aug-Oct 2025. Among these, 20 patients (16%) showed no neuropathic symptoms (non-PN), while 107 patients (84%) reported symptoms (PN). Patients without neuropathic symptoms exhibited a significantly higher global health status in comparison to those with symptoms (mean M (PN) = 62.54 (SD 22.27) vs. M (non-PN) = 81.25 (SD 19.09) $p < 0.001$). Even with moderate neuropathic symptoms, patients had a significantly lower global health status as compared to patients with mild symptoms (Dunn; $p < 0.001$) or no symptoms (Dunn; $p < 0.001$). Furthermore, female patients experienced higher levels of sensory and motor symptoms. The presence of peripheral neuropathic symptoms was independent of patient characteristics and disease-specific parameters such as m protein level. Patients with sensory symptoms also generally exhibited significant motor and autonomic impairments.

**Copyright Information:** (Online) ISSN: 2572-9241
©2026 The Author(s). HemaSphere is published by John Wiley & Sons Ltd on behalf of European Hematology Association. This is an open access Abstract Book distributed under the Attribution-NonCommercial-NoDerivs (CC BY-NC-ND), which allows third parties to download the articles and share them with others as long as they credit the author and the Abstract Book, but they cannot change the content in any way or use them commercially.
**Abstract Book Citations:** Authors, Title, HemaSphere, 2026;10:(S1):pages. The abstract book DOIs can be found on the “Author Information” page.
**Disclaimer:** Articles published in the journal HemaSphere exclusively reflect the opinions of the authors. The authors are responsible for all content in their abstracts including accuracy of the facts, statements, citing resources, etc.

**Summary/Conclusion**

The majority of SMM patients are affected by PN, and neuropathic symptoms in patients with SMM have a significant adverse impact on HR-QoL. HR-QoL questionnaires are key tools in identifying SMM patients with a high symptom burden in clinical routine, may guide clinical and therapeutic decision-making processes and prompt further neurological diagnostics. Further research in patients with SMM and neuropathic symptoms is needed to confirm the results and understand possible causal relationships and differential diagnoses.

**Copyright Information:** (Online) ISSN: 2572-9241
©2026 The Author(s). HemaSphere is published by John Wiley & Sons Ltd on behalf of European Hematology Association. This is an open access Abstract Book distributed under the Attribution-NonCommercial-NoDerivs (CC BY-NC-ND), which allows third parties to download the articles and share them with others as long as they credit the author and the Abstract Book, but they cannot change the content in any way or use them commercially.
**Abstract Book Citations:** Authors, Title, HemaSphere, 2026;10:(S1):pages. The abstract book DOIs can be found on the "Author Information" page.
**Disclaimer:** Articles published in the journal HemaSphere exclusively reflect the opinions of the authors. The authors are responsible for all content in their abstracts including accuracy of the facts, statements, citing resources, etc.

### (PB4430) PALLIATIVE CARE UTILIZATION AND HOSPITAL OUTCOMES IN HEMATOLOGIC MALIGNANCIES WITHOUT INPATIENT TREATMENT: A NATIONAL INPATIENT SAMPLE ANALYSIS

**Topic:** 36. Quality of life, ethics, supportive and palliative care

Ashish Samaddar*[1], Phyu Thin Naing[2]

*[1]Zucker/Northwell Hofstra School of Medicine, Internal Medicine, Manhasset, United States of America;[2]Vanderbilt University Medical Center, Department of Hematology/Oncology, Nashville, United States of America;*

**Background**

Palliative care is an established component of supportive oncology, yet its use in hematologic malignancies remains lower than in solid tumors. This difference is often attributed to the variable clinical course of hematologic cancers and frequent inpatient therapy. It remains unclear whether reduced use reflects treatment-related factors or care integration. We evaluated palliative care utilization among hospitalized patients with hematologic malignancies not receiving cancer-directed therapy.

**Aims**

To determine whether lower palliative care utilization in hematologic malignancies persists independent of inpatient cancer-directed therapy.

**Methods**

We performed a cohort study using the National Inpatient Sample (2020–2022). Adult hospitalizations with acute myeloid leukemia (excluding acute promyelocytic leukemia), lymphoma, and multiple myeloma were compared with metastatic solid tumor admissions. Analyses were restricted to hospitalizations without inpatient cancer-directed therapy. Palliative care consultation was identified using ICD-10 code Z51.5. Survey-weighted regression models with inverse probability weighting adjusted for demographics, socioeconomic factors, hospital characteristics, and illness severity were used to estimate the likelihood of consultation and its associations with in-hospital mortality, resource utilization (length of stay, and total charges).

**Results**

A total of 11,768 hospitalizations were included. Among admissions without inpatient oncologic treatment, hematologic malignancies were markedly less likely to receive inpatient palliative consultation than metastatic solid tumors including AML (OR:0.61,95% CI 0.50–0.74), lymphoma (OR:0.46, 95% CI 0.38–0.55), and myeloma (OR:0.44, 95% CI 0.37–0.53), all $p < 0.001$. When consultation occurred, it was strongly associated with in-hospital death across cancers (OR:6.5–11.2). Consultation was associated with lower hospital charges only in metastatic solid tumors (–17.8%, 95% CI –29.1% to –4.7%, $p = 0.009$) and not in AML, lymphoma, or myeloma.

**Summary/Conclusion**

Even among hospitalizations without inpatient cancer-directed therapy, multiple hematologic malignancies were less likely than metastatic solid tumors to receive palliative care consultation. Prior studies have reported lower palliative care utilization in hematologic malignancies; however, few have evaluated utilization specifically among hospitalizations without inpatient cancer-directed therapy. The persistence of this difference after excluding treatment-related admissions suggests variation in supportive care integration across cancer types rather than differences driven solely by active therapy.

**Copyright Information:** (Online) ISSN: 2572-9241
©2026 The Author(s). HemaSphere is published by John Wiley & Sons Ltd on behalf of European Hematology Association. This is an open access Abstract Book distributed under the Attribution-NonCommercial-NoDerivs (CC BY-NC-ND), which allows third parties to download the articles and share them with others as long as they credit the author and the Abstract Book, but they cannot change the content in any way or use them commercially.
**Abstract Book Citations:** Authors, Title, HemaSphere, 2026;10:(S1):pages. The abstract book DOIs can be found on the “Author Information” page.
**Disclaimer:** Articles published in the journal HemaSphere exclusively reflect the opinions of the authors. The authors are responsible for all content in their abstracts including accuracy of the facts, statements, citing resources, etc.

### (PB4431) REAL-WORLD IMPLEMENTATION OF COMPREHENSIVE GERIATRIC ASSESSMENT IN ONCOHEMATOLOGY REVEALS HIDDEN VULNERABILITY IN OLDER PATIENTS AT A REFERRAL CENTER.

**Topic:** 36. Quality of life, ethics, supportive and palliative care

Thiago Carneiro*[1, 2], Maria Clara Campelo[1], Antonia Evelyn Costa[1], Antônio Pereira[1], Ana Teles[1], Cauã Espírito Santo[1], João Pantoja[1], Livia Ramalho[2], Niele Moraes[1]

*[1]Universidade do Estado do Pará, Belém, Brazil; [2]Hospital Ophir Loyola / UEPA, Belém, Brazil;*

### Background

Comprehensive geriatric assessment (CGA) is widely recognized as the gold standard for evaluating frailty and vulnerability in older adults with cancer. Despite robust evidence linking CGA findings to clinically meaningful outcomes, its use in routine oncohematology practice remains limited due to time constraints, need for specialized teams, and lack of structured protocols. Consequently, most older patients are still assessed primarily through traditional performance scales, which may underestimate underlying vulnerability.

### Aims

To describe the real-world implementation of a structured CGA-based protocol in a hematologic oncology referral center and to characterize the multidimensional clinical and functional profile of older patients with hematologic malignancies identified through this approach.

### Methods

This observational study included patients aged ≥60 years with hematologic malignancies evaluated at a tertiary referral center in Northern Brazil. A structured CGA-based protocol was systematically applied at baseline and during follow-up, incorporating validated instruments for frailty (Fried phenotype, FRAIL scale, Rockwood Clinical Frailty Scale), functional status (Lawton and Katz scales), nutrition (Mini Nutritional Assessment), cognition, physical performance, and global clinical impression. Descriptive analyses were used to characterize the multidimensional patient profile identified through CGA in routine clinical practice.

### Results

Fifty-five patients were evaluated, with a mean age of 70.3 years. Diagnoses included multiple myeloma (32.3%), lymphomas (30.9%), chronic lymphocytic leukemia (25.5%), and other hematologic malignancies. Although 50.9% of patients were independent according to traditional functional criteria, CGA revealed that 72.7% were classified as pre-frail or frail. Overall, 32.7% were identified as frail based on combined frailty instruments. Cognitive impairment or complaints were detected in 14.5% of patients, frequently without prior clinical recognition. Nutritional abnormalities, including risk of malnutrition, sarcopenia, or established malnutrition, were present in a substantial proportion of patients despite apparently preserved body mass index in many cases.

### Summary/Conclusion

The systematic use of a CGA-based protocol in real-world oncohematology practice revealed a high burden of previously unrecognized vulnerability among older patients, even in those considered functionally fit by conventional assessments. These findings highlight the gap between CGA's established prognostic value and its limited use in daily practice. Broader implementation of CGA in hematologic oncology could substantially reshape the understanding of real-world patient profiles, support more individualized decision-making, and improve alignment between treatment strategies and patients' functional reserve.

**Copyright Information:** (Online) ISSN: 2572-9241
©2026 The Author(s). HemaSphere is published by John Wiley & Sons Ltd on behalf of European Hematology Association. This is an open access Abstract Book distributed under the Attribution-NonCommercial-NoDerivs (CC BY-NC-ND), which allows third parties to download the articles and share them with others as long as they credit the author and the Abstract Book, but they cannot change the content in any way or use them commercially.
**Abstract Book Citations:** Authors, Title, HemaSphere, 2026;10:(S1):pages. The abstract book DOIs can be found on the "Author Information" page.
**Disclaimer:** Articles published in the journal HemaSphere exclusively reflect the opinions of the authors. The authors are responsible for all content in their abstracts including accuracy of the facts, statements, citing resources, etc.

### Comprehensive geriatric assessment findings by hematologic malignancy

| CGA domain | Multiple Myeloma (n = 18) | Lymphoma (n = 17) | Chronic Lymphocytic Leukemia (n = 14) | Other hematologic malignancies (n = 6) |
|---|---|---|---|---|
| **Frailty status (combined CGA tools*)** | | | | |
| **Robust** | 4 (22.2%) | 5 (29.4%) | 3 (21.4%) | 2 (33.3%) |
| **Pre-frail** | 8 (44.4%) | 7 (41.2%) | 6 (42.9%) | 2 (33.3%) |
| **Frail** | 6 (33.3%) | 5 (29.4%) | 5 (35.7%) | 2 (33.3%) |
| **Functional status** | | | | |
| **Independent (ADL/IADL)** | 9 (50.0%) | 9 (52.9%) | 8 (57.1%) | 3 (50.0%) |
| **Partial dependence** | 6 (33.3%) | 5 (29.4%) | 4 (28.6%) | 2 (33.3%) |
| **Total dependence** | 3 (16.7%) | 3 (17.6%) | 2 (14.3%) | 1 (16.7%) |
| **Cognitive status** | | | | |
| **Preserved cognition** | 14 (77.8%) | 14 (82.4%) | 12 (85.7%) | 3 (50.0%) |
| **Cognitive complaint or impairment** | 4 (22.2%) | 3 (17.6%) | 2 (14.3%) | 3 (50.0%) |
| **Nutritional status** | | | | |
| **Eutrophic** | 8 (44.4%) | 7 (41.2%) | 6 (42.9%) | 2 (33.3%) |
| **Overweight / obesity** | 6 (33.3%) | 6 (35.3%) | 5 (35.7%) | 2 (33.3%) |
| **Nutritional risk malnutrition** | 4 (22.2%) | 4 (23.5%) | 3 (21.4%) | 2 (33.3%) |
| **Sarcopenia (screening positive)** | 2 (11.1%) | 1 (5.9%) | 1 (7.1%) | 1 (16.7%) |

* Frailty classification based on integrated interpretation of Fried frailty phenotype, FRAIL scale and Rockwood Clinical Frailty Scale.

Copyright Information: (Online) ISSN: 2572-9241
©2026 The Author(s). HemaSphere is published by John Wiley & Sons Ltd on behalf of European Hematology Association. This is an open access Abstract Book distributed under the Attribution-NonCommercial-NoDerivs (CC BY-NC-ND), which allows third parties to download the articles and share them with others as long as they credit the author and the Abstract Book, but they cannot change the content in any way or use them commercially.
Abstract Book Citations: Authors, Title, HemaSphere, 2026;10:(S1):pages. The abstract book DOIs can be found on the "Author Information" page.
Disclaimer: Articles published in the journal HemaSphere exclusively reflect the opinions of the authors. The authors are responsible for all content in their abstracts including accuracy of the facts, statements, citing resources, etc.

### (PB4432) BURNOUT AMONG HEALTHCARE PROFESSIONALS IN CLINICAL HEMATOLOGY :PREVALENCE AND ASSOCIATED ORGANIZATIONAL FACTORS

**Topic:** 36. Quality of life, ethics, supportive and palliative care

Nour Siala*[1], Faten Kallel[1], Imen Baati[2], Hanen Charfi[1], Jawaher Masmoudi[2], Moez Elloumi[1]

*[1]Hematology department Hedi Chaker Hospital, Sfax, Tunisia;[2]Psychiatry Department Hedi Chaker Hospital, Sfax, Tunisia;*

### Background

Burnout is a psychological syndrome resulting from chronic occupational stress and is particularly relevant in hematology, where healthcare professionals are exposed to severe diseases, intensive treatments, repeated suffering and death, and organizational constraints.

### Aims

We aim to assess the prevalence of burnout among healthcare professionals working in a clinical hematology department, identify associated factors, and propose context-adapted preventive strategies.

### Methods

We conducted a cross-sectional study in February 2025 among healthcare professionals in the Clinical Hematology Department of Hedi Chaker University Hospital, Sfax. Burnout was assessed using the Maslach Burnout Inventory (MBI). Sociodemographic, occupational, and satisfaction-related variables were collected and analyzed to identify factors associated with burnout.

### Results

Out of 47 distributed questionnaires, 43 were returned (response rate: 85%). The study population was predominantly female (62.5%), with a mean age of 33.65 ± 9.31 years. Participants included physicians (55%) and nurses (45%), with a mean seniority of 69.6 months in the department. Self-reported workload was high in physical (6.83/10), mental (8.2/10), and emotional (7.98/10) dimensions. Most participants reported dissatisfaction with working conditions (82.5%) and work organization (52.5%).
Overall, 72.5% of participants met criteria for burnout, with moderate-to-severe levels in 62% of cases. High emotional exhaustion was observed in 57.5%, high depersonalization in 45%, and low personal accomplishment in 30%. Burnout was significantly associated with age <40 years (p=0.043), higher physical workload (p=0.025), dissatisfaction with working conditions (p=0.021), and dissatisfaction with work organization (p=0.013). No significant associations were found with other sociodemographic variables, medical or psychiatric history, lifestyle habits, or general job characteristics.
Participants suggested several measures to improve job satisfaction and reduce burnout, including improved equipment and resources (88%), salary increases (58%), recruitment of additional staff (55%), and improved professional relationships and supervision (35–45%).

**Copyright Information:** (Online) ISSN: 2572-9241
©2026 The Author(s). HemaSphere is published by John Wiley & Sons Ltd on behalf of European Hematology Association. This is an open access Abstract Book distributed under the Attribution-NonCommercial-NoDerivs (CC BY-NC-ND), which allows third parties to download the articles and share them with others as long as they credit the author and the Abstract Book, but they cannot change the content in any way or use them commercially.
**Abstract Book Citations:** Authors, Title, HemaSphere, 2026;10:(S1):pages. The abstract book DOIs can be found on the “Author Information” page.
**Disclaimer:** Articles published in the journal HemaSphere exclusively reflect the opinions of the authors. The authors are responsible for all content in their abstracts including accuracy of the facts, statements, citing resources, etc.

**Summary/Conclusion**

The prevalence of burnout in our cohort (72%) is similar to that reported in a Tunisian study from Sousse (70%) involving nurses providing end-of-life care, but remains higher than international reports (42–50%). This highlights the significant burden of burnout among hematology healthcare professionals in our setting.
Burnout was significantly associated with age <40 years, higher physical workload, and dissatisfaction with working conditions and organization, in line with international findings. Other reported risk factors include fewer years of professional experience, frequent night shifts, and lack of choice of specialty or position.
Overall, burnout in clinical hematology appears to result from both individual and organizational factors. Interventions targeting workload regulation, improved work organization, and support for early-career professionals are essential. Occupational health services play a key role in prevention, early detection, and management through assessment of working conditions, psychological support, and preventive measures.

**Copyright Information:** (Online) ISSN: 2572-9241
©2026 The Author(s). HemaSphere is published by John Wiley & Sons Ltd on behalf of European Hematology Association. This is an open access Abstract Book distributed under the Attribution-NonCommercial-NoDerivs (CC BY-NC-ND), which allows third parties to download the articles and share them with others as long as they credit the author and the Abstract Book, but they cannot change the content in any way or use them commercially.
**Abstract Book Citations:** Authors, Title, HemaSphere, 2026;10:(S1):pages. The abstract book DOIs can be found on the “Author Information” page.
**Disclaimer:** Articles published in the journal HemaSphere exclusively reflect the opinions of the authors. The authors are responsible for all content in their abstracts including accuracy of the facts, statements, citing resources, etc.

### (PB4433) DEVELOPMENT AND IMPLEMENTATION OF A NOVEL CLINICAL TRIAL FOLLOW-UP MODEL WITHIN THE CLINICAL TRIALS NAVIGATOR PROGRAM: A CROSS-SECTIONAL ANALYSIS OF THE MD FOLLOWUP PROGRAM

**Topic:** 36. Quality of life, ethics, supportive and palliative care

Michael Touma*[1], Alfred Mina[2], Christina Di Carlo[1], Laurice Arayan[2], Renee Nassar[1], Mahmoud Hossami[3], Roaa Hirmiz[3], Swati Kalia[3], Batoul El Sayed Ali[3], Christina Trieu[4], Salah Alhajsaleh[4], Anaam Jaet[4], Matteo Frattaroli[4], Mohammad El-Hindawi[4], Hannah Chacko[4], Govana Sadik[5], Milica Paunic[6], Olla Hilal[7], Depen Sharma[8], Anthony Luginaah[9], Pratham Gupta[9], Ria Patel[9], Gregory Anagnostopoulos[9], Megan Delisle[10], Caroline Hamm[2]

*[1]Windsor Regional Hospital, Windsor, Canada;[2]Windsor Regional Hospital, Department of Oncology, Windsor, Canada;[3]Clinical Trials Navigator Inc., Windsor, Canada; [4]University of Windsor, Biomedical Sciences, Windsor, Canada;[5]University of Windsor, Biological Sciences, Windsor, Canada; [6]University of Toronto, Faculty of Medicine, Toronto, Canada;[7]Western University, Medical Sciences, London, Canada; [8]University of Ottawa, Faculty of Medicine, Ottawa, Canada;[9]Western University, Faculty of Medicine, London, Canada; [10]University of Manitoba, Department of Surgery, Winnipeg, Canada;*

### Background

The Clinical Trials Navigator Program (CTN) was developed to connect patients with clinical trials and improve accrual rates. Through this work, two key barriers were identified: limited patient literacy regarding clinical trials and challenges with trial follow-up. The MD FollowUp Program was created to address these gaps by explaining personalized trial reports and coordinating eligibility inquiries with trial sites, principal investigators, and pharmaceutical sponsors. The goal is to improve clinical trial accrual rates and patient literacy as well as reduce administrative burden for oncologists across clinical settings.

### Methods

A cross-sectional study was conducted between March 2025 and January 2026 to evaluate outcomes associated with the MD FollowUp program. During this period, 154 patient searches were analyzed. Primary outcomes included patient interest in contacting clinical trial sites, the number of external trial referrals sent, reasons for non-referral, clinical trial enrollment rates, and reasons for non-enrollment. Enrollment rates observed following MD FollowUp involvement were compared with historical enrollment rates prior to program implementation. Analyses were conducted at the search level; referral and enrolment outcomes are reported at the patient level. This analysis aimed to assess the impact of MD FollowUp on trial referral processes, patient engagement, and overall clinical trial accrual.

### Results

A total of 154 searches were included in the MD FollowUp program. Of the 154 searches, 124 (81%) had an available clinical trial and responded to initial outreach. Among these, 67 searches (54%) expressed interest in trial contact. Of the 67 searches expressing interest, 19 (28%) had an external referral sent, while 20 (30%) did not proceed to referral and 28 (42%) are still in the follow-up process. Reasons for non-referral were patient-specific (29%) or centre-specific (71%).
The patient specific reasons included: poor functional status (45%), patient refused (25%), alternative treatment (15%), no reply after initial contact (10%), and completeness of options (5%). Centre specific reasons included: ineligibility (44%), trial not enrolling (28%), all trial spots filled (8%), trial site closed (8%), trial site not open due to ethics review (6%), no response from site (4%), and trial arm closed (2%).
Among the 19 external referrals, four patients (21%) were declined by the trial site, four patients (21%) remain under review, and three patients (16%) declined participation. Eight patients were successfully enrolled, corresponding to an enrollment rate of 42%. In comparison, the pre-MD FollowUp enrollment rate was 18%.

**Copyright Information:** (Online) ISSN: 2572-9241
©2026 The Author(s). HemaSphere is published by John Wiley & Sons Ltd on behalf of European Hematology Association. This is an open access Abstract Book distributed under the Attribution-NonCommercial-NoDerivs (CC BY-NC-ND), which allows third parties to download the articles and share them with others as long as they credit the author and the Abstract Book, but they cannot change the content in any way or use them commercially.
**Abstract Book Citations:** Authors, Title, HemaSphere, 2026;10:(S1):pages. The abstract book DOIs can be found on the "Author Information" page.
**Disclaimer:** Articles published in the journal HemaSphere exclusively reflect the opinions of the authors. The authors are responsible for all content in their abstracts including accuracy of the facts, statements, citing resources, etc.

### Summary/Conclusion

The MD FollowUp program is a patient-centred initiative designed to guide patients through the clinical trial landscape. The program was associated with increased clinical trial enrolment rates, improved patient literacy, and an enhanced understanding of barriers to referral and enrolment.

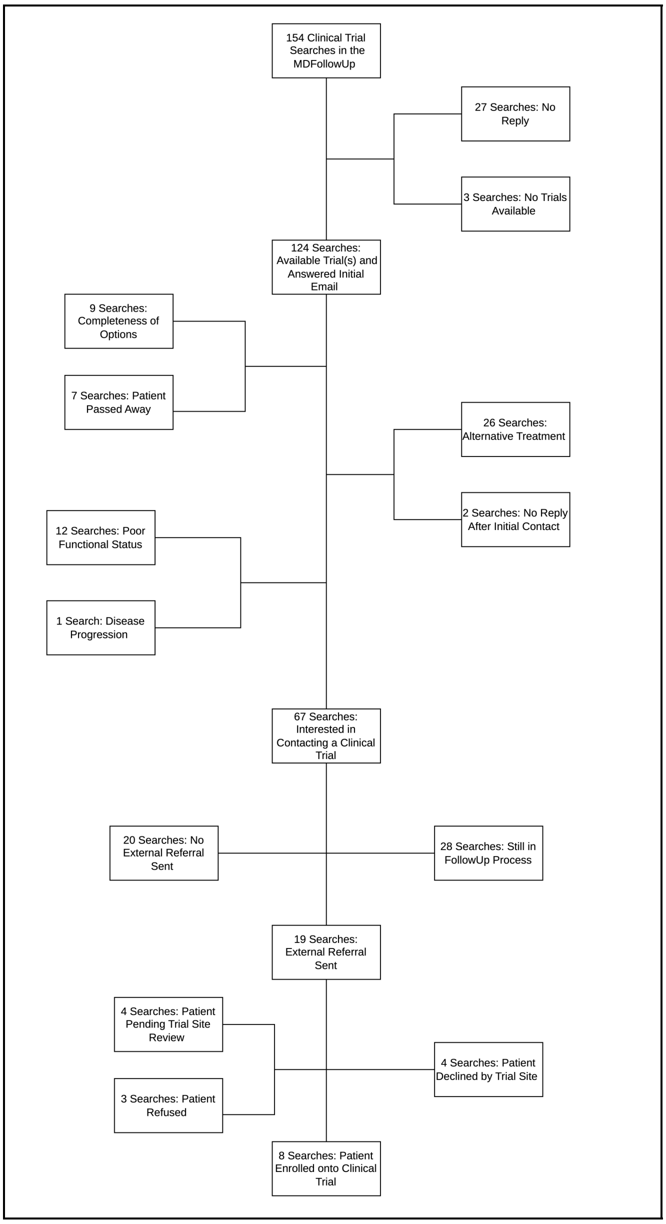

A flowchart representing the patients in the MDFollowUp Program from March 2025-January 2026

**Copyright Information:** (Online) ISSN: 2572-9241
©2026 The Author(s). HemaSphere is published by John Wiley & Sons Ltd on behalf of European Hematology Association. This is an open access Abstract Book distributed under the Attribution-NonCommercial-NoDerivs (CC BY-NC-ND), which allows third parties to download the articles and share them with others as long as they credit the author and the Abstract Book, but they cannot change the content in any way or use them commercially.
**Abstract Book Citations:** Authors, Title, HemaSphere, 2026;10:(S1):pages. The abstract book DOIs can be found on the “Author Information” page.
**Disclaimer:** Articles published in the journal HemaSphere exclusively reflect the opinions of the authors. The authors are responsible for all content in their abstracts including accuracy of the facts, statements, citing resources, etc.

### (PB4434) EFFECTIVENESS OF ACUTE GERIATRIC CARE IN HOSPITALIZED OLDER ADULTS WITH HEMATOLOGICAL MALIGNANCIES

**Topic:** 36. Quality of life, ethics, supportive and palliative care

Valentin Goede*[1], Tanja Pianta[1], Stefan Langenfeld[1], Hannah Dehnen[1], Janan Theeb[1], Marc Johnsen[1], Ralf-Joachim Schulz[1]

[1]*Cellitinnen-Hospital St. Marien, Dept. of Oncogeriatrics, Cologne, Germany;*

### Background

During a hospital stay, vulnerable elderly patients with hematological malignancies may develop geriatric disorders with uncontrolled age-related syndromes and diseases that compromise their ability to cope with everyday life and prevent their discharge or further outpatient treatment. Inpatient acute geriatric care with multidimensional assessment and management could be a suitable supportive measure for treating such disorders and restoring patient functionality. However, there are currently no data available on the effectiveness of acute geriatric care provided to hospitalized older adults with hematological malignancies for supportive purposes.

### Aims

To investigate the feasibility and outcomes of acute geriatric care in older adults with hematological neoplasms.

### Methods

In this single-center study, medical records of all patients diagnosed with hematological neoplasia who were referred over a period of 3 years to our Department of Oncogeriatrics for inpatient acute geriatric care were retrospectively analyzed with regard to numerous baseline and outcome variables. All patients underwent a comprehensive geriatric assessment (CGA) followed by ward-based targeted geriatric interventions performed by a multidisciplinary team. The primary endpoint was functional response as measured by the difference in the Barthel Index (BI) between the start and end of the intervention.

### Results

Of 142 patients (56% male, median age 78 years), 73% had lymphoid and 27% had myeloid neoplasia. Most common were multiple myeloma (MM, 18%), diffuse large B cell lymphoma (DLBCL, 16%), acute myeloid leukemia / myelodysplasia (AML/MDS, 16%), and chronic lymphocytic leukemia (CLL, 10%). The primary reason for hospitalization was the hematological disease itself in 57% and intercurrent illness unrelated to the blood disorder in 43% of cases. Most patients (86%) had an ECOG performance status of 3 or 4 at the start of the geriatric intervention; the median BI was 45. During the ward-based intervention (median duration: 19 days), 16% were receiving oral hematological therapies, 11% had grade 3-4 leukopenia, 29% required transfusions, and 44% antibiotic therapy; 8% of the patients died during acute geriatric treatment. On average, the BI increased by 8.9 points at the end of the geriatric intervention. The mean increase in BI was lower in AML/MDS (4.8) than in DLBCL (11.1), MM (9.6), and CLL (10.7). The median BI at discharge was 60; 60% of the patients were discharged to their homes or a rehabilitation facility, while 13% had to be institutionalized for nursing care or admitted to a hospice. Of 79 patients who were intended to continue or begin specific outpatient hematological systemic therapy after acute geriatric care, 51% achieved sufficient physical and mental fitness for this purpose.

### Summary/Conclusion

Inpatient acute geriatric care is feasible in hospitalized older adults with hematological malignancies. A majority of patients show a functional response and, following such ward-based supportive measures, are successfully discharged from hospital to receive further outpatient hematological treatment if necessary.

**Copyright Information:** (Online) ISSN: 2572-9241
©2026 The Author(s). HemaSphere is published by John Wiley & Sons Ltd on behalf of European Hematology Association. This is an open access Abstract Book distributed under the Attribution-NonCommercial-NoDerivs (CC BY-NC-ND), which allows third parties to download the articles and share them with others as long as they credit the author and the Abstract Book, but they cannot change the content in any way or use them commercially.
**Abstract Book Citations:** Authors, Title, HemaSphere, 2026;10:(S1):pages. The abstract book DOIs can be found on the “Author Information” page.
**Disclaimer:** Articles published in the journal HemaSphere exclusively reflect the opinions of the authors. The authors are responsible for all content in their abstracts including accuracy of the facts, statements, citing resources, etc.

### (PB4435) ASSESSMENT OF THE QUALITY OF LIFE OF CHILDREN WITH Β-THALASSEMIA WHO UNDERWENT ALLOGENENIC BONE MARROW TRANSPLANTATION IN AZERBAIJAN

**Topic:** 36. Quality of life, ethics, supportive and palliative care

Samira Hasanova*[1], Azer Kerimov[1], Nargiz Aliyeva[1], Parvana Safarova[1], Seher Ismayilova[1], Khanim Hamidova[1]

*[1]National Center of Haematology and Transfusiology, Baku, Azerbaijan;*

### Background

Allogenenic hematopoietic stem cell transplantation (HSCT) from a compatible donor is a feasible and curative treatment option for pediatric patients with β-thalassemia major (β-TM). However, data on health-related quality of life (HRQoL) in patients and their parents after transplantation remain limited. Therefore, we assessed HRQoL in children with β-TM who underwent sibling HSCT at the Thalassemia Center of Azerbaijan and compared their quality of life with that of patients receiving conventional therapy consisting of regular blood transfusions and iron chelation.

### Methods

The quality of life of 84 children with β-TM and 84 of their parents was assessed using the Pediatric Quality of Life Inventory (PedsQL).
Additional clinical data—including comorbidities (mainly endocrine disorders), acute or chronic graft-versus-host disease (GVHD), the need for blood transfusions, serum ferritin levels, and type of chelation therapy—were obtained from hospital medical records.
Patients were divided into three age groups: 5–7, 8–12, and 13–18 years. The median follow-up after HSCT was 5 years (2020–2024). The mean age at the time of the survey was 10.4 years in the transfusion-dependent group and 9.3 years in the transplant group.
Eighty-four patients underwent HSCT from an HLA-matched sibling donor, and one patient received HSCT from an HLA-matched father. Complete graft engraftment and transfusion independence were achieved in 82 patients. Graft rejection occurred in two patients: one in the 8–12 year age group and one in the 13–18 year age group.
The median age at transplantation was 10 years (range 2–15 years), and the median time between HSCT and completion of the PedsQL questionnaire was 2 years (range 1–5 years).

### Results

Compared with transplant recipients, patients receiving conventional therapy demonstrated a statistically significant decrease in HRQoL in the following PedsQL domains:

- Physical functioning of children and parents: $p = 0.006$ and $p = 0.004$, respectively
- Emotional functioning of children and parents: $p = 0.022$ and $p = 0.040$, respectively
- School functioning of children and parents: $p = 0.005$ and $p = 0.011$, respectively

Among transplant recipients, grade II acute cutaneous GVHD was observed in eight patients, two of whom subsequently developed chronic cutaneous GVHD.
After transplantation, 10 patients (12%) required venesection or oral deferasirox (8 patients, 10%) to reduce serum ferritin levels.
Patients receiving conventional therapy continued iron chelation treatment, most commonly oral deferasirox or combination chelation therapy.
The main comorbidities were osteoporosis and hypogonadism, particularly in the 13–18-year age group: osteoporosis was diagnosed in 16 patients (20%) and osteopenia in 17 patients (57%).

**Copyright Information:** (Online) ISSN: 2572-9241
©2026 The Author(s). HemaSphere is published by John Wiley & Sons Ltd on behalf of European Hematology Association. This is an open access Abstract Book distributed under the Attribution-NonCommercial-NoDerivs (CC BY-NC-ND), which allows third parties to download the articles and share them with others as long as they credit the author and the Abstract Book, but they cannot change the content in any way or use them commercially.
**Abstract Book Citations:** Authors, Title, HemaSphere, 2026;10:(S1):pages. The abstract book DOIs can be found on the "Author Information" page.
**Disclaimer:** Articles published in the journal HemaSphere exclusively reflect the opinions of the authors. The authors are responsible for all content in their abstracts including accuracy of the facts, statements, citing resources, etc.

**Summary/Conclusion**

HSCT in children with β-thalassemia major is associated with good overall health-related quality of life. Although objective indicators—particularly endocrine parameters—may worsen during the first years after transplantation, the long-term emotional well-being of patients who perceive themselves as cured is significantly better than that of patients receiving lifelong conventional therapy. During the first year after transplantation, quality of life may temporarily decline due to intensive conditioning therapy and early transplant-related complications. However, significant improvement is generally observed within 6–18 months after HSCT as recovery progresses and children regain physical activity and overall well-being.

**Copyright Information:** (Online) ISSN: 2572-9241
©2026 The Author(s). HemaSphere is published by John Wiley & Sons Ltd on behalf of European Hematology Association. This is an open access Abstract Book distributed under the Attribution-NonCommercial-NoDerivs (CC BY-NC-ND), which allows third parties to download the articles and share them with others as long as they credit the author and the Abstract Book, but they cannot change the content in any way or use them commercially.
**Abstract Book Citations:** Authors, Title, HemaSphere, 2026;10:(S1):pages. The abstract book DOIs can be found on the "Author Information" page.
**Disclaimer:** Articles published in the journal HemaSphere exclusively reflect the opinions of the authors. The authors are responsible for all content in their abstracts including accuracy of the facts, statements, citing resources, etc.

### (PB4436) THE EXPERIENCE OF FERTILITY PRESERVATION DECISION-MAKING IN HEMATOPOIETIC STEM CELL TRANSPLANTATION PATIENTS: A QUALITATIVE STUDY

**Topic:** 36. Quality of life, ethics, supportive and palliative care

Qianqian Zhang*[1], Yuebing Du[2], Xiaoqing Tang[2], Huijuan Zhang[3], Guiying Liu[2], Yuqiu Liu[4], Jie Zhang[2], Ying Wang[1], Donglin Yang[2], Erlie Jiang[4], Wenjun Xie[5]

*[1]Institute of Hematology and Blood Diseases Hospital, Chinese Academy of Medical Sciences & Peking Union Medical College, The International Medical Center, Tianjin, China; [2]Institute of Hematology & Blood Diseases Hospital, Chinese Academy of Medical Sciences & Peking Union Medical College, Tianjin, China; [3]State Key Laboratory of Experimental Hematology, National Clinical Research Center for Blood Diseases, Institute of Hematology & Blood Diseases Hospital, Chinese Academy of Medical Sciences & Peking Union Medical College, Tianjin, China; [4]Institute of Hematology and Blood Diseases Hospital, Chinese Academy of Medical Sciences & Peking Union Medical College, Stem Cell Transplantation Diagnosis and Treatment Center, Tianjin, China; [5]Institute of Hematology and Blood Diseases Hospital, Chinese Academy of Medical Sciences & Peking Union Medical College, Nursing Department, Tianjin, China;*

### Background

Hematopoietic stem cell transplantation (HSCT) is a curative treatment for malignant and non-malignant hematological diseases. It uses chemotherapy and/or radiotherapy to eliminate pathological cells and suppress immunity, followed by stem cell infusion to reconstruct hematopoietic and immune function. With improved techniques, long-term survival has increased, shifting clinical attention to survivorship.
However, HSCT severely compromises reproductive health through the underlying disease, prior chemotherapy, conditioning regimens, and complications such as GVHD. Alkylating agents, platinum drugs, and radiotherapy exert clear gonadotoxicity, and chronic GVHD may lead to irreversible reproductive damage.
Hematologic malignancies account for 28% of cancers in adolescents and young adults, with over 70% surviving long term after HSCT. This has made quality of life, especially fertility preservation, a major clinical concern.
Fertility preservation techniques have advanced greatly, and international guidelines recommend pretreatment fertility evaluation and counseling. Yet implementation remains poor, with few patients receiving adequate advice. Most research focuses on technical outcomes rather than patients' decision-making experiences and support needs.

### Aims

This study aims to employ a qualitative research approach to explore the experiences systematically and the influencing factors surrounding FP decision-making among HSCT patients. The findings are intended to provide an empirical foundation for developing a patient-centered decision-support framework.

### Methods

A qualitative study was conducted using semi-structured interviews with 12 patients of reproductive age at a tertiary hematology hospital in China. Data were analyzed through thematic analysis.

**Copyright Information:** (Online) ISSN: 2572-9241
©2026 The Author(s). HemaSphere is published by John Wiley & Sons Ltd on behalf of European Hematology Association. This is an open access Abstract Book distributed under the Attribution-NonCommercial-NoDerivs (CC BY-NC-ND), which allows third parties to download the articles and share them with others as long as they credit the author and the Abstract Book, but they cannot change the content in any way or use them commercially.
**Abstract Book Citations:** Authors, Title, HemaSphere, 2026;10:(S1):pages. The abstract book DOIs can be found on the "Author Information" page.
**Disclaimer:** Articles published in the journal HemaSphere exclusively reflect the opinions of the authors. The authors are responsible for all content in their abstracts including accuracy of the facts, statements, citing resources, etc.

**Results**

Three core themes were identified: (1) Survival as the absolute prerequisite – patients uniformly prioritized life-saving treatment over fertility preservation, particularly when facing critical illness; (2) Systemic resource constraints – limited information accessibility, financial burden, and healthcare system barriers significantly hindered decision-making; (3) Gender- and parenthood stage-driven motivations – female patients considered both reproductive and endocrine health, while male patients focused predominantly on biological continuity, with decisions varying based on family completion status.

**Summary/Conclusion**

This study shows that fertility preservation decision-making among hematopoietic stem cell transplantation patients is complex and shaped by multiple interacting factors, including survival priorities, disease pressure, information access, financial capacity, technology reliability, gender, and parental status, which together lead to decision dilemmas and value trade-offs. Based on the findings, clinical practice should establish a systematic decision-support pathway, proactively assess individual fertility intentions before treatment, and provide tailored fertility counselling. There is also an urgent need for structured decision aids to help patients make informed choices under time and information constraints, protecting their long-term quality of life and reproductive health rights while improving survival.

Table 3 Themes and subthemes

| **Themes** | **Subthemes** |
|---|---|
| The absolute prerequisite for decision-making: prioritizing survival | The absolute principle of survival first |
| | The realistic pressure of disease and treatment |
| Barriers to implementation: systemic constraints on resources | Accessibility of information and knowledge |
| | Economic and health system barriers |
| | Family and social support environment |
| The core logic of decision-making: will–role interaction based on gender and parenthood stage | Women's health–Fertility composite motivation |
| | Men's single motivation: biological continuity |
| | Parenthood goal achieved: psychological closure in decision-making |
| | Parenthood goal not yet achieved: an open state of decision-Making |

**Copyright Information:** (Online) ISSN: 2572-9241
©2026 The Author(s). HemaSphere is published by John Wiley & Sons Ltd on behalf of European Hematology Association. This is an open access Abstract Book distributed under the Attribution-NonCommercial-NoDerivs (CC BY-NC-ND), which allows third parties to download the articles and share them with others as long as they credit the author and the Abstract Book, but they cannot change the content in any way or use them commercially.
**Abstract Book Citations:** Authors, Title, HemaSphere, 2026;10:(S1):pages. The abstract book DOIs can be found on the "Author Information" page.
**Disclaimer:** Articles published in the journal HemaSphere exclusively reflect the opinions of the authors. The authors are responsible for all content in their abstracts including accuracy of the facts, statements, citing resources, etc.

### (PB4437) ANALYSIS OF RELATED FACTORS AND COUNTERMEASURES FOR LONELINESS IN HEMATOPOIETIC STEM CELL TRANSPLANTATION PATIENTS DURING PROTECTIVE ISOLATION

**Topic:** 36. Quality of life, ethics, supportive and palliative care

Yiran Yuan[1], Jianqiong Zhao[1], Ji Luo[1], Yongling Liang[1], Xiaoli Chen*[1]

[1]*the Second Affiliated Hospital of Army Medical University, Chongqing, China;*

### Background

Hematopoietic stem cell transplantation (HSCT) is one of the effective treatments for hematological malignancies. During protective isolation, HSCT patients live alone in sterile laminar airflow wards with limited activity space and reduced social interaction, which makes them prone to loneliness. This can affect patients' quality of life, treatment compliance, symptom burden, length of hospitalization, and survival rate. Therefore, healthcare professionals need to pay attention to the loneliness of HSCT patients during protective isolation.

### Aims

To analyze the related factors of loneliness in hematopoietic stem cell transplantation (HSCT) patients during protective isolation, and to provide evidence for developing effective nursing interventions.

### Methods

Using convenience sampling, 110 HSCT patients in protective isolation from a tertiary grade A general hospital in a certain city were selected as research subjects. General information questionnaires, the University of California at Los Angeles Loneliness Scale (UCLA), the de Jong Gierveld Loneliness Scale (DJGLS), and the Perception of Protective Isolation Scale (ISOLA) were used for assessment. Related factors of loneliness were analyzed.

### Results

A total of 110 patients completed the survey. The loneliness score of HSCT patients was (16.06±5.83) points. Pearson correlation analysis showed that loneliness was positively correlated with pain and fatigue ($r=0.597$, $P<0.01$), and negatively correlated with education level, social support, and chemotherapy duration ($r=-0.611$, $P<0.01$). Multiple stepwise linear regression analysis showed that gender, education level, pain, fatigue, social support, and chemotherapy duration were influencing factors of loneliness ($R^2=0.525$, adjusted $R^2=0.587$, $F=26.041$, $P<0.001$).

### Summary/Conclusion

The loneliness level of HSCT patients during protective isolation was at a moderate level, which should be taken seriously by nursing staff. Special attention should be paid to male patients, those with low education levels, those with fatigue accompanied by pain, those with short chemotherapy duration, and those with low social support levels. Early detection and early intervention should be implemented to alleviate their loneliness.

**Copyright Information:** (Online) ISSN: 2572-9241
©2026 The Author(s). HemaSphere is published by John Wiley & Sons Ltd on behalf of European Hematology Association. This is an open access Abstract Book distributed under the Attribution-NonCommercial-NoDerivs (CC BY-NC-ND), which allows third parties to download the articles and share them with others as long as they credit the author and the Abstract Book, but they cannot change the content in any way or use them commercially.
**Abstract Book Citations:** Authors, Title, HemaSphere, 2026;10:(S1):pages. The abstract book DOIs can be found on the "Author Information" page.
**Disclaimer:** Articles published in the journal HemaSphere exclusively reflect the opinions of the authors. The authors are responsible for all content in their abstracts including accuracy of the facts, statements, citing resources, etc.

### (PB4438) THE VALUE OF SHARED DECISION MAKING IN PAROXYSMAL NOCTURNAL HEMOGLOBINURIA: FINDINGS FROM A PATIENT ADVOCATE COUNCIL

**Topic:** 36. Quality of life, ethics, supportive and palliative care

Phillip Scheinberg[1], Gulin Kayserili[2], Ching Lum[3], Deepak Taneja[3], Umesh Chaudhari[3], Rosemarie Sellati*[3]

*[1]Hospital A Beneficência Portuguesa, Sao Paulo, Brazil; [2]Turkish Association of the Fight Against PNH and AHUS, Istanbul, Türkiye; [3]Regeneron Pharmaceuticals, Inc., Tarrytown, United States of America;*

### Background

Paroxysmal nocturnal hemoglobinuria (PNH) is an ultra-rare, life-threatening disease associated with chronic intravascular hemolysis and a high risk of thrombosis, and in some cases, with extravascular hemolysis. Treatment options for PNH can be complex, and include complement inhibitors that target the disease mechanism, and blood transfusions that can be necessary to address ongoing symptoms. Shared decision-making (SDM) is a collaborative approach in which people with PNH and their clinicians work together to evaluate treatment options and develop a personalized care plan, considering benefits, risks, and individual preferences. While SDM has demonstrated positive outcomes in other chronic conditions, its role in PNH remains unexplored.

### Aims

We report findings from a desktop analysis of PNH materials and subsequent review by a PNH Patient Advocate Council, exploring the existing and future role of SDM in the management of PNH.

### Methods

A desktop analysis of 50 publications across therapy areas and publication types, including medical journals, resource guides, conference materials, and accredited healthcare provider education, was conducted to identify best practices, tools, and frameworks in SDM. Sources were included if they discussed treatments or patient-provider dialogue, and provided actionable insights into facilitating patient understanding of treatment options, risks, benefits, or preferences. Findings were then assessed by an advisory PNH Patient Advocate Council, comprising eight people, five people living with PNH and three caregivers, all identified via patient advocacy groups. The council meeting aimed to examine the current state of patient–provider dialogue, including how treatment decisions are discussed and made, and to investigate key inputs and strategies to design, develop, and deploy SDM to advance individualized care plans in PNH.

### Results

This initiative was designed as an exploratory needs assessment to better understand shared decision-making in PNH and identify gaps, unmet needs, and opportunities to support more patient-centered treatment discussions. The PNH Patient Advocate Council emphasized that effective use of SDM sets treatment expectations, drives personalized care, and establishes a shared definition of success between the individual with PNH and their healthcare provider. They also highlighted that simple and concise SDM resources such as treatment comparison charts, question prompt lists, and personal values worksheets could help people with PNH identify their preferences, understand product information, and advance conversations with their healthcare providers in clinical practice. Key barriers to effective SDM identified included a disconnect in communicating care needs to healthcare professionals, regional and socioeconomic disparities, and decision-making fatigue.

**Copyright Information:** (Online) ISSN: 2572-9241
©2026 The Author(s). HemaSphere is published by John Wiley & Sons Ltd on behalf of European Hematology Association. This is an open access Abstract Book distributed under the Attribution-NonCommercial-NoDerivs (CC BY-NC-ND), which allows third parties to download the articles and share them with others as long as they credit the author and the Abstract Book, but they cannot change the content in any way or use them commercially.
**Abstract Book Citations:** Authors, Title, HemaSphere, 2026;10:(S1):pages. The abstract book DOIs can be found on the "Author Information" page.
**Disclaimer:** Articles published in the journal HemaSphere exclusively reflect the opinions of the authors. The authors are responsible for all content in their abstracts including accuracy of the facts, statements, citing resources, etc.

**Summary/Conclusion**

The implementation and benefits of SDM could be applicable to PNH, given the lifelong symptoms that people with PNH and their clinicians must manage. Increasing awareness and advocacy for the use of SDM in the PNH community could enable people with PNH to navigate the complex disease landscape and could enable them to align with their healthcare providers on values and preferences, problem-solve conflicts, and explore options regarding treatment choices.

**Copyright Information:** (Online) ISSN: 2572-9241
©2026 The Author(s). HemaSphere is published by John Wiley & Sons Ltd on behalf of European Hematology Association. This is an open access Abstract Book distributed under the Attribution-NonCommercial-NoDerivs (CC BY-NC-ND), which allows third parties to download the articles and share them with others as long as they credit the author and the Abstract Book, but they cannot change the content in any way or use them commercially.
**Abstract Book Citations:** Authors, Title, HemaSphere, 2026;10:(S1):pages. The abstract book DOIs can be found on the "Author Information" page.
**Disclaimer:** Articles published in the journal HemaSphere exclusively reflect the opinions of the authors. The authors are responsible for all content in their abstracts including accuracy of the facts, statements, citing resources, etc.

### (PB4439) PRELIMINARY ANALYSIS OF EDONOLA STUDY: RELATIONSHIP BETWEEN BASELINESOCIODEMOGRAPHIC VARIABLES, QUALITY OF LIFE AND FUNCTIONAL EXERCISE CAPACITY INPATIENTS WITH LYMPHOMA

**Topic:** 36. Quality of life, ethics, supportive and palliative care

Maren Martinez de Rituerto Zeberio*[1], Jon Madinabeitia Murillo[1], Begoña Mendibil Esquisabel[1, 2], Iñigo Olazabal Eizaguirre[1, 2], Mikel Perez Arrieta[1], Alejandra Maria Patiño Trives[1], Imanol Morante Sainz[3, 4], Sara Leturiaga Lersundi[3, 4], Mikel Tous Espelosin[3, 4, 5], Sara Maldonado Martin[3, 4], Ilargi Gorostegi Anduaga[3, 4, 5], Pablo Corres[3, 4], Izaskun Zeberio Etxetxipia[1, 2]

*[1]IIS Biogipuzkoa, San Sebastián, Spain;[2]Donostia University Hospital, Hematology, San Sebastián, Spain;[3]University of the Basque Country (UPV/EHU), Department of Physical Education and Sport, Faculty of Education and Sport, Vitoria-Gasteiz, Spain;[4]Instituto de Investigación Sanitaria Bioaraba, Vitoria-Gasteiz, Spain;[5]Research Network on Exercise and Quality of Life in Cancer Patients (REFICON), Zaragoza, Spain;*

### Background

Exercise interventions during chemotherapy may improve functional capacity and patient-reported outcomes in lymphoma. However, the relationship among objective
exercise performance, fatigue, and quality of life before treatment initiation remains insufficiently characterized.

### Aims

The main objective of this preliminary analysis from the EDONOLA study was to characterize the baseline variables of patients diagnosed with lymphoma and to explore
associations with quality-of-life questionnaires (HM-PRO, QLQ-FA 12) and functional capacity test (Modified Shuttle Walk Test [MSWT]).

### Methods

Fifty-nine patients (>18 yr old) with a lymphoma diagnosis requiring systemic treatment, and complete datasets were included (59.8 years old, 40.2% women, Table
1).Non-parametric tests and Spearman correlations were applied to analyze the relationship between the following clinical variables (age, sex, study level, hypertension, hyperuricaemia, diabetes and hyperlipidaemia), and HM-PRO, QLQ-FA12 and MSWT.

| **Category** | **N (%)** |
|---|---|
| Age<br><br>18-45<br>48.6-61<br>62-73<br>>74 | <br><br>15 (25.4)<br>15 (25.4)<br>15 (25.4)<br>14 (23.7) |
| **Sex** | |
| Man | 31 (52.5) |
| Woman | 28 (47.5) |
| **Medical history** | |
| Hypertension | 10 (16.9) |
| Diabetes | 1 (1.7) |
| Hyperuricemia | 3 (5.1) |
| Hyperlipemia | 13 (22) |

**Copyright Information:** (Online) ISSN: 2572-9241
©2026 The Author(s). HemaSphere is published by John Wiley & Sons Ltd on behalf of European Hematology Association. This is an open access Abstract Book distributed under the Attribution-NonCommercial-NoDerivs (CC BY-NC-ND), which allows third parties to download the articles and share them with others as long as they credit the author and the Abstract Book, but they cannot change the content in any way or use them commercially.
**Abstract Book Citations:** Authors, Title, HemaSphere, 2026;10:(S1):pages. The abstract book DOIs can be found on the "Author Information" page.
**Disclaimer:** Articles published in the journal HemaSphere exclusively reflect the opinions of the authors. The authors are responsible for all content in their abstracts including accuracy of the facts, statements, citing resources, etc.

### Results

There are no significant differences (p>0.05) between sexes in baseline clinical variables (Table 2).

MSWT performance declined significantly with age for both peak heart rate (HR) and distance (p<0.001), with no sex differences. Hypertension and dyslipidemia were associated with lower peak HR (p=0.02–0.04) and shorter walking distance (p≤0.01). Global fatigue (QLQ-FA12) was moderate (median 17) and did not differ by age or sex, but was significantly higher in patients with hyperuricemia (p=0.008). HM-PRO Part A differed by age (p=0.008), with worse scores in the 48–61-year group. Part B scores were associated with hypertension (p=0.008) and hyperuricemia (p=0.02). Peak HR correlated positively with worse HM-PRO Part A total (r=0.29; p=0.02) and higher global fatigue (r=0.27; p=0.03). No significant correlations were found between walking distance and patient-reported outcomes.

| | Total patients n=59 | 18-48.5 n=15 | 48.6-61 n=15 | 62-73 n=15 | >74 n=15 | P value | Man n=31 | Woman n=28 | P value |
|---|---|---|---|---|---|---|---|---|---|
| HM-PRO<br>Part A<br>Part B<br>(median+/-IQR) | 12(7-20)<br>5(2-8) | 14(9-20)<br>4(2-6) | 19(15-23)<br>5(2-11) | 7(5-12)<br>3(1-9) | 10(5-16)<br>6(2-8) | 0.008<br>0.82 | 10(6-21)<br>3(2-8) | 15(9-5)<br>3(8-19) | 0.27<br>0.22 |
| QLQ-FA12 (median +/-IQR) | 17(14-23) | 16(15-20) | 21(17-28) | 14(13-18) | 17(13-27) | 0.09 | 16(14-21) | 18(14-23) | 0.24 |
| MSWT distance (m) (median +/-IQR) | 560(360-780) | 880(730-885) | 780(730-885) | 510(375-615) | 355(343-400) | <0.0001 | 730(460-850) | 480(368-758) | 0.08 |

### Summary/Conclusion

Knowing patients' quality of life is essential to providing appropriate treatment. The baseline physical functions of a patient can influence the success of treatments and the risk of complications. The HM-PRO questionnaire can help professionals better understand the quality of life of these patients. It should be noted that the data presented in this analysis are preliminary and should be interpreted with caution. Some data may be biased due to the small sample size, and some groups are underrepresented.

**Copyright Information:** (Online) ISSN: 2572-9241
©2026 The Author(s). HemaSphere is published by John Wiley & Sons Ltd on behalf of European Hematology Association. This is an open access Abstract Book distributed under the Attribution-NonCommercial-NoDerivs (CC BY-NC-ND), which allows third parties to download the articles and share them with others as long as they credit the author and the Abstract Book, but they cannot change the content in any way or use them commercially.
**Abstract Book Citations:** Authors, Title, HemaSphere, 2026;10:(S1):pages. The abstract book DOIs can be found on the "Author Information" page.
**Disclaimer:** Articles published in the journal HemaSphere exclusively reflect the opinions of the authors. The authors are responsible for all content in their abstracts including accuracy of the facts, statements, citing resources, etc.

### (PB4440) RELATIONSHIP BETWEEN CHANGES IN QUALITY OF LIFE AND SOCIAL FUNCTION AND TREATMENT RESPONSE IN PATIENTS WITH IDIOPATHIC MULTICENTRIC CASTLEMAN DISEASE: A SINGLE-CENTER OBSERVATIONAL LONGITUDINAL SURVEY

**Topic:** 36. Quality of life, ethics, supportive and palliative care

Jia Chen*[1], Lu Zhang[1], Jian Li[1]

*[1]Peking Union Medical College Hospital, Chinese Academy of Medical Sciences & Peking Union Medical College, Hematology, Beijing, China;*

### Background

The majority of multicentric Castleman disease (MCD) patients in China are idiopathic MCD (iMCD) with systemic manifestations. The evaluation of symptomatic treatment response in iMCD currently relies on clinicians' assessments of patients' symptoms rather than patient-reported outcomes (PROs), which may be influenced by subjective judgments from physicians. PRO results in iMCD patients are rarely mentioned in current treatment response evaluations.

### Aims

This study aims to comprehensively describe changes in symptom burden, quality of life, psychological status, and social function in iMCD patients before and after treatment using PROs, and analyze their relationship with treatment responses.

### Methods

This single-center longitudinal observational study was conducted from October 2023 to April 2025 at Peking Union Medical College Hospital. Patients with a confirmed diagnosis of iMCD were included, based on the 2025 Chinese Guidelines for the Diagnosis and Treatment of Castleman Disease. PRO survey results (MCD-SS, SF-36, PHQ-9, and WPAI:GH) were collected from patients before and after treatment, along with clinical data, symptom, biochemical, and lymph node evaluation results. Paired analysis was used to observe changes in PRO results before and after treatment, which were then compared with treatment responses (biochemcial and symptomatic responses) evaluated according to Castleman Disease Collaborative Network (CDCN) criteria.

**Copyright Information:** (Online) ISSN: 2572-9241
©2026 The Author(s). HemaSphere is published by John Wiley & Sons Ltd on behalf of European Hematology Association. This is an open access Abstract Book distributed under the Attribution-NonCommercial-NoDerivs (CC BY-NC-ND), which allows third parties to download the articles and share them with others as long as they credit the author and the Abstract Book, but they cannot change the content in any way or use them commercially.
**Abstract Book Citations:** Authors, Title, HemaSphere, 2026;10:(S1):pages. The abstract book DOIs can be found on the "Author Information" page.
**Disclaimer:** Articles published in the journal HemaSphere exclusively reflect the opinions of the authors. The authors are responsible for all content in their abstracts including accuracy of the facts, statements, citing resources, etc.

### Results

A total of 19 iMCD patients were included. Two (10.5%) of them were classified as iMCD-TAFRO, 8 (42.1%) as iMCD-IPL, and the remaining as iMCD-NOS. Most of them (14/19, 73.7%) recieved IL-6-targeted therapy. After 4~6 months of treatment, 9 (47.4%) patients achieved symptomatic complete response (CR), 10 (52.6%) achieved biochemical CR, and none achieved oncological CR (Figure A). At the baseline, the MCD-SS reported a median of 11 MCD-related clinical symptoms, with a baseline median total score of 3.3 and a median fatigue dimension score of 5.5. After treatment, both the total MCD-SS score and fatigue score decreased significantly (P: 0.022~0.049). In terms of overall health status, the median “vitality” dimension score of the SF-36 increased significantly 8~12 months after treatment (55.0 vs. 75.0, P = 0.0055), and 78.9% of patients self-reported their health status as "slightly better" or "much better" compared to baseline. The PHQ-9 survey indicated a median baseline score of 8.0, with 13 patients (68.4%) exhibiting depressive symptoms and 7 patients (36.8%) potentially having moderate-to-severe depression. After treatment, the proportion of moderate/severe depressive symptoms decreased to 15.8% (Figure B). Regarding social function, the WPAI:GH survey showed that 47.7% of iMCD patients were unemployed before treatment, decreasing to 31.6% 4~6 months after treatment. PRO results were consistent with symptomatic and biochemical responses evaluated with CDCN criteria. For iMCD patients with clinical fatigue grades of G0, G1, and G2, the median MCD - SS fatigue scores were 2.0, 5.5, and 7.5 points respectively (P < 0.005). In patients who achieved complete symptomatic or biochemical remission, the depression - related scores, fatigue scores, and overall symptom scores decreased significantly (P: 0.0083~0.035) (Figure C).

### Summary/Conclusion

Patient-reported outcomes, as measurable indicators of patient self-report, demonstrate similarity to objective treatment response evaluations and can serve as a supplementary component in response assessment.

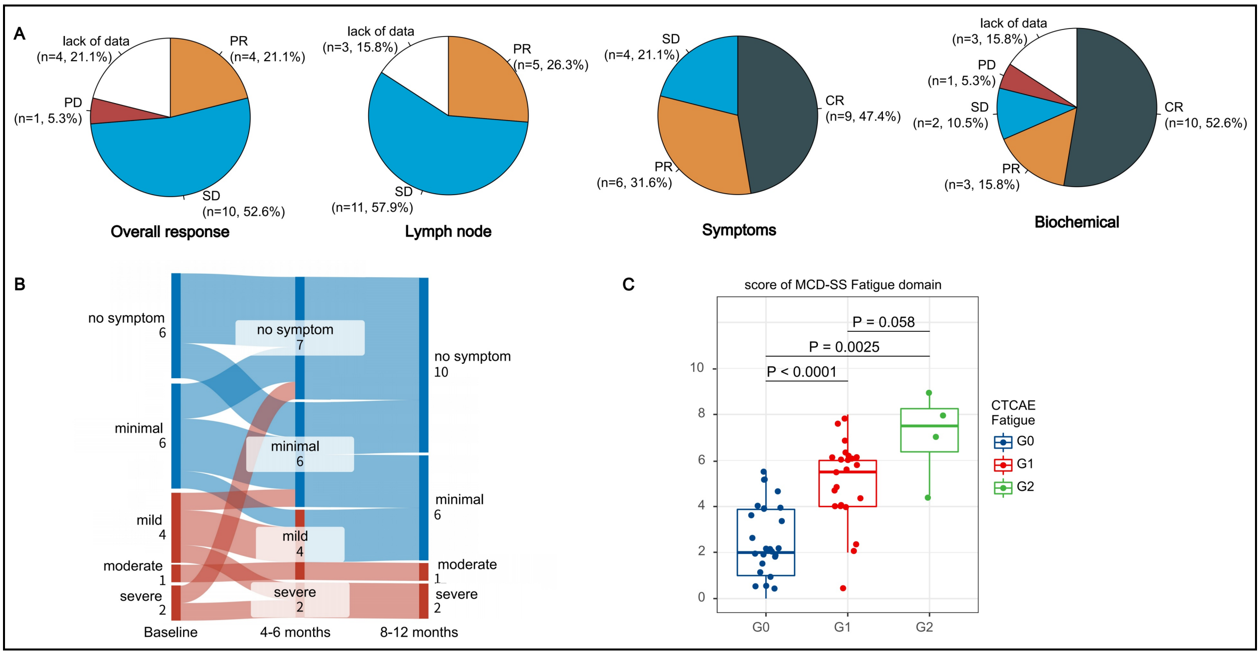

**Copyright Information:** (Online) ISSN: 2572-9241
©2026 The Author(s). HemaSphere is published by John Wiley & Sons Ltd on behalf of European Hematology Association. This is an open access Abstract Book distributed under the Attribution-NonCommercial-NoDerivs (CC BY-NC-ND), which allows third parties to download the articles and share them with others as long as they credit the author and the Abstract Book, but they cannot change the content in any way or use them commercially.
**Abstract Book Citations:** Authors, Title, HemaSphere, 2026;10:(S1):pages. The abstract book DOIs can be found on the “Author Information” page.
**Disclaimer:** Articles published in the journal HemaSphere exclusively reflect the opinions of the authors. The authors are responsible for all content in their abstracts including accuracy of the facts, statements, citing resources, etc.

### (PB4441) PATIENT NAVIGATION AND THERAPEUTIC EDUCATION IMPROVE SURVIVAL IN HEMATOLOGIC MALIGNANCIES: REAL – WORD EVIDENCE FROM A MIDDLE–INCOME COUNTRY.

**Topic:** 36. Quality of life, ethics, supportive and palliative care

Juanita Granados - Diaz[1], Adriana Aya -Porto[2], Carlos Gomez - Calcetero[1], Paola Omaña - Orduz[1], Jorge Daza - Buitrago[1], Virginia Abello - Polo*[1]

*[1]Luis Carlos Sarmiento Angulo Cancer Treatment and Research Centre (CTIC), Research Group GIGA, CTIC/Universidad El Bosque., Bogotá, Colombia; [2]Luis Carlos Sarmiento Angulo Cancer Treatment and Research Centre (CTIC), Research and Education Institute, Bogotá, Colombia;*

### Background

Treatment adherence is a critical determinant of clinical outcomes and survival in patients with hematologic malignancies. However, in low- and middle-income countries, adherence is often suboptimal due to geographic barriers, socioeconomic constraints, and treatment-related toxicities. In this context, nurses lead therapeutic education, integrated into patient-centered care models such as navigation programs, has emerged as a promising strategy to enhance treatment engagement, reduce interruptions, and optimize continuity of care. Nevertheless, evidence demonstrating a direct impact of these interventions on survival remains limited, representing an important gap in current knowledge.

### Aims

To evaluate the association between the number of therapeutic educational sessions delivered within a navigation model and overall survival in a cohort of patients with hematologic malignancies.

### Methods

We conducted a retrospective analytical cohort study including patients ≥18 years with an incident diagnosis of hematologic malignancies (Hodgkin lymphoma, non-Hodgkin lymphoma, multiple myeloma, and acute leukemias) treated at Fundación CTIC in Bogotá, Colombia, between January 2023 and December 2024. All patients were enrolled in the prospective institutional observational registry of EVA (Evidence–Verification–Analysis) and had provided informed consent.

Bivariate analysis between the number of educational sessions and vital status (alive/death) was performed using Pearson's $\chi^2$ test. Overall survival (OS) was estimated using Kaplan–Meier curves and compared across groups stratified by the number of educational sessions received using the log-rank test. A Cox proportional hazards regression model was subsequently applied to evaluate 24-month survival according to the number of educational sessions received, finally Hazard Ratio (HR) was calculated. Statistical significance was defined as a two-sided p-value <0.05. All analyses were conducted using Python version 3.11.

**Copyright Information:** (Online) ISSN: 2572-9241
©2026 The Author(s). HemaSphere is published by John Wiley & Sons Ltd on behalf of European Hematology Association. This is an open access Abstract Book distributed under the Attribution-NonCommercial-NoDerivs (CC BY-NC-ND), which allows third parties to download the articles and share them with others as long as they credit the author and the Abstract Book, but they cannot change the content in any way or use them commercially.
**Abstract Book Citations:** Authors, Title, HemaSphere, 2026;10:(S1):pages. The abstract book DOIs can be found on the "Author Information" page.
**Disclaimer:** Articles published in the journal HemaSphere exclusively reflect the opinions of the authors. The authors are responsible for all content in their abstracts including accuracy of the facts, statements, citing resources, etc.

**Results**

A total of 210 patients were included: 113 (53.8%) with lymphoma, 58 (27.6%) with acute leukemias, and 39 (18.6%) with multiple myeloma. Regarding the educational intervention, 153 patients (72.8%) received 1–2 sessions, whereas 60 (28.6%) received ≥3 sessions. The cohort was predominantly composed of older adults (> 60 years, 52%) and women (57%).
Contributory health insurance coverage was the most frequent (59%), and primary education was the most common educational level attained (32%). Additionally, 60% of patients lived outside of Bogotá (Table 1).
Patients who received ≥3 sessions demonstrated a significantly lower risk of death compared with those who received 1–2 sessions (HR = 0.54; 95% CI, 0.32–0.91), corresponding to a 46% relative reduction in mortality. This association was statistically significant in the Cox proportional hazards model (p = 0.020) and was supported by the log-rank test (p = 0.018), figure 1.

**Summary/Conclusion**

In low- and middle-income countries, where structural and socioeconomic barriers frequently compromise adherence and continuity of care, integrated educational support may represent a scalable strategy to optimize treatment delivery. Incorporating systematic, patient-centered education within Navigation programs could meaningfully contribute to improving survival outcomes in patients with hematologic malignancies.

**Copyright Information:** (Online) ISSN: 2572-9241
©2026 The Author(s). HemaSphere is published by John Wiley & Sons Ltd on behalf of European Hematology Association. This is an open access Abstract Book distributed under the Attribution-NonCommercial-NoDerivs (CC BY-NC-ND), which allows third parties to download the articles and share them with others as long as they credit the author and the Abstract Book, but they cannot change the content in any way or use them commercially.
**Abstract Book Citations:** Authors, Title, HemaSphere, 2026;10:(S1):pages. The abstract book DOIs can be found on the “Author Information” page.
**Disclaimer:** Articles published in the journal HemaSphere exclusively reflect the opinions of the authors. The authors are responsible for all content in their abstracts including accuracy of the facts, statements, citing resources, etc.

**Table 1 . Baseline patient characteristics**

| Categories | Total (n=210) | Alive (n=119) | Death(n=91) | p value |
|---|---|---|---|---|
| **Type hematological tumor, n(%)** | | | | |
| *Lymphoma* | 113(53,8) | 71(59,7) | 42(46,2) | |
| *Acute leukemias* | 58(27,6) | 21(17,6) | 37(40,7) | **0,0008** |
| *Multiple myeloma* | 39(18,6) | 27(22,7) | 12(13,2) | |
| **Risk, n(%)** | | | | |
| *High* | 37(17,6) | 16(13,4) | 21(23,1) | |
| *Intermediate* | 17(8,1) | 4(3,4) | 13(14,3) | 0,1325 |
| *Low* | 4(1,9) | 1(0,8) | 3(3,3) | |
| **Staging, n(%)** | | | | |
| *Advanced* | 120(57,1) | 77(64,7) | 43(47,3) | |
| *Early* | 24(11,4) | 18(15,1) | 6(6,6) | 0,1567 |
| *Unknown* | 8(3,8) | 3(2,5) | 5(5,5) | |
| **Number of educational sessions, n(%)** | | | | |
| *1 -- 2* | 135(64,3) | 77(64,7) | 58(63,7) | |
| *>3* | 60(28,6) | 41(34,5) | 19(20,9) | **0,0001** |
| *None* | 15(7,1) | 1(0,8) | 14(15,4) | |

**Figure 1. Survival curve at 24 months stratified by the number of education sessions received.**

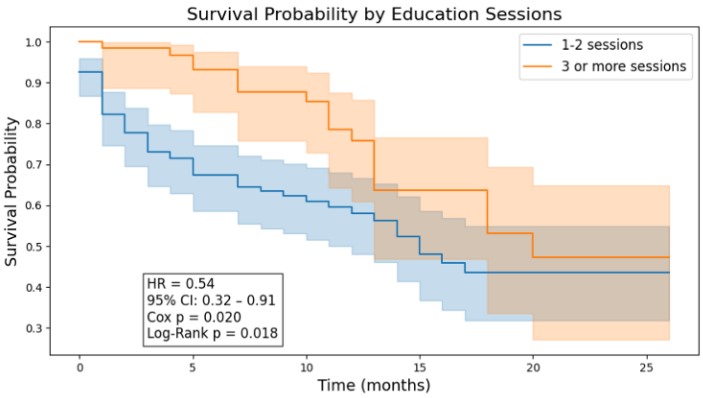

**Copyright Information:** (Online) ISSN: 2572-9241
©2026 The Author(s). HemaSphere is published by John Wiley & Sons Ltd on behalf of European Hematology Association. This is an open access Abstract Book distributed under the Attribution-NonCommercial-NoDerivs (CC BY-NC-ND), which allows third parties to download the articles and share them with others as long as they credit the author and the Abstract Book, but they cannot change the content in any way or use them commercially.
**Abstract Book Citations:** Authors, Title, HemaSphere, 2026;10:(S1):pages. The abstract book DOIs can be found on the "Author Information" page.
**Disclaimer:** Articles published in the journal HemaSphere exclusively reflect the opinions of the authors. The authors are responsible for all content in their abstracts including accuracy of the facts, statements, citing resources, etc.

### (PB4443) PATIENT-CONTROLLED SEDATION USING PROPOFOL DURING BONE MARROW BIOPSY

**Topic:** 36. Quality of life, ethics, supportive and palliative care

Maria Knutsson*[1], Lisa Gunnar Fahlgren[1], Carina Widmark[1]

[1]*Karolinska University Hospital, HUDDINGE, Sweden;*

### Background

Bone marrow sampling constitutes a central component in the diagnosis and follow-up of hematological malignancies. For the majority of patients, the procedure can be performed without major difficulties; however, for some patients, it is experienced as painful and anxiety-inducing. This may lead to increased apprehension prior to future examinations and, in some cases, reluctance to undergo necessary sampling.
To optimize both the patient experience and quality of care, there is a need to identify and implement strategies that reduce discomfort and anxiety in connection with bone marrow sampling. At the same time, such measures should be patient-safe, effective, and resource-sustainable.
Historically, the hematology clinic in Karolinska Huddinge has used oral anxiolytics, such as oxazepam, as well as intravenous sedation with midazolam in conjunction with bone marrow sampling. However, for some patients, these treatment options have been perceived as insufficient in terms of pain and anxiety relief.
In other clinical settings where procedural sedation is applied—such as endoscopic examinations, dental treatments, kidney stone procedures, and bronchoscopies—patient-controlled sedation (PCS) using propofol has been used with favorable results. Studies indicate that PCS may contribute to increased patient satisfaction, partly by allowing patients to regulate the level of sedation themselves and thereby experience greater control over the situation.

### Aims

The aim of the project was to introduce patient-controlled sedation with propofol for bone marrow sampling in order to achieve more effective, safe, and patient-centered sedation, as well as to improve patients' experience of the procedure.

### Methods

Patient-controlled sedation with propofol was introduced at the hematology outpatient clinic. The staff underwent training in the method, and a drug infusion pump intended for PCS was acquired. Local written protocols were developed to ensure standardized and patient-safe application.
The implementation was carried out gradually through a stepwise transition from intravenous midazolam to PCS with propofol.
Over a period of approximately 1.5 years, all patients undergoing bone marrow sampling with PCS were asked to complete an evaluation questionnaire following the procedure.

### Results

During the follow-up period, 19 evaluation questionnaires were collected. Patients rated perceived pain, level of sedation, sense of control, occurrence of side effects, and overall satisfaction. They were also given the opportunity to compare the experience with previous bone marrow procedures.
All patients reported being very satisfied with the experience and stated that they had full control over the sedation. The majority assessed that PCS with propofol functioned considerably better compared with previous bone marrow procedures. Most patients reported no side effects. The side effects that did occur were nausea, dizziness, and fatigue, which were consistently described as mild.

**Copyright Information:** (Online) ISSN: 2572-9241
©2026 The Author(s). HemaSphere is published by John Wiley & Sons Ltd on behalf of European Hematology Association. This is an open access Abstract Book distributed under the Attribution-NonCommercial-NoDerivs (CC BY-NC-ND), which allows third parties to download the articles and share them with others as long as they credit the author and the Abstract Book, but they cannot change the content in any way or use them commercially.
**Abstract Book Citations:** Authors, Title, HemaSphere, 2026;10:(S1):pages. The abstract book DOIs can be found on the "Author Information" page.
**Disclaimer:** Articles published in the journal HemaSphere exclusively reflect the opinions of the authors. The authors are responsible for all content in their abstracts including accuracy of the facts, statements, citing resources, etc.

**Summary/Conclusion**

Patient-controlled sedation with propofol is a patient-safe, effective, and symptom-relieving method for performing bone marrow sampling, particularly in patients with pronounced procedure-related anxiety. The method has improved the patient experience and thereby contributed to increased acceptance of repeated examinations within hematological care.

**Copyright Information:** (Online) ISSN: 2572-9241
©2026 The Author(s). HemaSphere is published by John Wiley & Sons Ltd on behalf of European Hematology Association. This is an open access Abstract Book distributed under the Attribution-NonCommercial-NoDerivs (CC BY-NC-ND), which allows third parties to download the articles and share them with others as long as they credit the author and the Abstract Book, but they cannot change the content in any way or use them commercially.
**Abstract Book Citations:** Authors, Title, HemaSphere, 2026;10:(S1):pages. The abstract book DOIs can be found on the "Author Information" page.
**Disclaimer:** Articles published in the journal HemaSphere exclusively reflect the opinions of the authors. The authors are responsible for all content in their abstracts including accuracy of the facts, statements, citing resources, etc.

### (PB4444) THE EFFECT OF PEER EDUCATION ON SELF-EFFICACY AND QUALITY OF LIFE IN PATIENTS WITH LYMPHOMA

**Topic:** 36. Quality of life, ethics, supportive and palliative care

Yang He*[1], Liyuan Qin[1]

[1]*Nursing Department of Ruijin Hospital, Shanghai Jiao Tong University School of Medicine, Shanghai, China;*

### Background

Lymphoma is a malignant tumor originating in lymph nodes or lymphoid tissues, clinically characterized primarily by painless, progressive lymphadenopathy. In recent years, the incidence of lymphoma has been increasing, ranking it among the top 10 most common malignancies. Treatment modalities for lymphoma include chemotherapy, targeted therapy, immunotherapy, biological therapy, stem cell transplantation, and radiotherapy. Patients often face challenges such as prolonged disease diagnosis and treatment, difficulties with repeated hospitalizations, numerous adverse effects of chemotherapy, and anxiety and fear due to concerns about disease recurrence, leading to reduced self-efficacy and significant impacts on their physical and mental health. Peer education is a novel and critical health education model that involves bringing together individuals of the same age group, similar living environments, life experiences, and equal cultural and social status, or gathering people with common interests due to shared circumstances, to share information, insights, or behavioral skills. This peer education approach enhances patient acceptance. Previous studies have demonstrated the high applicability of peer education in patients with malignant tumors such as breast cancer and colorectal cancer, but no research has explored its application in lymphoma patients. Based on the disease and treatment characteristics of lymphoma patients, this study developed a peer education model tailored to lymphoma and investigated its impact on patients' self-efficacy and quality of life.

### Aims

To observe the effects of peer education mode on the self-efficacy and quality of life of patients with lymphoma.

### Methods

Using a non-synchronous experimental design, 89 lymphoma patients from February 2018 to January 2019 were selected as the control group, and 89 patients from February 2019 to February 2020 were selected as the experimental group. The control group received routine nursing care, while the experimental group received peer education on the basis of routine nursing measures, with a nursing duration of 6 months for both groups. The quality of life, self-efficacy, and self-management level of two groups of patients before and after intervention were compared.

### Results

After intervention, the self-efficacy score of the experimental group (85.32±11.02) was significantly higher than that of the control group (72.54±9.61) (P<0.001)⊠IQR:50.00-83.33⊠was significantly higher than that in the control group [58.33 IQR:33.33-75.00] P=0.020. The standardized score of quality of life in the area of nausea and vomiting was 16.67⊠IQR:0.00-33.00⊠significantly lower than that in the control group (IQR: 0.00⊠50.00) P=0.024.

### Summary/Conclusion

Peer education model can significantly improve the quality of life and selfefficacy of patients with malignant tumor receiving chemotherapy.

**Copyright Information:** (Online) ISSN: 2572-9241
©2026 The Author(s). HemaSphere is published by John Wiley & Sons Ltd on behalf of European Hematology Association. This is an open access Abstract Book distributed under the Attribution-NonCommercial-NoDerivs (CC BY-NC-ND), which allows third parties to download the articles and share them with others as long as they credit the author and the Abstract Book, but they cannot change the content in any way or use them commercially.
**Abstract Book Citations:** Authors, Title, HemaSphere, 2026;10:(S1):pages. The abstract book DOIs can be found on the "Author Information" page.
**Disclaimer:** Articles published in the journal HemaSphere exclusively reflect the opinions of the authors. The authors are responsible for all content in their abstracts including accuracy of the facts, statements, citing resources, etc.

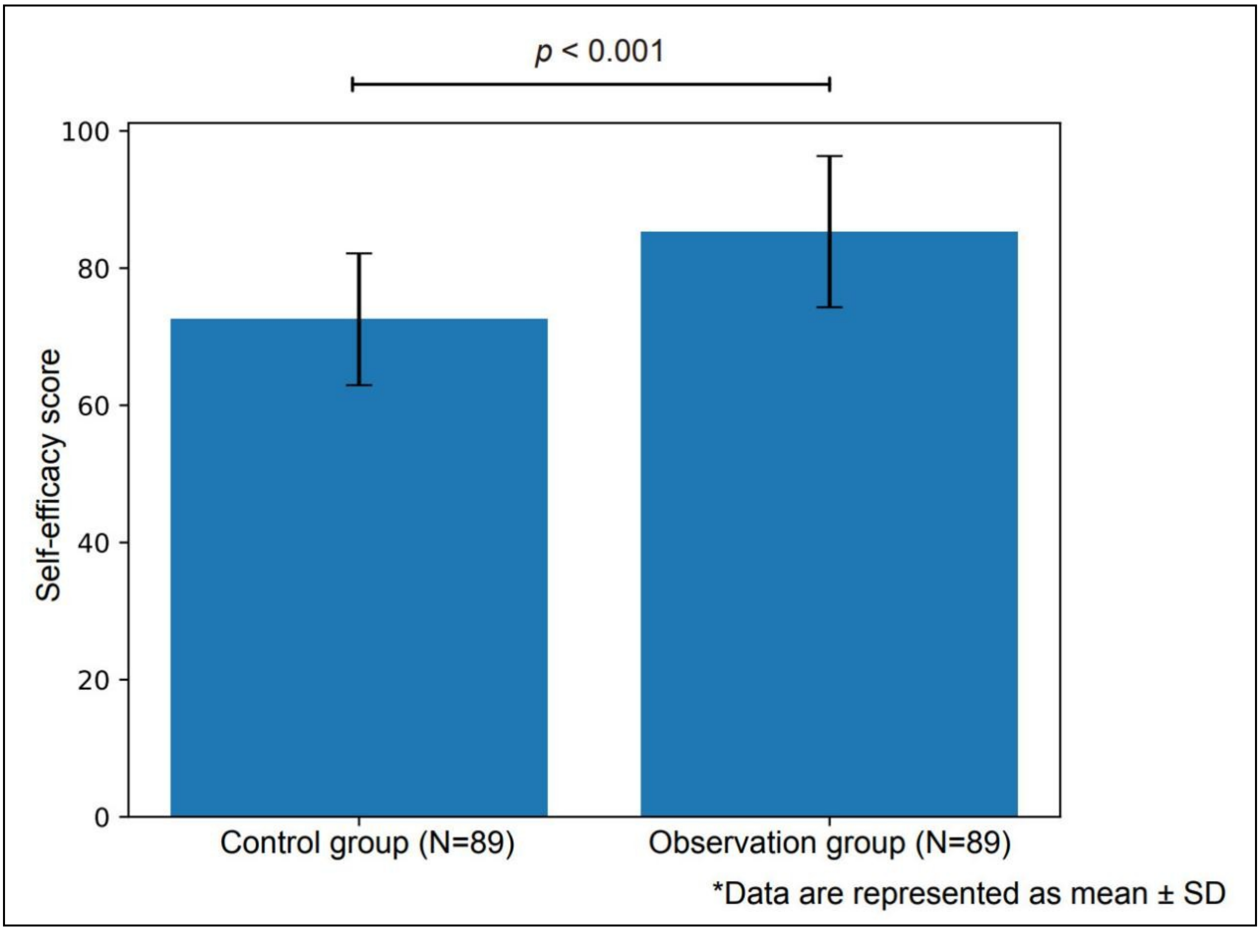

**Copyright Information:** (Online) ISSN: 2572-9241
©2026 The Author(s). HemaSphere is published by John Wiley & Sons Ltd on behalf of European Hematology Association. This is an open access Abstract Book distributed under the Attribution-NonCommercial-NoDerivs (CC BY-NC-ND), which allows third parties to download the articles and share them with others as long as they credit the author and the Abstract Book, but they cannot change the content in any way or use them commercially.
**Abstract Book Citations:** Authors, Title, HemaSphere, 2026;10:(S1):pages. The abstract book DOIs can be found on the "Author Information" page.
**Disclaimer:** Articles published in the journal HemaSphere exclusively reflect the opinions of the authors. The authors are responsible for all content in their abstracts including accuracy of the facts, statements, citing resources, etc.

### (PB4445) SOCIAL VULNERABILITY, TREATMENT COMPLETION AND DOSE INTENSITY TREATMENT: REAL – WORLD IMPACT OF A PATIENT –CENTERED NAVIGATION PROGRAM IN A MIDDLE – INCOME COUNTRY.

**Topic:** 36. Quality of life, ethics, supportive and palliative care

Adriana Aya -Porto[1], Juanita Granados - Diaz[2], Carlos Gomez - Calcetero[2], Giselle Sarmiento[2], Paola Omaña - Orduz[2], Jorge Daza - Buitrago[2], Virginia Abello - Polo*[2]

*[1]Luis Carlos Sarmiento Angulo Cancer Treatment and Research Centre (CTIC), Research and Education Institute, Bogotá, Colombia; [2]Luis Carlos Sarmiento Angulo Cancer Treatment and Research Centre (CTIC), Research Group GIGA, CTIC/Universidad El Bosque., Bogotá, Colombia;*

**Background**

In low- and middle-income countries, social vulnerability factors including economic dependency, geographic barriers, low educational attainment, and limited access to healthcare, compromise treatment adherence, leading to interruptions, dose reductions, and failure to achieve optimal relative dose intensity (RDI). Patient-centered navigation programs and multidisciplinary care models have been proposed to mitigate these barriers and enhance continuity of care. However, real-world evidence evaluating their impact on first-line treatment completion and RDI in middle-income countries remains limited, underscoring the need for studies addressing this gap.

**Aims**

To characterize social vulnerability factors in a cohort of patients with hematologic neoplasms and to evaluate their association withcompletion of first-line therapy as measured by RDI.

**Methods**

W e conducted a retrospective analytical cohort study including patients ≥18 years with an incident diagnosis of hematologic malignancies(Hodgkin lymphoma, non-Hodgkin lymphoma, multiple myeloma, and acute leukemias) treated at Fundación CTIC (Bogotá, Colombia) betweenJanuary 2023 and December 2024. All patients were enrolled in the prospective institutional observational registry EVA (Evidence–Verification–Analysis) and had provided informed consent.

Patients with complete data on first-line treatment, delivered dose intensity, vital status, and social variables were included. A non-probabilisticconvenience sampling strategy was applied. Patients were classified according to completion of first-line therapy and reasons for interruption(death, progression, treatment abandonment, or other causes). Care delivery included nurse-led clinical navigation and social work assessment to identify access barriers, vulnerability factors, and psychosocial needs. Statistical analysis comprised descriptive statistics and bivariate analysis using Pearson's chi-square test to assessassociations between social factors and treatment completion according to RDI ($p < 0.0001$). Analyses were performed using Python version 3.11.

**Results**

A total of 194 patients with incident hematologic malignancies were included (Table 1). Fifty-seven percent were male, and 60% resided outside of the capital of Colombia. Among patients with acute leukemias, 65% had high-risk cytogenetics; 74% of lymphomas were diagnosed at advanced stage; and 74% of multiple myeloma patients were classified as high risk. No statistically significant associations were observed between treatment completion according to RDI and social inequity variables ($p > 0.05$), although trends were noted among older patients and those with subsidizedinsurance coverage. Failure to complete treatment was significantly associated with higher mortality ($p < 0.001$).

**Copyright Information:** (Online) ISSN: 2572-9241
©2026 The Author(s). HemaSphere is published by John Wiley & Sons Ltd on behalf of European Hematology Association. This is an open access Abstract Book distributed under the Attribution-NonCommercial-NoDerivs (CC BY-NC-ND), which allows third parties to download the articles and share them with others as long as they credit the author and the Abstract Book, but they cannot change the content in any way or use them commercially.
**Abstract Book Citations:** Authors, Title, HemaSphere, 2026;10:(S1):pages. The abstract book DOIs can be found on the "Author Information" page.
**Disclaimer:** Articles published in the journal HemaSphere exclusively reflect the opinions of the authors. The authors are responsible for all content in their abstracts including accuracy of the facts, statements, citing resources, etc.

### Summary/Conclusion

Patient-centered navigation models are a promising strategy for mitigating the adverse impact of social vulnerability factors on treatment adherence and mortality in low- and middle-income countries.

Table 1. Baseline patient characteristics

| Categories | Total (n=194) | Treatment completed according to RDI (n=134) | Treatment incomplete according to RDI (n=60) | p value |
|---|---|---|---|---|
| **Social Demographics characteristics** | | | | |
| **Age, years, n (%)** | | | | 0,0668 |
| >60 | 95(49) | 60(40,8) | 35(58,3) | |
| 27-59 | 88(45,3) | 68(50,7) | 20(33,3) | |
| 18-26 | 11(5,7) | 6(4,5) | 5(8,3) | |
| **Sex, n(%)** | | | | 0,552 |
| Male | 108(55,7) | 77(57,5) | 31(51,7) | |
| Female | 86(44,3) | 57(42,5) | 29(48,3) | |
| **Assurance, n(%)** | | | | 0,0948 |
| Contributive | 114(58,9) | 80(59,7) | 34(56,7) | |
| Subsidized | 69(35,6) | 46(34,3) | 23(38,3) | |
| Special plans | 9(4,6) | 7(5,2) | 2(3,3) | |
| Not insured | 2(1) | 1(0,7) | 1(1,7) | |
| **Academic level, n(%)** | | | | 0,2919 |
| Primary | 61(31,4) | 43(32,1) | 18(30) | |
| Secundary | 54(27,8) | 39(29,1) | 15(25) | |
| Professional degree | 32(16,5) | 25(18,7) | 7(11,7) | |
| Technical studies | 25(12,9) | 14(10,4) | 11(18,3) | |
| None | 13(6,7) | 7(5,2) | 6(10) | |
| No data | 9(4,6) | 6(4,5) | 3(5) | |
| **Source of economic income, n(%)** | | | | 0,2912 |
| Dependent | 74(38,1) | 44(32,8) | 30(50) | |
| Employment | 40(20,6) | 32(23,9) | 8(13,3) | |
| Pension | 30(15,5) | 20(14,9) | 10(16,7) | |
| Self-employed | 24(12,4) | 19(14,2) | 5(8,3) | |
| No data | 9(4,6) | 6(4,5) | 3(5) | |
| None | 7(3,6) | 5(3,7) | 2(3,3) | |
| Informal work | 6(3,1) | 5(3,7) | 1(1,7) | |
| Subsidy | 4(2,1) | 3(2,2) | 1(1,7) | |
| **Support network, n(%)** | | | | 0,7542 |
| Yes | 148(76,3) | 103(76,9) | 45(75) | |
| Limited | 36(18,6) | 24(17,9) | 12(20) | |
| No data | 9(4,6) | 6(4,5) | 3(5) | |
| No | 1(0,5) | 1(0,7) | - | |
| **Diagnosis characteristics** | | | | |
| **Type hematological tumor, n(%)** | | | | 0,9754 |
| Lymphoma | 105(54,1) | 73(54,5) | 32(53,3) | |
| Multiple myeloma | 52(26,8) | 36(26,9) | 16(26,7) | |
| Acute leukemias | 37(19,1) | 25(18,7) | 12(20) | |
| **Mortality, n(%)** | | | | **0,000** |
| No | 113(58,2) | 100(74,6) | 13(21,7) | |
| Yes | 81(41,8) | 34(25,4) | 47(78,3) | |

**Copyright Information:** (Online) ISSN: 2572-9241
©2026 The Author(s). HemaSphere is published by John Wiley & Sons Ltd on behalf of European Hematology Association. This is an open access Abstract Book distributed under the Attribution-NonCommercial-NoDerivs (CC BY-NC-ND), which allows third parties to download the articles and share them with others as long as they credit the author and the Abstract Book, but they cannot change the content in any way or use them commercially.
**Abstract Book Citations:** Authors, Title, HemaSphere, 2026;10:(S1):pages. The abstract book DOIs can be found on the "Author Information" page.
**Disclaimer:** Articles published in the journal HemaSphere exclusively reflect the opinions of the authors. The authors are responsible for all content in their abstracts including accuracy of the facts, statements, citing resources, etc.

### (PB4446) LUSPATERCEPT IN CANCER-RELATED ANEMIA: PRELIMINARY CLINICAL OBSERVATIONS

**Topic:** 36. Quality of life, ethics, supportive and palliative care

Juan Zhang*[1], Miao Chen[2]

*[1]Beijing Sixth Hospital, department of hematopathology, Beijing, China;[2]Peking Union Medical College Hospital, Haematology, Beijing, China;*

### Background

Cancer-related anemia (CRA) frequently complicates malignancy, compromising quality of life, limiting treatment tolerance, and serving as an independent prognostic factor for reduced survival and increased morbidity.Current management remains largely confined to erythropoiesis-stimulating agents (ESAs), iron supplementation, and transfusions. However, concerns over ESA-associated thromboembolic risk and increased all-cause mortality in cancer patients restrict their use, highlighting an urgent need for safer alternatives. Luspatercept, a first-in-class erythroid maturation agent, promotes terminal erythroid differentiation via an erythropoietin (EPO) receptor-independent mechanism by targeting transforming growth factor-β (TGF-β) superfamily ligands, offering a potential novel approach for CRA management.

### Aims

To preliminarily evaluate the efficacy and safety of luspatercept in patients with CRA.

### Methods

We retrospectively reviewed patients with CRA secondary to solid tumors who received luspatercept between July 2025 and February 2026. Included patients were those receiving non-palliative chemotherapy who were either unsuitable for ESA therapy or had shown prior ESA treatment failure. Anemia etiologies such as bone marrow infiltration, blood loss, or folate/vitamin B12 deficiency were excluded. Patients with absolute or functional iron deficiency received intravenous iron supplementation prior to or during treatment. Luspatercept was administered subcutaneously at a starting dose of 1.0 mg/kg every 3 weeks (Q3W). The primary outcomes were erythroid hematologic improvement (HI-E, defined as a hemoglobin [Hb] increase ≥15 g/L from baseline) and absolute Hb change. Secondary outcomes included the proportion of patients achieving Hb ≥100 g/L, change in transfusion burden, and adverse events (AEs).

### Results

A total of 16 patients with CRA secondary to solid tumors were included. The median age was 66.5 years; 7 patients (43.8%) were male and 9 (56.3%) were female. Tumor types included breast (n=6), pancreatic (n=5), lung (n=3), ovarian (n=1), and colon cancer (n=1). Baseline median Hb was 76.5 g/L (range: 51–93 g/L), and 3 patients were transfusion-dependent (TD). Median baseline serum ferritin was 508 ng/mL (range: 12–6647 ng/mL). After a median follow-up of 10 weeks (range: 1–23 weeks), 11 patients were evaluable for response (defined as follow-up duration or survival >3 weeks). HI-E was observed in 8 patients (72.7%), with a median Hb increase of 24 g/L (range: 7–38 g/L). Six patients (54.5%) achieved Hb ≥100 g/L. Among the 3 TD patients at baseline, 1 became transfusion-independent. Treatment was generally well tolerated. No drug-related thromboembolic events or grade ≥3 AEs were observed. One death occurred due to end-stage disease with multiple metastases, and one case of chemotherapy-induced severe agranulocytosis with septic shock was recorded; both were considered unrelated to luspatercept.

**Copyright Information:** (Online) ISSN: 2572-9241
©2026 The Author(s). HemaSphere is published by John Wiley & Sons Ltd on behalf of European Hematology Association. This is an open access Abstract Book distributed under the Attribution-NonCommercial-NoDerivs (CC BY-NC-ND), which allows third parties to download the articles and share them with others as long as they credit the author and the Abstract Book, but they cannot change the content in any way or use them commercially.
**Abstract Book Citations:** Authors, Title, HemaSphere, 2026;10:(S1):pages. The abstract book DOIs can be found on the "Author Information" page.
**Disclaimer:** Articles published in the journal HemaSphere exclusively reflect the opinions of the authors. The authors are responsible for all content in their abstracts including accuracy of the facts, statements, citing resources, etc.

**Summary/Conclusion**

Luspatercept demonstrated promising efficacy and a favorable safety profile in this case series of patients with CRA secondary to solid tumors. These preliminary findings suggest it may represent a potential therapeutic option for CRA management and warrant further investigation in larger studies.

**Copyright Information:** (Online) ISSN: 2572-9241
©2026 The Author(s). HemaSphere is published by John Wiley & Sons Ltd on behalf of European Hematology Association. This is an open access Abstract Book distributed under the Attribution-NonCommercial-NoDerivs (CC BY-NC-ND), which allows third parties to download the articles and share them with others as long as they credit the author and the Abstract Book, but they cannot change the content in any way or use them commercially.
**Abstract Book Citations:** Authors, Title, HemaSphere, 2026;10:(S1):pages. The abstract book DOIs can be found on the "Author Information" page.
**Disclaimer:** Articles published in the journal HemaSphere exclusively reflect the opinions of the authors. The authors are responsible for all content in their abstracts including accuracy of the facts, statements, citing resources, etc.

### (PB4447) A CANADIAN LEUKEMIA STUDY GROUP (CLSG) CURRENT STATE ASSESSMENT OF HEMATOLOGY ASSOCIATED BIOBANKING IN CANADA

**Topic:** 36. Quality of life, ethics, supportive and palliative care

Shane Toolan*[1], Alexandra Misura[2], Tina Khazaee[3], Josee Hebert[4], Amy Trottier[5], Deepesh Lad[6], Florian Kuchenbauer[6], Ly Vu[6], Jongbok Lee[7], Sonia Cerquozzi[7], Bruce Ritchie[8], Tobias Berg[9], Dianne Chadwick[10], Katya Godard[11], Mitchell Sabloff[12], Jenny Ho[13], Anargyros Xenocostas[13], Andrea Arruda[1], Hubert Tsui[3], Signy Chow[3]

*[1]Princess Margaret Cancer Center, Leukemia, Toronto, Canada;[2]Sunnybrook Research Institute, Toronto, Canada; [3]Sunnybrook Health Sciences Center, Toronto, Canada; [4]Quebec Leukemia Cell Bank, Quebec City, Canada;[5]Dalhousie University, Halifax, Canada; [6]University of British Columbia, Vancouver, Canada;[7]University of Calgary, Alberta, Canada;[8]University of Alberta, Edmonton, Canada; [9]McMaster University, Hamilton, Canada; [10]Ontario Tumour Bank, Toronto, Canada; [11]Ottawa Hospital Tumour Bank, Ottawa, Canada; [12]The Ottawa Hospital, Ottawa, Canada; [13]London Health Sciences Center, London, Canada;*

### Background

The CLSG is committed to bringing world-class leukemia care to all Canadians throughout the discovery, translational and clinical phases of development.  Biobanks are essential for facilitating this progress; however, challenges regarding sample under-utilization, financial sustainability, and regulatory compliance are frequently cited. In Canada, these operational difficulties are compounded by a complex regulatory and legal environment. Of note, the First Nations Principles of OCAP (Ownership, Control, Access, and Possession) add a necessary but distinct layer of governance for Indigenous data. Although various bodies including the Canadian Tissue Repository Network (CTRNet) have attempted to standardize practice, their efforts are hampered by regulatory heterogeneity.

### Aims

While the challenges of biobanking are well documented globally, it remains unclear to what extent these apply in the Canadian context. To date, no comprehensive review of biobanking from the perspective of Canadian biobanking staff has been conducted. Therefore, the purpose of this study was to characterize the leukemia biobanking landscape, identify current operational practices and funding models, and establish barriers to future research collaboration.

### Methods

A cross-sectional survey was developed and distributed electronically via REDCap to biobank managers and lead clinician-scientists. Participants were identified through the CLSG. The survey captured data relating to legal and ethical frameworks, quality assurance standards, biobank operations and data sharing, commercialization, and challenges and opportunities in biobanking. Descriptive statistics were used to summarize survey responses.

**Copyright Information:** (Online) ISSN: 2572-9241
©2026 The Author(s). HemaSphere is published by John Wiley & Sons Ltd on behalf of European Hematology Association. This is an open access Abstract Book distributed under the Attribution-NonCommercial-NoDerivs (CC BY-NC-ND), which allows third parties to download the articles and share them with others as long as they credit the author and the Abstract Book, but they cannot change the content in any way or use them commercially.
**Abstract Book Citations:** Authors, Title, HemaSphere, 2026;10:(S1):pages. The abstract book DOIs can be found on the "Author Information" page.
**Disclaimer:** Articles published in the journal HemaSphere exclusively reflect the opinions of the authors. The authors are responsible for all content in their abstracts including accuracy of the facts, statements, citing resources, etc.

**Results**

Twelve biobanks across 5 provinces responded (median age of biobank: 15.5 years; median number of patients: 2617). Most (75%) are led by principal investigators rather than central repositories. All collect AML samples, with 73% storing ALL, MPN, and MDS specimens. Quality assurance is robust: 75% are CTRNet certified, 100% utilize standard operating procedures, and 100% de-identify stored data; however, only 25% utilize specific data annotation standards (e.g., MIABIS) and only 2 have active data catalogues. Regarding governance, one-third collect Indigenous samples, of which half follow Ownership, Control, Access, and Possession (OCAP) principles. 83% of respondents expressed dissatisfaction with sample distribution procedures, with complex legal agreements (67%) and REB reviews (42%) cited as major obstacles in this regard. Access procedures varied: 83% utilized MTAs/DTAs, but only 33% had formal data access committees. Financial sustainability is a major concern (92%), yet 50% lacked contingency plans in this regard. Funding relies heavily on grants (63%); only 33% allow industry use, though 40% view commercial funding as essential for future sustainability.

**Summary/Conclusion**

The Canadian leukemia biobanking ecosystem is characterised by heterogeneous access procedures, funding models, and a complex regulatory environment which threaten the long-term sustainability and societal impact of biobanks. A nationally coordinated biobanking model is required, with harmonized legal agreements, transparent access procedures, and diversified funding streams that include ethical commercial engagement. These will serve to improve sample utilization and research output.  For the CLSG, biobanking should be integrated into commitments towards a national acute leukemia registry.

**Copyright Information:** (Online) ISSN: 2572-9241
©2026 The Author(s). HemaSphere is published by John Wiley & Sons Ltd on behalf of European Hematology Association. This is an open access Abstract Book distributed under the Attribution-NonCommercial-NoDerivs (CC BY-NC-ND), which allows third parties to download the articles and share them with others as long as they credit the author and the Abstract Book, but they cannot change the content in any way or use them commercially.
**Abstract Book Citations:** Authors, Title, HemaSphere, 2026;10:(S1):pages. The abstract book DOIs can be found on the "Author Information" page.
**Disclaimer:** Articles published in the journal HemaSphere exclusively reflect the opinions of the authors. The authors are responsible for all content in their abstracts including accuracy of the facts, statements, citing resources, etc.

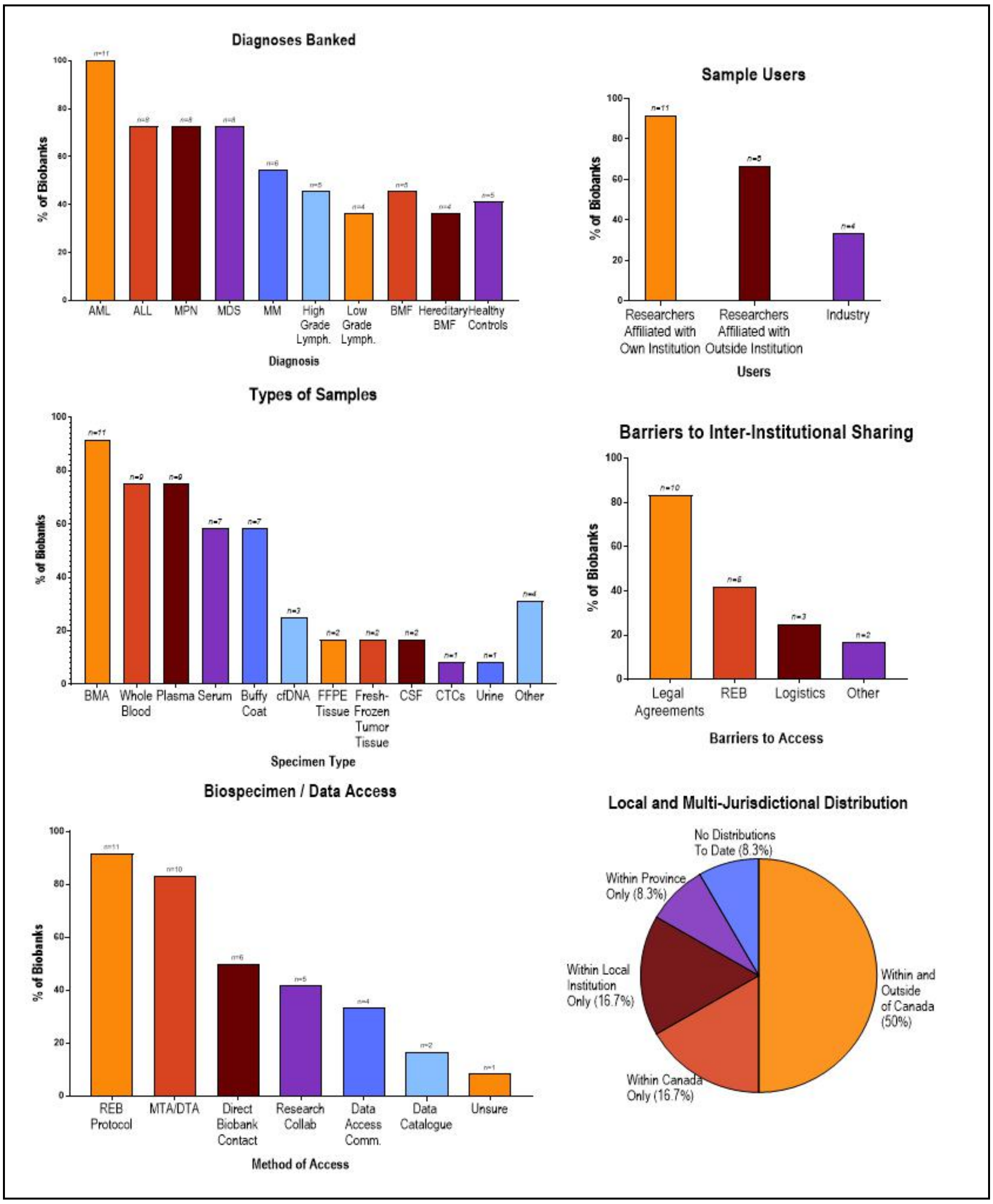

**Copyright Information:** (Online) ISSN: 2572-9241
©2026 The Author(s). HemaSphere is published by John Wiley & Sons Ltd on behalf of European Hematology Association. This is an open access Abstract Book distributed under the Attribution-NonCommercial-NoDerivs (CC BY-NC-ND), which allows third parties to download the articles and share them with others as long as they credit the author and the Abstract Book, but they cannot change the content in any way or use them commercially.
**Abstract Book Citations:** Authors, Title, HemaSphere, 2026;10:(S1):pages. The abstract book DOIs can be found on the "Author Information" page.
**Disclaimer:** Articles published in the journal HemaSphere exclusively reflect the opinions of the authors. The authors are responsible for all content in their abstracts including accuracy of the facts, statements, citing resources, etc.

### (PB4448) LATENT CLASSES OF EHEALTH LITERACY AND THEIR RELATED FACTORS IN PATIENTS UNDERGOING HEMATOPOIETIC STEM CELL TRANSPLANTATION: A CROSS-SECTIONAL STUDY

**Topic:** 36. Quality of life, ethics, supportive and palliative care

Yiming Gao*[1], Yuqiu Liu[1]

[1] *Institute of Hematology & Blood Diseases Hospital, Chinese Academy of Medical Sciences & Peking Union Medical College, Tianjin, China;*

### Background

In the current context of widespread digital information, good eHealth literacy can directly promote patients' active use of digital health service resources, and indirectly enhance patients' self-management abilities and health-promoting behaviors. In the long run, it also helps improve individuals' quality of life. However, few studies have investigated the level and influencing factors of eHealth literacy among patients undergoing hematopoietic stem cell transplantation (HSCT).

### Aims

To explore the latent classes of eHealth literacy and their influencing factors in patients undergoing HSCT, and to provide a reference for developing targeted intervention measures.

### Methods

A convenience sampling method was used to select patients undergoing their first HSCT at a specialized hematology hospital in Tianjin from January to December 2025. Participants were surveyed using a general information questionnaire, the eHealth Literacy Scale, the Information Self-Efficacy Scale, and the Social Support Scale. Latent profile analysis was employed to identify potential classes of patients' eHealth literacy, and logistic regression analysis was used to explore factors associated with these latent classes.

### Results

A total of 335 participants were included. Their eHealth literacy could be classified into three distinct classes, which were named based on their characteristics: the low literacy group (16.7%), the medium literacy group (55.2%), and the high literacy group (28.1%). Logistic regression analysis results showed that age, education level, subjective economic status, information self-efficacy, and social support were factors associated with the latent profile classes of eHealth literacy in these patients ($P < 0.05$).

### Summary/Conclusion

Heterogeneity exists in the eHealth literacy of patients undergoing HSCT. Healthcare professionals should provide targeted intervention measures based on the characteristics of patients with different eHealth literacy levels. Particular attention should be paid to the dual drivers of internal self-efficacy and external social support to improve patients' eHealth literacy levels and the utilization rate of digital health resources, thereby promoting individuals' self-management abilities and quality of life.

**Copyright Information:** (Online) ISSN: 2572-9241
©2026 The Author(s). HemaSphere is published by John Wiley & Sons Ltd on behalf of European Hematology Association. This is an open access Abstract Book distributed under the Attribution-NonCommercial-NoDerivs (CC BY-NC-ND), which allows third parties to download the articles and share them with others as long as they credit the author and the Abstract Book, but they cannot change the content in any way or use them commercially.
**Abstract Book Citations:** Authors, Title, HemaSphere, 2026;10:(S1):pages. The abstract book DOIs can be found on the "Author Information" page.
**Disclaimer:** Articles published in the journal HemaSphere exclusively reflect the opinions of the authors. The authors are responsible for all content in their abstracts including accuracy of the facts, statements, citing resources, etc.

### (PB4449) DEVELOPMENT OF BALANCED PSYCHOTHERAPY FOR PATIENTS WITH HEMATOLOGIC MALIGNANCIES: A NOVEL MODEL BASED ON EASTERN PHILOSOPHY

**Topic:** 36. Quality of life, ethics, supportive and palliative care

Xiaoyi Deng*[1], Ziying Jian[2], Yonggui Yuan[3], Zheng Ge[2]

*[1]Institute of Humanities, Department of Psychosomatic Medicine, Nanjing, China;[2]Zhongda Hospital Southeast University, Institute of Hematology Southeast University, Department of Hematology, Nanjing, China; [3]Institute of Psychosomatic Medicine Southeast University, Department of Psychosomatic Medicine, Nanjing, China;*

### Background

Given the limitations of previous intervention, this study developed an integrated psychotherapy model—Balanced Psychotherapy (BPT). BPT is based on Eastern philosophy, intended to improve patients' physical health, mental health and quality of life in a more comprehensive and targeted way.

### Aims

To investigate the current status of physical and mental health in patients with hematologic malignancies, develop a structured balanced psychotherapy program and preliminarily evaluate its feasibility.

### Methods

This study employed a single-center, two-phase mixed-methods design. In the investigation phase, a cross-sectional design was used to recruit patients from the hematology ward of Zhongda Hospital Affiliated to Southeast University between December 2025 and February 2026. Data were collected through semi-structured interviews, including: (1) demographic characteristics; (2) clinical characteristics; (3) psychological distress assessment: PHQ-9, GAD-7, and FoP-Q-SF; (4) psychological adaptation assessment: Mini-MAC; (5) fatigue assessment: FACT-F; (6) quality of life assessment: EORTC QLQ-C30.

Based on the survey findings, a targeted balanced psychotherapy program was developed, and a pilot trial was designed to evaluate its feasibility. The BPT group received six sessions of structured balanced psychotherapy (50 minutes per session, twice a week for 3 weeks) in addition to usual care. The control group received usual care only. All outcome measures were assessed at baseline and after the intervention. Primary outcome measures included depression (PHQ-9), fear of disease progression (FoP-Q-SF), and quality of life (EORTC QLQ-C30). Secondary outcome measures included fatigue (FACT-F), psychological adaptation (Mini-MAC), and post-traumatic growth (C-PTGI).

Descriptive statistics were used to summarize the survey data, and effect sizes (Hedge's g) were calculated for between-group differences in change.

**Copyright Information:** (Online) ISSN: 2572-9241
©2026 The Author(s). HemaSphere is published by John Wiley & Sons Ltd on behalf of European Hematology Association. This is an open access Abstract Book distributed under the Attribution-NonCommercial-NoDerivs (CC BY-NC-ND), which allows third parties to download the articles and share them with others as long as they credit the author and the Abstract Book, but they cannot change the content in any way or use them commercially.
**Abstract Book Citations:** Authors, Title, HemaSphere, 2026;10:(S1):pages. The abstract book DOIs can be found on the "Author Information" page.
**Disclaimer:** Articles published in the journal HemaSphere exclusively reflect the opinions of the authors. The authors are responsible for all content in their abstracts including accuracy of the facts, statements, citing resources, etc.

**Results**

34 patients were included in the investigation phase, with a mean age of 58.2 years (SD = 14.38), and 23 (67.6%) were male. Disease distribution included acute myeloid leukemia(50%), multiple myeloma(14.7%), lymphoma(11.8%) and others (23.5%). The mean PHQ-9 score was 7.3 (SD = 5.30), with 12 patients (35.3%) scoring ≥10. The mean GAD-7 score was 5.0 (SD = 4.67), with 4 patients (11.8%) scoring ≥10. The mean FoP-Q-SF score was 32.0 (SD = 9.75), with 15 patients (44.1%) scoring ≥34. The mean EORTC QLQ-C30 subscale scores were: global health status,60.1 (SD = 23.51); functioning status, 71.8 (SD = 16.24); and symptom status, 27.8 (SD = 18.59). The mean FACT-F score was 34.9 (SD = 9.05). The mean Mini-MAC subscale scores were: helplessness, 6.5 (SD = 3.56); anxious preoccupation, 6.4 (SD = 2.40); and positive attitude, 11.8 (SD = 3.57).

6 patients were included in the intervention phase(3 patients per group). The intervention results showed that, compared to the control group, the BPT group demonstrated greater improvements in depressive symptoms (g = -0.96), global quality of life (g = 1.96), and post-traumatic growth (g = 0.74). No significant differences or only small effects were observed for the other outcomes.

**Summary/Conclusion**

Balanced Psychotherapy (BPT) shows potential in improving the physical and psychological health, especially for depressive symptoms, quality of life and post-traumatic growth. However, these preliminary findings require confirmation through rigorous randomized controlled trials.

**Copyright Information:** (Online) ISSN: 2572-9241
©2026 The Author(s). HemaSphere is published by John Wiley & Sons Ltd on behalf of European Hematology Association. This is an open access Abstract Book distributed under the Attribution-NonCommercial-NoDerivs (CC BY-NC-ND), which allows third parties to download the articles and share them with others as long as they credit the author and the Abstract Book, but they cannot change the content in any way or use them commercially.
**Abstract Book Citations:** Authors, Title, HemaSphere, 2026;10:(S1):pages. The abstract book DOIs can be found on the "Author Information" page.
**Disclaimer:** Articles published in the journal HemaSphere exclusively reflect the opinions of the authors. The authors are responsible for all content in their abstracts including accuracy of the facts, statements, citing resources, etc.

### (PB4450) A SURVEY ON THE CURRENT STATUS OF QUALITY OF LIFE AND CAREGIVER BURDEN OF PATIENTS WITH ACUTE LEUKEMIA BASED ON SELF-REPORTS

**Topic:** 36. Quality of life, ethics, supportive and palliative care

Jia Deng*[1]

[1]*[illegible], [illegible], China;*

### Background

Acute leukemia is characterized by rapid onset, swift progression, and poor prognosis. The disease itself causes damage to the bone marrow hematopoietic system, while diagnostic and therapeutic factors such as nausea, vomiting, and poor appetite during chemotherapy, along with negative emotions like depression, anxiety, and sleep deprivation, significantly impair patients 'quality of life [1]. "Patient-centered care" has been a consistently emphasized principle in advancing high-quality medical services [2]. Patient-reported outcomes (PRO), as direct reports of patients' health status and treatment results, differ from the traditional model where physicians inquire about symptoms and assess their severity. PRO provides valuable insights from patients' perspectives for clinical research and practice, and has gained increasing recognition and advocacy [3].

### Aims

To understand the current status of patients' self-reported outcomes of quality of life and caregiver burden in acute leukemia, and to explore the factors influencing each other.

### Methods

Using random sampling, we recruited acute leukemia patients and their caregivers who received treatment at the Hematology Center of a Grade III-A hospital in Chongqing between March 2024 and March 2025. Data collection involved questionnaires including general information forms, patient self-reports, and caregiver burden scales. The study employed univariate analysis and multiple linear regression to investigate influencing factors.

### Results

The total score of the Patient Self-Reported Clinical Outcomes Scale (PSROCS) for acute leukemia patients was 36.31±21.25, with scores in physiological, psychological, social, and treatment domains: 10.67±6.77,12.47±8.59,5.59±4.38, and 7.58±6.99 respectively. The Caregiver Burden Scale (CBBS) total score was 42.99±12.196, with personal burden and responsibility burden scores at 27.88±7.437 and 12.32±5.216 respectively. Correlation analysis revealed that caregiver burden was significantly associated with patients' self-reported clinical outcomes in both physiological and psychological domains ($P<0.05$). Disease status was a significant risk factor for caregiver burden ($p=0.007$), while patient infection occurrence showed no correlation with age, disease risk stratification, or patient dependency levels ($P>0.05$).

### Summary/Conclusion

Acute leukemia patients report suboptimal self-reported health status, with generally low self-rated scores and caregivers bearing heavier burdens. Notably, none reported mild caregiving stress, while only 21% of caregivers experienced moderate stress levels, with a staggering 79% reporting severe caregiving burdens. Developing intervention strategies that support both patients and their family caregivers (FC pairs) could significantly reduce caregiving pressures and improve patient outcomes/life quality.

**Copyright Information:** (Online) ISSN: 2572-9241
©2026 The Author(s). HemaSphere is published by John Wiley & Sons Ltd on behalf of European Hematology Association. This is an open access Abstract Book distributed under the Attribution-NonCommercial-NoDerivs (CC BY-NC-ND), which allows third parties to download the articles and share them with others as long as they credit the author and the Abstract Book, but they cannot change the content in any way or use them commercially.
**Abstract Book Citations:** Authors, Title, HemaSphere, 2026;10:(S1):pages. The abstract book DOIs can be found on the "Author Information" page.
**Disclaimer:** Articles published in the journal HemaSphere exclusively reflect the opinions of the authors. The authors are responsible for all content in their abstracts including accuracy of the facts, statements, citing resources, etc.

### (PB4451) APPLICATION OF SELF-MANAGEMENT BASED ON DRUG LITERACY THEORY IN ORAL IMMUNOSUPPRESSANTS IN HEMATOPOIETIC STEM CELL TRANSPLANT PATIENTS

**Topic:** 36. Quality of life, ethics, supportive and palliative care

Qin Ren*[1], Zhenglan He[1], Jing Wen[1], Xiaoli Chen[1]

[1]*Xinqiao hospital Army Medical University, Chongqing, China;*

### Background

Hematopoietic stem cell transplantation is a critical treatment for hematologic malignancies and immune system disorders. Adherence to complex, disease-adjusted medication regimens is essential for preventing graft-versus-host disease (GVHD) and infections. Previous research by our team has shown that during self-management, patients often miss doses, reduce doses on their own, or take medications earlier or later than prescribed due to polypharmacy, forgetfulness, or adverse drug reactions. Poor adherence to oral immunosuppressants in transplant recipients may increase the incidence of GVHD, reduce quality of life, and elevate overall healthcare demands. Drug literacy theory refers to an individual's capacity to obtain, understand, and evaluate drug-related information and to use this information to make appropriate decisions and behaviors regarding medication use. A self-management approach grounded in drug literacy theory can help patients acquire the skills necessary for safe and appropriate medication use, thereby improving adherence to oral immunosuppressants, reducing the incidence of GVHD, and enhancing quality of life.

### Aims

To implement an intervention based on drug literacy theory aimed at strengthening self-management among hematopoietic stem cell transplant patients receiving oral immunosuppressants, with the goal of reducing or preventing adverse drug events, lowering the incidence of GVHD, improving patients' quality of life, and decreasing overall healthcare needs.

### Methods

A total of 92 patients who underwent hematopoietic stem cell transplantation in our department between May 2023 and October 2023 were enrolled and divided into an observation group and a control group, with 46 patients in each. The control group received routine nursing care, while the experimental group participated in a self-management intervention based on drug literacy theory. The intervention components included drug information acquisition, comprehension, evaluation, skill training, awareness enhancement, and consolidation exercises. The researcher assessed the type of immunosuppressant each patient was taking, evaluated their knowledge of the medication, provided one-on-one guidance, and developed medication cards to reinforce patient awareness. The Drug Literacy Assessment Scale was administered at baseline (pre-transplant). One month post-transplantation, drug literacy, medication adherence (using the Basel Assessment Scale for Immunosuppressants), and medication beliefs (using the Chinese version of the Beliefs about Medicines Questionnaire) were reassessed.

### Results

At baseline, the total drug literacy scores were (8.97 ± 3.90) in the control group and (8.90 ± 4.80) in the experimental group. After the intervention, the experimental group demonstrated significantly higher scores in drug literacy, medication adherence, and medication beliefs compared to the control group ($P < 0.05$).

**Copyright Information:** (Online) ISSN: 2572-9241
©2026 The Author(s). HemaSphere is published by John Wiley & Sons Ltd on behalf of European Hematology Association. This is an open access Abstract Book distributed under the Attribution-NonCommercial-NoDerivs (CC BY-NC-ND), which allows third parties to download the articles and share them with others as long as they credit the author and the Abstract Book, but they cannot change the content in any way or use them commercially.
**Abstract Book Citations:** Authors, Title, HemaSphere, 2026;10:(S1):pages. The abstract book DOIs can be found on the "Author Information" page.
**Disclaimer:** Articles published in the journal HemaSphere exclusively reflect the opinions of the authors. The authors are responsible for all content in their abstracts including accuracy of the facts, statements, citing resources, etc.

**Summary/Conclusion**

Drug literacy theory plays a significant predictive role in patients' safe medication practices. Its application can reduce or prevent adverse drug events, lower the incidence of GVHD, and improve patients' quality of life.

**Copyright Information:** (Online) ISSN: 2572-9241
©2026 The Author(s). HemaSphere is published by John Wiley & Sons Ltd on behalf of European Hematology Association. This is an open access Abstract Book distributed under the Attribution-NonCommercial-NoDerivs (CC BY-NC-ND), which allows third parties to download the articles and share them with others as long as they credit the author and the Abstract Book, but they cannot change the content in any way or use them commercially.
**Abstract Book Citations:** Authors, Title, HemaSphere, 2026;10:(S1):pages. The abstract book DOIs can be found on the "Author Information" page.
**Disclaimer:** Articles published in the journal HemaSphere exclusively reflect the opinions of the authors. The authors are responsible for all content in their abstracts including accuracy of the facts, statements, citing resources, etc.

### (PB4452) ANALYSIS OF THE EFFECT OF HOSPICE CARE ON PSYCHOLOGICAL AND SYMPTOM MANAGEMENT IN PEDIATRIC PATIENTS WITH TERMINAL TUMORS IN A PALLIATIVE CARE WARD

**Topic:** 36. Quality of life, ethics, supportive and palliative care

Xingwang Shi*[1], Jianming Fang[1]

[1]*Beijing Jingdu Children's Hospital, Beijing, China;*

### Background

Pediatric patients with terminal tumors often suffer from severe physical symptoms and psychological distress, while their families face enormous emotional pressure and bereavement challenges. Hospice care, as a core component of pediatric palliative care, focuses on relieving suffering and improving quality of life, but its application effect in symptom control and psychosocial support for this specific population needs further verification.

### Aims

To analyze the effect of hospice care on psychological status and symptom management in pediatric patients with terminal tumors and their families in a pediatric palliative care ward.

### Methods

A retrospective analysis was conducted on the clinical data of 20 pediatric patients with terminal tumors admitted to our hospital's pediatric palliative care ward from April 2023 to July 2025. All children received multidisciplinary hospice care, including pain control and family bereavement counseling. The PHQ-9 and GAD-7 scales were used to evaluate psychological status and symptom remission rates before and after the intervention.

### Results

- **Medical necessity:** Hospice care effectively relieved symptoms such as pain (e.g., improved pain control rate in children with neuroblastoma) and dyspnea in terminal tumor patients, and reduced ineffective medical interventions (e.g., invasive resuscitation and decreased ICU admission rate).
- **Psychosocial support:** Intervention by a multidisciplinary team (physicians, nurses, social workers, and psychologists) significantly alleviated anxiety and depression in children and their families. Family bereavement counseling was effectively implemented (e.g., anxiety scores of family members of children in the "Daisy House" ward were significantly reduced with the support of the German-Chinese medical social work team), with an effective rate of 90%.
- **Ethical and policy value:** This model balanced the ethical conflict between "life prolongation" and "quality of life" and promoted the transformation of the treatment paradigm toward "holistic care". However, it faces systemic challenges such as the absence of medical insurance coverage.

### Summary/Conclusion

Hospice care can effectively optimize symptom management and relieve the psychological burden of families in children with terminal tumors, serving as a core component of pediatric palliative care. Sustainable development requires dedicated medical insurance policies, professional talent training, and public life education.

**Copyright Information:** (Online) ISSN: 2572-9241
©2026 The Author(s). HemaSphere is published by John Wiley & Sons Ltd on behalf of European Hematology Association. This is an open access Abstract Book distributed under the Attribution-NonCommercial-NoDerivs (CC BY-NC-ND), which allows third parties to download the articles and share them with others as long as they credit the author and the Abstract Book, but they cannot change the content in any way or use them commercially.
**Abstract Book Citations:** Authors, Title, HemaSphere, 2026;10:(S1):pages. The abstract book DOIs can be found on the "Author Information" page.
**Disclaimer:** Articles published in the journal HemaSphere exclusively reflect the opinions of the authors. The authors are responsible for all content in their abstracts including accuracy of the facts, statements, citing resources, etc.

### (PB4453) PROPHYLACTIC HETROMBOPAG FOR PREVENTION OF CANCER THERAPY-INDUCED THROMBOCYTOPENIA IN GASTROINTESTINAL MALIGNANCIES: RESULTS FROM AN ONGOING SINGLE-ARM PROSPECTIVE STUDY

**Topic:** 36. Quality of life, ethics, supportive and palliative care

Xianbao Zhan[1], Longpei Chen*[1], Fang Yan[1], Yingyi Zhang[1], Xiaobo Peng[1], Mingzhen Ying[1]

[1]*Shanghai Changhai Hospital, Shanghai, China;*

### Background

Cancer therapy-induced thrombocytopenia (CTIT) is a frequent complication of anti-cancer therapy in patients with gastrointestinal (GI) malignancies and may lead to treatment delay or dose reduction, increased bleeding risk, and the need for rescue platelet support. Emerging evidence suggests that hetrombopag, an oral thrombopoietin receptor agonist (TPO-RA), may improve platelet recovery in patients with persistent CTIT and platelet counts <75×10^9/L. However, evidence remains limited in GI malignancies, particularly regarding secondary prophylaxis in patients with prior-cycle platelet nadirs of 75-125×10^9/L who may still be at risk of bleeding and chemotherapy disruption.

### Aims

To evaluate the efficacy and safety of prophylactic hetrombopag for prevention of CTIT in patients with GI malignancies who experienced a platelet nadir of 75-125×10^9/L during the prior chemotherapy cycle and had at least one bleeding high-risk factor.

### Methods

This single-center, single-arm, prospective study plans to enroll 40 patients with histologically confirmed GI malignancies receiving chemotherapy on 14- or 21-day cycles and scheduled for ≥1 additional cycle. Key eligibility criteria included a prior-cycle platelet nadir of 75-125×10^9/L with ≥1 bleeding high-risk factor and a platelet count ≥75×10^9/L on Day 1 of the index cycle. Hetrombopag 7.5 mg was administered orally once daily on Days 1-14, starting on the chemotherapy day. The primary endpoint was the proportion of patients with platelet count >75×10^9/L on Day 14. Secondary endpoints included chemotherapy delay ≥4 days in the subsequent cycle, platelet count >75×10^9/L and ≥100×10^9/L on Day 21, platelet count <50×10^9/L, and the proportion requiring platelet rescue therapy. This study was approved by the Ethics Committee of Shanghai Changhai Hospital. Trial registration: ChiCTR2500106644.

### Results

A total of 25 patients were included in the efficacy and safety analyses. The median baseline platelet count was 101×10^9/L, and 61% of patients had colorectal cancer. Regimens included XELOX, SOX, and other platinum-based combinations with targeted agents or immunotherapy (44%). The primary endpoint was achieved in 76% (19/25) of patients (platelet count >75×10^9/L on Day 14). On Day 21, 83% (20/24) had platelet count >75×10^9/L and 79% (19/24) had platelet count ≥100×10^9/L. Chemotherapy delay ≥4 days in the next cycle occurred in 4% (1/25). Platelet count <50×10^9/L occurred in 8% (2/25), and rescue therapy was required in 12% (3/25). Among 24 evaluable patients, 75% (18/24) had higher platelet counts at C1D21 versus baseline. Safety analysis reported 25 treatment-emergent adverse events, all grade 1-2 hematologic toxicities; no investigator-assessed treatment-related adverse events, no grade ≥3 TEAEs, no serious adverse events, and no treatment-related deaths were observed. Median follow-up was 1 month.

**Copyright Information:** (Online) ISSN: 2572-9241
©2026 The Author(s). HemaSphere is published by John Wiley & Sons Ltd on behalf of European Hematology Association. This is an open access Abstract Book distributed under the Attribution-NonCommercial-NoDerivs (CC BY-NC-ND), which allows third parties to download the articles and share them with others as long as they credit the author and the Abstract Book, but they cannot change the content in any way or use them commercially.
**Abstract Book Citations:** Authors, Title, HemaSphere, 2026;10:(S1):pages. The abstract book DOIs can be found on the “Author Information” page.
**Disclaimer:** Articles published in the journal HemaSphere exclusively reflect the opinions of the authors. The authors are responsible for all content in their abstracts including accuracy of the facts, statements, citing resources, etc.

**Summary/Conclusion**

Prophylactic hetrombopag (7.5 mg daily for 14 days starting on chemotherapy day) showed promising efficacy in maintaining platelet counts and minimizing chemotherapy delay in patients with GI malignancies at risk of CTIT, with a favorable short-term safety profile. Ongoing enrollment and longer follow-up are needed.

**Copyright Information:** (Online) ISSN: 2572-9241
©2026 The Author(s). HemaSphere is published by John Wiley & Sons Ltd on behalf of European Hematology Association. This is an open access Abstract Book distributed under the Attribution-NonCommercial-NoDerivs (CC BY-NC-ND), which allows third parties to download the articles and share them with others as long as they credit the author and the Abstract Book, but they cannot change the content in any way or use them commercially.
**Abstract Book Citations:** Authors, Title, HemaSphere, 2026;10:(S1):pages. The abstract book DOIs can be found on the "Author Information" page.
**Disclaimer:** Articles published in the journal HemaSphere exclusively reflect the opinions of the authors. The authors are responsible for all content in their abstracts including accuracy of the facts, statements, citing resources, etc.

### (PB4454) TRANSFORMATION OF THE PEDIATRIC HEMATOLOGY–ONCOLOGY SERVICE IN UZBEKISTAN: NATIONAL REFORM STRATEGY AND INTERNATIONAL COLLABORATION TO IMPROVE SURVIVAL

**Topic:** 36. Quality of life, ethics, supportive and palliative care

Khilola Rustamova*[1], Djamila Polatova[2], Guzal Mamedova[1]

*[1]Scientific and Practical Medical Center of Pediatric Oncology, Hematology and Immunology, Hematology oncology, Tashkent, Uzbekistan; [2]Scientific and Practical Medical Center of Pediatric Oncology, Hematology and Immunology, Tashkent, Uzbekistan;*

### Background

Uzbekistan, with a children and adolescents population of over 12.9 million (0–21 years), has initiated a nationwide reform of its pediatric hematology–oncology service to address mortality, late-stage presentation, and regional disparities in access to care. As of 2025, 16,284 patients are under active follow-up, with more than 4,000 newly diagnosed cases annually. Increased case detection in recent years reflects improved referral systems and registry strengthening.

### Aims

To present the structural transformation of pediatric hematology–oncology care in Uzbekistan, identify key systemic challenges, and outline the national strategy to improve survival by 2030.

### Methods

A health system reform was implemented under Presidential decrees establishing a three-tier vertically integrated model of care. The model includes: (1) primary-level early detection and rapid referral; (2) secondary-level regional centers providing diagnostics and initiation of specialized treatment; and (3) a tertiary National Scientific and Practical Medical Center of Oncology, Hematology and Immunology delivering high-complexity care, including intensive chemotherapy, hematopoietic stem cell transplantation, targeted therapy, immunotherapy, and multidisciplinary management. Parallel interventions included infrastructure modernization, workforce development, protocol harmonization with international standards, registry digitalization, and telemedicine expansion through international partnerships.

### Results

Optimization of patient routing reduced the time to treatment initiation to 10–24 days. Stage distribution analysis demonstrates a gradual increase in early-stage detection and a projected decline in advanced-stage disease by 2030. The integrated reform framework provides the basis for a projected 2.5-fold reduction in mortality with full implementation of the strategy. Key challenges remain, including regional workforce shortages, unequal resource distribution, limited access to high-technology diagnostics in some areas, and the need to expand rehabilitation and survivorship care. International collaboration with WHO, St. Jude Children's Research Hospital, Dana-Farber Cancer Institute, and other partners has supported protocol standardization, professional training, quality assurance, and research capacity building.The participation of countries in GICC, GPACCM projects also contributes to positive progress in the fight against childhood cancer.

**Copyright Information:** (Online) ISSN: 2572-9241
©2026 The Author(s). HemaSphere is published by John Wiley & Sons Ltd on behalf of European Hematology Association. This is an open access Abstract Book distributed under the Attribution-NonCommercial-NoDerivs (CC BY-NC-ND), which allows third parties to download the articles and share them with others as long as they credit the author and the Abstract Book, but they cannot change the content in any way or use them commercially.
**Abstract Book Citations:** Authors, Title, HemaSphere, 2026;10:(S1):pages. The abstract book DOIs can be found on the "Author Information" page.
**Disclaimer:** Articles published in the journal HemaSphere exclusively reflect the opinions of the authors. The authors are responsible for all content in their abstracts including accuracy of the facts, statements, citing resources, etc.

**Summary/Conclusion**

The systemic transformation of pediatric hematology–oncology services in Uzbekistan demonstrates how coordinated national policy, infrastructure investment, and international collaboration can strengthen care delivery in a middle-income setting. Continued implementation of the 2030 strategy is expected to improve survival, reduce regional inequities, and establish a sustainable, patient-centered pediatric hematology–oncology system aligned with global standards.

**Copyright Information:** (Online) ISSN: 2572-9241
©2026 The Author(s). HemaSphere is published by John Wiley & Sons Ltd on behalf of European Hematology Association. This is an open access Abstract Book distributed under the Attribution-NonCommercial-NoDerivs (CC BY-NC-ND), which allows third parties to download the articles and share them with others as long as they credit the author and the Abstract Book, but they cannot change the content in any way or use them commercially.
**Abstract Book Citations:** Authors, Title, HemaSphere, 2026;10:(S1):pages. The abstract book DOIs can be found on the "Author Information" page.
**Disclaimer:** Articles published in the journal HemaSphere exclusively reflect the opinions of the authors. The authors are responsible for all content in their abstracts including accuracy of the facts, statements, citing resources, etc.

### (PB4455) TREATMENT DECISION-MAKING AND END-OF-LIFE CARE IN OCTOGENARIANS WITH AGGRESSIVE LYMPHOMAS: A REAL-WORLD LATIN AMERICAN COHORT

**Topic:** 36. Quality of life, ethics, supportive and palliative care

Manuela Estrada-Schweineberg*[1], Juliana Castro[2], Dana Taub[2], Maria Uchima-Vera[1], Laura Ortiz[1], Juan Jaimes[2], Carmenza Camargo[1], Karen Milena Chaves Garcia[1], Guillermo Enrique Quintero Vega[1, 2], Beatriz Wills[1, 2], Angela Esparza[1], Claudia Agudelo-López[1, 2]

*[1]Fundación Santa Fe de Bogotá, Colombia, Colombia;[2]Universidad de los Andes, Facultad de Medicina, Bogota, Colombia;*

### Background

Patients aged ≥80 years with aggressive lymphomas represent a rapidly expanding yet clinically heterogeneous population. In this setting, therapeutic decisions require balancing disease control with age-related vulnerability, comorbidities, and functional reserve. However, real-world data describing how treatment intensity and patterns of care of end-of-life remain limited, particularly in Latin America.

### Aims

To characterize treatment decision-making, treatment intensity, and end-of-life care patterns in patients aged ≥80 years with aggressive lymphomas, and to explore the association between geriatric parameters and therapeutic limitation.

### Methods

We conducted a retrospective cohort study of patients aged ≥80 years with aggressive lymphomas treated between 2015 and 2025. Clinical, geriatric and end-of-life variables were collected. Functional status was assessed using ECOG, Barthel Index, and Lawton scale. Frailty was recorded based on clinical geriatric assessment. Treatment intensity was classified as standard or attenuated. Overall survival (OS) and progression-free survival (PFS) were estimated using the Kaplan–Meier method. Associations between geriatric parameters and treatment receipt or intensity were explored descriptively.

**Copyright Information:** (Online) ISSN: 2572-9241
©2026 The Author(s). HemaSphere is published by John Wiley & Sons Ltd on behalf of European Hematology Association. This is an open access Abstract Book distributed under the Attribution-NonCommercial-NoDerivs (CC BY-NC-ND), which allows third parties to download the articles and share them with others as long as they credit the author and the Abstract Book, but they cannot change the content in any way or use them commercially.
**Abstract Book Citations:** Authors, Title, HemaSphere, 2026;10:(S1):pages. The abstract book DOIs can be found on the "Author Information" page.
**Disclaimer:** Articles published in the journal HemaSphere exclusively reflect the opinions of the authors. The authors are responsible for all content in their abstracts including accuracy of the facts, statements, citing resources, etc.

### Results

Thirty-three patients were included (mean age 87 years, 54.5% female). Diffuse large B-cell lymphoma (DLBCL) was the most frequent histology (60.6%), followed by transformed follicular lymphoma and grade 3B disease (18.2%). Advanced-stage disease was frequent at diagnosis.
Most patients had preserved functional status (ECOG 0–1). Functional independence was observed in 69.7%, with a median Barthel Index of 95 (IQR 90–100) and Lawton score of 8 (IQR 3–8). Frailty was identified in 12.1% of patients.
Systemic therapy was administered in 93.9% of patients, predominantly anti-CD20–based regimens (90.6%). First-line treatment consisted mainly of R-CHOP (41.9%) or R-mini-CHOP (29%). Standard-intensity regimens were delivered in 79.3% of patients, while 20.7% received attenuated therapy. First-line therapy was completed in 84% of cases, with dose reductions required in four patients. Nearly half experienced no documented adverse events.
After a median follow-up of 61.3 months, median OS and PFS were not reached. Chronological age, frailty, functional dependence, and ECOG performance status were not significantly associated with treatment receipt, treatment intensity, or discontinuation, although the study was not powered to detect small differences.
Eleven patients (33.3%) received palliative care during their disease course, and seven (21.2%) had documented therapeutic effort limitation. Six patients (18.2%) died during follow-up. Among them, four (66%) were hospitalized in the last 30 days of life and one (16.7%) required ICU admission. No patients received chemotherapy in the last 30 days of life, and care during this period was predominantly palliative.

### Summary/Conclusion

In this real-world cohort of octogenarians with aggressive lymphomas, systemic therapy was frequently delivered with acceptable feasibility. Treatment decisions were not driven by chronological age alone, and end-of-life care patterns showed no chemotherapy use in the final 30 days of life, suggesting alignment with palliative-oriented management. These findings support individualized strategies guided by functional and clinical assessment rather than age alone, highlighting the need for larger studies to better define therapeutic approaches in this population.

**Figure I A-B. Kaplan–Meier Survival Curves in Octogenarian Patients with Aggressive Lymphoma. A: Overall Survival. B: Progression-Free Survival**

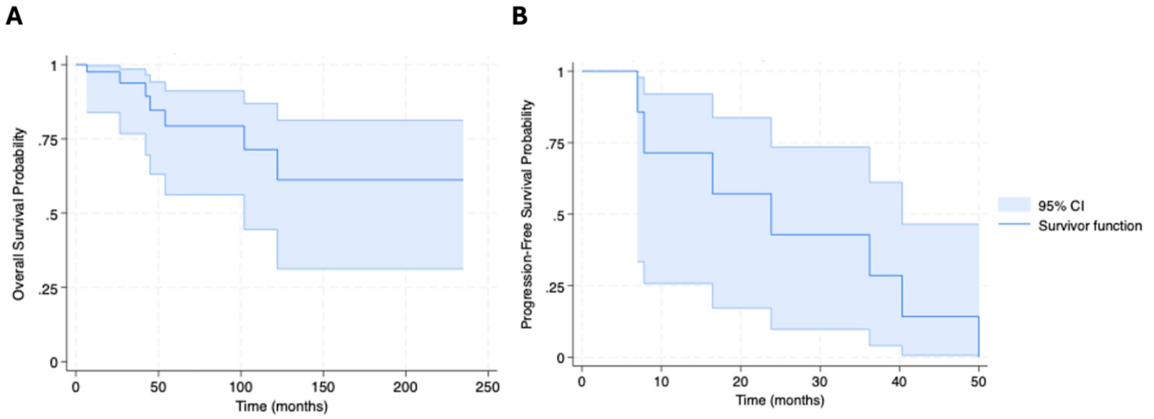

**Copyright Information:** (Online) ISSN: 2572-9241
©2026 The Author(s). HemaSphere is published by John Wiley & Sons Ltd on behalf of European Hematology Association. This is an open access Abstract Book distributed under the Attribution-NonCommercial-NoDerivs (CC BY-NC-ND), which allows third parties to download the articles and share them with others as long as they credit the author and the Abstract Book, but they cannot change the content in any way or use them commercially.
**Abstract Book Citations:** Authors, Title, HemaSphere, 2026;10:(S1):pages. The abstract book DOIs can be found on the "Author Information" page.
**Disclaimer:** Articles published in the journal HemaSphere exclusively reflect the opinions of the authors. The authors are responsible for all content in their abstracts including accuracy of the facts, statements, citing resources, etc.

### (PB4457) LATENT PROFILE ANALYSIS OF SYMPTOM AND FUNCTIONAL STATUS AND ASSOCIATED FACTORS IN PATIENTS WITH HEMATOLOGIC MALIGNANCIES

**Topic:** 36. Quality of life, ethics, supportive and palliative care

Qianqian Zhang*[1], Yujie Zhao[2], Jinying Zhao[2], Yating Liu[2], Zhongfan Kan[2], Yan Cui[2], Jie Zhang[2], Yun Wu[2], Fei Tian[2], Yanxia Liu[2], Zhixin Wang[2], Xiaoxu Ren[2], Jie Yan[2], Ying Wang[3], Wenjun Xie[2]

*[1]Institute of Hematology and Blood Diseases Hospital, Chinese Academy of Medical Sciences & Peking Union Medical College, The International Medical Center, Tianjin, China; [2]Institute of Hematology and Blood Diseases Hospital, Chinese Academy of Medical Sciences & Peking Union Medical College, Tianjin, China; [3]Institute of Hematology and Blood Diseases Hospital, Chinese Academy of Medical Sciences & Peking Union Medical College, Nursing Department, Tianjin, China;*

### Background

Hematological malignancies are a heterogeneous group of clonal disorders arising from hematopoietic or lymphoid tissues, mainly including leukemia, lymphoma, and multiple myeloma, with increasing incidence and mortality globally. Compared with solid tumors, patients often receive intensive treatments such as high-dose chemotherapy and hematopoietic stem cell transplantation, leading to heavy physical and psychological burdens such as fatigue, pain, infection risk, anxiety, and depression.
Most current studies only provide overall group-level summaries based on total symptom scores, neglecting within-population heterogeneity and failing to identify latent subgroups with distinct symptom and functional patterns. Although some subgroup analyses have been conducted in pediatric leukemia and multiple myeloma, adult patients show greater heterogeneity in age, disease biology, symptom burden, and psychosocial factors, making clinical management more challenging. Research on the latent classes and characteristics of symptoms and functional status in this population remains limited.
This study aims to identify latent subgroups of symptom and functional status among adult patients with hematological malignancies using latent profile analysis and to explore related influencing factors. By distinguishing subgroups with different clinical patterns, we aim to reveal diverse patient needs and support the development of targeted interventions and precision management strategies, ultimately improving patient-centered care, quality of life, and long-term rehabilitation.

### Aims

To identify latent classes of symptoms and functional characteristics in patients with hematological tumors using latent profile analysis (LPA), and to explore their associated influencing factors.

### Methods

This study recruited patients with hematological malignancies from four tertiary hospitals in Tianjin, Shandong, Anhui, and Jiangxi provinces during the period from June to August 2023. Data were collected using a general information questionnaire and the simplified Chinese version of PROMIS-29 V2.1. Latent profile analysis was conducted using the PROMIS-29 V2.1 domains as manifest indicators. Univariate analysis and multivariate logistic regression were performed to identify factors associated with latent class membership.

**Copyright Information:** (Online) ISSN: 2572-9241
©2026 The Author(s). HemaSphere is published by John Wiley & Sons Ltd on behalf of European Hematology Association. This is an open access Abstract Book distributed under the Attribution-NonCommercial-NoDerivs (CC BY-NC-ND), which allows third parties to download the articles and share them with others as long as they credit the author and the Abstract Book, but they cannot change the content in any way or use them commercially.
**Abstract Book Citations:** Authors, Title, HemaSphere, 2026;10:(S1):pages. The abstract book DOIs can be found on the "Author Information" page.
**Disclaimer:** Articles published in the journal HemaSphere exclusively reflect the opinions of the authors. The authors are responsible for all content in their abstracts including accuracy of the facts, statements, citing resources, etc.

**Results**

A total of 371 questionnaires were distributed, and 354 valid responses were collected, yielding an effective response rate of 95.4%. Latent profile analysis identified four distinct latent profiles of symptom and functional status among patients with hematologic malignancies: high functioning–low symptom group (33.3%), moderate functioning–high psychological symptom group (36.2%), moderate functioning–low psychological symptom group (11.9%), and low functioning–high-symptom group (18.6%). Multivariate logistic regression indicated that sex, household monthly income, treatment stage, diagnosis, and treatment modality were significant factors influencing patients' symptom and functional status profiles ($P < 0.05$).

**Summary/Conclusion**

Symptom and functional profiles of patients with hematological malignancies show significant heterogeneity. Healthcare providers may implement stratified management and targeted interventions based on class-specific influencing factors to optimize symptom control and functional status, thereby improving patients' long-term quality of life.

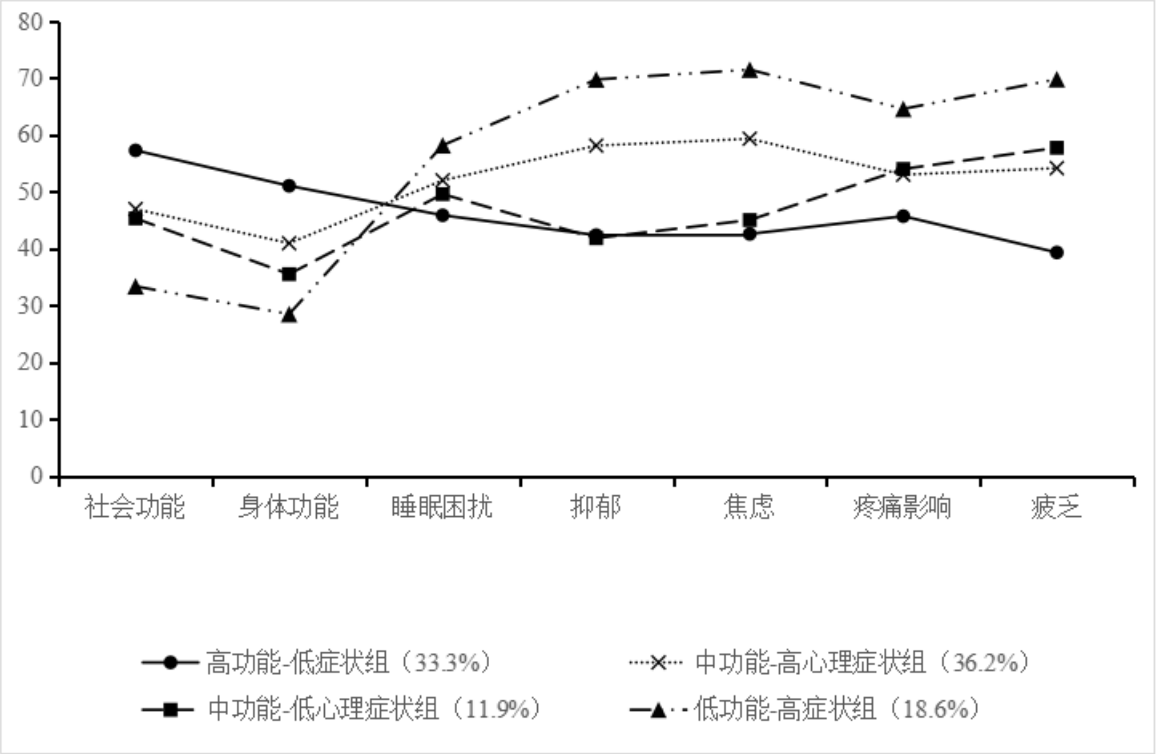

**图 2　HM 患者症状与功能状态潜在剖面类别特征图**

Copyright Information: (Online) ISSN: 2572-9241
©2026 The Author(s). HemaSphere is published by John Wiley & Sons Ltd on behalf of European Hematology Association. This is an open access Abstract Book distributed under the Attribution-NonCommercial-NoDerivs (CC BY-NC-ND), which allows third parties to download the articles and share them with others as long as they credit the author and the Abstract Book, but they cannot change the content in any way or use them commercially.
Abstract Book Citations: Authors, Title, HemaSphere, 2026;10:(S1):pages. The abstract book DOIs can be found on the "Author Information" page.
Disclaimer: Articles published in the journal HemaSphere exclusively reflect the opinions of the authors. The authors are responsible for all content in their abstracts including accuracy of the facts, statements, citing resources, etc.

### (PB4458) ACUPUNCTURE AT NEIGUAN (PC6) FOR BREAKTHROUGH NAUSEA AND VOMITING DURING HEMATOPOIETIC STEM CELL TRANSPLANT PRECONDITIONING: A RETROSPECTIVE COHORT STUDY

**Topic:** 36. Quality of life, ethics, supportive and palliative care

Xiao Wang*[1], Shan Zhang[1], Guangcui He[1], Xu-Pai Zhang[1], Yan Deng[1], Lei MA[1], Hai Yi[1]

[1]*The General Hospital of Western Theater Command, Chengdu, China;*

### Background

Nausea and vomiting are among the most common adverse effects in patients undergoing hematopoietic stem cell transplantation (HSCT), significantly compromising quality of life and treatment adherence. Although standard prophylactic regimens typically combine 5-$HT_3$ receptor antagonists with dexamethasone, a substantial proportion of patients still develop grade 2~3 breakthrough emesis, highlighting an urgent clinical need for effective rescue interventions.

### Aims

To investigate the safety and efficacy of acupuncture at Neiguan (PC6) as a rescue therapy for breakthrough nausea and vomiting during HSCT preconditioning.

### Methods

We conducted a retrospective analysis of patients who experienced grade 2~3 breakthrough nausea and vomiting during HSCT preconditioning at Western Theater General Hospital between January 2025 and January 2026. All patients had received standard antiemetic prophylaxis with a 5-$HT_3$ receptor antagonist plus dexamethasone. Participants were assigned to an observation group (n=20) or a control group (n=39) according to whether they received additional acupuncture at Neiguan. The control group continued conventional antiemetic therapy alone, while the observation group received acupuncture at Neiguan in addition to conventional therapy. The primary endpoints were nausea and vomiting grade on the following day (assessed by CTCAE v5.0) and the complete response rate (defined as grade 0 vomiting). The secondary endpoint was the modified Rhodes Index of Nausea, Vomiting, and Retching (R-INVR).

### Results

All patients experienced vomiting on day 1, and no complete responses (grade 0) were observed on day 2. In the observation group, 9 patients (45.0%) achieved grade 1 vomiting on day 2, compared with 7 patients (17.9%) in the control group, a difference that was statistically significant (P=0.03). Furthermore, the observation group demonstrated significantly lower R-INVR scores than the control group (19.21±2.56 *vs.* 24.82±3.87, P<0.01). No acupuncture-related adverse events were reported.

### Summary/Conclusion

Acupuncture at Neiguan (PC6) appears to be a safe and effective rescue therapy for alleviating breakthrough nausea and vomiting during HSCT preconditioning, thereby improving patient quality of life. These findings provide preliminary evidence supporting the integration of acupuncture into transplant supportive care and merit validation through prospective, large-scale studies.

**Copyright Information:** (Online) ISSN: 2572-9241
©2026 The Author(s). HemaSphere is published by John Wiley & Sons Ltd on behalf of European Hematology Association. This is an open access Abstract Book distributed under the Attribution-NonCommercial-NoDerivs (CC BY-NC-ND), which allows third parties to download the articles and share them with others as long as they credit the author and the Abstract Book, but they cannot change the content in any way or use them commercially.
**Abstract Book Citations:** Authors, Title, HemaSphere, 2026;10:(S1):pages. The abstract book DOIs can be found on the “Author Information” page.
**Disclaimer:** Articles published in the journal HemaSphere exclusively reflect the opinions of the authors. The authors are responsible for all content in their abstracts including accuracy of the facts, statements, citing resources, etc.

### (PB4459) ANALYSING THE IMPACT OF NURSING PRACTICE AND EDUCATION IN THE MANAGEMENT OF PATIENTS WITH PAROXYSMAL NOCTURNAL HAEMOGLOBINURIA

**Topic:** 36. Quality of life, ethics, supportive and palliative care

Louise Arnold*[1], Michael Brown[2]

*[1]Saint James' University Hospital, Haematology, Leeds, United Kingdom;[2]Peter Maccallum Cancer Centre, Haematology, Melbourne CBD, Australia;*

**Background**

Paroxysmal Nocturnal Haemoglobinuria (PNH) is a rare, chronic, life-threatening, complement-mediated haematologic disorder associated with haemolysis, thrombosis, and reduced quality of life. Although therapeutic advances, particularly complement inhibitors, have significantly improved outcomes, optimal disease control requires highly coordinated, multidisciplinary care. Nurses play a central role in PNH management; however, variability in disease awareness and educational resources may impact care consistency and patient outcomes.

**Aims**

To highlight the clinical and educational impact of nursing practice in patient management. Analyse and propose a structured education framework to support best practice across the patient care pathway based an international collaboration.

**Methods**

A narrative review of published literature, clinical guidance, and nursing best-practice post a recent educational speaker tour was undertaken to evaluate nursing roles, educational needs, and existing models of care in PNH.

**Results**

Nurses are integral to patient care in PNH. Leading patient education on symptom recognition and treatment adherence. Providing ongoing clinical assessment, monitoring for haemolysis and thromboses, management of infusion-based and other complement therapies. Evidence indicates that nurse-led education improves patient understanding, engagement, and self-management in rare haematologic diseases. This was evident through both patient feedback and a medical educational speaker tour for nursing professionals. Further analysis is proposed involving a PNH Nursing Education Framework that comprises of four core domains based on these insights: (1) Patient-centred education, addressing quality of life, psychosocial needs, and shared decision-making (2) Foundational disease knowledge, including pathophysiology and evolving treatment mechanisms; (3) Clinical vigilance, focusing on early detection of infection risk, haemolysis and thrombosis; and (4) Care coordination and advocacy, supporting transitions of care, long-term follow-up, and access to specialist services. This framework supports consistent, high-quality nursing practice across varied healthcare settings.

**Summary/Conclusion**

Nurses are pivotal in delivering safe, effective, and patient-centred care for individuals with PNH, with aim to improve quality of life. Structured and continuous nurse education is essential to maximise clinical outcomes and patient experience. Adoption of a standardised PNH nursing education framework may reduce variability in care and strengthen multidisciplinary management, underscoring the need for formalised educational strategies within PNH treatment models.

**Copyright Information:** (Online) ISSN: 2572-9241
©2026 The Author(s). HemaSphere is published by John Wiley & Sons Ltd on behalf of European Hematology Association. This is an open access Abstract Book distributed under the Attribution-NonCommercial-NoDerivs (CC BY-NC-ND), which allows third parties to download the articles and share them with others as long as they credit the author and the Abstract Book, but they cannot change the content in any way or use them commercially.
**Abstract Book Citations:** Authors, Title, HemaSphere, 2026;10:(S1):pages. The abstract book DOIs can be found on the "Author Information" page.
**Disclaimer:** Articles published in the journal HemaSphere exclusively reflect the opinions of the authors. The authors are responsible for all content in their abstracts including accuracy of the facts, statements, citing resources, etc.

### (PB4460) THE IMPACT OF AESTHETIC MEDICINE TREATMENTS ON QUALITY OF LIFE IN PATENTS WITH HEMATOLOGICAL DISEASES AND PATIENTS AFTER ALLOGENEIC HEMATOPOIETIC STEM CELL TRANSPLANTATION.

**Topic:** 36. Quality of life, ethics, supportive and palliative care

Anna Dmitrova*[1]

*[1]MaVie Clinique, Department of aesthetic medicine, Moscow, Russia;*

### Background

Despite improved overall survival in hematological patients and patients after allogeneic hematopoietic stem cell transplantation (allo-HSCT) there are still many issues affected their quality of life (QOL). Some of problems related to not only the immune, infectious or other complications after chemotherapy and allo-HSCT but also to the general well-being of the patients. For instance, there are many concerns about skin complications after chemotherapy, hormonal treatment especially among female patients such as dryness, rashes, hyperpigmentation, scarring, alopecia, premature skin aging etc. Other common issue includes increased duration of recovery after many interventions that may last for months due to immunosuppressive conditions after treatment. Moreover, there is another challenge – most of the centers provide treatments for blood diseases only but have no connections with rehabilitation centers, cosmetology and plastic surgery clinics and specialists in aesthetic medicine.

### Aims

The aim of this study is to evaluate the impact of treatment in aesthetic medicine on QOL in patents with blood diseases and patients after allo-HSCT and to expand the opportunities in aesthetic medicine for these patients.

### Methods

Aesthetic treatments combine injectable procedures such as different types of fillers, Botulinum neurotoxins injections and laser procedures (ablative, non-ablative, fractional etc.)
N=12 (100%) female patients with hematological diseases are included in the study. All of the patients are in complete remission and finished their treatment. All of them underwent one or several injectable and/or laser procedures with dermatologists/cosmetologists in aesthetic medicine clinics. Detailed patients' characteristics are presented in table 1.

All patients completed two questionnaires: The Global Aesthetic Improvement Scale (GAIS) and The Patient's Satisfaction Score (PSS) and also physicians completed The Physician Satisfaction Score (PhSS).

**Copyright Information:** (Online) ISSN: 2572-9241
©2026 The Author(s). HemaSphere is published by John Wiley & Sons Ltd on behalf of European Hematology Association. This is an open access Abstract Book distributed under the Attribution-NonCommercial-NoDerivs (CC BY-NC-ND), which allows third parties to download the articles and share them with others as long as they credit the author and the Abstract Book, but they cannot change the content in any way or use them commercially.
**Abstract Book Citations:** Authors, Title, HemaSphere, 2026;10:(S1):pages. The abstract book DOIs can be found on the "Author Information" page.
**Disclaimer:** Articles published in the journal HemaSphere exclusively reflect the opinions of the authors. The authors are responsible for all content in their abstracts including accuracy of the facts, statements, citing resources, etc.

### Results

The content of the questionnaires and overall results are shown in the table 2, 3.

**Table 2. The Global Aesthetic Improvement Scale (GAIS) and patients' evaluation.**

| Score | Description | Patient' score, n (%) |
|---|---|---|
| 3 | Very much improved | 4 (33,3) |
| 2 | Much improved | 3 (25) |
| 1 | Improved | 5 (41,7) |
| 0 | No change | - |
| -1 | Worse | - |
| -2 | Much worse | - |
| -3 | Very much worse | - |

**Table 3. The Patient's Satisfaction Score (PSS), The Physician Satisfaction Score (PhSS) and patients' and physicians' evaluation.**

| Description | PSS | PhSS | PSS score, n (%) | PhSS score, n (%) |
|---|---|---|---|---|
| Very bad outcome | 0 | 0 | - | - |
| | 1 | 1 | - | - |
| | 2 | 2 | - | - |
| | 3 | 3 | - | - |
| | 4 | 4 | - | - |
| | 5 | 5 | - | - |
| | 6 | 6 | 2 (16,7) | 1 (8,3) |
| | 7 | 7 | 4 (33,3) | 1 (8,3) |
| | 8 | 8 | 2 (16,7) | 5 (41,7) |
| | 9 | 9 | 1 (8,3) | 2 (16,7) |
| Very good outcome | 10 | 10 | 3 (25) | 3 (25) |

Depending on the results of GAIS all of the patients received positive results after aesthetic treatments, none of them noted suffocation of their appearance.
The summary of PSS showed the high satisfaction rating with patients' appearance after aesthetic medicine interventions which is also confirmed by a survey of physicians.
Moreover, none of the patients complained about infectious, immune, bleeding complications after aesthetic procedures.

### Summary/Conclusion

The survival rate of hematological patients has significantly increased due to novel available treatment methods. Otherwise, long-term consequences such as appearance changes affecting patients' QOL still remain a serious problem for majority ofpatients. Hopefully, expanding the opportunities in aesthetic medicine for these patients, awareness and education for hospital physicians will significantly improve the QOL of most patients.

**Copyright Information:** (Online) ISSN: 2572-9241
©2026 The Author(s). HemaSphere is published by John Wiley & Sons Ltd on behalf of European Hematology Association. This is an open access Abstract Book distributed under the Attribution-NonCommercial-NoDerivs (CC BY-NC-ND), which allows third parties to download the articles and share them with others as long as they credit the author and the Abstract Book, but they cannot change the content in any way or use them commercially.
**Abstract Book Citations:** Authors, Title, HemaSphere, 2026;10:(S1):pages. The abstract book DOIs can be found on the "Author Information" page.
**Disclaimer:** Articles published in the journal HemaSphere exclusively reflect the opinions of the authors. The authors are responsible for all content in their abstracts including accuracy of the facts, statements, citing resources, etc.

**Table 1. Patients' characteristics.**

| | |
|---|---|
| **Age, Median (IQR)** 36 (30–72) | |
| **Gender** | **n (%)** |
| Female | 12 (100) |
| Male | - |
| **Diagnosis** | **n (%)** |
| Acute lymphoblastic leukemia (ALL) | 1 (8,3) |
| Acute myeloid leukemia (AML) | 2 (16,7) |
| Chronic myelogenous leukemia (CML) | 6 (50) |
| Lymphoma | 3 (25) |
| **Patient underwent allo-HSCT** | **n (%)** |
| Yes | 9 (75) |
| No | 3 (25) |

**Copyright Information:** (Online) ISSN: 2572-9241
©2026 The Author(s). HemaSphere is published by John Wiley & Sons Ltd on behalf of European Hematology Association. This is an open access Abstract Book distributed under the Attribution-NonCommercial-NoDerivs (CC BY-NC-ND), which allows third parties to download the articles and share them with others as long as they credit the author and the Abstract Book, but they cannot change the content in any way or use them commercially.
**Abstract Book Citations:** Authors, Title, HemaSphere, 2026;10:(S1):pages. The abstract book DOIs can be found on the "Author Information" page.
**Disclaimer:** Articles published in the journal HemaSphere exclusively reflect the opinions of the authors. The authors are responsible for all content in their abstracts including accuracy of the facts, statements, citing resources, etc.

### (PB4461) ASSESSMENT OF QUALITY OF LIFE AND ANALYSIS OF INFLUENCING FACTORS IN ELDERLY PATIENTS WITH LEUKEMIA

**Topic:** 36. Quality of life, ethics, supportive and palliative care

Jun Tao*[1, 1], Yimei Feng[1]

[1]*Xinqiao Hospital of Army Medical University, CHONGQING, China;*

**Background**

Relying solely on incidence, prevalence, and mortality rates to reflect the effectiveness of clinical treatments and health prevention measures has certain limitations. Consequently, quality of life is increasingly being used as an e

**Aims**

This study aims to investigate and evaluate the quality of life and its influencing factors in elderly patients with leukemia.

**Methods**

Elderly patients with leukemia (including those undergoing chemotherapy, cellular therapy, and hematopoietic stem cell transplantation) treated at a tertiary hospital from June 2023 to December 2025 were selected. Sixty patients aged over 65 years with adequate comprehension of the Chinese language were included, comprising 46 males and 14 females. After obtaining patient consent, the quality of life of the 60 patients was investigated using the QLQ-C30 questionnaire. The QLQ-C30 (Version 3.0) consists of 30 questions across 3 subscales and 6 single-item measures, reflecting patients' global health status, physical function, role function, emotional function, cognitive function, social function, as well as symptoms such as fatigue, nausea and vomiting, pain, dyspnea, insomnia, appetite loss, constipation, diarrhea, and financial difficulties. Higher scores for functional scales and global health status indicate better quality of life, whereas higher scores for symptom scales indicate more severe symptoms. Thirty healthy elderly individuals were selected as a control group for comparison of domain scores. Correlation analysis and multiple regression models were used to analyze factors influencing patients' quality of life.

**Results**

All 90 subjects were included in the final analysis. The 60 patients had a moderate global health status. Compared to the general population, all functional statuses showed poor recovery, with the median score for social function being the lowest ($P < 0.01$). Among the symptom scales, fatigue was the most prominent ($P = 0.000$), and 56.9% of patients reported financial difficulties ($P = 0.000$). Logistic regression analysis showed that average monthly family income, presence of bleeding in the past week, stage of treatment, marital status, and borrowing money due to illness were correlated with the functional status of quality of life and significantly influenced functional domain scores ($P < 0.05$). Factors such as age and occupation did not significantly affect functional domain scores.

**Summary/Conclusion**

The quality of life in elderly patients with leukemia is poor, with social function being the most impaired and fatigue being a common symptom. Financial status is a major influencing factor on their quality of life.

**Copyright Information:** (Online) ISSN: 2572-9241
©2026 The Author(s). HemaSphere is published by John Wiley & Sons Ltd on behalf of European Hematology Association. This is an open access Abstract Book distributed under the Attribution-NonCommercial-NoDerivs (CC BY-NC-ND), which allows third parties to download the articles and share them with others as long as they credit the author and the Abstract Book, but they cannot change the content in any way or use them commercially.
**Abstract Book Citations:** Authors, Title, HemaSphere, 2026;10:(S1):pages. The abstract book DOIs can be found on the "Author Information" page.
**Disclaimer:** Articles published in the journal HemaSphere exclusively reflect the opinions of the authors. The authors are responsible for all content in their abstracts including accuracy of the facts, statements, citing resources, etc.

### (PB4462) INTEGRATING VIRTUAL REALITY INTO PAIN AND ANXIETY MANAGEMENT DURING BONE MARROW BIOPSIES

**Topic:** 36. Quality of life, ethics, supportive and palliative care

Yesmine Amara*[1, 2], Imen Frikha[1, 2], Lobna Ben Mahmod[2, 3], Moez Elloumi[1], Moez Medhaffar[1]

*[1]Hedi Chaker hospital of Sfax, Department of Clinical Hematology, Hedi Chaker Hospital of Sfax. University of Sfax, Tunisia, Sfax, Tunisia; [2]university of Sfax, Research Laboratory: LR19ES10 "Acute Medicine, Pharmacology, and Toxicology of Xenobiotics", faculty of medicine of Sfax, University of Sfax, Sfax, Tunisia., Sfax, Tunisia; [3]University of Sfax, Department of Pharmacology, Faculty of Medicine, University of Sfax, Sfax, Tunisia, Sfax, Tunisia;*

### Background

Bone marrow biopsy (BMB) is routinely performed procedures against patients with haematological malignancies; however, it is frequently perceived as invasive and distressing techniques despite the use of local anaesthesia with Lidocaine. Nonpharmacological approaches have been increasingly recognized as effective strategies for reducing procedural pain and anxiety, thereby improving patient comfort and overall experience.

### Aims

Our aim is to approve virtual reality (VR)'s efficacy in reducing pain and anxiety related to BMB for patients with haematological malignancies.

### Methods

It was a crossover study, associated with external control group, conducted in Haematology department, Hedi Chaker university hospital of Sfax, Tunisia. Participants from interventional group were undergo the biopsy, at first time, with standard care only, followed by washout out period of at least 7 days, and were undergo the procedure, at second time, with standard care (Lidocaine anaesthesia) associated to VR. Participants from external control group experienced the procedure with standard care only. Subjectively, pain and anxiety were assessed, respectively through "the numerical rating scale (NRS)", and "the Spielberger State-Trait Anxiety Inventory (STAI)". Objectively, team research gathered patients' satisfaction and acceptation of the intervention, patients' reactions to pain, and side effects through passive observation. Intervention's efficacy was analysed through within group comparison ($\Delta T1$ = Pre-Post anxiety scores/Post-Pre pain scores during the period without VR vs $\Delta T2$ = Pre-Post anxiety scores/Post-Pre pain scores during the period with VR), and between groups comparison ($\Delta T2$ = Pre-post anxiety scores/post-pre pain scores during the period with VR vs $\Delta$ = Pre-post anxiety scores/post-pre pain scores in control group). Non-parametric tests were used, with significance set at $p < 0.05$. Median values were reported. This study was registered at ClinicalTrials.gov (NCT07085065).

### Results forty-one patients, undergoing BMB, were allocated into interventional group (n = 17) and control group (n = 24). Baseline demographic and clinical characteristics were comparable between groups ($p>0.05$). The interventional group during the period with VR showed a significantly greater reduction in anxiety scores 'differences between the pre and post measurements compared to the period with standard care only ($\Delta T1$(median = 0) vs $\Delta T2$ (median = 30), $p < 0.001$), and to external control group ($\Delta T2$ (median = 30 vs $\Delta$ (median = 7), $p < 0.001$). Pain scores also decreased significantly in the interventional group during the intervention period, patients undergoing the procedure with VR, for both comparisons ($\Delta T1$(median = 8) vs $\Delta T2$ (median = 4), $p < 0.001$; $\Delta T2$ (median = 4) vs $\Delta$ (median = 6), $p = 0.002$). Patient satisfaction was higher in the interventional group. No adverse events related to the intervention were reported.

**Copyright Information:** (Online) ISSN: 2572-9241
©2026 The Author(s). HemaSphere is published by John Wiley & Sons Ltd on behalf of European Hematology Association. This is an open access Abstract Book distributed under the Attribution-NonCommercial-NoDerivs (CC BY-NC-ND), which allows third parties to download the articles and share them with others as long as they credit the author and the Abstract Book, but they cannot change the content in any way or use them commercially.
**Abstract Book Citations:** Authors, Title, HemaSphere, 2026;10:(S1):pages. The abstract book DOIs can be found on the "Author Information" page.
**Disclaimer:** Articles published in the journal HemaSphere exclusively reflect the opinions of the authors. The authors are responsible for all content in their abstracts including accuracy of the facts, statements, citing resources, etc.

**Summary/Conclusion**

our study approved VR's efficacy in pain and anxiety reduction during BMB, as a supportive strategy to enhance patient comfort during this procedure. Our findings align with another trial (Le Du et al., 2023), in which they compared the effectiveness of VR during BMB compared to Oxygen-nitrous oxide equimolar analgesia (MEOPA). Results showed that VR was safe, and could be an adjunctive non pharmacological treatment in case of contraindication or intolerance to MEOPA, as both interventions approved same level of efficacy.

**Copyright Information:** (Online) ISSN: 2572-9241
©2026 The Author(s). HemaSphere is published by John Wiley & Sons Ltd on behalf of European Hematology Association. This is an open access Abstract Book distributed under the Attribution-NonCommercial-NoDerivs (CC BY-NC-ND), which allows third parties to download the articles and share them with others as long as they credit the author and the Abstract Book, but they cannot change the content in any way or use them commercially.
**Abstract Book Citations:** Authors, Title, HemaSphere, 2026;10:(S1):pages. The abstract book DOIs can be found on the "Author Information" page.
**Disclaimer:** Articles published in the journal HemaSphere exclusively reflect the opinions of the authors. The authors are responsible for all content in their abstracts including accuracy of the facts, statements, citing resources, etc.

### (PB4463) FRAILTY PREDICTS MORTALITY IN OLDER PATIENTS WITH CHRONIC LYMPHOCYTIC LEUKEMIA: REAL-WORLD DATA FROM A COMPREHENSIVE GERIATRIC ASSESSMENT.

**Topic:** 36. Quality of life, ethics, supportive and palliative care

Thiago Carneiro*[1, 2], João Sales[2], Juliana Tavares[2], Cauã Espírito Santo[2], João Pantoja[2], Lucas Martins[2], Ana Teles[2], Luiza Souza[2], Antônio Pereira[2], Lucas Monteiro[2], Annanda Guedes[1], Niele Moraes[2]

*[1]Hospital Ophir Loyola / UEPA, Belém, Brazil;[2]Universidade do Estado do Pará, Belém, Brazil;*

### Background

Chronic lymphocytic leukemia (CLL) predominantly affects older adults. Chronological age alone is an insufficient predictor of outcomes in this population, and frailty has emerged as a key determinant of vulnerability, treatment tolerance, and survival. However, real-world data evaluating the association between frailty and mortality in older patients with CLL, particularly in middle-income settings, remain scarce.

### Aims

To assess frailty using comprehensive geriatric assessment (CGA) tools in older patients with CLL and to evaluate its association with mortality in a real-world clinical cohort.

### Methods

This observational real-world study included patients aged ≥60 years with confirmed CLL followed at two tertiary outpatient services in Northern Brazil. All participants underwent CGA, including functional, physical, and frailty assessment using the Frail Scale (Edmonton) and the Fried frailty phenotype. Clinical outcomes were obtained through medical record review after a two-year follow-up and classified as death due to disease-related complications or ongoing clinical follow-up. Patients lost to follow-up were excluded from outcome-based analyses. Categorical variables were compared using Fisher's exact test.

### Results

Sixteen patients were included (median age 71.5 years; 50% male). During follow-up, five patients (31.3%) died and nine (56.3%) remained under clinical follow-up. Frailty was strongly associated with mortality. According to the Fried frailty phenotype, 80.0% of deceased patients were classified as frail, compared with 22.2% among patients under clinical follow-up ($p = 0.04$). Non-frail status (pre-frail or non-frail) predominated among survivors (77.8%). Using the Frail Scale, frailty was also more frequent among patients who died (40.0% vs 22.2%), although this difference did not reach statistical significance ($p = 0.57$). Most deaths occurred in patients with multiple comorbidities and impaired physical performance.

### Summary/Conclusion

Frailty, particularly when assessed using the Fried frailty phenotype, was strongly associated with mortality in older patients with CLL in this real-world cohort. These findings highlight frailty as a clinically meaningful marker to support risk stratification, supportive care planning, and individualized decision-making in older patients with CLL.

**Copyright Information:** (Online) ISSN: 2572-9241
©2026 The Author(s). HemaSphere is published by John Wiley & Sons Ltd on behalf of European Hematology Association. This is an open access Abstract Book distributed under the Attribution-NonCommercial-NoDerivs (CC BY-NC-ND), which allows third parties to download the articles and share them with others as long as they credit the author and the Abstract Book, but they cannot change the content in any way or use them commercially.
**Abstract Book Citations:** Authors, Title, HemaSphere, 2026;10:(S1):pages. The abstract book DOIs can be found on the "Author Information" page.
**Disclaimer:** Articles published in the journal HemaSphere exclusively reflect the opinions of the authors. The authors are responsible for all content in their abstracts including accuracy of the facts, statements, citing resources, etc.

**Association between frailty status and mortality in older patients with chronic lymphocytic leukemia**

| Frailty status | Death (n = 5) | No death – (n = 9) | *p* value* |
|---|---|---|---|
| **Frail Scale (Edmonton)** | | | |
| **Frail** | 2 (40.0%) | 2 (22.2%) | 0.57 |
| **Non-frail†** | 3 (60.0%) | 7 (77.8%) | |
| **Fried Frailty Phenotype** | | | |
| **Frail** | 4 (80.0%) | 2 (22.2%) | 0.04 |
| **Non-frail‡** | 1 (20.0%) | 7 (77.8%) | |

** P values calculated using Fisher's exact test.*
† Non-frail = pre-frail + robust according to the Frail Scale.
‡ Non-frail = pre-frail + non-frail according to the Fried phenotype.

**Copyright Information:** (Online) ISSN: 2572-9241
©2026 The Author(s). HemaSphere is published by John Wiley & Sons Ltd on behalf of European Hematology Association. This is an open access Abstract Book distributed under the Attribution-NonCommercial-NoDerivs (CC BY-NC-ND), which allows third parties to download the articles and share them with others as long as they credit the author and the Abstract Book, but they cannot change the content in any way or use them commercially.
**Abstract Book Citations:** Authors, Title, HemaSphere, 2026;10:(S1):pages. The abstract book DOIs can be found on the "Author Information" page.
**Disclaimer:** Articles published in the journal HemaSphere exclusively reflect the opinions of the authors. The authors are responsible for all content in their abstracts including accuracy of the facts, statements, citing resources, etc.

### (PB4464) WHEN TWO MALIGNANCIES COLLIDE: THERAPEUTIC CHALLENGES AND SURVIVAL OUTCOMES IN PATIENTS WITH CONCURRENT SOLID AND HEMATOLOGIC CANCERS

**Topic:** 36. Quality of life, ethics, supportive and palliative care

Imane Chajai*[1], Maha EL Amani[1], Otman Abdelmoula[1], Zineb Afroukh[1], Souad Fennan[1], Othman Doghmi[1], Amal Terfai[1], Elmaachi Nora[1], Naoufal Benlachgar[1], Soukaina Haidouri[1], Zoubida Tazi Mezalek[1]

*[1]Ibn Sina University Hospital, Clinical Hematology, Rabat, Morocco;*

### Background

Population ageing is associated with an increasing incidence of multiple primary malignancies. In addition, advances in cancer therapy have paradoxically increased the risk of secondary malignancies related to prior treatments. The coexistence of solid tumors and hematologic cancers represents a growing clinical challenge, particularly in elderly and multimorbid patients. Currently, no specific guidelines address their simultaneous management, leading to individualized and often complex therapeutic decisions.

### Aims

To analyze clinical and organizational determinants influencing therapeutic strategies in patients presenting with concurrent solid and hematologic malignancies.

### Methods

We conducted a retrospective descriptive single-center study at Ibn Sina Hospital, Rabat, Morocco. Patients diagnosed with both a solid tumor and a hematologic malignancy were included. Clinical variables included age, sex, and Charlson Comorbidity Index, performans status, geriatric fitness assessment and treatment intent (curative versus palliative). Variables related to decision-making included therapeutic prioritization and treatment modifications. Organizational factors included multidisciplinary tumor board discussions. Overall survival was estimated using Kaplan–Meier analysis.

### Results

Twenty-nine patients were included. The median age at diagnosis was 66 years (range: 36-95). Sex distribution was balanced, with 15 females and 14 males (sex ratio M/F = 0.93). The median Charlson Comorbidity Index was 7, reflecting a highly comorbid population. Geriatric assessment showed that 27.6% of patients were classified as unfit, reflecting a non-negligible proportion of frail patients.
Breast and prostate cancers were the most frequent solid tumors, while diffuse large B-cell lymphoma and indolent lymphomas were the predominant hematologic malignancies. The solid tumor preceded the hematologic malignancy in 48.3% of cases, while 24.1% were diagnosed synchronously.
Therapeutic prioritization was heterogeneous: the solid tumor was prioritized in 37.9% of cases, the hematologic malignancy in 27.5%, and both malignancies were considered of equal priority in 34.5%. Treatment modification of the hematologic malignancy due to the presence of the solid tumor occurred in approximately one third of patients. Only 20.7% of cases were discussed in a dedicated multidisciplinary tumor board and two patients were managed with upfront palliative care due to the pre-existing hematologic malignancy.
Median overall survival was 112.8 months (95% CI: 45.6–244.7). The overall survival at one, three and five year was of 86.4%, 72.1%, and 56.7%, respectively.

**Copyright Information:** (Online) ISSN: 2572-9241
©2026 The Author(s). HemaSphere is published by John Wiley & Sons Ltd on behalf of European Hematology Association. This is an open access Abstract Book distributed under the Attribution-NonCommercial-NoDerivs (CC BY-NC-ND), which allows third parties to download the articles and share them with others as long as they credit the author and the Abstract Book, but they cannot change the content in any way or use them commercially.
**Abstract Book Citations:** Authors, Title, HemaSphere, 2026;10:(S1):pages. The abstract book DOIs can be found on the "Author Information" page.
**Disclaimer:** Articles published in the journal HemaSphere exclusively reflect the opinions of the authors. The authors are responsible for all content in their abstracts including accuracy of the facts, statements, citing resources, etc.

**Summary/Conclusion**

Concurrent solid and hematologic malignancies generate significant decision-making complexity in a predominantly elderly and comorbid population.
Our real-world data demonstrate frequent therapeutic adaptations, high comorbidity burden, and limited structured multidisciplinary coordination.
These findings underscore the need for integrated hematology-oncology tumor boards and optimized institutional care pathways to improve survival outcomes in this growing patient population.

**Copyright Information:** (Online) ISSN: 2572-9241
©2026 The Author(s). HemaSphere is published by John Wiley & Sons Ltd on behalf of European Hematology Association. This is an open access Abstract Book distributed under the Attribution-NonCommercial-NoDerivs (CC BY-NC-ND), which allows third parties to download the articles and share them with others as long as they credit the author and the Abstract Book, but they cannot change the content in any way or use them commercially.
**Abstract Book Citations:** Authors, Title, HemaSphere, 2026;10:(S1):pages. The abstract book DOIs can be found on the “Author Information” page.
**Disclaimer:** Articles published in the journal HemaSphere exclusively reflect the opinions of the authors. The authors are responsible for all content in their abstracts including accuracy of the facts, statements, citing resources, etc.

### (PB4465) DIAGNOSTIC AND TREATMENT DELAYS AND CARE PATHWAYS IN DIFFUSE LARGE B-CELL LYMPHOMA: A STUDY FROM AN EASTERN EUROPEAN CENTER

**Topic:** 37. Health economics

Gratiela-Elena Dobre*[1, 2], Ana-Maria Petraru[1, 2], Mihaela-Sabrina Herdea[1, 2], Adela-Sara Ionescu[1, 2], Alexandra-Diana Malenda[1, 2], Florina Patranoiu[1], Viola Maria Popov[1], Lidia Felicia Mihai[1], Geanina Ofiteru[1], Mihaela Popescu[1], Mihaela Andreescu[1, 2], Oana-Diana Dragoi-Cozmaciuc[1]

*[1]Colentina Clinical Hospital, Hematology I Department, Bucharest, Romania;[2]Titu Maiorescu University, Faculty of Medicine, Bucharest, Romania;*

### Background

There are no reports describing diagnostic and treatment delays and pathways for diffuse large B-cell lymphoma (DLBCL) in Eastern Europe. Such delays can have important health-economic implications.

### Aims

To describe diagnostic and treatment delays and patients' characteristics and outcomes in a Romanian DLBCL cohort.

### Methods

We evaluated 52 consecutive patients with a new diagnosis of DLBCL treated at our center between 2023 and 2025. Patient related, disease specific, and response to treatment data were retrospectively collected. Chi-squared test/Fisher's exact test were used to evaluate differences between categorical variables, and the Mann-Whitney U test was applied for continuous variables. Survival analyses were performed using the Kaplan–Meier method, and groups were compared using the log-rank test.

### Results

The median age was 65 years old, 46% were female. The median follow-up was 296 days. The median time from symptoms onset to treatment initiation was 106 days (IQR 71-216 days). Patients waited a median of 80 days (IQR 43-168 days) from the symptoms onset until the diagnostic biopsy was performed. A further median of 21 days (IQR 12-28 days) passed before first presentation to hematology. 8 patients had biopsy performed after the first presentation to hematology. From initial hematology consult, a median of 13 days (IQR 3-30 days) elapsed until chemotherapy initiation. B symptoms and palpable mass were the most common presenting complaints.
33% of patients lived more than 100 km away from our centre and were significantly more likely to present with advanced disease than those living closer (stage III/IV in 87% vs 58%, p=0.036), suggesting reduced access to routine medical services and likely resulting in higher treatment costs.
Patients with higher ECOG performance status had significantly shorter symptom-to-biopsy intervals (ECOG PS≥2 vs 0-1, median 66 vs 93 days, p=0.038), suggesting that the diagnostic pathway is accelerated for frailer patients. They also had higher IPI score (≥3 in 90% vs 51%, p=0.034) and inferior overall survival (1 year OS 66% vs 85%, p=0.031).
No associations were observed between diagnostic delays (>3 months) or treatment delays (>4 weeks) and other patient characteristics (sex, urban vs rural residency, distance to center, marital status), disease parameters (LDH, stage, IPI score, cell of origin, ki67, extranodal disease), response to treatment, or overall survival.

**Copyright Information:** (Online) ISSN: 2572-9241
©2026 The Author(s). HemaSphere is published by John Wiley & Sons Ltd on behalf of European Hematology Association. This is an open access Abstract Book distributed under the Attribution-NonCommercial-NoDerivs (CC BY-NC-ND), which allows third parties to download the articles and share them with others as long as they credit the author and the Abstract Book, but they cannot change the content in any way or use them commercially.
**Abstract Book Citations:** Authors, Title, HemaSphere, 2026;10:(S1):pages. The abstract book DOIs can be found on the "Author Information" page.
**Disclaimer:** Articles published in the journal HemaSphere exclusively reflect the opinions of the authors. The authors are responsible for all content in their abstracts including accuracy of the facts, statements, citing resources, etc.

**Summary/Conclusion**

Overall, the diagnostic and treatment intervals in our cohort were comparable to those reported internationally. While the delays were not associated with inferior outcomes, our findings suggest geographical disparities in access to medical care and inefficient resource allocation. The accelerated diagnostic pathway for frailer patients reflects a reactive healthcare system that prioritizes clinical deterioration over early detection, potentially increasing overall healthcare costs through emergency presentations and complications.

**Copyright Information:** (Online) ISSN: 2572-9241
©2026 The Author(s). HemaSphere is published by John Wiley & Sons Ltd on behalf of European Hematology Association. This is an open access Abstract Book distributed under the Attribution-NonCommercial-NoDerivs (CC BY-NC-ND), which allows third parties to download the articles and share them with others as long as they credit the author and the Abstract Book, but they cannot change the content in any way or use them commercially.
**Abstract Book Citations:** Authors, Title, HemaSphere, 2026;10:(S1):pages. The abstract book DOIs can be found on the "Author Information" page.
**Disclaimer:** Articles published in the journal HemaSphere exclusively reflect the opinions of the authors. The authors are responsible for all content in their abstracts including accuracy of the facts, statements, citing resources, etc.

### (PB4466) PREVALENCE AND TRENDS OF HEMATOLOGICAL MALIGNANCIES IN SAUDI ARABIA: A LONGITUDINAL ANALYSIS AND INTERNATIONAL COMPARISON (2000-2023)

**Topic:** 37. Health economics

Abdulrahman Nasiri*[1], Mostafa Mohammed Saleh[2], Manal Alshammari[3], Huda Alfattah[2], Reem Alkharras[4], Aljurf Mahmoud[2]

*[1]Imam Mohammad Ibn Saud Islamic University, College of Medicine, Riyadh, Saudi Arabia;[2]King Faisal Specialist Hospital & Research Centre, Department of Hematology, Riyadh, Saudi Arabia; [3]King Salman Specialist Hospital, Department of Hematology, Hail, Saudi Arabia; [4]Security Forces Hospital, Department of Medicine, Riyadh, Saudi Arabia;*

### Background

Hematological malignancies represent a major contributor to global cancer burden, yet their epidemiology varies substantially across regions due to differences in demographics, genetics, environmental exposures, and healthcare systems. Saudi Arabia has a distinctly young population structure compared with Western countries, which may influence disease distribution and age at diagnosis. Despite continuous reporting by the Saudi Cancer Registry, comprehensive longitudinal analyses examining national trends and subtype-specific patterns over extended periods remain limited.

### Aims

To characterize long-term incidence trends, demographic distribution, and subtype-specific burden of hematological malignancies in Saudi Arabia from 2000 to 2023, and to identify epidemiologic features relevant to national healthcare strategy.

### Methods

This retrospective, population-based longitudinal study analyzed data from the Saudi Cancer Registry between January 2000 and December 2023. Hematological malignancies were classified into leukemia, non-Hodgkin lymphoma (NHL), Hodgkin lymphoma (HL), and multiple myeloma (MM) according to ICD-O-3 coding. Annual case counts, sex distribution, and incidence patterns were evaluated. Age-standardized incidence rates were assessed where available. Temporal trends were analyzed using linear regression, and comparisons between early (2000–2011) and later (2012–2023) periods were performed to evaluate changes in incidence burden.

### Results

A total of 47,279 hematological malignancies were identified, accounting for 17.1% of all cancers nationally. Overall incidence increased significantly over the study period, with a mean rise of 62.36 additional cases annually ($p<0.001$). NHL was the most common malignancy (38.7%), followed by leukemia (34.6%), HL (19.2%), and MM (7.6%). All major malignancies demonstrated increasing incidence trends, with HL showing the largest relative increase of 61.8% between the early and later study periods ($p<0.001$). Leukemia and NHL also increased substantially, rising by 39.3% and 42.4%, respectively. Male predominance was observed consistently across all malignancy types, with male-to-female ratios ranging from 1.21 in MM to 1.49 in HL. Notably, the median age at diagnosis was substantially younger than that reported in Western populations, particularly for leukemia and HL. Age-standardized incidence rates increased significantly for leukemia, NHL, and HL, suggesting a true rise in disease burden beyond population growth alone, while MM rates remained relatively stable.

**Copyright Information:** (Online) ISSN: 2572-9241
©2026 The Author(s). HemaSphere is published by John Wiley & Sons Ltd on behalf of European Hematology Association. This is an open access Abstract Book distributed under the Attribution-NonCommercial-NoDerivs (CC BY-NC-ND), which allows third parties to download the articles and share them with others as long as they credit the author and the Abstract Book, but they cannot change the content in any way or use them commercially.
**Abstract Book Citations:** Authors, Title, HemaSphere, 2026;10:(S1):pages. The abstract book DOIs can be found on the "Author Information" page.
**Disclaimer:** Articles published in the journal HemaSphere exclusively reflect the opinions of the authors. The authors are responsible for all content in their abstracts including accuracy of the facts, statements, citing resources, etc.

**Summary/Conclusion**

Hematological malignancies in Saudi Arabia demonstrate a sustained and statistically significant increase in incidence over more than two decades, with NHL and leukemia contributing the largest absolute burden and HL showing the most pronounced relative growth. The consistently younger age at diagnosis compared with Western populations represents a distinct epidemiologic characteristic with important clinical and public health implications. These findings underscore the need for expanded national surveillance, targeted research into etiologic and genetic factors, and strategic healthcare planning to address the evolving burden of hematological malignancies in Saudi Arabia.

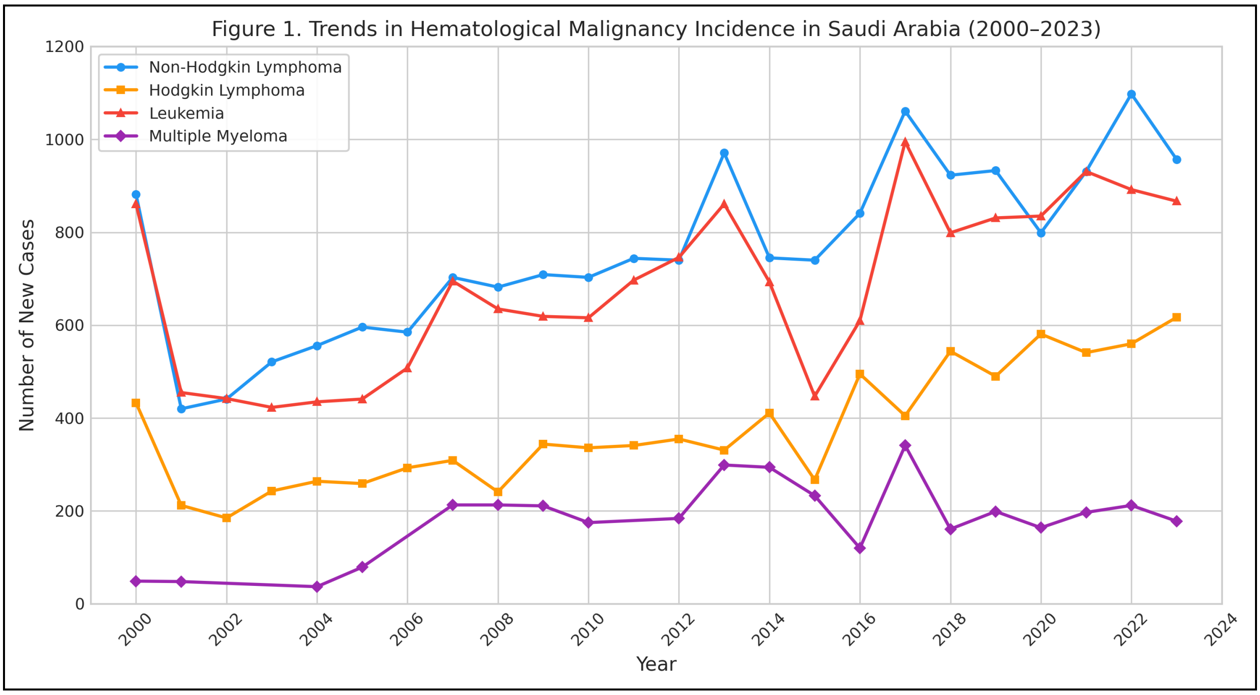

**Copyright Information:** (Online) ISSN: 2572-9241
©2026 The Author(s). HemaSphere is published by John Wiley & Sons Ltd on behalf of European Hematology Association. This is an open access Abstract Book distributed under the Attribution-NonCommercial-NoDerivs (CC BY-NC-ND), which allows third parties to download the articles and share them with others as long as they credit the author and the Abstract Book, but they cannot change the content in any way or use them commercially.
**Abstract Book Citations:** Authors, Title, HemaSphere, 2026;10:(S1):pages. The abstract book DOIs can be found on the "Author Information" page.
**Disclaimer:** Articles published in the journal HemaSphere exclusively reflect the opinions of the authors. The authors are responsible for all content in their abstracts including accuracy of the facts, statements, citing resources, etc.

### (PB4467) ASSESSING THE FINANCIAL IMPLICATIONS OF INTRODUCING SELINEXOR-BASED THERAPY FOR RELAPSED/REFRACTORY MULTIPLE MYELOMA IN QATAR

**Topic:** 37. Health economics

Anas Hamad*[1, 2], Ahmed Abdelmohsen[3], Pratik Ramesh Dhopte[4], Rita El Qjeil[5]

*[1]Pharmacy Department, National Center for Cancer Care and Research, Hamad Medical Corporation, Doha, Qatar;[2]College of Pharmacy, QU Health Sector, Qatar University, Doha, Qatar; [3]Menarini Group, Dubai, United Arab Emirates;[4]Carexso, Dubai, United Arab Emirates; [5]PDC-CRO, Dubai, United Arab Emirates;*

### Background

Relapsed/refractory multiple myeloma (RRMM) is a progressive hematologic malignancy that poses significant clinical and economic challenges. Selinexor is a first-in-class oral exportin-1 (XPO1) inhibitor with a unique mechanism of action. Regulatory approval of selinexor in the United States and European Union was based on the phase 3 BOSTON trial (NCT03110562) that showed significantly improved progression-free survival, overall response rate, and clinically improved overall survival with selinexor in combination with bortezomib and dexamethasone (SVd) versus bortezomib and dexamethasone (Vd) in patients with RRMM (Grosicki S, et al. *Lancet* 2020). In Qatar, the current treatment landscape for RRMM primarily relies on established therapies, while therapeutic options remain limited for patients who fail multiple lines of therapy. Hence, switching drug class to a new agent with the introduction of selinexor (in SVd) offers a potential opportunity to improve outcomes while addressing financial implications from a health system perspective.

### Aims

To assess the budgetary impact on the health system in Qatar of introducing selinexor into the treatment landscape for RRMM.

### Methods

A budget impact model was developed in accordance with ISPOR best practice guidelines (Sullivan SD, et al. *Value Health* 2014), using available data on key inputs such as patient population, treatment costs, and costs associated with complications and adverse events (AEs). These inputs were identified through an exploratory literature review and supplemented by primary market research. Current standard-of-care (SOC) regimens were identified as daratumumab[D]-carfilzomib-d, D-pomalidomide[P]-d, Pd, with market share derived from clinical opinions. The model estimated the total costs of current SOC, including drug acquisition, administration, AE management, disease state, subsequent therapies, and end-of-life, for scenarios with and without selinexor over a 5-year time horizon (2025 to 2029). AE costs were estimated using microcosting; AE management cost per regimen (including supportive medications such as antiemetics where clinically indicated) was calculated.

### Results

Introducing SVd for the management of RRMM in Qatar is projected to generate a substantial cost savings compared with current SOC regimens. Over a 5-year horizon, SVd as second-line therapy reduces total costs by QAR 472,630, primarily driven by lower drug costs (-QAR 153,300) and subsequent therapy costs (-QAR 329,443), which offset slight increases in administration (+QAR 1,357) and disease state (+QAR 21,966) costs. The most significant drug cost reduction occurs in the first year (-QAR 120,098), highlighting an early economic impact. Marginal increases in disease state and end-of-life costs reflect continued patient support. Similarly, using SVd as third-line therapy results in savings of QAR 237,763, mainly due to reductions in drug costs (-QAR 114,234) and subsequent therapy costs (-QAR 125,628), without additional administration or disease state expenditures.

**Copyright Information:** (Online) ISSN: 2572-9241
©2026 The Author(s). HemaSphere is published by John Wiley & Sons Ltd on behalf of European Hematology Association. This is an open access Abstract Book distributed under the Attribution-NonCommercial-NoDerivs (CC BY-NC-ND), which allows third parties to download the articles and share them with others as long as they credit the author and the Abstract Book, but they cannot change the content in any way or use them commercially.
**Abstract Book Citations:** Authors, Title, HemaSphere, 2026;10:(S1):pages. The abstract book DOIs can be found on the “Author Information” page.
**Disclaimer:** Articles published in the journal HemaSphere exclusively reflect the opinions of the authors. The authors are responsible for all content in their abstracts including accuracy of the facts, statements, citing resources, etc.

**Summary/Conclusion**

Results of this model show that introducing SVd for RRMM in Qatar is cost saving compared with current care, reducing overall treatment costs over 5 years primarily through lower drug and subsequent therapy expenses while optimizing downstream treatment requirements. These reductions in subsequent therapy costs are largely attributable to the documented efficacy and improved clinical outcomes of selinexor in RRMM patients.

**Copyright Information:** (Online) ISSN: 2572-9241
©2026 The Author(s). HemaSphere is published by John Wiley & Sons Ltd on behalf of European Hematology Association. This is an open access Abstract Book distributed under the Attribution-NonCommercial-NoDerivs (CC BY-NC-ND), which allows third parties to download the articles and share them with others as long as they credit the author and the Abstract Book, but they cannot change the content in any way or use them commercially.
**Abstract Book Citations:** Authors, Title, HemaSphere, 2026;10:(S1):pages. The abstract book DOIs can be found on the "Author Information" page.
**Disclaimer:** Articles published in the journal HemaSphere exclusively reflect the opinions of the authors. The authors are responsible for all content in their abstracts including accuracy of the facts, statements, citing resources, etc.

### (PB4468) WHO CARES FOR THE CAREGIVERS? PSYCHOLOGICAL, ORGANIZATIONAL, AND ECONOMIC BURDEN IN CHRONIC HEMATOLOGIC MALIGNANCIES

**Topic:** 37. Health economics

Salvatore Tallarico[1], Maria Zifaro[2], Ledjia Sijoni[3], Lorenzo Toscanelli[4], Maria Livia Del Giudice[5], Amedeo Votto[3], Ines Rina[3], Gaspare Tancredi[6], Matteo Pelosini[5], Gabriele Buda[3], Katia Belvedere[5], Giuliana Fabbri[5], Mojgan Azadegan[5], Simone Lazzini[1], Sara Galimberti*[3]

*[1]Department of Economics and Management, University of Pisa, PISA, Italy;[2]UNIMERCATORUM, ROMA, Italy; [3]DEPARTMENT OF CLINICAL AND EXPERIMENTAL MEDICINE, UNIVERSITY OF PISA, PISA, Italy; [4]SERVIZIO CIVILE UNIVERSALE, AOUP, PISA, Italy; [5]Azienda Ospedaliera Universitaria Pisana, PISA, Italy; [6]DOCTORAT GENOMEC, UNIVERSITY OF SIENA & DEPARTMENT OF CLINICAL AND EXPERIMENTAL MEDICINE, UNIVERSITY OF PISA, PISA, Italy;*

### Background

Advances in the treatment of hematologic malignancies have significantly improved survival, transforming conditions such as multiple myeloma, lymphomas, and myelodysplastic syndromes into long-term or chronic diseases. This therapeutic progress has reshaped the cancer trajectory into a prolonged experience marked by repeated treatment decisions, clinical uncertainty, and sustained psychosocial demands. Within this evolving landscape, informal caregivers play a central role. From a gender medicine perspective, understanding whether and how caregiver gender influences the multidimensional burden of care is essential to promote equitable and personalized health strategies.

### Aims

To evaluate the impact of caregiver gender on psychological, organizational, and economic burden within a comprehensive research framework shown in figure 1, and to explore the role of demographic and structural determinants in shaping caregiving strain.

### Methods

An ad hoc questionnaire was administered to caregivers recruited in the outpatient setting of the Hematology Unit in Pisa, Italy. The survey collected sociodemographic data and measured three latent burden dimensions: psychological (negative emotional states and loss of personal time), organizational (logistical demands and preferences for home-based services), and economic (work-related impact and increased expenses). The final sample included 207 caregivers, predominantly female (66.2%), with a mean age of 58.4 years. Patients presented heterogeneous hematologic conditions, most of them undergoing active treatment. The research framework was tested using Structural Equation Modeling (SEM).

**Copyright Information:** (Online) ISSN: 2572-9241
©2026 The Author(s). HemaSphere is published by John Wiley & Sons Ltd on behalf of European Hematology Association. This is an open access Abstract Book distributed under the Attribution-NonCommercial-NoDerivs (CC BY-NC-ND), which allows third parties to download the articles and share them with others as long as they credit the author and the Abstract Book, but they cannot change the content in any way or use them commercially.
**Abstract Book Citations:** Authors, Title, HemaSphere, 2026;10:(S1):pages. The abstract book DOIs can be found on the “Author Information” page.
**Disclaimer:** Articles published in the journal HemaSphere exclusively reflect the opinions of the authors. The authors are responsible for all content in their abstracts including accuracy of the facts, statements, citing resources, etc.

### Results

Descriptive findings indicated that caregivers experienced high levels of worry regarding disease outcomes, while anger, despair, depression, and insomnia were reported at low levels. Although the overall restriction in personal time and occupational functioning was modest, caregivers reported a moderate perception that the illness had changed their lives. Increased travel for medical reasons emerged as one of the most relevant organizational burdens. Economically, respondents reported moderate increases in overall, medical, and transportation-related expenses, whereas indicators of severe financial deprivation were infrequent. A strong preference for home-based healthcare services—such as home blood sampling, home therapies, medication delivery, and transportation support—highlighted the importance of decentralized care models. Contrary to expectations derived from gender medicine literature, caregiver gender was not a significant predictor of psychological, organizational, or economic burden in the structural model. In contrast, age was positively associated with psychological and economic burden, indicating greater vulnerability among older caregivers.

### Summary/Conclusion

The research findings suggest that in the context of chronic hematologic malignancies, caregiving strain may be more strongly influenced by life-course factors and healthcare organization than by gender differences per se. From a gender medicine perspective, the results underscore the need to move beyond binary assumptions and adopt a more nuanced approach that integrates demographic, structural, and contextual determinants of caregiving experience. Decentralized care pathways and telemedicine may represent cross-cutting strategies to mitigate caregiver burden irrespective of gender, fostering a more equitable, family-centered model of care in hematologic oncology.

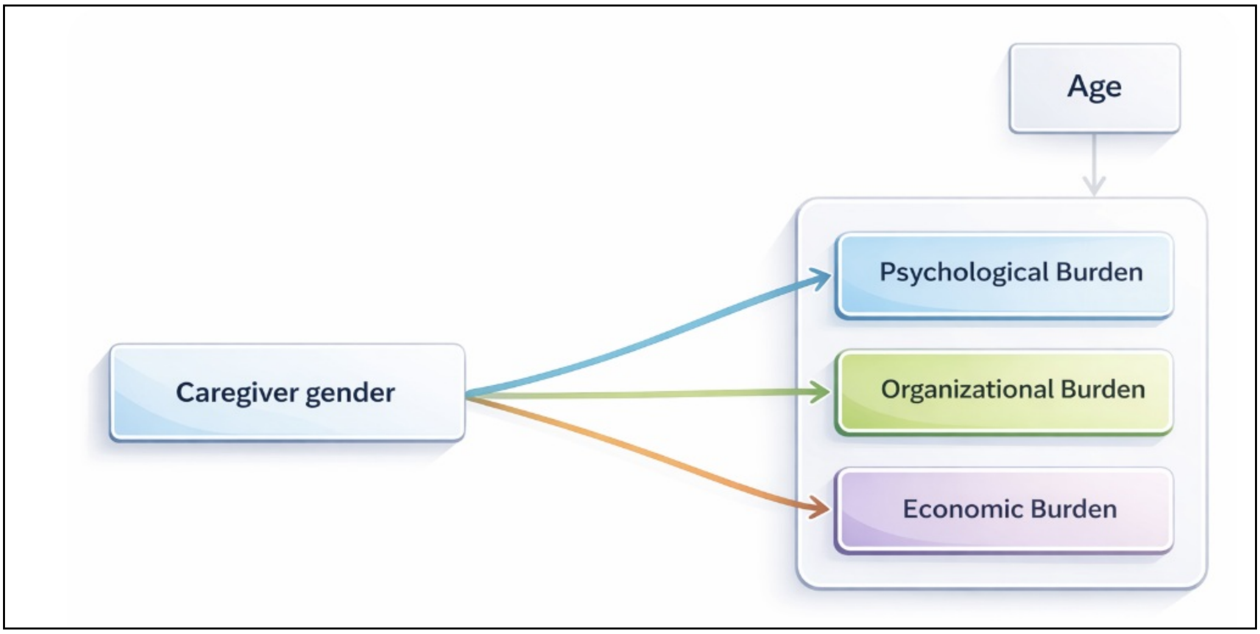

Figure 1. Research framework

**Copyright Information:** (Online) ISSN: 2572-9241
©2026 The Author(s). HemaSphere is published by John Wiley & Sons Ltd on behalf of European Hematology Association. This is an open access Abstract Book distributed under the Attribution-NonCommercial-NoDerivs (CC BY-NC-ND), which allows third parties to download the articles and share them with others as long as they credit the author and the Abstract Book, but they cannot change the content in any way or use them commercially.
**Abstract Book Citations:** Authors, Title, HemaSphere, 2026;10:(S1):pages. The abstract book DOIs can be found on the "Author Information" page.
**Disclaimer:** Articles published in the journal HemaSphere exclusively reflect the opinions of the authors. The authors are responsible for all content in their abstracts including accuracy of the facts, statements, citing resources, etc.

### (PB4469) FINANCIAL TOXICITY AND CARE DELIVERY GAPS IN HAEMATOLOGY: A FIVE-YEAR REAL-WORLD HEALTH SYSTEMS ANALYSIS FROM A NIGERIAN COMPREHENSIVE CANCER CENTRE (2020–2025)

**Topic:** 37. Health economics

Oluyemisi Osakwe[1], Oluwafemi Akinwunmi*[2], Samson Adegbe[2], Imoh Essien[2], Anu Matthew[2], Modupe Elebute-Odunsi[2]

*[1]Marcelle Ruth Cancer Centre & Specialist Hospital, Research & Development, Lagos, Nigeria;[2]Marcelle Ruth Cancer Centre & Specialist Hospital, Lagos, Nigeria;*

### Background

Haematological disorders impose a substantial clinical and economic burden in low- and middle-income countries (LMICs). However, detailed hospital-level data linking disease patterns, financing mechanisms, and long-term outcomes in LMIC settings remain limited. Critically, essential confirmatory investigations, including immunophenotyping and molecular genetic testing, remain unavailable in-country, necessitating expensive overseas referrals for definitive diagnosis. We analysed the first five years of haematology service delivery at a specialist cancer centre in Nigeria to characterise disease distribution, funding mechanisms, and follow-up integrity within a predominantly out-of-pocket health system.

### Aims

To characterise the spectrum of malignant and benign haematological conditions managed during the first five years of service delivery at a Nigerian specialist centre, and to evaluate the relationship between diagnostic complexity, financing mechanisms, and continuity of care within a predominantly out-of-pocket health system.

### Methods

A retrospective registry-based analysis was conducted of all patients managed for malignant and benign haematological conditions at Marcelle Ruth Cancer Centre and Specialist Hospital (MRCC), Lagos, between July 2020 and August 2025. Demographic characteristics, diagnostic spectrum, financing source, and follow-up status were analysed to assess patterns of access, financial exposure, and continuity of care.

### Results

Out of 5,494 total patient encounters, 223 patients met inclusion criteria: 170 (76.2%) malignant and 53 (23.8%) benign cases. Malignant disorders predominantly affected economically productive adults, with 68.9% aged 40–69 years and peak incidence in the sixth decade. The diagnostic profile was dominated by multiple myeloma (26.5%) and non-Hodgkin lymphoma (25.3%), followed by acute myeloid leukaemia (11.2%). A modest male predominance was observed (55.3%). At analysis, 39.4% were confirmed alive and 17.1% deceased; however, 43.5% had incomplete outcome documentation (unknown or lost to follow-up), indicating substantial longitudinal care gaps.

Benign conditions demonstrated a younger age profile (mean 44 years) and female predominance (64.2%). Sickle cell disease (HbSS/HbSC) accounted for 79.2% of benign cases. In contrast to the malignant cohort, 79.2% were alive at last follow-up.

Across both cohorts, more than 92% of patients financed care through direct out-of-pocket payment, with fewer than 8% covered by insurance mechanisms. This financing structure coincided with measurable attrition in follow-up among malignant cases, suggesting vulnerability in care continuity within a high-cost disease environment. All patients were resident in Nigeria, predominantly Lagos State, positioning the centre as a regional referral hub operating within a fragmented financing ecosystem.

**Copyright Information:** (Online) ISSN: 2572-9241
©2026 The Author(s). HemaSphere is published by John Wiley & Sons Ltd on behalf of European Hematology Association. This is an open access Abstract Book distributed under the Attribution-NonCommercial-NoDerivs (CC BY-NC-ND), which allows third parties to download the articles and share them with others as long as they credit the author and the Abstract Book, but they cannot change the content in any way or use them commercially.
**Abstract Book Citations:** Authors, Title, HemaSphere, 2026;10:(S1):pages. The abstract book DOIs can be found on the "Author Information" page.
**Disclaimer:** Articles published in the journal HemaSphere exclusively reflect the opinions of the authors. The authors are responsible for all content in their abstracts including accuracy of the facts, statements, citing resources, etc.

**Summary/Conclusion**

This five-year institutional analysis demonstrates a dual burden of adult-onset haematological malignancies and chronic hemoglobinopathies within a health system overwhelmingly reliant on private financing. The convergence of high-cost malignant disease, diagnostic infrastructure deficits, limited insurance penetration, and substantial follow-up attrition highlights structural weaknesses in haematology care delivery in LMIC settings. Strengthening financial risk protection and registry-linked survivorship tracking is critical to improving equitable access and measurable outcomes in African haematology.

**Copyright Information:** (Online) ISSN: 2572-9241
©2026 The Author(s). HemaSphere is published by John Wiley & Sons Ltd on behalf of European Hematology Association. This is an open access Abstract Book distributed under the Attribution-NonCommercial-NoDerivs (CC BY-NC-ND), which allows third parties to download the articles and share them with others as long as they credit the author and the Abstract Book, but they cannot change the content in any way or use them commercially.
**Abstract Book Citations:** Authors, Title, HemaSphere, 2026;10:(S1):pages. The abstract book DOIs can be found on the “Author Information” page.
**Disclaimer:** Articles published in the journal HemaSphere exclusively reflect the opinions of the authors. The authors are responsible for all content in their abstracts including accuracy of the facts, statements, citing resources, etc.

### (PB4470) COST PER COMPLETE RESPONDER FOR EPCORITAMAB + LENALIDOMIDE AND RITUXIMAB (R2) VERSUS TAFASITAMAB + R2 IN RELAPSED OR REFRACTORY FOLLICULAR LYMPHOMA: A US MEDICARE PERSPECTIVE

**Topic:** 37. Health economics

Taiji Wang[1], Taylor Wang[2], Wei Gao[2], Savreet Bains Chawla*[3], Tychell Branchcomb[3], Zhijie Ding[3], Monika Jun[3], Felipe Marques Goncalves[3], David Tybor[4], Abualbishr Alshreef[4], Elyse Swallow[2], Donald Moore[5]

*[1]Analysis Group, Menlo Park, United States of America; [2]Analysis Group, Boston, United States of America; [3]Genmab US Inc., Plainsboro, United States of America; [4]AbbVie, Inc., North Chicago, United States of America; [5]Atrium Health Levine Cancer Institute, Charlotte, United States of America;*

### Background

Follicular lymphoma (FL) is a common non-Hodgkin lymphoma characterized by recurrent cycles of remission and relapse, resulting in substantial clinical and economic burden. Novel immunotherapies have expanded treatment options for patients with relapsed or refractory (R/R) FL. Epcoritamab (epcor), a subcutaneous CD3×CD20 bispecific antibody, has demonstrated significant improvements in overall response rate, complete response (CR) rate, and progression-free survival in combination with lenalidomide and rituximab ($R^2$) (EPCORE FL-1; NCT05409066) and is FDA-approved for R/R FL. Tafasitamab (tafa), an Fc-engineered anti-CD19 monoclonal antibody, has also shown clinical benefit when combined with $R^2$ (inMIND; NCT04680052). Although no head-to-head trials have compared these regimens, anchored indirect comparisons demonstrate significantly higher response rates, including CR, with epcor+$R^2$ relative to tafa+$R^2$. Given this established clinical differentiation, cost-per-responder (CPR) analyses can assess whether improvements in depth of response translate into tangible economic value.

### Aims

To evaluate 1-year CPR outcomes for complete responders for epcor+$R^2$ versus tafa+$R^2$ in patients with R/R FL from a US Medicare payer perspective.

### Methods

A 1-year CPR model was developed. Response was defined as CR and informed by results from an anchored matching-adjusted indirect comparison (MAIC) of EPCORE FL-1 and inMIND trials. Costs included drug acquisition, administration, adverse event (AE) management, and subsequent treatment, and were adjusted to 2025 US dollars. The base case assumed a fixed treatment duration of 12 cycles for both regimens. Drug acquisition costs were estimated using Centers for Medicare & Medicaid Services (CMS) average sales price data and dosing schedules from US prescribing information. Drug administration costs comprised intravenous and subcutaneous administration, laboratory testing, and premedications, estimated based on CMS fee schedules and expert opinion. AE management costs were calculated using AE incidence rates from prescribing information and unit costs from published sources, including management of grade 1-4 cytokine release syndrome and neurotoxicity. Subsequent treatment costs following disease progression were estimated using data from an anchor-based MAIC for time-to-next-treatment (TTNT) and published Medicare claims analyses. CPR was calculated as total 1-year cost divided by CR rate.

**Copyright Information:** (Online) ISSN: 2572-9241
©2026 The Author(s). HemaSphere is published by John Wiley & Sons Ltd on behalf of European Hematology Association. This is an open access Abstract Book distributed under the Attribution-NonCommercial-NoDerivs (CC BY-NC-ND), which allows third parties to download the articles and share them with others as long as they credit the author and the Abstract Book, but they cannot change the content in any way or use them commercially.
**Abstract Book Citations:** Authors, Title, HemaSphere, 2026;10:(S1):pages. The abstract book DOIs can be found on the "Author Information" page.
**Disclaimer:** Articles published in the journal HemaSphere exclusively reflect the opinions of the authors. The authors are responsible for all content in their abstracts including accuracy of the facts, statements, citing resources, etc.

**Results**

In EPCORE FL-1 and inMIND, unadjusted CR rates were 82.7% for epcor+$R^2$ and 52.0% for tafa+$R^2$; corresponding MAIC-adjusted CR rates were 81.2% and 52.0%, respectively. The estimated CPR was 11.8% lower with epcor+$R^2$ compared with tafa+$R^2$, corresponding to a cost savings of $79,141 per complete responder at 1 year. This difference was primarily driven by the higher CR rate observed with epcor+$R^2$. Post-progression costs accrued within the 1-year accounted for 0.7% (epcor+$R^2$) and 1.1% (tafa+$R^2$) of total CPR, but these do not reflect full post-progression costs as median TTNT has not yet been reached.

**Summary/Conclusion**

This analysis suggests that epcor+$R^2$ is associated with a lower cost per complete responder in the first year compared with tafa+$R^2$, highlighting its dominant clinical and economic value for patients with R/R FL. As longer-term clinical data from EPCORE FL-1 and inMIND become available, further evaluation of CPR benefit for epcor+$R^2$ over extended time horizons is warranted.

**Copyright Information:** (Online) ISSN: 2572-9241
©2026 The Author(s). HemaSphere is published by John Wiley & Sons Ltd on behalf of European Hematology Association. This is an open access Abstract Book distributed under the Attribution-NonCommercial-NoDerivs (CC BY-NC-ND), which allows third parties to download the articles and share them with others as long as they credit the author and the Abstract Book, but they cannot change the content in any way or use them commercially.
**Abstract Book Citations:** Authors, Title, HemaSphere, 2026;10:(S1):pages. The abstract book DOIs can be found on the "Author Information" page.
**Disclaimer:** Articles published in the journal HemaSphere exclusively reflect the opinions of the authors. The authors are responsible for all content in their abstracts including accuracy of the facts, statements, citing resources, etc.

### (PB4471) ENVIRONMENTAL CONCERNS AMONG FRENCH HEMATOLOGISTS: A CROSS-SECTIONAL SURVEY TOWARD MORE SUSTAINABLE PRACTICE

**Topic:** 37. Health economics

Alya Perthus*[1], Arthur Dony[2], Olivier Decaux[1], Aurélie Cabannes-Hamy[3], Robin Noel[4], Aude Charbonnier[4], Emmanuel Gyan[5], Mathilde Chanut[3], Côme Bommier[6], Bérénice Schell[7], Katiuska Miliani Lugo Ascanio[8], Marie Herr[8], Caroline Besson[3]

*[1]CHU Pontchaillou, Department of Hematology, Rennes, France; [2]CH Métropole Savoie, Chambéry, France; [3]Hospital Center of Versailles, Le Chesnay, France; [4]Institut Paoli Calmettes, Marseille, France; [5]CHRU Tours, Tours, France; [6]Hôpital Saint-Louis, Paris, France; [7]Hôpital Henri Montdor, Créteil, France; [8]Hôpital Raymond Pointcarré, Garches, France;*

### Background

Ecosystem degradation, climate change, and pollution represent growing threats to human health. The healthcare sector itself contributes substantially to greenhouse gas (GHG) emissions, and hematology appears to be a particularly resource- and energy-intensive specialty. In this context, the collaborative group Hemato-Green, established in 2023, conducted a survey among hematologists.

### Aims

This study aimed to assess environmental awareness among hematologists and to evaluate their perceptions of the environmental impact of their clinical practice, as well as potential levers for more sustainable hematology care.

### Methods

An anonymous, cross-sectional survey was conducted using LimeSurvey and disseminated to hematologists and laboratory biologists through the French Society of Hematology (Société Française d'Hématologie) and the Association of Hematology Residents (Association des Internes d'Hématologie). The questionnaire comprised 27 items divided into five domains: general characteristics, professional practices, institutional levers for change, perceptions of the healthcare system's role in the environmental crisis, and personal positioning toward ecological issues. The study received approval from the Ethics Committee of Foch Hospital (Suresnes, France).

**Copyright Information:** (Online) ISSN: 2572-9241
©2026 The Author(s). HemaSphere is published by John Wiley & Sons Ltd on behalf of European Hematology Association. This is an open access Abstract Book distributed under the Attribution-NonCommercial-NoDerivs (CC BY-NC-ND), which allows third parties to download the articles and share them with others as long as they credit the author and the Abstract Book, but they cannot change the content in any way or use them commercially.
**Abstract Book Citations:** Authors, Title, HemaSphere, 2026;10:(S1):pages. The abstract book DOIs can be found on the "Author Information" page.
**Disclaimer:** Articles published in the journal HemaSphere exclusively reflect the opinions of the authors. The authors are responsible for all content in their abstracts including accuracy of the facts, statements, citing resources, etc.

### Results

Among the 238 responses included in the analysis, 183 were fully completed. Two-thirds of respondents were clinicians, and one quarter were laboratory biologists. Seventy percent were over 35 years of age, and approximately half practiced in medium-sized cities.
Some sustainability initiatives had been implemented in the majority of respondents' institutions; however, only 18.5% reported being satisfied with these measures. Among the 160 clinicians, 31% felt they overprescribed certain laboratory tests and 9% some imaging examinations. Concurrently, 27% of respondents reported adapting their test ordering practices with a view to ecological impact. Approximately two-thirds of the subjects in this study indicated that they were in favour of a reassessment of prescribed therapies from a sustainability perspective.
Carbon emissions related to travel for scientific conferences were considered a relevant issue by 64% of respondents. Approximately two-thirds believed that the pharmaceutical industry should communicate transparently about an environmental "eco-score" for treatments, reduce the number of in-person events, and promote "eco-designed" clinical trials.
In addition, 54% considered the development of parsimonious surveillance testing recommendations by cooperative groups to be a priority. Nearly 70% felt that the healthcare system should reduce its GHG emissions, play an active role in the ecological transition, and adapt professional practices in response to the climate emergency. Only 25% reported feeling sufficiently informed about the healthcare system's GHG emissions. Finally, around 70% expressed the need for dedicated working groups within scientific societies, clear eco-responsible practice guidelines, and access to structured information—such as data on the life cycle of medications.

### Summary/Conclusion

This survey highlights a strong level of environmental concern among hematologists, coupled with a willingness to adapt clinical practices when sustainable alternatives are available. The report emphasizes the necessity for a well-defined institutional and scientific framework to facilitate this transition. These findings provide a solid foundation for advancing toward more sustainable hematology practice.

**Copyright Information:** (Online) ISSN: 2572-9241
©2026 The Author(s). HemaSphere is published by John Wiley & Sons Ltd on behalf of European Hematology Association. This is an open access Abstract Book distributed under the Attribution-NonCommercial-NoDerivs (CC BY-NC-ND), which allows third parties to download the articles and share them with others as long as they credit the author and the Abstract Book, but they cannot change the content in any way or use them commercially.
**Abstract Book Citations:** Authors, Title, HemaSphere, 2026;10:(S1):pages. The abstract book DOIs can be found on the "Author Information" page.
**Disclaimer:** Articles published in the journal HemaSphere exclusively reflect the opinions of the authors. The authors are responsible for all content in their abstracts including accuracy of the facts, statements, citing resources, etc.

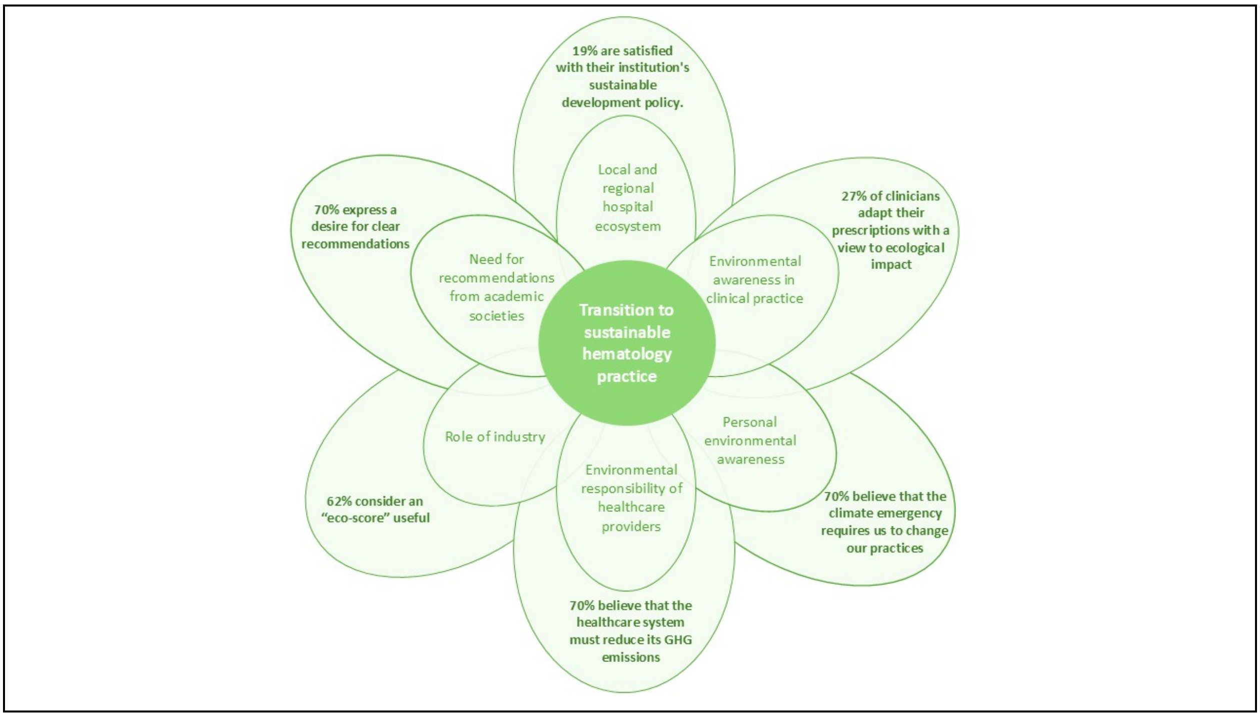

Conceptual framework for the transition to sustainable hematology practice

**Copyright Information:** (Online) ISSN: 2572-9241
©2026 The Author(s). HemaSphere is published by John Wiley & Sons Ltd on behalf of European Hematology Association. This is an open access Abstract Book distributed under the Attribution-NonCommercial-NoDerivs (CC BY-NC-ND), which allows third parties to download the articles and share them with others as long as they credit the author and the Abstract Book, but they cannot change the content in any way or use them commercially.
**Abstract Book Citations:** Authors, Title, HemaSphere, 2026;10:(S1):pages. The abstract book DOIs can be found on the "Author Information" page.
**Disclaimer:** Articles published in the journal HemaSphere exclusively reflect the opinions of the authors. The authors are responsible for all content in their abstracts including accuracy of the facts, statements, citing resources, etc.

### (PB4472) IMPACT OF SOCIOECONOMIC STATUS ON THE MANAGEMENT OF HEMATOLOGIC MALIGNANCIES ACCORDING TO GUIDELINES: A SINGLE-CENTER STUDY FROM ARMENIA

**Topic:** 37. Health economics

Astghik Voskanyan[1], Susanna Mikayelyan*[2], Nerses Ghahramanyan[1], Nare Martirosyan[1], Hayk Grigoryan[1], Miranush Saaryan[1], Yervand Hakobyan[1], Marine Melik-Andreasyan[1], Avetis Darbinyan[1], Gevorg Saghumyan[1], Marina Ghazaryan[1], Nshan Hovhannisyan[1], Anahit Ter-Grigoryan[1], Anahit Aynajyan[1], Iveta Mosesyan[1], Marieta Aleksanyan[2], Gayane Tsaturyan[2], Gevorg Tamamyan[2]

*[1]Yeolyan Hematology and Oncology Center, Yerevan, Armenia;[2]Yerevan State Medical University after Mkhitar Heratsi, Yerevan, Armenia;*

### Background

Beyond medical and clinical considerations, non-medical factors can significantly affect the implementation of recommended treatment guidelines in hematology.

### Aims

This study aims to assess the frequency and underlying causes of protocol deviations attributable to non-medical factors, including socioeconomic status, healthcare access, and patient support.
Protocol non-adherence was defined as any deviation from the initially recommended treatment regimen, omission of components or failure to complete recommended diagnostic or staging examinations.

### Methods

We performed a single-center observational study of adult patients treated in the hematology outpatient department at Yeolyan Hematology and Oncology Center. Socioeconomic and organizational variables were collected prospectively, including employment status, profession, marital status, and source of funding. All data were anonymized prior to analysis.

### Results

Among 181 patients, 19,88 % did not receive the recommended protocol due to non-medical reasons.
The most frequently missed components were rituximab (23%) and daratumumab (25%).
The most frequent causes were financial (67%) and social (15%).
Patients without stable employment or family financial support accounted for 61%.
In a subset of patients, protocol deviations were related to failure to complete PET-CT examination (17%).

### Summary/Conclusion

Non-medical barriers represent a substantial cause of protocol non-adherence in patients with hematologic malignancies.
Our findings emphasize that protocol deviations were particularly frequent among patients lacking stable employment, insurance, or financial coverage, underscoring the importance of targeted state and fund support.

**Copyright Information:** (Online) ISSN: 2572-9241
©2026 The Author(s). HemaSphere is published by John Wiley & Sons Ltd on behalf of European Hematology Association. This is an open access Abstract Book distributed under the Attribution-NonCommercial-NoDerivs (CC BY-NC-ND), which allows third parties to download the articles and share them with others as long as they credit the author and the Abstract Book, but they cannot change the content in any way or use them commercially.
**Abstract Book Citations:** Authors, Title, HemaSphere, 2026;10:(S1):pages. The abstract book DOIs can be found on the “Author Information” page.
**Disclaimer:** Articles published in the journal HemaSphere exclusively reflect the opinions of the authors. The authors are responsible for all content in their abstracts including accuracy of the facts, statements, citing resources, etc.

### (PB4473) RESOURCE UTILIZATION AND COSTS RELATED TO THE ADVERSE EVENTS MANAGEMENT OF RELAPSED/REFRACTORY MULTIPLE MYELOMA IN BRAZIL: MICROCOSTING FROM THE PRIVATE HEALTHCARE SYSTEM PERSPECTIVE

**Topic:** 37. Health economics

Straus Tanaka*[1], Marcella Alemar[1], Marcela Antonio[1], Thiago Bianco[1], Ana Padula[2], Gabriel Marasco[2], Graziela Bernardino[1]

*[1]GSK, São Paulo, Brazil; [2]Origin Health, São Paulo, Brazil;*

### Background

Relapsed/refractory multiple myeloma (RRMM) imposes a substantial clinical and economic burden. Beyond drug acquisition costs, the management of adverse events (AEs) significantly impacts total treatment expenditure, depending on their severity and frequency.

### Aims

This analysis aimed to quantify resource utilization and costs associated with AE management for second- and third-line RRMM treatments from the perspective of the Brazilian supplementary healthcare system.

### Methods

An expert panel of five hematologists with experience in treating MM in brazil validated a microcosting model based on hospital resource use that incorporated AEs from comercially approved second- and third-line treatments as of December 2025 for RRMM. Only AEs with significant clinical and economic impact were included. In this context, the most frequently reported AEs included cytokine release syndrome (CRS), immune effector cell-associated neurotoxicity syndrome (ICANS), infections, hypogammaglobulinemia, and hematologic toxicities such as anemia, neutropenia, and thrombocytopenia. Costs were estimated based on identified resources using prices from official Brazilian reimbursement lists (CMED, CBHPM and planserv); only direct medical costs were considered. Expenses related to drug acquisition and complications commonly associated with the CRAB criteria (hypercalcemia, renal impairment, anemia, and bone lesions) were excluded. All costs are reported in Brazilian Reais (BRL).

### Results

Infections, CRS, and anemia were the most costly conditions, with estimated management costs of 9,832.55 BRL, 7,822.56 BRL, and 7,079.87 BRL, respectively. Hematologists expressed concerns for increasing costs due to the association of CRS and infections with bispecifics.

### Summary/Conclusion

The Brazilian MM expert panel validated the microcosting model identifying management of infections, CRS and anemia as the most costly AE conditions for RRMM in Brazil and described costs related to bispecific antibodies, particularly due to the management of CRS and infections. The CRS and infections associated with bispecific antibodies was highlighted as an area of concern, as their management represented the highest direct medical costs generated in the microcosting model and may substantially influence overall treatment costs in multiple myeloma.
**Funding:** Study #300841 was funded by GSK.

**Copyright Information:** (Online) ISSN: 2572-9241
©2026 The Author(s). HemaSphere is published by John Wiley & Sons Ltd on behalf of European Hematology Association. This is an open access Abstract Book distributed under the Attribution-NonCommercial-NoDerivs (CC BY-NC-ND), which allows third parties to download the articles and share them with others as long as they credit the author and the Abstract Book, but they cannot change the content in any way or use them commercially.
**Abstract Book Citations:** Authors, Title, HemaSphere, 2026;10:(S1):pages. The abstract book DOIs can be found on the “Author Information” page.
**Disclaimer:** Articles published in the journal HemaSphere exclusively reflect the opinions of the authors. The authors are responsible for all content in their abstracts including accuracy of the facts, statements, citing resources, etc.

### (PB4474) EXPLORING THE PATIENT AND HEMATOLOGIST PREFERENCE OF TREATMENT CHARACTERISTICS FOR CHRONIC LYMPHOCYTIC LEUKEMIA (CLL): A QUALITATIVE STUDY IN EUROPE

**Topic:** 37. Health economics

Lydia Scarfò*[1, 2], Lianne Barnieh[3], Jennifer Fitzgerald[4], Silvy Mardiguian[5], Jan Rynne[6], Gereon Mänzel[7], Georgina Tickler[8], Sara Savar[8], Jacqui Bernarde[8], Kate Williams[8], Talha Munir[9]

*[1]Università Vita Salute San Raffaele, Milano, Italy; [2]IRCCS Ospedale San Raffaele, Milano, Italy; [3]BeOne Medicines Ltd, Paris, France; [4]BeOne Medicines Ltd, Basel, Switzerland; [5]BeOne Medicines Ltd, London, United Kingdom; [6]European CLL Association, Dublin, Ireland; [7]Leukämiehilfe RHEIN-MAIN e.V., Rüsselsheim, Germany; [8]Acaster Lloyd, London, United Kingdom; [9]Leeds Teaching Hospital, Leeds, United Kingdom;*

### Background

Chronic lymphocytic leukemia (CLL) is the most common adult leukemia. Novel targeted therapies, including Bruton tyrosine kinase (BTK) inhibitors (acalabrutinib, ibrutinib, zanubrutinib) and B-cell lymphoma 2 inhibitors (venetoclax) have greatly improved treatment outcomes for patients. While treatment choice is often personalized based on disease characteristics, genetic markers and patient fitness, other treatment characteristics, such as side effect profile or treatment duration, may affect patient preference.

### Aims

The study explored the most and least relevant treatment characteristics for treatment selection among patients with CLL and hematologists treating CLL in Europe.

### Methods

Qualitative interviews were conducted with independently recruited patients with CLL and hematologists with experience treating CLL in the United Kingdom, Ireland, Germany, France, Italy and Spain. Patients were required to be currently taking a continuous BTK inhibitor (acalabrutinib, ibrutinib or zanubrutinib for ≥6 months). Eligible participants completed a background questionnaire followed by a 60-minute qualitative interview. Interviews followed semi-structured guides that included open-ended questions on treatment experiences as well as a ranking exercise on the characteristics that were most and least relevant to treatment choice.

**Copyright Information:** (Online) ISSN: 2572-9241
©2026 The Author(s). HemaSphere is published by John Wiley & Sons Ltd on behalf of European Hematology Association. This is an open access Abstract Book distributed under the Attribution-NonCommercial-NoDerivs (CC BY-NC-ND), which allows third parties to download the articles and share them with others as long as they credit the author and the Abstract Book, but they cannot change the content in any way or use them commercially.
**Abstract Book Citations:** Authors, Title, HemaSphere, 2026;10:(S1):pages. The abstract book DOIs can be found on the “Author Information” page.
**Disclaimer:** Articles published in the journal HemaSphere exclusively reflect the opinions of the authors. The authors are responsible for all content in their abstracts including accuracy of the facts, statements, citing resources, etc.

**Results**

A total of 33 patients and 14 hematologists completed the ranking exercise. When patients ranked treatment characteristics from most to least relevant when choosing a treatment for CLL, ‘effectiveness and relief of symptoms’ was ranked first: “*First and foremost, a therapy must be effective – that is fundamental. If a treatment is administered, it should lead to a reduction in the signs of the disease.”* ‘Side effects’ and ‘impact on quality of life’ were tied as the second most relevant treatment characteristics for patients. ‘Duration of therapy’ and ‘dose frequency’ were ranked as least relevant for patients: “*…[duration of therapy] doesn’t matter so much to me because I don’t care…whatever is more effective for me, if I have to take it continuously, I take it, and if I have to do it another way, I do it.*” Hematologists also ranked ‘effectiveness and relief of symptoms’ as the most relevant treatment characteristic: *“It is really important for the patient – especially if they have symptoms – that the disease is treated first, and therefore effectiveness with alleviation of symptoms comes first.”* ‘Side effects’ and ‘impact on quality of life’ were ranked second and third, respectively. Some hematologists tied these characteristics to the effectiveness of the treatment: “*…[effectiveness and relief of symptoms] impacts on daily activities [and] quality of life. I don’t really think you can completely divorce those and call them separate characteristics because I think if patients’ symptoms are better, they’re able to do more and their quality of life improves.”* Unlike patients, hematologists ranked ‘duration of therapy’ higher than ‘mode of administration’ and ‘dose frequency’, which were ranked as least relevant.

**Summary/Conclusion**

Treatment effectiveness and symptom relief were ranked as the most relevant characteristic when choosing a CLL treatment from both the patient and hematologist perspective. These findings highlight the importance of communication regarding patient preferences in treatment choice to facilitate shared decision-making.

**Copyright Information:** (Online) ISSN: 2572-9241
©2026 The Author(s). HemaSphere is published by John Wiley & Sons Ltd on behalf of European Hematology Association. This is an open access Abstract Book distributed under the Attribution-NonCommercial-NoDerivs (CC BY-NC-ND), which allows third parties to download the articles and share them with others as long as they credit the author and the Abstract Book, but they cannot change the content in any way or use them commercially.
**Abstract Book Citations:** Authors, Title, HemaSphere, 2026;10:(S1):pages. The abstract book DOIs can be found on the “Author Information” page.
**Disclaimer:** Articles published in the journal HemaSphere exclusively reflect the opinions of the authors. The authors are responsible for all content in their abstracts including accuracy of the facts, statements, citing resources, etc.

### (PB4475) EVOLVING VALUE ASSESSMENT FOR CAR-T CELL THERAPIES: COMPARATIVE REIMBURSEMENT PATHWAYS IN JAPAN, THE UNITED STATES, THE UNITED KINGDOM, FRANCE, AND GERMANY

**Topic:** 37. Health economics

Saaya Tsutsué*[1], Kenshi Suzuki[2], Hideyuki Muromine[1], Keita Kato[1], George Papadopoulos[3], Surabhi Aggarwal[4], Parinita Barman[4], Amit Gupta[4], Michael Lopresti[5], Kittima Wattanakamolkul[1], Raf De Moor[1], Mami Kasahara-Kiritani[1], Celine Miyazaki[1]

*[1]Janssen Pharmaceutical K.K., Tokyo, Japan; [2]Japanese Red Cross Medical Center, Tokyo, Japan; [3]Lucid Health Consulting Pty Ltd, Sydney, Australia; [4]Skyward Analytics, India, Assam, India; [5]INTAGE Healthcare Inc, Tokyo, Japan;*

### Background

CAR-T cell therapies have transformed outcomes for patients with relapsed/refractory (R/R) B-cell malignancies, offering durable remissions where conventional treatments often fail. In Japan, a rapidly ageing population, limited autologous stem-cell transplantation (ASCT) eligibility due to treatment consensus, and hospital centric delivery with prolonged inpatient care led to poorer outcomes and higher hospitalization driven costs in pre-CAR-T era.

### Aims

We evaluated how pricing and reimbursement decisions relate to unmet need and evidence maturity and compared Japan's CAR-T cell therapy value assessment framework with referencing countries (the United States, the United Kingdom, France, and Germany) to outline pathways for Japan to shift from parity pricing toward value-aligned reimbursement.

### Methods

A targeted literature review was conducted across PubMed, the Cochrane Library, INAHTA, and ICHUSHI Web to identify records from database inception through 22–23 July 2025. Grey literature from Health Technology Assessment (HTA) and regulatory authorities (14–18 July 2025) was also screened. Records were screened against predefined criteria; 100 studies met inclusion. Primary outcomes concerned reimbursement and HTA; access was included only as a consequence.

### Results

Table 1 outlines the ways in which the United States (US), the United Kingdom (UK), France, and Germany shape value assessment for CAR-T cell therapies, as well as the approaches each system uses to manage uncertainty. Across the the UK (England), France and Germany, structured HTA processes tie appraisal directly to pricing and reimbursement, with uncertainty managed through access agreements and data-collection requirements. The US remains decentralized, producing more variable reimbursement frameworks. Japan applies national list pricing with selective cost-effectiveness evaluation; despite indication-specific reviews, prices generally stay uniform across indications, while budget impact varies by patient access and each country's demographic context.

### Summary/Conclusion

In consideration of European systems that offer mechanisms such as managed access, ongoing evidence development, and structured economic evaluation, Japan could recognize product- and indication- level differences in clinical value and evidence maturity rather than applying uniform prices across products within existing governance processes to support sustainability.

**Copyright Information:** (Online) ISSN: 2572-9241
©2026 The Author(s). HemaSphere is published by John Wiley & Sons Ltd on behalf of European Hematology Association. This is an open access Abstract Book distributed under the Attribution-NonCommercial-NoDerivs (CC BY-NC-ND), which allows third parties to download the articles and share them with others as long as they credit the author and the Abstract Book, but they cannot change the content in any way or use them commercially.
**Abstract Book Citations:** Authors, Title, HemaSphere, 2026;10:(S1):pages. The abstract book DOIs can be found on the "Author Information" page.
**Disclaimer:** Articles published in the journal HemaSphere exclusively reflect the opinions of the authors. The authors are responsible for all content in their abstracts including accuracy of the facts, statements, citing resources, etc.

**Table 1**

| Countries of reference | Value Assessment | Uncertainty management and notes |
|---|---|---|
| UK (England) | Structured HTA links clinical/economic evidence to pricing/reimbursement decisions | Managed access / coverage with evidence (e.g., Cancer Drugs Fund) |
| France | Structured HTA (clinical + economic) to pricing/ reimbursement | Data collection; post listing review *liste en sus* as hospital funding mechanism |
| Germany | Structured HTA (clinical) to pricing/ reimbursement | Post listing review; real world evidence Indication-specific ratings |
| United States | No single national HTA linked pricing; decentralized payer policies (e.g., CMS) | Heterogeneous, plan specific approaches Greater variability in reimbursement |
| Japan | National list pricing + selective cost-effectiveness evaluation | Handled mainly via post listing rules Indication-specific assessment + uniform list price within product |

**Copyright Information:** (Online) ISSN: 2572-9241
©2026 The Author(s). HemaSphere is published by John Wiley & Sons Ltd on behalf of European Hematology Association. This is an open access Abstract Book distributed under the Attribution-NonCommercial-NoDerivs (CC BY-NC-ND), which allows third parties to download the articles and share them with others as long as they credit the author and the Abstract Book, but they cannot change the content in any way or use them commercially.
**Abstract Book Citations:** Authors, Title, HemaSphere, 2026;10:(S1):pages. The abstract book DOIs can be found on the "Author Information" page.
**Disclaimer:** Articles published in the journal HemaSphere exclusively reflect the opinions of the authors. The authors are responsible for all content in their abstracts including accuracy of the facts, statements, citing resources, etc.

### (PB4476) ATTENTION-ENHANCED MULTI-OMICS MODELING ENABLES PAN-CANCER DRUG RESPONSE PREDICTION AND BIOMARKER DISCOVERY FOR ANTICANCER AND REPURPOSED COMPOUNDS

**Topic:** 38. Novel technologies, techniques and digital analytical tools in hematology

Yibo Wang*[1], Xiaoqi Wang[1], Xinwen Zhang[1], Yidan Ye[1], Xiaoli Liang[1], Changzheng Li[1], Xi Zhang[1]

[1]*Xinqiao Hospital of Army Medical University, Chongqing, China;*

### Background

Cancer drug discovery remains challenged by tumor heterogeneity and limited experimental scalability. Rencently virtual drug screening integrating machine learning algorithms has numerous research results, and some of them have even been successfully patented. Current machine learning models for virtual drug screening fail to effectively integrate multi-omics data or capture nonlinear cross-omics interactions, restricting predictive accuracy and biomarker discovery across diverse cancers with high heterogeneity.

### Aims

Based on the deep learning model that integrates multi-omics data, the initial screening of anti-cancer drugs and the exploration of drug action targets are achieved at the level of pan-cancer cell lines.

### Methods

We develop a deep multi-omics fusion model integrating mutation, methylation, transcriptomic, and metabolomic profiles from over 900 pan-cancer cell lines derived form database DepMap. Our framework uniquely synergizes random forest-based feature selection to prioritize biologically relevant omics features, feature compression fusion for cross-omics dimensionality reduction, and multi-head attention mechanisms to model nonlinear interactions between cellular multi-omics landscapes. Further biological analysis on selected features enables to decipher potential biomarkers related to drug effects.

### Results

Our multi-omics fusion model attained high-performance drug response prediction across over 900 cancer cell lines (median Pearson r=0.50 for all included drugs). Validation demonstrated robust accuracy for MEK inhibitor Trametinib (r=0.78, MAE=0.59) and non-oncology agent BMOV (r=0.73). The model identified BRAF-mutant melanoma sensitivity and PI3K/AKT bypass resistance, consistent with existing findings. Feature mining revealed TERT modulation and oxidative stress induction as BMOV's probable anticancer mechanisms, while Benzamide targeted metabolic vulnerabilities.

### Summary/Conclusion

In conclusion, this work established a robust ML-driven platform for virtual drug screening and biomarker discovery which is a valuable tool for accelerating precision oncology.

**Copyright Information:** (Online) ISSN: 2572-9241
©2026 The Author(s). HemaSphere is published by John Wiley & Sons Ltd on behalf of European Hematology Association. This is an open access Abstract Book distributed under the Attribution-NonCommercial-NoDerivs (CC BY-NC-ND), which allows third parties to download the articles and share them with others as long as they credit the author and the Abstract Book, but they cannot change the content in any way or use them commercially.
**Abstract Book Citations:** Authors, Title, HemaSphere, 2026;10:(S1):pages. The abstract book DOIs can be found on the “Author Information” page.
**Disclaimer:** Articles published in the journal HemaSphere exclusively reflect the opinions of the authors. The authors are responsible for all content in their abstracts including accuracy of the facts, statements, citing resources, etc.

### (PB4477) REDEFINING FATIGUE AS A MEASURABLE BIOLOGICAL BURDEN IN SICKLE CELL DISEASE THROUGH A LARGE-SCALE, REAL-WORLD DIGITAL COHORT

**Topic:** 38. Novel technologies, techniques and digital analytical tools in hematology

Kim Summers*[1], Orlando Agrippa[1], Muna Yusuf[1], Paul Telfer[2], Sanne Lugthart[3]

*[1]Sanius Health, London, United Kingdom; [2]Barts Health NHS Trust, London, United Kingdom; [3]University Hospitals Bristol and Weston NHS Foundation Trust, Bristol, United Kingdom;*

### Background

Fatigue is a prevalent and debilitating symptom in sickle cell disease (SCD), yet remains undercharacterised in routine care and clinical trials, despite its persistent burden often beyond episodic vaso-occlusive crises (VOC) and severe pain. Traditional periodic patient-reported outcome (PRO) measures may not capture daily variability and functional impairment. Continuous digital monitoring offers an opportunity to identify objective correlates of fatigue in real-world settings.

### Aims

To evaluate the utility of objective wearable-derived metrics through their association with daily fatigue burden in SCD, independent of pain and VOC status.

### Methods

Longitudinal analysis was conducted within a digital SCD ecosystem comprising 1,295 enrolled global patients. A UK subcohort of 365 high-data-density participants was selected for completeness and repeated-measures suitability (Jan 2023-Feb 2026). Daily ePROs captured fatigue and pain severity (0-10), and VOC status (Yes/Maybe/No) via a mobile application, alongside passive wearable-derived metrics. Measures were selected for interpretability, including steps, activity duration by metabolic equivalent of task (MET<3, 3-6, or >6), total sleep duration, deep sleep proportion, nightly $SpO_2$, and wake-up disturbances. Linear mixed models with patient-level random intercepts assessed associations between daily fatigue and wearable metrics, adjusting for pain, VOC, age, sex, and genotype.

### Results

122,543 total patient-day observations were captured, with median follow-up of 21 months (IQR 8-28). Participants were 70% female, median age 35 years (range 15-70), with genotype distribution of HbSS 74%, HbSC 18%, and 'Other' 8%.
Variance decomposition from a random intercept model (ICC=0.581) suggested 58.1% of fatigue variability was between patients and 41.9% reflected within-patient variation. Severe fatigue was defined as a score ≥7/10. Across observed days, 30% were severe-fatigue days, 77% occurring without self-reported VOC.
Across metric-specific adjusted models, higher pain was consistently associated with higher fatigue ($p<0.001$). Male sex was linked to lower reported fatigue ($p<0.05$), while age and genotype were not significant. VOC status showed an overall association with fatigue across models (Type III tests $p\leq0.009$), however, category-specific contrasts versus the reference group were not directionally consistent.
Significant but modest associations were observed between wearable metrics and fatigue. Higher step count ($\beta=-1.066\times10^{-5}$, $p<0.001$), intense (MET>6) activity ($\beta=-1.401\times10^{-5}$, $p=0.005$), longer sleep duration ($\beta=-4.305\times10^{-6}$, $p<0.001$), and higher $SpO_2$ ($\beta=-0.010$, $p=0.013$) were each independently associated with lower fatigue. In the combined multivariable model, step count, sleep duration, and $SpO_2$ remained independently linked to fatigue. Intense activity was not retained, possibly reflecting limited MET>6 variation and overlap with other activity metrics.

**Copyright Information:** (Online) ISSN: 2572-9241
©2026 The Author(s). HemaSphere is published by John Wiley & Sons Ltd on behalf of European Hematology Association. This is an open access Abstract Book distributed under the Attribution-NonCommercial-NoDerivs (CC BY-NC-ND), which allows third parties to download the articles and share them with others as long as they credit the author and the Abstract Book, but they cannot change the content in any way or use them commercially.
**Abstract Book Citations:** Authors, Title, HemaSphere, 2026;10:(S1):pages. The abstract book DOIs can be found on the "Author Information" page.
**Disclaimer:** Articles published in the journal HemaSphere exclusively reflect the opinions of the authors. The authors are responsible for all content in their abstracts including accuracy of the facts, statements, citing resources, etc.

### Summary/Conclusion

In the largest longitudinally monitored real-world SCD cohort, fatigue demonstrated significant associations with objective activity, sleep, and nightly $SpO_2$, independent of pain and VOC status. This supports fatigue as a measurable burden and suggests continuous digital biomarkers may help redefine endpoint development and lived impact assessment. Future analyses will explore temporal dynamics in depth to characterise the causal directionality between wearable metrics and fatigue burden, and the potential influence of treatment factors, including analgesia.

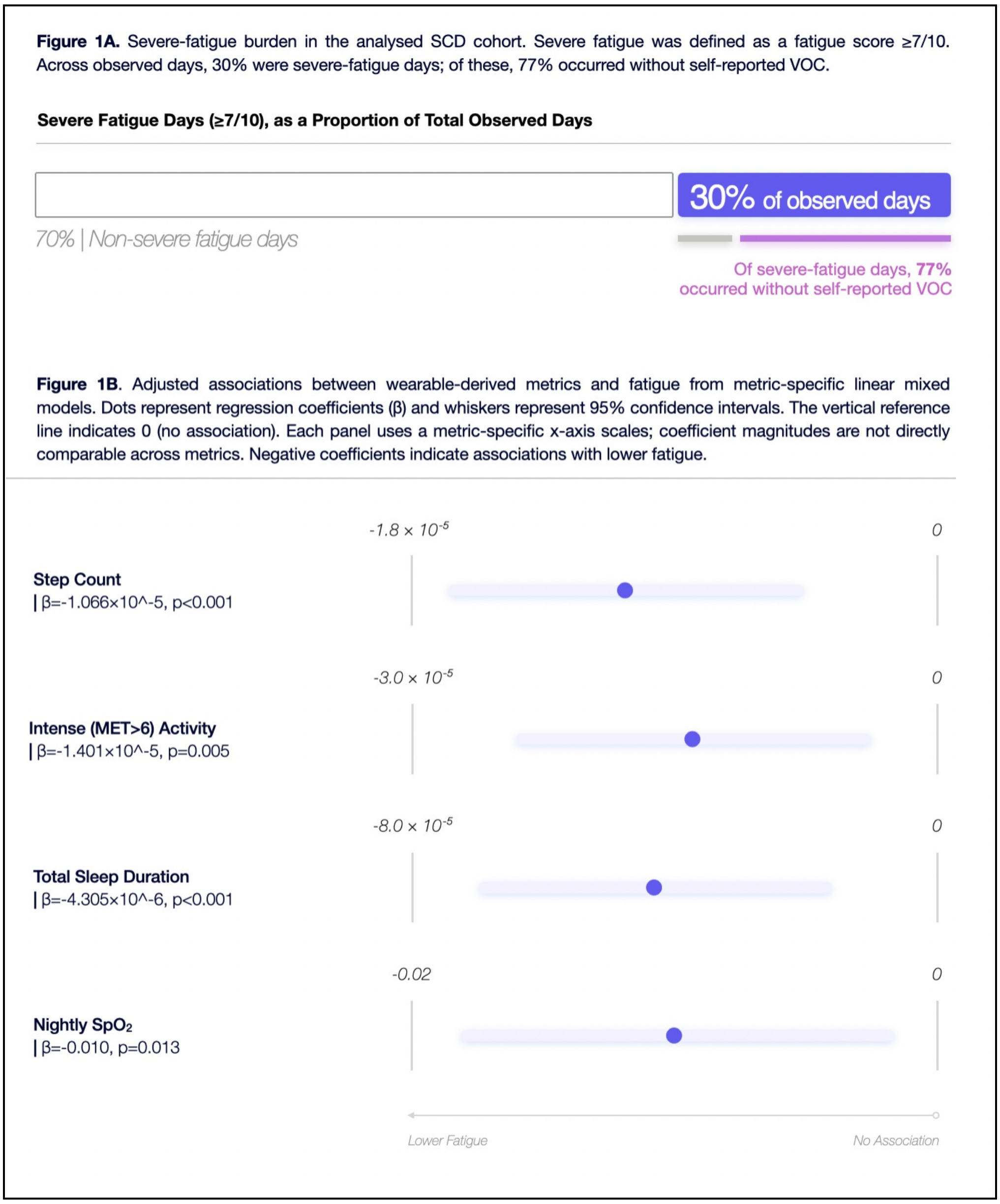

**Figure 1A.** Severe-fatigue burden in the analysed SCD cohort. Severe fatigue was defined as a fatigue score ≥7/10. Across observed days, 30% were severe-fatigue days; of these, 77% occurred without self-reported VOC.

**Figure 1B.** Adjusted associations between wearable-derived metrics and fatigue from metric-specific linear mixed models. Dots represent regression coefficients (β) and whiskers represent 95% confidence intervals. The vertical reference line indicates 0 (no association). Each panel uses a metric-specific x-axis scales; coefficient magnitudes are not directly comparable across metrics. Negative coefficients indicate associations with lower fatigue.

Figure 1. Summary of severe-fatigue burden and adjusted wearable-derived correlates of fatigue in SCD.

**Copyright Information:** (Online) ISSN: 2572-9241
©2026 The Author(s). HemaSphere is published by John Wiley & Sons Ltd on behalf of European Hematology Association. This is an open access Abstract Book distributed under the Attribution-NonCommercial-NoDerivs (CC BY-NC-ND), which allows third parties to download the articles and share them with others as long as they credit the author and the Abstract Book, but they cannot change the content in any way or use them commercially.
**Abstract Book Citations:** Authors, Title, HemaSphere, 2026;10:(S1):pages. The abstract book DOIs can be found on the “Author Information” page.
**Disclaimer:** Articles published in the journal HemaSphere exclusively reflect the opinions of the authors. The authors are responsible for all content in their abstracts including accuracy of the facts, statements, citing resources, etc.

### (PB4478) MONOCYTE DISTRIBUTION WIDTH (MDW): AS A PROMISING DIAGNOSTIC AND PROGNOSTIC TOOL IN EARLY SEPSIS DETECTION

**Topic:** 38. Novel technologies, techniques and digital analytical tools in hematology

Neirouz Assali[1], Salma Riahi*[1], Donia Mbarki[1], Yosra Dhaha[1], Amina Bouatay[1]

[1]*Sahloul University Hospital, Laboratory of hematology and Blood Bank, Sousse, Tunisia;*

### Background

Sepsis remains a global health challenge in emergency departments (ED), with diagnosis often delayed due to nonspecific clinical and biological features. Early risk stratification is crucial, as progression to septic shock leads to high mortality. Monocyte distribution width (MDW), is an emerging biomarker, derived from the complete blood count (CBC) analysis, that offers added value in early sepsis detection, monitoring, and assessing disease prognosis. Its rapid availability and cost-neutral integration make MDW a practical diagnostic and prognostic tool in the emergency department (ED).

### Aims

To evaluate MDW as a diagnostic tool for early sepsis detection and a prognostic tool for septic shock detection in EDs, determine the optimal cut-off for better management in our hospitals, and compare it with other biomarkers.

### Methods

This is a monocentric prospective cohort study performed at the Medical ED in collaboration with the Hematology laboratory of Sahloul University Hospital in Sousse, Tunisia, over 6 months (January-June 2024). Consenting adults consulting the ED for any reason and whose evaluation included a CBC with differential were included. The enrolled patients were stratified into three groups (sepsis , infection and control group); the sepsis group was further categorized into septic shock and non-shock patients according to sepsis-3 criteria. To determine the optimal cut-off values for MDW, we performed multiple receiver operating characteristic (ROC) analyses, including the calculation of sensitivity, specificity, likelihood ratios, and the area under the curve (AUC). Multivariate logistic regression adjusted for clinical and biological confounders was performed to assess the independent association between MDW and septic shock.

### Results

Our study included 592 patients: 253 controls, 175 with infection, and 164 with sepsis. Median MDW was higher in the sepsis group (25.5; IQR 22.5–30.7; **p < 0.001**). At a cutoff of 22.48 MDW showed a 81% sensitivity and 62% specificity while the calculated positive likelihood ratio (LR+) and negative likelihood ratio (LR-) were 2.13 and 0.31, respectively. The ROC Curve analysis showed that MDW achieved the highest diagnostic performance among the tested biomarkers in the sepsis group, with an AUC of 0.796 (95% CI: 0.736 – 0.856; **p < 0.001**). It was higher than CRP, PCT, and WBC. The sepsis group was further divided into patients with septic shock (n = 16, 9.7%) and those without septic shock (n = 148, 90.3%). MDW demonstrated significant prognostic performance in predicting septic shock. MDW levels were higher in the septic shock group (28.99, p = 0.002). ROC analysis showed an AUC of 0.701 (95% CI: 0.588–0.815; p = 0.001), concluding to a fair discriminatory ability of MDW for identifying septic shock. In the multivariate analysis, elevated MDW was an independent predictor of septic shock (adjusted OR = 1.13, 95% CI: 1.01–1.26; p = 0.032).

**Copyright Information:** (Online) ISSN: 2572-9241
©2026 The Author(s). HemaSphere is published by John Wiley & Sons Ltd on behalf of European Hematology Association. This is an open access Abstract Book distributed under the Attribution-NonCommercial-NoDerivs (CC BY-NC-ND), which allows third parties to download the articles and share them with others as long as they credit the author and the Abstract Book, but they cannot change the content in any way or use them commercially.
**Abstract Book Citations:** Authors, Title, HemaSphere, 2026;10:(S1):pages. The abstract book DOIs can be found on the "Author Information" page.
**Disclaimer:** Articles published in the journal HemaSphere exclusively reflect the opinions of the authors. The authors are responsible for all content in their abstracts including accuracy of the facts, statements, citing resources, etc.

**Summary/Conclusion**

MDW has a significant diagnostic and prognostic value for early sepsis detection and septic shock identification in the ED without additional sampling, reagents, or turnaround time, offering a practical advantage compared to traditional biomarkers. Thus, the integration of MDW into routine ED assessment may enhance early risk detection and support timely clinical decision-making.

**Copyright Information:** (Online) ISSN: 2572-9241
©2026 The Author(s). HemaSphere is published by John Wiley & Sons Ltd on behalf of European Hematology Association. This is an open access Abstract Book distributed under the Attribution-NonCommercial-NoDerivs (CC BY-NC-ND), which allows third parties to download the articles and share them with others as long as they credit the author and the Abstract Book, but they cannot change the content in any way or use them commercially.
**Abstract Book Citations:** Authors, Title, HemaSphere, 2026;10:(S1):pages. The abstract book DOIs can be found on the "Author Information" page.
**Disclaimer:** Articles published in the journal HemaSphere exclusively reflect the opinions of the authors. The authors are responsible for all content in their abstracts including accuracy of the facts, statements, citing resources, etc.

### (PB4479) BONE MARROW HEMODILUTION CALCULATOR: A TOOL FOR BONE MARROW ASPIRATE QUALITY ASSESSMENT

**Topic:** 38. Novel technologies, techniques and digital analytical tools in hematology

Tereza Podolská*[1, 2], Barbora Cabalková[1, 2], Ester Mejstrikova[1, 2], Michaela Reiterova[1, 2], Ondrej Hrusak[1, 2]

[1]*CLIP, Department of Paediatric Haematology and Oncology, Second Faculty of Medicine, Charles University, Prague, Czechia;* [2]*Department of Paediatric Haematology and Oncology, Motol and Homolka University Hospital, Prague, Czechia;*

### Background

Acute leukemia diagnostics relies heavily on the quality of bone marrow aspirates (BMA). Hemodilution, the contamination of BMA by peripheral blood (PB), is a common issue that can lead to misinterpretation of samples. The purity of BMA unaffected by chemotherapy or malignancy can be estimated by the presence of bone marrow (BM) specific cells, such as nucleated erythrocyte precursors. However, during chemotherapy, these populations may be depleted or prematurely released into PB, leading to a false interpretation of hemodilution.

### Aims

Develop a novel method for estimating bone marrow hemodilution based on quantitative differences between BMA and PB.

### Methods

We propose an alternative approach for estimating BM hemodilution that requires paired samples of BMA and PB measured at the same timepoint and analyzed by flow cytometry. Using equations describing the relationship between the degree of hemodilution and composition of cell populations in relevant fractions (BMA, PB, and undiluted BM), we apply logical constraints to define possible hemodilution intervals. We developed an interactive Shiny web application that compares up to five cell subsets between BMA and PB, calculates maximum hemodilution for each population, and predicts undiluted BM percentages of each population while considering experimental and analytical variability. The tool provides an interval estimate, which could lead to overinterpretation of upper-range values. To overcome this, we integrated interpretative guidelines to make the tool more accessible for routine use in laboratories analyzing BM specimens.

### Results

The calculator is available online at https://breakpoints.vm.cesnet.cz/calculator/ . The method was primarily designed to estimate hemodilution of MRD at day 15 of B-precursor acute lymphoblastic leukemia (BCP-ALL) treatment. At this timepoint, the probability of hemodilution is high and may influence patient risk stratification. We tested the tool using 266 paired BMA and PB samples from BCP-ALL patients taken at day 15. Using an identical flow cytometry tube to compare PB/BMA populations, we found that the BM hemodilution was below one log (<90%) in 78% of the BCP-ALL samples. We continue refining the tool by identifying consistent cell subsets that reliably differ between PB and BMA, which will improve the accuracy of hemodilution estimations. Moreover, this tool can be used at any other timepointsor in other scenarios of mixing biological fluids.

**Copyright Information:** (Online) ISSN: 2572-9241
©2026 The Author(s). HemaSphere is published by John Wiley & Sons Ltd on behalf of European Hematology Association. This is an open access Abstract Book distributed under the Attribution-NonCommercial-NoDerivs (CC BY-NC-ND), which allows third parties to download the articles and share them with others as long as they credit the author and the Abstract Book, but they cannot change the content in any way or use them commercially.
**Abstract Book Citations:** Authors, Title, HemaSphere, 2026;10:(S1):pages. The abstract book DOIs can be found on the "Author Information" page.
**Disclaimer:** Articles published in the journal HemaSphere exclusively reflect the opinions of the authors. The authors are responsible for all content in their abstracts including accuracy of the facts, statements, citing resources, etc.

**Summary/Conclusion**

This tool provides a quantitative and objective approach to estimating BM hemodilution, requiring paired BMA and PB samples measured at the same timepoint. It addresses a critical challenge in MRD monitoring. By reducing misclassification of samples, it has the potential to improve MRD evaluation.

Supported by UNCE/24/MED/003, AZV NU23-07-00220 and NU23-05-00353. Co-funded by the EU and the State Budget of Czechia OP JAC, project SALVAGE, No. CZ.02.01.01/00/22_008/0004644.

**Copyright Information:** (Online) ISSN: 2572-9241
©2026 The Author(s). HemaSphere is published by John Wiley & Sons Ltd on behalf of European Hematology Association. This is an open access Abstract Book distributed under the Attribution-NonCommercial-NoDerivs (CC BY-NC-ND), which allows third parties to download the articles and share them with others as long as they credit the author and the Abstract Book, but they cannot change the content in any way or use them commercially.
**Abstract Book Citations:** Authors, Title, HemaSphere, 2026;10:(S1):pages. The abstract book DOIs can be found on the "Author Information" page.
**Disclaimer:** Articles published in the journal HemaSphere exclusively reflect the opinions of the authors. The authors are responsible for all content in their abstracts including accuracy of the facts, statements, citing resources, etc.

### (PB4480) PEPTIDE-BASED THERANOSTICS FOR BCMA-POSITIVE MALIGNANCIES: A NOVEL STRATEGY TARGETING PLASMABLASTIC LYMPHOMA

**Topic:** 38. Novel technologies, techniques and digital analytical tools in hematology

Elisabetta Pingitore*[1], Khushboo Fatima[1], Selena Mimmi[2], Valentina Crapella[2], Anna Maria Zimbo[2], Antonio Lupia[3], Alessia Onali[3], Domenico Maisano[4], Alessandra Scagliola[5], Federica Melle[5], Maria Carmela Vegliante[5], Doriana Gramegna[5], Michele Guida[5], Sabino Ciavarella[5], Enrico Iaccino[1]

*[1]University Magna Graecia of Catanzaro, Department of Experimental and Clinical Medicine,, Catanzaro, Italy;[2]University "Magna Graecia" of Catanzaro, Department of Medical and Surgical Sciences, Catanzaro, Italy; [3]University of Cagliari, Department of Life and Enviromental Science, Cagliari, Italy; [4]Dana-Farber Cancer Institute and Harvard Medical School, Boston, United States of America; [5]Istituto Tumori Giovanni Paolo II, Bari, Italy;*

### Background

B-cell maturation antigen (BCMA, *TNFRSF17*) is an established target in multiple myeloma but remains underexploited in aggressive plasmablastic lymphomas (PBL and related DLBCL variants), which show high BCMA expression yet poor outcomes with conventional, Fc-dependent biologics due to extramedullary growth, poor vascularization and immunosuppression. Peptide ligands (≈1–5 kDa) offer rapid tissue penetration and flexible payload conjugation, potentially overcoming these limitations.

### Aims

Develop and validate a theranostic development pipeline to discover peptide-based ligands that bind native, membrane-embedded BCMA and are suitable for diagnostic and therapeutic payload delivery in BCMA-positive lymphomas, including PBL.

### Methods

We implemented a cell-based phage display selection against native BCMA on intact cells (preserving ectodomain conformation, N-linked glycosylation and membrane context) using large M13 combinatorial peptide libraries. Three iterative rounds of selection/counter-selection were performed on BCMA-positive versus induced BCMA-negative H929 cells. Lead peptides were profiled for binding specificity vs BCMA-negative controls and for biophysical features (size, cyclization/PEGylation/albumin-binding amenability, absence of Fc). In silico characterization included protein–peptide docking (HADDOCK), binding free energy estimation (PRODIGY) and three independent 200 ns MD simulations to assess complex stability. Binding was tested across a panel of BCMA-expressing multiple myeloma (H929, U266, IM9) and plasmablastic lymphoma (DB, Pfeiffer, Karpas 422) cell lines.

### Results

The cell-based pipeline yielded three BCMA-binding peptide scaffolds that recognize the native, glycosylated, membrane-anchored receptor with high specificity. These peptides retain binding despite native glycosylation and membrane anchoring, addressing limitations of ectodomain-only screens for TNF receptor family members. Their small size and lack of Fc domains make them compatible with rapid tumor accumulation/clearance and flexible conjugation for theranostics. The lead peptide, pBCMA_7 (GYAPQLDHWIAR), selected for clonal frequency and cell binding, showed a stable binding pose in a surface-exposed BCMA pocket by docking and MD, with favorable predicted free energy. pBCMA_7 bound robustly to multiple myeloma and molecularly distinct plasmablastic lymphoma models; binding correlated with BCMA expression and discriminated BCMA-high subpopulations with fidelity comparable to commercial anti-BCMA antibodies.

**Copyright Information:** (Online) ISSN: 2572-9241
©2026 The Author(s). HemaSphere is published by John Wiley & Sons Ltd on behalf of European Hematology Association. This is an open access Abstract Book distributed under the Attribution-NonCommercial-NoDerivs (CC BY-NC-ND), which allows third parties to download the articles and share them with others as long as they credit the author and the Abstract Book, but they cannot change the content in any way or use them commercially.
**Abstract Book Citations:** Authors, Title, HemaSphere, 2026;10:(S1):pages. The abstract book DOIs can be found on the "Author Information" page.
**Disclaimer:** Articles published in the journal HemaSphere exclusively reflect the opinions of the authors. The authors are responsible for all content in their abstracts including accuracy of the facts, statements, citing resources, etc.

**Summary/Conclusion**

We established a native-receptor-focused phage display platform that generates peptide ligands tailored to the structural and biological constraints of PBL and related entities. Targeting membrane-embedded, glycosylated BCMA enables discovery of high-affinity, therapeutically relevant binders for TNF receptor family members. Resulting peptide scaffolds constitute a modular theranostic toolbox for patient selection, monitoring and mechanism-independent delivery of imaging/therapeutic payloads, with potential to expand BCMA-directed strategies beyond plasma cell neoplasms to aggressive plasmablastic lymphomas.

**Copyright Information:** (Online) ISSN: 2572-9241
©2026 The Author(s). HemaSphere is published by John Wiley & Sons Ltd on behalf of European Hematology Association. This is an open access Abstract Book distributed under the Attribution-NonCommercial-NoDerivs (CC BY-NC-ND), which allows third parties to download the articles and share them with others as long as they credit the author and the Abstract Book, but they cannot change the content in any way or use them commercially.
**Abstract Book Citations:** Authors, Title, HemaSphere, 2026;10:(S1):pages. The abstract book DOIs can be found on the “Author Information” page.
**Disclaimer:** Articles published in the journal HemaSphere exclusively reflect the opinions of the authors. The authors are responsible for all content in their abstracts including accuracy of the facts, statements, citing resources, etc.

### (PB4481) AUTOMATED DEEP LEARNING-BASED BONE MARROW ANALYSIS IN PEDIATRIC LEUKEMIA

**Topic:** 38. Novel technologies, techniques and digital analytical tools in hematology

Tabita Ghete*[1], Farina Kock[2], Martina Pontones[1], David Pfrang[2], Max Westphal[2], Henning Höfener[2], Markus Metzler[1]

*[1]University Hospital Erlangen, Department of Pediatrics and Adolescent Medicine, Erlangen, Germany; [2]Fraunhofer Institute for Digital Medicine (MEVIS), Bremen, Germany;*

### Background

Morphological assessment of bone marrow aspirate (BMA) smears is crucial for detecting leukemia, and the current analogue process of classifying nucleated cells can be subjective and error-prone. In recent years, there has been a rapid growth in the use of deep learning (DL) algorithms for image recognition in the fields of hematology and oncology, which has the potential to overcome the limitations of the current approach.

### Aims

To streamline this process, we propose an automated BMA analysis pipeline, incorporating cell detection and classification, for the diagnosis prediction of pediatric acute lymphatic leukemia (ALL), acute myeloid leukemia (AML), and chronic myeloid leukemia (CML).

### Methods

BMA smears at the time of diagnosis from 246 pediatric patients with ALL, AML, or CML were digitized with 40x objective lens using a whole-slide scanner. Cells from the monolayer were annotated into 33 distinct cell classes using a custom classification tool, which generated a total of 28 000 cell class labels using a consensus approach. Additional data included 18 laboratory parameters, leukemia type and subtype, gender, and age. The dataset was divided into three sets for training, validation, and test (60%, 20%, 20% of patients, respectively). Cell detection was conducted by training CenterNet and Faster R-CNN, while for cell classification, we trained an ImageNet-pretrained ResNet-50 with modified final linear layer. The best model was chosen through the median F1-score on the validation set. To differentiate between the diagnoses of ALL, AML, and CML, machine learning models were trained with differential cell counts (DCC) generated by the cell classification pipeline or by hematologists. A further analysis incorporated the 18 laboratory parameters, and the performance of the models trained using only lab values was compared with that of the models trained with both DCC and lab values.

### Results

The models for cell detection obtained an average precision of 0.96, while those for cell classification obtained an area under the curve of 0.98 (95% CI [0.976-0.985) and an F1-score of 0.61 (95% CI [0.557-0.680]). Classes with fewer training samples tend to have lower F1-scores. Diagnostic models for leukemia prediction using predicted DCC achieved a mean F1-score of 0.90 (95% CI [0.745-0.971]). While ALL and CML were predicted accurately using only lab values, an accurate prediction of AML was achieved using only DCC. Interestingly, the combination of lab values and DCC do not improve diagnostic prediction.

**Copyright Information:** (Online) ISSN: 2572-9241
©2026 The Author(s). HemaSphere is published by John Wiley & Sons Ltd on behalf of European Hematology Association. This is an open access Abstract Book distributed under the Attribution-NonCommercial-NoDerivs (CC BY-NC-ND), which allows third parties to download the articles and share them with others as long as they credit the author and the Abstract Book, but they cannot change the content in any way or use them commercially.
**Abstract Book Citations:** Authors, Title, HemaSphere, 2026;10:(S1):pages. The abstract book DOIs can be found on the "Author Information" page.
**Disclaimer:** Articles published in the journal HemaSphere exclusively reflect the opinions of the authors. The authors are responsible for all content in their abstracts including accuracy of the facts, statements, citing resources, etc.

**Summary/Conclusion**

We present a publicly available, high-quality dataset of 246 digitized BMA smears with cell-level annotations and patient-level clinical data for leukemia classification. Our proposed pipeline shows strong potential as a decision-support tool to streamline diagnostic workflows, with future work needed to improve performance in underrepresented cell classes and validate generalizability in larger, multi-center cohorts.

**Copyright Information:** (Online) ISSN: 2572-9241
©2026 The Author(s). HemaSphere is published by John Wiley & Sons Ltd on behalf of European Hematology Association. This is an open access Abstract Book distributed under the Attribution-NonCommercial-NoDerivs (CC BY-NC-ND), which allows third parties to download the articles and share them with others as long as they credit the author and the Abstract Book, but they cannot change the content in any way or use them commercially.
**Abstract Book Citations:** Authors, Title, HemaSphere, 2026;10:(S1):pages. The abstract book DOIs can be found on the "Author Information" page.
**Disclaimer:** Articles published in the journal HemaSphere exclusively reflect the opinions of the authors. The authors are responsible for all content in their abstracts including accuracy of the facts, statements, citing resources, etc.

### (PB4482) MACHINE LEARNING–BASED DIAGNOSTIC DECISION SUPPORT FOR HEMATOLOGIC MALIGNANCIES IN FLOW CYTOMETRY

**Topic:** 38. Novel technologies, techniques and digital analytical tools in hematology

Naoko Senda*[1], Yoshikane Kikushige[2]

*[1]Hitachi, Ltd., Research & Development Group, Tokyo, Japan; [2]Kyushu University Hospital, Center for Cellular and Molecular Medicine, Fukuoka, Japan;*

### Background

Flow cytometry (FCM) is a cornerstone technique for the diagnosis of hematologic malignancies, enabling detailed immunophenotypic characterization of malignant and normal cell populations. However, interpretation of FCM data is inherently complex, requires substantial expertise, and may be influenced by inter-observer variability. With the increasing demand for flow cytometry in routine hematologic diagnostics, the analytical workload continues to rise. Therefore, the development of automated approaches to support FCM data interpretation and assist physicians in diagnostic decision-making is strongly warranted.

### Aims

We aimed to develop and validate a machine learning–based diagnostic decision support system capable of classifying hematologic malignancies, including leukemia, lymphoma, and multiple myeloma, using flow cytometry data.

### Methods

A machine learning–based diagnostic classification framework was developed using two distinct flow cytometry panels, one for leukemia and another for lymphoma and multiple myeloma. More than 500 previously acquired diagnostic flow cytometry datasets from patients with hematologic malignancies at a single tertiary care center were retrospectively analyzed for model training and validation. Flow cytometry data had been preprocessed and gated by expert hematologists as part of routine diagnostics, and models were trained on quantitative features derived from immunophenotypic cell populations identified by flow cytometry.

### Results

The proposed system achieved an overall area under the receiver operating characteristic curve (AUC) exceeding 0.80 for multi-class disease classification across leukemia, lymphoma, and multiple myeloma. In addition to single-label predictions, the system provides probability-ranked diagnostic candidates, allowing multiple high-confidence classes to be retained for differential diagnosis. This design enabled clinically meaningful diagnostic support, particularly in cases with overlapping immunophenotypic features. High classification performance was further achieved by incorporating clinically informed feature selection and hierarchical model design.

### Summary/Conclusion

This study demonstrates the feasibility of a machine learning–based diagnostic decision support system for hematologic malignancies using routine flow cytometry data. By combining robust overall classification performance with probability-ranked diagnostic outputs, the system supports differential diagnosis rather than enforcing single-label decisions. This approach highlights the potential of human-centered artificial intelligence to assist physicians and improve consistency in routine hematologic diagnostics without replacing clinical judgment.

**Copyright Information:** (Online) ISSN: 2572-9241
©2026 The Author(s). HemaSphere is published by John Wiley & Sons Ltd on behalf of European Hematology Association. This is an open access Abstract Book distributed under the Attribution-NonCommercial-NoDerivs (CC BY-NC-ND), which allows third parties to download the articles and share them with others as long as they credit the author and the Abstract Book, but they cannot change the content in any way or use them commercially.
**Abstract Book Citations:** Authors, Title, HemaSphere, 2026;10:(S1):pages. The abstract book DOIs can be found on the "Author Information" page.
**Disclaimer:** Articles published in the journal HemaSphere exclusively reflect the opinions of the authors. The authors are responsible for all content in their abstracts including accuracy of the facts, statements, citing resources, etc.

### (PB4483) ALEM-AI: AN EXPLAINABLE LOGISTIC REGRESSION–COX MODEL FOR INDIVIDUALIZED SURVIVAL PREDICTION IN ACUTE LEUKEMIA

**Topic:** 38. Novel technologies, techniques and digital analytical tools in hematology

Naveed Syed*[1], Shahrukh Hashmi[1, 2, 3], Mohamed Abu Haleeqa[4], Emadullah Raidullah[1], Imrana Afrooz[1], Afaf Almaskari[1], Azmat Khan[1], Aysheh Ismail[1], Waed Jaber[1]

*[1]Sheikh Shakbout Medical City, Hematology and Oncology, Abu Dhabi, United Arab Emirates;[2]Department of Health, Clinical Research, Abu Dhabi, United Arab Emirates; [3]Mayo Clinic, Hematology and Oncology, Rochester, United Arab Emirates;[4]Abu Dhabi Stem Cell Center, Abu Dhabi, United Arab Emirates;*

### Background

Overall survival is the key endpoint in acute leukemia, yet risk assessment remains fragmented and often lacks integrated prediction. Existing prognostic tools and many AI models are limited or "black-box," reducing clinical trust. Transparent, clinician-friendly approaches using routine data to provide individualized survival estimates are needed.

### Aims

To develop and internally validate an explainable, clinician-oriented framework that provides individualized overall survival probabilities and time-to-event risk estimates using routinely collected leukemia data

### Methods

This retrospective single-center pilot study included 114 consecutive patients with acute leukemia treated between June 2021 and May 2024. Predictors reflected routine practice and comprised demographics, baseline laboratory parameters (LDH, marrow blasts), ECOG status, leukemia subtype, post-induction MRD, induction response, date of first remission, and cytogenetic/molecular features. Common FISH fusions and recurrent NGS mutations were entered as structured variables, with rare alterations grouped.
Logistic regression and Cox models estimated mortality risk and overall survival. The platform provides real-time risk stratification, individualized survival curves, and predicted survival at 6 months, 1, 2, and 3 years. Internal validation used 1,000-sample bootstrapping, and SHAP explanations identified patient-specific risk drivers.

**Copyright Information:** (Online) ISSN: 2572-9241
©2026 The Author(s). HemaSphere is published by John Wiley & Sons Ltd on behalf of European Hematology Association. This is an open access Abstract Book distributed under the Attribution-NonCommercial-NoDerivs (CC BY-NC-ND), which allows third parties to download the articles and share them with others as long as they credit the author and the Abstract Book, but they cannot change the content in any way or use them commercially.
**Abstract Book Citations:** Authors, Title, HemaSphere, 2026;10:(S1):pages. The abstract book DOIs can be found on the "Author Information" page.
**Disclaimer:** Articles published in the journal HemaSphere exclusively reflect the opinions of the authors. The authors are responsible for all content in their abstracts including accuracy of the facts, statements, citing resources, etc.

### Results

Among 114 patients, 28 deaths occurred during follow-up. The model showed moderate discrimination (ROC AUC 0.74) with acceptable calibration (Brier 0.15). Bootstrap validation (1,000 iterations) demonstrated stable performance with consistently high specificity (~0.84), enabling reliable identification of high-risk patients, while sensitivity varied due to limited events. Wide confidence intervals reflected the small cohort without evidence of overfitting.
Survival modeling produced individualized curves and predicted survival at 6 months, 1, 2, and 3 years. Explainability analyses identified intuitive clinical drivers, including age, disease burden, MRD, induction response, LDH, and molecular risk features. The platform supports retraining on new datasets to update risk estimates and explanations, facilitating scalability and external validation.

| Metric | Mean | Median | 95% CI interval | Interpretation |
|---|---|---|---|---|
| ROC AUC | 0.743 | 0.748 | 0.575 – 0.871 | Moderate discrimination: uncertainty is wide (small dataset effect). |
| Average Precision (PR-AUC) | 0.530 | 0.517 | 0.291 – 0.794 | Moderate; strongly influenced by event prevalence. |
| Brier score | 0.145 | 0.142 | 0.087 – 0.208 | Reasonable overall probabilistic accuracy |
| Sensitivity | 0.656 | 0.667 | 0.200 – 1.000 | Highly variable; threshold dependent. |
| Specificity | 0.839 | 0.857 | 0.576 – 1.000 | Consistently high (good “rule-in” tendency). |
| F1 | 0.580 | 0.588 | 0.200 – 0.833 | Moderate, with notable spread. |
| Balanced Accuracy | 0.747 | 0.754 | 0.574 – 0.892 | Consistent with ROC AUC being moderate. |

### Summary/Conclusion

The ALEM-AI framework demonstrates the feasibility of an explainable, clinically deployable tool that integrates routine clinical and molecular data to generate personalized overall survival predictions at the bedside. Transparent feature explanations and individualized survival curves support clinician trust and decision-making. Bootstrap validation showed stable internal performance despite the small cohort. The platform supports retraining on new datasets to enhance scalability and external validation. Multicenter studies are ongoing to confirm generalizability and improve accuracy.

**Copyright Information:** (Online) ISSN: 2572-9241
©2026 The Author(s). HemaSphere is published by John Wiley & Sons Ltd on behalf of European Hematology Association. This is an open access Abstract Book distributed under the Attribution-NonCommercial-NoDerivs (CC BY-NC-ND), which allows third parties to download the articles and share them with others as long as they credit the author and the Abstract Book, but they cannot change the content in any way or use them commercially.
**Abstract Book Citations:** Authors, Title, HemaSphere, 2026;10:(S1):pages. The abstract book DOIs can be found on the “Author Information” page.
**Disclaimer:** Articles published in the journal HemaSphere exclusively reflect the opinions of the authors. The authors are responsible for all content in their abstracts including accuracy of the facts, statements, citing resources, etc.

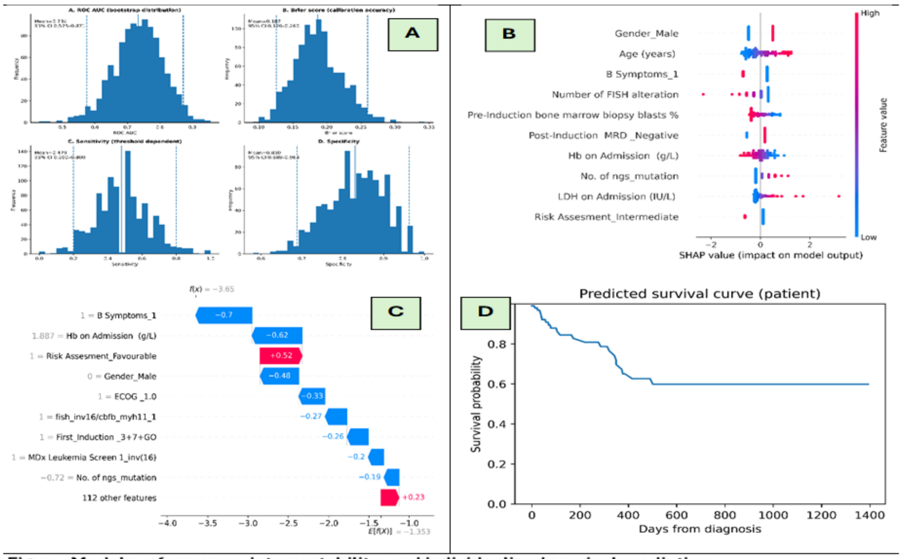

**Figure. Model performance, interpretability, and individualized survival prediction.**

**A)** Bootstrap internal validation (1,000 resamples) showing stable moderate discrimination and good calibration. **B)** Cumulative feature impact plot identifying the most important clinical and molecular predictors of survival. **C)** Instant individual patient explanation demonstrates how specific factors increase (red) or decrease (blue) predicted risk. **D)** Personalized survival curve displaying estimated probability of survival over time from diagnosis.

**Copyright Information:** (Online) ISSN: 2572-9241
©2026 The Author(s). HemaSphere is published by John Wiley & Sons Ltd on behalf of European Hematology Association. This is an open access Abstract Book distributed under the Attribution-NonCommercial-NoDerivs (CC BY-NC-ND), which allows third parties to download the articles and share them with others as long as they credit the author and the Abstract Book, but they cannot change the content in any way or use them commercially.
**Abstract Book Citations:** Authors, Title, HemaSphere, 2026;10:(S1):pages. The abstract book DOIs can be found on the “Author Information” page.
**Disclaimer:** Articles published in the journal HemaSphere exclusively reflect the opinions of the authors. The authors are responsible for all content in their abstracts including accuracy of the facts, statements, citing resources, etc.

### (PB4484) ARTIFICIAL INTELLIGENCE IN CANCER THERAPEUTICS: EVALUATING CLINICAL REASONING IN HEMATOLOGY AND ONCOLOGY PHARMACOLOGY AND PHARMACOTHERAPY

**Topic:** 38. Novel technologies, techniques and digital analytical tools in hematology

Ghaith Mansour*[1]

[1]*Alfaisal University, Riyadh, Saudi Arabia;*

### Background

Large language models (LLMs) are increasingly integrated into medical education and clinical decision support by healthcare professionals and trainees. Their rapid adoption has outpaced rigorous, domain-specific validation, particularly in high-stakes specialties such as hematology and oncology pharmacology and pharmacotherapy. Moreover, systematic comparisons between free and premium versions of widely used LLMs remain limited, leaving uncertainty regarding their relative accuracy, reliability, and suitability for advanced clinical learning.

### Aims

This study aimed to systematically evaluate and compare the clinical reasoning accuracy and reliability of multiple large language models (LLMs) in hematology and oncology pharmacology and pharmacotherapy. Specifically, it sought to assess performance differences between free and premium access tiers across multiple-choice and short-answer clinical questions, and to determine the suitability of these mdels for advanced clinical education and decision support in high-stakes oncology and hematology settings.

### Methods

A standardized assessment question bank comprising 150 English-language questions was developed, including 100 multiple-choice questions (MCQs; 50 hematology and 50 oncology) and 50 short-answer questions (SAQs) spanning both disciplines. SAQs were adapted from Tumor Board Review: Evidence-Based Case Reviews and Questions, while MCQs were derived from Cancer: Principles and Practice of Hematology and Oncology Review. MCQ responses were evaluated against authoritative textbook reference answers. SAQ responses were independently assessed by a panel of four experienced hematology–oncology clinical pharmacists using a structured Likert-scale scoring framework. Comparative analyses were conducted across AI models, access tiers, question formats, and subject domains.

### Results

Across 100 hematology and oncology MCQs, all six evaluated AI models demonstrated high overall accuracy. Paid Google Gemini 2.5 Pro achieved the highest total score (95/100), followed by paid ChatGPT-5 (93/100) and Microsoft Copilot (91/100). Performance was uniformly strong in hematology, whereas greater variability was observed in oncology, with premium models demonstrating a clear advantage. In SAQs, paid ChatGPT-5 and paid Google Gemini 2.5 Pro achieved the highest and identical mean scores (230.3/250), outperforming all standard models. Overall, premium models consistently demonstrated superior performance, particularly in oncology-focused and open-ended clinical reasoning tasks.

**Copyright Information:** (Online) ISSN: 2572-9241
©2026 The Author(s). HemaSphere is published by John Wiley & Sons Ltd on behalf of European Hematology Association. This is an open access Abstract Book distributed under the Attribution-NonCommercial-NoDerivs (CC BY-NC-ND), which allows third parties to download the articles and share them with others as long as they credit the author and the Abstract Book, but they cannot change the content in any way or use them commercially.
**Abstract Book Citations:** Authors, Title, HemaSphere, 2026;10:(S1):pages. The abstract book DOIs can be found on the “Author Information” page.
**Disclaimer:** Articles published in the journal HemaSphere exclusively reflect the opinions of the authors. The authors are responsible for all content in their abstracts including accuracy of the facts, statements, citing resources, etc.

**Summary/Conclusion**

Large language models exhibit strong performance in hematology and oncology pharmacology and pharmacotherapy; however, accuracy and reliability vary by model and access tier. Premium models provide greater consistency and depth, especially for oncology and complex reasoning tasks, highlighting both the educational potential of LLMs and the continued necessity for expert oversight in high-stakes clinical domains.

**Copyright Information:** (Online) ISSN: 2572-9241
©2026 The Author(s). HemaSphere is published by John Wiley & Sons Ltd on behalf of European Hematology Association. This is an open access Abstract Book distributed under the Attribution-NonCommercial-NoDerivs (CC BY-NC-ND), which allows third parties to download the articles and share them with others as long as they credit the author and the Abstract Book, but they cannot change the content in any way or use them commercially.
**Abstract Book Citations:** Authors, Title, HemaSphere, 2026;10:(S1):pages. The abstract book DOIs can be found on the "Author Information" page.
**Disclaimer:** Articles published in the journal HemaSphere exclusively reflect the opinions of the authors. The authors are responsible for all content in their abstracts including accuracy of the facts, statements, citing resources, etc.

### (PB4486) FROM FLAG TO RELEASE: CLINICAL VALIDATION OF ARTIFICIAL INTELLIGENCE–ASSISTED PERIPHERAL BLOOD MORPHOLOGY WITHIN AN INTEGRATED FULL BLOOD COUNT WORKFLOW

**Topic:** 38. Novel technologies, techniques and digital analytical tools in hematology

Ramlah Mohamed Ibrahim*[1, 2], Norlelawati A. Talib[1, 3], Anirawati Mohd Sahir[1], Nurul Athira Mohd Salim[1], Muhammad Faiz Mohd Ismail[4], Mohd Khairul Idzwan Othman[4]

*[1]Sultan Ahmad Shah Medical Centre @IIUM, Pathology and Laboratory Medicine, Kuantan, Malaysia;[2]Kulliyyah of Medicine, International Islamic University, Pathology, Kuantan, Malaysia; [3]Kulliyyah of Medicine, International Islamic University, Basic Science, Kuantan, Malaysia; [4]Utas Maju Sdn. Bhd., Petaling Jaya, Malaysia;*

### Background

Automated hematology analyzers generate review flags that frequently prompt peripheral blood film examination, increasing laboratory workload and delaying reporting. Artificial intelligence–assisted digital morphology has demonstrated analytical comparability with conventional microscopy. However, evidence supporting its application in consecutive real-world flagged samples within structured reporting workflows remains limited.

### Aims

This study aimed to determine whether artificial intelligence–assisted peripheral blood morphology can reliably validate flagged full blood count samples and support selective omission of routine conventional microscopy under predefined analytical criteria.

### Methods

A prospective validation was conducted on 100 consecutive flagged full blood count samples representing high white blood cell count (>20×10⁹/L), low white blood cell count (<3×10⁹/L), thrombocytopenia (<100×10⁹/L), platelet morphology flags, and analyzer review alerts. Samples were analyzed using an artificial intelligence–assisted digital morphology platform (MC-80, Mindray) and compared with dual-observer conventional microscopy with adjudication as reference. Analytical agreement was assessed using Pearson correlation and Bland–Altman analysis. Diagnostic performance was evaluated using confusion matrix metrics within a predefined risk-stratified release algorithm incorporating quantitative thresholds and morphological exclusion criteria.

### Results

Strong analytical agreement was observed for major leukocyte populations. Correlation between artificial intelligence and conventional microscopy was excellent for neutrophils (r=0.94; 95% CI 0.91–0.96) and lymphocytes (r=0.92; 95% CI 0.88–0.95), and moderate for monocytes (r=0.82; 95% CI 0.74–0.88). Minor populations showed greater variability, consistent with low absolute frequencies. Bland–Altman analysis demonstrated minimal systematic bias, with mean differences of +0.19% for neutrophils (–15.26 to +15.64), –1.52% for lymphocytes (–16.34 to +13.30), and +2.09% for monocytes (–6.02 to +10.20).
Concordance for high and low white blood cell counts and thrombocytopenia ranged from 89% to 92% when artificial intelligence classifications were compared with conventional microscopy, and agreement for platelet clumping detection exceeded 90%. When stratified according to release eligibility, overall diagnostic agreement between artificial intelligence–based reporting decisions and conventional microscopy was 90% (95% CI 83–95%), with sensitivity 88.2%, specificity 89.8%, and substantial agreement beyond chance (Cohen's kappa 0.78). Discordant cases between the two approaches accounted for fewer than 5% of samples and were primarily related to reactive differential shifts rather than overt malignant morphology.

**Copyright Information:** (Online) ISSN: 2572-9241
©2026 The Author(s). HemaSphere is published by John Wiley & Sons Ltd on behalf of European Hematology Association. This is an open access Abstract Book distributed under the Attribution-NonCommercial-NoDerivs (CC BY-NC-ND), which allows third parties to download the articles and share them with others as long as they credit the author and the Abstract Book, but they cannot change the content in any way or use them commercially.
**Abstract Book Citations:** Authors, Title, HemaSphere, 2026;10:(S1):pages. The abstract book DOIs can be found on the "Author Information" page.
**Disclaimer:** Articles published in the journal HemaSphere exclusively reflect the opinions of the authors. The authors are responsible for all content in their abstracts including accuracy of the facts, statements, citing resources, etc.

**Summary/Conclusion**

Artificial intelligence–assisted digital morphology demonstrates high analytical and categorical concordance in consecutive flagged samples. When applied within a structured risk-stratified framework, a substantial proportion of non-critical cases met predefined concordance criteria and could be safely reported without additional slide review in this cohort. These findings support integration of artificial intelligence as a complementary validation and triage tool to reduce review burden while maintaining diagnostic safety. Further evaluation in larger and diagnostically diverse populations is required before broader implementation.

**Copyright Information:** (Online) ISSN: 2572-9241
©2026 The Author(s). HemaSphere is published by John Wiley & Sons Ltd on behalf of European Hematology Association. This is an open access Abstract Book distributed under the Attribution-NonCommercial-NoDerivs (CC BY-NC-ND), which allows third parties to download the articles and share them with others as long as they credit the author and the Abstract Book, but they cannot change the content in any way or use them commercially.
**Abstract Book Citations:** Authors, Title, HemaSphere, 2026;10:(S1):pages. The abstract book DOIs can be found on the "Author Information" page.
**Disclaimer:** Articles published in the journal HemaSphere exclusively reflect the opinions of the authors. The authors are responsible for all content in their abstracts including accuracy of the facts, statements, citing resources, etc.

### (PB4488) A STREAMLINED SOMATIC VARIANT CLASSIFICATION FRAMEWORK FOR CLINICAL REPORTING IN MYELOID NEOPLASMS

**Topic:** 38. Novel technologies, techniques and digital analytical tools in hematology

Lucy Mathot[1, 2], Ahmed Al-Djaber*[1, 3], Simone Weström[1, 3, 4], Monica Hermansson[1, 3], Kristin Ayoola Gustafsson[1, 3], Anne Neddermeyer[1, 3], Panagiotis Baliakas[1, 3, 4], Tatjana Pandzic[1, 3, 4]

*[1]Uppsala University, Department of Immunology, Genetics and Pathology, Uppsala, Sweden; [2]Örebro University, Department of Clinical Genetics, Faculty of Medicine and Health, Örebro, Sweden; [3]Uppsala University Hospital, Department of Clinical Genetics, Uppsala, Sweden; [4]Science for Life Laboratory, Uppsala University, Clinical Genomics Uppsala, Uppsala, Sweden;*

### Background

Several recommendations for the classification of somatic variants in cancer have been proposed. However, none have achieved the widespread adoption seen with the ACMG guidelines in germline testing.

### Aims

To evaluate the performance of the various classification systems for somatic variants in myeloid neoplasms (MN) and present a novel scoring system, Uppsala Variant Classification (UVC). UVC was developed for rapid and reliable classification of somatic variants in MNs based on algorithms used both in the somatic and the germline context, with the addition of gene specific recommendations (Table 1).

### Methods

The UVC system was applied to classify 69 variants identified in routine clinical samples, and was compared against 3 existing guidelines: (1) the Variant Interpretation for Cancer Consortium (VICC); (2) the ComPerMed classification and; (3) the Association for Clinical Genomic Science (ACGS). The discrepancy between UVC and the other various systems was defined as: (1) minor when it did not alter the clinical decision (for example likely pathogenic (LP) versus pathogenic (P) or likely benign (LB) vs benign (B)); (2) moderate if the interpretation differed between UVC and one other system with regards to the clinical impact (P/LP versus variant of uncertain significance (VUS)), and; (3) major, if the interpretation differed between UVC and ≥2 other systems with regards to the clinical impact (P/LP versus VUS). We considered the discrepancy major even in the case of different classification between UVC and one of the other systems if the interpretation ranged from P/LP to B/LB.

### Results

UVC exhibited none or minor discrepancy in 21/69 (30%) and 24/69 (35%) variants respectively with the other systems reaching a total concordance regarding clinical impact up to 65%. A moderate discordance was observed in 21/69 (30%) of the variants, while a major discrepancy was observed in only 3/69 (4%). These three variants were *JAK2* p.R1063H, *SMC1A* p.R790Q and *CSF3R* p.Q749*. *JAK2* p.R1063H was classified as B/LB by UVC, ComPerMed and ACGS compared to LP by VICC. *SMC1A* p.R790Q was classified as P according to UVC but as VUS by all three other systems. Of note this variant is reported as P in the Cornelia de Lange syndrome. Finally, *CSF3R* p.Q749* was classified as LP by UVC and ACGS, whereas it was considered as VUS by ComPerMed and VICC. The number of VUS among these 69 variants differed significantly between UVC and ACGS compared to ComPerMed and VICC (13/69 and 14/69 vs 22/69 and 28/69 respectively, $p<0.05$) suggesting that UVC and ACGS follow similar approaches. Indeed, the total concordance between UVC and ACGS reached 88% with 61/69 (88%) variants being classified identically, while among the 8 remaining variants the discordance was minor in 5 and moderate in 3.

**Copyright Information:** (Online) ISSN: 2572-9241
©2026 The Author(s). HemaSphere is published by John Wiley & Sons Ltd on behalf of European Hematology Association. This is an open access Abstract Book distributed under the Attribution-NonCommercial-NoDerivs (CC BY-NC-ND), which allows third parties to download the articles and share them with others as long as they credit the author and the Abstract Book, but they cannot change the content in any way or use them commercially.
**Abstract Book Citations:** Authors, Title, HemaSphere, 2026;10:(S1):pages. The abstract book DOIs can be found on the "Author Information" page.
**Disclaimer:** Articles published in the journal HemaSphere exclusively reflect the opinions of the authors. The authors are responsible for all content in their abstracts including accuracy of the facts, statements, citing resources, etc.

### Summary/Conclusion

Application of different classifications systems for somatic variants may lead to discrepancies which in most cases will not be translated to different clinical action. There are however cases where major disparities can be observed depending on the system that is followed. ComPerMed and VICC are more prone to classify a variant as VUS compared to UVC and ACGS. UVC is a relatively user-friendly classification system tailored to MNs and performs similarly to ACGS.

| Criteria | Score | | | | | | |
|---|---|---|---|---|---|---|---|
| | + 2 | + 1.5 | + 1 | + 0.5 | - 0.5 | - 1 | - 3 |
| **Established P/LP germline variant**<br>Reported in ≥ 3 different families or 2 families with ≥ 6 meiosis. Interpreted as DM in HGMD. No conflicting results in ClinVar (≥ 2 stars). Expert-panel evaluation should be followed. | Variant established as P/LP germline | | | | | | |
| **Entries in databases**<br>Total no. of entries of the specific amino acid change in COSMIC and internal database for hematological neoplasms or solid cancers. For *TP53* all cancer types are considered. | ≥10 hematological or ≥50 solid cancer cases (*TP53* ≥ 50 total cases) | 5-9 hematological or 10-49 solid cancer cases (*TP53* 10-49 total cases) | | | | | |
| **Entries affecting the same amino acid in databases**<br>Total no. of entries affecting the same amino acid position in COSMIC and internal database for hematological neoplasms or solid cancers. For *TP53* all cancer types are considered. Entries with the same aa change should not be included if the criterion above is applied. | | ≥10 hematological or ≥50 solid cancer cases (*TP53* ≥ 50 total cases) | 5-9 hematological or 10-49 solid cancer cases (*TP53* 10-49 total cases) | | | | |
| **In silico prediction**<br>Revel score is used for missense variants. | | | | Revel ≥ 0.773 | Revel ≤ 0.290 | | |
| **Harmful in functional studies**<br>Well-established functional studies performed in vitro and/or in vivo. | | | | Harmful | | Not Harmful | |
| **Population database**<br>GnomAD database v4.1 (non-UKB) should be used | | | | 0% in normal population | | | Reported as homozygote and a population frequency ≥ 0.01%. |

Table 1. The Uppsala Variant Classification (UVC) scoring system. This table represents only one component of the UVC workflow and is specifically applied to missense variants. Variants are classified based on cumulative score as follows: ≥ 3 points, pathogenic (P); 2.5 points, likely pathogenic (LP); −1.5 to 2 points, variant of uncertain significance (VUS); −2 to −2.5 points, likely benign (LB); ≤ −3 points, benign (B).

**Copyright Information:** (Online) ISSN: 2572-9241
©2026 The Author(s). HemaSphere is published by John Wiley & Sons Ltd on behalf of European Hematology Association. This is an open access Abstract Book distributed under the Attribution-NonCommercial-NoDerivs (CC BY-NC-ND), which allows third parties to download the articles and share them with others as long as they credit the author and the Abstract Book, but they cannot change the content in any way or use them commercially.
**Abstract Book Citations:** Authors, Title, HemaSphere, 2026;10:(S1):pages. The abstract book DOIs can be found on the “Author Information” page.
**Disclaimer:** Articles published in the journal HemaSphere exclusively reflect the opinions of the authors. The authors are responsible for all content in their abstracts including accuracy of the facts, statements, citing resources, etc.

### (PB4489) PROGNOSTIC ROLE OF TUMOR MUTATIONAL BURDEN IN PATIENTS WITH HEMATOLOGIC MALIGNANCIES

**Topic:** 38. Novel technologies, techniques and digital analytical tools in hematology

Mariia Mikhaleva*[1], Evgenii Kunevich[2], Asiyat Radzhabova[1], Daria Chebykina[1], Alexey Kuvshinov[1], Irina Martynkevich[1], Sergey Sidorkevich[1], Sergey Voloshin[2]

*[1]Russian Research Institute Of Hematology And Transfusiology, St Petersburg, Russia;[2]Leningrad Regional Clinical Hospital, St Petersburg, Russia;*

### Background

While tumor mutational burden (TMB) serves as a unified biomarker for immunotherapy response in solid tumors, its prognostic implications for hematological malignancies remain under investigation.

### Aims

To assess the prognostic impact of TMB in patients with chronic lymphocytic leukemia (CLL), follicular lymphoma (FL), multiple myeloma (MM), and acute myeloid leukemia (AML).

### Methods

This study analyzed 155 patients, comprising 50 with CLL, 26 with FL, 40 with MM, and 39 with AML. Targeted next-generation sequencing (NGS) was performed on the NextSeq platform (Illumina) using a panel covering 118 genes. A standardized pipeline was applied for TMB calculation, filtering out benign variants and germline mutations. TMB was quantified as the number of somatic mutations per megabase (mut/Mb) of sequenced DNA using the formula: TMB = (Total Mutations per patient) / 0.603, with 0.603 Mb representing the coding region length of the panel. Statistical analysis was performed using Jamovi v.2.4.8.0 and R v.4.4.2.

**Copyright Information:** (Online) ISSN: 2572-9241
©2026 The Author(s). HemaSphere is published by John Wiley & Sons Ltd on behalf of European Hematology Association. This is an open access Abstract Book distributed under the Attribution-NonCommercial-NoDerivs (CC BY-NC-ND), which allows third parties to download the articles and share them with others as long as they credit the author and the Abstract Book, but they cannot change the content in any way or use them commercially.
**Abstract Book Citations:** Authors, Title, HemaSphere, 2026;10:(S1):pages. The abstract book DOIs can be found on the "Author Information" page.
**Disclaimer:** Articles published in the journal HemaSphere exclusively reflect the opinions of the authors. The authors are responsible for all content in their abstracts including accuracy of the facts, statements, citing resources, etc.

### Results

Distinct mutational landscapes were observed across different entities. In CLL, clinically significant genes frequently mutated included *NOTCH1* – 26.0%, *SF3B1* – 17.8%, *ATM* – 16.2%, *TP53* – 15.1%, and *BIRC3* – 5.5%. Also, mutations were found in *XPO1* – 17.8%, *EP300* – 11.0%, and *SYK* – 9.6%. The majority of variants (82.8%) exhibited a median variant allele frequency (VAF) ≥10%. For FL, mutations in epigenetic modifiers were predominant, specifically *KMT2D* – 48%, *KMT2C* – 30%, *CREBBP* – 26%, with significant co-occurrence patterns: *NOTCH2* and *FAT1* ($p$=0.0087), *CREBBP* and *EZH2* ($p$=0.0113), *IDH1* and *DEK* ($p$=0.01186). In MM, mutations were most common in *KMT2C* – 35%, *KMT2D* – 21%, and *DNMT3A* – 18%, with a limited co-mutations. The AML cohort displayed the highest mutational diversity (involving *ASXL1* – 25%, *DNMT3A* – 20%, *PTPN11* – 20%, etc.) and a high median VAF 34.1%.

Median TMB values differed significantly across groups ($p$<0.001), being highest in CLL (11.6 mut/Mb), followed by AML (8.29 mut/Mb), MM (5.81 mut/Mb), and FL (4.98 mut/Mb). Patient stratification into high/low TMB groups was performed using the 3rd quartile (Q3) and maximally selected log-rank statistics.

The most significant differences in overall survival (OS) and progression-free survival (PFS) were observed in AML patients. Using Q3 threshold (11.60 mut/Mb), patients with low TMB had a 1-year OS of 76.5%, whereas those with high TMB had 0% ( $p$=0.014). Similar trends were observed for PFS (67.4% vs 0%, $p$=0.032). Conversely, the log-rank method yielded a threshold of 9.95 mut/Mb, which did not reach statistical significance for survival outcomes in the AML group. In FL, both methods identified thresholds (7.88 and 6.63 mut/Mb) where high TMB correlated with worse 2-year OS (Q3: 80.8% vs 57.1%, $p$=0.031) and PFS (log-method: 76.9% vs 25.0%, $p$=0.011). For CLL, the log-method established a threshold of 13.27 mut/Mb, linking high TMB to a shorter time to first-line treatment (TTFT) with an HR of 2 (95% CI 1.1–4.0), $p$=0.05. No significant association between TMB and survival was observed in MM.

### Summary/Conclusion

TMB levels are notably higher in CLL and AML. A high TMB is associated with an adverse outcome in AML and FL, and is linked with a shorter TTFT in CLL. The prognostic significance of TMB in multiple myeloma remains uncertain.

**Copyright Information:** (Online) ISSN: 2572-9241
©2026 The Author(s). HemaSphere is published by John Wiley & Sons Ltd on behalf of European Hematology Association. This is an open access Abstract Book distributed under the Attribution-NonCommercial-NoDerivs (CC BY-NC-ND), which allows third parties to download the articles and share them with others as long as they credit the author and the Abstract Book, but they cannot change the content in any way or use them commercially.
**Abstract Book Citations:** Authors, Title, HemaSphere, 2026;10:(S1):pages. The abstract book DOIs can be found on the "Author Information" page.
**Disclaimer:** Articles published in the journal HemaSphere exclusively reflect the opinions of the authors. The authors are responsible for all content in their abstracts including accuracy of the facts, statements, citing resources, etc.

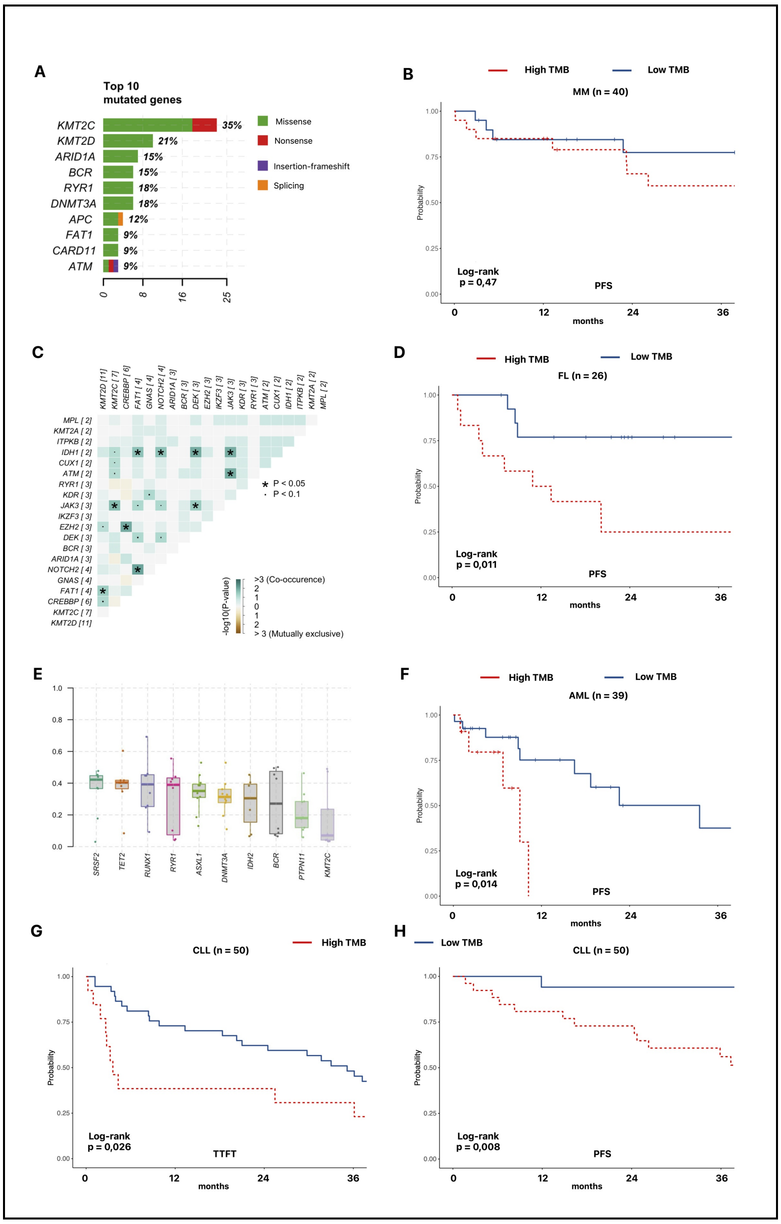

Figure 1. A) Mutation frequency of the 10 most common genes in MM and the corresponding mutation types. B) Progression-free survival of MM patients in subgroups with high and low TMB. C) Plot showing co-occurring and mutually exclusive mutations based on the results of pairwise Fisher's exact tests for each gene pair in patients with FL. Interaction values are presented as negative base-10 logarithms of the p-value. D) Progression-free survival of FL patients in subgroups with high and low TMB. E) VAF of the most frequently mutated genes in AML (horizontal line – median VAF; box boundaries – first and third quartiles of VAF distribution; whiskers – VAF outliers). F) Progression-free survival of AML patients in subgroups with high and low TMB. G) Time to first-line treatment in subgroups with high and low TMB of CLL cohort. H) Progression-free survival of CLL patients in subgroups with high and low TMB.

**Copyright Information:** (Online) ISSN: 2572-9241
©2026 The Author(s). HemaSphere is published by John Wiley & Sons Ltd on behalf of European Hematology Association. This is an open access Abstract Book distributed under the Attribution-NonCommercial-NoDerivs (CC BY-NC-ND), which allows third parties to download the articles and share them with others as long as they credit the author and the Abstract Book, but they cannot change the content in any way or use them commercially.
**Abstract Book Citations:** Authors, Title, HemaSphere, 2026;10:(S1):pages. The abstract book DOIs can be found on the “Author Information” page.
**Disclaimer:** Articles published in the journal HemaSphere exclusively reflect the opinions of the authors. The authors are responsible for all content in their abstracts including accuracy of the facts, statements, citing resources, etc.

### (PB4491) FROM GLASS SLIDE TO ALGORITHM: VALIDATING ARTIFICIAL INTELLIGENCE FOR PERIPHERAL BLOOD FILM REVIEW

**Topic:** 38. Novel technologies, techniques and digital analytical tools in hematology

Ramlah Mohamed Ibrahim*[1, 2], Hafiza Alauddin[3], Nor Farisah A. Razak2[3]

*[1]Sultan Ahmad Shah Medical Centre @IIUM, Pathology and Laboratory Medicine, Kuantan, Malaysia;[2]Kulliyyah of Medicine, International Islamic University, Pathology, Kuantan, Malaysia; [3]Universiti Kebangsaan Malaysia, Department of Pathology, Faculty of Medicine, CHERAS, Malaysia;*

### Background

Peripheral blood film (PBF) examination is crucial in haematology but is time-consuming and subject to observer variability, especially for atypical or complex cells. Artificial intelligence (AI) platforms, such as the SigTuple AI100, have been proposed to support PBF analysis by automating WBC differentials, RBC morphologies, and platelet counts, potentially improving efficiency and reproducibility.

### Aims

To evaluate the performance of SigTuple AI100 in assessing WBCs, RBCs, and platelets, using manual microscopy as the reference standard.

### Methods

A total of 100 PBFs were analyzed: 50 archived educational slides from the Royal College of Pathologists of Australasia (RCPA) and 50 routine clinical samples (20 normal, 30 abnormal). Each slide was independently reviewed by AI and manual microscopy. Agreement was assessed using correlation coefficients (r), Bland–Altman analysis, confusion matrices, and sensitivity/specificity metrics. Parameters included WBC differentials (neutrophils, lymphocytes, monocytes, eosinophils, basophils, blasts/atypical lymphoid cells), RBC morphologies (normocytes, microcytes, macrocytes, ovalocytes, elliptocytes, tear drop cells, target cells, fragmented RBCs, echinocytes), and platelet counts/morphology.

### Results

Strong correlations were observed for neutrophils (r = 0.92), lymphocytes (r = 0.89), and monocytes (r = 0.87), while eosinophils (r = 0.75), basophils (r = 0.68), and blasts (r = 0.65) showed moderate correlations. Normocytes were detected with 100% sensitivity; microcytes (78.8%) and macrocytes (22.9%) were underdetected, and tear drop/target cells were not detected. Fragmented RBCs and echinocytes showed moderate sensitivity (50%) and high specificity. Platelet counts correlated strongly with FBC analyzers (r = 0.98), with high sensitivity for normal and large/giant platelets. Platelet clumps were detected with good sensitivity (0.9) but lower specificity (0.39) due to debris misclassification.

### Summary/Conclusion

SigTuple AI100 showed strong agreement with manual microscopy for common WBCs, normocytic RBCs, and platelet counts, but sensitivity was lower for rare leukocytes, complex RBC morphologies, and platelet clumps. Expanded training datasets and refinement are needed to improve detection of less common hematologic features, supporting AI as a complementary tool in routine haematology workflows.

**Copyright Information:** (Online) ISSN: 2572-9241
©2026 The Author(s). HemaSphere is published by John Wiley & Sons Ltd on behalf of European Hematology Association. This is an open access Abstract Book distributed under the Attribution-NonCommercial-NoDerivs (CC BY-NC-ND), which allows third parties to download the articles and share them with others as long as they credit the author and the Abstract Book, but they cannot change the content in any way or use them commercially.
**Abstract Book Citations:** Authors, Title, HemaSphere, 2026;10:(S1):pages. The abstract book DOIs can be found on the “Author Information” page.
**Disclaimer:** Articles published in the journal HemaSphere exclusively reflect the opinions of the authors. The authors are responsible for all content in their abstracts including accuracy of the facts, statements, citing resources, etc.

### (PB4492) CYSTAINER: A TRANSFORMER-BASED VARIATIONAL AUTOENCODER FOR ROBUST MARKER IMPUTATION IN HIGH-PARAMETER CYTOMETRY

**Topic:** 38. Novel technologies, techniques and digital analytical tools in hematology

Konstantin Ivanov*[1], Mouhamad Al Moussawy[1], Samuli Rounioja[2], Olli Lohi[3, 4], Lars Olsen[5], Signe Modvig[5, 6], Ville Hautamäki[1], Merja Heinäniemi[1]

*[1]University of Eastern Finland, Kupio/Joensuu, Finland;[2]Fimlab Laboratories, Tampere, Finland;[3]Tampere University, Tampere, Finland;[4]Tampere University Hospital, Tampere, Finland;[5]University of Copenhagen, Copenhagen, Denmark;[6]Copenhagen University Hospital Rigshospitalet, Copenhagen, Denmark;*

### Background

High-parameter cytometry is a critical tool in hematology, but the number of markers analyzed in a single experiment remains limited, creating a need to merge different panels for unified analysis. Existing methods for marker imputation and panel merging often face challenges related to imputation quality, speed, and the ability to impute markers without having shared (backbone) markers.

### Aims

We aimed to develop a robust tool, cyStainer, to address cross-platform and cross-panel variability in surface protein profiling. The goal was to create a model capable of high-accuracy marker prediction without the need for backbone markers, achieving good performance in merging different panels for the same donors within one dataset, as well as predicting markers in out-of-distribution tasks.

### Methods

We designed cyStainer as a transformer-based variational autoencoder (VAE). The model uses a transformer encoder to model complex, non-linear relationships and co-expression patterns between markers, learned through masking operation, combined with a VAE to learn compressed latent representations of cell states. We benchmarked cyStainer against existing methods (cyCombine, cyMAE and cytoVI) using four datasets and three measurement platforms: healthy bone marrow cyTOF CytoBackBone (1 donor), COVID-19 cyTOF (105 donors), healthy bone marrow Infinity Flow (9 donors) and healthy bone marrow CITE-seq (9 donors). Performance was evaluated on held-out test data based on leave-one-out test, Pearson correlation, R2 scores, and Earth Mover's Distance (EMD) metrics.

**Copyright Information:** (Online) ISSN: 2572-9241
©2026 The Author(s). HemaSphere is published by John Wiley & Sons Ltd on behalf of European Hematology Association. This is an open access Abstract Book distributed under the Attribution-NonCommercial-NoDerivs (CC BY-NC-ND), which allows third parties to download the articles and share them with others as long as they credit the author and the Abstract Book, but they cannot change the content in any way or use them commercially.
**Abstract Book Citations:** Authors, Title, HemaSphere, 2026;10:(S1):pages. The abstract book DOIs can be found on the "Author Information" page.
**Disclaimer:** Articles published in the journal HemaSphere exclusively reflect the opinions of the authors. The authors are responsible for all content in their abstracts including accuracy of the facts, statements, citing resources, etc.

**Results**

cyStainer accurately imputes markers on the healthy bone marrow cyTOF dataset, even when backbone markers are missing in the test split but present in the training split. The model achieved median metric scores of Pearson correlation 0.43, EMD 0.22, and R2 0.19 across all markers, while other established methods failed to provide results.
On the same dataset, cyStainer was evaluated for a panel merging task, combining partially overlapping panels from the same donor by imputing missing markers. In a leave-one-out test using a master panel, cyStainer outperformed cyCombine in median Pearson correlation (0.52 vs 0.23) and R2 scores (0.25 vs -0.49), although cyCombine performed better in EMD (0.11 vs 0.24). Furthermore, on the Infinity Flow healthy bone marrow data (143 markers), cyStainer's Pearson correlation (0.61) was comparable to gradient boosting regressor (0.63) and cytoVI (0.63).
When predicting out-of-distribution missing markers using COVID-19 datasets, cyStainer achieved better Pearson correlation (0.76 vs 0.74), R2 scores (0.55 vs 0.50), and EMD (0.34 vs 0.58) compared to cyMAE across all cell types.
Finally, we demonstrated the model's cross-platform functionality. Pre-trained on the Infinity Flow dataset and tested on the protein modality of a CITE-seq dataset, cyStainer was the only method that successfully imputed markers without backbone markers, achieving a median Pearson correlation of 0.57, R2 score of -0.2, and EMD of 0.31.

**Summary/Conclusion**

CyStainer demonstrates competitive or superior performance to existing methods for marker imputation and panel merging. Its ability to impute markers without need of backbone markers makes it a valuable tool for comprehensive big data analysis in hematology, allowing for the integration of diverse datasets without losing unique marker information.

**Copyright Information:** (Online) ISSN: 2572-9241
©2026 The Author(s). HemaSphere is published by John Wiley & Sons Ltd on behalf of European Hematology Association. This is an open access Abstract Book distributed under the Attribution-NonCommercial-NoDerivs (CC BY-NC-ND), which allows third parties to download the articles and share them with others as long as they credit the author and the Abstract Book, but they cannot change the content in any way or use them commercially.
**Abstract Book Citations:** Authors, Title, HemaSphere, 2026;10:(S1):pages. The abstract book DOIs can be found on the “Author Information” page.
**Disclaimer:** Articles published in the journal HemaSphere exclusively reflect the opinions of the authors. The authors are responsible for all content in their abstracts including accuracy of the facts, statements, citing resources, etc.

### (PB4493) GENETIC ENCODING OF PER NUCLEOTIDE SINGLE BASE SUBSTITUTION PROBABILITIES FOR INDIVIDUAL PATIENTS WITH MYELOID MALIGNANCIES.

**Topic:** 38. Novel technologies, techniques and digital analytical tools in hematology

Mario Aguilar-Herrador[1, 2], Anna Poetsch*[3, 4]

*[1]TU Dresden, Dreden, Germany; [2]Helmholtz Center Dresden-Rossendorf (HZDR), Center of Advanced Systems Understanding (CASUS), Görlitz, Germany; [3]University of Cologne, Institute of Genetics, CECAD, Cologne, Germany; [4]TU Dresden, Dresden, Germany;*

### Background

Cancer mutations distribute non-randomly over genomes. To understand the genome specificity of mutagenesis, we use DNA language models to investigate how stability is encoded in the DNA.

### Methods

EAGLE-MUT (<u>E</u>fficient <u>A</u>nalysis with a <u>G</u>enome-wide <u>L</u>STM to <u>E</u>valuate <u>MUT</u>ation probabilities) learns sequence context of susceptibilities to somatic single base substitutions in individual patients. It is based on a bidirectional recurrent neural network with long short-term memory (LSTM). In 500 nt windows it calculates the mutated genome from the unmutated genome. First, the model is trained on single base substitutions pooled from multiple tumors and then fine-tuned for individual patients. From the model we can extract the probabilities for each nucleotide to have mutated *en route* to the mutational pattern we see. With a model pooled over samples from myeloid malignancies and finetuned on individual samples, we achieve about 17-fold performance over a model that takes only the trinucleotide context of mutations into consideration. Since selection has limited impact on the imputed probabilities, the model output represents a background profile of mutagenic sensitivities for each nucleotide, which is computed purely on the larger sequence context in combination with the mutations that actually happened.

### Results

The obtained profiles of mutation probability reveal very heterogenous patterns. While some mutation types follow genome elements and epigenetic marks, others show distinct hotspots that are defined by currently undescribed sequence contexts and also distinct sequence patterns that related to presumed variant calling artefacts. Relating the mutation probabilities to known driver mutations at different locations within the respective genes, we can differentiate between mutations that were selected despite relatively lower probability of mutagenesis and mutations that are enriched in specific patient subgroups due to favorable mechanisms of mutagenesis. Thus, EAGLE-MUT can also serve as a background model to infer fitness of individual clones.

### Summary/Conclusion

Taken together, EAGLE-MUT shows that heterogeneity of mutagenesis over genomes is to a large extent encoded in the sequence, a code that is interpreted context dependently and thus leads to patient specific mutation probabilities for each nucleotide in the genome.

**Copyright Information:** (Online) ISSN: 2572-9241
©2026 The Author(s). HemaSphere is published by John Wiley & Sons Ltd on behalf of European Hematology Association. This is an open access Abstract Book distributed under the Attribution-NonCommercial-NoDerivs (CC BY-NC-ND), which allows third parties to download the articles and share them with others as long as they credit the author and the Abstract Book, but they cannot change the content in any way or use them commercially.
**Abstract Book Citations:** Authors, Title, HemaSphere, 2026;10:(S1):pages. The abstract book DOIs can be found on the "Author Information" page.
**Disclaimer:** Articles published in the journal HemaSphere exclusively reflect the opinions of the authors. The authors are responsible for all content in their abstracts including accuracy of the facts, statements, citing resources, etc.

### (PB4494) INTEGRATED 5MC/5HMC MICROARRAY PLATFORM FOR BLOOD-BASED EPIGENETIC CLASSIFICATION OF LYMPHOMA

**Topic:** 38. Novel technologies, techniques and digital analytical tools in hematology

Tahir Detinis Zur*[1], Jasline Deek[1], Rita Haldar[1], Miriam Neaman[1, 2], Irit Avivi Mazza[2, 3], Karolis Šablauskas[4, 5], Efstathios Kastritis[6], Tomas Papajik[7], Eva Kriegova[8], Yael Michaeli[9], Shahar Zikrin[9], Lynne Itelson[9], Noa Gilat[9], Yuval Ebenstein[1]

*[1]Tel Aviv University, Physical Chemistry, Tel Aviv, Israel; [2]Tel Aviv Sourasky Medical Center, Department of Hematology, Tel Aviv, Israel; [3]Tel Aviv University, Faculty of Medicine, Tel Aviv, Israel; [4]Vilnius University, Institute of Data Science and Digital Technologies, Vilnius, Lithuania; [5]Vilnius University Hospital Santaros Klinikos, Hematology, Oncology and Transfusion Medicine Center, Vilnius, Lithuania; [6]National and Kapodistrian University of Athens, School of Medicine, Department of Clinical Therapeutics, Athens, Greece; [7]Palacky University and University Hospital, Department of Hemato-Oncology, Faculty of Medicine and Dentistry, Olomouc, Czechia; [8]Palacky University and University Hospital, Department of Immunology, Faculty of Medicine and Dentistry, Olomouc, Czechia; [9]JaxBio Technologies Ltd., Netanya, Israel;*

### Background

Aberrant DNA methylation (5mC) and hydroxymethylation (5hmC) are key epigenetic modifications implicated in lymphoma pathogenesis, contributing to tumor development and subtype heterogeneity. Despite their diagnostic potential, these markers are not yet integrated into routine clinical workflows, which still predominantly rely on invasive tissue biopsies. We introduce a novel microarray-based epigenetic profiling strategy optimized for peripheral blood analysis, providing a scalable and non-invasive tool for lymphoma classification. Within a large multicenter trial funded by the Horizon Cancer Mission (NCT05735704), this study focuses on lymphoma, aiming to develop a simple, peripheral blood-based tool for lymphoma detection and monitoring using epigenetic markers.

### Aims

To establish and validate an integrated 5mC/5hmC microarray platform for peripheral blood epigenetic profiling, enabling non-invasive lymphoma detection, subtype discrimination, and future clinical deployment as a cost-efficient diagnostic tool.

### Methods

We employed a new fluorescence-based epigenetic labeling method for microarray analysis. Peripheral blood samples from patients with follicular lymphoma (FL), diffuse large B-cell lymphoma (DLBCL), and healthy controls were processed. Genomic DNA was extracted and fragmented into uniform 200–300 bp segments for optimal hybridization. DNA modifications were labeled using a novel approach, including the labeling of 5mC with specific cofactors for inverse quantification and 5hmC via glucosylation and strain-promoted click chemistry for fluorophore conjugation. The labeled DNA was then hybridized onto high-density 60K CGH microarrays for optical detection. Data were processed with a custom computational platform to normalize signals, select informative loci, and perform multivariate modeling for classification. The study was conducted in accordance with institutional review board approval, and informed consent was obtained from all participants.

**Copyright Information:** (Online) ISSN: 2572-9241
©2026 The Author(s). HemaSphere is published by John Wiley & Sons Ltd on behalf of European Hematology Association. This is an open access Abstract Book distributed under the Attribution-NonCommercial-NoDerivs (CC BY-NC-ND), which allows third parties to download the articles and share them with others as long as they credit the author and the Abstract Book, but they cannot change the content in any way or use them commercially.
**Abstract Book Citations:** Authors, Title, HemaSphere, 2026;10:(S1):pages. The abstract book DOIs can be found on the "Author Information" page.
**Disclaimer:** Articles published in the journal HemaSphere exclusively reflect the opinions of the authors. The authors are responsible for all content in their abstracts including accuracy of the facts, statements, citing resources, etc.

### Results

Proof-of-concept analysis based on 5mC profiling demonstrated clear separation between lymphoma samples and healthy controls. The classifier correctly identified 15/15 DLBCL samples and 16/16 healthy controls. For FL versus healthy controls, 16/17 FL samples and 15/16 healthy controls were correctly classified. In subtype discrimination between DLBCL and FL, the model correctly identified 11/15 DLBCL samples and 16/17 FL samples. While partial overlap reflected shared epigenetic features, distinct subtype-specific epigenetic signatures were preserved. The method demonstrated high reproducibility and stability across arrays and was fully compatible with DNA extracted directly from whole blood. The modular microarray design enables conversion into single-sample units, supporting rapid clinical implementation and reduced per-sample cost.

### Summary/Conclusion

We present a scalable, blood-based epigenetic profiling platform for lymphoma classification, designed for integration into clinical workflows. This study establishes proof-of-concept for the dual-modification LymphoChip assay, offering modular, single-sample operation, cost-efficient processing, and potential for routine clinical implementation.

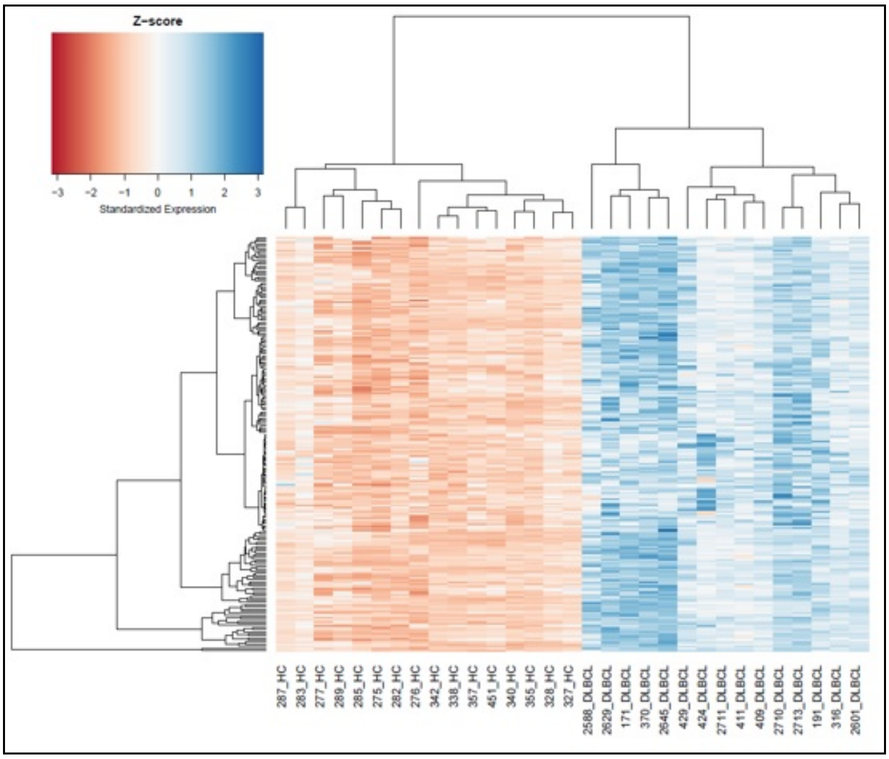

Figure 1. Heatmap showing DNA methylation levels of the 200 most differentially methylated probes in DLBCL compared to healthy whole blood samples. The legend represents Z-scores, where blue signifies higher unmodified-CpG labeling and red indicates lower unmodified-CpG labeling. Classification Performance: 100% accuracy in distinguishing DLBCL from healthy (FN = 0, FP = 0).

**Copyright Information:** (Online) ISSN: 2572-9241
©2026 The Author(s). HemaSphere is published by John Wiley & Sons Ltd on behalf of European Hematology Association. This is an open access Abstract Book distributed under the Attribution-NonCommercial-NoDerivs (CC BY-NC-ND), which allows third parties to download the articles and share them with others as long as they credit the author and the Abstract Book, but they cannot change the content in any way or use them commercially.
**Abstract Book Citations:** Authors, Title, HemaSphere, 2026;10:(S1):pages. The abstract book DOIs can be found on the "Author Information" page.
**Disclaimer:** Articles published in the journal HemaSphere exclusively reflect the opinions of the authors. The authors are responsible for all content in their abstracts including accuracy of the facts, statements, citing resources, etc.

### (PB4495) A MACHINE LEARNING MODEL INCORPORATING HBV INFECTION STATUS TO PREDICT HISTOLOGIC TRANSFORMATION IN FOLLICULAR LYMPHOMA

**Topic:** 38. Novel technologies, techniques and digital analytical tools in hematology

Xiao Luo[1], Huilin Peng[1], Xi Chen[1], Weihao Zhuang[1], Zucheng Xie[1], Yangjie Ru[2], Xiaowu Dong[2], Tao Lei[1], Jialiang Lu[2], Haiyan Yang*[3]

*[1]Zhejiang Cancer Hospital, Hangzhou, China;[2]College of Pharmaceutical Sciences, Zhejiang University, Hangzhou, China; [3]Zhejiang Cancer Hospital, Lymphoma department, Hangzhou, China;*

### Background

Follicular lymphoma (FL) is a common indolent lymphoma, however, a subset of patients experiences transformation to an aggressive lymphoma, leading to dramatically worsened prognosis. Existing prognostic models have not adequately accounted for the distinct features of hepatitis B surface antigen (HBsAg) positive FL patients in hepatitis B virus (HBV)-endemic regions.

### Aims

This study aimed to develop a machine learning model incorporating HBV infection status to predict the risk of transformation in FL patients.

### Methods

This retrospective study enrolled 357 FL patients diagnosed at Zhejiang Cancer Hospital between January 2015 and December 2023. Multidimensional data encompassing clinical, laboratory, imaging, and pathological features were collected. Feature selection was performed by identifying the intersection of features selected through both LASSO regression and the Boruta algorithm, aiming to ensure the robustness and specificity of the selected feature set. The predictive performance of eight machine learning models, including Logistic Regression, Support Vector Machine, Gradient Boosting Machine, Neural Network, XGBoost, K-Nearest Neighbors, AdaBoost, and CatBoost, was compared. Model efficacy was evaluated using the area under the receiver operating characteristic curve (AUC), decision curve analysis (DCA), and others. SHapley additive explanations (SHAP) values were employed for model interpretation. The optimal model was subsequently deployed as an online tool and used to construct a clinical scoring system.

### Results

With a median follow-up of 46.7 months, histological transformation (HT) occurred in 19 patients (5.3%). Weights were determined based on the importance of each feature, ranked from highest to lowest as follows: HBsAg, lactate dehydrogenase (LDH), neutrophil-to-lymphocyte ratio (NLR) and SUVmax. The CatBoost model demonstrated the best predictive performance in the comparative analysis, achieving an AUC of 0.939 (95% CI: 0.895–0.984) on the training set and 0.863 (95% CI: 0.660–1.000) on the validation set, thus demonstrating strong generalizability. DCA confirmed its superior clinical net benefit within the 0.0-0.4 high-risk threshold probability range compared to other models and traditional scoring systems. A novel HT scoring system based on these core features achieved an AUC of 0.723, significantly outperforming the traditional FLIPI (AUC=0.63) and FLIPI-2 (AUC=0.538). Survival analysis demonstrated that the scoring system (low: 0-3, intermediate: 4-6, high: 7-9 points) effectively stratified patients into distinct risk groups for both progression free survival and overall survival. Furthermore, an online prediction tool (https://zjcancerfl.shinyapps.io/make_web/) was established, allowing convenient calculation of individual patient HT risk by inputting the core feature values.

**Copyright Information:** (Online) ISSN: 2572-9241
©2026 The Author(s). HemaSphere is published by John Wiley & Sons Ltd on behalf of European Hematology Association. This is an open access Abstract Book distributed under the Attribution-NonCommercial-NoDerivs (CC BY-NC-ND), which allows third parties to download the articles and share them with others as long as they credit the author and the Abstract Book, but they cannot change the content in any way or use them commercially.
**Abstract Book Citations:** Authors, Title, HemaSphere, 2026;10:(S1):pages. The abstract book DOIs can be found on the "Author Information" page.
**Disclaimer:** Articles published in the journal HemaSphere exclusively reflect the opinions of the authors. The authors are responsible for all content in their abstracts including accuracy of the facts, statements, citing resources, etc.

### Summary/Conclusion

This study successfully developed and validated an interpretable machine learning model with high predictive performance for FL transformation. The derived risk scoring system and its readily applicable online tool, which integrate HBsAg status, facilitate personalized risk assessment and timely intervention, particularly in HBV-endemic areas such as China.

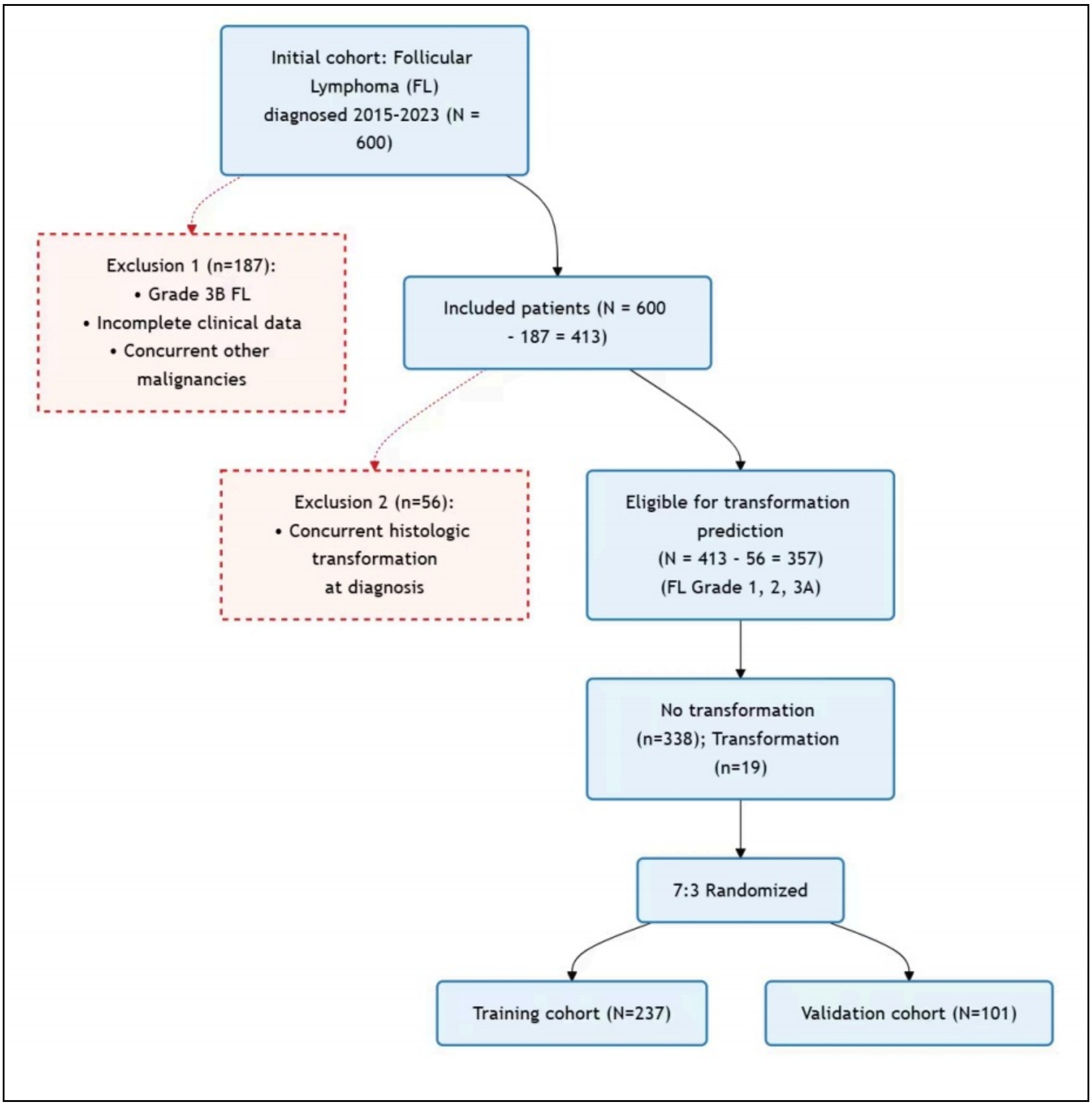

Flowchart of screening

**Copyright Information:** (Online) ISSN: 2572-9241
©2026 The Author(s). HemaSphere is published by John Wiley & Sons Ltd on behalf of European Hematology Association. This is an open access Abstract Book distributed under the Attribution-NonCommercial-NoDerivs (CC BY-NC-ND), which allows third parties to download the articles and share them with others as long as they credit the author and the Abstract Book, but they cannot change the content in any way or use them commercially.
**Abstract Book Citations:** Authors, Title, HemaSphere, 2026;10:(S1):pages. The abstract book DOIs can be found on the "Author Information" page.
**Disclaimer:** Articles published in the journal HemaSphere exclusively reflect the opinions of the authors. The authors are responsible for all content in their abstracts including accuracy of the facts, statements, citing resources, etc.

### (PB4496) ONE ASSAY, COMPLETE INSIGHT: BREAKING BOUNDARIES IN AML DIAGNOSTICS WITH AN INTEGRATED DNA-BASED NGS APPROACH FOR MOLECULAR AND CYTOGENETIC PROFILING

**Topic:** 38. Novel technologies, techniques and digital analytical tools in hematology

Alessandra Potenza*[1], Alessandra Galdiero[1], Roberta Visconti[1], Santa Errichiello[1], Angelo Zanniti[1], Fabrizio Quarantelli[1], Ciro Del Prete[1], Teresa Femiano[1], Cosimo Barbato[1], Giuseppina Esposito[1], Mariangela Capone[1], Giuliano Pennacchio[1], Veronica Pallotta[1], Rosa Farina[1], Aurora Siniscalchi[1], Pierluigi Iapicca[2], Barbara Izzo[1, 3]

*[1]Ceinge Biotecnologie Avanzate Franco Salvatore, Napoli, Italy; [2]SOPHiA GENETICS, Rolle, Switzerland; [3]University of Naples "Federico II", Department of Molecular Medicine and Medical Biotechnology, Napoli, Italy;*

### Background

According to the World Health Organization (WHO, 5th edition) and the International Consensus Classification (ICC), comprehensive genetic characterization of Acute Myeloid Leukemia (AML) is essential for accurate diagnosis, risk stratification, and treatment selection. This requires integrating molecular assays for point mutations and copy number variations (CNVs) with cytogenetic analyses to detect structural and numerical chromosomal abnormalities. However, multiple complementary tests often extend turnaround time (TAT) and increase laboratory workload.

### Aims

To evaluate a novel Next-Generation Sequencing (NGS)-based approach for comprehensive genomic profiling in a single assay, enabling simultaneous detection of point mutations and prediction of chromosomal abnormalities, thereby optimizing laboratory resources and TAT.

### Methods

A total of 29 unique samples were analyzed, including one reference and 28 AML patient samples with previously characterized genetic profiles. DNA was extracted from bone marrow or peripheral blood using the Maxwell CSC 48 system. Sequencing was performed using SOPHiA DDM™ Community Myeloid Solution v2 (MYS2) on Illumina NextSeq 550 platform. The assay incorporates UMI-based adapters to achieve high molecular sensitivity, detecting single nucleotide variants (SNVs), CNVs, internal/partial tandem duplications, and fusion exclusively from DNA (Figure 1A). The application infers also chromosomal aberrations by targeting commonly deleted/duplicated regions, clustering multiple gene-level losses/gains by chromosomes (Figure 1B). Pathogenic variants per ClinVar, COSMIC, and Franklin were analyzed. All testing was performed at the CEINGE Onco-Hematology Laboratory.

### Results

Four sequencing runs were performed, with selected replicates to assess repeatability and reproducibility. All runs generated high-quality data (29–44 million reads per run; >99% mapping rate) with robust coverage and uniformity. All expected variants in the reference sample were detected (100% sensitivity). All previously identified SNVs were confirmed in patient samples (100% concordance in repeatability testing), with no false negatives observed. Previously identified CNVs were confirmed, and additional CNVs were detected, reflecting the expanded panel design and increased sensitivity enabled by UMI-based sequencing. These additional events were consistent with cytogenetic findings, such as *Del5q, Del7q, trisomy 8 and Del17p*, including *monosomy 18*, although the latter was not fully covered by the MYS2 assay (Figure 1C). DNA-based fusion analysis in these records confirmed the expected lower sensitivity compared with RNA-based assays.

**Copyright Information:** (Online) ISSN: 2572-9241
©2026 The Author(s). HemaSphere is published by John Wiley & Sons Ltd on behalf of European Hematology Association. This is an open access Abstract Book distributed under the Attribution-NonCommercial-NoDerivs (CC BY-NC-ND), which allows third parties to download the articles and share them with others as long as they credit the author and the Abstract Book, but they cannot change the content in any way or use them commercially.
**Abstract Book Citations:** Authors, Title, HemaSphere, 2026;10:(S1):pages. The abstract book DOIs can be found on the "Author Information" page.
**Disclaimer:** Articles published in the journal HemaSphere exclusively reflect the opinions of the authors. The authors are responsible for all content in their abstracts including accuracy of the facts, statements, citing resources, etc.

### Summary/Conclusion

These preliminary results show how this NGS panel enables simultaneous detection of point mutations, CNVs, rearrangements, and selected chromosomal abnormalities in a single assay, integrating molecular and cytogenetic information for comprehensive AML characterization. An investigation on a larger cohort is ongoing. Although fusion detection sensitivity remains limited and should be complemented by RNA-based analysis, targeted CNV profiling showed strong concordance with conventional cytogenetics. Importantly, this approach complements conventional karyotyping, which remains essential for genome-wide assessment of chromosomal architecture. When properly integrated, these two approaches provide potentially novel insights into leukemic genomic landscape, refining diagnostic and biological interpretation in AML.

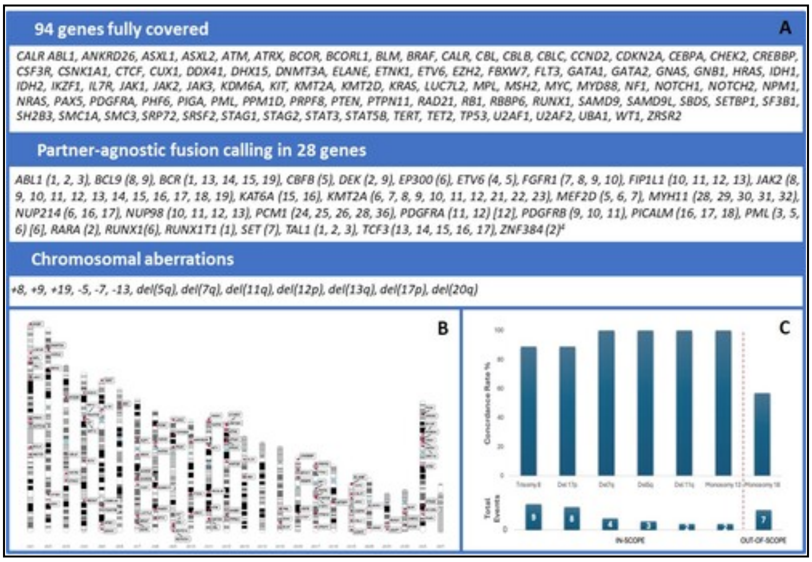

Figure 1. (A) SOPHiA DDM™ Community Myeloid Solution v2 (MYS2) Gene Panel; (B) Distribution of targeted genes across the chromosomes inferred for cytogenetic alterations; (C) Comparison study between Conventional Karyotyping analyses and Chromosomal alteration identified by the NGS assay (MYS2).

**Copyright Information:** (Online) ISSN: 2572-9241
©2026 The Author(s). HemaSphere is published by John Wiley & Sons Ltd on behalf of European Hematology Association. This is an open access Abstract Book distributed under the Attribution-NonCommercial-NoDerivs (CC BY-NC-ND), which allows third parties to download the articles and share them with others as long as they credit the author and the Abstract Book, but they cannot change the content in any way or use them commercially.
**Abstract Book Citations:** Authors, Title, HemaSphere, 2026;10:(S1):pages. The abstract book DOIs can be found on the "Author Information" page.
**Disclaimer:** Articles published in the journal HemaSphere exclusively reflect the opinions of the authors. The authors are responsible for all content in their abstracts including accuracy of the facts, statements, citing resources, etc.

### (PB4497) A TIERED WORKFLOW FOR RAPID KMT2A FUSION SCREENING AND PATIENT-SPECIFIC GENOMIC BREAKPOINT MRD ASSAY DEVELOPMENT

**Topic:** 38. Novel technologies, techniques and digital analytical tools in hematology

Minenori Eguchi-Ishimae*[1, 1], Mariko Eguchi[1]

[1]*Ehime University Graduate School of Medicine, Pediatrics, Shitsukawa, Toon, Ehime, Japan;*

### Background

Genomic DNA (gDNA) breakpoint–based measurable residual disease (MRD) monitoring can overcome limitations of RNA-based RT-qPCR, including variable transcript abundance and RNA quality. However, clinical implementation is hindered by the time and complexity required to identify patient-specific genomic fusion junctions and to establish validated MRD assays. *KMT2A*-rearranged (*KMT2A*-r) leukemias are an ideal test case because of diverse fusion partners and the need for robust, patient-specific MRD markers.

### Aims

To develop a tiered workflow that enables rapid construction of patient-specific gDNA breakpoint MRD assays within a turnaround time (TAT) compatible with clinical practice, focusing on *KMT2A* fusion–positive cases.

### Methods

We propose a frequency-informed, tiered diagnostic-to-MRD pipeline based on the *KMT2A* recombinome dataset (Meyer C., Leukemia 2023:37:988). **Tier-1** used a multiplex RT-PCR panel targeting the top 18 *KMT2A* fusion partners (*AFF1–CREBBP* including KMT2A-PTD). These top 18 events represent approximately 95% of reported *KMT2A* abnormalities, enabling rapid screening in most patients. For validation of the multiplex RT-PCR assay, we used diagnostic or relapse samples for relatively frequent fusion genes, whereas for low-frequency fusion genes we used artificial fusion constructs generated by PCR. Nested RT-PCR was incorporated to maximize sensitivity. **Tier-2** (triggered by Tier-1 positivity) defined fusion exons by RT-PCR (optional singleplex PCR/Sanger) and then attempted rapid genomic breakpoint identification using a DNA-based multiplex PCR strategy with multiple primers placed within the *KMT2A* breakpoint cluster region (BCR) and candidate intronic regions of partner genes, avoiding technically complex and time-consuming inverse PCR. **Tier-3** rescue testing for Tier-1 negative/indeterminate cases used anchored multiplex PCR (AMP)-based strategy to identify fusion partners and junctions, followed by Tier-2. The study was performed in accordance with the Declaration of Helsinki and approved by the Ehime University Institutional Review Board.

### Results

In Tier-1, nested RT-PCR enabled detection of all targeted fusions with ~$10^{-4}$ sensitivity. Multiple primer placement across *KMT2A*-BCR and partner genes accommodated exon-junction heterogeneity and enabled detection of atypical fusion transcripts. TAT for Tier-1 positive cases was consistently 1–2 days. In Tier-2, pre-defining fusion exons and narrowing candidate introns enabled genomic breakpoint identification within one week in most evaluable leukemia samples. Remaining challenges included primer/probe design for junctional amplification when the genomic junction resided within repetitive elements (e.g., Alu repeat) and the time required for formal and personalized determination of analytical sensitivity and limit of detection which contains multiple and complex procedures.

**Copyright Information:** (Online) ISSN: 2572-9241
©2026 The Author(s). HemaSphere is published by John Wiley & Sons Ltd on behalf of European Hematology Association. This is an open access Abstract Book distributed under the Attribution-NonCommercial-NoDerivs (CC BY-NC-ND), which allows third parties to download the articles and share them with others as long as they credit the author and the Abstract Book, but they cannot change the content in any way or use them commercially.
**Abstract Book Citations:** Authors, Title, HemaSphere, 2026;10:(S1):pages. The abstract book DOIs can be found on the “Author Information” page.
**Disclaimer:** Articles published in the journal HemaSphere exclusively reflect the opinions of the authors. The authors are responsible for all content in their abstracts including accuracy of the facts, statements, citing resources, etc.

**Summary/Conclusion**

This tiered strategy enables rapid *KMT2A* fusion screening and early establishment of patient-specific gDNA breakpoint MRD infrastructure with clinically feasible TAT. For Tier-1 positive cases, MRD primer/probe design within ~2 weeks appears achievable, supporting earlier deployment of gDNA-based MRD monitoring.

**Copyright Information:** (Online) ISSN: 2572-9241
©2026 The Author(s). HemaSphere is published by John Wiley & Sons Ltd on behalf of European Hematology Association. This is an open access Abstract Book distributed under the Attribution-NonCommercial-NoDerivs (CC BY-NC-ND), which allows third parties to download the articles and share them with others as long as they credit the author and the Abstract Book, but they cannot change the content in any way or use them commercially.
**Abstract Book Citations:** Authors, Title, HemaSphere, 2026;10:(S1):pages. The abstract book DOIs can be found on the "Author Information" page.
**Disclaimer:** Articles published in the journal HemaSphere exclusively reflect the opinions of the authors. The authors are responsible for all content in their abstracts including accuracy of the facts, statements, citing resources, etc.

### (PB4498) QUANTITATIVE COMPARISON OF AN AUTOMATED RT-QPCR (GENEXPERT) AND CONVENTIONAL RT-QPCR FOR NPM1 MRD DETECTION IN AML

**Topic:** 38. Novel technologies, techniques and digital analytical tools in hematology

Ing Soo Tiong*[1, 2], Michelle McBean[1], Cheryl Smith[3], Samuel Humphrey[3], Ashleigh De Klerk[4], Wendi Lin[5], Rishu Agarwal[5], Alison Louw[6], Courtney Tate[4], Clare Gould[1, 2, 4], Adam Ivey[7], Harry Iland[3]

*[1]Peter MacCallum Cancer Centre, Melbourne, Australia; [2]University of Melbourne, Melbourne, Australia; [3]Royal Prince Alfred Hospital, Camperdown, Australia; [4]Pathology Queensland, Brisbane, Australia; [5]Austin Health, Melbourne, Australia; [6]PathWest, Nedlands, Australia; [7]Alfred Hospital, Melbourne, Australia;*

### Background

Molecular measurable residual disease (MRD) testing is vital for prognosis and monitoring in *NPM1*-mutated acute myeloid leukaemia (AML). RT-qPCR, the current standard, is time-consuming and variable across labs. A closed-system, automated cartridge-based RT-qPCR assay (GeneXpert® system; GX) can significantly reduce turnaround time (~3 hours) and potentially improve interlaboratory concordance. A previous study (PMID: 39063154) evaluated the GX with the Ipsogen assay, but it was limited to derived lysates from 40 peripheral blood (PB) samples.

### Aims

We aimed to validate the *NPM1* GX assay across sample types by comparing it with conventional RT-qPCR assays for *NPM1* mutation detection, and assess interlaboratory concordance.

### Methods

Samples tested for *NPM1* mutant isoforms A, B, and D by six participating labs were included. RT-qPCR was performed using an in-house assay (Alfred Hospital; PMID: 26789727) or the Ipsogen NPM1 MutaScreen Kit (QIAGEN, Germany). Xpert® NPM1 Mutation (Cepheid, U.S.) assay was performed as per manufacturer's instructions (fresh 4 ml PB). Additional sample types assessed included bone marrow aspirate (BM, 50–600 µL) and RNA (median 750 ng, range 620–1350 ng). Results were expressed as *NPM1* mutant transcript /100% ABL1. When the level was above the limit of quantification (>500%) or below the limit of detection (0.03%) for the GX assay, manufacturer-provided lot-specific scaling and efficiency factors were used to calculate the value. This study was conducted in accordance with the Declaration of Helsinki and was approved by the Human Research Ethics Committee (PMCC No: 03/90).

**Copyright Information:** (Online) ISSN: 2572-9241
©2026 The Author(s). HemaSphere is published by John Wiley & Sons Ltd on behalf of European Hematology Association. This is an open access Abstract Book distributed under the Attribution-NonCommercial-NoDerivs (CC BY-NC-ND), which allows third parties to download the articles and share them with others as long as they credit the author and the Abstract Book, but they cannot change the content in any way or use them commercially.
**Abstract Book Citations:** Authors, Title, HemaSphere, 2026;10:(S1):pages. The abstract book DOIs can be found on the "Author Information" page.
**Disclaimer:** Articles published in the journal HemaSphere exclusively reflect the opinions of the authors. The authors are responsible for all content in their abstracts including accuracy of the facts, statements, citing resources, etc.

**Results**

A total of 491 samples were analysed using RT-qPCR and GX, comprising 416 fresh samples and 75 extracted RNA. The *NPM1* mutant isoforms included types A (n=426), B (n=38), and D (n=27).
321 fresh samples were tested with Ipsogen and GX assays (71 PB + 250 BM). Qualitative MRD results matched in 283 samples (88.2%), with 18 detected only by GX and 20 by Ipsogen. Pearson correlations were 0.954 and 0.866 for PB and BM (overall r=0.878). An MRD of 0.01% by RT-qPCR equated to 0.008% and 0.048% in PB and BM, respectively, while 0.1% equated to 0.093% and 0.322% (Figures A and B).
95 samples (41 PB + 54 BM) tested by Alfred RT-qPCR and GX assays showed 88.4% concordance, with 8 detected only by GX and 3 only by Alfred RT-qPCR. Results were highly correlated (overall r=0.941; PB r=0.96; BM r=0.94). An MRD of 0.01% and 0.1% by RT-qPCR equals 0.029% and 0.257% (Figures C and D).
75 RNA samples included 20 PB (all Ipsogen) and 55 BM (29 Ipsogen, 26 Alfred). 84% (63) were concordant; 3 detected only by Xpert and 9 only by RT-qPCR. Results correlated linearly (r=0.857): log10(Xpert) = 0.96 * log10(RT-qPCR) + 0.282.
Regarding interlaboratory concordance, 4 serial RNA samples from a patient's PB were sent to 3 labs performing the *NPM1* GX assay. RT-qPCR results were 250.4%, 0.976%, 0%, and 0.126%. The corresponding mean values (CV %) from GX were 342.5% (3.2), 1.8% (10.4), not detectable (3 out of 3), and 0.32% (9.7).

**Summary/Conclusion**

*NPM1* MRD results by GX show high concordance (84–88%) and strong linear correlation (r = 0.86–0.96) across comparisons, including fresh material and extracted RNA from both PB and BM. Interlaboratory concordance is high (CV 10% at MRD levels). Key GX MRD values equivalent to the MRD thresholds from the European LeukemiaNet (ELN 2025 recommendations (0.01% in PB, 0.1% in BM) may guide labs using the GX automated RT-qPCR assay.

**Copyright Information:** (Online) ISSN: 2572-9241
©2026 The Author(s). HemaSphere is published by John Wiley & Sons Ltd on behalf of European Hematology Association. This is an open access Abstract Book distributed under the Attribution-NonCommercial-NoDerivs (CC BY-NC-ND), which allows third parties to download the articles and share them with others as long as they credit the author and the Abstract Book, but they cannot change the content in any way or use them commercially.
**Abstract Book Citations:** Authors, Title, HemaSphere, 2026;10:(S1):pages. The abstract book DOIs can be found on the “Author Information” page.
**Disclaimer:** Articles published in the journal HemaSphere exclusively reflect the opinions of the authors. The authors are responsible for all content in their abstracts including accuracy of the facts, statements, citing resources, etc.

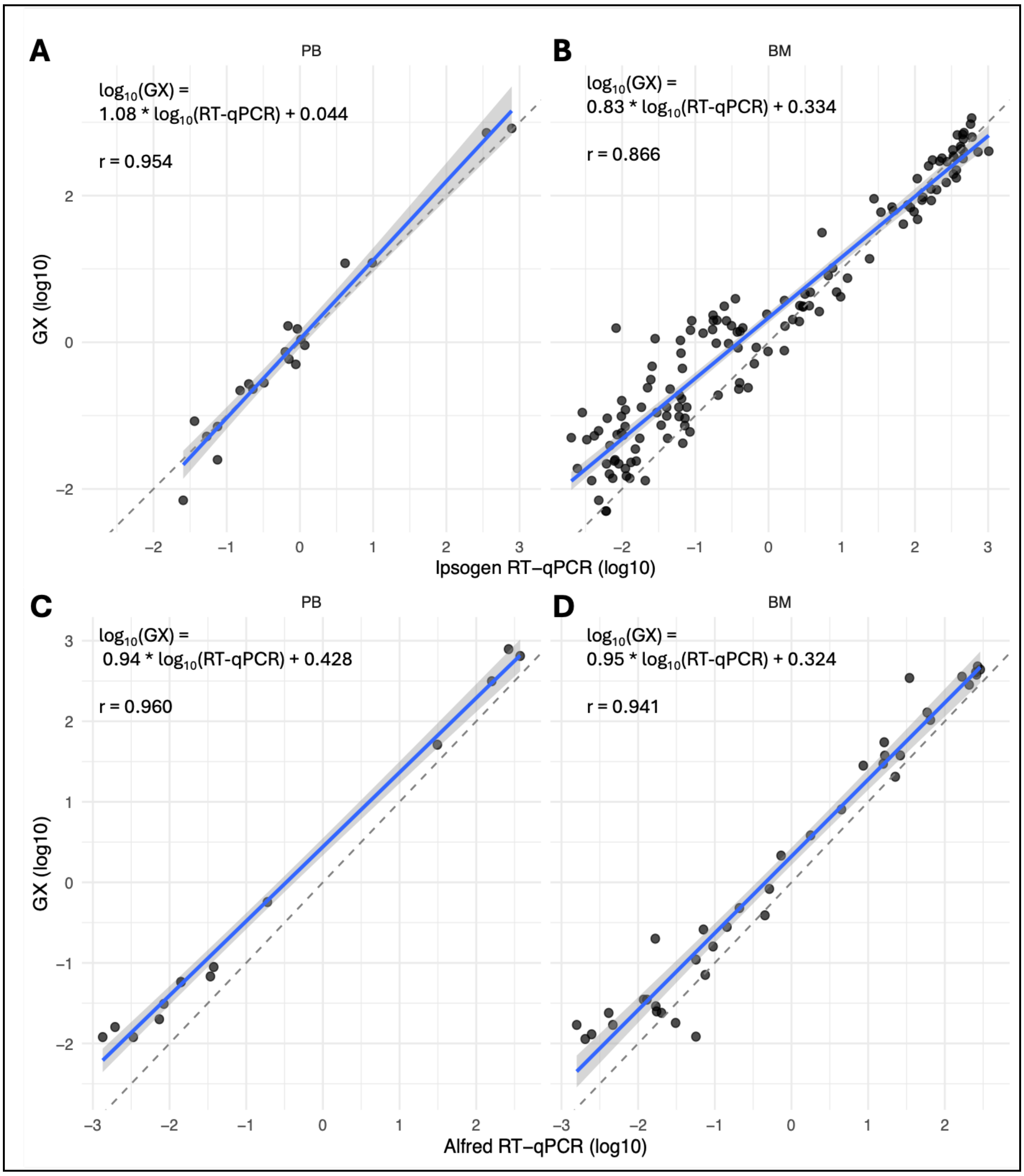

Correlation of NPM1 MRD levels between GeneXpert (GX) and RT-qPCR assays. (A+B) Comparison with the Ipsogen RT-qPCR assay. (C+D) Comparison with the in-house Alfred RT-qPCR assay.

**Copyright Information:** (Online) ISSN: 2572-9241
©2026 The Author(s). HemaSphere is published by John Wiley & Sons Ltd on behalf of European Hematology Association. This is an open access Abstract Book distributed under the Attribution-NonCommercial-NoDerivs (CC BY-NC-ND), which allows third parties to download the articles and share them with others as long as they credit the author and the Abstract Book, but they cannot change the content in any way or use them commercially.
**Abstract Book Citations:** Authors, Title, HemaSphere, 2026;10:(S1):pages. The abstract book DOIs can be found on the “Author Information” page.
**Disclaimer:** Articles published in the journal HemaSphere exclusively reflect the opinions of the authors. The authors are responsible for all content in their abstracts including accuracy of the facts, statements, citing resources, etc.

### (PB4499) PRODUCTION-SCALE ENGINEERING OF A CLOUD-NATIVE, DOMAIN-ADAPTED GENOMIC DATA COMMONS FOR PRECISION HEMATOLOGY IN MULTIPLE MYELOMA

**Topic:** 38. Novel technologies, techniques and digital analytical tools in hematology

Jessica Schulman[1], Craig Barnes[2], Rita Mormando[1], Jon Rodiger[1], Andrew Prokhorenkov[2], Sara Volk de Garcia[2], Edward Malinowski[2], Edgar Sioson[3], Alex Acic[3], Arianna Haynes[1], Jordan Miller[4], Brendan Coli[4], Arif Khan[4], Steven Foltz[1], Chaitanya Acharya[1], Jeff Knight[2], Michael Andreini[1], Michael Fitzsimons[2], George Mulligan[1], Xin Zhou[3], Robert Grossman[2], Alexander Gout*[1]

*[1]Multiple Myeloma Research Foundation, Norwalk, United States of America;[2]Center for Translational Data Science, The University of Chicago, Chicago, United States of America; [3]St. Jude Children's Research Hospital, Memphis, United States of America; [4]Amazon Web Services, Seattle, United States of America;*

### Background

Medical centers and foundations, including MMRF, invest in large-scale clinical genomic datasets to characterize patient biology and prognosis to improve outcomes. However, multi-omic data utilization is often limited by restricted access and fragmentation, constraining interoperable reuse and reproducible computational analysis. Gen3 provides a modular open-source framework for building data commons, supporting disease-focused translational research via domain-specific schemas, specialized indexing, and integrated governance. We report the engineering and production deployment of a cloud-native multiple myeloma (MM) data ecosystem (virtuallab.themmrf.org) extending core Gen3 services and aligning with the NCI Genomic Data Commons (GDC) user experience while introducing scalable, disease-adapted computational architecture.

### Aims

To architect and operationalize a production-scale MM data commons by 1) extending Gen3 metadata model with a harmonized MM-specific GDC data dictionary compatible graph schema 2) implementing structured linkage of patients, therapies, outcomes, biospecimens, and multi-omic data 3) developing a customized mutation indexing and transformation pipeline enabling real-time variant-level querying 4) deploying scalable cloud-native infrastructure with API-driven interoperability 5) integrating governed role-based access control with reproducible analytical workspaces.

### Methods

The system is deployed on AWS using Elastic Kubernetes Service with raw/processed omic data stored in AWS S3. Harmonized metadata is modeled in PostgreSQL using a MM-specific graph schema extending the GDC data model. Using Gen3 metadata services, Elasticsearch-backed faceted search and GraphQL APIs enable low-latency cohort and attribute queries across clinical/molecular entities. A domain-specific mutation indexing pipeline transforms variant/omic-derived metadata into structured Elasticsearch indices optimized for multi-omic querying across WGS, WES, RNAseq, scRNAseq, ATACseq, and WGBS assays. A unified schema links 1500 patients across SMM/NDMM/RRMM with clinical and outcome attributes. Governance via Cognito and Fence role-based access control is integrated with Gen3 workspace services for secure, reproducible analysis within controlled-access environments.

**Copyright Information:** (Online) ISSN: 2572-9241
©2026 The Author(s). HemaSphere is published by John Wiley & Sons Ltd on behalf of European Hematology Association. This is an open access Abstract Book distributed under the Attribution-NonCommercial-NoDerivs (CC BY-NC-ND), which allows third parties to download the articles and share them with others as long as they credit the author and the Abstract Book, but they cannot change the content in any way or use them commercially.
**Abstract Book Citations:** Authors, Title, HemaSphere, 2026;10:(S1):pages. The abstract book DOIs can be found on the "Author Information" page.
**Disclaimer:** Articles published in the journal HemaSphere exclusively reflect the opinions of the authors. The authors are responsible for all content in their abstracts including accuracy of the facts, statements, citing resources, etc.

**Results**

The infrastructure hosts three harmonized multi-institutional cohort datasets, publication data, and is built for clinical trial integration. Over 500 active users access API-mediated query services, customized clinico-genomic visualizations, and governed analytical workspaces. The Gen3 microservice architecture, MM-specific schema, and mutation-level indexing provide scalable performance across >200 TB of integrated data. The platform supports variant-level query execution across tens of millions of indexed variants and reproducible re-execution of previously reported MM analyses within controlled-access workspaces.

**Summary/Conclusion**

This work demonstrates that a modular open-source data commons framework for MM can be engineered into a production-scale, domain-adapted computational infrastructure for precision hematology. The schema-driven architecture is adaptable to other heme malignancies without core service modification. By integrating graph-based metadata modeling, customized indexing pipelines, API-driven search, governed access control, and elastic cloud-native compute, this implementation provides a transferable blueprint for scalable digital ecosystems supporting collaborative translational and clinical research

**Copyright Information:** (Online) ISSN: 2572-9241
©2026 The Author(s). HemaSphere is published by John Wiley & Sons Ltd on behalf of European Hematology Association. This is an open access Abstract Book distributed under the Attribution-NonCommercial-NoDerivs (CC BY-NC-ND), which allows third parties to download the articles and share them with others as long as they credit the author and the Abstract Book, but they cannot change the content in any way or use them commercially.
**Abstract Book Citations:** Authors, Title, HemaSphere, 2026;10:(S1):pages. The abstract book DOIs can be found on the “Author Information” page.
**Disclaimer:** Articles published in the journal HemaSphere exclusively reflect the opinions of the authors. The authors are responsible for all content in their abstracts including accuracy of the facts, statements, citing resources, etc.

### (PB4500) APPLICATION OF UMAP-BASED DIMENSIONALITY REDUCTION AND MACHINE LEARNING FOR AUTOMATED BONE MARROW IMMUNOPHENOTYPING IN LYMPHOPROLIFERATIVE DISORDERS

**Topic:** 38. Novel technologies, techniques and digital analytical tools in hematology

Vsevolod Misyurin*[1, 2, 3], Anastasiya Yuzhakova[3, 4], Alexey Kopylov[3, 5]

*[1]Karolinska Institutet, CMB, Stockholm, Sweden;[2]N.I. Vavilov Institute of General Genetics, Moscow, Russia;[3]GeneTechnology LLC, Moscow, Russia; [4]Morozov Children's City Clinical Hospital of the Moscow City Department of Healthcare, Moscow, Russia; [5]National Research Nuclear University MEPhI (Moscow Engineering Physics Institute), Moscow, Russia;*

### Background

Routine diagnosis of chronic lymphoproliferative disorders (LPDs) and multiple myeloma (MM) relies on flow cytometry, yet detection of rare malignant events remains challenging. Machine learning methods, particularly dimensionality reduction algorithms, are increasingly applied to flow cytometry data, enabling transformation of high-dimensional fluorescence measurements into an interpretable two-dimensional space. Unlike conventional pairwise scatter plots, these approaches allow simultaneous visualization of all events based on the full marker panel. As a result, major bone marrow populations form distinct clusters, while pathological populations—including MM cells, B-cell chronic lymphocytic leukemia (B-CLL), and other LPD-associated cells—appear as separate clusters. UMAP is especially suitable for this task, as it preserves topological relationships and supports projection of new samples.

### Aims

To develop and evaluate a method for flow cytometry data analysis based on UMAP transformation and a CatBoost classifier for automated identification of cellular populations in patients with LPDs and MM.

### Methods

The study included 925 bone marrow samples representing B-CLL, marginal zone lymphoma, hairy cell leukemia, mantle cell lymphoma, reactive and non-LPD cases, as well as 68 samples with confirmed MM and 143 without MM. Multicolor panels included lineage, maturation, and clonality markers. Data were transformed using UMAP, and manually annotated regions on the projection were used to quantify cellular populations. These features served as input for a CatBoost classifier trained with stratified cross-validation and evaluated on a held-out test set. The workflow included normalization, outlier removal, model training in Python, and application to independent samples. Abnormal cells in test samples projected to regions corresponding to malignant clusters defined in the training set.

### Results

Application of the proposed method enabled identification of MM cells and LPD-associated cells in bone marrow samples. On the UMAP projection, test samples containing malignant cells mapped to the same region as the corresponding clusters formed in the training dataset. Events corresponding to dead cells or cellular aggregates that typically interfere with routine analysis formed an independent cluster and did not affect quantification of abnormal cells. Thus, the algorithm successfully isolates rare events corresponding to malignant cells by forming a distinct cluster. This approach addresses the issue of contamination of the analyzed population by unwanted events, thereby significantly improving diagnostic accuracy. Training of the CatBoost classifier on UMAP-transformed data yielded a mean cross-validation accuracy of 0.71 and a test set accuracy of 0.69. In 37% of cases, the model generated high-confidence predictions ($p > 0.95$), achieving an accuracy exceeding 94% within this subgroup. Overall, the use of UMAP transformation provides a foundation for automated detection and classification of cellular populations, highlighting the potential of such algorithms for clinical diagnostics.

**Copyright Information:** (Online) ISSN: 2572-9241
©2026 The Author(s). HemaSphere is published by John Wiley & Sons Ltd on behalf of European Hematology Association. This is an open access Abstract Book distributed under the Attribution-NonCommercial-NoDerivs (CC BY-NC-ND), which allows third parties to download the articles and share them with others as long as they credit the author and the Abstract Book, but they cannot change the content in any way or use them commercially.
**Abstract Book Citations:** Authors, Title, HemaSphere, 2026;10:(S1):pages. The abstract book DOIs can be found on the “Author Information” page.
**Disclaimer:** Articles published in the journal HemaSphere exclusively reflect the opinions of the authors. The authors are responsible for all content in their abstracts including accuracy of the facts, statements, citing resources, etc.

**Summary/Conclusion**

The proposed approach combining UMAP transformation and a CatBoost classifier demonstrated the feasibility of reliable automated identification of bone marrow cellular populations. Notably, in approximately one-third of cases, high-confidence and highly accurate predictions were achieved, supporting the potential for further automation of diagnostics in lymphoproliferative disorders.

**Copyright Information:** (Online) ISSN: 2572-9241
©2026 The Author(s). HemaSphere is published by John Wiley & Sons Ltd on behalf of European Hematology Association. This is an open access Abstract Book distributed under the Attribution-NonCommercial-NoDerivs (CC BY-NC-ND), which allows third parties to download the articles and share them with others as long as they credit the author and the Abstract Book, but they cannot change the content in any way or use them commercially.
**Abstract Book Citations:** Authors, Title, HemaSphere, 2026;10:(S1):pages. The abstract book DOIs can be found on the "Author Information" page.
**Disclaimer:** Articles published in the journal HemaSphere exclusively reflect the opinions of the authors. The authors are responsible for all content in their abstracts including accuracy of the facts, statements, citing resources, etc.

### (PB4501) ANALYTICAL AND MORPHOLOGICAL PERFORMANCE OF A DIGITAL IMAGE-BASED MORPHOLOGY ANALYZER VERSUS A CONVENTIONAL FLOW-CYTOMETRY-BASED AUTOMATED HEMATOLOGY ANALYZER

**Topic:** 38. Novel technologies, techniques and digital analytical tools in hematology

Jiyeon Kim*[1], Young-Uk Cho[1]

[1]*Asan Medical Center, University of Ulsan College of Medicine, Department of Laboratory Medicine, Seoul, South Korea;*

### Background

Conventional flow-cytometry-based automated hematology analyzers (automated analyzers) often trigger manual smear reviews for abnormal samples. Digital morphology analyzers (digital analyzers) integrating CBC and morphological analysis may reduce this burden, but real-world validation data remain limited.

### Aims

To evaluate the CBC accuracy, WBC differential agreement, and blast screening performance of a digital analyzer versus a conventional automated analyzer and expert manual review.

### Methods

We analyzed 417 consecutive routine CBC samples, including 92 abnormal cases (blasts or left shifts). The digital analyzer (miLab BCM) integrates a fully automated workflow (smear preparation, staining, and AI-driven imaging). It employs a dual-imaging strategy: major RBC parameters are derived from unstained images, while WBC and PLT data are extracted from stained images, enabling simultaneous quantitative CBC and morphological classification. WBC differentials were compared against the automated analyzer (Sysmex XN-series, n=417) and a subset of expert manual reviews (n=361). Blast detection was evaluated in 72 samples using expert microscopy as the reference.

**Copyright Information:** (Online) ISSN: 2572-9241
©2026 The Author(s). HemaSphere is published by John Wiley & Sons Ltd on behalf of European Hematology Association. This is an open access Abstract Book distributed under the Attribution-NonCommercial-NoDerivs (CC BY-NC-ND), which allows third parties to download the articles and share them with others as long as they credit the author and the Abstract Book, but they cannot change the content in any way or use them commercially.
**Abstract Book Citations:** Authors, Title, HemaSphere, 2026;10:(S1):pages. The abstract book DOIs can be found on the "Author Information" page.
**Disclaimer:** Articles published in the journal HemaSphere exclusively reflect the opinions of the authors. The authors are responsible for all content in their abstracts including accuracy of the facts, statements, citing resources, etc.

### Results

The digital analyzer showed high correlation with the automated analyzer for major CBC parameters (r > 0.97) with small mean biases (Table 1). For WBC differentials, agreement with expert morphology was higher for lymphocytes (r=0.9372 vs. 0.8965) and monocytes (r=0.8911 vs. 0.8603) than with the automated analyzer alone (Figure 1A). The higher concordance for lymphocytes and monocytes with manual review suggests that the digital analyzer's AI-driven morphological classification aligns more closely with expert visual identification than traditional flow-cytometry-based flagging. In blast detection (n=72), correlation was high (r=0.9205), despite quantitative underestimation (slope 0.54) (Figure 1B). Sensitivity, specificity, and NPV were 88%, 80%, and 96%, respectively, supporting its role in ruling out blast-negative samples. Performance for basophils and immature granulocytes was lower.

**Table 1.** Correlation of key CBC and WBC differential parameters

| Parameter | Sample size | Correlation (r) | Slope (95% CI) | Intercept | Mean Bias |
|---|---|---|---|---|---|
| RBC (106/μL) | 417 | 0.9746 | 0.9847 (0.9622–1.0075) | 0.1210 | 0.0616 |
| WBC (103/μL) | 395 | 0.9852 | 0.9533 (0.9231–0.9952) | 0.5473 | 0.6539 |
| PLT (103/μL) | 395 | 0.9718 | 0.9476 (0.9241–0.9725) | 2.3516 | -9.5544 |
| Hgb (g/dL) | 417 | 0.9768 | 1.0000 (1.0000–1.0270) | 0.1000 | 0.1847 |
| Hct (%) | 417 | 0.9808 | 1.0000 (0.9783–1.0140) | 0.3000 | 0.2468 |
| RDW-CV (%) | 382 | 0.8881 | 0.8081 (0.7619–0.8621) | 2.6556 | -0.2145 |
| Neutrophil | 361 | 0.9531 | 1.0063 (0.9776–1.0343) | 3.5127 | 3.8789 |
| Lymphocyte | 361 | 0.8965 | 0.9833 (0.9557–1.0117) | -2.3717 | -2.7138 |
| Monocyte | 361 | 0.8603 | 1.0122 (0.9597–1.0870) | -1.2696 | -2.0029 |
| Eosinophil | 361 | 0.9483 | 1.0006 (0.9615–1.0811) | -0.1007 | -0.1356 |
| Basophil | 228 | 0.6574 | 2.4429 (1.6667–2.7500) | -0.3879 | 0.1916 |

### Summary/Conclusion

The digital analyzer provides high analytical agreement for CBC parameters and improved concordance with expert morphology for key WBC subsets. Despite quantitative blast underestimation, its high sensitivity and NPV support its role as a robust screening tool. miLab's PoCT-based integration of automated smear and dual-imaging creates a diagnostic synergy with conventional analyzers, serving as an efficient verification layer that optimizes workflow efficiency while maintaining diagnostic safety.

**Copyright Information:** (Online) ISSN: 2572-9241
©2026 The Author(s). HemaSphere is published by John Wiley & Sons Ltd on behalf of European Hematology Association. This is an open access Abstract Book distributed under the Attribution-NonCommercial-NoDerivs (CC BY-NC-ND), which allows third parties to download the articles and share them with others as long as they credit the author and the Abstract Book, but they cannot change the content in any way or use them commercially.
**Abstract Book Citations:** Authors, Title, HemaSphere, 2026;10:(S1):pages. The abstract book DOIs can be found on the "Author Information" page.
**Disclaimer:** Articles published in the journal HemaSphere exclusively reflect the opinions of the authors. The authors are responsible for all content in their abstracts including accuracy of the facts, statements, citing resources, etc.

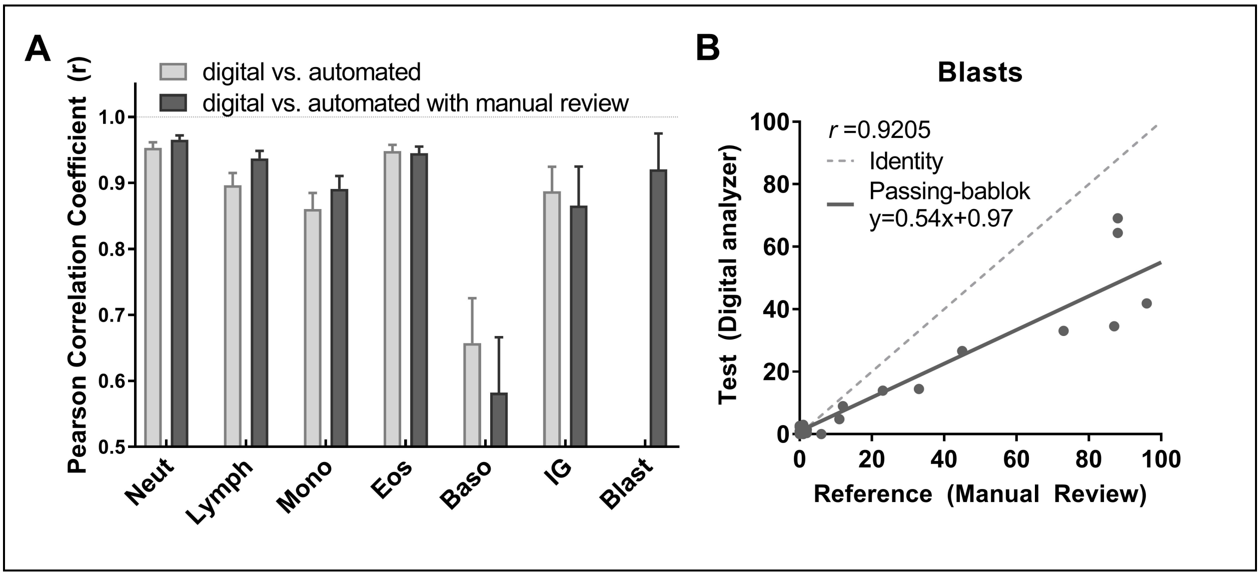

Figure 1. WBC differential correlation (r) and blast detection performance of the digital analyzer. Error bars: 95% CI. The digital analyzer showed improved lymphocyte and monocyte concordance with expert manual review compared to the automated analyzer alone.

**Copyright Information:** (Online) ISSN: 2572-9241
©2026 The Author(s). HemaSphere is published by John Wiley & Sons Ltd on behalf of European Hematology Association. This is an open access Abstract Book distributed under the Attribution-NonCommercial-NoDerivs (CC BY-NC-ND), which allows third parties to download the articles and share them with others as long as they credit the author and the Abstract Book, but they cannot change the content in any way or use them commercially.
**Abstract Book Citations:** Authors, Title, HemaSphere, 2026;10:(S1):pages. The abstract book DOIs can be found on the "Author Information" page.
**Disclaimer:** Articles published in the journal HemaSphere exclusively reflect the opinions of the authors. The authors are responsible for all content in their abstracts including accuracy of the facts, statements, citing resources, etc.

### (PB4502) DIAGNOSTIC TEST ACCURACY OF SMARTPHONE-BASED ARTIFICIAL INTELLIGENCE FOR THE SCREENING OF HEMOGLOBINOPATHIES: A SYSTEMATIC REVIEW AND META-ANALYSIS

**Topic:** 38. Novel technologies, techniques and digital analytical tools in hematology

Rahul Falodia*[1]

[1]*All India Institute of Medical Sciences, Jodhpur, India;*

### Background

Hemoglobinopathies, including Sickle Cell Disease (SCD) and Thalassemia, present a profound global health burden. In resource-limited, high-volume regions, early carrier detection and diagnosis are critically hindered by the cost and infrastructure requirements of traditional benchtop microscopy and high-performance liquid chromatography. Artificial intelligence (AI) combined with smartphone technology offers a universally accessible point-of-care (POC) alternative. However, the definitive diagnostic test accuracy (DTA) of strictly smartphone-based modalities remains unquantified across distinct hardware and software architectures.

### Aims

To evaluate the aggregate diagnostic accuracy, risk of bias, and certainty of evidence of smartphone-based AI modalities, comparing hardware-attached optical modules against non-invasive, software-only applications, for the screening of SCD and Thalassemia.

### Methods

A PRISMA-DTA compliant systematic review and meta-analysis was conducted. Literature from inception to February 2026 was screened for studies reporting explicit diagnostic contingency data for strictly smartphone-based hemoglobinopathy screening. A generalized linear mixed model (GLMM) via the Reitsma bivariate approach was utilized to pool sensitivity, specificity, and Area Under the Curve (AUC). Methodological quality was appraised via QUADAS-2, and evidence certainty via the GRADE approach. Subgroup analyses evaluated hardware-attached smartphone microscopes versus native, non-invasive smartphone AI applications.

### Results

Out of 6 systematically identified primary modalities strictly targeting SCD and Thalassemia, 4 validation cohorts provided fully extractable quantitative contingency data. The overall pooled sensitivity was 86.3% (95% CI: 76.5-92.4%) and pooled specificity was 85.0% (95% CI: 77.8-90.1%), with a summary AUC of 0.93. Subgroup GLMM analysis revealed a distinct diagnostic dichotomy. Hardware-attached smartphone microscopes (e.g., low-cost 3D-printed optical modules) achieved elite diagnostic non-inferiority to laboratory benchtop microscopes, yielding sensitivities >96% and specificities >98% (AUC >0.99). Conversely, software-only, non-invasive modalities (analyzing fingernail pallor or using optoelectronic analysis) demonstrated exceptional utility as initial screening filters- reaching 100% sensitivity in high-risk pediatric cohorts (<5 years)- but with lower specificity (~64-76%). QUADAS-2 appraisal indicated a low risk of bias across the index test domains, and the GRADE framework established a moderate-to-high certainty of evidence for POC deployment.

**Copyright Information:** (Online) ISSN: 2572-9241
©2026 The Author(s). HemaSphere is published by John Wiley & Sons Ltd on behalf of European Hematology Association. This is an open access Abstract Book distributed under the Attribution-NonCommercial-NoDerivs (CC BY-NC-ND), which allows third parties to download the articles and share them with others as long as they credit the author and the Abstract Book, but they cannot change the content in any way or use them commercially.
**Abstract Book Citations:** Authors, Title, HemaSphere, 2026;10:(S1):pages. The abstract book DOIs can be found on the "Author Information" page.
**Disclaimer:** Articles published in the journal HemaSphere exclusively reflect the opinions of the authors. The authors are responsible for all content in their abstracts including accuracy of the facts, statements, citing resources, etc.

**Summary/Conclusion**

Smartphone-based AI diagnostics provide a highly rigorous, cost-effective screening mechanism for hemoglobinopathies. While low-cost smartphone-attached microscopes deliver definitive, benchtop-equivalent diagnostic accuracy, zero-cost non-invasive AI applications offer unprecedented, highly sensitive "rule-out" triage capabilities. By definitively quantifying this performance, this meta-analysis justifies the immediate funding and deployment of prospective implementation trials in high-volume, resource-constrained clinical settings to democratize hemoglobinopathy screening globally.

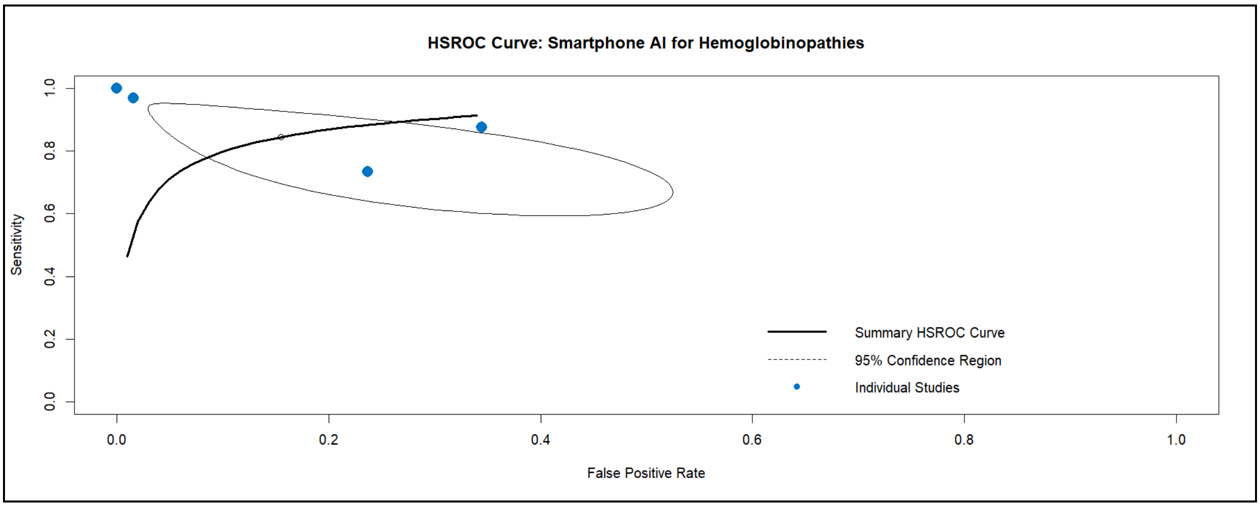

**Copyright Information:** (Online) ISSN: 2572-9241
©2026 The Author(s). HemaSphere is published by John Wiley & Sons Ltd on behalf of European Hematology Association. This is an open access Abstract Book distributed under the Attribution-NonCommercial-NoDerivs (CC BY-NC-ND), which allows third parties to download the articles and share them with others as long as they credit the author and the Abstract Book, but they cannot change the content in any way or use them commercially.
**Abstract Book Citations:** Authors, Title, HemaSphere, 2026;10:(S1):pages. The abstract book DOIs can be found on the "Author Information" page.
**Disclaimer:** Articles published in the journal HemaSphere exclusively reflect the opinions of the authors. The authors are responsible for all content in their abstracts including accuracy of the facts, statements, citing resources, etc.

### (PB4504) PHASE 1 STUDY TO EVALUATE SUBCUTANEOUS BUDOPRUTUG, A LOW-FUCOSYLATED ANTI-CD19 MAB, IN HEALTHY VOLUNTEERS, AS A POTENTIAL TREATMENT FOR IMMUNE-MEDIATED DISEASES WITH ADMINISTRATION OPTIONALITY

**Topic:** 38. Novel technologies, techniques and digital analytical tools in hematology

Stacey Luo*[1], Michael Tagen[2], Edgar Charles[1]

*[1]Climb Bio Inc., Wellesley, United States of America;[2]Verdient Science, Denver, United States of America;*

### Background

Budoprutug (TNT119) is an anti-CD19 mAb bioengineered with picomolar binding affinity for CD19 and a low-fucosylated Fc region for enhanced antibody-dependent cell cytotoxicity. CD19 is expressed from the early pro-B stage through maturation and differentiation to plasmablasts and on subsets of plasma cells, providing the potential for budoprutug to provide deep and sustained depletion of pathogenic B cells, including autoantibody-producing cells. Intravenous (IV) budoprutug was previously evaluated in two Phase 1 studies, one of which was a study of advanced CD19+ indolent B-cell lymphoma or chronic lymphocytic leukemia (CRUKD/12/003). It is currently being evaluated in several studies in immune-mediated diseases, including a Phase 1b/2a study in immune thrombocytopenia (NCT07043946). A low viscosity, high concentration (>175 mg/mL) formulation of budoprutug was developed to enable a subcutaneous (SC) administration, which could provide route-of-administration optionality. In non-human primates, SC budoprutug demonstrated high bioavailability without safety or local tolerability concerns.

### Aims

To assess the safety, pharmacokinetics (PK) and pharmacodynamics (PD) of SC budoprutug in adult healthy volunteers (HV) and to assess feasibility for clinical trials in immune-mediated diseases.

### Methods

This was a Phase 1, randomized, double-blind, placebo-controlled, single-ascending-dose study (NCT07090655) in HV. Primary endpoints included incidence and severity of treatment-emergent adverse events and incidence of infusion/injection-related reactions (IRRs). Secondary and exploratory endpoints included PK parameters, bioavailability, B-cell counts, and serum immunoglobulin levels. Participants received SC budoprutug (3 mg or 6 mg), IV budoprutug (6 mg), or placebo. SC cohorts were randomized 6:2 (active:placebo); IV cohort was randomized 12:2. Doses were selected to produce measurable but submaximal B-cell depletion (<50% predicted). Acetaminophen/paracetamol and diphenhydramine were given as pre-medications. Pts were followed for 8 weeks.

### Results

The study enrolled a total of 30 pts (SC 3mg, n=6; SC 6mg, n=6; IV 6mg, n=12; placebo, n=6). Safety profiles were comparable between SC and IV groups. IRRs occurred in 0/6, 3/6, 4/12, and 0/6 pts in the SC 3mg, SC 6mg, IV 6mg, and placebo groups, respectively. All were Grade 1 except a single Grade 2 postural hypotension (IV group), and all resolved within 48 hours. No injection/infusion-site reactions were observed. PK interpretation for SC doses was limited as most of the concentrations were below the limit of quantification; however robust PD effects were observed. All active groups demonstrated rapid B-cell depletion, reaching approximately 85% reduction from baseline (Figure 1). B-cell depletion was similar between SC and IV administration, suggesting adequate systemic exposure to achieve robust PD activity. Higher baseline natural killer (NK) cell counts were associated with greater depth of CD20+ B-cell depletion.

**Copyright Information:** (Online) ISSN: 2572-9241
©2026 The Author(s). HemaSphere is published by John Wiley & Sons Ltd on behalf of European Hematology Association. This is an open access Abstract Book distributed under the Attribution-NonCommercial-NoDerivs (CC BY-NC-ND), which allows third parties to download the articles and share them with others as long as they credit the author and the Abstract Book, but they cannot change the content in any way or use them commercially.
**Abstract Book Citations:** Authors, Title, HemaSphere, 2026;10:(S1):pages. The abstract book DOIs can be found on the “Author Information” page.
**Disclaimer:** Articles published in the journal HemaSphere exclusively reflect the opinions of the authors. The authors are responsible for all content in their abstracts including accuracy of the facts, statements, citing resources, etc.

**Summary/Conclusion**

To our knowledge, these are the first presented data of a B-cell depleting anti-CD19 mAb in HV. SC budoprutug showed robust effects on B-cell depletion with similar tolerability to matched-dose IV budoprutug, despite being studied at doses predicted to produce modest impact on B cells. The comparable PD between routes supports the feasibility of SC administration and provides flexibility for continued development in immune-mediated diseases.

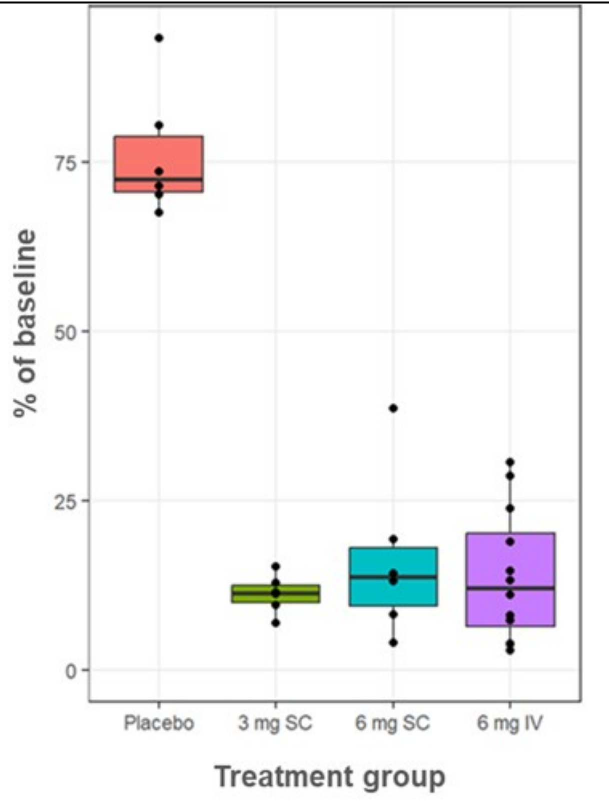

**Figure 1.** *B-cell Nadir as % of Baseline*

**Copyright Information:** (Online) ISSN: 2572-9241
©2026 The Author(s). HemaSphere is published by John Wiley & Sons Ltd on behalf of European Hematology Association. This is an open access Abstract Book distributed under the Attribution-NonCommercial-NoDerivs (CC BY-NC-ND), which allows third parties to download the articles and share them with others as long as they credit the author and the Abstract Book, but they cannot change the content in any way or use them commercially.
**Abstract Book Citations:** Authors, Title, HemaSphere, 2026;10:(S1):pages. The abstract book DOIs can be found on the "Author Information" page.
**Disclaimer:** Articles published in the journal HemaSphere exclusively reflect the opinions of the authors. The authors are responsible for all content in their abstracts including accuracy of the facts, statements, citing resources, etc.

### (PB4505) ANALYSIS OF THE BLOOD ROUTINE CHARACTERISTICS AND CLINICAL SIGNIFICANCE OF THE POPULATION WITH DECREASED MCV IN THE 4300-METER ALTITUDE AREA

**Topic:** 38. Novel technologies, techniques and digital analytical tools in hematology

Miao He*[1], Tao Wu[1]

[1] *The 940th Hospital of Joint Logistics Support force of Chinese People's Liberation Army, hematology, Gansu, China;*

### Background

In the hypoxic environment at high altitudes, the human body compensates for hypoxia through erythrocytosis, yet high-burden hematopoiesis may induce hematopoietic abnormalities such as decreased mean corpuscular volume (MCV). The current diagnostic criteria for anemia are formulated based on the plain population, which fail to take into account the physiological compensation mechanisms under high-altitude conditions and thus tend to result in the missed diagnosis of such abnormalities. At present, there are extremely few studies on the population with decreased MCV in high-altitude regions, making it imperative to conduct targeted research to improve the evaluation system for hematopoietic function in high-altitude areas.

### Aims

To investigate the blood routine characteristics of the population with decreased mean corpuscular volume (MCV) in the 4300-meter altitude environment, analyze the applicability limitations of the current diagnostic criteria for anemia in the plains, and provide data support for establishing an exclusive hematopoietic function assessment system for the plateau.

### Methods

A total of 60 healthy people from the plains (plain control group), 60 healthy people from the plateau (plateau control group, altitude 4300m, residence ≥6months) and 60 people with decreased MCV from the plateau (MCV<80fL, residence ≥6months) were selected as the research objects. The blood routine indicators of the three groups were detected, the peripheral blood red blood cell morphology of the group with decreased MCV was observed, and statistical analysis was performed.

### Results

There was no significant difference in age baseline among the three groups ($P>0.05$); the RBC of the MCV-decreased group was significantly higher than that of the plains and plateau control groups ($P<0.05$), and the HGB was significantly lower than that of the plateau control group ($P<0.05$), but still higher than that of the plains control group ($P<0.05$); MCV, MCH, and MCHC were significantly lower than those of the plateau control group and the plains control group ($P<0.05$). There was no difference in PLT between the MCV-decreased group and the plains control group, while PLT was significantly lower in the plateau control group ($P<0.05$). 90% of the population in the MCV-decreased group had red blood cell deformities, and 50% showed pale staining and enlarged pale staining areas. According to the plain anemia standard (HGB<130 g/L for adult males), this group had no anemia diagnosis. After 2024 WHO altitude correction, the anemia rate was 38.3% (23/60).

**Copyright Information:** (Online) ISSN: 2572-9241
©2026 The Author(s). HemaSphere is published by John Wiley & Sons Ltd on behalf of European Hematology Association. This is an open access Abstract Book distributed under the Attribution-NonCommercial-NoDerivs (CC BY-NC-ND), which allows third parties to download the articles and share them with others as long as they credit the author and the Abstract Book, but they cannot change the content in any way or use them commercially.
**Abstract Book Citations:** Authors, Title, HemaSphere, 2026;10:(S1):pages. The abstract book DOIs can be found on the "Author Information" page.
**Disclaimer:** Articles published in the journal HemaSphere exclusively reflect the opinions of the authors. The authors are responsible for all content in their abstracts including accuracy of the facts, statements, citing resources, etc.

**Summary/Conclusion**

The MCV-decreased group at the plateau presents the characteristic of increased red blood cell quantity but decreased quality. Essentially, it is a comprehensive manifestation of the plateau's specific compensatory hematopoiesis and iron metabolism imbalance; the current diagnostic criteria for anemia in the plains are prone to miss this type of hematopoietic abnormality, and an assessment system adapted to the plateau environment needs to be established.

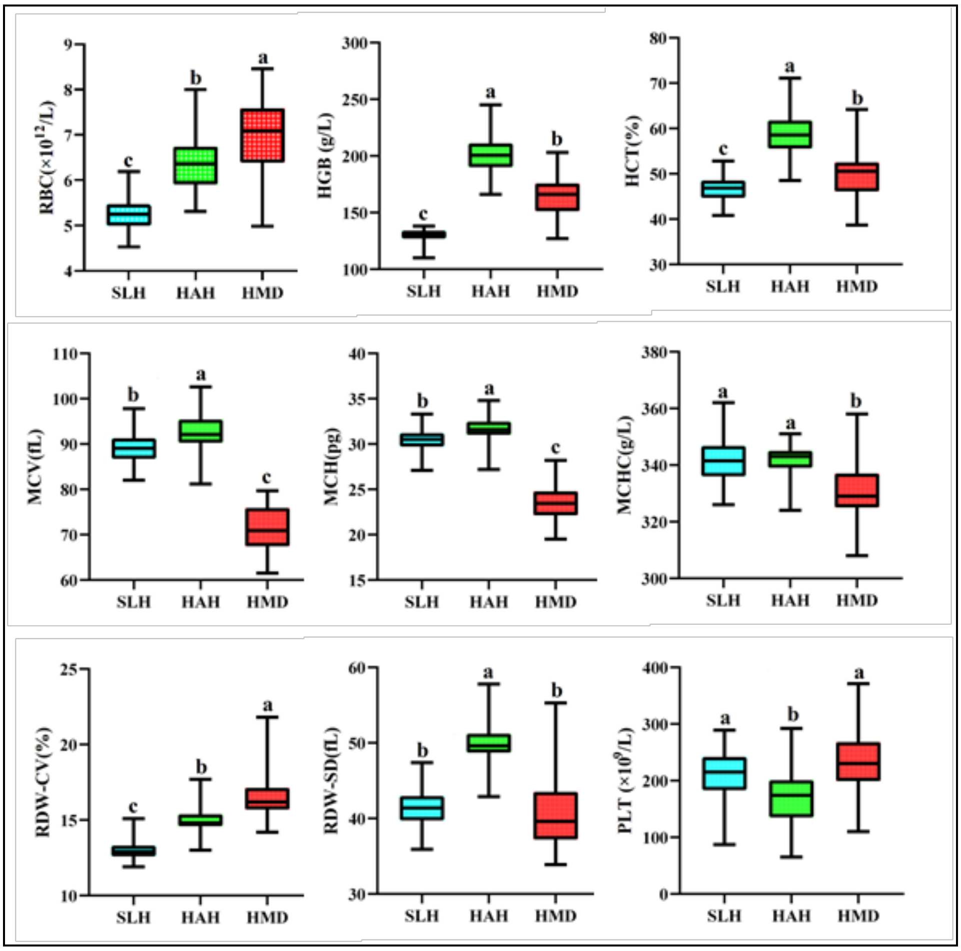

**Copyright Information:** (Online) ISSN: 2572-9241
©2026 The Author(s). HemaSphere is published by John Wiley & Sons Ltd on behalf of European Hematology Association. This is an open access Abstract Book distributed under the Attribution-NonCommercial-NoDerivs (CC BY-NC-ND), which allows third parties to download the articles and share them with others as long as they credit the author and the Abstract Book, but they cannot change the content in any way or use them commercially.
**Abstract Book Citations:** Authors, Title, HemaSphere, 2026;10:(S1):pages. The abstract book DOIs can be found on the “Author Information” page.
**Disclaimer:** Articles published in the journal HemaSphere exclusively reflect the opinions of the authors. The authors are responsible for all content in their abstracts including accuracy of the facts, statements, citing resources, etc.

### (PB4506) EASYGEN: A EUROPEAN IHI CONSORTIUM DEVELOPING A NOVEL PROCESS TO ENABLE HOSPITAL-BASED DECENTRALIZED CAR-T MANUFACTURING

**Topic:** 38. Novel technologies, techniques and digital analytical tools in hematology

Rebecca Scheiwe*[1], Dominik Narres[1], Carmen Sanges[2, 3], Ulrike Köhl[2], David Krones[2], Michael Hudecek[2, 3], Bertram Glass[4], Katrin Mestermann[2], Chris Wegener[5], Vivienne Williams[6], Jens Otto Brunner[7], Patrick Mennig[8], Thomas Brzoska[9], Sonja Steppan[1]

*[1]Fresenius SE, Bad Homburg, Germany;[2]Fraunhofer Institute for Cell Therapy and Immunology (IZI), Leipzig, Germany; [3]University Hospital Würzburg, Würzburg, Germany; [4]Helios Hospital Berlin-Buch, Berlin, Germany; [5]Fresenius Kabi USA, IL, United States of America; [6]Cellix Limited, Dublin, Ireland; [7]Danmarks Tekniske Universitet, Copenhagen, Denmark; [8]Fraunhofer-Institut für Experimentelles Software Engineering (IESE), Kaiserslautern, Germany; [9]Pro-Liance Global Solutions GmbH, Münster, Germany;*

### Background

Liquid hematologic malignancies are projected to increase substantially over the next decade, with estimated rises of 12.3% in Europe and 18.4% in the United States between 2025 and 2035. This burden will intensify demand for advanced therapies and further expose limitations in healthcare systems' capacity to deliver complex treatments at scale.

Access to chimeric antigen receptor T-cell (CAR-T) therapies remains restricted due to prolonged, resource-intensive manufacturing processes, reliance on centralized production models, and limited availability of qualified cleanroom infrastructure. Based on current paradigms, treating patients with hematologic malignancies alone would require approximately half of the available cleanroom capacity in Germany, highlighting a critical infrastructure bottleneck and the importance of room quality and regulatory qualification for manufacturing feasibility and patient safety.

Automated, closed manufacturing systems may reduce operational complexity but require robust data to demonstrate safety and process reliability. In parallel, decentralizing manufacturing to hospital-based settings could reduce logistical complexity and associated risks while enabling better integration into clinical workflows. These challenges are expected to be amplified by the expansion of CAR-T therapies to solid tumors and autoimmune diseases, which involve patient populations approximately one order of magnitude larger.

### Aims

EASYGEN (EASY workflow integration for GENe therapy) is a newly established European public–private consortium funded under the Innovative Health Initiative (IHI) Call 7.2 (Project No. 101194710). The initiative brings together 18 partners across academia, industry, clinical networks, and healthcare innovation, with the shared goal of driving a paradigm shift toward decentralized, point-of-care manufacturing of cellular therapies within hospitals.
EASYGEN aims to develop a novel process framework to support hospital-based implementation of decentralized CAR-T manufacturing through an automated, modular, closed-loop "one-stop" solution. This concept is intended to reduce operational complexity and contribute to future scalability of access.

**Copyright Information:** (Online) ISSN: 2572-9241
©2026 The Author(s). HemaSphere is published by John Wiley & Sons Ltd on behalf of European Hematology Association. This is an open access Abstract Book distributed under the Attribution-NonCommercial-NoDerivs (CC BY-NC-ND), which allows third parties to download the articles and share them with others as long as they credit the author and the Abstract Book, but they cannot change the content in any way or use them commercially.
**Abstract Book Citations:** Authors, Title, HemaSphere, 2026;10:(S1):pages. The abstract book DOIs can be found on the "Author Information" page.
**Disclaimer:** Articles published in the journal HemaSphere exclusively reflect the opinions of the authors. The authors are responsible for all content in their abstracts including accuracy of the facts, statements, citing resources, etc.

**Methods**

The consortium is developing this process innovation to facilitate integration of key steps from leukapheresis to infusion within coordinated clinical pathways. EASYGEN focuses on workflow standardization, automation of critical manufacturing components, and alignment with multidisciplinary hospital teams. The process is designed to support adaptability across diverse European healthcare settings and potentially enable delivery beyond highly specialized centers.

**Results**

EASYGEN represents a European consortium-based response to a rapidly increasing hematologic cancer burden and the urgent need for more accessible cellular therapy models. By advancing a novel process for hospital implementation of decentralized manufacturing, the project aims to strengthen healthcare systems' long-term preparedness and support broader, equitable access to innovative immunotherapies.

**Summary/Conclusion**

With liquid hematologic malignancies expected to rise significantly over the next decade, innovation in decentralized manufacturing and clinical implementation processes will become increasingly essential. EASYGEN, funded under IHI Call 7.2, provides a collaborative European framework to develop a novel hospital-oriented process concept that may enable more scalable delivery of CAR-T therapies for a broader patient population.

**Copyright Information:** (Online) ISSN: 2572-9241
©2026 The Author(s). HemaSphere is published by John Wiley & Sons Ltd on behalf of European Hematology Association. This is an open access Abstract Book distributed under the Attribution-NonCommercial-NoDerivs (CC BY-NC-ND), which allows third parties to download the articles and share them with others as long as they credit the author and the Abstract Book, but they cannot change the content in any way or use them commercially.
**Abstract Book Citations:** Authors, Title, HemaSphere, 2026;10:(S1):pages. The abstract book DOIs can be found on the “Author Information” page.
**Disclaimer:** Articles published in the journal HemaSphere exclusively reflect the opinions of the authors. The authors are responsible for all content in their abstracts including accuracy of the facts, statements, citing resources, etc.

### (PB4507) WEAKLY SUPERVISED AND ATTENTION-BASED MULTI-INSTANCE-LEARNING PREDICTS IDH1 MUTATION STATUS IN ACUTE MYELOID LEUKEMIA FROM BONE MARROW SMEARS

**Topic:** 38. Novel technologies, techniques and digital analytical tools in hematology

Jan-Niklas Eckardt*[1, 2, 3, 4], Chethan Babu V. Reddy[4], Andrew Srisuwananukorn[5], Christian Pohlkamp[6], Sebastian Riechert[4], Tim Schmittmann[4], Martin M.K. Schneider[1], Maryam Schübel[7], Miriam Stoerk[7], Klaus Metzeler[8, 9], Karsten Spiekermann[7], Tobias Herold[7, 8], Markus Badstübner[4], Ishan Srivastava[1], Susann Winter[1], Katja Sockel[1], Frank Kroschinsky[1], Christoph Röllig[1], Martin Bornhauser[1, 10, 11], Ann-Kathrin Eisfeld[5], Torsten Haferlach[6], Jan Moritz Middeke[1, 2, 3, 4]

*[1]Department of Internal Medicine I, University Hospital Carl Gustav Carus, Technical University Dresden, Dresden, Germany; [2]Else Kröner Fresenius Center for Digital Health, Technical University Dresden, Dresden, Germany;[3]Mildred Scheel Cancer Center, University Hospital Dresden, Technical University Dresden, Dresden, Germany; [4]Cancilico GmbH, Dresden, Germany; [5]Division of Hematology, Department of Internal Medicine, The Ohio State University Comprehensive Cancer Center, Columbus, OH, United States of America; [6]MLL Munich Leukemia Laboratory, Munich, Germany; [7]Department of Medicine III, University Hospital, LMU, Munich, Germany; [8]Department of Hematology, Oncology, and Stem-Cell Transplantation, Munich Hospital Bogenhausen, Munich Municipal Hospital Group, Munich, Germany; [9]Department of Hematology, Cellular Therapy, Hemostaseology and Infectious Diseases, University Hospital Leipzig, Leipzig, Germany; [10]German Cancer Consortium (DKTK), Partner Site Dresden, and German Cancer Research Center (DKFZ), Heidelberg, Germany; [11]National Center for Tumor Diseases Dresden (NCT/UCC), a partnership between DKFZ, Faculty of Medicine and University Hospital Dresden, TUD Dresden University of Technology, and Helmholtz-Zentrum Dresden-Rossendorf (HZDR), Dresden, Germany;*

### Background

Alterations of the *isocitrate dehydrogenase 1 (IDH1)* gene are recurrent events in AML, occur in approximately 8% of patients, and are targetable via ivosidenib or olutasidenib. Deep learning-based computer vision has been reported to accurately predict the mutation status of genetic alterations in AML based on bone marrow whole slide images (WSI).

### Aims

To predict *IDH1* mutation status in AML from bone marrow smears (BMS)

### Methods

WSI of 436 adult AML patients were digitized at initial diagnosis from four centers in Germany and the United States (186*IDH1*-mutated). WSI were segmented into regions of interest using a customized U-Net model. Tiles were extracted using SlideFlow and ranked for evaluability using a ResNet model. A hierarchical classification framework was developed: First, a vision transformer was trained in a weakly supervised manner to extract tile-level feature representations, where slide-level labels (*IDH1* mutated, *IDH1* wildtype) were assigned to individual tiles per WSI. The extracted feature vectors were subsequently used as an input for an attention-based multi-instance-learning (MIL) model which assigns attention weights to tiles according to their importance for *IDH1* mutation status prediction, thereby providing a slide-level aggregated prediction (Figure 1: End-to-end *IDH1* mutation prediction on bone marrow WSIs). Model performance was evaluated using three-fold cross-validation with patient-level splits. All models were trained using the AdamW optimizer with cross-entropy loss.

**Copyright Information:** (Online) ISSN: 2572-9241
©2026 The Author(s). HemaSphere is published by John Wiley & Sons Ltd on behalf of European Hematology Association. This is an open access Abstract Book distributed under the Attribution-NonCommercial-NoDerivs (CC BY-NC-ND), which allows third parties to download the articles and share them with others as long as they credit the author and the Abstract Book, but they cannot change the content in any way or use them commercially.
**Abstract Book Citations:** Authors, Title, HemaSphere, 2026;10:(S1):pages. The abstract book DOIs can be found on the "Author Information" page.
**Disclaimer:** Articles published in the journal HemaSphere exclusively reflect the opinions of the authors. The authors are responsible for all content in their abstracts including accuracy of the facts, statements, citing resources, etc.

### Results

In preliminary experiments using only the weakly supervised tile-level classifier, slide-level predictions were obtained by aggregating tile-level probabilities using mean pooling. This operated under the assumption that visual cues informative for *IDH1* classification are abundantly present across all tiles. This initial approach showed a macro-average F1 score of 0.6769 (std: ± 0.0555), accuracy of 0.7096 (std: ± 0.0258), recall of 0.6867 (std: ± 0.0361), and precision of 0.7542 (std: ±0.0558). We concluded that visual cues informative of *IDH1* mutation status are likely sparse and not present in all tiles equally. To address this limitation, the*classify (CLS)* token embedding from the final transformer layer was extracted, resulting in a 768-dimensional feature vector per tile, which served as an input to an attention-based MIL model. This mechanism enables the model to focus on informative tiles while down-weighing non-discriminative or noisy tiles. Using attention-based MIL, we obtained a macro-averaged F1 score of 0.7249 (std: ± 0.0210), accuracy of 0.7413 (std: ±0.0231), recall of 0.7223 (std: ±0.0209), and precision of 0.7538 (std: ±0.0471). Mean area under the curve for the receiver operating characteristc (ROC-AUC) was 0.8057 (std: ±0.0282), with fold-wise ROC-AUC values ranging from 0.7735 to 0.8285, arguing against reliance on a small number of highly informative tiles and instead suggesting that *IDH1* mutation status is associated with a broadly distributed, albeit subtle, digital biomarker in bone marrow morphology.

### Summary/Conclusion

Our findings indicate a digital biomarker for *IDH1* mutation status in AML that is latently embedded in bone marrow morphology. This is supported by the substantial performance gain of attention-based MIL over tile-wise classification which suggests subtle morphological changes detectable by computer vision that are robustly identifiable across a multicenter cohort with variances in staining procedure and imaging hardware.

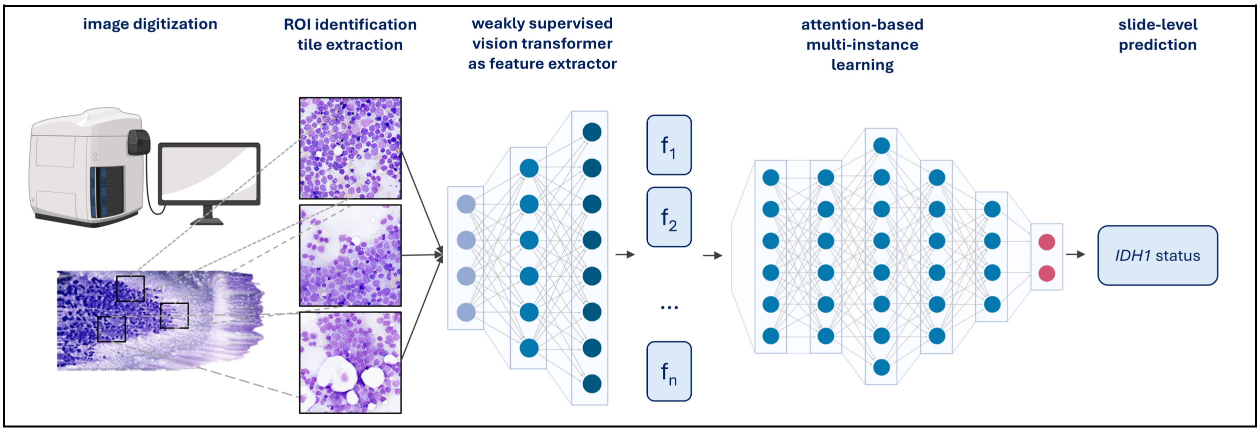

**Copyright Information:** (Online) ISSN: 2572-9241
©2026 The Author(s). HemaSphere is published by John Wiley & Sons Ltd on behalf of European Hematology Association. This is an open access Abstract Book distributed under the Attribution-NonCommercial-NoDerivs (CC BY-NC-ND), which allows third parties to download the articles and share them with others as long as they credit the author and the Abstract Book, but they cannot change the content in any way or use them commercially.
**Abstract Book Citations:** Authors, Title, HemaSphere, 2026;10:(S1):pages. The abstract book DOIs can be found on the "Author Information" page.
**Disclaimer:** Articles published in the journal HemaSphere exclusively reflect the opinions of the authors. The authors are responsible for all content in their abstracts including accuracy of the facts, statements, citing resources, etc.

### (PB4508) CLINICAL UTILITY OF HF-MCV IN SCREENING FOR VITAMIN B12 DEFICIENCY: A RETROSPECTIVE STUDY

**Topic:** 38. Novel technologies, techniques and digital analytical tools in hematology

Malek Haouari[1], Mohamed Yassine Kaabar[2], Ikbel Ghachem*[3], Selma Ben Cheick[1], Wala Chabchoub[4], Lamia Sghair[5], Asma Bachali[3]

*[1]Faculty of pharmacy of Monastir, Nabeul, Tunisia;[2]University of Monastir, Faculty of Pharmacy of Monastir, Laboratory of Biology, Tahar Maamouri Hospital,, Nabeul, Tunisia; [3]University of Tunis El Manar, Faculty of Medicine of Tunis, Laboratory of Biology, Tahar Maamouri Hospital, Nabeul, Tunisia; [4]Faculty of pharmacy,, Monastir, Tunisia; [5]Laboratory of Biology, Taher Maamouri Hospital, Nabeul, Tunisia;*

### Background

Vitamin B12 deficiency is a primary etiology of macrocytic anemia, often presenting alongside megaloblastic changes due to impaired DNA synthesis. A characteristic feature of this deficiency is the presence of fragmented red blood cells, which can lead to a "pseudo-reduction" of the Mean Corpuscular Volume (MCV), a phenomenon less frequent in differential diagnoses such as folate deficiency or MDS. Modern hematology analyzers, such as the Mindray BC-6200, now provide the high-frequency corpuscular volume (HF-MCV), representing the most frequent cell volume.

### Aims

This study aims to evaluate the diagnostic value of the "HF-MCV - MCV" parameter as a supplementary tool for screening Vitamin B12 deficiency in patients with macrocytic anomalies.

### Methods

This retrospective study was conducted at Mohamed Taher Maamouri Hospital in Nabeul between January 2025 and February 2026. Hematological parameters (Hb, MCV, HF-MCV) were obtained via the Mindray BC-6200, while Vitamin B12 assays were performed on the Cobas e 402 platform. Vitamin B12 deficiency was defined as a serum level < 140 pmol/L. The diagnostic performance of the difference between HF-MCV and MCV was assessed using ROC curve analysis, with statistical significance defined as p < 0.05.

### Results

We analyzed 120 subjects, including a deficient group (n=84, anemic patients with vitamin B12 deficiency) and a control group (n=36, non-anemic patients with normal vitamin B12 levels). The study population had a mean age of 59.9 years with a M/F sex ratio of 0.74. ROC analysis demonstrated an excellent discriminatory capacity for the "HF-MCV - MCV" parameter, yielding an Area Under the Curve (AUC) of 0.81 (p < 0.0001). We observed that Specificity increased proportionally with higher "HF-MCV - MCV" values, while sensitivity decreased inversely. Positive predictive value (PPV) improved with increasing thresholds: a threshold > 0.5 identified deficiency with a Positive Predictive Value (PPV) of 82.9% (Se=81%, Sp=61.1%), while a threshold > 1.1 raised the PPV to 87.0% (Se = 71,4% ; Sp = 75,0%).. Higher thresholds of > 2.1 and > 3.2 yielded PPVs of 90.2% (Se= 40,5% ; Sp = 88,9%) and 95.5% (Se= 25% ; Sp = 97,2%) respectively, reaching 100% for values > 5.3. Conversely, the Negative Predictive Value (NPV) remained insufficient for exclusion (max NPV 57.9%), indicating that a low delta variation cannot rule out a confirmed deficiency.

**Copyright Information:** (Online) ISSN: 2572-9241
©2026 The Author(s). HemaSphere is published by John Wiley & Sons Ltd on behalf of European Hematology Association. This is an open access Abstract Book distributed under the Attribution-NonCommercial-NoDerivs (CC BY-NC-ND), which allows third parties to download the articles and share them with others as long as they credit the author and the Abstract Book, but they cannot change the content in any way or use them commercially.
**Abstract Book Citations:** Authors, Title, HemaSphere, 2026;10:(S1):pages. The abstract book DOIs can be found on the "Author Information" page.
**Disclaimer:** Articles published in the journal HemaSphere exclusively reflect the opinions of the authors. The authors are responsible for all content in their abstracts including accuracy of the facts, statements, citing resources, etc.

**Summary/Conclusion**

The HF-MCV is a valuable high-specificity marker that complements traditional biological screening for Vitamin B12 deficiency. A "HF-MCV - MCV" threshold > 2.1 serves as a robust predictive indicator, systematically justifying a serum B12 assay.

**Copyright Information:** (Online) ISSN: 2572-9241
©2026 The Author(s). HemaSphere is published by John Wiley & Sons Ltd on behalf of European Hematology Association. This is an open access Abstract Book distributed under the Attribution-NonCommercial-NoDerivs (CC BY-NC-ND), which allows third parties to download the articles and share them with others as long as they credit the author and the Abstract Book, but they cannot change the content in any way or use them commercially.
**Abstract Book Citations:** Authors, Title, HemaSphere, 2026;10:(S1):pages. The abstract book DOIs can be found on the "Author Information" page.
**Disclaimer:** Articles published in the journal HemaSphere exclusively reflect the opinions of the authors. The authors are responsible for all content in their abstracts including accuracy of the facts, statements, citing resources, etc.

### (PB4509) MEDNEXT-BASED AUTOMATIC SEGMENTATION OF 18F-FDG PET IMAGES IN ANGIOIMMUNOBLASTIC T-CELL LYMPHOMA AND PROGNOSTIC VALUE EXPLORATION OF PREDICTED TOTAL METABOLIC TUMOR VOLUME

**Topic:** 38. Novel technologies, techniques and digital analytical tools in hematology

Jing-Yan Xu*[1], Xinyuan Chen[1]

[1]*Nanjing Drum Tower Hospital, nanjing, China;*

### Background

Angioimmunoblastic T-cell lymphoma (AITL), a distinct pathologic entity within peripheral T-cell lymphoma (PTCL), represents 15-20% of PTCL cases, ranking as the second most common subtype after PTCL-not otherwise specified (PTCL-NOS).Prognostic stratification remains suboptimal, as conventional indices like the International Prognostic Index (IPI) exhibit limited discriminative power in AITL. Although refined systems such as the PTCL Prognostic Index (PIT) and International Peripheral T-Cell Lymphoma Project (IPTCLP) criteria show improved risk stratification for PTCL-NOS, their clinical utility in identifying high-risk AITL cohorts requires further validation.

### Aims

To evaluate MedNeXt, a deep learning network, for automated segmentation of 18F-FDG PET images in angioimmunoblastic T-cell lymphoma (AITL) and validate the prognostic utility of predicted total metabolic tumor volume (pTMTV).

### Methods

A multicenter cohort of 84 AITL patients was stratified into training (n=68) and validation (n=16) sets (8:2 ratio). MedNeXt was trained using a Dice-cross-entropy dual-loss function. Segmentation accuracy was quantified through seven metrics: Dice similarity coefficient (DSC), Jaccard index (JSC), sensitivity (SEN), positive predictive value (PPV), false discovery rate (FDR), 95% Hausdorff distance (HD95), and average symmetric surface distance (ASSD). Prognostic significance of pTMTV was assessed via Cox proportional hazards models.

### Results

Validation cohort performance metrics (mean±SD): DSC=0.769±0.153, JSC=0.645±0.173, PPV=0.773±0.201, FDR=0.227±0.201, SEN=0.799±0.110, ASSD=1.903±2.288 mm, HD95=32.183±44.664 mm. Ground truth TMTV (gtTMTV=949.4±734.5 $cm^3$) and pTMTV (934.1±617.7 $cm^3$) demonstrated strong agreement in Bland-Altman analysis and linear regression ($R^2$=0.868, p<0.001). Elevated pTMTV (≥606.9 $cm^3$) independently predicted reduced progression-free survival (HR=1.781, 95% CI:1.057-3.002; p=0.030) and overall survival (HR=2.190, 95% CI:1.042-4.600; p=0.038).

### Summary/Conclusion

MedNeXt achieves precise lesion delineation and automated quantification of TMTV in AITL PET imaging. pTMTV emerges as an independent prognostic predictor for survival outcomes, demonstrating clinical viability for lymphoma management.

**Copyright Information:** (Online) ISSN: 2572-9241
©2026 The Author(s). HemaSphere is published by John Wiley & Sons Ltd on behalf of European Hematology Association. This is an open access Abstract Book distributed under the Attribution-NonCommercial-NoDerivs (CC BY-NC-ND), which allows third parties to download the articles and share them with others as long as they credit the author and the Abstract Book, but they cannot change the content in any way or use them commercially.
**Abstract Book Citations:** Authors, Title, HemaSphere, 2026;10:(S1):pages. The abstract book DOIs can be found on the "Author Information" page.
**Disclaimer:** Articles published in the journal HemaSphere exclusively reflect the opinions of the authors. The authors are responsible for all content in their abstracts including accuracy of the facts, statements, citing resources, etc.

### (PB4510) DIGITAL MORPHOLOGY: A PROPOSAL GIMEMA GROUP OF NEW LEARNING FOR ITALIAN MORPHOLOGISTS

**Topic:** 38. Novel technologies, techniques and digital analytical tools in hematology

Gianni Binotto*[1], Susanna Fenu[2], Rosalba Cucci[3], Giulia Polese[3], Silvio Canino[4], Elena Rossi[5], Luca Pizzato[3], Giulio Pandolfelli[3], Alfonso Piciocchi[3], Paola Fazi[3], Marco Vignetti[3], Gina Zini[5]

*[1]PADUA UNIVERSITY CITY HOSPITAL, Padova, Italy; [2]San Giovanni Addolorata Hospital, Roma, Italy; [3]GIMEMA FOUNDATION, Rome, Italy; [4]Università di Perugia, Perugia, Italy; [5]Fond. Pol. Univ. A.Gemelli IRCCS, Rome, Italy;*

### Background

Morphology continues to play a fundamental role in the diagnosis of hematological pathologies, even though training in this discipline is often insufficient among young professionals. In 2022, a panel of morphologists collaborated with the GIMEMA Foundation to launch the GIMEMA Virtual Microscope (GVM) project. This innovative e-learning platform offers an interactive web-accessible virtual microscope, designed for the cytomorphological analysis of peripheral blood and bone marrow aspirates from patients with both hematological and non-hematological neoplasms.
The GVM project consists of two main components.

### Aims

The first is a digital software called GIMEMA-microscope, developed by GIMEMA which allows users to explore high-resolution images of hematological cells online (see Image 1). Users can manipulate different layers and fields of view (FOVs) as well as adjust magnification levels for more detailed analysis. The second component is a comprehensive training program that includes the study of four exemplary clinical cases per year, available on a dedicated online platform (https://elearning.gimema.it).

### Methods

The training is designed to be interactive, incorporating a questionnaire of ten questions for each clinical case. Each question presents four possible answers related to the specific FOV under examination. Participants can save their progress and resume later. If they do not pass the test on their first attempt, they may retake it up to three times. Upon completion, participants receive feedback from the scientific board, including comments and clarification correct and incorrect answers. A passing score of 70% is required to pass.

### Results

Since its launch, the GVM project has produced twelve exercises over its last three editions. To date, 694 participants have registered for the training program, including 331 doctors, 169 medical residents, 116 biologists and clinical pathology residents and other professionals from other fields.

**Copyright Information:** (Online) ISSN: 2572-9241
©2026 The Author(s). HemaSphere is published by John Wiley & Sons Ltd on behalf of European Hematology Association. This is an open access Abstract Book distributed under the Attribution-NonCommercial-NoDerivs (CC BY-NC-ND), which allows third parties to download the articles and share them with others as long as they credit the author and the Abstract Book, but they cannot change the content in any way or use them commercially.
**Abstract Book Citations:** Authors, Title, HemaSphere, 2026;10:(S1):pages. The abstract book DOIs can be found on the "Author Information" page.
**Disclaimer:** Articles published in the journal HemaSphere exclusively reflect the opinions of the authors. The authors are responsible for all content in their abstracts including accuracy of the facts, statements, citing resources, etc.

**Summary/Conclusion**

The project has generated significant interest, with participants achieving correct answers in 57,29 % of the presented cases (see Table1). The GVM initiative has effectively raised awareness about the importance of morphological and cytochemical diagnostics. Overall, this project represents a meaningful advancement in medical education and diagnostic training, helping healthcare professionals improve their skills in analyzing complex hematological conditions. Participants' feedback highlighted a strong interest in studying morphology and emphasized the need for further, more comprehensive exploration of the topic.

| Results twelve exercises | | **Users who have taken the test** | **Users who have passed the test** | **% of users who have passed the test** |
|---|---|---|---|---|
| **1** | Acute myeloid leukemia near-tetraploid. | 91 | 31 | 34.7 |
| **2** | Megaloblastic anemia due to vitamin B12 deficiency | 87 | 65 | 74.71 |
| **3** | Prolymphocytic leukemia | 79 | 52 | 65.82 |
| **4** | Acute microgranular promyelocytic leukemia | 76 | 74 | 97.37 |
| **5** | Acute monoblastic leukemia classified in the WHO as NOS leukemias | 44 | 20 | 45.45 |
| **6** | Amyloidosis secondary to micromolecular kappa multiple myeloma | 57 | 33 | 57.89 |
| **7** | Systemic Leishmania infection | 45 | 33 | 73.33 |
| **8** | MDS with ringed sideroblasts | 43 | 6 | 13.95 |
| **9** | Slide 1: Double malaria infection by *Plasmodium Falciparum* and *Plasmodium Vivax* , Slide 2: Systemic candidiasis, Slide 3: Expansion of LGLs associated with the presence of some activated lymphocytes: clonal evaluation required | 96 | 31 | 32.29 |
| **10** | Mastocytosis | 60 | 38 | 63.33 |
| **11** | Marginal zone non-Hodgkin lymphoma with villosus lymphocytes | 45 | 35 | 77.77 |
| **12** | Acute monocytic Leukemia | 53 | 27 | 50.94 |

**Copyright Information:** (Online) ISSN: 2572-9241
©2026 The Author(s). HemaSphere is published by John Wiley & Sons Ltd on behalf of European Hematology Association. This is an open access Abstract Book distributed under the Attribution-NonCommercial-NoDerivs (CC BY-NC-ND), which allows third parties to download the articles and share them with others as long as they credit the author and the Abstract Book, but they cannot change the content in any way or use them commercially.
**Abstract Book Citations:** Authors, Title, HemaSphere, 2026;10:(S1):pages. The abstract book DOIs can be found on the "Author Information" page.
**Disclaimer:** Articles published in the journal HemaSphere exclusively reflect the opinions of the authors. The authors are responsible for all content in their abstracts including accuracy of the facts, statements, citing resources, etc.

### (PB4511) CYTOKINE PROFILING IN THE DLBCL MICROENVIRONMENT REVEALS UPREGULATION OF THE NFKB1 TRANSCRIPTION FACTOR CORRELATED WITH IL6, IL15 AND IFNG OVEREXPRESSION

**Topic:** 38. Novel technologies, techniques and digital analytical tools in hematology

E Carrera*[1], M Resanoa[2], Ms Braza[3]

*[1]University of the Basque Country, Bilbao, Spain; [2]Department of Surgical Pathology. University Hospital of Navarra, Pamplona, Spain; [3]Biogipuzkoa IIS, Ikerbasque Foundation for Science, and Icahn School of Medicine at Mount Sinai, San Sebastian, Spain;*

### Background

Diffuse large B-cell lymphoma (DLBCL) is the most frequent non-Hodgkin lymphoma of B-cell origin. In DLBCL, inflammation drives continuous immune stimulation (Cvetković et al., 2025), and shapes a complex tumor microenvironment (TME) rich in inflammatory macrophages and cytokines (Tamma et al., 2020) (Serna et al., 2023) (Resanoa et al., 2025) (Resanoa et al., 2026). Macrophages are innate immune cells that can display pro- or anti-tumor functions depending on their cytokine profile and adapt to specific contexts through reprogramming of key pathways, such as inflammation. In DLBCL, the expression profile of the key cytokines IL-6, IL-15 and interferon (IFN)-g have not been thoroughly studied, and little is known about their link with key transcription factors (such as NF-kB1) in the rich macrophage infiltrate.

### Aims

-To determine the overall expression of cytokines and NF-kB1 in the DLBCL microenvironment.
-To assess the expression profile of the key IFN-g, IL-6, IL-15 cytokines and their possible link with NF-kB1 in the rich-macrophage DLBCL microenvironment.

### Methods

We used bioinformatics tools (e.g. Gepia2, Timer2.0...) and algorithms (EPIC, CYBERSORT) to perform gene expression profiling, correlation and deconvolution analyses of a TCGA dataset of 48 DLBCL samples and of a GTEx dataset of 337 control samples. In addition, to validate our results at the protein level, we performed immunohistochemistry (IHC) and immunofluorescence (IF) (ongoig) analyses using antibodies against CD68, CD86, IFN-g, IL-6, IL-15 and NF-kB1 and tissue microarrays containing 128 DLBCL samples and 20 controls.

**Copyright Information:** (Online) ISSN: 2572-9241
©2026 The Author(s). HemaSphere is published by John Wiley & Sons Ltd on behalf of European Hematology Association. This is an open access Abstract Book distributed under the Attribution-NonCommercial-NoDerivs (CC BY-NC-ND), which allows third parties to download the articles and share them with others as long as they credit the author and the Abstract Book, but they cannot change the content in any way or use them commercially.
**Abstract Book Citations:** Authors, Title, HemaSphere, 2026;10:(S1):pages. The abstract book DOIs can be found on the "Author Information" page.
**Disclaimer:** Articles published in the journal HemaSphere exclusively reflect the opinions of the authors. The authors are responsible for all content in their abstracts including accuracy of the facts, statements, citing resources, etc.

### Results

Using TCGA and GTEx, public datasets, we investigated in DLBCL vs control samples, the expression profiles of genes encoding different cytokines. Many genes that encode pro-inflammatory cytokines (e.g. *IFNG*, *IL6*, *IL7*, *IL15*, *IL18* and *TNFA*) were upregulated in the DLBCL TME (Figure 1A). Among them, *IFNG*, *IL6* and *IL15* were the most significantly correlated with the expression levels of *CD86*, which is considered as M1 macrophage marker (Figure 1B). In addition, the expression levels of*IFNG*, *IL6* and *IL15* were positively and significantly correlated with M1 macrophage infiltration levels in the DLBCL TME (Figure 1C). These results indicate a strong presence of pro-inflammatory cytokines in DLBCL linked to its macrophage-rich microenvironment. Moreover, we found that *NFKB1*, a gene encoding a transcription factor involved in inflammation, was upregulated in DLBCL samples vs controls (Figure 1D) and that its expression levels were positively correlated with those of *IFNG*, *IL6*, and *IL15* (genes encoding pro-inflammation cytokines) (data not shown). *NFKB1* expression level could also be used to stratify overall survival in patients with DLBCL (Figure 1E). Lastly, we confirmed by IHC, in an independent cohort of 128 DLBCL vs 20 controls, that NF-kB1 is strongly expressed in DLBCL compared with controls, in term of staining intensity (histoscore values: 6.1 ± 0.8 vs 2.8 ± 0.4, p= 4e-6) and percentage of positive cells (74.3 ± 6.9 vs 60.1 ± 4.7, p= 0.003).

### Summary/Conclusion

Our results highlight that in the DLBCL TME, the upregulation of the pro-inflammatory cytokines*IFNG*, *IL6* and *IL15* is associated with *NFKB1* upregulation. These results suggest that IFN-g, IL-6 and IL-15 could be potential therapeutic targets to promote the anti-tumor immune response in DLBCL.

*Disclosures:*

This work was supported by the Spanish Ministry of Science and Innovation - ISCIII (PI24/01691), and by the European Union.

Figure 1. Summary of the main results. DLBCL: Diffuse Large B-cell Lymphoma, LN: lymph node, TMA: tissue microarray. p-values ≤0.05 is considered significant. Scale bars = 100 um.

**Copyright Information:** (Online) ISSN: 2572-9241
©2026 The Author(s). HemaSphere is published by John Wiley & Sons Ltd on behalf of European Hematology Association. This is an open access Abstract Book distributed under the Attribution-NonCommercial-NoDerivs (CC BY-NC-ND), which allows third parties to download the articles and share them with others as long as they credit the author and the Abstract Book, but they cannot change the content in any way or use them commercially.
**Abstract Book Citations:** Authors, Title, HemaSphere, 2026;10:(S1):pages. The abstract book DOIs can be found on the "Author Information" page.
**Disclaimer:** Articles published in the journal HemaSphere exclusively reflect the opinions of the authors. The authors are responsible for all content in their abstracts including accuracy of the facts, statements, citing resources, etc.

### (PB4512) ASSOCIATION OF A COMPOSITE RESPONSE METRIC (ORR×DOR) WITH MEDIAN PROGRESSION FREE SURVIVAL AND OVERALL SURVIVAL ACROSS HEMATOLOGIC MALIGNANCY CLINICAL TRIALS

**Topic:** 38. Novel technologies, techniques and digital analytical tools in hematology

Ankit Kalucha[1], Judith Pérez-Granado*[1], Bruno Larvol[1], Mark Gramling[1]

[1]*The Larvol Group LLC, San Francisco, United States of America;*

### Background

Objective Response Rate (ORR) and Duration of Response (DoR) are often reported prior to median Progression-Free Survival (mPFS) and median Overall Survival (mOS) to assess antitumor activity. Prior surrogate analyses have typically evaluated single response endpoints and have been restricted to tumour specific or regimen-specific settings, limiting generalizability. A composite metric integrating the response (ORR) and its durability (DoR)—calculated as ORR×DoR—captures a cumulative treatment effect, reflecting both how many patients respond and how long benefit persists. This composite may more fully represent the clinical impact on disease control and provide a more robust early signal of survival benefit across diverse clinical contexts.

### Aims

To evaluate whether ORR×DoR, a composite response metric reflecting both response rate and duration, is associated with mPFS and mOS in hematologic malignancy clinical trials. This analysis included both experimental and control arms to strengthen generalizability. The dataset spanned multiple indications, therapy classes, and treatment settings.

### Methods

Data were extracted from LARVOL CLIN, a curated outcomes database, comprising phase I–III hematologic malignancy trials. Arms were required to report ORR (%), median DoR (months), and arm population size (Arm N). The initial dataset included 144 trial- mPFS was reported in 124 trials (157 arms) and mOS in 74 trials (98 arms). Multivariate outliers were removed using Mahalanobis distance with an IQR-based threshold, yielding final cohorts of 148 arms across 115 trials formPFS and 93 arms across 69 trials for mOS. Associations between ORR×DoR and mPFS or mOS were evaluated separately using linear mixed-effects regression with trial-specific random intercepts to account for within-trial correlation. Generalizability was assessed using 5-fold trial-level cross-validation, keeping all arms from each trial together. Model performance was summarized using cross-validated $R^2$, MAE, and RMSE (months).

### Results

ORR×DoR demonstrated a strong positive association with mPFS ($\beta = 0.0102$, $p < 0.001$; $R^2 = 0.851$), with low prediction error in cross-validation (MAE = 2.88 months; RMSE = 3.85 months). For mOS, the association remained positive ($\beta = 0.0146$, $p < 0.001$; $R^2 = 0.482$) but with higher prediction error (MAE = 7.31 months; RMSE = 9.32 months) (Table 1and Figure 1).

**Copyright Information:** (Online) ISSN: 2572-9241
©2026 The Author(s). HemaSphere is published by John Wiley & Sons Ltd on behalf of European Hematology Association. This is an open access Abstract Book distributed under the Attribution-NonCommercial-NoDerivs (CC BY-NC-ND), which allows third parties to download the articles and share them with others as long as they credit the author and the Abstract Book, but they cannot change the content in any way or use them commercially.
**Abstract Book Citations:** Authors, Title, HemaSphere, 2026;10:(S1):pages. The abstract book DOIs can be found on the "Author Information" page.
**Disclaimer:** Articles published in the journal HemaSphere exclusively reflect the opinions of the authors. The authors are responsible for all content in their abstracts including accuracy of the facts, statements, citing resources, etc.

### Summary/Conclusion

Across hematologic malignancy trial arms, ORR×DoR demonstrated a strong association with mPFS ($R^2$=0.85) and a moderate association with mOS ($R^2$=0.48) under trial-level cross-validation. These findings suggest ORR×DoR may serve as an early quantifiable signal of survival benefit, potentially informing clinical decision-making and trial endpoint selection. Incomplete reporting of DoR across trials restricted the analysable sample size, potentially limiting statistical power and generalizability.

**Table 1. Association of ORR×DoR with Survival Outcomes (Trial-Level Cross-Validation)**

| Endpoint | $R^2$ (CV) | MAE (months) | RMSE (months) | β | SE | p-value |
|---|---|---|---|---|---|---|
| **mPFS** *(115 trials; 148 arms)* | 0.851 | 2.88 | 3.85 | 0.0102 | 0.0003 | < 0.001 |
| **mOS** *(69 trials; 93 arms)* | 0.482 | 7.31 | 9.32 | 0.0146 | 0.0015 | < 0.001 |

$R^2$ = Coefficient of Determination; CV = cross-validation; MAE = mean absolute error; RMSE = root mean squared error; β = regression slope from linear mixed-effects model; SE = standard error; mPFS = median progression-free survival; mOS = median overall survival.

**Copyright Information:** (Online) ISSN: 2572-9241
©2026 The Author(s). HemaSphere is published by John Wiley & Sons Ltd on behalf of European Hematology Association. This is an open access Abstract Book distributed under the Attribution-NonCommercial-NoDerivs (CC BY-NC-ND), which allows third parties to download the articles and share them with others as long as they credit the author and the Abstract Book, but they cannot change the content in any way or use them commercially.
**Abstract Book Citations:** Authors, Title, HemaSphere, 2026;10:(S1):pages. The abstract book DOIs can be found on the "Author Information" page.
**Disclaimer:** Articles published in the journal HemaSphere exclusively reflect the opinions of the authors. The authors are responsible for all content in their abstracts including accuracy of the facts, statements, citing resources, etc.

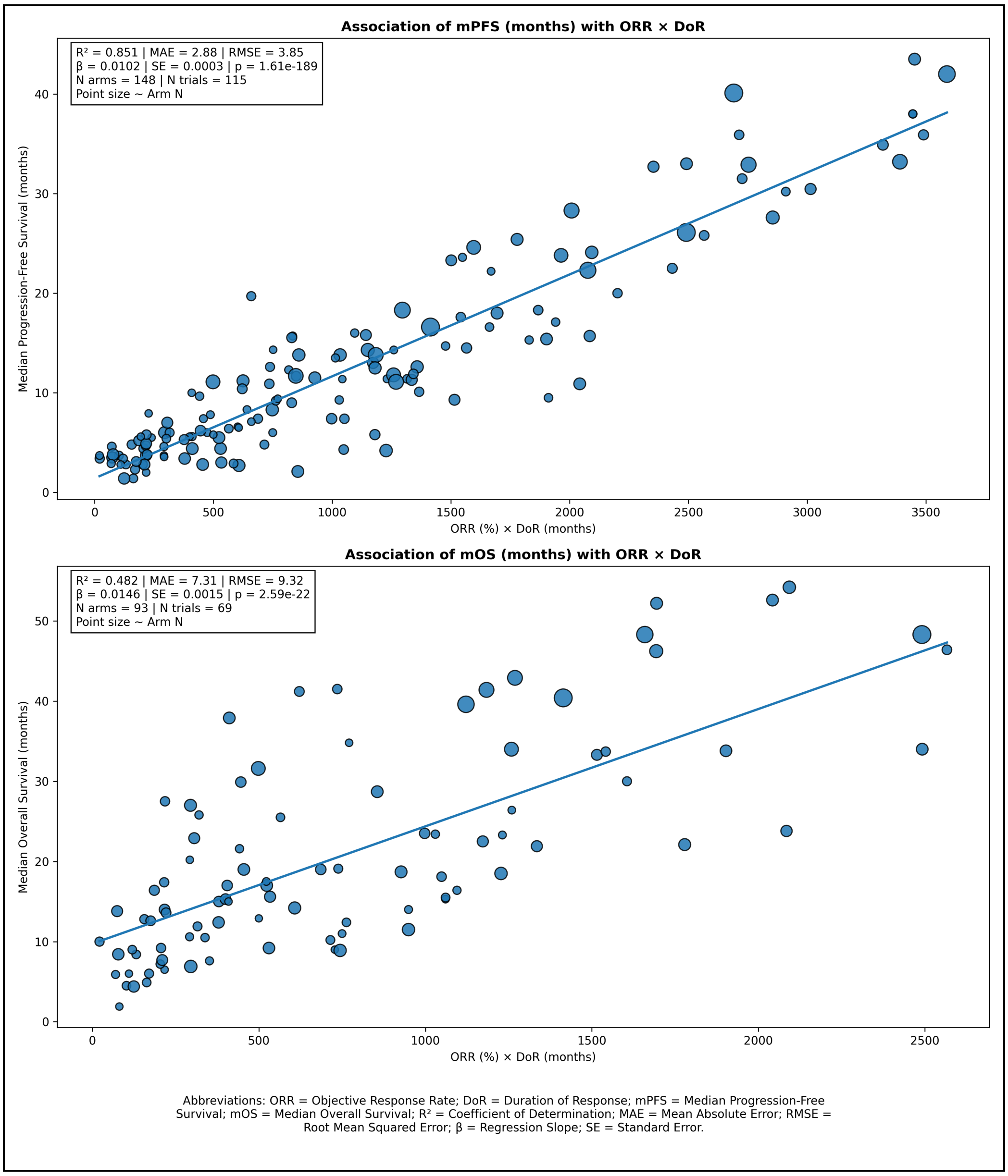

Figure 1: Association Between ORR×DoR and Survival Outcomes: Scatter Plots with Model Performance Metrics

**Copyright Information:** (Online) ISSN: 2572-9241
©2026 The Author(s). HemaSphere is published by John Wiley & Sons Ltd on behalf of European Hematology Association. This is an open access Abstract Book distributed under the Attribution-NonCommercial-NoDerivs (CC BY-NC-ND), which allows third parties to download the articles and share them with others as long as they credit the author and the Abstract Book, but they cannot change the content in any way or use them commercially.
**Abstract Book Citations:** Authors, Title, HemaSphere, 2026;10:(S1):pages. The abstract book DOIs can be found on the "Author Information" page.
**Disclaimer:** Articles published in the journal HemaSphere exclusively reflect the opinions of the authors. The authors are responsible for all content in their abstracts including accuracy of the facts, statements, citing resources, etc.

### (PB4513) USING HAIR-FOLLICLE VERSUS ORAL-MUCOSA DERIVED DNA IN A DEDICATED UNIT FOR GERMLINE HEMATOLOGIC NEOPLASMS

**Topic:** 38. Novel technologies, techniques and digital analytical tools in hematology

Ferran Balbastre-Ubeda*[1], Maria Gabarrós[1, 2], Francisco Beas[1], Sara Torres-Esquius[1, 2], Cristina Sanchez-Morales[2], Susana Urban-Giral[2], Meritxell Tena-Alegre[2], Maria Jesús Casas-Tio[2], Ana Garrido[1], Pamela Acha[1], Adoración Blanco[2], David Valcárcel[1, 2], Francisco Bosch Albareda[1, 2], Maria Julia Montoro Gomez[1, 2], Andres Jerez[1, 2]

*[1]Vall d'Hebron Institute of Oncology, Hematology, Barcelona, Spain;[2]Vall d'Hebron University Hospital, Hematology, Barcelona, Spain;*

### Background

At least 10% of patients with myeloid neoplasms harbor pathogenic germline variants in cancer predisposition genes. Early detection of these variants has major implications in the clinical management of individuals, such as therapeutic decision-making or donor selection. Cultured skin fibroblasts remain the gold standard DNA for germline testing. However, this approach is costly, technically demanding, and significantly extends turnaround time. Therefore, alternative DNA sources are needed.

### Aims

Here, we describe our institutional experience using hair follicle and oral mucosa-derived DNA as a routine source of germline DNA.

### Methods

The study population included individuals referred to Vall d'Hebron UH for germline genetic testing between April 2022 and February 2026, following CatSalut genetic testing criteria. Genomic DNA was extracted from hair follicle samples and/or oral mucosa swabs within 48h of collection using in-house protocols based on QIAamp DNA Kit Mini and Puregene Blood Kit (QIAGEN). A minimum of 25 hair follicles was required to proceed with DNA extraction. DNA quantity, purity and integrity were assessed using Qubit fluorometry, Nanodrop spectrophotometry and TapeStation analysis, respectively. Relevant clinical data was retrieved from electronic medical records. DNA samples were studied by Sanger sequencing and/or Whole Exome Sequencing (WES).

### Results

A total of 122 hair follicle DNA (hfDNA) and 70 oral mucosa DNA (omDNA) samples were included in the study. DNA concentration measured by Qubit revealed a median of 56.0 ng/µl (IQR, 24.4-106.5) for hfDNA and 53.6 ng/µl (IQR, 15.7-123.5) for omDNA. hfDNA yield was compared between chemotherapy-treated (n=13) vs untreated (n=76) patients. A significant higher yield was obtained for the untreated group ($p<0.001$), with a median of 59.1 ng/µl in contrast to 14.7 ng/µl on the treated group. Not statistically significant differences ($p=0.490$) were found in omDNA samples according to the therapy received. The median A260/A280 ratio assessed by Nanodrop was 2.06 ng/µl (IQR, 2.03-2.08) for hfDNA and 1.86 (IQR, 1.77-1.93) for omDNA samples. The median A260/A230 ratio was 1.71 (IQR, 6.8-7.5) for hfDNA and 1.19 (IQR, 0.71-1.62) for omDNA. DNA integrity analysis by TapeStation showed significantly better integrity ($p<0.001$) in hfDNA, with a median DIN value of 7.1 (IQR, 6.8-7.5) against a median value of 6.2 (IQR, 4.3-6.4) obtained for omDNA. Targeted Sanger sequencing achieved a 100% success rate in both hfDNA (n=38) and omDNA (n=5). WES showed high success rates as well, reaching 93.0% (53/57) in hfDNA and 94.7% (18/19) in omDNA.

**Copyright Information:** (Online) ISSN: 2572-9241
©2026 The Author(s). HemaSphere is published by John Wiley & Sons Ltd on behalf of European Hematology Association. This is an open access Abstract Book distributed under the Attribution-NonCommercial-NoDerivs (CC BY-NC-ND), which allows third parties to download the articles and share them with others as long as they credit the author and the Abstract Book, but they cannot change the content in any way or use them commercially.
**Abstract Book Citations:** Authors, Title, HemaSphere, 2026;10:(S1):pages. The abstract book DOIs can be found on the "Author Information" page.
**Disclaimer:** Articles published in the journal HemaSphere exclusively reflect the opinions of the authors. The authors are responsible for all content in their abstracts including accuracy of the facts, statements, citing resources, etc.

**Summary/Conclusion**

Hair follicle-derived DNA consistently provides adequate yield for germline genetic analyses, including WES. In cases where chemotherapeutic treatment is applied, the early collection of specimens (no later than the fourth day of treatment) is essential to ensure optimal performance. When this may not be feasible, oral mucosa represents a practical alternative. The short turnaround time, non-invasiveness and success rate on sequencing supports its implementation in time-demanding scenarios, such as hematopoietic stem cell transplantation donor screening.

**Copyright Information:** (Online) ISSN: 2572-9241
©2026 The Author(s). HemaSphere is published by John Wiley & Sons Ltd on behalf of European Hematology Association. This is an open access Abstract Book distributed under the Attribution-NonCommercial-NoDerivs (CC BY-NC-ND), which allows third parties to download the articles and share them with others as long as they credit the author and the Abstract Book, but they cannot change the content in any way or use them commercially.
**Abstract Book Citations:** Authors, Title, HemaSphere, 2026;10:(S1):pages. The abstract book DOIs can be found on the "Author Information" page.
**Disclaimer:** Articles published in the journal HemaSphere exclusively reflect the opinions of the authors. The authors are responsible for all content in their abstracts including accuracy of the facts, statements, citing resources, etc.

### (PB4514) ARTIFICIAL INTELLIGENCE MODELS TO PREDICT HIGH-GRADE CYTOKINE RELEASE SYNDROME IN CELLULAR AND BISPECIFIC ANTIBODY THERAPIES: AN INTEGRATED MULTI-SOURCE SIMULATION FRAMEWORK

**Topic:** 38. Novel technologies, techniques and digital analytical tools in hematology

Walter Dibenedetto[1, 2], Francesca Galeone[1, 2], Alessandro Massaro[2], Nicola Epicopo[2], Stefano Martinotti[1, 3], Francesco Gaudio*[1, 3]

*[1]Department of Medicine and Surgery, LUM University "Giuseppe Degennaro", casamassima, Italy; [2]Department of Engineering, LUM University "Giuseppe Degennaro", Casamassima (BA), Italy; [3]Ecclesiastical Entity Regional Generale Hospital "F. Miulli", Acquaviva delle Fonti (BA), Italy;*

### Background

Cytokine release syndrome (CRS) remains one of the most relevant toxicities associated with CAR-T cell treatments and CD3×CD20 bispecific antibodies. Recent studies have demonstrated the feasibility of predictive algorithms based on machine learning models and transfer learning based deep learning approaches, as well as nomograms identifying clinical predictors of CRS. However, model development is often limited by the scarcity of harmonized, high-quality clinical datasets and by the ethical constraints surrounding real-world patient data.

### Aims

To design a simulation-based framework reflecting the organizational and clinical workflow of a European hematology center, aimed at developing CRS predictive models without using real patient data. The objective was to generate realistic, multi-modal synthetic datasets enabling the training, comparison, and validation of machine learning and deep learning models for high-grade CRS prediction in cellular and bispecific antibody therapies.

### Methods

We created a multi-layer synthetic dataset (N=100 virtual patients) using parametric simulation and distribution modeling. Variable distributions were informed by published CRS predictive studies involving XGBoost-based classifiers, meta-analysis-informed models, nomograms for CD3×CD20 bispecific antibodies, and deep learning architectures employing transfer learning from COVID-19 patient datasets.
The dataset included simulated demographics, disease burden, inflammatory markers, coagulation parameters, complete blood counts, vital signs, and treatment-related variables.
Three models were developed: XGBoost-CRS baseline model, ML-Nomogram, and DeepSim-CRS, a transformer-based architecture incorporating sequential laboratory data. Performance was evaluated through 10-fold cross-validation and calibration analysis.

### Results

Synthetic data distributions successfully replicated patterns described in clinical CRS cohorts, including the relevance of $SpO_2$, D-dimer, diastolic blood pressure, INR, monocyte-to-lymphocyte ratio, and platelet count as key CRS predictors.
Model performance was as follows:  XGBoost-CRS: AUC 0.82; ML-Nomogram: AUC 0.78; DeepSim-CRS: AUC 0.89, with early-warning capability up to 48 hours before severe CRS onset, consistent with previously published transfer-learning approaches.
SHAP analysis highlighted inflammatory markers, hematologic indices, and simulated tumor burden as the most influential predictors across models.

**Copyright Information:** (Online) ISSN: 2572-9241
©2026 The Author(s). HemaSphere is published by John Wiley & Sons Ltd on behalf of European Hematology Association. This is an open access Abstract Book distributed under the Attribution-NonCommercial-NoDerivs (CC BY-NC-ND), which allows third parties to download the articles and share them with others as long as they credit the author and the Abstract Book, but they cannot change the content in any way or use them commercially.
**Abstract Book Citations:** Authors, Title, HemaSphere, 2026;10:(S1):pages. The abstract book DOIs can be found on the "Author Information" page.
**Disclaimer:** Articles published in the journal HemaSphere exclusively reflect the opinions of the authors. The authors are responsible for all content in their abstracts including accuracy of the facts, statements, citing resources, etc.

**Summary/Conclusion**

We developed a fully simulated, privacy-preserving framework for CRS prediction that emulates the operational structure of a real hematology department while avoiding the use of identifiable clinical data. This approach enables safe methodological experimentation, facilitates rapid prototyping of CRS prediction tools, and provides a scalable foundation for future validation on real-world datasets following ethical approval. Simulation-based modeling may accelerate the deployment of AI-driven risk stratification in immunotherapy and support safer, more personalized patient management.

**Copyright Information:** (Online) ISSN: 2572-9241
©2026 The Author(s). HemaSphere is published by John Wiley & Sons Ltd on behalf of European Hematology Association. This is an open access Abstract Book distributed under the Attribution-NonCommercial-NoDerivs (CC BY-NC-ND), which allows third parties to download the articles and share them with others as long as they credit the author and the Abstract Book, but they cannot change the content in any way or use them commercially.
**Abstract Book Citations:** Authors, Title, HemaSphere, 2026;10:(S1):pages. The abstract book DOIs can be found on the "Author Information" page.
**Disclaimer:** Articles published in the journal HemaSphere exclusively reflect the opinions of the authors. The authors are responsible for all content in their abstracts including accuracy of the facts, statements, citing resources, etc.

### (PB4515) EX VIVO DRUG SENSITIVITY PROFILING OF BONE MARROW CELLS OBTAINED AT DIAGNOSIS FROM PATIENTS WITH CML USING AN AI-ASSISTED MICROFLUIDIC PLATFORM

**Topic:** 38. Novel technologies, techniques and digital analytical tools in hematology

Hwisu Jeon[1], Eujin Um[2, 3], Chang-Yeol Jung[2, 4], Hongtae Kim[2, 5], Ji Hyun Lee[6], Sung-Hyun Kim[6], Taesung Kim*[1, 2]

*[1]Ulsan National Institute of Science and Technology, Department of Mechanical Engineering, Ulsan, South Korea;[2]TKMS, Inc, Ulsan, South Korea; [3]Ulsan National Institute of Science and Technology, Department of Physics, Ulsan, South Korea;[4]Ulsan National Institute of Science and Technology, Department of Mathematical Sciences, Ulsan, South Korea; [5]Ulsan National Institute of Science and Technology, Department of Biological Sciences, Ulsan, South Korea; [6]Dong-A University College of Medicine, Division of Hematology-Oncology, Department of Internal Medicine, Busan, South Korea;*

### Background

*BCR::ABL1* tyrosine kinase inhibitors (TKIs) used for the treatment of chronic myeloid leukemia (CML) exhibit heterogeneous efficacy depending on patient-specific genetic or biological factors. Although genomic profiling provides valuable therapeutic guidance, comprehensive assessments of drug responses using directly patient-derived cells offer complementary phenotype-based information that may enhance personalized treatment strategies. We developed a high-density microfluidic diagnostic platform containing approximately 1,000 culture chambers capable of testing multiple TKIs across 6-log-scale concentrations. In addition, we established an AI-assisted image analysis algorithm to quantitatively evaluate drug responses of bone marrow mononuclear cells obtained from patients with CML.

### Aims

We aimed to evaluate whether *ex vivo* drug sensitivity metrics derived from freshly isolated CML bone marrow cells correlated with clinical treatment responses or not. This study was designed as a translational exploratory investigation; platform results were not yet used to guide real-time therapeutic decisions.

### Methods

Bone marrow mononuclear cells were directly collected from CML patients at the time of diagnosis and loaded into the microfluidic cell culture array designed to stably confine suspension leukemia cells and enhance imaging accuracy (Fig. 1a). To accommodate variability and heterogeneous subpopulations in patient-derived samples, chamber geometry, loading strategy, and culture conditions were optimized beyond those used for established cell lines. Discrete concentration gradients of multiple TKIs (imatinib, dasatinib, radotinib, and asciminib) were generated using a programmable pneumatic pumping system, delivering each concentration to 100 chambers. An automated imaging system continuously monitored cell viability and drug response over 48 hours. Images were analyzed using our AI-based algorithm to quantify drug response metrics, including $E_{max}$ (maximum effect) and AUC (area under the inhibition curve). This integrated workflow, spanning sample processing to analysis, is optimized to deliver patient-specific results within a 72 hours. Clinical data, including disease phase, prior TKI exposure, and major molecular response, were collected. *Ex vivo* drug response profiles were analyzed in parallel with longitudinal clinical outcomes.

**Copyright Information:** (Online) ISSN: 2572-9241
©2026 The Author(s). HemaSphere is published by John Wiley & Sons Ltd on behalf of European Hematology Association. This is an open access Abstract Book distributed under the Attribution-NonCommercial-NoDerivs (CC BY-NC-ND), which allows third parties to download the articles and share them with others as long as they credit the author and the Abstract Book, but they cannot change the content in any way or use them commercially.
**Abstract Book Citations:** Authors, Title, HemaSphere, 2026;10:(S1):pages. The abstract book DOIs can be found on the “Author Information” page.
**Disclaimer:** Articles published in the journal HemaSphere exclusively reflect the opinions of the authors. The authors are responsible for all content in their abstracts including accuracy of the facts, statements, citing resources, etc.

**Results**

The microfluidic platform, powered by our proprietary AI-assisted image analysis algorithm, successfully supported the culture of patient bone marrow cells and distinguished the varying efficacies of different TKIs among individual CML patients (Fig. 1b). Quantitative comparison of $E_{max}$ and AUC values revealed patient-specific drug sensitivity patterns (Fig. 1c). In the representative cases, imatinib, dasatinib, and asciminib demonstrated superior efficacy compared to radotinib across both patients, with the relative magnitude of response showing critical inter-individual variability. Ongoing cohort expansion aims to further evaluate associations between *ex vivo* sensitivity metrics and subsequent molecular response.

**Summary/Conclusion**

We established an AI-assisted microfluidic platform enabling quantitative *ex vivo* profiling of TKI sensitivity in CML bone marrow cells within 72 hours. This translational approach may complement molecular diagnostics by providing timely phenotype-based drug response information. Prospective validation in larger cohorts is warranted.

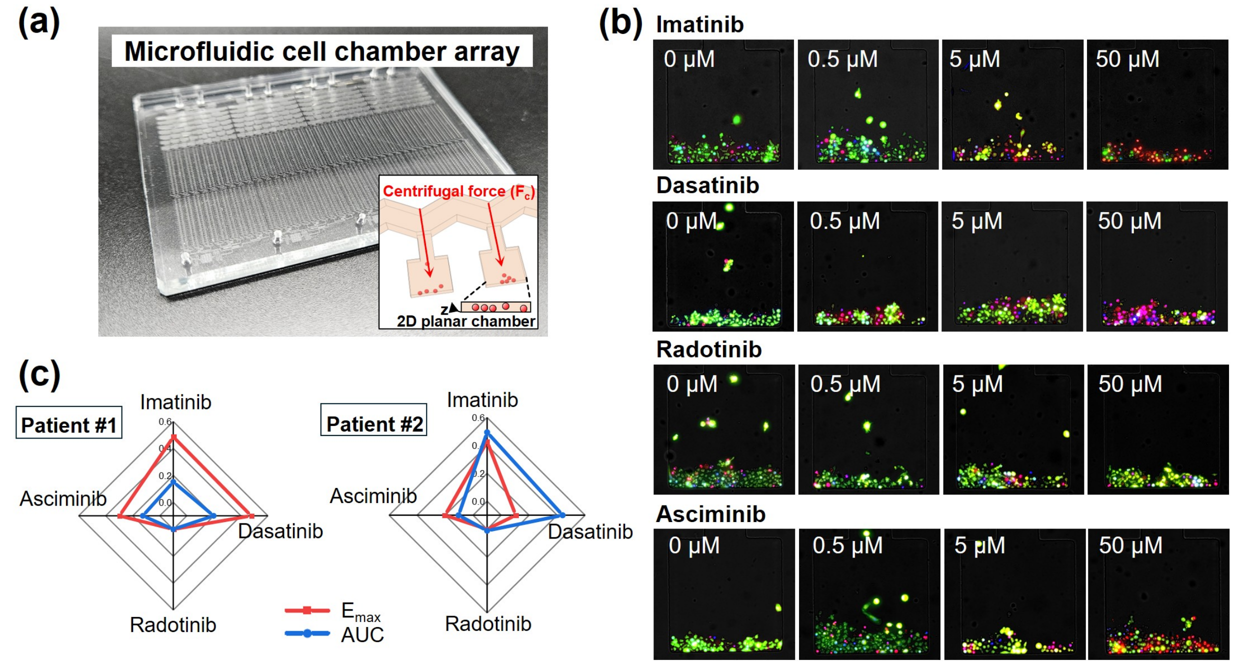

**Figure 1**. The microfluidic drug screening platform for personalized TKI evaluation. (a) The microfluidic cell chamber array to deliver freshly isolated patient bone marrow cells and multiple TKI concentrations. (b) Representative images from individual microfluidic chambers containing patient cells after 48-hour drug treatment at varying TKI concentrations. Drug responses were assessed using live/dead staining with calcein AM (green, live) and propidium iodide (red, dead). (c) Comparative analysis of drug response metrics ($E_{max}$ and AUC) across four different TKIs for each patient, showing individual sensitivity patterns.

Copyright Information: (Online) ISSN: 2572-9241
©2026 The Author(s). HemaSphere is published by John Wiley & Sons Ltd on behalf of European Hematology Association. This is an open access Abstract Book distributed under the Attribution-NonCommercial-NoDerivs (CC BY-NC-ND), which allows third parties to download the articles and share them with others as long as they credit the author and the Abstract Book, but they cannot change the content in any way or use them commercially.
**Abstract Book Citations:** Authors, Title, HemaSphere, 2026;10:(S1):pages. The abstract book DOIs can be found on the "Author Information" page.
**Disclaimer:** Articles published in the journal HemaSphere exclusively reflect the opinions of the authors. The authors are responsible for all content in their abstracts including accuracy of the facts, statements, citing resources, etc.

### (PB4516) VISION LANGUAGE MODEL PERFORMANCE IN ACUTE MYELOID LEUKEMIA DIAGNOSTICS: MULTI-MODEL BENCHMARK REVEALS CRITICAL GAPS IN CLINICAL REASONING

**Topic:** 38. Novel technologies, techniques and digital analytical tools in hematology

Freya Schulze*[1, 2, 3], Chiara Löffler[1, 2, 3], Martina Radoynova[1], Susann Winter[1, 3], Katja Sockel[1], Frank Kroschinsky[1], Martin Bornhäuser[1, 2], Jan Moritz Middeke[1, 3, 4], Jakob Kather[1, 3, 5, 6], Jan-Niklas Eckardt[1, 3, 4], Narmin Ghaffari Laleh[3]

*[1]Department of Internal Medicine I, University Hospital Carl Gustav Carus, Technical University Dresden, Department of Internal Medicine I, Dresden, Germany; [2]National Center for Tumor Diseases Dresden (NCT/UCC), a partnership between DKFZ, Faculty of Medicine and University Hospital Carl Gustav Carus, TUD Dresden University of Technology, and Helmholtz-Zentrum Dresden - Rossendorf (HZDR), Dresden, Germany; [3]Else Kröner Fresenius Center for Digital Health, Technical University Dresden, Dresden, Germany; [4]Mildred Scheel Early Career Center, National Center for Tumor Diseases (NCT/UCC) Dresden, Faculty of Medicine and University Hospital Carl Gustav Carus, TU Dresden University of Technology, Dresden, Germany; [5]Medical Oncology, National Center for Tumor Diseases (NCT), University Hospital Heidelberg, Heidelberg, Germany; [6]Pathology & Data Analytics, Leeds Institute of Medical Research at St James's, University of Leeds, Leeds, United Kingdom;*

### Background

Hematologic diagnostics and especially cytomorphologic assessment are time-intensive and require high levels of expertise. Vision Language Models (VLM) show promise in medical image analysis in radiology and histopathology, while an evaluation on hematological microscopy is lacking.

### Aims

To evaluate three state-of-the-art (SOTA) Vision Language Models regarding their diagnostic accuracy and safety in clinical decision support in detecting acute myeloid leukemia (AML) from digitized bone marrow smears (BMS).

### Methods

Whole slide images were obtained from bone marrow smears of 50 AML patients and 50 bone marrow donors. Ten representative fields of view per sample were extracted manually. Three VLMs were used, two of which are considered generalist models (Qwen3.5-397B-A17B-FP8, GLM-4.6V-FP8), while the third one is a medically adapted model (Medgemma-27b-it). All models performed zero-shot analysis using two prompting strategies: First, a context-rich prompt requesting reporting of WHO/FAB diagnostic criteria in a structured manner, and secondly, a minimal prompt without specific hematologic context. Model outputs were evaluated against ground-truth cytomorphologic reports using accuracy, sensitivity, specificity and Cohen's Kappa (Cohen's κ).

**Copyright Information:** (Online) ISSN: 2572-9241
©2026 The Author(s). HemaSphere is published by John Wiley & Sons Ltd on behalf of European Hematology Association. This is an open access Abstract Book distributed under the Attribution-NonCommercial-NoDerivs (CC BY-NC-ND), which allows third parties to download the articles and share them with others as long as they credit the author and the Abstract Book, but they cannot change the content in any way or use them commercially.
**Abstract Book Citations:** Authors, Title, HemaSphere, 2026;10:(S1):pages. The abstract book DOIs can be found on the "Author Information" page.
**Disclaimer:** Articles published in the journal HemaSphere exclusively reflect the opinions of the authors. The authors are responsible for all content in their abstracts including accuracy of the facts, statements, citing resources, etc.

**Results**

Overall diagnostic accuracy was poor for all SOTA models as they exhibited the overwhelming tendency to classify the majority of samples as leukemic: With context-rich prompts, GLM4.6 identified 90% of leukemic samples while also labeling 92% of bone marrow donors as AML. The medical specialist model MedGemma-27b showed similar failure, misclassifying 86% of healthy donors and correctly detecting AML in only 66% of cases. Qwen3.5 performed best under detailed prompting, achieving a specificity of 0.26 and accuracy of 0.51. Accuracy of all models improved with context-free prompts (range 0.47-0.79), yet they still lacked the ability to correctly distinguish between leukemia and healthy bone marrow. GLM-4.6 misclassified 80% of healthy donors as leukemic while MedGemma-27b hallucinated blast cells for all cases, falsely diagnosing them as AML. Qwen3.5 was the only model to maintain meaningful specificity (0.64) and correctly identified 94% of AML, yielding an overall accuracy of 0.79. Morphologic feature-level agreement with human expert reports was poor across all models (mean Cohen's $\kappa = 0.025$; range: 0.01 to 0.04), indicating poor recognition of cell-level morphologies.

**Summary/Conclusion**

While SOTA VLMs demonstrate promising performance in radiology and histopathology, they struggled to discriminate between leukemic and healthy bone marrow. Notably, Qwen3.5 exhibited a comparatively more balanced performance profile, suggesting that general-purpose large-scale VLMs may have a marginal advantage over smaller models, even though all tested models remain far below the threshold required for safe clinical application. This failure is likely driven by the fact that pathology imaging archives are vastly scraped during model training while hematological samples are not as widely available and therefore, hematology is an out-of-bounds use-case for these models, rendering them currently unsuitable for clinical decision support in hematology. Any future consideration of clinical deployment would first require substantial domain-specific adaptation on hematological datasets and rigorous prospective benchmarking.

**Copyright Information:** (Online) ISSN: 2572-9241
©2026 The Author(s). HemaSphere is published by John Wiley & Sons Ltd on behalf of European Hematology Association. This is an open access Abstract Book distributed under the Attribution-NonCommercial-NoDerivs (CC BY-NC-ND), which allows third parties to download the articles and share them with others as long as they credit the author and the Abstract Book, but they cannot change the content in any way or use them commercially.
**Abstract Book Citations:** Authors, Title, HemaSphere, 2026;10:(S1):pages. The abstract book DOIs can be found on the "Author Information" page.
**Disclaimer:** Articles published in the journal HemaSphere exclusively reflect the opinions of the authors. The authors are responsible for all content in their abstracts including accuracy of the facts, statements, citing resources, etc.

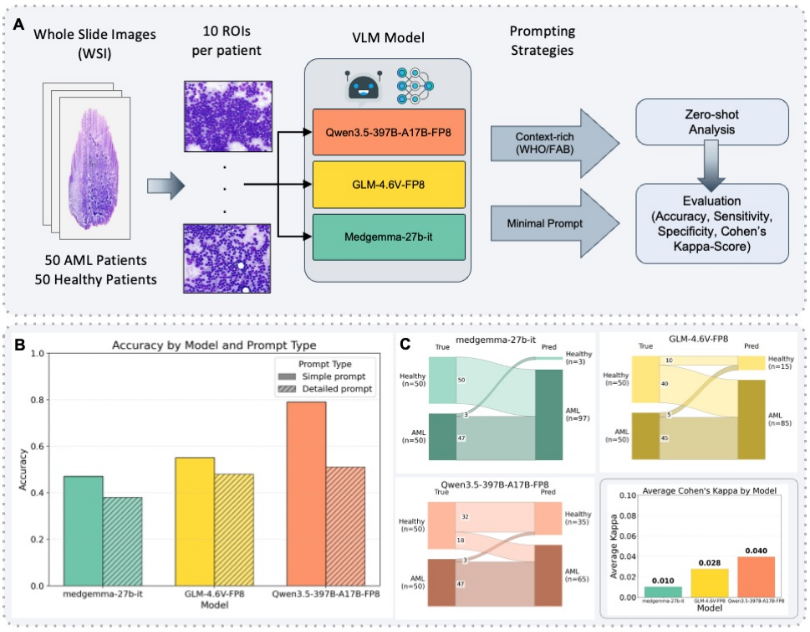

Figure 1: Overview of the study. A) Methods B) Results showing model accuracy using detailed or minimal prompting strategy C) Diagnostic performance per model using minimal prompt and average Cohen‘s kappa for inter-rater agreement across all morphologic features. AML= Acute myeloid leukemia, FAB= French American British Group, Pred= Diagnosis per model’s examination, ROI= Regions of Interest, True= Ground Truth (expert cytomorphologic report), VLM= Vision Language Model, WHO= World Health Organization

**Copyright Information:** (Online) ISSN: 2572-9241
©2026 The Author(s). HemaSphere is published by John Wiley & Sons Ltd on behalf of European Hematology Association. This is an open access Abstract Book distributed under the Attribution-NonCommercial-NoDerivs (CC BY-NC-ND), which allows third parties to download the articles and share them with others as long as they credit the author and the Abstract Book, but they cannot change the content in any way or use them commercially.
**Abstract Book Citations:** Authors, Title, HemaSphere, 2026;10:(S1):pages. The abstract book DOIs can be found on the “Author Information” page.
**Disclaimer:** Articles published in the journal HemaSphere exclusively reflect the opinions of the authors. The authors are responsible for all content in their abstracts including accuracy of the facts, statements, citing resources, etc.

### (PB4517) DURABLE VIROLOGICAL RESPONSE CORRELATES WITH IMPROVED SURVIVAL IN EBV-DRIVEN HEMOPHAGOCYTIC LYMPHOHISTIOCYTOSIS AMONG ADOLESCENTS AND ADULTS

**Topic:** 38. Novel technologies, techniques and digital analytical tools in hematology

Qiaolin Zhou*[1, 2, 3, 4], Yang Dai[1, 5, 6], Xushu Zhong[1, 5, 6], Yongqian Jia[1, 5, 6], Liping Xie[1, 5, 6], Luocheng Zhang[1, 4], Yushuang Xiao[1, 4], Wenyi Liang[1, 4], Zhuohang Zou[1, 4], Mengting Yang[1, 4], Jie Huang[1, 5, 6], Pu Kuang[1, 5, 6], Ting Niu[1, 5, 6]

*[1]Department of Hematology, Institute of Hematology, and Center for High Altitude Medicine, West China Hospital, Sichuan University., Chengdu, China; [2]West China Lecheng Hospital of Sichuan University, Hainan, China;[3]Department of Hematology, Mianyang Central Hospital School of Medicine. University of Electronic Science and Technology of China, Mianyang, China; [4]West China Medical School, West China Hospital, Sichuan University., Chengdu, China;[5]State Key Laboratory of Biotherapy, Collaborative Innovation Center of Biotherapy, West China Hospital, Sichuan University., Chengdu, China; [6]National Facility for Translational Medicine (Sichuan), West China Hospital, Sichuan University., Chengdu, China;*

### Background

Epstein–Barr virus–associated hemophagocytic lymphohistiocytosis (EBV-HLH) is recognized as a fulminant hyperinflammatory syndrome with particularly poor outcomes in adolescents and adults. Plasma EBV-DNA is routinely applied for diagnosis and disease monitoring; however, most prognostic models have been constructed primarily on baseline parameters and short-term clinical response. The prognostic relevance of longitudinal viral kinetics in adult EBV-HLH has not been systematically established.

### Aims

To determine the prognostic impact of longitudinal EBV-DNA dynamics in adolescents and adults with EBV-HLH and to develop a dynamic risk stratification model integrating virological and clinical variables.

### Methods

A total of 214 adolescents and adults with EBV-HLH who received standardized induction therapy were retrospectively analyzed. Serial plasma EBV-DNA measurements were collected during follow-up to characterize viral kinetics. Overall survival (OS) was estimated using the Kaplan–Meier method and evaluated with multivariable Cox proportional hazards regression. Landmark analyses with stabilized inverse probability weighting were performed to address time-dependent treatment exposure. Independent prognostic factors were incorporated into a nomogram, which was internally validated using bootstrap resampling to assess discrimination and calibration.

**Copyright Information:** (Online) ISSN: 2572-9241
©2026 The Author(s). HemaSphere is published by John Wiley & Sons Ltd on behalf of European Hematology Association. This is an open access Abstract Book distributed under the Attribution-NonCommercial-NoDerivs (CC BY-NC-ND), which allows third parties to download the articles and share them with others as long as they credit the author and the Abstract Book, but they cannot change the content in any way or use them commercially.
**Abstract Book Citations:** Authors, Title, HemaSphere, 2026;10:(S1):pages. The abstract book DOIs can be found on the "Author Information" page.
**Disclaimer:** Articles published in the journal HemaSphere exclusively reflect the opinions of the authors. The authors are responsible for all content in their abstracts including accuracy of the facts, statements, citing resources, etc.

### Results

The median age at diagnosis was 36 years, and the median OS was 7.53 months. Longitudinal EBV-DNA kinetics demonstrated marked time-dependent prognostic discrimination. Sustained EBV-DNA negativity during follow-up was identified as the strongest protective factor for long-term survival, with near-complete separation of survival curves at both 6 months and 1 year (both log-rank $P < 0.001$). In contrast, baseline viral burden was not retained as an independent predictor of OS after adjustment for dynamic viral status. EBV-DNA positivity at 1 year was associated with a substantially increased mortality risk (HR 160.45; 95% CI, 21.29–1209.35), indicating the dominant impact of persistent viral replication.
Central nervous system involvement was independently associated with inferior OS (HR 2.39; 95% CI, 1.40–4.07). Although PD-1 inhibitor exposure was associated with improved OS in conventional analyses, this association was attenuated after landmark analysis with stabilized inverse probability weighting, suggesting that survival benefit may have been mediated through downstream viral control rather than treatment exposure itself. Early induction response (≥PR) and earlier treatment initiation were associated with improved survival in univariate analyses but did not retain independent prognostic significance once longitudinal EBV-DNA status was incorporated into multivariable models.
A dynamic prognostic nomogram integrating 1-year EBV-DNA status and key clinical variables demonstrated favorable discrimination (C-index 0.835) and good calibration, with high predictive accuracy for 1-, 2-, and 3-year OS.

### Summary/Conclusion

Longitudinal EBV-DNA kinetics were identified as the principal determinant of long-term survival in adolescent and adult EBV-HLH. Persistent viral replication defined a high-risk biological phenotype with markedly increased mortality. Integration of serial EBV-DNA monitoring into prognostic modeling enabled robust risk stratification and may support response-adapted therapeutic strategies in EBV-HLH.

**Copyright Information:** (Online) ISSN: 2572-9241
©2026 The Author(s). HemaSphere is published by John Wiley & Sons Ltd on behalf of European Hematology Association. This is an open access Abstract Book distributed under the Attribution-NonCommercial-NoDerivs (CC BY-NC-ND), which allows third parties to download the articles and share them with others as long as they credit the author and the Abstract Book, but they cannot change the content in any way or use them commercially.
**Abstract Book Citations:** Authors, Title, HemaSphere, 2026;10:(S1):pages. The abstract book DOIs can be found on the "Author Information" page.
**Disclaimer:** Articles published in the journal HemaSphere exclusively reflect the opinions of the authors. The authors are responsible for all content in their abstracts including accuracy of the facts, statements, citing resources, etc.

### (PB4518) AUTOMATED NATURAL LANGUAGE PROCESSING OF PHYSICIAN HANDOVER NOTES REVEALS REAL-WORLD ANTIBIOTIC STEWARDSHIP PATTERNS IN A HEMATOLOGY WARD

**Topic:** 38. Novel technologies, techniques and digital analytical tools in hematology

Federico De Marchi*[1], Alessandro Atanasio[1], Giulia Ciotti[1], Roberta de Marchi[1], Alessandra Sperotto[1, 1], Michele Gottardi[1]

[1]*Istituto Oncologico veneto (IOV), Oncohematology, Castelfranco Veneto, Italy;*

### Background

Patients with hematological malignancies face high infection risk due to disease- and treatment-related immunosuppression. While guidelines recommend antibiotic stewardship, real-world prescribing data at the ward level remain scarce. Daily physician handover documents, routinely written for shift communication, are an untapped source of longitudinal clinical information that could bridge this evidence gap without requiring dedicated data collection infrastructure.

### Aims

To develop a fully automated natural language processing (NLP) pipeline converting unstructured daily handover documents into a structured longitudinal database, and to characterize real-world antibiotic prescribing, escalation dynamics, and multidrug-resistant organism (MDRO) burden in a hematology ward over 5 years.

### Methods

We collected 1,754 daily handover Word documents from a single hematology ward (October 2020-February 2026). A Python-based NLP pipeline was built to process all files without manual data entry. Key methodological features include: (1) patient deduplication via fuzzy string matching (threshold ≥0.90); (2) diagnosis harmonization through 50+ regex rules mapping Italian free-text to standardized categories; (3) a Zone A/Zone B text-splitting algorithm distinguishing active treatments from conditional plans (e.g., "if fever, start meropenem"), preventing misclassification of planned-but-not-administered drugs as active therapy; (4) negation-aware context matching for MDRO colonization and clinical worsening detection, excluding negated mentions; (5) admission reconstruction via a >2-day gap rule; and (6) baseline-aware escalation tracking counting only new antibacterials from day ≥2. Multivariable logistic regression assessed the independent association between MDRO colonization and clinical worsening, adjusting for diagnosis, length of stay, and calendar year.

### Results

The pipeline extracted 24,483 patient-days from 943 patients across 2,596 admissions. Case mix was AML/APL-dominant (43.7%), followed by aggressive (18.1%) and indolent NHL (16.8%). Median LOS was 7 days (IQR 4-12). Overall, 38.5% of admissions received antibacterials; 23.1% had ≥1 true escalation event (1,066 total; median day 8). Piperacillin-tazobactam was the most prescribed agent (29.9%) and leading escalation target. MDRO colonization (9.4%; predominantly VRE, 62.4%) was independently associated with clinical worsening on multivariable analysis (adjusted OR 2.31; 95%CI 1.68-3.17; p<0.0001). A declining temporal trend in worsening was observed (aOR 0.89/year; p=0.004). Conditional plans were documented in 79.4% of admissions; 18.3% were subsequently executed.

**Copyright Information:** (Online) ISSN: 2572-9241
©2026 The Author(s). HemaSphere is published by John Wiley & Sons Ltd on behalf of European Hematology Association. This is an open access Abstract Book distributed under the Attribution-NonCommercial-NoDerivs (CC BY-NC-ND), which allows third parties to download the articles and share them with others as long as they credit the author and the Abstract Book, but they cannot change the content in any way or use them commercially.
**Abstract Book Citations:** Authors, Title, HemaSphere, 2026;10:(S1):pages. The abstract book DOIs can be found on the "Author Information" page.
**Disclaimer:** Articles published in the journal HemaSphere exclusively reflect the opinions of the authors. The authors are responsible for all content in their abstracts including accuracy of the facts, statements, citing resources, etc.

**Summary/Conclusion**

Automated NLP of routine handover documents generates robust real-world evidence on antibiotic stewardship without dedicated data collection. This approach revealed persistent broad-spectrum escalation pressure and an independent association between MDRO colonization and clinical worsening. The methodology is reproducible, scalable to any ward generating handover notes, and offers a novel paradigm for continuous stewardship surveillance in high-risk hematology settings.

**Copyright Information:** (Online) ISSN: 2572-9241
©2026 The Author(s). HemaSphere is published by John Wiley & Sons Ltd on behalf of European Hematology Association. This is an open access Abstract Book distributed under the Attribution-NonCommercial-NoDerivs (CC BY-NC-ND), which allows third parties to download the articles and share them with others as long as they credit the author and the Abstract Book, but they cannot change the content in any way or use them commercially.
**Abstract Book Citations:** Authors, Title, HemaSphere, 2026;10:(S1):pages. The abstract book DOIs can be found on the “Author Information” page.
**Disclaimer:** Articles published in the journal HemaSphere exclusively reflect the opinions of the authors. The authors are responsible for all content in their abstracts including accuracy of the facts, statements, citing resources, etc.

### (PB4519) DEEP MOLECULAR INQUIRY BY ARTIFICIAL INTELLIGENCE ALGORITHMS UNVEILS THE HIGH DIAGNOSTIC INFORMATIVE YIELD OF SRSF2, RAS AND CHIPS ALLELIC BURDEN.

**Topic:** 38. Novel technologies, techniques and digital analytical tools in hematology

Micol Caccia*[1], Emanuela Deponti[2], Nawar Maher[1], Monia Lunghi[2], Monia Marchetti[3]

*[1]UNIUPO, Alessandria, Italy; [2]Division of Hematology, Department of Translational Medicine, Università del Piemonte Orientale and AOU Maggiore della Carità di Novara, NOVARA, Italy; [3]Azienda Ospedaliera Universitaria SS Antonio e Biagio e Cesare Arrigo, Alessandria, Italy;*

### Background

Diagnostic classification of patients with myeloid neoplasms i(MN) s continuously evolving towards fuzzy classes defined by molecular profiles, rather than by morphology items. However, some categories, e.g. myelodysplatic/myeloproliferative neoplasms (MDS/MPN) and myelodysplastic syndromes (MDS), might still be diagnosed mostly based on morphology patterns. In addition, some patients carrying non-neoplastic cytopenias often struggle with diagnostic discrimination. Finally, molecular patterns often better track and forecast patient trajectories than morophology features. Therefore, molecular patterns deserve to be further investigated in order to support the diagnostic and prognostic workup.

### Aims

The DEEP MOLECULAR INSIGHT (DMI) study aimed at ascertaining the diagnostic value of molecular features in patients receiving a full work-up for suspected MN. The study used artificial intelligence (AI) algorithms for unveiling the complex interactions among pieces of information, included the allelic burden (VAF) of NGS-assessed mutations.

### Methods

We collected 32-gene NGS and karyotype features of consecutive patients receiving bone marrow aspirate at AOUAL and AOUNO since Jan 2022. Final WHO 2022 diagnosis was also recorded. Five supervised machine learning algortihms were applied to the original dataset: Gradient Boost (GF), Ada Boost, Neural Network, Classification Tree, Random Forest (RF). Ten-fold cross-validation was requested. The informative value was estimated by the Area Under the Curve (AUC). Orange TM 3.39 software was used for the analysis. The study was approved by Novara EC.

**Copyright Information:** (Online) ISSN: 2572-9241
©2026 The Author(s). HemaSphere is published by John Wiley & Sons Ltd on behalf of European Hematology Association. This is an open access Abstract Book distributed under the Attribution-NonCommercial-NoDerivs (CC BY-NC-ND), which allows third parties to download the articles and share them with others as long as they credit the author and the Abstract Book, but they cannot change the content in any way or use them commercially.
**Abstract Book Citations:** Authors, Title, HemaSphere, 2026;10:(S1):pages. The abstract book DOIs can be found on the "Author Information" page.
**Disclaimer:** Articles published in the journal HemaSphere exclusively reflect the opinions of the authors. The authors are responsible for all content in their abstracts including accuracy of the facts, statements, citing resources, etc.

### Results

The analyzed cohort included 757 patients (55% males, median age 64 years) who received a WHO2022 diagnosis of MDS (17%), acute myeloid leuemia (28%), myeloproliferative neoplasms (25%), MDS/MPN (6%), or no MN diagnosis (24%). Karyotype was normal in 57.2% and complex in 6-5%. A median of 2 genes (range 0-8) per sample was mutated, but 25% of the patients reported abnormalities in > 4 genes. the most frequently mutated genes were JAK2 (27.8%), ASXL1 (23.7%), TET2 (23.6%), DNMT3A (17.45), RUNX1 (10.7%), and TP53 (8.8%).
MN were efficiently ruled out by RF and GB (0.74 AUC): the number of mutated genes was the single most powerful information, while RUNX1, SRSF2, and HRAS mutations, CEBPA, JAK2, TET2 and DNMT3A VAFs were the most informative features. However, the informative value of NGS features varied according to patient age and normal karyotype also efficiently ruled out MN. The AI algorithms were also effective in ruling in and out MDS/MPN (0.69 AUC) and highlighted unexpected informative NGS features (Figure). A high H-RAS mutation VAF was highly predictive for ruling in MDS/MPN (AUC decrease 0.062). However, the alleic burden of other gene mutations, namely KIT, SF3B1, DNMT3A, IDH2, also reported a high informative value.
Finally, the 5 AI algorithms were  involved in detecting MDS (0.69 AUC). Most of the aalgorithms selected SRSF2 VAF as the single most powerful piece of information. JAK2 VAF was also useful for ruling-out MDS. Detection of other gene mutation VAFs also helped ruling-in MDS, namely ZRSR2, CSF3R, DNMT3A, SF3B1, SETBP1.

### Summary/Conclusion

Diagnostic assessment of MN might be significantly enhanced by AI-based algorithms assessing patients' molecular profile. In particular, the allelic burden of detected mutations often has a relevant informative value, which deserves further validation.

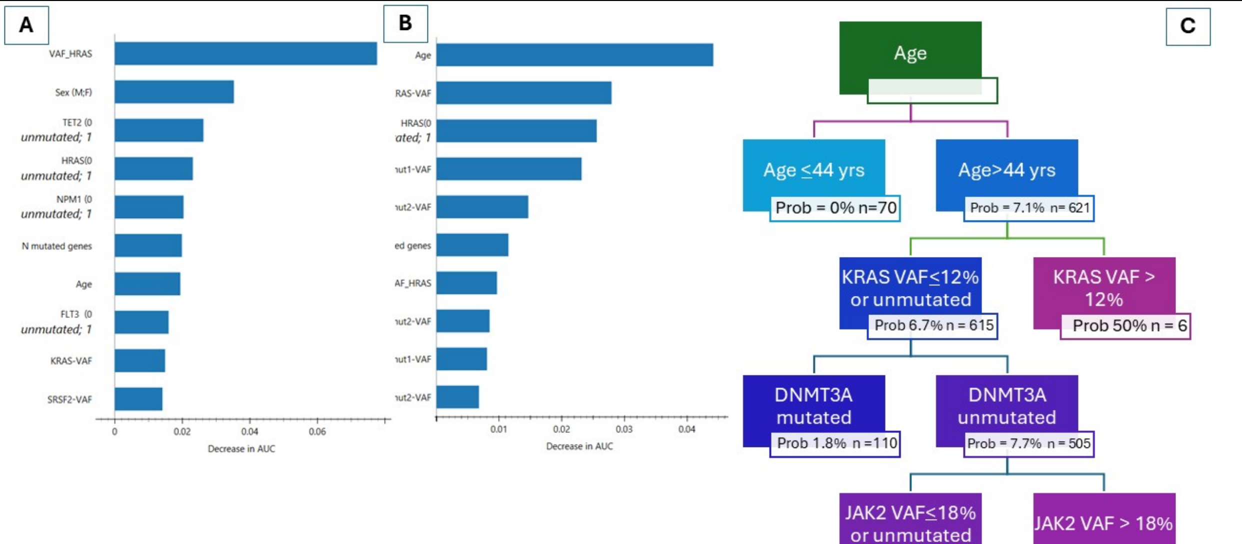

**MDS/MPN Informative value.**
A. Random Forest. B. Gradient boosting. C. Classification tree.

Legend: Prob = chance of MDS/MPN; n = number of patients

Copyright Information: (Online) ISSN: 2572-9241
©2026 The Author(s). HemaSphere is published by John Wiley & Sons Ltd on behalf of European Hematology Association. This is an open access Abstract Book distributed under the Attribution-NonCommercial-NoDerivs (CC BY-NC-ND), which allows third parties to download the articles and share them with others as long as they credit the author and the Abstract Book, but they cannot change the content in any way or use them commercially.
Abstract Book Citations: Authors, Title, HemaSphere, 2026;10:(S1):pages. The abstract book DOIs can be found on the “Author Information” page.
Disclaimer: Articles published in the journal HemaSphere exclusively reflect the opinions of the authors. The authors are responsible for all content in their abstracts including accuracy of the facts, statements, citing resources, etc.

### (PB4520) AN ULTRA-DEEP NGS-BASED MRD APPROACH FOR LONGITUDINAL MULTI-MARKER MONITORING IN AML

**Topic:** 38. Novel technologies, techniques and digital analytical tools in hematology

Stephanie Sungalee*[1], Juan Gomez-Gelvez[2], Rieke Kempfer[1], Jonathan Bieler[1], Yuanlong Liu[1], Slawomir Kubik[1], Zhenyu Xu[1]

*[1]SOPHiA GENETICS SA, Rolle, Switzerland;[2]Henry Ford Health, Department of Pathology and Laboratory Medicine, Detroit, United States of America;*

### Background

Measurable residual disease (MRD) is a validated prognostic factor in acute myeloid leukemia (AML) and guides post-remission management. MRD is assessed by longitudinal detection of leukemia-specific molecular or immunophenotypic biomarkers identified at diagnosis. Current MRD strategies rely on multiparameter flow cytometry and quantitative PCR for selected molecular targets such as mutated *NPM1*, while FLT3-ITD is typically detected at diagnosis by fragment analysis. Although these approaches are well established, their biomarker-specific and assay-dependent nature requires complementary technologies and may limit harmonized, integrated longitudinal monitoring across genetically heterogeneous AML. To address these methodological constraints, we developed an ultra-deep targeted DNA-based NGS assay using our proprietary CUMIN© barcoding technology to enable simultaneous multi-marker tracking while distinguishing true biological signal from technical noise.

### Aims

In this study, we aim at establishing the analytical performance characteristics, including sensitivity, and validating the technical robustness and clinical validity of our NGS-based MRD approach.

### Methods

A curated panel of 23 AML-associated gene biomarkers relevant for MRD monitoring was selected in accordance with European LeukemiaNet (ELN) recommendations for NGS-based MRD assessment. DNA input parameters were optimized to ensure adequate molecular coverage, enabling detection of variants at MRD-relevant sensitivity thresholds. Analytical performance was evaluated using reference cell lines and clinical samples to establish the limit of blank, limit of detection, analytical sensitivity, precision, and robustness, including inter-site reproducibility. Clinical validation assessed MRD detection in peripheral blood and bone marrow samples from AML patients at diagnosis and during longitudinal follow-ups.

### Results

Analytical validation demonstrated high sensitivity and precision for detection of confirmed variants, including FLT3-ITD, across technical replicates and inter-site testing. The limit of blank remained below the predefined limit of detection, supporting reliable low-frequency variant identification at variant allele fractions (VAF) down to 0.01%. Robust analytical performance was confirmed through consistent results across replicates, independent runs, and inter-site evaluation. Analysis of longitudinal clinical specimens demonstrated consistent detection of pathogenic variants, including FLT3-ITD when present, without observable background signal at corresponding genomic positions, supporting effective control of technical noise. Longitudinal tracking was enabled by a dedicated bioinformatic framework (Oncoportal™ Mutation Tracker; Patent pending), allowing simultaneous monitoring of multiple diagnostic variants and computation of a composite clonal likelihood score to distinguish true residual clones from background noise.

**Copyright Information:** (Online) ISSN: 2572-9241
©2026 The Author(s). HemaSphere is published by John Wiley & Sons Ltd on behalf of European Hematology Association. This is an open access Abstract Book distributed under the Attribution-NonCommercial-NoDerivs (CC BY-NC-ND), which allows third parties to download the articles and share them with others as long as they credit the author and the Abstract Book, but they cannot change the content in any way or use them commercially.
**Abstract Book Citations:** Authors, Title, HemaSphere, 2026;10:(S1):pages. The abstract book DOIs can be found on the “Author Information” page.
**Disclaimer:** Articles published in the journal HemaSphere exclusively reflect the opinions of the authors. The authors are responsible for all content in their abstracts including accuracy of the facts, statements, citing resources, etc.

**Summary/Conclusion**

Ultra-deep, targeted DNA-based NGS enables sensitive and reproducible multi-marker MRD assessment in AML, including FLT3-ITD, with robust longitudinal tracking and effective discrimination of biological signal from technical noise. This integrated approach supports harmonized MRD monitoring within a single workflow.

**Copyright Information:** (Online) ISSN: 2572-9241
©2026 The Author(s). HemaSphere is published by John Wiley & Sons Ltd on behalf of European Hematology Association. This is an open access Abstract Book distributed under the Attribution-NonCommercial-NoDerivs (CC BY-NC-ND), which allows third parties to download the articles and share them with others as long as they credit the author and the Abstract Book, but they cannot change the content in any way or use them commercially.
**Abstract Book Citations:** Authors, Title, HemaSphere, 2026;10:(S1):pages. The abstract book DOIs can be found on the "Author Information" page.
**Disclaimer:** Articles published in the journal HemaSphere exclusively reflect the opinions of the authors. The authors are responsible for all content in their abstracts including accuracy of the facts, statements, citing resources, etc.

### (PB4521) RADEEP: ELEVATING THE QUALITY OF REAL-WORLD DATA IN A EUROPEAN REGISTRY TO GENERATE REAL-WORLD EVIDENCE IN RARE ANEMIAS DISORDERS

**Topic:** 38. Novel technologies, techniques and digital analytical tools in hematology

Sara Isabel Reidel*[1, 2], Anna Collado Gimbert[1, 3], Nuria Torquet[1], Maximo Tartaglia[1], Beatrice Gulbis[4], Maria del Mar Mañú Pereira[1]

*[1]Vall d'Hebron Institut de Recerca, Barcelona, Spain;[2]Universitat Politècnica de Catalunya - BarcelonaTech, Barcelona, Spain; [3]Hospital Universitari Vall d'Hebron, Barcelona, Spain;[4]Hôpital Universitaire de Bruxelles - Université Libre de Bruxelles, Brussels, Belgium;*

### Background

Real-world data (RWD) are increasingly used to generate real-world evidence (RWE) for clinical and regulatory decision-making; however, they are often heterogeneous, incomplete and partially structured. Variability in information systems, workflows and documentation across centers and countries compromises data quality and limits the validity and generalizability of RWE. Patient registries are a key RWD source in rare diseases, but lack of harmonization and interoperability remains a major barrier. In rare anemia disorders (RADs), fragmented datasets and limited adoption of FAIR (Findable, Accessible, Interoperable, Reusable) principles have hindered cross-country analyses and robust evidence generation. The Rare Anemia Disorders European Epidemiological Platform (RADeep), established in 2017, is a GDPR-compliant European registry designed to maximize the public health value of standardized longitudinal RWD aligned with FAIR principles.

### Aims

To align RADeep with the European Medicines Agency (EMA) Data Quality Framework for regulatory use of RWD by: (i) strengthening data governance and operational maturity; and (ii) implementing quantitative metrics across key data quality dimensions.

### Methods

The EMA Data Quality Framework was operationalized by formalizing governance and quality procedures, defining registry study populations, and mapping the electronic case report form (eCRF) to the OMOP Common Data Model. Data quality controls addressing reliability, extensiveness, coherence, timeliness and relevance were implemented through validation rules, completeness and coverage metrics, and a three-scenario data capture strategy: direct manual entry, local REDCap installations with automated batch transfers, and semi-automated ETL from institutional systems. These processes were supported by a REDCap module for pseudonymization and duplicate detection.

### Results

A standardized REDCap-based eCRF is currently implemented in 10 European countries, with 6,456 registered patients (66.1% sickle cell disease, 21.1% thalassemia, 12.8% other RADs); 78.7% have a follow-up within the last two years. Interoperability has been achieved with national registries in Spain, Belgium and the Netherlands, with most data providers operating under automated or semi-automated batch transfer scenarios. Registry population coverage was highest in Spain and Belgium. CRF coverage exceeded 85% across providers, and 60% of variables achieved over 75% longitudinal completeness. A structured data query management process is in place.

**Copyright Information:** (Online) ISSN: 2572-9241
©2026 The Author(s). HemaSphere is published by John Wiley & Sons Ltd on behalf of European Hematology Association. This is an open access Abstract Book distributed under the Attribution-NonCommercial-NoDerivs (CC BY-NC-ND), which allows third parties to download the articles and share them with others as long as they credit the author and the Abstract Book, but they cannot change the content in any way or use them commercially.
**Abstract Book Citations:** Authors, Title, HemaSphere, 2026;10:(S1):pages. The abstract book DOIs can be found on the "Author Information" page.
**Disclaimer:** Articles published in the journal HemaSphere exclusively reflect the opinions of the authors. The authors are responsible for all content in their abstracts including accuracy of the facts, statements, citing resources, etc.

**Summary/Conclusion**

RADeep has implemented a registry-wide data quality and governance framework aligned with the EMA Data Quality Framework, progressively advancing its maturity from documented procedures towards systematically implemented and partially automated controls. This continuous work has already translated into interoperable, quality-assessed RWD supported by standardized validation, pseudonymization and monitoring processes, positioning RADeep as an infrastructure capable of generating real-world evidence with potential regulatory relevance. Ongoing developments will further strengthen automation, auditability and representativeness, with the explicit goal of qualifying RADeep data for use in EMA-regulated contexts.

**Copyright Information:** (Online) ISSN: 2572-9241
©2026 The Author(s). HemaSphere is published by John Wiley & Sons Ltd on behalf of European Hematology Association. This is an open access Abstract Book distributed under the Attribution-NonCommercial-NoDerivs (CC BY-NC-ND), which allows third parties to download the articles and share them with others as long as they credit the author and the Abstract Book, but they cannot change the content in any way or use them commercially.
**Abstract Book Citations:** Authors, Title, HemaSphere, 2026;10:(S1):pages. The abstract book DOIs can be found on the "Author Information" page.
**Disclaimer:** Articles published in the journal HemaSphere exclusively reflect the opinions of the authors. The authors are responsible for all content in their abstracts including accuracy of the facts, statements, citing resources, etc.

### (PB4522) BLINDED MULTI-EXPERT VALIDATION OF A BAYESIAN CDSS FOR CBC INTERPRETATION AND CRITICAL-LEVEL EMERGENCY ALERTING IN 3,091 REAL-WORLD CASES

**Topic:** 38. Novel technologies, techniques and digital analytical tools in hematology

Abel Costa*[1, 2], Dalila Cysne[2], Anderson Costa[3], Rafaela Nalesso[2], Roxana Cavalcanti[4], Carlos Rolemberg[1], Raphael Xavier[1], Lucyo Diniz[1, 5]

*[1]Hemodoctor Healthtech, São Paulo, Brazil; [2]D'Or Institute for Research and Education, São Paulo, Brazil; [3]Hospital Regional de Juazeiro, Juazeiro, Brazil; [4]Hospital Unimed, Petrolina, Brazil; [5]Hospital Universitário - UNIVASF, Petrolina, Brazil;*

### Background

The complete blood count (CBC) is the most frequently ordered laboratory test, yet its interpretation remains manual and operator-dependent. Delayed recognition of safety-critical patterns (e.g., severe cytopenias or blast-patterns presentations) can compromise patient safety. While AI in hematology has focused on morphological image analysis, decision support for structured CBC data remains underexplored. We developed an interpretable Bayesian clinical decision support system (CDSS), designed as a software medical device (SaMD), to aid systematic CBC interpretation.

### Aims

To validate abnormal-case differential concordance and critical-level emergency alerting of a Bayesian CDSS for CBC interpretation against blinded expert hematologist review on real-world laboratory data.

### Methods

We retrospectively analyzed 3,091 cases stratified from 64,038 consecutive de-identified adult (≥18 y) CBCs from a private healthcare laboratory in Petrolina, Brazil (Jan 2025–Feb 2026). The CDSS produces ranked differential hypotheses and a binary emergency alert from CBC parameters, sex, and age. Three specialist hematologists independently reviewed each case blinded to CDSS output, recording: normal/abnormal classification, up to three differential diagnoses, and urgency tier (routine/urgent/critical). Two experts completed all 3,091 cases; the third completed 1,202. The stratified sample preserved the demographic profile of the source cohort (median age 42 y, IQR 32–54; 68% female). Primary endpoints against majority-vote consensus (≥2/3 experts): (i) critical-level emergency sensitivity (Wilson 95% CI) and (ii) abnormal-case Top-3 concordance against consensus differential codes (diagnostic codes present in ≥2 of 3 expert lists). Per-expert concordance served as sensitivity analysis. Pairwise agreement was assessed with Cohen's κ (asymptotic 95% CI) on all evaluable cases (n = 3,055); multi-rater reliability with Fleiss' κ (bootstrap 95% CI) in the 3-expert subset.

### Results

Majority-vote consensus classified 879 of 3,091 hemograms as abnormal. Against consensus differential codes (n = 814), CDSS Top-3 concordance was 85.9% (95% CI 83.3–88.1; Figure 1A); per-expert analysis confirmed robustness at 89.6% (Expert A, n = 866) and 83.2% (Expert B, n = 1,259). Consensus critical-level sensitivity was 100% (61/61; 95% CI 94.1–100.0; Figure 1B) with specificity 97.3%; per-expert sensitivity was 100% (65/65) against Expert A and 92.2% (71/77) against Expert B, where the six discordant cases were all borderline. Pairwise normal/abnormal agreement (Cohen's κ, n = 3,055) was 0.67 (95% CI 0.63–0.70); in the 3-expert subset (Figure 1C), Fleiss' κ ranged from substantial for normal/abnormal (0.72, n = 1,167) and critical-binary classification (0.69, n = 1,194) to moderate for diagnosis across 93 categories (0.48, n = 1,201) and urgency tiers (0.41, n = 1,194).

**Copyright Information:** (Online) ISSN: 2572-9241
©2026 The Author(s). HemaSphere is published by John Wiley & Sons Ltd on behalf of European Hematology Association. This is an open access Abstract Book distributed under the Attribution-NonCommercial-NoDerivs (CC BY-NC-ND), which allows third parties to download the articles and share them with others as long as they credit the author and the Abstract Book, but they cannot change the content in any way or use them commercially.
**Abstract Book Citations:** Authors, Title, HemaSphere, 2026;10:(S1):pages. The abstract book DOIs can be found on the “Author Information” page.
**Disclaimer:** Articles published in the journal HemaSphere exclusively reflect the opinions of the authors. The authors are responsible for all content in their abstracts including accuracy of the facts, statements, citing resources, etc.

**Summary/Conclusion**

In a real-world blinded multi-expert evaluation of 3,091 adult CBCs, a Bayesian CDSS achieved 85.9% consensus Top-3 concordance and 100% consensus critical-level sensitivity (61/61), confirmed by per-expert analysis (Top-3: 83–90%; critical: 92–100%). Moderate inter-rater reliability underscores inherent ambiguity of CBC-only adjudication and supports reporting both consensus and per-expert endpoints. These findings support clinical validity of an interpretable decision support approach for CBC interpretation and triage.

**Copyright Information:** (Online) ISSN: 2572-9241
©2026 The Author(s). HemaSphere is published by John Wiley & Sons Ltd on behalf of European Hematology Association. This is an open access Abstract Book distributed under the Attribution-NonCommercial-NoDerivs (CC BY-NC-ND), which allows third parties to download the articles and share them with others as long as they credit the author and the Abstract Book, but they cannot change the content in any way or use them commercially.
**Abstract Book Citations:** Authors, Title, HemaSphere, 2026;10:(S1):pages. The abstract book DOIs can be found on the "Author Information" page.
**Disclaimer:** Articles published in the journal HemaSphere exclusively reflect the opinions of the authors. The authors are responsible for all content in their abstracts including accuracy of the facts, statements, citing resources, etc.

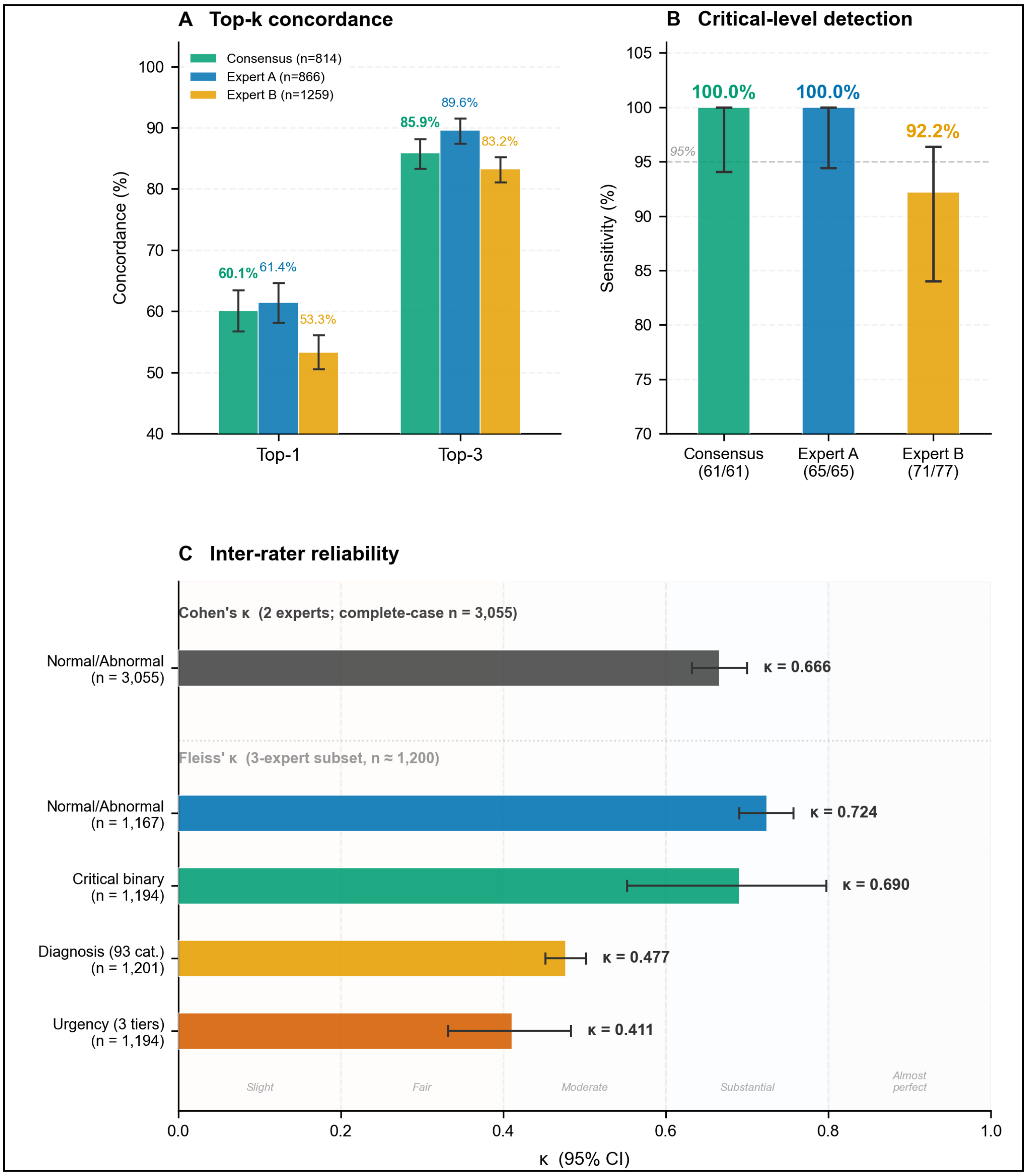

Figure 1. Validation of a Bayesian CDSS for CBC interpretation against expert hematologist review (3,091 real-world cases). (A) Top-1 and Top-3 diagnostic concordance for abnormal cases: consensus-based (majority vote ≥2/3 experts; primary endpoint) and per-expert sensitivity analysis. Error bars: Wilson 95% CI. (B) Critical-level emergency detection sensitivity against consensus and per-expert reference. Dashed line: 95% threshold. Consensus: 100% (61/61); Expert A: 100% (65/65); Expert B: 92.2% (71/77; 6 discordant cases, all borderline). Error bars: Wilson 95% CI. (C) Inter-rater reliability: pairwise agreement (Cohen's κ, 2 experts, n = 3,055) and multi-rater agreement (Fleiss' κ, 3-expert subset, n = 1,167–1,201) across four classification dimensions. Background shading indicates Landis & Koch interpretation zones.

**Copyright Information:** (Online) ISSN: 2572-9241
©2026 The Author(s). HemaSphere is published by John Wiley & Sons Ltd on behalf of European Hematology Association. This is an open access Abstract Book distributed under the Attribution-NonCommercial-NoDerivs (CC BY-NC-ND), which allows third parties to download the articles and share them with others as long as they credit the author and the Abstract Book, but they cannot change the content in any way or use them commercially.
**Abstract Book Citations:** Authors, Title, HemaSphere, 2026;10:(S1):pages. The abstract book DOIs can be found on the "Author Information" page.
**Disclaimer:** Articles published in the journal HemaSphere exclusively reflect the opinions of the authors. The authors are responsible for all content in their abstracts including accuracy of the facts, statements, citing resources, etc.

### (PB4523) EXPLAINABLE AI-ENABLED WEARABLE SENSORS FOR REAL-TIME THROMBOTIC EVENT EARLY WARNING IN CONNECTED HEALTH ENVIRONMENTS

**Topic:** 38. Novel technologies, techniques and digital analytical tools in hematology

Zi Wang*[1], Yufan Hu[2], Qinglin Hu[3], Destin Bai[4]

*[1]Peking Union Medical College Hospital, Chinese Academy of Medical Science and Peking Union Medical College, Hematology,, Beijing, China; [2]Peking Union Medical College Hospital, Chinese Academy of Medical Science and Peking Union Medical College, Beijing, China; [3]Peking University Third Hospital, Beijing, China; [4]College of Humanities and Social Sciences, Keele University, Keele, United Kingdom;*

### Background

Thrombotic events have multiple complications that remain difficult to identify early unless continuous non-invasive monitoring solutions are available to consumers; however, de- veloping such products has been very restrictive thus far.

### Aims

In this article we provide an overview about a novel Explainable Artificial Intelligence (XAI) framework for wearable sensorsand the earliest warning systems for thrombotic events in a connect health environment. We developed a method that integrates multiple types of multimodal physiological signals from ECG- derived heart rate variability (HRV) as well as dynamics of skin temperature into a hierarchical attention based model that provides risk assessments that are interpretable and clinically relevant; in this manner, we offer clinicians a clinical tool that allows them to support their clinical practice by combining the strengths of artificial intelligence with advancements made in wearable technology for consumers.

### Methods

We describe a Temporal- Spatial Attention Attribution Network (TSAAN) that combines temporal convolutional layers with multi-head self-attention lay- ers to enable the accurate identification of physiological patterns associated with thrombotic risk while still using an efficient edge- optimized architecture. The clinical relevance guided attention (CRGA) module, is a proprietary feature using clinically based guidelines and the CRGA is intended to improve the trust clinicians have in the correct interpretation of the features and can also facilitate the successful integration of the TSAAN framework into the existing clinical workflow.

### Results

Our extensive experiments show that the predictive accuracy of ThrombXAIis at 91.3% using an AUC value of 0.936 and represents improvements of 4.2% and 2.8% respectively to the current best available state-of-the-art methods for thrombotic event predic- tion. In addition, the ThrombXAI framework has a prediction time of less than 850 milliseconds in resource constrained ARM Cortex-M7 microcontrollers to facilitate easy integration into existing commercialized wearable devices. We further validated our results in a prospective study on 156 participants and compared the results to traditional screening approaches, in which we discovered statistically significant improvements;89.3% sensitivity and 91.5% specificity for the early identification of thrombotic events.

### Summary/Conclusion

We provide the way forward with new paradigms of personalized preventative cardiovascular medicine using consumer wearables integrated with clinically actionable XAI technology.

**Copyright Information:** (Online) ISSN: 2572-9241
©2026 The Author(s). HemaSphere is published by John Wiley & Sons Ltd on behalf of European Hematology Association. This is an open access Abstract Book distributed under the Attribution-NonCommercial-NoDerivs (CC BY-NC-ND), which allows third parties to download the articles and share them with others as long as they credit the author and the Abstract Book, but they cannot change the content in any way or use them commercially.
**Abstract Book Citations:** Authors, Title, HemaSphere, 2026;10:(S1):pages. The abstract book DOIs can be found on the "Author Information" page.
**Disclaimer:** Articles published in the journal HemaSphere exclusively reflect the opinions of the authors. The authors are responsible for all content in their abstracts including accuracy of the facts, statements, citing resources, etc.

### (PB4525) ESTABLISHING FUNCTIONAL PHOSPHO-SIGNALING PROFILING FOR PRECISION THERAPEUTIC ASSESSMENT IN SOUTH AFRICAN LEUKEMIA

**Topic:** 38. Novel technologies, techniques and digital analytical tools in hematology

Phumuzile Dube*[1, 2], Chrisna Durandt[3], Mutsa Takundwa[1, 2], Bernice Monchusi[1, 4], Johan Potgieter[2], Richard Khanyile[5], Jeanette Lötter[5], Deepak Thimiri Govinda Raj[1, 4]

*[1]Council for Scientific and Industrial Research, Future production Chemicals Cluster, Pretoria, South Africa;[2]University of Pretoria, Department of Haematology, Pretoria, South Africa; [3]University of Pretoria, Department of Immunology, Pretoria, South Africa; [4]University of the Witwatersrand, Department of Surgery, Johannesburg, South Africa;[5]University of Pretoria, Department of Medical Oncology, Pretoria, South Africa;*

### Background

Aberrant activation of the phosphorylation signaling pathways contributes to leukemogenesis, disease progression, and therapeutic resistance in chronic lymphocytic leukemia (CLL), acute myeloid leukemia (AML), and acute lymphoblastic leukemia (ALL). Phospho-flow cytometry enables single-cell quantification of intracellular phosphorylation events and provides functional assessment of signaling heterogeneity in primary patient samples. Despite its relevance to precision oncology, implementation in resource-constrained settings such as South Africa remains limited due to technical variability, lack of standardized protocols for fresh peripheral blood mononuclear cells (PBMCs), and logistical constraints in clinical workflows. Functional phospho-signaling profiling can bridge this gap by enabling rapid pharmacodynamic assessment and supporting individualized therapeutic decision-making within a precision medicine framework.

### Aims

To establish and optimize a reproducible phospho-flow cytometry platform for phosphorylation signaling analysis in PBMCs from South African patients with CLL, AML, and ALL, and to evaluate ex vivo phospho-signal modulation following exposure to repurposed therapeutic agents as a proof-of-concept for precision therapeutic assessment.

### Methods

PBMCs were isolated from peripheral blood samples obtained from 5 healthy donors and 8 patients diagnosed with CLL, AML, or ALL following informed consent and institutional ethical approval. Fixation and permeabilization conditions were optimized to enable intracellular detection of Phospho-p44/42 MAPK (Erk1/2) (Thr202/Tyr204) and Phospho-Akt (Ser473). Antibody titration, fluorescence-minus-one (FMO) controls, and appropriate compensation controls were implemented to ensure assay specificity, sensitivity, and reproducibility. Selected samples were exposed ex vivo to repurposed therapeutic agents chosen based on current standard-of-care regimens used in South African hospitals and supported by prior ex vivo drug-response studies conducted within our research group. Phospho-signal changes were quantified before and after treatment using multiparameter flow cytometry.

### Results

A robust and reproducible phospho-flow assay was successfully established for freshly isolated PBMCs. Basal ERK1/2 and Akt phosphorylation was detectable in both healthy donor and patient samples, with inter-patient variability observed across disease subtypes. Ex vivo exposure to repurposed agents resulted in measurable modulation of phospho-ERK1/2 and phospho-Akt signaling, demonstrating functional pathway responsiveness and assay sensitivity in limited clinical samples.

**Copyright Information:** (Online) ISSN: 2572-9241
©2026 The Author(s). HemaSphere is published by John Wiley & Sons Ltd on behalf of European Hematology Association. This is an open access Abstract Book distributed under the Attribution-NonCommercial-NoDerivs (CC BY-NC-ND), which allows third parties to download the articles and share them with others as long as they credit the author and the Abstract Book, but they cannot change the content in any way or use them commercially.
**Abstract Book Citations:** Authors, Title, HemaSphere, 2026;10:(S1):pages. The abstract book DOIs can be found on the "Author Information" page.
**Disclaimer:** Articles published in the journal HemaSphere exclusively reflect the opinions of the authors. The authors are responsible for all content in their abstracts including accuracy of the facts, statements, citing resources, etc.

**Summary/Conclusion**

This study demonstrates the feasibility of implementing functional phospho-signaling profiling in South African hematological malignancies. The platform supports dynamic assessment of therapeutic pathway modulation and provides a foundation for integrating single-cell signaling analysis into precision medicine strategies and pharmacodynamic-guided drug repurposing in resource-limited settings.

**Copyright Information:** (Online) ISSN: 2572-9241
©2026 The Author(s). HemaSphere is published by John Wiley & Sons Ltd on behalf of European Hematology Association. This is an open access Abstract Book distributed under the Attribution-NonCommercial-NoDerivs (CC BY-NC-ND), which allows third parties to download the articles and share them with others as long as they credit the author and the Abstract Book, but they cannot change the content in any way or use them commercially.
**Abstract Book Citations:** Authors, Title, HemaSphere, 2026;10:(S1):pages. The abstract book DOIs can be found on the "Author Information" page.
**Disclaimer:** Articles published in the journal HemaSphere exclusively reflect the opinions of the authors. The authors are responsible for all content in their abstracts including accuracy of the facts, statements, citing resources, etc.

### (PB4526) FROM VISUAL READS TO VISION ALGORITHMS: AI-POWERED INTERPRETATION OF COLUMN AGGLUTINATION FOR RED BLOOD CELL ALLOANTIBODY DETECTION

**Topic:** 38. Novel technologies, techniques and digital analytical tools in hematology

María Fernández López*[1], Cristina Anton[1], Nataly Ibarra[1], Beatriz Sánchez-Quiñones[1], Pedro Gonzalez Cabezas[2], Rafael Carrasco[2], Amparo Santamaria[1]

*[1]Vinalopó University Hospital, Elche, Spain; [2]Vinalopó University Hospital, Management, Elche, Spain;*

### Background

The interpretation of gel-card agglutination in immunohematology remains manual and partly subjective, with variability among observers that can impact transfusion safety. Automated image-based analysis using deep learning offers the potential to standardize grading, reduce operator-dependent variation, and streamline laboratory workflows.

### Aims

To assess the technical feasibility of deep learning models for:

1. Classifying the six standard agglutination grades in gel-card images;
2. Performing reliable binary discrimination between negative and positive reactions;
3. Integrating model outputs into a prototype workflow to support alloantibody identification.

### Methods

A dataset of 770 anonymized gel-card images from 77 patients was curated and expert-labeled into six agglutination categories. Five convolutional neural network (CNN) architectures were evaluated using transfer learning. To address marked class imbalance (e.g., only five images in the 4+ category), weighted sampling and controlled synthetic augmentation were applied. After model benchmarking, the best-performing network was incorporated into a deterministic workflow that combines predicted agglutination grades with conventional reaction-table logic for antibody assessment.

### Results

ResNet-50 achieved the highest performance, with a **balanced accuracy of 96%**, and errors occurring predominantly between adjacent agglutination grades, indicating a safe and clinically acceptable error pattern. The binary model (positive vs. negative) achieved **~99% accuracy**. The resulting prototype demonstrates the capacity to reduce subjectivity, provide consistent grading, and accelerate interpretation of gel-card immunohematology assays.

### Summary/Conclusion

This preliminary study demonstrates that deep learning is a feasible and promising approach for automated interpretation of gel-card agglutination in alloantibody testing. The models show high accuracy and stability despite class imbalance. Expansion of the dataset, multicenter validation, and integration into laboratory information systems are key next steps toward clinical deployment of an AI-assisted decision-support tool in immunohematology.

**Copyright Information:** (Online) ISSN: 2572-9241
©2026 The Author(s). HemaSphere is published by John Wiley & Sons Ltd on behalf of European Hematology Association. This is an open access Abstract Book distributed under the Attribution-NonCommercial-NoDerivs (CC BY-NC-ND), which allows third parties to download the articles and share them with others as long as they credit the author and the Abstract Book, but they cannot change the content in any way or use them commercially.
**Abstract Book Citations:** Authors, Title, HemaSphere, 2026;10:(S1):pages. The abstract book DOIs can be found on the "Author Information" page.
**Disclaimer:** Articles published in the journal HemaSphere exclusively reflect the opinions of the authors. The authors are responsible for all content in their abstracts including accuracy of the facts, statements, citing resources, etc.

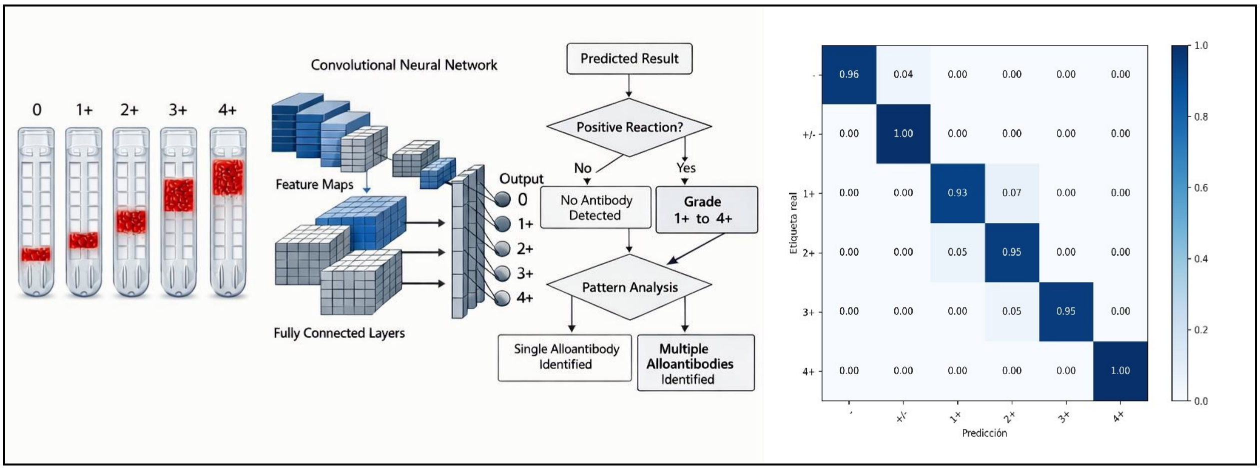

Pattern analysis and ResNet-50 performance as demonstrated by the confusion matrix.

**Copyright Information:** (Online) ISSN: 2572-9241
©2026 The Author(s). HemaSphere is published by John Wiley & Sons Ltd on behalf of European Hematology Association. This is an open access Abstract Book distributed under the Attribution-NonCommercial-NoDerivs (CC BY-NC-ND), which allows third parties to download the articles and share them with others as long as they credit the author and the Abstract Book, but they cannot change the content in any way or use them commercially.
**Abstract Book Citations:** Authors, Title, HemaSphere, 2026;10:(S1):pages. The abstract book DOIs can be found on the "Author Information" page.
**Disclaimer:** Articles published in the journal HemaSphere exclusively reflect the opinions of the authors. The authors are responsible for all content in their abstracts including accuracy of the facts, statements, citing resources, etc.

### (PB4527) OPTIMIZATION OF BLAST-RELATED FLAGGING RULES ON THE MINDRAY BC-6800PLUS TO BALANCE BLAST DETECTION AND SMEAR WORKLOAD: A SINGLE-CENTER EXPLORATORY STUDY

**Topic:** 38. Novel technologies, techniques and digital analytical tools in hematology

Sholhui Park*[1]

[1]*Ewha Womans University College of Medicine, Laboratory medicine, Seoul, South Korea;*

### Background

Automated hematology analyzers generate blast flags using multi-parameter algorithms, however factory thresholds often result in many false positives and unnecessary smear reviews. For the Mindray BC-6800Plus, which uses SF Cube and DUO-channel technology, optimal use of "Blasts?" and lymphocyte-related flags in daily practice is not fully provided.

### Aims

To assess the performance of blast-related flags on the BC-6800Plus and to explore a flagging strategy that preserves high sensitivity for smear-confirmed blasts while reducing smear workload.

### Methods

We retrospectively analyzed 292 consecutive peripheral blood samples with manual differential counts between 15 and 22 December 2025 at a tertiary hospital using the BC-6800Plus (Mindray, Shenzhen, China). Flag scores (0–100) and smear findings (blasts, atypical/abnormal lymphocytes, immature granulocytes, nucleated red blood cells) were collected; blast positivity was defined as blasts ≥1%.We focused on four flags ("Blasts?", "Abn Lymph/blasts?", "Immature Gran?", "Atypical Lymph?"). A composite blast score was defined as the maximum of "Blasts?" and "Abn Lymph/blasts?".
Receiver operating characteristic (ROC) analysis of the composite score provided and area under the curve (AUC). Sensitivity, specificity, predictive values, and flag-positive rate (workload) were calculated from 2×2 tables at thresholds of 20, 25, 30, 35, and 40. Multivariable logistic regression (per 10-point increase) was used to evaluate independent associations between the four flag scores and blast positivity. In a secondary analysis, we examined a combined rule defining blast suspicion as "Abn Lymph/blasts?" or "Immature Gran?" ≥35 and compared it with the local rule (Blasts? or Abn Lymph/blasts? ≥30) and a threshold of 40.

### Results

Blasts were present in 29 of 292 samples (9.9%). The composite score showed good discrimination for blasts (AUC 0.82). With the current rule (Blasts? or Abn Lymph/blasts? ≥30), sensitivity was 96.6% (28/29) and specificity 49.4%, and 55.1% of samples triggered blast-related flags. At 40, sensitivity fell to 86.2% (25/29), but specificity rose to 70.3%, and the flag-positive rate fell to 35.3%, with four missed blast-positive samples. At 25, sensitivity was 100%, but 66.1% of samples were flagged, whereas 35 gave sensitivity 89.7%, specificity 60.5%, and a flag-positive rate of 44.5%. In logistic regression, "Abn Lymph/blasts?" (coefficient 0.46 per 10 points; 95% CI 0.26–0.66) and "Immature Gran?" (0.24; 95% CI 0.11–0.37) were independently associated with blast positivity (both $p < 0.001$), while "Blasts?" and "Atypical Lymph?" were not significant. The combined rule "Abn Lymph/blasts? or Immature Gran? ≥35" maintained sensitivity close to 90% with fewer false positives than the current rule, in line with the analyzer's high fluorescent cell–based design.

**Copyright Information:** (Online) ISSN: 2572-9241
©2026 The Author(s). HemaSphere is published by John Wiley & Sons Ltd on behalf of European Hematology Association. This is an open access Abstract Book distributed under the Attribution-NonCommercial-NoDerivs (CC BY-NC-ND), which allows third parties to download the articles and share them with others as long as they credit the author and the Abstract Book, but they cannot change the content in any way or use them commercially.
**Abstract Book Citations:** Authors, Title, HemaSphere, 2026;10:(S1):pages. The abstract book DOIs can be found on the "Author Information" page.
**Disclaimer:** Articles published in the journal HemaSphere exclusively reflect the opinions of the authors. The authors are responsible for all content in their abstracts including accuracy of the facts, statements, citing resources, etc.

**Summary/Conclusion**

On the BC-6800Plus, this exploratory single-center study suggests that a cut-off around 35, applied particularly to the "Abn Lymph/blasts?" and "Immature Gran?" flags, offers a more favorable balance between sensitivity and specificity than lower (25–30) or higher (40) settings, while avoiding most missed blasts. Reconfiguring re-examination rules to focus on these flags may allow laboratories to reduce smear volume without compromising early detection of hematologic malignancy; the proposed thresholds require validation in larger, prospective cohorts and with different software versions.

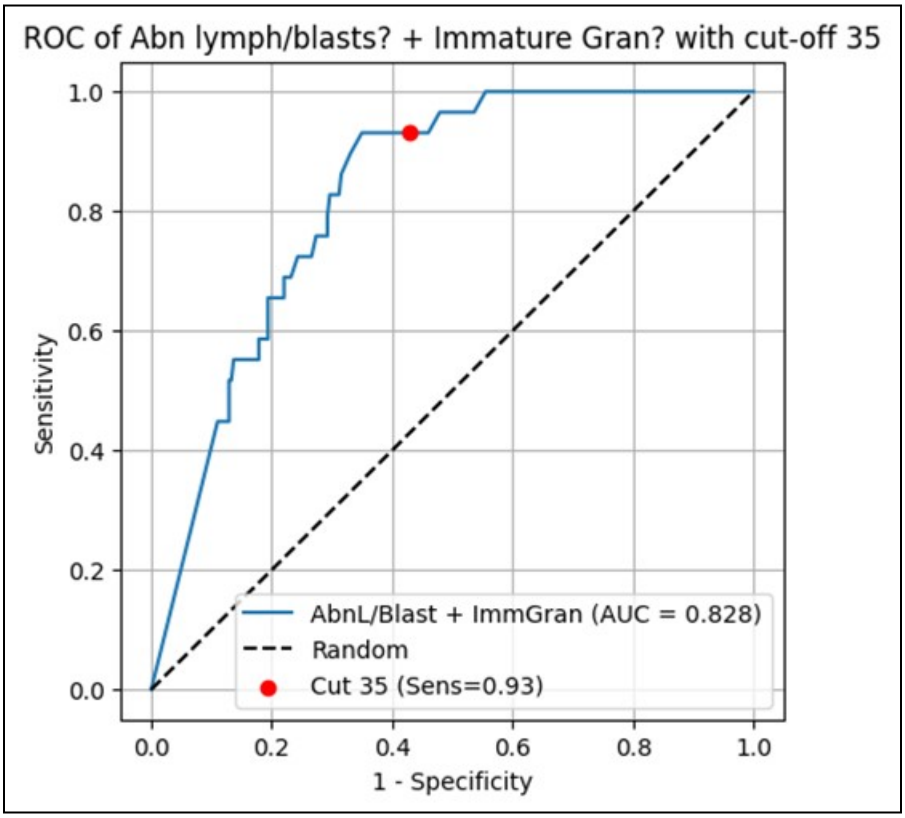

Figure 1. ROC curve of 'Abn Lymph/blasts?' plus 'Immature Gran?' for detecting smear confirmed blasts

**Copyright Information:** (Online) ISSN: 2572-9241
©2026 The Author(s). HemaSphere is published by John Wiley & Sons Ltd on behalf of European Hematology Association. This is an open access Abstract Book distributed under the Attribution-NonCommercial-NoDerivs (CC BY-NC-ND), which allows third parties to download the articles and share them with others as long as they credit the author and the Abstract Book, but they cannot change the content in any way or use them commercially.
**Abstract Book Citations:** Authors, Title, HemaSphere, 2026;10:(S1):pages. The abstract book DOIs can be found on the "Author Information" page.
**Disclaimer:** Articles published in the journal HemaSphere exclusively reflect the opinions of the authors. The authors are responsible for all content in their abstracts including accuracy of the facts, statements, citing resources, etc.

### (PB4528) UTILITY OF MYELOID NGS PANELS FOR IDENTIFYING GERMLINE VARIANTS ASSOCIATED WITH MYELOID NEOPLASMS WHICH GERMLINE PREDISPOSITION

**Topic:** 38. Novel technologies, techniques and digital analytical tools in hematology

Andrea Bender*[1], Cecilia Lang[1], Federico Torreguitart[1], Eugenia Bender[1], MacArena Giuliani[1], Micaela Quarchioni[2], Silvina Palmer[2], Leticia Rapan[3], Moira Bolesino[4], Daniela Andrea Bruno[4], Mariana Kalmus[4], Magali Barrenechea[4], Anabella Lopez[4], Gomez Sergio[5], Luciana Ferrari[6], Isolda Isabel Fernandez[6], Maria Marta Rivas[7], Ines Paganini[7], Lucia Bolzico[7], Jorge Valenzuela[7], Julio Pose[8], Evelina Babuin[9], Santiago Cranco[9], Juan Pablo Arriola[10], Juan Garcia Altuve[11], Santiago Boughen[12], Martin Beccacece[12], Ruben Kowalyszyn[13], Andrés Furque[14], Lisa Lopez Ares[15], Diego Sanhueza Carrasco[15], Paula Iommi[1], Evangelina Agriello[1, 16]

*[1]LEB, Bahía Blanca, Buenos Aires, Argentina; [2]Hosp. Británico, CABA, Buenos Aires, Argentina; [3]Srio. Sagrado Corazón, CABA, Buenos Aires, Argentina; [4]Srio. Las Lomas, Buenos Aires, Argentina; [5]Nueva Clínica del Niño de La Plata, La Plata, Argentina; [6]Fundaleu, CABA, Buenos Aires, Argentina; [7]Hosp. Austral, Buenos Aires, Argentina; [8]Consultorio hematológico, CABA, Buenos Aires, Argentina; [9]Instituto Alexander Fleming- IAF, CABA, Buenos Aires, Argentina; [10]Instituto Argentino de Diagnóstico y Tratamiento (IADT), CABA, Buenos Aires, Argentina; [11]CEMIC, CABA, Buenos Aires, Argentina; [12]Hospital Italiano Regional del Sur, Bahía Blanca, Buenos Aires, Argentina; [13]IMO, Viedma, Rio Negro, Argentina; [14]Srio. San Crlos, Bariloche, Rio Negro, Argentina; [15]Srio. Juan XXIII, Gral. Roca, Rio Negro, Argentina; [16]Universidad Nacional del Sur, Catedra de Hematología Clínica, Bahía Blanca, Argentina;*

### Background

Myeloid neoplasms with germline predisposition (MNPG) arise in individuals with a genetic alteration that confers an increased risk of developing a myeloid neoplasm (MN). Myeloid NGS panels designed for the study of somatic mutations may incidentally detect sequence variants (var) of germline origin (GO). Certain findings have a high pre-test probability of being of GO, namely: var with an allele frequency (VAF) close to 50% (heterozygous) or 100% (homozygous), those presenting with allelic inactivation (CEBPA, DDX41, RUNX1 and TP53), those that persist during disease remission, or those reported only in germline alterations. . Considering age, and personal and familiar history, suspicion of a germline variant should be confirmed by identifying it in a non-hematopoietic tissue (NHT).

### Aims

1)To demonstrate the utility of myeloid NGS panels in identifying variants with potential GO in CEBPA, DDX41, TP53, RUNX1, ANKRD21, ETV6, GATA2, NRAS, KRAS, CBL, and NF1. 2)To evaluate the strategies available in our setting and types of samples used to confirm the GO of the variants.

### Methods

A cohort of patients with different MN diagnosed between 2019 and 2025, were selected based on the presence of variants in genes related to MNPG with high pre-test probability of being germline (Table 1). DNA was obtained from peripheral blood (PB) or bone marrow (BM) and studied using a 63-target gene panel (SOPHiA Genetics), sequencing on MiSeq (Illumina), and bioinformatic analysis (DDM-SOPHiA Genetics). In order to confirm the GO of the variants, they were looked for in a NHT: hair follicle, nails, or CD3+ cells separated by sorting, by amplification followed by Sanger sequencing.

**Copyright Information:** (Online) ISSN: 2572-9241
©2026 The Author(s). HemaSphere is published by John Wiley & Sons Ltd on behalf of European Hematology Association. This is an open access Abstract Book distributed under the Attribution-NonCommercial-NoDerivs (CC BY-NC-ND), which allows third parties to download the articles and share them with others as long as they credit the author and the Abstract Book, but they cannot change the content in any way or use them commercially.
**Abstract Book Citations:** Authors, Title, HemaSphere, 2026;10:(S1):pages. The abstract book DOIs can be found on the "Author Information" page.
**Disclaimer:** Articles published in the journal HemaSphere exclusively reflect the opinions of the authors. The authors are responsible for all content in their abstracts including accuracy of the facts, statements, citing resources, etc.

### Results

Forty-seven pts presented potential germline variants: CEBPA=5, DDX41=2, ETV6=5, GATA2=6, RUNX1=8, TP53=20, and NF1=1 (Table 1). In 15 cases, suspected variant was subsequently evaluated in PB or BM in clinical remission (n=3), in PB from an asymptomatic family member (n=3), or in NHT samples from the patient (n=7). CEBPA variant was ruled out in 1 pt because not found in disease remission. Of the two pts with DDX41 variants, one was detected in a sibling, increasing suspicion of GO. In 2/6 cases with GATA2, suspected variant was detected in hair follicle and nails samples, one other patient was identified in a first-degree relative also diagnosed with MN, and another was not detected in NHT; two died before being studied. Of the 2/8 pts with RUNX1, GO was ruled out because not found in CD3+ cells in one of them, and  because  negative in a first-degree relative in the other. In 3/19 pts with TP53, the presence of the variants was studied in NHT (hair, nails, and CD3+ cells), ruling out MNPG in two and confirming it in one. In one pt, the variant was detected after hematopoietic cell transplantation with an HLA-identical donor.

### Summary/Conclusion

This is the first study in Argentine proving that NGS using a myeloid gene panel including genes associated with MNPG is a reliable tool for identifying variants with possible GO. Analysis of these variants in non-hematopoietic tissue (NHT) allowed confirmation/exclusion of the germline nature of the neoplasm. Although the gold standard NHT is fibroblast culture, other samples have proven to be useful. The diagnosis of MNPG is crucial to understand the neoplastic process, define the therapeutic strategy, select the BM donor, and provide proper genetic counseling to the family.

**Copyright Information:** (Online) ISSN: 2572-9241
©2026 The Author(s). HemaSphere is published by John Wiley & Sons Ltd on behalf of European Hematology Association. This is an open access Abstract Book distributed under the Attribution-NonCommercial-NoDerivs (CC BY-NC-ND), which allows third parties to download the articles and share them with others as long as they credit the author and the Abstract Book, but they cannot change the content in any way or use them commercially.
**Abstract Book Citations:** Authors, Title, HemaSphere, 2026;10:(S1):pages. The abstract book DOIs can be found on the "Author Information" page.
**Disclaimer:** Articles published in the journal HemaSphere exclusively reflect the opinions of the authors. The authors are responsible for all content in their abstracts including accuracy of the facts, statements, citing resources, etc.

Table 1. Characteristics of suspected variants

| N | Gen | Second Hit | Genomic Position | Transcript | Nucleotide change | Aminoacid change. | VAF (%) |
|---|---|---|---|---|---|---|---|
| 1 | CEBPA | CEBPA bzip | 19:33793079 | NM_004364.4 | c.241del | p.(Leu81Cysfs*79) | 34.2 |
| 2 | CEBPA | CEBPA monoallelic | 19:33793252 | NM_004364.4 | c.68dup | p.(His24Alafs*84) | 95.1 |
| 3 | CEBPA | | 19:33793153 | NM_004364.4 | c.155_167del | p.(Leu52Profs*104) | 51.0 |
| 4 | CEBPA | CEBPA bzip | 19:33793088 | NM_004364.4 | c.232del | p.(Leu78Trpfs*82) | 37.1 |
| 5 | CEBPA | CEBPA | 19:33793203 | NM_004364.4 | c.102_117del | p.(Gly36Serfs*119) | 41.7 |
| 6 | DDX41 | DDX41 | 5:176943354 | NM_016222,3 | c.232_233insAA | p.(Pro78Glnfs*3) | 50.4 |
| 7 | DDX41 | DDX41 | 5:176943129 | NM_016222,3 | c.364G>T | p.(Glu122*) | 46.8 |
| 8 | ETV6 | | 12:12006380 | NM_001987.4 | c.348_349insTT | p.(Leu117Phefs*6) | 43.1 |
| 9 | ETV6 | | 12:11992138 | NM_001987.4 | c.231_232del | p.(Ser78Phefs*5) | 34.7 |
| 10 | ETV6 | | 12:12022870 | NM_001987.4 | c.976G>T | p.(Glu326*) | 40.9 |
| 11 | ETV6 | | 12:12037508 | NM_001987.4 | c.1139G>A | p.(Trp380*) | 37.0 |
| 12 | ETV6 | | 12:11992223 | NM_001987.4 | c.313C>T | p.(Arg105*) | 42.0 |
| 13 | GATA2 | | 3:12820208 | NM_001145661.1 | c.911del | p.(Pro304Leufs*22) | 39.6 |
| 14 | GATA2 | | 3:128204872 | NM_001145661.1 | c.568del | p.(Ala190Leufs*28) | 49.9 |
| 15 | GATA2 | | 3:128202711 | NM_001145661.1 | c.1009C>T | p.(Arg337*) | 44.8 |
| 16 | GATA2 | | 3:128202711 | NM_032638,5 | c.351C>G | p.(Thr117=) | 50.5 |
| 17 | GATA2 | | 3:128200113 | NM_001145661.1 | c.1192C>T | p.(Arg398Trp) | 45.7 |
| 18 | GATA2 | | 3:128204767 | NM_001145661.1 | c.673del | p.(Ser225Valfs*9) | 53.4 |
| 19 | NF1 | NF1 | 17:29585433 | NM_001042492.3 | c.4245T>A | p.(Asn1415Lys) | 43.6 |
| | | | 17:29562766 | | c.3846T>G | p.(Ser1282Arg) | 43,3 |
| 20 | RUNX1 | RUNX1 | 21:36252877 | NM_001754.4 | c.485G>A | p.(Arg135Lys) | 47.7 |
| 21 | RUNX1 | WT1 | 21:36171700 | NM_001754.4 | c.865G>T | p.(Gly262*) | 49.2 |
| 22 | RUNX1 | WT1 | 21:36259205 | NM_001754.4 | c.285del | p.(Asn69Thrfs*26) | 37.9 |
| 23 | RUNX1 | RUNX1 | 21:36252856 | NM_001754.4 | c.506G>T | p.(Arg142Ile) / p.? | 29.8 |
| | | | 21:36231770 | | c.613+1G>A | | 35.2 |
| 24 | RUNX1 | RUNX1 | 21:36259160 | NM_001754.4 | c.592G>A | p.(Asp171Asn) | 61.5 |
| | | | 21:36231792 | | | | |
| 25 | RUNX1 | TET2 | 21:36171600 | NM_001754.4 | c.965C>G | p.(Ser295*) | 50.3 |
| 26 | RUNX1 | RUNX1 | 21:36252939 | NM_001754.4 | c.422_423insT | p.(Ala142Glyfs*2) | 40.7 |
| | | | 21:36171717 | NM_001754.4 | c.847del | p.(Gln283Serfs*28) | 42.6 |
| 27 | RUNX1 | TP53 LOH | 21:36252857 | NM_001754.4 | c.505ª>G | p.(Arg142Gly) | 44.1 |
| 28 | TP53 | TP53 del 17p | 17:7578190 | NM_000546.6 | c.659ª>G | p.(Tyr220Cys) | 71.0 |
| 29 | TP53 | TP53 mut | 17:7578403 | NM_000546.5 | c.527G>T | p.(Cys176Phe) | 35.0 |
| | | | 17:7577572 | NM_000546.5 | c.709A>G | p.(Met237Val) | 35.1 |
| 30 | TP53 | TP53 mut | 17:7577574 | NM_000546.5 | c.707A>G | p.(Tyr236Cys) | 72.7 |
| 31 | TP53 | TP53 mut | 17:7577508 | NM_000546.5 | c.772dupG | p.(Glu258Glyfs*6) | 37.0 |
| | | | 17:7577098 | NM_000546.5 | c.840A>C | p.(Arg280Ser) | 40.7 |
| 32 | TP53 | TP53 mut | 17:7578190 | NM_000546.5 | c.659A>G | p.(Tyr220Cys) | 30.4 |
| | | | /17:7579349 | NM_000546.5 | c.338T>G | p.(Phe113Cys) | 31.3 |
| 33 | TP53 | TP53 LOH | 17:7578535 | NM_000546.5 | c.395ª>G | p.(Lys132Arg) | 81.1 |
| 34 | TP53 | TP53 del 17p | 17:7577120 | NM_000546.5 | c.818G>A | p.(Arg273His) | 60.8 |
| 35 | TP53 | TP53 del 17P | 17:7578275 | NM_000546.5 | c.574C>T | p.(Gln192*) | 96.2 |
| 36 | TP53 | TP53 LOH | 17:7578554 | NM_000546.5 | c.376T>G | p.(Tyr126Asp) | 76.2 |
| 37 | TP53 | TP53 LOH | 17:7578553 | NM_000546.5 | c.377ª>G | p.(Tyr126Cys) | 70.6 |
| 38 | TP53 | TP53 LOH | 17:7578394 | NM_000546.5 | c.536ª>G | p.(His179Arg) | 87.8 |
| 39 | TP53 | TP53 mut | 17:7577538 | NM_000546.5 | c.743G>A | p.(Arg248Gln) | 41.7 |
| | | | 17:7578449 | NM_000546.5 | c.481G>A | p.(Ala161Thr) | 41.7 |
| 40 | TP53 | TP53 LOH | 17:7576882 | NM_000546.5 | c.963del | p.(Lys321Asnfs*24) | 77.8 |
| 41 | TP53 | TP53 LOH | 17:7577538 | NM_000546.6 | c.743G>A | p.(Arg248Gln) | 58.2 |
| 42 | TP53 | TP53 del 17P | 17:7578445 | NM_000546.5 | c.485T>A | p.(Ile162Asn) | 77.7 |
| 43 | TP53 | TP53 del 17P | 17:7577538 | NM_000546.6 | c.743G>A | p.(Arg248Gln) | 44.6 |
| 44 | TP53 | TP53 del 17P | 17:7578553 | NM_000546.5 | c.377ª>G | p.(Tyr126Cys) | 70.6 |
| 45 | TP53 | TP53 LOH | 17:7577139 | NM_000546.5 | c.799C>T | p.(Arg267Trp) | 55.6 |
| 46 | TP53 | TP53 LOH | 17:7579529 | NM 000546.5 | c.158G>A | p.(Trp53*) | 70.4 |

**Copyright Information:** (Online) ISSN: 2572-9241
©2026 The Author(s). HemaSphere is published by John Wiley & Sons Ltd on behalf of European Hematology Association. This is an open access Abstract Book distributed under the Attribution-NonCommercial-NoDerivs (CC BY-NC-ND), which allows third parties to download the articles and share them with others as long as they credit the author and the Abstract Book, but they cannot change the content in any way or use them commercially.
**Abstract Book Citations:** Authors, Title, HemaSphere, 2026;10:(S1):pages. The abstract book DOIs can be found on the "Author Information" page.
**Disclaimer:** Articles published in the journal HemaSphere exclusively reflect the opinions of the authors. The authors are responsible for all content in their abstracts including accuracy of the facts, statements, citing resources, etc.

### (PB4529) THE DIGITAL PATIENT WALLET: CONTINUOUS REAL-WORLD FUNCTIONAL MONITORING IN MULTIPLE MYELOMA ACROSS LINES OF THERAPY

**Topic:** 38. Novel technologies, techniques and digital analytical tools in hematology

Shivani Shrivastava[1], Kim Summers*[1], Muna Yusuf[1], Tushar Singh[1], Eric Low[2], Orlando Agrippa[1]

*[1]Sanius Health, London, United Kingdom; [2]Eric Low Consulting, Haddington, United Kingdom;*

### Background

Multiple myeloma (MM) remains largely incurable. Although newer therapies have extended survival, substantial symptom burden and treatment-related toxicity persist. Survival gains in MM are not matched by equivalent real-world functional data capture, and health-related quality of life (HRQoL) is frequently impaired, yet continuous real-world measurement of how patients feel and function remains limited outside clinical trials. Integrating patient-reported outcomes (PROs) with objective wearable data enables continuous multimodal digital phenotyping and may better characterise functional state across treatment lines.

### Aims

To determine whether continuous multimodal digital monitoring, integrating PROs and wearable-derived biometric data, differentiates functional state across treatment lines in MM using MyMM, a digital patient wallet enabling real-time recording of symptom burden, QoL, and supportive medication use.

### Methods

The Digital Patient Wallet (MyMM module) was co-developed with MM patients. PROs (MyPOS, EQ-5D-5L) and wearable-derived activity and sleep data were collected from 99 UK patients over eleven months (January 2025-February 2026). Linear mixed-effects models assessed associations with treatment line and outcomes using pairwise contrasts. Effect sizes are reported as estimated mean differences (EMD) and standard error (SE), with False Discovery Rate (FDR)-adjusted p-values ($p<0.05$).

### Results

The cohort (48.5% female; median age 60, range 37-86) was monitored for a median of 247 days (range 23-396). At the time of analysis, 31 patients were receiving first-line therapy, 10 second-line, 5 third-line, 17 fourth-line or later therapy, and 4 were on maintenance treatment. 18 patients were not receiving active treatment. A total of 45,383 datapoints were collected (17,707 activity; 14,849 sleep; 12,827 PROs). Across the cohort, the highest symptom severity (1-5 scale) was weakness/lack of energy (4.5), followed by drowsiness (2.5) and poor mobility (2.5).

Third-line patients demonstrated the highest mean step count (6,950), significantly higher than first-line patients (EMD: 3,251 steps, SE: 801; FDR-adjusted $p=0.0028$). Sleep duration was lowest in fourth-line patients (6h12min) and highest in third-line and untreated groups (7h42min; 24% difference). Self-reported health state was lowest in maintenance (40/100) and second-line (53/100) groups and highest in third- and fourth-line patients (70/100; 75% higher vs. maintenance). EQ-5D-5L peaked in third-line patients (0.82). Weakness severity was highest in maintenance (5.0) and second-line (4.8) groups, while third-line patients showed lower overall symptom burden, with weakness severity of 2.5. Findings are summarised in Figure 1.

**Copyright Information:** (Online) ISSN: 2572-9241
©2026 The Author(s). HemaSphere is published by John Wiley & Sons Ltd on behalf of European Hematology Association. This is an open access Abstract Book distributed under the Attribution-NonCommercial-NoDerivs (CC BY-NC-ND), which allows third parties to download the articles and share them with others as long as they credit the author and the Abstract Book, but they cannot change the content in any way or use them commercially.
**Abstract Book Citations:** Authors, Title, HemaSphere, 2026;10:(S1):pages. The abstract book DOIs can be found on the "Author Information" page.
**Disclaimer:** Articles published in the journal HemaSphere exclusively reflect the opinions of the authors. The authors are responsible for all content in their abstracts including accuracy of the facts, statements, citing resources, etc.

### Summary/Conclusion

The Digital Patient Wallet establishes a scalable framework for continuous MM functional monitoring and early functional phenotyping across treatment lines. This represents the first continuous multimodal UK MM dataset integrating wearables and PROs longitudinally across lines of therapy.
While current outputs are exploratory and caveated by small subgroup sizes, observed patterns suggest potential utility for treatment tolerance assessment and supportive care optimisation. Future work will expand recruitment, explore individual patient-level variation, incorporate covariate-adjusted analyses, and support predictive modelling for toxicity forecasting, relapse detection, and integration into adaptive care pathways and digitally enabled clinical trials.

**Figure 1. Functional outcomes across treatment lines.** Wearable-derived activity and sleep metrics, patient-reported outcomes, and symptom burden are shown by treatment line. Overall, third-line patients showed the highest activity, fourth-line patients the shortest sleep duration, and maintenance/second-line groups poorer self-reported health on selected measures.

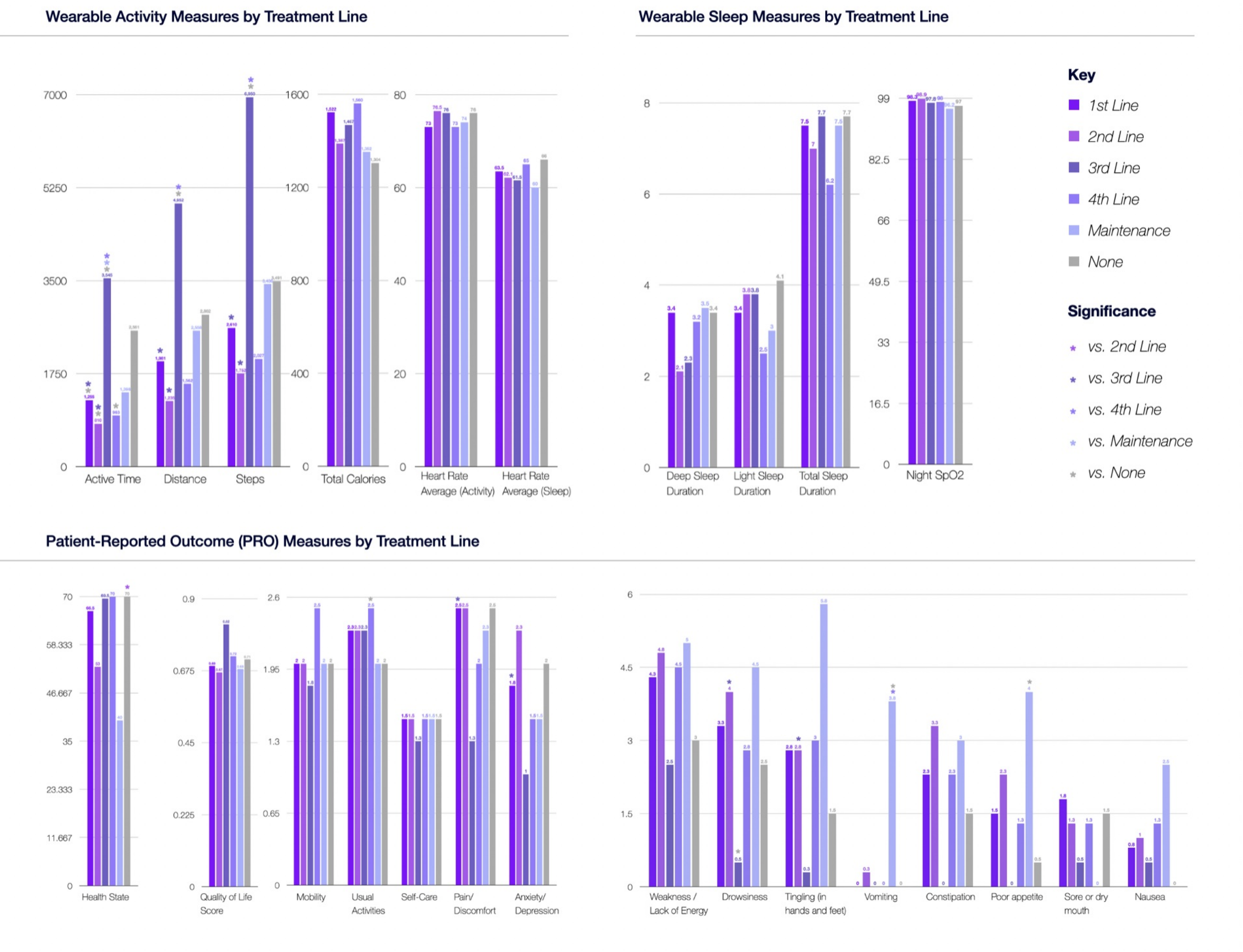

Figure 1. Differences in Cohort Average Wearables-Derived Biometrics and Patient-Reported Outcomes by Treatment Group

**Copyright Information:** (Online) ISSN: 2572-9241
©2026 The Author(s). HemaSphere is published by John Wiley & Sons Ltd on behalf of European Hematology Association. This is an open access Abstract Book distributed under the Attribution-NonCommercial-NoDerivs (CC BY-NC-ND), which allows third parties to download the articles and share them with others as long as they credit the author and the Abstract Book, but they cannot change the content in any way or use them commercially.
**Abstract Book Citations:** Authors, Title, HemaSphere, 2026;10:(S1):pages. The abstract book DOIs can be found on the "Author Information" page.
**Disclaimer:** Articles published in the journal HemaSphere exclusively reflect the opinions of the authors. The authors are responsible for all content in their abstracts including accuracy of the facts, statements, citing resources, etc.

### (PB4530) LONGITUDINAL HAEMOGLOBIN AS A DYNAMIC MARKER OF CLINICAL BURDEN AND PATIENT QUALITY OF LIFE IN SICKLE CELL DISEASE: EVIDENCE FROM A REAL-WORLD COHORT

**Topic:** 38. Novel technologies, techniques and digital analytical tools in hematology

Kim Summers*[1], Orlando Agrippa[1], Jay Corr[1], Muna Yusuf[1], Tushar Singh[1], Paul Telfer[2], Sanne Lugthart[3]

*[1]Sanius Health, London, United Kingdom;[2]Barts Health NHS Trust, London, United Kingdom;[3]University Hospitals Bristol and Weston NHS Foundation Trust, Bristol, United Kingdom;*

### Background

Despite the central role of haemoglobin (Hb) in sickle cell disease (SCD) biology and novel therapeutic development, longitudinal real-world evidence linking Hb to downstream clinical burden and patient experience remains limited.

### Aims

To assess the feasibility of deriving longitudinal Hb and clinically relevant endpoints from a real-world SCD cohort, and to explore whether lower Hb is associated with greater subsequent clinical burden and patient-reported quality of life.

### Methods

Data were extracted from a real-world SCD ecosystem integrating app-based electronic patient-reported outcome (ePRO) data with transcribed medical records. Analysis focused on a UK subcohort with linked Hb and clinical-event data (n=372), with direct Hb-ePRO linkage assessed in a prospectively linked subset (n=85). Medical record data were processed using optical character recognition (OCR) and an augmented large language model (LLM) workflow to extract structured clinical data.
Vaso-occlusive crises (VOCs), transfusions, healthcare resource use (HCRU) contacts, and comorbidities were identified from medical records. VOCs separated by <5 days were treated as a single episode. Hb was analysed by patient-level median Hb band (<7, 7 to <9, and ≥9 g/dL) and by row-level 30- and 365-day windows for downstream burden. Sensitivity analyses excluded same-day and within 7 days of transfusion Hb rows. In the prospectively linked subset, matched-day, -week, and -month analyses were performed between Hb and ePRO.

### Results

The cohort had a median age of 35 (range 16-73) years, with 79% HbSS, HU exposure in 49%, and a median follow-up of 7 (IQR 10) years. This yielded 16,337 Hb rows and a median of 16 Hb measurements per patient, with a median Hb of 8.8 g/dL (IQR 2). VOCs were recorded in 79%, transfusion in 44%, any comorbidity history in 52%, presenting complaint-linked comorbidities in 21%, and any HCRU in 93% of patients.
At a patient level, lower Hb was associated with higher disease burden, with any VOC recorded in 92% of patients in the <7 g/dL Hb band, compared with 82% in the 7 to <9 and 78% in the ≥9 groups. Transfusion exposure occurred in 54%, 51%, and 40%, respectively.
At an Hb-row level, VOCs within 30 days followed 31% of Hb rows <7 g/dL, in comparison with 19% and 16% in the 7 to <9 and ≥9 groups. Corresponding 365-day VOC rates were 66%, 56%, and 46%, respectively. 30-day HCRU followed 53%, 42%, and 41% of Hb rows, respectively, with corresponding 365-day HCRU rates of 88%, 83%, and 80%. These patterns were maintained in sensitivity analyses excluding same-day and within 7-day transfusion Hb rows.
In the prospectively linked subset, higher Hb correlated with lower matched-day EQ-5D-5L 'Mobility' severity (n=170 datapoints, r=-0.16, p=0.033) and with higher matched-week numeric rating scale 'Mood' score (n=146, r=0.18, p=0.026). No significant matched-month associations were observed.

**Copyright Information:** (Online) ISSN: 2572-9241
©2026 The Author(s). HemaSphere is published by John Wiley & Sons Ltd on behalf of European Hematology Association. This is an open access Abstract Book distributed under the Attribution-NonCommercial-NoDerivs (CC BY-NC-ND), which allows third parties to download the articles and share them with others as long as they credit the author and the Abstract Book, but they cannot change the content in any way or use them commercially.
**Abstract Book Citations:** Authors, Title, HemaSphere, 2026;10:(S1):pages. The abstract book DOIs can be found on the "Author Information" page.
**Disclaimer:** Articles published in the journal HemaSphere exclusively reflect the opinions of the authors. The authors are responsible for all content in their abstracts including accuracy of the facts, statements, citing resources, etc.

### Summary/Conclusion

This analysis demonstrates the feasibility of a real-world, multi-modal data approach in supporting longitudinal capture of repeated Hb measurements alongside clinically relevant downstream endpoints. Lower Hb was consistently associated with greater subsequent VOC burden and healthcare use across both patient-level and Hb-row-level analyses, with directional relationships maintained in transfusion-sensitive analyses. Ultimately, this supports the potential utility of integrated real-world data to enable future stratified analyses and more detailed mapping of patient journeys over time in SCD.

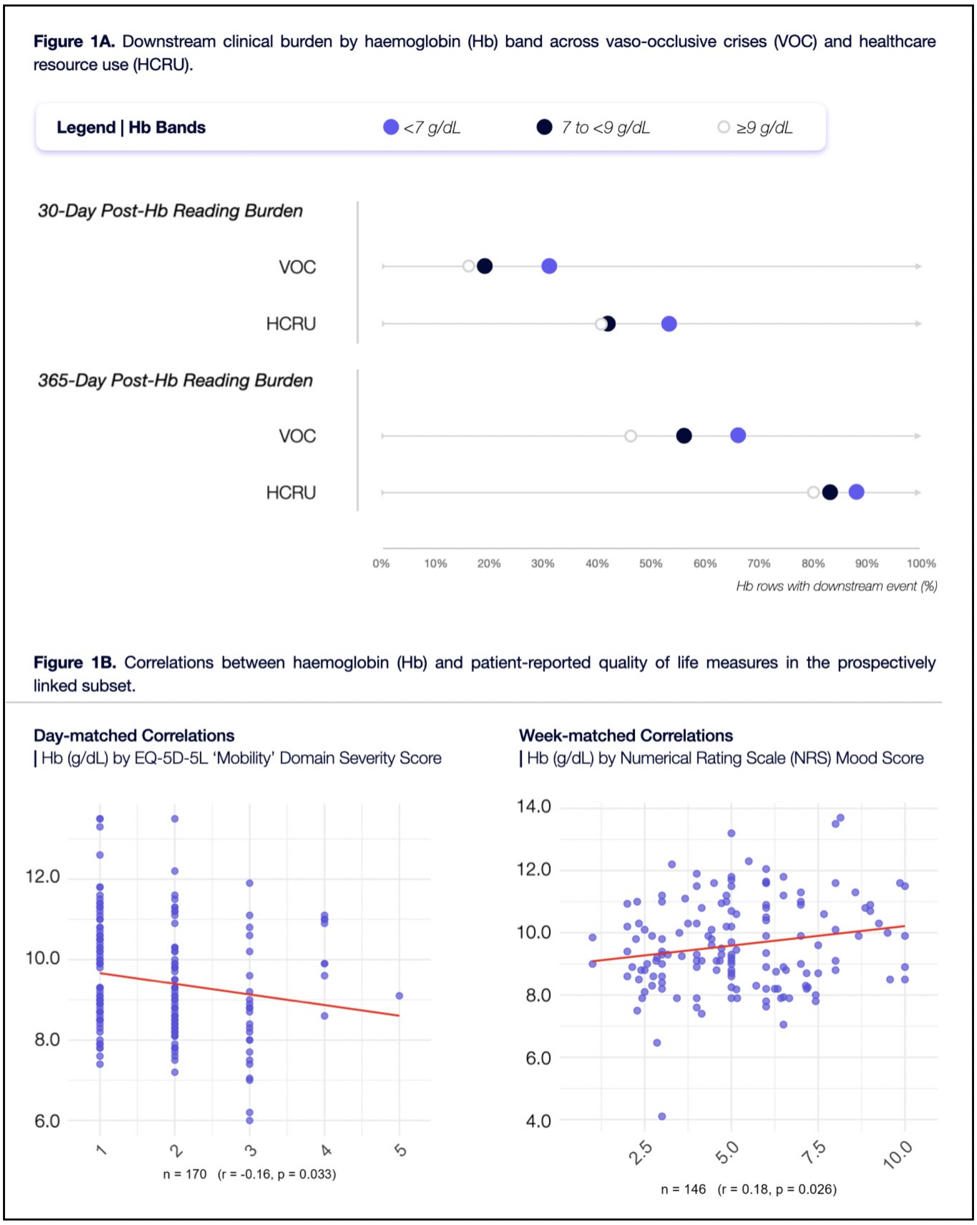

Figure 1. Visual summary of the key findings, showing greater downstream clinical burden following lower Hb readings and exploratory correlations between Hb and patient-reported outcomes in the prospectively linked subset.

**Copyright Information:** (Online) ISSN: 2572-9241
©2026 The Author(s). HemaSphere is published by John Wiley & Sons Ltd on behalf of European Hematology Association. This is an open access Abstract Book distributed under the Attribution-NonCommercial-NoDerivs (CC BY-NC-ND), which allows third parties to download the articles and share them with others as long as they credit the author and the Abstract Book, but they cannot change the content in any way or use them commercially.
**Abstract Book Citations:** Authors, Title, HemaSphere, 2026;10:(S1):pages. The abstract book DOIs can be found on the "Author Information" page.
**Disclaimer:** Articles published in the journal HemaSphere exclusively reflect the opinions of the authors. The authors are responsible for all content in their abstracts including accuracy of the facts, statements, citing resources, etc.

### (PB4531) AI-ASSISTED IDENTIFICATION MODEL FOR T(9;22) TRANSLOCATION CHROMOSOMES

**Topic:** 38. Novel technologies, techniques and digital analytical tools in hematology

Siyu Chen[1], Xi Zhang[1], Hao Wang*[1], Xigui Peng[1]

[1]*Xinqiao Hospital of Army Medical University, CHONGQING, China;*

### Aims

To construct a discriminative model based on artificial intelligence for the identification of common t(9;22) chromosomal translocations in hematologic neoplasms, and promote the intelligent recognition of abnormal karyotypes associated with hematologic malignancies.

### Methods

A total of 3000 images of normal metaphase chromosomes and 1500 images of chromosomes with t(9;22) translocation were used as input data. Self-supervised pre-training model using a Masked Autoencoder (MAE) algorithm was performed. Briefly, normal chromosome images were used as internal references and randomly masked before reconstructed. After iterative optimization, the model learned the morphological and distributional features of normal chromosomes.The model was then applied to detect and identify t(9;22) translocation in 1500 chromosome images, and the sensitivity and specificity of the model were calculated.

### Results

In validation experiments using t(9;22) translocation samples, the model achieved a sensitivity and specificity of 0.90, demonstrating favorable accuracy and stability, as well as promising clinical application prospects. Compared with manual karyotyping, the AI model can significantly improve the efficiency of chromosome karyotype analysis (6.5±0.5 VS 33.9±2.1min).

### Summary/Conclusion

This method demonstrates the application potential of self-supervised learning in the recognition of chromosomal abnormalities.It provides a novel strategy for the development of efficient and widely applicable auxiliary diagnostic tools for abnormal karyotypes associated with other hematologic neoplasms.

**Copyright Information:** (Online) ISSN: 2572-9241
©2026 The Author(s). HemaSphere is published by John Wiley & Sons Ltd on behalf of European Hematology Association. This is an open access Abstract Book distributed under the Attribution-NonCommercial-NoDerivs (CC BY-NC-ND), which allows third parties to download the articles and share them with others as long as they credit the author and the Abstract Book, but they cannot change the content in any way or use them commercially.
**Abstract Book Citations:** Authors, Title, HemaSphere, 2026;10:(S1):pages. The abstract book DOIs can be found on the "Author Information" page.
**Disclaimer:** Articles published in the journal HemaSphere exclusively reflect the opinions of the authors. The authors are responsible for all content in their abstracts including accuracy of the facts, statements, citing resources, etc.

### (PB4532) HOME ADMINISTRATION OF THERAPIES FOR HEMATOLOGIC MALIGNANCIES IN SWEDEN: INSIGHTS FROM A MIXED-METHODS STUDY OF REGISTERED NURSES

**Topic:** 38. Novel technologies, techniques and digital analytical tools in hematology

Bilguun Elbegzaya*[1], Björn Wettermark[1], David Hjelm Sempaya[2], Jon A Tsai[2], Margareta Westerbotn[3]

*[1]Department of Pharmacy, Uppsala University,, Uppsala, Sweden;[2]Sanofi AB, Medical, Stockholm, Sweden;[3]Department of Nursing Science, Sophiahemmet University, Stockholm, Sweden;*

### Background

Frequent hospital visits are common for patients with haematological malignancies and may negatively affect quality of life while placing increasing demands on healthcare services, because of a higher prevalence of these diseases due to better survival. Providing treatment in the home setting has emerged as a potentially valuable approach to enhancing patient wellbeing and reducing system strain.

### Aims

To explore how home administration of treatments for haematological malignancies is currently implemented in Sweden and to examine nurses' experiences, opportunities, and challenges associated with this model of care.

### Methods

A sequential explanatory mixed-methods design was employed, comprising a cross-sectional online survey followed by semi-structured interviews. Twenty-four registered nurses from six Swedish healthcare regions completed the survey, and data were analysed using descriptive and inferential statistics. Seven nurses subsequently participated in interviews, which were analysed using content analysis. Ethical approval was obtained from the Swedish Ethical Review Authority (Dnr.: 2025-06516-01).

### Results

Home administration of therapies for haematological malignancies was reported in 86.4% of the six healthcare regions, with approximately 25.0% of patients currently receiving treatment at home. Subcutaneous injections and infusions were the most frequently used administration routes, followed by intravenous therapy, and 68.0% responded that local protocols were in place. The most common conditions managed at home were acute myeloid leukemia and myelodysplastic syndrome, each reported by 89.5% of the respondents, followed by multiple myeloma which was reported by 78.9%. Medicine picked up by the patient was the most frequently applied method of delivery, reported by 78.9% of the respondents. Marked variation was identified in organisational structures and procedures across regions and hospitals. Key drivers for offering home administration included patient convenience, increased flexibility, and reduced pressure on hospital services. Content analysis yielded three themes: Enhanced quality of life and wellbeing through home administration; person-centered and safe care in the home environment; Organisational opportunities and challenges. Despite some organisational barriers, most nurses expressed strong support for home administration and valued being able to offer this option to patients.

### Summary/Conclusion

Home administration of treatments for haematological malignancies appears to be a feasible and advantageous model of care that may alleviate pressure on healthcare services while improving patients' quality of life. The development of standardised national guidelines is warranted to ensure equitable access to home-based cancer care across Sweden.

**Copyright Information:** (Online) ISSN: 2572-9241
©2026 The Author(s). HemaSphere is published by John Wiley & Sons Ltd on behalf of European Hematology Association. This is an open access Abstract Book distributed under the Attribution-NonCommercial-NoDerivs (CC BY-NC-ND), which allows third parties to download the articles and share them with others as long as they credit the author and the Abstract Book, but they cannot change the content in any way or use them commercially.
**Abstract Book Citations:** Authors, Title, HemaSphere, 2026;10:(S1):pages. The abstract book DOIs can be found on the "Author Information" page.
**Disclaimer:** Articles published in the journal HemaSphere exclusively reflect the opinions of the authors. The authors are responsible for all content in their abstracts including accuracy of the facts, statements, citing resources, etc.

### (PB4533) DEVELOPMENT OF A QUPATH EXTENSION FOR AUTOMATED CELL ANNOTATION IN BONE MARROW SMEARS TO ENABLE ACTIVE LEARNING–DRIVEN CELL DETECTION

**Topic:** 38. Novel technologies, techniques and digital analytical tools in hematology

Jon Kristian Nilssen*[1], Bjørn-Jostein Singstad[2], Vimala Nunavath[1], Martin Paulson[2]

*[1]University of South-Eastern Norway, Department of Science and Industry systems, Kongsberg, Norway;[2]Vestfold Hospital Trust, Department of Radiology, Tønsberg, Norway;*

### Background

The evaluation of blood- and bone marrow aspirate (BMA)-smears remains a cornerstone diagnostic process in hematology. The process of microscopy is labor-intensive, prone to inter- and intra-observer variability, and requires specialized expertise. Artificial intelligence (AI) can support automated cell detection in microscopy images, but model development usually requires large datasets. Active learning can increase annotation throughput by using AI-generated pre-annotations that are subsequently corrected and verified by specialists.

### Aims

We aimed to develop a QuPath (v0.6.0) extension to facilitate review and refinement of pre-annotations by a custom you-only-look-once (YOLO) model on whole-slide images (WSI) of BMA-smears to support active learning.

### Methods

Deep Java Library v0.33.0 is used for inference within user-defined regions of interest (ROIs), after the model path and target image size are specified. Each ROI is processed in tiles according to the image size, and post-processing displays the results in the QuPath viewer. Bounding boxes are colored by confidence score: red (0.40–0.60), orange (0.61–0.80), yellow (0.81–0.90), and green (≥0.91). The active learning window (Figure 1) retrieves all model predictions and displays their bounding boxes with its surrounding image context in a 5×5 grid. The top-left grid cell always shows the prediction with the lowest confidence score in the selected region, while the remaining grid cells contain its closest spatial neighbors, enabling contextual inspection. Users can select any subset of cells and click “Verify”, labelling the selected cells as “Verified plasma cell” and all others as “Not plasma cell”.
To assess the performance of the proposed tool, we carried out a timed annotation study on WSIs, comparing model inference time plus expert review using the cell matrix against a fully manual review process without the extension. In both cases, inference and annotation was performed on user-defined ROIs.

**Copyright Information:** (Online) ISSN: 2572-9241
©2026 The Author(s). HemaSphere is published by John Wiley & Sons Ltd on behalf of European Hematology Association. This is an open access Abstract Book distributed under the Attribution-NonCommercial-NoDerivs (CC BY-NC-ND), which allows third parties to download the articles and share them with others as long as they credit the author and the Abstract Book, but they cannot change the content in any way or use them commercially.
**Abstract Book Citations:** Authors, Title, HemaSphere, 2026;10:(S1):pages. The abstract book DOIs can be found on the “Author Information” page.
**Disclaimer:** Articles published in the journal HemaSphere exclusively reflect the opinions of the authors. The authors are responsible for all content in their abstracts including accuracy of the facts, statements, citing resources, etc.

### Results

We used a trained YOLOv12n to do inference on 10 BMA smears of patients with multiple myeloma (ICD-10: C90.0, n=7) and monoclonal gammopathy (ICD-10: D47.2, n=3) of uncertain significance. The results from the timed annotation study arepresented in Table 1, where the AI-assisted workflow required a mean processing time of 82 (95% CI, 65-98) seconds and was significantly faster (p<0.001) than the manual annotation workflow, which took 700 (95% CI, 420-982) seconds on average.

Table 1. Results from comparing manual annotation and YOLO detection plus expert review

| WSI ID | ICD-10 | YOLO Inference time (s) | YOLO detection count (n) | YOLO-detectiontime (s per 100 cells) | Expert review time (s per 100 cells) | Inference + expert review time (s per 100 cells) | Manual annotation time (s per 100 cells) | Time ratio (Manual vs AI) |
|---|---|---|---|---|---|---|---|---|
| 1 | C90.0 | 48* | 1468 | 3.3 | 66.8 | 70.1 | 300.0 | 4.3 |
| 2 | D47.2 | 493* | 1517 | 32.3 | 55.8 | 88.1 | 682.5 | 7.7 |
| 3 | C90.0 | 183* | 824 | 22.2 | 62.3 | 84.5 | 941.2 | 11.1 |
| 4 | D47.2 | 2124* | 2240 | 91.0 | 49.3 | 140.3 | 1166.0 | 8.3 |
| 5 | C90.0 | 77** | 1059 | 7.2 | 66.9 | 74.1 | 387.1 | 5.2 |
| 6 | C90.0 | 894,0* | 3463 | 25.6 | 50.0 | 75.6 | 962.0 | 12.7 |
| 7 | C90.0 | 360,0** | 900 | 40.0 | 54.7 | 94.7 | 606.9 | 6.4 |
| 8 | C90.0 | 9.5* | 342 | 2.8 | 61.1 | 63.9 | 283.5 | 4.4 |
| 9 | D47.2 | 710* | 2603 | 27.3 | 38.6 | 65.9 | 1372.0 | 20.8 |
| 10 | C90.0 | 109* | 898 | 12.1 | 48.8 | 60.9 | 307.7 | 5.1 |

**Tests performed on* 11th Gen i7-11700 @2.5 GHz CPU
**Tests performed on AMD Ryzen 5 7520U @2.8 GHz

### Summary/Conclusion

We developed a QuPath extension that incorporates customized YOLO models to automatically identify PCs, combined with expert-guided verification. Using this extension significantly reduced the time needed for annotation compared with entirely manual labeling in QuPath.

**Copyright Information:** (Online) ISSN: 2572-9241
©2026 The Author(s). HemaSphere is published by John Wiley & Sons Ltd on behalf of European Hematology Association. This is an open access Abstract Book distributed under the Attribution-NonCommercial-NoDerivs (CC BY-NC-ND), which allows third parties to download the articles and share them with others as long as they credit the author and the Abstract Book, but they cannot change the content in any way or use them commercially.
**Abstract Book Citations:** Authors, Title, HemaSphere, 2026;10:(S1):pages. The abstract book DOIs can be found on the "Author Information" page.
**Disclaimer:** Articles published in the journal HemaSphere exclusively reflect the opinions of the authors. The authors are responsible for all content in their abstracts including accuracy of the facts, statements, citing resources, etc.

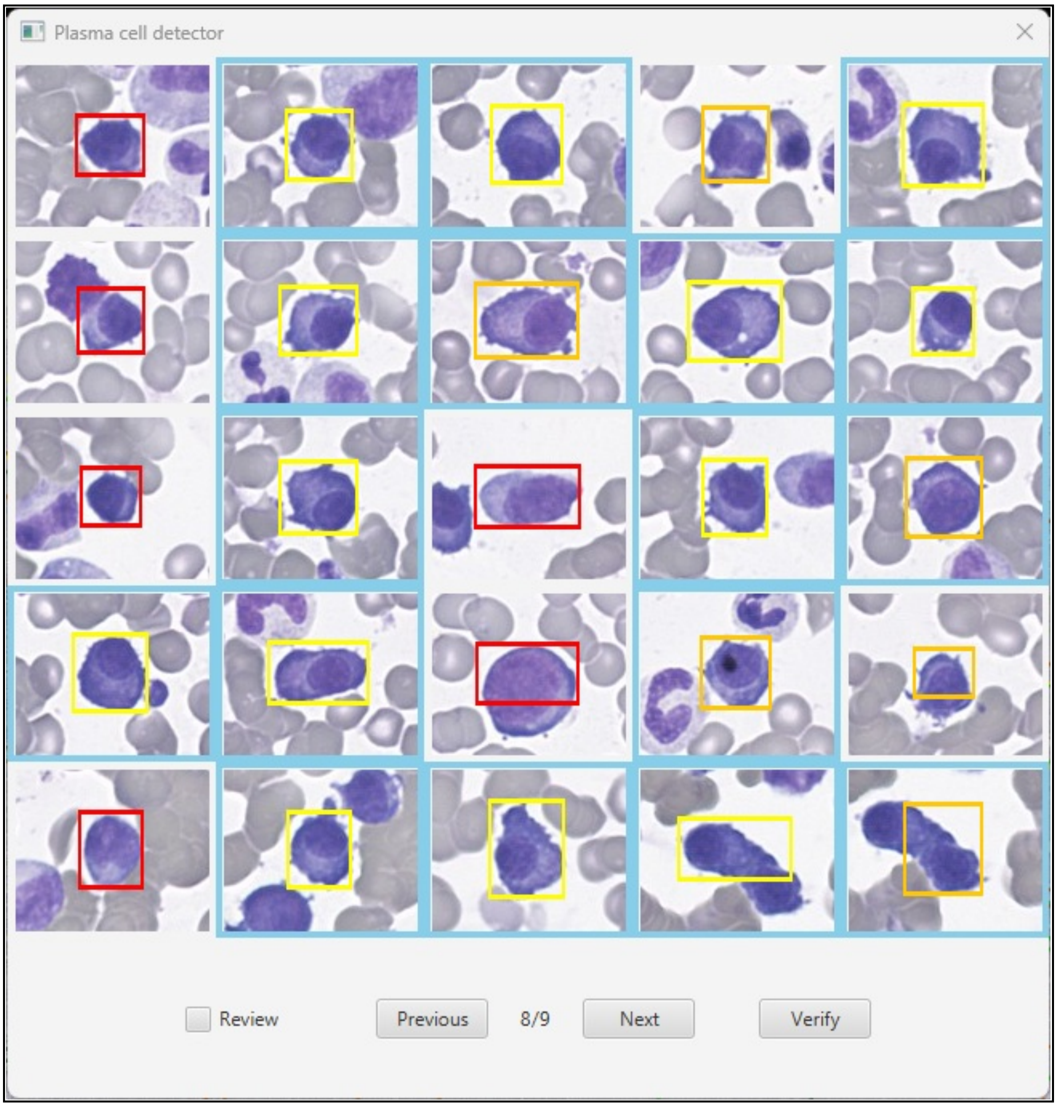

Figure 1. Active learning interface displaying the cell matrix view with predicted bounding boxes for each cell, color-coded according to model confidence. The light blue border around a grid cell indicates selection status.

**Copyright Information:** (Online) ISSN: 2572-9241
©2026 The Author(s). HemaSphere is published by John Wiley & Sons Ltd on behalf of European Hematology Association. This is an open access Abstract Book distributed under the Attribution-NonCommercial-NoDerivs (CC BY-NC-ND), which allows third parties to download the articles and share them with others as long as they credit the author and the Abstract Book, but they cannot change the content in any way or use them commercially.
**Abstract Book Citations:** Authors, Title, HemaSphere, 2026;10:(S1):pages. The abstract book DOIs can be found on the "Author Information" page.
**Disclaimer:** Articles published in the journal HemaSphere exclusively reflect the opinions of the authors. The authors are responsible for all content in their abstracts including accuracy of the facts, statements, citing resources, etc.

### (PB4534) DEEP LEARNING DETECTS DOPING: IMAGE-BASED DETECTION OF ERYTHROPOIETIN USE IN ATHLETES VIA WEAKLY SUPERVISED DEEP LEARNING

**Topic:** 38. Novel technologies, techniques and digital analytical tools in hematology

Martin Schneider*[1], Ishan Srivastava[1], Jan Venzke[2], Markus de Marées[2], Annekathrin Keiler[3, 4], Susann Winter[1, 5], Frank Kroschinsky[1], Martin Bornhäuser[1, 6, 7], Jan-Niklas Eckardt[1, 5, 8], Petra Platen[2], Sven Voss[3], Jan Moritz Middeke[1, 5, 8]

*[1]Department of Internal Medicine I, University Hospital Carl Gustav Carus, TUD Dresden University of Technology, Dresden, Germany; [2]Department of Sports Medicine and Sports Nutrition, Faculty of Sport Science, Ruhr University Bochum, Bochum, Germany; [3]Institute of Doping Analysis and Sports Biochemistry Dresden, Kreischa, Germany;[4]Environmental Monitoring & Endocrinology, Faculty of Biology, TUD Dresden University of Technology, Dresden, Germany; [5]Else Kröner Fresenius Center for Digital Health, TUD Dresden University of Technology, Dresden, Germany; [6]German Cancer Consortium (DKTK), Partner Site Dresden, and German Cancer Research Center (DKFZ), Heidelberg, Germany; [7]National Center for Tumor Diseases Dresden (NCT/UCC), a partnership between DKFZ, Faculty of Medicine and University Hospital Carl Gustav Carus, TUD Dresden University of Technology, and Helmholtz-Zentrum Dresden-Rossendorf (HZDR), Dresden, Germany; [8]Mildred Scheel Early Career Center, National Center for Tumor Diseases (NCT/UCC) Dresden, Faculty of Medicine and University Hospital Carl Gustav Carus, TUD Dresden University of Technology, Dresden, Germany;*

### Background

Recombinant erythropoietin (rEPO) remains a widely used performance-enhancing agent in endurance sports, yet current testing strategies are limited by short detection windows and high biological variability. rEPO-induced stimulation of erythropoiesis may lead to distinct alterations in red blood cell (RBC) morphology, providing exploitable signatures for deep learning (DL)-based analysis of peripheral blood smears (PBS), potentially extending existing anti-doping methods.

### Aims

To detect rEPO administration in athletes using DL-based morphological analysis of digitized PBS.

### Methods

In a randomized, double-blind study, thirty healthy, physically active female and male volunteers received either subcutaneous 3,000 IU rEPO (n = 12) or saline as a placebo (n = 18) twice weekly for three weeks, with blood sampling performed at 13 time points across an 11-week period encompassing pre-, during, and post-administration phases. Ferritin levels were monitored regularly, and intravenous iron supplementation was administered according to predefined sex-specific thresholds to prevent iron deficiency. Complete blood counts, including reticulocyte counts, were measured longitudinally. PBS were prepared at each time point, Pappenheim-stained, and digitized as whole-slide images (WSIs). WSIs were analysed using the Haemorasis pipeline (de Almeida et al., *Nat Commun*, 2023), adapted to detect extended (1500 × 1500 pixels), non-overlapping monolayer regions, combined with an in-house classification framework. A five-fold binary classification model, stratified for rEPO use and ensuring slide-level separation, was applied to prevent data leakage. Cell-level segmentation masks were added as an input channel to delineate cells and provide a spatial prior. Hyperparameters were optimized on one predefined fold, subsequently fixed and used to train all five cross-validation folds for final performance estimation.

**Copyright Information:** (Online) ISSN: 2572-9241
©2026 The Author(s). HemaSphere is published by John Wiley & Sons Ltd on behalf of European Hematology Association. This is an open access Abstract Book distributed under the Attribution-NonCommercial-NoDerivs (CC BY-NC-ND), which allows third parties to download the articles and share them with others as long as they credit the author and the Abstract Book, but they cannot change the content in any way or use them commercially.
**Abstract Book Citations:** Authors, Title, HemaSphere, 2026;10:(S1):pages. The abstract book DOIs can be found on the "Author Information" page.
**Disclaimer:** Articles published in the journal HemaSphere exclusively reflect the opinions of the authors. The authors are responsible for all content in their abstracts including accuracy of the facts, statements, citing resources, etc.

### Results

Reticulocyte counts showed a transient increase during rEPO administration, peaking shortly after the initiation of rEPO administration, whereas no changes were observed in the placebo group. Comparing PBS collected during the peak rEPO response from the intervention group with PBS obtained prior to rEPO administration, but after potential iron supplementation in all athletes, the DL model achieved a mean five-fold cross-validated accuracy of 0.92 (standard deviation [SD] ± 0.01) and an area under the receiver operating characteristic curve (AUROC) of 0.92 (SD ± 0.04) in the combined cohort of male and female athletes. Stratified by sex, the model achieved an AUROC of 0.92 (SD ± 0.04) in male athletes and 0.93 (SD ± 0.06) in female athletes at the same time points. Reliability map computation showed no evidence of overfitting. For the qualitative assessment, we generated occlusion sensitivity maps and gradient-weighted class activation maps (Grad-CAM) which demonstrated that the model's attention was directed mostly at cells, mainly RBCs (figure 1).

### Summary/Conclusion

This study provides proof of concept that DL-based morphological analysis of PBS could have the potential to detect rEPO administration in athletes. The approach captures rEPO-associated morphological signatures and may serve as a complementary tool for anti-doping applications. External validation in larger and more diverse cohorts is required to improve robustness, interpretability, and generalizability before routine implementation can be considered. This study was funded by the World Anti-Doping Agency (Grant 23D05SV).

**Copyright Information:** (Online) ISSN: 2572-9241
©2026 The Author(s). HemaSphere is published by John Wiley & Sons Ltd on behalf of European Hematology Association. This is an open access Abstract Book distributed under the Attribution-NonCommercial-NoDerivs (CC BY-NC-ND), which allows third parties to download the articles and share them with others as long as they credit the author and the Abstract Book, but they cannot change the content in any way or use them commercially.
**Abstract Book Citations:** Authors, Title, HemaSphere, 2026;10:(S1):pages. The abstract book DOIs can be found on the "Author Information" page.
**Disclaimer:** Articles published in the journal HemaSphere exclusively reflect the opinions of the authors. The authors are responsible for all content in their abstracts including accuracy of the facts, statements, citing resources, etc.

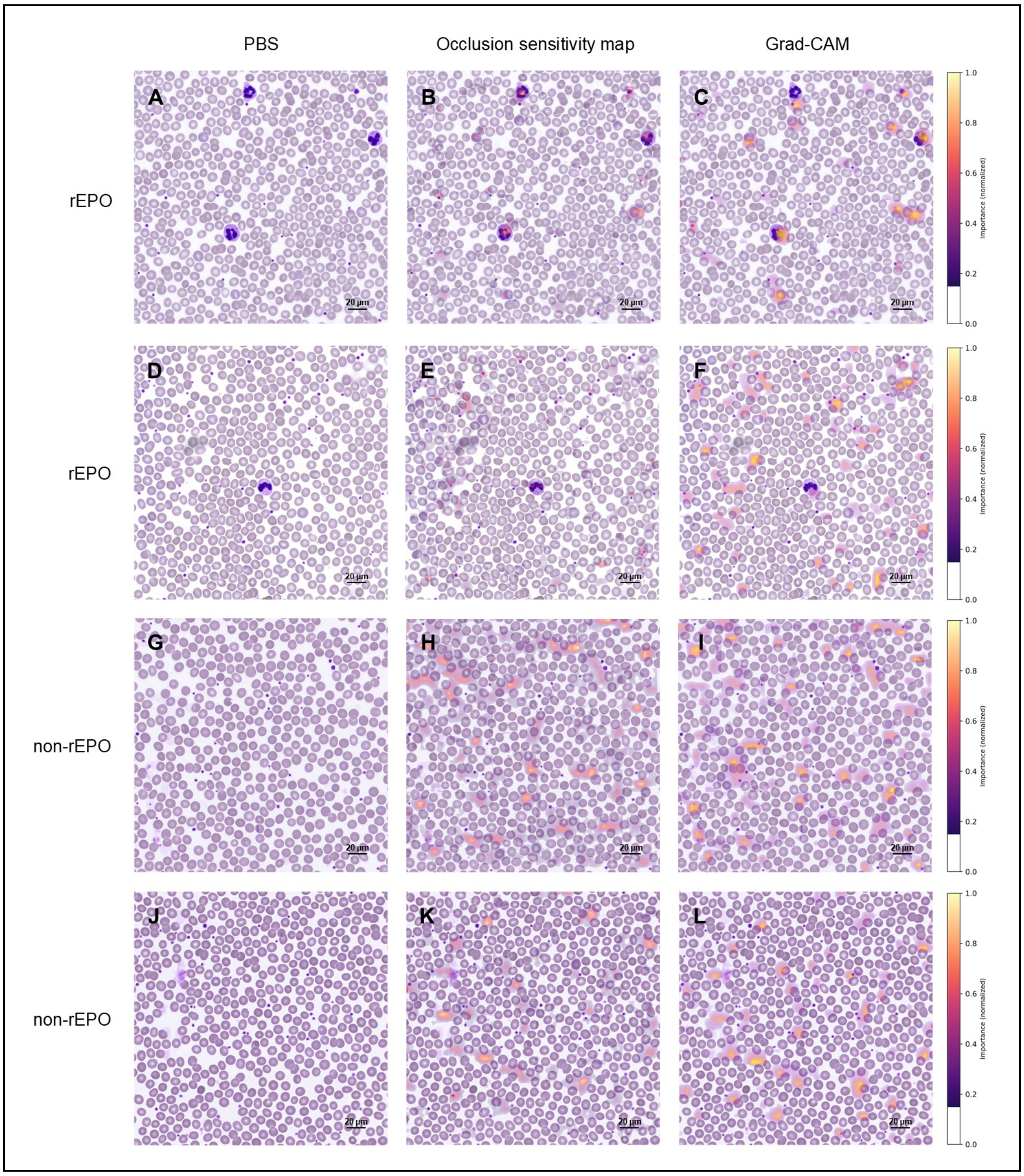

Figure 1: Representative PBS with corresponding occlusion sensitivity maps and Grad-CAMs highlighting morphological features relevant to the DL model. A and D show PBS correctly classified as rEPO samples with corresponding occlusion maps (B, E) and Grad-CAMs (C, F). G and J show correctly classified non-rEPO samples with corresponding occlusion maps (H, K) and Grad-CAMs (I, L). The color scale indicates the relative intensity of the model's attention across the morphological structures. DL: deep learning; Grad-CAM: gradient-weighted class activation map; PBS: peripheral blood smear; rEPO: recombinant erythropoietin.

**Copyright Information:** (Online) ISSN: 2572-9241
©2026 The Author(s). HemaSphere is published by John Wiley & Sons Ltd on behalf of European Hematology Association. This is an open access Abstract Book distributed under the Attribution-NonCommercial-NoDerivs (CC BY-NC-ND), which allows third parties to download the articles and share them with others as long as they credit the author and the Abstract Book, but they cannot change the content in any way or use them commercially.
**Abstract Book Citations:** Authors, Title, HemaSphere, 2026;10:(S1):pages. The abstract book DOIs can be found on the "Author Information" page.
**Disclaimer:** Articles published in the journal HemaSphere exclusively reflect the opinions of the authors. The authors are responsible for all content in their abstracts including accuracy of the facts, statements, citing resources, etc.

### (PB4535) DIAGNOSTIC TEST ACCURACY OF DEEP LEARNING MODELS FOR THE AUTOMATED CLASSIFICATION OF ACUTE LEUKEMIA FROM BONE MARROW ASPIRATES AND BIOPSY: A SYSTEMATIC REVIEW AND META-ANALYSIS

**Topic:** 38. Novel technologies, techniques and digital analytical tools in hematology

Rahul Falodia*[1]

[1]*All India Institute of Medical Sciences, Jodhpur, India;*

**Background**

Cytomorphological evaluation of bone marrow aspirates (BMA) remains the gold standard for diagnosing acute leukemias. However, manual microscopic review is hindered by inter-observer variability, heavy pathologist workloads, and the morphological ambiguity of atypical blasts, potentially delaying time-to-treatment. While deep learning (DL) has proven effective in peripheral blood screening, its diagnostic test accuracy (DTA) within the highly complex, densely packed cellular microenvironment of BMA remains underexplored.

**Aims**

This systematic review and meta-analysis quantifies the diagnostic accuracy of advanced DL architectures specifically engineered for automated leukemia classification in bone marrow smears, evaluating their readiness for real-world clinical deployment.

**Methods**

A systematic literature search was conducted across PubMed, Embase, and IEEE Xplore from inception to February 2026 in strict adherence to PRISMA-DTA guidelines. Primary studies were eligible if they evaluated DL models for the binary or multi-class detection of acute leukemias using exclusively BMA imaging. Risk of bias was assessed using QUADAS-2, with a specific focus on patient overlap and data leakage. True positives, false positives, true negatives, and false negatives were extracted. A bivariate mixed-effects regression model was utilized to calculate pooled sensitivity, specificity, and to generate a Hierarchical Summary Receiver Operating Characteristic (HSROC) curve.

**Results**

A total of 6 robust primary datasets were included, capturing a massive pooled dataset of over 770,000 single-cell and whole-slide BMA images. The evaluated architectures demonstrated a paradigm shift from traditional CNNs like ResNet-50 to advanced attention-based mechanisms like EfficientNetV2-B3 and Vision Transformers like ReLViT.
The bivariate meta-analysis yielded a random-effects pooled sensitivity of **86.5%** (95% CI: 72.0%-94.5%) and a pooled specificity of **96.8%** (95% CI: 94.2%-98.4%). The area under the HSROC curve was exceptionally high at**0.954**.
Significant statistical heterogeneity was observed across the studies for both pooled sensitivity and specificity ($p < 0.001$). Subgroup analyses revealed that this variance was primarily driven by the differing levels of clinical task analysis. Cell-level morphological models designed to flag individual myeloblasts and lymphoblasts prioritized specificity, achieving near-perfect negative predictive values (>98.8%), whereas patient-level diagnostic models demonstrated slightly lower specificities (~94.0%) but higher clinical utility for overall diagnosis. Extensively externally validated foundation models maintained robust accuracy even when differentiating AML from complex confounding dysplasias, such as myelodysplastic syndromes and aplastic anemia.

**Copyright Information:** (Online) ISSN: 2572-9241
©2026 The Author(s). HemaSphere is published by John Wiley & Sons Ltd on behalf of European Hematology Association. This is an open access Abstract Book distributed under the Attribution-NonCommercial-NoDerivs (CC BY-NC-ND), which allows third parties to download the articles and share them with others as long as they credit the author and the Abstract Book, but they cannot change the content in any way or use them commercially.
**Abstract Book Citations:** Authors, Title, HemaSphere, 2026;10:(S1):pages. The abstract book DOIs can be found on the "Author Information" page.
**Disclaimer:** Articles published in the journal HemaSphere exclusively reflect the opinions of the authors. The authors are responsible for all content in their abstracts including accuracy of the facts, statements, citing resources, etc.

### Summary/Conclusion

Deep learning architectures demonstrate exceptional, expert-level diagnostic accuracy for the automated classification of acute leukemias from bone marrow aspirates. The integration of Vision Transformers and multi-magnification ensembles has largely resolved historical challenges associated with BMA cellular density. The presence of significant statistical heterogeneity highlights a clinical reality: AI models are highly task-specific. Future prospective trials should prioritize end-to-end integration into hematopathology laboratory information systems (LIS) to evaluate real-world impacts on patient survival outcomes.

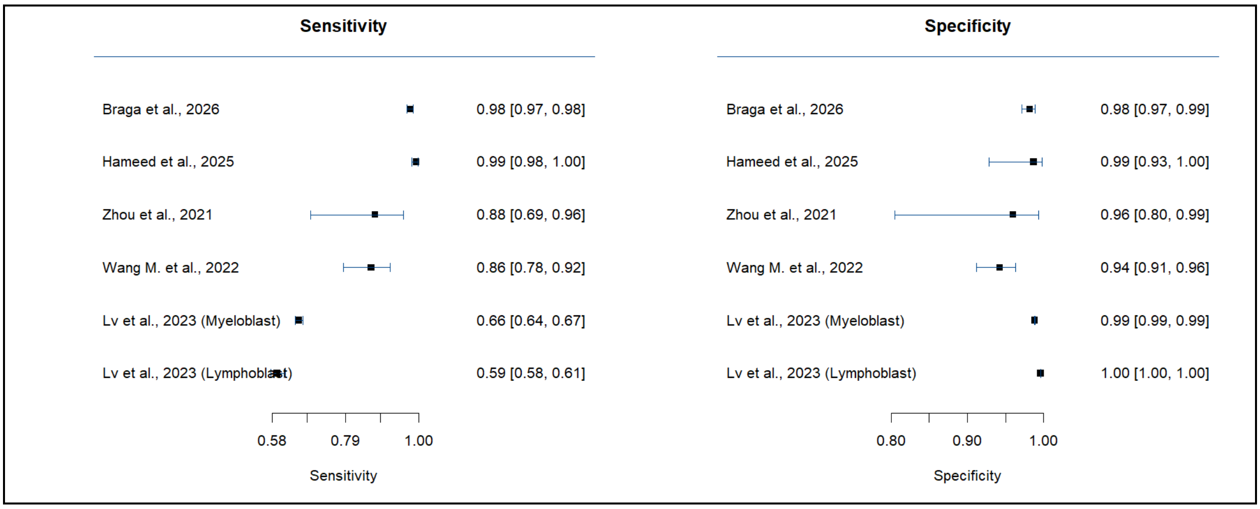

**Copyright Information:** (Online) ISSN: 2572-9241
©2026 The Author(s). HemaSphere is published by John Wiley & Sons Ltd on behalf of European Hematology Association. This is an open access Abstract Book distributed under the Attribution-NonCommercial-NoDerivs (CC BY-NC-ND), which allows third parties to download the articles and share them with others as long as they credit the author and the Abstract Book, but they cannot change the content in any way or use them commercially.
**Abstract Book Citations:** Authors, Title, HemaSphere, 2026;10:(S1):pages. The abstract book DOIs can be found on the "Author Information" page.
**Disclaimer:** Articles published in the journal HemaSphere exclusively reflect the opinions of the authors. The authors are responsible for all content in their abstracts including accuracy of the facts, statements, citing resources, etc.

### (PB4536) ANALYTICAL PERFORMANCE EVALUATION OF THE CELLAVISION DI-60 INTEGRATED WITH SYSMEX SP-50 USING CELLAVISION RAL WRIGHT STAIN AND SYSMEX BUFFER FOR DIGITAL WBC DIFFERENTIALS

**Topic:** 38. Novel technologies, techniques and digital analytical tools in hematology

Vishnu Prasad*[1], Frederick Ilagan[1], Kieren Cheng[1], Niythiya Mohan[1]

[1]*Khoo Teck Puat Hospital, Singapore, Singapore;*

### Background

Automation of peripheral blood smear preparation, staining, and digital morphology is increasingly adopted to enhance standardization, turnaround time, and reproducibility in laboratory hematology. The Sysmex SP-50 slide maker/stainer, when integrated with Cellavision DI-60 digital morphology analyzer, offers a fully automated workflow. However, the analytical performance of this configuration using Cellavision RAL Diagnostic Wright stain with Sysmex phosphate buffer (pH 6.8) requires validation.

### Aims

This study evaluates the accuracy, precision, and smear quality of DI-60 leukocyte differentials when slides are prepared and stained by the SP-50 using Cellavision RAL Diagnostic Wright stain with Sysmex phosphate buffer combination across broad range of clinical conditions encountered in routine practice.

### Methods

More than 50 smears underwent qualitative evaluation to ensure suitability for digital analysis, including assessment of working area, smear length (2.5 cm), gradual transition in thickness, acceptable morphology within the working area, minimal artefacts, minimal distributional distortion, reproducible stain quality, acceptable WBC distortion, and appropriate Wright stain color reactions. Differential accuracy was assessed by comparing DI-60 leukocyte differentials with the automated WBC differential results generated from Sysmex XN-10 analyzer. Rumke's statistical rule for allowable error was applied to determine acceptability for each leukocyte subtype. Precision studies included total imprecision (10-day repeated analysis of a single slide), within-slide imprecision, 10-slide reproducibility, and between-staff variability, with SDs calculated for neutrophils, lymphocytes, monocytes, eosinophils and basophils.

### Results

All SP-50-prepared smears demonstrated satisfactory film quality, confirming that the stain/buffer combination provides consistent and high-quality preparation for digital morphology analysis. Accuracy evaluation showed that all DI-60 leukocyte differential results fell within Rumke's allowable limits when compared with the XN-10 analyzer differential, demonstrating high concordance across all leukocytes categories. The 10-day total imprecision study revealed highly stable results, with SDs for all leukocyte types well within Cellavision's performance specification limits. The 1-slide repeatability study similarly produced consistent values across ten sequential runs. In the 10-slide reproducibility analysis, independently prepared and stained slides demonstrated strong agreement, again meeting all manufacturer performance limits. The between-staff imprecision study, involving ten medical technologists reviewing the same slide, showed robust inter-operator consistency, with SDs well within acceptable limits for all parameters

**Copyright Information:** (Online) ISSN: 2572-9241
©2026 The Author(s). HemaSphere is published by John Wiley & Sons Ltd on behalf of European Hematology Association. This is an open access Abstract Book distributed under the Attribution-NonCommercial-NoDerivs (CC BY-NC-ND), which allows third parties to download the articles and share them with others as long as they credit the author and the Abstract Book, but they cannot change the content in any way or use them commercially.
**Abstract Book Citations:** Authors, Title, HemaSphere, 2026;10:(S1):pages. The abstract book DOIs can be found on the "Author Information" page.
**Disclaimer:** Articles published in the journal HemaSphere exclusively reflect the opinions of the authors. The authors are responsible for all content in their abstracts including accuracy of the facts, statements, citing resources, etc.

**Summary/Conclusion**

Overall, the combined SP-50 and DI-60 platform using Cellavisoion RAL Wright stain and Sysmex buffer (pH 6.8) provides high-quality smear preparation, stable staining, accurate classification, and reproducible leukocyte differential counts. These findings confirm the system's suitability for routine hematology diagnostics and support its broader implementation in laboratories adopting fully automated digital morphology workflow.

**Copyright Information:** (Online) ISSN: 2572-9241
©2026 The Author(s). HemaSphere is published by John Wiley & Sons Ltd on behalf of European Hematology Association. This is an open access Abstract Book distributed under the Attribution-NonCommercial-NoDerivs (CC BY-NC-ND), which allows third parties to download the articles and share them with others as long as they credit the author and the Abstract Book, but they cannot change the content in any way or use them commercially.
**Abstract Book Citations:** Authors, Title, HemaSphere, 2026;10:(S1):pages. The abstract book DOIs can be found on the "Author Information" page.
**Disclaimer:** Articles published in the journal HemaSphere exclusively reflect the opinions of the authors. The authors are responsible for all content in their abstracts including accuracy of the facts, statements, citing resources, etc.

### (PB4537) FUNCTIONAL DRUG RESPONSE PROFILING USING PATIENT-DERIVED 3D LEUKEMIA SPHEROIDS FROM A SOUTH AFRICAN COHORT

**Topic:** 38. Novel technologies, techniques and digital analytical tools in hematology

Bernice Monchusi*[1, 2], Phumuzile Dube[1, 3], Mutsa Takundwa[1, 3], Ekene Nweke[4], Clement Penny[2], Aurelie Deroubaix[2], Atul Lakha[5], Vinitha Philip[5], Deepak Thimiri Govinda Raj[1]

*[1]Council for Scientific and Industrial Research (CSIR), Synthetic Biology and Precision Medicine Centre, Pretoria, South Africa; [2]University of the Witwatersrand, Internal Medicine, Johannesburg, South Africa; [3]University of Pretoria, Pretoria, South Africa; [4]University of the Witwatersrand, Surgery, Johannesburg, South Africa; [5]Chris Hani Baragwanath Academic Hospital, Hematology, Johannesburg, South Africa;*

### Background

The clinical burden of leukemia in South Africa highlights an urgent demand for more effective and individualized treatment strategies. Existing standard-of-care therapies frequently lead to relapses and suboptimal long-term survival, largely because of the pronounced genetic and clinical diversity among patients. Traditional 2D cell culture models do not adequately reproduce the intricacies of the tumor microenvironment, which limits their ability to predict clinical drug responses. In contrast, 3D spheroid models offer a more physiologically relevant platform that more accurately represents cell–cell interactions, proliferation behavior, and mechanisms underlying therapy resistance.

### Aims

In this study, we developed patient-derived 3D leukemia spheroid models using peripheral blood mononuclear cells (PBMCs) collected from 24 South African patients encompassing a range of leukemia subtypes.

### Methods

We characterized these spheroids via immunofluorescence imaging to evaluate their cellular composition, proliferative capacity, and baseline sensitivity to therapeutic agents. We further performed functional drug response profiling with 10 FDA-approved anticancer drugs, tested both individually and in combination, to pinpoint synergistic drug pairs capable of overcoming resistance.

### Results

Preliminary findings demonstrate subtype-specific differences in drug responses that parallel inter-patient heterogeneity and reflect the extensive genetic diversity typical of African populations. These results highlight the importance of utilizing physiologically relevant 3D models to capture the subtleties of patient-specific drug responsiveness.

### Summary/Conclusion

By delivering a customized platform for functional drug screening, this strategy may facilitate the discovery of new therapeutic combinations and help forecast clinically effective regimens. Ultimately, this work seeks to narrow the gap between bench research and real-world clinical application, thereby advancing precision medicine approaches for leukemia patients in the South African setting.

**Copyright Information:** (Online) ISSN: 2572-9241
©2026 The Author(s). HemaSphere is published by John Wiley & Sons Ltd on behalf of European Hematology Association. This is an open access Abstract Book distributed under the Attribution-NonCommercial-NoDerivs (CC BY-NC-ND), which allows third parties to download the articles and share them with others as long as they credit the author and the Abstract Book, but they cannot change the content in any way or use them commercially.
**Abstract Book Citations:** Authors, Title, HemaSphere, 2026;10:(S1):pages. The abstract book DOIs can be found on the "Author Information" page.
**Disclaimer:** Articles published in the journal HemaSphere exclusively reflect the opinions of the authors. The authors are responsible for all content in their abstracts including accuracy of the facts, statements, citing resources, etc.

### (PB4538) ACCELERATING REAL-WORLD DATA: A RANDOMIZED CROSSOVER STUDY COMPARING AI DOCTOR-IN-THE-LOOP VS. MANUAL ABSTRACTION OF FLOW CYTOMETRY DATA IN THE HEALTHTREE REGISTRY

**Topic:** 38. Novel technologies, techniques and digital analytical tools in hematology

Martha Paola Anchondo Commesse*[1], Jessica Pamela Salinas Espinosa[2], Alejandro López Ochoa[1], Andrea Victoria Pacheco[1], Ana Echenique Alcázar[1], Diego Franco Hernández[1], Diego Jose Fuentes Cañas[1], Edgar Ricardo Olivas-Domínguez[1], Magaly Valeria Escobedo Cruz[1], Karla Mariana Castro Bórquez[1], Miranda de la Luz Franco[1], Jorge Arturo Hurtado Martinez[1], Juan Capdevila[1]

*[1]HealthTree Foundation, South Jordan, United States of America;[2]HealthTree Foundation, Lehi, United States of America;*

### Background

Flow cytometry is a common method for diagnosing and monitoring hematological malignancies, and is increasingly useful for T-cell redirecting therapies. However, routine reports are often stored in unstructured files, leading to data collection challenges and errors from manual abstraction. To resolve these issues, we developed a dual-interface abstraction tool with both manual entry and an AI-assisted 'Doctor-in-the-Loop' (DITL) workflow, which organizes entries to clearly represent biological hierarchy by explicitly capturing relational data points within a single entry. This includes: Parent Gate, Identification Gate, Cell Population, Cell Behavior, % Cells Analyzed, and all Phenotype Markers expressed by or not expressed in the cell population.

### Aims

This study aimed to evaluate the feasibility of our AI-DITL tool for extracting flow cytometry data, quantify the manual correction rate required to finalize AI-generated drafts, and measure reductions in processing time and validation costs.

### Methods

We conducted a prospective, randomized crossover study to compare AI-DITL and manual data abstraction across 100 flow cytometry reports from various hematological malignancies within the HealthTree Registry. Seven expert clinical data abstractors were randomized to process the reports. To eliminate memory bias, no abstractor evaluated the same report using both modalities. The AI workflow utilized Gemini's 3.1 Pro LLM via Google Cloud Vertex AI. Primary endpoints included validation burden, time per extracted data point, and total operational cost. Validation burden was assessed by the manual edits needed to correct AI-generated drafts. Time per extracted data point was calculated by dividing total processing time by the validated data points. The total operational cost per AI-DITL report combines the national Chart Abstractor wages ($25/hour) with LLM API compute costs to assess the financial impact.

### Results

The AI-DITL workflow showed high fidelity, needing minimal human intervention for data extraction. The mean agreement score between preliminary AI results and final DITL validation was 81.90%, with a mean of 9.27 corrections (18%) per report. Validation took 8 seconds (CI 95%: 7.4-10.3) per data point, compared to 11 seconds (CI 95%: 11.4-15.7) by manual entry ($p<0.01$), resulting in a 37.5% slower manual process. 65% of manual reports lacked data, which the AI-DITL validation recovered. The AI-DITL method extracted an average of 51.22 data points per record, a 21% increase over the manual method's 42.32. Operational costs decreased by 26.3%, with AI-DITL validation costing $2.89 per report compared to $3.21 for manual validation. Direct compute costs for the LLM averaged $0.015 per report.

**Copyright Information:** (Online) ISSN: 2572-9241
©2026 The Author(s). HemaSphere is published by John Wiley & Sons Ltd on behalf of European Hematology Association. This is an open access Abstract Book distributed under the Attribution-NonCommercial-NoDerivs (CC BY-NC-ND), which allows third parties to download the articles and share them with others as long as they credit the author and the Abstract Book, but they cannot change the content in any way or use them commercially.
**Abstract Book Citations:** Authors, Title, HemaSphere, 2026;10:(S1):pages. The abstract book DOIs can be found on the "Author Information" page.
**Disclaimer:** Articles published in the journal HemaSphere exclusively reflect the opinions of the authors. The authors are responsible for all content in their abstracts including accuracy of the facts, statements, citing resources, etc.

**Summary/Conclusion**

Our proprietary AI tool fundamentally outperforms manual extraction in flow cytometry, achieving 81.9% agreement with the AI-generated draft and resulting in a fully validated, research-grade dataset following DITL review. It operates faster, effectively addressing manual data omissions, resulting in greater fidelity and lower costs. For a 10,000-report backlog, the AI-DITL workflow reduces processing time by 1,140 hours and total operational costs to $28,650, recovering 157 hours of chart abstractor time while extracting an additional 89,000 data points. Ultimately, this high-resolution data capture will enable faster, more accurate patient matching to novel therapeutics and significantly enhance retrospective analyses of disease progression.

**Copyright Information:** (Online) ISSN: 2572-9241
©2026 The Author(s). HemaSphere is published by John Wiley & Sons Ltd on behalf of European Hematology Association. This is an open access Abstract Book distributed under the Attribution-NonCommercial-NoDerivs (CC BY-NC-ND), which allows third parties to download the articles and share them with others as long as they credit the author and the Abstract Book, but they cannot change the content in any way or use them commercially.
**Abstract Book Citations:** Authors, Title, HemaSphere, 2026;10:(S1):pages. The abstract book DOIs can be found on the "Author Information" page.
**Disclaimer:** Articles published in the journal HemaSphere exclusively reflect the opinions of the authors. The authors are responsible for all content in their abstracts including accuracy of the facts, statements, citing resources, etc.

### (PB4539) CONSISTENCY ANALYSIS OF AI CELL RECOGNITION RESULTS BETWEEN NON ANTICOAGULANT AND ANTICOAGULANT MARROW SMEAR AFTER WRIGHT'S STAINING

**Topic:** 38. Novel technologies, techniques and digital analytical tools in hematology

Siheng Liu[1], Xiangui Peng[1], Xi Zhang[1], Cheng Zhang*[1]

[1]*Army Medical University Affiliated Xinqiao Hospital, Chongqing, China;*

### Background

The integration of artificial intelligence with bone marrow cytology represents a significant trend in the application of AI image recognition technology within the medical sector. Despite the current high accuracy of AI in cell identification, there remains a clinical need for fully automated AI cell recognition equipment that spans from sample processing to result generation. The source of the sample's origin as an influencing factor on the final results is a crucial consideration in equipment design.

### Aims

Exploring the consistency of results between direct bone marrow smear and 4 commonly used anticoagulants anticoagulated smear after Wright staining for artificial intelligence cell recognition.

### Methods

Randomly select 36 patients with roughly normal bone marrow. When the patient undergoes bone marrow puncture, 0.2ml of bone marrow fluid is extracted for direct smear. Then continue to extract 1ml of bone marrow fluid from the same area and inject it into purple-EDTAK$_2$(purple group,PG), blue-sodium citrate (1:10)(blue group,BluG), green-lithium heparin(green group,GG), and black-sodium citrate (1:5)(black group,BlaG) anticoagulant tubes, each tube containing approximately 0.2ml. The automatic smear machine will prepare the slides. Each patient's non anticoagulated direct smear and 4 anticoagulated smears, totaling 5 smears, were stained with Wright's stain and identified and classified by an artificial intelligence cell recognition platform. The consistency analysis of the results was conducted using the intra class correlation coefficient (ICC) method, and the analysis indicators included 13 common clinical cell types such as promyelocytes and erythroblasts.

### Results

After recognition by the artificial intelligence cell recognition platform, the proportion of 500 cells identified in the non anticoagulant group, PG, BluG, GG, and BlaG of 36 patient samples was 94.4%, 97.2%, 63.9%, 44.4%, and 58.3%, respectively; The number of items with significant consistency between the non anticoagulant group and the PG, BluG, GG, and BlaG in the 13 common cells is 7 items (53.8%), 5 items (38.5%), 5 items (38.5%), and 4 items (30.7%), respectively.

### Summary/Conclusion

When bone marrow smears anticoagulated with four commonly used anticoagulants and direct bone marrow smears are stained with Wright's stain and then recognized by an artificial intelligence cell recognition platform, the consistency between them is generally poor. This factor should be taken into account in the design of a fully automated artificial intelligence bone marrow cell recognition device.

**Copyright Information:** (Online) ISSN: 2572-9241
©2026 The Author(s). HemaSphere is published by John Wiley & Sons Ltd on behalf of European Hematology Association. This is an open access Abstract Book distributed under the Attribution-NonCommercial-NoDerivs (CC BY-NC-ND), which allows third parties to download the articles and share them with others as long as they credit the author and the Abstract Book, but they cannot change the content in any way or use them commercially.
**Abstract Book Citations:** Authors, Title, HemaSphere, 2026;10:(S1):pages. The abstract book DOIs can be found on the "Author Information" page.
**Disclaimer:** Articles published in the journal HemaSphere exclusively reflect the opinions of the authors. The authors are responsible for all content in their abstracts including accuracy of the facts, statements, citing resources, etc.

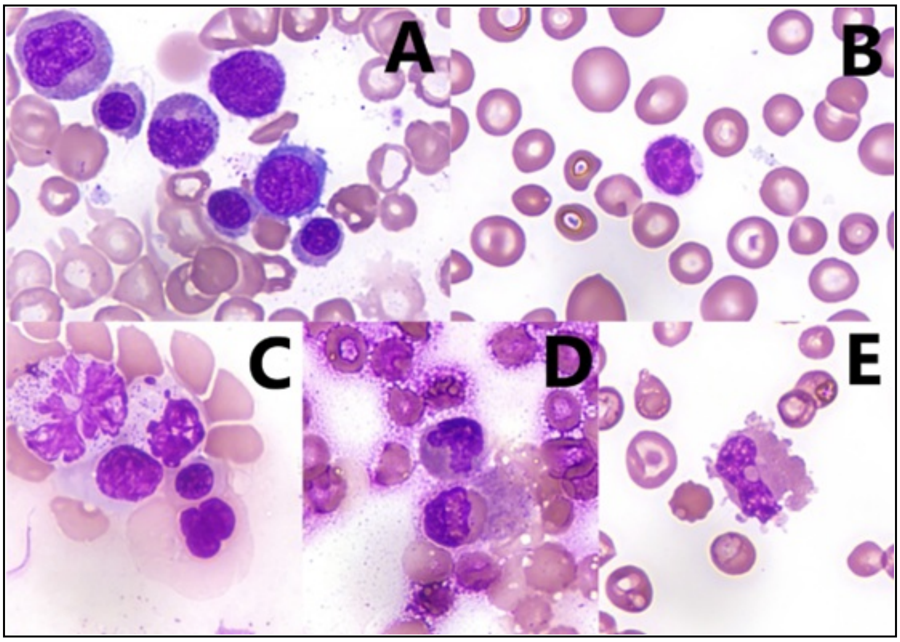

The cell integrity of non-anticoagulant bone marrow smear and four types of anticoagulant bone marrow smear in the ROI area. The magnification is 1000×. (A) non anticoagulant group; (B) purple group; (C) blue group ;(D) green group; (E) black group

**Copyright Information:** (Online) ISSN: 2572-9241
©2026 The Author(s). HemaSphere is published by John Wiley & Sons Ltd on behalf of European Hematology Association. This is an open access Abstract Book distributed under the Attribution-NonCommercial-NoDerivs (CC BY-NC-ND), which allows third parties to download the articles and share them with others as long as they credit the author and the Abstract Book, but they cannot change the content in any way or use them commercially.
**Abstract Book Citations:** Authors, Title, HemaSphere, 2026;10:(S1):pages. The abstract book DOIs can be found on the "Author Information" page.
**Disclaimer:** Articles published in the journal HemaSphere exclusively reflect the opinions of the authors. The authors are responsible for all content in their abstracts including accuracy of the facts, statements, citing resources, etc.

### (PB4540) DYNAMIC PLASMA EBV-DNA LEVELS CORRELATE WITH SURVIVAL OUTCOMES IN ADOLESCENT AND ADULTEBV-HLH PATIENTS RECEIVING PD-1 BLOCKADE THERAPY

**Topic:** 38. Novel technologies, techniques and digital analytical tools in hematology

Qiaolin Zhou*[1, 2, 3, 4], Yang Dai[1, 5, 6], Yongqian Jia[1, 5, 6], Liping Xie[1, 5, 6], Xushu Zhong[1, 5, 6], Luocheng Zhang[1, 4], Yushuang Xiao[1, 4], Wenyi Liang[1, 4], Zhuohang Zou[1, 4], Mengting Yang[1, 4], He Li[1, 5, 6], Ting Niu[1, 5, 6]

*[1]Department of Hematology, Institute of Hematology, and Center for High Altitude Medicine, West China Hospital, Sichuan University., Chengdu, China; [2]Department of Hematology, Mianyang Central Hospital School of Medicine. University of Electronic Science and Technology of China, Mianyang, China; [3]West China Lecheng Hospital of Sichuan University., Hainan, China;[4]West China Medical School, West China Hospital, Sichuan University., Chengdu, China; [5]State Key Laboratory of Biotherapy, Collaborative Innovation Center of Biotherapy, West China Hospital, Sichuan University., Chengdu, China; [6]National Facility for Translational Medicine (Sichuan), West China Hospital, Sichuan University., Chengdu, China;*

### Background

Epstein–Barr virus–associated hemophagocytic lymphohistiocytosis (EBV-HLH) is a life-threatening hyperinflammatory syndrome characterized by excessive immune activation and high mortality, particularly in adolescents and adults. Although programmed cell death protein-1 (PD-1) inhibitor–based therapy has shown encouraging efficacy in relapsed or refractory settings, in previous pilot study, determinants of durable survival in broader cohorts recurrent and refractory patients remain incompletely defined. In particular, the relative prognostic importance of sustained virological control compared with early clinical response has not been systematically evaluated.

### Aims

To investigate the prognostic value of longitudinal plasma EBV-DNA dynamics in adolescents and adults with EBV-HLH treated with PD-1 inhibitor–based therapy, and to construct clinically applicable prognostic models integrating virological and clinical variables.

### Methods

We conducted a retrospective cohort study including 85 recurrent and refractory adolescents and adults with EBV-HLH who received PD-1 inhibitor–based therapy at our center. Plasma EBV-DNA levels were serially monitored throughout follow-up. Overall survival (OS) was estimated using the Kaplan–Meier method and compared by log-rank test. Multivariable Cox proportional hazards regression was applied to identify independent predictors of OS. Logistic regression analysis was performed to determine factors associated with failure of EBV-DNA clearance. Prognostic nomograms were constructed based on independent variables and internally validated using concordance indices and calibration plots.

### Results

The median OS was 27.03 months. Persistent EBV-DNA positivity at 1 year was the strongest independent predictor of inferior survival (hazard ratio [HR] 8.037), demonstrating near-complete separation of survival curves. In contrast, early induction response did not retain independent prognostic significance for long-term OS after multivariable adjustment. Elevated globulin levels and lower lactate dehydrogenase at baseline were independently associated with failure to achieve sustained EBV-DNA clearance, suggesting that host inflammatory and immune status may influence viral persistence. Prognostic nomograms incorporating 1-year EBV-DNA status and key clinical variables demonstrated favorable discrimination and good calibration in internal validation.

**Copyright Information:** (Online) ISSN: 2572-9241
©2026 The Author(s). HemaSphere is published by John Wiley & Sons Ltd on behalf of European Hematology Association. This is an open access Abstract Book distributed under the Attribution-NonCommercial-NoDerivs (CC BY-NC-ND), which allows third parties to download the articles and share them with others as long as they credit the author and the Abstract Book, but they cannot change the content in any way or use them commercially.
**Abstract Book Citations:** Authors, Title, HemaSphere, 2026;10:(S1):pages. The abstract book DOIs can be found on the “Author Information” page.
**Disclaimer:** Articles published in the journal HemaSphere exclusively reflect the opinions of the authors. The authors are responsible for all content in their abstracts including accuracy of the facts, statements, citing resources, etc.

**Summary/Conclusion**

Durable virological control, rather than transient early clinical improvement, represents the principal determinant of long-term survival in adolescents and adults with EBV-HLH receiving PD-1 inhibitor–based therapy. Longitudinal EBV-DNA monitoring provides robust prognostic stratification and may facilitate response-adapted management strategies. These findings underscore the central role of viral dynamics in guiding therapeutic decision-making in EBV-HLH.

**Copyright Information:** (Online) ISSN: 2572-9241
©2026 The Author(s). HemaSphere is published by John Wiley & Sons Ltd on behalf of European Hematology Association. This is an open access Abstract Book distributed under the Attribution-NonCommercial-NoDerivs (CC BY-NC-ND), which allows third parties to download the articles and share them with others as long as they credit the author and the Abstract Book, but they cannot change the content in any way or use them commercially.
**Abstract Book Citations:** Authors, Title, HemaSphere, 2026;10:(S1):pages. The abstract book DOIs can be found on the "Author Information" page.
**Disclaimer:** Articles published in the journal HemaSphere exclusively reflect the opinions of the authors. The authors are responsible for all content in their abstracts including accuracy of the facts, statements, citing resources, etc.

### (PB4541) LARGE LANGUAGE MODEL–ASSISTED OUTPATIENT TRIAGE IN A NATIONAL HEMOPHILIA CENTER: A PROSPECTIVE IMPLEMENTATION STUDY

**Topic:** 38. Novel technologies, techniques and digital analytical tools in hematology

Nan Zhang[1], Qing Xu[1], Teng Wang[1], Hang Xiao[1], Jian-Chuan Deng*[1]

[1] *The Second Affiliated Hospital of Chongqing Medical University, Department of hematology, Chongqing, China;*

### Background

Timely and structured assessment of bleeding symptoms is essential in hemophilia management, particularly in high-volume comprehensive care centers. In routine outpatient practice, symptom intake and triage depend largely on manual interviews and documentation, which may introduce variability and increase workflow burden. Large language models (LLMs) offer the potential to standardize structured symptom intake and support safety-oriented triage within clinical settings.

### Aims

To implement and evaluate an LLM-assisted outpatient triage workstation integrated into routine clinical workflow in a national comprehensive hemophilia management center in China.

### Methods

This prospective implementation study was conducted in a national comprehensive hemophilia management center in China providing standardized multidisciplinary long-term care and maintaining a registry of 1,187 patients. An on-site digital triage workstation was deployed in the outpatient clinic, allowing patients to complete structured symptom intake prior to physician consultation. The system integrated predefined red-flag safety criteria with a large language model–based module capable of extracting and structuring bleeding-related variables from patient-reported input. Encounters were categorized into routine follow-up, expedited specialist review, or urgent evaluation. System-generated triage outputs were prospectively compared with hematologist assessment. Evaluation metrics included triage agreement, sensitivity for urgent presentations, structured data completeness, and workflow feasibility.

### Results

During early implementation, the LLM-assisted triage workstation showed general agreement with specialist assessment across routine outpatient encounters while maintaining sensitivity for predefined urgent red-flag scenarios. Deterministic safety safeguards ensured that potentially high-risk presentations were appropriately escalated. Compared with conventional manual intake, the system improved the consistency and completeness of structured bleeding-related information and reduced missing key variables. Automated generation of standardized triage summaries supported clinical documentation and reduced variability in reporting. Integration into outpatient workflow was operationally feasible and did not require modification of established clinical management pathways.

### Summary/Conclusion

LLM-assisted outpatient triage is feasible and operationally practical in comprehensive hemophilia management. By combining rule-based safety safeguards with structured data extraction, this approach supports standardized bleeding assessment and workflow optimization within routine outpatient care. Further prospective evaluation will clarify its long-term clinical and operational impact.

**Copyright Information:** (Online) ISSN: 2572-9241
©2026 The Author(s). HemaSphere is published by John Wiley & Sons Ltd on behalf of European Hematology Association. This is an open access Abstract Book distributed under the Attribution-NonCommercial-NoDerivs (CC BY-NC-ND), which allows third parties to download the articles and share them with others as long as they credit the author and the Abstract Book, but they cannot change the content in any way or use them commercially.
**Abstract Book Citations:** Authors, Title, HemaSphere, 2026;10:(S1):pages. The abstract book DOIs can be found on the "Author Information" page.
**Disclaimer:** Articles published in the journal HemaSphere exclusively reflect the opinions of the authors. The authors are responsible for all content in their abstracts including accuracy of the facts, statements, citing resources, etc.

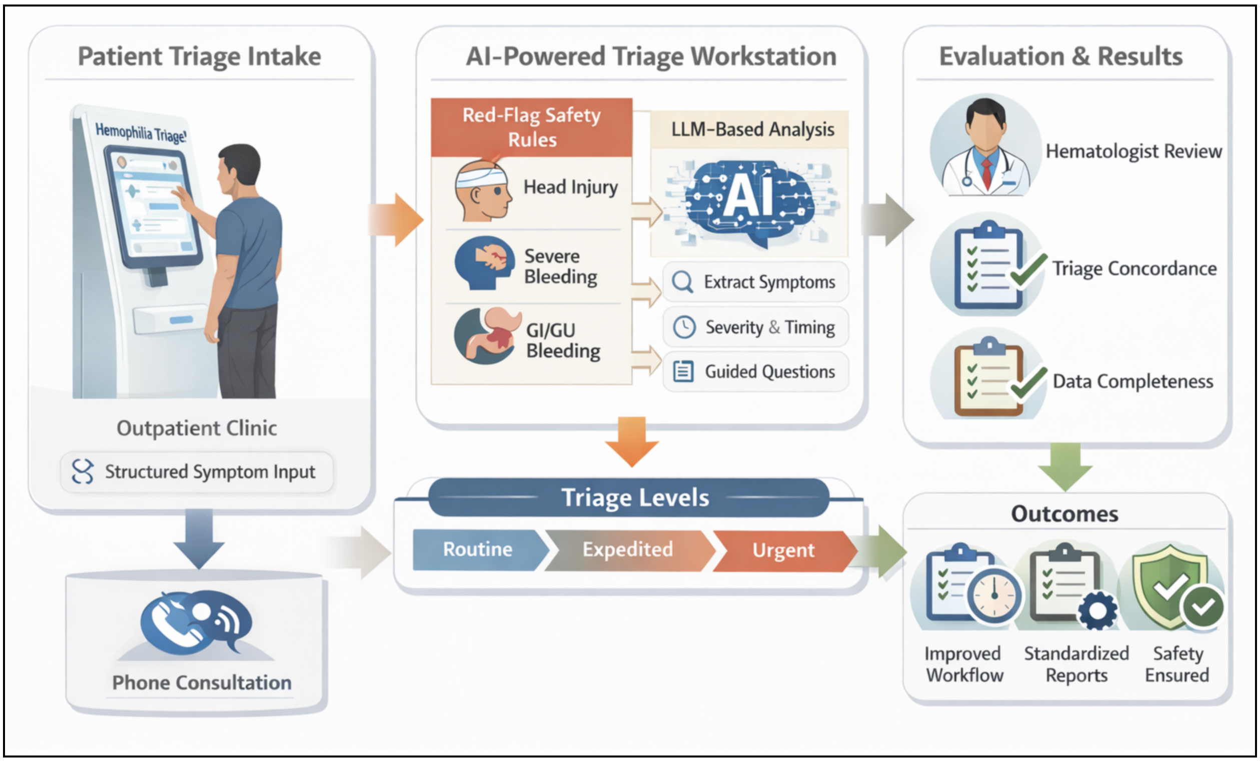

Schematic overview of the LLM-assisted outpatient triage workflow in a comprehensive hemophilia center, integrating rule-based safety screening and structured data extraction to support standardized triage and clinical workflow optimization.

**Copyright Information:** (Online) ISSN: 2572-9241
©2026 The Author(s). HemaSphere is published by John Wiley & Sons Ltd on behalf of European Hematology Association. This is an open access Abstract Book distributed under the Attribution-NonCommercial-NoDerivs (CC BY-NC-ND), which allows third parties to download the articles and share them with others as long as they credit the author and the Abstract Book, but they cannot change the content in any way or use them commercially.
**Abstract Book Citations:** Authors, Title, HemaSphere, 2026;10:(S1):pages. The abstract book DOIs can be found on the “Author Information” page.
**Disclaimer:** Articles published in the journal HemaSphere exclusively reflect the opinions of the authors. The authors are responsible for all content in their abstracts including accuracy of the facts, statements, citing resources, etc.

### (PB4542) DIGITAL CAMPUS HEMATOLOGY (DCH) – A TEACHING PLATFORM FOR INTERACTIVE LEARNING OF PERIPHERAL BLOOD AND BONE MARROW MORPHOLOGY FOR EDUCATION IN HEMATOLOGY

**Topic:** 38. Novel technologies, techniques and digital analytical tools in hematology

Anne Marie Asemissen*[1], Sabine Haase[1], Corina Turner[2], Martin Weihrauch[2], Aristoteles Giagounidis[1]

*[1]Marien Hospital Duesseldorf, Hematology, Oncology and Palliative Care, Duesseldorf, Germany; [2]Smart In Media AG, Cologne, Germany;*

### Background

Cytomorphology is fundamental in diagnosis of reactive, benign, and malignant hematologic disorders. It is rapid, cost-effective, and can be performed at the bedside; however, diagnostic accuracy is highly dependent on individual expertise and is associated with significant inter-observer variability. At the same time, structured training opportunities in cytomorphology are limited, leading to substantial gaps in postgraduate education. To address this unmet educational need, we developed the Digital Campus Hematology (DCH), a comprehensive digital microscopy–based e-learning platform. DCH evolved from the German pilot “eLearning Curriculum Hematology” (eLCH), funded by DGHO and Deutsche Krebshilfe. DCH will be launched internationally in May 2026.

### Aims

We aim to establish a globally accessible, high-quality digital education platform that standardizes and advances postgraduate training in hematologic cytomorphology.

### Methods

High-resolution slide digitization was performed using a high-speed camera and manual scanning to ensure optimal color fidelity and high-resolution visualization of subcellular structures. Only slides of superior quality, independently reviewed by at least two expert morphologists, were included. The commercially available teaching platform for microscopy slides SmartZoom® ClassRoom was customized to meet the specific requirements of DCH.

### Results

DCH comprises 10 structured courses covering the full spectrum of benign and malignant hematology. Furthermore, the platform integrates on-demand webinars, interactive eLearning modules, quizzes, and case-based teaching tools. To date, more than 1,000 images have been digitized, including high-resolution images of selected regions and whole-slide images (WSI) of peripheral blood smears, bone marrow aspirates, cerebrospinal fluid, and body effusions. Additionally, over 50 fully annotated patient cases combine cytomorphology with clinical and laboratory findings, wsi bone marrow histology, radiological images, FISH, cytogenetics, and flow cytometry data. Every slide is annotated at the single-cell level, including also diagnostic information, classification criteria, clinical and prognostic information, and myelogram parameters. The platform is device-independent and accessible worldwide.

### Summary/Conclusion

To our knowledge, DCH represents the first initiative to define structured educational goals in hematologic cytomorphology. By combining expert-validated digital microscopy with integrated multimodal case data, DCH aims to standardize training, reduce inter-observer variability, and close critical educational gaps in residency programs. Beyond education, the curated high-quality dataset provides a scalable foundation for collaborative research and future artificial intelligence applications in hematology.

**Copyright Information:** (Online) ISSN: 2572-9241
©2026 The Author(s). HemaSphere is published by John Wiley & Sons Ltd on behalf of European Hematology Association. This is an open access Abstract Book distributed under the Attribution-NonCommercial-NoDerivs (CC BY-NC-ND), which allows third parties to download the articles and share them with others as long as they credit the author and the Abstract Book, but they cannot change the content in any way or use them commercially.
**Abstract Book Citations:** Authors, Title, HemaSphere, 2026;10:(S1):pages. The abstract book DOIs can be found on the “Author Information” page.
**Disclaimer:** Articles published in the journal HemaSphere exclusively reflect the opinions of the authors. The authors are responsible for all content in their abstracts including accuracy of the facts, statements, citing resources, etc.

### (PB4543) COMPARATIVE EVALUATION OF MACHINE LEARNING MODELS AND DISCRIMINANT FORMULAE FOR β-THALASSEMIA TRAIT DETECTION IN AN INDIAN CLINICAL COHORT

**Topic:** 38. Novel technologies, techniques and digital analytical tools in hematology

Frederik Christensen*[1], Samir Maity[1], Alexander Fuglkjær[1], Prashant Sharma[2], Manu Jamwal[2], Jesper Brix Petersen[3], Andreas Glenthøj[3, 4], Jens Helby[3, 4], Tarec El-Galaly[5, 6, 7, 8], Reena Das[2], Izabela Nielsen[1]

*[1]Aalborg University, Department of Materials And Production, Ålborg Øst, Denmark;[2]Postgraduate Institute of Medical Education and Research, Department of Hematology, Chandigarh, Denmark; [3]Copenhagen University Hospital, Danish Red Blood Cell Centre, Department of Haematology, København Ø, Denmark; [4]University of Copenhagen, Department of Clinical Medicine, København K, Denmark; [5]Aarhus University Hospital, Department of Clinical Epidemiology, Aarhus, Denmark;[6]Aarhus University Hospital, Department of Molecular Medicine, Aarhus, Denmark; [7]Aarhus University Hospital, Department of Hematology, Aarhus, Denmark; [8]Aarhus University Hospital, Department of Clinical Medicine, Aarhus, Denmark;*

### Background

β-thalassemia trait (BTT) is highly prevalent in India, with >30 million carriers. Early carrier detection is essential to prevent severe hemoglobinopathies. Numerous discriminant formulae (DFs) based on complete blood count indices have been developed as alternative low-cost screening tools, but diagnostic performance varies across populations. Machine learning (ML) approaches may offer improved accuracy while maintaining reliance on routinely available hematological parameters.

### Aims

To compare the diagnostic performance of 26 published DFs and 9 ML classifiers for identifying BTT in an Indian clinical cohort, prioritizing high sensitivity suitable for screening.

**Copyright Information:** (Online) ISSN: 2572-9241
©2026 The Author(s). HemaSphere is published by John Wiley & Sons Ltd on behalf of European Hematology Association. This is an open access Abstract Book distributed under the Attribution-NonCommercial-NoDerivs (CC BY-NC-ND), which allows third parties to download the articles and share them with others as long as they credit the author and the Abstract Book, but they cannot change the content in any way or use them commercially.
**Abstract Book Citations:** Authors, Title, HemaSphere, 2026;10:(S1):pages. The abstract book DOIs can be found on the "Author Information" page.
**Disclaimer:** Articles published in the journal HemaSphere exclusively reflect the opinions of the authors. The authors are responsible for all content in their abstracts including accuracy of the facts, statements, citing resources, etc.

### Methods

We analysed data from 554 individuals referred for haematological evaluation at the Postgraduate Institute of Medical Education and Research, Chandigarh, India to develop ML models capable of identifying BTT individuals using routine blood test results.

**Genotype:**

Each participant was assigned to one of two classes based on results from High-Performance Liquid Chromatography, identifying 60 individuals with BTT (male:27, non-pregnant female:17, pregnant female:16) and 494 non-carriers (male: 57, non-pregnant female:103, pregnant female:334). In instances suggestive of HbS, standard sickling tests (solubility and microscopic induction methods) using sodium metabisulfite or sodium dithionite were conducted.

**Inclusion/Exclusion:**

The dataset excluded:

1. Age <1 year
2. Recent blood transfusions
3. [illegible]-thalassemia intermedia or major
4. Hemoglobin <6 g/dL or >17 g/dL and/or MCV <45 fL or >130 fL

Additionally, patients with structural variants such as HbS or HbC were excluded due to the very low prevalence in the data.

**Blood tests:**

- Hemoglobin, hematocrit, red blood cell (count, MCV, MCH, MCHC, RDW)

**Demographical covariates:** In addition to blood test results, the models were developed on age, sex and pregnancy status of the women.

**Models:** Extra Tree, Random Forest, and XGBoost trained using seven routine blood tests, age, sex and pregnancy status.

**Evaluation metrics:**

- Sensitivity and specificity
- Area under the receiver operating characteristic curve (ROC-AUC)
- Feature importance assessed via Shapley values (SHAP)

### Results

After calibrating the DF's only 7 out of 26 achieved acceptable performance:

- ROC-AUC: 0.949 (DF4), 0.922 (DF13), 0.938 (DF16), 0.919 (DF21), 0.930 (DF23), 0.948 (DF25), 0.948 (DF26)
- Specificities at 90% sensitivity: 87%(DF4,DF25,DF26), 86% (DF13,DF16,DF21), 83 (DF23)
- Specificities at 95% sensitivity: 76%(DF4), 49%(DF13), 67%(DF16), 67%(DF21), 65%(DF23), 75%(DF25), 81%(DF26)
- Key predictive features: Mean cell volume (MCV), mean cell haemoglobin (MCH) and red blood cell count (RBC)

All ML models exhibited strong discriminatory ability for identifying individuals with BTT in the population (Figure 1):

- ROC-AUC: 0.961 (Extra Tree), 0.961 (Random Forest), 0.954 (XGBoost)
- Specificities at 90% sensitivity: 89% (Extra Tree), 88% (Random Forest), 88% (XGBoost)
- Specificities at 95% sensitivity: 82% (Extra Tree), 82% (Random Forest), 80% (XGBoost)
- Key predictive features: Mean cell volume (MCV), mean cell haemoglobin (MCH) and red blood cell count (RBC)

**Copyright Information:** (Online) ISSN: 2572-9241
©2026 The Author(s). HemaSphere is published by John Wiley & Sons Ltd on behalf of European Hematology Association. This is an open access Abstract Book distributed under the Attribution-NonCommercial-NoDerivs (CC BY-NC-ND), which allows third parties to download the articles and share them with others as long as they credit the author and the Abstract Book, but they cannot change the content in any way or use them commercially.
**Abstract Book Citations:** Authors, Title, HemaSphere, 2026;10:(S1):pages. The abstract book DOIs can be found on the "Author Information" page.
**Disclaimer:** Articles published in the journal HemaSphere exclusively reflect the opinions of the authors. The authors are responsible for all content in their abstracts including accuracy of the facts, statements, citing resources, etc.

### Summary/Conclusion

ML classifiers outperformed most traditional discriminant formulae for BTT screening in this Indian cohort, especially at 95% sensitivity, where only DF26, an ML-derived formula, achieved comparable performance. With external validation, these ML models may offer a clinically effective, interpretable, and resource-efficient approach to large-scale carrier screening.

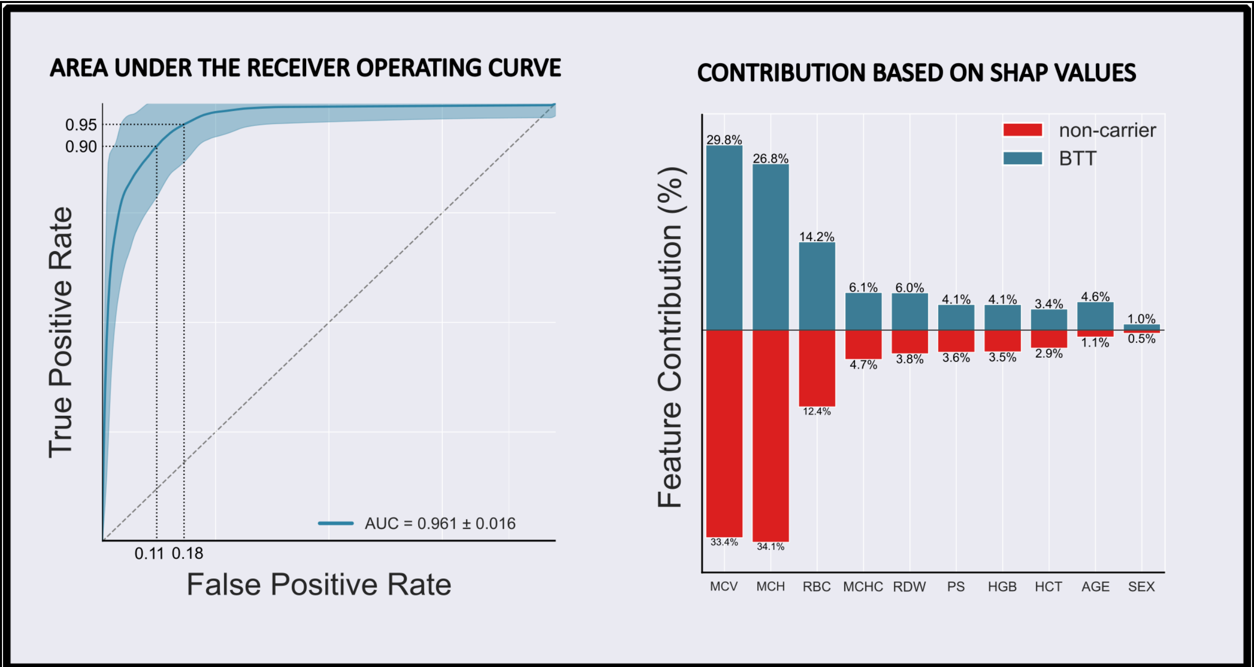

**Figure 1**: Performance of top performing machine learning model (Extra Tree Classifier).
**Left**: Area Under the Receiver Operating Characteristic Curve with 90% and 05% Sensitvitiy thresholds..
**Right**: Feature influence (%) on machine learning model predictions, seperated by class.

**Copyright Information:** (Online) ISSN: 2572-9241
©2026 The Author(s). HemaSphere is published by John Wiley & Sons Ltd on behalf of European Hematology Association. This is an open access Abstract Book distributed under the Attribution-NonCommercial-NoDerivs (CC BY-NC-ND), which allows third parties to download the articles and share them with others as long as they credit the author and the Abstract Book, but they cannot change the content in any way or use them commercially.
**Abstract Book Citations:** Authors, Title, HemaSphere, 2026;10:(S1):pages. The abstract book DOIs can be found on the "Author Information" page.
**Disclaimer:** Articles published in the journal HemaSphere exclusively reflect the opinions of the authors. The authors are responsible for all content in their abstracts including accuracy of the facts, statements, citing resources, etc.

### (PB4544) THE AGENTIC HEMATOLOGIST: A PATIENT-CENTRIC AI SYSTEM FOR INTEGRATED REASONING IN COMPLEX HEMATOLOGY

**Topic:** 38. Novel technologies, techniques and digital analytical tools in hematology

Miguel Luengo-Oroz*[1], David Bermejo-Pelaez[1], David Brau-Queralt[1], Joaquín Martinez-Lopez[2, 3, 4]

*[1]Spotlab, Madrid, Spain; [2]Hospital Universitario 12 de Octubre, Madrid, Spain; [3]Universidad Complutense de Madrid, Madrid, Spain; [4]Cnio - Centro Nacional de Investigaciones Oncologicas, Madrid, Spain;*

### Background

Hematology decision-making demands synthesis and integration of multiple data sources including laboratory data, cytology, treatments, and toxicities. Many times information is dispersed across systems and existing digital solutions mainly store data (EHR) or solve narrow tasks. AI has shown ability to answer medical exam questions and support diagnostics from individual data sources, yet few AI systems have been proposed to support the specialist's integrated reasoning across the full complexity of a case.

### Aims

To describe the vision and proof-of-concept of "The Agentic Hematologist", an AI system accessible as a web application that helps hematologists think through complex cases by transforming disperse patient data into an interactive reasoning workspace.

### Methods

We used the AI environement Claude Code & Claude Cowork to create an application that ingests a patient's clinical history (eg. PDFs) and structures it into a timeline of evidence blocks (diagnosis, lab, pathology/genetics, therapies, events). An agentic thinking engine combines a large language AI model (in this prototype Opus 4.6, Antrophic with feedback loops from ChatGPT5.2 Pro, OpenAI) with tools that analyze these blocks to dynamically generate and show the clinical questions a hematologist would ask next and the proposed answers. It also detects missing or conflicting data, computes risks scores, compares management strategies, flags potential safety issues, and relevant clinical trials. Users explore the case through a dashboard with three interactions: dynamically suggested questions specific to each case acting as cognitive nudges, an open-ended chat with the reasoning engine, and a structured overview. All outputs are linked to patient data and published references. The architecture is modular and MCP-ready enabling future connections to specialized AI agents (eg. AI bone-marrow morphology analysis).

### Results

We created the software application prototype that was tested with de-identified cases: a JAK2 V617F+ myeloproliferative neoplasm with cardiovascular complications and interferon toxicity, a post-transplant high-risk B-ALL with immunotherapy-related neurotoxicity, and a high-risk, multi-relapsed multiple myeloma case. The system generated patient summaries, prioritized actionable queries, and grounded responses. It identified clinically significant issues including rising MRD triggering therapy escalation, interferon dose-limiting toxicities, grade 3 ICANS requiring structured re-escalation, iron overload without chelation, severe malnutrition affecting pharmacokinetics, age-specific CAR-T eligibility or latent tuberculosis requiring clearance before immunosuppression. The engine surfaced data-quality gaps (eg, pending molecular monitoring) and generated treatment strategy comparisons. Dynamic questions covered 61 clinical scenarios across the 3 cases.

**Copyright Information:** (Online) ISSN: 2572-9241
©2026 The Author(s). HemaSphere is published by John Wiley & Sons Ltd on behalf of European Hematology Association. This is an open access Abstract Book distributed under the Attribution-NonCommercial-NoDerivs (CC BY-NC-ND), which allows third parties to download the articles and share them with others as long as they credit the author and the Abstract Book, but they cannot change the content in any way or use them commercially.
**Abstract Book Citations:** Authors, Title, HemaSphere, 2026;10:(S1):pages. The abstract book DOIs can be found on the "Author Information" page.
**Disclaimer:** Articles published in the journal HemaSphere exclusively reflect the opinions of the authors. The authors are responsible for all content in their abstracts including accuracy of the facts, statements, citing resources, etc.

### Summary/Conclusion

This work conceptualises a patient-grounded system that augments specialist reasoning in complex cases by leveraging AI agents that continuously organise evidence, surfacing discrepancies and proposing targeted next questions and answers. The platform is a thinking aid and is designed to be complementary to the EHR (not a replacement) and to keep clinicians in control. Its modular architecture and simplicity provides a pathway to integrate future diagnostic and quantitative modules. Next steps include transforming this proof of concept into a re-usable system with prospective usability studies and formal clinical evaluation.

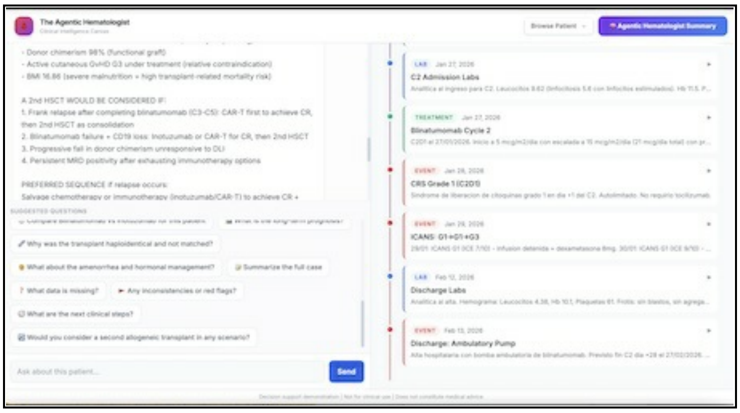

Screenshot of The Agentic Hematologist prototype

**Copyright Information:** (Online) ISSN: 2572-9241
©2026 The Author(s). HemaSphere is published by John Wiley & Sons Ltd on behalf of European Hematology Association. This is an open access Abstract Book distributed under the Attribution-NonCommercial-NoDerivs (CC BY-NC-ND), which allows third parties to download the articles and share them with others as long as they credit the author and the Abstract Book, but they cannot change the content in any way or use them commercially.
**Abstract Book Citations:** Authors, Title, HemaSphere, 2026;10:(S1):pages. The abstract book DOIs can be found on the "Author Information" page.
**Disclaimer:** Articles published in the journal HemaSphere exclusively reflect the opinions of the authors. The authors are responsible for all content in their abstracts including accuracy of the facts, statements, citing resources, etc.

### (PB4545) AI EMBEDDING MODEL CHOICE HAS MINIMAL IMPACT ON WHOLE-SLIDE CLASSIFICATION PERFORMANCE IN CASTLEMAN DISEASE

**Topic:** 38. Novel technologies, techniques and digital analytical tools in hematology

Muir Morrison*[1], Brendan O'fallon[1], Ashley Hutchings[1], Mark Dewey[1], Paul English[1], Alexandra Rangel[1], Lauren Zuromski[1], Katie Knight[1], Anna Bowen[1], Kiera Kearns[1], Kristin Shaw[1], Janini Sankar[1], Fnu Alnoor[2], Oscar Silva[3], Peyman Samghabadi[4], Olga Weinberg[5], Miguel Cantu[5], Yidan Xu-Monette[6], Archana Agarwal[1], Tim Hanley[1], Kristin H. Karner[1], Madhu Menon[1], Rodney Miles[1], Jay L. Patel[1], Anna Shestakova[1], Peng Li[1], Nicholas Spies[1], Ken Young[6], David Ng[1], Robert Ohgami[1]

*[1]ARUP Laboratories, Research and Innovation, Salt Lake City, United States of America;[2]University of Miami Miller School of Medicine, Miami, United States of America; [3]Stanford University, Stanford, United States of America; [4]University of California San Francisco, San Francisco, United States of America; [5]University of Texas Southwestern Medical Center, Dallas, United States of America; [6]Duke University, Durham, United States of America;*

### Background

Pathology foundation models generate tile embeddings for whole-slide image (WSI) classification. Although these models differ in architecture and pretraining data, it remains unclear whether embedding backbone selection meaningfully affects downstream performance in rare lymphoproliferative disorders such as Castleman disease (CD). We evaluated whether tile embedder choice influences CD grading classification accuracy in a multi-institutional WSI cohort.

### Aims

To determine the extent to which foundation model backbone selection impacts WSI-based CD grading classification when paired with an identical attention-based multiple instance learning (MIL) framework.

### Methods

Formalin-fixed paraffin-embedded lymph node WSIs from histologically confirmed CD and reactive controls were digitized at 40×. Foreground tissue was divided into 224×224 pixel tiles, and embeddings were extracted using five pretrained foundation models: Virchow-1, Virchow-2, H-Optimus-0, Hibou-L, and GigaPath. Embeddings were aggregated using an identical attention-based multiple instance learning (MIL) framework across models with patient-level training and validation splits. Performance was assessed using accuracy and Krippendorff's alpha. We also evaluated mixed-magnification inputs to model cell-level and follicle-level features.

### Results

With in-distribution magnification, classification performance was similar across embedding backbones. Mean Krippendorff's alpha ranged narrowly from 0.66 to 0.71, a smaller difference than typical inter-pathologist variability (Figure 1). Out-of-distribution magnification substantially reduced performance. Virchow-2, pretrained on multiple magnifications, better tolerated mixed inputs than models trained exclusively at 20×. Off-by-two-or-greater disagreements were uncommon (Table 1) and paralleled alpha trends. Attention heatmaps generally demonstrated concordant spatial attribution.

**Copyright Information:** (Online) ISSN: 2572-9241
©2026 The Author(s). HemaSphere is published by John Wiley & Sons Ltd on behalf of European Hematology Association. This is an open access Abstract Book distributed under the Attribution-NonCommercial-NoDerivs (CC BY-NC-ND), which allows third parties to download the articles and share them with others as long as they credit the author and the Abstract Book, but they cannot change the content in any way or use them commercially.
**Abstract Book Citations:** Authors, Title, HemaSphere, 2026;10:(S1):pages. The abstract book DOIs can be found on the "Author Information" page.
**Disclaimer:** Articles published in the journal HemaSphere exclusively reflect the opinions of the authors. The authors are responsible for all content in their abstracts including accuracy of the facts, statements, citing resources, etc.

**Summary/Conclusion**

In this CD WSI grading classification task, modern pathology foundation models yielded comparable performance when paired with an identical MIL framework. For CD morphologic discrimination, classifier architecture, magnification distribution, and dataset composition appear more influential than embedding backbone selection. Consistent results across models support embedding-agnostic robustness and reduced dependence on a single proprietary pipeline.

**Figure 1:** Krippendorff's alpha averaged over histologic features for each combination of foundation model backbone and input data magnification. 40x/20x/5x correspond to 0.25, 0.5, and 2 microns per pixel, respectively.

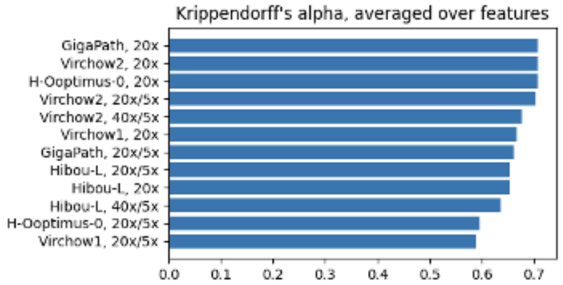

**Table 1:** Fraction of predictions differing from ground-truth label by more than 1 grade, for each model and input data mix.

| Model | Tile Magnification(s) | Fraction of predictions off-by-2 or more |
|---|---|---|
| Virchow2 | 20x/5x | 0.031 |
| GigaPath | 20x | 0.034 |
| Virchow2 | 20x | 0.036 |
| H-Ooptimus-0 | 20x | 0.040 |
| Virchow1 | 20x | 0.045 |
| Hibou-L | 40x/5x | 0.048 |
| Virchow2 | 40x/5x | 0.049 |
| Hibou-L | 20x | 0.052 |
| GigaPath | 20x/5x | 0.054 |
| Hibou-L | 20x/5x | 0.059 |
| Virchow1 | 20x/5x | 0.060 |
| H-Ooptimus-0 | 20x/5x | 0.063 |

**Copyright Information:** (Online) ISSN: 2572-9241
©2026 The Author(s). HemaSphere is published by John Wiley & Sons Ltd on behalf of European Hematology Association. This is an open access Abstract Book distributed under the Attribution-NonCommercial-NoDerivs (CC BY-NC-ND), which allows third parties to download the articles and share them with others as long as they credit the author and the Abstract Book, but they cannot change the content in any way or use them commercially.
**Abstract Book Citations:** Authors, Title, HemaSphere, 2026;10:(S1):pages. The abstract book DOIs can be found on the "Author Information" page.
**Disclaimer:** Articles published in the journal HemaSphere exclusively reflect the opinions of the authors. The authors are responsible for all content in their abstracts including accuracy of the facts, statements, citing resources, etc.

### (PB4546) IMPACT OF A 30-DAY DIGITAL GUIDED SELF-MANAGEMENT CHALLENGE ON SYMPTOM BURDEN IN PATIENTS WITH MYELOPROLIFERATIVE NEOPLASMS: A PROSPECTIVE REAL-WORLD COHORT STUDY

**Topic:** 38. Novel technologies, techniques and digital analytical tools in hematology

John Hoogland*[1], Nick van Dort[1]

[1]*Universitair Medisch Centrum Utrecht, Utrecht, The Netherlands;*

### Background

Many patients with myeloproliferative neoplasms (MPN) experience substantial symptom burden affecting quality of life. Digital self-management interventions may support patients in improving lifestyle behaviors and symptom control. In November 2025, a 30-day digital guided self-management lifestyle improvement challenge was offered to users of MPN Journal, a web-based patient-reported outcome platform.

### Aims

To evaluate the impact of a 30-day digital guided self-management challenge on total symptom burden and lifestyle behavior in patients with MPN.

### Methods

Patients were offered exercises and theoretical information to help realize sustainable lifestyle improvement interventions. Patients repeatedly reported 18 symptoms (0–10 scale) and lifestyle behaviors (0–5 scale). Composite scores were standardized to 0–100. Longitudinal analyses were conducted in patients with ≥7 (n=99), ≥14 (n=63), ≥21 (n=42), ≥28 (n=20), and 30 entries (n=10). For paired analyses, baseline and end values were defined as the mean of the first and last three measurements per patient. Paired change (Δ) was calculated as the median of individual end–start differences. Wilcoxon signed-rank tests were applied.

### Results

Lifestyle scores showed modest improvement across cohorts. In the ≥7 and ≥14 groups, median scores rose from 68.0 to 76.0 (Δ=+4.0; p=0.658) and from 72.0 to 76.0 (Δ=+4.0; p=0.648), respectively. In ≥21 and ≥28 cohorts, changes were +1.0 (p=0.751) and +4.0 (p=0.111). In the 30-entry cohort, lifestyle improved significantly from 64.5 to 80.0 (Δ=+7.5; p=0.033).

Symptom burden decreased significantly across all cohorts. Median scores declined from 59.4 to 41.3 (Δ=−1.2; p=0.009) in ≥7 entries and from 48.6 to 41.3 (Δ=−2.4; p=0.016) in ≥14 entries. Larger reductions were observed with increasing reporting frequency: Δ=−5.1 (p=0.030) in ≥21 entries, Δ=−7.7 (p=0.041) in ≥28 entries, and Δ=−25.6 (p=0.036) in 30 entries.

### Summary/Conclusion

In this real-world PRO study, participation in a 30-day digital self-management lifestyle improvement challenge was associated with a significant and sustained reduction in self-reported symptom burden. Aggregated lifestyle scores showed modest improvements overall, reaching statistical significance among the most engaged participants. All patient groups reported early and sustained improvements in perceived quality of life. Higher engagement was associated with greater lifestyle improvements and correspondingly greater improvements in reported quality of life.

**Copyright Information:** (Online) ISSN: 2572-9241
©2026 The Author(s). HemaSphere is published by John Wiley & Sons Ltd on behalf of European Hematology Association. This is an open access Abstract Book distributed under the Attribution-NonCommercial-NoDerivs (CC BY-NC-ND), which allows third parties to download the articles and share them with others as long as they credit the author and the Abstract Book, but they cannot change the content in any way or use them commercially.
**Abstract Book Citations:** Authors, Title, HemaSphere, 2026;10:(S1):pages. The abstract book DOIs can be found on the “Author Information” page.
**Disclaimer:** Articles published in the journal HemaSphere exclusively reflect the opinions of the authors. The authors are responsible for all content in their abstracts including accuracy of the facts, statements, citing resources, etc.

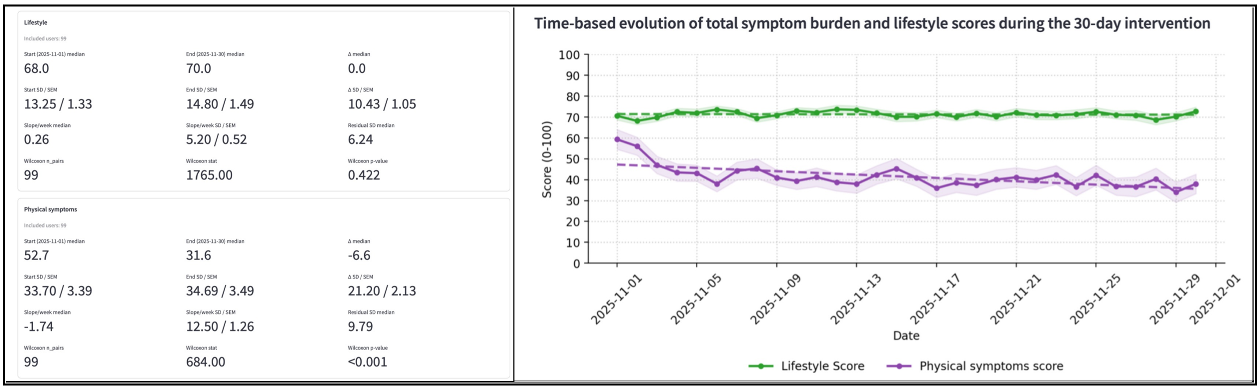

**Copyright Information:** (Online) ISSN: 2572-9241
©2026 The Author(s). HemaSphere is published by John Wiley & Sons Ltd on behalf of European Hematology Association. This is an open access Abstract Book distributed under the Attribution-NonCommercial-NoDerivs (CC BY-NC-ND), which allows third parties to download the articles and share them with others as long as they credit the author and the Abstract Book, but they cannot change the content in any way or use them commercially.
**Abstract Book Citations:** Authors, Title, HemaSphere, 2026;10:(S1):pages. The abstract book DOIs can be found on the "Author Information" page.
**Disclaimer:** Articles published in the journal HemaSphere exclusively reflect the opinions of the authors. The authors are responsible for all content in their abstracts including accuracy of the facts, statements, citing resources, etc.

### (PB4547) COMPARING NURSING EXPERIENCE WITH MANUAL VERSUS MECHANICAL PUMP-ASSISTED SUBCUTANEOUS MONOCLONAL ANTIBODY ADMINISTRATION: A TWO-CENTER PILOT SURVEY

**Topic:** 38. Novel technologies, techniques and digital analytical tools in hematology

Pamela Jones McIntyre*[1], Devin Wall[2], Anna Majapuro-Hirvonen[2], Brent Rutland[2]

[1]*Expert Infusion Nurse Consulting LLC, Celina, United States of America;* [2]*KORU Medical Systems, Global Medical and Clinical Affairs, Mahwah, United States of America;*

### Background

Large-volume (over 5 ml) subcutaneous (SC) monoclonal antibody injections are commonly administered over approximately five (5) minutes by manual syringe push, requiring continuous nurse presence and potentially contributing to ergonomic strain. Mechanical SC infusion pumps may standardize delivery and reduce hands-on time; however, comparative real-world data on nursing experience data are limited.

### Aims

To compare nursing workflow, workload, ergonomics, and satisfaction between manual syringe push administration (Site A) and mechanical pump-assisted subcutaneous administration (Site B).

### Methods

Nurses completed a standardized survey immediately after each SC monoclonal antibody injection. Measures included hands-on nursing time (minutes), multitasking feasibility (yes/no), workload (1=very low; 5=very high), physical strain/pain (1=lowest; 10=highest), satisfaction (1=very dissatisfied; 5=very satisfied), and free-text comments. Outcomes were reported descriptively using medians and interquartile ranges (IQR).

### Results

Thirty-nine injections were evaluated (Site A n=20; Site B n=19) between June-October 2025. Hands-on time was recorded in 18 administrations. Median hands-on nurse time was 8.5 minutes (IQR 8.0-12.8) with manual injection versus 5.0 minutes (IQR 4.3-7.5) with pump administration (~41% reduction in median time). Multitasking was feasible in 3/20 (15%) manual versus 17/19 (89%) pump administrations (absolute difference 74 percentage points; risk ratio 5.9). Median workload was low at both sites (2/5). Procedure-related physical strain was minimal (median 1/10 at both sites; IQR 1-2 for manual and 1-1 for pump). Retrospective strain ratings from prior routine manual injections were higher and more variable at Site A (median 5/10, IQR 1.8-6.3) than at Site B (median 1/10, IQR 1-4). Satisfaction was high across sites (median 5/5). Free-text comments at Site A emphasized improved ergonomics (e.g., “easier on back/hands”), while Site B feedback highlighted workflow standardization and ability to assist other patients, with usability concerns including setup time.

### Summary/Conclusion

In this two-center pilot study, mechanical pump-assisted SC administration was associated with shorter hands-on time and greater multitasking feasibility compared with manual injection, while perceived workload and procedure-related strain remained low with both approaches. These findings suggest potential operational efficiencies in infusion centers, including improved nursing resource allocation and capacity optimization. Larger studies are warranted to confirm these observations and assess implementation impact.

**Copyright Information:** (Online) ISSN: 2572-9241
©2026 The Author(s). HemaSphere is published by John Wiley & Sons Ltd on behalf of European Hematology Association. This is an open access Abstract Book distributed under the Attribution-NonCommercial-NoDerivs (CC BY-NC-ND), which allows third parties to download the articles and share them with others as long as they credit the author and the Abstract Book, but they cannot change the content in any way or use them commercially.
**Abstract Book Citations:** Authors, Title, HemaSphere, 2026;10:(S1):pages. The abstract book DOIs can be found on the “Author Information” page.
**Disclaimer:** Articles published in the journal HemaSphere exclusively reflect the opinions of the authors. The authors are responsible for all content in their abstracts including accuracy of the facts, statements, citing resources, etc.

### (PB4548) USAGE OF POINT-OF-CARE DIRECT ORAL ANTICOAGULANT URINE DIPSTICK ASSAY IN AN AUSTRALIAN STATE-WIDE ACUTE TELE-STROKE SERVICE.

**Topic:** 38. Novel technologies, techniques and digital analytical tools in hematology

Hannah Charles*[1], Edward Callaly[1], Dearne Stewart[2], Chris Bladin[2, 3, 4], Philip Choi[1, 2, 3, 4]

*[1]Austin Hospital, Neurology, Melbourne, Australia;[2]Ambulance Victoria, Victoria Stroke Telemedicine, Melbourne, Australia; [3]Eastern Health, Neuroscience, Box Hill, Australia;[4]Monash University, Eastern Health Clinical School, Melbourne, Australia;*

### Background

Intravenous thrombolysis remains the first-line therapy for patients presenting with acute ischemic stroke with or without proximal large vessel occlusion (LVO). Up to 25% may have an LVO suitable for treatment by mechanical thrombectomy (MT), yet thrombolysis alone achieves recanalization in up to 20% prior to MT, enabling earlier reperfusion and potentially avoiding an invasive procedure. Current guidelines recommend against thrombolysis where of stroke onset is within 48 hours of Direct Oral Anti-Coagulant (DOAC) ingestion unless low serum DOAC levels can be confirmed. This guidance stems from historical data demonstrating increased rates of thrombolysis-related cerebral haemorrhage in patients on warfarin.

DOAC plasma levels testing is unavailable across the 22 regional hospitals served by the Victorian Stroke Telemedicine (VST) network (catchment ~2.6 million). A point of care (POC) urine DOAC assay (validated to correlate with serum levels below 30ng/mL for dabigatran, apixaban and rivaroxaban) was rolled out in July 2025.

### Aims

To compare the clinical characteristics and thrombolysis rate between patients treated and not treated with a DOAC. To evaluate of the uptake, distribution, and operational feasibility of the point-of-care DOAC assay across the network. To determine clinical impact, specifically whether assay enabled thrombolysis in otherwise ineligible patients.

### Methods

The VST database was interrogated for patients diagnosed with ischemic stroke and TIA by VST physicians from July 2025 to February 2026. National Institute of Health Stroke Scale (NIHSS) is used to assess stroke severity. Good pre-stroke functional status is defined as modified Rankin Score (mRS) of 0 or 1. T-tests and Chi-square tests are used as appropriate.

**Copyright Information:** (Online) ISSN: 2572-9241
©2026 The Author(s). HemaSphere is published by John Wiley & Sons Ltd on behalf of European Hematology Association. This is an open access Abstract Book distributed under the Attribution-NonCommercial-NoDerivs (CC BY-NC-ND), which allows third parties to download the articles and share them with others as long as they credit the author and the Abstract Book, but they cannot change the content in any way or use them commercially.
**Abstract Book Citations:** Authors, Title, HemaSphere, 2026;10:(S1):pages. The abstract book DOIs can be found on the "Author Information" page.
**Disclaimer:** Articles published in the journal HemaSphere exclusively reflect the opinions of the authors. The authors are responsible for all content in their abstracts including accuracy of the facts, statements, citing resources, etc.

### Results

Patients taking a DOAC prior to stroke onset were older, had slightly more severe stroke, were less likely to have good pre-stroke functional status and had lower thrombolysis rates compared to non DOAC patients (p<0.05).

| Median (IQR) | DOAC N=208 | No DOAC N=1333 | P value |
|---|---|---|---|
| **Age** | 79 (73-85) | 73 (63-82) | <0.05 |
| **Male sex** | 58% | 55% | 0.35 |
| **Good pre-stroke functional status** | 56% | 70% | <0.05 |
| **NIHSS** | 4 (2-8) | 3 (1-6) | <0.05 |
| **Ischemic stroke vs intracerebral bleed** | 89% vs 11% | 93% vs 7% | 0.09 |
| **Thrombolysis** | 5.9% | 14% | <0.05 |

The POC assay was performed on 15 patients across eight hospitals. One patient was not on a DOAC, and it was unclear why the assay was used. Three did not have a final diagnosis of stroke (seizure, migraine, unknown). Of the remaining 11 patients with documented history of DOAC ingestion within 12 hours of stroke onset (10 apixaban, one dabigatran), two returned negative POC results and both received thrombolysis for severe stroke (NIHSS 13 and 16). One patient's arterial occlusion recanalized on first run angiography on arrival at the thrombectomy centre.
Separately, five other patients with recent DOAC ingestion (within 48 hours) received thrombolysis without DOAC level assessment. Two were on dabigatran with one receiving idarucizumab prior to thrombolysis. Two were on rivaroxaban and one apixaban. Ther details of the factors that guided omission of the POC assay or thrombolysis on DOAC therapy were not documented.

### Summary/Conclusion

Patients on DOAC therapy were older and had lower thrombolysis rate. The POC assay was used in fewer than half the hospitals, yet two patients with negative results received thrombolysis, supporting potential clinical value. Increased awareness and education on the utility of the POC assay in acute stroke within the VST network is required.

**Copyright Information:** (Online) ISSN: 2572-9241
©2026 The Author(s). HemaSphere is published by John Wiley & Sons Ltd on behalf of European Hematology Association. This is an open access Abstract Book distributed under the Attribution-NonCommercial-NoDerivs (CC BY-NC-ND), which allows third parties to download the articles and share them with others as long as they credit the author and the Abstract Book, but they cannot change the content in any way or use them commercially.
**Abstract Book Citations:** Authors, Title, HemaSphere, 2026;10:(S1):pages. The abstract book DOIs can be found on the “Author Information” page.
**Disclaimer:** Articles published in the journal HemaSphere exclusively reflect the opinions of the authors. The authors are responsible for all content in their abstracts including accuracy of the facts, statements, citing resources, etc.

### (PB4549) IMPLEMENTING OUTPATIENT BISPECIFIC ANTIBODY THERAPY IN COMMUNITY ONCOLOGY PRACTICE

**Topic:** 38. Novel technologies, techniques and digital analytical tools in hematology

Tara Graff*[1], Maddie McManus[1], Kerry Mann[1], Brooke Walter[1], Triston Behrend[2]

[1]*Mission Cancer and Blood/UIHSMG, Oncology, Waukee, United States of America;* [2]*University of Wisconsin, Madison, United States of America;*

### Background

Bispecific antibodies (BsAbs) are altering treatment paradigms for B-cell non-Hodgkin lymphoma (BNHL) and multiple myeloma (MM). As BsAbs move into earlier lines of therapy, scalable delivery beyond academic centers is critical to ensure equitable access. Outpatient implementation has been limited by concerns regarding T-cell–mediated toxicities, particularly cytokine release syndrome (CRS) and immune effector cell–associated neurotoxicity syndrome (ICANS). Prior reports have focused on inpatient or academic-center experiences, leaving limited guidance for safe, reproducible outpatient BsAb delivery in community oncology practices.

### Aims

1. Describe the development and execution of a standardized, multidisciplinary outpatient BsAb program in a community oncology network.
2. Demonstrate the feasibility and safety of outpatient CRS and ICANS management.
3. Define real-world CRS and ICANS incidence across BsAb agents.

### Methods

A multidisciplinary BsAb team composed of a physician champion, pharmacist, nurses, and advanced practice providers developed standardized outpatient administration and toxicity management protocols. Comprehensive education was provided to teams, supported by a mandatory pre-treatment checklist to ensure protocol adherence.
Patients and caregivers completed a 60-minute education session and received a medical alert bracelet and home monitoring equipment. Caregivers were trained to recognize CRS and ICANS and instructed in use of the Immune Effector Cell–Associated Encephalopathy (ICE) assessment. Two outpatient sites were designated for 24-hour patient evaluation. Eligibility for outpatient treatment required continuous caregiver availability, residence within 60 miles of the treatment center with access to tocilizumab, and absence of significant comorbidities.
During step-up dosing (SUD), patients were monitored by the BsAb team with nurse-led assessments every 6 hours. Suspected CRS or ICANS was managed using predefined escalation algorithms as outlined in Figure 1. Prophylactic measures included intravenous hydration during premedication and observation, and provision of dexamethasone 16 mg for home use, along with antipyretics, verified prior to treatment.

**Copyright Information:** (Online) ISSN: 2572-9241
©2026 The Author(s). HemaSphere is published by John Wiley & Sons Ltd on behalf of European Hematology Association. This is an open access Abstract Book distributed under the Attribution-NonCommercial-NoDerivs (CC BY-NC-ND), which allows third parties to download the articles and share them with others as long as they credit the author and the Abstract Book, but they cannot change the content in any way or use them commercially.
**Abstract Book Citations:** Authors, Title, HemaSphere, 2026;10:(S1):pages. The abstract book DOIs can be found on the "Author Information" page.
**Disclaimer:** Articles published in the journal HemaSphere exclusively reflect the opinions of the authors. The authors are responsible for all content in their abstracts including accuracy of the facts, statements, citing resources, etc.

### Results

Using standardized outpatient protocols, 32 patients (21 follicular lymphoma/diffuse large B-cell lymphoma; 11 MM) received BsAb therapy in an outpatient setting. CRS occurred in 8 patients (4 grade 1, 4 grade 2). No ICANS events were observed. No outpatient-treated patient required hospitalization or escalation to inpatient care. Toxicity occurrence compared to product inserts across BsAb agents is outlined in Table 1.

| | Mission Cancer | | | | | Product Inserts | | | | |
|---|---|---|---|---|---|---|---|---|---|---|
| | | CRS (%) | | ICANS (%) | | | CRS (%) | | ICANS (%) | |
| | n | Any | G3+ | Any | G3+ | n | Any | G3+ | Any | G3+ |
| **DLBCL** | | | | | | | | | | |
| Epcoritamab | 8 | 25 | 0 | 0 | 0 | 157 | 51 | 2.5 | 6 | 0.6 |
| Glofitamab | 2 | 0 | 0 | 0 | 0 | 145 | 70 | 4.2 | 4.8 | 0 |
| **FL** | | | | | | | | | | |
| Epcoritamab | 4 | 25 | 0 | 0 | 0 | 86 | 49 | 0 | 0 | 0 |
| Epcoritamab $R^{2}$* | 4 | 0 | 0 | 0 | 0 | 131 | 24 | 0 | 0.8 | 0 |
| Mosunetuzumab | 3 | 100 | 0 | 0 | 0 | 94 | 30 | 2.1 | 3.1 | 0 |
| **MM** | | | | | | | | | | |
| Teclistamab | 7 | 28.6 | 0 | 0 | 0 | 165 | 72 | 0.6 | 6 | 1.8 |
| Talquetamab | 4 | 0 | 0 | 0 | 0 | 339 | 76 | 1.5 | 9 | 0 |

*First-line trial at Mission Cancer

**Table 1** CRS/ICANS occurrence compared to product insert trials across BsAB agents

### Summary/Conclusion

This real-world experience demonstrates that outpatient BsAb therapy in the community setting is feasible, safe, and reproducible when supported by structured education, proactive monitoring, and standardized toxicity management pathways. These findings challenge the prevailing reliance on inpatient or academic-center administration and provide a scalable framework with immediate relevance as BsAbs expand into earlier treatment lines.

**Copyright Information:** (Online) ISSN: 2572-9241
©2026 The Author(s). HemaSphere is published by John Wiley & Sons Ltd on behalf of European Hematology Association. This is an open access Abstract Book distributed under the Attribution-NonCommercial-NoDerivs (CC BY-NC-ND), which allows third parties to download the articles and share them with others as long as they credit the author and the Abstract Book, but they cannot change the content in any way or use them commercially.
**Abstract Book Citations:** Authors, Title, HemaSphere, 2026;10:(S1):pages. The abstract book DOIs can be found on the “Author Information” page.
**Disclaimer:** Articles published in the journal HemaSphere exclusively reflect the opinions of the authors. The authors are responsible for all content in their abstracts including accuracy of the facts, statements, citing resources, etc.

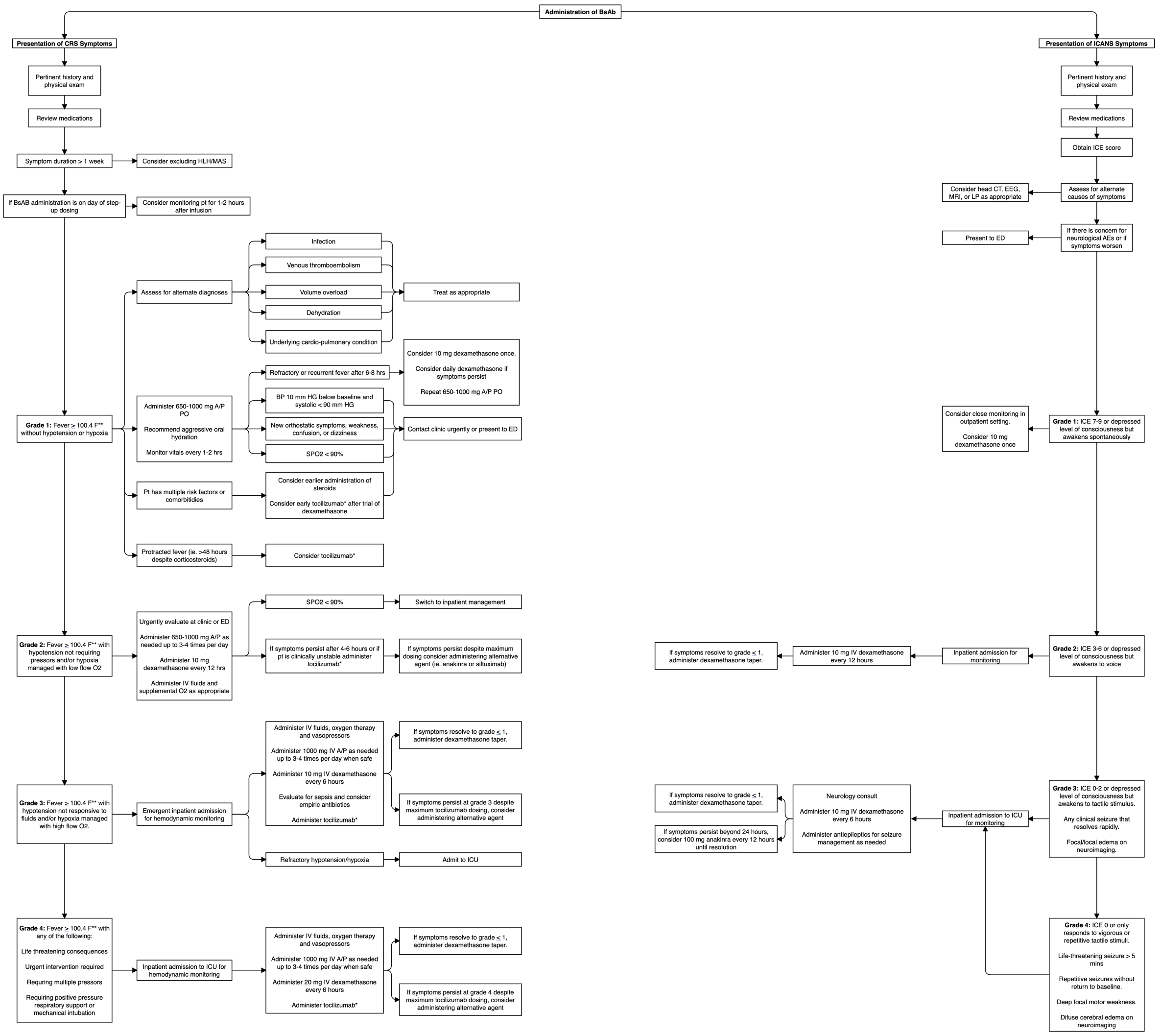

Abbreviations: A/P: Acetaminophen/Paracetamol, HLH/MAS: hemophagocytic lymphohistiocytosis/macrophage activation syndrome, CT: computed tomography, EEG: electroencephalogram, MRI: magnetic resonance imaging, LP: lumbar puncture
*Tocilizumab dosing: 8mg/kg IV. Tocilizumab should not be administered more than twice per CRS event (at least eight hours apart) or three times within a six-week period.
**Patients treated with antipyretics or corticosteroids in rare instances may not experience fever as a presenting symptom of CRS

**Copyright Information:** (Online) ISSN: 2572-9241
©2026 The Author(s). HemaSphere is published by John Wiley & Sons Ltd on behalf of European Hematology Association. This is an open access Abstract Book distributed under the Attribution-NonCommercial-NoDerivs (CC BY-NC-ND), which allows third parties to download the articles and share them with others as long as they credit the author and the Abstract Book, but they cannot change the content in any way or use them commercially.
**Abstract Book Citations:** Authors, Title, HemaSphere, 2026;10:(S1):pages. The abstract book DOIs can be found on the "Author Information" page.
**Disclaimer:** Articles published in the journal HemaSphere exclusively reflect the opinions of the authors. The authors are responsible for all content in their abstracts including accuracy of the facts, statements, citing resources, etc.

### (PB4550) VALIDATION OF KTX: A NOVEL METHOD OF KARYOTYPING ELIMINATES LABORATORY CULTURE INITIATION AND ENABLES RAPID, COST-EFFECTIVE, HIGHLY CONCORDANT CYTOGENETIC ANALYSIS.

**Topic:** 38. Novel technologies, techniques and digital analytical tools in hematology

Poonam Jain*[1], Shilpa Tiwari[1], Punit Jain[2, 3], Naziya Shaikh[1], Sandeep Nemani[4], Bhumi Nemani[5]

*[1]Helix Genetic and Pathology Laboratory (OPC) Pvt Ltd, Cytogenetics, Mumbai, India;[2]Apollo Hospitals, Navi Mumbai, India; [3]Hematcare speciality clinic, Mumbai, India;[4]Usha Haematology Centre, Sangli, India;[5]Nihira Diagnostics Laboratory, Sangli, India;*

### Background

Conventional G-banding karyotyping (CKT) requires samples within 24 hours, followed by a seven-step culture initiation protocol in a purpose-built environment, imposing logistical, infrastructure, and cost barriers in remote and resource-limited settings. The patented Helix Leukemia Transport Tube (HLTT) reduces culture failure rates and improves turnaround time (TAT) in acute leukaemia (Jain P. et al., Blood 2024;144:7639–7640). The KTX test, a novel karyotyping method, extends this innovation by eliminating laboratory-based culture initiation and its infrastructure requirements entirely.

### Aims

To validate KTX test by evaluating cytogenetic yield (metaphase index, morphology, slide redropping), case completion quality and concordance, TAT, and cost-effectiveness of KTX versus conventional karyotyping (CKT) across haematological disorders.

### Methods

Samples from five centres (November 2025–February 2026) were processed in parallel by KTX and CKT. CKT used overnight (ON) and overnight-with-colcemid (ONC) cultures; KTX used HLTT (16–18 hr at 25–40°C) with direct harvest on receipt (Figure 1). Metaphase index and morphology were graded; slide redropping was recorded as attempts of 4 slides each. Case Completion was graded as A (good: index 3/4, morphology 1/2, redrop ≤2); B (poor/reportable); C (failure: index=1 or morphology=4). Pre-harvest cost included consumables, equipment, area, and personnel. Grade distribution compared by Chi-square; concordance by McNemar's test.

**Copyright Information:** (Online) ISSN: 2572-9241
©2026 The Author(s). HemaSphere is published by John Wiley & Sons Ltd on behalf of European Hematology Association. This is an open access Abstract Book distributed under the Attribution-NonCommercial-NoDerivs (CC BY-NC-ND), which allows third parties to download the articles and share them with others as long as they credit the author and the Abstract Book, but they cannot change the content in any way or use them commercially.
**Abstract Book Citations:** Authors, Title, HemaSphere, 2026;10:(S1):pages. The abstract book DOIs can be found on the "Author Information" page.
**Disclaimer:** Articles published in the journal HemaSphere exclusively reflect the opinions of the authors. The authors are responsible for all content in their abstracts including accuracy of the facts, statements, citing resources, etc.

### Results

There were 25 samples (24 bone marrow, 1 blood). The diagnoses were MDS/MPN (n=7), acute leukaemia/ALL/AML (n=8), CML (n=1), ITP/cytopenias (n=5), other (n=4). Two KTX pre-analytical failures (clotted samples) and one peripheral blood (extreme hyperleukocytosis, AML) failing both methods equally. Excluding pre-analytical failures (n=23), completion concordance is 100% for both.

| Outcome Parameter | Conventional KT | | KTX |
|---|---|---|---|
| **Culture initiation** | **7 steps required** | | **eliminated** |
| Transport time | Up to 24 hours | | **Up to 72 hours (3×)** |
| | ON | ONC | |
| **Metaphase Index grade (mode)** *(1=none → 4=good)* | 3 | 4 | 3 |
| **Morphology grade (mode) (1=good → 4=not analysable** | 1 | 2 | 2 |
| **Slide redropping (cases)** | 10/25 | | 10/25 |
| **Avg. redropping attempts** | 2 | | 2 |
| **Case Completion (A+B)** | **24/25 (96%)** | | **22/25 (88%)** p = 0.409 ($\chi^2$) |
| Grade A (good) | 18/25 (72%) | | 14/25 (56%) |
| Grade B (poor/reportable) | 6/25 (24%) | | 8/25 (32%) |
| **Grade C (failure)** | **1/25 (4%)** | | **3/25(12%)** |
| Grade concordance | **20/25 (80%)** | | p = 0.062 (McNemar) |
| Turnaround time (TAT) | 10 days (7-12 days) | | **8 days (6-12 days)** |
| | COST (pre-harvest/sample) | | |
| Equipment cost | ₹673 ($7.39) | | **₹0 (100% saving)** |
| Resources (consumables, personnel, area) | ₹1500 ($16.47) | | ₹1,300 ($14.27) |
| TOTAL | **₹2173 ($23.86)** | | **₹1300 ($14.27) (↓40%)** |

One KTX-superior case: MDS,-7 (grade B conventional vs A KTX). Grade distribution ($\chi^2$=1.79, p=0.41) and ordinal concordance (McNemar p=0.062) were not significantly different, confirming KTX performs comparably across the quality spectrum.

### Summary/Conclusion

KTX eliminates laboratory culture initiation by utilising the patented HLTT. Across haematological conditions, when compared with the gold standard method of karyotyping, KTX achieved equivalent case completion (88% vs 96%, p=0.41), concordant grade quality (p=0.062), and shorter TAT. Pre-harvest cost reduced by 40% (₹1300 vs ₹2173/sample) with 100% saving on equipment. The sample transport time is extended threefold (≤72 vs ≤24 hrs). Combined with HLTT data in 125 leukaemia patients (ASH 2024), the KTX is a streamlined, cost-efficient, quality-concordant alternative with transformative impact for decentralised and resource-limited haematology.

**Copyright Information:** (Online) ISSN: 2572-9241
©2026 The Author(s). HemaSphere is published by John Wiley & Sons Ltd on behalf of European Hematology Association. This is an open access Abstract Book distributed under the Attribution-NonCommercial-NoDerivs (CC BY-NC-ND), which allows third parties to download the articles and share them with others as long as they credit the author and the Abstract Book, but they cannot change the content in any way or use them commercially.
**Abstract Book Citations:** Authors, Title, HemaSphere, 2026;10:(S1):pages. The abstract book DOIs can be found on the "Author Information" page.
**Disclaimer:** Articles published in the journal HemaSphere exclusively reflect the opinions of the authors. The authors are responsible for all content in their abstracts including accuracy of the facts, statements, citing resources, etc.

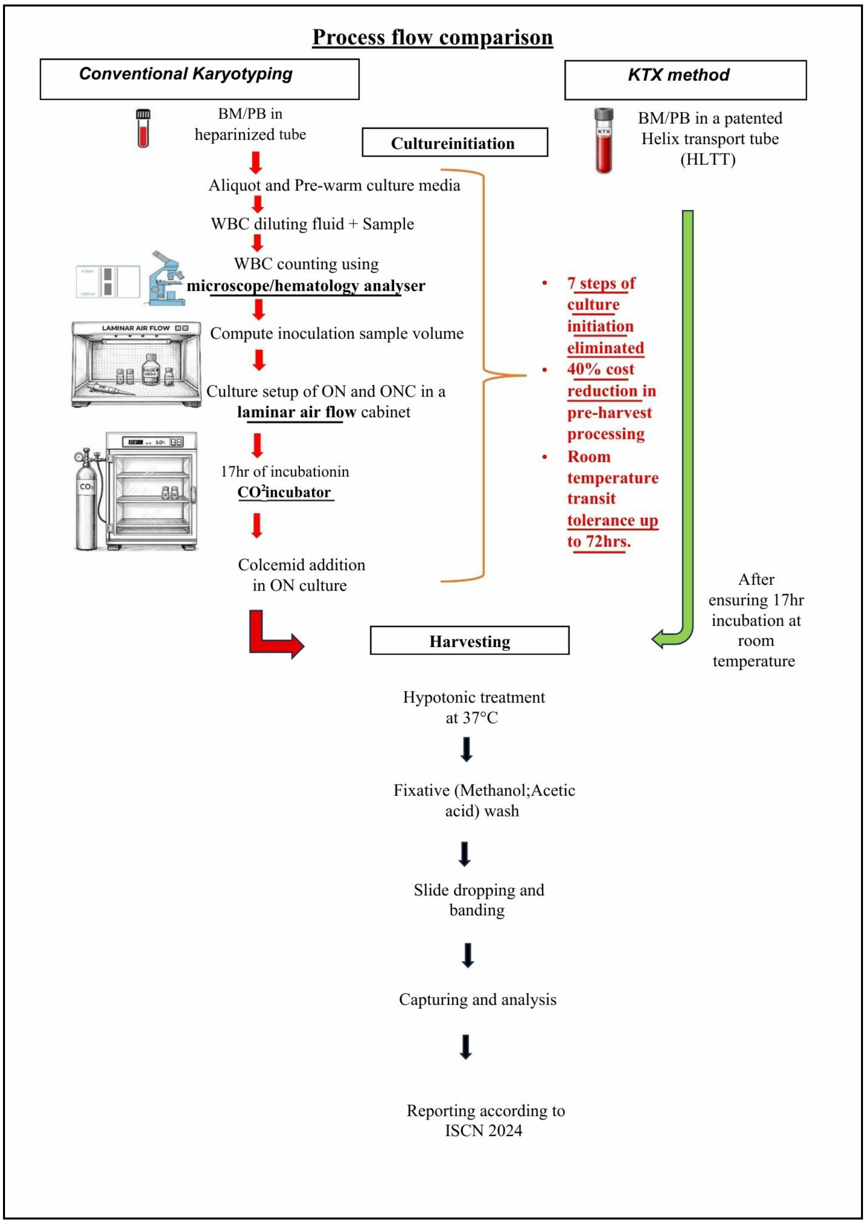

Figure 1 Process flow comparison: Conventional Karyotyping vs KTX

Copyright Information: (Online) ISSN: 2572-9241
©2026 The Author(s). HemaSphere is published by John Wiley & Sons Ltd on behalf of European Hematology Association. This is an open access Abstract Book distributed under the Attribution-NonCommercial-NoDerivs (CC BY-NC-ND), which allows third parties to download the articles and share them with others as long as they credit the author and the Abstract Book, but they cannot change the content in any way or use them commercially.
Abstract Book Citations: Authors, Title, HemaSphere, 2026;10:(S1):pages. The abstract book DOIs can be found on the "Author Information" page.
Disclaimer: Articles published in the journal HemaSphere exclusively reflect the opinions of the authors. The authors are responsible for all content in their abstracts including accuracy of the facts, statements, citing resources, etc.

### (PB4551) EXPLORING THE IMPACT OF HERPES ZOSTER AND ITS COMPLICATIONS IN CANCER PATIENTS IN SPAIN. THE HORION OBSERVATIONAL STUDY: DESIGN AND PRELIMINARY RESULTS

**Topic:** 38. Novel technologies, techniques and digital analytical tools in hematology

Lucrecia Yáñez*[1], Rodrigo Martino[2], Rodrigo Sánchez Bayona[3], Juan Carlos Cámara Vicario[4], Mónica Guillot Morales[5], Martín Mejuto-Díez[6], M del Rosario Cambronero-Martínez[6], Ester De la Cruz[7], Wilson A. Quezada Cumbicos[7], Gabriel de Maeztu[8], Natalia Mc Cormick De La Cruz[6]

*[1]Hospital Universitario Marqués de Valdecilla, Hematology Department, Santander, Spain;[2]Hospital de la Santa Creu i Sant Pau, Hematology Department, Barcelona, Spain; [3]Hospital Universitario 12 de Octubre, Department of Medical Oncology, Madrid, Spain; [4]Hospital Universitario Fundación Alcorcón, Oncology Department, Madrid, Spain;[5]Son Espases University Hospital, Department of Medical Oncology, Palma de Mallorca, Spain; [6]GSK, Madrid, Spain; [7]Outcomes'10 (a ProductLife Group Company), Castellón de la Plana, Spain; [8]IOMED, Barcelona, Spain;*

### Background

The reactivation of a latent infection of the varicella-zoster virus (VVZ) –known as Herpes Zoster (HZ) or shingles- is recognized as a significant public health issue, especially in the elderly due to immunosenescence and in patients with immunosuppressive conditions, such as solid or hematological malignancies (HM)[1, 2]. The incidence of HZ is higher in these cancer patients (10.97 and 11.99 vs. 4.65 cases/1,000 person years, respectively) compared to immunocompetent individuals[3]. Nevertheless, data on its incidence per cancer type and per oncological therapy, and impact on cancer patients are scarce. Expanding this information through conventional observational studies is challenging due to the limitations in data availability and the difficulties in analyzing diverse real-world data sources. In this context, artificial intelligence (AI) presents opportunities to maximize the extraction of available data from a real-world setting and enhance insights into the disease.

### Aims

The HORION (*Herpes zOster RIsk in ONcohematology patients*) study aims to estimate the incidence of HZ and HZ complications (overall and by type of tumor and oncological therapy), describe the patient profile, and assess the risk factors and impact of HZ on oncohematological patients in routine clinical practice in Spain.

### Methods

A multicenter, retrospective observational study has been conducted using both structured data and unstructured clinical notes from patients' electronic health records (EHRs) of adult patients without previous history of HZ and with a first solid tumor (colorectal, lung, breast, prostate, urinary bladder) or HM (Hodgkin's and non-Hodgkin's lymphoma, multiple myeloma, leukemia) between January 2017 and December 2018. EHRs' from 7 Spanish hospitals and primary care data from the Cantabria health system have been integrated into a federated database following the Observational Medical Outcomes Partnership Common Data Model (Figure 1). Unstructured data have been extracted using AI-driven natural language processing (NLP) models and verification and validation processes are being performed to ensure data quality[4]. Two cohorts will be studied: one with HZ diagnosis within three years post-cancer diagnosis, and one without HZ. The incidence of HZ, HZ complications and recurrent HZ will be assessed for the study period, both for the overall population and by cancer type and oncological therapy. Sociodemographic, clinical, and healthcare resource utilization including cancer and HZ treatments will be extracted. The study has been approved by the reference Ethics Committee (all data were anonymized previous extraction).

**Copyright Information:** (Online) ISSN: 2572-9241
©2026 The Author(s). HemaSphere is published by John Wiley & Sons Ltd on behalf of European Hematology Association. This is an open access Abstract Book distributed under the Attribution-NonCommercial-NoDerivs (CC BY-NC-ND), which allows third parties to download the articles and share them with others as long as they credit the author and the Abstract Book, but they cannot change the content in any way or use them commercially.
**Abstract Book Citations:** Authors, Title, HemaSphere, 2026;10:(S1):pages. The abstract book DOIs can be found on the "Author Information" page.
**Disclaimer:** Articles published in the journal HemaSphere exclusively reflect the opinions of the authors. The authors are responsible for all content in their abstracts including accuracy of the facts, statements, citing resources, etc.

### Results

Preliminarily, a cohort of 23,713 oncological patients has been identified, with a mean age of 66.5 (SD 15.0) years and 53.2% male. The study is currently in the data processing, quality assurance, and analysis phase.

### Summary/Conclusion

This study will provide valuable evidence to enhance the understanding and impact of HZ on patients with solid tumors or HM in Spanish clinical practice, being the first to use AI-driven NLP to generate such data, considering tumor and therapy types. This approach will analyze large volumes of data, increasing the robustness and reliability of the findings.

**References:**[1]Pedrazzoli P et al. ESMO Open. 2022;7(4):100548. [2]Habel et al. Cancer Epidemiol Biomarkers Prev. 2013;22(1):82-90. [3]Muñoz-Quiles et al. BMC Infect Dis. 2020;20(1):905. [4]de Maeztu et al. Zenodo. 2025.
**Funding:** The study is funded by GSK. GSK study identifier: VEO-001231.

Figure 1. Diagram of data extraction from electronic health records using Natural Language

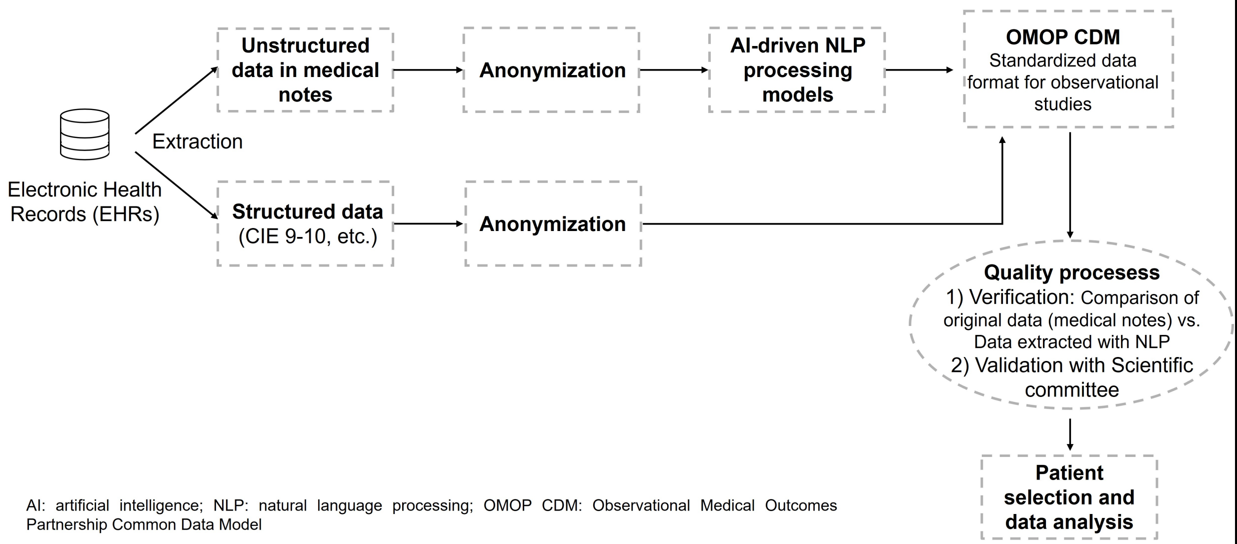

AI: artificial intelligence; NLP: natural language processing; OMOP CDM: Observational Medical Outcomes Partnership Common Data Model

**Copyright Information:** (Online) ISSN: 2572-9241
©2026 The Author(s). HemaSphere is published by John Wiley & Sons Ltd on behalf of European Hematology Association. This is an open access Abstract Book distributed under the Attribution-NonCommercial-NoDerivs (CC BY-NC-ND), which allows third parties to download the articles and share them with others as long as they credit the author and the Abstract Book, but they cannot change the content in any way or use them commercially.
**Abstract Book Citations:** Authors, Title, HemaSphere, 2026;10:(S1):pages. The abstract book DOIs can be found on the "Author Information" page.
**Disclaimer:** Articles published in the journal HemaSphere exclusively reflect the opinions of the authors. The authors are responsible for all content in their abstracts including accuracy of the facts, statements, citing resources, etc.

### (PB4552) GENERATIVE ARTIFICIAL INTELLIGENCE FOR SYNTHETIC DATA CREATION: APPLICATION TO ACUTE MYELOID LEUKEMIA

**Topic:** 38. Novel technologies, techniques and digital analytical tools in hematology

Valentin Andral*[1, 2, 3], Raphaël Fontaine[4], Elmehdi Ellanoui[4], Philippe Leleux[3, 5, 6], Suzanne Tavitian[7], Leopoldine Lapierre[7], Jean-Baptiste Rieu[7], Alban Canali[7], Isabelle Luquet[7], Lucie Rigolot[7], Francois Vergez[2, 3, 7], Véronique De Mas[2, 3, 7], Audrey Sarry[7], Emilie Bérard[7], Christian Recher[2, 3, 7], Olivier Thuillart[4], Jean-Louis Fraysse[4], Sandrine Mouysset[1, 2, 3], Sarah Bertoli[2, 3, 7]

*[1]Institut de Recherche en Informatique de Toulouse, Toulouse, France;[2]Université Toulouse III - Paul Sabatier, Toulouse, France; [3]Centre de Recherches en Cancérologie de Toulouse, Toulouse, France;[4]Botdesign, Toulouse, France;[5]Laboratoire d'Analyse et d'Architecture des Systèmes, Toulouse, France;[6]Institut National des Sciences Appliquées, Toulouse, France;[7]Centre Hospitalier Universitaire de Toulouse, Toulouse, France;*

### Background

Acute myeloid leukemia (AML) is an aggressive hematological cancer, characterized by high clinical, biological, genetic, molecular and prognostic heterogeneity. This variability requires robust analytical approaches to better understand molecular subtypes and optimize its management. However, the limited availability of data for certain rare profiles is a major obstacle to the development of reliable statistical models.

### Aims

Generative artificial intelligence, in particular Variational AutoEncoders (VAEs), offers an opportunity to expand cohorts while preserving their medical plausibility. We propose to generate synthetic data from our DATAML real AML database.

### Methods

In this study, we apply a Riemannian VAE (RHVAE) to a cohort of AML included in the French DATAML registry, including 1,693 patients and 243 demographic, biological, cytogenetic and molecular variables, with a missing data rate of 19.0%. The model allows for a ten-fold increase in the number of available patients The validation of artificial data is based on three complementary approaches: (1) the assessment of distributional conformity (means, variances, asymmetry and kurtosis), with a maximum deviation threshold set at 5% between real and artificial data; (2) the verification of rules derived from the pathophysiology of AML, such as the associations between *FLT3*-ITD and hyperleukocytosis, or between *NPM1*, CD34-negative and normal karyotype; (3) the use of visual validations including the comparison of correlation matrices, visualization in latent space with a Riemannian metric, as well as the analysis of the overlap of molecular clusters.

**Copyright Information:** (Online) ISSN: 2572-9241
©2026 The Author(s). HemaSphere is published by John Wiley & Sons Ltd on behalf of European Hematology Association. This is an open access Abstract Book distributed under the Attribution-NonCommercial-NoDerivs (CC BY-NC-ND), which allows third parties to download the articles and share them with others as long as they credit the author and the Abstract Book, but they cannot change the content in any way or use them commercially.
**Abstract Book Citations:** Authors, Title, HemaSphere, 2026;10:(S1):pages. The abstract book DOIs can be found on the "Author Information" page.
**Disclaimer:** Articles published in the journal HemaSphere exclusively reflect the opinions of the authors. The authors are responsible for all content in their abstracts including accuracy of the facts, statements, citing resources, etc.

### Results

The results show that all statistical distances used to measure the gap between actual and generated data remain below the overall threshold of 5%. For example, the median age of the real cohort is 60 years (IQR: 49-68) and that of the synthetic cohort is 59 years (IQR: 48-68); the median white blood cell count is 9.8 G/L (IQR: 2.8-43.5) VS. 9.44 G/L (IQR: 2.97-42.14); the normal karyotype rate is 89.6% vs. 90.0%; the mutation rate of *NPM1* is 34.0% vs. 28.6% and the mutation rate of *FLT3-ITD* is 22.2% vs. 20.0%. Similarly, correlation matrices, which identify the links between variables, maintain the same association profiles between real and artificial data, while subgroup identification algorithms, used here based on molecular markers, make it possible to obtain clusters aligned between the two different cohorts. The data generation also makes it possible to improve the representativeness of rare subgroups such as AML with *IDH1/2* (N=242 vs. 2319) or *CEBPA* (N=78 vs. 741) mutations, making it possible to carry out specific analyses previously limited by small numbers.

### Summary/Conclusion

These results demonstrate that the RHVAE can generate statistically, clinically and visually consistent artificial data, constituting a methodological lever to strengthen the study of rare AML subtypes, impute missing data (molecular biology for example), develop more robust predictive models, and even serve as a basis for synthetic cohorts for future clinical trials.

**Copyright Information:** (Online) ISSN: 2572-9241
©2026 The Author(s). HemaSphere is published by John Wiley & Sons Ltd on behalf of European Hematology Association. This is an open access Abstract Book distributed under the Attribution-NonCommercial-NoDerivs (CC BY-NC-ND), which allows third parties to download the articles and share them with others as long as they credit the author and the Abstract Book, but they cannot change the content in any way or use them commercially.
**Abstract Book Citations:** Authors, Title, HemaSphere, 2026;10:(S1):pages. The abstract book DOIs can be found on the "Author Information" page.
**Disclaimer:** Articles published in the journal HemaSphere exclusively reflect the opinions of the authors. The authors are responsible for all content in their abstracts including accuracy of the facts, statements, citing resources, etc.

### (PB4553) EFFECTS OF STORAGE DURATION AND TEMPERATURE ON RETICULOCYTE PARAMETERS STABILITY

**Topic:** 38. Novel technologies, techniques and digital analytical tools in hematology

Asma Ouahada[1], Mohamed Yassine Kaabar[2], Ikbel Ghachem*[3], Hamida Khémiri Guerbouj[4], Zeineb Alaya[4], Roua Mankai[4], Wafa Skouri[4], Haifa Tounsi[4], Raja Elamri[4], Asma Bachali[3]

*[1]Faculty of pharmacy,, Monastir, Tunisia; [2]University of Monastir, Faculty of Pharmacy of Monastir, Laboratory of Biology, Tahar Maamouri Hospital,, Nabeul, Tunisia; [3]University of Tunis El Manar, Faculty of Medicine of Tunis, Laboratory of Biology, Tahar Maamouri Hospital, Nabeul, Tunisia; [4]University of Tunis El Manar, Faculty of medicine of Tunis, Internal medicine Department, Taher Maamouri Hospital, Nabeul, Tunisia;*

### Background

Reticulocyte analysis is important for evaluating erythropoiesis. Delayed sample analysis necessitates robust stability data.

### Aims

This study assessed the stability of advanced reticulocyte parameters over 48 hours under two storage conditions.

### Methods

Sixty venous blood samples were analyzed at baseline (D0). Each sample was aliquoted and stored either at +4°C (refrigerated, n=30) or +25°C (room temperature, n=30). Stability was assessed by re-analyzing samples at 24h (D1) and 48h (D2) on a Mindray BC-6200 analyzer. The mean percentage deviation from D0 was calculated for each parameter.

### Results

In the vast majority of cases, the mean percentage deviation from baseline was lower at 4°C than at 25°C at both 24h (D1) and 48h (D2), demonstrating superior preservation by refrigeration. However, specific parameters showed notable temperature-dependent behaviors. At 25°C, the reticulocyte count (RET#) and fraction (FRC%) decreased significantly by D2 (-12.3% and -4.2%, respectively), while platelet count (PLT-O) showed a marked decline of -17.7%. These degradations were substantially mitigated at 4°C, with RET# at -8.7%, PLT-O at -3.0%, and FRC% showing an increase of +33.6% at D2. Exceptions to the overall trend were observed for the hypochromic reticulocyte fraction (HYPO-CH%), the reticulocyte fraction (FRC%), and the immature reticulocyte fraction (IRF). HYPO-CH% showed a higher percentage deviation at 4°C (16.8%) than at 25°C (13.0%) by D2, and IRF increased at 4°C (+10.7%) but decreased at 25°C (-10.2%) at the same timepoint.
The observed inconsistencies, particularly for HYPO-CH%, IRF, and FRC%, are largely attributed to their very low baseline values. A minimal absolute change in these parameters results in a disproportionately high percentage variation, amplifying the perceived instability.

### Summary/Conclusion

For reliable reticulocyte analysis delayed up to 48 hours, refrigeration at 4°C is strongly recommended to preserve cellularity (RET#), maturity (IRF), and volume indices (MCVr). While core hematological parameters are stable at both temperatures, the interpretation of reticulocyte hemoglobinization indices (HYPO/HYPER-CH%) is highly time- and temperature-sensitive. Their significant percentage changes, influenced by low baseline concentrations, limit their clinical reliability in delayed testing protocols.

**Copyright Information:** (Online) ISSN: 2572-9241
©2026 The Author(s). HemaSphere is published by John Wiley & Sons Ltd on behalf of European Hematology Association. This is an open access Abstract Book distributed under the Attribution-NonCommercial-NoDerivs (CC BY-NC-ND), which allows third parties to download the articles and share them with others as long as they credit the author and the Abstract Book, but they cannot change the content in any way or use them commercially.
**Abstract Book Citations:** Authors, Title, HemaSphere, 2026;10:(S1):pages. The abstract book DOIs can be found on the “Author Information” page.
**Disclaimer:** Articles published in the journal HemaSphere exclusively reflect the opinions of the authors. The authors are responsible for all content in their abstracts including accuracy of the facts, statements, citing resources, etc.

### (PB4554) DIAGNOSTIC ACCURACY OF DEEP LEARNING FOR BONE MARROW AND PERIPHERAL BLOOD SMEAR ANALYSIS IN LEUKAEMIA: A SYSTEMATIC REVIEW AND META-ANALYSIS

**Topic:** 38. Novel technologies, techniques and digital analytical tools in hematology

Ahmed Elgebaly*[1]

[1]*University of East London, London, United Kingdom;*

### Background

Deep learning (DL) systems are increasingly being developed to support smear-based leukaemia diagnostics using bone marrow smear (BMS) and peripheral blood smear (PBS) images. However, reported performance varies across leukaemia subtypes, specimen types, and validation settings, and the overall diagnostic accuracy of these models has not been clearly synthesised

### Aims

To systematically review the literature on DL-based analysis of BMS and PBS images for leukaemia detection and classification, and to quantitatively synthesise diagnostic accuracy where binary 2×2 data were available or could be reliably reconstructed

### Methods

A systematic review was conducted in accordance with PRISMA 2020 reporting principles. Studies evaluating DL models on BMS or PBS images for leukaemia diagnosis or classification were considered eligible. Data extracted included specimen type, target disease, model architecture, validation setting, and diagnostic performance metrics. For meta-analysis, only studies with exact or reconstructable binary 2×2 data were included in the primary pooling. Because hierarchical bivariate models are preferred for diagnostic test accuracy reviews but require a broader study base and adequately reported thresholds, a constrained random-effects logit meta-analysis of sensitivity and specificity was performed using the available binary datasets. An exploratory analysis additionally included correlated outcomes from the same study to assess robustness

### Results

The qualitative review identified DL studies across both BMS and PBS platforms, covering acute lymphoblastic leukaemia (ALL), acute myeloid leukaemia (AML), acute promyelocytic leukaemia (APL), and broader leukaemia classification tasks. Quantitative synthesis was limited by incomplete reporting of confusion matrices in several otherwise eligible studies. The primary meta-analysis included 2 independent binary datasets: Eckardt et al. (AML vs healthy BMS donors; TP 223, FP 5, FN 28, TN 43) and Syed et al. (external patient-level PBS pipeline, collapsed to leukaemia vs no leukaemia; TP 36, FP 4, FN 0, TN 24). The pooled sensitivity was 93.9% (95% CI, 67.4%–99.1%) and the pooled specificity was 87.0% (95% CI, 77.5%–92.9%). The pooled diagnostic odds ratio was 90.5 (95% CI, 20.9–392.0). In an exploratory synthesis that included additional correlated internal-validation outcomes from the same PBS study, pooled sensitivity was 93.9% (95% CI, 87.9%–97.0%) and pooled specificity was 95.2% (95% CI, 89.1%–98.0%), although these findings should be interpreted cautiously because study independence was not preserved

**Copyright Information:** (Online) ISSN: 2572-9241
©2026 The Author(s). HemaSphere is published by John Wiley & Sons Ltd on behalf of European Hematology Association. This is an open access Abstract Book distributed under the Attribution-NonCommercial-NoDerivs (CC BY-NC-ND), which allows third parties to download the articles and share them with others as long as they credit the author and the Abstract Book, but they cannot change the content in any way or use them commercially.
**Abstract Book Citations:** Authors, Title, HemaSphere, 2026;10:(S1):pages. The abstract book DOIs can be found on the "Author Information" page.
**Disclaimer:** Articles published in the journal HemaSphere exclusively reflect the opinions of the authors. The authors are responsible for all content in their abstracts including accuracy of the facts, statements, citing resources, etc.

**Summary/Conclusion**

DL-based analysis of BMS and PBS images shows high apparent diagnostic accuracy for leukaemia detection, with pooled sensitivity approaching 94% in the primary synthesis. Nevertheless, the evidence base remains limited by sparse independent validation datasets, inconsistent reporting of threshold-specific 2×2 data, and heterogeneity in outcome definitions and validation design. Larger, prospectively validated, externally tested studies with standardised reporting are needed before these tools can be robustly compared and translated into routine haematology workflows

**Copyright Information:** (Online) ISSN: 2572-9241
©2026 The Author(s). HemaSphere is published by John Wiley & Sons Ltd on behalf of European Hematology Association. This is an open access Abstract Book distributed under the Attribution-NonCommercial-NoDerivs (CC BY-NC-ND), which allows third parties to download the articles and share them with others as long as they credit the author and the Abstract Book, but they cannot change the content in any way or use them commercially.
**Abstract Book Citations:** Authors, Title, HemaSphere, 2026;10:(S1):pages. The abstract book DOIs can be found on the "Author Information" page.
**Disclaimer:** Articles published in the journal HemaSphere exclusively reflect the opinions of the authors. The authors are responsible for all content in their abstracts including accuracy of the facts, statements, citing resources, etc.

### (PB4555) ADVANCING BONE MARROW BIOPSY PRACTICE: A SINGLE-CENTER QUALITY IMPROVEMENT STUDY

**Topic:** 38. Novel technologies, techniques and digital analytical tools in hematology

Ammar Abdullah*[1], Ahlam Almasari[1], Abdullah Alghamdi[1], Maram Ismaeil[1], Anitha Mathew[1], Ahmed Omari[1], Masud Nabi[1], Salmin Muftah[1], Noha Eisa[1, 2]

*[1]King Faisal Specialist Hospital and research center, Jeddah, Saudi Arabia, Department of Oncology, Jeddah, Saudi Arabia;*
*[2]Oncology Center-Mansoura University, Internal medicine, Mansoura, Egypt;*

### Background

High-quality bone marrow aspirates and trephine biopsies are essential for accurate diagnosis and monitoring of hematologic diseases; however, variability in specimen adequacy at our institution has affected diagnostic efficiency and occasionally required repeat procedures. To address this, we initiated an approved quality improvement project to evaluate baseline sampling success rates, identify gaps in collection practices, implement targeted interventions, and assess their impact on sample quality and patient outcomes.

### Aims

To enhance the effectiveness and reliability of bone marrow aspiration and biopsy procedures

### Methods

This is a single-center, pre–post interventional quality improvement study designed to enhance bone marrow aspiration and biopsy outcomes. To identify the current state, we assessed the quality of 132  bone marrow samples collected  Our system analysis identified the following issues: Non-standardized collection technique, communication errors between technicians and providers in terms of core length and aspirate adequacy, lower adquacy for both bone marrow aspiration and biopsy compared to international data. the intervention included standardization of technique through updated protocol training, equipment upgrade with new biopsy needle sets, touch imprints for all biopsies, structured bedside and post-procedure documentation forms, regular training and competency sessions, and improved team communication via pre-procedure briefings. Data are collected retrospectively from ICIS and medical records and prospectively from the newly implemented documentation forms for analysis. We set apre-defined goal of success as 70% adequacy for bone marrow biopsy aspirates and 90% adequacy for core biopsies. Following our interventions, we started collecting post-intervention bone marrow biopsies in May2025 and evaluated the adequacy of 93 bone marrow samples

### Results

Implementation of this quality improvement measures significantly enhanced bone marrow sample quality, with marked increases in aspirate adequacy (59.8%→74.2%) and biopsy adequacy (65.2%→88.2%), reduced dry taps (9.1%→3.2%) and inadequate biopsies (21.2%→6.5%), longer core samples, improved clot preparation and touch imprint performance (0%→61.3%), fewer repeat procedures(from 9.8% to 7.5%), more first-attempt success (13.6%→81.7%), and substantially better documentation, while diagnostic yield improved numerically without statistical significance.

**Copyright Information:** (Online) ISSN: 2572-9241
©2026 The Author(s). HemaSphere is published by John Wiley & Sons Ltd on behalf of European Hematology Association. This is an open access Abstract Book distributed under the Attribution-NonCommercial-NoDerivs (CC BY-NC-ND), which allows third parties to download the articles and share them with others as long as they credit the author and the Abstract Book, but they cannot change the content in any way or use them commercially.
**Abstract Book Citations:** Authors, Title, HemaSphere, 2026;10:(S1):pages. The abstract book DOIs can be found on the "Author Information" page.
**Disclaimer:** Articles published in the journal HemaSphere exclusively reflect the opinions of the authors. The authors are responsible for all content in their abstracts including accuracy of the facts, statements, citing resources, etc.

### Summary/Conclusion

The implementation of targeted quality interventions resulted in a marked improvement in the adequacy of both bone marrow aspirate and core biopsy specimens, reflecting enhanced procedural performance and sample quality. Sustained monitoring and long-term follow-up are essential to confirm durability of these improvements and to prevent regression to baseline variability. We advocate for broader adoption of structured quality optimization strategies across centers performing bone marrow procedures to strengthen diagnostic accuracy, reduce repeat interventions, and improve overall patient care and monitoring outcomes

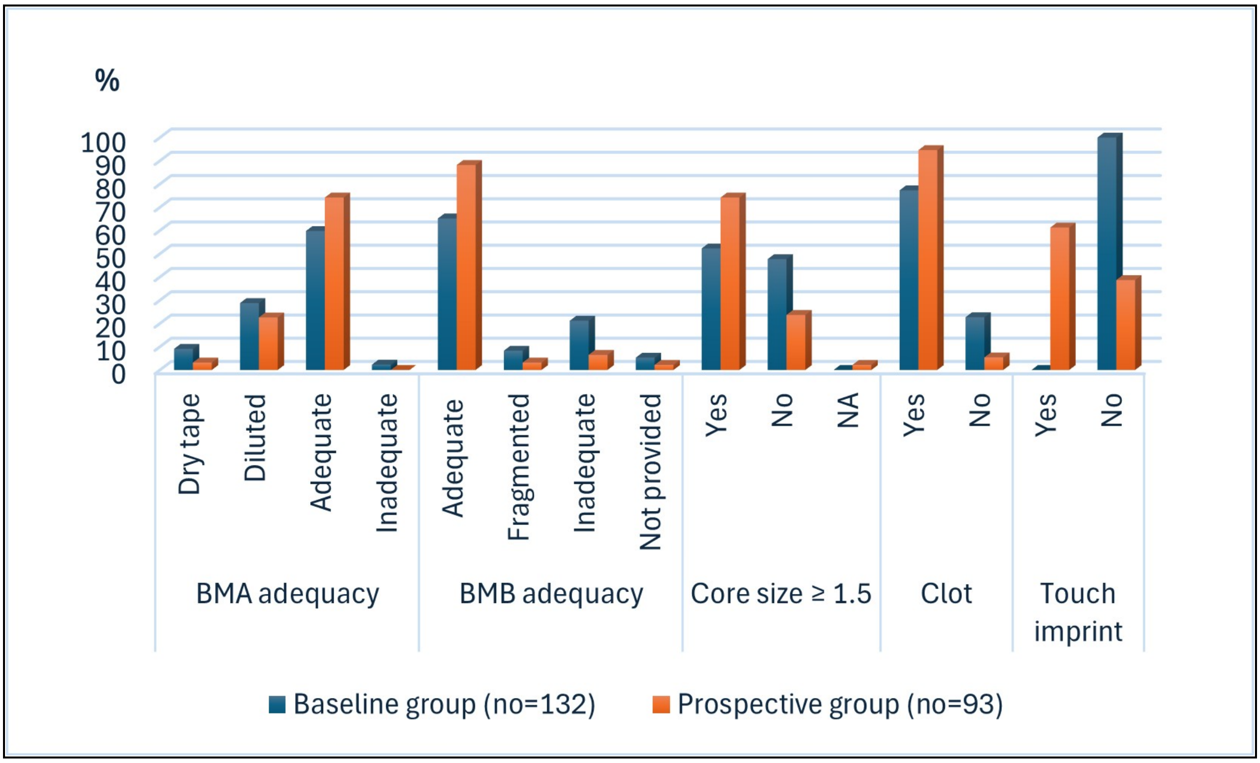

Figure1: Showing the outcome results between the baseline group and prospective group[post-intervention]

**Copyright Information:** (Online) ISSN: 2572-9241
©2026 The Author(s). HemaSphere is published by John Wiley & Sons Ltd on behalf of European Hematology Association. This is an open access Abstract Book distributed under the Attribution-NonCommercial-NoDerivs (CC BY-NC-ND), which allows third parties to download the articles and share them with others as long as they credit the author and the Abstract Book, but they cannot change the content in any way or use them commercially.
**Abstract Book Citations:** Authors, Title, HemaSphere, 2026;10:(S1):pages. The abstract book DOIs can be found on the "Author Information" page.
**Disclaimer:** Articles published in the journal HemaSphere exclusively reflect the opinions of the authors. The authors are responsible for all content in their abstracts including accuracy of the facts, statements, citing resources, etc.

### (PB4557) PREDICTIVE VALUE OF AI-ENHANCED ECG METRICS FOR CARDIAC DYSFUNCTION IN TELOMERE BIOLOGY DISORDERS

**Topic:** 38. Novel technologies, techniques and digital analytical tools in hematology

Madeline Franke*[1], Alejandro Ferrer[2, 3], Abhishek Mangaonkar[2], Eva Carmona[3], Douglas Simonetto[4], Laura Ongie[5], Terra Lasho[2], Jenna Fernandez[2], Abhishek Deshmukh[6], Mrinal Patnaik[2]

*[1]Mayo Clinic, Internal Medicine, Rochester, Minnesota, United States of America;[2]Mayo Clinic, Division of Hematology, Department of Internal Medicine, Rochester, Minnesota, United States of America; [3]Mayo Clinic, Pulmonary and Critical Care Medicine, Rochester, Minnesota, United States of America; [4]Mayo Clinic, Gastroenterology and Hepatology, Rochester, United States of America; [5]Mayo Clinic, Clinical Genomics, Rochester, United States of America;[6]Mayo Clinic, Cardiovascular Medicine, Rochester, United States of America;*

### Background

Telomere biology disorders (TBDs) are defined by shortened telomere lengths (TLs) and a spectrum of manifestations, including bone marrow failure and lung fibrosis. TL has been studied as a clinically relevant cardiac biomarker due to links between cardiac dysfunction and cellular senescence, and maladaptive remodeling, however little is known about cardiac disease in patients with TBDs. Novel AI-based ECG technologies analyze patterns predictive of cardiovascular conditions and outcomes, including LV dysfunction, atrial fibrillation (AF), structural heart disease, and future events [Armoundas et al., 2024].

### Aims

Our study investigates whether AI-based ECG can detect accelerated physiological or cardiac aging in TBD patients.

### Methods

We conducted a retrospective analysis of TBD patients with lymphocyte TL ≤10th percentile versus age-matched controls using flowFISH. Following IRB approval, cardiac data were obtained from the EMR. TBD patients were included if they had ≥1 ECG in the Mayo Clinic AI-ECG dashboard. Statistical comparisons were made with controls having TL >10th percentile. Parameters included AI-ECG–predicted age, probability of low ejection fraction (EF), and probability of AF.

**Copyright Information:** (Online) ISSN: 2572-9241
©2026 The Author(s). HemaSphere is published by John Wiley & Sons Ltd on behalf of European Hematology Association. This is an open access Abstract Book distributed under the Attribution-NonCommercial-NoDerivs (CC BY-NC-ND), which allows third parties to download the articles and share them with others as long as they credit the author and the Abstract Book, but they cannot change the content in any way or use them commercially.
**Abstract Book Citations:** Authors, Title, HemaSphere, 2026;10:(S1):pages. The abstract book DOIs can be found on the "Author Information" page.
**Disclaimer:** Articles published in the journal HemaSphere exclusively reflect the opinions of the authors. The authors are responsible for all content in their abstracts including accuracy of the facts, statements, citing resources, etc.

**Results**

Seventy TBD patients [47 males (67%), mean age 70.5 years] had 763 ECGs (662 with TL 1–10th percentile, 94 with TL <1st percentile). The control group included 27 individuals [12 males (44%), mean age 56.7 years] with 186 ECGs. Mean AI-predicted ECG age minus chronological age was +2.95 years in TBD (95% CI 2.24–3.65; $p < 0.001$) versus −0.29 years in controls (95% CI −1.72–1.13; $p = 0.699$). Post hoc analysis showed TBD patients had predicted ages 3.24 years higher than controls ($p = 6.77\times10^{-5}$). AI-ECG predicted low EF was higher in TBD patients (10.4%, 95% CI 8.92–11.89) than controls (6.3%, 95% CI 3.27–9.28), with a post hoc difference of 4.13% ($p = 0.0157$). Median EF (%) in the TBD group (234 echocardiograms from 59 TBD patients) was found to be at 61.13% (range 22%-73%) vs 59.5% (range 38%-75%) in controls (59 echos from 21 patients) at a level that did not show significance (delta 1.6%, $p = 0.077$).
Significance was found between TBD and control groups when investigating the AI-ECG predictions regarding AF. The probability of AF in the TBD group was 21.86% (95% CI 20.072–23.639) and the control group was 8.06% (95% CI 4.486–11.6405). The difference between the two groups being 13.79%, with the TBD group having a significantly higher predicted probability than the control group ($p = 2.3025e-11$). Comparing these findings to actual ECG rhythms, 44 out of the 763 ECGs (5.8%) had documented AF in the TBD group (9 out of 70 individuals (12.9%)), whereas 8 out of 186 ECGs (4.3%) had documented AF (4 out of 27 individuals (14.8%)) in the control group.

**Summary/Conclusion**

Our analysis found statistical differences in all AI-predicted parameters between the TBD cohort and the control group. TBD patients on average demonstrated an AI-ECG age 3 years greater than their chronological age. Additionally, the TBD group had a 4.13% higher probability of having a low EF and a 13.79% higher probability of having AF compared to controls. Notably, non-AI measurements, such as actual EF from patient echocardiograms, did not show significant difference between the groups. These findings suggest AI-ECG analysis may detect subclinical physiological or cardiac aging, consistent with telomere attrition and premature senescence. Further prospective studies are needed to expand upon these findings.

**Copyright Information:** (Online) ISSN: 2572-9241
©2026 The Author(s). HemaSphere is published by John Wiley & Sons Ltd on behalf of European Hematology Association. This is an open access Abstract Book distributed under the Attribution-NonCommercial-NoDerivs (CC BY-NC-ND), which allows third parties to download the articles and share them with others as long as they credit the author and the Abstract Book, but they cannot change the content in any way or use them commercially.
**Abstract Book Citations:** Authors, Title, HemaSphere, 2026;10:(S1):pages. The abstract book DOIs can be found on the "Author Information" page.
**Disclaimer:** Articles published in the journal HemaSphere exclusively reflect the opinions of the authors. The authors are responsible for all content in their abstracts including accuracy of the facts, statements, citing resources, etc.

### (PB4559) A COST-EFFECTIVE SCALABLE WORKFLOW FOR CLINICAL BLOOD MONITORING USING GEM-X FLEX V2 (APEX) SINGLE-CELL TRANSCRIPTOMIC PROFILING

**Topic:** 38. Novel technologies, techniques and digital analytical tools in hematology

Tingsheng Drennon*[1], Davina Sin[1], Jawad Abousoud[1], Yinzhou Zhu[1], Xue Wu[1], Andrew Kohlway[1], Peter Smibert[1]

[1] *10x Genomics, Pleasanton, United States of America;*

### Background

Peripheral blood sampling is fundamental for minimally invasive disease monitoring and therapeutic development. However, the requirement to process blood within hours of collection to prevent transcriptional drift is often impractical, particularly in multi-site clinical trials. While bulk RNA-Seq is widely used to profile transcriptomic changes, it masks cell-type-specific responses critical to understanding complex disease mechanisms.

### Aims

We provide a cost-effective, high-throughput workflow integrating commercially available blood collection tubes (BCTs) with the GEM-X Flex v2 (Apex) assay for multiplexing up to 384 samples per pool. By stabilizing *in vivo* gene expression at the point of collection, this approach supports room temperature (RT) shipping, offers processing flexibility, and delivers scalable, high-resolution single-cell transcriptomic profiling.

### Methods

We evaluated several specialized BCTs, originally designed for cell-free DNA and circulating tumor cells, against K2 EDTA controls. Healthy donor blood was collected and stored at RT for up to seven days. To best preserve biology, blood samples were first fixed with 4% PFA followed by PBMC or whole leukocyte isolation and then processed using the GEM-X Flex v2 (Apex) assay. Performance benchmarks included complexity (median genes/UMIs), cell type proportion, alongside assessment of stress-induced artifacts and differentially expressed genes (DEGs) relative to fresh samples.

### Results

We identified four BCTs that can stabilize blood transcriptome for up to 2 days at RT, providing a critical window for sample processing and transport. While EDTA controls exhibited significant transcriptional drift within 24 hours, the stabilizing BCTs preserved the biological state, effectively suppressing stress signatures (*EGR1, FOS, JUN, DUSP1)* and maintaining minimal DEGs. Flex assay data complexity and cell-type distributions remained consistent across the 2-day window, demonstrating assay robustness while providing granular insights into various cell populations, compared to traditional bulk sequencing. Successful long-term storage of fixed blood at -80°C highlighted the compatibility with sample batching and dry ice shipping. Preliminary results also demonstrate the feasibility of plate-based, high-throughput isolation protocols, supporting future automated scalability.

### Summary/Conclusion

In summary, integrating stabilizing BCTs with the GEM-X Flex v2 (Apex) assay overcomes logistical barriers and provides a cost-effective path to clinical scRNA-seq. By enabling 2-day RT stability, this workflow allows transition from bulk RNA-seq to high-resolution single-cell analysis using existing clinical trial infrastructure. When combined with plate-based isolation, this approach offers  a scalable, clinical-grade solution for global biobanking and longitudinal immune monitoring for therapeutic development.

**Copyright Information:** (Online) ISSN: 2572-9241
©2026 The Author(s). HemaSphere is published by John Wiley & Sons Ltd on behalf of European Hematology Association. This is an open access Abstract Book distributed under the Attribution-NonCommercial-NoDerivs (CC BY-NC-ND), which allows third parties to download the articles and share them with others as long as they credit the author and the Abstract Book, but they cannot change the content in any way or use them commercially.
**Abstract Book Citations:** Authors, Title, HemaSphere, 2026;10:(S1):pages. The abstract book DOIs can be found on the “Author Information” page.
**Disclaimer:** Articles published in the journal HemaSphere exclusively reflect the opinions of the authors. The authors are responsible for all content in their abstracts including accuracy of the facts, statements, citing resources, etc.

### (PB4560) PREPARING A SOUTHERN TUNISIAN HEMOPHILIA REGISTRY FOR AI-DRIVEN CLINICAL KNOWLEDGE RETRIEVAL

**Topic:** 38. Novel technologies, techniques and digital analytical tools in hematology

Lobna Kammoun*[1], Maha Charfi[2], Fatma Megdiche[3], Faten Kallel[2], Hatem Bellaaj[4], Moez Elloumi[2]

*[1]Hôpital Robert Balanger, Villepint, France; [2]CHU Hédi Chaker, Hématologie clinique, sfax, Tunisia; [3]Clinical Medical Laboratory, Military University Hospital of Sfax, sfax, Tunisia; [4]REDCAD Laboratory, Sfax, Tunisia;*

### Background

Hemophilia requires lifelong monitoring of coagulation parameters, treatment exposure, and bleeding complications. Regional registries are essential for rare disease management, particularly in resource-limited settings, yet their hybrid structure, combining structured variables and unstructured narratives, limits interoperability and advanced analytical use. In Southern Tunisia, a regional hemophilia registry collects longitudinal demographic, biological, therapeutic, and complication data. Retrieval-Augmented Generation (RAG) offers a framework for traceable, context-aware knowledge retrieval from complex clinical datasets.

### Aims

To develop a structured digital framework transforming a regional hemophilia registry in Southern Tunisia into an AI-ready analytical platform compatible with RAG-based clinical knowledge retrieval.

### Methods

A retrospective registry of hemophilia patients was analyzed, including demographics, severity, factor levels, inhibitor titers, treatment modalities, bleeding events, orthopedic outcomes, and free text notes.
An AI-oriented transformation pipeline was implemented (Figure 1). Data harmonization included missing data analysis, unit normalization, categorical standardization, and temporal alignment. Severity grading was unified across heterogeneous formats. Numeric variables were converted into standardized clinical statements (e.g., “Factor VIII <1%” → “Severe hemophilia A”). Free-text entries were semantically enriched through entity extraction (bleeding type, anatomical site, inhibitor status) and temporal tagging. Structured and enriched data were merged into longitudinal clinical documents segmented into semantically coherent chunks. Biomedical embeddings were generated to build a vector database synchronized with the relational registry. Retrieval performance was assessed using predefined clinical queries and Recall@k metrics within a RAG framework.

### Results

The registry included 83 patients and 165 variables. Overall structural completeness was 61.5%, with inactive fields accounting for most missing data.
Numeric data coverage reached 77.6%, while 45.9% of textual fields contained clinically meaningful content. Factor VIII values were available in 72 patients, with severity distribution of 31.9% severe, 50.0% moderate, and 18.1% mild. Inhibitor status was documented in 100% of patients. Bleeding frequency over six months was recorded in 28.9%.
Preprocessing harmonized heterogeneous severity and laboratory reporting. Numeric fields were systematically converted into standardized clinical language, and narrative entries were structured into categorized, temporally ordered events. The hybrid relational vector transformation generated AI-ready longitudinal documents enabling context-aware retrieval across severity, inhibitor status, bleeding phenotype, and treatment exposure.

**Copyright Information:** (Online) ISSN: 2572-9241
©2026 The Author(s). HemaSphere is published by John Wiley & Sons Ltd on behalf of European Hematology Association. This is an open access Abstract Book distributed under the Attribution-NonCommercial-NoDerivs (CC BY-NC-ND), which allows third parties to download the articles and share them with others as long as they credit the author and the Abstract Book, but they cannot change the content in any way or use them commercially.
**Abstract Book Citations:** Authors, Title, HemaSphere, 2026;10:(S1):pages. The abstract book DOIs can be found on the “Author Information” page.
**Disclaimer:** Articles published in the journal HemaSphere exclusively reflect the opinions of the authors. The authors are responsible for all content in their abstracts including accuracy of the facts, statements, citing resources, etc.

### Summary/Conclusion

Transformation of a regional hemophilia registry into an AI-ready analytical platform is feasible and reproducible. Structured normalization and semantic enrichment within a hybrid relational vector architecture enable traceable, advanced clinical knowledge retrieval. This approach represents a novel digital analytical tool in hematology and supports secondary use of regional rare disease registries.

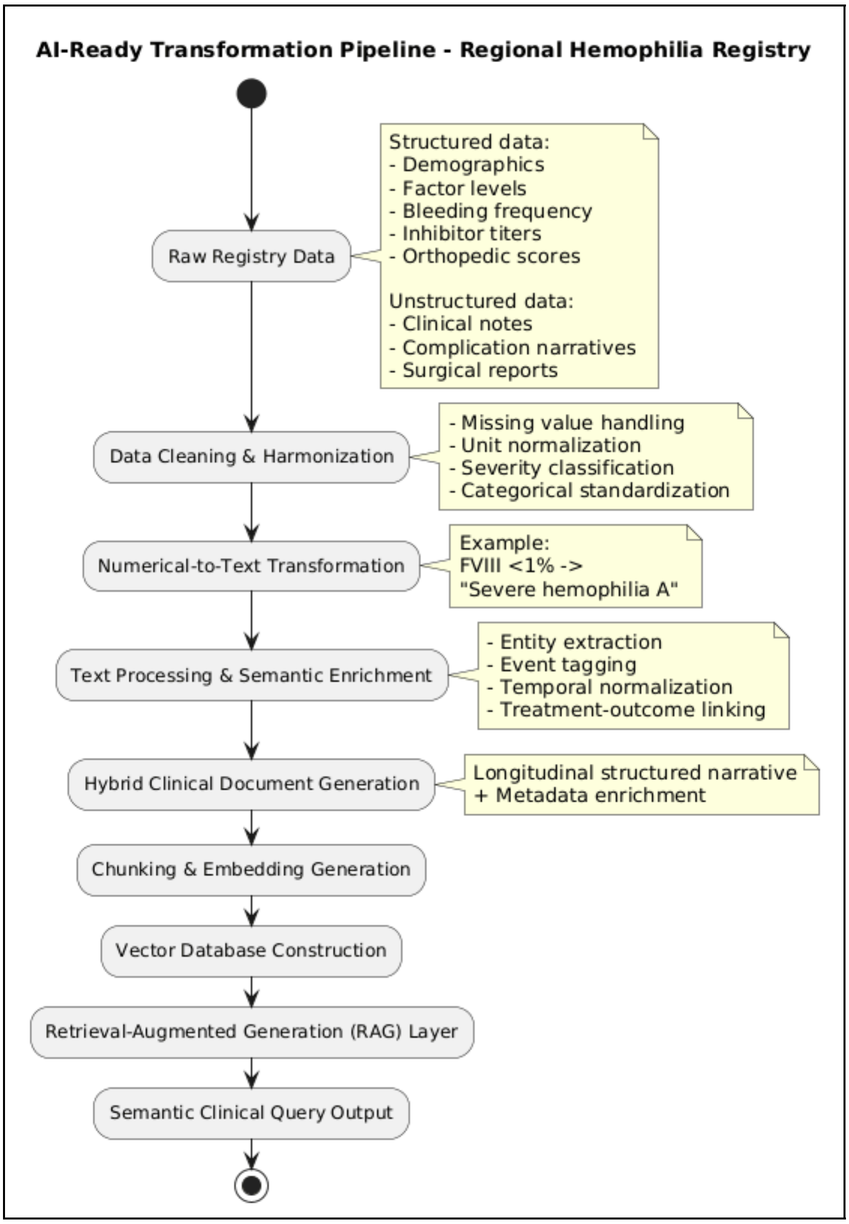

**Copyright Information:** (Online) ISSN: 2572-9241
©2026 The Author(s). HemaSphere is published by John Wiley & Sons Ltd on behalf of European Hematology Association. This is an open access Abstract Book distributed under the Attribution-NonCommercial-NoDerivs (CC BY-NC-ND), which allows third parties to download the articles and share them with others as long as they credit the author and the Abstract Book, but they cannot change the content in any way or use them commercially.
**Abstract Book Citations:** Authors, Title, HemaSphere, 2026;10:(S1):pages. The abstract book DOIs can be found on the "Author Information" page.
**Disclaimer:** Articles published in the journal HemaSphere exclusively reflect the opinions of the authors. The authors are responsible for all content in their abstracts including accuracy of the facts, statements, citing resources, etc.

### (PB4561) STABILITY OF COMPLETE BLOOD COUNT PARAMETERS STORED AT 4°C

**Topic:** 38. Novel technologies, techniques and digital analytical tools in hematology

Zeineb Landolsi[1], Mohamed Yassine Kaabar[2], Asma Ouahada[1], Ikbel Ghachem*[3], Amira Ben Hriz[1], Sonia Chouaieb[4], Haifa Tounsi[5], Wafa Skouri[5], Hamida Khémiri Guerbouj[5], Rabeb Berred[6], Asma Bachali[3]

*[1]Faculty of pharmacy of Monastir, Nabeul, Tunisia;[2]University of Monastir, Faculty of Pharmacy of Monastir, Laboratory of Biology, Tahar Maamouri Hospital,, Nabeul, Tunisia; [3]University of Tunis El Manar, Faculty of Medicine of Tunis, Laboratory of Biology, Tahar Maamouri Hospital, Nabeul, Tunisia; [4]University of Monastir, Faculty of Pharmacy of Monastir, Laboratory of Biology, Habib Thamer Hospital, Tunis, Tunisia; [5]University of Tunis El Manar, Faculty of medicine of Tunis, Internal medicine Department, Taher Maamouri Hospital, Nabeul, Tunisia; [6]University of Tunis El Manar, Faculty of Medicine of Tunis, Department of Hematology, Aziza Othmana Hospital, Tunis, Tunisia;*

### Background

Clinical biology practice occasionally encounters situations where performing a complete blood count (CBC) on a sample significantly after collection is necessary. This requirement, however, is constrained by sample stability, necessitating an evaluation of optimal storage conditions.

### Aims

This study aims to assess the stability of CBC parameters in samples stored at +4°C.

### Methods

Thirty-eight randomly selected CBC samples were stored at +4°C immediately after initial analysis on Day 0 (D0). They were reanalyzed on Days 1, 2, and 3 (D1, D2, D3) using a Mindray® BC-6200 analyzer. Parameters assessed included leukocytes, red blood cells (RBC), hemoglobin (Hb), hematocrit (Hct), mean corpuscular volume (MCV), mean corpuscular hemoglobin (MCH), mean corpuscular hemoglobin concentration (MCHC), red cell distribution width (RDW-CV), and platelets (PLT). Results were compared to D0 values, with deviations evaluated against acceptable limits derived from the Ricos biological variation database.

### Results

By D3, leukocyte, RBC, Hb, and RDW-CV parameters demonstrated stability within acceptable limits in over 88% of samples. Parameters related to erythrocyte volume were less stable, with Hct, MCV, MCH, and MCHC remaining stable in only 50%, 58%, 61%, and 26% of samples, respectively. MCHC and PLT counts showed markedly reduced stability as early as D1, with only 58% and 76% of results remaining stable, respectively. Median percentage changes between D0 and D3 for all parameters ranged from -2.4% (Hct) to 3.3% (PLT). Platelets were stable in 71% of cases by D3 but exhibited the highest variability, with extreme changes ranging from -18.1% to +20.3% relative to D0 values.

### Summary/Conclusion

Clinically critical parameters, including leukocyte count, RBC, and hemoglobin, exhibit satisfactory stability under refrigeration at +4°C when judged against stringent Ricos-based criteria. Although platelet counts demonstrate greater variability, they retain clinically interpretable values up to D3. Overall, CBC parameters show enhanced stability at 4°C compared to room temperature storage.

**Copyright Information:** (Online) ISSN: 2572-9241
©2026 The Author(s). HemaSphere is published by John Wiley & Sons Ltd on behalf of European Hematology Association. This is an open access Abstract Book distributed under the Attribution-NonCommercial-NoDerivs (CC BY-NC-ND), which allows third parties to download the articles and share them with others as long as they credit the author and the Abstract Book, but they cannot change the content in any way or use them commercially.
**Abstract Book Citations:** Authors, Title, HemaSphere, 2026;10:(S1):pages. The abstract book DOIs can be found on the "Author Information" page.
**Disclaimer:** Articles published in the journal HemaSphere exclusively reflect the opinions of the authors. The authors are responsible for all content in their abstracts including accuracy of the facts, statements, citing resources, etc.

### (PB4562) A COMPARATIVE STUDY OF GENERATIVE AI IN HEALTH EDUCATION SUPPORT FOR HEMATOPOIETIC STEM CELL TRANSPLANTATION PATIENTS: CHATGPT-4O VS. DEEPSEEK-V3

**Topic:** 38. Novel technologies, techniques and digital analytical tools in hematology

Shashali Shasha*[1]

[1]*Blood Disease Medicine Center, Second Affiliated Hospital of Army Medical University, Blood Disease Medicine Center, Second Affiliated Hospital of Army Medical University, Chongqing, China;*

### Background

To compare and analyze the quality of information generated by generative artificial intelligence (AI) systems, represented by ChatGPT-4o and DeepSeek-V3, in patient education for hematopoietic stem cell transplantation (HSCT).

### Aims

The analysis covers core content such as post-transplant self-management, identification and response to graft-versus-host disease (GVHD), disease recurrence monitoring, and psychological support, aiming to evaluate their applicability and limitations in transplant care health education. This study provides a basis for the development of intelligent patient education tools

### Methods

Through literature review and clinical interviews, the research team systematically identified 34 common nursing issues encountered by hematopoietic stem cell transplantation patients during in-hospital and out-of-hospital rehabilitation, categorizing them into three major types: transplantation basics (e.g., transplantation principles, immune reconstitution process), lifestyle guidance (e.g., diet, activity, infection prevention), and professional response (e.g., GVHD recognition, recurrence signs, management of drug side effects). Each issue was structured into instructions and sequentially input into the ChatGPT-4o and DeepSeek-V3 models for interaction. Six clinical experts with over five years of transplantation nursing experience conducted double-blind evaluations of the model outputs, using the "China Hematopoietic Stem Cell Transplantation Nursing Practice Guidelines" as the standard to assess information accuracy. Additionally, the generated content was systematically evaluated using the modified "Ensure Quality of Patient Information" (EQIP) tool, covering dimensions such as information completeness, readability, structural logic, and source transparency. Scores ≥19 were considered high-quality models.

### Results

ChatGPT-4o generated 28 accurate responses across 34 questions, achieving an accuracy rate of 82.4%, while DeepSeek-V3 produced 23 accurate responses with an accuracy rate of 67.6%. In terms of classification performance, both models demonstrated optimal results in life guidance questions (100% accuracy), but showed relatively weaker performance in professional response questions such as GVHD identification and relapse monitoring. Regarding EQIP scores, ChatGPT-4o and DeepSeek-V3 achieved 22 and 24 points respectively, both meeting high-quality standards. However, both models exhibited deficiencies in information source transparency and encountered varying degrees of factual errors or "AI hallucinations," such as providing incomplete differential diagnostic points when answering "acute GVHD skin manifestations."

**Copyright Information:** (Online) ISSN: 2572-9241
©2026 The Author(s). HemaSphere is published by John Wiley & Sons Ltd on behalf of European Hematology Association. This is an open access Abstract Book distributed under the Attribution-NonCommercial-NoDerivs (CC BY-NC-ND), which allows third parties to download the articles and share them with others as long as they credit the author and the Abstract Book, but they cannot change the content in any way or use them commercially.
**Abstract Book Citations:** Authors, Title, HemaSphere, 2026;10:(S1):pages. The abstract book DOIs can be found on the "Author Information" page.
**Disclaimer:** Articles published in the journal HemaSphere exclusively reflect the opinions of the authors. The authors are responsible for all content in their abstracts including accuracy of the facts, statements, citing resources, etc.

**Summary/Conclusion**

ChatGPT-4o and DeepSeek-V3 demonstrate certain auxiliary potential in health education for hematopoietic stem cell transplantation (HSCT) patients, particularly suitable for disseminating basic transplantation knowledge and providing daily life guidance. However, in complex scenarios requiring highly specialized knowledge and individualized judgment, such as GVHD assessment and recurrence risk evaluation, the accuracy and completeness of their generated content remain insufficient. Future efforts should focus on developing large language models tailored for transplantation fields, incorporating knowledge graphs and clinically validated data, while integrating professional review mechanisms by healthcare teams to establish a credible, accessible, and personalized intelligent patient education support system.

**Copyright Information:** (Online) ISSN: 2572-9241
©2026 The Author(s). HemaSphere is published by John Wiley & Sons Ltd on behalf of European Hematology Association. This is an open access Abstract Book distributed under the Attribution-NonCommercial-NoDerivs (CC BY-NC-ND), which allows third parties to download the articles and share them with others as long as they credit the author and the Abstract Book, but they cannot change the content in any way or use them commercially.
**Abstract Book Citations:** Authors, Title, HemaSphere, 2026;10:(S1):pages. The abstract book DOIs can be found on the “Author Information” page.
**Disclaimer:** Articles published in the journal HemaSphere exclusively reflect the opinions of the authors. The authors are responsible for all content in their abstracts including accuracy of the facts, statements, citing resources, etc.

### (PB4563) ARTIFICIAL INTELLIGENCE FOR BONE MARROW IMAGE–BASED DIAGNOSIS OF MYELODYSPLASTIC NEOPLASMS: A SYSTEMATIC REVIEW AND META-ANALYSIS

**Topic:** 38. Novel technologies, techniques and digital analytical tools in hematology

Ahmed Elgebaly*[1]

[1] *University of East London, London, United Kingdom;*

### Background

Artificial intelligence (AI)-based morphology tools for myelodysplastic neoplasms (MDS) are emerging, but their diagnostic performance across bone marrow image studies has not been synthesized

### Aims

To systematically review AI models applied to bone marrow smears/biopsies for MDS diagnosis or dysplasia detection and to meta-analyse compatible patient-level external-validation datasets.

### Methods

We performed a rapid systematic search of PubMed and publisher-hosted literature from inception to February 1, 2026. Eligible studies were original human studies using AI/machine learning/deep learning on bone marrow smear or biopsy images for MDS diagnosis or MDS-defining dysplasia. Reviews, peripheral-blood-only studies, genomics-only studies, and non-diagnostic studies were excluded. We extracted study design, specimen type, task, validation strategy, and performance metrics. Quantitative synthesis used random-effects pooling of external-validation accuracy for compatible patient-level classifiers.

### Results

Six studies met the inclusion criteria. Three used bone marrow smears for direct case-level classification, two focused mainly on cell-level dysplasia recognition, and one used marrow biopsy/megakaryocyte-based classification. Reported performance was generally high. In the largest externally validated smear study, Wang et al. reported an external-validation AUC of 0.942 (95% CI, 0.918–0.967) and accuracy of 0.921 (95% CI, 0.901–0.942) for MDS classification. In a recent end-to-end smear study, Eckardt et al. reported external-validation accuracy of 0.9902 (95% CI, 0.9830–0.9974) for MDS vs donor and 0.9210 (95% CI, 0.8919–0.9501) for MDS vs AML. Exploratory inverse-variance pooling of the two external-validation accuracy estimates most suitable for quantitative synthesis yielded a pooled accuracy of 0.921 (95% CI, 0.904–0.938).

### Summary/Conclusion

AI-based bone marrow image analysis shows promising diagnostic performance for MDS, but current evidence remains small, heterogeneous, and predominantly retrospective. The field appears ready for clinical-assistive use studies. Standardized external validation against real-world data is needed.

**Copyright Information:** (Online) ISSN: 2572-9241
©2026 The Author(s). HemaSphere is published by John Wiley & Sons Ltd on behalf of European Hematology Association. This is an open access Abstract Book distributed under the Attribution-NonCommercial-NoDerivs (CC BY-NC-ND), which allows third parties to download the articles and share them with others as long as they credit the author and the Abstract Book, but they cannot change the content in any way or use them commercially.
**Abstract Book Citations:** Authors, Title, HemaSphere, 2026;10:(S1):pages. The abstract book DOIs can be found on the "Author Information" page.
**Disclaimer:** Articles published in the journal HemaSphere exclusively reflect the opinions of the authors. The authors are responsible for all content in their abstracts including accuracy of the facts, statements, citing resources, etc.

### (PB4564) NEW MULTIPARAMETRIC FLOW CYTOMETRY DATA ANALYSIS USING A COMBINED FLOWSOM AND BAYESIAN PROCESS. APPLICATION TO PERIPHERAL BLOOD LEUKOCYTE DIFFERENTIAL.BACKGROUND.

**Topic:** 38. Novel technologies, techniques and digital analytical tools in hematology

Francis Lacombe[1], Gilbert Carron[2], Marie Christine Béné*[3]

*[1]Bordeaux University Hospital, Pessac, France; [2]Laboratoires Carron, Montceau les Mines, France; [3]Nantes University, Nantes, France;*

### Background

Multiparameter flow cytometry generates high-dimensional files from single-cell analysis of protein marker expression that still mostly rely on expert-depending gating strategies. These approaches raise major concerns of reproducibility, analytical precision and time efficiency, with little explicit modeling of probabilistic uncertainty. Current unsupervised strategies are liable to increase the precision of cell subsets characterization. Yet, they still rely on supervised validation for quantitative analysis.

### Aims

To develop unsupervised structuration methods providing a formal decisional framework for cell classification and quantitation.

### Methods

The LBC-Flow solution proposed here is a structured probabilistic framework for automatized and reproducible assessment of cell subsets, in the exemplified application of peripheral blood leukocyte subsets differential. This strategy first requires unsupervised data clustering using the self-organizing map strategy with FlowSOM. The latter is built from the merge of a series of normal and relevant pathological blood samples. This yields a reference minimal spanning tree (MST) of 64 nodes. Supervised expert analysis of each node allows to characterize cell subsets. In the LBC-Flow panel presented here, 26 cell subsets were characterized, with the addition of potentially unclassifiable events in a patient blood. From this annotation, providing multidimensional immunophenotypic characteristics of each subset, a naïve gaussian Bayesian model can be trained. For upcoming new samples, this Bayesian model will assign to each leukocyte a posterior probability of belonging to a given pre-defined cell subset. Classification decisions rely then on probabilistic acceptance criteria allowing for a strong assignment of cell subset populations.

### Results

This method was validated on a series of 30 blood samples that had not raised any alarm on a hematology analyzer nor in digital morphology. Manual gating, FlowSOM-based quantification and Bayesian classification methods were compared. A high concordance was evidenced between these approaches, the Bayesian cell-based gaussian strategy providing the best performance, yielding probabilistic results in less than 30 seconds per sample for the 27 cell subsets identified by this screening flow cytometry panel.

### Summary/Conclusion

This study demonstrated the LBC-Flow approach to be a robust and reliable tool for the automated assignment and quantification of blood leukocyte subsets.
This interpretable decisional probabilistic framework is applicable to any flow cytometry panel in a broad field of clinical and biological contexts.

**Copyright Information:** (Online) ISSN: 2572-9241
©2026 The Author(s). HemaSphere is published by John Wiley & Sons Ltd on behalf of European Hematology Association. This is an open access Abstract Book distributed under the Attribution-NonCommercial-NoDerivs (CC BY-NC-ND), which allows third parties to download the articles and share them with others as long as they credit the author and the Abstract Book, but they cannot change the content in any way or use them commercially.
**Abstract Book Citations:** Authors, Title, HemaSphere, 2026;10:(S1):pages. The abstract book DOIs can be found on the "Author Information" page.
**Disclaimer:** Articles published in the journal HemaSphere exclusively reflect the opinions of the authors. The authors are responsible for all content in their abstracts including accuracy of the facts, statements, citing resources, etc.

### (PB4565) MISCLUS: MISASSEMBLY CLUSTERING BASED ON PAIRED-END READS

**Topic:** 38. Novel technologies, techniques and digital analytical tools in hematology

Junqing Xu*[1], Xiangyan Feng[1], Xiao Zhu[2]

*[1]Yantai Yuhuangding Hospital Affiliated to Qingdao University, Hematology, Yantai, China;[2]Scool of Computer and Control Engineering, Yantai University, Yantai, China;*

### Background

Along with the development of genome sequence technologies, dozens of assembly algorithms have advent. It remains crucial to reconstruct genome sequences perfectly, there may be misassembly errors in assembled sequences and the errors may have an adverse impact on subsequent analysis, some methods are proposed to detect misassembly errors, whereas there may be many correct sequences mistaken for misassemblies in reported results.

### Aims

To solve the high false positive rate problem and enable the correction of assembly errors, we propose a new method called MisClus. We observed that genuine assembly errors and false positives exhibit distinct characteristics in their read alignment patterns. These features include non-uniform read coverage, abnormal insertions/deletions (indels), and unusual cross-contig read pairs. By extracting these features and utilizing a K-means clustering algorithm, our method can effectively distinguish true assembly errors from false positives, thereby significantly reducing the false positive rate and improving detection accuracy. Furthermore, MisClus is designed to identify error breakpoints and attempt sequence correction, which can further enhance the results of downstream scaffolding and binning.

### Methods

MisClus uses the alignment information of paired-end reads onto assembled sequences to inspect the candidate misassemblies, and the alignment information can be generated by different tools. It consists of the following steps: (1) Extract and normalize misassembly signals including abnormal coverage, abundant indels and clipping reads (IDC), incorrectly paired orientation, unreasonable insert size and inter-contig read pairs; (2) Carry out cluster analysis for these signals, and cluster candidates into misassembly and correct assembly according to different kinds of signals, respectively; (3) Analyze the signals of candidates comprehensively and make sure the misassembly cluster and the correct assembly cluster. An overview of the MisClus pipeline is given in Fig. 1.

### Results we present MisClus, a misassembly clustering method that distinguishes assembly errors identified by misassembly detection methods to determine whether they are true or false by using the alignment information of paired-end reads onto contigs which are assembled by assembly methods. Some signals extracted from the alignment information can be used to estimate candidate misassemblies which are detected by misassembly identification methods. MisClus applies K-means to analyze the characteristic of candidate misassemblies to determine whether they are true or false. MisClus was tested on both bacterial genomes and the human genome.

### Summary/Conclusion

The results demonstrate that MisClus has a good result on misassembly clustering, and we expect MisClus can be used to benefit misassembly work.

**Copyright Information:** (Online) ISSN: 2572-9241
©2026 The Author(s). HemaSphere is published by John Wiley & Sons Ltd on behalf of European Hematology Association. This is an open access Abstract Book distributed under the Attribution-NonCommercial-NoDerivs (CC BY-NC-ND), which allows third parties to download the articles and share them with others as long as they credit the author and the Abstract Book, but they cannot change the content in any way or use them commercially.
**Abstract Book Citations:** Authors, Title, HemaSphere, 2026;10:(S1):pages. The abstract book DOIs can be found on the "Author Information" page.
**Disclaimer:** Articles published in the journal HemaSphere exclusively reflect the opinions of the authors. The authors are responsible for all content in their abstracts including accuracy of the facts, statements, citing resources, etc.

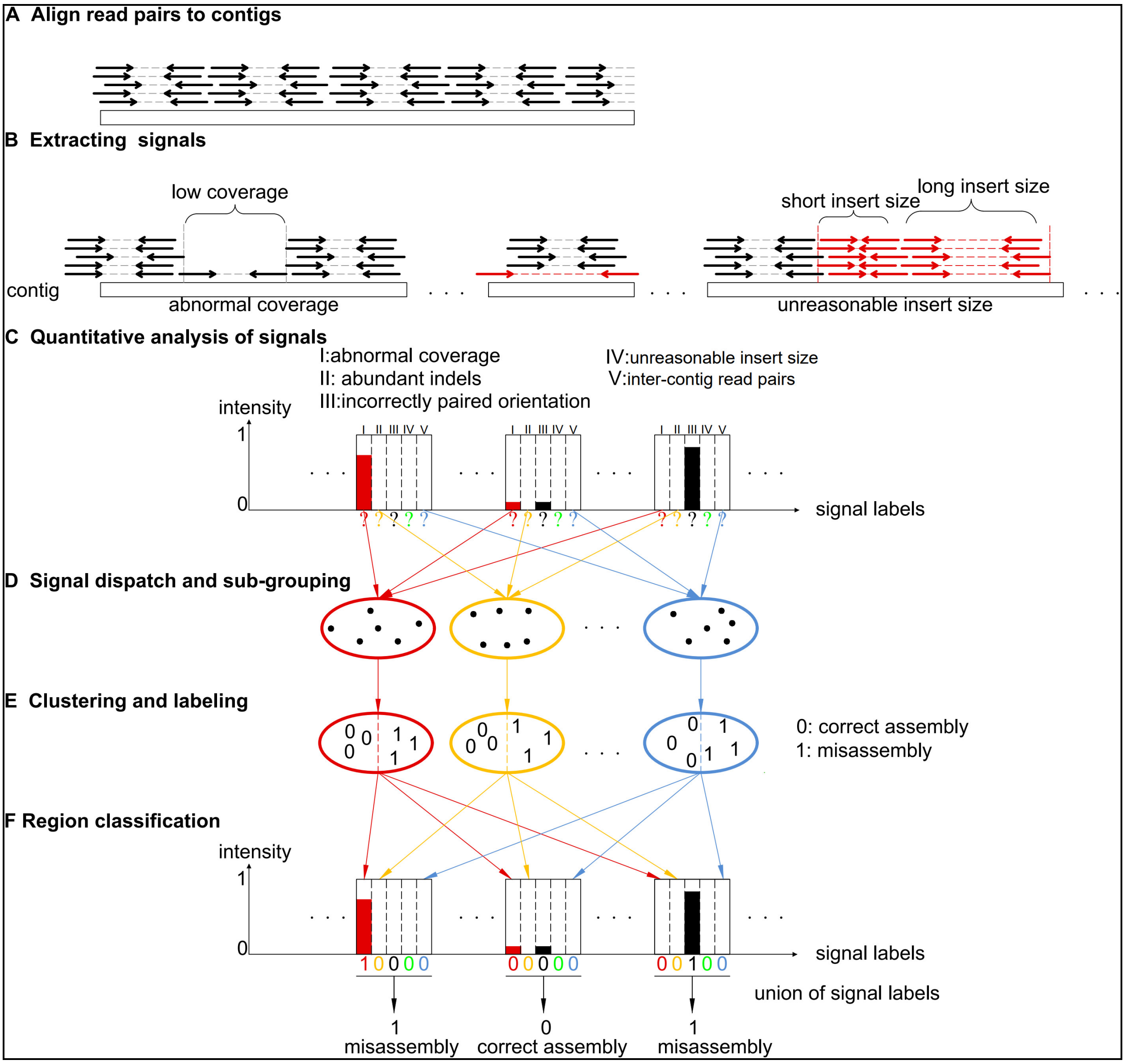

**Copyright Information:** (Online) ISSN: 2572-9241
©2026 The Author(s). HemaSphere is published by John Wiley & Sons Ltd on behalf of European Hematology Association. This is an open access Abstract Book distributed under the Attribution-NonCommercial-NoDerivs (CC BY-NC-ND), which allows third parties to download the articles and share them with others as long as they credit the author and the Abstract Book, but they cannot change the content in any way or use them commercially.
**Abstract Book Citations:** Authors, Title, HemaSphere, 2026;10:(S1):pages. The abstract book DOIs can be found on the "Author Information" page.
**Disclaimer:** Articles published in the journal HemaSphere exclusively reflect the opinions of the authors. The authors are responsible for all content in their abstracts including accuracy of the facts, statements, citing resources, etc.

### (PB4566) OPTIMIZING SUBCUTANEOUS SYSTEMIC ANTICANCER THERAPY (SACT) DELIVERY: UNITED KINGDOM NURSE AND PHARMACIST PERSPECTIVES ON ON-BODY INJECTORS VERSUS MANUAL SYRINGES FOR LARGE-VOLUME ADMINISTRATION

**Topic:** 38. Novel technologies, techniques and digital analytical tools in hematology

Catherine Loughran[1], Hannah Miller[2], Rachel Senior[3], Jackie Quinn[4], Mehul Desai[5], Haydon Dumond*[5]

*[1]University Hospitals of Leicester NHS Trust, Leicester, United Kingdom;[2]The Christie NHS Foundation Trust @ Macclesfield, Manchester, United Kingdom;[3]Leeds Teaching Hospitals NHS Trust, Leeds, United Kingdom;[4]Belfast Health and Social Care Trust, Belfast, United Kingdom;[5]Enable Injections Inc., Cincinnati, United States of America;*

### Background

Across the United Kingdom (UK), the expanded use of large-volume subcutaneous (LVSC) biologics combined with rising demand for systemic anti-cancer therapies (SACT) is placing increasing operational pressure on oncology and hematology services and constraining the delivery of timely, efficient care. Innovative solutions are therefore needed to optimize resources and enhance efficiency. On-body injector (OBI) systems may help address these pressures by simplifying preparation steps, reducing hands-on workload, and supporting more flexible administration models, including HCP-assisted home delivery.

### Aims

To evaluate nurse and pharmacist preferences for OBI technology compared with conventional manual syringe preparation for LVSC therapies.

### Methods

Nurses and pharmacists from multiple European healthcare centers viewed a standardized instructional video and 3-minute presentation demonstrating an OBI (Product X). Participants then completed an online questionnaire assessing ease of use, training requirements, perceived safety, and applicability in hospital and home-care models. Descriptive statistics were used to summarize responses.

### Results

A total of 162 respondents participated in the survey, comprising 81 nurses and 81 pharmacists, with an average professional experience of 13 years. Respondents reported strong familiarity with current LVSC preparation processes (mean rating 4.1/5) and widespread recognition of the SACT crisis in the UK (4.2/5). After viewing the demonstration of the on-body injector (Product X), nearly all respondents rated the preparation process as easy to use (96.9%) and easy to learn (95.7%). When asked to compare preparation methods for LVSC biologics and for treating multiple myeloma, 93.8% favored the hands-free OBI over traditional manual syringe preparation. Perceived advantages included reduced preparation time, lower physical effort, improved operator safety, and less complexity/supplies needed. Anticipated workflow benefits were also highly rated: 94.4% expected efficiency gains, 91.4% anticipated fewer preparation-related errors due to simplified steps and reduced supply requirements, and 96.3% believed the OBI would decrease physical and mental workload for hospital staff. Occupational safety concerns were prominent. A history of needlestick injuries during sterile compounding was reported by 87% of participants, and 96.9% indicated that the needle-free preparation pathway of Product X would eliminate this risk (weighted average 1.78% occurrence). Finally, respondents rated the device as well suited for administration outside of the clinic across user groups, including providers (4.4/5), caregivers (4.1/5), and patients (4.1/5).

**Copyright Information:** (Online) ISSN: 2572-9241
©2026 The Author(s). HemaSphere is published by John Wiley & Sons Ltd on behalf of European Hematology Association. This is an open access Abstract Book distributed under the Attribution-NonCommercial-NoDerivs (CC BY-NC-ND), which allows third parties to download the articles and share them with others as long as they credit the author and the Abstract Book, but they cannot change the content in any way or use them commercially.
**Abstract Book Citations:** Authors, Title, HemaSphere, 2026;10:(S1):pages. The abstract book DOIs can be found on the “Author Information” page.
**Disclaimer:** Articles published in the journal HemaSphere exclusively reflect the opinions of the authors. The authors are responsible for all content in their abstracts including accuracy of the facts, statements, citing resources, etc.

**Summary/Conclusion**

Nurses and pharmacists working within the UK clinical environment expressed a strong preference for OBI technology over manual syringe preparation for LVSC therapies. Anticipated advantages included improved efficiency, enhanced operator safety, and reduced physical workload, alongside perceived feasibility for administration outside of traditional clinical settings. These findings suggest that OBI systems may help address ongoing operational challenges across UK oncology and hematology services.

**Copyright Information:** (Online) ISSN: 2572-9241
©2026 The Author(s). HemaSphere is published by John Wiley & Sons Ltd on behalf of European Hematology Association. This is an open access Abstract Book distributed under the Attribution-NonCommercial-NoDerivs (CC BY-NC-ND), which allows third parties to download the articles and share them with others as long as they credit the author and the Abstract Book, but they cannot change the content in any way or use them commercially.
**Abstract Book Citations:** Authors, Title, HemaSphere, 2026;10:(S1):pages. The abstract book DOIs can be found on the "Author Information" page.
**Disclaimer:** Articles published in the journal HemaSphere exclusively reflect the opinions of the authors. The authors are responsible for all content in their abstracts including accuracy of the facts, statements, citing resources, etc.

### (PB4567) DIAGNOSTIC VALUE OF BONE MARROW BIOPSY IN POST-TRANSPLANTATION RELAPSE OF PATIENTS WITH ACUTE LEUKEMIA

**Topic:** 38. Novel technologies, techniques and digital analytical tools in hematology

Miao He*[1], Tao Wu[1]

[1] *The 940th Hospital of Joint Logistics Support force of Chinese People's Liberation Army, hematology, Gansu, China;*

### Background

Cell morphology (bone marrow smears and bone marrow biopsy), immunology (flow cytometry), cytogenetics and molecular biology (MICM) have become routine diagnostic methods for leukemia. The combination of these four approaches enables accurate diagnosis of patients and provides critical information for treatment implementation and prognostic assessment. Hematopoietic stem cell transplantation (HSCT) is one of the effective therapeutic modalities for hematologic diseases, and post-transplant relapse remains the primary clinical concern [1-3]. Currently, the commonly used methods for evaluating post-transplant relapse in patients include hematologic relapse, cytogenetic relapse and molecular relapse, while bone marrow biopsy has not yet been incorporated into routine assessment protocols. In this study, we identified that bone marrow biopsy plays an irreplaceable role in evaluating post-transplant relapse and treatment efficacy in patients, and we now report the relevant findings through two case reports.

### Aims

To explore the role of bone marrow biopsy in assessing relapse in patients with acute leukemia after allogeneic hematopoietic stem cell transplantation (allo-HSCT), addressing the diagnostic limitations of conventional monitoring methods.

### Methods

Clinical data of two acute lymphoblastic leukemia(ALL) patients post-allo-HSCT were reviewed. Routine monitoring included bone marrow smears, flow cytometry minimal residual disease (MRD), and chimerism testing. Bone marrow biopsy was performed for patients presenting symptoms but with inconclusive conventional monitoring results.

### Results

Both patients developed symptoms such as bone pain and joint discomfort several months post-transplantation. Bone marrow smears indicated remission, and flow cytometry MRD showed no clear abnormalities. However, bone marrow biopsy confirmed relapse with abundant primitive immature cells, ultimately leading to treatment failure and death.

### Summary/Conclusion

In assessing relapse in ALL patients post-allo-HSCT, bone marrow biopsy compensates for the limitations of bone marrow smears (susceptible to dilution/fibrosis) and flow cytometry MRD (insufficient sensitivity in early stages). Particularly when conventional monitoring fails to clarify relapse but patients exhibit clinical symptoms, bone marrow biopsy provides definitive diagnosis through complete histological features, offering irreplaceable diagnostic value. Clinically, synchronized monitoring of bone marrow smears and biopsies is crucial to avoid delays in relapse diagnosis and treatment.

**Copyright Information:** (Online) ISSN: 2572-9241
©2026 The Author(s). HemaSphere is published by John Wiley & Sons Ltd on behalf of European Hematology Association. This is an open access Abstract Book distributed under the Attribution-NonCommercial-NoDerivs (CC BY-NC-ND), which allows third parties to download the articles and share them with others as long as they credit the author and the Abstract Book, but they cannot change the content in any way or use them commercially.
**Abstract Book Citations:** Authors, Title, HemaSphere, 2026;10:(S1):pages. The abstract book DOIs can be found on the "Author Information" page.
**Disclaimer:** Articles published in the journal HemaSphere exclusively reflect the opinions of the authors. The authors are responsible for all content in their abstracts including accuracy of the facts, statements, citing resources, etc.
